# Automated Platform for the Plasmid Construction Process

**DOI:** 10.1021/acssynbio.3c00292

**Published:** 2023-11-10

**Authors:** Alberto A. Nava, Anna Lisa Fear, Namil Lee, Peter Mellinger, Guangxu Lan, Joshua McCauley, Stephen Tan, Nurgul Kaplan, Garima Goyal, R. Cameron Coates, Jacob Roberts, Zahmiria Johnson, Romina Hu, Bryan Wu, Jared Ahn, Woojoo E. Kim, Yao Wan, Kevin Yin, Nathan Hillson, Robert W. Haushalter, Jay D. Keasling

**Affiliations:** † Joint BioEnergy Institute, Lawrence Berkeley National Laboratory, Emeryville, California 94608, United States; ‡ Biological Systems and Engineering Division, Lawrence Berkeley National Laboratory, Berkeley, California 94720, United States; § Department of Chemical and Biomolecular Engineering, University of California, Berkeley, Berkeley, California 94720, United States; ∥ DOE Agile BioFoundry, Emeryville, California 94608, United States; ⊥ Department of Bioengineering, University of California, Berkeley, Berkeley, California 94720, United States; # Department of Plant and Microbial Biology, University of California, Berkeley, Berkeley, California 94720, United States; ¶ Center for Synthetic Biochemistry, Shenzhen Institutes for Advanced Technologies, Shenzhen 518055, P.R. China; ▽ The Novo Nordisk Foundation Center for Biosustainability, Technical University Denmark, Kemitorvet, Building 220, Kongens Lyngby 2800, Denmark

**Keywords:** synthetic biology, automation, plasmid construction, polyketide synthases

## Abstract

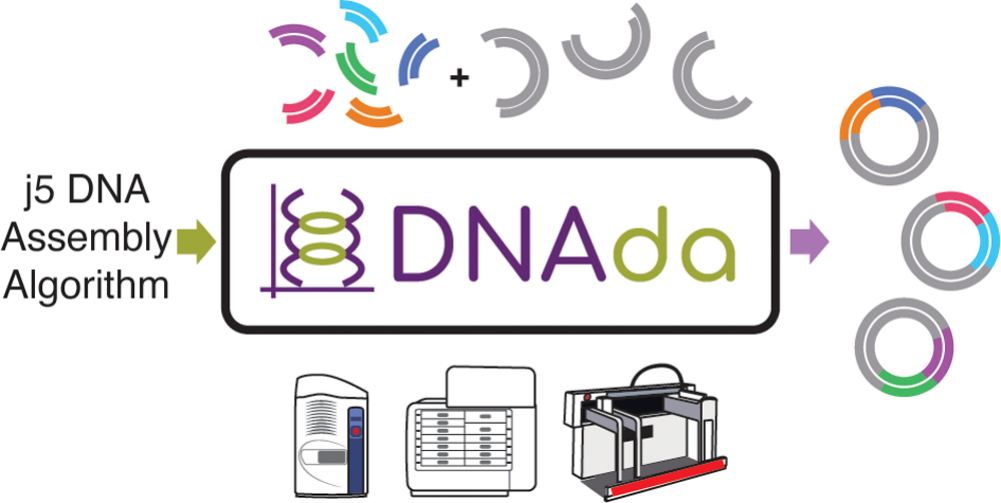

There is a growing need for applications capable of handling large synthesis biology experiments. At the core of synthetic biology is the process of cloning and manipulating DNA as plasmids. Here, we report the development of an application named DNAda capable of writing automation instructions for any given DNA construct design generated by the J5 DNA assembly program. We also describe the automation pipeline and several useful features. The pipeline is particularly useful for the construction of combinatorial DNA assemblies. Furthermore, we demonstrate the platform by constructing a library of polyketide synthase parts, which includes 120 plasmids ranging in size from 7 to 14 kb from 4 to 7 DNA fragments.

## Introduction

Synthetic biology is a field of engineering predicated on the central dogma of biology: that of characterizing and manipulating DNA to alter the structure and function of proteins, cells, and organisms. Metabolic engineering employs the principles of synthetic biology for the exploitation of natural and new-to-nature biosynthetic pathways to produce the desired natural and unnatural products. Technological advances in DNA sequencing and molecular biology have enabled impressive results, such as the reengineering of artemisinic acid biosynthesis in yeast^1^ and the production of biofuels and bulk chemicals in microorganisms.^2−4^ As metabolic pathways become larger and more complicated, so too grows the demand for solutions for larger and more complicated DNA assemblies.

The j5 algorithm^5^ and its associated software packages have played a significant role in the advancement of synthetic biology, particularly in the context of DNA assembly. The algorithm is a computational tool that automates the design of DNA assembly protocols to allow for the efficient combination of DNA fragments into a single construct. Its development was inspired by the need to streamline the assembly process and reduce the time and resources required in the rapidly expanding field of synthetic biology. Over the years, the j5 algorithm has been integrated into a user-friendly software package, DeviceEditor,^6^ a visual design tool for DNA assembly. The J5 algorithm has expanded to include a variety of assembly strategies, including type IIS restriction enzyme-based Golden Gate assembly and homology-based strategies, such as sequence and ligation-independent cloning, isothermal Gibson assembly, and circular polymerase extension cloning. These advancements have had a profound impact on the field and have enabled researchers to efficiently tackle increasingly complex challenges in synthetic biology and metabolic engineering.

The rise of biofoundries as advanced laboratories equipped with automation resources has increased the accessibility of high-throughput experiments.^7^ As biofoundries and other automation laboratories have grown, bioinformatic infrastructure has been developed to handle higher-volume experiments.^8−13^ Standards, such as the Synthetic Biology Open Language (SBOL), have been developed to aid in the communication of experimental designs.^14,15^ New programming languages and communication standards (SiLA2) have even been developed for compiling automation instructions across different instruments.^16^ Many of the software tools developed are deployed in complementary web applications that improve adoption by allowing any organization to host the tool. For example, PlasmidMaker^17^ is a powerful web application that provides automation worklists for the end-to-end construction of plasmids using a novel *Pyrococcus furiosus* Argonaute-based artificial restriction enzyme DNA assembly strategy. Additionally, the CUBA web service provided by the Edinburgh Genome Foundry (cuba.genomefoundry.org) provides a suite of powerful DNA design and assembly tools. However, to our knowledge, there are no publicly available software tools that integrate the powerful j5 design algorithm with automation instruments, which leaves a gap in the end-to-end workflow for high-throughput plasmid construction experiments.

In this study, we present DNAda, a novel application developed to bridge the gap between j5 algorithm-based DNA construct designs and automated laboratory workflows. DNAda (pronounced deh-nah-dah) is a user-friendly web application that seamlessly integrates with automation infrastructure to facilitate the construction of DNA assemblies designed using the j5 algorithm. By generating customized automation instructions for any given DNA construct design, DNAda streamlines the entire process from design to assembly. Moreover, DNAda offers additional downstream functionalities, such as active sample tracking and handling, which further enhances the efficiency and reliability of high-throughput plasmid construction experiments. As a proof of concept, we constructed a polyketide synthase (PKS) part library consisting of 120 plasmids in a single batch through the application of J5 and DNAda in our automation facility.

## Results and Discussion

### Automated Build Workflow

**Figure 1:**
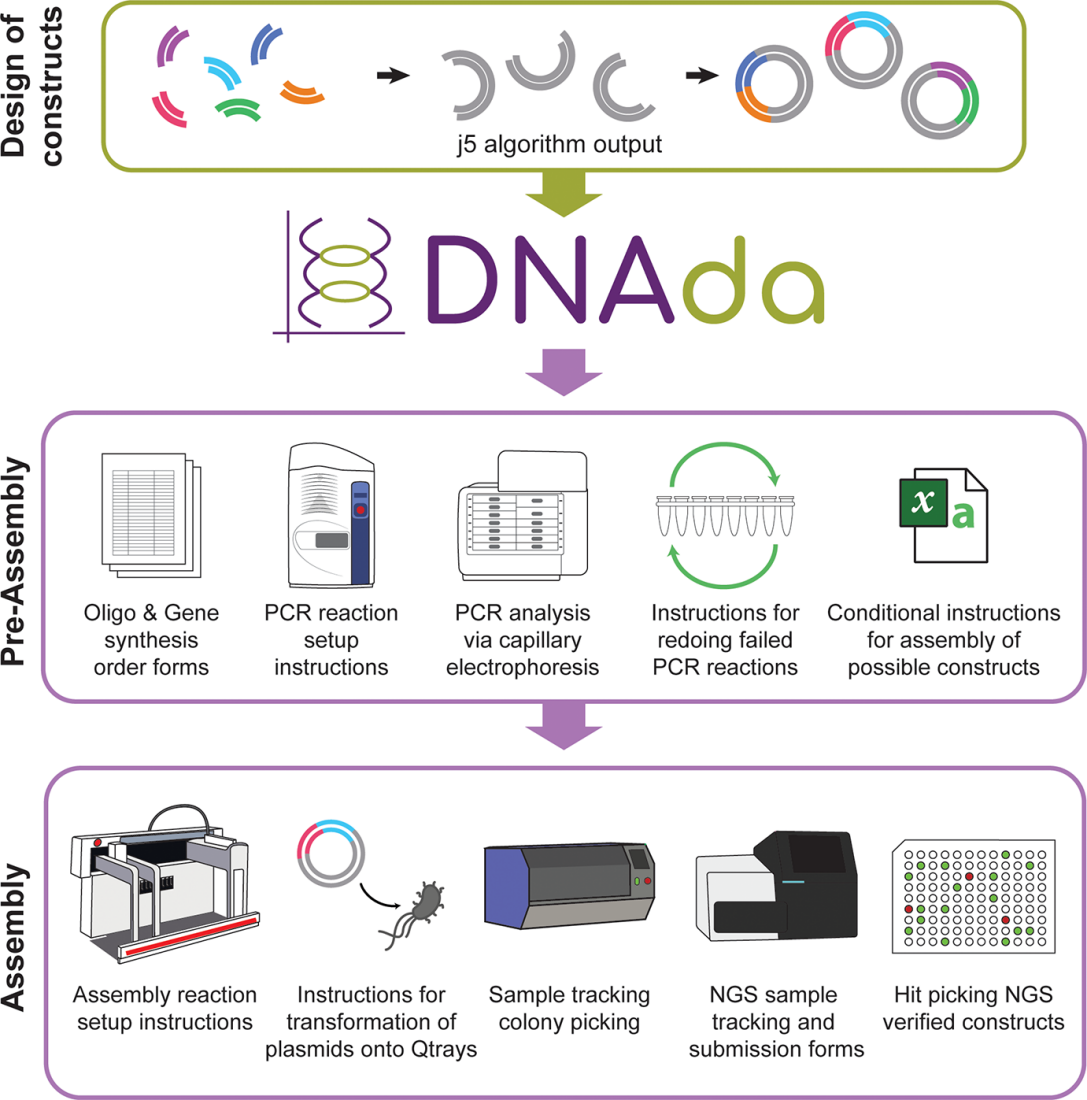
DNAda build workflow. DNA constructs are designed using the computer-aided design tool DeviceEditor, and build instructions are generated through the j5 DNA assembly algorithm. Those build instructions are then translated into step-by-step instructions for automated liquid handlers by DNAda.

Overall, the workflow consists of creating one or more designs with DeviceEditor,^6^ using j5^5^ and DNAda to generate automated workflow instructions, creating homologous regions between DNA parts by running polymerase chain reactions (PCRs), utilizing yeast-assisted homologous recombination^18^ to assemble plasmids, shuttling plasmids into *Escherichia coli* for next-generation sequencing (NGS) verification, and finally consolidating successful constructs for archival and downstream applications (Figure 1).

The workflow starts by using the biological computer-aided design (CAD) tool DeviceEditor to set up one or more combinatorial designs of DNA fragments, which are sourced from ICE,^19^ an online registry of DNA parts. Afterward, each design is processed using the integrated primer design tool j5, which will optimally design oligos for each polymerase chain reaction (PCR) required to produce a user-defined minimum of homologous overlap with neighboring DNA parts. The output of j5 can be directly passed to the web application DNAda to create customized cloning automation instructions for any given design.

DNAda provides a purchasing order sheet that can be directly used to order oligos and genes in 96-well plates from third-party DNA synthesis vendors. DNAda also provides instructions for preparing PCR templates in Echo Acoustic Liquid Handler (Beckman Coulter, 5350 Lakeview Pkwy S Drive Indianapolis, IN 46268, United States)-compatible 384-well plates. DNAda furthermore provides instructions that Echo can use to dispense appropriate oligomers and templates into 96-well plates for PCR preparation. Additionally, DNAda provides optimal reaction conditions for each PCR. The results of each PCR are analyzed on a zero agarose gel (ZAG) DNA electrophoresis instrument (Agilent Technologies, 5301 Stevens Creek Blvd., Santa Clara, CA 95051, United States), which can characterize the size in base pairs of all DNA fragments in a sample. The raw ZAG data are uploaded to DNAda where each PCR product is automatically compared with its expected product size. After analysis of a round of PCR data, DNAda is able to produce instructions for a subsequent round of PCRs from failed reactions in the previous round or it can produce assembly instructions for all of the possible constructs given the PCR reactions that succeeded.

If the user chooses to proceed to the assembly step, DNAda provides custom liquid handler instructions for consolidating each round of PCRs (in our case, 8-channel Biomek NX), performing magnetic bead-based DNA purification (in our case, 96-channel Biomek FXp), performing DpnI digestion of DNA samples (in our case, 96-channel Biomek FXp), and mixing appropriate DNA parts for each construct (in our case, Echo). At this point, the user follows a 96-well-compatible yeast-assisted homologous recombination protocol for each construct. After 3 days of growth, the assembled plasmids are extracted from yeast using a 96-well yeast plasmid extraction kit from Zymo Research. DNAda provides Echo and Biomek FXp instructions for those plasmids to then be transformed into *E. coli* DH5-alpha (NEB) on 48-well agar QTrays. A QPix colony picker (Molecular Devices, 3860 N First Street San Jose, CA 95134, United States) picks three colonies from each successful construct into 1 mL of medium for overnight growth at 37 °C. The overnight cultures are split for archival and NGS library preparation. The output of the QPix is uploaded to DNAda, which tracks which construct is in each well and creates a sample submission form for an NGS service. The NGS service performs library prep, sequencing, and subsequent read alignment for each sample. The analysis of the NGS data is left up to the user or NGS service with DNAda supplying the expected sequence for each sample submitted. The NGS results are consolidated into a list of correctly assembled constructs and supplied to DNAda, which provides instructions to consolidate at most one sample for each correct construct into a minimal amount of plates. Those successfully consolidated constructs are then archived at −80 °C for downstream applications.

### Core DNAda Features

Web applications have become an integral part of distributing open-source scientific tools to the wider scientific community. DNAda is a user-friendly web application developed for creating customized automation instructions that automate the plasmid building process. It consists of a user-friendly web interface built with the typescript framework Vue.js served by NGINX, a REST API served by an asynchronous Python service based on FastAPI, and a PostgreSQL database with custom schema types used to store the information and results of a build process. The entire package is contained within microservice docker containers, and the use of docker-compose scripts allow for seamless deployment in a docker swarm configuration (see schematic of the microservices in DNAda in Supplementary Figure 1).

**Figure 2:**
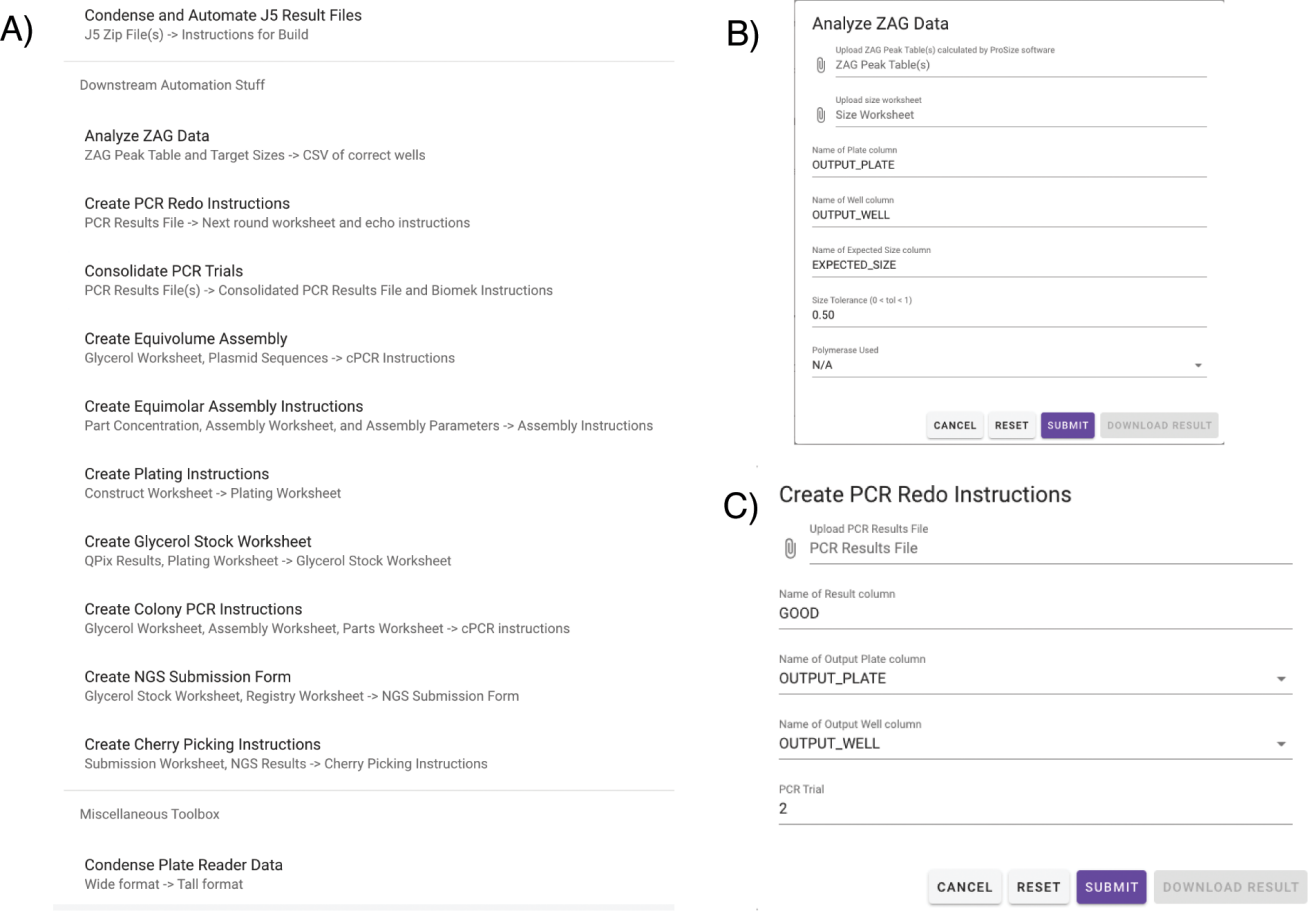
DNAda core features. (A) DNAda contains numerous functions that automatically create customized liquid handler instructions given design files and result files. (B) An example intermediate workflow involves analyzing uploaded PCR result data from the ZAG. (C) DNAda can then use that uploaded PCR data in order to generate automated redo PCR instructions for the failed reactions.

By offering a user-friendly web interface with intuitive navigation and clear instructions, DNAda enables users to complete tasks more efficiently (Figure 2). This interface features straightforward forms for executing and analyzing PCR reactions, as well as for conducting equivolume or equimolar assembly reactions. It also supports downstream processes, such as colony picking and glycerol stock cherry picking (Supplementary Table 1). DNAda integrates the autoprotocol standard (autoprotocol.org) into many automated processes, thereby allowing for compatibility with generalized hardware. The platform offers two modes of operation: (1) a standalone mode in which all analyses can be performed ad hoc without requiring prior information about the build, and (2) a project mode that stores design information and the current progress, thereby enabling users to resume work where they left off. For those who prefer not to use the web application, a command-line interface is also available through the Python Package Index (PyPI).

### High-Throughput Plasmid Construction

To validate the DNAda platform, we constructed a diverse library of PKS parts. Since their first elucidation as modular enzymatic assembly lines in the early 1990s, PKSs have been heralded as a potential foundation for retrobiosynthesis.^20,21^ The potential in engineering PKSs comes from their colinear biosynthetic logic, which means that their genetic organization matches the order of the enzymatic events. PKSs are composed of modules that act as parts of an assembly line. Each module performs a catalytic addition to the product from the upstream module before passing it on to a downstream module. Notably, many of the host organisms containing PKSs are genetically intractable or very difficult to cultivate in the laboratory; therefore, heterologous expression is very much desired. However, the large size of PKSs, high guanine–cytosine (GC) content, and frequent sequence repeats make cloning nontrivial. The construction of a library of PKS parts should serve as a challenging yet sensible proof of concept with substantial applicability.

**Figure 3:**
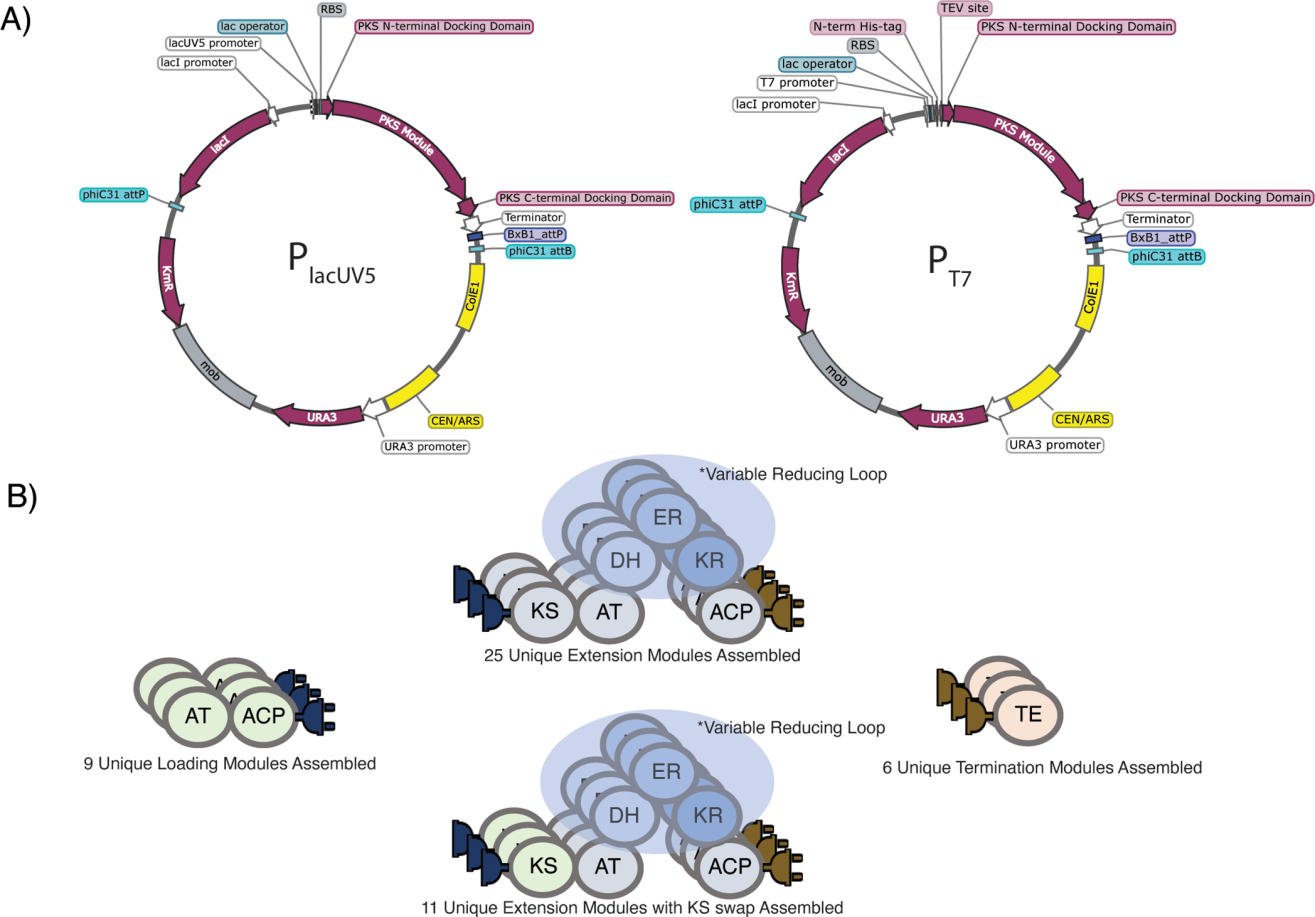
PKS part library architecture. (A) There are two versions of yeast-assembly-modified Serine Recombinate-assisted Genome Engineering (SAGE) vectors in which PKS parts were assembled. The first drives PKS expression under a P_lacUV5_ promoter and is suited for inducible expression in a variety of hosts. The second drives PKS expression under a P_T7_ promoter and links each PKS part to an N-terminal 6X-His Tag and a TEV protease cleavage site. This second vector is suited for high expression in *E. coli* and could serve a variety of applications, including protein purification and rapid cell-free assays. (B) The architecture of PKS parts that were successfully assembled. In total, we assembled a library of 120 plasmids. Within those 120 plasmids, there are 9 unique loading modules linked to a variety of C-terminal docking domains, 25 unique extension modules flanked by a variety of docking domains, 11 unique extension modules with the KS domain swapped with that of another extension module, and 6 unique termination modules that consist of an N-terminal docking domain fused to a thioesterase.

We made four PKS part designs in DeviceEditor that were consolidated into one batch plasmid construction design (Figure 3B). Each PKS module was flanked by docking domains, thereby enabling combinatorial communication between modules. Additionally, by limiting the size of each PKS part to an individual module, we attempted to improve cloning efficiency and heterologous expression. We also included a few designs of PKS modules that had the KS domain swapped for an alternative KS domain to enable further exploration of the optimal module boundaries. However, as docking domains natively exist at the C-terminus of acyl carrier protein (ACP) domains and the N-terminus of KS domains, the modules with KS domains swapped limit the full combinatorial communication potential of the library, as those particular modules are forced to communicate with a particular upstream module. For instance, one can imagine a cell-free transcription–translation experiment in which each individual PKS part is heterologously expressed, and cell lysates with active protein are prepared. These lysates can then be mixed in various combinations to evaluate the efficiency and specificity of intermodule communication. The only theoretical limitation is compatibility between the docking domains. While docking domain compatibility is not the only factor in intermodule communication, the success of such an experiment could give insights into the adaptability and flexibility of the modified PKS modules, thereby potentially paving the way for the tailored synthesis of complex molecules. Our design aims not only to increase the efficiency of cloning and expression but also to provide a robust platform for high-throughput plasmid construction endeavors, thereby allowing researchers to reconfigure and optimize PKS pathways for the desired outcomes.

The vector selected was a modified version of a Serine Recombinate-assisted Genome Engineering (SAGE)^22^ plasmid containing a Bxb1 attP site allowing for serine integrase-mediated integration into the genome of a compatible host organism. The main modifications included the insertion of a *Saccharomyces cerevisiae* uracil auxotrophic marker URA3, an origin of replication CEN/ARS, and a conjugative mobilization element Mob (Figure 3A). This vector thus enables episomal replication in *S. cerevisiae* for DNA assembly, episomal replication in *E. coli* for cloning and potentially rapid expression profiling, and reversible chromosomal integration into a compatible host organism with an integrated Bxb1 attB site. As Elmore et al. demonstrated, there is a wide variety of organisms that can potentially be made SAGE -compatible with the integration of a Bxb1 attB site, which means that cross-species comparison of our assembled PKSs is feasible.^22^ We additionally incorporated two promoters into the design for each possible PKS part—P_lacUV5_ and P_T7_ with a linked N-terminal 6x-His Tag—to enable two different applications, namely, cross-species isopropyl-β-d-thiogalactopyranoside (IPTG)-inducible expression with P_lacUV5_ and high inducible expression in *E. coli* with P_T7_ for protein purification and cell-free assay purposes.

Overall, our consolidated DNA assembly design included designs for a total of 882 unique plasmids. The J5 DNA assembly algorithm was performed with default settings with the exception of the homology overlap between parts, which was set to 60 base pairs, and the maximum primer size was set to 20 base pairs. These 882 designs required the use of 422 unique single-stranded DNA oligonucleotides to PCR-amplify 502 unique DNA fragments. DNA oligonucleotides were ordered from IDT under standard purification procedures normalized in 96-well plates. Because of the combinatorial nature of the assembly, there were some high-use parts used in many reactions, which required replicates in order to fulfill the design requirements. DNAda is able to evaluate the usage of each part to determine the amount of each fragment required for the full build, which meant that 706 total PCR reactions were required to fulfill the demand of the 502 unique DNA fragments. After six rounds of PCR amplification, we were able to verify 623 of the 706 DNA fragments using capillary electrophoresis. From those 623 fragments, we could theoretically assemble 715 of the 882 plasmid constructs. We used yeast (*S. cerevisae BY4742*)-assisted homologous recombination to attempt the assembly of those 715 constructs, and after 3 days of growth in uracil-deficient media, observed live yeast in 471 of those 715 constructs. The assemblies without live yeast were presumably unable to assemble any plasmid. After DNA extraction and transformation into *E. coli* DH5-alpha F’*I*^*q*^ (NEB no. C2992I), we observed colonies in 332 of the 471 transformations. There are several possible reasons for the failed *E. coli* transformations, including low DNA extraction yields and inefficient transformation protocol execution. We picked 1018 total colonies from the 332 transformations and split the corresponding cultures for archival and for colony PCR validation. For colony PCR validation of the 1018 colonies, we utilized LongAmp Taq (NEB #M0287L) to amplify the full PKS coding sequence (CDS). We observed expected CDS sizes in 417 out of 1018 colonies using capillary electrophoresis. The most likely reason for failed colony PCRs is improper assembly of the PKS parts. We sequenced 417 colonies, which consisted of 182 unique plasmid constructs that passed colony PCR validation. We observed full coverage of the PKS CDS in 76% of the 417 samples. Of those 76% of samples that had full PKS coverage, 65% had zero mutations. In those 65% that had zero mutations and full PKS coverage there were 120 unique constructs. The full list of successfully sequenced plasmids is available in Supplementary Table 2. The J5 designs, DNAda workflow, and corresponding plasmid construction data files are available in the Supporting Information. Overall, with a success rate of 14% in terms of the total number of successfully sequenced unique constructs out of the total number of constructs designed, there is definite room for improvement in the efficiency of processes throughout the plasmid construction process. Ultimately, to our knowledge, this is still one of the largest libraries of PKS parts, and with it being accomplished in approximately 10 weeks, it represents a significant milestone.

## Conclusion

DNAda is a fast and easy-to-use web application enabling users to quickly develop customized build instructions for their plasmid assembly designs. This application is particularly useful for groups that are able to leverage automation capabilities to improve the throughput and reproducibility of their workflows. The architecture of DNAda has been designed such that it can easily be deployed both locally on a personal desktop workstation or within a docker swarm, thereby enabling a robust and secure distributed production application. The intuitive user interface enables users without programming experience to utilize powerful automation capabilities without a large learning curve.

There is a growing ecosystem of automation tools that have been created to accelerate the synthetic biology experiments. The plasmid construction process remains a challenging step. With J5 still being one of the most capable and flexible DNA assembly programs available, DNAda serves as a means of executing any J5 design on automated liquid handlers. With definite room for improvement in automated plasmid construction processes, it is clear that small errors or inefficiencies result in substantial reductions in success that are amplified by the scale of the batch. Furthermore, it is likely that there is room for improvement in the design phase, which can reduce the probability of incorrect assemblies and unsuccessful PCR reactions.

Lastly, we provide a PKS part library to the community to accelerate the rate at which novel insights can be generated into the behavior of PKS enzymes. By the utilization of advances in automation and synthetic biology, data-driven approaches to PKS exploration can be leveraged and used to fuel modern machine learning algorithms. We hope that this library serves as a useful proof-of-concept and encourages the community to further invest into automated synthetic biology.

## Data Availability

DNAda is composed as a Docker (https://github.com/docker) microservice stack that can be easily deployed as a personal use application or production-ready service. Alternatively, a command line interface is packaged on Pypi under the name ′dnada.′ The source code of DNAda is freely available to all users and can be accessed on GitHub (https://github.com/JBEI/dnada) under an open source license.

## Supporting Information Available

Schematic of DNAda microservice framework (Figure S1), table of steps implemented in DNAda workflow (Table S1), and table of constructed plasmids (Table S2) (PDF)
PKS library part build J5 Designs, DNAda workflow, and construction data files (ZIP)
