# Supplementary material for: Automated Platform for the Plasmid Construction Process
Source: ACS Synth Biol. 2023 Nov 10;12(12):3506–13. doi: 10.1021/acssynbio.3c00292 (PMC10729297; doi:10.1021/acssynbio.3c00292)
Supplement: Supplementary file 1 — sb3c00292_si_001.pdf [file sb3c00292_si_001.pdf]

Supporting Information

An automated platform for the plasmid construction process

Authors:

Alberto A. Nava[1,2,3], Anna Lisa Fear[1,2], Namil Lee[1,2], Peter Mellinger[1,2], Guangxu Lan[1,2], Joshua McCauley[1,2,4], Stephen Tan[1,2,4], Nurgul Kaplan[1,2,4], Garima Goyal[1,2,4], R. Cameron Coates[1,2,4], Jacob Roberts[1,2,5], Zahmiria Johnson[3], Romina Hu[5], Bryan Wu[5], Jared Ahn[5], Woojoo E. Kim[1,2], Yao Wan[1,2], Kevin Yin[1,2,6], Nathan Hillson[1,2,4], Robert W. Haushalter[1,2], Jay D. Keasling[1,2,3,5,7,8]

1. Joint BioEnergy Institute, Lawrence Berkeley National Laboratory, Emeryville, CA 94608, USA
2. Biological Systems and Engineering Division, Lawrence Berkeley National Laboratory, Berkeley, California 94720, United States
3. Department of Chemical and Biomolecular Engineering, University of California, Berkeley, Berkeley, CA 94720, USA
4. DOE Agile BioFoundry, Emeryville, CA 94608, USA
5. Department of Bioengineering, University of California, Berkeley, Berkeley, CA 94720, USA
6. Department of Plant and Microbial Biology, University of California, Berkeley, Berkeley, CA 94720, USA
7. Center for Synthetic Biochemistry, Shenzhen Institutes for Advanced Technologies, Shenzhen 518055, P.R. China
8. The Novo Nordisk Foundation Center for Biosustainability, Technical University Denmark, Kemitorvet, Building 220, Kongens Lyngby 2800, Denmark

Supplementary Information

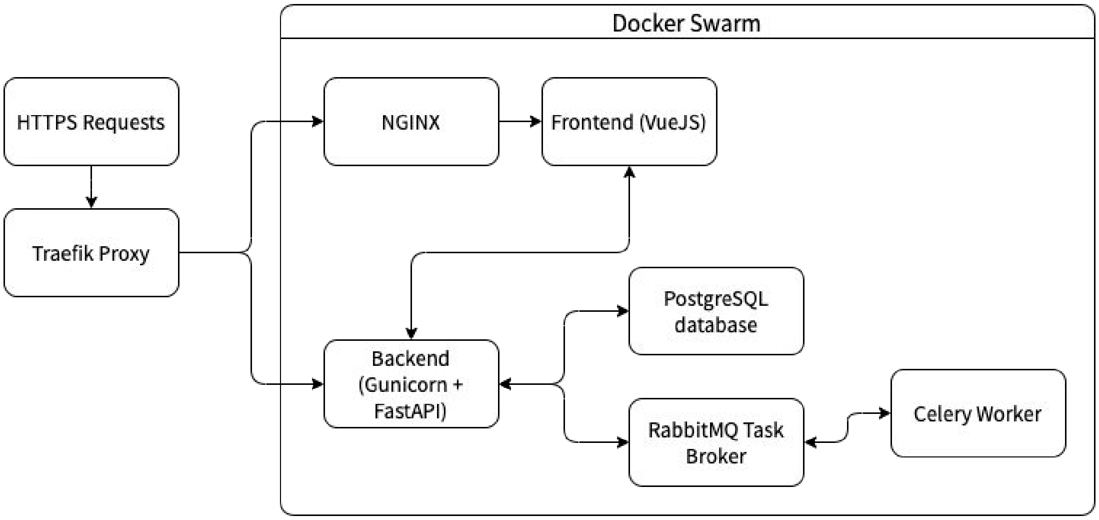

Supplementary Figure S1: DNAda microservice framework schematic.

Supplementary Table 1. Build Pipeline Instruction Manual

Task ID	Task	Human Instructions	Robot Instructions
1	Order Genes	• Order sequences in synths_plate.csv from favorite DNA synthesis company. • Move synthesized parts from order plates into 384-well echo synth source plates • Note, synths_plate.csv includes location information for each part in the design	• Biomek: 96-to-384-stamp or 384-to-384-stamp
2	Order Oligos	• Order sequences in oligos_order_96.csv or oligos_order_384.csv • Move oligos from order plates into 384-well echo oligo source plates according to oligos_plate.csv. • Note, Oligo synthesis companies (IDT) are able to process orders faster in 96-well format vs 384-well format. • Note, Oligo synthesis companies (IDT) sometimes heat seal 384-well plates which leads to higher error rate in acoustic liquid handlers.	• Biomek: 96-to-384-stamp or 384-to-384-stamp
3	Order Templates	• Order sequences from registry of parts • Move DNA templates into 384-well echo template source plates according to templates_plate.csv • Note, this step difficult to automate. Focus as you transfer DNA templates into correct wells	N/A
4	Perform PCRs	• Utilize echo to distribute oligos and templates • Utilize mantis to distribute master mix and water • Utilize thermocyclers with block zones set to optimal annealing temperatures.	For the echo use pcr_echo_instructions.csv. For the thermocycler use pcr_thermocycler_instructions.csv
5	Analyze PCRs	• Utilize biomek to prepare TE buffer 96-well plates • Utilize a 96-pin replicator to add 1 uL of PCR reactions to buffer plates • Utilize ZAG to collect data on PCR results.	• Biomek: reservoir-to-96-stamp
6	(optional) Redo Failed PCRs	• Upload ZAG data to DNAda to create redo pcr instructions for failed reactions • Redo previous 2 steps.	• Upload ZAG data to DNAda to create redo pcr instructions for failed reactions • Redo previous 2 steps. Note, Use clean_pcr_worksheet.csv as size worksheet and ZAG peak

			tables as measured data
7	(if necessary) Consolidate PCRs	• Upload results of all PCR runs to DNAda to create consolidation instructions.	• Biomek: cherry-picking (consolidate_pcrs.csv)
8	Perform Restriction digest	• Utilize mantis to distribute 10X buffer and restriction enzymes to reactions.	N/A
9	Perform DNA cleanup	• Utilize magbead based automation method on biomek for each plate of DNA.	• Biomek: magbead-purification
10	(optional) Quantify part concentration	• Utilize mantis to prepare DNA quantification buffer in 96-well plates • Utilize 96-pin replicator to stamp DNA parts into 96-well plates • Use spectrophotometer to measure DNA concentration • Upload results to DNAda to get equimolar assembly instructions.	N/A
11	Perform Assembly	• Utilize echo to distribute DNA parts • Utilize mantis to distribute gibson/golden-gate master mix and water • Utilize thermocycler to incubate assembly.	N/A
		• Utilize echo to distribute DNA parts • Utilize biomek to perform assembly • Incubate assembly for 3 days at 30C • Utilize zymoprep kit to extract plasmids from yeast.	• Biomek: yeast-assembly
12	E. coli Transformation	• Utilize mantis to distribute commercial pir2 E. coli competent cells into plates • Use biomek to stamp assembly products into comp cell plate • Utilize biomek to plate transformation onto 48-well agar plates • Utilize whisper flow cabinet to speed up drying process • Incubate overnight.	• Biomek: 96-to-96-stamp, q-tray-plating

13	Colony Picking	• Use qpix to pick colonies from 48-well agar plates into 96-well plates • Upload picking results to DNAda to create NGS submission form.	N/A
14	NGS Sequencin g	• Use biomek to dilute cultures into water and to prepare glycerol stocks of each colony • Use thermocycler to boil diluted cultures • Submit boil preps to NGS.	N/A
15	Cherry-pick successful constructs	• Upload results to DNAda to obtain cherry-picking instructions • Use cherry picking worksheet to consolidate and inoculate successful assemblies from glycerol stock	• Biomek: 96-to-96-cherry-picking (cherry_picking_worksheet.csv)
16	Prepare for archival	• Submit samples to archival team	N/A

Supplementary Table 2. List of constructed PKS plasmids. All plasmids are available on the JBEI public registry at https://public-registry.jbei.org.

Index	Plasmid ID	Strain ID	Parts
1	JPUB_023125	JPUB_023124	borA2_Ncom + borM1 + mlsA8_Ccom + pPM021_MfeI_XhoI + lacI_lacUV5
2	JPUB_023127	JPUB_023126	borA2_Ncom + eryM6_epoAT + pks12_Ccom + pPM021_MfeI_XhoI + lacI_lacUV5
3	JPUB_023129	JPUB_023128	borA2_Ncom + hisM2 + mlsA8_Ccom + pPM021_MfeI_XhoI + lacI_T7_6xHis_TEV
4	JPUB_023131	JPUB_023130	borA2_Ncom + MASmm + mlsA8_Ccom + pPM021_MfeI_XhoI + lacI_T7_6xHis_TEV
5	JPUB_023133	JPUB_023132	mlsA9_Ncom + pikM6 + borA1_Ccom + pPM021_MfeI_XhoI + lacI_lacUV5
6	JPUB_023135	JPUB_023134	mlsA9_Ncom + hygM3 + pks12_Ccom + pPM021_MfeI_XhoI + lacI_lacUV5
7	JPUB_023137	JPUB_023136	mlsA9_Ncom + ctgM2 + borA1_Ccom + pPM021_MfeI_XhoI + lacI_lacUV5
8	JPUB_023139	JPUB_023138	mlsA9_Ncom + ctgM2 + pks12_Ccom + pPM021_MfeI_XhoI + lacI_T7_6xHis_TEV
9	JPUB_023141	JPUB_023140	mlsA9_Ncom + rifM2 + borA1_Ccom + pPM021_MfeI_XhoI + lacI_lacUV5

10	JPUB_023143	JPUB_023142	mlsA9_Ncom + rifM2 + pks12_Ccom + pPM021_MfeI_XhoI + lacI_T7_6xHis_TEV
11	JPUB_023145	JPUB_023144	mlsA9_Ncom + pks6tb + borA1_Ccom + pPM021_MfeI_XhoI + lacI_T7_6xHis_TEV
12	JPUB_023147	JPUB_023146	mlsA9_Ncom + pks6tb + borA1_Ccom + pPM021_MfeI_XhoI + lacI_lacUV5
13	JPUB_023149	JPUB_023148	pks12_Ncom + borM5 + borA1_Ccom + pPM021_MfeI_XhoI + lacI_lacUV5
14	JPUB_023151	JPUB_023150	pks12_Ncom + borM5 + mlsA8_Ccom + pPM021_MfeI_XhoI + lacI_lacUV5
15	JPUB_023153	JPUB_023152	pks12_Ncom + eryM1 + borA1_Ccom + pPM021_MfeI_XhoI + lacI_lacUV5
16	JPUB_023155	JPUB_023154	pks12_Ncom + eryM1 + mlsA8_Ccom + pPM021_MfeI_XhoI + lacI_lacUV5
17	JPUB_023157	JPUB_023156	pks12_Ncom + eryM2 + mlsA8_Ccom + pPM021_MfeI_XhoI + lacI_T7_6xHis_TEV
18	JPUB_023159	JPUB_023158	pks12_Ncom + eryM2 + mlsA8_Ccom + pPM021_MfeI_XhoI + lacI_lacUV5
19	JPUB_023161	JPUB_023160	pks12_Ncom + eryM6 + borA1_Ccom + pPM021_MfeI_XhoI + lacI_T7_6xHis_TEV
20	JPUB_023163	JPUB_023162	pks12_Ncom + eryM6_epoAT + mlsA8_Ccom + pPM021_MfeI_XhoI + lacI_T7_6xHis_TEV
21	JPUB_023165	JPUB_023164	pks12_Ncom + lipM1_YtoF + mlsA8_Ccom + pPM021_MfeI_XhoI + lacI_lacUV5
22	JPUB_023167	JPUB_023166	pks12_Ncom + lipM1_YtoF_borAT + borA1_Ccom + pPM021_MfeI_XhoI + lacI_T7_6xHis_TEV
23	JPUB_023169	JPUB_023168	pks12_Ncom + lipM1_YtoF_borAT + borA1_Ccom + pPM021_MfeI_XhoI + lacI_lacUV5
24	JPUB_023171	JPUB_023170	pks12_Ncom + pikM6 + borA1_Ccom + pPM021_MfeI_XhoI + lacI_lacUV5
25	JPUB_023173	JPUB_023172	pks12_Ncom + hygM3 + borA1_Ccom + pPM021_MfeI_XhoI + lacI_lacUV5
26	JPUB_023175	JPUB_023174	pks12_Ncom + nigM1 + borA1_Ccom + pPM021_MfeI_XhoI + lacI_T7_6xHis_TEV
27	JPUB_023177	JPUB_023176	pks12_Ncom + nigM1 + mlsA8_Ccom + pPM021_MfeI_XhoI + lacI_T7_6xHis_TEV
28	JPUB_023179	JPUB_023178	pks12_Ncom + blv75M2 + borA1_Ccom + pPM021_MfeI_XhoI + lacI_T7_6xHis_TEV
29	JPUB_023181	JPUB_023180	pks12_Ncom + blv75M2 + mlsA8_Ccom + pPM021_MfeI_XhoI + lacI_T7_6xHis_TEV

30	JPUB_023183	JPUB_023182	pks12_Ncom + ctgM2 + borA1_Ccom + pPM021_MfeI_XhoI + lacI_T7_6xHis_TEV
31	JPUB_023185	JPUB_023184	pks12_Ncom + ctgM2 + borA1_Ccom + pPM021_MfeI_XhoI + lacI_lacUV5
32	JPUB_023187	JPUB_023186	pks12_Ncom + MASmm + borA1_Ccom + pPM021_MfeI_XhoI + lacI_T7_6xHis_TEV
33	JPUB_023189	JPUB_023188	pks12_Ncom + mlsA8 + mlsA8_Ccom + pPM021_MfeI_XhoI + lacI_lacUV5
34	JPUB_023191	JPUB_023190	pks12_Ncom + mlsA9 + borA1_Ccom + pPM021_MfeI_XhoI + lacI_lacUV5
35	JPUB_023193	JPUB_023192	pks12_Ncom + mlsB1 + borA1_Ccom + pPM021_MfeI_XhoI + lacI_T7_6xHis_TEV
36	JPUB_023195	JPUB_023194	pks12_Ncom + mlsB1 + mlsA8_Ccom + pPM021_MfeI_XhoI + lacI_T7_6xHis_TEV
37	JPUB_023197	JPUB_023196	pks12_Ncom + mlsB1 + mlsA8_Ccom + pPM021_MfeI_XhoI + lacI_lacUV5
38	JPUB_023199	JPUB_023198	pks12_Ncom + ppsAtb + borA1_Ccom + pPM021_MfeI_XhoI + lacI_T7_6xHis_TEV
39	JPUB_023201	JPUB_023200	pks12_Ncom + ppsBtb + mlsA8_Ccom + pPM021_MfeI_XhoI + lacI_lacUV5
40	JPUB_023203	JPUB_023202	pks12_Ncom + spnM1 + borA1_Ccom + pPM021_MfeI_XhoI + lacI_T7_6xHis_TEV
41	JPUB_023205	JPUB_023204	pks12_Ncom + spnM2 + borA1_Ccom + pPM021_MfeI_XhoI + lacI_lacUV5
42	JPUB_023207	JPUB_023206	pks12_Ncom + pks15mm + borA1_Ccom + pPM021_MfeI_XhoI + lacI_T7_6xHis_TEV
43	JPUB_023209	JPUB_023208	pks12_Ncom + pks6tb + mlsA8_Ccom + pPM021_MfeI_XhoI + lacI_lacUV5
44	JPUB_023211	JPUB_023210	lipLM + mlsA8_Ccom + pPM021_MfeI_XhoI + lacI_T7_6xHis_TEV
45	JPUB_023213	JPUB_023212	lipLM + mlsA8_Ccom + pPM021_MfeI_XhoI + lacI_lacUV5
46	JPUB_023215	JPUB_023214	cndLM + borA1_Ccom + pPM021_MfeI_XhoI + lacI_T7_6xHis_TEV
47	JPUB_023217	JPUB_023216	cndLM + borA1_Ccom + pPM021_MfeI_XhoI + lacI_lacUV5
48	JPUB_023219	JPUB_023218	cndLM + mlsA8_Ccom + pPM021_MfeI_XhoI + lacI_T7_6xHis_TEV
49	JPUB_023221	JPUB_023220	cndLM + mlsA8_Ccom + pPM021_MfeI_XhoI + lacI_lacUV5
50	JPUB_023223	JPUB_023222	cndLM + pks12_Ccom + pPM021_MfeI_XhoI + lacI_lacUV5
51	JPUB_023225	JPUB_023224	jamC + borA1_Ccom + pPM021_MfeI_XhoI + lacI_lacUV5

52	JPUB_023227	JPUB_023226	jamC + mlsA8_Ccom + pPM021_MfeI_XhoI + lacI_T7_6xHis_TEV
53	JPUB_023229	JPUB_023228	jamC + mlsA8_Ccom + pPM021_MfeI_XhoI + lacI_lacUV5
54	JPUB_023231	JPUB_023230	jamC + pks12_Ccom + pPM021_MfeI_XhoI + lacI_T7_6xHis_TEV
55	JPUB_023233	JPUB_023232	mycLM + borA1_Ccom + pPM021_MfeI_XhoI + lacI_T7_6xHis_TEV
56	JPUB_023235	JPUB_023234	mycLM + mlsA8_Ccom + pPM021_MfeI_XhoI + lacI_T7_6xHis_TEV
57	JPUB_023237	JPUB_023236	mycLM + pks12_Ccom + pPM021_MfeI_XhoI + lacI_T7_6xHis_TEV
58	JPUB_023239	JPUB_023238	mycLM + pks12_Ccom + pPM021_MfeI_XhoI + lacI_lacUV5
59	JPUB_023241	JPUB_023240	cylB + borA1_Ccom + pPM021_MfeI_XhoI + lacI_T7_6xHis_TEV
60	JPUB_023243	JPUB_023242	cylB + borA1_Ccom + pPM021_MfeI_XhoI + lacI_lacUV5
61	JPUB_023245	JPUB_023244	cylB + mlsA8_Ccom + pPM021_MfeI_XhoI + lacI_T7_6xHis_TEV
62	JPUB_023247	JPUB_023246	cylB + mlsA8_Ccom + pPM021_MfeI_XhoI + lacI_lacUV5
63	JPUB_023249	JPUB_023248	cylB + pks12_Ccom + pPM021_MfeI_XhoI + lacI_T7_6xHis_TEV
64	JPUB_023251	JPUB_023250	cylB + pks12_Ccom + pPM021_MfeI_XhoI + lacI_lacUV5
65	JPUB_023253	JPUB_023252	hctB + borA1_Ccom + pPM021_MfeI_XhoI + lacI_T7_6xHis_TEV
66	JPUB_023255	JPUB_023254	hctB + borA1_Ccom + pPM021_MfeI_XhoI + lacI_lacUV5
67	JPUB_023257	JPUB_023256	hctB + mlsA8_Ccom + pPM021_MfeI_XhoI + lacI_T7_6xHis_TEV
68	JPUB_023259	JPUB_023258	hctB + mlsA8_Ccom + pPM021_MfeI_XhoI + lacI_lacUV5
69	JPUB_023261	JPUB_023260	hctB + pks12_Ccom + pPM021_MfeI_XhoI + lacI_T7_6xHis_TEV
70	JPUB_023263	JPUB_023262	fluP1 + borA1_Ccom + pPM021_MfeI_XhoI + lacI_T7_6xHis_TEV
71	JPUB_023265	JPUB_023264	fluP1 + borA1_Ccom + pPM021_MfeI_XhoI + lacI_lacUV5
72	JPUB_023267	JPUB_023266	fluP1 + mlsA8_Ccom + pPM021_MfeI_XhoI + lacI_T7_6xHis_TEV
73	JPUB_023269	JPUB_023268	fluP1 + mlsA8_Ccom + pPM021_MfeI_XhoI + lacI_lacUV5
74	JPUB_023271	JPUB_023270	fluP1 + pks12_Ccom + pPM021_MfeI_XhoI + lacI_T7_6xHis_TEV
75	JPUB_023273	JPUB_023272	fluP1 + pks12_Ccom + pPM021_MfeI_XhoI + lacI_lacUV5

76	JPUB_023275	JPUB_023274	spnLM + pks12_Ccom + pPM021_MfeI_XhoI + lacI_T7_6xHis_TEV
77	JPUB_023277	JPUB_023276	ppsLM + borA1_Ccom + pPM021_MfeI_XhoI + lacI_lacUV5
78	JPUB_023279	JPUB_023278	ppsLM + mlsA8_Ccom + pPM021_MfeI_XhoI + lacI_lacUV5
79	JPUB_023281	JPUB_023280	ppsLM + pks12_Ccom + pPM021_MfeI_XhoI + lacI_T7_6xHis_TEV
80	JPUB_023283	JPUB_023282	ppsLM + pks12_Ccom + pPM021_MfeI_XhoI + lacI_lacUV5
81	JPUB_023285	JPUB_023284	borA2_Ncom + debsTE + pPM021_MfeI_XhoI + lacI_T7_6xHis_TEV
82	JPUB_023287	JPUB_023286	borA2_Ncom + debsTE + pPM021_MfeI_XhoI + lacI_lacUV5
83	JPUB_023289	JPUB_023288	borA2_Ncom + pikTE + pPM021_MfeI_XhoI + lacI_T7_6xHis_TEV
84	JPUB_023291	JPUB_023290	borA2_Ncom + borTE + pPM021_MfeI_XhoI + lacI_T7_6xHis_TEV
85	JPUB_023293	JPUB_023292	borA2_Ncom + borTE + pPM021_MfeI_XhoI + lacI_lacUV5
86	JPUB_023295	JPUB_023294	borA2_Ncom + debsTEshort + pPM021_MfeI_XhoI + lacI_T7_6xHis_TEV
87	JPUB_023297	JPUB_023296	borA2_Ncom + debsTEshort + pPM021_MfeI_XhoI + lacI_lacUV5
88	JPUB_023299	JPUB_023298	mlsA9_Ncom + debsTE + pPM021_MfeI_XhoI + lacI_T7_6xHis_TEV
89	JPUB_023301	JPUB_023300	mlsA9_Ncom + zeaTE + pPM021_MfeI_XhoI + lacI_lacUV5
90	JPUB_023303	JPUB_023302	mlsA9_Ncom + flvTE + pPM021_MfeI_XhoI + lacI_T7_6xHis_TEV
91	JPUB_023305	JPUB_023304	mlsA9_Ncom + flvTE + pPM021_MfeI_XhoI + lacI_lacUV5
92	JPUB_023307	JPUB_023306	mlsA9_Ncom + borTE + pPM021_MfeI_XhoI + lacI_T7_6xHis_TEV
93	JPUB_023309	JPUB_023308	mlsA9_Ncom + borTE + pPM021_MfeI_XhoI + lacI_lacUV5
94	JPUB_023311	JPUB_023310	mlsA9_Ncom + debsTEshort + pPM021_MfeI_XhoI + lacI_T7_6xHis_TEV
95	JPUB_023313	JPUB_023312	mlsA9_Ncom + debsTEshort + pPM021_MfeI_XhoI + lacI_lacUV5
96	JPUB_023315	JPUB_023314	pks12_Ncom + debsTE + pPM021_MfeI_XhoI + lacI_T7_6xHis_TEV
97	JPUB_023317	JPUB_023316	pks12_Ncom + debsTE + pPM021_MfeI_XhoI + lacI_lacUV5
98	JPUB_023319	JPUB_023318	pks12_Ncom + pikTE + pPM021_MfeI_XhoI + lacI_T7_6xHis_TEV
99	JPUB_023321	JPUB_023320	pks12_Ncom + pikTE + pPM021_MfeI_XhoI + lacI_lacUV5

100	JPUB_023323	JPUB_023322	pks12_Ncom + zeaTE + pPM021_MfeI_XhoI + lacI_lacUV5
101	JPUB_023325	JPUB_023324	pks12_Ncom + flvTE + pPM021_MfeI_XhoI + lacI_T7_6xHis_TEV
102	JPUB_023327	JPUB_023326	pks12_Ncom + flvTE + pPM021_MfeI_XhoI + lacI_lacUV5
103	JPUB_023329	JPUB_023328	pks12_Ncom + borTE + pPM021_MfeI_XhoI + lacI_T7_6xHis_TEV
104	JPUB_023331	JPUB_023330	pks12_Ncom + borTE + pPM021_MfeI_XhoI + lacI_lacUV5
105	JPUB_023333	JPUB_023332	pks12_Ncom + debsTEshort + pPM021_MfeI_XhoI + lacI_T7_6xHis_TEV
106	JPUB_023335	JPUB_023334	pks12_Ncom + debsTEshort + pPM021_MfeI_XhoI + lacI_lacUV5
107	JPUB_023337	JPUB_023336	pks12_Ncom + eryM1_KS + eryM6_AT_ACP + mlsA8_Ccom + pPM021_MfeI_XhoI + lacI_T7_6xHis_TEV
108	JPUB_023339	JPUB_023338	pks12_Ncom + eryM1_KS + eryM6_AT_ACP + mlsA8_Ccom + pPM021_MfeI_XhoI + lacI_lacUV5
109	JPUB_023341	JPUB_023340	pks12_Ncom + lipM1_KS + eryM1_AT_ACP + mlsA8_Ccom + pPM021_MfeI_XhoI + lacI_T7_6xHis_TEV
110	JPUB_023343	JPUB_023342	pks12_Ncom + lipM1_KS + pikM1_AT_ACP + mlsA8_Ccom + pPM021_MfeI_XhoI + lacI_T7_6xHis_TEV
111	JPUB_023345	JPUB_023344	pks12_Ncom + pikM1_KS + mlsB1_AT_ACP + mlsA8_Ccom + pPM021_MfeI_XhoI + lacI_T7_6xHis_TEV
112	JPUB_023347	JPUB_023346	mlsA9_Ncom + eryM1_KS + eryM6_AT_ACP + pks12_Ccom + pPM021_MfeI_XhoI + lacI_lacUV5
113	JPUB_023349	JPUB_023348	mlsA9_Ncom + spnM1_KS + lipM1_AT_ACP + pks12_Ccom + pPM021_MfeI_XhoI + lacI_lacUV5
114	JPUB_023351	JPUB_023350	mlsA9_Ncom + mlsB1_KS + eryM1_AT_ACP + pks12_Ccom + pPM021_MfeI_XhoI + lacI_lacUV5
115	JPUB_023353	JPUB_023352	mlsA9_Ncom + mlsB1_KS + lipM1_AT_ACP + pks12_Ccom + pPM021_MfeI_XhoI + lacI_lacUV5
116	JPUB_023355	JPUB_023354	mlsA9_Ncom + mlsB1_KS + pikM1_AT_ACP + pks12_Ccom + pPM021_MfeI_XhoI + lacI_T7_6xHis_TEV
117	JPUB_023357	JPUB_023356	mlsA9_Ncom + mlsB1_KS + pikM1_AT_ACP + pks12_Ccom + pPM021_MfeI_XhoI + lacI_lacUV5
118	JPUB_023359	JPUB_023358	mlsA9_Ncom + pks12M1_KS + eryM1_AT_ACP + pks12_Ccom + pPM021_MfeI_XhoI + lacI_T7_6xHis_TEV
119	JPUB_023361	JPUB_023360	mlsA9_Ncom + eryM6_KS + eryM1_AT_ACP + pks12_Ccom + pPM021_MfeI_XhoI + lacI_T7_6xHis_TEV
120	JPUB_023363	JPUB_023362	mlsA9_Ncom + eryM6_KS + lipM1_AT_ACP + pks12_Ccom + pPM021_MfeI_XhoI + lacI_T7_6xHis_TEV
