# Supplementary material for: Automated Platform for the Plasmid Construction Process
Source: ACS Synth Biol. 2023 Nov 10;12(12):3506–13. doi: 10.1021/acssynbio.3c00292 (PMC10729297; doi:10.1021/acssynbio.3c00292)
Supplement: Supplementary file 2 — sb3c00292_si_002.zip [file sb3c00292_si_002.zip › dnada_supplementary_material_pks_library_build/plate_visualizations/assemblytoautomate_PCR_plate_6-preview.pdf]

assemblytoautomate_PCR_plate_6

ROW	1	2	3	4	5	6	7	8	9	10	11	12
A	PCRRXN-39	PCRRXN-49	PCRRXN-144	PCRRXN-11	PCRRXN-25	PCRRXN-466	PCRRXN-164	PCRRXN-429	PCRRXN-500	PCRRXN-282	PCRRXN-75	PCRRXN-420
B	PCRRXN-44	PCRRXN-293	PCRRXN-239	PCRRXN-203	PCRRXN-410	PCRRXN-473	PCRRXN-255	PCRRXN-444	PCRRXN-62	PCRRXN-88	PCRRXN-117	PCRRXN-427
C	PCRRXN-45	PCRRXN-294	PCRRXN-240	PCRRXN-204	PCRRXN-417	PCRRXN-480	PCRRXN-256	PCRRXN-451	PCRRXN-63	PCRRXN-89	PCRRXN-118	PCRRXN-434
D	PCRRXN-145	PCRRXN-295	PCRRXN-46	PCRRXN-121	PCRRXN-424	PCRRXN-487	PCRRXN-259	PCRRXN-457	PCRRXN-66	PCRRXN-171	PCRRXN-20	PCRRXN-441
E	PCRRXN-146	PCRRXN-296	PCRRXN-47	PCRRXN-122	PCRRXN-431	PCRRXN-494	PCRRXN-260	PCRRXN-464	PCRRXN-67	PCRRXN-172	PCRRXN-21	PCRRXN-448
F	PCRRXN-241	PCRRXN-297	PCRRXN-10	PCRRXN-217	PCRRXN-438	PCRRXN-159	PCRRXN-408	PCRRXN-471	PCRRXN-185	PCRRXN-267	PCRRXN-213	PCRRXN-454
G	PCRRXN-242	PCRRXN-298	PCRRXN-107	PCRRXN-218	PCRRXN-445	PCRRXN-160	PCRRXN-415	PCRRXN-478	PCRRXN-186	PCRRXN-268	PCRRXN-214	PCRRXN-469
H	PCRRXN-48	PCRRXN-143	PCRRXN-108	PCRRXN-24	PCRRXN-459	PCRRXN-163	PCRRXN-422	PCRRXN-493	PCRRXN-281	PCRRXN-74	PCRRXN-405	PCRRXN-476

COLUMN
