# Supplementary material for: Automated Platform for the Plasmid Construction Process
Source: ACS Synth Biol. 2023 Nov 10;12(12):3506–13. doi: 10.1021/acssynbio.3c00292 (PMC10729297; doi:10.1021/acssynbio.3c00292)
Supplement: Supplementary file 2 — sb3c00292_si_002.zip [file sb3c00292_si_002.zip › dnada_supplementary_material_pks_library_build/plate_visualizations/assemblytoautomate_PCR_plate_5-preview.pdf]

assemblytoautomate_PCR_plate_5

ROW	1	2	3	4	5	6	7	8	9	10	11	12
A	PCRRXN-235	PCRRXN-72	PCRRXN-385	PCRRXN-304	PCRRXN-154	PCRRXN-15	PCRRXN-37	PCRRXN-396	PCRRXN-128	PCRRXN-223	PCRRXN-31	PCRRXN-136
B	PCRRXN-236	PCRRXN-73	PCRRXN-394	PCRRXN-181	PCRRXN-249	PCRRXN-207	PCRRXN-418	PCRRXN-119	PCRRXN-215	PCRRXN-224	PCRRXN-137	PCRRXN-141
C	PCRRXN-42	PCRRXN-317	PCRRXN-403	PCRRXN-182	PCRRXN-250	PCRRXN-208	PCRRXN-467	PCRRXN-120	PCRRXN-216	PCRRXN-23	PCRRXN-138	PCRRXN-142
D	PCRRXN-43	PCRRXN-318	PCRRXN-299	PCRRXN-277	PCRRXN-56	PCRRXN-133	PCRRXN-377	PCRRXN-123	PCRRXN-219	PCRRXN-26	PCRRXN-233	PCRRXN-231
E	PCRRXN-169	PCRRXN-319	PCRRXN-300	PCRRXN-278	PCRRXN-57	PCRRXN-134	PCRRXN-386	PCRRXN-124	PCRRXN-22	PCRRXN-27	PCRRXN-234	PCRRXN-232
F	PCRRXN-170	PCRRXN-320	PCRRXN-301	PCRRXN-84	PCRRXN-111	PCRRXN-229	PCRRXN-395	PCRRXN-125	PCRRXN-220	PCRRXN-28	PCRRXN-40	PCRRXN-237
G	PCRRXN-265	PCRRXN-321	PCRRXN-302	PCRRXN-85	PCRRXN-112	PCRRXN-230	PCRRXN-378	PCRRXN-126	PCRRXN-221	PCRRXN-29	PCRRXN-41	PCRRXN-238
H	PCRRXN-266	PCRRXN-322	PCRRXN-303	PCRRXN-153	PCRRXN-14	PCRRXN-36	PCRRXN-387	PCRRXN-127	PCRRXN-222	PCRRXN-30	PCRRXN-135	PCRRXN-38

COLUMN
