# Supplementary material for: Automated Platform for the Plasmid Construction Process
Source: ACS Synth Biol. 2023 Nov 10;12(12):3506–13. doi: 10.1021/acssynbio.3c00292 (PMC10729297; doi:10.1021/acssynbio.3c00292)
Supplement: Supplementary file 2 — sb3c00292_si_002.zip [file sb3c00292_si_002.zip › dnada_supplementary_material_pks_library_build/plate_visualizations/assemblytoautomate_PCR_plate_4-preview.pdf]

assemblytoautomate_PCR_plate_4

ROW	1	2	3	4	5	6	7	8	9	10	11	12
A	PCRRXN-199	PCRRXN-199	PCRRXN-423	PCRRXN-486	PCRRXN-263	PCRRXN-313	PCRRXN-327	PCRRXN-461	PCRRXN-18	PCRRXN-104	PCRRXN-351	PCRRXN-371
B	PCRRXN-199	PCRRXN-199	PCRRXN-430	PCRRXN-501	PCRRXN-264	PCRRXN-314	PCRRXN-328	PCRRXN-468	PCRRXN-19	PCRRXN-198	PCRRXN-352	PCRRXN-372
C	PCRRXN-199	PCRRXN-199	PCRRXN-437	PCRRXN-165	PCRRXN-68	PCRRXN-315	PCRRXN-412	PCRRXN-475	PCRRXN-211	PCRRXN-200	PCRRXN-113	PCRRXN-373
D	PCRRXN-199	PCRRXN-381	PCRRXN-452	PCRRXN-166	PCRRXN-69	PCRRXN-316	PCRRXN-419	PCRRXN-482	PCRRXN-212	PCRRXN-6	PCRRXN-114	PCRRXN-374
E	PCRRXN-199	PCRRXN-390	PCRRXN-458	PCRRXN-167	PCRRXN-70	PCRRXN-323	PCRRXN-426	PCRRXN-489	PCRRXN-411	PCRRXN-347	PCRRXN-16	PCRRXN-375
F	PCRRXN-199	PCRRXN-399	PCRRXN-465	PCRRXN-168	PCRRXN-71	PCRRXN-324	PCRRXN-433	PCRRXN-496	PCRRXN-460	PCRRXN-348	PCRRXN-17	PCRRXN-376
G	PCRRXN-199	PCRRXN-409	PCRRXN-472	PCRRXN-261	PCRRXN-311	PCRRXN-325	PCRRXN-440	PCRRXN-115	PCRRXN-1	PCRRXN-349	PCRRXN-209	PCRRXN-139
H	PCRRXN-199	PCRRXN-416	PCRRXN-479	PCRRXN-262	PCRRXN-312	PCRRXN-326	PCRRXN-447	PCRRXN-116	PCRRXN-101	PCRRXN-350	PCRRXN-210	PCRRXN-140

COLUMN
