# Supplementary figures and images for: Automated Platform for the Plasmid Construction Process (part 2 of 2)
Source: ACS Synth Biol. 2023 Nov 10;12(12):3506–13. doi: 10.1021/acssynbio.3c00292 (PMC10729297; doi:10.1021/acssynbio.3c00292)

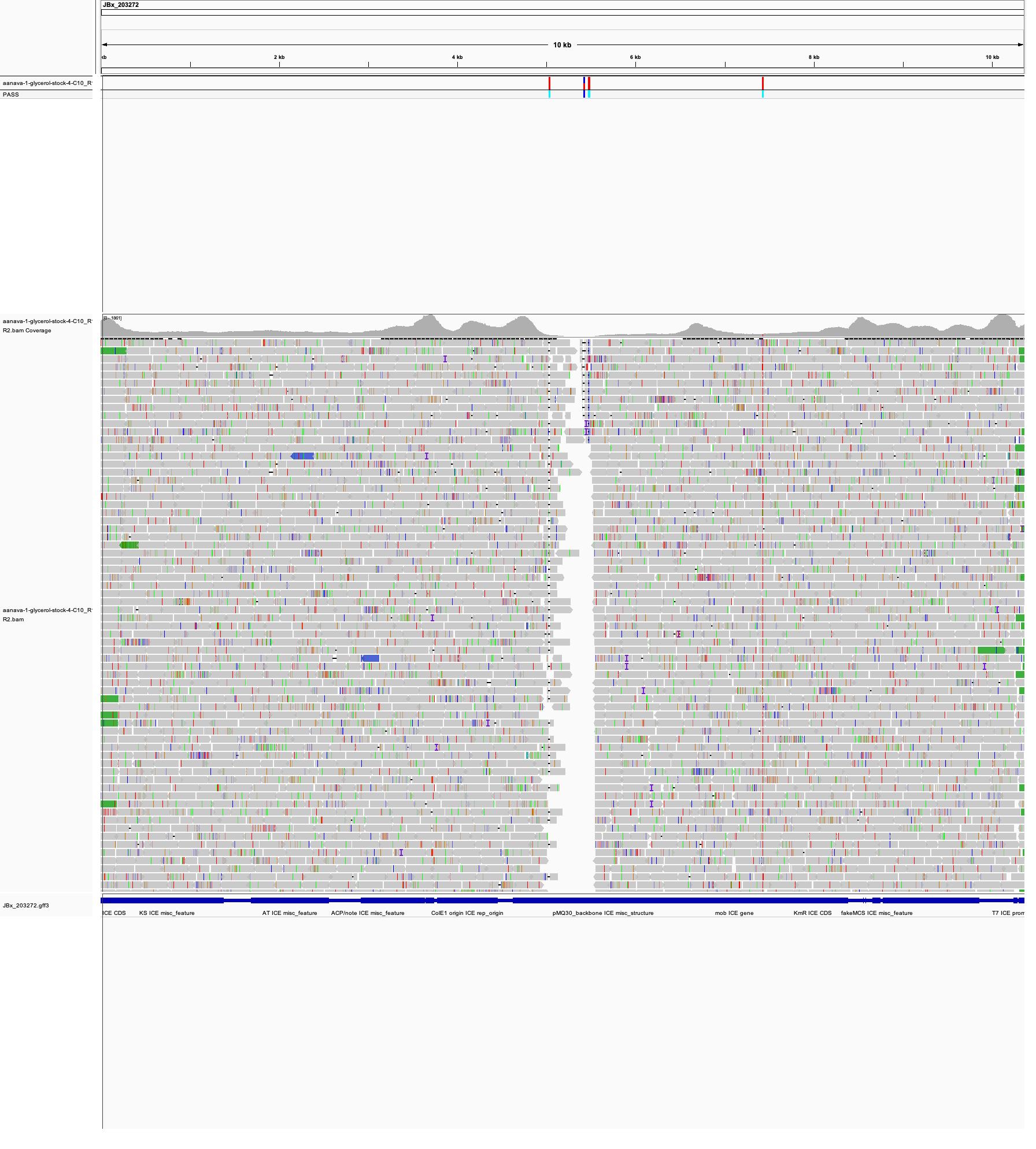

Supplement: Supplementary file 2 — sb3c00292_si_002.zip [file sb3c00292_si_002.zip › dnada_supplementary_material_pks_library_build/divaseq/211117_divaseq_analysis/alberto/snapshots/JBx_203272_nava-1-glycerol-stock-4-C10_R1R2.jpg]

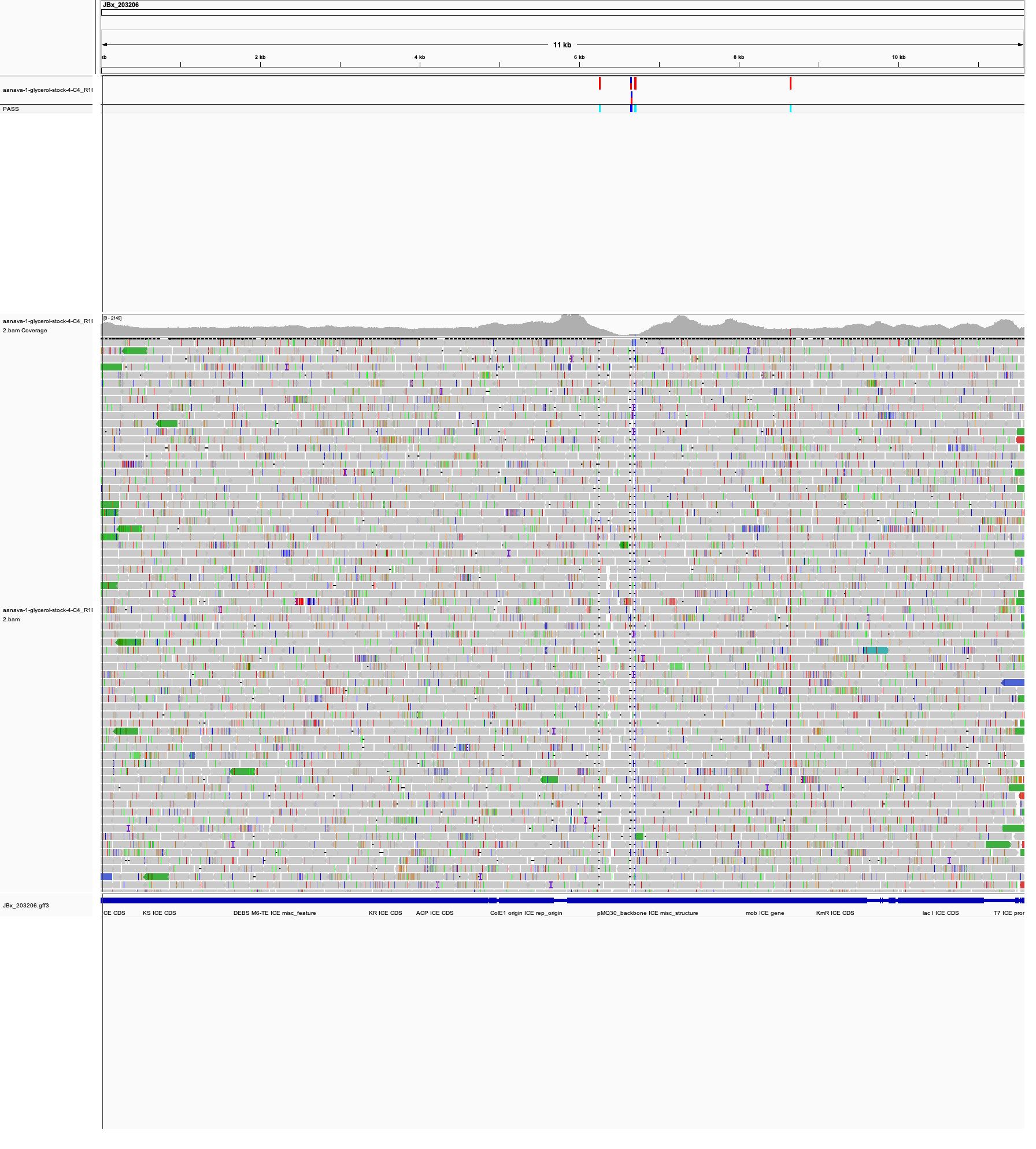

Supplement: Supplementary file 2 — sb3c00292_si_002.zip [file sb3c00292_si_002.zip › dnada_supplementary_material_pks_library_build/divaseq/211117_divaseq_analysis/alberto/snapshots/JBx_203206_nava-1-glycerol-stock-4-C4_R1R2.jpg]

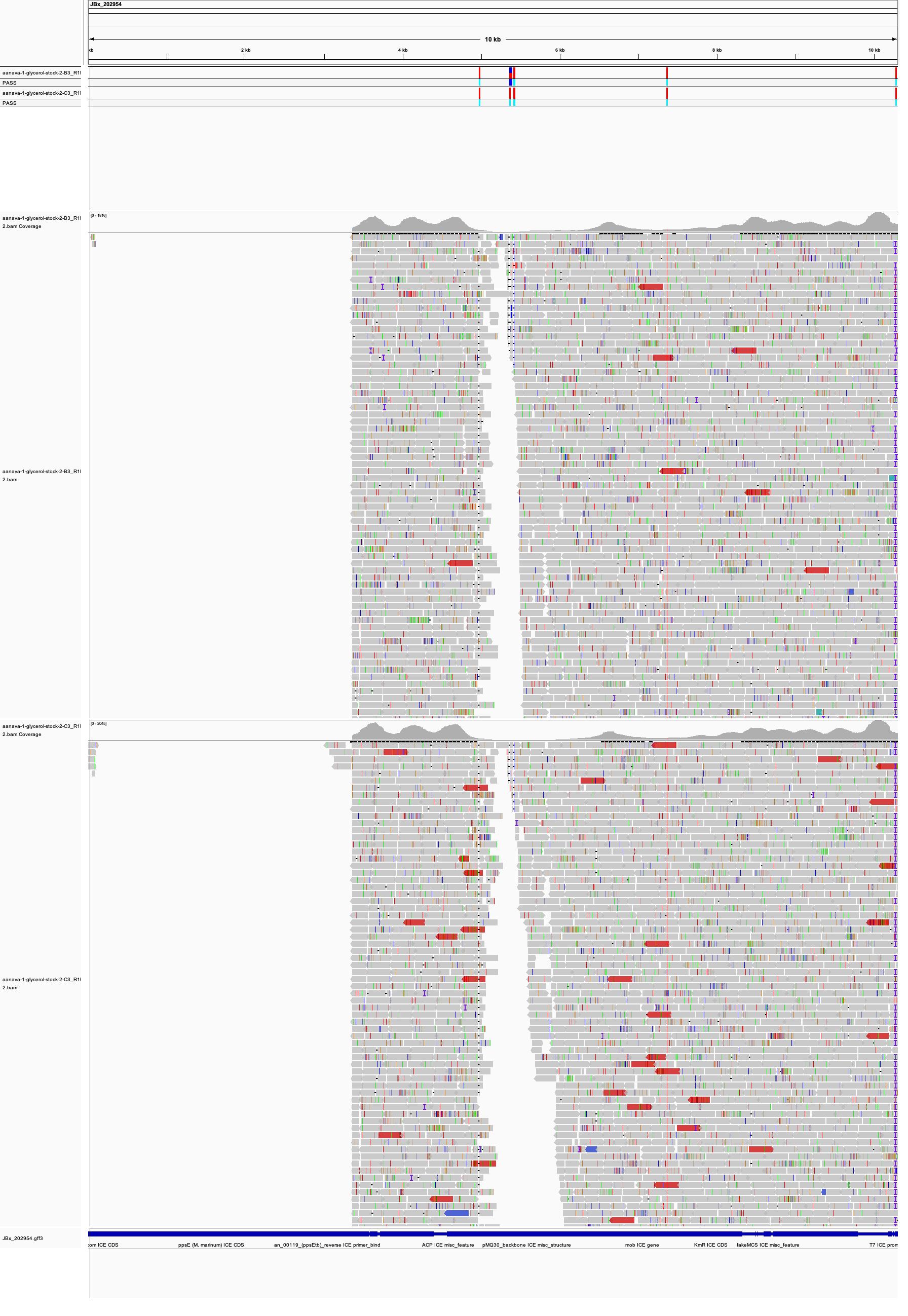

Supplement: Supplementary file 2 — sb3c00292_si_002.zip [file sb3c00292_si_002.zip › dnada_supplementary_material_pks_library_build/divaseq/211117_divaseq_analysis/alberto/snapshots/JBx_202954_nava-1-glycerol-stock-2-C3_R1R2.jpg]

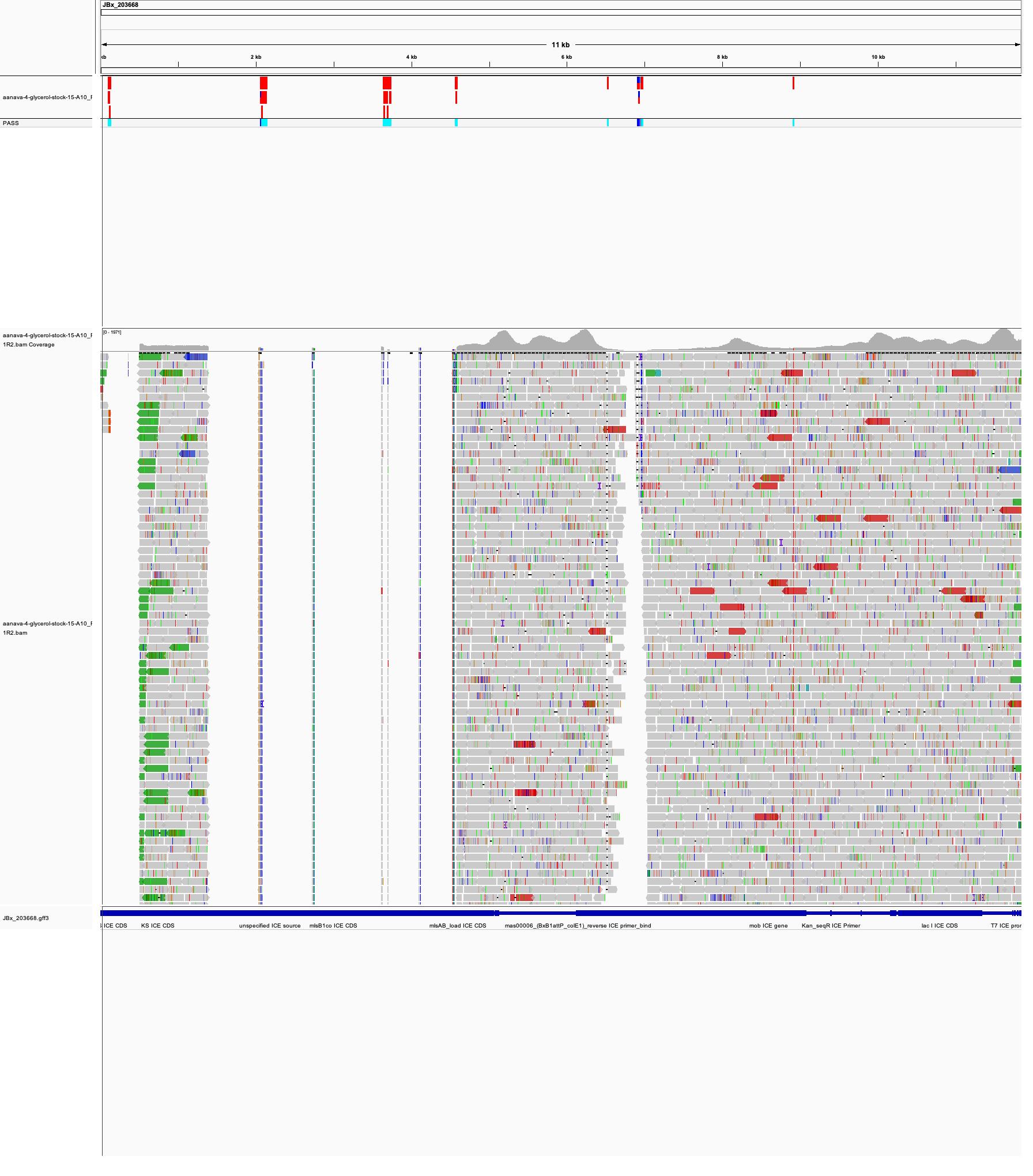

Supplement: Supplementary file 2 — sb3c00292_si_002.zip [file sb3c00292_si_002.zip › dnada_supplementary_material_pks_library_build/divaseq/211117_divaseq_analysis/alberto/snapshots/JBx_203668_nava-4-glycerol-stock-15-A10_R1R2.jpg]

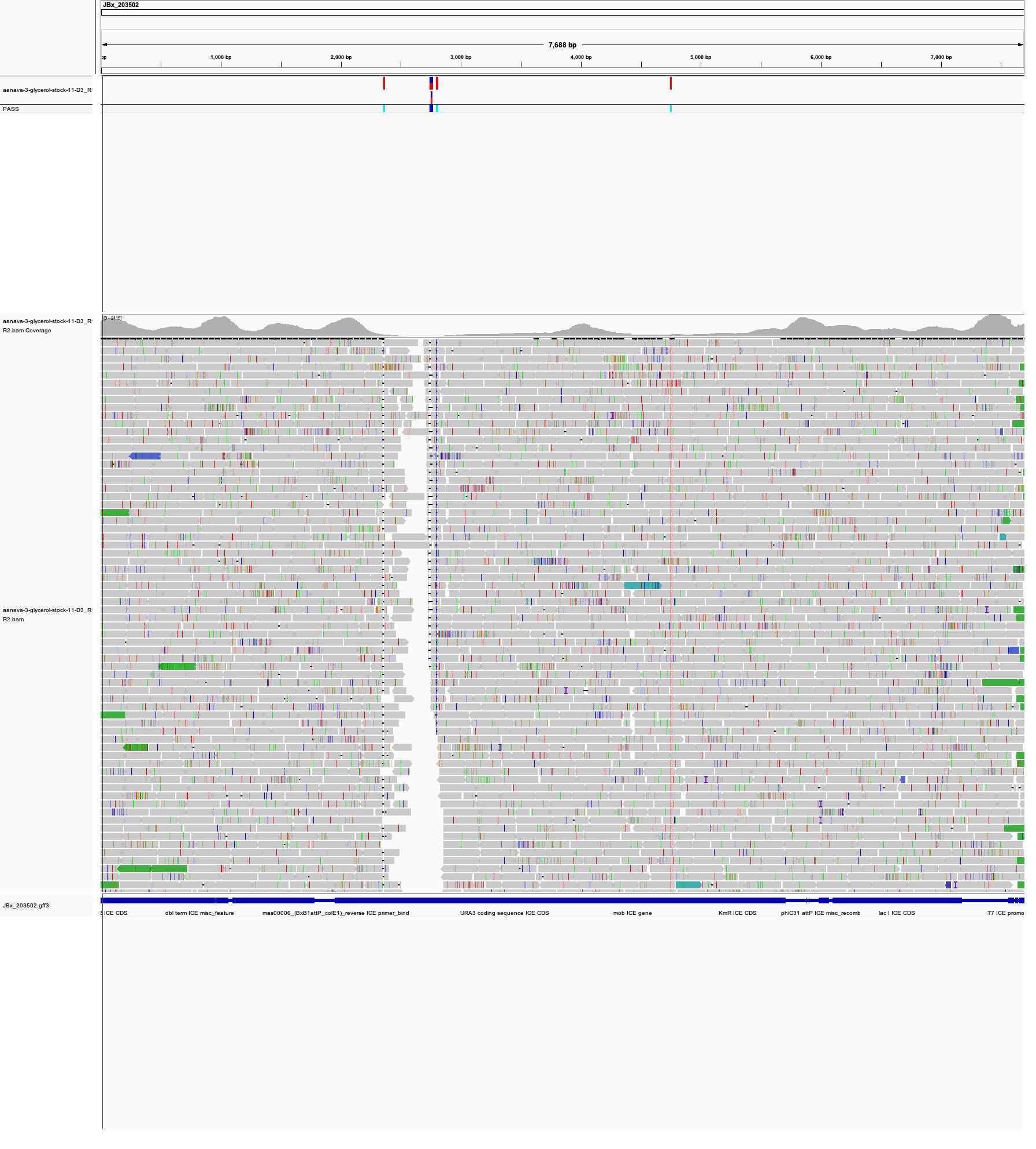

Supplement: Supplementary file 2 — sb3c00292_si_002.zip [file sb3c00292_si_002.zip › dnada_supplementary_material_pks_library_build/divaseq/211117_divaseq_analysis/alberto/snapshots/JBx_203502_nava-3-glycerol-stock-11-D3_R1R2.jpg]

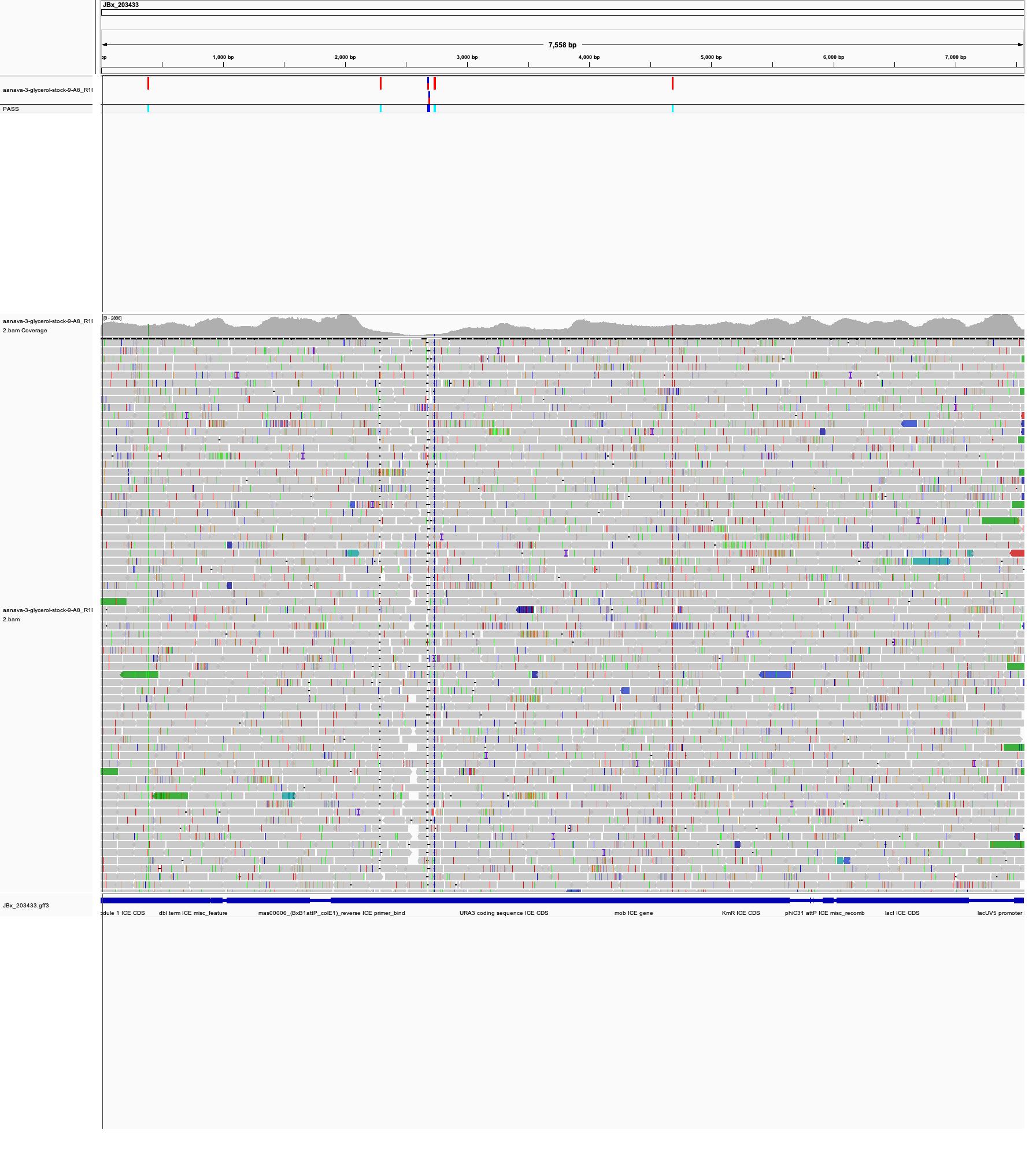

Supplement: Supplementary file 2 — sb3c00292_si_002.zip [file sb3c00292_si_002.zip › dnada_supplementary_material_pks_library_build/divaseq/211117_divaseq_analysis/alberto/snapshots/JBx_203433_nava-3-glycerol-stock-9-A8_R1R2.jpg]

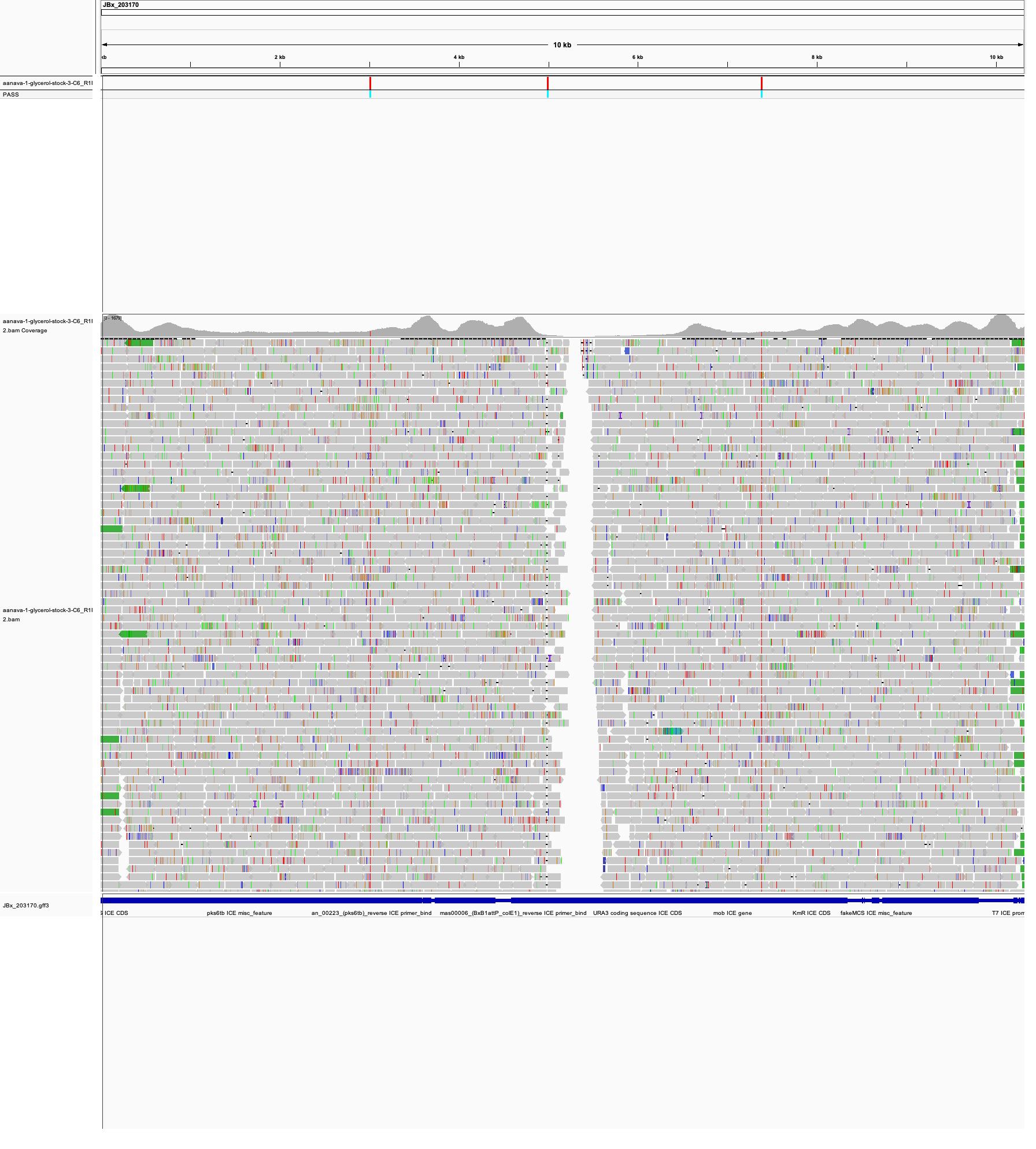

Supplement: Supplementary file 2 — sb3c00292_si_002.zip [file sb3c00292_si_002.zip › dnada_supplementary_material_pks_library_build/divaseq/211117_divaseq_analysis/alberto/snapshots/JBx_203170_nava-1-glycerol-stock-3-C6_R1R2.jpg]

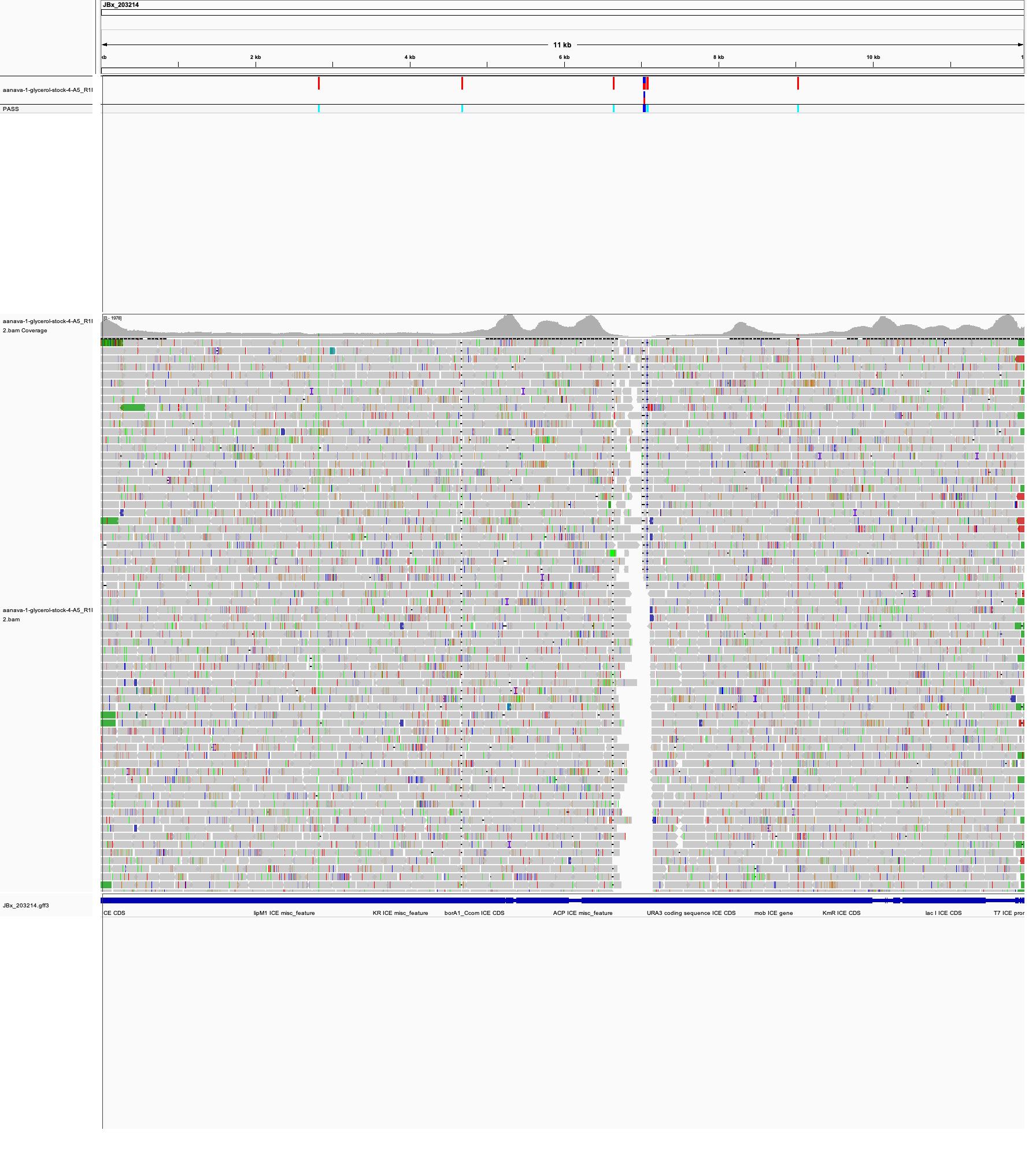

Supplement: Supplementary file 2 — sb3c00292_si_002.zip [file sb3c00292_si_002.zip › dnada_supplementary_material_pks_library_build/divaseq/211117_divaseq_analysis/alberto/snapshots/JBx_203214_nava-1-glycerol-stock-4-A5_R1R2.jpg]

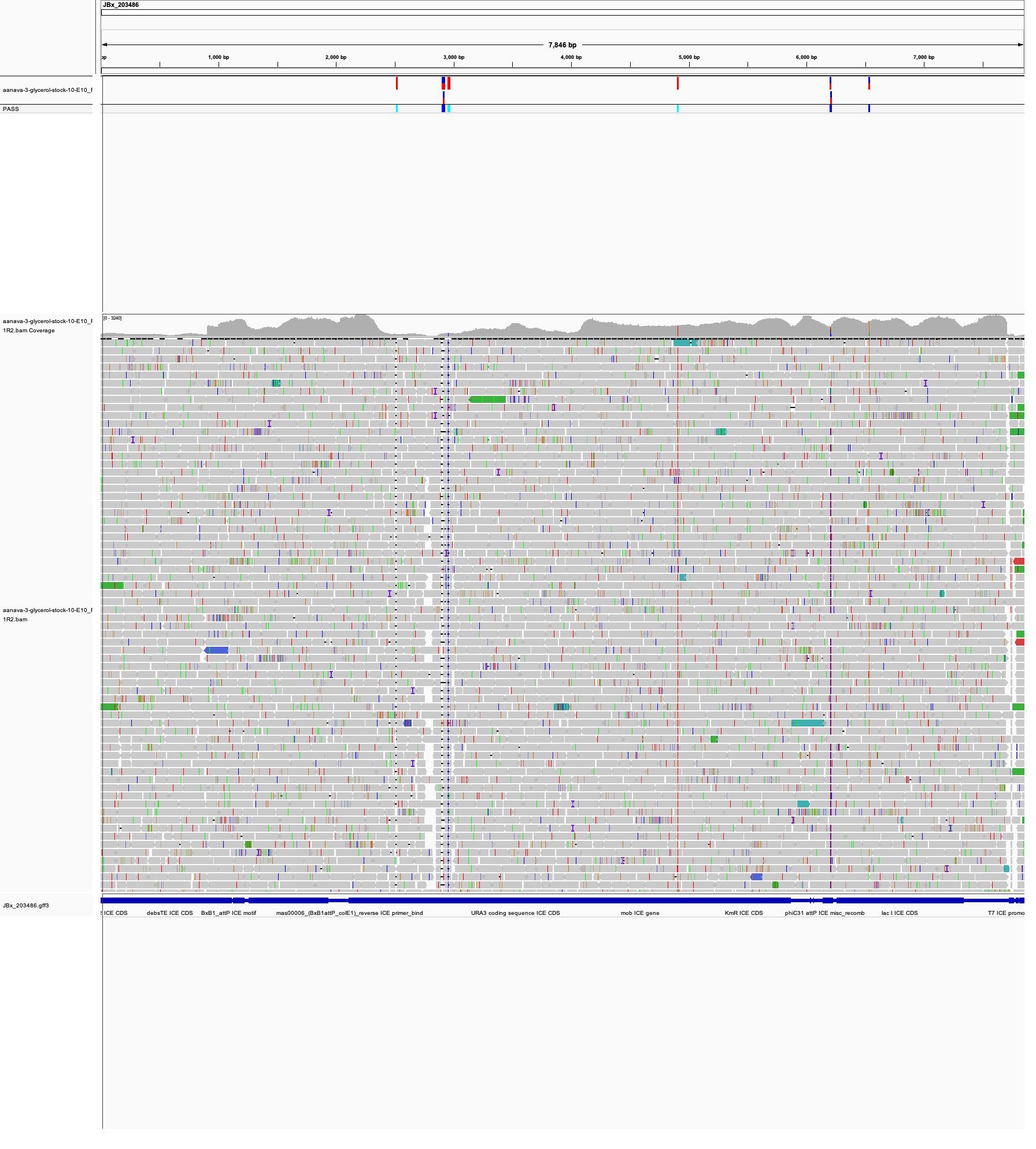

Supplement: Supplementary file 2 — sb3c00292_si_002.zip [file sb3c00292_si_002.zip › dnada_supplementary_material_pks_library_build/divaseq/211117_divaseq_analysis/alberto/snapshots/JBx_203486_nava-3-glycerol-stock-10-E10_R1R2.jpg]

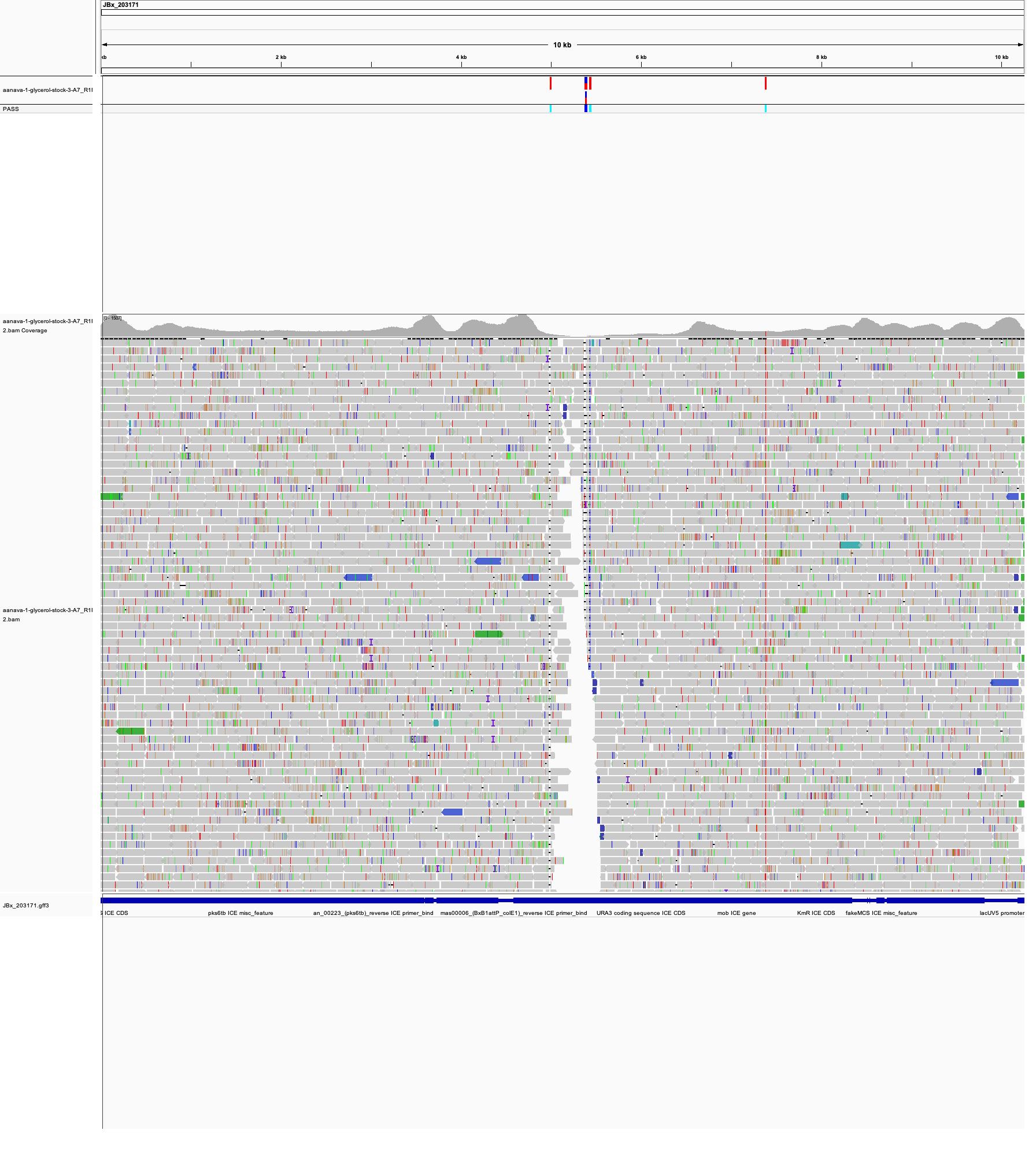

Supplement: Supplementary file 2 — sb3c00292_si_002.zip [file sb3c00292_si_002.zip › dnada_supplementary_material_pks_library_build/divaseq/211117_divaseq_analysis/alberto/snapshots/JBx_203171_nava-1-glycerol-stock-3-A7_R1R2.jpg]

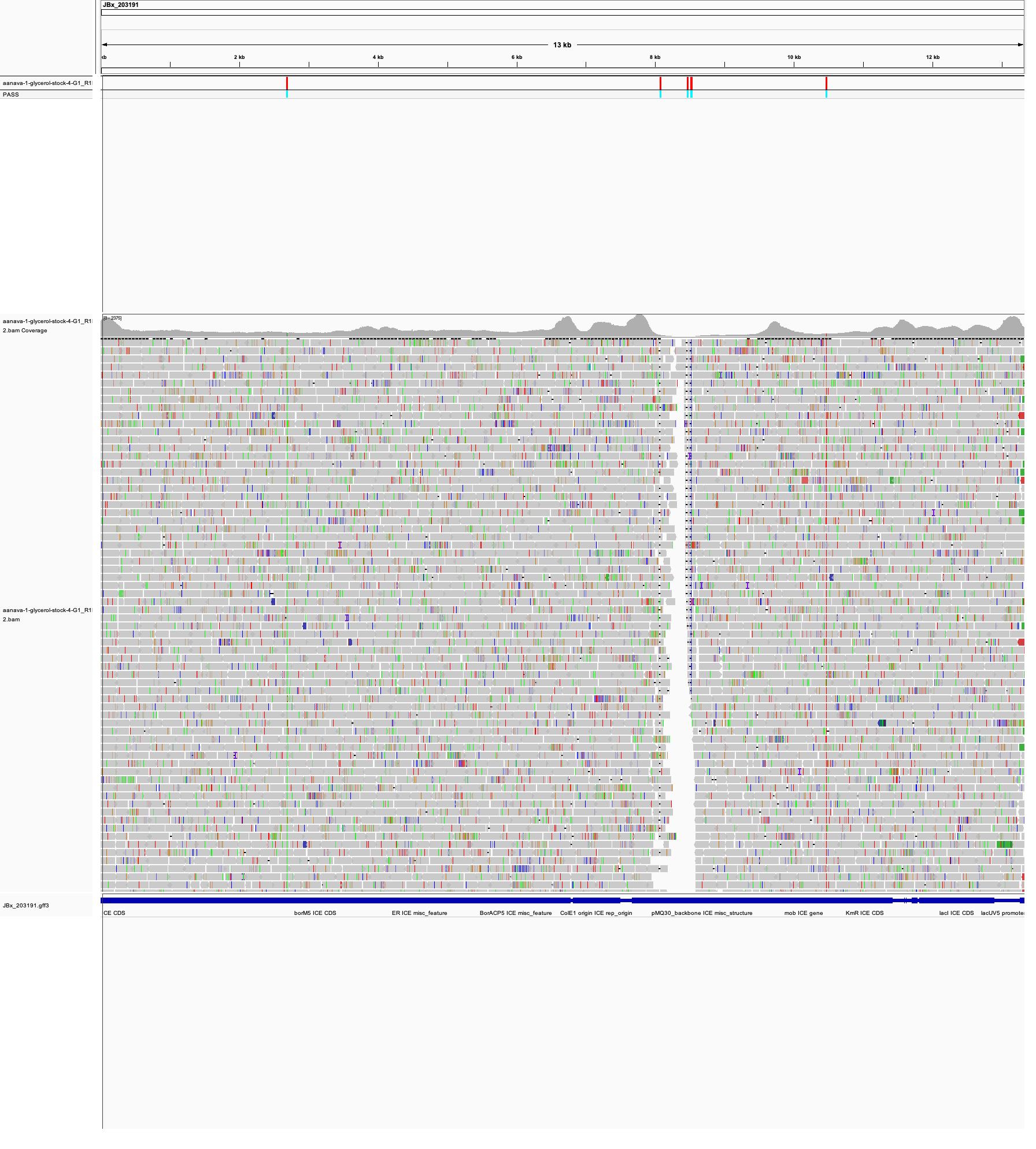

Supplement: Supplementary file 2 — sb3c00292_si_002.zip [file sb3c00292_si_002.zip › dnada_supplementary_material_pks_library_build/divaseq/211117_divaseq_analysis/alberto/snapshots/JBx_203191_nava-1-glycerol-stock-4-G1_R1R2.jpg]

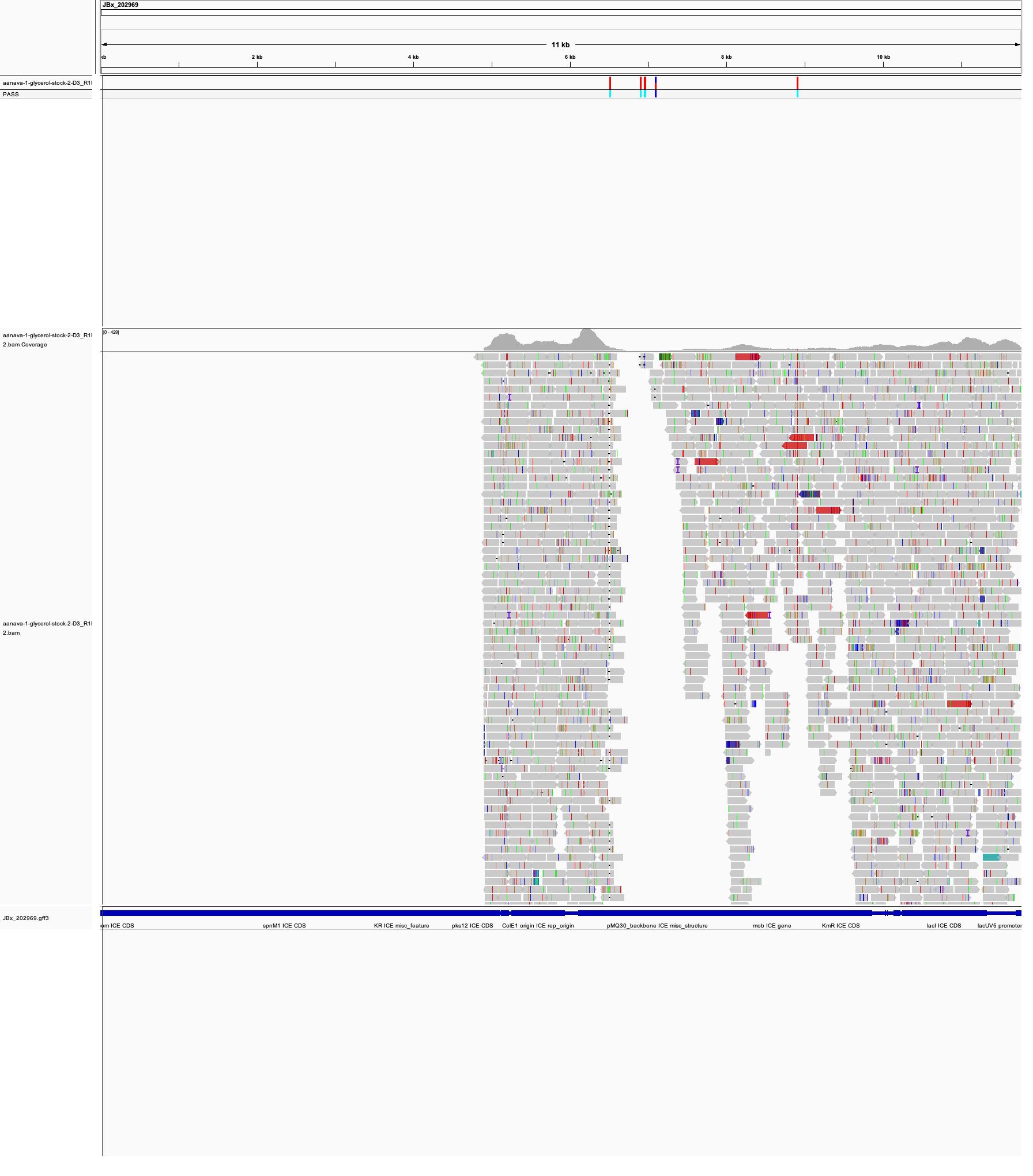

Supplement: Supplementary file 2 — sb3c00292_si_002.zip [file sb3c00292_si_002.zip › dnada_supplementary_material_pks_library_build/divaseq/211117_divaseq_analysis/alberto/snapshots/JBx_202969_nava-1-glycerol-stock-2-D3_R1R2.jpg]

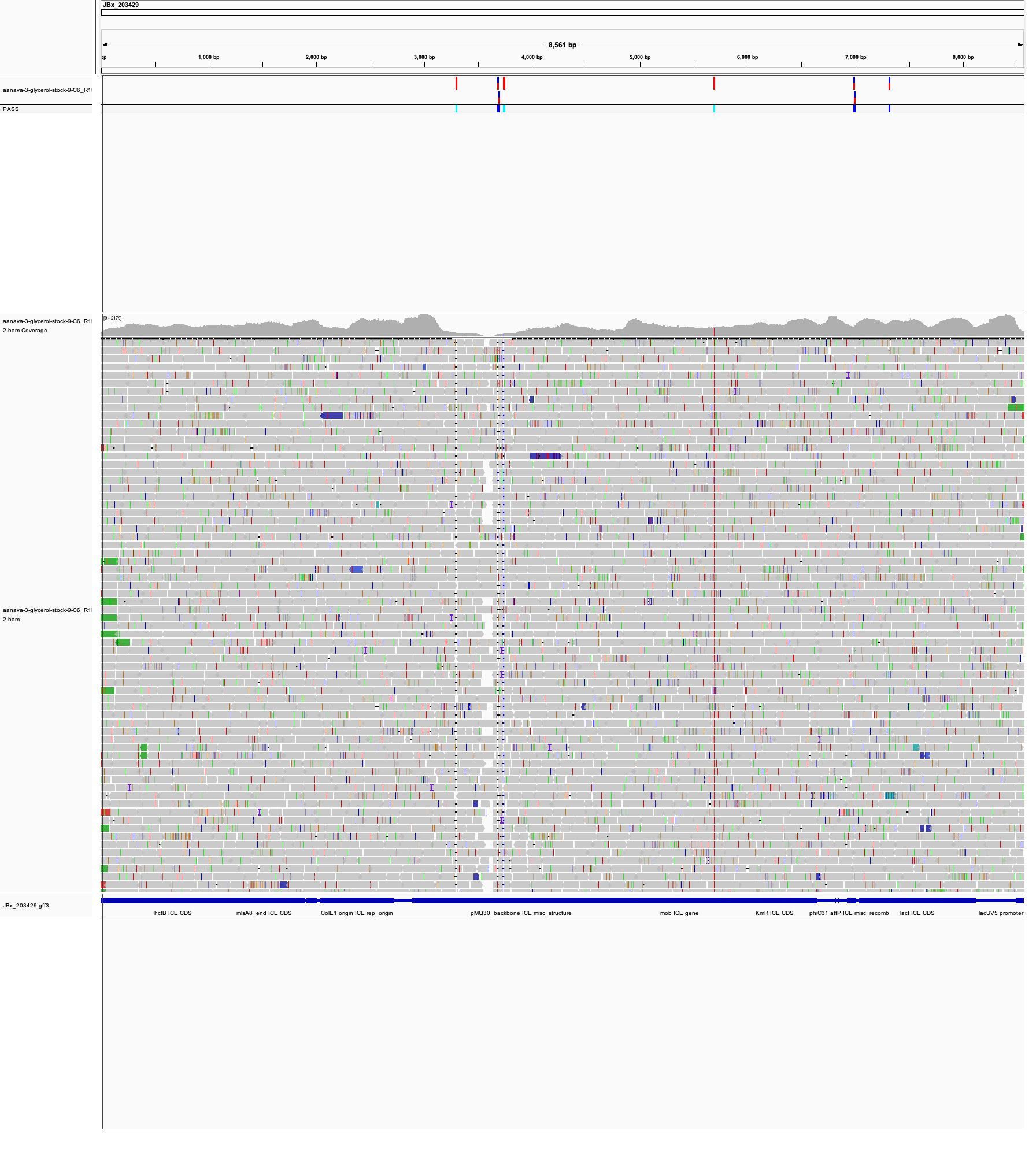

Supplement: Supplementary file 2 — sb3c00292_si_002.zip [file sb3c00292_si_002.zip › dnada_supplementary_material_pks_library_build/divaseq/211117_divaseq_analysis/alberto/snapshots/JBx_203429_nava-3-glycerol-stock-9-C6_R1R2.jpg]

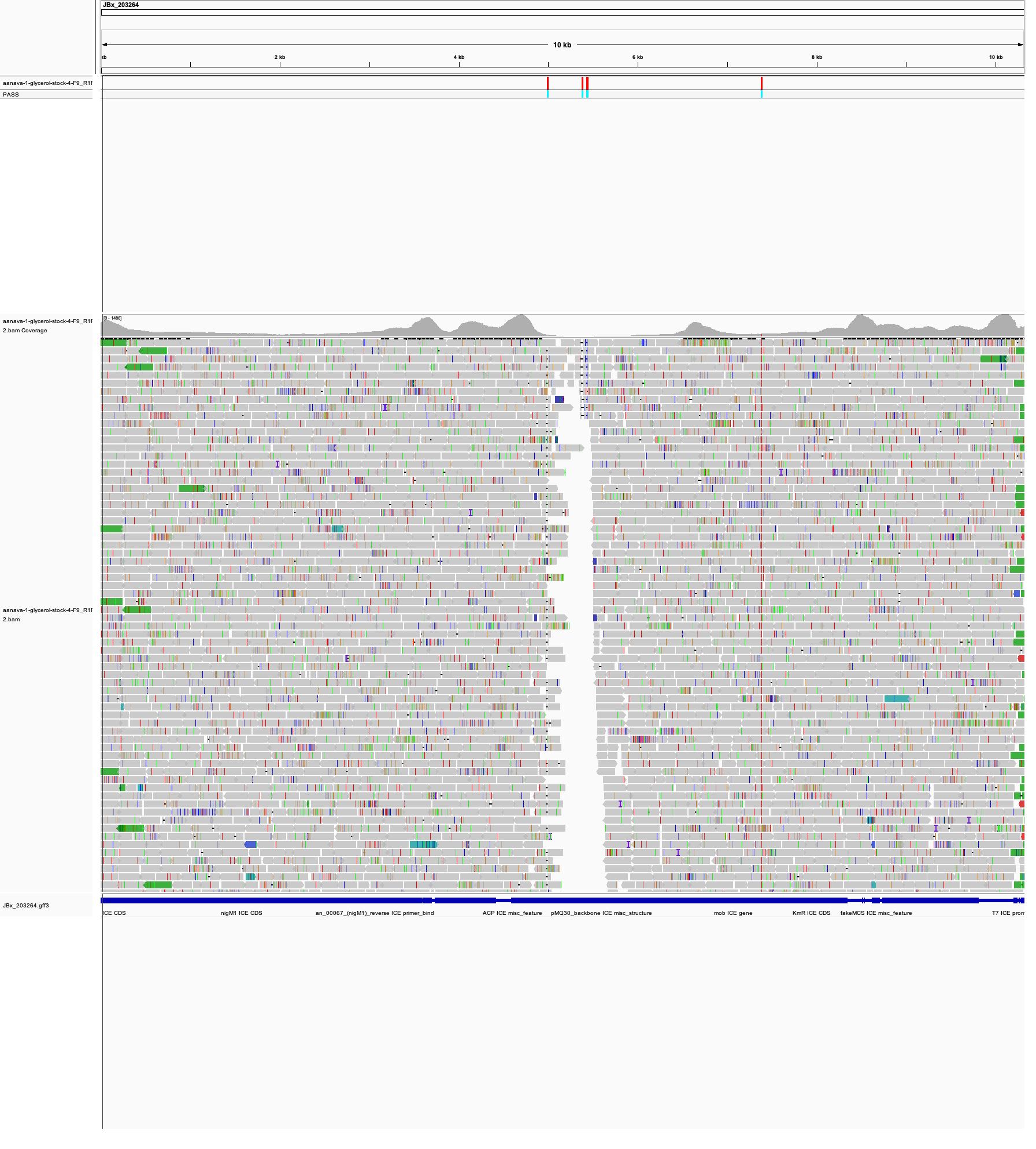

Supplement: Supplementary file 2 — sb3c00292_si_002.zip [file sb3c00292_si_002.zip › dnada_supplementary_material_pks_library_build/divaseq/211117_divaseq_analysis/alberto/snapshots/JBx_203264_nava-1-glycerol-stock-4-F9_R1R2.jpg]

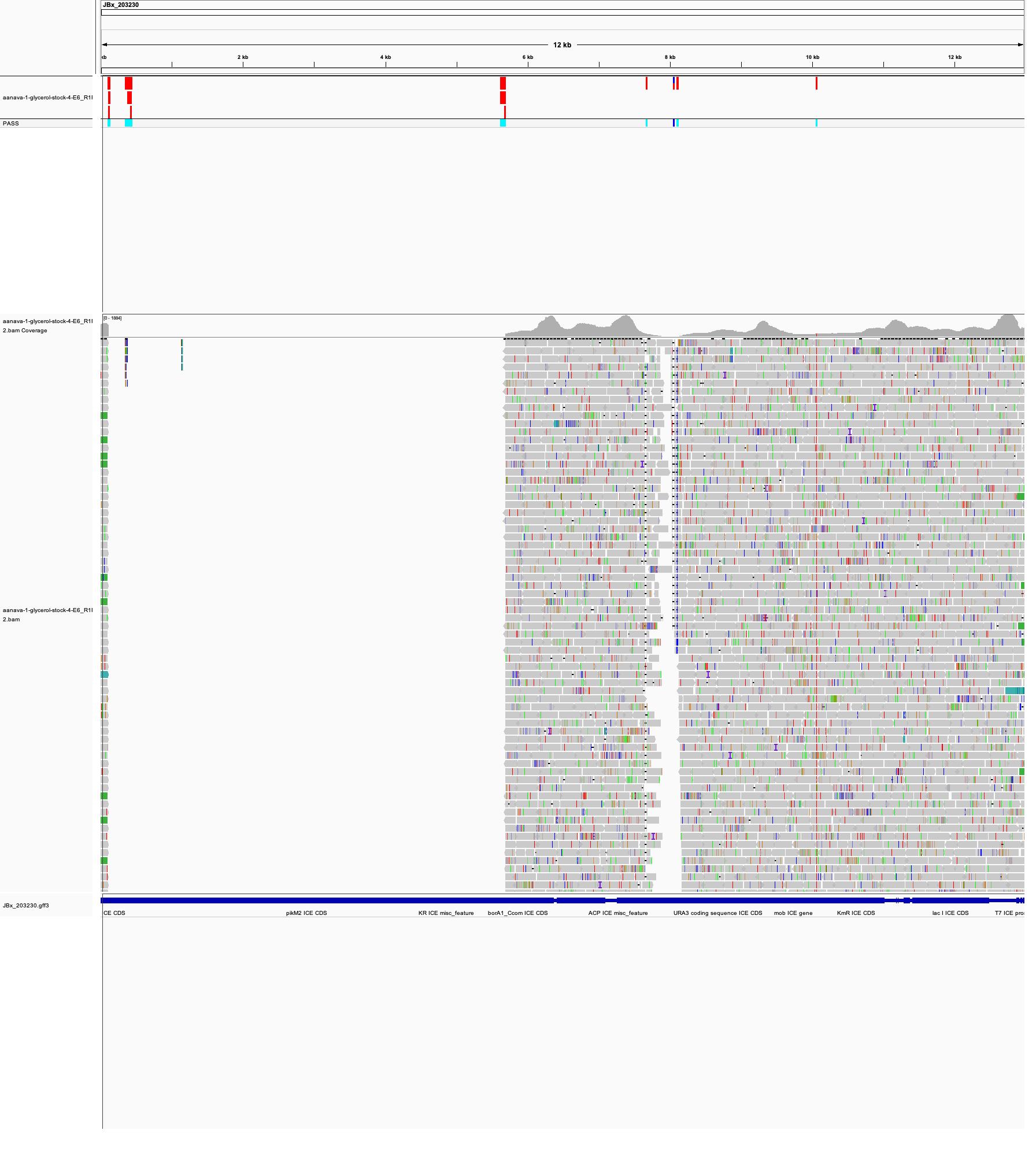

Supplement: Supplementary file 2 — sb3c00292_si_002.zip [file sb3c00292_si_002.zip › dnada_supplementary_material_pks_library_build/divaseq/211117_divaseq_analysis/alberto/snapshots/JBx_203230_nava-1-glycerol-stock-4-E6_R1R2.jpg]

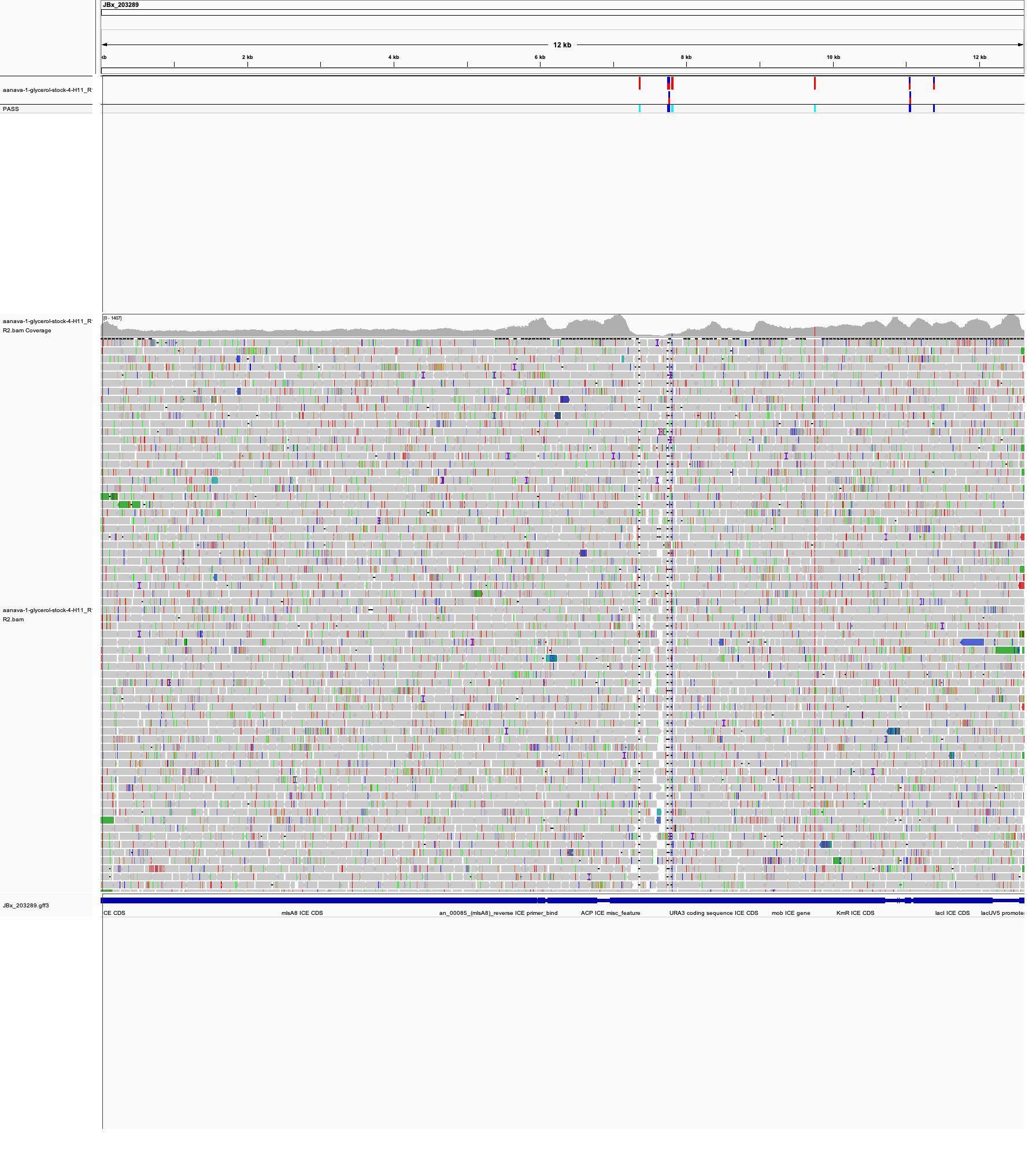

Supplement: Supplementary file 2 — sb3c00292_si_002.zip [file sb3c00292_si_002.zip › dnada_supplementary_material_pks_library_build/divaseq/211117_divaseq_analysis/alberto/snapshots/JBx_203289_nava-1-glycerol-stock-4-H11_R1R2.jpg]

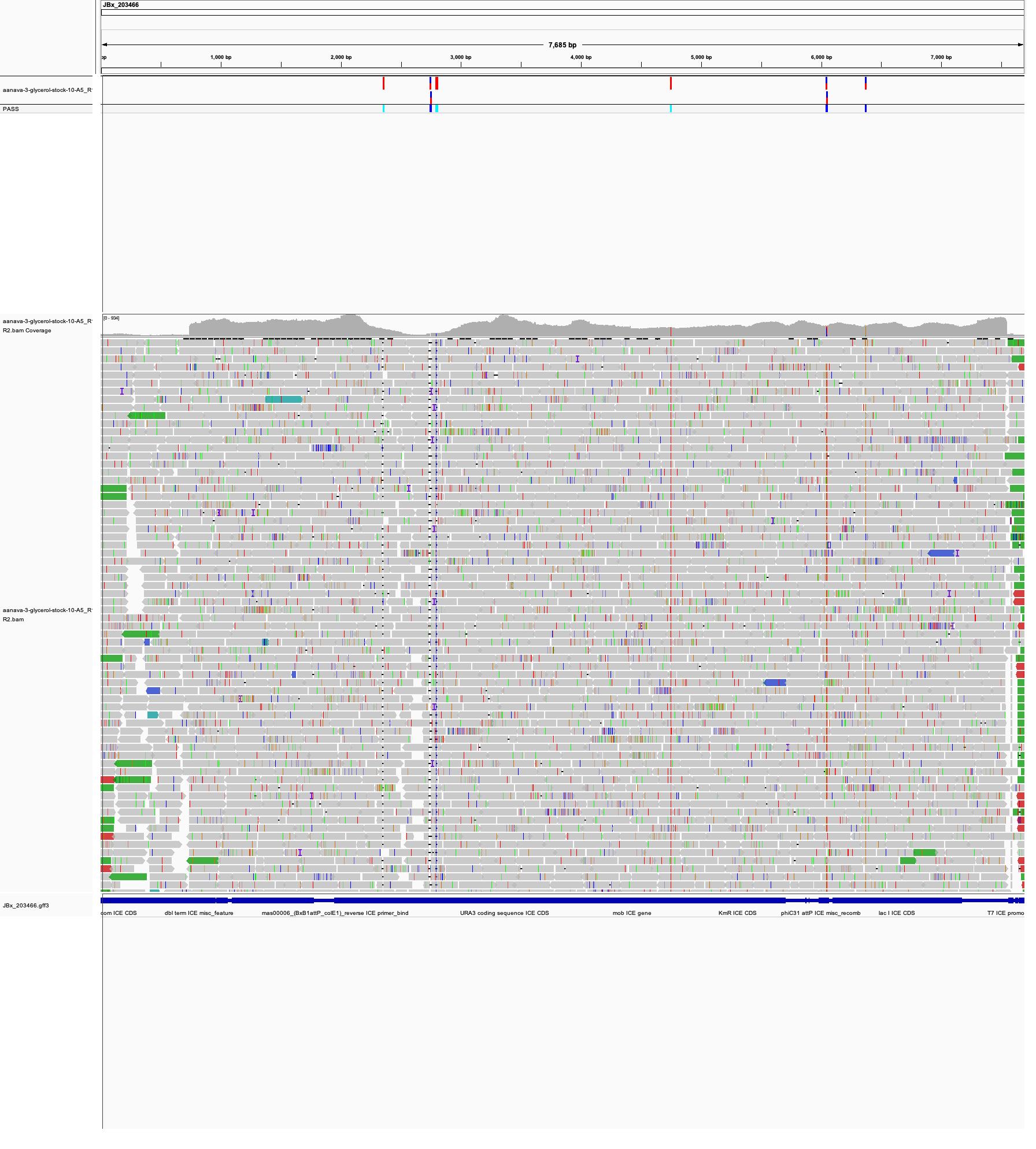

Supplement: Supplementary file 2 — sb3c00292_si_002.zip [file sb3c00292_si_002.zip › dnada_supplementary_material_pks_library_build/divaseq/211117_divaseq_analysis/alberto/snapshots/JBx_203466_nava-3-glycerol-stock-10-A5_R1R2.jpg]

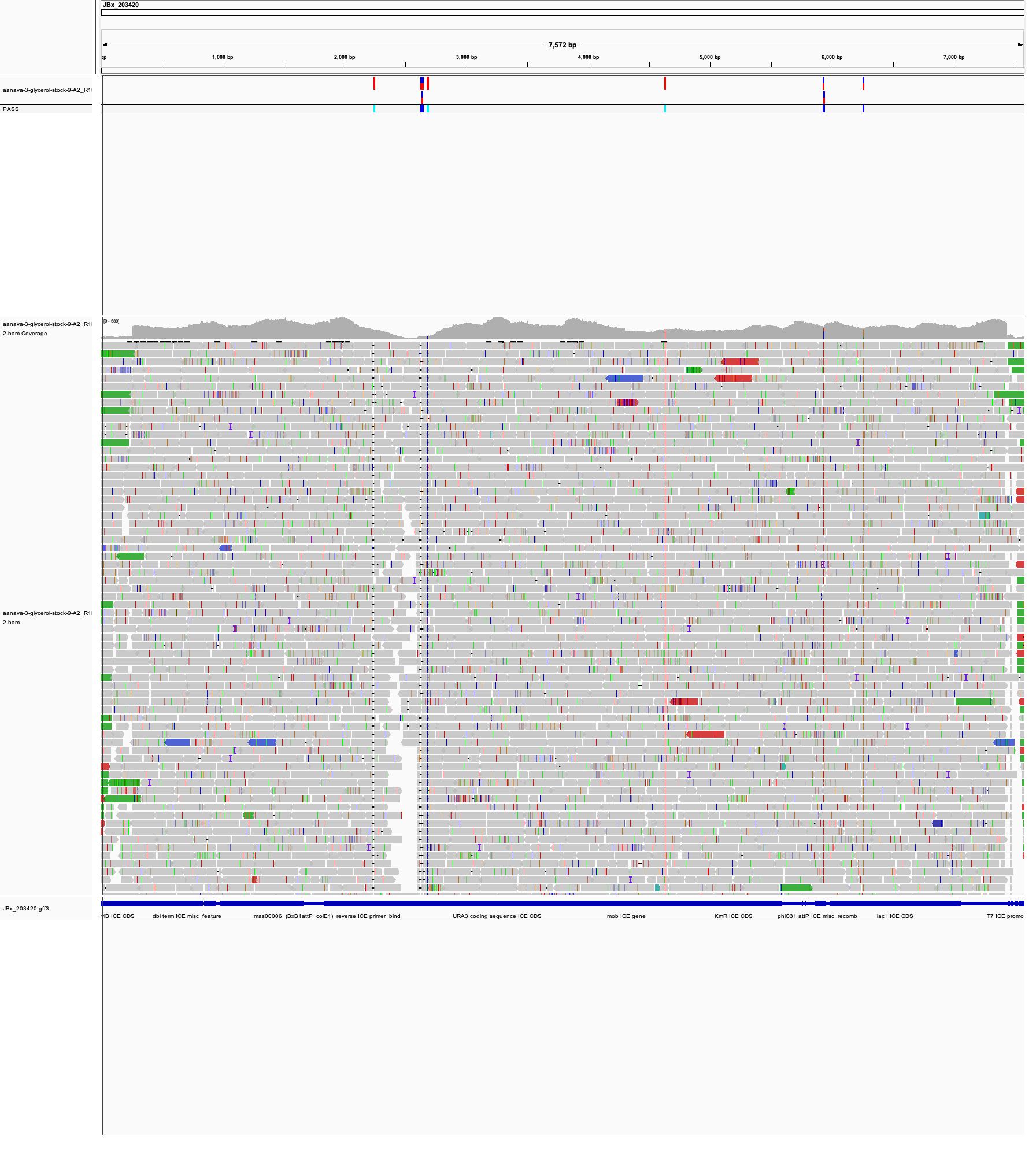

Supplement: Supplementary file 2 — sb3c00292_si_002.zip [file sb3c00292_si_002.zip › dnada_supplementary_material_pks_library_build/divaseq/211117_divaseq_analysis/alberto/snapshots/JBx_203420_nava-3-glycerol-stock-9-A2_R1R2.jpg]

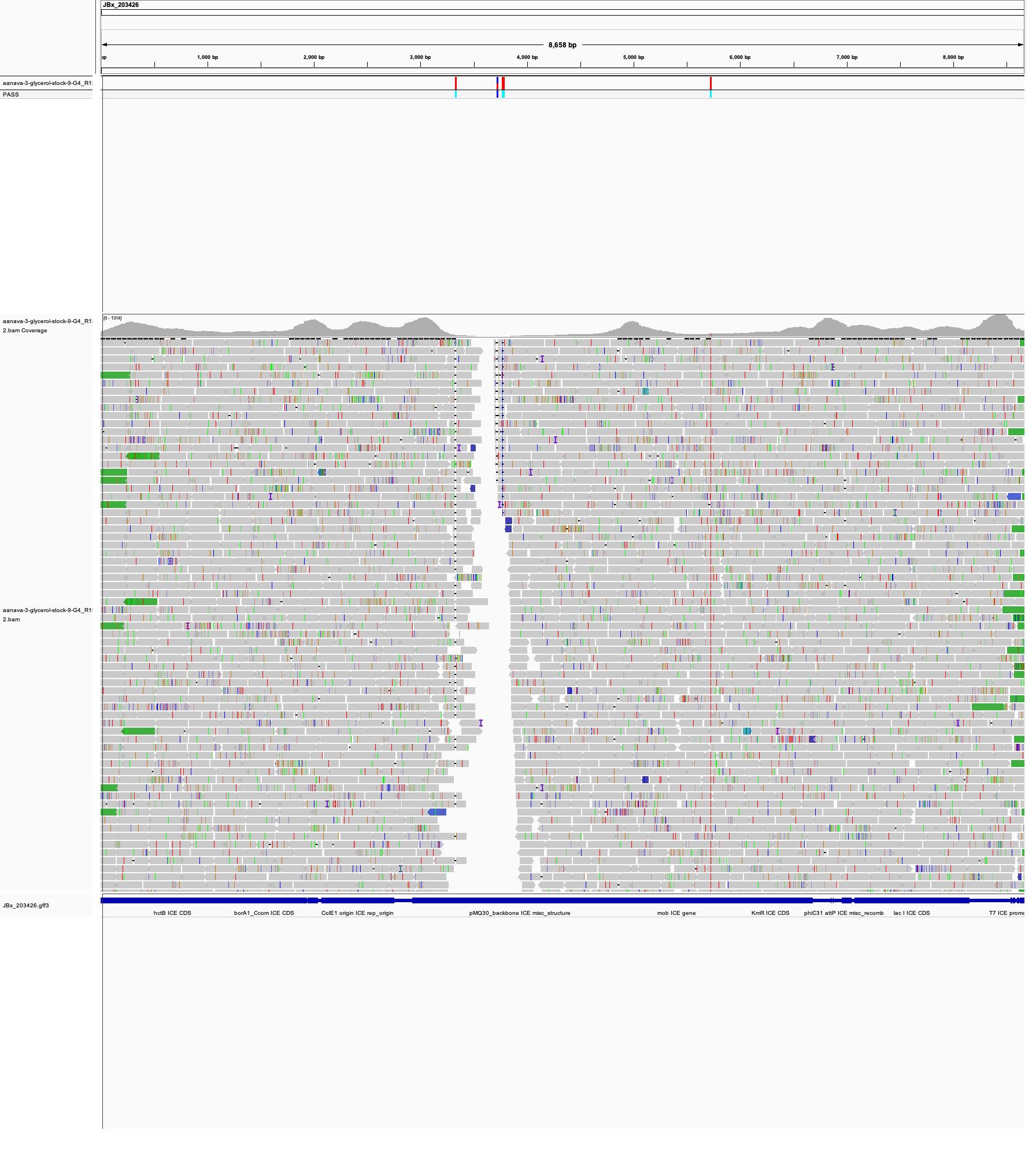

Supplement: Supplementary file 2 — sb3c00292_si_002.zip [file sb3c00292_si_002.zip › dnada_supplementary_material_pks_library_build/divaseq/211117_divaseq_analysis/alberto/snapshots/JBx_203426_nava-3-glycerol-stock-9-G4_R1R2.jpg]

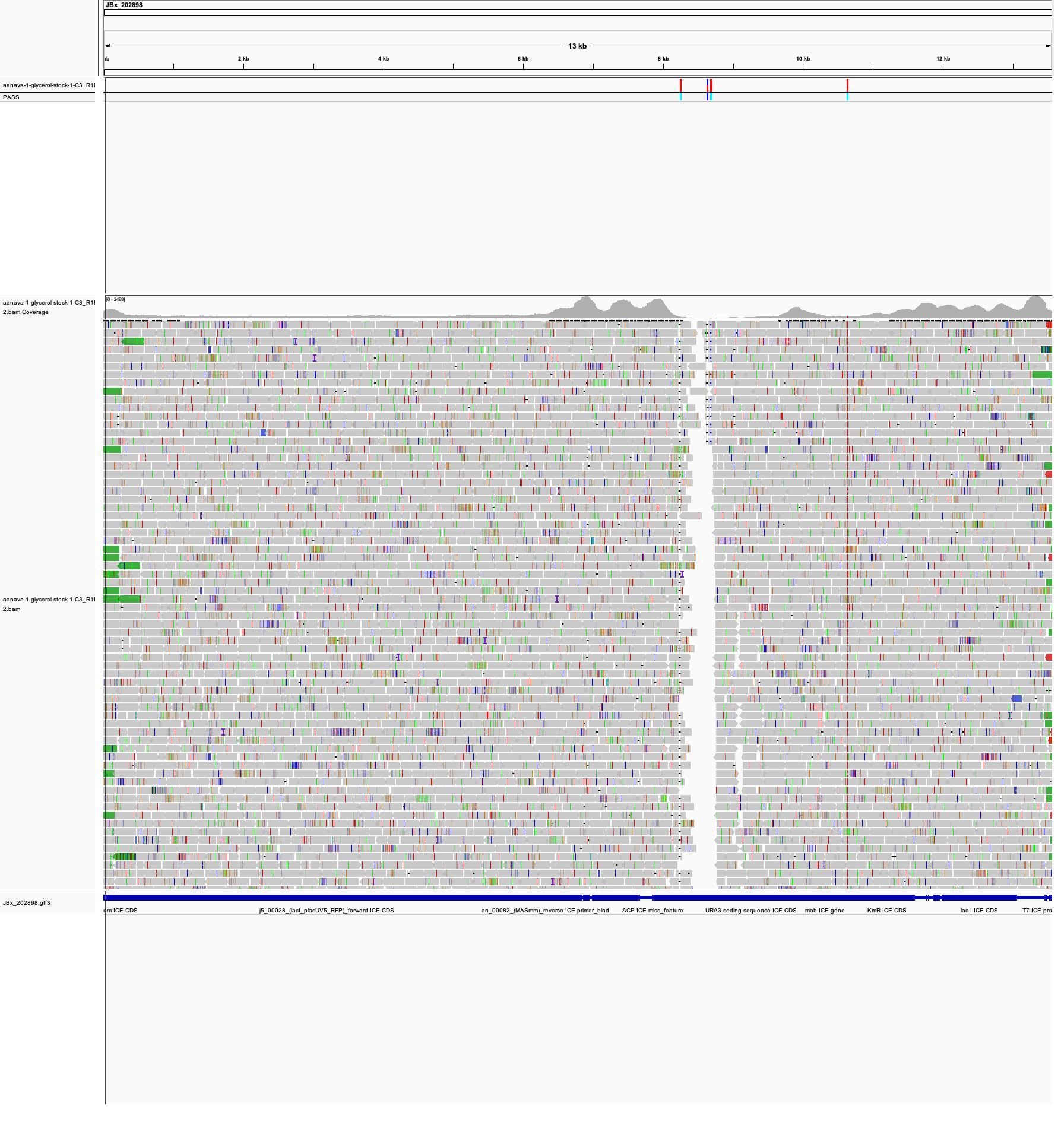

Supplement: Supplementary file 2 — sb3c00292_si_002.zip [file sb3c00292_si_002.zip › dnada_supplementary_material_pks_library_build/divaseq/211117_divaseq_analysis/alberto/snapshots/JBx_202898_nava-1-glycerol-stock-1-C3_R1R2.jpg]
