# Supplementary figures and images for: Automated Platform for the Plasmid Construction Process (part 1 of 2)
Source: ACS Synth Biol. 2023 Nov 10;12(12):3506–13. doi: 10.1021/acssynbio.3c00292 (PMC10729297; doi:10.1021/acssynbio.3c00292)

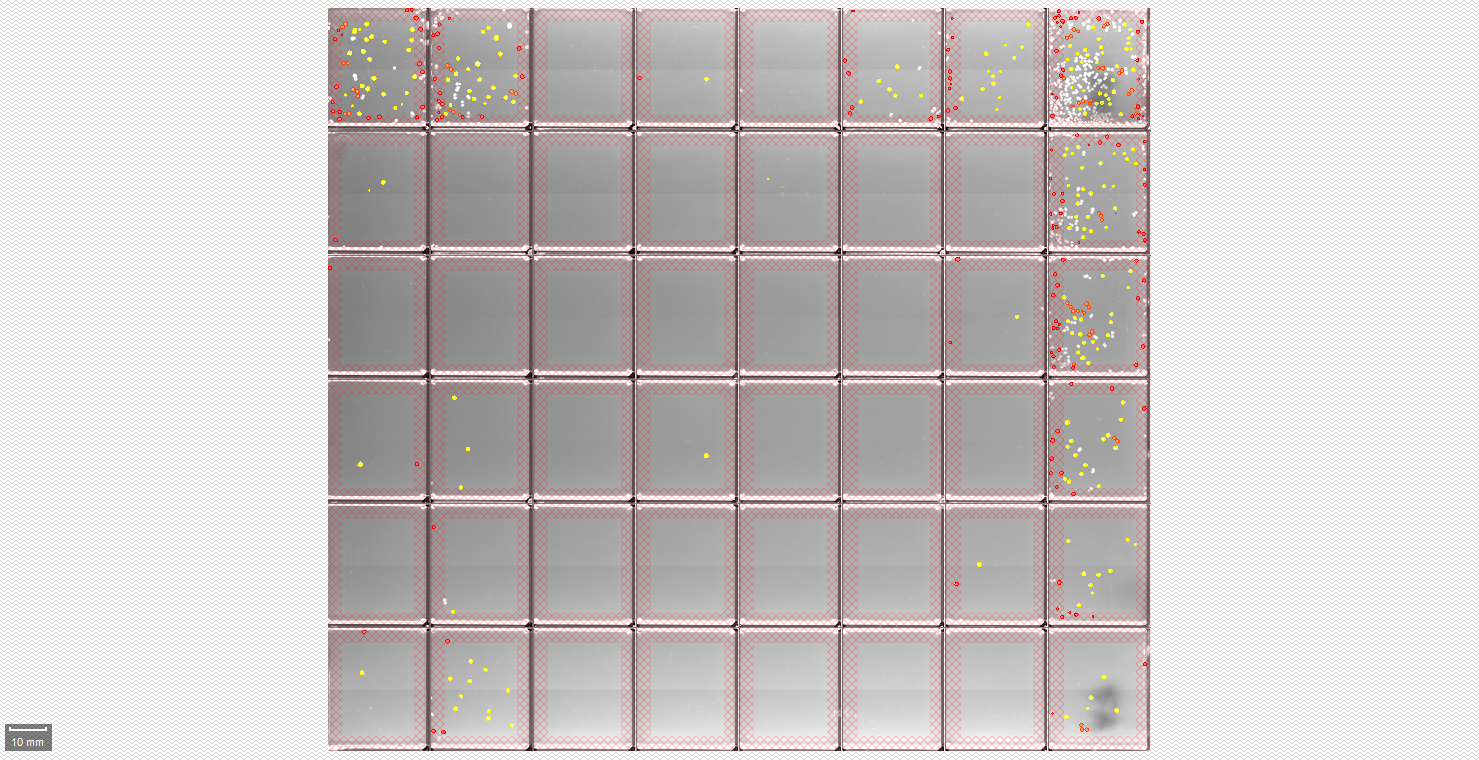

Supplement: Supplementary file 2 — sb3c00292_si_002.zip [file sb3c00292_si_002.zip › dnada_supplementary_material_pks_library_build/qpix/AlbertoNava/211013_plate4b.png]

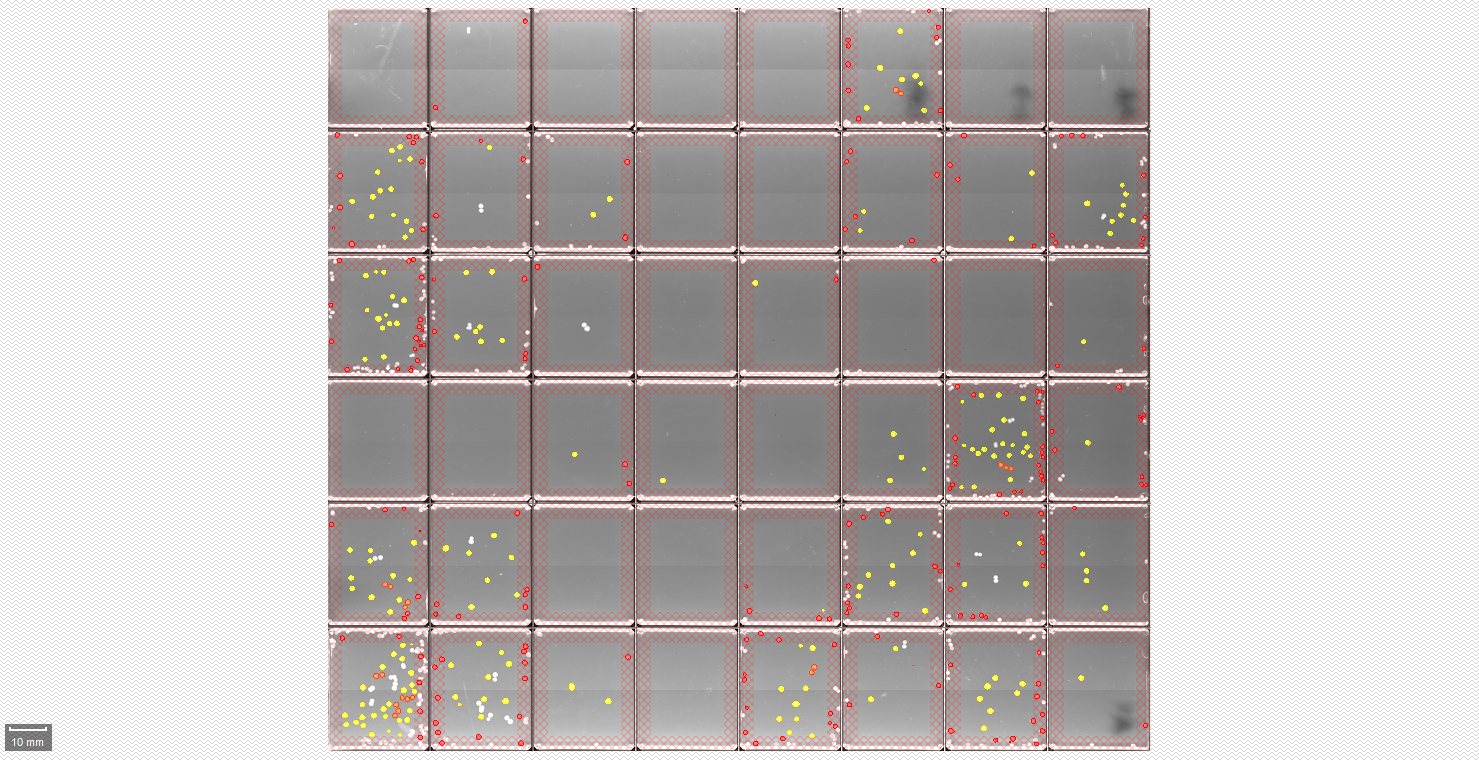

Supplement: Supplementary file 2 — sb3c00292_si_002.zip [file sb3c00292_si_002.zip › dnada_supplementary_material_pks_library_build/qpix/AlbertoNava/211013_plate8a.png]

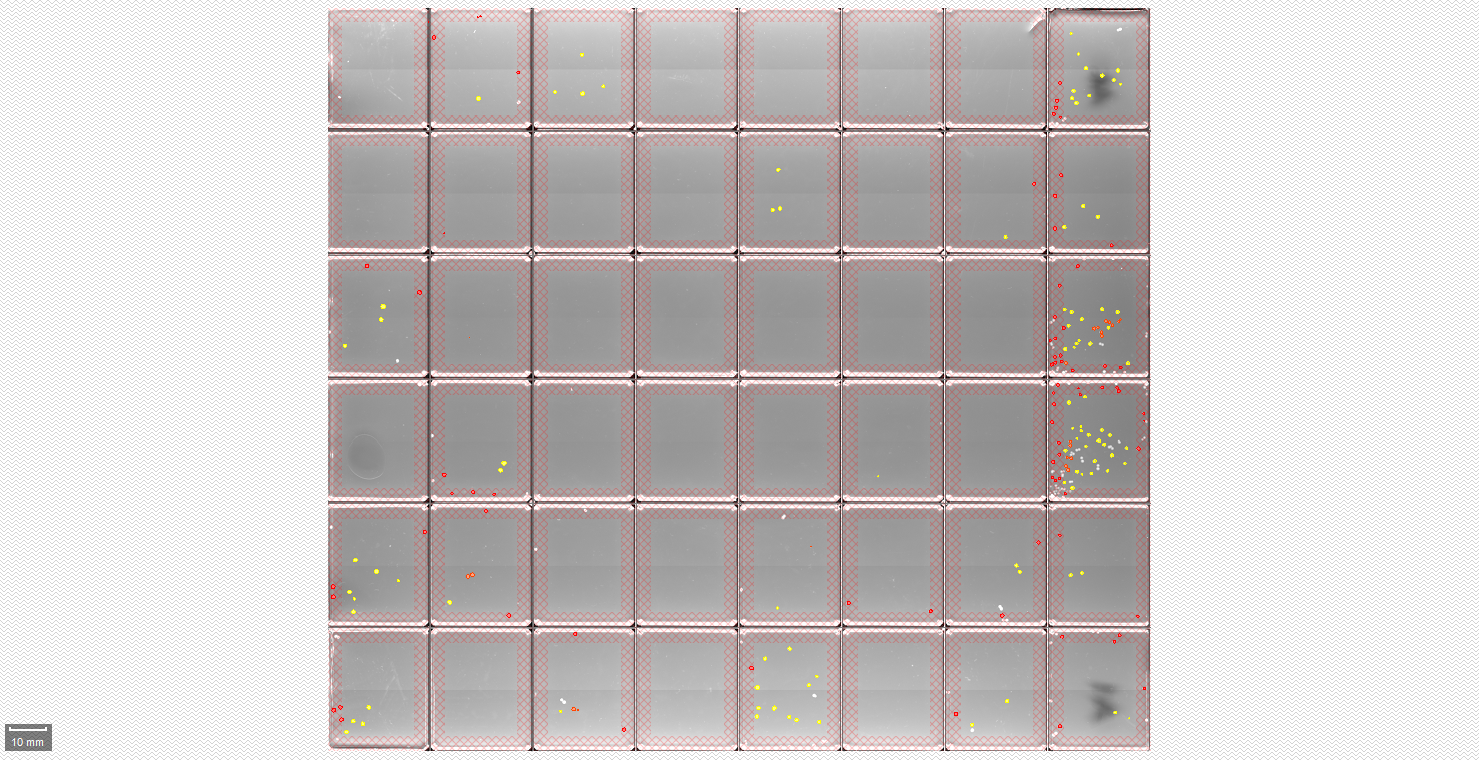

Supplement: Supplementary file 2 — sb3c00292_si_002.zip [file sb3c00292_si_002.zip › dnada_supplementary_material_pks_library_build/qpix/AlbertoNava/211013_plate4a.png]

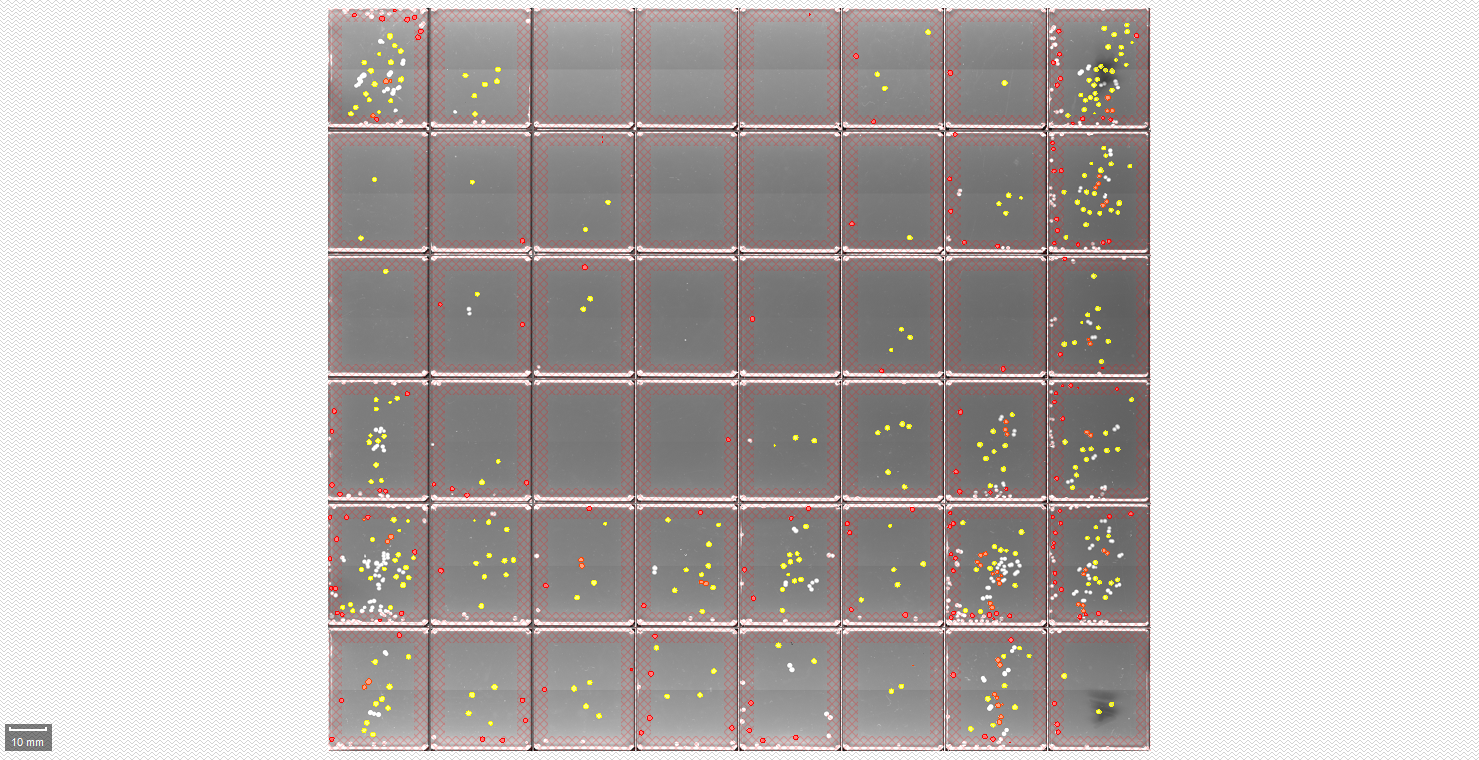

Supplement: Supplementary file 2 — sb3c00292_si_002.zip [file sb3c00292_si_002.zip › dnada_supplementary_material_pks_library_build/qpix/AlbertoNava/211013_plate5a.png]

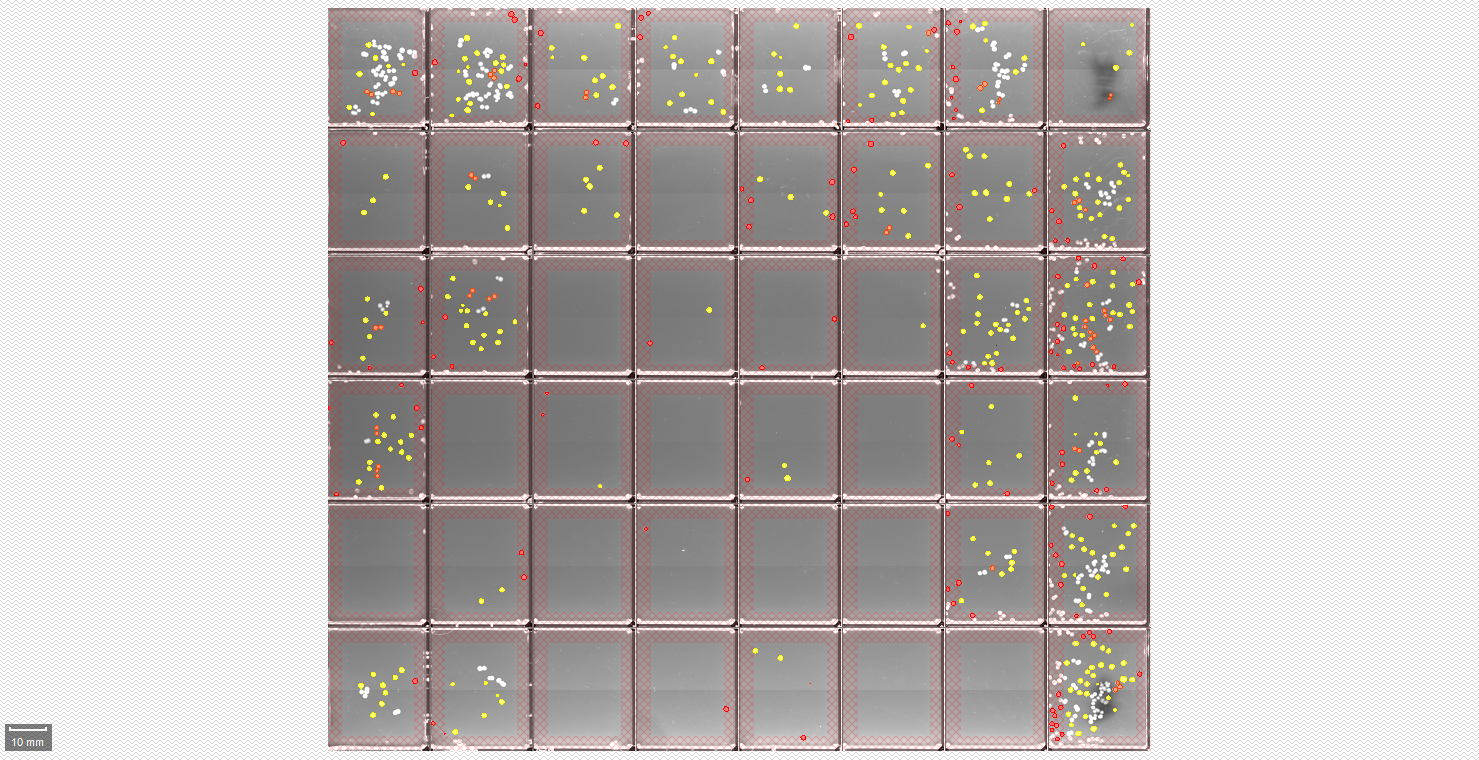

Supplement: Supplementary file 2 — sb3c00292_si_002.zip [file sb3c00292_si_002.zip › dnada_supplementary_material_pks_library_build/qpix/AlbertoNava/211013_plate5b.png]

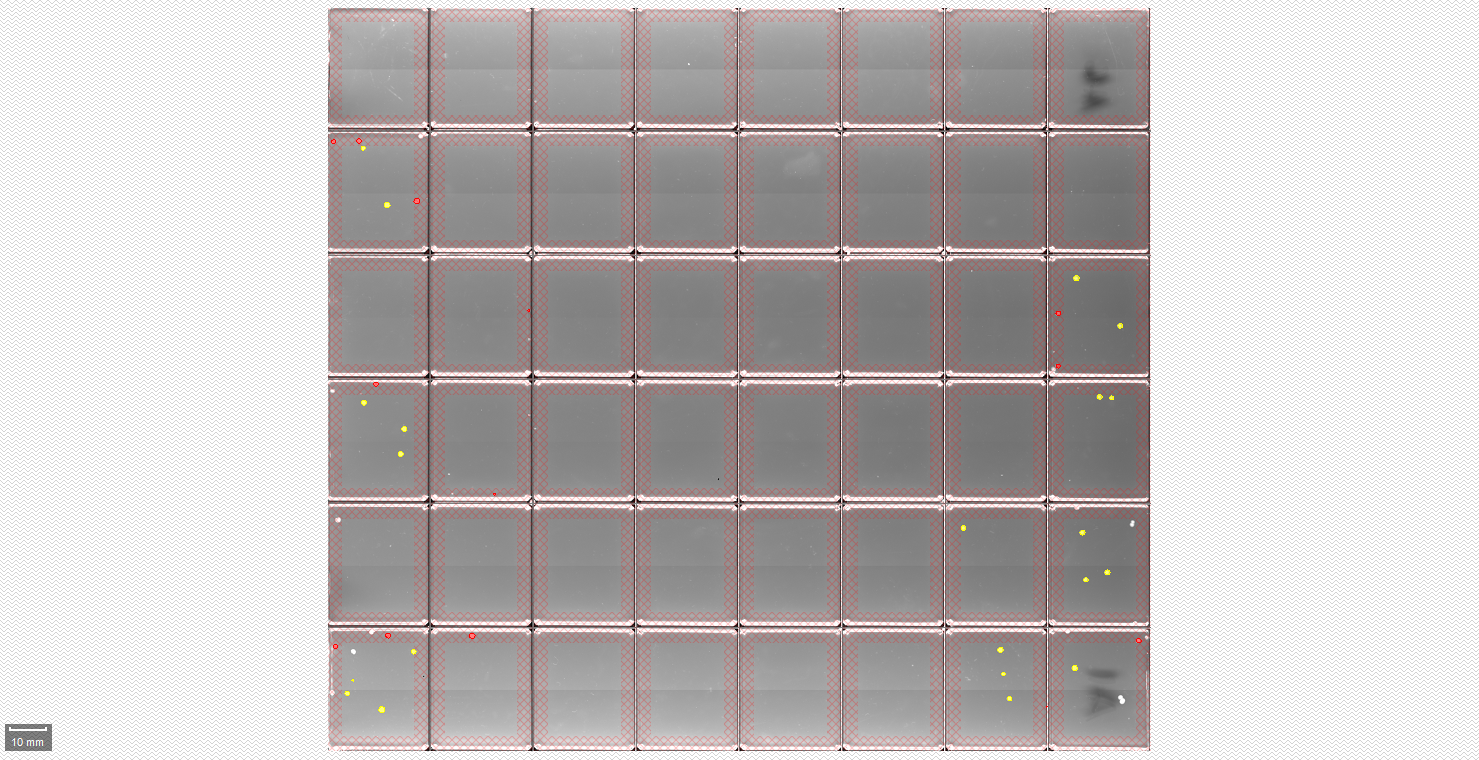

Supplement: Supplementary file 2 — sb3c00292_si_002.zip [file sb3c00292_si_002.zip › dnada_supplementary_material_pks_library_build/qpix/AlbertoNava/211013_plate2a.png]

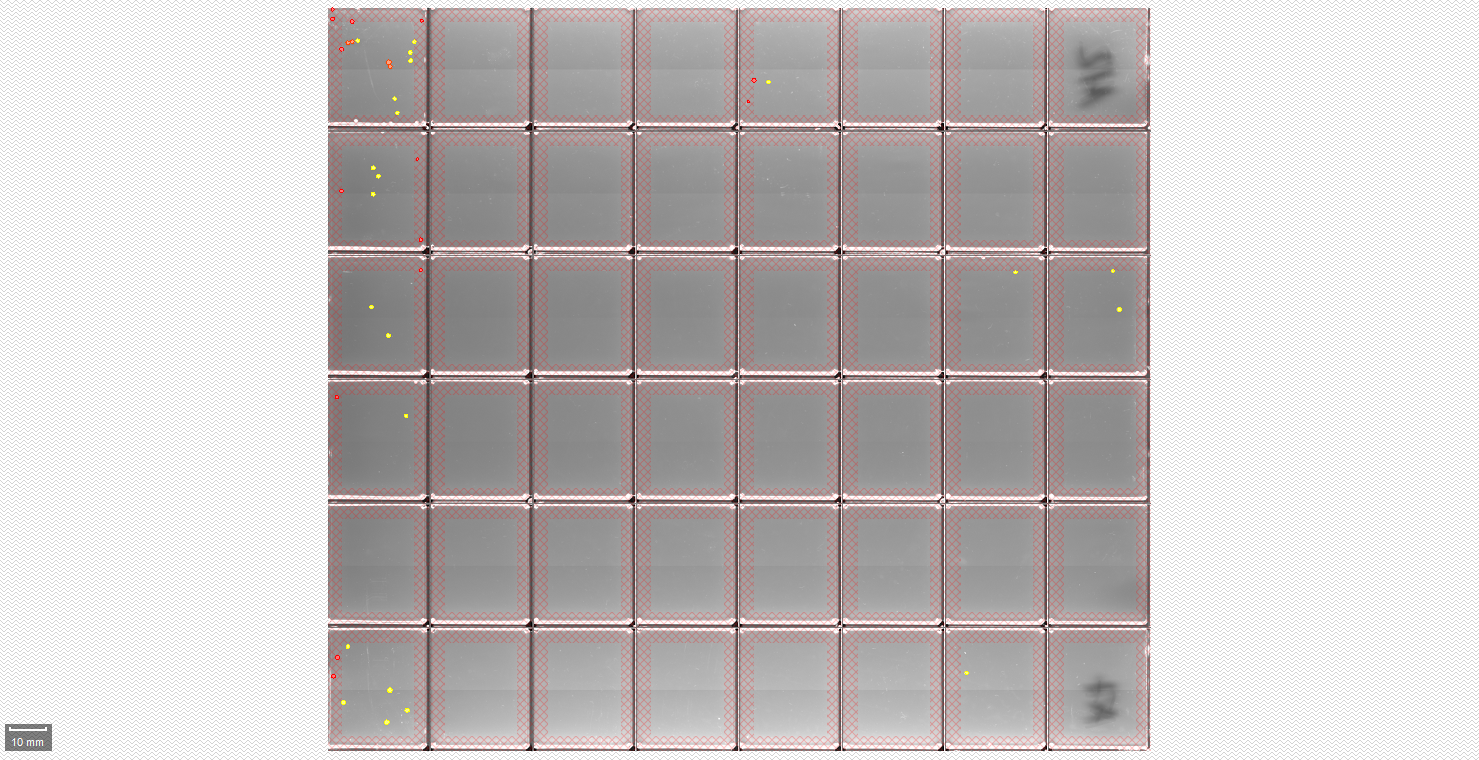

Supplement: Supplementary file 2 — sb3c00292_si_002.zip [file sb3c00292_si_002.zip › dnada_supplementary_material_pks_library_build/qpix/AlbertoNava/211013_plate2b.png]

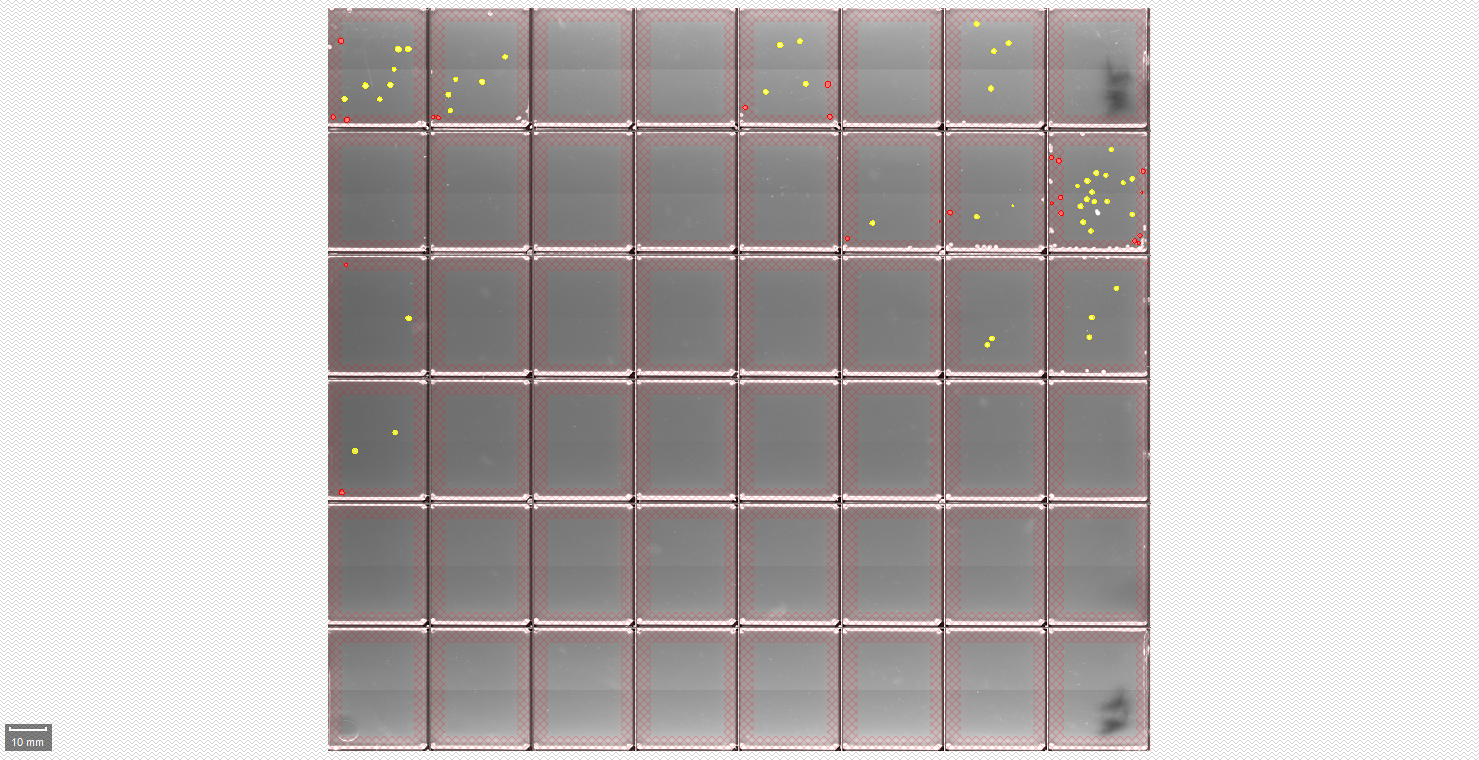

Supplement: Supplementary file 2 — sb3c00292_si_002.zip [file sb3c00292_si_002.zip › dnada_supplementary_material_pks_library_build/qpix/AlbertoNava/211013_plate3b.png]

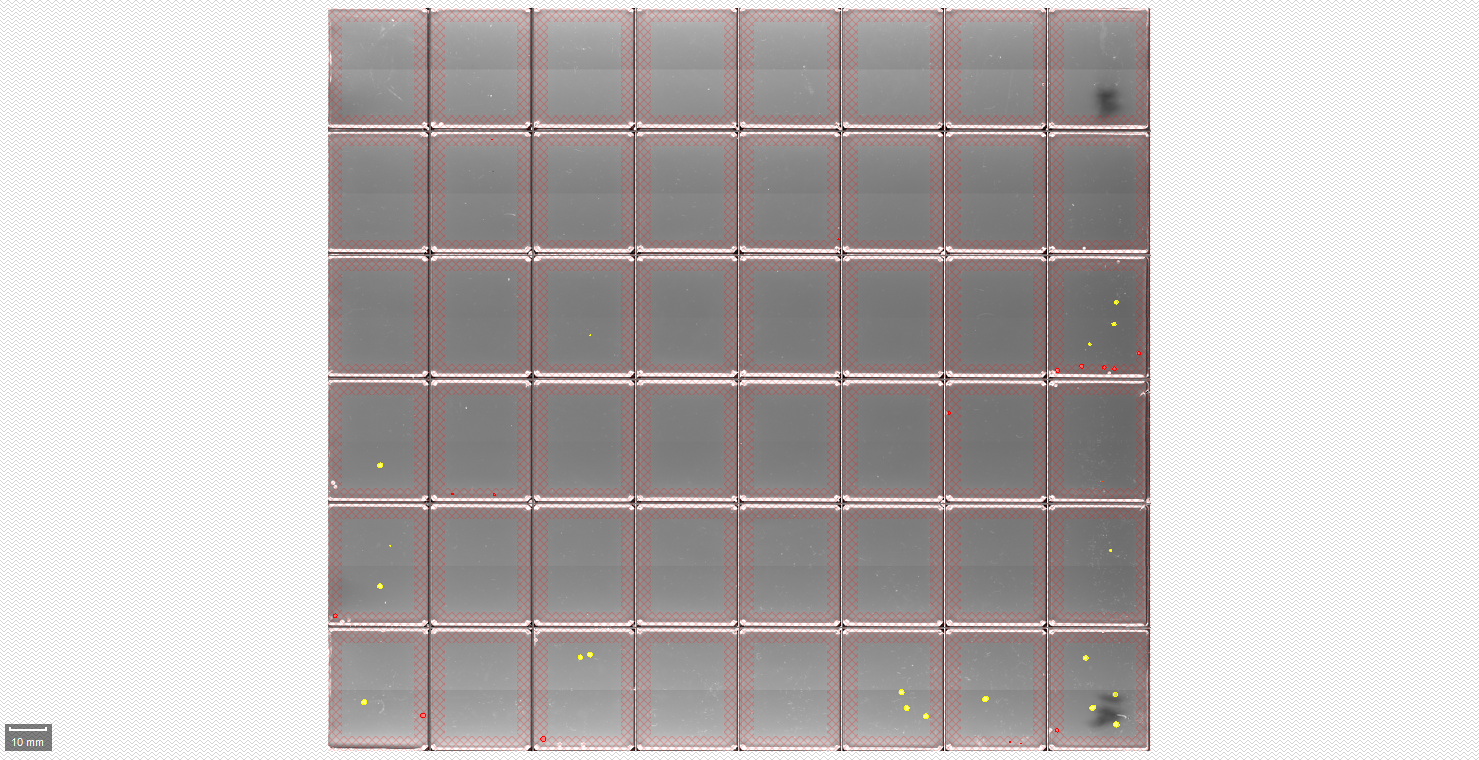

Supplement: Supplementary file 2 — sb3c00292_si_002.zip [file sb3c00292_si_002.zip › dnada_supplementary_material_pks_library_build/qpix/AlbertoNava/211013_plate3a.png]

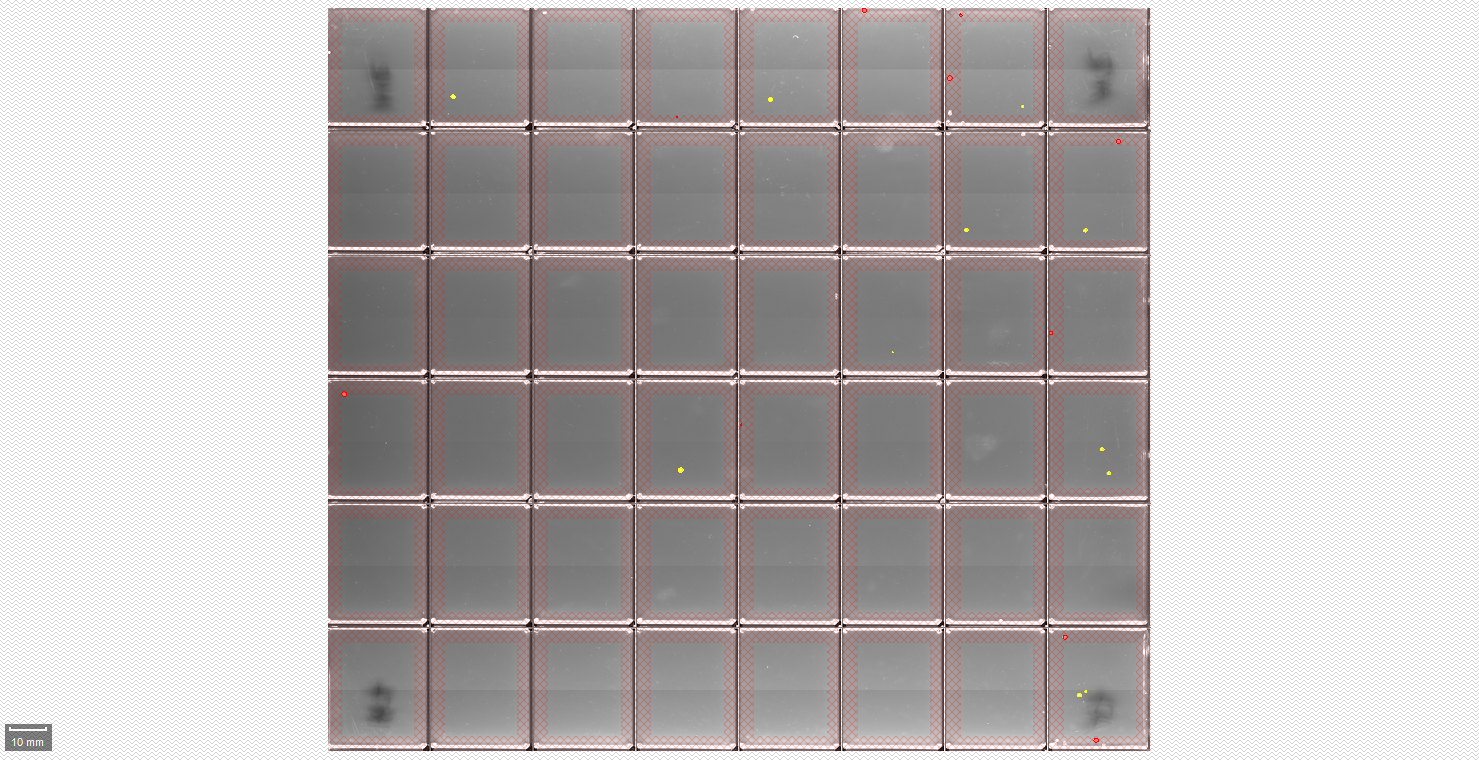

Supplement: Supplementary file 2 — sb3c00292_si_002.zip [file sb3c00292_si_002.zip › dnada_supplementary_material_pks_library_build/qpix/AlbertoNava/211013_plate1b.png]

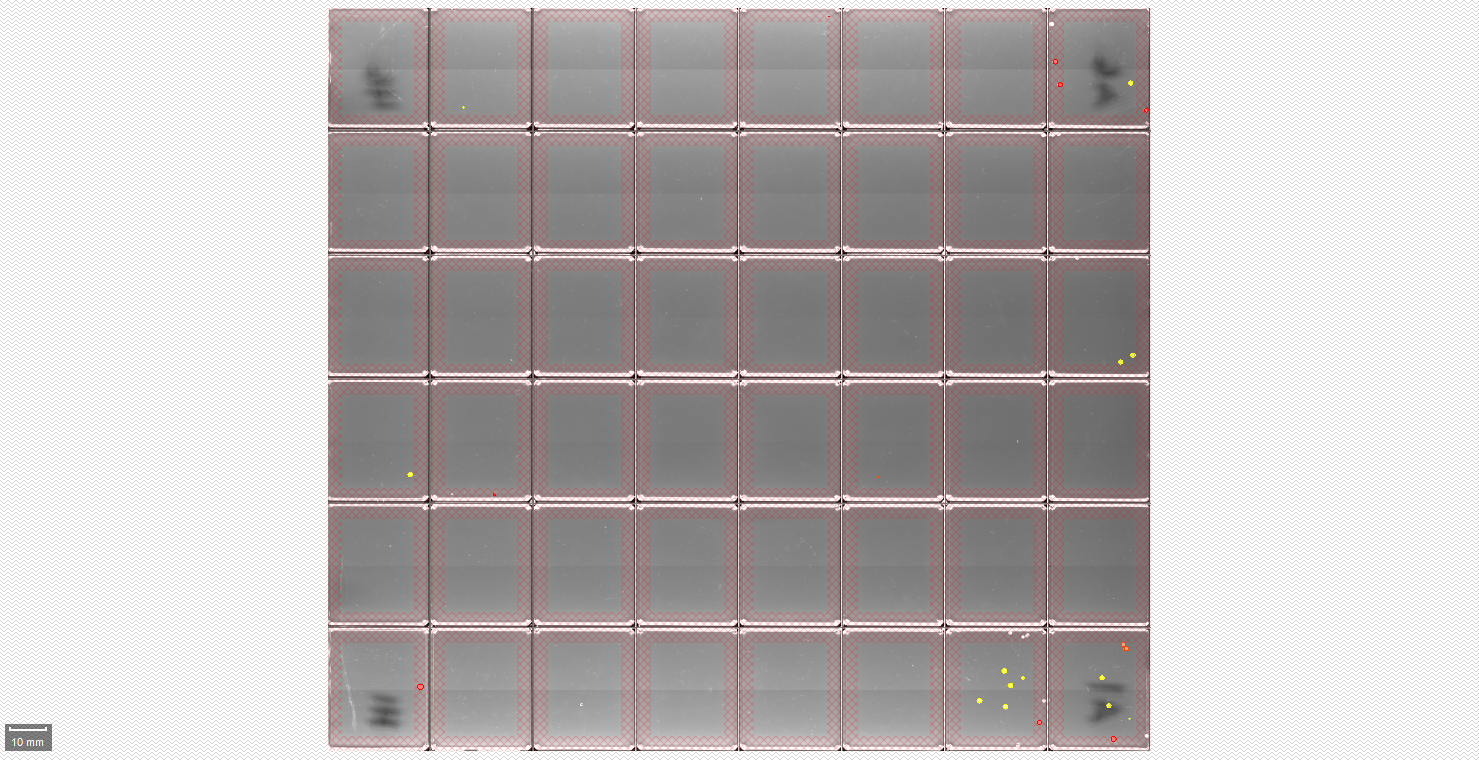

Supplement: Supplementary file 2 — sb3c00292_si_002.zip [file sb3c00292_si_002.zip › dnada_supplementary_material_pks_library_build/qpix/AlbertoNava/211013_plate1a.png]

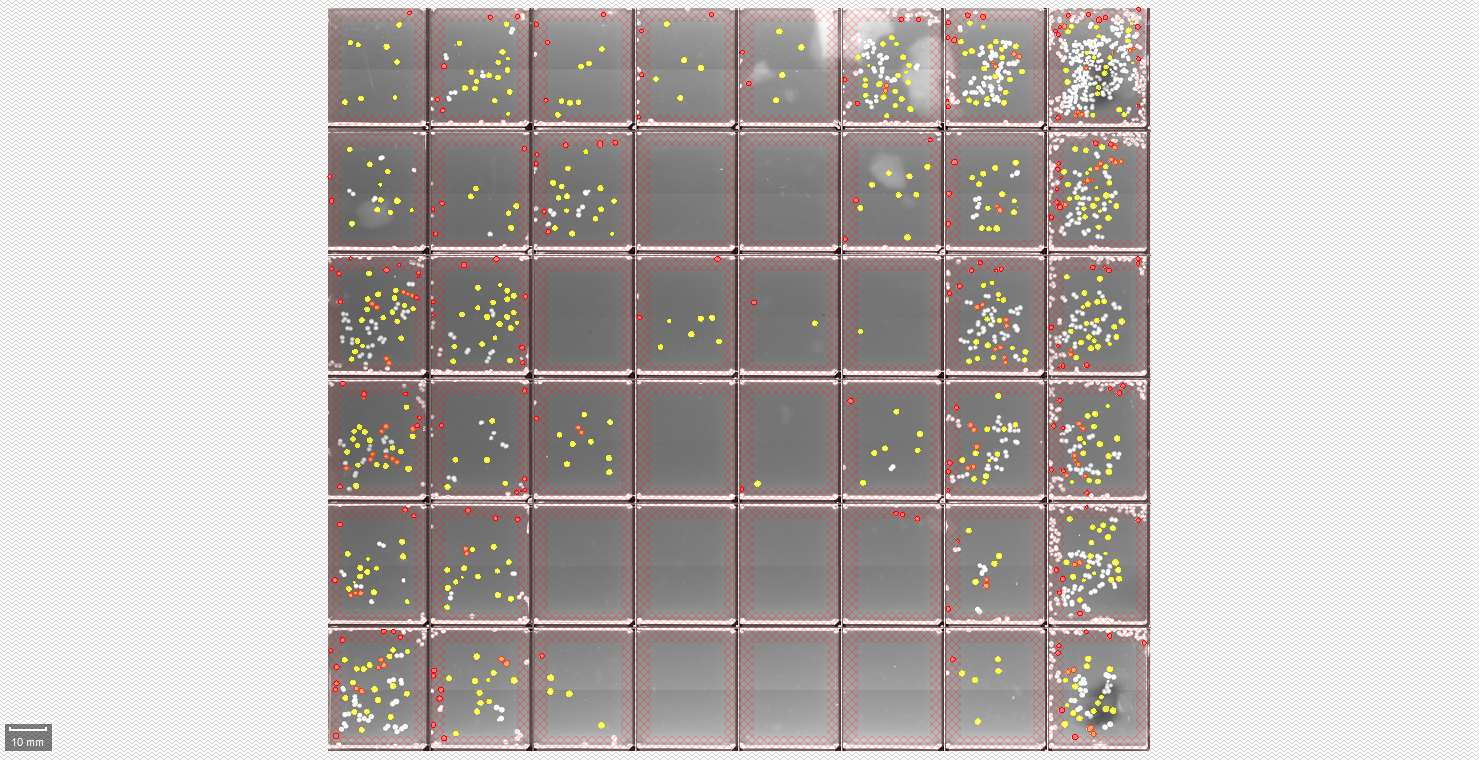

Supplement: Supplementary file 2 — sb3c00292_si_002.zip [file sb3c00292_si_002.zip › dnada_supplementary_material_pks_library_build/qpix/AlbertoNava/211013_plate6b.png]

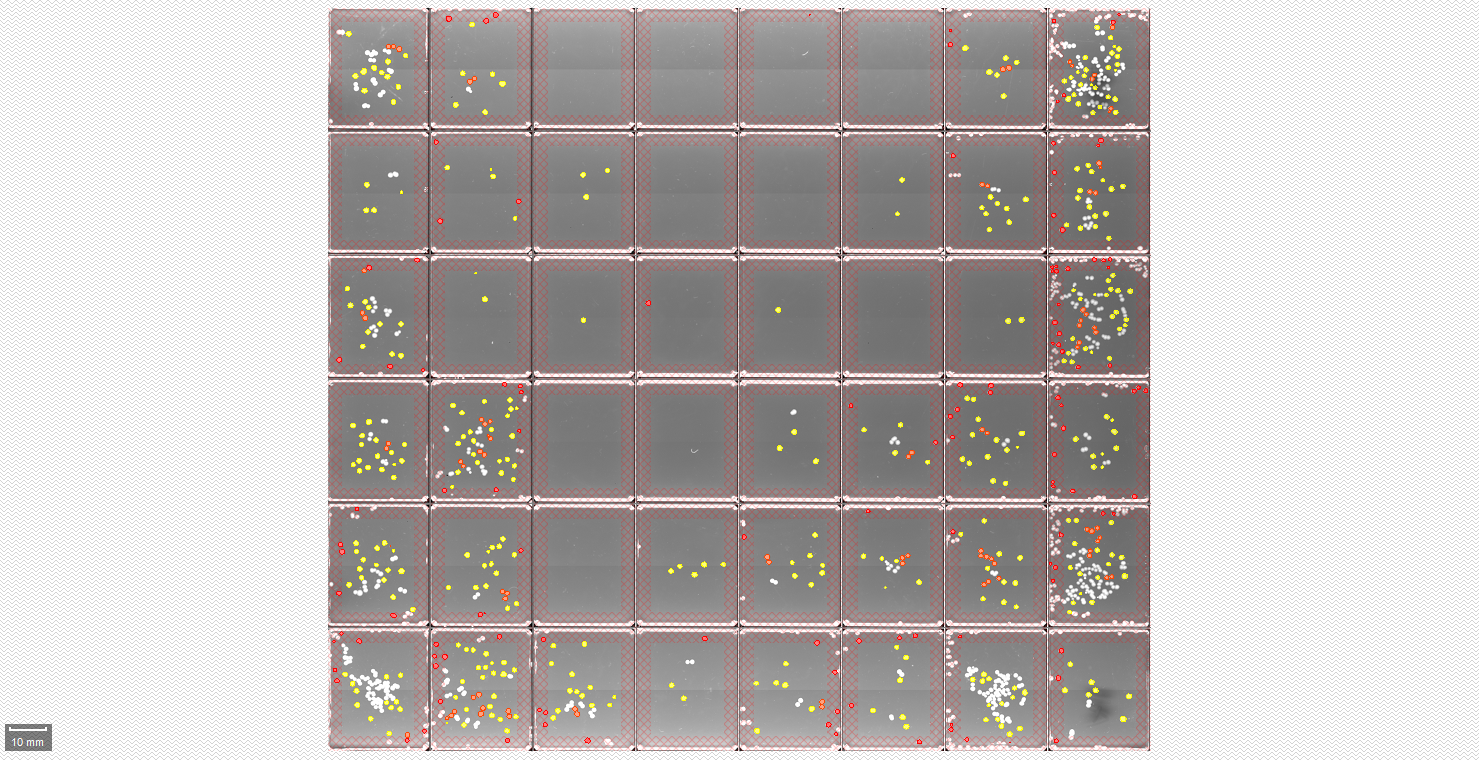

Supplement: Supplementary file 2 — sb3c00292_si_002.zip [file sb3c00292_si_002.zip › dnada_supplementary_material_pks_library_build/qpix/AlbertoNava/211013_plate6a.png]

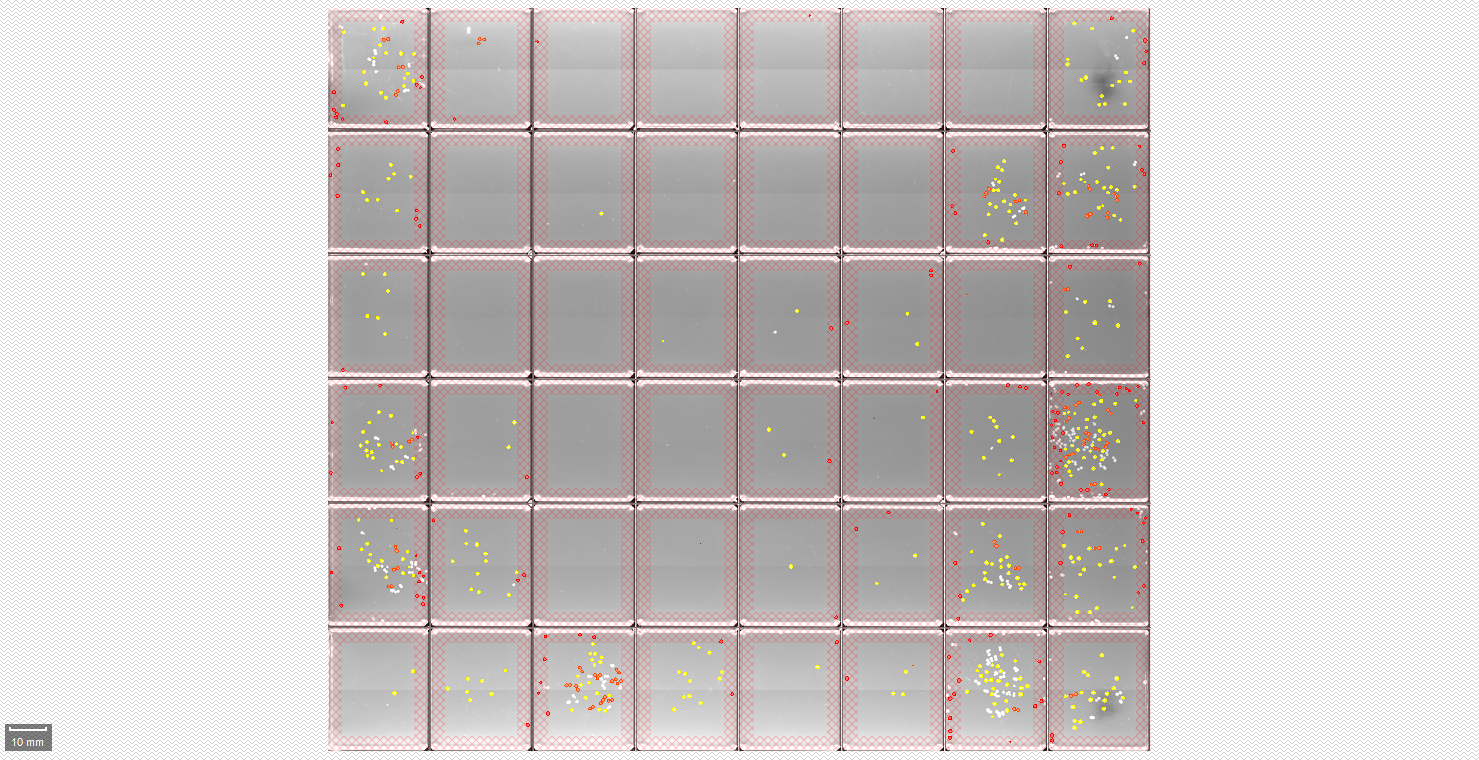

Supplement: Supplementary file 2 — sb3c00292_si_002.zip [file sb3c00292_si_002.zip › dnada_supplementary_material_pks_library_build/qpix/AlbertoNava/211013_plate7a.png]

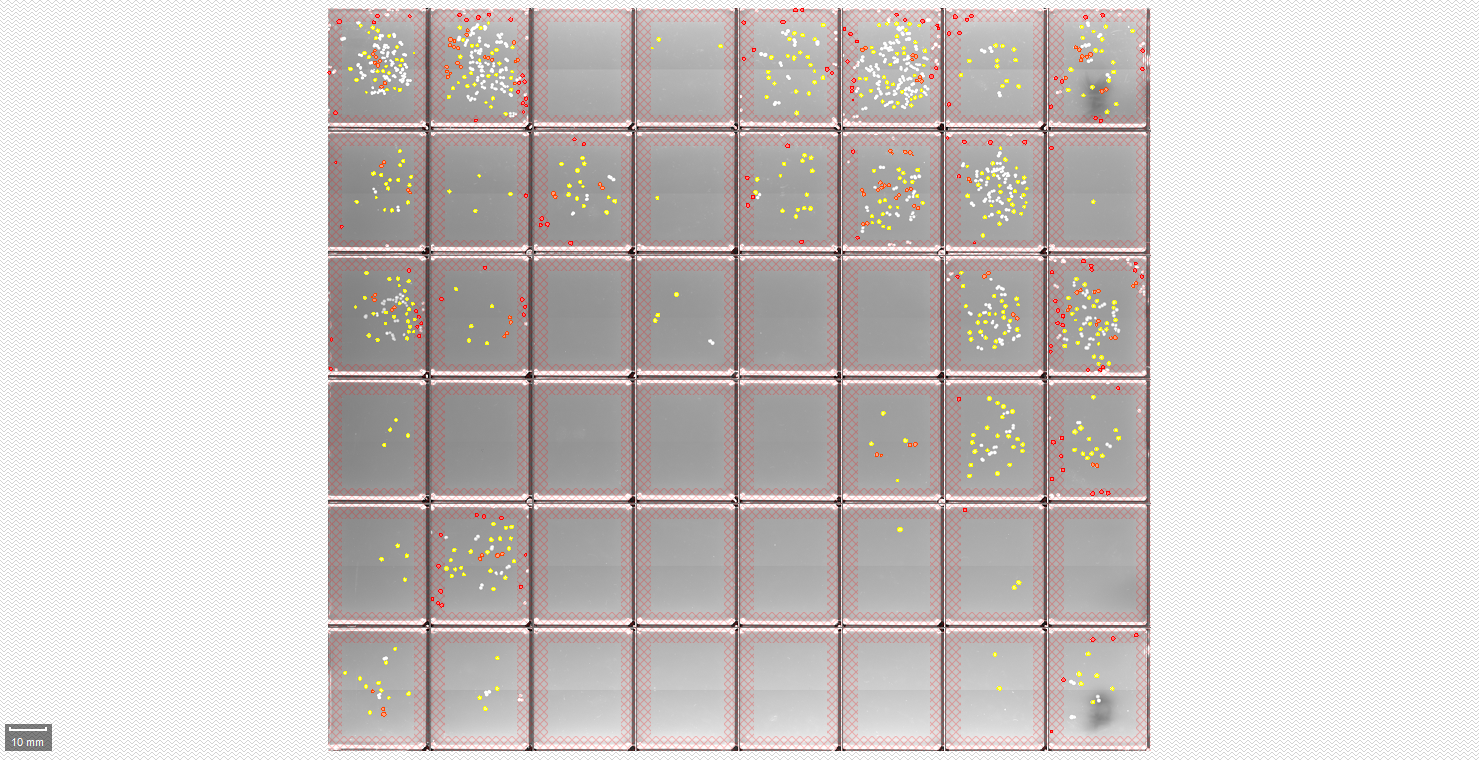

Supplement: Supplementary file 2 — sb3c00292_si_002.zip [file sb3c00292_si_002.zip › dnada_supplementary_material_pks_library_build/qpix/AlbertoNava/211013_plate7b.png]

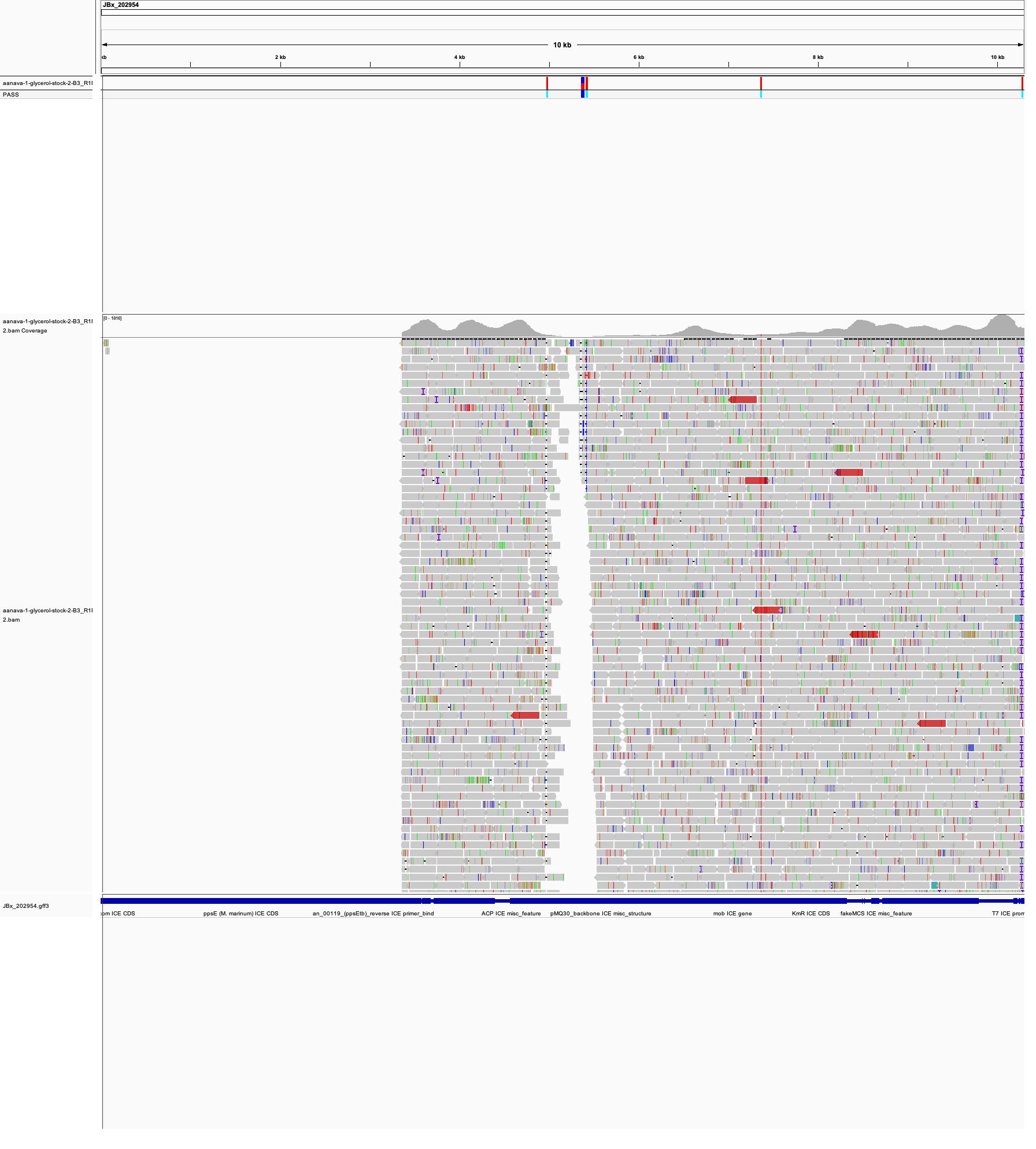

Supplement: Supplementary file 2 — sb3c00292_si_002.zip [file sb3c00292_si_002.zip › dnada_supplementary_material_pks_library_build/divaseq/211117_divaseq_analysis/alberto/snapshots/JBx_202954_nava-1-glycerol-stock-2-B3_R1R2.jpg]

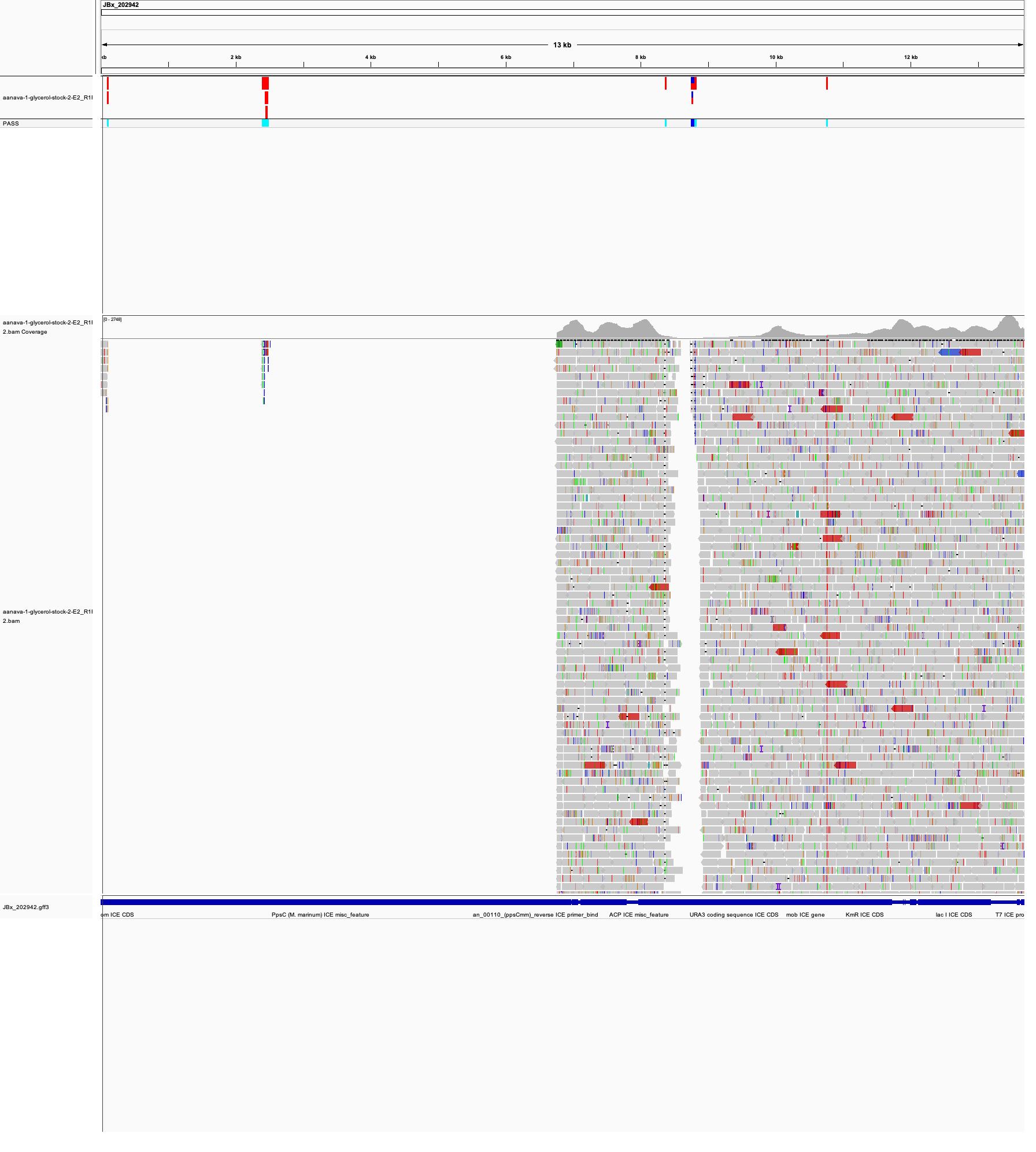

Supplement: Supplementary file 2 — sb3c00292_si_002.zip [file sb3c00292_si_002.zip › dnada_supplementary_material_pks_library_build/divaseq/211117_divaseq_analysis/alberto/snapshots/JBx_202942_nava-1-glycerol-stock-2-E2_R1R2.jpg]

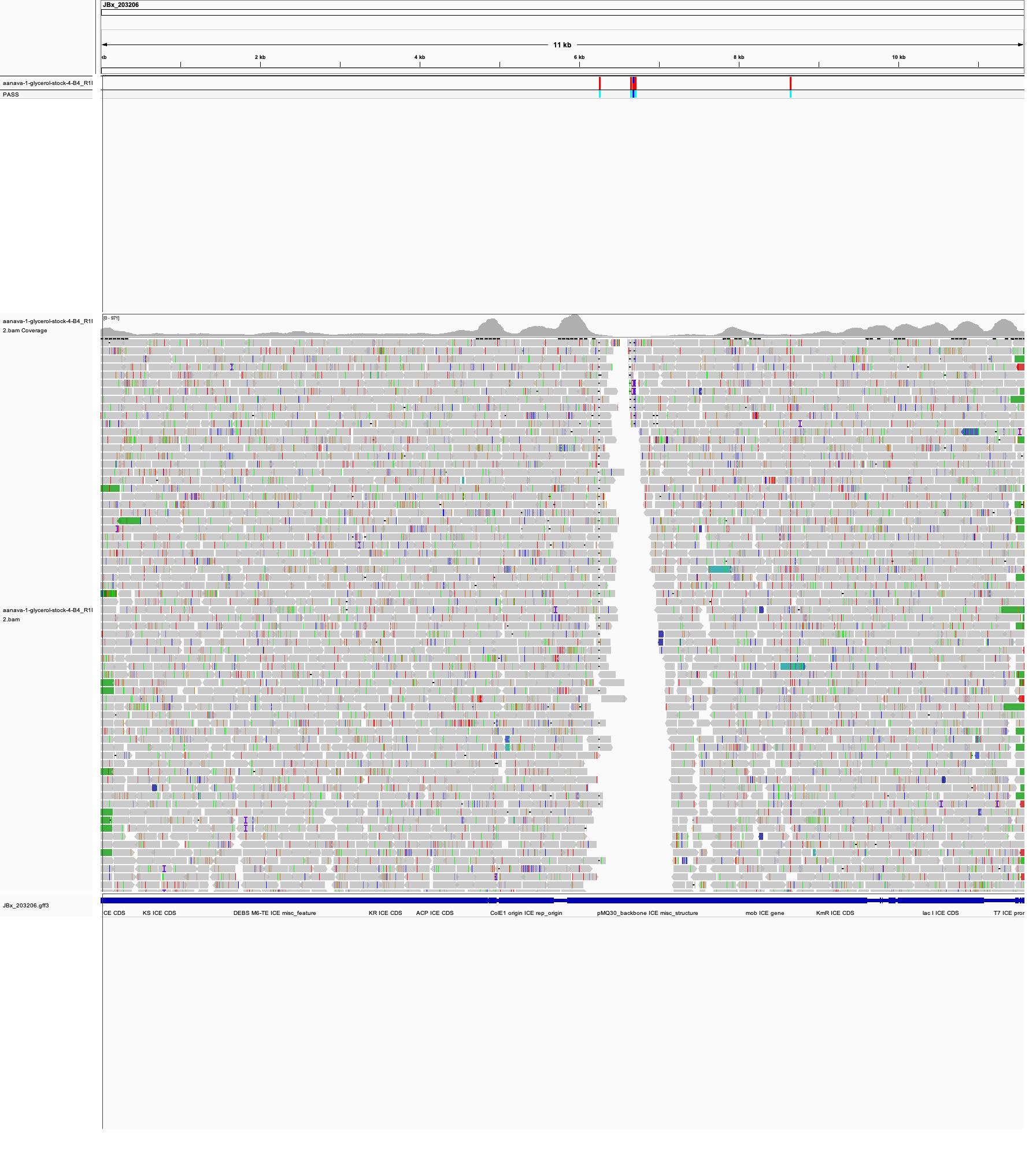

Supplement: Supplementary file 2 — sb3c00292_si_002.zip [file sb3c00292_si_002.zip › dnada_supplementary_material_pks_library_build/divaseq/211117_divaseq_analysis/alberto/snapshots/JBx_203206_nava-1-glycerol-stock-4-B4_R1R2.jpg]

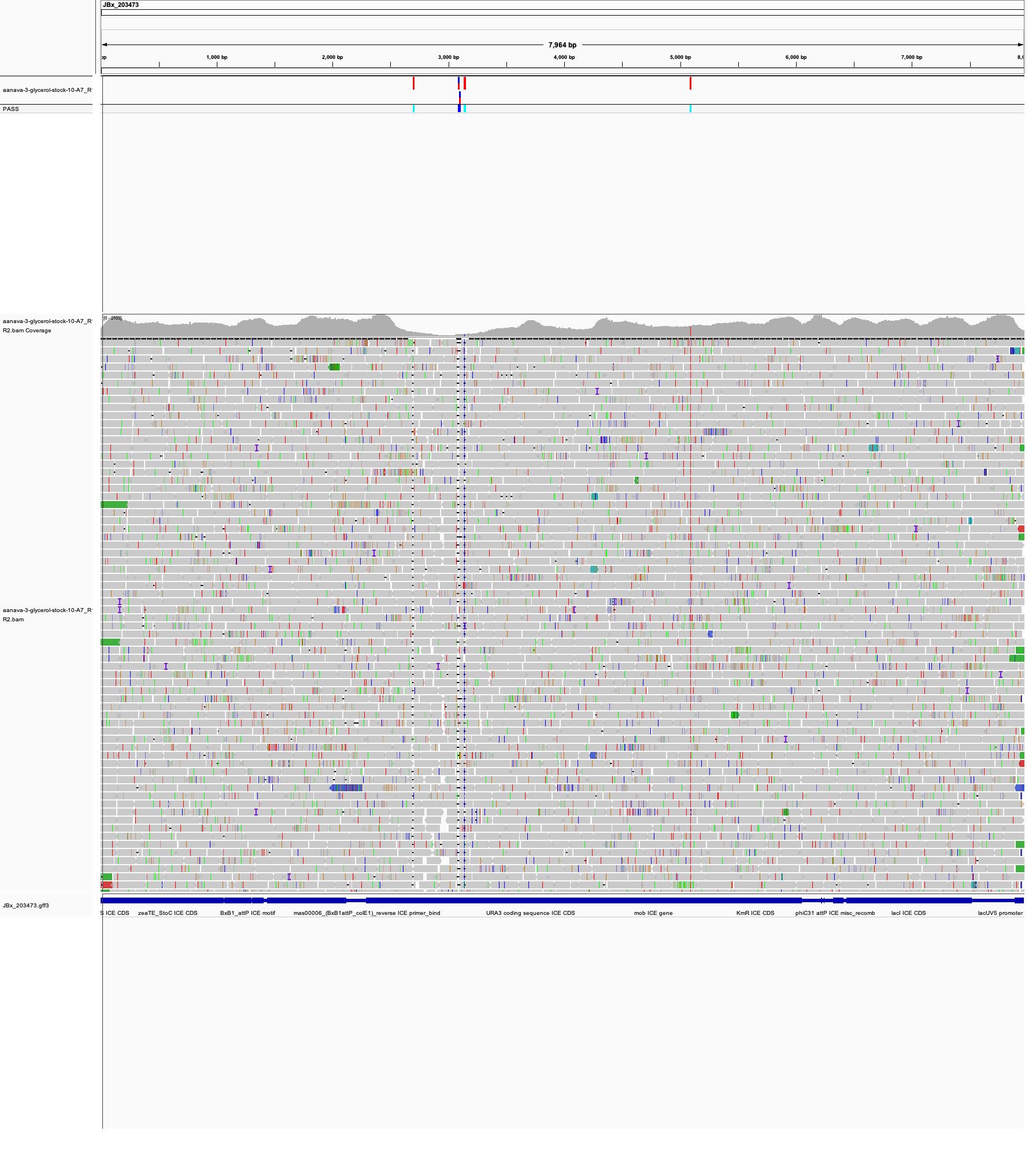

Supplement: Supplementary file 2 — sb3c00292_si_002.zip [file sb3c00292_si_002.zip › dnada_supplementary_material_pks_library_build/divaseq/211117_divaseq_analysis/alberto/snapshots/JBx_203473_nava-3-glycerol-stock-10-A7_R1R2.jpg]

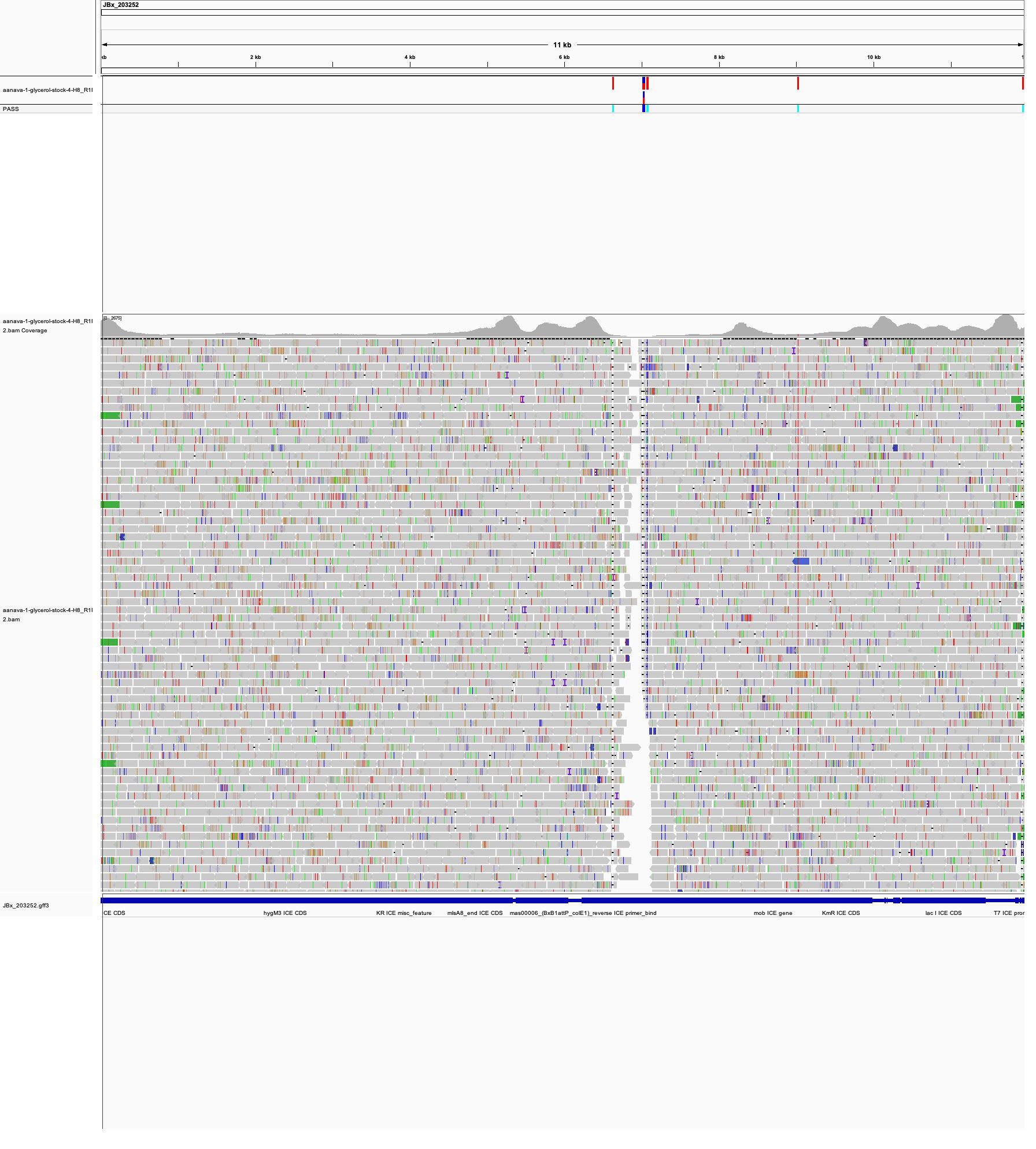

Supplement: Supplementary file 2 — sb3c00292_si_002.zip [file sb3c00292_si_002.zip › dnada_supplementary_material_pks_library_build/divaseq/211117_divaseq_analysis/alberto/snapshots/JBx_203252_nava-1-glycerol-stock-4-H8_R1R2.jpg]

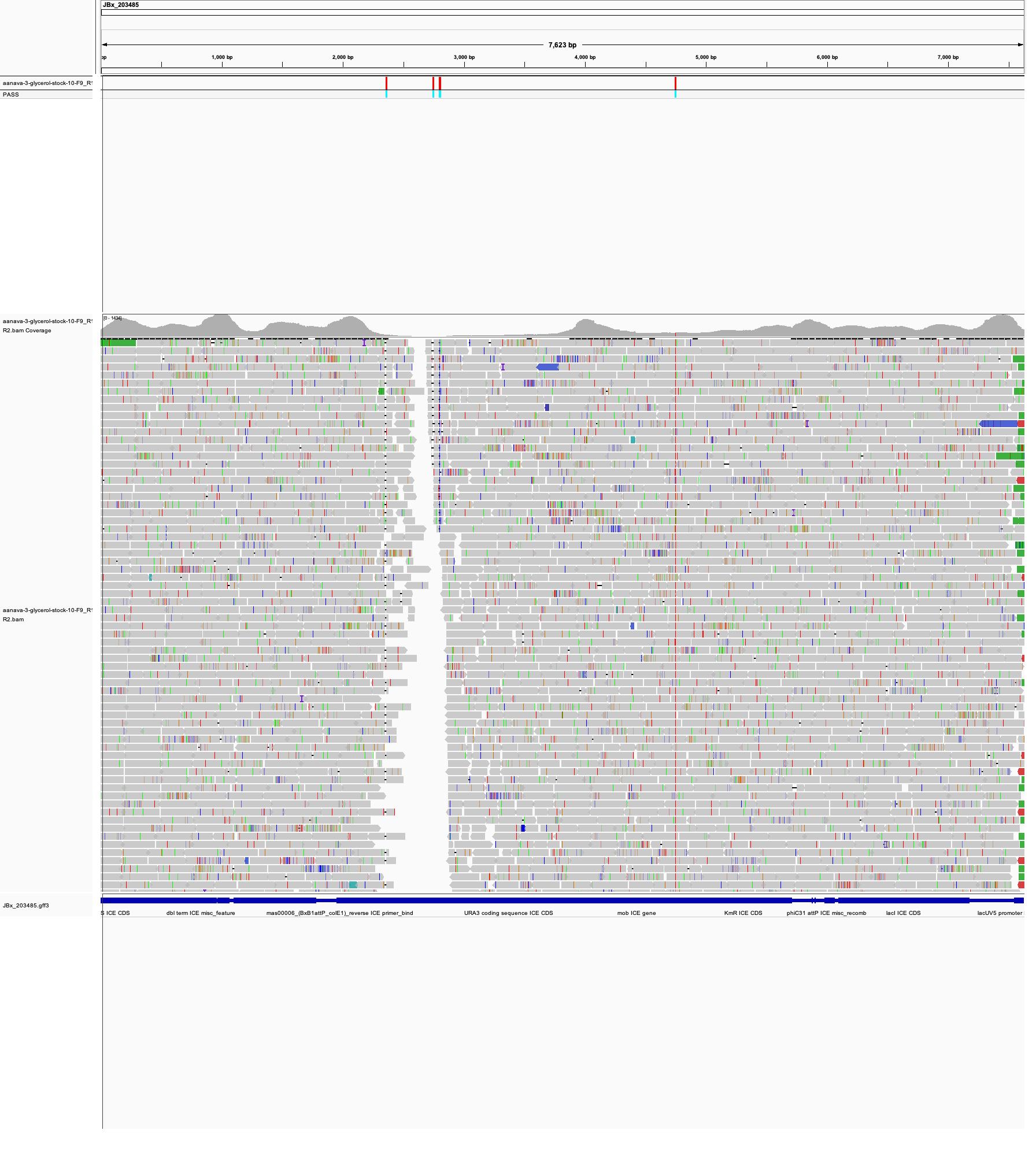

Supplement: Supplementary file 2 — sb3c00292_si_002.zip [file sb3c00292_si_002.zip › dnada_supplementary_material_pks_library_build/divaseq/211117_divaseq_analysis/alberto/snapshots/JBx_203485_nava-3-glycerol-stock-10-F9_R1R2.jpg]

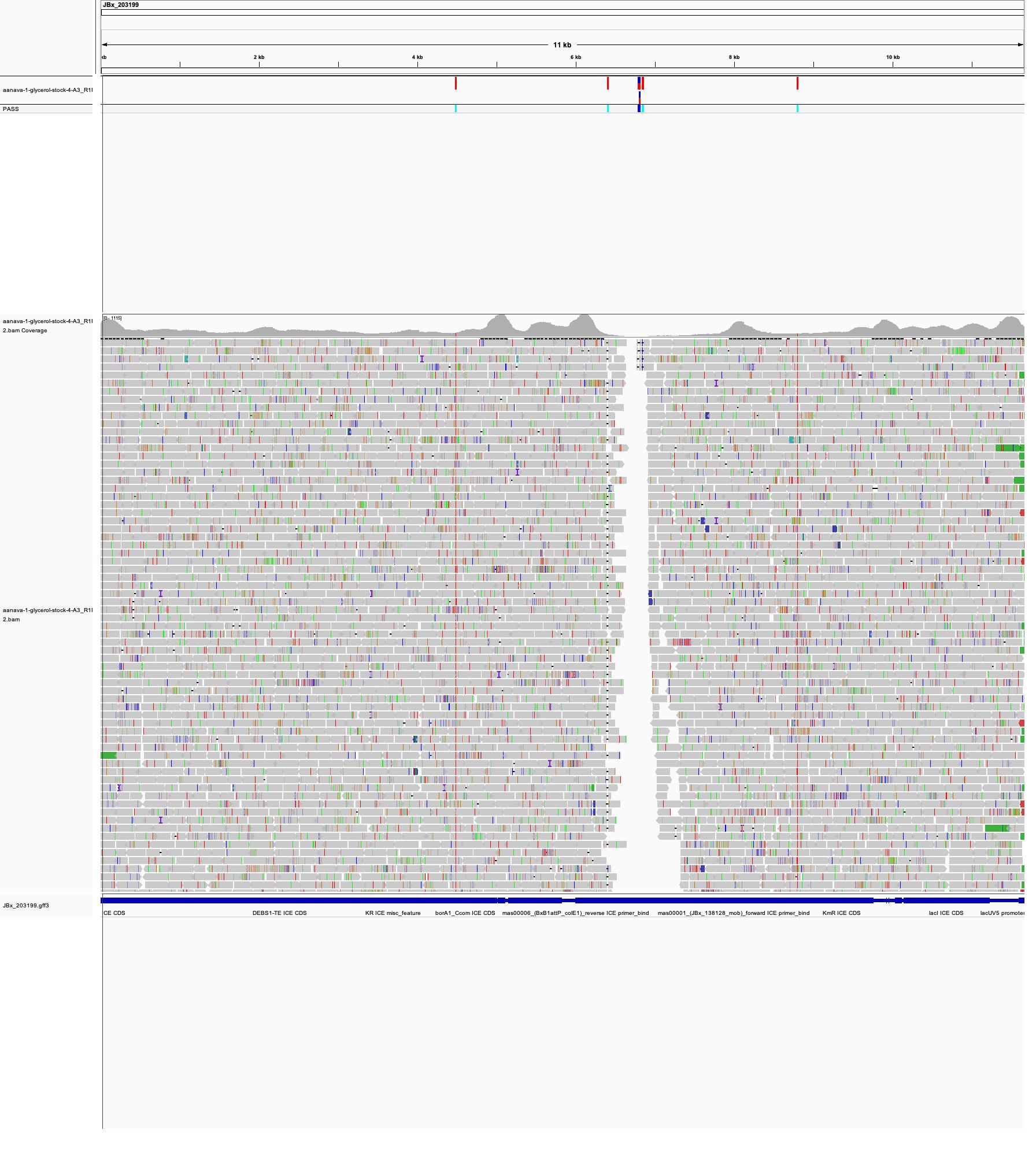

Supplement: Supplementary file 2 — sb3c00292_si_002.zip [file sb3c00292_si_002.zip › dnada_supplementary_material_pks_library_build/divaseq/211117_divaseq_analysis/alberto/snapshots/JBx_203199_nava-1-glycerol-stock-4-A3_R1R2.jpg]

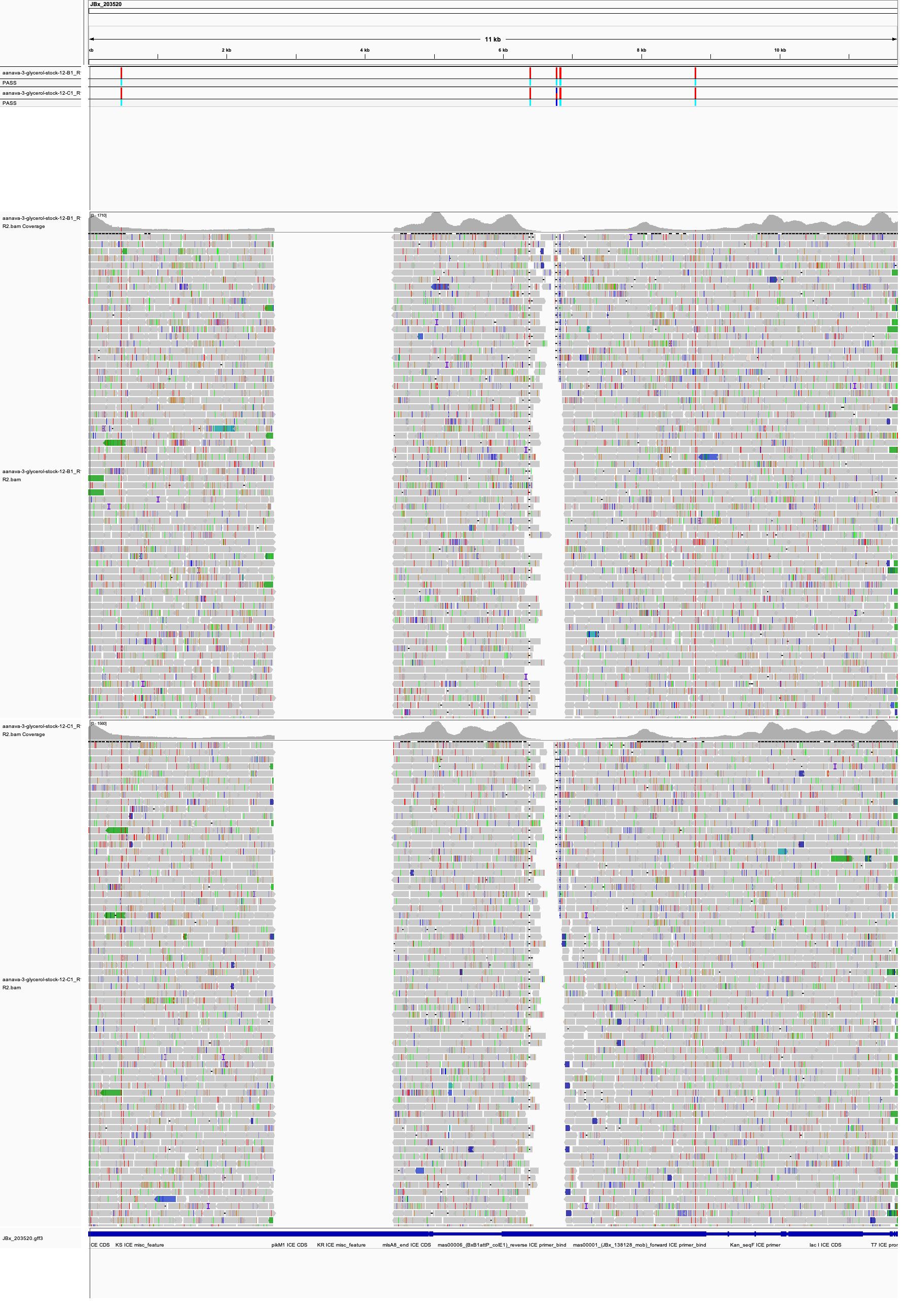

Supplement: Supplementary file 2 — sb3c00292_si_002.zip [file sb3c00292_si_002.zip › dnada_supplementary_material_pks_library_build/divaseq/211117_divaseq_analysis/alberto/snapshots/JBx_203520_nava-3-glycerol-stock-12-C1_R1R2.jpg]

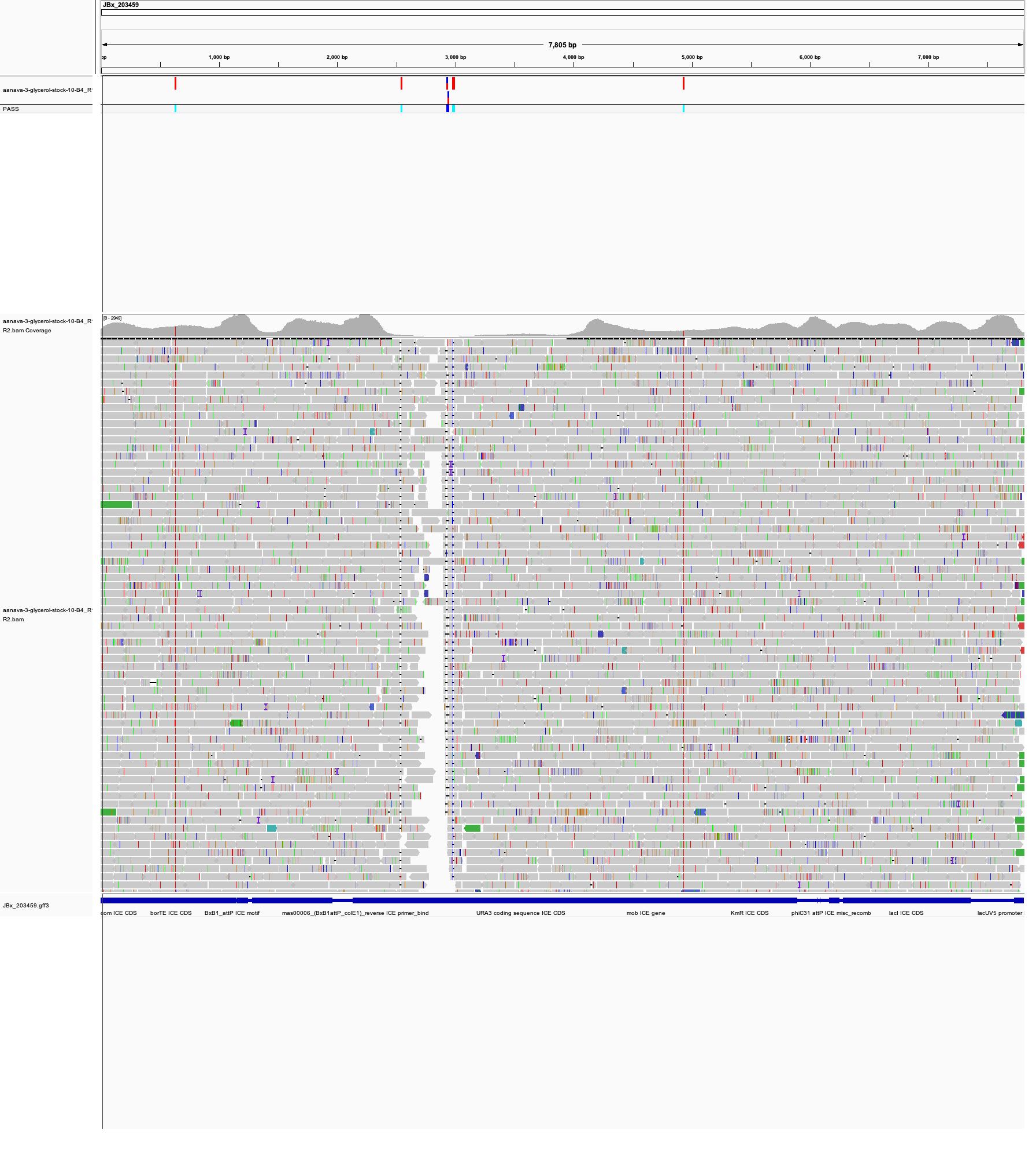

Supplement: Supplementary file 2 — sb3c00292_si_002.zip [file sb3c00292_si_002.zip › dnada_supplementary_material_pks_library_build/divaseq/211117_divaseq_analysis/alberto/snapshots/JBx_203459_nava-3-glycerol-stock-10-B4_R1R2.jpg]

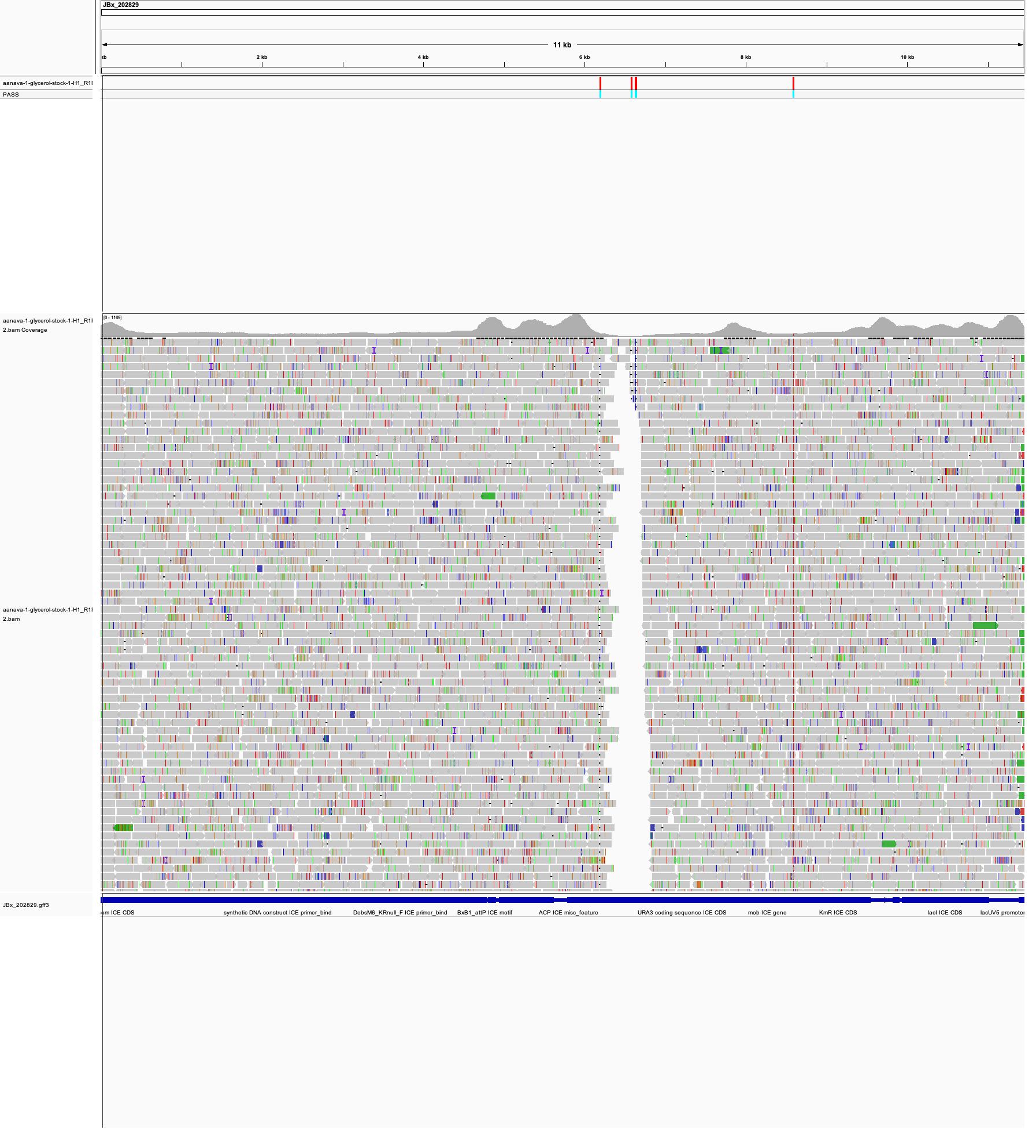

Supplement: Supplementary file 2 — sb3c00292_si_002.zip [file sb3c00292_si_002.zip › dnada_supplementary_material_pks_library_build/divaseq/211117_divaseq_analysis/alberto/snapshots/JBx_202829_nava-1-glycerol-stock-1-H1_R1R2.jpg]

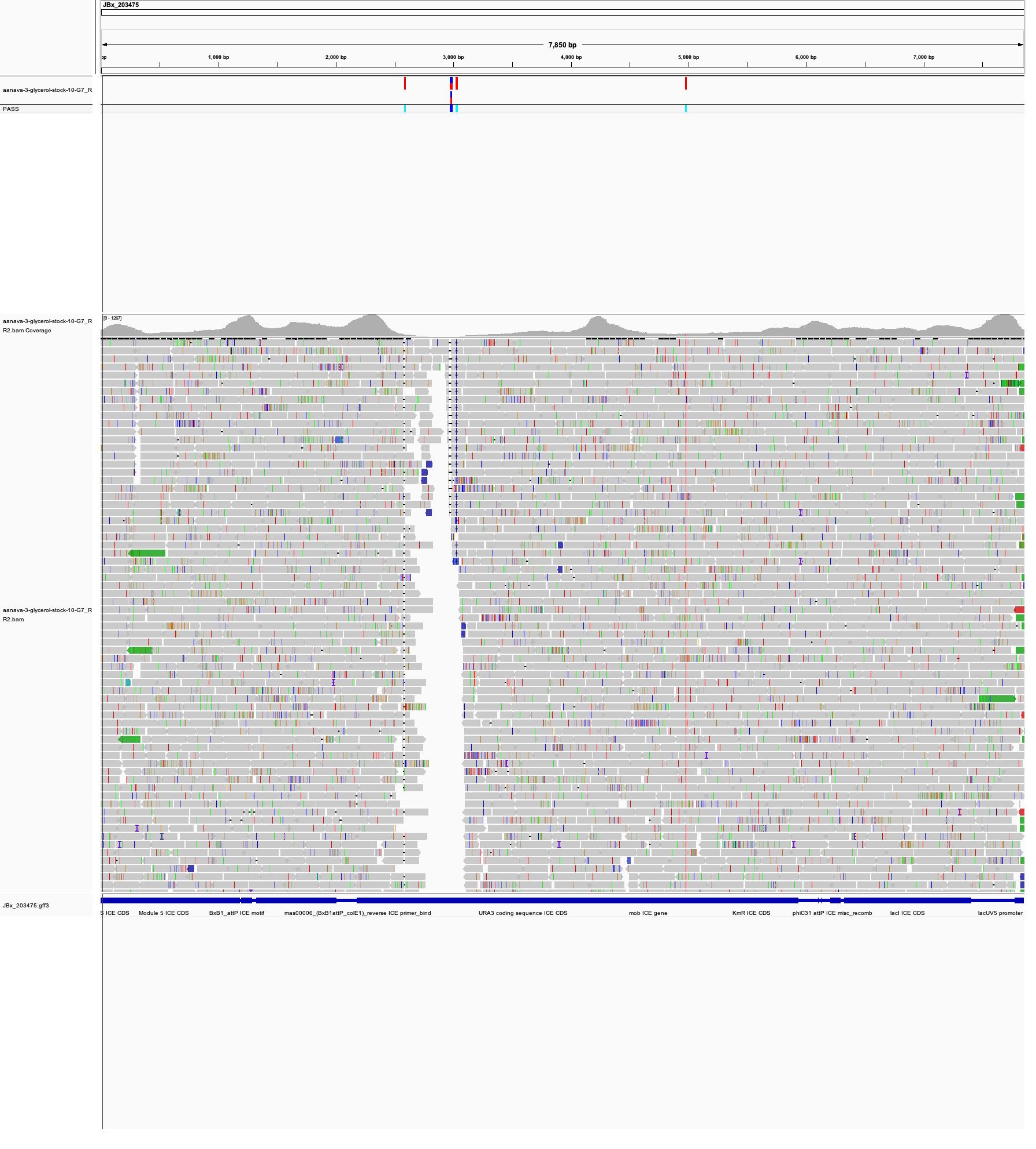

Supplement: Supplementary file 2 — sb3c00292_si_002.zip [file sb3c00292_si_002.zip › dnada_supplementary_material_pks_library_build/divaseq/211117_divaseq_analysis/alberto/snapshots/JBx_203475_nava-3-glycerol-stock-10-G7_R1R2.jpg]

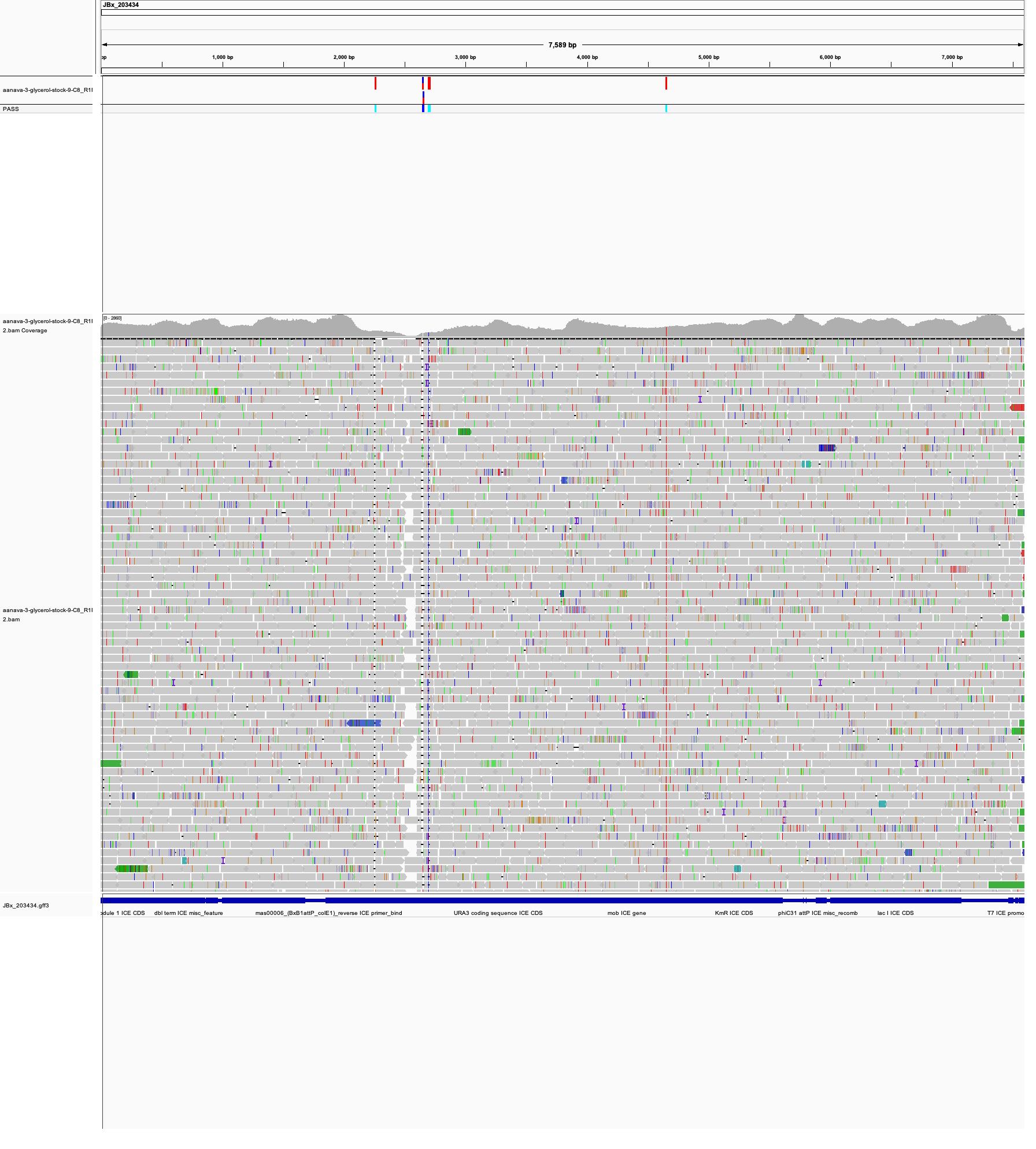

Supplement: Supplementary file 2 — sb3c00292_si_002.zip [file sb3c00292_si_002.zip › dnada_supplementary_material_pks_library_build/divaseq/211117_divaseq_analysis/alberto/snapshots/JBx_203434_nava-3-glycerol-stock-9-C8_R1R2.jpg]

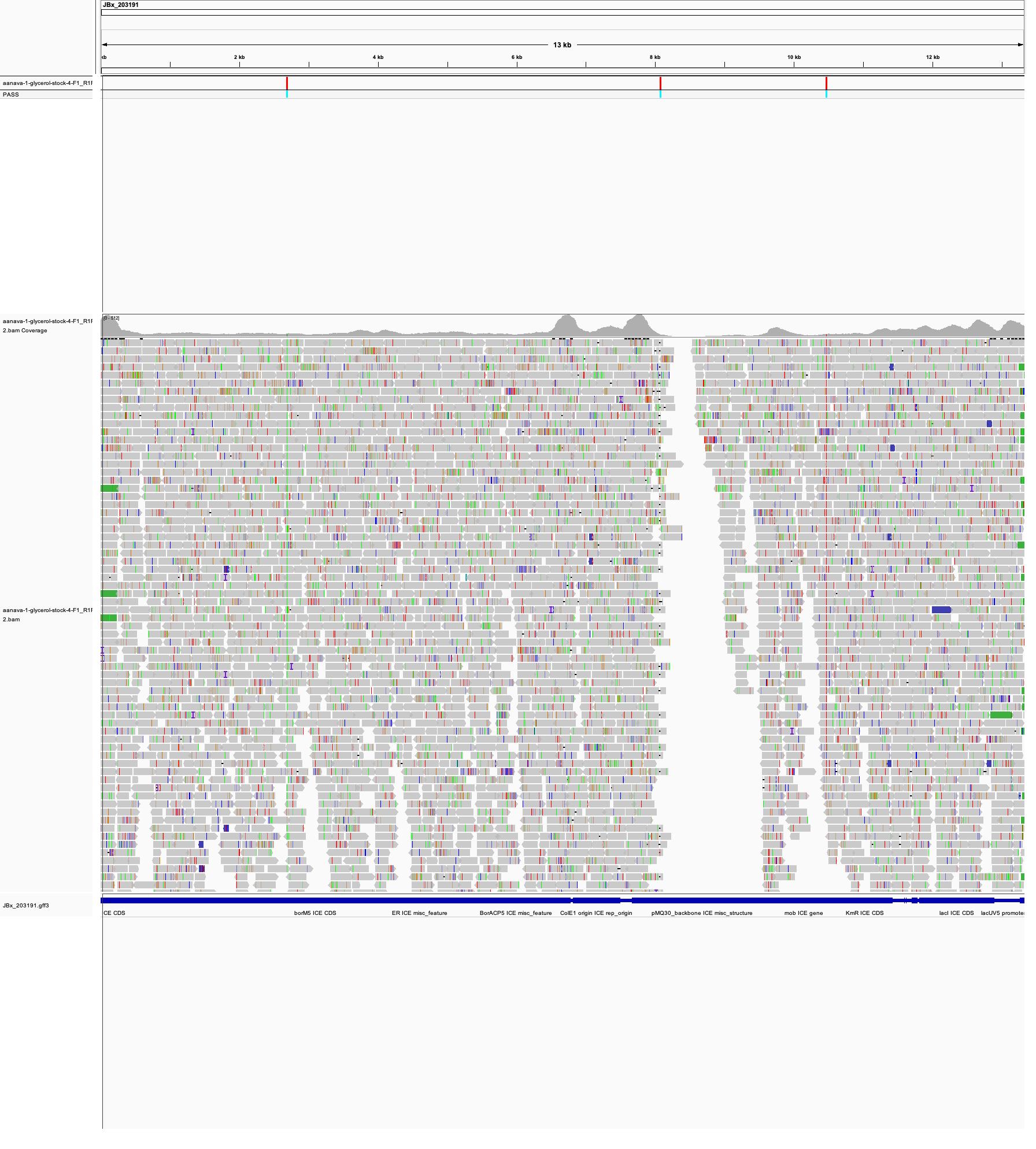

Supplement: Supplementary file 2 — sb3c00292_si_002.zip [file sb3c00292_si_002.zip › dnada_supplementary_material_pks_library_build/divaseq/211117_divaseq_analysis/alberto/snapshots/JBx_203191_nava-1-glycerol-stock-4-F1_R1R2.jpg]

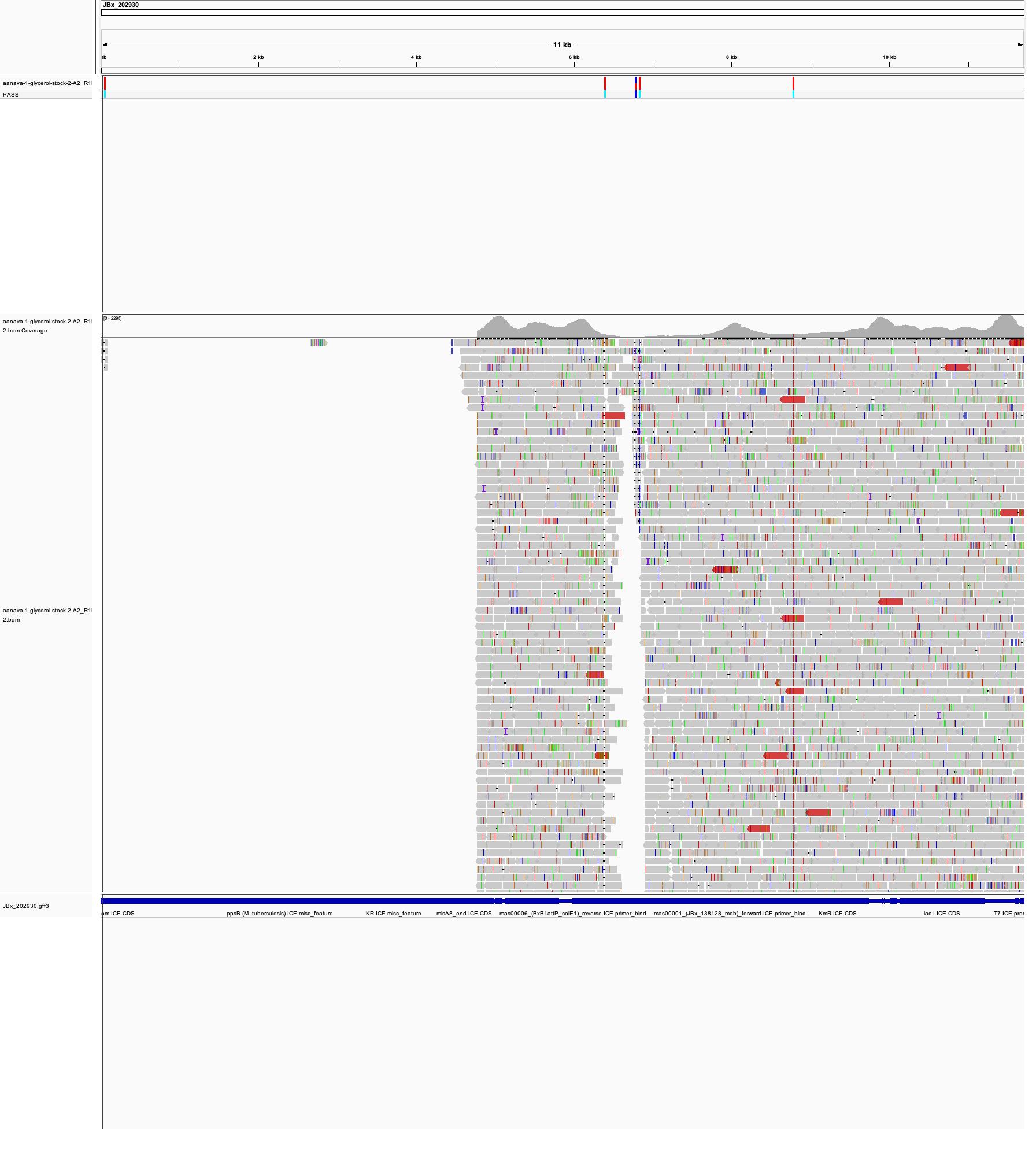

Supplement: Supplementary file 2 — sb3c00292_si_002.zip [file sb3c00292_si_002.zip › dnada_supplementary_material_pks_library_build/divaseq/211117_divaseq_analysis/alberto/snapshots/JBx_202930_nava-1-glycerol-stock-2-A2_R1R2.jpg]

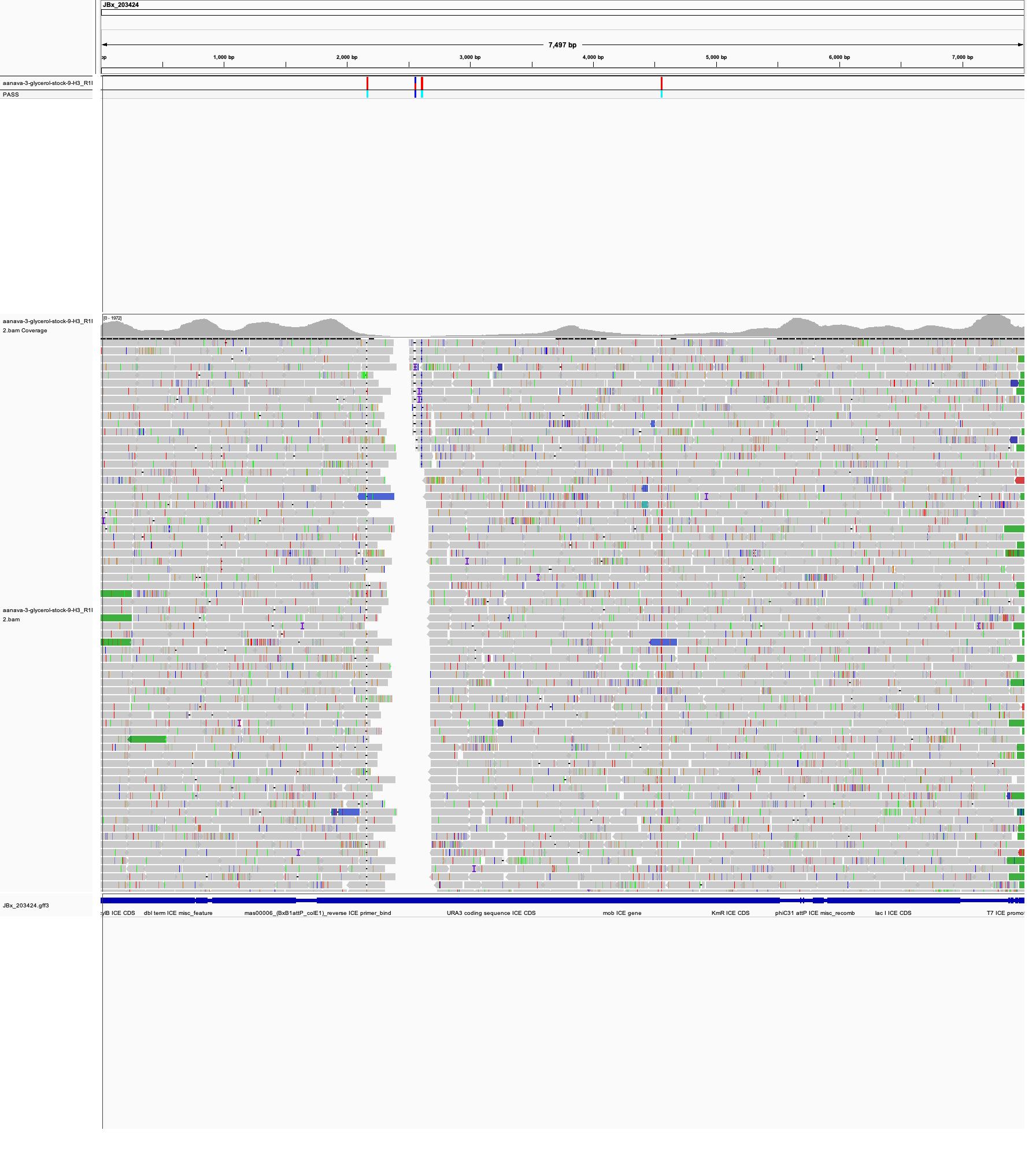

Supplement: Supplementary file 2 — sb3c00292_si_002.zip [file sb3c00292_si_002.zip › dnada_supplementary_material_pks_library_build/divaseq/211117_divaseq_analysis/alberto/snapshots/JBx_203424_nava-3-glycerol-stock-9-H3_R1R2.jpg]

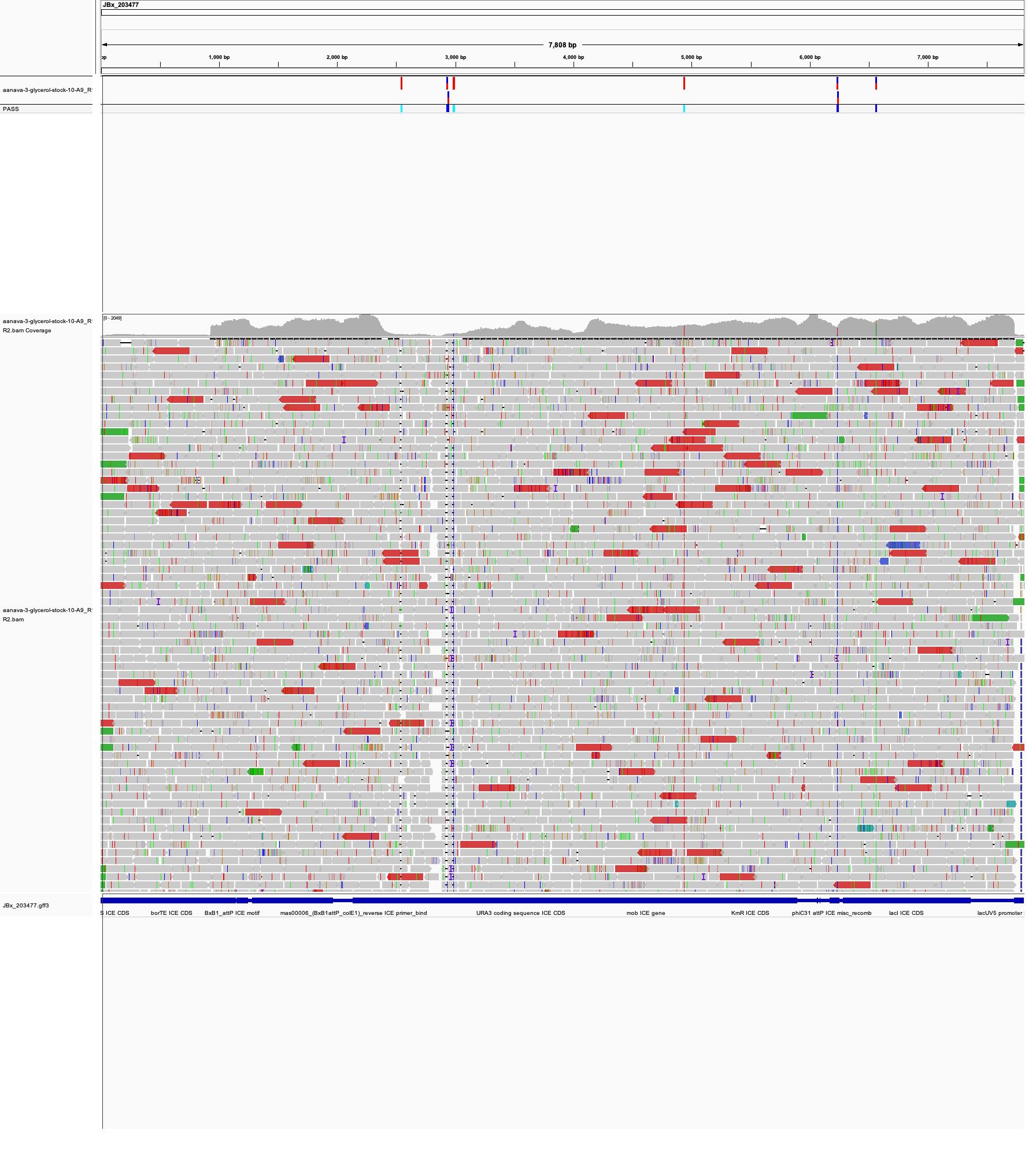

Supplement: Supplementary file 2 — sb3c00292_si_002.zip [file sb3c00292_si_002.zip › dnada_supplementary_material_pks_library_build/divaseq/211117_divaseq_analysis/alberto/snapshots/JBx_203477_nava-3-glycerol-stock-10-A9_R1R2.jpg]

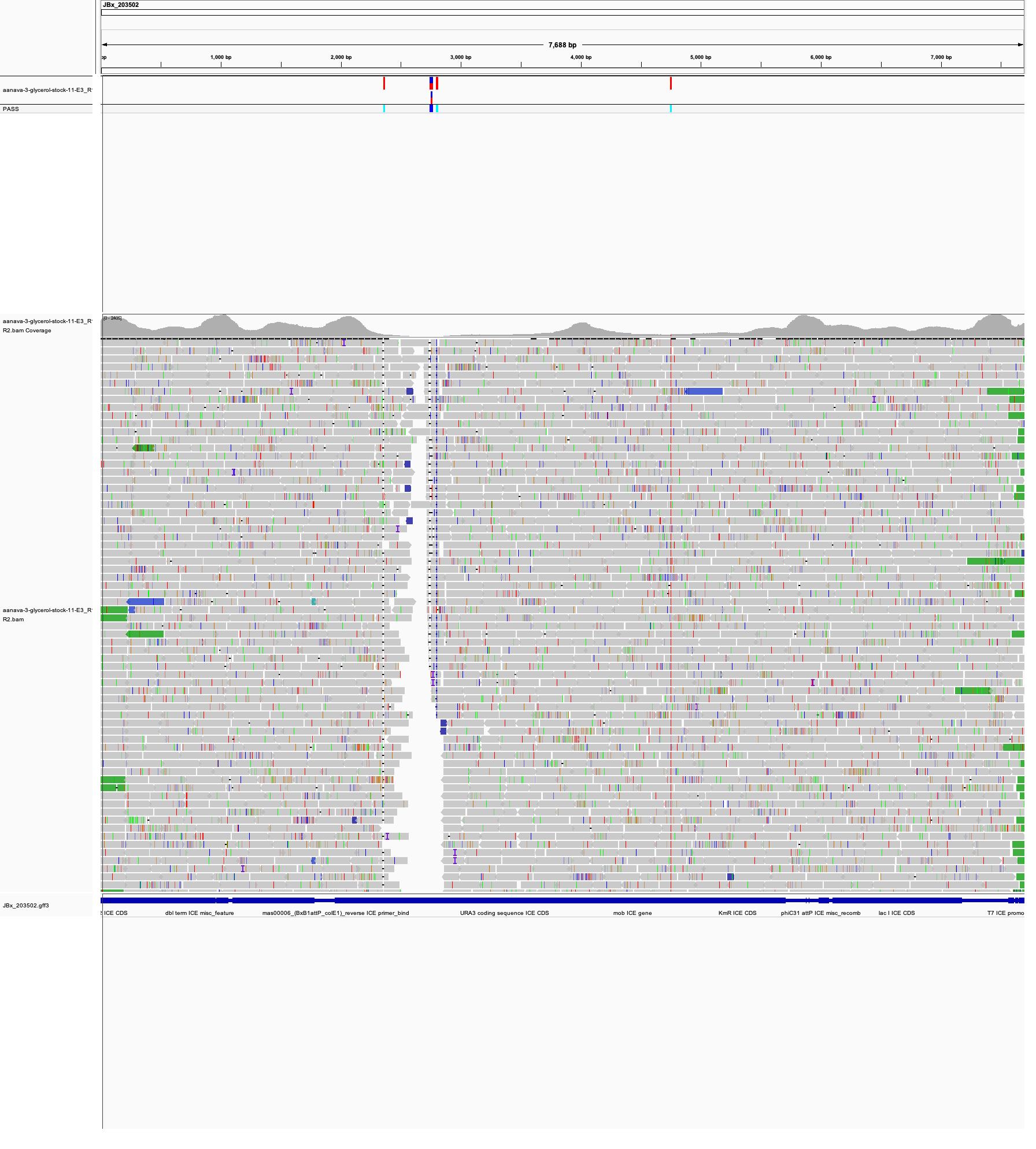

Supplement: Supplementary file 2 — sb3c00292_si_002.zip [file sb3c00292_si_002.zip › dnada_supplementary_material_pks_library_build/divaseq/211117_divaseq_analysis/alberto/snapshots/JBx_203502_nava-3-glycerol-stock-11-E3_R1R2.jpg]

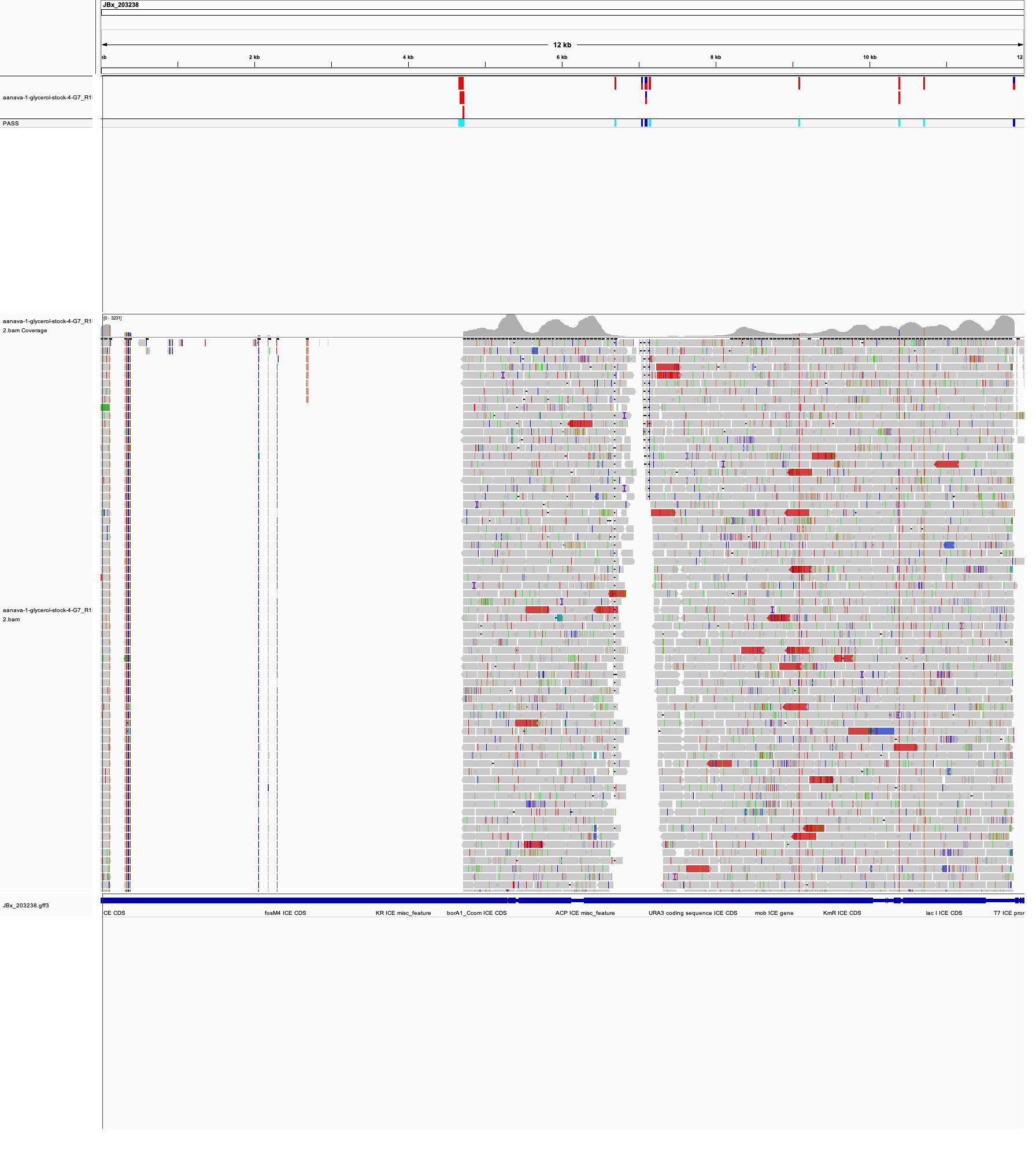

Supplement: Supplementary file 2 — sb3c00292_si_002.zip [file sb3c00292_si_002.zip › dnada_supplementary_material_pks_library_build/divaseq/211117_divaseq_analysis/alberto/snapshots/JBx_203238_nava-1-glycerol-stock-4-G7_R1R2.jpg]

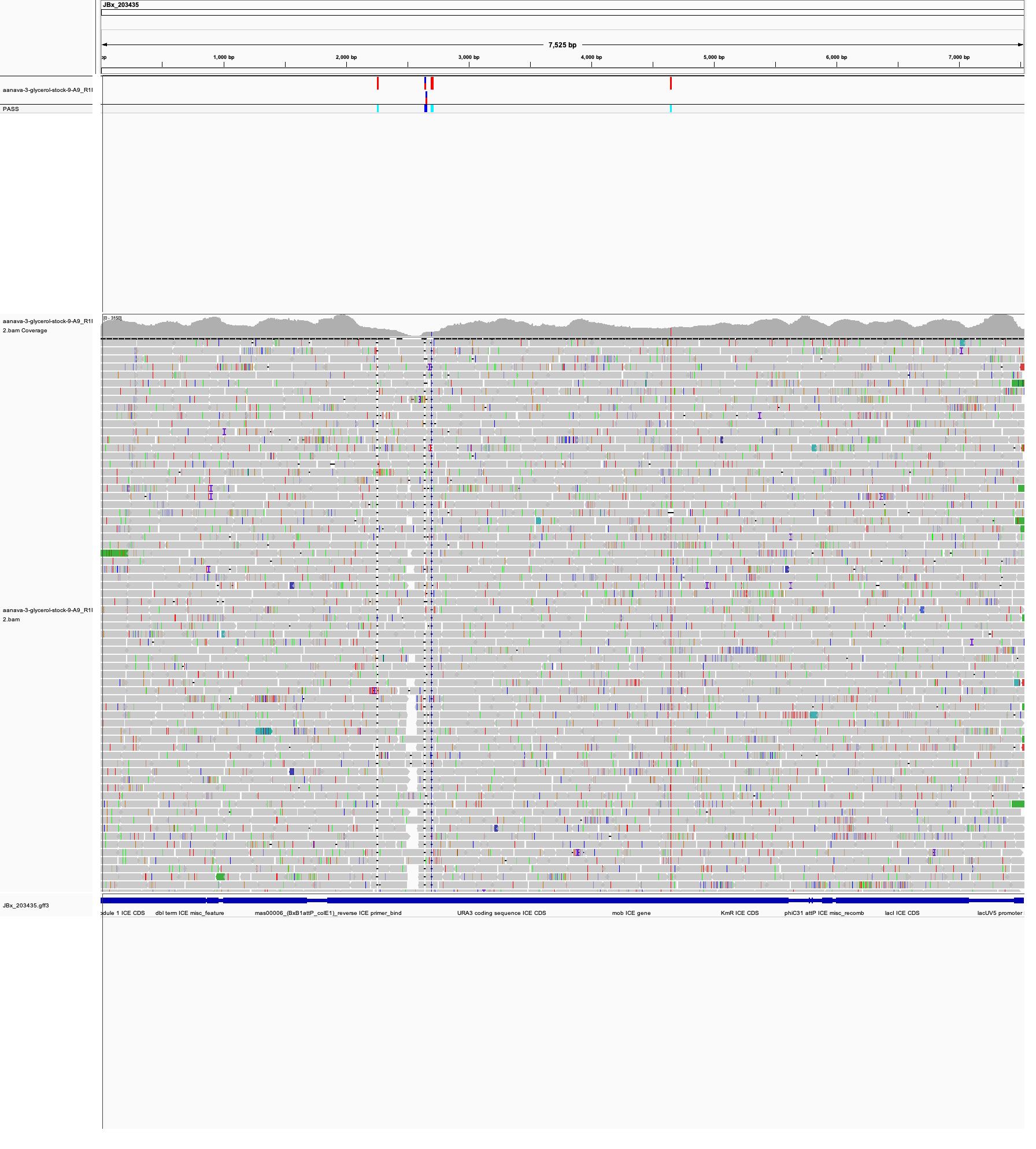

Supplement: Supplementary file 2 — sb3c00292_si_002.zip [file sb3c00292_si_002.zip › dnada_supplementary_material_pks_library_build/divaseq/211117_divaseq_analysis/alberto/snapshots/JBx_203435_nava-3-glycerol-stock-9-A9_R1R2.jpg]

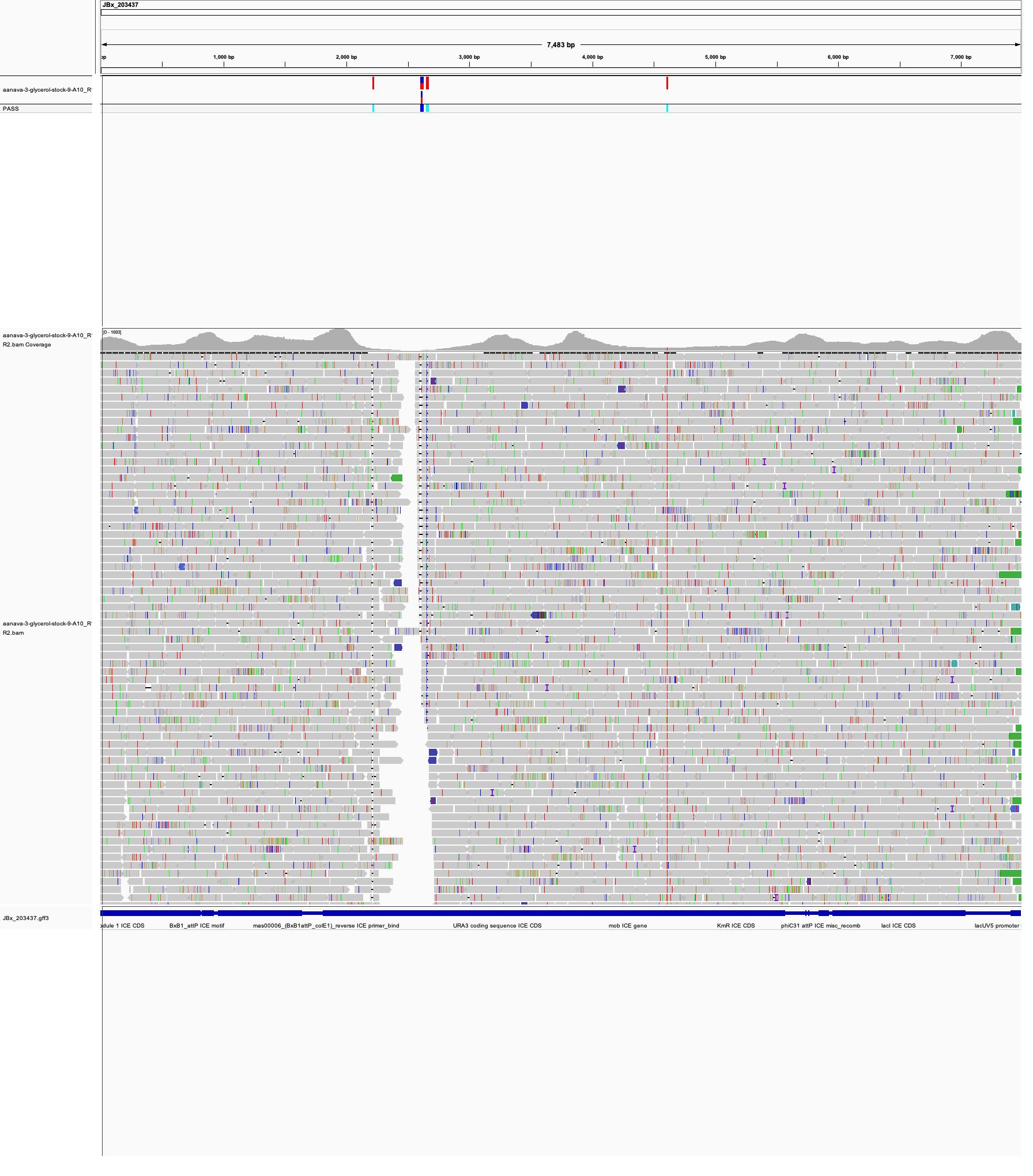

Supplement: Supplementary file 2 — sb3c00292_si_002.zip [file sb3c00292_si_002.zip › dnada_supplementary_material_pks_library_build/divaseq/211117_divaseq_analysis/alberto/snapshots/JBx_203437_nava-3-glycerol-stock-9-A10_R1R2.jpg]

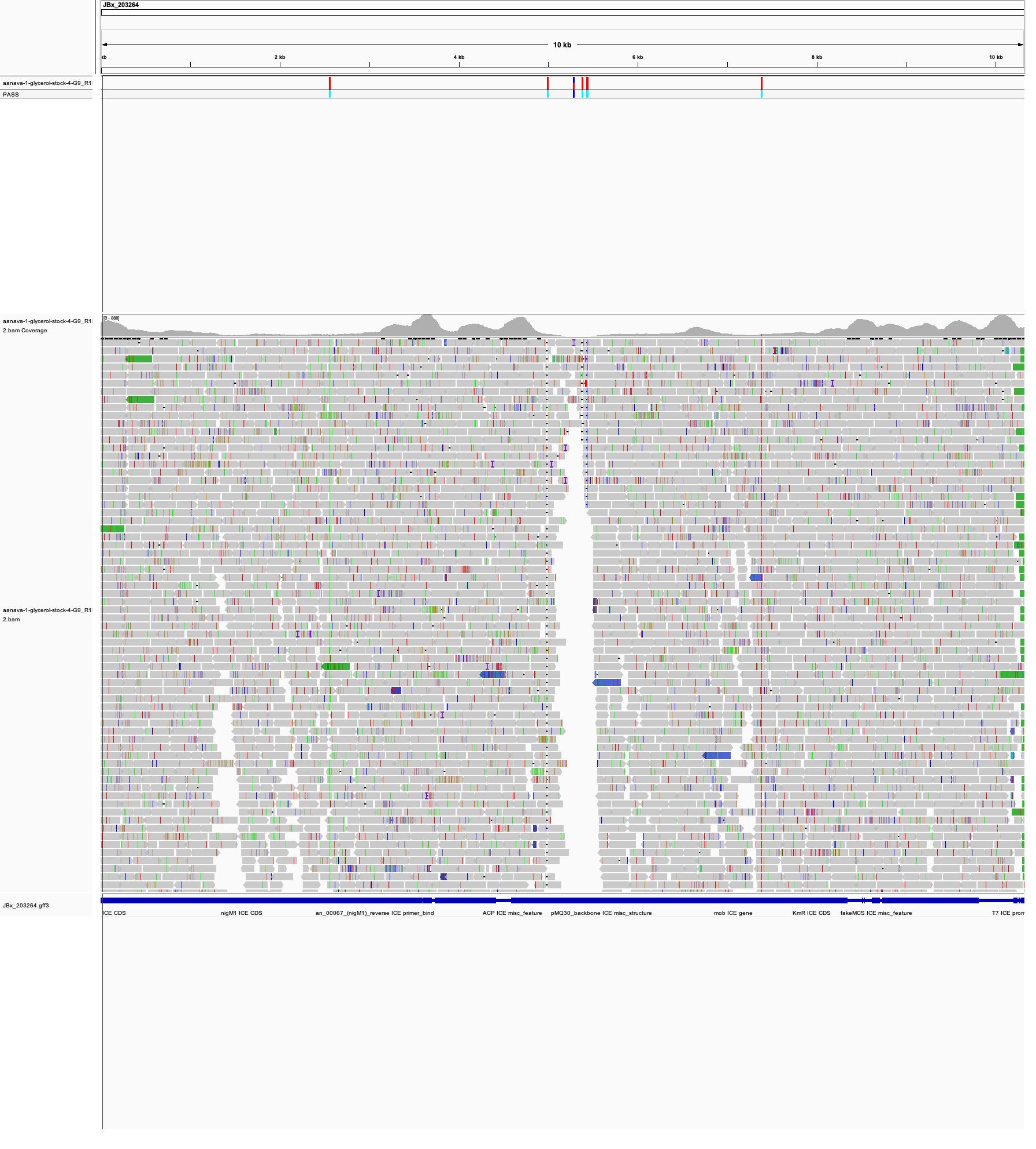

Supplement: Supplementary file 2 — sb3c00292_si_002.zip [file sb3c00292_si_002.zip › dnada_supplementary_material_pks_library_build/divaseq/211117_divaseq_analysis/alberto/snapshots/JBx_203264_nava-1-glycerol-stock-4-G9_R1R2.jpg]

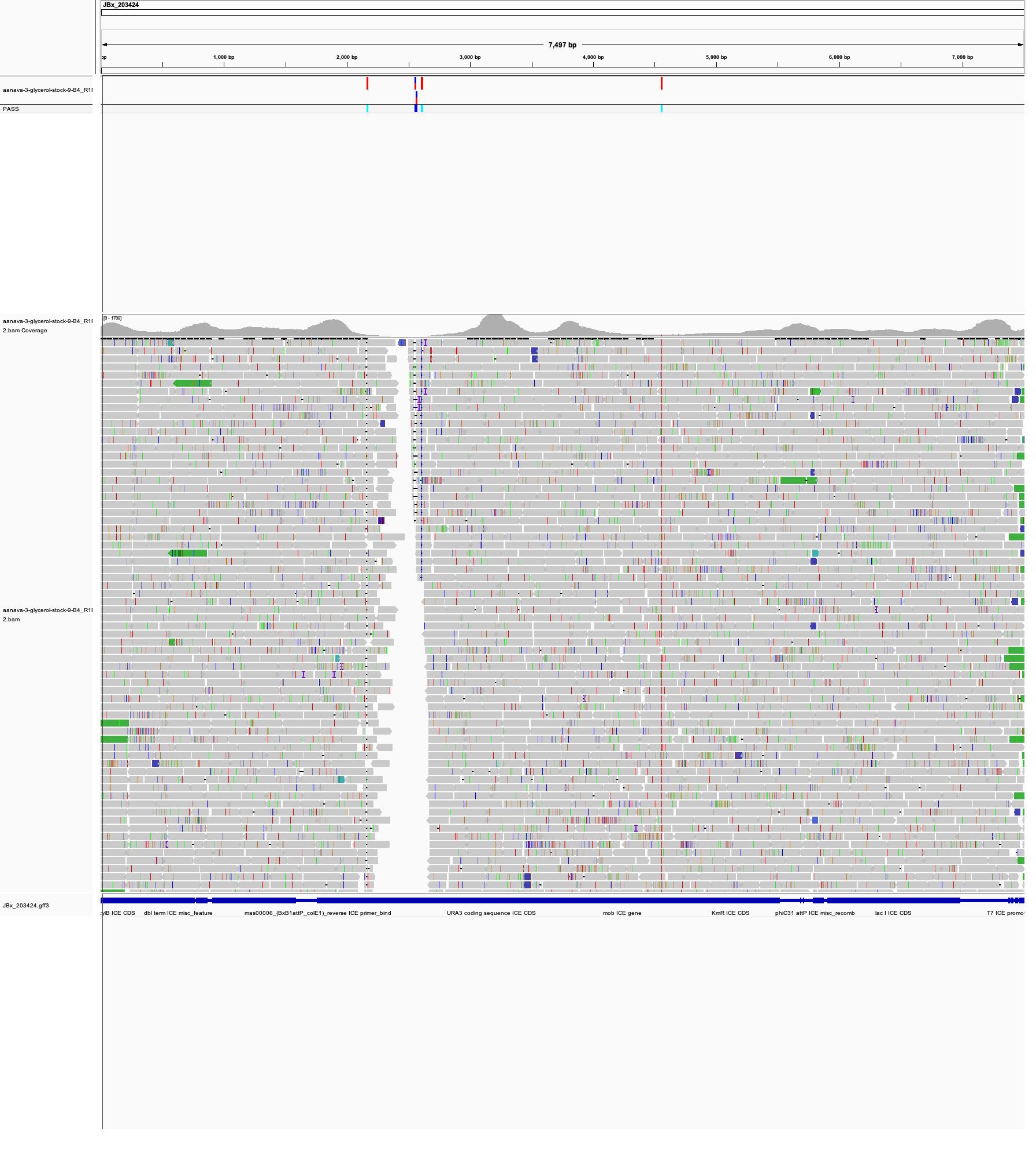

Supplement: Supplementary file 2 — sb3c00292_si_002.zip [file sb3c00292_si_002.zip › dnada_supplementary_material_pks_library_build/divaseq/211117_divaseq_analysis/alberto/snapshots/JBx_203424_nava-3-glycerol-stock-9-B4_R1R2.jpg]

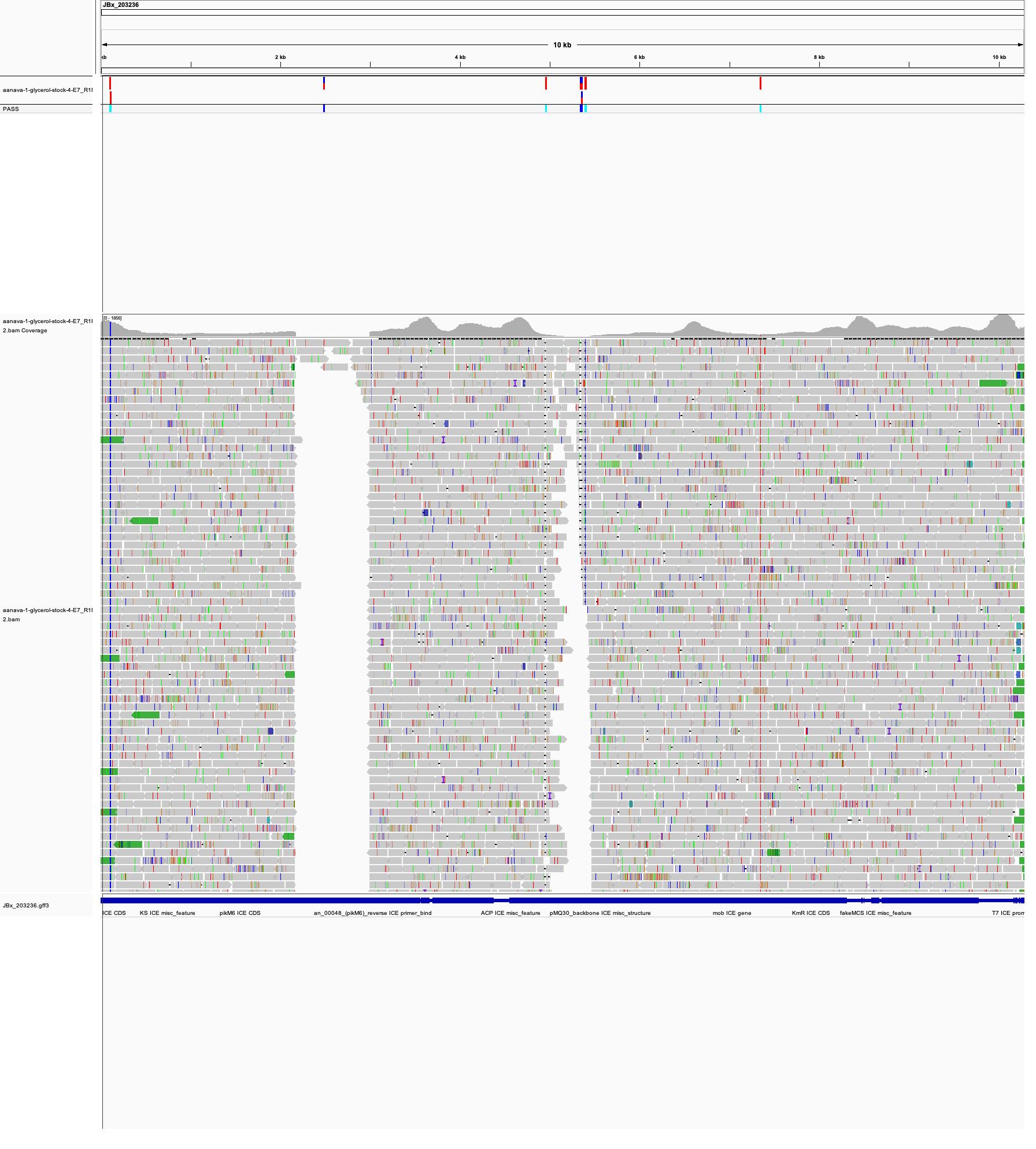

Supplement: Supplementary file 2 — sb3c00292_si_002.zip [file sb3c00292_si_002.zip › dnada_supplementary_material_pks_library_build/divaseq/211117_divaseq_analysis/alberto/snapshots/JBx_203236_nava-1-glycerol-stock-4-E7_R1R2.jpg]

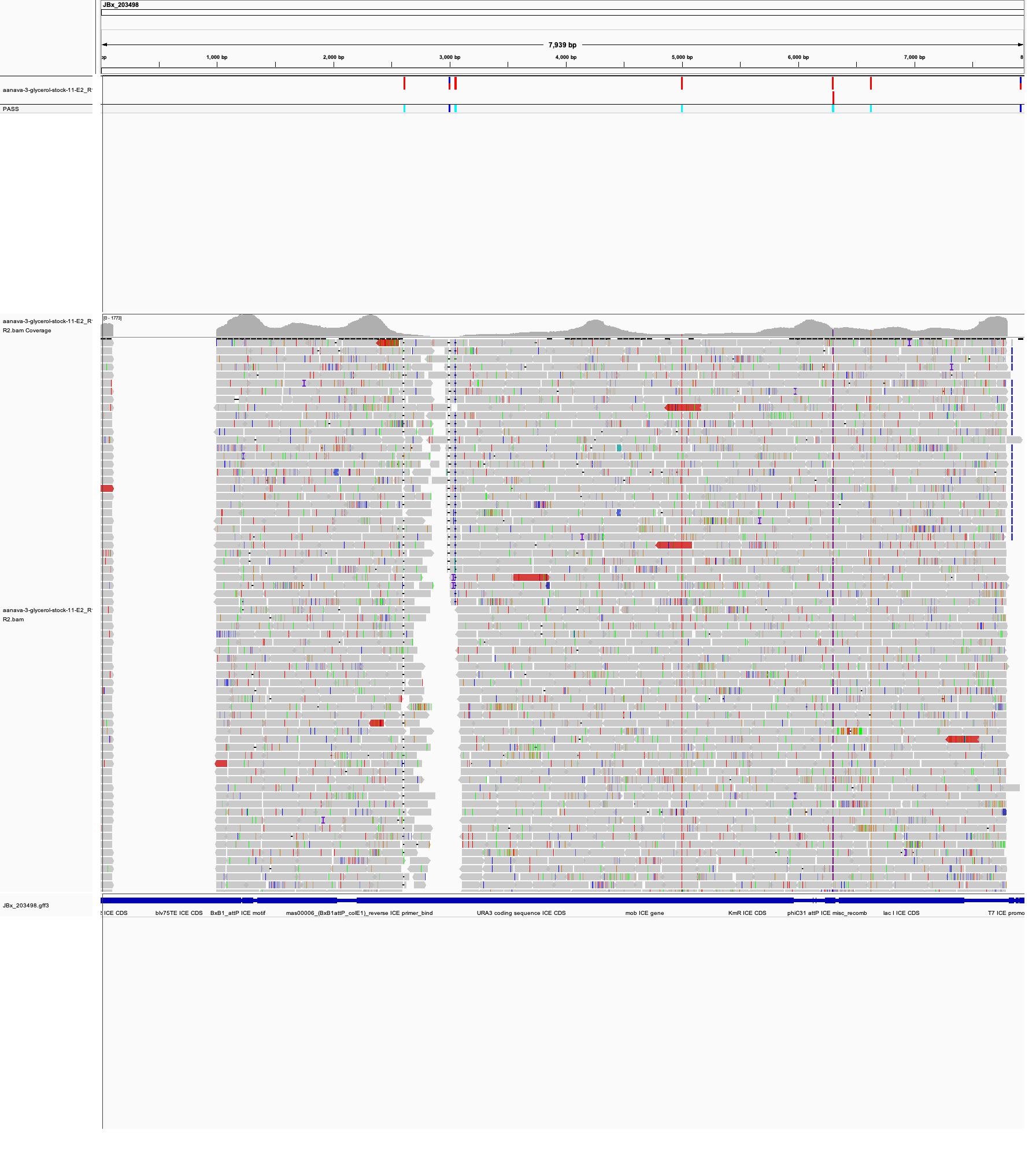

Supplement: Supplementary file 2 — sb3c00292_si_002.zip [file sb3c00292_si_002.zip › dnada_supplementary_material_pks_library_build/divaseq/211117_divaseq_analysis/alberto/snapshots/JBx_203498_nava-3-glycerol-stock-11-E2_R1R2.jpg]

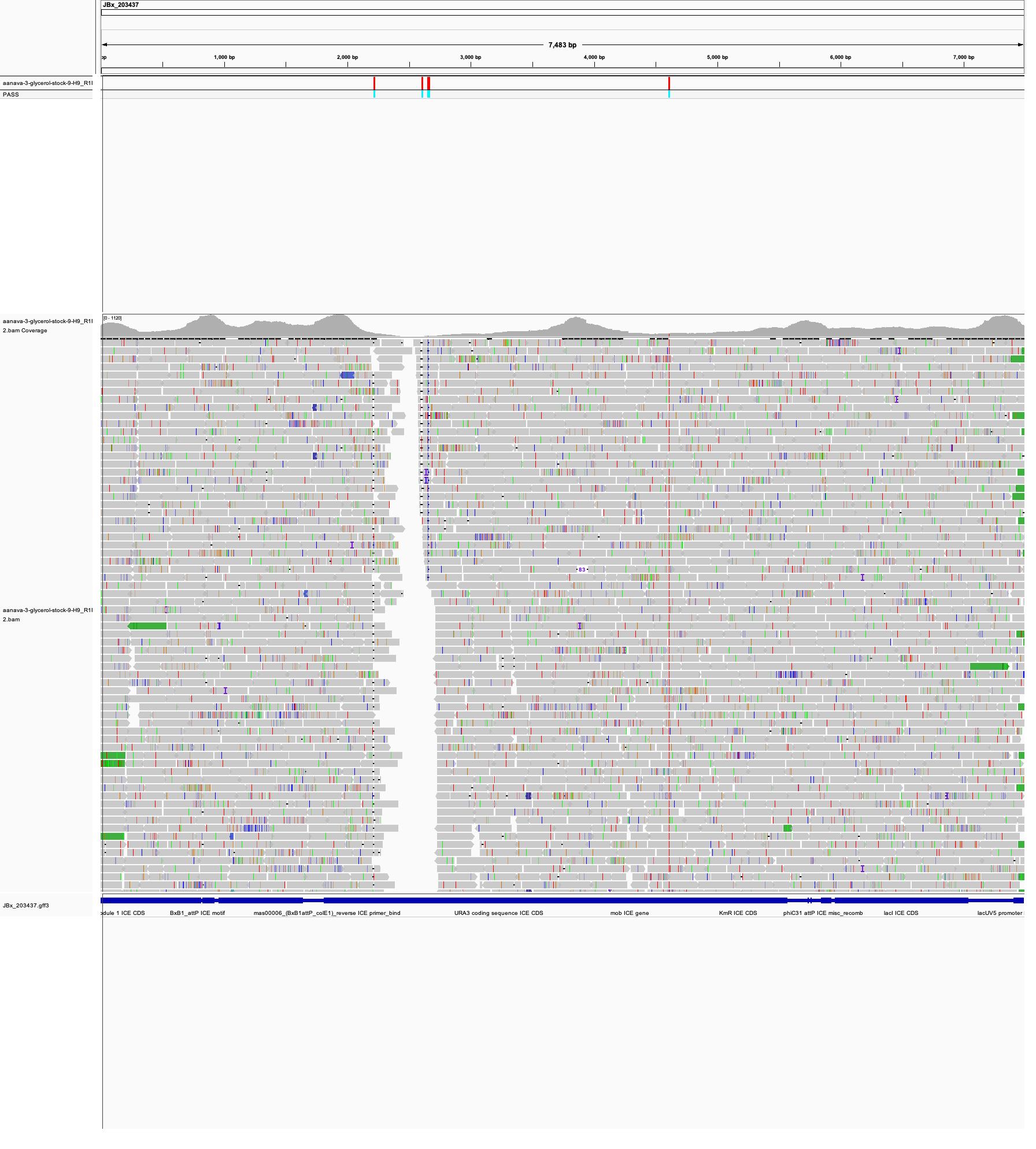

Supplement: Supplementary file 2 — sb3c00292_si_002.zip [file sb3c00292_si_002.zip › dnada_supplementary_material_pks_library_build/divaseq/211117_divaseq_analysis/alberto/snapshots/JBx_203437_nava-3-glycerol-stock-9-H9_R1R2.jpg]

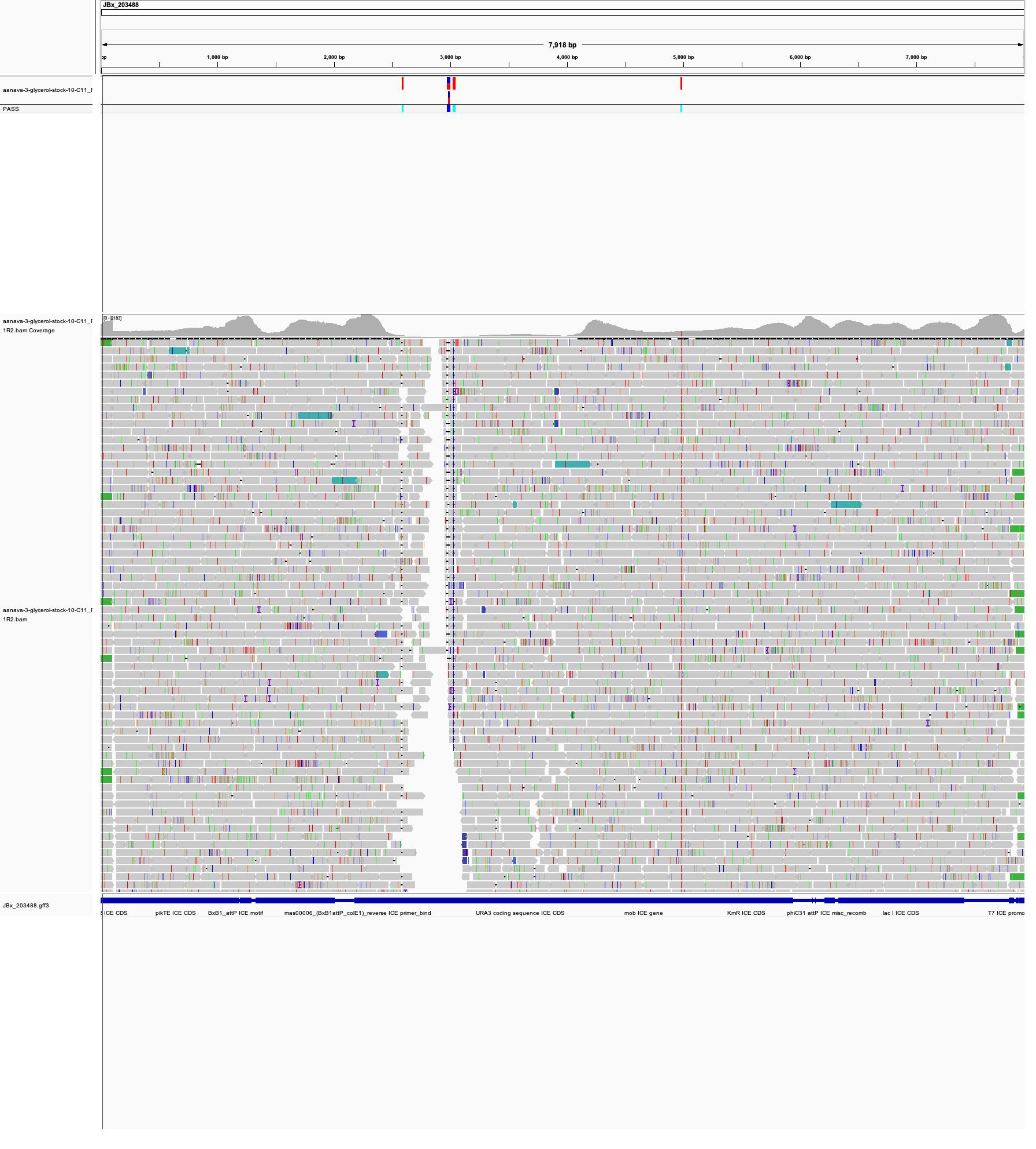

Supplement: Supplementary file 2 — sb3c00292_si_002.zip [file sb3c00292_si_002.zip › dnada_supplementary_material_pks_library_build/divaseq/211117_divaseq_analysis/alberto/snapshots/JBx_203488_nava-3-glycerol-stock-10-C11_R1R2.jpg]

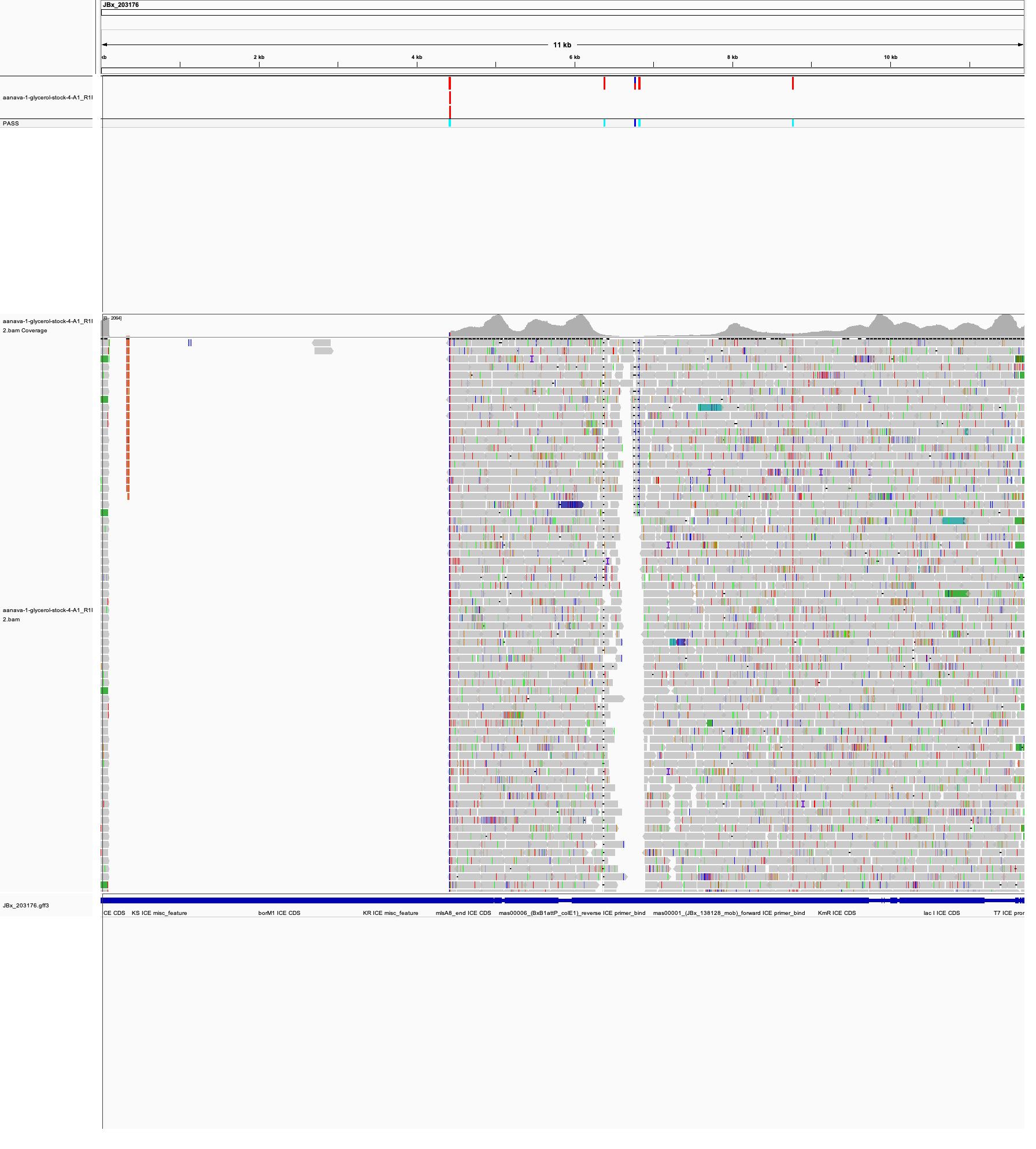

Supplement: Supplementary file 2 — sb3c00292_si_002.zip [file sb3c00292_si_002.zip › dnada_supplementary_material_pks_library_build/divaseq/211117_divaseq_analysis/alberto/snapshots/JBx_203176_nava-1-glycerol-stock-4-A1_R1R2.jpg]

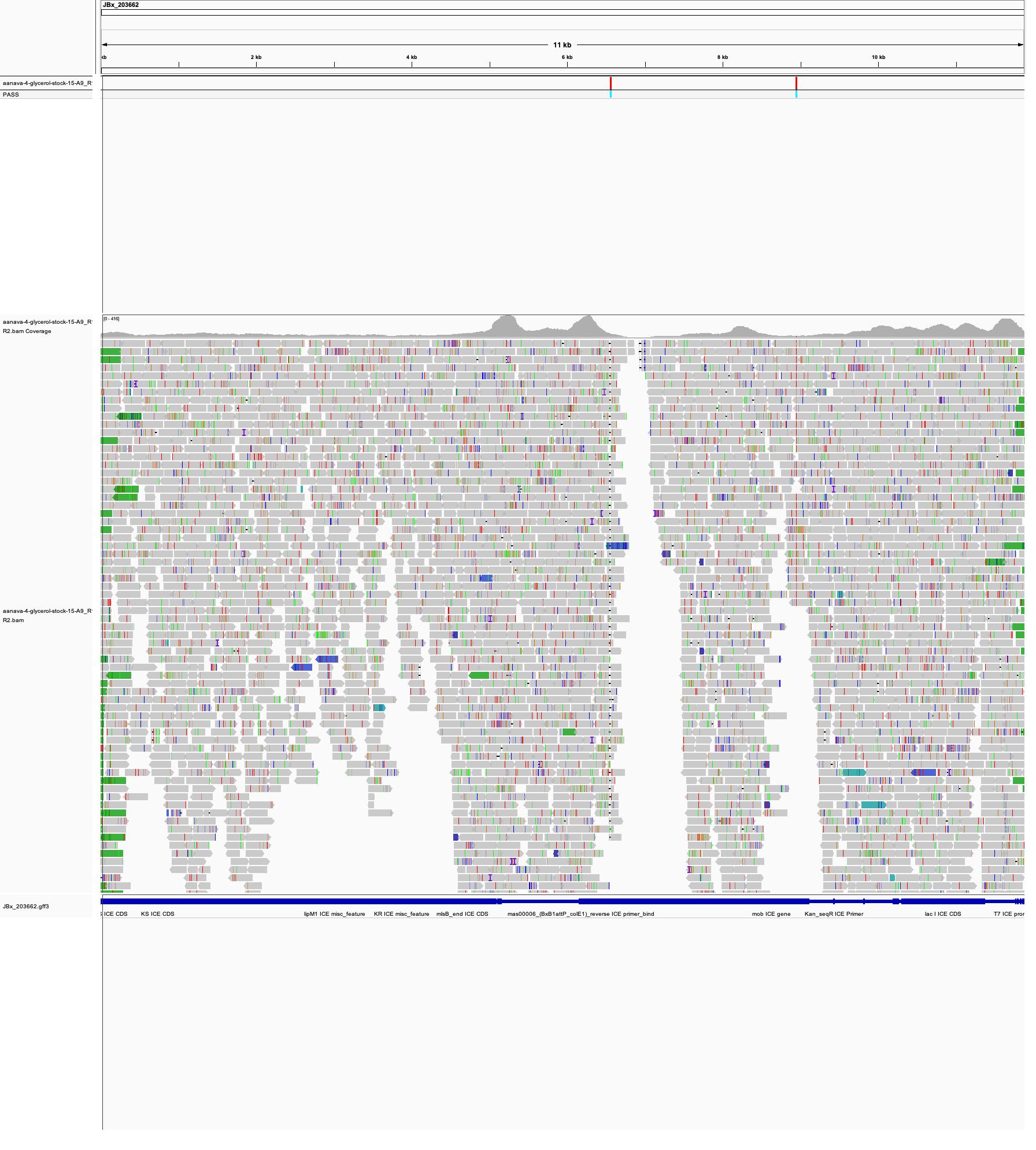

Supplement: Supplementary file 2 — sb3c00292_si_002.zip [file sb3c00292_si_002.zip › dnada_supplementary_material_pks_library_build/divaseq/211117_divaseq_analysis/alberto/snapshots/JBx_203662_nava-4-glycerol-stock-15-A9_R1R2.jpg]

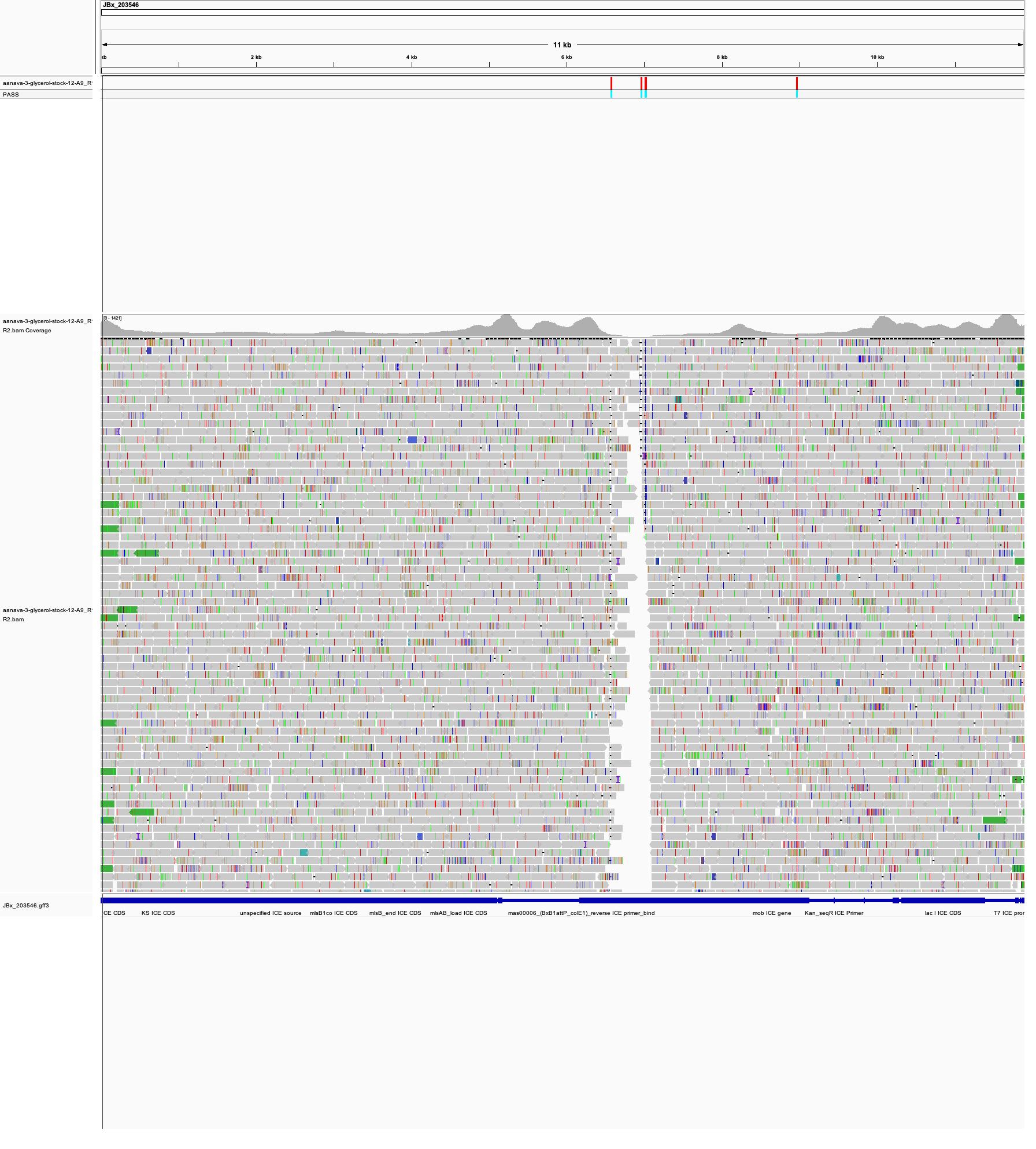

Supplement: Supplementary file 2 — sb3c00292_si_002.zip [file sb3c00292_si_002.zip › dnada_supplementary_material_pks_library_build/divaseq/211117_divaseq_analysis/alberto/snapshots/JBx_203546_nava-3-glycerol-stock-12-A9_R1R2.jpg]

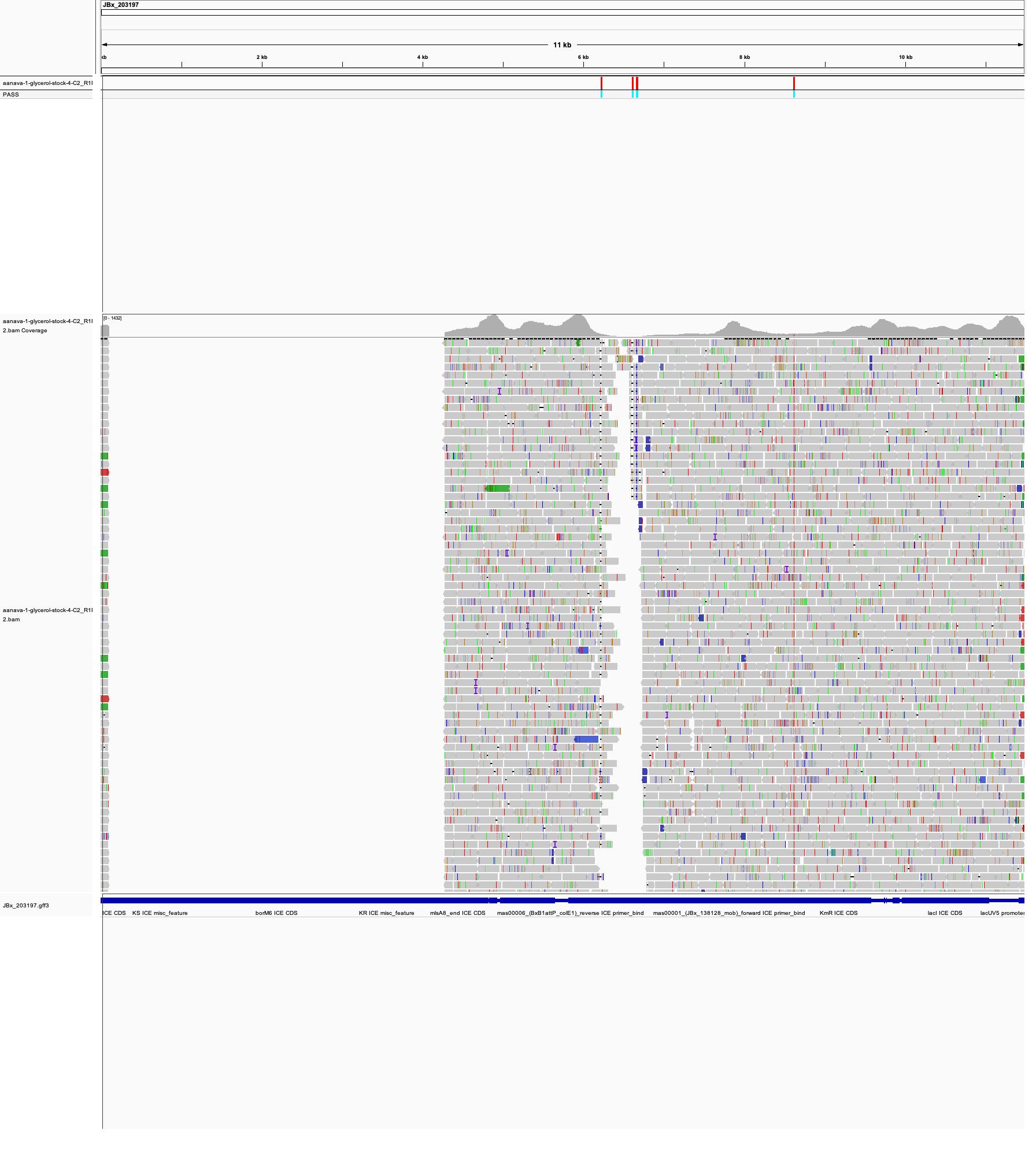

Supplement: Supplementary file 2 — sb3c00292_si_002.zip [file sb3c00292_si_002.zip › dnada_supplementary_material_pks_library_build/divaseq/211117_divaseq_analysis/alberto/snapshots/JBx_203197_nava-1-glycerol-stock-4-C2_R1R2.jpg]

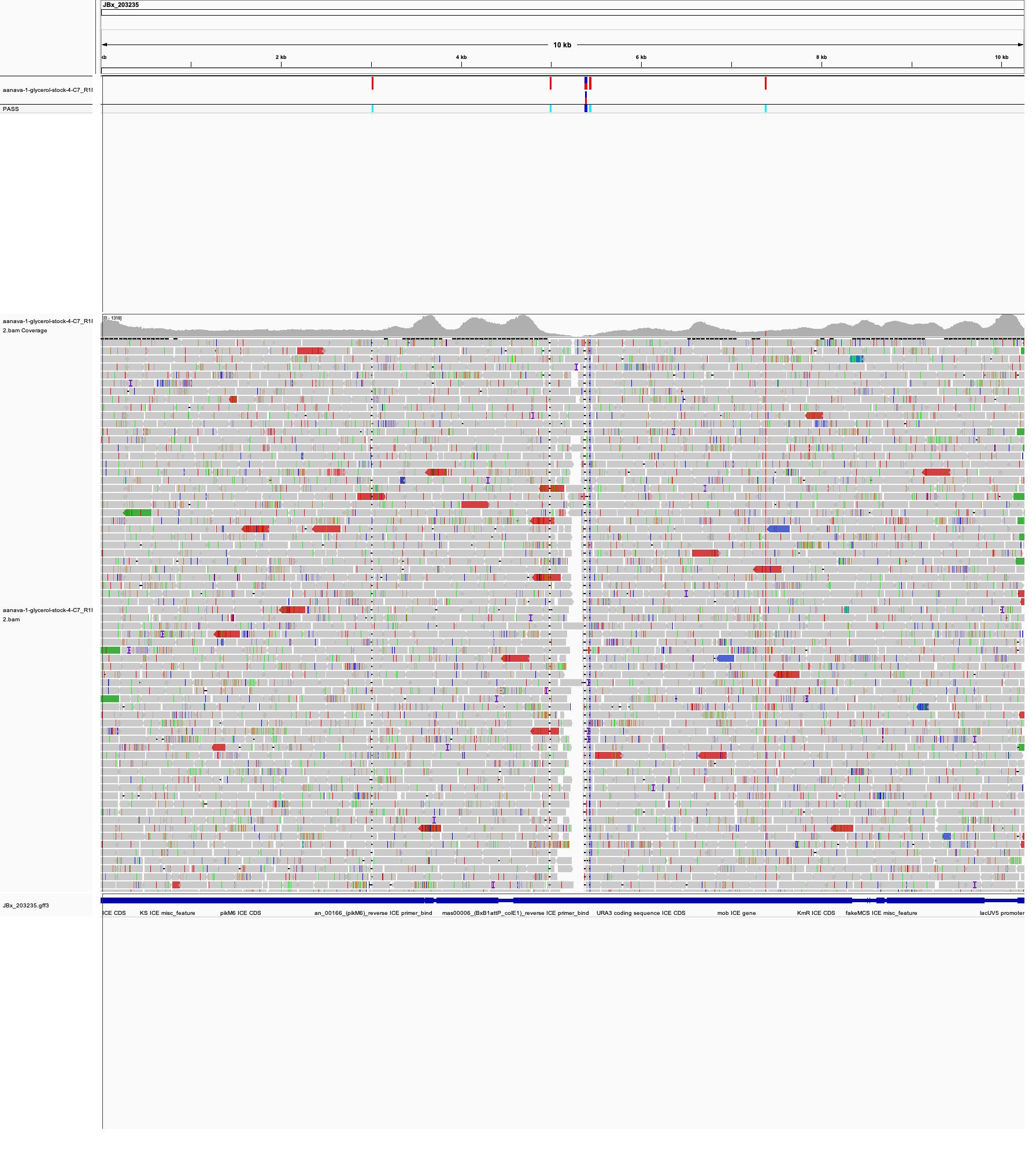

Supplement: Supplementary file 2 — sb3c00292_si_002.zip [file sb3c00292_si_002.zip › dnada_supplementary_material_pks_library_build/divaseq/211117_divaseq_analysis/alberto/snapshots/JBx_203235_nava-1-glycerol-stock-4-C7_R1R2.jpg]

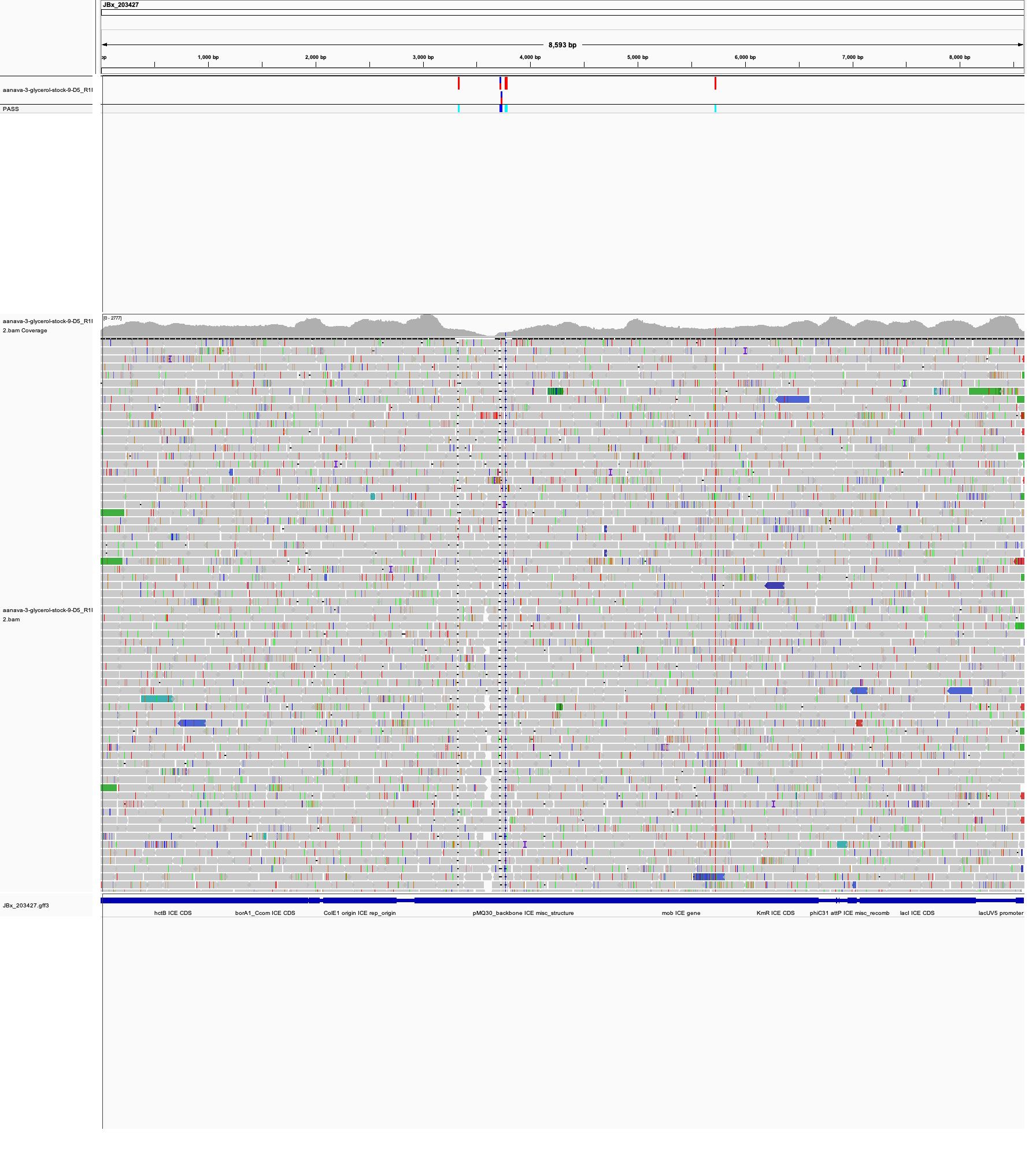

Supplement: Supplementary file 2 — sb3c00292_si_002.zip [file sb3c00292_si_002.zip › dnada_supplementary_material_pks_library_build/divaseq/211117_divaseq_analysis/alberto/snapshots/JBx_203427_nava-3-glycerol-stock-9-D5_R1R2.jpg]

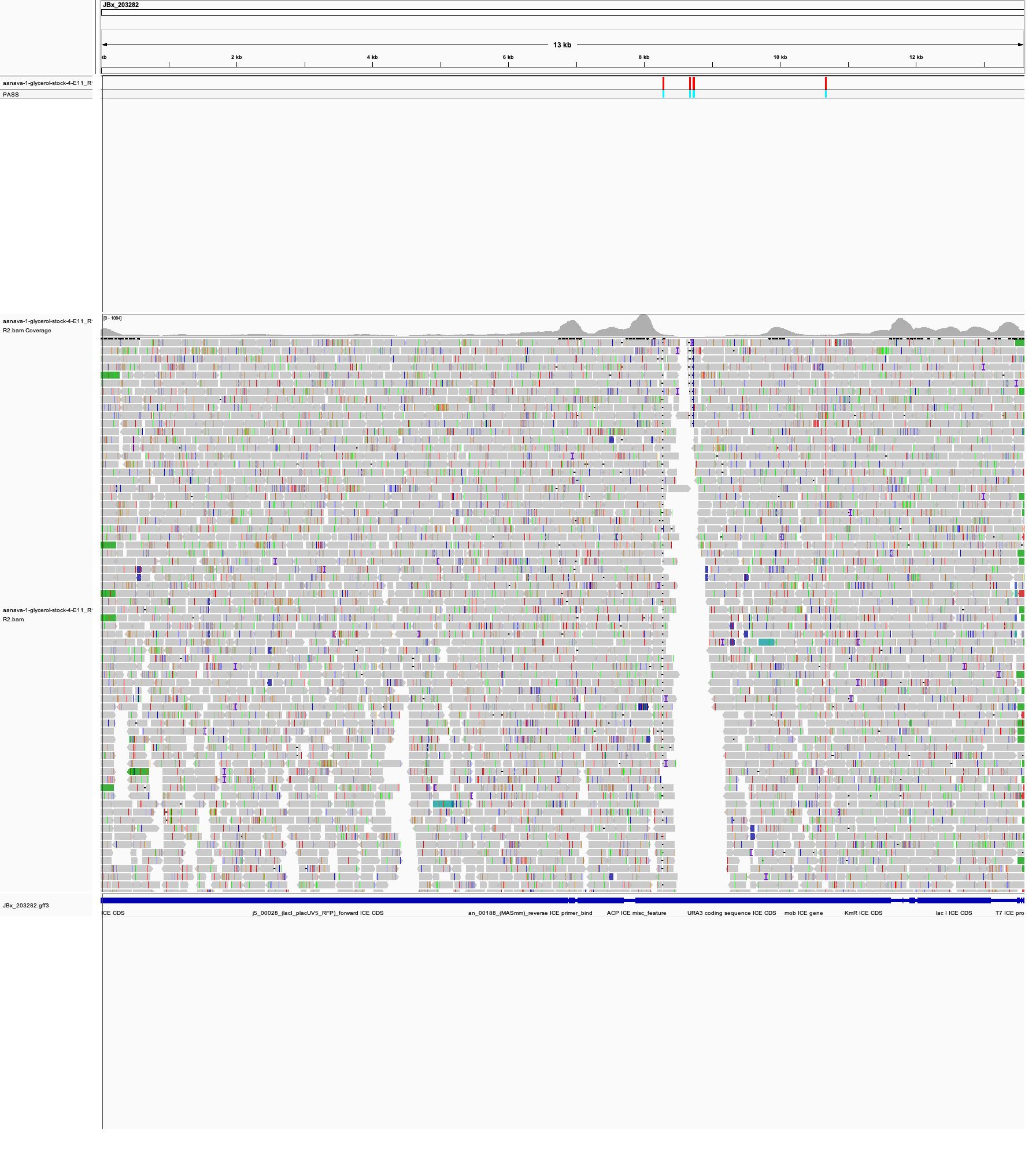

Supplement: Supplementary file 2 — sb3c00292_si_002.zip [file sb3c00292_si_002.zip › dnada_supplementary_material_pks_library_build/divaseq/211117_divaseq_analysis/alberto/snapshots/JBx_203282_nava-1-glycerol-stock-4-E11_R1R2.jpg]

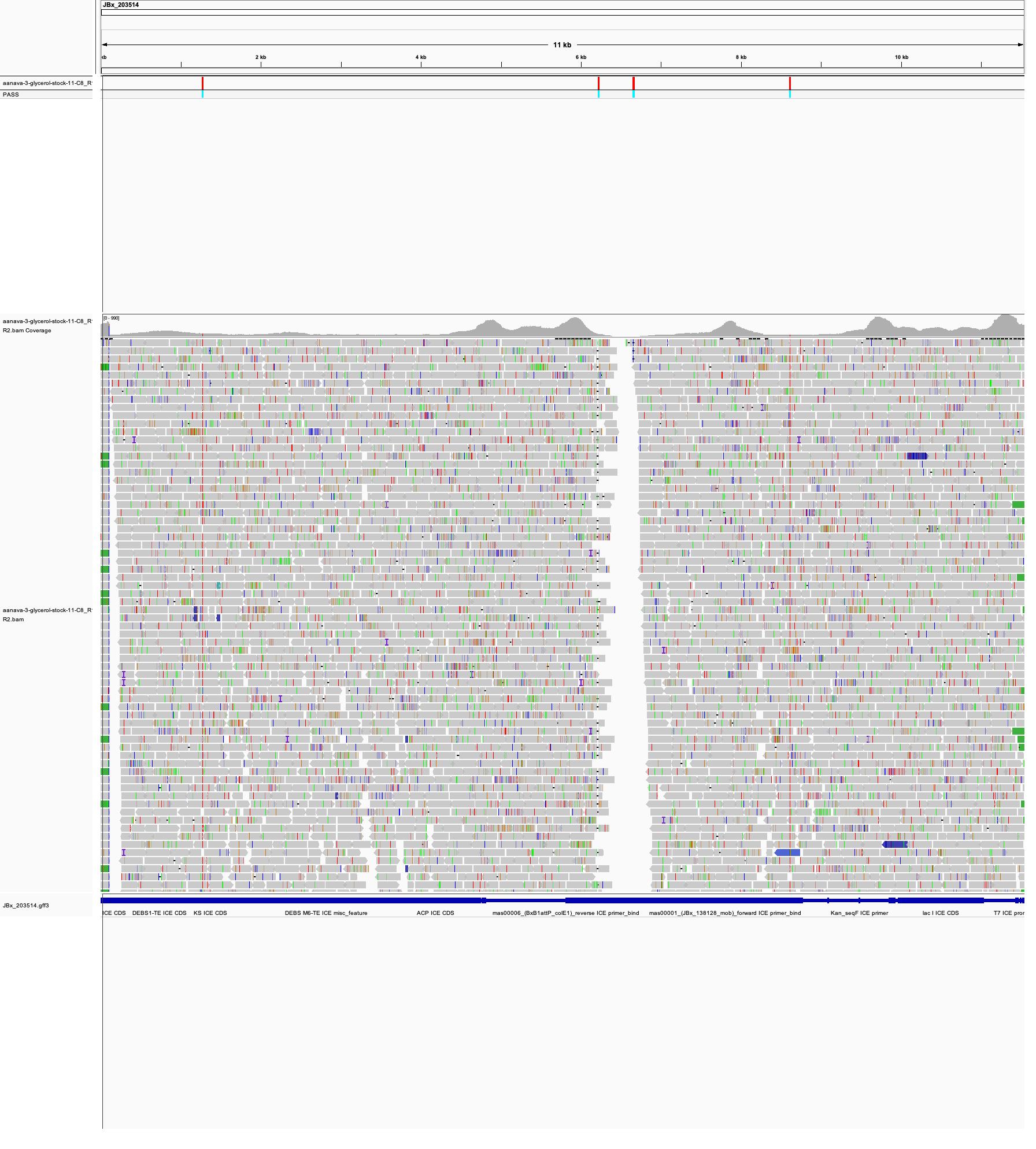

Supplement: Supplementary file 2 — sb3c00292_si_002.zip [file sb3c00292_si_002.zip › dnada_supplementary_material_pks_library_build/divaseq/211117_divaseq_analysis/alberto/snapshots/JBx_203514_nava-3-glycerol-stock-11-C8_R1R2.jpg]

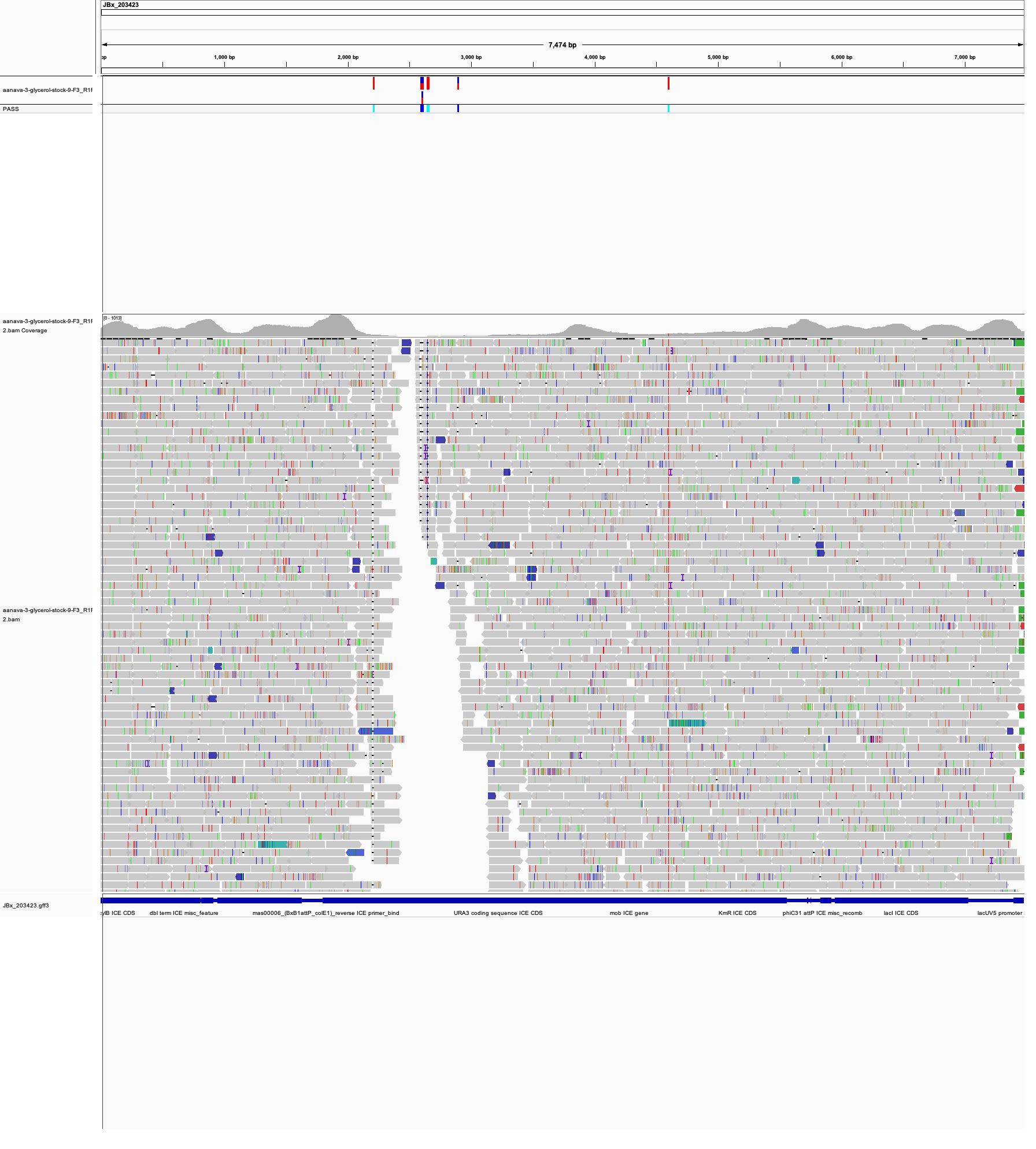

Supplement: Supplementary file 2 — sb3c00292_si_002.zip [file sb3c00292_si_002.zip › dnada_supplementary_material_pks_library_build/divaseq/211117_divaseq_analysis/alberto/snapshots/JBx_203423_nava-3-glycerol-stock-9-F3_R1R2.jpg]

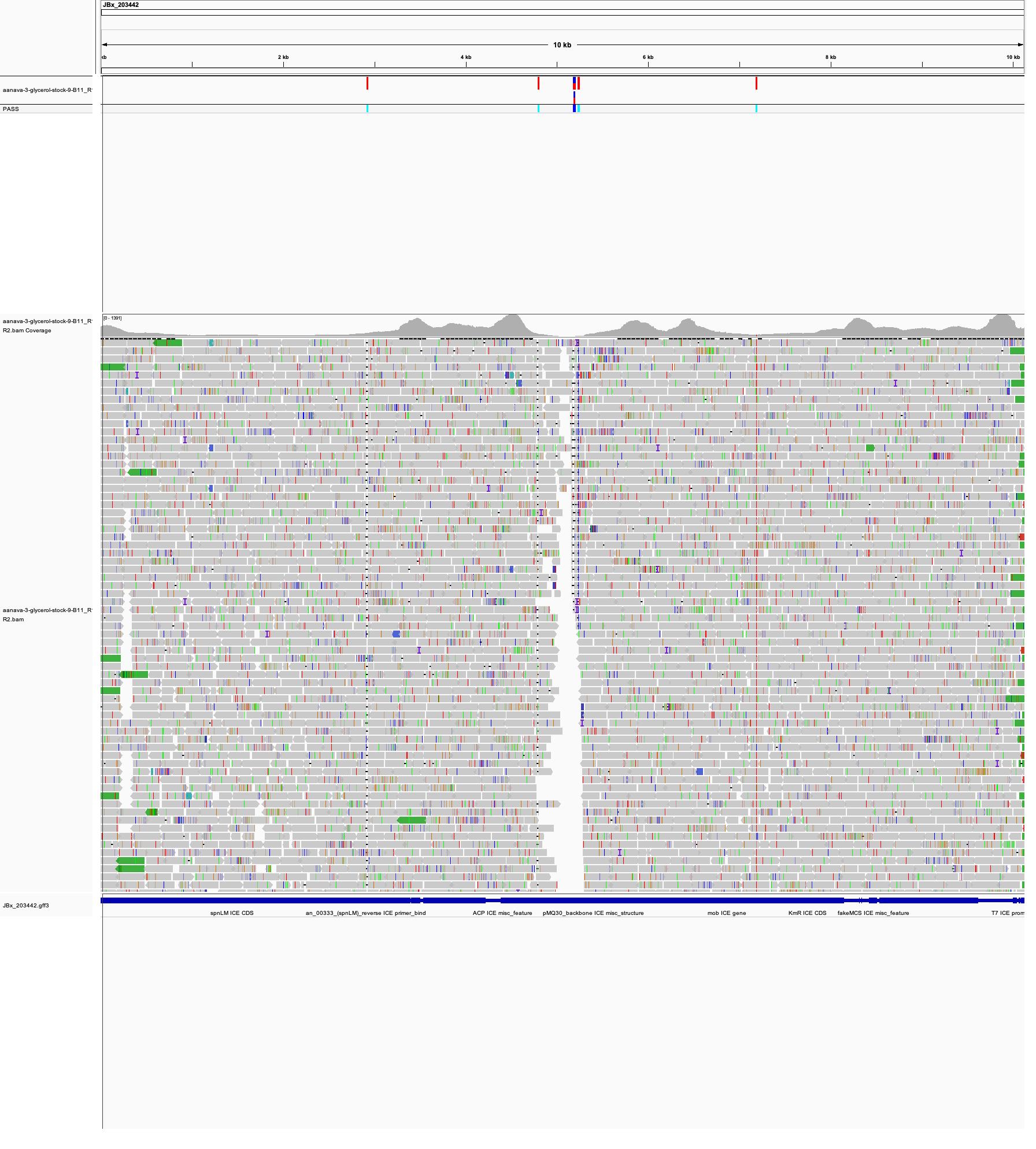

Supplement: Supplementary file 2 — sb3c00292_si_002.zip [file sb3c00292_si_002.zip › dnada_supplementary_material_pks_library_build/divaseq/211117_divaseq_analysis/alberto/snapshots/JBx_203442_nava-3-glycerol-stock-9-B11_R1R2.jpg]

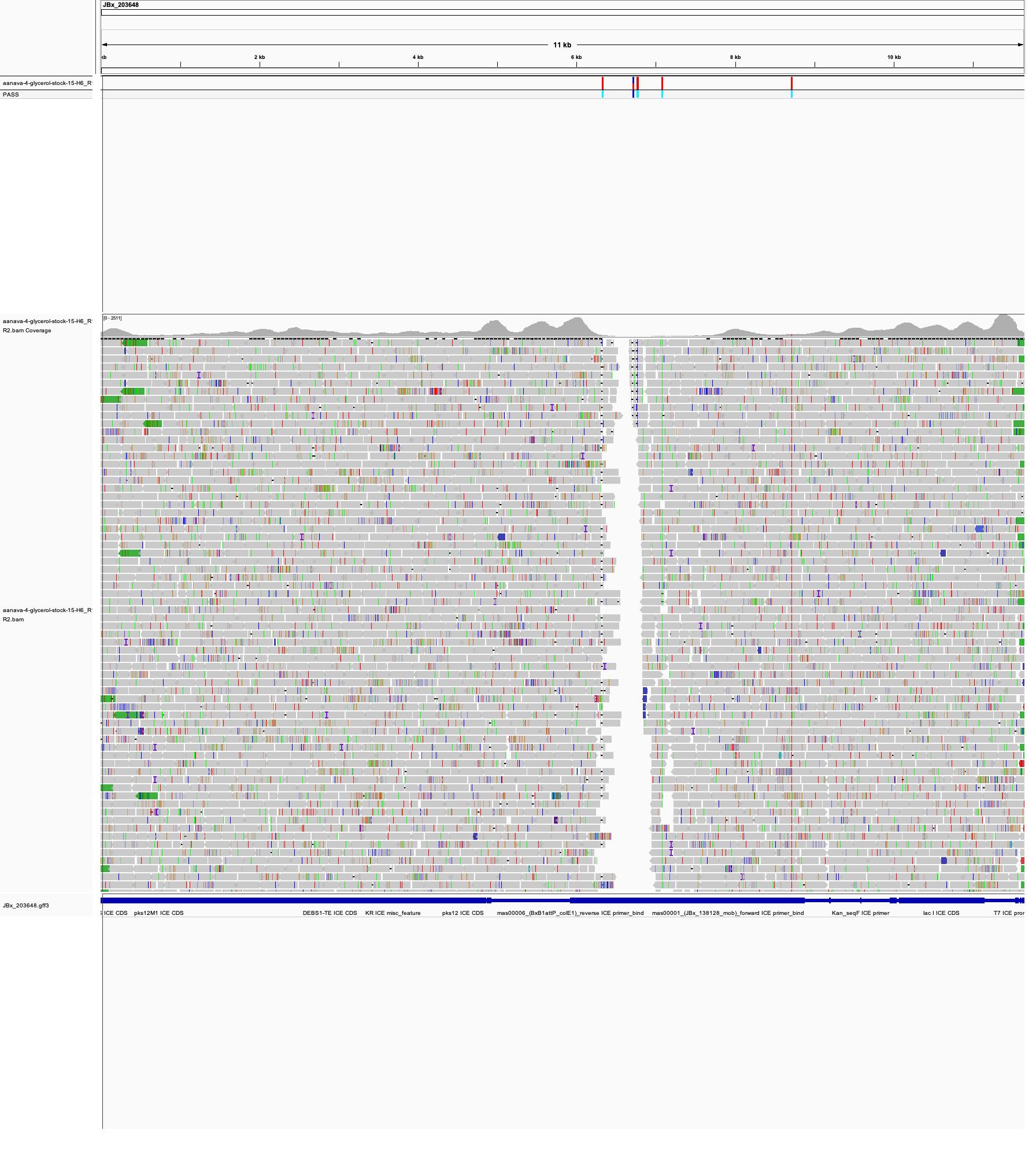

Supplement: Supplementary file 2 — sb3c00292_si_002.zip [file sb3c00292_si_002.zip › dnada_supplementary_material_pks_library_build/divaseq/211117_divaseq_analysis/alberto/snapshots/JBx_203648_nava-4-glycerol-stock-15-H6_R1R2.jpg]

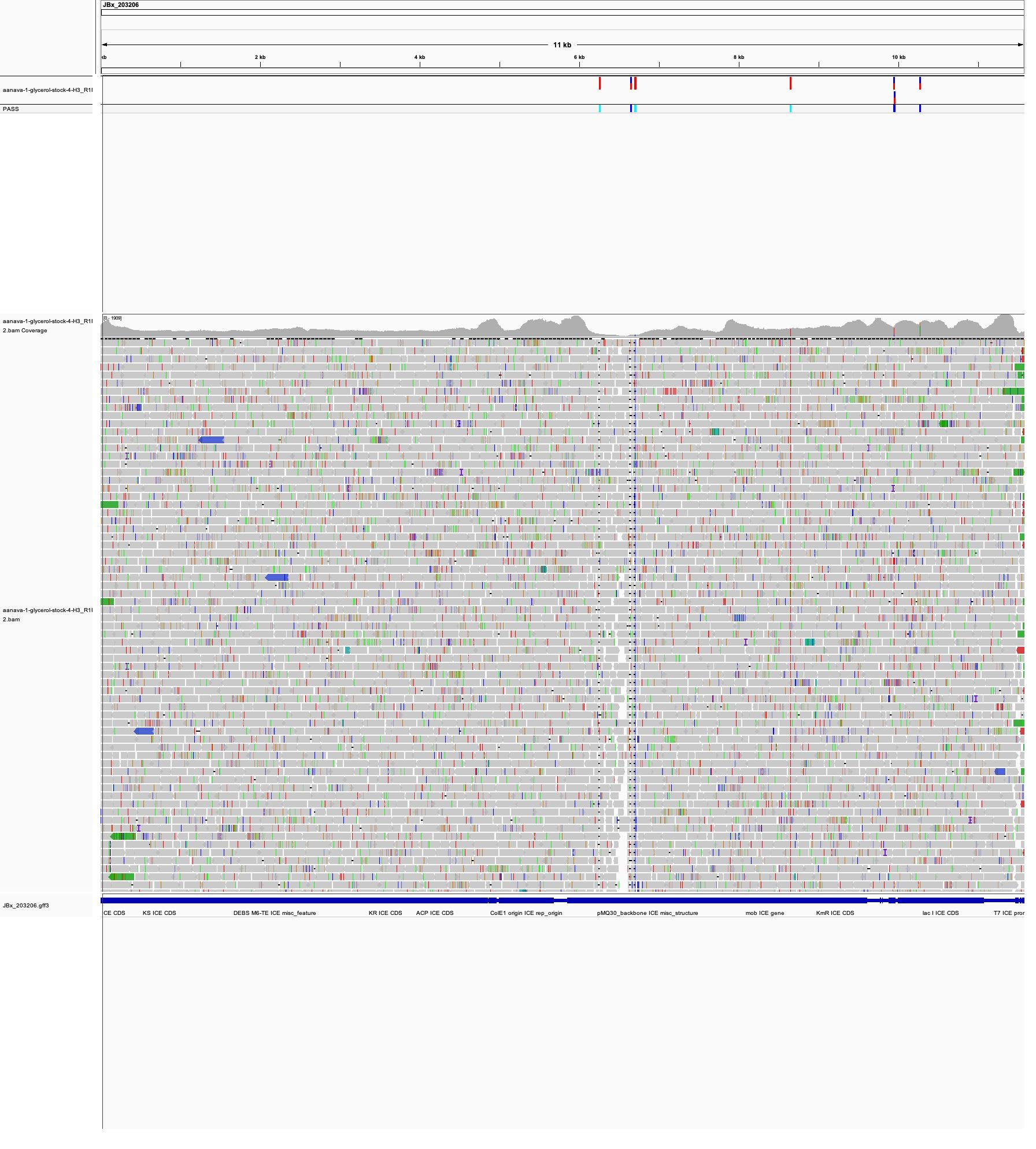

Supplement: Supplementary file 2 — sb3c00292_si_002.zip [file sb3c00292_si_002.zip › dnada_supplementary_material_pks_library_build/divaseq/211117_divaseq_analysis/alberto/snapshots/JBx_203206_nava-1-glycerol-stock-4-H3_R1R2.jpg]

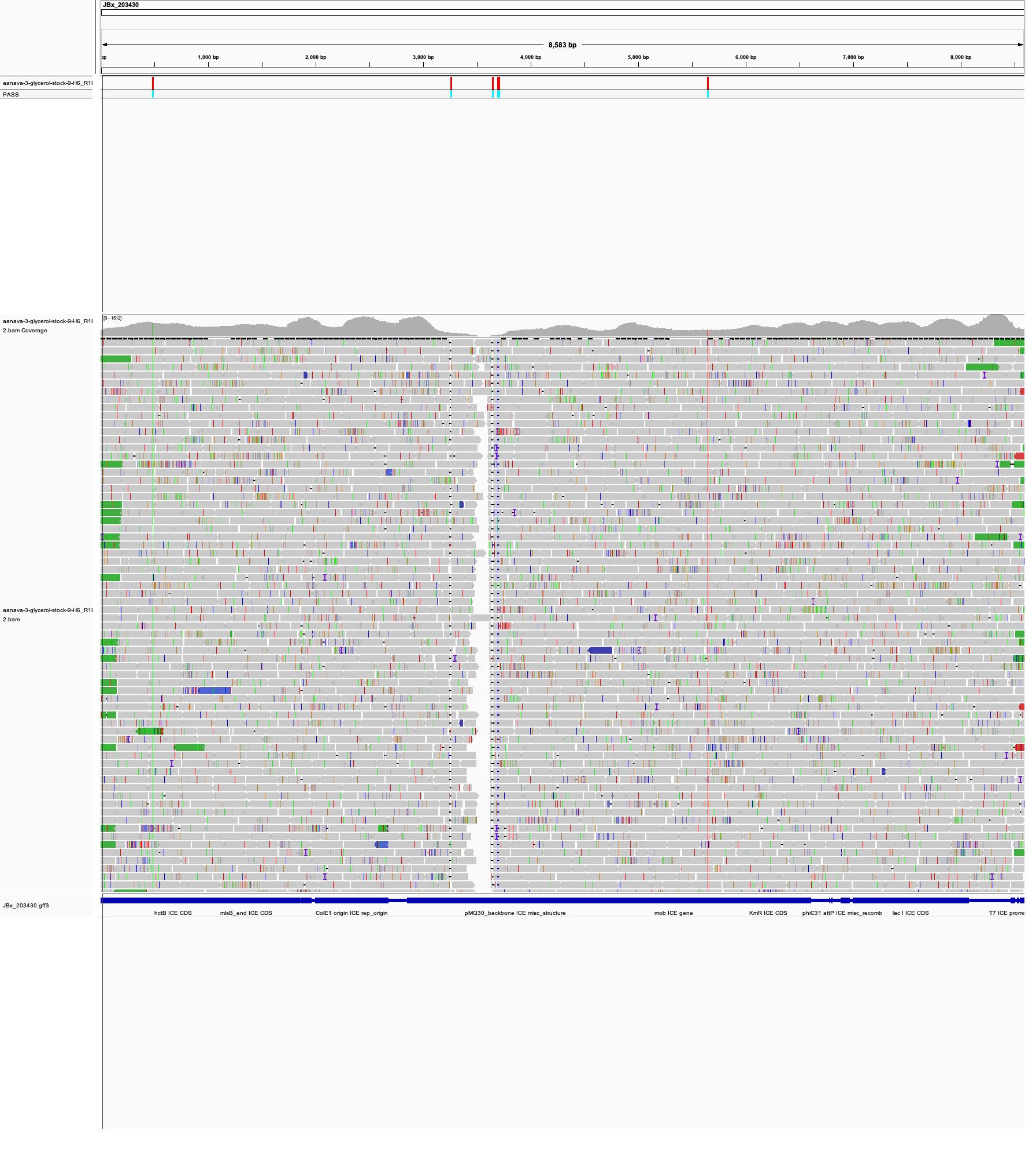

Supplement: Supplementary file 2 — sb3c00292_si_002.zip [file sb3c00292_si_002.zip › dnada_supplementary_material_pks_library_build/divaseq/211117_divaseq_analysis/alberto/snapshots/JBx_203430_nava-3-glycerol-stock-9-H6_R1R2.jpg]

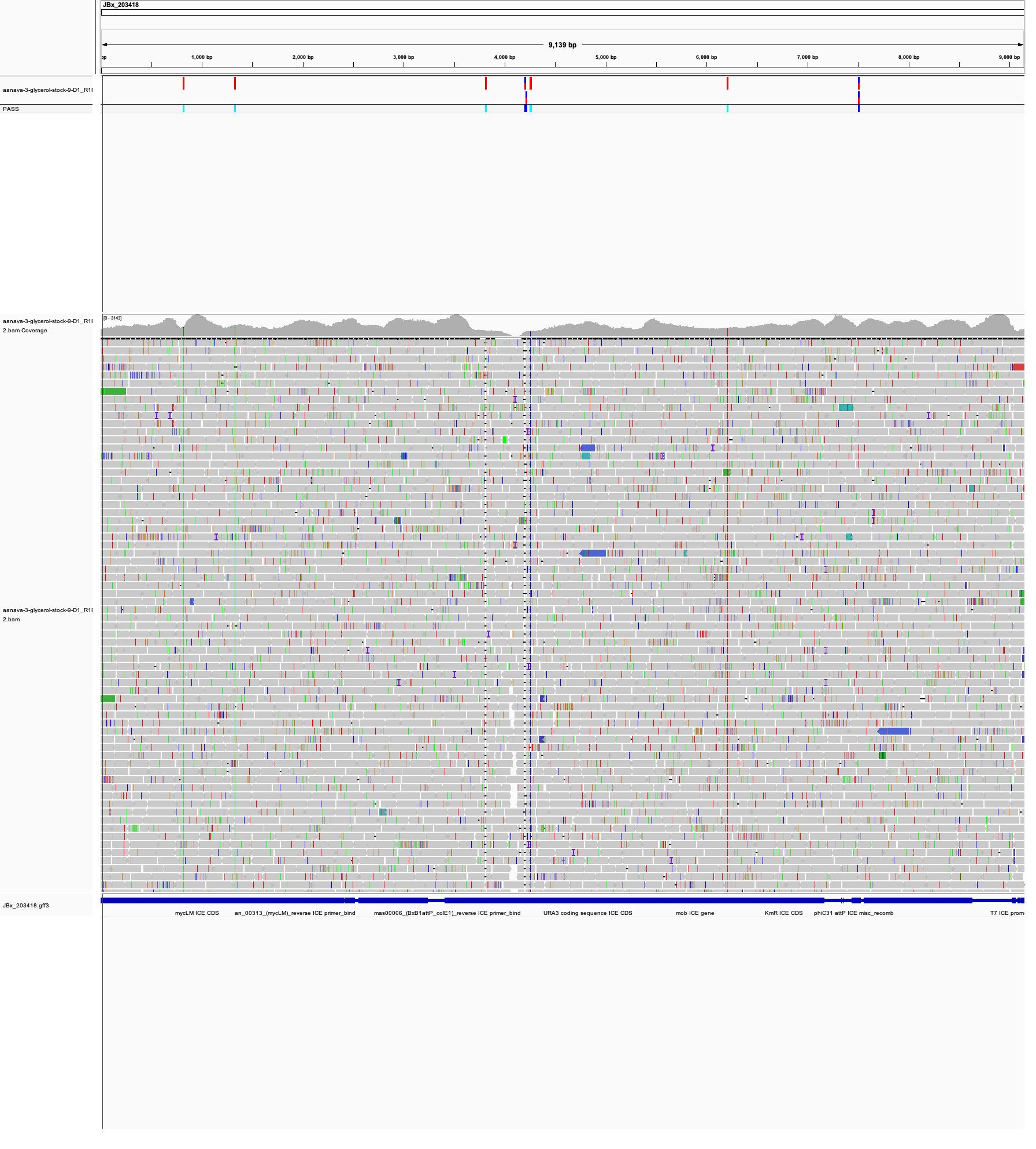

Supplement: Supplementary file 2 — sb3c00292_si_002.zip [file sb3c00292_si_002.zip › dnada_supplementary_material_pks_library_build/divaseq/211117_divaseq_analysis/alberto/snapshots/JBx_203418_nava-3-glycerol-stock-9-D1_R1R2.jpg]

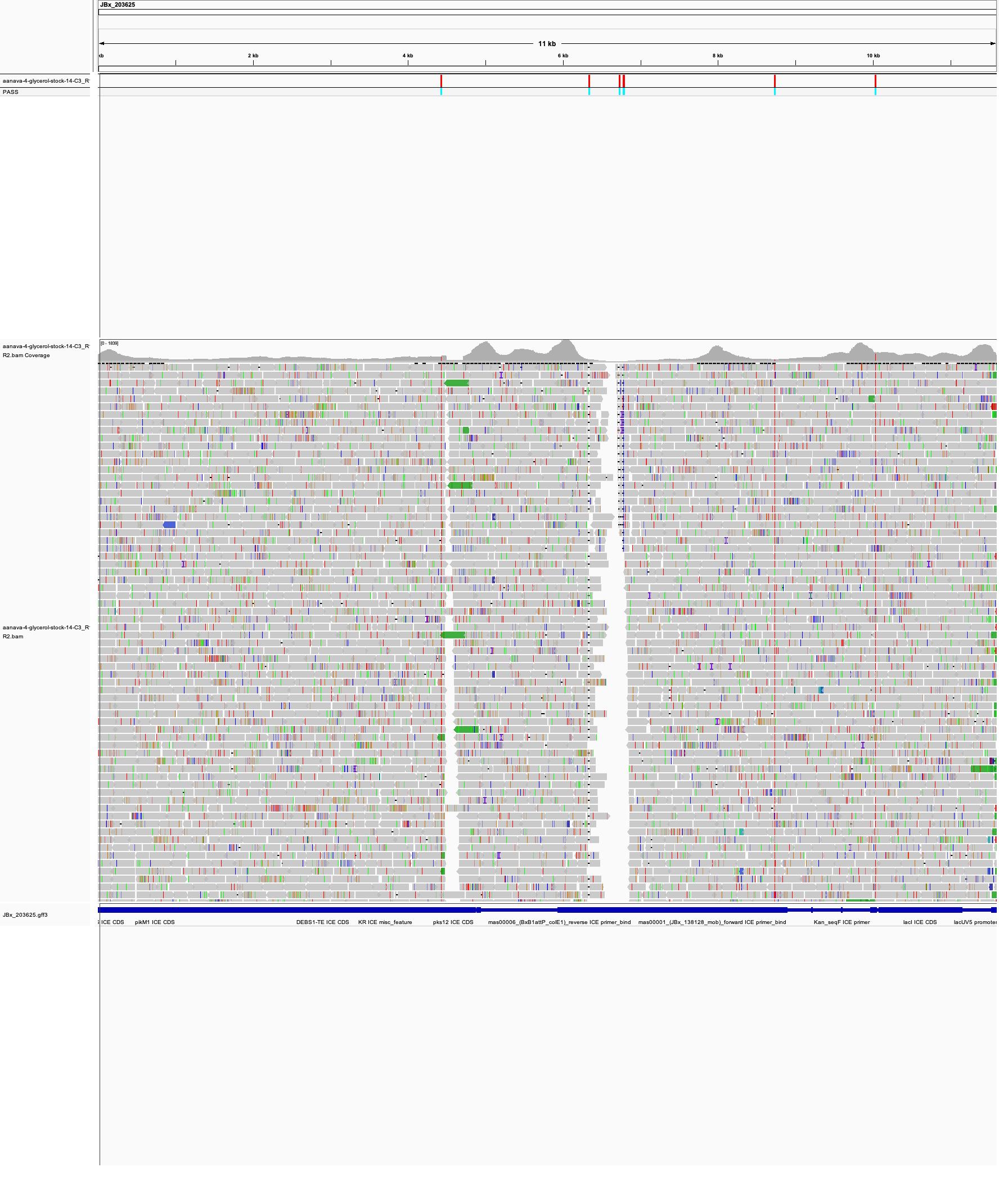

Supplement: Supplementary file 2 — sb3c00292_si_002.zip [file sb3c00292_si_002.zip › dnada_supplementary_material_pks_library_build/divaseq/211117_divaseq_analysis/alberto/snapshots/JBx_203625_nava-4-glycerol-stock-14-C3_R1R2.jpg]

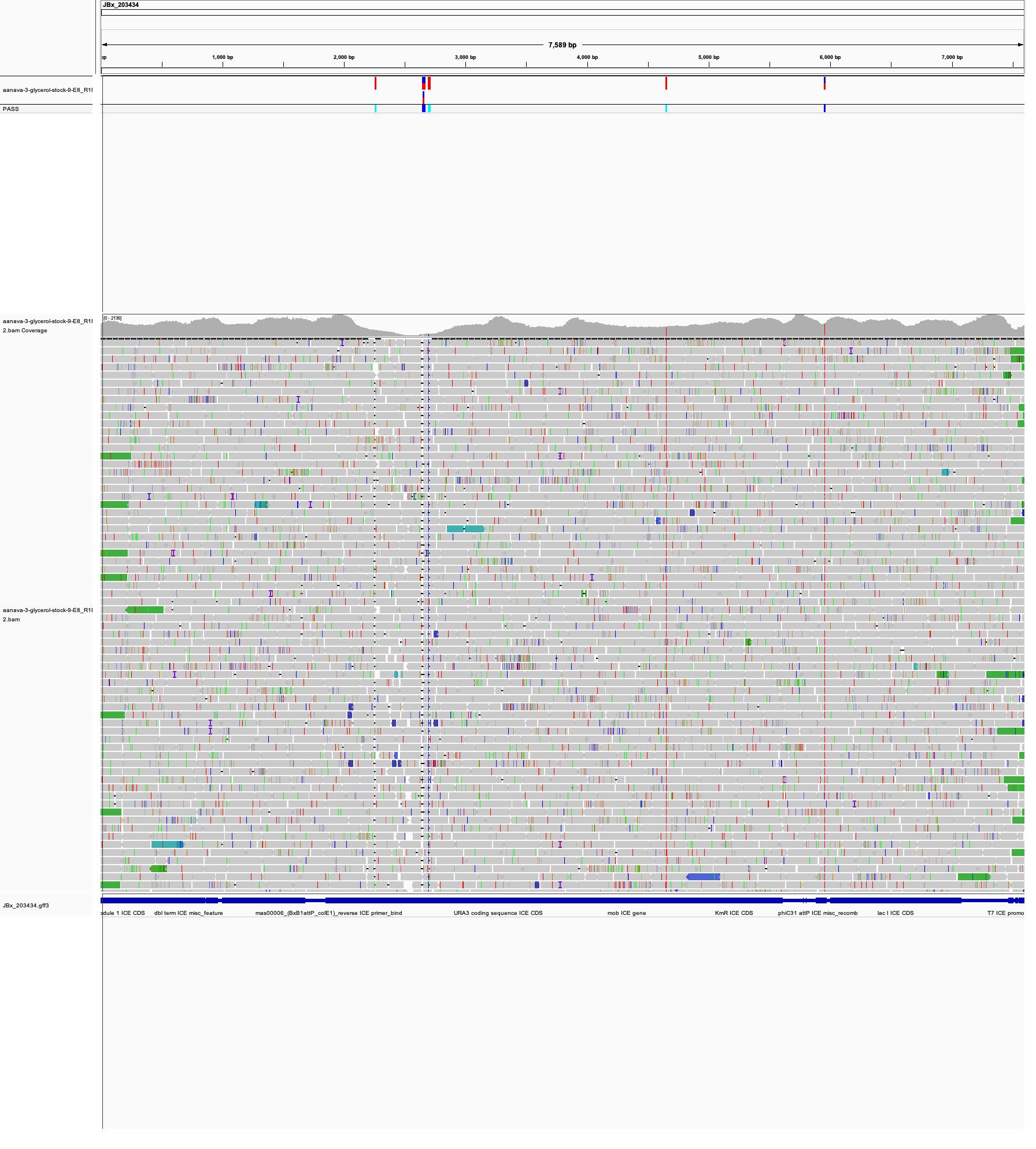

Supplement: Supplementary file 2 — sb3c00292_si_002.zip [file sb3c00292_si_002.zip › dnada_supplementary_material_pks_library_build/divaseq/211117_divaseq_analysis/alberto/snapshots/JBx_203434_nava-3-glycerol-stock-9-E8_R1R2.jpg]

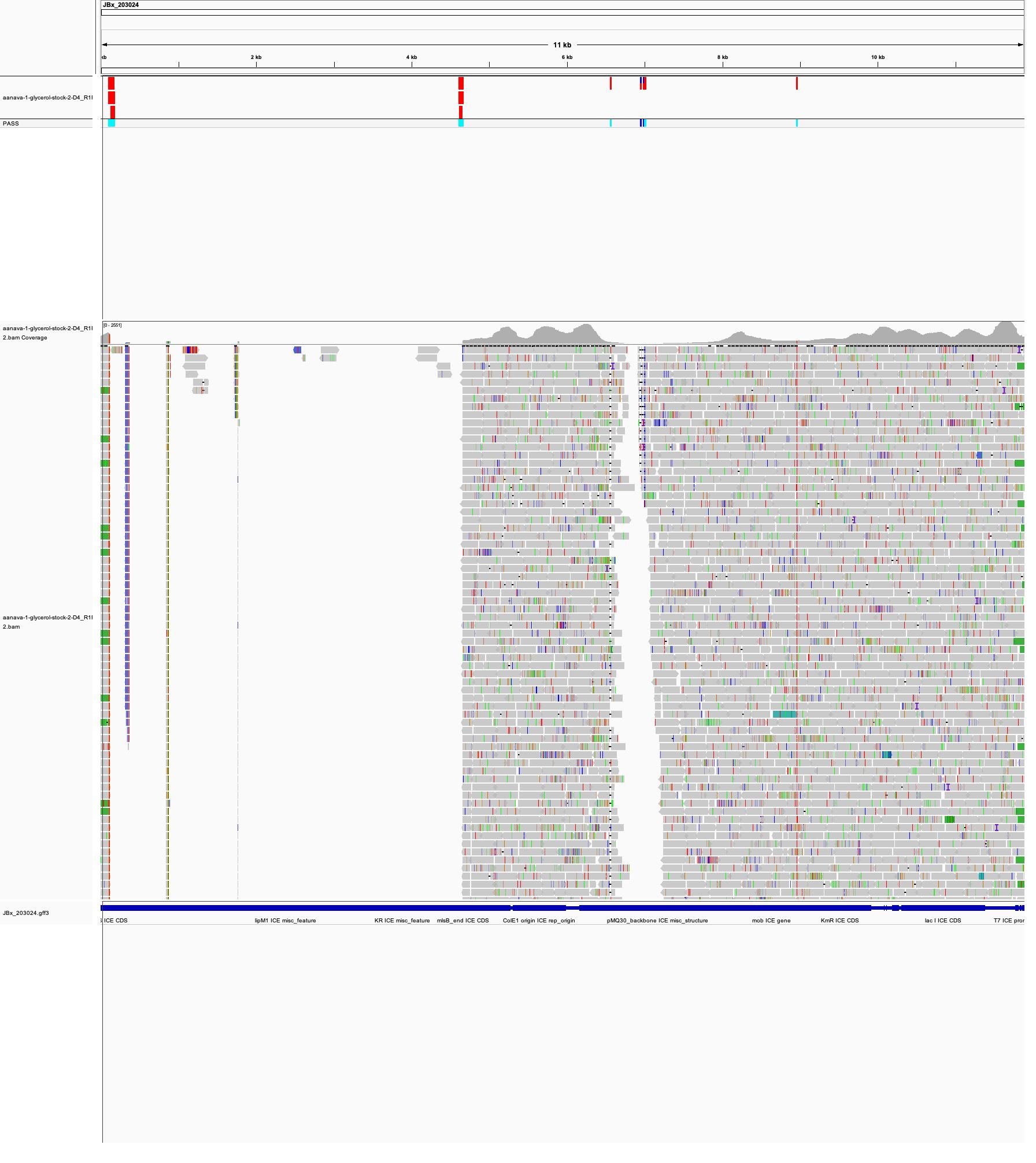

Supplement: Supplementary file 2 — sb3c00292_si_002.zip [file sb3c00292_si_002.zip › dnada_supplementary_material_pks_library_build/divaseq/211117_divaseq_analysis/alberto/snapshots/JBx_203024_nava-1-glycerol-stock-2-D4_R1R2.jpg]

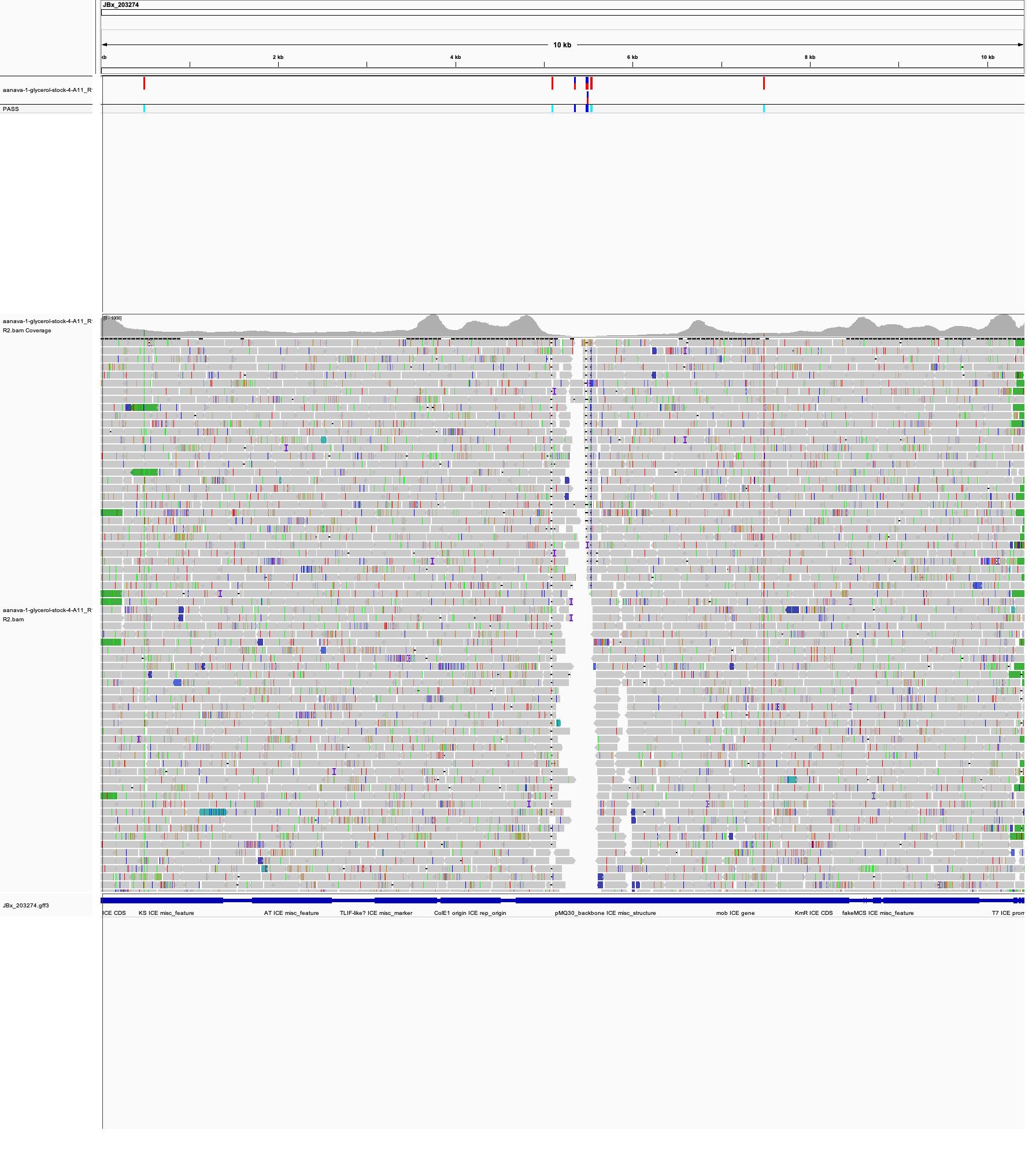

Supplement: Supplementary file 2 — sb3c00292_si_002.zip [file sb3c00292_si_002.zip › dnada_supplementary_material_pks_library_build/divaseq/211117_divaseq_analysis/alberto/snapshots/JBx_203274_nava-1-glycerol-stock-4-A11_R1R2.jpg]

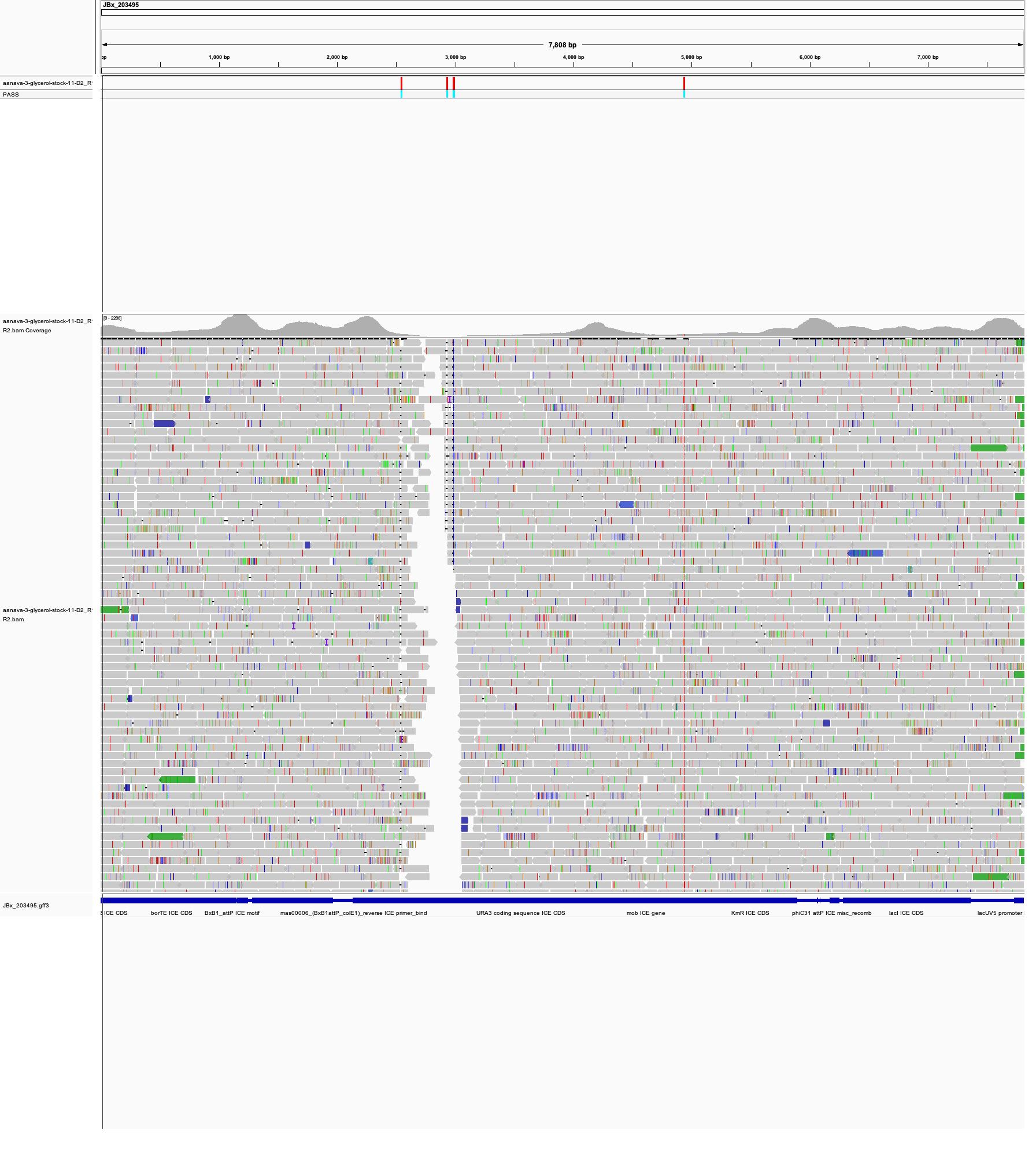

Supplement: Supplementary file 2 — sb3c00292_si_002.zip [file sb3c00292_si_002.zip › dnada_supplementary_material_pks_library_build/divaseq/211117_divaseq_analysis/alberto/snapshots/JBx_203495_nava-3-glycerol-stock-11-D2_R1R2.jpg]

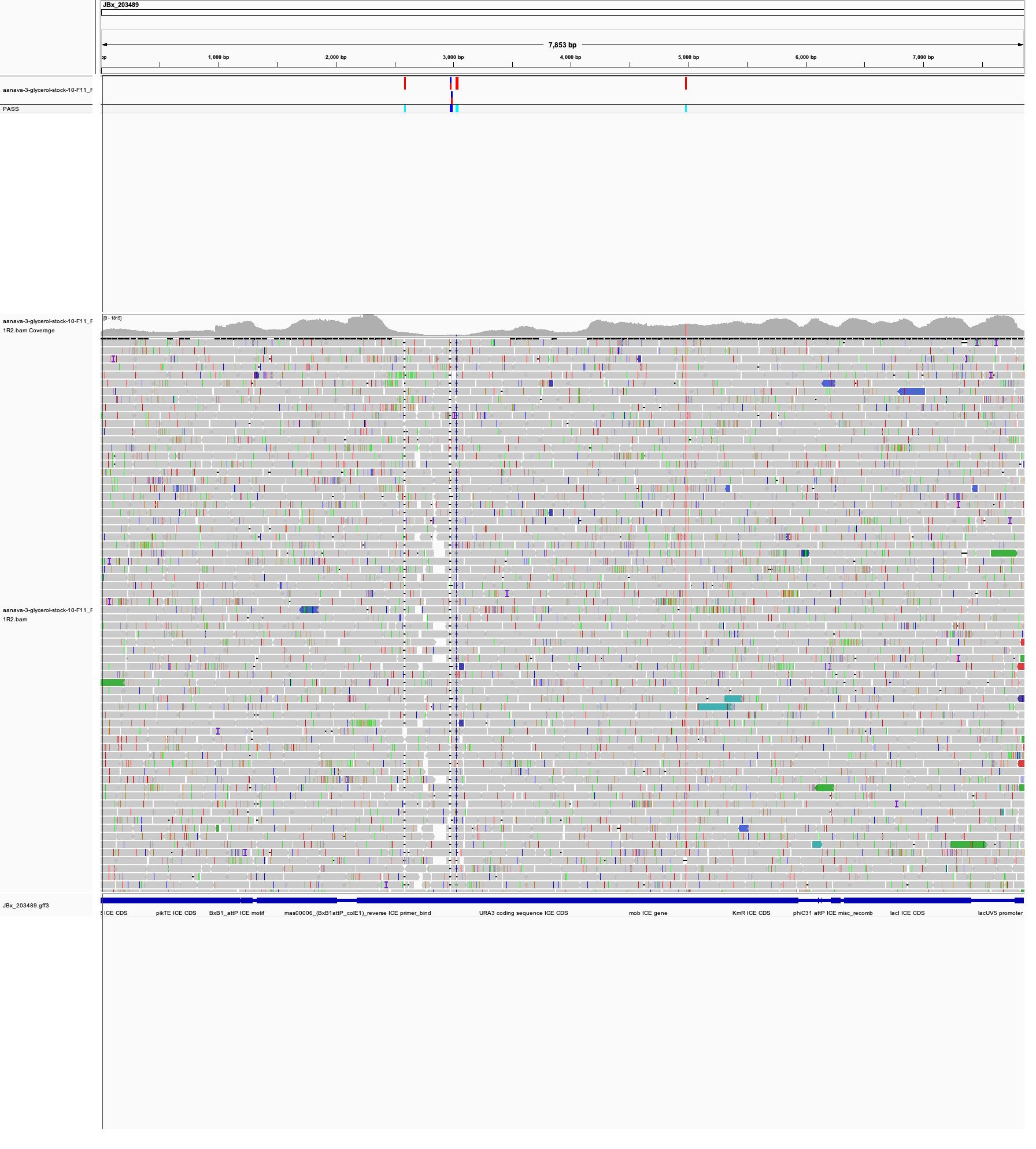

Supplement: Supplementary file 2 — sb3c00292_si_002.zip [file sb3c00292_si_002.zip › dnada_supplementary_material_pks_library_build/divaseq/211117_divaseq_analysis/alberto/snapshots/JBx_203489_nava-3-glycerol-stock-10-F11_R1R2.jpg]

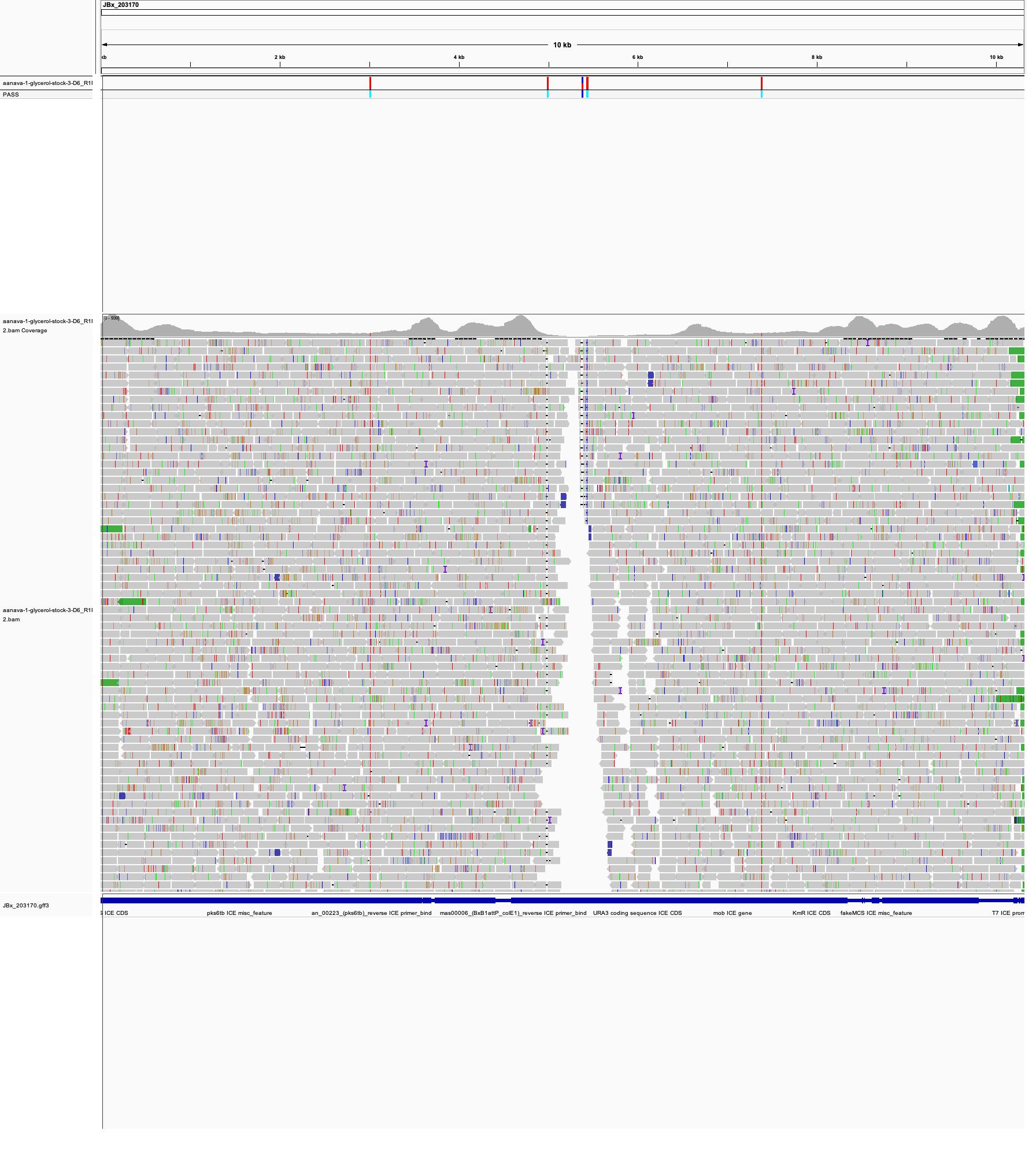

Supplement: Supplementary file 2 — sb3c00292_si_002.zip [file sb3c00292_si_002.zip › dnada_supplementary_material_pks_library_build/divaseq/211117_divaseq_analysis/alberto/snapshots/JBx_203170_nava-1-glycerol-stock-3-D6_R1R2.jpg]

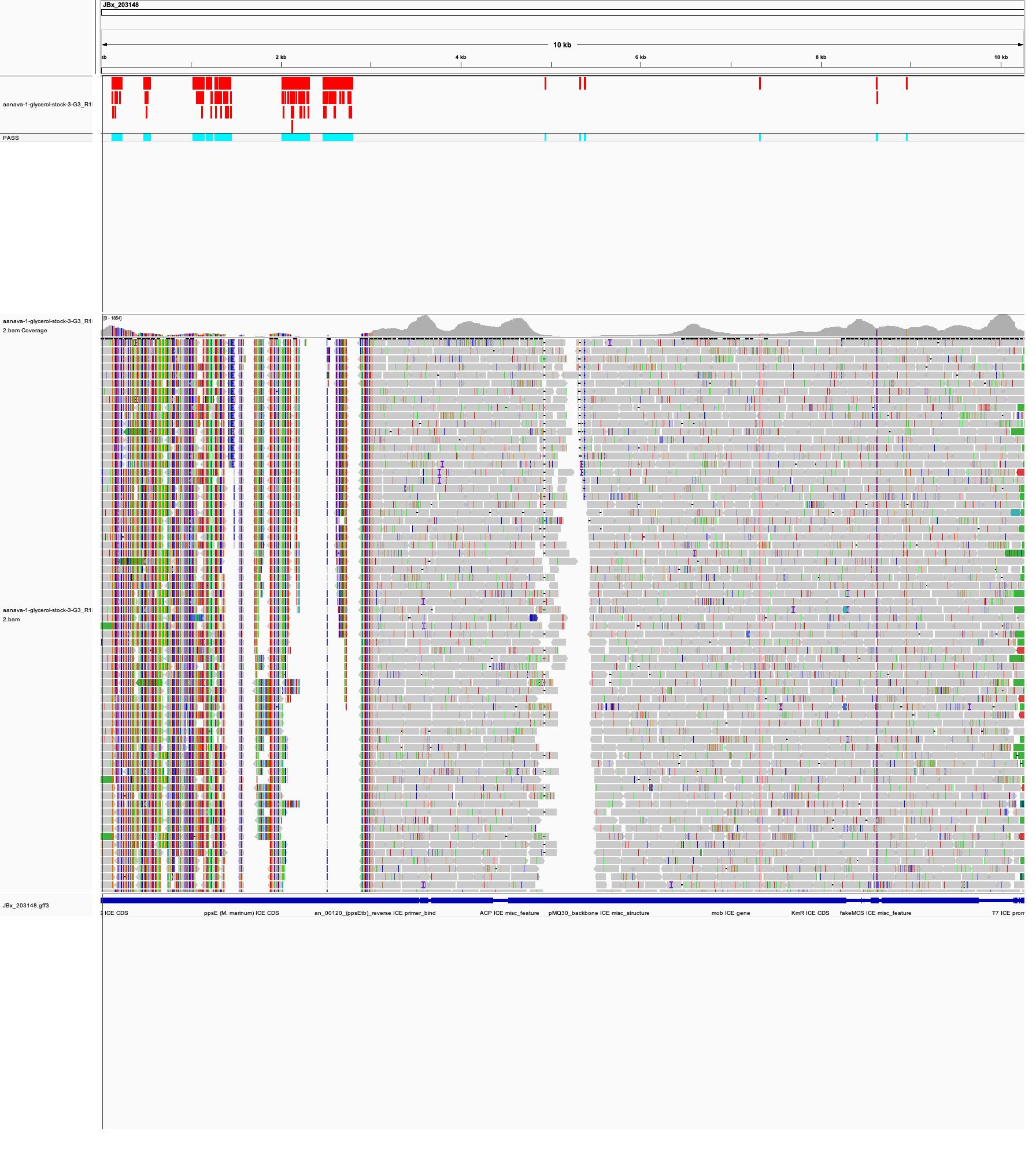

Supplement: Supplementary file 2 — sb3c00292_si_002.zip [file sb3c00292_si_002.zip › dnada_supplementary_material_pks_library_build/divaseq/211117_divaseq_analysis/alberto/snapshots/JBx_203148_nava-1-glycerol-stock-3-G3_R1R2.jpg]

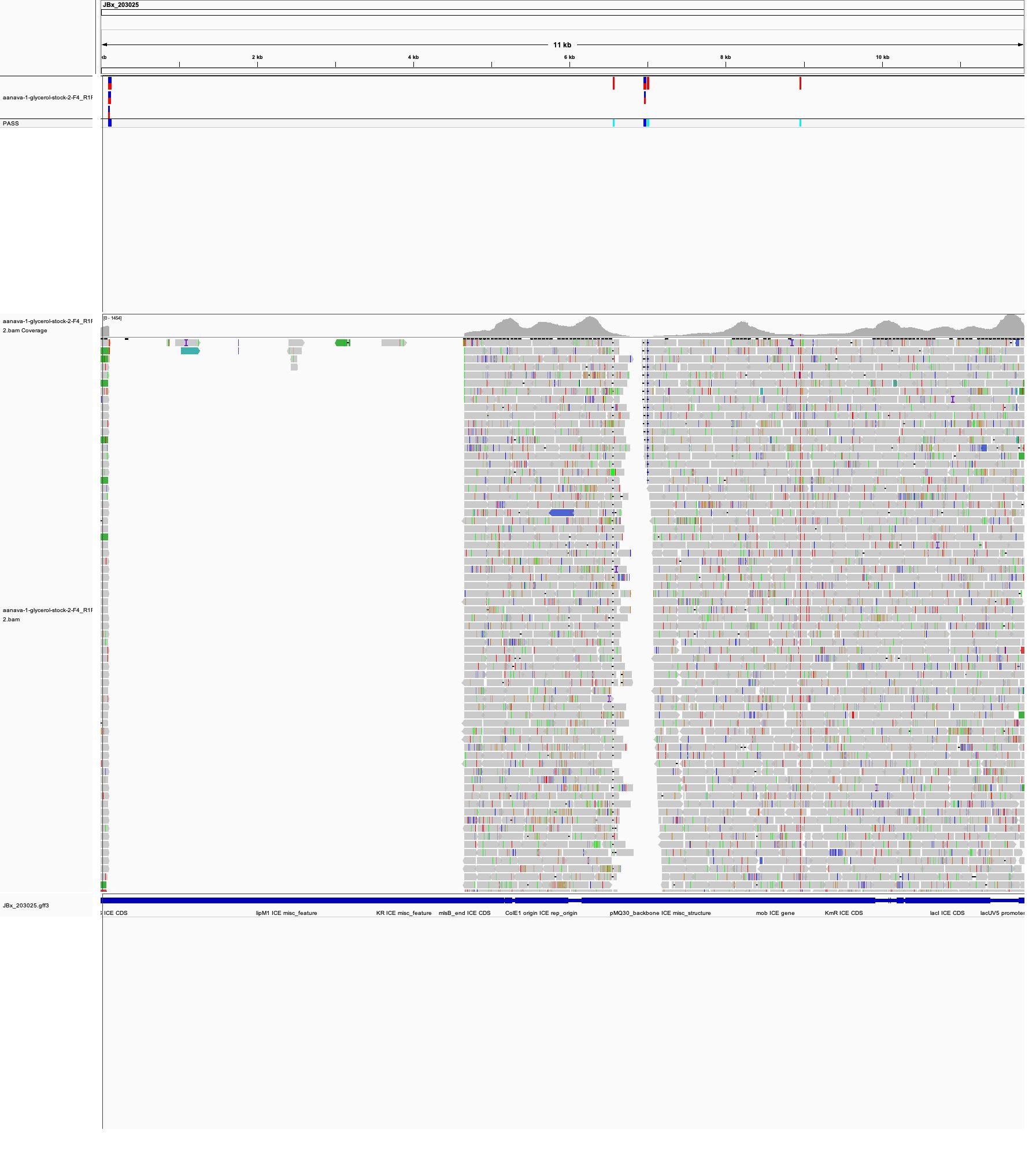

Supplement: Supplementary file 2 — sb3c00292_si_002.zip [file sb3c00292_si_002.zip › dnada_supplementary_material_pks_library_build/divaseq/211117_divaseq_analysis/alberto/snapshots/JBx_203025_nava-1-glycerol-stock-2-F4_R1R2.jpg]

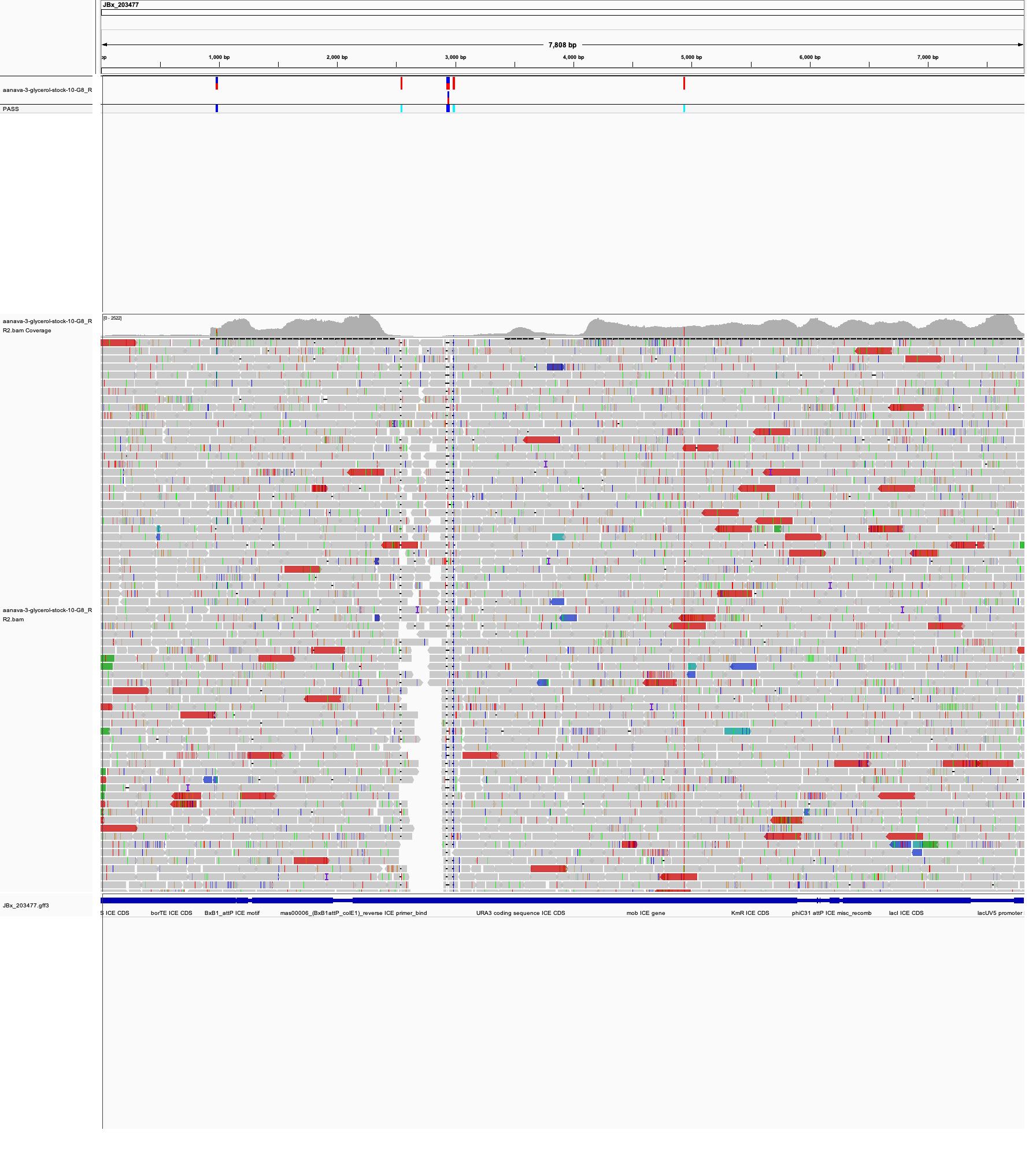

Supplement: Supplementary file 2 — sb3c00292_si_002.zip [file sb3c00292_si_002.zip › dnada_supplementary_material_pks_library_build/divaseq/211117_divaseq_analysis/alberto/snapshots/JBx_203477_nava-3-glycerol-stock-10-G8_R1R2.jpg]

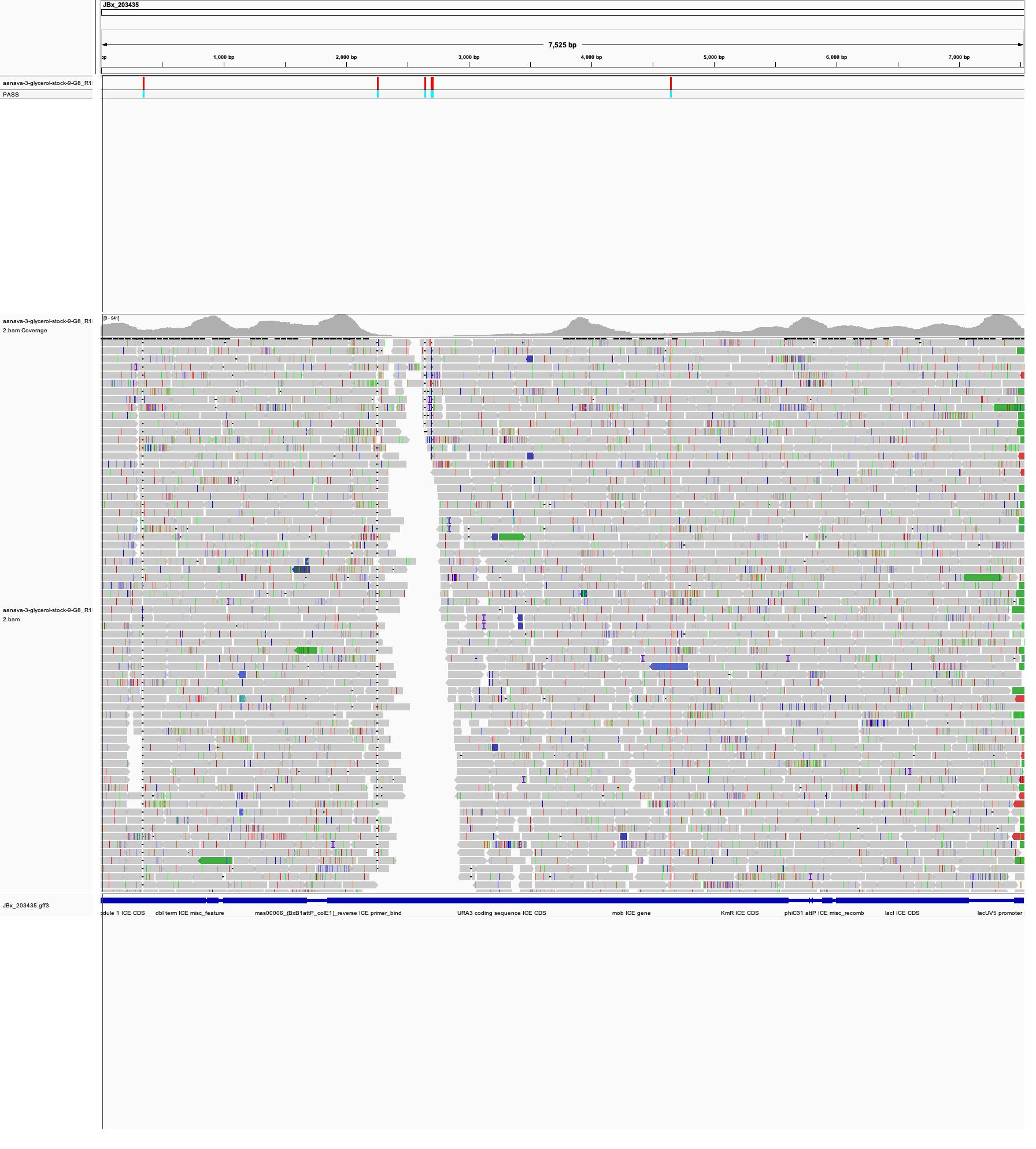

Supplement: Supplementary file 2 — sb3c00292_si_002.zip [file sb3c00292_si_002.zip › dnada_supplementary_material_pks_library_build/divaseq/211117_divaseq_analysis/alberto/snapshots/JBx_203435_nava-3-glycerol-stock-9-G8_R1R2.jpg]

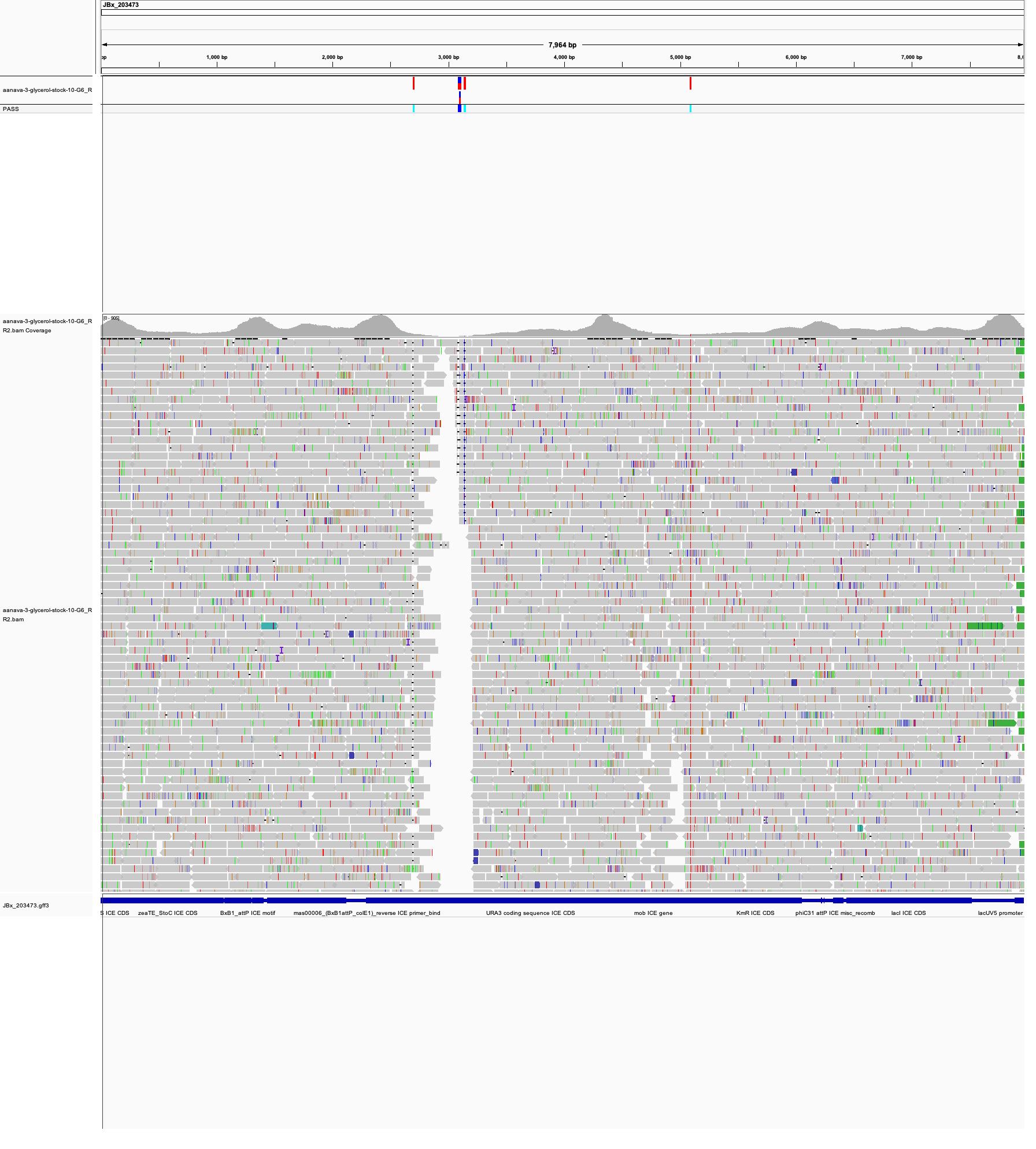

Supplement: Supplementary file 2 — sb3c00292_si_002.zip [file sb3c00292_si_002.zip › dnada_supplementary_material_pks_library_build/divaseq/211117_divaseq_analysis/alberto/snapshots/JBx_203473_nava-3-glycerol-stock-10-G6_R1R2.jpg]

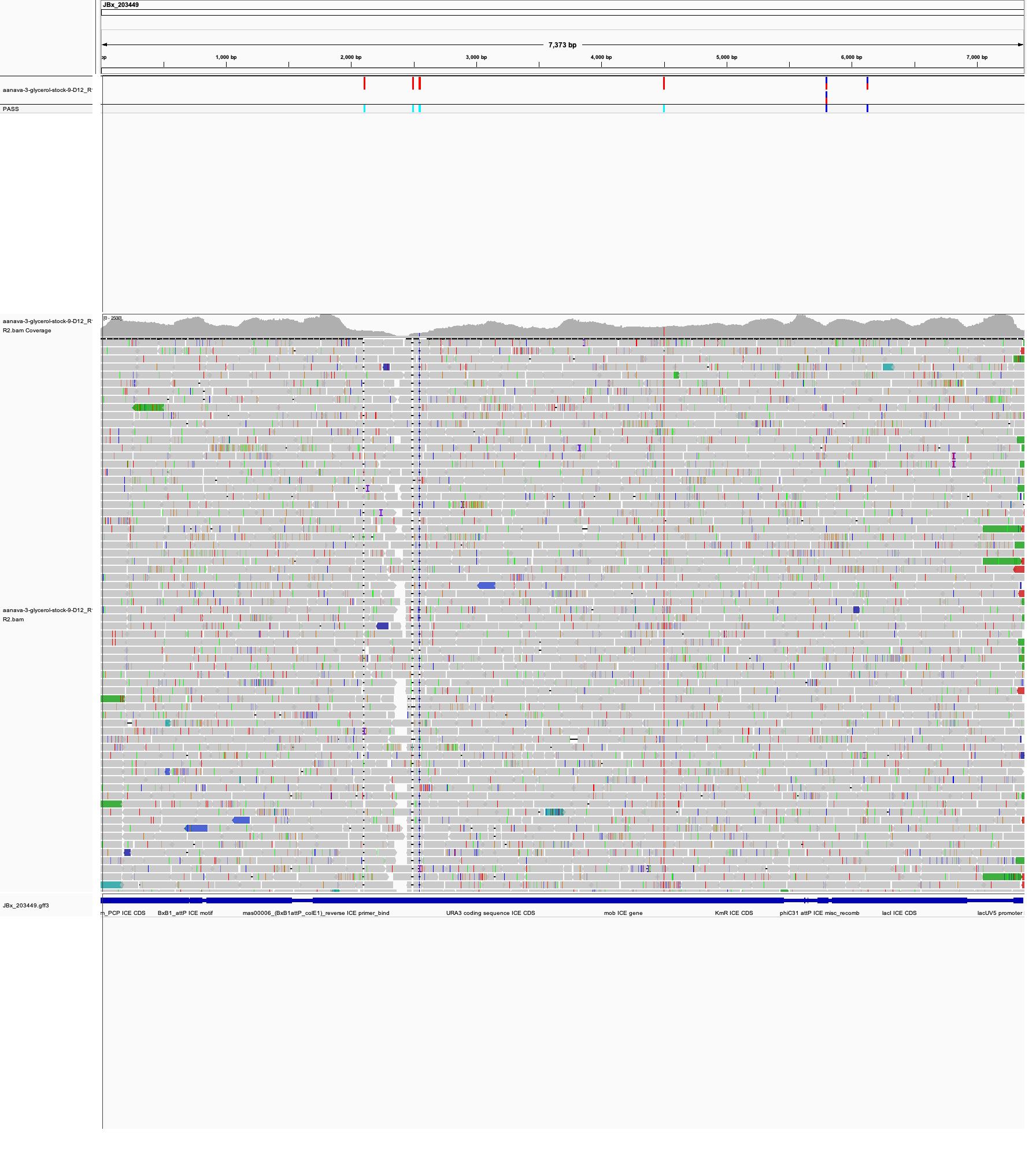

Supplement: Supplementary file 2 — sb3c00292_si_002.zip [file sb3c00292_si_002.zip › dnada_supplementary_material_pks_library_build/divaseq/211117_divaseq_analysis/alberto/snapshots/JBx_203449_nava-3-glycerol-stock-9-D12_R1R2.jpg]

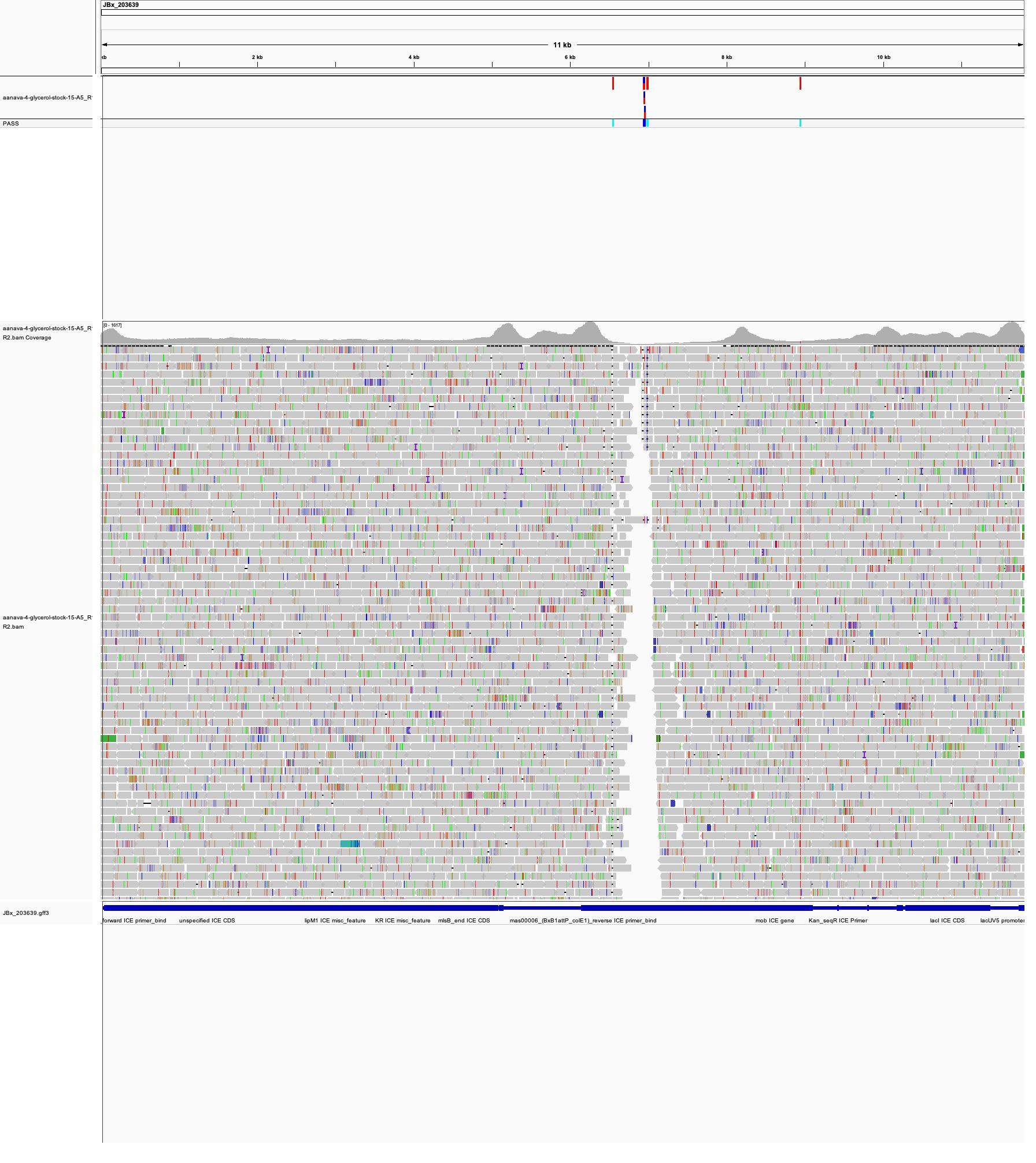

Supplement: Supplementary file 2 — sb3c00292_si_002.zip [file sb3c00292_si_002.zip › dnada_supplementary_material_pks_library_build/divaseq/211117_divaseq_analysis/alberto/snapshots/JBx_203639_nava-4-glycerol-stock-15-A5_R1R2.jpg]

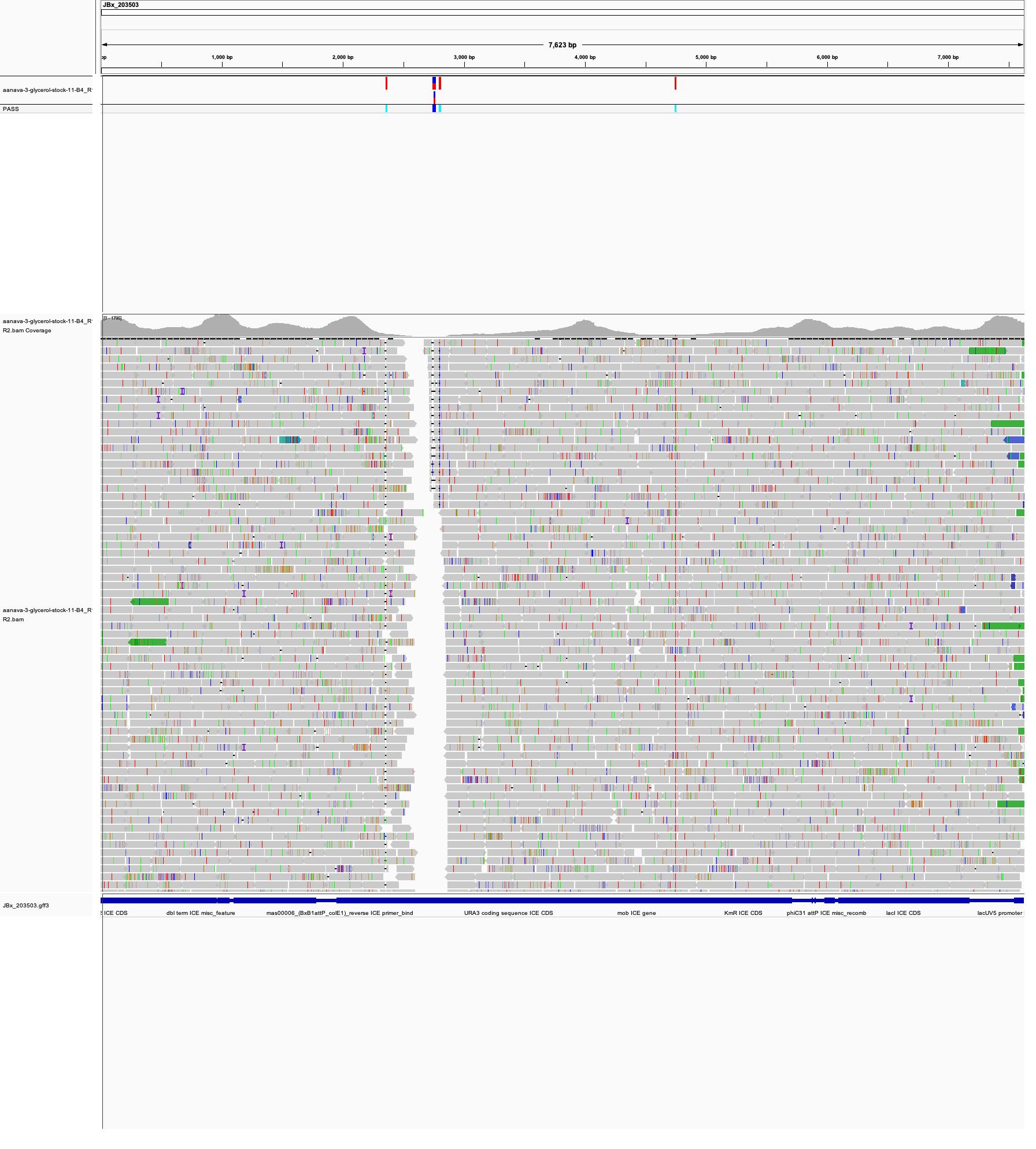

Supplement: Supplementary file 2 — sb3c00292_si_002.zip [file sb3c00292_si_002.zip › dnada_supplementary_material_pks_library_build/divaseq/211117_divaseq_analysis/alberto/snapshots/JBx_203503_nava-3-glycerol-stock-11-B4_R1R2.jpg]

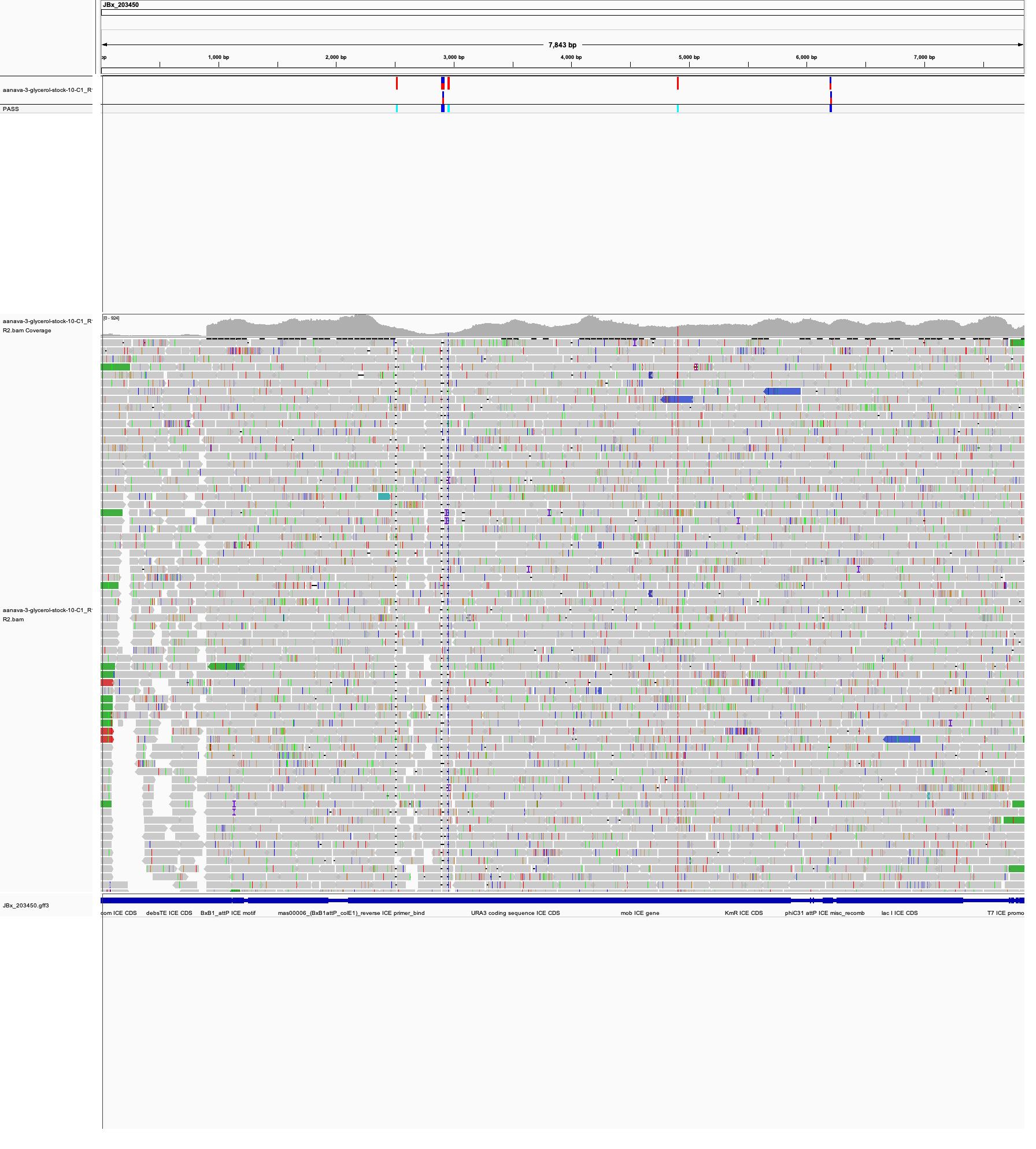

Supplement: Supplementary file 2 — sb3c00292_si_002.zip [file sb3c00292_si_002.zip › dnada_supplementary_material_pks_library_build/divaseq/211117_divaseq_analysis/alberto/snapshots/JBx_203450_nava-3-glycerol-stock-10-C1_R1R2.jpg]

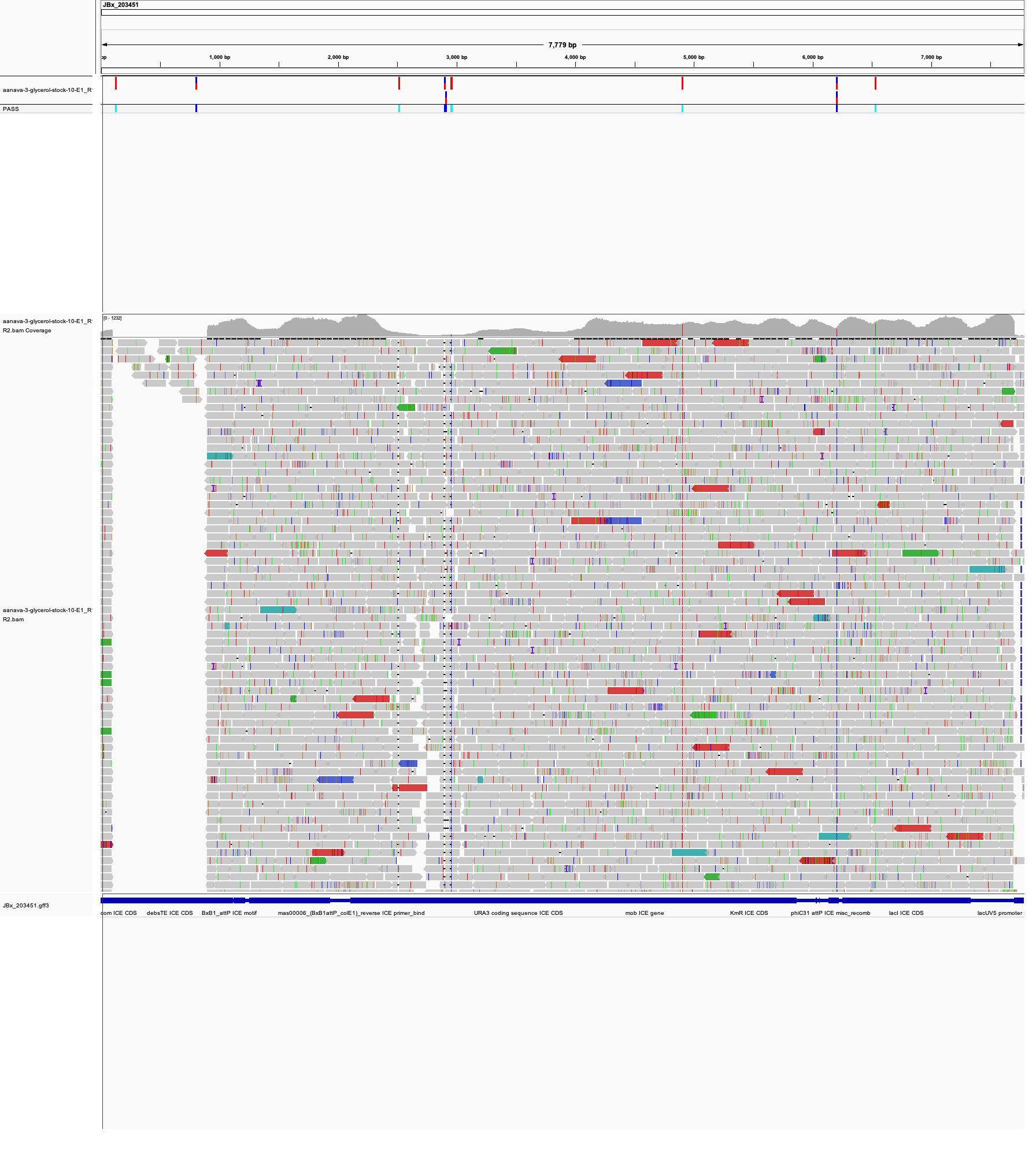

Supplement: Supplementary file 2 — sb3c00292_si_002.zip [file sb3c00292_si_002.zip › dnada_supplementary_material_pks_library_build/divaseq/211117_divaseq_analysis/alberto/snapshots/JBx_203451_nava-3-glycerol-stock-10-E1_R1R2.jpg]

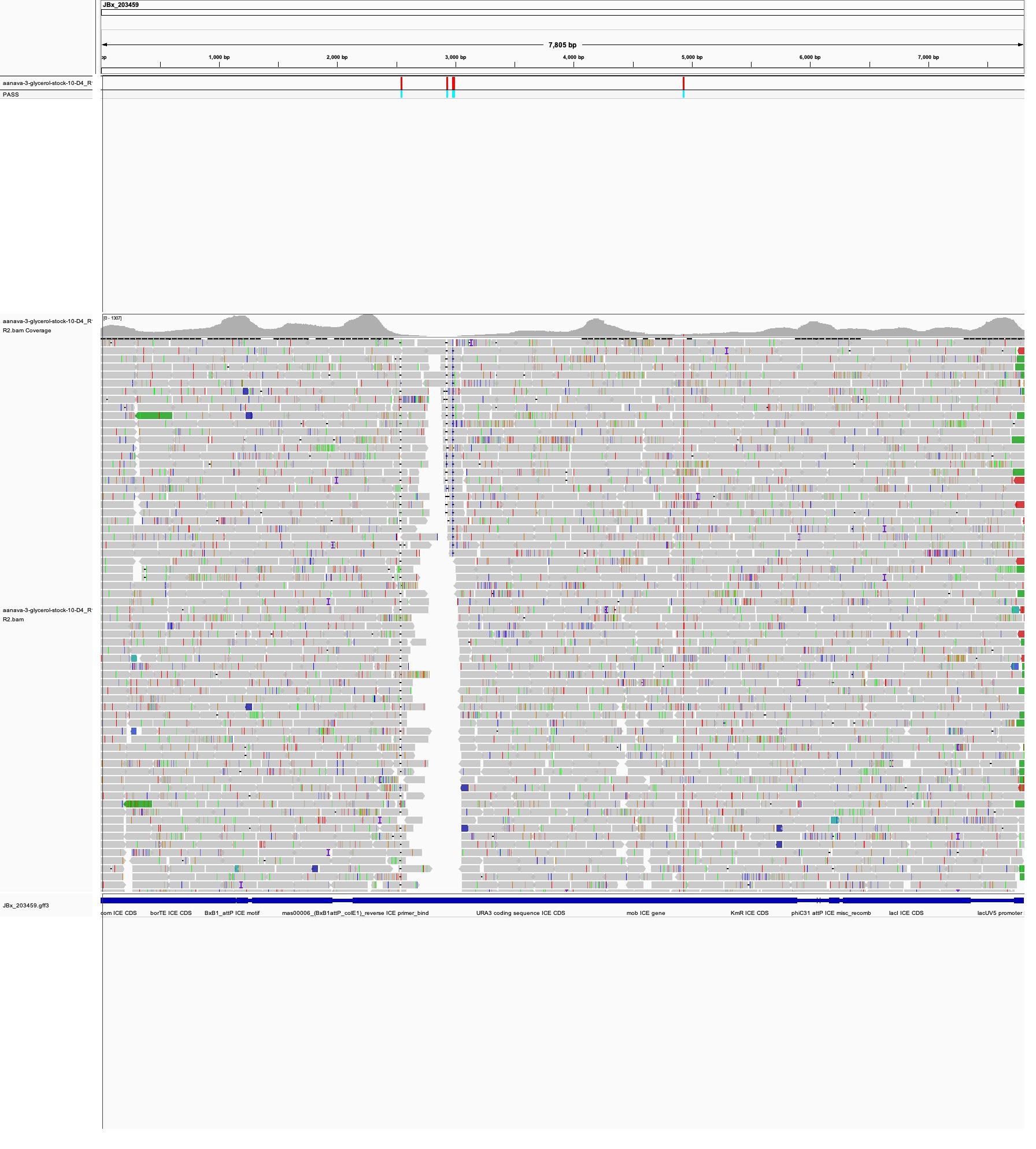

Supplement: Supplementary file 2 — sb3c00292_si_002.zip [file sb3c00292_si_002.zip › dnada_supplementary_material_pks_library_build/divaseq/211117_divaseq_analysis/alberto/snapshots/JBx_203459_nava-3-glycerol-stock-10-D4_R1R2.jpg]

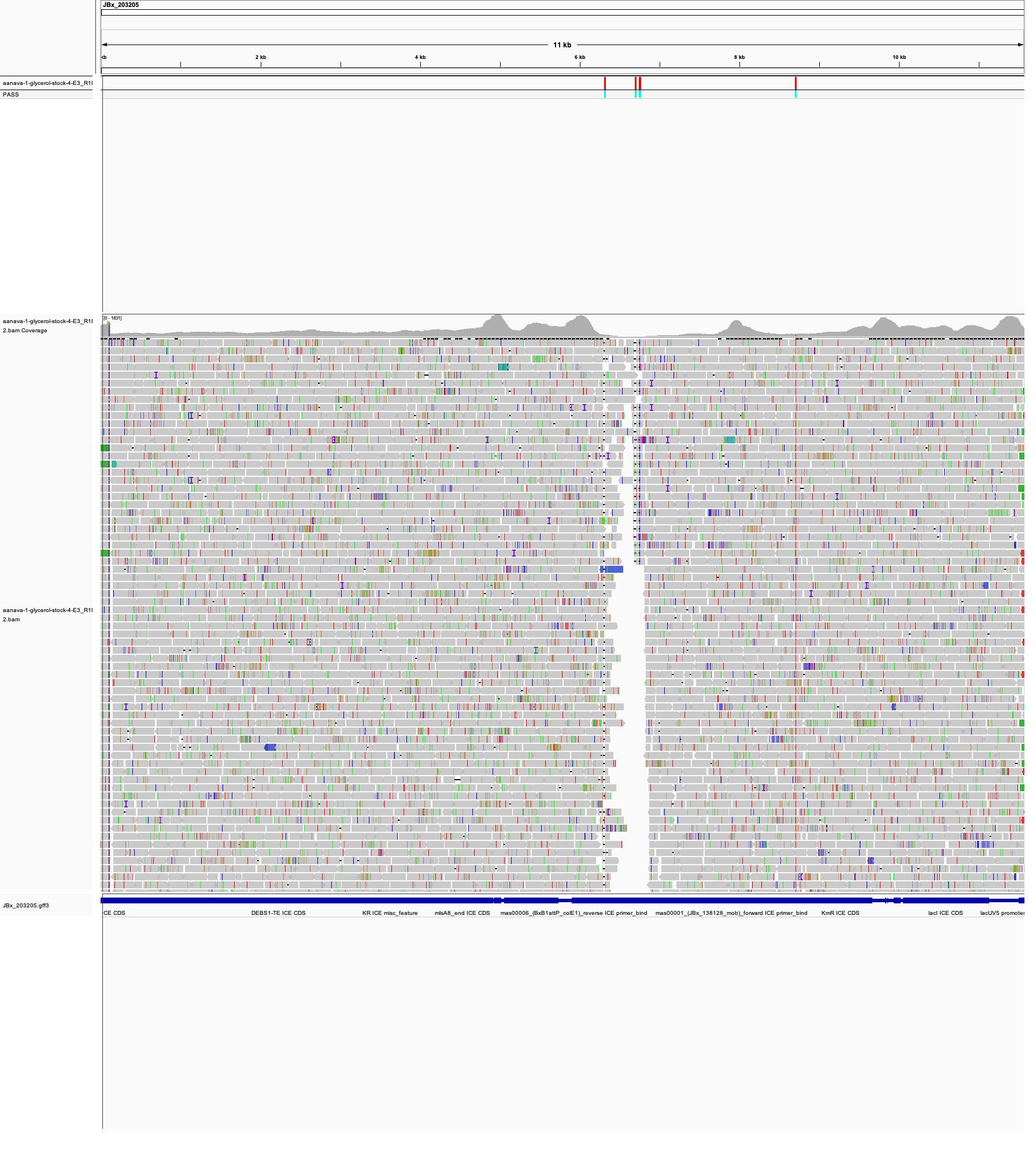

Supplement: Supplementary file 2 — sb3c00292_si_002.zip [file sb3c00292_si_002.zip › dnada_supplementary_material_pks_library_build/divaseq/211117_divaseq_analysis/alberto/snapshots/JBx_203205_nava-1-glycerol-stock-4-E3_R1R2.jpg]

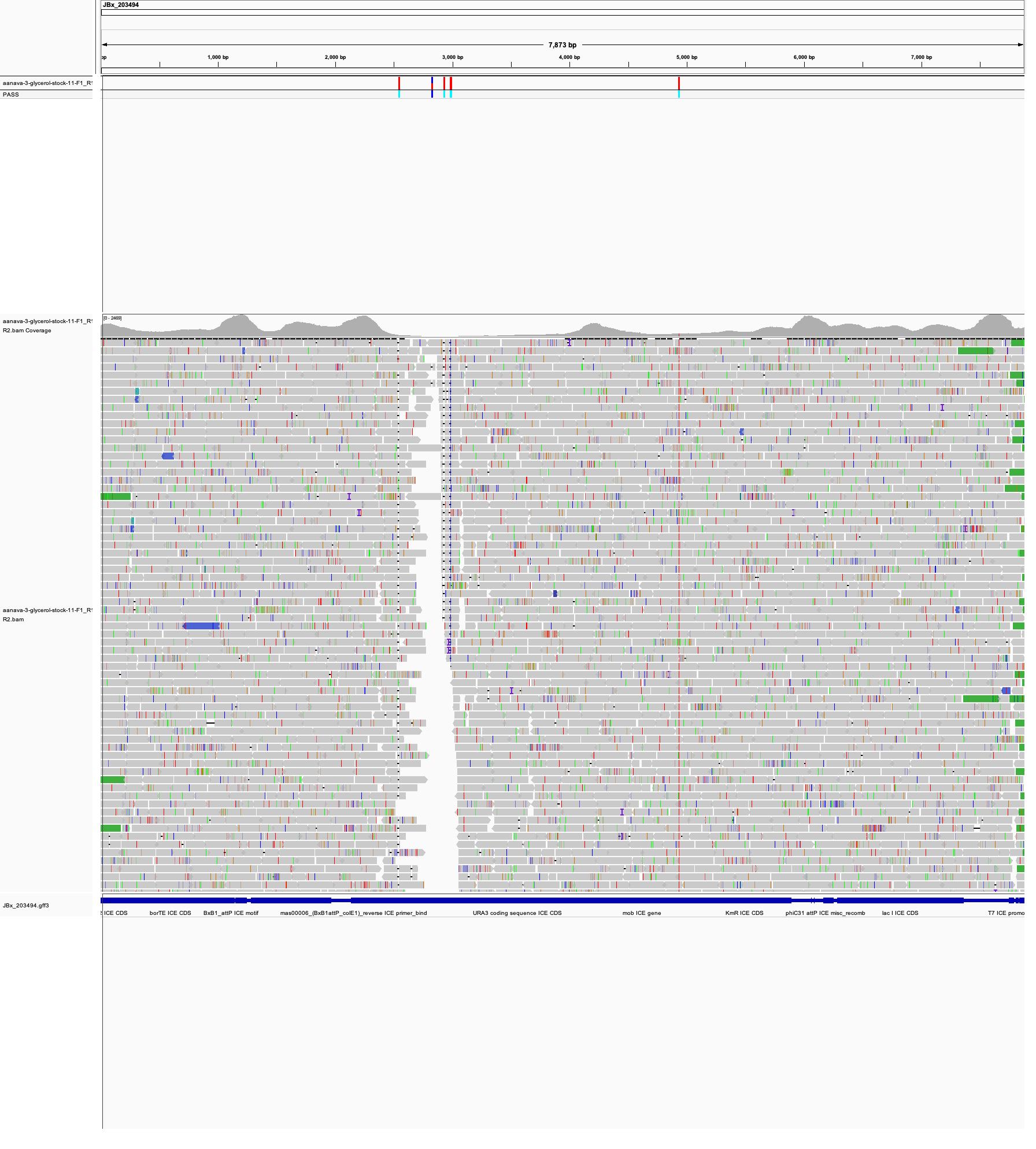

Supplement: Supplementary file 2 — sb3c00292_si_002.zip [file sb3c00292_si_002.zip › dnada_supplementary_material_pks_library_build/divaseq/211117_divaseq_analysis/alberto/snapshots/JBx_203494_nava-3-glycerol-stock-11-F1_R1R2.jpg]

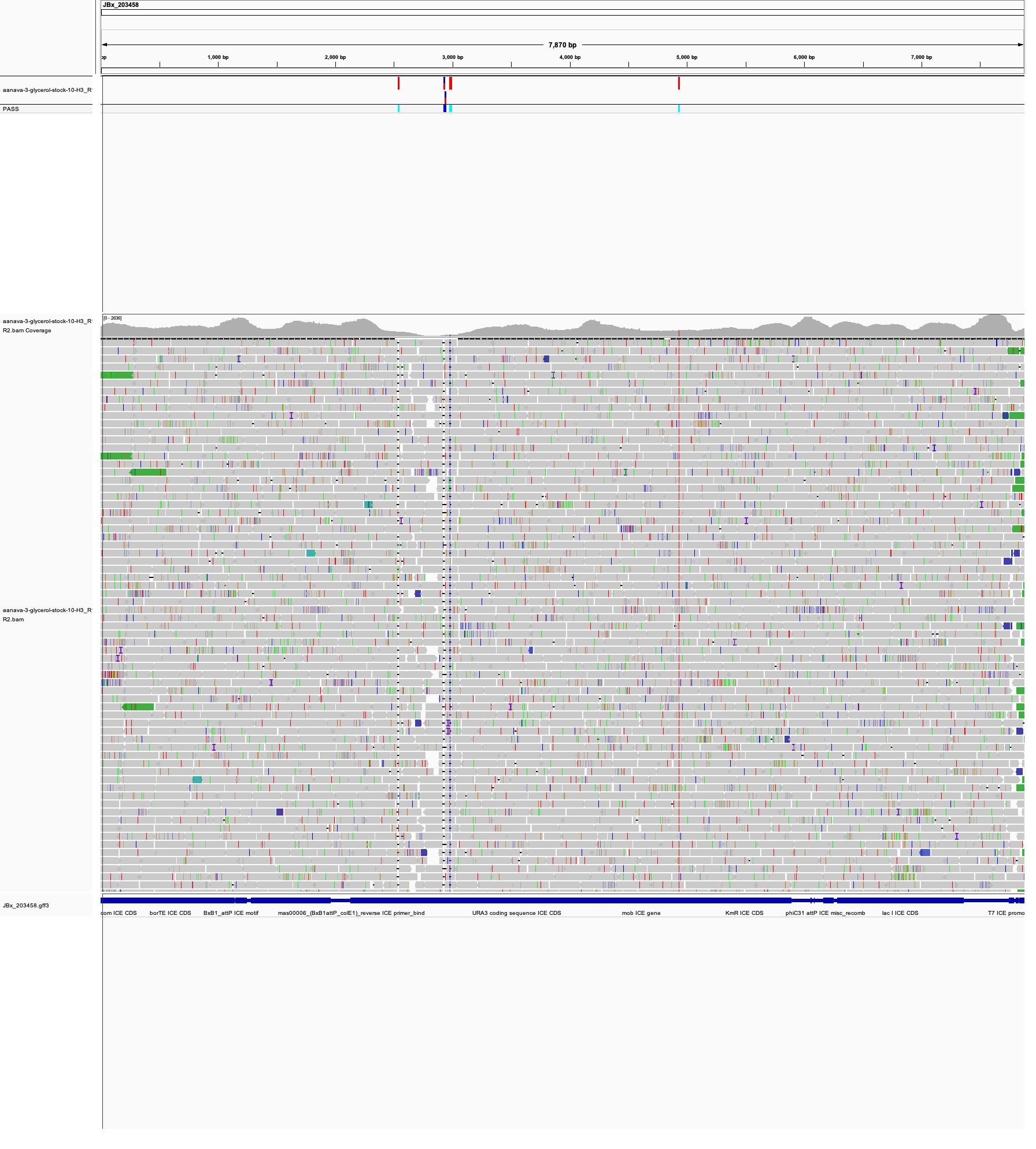

Supplement: Supplementary file 2 — sb3c00292_si_002.zip [file sb3c00292_si_002.zip › dnada_supplementary_material_pks_library_build/divaseq/211117_divaseq_analysis/alberto/snapshots/JBx_203458_nava-3-glycerol-stock-10-H3_R1R2.jpg]

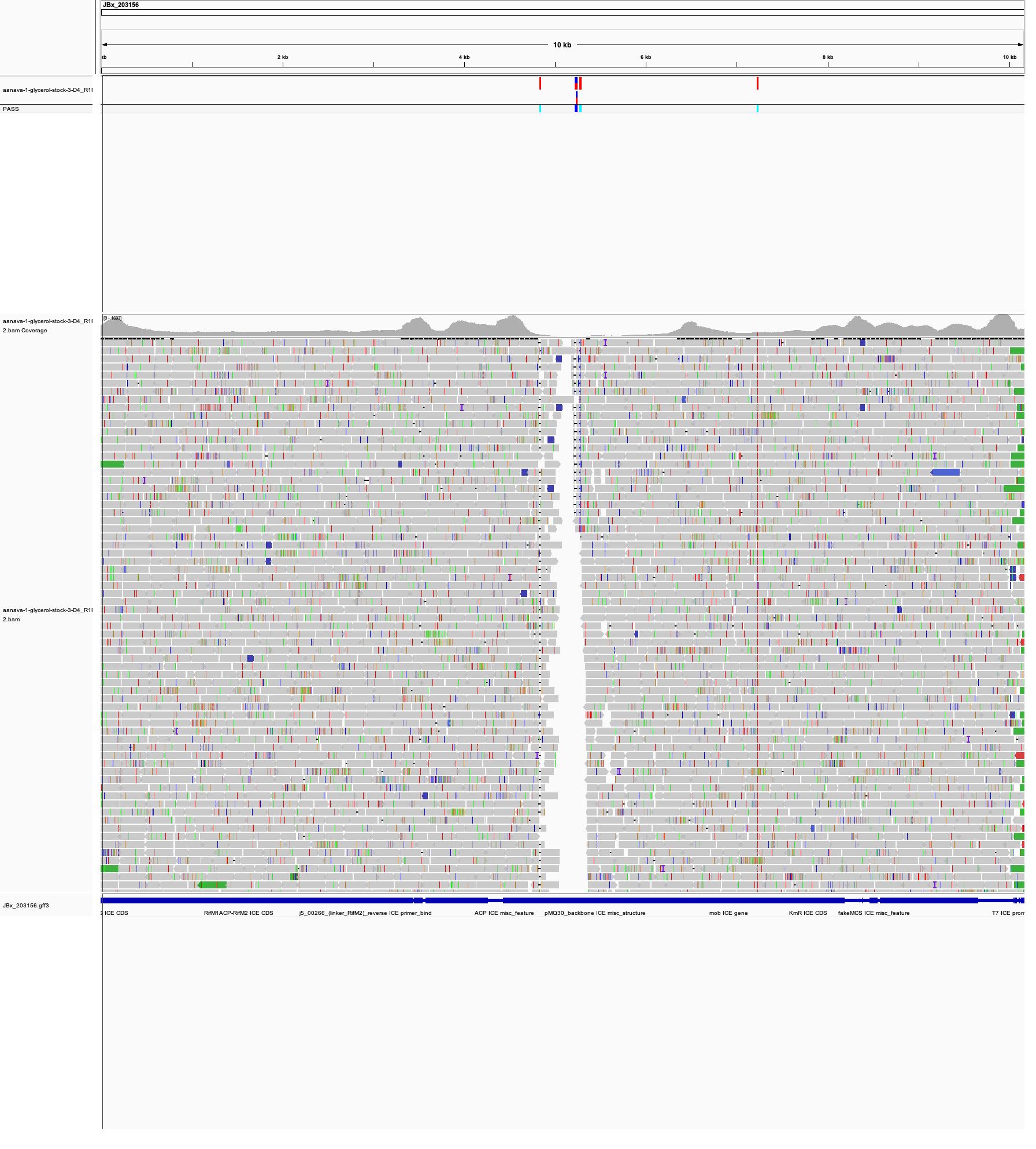

Supplement: Supplementary file 2 — sb3c00292_si_002.zip [file sb3c00292_si_002.zip › dnada_supplementary_material_pks_library_build/divaseq/211117_divaseq_analysis/alberto/snapshots/JBx_203156_nava-1-glycerol-stock-3-D4_R1R2.jpg]

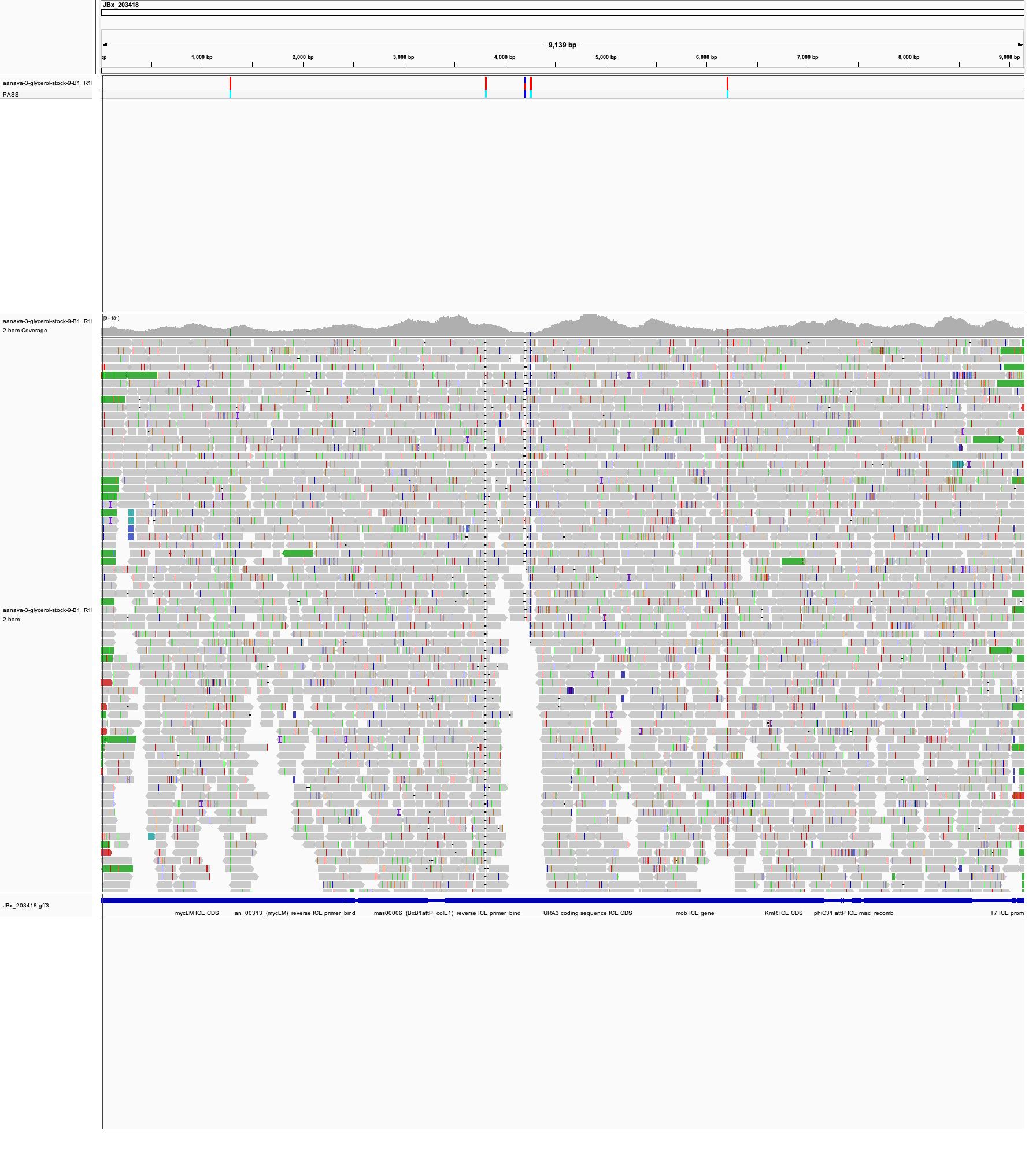

Supplement: Supplementary file 2 — sb3c00292_si_002.zip [file sb3c00292_si_002.zip › dnada_supplementary_material_pks_library_build/divaseq/211117_divaseq_analysis/alberto/snapshots/JBx_203418_nava-3-glycerol-stock-9-B1_R1R2.jpg]

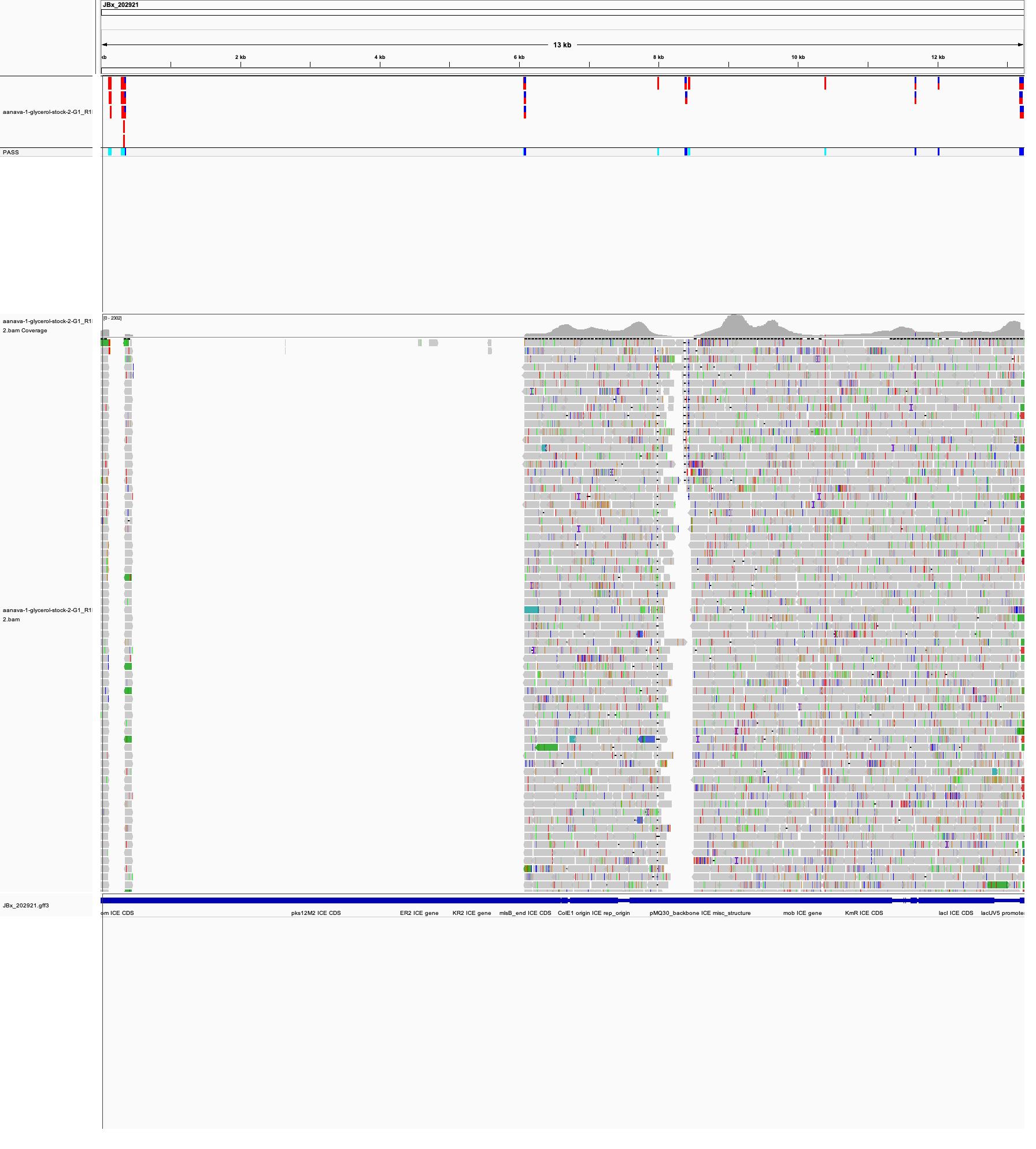

Supplement: Supplementary file 2 — sb3c00292_si_002.zip [file sb3c00292_si_002.zip › dnada_supplementary_material_pks_library_build/divaseq/211117_divaseq_analysis/alberto/snapshots/JBx_202921_nava-1-glycerol-stock-2-G1_R1R2.jpg]

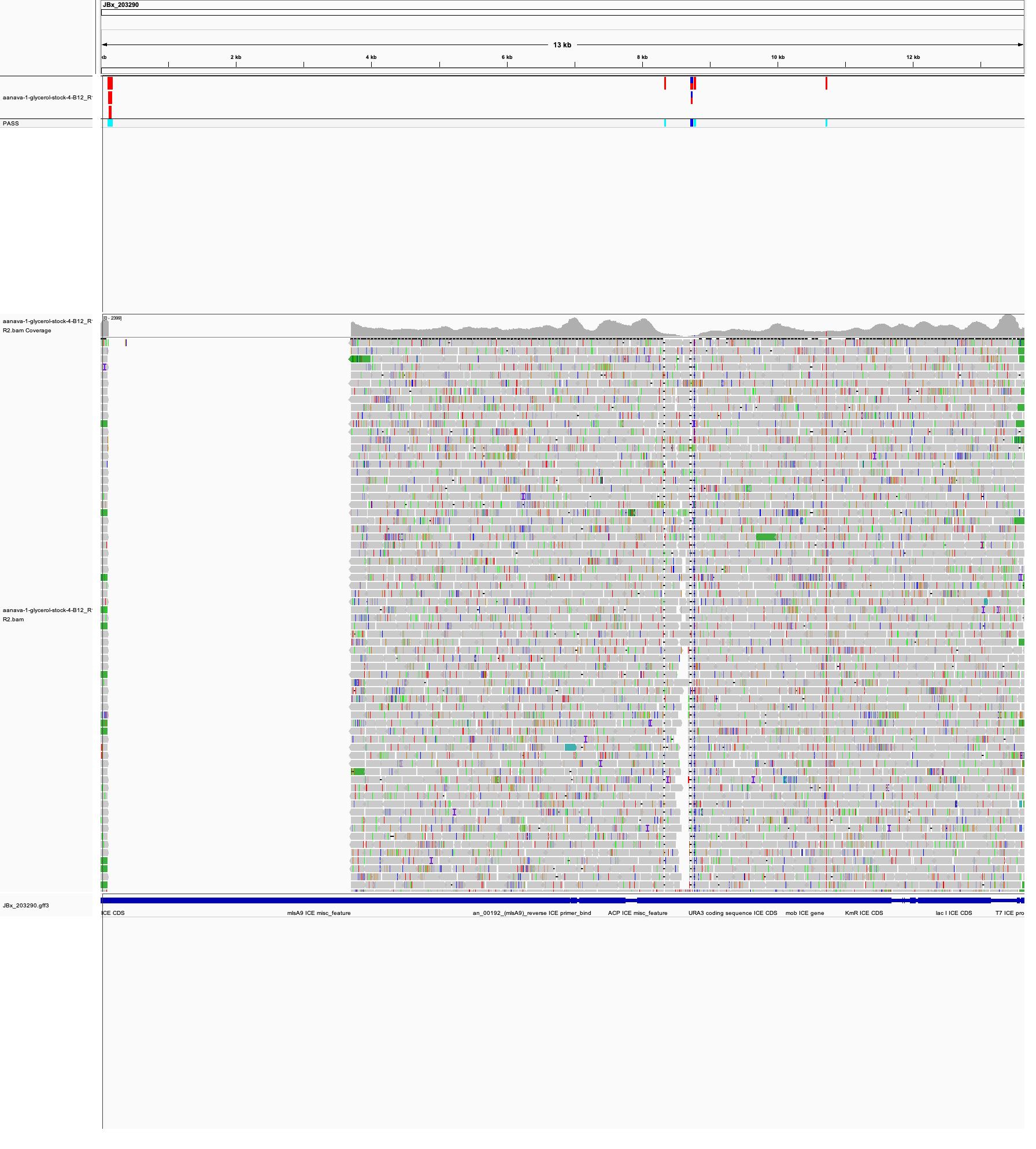

Supplement: Supplementary file 2 — sb3c00292_si_002.zip [file sb3c00292_si_002.zip › dnada_supplementary_material_pks_library_build/divaseq/211117_divaseq_analysis/alberto/snapshots/JBx_203290_nava-1-glycerol-stock-4-B12_R1R2.jpg]

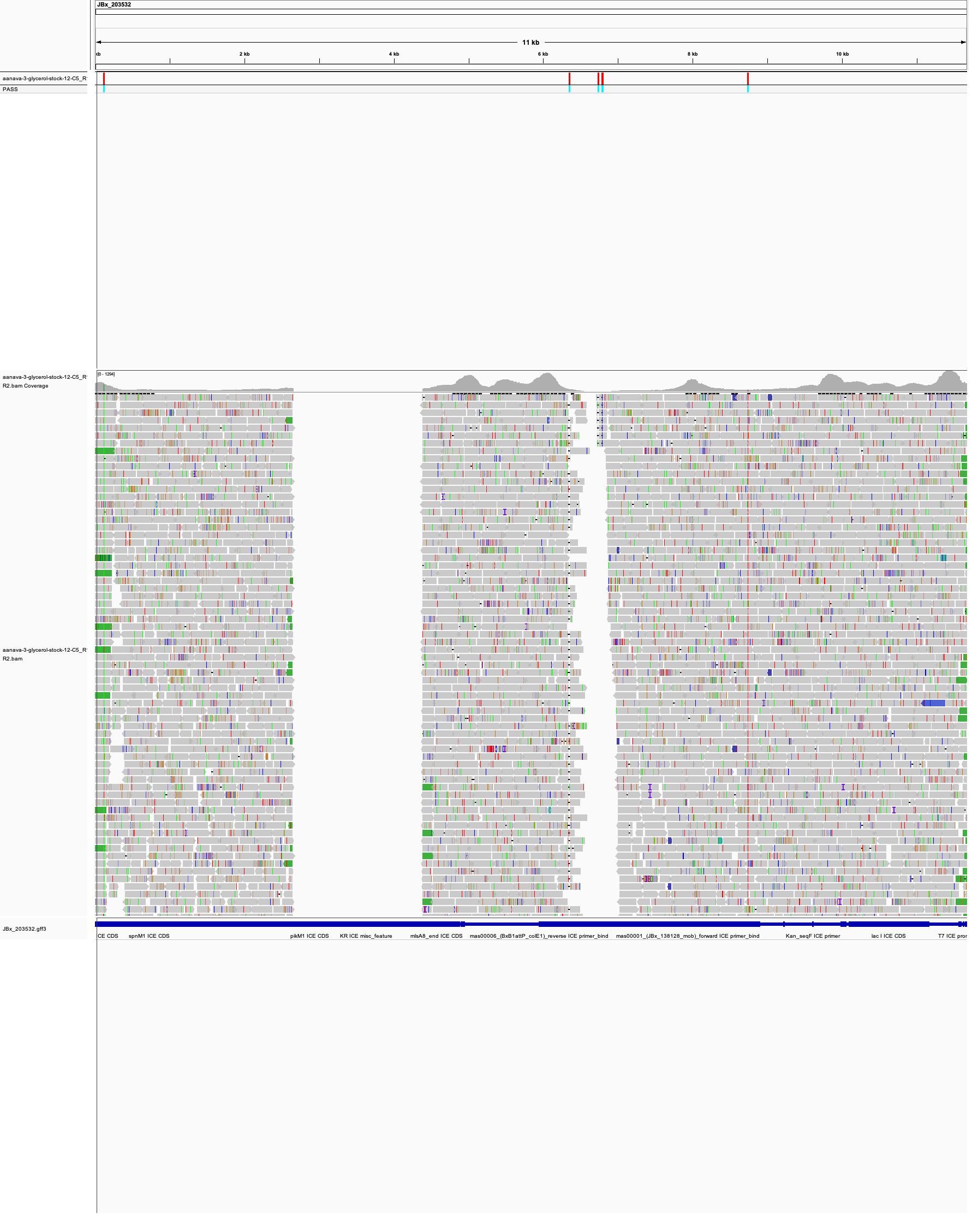

Supplement: Supplementary file 2 — sb3c00292_si_002.zip [file sb3c00292_si_002.zip › dnada_supplementary_material_pks_library_build/divaseq/211117_divaseq_analysis/alberto/snapshots/JBx_203532_nava-3-glycerol-stock-12-C5_R1R2.jpg]

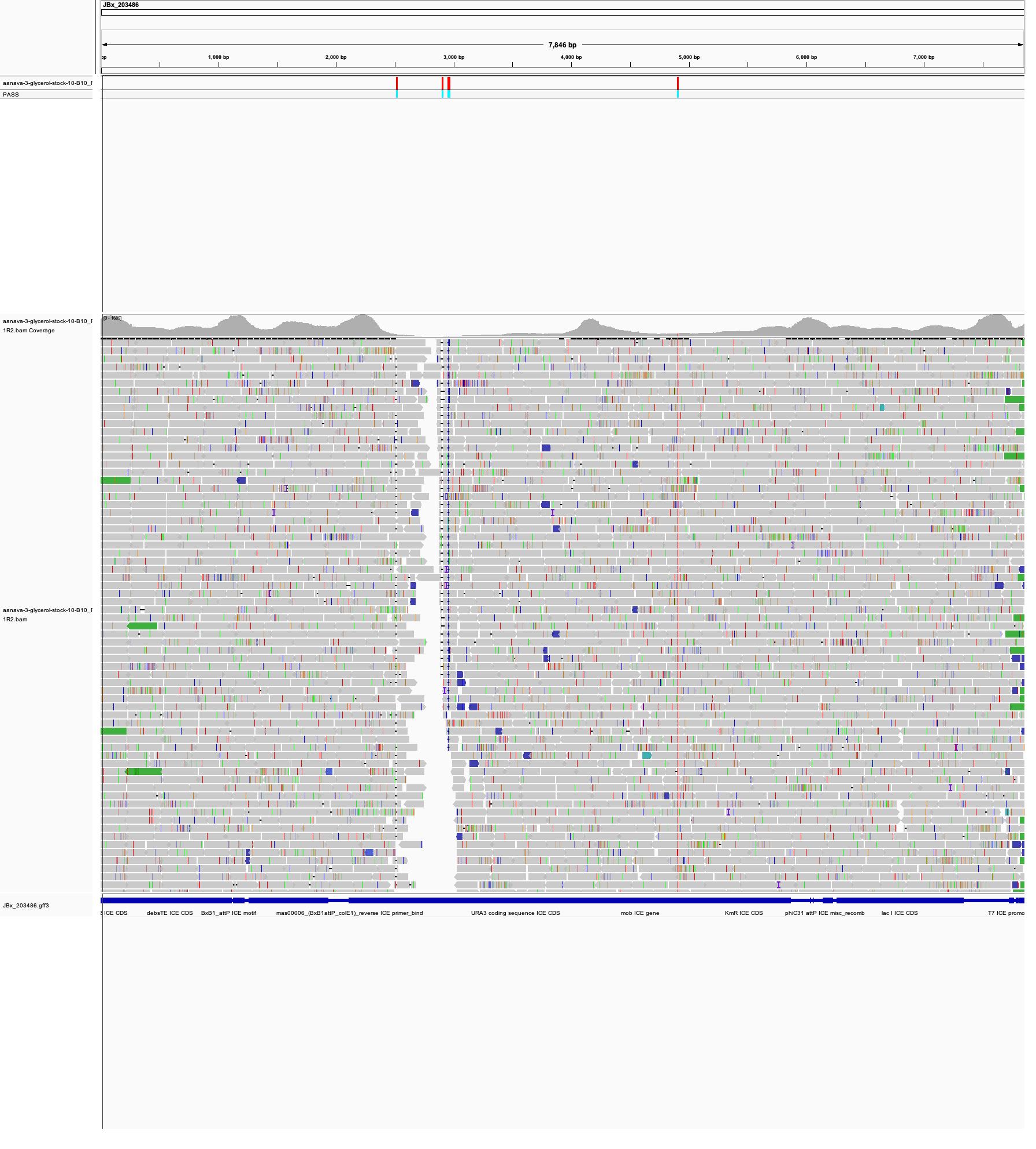

Supplement: Supplementary file 2 — sb3c00292_si_002.zip [file sb3c00292_si_002.zip › dnada_supplementary_material_pks_library_build/divaseq/211117_divaseq_analysis/alberto/snapshots/JBx_203486_nava-3-glycerol-stock-10-B10_R1R2.jpg]

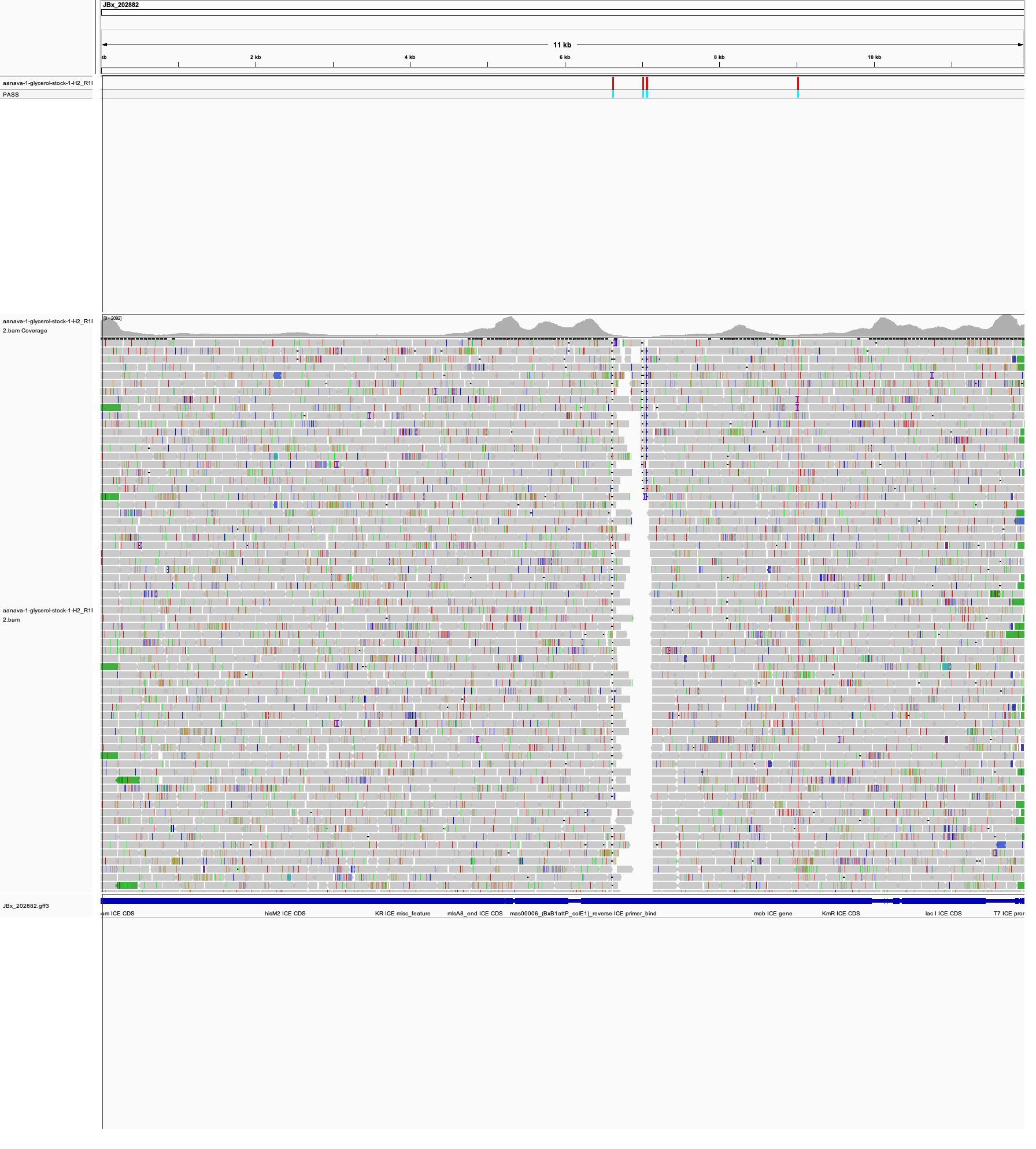

Supplement: Supplementary file 2 — sb3c00292_si_002.zip [file sb3c00292_si_002.zip › dnada_supplementary_material_pks_library_build/divaseq/211117_divaseq_analysis/alberto/snapshots/JBx_202882_nava-1-glycerol-stock-1-H2_R1R2.jpg]

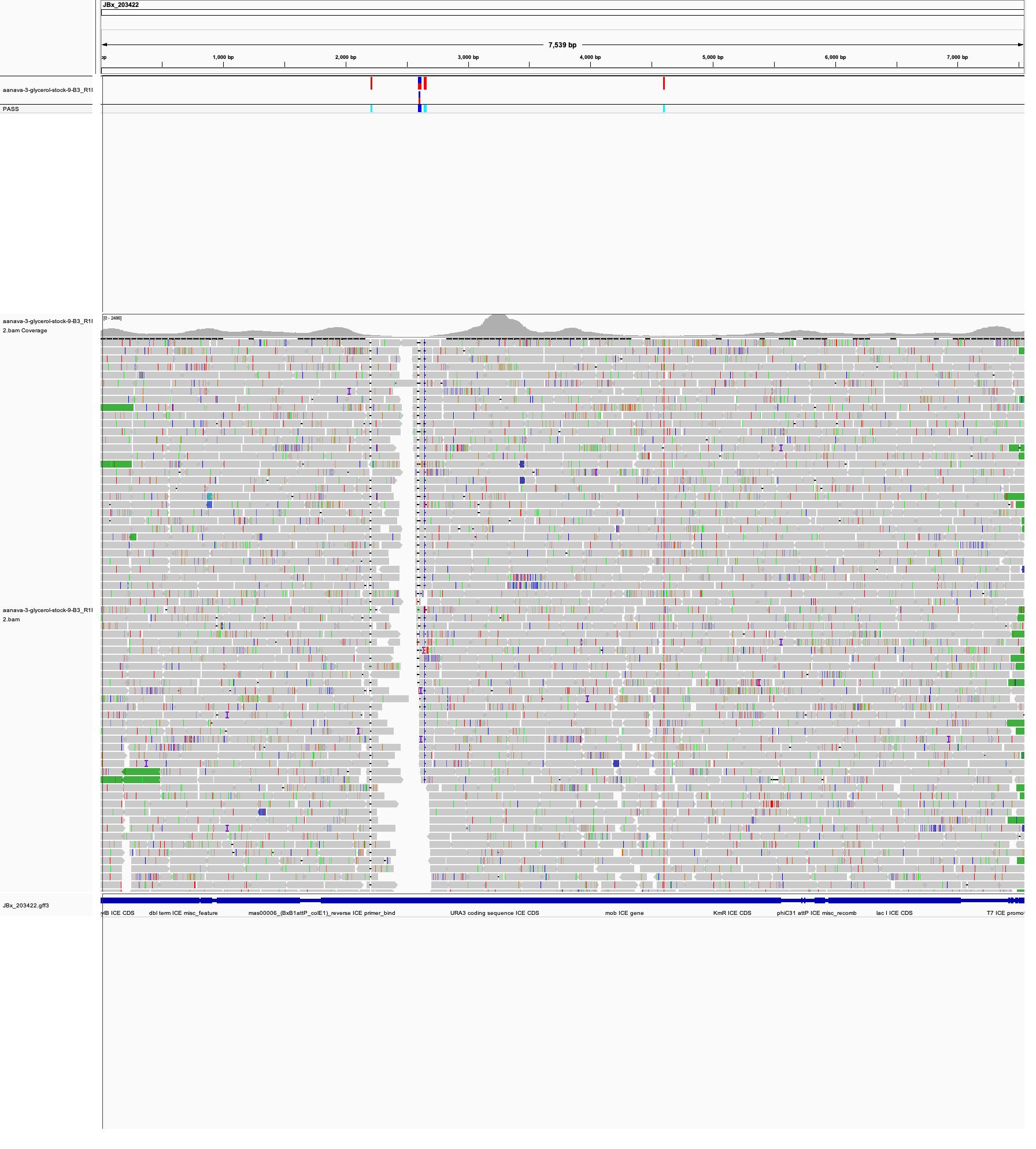

Supplement: Supplementary file 2 — sb3c00292_si_002.zip [file sb3c00292_si_002.zip › dnada_supplementary_material_pks_library_build/divaseq/211117_divaseq_analysis/alberto/snapshots/JBx_203422_nava-3-glycerol-stock-9-B3_R1R2.jpg]

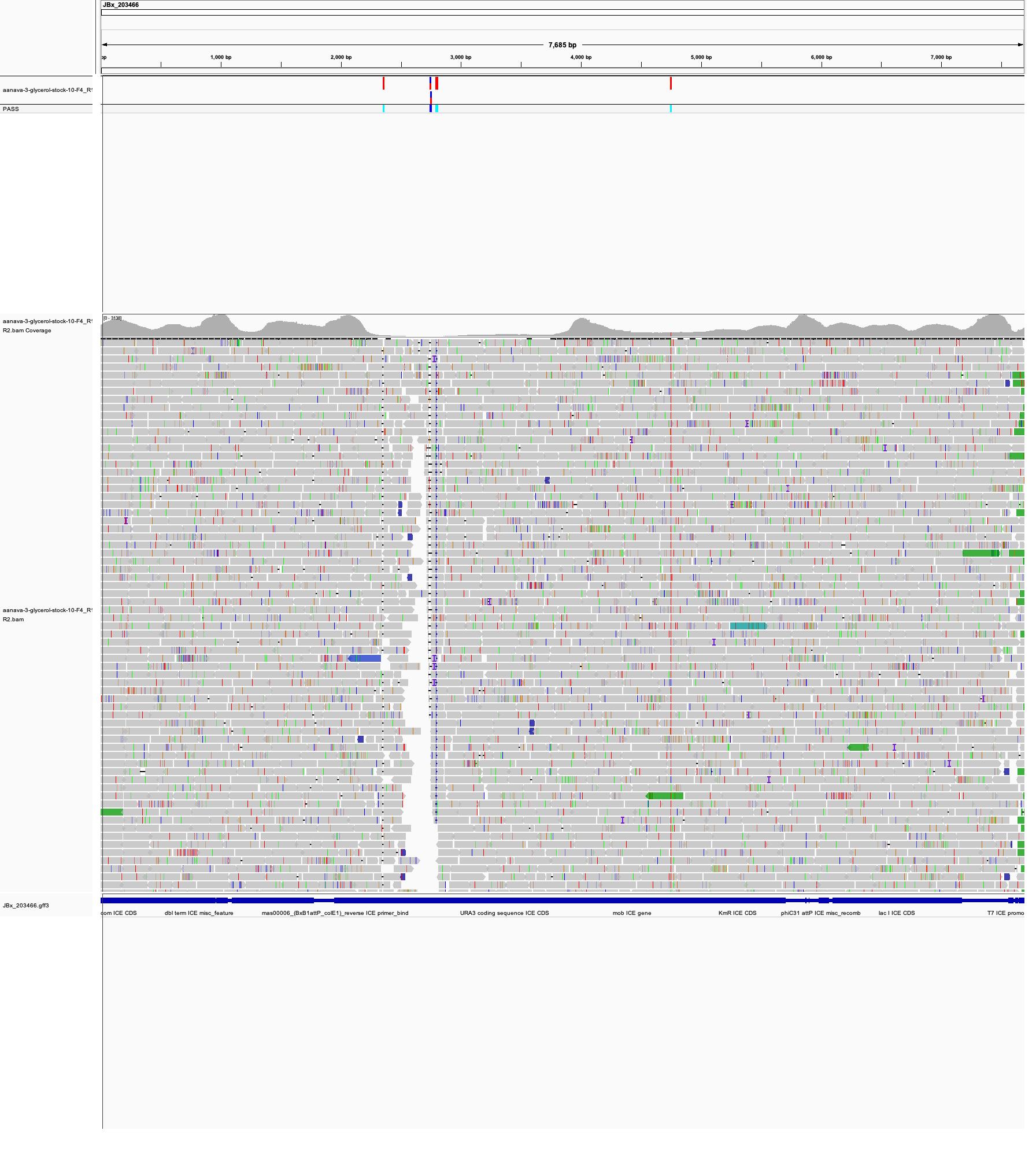

Supplement: Supplementary file 2 — sb3c00292_si_002.zip [file sb3c00292_si_002.zip › dnada_supplementary_material_pks_library_build/divaseq/211117_divaseq_analysis/alberto/snapshots/JBx_203466_nava-3-glycerol-stock-10-F4_R1R2.jpg]

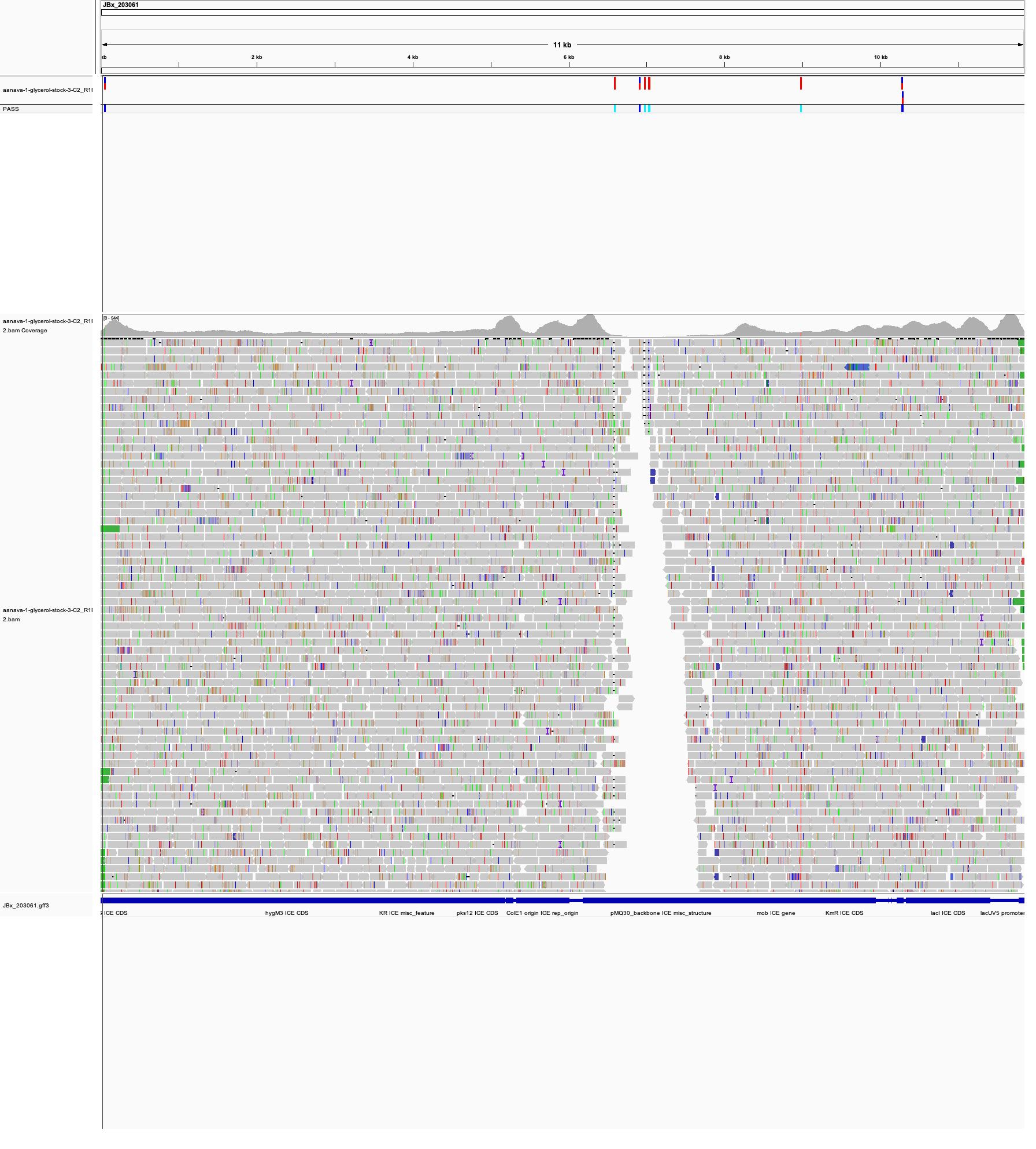

Supplement: Supplementary file 2 — sb3c00292_si_002.zip [file sb3c00292_si_002.zip › dnada_supplementary_material_pks_library_build/divaseq/211117_divaseq_analysis/alberto/snapshots/JBx_203061_nava-1-glycerol-stock-3-C2_R1R2.jpg]

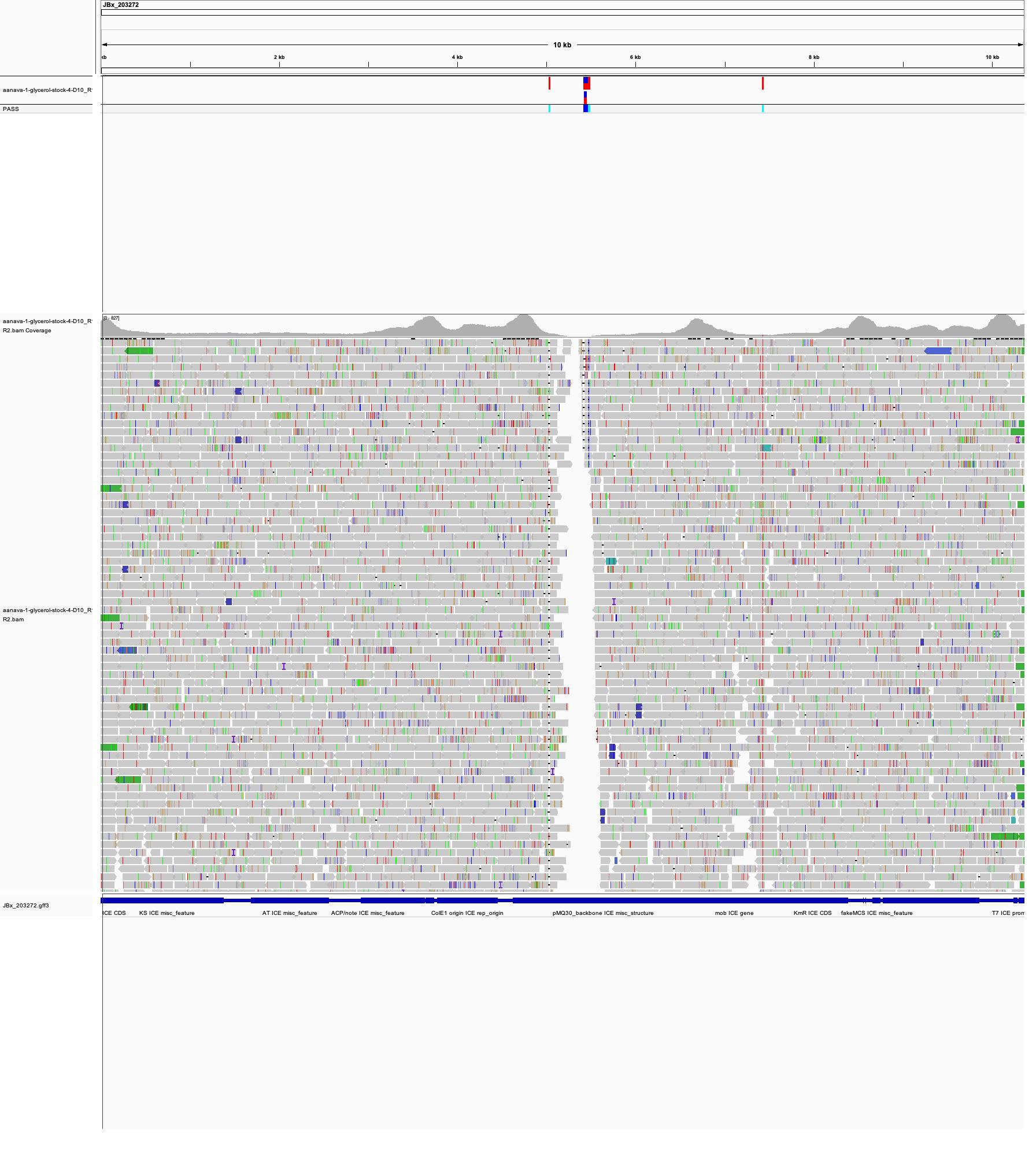

Supplement: Supplementary file 2 — sb3c00292_si_002.zip [file sb3c00292_si_002.zip › dnada_supplementary_material_pks_library_build/divaseq/211117_divaseq_analysis/alberto/snapshots/JBx_203272_nava-1-glycerol-stock-4-D10_R1R2.jpg]

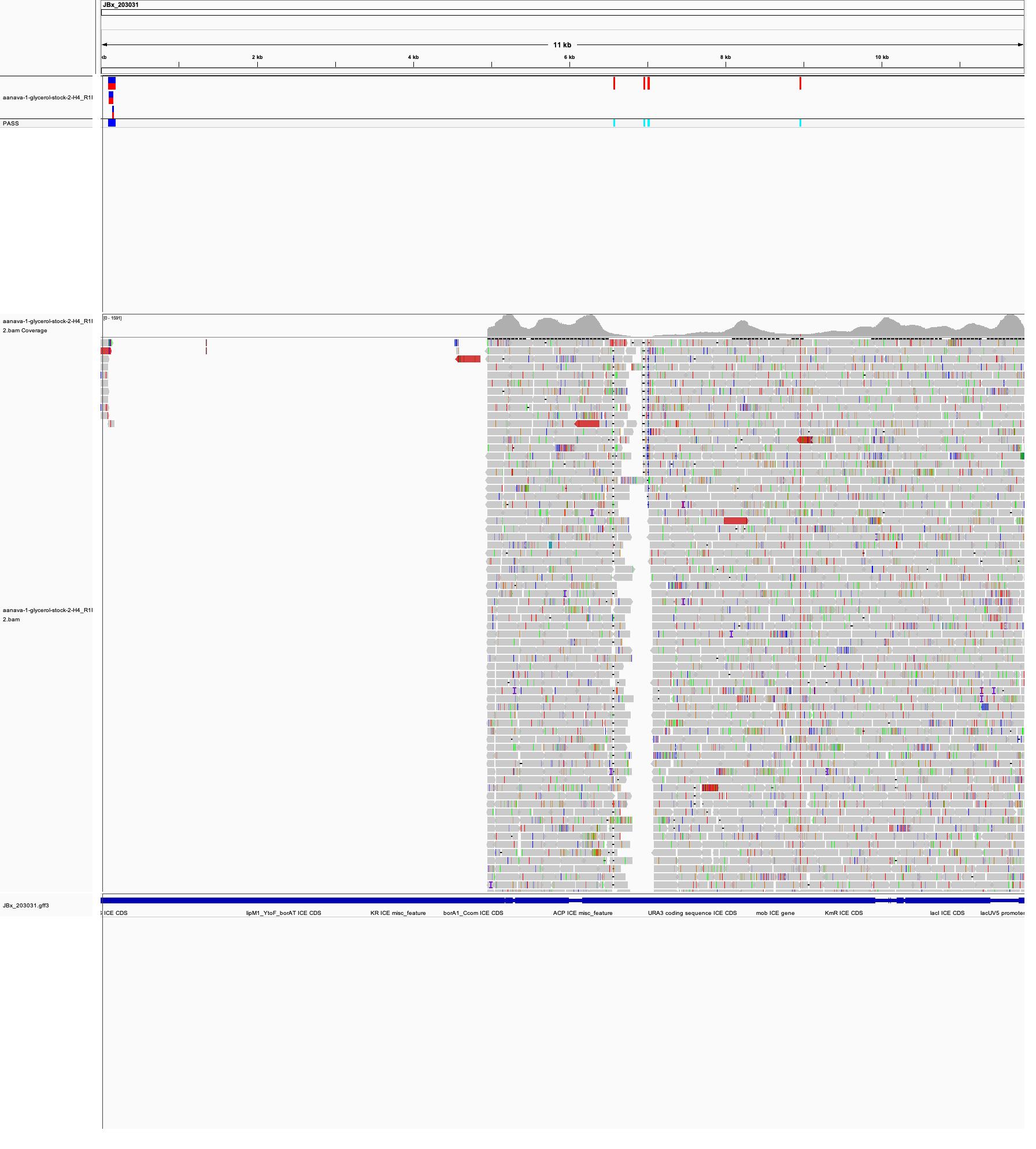

Supplement: Supplementary file 2 — sb3c00292_si_002.zip [file sb3c00292_si_002.zip › dnada_supplementary_material_pks_library_build/divaseq/211117_divaseq_analysis/alberto/snapshots/JBx_203031_nava-1-glycerol-stock-2-H4_R1R2.jpg]

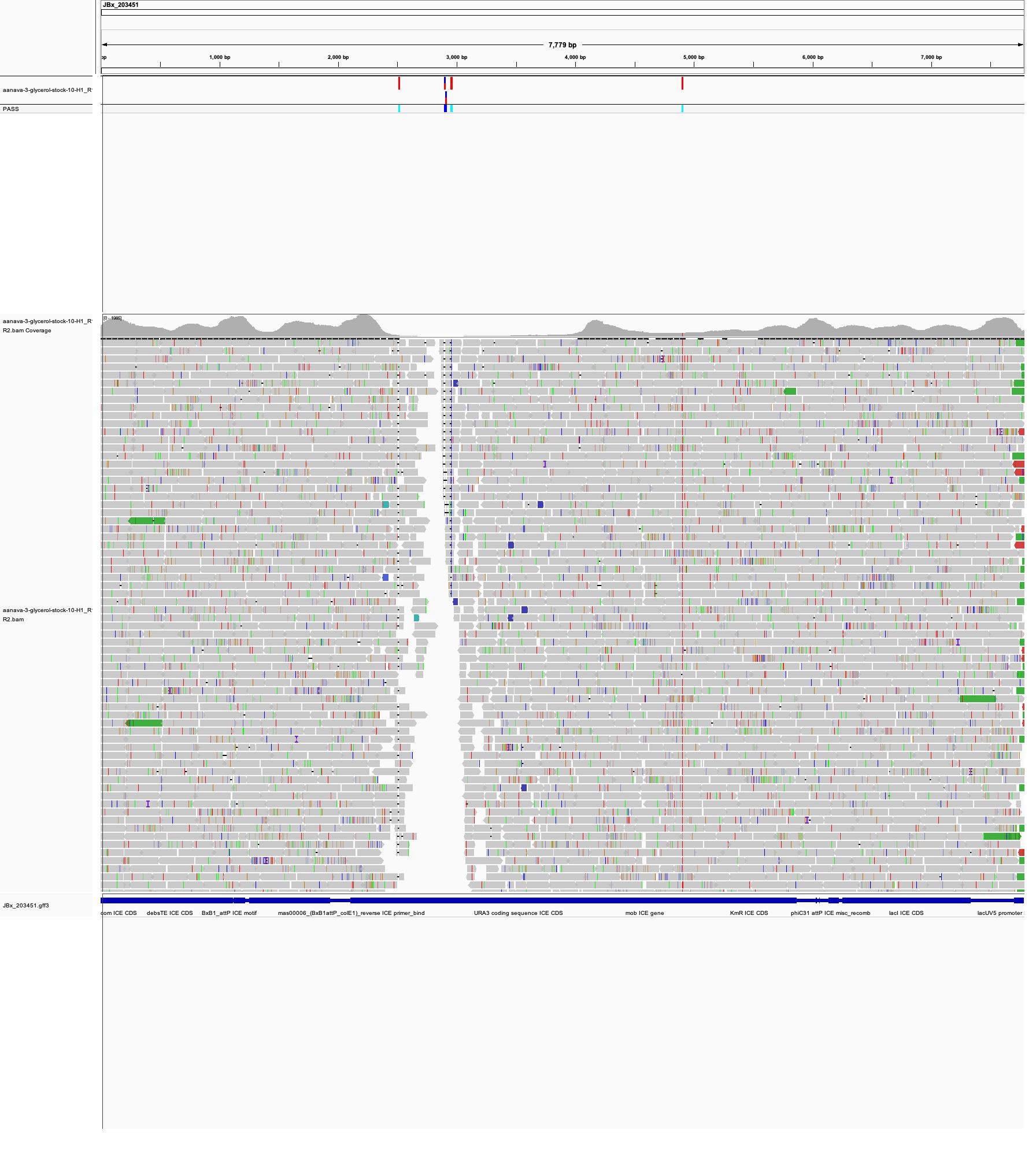

Supplement: Supplementary file 2 — sb3c00292_si_002.zip [file sb3c00292_si_002.zip › dnada_supplementary_material_pks_library_build/divaseq/211117_divaseq_analysis/alberto/snapshots/JBx_203451_nava-3-glycerol-stock-10-H1_R1R2.jpg]

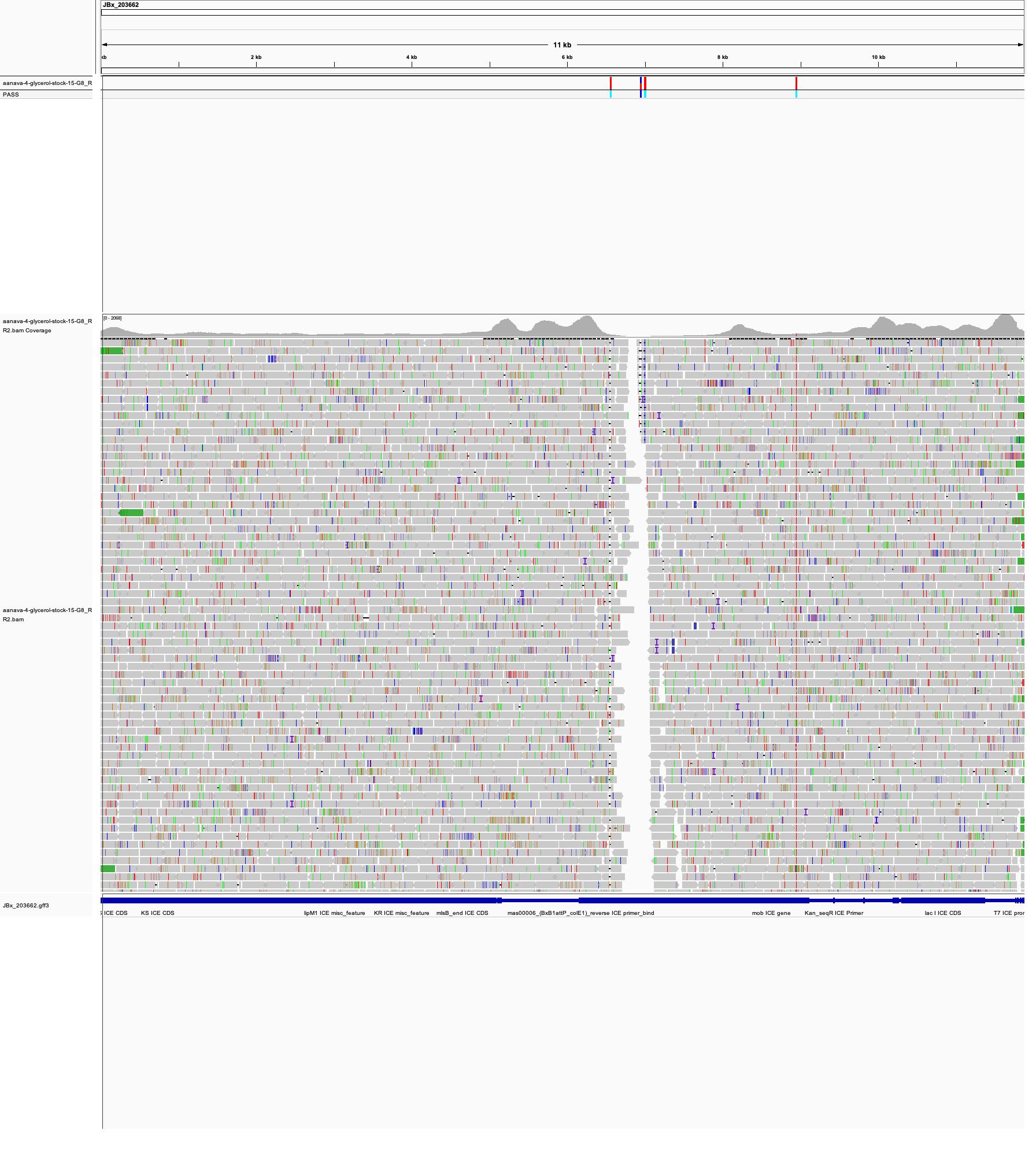

Supplement: Supplementary file 2 — sb3c00292_si_002.zip [file sb3c00292_si_002.zip › dnada_supplementary_material_pks_library_build/divaseq/211117_divaseq_analysis/alberto/snapshots/JBx_203662_nava-4-glycerol-stock-15-G8_R1R2.jpg]

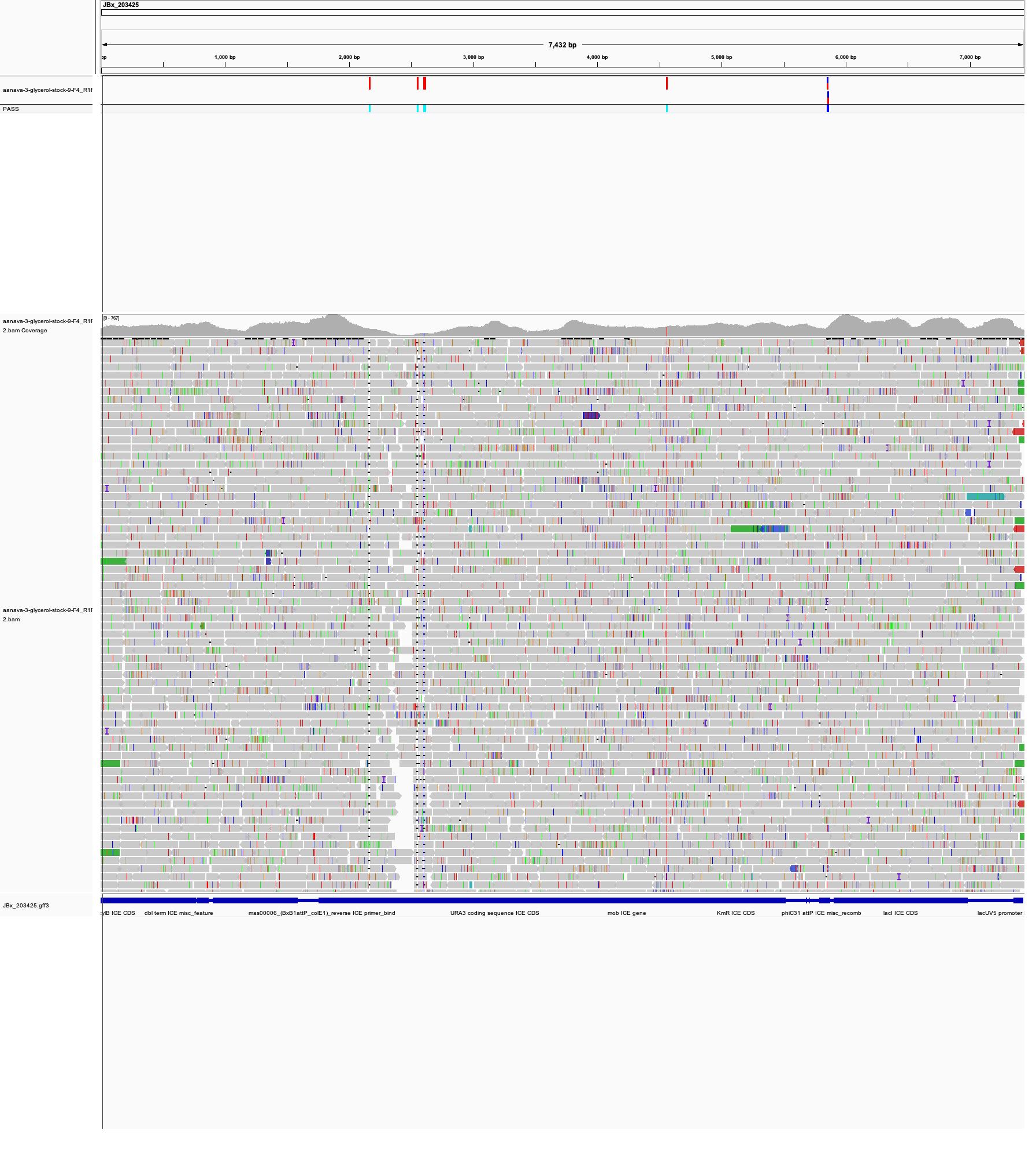

Supplement: Supplementary file 2 — sb3c00292_si_002.zip [file sb3c00292_si_002.zip › dnada_supplementary_material_pks_library_build/divaseq/211117_divaseq_analysis/alberto/snapshots/JBx_203425_nava-3-glycerol-stock-9-F4_R1R2.jpg]

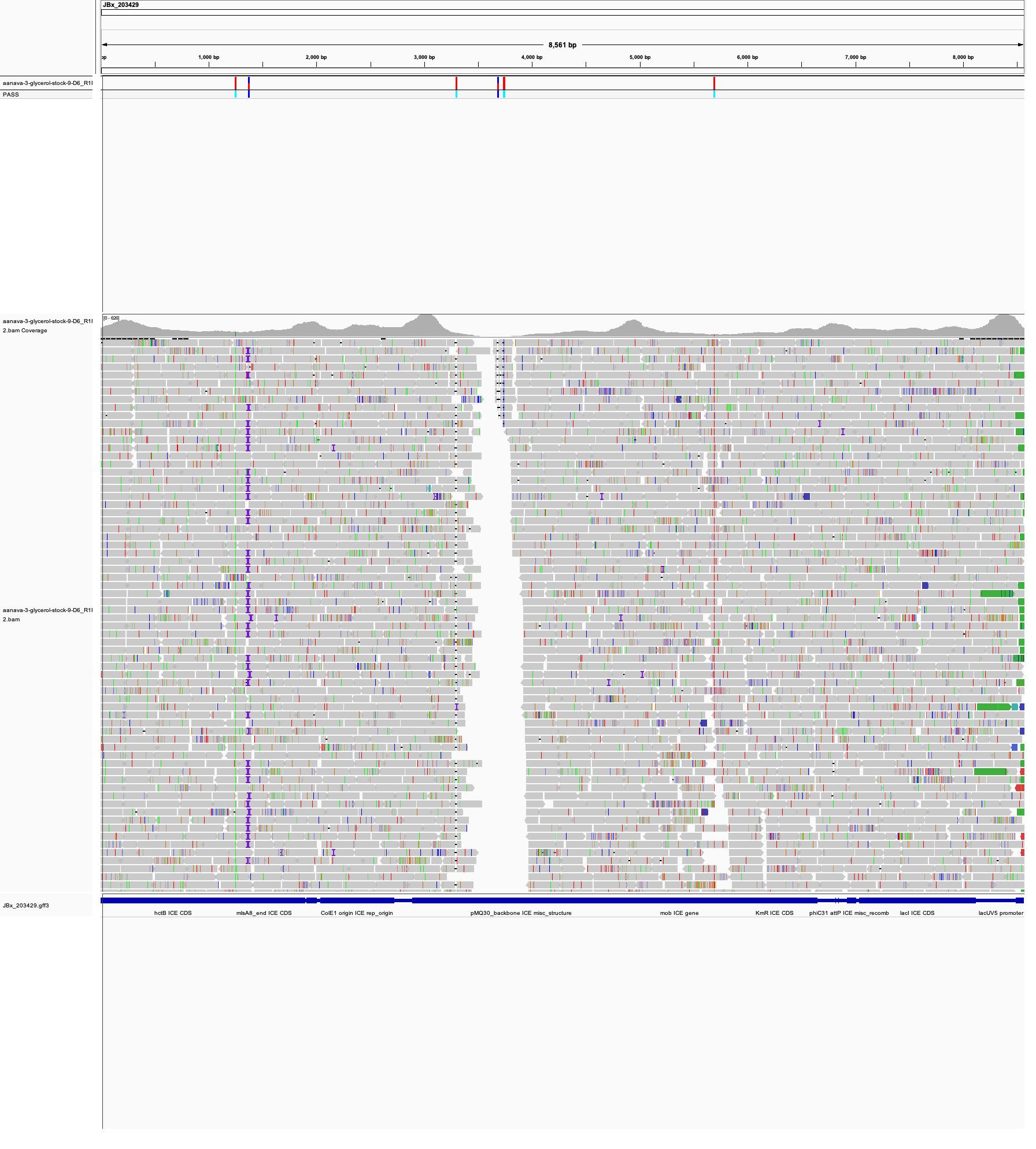

Supplement: Supplementary file 2 — sb3c00292_si_002.zip [file sb3c00292_si_002.zip › dnada_supplementary_material_pks_library_build/divaseq/211117_divaseq_analysis/alberto/snapshots/JBx_203429_nava-3-glycerol-stock-9-D6_R1R2.jpg]

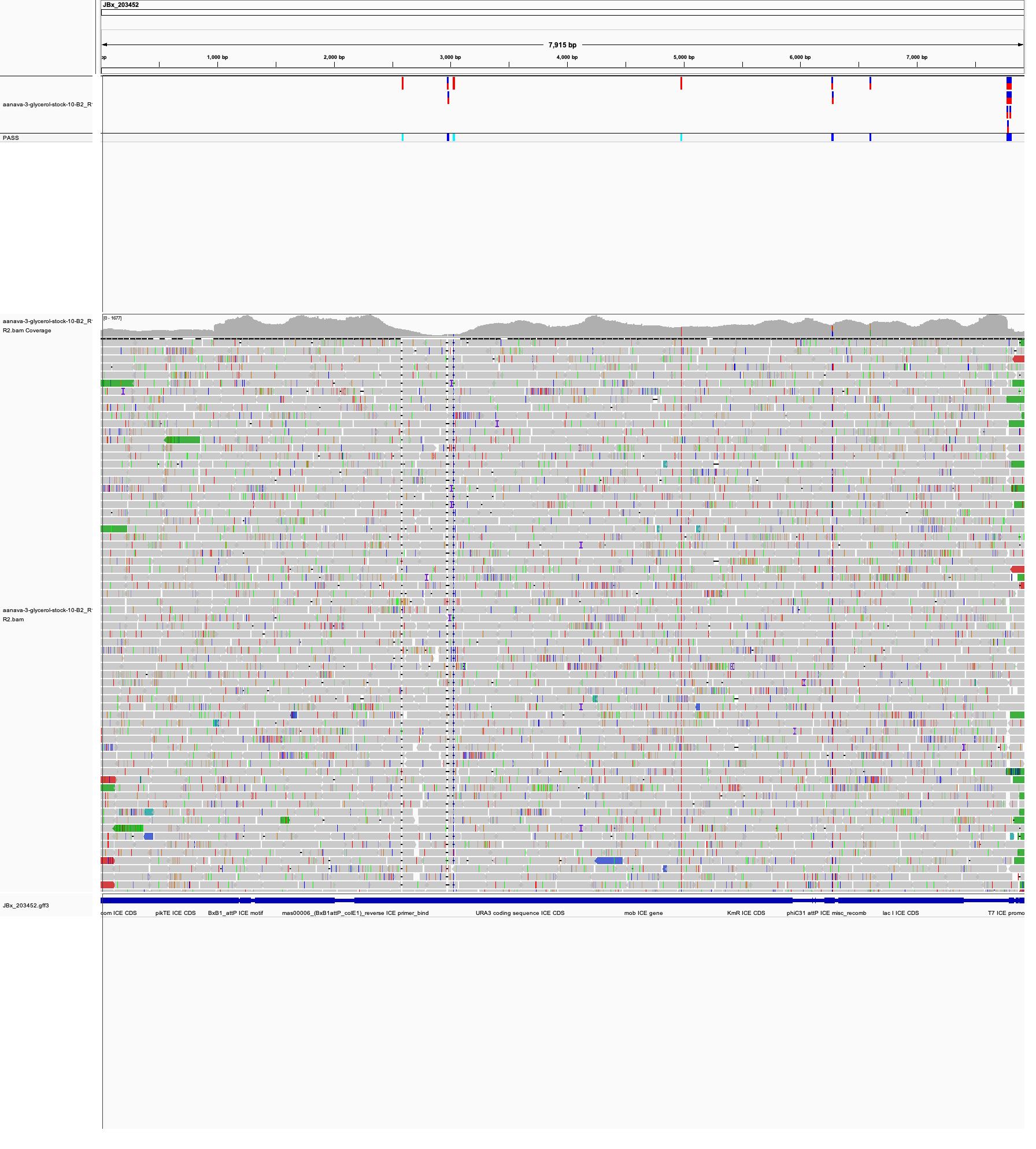

Supplement: Supplementary file 2 — sb3c00292_si_002.zip [file sb3c00292_si_002.zip › dnada_supplementary_material_pks_library_build/divaseq/211117_divaseq_analysis/alberto/snapshots/JBx_203452_nava-3-glycerol-stock-10-B2_R1R2.jpg]

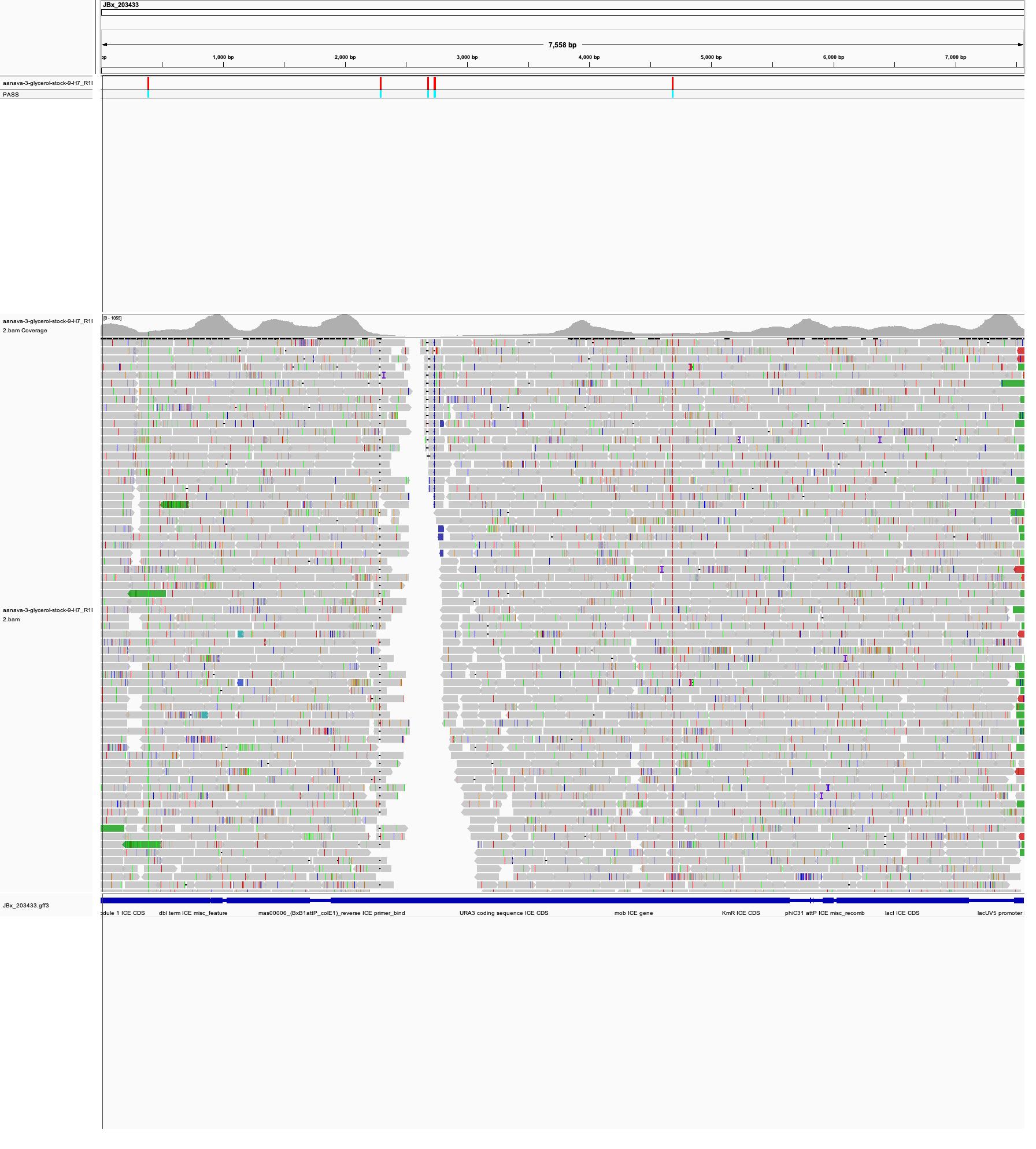

Supplement: Supplementary file 2 — sb3c00292_si_002.zip [file sb3c00292_si_002.zip › dnada_supplementary_material_pks_library_build/divaseq/211117_divaseq_analysis/alberto/snapshots/JBx_203433_nava-3-glycerol-stock-9-H7_R1R2.jpg]

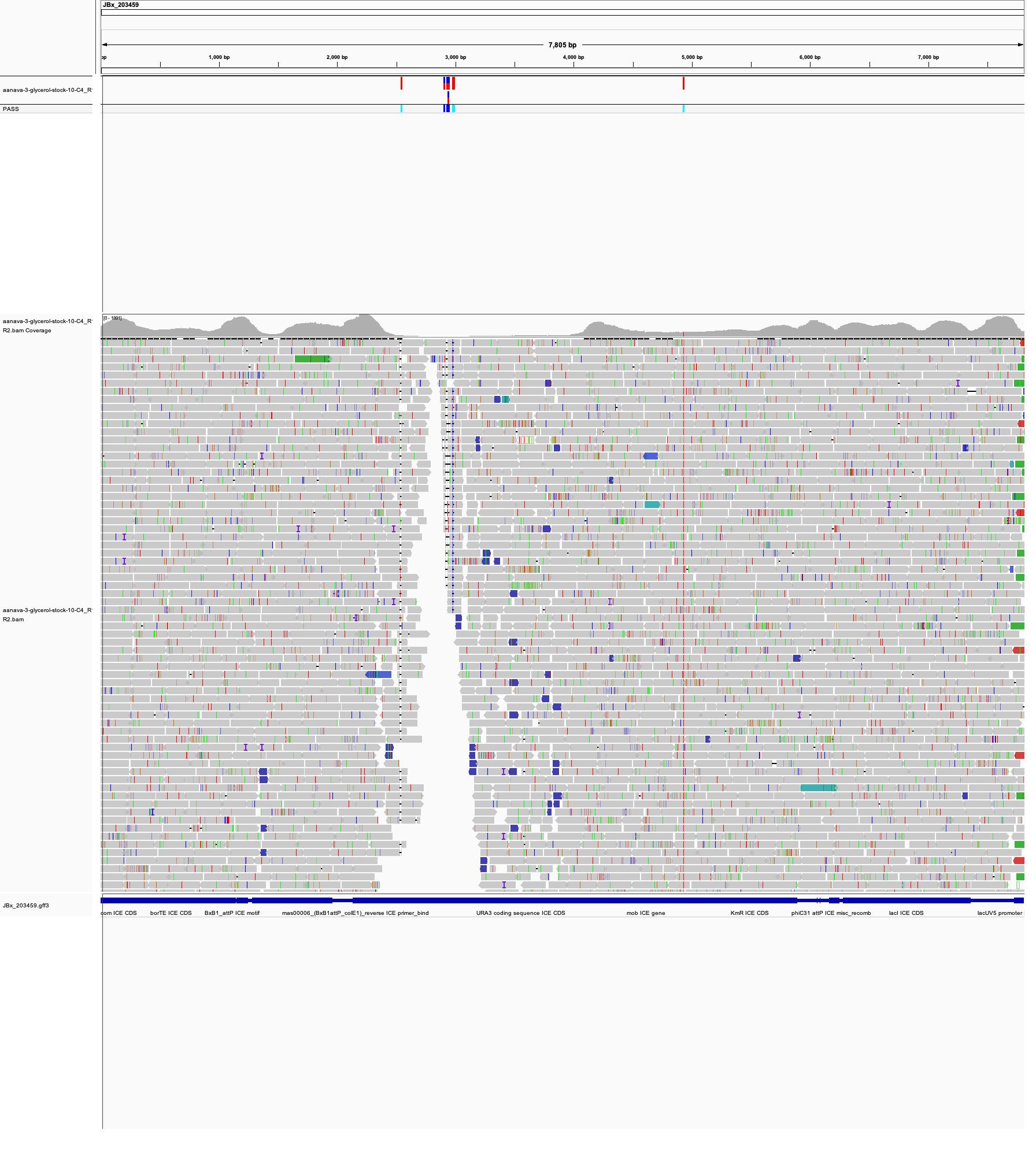

Supplement: Supplementary file 2 — sb3c00292_si_002.zip [file sb3c00292_si_002.zip › dnada_supplementary_material_pks_library_build/divaseq/211117_divaseq_analysis/alberto/snapshots/JBx_203459_nava-3-glycerol-stock-10-C4_R1R2.jpg]

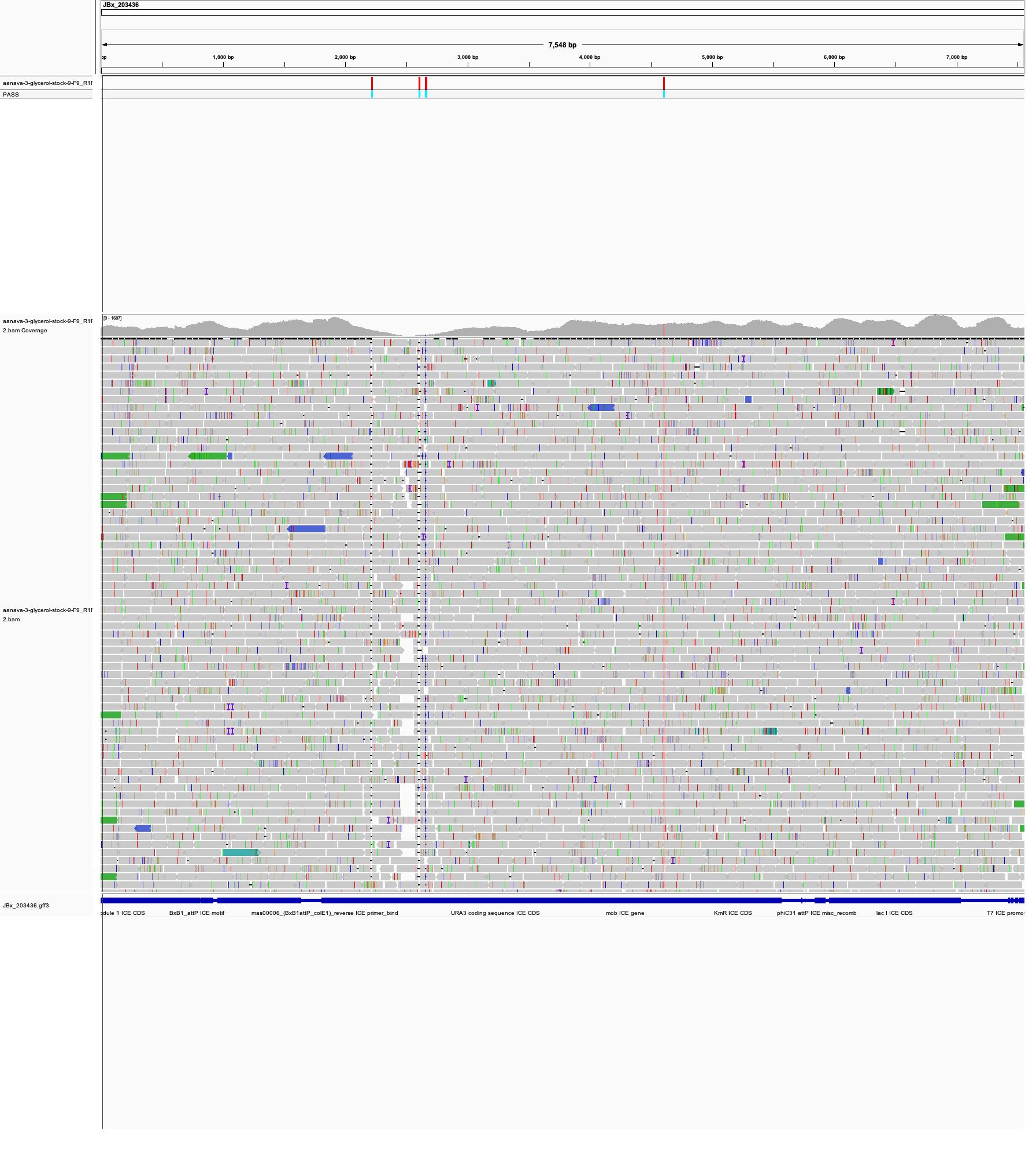

Supplement: Supplementary file 2 — sb3c00292_si_002.zip [file sb3c00292_si_002.zip › dnada_supplementary_material_pks_library_build/divaseq/211117_divaseq_analysis/alberto/snapshots/JBx_203436_nava-3-glycerol-stock-9-F9_R1R2.jpg]

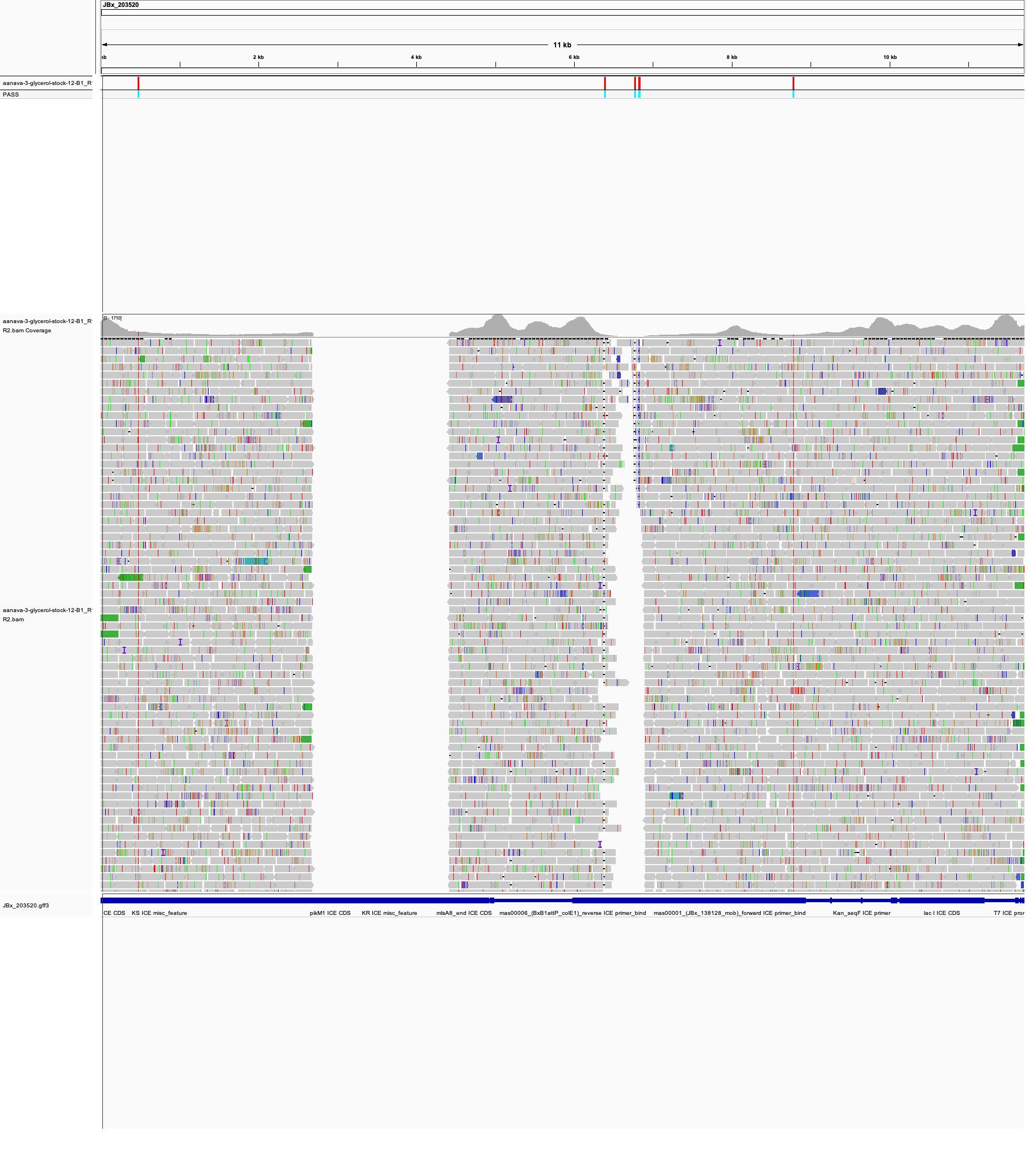

Supplement: Supplementary file 2 — sb3c00292_si_002.zip [file sb3c00292_si_002.zip › dnada_supplementary_material_pks_library_build/divaseq/211117_divaseq_analysis/alberto/snapshots/JBx_203520_nava-3-glycerol-stock-12-B1_R1R2.jpg]

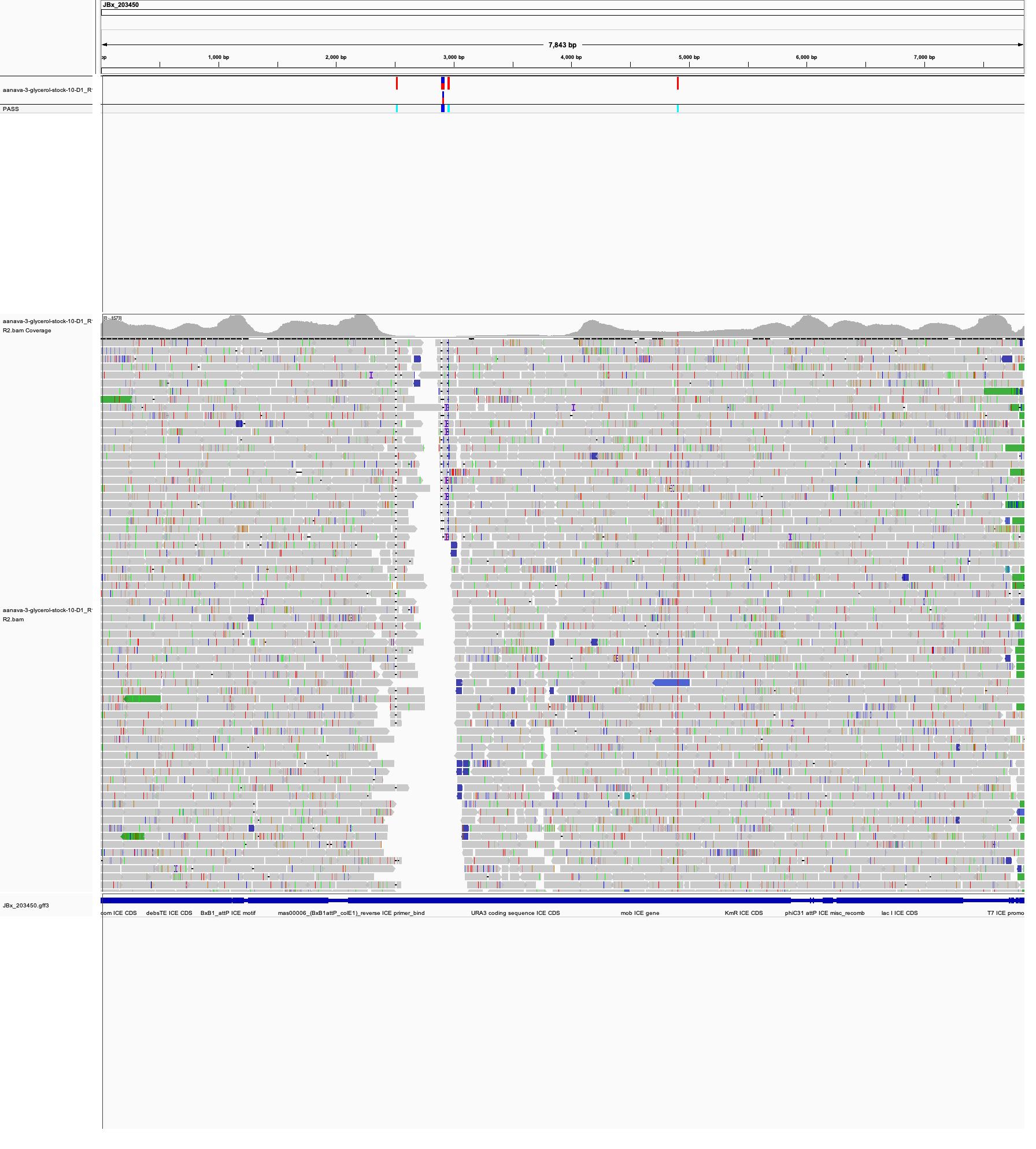

Supplement: Supplementary file 2 — sb3c00292_si_002.zip [file sb3c00292_si_002.zip › dnada_supplementary_material_pks_library_build/divaseq/211117_divaseq_analysis/alberto/snapshots/JBx_203450_nava-3-glycerol-stock-10-D1_R1R2.jpg]

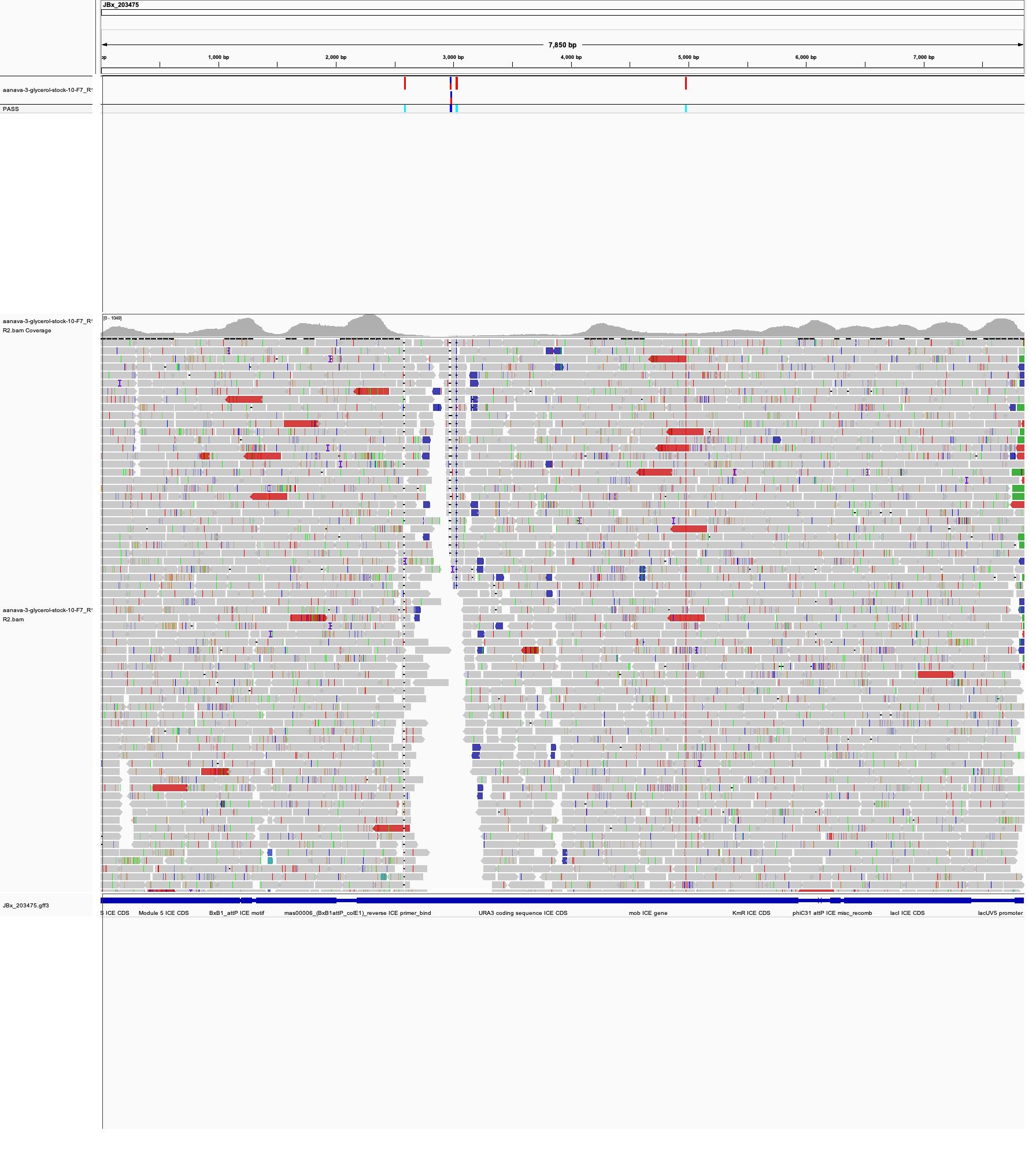

Supplement: Supplementary file 2 — sb3c00292_si_002.zip [file sb3c00292_si_002.zip › dnada_supplementary_material_pks_library_build/divaseq/211117_divaseq_analysis/alberto/snapshots/JBx_203475_nava-3-glycerol-stock-10-F7_R1R2.jpg]

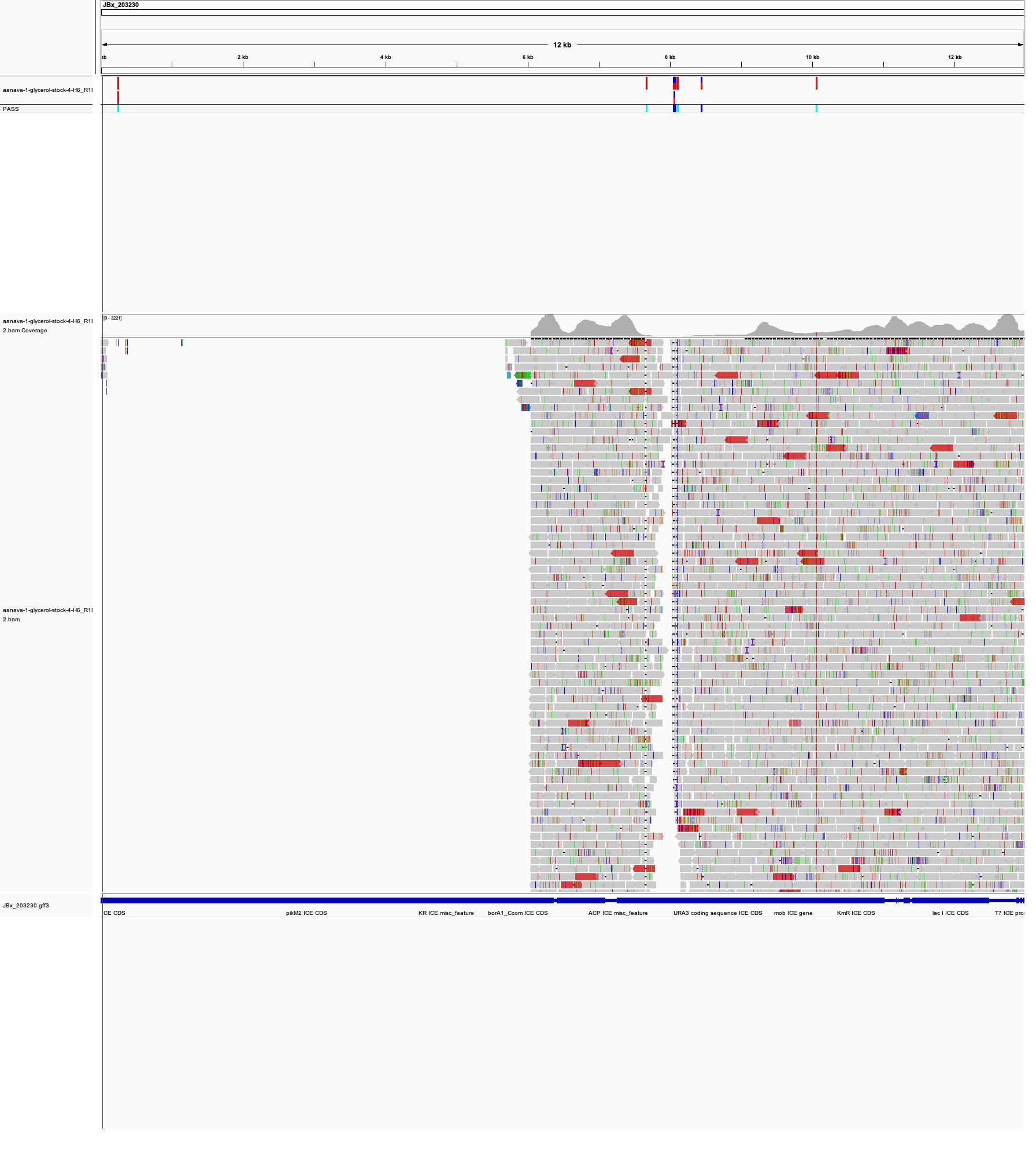

Supplement: Supplementary file 2 — sb3c00292_si_002.zip [file sb3c00292_si_002.zip › dnada_supplementary_material_pks_library_build/divaseq/211117_divaseq_analysis/alberto/snapshots/JBx_203230_nava-1-glycerol-stock-4-H6_R1R2.jpg]
