# Supplementary material for: Automated Platform for the Plasmid Construction Process
Source: ACS Synth Biol. 2023 Nov 10;12(12):3506–13. doi: 10.1021/acssynbio.3c00292 (PMC10729297; doi:10.1021/acssynbio.3c00292)
Supplement: Supplementary file 2 — sb3c00292_si_002.zip [file assemblytoautomate_PCR_plate_3-preview.pdf › dnada_supplementary_material_pks_library_build/plate_visualizations/assemblytoautomate_PCR_plate_3-preview.pdf]

assemblytoautomate_PCR_plate_3

ROW	1	2	3	4	5	6	7	8	9	10	11	12
A	PCRRXN-103	PCRRXN-329	PCRRXN-404	PCRRXN-7	PCRRXN-7	PCRRXN-7	PCRRXN-3	PCRRXN-3	PCRRXN-3	PCRRXN-3	PCRRXN-3	PCRRXN-197
B	PCRRXN-103	PCRRXN-330	PCRRXN-453	PCRRXN-7	PCRRXN-7	PCRRXN-7	PCRRXN-3	PCRRXN-3	PCRRXN-3	PCRRXN-3	PCRRXN-197	PCRRXN-197
C	PCRRXN-103	PCRRXN-331	PCRRXN-7	PCRRXN-7	PCRRXN-7	PCRRXN-7	PCRRXN-3	PCRRXN-3	PCRRXN-3	PCRRXN-3	PCRRXN-197	PCRRXN-197
D	PCRRXN-103	PCRRXN-332	PCRRXN-7	PCRRXN-7	PCRRXN-7	PCRRXN-7	PCRRXN-3	PCRRXN-3	PCRRXN-3	PCRRXN-3	PCRRXN-197	PCRRXN-197
E	PCRRXN-103	PCRRXN-333	PCRRXN-7	PCRRXN-7	PCRRXN-7	PCRRXN-3	PCRRXN-3	PCRRXN-3	PCRRXN-3	PCRRXN-3	PCRRXN-197	PCRRXN-197
F	PCRRXN-103	PCRRXN-334	PCRRXN-7	PCRRXN-7	PCRRXN-7	PCRRXN-3	PCRRXN-3	PCRRXN-3	PCRRXN-3	PCRRXN-3	PCRRXN-197	PCRRXN-197
G	PCRRXN-103	PCRRXN-446	PCRRXN-7	PCRRXN-7	PCRRXN-7	PCRRXN-3	PCRRXN-3	PCRRXN-3	PCRRXN-3	PCRRXN-3	PCRRXN-197	PCRRXN-199
H	PCRRXN-103	PCRRXN-495	PCRRXN-7	PCRRXN-7	PCRRXN-7	PCRRXN-3	PCRRXN-3	PCRRXN-3	PCRRXN-3	PCRRXN-3	PCRRXN-197	PCRRXN-199

COLUMN
