# Supplementary material for: Automated Platform for the Plasmid Construction Process
Source: ACS Synth Biol. 2023 Nov 10;12(12):3506–13. doi: 10.1021/acssynbio.3c00292 (PMC10729297; doi:10.1021/acssynbio.3c00292)
Supplement: Supplementary file 2 — sb3c00292_si_002.zip [file assemblytoautomate_PCR_plate_7-preview.pdf › dnada_supplementary_material_pks_library_build/plate_visualizations/assemblytoautomate_PCR_plate_7-preview.pdf]

assemblytoautomate_PCR_plate_7

ROW	1	2	3	4	5	6	7	8	9	10	11	12
A	PCRRXN-483	PCRRXN-0	PCRRXN-4	PCRRXN-202	PCRRXN-61	PCRRXN-156	PCRRXN-290	PCRRXN-81	PCRRXN-132	PCRRXN-226	PCRRXN-248	PCRRXN-450
B	PCRRXN-490	PCRRXN-0	PCRRXN-4	PCRRXN-8	PCRRXN-359	PCRRXN-251	PCRRXN-96	PCRRXN-365	PCRRXN-227	PCRRXN-32	PCRRXN-54	PCRRXN-456
C	PCRRXN-497	PCRRXN-0	PCRRXN-4	PCRRXN-9	PCRRXN-360	PCRRXN-252	PCRRXN-97	PCRRXN-366	PCRRXN-228	PCRRXN-33	PCRRXN-55	PCRRXN-463
D	PCRRXN-0	PCRRXN-0	PCRRXN-4	PCRRXN-157	PCRRXN-361	PCRRXN-58	PCRRXN-177	PCRRXN-367	PCRRXN-34	PCRRXN-425	PCRRXN-407	PCRRXN-470
E	PCRRXN-0	PCRRXN-4	PCRRXN-4	PCRRXN-158	PCRRXN-362	PCRRXN-59	PCRRXN-178	PCRRXN-368	PCRRXN-35	PCRRXN-474	PCRRXN-414	PCRRXN-485
F	PCRRXN-0	PCRRXN-4	PCRRXN-105	PCRRXN-253	PCRRXN-363	PCRRXN-193	PCRRXN-273	PCRRXN-369	PCRRXN-129	PCRRXN-151	PCRRXN-421	PCRRXN-492
G	PCRRXN-0	PCRRXN-4	PCRRXN-106	PCRRXN-254	PCRRXN-364	PCRRXN-194	PCRRXN-274	PCRRXN-370	PCRRXN-130	PCRRXN-152	PCRRXN-436	PCRRXN-499
H	PCRRXN-0	PCRRXN-4	PCRRXN-201	PCRRXN-60	PCRRXN-155	PCRRXN-289	PCRRXN-80	PCRRXN-131	PCRRXN-225	PCRRXN-247	PCRRXN-443	PCRRXN-179

COLUMN
