# Supplementary material for: Automated Platform for the Plasmid Construction Process
Source: ACS Synth Biol. 2023 Nov 10;12(12):3506–13. doi: 10.1021/acssynbio.3c00292 (PMC10729297; doi:10.1021/acssynbio.3c00292)
Supplement: Supplementary file 2 — sb3c00292_si_002.zip [file assemblytoautomate_PCR_plate_2-preview.pdf › dnada_supplementary_material_pks_library_build/plate_visualizations/assemblytoautomate_PCR_plate_2-preview.pdf]

assemblytoautomate_PCR_plate_2

ROW \ COLUMN	1	2	3	4	5	6	7	8	9	10	11	12
A	PCRRXN-428	PCRRXN-491	PCRRXN-335	PCRRXN-343	PCRRXN-353		PCRRXN-109		PCRRXN-187	PCRRXN-307	PCRRXN-100	PCRRXN-100
B	PCRRXN-435	PCRRXN-498	PCRRXN-336	PCRRXN-344	PCRRXN-354		PCRRXN-110		PCRRXN-188	PCRRXN-308	PCRRXN-100	PCRRXN-100
C	PCRRXN-442	PCRRXN-189	PCRRXN-337	PCRRXN-345	PCRRXN-355		PCRRXN-12		PCRRXN-283	PCRRXN-309	PCRRXN-100	PCRRXN-100
D	PCRRXN-449	PCRRXN-190	PCRRXN-338	PCRRXN-346	PCRRXN-356		PCRRXN-13		PCRRXN-284	PCRRXN-310	PCRRXN-100	PCRRXN-103
E	PCRRXN-455	PCRRXN-285	PCRRXN-339		PCRRXN-357		PCRRXN-205		PCRRXN-90	PCRRXN-439	PCRRXN-100	PCRRXN-103
F	PCRRXN-462	PCRRXN-286	PCRRXN-340		PCRRXN-358		PCRRXN-206		PCRRXN-91	PCRRXN-488	PCRRXN-100	PCRRXN-103
G	PCRRXN-477	PCRRXN-92	PCRRXN-341						PCRRXN-305	PCRRXN-100	PCRRXN-100	PCRRXN-103
H	PCRRXN-484	PCRRXN-93	PCRRXN-342						PCRRXN-306	PCRRXN-100	PCRRXN-100	PCRRXN-103
