# Supplementary material for: Automated Platform for the Plasmid Construction Process
Source: ACS Synth Biol. 2023 Nov 10;12(12):3506–13. doi: 10.1021/acssynbio.3c00292 (PMC10729297; doi:10.1021/acssynbio.3c00292)
Supplement: Supplementary file 2 — sb3c00292_si_002.zip [file assemblytoautomate_PCR_plate_1-preview.pdf › dnada_supplementary_material_pks_library_build/plate_visualizations/assemblytoautomate_PCR_plate_1-preview.pdf]

assemblytoautomate_PCR_plate_1

ROW	1	2	3	4	5	6	7	8	9	10	11	12
A	PCRRXN-5	PCRRXN-5	PCRRXN-5	PCRRXN-5	PCRRXN-5				PCRRXN-102	PCRRXN-102	PCRRXN-102	
B	PCRRXN-5	PCRRXN-5	PCRRXN-5	PCRRXN-5	PCRRXN-5				PCRRXN-102	PCRRXN-102	PCRRXN-102	
C	PCRRXN-5	PCRRXN-5	PCRRXN-5	PCRRXN-5	PCRRXN-5				PCRRXN-102	PCRRXN-102	PCRRXN-102	
D	PCRRXN-5	PCRRXN-5	PCRRXN-5	PCRRXN-5	PCRRXN-5				PCRRXN-102	PCRRXN-102	PCRRXN-406	
E	PCRRXN-5	PCRRXN-5	PCRRXN-5	PCRRXN-5	PCRRXN-5				PCRRXN-102	PCRRXN-102	PCRRXN-413	
F	PCRRXN-5	PCRRXN-5	PCRRXN-5	PCRRXN-5					PCRRXN-102	PCRRXN-102		
G	PCRRXN-5	PCRRXN-5	PCRRXN-5	PCRRXN-5					PCRRXN-102	PCRRXN-102		
H	PCRRXN-5	PCRRXN-5	PCRRXN-5	PCRRXN-5					PCRRXN-102	PCRRXN-102		

COLUMN
