# Supplementary material for: Automated Platform for the Plasmid Construction Process
Source: ACS Synth Biol. 2023 Nov 10;12(12):3506–13. doi: 10.1021/acssynbio.3c00292 (PMC10729297; doi:10.1021/acssynbio.3c00292)
Supplement: Supplementary file 2 — sb3c00292_si_002.zip [file assemblytoautomate_PCR_plate_8-preview.pdf › dnada_supplementary_material_pks_library_build/plate_visualizations/assemblytoautomate_PCR_plate_8-preview.pdf]

assemblytoautomate_PCR_plate_8

ROW	1	2	3	4	5	6	7	8	9	10	11	12
A	PCRRXN-180	PCRRXN-183	PCRRXN-382	PCRRXN-95	PCRRXN-162	PCRRXN-246	PCRRXN-79	PCRRXN-389	PCRRXN-292	PCRRXN-2	PCRRXN-2	PCRRXN-2
B	PCRRXN-275	PCRRXN-184	PCRRXN-391	PCRRXN-147	PCRRXN-257	PCRRXN-52	PCRRXN-173	PCRRXN-398	PCRRXN-98	PCRRXN-2	PCRRXN-2	PCRRXN-2
C	PCRRXN-276	PCRRXN-279	PCRRXN-400	PCRRXN-148	PCRRXN-258	PCRRXN-53	PCRRXN-174	PCRRXN-379	PCRRXN-99	PCRRXN-2	PCRRXN-2	PCRRXN-2
D	PCRRXN-82	PCRRXN-280	PCRRXN-191	PCRRXN-243	PCRRXN-64	PCRRXN-175	PCRRXN-269	PCRRXN-388	PCRRXN-384	PCRRXN-2	PCRRXN-2	PCRRXN-2
E	PCRRXN-83	PCRRXN-86	PCRRXN-192	PCRRXN-244	PCRRXN-65	PCRRXN-176	PCRRXN-270	PCRRXN-397	PCRRXN-393	PCRRXN-2	PCRRXN-2	PCRRXN-2
F	PCRRXN-383	PCRRXN-87	PCRRXN-287	PCRRXN-50	PCRRXN-149	PCRRXN-271	PCRRXN-76	PCRRXN-195	PCRRXN-402	PCRRXN-2	PCRRXN-2	PCRRXN-2
G	PCRRXN-392	PCRRXN-432	PCRRXN-288	PCRRXN-51	PCRRXN-150	PCRRXN-272	PCRRXN-77	PCRRXN-196	PCRRXN-2	PCRRXN-2	PCRRXN-2	PCRRXN-2
H	PCRRXN-401	PCRRXN-481	PCRRXN-94	PCRRXN-161	PCRRXN-245	PCRRXN-78	PCRRXN-380	PCRRXN-291	PCRRXN-2	PCRRXN-2	PCRRXN-2	PCRRXN-2

COLUMN
