# Supplementary material for: Automated Platform for the Plasmid Construction Process
Source: ACS Synth Biol. 2023 Nov 10;12(12):3506–13. doi: 10.1021/acssynbio.3c00292 (PMC10729297; doi:10.1021/acssynbio.3c00292)

Instrument controller software run summary:

Filename and data path: C:\AATI\Data\2021 09 14\17-24-14\2021 09 14 17H 24M..raw
Created: Tuesday, September 14, 2021 5:34:08 PM
Number of capillaries: 96
Array serial number: 111920-09SFS
Effect length: 33 cm
Array usage count: 214
Instrument type: ZAG DNA Analyzer
Instrument controller software version: 1.0.1.9
Device serial number: Z7597

Method Information

Method name: ZAG130GP33 - DNA 75-20000bp - Gel Prime Only.mthds
Gel prime: Yes
Full conditioning: No
Gel prime to buffer: No
Gel selection: Gel 1
Perform prerun: 8.0 kV, 30 sec.
Rinse: No
Marker 1: Row: A, 5.0 kV, 5 sec.
Rinse: No
Sample injection: 5.0 kV, 5 sec.
Separation: 8.0 kV, 30.0 min.
Tray name: Sample Tray-2

Analysis mode: DNA

Notes

Gel Image

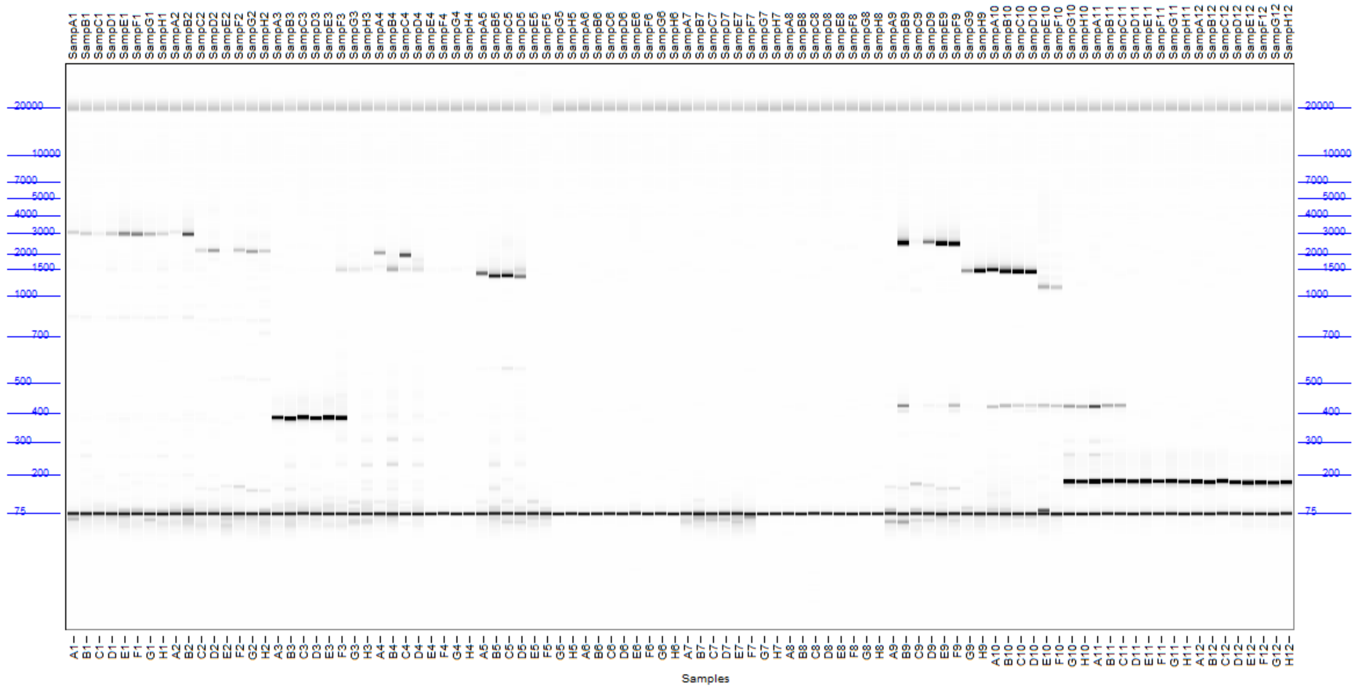
20000
10000
7000
5000
4000
3000
2000
1500
1000
700
500
400
300
200
75
Samples

Filename and data path: C:\AATI\Data\2021 09 14\17-24-14\2021 09 14 17H 24M..raw

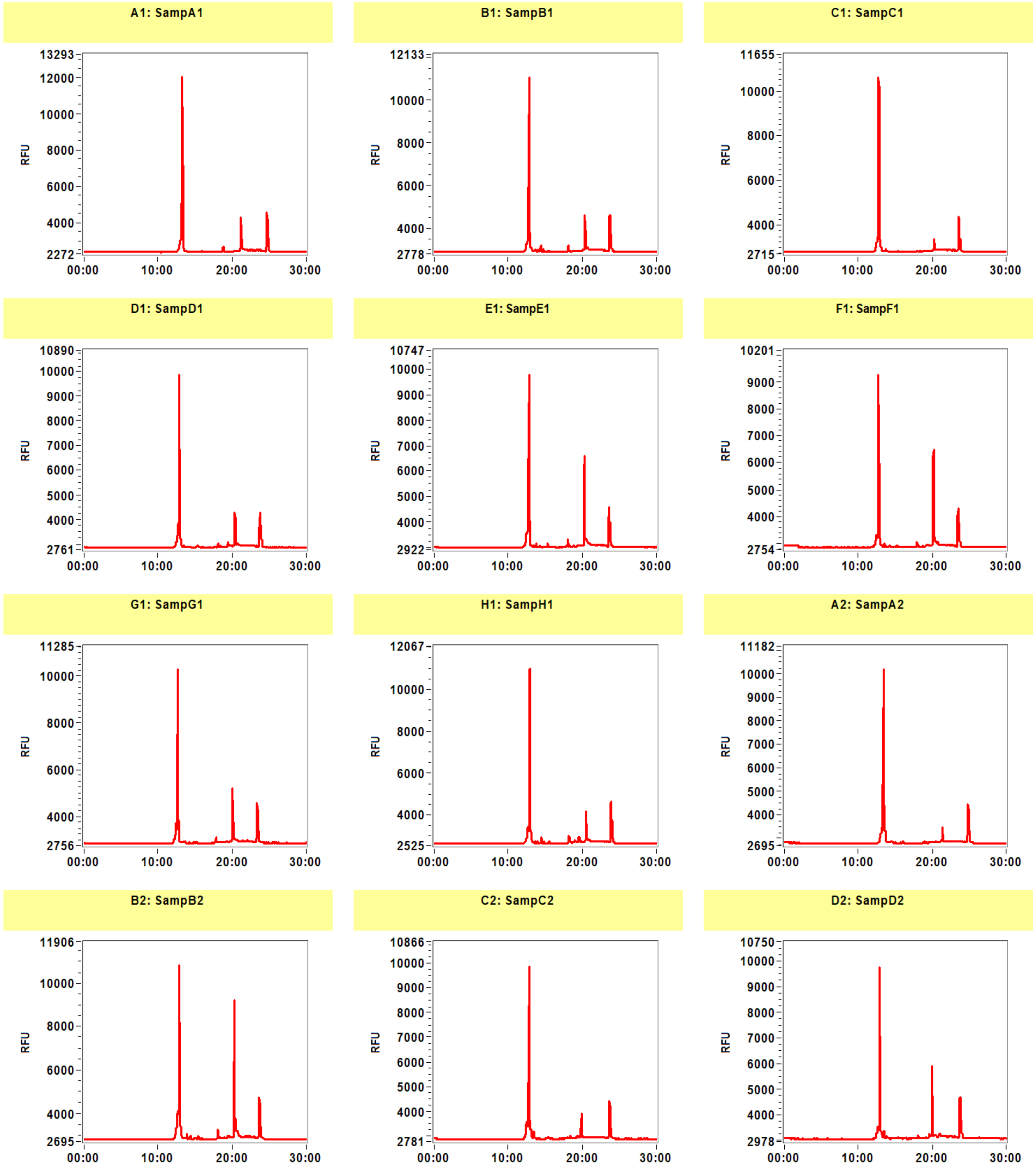

Filename and data path: C:\AATI\Data\2021 09 14\17-24-14\2021 09 14 17H 24M..raw

E2: SampE2

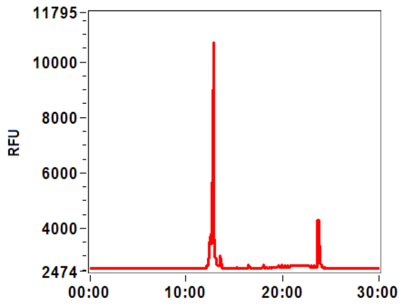

F2: SampF2

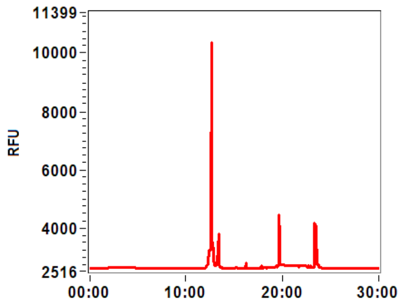

G2: SampG2

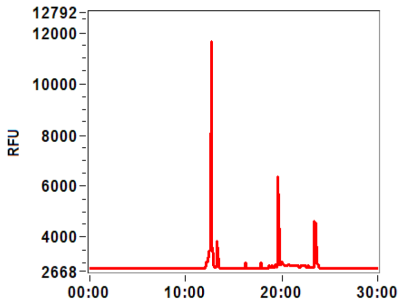

H2: SampH2

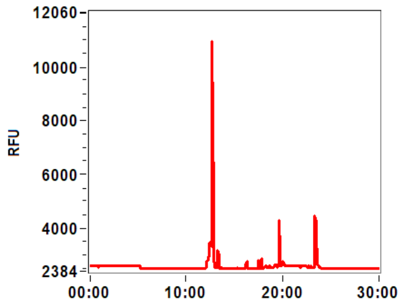

A3: SampA3

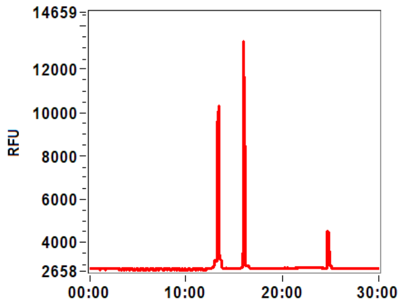

B3: SampB3

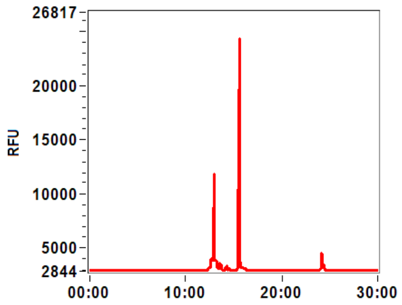

C3: SampC3

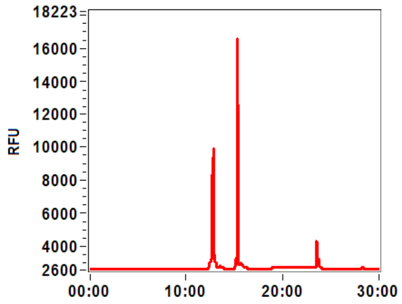

D3: SampD3

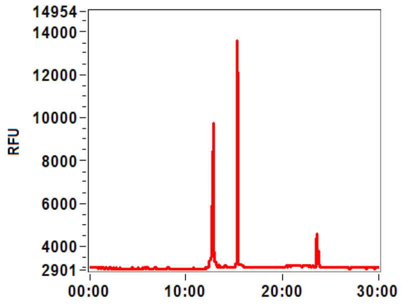

E3: SampE3

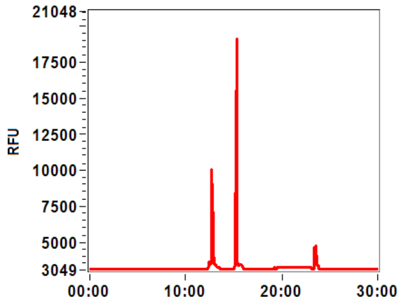

F3: SampF3

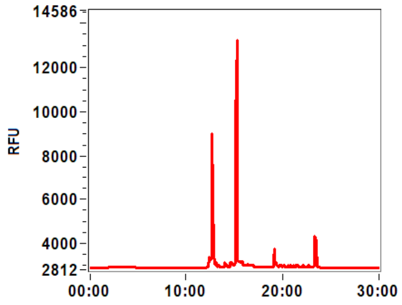

G3: SampG3

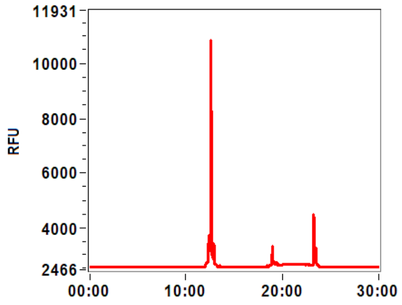

H3: SampH3

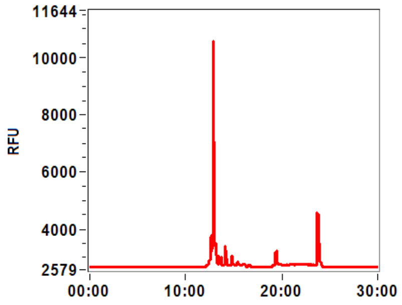

Filename and data path: C:\AATI\Data\2021 09 14\17-24-14\2021 09 14 17H 24M..raw

A4: SampA4

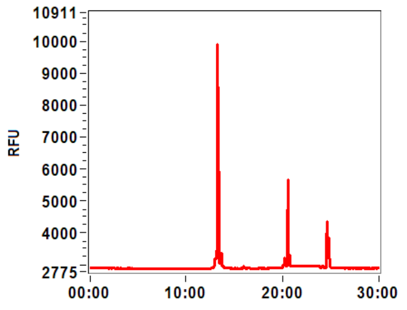

B4: SampB4

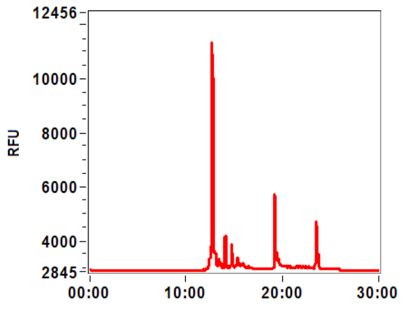

C4: SampC4

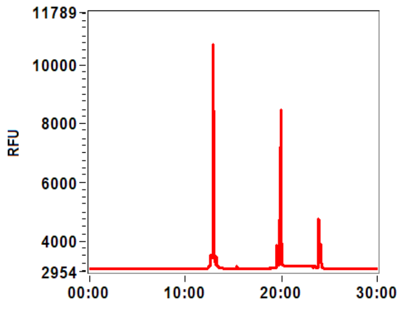

D4: SampD4

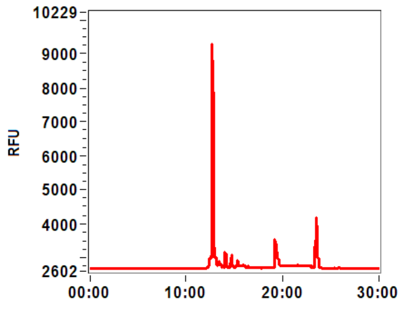

E4: SampE4

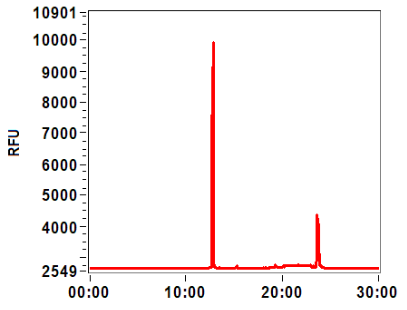

F4: SampF4

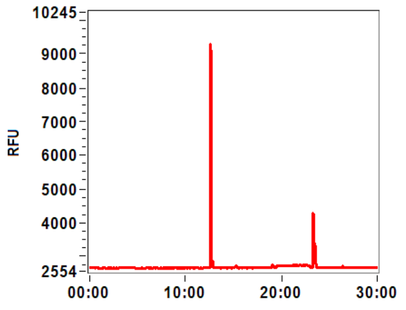

G4: SampG4

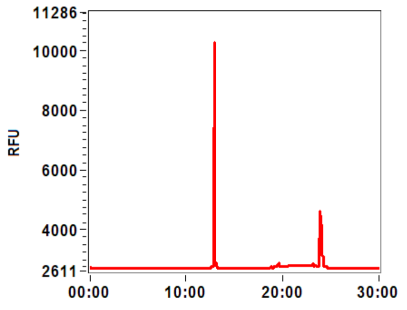

H4: SampH4

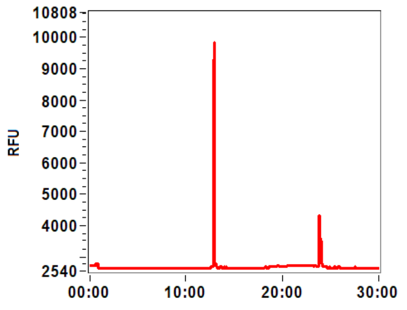

A5: SampA5

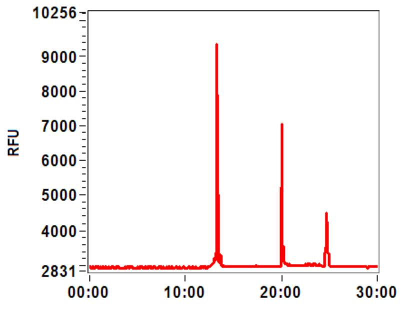

B5: SampB5

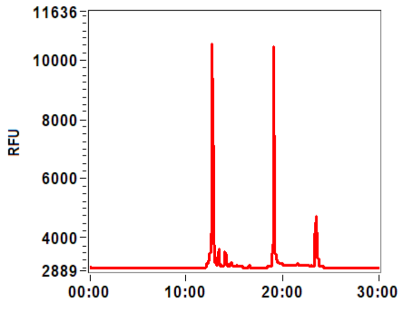

C5: SampC5

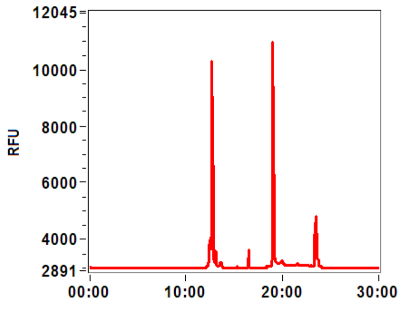

D5: SampD5

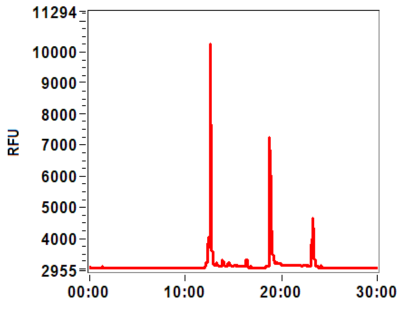

Filename and data path: C:\AATI\Data\2021 09 14\17-24-14\2021 09 14 17H 24M..raw

E5: SampE5

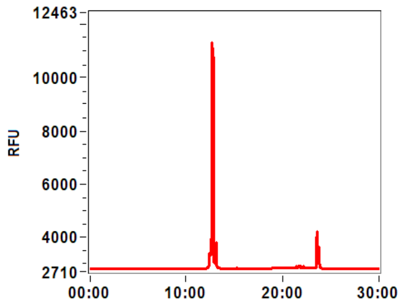

F5: SampF5

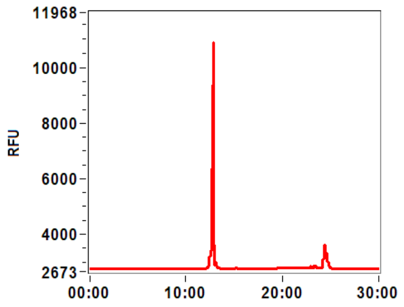

G5: SampG5

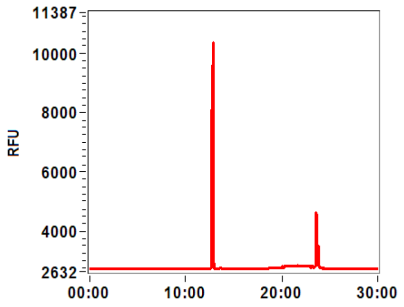

H5: SampH5

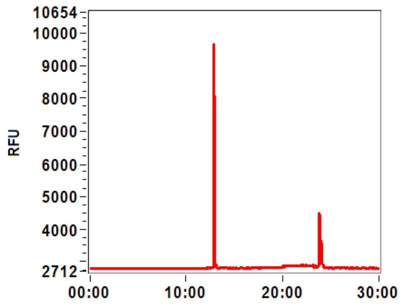

A6: SampA6

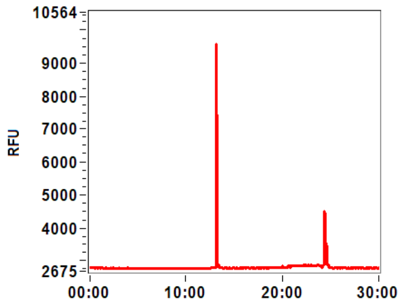

B6: SampB6

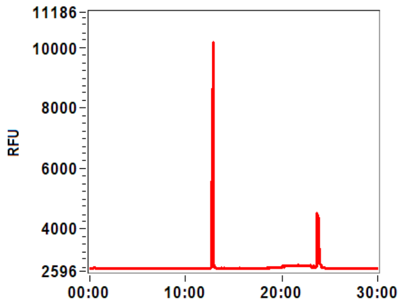

C6: SampC6

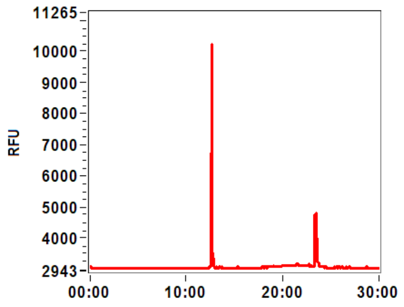

D6: SampD6

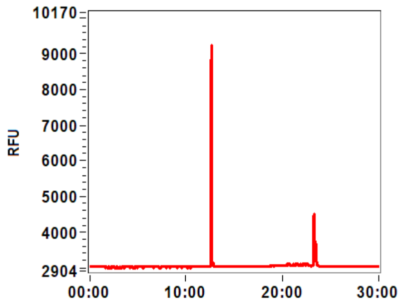

E6: SampE6

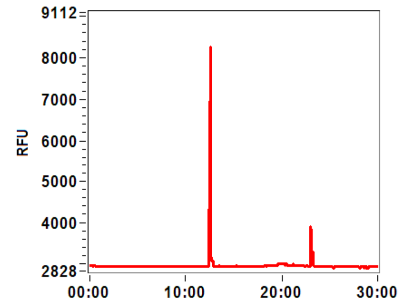

F6: SampF6

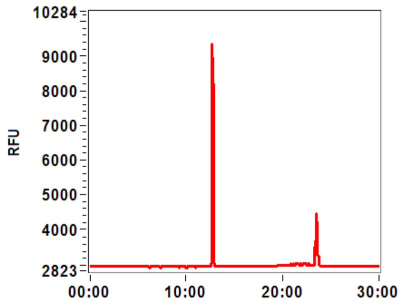

G6: SampG6

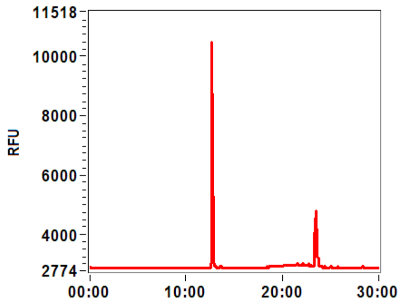

H6: SampH6

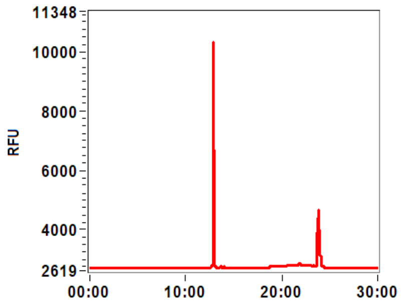

Filename and data path: C:\AATI\Data\2021 09 14\17-24-14\2021 09 14 17H 24M..raw

A7: SampA7

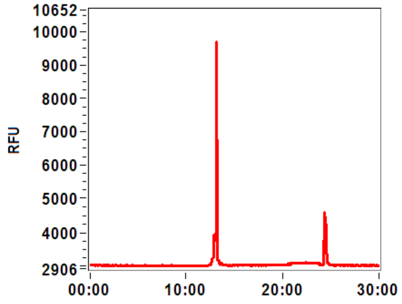

B7: SampB7

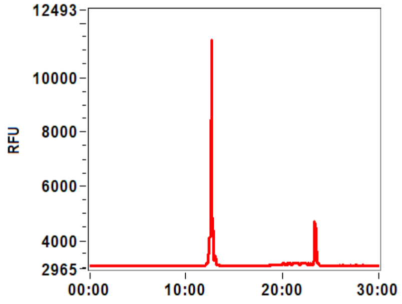

C7: SampC7

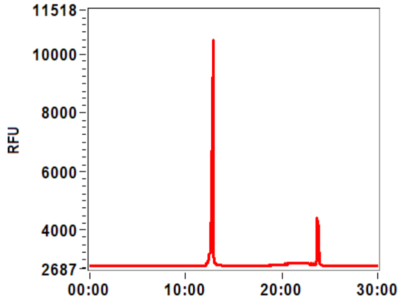

D7: SampD7

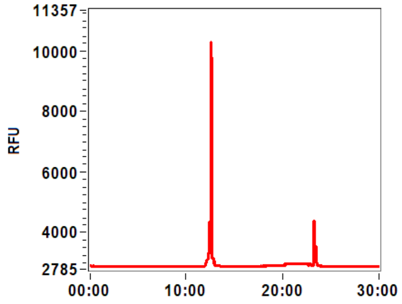

E7: SampE7

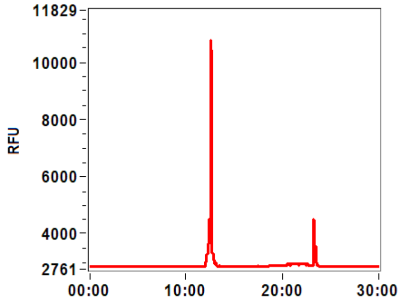

F7: SampF7

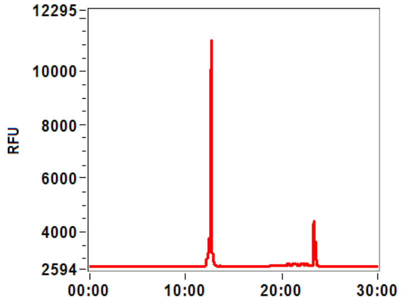

G7: SampG7

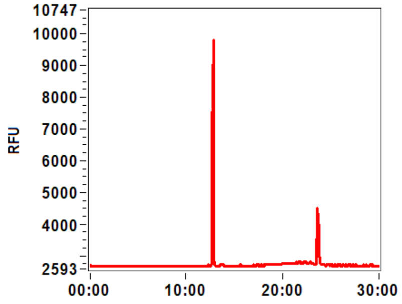

H7: SampH7

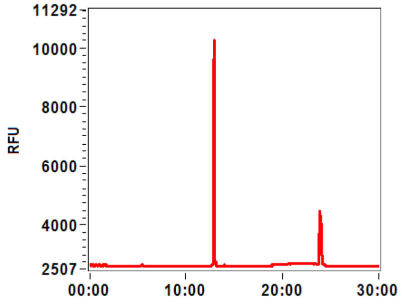

A8: SampA8

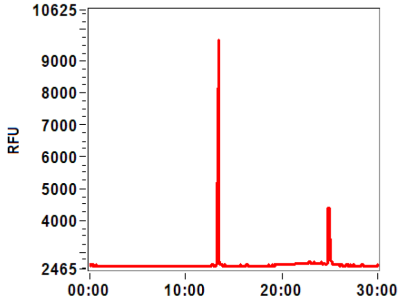

B8: SampB8

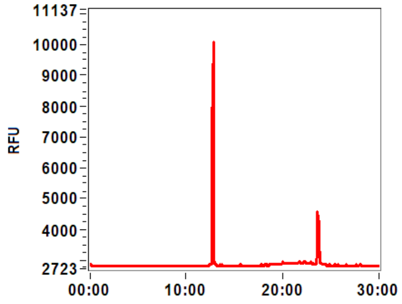

C8: SampC8

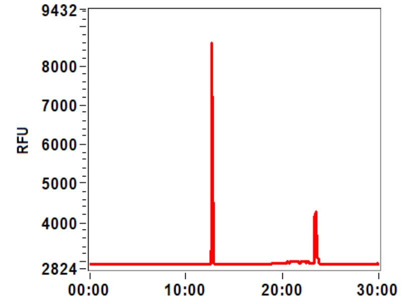

D8: SampD8

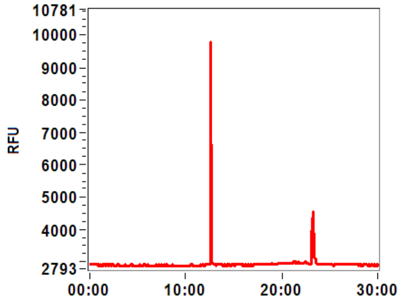

Filename and data path: C:\AATI\Data\2021 09 14\17-24-14\2021 09 14 17H 24M..raw

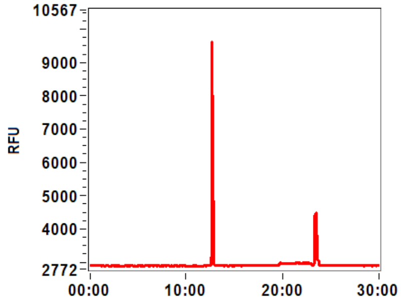

E8: SampE8

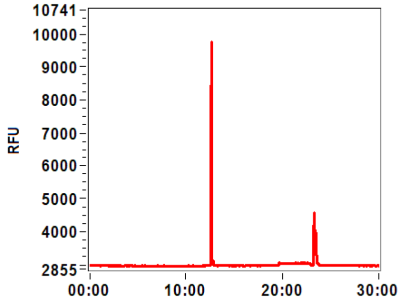

F8: SampF8

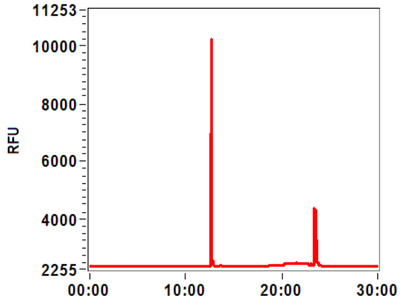

G8: SampG8

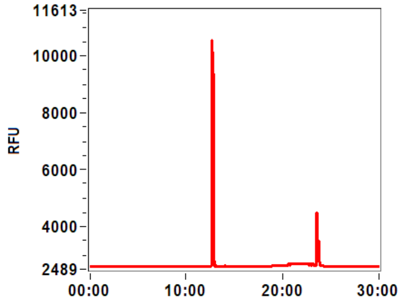

H8: SampH8

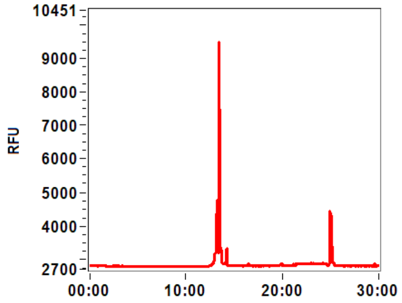

A9: SampA9

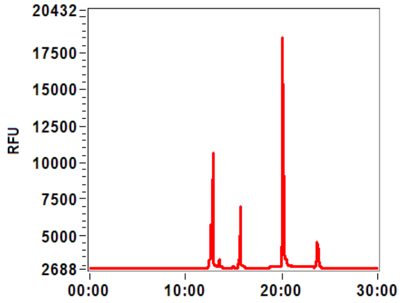

B9: SampB9

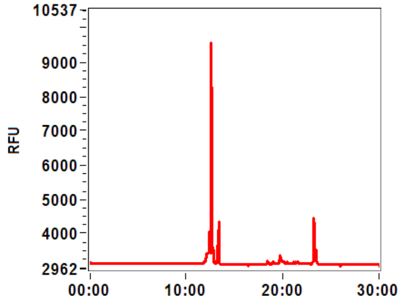

C9: SampC9

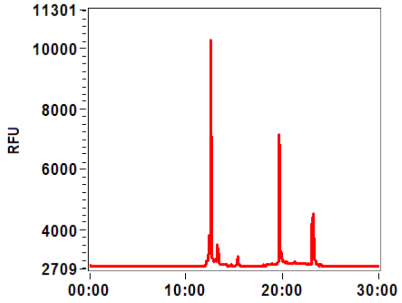

D9: SampD9

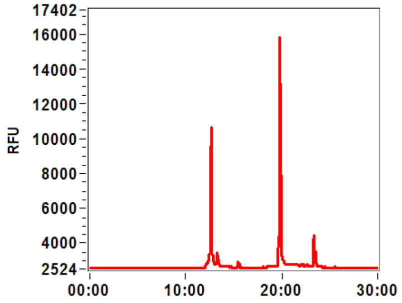

E9: SampE9

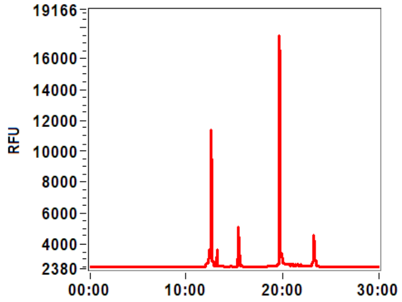

F9: SampF9

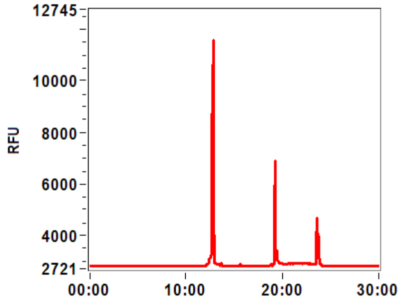

G9: SampG9

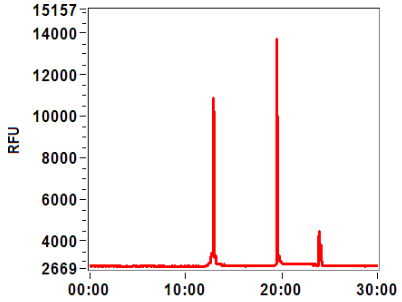

H9: SampH9

Filename and data path: C:\AATI\Data\2021 09 14\17-24-14\2021 09 14 17H 24M..raw

A10: SampA10

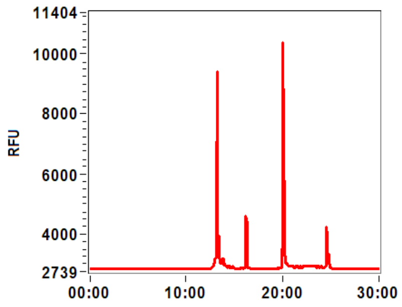

B10: SampB10

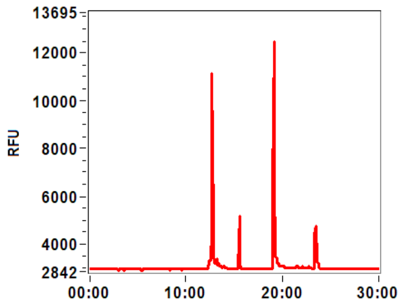

C10: SampC10

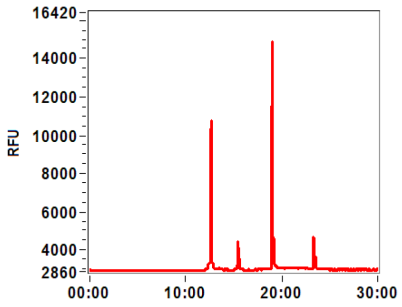

D10: SampD10

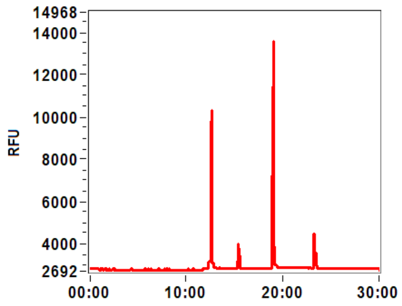

E10: SampE10

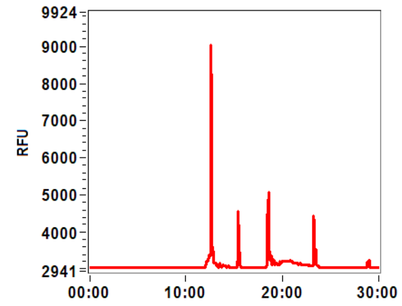

F10: SampF10

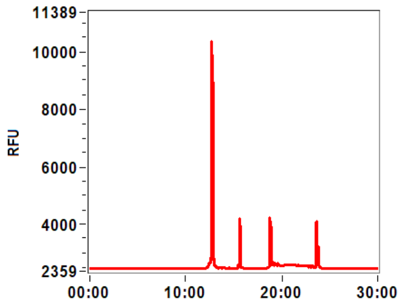

G10: SampG10

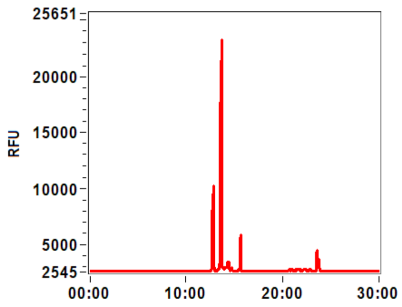

H10: SampH10

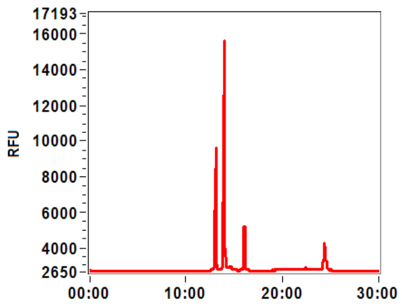

A11: SampA11

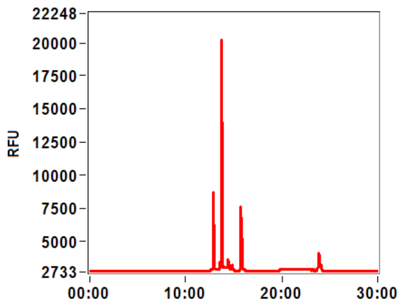

B11: SampB11

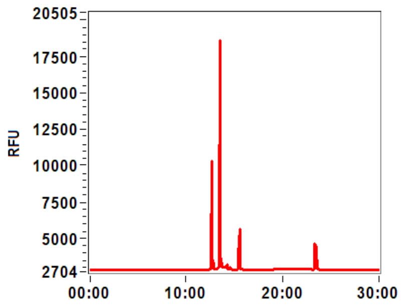

C11: SampC11

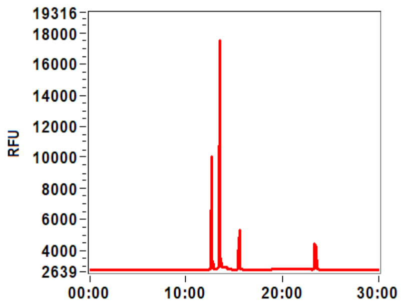

D11: SampD11

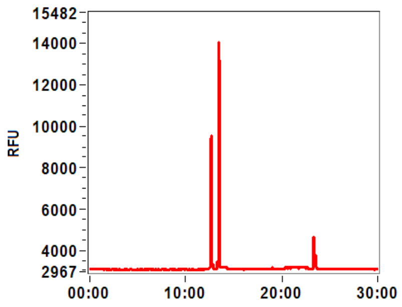

Filename and data path: C:\AATI\Data\2021 09 14\17-24-14\2021 09 14 17H 24M..raw

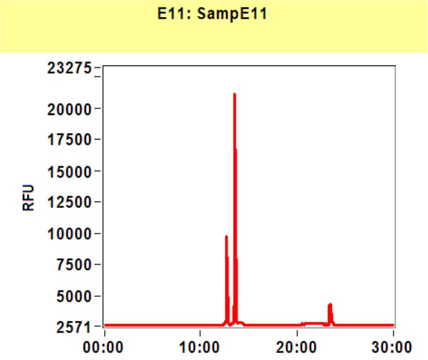

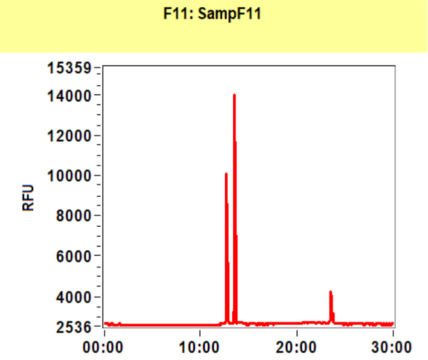

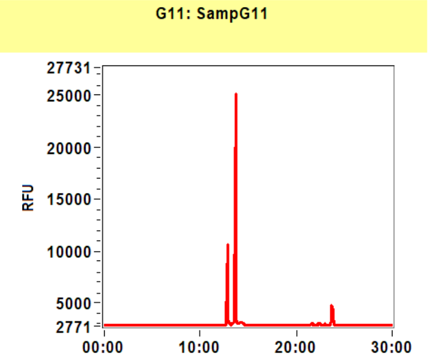

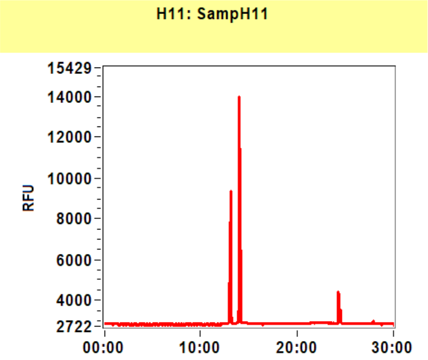

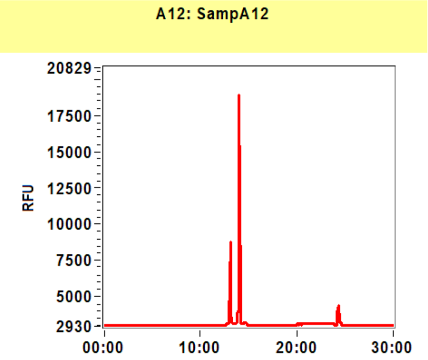

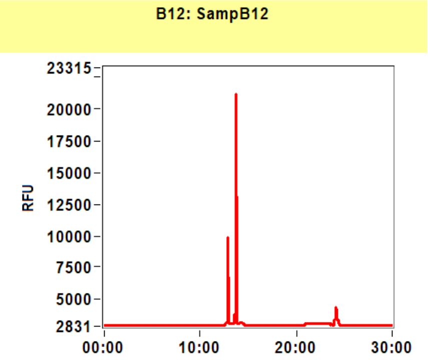

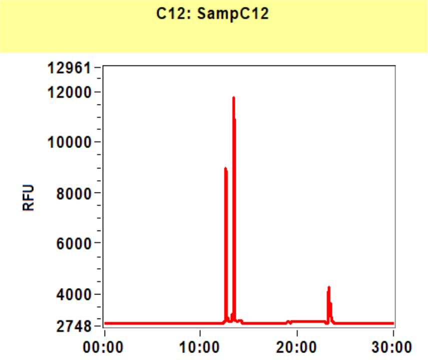

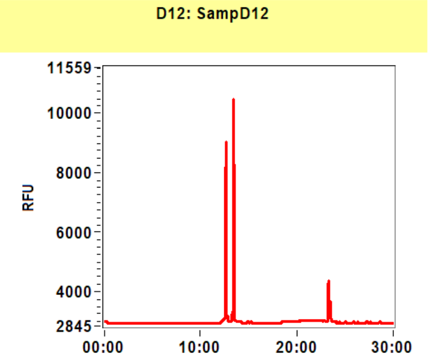

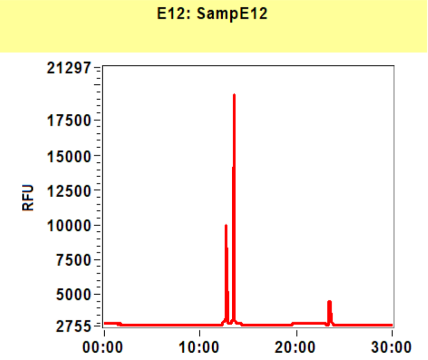

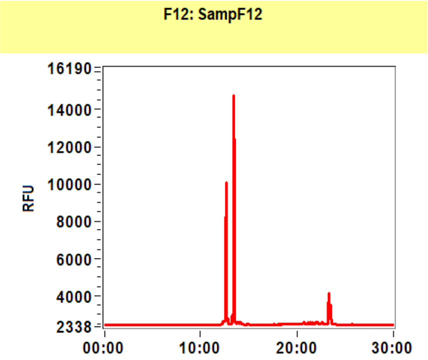

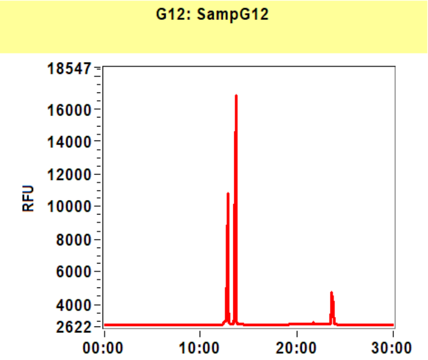

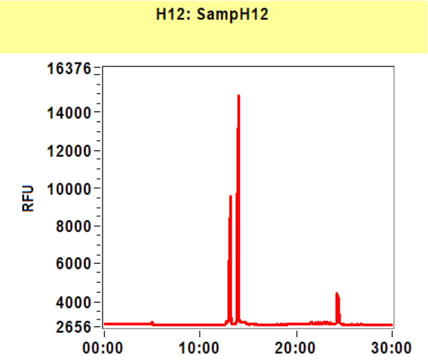

Sample: SampA1
Well location: A1
Created: Tuesday, September 14, 2021 5:34:08 PM

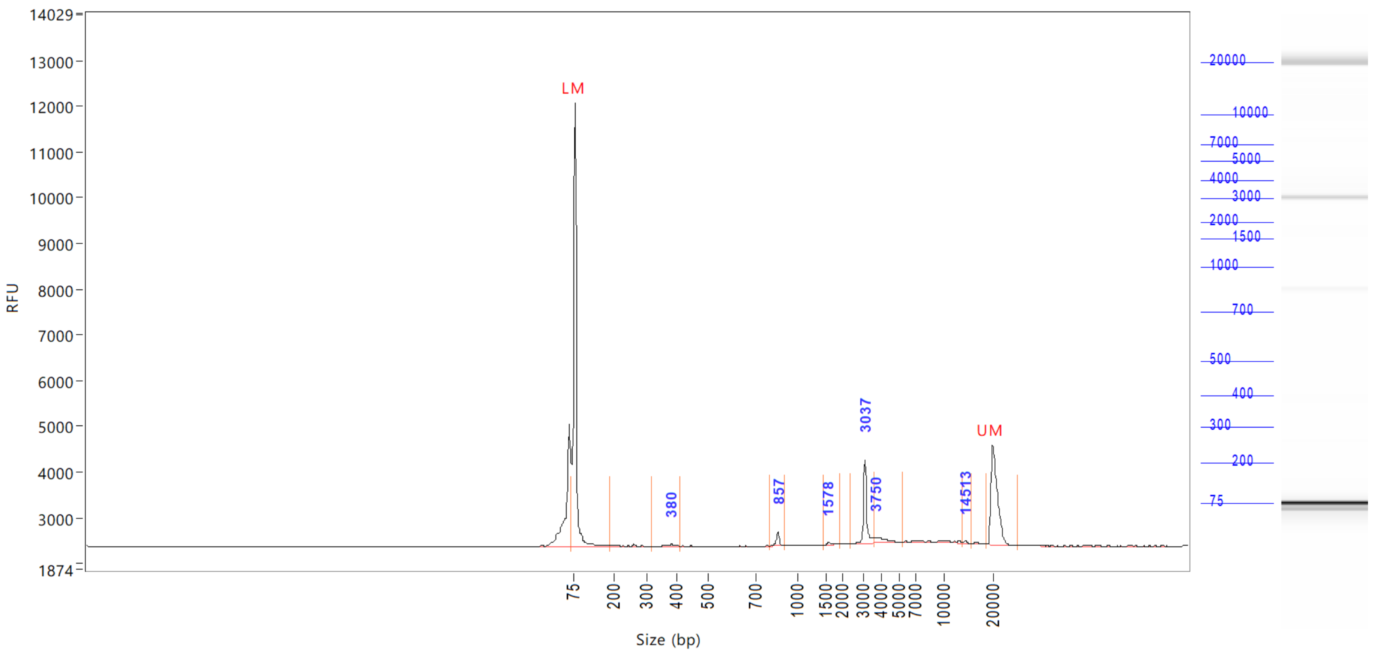

Peak	Size (bp)	Concentration (ng/uL)	From (bp)	To (bp)	Average size (bp)	CV%	RFU	Corrected peak area
1	75 (LM)	0.5000	66	185	76	14.33	9715	66.532
2	380	0.0668	313	409	373	5.43	54	0.740
3	857	0.1540	788	900	848	1.81	319	1.708
4	1578	0.0461	1478	1923	1671	6.84	52	0.511
5	3037	0.8080	2372	3572	3063	4.72	1827	8.960
6	3750	0.1720	3572	5371	4018	8.26	116	1.907
7	14513	0.0229	13903	15732	14392	2.04	56	0.254
8	20000 (UM)	0.1384	18659	25078	20392	3.52	2176	18.420

TIC:	1.2697	ng/uL
TIM:	1.1461	nmole/L
Total concentration:	1.5416	ng/uL

```
Sample peak width (sec): 5    Sample min peak height: 50    Sample baseline V to V?: Y    Sample baseline V to V points: 3
Sample filter: Binomial       Number of points for filter: 3  Sample start region (min): 0  Sample end region (min): 30
Marker peak width (sec): 5    Marker min peak height: 200   Marker baseline V to V?: Y    Marker baseline V to V points: 3
Lower marker selection: First peak > 500 RFU                Upper marker selection: Last peak > 200 RFU
Ladder size (bp)75, 200, 300, 400, 500, 700, 1000, 1500, 2000, 3000, 4000, 5000, 7000, 10000, 20000
Quantification using: Lower Marker          Final concentration (ng/uL): 0.5000          Dilution factor: 12.0
```

Sample: SampB1
Well location: B1
Created: Tuesday, September 14, 2021 5:34:08 PM

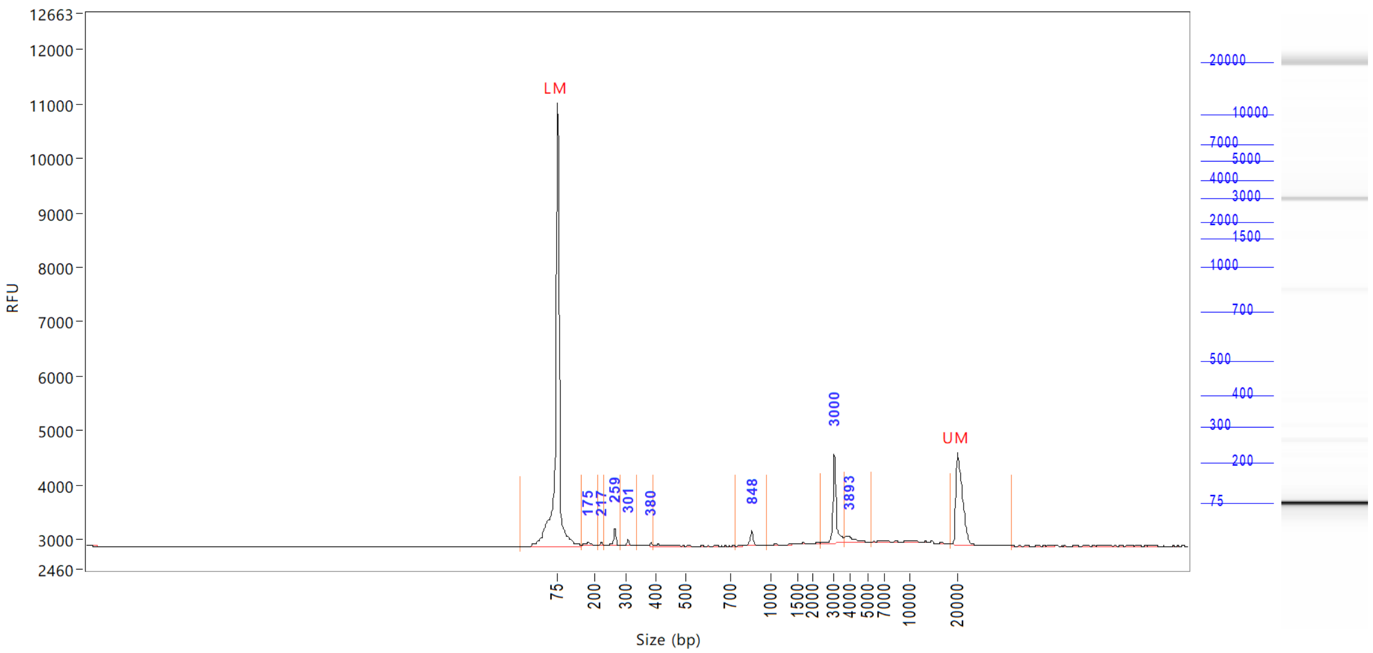

Peak	Size (bp)	Concentration (ng/uL)	From (bp)	To (bp)	Average size (bp)	CV%	RFU	Corrected peak area
1	75 (LM)	0.5000	0	154	68	28.36	8150	75.352
2	175	0.0889	154	208	175	6.98	69	1.116
3	217	0.0337	208	226	216	1.93	71	0.424
4	259	0.1468	226	280	256	3.20	304	1.844
5	301	0.0475	280	333	300	2.11	116	0.596
6	380	0.0321	333	392	375	3.13	58	0.403
7	848	0.1422	731	970	848	3.44	288	1.786
8	3000	0.6980	2315	3572	3021	5.63	1634	8.766
9	3893	0.1682	3572	5371	4088	9.85	121	2.113
10	20000 (UM)	0.0970	18415	31244	20448	4.54	1693	14.623

TIC:	1.3575	ng/uL
TIM:	3.1604	nmole/L
Total concentration:	1.5735	ng/uL

```
Sample peak width (sec): 5    Sample min peak height: 50    Sample baseline V to V?: Y    Sample baseline V to V points: 3
Sample filter: Binomial       Number of points for filter: 3  Sample start region (min): 0  Sample end region (min): 30
Marker peak width (sec): 5    Marker min peak height: 200   Marker baseline V to V?: Y    Marker baseline V to V points: 3
Lower marker selection: First peak > 500 RFU                Upper marker selection: Last peak > 200 RFU
Ladder size (bp)75, 200, 300, 400, 500, 700, 1000, 1500, 2000, 3000, 4000, 5000, 7000, 10000, 20000
Quantification using: Lower Marker          Final concentration (ng/uL): 0.5000          Dilution factor: 12.0
```

Sample: SampC1
Well location: C1
Created: Tuesday, September 14, 2021 5:34:08 PM

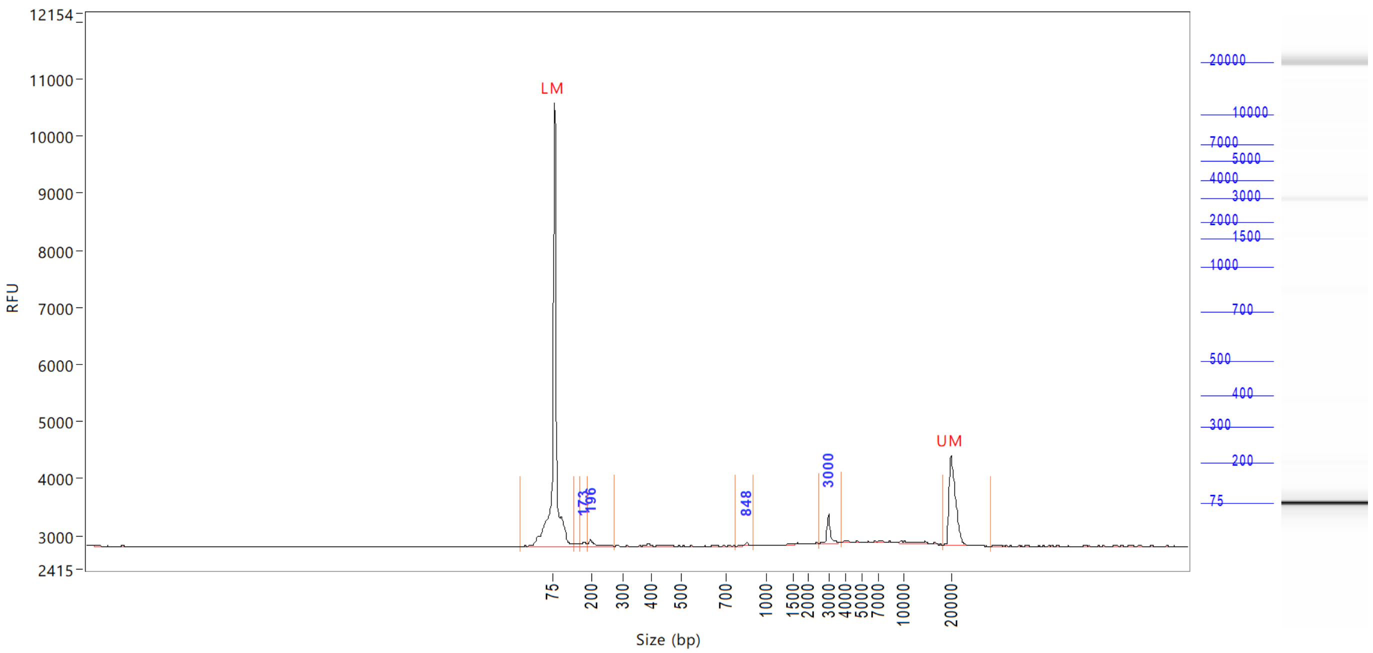

Peak	Size (bp)	Concentration (ng/uL)	From (bp)	To (bp)	Average size (bp)	CV%	RFU	Corrected peak area
1	75 (LM)	0.5000	0	141	70	26.68	7781	71.017
2	173	0.0662	160	185	173	4.14	63	0.783
3	196	0.1314	185	273	207	9.90	120	1.556
4	848	0.0316	766	900	839	2.57	54	0.374
5	3000	0.2478	2515	3822	3028	6.81	506	2.933
6	20000 (UM)	0.0956	18293	28342	20414	4.07	1569	13.578

TIC:	0.4770	ng/uL
TIM:	1.8696	nmole/L
Total concentration:	0.8245	ng/uL

Sample peak width (sec): 5   Sample min peak height: 50   Sample baseline V to V?: Y   Sample baseline V to V points: 3
Sample filter: Binomial   Number of points for filter: 3   Sample start region (min): 0   Sample end region (min): 30
Marker peak width (sec): 5   Marker min peak height: 200   Marker baseline V to V?: Y   Marker baseline V to V points: 3
Lower marker selection: First peak > 500 RFU   Upper marker selection: Last peak > 200 RFU
Ladder size (bp)75, 200, 300, 400, 500, 700, 1000, 1500, 2000, 3000, 4000, 5000, 7000, 10000, 20000
Quantification using: Lower Marker   Final concentration (ng/uL): 0.5000   Dilution factor: 12.0

Sample: SampD1
Well location: D1
Created: Tuesday, September 14, 2021 5:34:08 PM

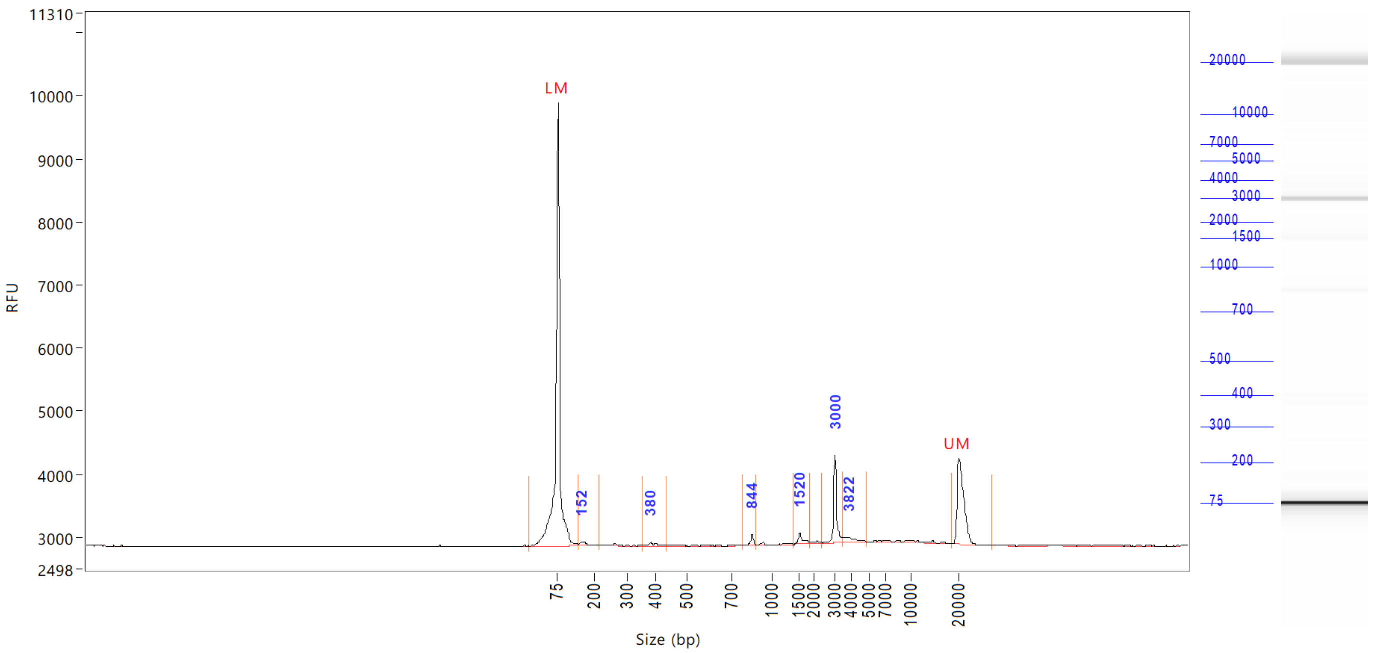

Peak	Size (bp)	Concentration (ng/uL)	From (bp)	To (bp)	Average size (bp)	CV%	RFU	Corrected peak area
1	75 (LM)	0.5000	0	141	69	25.54	7037	64.461
2	152	0.0915	141	210	160	9.16	57	0.983
3	380	0.0787	349	433	387	4.66	52	0.845
4	844	0.0811	770	883	841	1.61	170	0.872
5	1520	0.1262	1411	1846	1579	6.77	174	1.355
6	3000	0.6336	2372	3465	2995	4.33	1374	6.807
7	3822	0.1337	3465	4862	3947	8.74	81	1.437
8	20000 (UM)	0.0940	18415	27012	20497	3.84	1373	12.123

TIC:	1.1447	ng/uL
TIM:	1.9685	nmole/L
Total concentration:	1.4530	ng/uL

```
Sample peak width (sec): 5     Sample min peak height: 50     Sample baseline V to V?: Y     Sample baseline V to V points: 3
Sample filter: Binomial        Number of points for filter: 3  Sample start region (min): 0  Sample end region (min): 30
Marker peak width (sec): 5     Marker min peak height: 200    Marker baseline V to V?: Y     Marker baseline V to V points: 3
Lower marker selection: First peak > 500 RFU                  Upper marker selection: Last peak > 200 RFU
Ladder size (bp)75, 200, 300, 400, 500, 700, 1000, 1500, 2000, 3000, 4000, 5000, 7000, 10000, 20000
Quantification using: Lower Marker          Final concentration (ng/uL): 0.5000          Dilution factor: 12.0
```

Sample: SampE1
Well location: E1
Created: Tuesday, September 14, 2021 5:34:08 PM

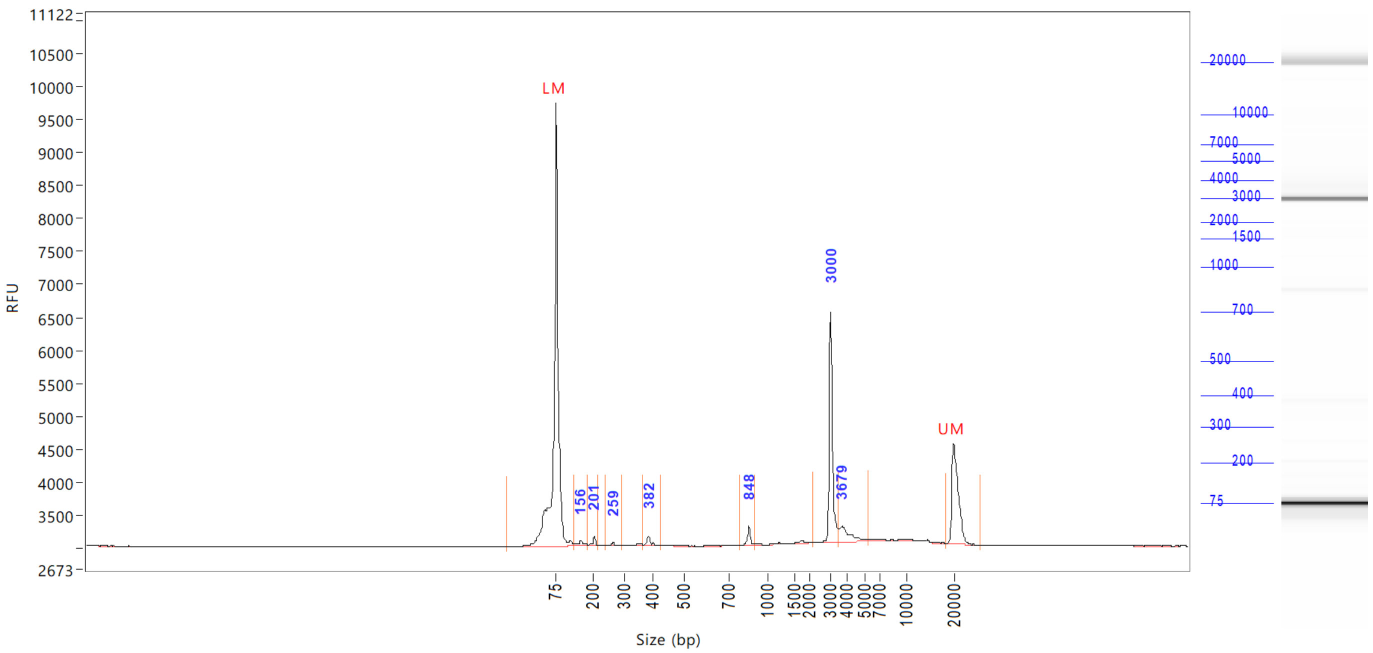

Peak	Size (bp)	Concentration (ng/uL)	From (bp)	To (bp)	Average size (bp)	CV%	RFU	Corrected peak area
1	75 (LM)	0.5000	0	135	66	31.32	6747	71.260
2	156	0.0956	135	181	156	7.51	76	1.135
3	201	0.0751	181	212	197	3.04	135	0.892
4	259	0.0333	237	287	257	3.56	54	0.396
5	382	0.1022	362	424	381	2.61	157	1.214
6	848	0.1322	783	896	844	1.95	293	1.570
7	3000	1.4727	2087	3500	2999	4.67	3501	17.491
8	3679	0.3220	3500	5371	4019	10.56	235	3.824
9	20000 (UM)	0.0928	18415	25561	20451	3.70	1530	13.229

TIC:	2.2331	ng/uL
TIM:	3.4870	nmole/L
Total concentration:	2.5084	ng/uL

```
Sample peak width (sec): 5    Sample min peak height: 50    Sample baseline V to V?: Y    Sample baseline V to V points: 3
Sample filter: Binomial       Number of points for filter: 3  Sample start region (min): 0  Sample end region (min): 30
Marker peak width (sec): 5    Marker min peak height: 200   Marker baseline V to V?: Y    Marker baseline V to V points: 3
Lower marker selection: First peak > 500 RFU                Upper marker selection: Last peak > 200 RFU
Ladder size (bp)75, 200, 300, 400, 500, 700, 1000, 1500, 2000, 3000, 4000, 5000, 7000, 10000, 20000
Quantification using: Lower Marker          Final concentration (ng/uL): 0.5000          Dilution factor: 12.0
```

Sample: SampF1
Well location: F1
Created: Tuesday, September 14, 2021 5:34:08 PM

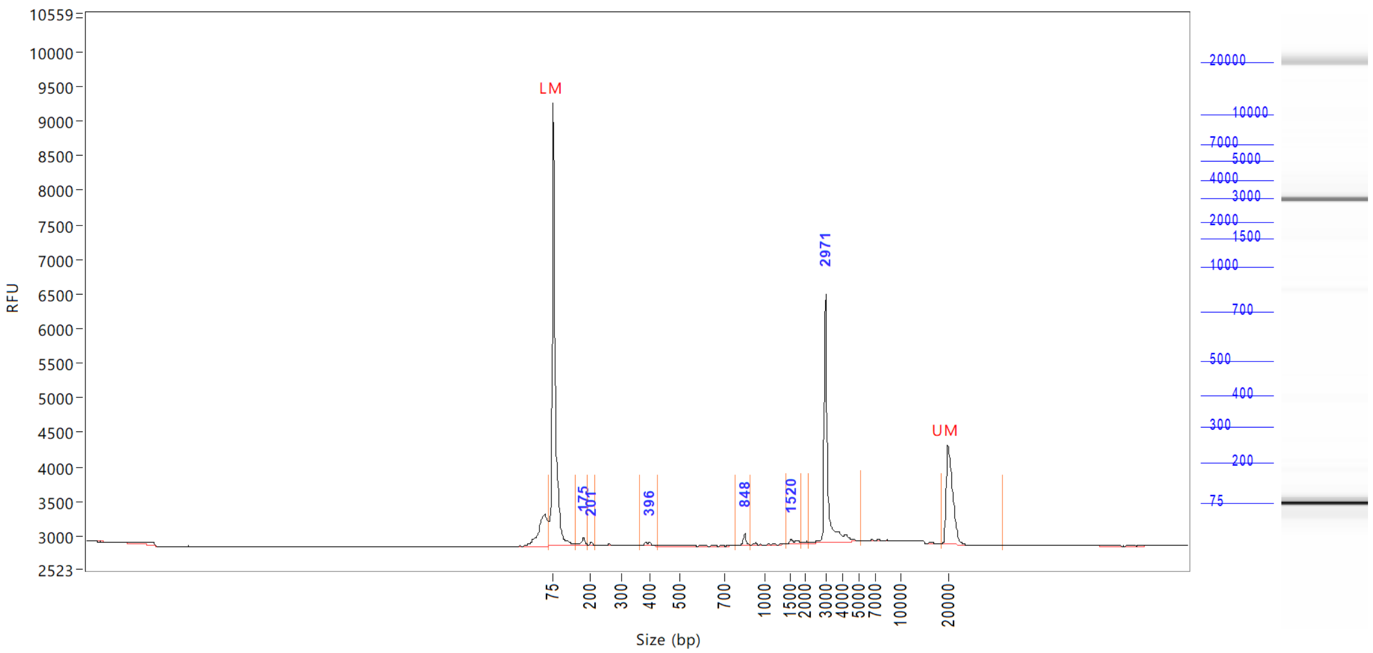

Peak	Size (bp)	Concentration (ng/uL)	From (bp)	To (bp)	Average size (bp)	CV%	RFU	Corrected peak area
1	75 (LM)	0.5000	60	152	77	14.82	6415	50.279
2	175	0.1418	152	192	172	5.07	121	1.188
3	201	0.0404	192	215	200	1.92	57	0.338
4	396	0.0753	362	422	386	2.93	54	0.631
5	848	0.1082	783	892	843	1.98	165	0.907
6	1520	0.0911	1411	1846	1604	6.93	75	0.763
7	2971	2.4301	2115	5075	3091	11.89	3590	20.364
8	20000 (UM)	0.1226	18537	31607	20481	4.76	1435	12.326

TIC:	2.8868	ng/uL
TIM:	3.6066	nmole/L
Total concentration:	3.2133	ng/uL

Sample peak width (sec): 5 Sample min peak height: 50 Sample baseline V to V?: Y Sample baseline V to V points: 3
Sample filter: Binomial Number of points for filter: 3 Sample start region (min): 0 Sample end region (min): 30
Marker peak width (sec): 5 Marker min peak height: 200 Marker baseline V to V?: Y Marker baseline V to V points: 3
Lower marker selection: First peak > 500 RFU Upper marker selection: Last peak > 200 RFU
Ladder size (bp)75, 200, 300, 400, 500, 700, 1000, 1500, 2000, 3000, 4000, 5000, 7000, 10000, 20000
Quantification using: Lower Marker Final concentration (ng/uL): 0.5000 Dilution factor: 12.0

Sample: SampG1
Well location: G1
Created: Tuesday, September 14, 2021 5:34:08 PM

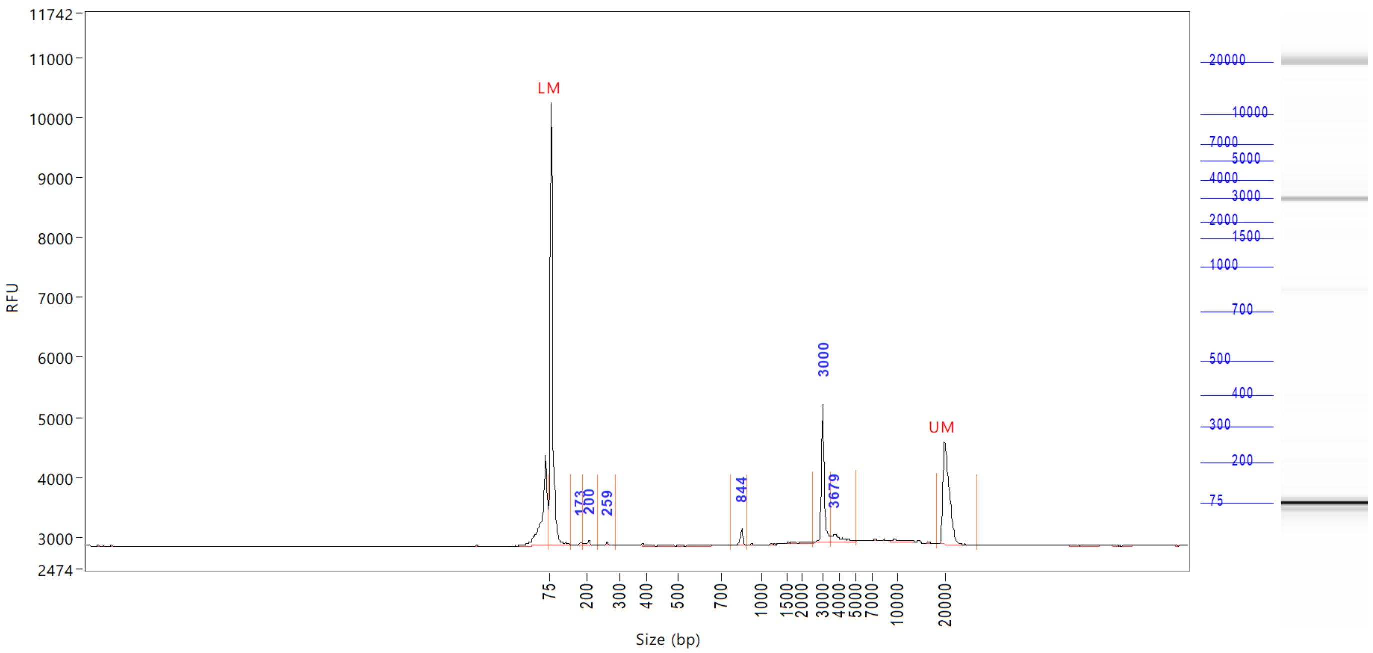

Peak	Size (bp)	Concentration (ng/uL)	From (bp)	To (bp)	Average size (bp)	CV%	RFU	Corrected peak area
1	75 (LM)	0.5000	66	143	76	11.44	7396	50.371
2	173	0.0925	143	185	167	7.12	58	0.777
3	200	0.0941	185	232	198	4.15	85	0.790
4	259	0.0476	232	286	256	3.40	52	0.399
5	844	0.1750	766	892	839	2.02	276	1.469
6	3000	1.2872	2515	3465	2977	4.05	2295	10.806
7	3679	0.2344	3465	5000	3922	8.96	129	1.968
8	20000 (UM)	0.1481	18415	26891	20449	4.07	1716	14.920

TIC:	1.9308	ng/uL
TIM:	3.1513	nmole/L
Total concentration:	2.3313	ng/uL

```
Sample peak width (sec): 5    Sample min peak height: 50    Sample baseline V to V?: Y    Sample baseline V to V points: 3
Sample filter: Binomial       Number of points for filter: 3  Sample start region (min): 0  Sample end region (min): 30
Marker peak width (sec): 5    Marker min peak height: 200   Marker baseline V to V?: Y    Marker baseline V to V points: 3
Lower marker selection: First peak > 500 RFU                Upper marker selection: Last peak > 200 RFU
Ladder size (bp)75, 200, 300, 400, 500, 700, 1000, 1500, 2000, 3000, 4000, 5000, 7000, 10000, 20000
Quantification using: Lower Marker          Final concentration (ng/uL): 0.5000          Dilution factor: 12.0
```

Sample: SampH1
Well location: H1
Created: Tuesday, September 14, 2021 5:34:08 PM

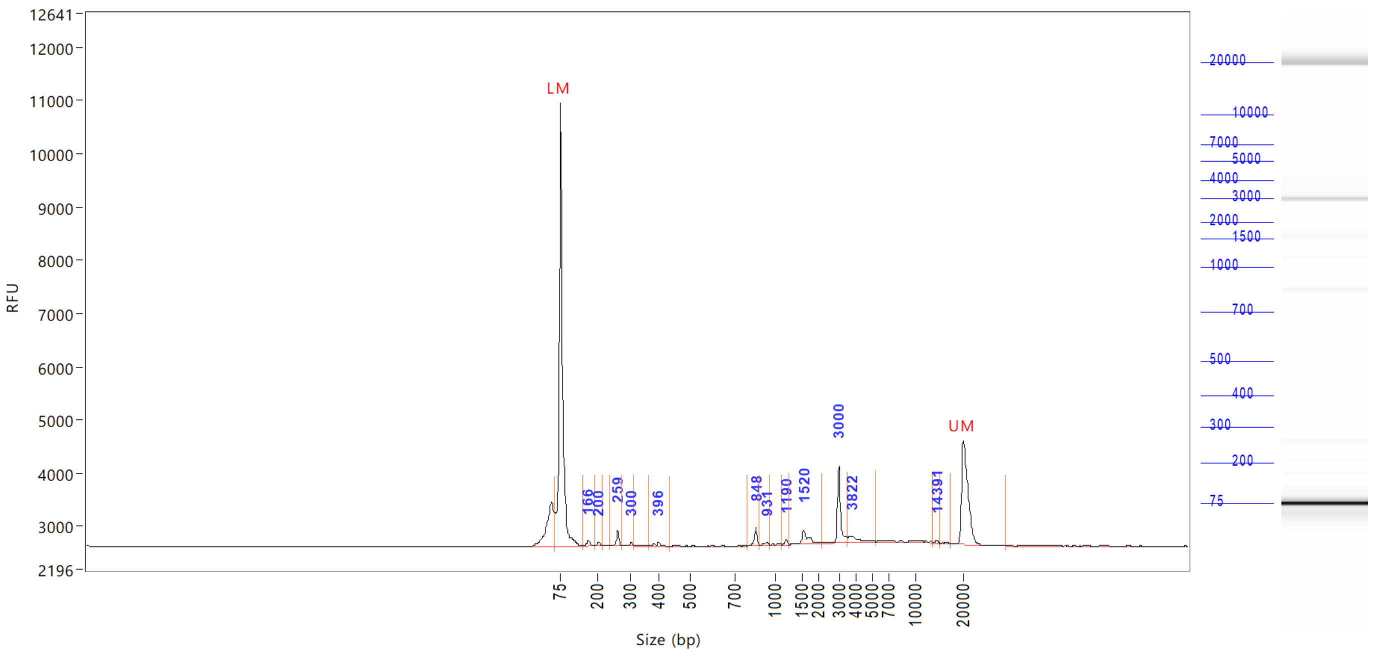

Peak	Size (bp)	Concentration (ng/uL)	From (bp)	To (bp)	Average size (bp)	CV%	RFU	Corrected peak area
1	75 (LM)	0.5000	60	147	76	13.35	8342	67.816
2	166	0.0798	147	190	164	5.38	95	0.902
3	200	0.0353	190	212	198	1.62	72	0.399
4	259	0.1426	237	271	256	1.93	303	1.611
5	300	0.0359	271	309	297	1.94	76	0.406
6	396	0.0717	364	433	388	3.21	82	0.811
7	848	0.1461	783	870	840	1.59	339	1.652
8	931	0.0401	870	952	912	2.23	59	0.453
9	1190	0.0389	1112	1245	1173	1.72	105	0.439
10	1520	0.2267	1245	2201	1621	10.02	267	2.562
11	3000	0.6547	2201	3500	3005	5.29	1451	7.400
12	3822	0.1639	3500	5297	3945	8.81	124	1.852
13	14391	0.0236	13659	15244	14271	2.26	58	0.267
14	20000 (UM)	0.1247	17439	28826	20430	4.08	1961	16.912

TIC:	1.6593	ng/uL
TIM:	3.5856	nmole/L
Total concentration:	1.8916	ng/uL

Sample peak width (sec): 5 Sample min peak height: 50 Sample baseline V to V?: Y Sample baseline V to V points: 3
Sample filter: Binomial Number of points for filter: 3 Sample start region (min): 0 Sample end region (min): 30
Marker peak width (sec): 5 Marker min peak height: 200 Marker baseline V to V?: Y Marker baseline V to V points: 3
Lower marker selection: First peak > 500 RFU Upper marker selection: Last peak > 200 RFU
Ladder size (bp)75, 200, 300, 400, 500, 700, 1000, 1500, 2000, 3000, 4000, 5000, 7000, 10000, 20000
Quantification using: Lower Marker Final concentration (ng/uL): 0.5000 Dilution factor: 12.0

Sample: SampA2
Well location: A2
Created: Tuesday, September 14, 2021 5:34:08 PM

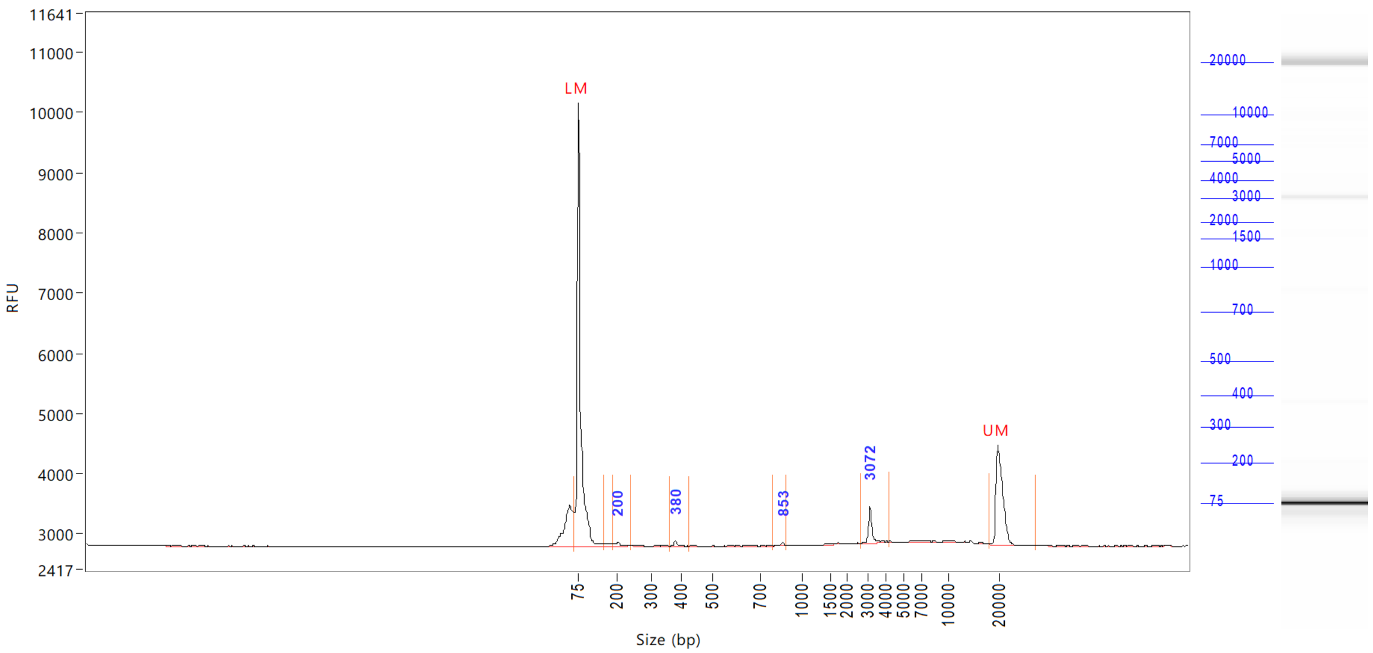

Peak	Size (bp)	Concentration (ng/uL)	From (bp)	To (bp)	Average size (bp)	CV%	RFU	Corrected peak area
1	75 (LM)	0.5000	62	158	78	15.53	7369	60.232
2	200	0.0761	187	239	201	5.40	63	0.764
3	380	0.0626	362	422	381	2.88	95	0.629
4	853	0.0387	788	874	839	2.02	64	0.388
5	3072	0.3349	2657	4139	3140	8.33	609	3.362
6	20000 (UM)	0.1166	18293	27375	20315	3.63	1647	14.045

TIC:	0.5123	ng/uL
TIM:	1.1445	nmole/L
Total concentration:	0.8575	ng/uL

Sample peak width (sec): 5 Sample min peak height: 50 Sample baseline V to V?: Y Sample baseline V to V points: 3
Sample filter: Binomial Number of points for filter: 3 Sample start region (min): 0 Sample end region (min): 30
Marker peak width (sec): 5 Marker min peak height: 200 Marker baseline V to V?: Y Marker baseline V to V points: 3
Lower marker selection: First peak > 500 RFU Upper marker selection: Last peak > 200 RFU
Ladder size (bp)75, 200, 300, 400, 500, 700, 1000, 1500, 2000, 3000, 4000, 5000, 7000, 10000, 20000
Quantification using: Lower Marker Final concentration (ng/uL): 0.5000 Dilution factor: 12.0

Sample: SampB2
Well location: B2
Created: Tuesday, September 14, 2021 5:34:08 PM

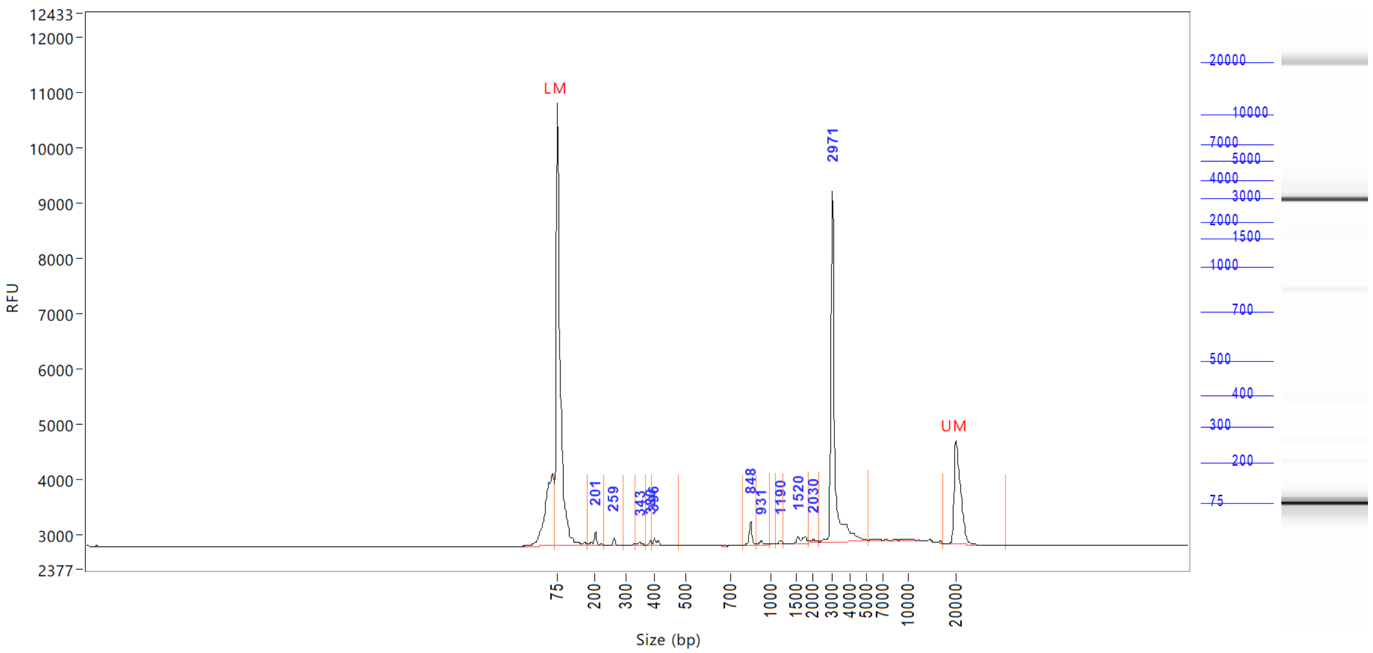

Peak	Size (bp)	Concentration (ng/uL)	From (bp)	To (bp)	Average size (bp)	CV%	RFU	Corrected peak area
1	75 (LM)	0.5000	66	177	80	18.17	8024	76.695
2	201	0.1581	177	228	198	5.03	252	2.021
3	259	0.0746	228	287	254	4.00	136	0.954
4	343	0.0452	329	368	346	2.74	53	0.578
5	380	0.0408	368	388	379	1.30	88	0.521
6	396	0.1040	388	477	409	5.29	132	1.329
7	848	0.1780	783	896	845	1.81	443	2.275
8	931	0.0395	896	996	928	2.21	74	0.505
9	1190	0.0218	1101	1245	1173	1.66	68	0.279
10	1520	0.1196	1245	1866	1618	7.93	118	1.529
11	2030	0.0460	1866	2344	2049	6.18	59	0.588
12	2971	2.8506	2344	5149	3116	12.15	6338	36.437
13	20000 (UM)	0.1054	17439	30519	20430	4.21	1867	16.165

TIC:	3.6782	ng/uL
TIM:	4.7181	nmole/L
Total concentration:	3.8262	ng/uL

```
Sample peak width (sec): 5     Sample min peak height: 50     Sample baseline V to V?: Y     Sample baseline V to V points: 3
Sample filter: Binomial        Number of points for filter: 3  Sample start region (min): 0  Sample end region (min): 30
Marker peak width (sec): 5     Marker min peak height: 200    Marker baseline V to V?: Y     Marker baseline V to V points: 3
Lower marker selection: First peak > 500 RFU                  Upper marker selection: Last peak > 200 RFU
Ladder size (bp)75, 200, 300, 400, 500, 700, 1000, 1500, 2000, 3000, 4000, 5000, 7000, 10000, 20000
Quantification using: Lower Marker          Final concentration (ng/uL): 0.5000          Dilution factor: 12.0
```

Sample: SampC2
Well location: C2
Created: Tuesday, September 14, 2021 5:34:08 PM

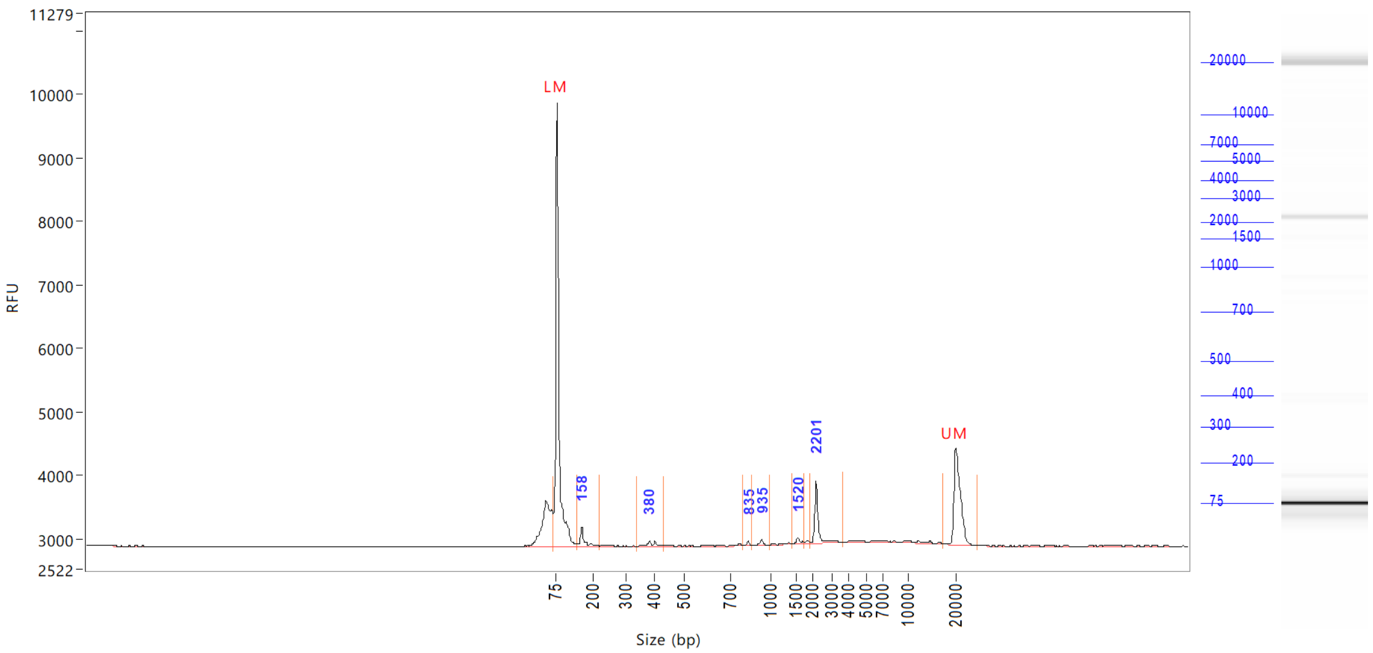

Peak	Size (bp)	Concentration (ng/uL)	From (bp)	To (bp)	Average size (bp)	CV%	RFU	Corrected peak area
1	75 (LM)	0.5000	64	141	78	15.64	6996	52.373
2	158	0.3146	141	214	161	8.31	302	2.746
3	380	0.1259	335	428	385	4.53	79	1.099
4	835	0.0383	788	866	825	1.51	67	0.334
5	935	0.0656	866	991	926	2.12	91	0.573
6	1520	0.0797	1411	1750	1557	5.53	91	0.696
7	2201	0.6201	1942	3643	2249	11.61	981	5.413
8	20000 (UM)	0.1250	17439	24594	20357	3.66	1529	13.095

TIC:	1.2443	ng/uL
TIM:	4.4810	nmole/L
Total concentration:	1.6769	ng/uL

Sample peak width (sec): 5 Sample min peak height: 50 Sample baseline V to V?: Y Sample baseline V to V points: 3
Sample filter: Binomial Number of points for filter: 3 Sample start region (min): 0 Sample end region (min): 30
Marker peak width (sec): 5 Marker min peak height: 200 Marker baseline V to V?: Y Marker baseline V to V points: 3
Lower marker selection: First peak > 500 RFU Upper marker selection: Last peak > 200 RFU
Ladder size (bp)75, 200, 300, 400, 500, 700, 1000, 1500, 2000, 3000, 4000, 5000, 7000, 10000, 20000
Quantification using: Lower Marker Final concentration (ng/uL): 0.5000 Dilution factor: 12.0

Sample: SampD2
Well location: D2
Created: Tuesday, September 14, 2021 5:34:08 PM

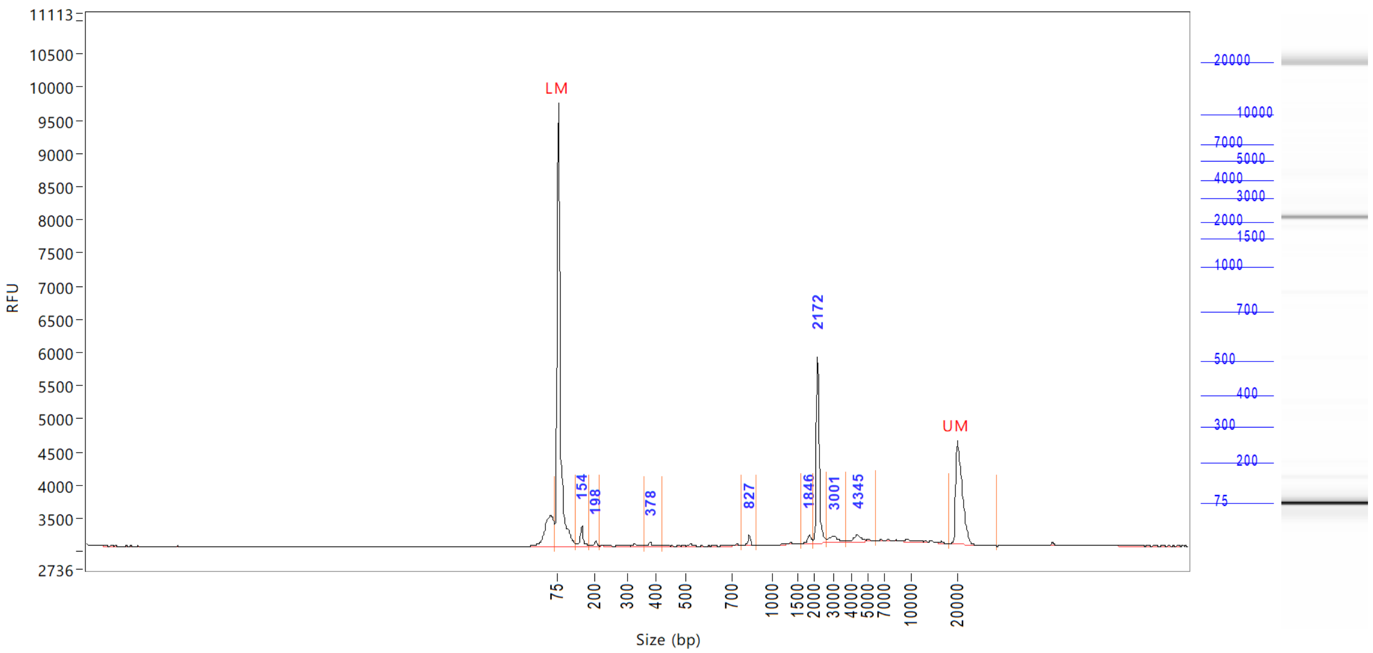

Peak	Size (bp)	Concentration (ng/uL)	From (bp)	To (bp)	Average size (bp)	CV%	RFU	Corrected peak area
1	75 (LM)	0.5000	64	131	78	13.66	6692	49.748
2	154	0.2850	131	177	151	5.56	297	2.363
3	198	0.0722	177	210	194	3.46	83	0.599
4	378	0.0590	360	418	380	3.31	62	0.489
5	827	0.0935	770	887	822	1.74	154	0.776
6	1846	0.1029	1597	1981	1842	4.24	132	0.853
7	2172	1.5448	1981	2629	2171	4.64	2798	12.808
8	3001	0.1738	2629	3715	2979	8.18	95	1.441
9	4345	0.1760	3715	6038	4499	10.03	106	1.459
10	20000 (UM)	0.1353	18171	28463	20413	4.31	1553	13.464

TIC:	2.5073	ng/uL
TIM:	5.5805	nmole/L
Total concentration:	2.8818	ng/uL

```
Sample peak width (sec): 5    Sample min peak height: 50    Sample baseline V to V?: Y    Sample baseline V to V points: 3
Sample filter: Binomial       Number of points for filter: 3  Sample start region (min): 0  Sample end region (min): 30
Marker peak width (sec): 5    Marker min peak height: 200   Marker baseline V to V?: Y    Marker baseline V to V points: 3
Lower marker selection: First peak > 500 RFU                Upper marker selection: Last peak > 200 RFU
Ladder size (bp)75, 200, 300, 400, 500, 700, 1000, 1500, 2000, 3000, 4000, 5000, 7000, 10000, 20000
Quantification using: Lower Marker          Final concentration (ng/uL): 0.5000          Dilution factor: 12.0
```

Sample: SampE2
Well location: E2
Created: Tuesday, September 14, 2021 5:34:08 PM

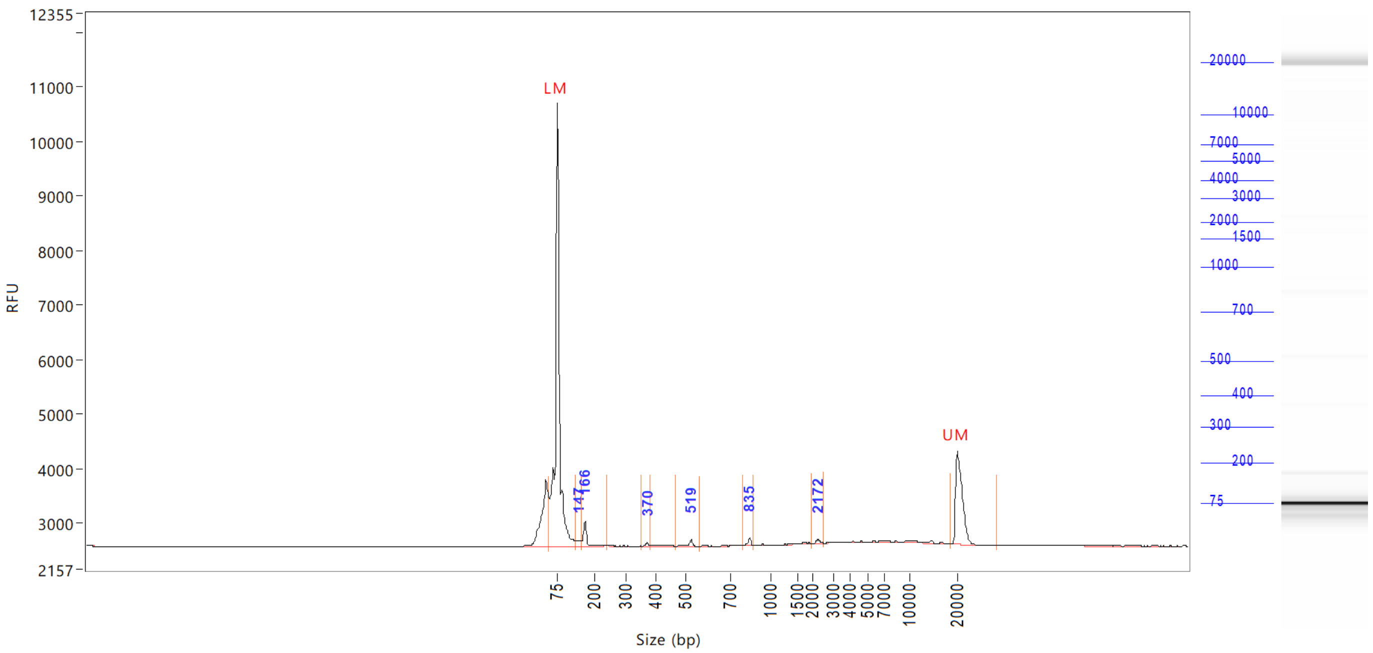

Peak	Size (bp)	Concentration (ng/uL)	From (bp)	To (bp)	Average size (bp)	CV%	RFU	Corrected peak area
1	75 (LM)	0.5000	48	137	74	18.68	8144	75.246
2	147	0.0999	137	156	147	4.06	108	1.253
3	166	0.2366	156	239	166	6.85	452	2.967
4	370	0.0308	349	380	366	1.36	65	0.386
5	519	0.0633	464	557	511	2.66	126	0.794
6	835	0.0584	779	861	825	1.79	134	0.733
7	2172	0.0330	1962	2515	2166	4.14	76	0.414
8	20000 (UM)	0.1002	18415	28221	20388	3.98	1711	15.084

TIC:	0.5220	ng/uL
TIM:	3.9431	nmole/L
Total concentration:	0.7930	ng/uL

```
Sample peak width (sec): 5    Sample min peak height: 50    Sample baseline V to V?: Y    Sample baseline V to V points: 3
Sample filter: Binomial       Number of points for filter: 3  Sample start region (min): 0  Sample end region (min): 30
Marker peak width (sec): 5    Marker min peak height: 200   Marker baseline V to V?: Y    Marker baseline V to V points: 3
Lower marker selection: First peak > 500 RFU                Upper marker selection: Last peak > 200 RFU
Ladder size (bp)75, 200, 300, 400, 500, 700, 1000, 1500, 2000, 3000, 4000, 5000, 7000, 10000, 20000
Quantification using: Lower Marker          Final concentration (ng/uL): 0.5000          Dilution factor: 12.0
```

Sample: SampF2
Well location: F2
Created: Tuesday, September 14, 2021 5:34:08 PM

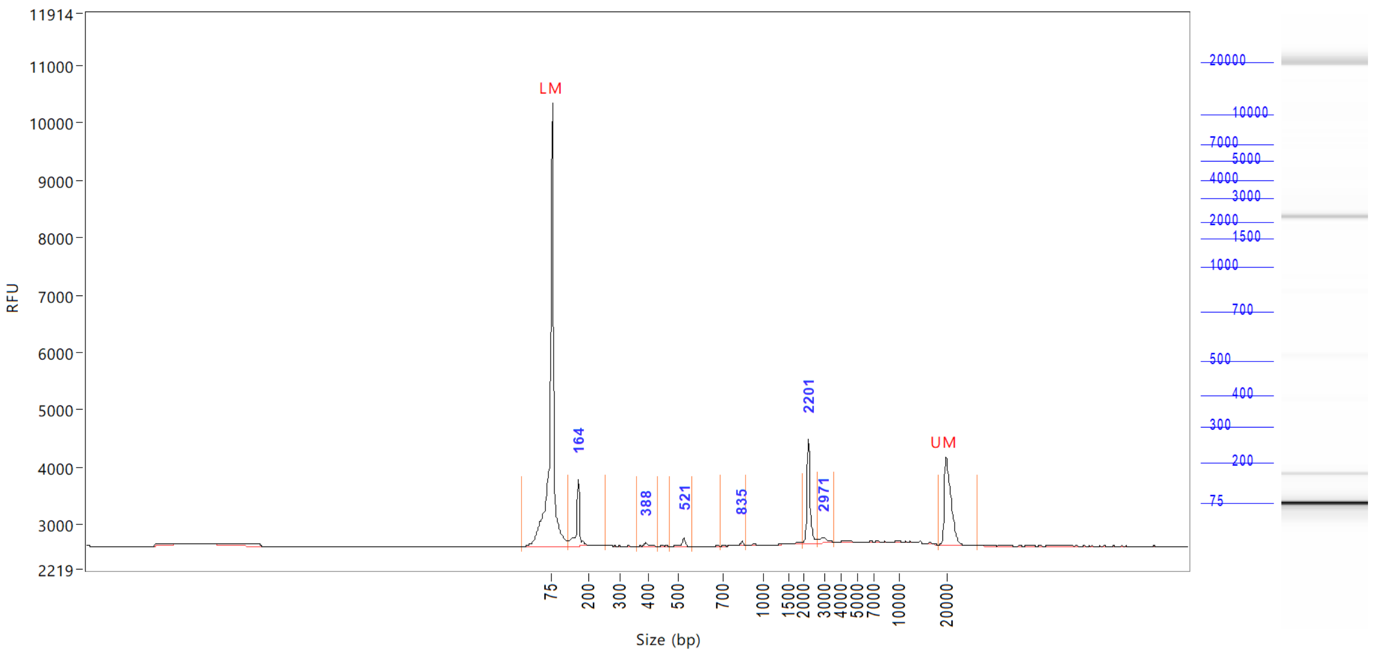

Peak	Size (bp)	Concentration (ng/uL)	From (bp)	To (bp)	Average size (bp)	CV%	RFU	Corrected peak area
1	75 (LM)	0.5000	0	129	68	27.63	7743	75.792
2	164	0.7231	129	255	159	8.52	1160	9.134
3	388	0.0392	360	428	390	2.74	60	0.495
4	521	0.0850	468	562	516	2.53	161	1.074
5	835	0.0392	685	874	820	4.40	78	0.495
6	2201	0.7563	1942	2629	2194	5.05	1812	9.554
7	2971	0.1019	2629	3608	2959	7.69	84	1.287
8	20000 (UM)	0.0906	18293	26529	20496	4.11	1538	13.732

TIC:	1.7448	ng/uL
TIM:	8.6146	nmole/L
Total concentration:	1.9588	ng/uL

```
Sample peak width (sec): 5    Sample min peak height: 50    Sample baseline V to V?: Y    Sample baseline V to V points: 3
Sample filter: Binomial       Number of points for filter: 3  Sample start region (min): 0  Sample end region (min): 30
Marker peak width (sec): 5    Marker min peak height: 200   Marker baseline V to V?: Y    Marker baseline V to V points: 3
Lower marker selection: First peak > 500 RFU                Upper marker selection: Last peak > 200 RFU
Ladder size (bp)75, 200, 300, 400, 500, 700, 1000, 1500, 2000, 3000, 4000, 5000, 7000, 10000, 20000
Quantification using: Lower Marker          Final concentration (ng/uL): 0.5000          Dilution factor: 12.0
```

Sample: SampG2
Well location: G2
Created: Tuesday, September 14, 2021 5:34:08 PM

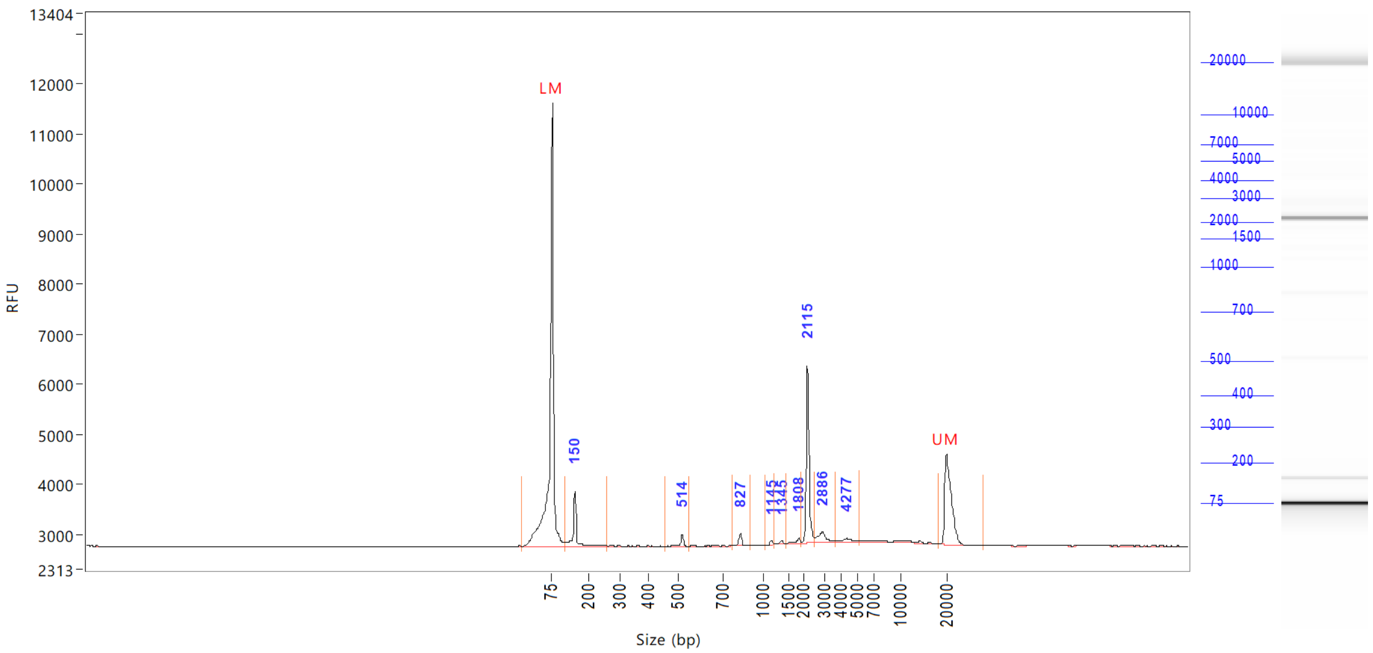

Peak	Size (bp)	Concentration (ng/uL)	From (bp)	To (bp)	Average size (bp)	CV%	RFU	Corrected peak area
1	75 (LM)	0.5000	0	118	64	31.01	8863	81.612
2	150	0.6398	118	259	152	13.87	1104	8.703
3	514	0.1027	452	544	506	2.53	243	1.397
4	827	0.0951	766	900	821	2.37	239	1.293
5	1145	0.0293	1023	1201	1134	2.36	88	0.398
6	1345	0.0272	1201	1433	1331	3.07	63	0.370
7	1808	0.0565	1433	1904	1717	7.25	101	0.768
8	2115	1.2261	1904	2515	2124	4.43	3522	16.678
9	2886	0.1968	2515	3679	2864	8.01	209	2.677
10	4277	0.0682	3679	5149	4357	7.47	68	0.928
11	20000 (UM)	0.0993	18171	27859	20458	4.30	1811	16.209

TIC:	2.4417	ng/uL
TIM:	8.6625	nmole/L
Total concentration:	2.6419	ng/uL

Sample peak width (sec): 5 Sample min peak height: 50 Sample baseline V to V?: Y Sample baseline V to V points: 3
Sample filter: Binomial Number of points for filter: 3 Sample start region (min): 0 Sample end region (min): 30
Marker peak width (sec): 5 Marker min peak height: 200 Marker baseline V to V?: Y Marker baseline V to V points: 3
Lower marker selection: First peak > 500 RFU Upper marker selection: Last peak > 200 RFU
Ladder size (bp)75, 200, 300, 400, 500, 700, 1000, 1500, 2000, 3000, 4000, 5000, 7000, 10000, 20000
Quantification using: Lower Marker Final concentration (ng/uL): 0.5000 Dilution factor: 12.0

Sample: SampH2
Well location: H2
Created: Tuesday, September 14, 2021 5:34:08 PM

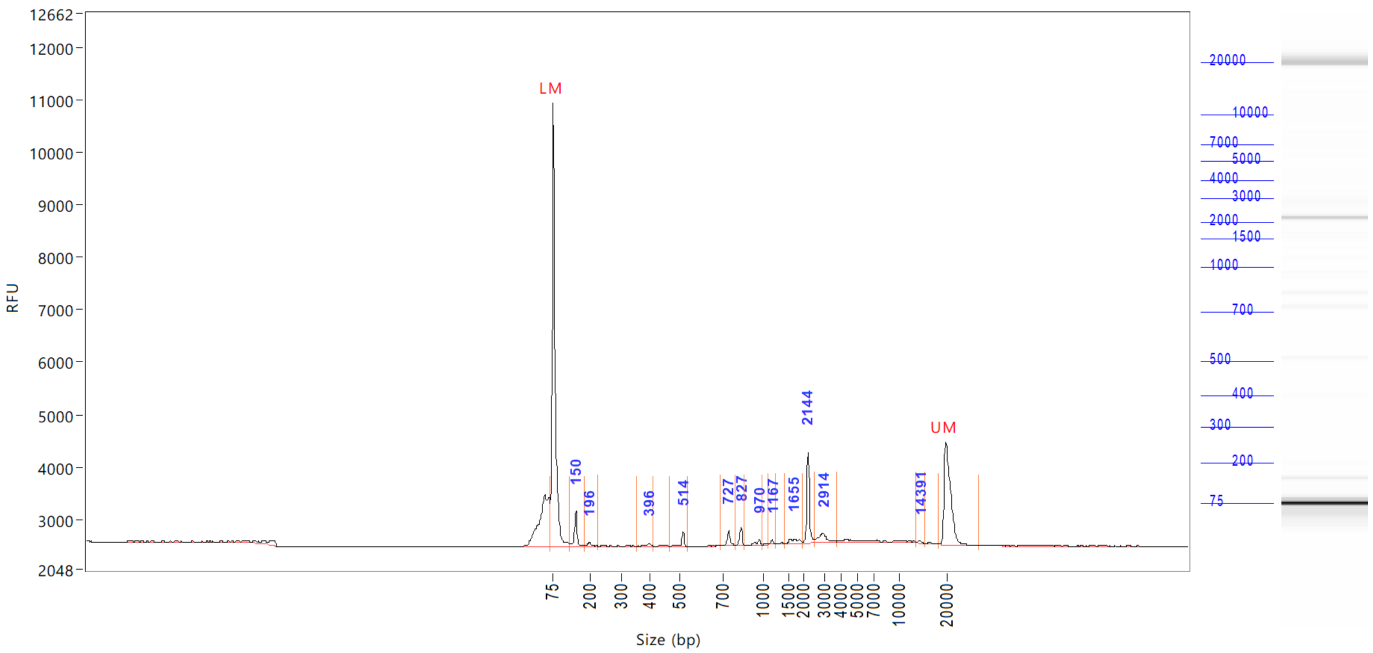

Peak	Size (bp)	Concentration (ng/uL)	From (bp)	To (bp)	Average size (bp)	CV%	RFU	Corrected peak area
1	75 (LM)	0.5000	66	131	76	11.39	8481	62.877
2	150	0.4081	131	181	148	4.81	668	4.277
3	196	0.0549	181	226	194	3.95	62	0.576
4	396	0.0382	352	414	389	3.19	58	0.401
5	514	0.1346	466	539	508	1.96	279	1.410
6	727	0.1490	682	783	728	2.06	296	1.561
7	827	0.1487	783	857	820	1.26	347	1.559
8	970	0.0675	857	987	942	2.78	94	0.707
9	1167	0.0362	1090	1234	1151	2.20	84	0.379
10	1655	0.1279	1411	1923	1649	8.12	94	1.341
11	2144	0.7464	1923	2515	2141	4.34	1741	7.822
12	2914	0.2194	2515	3715	2894	8.30	175	2.299
13	14391	0.0221	13659	15488	14234	2.07	53	0.232
14	20000 (UM)	0.1358	18293	27133	20411	4.22	1947	17.080

TIC:	2.1530	ng/uL
TIM:	7.2286	nmole/L
Total concentration:	2.4604	ng/uL

```
Sample peak width (sec): 5    Sample min peak height: 50    Sample baseline V to V?: Y    Sample baseline V to V points: 3
Sample filter: Binomial       Number of points for filter: 3  Sample start region (min): 0  Sample end region (min): 30
Marker peak width (sec): 5    Marker min peak height: 200   Marker baseline V to V?: Y    Marker baseline V to V points: 3
Lower marker selection: First peak > 500 RFU                Upper marker selection: Last peak > 200 RFU
Ladder size (bp)75, 200, 300, 400, 500, 700, 1000, 1500, 2000, 3000, 4000, 5000, 7000, 10000, 20000
Quantification using: Lower Marker          Final concentration (ng/uL): 0.5000          Dilution factor: 12.0
```

Sample: SampA3
Well location: A3
Created: Tuesday, September 14, 2021 5:34:08 PM

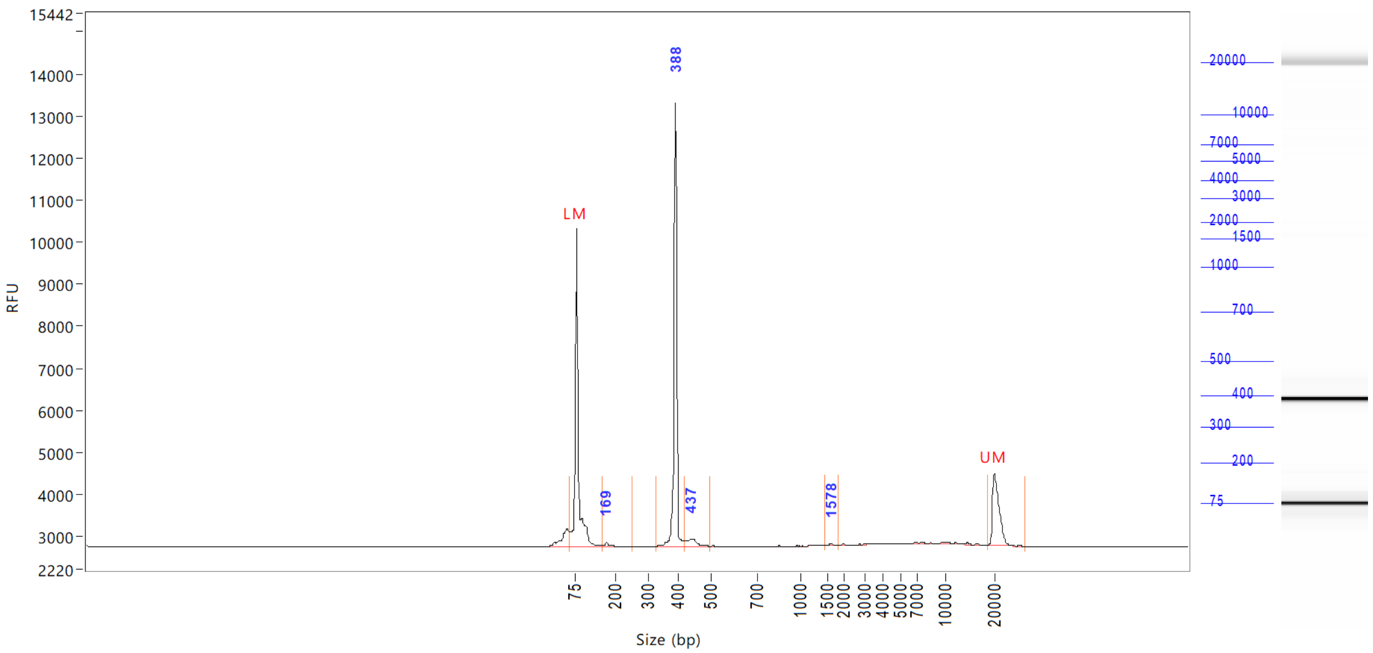

Peak	Size (bp)	Concentration (ng/uL)	From (bp)	To (bp)	Average size (bp)	CV%	RFU	Corrected peak area
1	75 (LM)	0.5000	54	158	77	17.29	7562	56.549
2	169	0.1062	158	248	175	9.02	96	1.001
3	388	5.8890	321	414	384	2.03	10565	55.503
4	437	0.3975	414	498	439	3.82	175	3.746
5	1578	0.0328	1456	1846	1586	5.00	51	0.310
6	20000 (UM)	0.1293	18537	26045	20353	3.65	1726	14.624

TIC:	6.4256	ng/uL
TIM:	27.7530	nmole/L
Total concentration:	6.7293	ng/uL

Sample peak width (sec): 5   Sample min peak height: 50   Sample baseline V to V?: Y   Sample baseline V to V points: 3
Sample filter: Binomial   Number of points for filter: 3 Sample start region (min): 0 Sample end region (min): 30
Marker peak width (sec): 5   Marker min peak height: 200   Marker baseline V to V?: Y   Marker baseline V to V points: 3
Lower marker selection: First peak > 500 RFU   Upper marker selection: Last peak > 200 RFU
Ladder size (bp)75, 200, 300, 400, 500, 700, 1000, 1500, 2000, 3000, 4000, 5000, 7000, 10000, 20000
Quantification using: Lower Marker   Final concentration (ng/uL): 0.5000   Dilution factor: 12.0

Sample: SampB3
Well location: B3
Created: Tuesday, September 14, 2021 5:34:08 PM

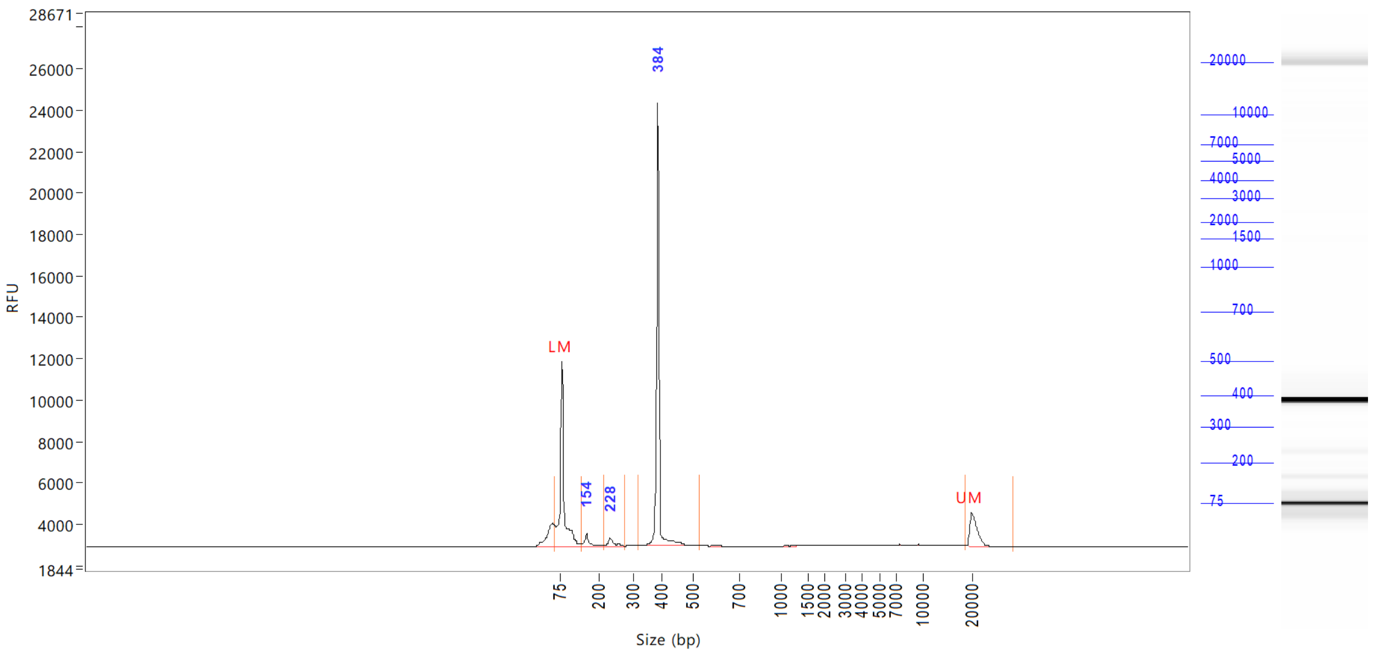

Peak	Size<br>(bp)	Concentration<br>(ng/uL)	From<br>(bp)	To<br>(bp)	Average size<br>(bp)	CV%	RFU	Corrected peak area
1	75 (LM)	0.5000	56	137	78	18.09	8921	80.357
2	154	0.4945	137	210	160	10.16	602	6.623
3	228	0.3433	210	273	232	4.87	390	4.598
4	384	8.5500	317	527	384	3.90	21400	114.508
5	20000 (UM)	0.0946	18781	28584	20516	4.38	1610	15.208

TIC:	9.3878	ng/uL
TIM:	44.1459	nmole/L
Total concentration:	9.6732	ng/uL

Sample peak width (sec): 5   Sample min peak height: 50   Sample baseline V to V?: Y   Sample baseline V to V points: 3
Sample filter: Binomial   Number of points for filter: 3 Sample start region (min): 0 Sample end region (min): 30
Marker peak width (sec): 5   Marker min peak height: 200   Marker baseline V to V?: Y   Marker baseline V to V points: 3
Lower marker selection: First peak > 500 RFU   Upper marker selection: Last peak > 200 RFU
Ladder size (bp)75, 200, 300, 400, 500, 700, 1000, 1500, 2000, 3000, 4000, 5000, 7000, 10000, 20000
Quantification using: Lower Marker   Final concentration (ng/uL): 0.5000   Dilution factor: 12.0

Sample: SampC3
Well location: C3
Created: Tuesday, September 14, 2021 5:34:08 PM

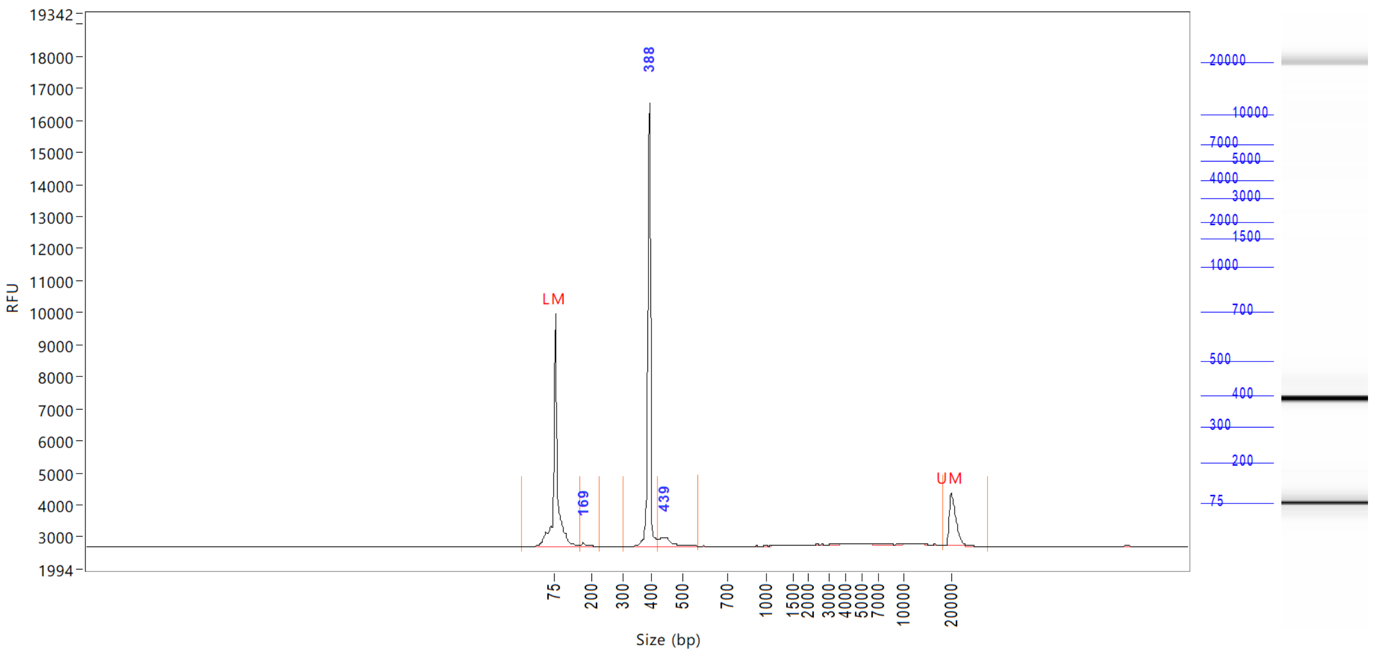

Peak	Size (bp)	Concentration (ng/uL)	From (bp)	To (bp)	Average size (bp)	CV%	RFU	Corrected peak area
1	75 (LM)	0.5000	0	158	75	26.53	7271	70.498
2	169	0.1344	158	221	178	8.92	117	1.579
3	388	6.4577	296	418	385	2.12	13861	75.876
4	439	0.5643	418	570	446	5.01	273	6.631
5	20000 (UM)	0.1038	18415	27859	20485	4.12	1665	14.630

TIC:	7.1564	ng/uL
TIM:	30.9195	nmole/L
Total concentration:	7.4249	ng/uL

Sample peak width (sec): 5   Sample min peak height: 50   Sample baseline V to V?: Y   Sample baseline V to V points: 3
Sample filter: Binomial   Number of points for filter: 3 Sample start region (min): 0 Sample end region (min): 30
Marker peak width (sec): 5   Marker min peak height: 200   Marker baseline V to V?: Y   Marker baseline V to V points: 3
Lower marker selection: First peak > 500 RFU   Upper marker selection: Last peak > 200 RFU
Ladder size (bp)75, 200, 300, 400, 500, 700, 1000, 1500, 2000, 3000, 4000, 5000, 7000, 10000, 20000
Quantification using: Lower Marker   Final concentration (ng/uL): 0.5000   Dilution factor: 12.0

Sample: SampD3
Well location: D3
Created: Tuesday, September 14, 2021 5:34:08 PM

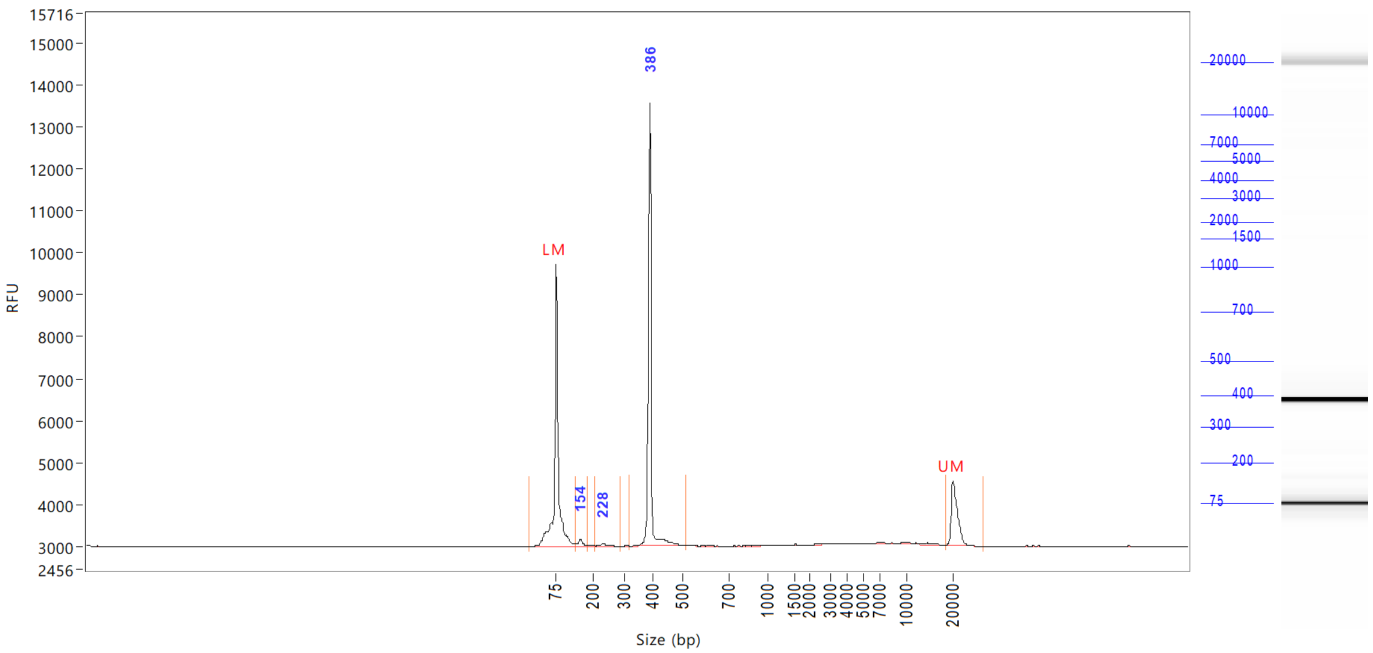

Peak	Size (bp)	Concentration (ng/uL)	From (bp)	To (bp)	Average size (bp)	CV%	RFU	Corrected peak area
1	75 (LM)	0.5000	0	143	72	25.86	6745	60.670
2	154	0.1796	143	181	155	5.40	183	1.816
3	228	0.1053	205	286	234	6.64	69	1.065
4	386	5.9232	317	511	386	4.01	10582	59.893
5	20000 (UM)	0.1079	18415	26649	20428	3.72	1527	13.089

TIC:	6.2081	ng/uL
TIM:	27.8909	nmole/L
Total concentration:	6.5695	ng/uL

Sample peak width (sec): 5   Sample min peak height: 50   Sample baseline V to V?: Y   Sample baseline V to V points: 3
Sample filter: Binomial   Number of points for filter: 3 Sample start region (min): 0 Sample end region (min): 30
Marker peak width (sec): 5   Marker min peak height: 200   Marker baseline V to V?: Y   Marker baseline V to V points: 3
Lower marker selection: First peak > 500 RFU   Upper marker selection: Last peak > 200 RFU
Ladder size (bp)75, 200, 300, 400, 500, 700, 1000, 1500, 2000, 3000, 4000, 5000, 7000, 10000, 20000
Quantification using: Lower Marker   Final concentration (ng/uL): 0.5000   Dilution factor: 12.0

Sample: SampE3
Well location: E3
Created: Tuesday, September 14, 2021 5:34:08 PM

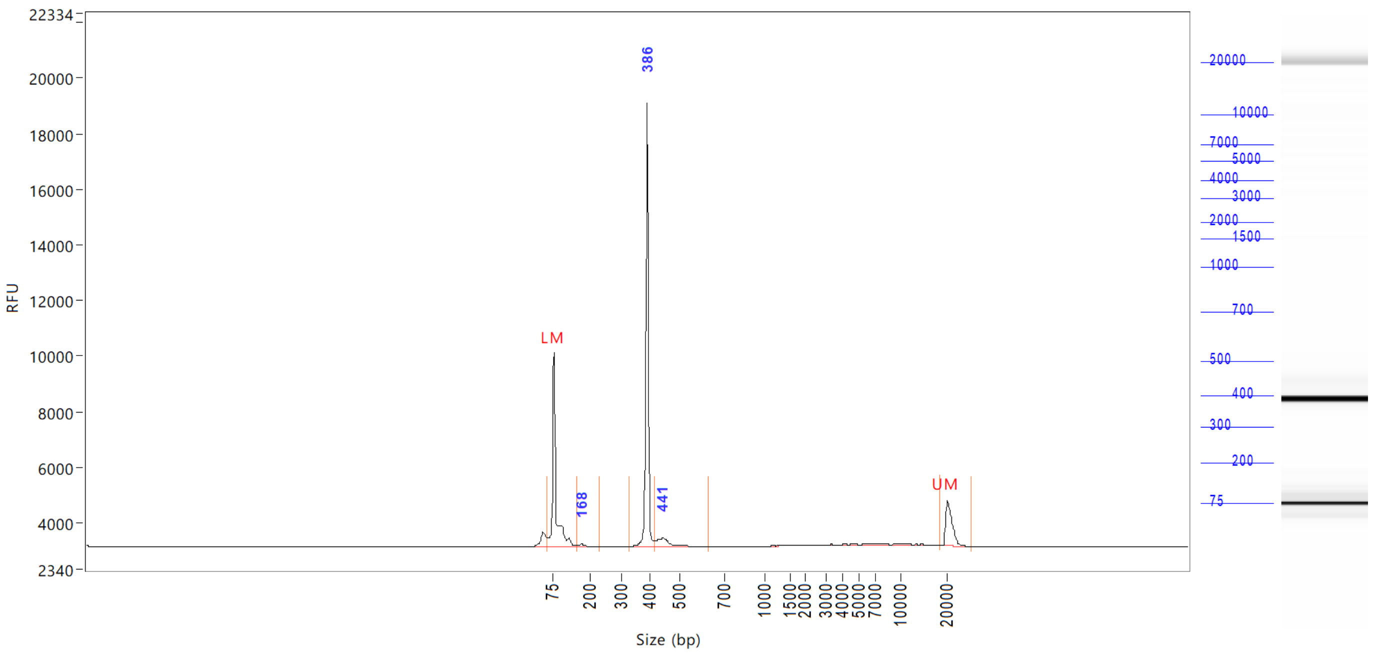

Peak	Size (bp)	Concentration (ng/uL)	From (bp)	To (bp)	Average size (bp)	CV%	RFU	Corrected peak area
1	75 (LM)	0.5000	54	154	81	20.62	6963	58.731
2	168	0.1350	154	230	174	9.32	104	1.322
3	386	8.7608	327	412	385	1.78	15981	85.755
4	441	0.7591	412	629	446	6.83	291	7.431
5	20000 (UM)	0.1176	18293	25199	20460	3.58	1613	13.816

TIC:	9.6550	ng/uL
TIM:	41.5147	nmole/L
Total concentration:	9.9358	ng/uL

Sample peak width (sec): 5   Sample min peak height: 50   Sample baseline V to V?: Y   Sample baseline V to V points: 3
Sample filter: Binomial   Number of points for filter: 3   Sample start region (min): 0   Sample end region (min): 30
Marker peak width (sec): 5   Marker min peak height: 200   Marker baseline V to V?: Y   Marker baseline V to V points: 3
Lower marker selection: First peak > 500 RFU   Upper marker selection: Last peak > 200 RFU
Ladder size (bp)75, 200, 300, 400, 500, 700, 1000, 1500, 2000, 3000, 4000, 5000, 7000, 10000, 20000
Quantification using: Lower Marker   Final concentration (ng/uL): 0.5000   Dilution factor: 12.0

Sample: SampF3
Well location: F3
Created: Tuesday, September 14, 2021 5:34:08 PM

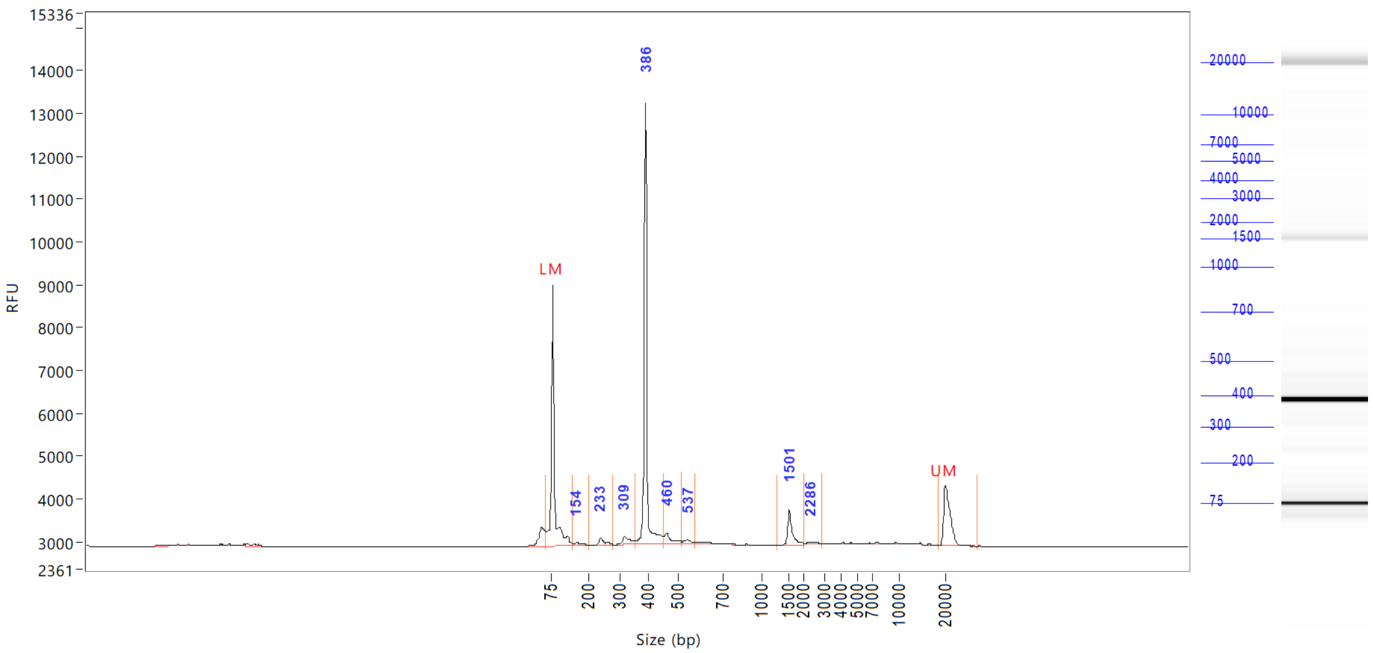

Peak	Size (bp)	Concentration (ng/uL)	From (bp)	To (bp)	Average size (bp)	CV%	RFU	Corrected peak area
1	75 (LM)	0.5000	52	145	78	19.50	6075	47.067
2	154	0.1542	145	196	162	7.24	80	1.209
3	233	0.2819	196	277	239	5.40	177	2.212
4	309	0.3852	277	349	320	4.67	176	3.022
5	386	7.6638	349	447	387	3.25	10312	60.119
6	460	0.4178	447	511	466	3.54	249	3.278
7	537	0.1313	511	575	536	2.82	78	1.030
8	1501	0.7893	1289	2030	1564	7.83	836	6.192
9	2286	0.1200	2030	2914	2399	9.69	58	0.941
10	20000 (UM)	0.1298	18415	26891	20418	3.77	1416	12.214

TIC:	9.9435	ng/uL
TIM:	40.8572	nmole/L
Total concentration:	10.3058	ng/uL

```
Sample peak width (sec): 5    Sample min peak height: 50    Sample baseline V to V?: Y    Sample baseline V to V points: 3
Sample filter: Binomial       Number of points for filter: 3  Sample start region (min): 0  Sample end region (min): 30
Marker peak width (sec): 5    Marker min peak height: 200    Marker baseline V to V?: Y    Marker baseline V to V points: 3
Lower marker selection: First peak > 500 RFU                 Upper marker selection: Last peak > 200 RFU
Ladder size (bp)75, 200, 300, 400, 500, 700, 1000, 1500, 2000, 3000, 4000, 5000, 7000, 10000, 20000
Quantification using: Lower Marker          Final concentration (ng/uL): 0.5000          Dilution factor: 12.0
```

Sample: SampG3
Well location: G3
Created: Tuesday, September 14, 2021 5:34:08 PM

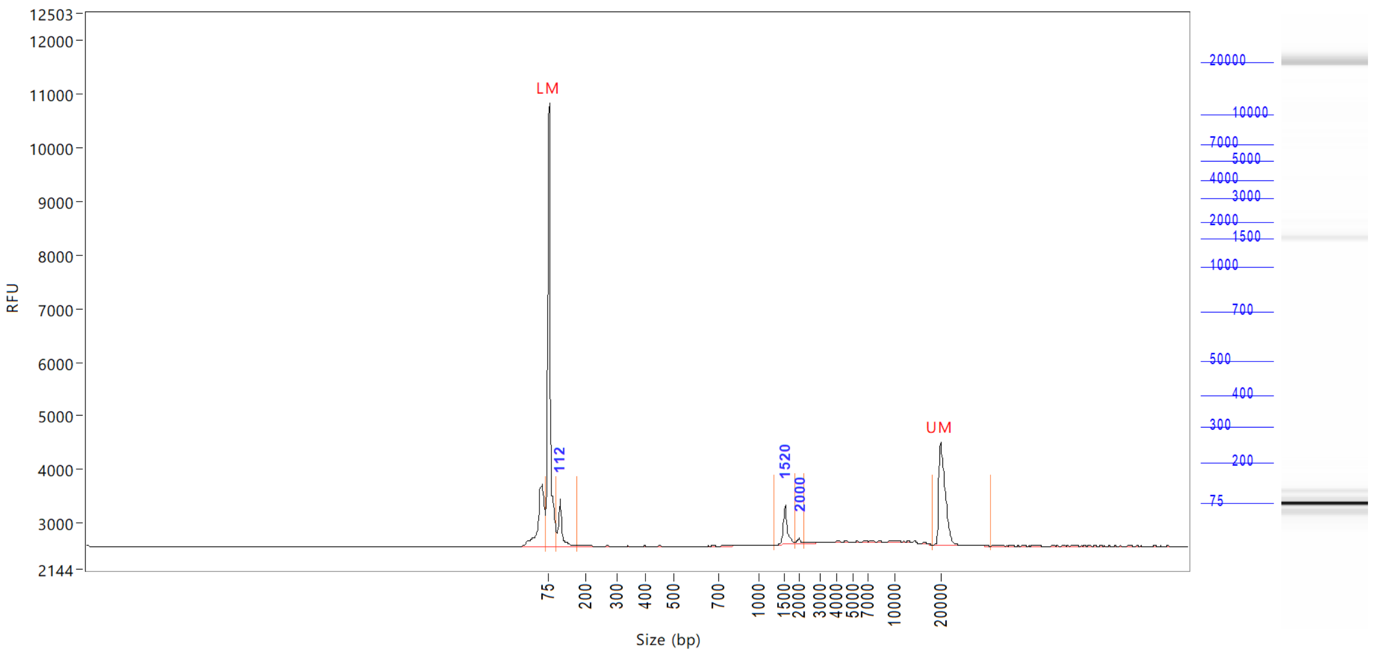

Peak	Size (bp)	Concentration (ng/uL)	From (bp)	To (bp)	Average size (bp)	CV%	RFU	Corrected peak area
1	75 (LM)	0.5000	62	101	74	9.21	8279	57.145
2	112	0.8175	101	171	114	11.29	875	7.786
3	1520	0.5189	1311	1846	1532	5.29	725	4.943
4	2000	0.0675	1846	2258	1980	4.36	98	0.643
5	20000 (UM)	0.1491	18293	30639	20454	4.65	1904	17.045

TIC:	1.4039	ng/uL
TIM:	12.3931	nmole/L
Total concentration:	1.7836	ng/uL

Sample peak width (sec): 5   Sample min peak height: 50   Sample baseline V to V?: Y   Sample baseline V to V points: 3
Sample filter: Binomial   Number of points for filter: 3 Sample start region (min): 0 Sample end region (min): 30
Marker peak width (sec): 5   Marker min peak height: 200   Marker baseline V to V?: Y   Marker baseline V to V points: 3
Lower marker selection: First peak > 500 RFU   Upper marker selection: Last peak > 200 RFU
Ladder size (bp)75, 200, 300, 400, 500, 700, 1000, 1500, 2000, 3000, 4000, 5000, 7000, 10000, 20000
Quantification using: Lower Marker   Final concentration (ng/uL): 0.5000   Dilution factor: 12.0

Sample: SampH3
Well location: H3
Created: Tuesday, September 14, 2021 5:34:08 PM

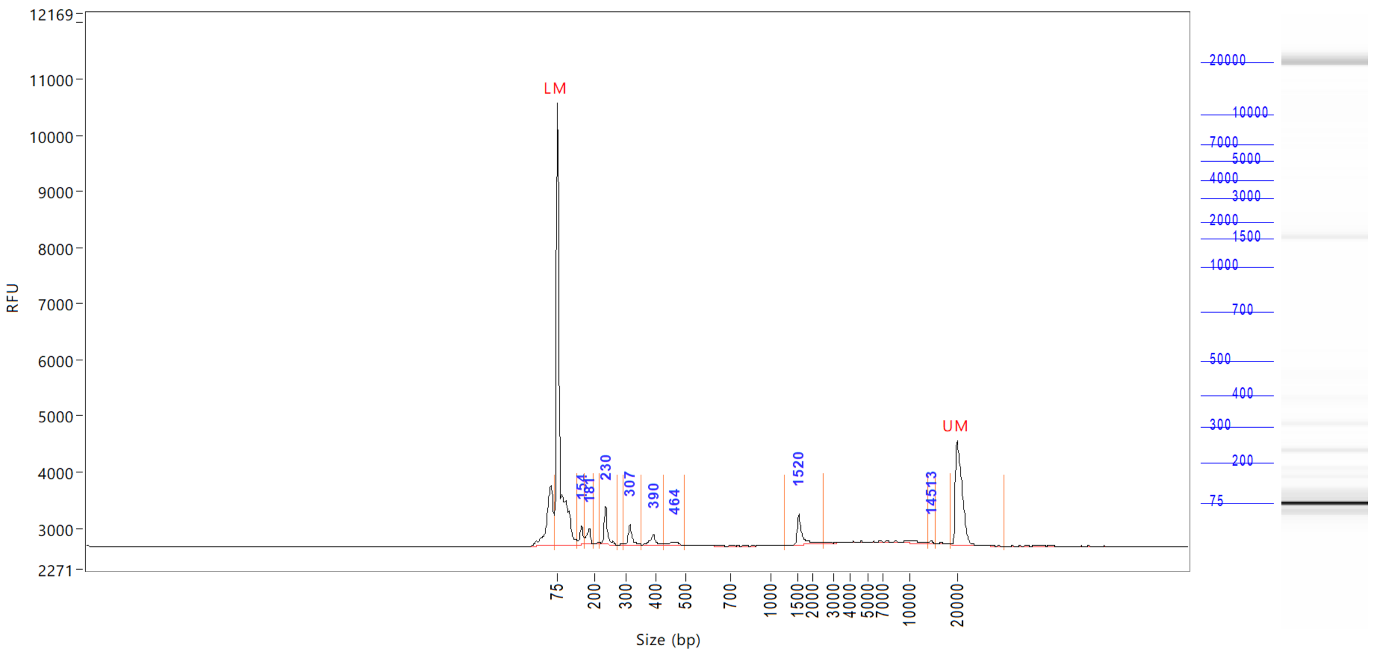

Peak	Size (bp)	Concentration (ng/uL)	From (bp)	To (bp)	Average size (bp)	CV%	RFU	Corrected peak area
1	75 (LM)	0.5000	66	141	81	18.25	7889	62.955
2	154	0.2493	141	164	153	3.79	331	2.616
3	181	0.2470	164	196	175	3.38	280	2.591
4	230	0.4731	215	271	232	3.31	678	4.964
5	307	0.2936	289	352	309	3.23	375	3.080
6	390	0.2206	352	428	387	3.89	179	2.315
7	464	0.1178	428	498	458	3.13	67	1.236
8	1520	0.4382	1256	2543	1626	12.88	562	4.598
9	14513	0.0218	13903	15244	14365	1.92	53	0.229
10	20000 (UM)	0.1305	18415	30035	20477	4.32	1865	16.431

TIC:	2.0614	ng/uL
TIM:	11.7219	nmole/L
Total concentration:	2.3660	ng/uL

Sample peak width (sec): 5 Sample min peak height: 50 Sample baseline V to V?: Y Sample baseline V to V points: 3
Sample filter: Binomial Number of points for filter: 3 Sample start region (min): 0 Sample end region (min): 30
Marker peak width (sec): 5 Marker min peak height: 200 Marker baseline V to V?: Y Marker baseline V to V points: 3
Lower marker selection: First peak > 500 RFU Upper marker selection: Last peak > 200 RFU
Ladder size (bp)75, 200, 300, 400, 500, 700, 1000, 1500, 2000, 3000, 4000, 5000, 7000, 10000, 20000
Quantification using: Lower Marker Final concentration (ng/uL): 0.5000 Dilution factor: 12.0

Sample: SampA4
Well location: A4
Created: Tuesday, September 14, 2021 5:34:08 PM

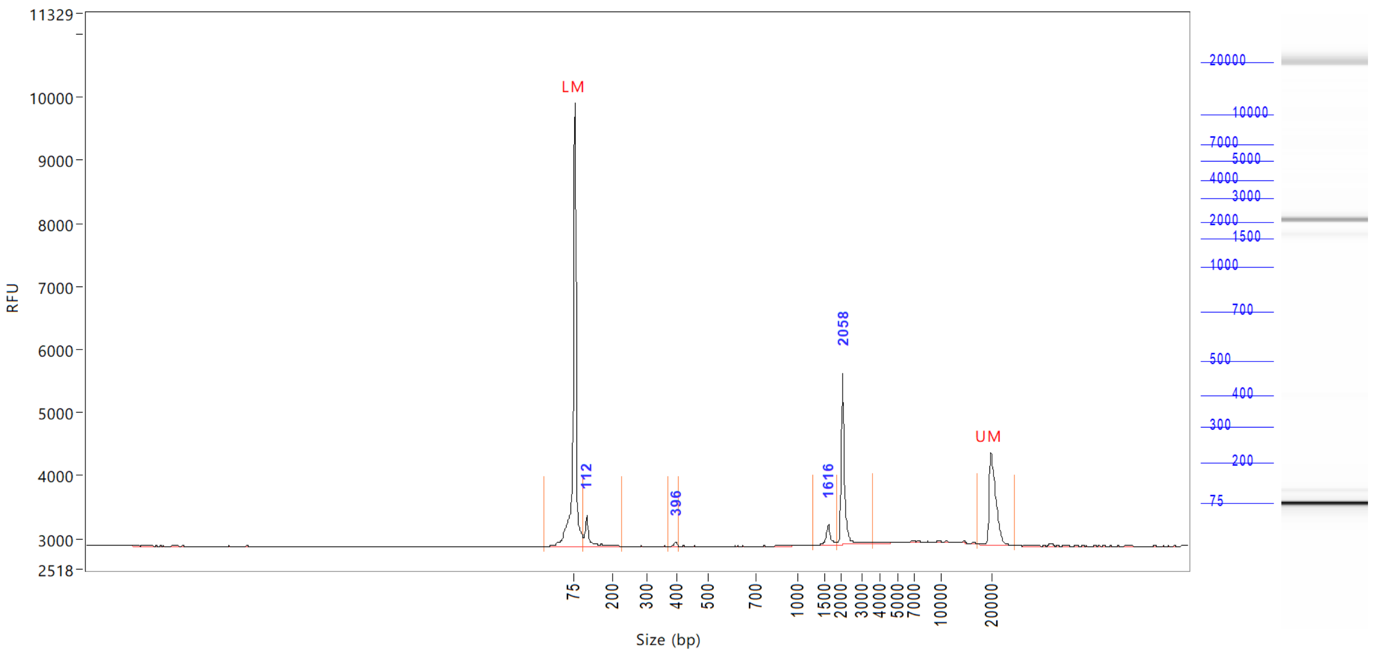

Peak	Size (bp)	Concentration (ng/uL)	From (bp)	To (bp)	Average size (bp)	CV%	RFU	Corrected peak area
1	75 (LM)	0.5000	0	101	69	18.81	7044	54.139
2	112	0.5699	101	223	123	19.54	483	5.142
3	396	0.0473	372	407	392	1.59	70	0.427
4	1616	0.2604	1278	1866	1594	6.11	322	2.349
5	2058	1.5311	1866	3643	2109	10.40	2717	13.815
6	20000 (UM)	0.1169	17317	24957	20453	3.85	1454	12.661

TIC:	2.4086	ng/uL
TIM:	9.2745	nmole/L
Total concentration:	2.7205	ng/uL

```
Sample peak width (sec): 5    Sample min peak height: 50    Sample baseline V to V?: Y    Sample baseline V to V points: 3
Sample filter: Binomial       Number of points for filter: 3  Sample start region (min): 0  Sample end region (min): 30
Marker peak width (sec): 5    Marker min peak height: 200   Marker baseline V to V?: Y    Marker baseline V to V points: 3
Lower marker selection: First peak > 500 RFU                Upper marker selection: Last peak > 200 RFU
Ladder size (bp)75, 200, 300, 400, 500, 700, 1000, 1500, 2000, 3000, 4000, 5000, 7000, 10000, 20000
Quantification using: Lower Marker          Final concentration (ng/uL): 0.5000          Dilution factor: 12.0
```

Sample: SampB4
Well location: B4
Created: Tuesday, September 14, 2021 5:34:08 PM

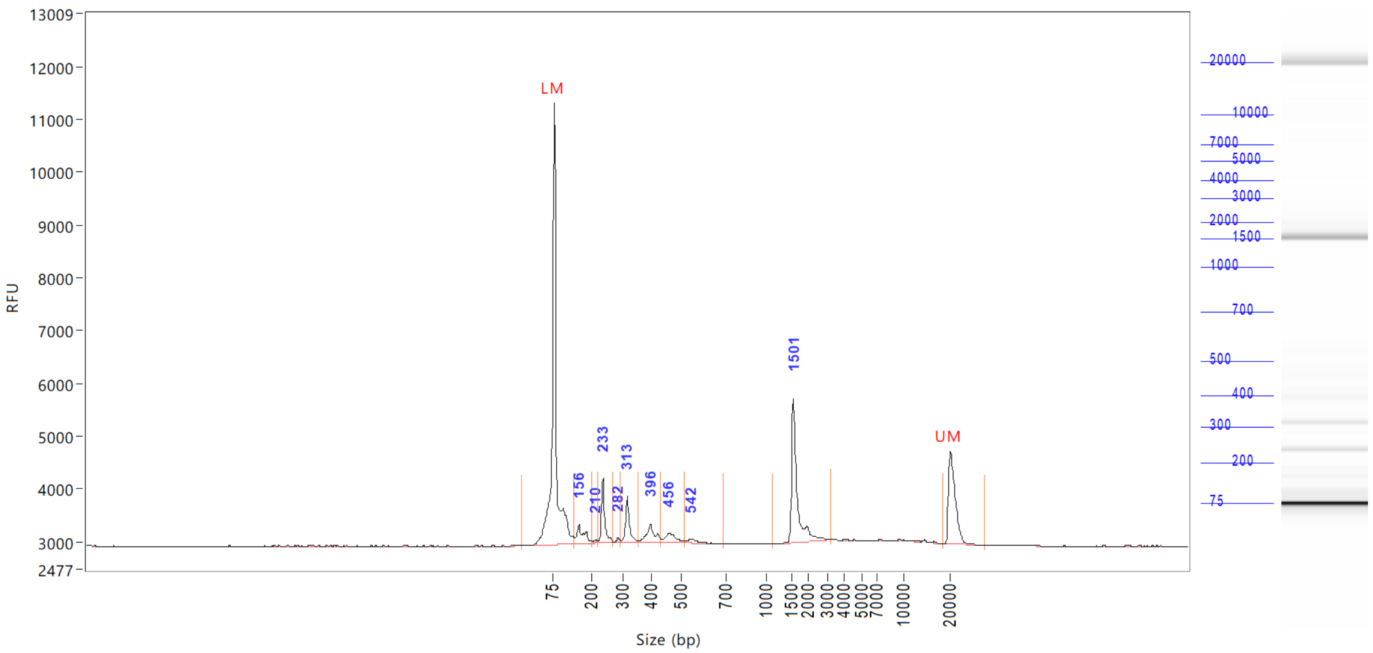

Peak	Size (bp)	Concentration (ng/uL)	From (bp)	To (bp)	Average size (bp)	CV%	RFU	Corrected peak area
1	75 (LM)	0.5000	0	143	74	26.37	8382	85.897
2	156	0.4052	143	201	165	8.43	358	5.800
3	210	0.0340	201	219	210	2.46	58	0.487
4	233	0.6400	219	269	234	3.27	1224	9.162
5	282	0.0338	269	291	282	1.73	74	0.484
6	313	0.4936	291	352	312	3.04	871	7.066
7	396	0.3774	352	431	394	4.39	344	5.403
8	456	0.2541	431	511	464	3.95	159	3.638
9	542	0.1098	511	687	558	6.60	78	1.572
10	1501	1.6683	1134	3144	1638	14.81	2732	23.884
11	20000 (UM)	0.0915	18415	27496	20472	4.23	1759	15.720

TIC:	4.0162	ng/uL
TIM:	16.0775	nmole/L
Total concentration:	4.1261	ng/uL

Sample peak width (sec): 5 Sample min peak height: 50 Sample baseline V to V?: Y Sample baseline V to V points: 3
Sample filter: Binomial Number of points for filter: 3 Sample start region (min): 0 Sample end region (min): 30
Marker peak width (sec): 5 Marker min peak height: 200 Marker baseline V to V?: Y Marker baseline V to V points: 3
Lower marker selection: First peak > 500 RFU Upper marker selection: Last peak > 200 RFU
Ladder size (bp)75, 200, 300, 400, 500, 700, 1000, 1500, 2000, 3000, 4000, 5000, 7000, 10000, 20000
Quantification using: Lower Marker Final concentration (ng/uL): 0.5000 Dilution factor: 12.0

Sample: SampC4
Well location: C4
Created: Tuesday, September 14, 2021 5:34:08 PM

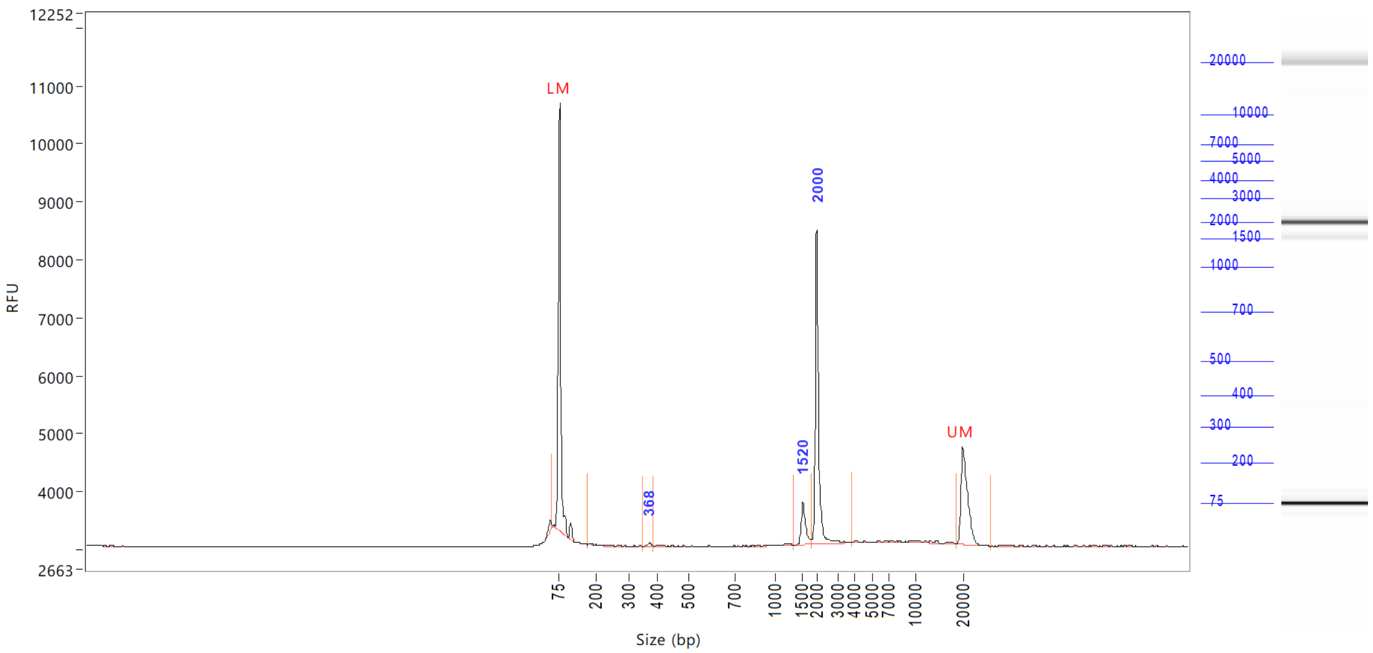

Peak	Size (bp)	Concentration (ng/uL)	From (bp)	To (bp)	Average size (bp)	CV%	RFU	Corrected peak area
1	75 (LM)	0.5000	52	168	75	12.65	7393	46.157
2	368	0.0588	345	384	365	1.93	64	0.452
3	1520	0.6745	1334	1808	1552	5.35	748	5.189
4	2000	3.5774	1808	3857	2019	10.48	5409	27.520
5	20000 (UM)	0.1609	18537	25803	20452	3.83	1679	14.854

TIC:	4.3107	ng/uL
TIM:	3.8972	nmole/L
Total concentration:	4.8101	ng/uL

Sample peak width (sec): 5   Sample min peak height: 50   Sample baseline V to V?: Y   Sample baseline V to V points: 3
Sample filter: Binomial   Number of points for filter: 3   Sample start region (min): 0   Sample end region (min): 30
Marker peak width (sec): 5   Marker min peak height: 200   Marker baseline V to V?: Y   Marker baseline V to V points: 3
Lower marker selection: First peak > 500 RFU   Upper marker selection: Last peak > 200 RFU
Ladder size (bp)75, 200, 300, 400, 500, 700, 1000, 1500, 2000, 3000, 4000, 5000, 7000, 10000, 20000
Quantification using: Lower Marker   Final concentration (ng/uL): 0.5000   Dilution factor: 12.0

Sample: SampD4
Well location: D4
Created: Tuesday, September 14, 2021 5:34:08 PM

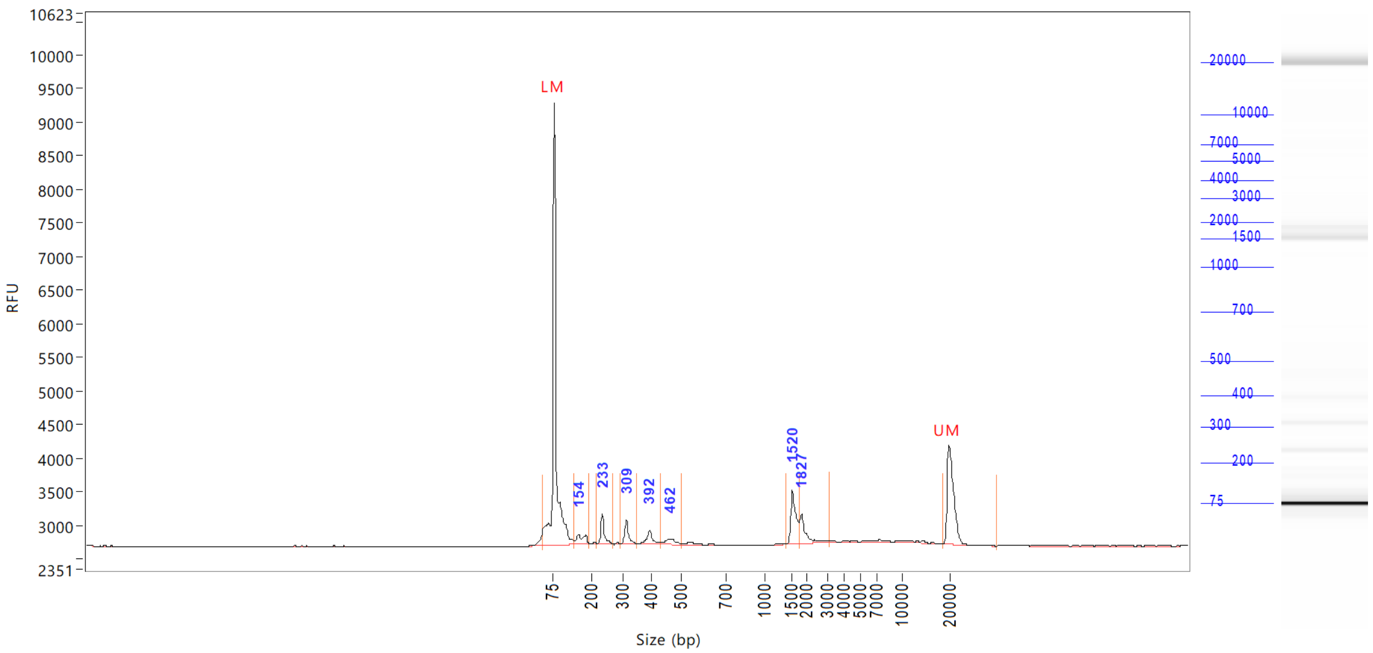

Peak	Size (bp)	Concentration (ng/uL)	From (bp)	To (bp)	Average size (bp)	CV%	RFU	Corrected peak area
1	75 (LM)	0.5000	37	143	76	20.57	6590	55.657
2	154	0.2796	143	192	164	7.46	147	2.593
3	233	0.3582	217	269	233	3.54	438	3.323
4	309	0.3232	289	349	311	3.08	359	2.998
5	392	0.2783	349	433	393	4.12	211	2.581
6	462	0.1822	433	498	462	3.30	84	1.690
7	1520	0.7262	1400	1731	1557	5.08	813	6.737
8	1827	0.4654	1731	3144	1975	15.00	442	4.317
9	20000 (UM)	0.1143	18537	30277	20436	4.50	1486	12.724

TIC:	2.6130	ng/uL
TIM:	10.0091	nmole/L
Total concentration:	2.9799	ng/uL

```
Sample peak width (sec): 5    Sample min peak height: 50    Sample baseline V to V?: Y    Sample baseline V to V points: 3
Sample filter: Binomial       Number of points for filter: 3  Sample start region (min): 0  Sample end region (min): 30
Marker peak width (sec): 5    Marker min peak height: 200   Marker baseline V to V?: Y    Marker baseline V to V points: 3
Lower marker selection: First peak > 500 RFU                Upper marker selection: Last peak > 200 RFU
Ladder size (bp)75, 200, 300, 400, 500, 700, 1000, 1500, 2000, 3000, 4000, 5000, 7000, 10000, 20000
Quantification using: Lower Marker          Final concentration (ng/uL): 0.5000          Dilution factor: 12.0
```

Sample: SampE4
Well location: E4
Created: Tuesday, September 14, 2021 5:34:08 PM

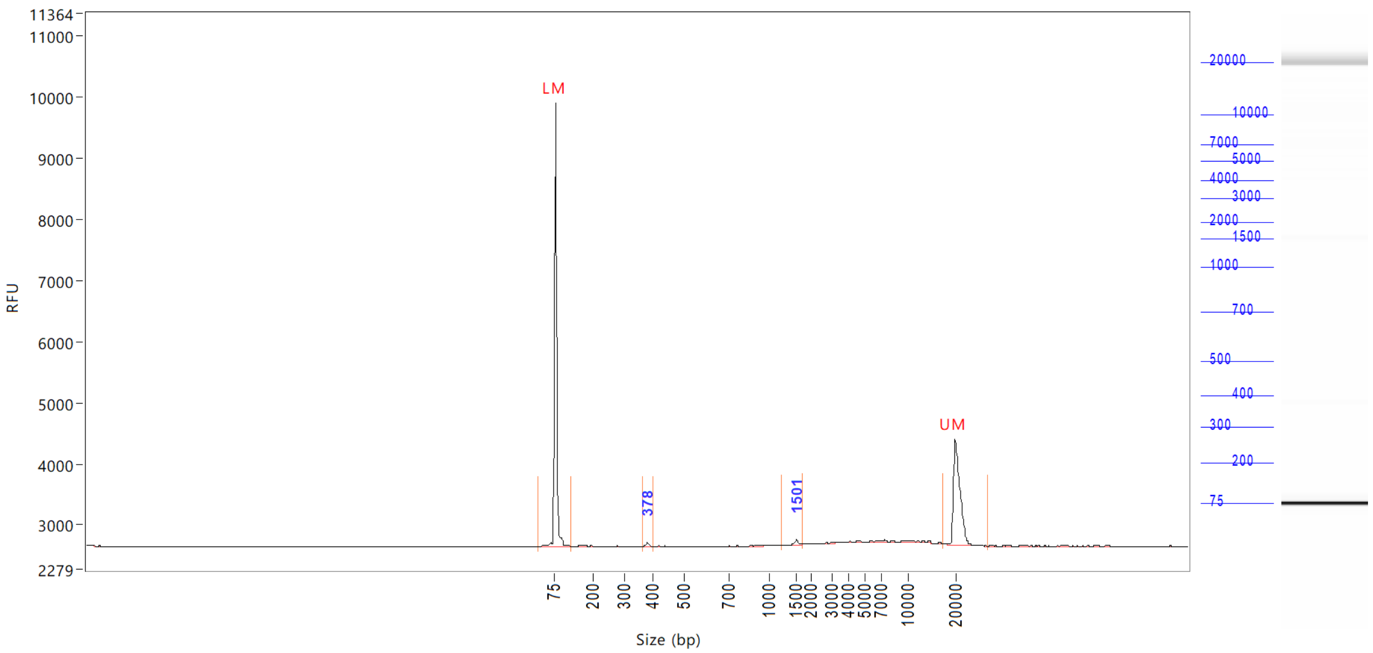

Peak	Size (bp)	Concentration (ng/uL)	From (bp)	To (bp)	Average size (bp)	CV%	RFU	Corrected peak area
1	75 (LM)	0.5000	18	128	73	8.89	7262	41.460
2	378	0.0580	360	400	375	1.80	65	0.401
3	1501	0.0792	1234	1712	1490	5.22	87	0.547
4	20000 (UM)	0.1824	17561	26891	20415	3.89	1715	15.128

TIC:	0.1372	ng/uL
TIM:	0.3421	nmole/L
Total concentration:	0.6340	ng/uL

Sample peak width (sec): 5   Sample min peak height: 50   Sample baseline V to V?: Y   Sample baseline V to V points: 3
Sample filter: Binomial   Number of points for filter: 3 Sample start region (min): 0 Sample end region (min): 30
Marker peak width (sec): 5   Marker min peak height: 200   Marker baseline V to V?: Y   Marker baseline V to V points: 3
Lower marker selection: First peak > 500 RFU   Upper marker selection: Last peak > 200 RFU
Ladder size (bp)75, 200, 300, 400, 500, 700, 1000, 1500, 2000, 3000, 4000, 5000, 7000, 10000, 20000
Quantification using: Lower Marker   Final concentration (ng/uL): 0.5000   Dilution factor: 12.0

Sample: SampF4
Well location: F4
Created: Tuesday, September 14, 2021 5:34:08 PM

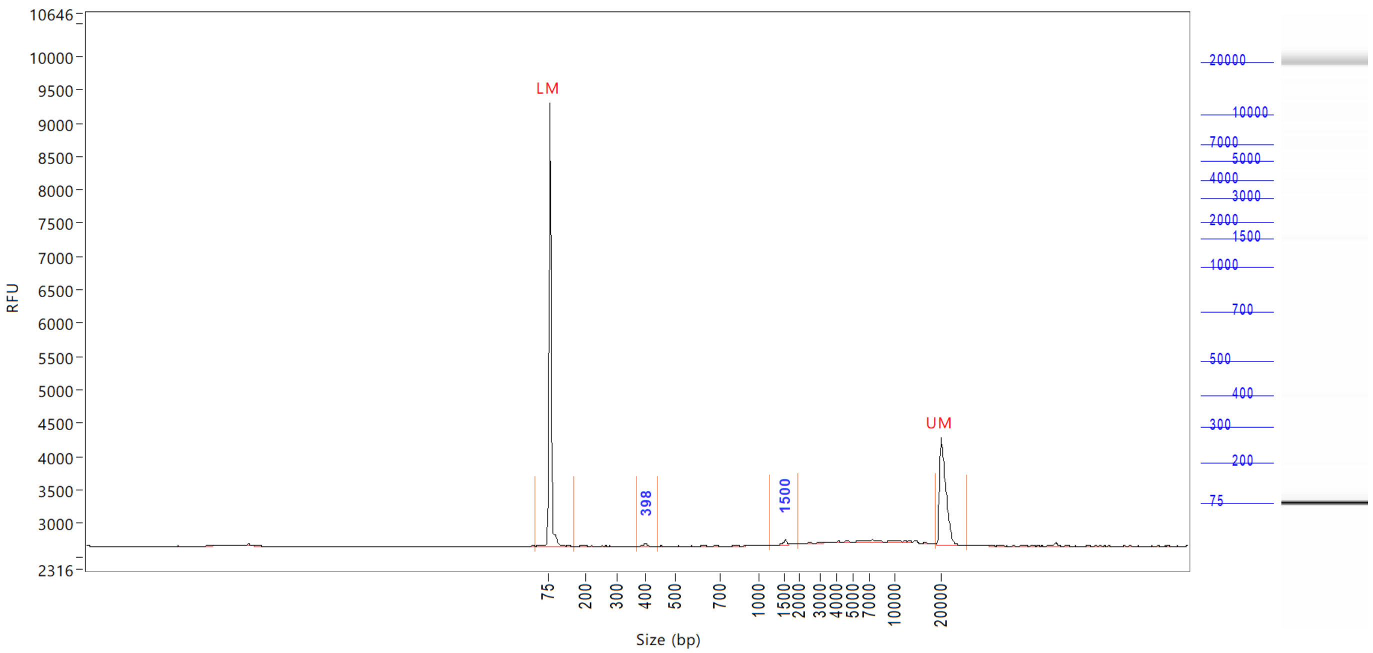

Peak	Size (bp)	Concentration (ng/uL)	From (bp)	To (bp)	Average size (bp)	CV%	RFU	Corrected peak area
1	75 (LM)	0.5000	26	160	75	9.97	6658	39.209
2	398	0.0686	366	437	397	3.20	50	0.449
3	1500	0.0813	1190	1962	1524	7.27	78	0.531
4	20000 (UM)	0.1783	18781	25561	20434	3.78	1593	13.979

TIC:	0.1499	ng/uL
TIM:	0.3723	nmole/L
Total concentration:	0.5840	ng/uL

Sample peak width (sec): 5   Sample min peak height: 50   Sample baseline V to V?: Y   Sample baseline V to V points: 3
Sample filter: Binomial   Number of points for filter: 3 Sample start region (min): 0 Sample end region (min): 30
Marker peak width (sec): 5   Marker min peak height: 200   Marker baseline V to V?: Y   Marker baseline V to V points: 3
Lower marker selection: First peak > 500 RFU   Upper marker selection: Last peak > 200 RFU
Ladder size (bp)75, 200, 300, 400, 500, 700, 1000, 1500, 2000, 3000, 4000, 5000, 7000, 10000, 20000
Quantification using: Lower Marker   Final concentration (ng/uL): 0.5000   Dilution factor: 12.0

Sample: SampG4
Well location: G4
Created: Tuesday, September 14, 2021 5:34:08 PM

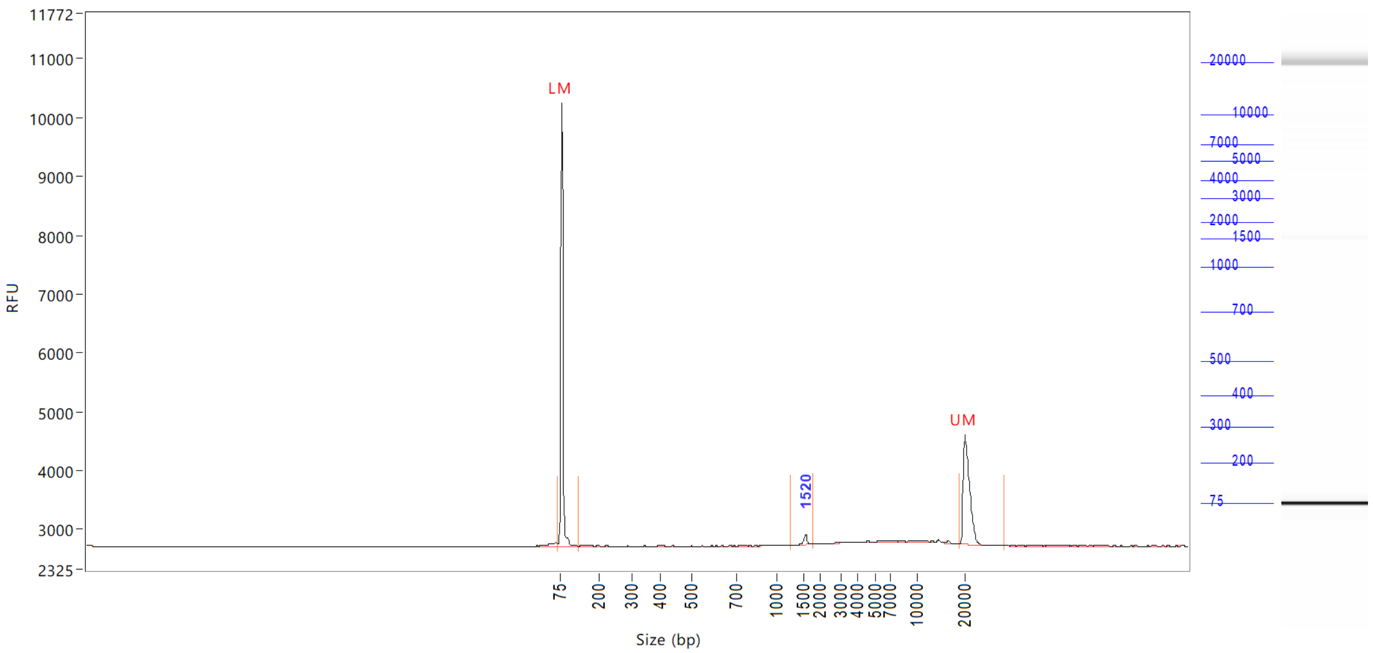

Peak	Size (bp)	Concentration (ng/uL)	From (bp)	To (bp)	Average size (bp)	CV%	RFU	Corrected peak area
1	75 (LM)	0.5000	64	129	73	7.20	7550	42.037
2	1520	0.1260	1256	1770	1521	4.23	166	0.883
3	20000 (UM)	0.1902	18659	28342	20380	3.85	1859	15.990

TIC:	0.1260	ng/uL
TIM:	0.1364	nmole/L
Total concentration:	0.5873	ng/uL

Sample peak width (sec): 5 Sample min peak height: 50 Sample baseline V to V?: Y Sample baseline V to V points: 3
Sample filter: Binomial Number of points for filter: 3 Sample start region (min): 0 Sample end region (min): 30
Marker peak width (sec): 5 Marker min peak height: 200 Marker baseline V to V?: Y Marker baseline V to V points: 3
Lower marker selection: First peak > 500 RFU Upper marker selection: Last peak > 200 RFU
Ladder size (bp)75, 200, 300, 400, 500, 700, 1000, 1500, 2000, 3000, 4000, 5000, 7000, 10000, 20000
Quantification using: Lower Marker Final concentration (ng/uL): 0.5000 Dilution factor: 12.0

Sample: SampH4
Well location: H4
Created: Tuesday, September 14, 2021 5:34:08 PM

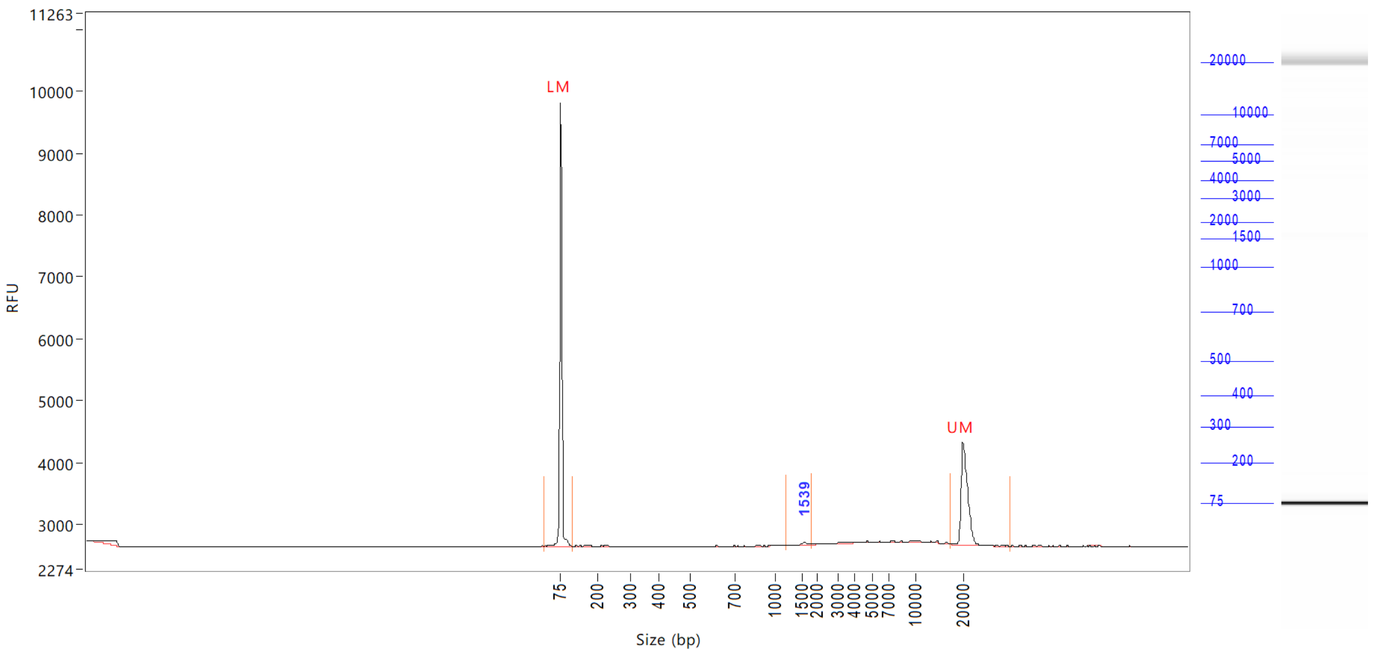

Peak	Size<br>(bp)	Concentration<br>(ng/uL)	From<br>(bp)	To<br>(bp)	Average size<br>(bp)	CV%	RFU	Corrected peak area
1	75 (LM)	0.5000	20	116	73	7.66	7183	39.647
2	1539	0.0690	1190	1808	1530	8.14	50	0.456
3	20000 (UM)	0.1848	17561	29914	20472	4.20	1659	14.650

TIC:	0.0690	ng/uL
TIM:	0.0742	nmole/L
Total concentration:	0.6100	ng/uL

Sample peak width (sec): 5   Sample min peak height: 50   Sample baseline V to V?: Y   Sample baseline V to V points: 3
Sample filter: Binomial   Number of points for filter: 3 Sample start region (min): 0 Sample end region (min): 30
Marker peak width (sec): 5   Marker min peak height: 200   Marker baseline V to V?: Y   Marker baseline V to V points: 3
Lower marker selection: First peak > 500 RFU   Upper marker selection: Last peak > 200 RFU
Ladder size (bp)75, 200, 300, 400, 500, 700, 1000, 1500, 2000, 3000, 4000, 5000, 7000, 10000, 20000
Quantification using: Lower Marker   Final concentration (ng/uL): 0.5000   Dilution factor: 12.0

Sample: SampA5
Well location: A5
Created: Tuesday, September 14, 2021 5:34:08 PM

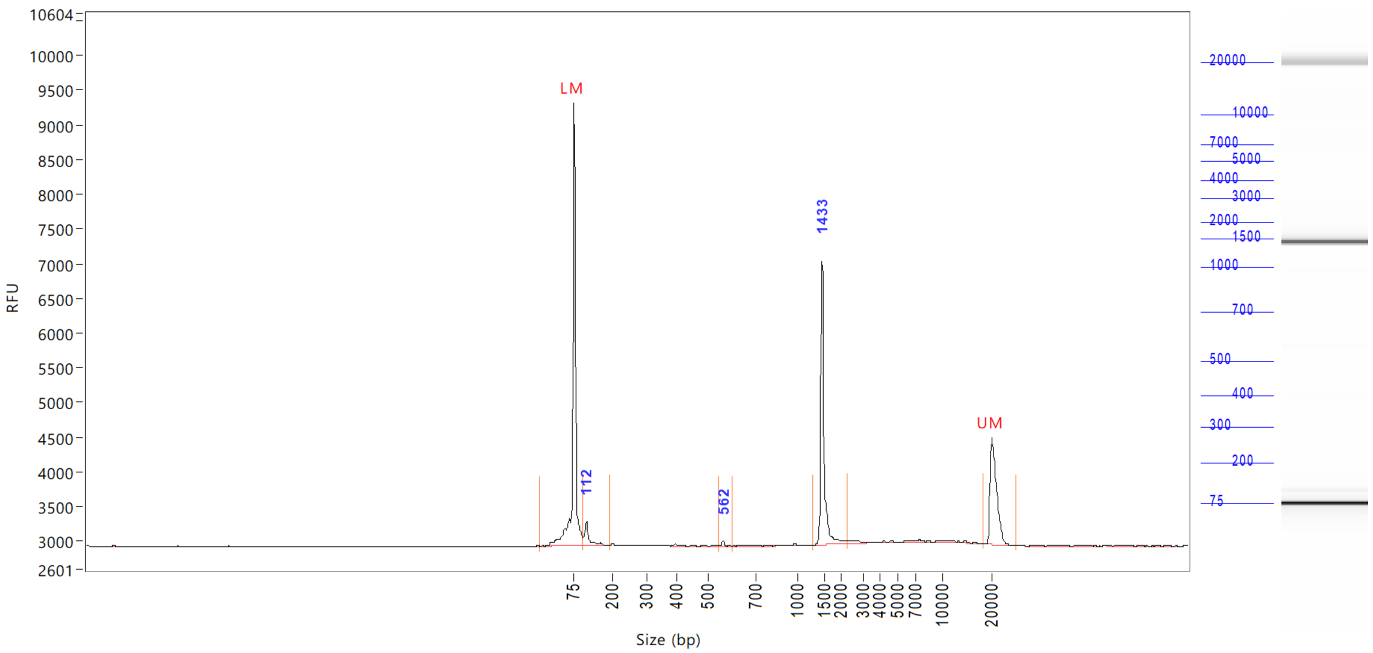

Peak	Size (bp)	Concentration (ng/uL)	From (bp)	To (bp)	Average size (bp)	CV%	RFU	Corrected peak area
1	75 (LM)	0.5000	0	103	69	22.88	6383	45.428
2	112	0.4415	103	189	118	12.20	343	3.343
3	562	0.0556	542	598	561	1.77	75	0.421
4	1433	2.9008	1267	2286	1467	7.86	4072	21.963
5	20000 (UM)	0.1439	18415	24957	20341	3.55	1537	13.076

TIC:	3.3979	ng/uL
TIM:	9.5646	nmole/L
Total concentration:	3.9077	ng/uL

```
Sample peak width (sec): 5    Sample min peak height: 50    Sample baseline V to V?: Y    Sample baseline V to V points: 3
Sample filter: Binomial       Number of points for filter: 3  Sample start region (min): 0  Sample end region (min): 30
Marker peak width (sec): 5    Marker min peak height: 200   Marker baseline V to V?: Y    Marker baseline V to V points: 3
Lower marker selection: First peak > 500 RFU                Upper marker selection: Last peak > 200 RFU
Ladder size (bp)75, 200, 300, 400, 500, 700, 1000, 1500, 2000, 3000, 4000, 5000, 7000, 10000, 20000
Quantification using: Lower Marker          Final concentration (ng/uL): 0.5000          Dilution factor: 12.0
```

Sample: SampB5
Well location: B5
Created: Tuesday, September 14, 2021 5:34:08 PM

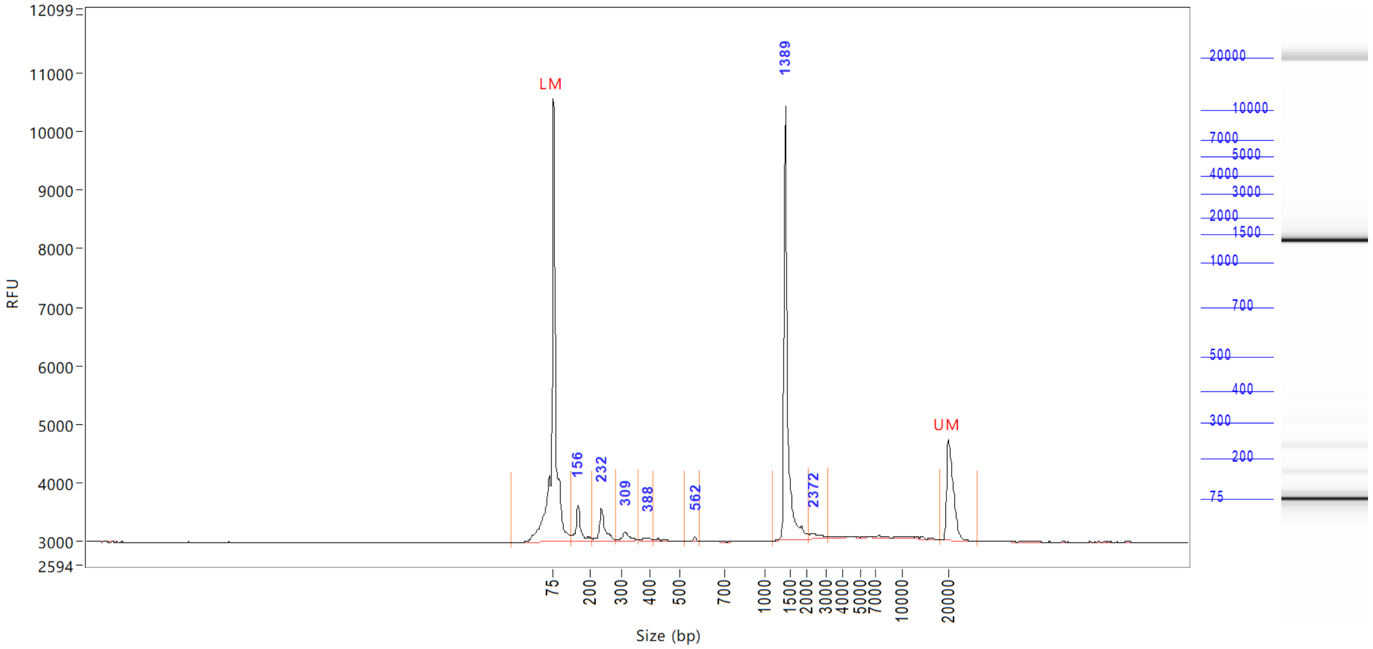

Peak	Size (bp)	Concentration (ng/uL)	From (bp)	To (bp)	Average size (bp)	CV%	RFU	Corrected peak area
1	75 (LM)	0.5000	0	133	70	29.51	7584	80.395
2	156	0.5565	133	205	160	9.25	626	7.456
3	232	0.5145	205	280	237	5.49	546	6.894
4	309	0.1575	280	356	312	4.74	139	2.110
5	388	0.0583	356	407	383	3.09	51	0.781
6	562	0.0389	521	588	557	1.89	94	0.522
7	1389	3.3991	1167	2115	1433	8.55	7427	45.545
8	2372	0.1153	2115	3037	2480	10.03	88	1.545
9	20000 (UM)	0.0938	18293	26045	20453	3.99	1715	15.080

TIC:	4.8402	ng/uL
TIM:	14.4638	nmole/L
Total concentration:	5.0768	ng/uL

Sample peak width (sec): 5   Sample min peak height: 50   Sample baseline V to V?: Y   Sample baseline V to V points: 3
Sample filter: Binomial   Number of points for filter: 3   Sample start region (min): 0   Sample end region (min): 30
Marker peak width (sec): 5   Marker min peak height: 200   Marker baseline V to V?: Y   Marker baseline V to V points: 3
Lower marker selection: First peak > 500 RFU   Upper marker selection: Last peak > 200 RFU
Ladder size (bp)75, 200, 300, 400, 500, 700, 1000, 1500, 2000, 3000, 4000, 5000, 7000, 10000, 20000
Quantification using: Lower Marker   Final concentration (ng/uL): 0.5000   Dilution factor: 12.0

Sample: SampC5
Well location: C5
Created: Tuesday, September 14, 2021 5:34:08 PM

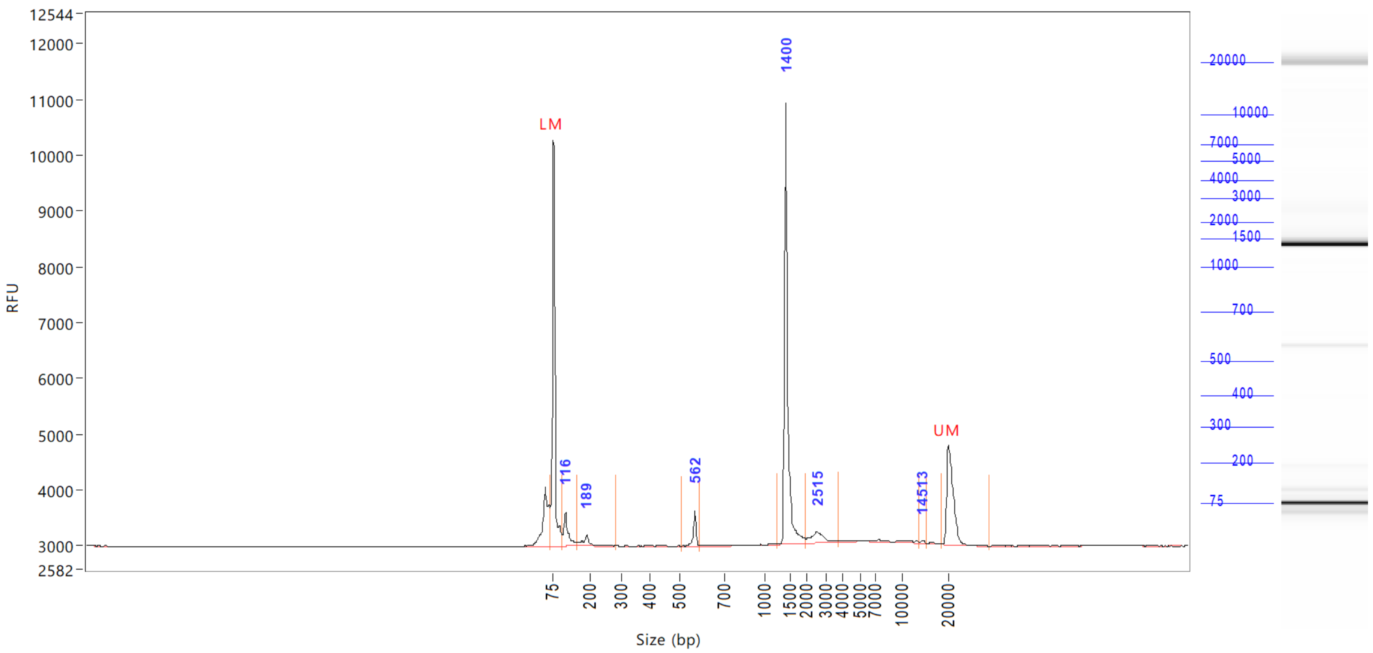

Peak	Size (bp)	Concentration (ng/uL)	From (bp)	To (bp)	Average size (bp)	CV%	RFU	Corrected peak area
1	75 (LM)	0.5000	66	105	75	8.80	7273	48.154
2	116	0.8216	105	154	120	9.88	615	6.593
3	189	0.3804	154	278	186	12.19	208	3.053
4	562	0.4300	501	585	557	1.85	641	3.451
5	1400	5.4047	1234	2000	1428	6.87	7921	43.376
6	2515	0.5104	2000	3750	2582	14.46	195	4.096
7	14513	0.0320	13781	15366	14368	2.52	52	0.256
8	20000 (UM)	0.1602	18537	28584	20465	4.28	1777	15.428

TIC:	7.5791	ng/uL
TIM:	22.4393	nmole/L
Total concentration:	8.0132	ng/uL

```
Sample peak width (sec): 5    Sample min peak height: 50    Sample baseline V to V?: Y    Sample baseline V to V points: 3
Sample filter: Binomial       Number of points for filter: 3  Sample start region (min): 0  Sample end region (min): 30
Marker peak width (sec): 5    Marker min peak height: 200   Marker baseline V to V?: Y    Marker baseline V to V points: 3
Lower marker selection: First peak > 500 RFU                Upper marker selection: Last peak > 200 RFU
Ladder size (bp)75, 200, 300, 400, 500, 700, 1000, 1500, 2000, 3000, 4000, 5000, 7000, 10000, 20000
Quantification using: Lower Marker          Final concentration (ng/uL): 0.5000          Dilution factor: 12.0
```

Sample: SampD5
Well location: D5
Created: Tuesday, September 14, 2021 5:34:08 PM

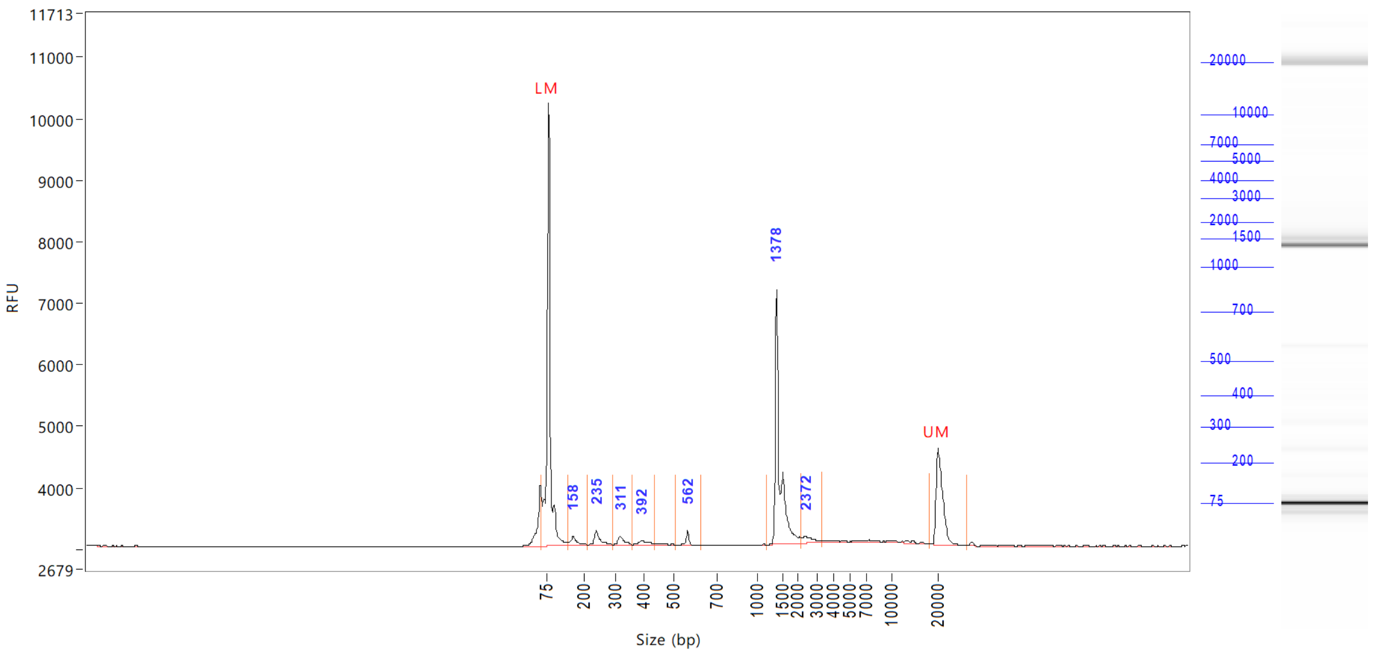

Peak	Size (bp)	Concentration (ng/uL)	From (bp)	To (bp)	Average size (bp)	CV%	RFU	Corrected peak area
1	75 (LM)	0.5000	54	145	76	16.59	7211	56.802
2	158	0.2261	145	206	164	8.34	149	2.141
3	235	0.3248	206	289	240	5.92	247	3.075
4	311	0.2154	289	356	318	4.24	143	2.039
5	392	0.1670	356	437	397	4.59	78	1.581
6	562	0.1632	506	621	558	3.14	238	1.545
7	1378	3.2833	1178	2172	1460	10.39	4132	31.082
8	2372	0.1943	2172	3251	2553	10.78	105	1.839
9	20000 (UM)	0.1230	18171	26408	20432	4.14	1577	13.971

TIC:	4.5741	ng/uL
TIM:	10.6070	nmole/L
Total concentration:	4.9331	ng/uL

```
Sample peak width (sec): 5    Sample min peak height: 50    Sample baseline V to V?: Y    Sample baseline V to V points: 3
Sample filter: Binomial       Number of points for filter: 3  Sample start region (min): 0  Sample end region (min): 30
Marker peak width (sec): 5    Marker min peak height: 200   Marker baseline V to V?: Y    Marker baseline V to V points: 3
Lower marker selection: First peak > 500 RFU                Upper marker selection: Last peak > 200 RFU
Ladder size (bp)75, 200, 300, 400, 500, 700, 1000, 1500, 2000, 3000, 4000, 5000, 7000, 10000, 20000
Quantification using: Lower Marker          Final concentration (ng/uL): 0.5000          Dilution factor: 12.0
```

Sample: SampE5
Well location: E5
Created: Tuesday, September 14, 2021 5:34:08 PM

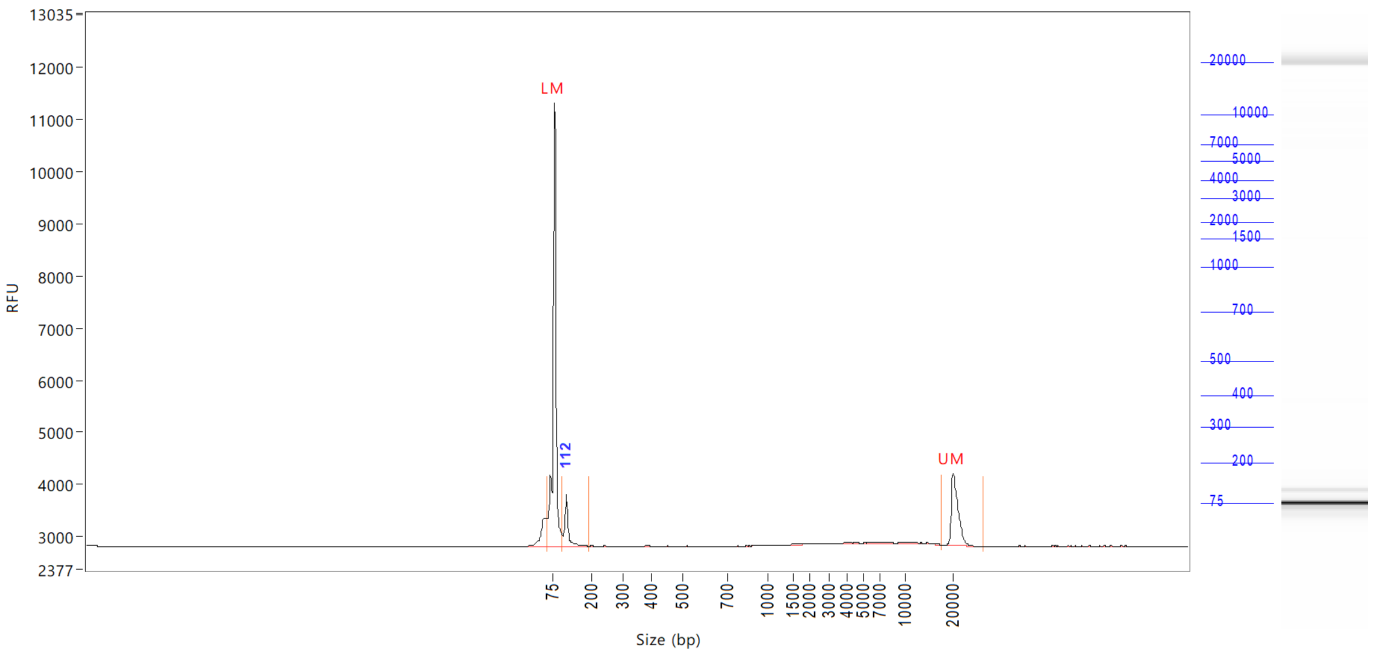

Peak	Size (bp)	Concentration (ng/uL)	From (bp)	To (bp)	Average size (bp)	CV%	RFU	Corrected peak area
1	75 (LM)	0.5000	50	103	72	11.66	8520	66.523
2	112	0.7824	103	189	118	12.59	1000	8.675
3	20000 (UM)	0.0941	17561	26408	20513	3.98	1383	12.524

TIC:	0.7824	ng/uL
TIM:	10.8926	nmole/L
Total concentration:	1.1425	ng/uL

Sample peak width (sec): 5   Sample min peak height: 50   Sample baseline V to V?: Y   Sample baseline V to V points: 3
Sample filter: Binomial   Number of points for filter: 3   Sample start region (min): 0   Sample end region (min): 30
Marker peak width (sec): 5   Marker min peak height: 200   Marker baseline V to V?: Y   Marker baseline V to V points: 3
Lower marker selection: First peak > 500 RFU   Upper marker selection: Last peak > 200 RFU
Ladder size (bp)75, 200, 300, 400, 500, 700, 1000, 1500, 2000, 3000, 4000, 5000, 7000, 10000, 20000
Quantification using: Lower Marker   Final concentration (ng/uL): 0.5000   Dilution factor: 12.0

Sample: SampF5
Well location: F5
Created: Tuesday, September 14, 2021 5:34:08 PM

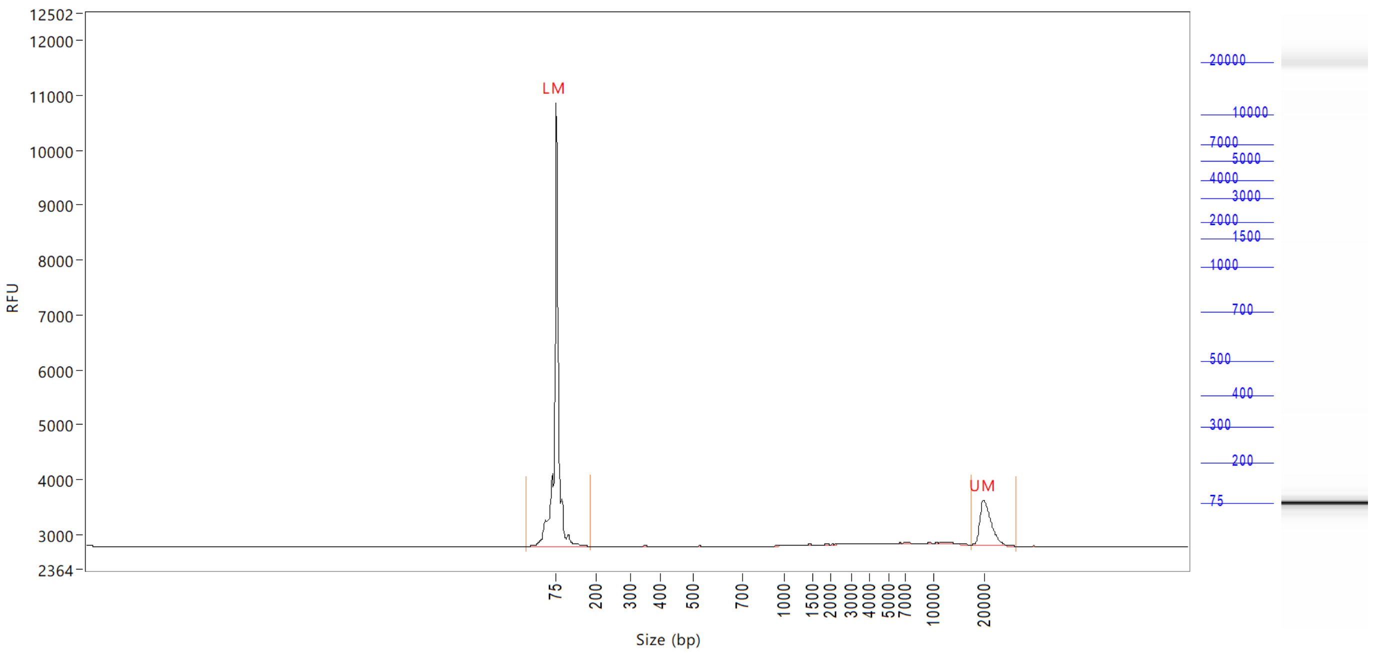

Peak	Size (bp)	Concentration (ng/uL)	From (bp)	To (bp)	Average size (bp)	CV%	RFU	Corrected peak area
1	75 (LM)	0.5000	0	181	71	24.12	8102	79.279
2	20000 (UM)	0.0751	17439	26408	20442	5.76	826	11.900

TIC:	0.0000	ng/uL
TIM:	0.0000	nmole/L
Total concentration:	0.2345	ng/uL

Sample peak width (sec): 5 Sample min peak height: 50 Sample baseline V to V?: Y Sample baseline V to V points: 3
Sample filter: Binomial Number of points for filter: 3 Sample start region (min): 0 Sample end region (min): 30
Marker peak width (sec): 5 Marker min peak height: 200 Marker baseline V to V?: Y Marker baseline V to V points: 3
Lower marker selection: First peak > 500 RFU Upper marker selection: Last peak > 200 RFU
Ladder size (bp)75, 200, 300, 400, 500, 700, 1000, 1500, 2000, 3000, 4000, 5000, 7000, 10000, 20000
Quantification using: Lower Marker Final concentration (ng/uL): 0.5000 Dilution factor: 12.0

Sample: SampG5
Well location: G5
Created: Tuesday, September 14, 2021 5:34:08 PM

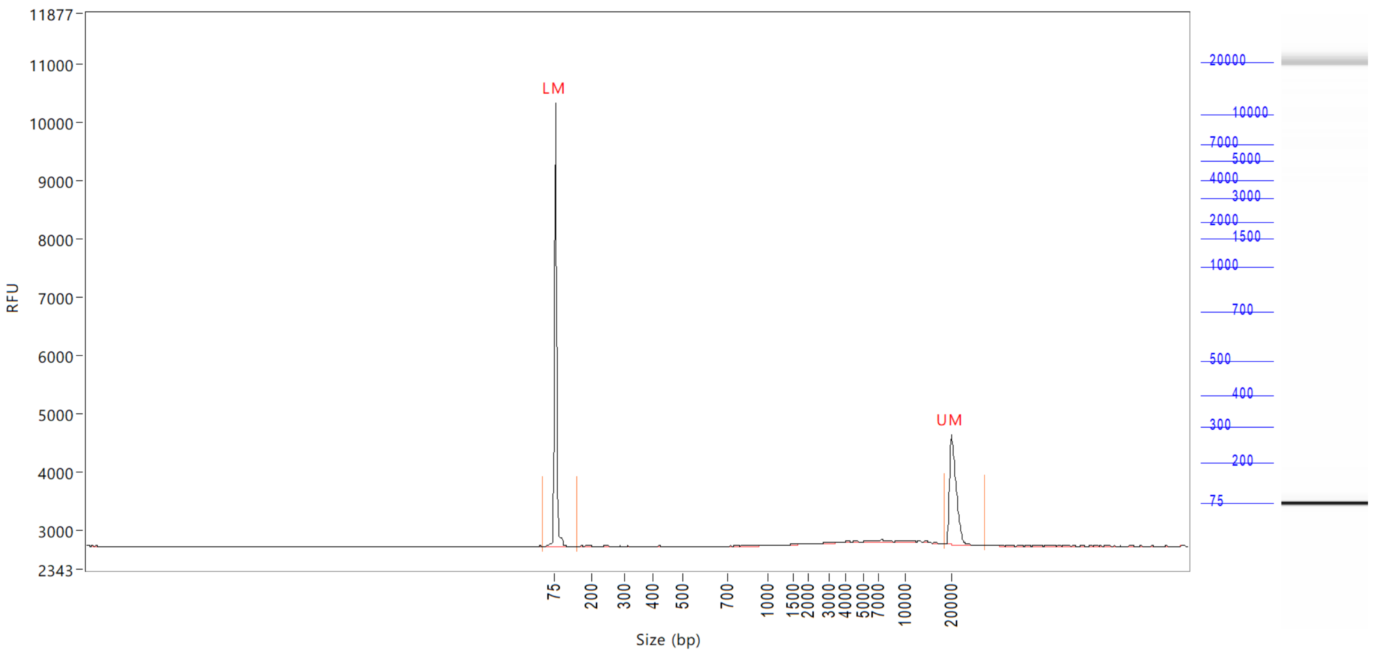

Peak	Size (bp)	Concentration (ng/uL)	From (bp)	To (bp)	Average size (bp)	CV%	RFU	Corrected peak area
1	75 (LM)	0.5000	31	148	73	7.86	7619	43.377
2	20000 (UM)	0.1913	18415	27012	20382	3.89	1888	16.598

TIC:	0.0000	ng/uL
TIM:	0.0000	nmole/L
Total concentration:	0.5034	ng/uL

Sample peak width (sec): 5 Sample min peak height: 50 Sample baseline V to V?: Y Sample baseline V to V points: 3
Sample filter: Binomial Number of points for filter: 3 Sample start region (min): 0 Sample end region (min): 30
Marker peak width (sec): 5 Marker min peak height: 200 Marker baseline V to V?: Y Marker baseline V to V points: 3
Lower marker selection: First peak > 500 RFU Upper marker selection: Last peak > 200 RFU
Ladder size (bp)75, 200, 300, 400, 500, 700, 1000, 1500, 2000, 3000, 4000, 5000, 7000, 10000, 20000
Quantification using: Lower Marker Final concentration (ng/uL): 0.5000 Dilution factor: 12.0

Sample: SampH5
Well location: H5
Created: Tuesday, September 14, 2021 5:34:08 PM

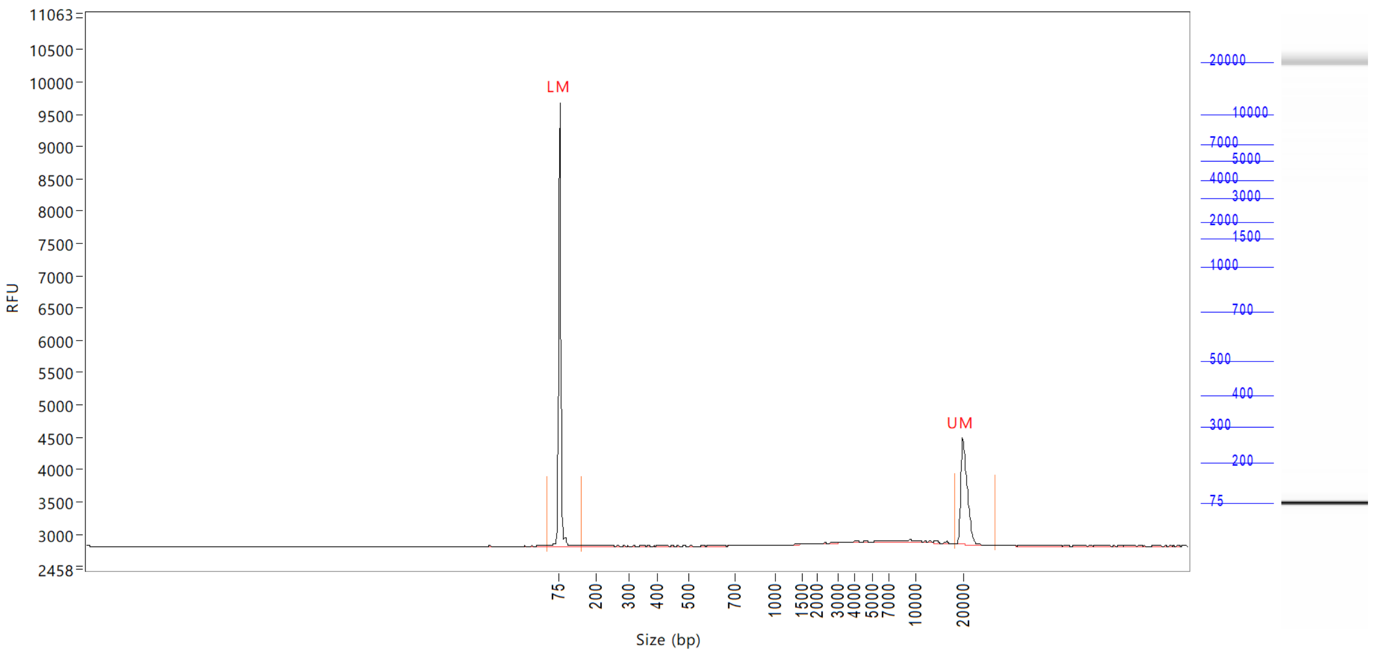

Peak	Size (bp)	Concentration (ng/uL)	From (bp)	To (bp)	Average size (bp)	CV%	RFU	Corrected peak area
1	75 (LM)	0.5000	35	148	74	8.43	6874	38.536
2	20000 (UM)	0.1861	18293	26770	20381	3.75	1654	14.342

TIC:	0.0000	ng/uL
TIM:	0.0000	nmole/L
Total concentration:	0.5910	ng/uL

Sample peak width (sec): 5 Sample min peak height: 50 Sample baseline V to V?: Y Sample baseline V to V points: 3
Sample filter: Binomial Number of points for filter: 3 Sample start region (min): 0 Sample end region (min): 30
Marker peak width (sec): 5 Marker min peak height: 200 Marker baseline V to V?: Y Marker baseline V to V points: 3
Lower marker selection: First peak > 500 RFU Upper marker selection: Last peak > 200 RFU
Ladder size (bp)75, 200, 300, 400, 500, 700, 1000, 1500, 2000, 3000, 4000, 5000, 7000, 10000, 20000
Quantification using: Lower Marker Final concentration (ng/uL): 0.5000 Dilution factor: 12.0

Sample: SampA6
Well location: A6
Created: Tuesday, September 14, 2021 5:34:08 PM

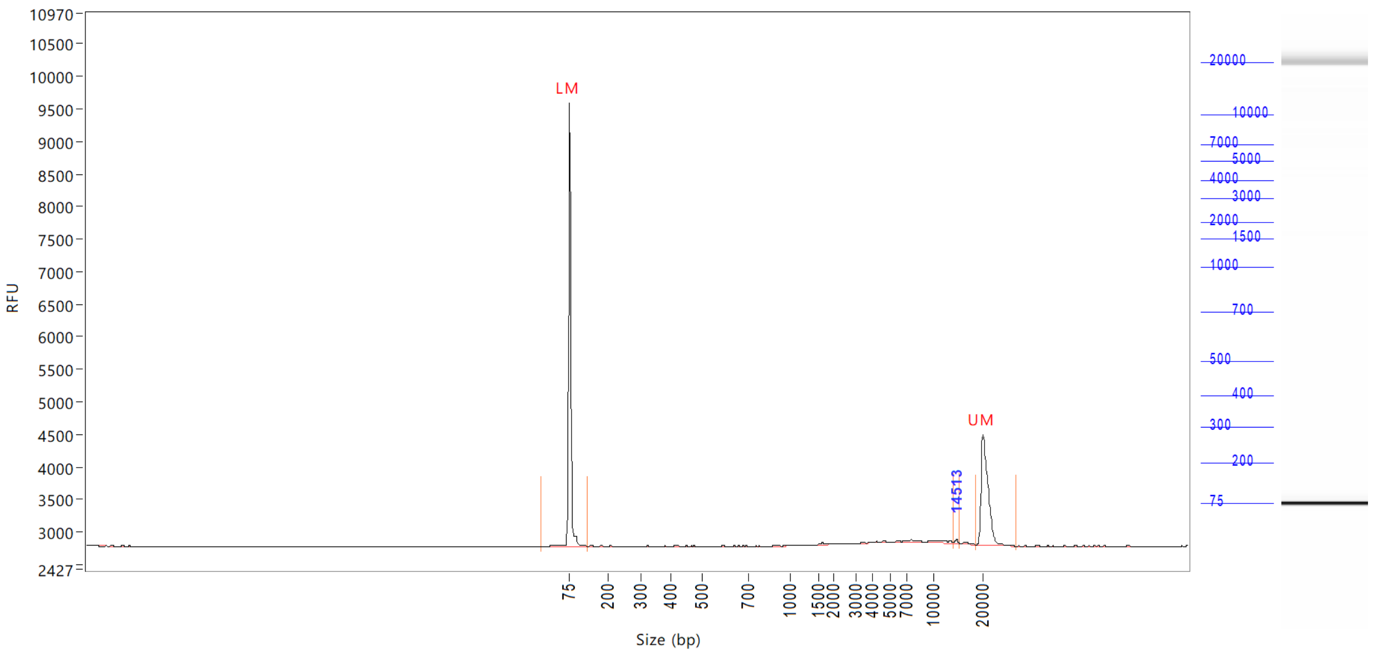

Peak	Size (bp)	Concentration (ng/uL)	From (bp)	To (bp)	Average size (bp)	CV%	RFU	Corrected peak area
1	75 (LM)	0.5000	0	133	73	10.66	6828	38.555
2	14513	0.0449	13903	15366	14507	2.39	61	0.288
3	20000 (UM)	0.1976	18659	26770	20497	4.21	1703	15.236

TIC:	0.0449	ng/uL
TIM:	0.0051	nmole/L
Total concentration:	0.6737	ng/uL

```
Sample peak width (sec): 5    Sample min peak height: 50    Sample baseline V to V?: Y    Sample baseline V to V points: 3
Sample filter: Binomial       Number of points for filter: 3  Sample start region (min): 0  Sample end region (min): 30
Marker peak width (sec): 5    Marker min peak height: 200   Marker baseline V to V?: Y    Marker baseline V to V points: 3
Lower marker selection: First peak > 500 RFU                Upper marker selection: Last peak > 200 RFU
Ladder size (bp)75, 200, 300, 400, 500, 700, 1000, 1500, 2000, 3000, 4000, 5000, 7000, 10000, 20000
Quantification using: Lower Marker         Final concentration (ng/uL): 0.5000         Dilution factor: 12.0
```

Sample: SampB6
Well location: B6
Created: Tuesday, September 14, 2021 5:34:08 PM

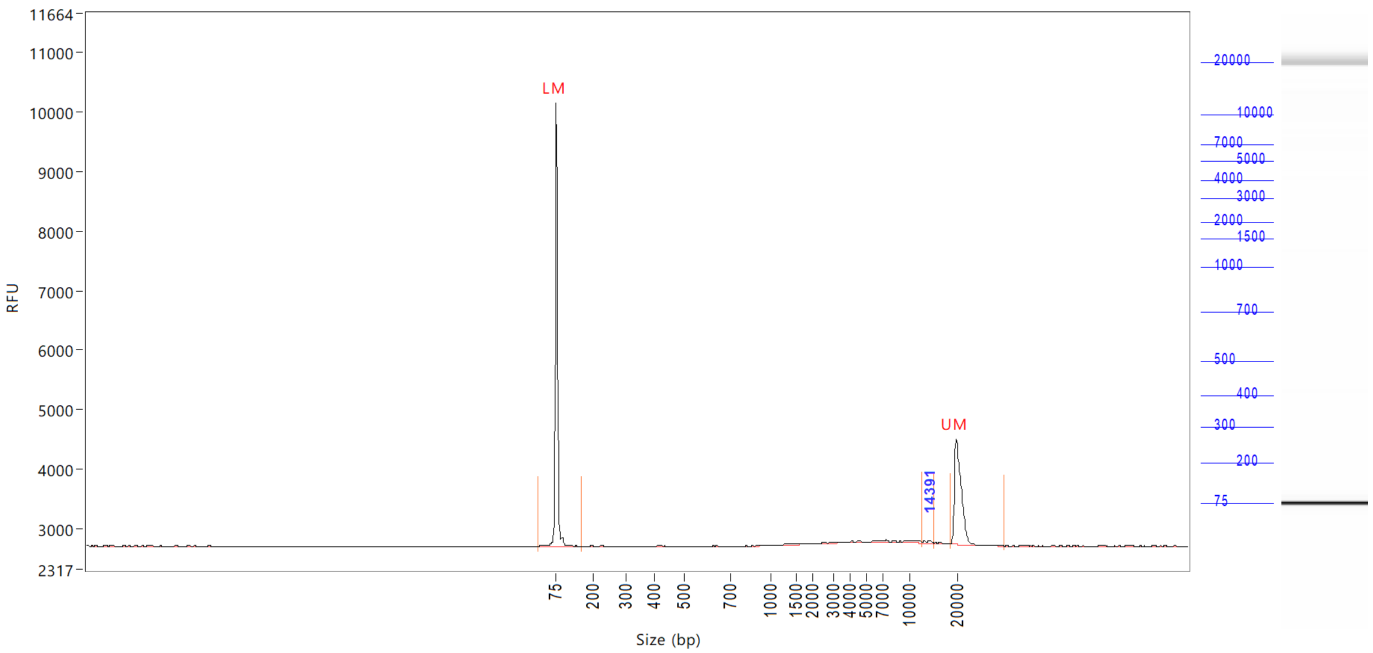

Peak	Size (bp)	Concentration (ng/uL)	From (bp)	To (bp)	Average size (bp)	CV%	RFU	Corrected peak area
1	75 (LM)	0.5000	16	158	73	10.07	7471	42.036
2	14391	0.0654	12684	15122	13839	4.65	57	0.458
3	20000 (UM)	0.1904	18537	30156	20422	4.37	1775	16.005

TIC:	0.0654	ng/uL
TIM:	0.0078	nmole/L
Total concentration:	0.6602	ng/uL

```
Sample peak width (sec): 5    Sample min peak height: 50    Sample baseline V to V?: Y    Sample baseline V to V points: 3
Sample filter: Binomial       Number of points for filter: 3  Sample start region (min): 0  Sample end region (min): 30
Marker peak width (sec): 5    Marker min peak height: 200   Marker baseline V to V?: Y    Marker baseline V to V points: 3
Lower marker selection: First peak > 500 RFU                Upper marker selection: Last peak > 200 RFU
Ladder size (bp)75, 200, 300, 400, 500, 700, 1000, 1500, 2000, 3000, 4000, 5000, 7000, 10000, 20000
Quantification using: Lower Marker          Final concentration (ng/uL): 0.5000          Dilution factor: 12.0
```

Sample: SampC6
Well location: C6
Created: Tuesday, September 14, 2021 5:34:08 PM

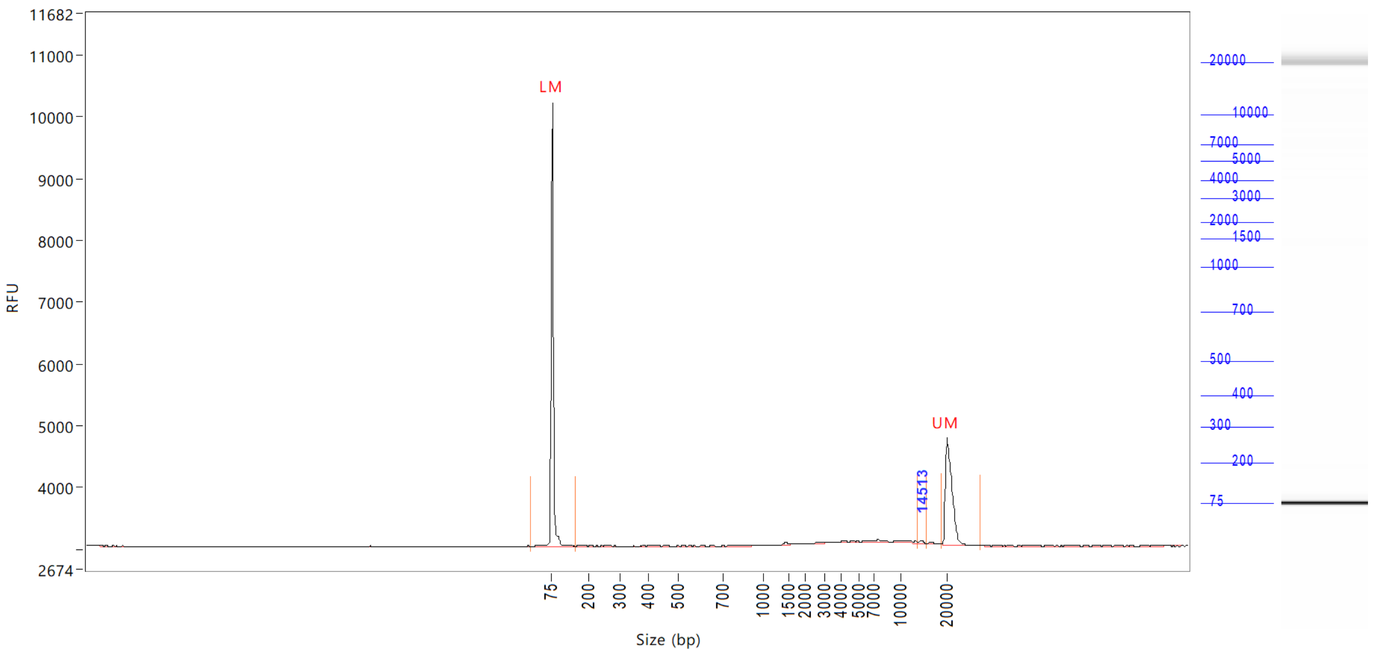

Peak	Size (bp)	Concentration (ng/uL)	From (bp)	To (bp)	Average size (bp)	CV%	RFU	Corrected peak area
1	75 (LM)	0.5000	3	152	74	10.30	7197	41.351
2	14513	0.0415	13659	15366	14308	2.65	54	0.286
3	20000 (UM)	0.1824	18659	26891	20441	4.05	1723	15.089

TIC:	0.0415	ng/uL
TIM:	0.0048	nmole/L
Total concentration:	0.7037	ng/uL

Sample peak width (sec): 5   Sample min peak height: 50   Sample baseline V to V?: Y   Sample baseline V to V points: 3
Sample filter: Binomial   Number of points for filter: 3   Sample start region (min): 0   Sample end region (min): 30
Marker peak width (sec): 5   Marker min peak height: 200   Marker baseline V to V?: Y   Marker baseline V to V points: 3
Lower marker selection: First peak > 500 RFU   Upper marker selection: Last peak > 200 RFU
Ladder size (bp)75, 200, 300, 400, 500, 700, 1000, 1500, 2000, 3000, 4000, 5000, 7000, 10000, 20000
Quantification using: Lower Marker   Final concentration (ng/uL): 0.5000   Dilution factor: 12.0

Sample: SampD6
Well location: D6
Created: Tuesday, September 14, 2021 5:34:08 PM

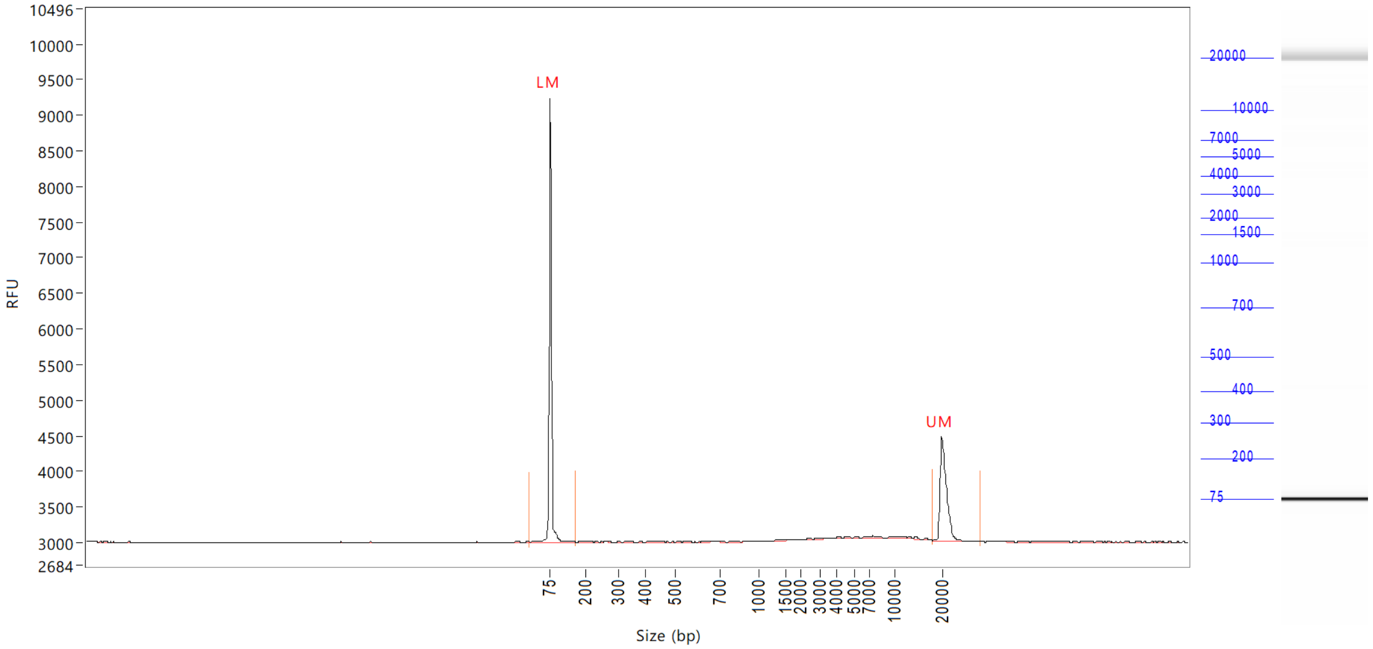

Peak	Size (bp)	Concentration (ng/uL)	From (bp)	To (bp)	Average size (bp)	CV%	RFU	Corrected peak area
1	75 (LM)	0.5000	7	162	73	11.19	6240	36.288
2	20000 (UM)	0.1748	18049	28221	20446	4.19	1455	12.687

TIC:	0.0000	ng/uL
TIM:	0.0000	nmole/L
Total concentration:	0.6545	ng/uL

```
Sample peak width (sec): 5    Sample min peak height: 50    Sample baseline V to V?: Y    Sample baseline V to V points: 3
Sample filter: Binomial       Number of points for filter: 3  Sample start region (min): 0  Sample end region (min): 30
Marker peak width (sec): 5    Marker min peak height: 200   Marker baseline V to V?: Y    Marker baseline V to V points: 3
Lower marker selection: First peak > 500 RFU                Upper marker selection: Last peak > 200 RFU
Ladder size (bp)75, 200, 300, 400, 500, 700, 1000, 1500, 2000, 3000, 4000, 5000, 7000, 10000, 20000
Quantification using: Lower Marker          Final concentration (ng/uL): 0.5000          Dilution factor: 12.0
```

Sample: SampE6
Well location: E6
Created: Tuesday, September 14, 2021 5:34:08 PM

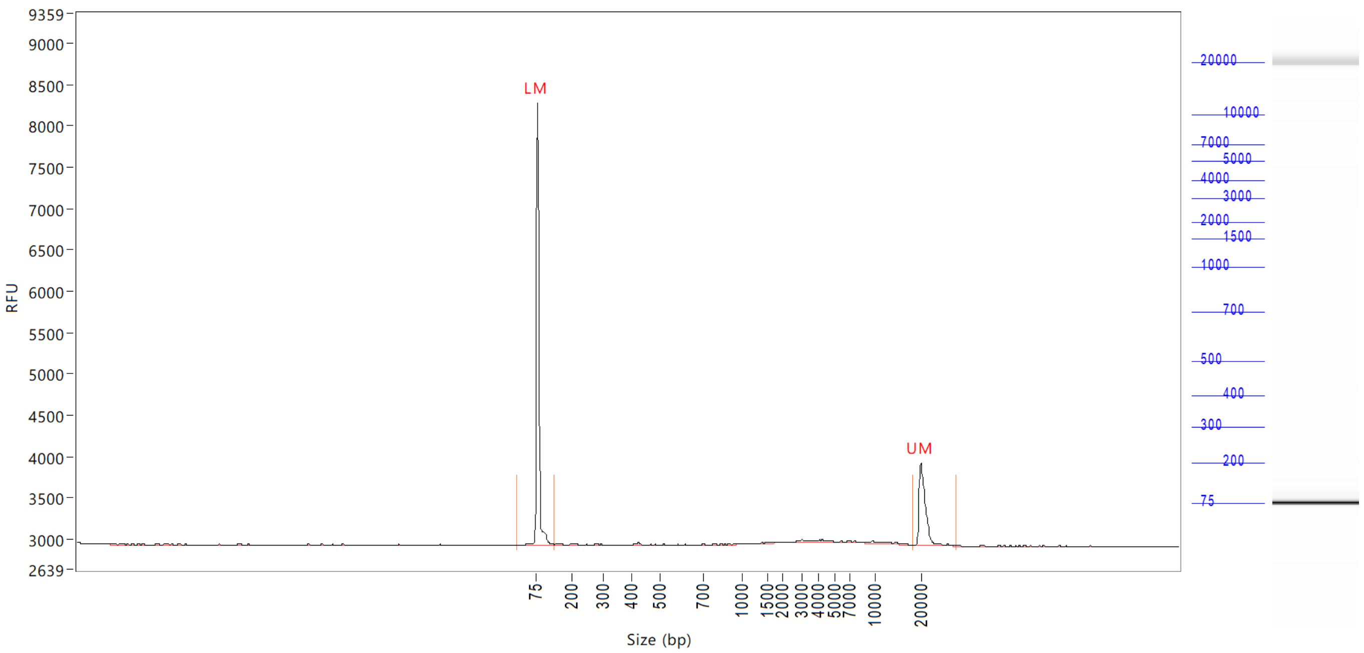

Peak	Size (bp)	Concentration (ng/uL)	From (bp)	To (bp)	Average size (bp)	CV%	RFU	Corrected peak area
1	75 (LM)	0.5000	5	133	75	11.20	5355	33.079
2	20000 (UM)	0.1327	18415	27859	20520	5.55	987	8.781

TIC:	0.0000	ng/uL
TIM:	0.0000	nmole/L
Total concentration:	0.7658	ng/uL

```
Sample peak width (sec): 5    Sample min peak height: 50    Sample baseline V to V?: Y    Sample baseline V to V points: 3
Sample filter: Binomial       Number of points for filter: 3  Sample start region (min): 0  Sample end region (min): 30
Marker peak width (sec): 5    Marker min peak height: 200   Marker baseline V to V?: Y    Marker baseline V to V points: 3
Lower marker selection: First peak > 500 RFU                Upper marker selection: Last peak > 200 RFU
Ladder size (bp)75, 200, 300, 400, 500, 700, 1000, 1500, 2000, 3000, 4000, 5000, 7000, 10000, 20000
Quantification using: Lower Marker          Final concentration (ng/uL): 0.5000          Dilution factor: 12.0
```

Sample: SampF6
Well location: F6
Created: Tuesday, September 14, 2021 5:34:08 PM

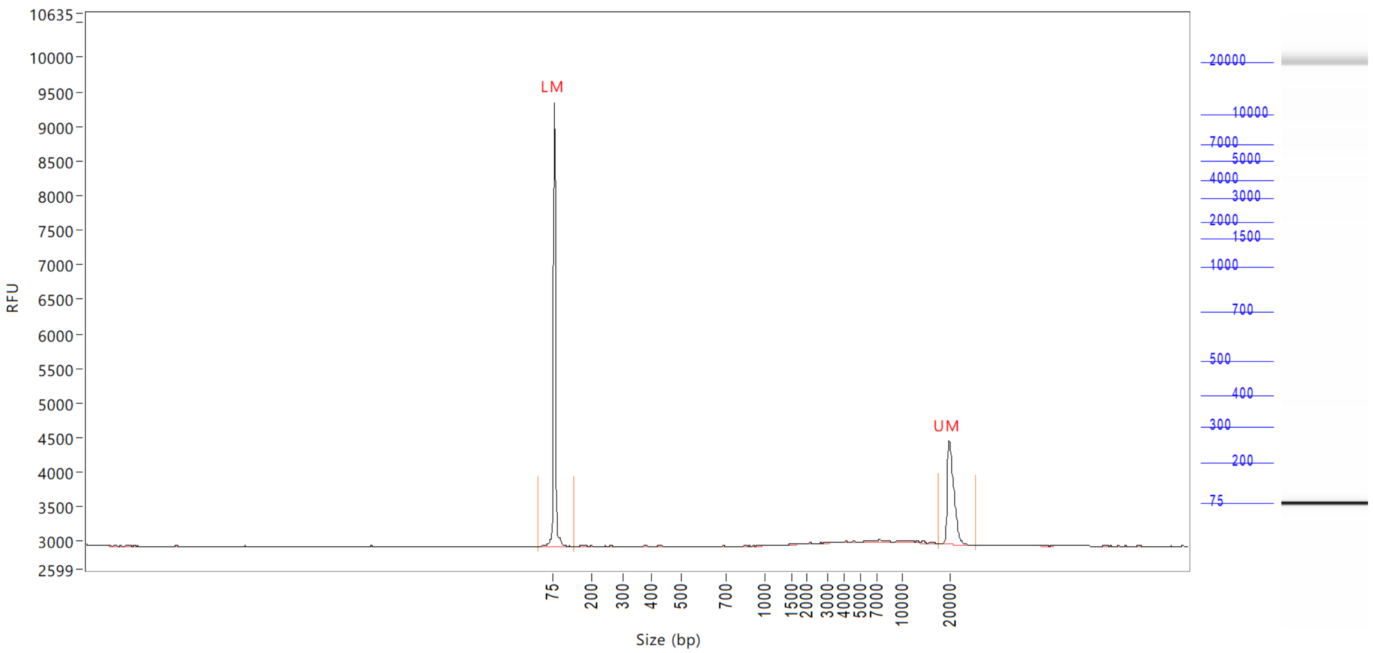

Peak	Size (bp)	Concentration (ng/uL)	From (bp)	To (bp)	Average size (bp)	CV%	RFU	Corrected peak area
1	75 (LM)	0.5000	22	141	73	8.76	6422	36.736
2	20000 (UM)	0.1770	17805	25803	20431	3.84	1506	13.006

TIC:	0.0000	ng/uL
TIM:	0.0000	nmole/L
Total concentration:	0.6461	ng/uL

Sample peak width (sec): 5 Sample min peak height: 50 Sample baseline V to V?: Y Sample baseline V to V points: 3
Sample filter: Binomial Number of points for filter: 3 Sample start region (min): 0 Sample end region (min): 30
Marker peak width (sec): 5 Marker min peak height: 200 Marker baseline V to V?: Y Marker baseline V to V points: 3
Lower marker selection: First peak > 500 RFU Upper marker selection: Last peak > 200 RFU
Ladder size (bp)75, 200, 300, 400, 500, 700, 1000, 1500, 2000, 3000, 4000, 5000, 7000, 10000, 20000
Quantification using: Lower Marker Final concentration (ng/uL): 0.5000 Dilution factor: 12.0

Sample: SampG6
Well location: G6
Created: Tuesday, September 14, 2021 5:34:08 PM

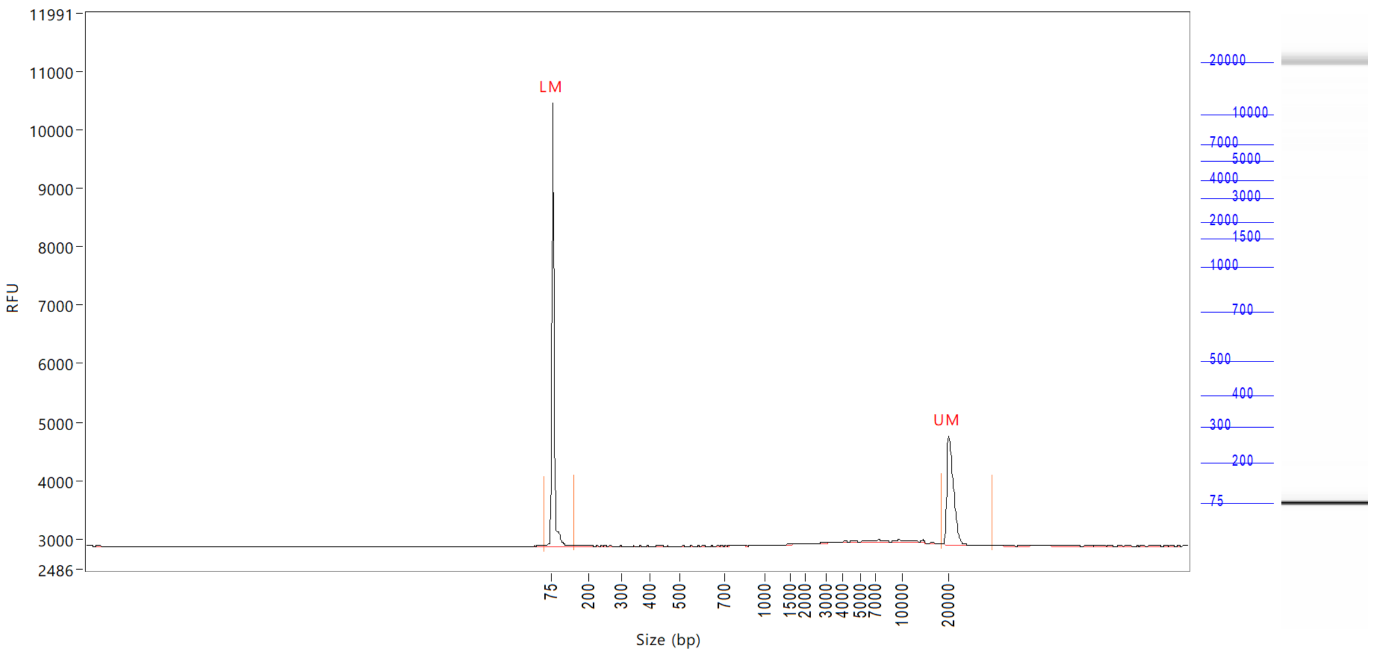

Peak	Size (bp)	Concentration (ng/uL)	From (bp)	To (bp)	Average size (bp)	CV%	RFU	Corrected peak area
1	75 (LM)	0.5000	47	148	75	9.36	7592	44.589
2	20000 (UM)	0.1832	18537	29309	20482	4.09	1862	16.340

TIC:	0.0000	ng/uL
TIM:	0.0000	nmole/L
Total concentration:	0.5173	ng/uL

Sample peak width (sec): 5 Sample min peak height: 50 Sample baseline V to V?: Y Sample baseline V to V points: 3
Sample filter: Binomial Number of points for filter: 3 Sample start region (min): 0 Sample end region (min): 30
Marker peak width (sec): 5 Marker min peak height: 200 Marker baseline V to V?: Y Marker baseline V to V points: 3
Lower marker selection: First peak > 500 RFU Upper marker selection: Last peak > 200 RFU
Ladder size (bp)75, 200, 300, 400, 500, 700, 1000, 1500, 2000, 3000, 4000, 5000, 7000, 10000, 20000
Quantification using: Lower Marker Final concentration (ng/uL): 0.5000 Dilution factor: 12.0

Sample: SampH6
Well location: H6
Created: Tuesday, September 14, 2021 5:34:08 PM

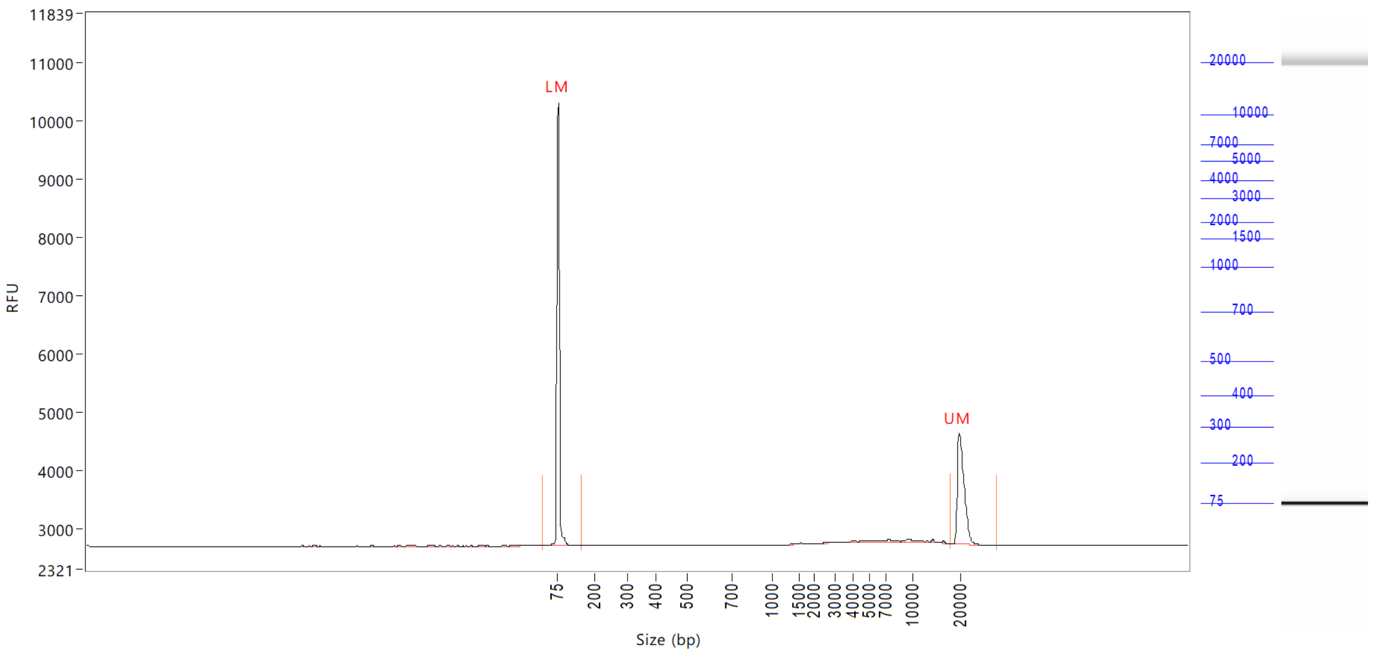

Peak	Size (bp)	Concentration (ng/uL)	From (bp)	To (bp)	Average size (bp)	CV%	RFU	Corrected peak area
1	75 (LM)	0.5000	26	154	73	8.97	7596	43.582
2	20000 (UM)	0.1937	18171	27738	20358	3.75	1904	16.881

TIC:	0.0000	ng/uL
TIM:	0.0000	nmole/L
Total concentration:	0.6163	ng/uL

Sample peak width (sec): 5 Sample min peak height: 50 Sample baseline V to V?: Y Sample baseline V to V points: 3
Sample filter: Binomial Number of points for filter: 3 Sample start region (min): 0 Sample end region (min): 30
Marker peak width (sec): 5 Marker min peak height: 200 Marker baseline V to V?: Y Marker baseline V to V points: 3
Lower marker selection: First peak > 500 RFU Upper marker selection: Last peak > 200 RFU
Ladder size (bp)75, 200, 300, 400, 500, 700, 1000, 1500, 2000, 3000, 4000, 5000, 7000, 10000, 20000
Quantification using: Lower Marker Final concentration (ng/uL): 0.5000 Dilution factor: 12.0

Sample: SampA7
Well location: A7
Created: Tuesday, September 14, 2021 5:34:08 PM

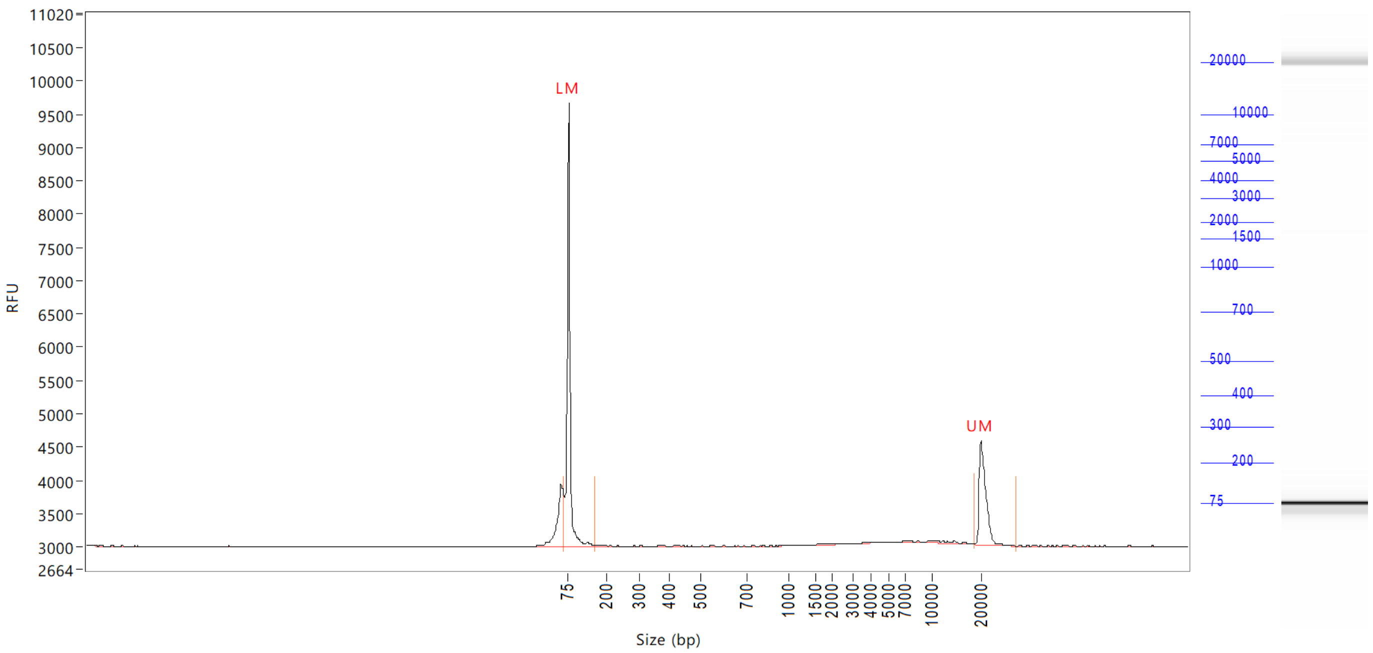

Peak	Size (bp)	Concentration (ng/uL)	From (bp)	To (bp)	Average size (bp)	CV%	RFU	Corrected peak area
1	75 (LM)	0.5000	62	162	76	15.08	6677	45.833
2	20000 (UM)	0.1484	18781	27254	20475	4.46	1559	13.607

TIC:	0.0000	ng/uL
TIM:	0.0000	nmole/L
Total concentration:	0.5133	ng/uL

Sample peak width (sec): 5   Sample min peak height: 50   Sample baseline V to V?: Y   Sample baseline V to V points: 3
Sample filter: Binomial   Number of points for filter: 3 Sample start region (min): 0 Sample end region (min): 30
Marker peak width (sec): 5   Marker min peak height: 200   Marker baseline V to V?: Y   Marker baseline V to V points: 3
Lower marker selection: First peak > 500 RFU   Upper marker selection: Last peak > 200 RFU
Ladder size (bp)75, 200, 300, 400, 500, 700, 1000, 1500, 2000, 3000, 4000, 5000, 7000, 10000, 20000
Quantification using: Lower Marker   Final concentration (ng/uL): 0.5000   Dilution factor: 12.0

Sample: SampB7
Well location: B7
Created: Tuesday, September 14, 2021 5:34:08 PM

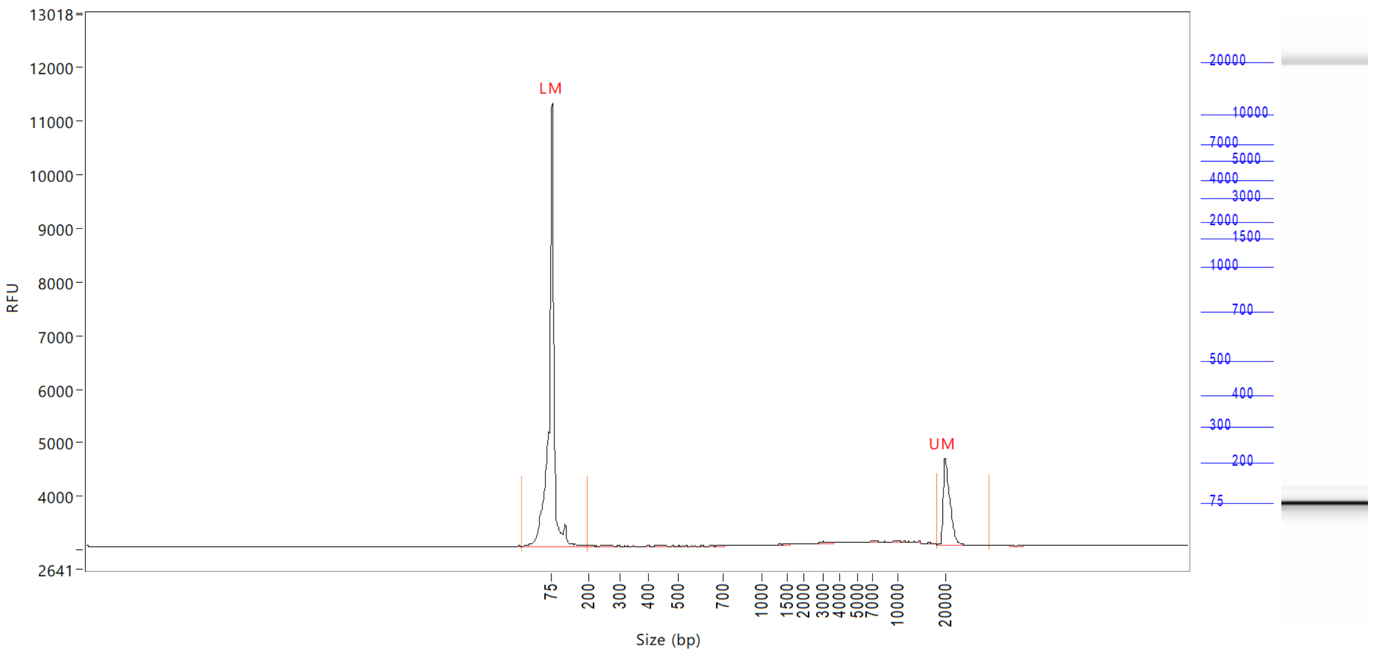

Peak	Size (bp)	Concentration (ng/uL)	From (bp)	To (bp)	Average size (bp)	CV%	RFU	Corrected peak area
1	75 (LM)	0.5000	0	196	69	30.18	8292	101.181
2	20000 (UM)	0.0717	18293	29551	20542	4.60	1603	14.505

TIC:	0.0000	ng/uL
TIM:	0.0000	nmole/L
Total concentration:	0.2609	ng/uL

Sample peak width (sec): 5 Sample min peak height: 50 Sample baseline V to V?: Y Sample baseline V to V points: 3
Sample filter: Binomial Number of points for filter: 3 Sample start region (min): 0 Sample end region (min): 30
Marker peak width (sec): 5 Marker min peak height: 200 Marker baseline V to V?: Y Marker baseline V to V points: 3
Lower marker selection: First peak > 500 RFU Upper marker selection: Last peak > 200 RFU
Ladder size (bp)75, 200, 300, 400, 500, 700, 1000, 1500, 2000, 3000, 4000, 5000, 7000, 10000, 20000
Quantification using: Lower Marker Final concentration (ng/uL): 0.5000 Dilution factor: 12.0

Sample: SampC7
Well location: C7
Created: Tuesday, September 14, 2021 5:34:08 PM

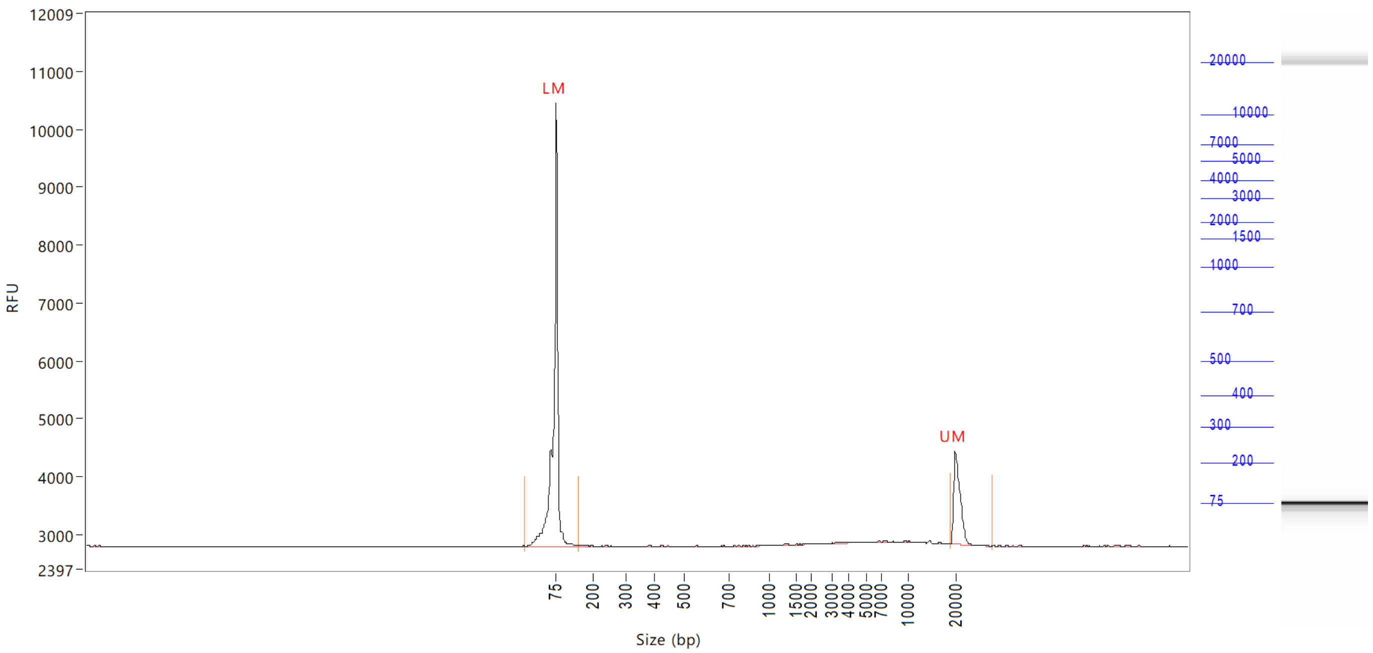

Peak	Size (bp)	Concentration (ng/uL)	From (bp)	To (bp)	Average size (bp)	CV%	RFU	Corrected peak area
1	75 (LM)	0.5000	0	150	64	29.76	7683	78.075
2	20000 (UM)	0.0875	18781	27859	20443	3.64	1596	13.662

TIC:	0.0000	ng/uL
TIM:	0.0000	nmole/L
Total concentration:	0.3081	ng/uL

Sample peak width (sec): 5 Sample min peak height: 50 Sample baseline V to V?: Y Sample baseline V to V points: 3
Sample filter: Binomial Number of points for filter: 3 Sample start region (min): 0 Sample end region (min): 30
Marker peak width (sec): 5 Marker min peak height: 200 Marker baseline V to V?: Y Marker baseline V to V points: 3
Lower marker selection: First peak > 500 RFU Upper marker selection: Last peak > 200 RFU
Ladder size (bp)75, 200, 300, 400, 500, 700, 1000, 1500, 2000, 3000, 4000, 5000, 7000, 10000, 20000
Quantification using: Lower Marker Final concentration (ng/uL): 0.5000 Dilution factor: 12.0

Sample: SampD7
Well location: D7
Created: Tuesday, September 14, 2021 5:34:08 PM

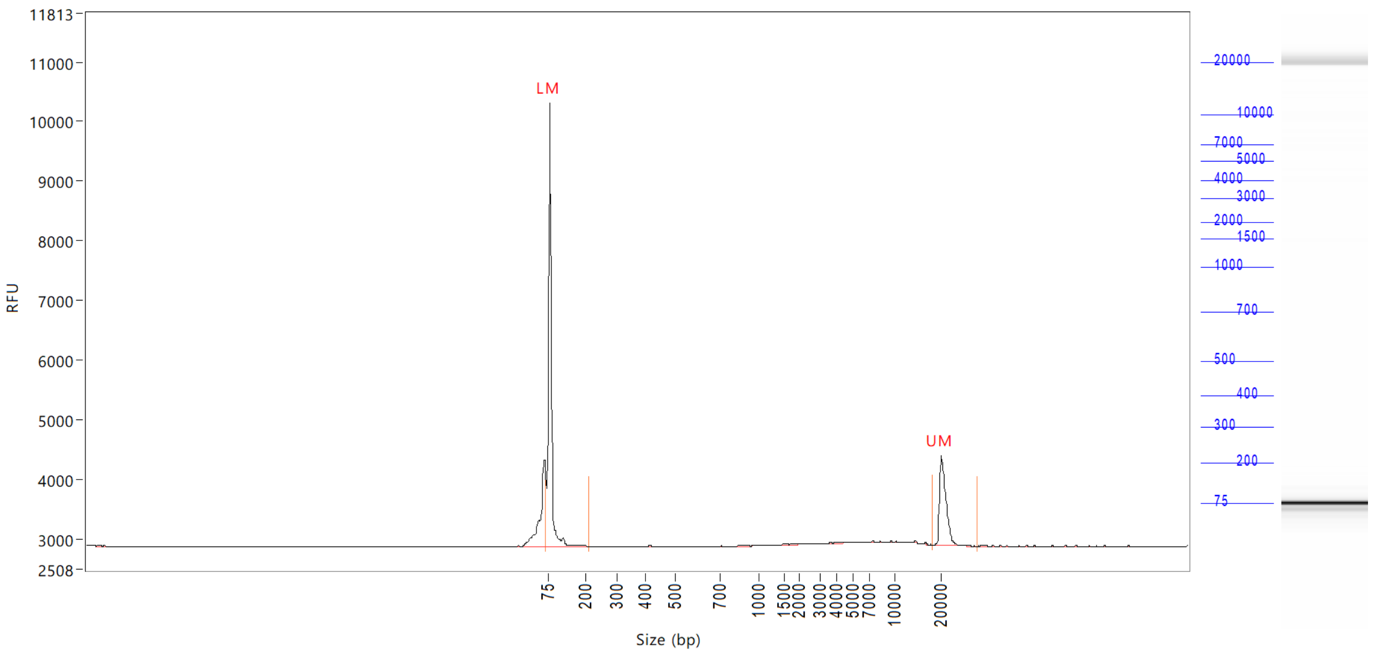

Peak	Size (bp)	Concentration (ng/uL)	From (bp)	To (bp)	Average size (bp)	CV%	RFU	Corrected peak area
1	75 (LM)	0.5000	66	208	77	17.20	7437	54.424
2	20000 (UM)	0.1218	18293	27617	20462	4.16	1504	13.258

TIC:	0.0000	ng/uL
TIM:	0.0000	nmole/L
Total concentration:	0.3436	ng/uL

```
Sample peak width (sec): 5    Sample min peak height: 50    Sample baseline V to V?: Y    Sample baseline V to V points: 3
Sample filter: Binomial       Number of points for filter: 3  Sample start region (min): 0  Sample end region (min): 30
Marker peak width (sec): 5    Marker min peak height: 200   Marker baseline V to V?: Y    Marker baseline V to V points: 3
Lower marker selection: First peak > 500 RFU                Upper marker selection: Last peak > 200 RFU
Ladder size (bp)75, 200, 300, 400, 500, 700, 1000, 1500, 2000, 3000, 4000, 5000, 7000, 10000, 20000
Quantification using: Lower Marker          Final concentration (ng/uL): 0.5000          Dilution factor: 12.0
```

Sample: SampE7
Well location: E7
Created: Tuesday, September 14, 2021 5:34:08 PM

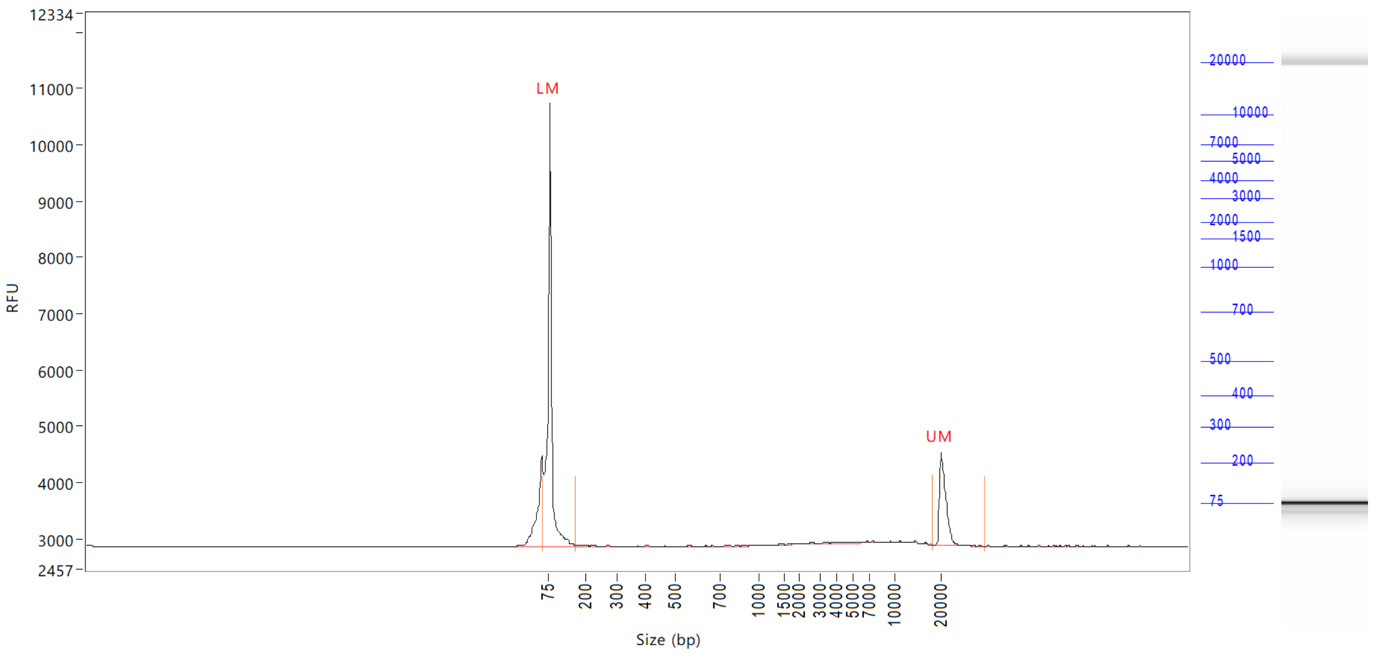

Peak	Size (bp)	Concentration (ng/uL)	From (bp)	To (bp)	Average size (bp)	CV%	RFU	Corrected peak area
1	75 (LM)	0.5000	54	164	75	19.41	7891	72.722
2	20000 (UM)	0.0991	18293	29551	20474	4.56	1632	14.416

TIC:	0.0000	ng/uL
TIM:	0.0000	nmole/L
Total concentration:	0.3619	ng/uL

Sample peak width (sec): 5 Sample min peak height: 50 Sample baseline V to V?: Y Sample baseline V to V points: 3
Sample filter: Binomial Number of points for filter: 3 Sample start region (min): 0 Sample end region (min): 30
Marker peak width (sec): 5 Marker min peak height: 200 Marker baseline V to V?: Y Marker baseline V to V points: 3
Lower marker selection: First peak > 500 RFU Upper marker selection: Last peak > 200 RFU
Ladder size (bp)75, 200, 300, 400, 500, 700, 1000, 1500, 2000, 3000, 4000, 5000, 7000, 10000, 20000
Quantification using: Lower Marker Final concentration (ng/uL): 0.5000 Dilution factor: 12.0

Sample: SampF7
Well location: F7
Created: Tuesday, September 14, 2021 5:34:08 PM

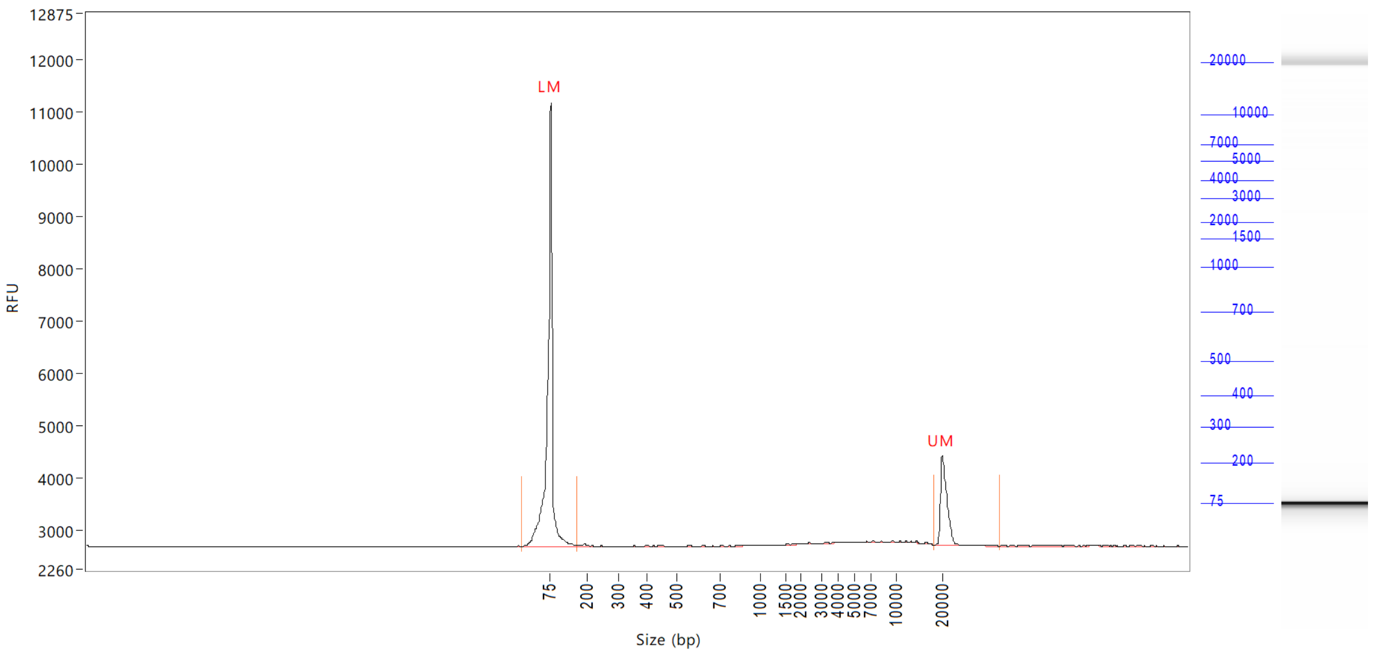

Peak	Size (bp)	Concentration (ng/uL)	From (bp)	To (bp)	Average size (bp)	CV%	RFU	Corrected peak area
1	75 (LM)	0.5000	0	164	65	29.33	8481	95.953
2	20000 (UM)	0.0785	18293	32332	20439	5.03	1698	15.069

TIC:	0.0000	ng/uL
TIM:	0.0000	nmole/L
Total concentration:	0.2643	ng/uL

```
Sample peak width (sec): 5    Sample min peak height: 50    Sample baseline V to V?: Y    Sample baseline V to V points: 3
Sample filter: Binomial       Number of points for filter: 3  Sample start region (min): 0  Sample end region (min): 30
Marker peak width (sec): 5    Marker min peak height: 200   Marker baseline V to V?: Y    Marker baseline V to V points: 3
Lower marker selection: First peak > 500 RFU                Upper marker selection: Last peak > 200 RFU
Ladder size (bp)75, 200, 300, 400, 500, 700, 1000, 1500, 2000, 3000, 4000, 5000, 7000, 10000, 20000
Quantification using: Lower Marker          Final concentration (ng/uL): 0.5000          Dilution factor: 12.0
```

Sample: SampG7
Well location: G7
Created: Tuesday, September 14, 2021 5:34:08 PM

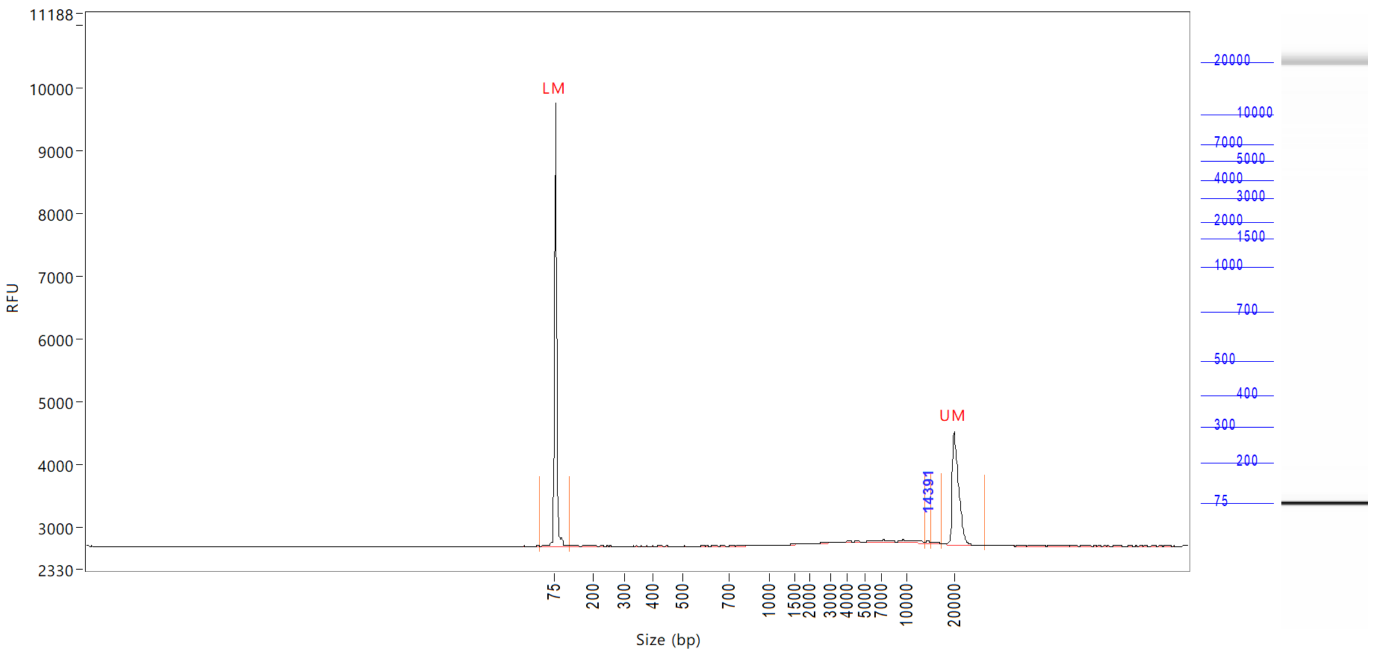

Peak	Size (bp)	Concentration (ng/uL)	From (bp)	To (bp)	Average size (bp)	CV%	RFU	Corrected peak area
1	75 (LM)	0.5000	24	122	73	7.66	7078	40.010
2	14391	0.0416	13659	15122	14320	2.52	55	0.278
3	20000 (UM)	0.1959	17439	26529	20399	3.91	1809	15.673

TIC:	0.0416	ng/uL
TIM:	0.0048	nmole/L
Total concentration:	0.6765	ng/uL

Sample peak width (sec): 5 Sample min peak height: 50 Sample baseline V to V?: Y Sample baseline V to V points: 3
Sample filter: Binomial Number of points for filter: 3 Sample start region (min): 0 Sample end region (min): 30
Marker peak width (sec): 5 Marker min peak height: 200 Marker baseline V to V?: Y Marker baseline V to V points: 3
Lower marker selection: First peak > 500 RFU Upper marker selection: Last peak > 200 RFU
Ladder size (bp)75, 200, 300, 400, 500, 700, 1000, 1500, 2000, 3000, 4000, 5000, 7000, 10000, 20000
Quantification using: Lower Marker Final concentration (ng/uL): 0.5000 Dilution factor: 12.0

Sample: SampH7
Well location: H7
Created: Tuesday, September 14, 2021 5:34:08 PM

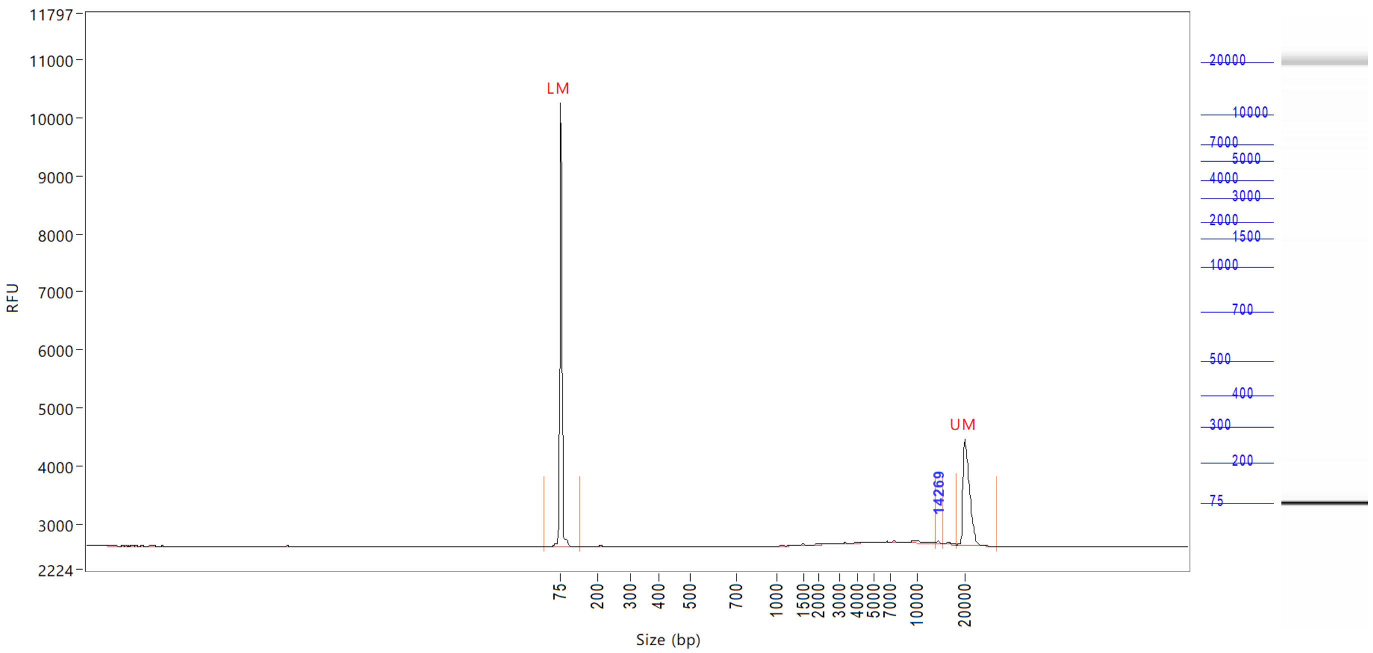

Peak	Size (bp)	Concentration (ng/uL)	From (bp)	To (bp)	Average size (bp)	CV%	RFU	Corrected peak area
1	75 (LM)	0.5000	24	141	73	7.53	7656	42.182
2	14269	0.0354	13659	15244	14231	2.23	51	0.249
3	20000 (UM)	0.1887	18293	26649	20350	3.68	1825	15.920

TIC:	0.0354	ng/uL
TIM:	0.0041	nmole/L
Total concentration:	0.5631	ng/uL

Sample peak width (sec): 5 Sample min peak height: 50 Sample baseline V to V?: Y Sample baseline V to V points: 3
Sample filter: Binomial Number of points for filter: 3 Sample start region (min): 0 Sample end region (min): 30
Marker peak width (sec): 5 Marker min peak height: 200 Marker baseline V to V?: Y Marker baseline V to V points: 3
Lower marker selection: First peak > 500 RFU Upper marker selection: Last peak > 200 RFU
Ladder size (bp)75, 200, 300, 400, 500, 700, 1000, 1500, 2000, 3000, 4000, 5000, 7000, 10000, 20000
Quantification using: Lower Marker Final concentration (ng/uL): 0.5000 Dilution factor: 12.0

Sample: SampA8
Well location: A8
Created: Tuesday, September 14, 2021 5:34:08 PM

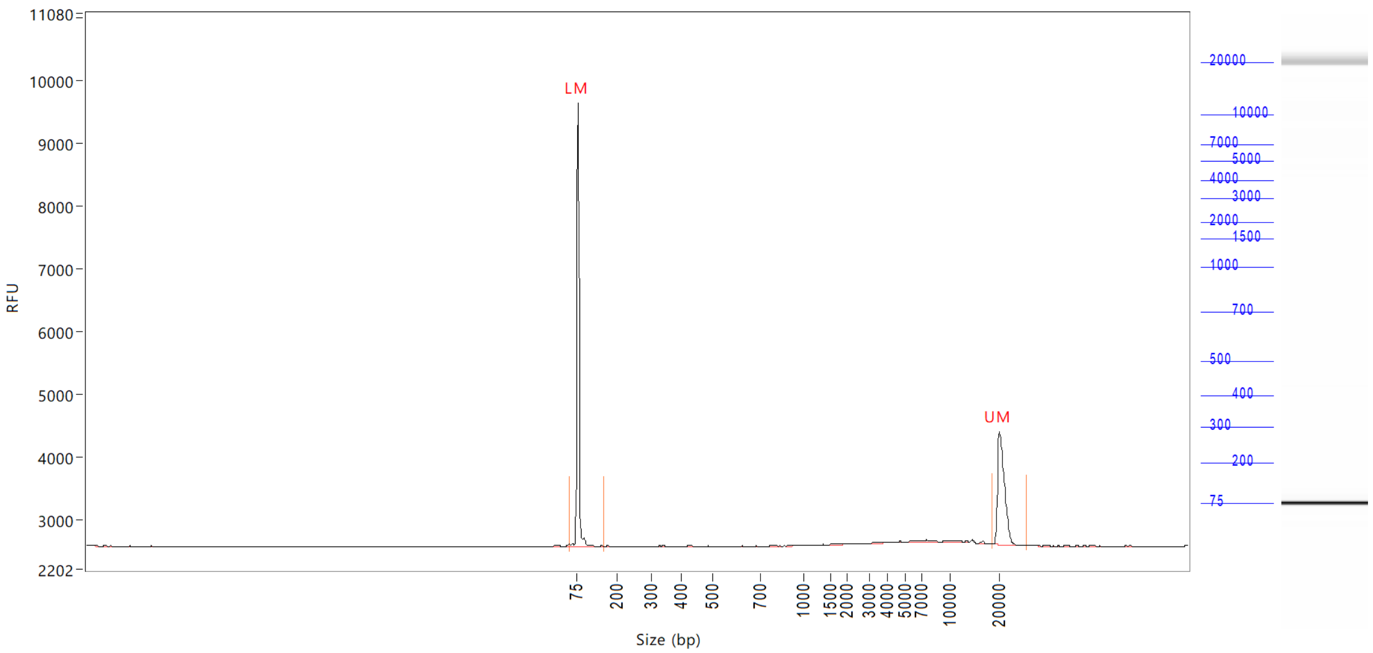

Peak	Size (bp)	Concentration (ng/uL)	From (bp)	To (bp)	Average size (bp)	CV%	RFU	Corrected peak area
1	75 (LM)	0.5000	52	158	74	8.52	7097	38.335
2	20000 (UM)	0.2007	18537	25440	20415	3.47	1796	15.385

TIC:	0.0000	ng/uL
TIM:	0.0000	nmole/L
Total concentration:	0.5772	ng/uL

Sample peak width (sec): 5 Sample min peak height: 50 Sample baseline V to V?: Y Sample baseline V to V points: 3
Sample filter: Binomial Number of points for filter: 3 Sample start region (min): 0 Sample end region (min): 30
Marker peak width (sec): 5 Marker min peak height: 200 Marker baseline V to V?: Y Marker baseline V to V points: 3
Lower marker selection: First peak > 500 RFU Upper marker selection: Last peak > 200 RFU
Ladder size (bp)75, 200, 300, 400, 500, 700, 1000, 1500, 2000, 3000, 4000, 5000, 7000, 10000, 20000
Quantification using: Lower Marker Final concentration (ng/uL): 0.5000 Dilution factor: 12.0

Sample: SampB8
Well location: B8
Created: Tuesday, September 14, 2021 5:34:08 PM

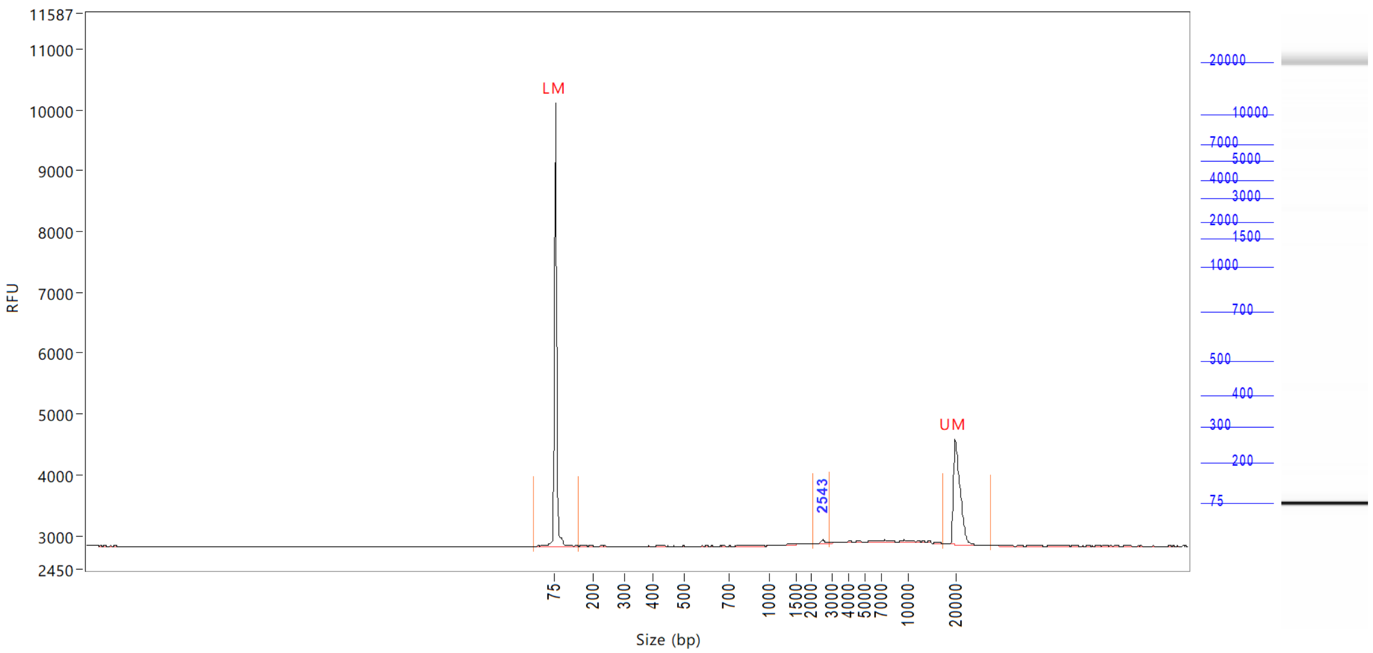

Peak	Size (bp)	Concentration (ng/uL)	From (bp)	To (bp)	Average size (bp)	CV%	RFU	Corrected peak area
1	75 (LM)	0.5000	1	152	73	10.74	7302	42.377
2	2543	0.0628	2087	2914	2546	6.04	63	0.443
3	20000 (UM)	0.1844	17439	27375	20439	4.16	1734	15.627

TIC:	0.0628	ng/uL
TIM:	0.0406	nmole/L
Total concentration:	0.6013	ng/uL

```
Sample peak width (sec): 5    Sample min peak height: 50    Sample baseline V to V?: Y    Sample baseline V to V points: 3
Sample filter: Binomial       Number of points for filter: 3  Sample start region (min): 0  Sample end region (min): 30
Marker peak width (sec): 5    Marker min peak height: 200   Marker baseline V to V?: Y    Marker baseline V to V points: 3
Lower marker selection: First peak > 500 RFU                Upper marker selection: Last peak > 200 RFU
Ladder size (bp)75, 200, 300, 400, 500, 700, 1000, 1500, 2000, 3000, 4000, 5000, 7000, 10000, 20000
Quantification using: Lower Marker          Final concentration (ng/uL): 0.5000          Dilution factor: 12.0
```

Sample: SampC8
Well location: C8
Created: Tuesday, September 14, 2021 5:34:08 PM

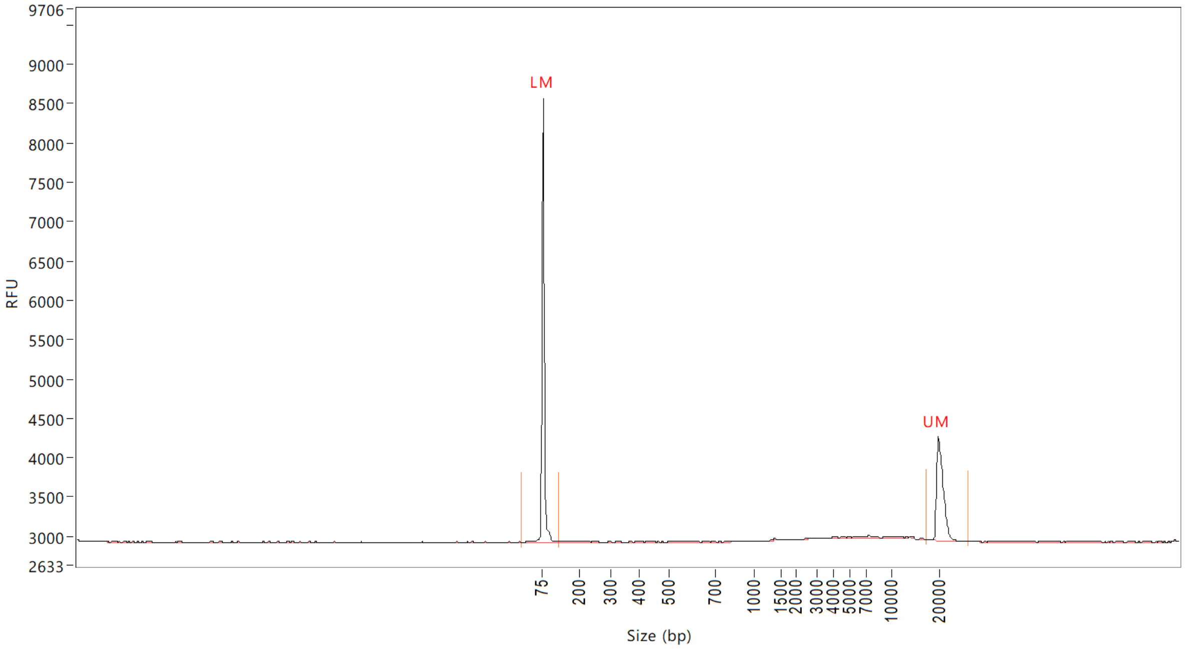

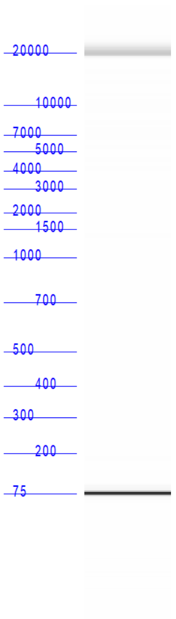

Peak	Size (bp)	Concentration (ng/uL)	From (bp)	To (bp)	Average size (bp)	CV%	RFU	Corrected peak area
1	75 (LM)	0.5000	3	129	74	9.26	5651	33.766
2	20000 (UM)	0.1707	17195	26287	20334	3.93	1316	11.527

TIC:	0.0000	ng/uL
TIM:	0.0000	nmole/L
Total concentration:	0.6173	ng/uL

Sample peak width (sec): 5   Sample min peak height: 50   Sample baseline V to V?: Y   Sample baseline V to V points: 3
Sample filter: Binomial   Number of points for filter: 3 Sample start region (min): 0 Sample end region (min): 30
Marker peak width (sec): 5   Marker min peak height: 200   Marker baseline V to V?: Y   Marker baseline V to V points: 3
Lower marker selection: First peak > 500 RFU   Upper marker selection: Last peak > 200 RFU
Ladder size (bp)75, 200, 300, 400, 500, 700, 1000, 1500, 2000, 3000, 4000, 5000, 7000, 10000, 20000
Quantification using: Lower Marker   Final concentration (ng/uL): 0.5000   Dilution factor: 12.0

Sample: SampD8
Well location: D8
Created: Tuesday, September 14, 2021 5:34:08 PM

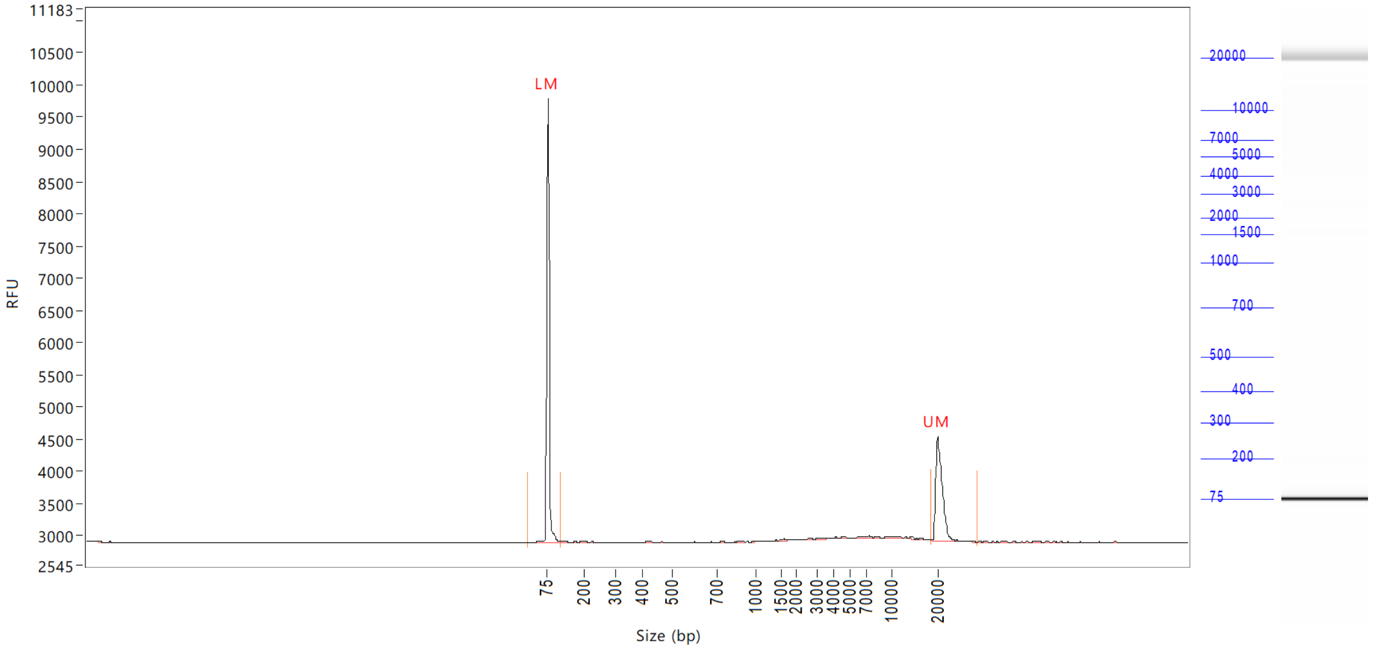

Peak	Size	Concentration	From	To	Average size	CV%	RFU	Corrected peak area
	(bp)	(ng/uL)	(bp)	(bp)	(bp)			
1	75 (LM)	0.5000	7	120	74	8.60	6903	39.738
2	20000 (UM)	0.1784	18659	28463	20488	4.39	1613	14.178

TIC:	0.0000	ng/uL
TIM:	0.0000	nmole/L
Total concentration:	0.7292	ng/uL

Sample peak width (sec): 5   Sample min peak height: 50   Sample baseline V to V?: Y   Sample baseline V to V points: 3
Sample filter: Binomial   Number of points for filter: 3   Sample start region (min): 0   Sample end region (min): 30
Marker peak width (sec): 5   Marker min peak height: 200   Marker baseline V to V?: Y   Marker baseline V to V points: 3
Lower marker selection: First peak > 500 RFU   Upper marker selection: Last peak > 200 RFU
Ladder size (bp)75, 200, 300, 400, 500, 700, 1000, 1500, 2000, 3000, 4000, 5000, 7000, 10000, 20000
Quantification using: Lower Marker   Final concentration (ng/uL): 0.5000   Dilution factor: 12.0

Sample: SampE8
Well location: E8
Created: Tuesday, September 14, 2021 5:34:08 PM

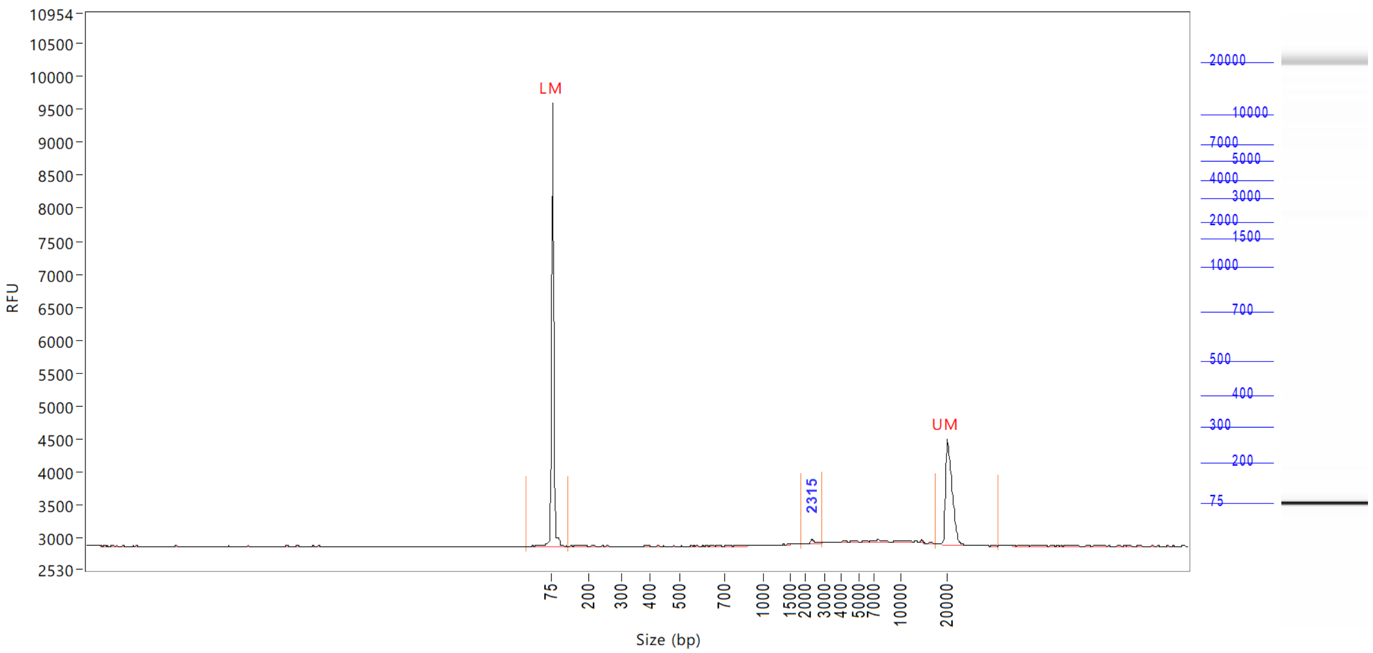

Peak	Size (bp)	Concentration (ng/uL)	From (bp)	To (bp)	Average size (bp)	CV%	RFU	Corrected peak area
1	75 (LM)	0.5000	0	126	73	10.95	6733	38.642
2	2315	0.0644	1866	2829	2338	7.53	57	0.415
3	20000 (UM)	0.1790	17439	31002	20461	4.31	1593	13.837

TIC:	0.0644	ng/uL
TIM:	0.0453	nmole/L
Total concentration:	0.6449	ng/uL

```
Sample peak width (sec): 5    Sample min peak height: 50    Sample baseline V to V?: Y    Sample baseline V to V points: 3
Sample filter: Binomial       Number of points for filter: 3  Sample start region (min): 0  Sample end region (min): 30
Marker peak width (sec): 5    Marker min peak height: 200   Marker baseline V to V?: Y    Marker baseline V to V points: 3
Lower marker selection: First peak > 500 RFU                Upper marker selection: Last peak > 200 RFU
Ladder size (bp)75, 200, 300, 400, 500, 700, 1000, 1500, 2000, 3000, 4000, 5000, 7000, 10000, 20000
Quantification using: Lower Marker          Final concentration (ng/uL): 0.5000          Dilution factor: 12.0
```

Sample: SampF8
Well location: F8
Created: Tuesday, September 14, 2021 5:34:08 PM

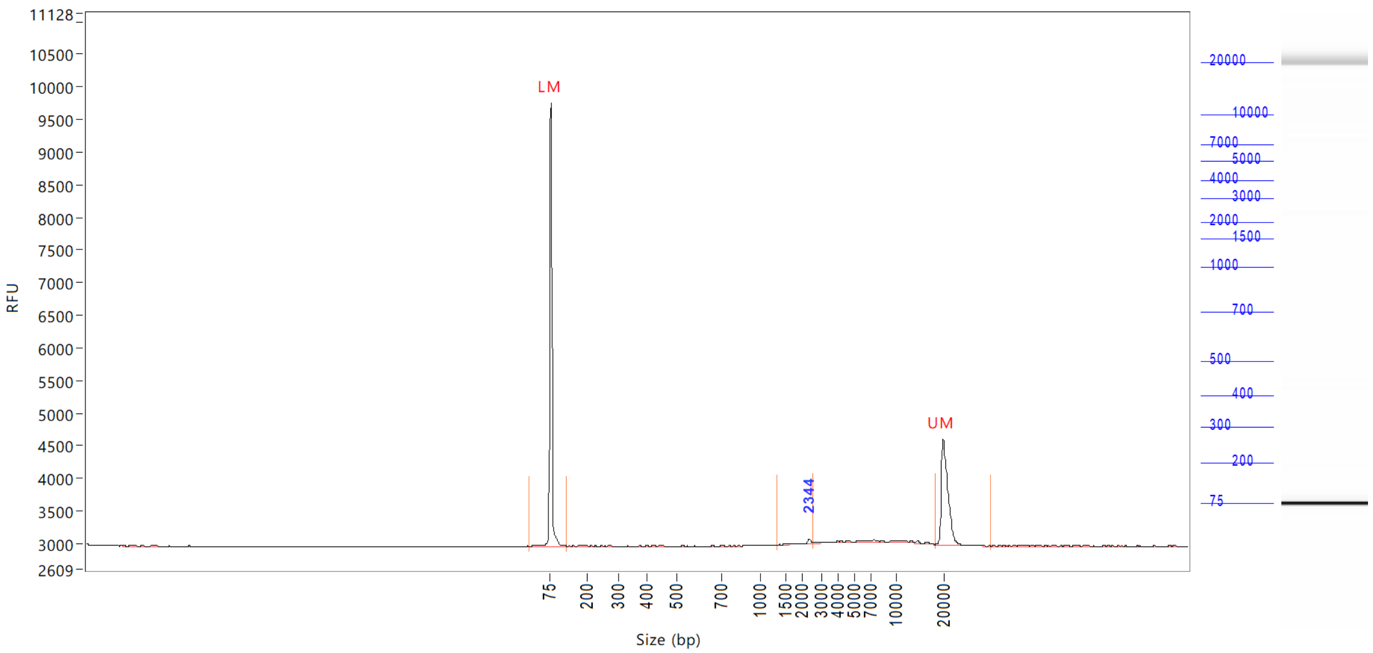

Peak	Size (bp)	Concentration (ng/uL)	From (bp)	To (bp)	Average size (bp)	CV%	RFU	Corrected peak area
1	75 (LM)	0.5000	3	128	73	9.70	6807	39.672
2	2344	0.0786	1311	2572	2115	17.68	68	0.520
3	20000 (UM)	0.1846	18293	30398	20507	4.67	1619	14.651

TIC:	0.0786	ng/uL
TIM:	0.0612	nmole/L
Total concentration:	0.6741	ng/uL

Sample peak width (sec): 5 Sample min peak height: 50 Sample baseline V to V?: Y Sample baseline V to V points: 3
Sample filter: Binomial Number of points for filter: 3 Sample start region (min): 0 Sample end region (min): 30
Marker peak width (sec): 5 Marker min peak height: 200 Marker baseline V to V?: Y Marker baseline V to V points: 3
Lower marker selection: First peak > 500 RFU Upper marker selection: Last peak > 200 RFU
Ladder size (bp)75, 200, 300, 400, 500, 700, 1000, 1500, 2000, 3000, 4000, 5000, 7000, 10000, 20000
Quantification using: Lower Marker Final concentration (ng/uL): 0.5000 Dilution factor: 12.0

Sample: SampG8
Well location: G8
Created: Tuesday, September 14, 2021 5:34:08 PM

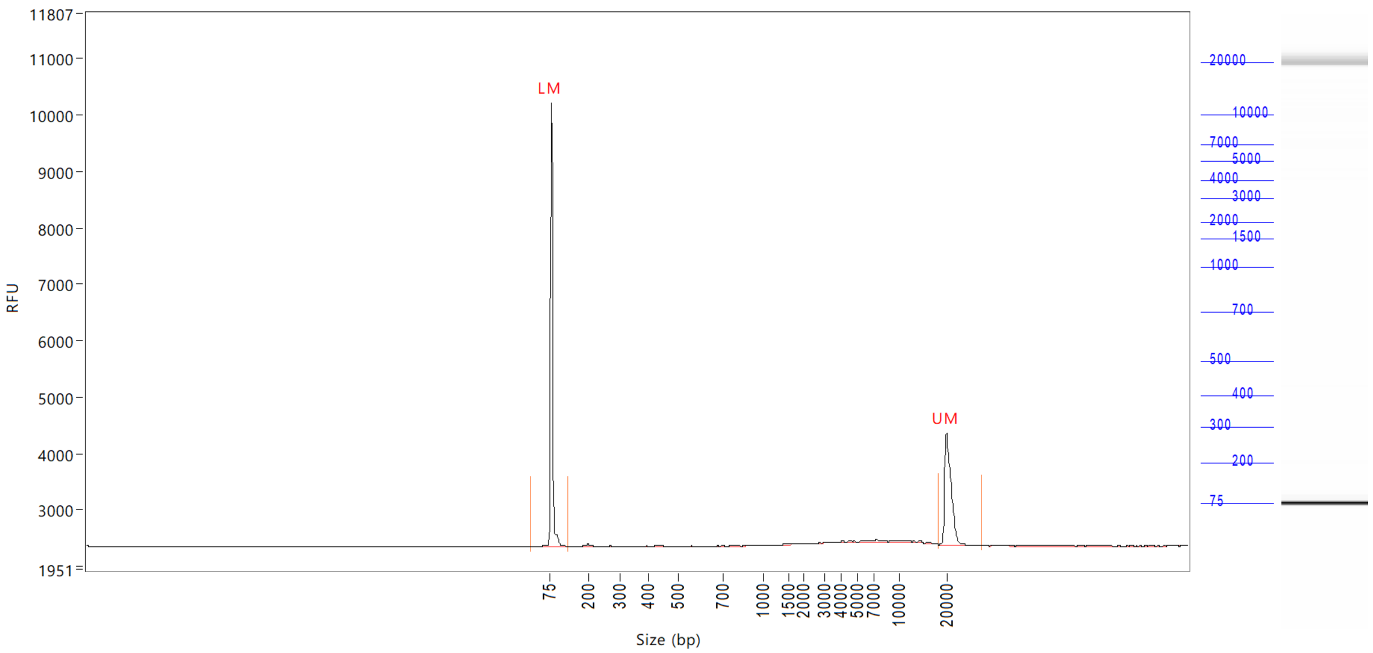

Peak	Size (bp)	Concentration (ng/uL)	From (bp)	To (bp)	Average size (bp)	CV%	RFU	Corrected peak area
1	75 (LM)	0.5000	5	133	74	8.20	7875	44.101
2	20000 (UM)	0.1935	18293	27496	20375	3.84	1974	17.066

TIC:	0.0000	ng/uL
TIM:	0.0000	nmole/L
Total concentration:	0.4857	ng/uL

```
Sample peak width (sec): 5    Sample min peak height: 50    Sample baseline V to V?: Y    Sample baseline V to V points: 3
Sample filter: Binomial       Number of points for filter: 3  Sample start region (min): 0  Sample end region (min): 30
Marker peak width (sec): 5    Marker min peak height: 200   Marker baseline V to V?: Y    Marker baseline V to V points: 3
Lower marker selection: First peak > 500 RFU                Upper marker selection: Last peak > 200 RFU
Ladder size (bp)75, 200, 300, 400, 500, 700, 1000, 1500, 2000, 3000, 4000, 5000, 7000, 10000, 20000
Quantification using: Lower Marker          Final concentration (ng/uL): 0.5000          Dilution factor: 12.0
```

Sample: SampH8
Well location: H8
Created: Tuesday, September 14, 2021 5:34:08 PM

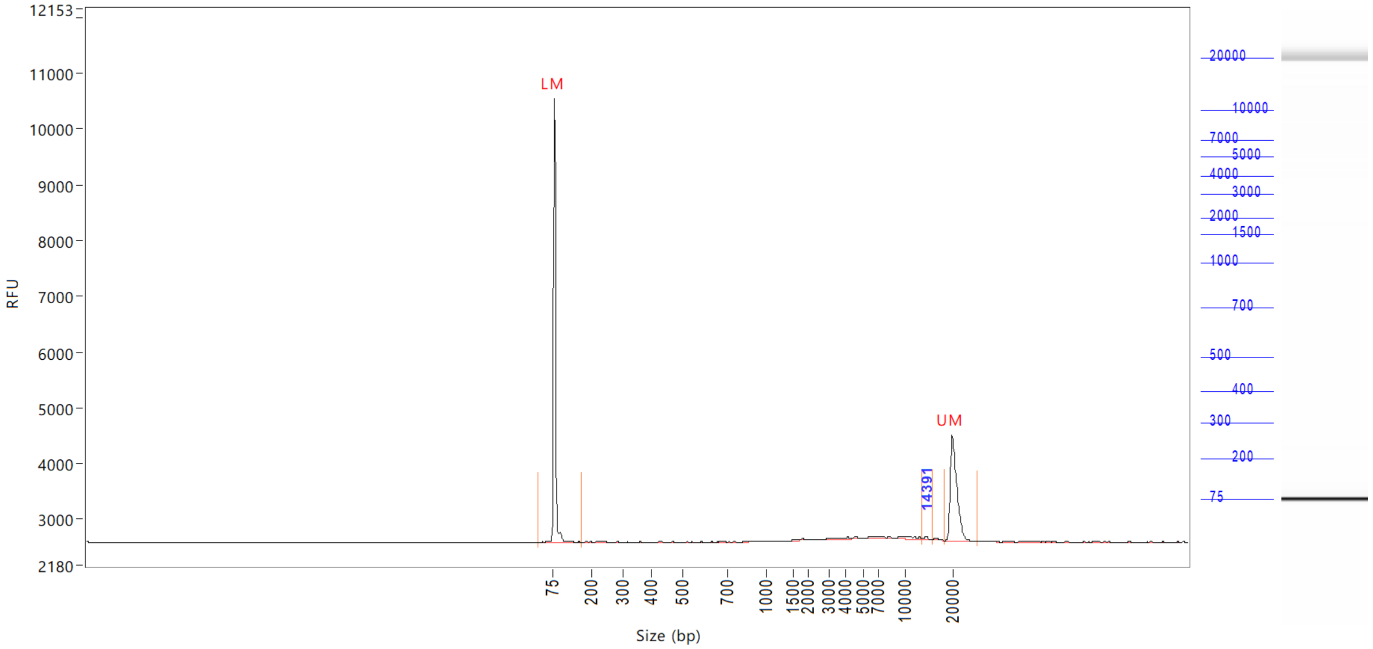

Peak	Size (bp)	Concentration (ng/uL)	From (bp)	To (bp)	Average size (bp)	CV%	RFU	Corrected peak area
1	75 (LM)	0.5000	24	164	74	9.41	7968	44.448
2	14391	0.0412	13659	15854	14491	3.89	56	0.305
3	20000 (UM)	0.1883	18415	25320	20405	3.84	1894	16.738

TIC:	0.0412	ng/uL
TIM:	0.0047	nmole/L
Total concentration:	0.7195	ng/uL

```
Sample peak width (sec): 5    Sample min peak height: 50    Sample baseline V to V?: Y    Sample baseline V to V points: 3
Sample filter: Binomial       Number of points for filter: 3  Sample start region (min): 0  Sample end region (min): 30
Marker peak width (sec): 5    Marker min peak height: 200   Marker baseline V to V?: Y    Marker baseline V to V points: 3
Lower marker selection: First peak > 500 RFU                Upper marker selection: Last peak > 200 RFU
Ladder size (bp)75, 200, 300, 400, 500, 700, 1000, 1500, 2000, 3000, 4000, 5000, 7000, 10000, 20000
Quantification using: Lower Marker          Final concentration (ng/uL): 0.5000          Dilution factor: 12.0
```

Sample: SampA9
Well location: A9
Created: Tuesday, September 14, 2021 5:34:08 PM

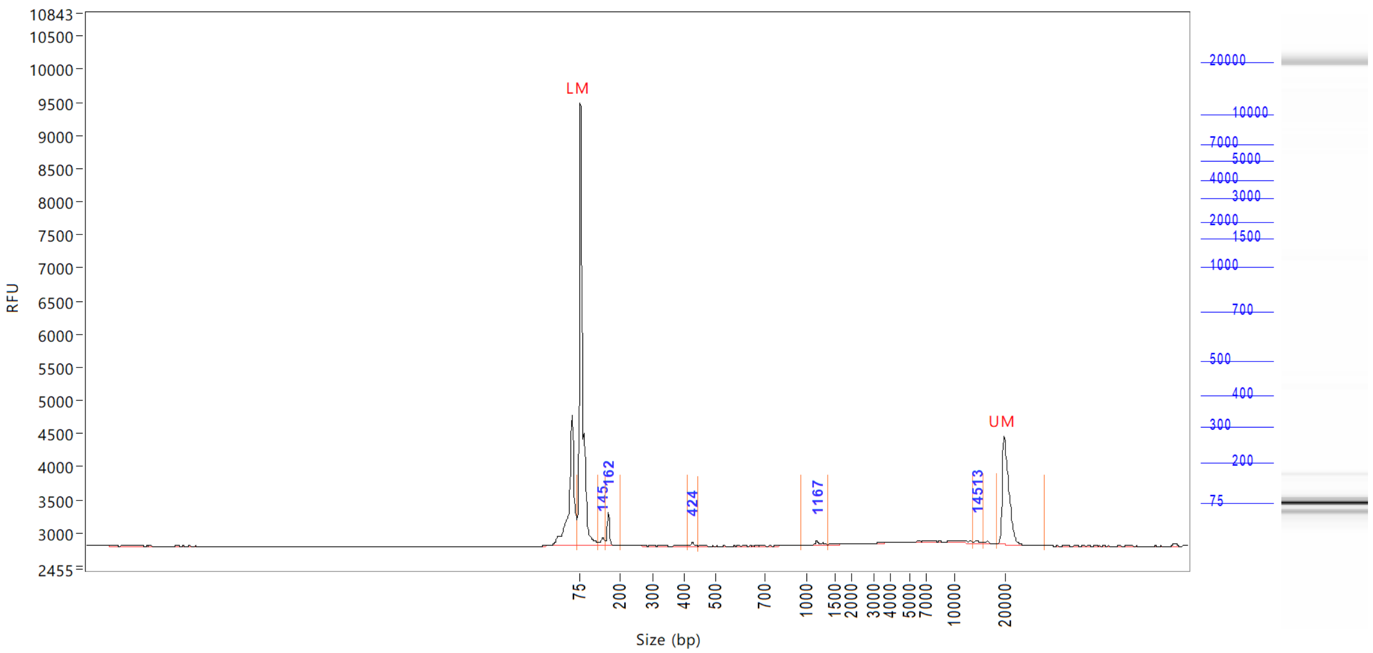

Peak	Size (bp)	Concentration (ng/uL)	From (bp)	To (bp)	Average size (bp)	CV%	RFU	Corrected peak area
1	75 (LM)	0.5000	66	131	78	11.68	6694	51.652
2	145	0.1374	131	152	142	4.08	125	1.183
3	162	0.3324	152	201	161	4.02	494	2.861
4	424	0.0390	412	443	423	1.35	60	0.336
5	1167	0.0641	957	1356	1180	6.80	62	0.551
6	14513	0.0356	13903	15854	14644	3.54	50	0.306
7	20000 (UM)	0.1327	18537	28221	20368	3.92	1630	13.707

TIC:	0.6085	ng/uL
TIM:	5.2290	nmole/L
Total concentration:	0.9884	ng/uL

Sample peak width (sec): 5 Sample min peak height: 50 Sample baseline V to V?: Y Sample baseline V to V points: 3
Sample filter: Binomial Number of points for filter: 3 Sample start region (min): 0 Sample end region (min): 30
Marker peak width (sec): 5 Marker min peak height: 200 Marker baseline V to V?: Y Marker baseline V to V points: 3
Lower marker selection: First peak > 500 RFU Upper marker selection: Last peak > 200 RFU
Ladder size (bp)75, 200, 300, 400, 500, 700, 1000, 1500, 2000, 3000, 4000, 5000, 7000, 10000, 20000
Quantification using: Lower Marker Final concentration (ng/uL): 0.5000 Dilution factor: 12.0

Sample: SampB9
Well location: B9
Created: Tuesday, September 14, 2021 5:34:08 PM

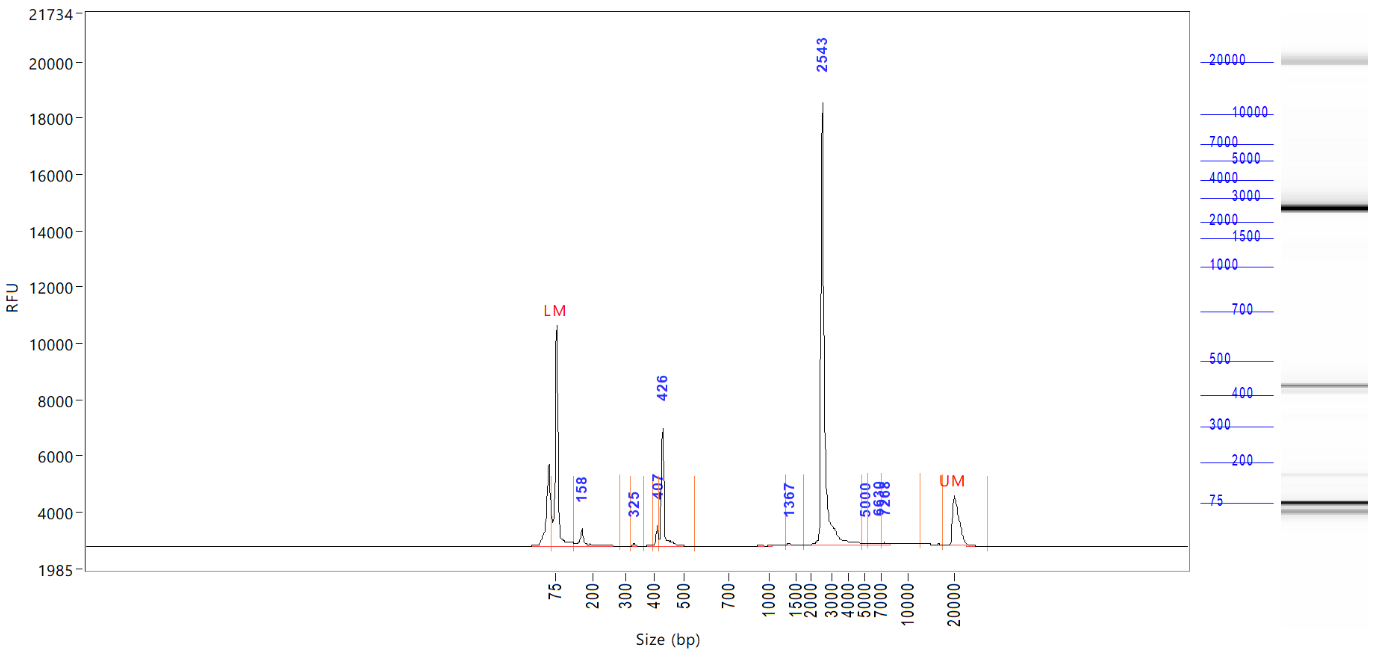

Peak	Size (bp)	Concentration (ng/uL)	From (bp)	To (bp)	Average size (bp)	CV%	RFU	Corrected peak area
1	75 (LM)	0.5000	60	133	75	15.63	7851	55.174
2	158	0.7614	133	282	166	15.58	601	7.001
3	325	0.0531	315	362	327	2.05	78	0.488
4	407	0.4038	396	416	407	1.19	707	3.713
5	426	2.5148	416	544	429	3.27	4178	23.125
6	1367	0.0634	1300	1789	1410	6.48	64	0.583
7	2543	9.8998	1789	4828	2632	12.34	15731	91.035
8	5000	0.0410	4828	5223	4991	2.34	52	0.377
9	6630	0.0896	5223	7000	6080	8.67	53	0.824
10	7268	0.1389	7000	12684	8871	15.87	57	1.277
11	20000 (UM)	0.1416	17317	27012	20413	3.96	1783	15.625

TIC:	13.9657	ng/uL
TIM:	25.4133	nmole/L
Total concentration:	14.0898	ng/uL

```
Sample peak width (sec): 5    Sample min peak height: 50    Sample baseline V to V?: Y    Sample baseline V to V points: 3
Sample filter: Binomial       Number of points for filter: 3  Sample start region (min): 0  Sample end region (min): 30
Marker peak width (sec): 5    Marker min peak height: 200   Marker baseline V to V?: Y    Marker baseline V to V points: 3
Lower marker selection: First peak > 500 RFU                Upper marker selection: Last peak > 200 RFU
Ladder size (bp)75, 200, 300, 400, 500, 700, 1000, 1500, 2000, 3000, 4000, 5000, 7000, 10000, 20000
Quantification using: Lower Marker          Final concentration (ng/uL): 0.5000          Dilution factor: 12.0
```

Sample: SampC9
Well location: C9
Created: Tuesday, September 14, 2021 5:34:08 PM

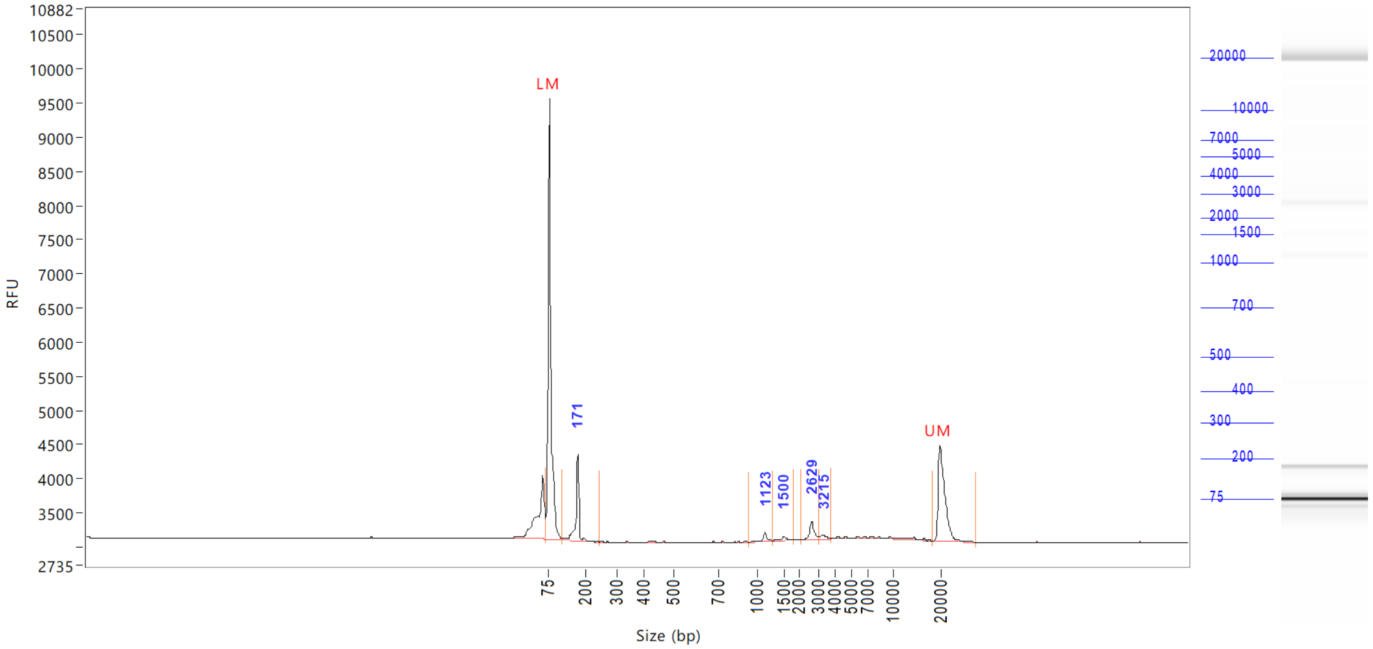

Peak	Size (bp)	Concentration (ng/uL)	From (bp)	To (bp)	Average size (bp)	CV%	RFU	Corrected peak area
1	75 (LM)	0.5000	66	118	77	9.57	6464	46.213
2	171	1.1967	118	242	167	8.15	1258	9.217
3	1123	0.1080	931	1278	1110	4.89	133	0.832
4	1500	0.0681	1278	1846	1508	7.08	64	0.524
5	2629	0.2576	2115	2971	2621	5.36	271	1.984
6	3215	0.0834	2971	3750	3265	6.48	52	0.642
7	20000 (UM)	0.1393	18415	27859	20560	4.98	1393	12.877

TIC:	1.7137	ng/uL
TIM:	12.2173	nmole/L
Total concentration:	2.1589	ng/uL

Sample peak width (sec): 5   Sample min peak height: 50   Sample baseline V to V?: Y   Sample baseline V to V points: 3
Sample filter: Binomial   Number of points for filter: 3 Sample start region (min): 0 Sample end region (min): 30
Marker peak width (sec): 5   Marker min peak height: 200   Marker baseline V to V?: Y   Marker baseline V to V points: 3
Lower marker selection: First peak > 500 RFU   Upper marker selection: Last peak > 200 RFU
Ladder size (bp)75, 200, 300, 400, 500, 700, 1000, 1500, 2000, 3000, 4000, 5000, 7000, 10000, 20000
Quantification using: Lower Marker   Final concentration (ng/uL): 0.5000   Dilution factor: 12.0

Sample: SampD9
Well location: D9
Created: Tuesday, September 14, 2021 5:34:08 PM

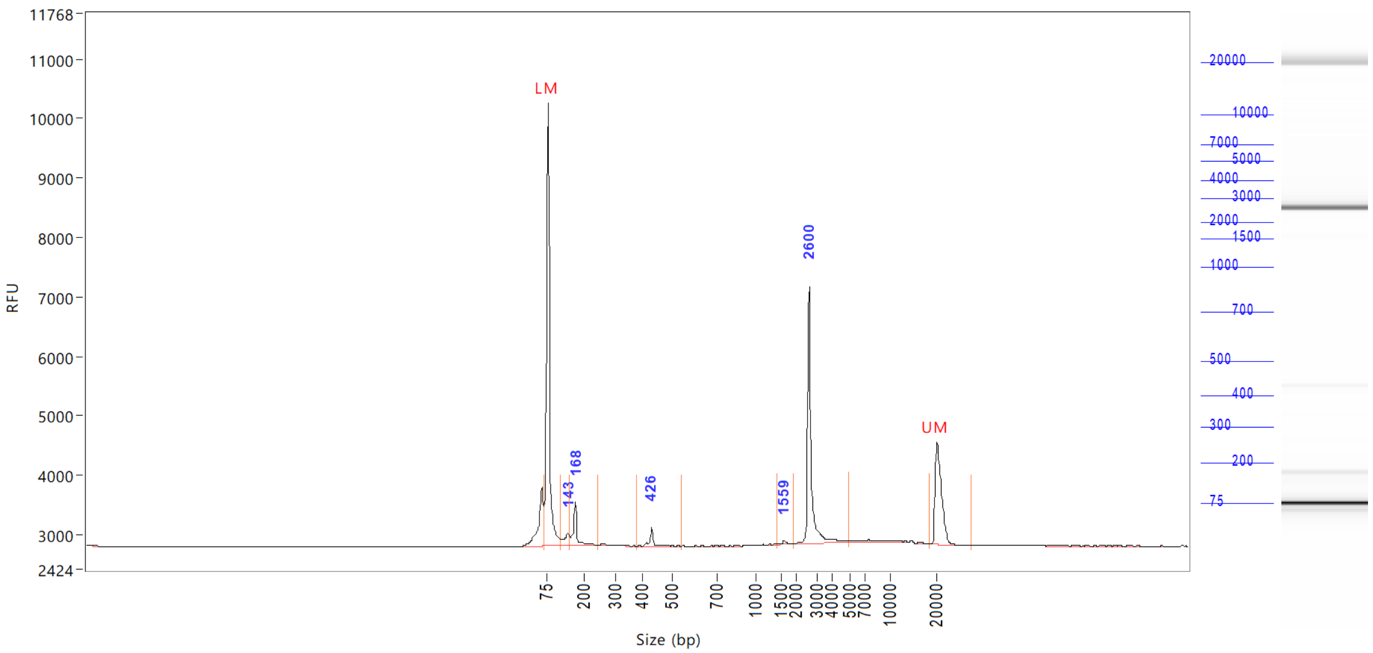

Peak	Size (bp)	Concentration (ng/uL)	From (bp)	To (bp)	Average size (bp)	CV%	RFU	Corrected peak area
1	75 (LM)	0.5000	62	120	76	11.68	7458	53.936
2	143	0.3289	120	152	137	6.70	205	2.957
3	168	0.6976	152	244	171	8.93	723	6.271
4	426	0.2582	374	542	428	4.81	315	2.321
5	1559	0.0502	1411	1904	1579	6.29	58	0.452
6	2600	2.8418	1904	4966	2718	13.95	4303	25.546
7	20000 (UM)	0.1368	18293	27496	20440	3.92	1709	14.753

TIC:	4.1768	ng/uL
TIM:	13.4180	nmole/L
Total concentration:	4.4269	ng/uL

```
Sample peak width (sec): 5    Sample min peak height: 50    Sample baseline V to V?: Y    Sample baseline V to V points: 3
Sample filter: Binomial       Number of points for filter: 3  Sample start region (min): 0  Sample end region (min): 30
Marker peak width (sec): 5    Marker min peak height: 200   Marker baseline V to V?: Y    Marker baseline V to V points: 3
Lower marker selection: First peak > 500 RFU                Upper marker selection: Last peak > 200 RFU
Ladder size (bp)75, 200, 300, 400, 500, 700, 1000, 1500, 2000, 3000, 4000, 5000, 7000, 10000, 20000
Quantification using: Lower Marker          Final concentration (ng/uL): 0.5000          Dilution factor: 12.0
```

Sample: SampE9
Well location: E9
Created: Tuesday, September 14, 2021 5:34:08 PM

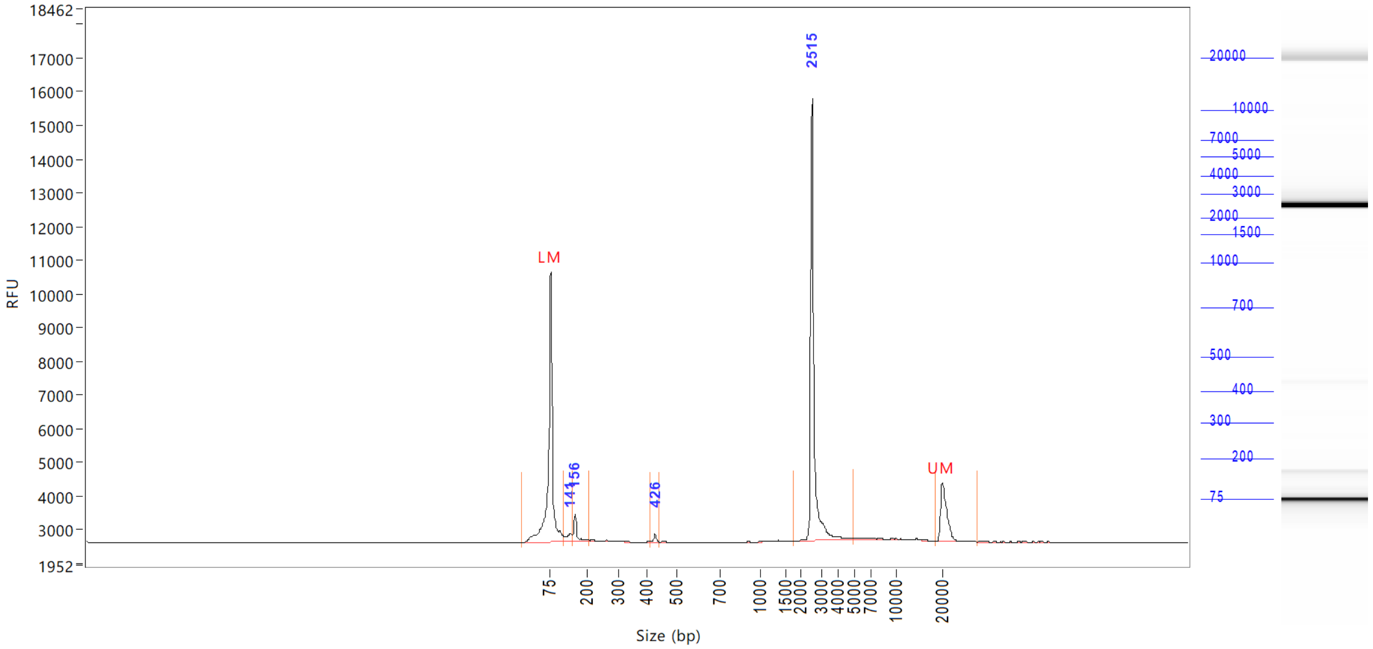

Peak	Size (bp)	Concentration (ng/uL)	From (bp)	To (bp)	Average size (bp)	CV%	RFU	Corrected peak area
1	75 (LM)	0.5000	0	120	67	28.29	8031	81.076
2	141	0.2368	120	147	135	5.90	242	3.200
3	156	0.4669	147	205	160	7.73	802	6.308
4	426	0.0974	412	441	423	1.16	252	1.316
5	2515	5.3998	1750	4931	2602	12.02	13138	72.966
6	20000 (UM)	0.0925	18537	27496	20390	3.89	1754	15.001

TIC:	6.2009	ng/uL
TIM:	11.4737	nmole/L
Total concentration:	6.4637	ng/uL

Sample peak width (sec): 5 Sample min peak height: 50 Sample baseline V to V?: Y Sample baseline V to V points: 3
Sample filter: Binomial Number of points for filter: 3 Sample start region (min): 0 Sample end region (min): 30
Marker peak width (sec): 5 Marker min peak height: 200 Marker baseline V to V?: Y Marker baseline V to V points: 3
Lower marker selection: First peak > 500 RFU Upper marker selection: Last peak > 200 RFU
Ladder size (bp)75, 200, 300, 400, 500, 700, 1000, 1500, 2000, 3000, 4000, 5000, 7000, 10000, 20000
Quantification using: Lower Marker Final concentration (ng/uL): 0.5000 Dilution factor: 12.0

Sample: SampF9
Well location: F9
Created: Tuesday, September 14, 2021 5:34:08 PM

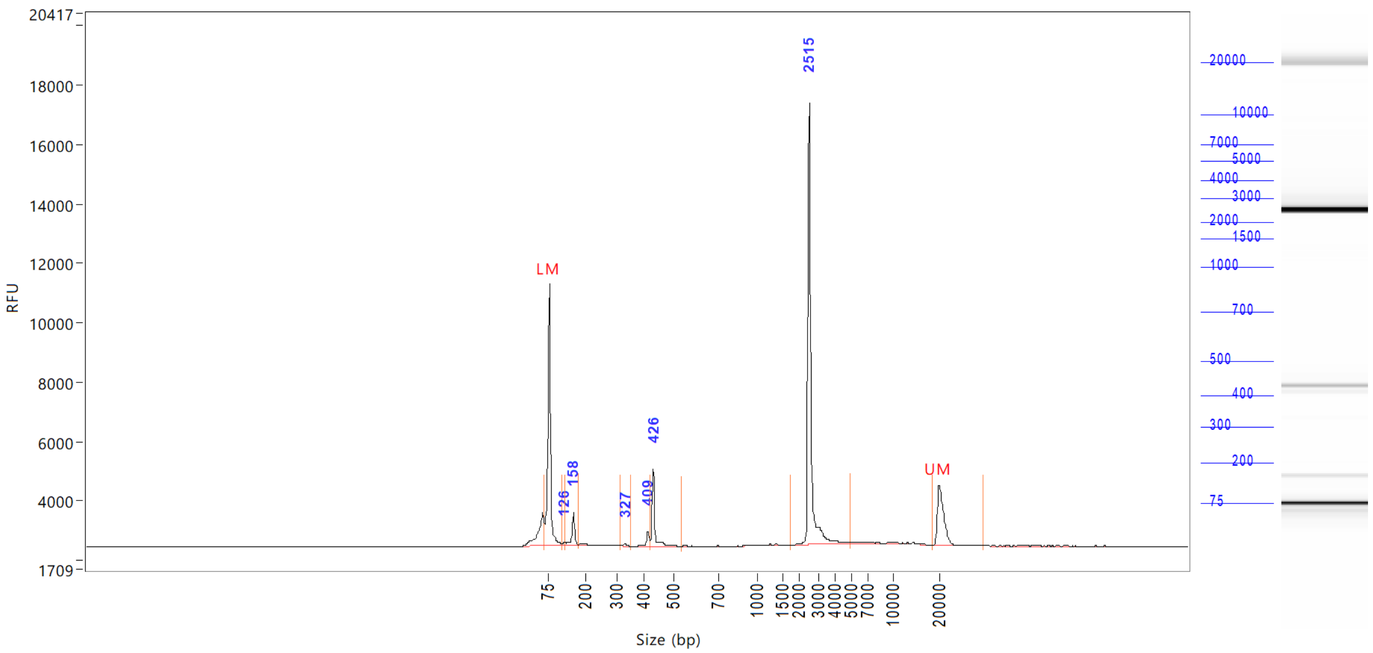

Peak	Size (bp)	Concentration (ng/uL)	From (bp)	To (bp)	Average size (bp)	CV%	RFU	Corrected peak area
1	75 (LM)	0.5000	60	118	74	9.70	8829	59.819
2	126	0.0668	118	133	126	3.82	73	0.666
3	158	0.6929	133	175	154	3.90	1098	6.908
4	327	0.0461	311	349	326	1.50	77	0.459
5	409	0.2835	349	416	406	2.13	496	2.826
6	426	1.4572	416	529	429	3.10	2574	14.528
7	2515	7.8786	1750	4931	2595	12.94	14884	78.549
8	20000 (UM)	0.1483	18537	29551	20401	4.40	2029	17.743

TIC:	10.4250	ng/uL
TIM:	20.2342	nmole/L
Total concentration:	10.7186	ng/uL

```
Sample peak width (sec): 5    Sample min peak height: 50    Sample baseline V to V?: Y    Sample baseline V to V points: 3
Sample filter: Binomial       Number of points for filter: 3  Sample start region (min): 0  Sample end region (min): 30
Marker peak width (sec): 5    Marker min peak height: 200   Marker baseline V to V?: Y    Marker baseline V to V points: 3
Lower marker selection: First peak > 500 RFU                Upper marker selection: Last peak > 200 RFU
Ladder size (bp)75, 200, 300, 400, 500, 700, 1000, 1500, 2000, 3000, 4000, 5000, 7000, 10000, 20000
Quantification using: Lower Marker          Final concentration (ng/uL): 0.5000          Dilution factor: 12.0
```

Sample: SampG9
Well location: G9
Created: Tuesday, September 14, 2021 5:34:08 PM

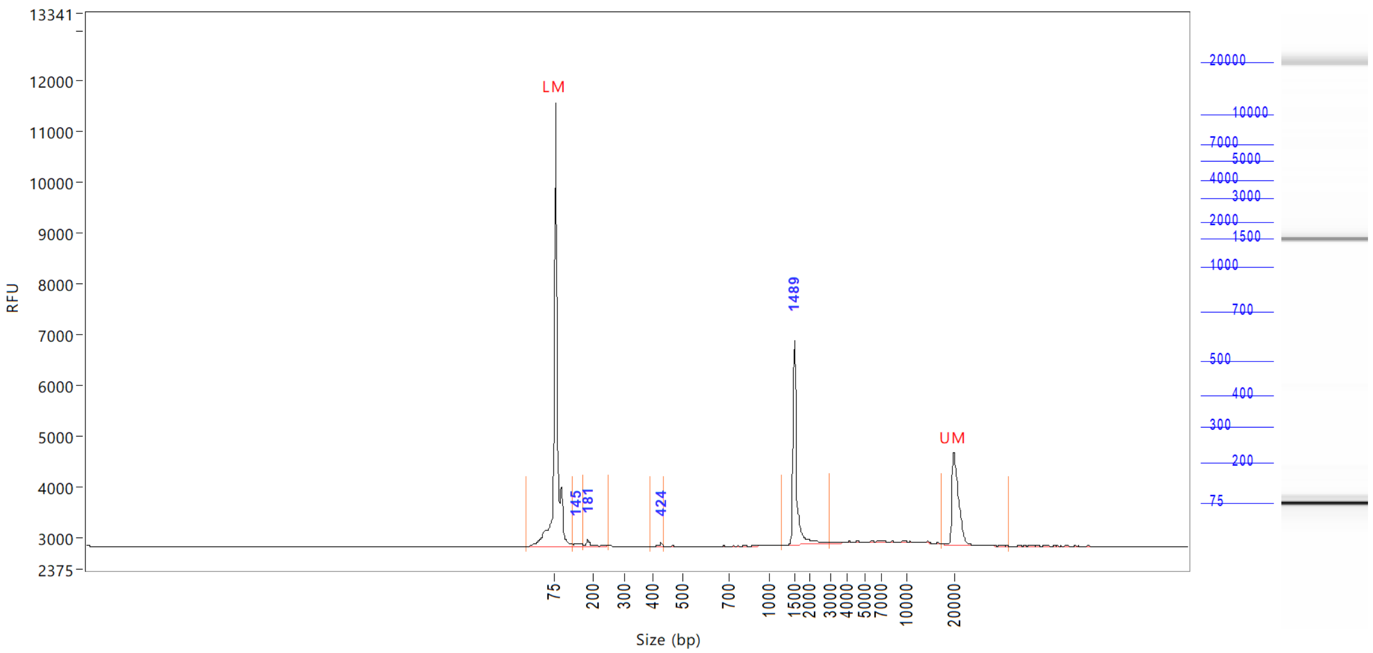

Peak	Size (bp)	Concentration (ng/uL)	From (bp)	To (bp)	Average size (bp)	CV%	RFU	Corrected peak area
1	75 (LM)	0.5000	0	131	71	23.90	8762	73.816
2	145	0.0758	131	168	148	6.87	60	0.932
3	181	0.0912	168	248	184	6.86	122	1.122
4	424	0.0415	386	435	416	2.32	68	0.511
5	1489	1.8998	1245	2943	1556	13.44	4041	23.373
6	20000 (UM)	0.1101	17439	31728	20419	5.11	1834	16.252

TIC:	2.1083	ng/uL
TIM:	3.8300	nmole/L
Total concentration:	2.3683	ng/uL

Sample peak width (sec): 5   Sample min peak height: 50   Sample baseline V to V?: Y   Sample baseline V to V points: 3
Sample filter: Binomial   Number of points for filter: 3   Sample start region (min): 0   Sample end region (min): 30
Marker peak width (sec): 5   Marker min peak height: 200   Marker baseline V to V?: Y   Marker baseline V to V points: 3
Lower marker selection: First peak > 500 RFU   Upper marker selection: Last peak > 200 RFU
Ladder size (bp)75, 200, 300, 400, 500, 700, 1000, 1500, 2000, 3000, 4000, 5000, 7000, 10000, 20000
Quantification using: Lower Marker   Final concentration (ng/uL): 0.5000   Dilution factor: 12.0

Sample: SampH9
Well location: H9
Created: Tuesday, September 14, 2021 5:34:08 PM

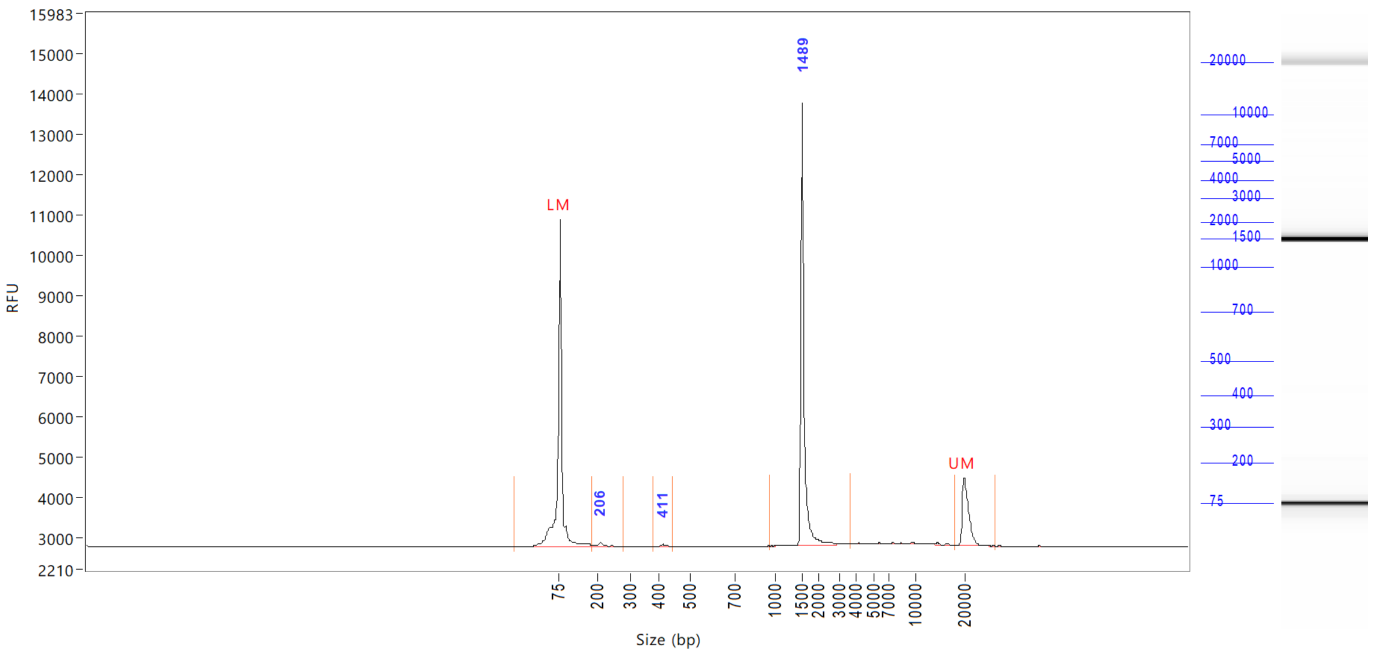

Peak	Size (bp)	Concentration (ng/uL)	From (bp)	To (bp)	Average size (bp)	CV%	RFU	Corrected peak area
1	75 (LM)	0.5000	0	183	70	33.77	8120	76.744
2	206	0.1134	183	280	211	9.74	105	1.451
3	411	0.0408	380	445	410	3.06	51	0.522
4	1489	4.4978	952	3643	1540	13.52	10980	57.529
5	20000 (UM)	0.0929	18171	26649	20450	3.90	1674	14.260

TIC:	4.6520	ng/uL
TIM:	5.8554	nmole/L
Total concentration:	4.8559	ng/uL

Sample peak width (sec): 5   Sample min peak height: 50   Sample baseline V to V?: Y   Sample baseline V to V points: 3
Sample filter: Binomial   Number of points for filter: 3 Sample start region (min): 0 Sample end region (min): 30
Marker peak width (sec): 5   Marker min peak height: 200   Marker baseline V to V?: Y   Marker baseline V to V points: 3
Lower marker selection: First peak > 500 RFU   Upper marker selection: Last peak > 200 RFU
Ladder size (bp)75, 200, 300, 400, 500, 700, 1000, 1500, 2000, 3000, 4000, 5000, 7000, 10000, 20000
Quantification using: Lower Marker   Final concentration (ng/uL): 0.5000   Dilution factor: 12.0

Sample: SampA10
Well location: A10
Created: Tuesday, September 14, 2021 5:34:08 PM

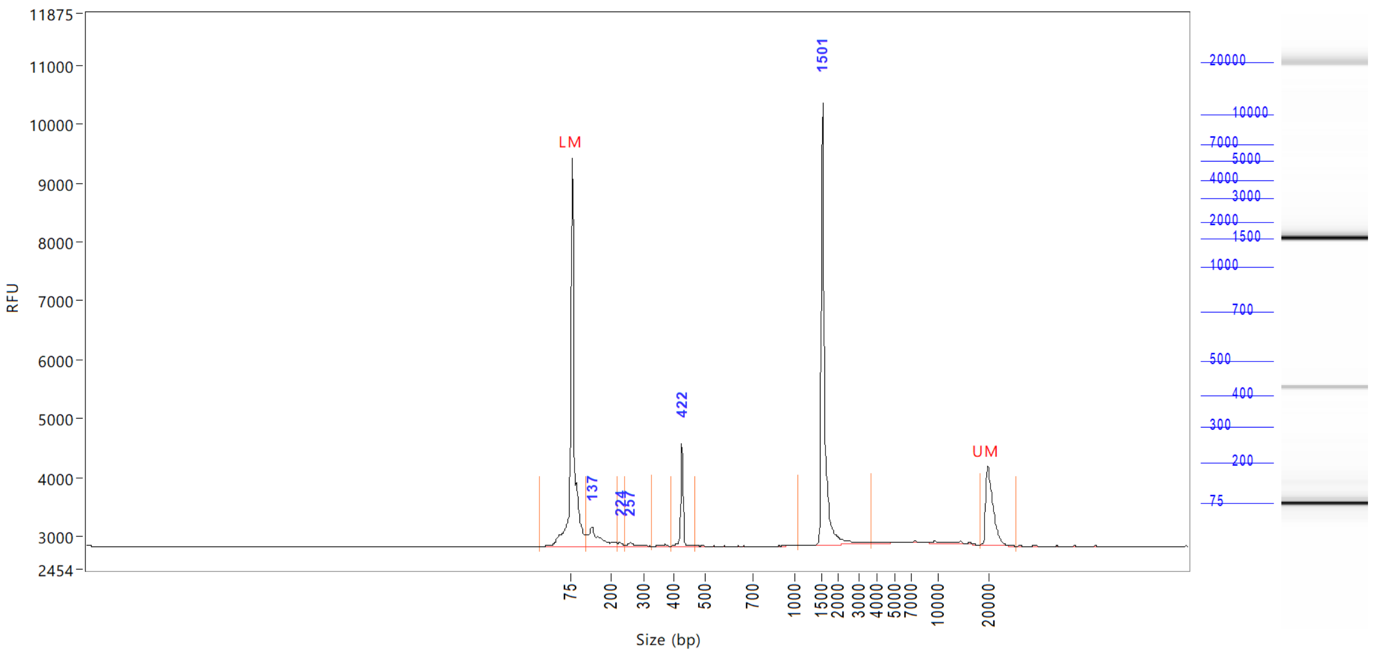

Peak	Size (bp)	Concentration (ng/uL)	From (bp)	To (bp)	Average size (bp)	CV%	RFU	Corrected peak area
1	75 (LM)	0.5000	0	122	73	23.26	6571	58.736
2	137	0.8327	122	217	154	16.54	327	8.152
3	224	0.0735	217	242	227	3.32	67	0.720
4	257	0.0985	242	325	263	6.16	70	0.964
5	422	0.8809	388	468	421	1.98	1727	8.623
6	1501	4.3645	1056	3643	1580	14.30	7506	42.725
7	20000 (UM)	0.1050	18415	25682	20522	4.35	1351	12.331

TIC:	6.2501	ng/uL
TIM:	18.0254	nmole/L
Total concentration:	6.5411	ng/uL

Sample peak width (sec): 5 Sample min peak height: 50 Sample baseline V to V?: Y Sample baseline V to V points: 3
Sample filter: Binomial Number of points for filter: 3 Sample start region (min): 0 Sample end region (min): 30
Marker peak width (sec): 5 Marker min peak height: 200 Marker baseline V to V?: Y Marker baseline V to V points: 3
Lower marker selection: First peak > 500 RFU Upper marker selection: Last peak > 200 RFU
Ladder size (bp)75, 200, 300, 400, 500, 700, 1000, 1500, 2000, 3000, 4000, 5000, 7000, 10000, 20000
Quantification using: Lower Marker Final concentration (ng/uL): 0.5000 Dilution factor: 12.0

Sample: SampB10
Well location: B10
Created: Tuesday, September 14, 2021 5:34:08 PM

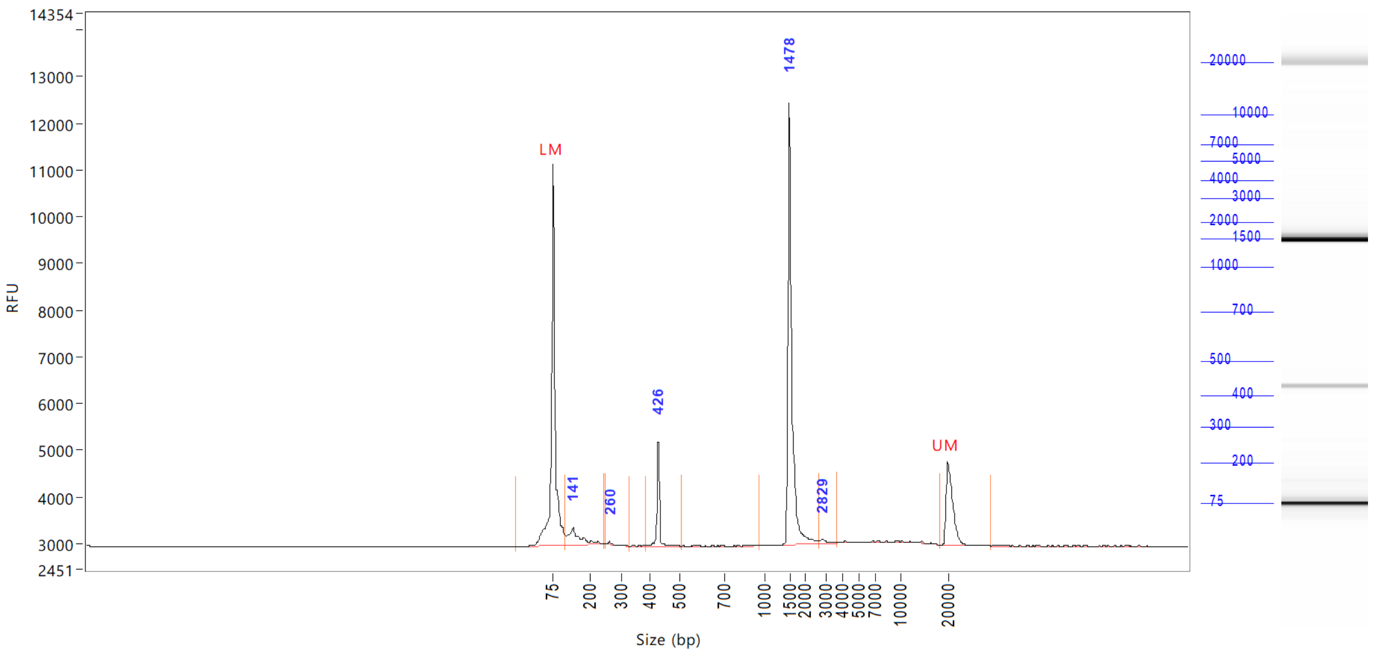

Peak	Size (bp)	Concentration (ng/uL)	From (bp)	To (bp)	Average size (bp)	CV%	RFU	Corrected peak area
1	75 (LM)	0.5000	0	118	72	21.78	8156	75.808
2	141	0.7668	118	244	155	17.91	366	9.688
3	260	0.0381	248	325	266	6.58	62	0.482
4	426	0.9489	386	501	425	2.30	2226	11.989
5	1478	4.8355	965	2657	1530	10.27	9475	61.096
6	2829	0.0853	2657	3679	2978	8.75	97	1.077
7	20000 (UM)	0.1034	18171	29189	20490	4.39	1773	15.675

TIC:	6.6747	ng/uL
TIM:	17.2901	nmole/L
Total concentration:	6.8610	ng/uL

Sample peak width (sec): 5   Sample min peak height: 50   Sample baseline V to V?: Y   Sample baseline V to V points: 3
Sample filter: Binomial   Number of points for filter: 3 Sample start region (min): 0 Sample end region (min): 30
Marker peak width (sec): 5   Marker min peak height: 200   Marker baseline V to V?: Y   Marker baseline V to V points: 3
Lower marker selection: First peak > 500 RFU   Upper marker selection: Last peak > 200 RFU
Ladder size (bp)75, 200, 300, 400, 500, 700, 1000, 1500, 2000, 3000, 4000, 5000, 7000, 10000, 20000
Quantification using: Lower Marker   Final concentration (ng/uL): 0.5000   Dilution factor: 12.0

Sample: SampC10
Well location: C10
Created: Tuesday, September 14, 2021 5:34:08 PM

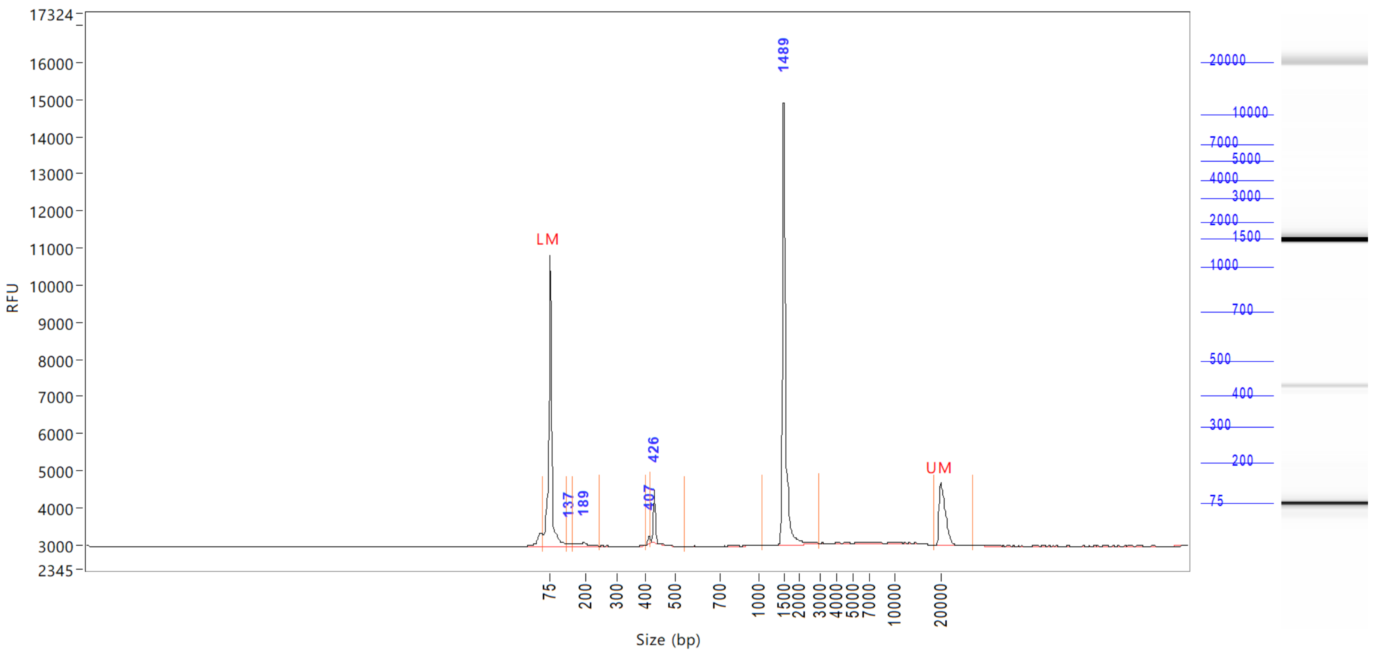

Peak	Size (bp)	Concentration (ng/uL)	From (bp)	To (bp)	Average size (bp)	CV%	RFU	Corrected peak area
1	75 (LM)	0.5000	52	131	74	14.30	7828	58.920
2	137	0.0979	131	154	142	4.99	71	0.962
3	189	0.2586	154	239	184	9.94	114	2.540
4	407	0.0897	398	416	407	0.90	217	0.881
5	426	0.6627	416	537	426	1.83	1436	6.508
6	1489	6.6525	1056	2971	1513	10.41	11932	65.328
7	20000 (UM)	0.1271	18537	26891	20500	3.95	1699	14.983

TIC:	7.7615	ng/uL
TIM:	13.6040	nmole/L
Total concentration:	8.0838	ng/uL

```
Sample peak width (sec): 5     Sample min peak height: 50     Sample baseline V to V?: Y     Sample baseline V to V points: 3
Sample filter: Binomial        Number of points for filter: 3  Sample start region (min): 0  Sample end region (min): 30
Marker peak width (sec): 5     Marker min peak height: 200    Marker baseline V to V?: Y     Marker baseline V to V points: 3
Lower marker selection: First peak > 500 RFU                  Upper marker selection: Last peak > 200 RFU
Ladder size (bp)75, 200, 300, 400, 500, 700, 1000, 1500, 2000, 3000, 4000, 5000, 7000, 10000, 20000
Quantification using: Lower Marker          Final concentration (ng/uL): 0.5000          Dilution factor: 12.0
```

Sample: SampD10
Well location: D10
Created: Tuesday, September 14, 2021 5:34:08 PM

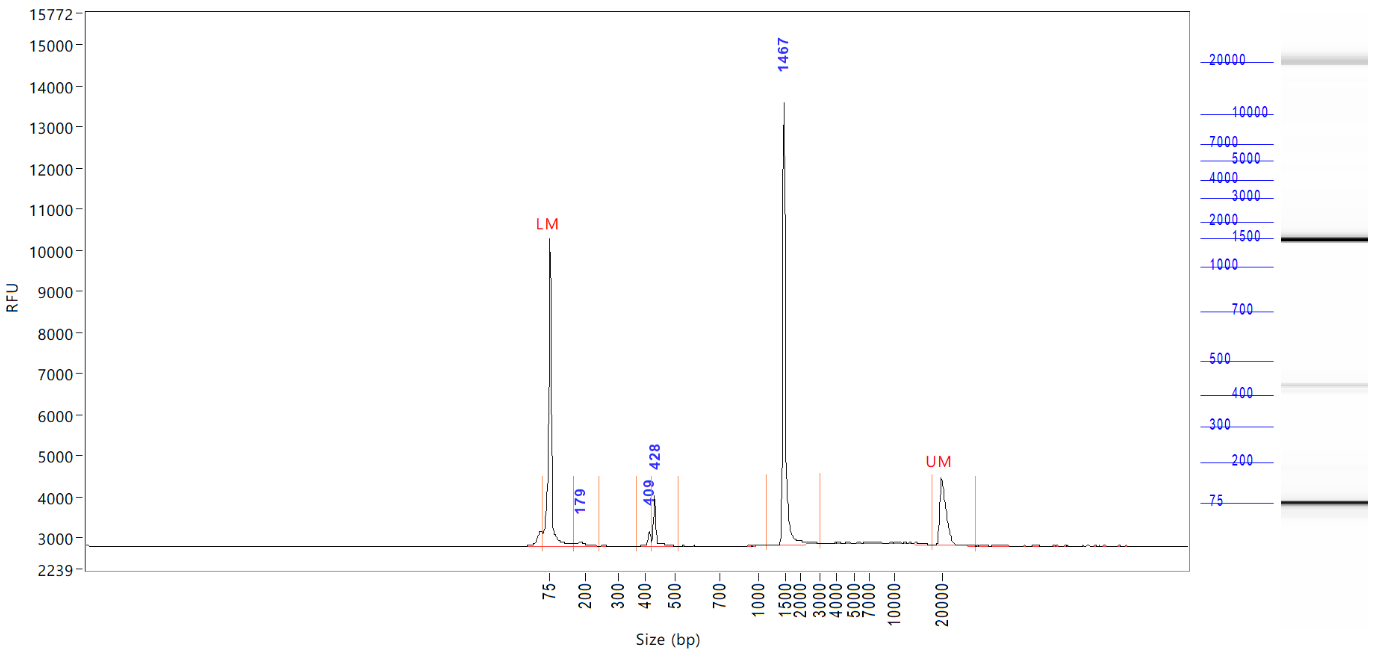

Peak	Size (bp)	Concentration (ng/uL)	From (bp)	To (bp)	Average size (bp)	CV%	RFU	Corrected peak area
1	75 (LM)	0.5000	50	156	74	18.77	7494	58.166
2	179	0.2501	156	239	184	10.43	114	2.425
3	409	0.2002	366	416	407	1.74	339	1.941
4	428	0.7233	416	509	429	2.99	1225	7.012
5	1467	5.5566	1123	2971	1506	10.75	10784	53.868
6	20000 (UM)	0.1229	18049	27375	20445	4.43	1641	14.300

TIC:	6.7302	ng/uL
TIM:	11.8914	nmole/L
Total concentration:	7.0364	ng/uL

Sample peak width (sec): 5 Sample min peak height: 50 Sample baseline V to V?: Y Sample baseline V to V points: 3
Sample filter: Binomial Number of points for filter: 3 Sample start region (min): 0 Sample end region (min): 30
Marker peak width (sec): 5 Marker min peak height: 200 Marker baseline V to V?: Y Marker baseline V to V points: 3
Lower marker selection: First peak > 500 RFU Upper marker selection: Last peak > 200 RFU
Ladder size (bp)75, 200, 300, 400, 500, 700, 1000, 1500, 2000, 3000, 4000, 5000, 7000, 10000, 20000
Quantification using: Lower Marker Final concentration (ng/uL): 0.5000 Dilution factor: 12.0

Sample: SampE10
Well location: E10
Created: Tuesday, September 14, 2021 5:34:08 PM

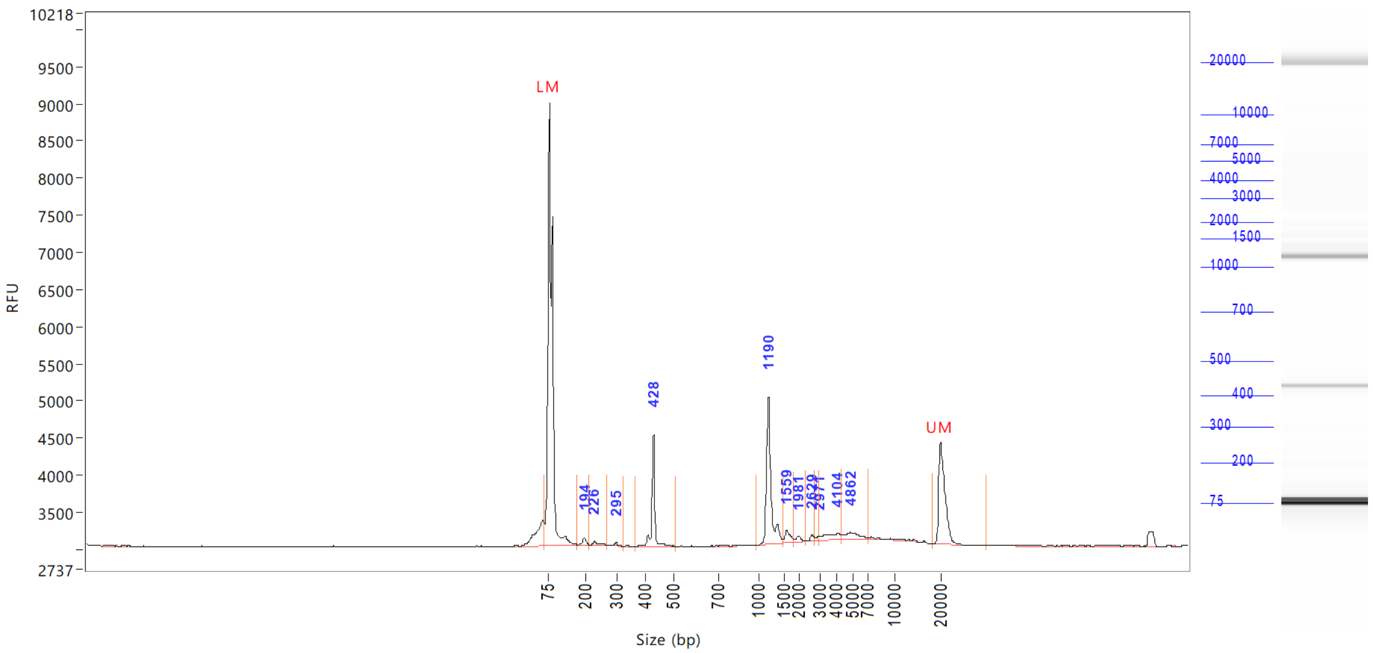

Peak	Size (bp)	Concentration (ng/uL)	From (bp)	To (bp)	Average size (bp)	CV%	RFU	Corrected peak area
1	75 (LM)	0.5000	60	171	80	15.98	5971	65.008
2	194	0.0889	171	212	191	3.96	110	0.963
3	226	0.0737	212	269	236	5.89	54	0.798
4	295	0.0406	269	319	290	3.31	51	0.440
5	428	0.8530	366	501	424	3.08	1510	9.242
6	1190	1.3805	987	1467	1206	6.24	1982	14.957
7	1559	0.1477	1467	1827	1599	5.73	171	1.600
8	1981	0.0595	1827	2286	1952	3.79	77	0.645
9	2629	0.0461	2286	2772	2609	3.48	87	0.500
10	2971	0.0317	2772	3000	2896	2.50	62	0.344
11	4104	0.1956	3000	4311	3653	10.63	82	2.120
12	4862	0.1916	4311	7000	5123	12.72	90	2.076
13	20000 (UM)	0.0917	18415	29914	20415	4.42	1370	11.927

TIC:	3.1089	ng/uL
TIM:	7.1020	nmole/L
Total concentration:	3.2146	ng/uL

```
Sample peak width (sec): 5    Sample min peak height: 50    Sample baseline V to V?: Y    Sample baseline V to V points: 3
Sample filter: Binomial       Number of points for filter: 3  Sample start region (min): 0  Sample end region (min): 30
Marker peak width (sec): 5    Marker min peak height: 200   Marker baseline V to V?: Y    Marker baseline V to V points: 3
Lower marker selection: First peak > 500 RFU                Upper marker selection: Last peak > 200 RFU
Ladder size (bp)75, 200, 300, 400, 500, 700, 1000, 1500, 2000, 3000, 4000, 5000, 7000, 10000, 20000
Quantification using: Lower Marker          Final concentration (ng/uL): 0.5000         Dilution factor: 12.0
```

Sample: SampF10
Well location: F10
Created: Tuesday, September 14, 2021 5:34:08 PM

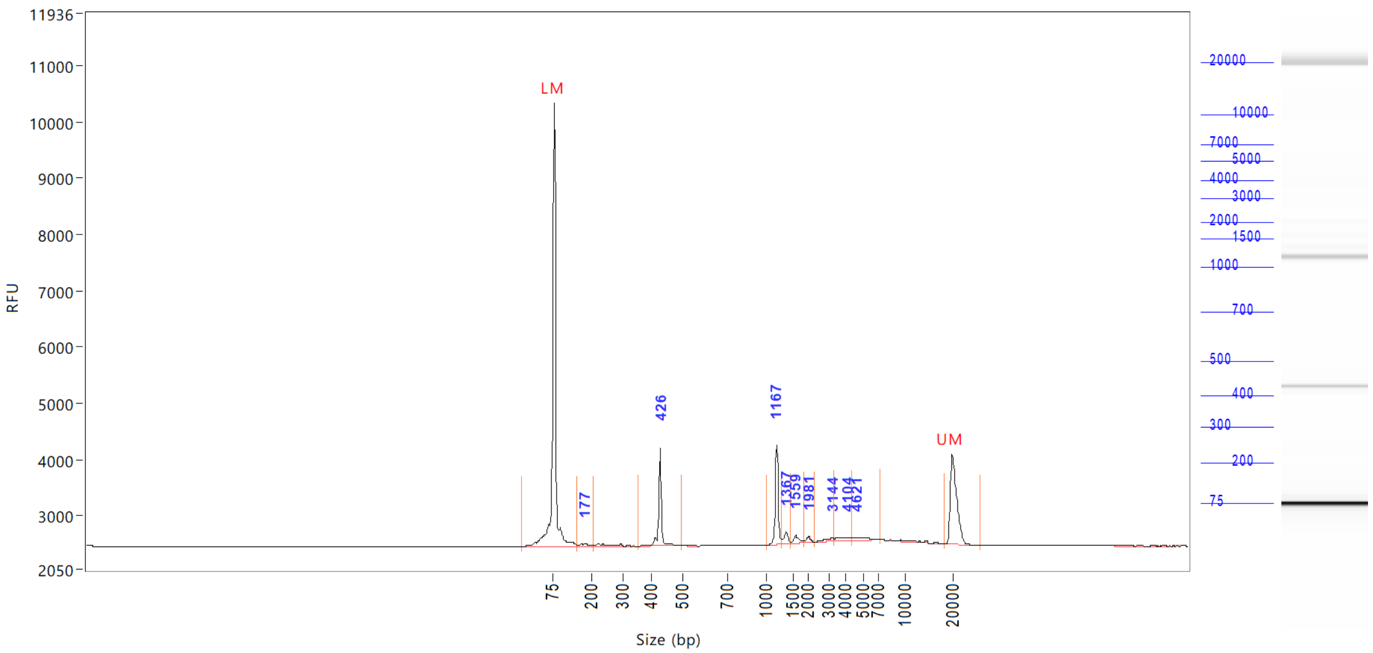

Peak	Size (bp)	Concentration (ng/uL)	From (bp)	To (bp)	Average size (bp)	CV%	RFU	Corrected peak area
1	75 (LM)	0.5000	0	152	70	24.37	7898	64.557
2	177	0.0769	152	205	174	7.59	53	0.828
3	426	0.9280	352	498	423	3.00	1744	9.985
4	1167	0.8569	991	1278	1167	2.96	1756	9.219
5	1367	0.1416	1278	1445	1346	2.83	212	1.523
6	1559	0.1130	1445	1827	1587	5.82	142	1.216
7	1981	0.0667	1827	2286	1970	3.61	101	0.718
8	3144	0.0762	2286	3322	2940	8.29	54	0.820
9	4104	0.1189	3322	4380	3852	7.74	61	1.279
10	4621	0.1005	4380	7134	5066	11.62	52	1.082
11	20000 (UM)	0.1079	18293	25803	20376	3.66	1611	13.934

TIC:	2.4787	ng/uL
TIM:	6.0172	nmole/L
Total concentration:	2.7011	ng/uL

Sample peak width (sec): 5 Sample min peak height: 50 Sample baseline V to V?: Y Sample baseline V to V points: 3
Sample filter: Binomial Number of points for filter: 3 Sample start region (min): 0 Sample end region (min): 30
Marker peak width (sec): 5 Marker min peak height: 200 Marker baseline V to V?: Y Marker baseline V to V points: 3
Lower marker selection: First peak > 500 RFU Upper marker selection: Last peak > 200 RFU
Ladder size (bp)75, 200, 300, 400, 500, 700, 1000, 1500, 2000, 3000, 4000, 5000, 7000, 10000, 20000
Quantification using: Lower Marker Final concentration (ng/uL): 0.5000 Dilution factor: 12.0

Sample: SampG10
Well location: G10
Created: Tuesday, September 14, 2021 5:34:08 PM

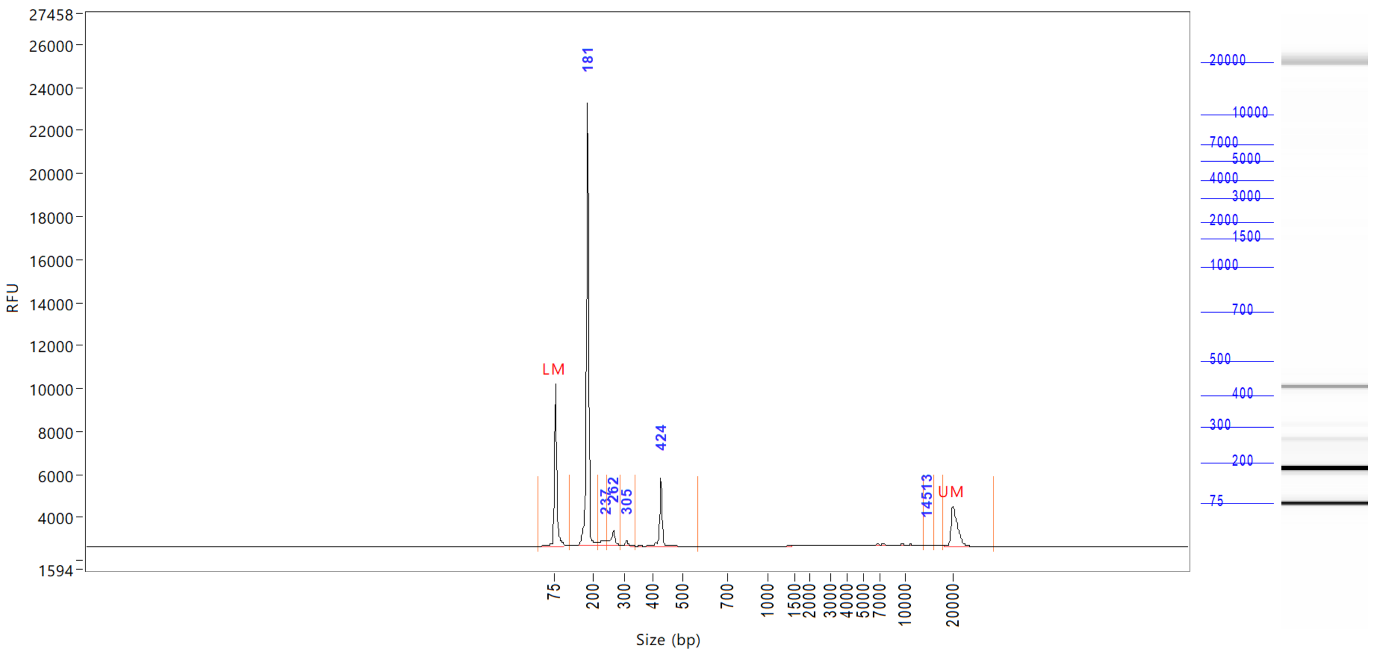

Peak	Size (bp)	Concentration (ng/uL)	From (bp)	To (bp)	Average size (bp)	CV%	RFU	Corrected peak area
1	75 (LM)	0.5000	20	122	73	11.51	7521	47.801
2	181	15.0160	122	215	178	4.20	20633	119.630
3	237	0.4944	215	246	232	3.90	241	3.939
4	262	0.8435	246	287	261	3.07	745	6.720
5	305	0.2427	287	337	304	2.82	227	1.934
6	424	2.1858	337	565	423	3.84	3182	17.414
7	14513	0.0357	13781	15976	14560	3.82	51	0.285
8	20000 (UM)	0.1673	17927	28584	20442	4.08	1855	15.992

TIC:	18.8182	ng/uL
TIM:	157.3237	nmole/L
Total concentration:	19.1750	ng/uL

```
Sample peak width (sec): 5     Sample min peak height: 50     Sample baseline V to V?: Y     Sample baseline V to V points: 3
Sample filter: Binomial        Number of points for filter: 3  Sample start region (min): 0  Sample end region (min): 30
Marker peak width (sec): 5     Marker min peak height: 200    Marker baseline V to V?: Y     Marker baseline V to V points: 3
Lower marker selection: First peak > 500 RFU                  Upper marker selection: Last peak > 200 RFU
Ladder size (bp)75, 200, 300, 400, 500, 700, 1000, 1500, 2000, 3000, 4000, 5000, 7000, 10000, 20000
Quantification using: Lower Marker          Final concentration (ng/uL): 0.5000          Dilution factor: 12.0
```

Sample: SampH10
Well location: H10
Created: Tuesday, September 14, 2021 5:34:08 PM

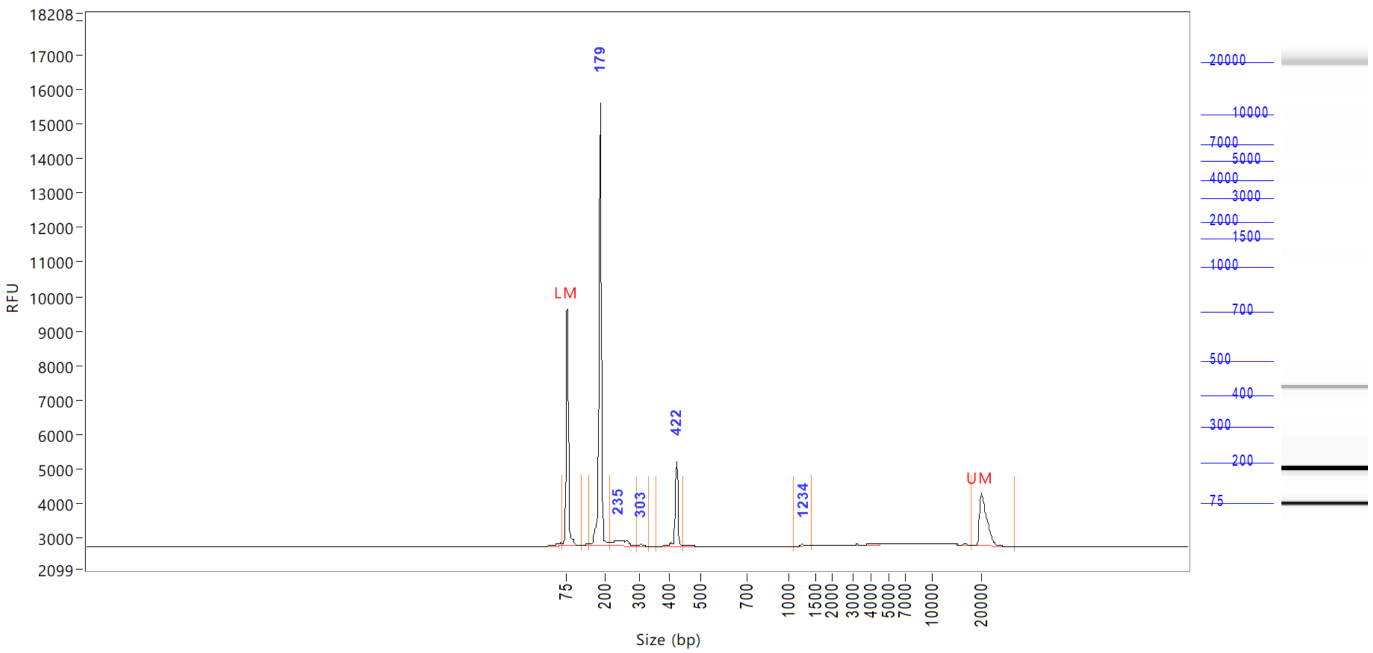

Peak	Size (bp)	Concentration (ng/uL)	From (bp)	To (bp)	Average size (bp)	CV%	RFU	Corrected peak area
1	75 (LM)	0.5000	60	120	73	6.51	6840	40.516
2	179	10.8789	147	208	177	3.46	12848	73.462
3	235	0.7949	208	295	241	7.92	160	5.367
4	303	0.0689	295	329	303	1.95	61	0.466
5	422	1.9453	356	445	419	2.19	2439	13.136
6	1234	0.0507	1090	1422	1247	4.19	55	0.342
7	20000 (UM)	0.1792	17927	26891	20466	4.13	1536	14.518

TIC:	13.7387	ng/uL
TIM:	114.5458	nmole/L
Total concentration:	14.2325	ng/uL

Sample peak width (sec): 5 Sample min peak height: 50 Sample baseline V to V?: Y Sample baseline V to V points: 3
Sample filter: Binomial Number of points for filter: 3 Sample start region (min): 0 Sample end region (min): 30
Marker peak width (sec): 5 Marker min peak height: 200 Marker baseline V to V?: Y Marker baseline V to V points: 3
Lower marker selection: First peak > 500 RFU Upper marker selection: Last peak > 200 RFU
Ladder size (bp)75, 200, 300, 400, 500, 700, 1000, 1500, 2000, 3000, 4000, 5000, 7000, 10000, 20000
Quantification using: Lower Marker Final concentration (ng/uL): 0.5000 Dilution factor: 12.0

Sample: SampA11
Well location: A11
Created: Tuesday, September 14, 2021 5:34:08 PM

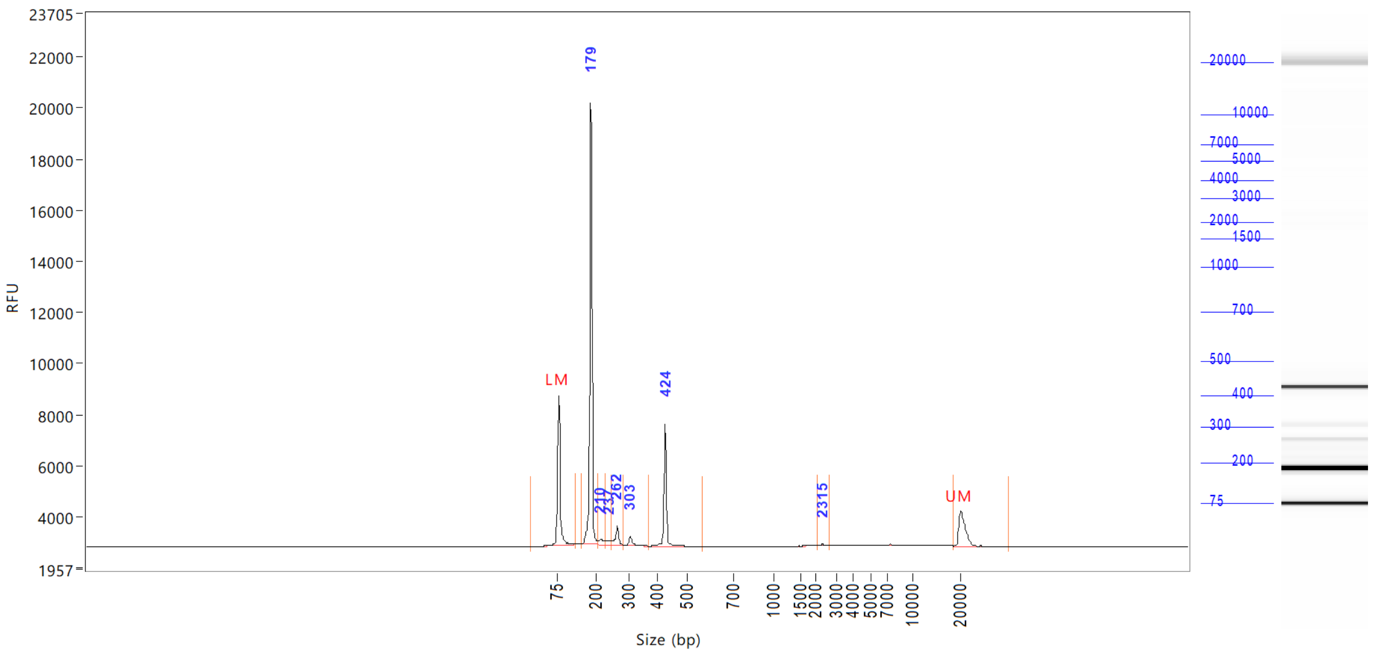

Peak	Size (bp)	Concentration (ng/uL)	From (bp)	To (bp)	Average size (bp)	CV%	RFU	Corrected peak area
1	75 (LM)	0.5000	0	131	74	12.10	5856	36.962
2	179	15.7312	148	203	178	2.92	17299	96.910
3	210	0.4104	203	226	213	3.24	220	2.528
4	237	0.3532	226	248	237	2.76	173	2.176
5	262	0.8777	248	284	260	2.21	752	5.407
6	303	0.4221	284	370	304	3.16	351	2.601
7	424	4.1311	370	562	423	3.08	4799	25.449
8	2315	0.0712	2087	2657	2311	6.15	50	0.439
9	20000 (UM)	0.1592	18415	30156	20465	4.94	1375	11.772

TIC:	21.9970	ng/uL
TIM:	174.8643	nmole/L
Total concentration:	22.4194	ng/uL

Sample peak width (sec): 5 Sample min peak height: 50 Sample baseline V to V?: Y Sample baseline V to V points: 3
Sample filter: Binomial Number of points for filter: 3 Sample start region (min): 0 Sample end region (min): 30
Marker peak width (sec): 5 Marker min peak height: 200 Marker baseline V to V?: Y Marker baseline V to V points: 3
Lower marker selection: First peak > 500 RFU Upper marker selection: Last peak > 200 RFU
Ladder size (bp)75, 200, 300, 400, 500, 700, 1000, 1500, 2000, 3000, 4000, 5000, 7000, 10000, 20000
Quantification using: Lower Marker Final concentration (ng/uL): 0.5000 Dilution factor: 12.0

Sample: SampB11
Well location: B11
Created: Tuesday, September 14, 2021 5:34:08 PM

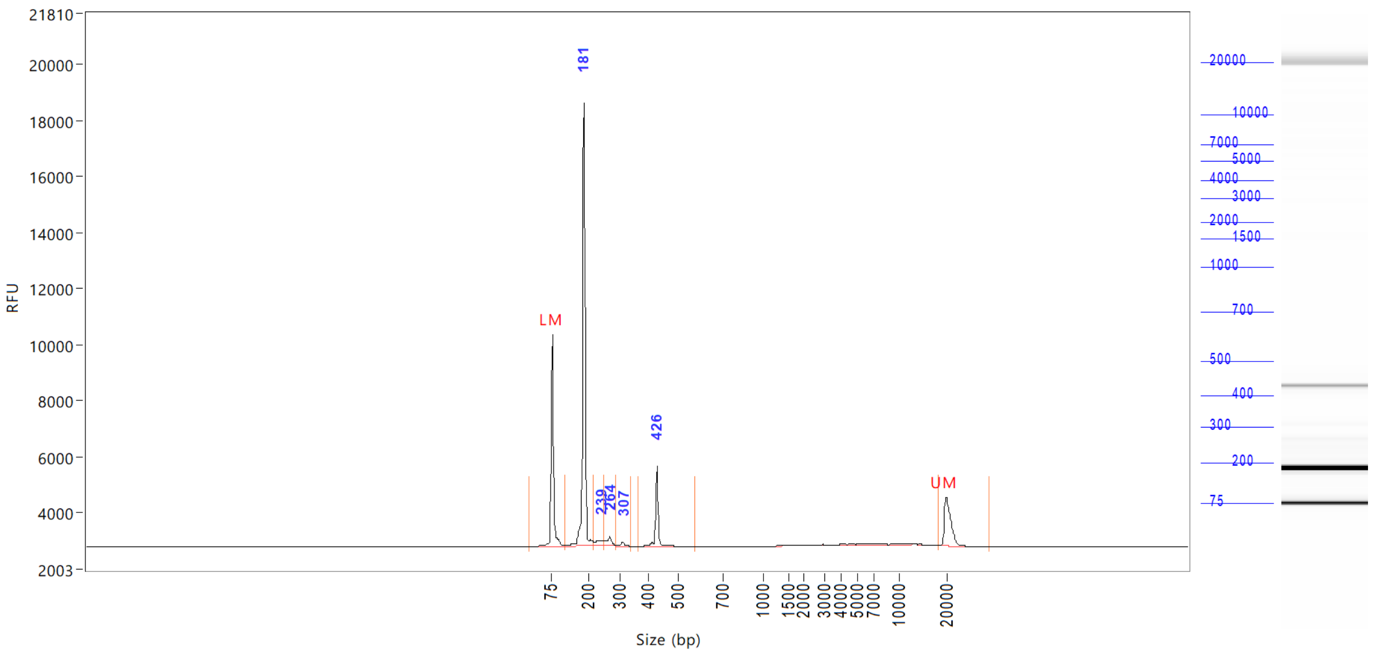

Peak	Size (bp)	Concentration (ng/uL)	From (bp)	To (bp)	Average size (bp)	CV%	RFU	Corrected peak area
1	75 (LM)	0.5000	0	122	74	12.24	7521	46.593
2	181	11.6891	122	215	179	4.31	15809	90.772
3	239	0.4497	215	248	232	4.14	192	3.492
4	264	0.4591	248	287	261	3.29	334	3.565
5	307	0.1666	287	335	305	2.68	152	1.294
6	426	2.0394	364	570	426	3.32	2850	15.837
7	20000 (UM)	0.1622	18293	29309	20473	4.38	1752	15.119

TIC:	14.8038	ng/uL
TIM:	122.2109	nmole/L
Total concentration:	15.1602	ng/uL

Sample peak width (sec): 5 Sample min peak height: 50 Sample baseline V to V?: Y Sample baseline V to V points: 3
Sample filter: Binomial Number of points for filter: 3 Sample start region (min): 0 Sample end region (min): 30
Marker peak width (sec): 5 Marker min peak height: 200 Marker baseline V to V?: Y Marker baseline V to V points: 3
Lower marker selection: First peak > 500 RFU Upper marker selection: Last peak > 200 RFU
Ladder size (bp)75, 200, 300, 400, 500, 700, 1000, 1500, 2000, 3000, 4000, 5000, 7000, 10000, 20000
Quantification using: Lower Marker Final concentration (ng/uL): 0.5000 Dilution factor: 12.0

Sample: SampC11
Well location: C11
Created: Tuesday, September 14, 2021 5:34:08 PM

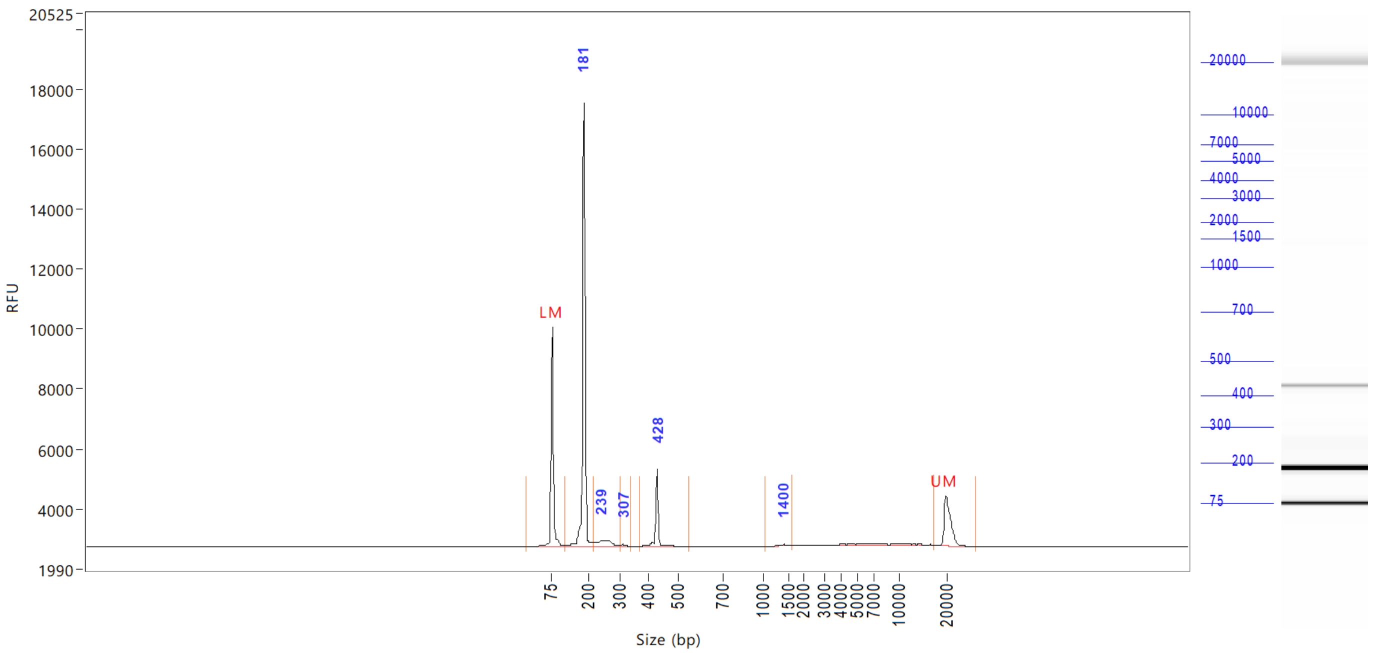

Peak	Size (bp)	Concentration (ng/uL)	From (bp)	To (bp)	Average size (bp)	CV%	RFU	Corrected peak area
1	75 (LM)	0.5000	0	122	74	13.19	7298	44.855
2	181	11.3659	122	215	179	4.50	14815	84.970
3	239	0.8930	215	296	247	7.70	201	6.676
4	307	0.0835	296	335	308	2.56	71	0.624
5	428	1.9631	370	547	425	3.60	2561	14.676
6	1400	0.0549	1034	1616	1395	6.66	50	0.411
7	20000 (UM)	0.1618	17317	26287	20411	3.98	1681	14.519

TIC:	14.3604	ng/uL
TIM:	118.4434	nmole/L
Total concentration:	14.6955	ng/uL

Sample peak width (sec): 5 Sample min peak height: 50 Sample baseline V to V?: Y Sample baseline V to V points: 3
Sample filter: Binomial Number of points for filter: 3 Sample start region (min): 0 Sample end region (min): 30
Marker peak width (sec): 5 Marker min peak height: 200 Marker baseline V to V?: Y Marker baseline V to V points: 3
Lower marker selection: First peak > 500 RFU Upper marker selection: Last peak > 200 RFU
Ladder size (bp)75, 200, 300, 400, 500, 700, 1000, 1500, 2000, 3000, 4000, 5000, 7000, 10000, 20000
Quantification using: Lower Marker Final concentration (ng/uL): 0.5000 Dilution factor: 12.0

Sample: SampD11
Well location: D11
Created: Tuesday, September 14, 2021 5:34:08 PM

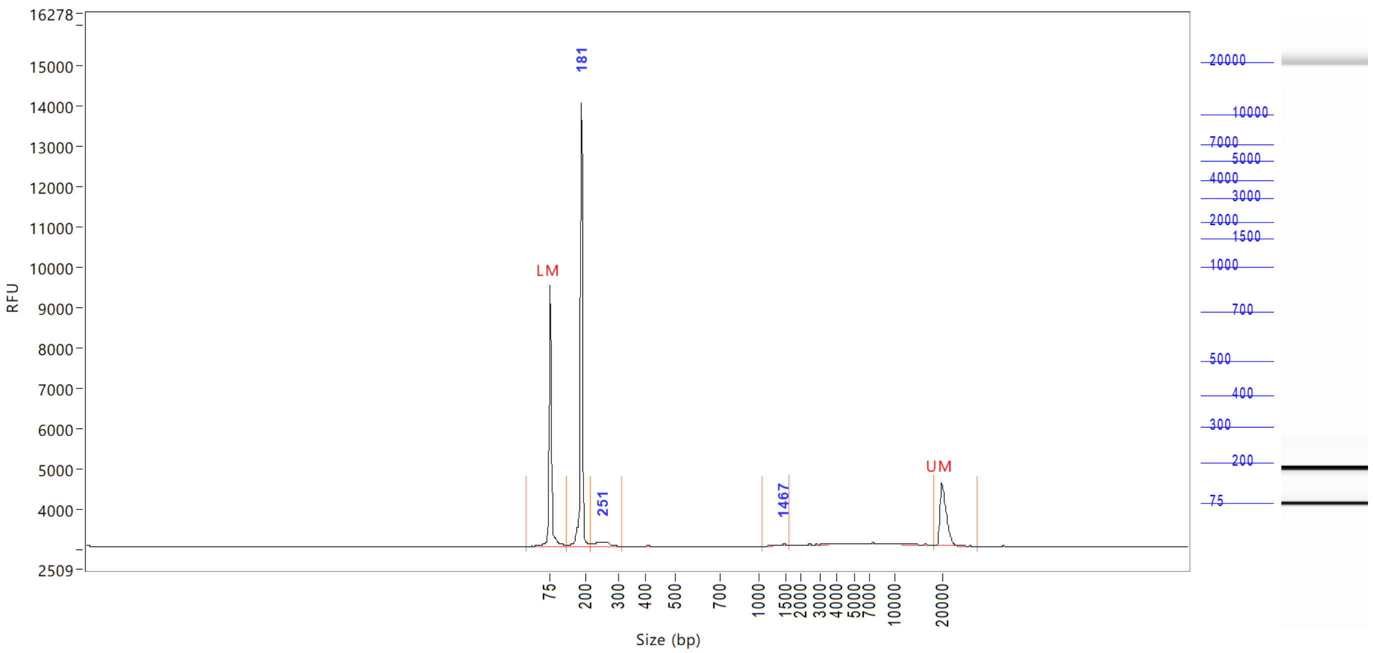

Peak	Size (bp)	Concentration (ng/uL)	From (bp)	To (bp)	Average size (bp)	CV%	RFU	Corrected peak area
1	75 (LM)	0.5000	0	129	73	14.48	6461	41.361
2	181	9.1013	129	210	178	4.08	11006	62.741
3	251	0.5925	210	311	245	8.36	116	4.084
4	1467	0.0691	1034	1635	1411	8.61	57	0.476
5	20000 (UM)	0.1646	18293	27617	20423	4.23	1564	13.614

TIC:	9.7629	ng/uL
TIM:	88.1151	nmole/L
Total concentration:	10.2519	ng/uL

Sample peak width (sec): 5 Sample min peak height: 50 Sample baseline V to V?: Y Sample baseline V to V points: 3
Sample filter: Binomial Number of points for filter: 3 Sample start region (min): 0 Sample end region (min): 30
Marker peak width (sec): 5 Marker min peak height: 200 Marker baseline V to V?: Y Marker baseline V to V points: 3
Lower marker selection: First peak > 500 RFU Upper marker selection: Last peak > 200 RFU
Ladder size (bp)75, 200, 300, 400, 500, 700, 1000, 1500, 2000, 3000, 4000, 5000, 7000, 10000, 20000
Quantification using: Lower Marker Final concentration (ng/uL): 0.5000 Dilution factor: 12.0

Sample: SampE11
Well location: E11
Created: Tuesday, September 14, 2021 5:34:08 PM

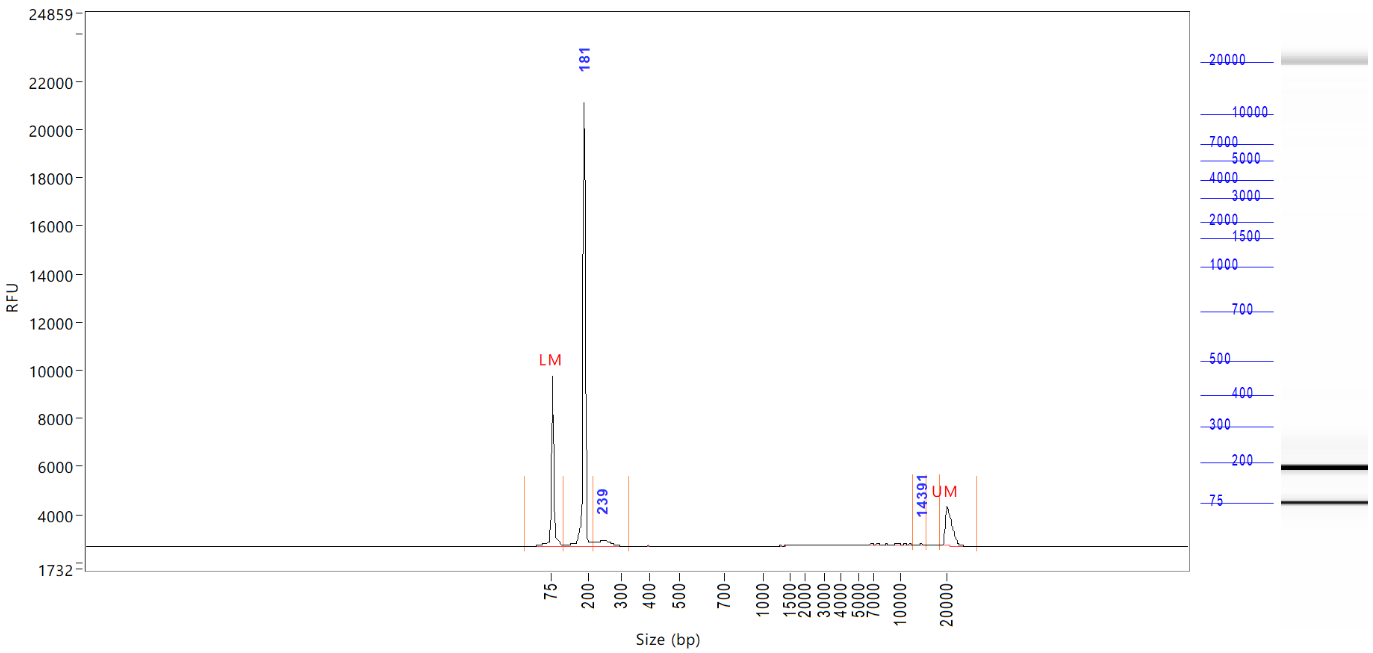

Peak	Size (bp)	Concentration (ng/uL)	From (bp)	To (bp)	Average size (bp)	CV%	RFU	Corrected peak area
1	75 (LM)	0.5000	0	112	72	15.74	7112	46.302
2	181	13.9181	112	214	178	4.93	18490	107.407
3	239	1.0420	214	331	245	8.14	245	8.041
4	14391	0.0537	12562	15488	13837	5.02	51	0.415
5	20000 (UM)	0.1548	18293	26287	20470	3.77	1647	14.334

TIC:	15.0138	ng/uL
TIM:	135.5446	nmole/L
Total concentration:	15.4086	ng/uL

Sample peak width (sec): 5 Sample min peak height: 50 Sample baseline V to V?: Y Sample baseline V to V points: 3
Sample filter: Binomial Number of points for filter: 3 Sample start region (min): 0 Sample end region (min): 30
Marker peak width (sec): 5 Marker min peak height: 200 Marker baseline V to V?: Y Marker baseline V to V points: 3
Lower marker selection: First peak > 500 RFU Upper marker selection: Last peak > 200 RFU
Ladder size (bp)75, 200, 300, 400, 500, 700, 1000, 1500, 2000, 3000, 4000, 5000, 7000, 10000, 20000
Quantification using: Lower Marker Final concentration (ng/uL): 0.5000 Dilution factor: 12.0

Sample: SampF11
Well location: F11
Created: Tuesday, September 14, 2021 5:34:08 PM

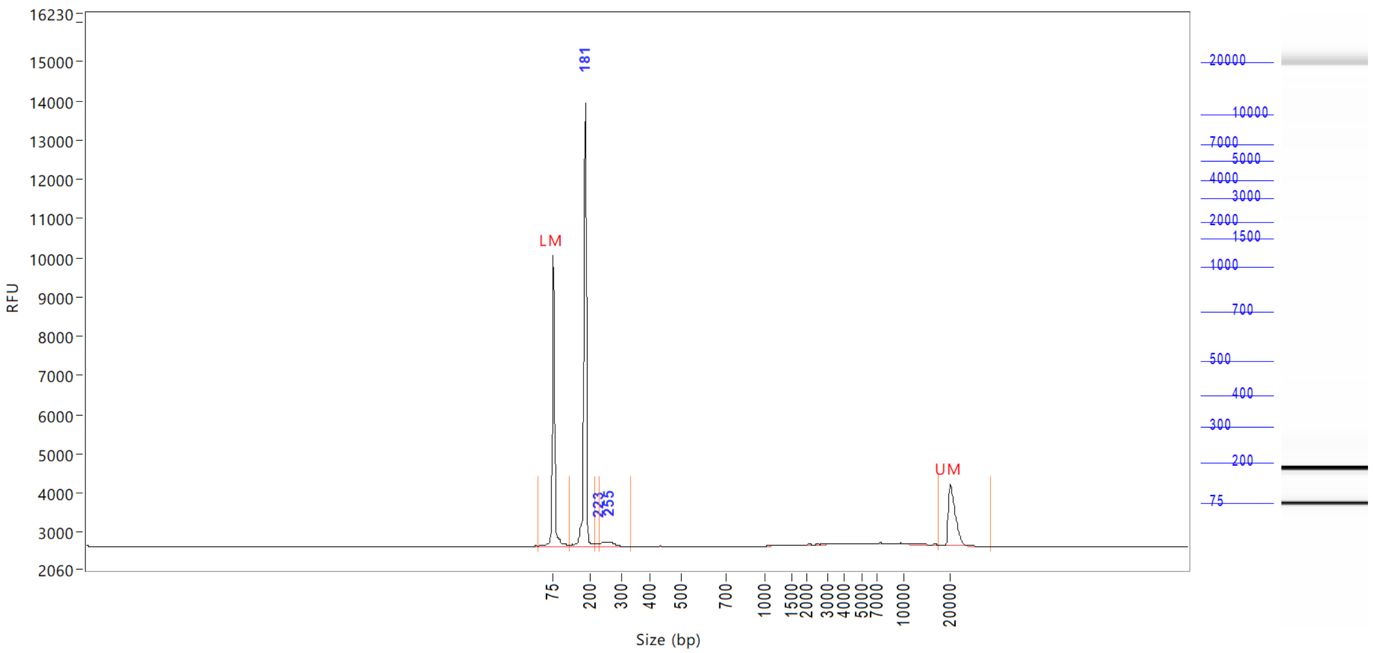

Peak	Size (bp)	Concentration (ng/uL)	From (bp)	To (bp)	Average size (bp)	CV%	RFU	Corrected peak area
1	75 (LM)	0.5000	24	131	74	11.67	7406	44.430
2	181	8.5879	131	212	178	3.95	11320	63.593
3	223	0.1035	212	228	220	2.32	77	0.767
4	255	0.4244	228	329	254	6.59	111	3.143
5	20000 (UM)	0.1608	17561	28463	20479	4.26	1560	14.290

TIC:	9.1158	ng/uL
TIM:	82.8371	nmole/L
Total concentration:	9.5825	ng/uL

Sample peak width (sec): 5   Sample min peak height: 50   Sample baseline V to V?: Y   Sample baseline V to V points: 3
Sample filter: Binomial   Number of points for filter: 3 Sample start region (min): 0 Sample end region (min): 30
Marker peak width (sec): 5   Marker min peak height: 200   Marker baseline V to V?: Y   Marker baseline V to V points: 3
Lower marker selection: First peak > 500 RFU   Upper marker selection: Last peak > 200 RFU
Ladder size (bp)75, 200, 300, 400, 500, 700, 1000, 1500, 2000, 3000, 4000, 5000, 7000, 10000, 20000
Quantification using: Lower Marker   Final concentration (ng/uL): 0.5000   Dilution factor: 12.0

Sample: SampG11
Well location: G11
Created: Tuesday, September 14, 2021 5:34:08 PM

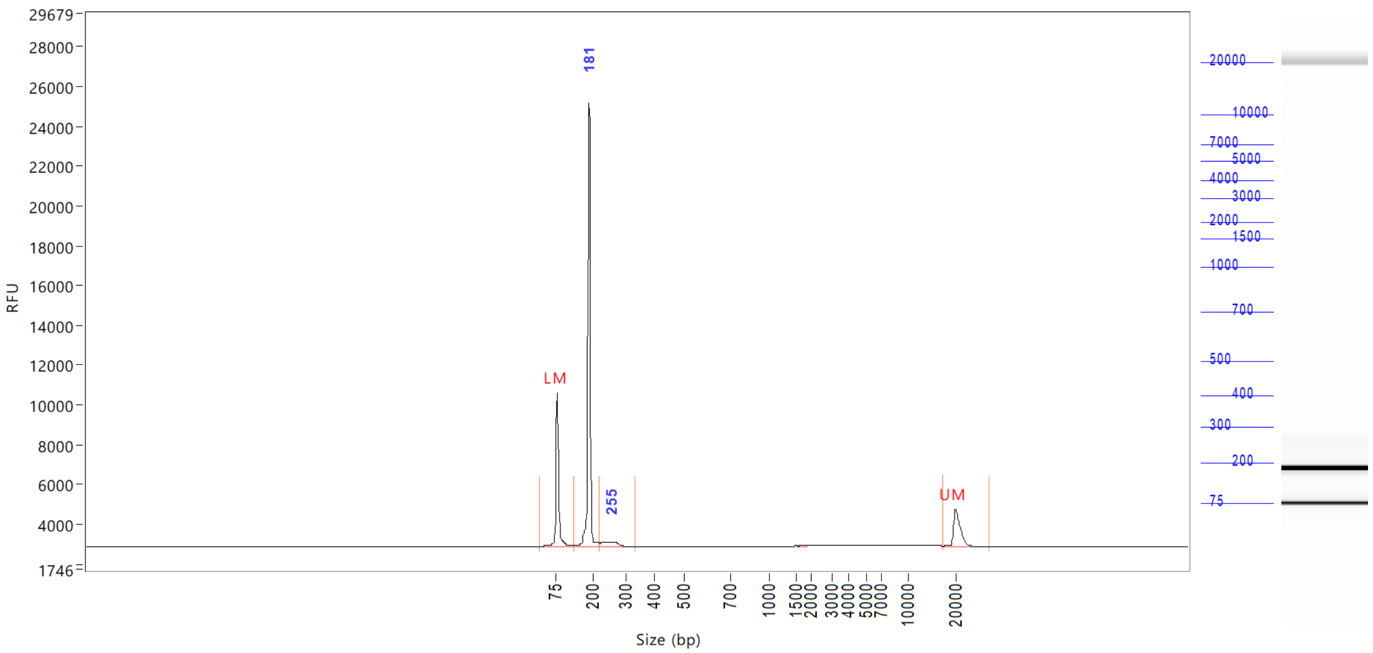

Peak	Size (bp)	Concentration (ng/uL)	From (bp)	To (bp)	Average size (bp)	CV%	RFU	Corrected peak area
1	75 (LM)	0.5000	20	131	75	15.48	7755	50.900
2	181	15.3748	131	215	179	4.22	22335	130.429
3	255	1.0332	215	329	248	7.96	254	8.765
4	20000 (UM)	0.1617	17439	27254	20450	3.86	1874	16.465

TIC:	16.4080	ng/uL
TIM:	148.0570	nmole/L
Total concentration:	16.8050	ng/uL

Sample peak width (sec): 5 Sample min peak height: 50 Sample baseline V to V?: Y Sample baseline V to V points: 3
Sample filter: Binomial Number of points for filter: 3 Sample start region (min): 0 Sample end region (min): 30
Marker peak width (sec): 5 Marker min peak height: 200 Marker baseline V to V?: Y Marker baseline V to V points: 3
Lower marker selection: First peak > 500 RFU Upper marker selection: Last peak > 200 RFU
Ladder size (bp)75, 200, 300, 400, 500, 700, 1000, 1500, 2000, 3000, 4000, 5000, 7000, 10000, 20000
Quantification using: Lower Marker Final concentration (ng/uL): 0.5000 Dilution factor: 12.0

Sample: SampH11
Well location: H11
Created: Tuesday, September 14, 2021 5:34:08 PM

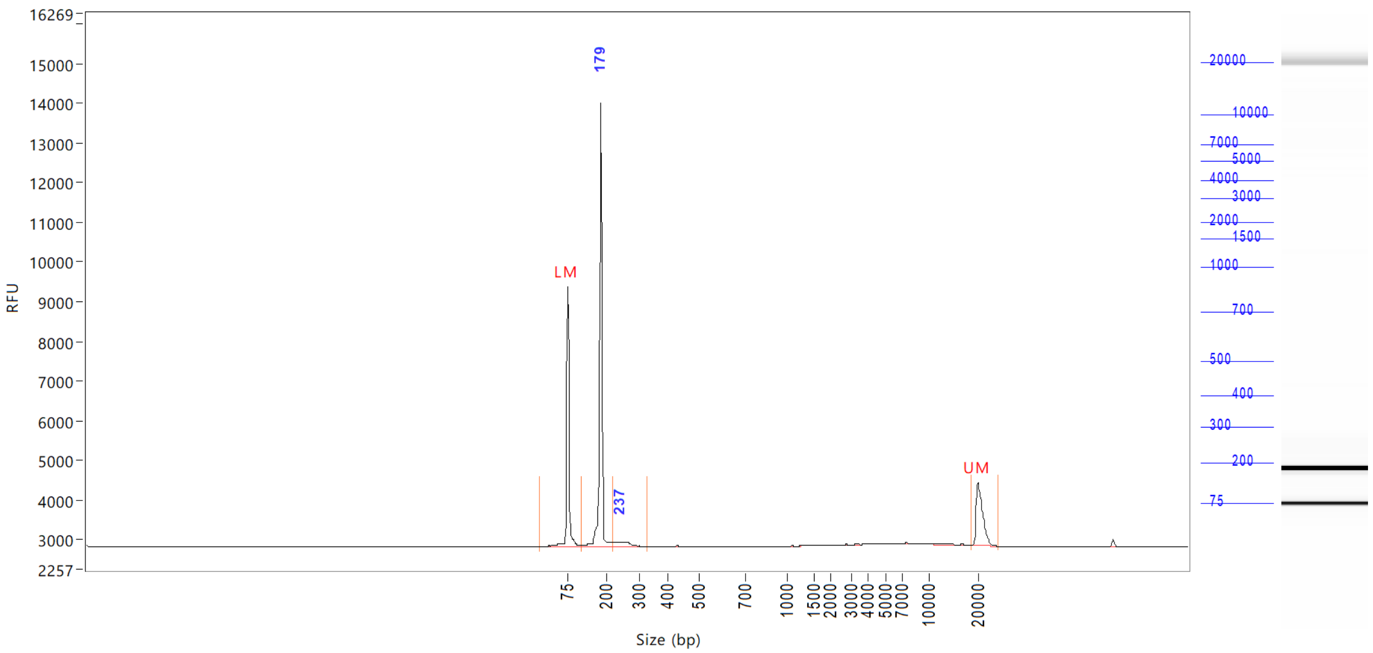

Peak	Size (bp)	Concentration (ng/uL)	From (bp)	To (bp)	Average size (bp)	CV%	RFU	Corrected peak area
1	75 (LM)	0.5000	0	120	72	14.15	6562	40.212
2	179	9.7372	120	217	177	4.94	11200	65.259
3	237	0.5974	217	329	247	7.85	122	4.004
4	20000 (UM)	0.1702	18659	24110	20405	3.57	1593	13.688

TIC:	10.3346	ng/uL
TIM:	94.4154	nmole/L
Total concentration:	10.8610	ng/uL

```
Sample peak width (sec): 5    Sample min peak height: 50    Sample baseline V to V?: Y    Sample baseline V to V points: 3
Sample filter: Binomial       Number of points for filter: 3  Sample start region (min): 0  Sample end region (min): 30
Marker peak width (sec): 5    Marker min peak height: 200   Marker baseline V to V?: Y    Marker baseline V to V points: 3
Lower marker selection: First peak > 500 RFU                Upper marker selection: Last peak > 200 RFU
Ladder size (bp)75, 200, 300, 400, 500, 700, 1000, 1500, 2000, 3000, 4000, 5000, 7000, 10000, 20000
Quantification using: Lower Marker          Final concentration (ng/uL): 0.5000          Dilution factor: 12.0
```

Sample: SampA12
Well location: A12
Created: Tuesday, September 14, 2021 5:34:08 PM

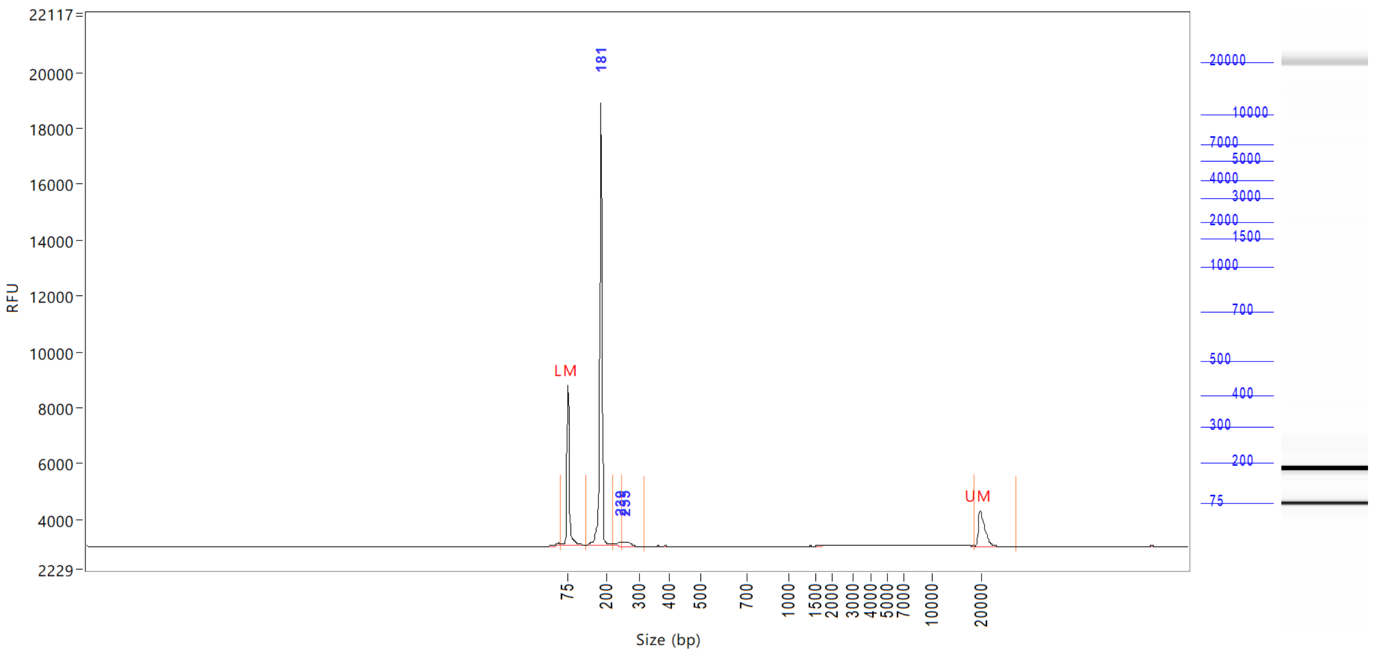

Peak	Size<br>(bp)	Concentration<br>(ng/uL)	From<br>(bp)	To<br>(bp)	Average size<br>(bp)	CV%	RFU	Corrected peak area
1	75 (LM)	0.5000	52	131	75	10.03	5737	34.545
2	181	15.6206	131	217	178	3.76	15861	89.936
3	239	0.3221	217	244	232	3.50	123	1.854
4	255	0.4502	244	315	260	4.41	137	2.592
5	20000 (UM)	0.1551	18903	27375	20375	4.07	1265	10.713

TIC:	16.3928	ng/uL
TIM:	149.3984	nmole/L
Total concentration:	16.9793	ng/uL

Sample peak width (sec): 5   Sample min peak height: 50   Sample baseline V to V?: Y   Sample baseline V to V points: 3
Sample filter: Binomial   Number of points for filter: 3 Sample start region (min): 0 Sample end region (min): 30
Marker peak width (sec): 5   Marker min peak height: 200   Marker baseline V to V?: Y   Marker baseline V to V points: 3
Lower marker selection: First peak > 500 RFU   Upper marker selection: Last peak > 200 RFU
Ladder size (bp)75, 200, 300, 400, 500, 700, 1000, 1500, 2000, 3000, 4000, 5000, 7000, 10000, 20000
Quantification using: Lower Marker   Final concentration (ng/uL): 0.5000   Dilution factor: 12.0

Sample: SampB12
Well location: B12
Created: Tuesday, September 14, 2021 5:34:08 PM

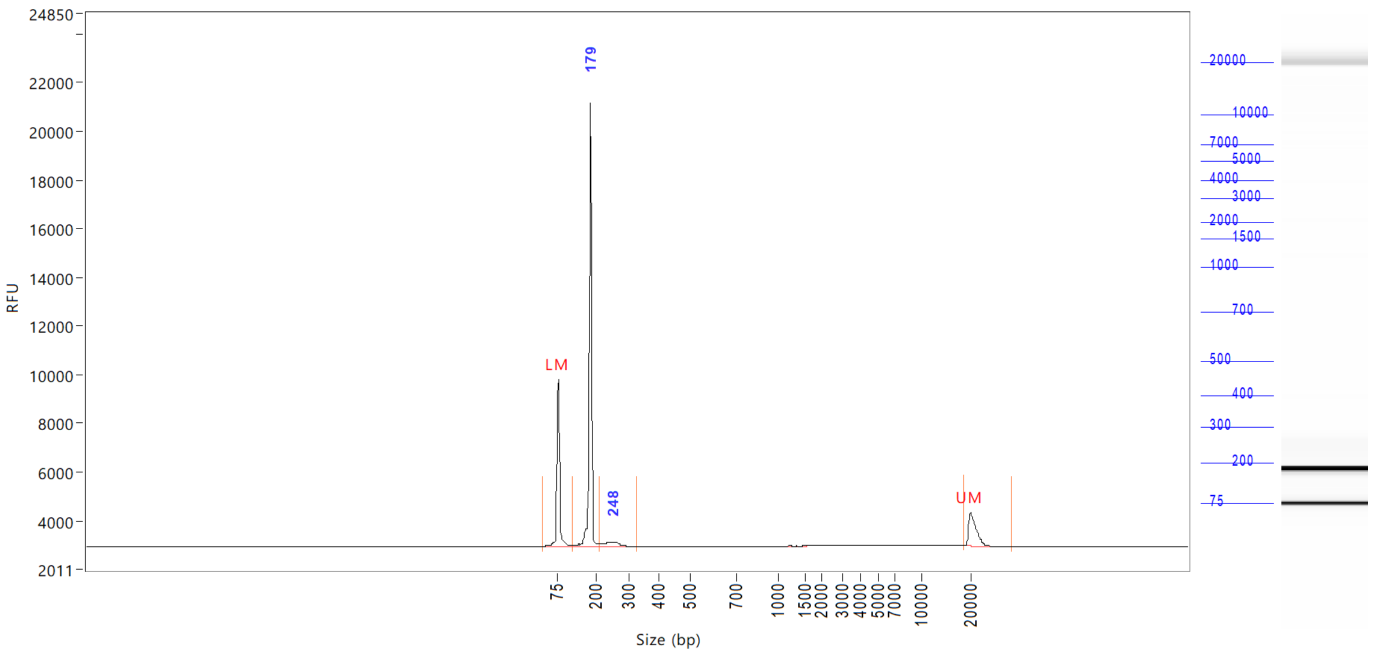

Peak	Size (bp)	Concentration (ng/uL)	From (bp)	To (bp)	Average size (bp)	CV%	RFU	Corrected peak area
1	75 (LM)	0.5000	26	122	74	13.40	6906	44.617
2	179	14.5099	122	208	175	4.37	18264	107.899
3	248	0.9420	208	325	242	8.12	191	7.005
4	20000 (UM)	0.1524	18781	28584	20583	4.91	1365	13.600

TIC:	15.4518	ng/uL
TIM:	142.7045	nmole/L
Total concentration:	15.9194	ng/uL

```
Sample peak width (sec): 5    Sample min peak height: 50    Sample baseline V to V?: Y    Sample baseline V to V points: 3
Sample filter: Binomial       Number of points for filter: 3  Sample start region (min): 0  Sample end region (min): 30
Marker peak width (sec): 5    Marker min peak height: 200   Marker baseline V to V?: Y    Marker baseline V to V points: 3
Lower marker selection: First peak > 500 RFU                Upper marker selection: Last peak > 200 RFU
Ladder size (bp)75, 200, 300, 400, 500, 700, 1000, 1500, 2000, 3000, 4000, 5000, 7000, 10000, 20000
Quantification using: Lower Marker          Final concentration (ng/uL): 0.5000          Dilution factor: 12.0
```

Sample: SampC12
Well location: C12
Created: Tuesday, September 14, 2021 5:34:08 PM

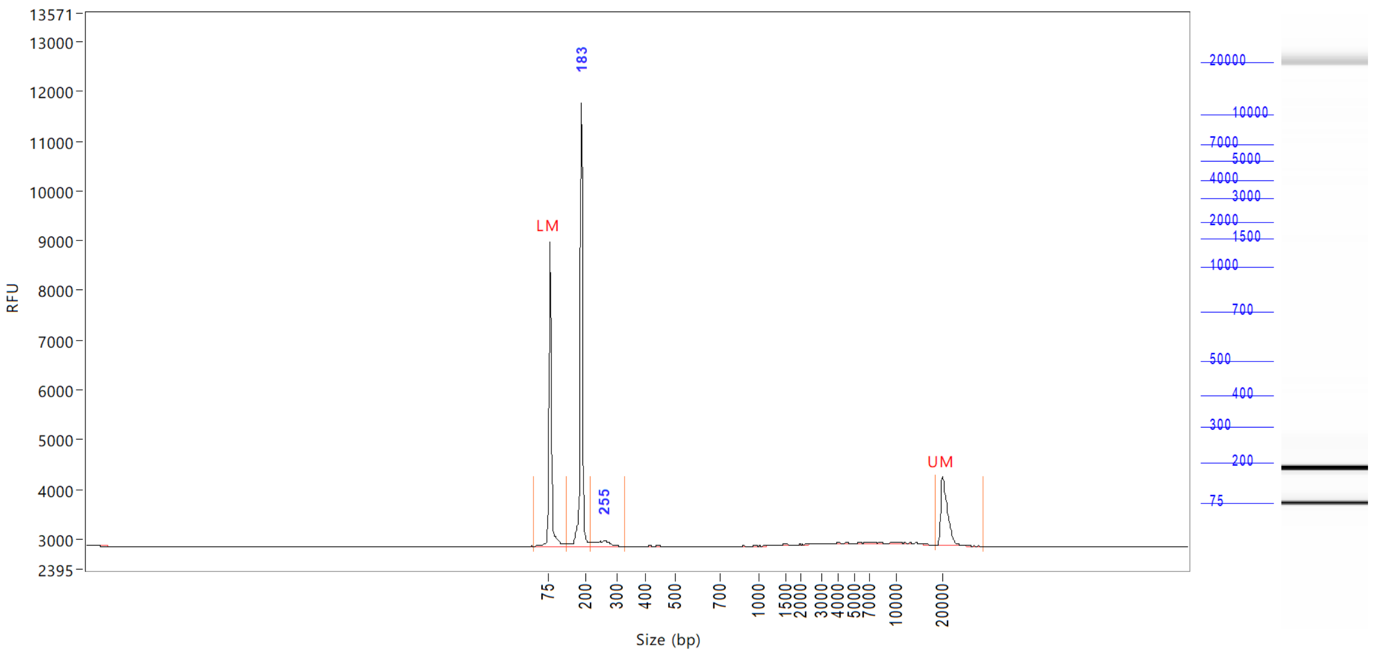

Peak	Size (bp)	Concentration (ng/uL)	From (bp)	To (bp)	Average size (bp)	CV%	RFU	Corrected peak area
1	75 (LM)	0.5000	22	131	75	13.46	6134	40.905
2	183	8.1661	131	212	179	4.23	8936	55.672
3	255	0.5942	212	323	247	8.54	111	4.051
4	20000 (UM)	0.1555	18537	28947	20554	4.66	1390	12.723

TIC:	8.7602	ng/uL
TIM:	78.9550	nmole/L
Total concentration:	9.1628	ng/uL

Sample peak width (sec): 5 Sample min peak height: 50 Sample baseline V to V?: Y Sample baseline V to V points: 3
Sample filter: Binomial Number of points for filter: 3 Sample start region (min): 0 Sample end region (min): 30
Marker peak width (sec): 5 Marker min peak height: 200 Marker baseline V to V?: Y Marker baseline V to V points: 3
Lower marker selection: First peak > 500 RFU Upper marker selection: Last peak > 200 RFU
Ladder size (bp)75, 200, 300, 400, 500, 700, 1000, 1500, 2000, 3000, 4000, 5000, 7000, 10000, 20000
Quantification using: Lower Marker Final concentration (ng/uL): 0.5000 Dilution factor: 12.0

Sample: SampD12
Well location: D12
Created: Tuesday, September 14, 2021 5:34:08 PM

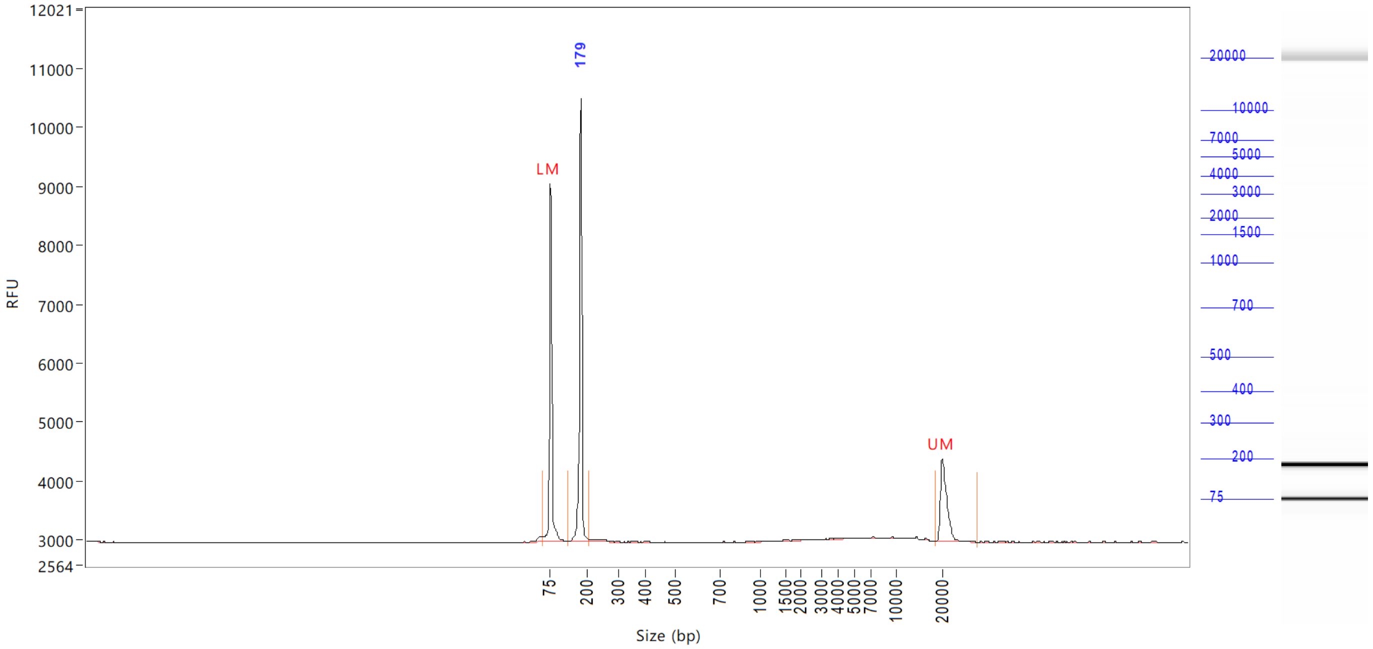

Peak	Size (bp)	Concentration (ng/uL)	From (bp)	To (bp)	Average size (bp)	CV%	RFU	Corrected peak area
1	75 (LM)	0.5000	50	139	75	9.11	6073	37.619
2	179	7.0218	139	206	175	3.33	7539	44.026
3	20000 (UM)	0.1639	18415	27617	20395	4.31	1397	12.333

TIC:	7.0218	ng/uL
TIM:	65.9618	nmole/L
Total concentration:	7.7697	ng/uL

Sample peak width (sec): 5 Sample min peak height: 50 Sample baseline V to V?: Y Sample baseline V to V points: 3
Sample filter: Binomial Number of points for filter: 3 Sample start region (min): 0 Sample end region (min): 30
Marker peak width (sec): 5 Marker min peak height: 200 Marker baseline V to V?: Y Marker baseline V to V points: 3
Lower marker selection: First peak > 500 RFU Upper marker selection: Last peak > 200 RFU
Ladder size (bp)75, 200, 300, 400, 500, 700, 1000, 1500, 2000, 3000, 4000, 5000, 7000, 10000, 20000
Quantification using: Lower Marker Final concentration (ng/uL): 0.5000 Dilution factor: 12.0

Sample: SampE12
Well location: E12
Created: Tuesday, September 14, 2021 5:34:08 PM

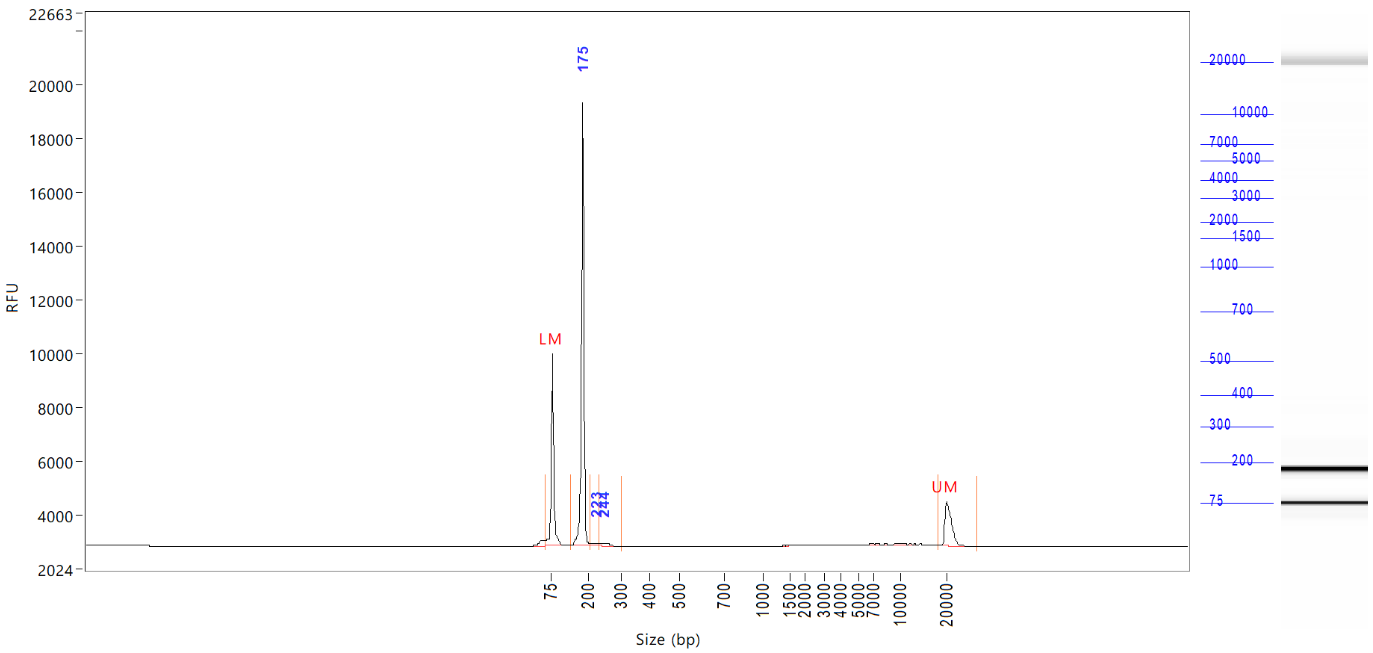

Peak	Size (bp)	Concentration (ng/uL)	From (bp)	To (bp)	Average size (bp)	CV%	RFU	Corrected peak area
1	75 (LM)	0.5000	50	137	73	9.72	7124	45.635
2	175	12.9469	137	203	174	3.36	16462	98.472
3	223	0.1763	203	228	216	3.54	89	1.341
4	244	0.2859	228	300	247	5.13	103	2.174
5	20000 (UM)	0.1563	18171	26408	20368	3.73	1657	14.263

TIC:	13.4091	ng/uL
TIM:	125.5648	nmole/L
Total concentration:	13.8292	ng/uL

Sample peak width (sec): 5   Sample min peak height: 50   Sample baseline V to V?: Y   Sample baseline V to V points: 3
Sample filter: Binomial   Number of points for filter: 3 Sample start region (min): 0 Sample end region (min): 30
Marker peak width (sec): 5   Marker min peak height: 200   Marker baseline V to V?: Y   Marker baseline V to V points: 3
Lower marker selection: First peak > 500 RFU   Upper marker selection: Last peak > 200 RFU
Ladder size (bp)75, 200, 300, 400, 500, 700, 1000, 1500, 2000, 3000, 4000, 5000, 7000, 10000, 20000
Quantification using: Lower Marker   Final concentration (ng/uL): 0.5000   Dilution factor: 12.0

Sample: SampF12
Well location: F12
Created: Tuesday, September 14, 2021 5:34:08 PM

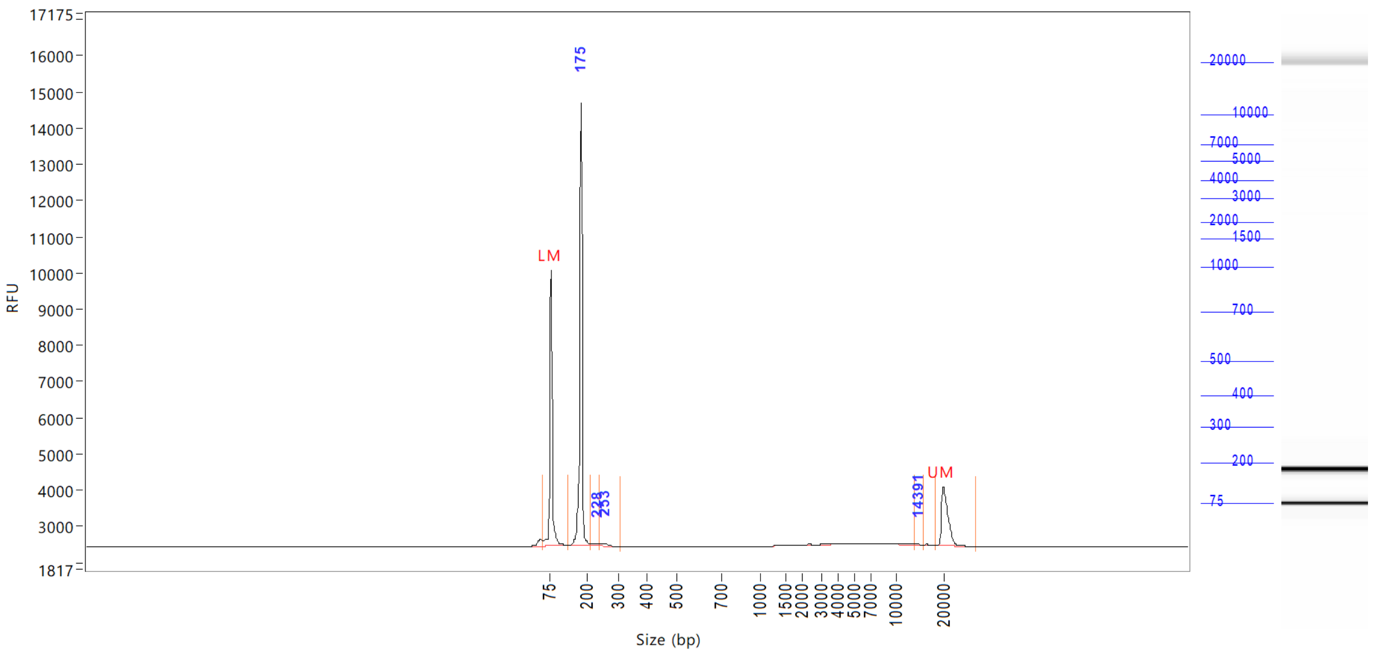

Peak	Size (bp)	Concentration (ng/uL)	From (bp)	To (bp)	Average size (bp)	CV%	RFU	Corrected peak area
1	75 (LM)	0.5000	50	135	74	9.61	7605	47.368
2	175	9.3386	135	208	174	3.49	12250	73.724
3	228	0.1430	208	235	222	3.70	68	1.129
4	253	0.1846	235	305	251	4.66	84	1.457
5	14391	0.0317	13903	15732	14475	3.00	51	0.250
6	20000 (UM)	0.1522	18293	27133	20493	3.98	1642	14.421

TIC:	9.6979	ng/uL
TIM:	90.5012	nmole/L
Total concentration:	10.0957	ng/uL

```
Sample peak width (sec): 5    Sample min peak height: 50    Sample baseline V to V?: Y    Sample baseline V to V points: 3
Sample filter: Binomial       Number of points for filter: 3  Sample start region (min): 0  Sample end region (min): 30
Marker peak width (sec): 5    Marker min peak height: 200   Marker baseline V to V?: Y    Marker baseline V to V points: 3
Lower marker selection: First peak > 500 RFU                Upper marker selection: Last peak > 200 RFU
Ladder size (bp)75, 200, 300, 400, 500, 700, 1000, 1500, 2000, 3000, 4000, 5000, 7000, 10000, 20000
Quantification using: Lower Marker          Final concentration (ng/uL): 0.5000          Dilution factor: 12.0
```

Sample: SampG12
Well location: G12
Created: Tuesday, September 14, 2021 5:34:08 PM

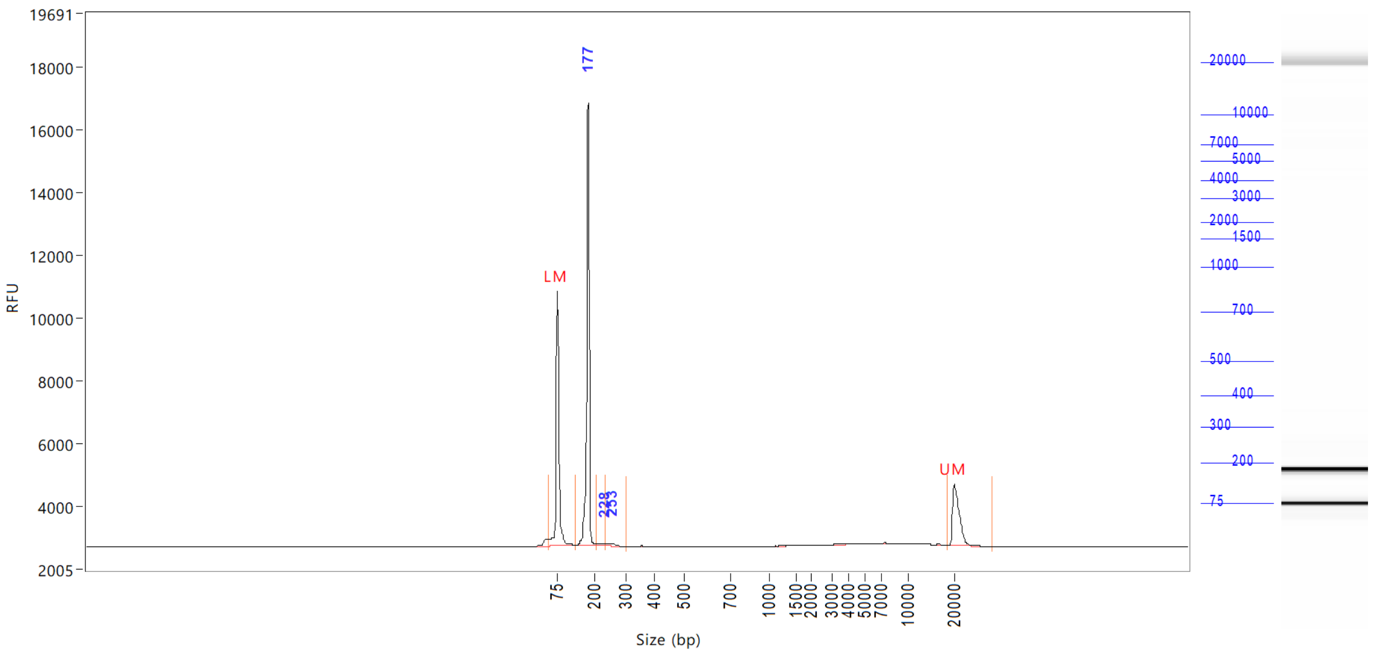

Peak	Size (bp)	Concentration (ng/uL)	From (bp)	To (bp)	Average size (bp)	CV%	RFU	Corrected peak area
1	75 (LM)	0.5000	48	133	73	10.73	8079	53.608
2	177	9.4792	133	203	173	3.38	14102	84.693
3	228	0.1241	203	233	219	4.16	62	1.109
4	253	0.1756	233	298	250	4.55	93	1.569
5	20000 (UM)	0.1652	18415	27979	20427	4.29	1963	17.716

TIC:	9.7789	ng/uL
TIM:	92.1607	nmole/L
Total concentration:	10.1711	ng/uL

Sample peak width (sec): 5 Sample min peak height: 50 Sample baseline V to V?: Y Sample baseline V to V points: 3
Sample filter: Binomial Number of points for filter: 3 Sample start region (min): 0 Sample end region (min): 30
Marker peak width (sec): 5 Marker min peak height: 200 Marker baseline V to V?: Y Marker baseline V to V points: 3
Lower marker selection: First peak > 500 RFU Upper marker selection: Last peak > 200 RFU
Ladder size (bp)75, 200, 300, 400, 500, 700, 1000, 1500, 2000, 3000, 4000, 5000, 7000, 10000, 20000
Quantification using: Lower Marker Final concentration (ng/uL): 0.5000 Dilution factor: 12.0

Sample: SampH12
Well location: H12
Created: Tuesday, September 14, 2021 5:34:08 PM

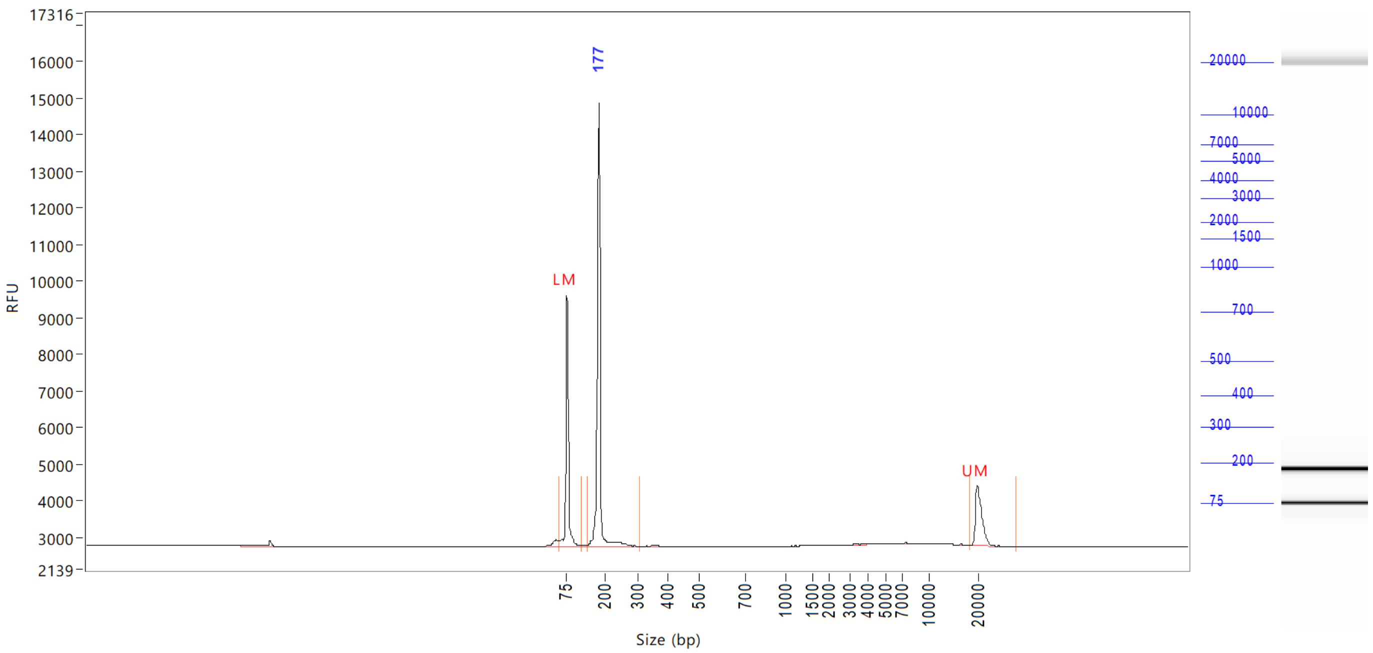

Peak	Size (bp)	Concentration (ng/uL)	From (bp)	To (bp)	Average size (bp)	CV%	RFU	Corrected peak area
1	75 (LM)	0.5000	50	122	75	10.13	6832	43.126
2	177	10.4567	141	303	178	8.50	12128	75.160
3	20000 (UM)	0.1621	18293	27979	20382	3.92	1658	13.979

TIC:	10.4567	ng/uL
TIM:	96.5756	nmole/L
Total concentration:	10.9829	ng/uL

Sample peak width (sec): 5   Sample min peak height: 200   Sample baseline V to V?: Y   Sample baseline V to V points: 3
Sample filter: Binomial   Number of points for filter: 3   Sample start region (min): 0   Sample end region (min): 30
Marker peak width (sec): 5   Marker min peak height: 200   Marker baseline V to V?: Y   Marker baseline V to V points: 3
Lower marker selection: First peak > 500 RFU   Upper marker selection: Last peak > 200 RFU
Ladder size (bp)75, 200, 300, 400, 500, 700, 1000, 1500, 2000, 3000, 4000, 5000, 7000, 10000, 20000
Quantification using: Lower Marker   Final concentration (ng/uL): 0.5000   Dilution factor: 12.0

Sample: DNA size ladder
Well location: A7
Created: Tuesday, September 14, 2021 5:34:08 PM
Import from: C:\Users\admin\Desktop\210810_alberto_ladder.SCAL
Fit type: Point to point

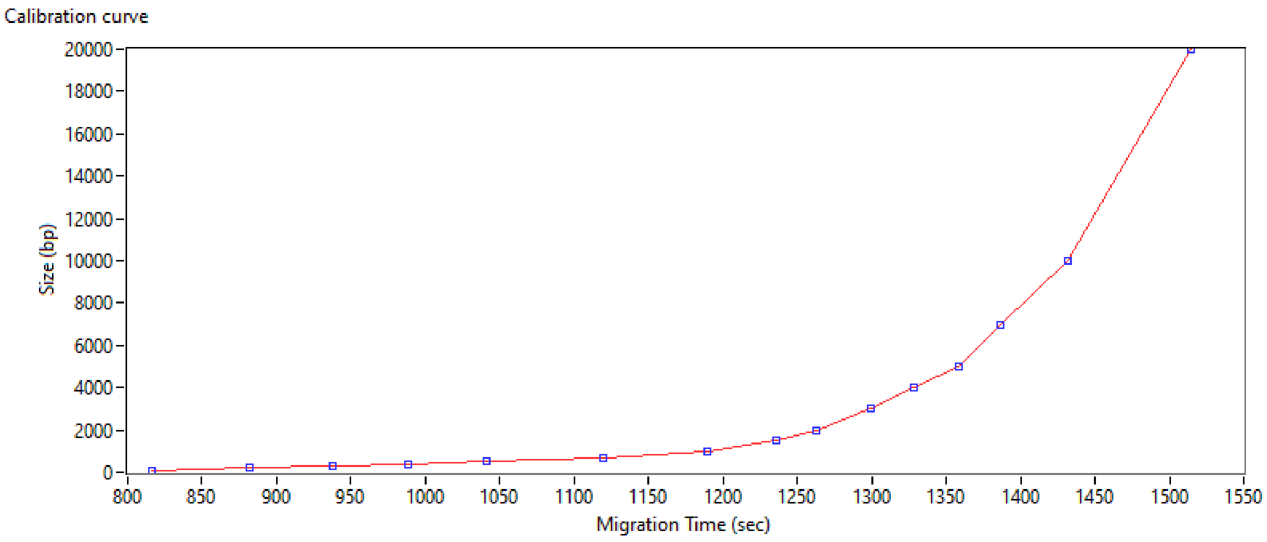

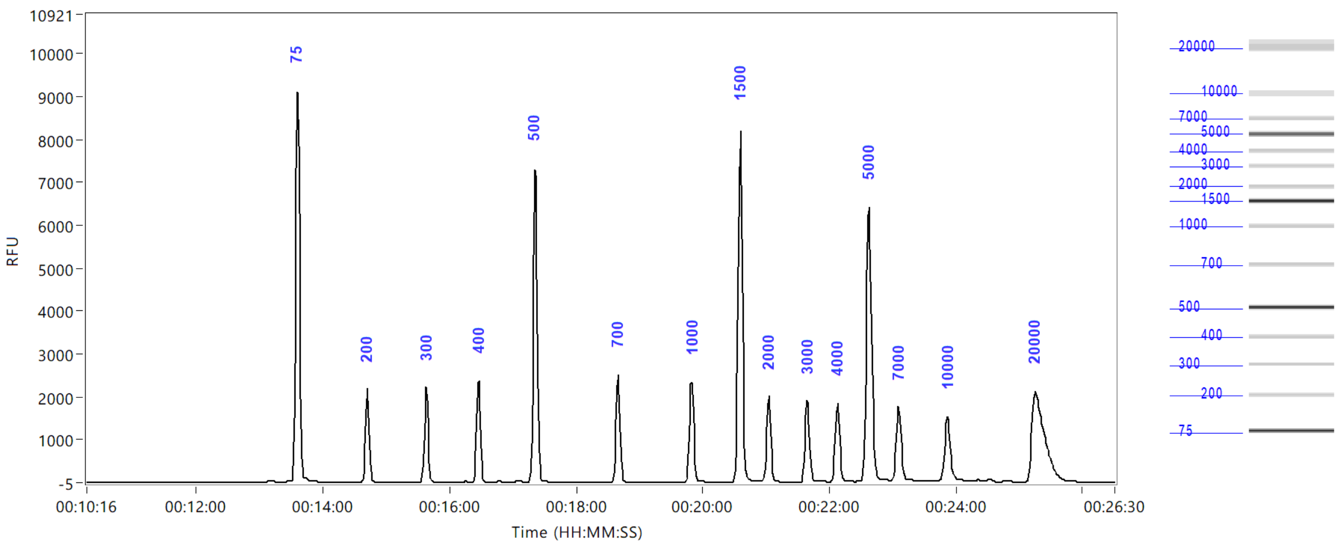

Supplement: Supplementary file 2 — sb3c00292_si_002.zip [file sb3c00292_si_002.zip › dnada_supplementary_material_pks_library_build/ZAG_trials/ZAG_Trial_5/17-24-14_plate2/plate2-t1.pdf]
