# Supplementary material for: Automated Platform for the Plasmid Construction Process
Source: ACS Synth Biol. 2023 Nov 10;12(12):3506–13. doi: 10.1021/acssynbio.3c00292 (PMC10729297; doi:10.1021/acssynbio.3c00292)

Instrument controller software run summary:

Filename and data path: C:\AATI\Data\2021 09 14\19-24-11\2021 09 14 19H 24M..raw
Created: Tuesday, September 14, 2021 7:33:54 PM
Number of capillaries: 96
Array serial number: 111920-09SFS
Effect length: 33 cm
Array usage count: 217
Instrument type: ZAG DNA Analyzer
Instrument controller software version: 1.0.1.9
Device serial number: Z7597

Method Information

Method name: ZAG130GP33 - DNA 75-20000bp - Gel Prime Only.mthds
Gel prime: Yes
Full conditioning: No
Gel prime to buffer: No
Gel selection: Gel 1
Perform prerun: 8.0 kV, 30 sec.
Rinse: No
Marker 1: Row: A, 5.0 kV, 5 sec.
Rinse: No
Sample injection: 5.0 kV, 5 sec.
Separation: 8.0 kV, 30.0 min.
Tray name: Sample Tray-5

Analysis mode: DNA

Notes

Gel Image

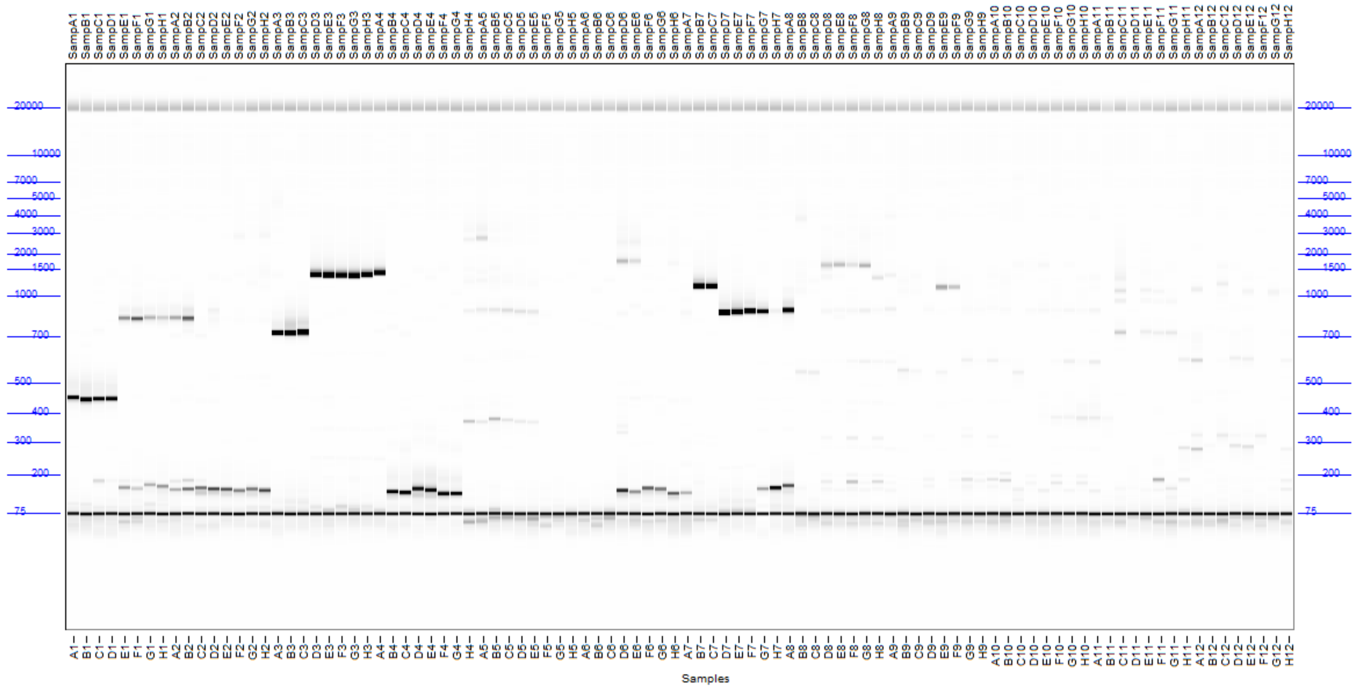
20000
10000
7000
5000
4000
3000
2000
1500
1000
700
500
400
300
200
75
Samples

Filename and data path: C:\AATI\Data\2021 09 14\19-24-11\2021 09 14 19H 24M..raw

A1: SampA1

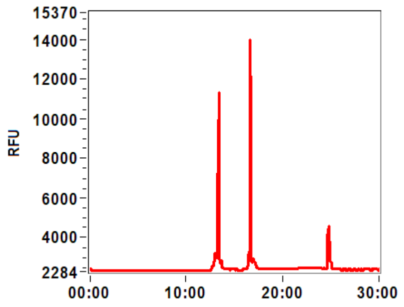

B1: SampB1

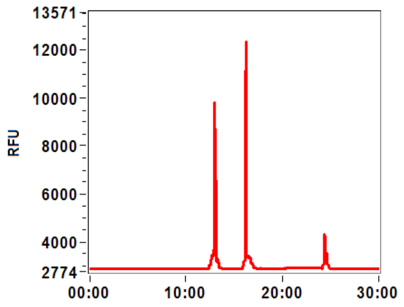

C1: SampC1

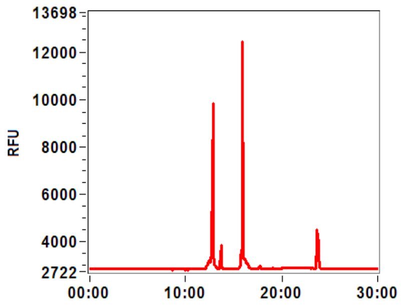

D1: SampD1

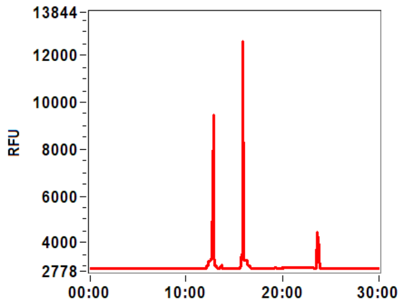

E1: SampE1

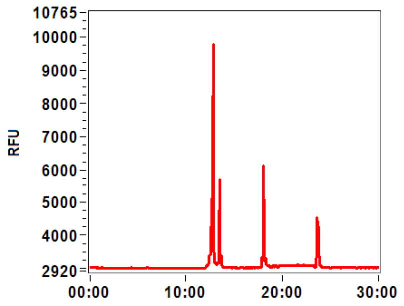

F1: SampF1

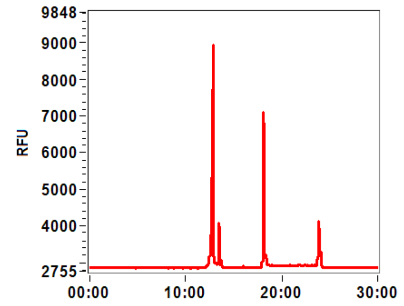

G1: SampG1

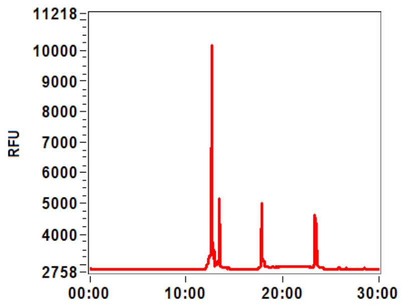

H1: SampH1

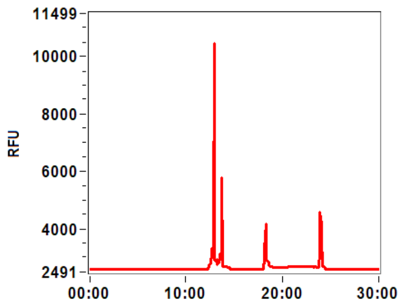

A2: SampA2

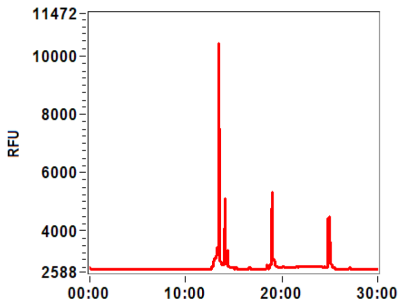

B2: SampB2

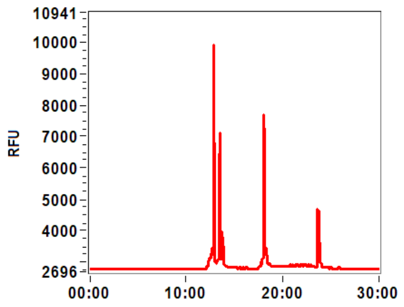

C2: SampC2

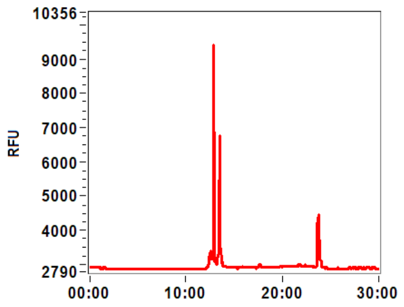

D2: SampD2

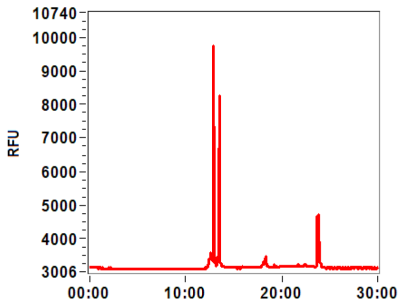

Filename and data path: C:\AATI\Data\2021 09 14\19-24-11\2021 09 14 19H 24M..raw

E2: SampE2

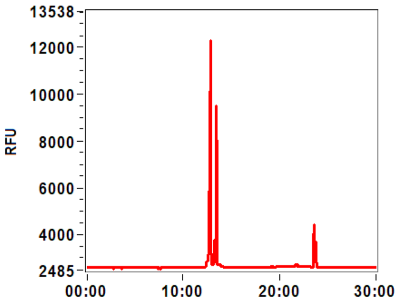

F2: SampF2

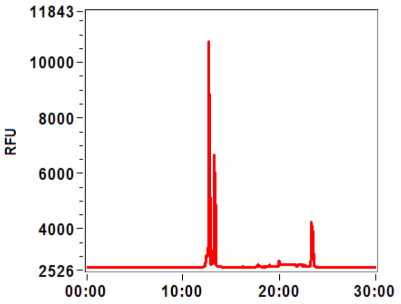

G2: SampG2

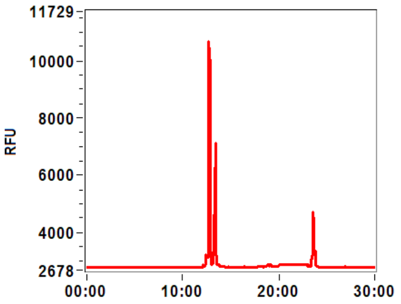

H2: SampH2

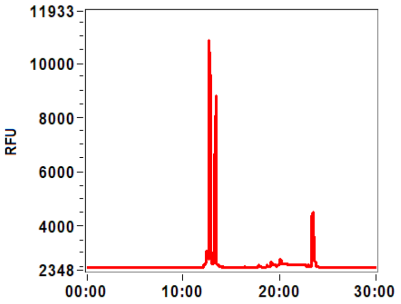

A3: SampA3

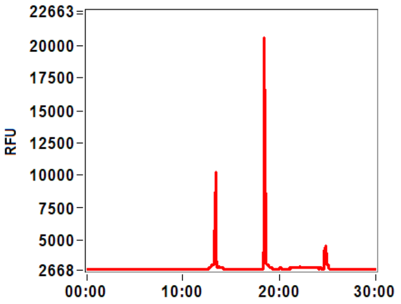

B3: SampB3

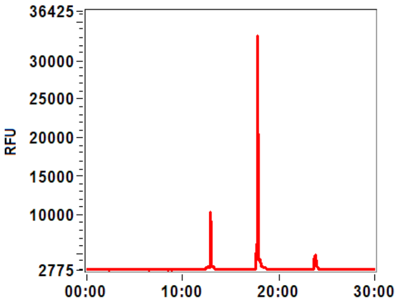

C3: SampC3

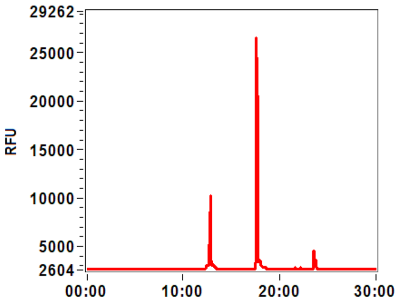

D3: SampD3

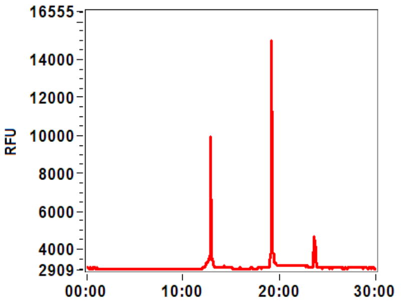

E3: SampE3

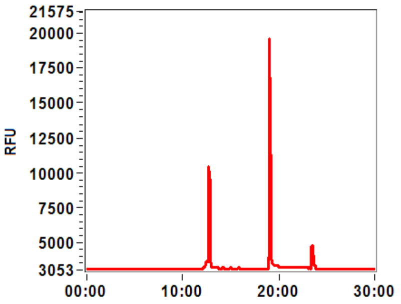

F3: SampF3

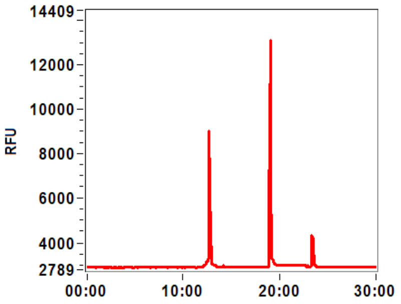

G3: SampG3

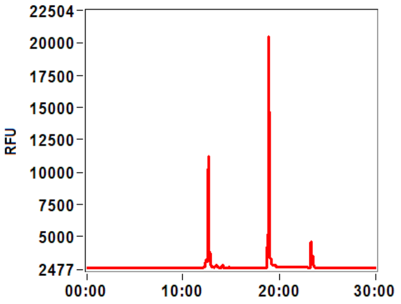

H3: SampH3

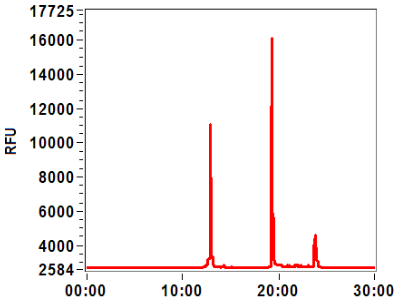

Filename and data path: C:\AATI\Data\2021 09 14\19-24-11\2021 09 14 19H 24M..raw

A4: SampA4

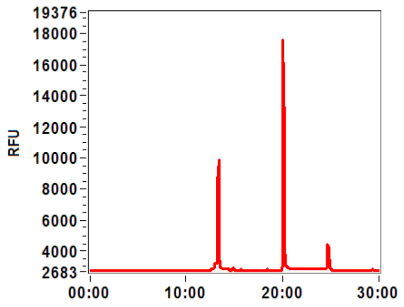

B4: SampB4

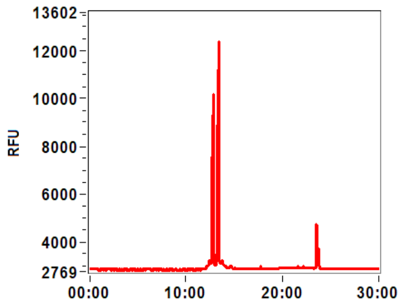

C4: SampC4

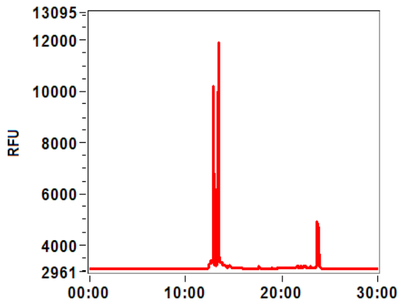

D4: SampD4

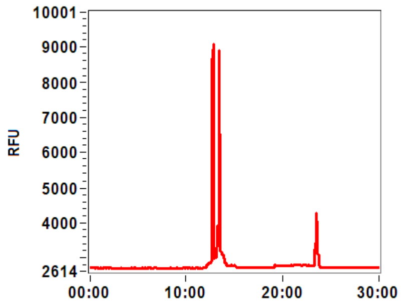

E4: SampE4

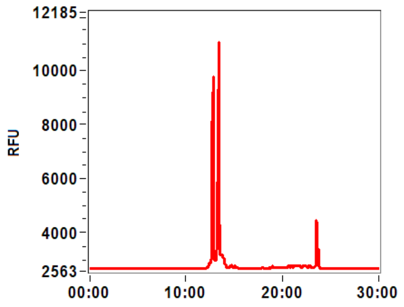

F4: SampF4

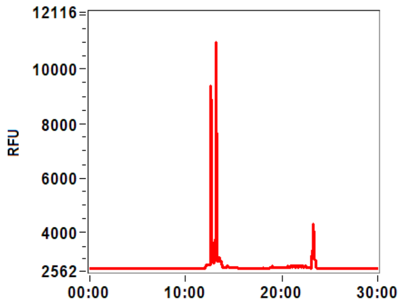

G4: SampG4

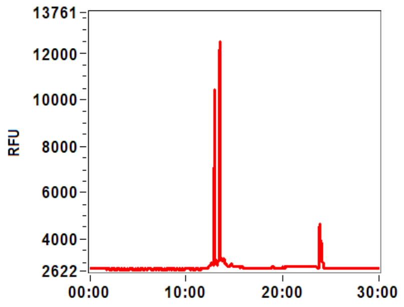

H4: SampH4

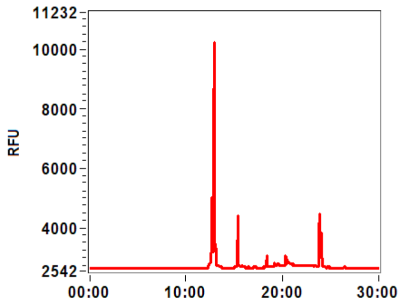

A5: SampA5

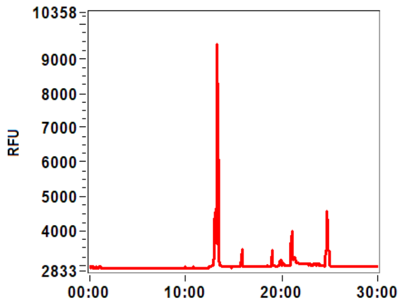

B5: SampB5

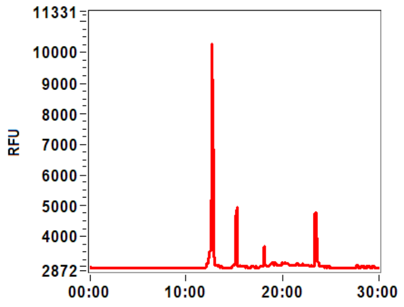

C5: SampC5

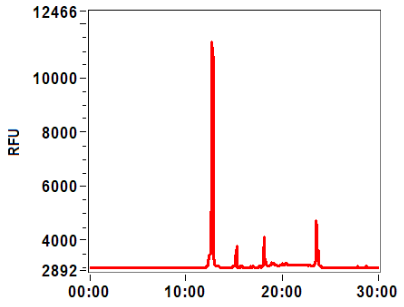

D5: SampD5

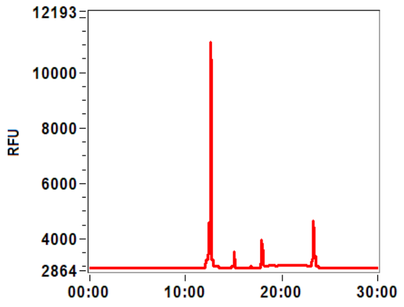

Filename and data path: C:\AATI\Data\2021 09 14\19-24-11\2021 09 14 19H 24M..raw

E5: SampE5

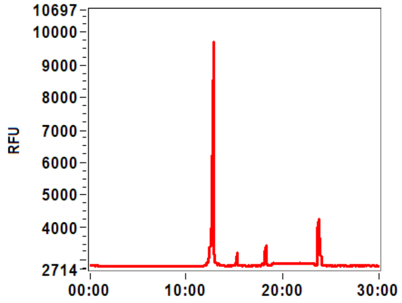

F5: SampF5

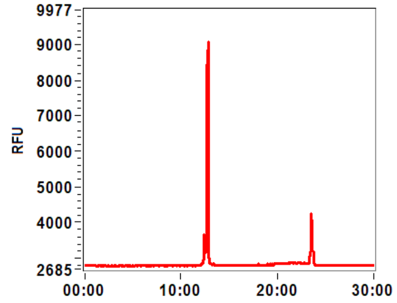

G5: SampG5

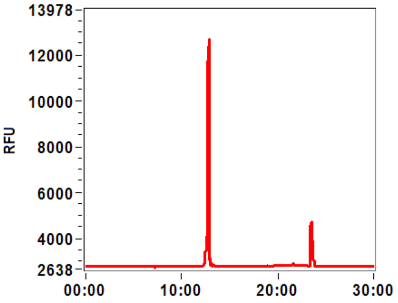

H5: SampH5

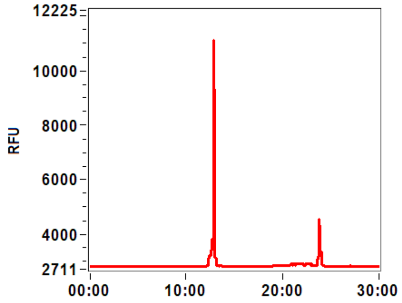

A6: SampA6

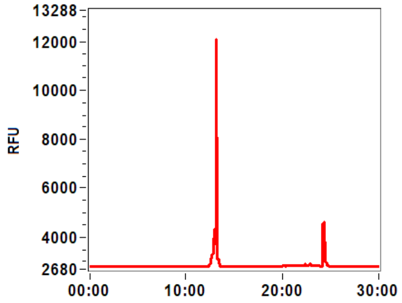

B6: SampB6

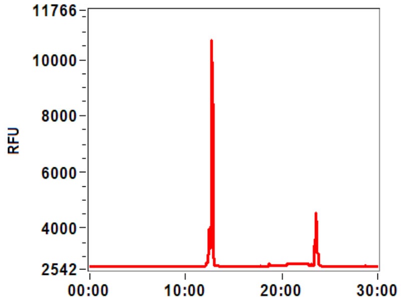

C6: SampC6

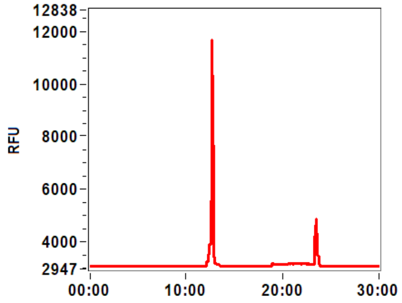

D6: SampD6

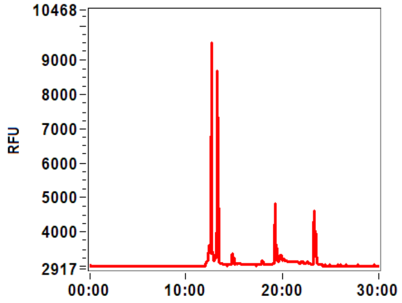

E6: SampE6

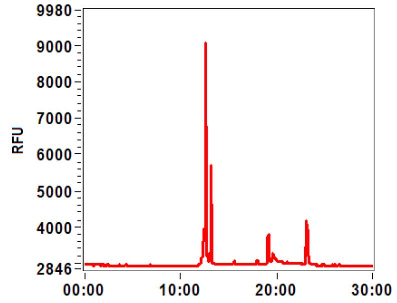

F6: SampF6

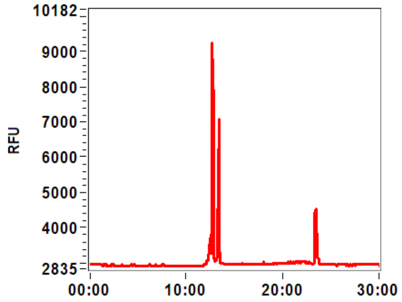

G6: SampG6

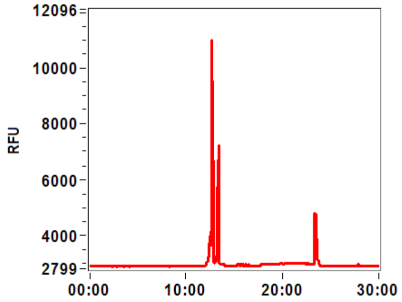

H6: SampH6

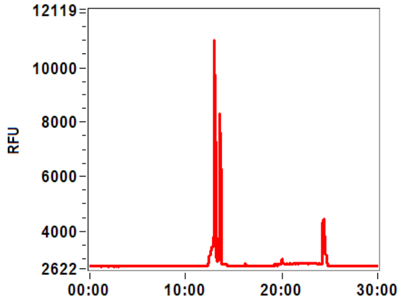

Filename and data path: C:\AATI\Data\2021 09 14\19-24-11\2021 09 14 19H 24M..raw

A7: SampA7

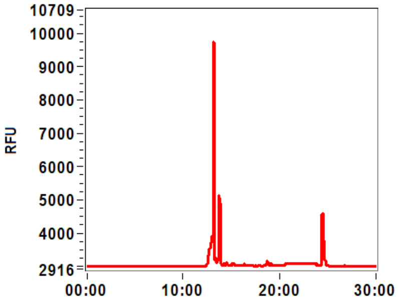

B7: SampB7

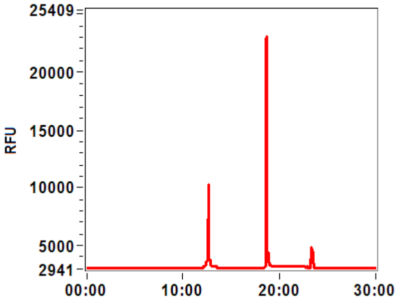

C7: SampC7

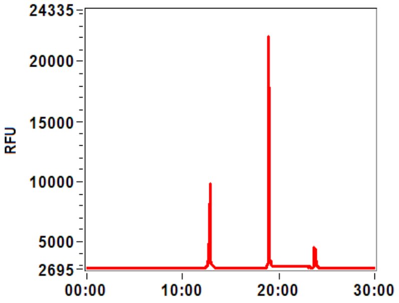

D7: SampD7

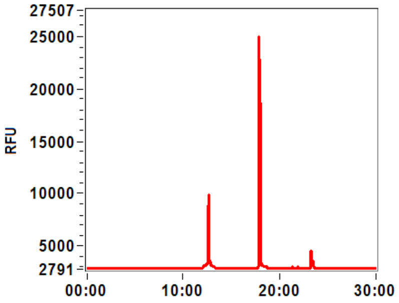

E7: SampE7

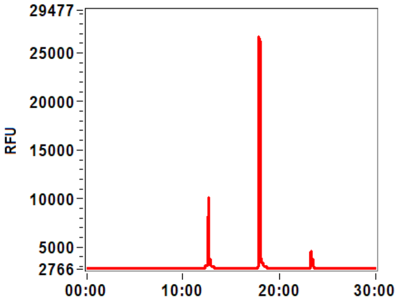

F7: SampF7

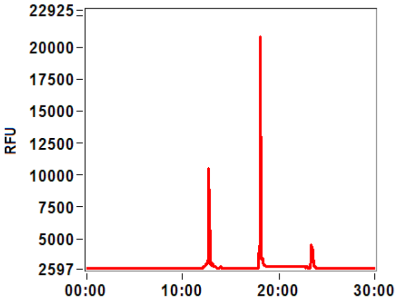

G7: SampG7

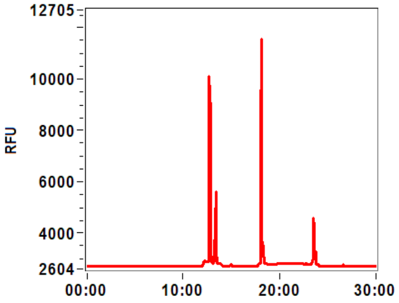

H7: SampH7

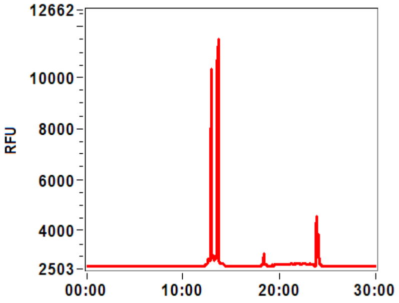

A8: SampA8

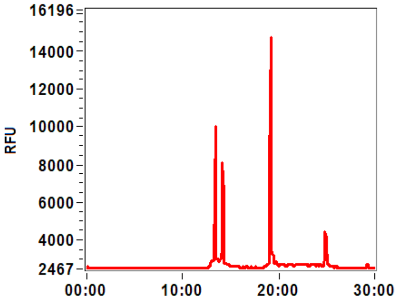

B8: SampB8

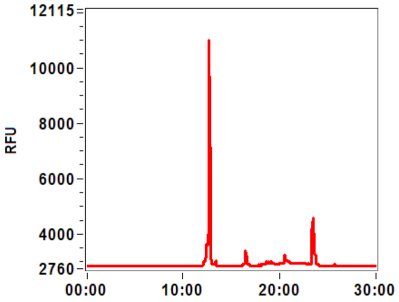

C8: SampC8

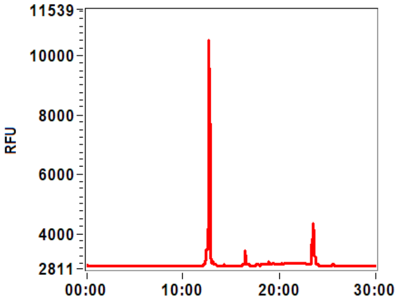

D8: SampD8

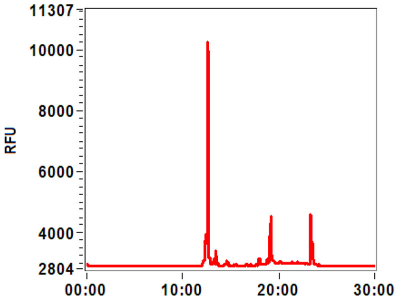

Filename and data path: C:\AATI\Data\2021 09 14\19-24-11\2021 09 14 19H 24M..raw

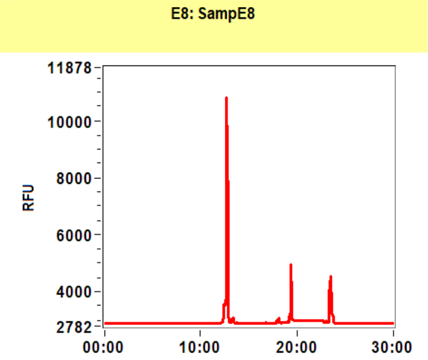

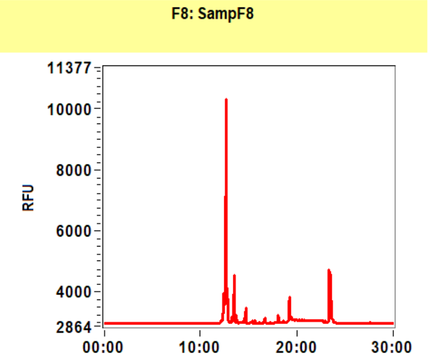

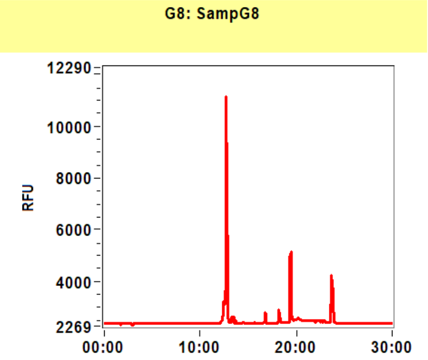

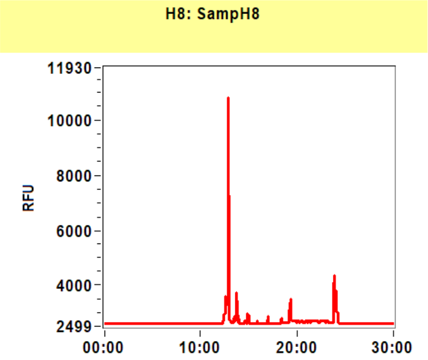

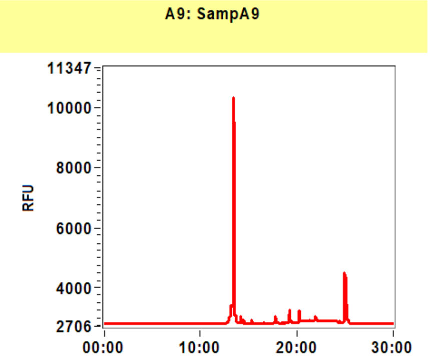

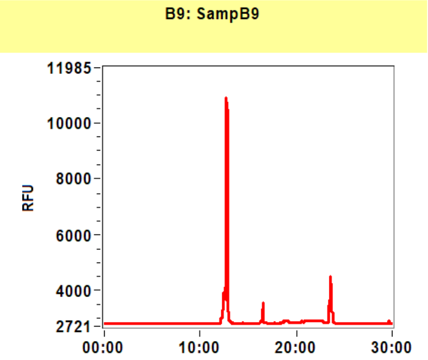

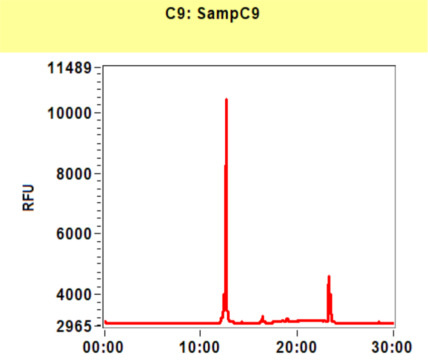

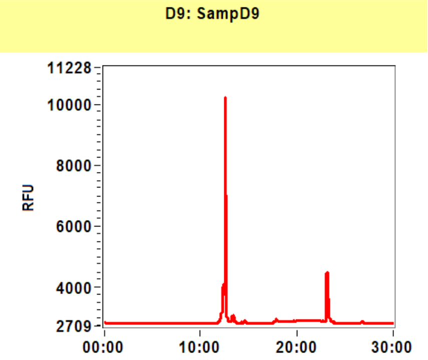

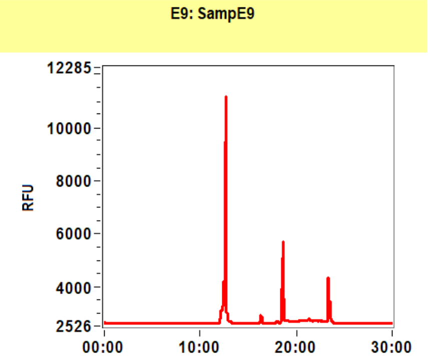

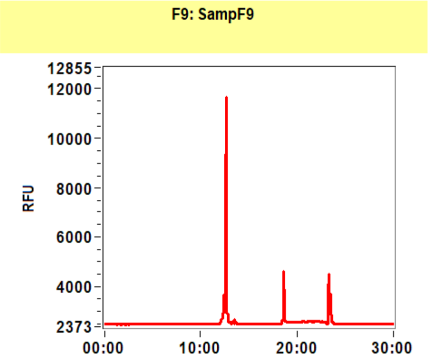

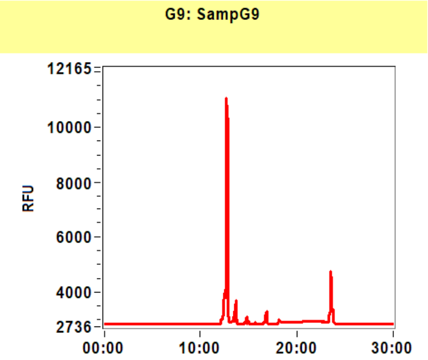

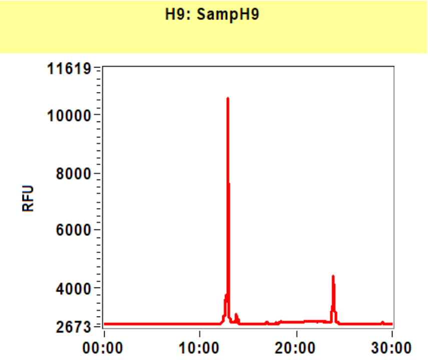

Filename and data path: C:\AATI\Data\2021 09 14\19-24-11\2021 09 14 19H 24M..raw

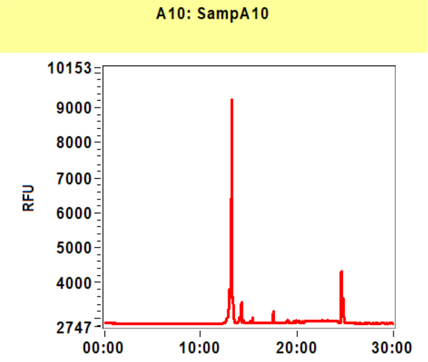

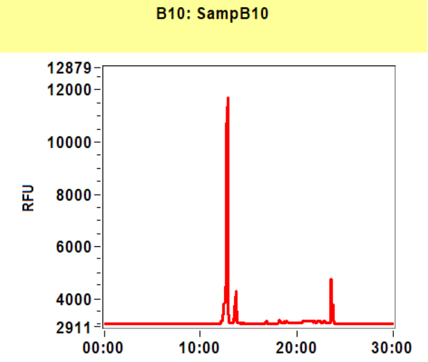

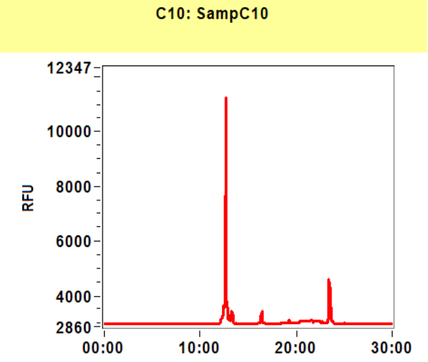

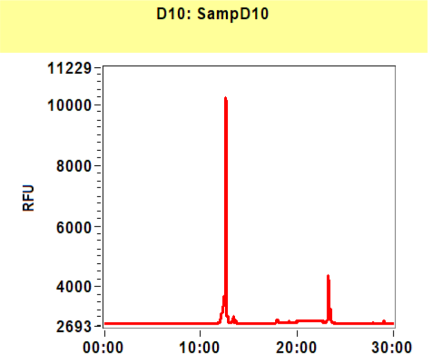

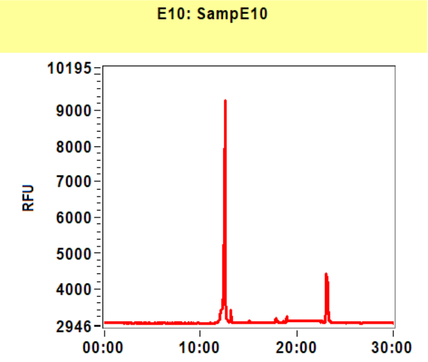

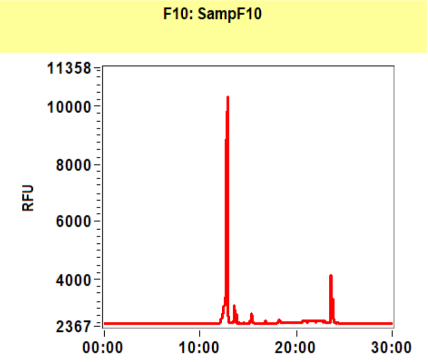

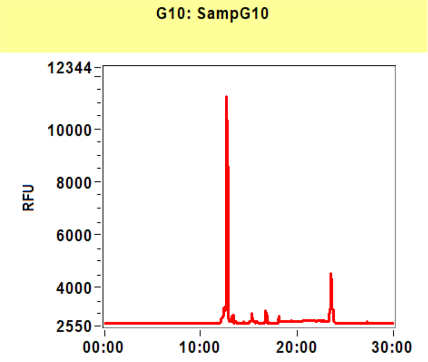

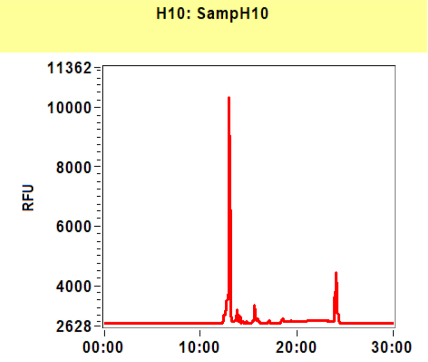

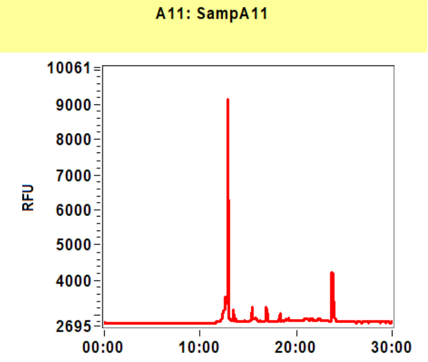

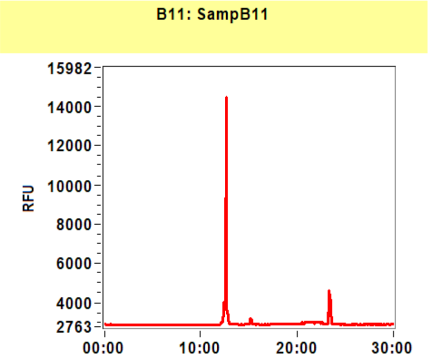

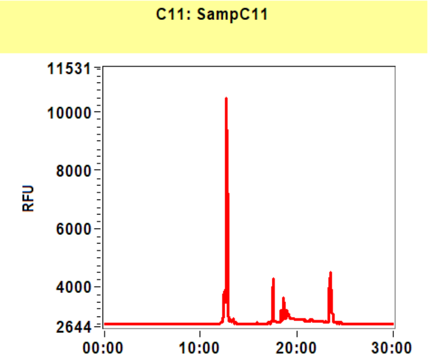

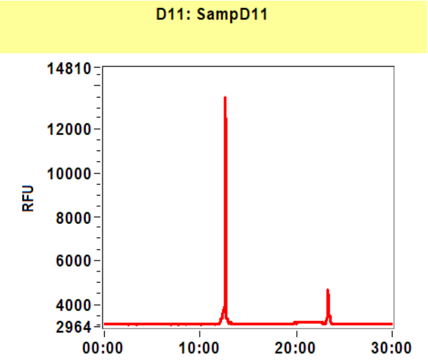

Filename and data path: C:\AATI\Data\2021 09 14\19-24-11\2021 09 14 19H 24M..raw

E11: SampE11

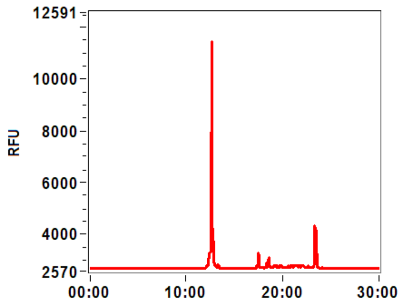

F11: SampF11

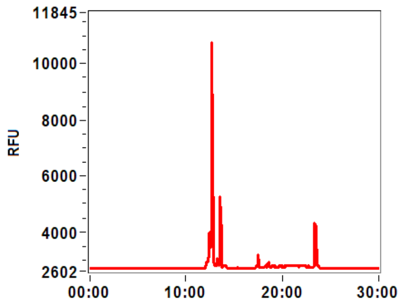

G11: SampG11

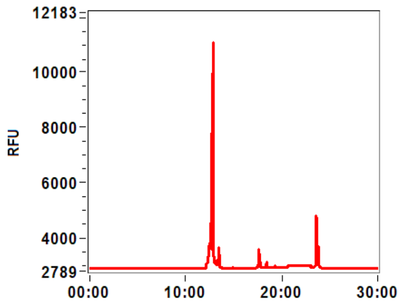

H11: SampH11

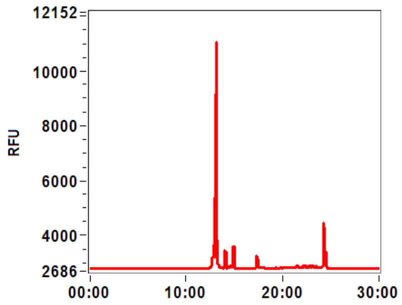

A12: SampA12

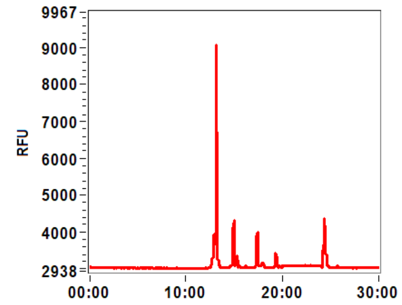

B12: SampB12

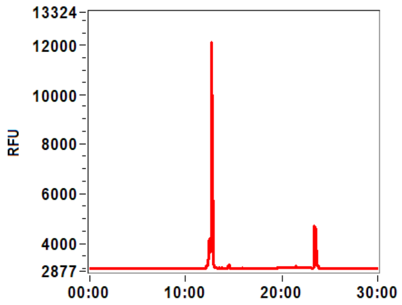

C12: SampC12

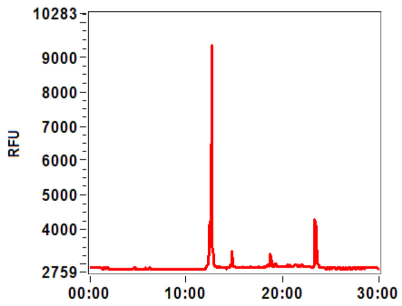

D12: SampD12

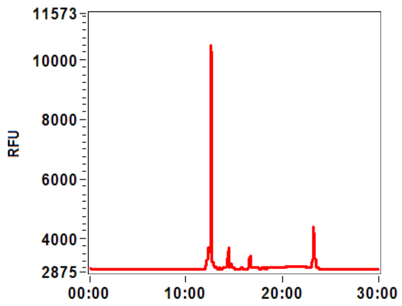

E12: SampE12

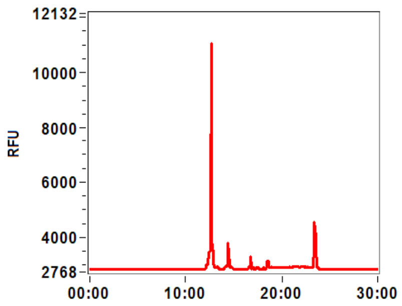

F12: SampF12

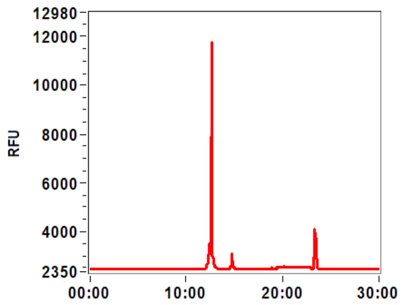

G12: SampG12

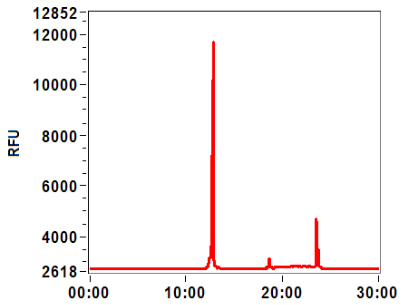

H12: SampH12

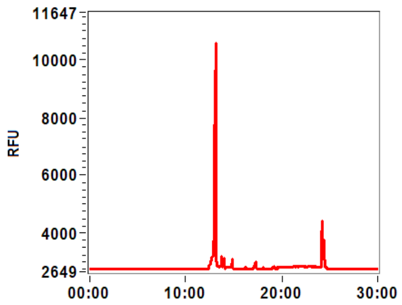

Sample: SampA1
Well location: A1
Created: Tuesday, September 14, 2021 7:33:54 PM

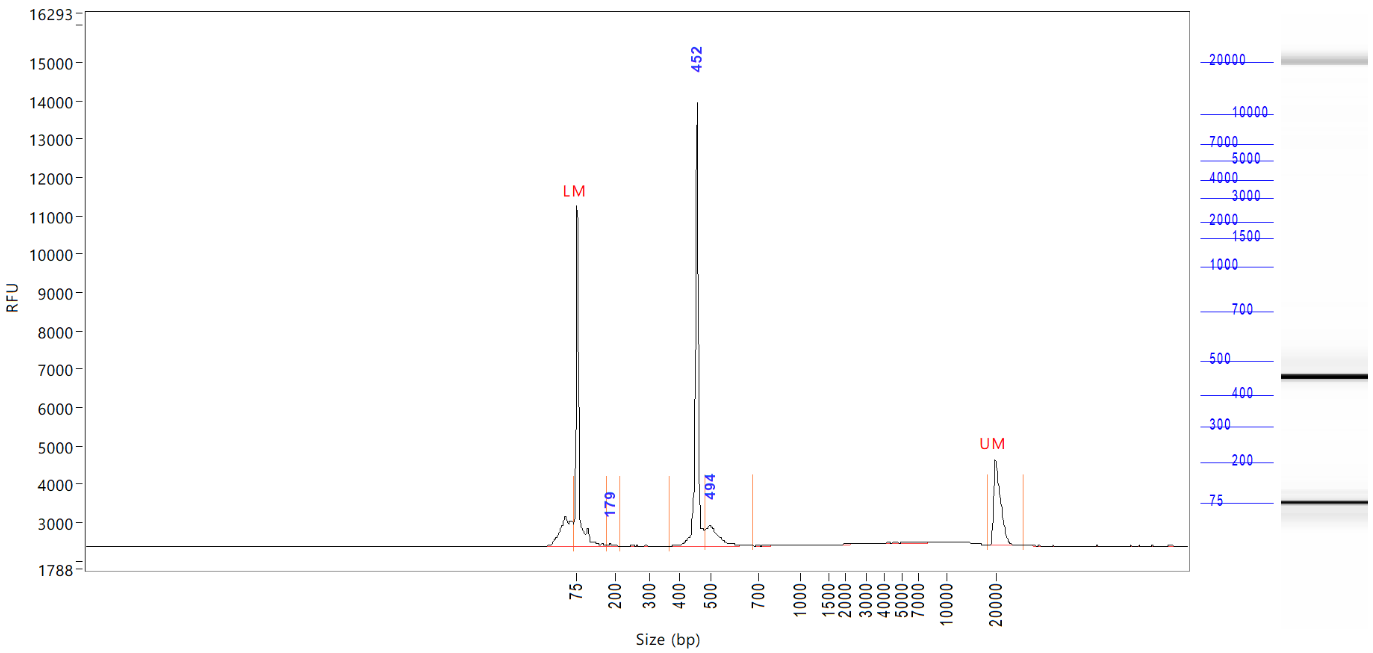

Peak	Size (bp)	Concentration (ng/uL)	From (bp)	To (bp)	Average size (bp)	CV%	RFU	Corrected peak area
1	75 (LM)	0.5000	66	171	79	18.77	8901	60.909
2	179	0.0560	171	214	183	4.88	52	0.568
3	452	6.7785	366	479	450	2.18	11584	68.812
4	494	1.2377	479	674	513	6.21	520	12.565
5	20000 (UM)	0.1591	18415	25561	20467	3.81	2202	19.380

TIC:	8.0722	ng/uL
TIM:	29.2585	nmole/L
Total concentration:	8.3266	ng/uL

Sample peak width (sec): 5 Sample min peak height: 50 Sample baseline V to V?: Y Sample baseline V to V points: 3
Sample filter: Binomial Number of points for filter: 3 Sample start region (min): 0 Sample end region (min): 30
Marker peak width (sec): 5 Marker min peak height: 200 Marker baseline V to V?: Y Marker baseline V to V points: 3
Lower marker selection: First peak > 500 RFU Upper marker selection: Last peak > 200 RFU
Ladder size (bp)75, 200, 300, 400, 500, 700, 1000, 1500, 2000, 3000, 4000, 5000, 7000, 10000, 20000
Quantification using: Lower Marker Final concentration (ng/uL): 0.5000 Dilution factor: 12.0

Sample: SampB1
Well location: B1
Created: Tuesday, September 14, 2021 7:33:54 PM

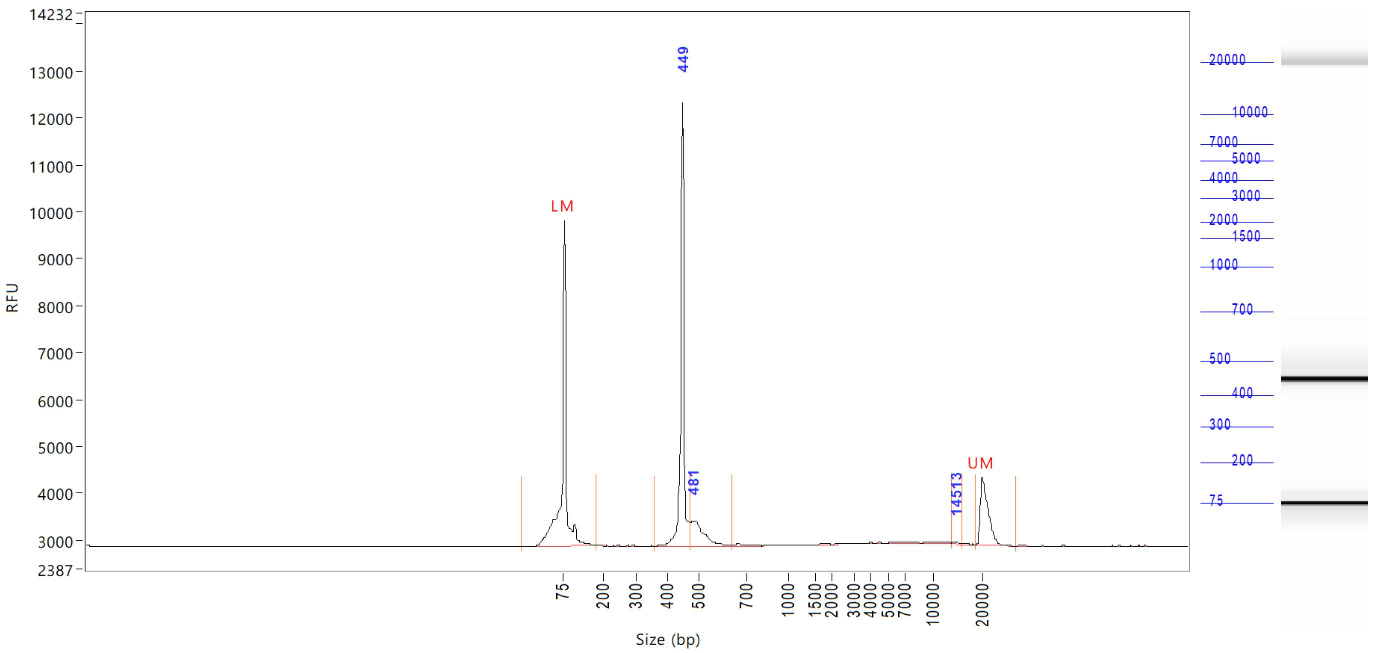

Peak	Size (bp)	Concentration (ng/uL)	From (bp)	To (bp)	Average size (bp)	CV%	RFU	Corrected peak area
1	75 (LM)	0.5000	0	175	66	36.04	6922	71.469
2	449	5.1989	358	473	444	2.33	9459	61.926
3	481	1.1045	473	641	507	6.19	533	13.156
4	14513	0.0364	13781	15854	14660	3.91	53	0.434
5	20000 (UM)	0.1043	18537	26649	20514	4.56	1447	14.911

TIC:	6.3398	ng/uL
TIM:	22.8551	nmole/L
Total concentration:	6.6483	ng/uL

```
Sample peak width (sec): 5    Sample min peak height: 50    Sample baseline V to V?: Y    Sample baseline V to V points: 3
Sample filter: Binomial       Number of points for filter: 3  Sample start region (min): 0  Sample end region (min): 30
Marker peak width (sec): 5    Marker min peak height: 200   Marker baseline V to V?: Y    Marker baseline V to V points: 3
Lower marker selection: First peak > 500 RFU                Upper marker selection: Last peak > 200 RFU
Ladder size (bp)75, 200, 300, 400, 500, 700, 1000, 1500, 2000, 3000, 4000, 5000, 7000, 10000, 20000
Quantification using: Lower Marker          Final concentration (ng/uL): 0.5000          Dilution factor: 12.0
```

Sample: SampC1
Well location: C1
Created: Tuesday, September 14, 2021 7:33:54 PM

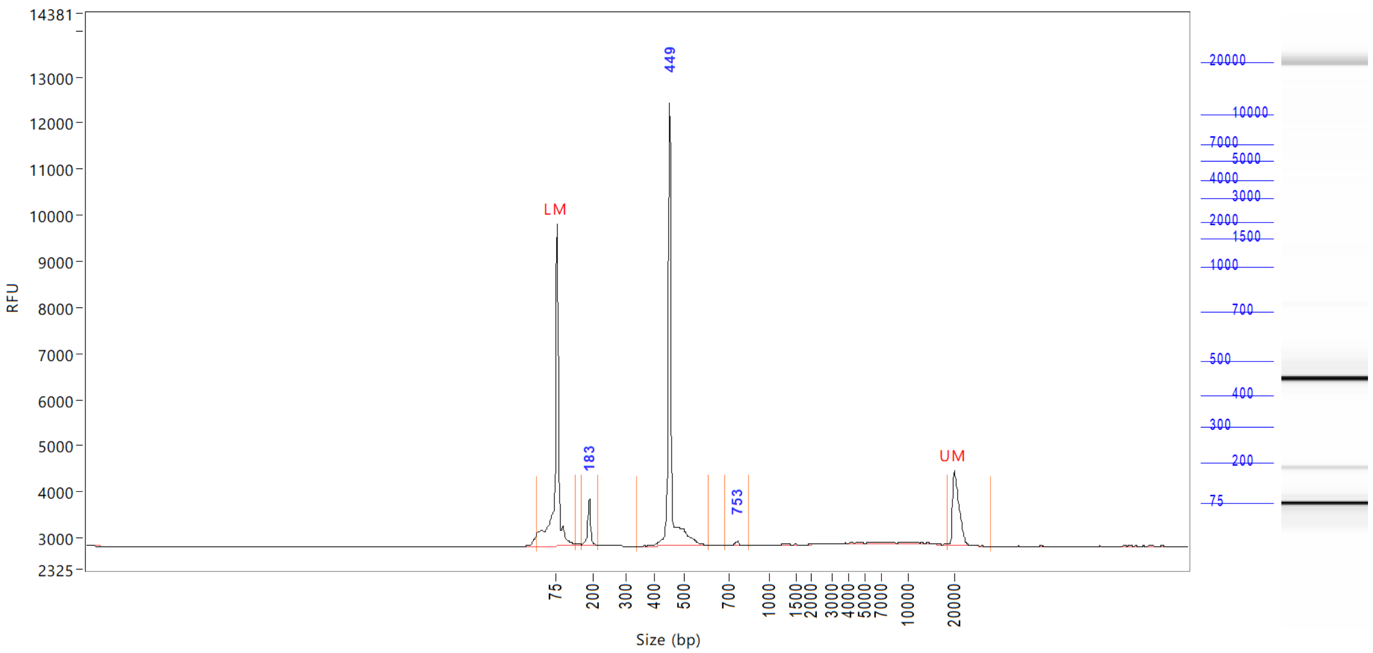

Peak	Size (bp)	Concentration (ng/uL)	From (bp)	To (bp)	Average size (bp)	CV%	RFU	Corrected peak area
1	75 (LM)	0.5000	11	139	67	29.14	6973	62.407
2	183	0.7174	156	212	179	3.93	1017	7.462
3	449	6.2254	339	608	454	5.10	9621	64.751
4	753	0.0739	682	848	748	2.96	100	0.768
5	20000 (UM)	0.1130	18415	27617	20364	3.85	1610	14.108

TIC:	7.0167	ng/uL
TIM:	29.3139	nmole/L
Total concentration:	7.3540	ng/uL

```
Sample peak width (sec): 5    Sample min peak height: 50    Sample baseline V to V?: Y    Sample baseline V to V points: 3
Sample filter: Binomial       Number of points for filter: 3  Sample start region (min): 0  Sample end region (min): 30
Marker peak width (sec): 5    Marker min peak height: 200   Marker baseline V to V?: Y    Marker baseline V to V points: 3
Lower marker selection: First peak > 500 RFU                Upper marker selection: Last peak > 200 RFU
Ladder size (bp)75, 200, 300, 400, 500, 700, 1000, 1500, 2000, 3000, 4000, 5000, 7000, 10000, 20000
Quantification using: Lower Marker          Final concentration (ng/uL): 0.5000          Dilution factor: 12.0
```

Sample: SampD1
Well location: D1
Created: Tuesday, September 14, 2021 7:33:54 PM

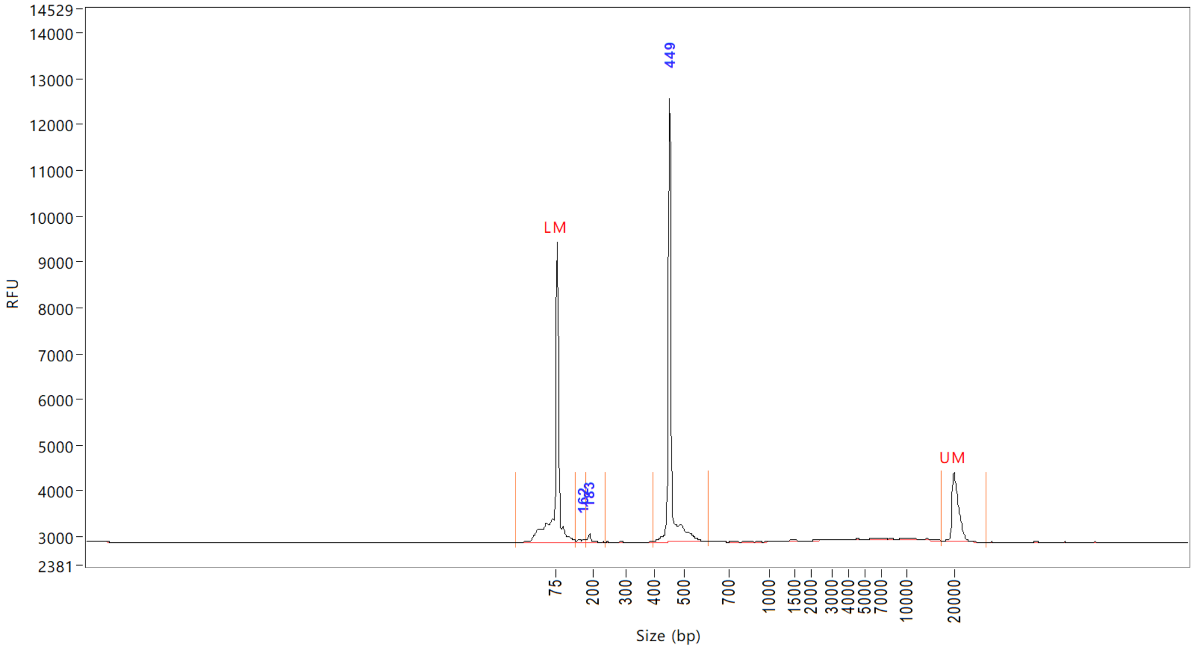

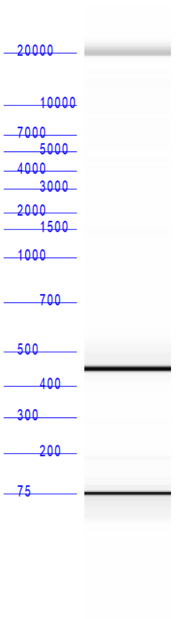

Peak	Size (bp)	Concentration (ng/uL)	From (bp)	To (bp)	Average size (bp)	CV%	RFU	Corrected peak area
1	75 (LM)	0.5000	0	139	64	40.00	6576	59.824
2	162	0.0962	139	171	156	6.24	60	0.959
3	183	0.1544	171	233	186	7.27	178	1.540
4	449	6.2393	394	603	454	4.80	9697	62.211
5	20000 (UM)	0.1107	17195	26891	20365	3.91	1504	13.247

TIC:	6.4899	ng/uL
TIM:	24.9910	nmole/L
Total concentration:	6.8639	ng/uL

Sample peak width (sec): 5   Sample min peak height: 50   Sample baseline V to V?: Y   Sample baseline V to V points: 3
Sample filter: Binomial   Number of points for filter: 3 Sample start region (min): 0 Sample end region (min): 30
Marker peak width (sec): 5   Marker min peak height: 200   Marker baseline V to V?: Y   Marker baseline V to V points: 3
Lower marker selection: First peak > 500 RFU   Upper marker selection: Last peak > 200 RFU
Ladder size (bp)75, 200, 300, 400, 500, 700, 1000, 1500, 2000, 3000, 4000, 5000, 7000, 10000, 20000
Quantification using: Lower Marker   Final concentration (ng/uL): 0.5000   Dilution factor: 12.0

Sample: SampE1
Well location: E1
Created: Tuesday, September 14, 2021 7:33:54 PM

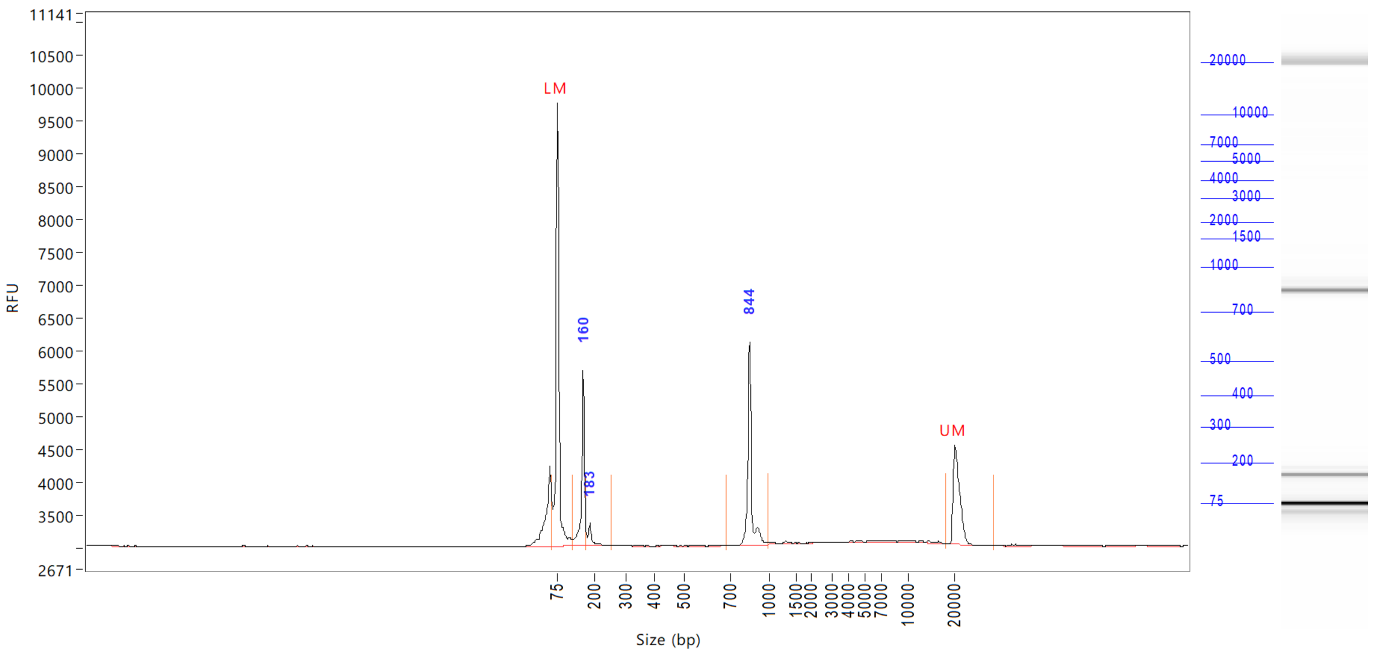

Peak	Size (bp)	Concentration (ng/uL)	From (bp)	To (bp)	Average size (bp)	CV%	RFU	Corrected peak area
1	75 (LM)	0.5000	58	124	74	13.24	6763	47.183
2	160	2.3105	124	173	155	5.60	2680	18.170
3	183	0.3912	173	253	188	8.46	340	3.077
4	844	2.5858	685	987	847	3.67	3088	20.334
5	20000 (UM)	0.1401	18293	28463	20488	3.98	1506	13.224

TIC:	5.2876	ng/uL
TIM:	32.9470	nmole/L
Total concentration:	5.7260	ng/uL

Sample peak width (sec): 5   Sample min peak height: 50   Sample baseline V to V?: Y   Sample baseline V to V points: 3
Sample filter: Binomial   Number of points for filter: 3   Sample start region (min): 0   Sample end region (min): 30
Marker peak width (sec): 5   Marker min peak height: 200   Marker baseline V to V?: Y   Marker baseline V to V points: 3
Lower marker selection: First peak > 500 RFU   Upper marker selection: Last peak > 200 RFU
Ladder size (bp)75, 200, 300, 400, 500, 700, 1000, 1500, 2000, 3000, 4000, 5000, 7000, 10000, 20000
Quantification using: Lower Marker   Final concentration (ng/uL): 0.5000   Dilution factor: 12.0

Sample: SampF1
Well location: F1
Created: Tuesday, September 14, 2021 7:33:54 PM

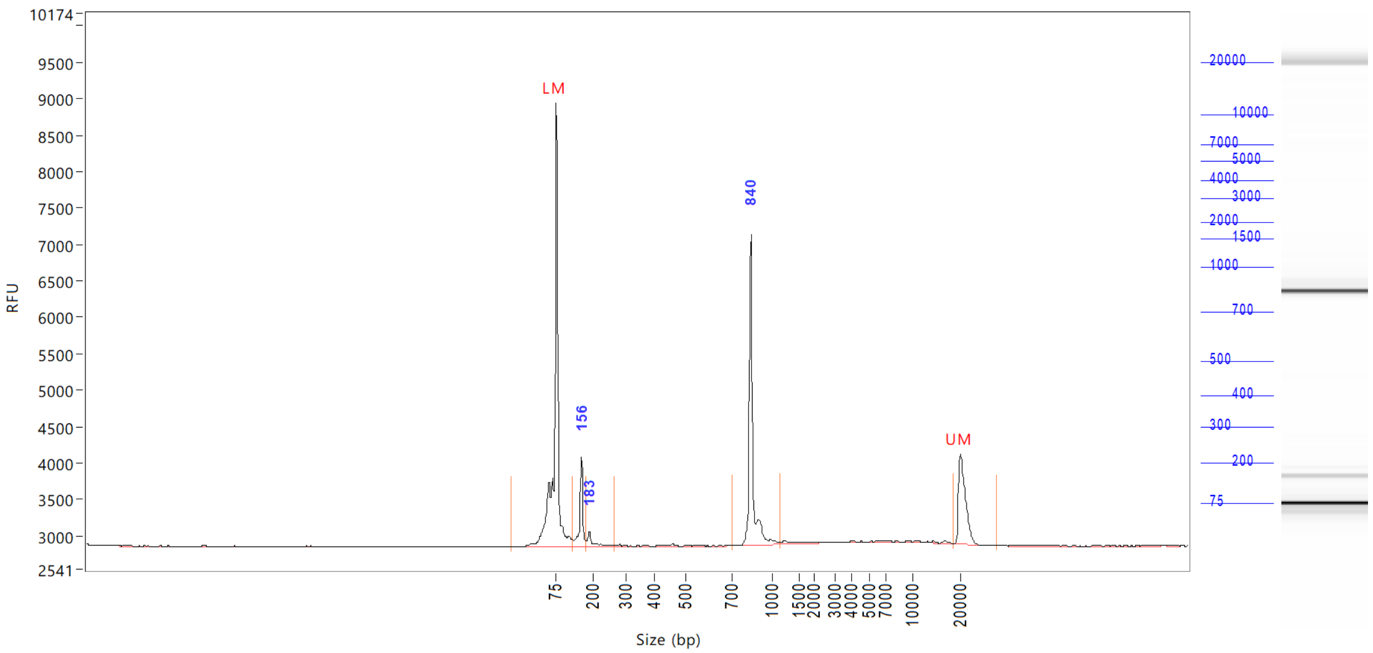

Peak	Size (bp)	Concentration (ng/uL)	From (bp)	To (bp)	Average size (bp)	CV%	RFU	Corrected peak area
1	75 (LM)	0.5000	0	131	66	31.60	6100	57.251
2	156	0.9068	131	173	154	4.93	1222	8.653
3	183	0.2505	173	262	197	11.71	202	2.390
4	840	2.8608	701	1156	850	5.32	4263	27.297
5	20000 (UM)	0.0988	18415	27859	20529	4.08	1238	11.315

TIC:	4.0181	ng/uL
TIM:	17.3079	nmole/L
Total concentration:	4.4205	ng/uL

Sample peak width (sec): 5 Sample min peak height: 50 Sample baseline V to V?: Y Sample baseline V to V points: 3
Sample filter: Binomial Number of points for filter: 3 Sample start region (min): 0 Sample end region (min): 30
Marker peak width (sec): 5 Marker min peak height: 200 Marker baseline V to V?: Y Marker baseline V to V points: 3
Lower marker selection: First peak > 500 RFU Upper marker selection: Last peak > 200 RFU
Ladder size (bp)75, 200, 300, 400, 500, 700, 1000, 1500, 2000, 3000, 4000, 5000, 7000, 10000, 20000
Quantification using: Lower Marker Final concentration (ng/uL): 0.5000 Dilution factor: 12.0

Sample: SampG1
Well location: G1
Created: Tuesday, September 14, 2021 7:33:54 PM

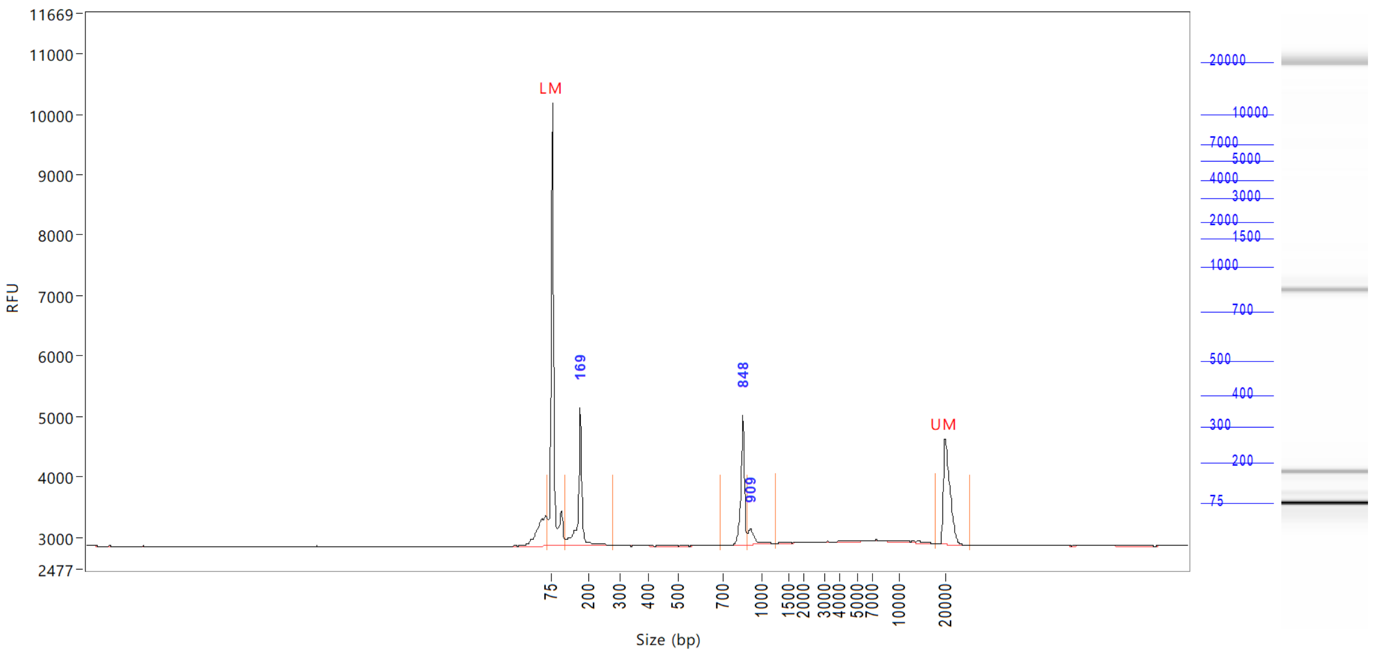

Peak	Size (bp)	Concentration (ng/uL)	From (bp)	To (bp)	Average size (bp)	CV%	RFU	Corrected peak area
1	75 (LM)	0.5000	60	118	77	13.44	7337	52.005
2	169	2.3618	118	277	166	11.70	2279	20.471
3	848	1.5703	690	883	841	2.13	2141	13.611
4	909	0.3727	883	1256	933	6.12	259	3.230
5	20000 (UM)	0.1498	18049	25078	20384	3.82	1749	15.575

TIC:	4.3048	ng/uL
TIM:	27.1179	nmole/L
Total concentration:	4.6346	ng/uL

Sample peak width (sec): 5 Sample min peak height: 50 Sample baseline V to V?: Y Sample baseline V to V points: 3
Sample filter: Binomial Number of points for filter: 3 Sample start region (min): 0 Sample end region (min): 30
Marker peak width (sec): 5 Marker min peak height: 200 Marker baseline V to V?: Y Marker baseline V to V points: 3
Lower marker selection: First peak > 500 RFU Upper marker selection: Last peak > 200 RFU
Ladder size (bp)75, 200, 300, 400, 500, 700, 1000, 1500, 2000, 3000, 4000, 5000, 7000, 10000, 20000
Quantification using: Lower Marker Final concentration (ng/uL): 0.5000 Dilution factor: 12.0

Sample: SampH1
Well location: H1
Created: Tuesday, September 14, 2021 7:33:54 PM

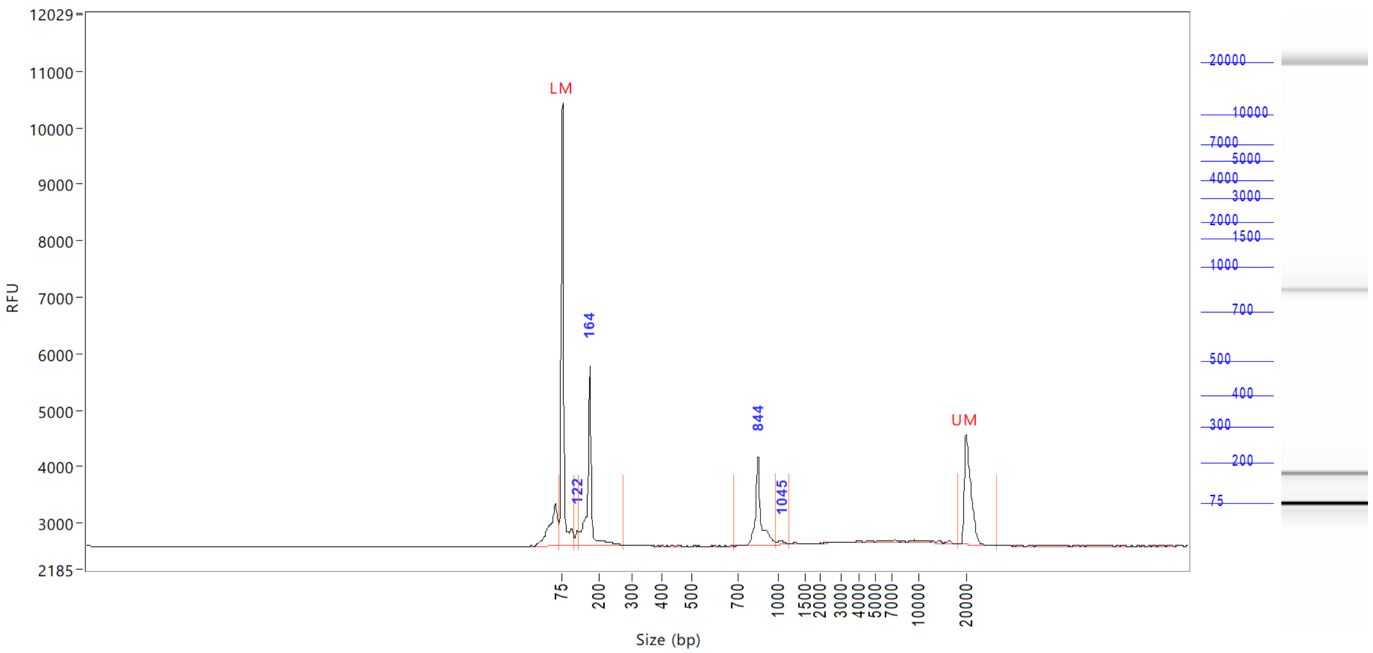

Peak	Size (bp)	Concentration (ng/uL)	From (bp)	To (bp)	Average size (bp)	CV%	RFU	Corrected peak area
1	75 (LM)	0.5000	64	112	75	11.11	7858	50.702
2	122	0.2886	112	129	121	4.27	266	2.438
3	164	3.3558	129	271	164	12.27	3176	28.357
4	844	1.7616	685	987	854	4.74	1558	14.886
5	1045	0.0935	987	1212	1052	5.40	65	0.790
6	20000 (UM)	0.1684	18171	26529	20394	3.82	1936	17.078

TIC:	5.4994	ng/uL
TIM:	41.0935	nmole/L
Total concentration:	5.8759	ng/uL

Sample peak width (sec): 5 Sample min peak height: 50 Sample baseline V to V?: Y Sample baseline V to V points: 3
Sample filter: Binomial Number of points for filter: 3 Sample start region (min): 0 Sample end region (min): 30
Marker peak width (sec): 5 Marker min peak height: 200 Marker baseline V to V?: Y Marker baseline V to V points: 3
Lower marker selection: First peak > 500 RFU Upper marker selection: Last peak > 200 RFU
Ladder size (bp)75, 200, 300, 400, 500, 700, 1000, 1500, 2000, 3000, 4000, 5000, 7000, 10000, 20000
Quantification using: Lower Marker Final concentration (ng/uL): 0.5000 Dilution factor: 12.0

Sample: SampA2
Well location: A2
Created: Tuesday, September 14, 2021 7:33:54 PM

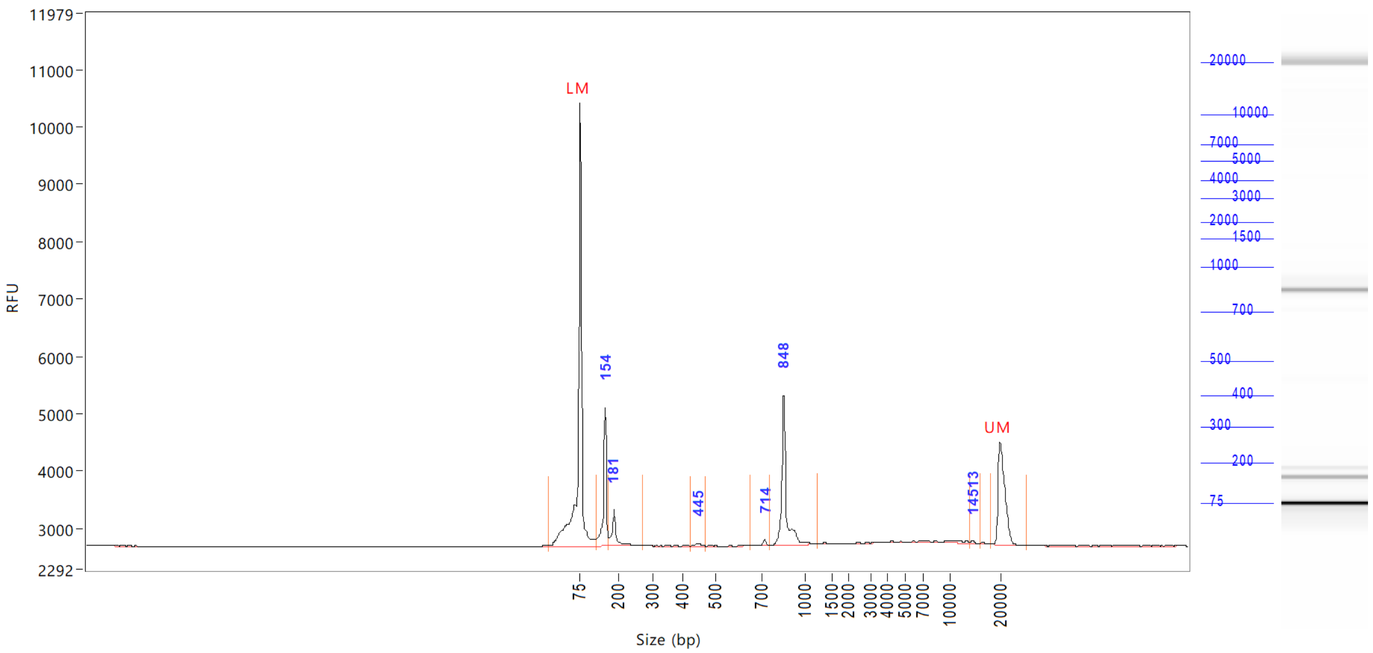

Peak	Size<br>(bp)	Concentration<br>(ng/uL)	From<br>(bp)	To<br>(bp)	Average size<br>(bp)	CV%	RFU	Corrected peak area
1	75 (LM)	0.5000	0	124	64	34.26	7737	68.148
2	154	1.4484	124	166	149	5.10	2410	16.451
3	181	0.5152	166	269	186	9.27	631	5.852
4	445	0.0509	420	471	444	2.29	55	0.578
5	714	0.0470	649	744	707	1.57	99	0.534
6	848	1.7390	744	1223	858	5.22	2611	19.752
7	14513	0.0294	13781	15854	14558	3.83	52	0.334
8	20000 (UM)	0.1118	18049	25440	20390	3.56	1785	15.233

TIC:	3.8300	ng/uL
TIM:	24.1680	nmole/L
Total concentration:	4.0747	ng/uL

Sample peak width (sec): 5   Sample min peak height: 50   Sample baseline V to V?: Y   Sample baseline V to V points: 3
Sample filter: Binomial   Number of points for filter: 3 Sample start region (min): 0 Sample end region (min): 30
Marker peak width (sec): 5   Marker min peak height: 200   Marker baseline V to V?: Y   Marker baseline V to V points: 3
Lower marker selection: First peak > 500 RFU   Upper marker selection: Last peak > 200 RFU
Ladder size (bp)75, 200, 300, 400, 500, 700, 1000, 1500, 2000, 3000, 4000, 5000, 7000, 10000, 20000
Quantification using: Lower Marker   Final concentration (ng/uL): 0.5000   Dilution factor: 12.0

Sample: SampB2
Well location: B2
Created: Tuesday, September 14, 2021 7:33:54 PM

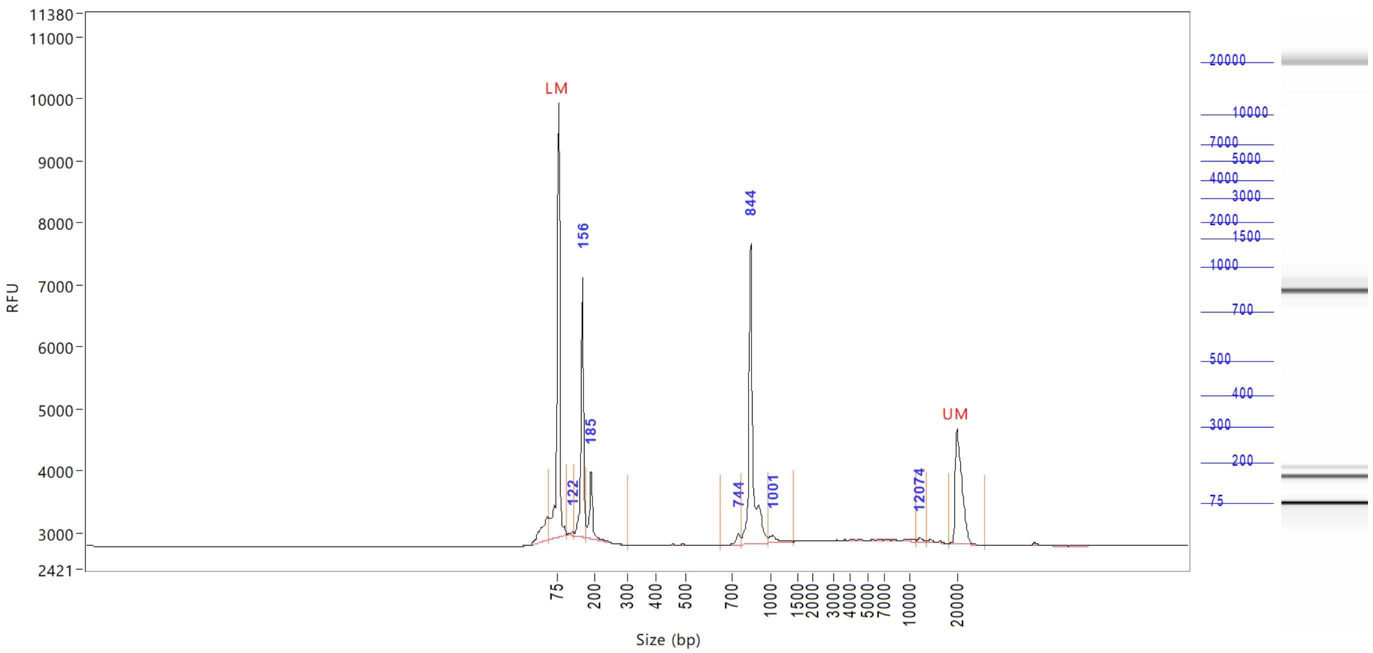

Peak	Size (bp)	Concentration (ng/uL)	From (bp)	To (bp)	Average size (bp)	CV%	RFU	Corrected peak area
1	75 (LM)	0.5000	45	103	72	10.83	7008	46.581
2	122	0.0719	103	128	117	5.72	53	0.558
3	156	3.4354	128	168	152	3.96	4186	26.671
4	185	1.0886	168	300	185	8.04	1078	8.452
5	744	0.1936	649	770	739	2.96	183	1.503
6	844	4.6676	770	974	848	3.96	4835	36.237
7	1001	0.2212	974	1411	1057	8.58	119	1.718
8	12074	0.0685	11342	13537	12304	4.86	65	0.532
9	20000 (UM)	0.1756	18171	25803	20434	3.79	1853	16.364

TIC:	9.7469	ng/uL
TIM:	57.6743	nmole/L
Total concentration:	10.0725	ng/uL

```
Sample peak width (sec): 5    Sample min peak height: 50    Sample baseline V to V?: Y    Sample baseline V to V points: 3
Sample filter: Binomial       Number of points for filter: 3  Sample start region (min): 0  Sample end region (min): 30
Marker peak width (sec): 5    Marker min peak height: 200   Marker baseline V to V?: Y    Marker baseline V to V points: 3
Lower marker selection: First peak > 500 RFU                Upper marker selection: Last peak > 200 RFU
Ladder size (bp)75, 200, 300, 400, 500, 700, 1000, 1500, 2000, 3000, 4000, 5000, 7000, 10000, 20000
Quantification using: Lower Marker          Final concentration (ng/uL): 0.5000          Dilution factor: 12.0
```

Sample: SampC2
Well location: C2
Created: Tuesday, September 14, 2021 7:33:54 PM

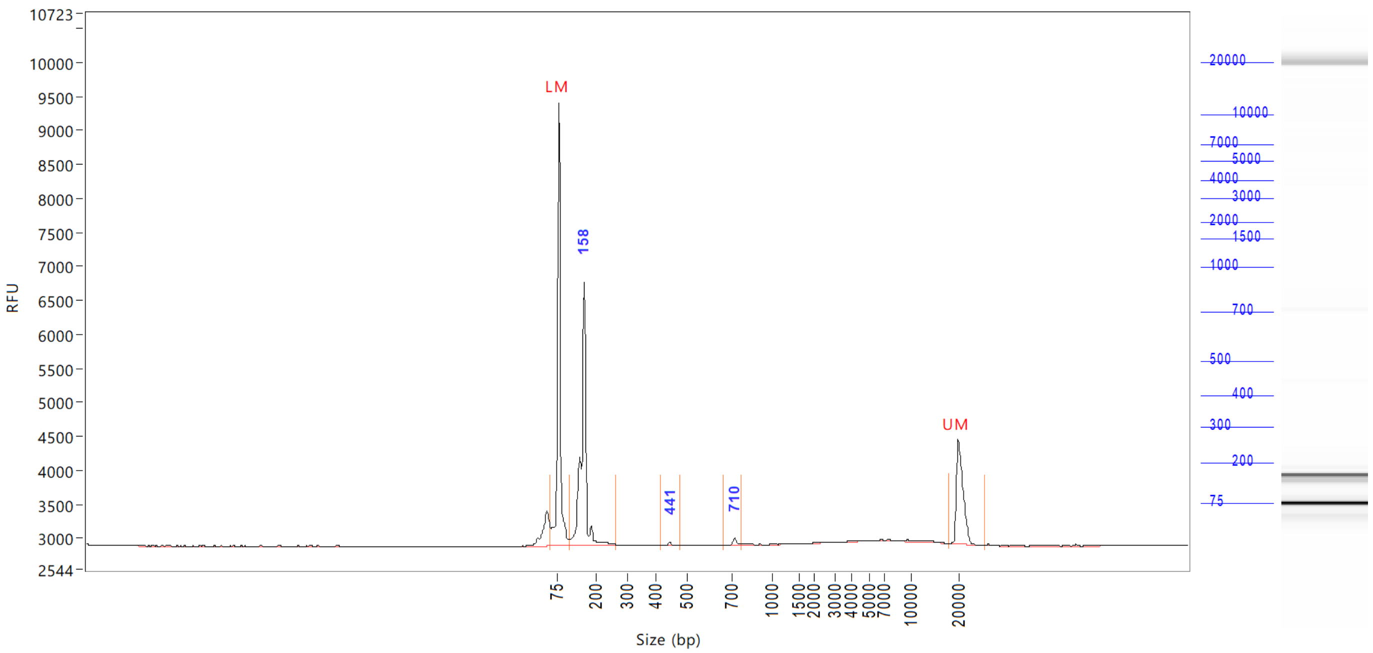

Peak	Size (bp)	Concentration (ng/uL)	From (bp)	To (bp)	Average size (bp)	CV%	RFU	Corrected peak area
1	75 (LM)	0.5000	48	110	74	11.37	6527	44.089
2	158	5.5475	110	264	154	11.21	3866	40.764
3	441	0.0677	414	477	443	2.76	58	0.498
4	710	0.1121	659	762	710	2.57	118	0.824
5	20000 (UM)	0.1514	17927	25561	20386	3.62	1544	13.354

TIC:	5.7273	ng/uL
TIM:	59.7183	nmole/L
Total concentration:	6.1395	ng/uL

```
Sample peak width (sec): 5    Sample min peak height: 50    Sample baseline V to V?: Y    Sample baseline V to V points: 3
Sample filter: Binomial       Number of points for filter: 3  Sample start region (min): 0  Sample end region (min): 30
Marker peak width (sec): 5    Marker min peak height: 200   Marker baseline V to V?: Y    Marker baseline V to V points: 3
Lower marker selection: First peak > 500 RFU                Upper marker selection: Last peak > 200 RFU
Ladder size (bp)75, 200, 300, 400, 500, 700, 1000, 1500, 2000, 3000, 4000, 5000, 7000, 10000, 20000
Quantification using: Lower Marker          Final concentration (ng/uL): 0.5000          Dilution factor: 12.0
```

Sample: SampD2
Well location: D2
Created: Tuesday, September 14, 2021 7:33:54 PM

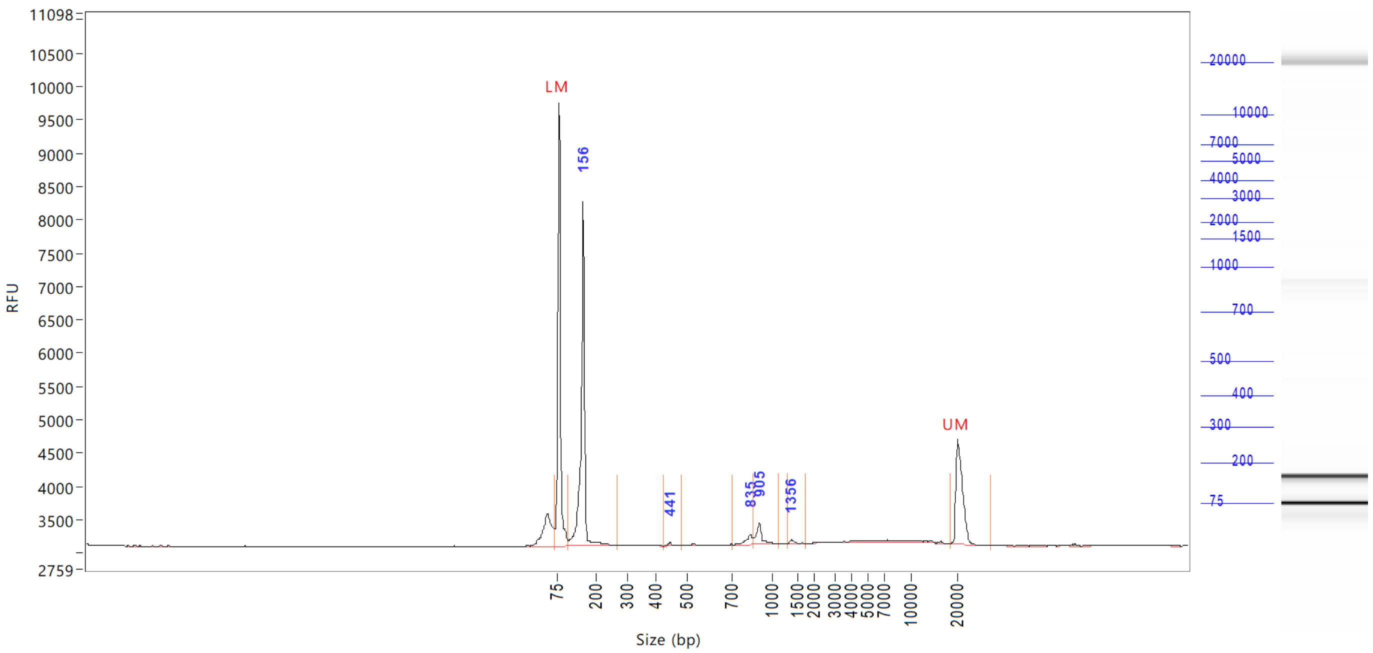

Peak	Size (bp)	Concentration (ng/uL)	From (bp)	To (bp)	Average size (bp)	CV%	RFU	Corrected peak area
1	75 (LM)	0.5000	64	105	75	7.90	6658	41.462
2	156	6.1040	105	268	152	10.92	5157	42.181
3	441	0.0713	420	479	446	3.12	50	0.493
4	835	0.2526	705	857	814	3.86	157	1.745
5	905	0.4431	857	1112	903	3.69	326	3.062
6	1356	0.0711	1289	1712	1392	5.25	61	0.492
7	20000 (UM)	0.1648	18415	27012	20422	3.87	1574	13.668

TIC:	6.9421	ng/uL
TIM:	67.6673	nmole/L
Total concentration:	7.3672	ng/uL

Sample peak width (sec): 5 Sample min peak height: 50 Sample baseline V to V?: Y Sample baseline V to V points: 3
Sample filter: Binomial Number of points for filter: 3 Sample start region (min): 0 Sample end region (min): 30
Marker peak width (sec): 5 Marker min peak height: 200 Marker baseline V to V?: Y Marker baseline V to V points: 3
Lower marker selection: First peak > 500 RFU Upper marker selection: Last peak > 200 RFU
Ladder size (bp)75, 200, 300, 400, 500, 700, 1000, 1500, 2000, 3000, 4000, 5000, 7000, 10000, 20000
Quantification using: Lower Marker Final concentration (ng/uL): 0.5000 Dilution factor: 12.0

Sample: SampE2
Well location: E2
Created: Tuesday, September 14, 2021 7:33:54 PM

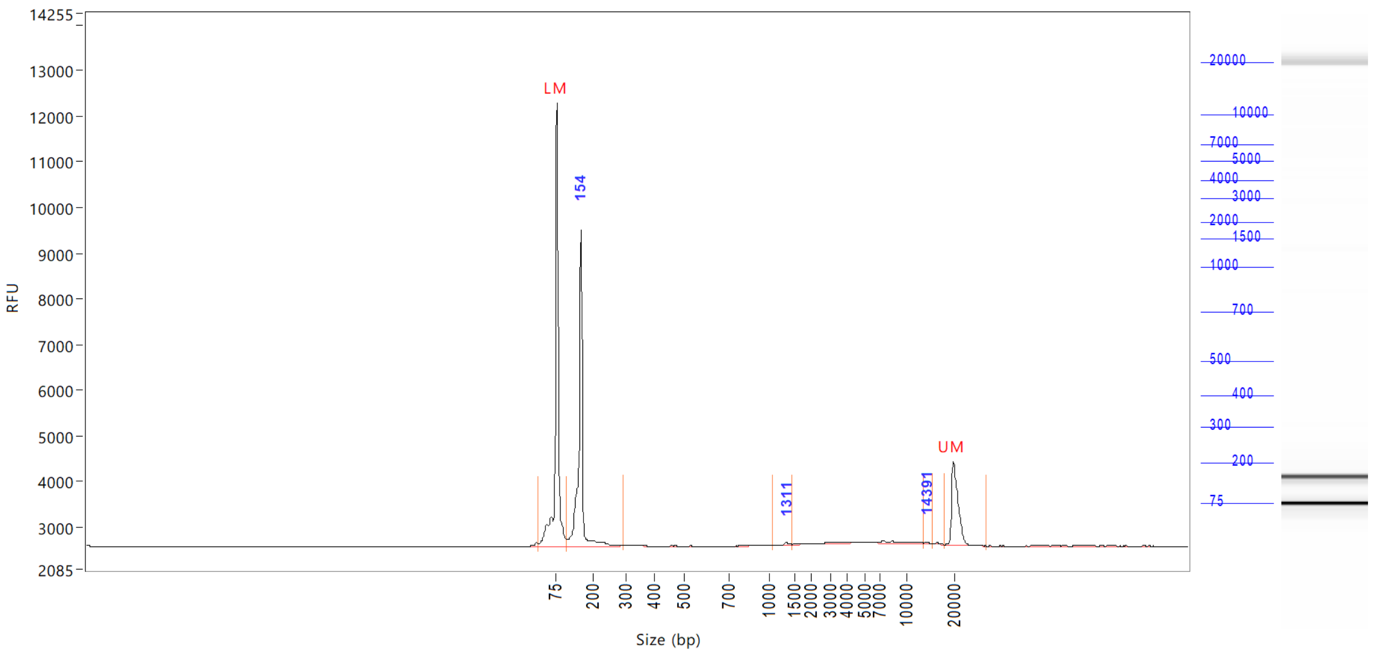

Peak	Size (bp)	Concentration (ng/uL)	From (bp)	To (bp)	Average size (bp)	CV%	RFU	Corrected peak area
1	75 (LM)	0.5000	14	108	69	19.76	9724	73.947
2	154	4.8705	108	293	152	13.55	6914	60.026
3	1311	0.0367	1068	1445	1315	4.46	63	0.453
4	14391	0.0250	13537	15610	14333	3.37	52	0.308
5	20000 (UM)	0.1069	18049	27012	20401	3.69	1816	15.804

TIC:	4.9322	ng/uL
TIM:	52.7123	nmole/L
Total concentration:	5.2852	ng/uL

```
Sample peak width (sec): 5    Sample min peak height: 50    Sample baseline V to V?: Y    Sample baseline V to V points: 3
Sample filter: Binomial       Number of points for filter: 3  Sample start region (min): 0  Sample end region (min): 30
Marker peak width (sec): 5    Marker min peak height: 200   Marker baseline V to V?: Y    Marker baseline V to V points: 3
Lower marker selection: First peak > 500 RFU               Upper marker selection: Last peak > 200 RFU
Ladder size (bp)75, 200, 300, 400, 500, 700, 1000, 1500, 2000, 3000, 4000, 5000, 7000, 10000, 20000
Quantification using: Lower Marker          Final concentration (ng/uL): 0.5000          Dilution factor: 12.0
```

Sample: SampF2
Well location: F2
Created: Tuesday, September 14, 2021 7:33:54 PM

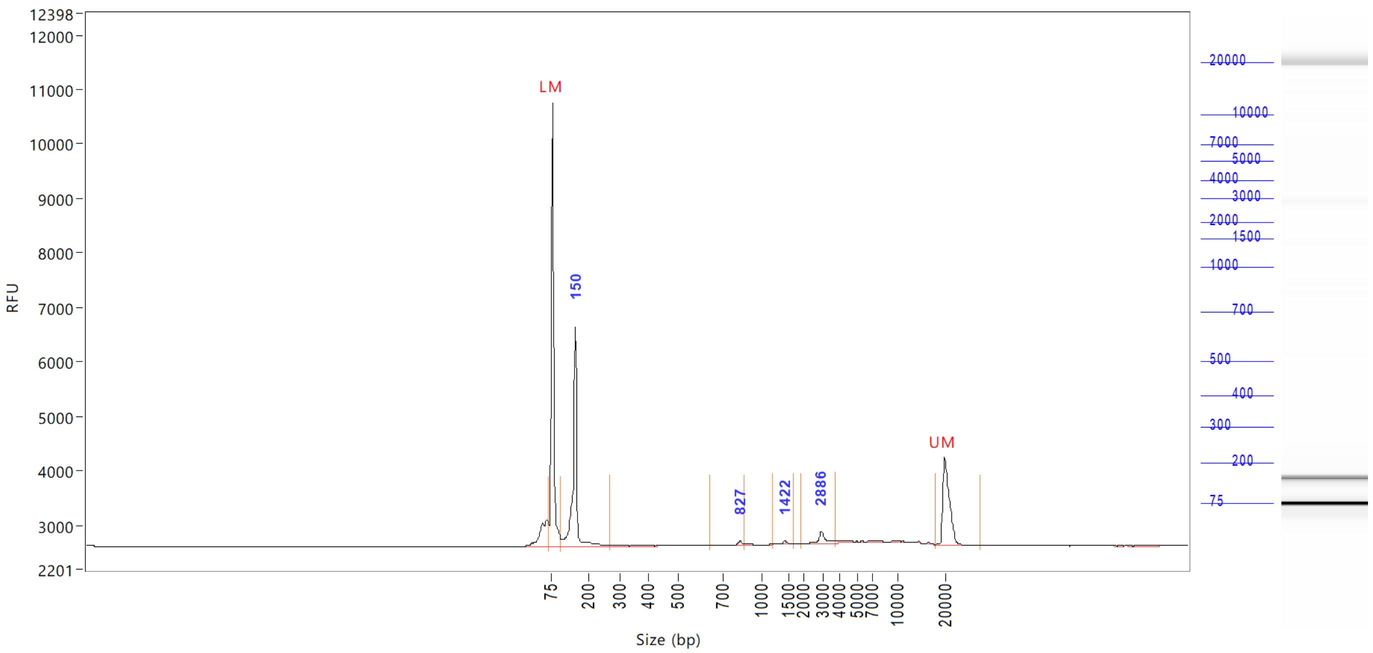

Peak	Size (bp)	Concentration (ng/uL)	From (bp)	To (bp)	Average size (bp)	CV%	RFU	Corrected peak area
1	75 (LM)	0.5000	62	103	73	8.05	8147	52.261
2	150	3.9620	103	262	148	12.47	4017	34.510
3	827	0.0718	641	857	802	5.74	71	0.625
4	1422	0.0471	1212	1655	1427	3.63	54	0.410
5	2886	0.2556	1923	3715	2948	10.30	218	2.227
6	20000 (UM)	0.1366	18049	27496	20392	4.07	1600	14.273

TIC:	4.3365	ng/uL
TIM:	44.3410	nmole/L
Total concentration:	4.7627	ng/uL

```
Sample peak width (sec): 5    Sample min peak height: 50    Sample baseline V to V?: Y    Sample baseline V to V points: 3
Sample filter: Binomial       Number of points for filter: 3  Sample start region (min): 0  Sample end region (min): 30
Marker peak width (sec): 5    Marker min peak height: 200   Marker baseline V to V?: Y    Marker baseline V to V points: 3
Lower marker selection: First peak > 500 RFU                Upper marker selection: Last peak > 200 RFU
Ladder size (bp)75, 200, 300, 400, 500, 700, 1000, 1500, 2000, 3000, 4000, 5000, 7000, 10000, 20000
Quantification using: Lower Marker          Final concentration (ng/uL): 0.5000          Dilution factor: 12.0
```

Sample: SampG2
Well location: G2
Created: Tuesday, September 14, 2021 7:33:54 PM

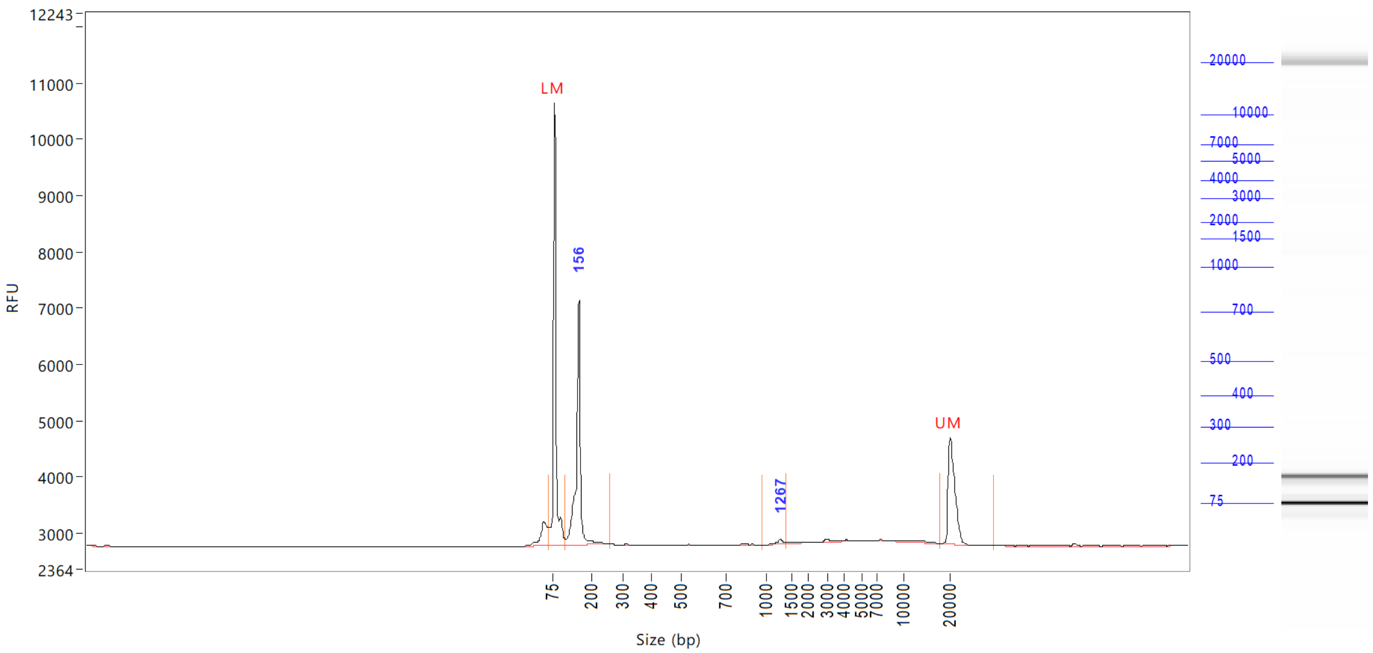

Peak	Size (bp)	Concentration (ng/uL)	From (bp)	To (bp)	Average size (bp)	CV%	RFU	Corrected peak area
1	75 (LM)	0.5000	54	110	75	10.73	7876	52.423
2	156	4.7148	110	257	151	10.14	4333	41.194
3	1267	0.1037	970	1378	1240	6.40	85	0.906
4	20000 (UM)	0.1624	17683	29309	20362	3.99	1894	17.032

TIC:	4.8185	ng/uL
TIM:	51.4549	nmole/L
Total concentration:	5.3855	ng/uL

Sample peak width (sec): 5   Sample min peak height: 50   Sample baseline V to V?: Y   Sample baseline V to V points: 3
Sample filter: Binomial   Number of points for filter: 3 Sample start region (min): 0 Sample end region (min): 30
Marker peak width (sec): 5   Marker min peak height: 200   Marker baseline V to V?: Y   Marker baseline V to V points: 3
Lower marker selection: First peak > 500 RFU   Upper marker selection: Last peak > 200 RFU
Ladder size (bp)75, 200, 300, 400, 500, 700, 1000, 1500, 2000, 3000, 4000, 5000, 7000, 10000, 20000
Quantification using: Lower Marker   Final concentration (ng/uL): 0.5000   Dilution factor: 12.0

Sample: SampH2
Well location: H2
Created: Tuesday, September 14, 2021 7:33:54 PM

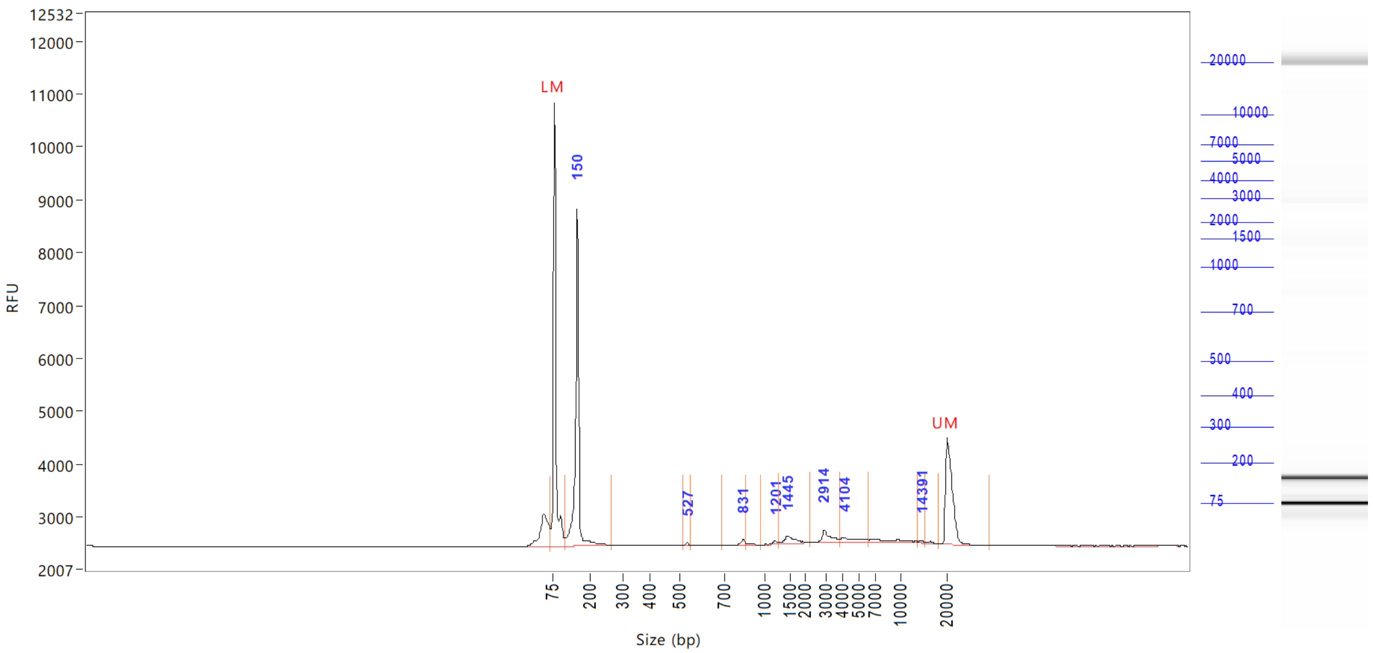

Peak	Size (bp)	Concentration (ng/uL)	From (bp)	To (bp)	Average size (bp)	CV%	RFU	Corrected peak area
1	75 (LM)	0.5000	62	112	75	10.83	8406	54.721
2	150	5.4530	112	262	149	10.39	6375	49.732
3	527	0.0409	511	547	524	1.35	62	0.373
4	831	0.0896	685	861	819	3.31	112	0.817
5	1201	0.0582	974	1256	1168	5.40	60	0.531
6	1445	0.3021	1256	2229	1540	12.55	144	2.755
7	2914	0.3390	2229	3822	3080	10.91	238	3.092
8	4104	0.2246	3822	6260	4660	13.87	76	2.048
9	14391	0.0334	13659	15244	14292	2.70	55	0.305
10	20000 (UM)	0.1634	18049	28947	20489	4.17	2017	17.884

TIC:	6.5409	ng/uL
TIM:	61.1255	nmole/L
Total concentration:	6.8861	ng/uL

Sample peak width (sec): 5 Sample min peak height: 50 Sample baseline V to V?: Y Sample baseline V to V points: 3
Sample filter: Binomial Number of points for filter: 3 Sample start region (min): 0 Sample end region (min): 30
Marker peak width (sec): 5 Marker min peak height: 200 Marker baseline V to V?: Y Marker baseline V to V points: 3
Lower marker selection: First peak > 500 RFU Upper marker selection: Last peak > 200 RFU
Ladder size (bp)75, 200, 300, 400, 500, 700, 1000, 1500, 2000, 3000, 4000, 5000, 7000, 10000, 20000
Quantification using: Lower Marker Final concentration (ng/uL): 0.5000 Dilution factor: 12.0

Sample: SampA3
Well location: A3
Created: Tuesday, September 14, 2021 7:33:54 PM

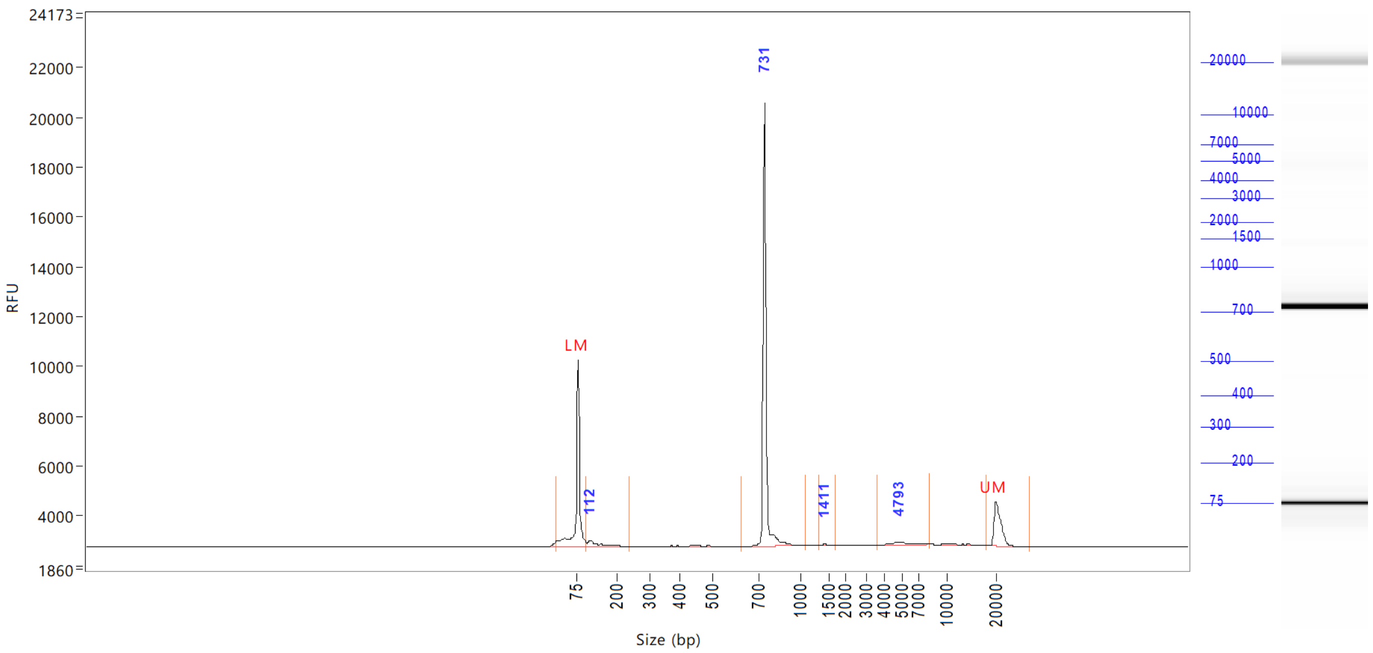

Peak	Size (bp)	Concentration (ng/uL)	From (bp)	To (bp)	Average size (bp)	CV%	RFU	Corrected peak area
1	75 (LM)	0.5000	9	103	68	25.37	7521	59.962
2	112	0.5671	103	235	135	20.29	231	5.668
3	731	9.5375	621	1079	737	4.34	17818	95.314
4	1411	0.0533	1311	1693	1421	3.80	85	0.533
5	4793	0.2647	3572	8201	5093	17.12	115	2.646
6	20000 (UM)	0.1304	18049	26891	20464	3.80	1779	15.638

TIC:	10.4227	ng/uL
TIM:	28.3487	nmole/L
Total concentration:	10.6420	ng/uL

```
Sample peak width (sec): 5    Sample min peak height: 50    Sample baseline V to V?: Y    Sample baseline V to V points: 3
Sample filter: Binomial       Number of points for filter: 3  Sample start region (min): 0  Sample end region (min): 30
Marker peak width (sec): 5    Marker min peak height: 200   Marker baseline V to V?: Y    Marker baseline V to V points: 3
Lower marker selection: First peak > 500 RFU                Upper marker selection: Last peak > 200 RFU
Ladder size (bp)75, 200, 300, 400, 500, 700, 1000, 1500, 2000, 3000, 4000, 5000, 7000, 10000, 20000
Quantification using: Lower Marker          Final concentration (ng/uL): 0.5000          Dilution factor: 12.0
```

Sample: SampB3
Well location: B3
Created: Tuesday, September 14, 2021 7:33:54 PM

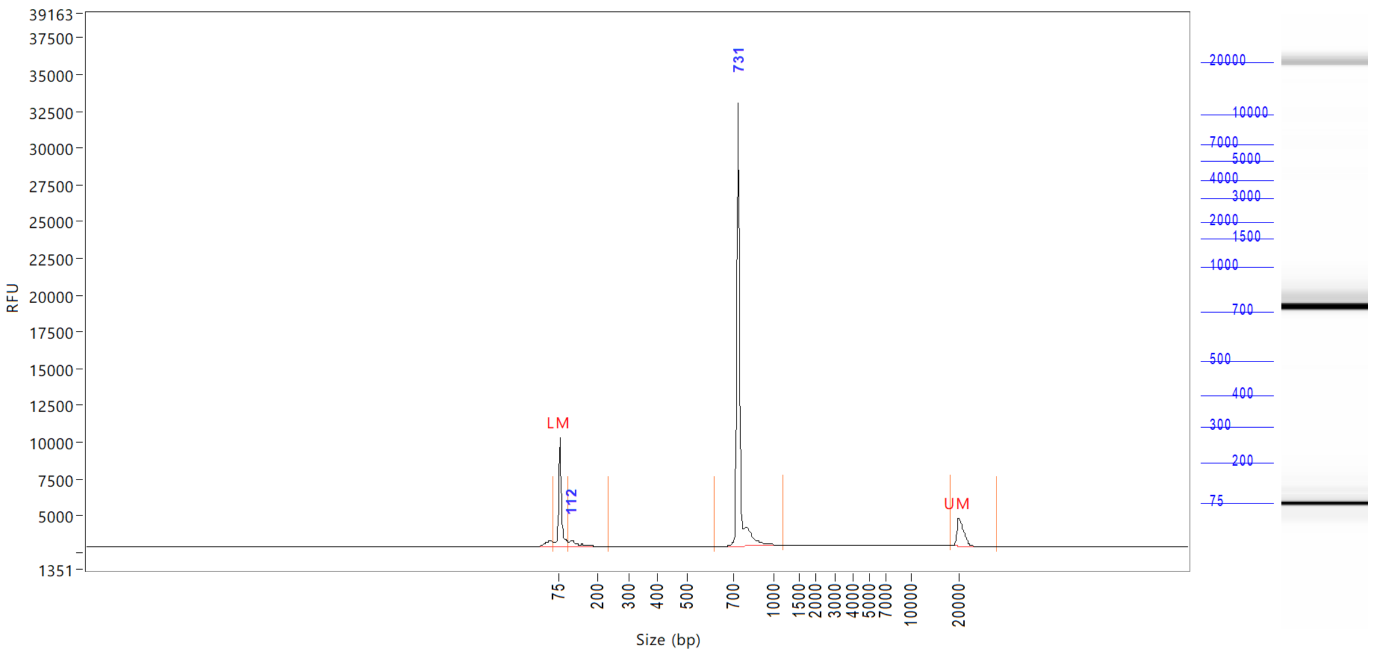

Peak	Size (bp)	Concentration (ng/uL)	From (bp)	To (bp)	Average size (bp)	CV%	RFU	Corrected peak area
1	75 (LM)	0.5000	54	103	74	10.69	7387	53.563
2	112	1.0246	103	235	133	19.93	403	9.146
3	731	19.3459	613	1167	742	5.59	30196	172.703
4	20000 (UM)	0.1541	18293	27859	20405	3.98	1912	16.512

TIC:	20.3705	ng/uL
TIM:	55.5680	nmole/L
Total concentration:	20.6645	ng/uL

```
Sample peak width (sec): 5    Sample min peak height: 50    Sample baseline V to V?: Y    Sample baseline V to V points: 3
Sample filter: Binomial       Number of points for filter: 3  Sample start region (min): 0  Sample end region (min): 30
Marker peak width (sec): 5    Marker min peak height: 200   Marker baseline V to V?: Y    Marker baseline V to V points: 3
Lower marker selection: First peak > 500 RFU                Upper marker selection: Last peak > 200 RFU
Ladder size (bp)75, 200, 300, 400, 500, 700, 1000, 1500, 2000, 3000, 4000, 5000, 7000, 10000, 20000
Quantification using: Lower Marker          Final concentration (ng/uL): 0.5000          Dilution factor: 12.0
```

Sample: SampC3
Well location: C3
Created: Tuesday, September 14, 2021 7:33:54 PM

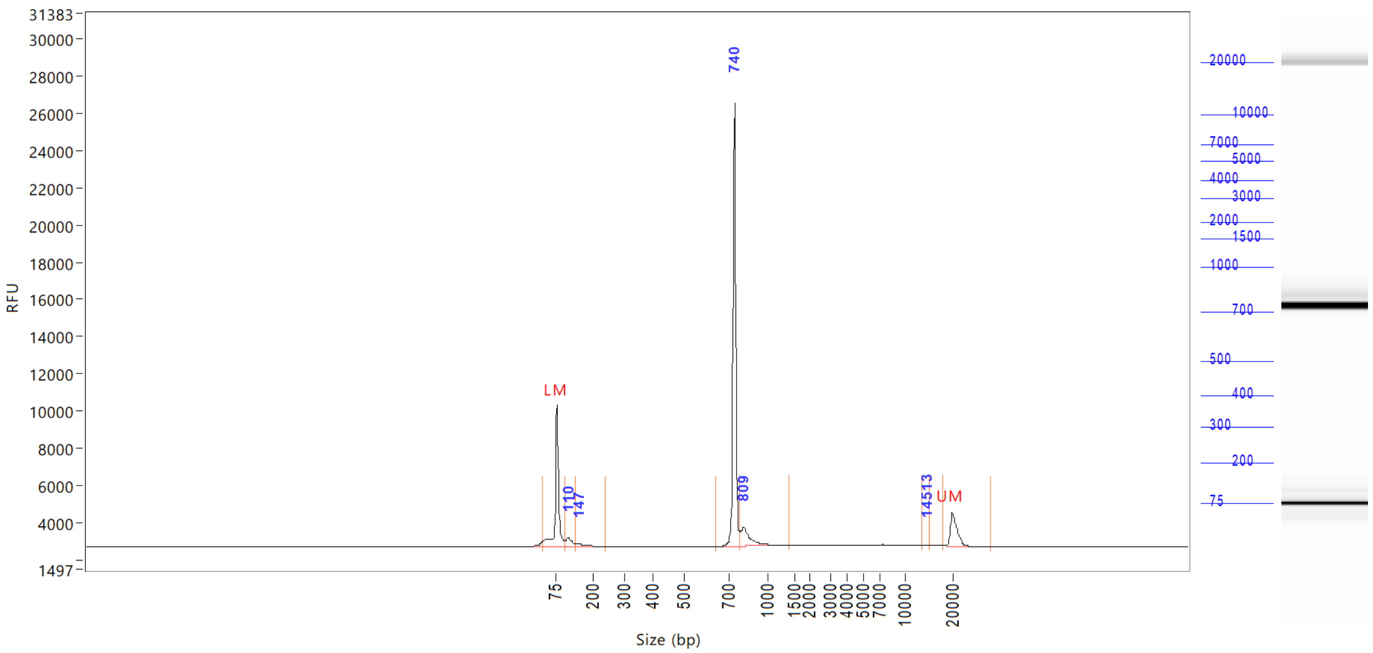

Peak	Size (bp)	Concentration (ng/uL)	From (bp)	To (bp)	Average size (bp)	CV%	RFU	Corrected peak area
1	75 (LM)	0.5000	30	103	71	18.14	7647	60.450
2	110	0.6676	103	141	117	8.79	481	6.726
3	147	0.3385	141	237	165	11.80	172	3.411
4	740	12.5501	644	783	735	1.86	23869	126.442
5	809	1.7334	783	1400	852	8.77	993	17.464
6	14513	0.0275	13659	15244	14292	2.58	52	0.277
7	20000 (UM)	0.1297	18171	28221	20429	3.95	1800	15.677

TIC:	15.3171	ng/uL
TIM:	44.1995	nmole/L
Total concentration:	15.5215	ng/uL

Sample peak width (sec): 5   Sample min peak height: 50   Sample baseline V to V?: Y   Sample baseline V to V points: 3
Sample filter: Binomial   Number of points for filter: 3 Sample start region (min): 0 Sample end region (min): 30
Marker peak width (sec): 5   Marker min peak height: 200   Marker baseline V to V?: Y   Marker baseline V to V points: 3
Lower marker selection: First peak > 500 RFU   Upper marker selection: Last peak > 200 RFU
Ladder size (bp)75, 200, 300, 400, 500, 700, 1000, 1500, 2000, 3000, 4000, 5000, 7000, 10000, 20000
Quantification using: Lower Marker   Final concentration (ng/uL): 0.5000   Dilution factor: 12.0

Sample: SampD3
Well location: D3
Created: Tuesday, September 14, 2021 7:33:54 PM

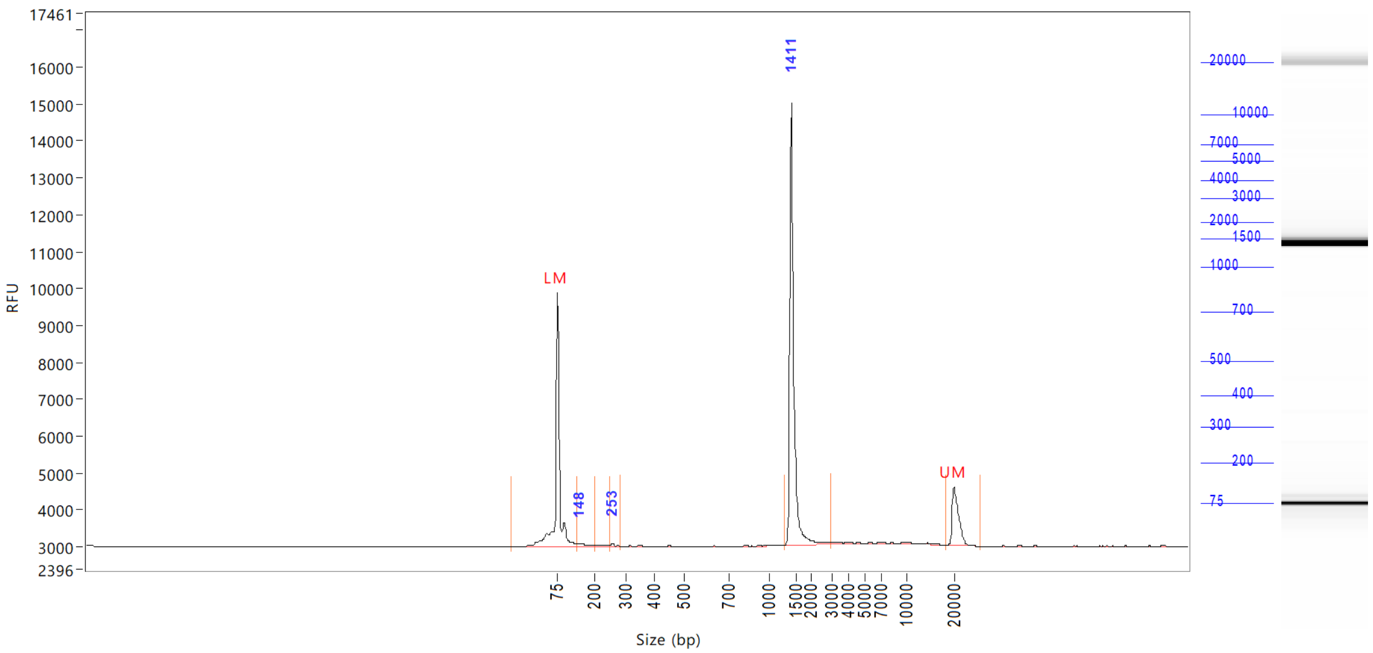

Peak	Size<br>(bp)	Concentration<br>(ng/uL)	From<br>(bp)	To<br>(bp)	Average size<br>(bp)	CV%	RFU	Corrected peak area
1	75 (LM)	0.5000	0	141	68	35.09	6892	61.564
2	148	0.1594	141	201	164	10.29	73	1.635
3	253	0.0487	246	280	254	2.45	86	0.499
4	1411	7.7066	1278	3000	1459	11.94	12004	79.075
5	20000 (UM)	0.1118	18293	25440	20360	3.62	1587	13.763

TIC:	7.9146	ng/uL
TIM:	10.6073	nmole/L
Total concentration:	8.3612	ng/uL

Sample peak width (sec): 5   Sample min peak height: 50   Sample baseline V to V?: Y   Sample baseline V to V points: 3
Sample filter: Binomial   Number of points for filter: 3 Sample start region (min): 0 Sample end region (min): 30
Marker peak width (sec): 5   Marker min peak height: 200   Marker baseline V to V?: Y   Marker baseline V to V points: 3
Lower marker selection: First peak > 500 RFU   Upper marker selection: Last peak > 200 RFU
Ladder size (bp)75, 200, 300, 400, 500, 700, 1000, 1500, 2000, 3000, 4000, 5000, 7000, 10000, 20000
Quantification using: Lower Marker   Final concentration (ng/uL): 0.5000   Dilution factor: 12.0

Sample: SampE3
Well location: E3
Created: Tuesday, September 14, 2021 7:33:54 PM

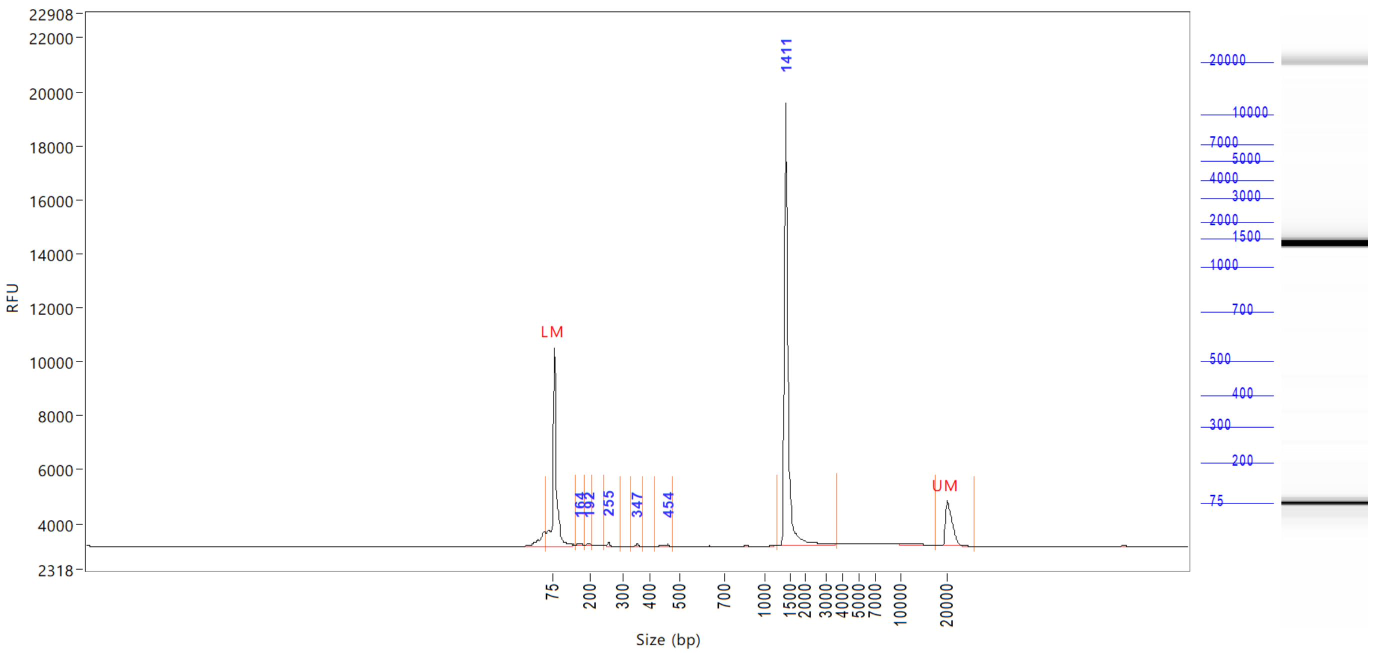

Peak	Size (bp)	Concentration (ng/uL)	From (bp)	To (bp)	Average size (bp)	CV%	RFU	Corrected peak area
1	75 (LM)	0.5000	47	147	75	17.31	7341	61.979
2	164	0.1005	147	175	160	5.04	71	1.038
3	192	0.0757	175	205	187	3.45	79	0.781
4	255	0.0677	241	295	253	2.63	126	0.699
5	347	0.0422	327	370	346	1.71	75	0.436
6	454	0.0801	412	471	443	3.10	77	0.827
7	1411	9.9410	1234	3715	1451	14.52	16426	102.689
8	20000 (UM)	0.1191	17317	25924	20451	3.91	1674	14.758

TIC:	10.3071	ng/uL
TIM:	13.9129	nmole/L
Total concentration:	10.6502	ng/uL

Sample peak width (sec): 5 Sample min peak height: 50 Sample baseline V to V?: Y Sample baseline V to V points: 3
Sample filter: Binomial Number of points for filter: 3 Sample start region (min): 0 Sample end region (min): 30
Marker peak width (sec): 5 Marker min peak height: 200 Marker baseline V to V?: Y Marker baseline V to V points: 3
Lower marker selection: First peak > 500 RFU Upper marker selection: Last peak > 200 RFU
Ladder size (bp)75, 200, 300, 400, 500, 700, 1000, 1500, 2000, 3000, 4000, 5000, 7000, 10000, 20000
Quantification using: Lower Marker Final concentration (ng/uL): 0.5000 Dilution factor: 12.0

Sample: SampF3
Well location: F3
Created: Tuesday, September 14, 2021 7:33:54 PM

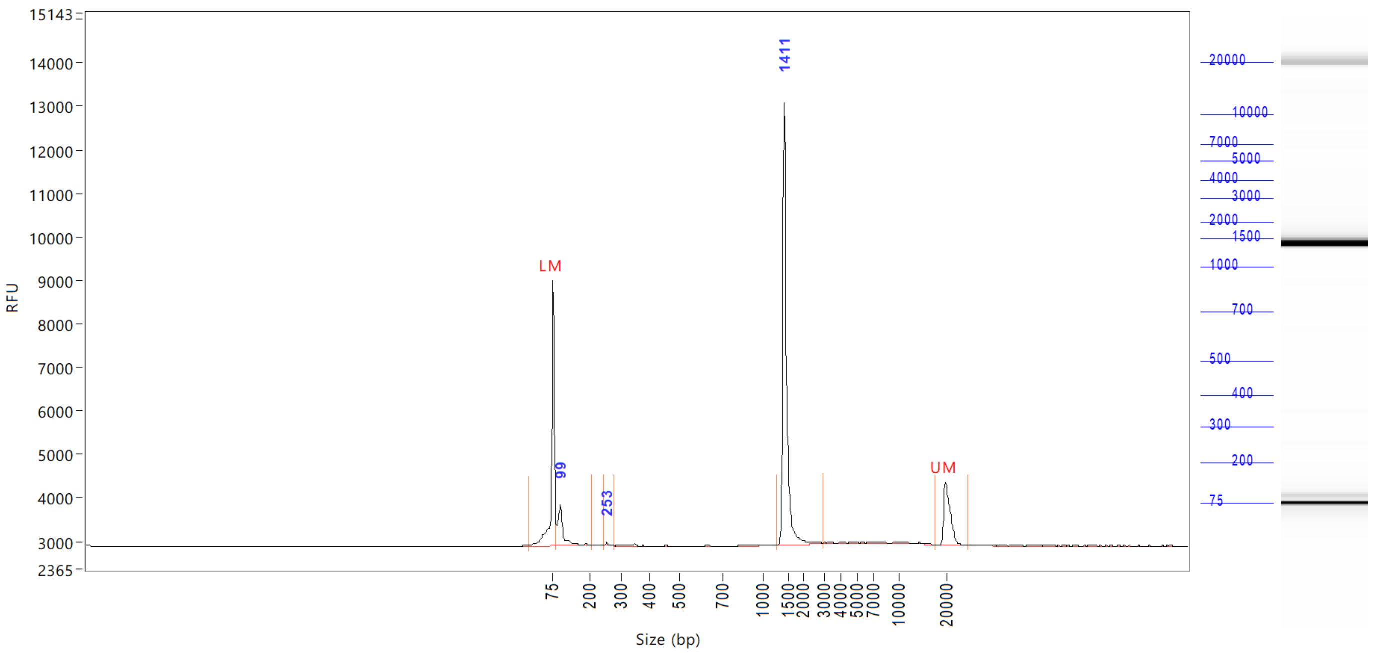

Peak	Size (bp)	Concentration (ng/uL)	From (bp)	To (bp)	Average size (bp)	CV%	RFU	Corrected peak area
1	75 (LM)	0.5000	0	87	68	21.35	6089	44.106
2	99	1.6473	87	208	106	19.72	935	12.109
3	253	0.0546	242	278	251	2.12	74	0.401
4	1411	9.0496	1278	2914	1437	10.34	10183	66.523
5	20000 (UM)	0.1431	17683	24836	20346	3.77	1437	12.625

TIC:	10.7514	ng/uL
TIM:	36.2460	nmole/L
Total concentration:	11.2710	ng/uL

```
Sample peak width (sec): 5    Sample min peak height: 50    Sample baseline V to V?: Y    Sample baseline V to V points: 3
Sample filter: Binomial       Number of points for filter: 3  Sample start region (min): 0  Sample end region (min): 30
Marker peak width (sec): 5    Marker min peak height: 200   Marker baseline V to V?: Y    Marker baseline V to V points: 3
Lower marker selection: First peak > 500 RFU                Upper marker selection: Last peak > 200 RFU
Ladder size (bp)75, 200, 300, 400, 500, 700, 1000, 1500, 2000, 3000, 4000, 5000, 7000, 10000, 20000
Quantification using: Lower Marker          Final concentration (ng/uL): 0.5000          Dilution factor: 12.0
```

Sample: SampG3
Well location: G3
Created: Tuesday, September 14, 2021 7:33:54 PM

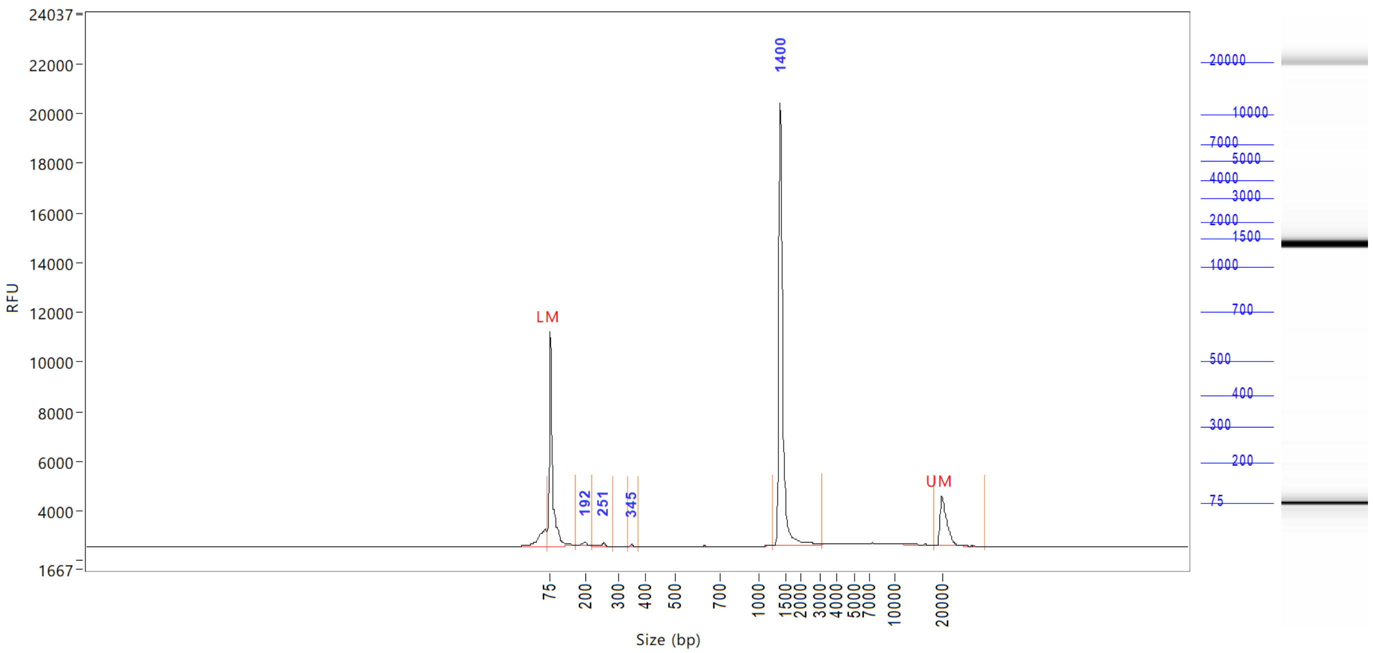

Peak	Size (bp)	Concentration (ng/uL)	From (bp)	To (bp)	Average size (bp)	CV%	RFU	Corrected peak area
1	75 (LM)	0.5000	66	160	79	16.55	8624	67.805
2	192	0.1933	160	217	186	6.89	149	2.184
3	251	0.1158	217	282	243	4.67	152	1.309
4	345	0.0409	333	370	344	1.50	83	0.462
5	1400	10.0061	1234	3037	1433	11.57	17844	113.077
6	20000 (UM)	0.1343	18293	29189	20511	4.50	1999	18.209

TIC:	10.3561	ng/uL
TIM:	14.1815	nmole/L
Total concentration:	10.7283	ng/uL

Sample peak width (sec): 5 Sample min peak height: 50 Sample baseline V to V?: Y Sample baseline V to V points: 3
Sample filter: Binomial Number of points for filter: 3 Sample start region (min): 0 Sample end region (min): 30
Marker peak width (sec): 5 Marker min peak height: 200 Marker baseline V to V?: Y Marker baseline V to V points: 3
Lower marker selection: First peak > 500 RFU Upper marker selection: Last peak > 200 RFU
Ladder size (bp)75, 200, 300, 400, 500, 700, 1000, 1500, 2000, 3000, 4000, 5000, 7000, 10000, 20000
Quantification using: Lower Marker Final concentration (ng/uL): 0.5000 Dilution factor: 12.0

Sample: SampH3
Well location: H3
Created: Tuesday, September 14, 2021 7:33:54 PM

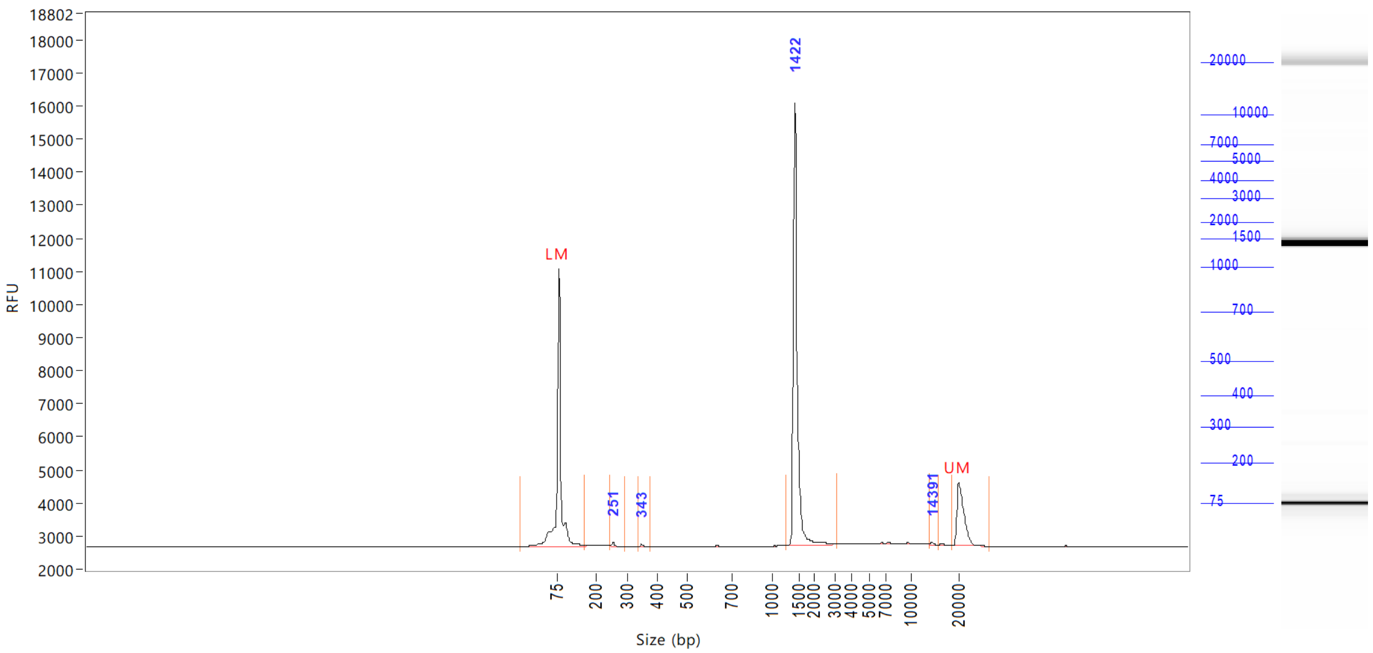

Peak	Size (bp)	Concentration (ng/uL)	From (bp)	To (bp)	Average size (bp)	CV%	RFU	Corrected peak area
1	75 (LM)	0.5000	0	160	71	28.96	8375	72.250
2	251	0.0504	242	287	251	2.78	110	0.606
3	343	0.0298	333	376	344	2.15	60	0.359
4	1422	6.8947	1245	3072	1448	10.92	13398	83.023
5	14391	0.0247	13659	15610	14279	3.00	55	0.297
6	20000 (UM)	0.1186	18415	26408	20452	3.96	1900	17.132

TIC:	6.9995	ng/uL
TIM:	8.3130	nmole/L
Total concentration:	7.3537	ng/uL

```
Sample peak width (sec): 5    Sample min peak height: 50    Sample baseline V to V?: Y    Sample baseline V to V points: 3
Sample filter: Binomial       Number of points for filter: 3  Sample start region (min): 0  Sample end region (min): 30
Marker peak width (sec): 5    Marker min peak height: 200   Marker baseline V to V?: Y    Marker baseline V to V points: 3
Lower marker selection: First peak > 500 RFU                Upper marker selection: Last peak > 200 RFU
Ladder size (bp)75, 200, 300, 400, 500, 700, 1000, 1500, 2000, 3000, 4000, 5000, 7000, 10000, 20000
Quantification using: Lower Marker          Final concentration (ng/uL): 0.5000         Dilution factor: 12.0
```

Sample: SampA4
Well location: A4
Created: Tuesday, September 14, 2021 7:33:54 PM

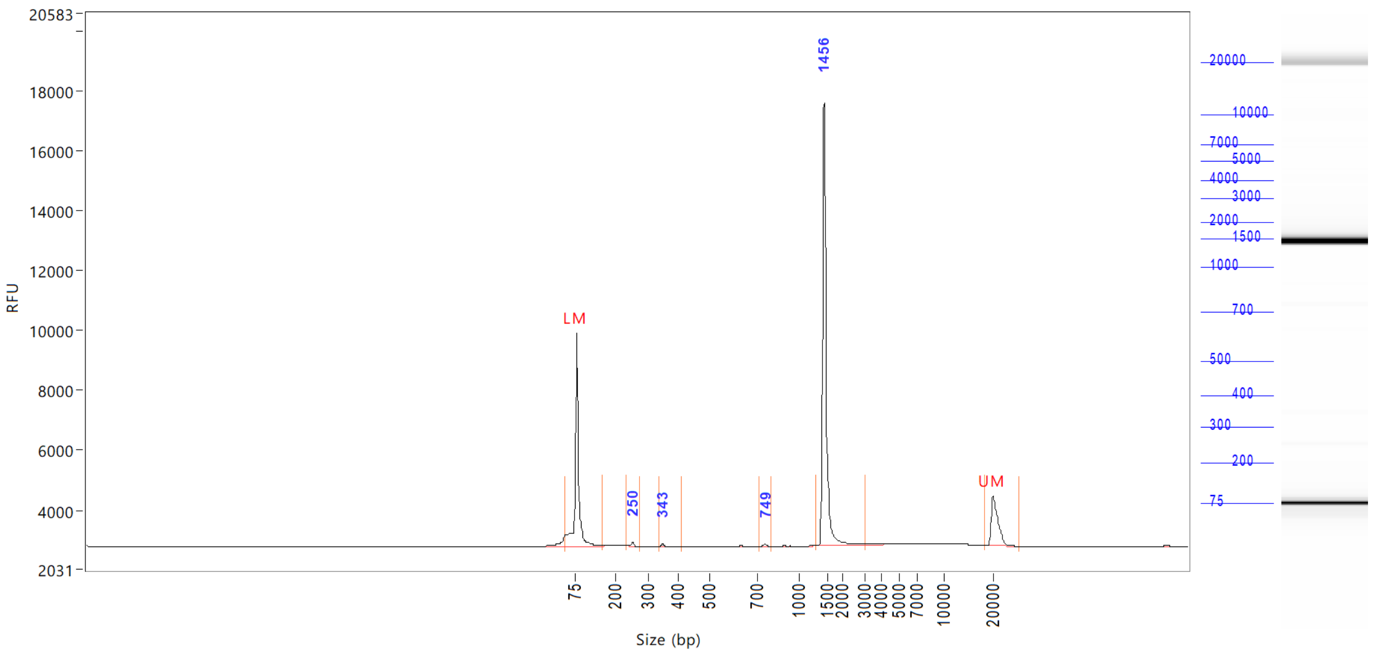

Peak	Size (bp)	Concentration (ng/uL)	From (bp)	To (bp)	Average size (bp)	CV%	RFU	Corrected peak area
1	75 (LM)	0.5000	41	156	74	18.37	7108	56.351
2	250	0.0797	233	271	248	2.04	133	0.749
3	343	0.0484	333	407	347	3.20	71	0.455
4	749	0.0510	714	792	746	1.85	80	0.479
5	1456	9.1907	1300	3000	1480	10.16	14801	86.318
6	20000 (UM)	0.1270	18293	25320	20389	3.73	1652	14.310

TIC:	9.3699	ng/uL
TIM:	11.0929	nmole/L
Total concentration:	9.7403	ng/uL

```
Sample peak width (sec): 5    Sample min peak height: 50    Sample baseline V to V?: Y    Sample baseline V to V points: 3
Sample filter: Binomial       Number of points for filter: 3  Sample start region (min): 0  Sample end region (min): 30
Marker peak width (sec): 5    Marker min peak height: 200   Marker baseline V to V?: Y    Marker baseline V to V points: 3
Lower marker selection: First peak > 500 RFU                Upper marker selection: Last peak > 200 RFU
Ladder size (bp)75, 200, 300, 400, 500, 700, 1000, 1500, 2000, 3000, 4000, 5000, 7000, 10000, 20000
Quantification using: Lower Marker          Final concentration (ng/uL): 0.5000          Dilution factor: 12.0
```

Sample: SampB4
Well location: B4
Created: Tuesday, September 14, 2021 7:33:54 PM

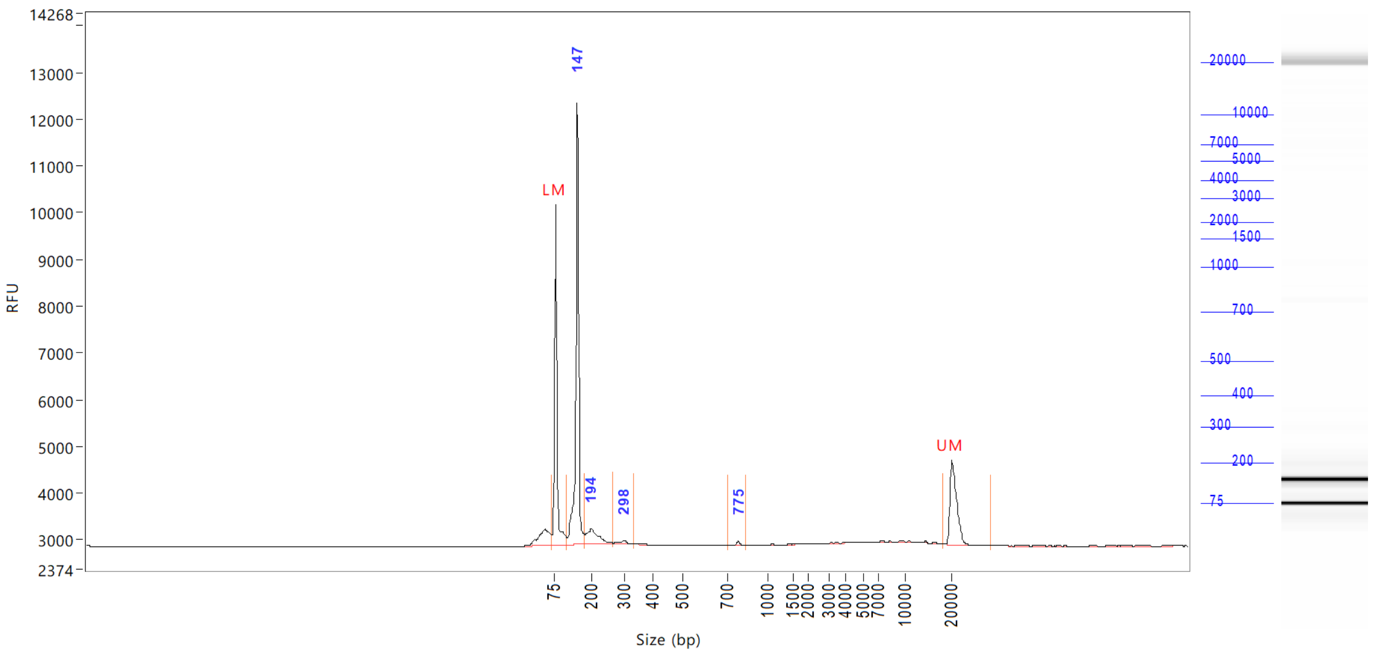

Peak	Size (bp)	Concentration (ng/uL)	From (bp)	To (bp)	Average size (bp)	CV%	RFU	Corrected peak area
1	75 (LM)	0.5000	64	114	75	11.23	7288	45.237
2	147	8.8902	114	173	143	5.91	9461	67.028
3	194	1.0627	173	260	199	8.63	320	8.013
4	298	0.1119	260	333	293	3.87	59	0.844
5	775	0.0930	705	840	776	2.73	103	0.701
6	20000 (UM)	0.1786	18293	28463	20463	3.84	1815	16.157

TIC:	10.1579	ng/uL
TIM:	111.7741	nmole/L
Total concentration:	10.5208	ng/uL

Sample peak width (sec): 5   Sample min peak height: 50   Sample baseline V to V?: Y   Sample baseline V to V points: 3
Sample filter: Binomial   Number of points for filter: 3   Sample start region (min): 0   Sample end region (min): 30
Marker peak width (sec): 5   Marker min peak height: 200   Marker baseline V to V?: Y   Marker baseline V to V points: 3
Lower marker selection: First peak > 500 RFU   Upper marker selection: Last peak > 200 RFU
Ladder size (bp)75, 200, 300, 400, 500, 700, 1000, 1500, 2000, 3000, 4000, 5000, 7000, 10000, 20000
Quantification using: Lower Marker   Final concentration (ng/uL): 0.5000   Dilution factor: 12.0

Sample: SampC4
Well location: C4
Created: Tuesday, September 14, 2021 7:33:54 PM

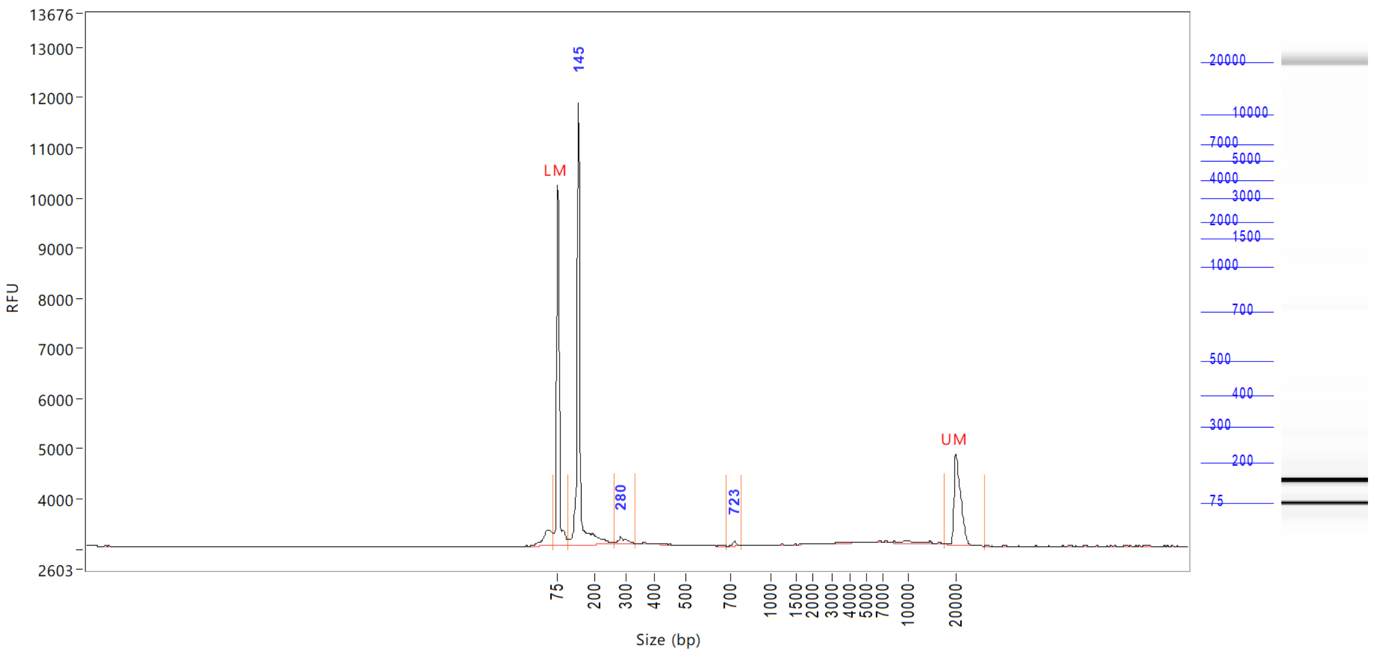

Peak	Size (bp)	Concentration (ng/uL)	From (bp)	To (bp)	Average size (bp)	CV%	RFU	Corrected peak area
1	75 (LM)	0.5000	64	112	76	9.59	7166	44.258
2	145	8.7922	112	262	148	14.11	8820	64.853
3	280	0.3033	262	327	285	4.60	142	2.237
4	723	0.0881	682	779	724	2.28	99	0.650
5	20000 (UM)	0.1823	17683	26045	20417	3.76	1811	16.133

TIC:	9.1836	ng/uL
TIM:	99.5839	nmole/L
Total concentration:	9.7737	ng/uL

```
Sample peak width (sec): 5    Sample min peak height: 50    Sample baseline V to V?: Y    Sample baseline V to V points: 3
Sample filter: Binomial       Number of points for filter: 3  Sample start region (min): 0  Sample end region (min): 30
Marker peak width (sec): 5    Marker min peak height: 200   Marker baseline V to V?: Y    Marker baseline V to V points: 3
Lower marker selection: First peak > 500 RFU                Upper marker selection: Last peak > 200 RFU
Ladder size (bp)75, 200, 300, 400, 500, 700, 1000, 1500, 2000, 3000, 4000, 5000, 7000, 10000, 20000
Quantification using: Lower Marker          Final concentration (ng/uL): 0.5000          Dilution factor: 12.0
```

Sample: SampD4
Well location: D4
Created: Tuesday, September 14, 2021 7:33:54 PM

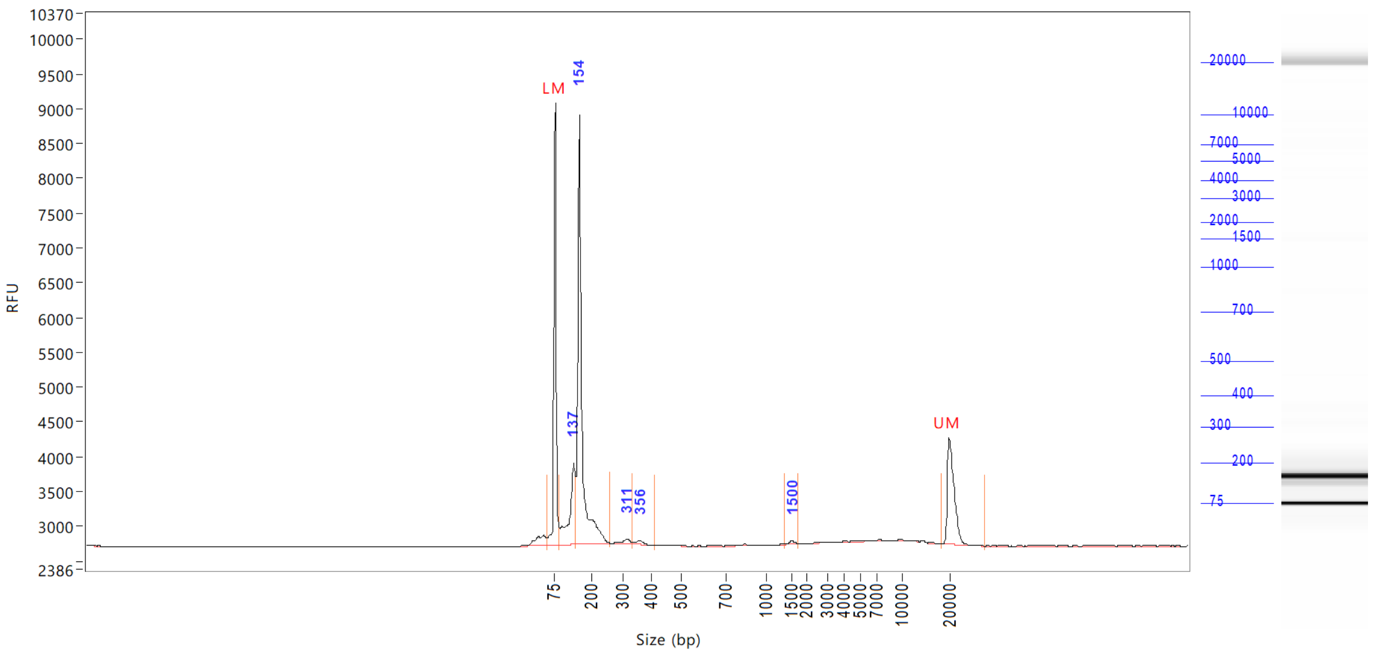

Peak	Size (bp)	Concentration (ng/uL)	From (bp)	To (bp)	Average size (bp)	CV%	RFU	Corrected peak area
1	75 (LM)	0.5000	48	89	72	7.32	6360	37.695
2	137	2.8397	89	145	126	12.06	1163	17.840
3	154	9.4134	145	259	163	11.51	6160	59.139
4	311	0.1746	259	331	305	4.83	67	1.097
5	356	0.1345	331	409	354	3.84	51	0.845
6	1500	0.0636	1367	1693	1507	4.14	51	0.400
7	20000 (UM)	0.1798	18293	27617	20488	4.05	1526	13.556

TIC:	12.6258	ng/uL
TIM:	133.5913	nmole/L
Total concentration:	12.8117	ng/uL

Sample peak width (sec): 5 Sample min peak height: 50 Sample baseline V to V?: Y Sample baseline V to V points: 3
Sample filter: Binomial Number of points for filter: 3 Sample start region (min): 0 Sample end region (min): 30
Marker peak width (sec): 5 Marker min peak height: 200 Marker baseline V to V?: Y Marker baseline V to V points: 3
Lower marker selection: First peak > 500 RFU Upper marker selection: Last peak > 200 RFU
Ladder size (bp)75, 200, 300, 400, 500, 700, 1000, 1500, 2000, 3000, 4000, 5000, 7000, 10000, 20000
Quantification using: Lower Marker Final concentration (ng/uL): 0.5000 Dilution factor: 12.0

Sample: SampE4
Well location: E4
Created: Tuesday, September 14, 2021 7:33:54 PM

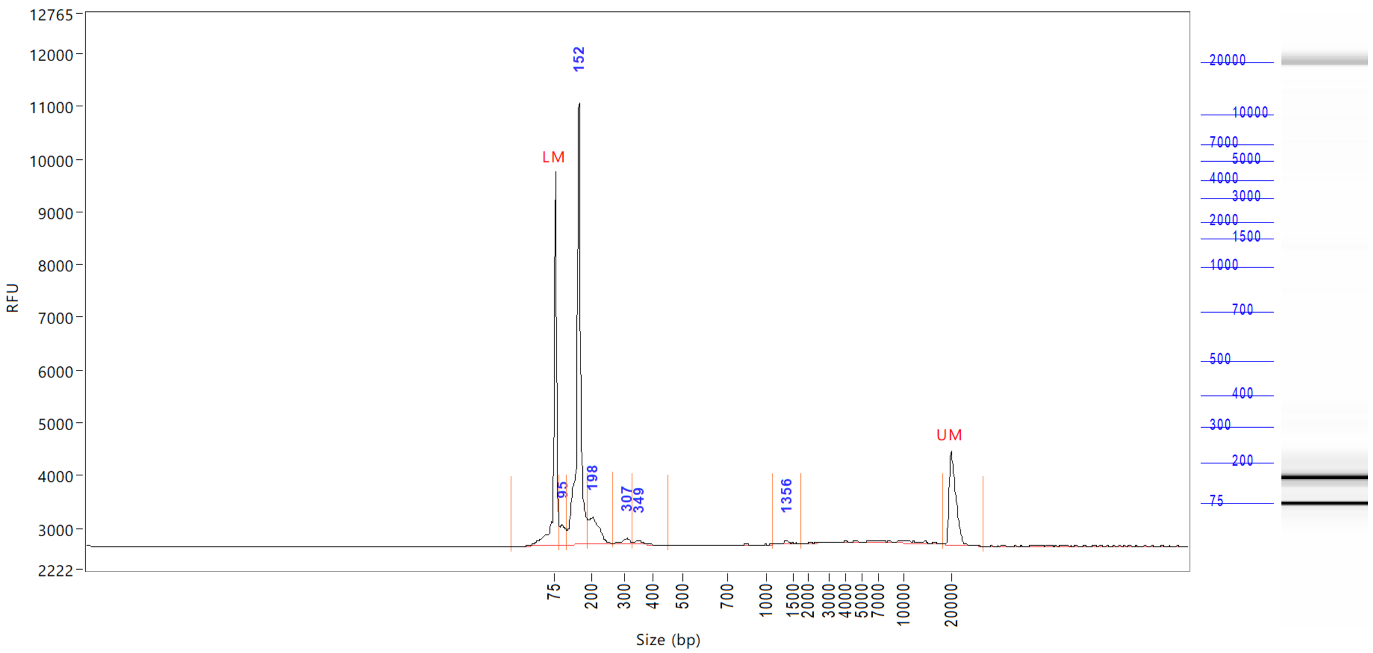

Peak	Size (bp)	Concentration (ng/uL)	From (bp)	To (bp)	Average size (bp)	CV%	RFU	Corrected peak area
1	75 (LM)	0.5000	0	89	67	23.45	7089	50.609
2	95	0.6060	89	110	99	6.57	386	5.111
3	152	9.5756	110	183	149	8.42	8385	80.768
4	198	1.4184	183	266	204	7.10	499	11.964
5	307	0.1863	266	325	302	4.08	100	1.571
6	349	0.1696	325	452	356	7.08	76	1.430
7	1356	0.0834	1112	1750	1406	8.12	75	0.704
8	20000 (UM)	0.1545	18293	27012	20441	3.81	1771	15.636

TIC:	12.0392	ng/uL
TIM:	128.9994	nmole/L
Total concentration:	12.3922	ng/uL

Sample peak width (sec): 5 Sample min peak height: 50 Sample baseline V to V?: Y Sample baseline V to V points: 3
Sample filter: Binomial Number of points for filter: 3 Sample start region (min): 0 Sample end region (min): 30
Marker peak width (sec): 5 Marker min peak height: 200 Marker baseline V to V?: Y Marker baseline V to V points: 3
Lower marker selection: First peak > 500 RFU Upper marker selection: Last peak > 200 RFU
Ladder size (bp)75, 200, 300, 400, 500, 700, 1000, 1500, 2000, 3000, 4000, 5000, 7000, 10000, 20000
Quantification using: Lower Marker Final concentration (ng/uL): 0.5000 Dilution factor: 12.0

Sample: SampF4
Well location: F4
Created: Tuesday, September 14, 2021 7:33:54 PM

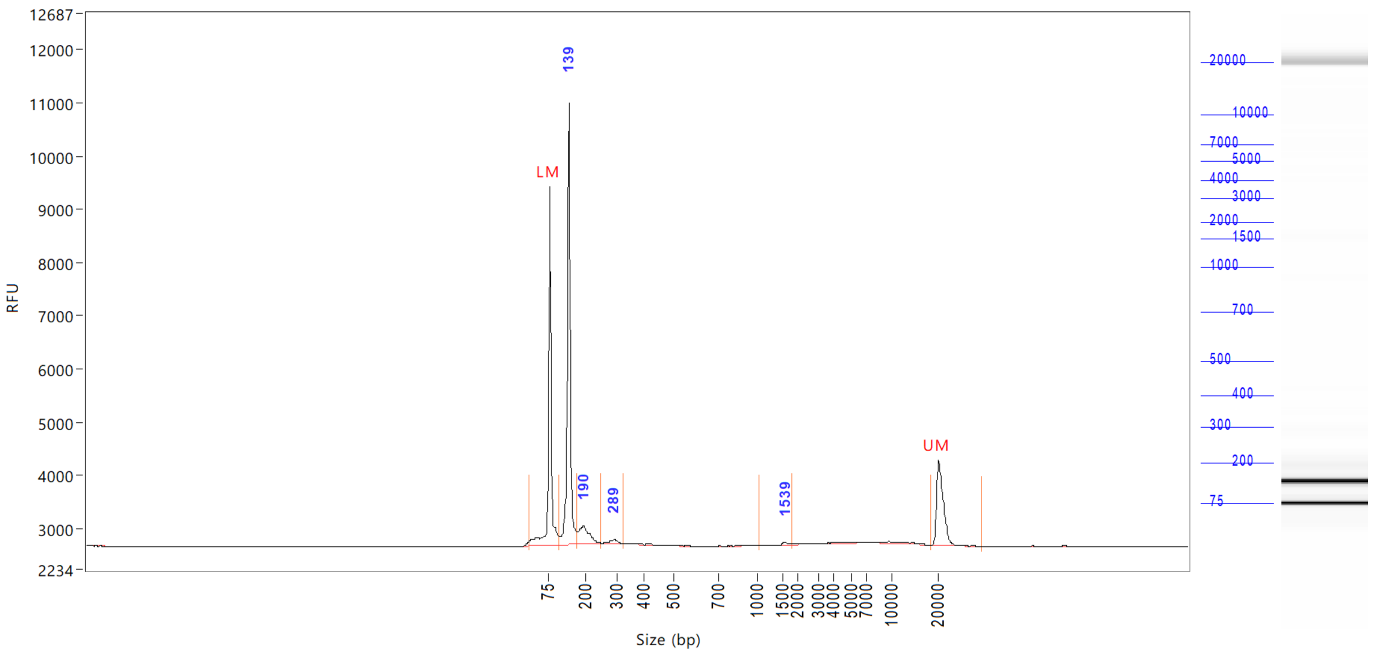

Peak	Size (bp)	Concentration (ng/uL)	From (bp)	To (bp)	Average size (bp)	CV%	RFU	Corrected peak area
1	75 (LM)	0.5000	9	107	71	21.08	6732	46.622
2	139	7.8094	107	169	138	6.13	8317	60.682
3	190	1.0490	169	250	193	7.96	341	8.151
4	289	0.1710	250	323	286	4.55	74	1.328
5	1539	0.0765	1012	1789	1491	10.19	55	0.594
6	20000 (UM)	0.1563	18537	29551	20526	4.44	1606	14.576

TIC:	9.1058	ng/uL
TIM:	102.9958	nmole/L
Total concentration:	9.5863	ng/uL

Sample peak width (sec): 5   Sample min peak height: 50   Sample baseline V to V?: Y   Sample baseline V to V points: 3
Sample filter: Binomial   Number of points for filter: 3   Sample start region (min): 0   Sample end region (min): 30
Marker peak width (sec): 5   Marker min peak height: 200   Marker baseline V to V?: Y   Marker baseline V to V points: 3
Lower marker selection: First peak > 500 RFU   Upper marker selection: Last peak > 200 RFU
Ladder size (bp)75, 200, 300, 400, 500, 700, 1000, 1500, 2000, 3000, 4000, 5000, 7000, 10000, 20000
Quantification using: Lower Marker   Final concentration (ng/uL): 0.5000   Dilution factor: 12.0

Sample: SampG4
Well location: G4
Created: Tuesday, September 14, 2021 7:33:54 PM

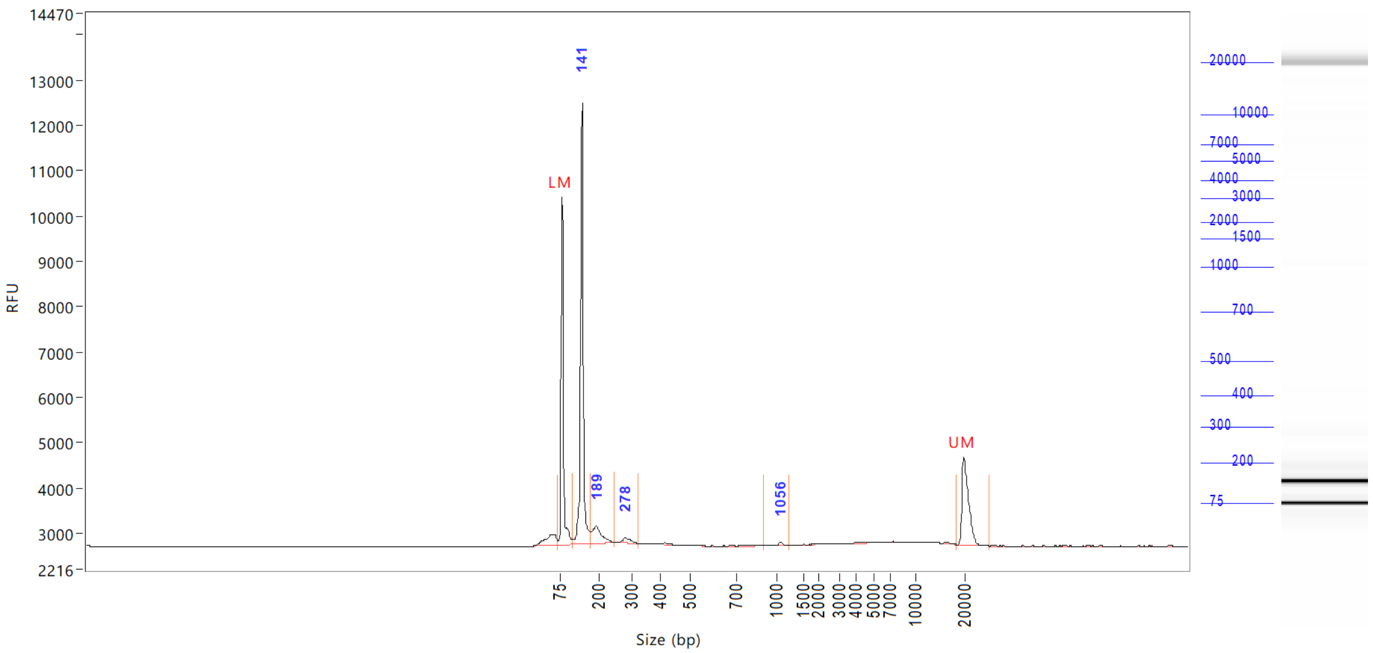

Peak	Size (bp)	Concentration (ng/uL)	From (bp)	To (bp)	Average size (bp)	CV%	RFU	Corrected peak area
1	75 (LM)	0.5000	64	110	75	9.18	7651	45.635
2	141	8.5016	110	169	139	5.50	9739	64.662
3	189	1.0744	169	248	192	8.05	383	8.172
4	278	0.2527	248	321	282	4.61	121	1.922
5	1056	0.0651	900	1234	1055	4.95	70	0.495
6	20000 (UM)	0.1864	18537	25440	20395	3.67	1924	17.012

TIC:	9.8938	ng/uL
TIM:	111.2837	nmole/L
Total concentration:	10.2336	ng/uL

Sample peak width (sec): 5   Sample min peak height: 50   Sample baseline V to V?: Y   Sample baseline V to V points: 3
Sample filter: Binomial   Number of points for filter: 3 Sample start region (min): 0 Sample end region (min): 30
Marker peak width (sec): 5   Marker min peak height: 200   Marker baseline V to V?: Y   Marker baseline V to V points: 3
Lower marker selection: First peak > 500 RFU   Upper marker selection: Last peak > 200 RFU
Ladder size (bp)75, 200, 300, 400, 500, 700, 1000, 1500, 2000, 3000, 4000, 5000, 7000, 10000, 20000
Quantification using: Lower Marker   Final concentration (ng/uL): 0.5000   Dilution factor: 12.0

Sample: SampH4
Well location: H4
Created: Tuesday, September 14, 2021 7:33:54 PM

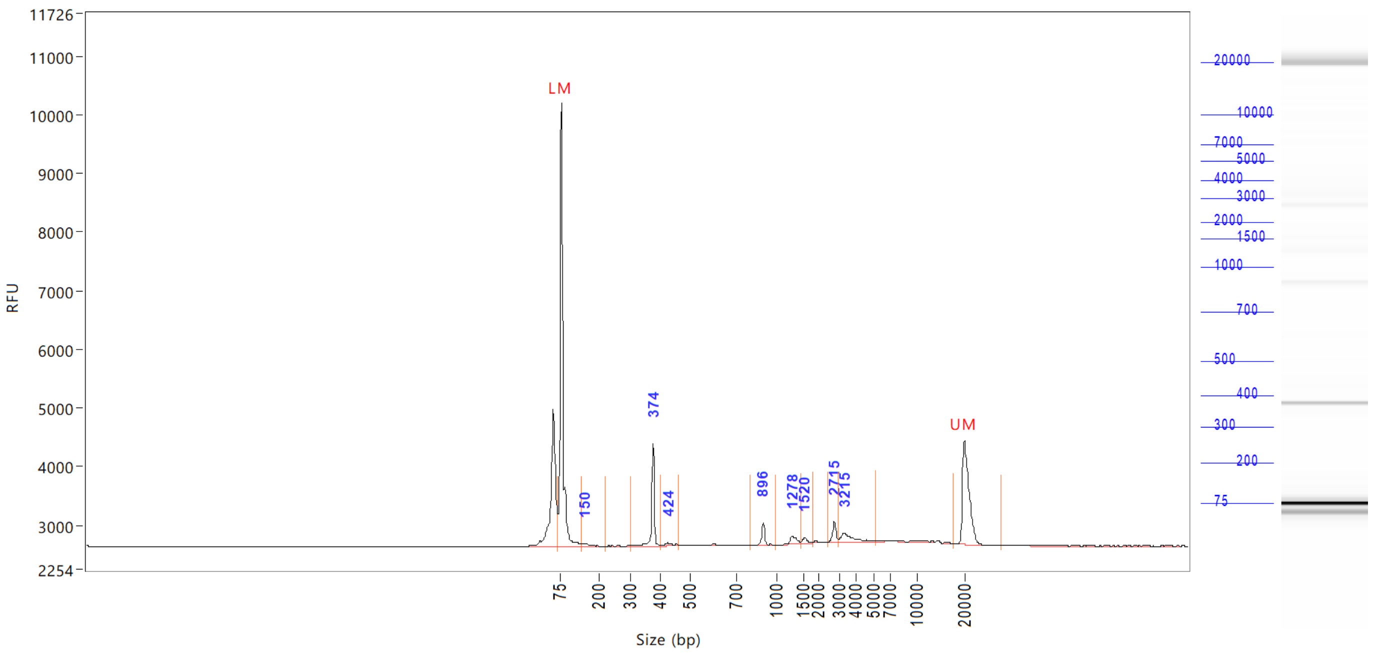

Peak	Size (bp)	Concentration (ng/uL)	From (bp)	To (bp)	Average size (bp)	CV%	RFU	Corrected peak area
1	75 (LM)	0.5000	66	143	76	13.55	7570	51.748
2	150	0.0983	143	221	161	10.26	54	0.848
3	374	1.1290	300	401	369	3.25	1738	9.737
4	424	0.0951	401	462	427	3.43	57	0.820
5	896	0.2522	801	987	893	2.07	376	2.175
6	1278	0.1801	987	1456	1299	5.70	146	1.553
7	1520	0.0890	1456	1789	1558	5.71	95	0.767
8	2715	0.2294	2458	2914	2700	3.28	353	1.978
9	3215	0.3325	2914	5223	3581	14.10	151	2.867
10	20000 (UM)	0.1531	17561	27859	20384	4.00	1761	15.850

TIC:	2.4055	ng/uL
TIM:	7.4837	nmole/L
Total concentration:	2.6608	ng/uL

Sample peak width (sec): 5 Sample min peak height: 50 Sample baseline V to V?: Y Sample baseline V to V points: 3
Sample filter: Binomial Number of points for filter: 3 Sample start region (min): 0 Sample end region (min): 30
Marker peak width (sec): 5 Marker min peak height: 200 Marker baseline V to V?: Y Marker baseline V to V points: 3
Lower marker selection: First peak > 500 RFU Upper marker selection: Last peak > 200 RFU
Ladder size (bp)75, 200, 300, 400, 500, 700, 1000, 1500, 2000, 3000, 4000, 5000, 7000, 10000, 20000
Quantification using: Lower Marker Final concentration (ng/uL): 0.5000 Dilution factor: 12.0

Sample: SampA5
Well location: A5
Created: Tuesday, September 14, 2021 7:33:54 PM

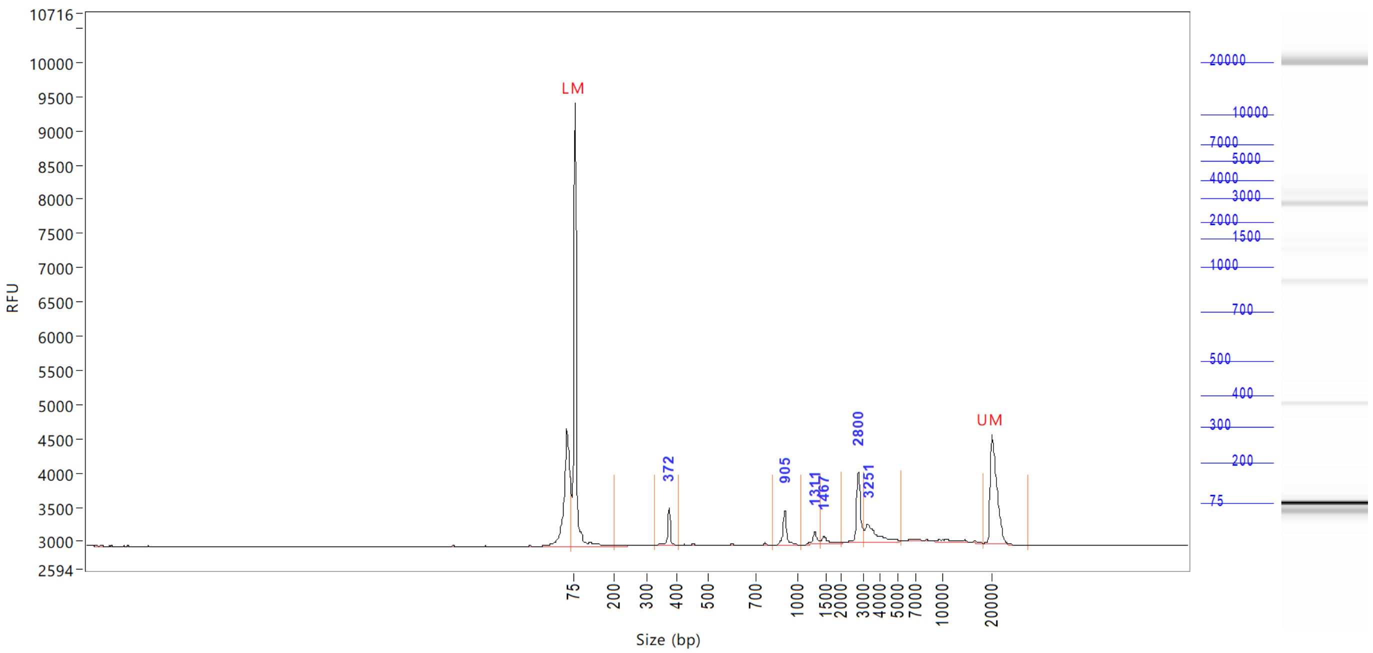

Peak	Size (bp)	Concentration (ng/uL)	From (bp)	To (bp)	Average size (bp)	CV%	RFU	Corrected peak area
1	75 (LM)	0.5000	66	203	77	18.10	6487	41.899
2	372	0.4638	325	403	368	2.85	546	3.238
3	905	0.4854	818	1045	904	2.64	507	3.390
4	1311	0.1883	1045	1400	1292	4.38	178	1.315
5	1467	0.1701	1400	2001	1565	10.24	105	1.188
6	2800	0.9002	2001	3037	2762	5.06	1024	6.286
7	3251	0.6423	3037	5223	3646	13.97	259	4.485
8	20000 (UM)	0.1657	18415	27375	20411	3.86	1596	13.885

TIC:	2.8500	ng/uL
TIM:	4.2023	nmole/L
Total concentration:	3.3351	ng/uL

Sample peak width (sec): 5   Sample min peak height: 50   Sample baseline V to V?: Y   Sample baseline V to V points: 3
Sample filter: Binomial   Number of points for filter: 3 Sample start region (min): 0 Sample end region (min): 30
Marker peak width (sec): 5   Marker min peak height: 200   Marker baseline V to V?: Y   Marker baseline V to V points: 3
Lower marker selection: First peak > 500 RFU   Upper marker selection: Last peak > 200 RFU
Ladder size (bp)75, 200, 300, 400, 500, 700, 1000, 1500, 2000, 3000, 4000, 5000, 7000, 10000, 20000
Quantification using: Lower Marker   Final concentration (ng/uL): 0.5000   Dilution factor: 12.0

Sample: SampB5
Well location: B5
Created: Tuesday, September 14, 2021 7:33:54 PM

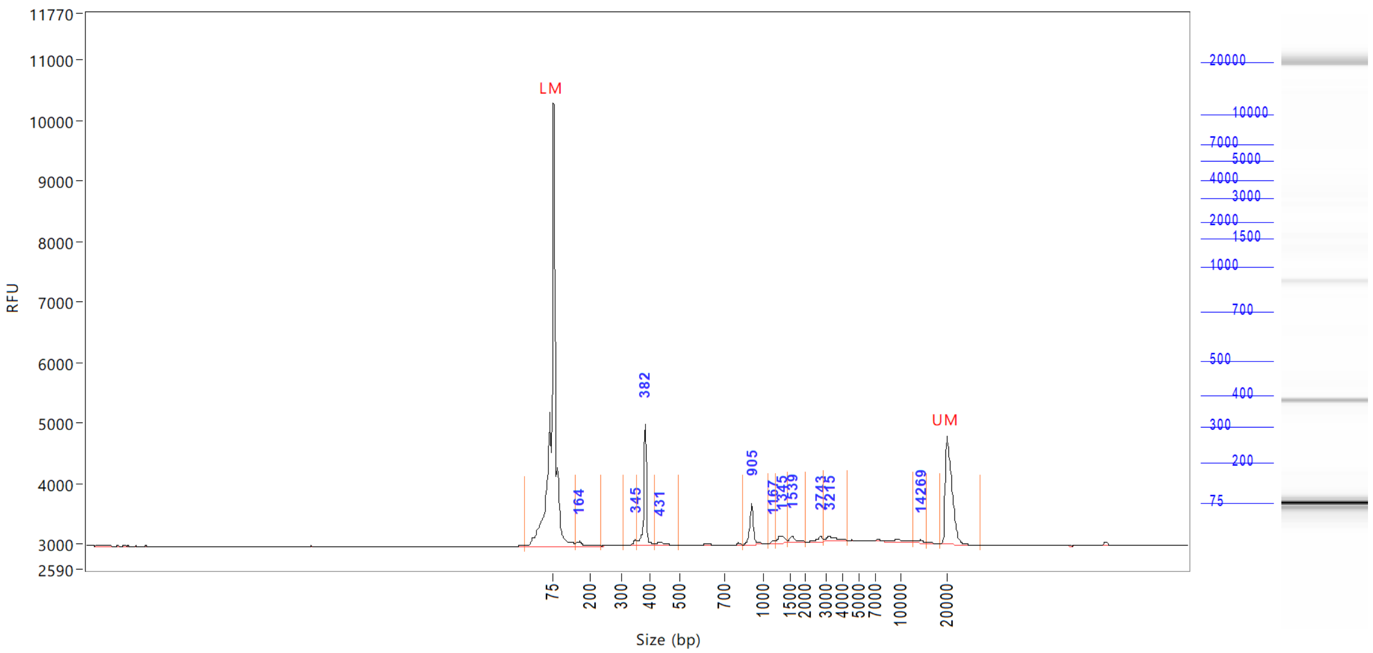

Peak	Size (bp)	Concentration (ng/uL)	From (bp)	To (bp)	Average size (bp)	CV%	RFU	Corrected peak area
1	75 (LM)	0.5000	0	148	68	29.89	7334	84.109
2	164	0.0954	148	235	170	11.56	78	1.337
3	345	0.0744	305	356	340	3.64	96	1.044
4	382	0.8438	356	416	380	2.18	1998	11.829
5	431	0.0793	416	496	443	4.47	53	1.111
6	905	0.3229	831	1068	903	2.52	689	4.526
7	1167	0.0286	1068	1223	1162	3.24	55	0.402
8	1345	0.1006	1223	1456	1329	4.55	113	1.410
9	1539	0.0811	1456	2001	1600	7.84	114	1.137
10	2743	0.0538	2001	2914	2647	6.37	92	0.754
11	3215	0.1080	2914	4345	3462	10.71	88	1.514
12	14269	0.0368	12562	15366	13884	5.23	53	0.516
13	20000 (UM)	0.0948	18415	27254	20435	4.14	1787	15.945

TIC:	1.8246	ng/uL
TIM:	6.1563	nmole/L
Total concentration:	2.0157	ng/uL

```
Sample peak width (sec): 5    Sample min peak height: 50    Sample baseline V to V?: Y    Sample baseline V to V points: 3
Sample filter: Binomial       Number of points for filter: 3  Sample start region (min): 0  Sample end region (min): 30
Marker peak width (sec): 5    Marker min peak height: 200   Marker baseline V to V?: Y    Marker baseline V to V points: 3
Lower marker selection: First peak > 500 RFU                Upper marker selection: Last peak > 200 RFU
Ladder size (bp)75, 200, 300, 400, 500, 700, 1000, 1500, 2000, 3000, 4000, 5000, 7000, 10000, 20000
Quantification using: Lower Marker          Final concentration (ng/uL): 0.5000          Dilution factor: 12.0
```

Sample: SampC5
Well location: C5
Created: Tuesday, September 14, 2021 7:33:54 PM

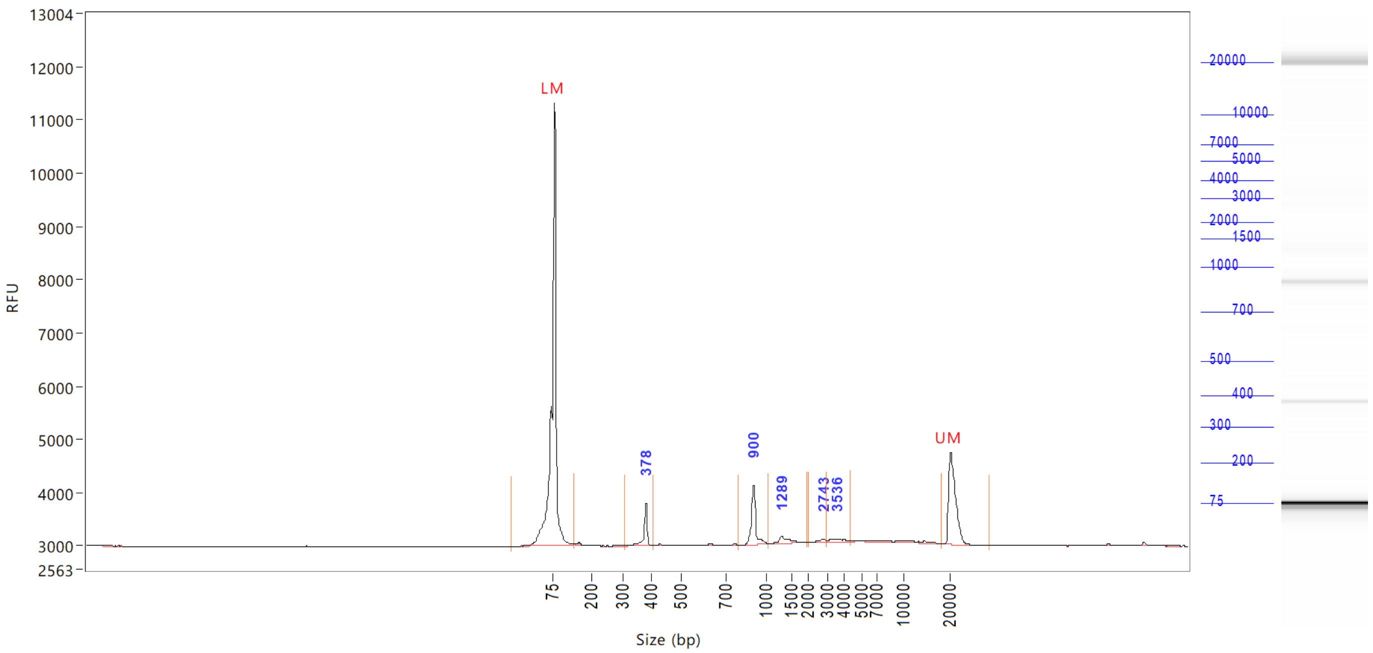

Peak	Size (bp)	Concentration (ng/uL)	From (bp)	To (bp)	Average size (bp)	CV%	RFU	Corrected peak area
1	75 (LM)	0.5000	0	143	66	25.30	8320	85.823
2	378	0.3909	305	403	371	3.88	810	5.591
3	900	0.5712	788	1034	902	3.01	1114	8.171
4	1289	0.1575	1034	1962	1353	10.07	132	2.252
5	2743	0.0425	2001	2914	2632	7.07	64	0.607
6	3536	0.0889	2914	4345	3485	10.32	60	1.271
7	20000 (UM)	0.0932	18171	28221	20520	4.12	1733	16.004

TIC:	1.2509	ng/uL
TIM:	3.0359	nmole/L
Total concentration:	1.5265	ng/uL

Sample peak width (sec): 5   Sample min peak height: 50   Sample baseline V to V?: Y   Sample baseline V to V points: 3
Sample filter: Binomial   Number of points for filter: 3   Sample start region (min): 0   Sample end region (min): 30
Marker peak width (sec): 5   Marker min peak height: 200   Marker baseline V to V?: Y   Marker baseline V to V points: 3
Lower marker selection: First peak > 500 RFU   Upper marker selection: Last peak > 200 RFU
Ladder size (bp)75, 200, 300, 400, 500, 700, 1000, 1500, 2000, 3000, 4000, 5000, 7000, 10000, 20000
Quantification using: Lower Marker   Final concentration (ng/uL): 0.5000   Dilution factor: 12.0

Sample: SampD5
Well location: D5
Created: Tuesday, September 14, 2021 7:33:54 PM

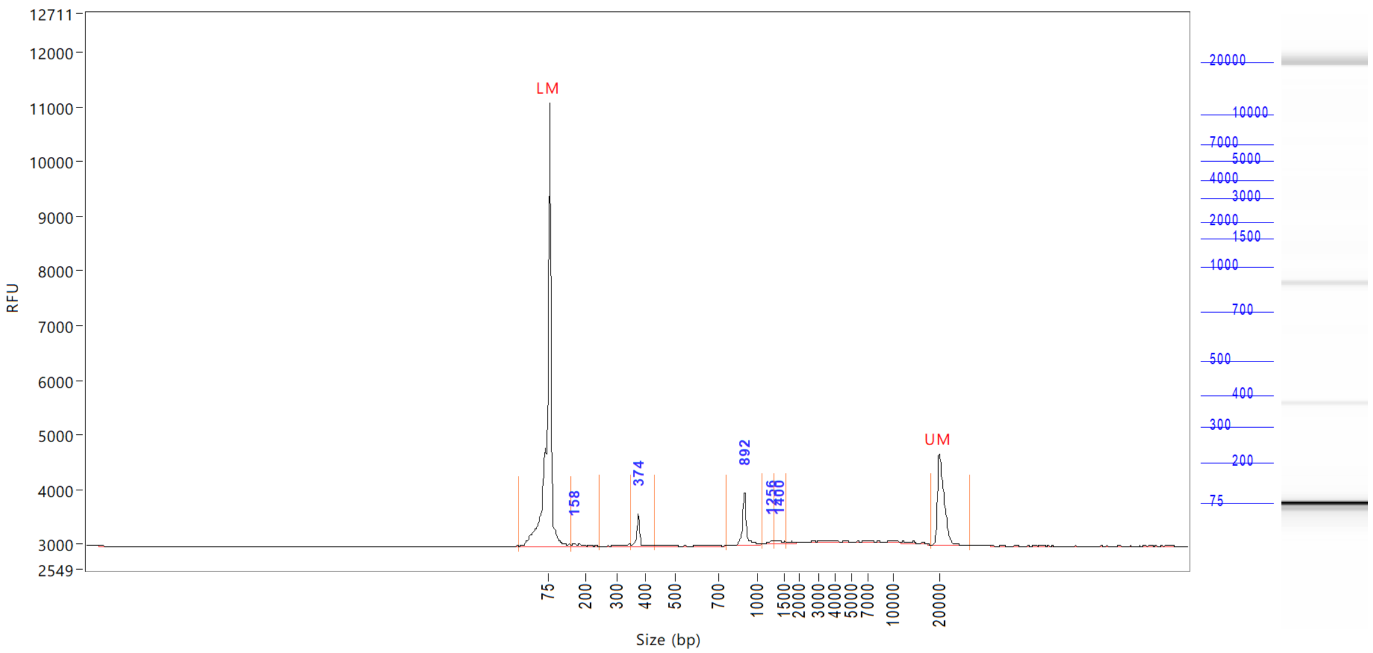

Peak	Size (bp)	Concentration (ng/uL)	From (bp)	To (bp)	Average size (bp)	CV%	RFU	Corrected peak area
1	75 (LM)	0.5000	0	147	64	31.23	8120	83.239
2	158	0.0762	147	244	172	11.80	56	1.057
3	374	0.2746	349	433	373	3.27	597	3.809
4	892	0.4900	757	1068	893	2.88	962	6.798
5	1256	0.0428	1068	1311	1231	4.50	64	0.594
6	1400	0.0609	1311	1597	1419	5.29	60	0.846
7	20000 (UM)	0.0918	18171	26649	20458	4.25	1666	15.282

TIC:	0.9445	ng/uL
TIM:	2.9701	nmole/L
Total concentration:	1.2985	ng/uL

Sample peak width (sec): 5   Sample min peak height: 50   Sample baseline V to V?: Y   Sample baseline V to V points: 3
Sample filter: Binomial   Number of points for filter: 3 Sample start region (min): 0 Sample end region (min): 30
Marker peak width (sec): 5   Marker min peak height: 200   Marker baseline V to V?: Y   Marker baseline V to V points: 3
Lower marker selection: First peak > 500 RFU   Upper marker selection: Last peak > 200 RFU
Ladder size (bp)75, 200, 300, 400, 500, 700, 1000, 1500, 2000, 3000, 4000, 5000, 7000, 10000, 20000
Quantification using: Lower Marker   Final concentration (ng/uL): 0.5000   Dilution factor: 12.0

Sample: SampE5
Well location: E5
Created: Tuesday, September 14, 2021 7:33:54 PM

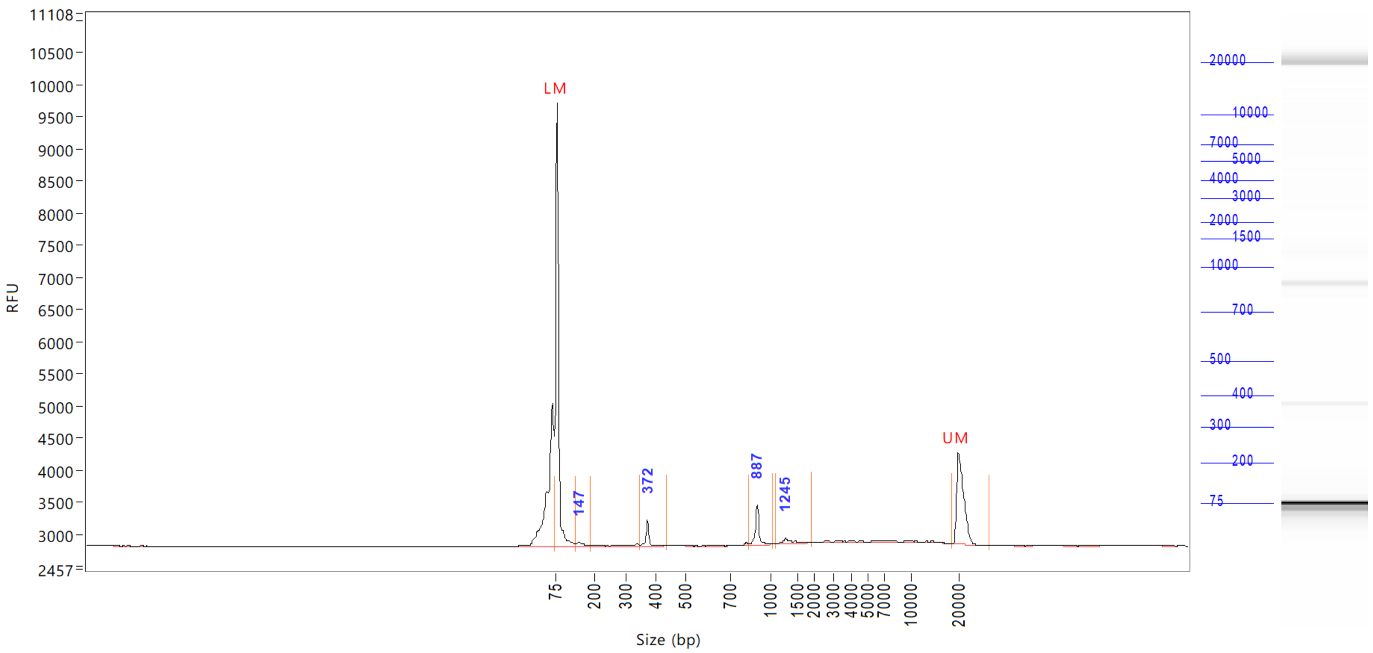

Peak	Size (bp)	Concentration (ng/uL)	From (bp)	To (bp)	Average size (bp)	CV%	RFU	Corrected peak area
1	75 (LM)	0.5000	67	135	76	12.46	6913	46.576
2	147	0.1379	135	189	153	8.83	68	1.070
3	372	0.3230	343	437	371	3.69	397	2.507
4	887	0.5202	831	1012	889	2.74	616	4.038
5	1245	0.1617	1090	1942	1322	10.13	85	1.255
6	20000 (UM)	0.1413	18659	26408	20535	3.98	1415	13.165

TIC:	1.1427	ng/uL
TIM:	4.0777	nmole/L
Total concentration:	1.6709	ng/uL

Sample peak width (sec): 5 Sample min peak height: 50 Sample baseline V to V?: Y Sample baseline V to V points: 3
Sample filter: Binomial Number of points for filter: 3 Sample start region (min): 0 Sample end region (min): 30
Marker peak width (sec): 5 Marker min peak height: 200 Marker baseline V to V?: Y Marker baseline V to V points: 3
Lower marker selection: First peak > 500 RFU Upper marker selection: Last peak > 200 RFU
Ladder size (bp)75, 200, 300, 400, 500, 700, 1000, 1500, 2000, 3000, 4000, 5000, 7000, 10000, 20000
Quantification using: Lower Marker Final concentration (ng/uL): 0.5000 Dilution factor: 12.0

Sample: SampF5
Well location: F5
Created: Tuesday, September 14, 2021 7:33:54 PM

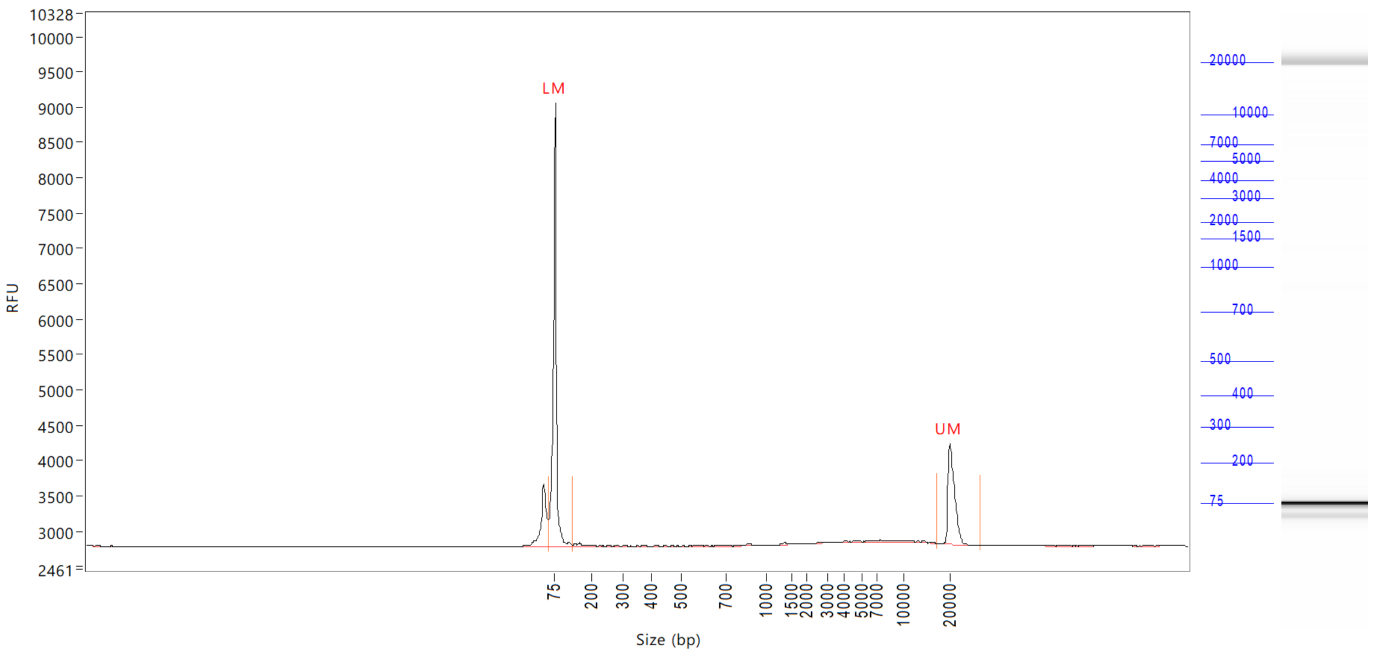

Peak	Size	Concentration	From	To	Average size	CV%	RFU	Corrected peak area
	(bp)	(ng/uL)	(bp)	(bp)	(bp)			
1	75 (LM)	0.5000	52	131	72	12.87	6284	48.309
2	20000 (UM)	0.1302	17195	26649	20454	4.03	1417	12.575

TIC:	0.0000	ng/uL
TIM:	0.0000	nmole/L
Total concentration:	0.4955	ng/uL

Sample peak width (sec): 5 Sample min peak height: 50 Sample baseline V to V?: Y Sample baseline V to V points: 3
Sample filter: Binomial Number of points for filter: 3 Sample start region (min): 0 Sample end region (min): 30
Marker peak width (sec): 5 Marker min peak height: 200 Marker baseline V to V?: Y Marker baseline V to V points: 3
Lower marker selection: First peak > 500 RFU Upper marker selection: Last peak > 200 RFU
Ladder size (bp)75, 200, 300, 400, 500, 700, 1000, 1500, 2000, 3000, 4000, 5000, 7000, 10000, 20000
Quantification using: Lower Marker Final concentration (ng/uL): 0.5000 Dilution factor: 12.0

Sample: SampG5
Well location: G5
Created: Tuesday, September 14, 2021 7:33:54 PM

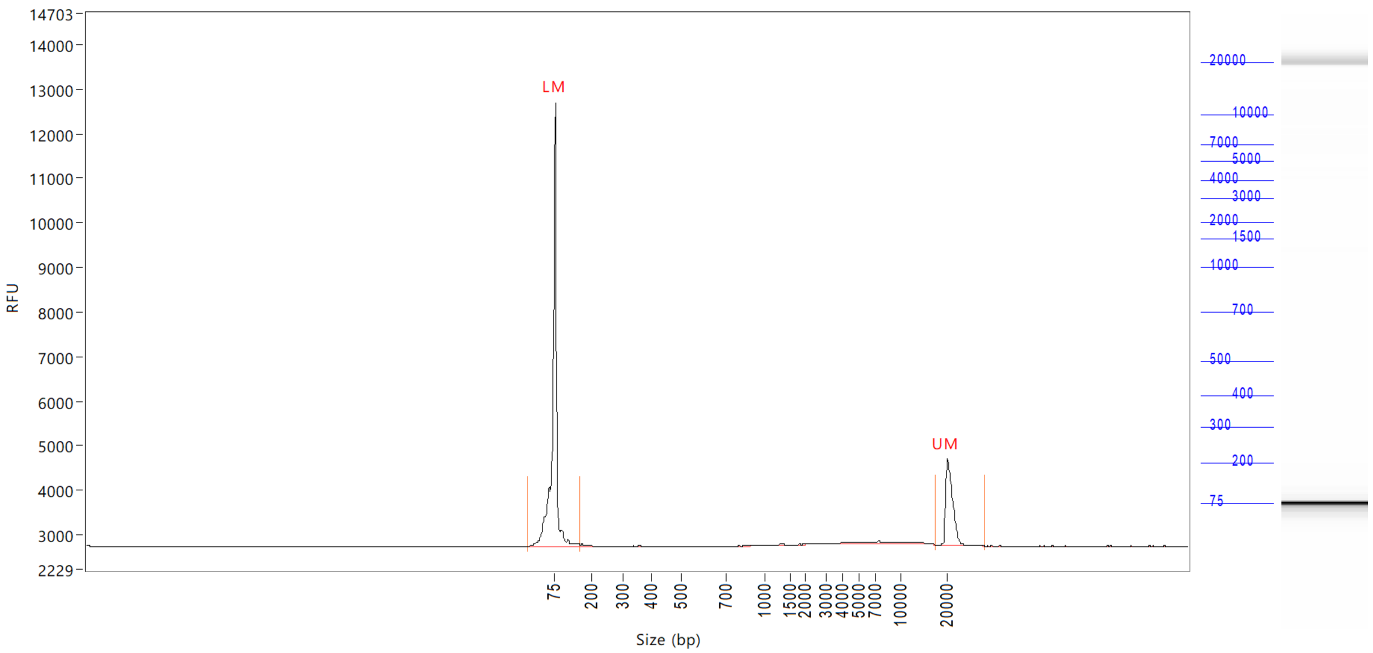

Peak	Size (bp)	Concentration (ng/uL)	From (bp)	To (bp)	Average size (bp)	CV%	RFU	Corrected peak area
1	75 (LM)	0.5000	0	160	67	27.16	9970	95.398
2	20000 (UM)	0.0933	17439	28100	20520	4.11	1935	17.811

TIC:	0.0000	ng/uL
TIM:	0.0000	nmole/L
Total concentration:	0.3448	ng/uL

Sample peak width (sec): 5   Sample min peak height: 50   Sample baseline V to V?: Y   Sample baseline V to V points: 3
Sample filter: Binomial   Number of points for filter: 3 Sample start region (min): 0 Sample end region (min): 30
Marker peak width (sec): 5   Marker min peak height: 200   Marker baseline V to V?: Y   Marker baseline V to V points: 3
Lower marker selection: First peak > 500 RFU   Upper marker selection: Last peak > 200 RFU
Ladder size (bp)75, 200, 300, 400, 500, 700, 1000, 1500, 2000, 3000, 4000, 5000, 7000, 10000, 20000
Quantification using: Lower Marker   Final concentration (ng/uL): 0.5000   Dilution factor: 12.0

Sample: SampH5
Well location: H5
Created: Tuesday, September 14, 2021 7:33:54 PM

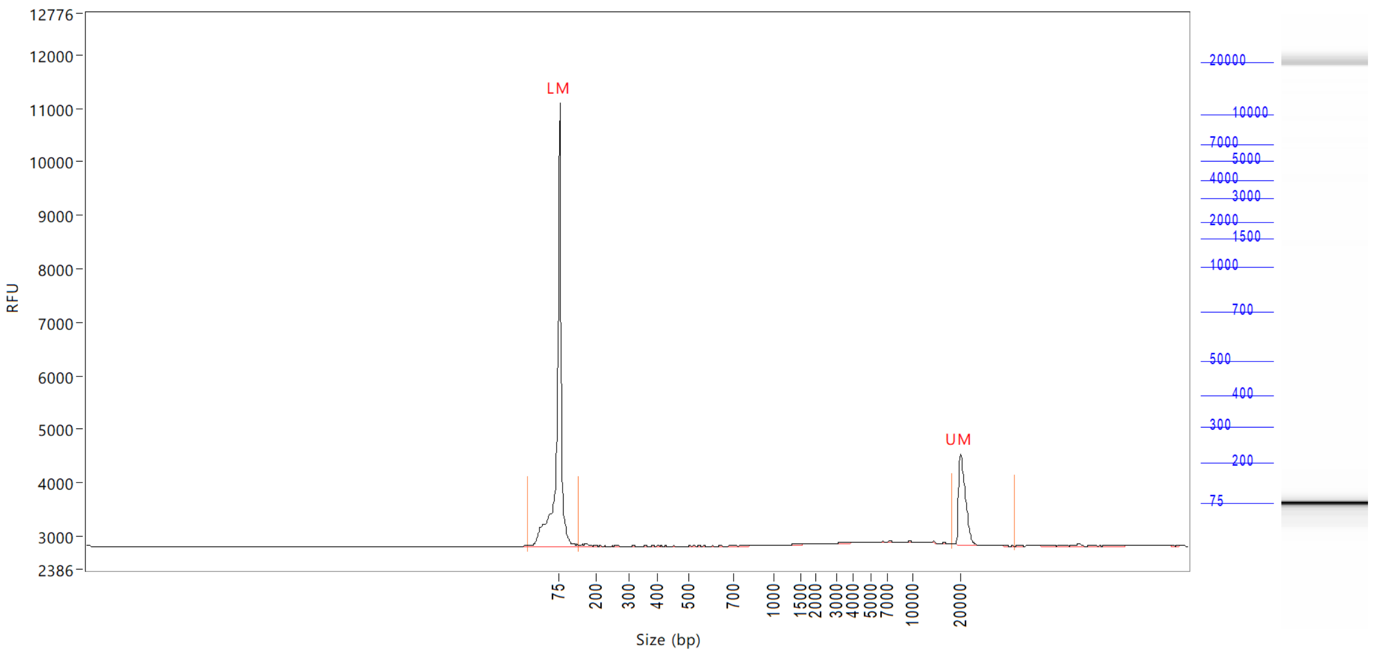

Peak	Size (bp)	Concentration (ng/uL)	From (bp)	To (bp)	Average size (bp)	CV%	RFU	Corrected peak area
1	75 (LM)	0.5000	0	141	64	33.72	8304	85.041
2	20000 (UM)	0.0898	18171	31365	20439	4.96	1701	15.276

TIC:	0.0000	ng/uL
TIM:	0.0000	nmole/L
Total concentration:	0.3083	ng/uL

Sample peak width (sec): 5 Sample min peak height: 50 Sample baseline V to V?: Y Sample baseline V to V points: 3
Sample filter: Binomial Number of points for filter: 3 Sample start region (min): 0 Sample end region (min): 30
Marker peak width (sec): 5 Marker min peak height: 200 Marker baseline V to V?: Y Marker baseline V to V points: 3
Lower marker selection: First peak > 500 RFU Upper marker selection: Last peak > 200 RFU
Ladder size (bp)75, 200, 300, 400, 500, 700, 1000, 1500, 2000, 3000, 4000, 5000, 7000, 10000, 20000
Quantification using: Lower Marker Final concentration (ng/uL): 0.5000 Dilution factor: 12.0

Sample: SampA6
Well location: A6
Created: Tuesday, September 14, 2021 7:33:54 PM

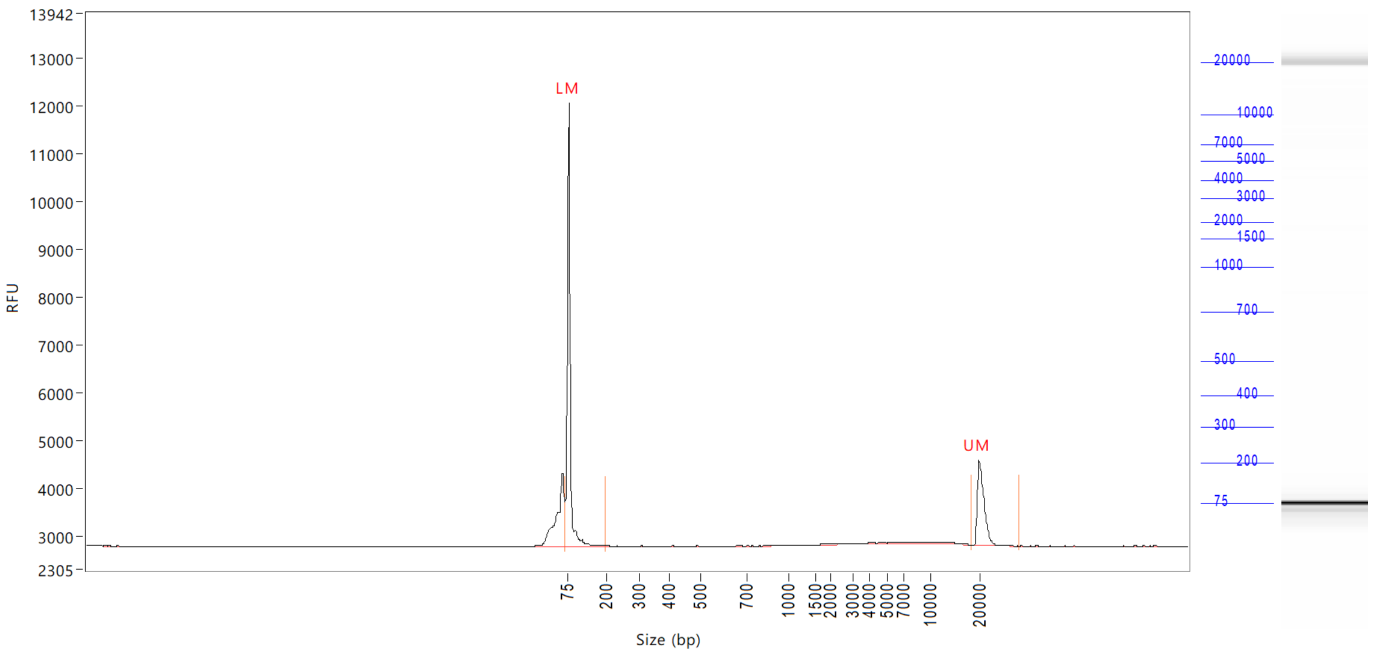

Peak	Size	Concentration	From	To	Average size	CV%	RFU	Corrected peak area
	(bp)	(ng/uL)	(bp)	(bp)	(bp)			
1	75 (LM)	0.5000	64	196	77	17.21	9300	64.873
2	20000 (UM)	0.1209	18293	28342	20449	4.16	1776	15.691

TIC:	0.0000	ng/uL
TIM:	0.0000	nmole/L
Total concentration:	0.4647	ng/uL

Sample peak width (sec): 5   Sample min peak height: 50   Sample baseline V to V?: Y   Sample baseline V to V points: 3
Sample filter: Binomial   Number of points for filter: 3 Sample start region (min): 0 Sample end region (min): 30
Marker peak width (sec): 5   Marker min peak height: 200   Marker baseline V to V?: Y   Marker baseline V to V points: 3
Lower marker selection: First peak > 500 RFU   Upper marker selection: Last peak > 200 RFU
Ladder size (bp)75, 200, 300, 400, 500, 700, 1000, 1500, 2000, 3000, 4000, 5000, 7000, 10000, 20000
Quantification using: Lower Marker   Final concentration (ng/uL): 0.5000   Dilution factor: 12.0

Sample: SampB6
Well location: B6
Created: Tuesday, September 14, 2021 7:33:54 PM

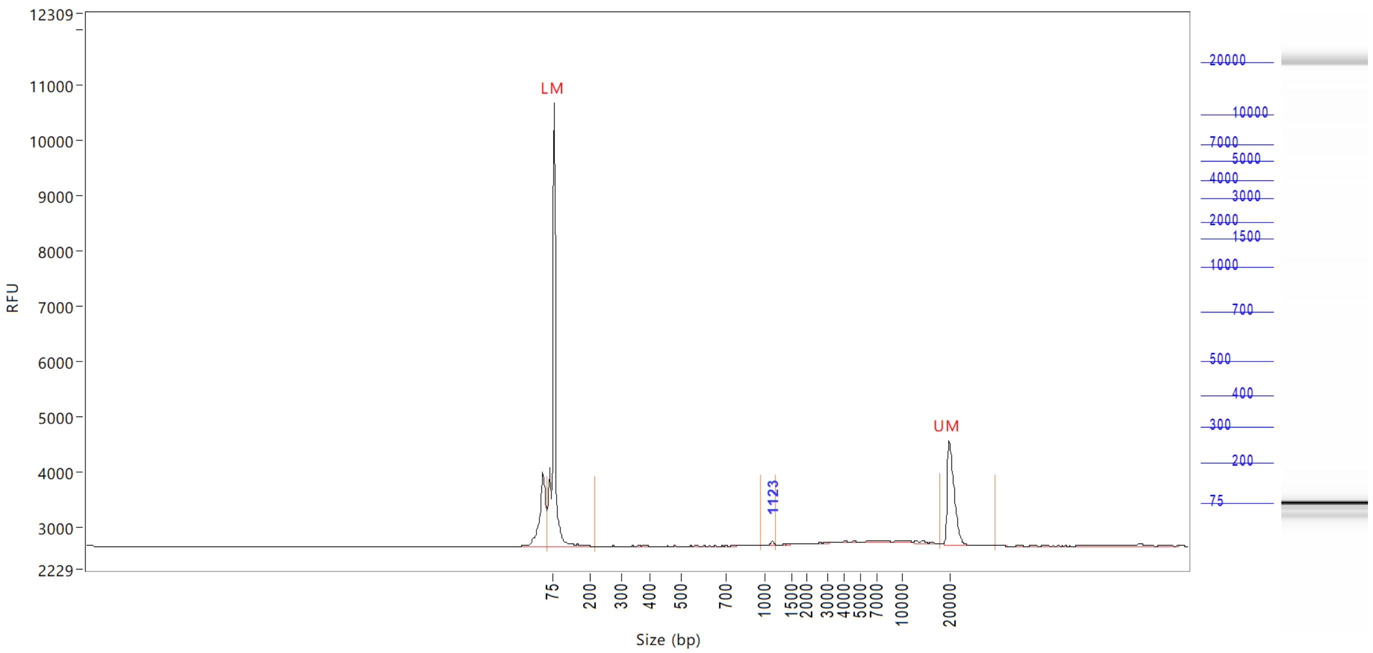

Peak	Size (bp)	Concentration (ng/uL)	From (bp)	To (bp)	Average size (bp)	CV%	RFU	Corrected peak area
1	75 (LM)	0.5000	52	214	74	19.29	8055	63.021
2	1123	0.0436	965	1212	1116	3.25	77	0.457
3	20000 (UM)	0.1351	18171	29914	20485	4.28	1876	17.030

TIC:	0.0436	ng/uL
TIM:	0.0642	nmole/L
Total concentration:	0.4320	ng/uL

Sample peak width (sec): 5   Sample min peak height: 50   Sample baseline V to V?: Y   Sample baseline V to V points: 3
Sample filter: Binomial   Number of points for filter: 3   Sample start region (min): 0   Sample end region (min): 30
Marker peak width (sec): 5   Marker min peak height: 200   Marker baseline V to V?: Y   Marker baseline V to V points: 3
Lower marker selection: First peak > 500 RFU   Upper marker selection: Last peak > 200 RFU
Ladder size (bp)75, 200, 300, 400, 500, 700, 1000, 1500, 2000, 3000, 4000, 5000, 7000, 10000, 20000
Quantification using: Lower Marker   Final concentration (ng/uL): 0.5000   Dilution factor: 12.0

Sample: SampC6
Well location: C6
Created: Tuesday, September 14, 2021 7:33:54 PM

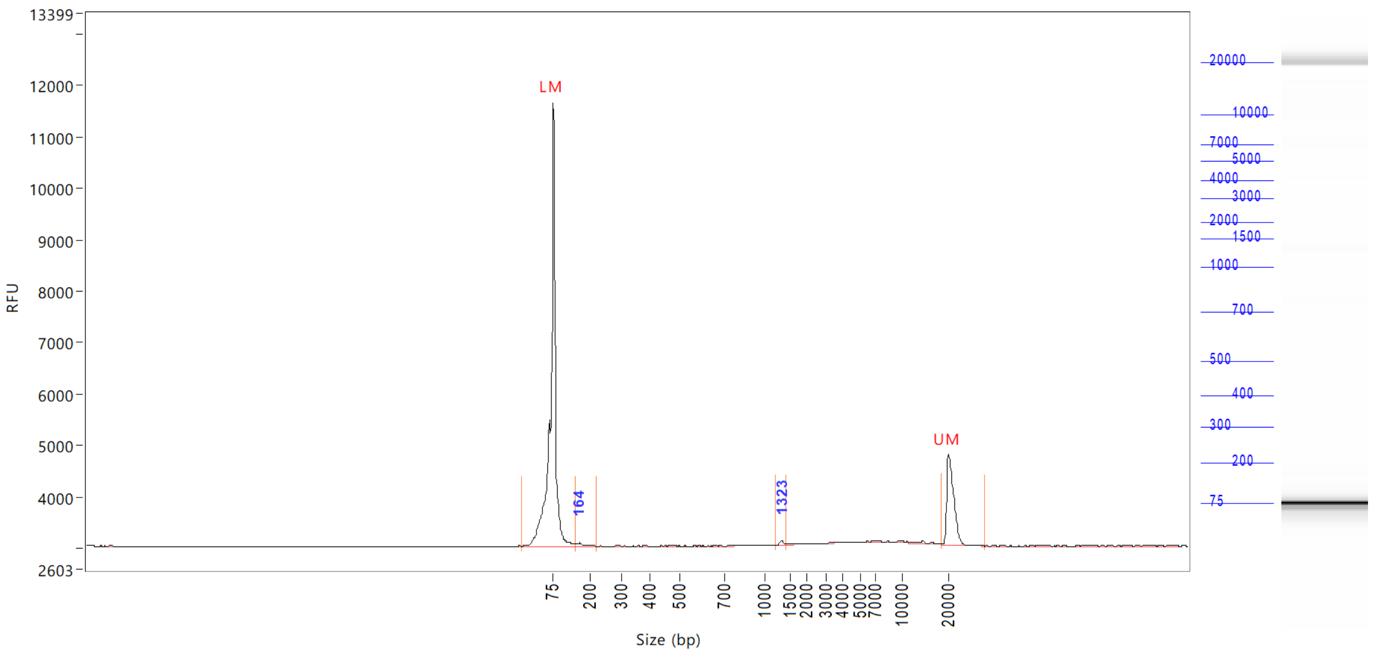

Peak	Size (bp)	Concentration (ng/uL)	From (bp)	To (bp)	Average size (bp)	CV%	RFU	Corrected peak area
1	75 (LM)	0.5000	0	148	66	30.11	8627	103.358
2	164	0.0678	148	217	169	9.80	67	1.169
3	1323	0.0296	1212	1411	1312	2.69	87	0.511
4	20000 (UM)	0.0783	18415	27738	20490	3.97	1769	16.177

TIC:	0.0975	ng/uL
TIM:	0.6970	nmole/L
Total concentration:	0.3349	ng/uL

Sample peak width (sec): 5 Sample min peak height: 50 Sample baseline V to V?: Y Sample baseline V to V points: 3
Sample filter: Binomial Number of points for filter: 3 Sample start region (min): 0 Sample end region (min): 30
Marker peak width (sec): 5 Marker min peak height: 200 Marker baseline V to V?: Y Marker baseline V to V points: 3
Lower marker selection: First peak > 500 RFU Upper marker selection: Last peak > 200 RFU
Ladder size (bp)75, 200, 300, 400, 500, 700, 1000, 1500, 2000, 3000, 4000, 5000, 7000, 10000, 20000
Quantification using: Lower Marker Final concentration (ng/uL): 0.5000 Dilution factor: 12.0

Sample: SampD6
Well location: D6
Created: Tuesday, September 14, 2021 7:33:54 PM

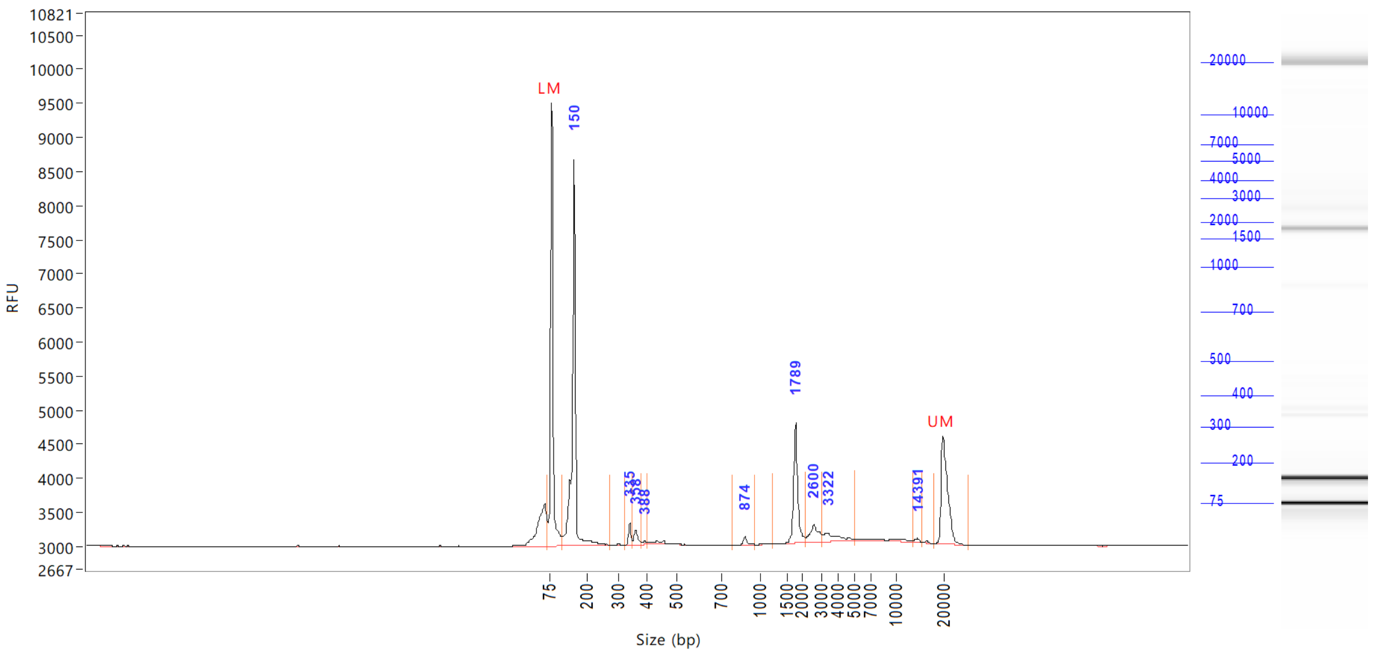

Peak	Size (bp)	Concentration (ng/uL)	From (bp)	To (bp)	Average size (bp)	CV%	RFU	Corrected peak area
1	75 (LM)	0.5000	62	112	75	10.23	6511	41.886
2	150	6.3990	112	269	149	10.88	5662	44.672
3	335	0.2409	317	343	333	1.35	326	1.682
4	358	0.2594	343	380	357	2.39	213	1.811
5	388	0.0660	380	401	390	1.65	52	0.461
6	874	0.1243	779	957	872	2.85	128	0.868
7	1789	1.6802	1223	2172	1784	5.73	1760	11.729
8	2600	0.4947	2172	3001	2601	8.34	255	3.454
9	3322	0.4155	3001	5001	3710	14.07	132	2.900
10	14391	0.0554	13415	15610	14289	3.45	59	0.387
11	20000 (UM)	0.1680	18171	25561	20405	3.92	1576	14.077

TIC:	9.7353	ng/uL
TIM:	75.5341	nmole/L
Total concentration:	10.1898	ng/uL

```
Sample peak width (sec): 5     Sample min peak height: 50    Sample baseline V to V?: Y    Sample baseline V to V points: 3
Sample filter: Binomial        Number of points for filter: 3  Sample start region (min): 0  Sample end region (min): 30
Marker peak width (sec): 5     Marker min peak height: 200   Marker baseline V to V?: Y    Marker baseline V to V points: 3
Lower marker selection: First peak > 500 RFU                 Upper marker selection: Last peak > 200 RFU
Ladder size (bp)75, 200, 300, 400, 500, 700, 1000, 1500, 2000, 3000, 4000, 5000, 7000, 10000, 20000
Quantification using: Lower Marker          Final concentration (ng/uL): 0.5000         Dilution factor: 12.0
```

Sample: SampE6
Well location: E6
Created: Tuesday, September 14, 2021 7:33:54 PM

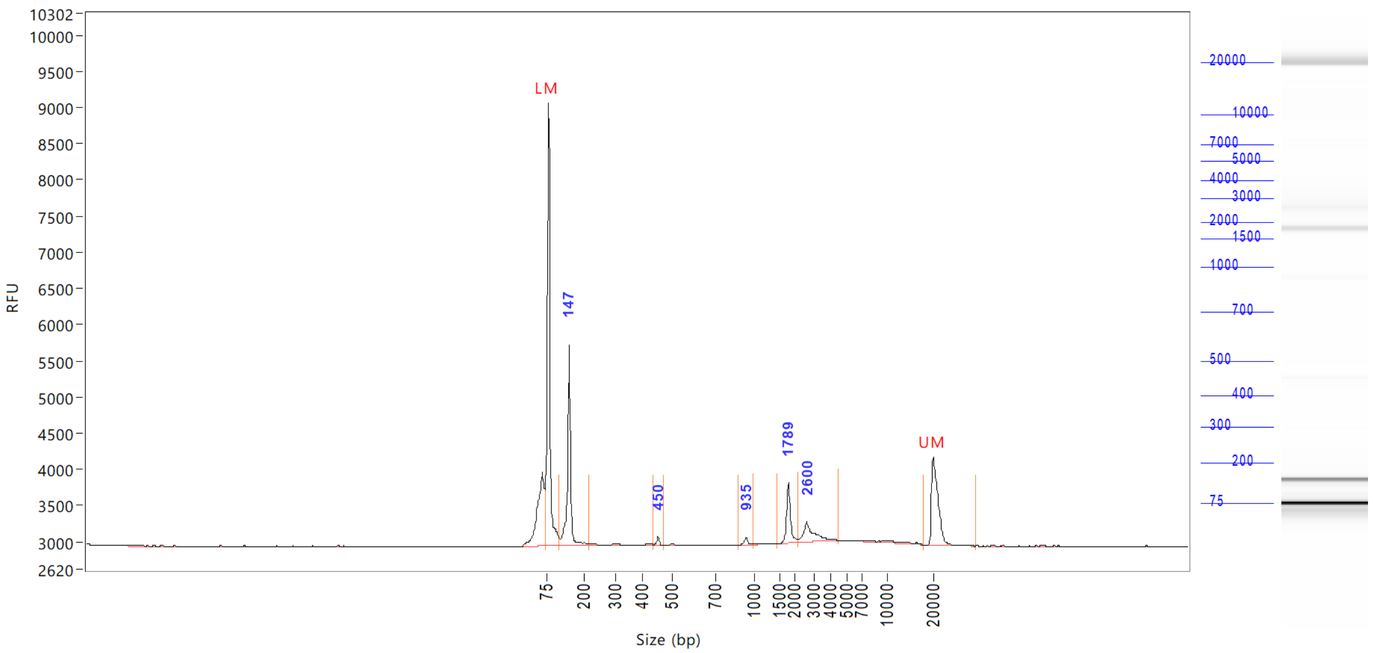

Peak	Size (bp)	Concentration (ng/uL)	From (bp)	To (bp)	Average size (bp)	CV%	RFU	Corrected peak area
1	75 (LM)	0.5000	64	114	75	10.78	6130	41.109
2	147	3.0166	114	217	144	8.85	2773	20.668
3	450	0.0922	435	470	448	1.05	117	0.632
4	935	0.1031	870	996	930	2.01	106	0.706
5	1789	0.8064	1456	2144	1788	5.33	828	5.525
6	2600	0.6473	2144	4518	2842	15.61	276	4.435
7	20000 (UM)	0.1326	17805	29309	20510	4.68	1223	10.898

TIC:	4.6655	ng/uL
TIM:	36.0651	nmole/L
Total concentration:	5.0478	ng/uL

Sample peak width (sec): 5 Sample min peak height: 50 Sample baseline V to V?: Y Sample baseline V to V points: 3
Sample filter: Binomial Number of points for filter: 3 Sample start region (min): 0 Sample end region (min): 30
Marker peak width (sec): 5 Marker min peak height: 200 Marker baseline V to V?: Y Marker baseline V to V points: 3
Lower marker selection: First peak > 500 RFU Upper marker selection: Last peak > 200 RFU
Ladder size (bp)75, 200, 300, 400, 500, 700, 1000, 1500, 2000, 3000, 4000, 5000, 7000, 10000, 20000
Quantification using: Lower Marker Final concentration (ng/uL): 0.5000 Dilution factor: 12.0

Sample: SampF6
Well location: F6
Created: Tuesday, September 14, 2021 7:33:54 PM

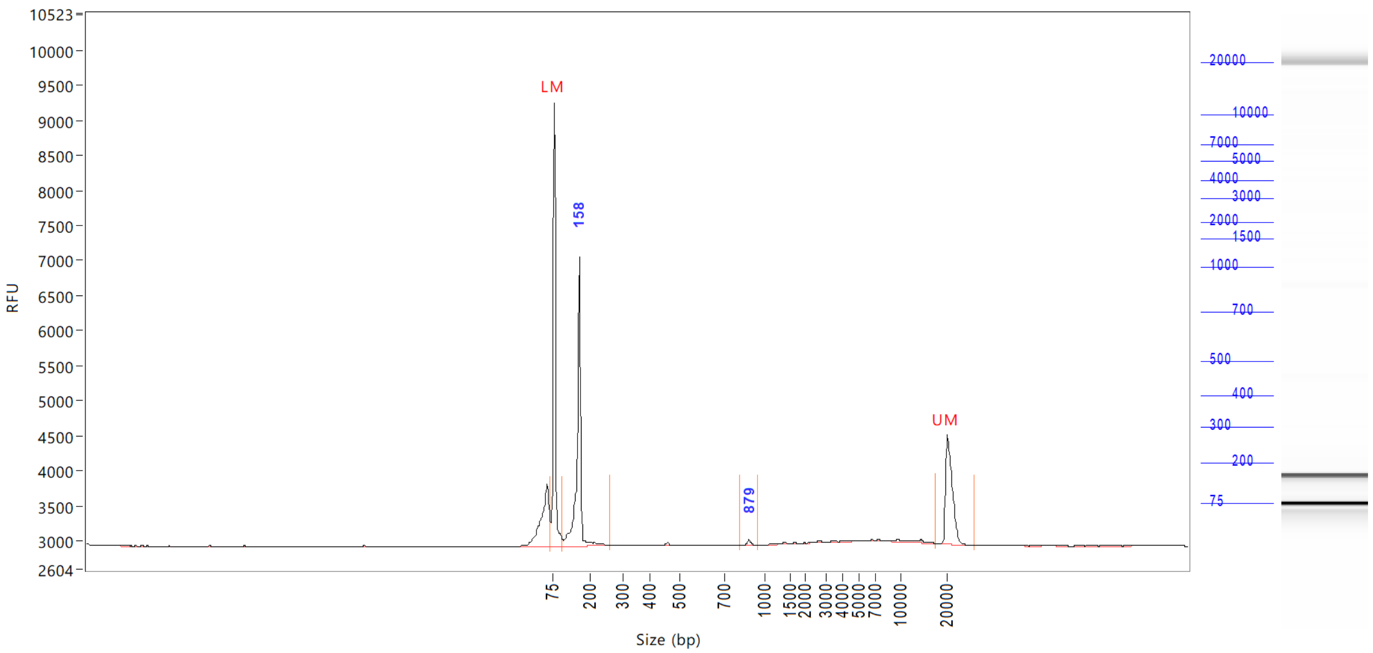

Peak	Size (bp)	Concentration (ng/uL)	From (bp)	To (bp)	Average size (bp)	CV%	RFU	Corrected peak area
1	75 (LM)	0.5000	62	103	73	7.71	6327	39.219
2	158	5.3665	103	257	153	11.00	4128	35.078
3	879	0.0852	809	952	877	2.50	80	0.557
4	20000 (UM)	0.1767	17439	25803	20460	3.87	1565	13.863

TIC:	5.4516	ng/uL
TIM:	57.8078	nmole/L
Total concentration:	6.1345	ng/uL

Sample peak width (sec): 5 Sample min peak height: 50 Sample baseline V to V?: Y Sample baseline V to V points: 3
Sample filter: Binomial Number of points for filter: 3 Sample start region (min): 0 Sample end region (min): 30
Marker peak width (sec): 5 Marker min peak height: 200 Marker baseline V to V?: Y Marker baseline V to V points: 3
Lower marker selection: First peak > 500 RFU Upper marker selection: Last peak > 200 RFU
Ladder size (bp)75, 200, 300, 400, 500, 700, 1000, 1500, 2000, 3000, 4000, 5000, 7000, 10000, 20000
Quantification using: Lower Marker Final concentration (ng/uL): 0.5000 Dilution factor: 12.0

Sample: SampG6
Well location: G6
Created: Tuesday, September 14, 2021 7:33:54 PM

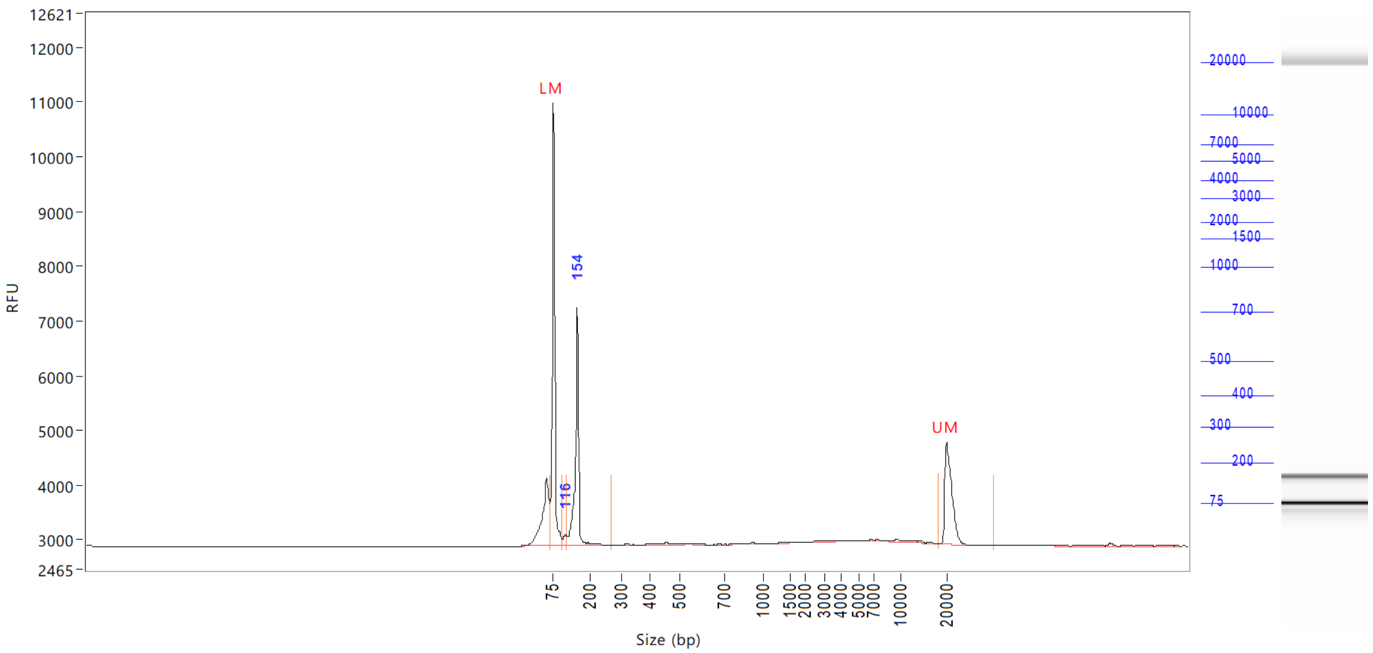

Peak	Size	Concentration	From	To	Average size	CV%	RFU	Corrected peak area
	(bp)	(ng/uL)	(bp)	(bp)	(bp)			
1	75 (LM)	0.5000	66	103	75	7.57	8104	51.961
2	116	0.2430	103	122	113	5.17	188	2.104
3	154	3.9507	122	268	152	9.05	4339	34.214
4	20000 (UM)	0.1694	18293	30156	20545	4.86	1869	17.609

TIC:	4.1937	ng/uL
TIM:	46.2505	nmole/L
Total concentration:	4.7819	ng/uL

Sample peak width (sec): 5 Sample min peak height: 50 Sample baseline V to V?: Y Sample baseline V to V points: 3
Sample filter: Binomial Number of points for filter: 3 Sample start region (min): 0 Sample end region (min): 30
Marker peak width (sec): 5 Marker min peak height: 200 Marker baseline V to V?: Y Marker baseline V to V points: 3
Lower marker selection: First peak > 500 RFU Upper marker selection: Last peak > 200 RFU
Ladder size (bp)75, 200, 300, 400, 500, 700, 1000, 1500, 2000, 3000, 4000, 5000, 7000, 10000, 20000
Quantification using: Lower Marker Final concentration (ng/uL): 0.5000 Dilution factor: 12.0

Sample: SampH6
Well location: H6
Created: Tuesday, September 14, 2021 7:33:54 PM

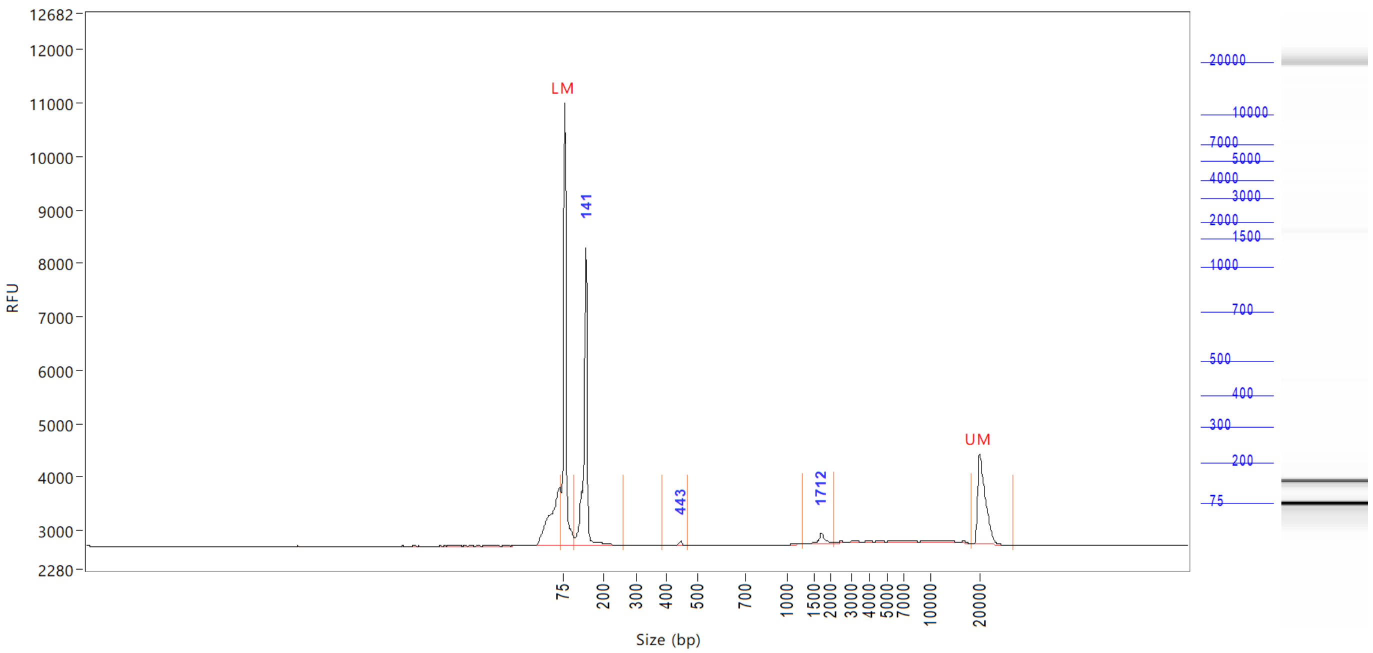

Peak	Size (bp)	Concentration (ng/uL)	From (bp)	To (bp)	Average size (bp)	CV%	RFU	Corrected peak area
1	75 (LM)	0.5000	64	103	73	8.13	8300	55.520
2	141	4.7124	103	257	138	10.92	5577	43.606
3	443	0.0873	384	468	430	4.12	86	0.808
4	1712	0.2195	1289	2201	1734	9.53	208	2.031
5	20000 (UM)	0.1521	18415	26891	20526	4.37	1678	16.888

TIC:	5.0192	ng/uL
TIM:	56.6566	nmole/L
Total concentration:	5.4372	ng/uL

```
Sample peak width (sec): 5    Sample min peak height: 50    Sample baseline V to V?: Y    Sample baseline V to V points: 3
Sample filter: Binomial       Number of points for filter: 3  Sample start region (min): 0  Sample end region (min): 30
Marker peak width (sec): 5    Marker min peak height: 200   Marker baseline V to V?: Y    Marker baseline V to V points: 3
Lower marker selection: First peak > 500 RFU                Upper marker selection: Last peak > 200 RFU
Ladder size (bp)75, 200, 300, 400, 500, 700, 1000, 1500, 2000, 3000, 4000, 5000, 7000, 10000, 20000
Quantification using: Lower Marker          Final concentration (ng/uL): 0.5000          Dilution factor: 12.0
```

Sample: SampA7
Well location: A7
Created: Tuesday, September 14, 2021 7:33:54 PM

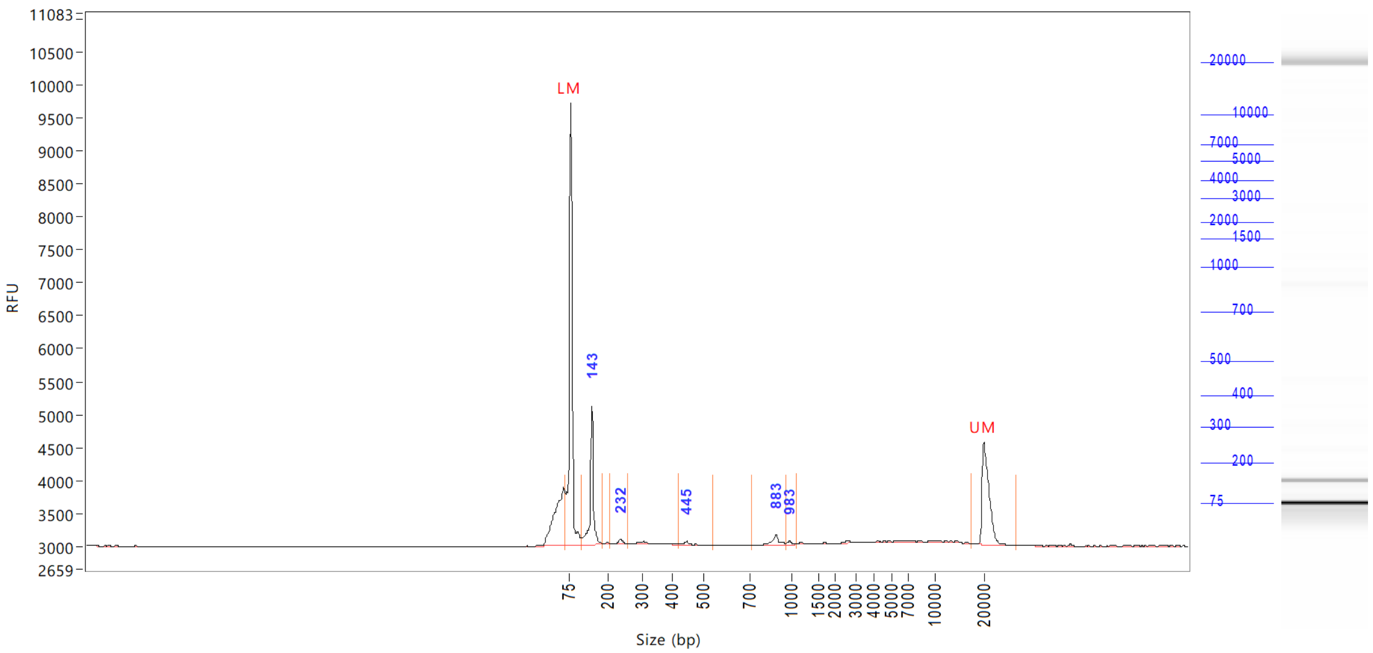

Peak	Size (bp)	Concentration (ng/uL)	From (bp)	To (bp)	Average size (bp)	CV%	RFU	Corrected peak area
1	75 (LM)	0.5000	60	112	74	10.07	6713	46.574
2	143	1.9844	112	179	140	6.63	2089	15.403
3	232	0.1018	203	257	232	3.59	78	0.790
4	445	0.0677	420	539	452	5.13	50	0.525
5	883	0.2239	714	957	874	4.30	158	1.738
6	983	0.0565	957	1101	991	3.23	57	0.438
7	20000 (UM)	0.1486	17439	26649	20446	4.09	1556	13.842

TIC:	2.4342	ng/uL
TIM:	24.7759	nmole/L
Total concentration:	2.8984	ng/uL

```
Sample peak width (sec): 5    Sample min peak height: 50    Sample baseline V to V?: Y    Sample baseline V to V points: 3
Sample filter: Binomial       Number of points for filter: 3  Sample start region (min): 0  Sample end region (min): 30
Marker peak width (sec): 5    Marker min peak height: 200   Marker baseline V to V?: Y    Marker baseline V to V points: 3
Lower marker selection: First peak > 500 RFU                Upper marker selection: Last peak > 200 RFU
Ladder size (bp)75, 200, 300, 400, 500, 700, 1000, 1500, 2000, 3000, 4000, 5000, 7000, 10000, 20000
Quantification using: Lower Marker          Final concentration (ng/uL): 0.5000          Dilution factor: 12.0
```

Sample: SampB7
Well location: B7
Created: Tuesday, September 14, 2021 7:33:54 PM

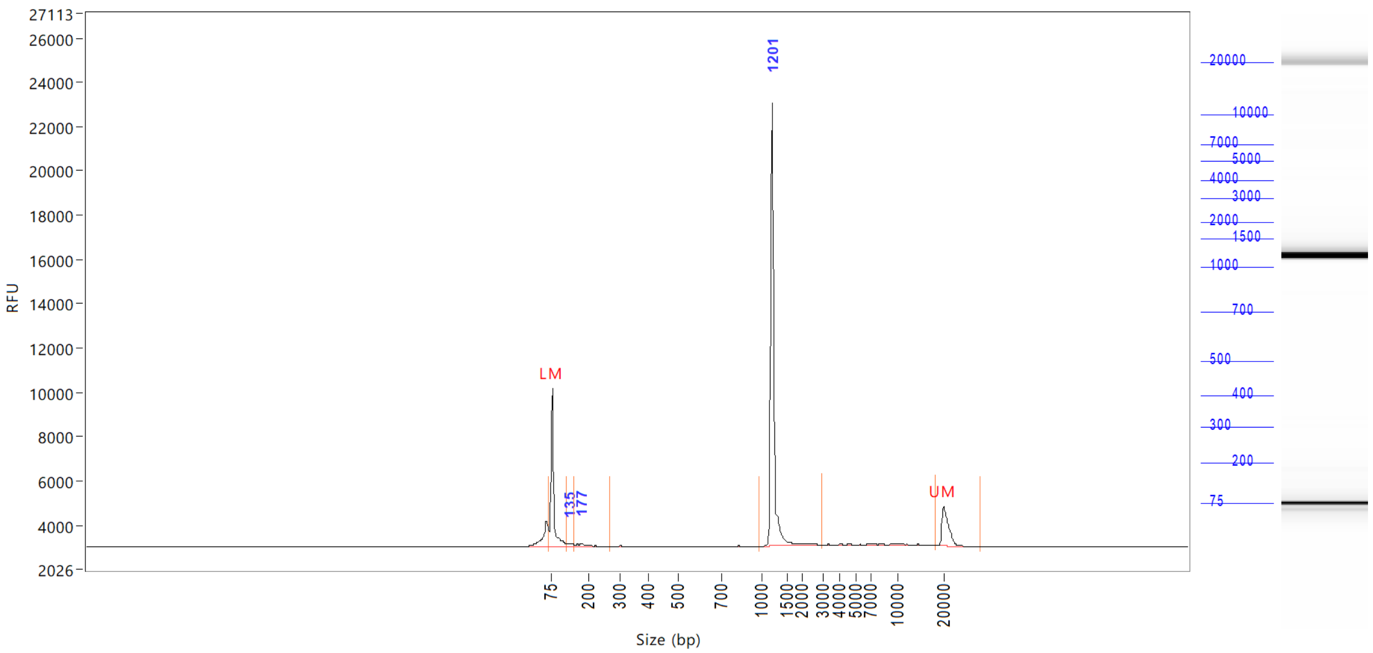

Peak	Size (bp)	Concentration (ng/uL)	From (bp)	To (bp)	Average size (bp)	CV%	RFU	Corrected peak area
1	75 (LM)	0.5000	66	124	77	13.57	7177	49.895
2	135	0.2259	124	150	136	5.80	123	1.878
3	177	0.3747	150	266	180	12.33	148	3.116
4	1201	13.9520	978	3000	1217	9.92	20023	116.022
5	20000 (UM)	0.1605	18293	28100	20487	4.13	1787	16.019

TIC:	14.5525	ng/uL
TIM:	25.0213	nmole/L
Total concentration:	14.9811	ng/uL

```
Sample peak width (sec): 5    Sample min peak height: 50    Sample baseline V to V?: Y    Sample baseline V to V points: 3
Sample filter: Binomial       Number of points for filter: 3  Sample start region (min): 0  Sample end region (min): 30
Marker peak width (sec): 5    Marker min peak height: 200   Marker baseline V to V?: Y    Marker baseline V to V points: 3
Lower marker selection: First peak > 500 RFU                Upper marker selection: Last peak > 200 RFU
Ladder size (bp)75, 200, 300, 400, 500, 700, 1000, 1500, 2000, 3000, 4000, 5000, 7000, 10000, 20000
Quantification using: Lower Marker          Final concentration (ng/uL): 0.5000          Dilution factor: 12.0
```

Sample: SampC7
Well location: C7
Created: Tuesday, September 14, 2021 7:33:54 PM

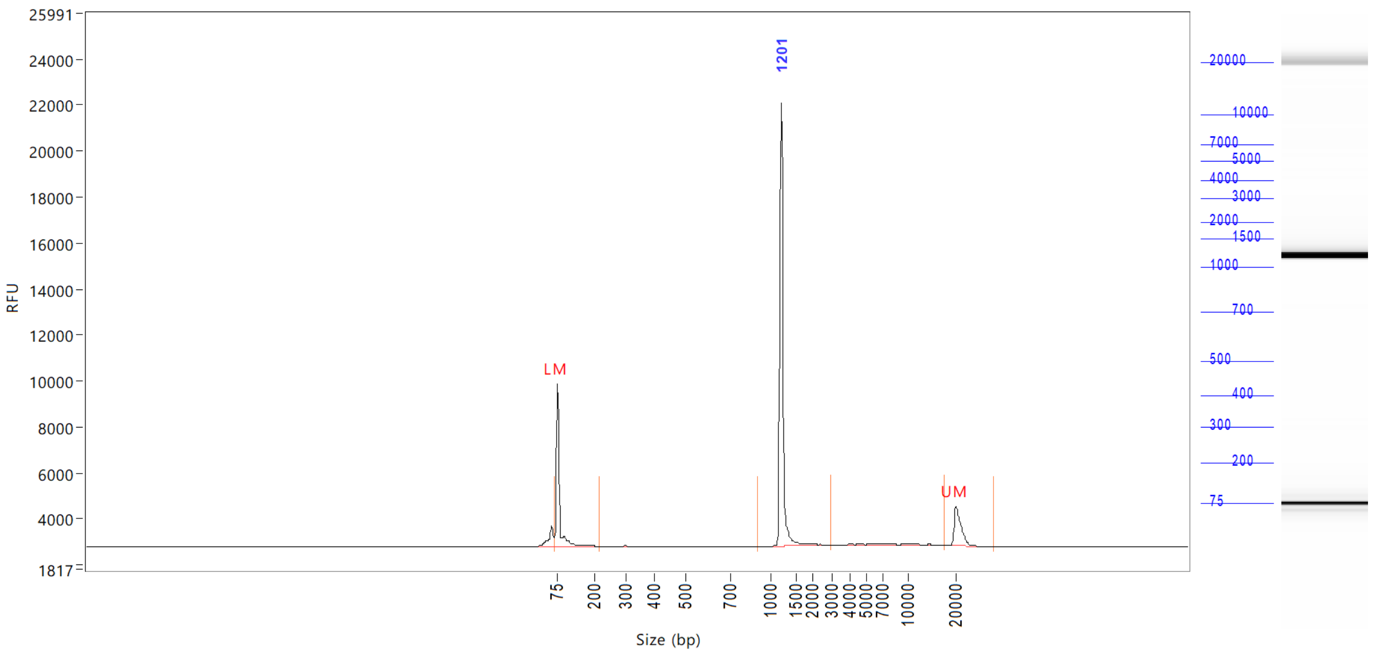

Peak	Size (bp)	Concentration (ng/uL)	From (bp)	To (bp)	Average size (bp)	CV%	RFU	Corrected peak area
1	75 (LM)	0.5000	66	212	80	23.68	7064	48.246
2	1201	12.9409	900	2886	1216	10.73	19301	104.057
3	20000 (UM)	0.1598	17683	28100	20512	4.32	1699	15.415

TIC:	12.9409	ng/uL
TIM:	17.5172	nmole/L
Total concentration:	13.4123	ng/uL

```
Sample peak width (sec): 5    Sample min peak height: 50    Sample baseline V to V?: Y    Sample baseline V to V points: 3
Sample filter: Binomial       Number of points for filter: 3  Sample start region (min): 0  Sample end region (min): 30
Marker peak width (sec): 5    Marker min peak height: 200   Marker baseline V to V?: Y    Marker baseline V to V points: 3
Lower marker selection: First peak > 500 RFU                Upper marker selection: Last peak > 200 RFU
Ladder size (bp)75, 200, 300, 400, 500, 700, 1000, 1500, 2000, 3000, 4000, 5000, 7000, 10000, 20000
Quantification using: Lower Marker          Final concentration (ng/uL): 0.5000          Dilution factor: 12.0
```

Sample: SampD7
Well location: D7
Created: Tuesday, September 14, 2021 7:33:54 PM

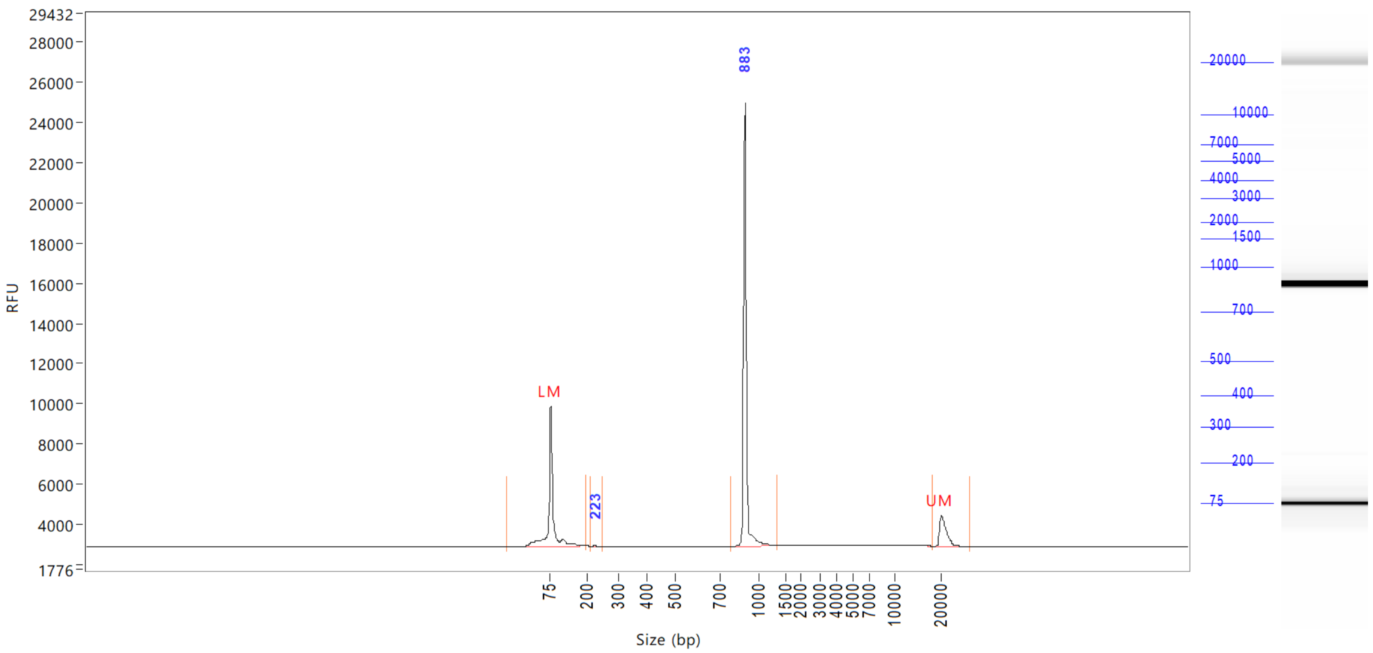

Peak	Size (bp)	Concentration (ng/uL)	From (bp)	To (bp)	Average size (bp)	CV%	RFU	Corrected peak area
1	75 (LM)	0.5000	0	198	72	38.37	6983	71.440
2	223	0.0307	210	246	221	2.14	72	0.366
3	883	9.7000	779	1345	888	4.26	22090	115.494
4	20000 (UM)	0.0984	18049	26408	20523	4.05	1543	14.064

TIC:	9.7307	ng/uL
TIM:	18.2071	nmole/L
Total concentration:	10.0842	ng/uL

Sample peak width (sec): 5 Sample min peak height: 50 Sample baseline V to V?: Y Sample baseline V to V points: 3
Sample filter: Binomial Number of points for filter: 3 Sample start region (min): 0 Sample end region (min): 30
Marker peak width (sec): 5 Marker min peak height: 200 Marker baseline V to V?: Y Marker baseline V to V points: 3
Lower marker selection: First peak > 500 RFU Upper marker selection: Last peak > 200 RFU
Ladder size (bp)75, 200, 300, 400, 500, 700, 1000, 1500, 2000, 3000, 4000, 5000, 7000, 10000, 20000
Quantification using: Lower Marker Final concentration (ng/uL): 0.5000 Dilution factor: 12.0

Sample: SampE7
Well location: E7
Created: Tuesday, September 14, 2021 7:33:54 PM

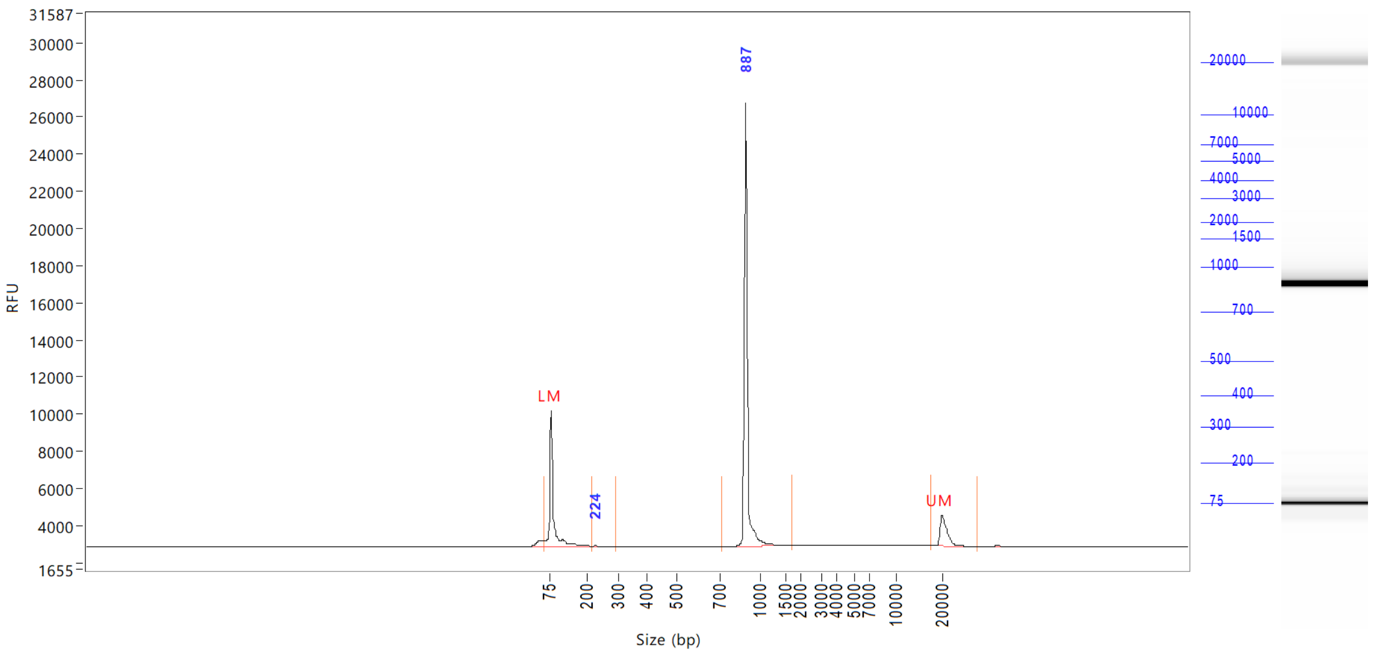

Peak	Size (bp)	Concentration (ng/uL)	From (bp)	To (bp)	Average size (bp)	CV%	RFU	Corrected peak area
1	75 (LM)	0.5000	54	214	84	29.04	7349	64.191
2	224	0.0614	214	291	229	6.51	86	0.657
3	887	12.9146	710	1712	900	7.14	23907	138.168
4	20000 (UM)	0.1204	17683	27617	20527	4.36	1678	15.462

TIC:	12.9761	ng/uL
TIM:	24.0591	nmole/L
Total concentration:	13.2682	ng/uL

Sample peak width (sec): 5 Sample min peak height: 50 Sample baseline V to V?: Y Sample baseline V to V points: 3
Sample filter: Binomial Number of points for filter: 3 Sample start region (min): 0 Sample end region (min): 30
Marker peak width (sec): 5 Marker min peak height: 200 Marker baseline V to V?: Y Marker baseline V to V points: 3
Lower marker selection: First peak > 500 RFU Upper marker selection: Last peak > 200 RFU
Ladder size (bp)75, 200, 300, 400, 500, 700, 1000, 1500, 2000, 3000, 4000, 5000, 7000, 10000, 20000
Quantification using: Lower Marker Final concentration (ng/uL): 0.5000 Dilution factor: 12.0

Sample: SampF7
Well location: F7
Created: Tuesday, September 14, 2021 7:33:54 PM

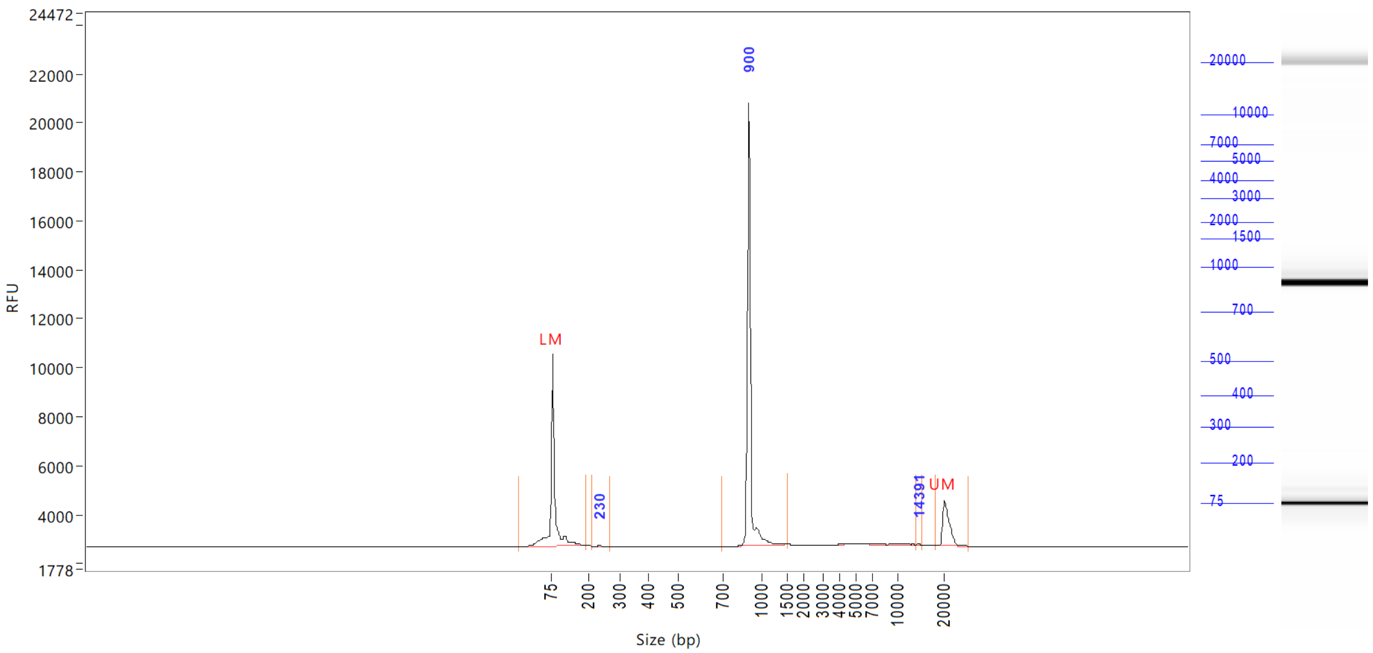

Peak	Size (bp)	Concentration (ng/uL)	From (bp)	To (bp)	Average size (bp)	CV%	RFU	Corrected peak area
1	75 (LM)	0.5000	0	189	75	32.01	7843	71.716
2	230	0.0254	206	266	231	3.31	56	0.304
3	900	8.7032	700	1489	902	4.69	18106	104.026
4	14391	0.0240	13781	15366	14391	2.73	53	0.287
5	20000 (UM)	0.1156	18171	25320	20489	3.82	1847	16.585

TIC:	8.7526	ng/uL
TIM:	16.0646	nmole/L
Total concentration:	8.9927	ng/uL

Sample peak width (sec): 5   Sample min peak height: 50   Sample baseline V to V?: Y   Sample baseline V to V points: 3
Sample filter: Binomial   Number of points for filter: 3 Sample start region (min): 0 Sample end region (min): 30
Marker peak width (sec): 5   Marker min peak height: 200   Marker baseline V to V?: Y   Marker baseline V to V points: 3
Lower marker selection: First peak > 500 RFU   Upper marker selection: Last peak > 200 RFU
Ladder size (bp)75, 200, 300, 400, 500, 700, 1000, 1500, 2000, 3000, 4000, 5000, 7000, 10000, 20000
Quantification using: Lower Marker   Final concentration (ng/uL): 0.5000   Dilution factor: 12.0

Sample: SampG7
Well location: G7
Created: Tuesday, September 14, 2021 7:33:54 PM

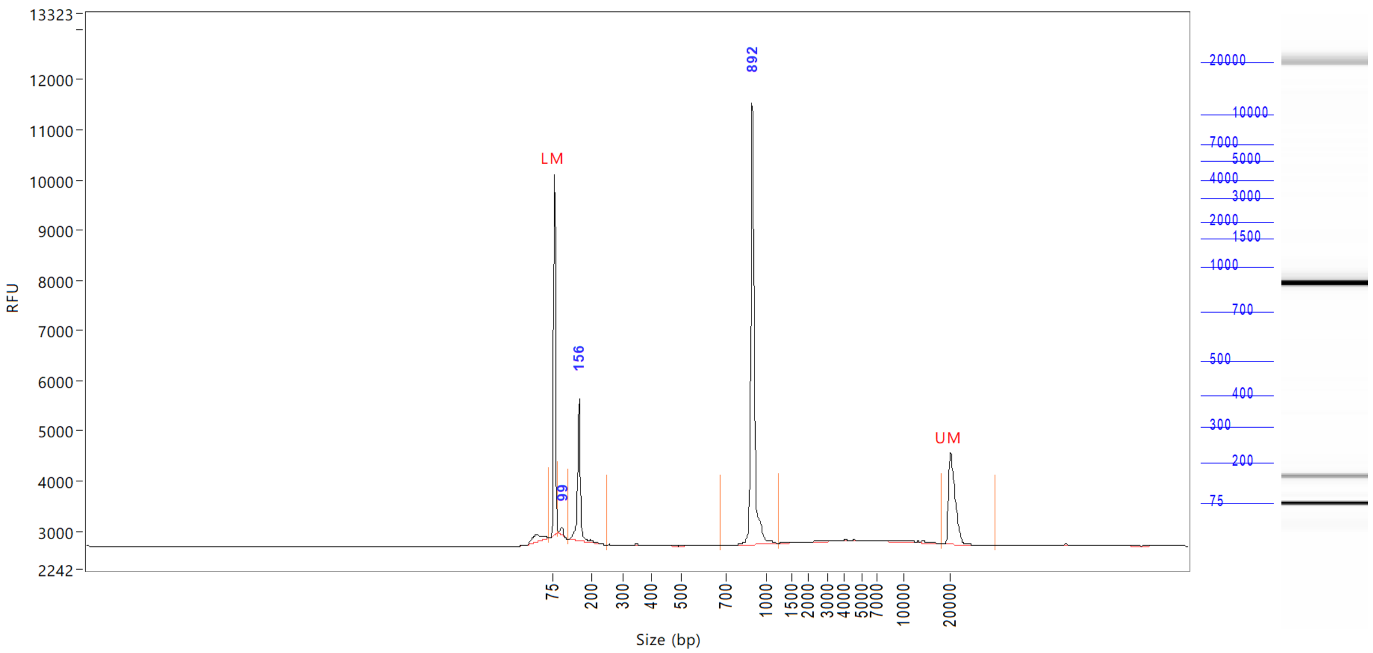

Peak	Size (bp)	Concentration (ng/uL)	From (bp)	To (bp)	Average size (bp)	CV%	RFU	Corrected peak area
1	75 (LM)	0.5000	54	86	73	3.83	7175	37.618
2	99	0.1686	86	120	96	4.48	152	1.057
3	156	3.0981	120	250	156	7.72	2826	19.424
4	892	8.6765	674	1223	896	3.39	8819	54.398
5	20000 (UM)	0.2130	18171	29672	20466	4.41	1843	16.023

TIC:	11.9431	ng/uL
TIM:	51.4629	nmole/L
Total concentration:	12.6101	ng/uL

Sample peak width (sec): 5   Sample min peak height: 50   Sample baseline V to V?: Y   Sample baseline V to V points: 3
Sample filter: Binomial   Number of points for filter: 3 Sample start region (min): 0 Sample end region (min): 30
Marker peak width (sec): 5   Marker min peak height: 200   Marker baseline V to V?: Y   Marker baseline V to V points: 3
Lower marker selection: First peak > 500 RFU   Upper marker selection: Last peak > 200 RFU
Ladder size (bp)75, 200, 300, 400, 500, 700, 1000, 1500, 2000, 3000, 4000, 5000, 7000, 10000, 20000
Quantification using: Lower Marker   Final concentration (ng/uL): 0.5000   Dilution factor: 12.0

Sample: SampH7
Well location: H7
Created: Tuesday, September 14, 2021 7:33:54 PM

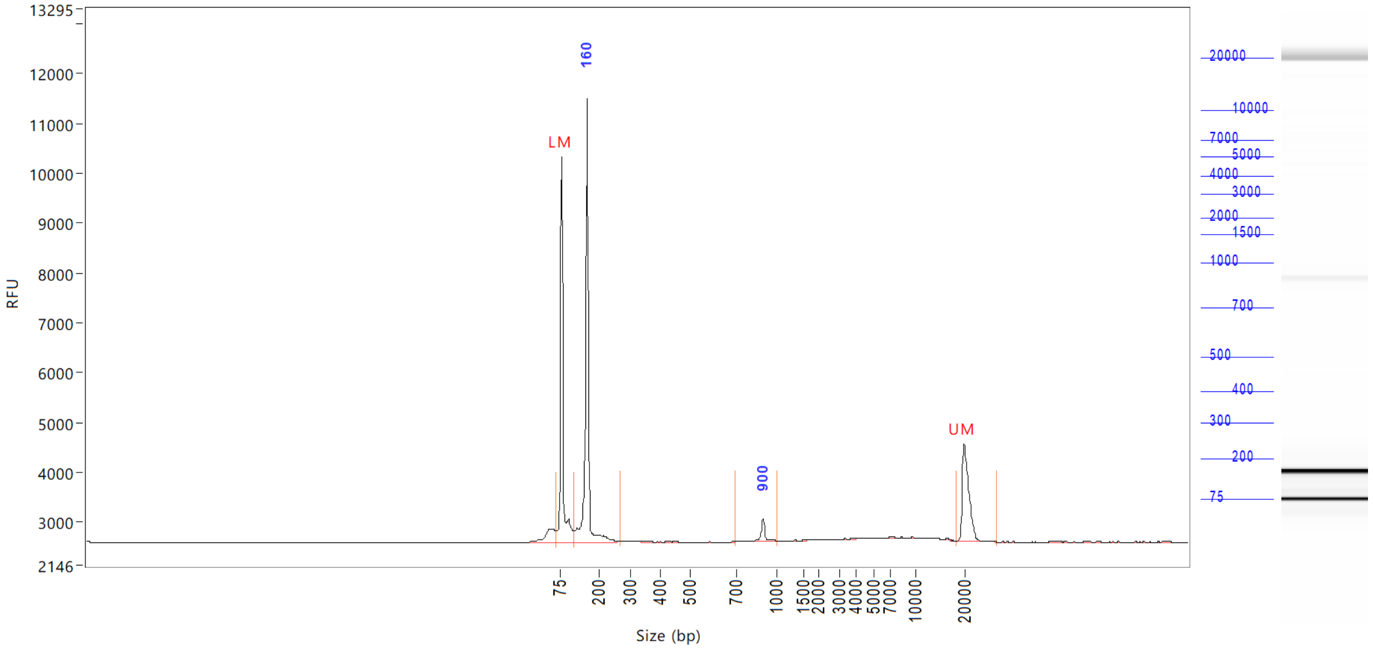

Peak	Size (bp)	Concentration (ng/uL)	From (bp)	To (bp)	Average size (bp)	CV%	RFU	Corrected peak area
1	75 (LM)	0.5000	60	118	77	13.52	7732	51.668
2	160	7.1989	118	268	158	10.03	8905	61.993
3	900	0.4192	700	1012	888	4.83	464	3.610
4	20000 (UM)	0.1669	18293	27012	20471	3.90	1933	17.249

TIC:	7.6181	ng/uL
TIM:	75.6668	nmole/L
Total concentration:	7.9437	ng/uL

Sample peak width (sec): 5 Sample min peak height: 50 Sample baseline V to V?: Y Sample baseline V to V points: 3
Sample filter: Binomial Number of points for filter: 3 Sample start region (min): 0 Sample end region (min): 30
Marker peak width (sec): 5 Marker min peak height: 200 Marker baseline V to V?: Y Marker baseline V to V points: 3
Lower marker selection: First peak > 500 RFU Upper marker selection: Last peak > 200 RFU
Ladder size (bp)75, 200, 300, 400, 500, 700, 1000, 1500, 2000, 3000, 4000, 5000, 7000, 10000, 20000
Quantification using: Lower Marker Final concentration (ng/uL): 0.5000 Dilution factor: 12.0

Sample: SampA8
Well location: A8
Created: Tuesday, September 14, 2021 7:33:54 PM

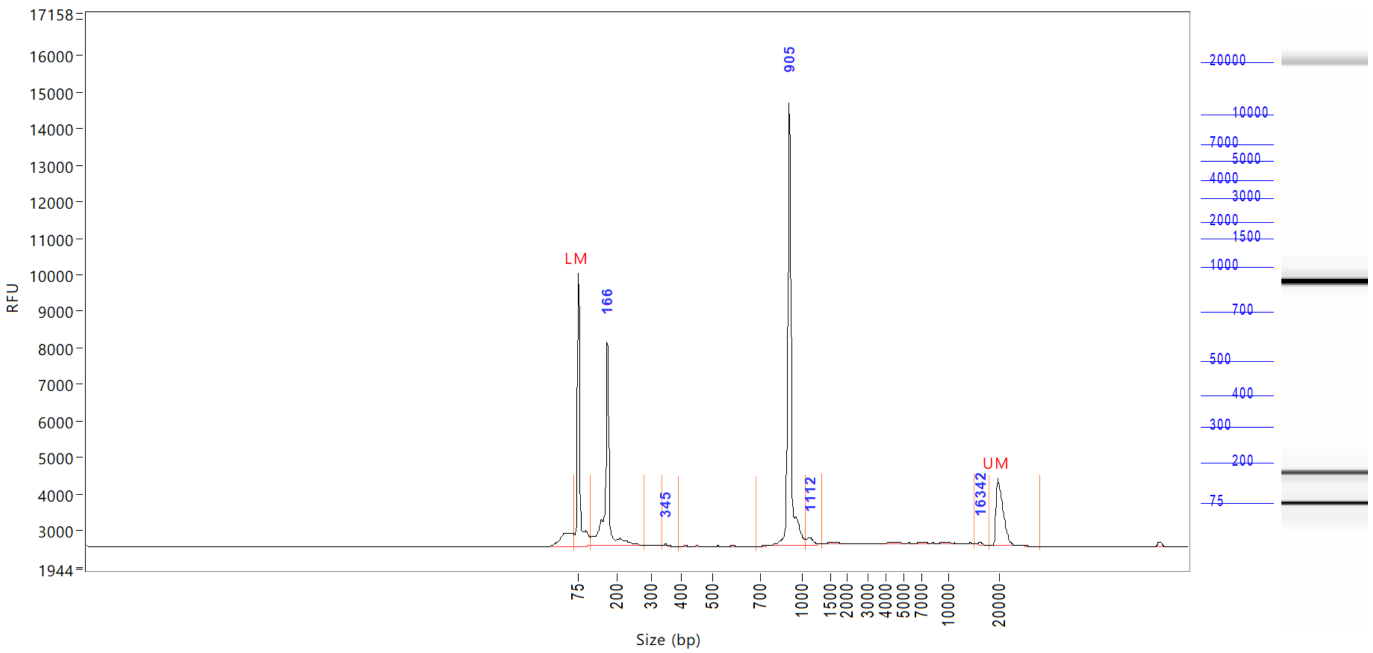

Peak	Size (bp)	Concentration (ng/uL)	From (bp)	To (bp)	Average size (bp)	CV%	RFU	Corrected peak area
1	75 (LM)	0.5000	64	112	76	12.10	7485	47.532
2	166	6.4497	112	278	164	13.24	5566	51.095
3	345	0.0402	337	390	348	2.94	54	0.318
4	905	9.1907	677	1034	903	3.11	12127	72.808
5	1112	0.2944	1034	1345	1126	6.35	195	2.332
6	16342	0.0354	15244	18171	16349	2.86	50	0.280
7	20000 (UM)	0.1692	18171	28463	20412	3.98	1828	16.086

TIC:	16.0103	ng/uL
TIM:	82.0207	nmole/L
Total concentration:	16.4519	ng/uL

Sample peak width (sec): 5 Sample min peak height: 50 Sample baseline V to V?: Y Sample baseline V to V points: 3
Sample filter: Binomial Number of points for filter: 3 Sample start region (min): 0 Sample end region (min): 30
Marker peak width (sec): 5 Marker min peak height: 200 Marker baseline V to V?: Y Marker baseline V to V points: 3
Lower marker selection: First peak > 500 RFU Upper marker selection: Last peak > 200 RFU
Ladder size (bp)75, 200, 300, 400, 500, 700, 1000, 1500, 2000, 3000, 4000, 5000, 7000, 10000, 20000
Quantification using: Lower Marker Final concentration (ng/uL): 0.5000 Dilution factor: 12.0

Sample: SampB8
Well location: B8
Created: Tuesday, September 14, 2021 7:33:54 PM

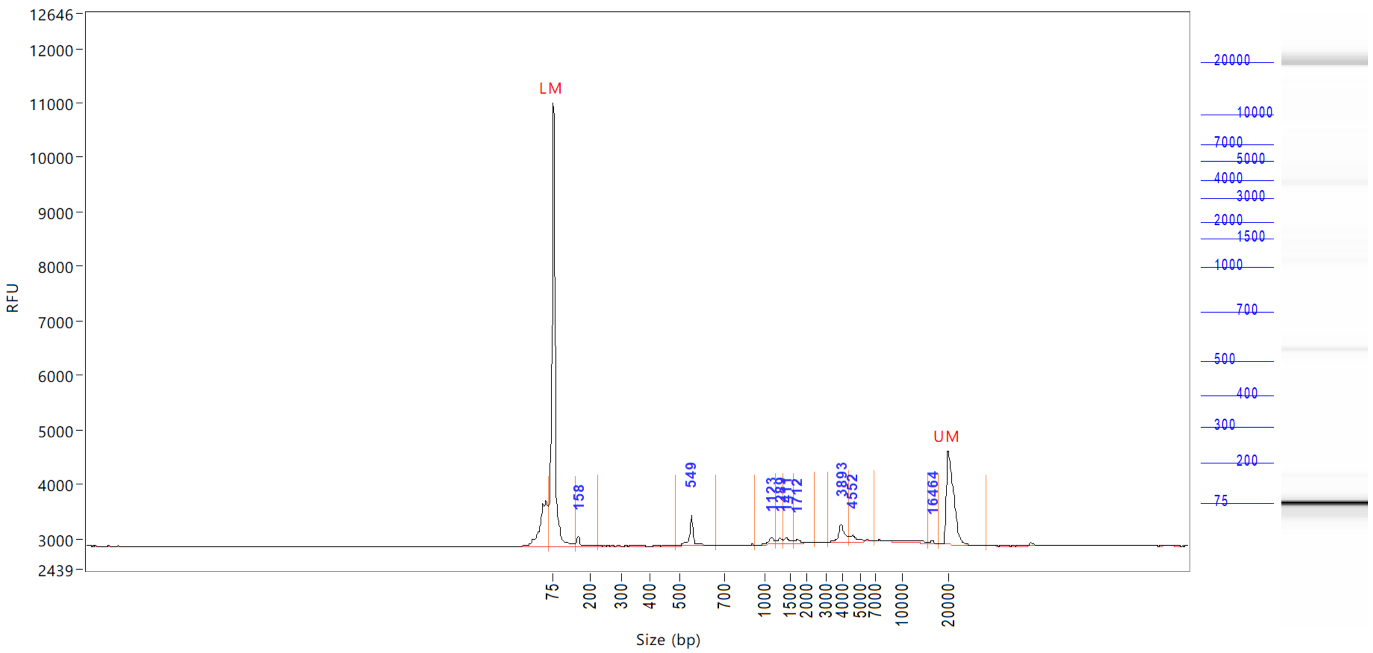

Peak	Size (bp)	Concentration (ng/uL)	From (bp)	To (bp)	Average size (bp)	CV%	RFU	Corrected peak area
1	75 (LM)	0.5000	58	148	75	14.77	8156	67.630
2	158	0.1450	148	223	163	9.58	188	1.635
3	549	0.3920	483	659	545	4.11	568	4.419
4	1123	0.1193	922	1223	1107	5.74	126	1.345
5	1289	0.0656	1223	1345	1285	2.72	102	0.740
6	1411	0.1032	1345	1559	1429	3.90	110	1.164
7	1712	0.0677	1559	2401	1751	9.02	70	0.763
8	3893	0.2729	3072	4345	3897	5.65	331	3.076
9	4552	0.1482	4345	6852	4966	12.90	113	1.671
10	16464	0.0241	15610	17927	16329	2.20	54	0.272
11	20000 (UM)	0.1229	17927	27979	20480	4.39	1713	16.626

TIC:	1.3382	ng/uL
TIM:	3.2570	nmole/L
Total concentration:	1.5441	ng/uL

Sample peak width (sec): 5 Sample min peak height: 50 Sample baseline V to V?: Y Sample baseline V to V points: 3
Sample filter: Binomial Number of points for filter: 3 Sample start region (min): 0 Sample end region (min): 30
Marker peak width (sec): 5 Marker min peak height: 200 Marker baseline V to V?: Y Marker baseline V to V points: 3
Lower marker selection: First peak > 500 RFU Upper marker selection: Last peak > 200 RFU
Ladder size (bp)75, 200, 300, 400, 500, 700, 1000, 1500, 2000, 3000, 4000, 5000, 7000, 10000, 20000
Quantification using: Lower Marker Final concentration (ng/uL): 0.5000 Dilution factor: 12.0

Sample: SampC8
Well location: C8
Created: Tuesday, September 14, 2021 7:33:54 PM

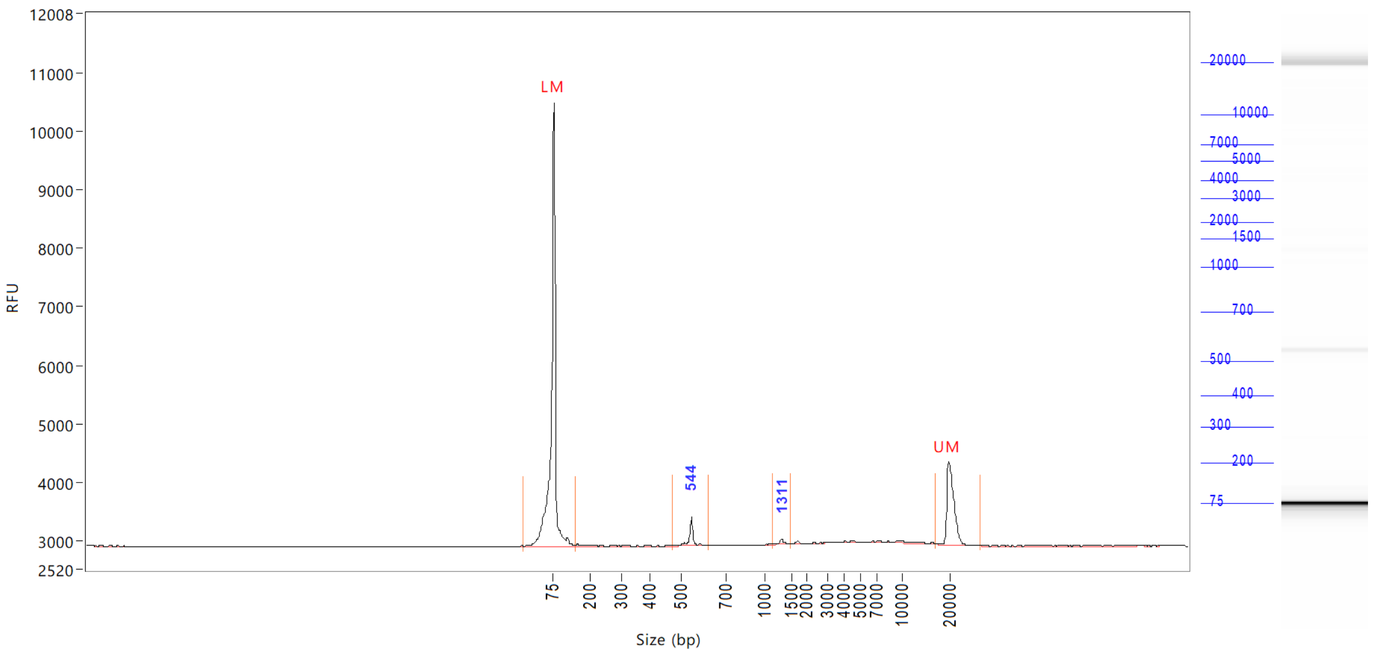

Peak	Size (bp)	Concentration (ng/uL)	From (bp)	To (bp)	Average size (bp)	CV%	RFU	Corrected peak area
1	75 (LM)	0.5000	0	147	67	26.37	7580	76.560
2	544	0.2865	471	623	540	3.91	495	3.656
3	1311	0.0503	1145	1489	1300	4.96	83	0.642
4	20000 (UM)	0.0853	17195	26529	20381	4.07	1424	13.058

TIC:	0.3368	ng/uL
TIM:	0.9369	nmole/L
Total concentration:	0.6672	ng/uL

Sample peak width (sec): 5 Sample min peak height: 50 Sample baseline V to V?: Y Sample baseline V to V points: 3
Sample filter: Binomial Number of points for filter: 3 Sample start region (min): 0 Sample end region (min): 30
Marker peak width (sec): 5 Marker min peak height: 200 Marker baseline V to V?: Y Marker baseline V to V points: 3
Lower marker selection: First peak > 500 RFU Upper marker selection: Last peak > 200 RFU
Ladder size (bp)75, 200, 300, 400, 500, 700, 1000, 1500, 2000, 3000, 4000, 5000, 7000, 10000, 20000
Quantification using: Lower Marker Final concentration (ng/uL): 0.5000 Dilution factor: 12.0

Sample: SampD8
Well location: D8
Created: Tuesday, September 14, 2021 7:33:54 PM

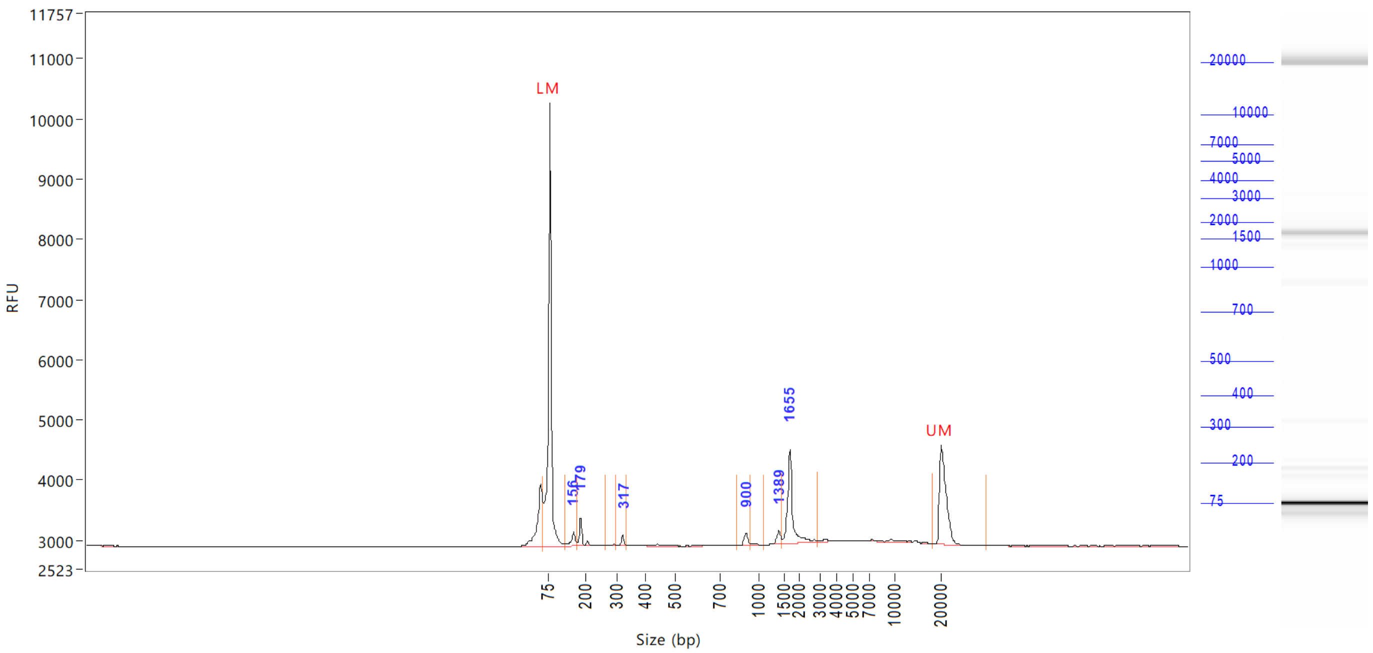

Peak	Size (bp)	Concentration (ng/uL)	From (bp)	To (bp)	Average size (bp)	CV%	RFU	Corrected peak area
1	75 (LM)	0.5000	54	128	73	12.03	7375	55.704
2	156	0.2400	128	168	151	6.11	229	2.228
3	179	0.3637	168	259	182	6.68	464	3.377
4	317	0.1160	295	333	313	1.87	185	1.077
5	900	0.1527	831	935	893	1.72	203	1.418
6	1389	0.1584	1079	1445	1367	3.11	222	1.471
7	1655	1.4215	1445	2886	1713	13.50	1560	13.197
8	20000 (UM)	0.1362	18293	29793	20478	4.31	1659	15.178

TIC:	2.4524	ng/uL
TIM:	8.3457	nmole/L
Total concentration:	2.8069	ng/uL

```
Sample peak width (sec): 5    Sample min peak height: 50    Sample baseline V to V?: Y    Sample baseline V to V points: 3
Sample filter: Binomial       Number of points for filter: 3  Sample start region (min): 0  Sample end region (min): 30
Marker peak width (sec): 5    Marker min peak height: 200   Marker baseline V to V?: Y    Marker baseline V to V points: 3
Lower marker selection: First peak > 500 RFU                Upper marker selection: Last peak > 200 RFU
Ladder size (bp)75, 200, 300, 400, 500, 700, 1000, 1500, 2000, 3000, 4000, 5000, 7000, 10000, 20000
Quantification using: Lower Marker          Final concentration (ng/uL): 0.5000          Dilution factor: 12.0
```

Sample: SampE8
Well location: E8
Created: Tuesday, September 14, 2021 7:33:54 PM

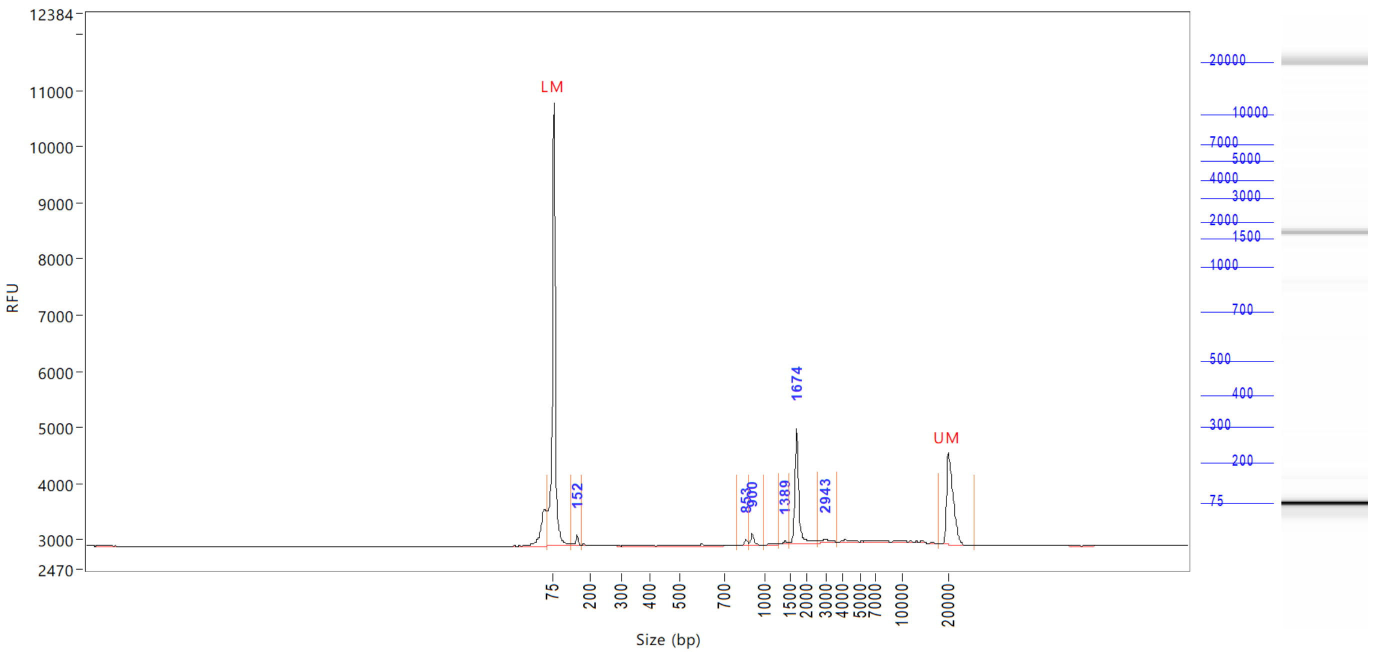

Peak	Size (bp)	Concentration (ng/uL)	From (bp)	To (bp)	Average size (bp)	CV%	RFU	Corrected peak area
1	75 (LM)	0.5000	52	133	73	12.50	7915	61.500
2	152	0.1457	133	168	150	4.84	193	1.493
3	853	0.0711	783	879	849	2.10	111	0.729
4	900	0.1205	879	996	903	1.99	202	1.235
5	1389	0.0439	1256	1478	1384	3.40	55	0.450
6	1674	1.3140	1478	2572	1705	9.37	2067	13.469
7	2943	0.0980	2572	3608	2990	8.57	60	1.004
8	20000 (UM)	0.1156	17927	25682	20400	3.72	1626	14.224

TIC:	1.7932	ng/uL
TIM:	3.3287	nmole/L
Total concentration:	2.1261	ng/uL

Sample peak width (sec): 5   Sample min peak height: 50   Sample baseline V to V?: Y   Sample baseline V to V points: 3
Sample filter: Binomial   Number of points for filter: 3   Sample start region (min): 0   Sample end region (min): 30
Marker peak width (sec): 5   Marker min peak height: 200   Marker baseline V to V?: Y   Marker baseline V to V points: 3
Lower marker selection: First peak > 500 RFU   Upper marker selection: Last peak > 200 RFU
Ladder size (bp)75, 200, 300, 400, 500, 700, 1000, 1500, 2000, 3000, 4000, 5000, 7000, 10000, 20000
Quantification using: Lower Marker   Final concentration (ng/uL): 0.5000   Dilution factor: 12.0

Sample: SampF8
Well location: F8
Created: Tuesday, September 14, 2021 7:33:54 PM

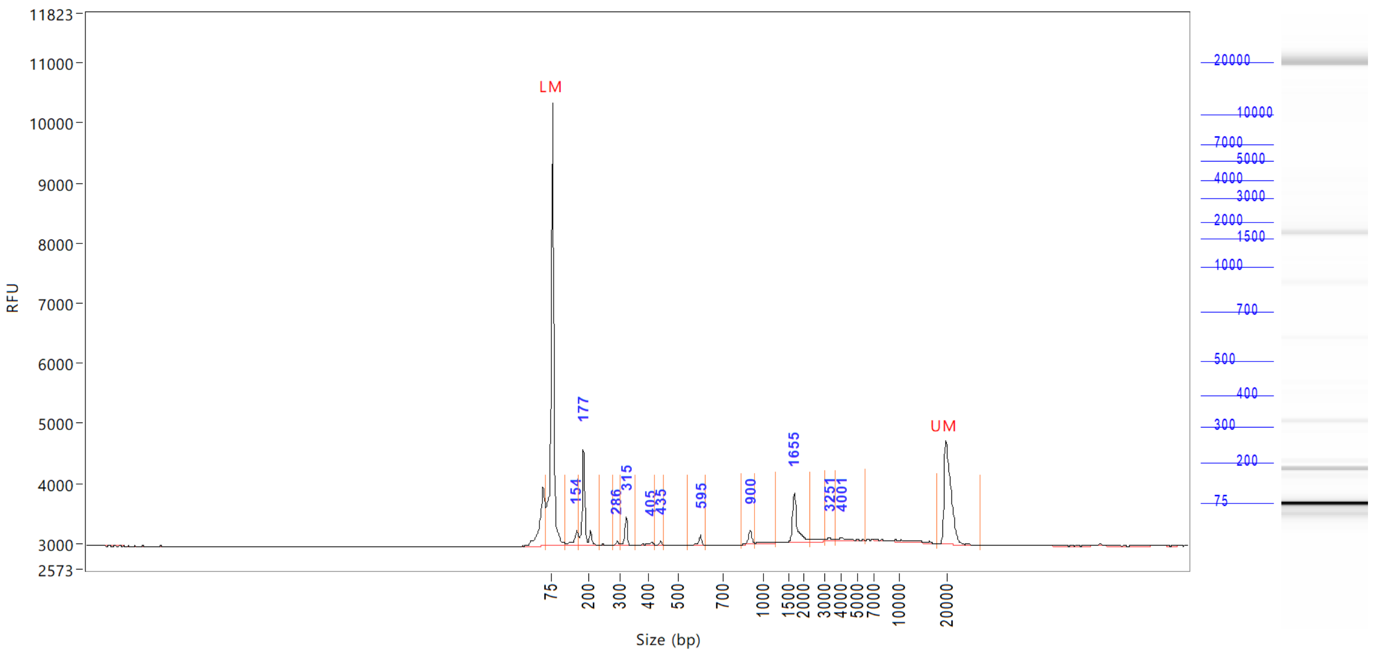

Peak	Size (bp)	Concentration (ng/uL)	From (bp)	To (bp)	Average size (bp)	CV%	RFU	Corrected peak area
1	75 (LM)	0.5000	52	120	72	11.41	7381	54.362
2	154	0.3188	120	166	150	7.63	245	2.889
3	177	1.1970	166	233	180	5.13	1597	10.845
4	286	0.0489	277	296	286	1.66	72	0.443
5	315	0.2770	296	349	313	1.74	468	2.510
6	405	0.0677	349	420	394	3.74	57	0.613
7	435	0.0405	420	447	434	1.03	72	0.367
8	595	0.1163	534	618	588	2.11	172	1.054
9	900	0.1670	831	939	895	1.77	222	1.513
10	1655	0.6988	1245	2315	1716	9.32	821	6.331
11	3251	0.0570	3037	3679	3303	5.25	56	0.517
12	4001	0.0824	3679	6038	4241	9.60	51	0.746
13	20000 (UM)	0.1453	18171	27496	20480	4.43	1721	15.795

TIC:	3.0714	ng/uL
TIM:	17.9626	nmole/L
Total concentration:	3.4136	ng/uL

Sample peak width (sec): 5 Sample min peak height: 50 Sample baseline V to V?: Y Sample baseline V to V points: 3
Sample filter: Binomial Number of points for filter: 3 Sample start region (min): 0 Sample end region (min): 30
Marker peak width (sec): 5 Marker min peak height: 200 Marker baseline V to V?: Y Marker baseline V to V points: 3
Lower marker selection: First peak > 500 RFU Upper marker selection: Last peak > 200 RFU
Ladder size (bp)75, 200, 300, 400, 500, 700, 1000, 1500, 2000, 3000, 4000, 5000, 7000, 10000, 20000
Quantification using: Lower Marker Final concentration (ng/uL): 0.5000 Dilution factor: 12.0

Sample: SampG8
Well location: G8
Created: Tuesday, September 14, 2021 7:33:54 PM

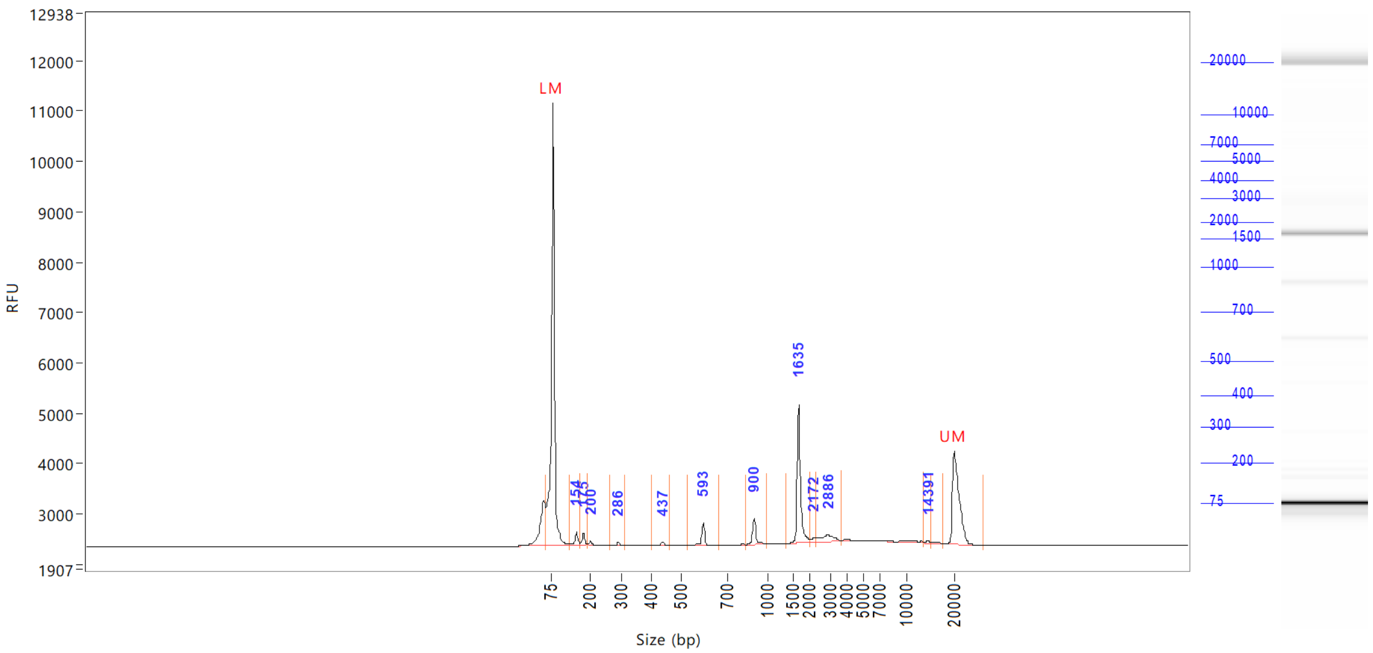

Peak	Size (bp)	Concentration (ng/uL)	From (bp)	To (bp)	Average size (bp)	CV%	RFU	Corrected peak area
1	75 (LM)	0.5000	52	133	73	11.99	8791	69.736
2	154	0.1535	133	166	151	4.11	253	1.785
3	175	0.1117	166	190	174	2.20	229	1.299
4	200	0.0318	190	260	201	5.93	71	0.370
5	286	0.0393	260	307	283	2.35	76	0.457
6	437	0.0460	401	462	432	2.14	80	0.534
7	593	0.2323	529	662	590	2.70	442	2.700
8	900	0.2683	835	996	900	2.39	508	3.119
9	1635	1.3366	1356	2058	1652	5.62	2729	15.535
10	2172	0.0487	2058	2286	2172	3.35	79	0.566
11	2886	0.2412	2286	3679	2833	10.78	144	2.803
12	14391	0.0235	13659	15244	14330	2.66	52	0.273
13	20000 (UM)	0.1209	17561	26166	20507	4.08	1839	16.859

TIC:	2.5330	ng/uL
TIM:	6.0408	nmole/L
Total concentration:	2.7241	ng/uL

```
Sample peak width (sec): 5     Sample min peak height: 50     Sample baseline V to V?: Y     Sample baseline V to V points: 3
Sample filter: Binomial        Number of points for filter: 3  Sample start region (min): 0  Sample end region (min): 30
Marker peak width (sec): 5     Marker min peak height: 200    Marker baseline V to V?: Y     Marker baseline V to V points: 3
Lower marker selection: First peak > 500 RFU                  Upper marker selection: Last peak > 200 RFU
Ladder size (bp)75, 200, 300, 400, 500, 700, 1000, 1500, 2000, 3000, 4000, 5000, 7000, 10000, 20000
Quantification using: Lower Marker          Final concentration (ng/uL): 0.5000          Dilution factor: 12.0
```

Sample: SampH8
Well location: H8
Created: Tuesday, September 14, 2021 7:33:54 PM

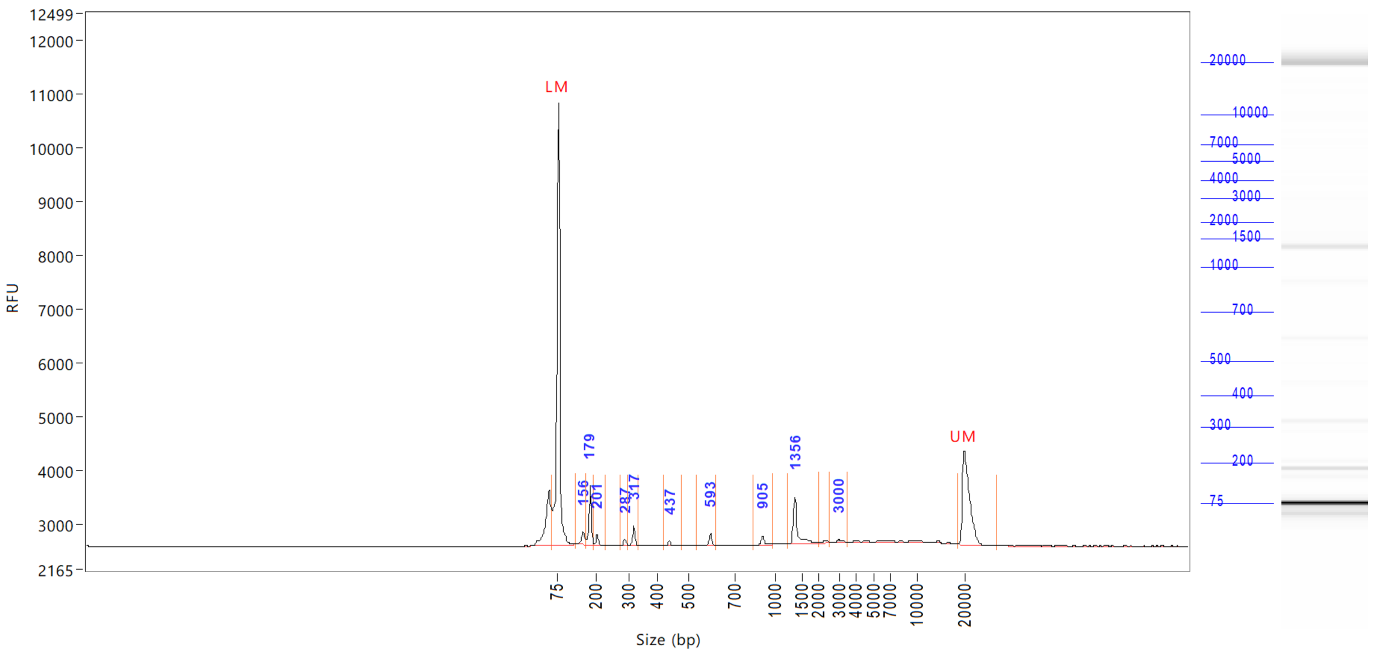

Peak	Size (bp)	Concentration (ng/uL)	From (bp)	To (bp)	Average size (bp)	CV%	RFU	Corrected peak area
1	75 (LM)	0.5000	54	129	73	9.81	8232	58.105
2	156	0.2048	129	164	153	4.40	241	1.983
3	179	0.6473	164	192	177	2.62	1088	6.269
4	201	0.1057	192	228	200	2.25	187	1.023
5	287	0.0634	275	296	286	1.52	109	0.614
6	317	0.1994	296	333	313	1.56	364	1.931
7	437	0.0505	416	475	434	1.64	85	0.489
8	593	0.1359	532	621	586	2.39	230	1.316
9	905	0.1089	840	987	905	2.46	173	1.054
10	1356	0.5982	1234	2001	1414	10.02	868	5.793
11	3000	0.0617	2543	3500	2962	7.45	53	0.598
12	20000 (UM)	0.1447	18537	26770	20460	4.21	1752	16.811

TIC:	2.1756	ng/uL
TIM:	11.9946	nmole/L
Total concentration:	2.4707	ng/uL

```
Sample peak width (sec): 5    Sample min peak height: 50    Sample baseline V to V?: Y    Sample baseline V to V points: 3
Sample filter: Binomial       Number of points for filter: 3  Sample start region (min): 0  Sample end region (min): 30
Marker peak width (sec): 5    Marker min peak height: 200   Marker baseline V to V?: Y    Marker baseline V to V points: 3
Lower marker selection: First peak > 500 RFU                Upper marker selection: Last peak > 200 RFU
Ladder size (bp)75, 200, 300, 400, 500, 700, 1000, 1500, 2000, 3000, 4000, 5000, 7000, 10000, 20000
Quantification using: Lower Marker          Final concentration (ng/uL): 0.5000          Dilution factor: 12.0
```

Sample: SampA9
Well location: A9
Created: Tuesday, September 14, 2021 7:33:54 PM

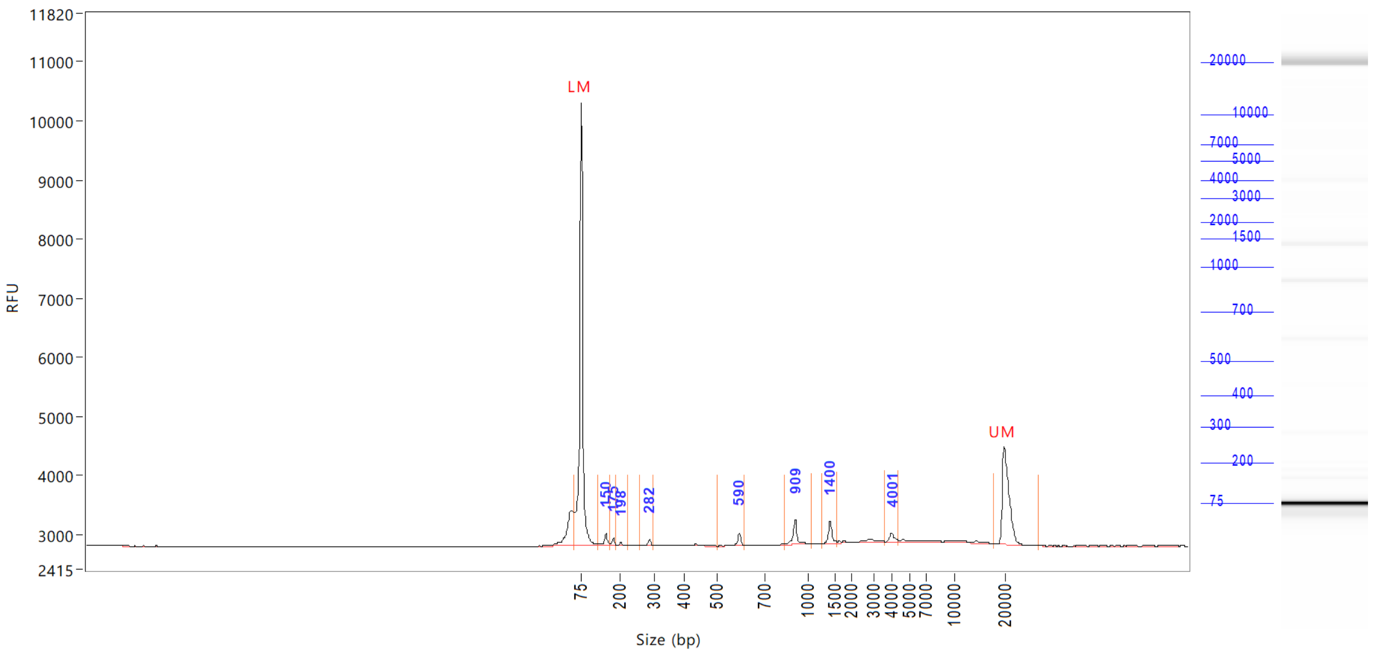

Peak	Size (bp)	Concentration (ng/uL)	From (bp)	To (bp)	Average size (bp)	CV%	RFU	Corrected peak area
1	75 (LM)	0.5000	54	128	72	10.66	7501	55.512
2	150	0.1545	128	166	148	5.10	198	1.429
3	175	0.0756	166	187	173	1.97	132	0.700
4	198	0.0294	187	219	197	2.39	52	0.272
5	282	0.0649	257	296	278	2.24	106	0.601
6	590	0.1507	500	611	582	2.87	220	1.394
7	909	0.3006	840	1056	909	3.03	419	2.781
8	1400	0.2267	1267	1539	1402	2.92	369	2.098
9	4001	0.1057	3679	4414	4044	3.47	137	0.978
10	20000 (UM)	0.1290	18049	26770	20373	3.78	1652	14.323

TIC:	1.1081	ng/uL
TIM:	4.3428	nmole/L
Total concentration:	1.6132	ng/uL

Sample peak width (sec): 5   Sample min peak height: 50   Sample baseline V to V?: Y   Sample baseline V to V points: 3
Sample filter: Binomial   Number of points for filter: 3   Sample start region (min): 0   Sample end region (min): 30
Marker peak width (sec): 5   Marker min peak height: 200   Marker baseline V to V?: Y   Marker baseline V to V points: 3
Lower marker selection: First peak > 500 RFU   Upper marker selection: Last peak > 200 RFU
Ladder size (bp)75, 200, 300, 400, 500, 700, 1000, 1500, 2000, 3000, 4000, 5000, 7000, 10000, 20000
Quantification using: Lower Marker   Final concentration (ng/uL): 0.5000   Dilution factor: 12.0

Sample: SampB9
Well location: B9
Created: Tuesday, September 14, 2021 7:33:54 PM

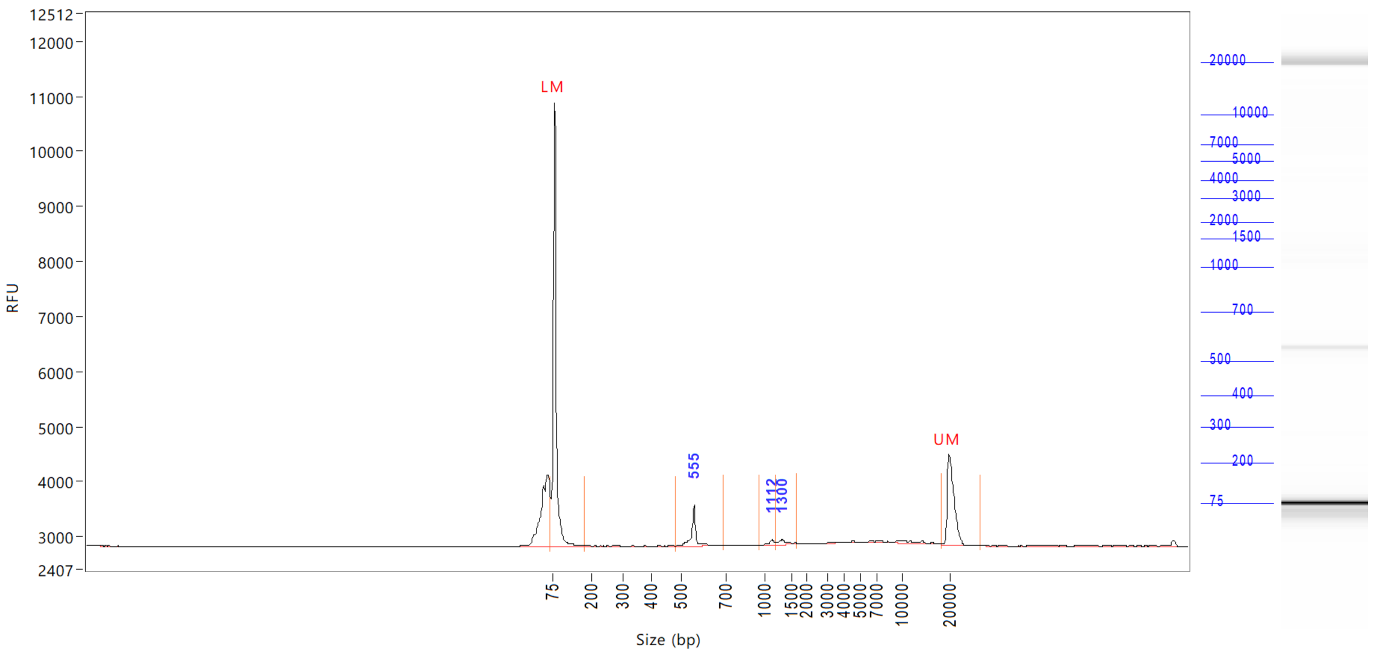

Peak	Size (bp)	Concentration (ng/uL)	From (bp)	To (bp)	Average size (bp)	CV%	RFU	Corrected peak area
1	75 (LM)	0.5000	62	177	76	15.47	8078	60.438
2	555	0.6048	481	687	552	4.84	751	6.092
3	1112	0.0811	948	1190	1093	5.14	92	0.817
4	1300	0.1308	1190	1674	1353	8.11	85	1.317
5	20000 (UM)	0.1221	18293	26529	20457	3.85	1653	14.758

TIC:	0.8167	ng/uL
TIM:	2.0841	nmole/L
Total concentration:	1.2386	ng/uL

Sample peak width (sec): 5   Sample min peak height: 50   Sample baseline V to V?: Y   Sample baseline V to V points: 3
Sample filter: Binomial   Number of points for filter: 3   Sample start region (min): 0   Sample end region (min): 30
Marker peak width (sec): 5   Marker min peak height: 200   Marker baseline V to V?: Y   Marker baseline V to V points: 3
Lower marker selection: First peak > 500 RFU   Upper marker selection: Last peak > 200 RFU
Ladder size (bp)75, 200, 300, 400, 500, 700, 1000, 1500, 2000, 3000, 4000, 5000, 7000, 10000, 20000
Quantification using: Lower Marker   Final concentration (ng/uL): 0.5000   Dilution factor: 12.0

Sample: SampC9
Well location: C9
Created: Tuesday, September 14, 2021 7:33:54 PM

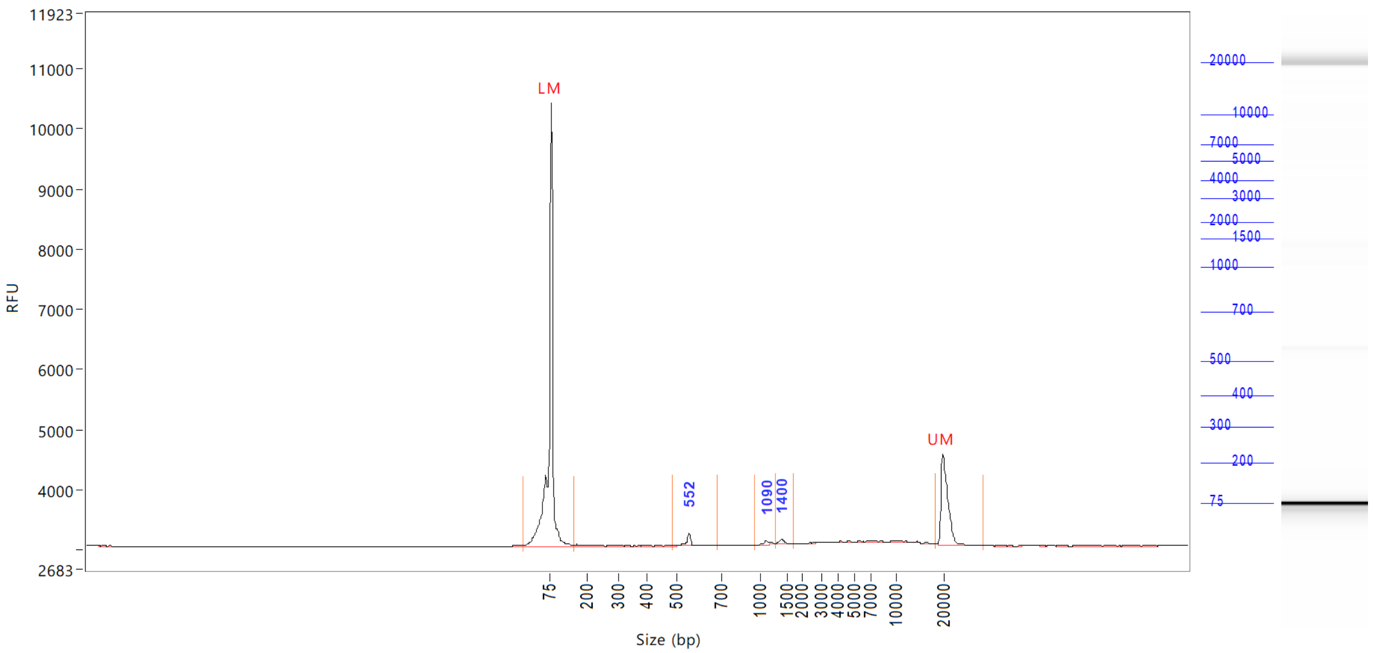

Peak	Size	Concentration	From	To	Average size	CV%	RFU	Corrected peak area
	(bp)	(ng/uL)	(bp)	(bp)	(bp)			
1	75 (LM)	0.5000	0	150	65	29.73	7383	72.865
2	552	0.1588	483	680	548	5.40	211	1.928
3	1090	0.0547	952	1278	1114	7.03	62	0.664
4	1400	0.0659	1278	1693	1404	5.36	86	0.801
5	20000 (UM)	0.0958	18293	28584	20465	4.62	1499	13.962

TIC:	0.2794	ng/uL
TIM:	0.6349	nmole/L
Total concentration:	0.5632	ng/uL

Sample peak width (sec): 5 Sample min peak height: 50 Sample baseline V to V?: Y Sample baseline V to V points: 3
Sample filter: Binomial Number of points for filter: 3 Sample start region (min): 0 Sample end region (min): 30
Marker peak width (sec): 5 Marker min peak height: 200 Marker baseline V to V?: Y Marker baseline V to V points: 3
Lower marker selection: First peak > 500 RFU Upper marker selection: Last peak > 200 RFU
Ladder size (bp)75, 200, 300, 400, 500, 700, 1000, 1500, 2000, 3000, 4000, 5000, 7000, 10000, 20000
Quantification using: Lower Marker Final concentration (ng/uL): 0.5000 Dilution factor: 12.0

Sample: SampD9
Well location: D9
Created: Tuesday, September 14, 2021 7:33:54 PM

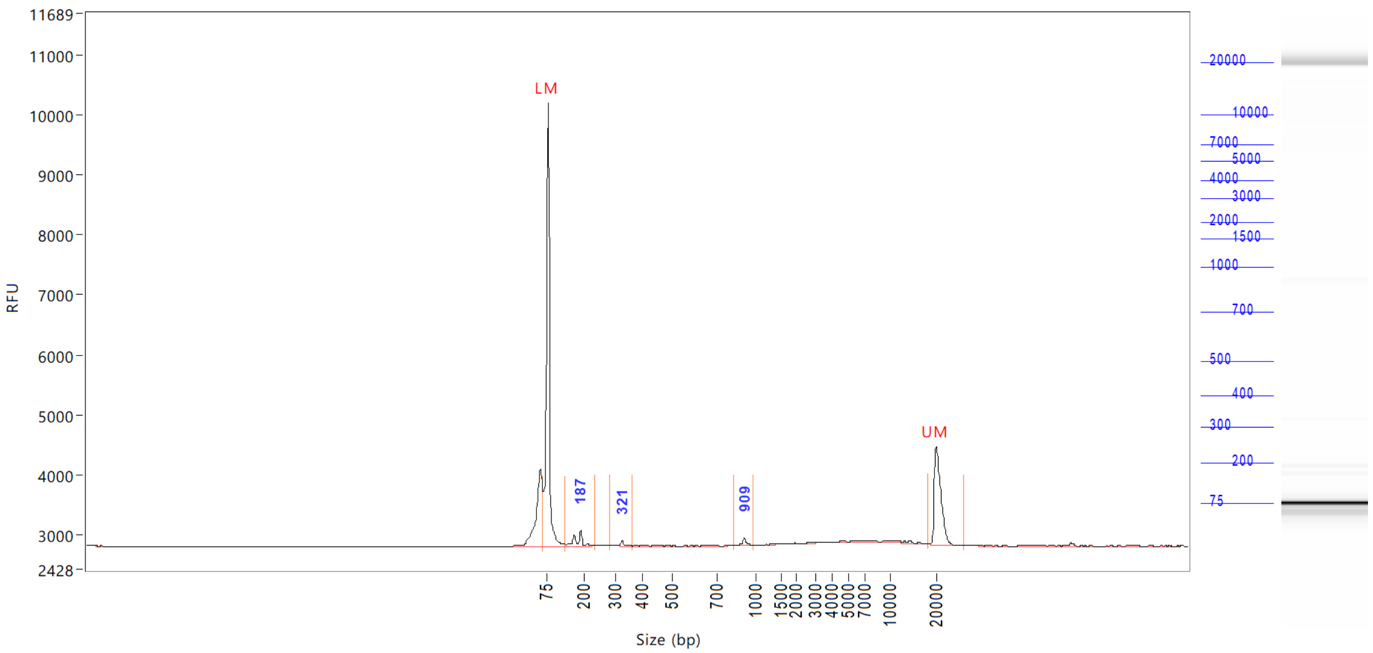

Peak	Size (bp)	Concentration (ng/uL)	From (bp)	To (bp)	Average size (bp)	CV%	RFU	Corrected peak area
1	75 (LM)	0.5000	58	135	74	12.05	7400	55.060
2	187	0.4000	135	235	175	10.28	261	3.671
3	321	0.0738	284	360	316	3.66	95	0.677
4	909	0.1054	835	987	908	2.66	125	0.967
5	20000 (UM)	0.1332	18293	25803	20414	3.96	1638	14.667

TIC:	0.5792	ng/uL
TIM:	4.3329	nmole/L
Total concentration:	0.9691	ng/uL

Sample peak width (sec): 5   Sample min peak height: 50   Sample baseline V to V?: Y   Sample baseline V to V points: 3
Sample filter: Binomial   Number of points for filter: 3 Sample start region (min): 0 Sample end region (min): 30
Marker peak width (sec): 5   Marker min peak height: 200   Marker baseline V to V?: Y   Marker baseline V to V points: 3
Lower marker selection: First peak > 500 RFU   Upper marker selection: Last peak > 200 RFU
Ladder size (bp)75, 200, 300, 400, 500, 700, 1000, 1500, 2000, 3000, 4000, 5000, 7000, 10000, 20000
Quantification using: Lower Marker   Final concentration (ng/uL): 0.5000   Dilution factor: 12.0

Sample: SampE9
Well location: E9
Created: Tuesday, September 14, 2021 7:33:54 PM

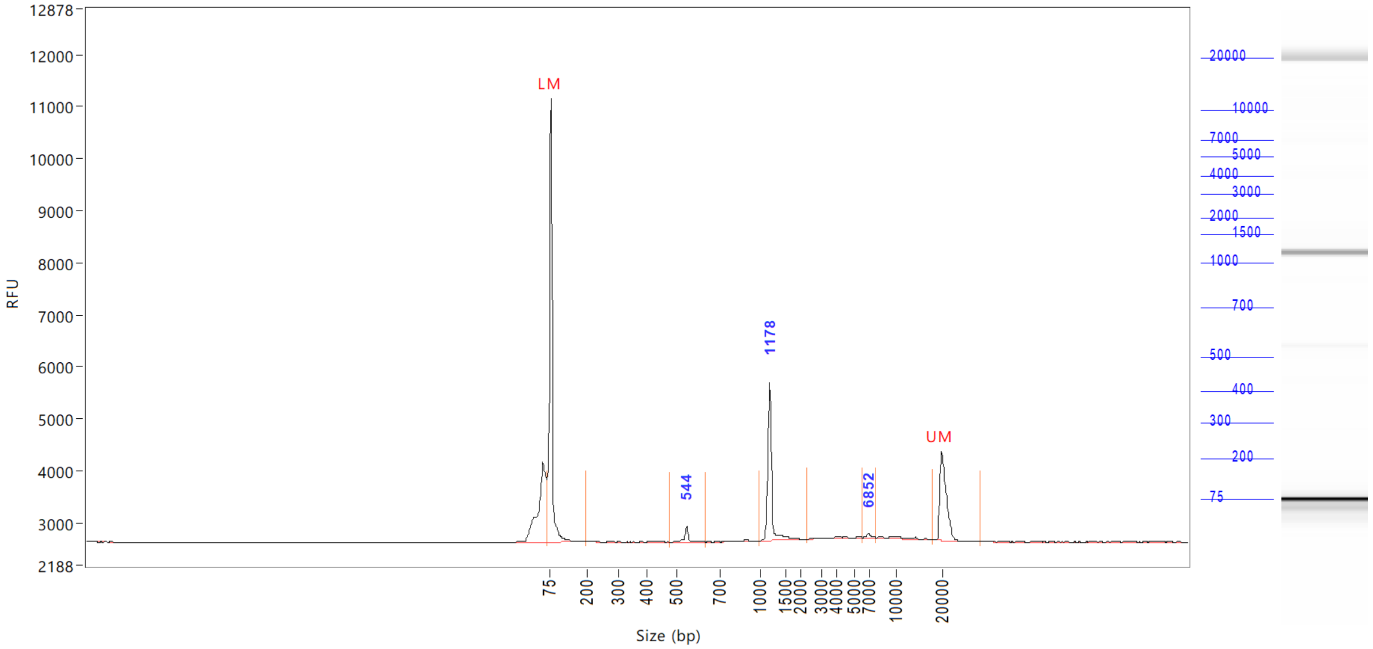

Peak	Size (bp)	Concentration (ng/uL)	From (bp)	To (bp)	Average size (bp)	CV%	RFU	Corrected peak area
1	75 (LM)	0.5000	62	198	75	14.58	8537	60.584
2	544	0.2506	477	631	541	4.50	313	2.530
3	1178	1.9978	996	2286	1195	8.83	3033	20.172
4	6852	0.0599	6038	7734	6912	5.08	85	0.605
5	20000 (UM)	0.1256	18049	28342	20393	4.00	1702	15.222

TIC:	2.3083	ng/uL
TIM:	3.5282	nmole/L
Total concentration:	2.6785	ng/uL

Sample peak width (sec): 5   Sample min peak height: 50   Sample baseline V to V?: Y   Sample baseline V to V points: 3
Sample filter: Binomial   Number of points for filter: 3 Sample start region (min): 0 Sample end region (min): 30
Marker peak width (sec): 5   Marker min peak height: 200   Marker baseline V to V?: Y   Marker baseline V to V points: 3
Lower marker selection: First peak > 500 RFU   Upper marker selection: Last peak > 200 RFU
Ladder size (bp)75, 200, 300, 400, 500, 700, 1000, 1500, 2000, 3000, 4000, 5000, 7000, 10000, 20000
Quantification using: Lower Marker   Final concentration (ng/uL): 0.5000   Dilution factor: 12.0

Sample: SampF9
Well location: F9
Created: Tuesday, September 14, 2021 7:33:54 PM

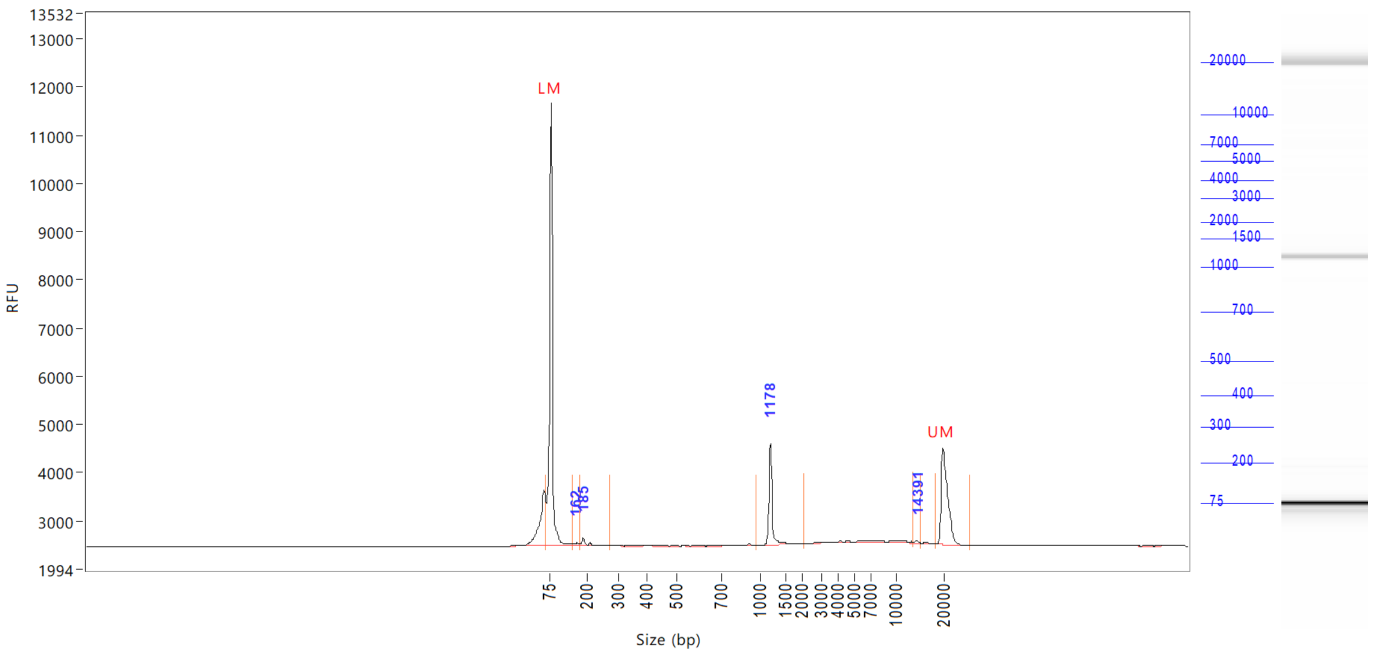

Peak	Size (bp)	Concentration (ng/uL)	From (bp)	To (bp)	Average size (bp)	CV%	RFU	Corrected peak area
1	75 (LM)	0.5000	60	147	74	12.48	9207	67.265
2	162	0.0718	147	173	161	4.72	64	0.805
3	185	0.1539	173	269	194	9.42	169	1.726
4	1178	1.0704	961	2087	1196	8.60	2094	12.000
5	14391	0.0260	13659	15244	14256	2.57	52	0.292
6	20000 (UM)	0.1318	18415	25682	20411	3.80	1991	17.727

TIC:	1.3222	ng/uL
TIM:	3.5137	nmole/L
Total concentration:	1.6652	ng/uL

```
Sample peak width (sec): 5    Sample min peak height: 50    Sample baseline V to V?: Y    Sample baseline V to V points: 3
Sample filter: Binomial       Number of points for filter: 3  Sample start region (min): 0  Sample end region (min): 30
Marker peak width (sec): 5    Marker min peak height: 200   Marker baseline V to V?: Y    Marker baseline V to V points: 3
Lower marker selection: First peak > 500 RFU                Upper marker selection: Last peak > 200 RFU
Ladder size (bp)75, 200, 300, 400, 500, 700, 1000, 1500, 2000, 3000, 4000, 5000, 7000, 10000, 20000
Quantification using: Lower Marker          Final concentration (ng/uL): 0.5000          Dilution factor: 12.0
```

Sample: SampG9
Well location: G9
Created: Tuesday, September 14, 2021 7:33:54 PM

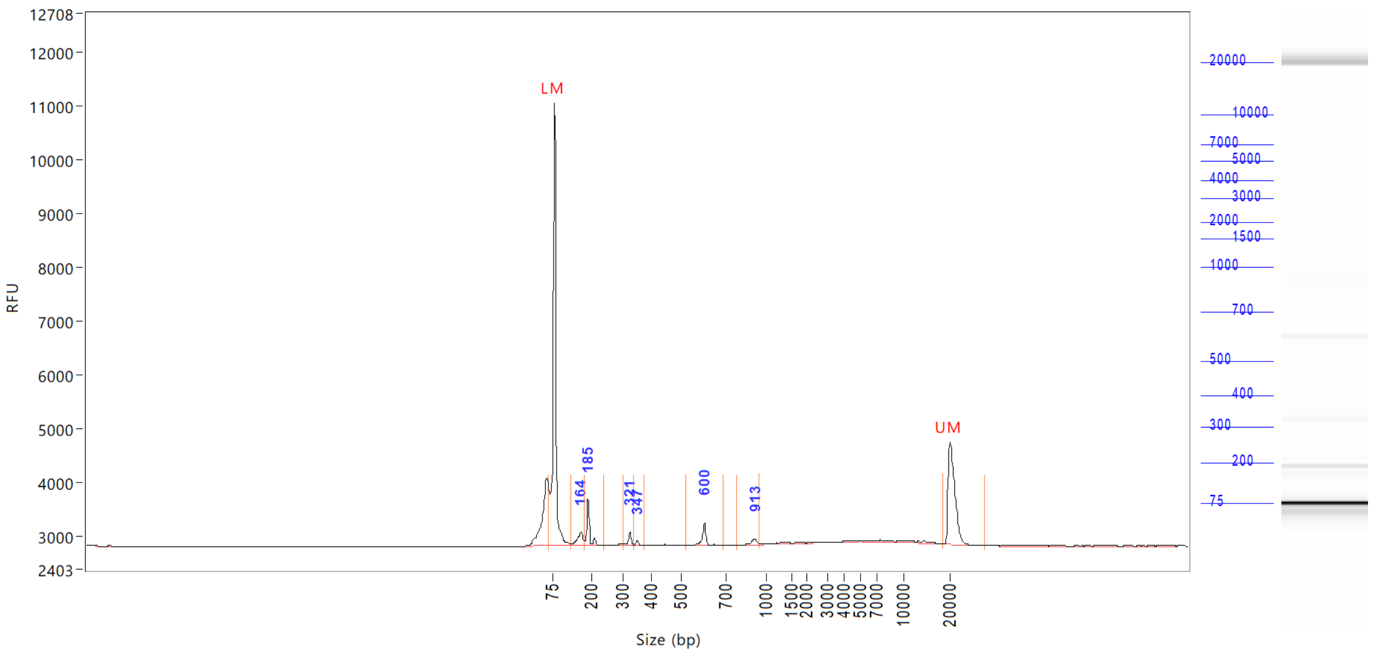

Peak	Size (bp)	Concentration (ng/uL)	From (bp)	To (bp)	Average size (bp)	CV%	RFU	Corrected peak area
1	75 (LM)	0.5000	58	129	74	11.54	8222	58.831
2	164	0.3551	129	175	157	6.89	260	3.481
3	185	0.5847	175	241	188	4.74	853	5.733
4	321	0.1515	300	337	319	2.17	241	1.485
5	347	0.0522	337	374	347	1.65	92	0.512
6	600	0.3224	519	687	597	3.35	433	3.162
7	913	0.1122	775	948	896	3.50	113	1.100
8	20000 (UM)	0.1447	18537	27617	20502	3.94	1891	17.029

TIC:	1.5780	ng/uL
TIM:	10.9534	nmole/L
Total concentration:	2.0054	ng/uL

Sample peak width (sec): 5 Sample min peak height: 50 Sample baseline V to V?: Y Sample baseline V to V points: 3
Sample filter: Binomial Number of points for filter: 3 Sample start region (min): 0 Sample end region (min): 30
Marker peak width (sec): 5 Marker min peak height: 200 Marker baseline V to V?: Y Marker baseline V to V points: 3
Lower marker selection: First peak > 500 RFU Upper marker selection: Last peak > 200 RFU
Ladder size (bp)75, 200, 300, 400, 500, 700, 1000, 1500, 2000, 3000, 4000, 5000, 7000, 10000, 20000
Quantification using: Lower Marker Final concentration (ng/uL): 0.5000 Dilution factor: 12.0

Sample: SampH9
Well location: H9
Created: Tuesday, September 14, 2021 7:33:54 PM

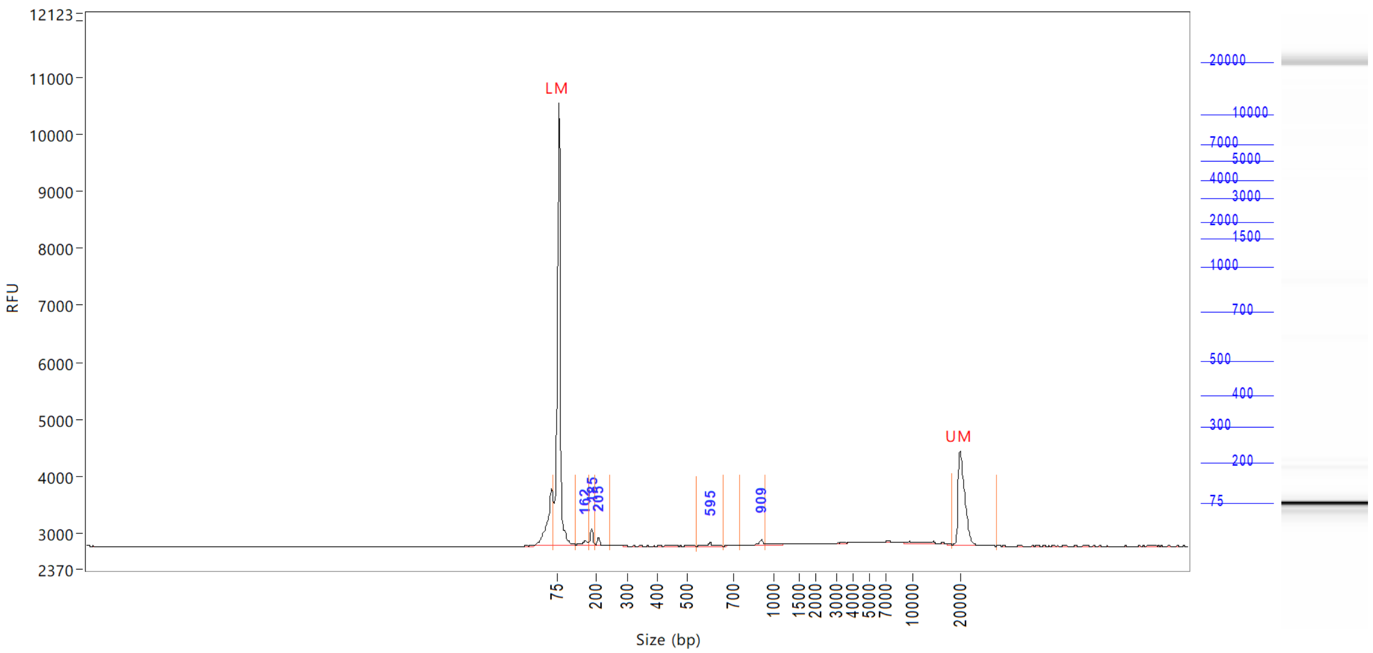

Peak	Size (bp)	Concentration (ng/uL)	From (bp)	To (bp)	Average size (bp)	CV%	RFU	Corrected peak area
1	75 (LM)	0.5000	58	129	73	10.12	7775	55.922
2	162	0.0944	129	173	159	6.87	65	0.880
3	185	0.1854	173	196	181	2.38	292	1.728
4	205	0.0781	196	239	204	3.21	132	0.728
5	595	0.0679	539	657	592	4.03	62	0.633
6	909	0.0793	749	939	889	4.26	90	0.739
7	20000 (UM)	0.1291	18293	27859	20402	3.90	1644	14.441

TIC:	0.5052	ng/uL
TIM:	3.6258	nmole/L
Total concentration:	0.8915	ng/uL

Sample peak width (sec): 5   Sample min peak height: 50   Sample baseline V to V?: Y   Sample baseline V to V points: 3
Sample filter: Binomial   Number of points for filter: 3 Sample start region (min): 0 Sample end region (min): 30
Marker peak width (sec): 5   Marker min peak height: 200   Marker baseline V to V?: Y   Marker baseline V to V points: 3
Lower marker selection: First peak > 500 RFU   Upper marker selection: Last peak > 200 RFU
Ladder size (bp)75, 200, 300, 400, 500, 700, 1000, 1500, 2000, 3000, 4000, 5000, 7000, 10000, 20000
Quantification using: Lower Marker   Final concentration (ng/uL): 0.5000   Dilution factor: 12.0

Sample: SampA10
Well location: A10
Created: Tuesday, September 14, 2021 7:33:54 PM

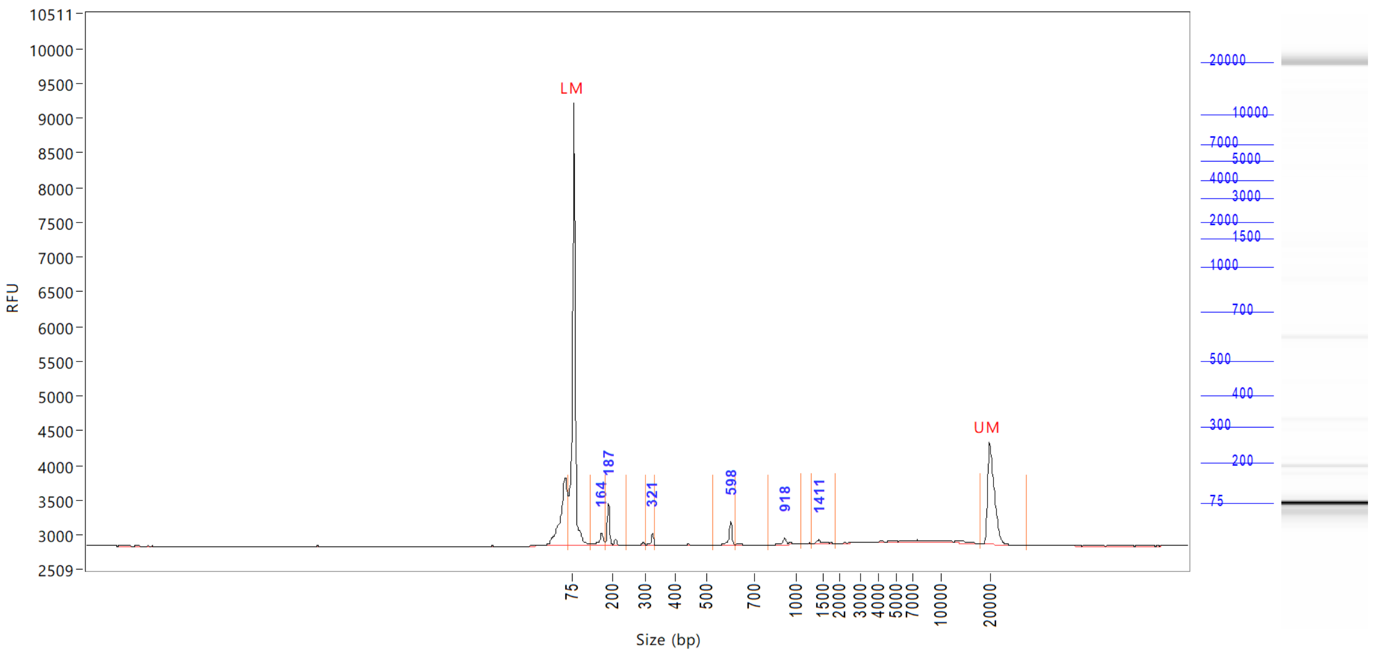

Peak	Size (bp)	Concentration (ng/uL)	From (bp)	To (bp)	Average size (bp)	CV%	RFU	Corrected peak area
1	75 (LM)	0.5000	58	131	74	11.33	6383	46.399
2	164	0.2483	131	175	158	6.79	179	1.920
3	187	0.4961	175	239	188	4.94	610	3.837
4	321	0.1138	300	331	317	1.78	168	0.880
5	598	0.2697	521	616	590	2.32	341	2.086
6	918	0.1267	796	1090	930	5.53	88	0.979
7	1411	0.0684	1289	1866	1452	7.46	55	0.529
8	20000 (UM)	0.1373	18171	27496	20390	3.97	1462	12.743

TIC:	1.3229	ng/uL
TIM:	8.5658	nmole/L
Total concentration:	1.7779	ng/uL

```
Sample peak width (sec): 5    Sample min peak height: 50    Sample baseline V to V?: Y    Sample baseline V to V points: 3
Sample filter: Binomial       Number of points for filter: 3  Sample start region (min): 0  Sample end region (min): 30
Marker peak width (sec): 5    Marker min peak height: 200   Marker baseline V to V?: Y    Marker baseline V to V points: 3
Lower marker selection: First peak > 500 RFU                Upper marker selection: Last peak > 200 RFU
Ladder size (bp)75, 200, 300, 400, 500, 700, 1000, 1500, 2000, 3000, 4000, 5000, 7000, 10000, 20000
Quantification using: Lower Marker          Final concentration (ng/uL): 0.5000          Dilution factor: 12.0
```

Sample: SampB10
Well location: B10
Created: Tuesday, September 14, 2021 7:33:54 PM

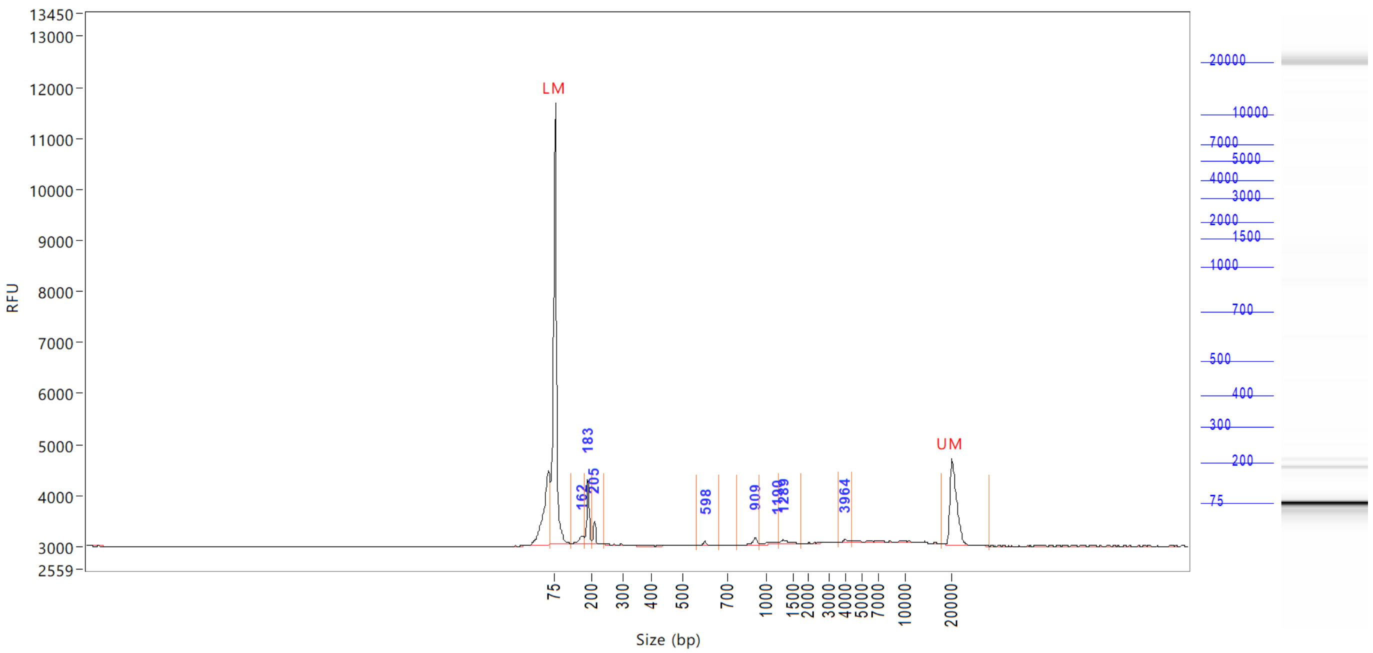

Peak	Size (bp)	Concentration (ng/uL)	From (bp)	To (bp)	Average size (bp)	CV%	RFU	Corrected peak area
1	75 (LM)	0.5000	58	129	72	9.74	8660	69.497
2	162	0.1678	129	173	160	5.89	155	1.944
3	183	0.6284	173	198	182	2.47	1249	7.279
4	205	0.2133	198	235	204	2.01	458	2.471
5	598	0.0545	560	662	594	2.60	86	0.632
6	909	0.1012	770	935	891	3.49	151	1.172
7	1190	0.0937	935	1223	1066	8.34	52	1.085
8	1289	0.0957	1223	1770	1352	7.21	83	1.109
9	3964	0.0410	3608	4380	4027	3.98	52	0.475
10	20000 (UM)	0.1084	17805	28100	20468	4.01	1686	15.073

TIC:	1.3957	ng/uL
TIM:	9.7362	nmole/L
Total concentration:	1.5852	ng/uL

Sample peak width (sec): 5   Sample min peak height: 50   Sample baseline V to V?: Y   Sample baseline V to V points: 3
Sample filter: Binomial   Number of points for filter: 3   Sample start region (min): 0   Sample end region (min): 30
Marker peak width (sec): 5   Marker min peak height: 200   Marker baseline V to V?: Y   Marker baseline V to V points: 3
Lower marker selection: First peak > 500 RFU   Upper marker selection: Last peak > 200 RFU
Ladder size (bp)75, 200, 300, 400, 500, 700, 1000, 1500, 2000, 3000, 4000, 5000, 7000, 10000, 20000
Quantification using: Lower Marker   Final concentration (ng/uL): 0.5000   Dilution factor: 12.0

Sample: SampC10
Well location: C10
Created: Tuesday, September 14, 2021 7:33:54 PM

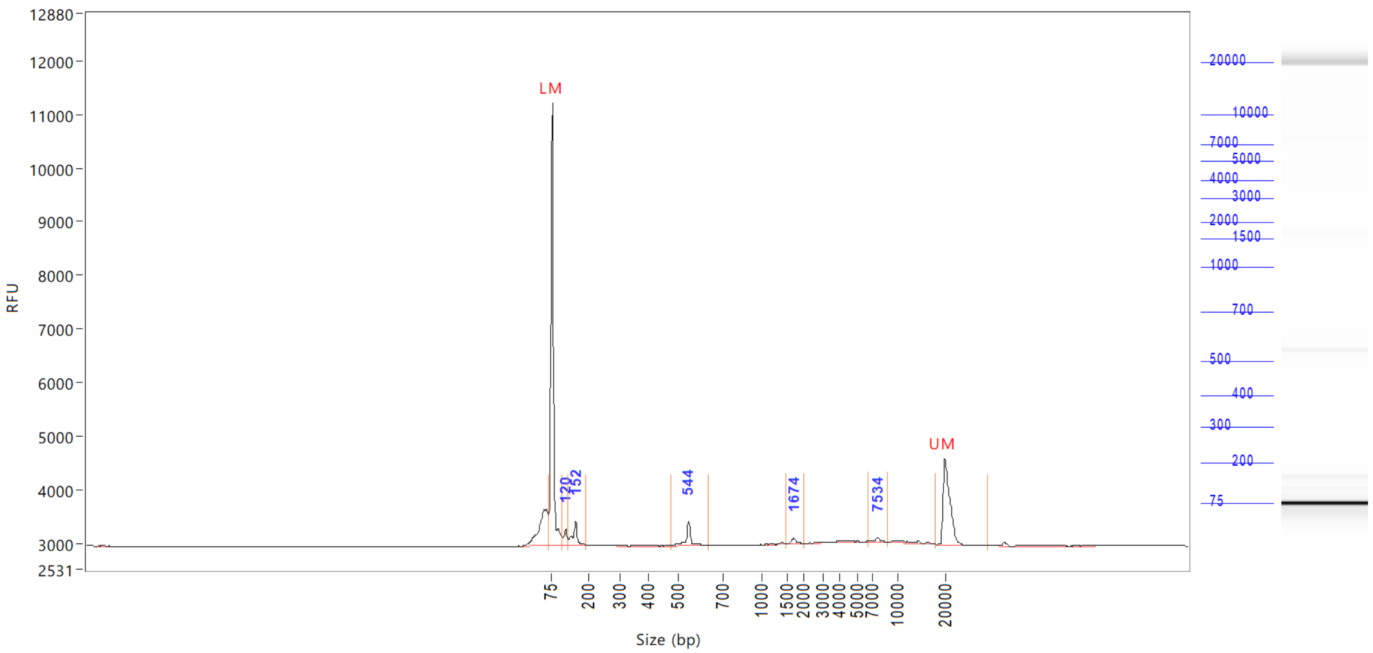

Peak	Size (bp)	Concentration (ng/uL)	From (bp)	To (bp)	Average size (bp)	CV%	RFU	Corrected peak area
1	75 (LM)	0.5000	62	112	75	10.28	8254	55.906
2	120	0.2557	112	129	120	3.98	304	2.383
3	152	0.4807	129	189	149	7.00	435	4.479
4	544	0.4519	477	639	540	5.00	463	4.210
5	1674	0.0961	1456	2058	1675	7.02	102	0.896
6	7534	0.0896	6556	8734	7476	6.16	87	0.835
7	20000 (UM)	0.1325	18171	29068	20543	4.66	1610	14.813

TIC:	1.3741	ng/uL
TIM:	10.2947	nmole/L
Total concentration:	1.8199	ng/uL

Sample peak width (sec): 5 Sample min peak height: 50 Sample baseline V to V?: Y Sample baseline V to V points: 3
Sample filter: Binomial Number of points for filter: 3 Sample start region (min): 0 Sample end region (min): 30
Marker peak width (sec): 5 Marker min peak height: 200 Marker baseline V to V?: Y Marker baseline V to V points: 3
Lower marker selection: First peak > 500 RFU Upper marker selection: Last peak > 200 RFU
Ladder size (bp)75, 200, 300, 400, 500, 700, 1000, 1500, 2000, 3000, 4000, 5000, 7000, 10000, 20000
Quantification using: Lower Marker Final concentration (ng/uL): 0.5000 Dilution factor: 12.0

Sample: SampD10
Well location: D10
Created: Tuesday, September 14, 2021 7:33:54 PM

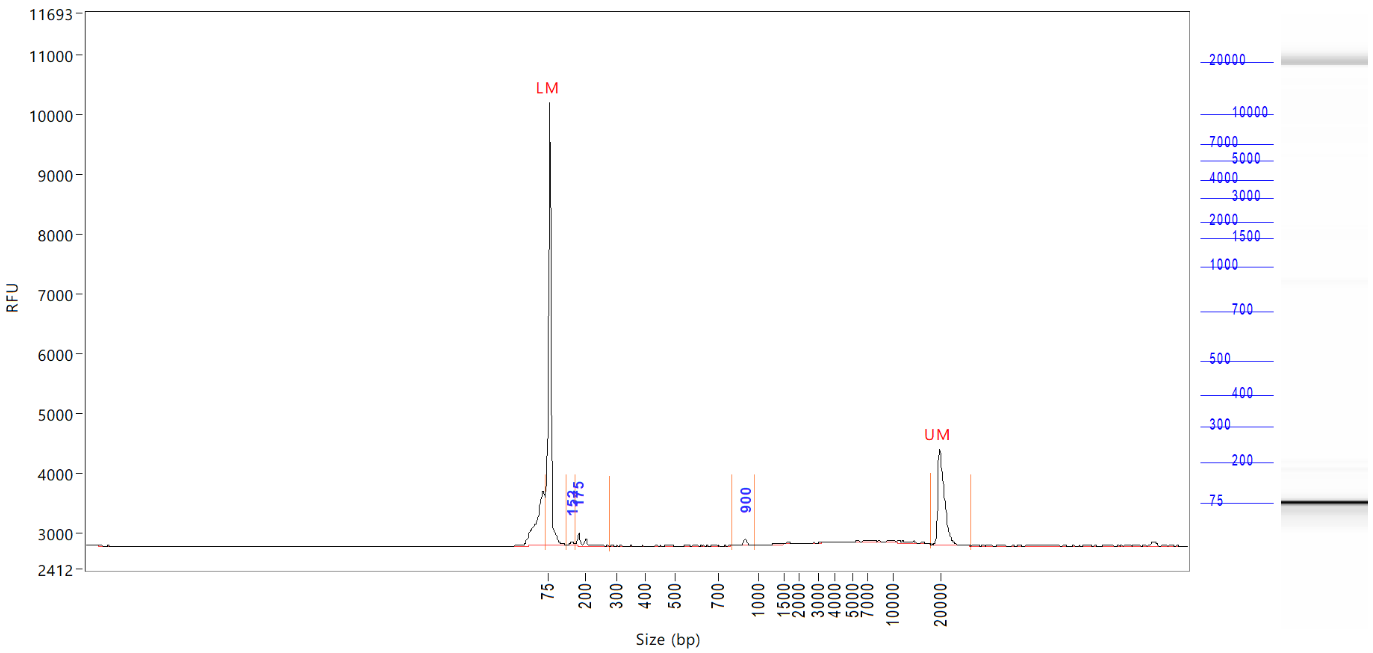

Peak	Size (bp)	Concentration (ng/uL)	From (bp)	To (bp)	Average size (bp)	CV%	RFU	Corrected peak area
1	75 (LM)	0.5000	60	135	74	10.53	7407	53.920
2	152	0.0772	135	162	149	4.74	67	0.694
3	175	0.2462	162	275	185	10.90	208	2.213
4	900	0.0909	801	974	895	2.62	111	0.817
5	20000 (UM)	0.1336	18171	26770	20457	4.24	1583	14.412

TIC:	0.4143	ng/uL
TIM:	3.2068	nmole/L
Total concentration:	0.8144	ng/uL

Sample peak width (sec): 5 Sample min peak height: 50 Sample baseline V to V?: Y Sample baseline V to V points: 3
Sample filter: Binomial Number of points for filter: 3 Sample start region (min): 0 Sample end region (min): 30
Marker peak width (sec): 5 Marker min peak height: 200 Marker baseline V to V?: Y Marker baseline V to V points: 3
Lower marker selection: First peak > 500 RFU Upper marker selection: Last peak > 200 RFU
Ladder size (bp)75, 200, 300, 400, 500, 700, 1000, 1500, 2000, 3000, 4000, 5000, 7000, 10000, 20000
Quantification using: Lower Marker Final concentration (ng/uL): 0.5000 Dilution factor: 12.0

Sample: SampE10
Well location: E10
Created: Tuesday, September 14, 2021 7:33:54 PM

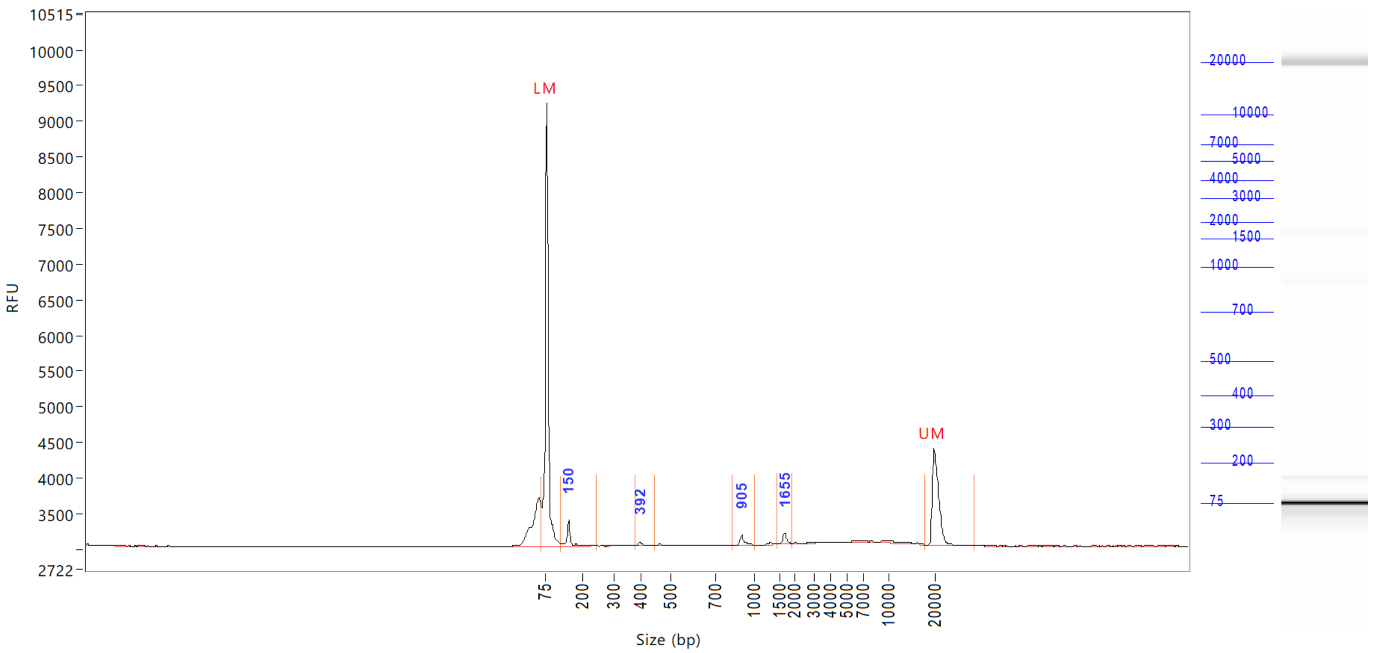

Peak	Size (bp)	Concentration (ng/uL)	From (bp)	To (bp)	Average size (bp)	CV%	RFU	Corrected peak area
1	75 (LM)	0.5000	58	124	75	11.45	6227	45.951
2	150	0.3981	124	241	152	11.53	360	3.049
3	392	0.0969	378	443	402	4.33	60	0.742
4	905	0.1521	835	1012	909	3.03	142	1.165
5	1655	0.1307	1456	1923	1643	4.70	151	1.001
6	20000 (UM)	0.1260	17805	28826	20367	4.56	1348	11.576

TIC:	0.7779	ng/uL
TIM:	5.1082	nmole/L
Total concentration:	1.2043	ng/uL

Sample peak width (sec): 5 Sample min peak height: 50 Sample baseline V to V?: Y Sample baseline V to V points: 3
Sample filter: Binomial Number of points for filter: 3 Sample start region (min): 0 Sample end region (min): 30
Marker peak width (sec): 5 Marker min peak height: 200 Marker baseline V to V?: Y Marker baseline V to V points: 3
Lower marker selection: First peak > 500 RFU Upper marker selection: Last peak > 200 RFU
Ladder size (bp)75, 200, 300, 400, 500, 700, 1000, 1500, 2000, 3000, 4000, 5000, 7000, 10000, 20000
Quantification using: Lower Marker Final concentration (ng/uL): 0.5000 Dilution factor: 12.0

Sample: SampF10
Well location: F10
Created: Tuesday, September 14, 2021 7:33:54 PM

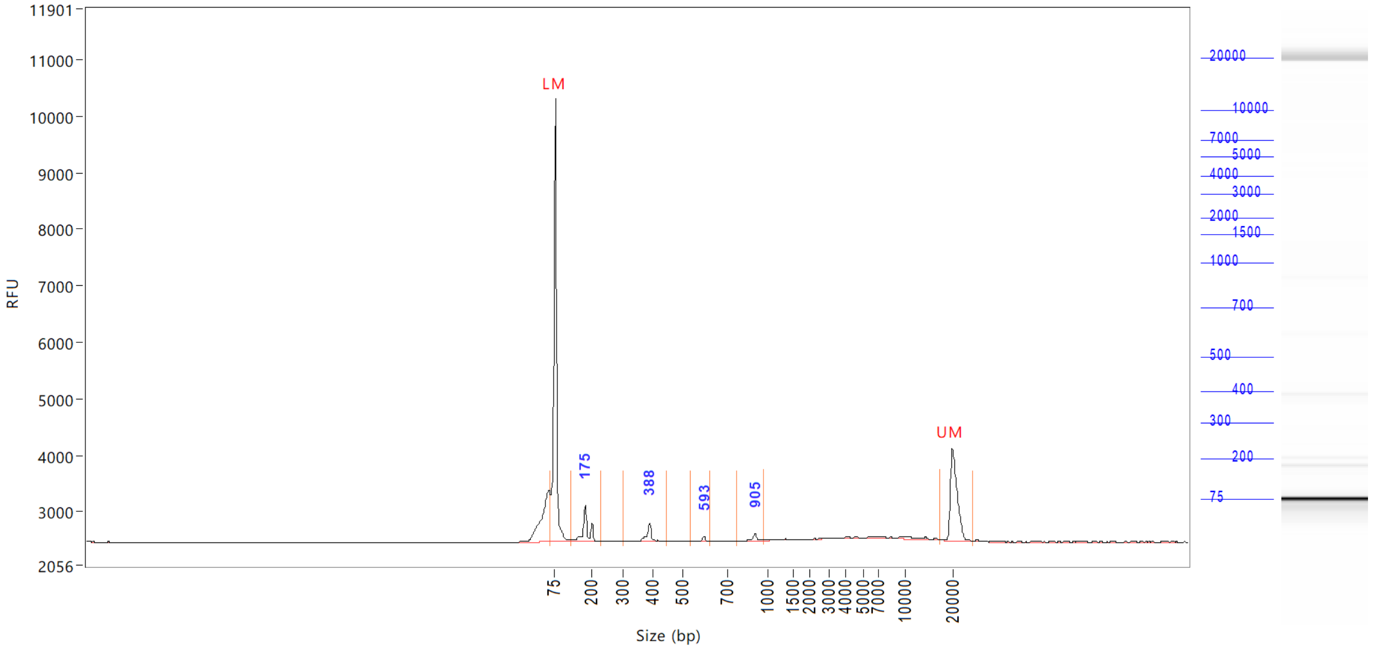

Peak	Size (bp)	Concentration (ng/uL)	From (bp)	To (bp)	Average size (bp)	CV%	RFU	Corrected peak area
1	75 (LM)	0.5000	60	129	74	9.88	7856	55.177
2	175	0.7514	129	228	176	9.87	636	6.910
3	388	0.3674	296	445	382	5.10	337	3.379
4	593	0.0686	532	623	587	2.58	90	0.631
5	905	0.1209	770	970	893	4.21	118	1.112
6	20000 (UM)	0.1363	17195	24594	20413	3.85	1644	15.042

TIC:	1.3083	ng/uL
TIM:	9.0163	nmole/L
Total concentration:	1.7493	ng/uL

Sample peak width (sec): 5 Sample min peak height: 50 Sample baseline V to V?: Y Sample baseline V to V points: 3
Sample filter: Binomial Number of points for filter: 3 Sample start region (min): 0 Sample end region (min): 30
Marker peak width (sec): 5 Marker min peak height: 200 Marker baseline V to V?: Y Marker baseline V to V points: 3
Lower marker selection: First peak > 500 RFU Upper marker selection: Last peak > 200 RFU
Ladder size (bp)75, 200, 300, 400, 500, 700, 1000, 1500, 2000, 3000, 4000, 5000, 7000, 10000, 20000
Quantification using: Lower Marker Final concentration (ng/uL): 0.5000 Dilution factor: 12.0

Sample: SampG10
Well location: G10
Created: Tuesday, September 14, 2021 7:33:54 PM

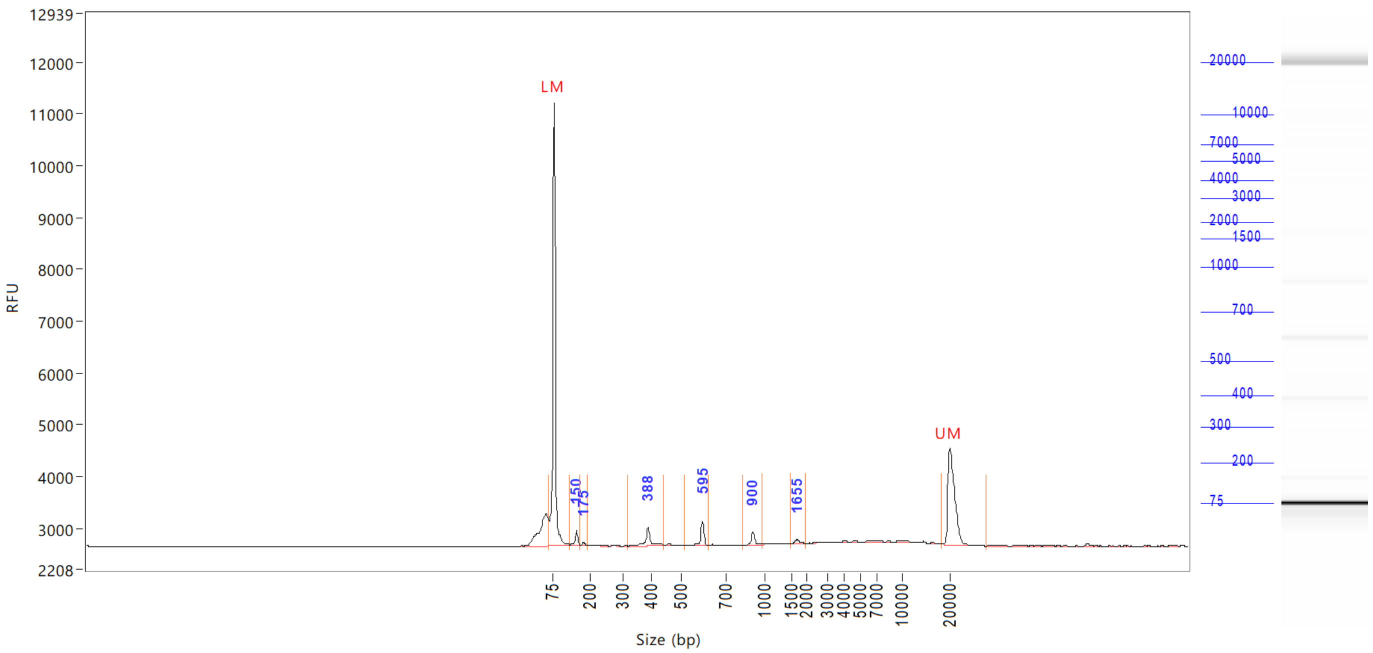

Peak	Size (bp)	Concentration (ng/uL)	From (bp)	To (bp)	Average size (bp)	CV%	RFU	Corrected peak area
1	75 (LM)	0.5000	58	129	74	9.45	8564	56.071
2	150	0.1970	129	166	147	4.11	283	1.841
3	175	0.0307	166	187	172	1.95	51	0.287
4	388	0.3936	319	439	387	5.09	354	3.678
5	595	0.3101	511	621	588	2.11	484	2.898
6	900	0.1654	822	970	901	2.20	246	1.546
7	1655	0.0559	1489	1942	1649	4.58	79	0.522
8	20000 (UM)	0.1492	18293	27738	20448	3.96	1871	16.736

TIC:	1.1527	ng/uL
TIM:	5.3954	nmole/L
Total concentration:	1.4892	ng/uL

Sample peak width (sec): 5   Sample min peak height: 50   Sample baseline V to V?: Y   Sample baseline V to V points: 3
Sample filter: Binomial   Number of points for filter: 3 Sample start region (min): 0 Sample end region (min): 30
Marker peak width (sec): 5   Marker min peak height: 200   Marker baseline V to V?: Y   Marker baseline V to V points: 3
Lower marker selection: First peak > 500 RFU   Upper marker selection: Last peak > 200 RFU
Ladder size (bp)75, 200, 300, 400, 500, 700, 1000, 1500, 2000, 3000, 4000, 5000, 7000, 10000, 20000
Quantification using: Lower Marker   Final concentration (ng/uL): 0.5000   Dilution factor: 12.0

Sample: SampH10
Well location: H10
Created: Tuesday, September 14, 2021 7:33:54 PM

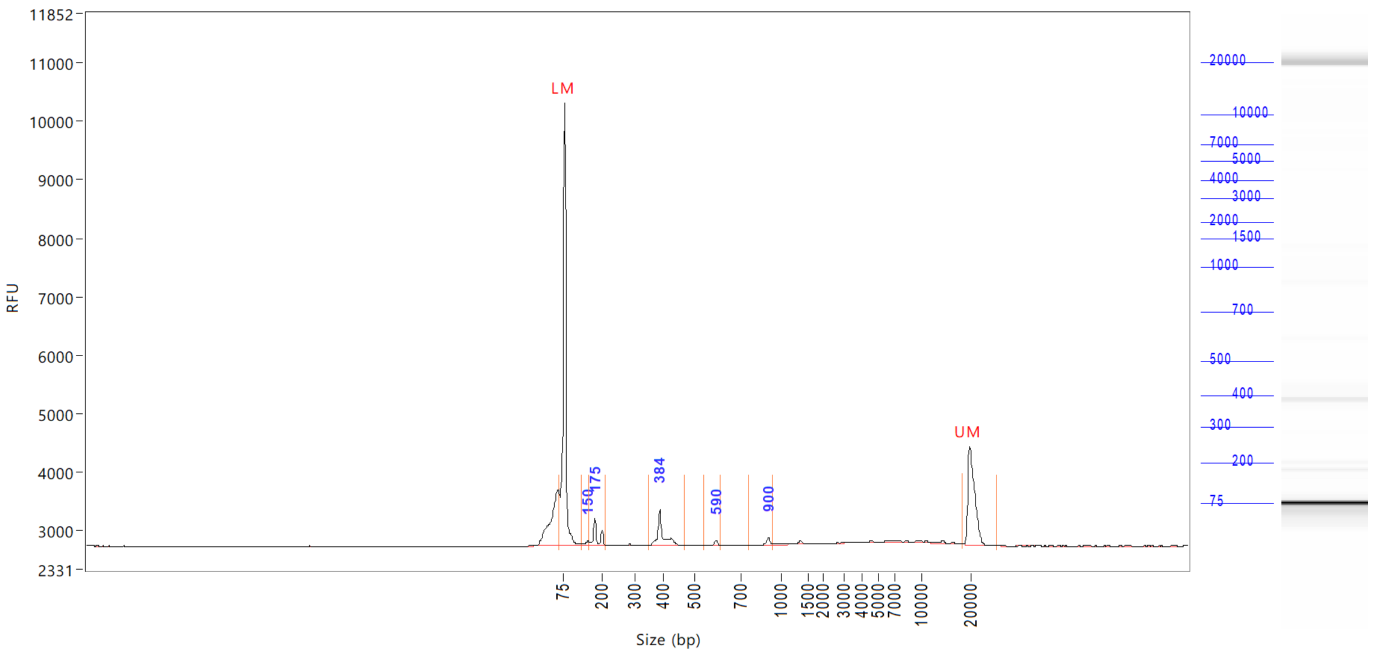

Peak	Size (bp)	Concentration (ng/uL)	From (bp)	To (bp)	Average size (bp)	CV%	RFU	Corrected peak area
1	75 (LM)	0.5000	62	133	74	9.79	7595	53.785
2	150	0.0895	133	158	148	4.52	78	0.802
3	175	0.4831	158	210	180	6.78	451	4.331
4	384	0.7859	345	468	392	5.09	614	7.044
5	590	0.0738	539	611	585	2.20	92	0.662
6	900	0.0914	757	939	893	2.87	117	0.819
7	20000 (UM)	0.1391	18415	25682	20376	3.67	1680	14.964

TIC:	1.5237	ng/uL
TIM:	9.0809	nmole/L
Total concentration:	1.9600	ng/uL

Sample peak width (sec): 5   Sample min peak height: 50   Sample baseline V to V?: Y   Sample baseline V to V points: 3
Sample filter: Binomial   Number of points for filter: 3 Sample start region (min): 0 Sample end region (min): 30
Marker peak width (sec): 5   Marker min peak height: 200   Marker baseline V to V?: Y   Marker baseline V to V points: 3
Lower marker selection: First peak > 500 RFU   Upper marker selection: Last peak > 200 RFU
Ladder size (bp)75, 200, 300, 400, 500, 700, 1000, 1500, 2000, 3000, 4000, 5000, 7000, 10000, 20000
Quantification using: Lower Marker   Final concentration (ng/uL): 0.5000   Dilution factor: 12.0

Sample: SampA11
Well location: A11
Created: Tuesday, September 14, 2021 7:33:54 PM

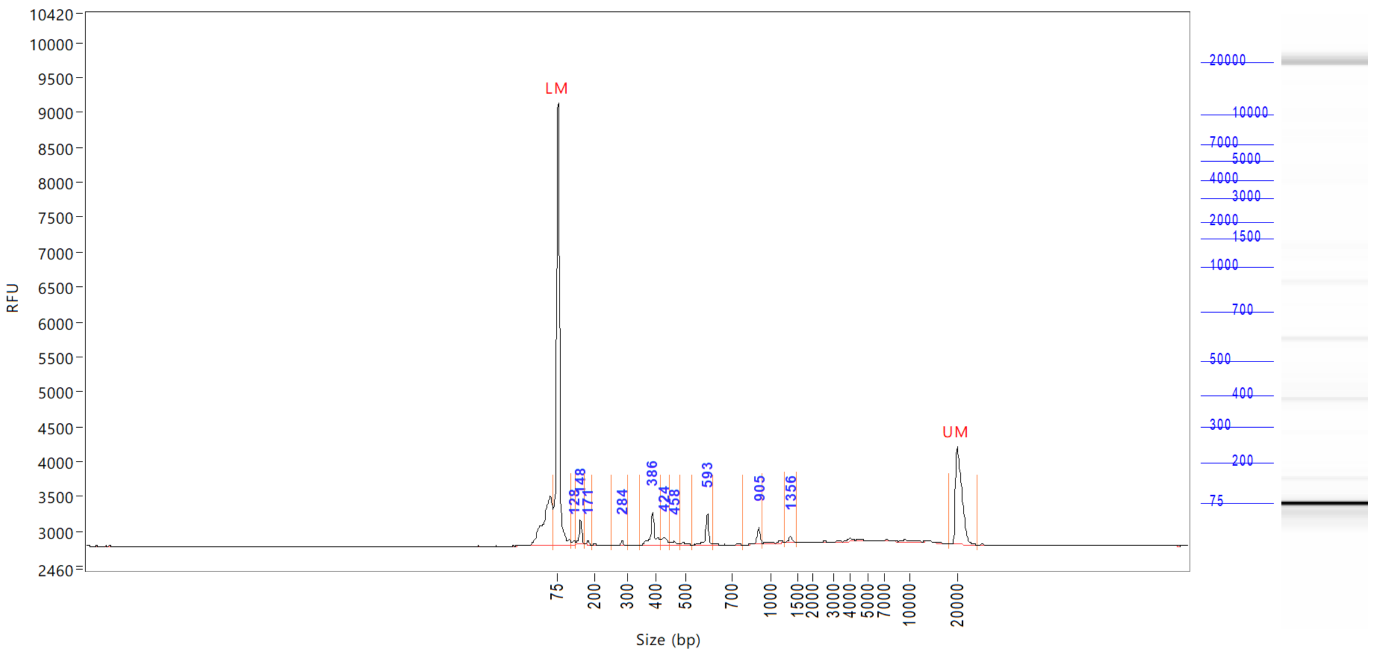

Peak	Size (bp)	Concentration (ng/uL)	From (bp)	To (bp)	Average size (bp)	CV%	RFU	Corrected peak area
1	75 (LM)	0.5000	58	120	73	10.38	6338	44.752
2	128	0.0620	120	133	126	3.31	56	0.463
3	148	0.3046	133	164	146	3.45	344	2.272
4	171	0.0419	164	189	171	2.28	61	0.312
5	284	0.0539	248	301	278	2.81	61	0.402
6	386	0.5663	341	412	385	3.53	462	4.224
7	424	0.1844	412	445	426	2.01	107	1.375
8	458	0.0745	445	477	458	1.84	54	0.555
9	593	0.3389	524	618	585	1.91	439	2.528
10	905	0.1753	788	931	894	1.81	234	1.307
11	1356	0.0822	1245	1478	1349	3.44	86	0.613
12	20000 (UM)	0.1320	18293	24231	20407	3.48	1406	11.816

TIC:	1.8840	ng/uL
TIM:	9.7358	nmole/L
Total concentration:	2.2753	ng/uL

```
Sample peak width (sec): 5    Sample min peak height: 50    Sample baseline V to V?: Y    Sample baseline V to V points: 3
Sample filter: Binomial       Number of points for filter: 3  Sample start region (min): 0  Sample end region (min): 30
Marker peak width (sec): 5    Marker min peak height: 200   Marker baseline V to V?: Y    Marker baseline V to V points: 3
Lower marker selection: First peak > 500 RFU                Upper marker selection: Last peak > 200 RFU
Ladder size (bp)75, 200, 300, 400, 500, 700, 1000, 1500, 2000, 3000, 4000, 5000, 7000, 10000, 20000
Quantification using: Lower Marker          Final concentration (ng/uL): 0.5000          Dilution factor: 12.0
```

Sample: SampB11
Well location: B11
Created: Tuesday, September 14, 2021 7:33:54 PM

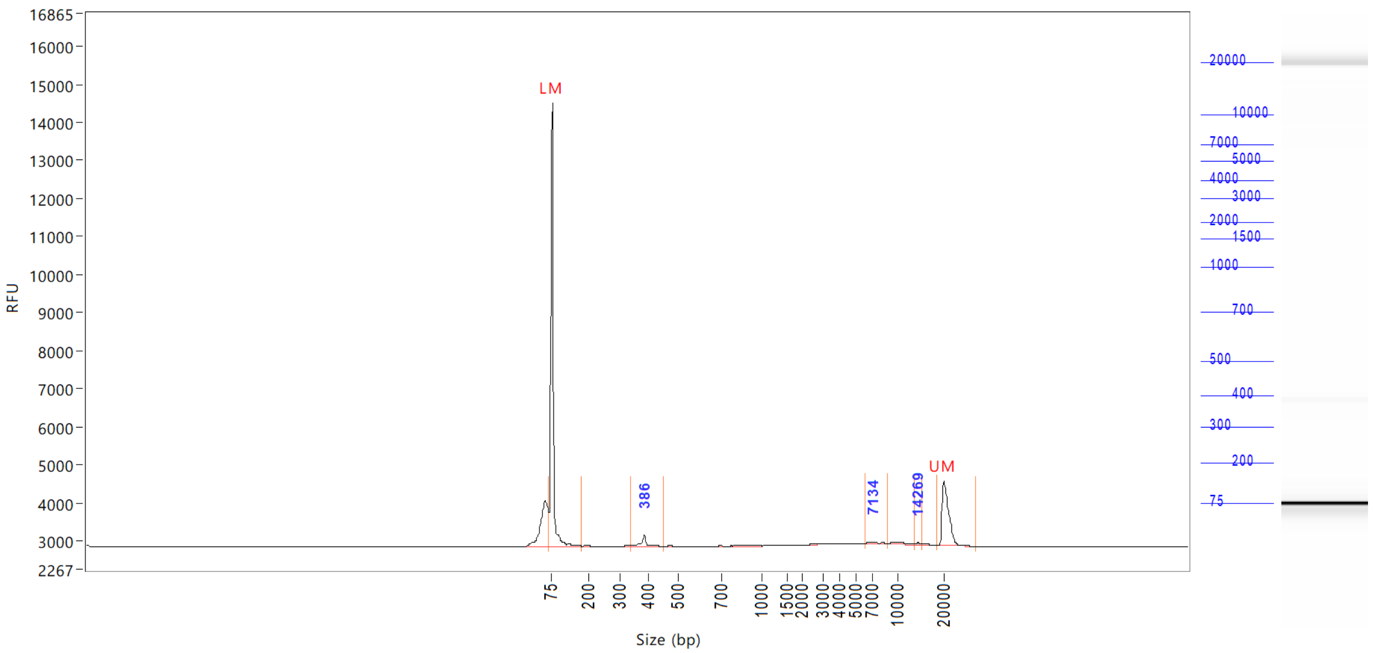

Peak	Size (bp)	Concentration (ng/uL)	From (bp)	To (bp)	Average size (bp)	CV%	RFU	Corrected peak area
1	75 (LM)	0.5000	62	175	75	14.65	11668	74.817
2	386	0.2518	337	449	381	5.23	306	3.140
3	7134	0.0655	6112	8800	7323	8.99	57	0.817
4	14269	0.0286	13537	15366	14263	3.29	57	0.357
5	20000 (UM)	0.1028	18415	26770	20374	4.01	1711	15.379

TIC:	0.3460	ng/uL
TIM:	1.1054	nmole/L
Total concentration:	0.6810	ng/uL

```
Sample peak width (sec): 5    Sample min peak height: 50    Sample baseline V to V?: Y    Sample baseline V to V points: 3
Sample filter: Binomial       Number of points for filter: 3  Sample start region (min): 0  Sample end region (min): 30
Marker peak width (sec): 5    Marker min peak height: 200   Marker baseline V to V?: Y    Marker baseline V to V points: 3
Lower marker selection: First peak > 500 RFU                Upper marker selection: Last peak > 200 RFU
Ladder size (bp)75, 200, 300, 400, 500, 700, 1000, 1500, 2000, 3000, 4000, 5000, 7000, 10000, 20000
Quantification using: Lower Marker          Final concentration (ng/uL): 0.5000          Dilution factor: 12.0
```

Sample: SampC11
Well location: C11
Created: Tuesday, September 14, 2021 7:33:54 PM

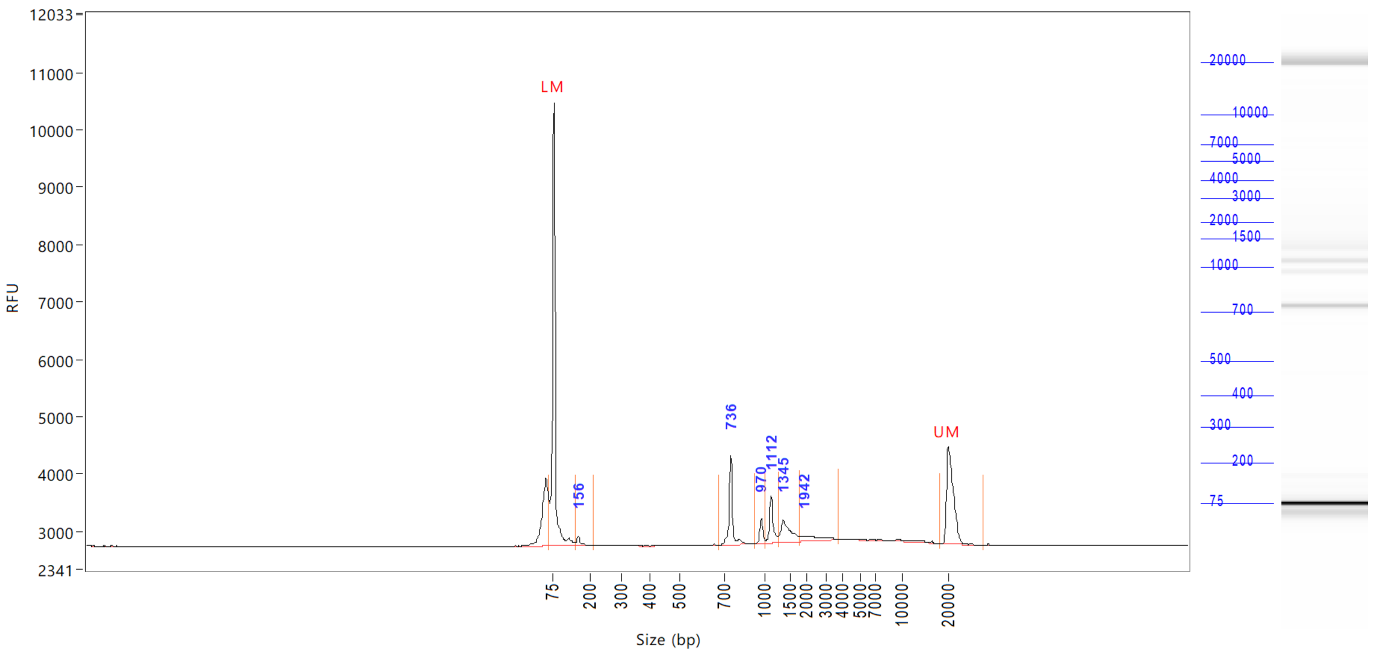

Peak	Size (bp)	Concentration (ng/uL)	From (bp)	To (bp)	Average size (bp)	CV%	RFU	Corrected peak area
1	75 (LM)	0.5000	60	147	76	16.70	7736	54.972
2	156	0.1842	147	206	161	7.92	176	1.687
3	736	1.0767	669	918	738	3.97	1544	9.864
4	970	0.2720	918	1001	969	1.36	443	2.492
5	1112	0.5944	1001	1256	1115	4.75	823	5.445
6	1345	0.6899	1256	1789	1435	8.96	406	6.321
7	1942	0.3244	1789	3750	2360	19.90	99	2.972
8	20000 (UM)	0.1403	18293	27617	20451	4.04	1706	15.421

TIC:	3.1415	ng/uL
TIM:	6.6385	nmole/L
Total concentration:	3.4320	ng/uL

```
Sample peak width (sec): 5    Sample min peak height: 50    Sample baseline V to V?: Y    Sample baseline V to V points: 3
Sample filter: Binomial       Number of points for filter: 3  Sample start region (min): 0  Sample end region (min): 30
Marker peak width (sec): 5    Marker min peak height: 200   Marker baseline V to V?: Y    Marker baseline V to V points: 3
Lower marker selection: First peak > 500 RFU                Upper marker selection: Last peak > 200 RFU
Ladder size (bp)75, 200, 300, 400, 500, 700, 1000, 1500, 2000, 3000, 4000, 5000, 7000, 10000, 20000
Quantification using: Lower Marker          Final concentration (ng/uL): 0.5000          Dilution factor: 12.0
```

Sample: SampD11
Well location: D11
Created: Tuesday, September 14, 2021 7:33:54 PM

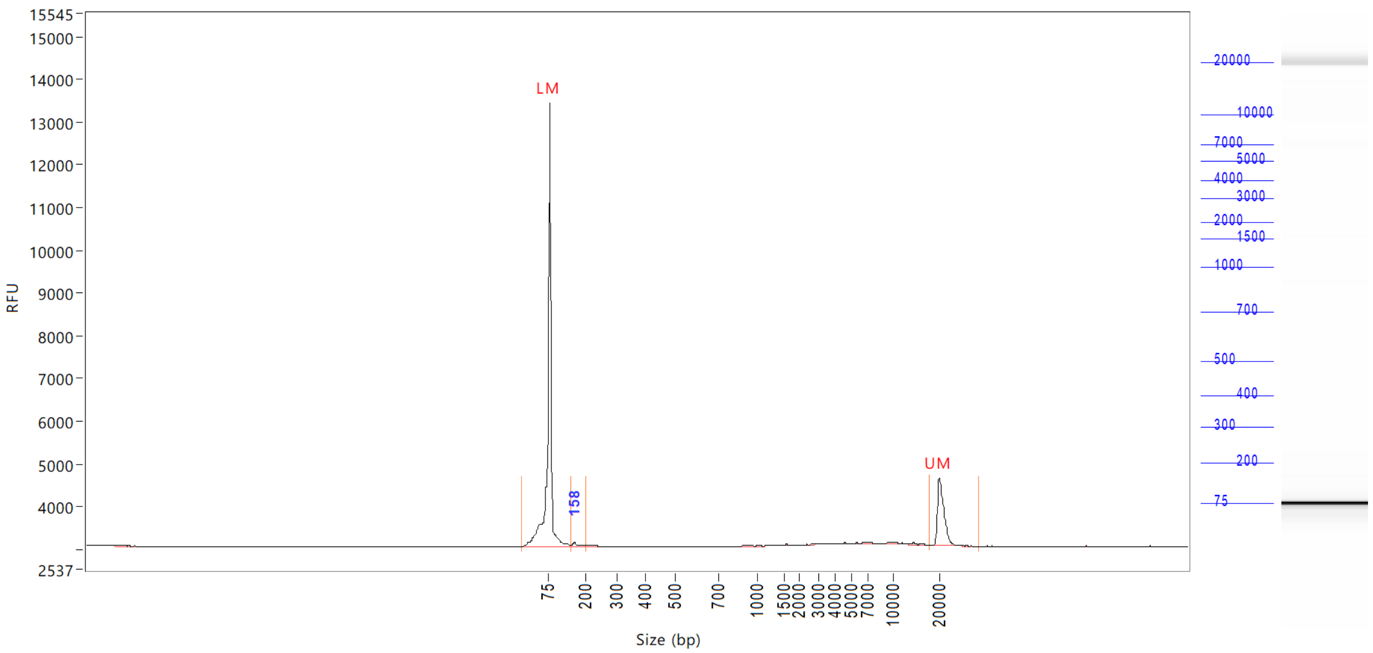

Peak	Size	Concentration	From	To	Average size	CV%	RFU	Corrected peak area
	(bp)	(ng/uL)	(bp)	(bp)	(bp)			
1	75 (LM)	0.5000	0	147	67	28.26	10401	85.299
2	158	0.0980	147	198	166	8.74	106	1.394
3	20000 (UM)	0.0828	18049	28463	20441	4.35	1565	14.121

TIC:	0.0980	ng/uL
TIM:	0.9707	nmole/L
Total concentration:	0.4488	ng/uL

```
Sample peak width (sec): 5    Sample min peak height: 50    Sample baseline V to V?: Y    Sample baseline V to V points: 3
Sample filter: Binomial       Number of points for filter: 3  Sample start region (min): 0  Sample end region (min): 30
Marker peak width (sec): 5    Marker min peak height: 200   Marker baseline V to V?: Y    Marker baseline V to V points: 3
Lower marker selection: First peak > 500 RFU                Upper marker selection: Last peak > 200 RFU
Ladder size (bp)75, 200, 300, 400, 500, 700, 1000, 1500, 2000, 3000, 4000, 5000, 7000, 10000, 20000
Quantification using: Lower Marker          Final concentration (ng/uL): 0.5000          Dilution factor: 12.0
```

Sample: SampE11
Well location: E11
Created: Tuesday, September 14, 2021 7:33:54 PM

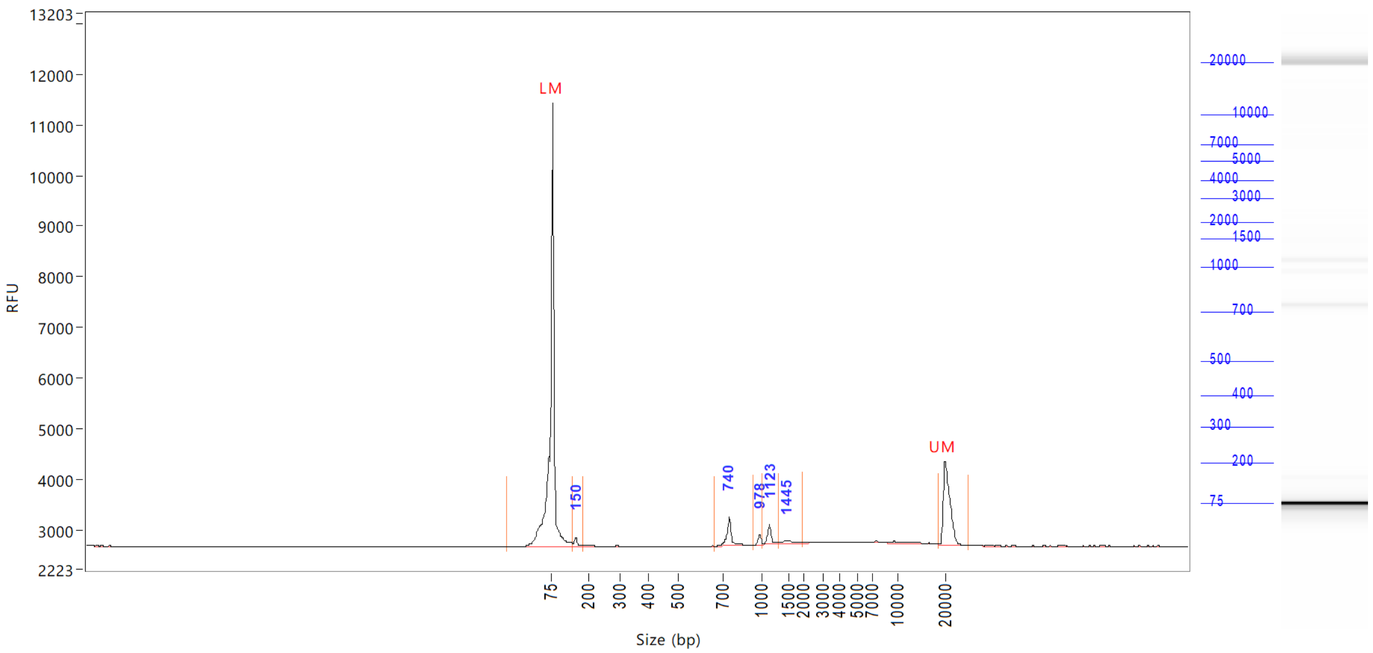

Peak	Size (bp)	Concentration (ng/uL)	From (bp)	To (bp)	Average size (bp)	CV%	RFU	Corrected peak area
1	75 (LM)	0.5000	0	143	66	30.98	8778	85.132
2	150	0.1064	143	175	153	4.68	185	1.509
3	740	0.3302	664	931	743	4.96	575	4.685
4	978	0.0772	931	1001	972	1.27	190	1.095
5	1123	0.1830	1001	1311	1125	5.02	393	2.597
6	1445	0.0811	1311	1962	1542	10.40	66	1.151
7	20000 (UM)	0.0857	18537	25199	20477	3.59	1641	14.583

TIC:	0.7779	ng/uL
TIM:	2.3590	nmole/L
Total concentration:	1.0286	ng/uL

```
Sample peak width (sec): 5    Sample min peak height: 50    Sample baseline V to V?: Y    Sample baseline V to V points: 3
Sample filter: Binomial       Number of points for filter: 3  Sample start region (min): 0  Sample end region (min): 30
Marker peak width (sec): 5    Marker min peak height: 200   Marker baseline V to V?: Y    Marker baseline V to V points: 3
Lower marker selection: First peak > 500 RFU                Upper marker selection: Last peak > 200 RFU
Ladder size (bp)75, 200, 300, 400, 500, 700, 1000, 1500, 2000, 3000, 4000, 5000, 7000, 10000, 20000
Quantification using: Lower Marker          Final concentration (ng/uL): 0.5000          Dilution factor: 12.0
```

Sample: SampF11
Well location: F11
Created: Tuesday, September 14, 2021 7:33:54 PM

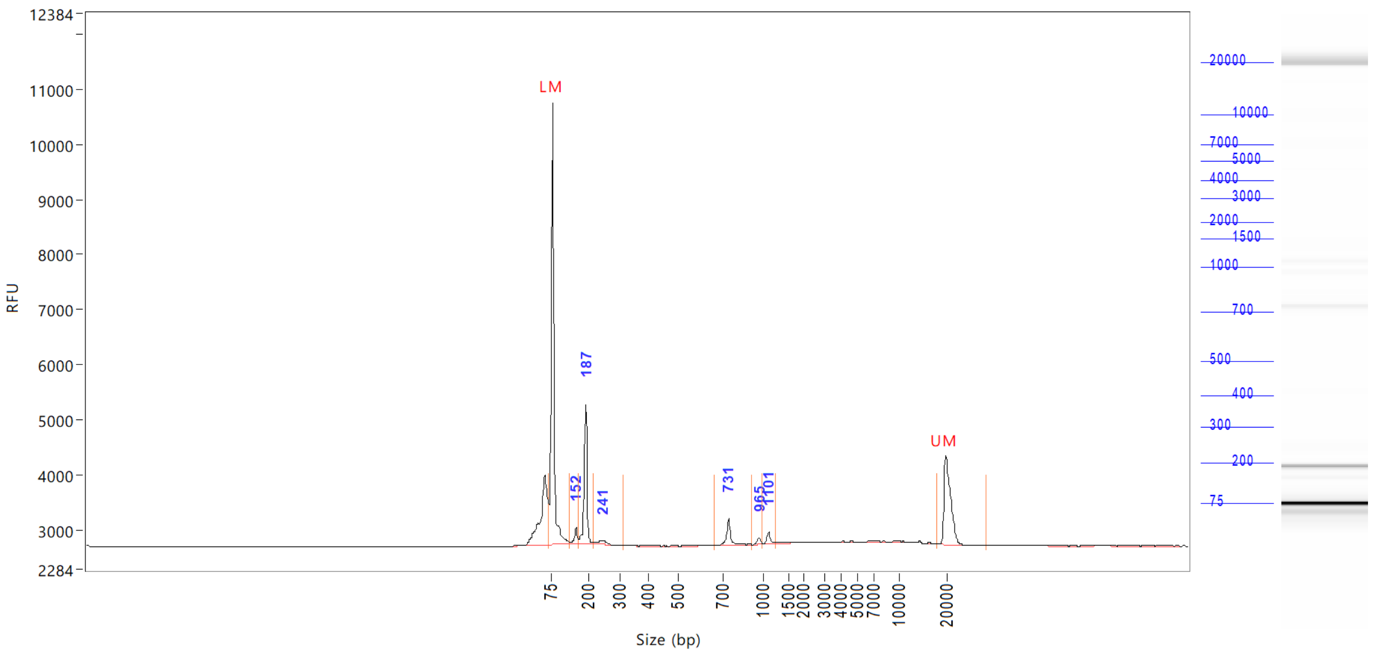

Peak	Size (bp)	Concentration (ng/uL)	From (bp)	To (bp)	Average size (bp)	CV%	RFU	Corrected peak area
1	75 (LM)	0.5000	62	135	75	12.43	8035	55.126
2	152	0.2102	135	162	151	3.87	288	1.931
3	187	1.7485	162	212	183	3.32	2529	16.064
4	241	0.1444	212	307	239	5.81	70	1.326
5	731	0.4129	657	918	736	5.15	494	3.793
6	965	0.0793	918	1000	963	1.29	127	0.729
7	1101	0.1416	1000	1256	1096	4.14	223	1.301
8	20000 (UM)	0.1309	18049	28705	20408	4.00	1617	14.431

TIC:	2.7368	ng/uL
TIM:	20.2600	nmole/L
Total concentration:	3.0445	ng/uL

Sample peak width (sec): 5 Sample min peak height: 50 Sample baseline V to V?: Y Sample baseline V to V points: 3
Sample filter: Binomial Number of points for filter: 3 Sample start region (min): 0 Sample end region (min): 30
Marker peak width (sec): 5 Marker min peak height: 200 Marker baseline V to V?: Y Marker baseline V to V points: 3
Lower marker selection: First peak > 500 RFU Upper marker selection: Last peak > 200 RFU
Ladder size (bp)75, 200, 300, 400, 500, 700, 1000, 1500, 2000, 3000, 4000, 5000, 7000, 10000, 20000
Quantification using: Lower Marker Final concentration (ng/uL): 0.5000 Dilution factor: 12.0

Sample: SampG11
Well location: G11
Created: Tuesday, September 14, 2021 7:33:54 PM

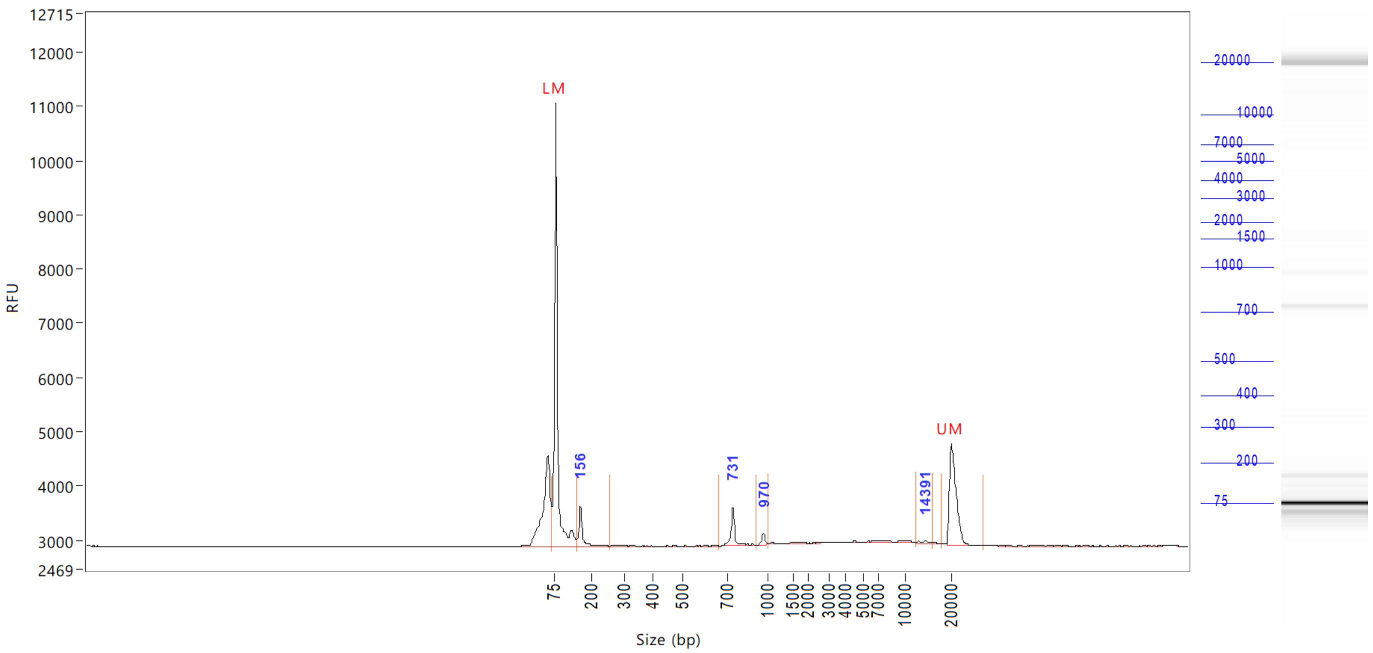

Peak	Size (bp)	Concentration (ng/uL)	From (bp)	To (bp)	Average size (bp)	CV%	RFU	Corrected peak area
1	75 (LM)	0.5000	60	147	79	20.32	8187	62.483
2	156	0.5816	147	251	162	9.74	744	6.057
3	731	0.4769	659	909	734	4.31	709	4.967
4	970	0.1198	909	1001	966	1.47	207	1.248
5	14391	0.0519	12318	15732	13930	6.48	52	0.540
6	20000 (UM)	0.1347	17927	26891	20382	3.96	1870	16.830

TIC:	1.2303	ng/uL
TIM:	7.1811	nmole/L
Total concentration:	1.5602	ng/uL

```
Sample peak width (sec): 5    Sample min peak height: 50    Sample baseline V to V?: Y    Sample baseline V to V points: 3
Sample filter: Binomial       Number of points for filter: 3  Sample start region (min): 0  Sample end region (min): 30
Marker peak width (sec): 5    Marker min peak height: 200   Marker baseline V to V?: Y    Marker baseline V to V points: 3
Lower marker selection: First peak > 500 RFU                Upper marker selection: Last peak > 200 RFU
Ladder size (bp)75, 200, 300, 400, 500, 700, 1000, 1500, 2000, 3000, 4000, 5000, 7000, 10000, 20000
Quantification using: Lower Marker          Final concentration (ng/uL): 0.5000          Dilution factor: 12.0
```

Sample: SampH11
Well location: H11
Created: Tuesday, September 14, 2021 7:33:54 PM

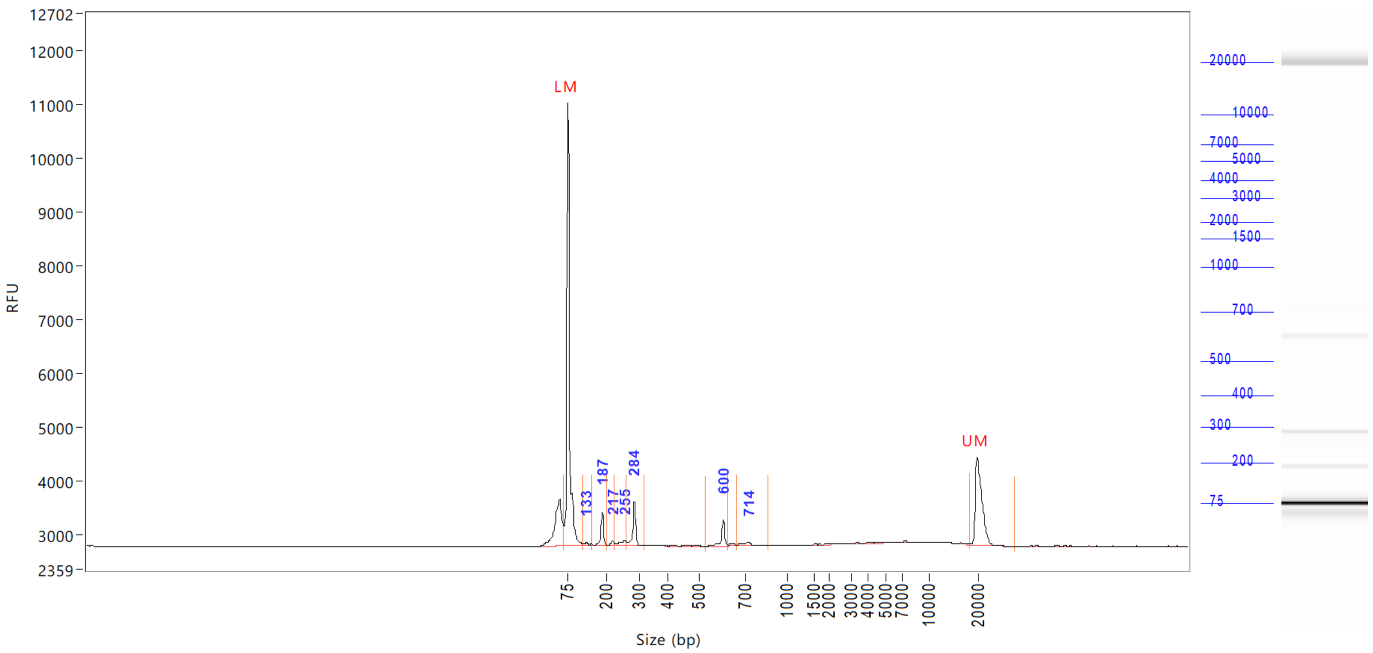

Peak	Size (bp)	Concentration (ng/uL)	From (bp)	To (bp)	Average size (bp)	CV%	RFU	Corrected peak area
1	75 (LM)	0.5000	62	124	75	10.08	8253	54.755
2	133	0.0616	124	156	135	6.27	58	0.562
3	187	0.4084	156	203	183	3.09	619	3.727
4	217	0.0433	203	224	216	1.84	69	0.395
5	255	0.1373	224	262	247	4.01	78	1.253
6	284	0.5539	262	317	282	2.62	810	5.055
7	600	0.3258	524	621	594	2.42	477	2.973
8	714	0.1225	659	861	706	4.92	63	1.118
9	20000 (UM)	0.1302	18415	27617	20399	3.99	1625	14.263

TIC:	1.6527	ng/uL
TIM:	10.0807	nmole/L
Total concentration:	2.0048	ng/uL

Sample peak width (sec): 5   Sample min peak height: 50   Sample baseline V to V?: Y   Sample baseline V to V points: 3
Sample filter: Binomial   Number of points for filter: 3 Sample start region (min): 0 Sample end region (min): 30
Marker peak width (sec): 5   Marker min peak height: 200   Marker baseline V to V?: Y   Marker baseline V to V points: 3
Lower marker selection: First peak > 500 RFU   Upper marker selection: Last peak > 200 RFU
Ladder size (bp)75, 200, 300, 400, 500, 700, 1000, 1500, 2000, 3000, 4000, 5000, 7000, 10000, 20000
Quantification using: Lower Marker   Final concentration (ng/uL): 0.5000   Dilution factor: 12.0

Sample: SampA12
Well location: A12
Created: Tuesday, September 14, 2021 7:33:54 PM

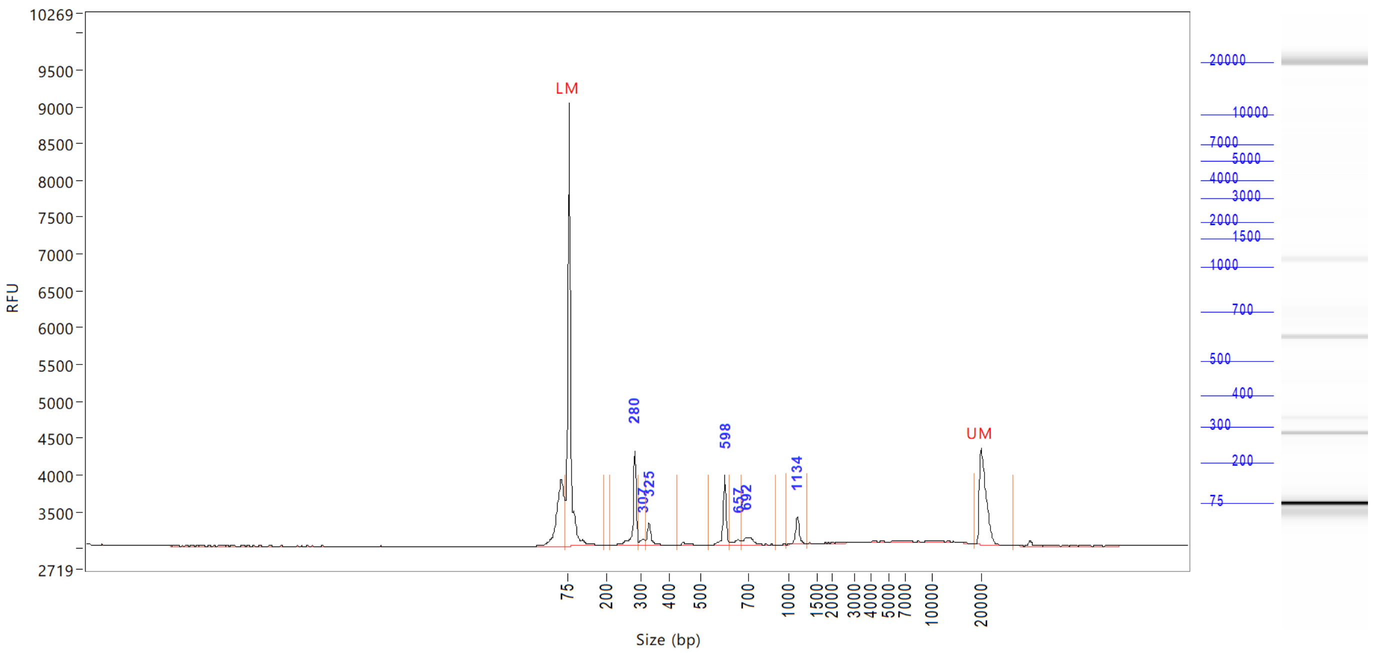

Peak	Size (bp)	Concentration (ng/uL)	From (bp)	To (bp)	Average size (bp)	CV%	RFU	Corrected peak area
1	75 (LM)	0.5000	64	189	76	16.65	6028	42.202
2	280	1.1832	206	291	274	4.84	1288	8.322
3	307	0.1361	291	315	303	2.35	79	0.957
4	325	0.3603	315	424	332	5.50	310	2.535
5	598	0.7704	532	618	593	1.83	947	5.419
6	657	0.1363	618	669	647	2.20	73	0.958
7	692	0.2823	669	905	713	5.80	108	1.985
8	1134	0.3371	978	1323	1131	4.00	374	2.371
9	20000 (UM)	0.1369	18659	26649	20505	4.13	1313	11.559

TIC:	3.2056	ng/uL
TIM:	13.2534	nmole/L
Total concentration:	3.5637	ng/uL

```
Sample peak width (sec): 5     Sample min peak height: 50     Sample baseline V to V?: Y     Sample baseline V to V points: 3
Sample filter: Binomial        Number of points for filter: 3  Sample start region (min): 0  Sample end region (min): 30
Marker peak width (sec): 5     Marker min peak height: 200    Marker baseline V to V?: Y     Marker baseline V to V points: 3
Lower marker selection: First peak > 500 RFU                  Upper marker selection: Last peak > 200 RFU
Ladder size (bp)75, 200, 300, 400, 500, 700, 1000, 1500, 2000, 3000, 4000, 5000, 7000, 10000, 20000
Quantification using: Lower Marker          Final concentration (ng/uL): 0.5000          Dilution factor: 12.0
```

Sample: SampB12
Well location: B12
Created: Tuesday, September 14, 2021 7:33:54 PM

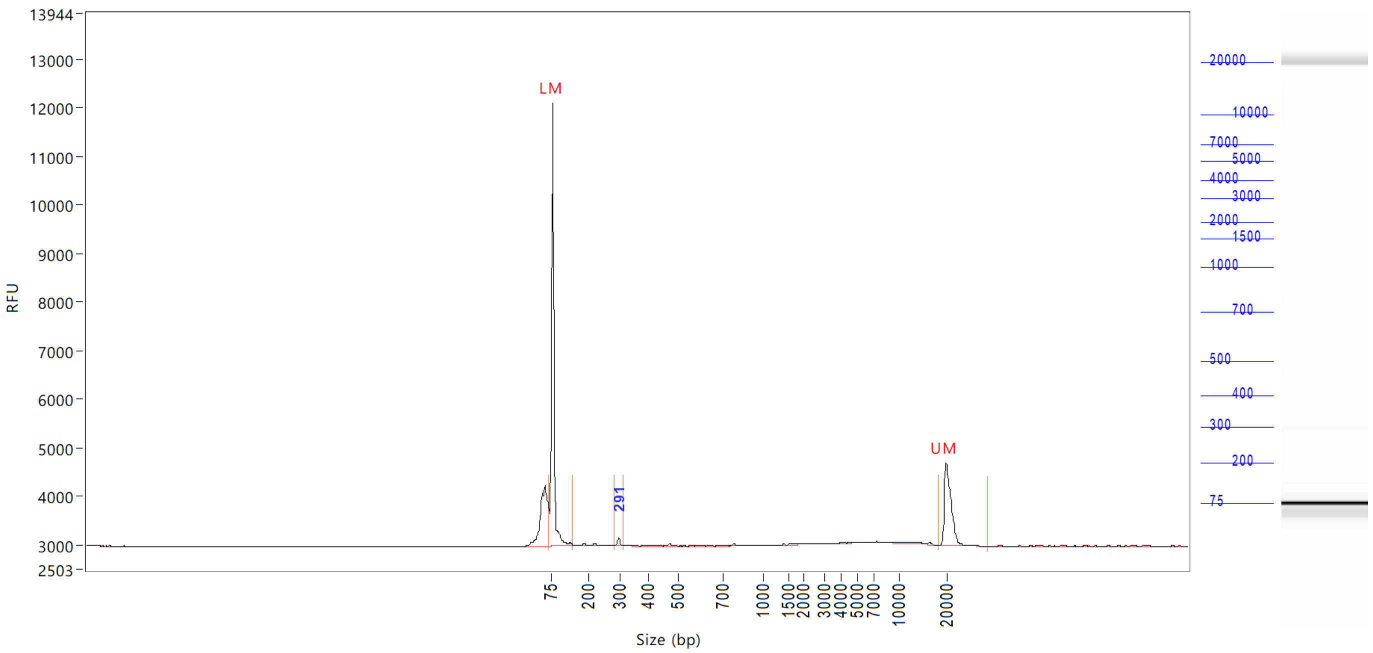

Peak	Size (bp)	Concentration (ng/uL)	From (bp)	To (bp)	Average size (bp)	CV%	RFU	Corrected peak area
1	75 (LM)	0.5000	66	143	75	12.27	9132	56.861
2	291	0.0940	280	305	290	1.34	162	0.890
3	20000 (UM)	0.1336	18415	28826	20497	4.23	1697	15.199

TIC:	0.0940	ng/uL
TIM:	0.5329	nmole/L
Total concentration:	0.6306	ng/uL

```
Sample peak width (sec): 5    Sample min peak height: 50    Sample baseline V to V?: Y    Sample baseline V to V points: 3
Sample filter: Binomial       Number of points for filter: 3  Sample start region (min): 0  Sample end region (min): 30
Marker peak width (sec): 5    Marker min peak height: 200   Marker baseline V to V?: Y    Marker baseline V to V points: 3
Lower marker selection: First peak > 500 RFU                Upper marker selection: Last peak > 200 RFU
Ladder size (bp)75, 200, 300, 400, 500, 700, 1000, 1500, 2000, 3000, 4000, 5000, 7000, 10000, 20000
Quantification using: Lower Marker          Final concentration (ng/uL): 0.5000          Dilution factor: 12.0
```

Sample: SampC12
Well location: C12
Created: Tuesday, September 14, 2021 7:33:54 PM

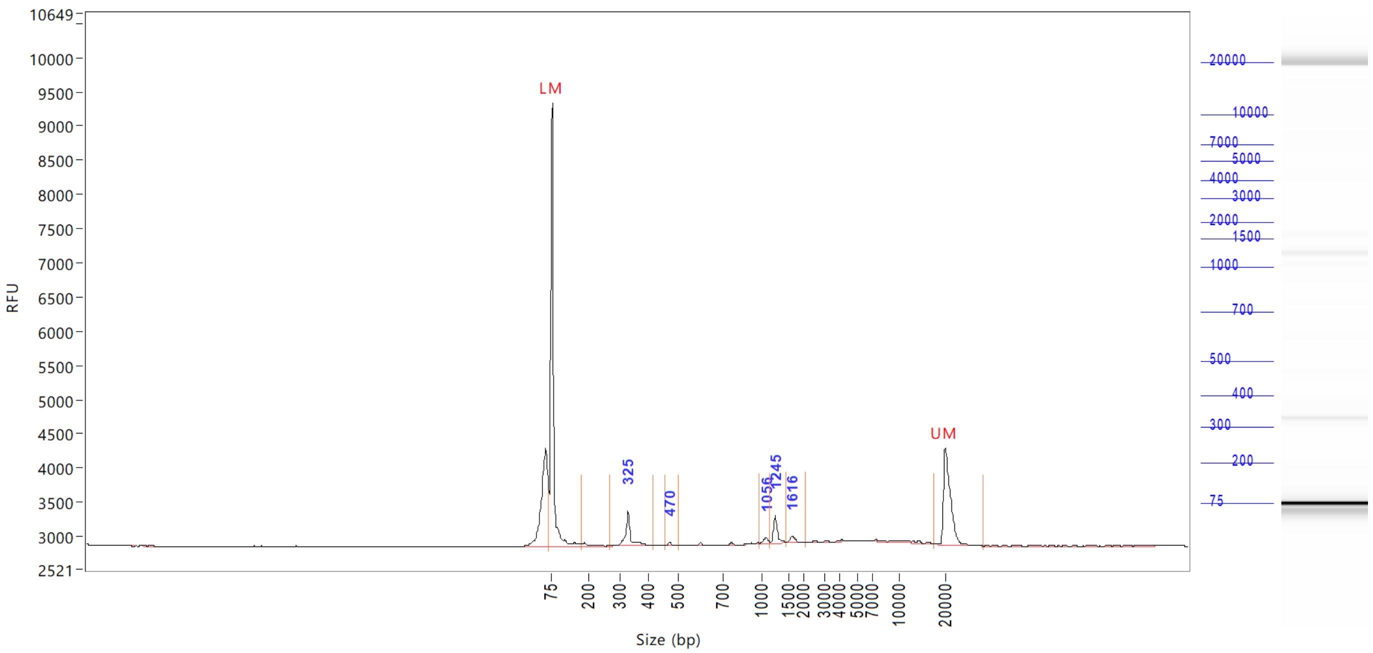

Peak	Size (bp)	Concentration (ng/uL)	From (bp)	To (bp)	Average size (bp)	CV%	RFU	Corrected peak area
1	75 (LM)	0.5000	66	173	77	18.98	6493	45.936
2	325	0.7269	264	418	329	7.02	509	5.565
3	470	0.0546	454	500	471	1.95	53	0.418
4	1056	0.0915	983	1145	1048	3.97	80	0.701
5	1245	0.3579	1145	1445	1246	4.29	399	2.740
6	1616	0.1117	1445	2058	1625	6.73	98	0.855
7	20000 (UM)	0.1435	17561	28221	20524	4.77	1417	13.186

TIC:	1.3426	ng/uL
TIM:	4.5550	nmole/L
Total concentration:	1.8811	ng/uL

Sample peak width (sec): 5 Sample min peak height: 50 Sample baseline V to V?: Y Sample baseline V to V points: 3
Sample filter: Binomial Number of points for filter: 3 Sample start region (min): 0 Sample end region (min): 30
Marker peak width (sec): 5 Marker min peak height: 200 Marker baseline V to V?: Y Marker baseline V to V points: 3
Lower marker selection: First peak > 500 RFU Upper marker selection: Last peak > 200 RFU
Ladder size (bp)75, 200, 300, 400, 500, 700, 1000, 1500, 2000, 3000, 4000, 5000, 7000, 10000, 20000
Quantification using: Lower Marker Final concentration (ng/uL): 0.5000 Dilution factor: 12.0

Sample: SampD12
Well location: D12
Created: Tuesday, September 14, 2021 7:33:54 PM

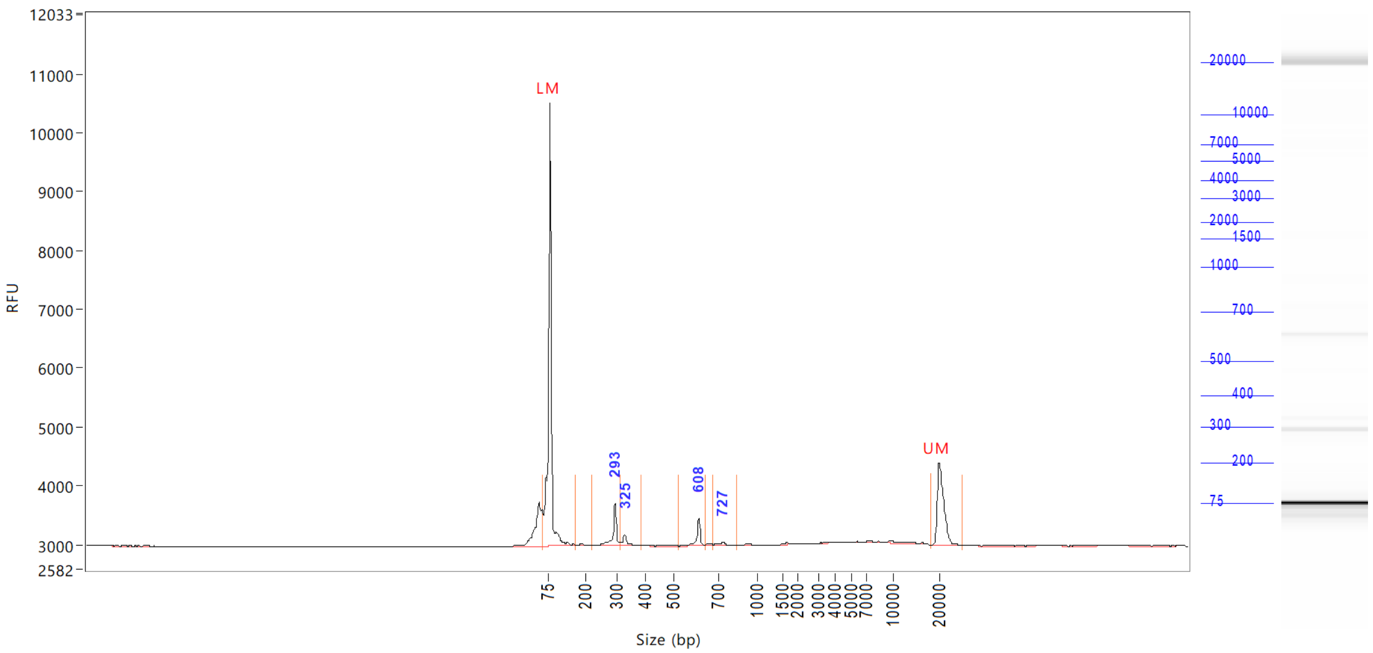

Peak	Size (bp)	Concentration (ng/uL)	From (bp)	To (bp)	Average size (bp)	CV%	RFU	Corrected peak area
1	75 (LM)	0.5000	54	166	74	16.58	7545	56.713
2	293	0.6136	217	313	284	5.29	716	5.800
3	325	0.1925	313	386	331	4.79	195	1.820
4	608	0.3366	519	636	600	2.78	462	3.182
5	727	0.0906	672	835	721	4.67	52	0.856
6	20000 (UM)	0.1105	18415	25199	20504	3.97	1400	12.534

TIC:	1.2334	ng/uL
TIM:	5.6409	nmole/L
Total concentration:	1.6385	ng/uL

Sample peak width (sec): 5   Sample min peak height: 50   Sample baseline V to V?: Y   Sample baseline V to V points: 3
Sample filter: Binomial   Number of points for filter: 3 Sample start region (min): 0 Sample end region (min): 30
Marker peak width (sec): 5   Marker min peak height: 200   Marker baseline V to V?: Y   Marker baseline V to V points: 3
Lower marker selection: First peak > 500 RFU   Upper marker selection: Last peak > 200 RFU
Ladder size (bp)75, 200, 300, 400, 500, 700, 1000, 1500, 2000, 3000, 4000, 5000, 7000, 10000, 20000
Quantification using: Lower Marker   Final concentration (ng/uL): 0.5000   Dilution factor: 12.0

Sample: SampE12
Well location: E12
Created: Tuesday, September 14, 2021 7:33:54 PM

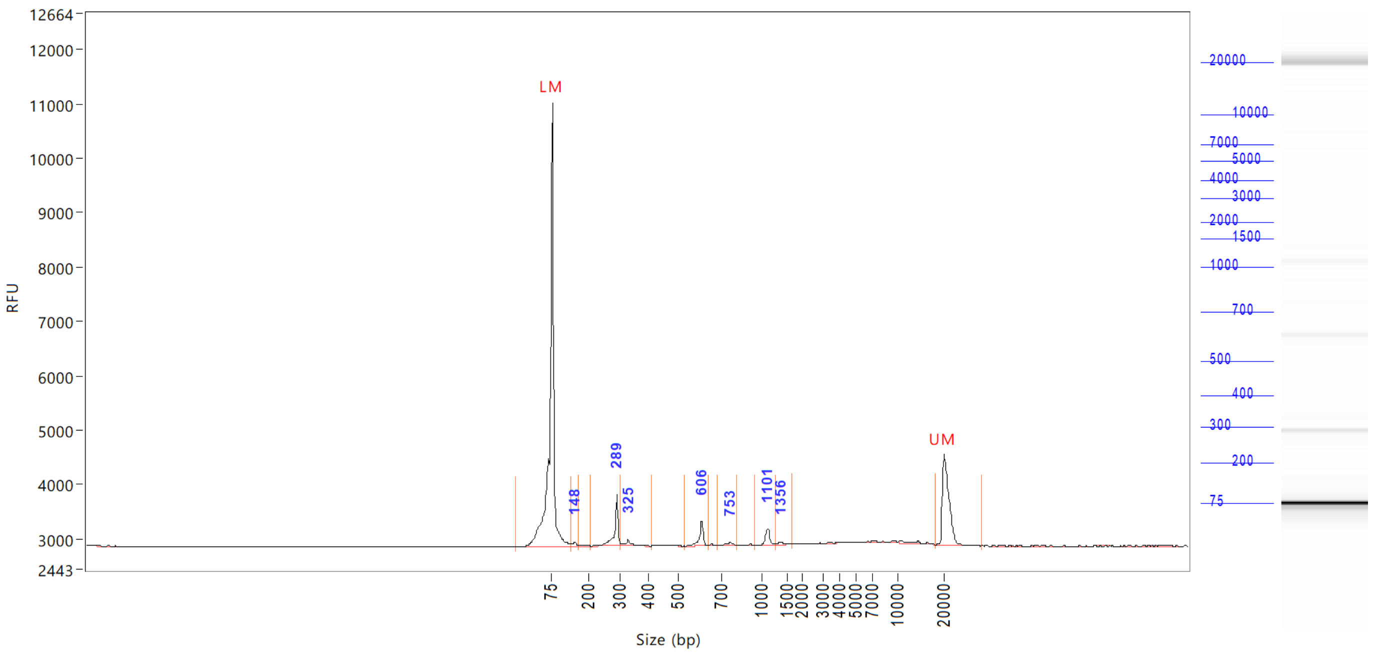

Peak	Size (bp)	Concentration (ng/uL)	From (bp)	To (bp)	Average size (bp)	CV%	RFU	Corrected peak area
1	75 (LM)	0.5000	0	141	66	29.30	8166	78.412
2	148	0.0563	141	168	150	4.43	85	0.735
3	289	0.5530	206	301	279	5.15	939	7.227
4	325	0.1066	301	411	332	6.12	110	1.393
5	606	0.2569	532	634	597	2.45	459	3.357
6	753	0.0708	680	805	736	4.23	53	0.925
7	1101	0.1740	948	1267	1088	4.49	302	2.273
8	1356	0.0420	1267	1655	1392	6.17	57	0.549
9	20000 (UM)	0.0948	18171	28100	20441	4.12	1667	14.866

TIC:	1.2595	ng/uL
TIM:	5.5841	nmole/L
Total concentration:	1.5240	ng/uL

```
Sample peak width (sec): 5    Sample min peak height: 50    Sample baseline V to V?: Y    Sample baseline V to V points: 3
Sample filter: Binomial       Number of points for filter: 3  Sample start region (min): 0  Sample end region (min): 30
Marker peak width (sec): 5    Marker min peak height: 200   Marker baseline V to V?: Y    Marker baseline V to V points: 3
Lower marker selection: First peak > 500 RFU                Upper marker selection: Last peak > 200 RFU
Ladder size (bp)75, 200, 300, 400, 500, 700, 1000, 1500, 2000, 3000, 4000, 5000, 7000, 10000, 20000
Quantification using: Lower Marker          Final concentration (ng/uL): 0.5000          Dilution factor: 12.0
```

Sample: SampF12
Well location: F12
Created: Tuesday, September 14, 2021 7:33:54 PM

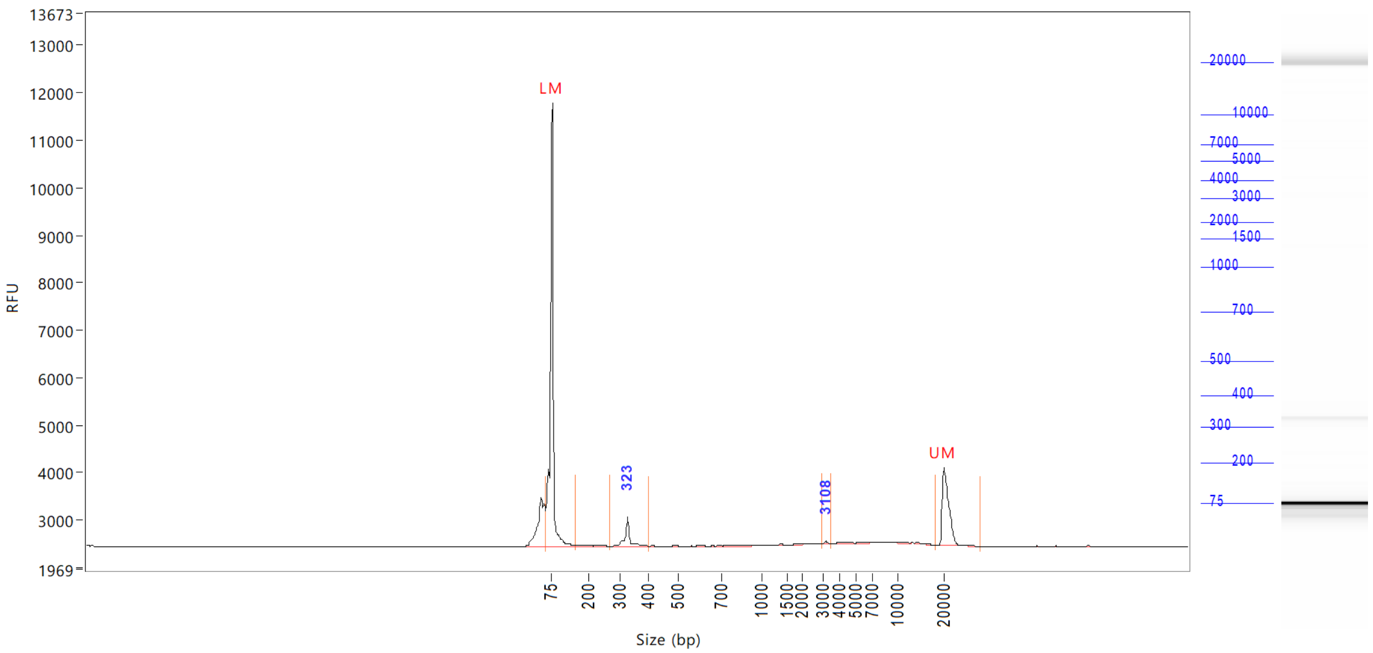

Peak	Size (bp)	Concentration (ng/uL)	From (bp)	To (bp)	Average size (bp)	CV%	RFU	Corrected peak area
1	75 (LM)	0.5000	54	152	72	14.78	9350	69.097
2	323	0.4935	268	400	324	5.89	614	5.683
3	3108	0.0244	2943	3465	3137	3.35	53	0.281
4	20000 (UM)	0.1058	18171	27617	20399	4.10	1649	14.621

TIC:	0.5179	ng/uL
TIM:	2.5184	nmole/L
Total concentration:	0.8252	ng/uL

Sample peak width (sec): 5 Sample min peak height: 50 Sample baseline V to V?: Y Sample baseline V to V points: 3
Sample filter: Binomial Number of points for filter: 3 Sample start region (min): 0 Sample end region (min): 30
Marker peak width (sec): 5 Marker min peak height: 200 Marker baseline V to V?: Y Marker baseline V to V points: 3
Lower marker selection: First peak > 500 RFU Upper marker selection: Last peak > 200 RFU
Ladder size (bp)75, 200, 300, 400, 500, 700, 1000, 1500, 2000, 3000, 4000, 5000, 7000, 10000, 20000
Quantification using: Lower Marker Final concentration (ng/uL): 0.5000 Dilution factor: 12.0

Sample: SampG12
Well location: G12
Created: Tuesday, September 14, 2021 7:33:54 PM

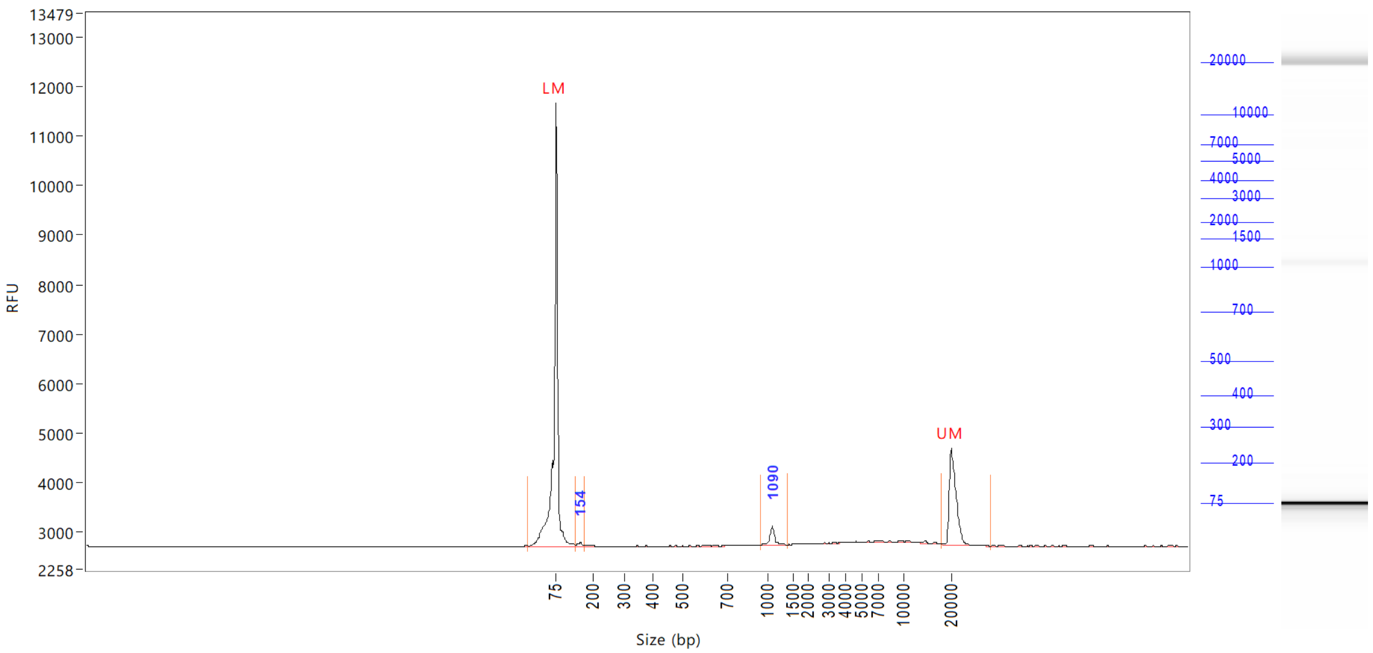

Peak	Size (bp)	Concentration (ng/uL)	From (bp)	To (bp)	Average size (bp)	CV%	RFU	Corrected peak area
1	75 (LM)	0.5000	0	139	65	27.62	8966	82.213
2	154	0.0591	139	171	152	4.96	81	0.810
3	1090	0.2350	948	1389	1077	5.73	379	3.220
4	20000 (UM)	0.1068	18049	28342	20516	4.22	1947	17.560

TIC:	0.2941	ng/uL
TIM:	0.9982	nmole/L
Total concentration:	0.5469	ng/uL

Sample peak width (sec): 5 Sample min peak height: 50 Sample baseline V to V?: Y Sample baseline V to V points: 3
Sample filter: Binomial Number of points for filter: 3 Sample start region (min): 0 Sample end region (min): 30
Marker peak width (sec): 5 Marker min peak height: 200 Marker baseline V to V?: Y Marker baseline V to V points: 3
Lower marker selection: First peak > 500 RFU Upper marker selection: Last peak > 200 RFU
Ladder size (bp)75, 200, 300, 400, 500, 700, 1000, 1500, 2000, 3000, 4000, 5000, 7000, 10000, 20000
Quantification using: Lower Marker Final concentration (ng/uL): 0.5000 Dilution factor: 12.0

Sample: SampH12
Well location: H12
Created: Tuesday, September 14, 2021 7:33:54 PM

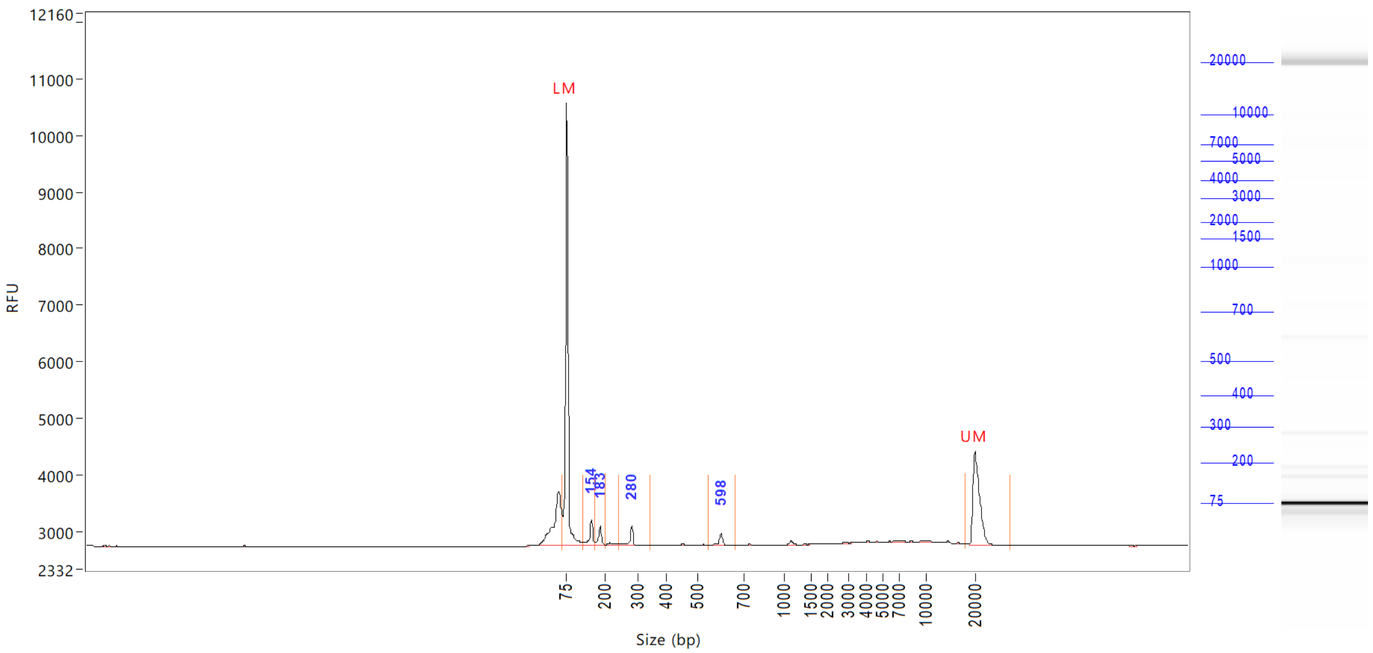

Peak	Size (bp)	Concentration (ng/uL)	From (bp)	To (bp)	Average size (bp)	CV%	RFU	Corrected peak area
1	75 (LM)	0.5000	64	126	74	10.12	7831	48.351
2	154	0.4144	126	166	149	5.76	438	3.339
3	183	0.2725	166	201	180	2.92	346	2.196
4	280	0.2807	242	341	276	5.05	328	2.262
5	598	0.2037	544	659	594	3.02	226	1.641
6	20000 (UM)	0.1472	18049	27254	20428	3.92	1651	14.239

TIC:	1.1712	ng/uL
TIM:	9.2965	nmole/L
Total concentration:	1.6175	ng/uL

Sample peak width (sec): 5   Sample min peak height: 200   Sample baseline V to V?: Y   Sample baseline V to V points: 3
Sample filter: Binomial   Number of points for filter: 3   Sample start region (min): 0   Sample end region (min): 30
Marker peak width (sec): 5   Marker min peak height: 200   Marker baseline V to V?: Y   Marker baseline V to V points: 3
Lower marker selection: First peak > 500 RFU   Upper marker selection: Last peak > 200 RFU
Ladder size (bp)75, 200, 300, 400, 500, 700, 1000, 1500, 2000, 3000, 4000, 5000, 7000, 10000, 20000
Quantification using: Lower Marker   Final concentration (ng/uL): 0.5000   Dilution factor: 12.0

Sample: DNA size ladder
Well location: A7
Created: Tuesday, September 14, 2021 7:33:54 PM
Import from: C:\Users\admin\Desktop\210810_alberto_ladder.SCAL
Fit type: Point to point

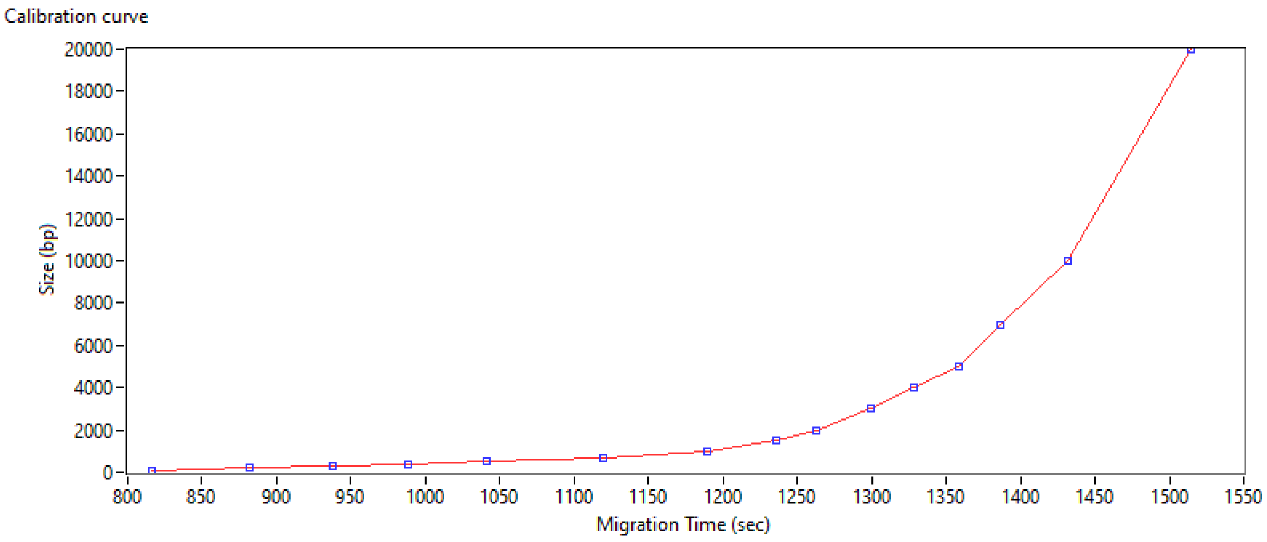

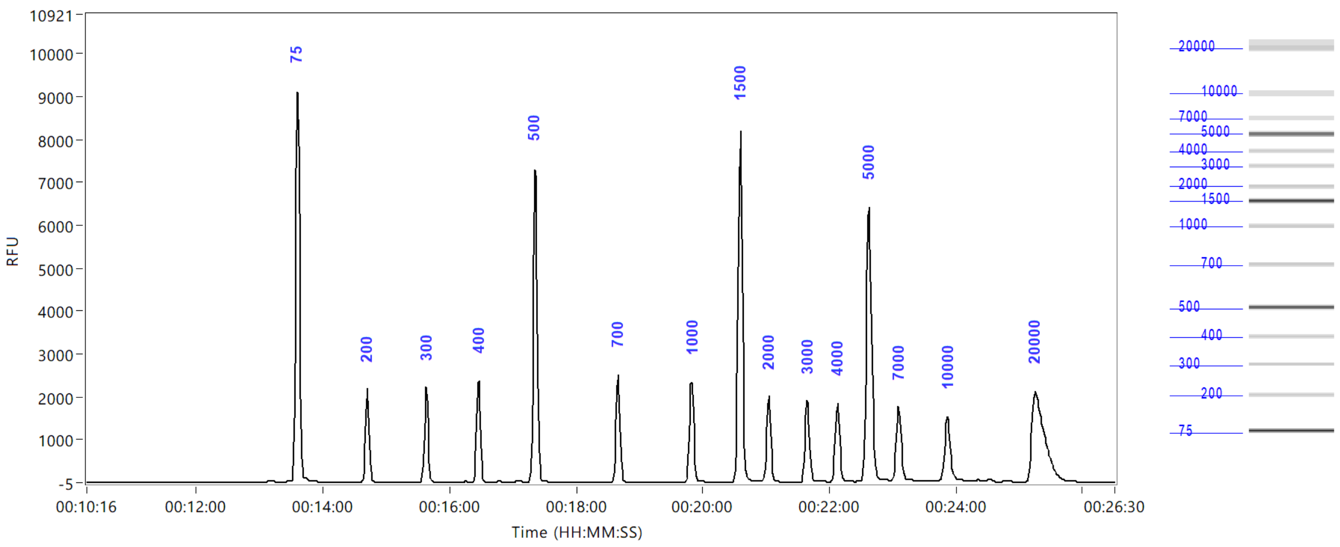

Supplement: Supplementary file 2 — sb3c00292_si_002.zip [file sb3c00292_si_002.zip › dnada_supplementary_material_pks_library_build/ZAG_trials/ZAG_Trial_5/19-24-11_plate5/plate5-t1.pdf]
