# Supplementary material for: Automated Platform for the Plasmid Construction Process
Source: ACS Synth Biol. 2023 Nov 10;12(12):3506–13. doi: 10.1021/acssynbio.3c00292 (PMC10729297; doi:10.1021/acssynbio.3c00292)

Instrument controller software run summary:

Filename and data path: C:\AATI\Data\2021 09 14\18-04-19\2021 09 14 18H 04M..raw
Created: Tuesday, September 14, 2021 6:14:00 PM
Number of capillaries: 96
Array serial number: 111920-09SFS
Effect length: 33 cm
Array usage count: 215
Instrument type: ZAG DNA Analyzer
Instrument controller software version: 1.0.1.9
Device serial number: Z7597

Method Information

Method name: ZAG130GP33 - DNA 75-20000bp - Gel Prime Only.mthds
Gel prime: Yes
Full conditioning: No
Gel prime to buffer: No
Gel selection: Gel 1
Perform prerun: 8.0 kV, 30 sec.
Rinse: No
Marker 1: Row: A, 5.0 kV, 5 sec.
Rinse: No
Sample injection: 5.0 kV, 5 sec.
Separation: 8.0 kV, 30.0 min.
Tray name: Sample Tray-3

Analysis mode: DNA

Notes

Gel Image

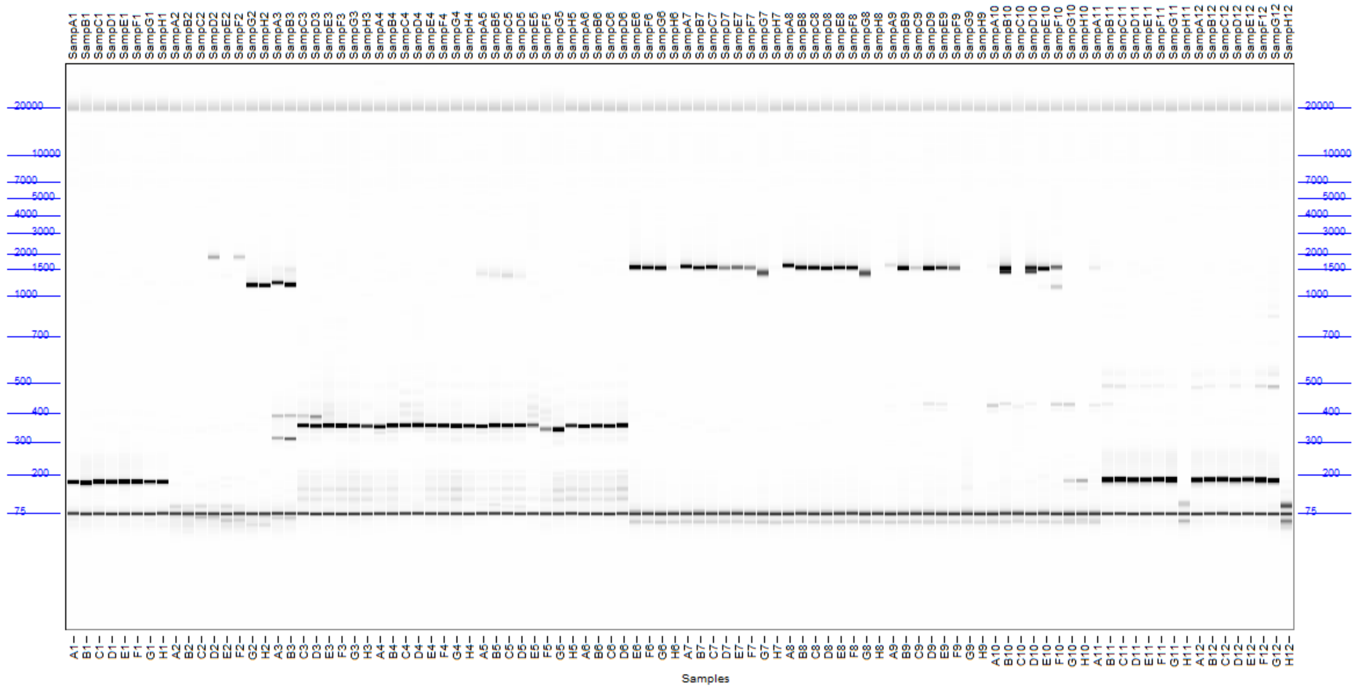
20000
10000
7000
5000
4000
3000
2000
1500
1000
700
500
400
300
200
75
Samples

Filename and data path: C:\AATI\Data\2021 09 14\18-04-19\2021 09 14 18H 04M..raw

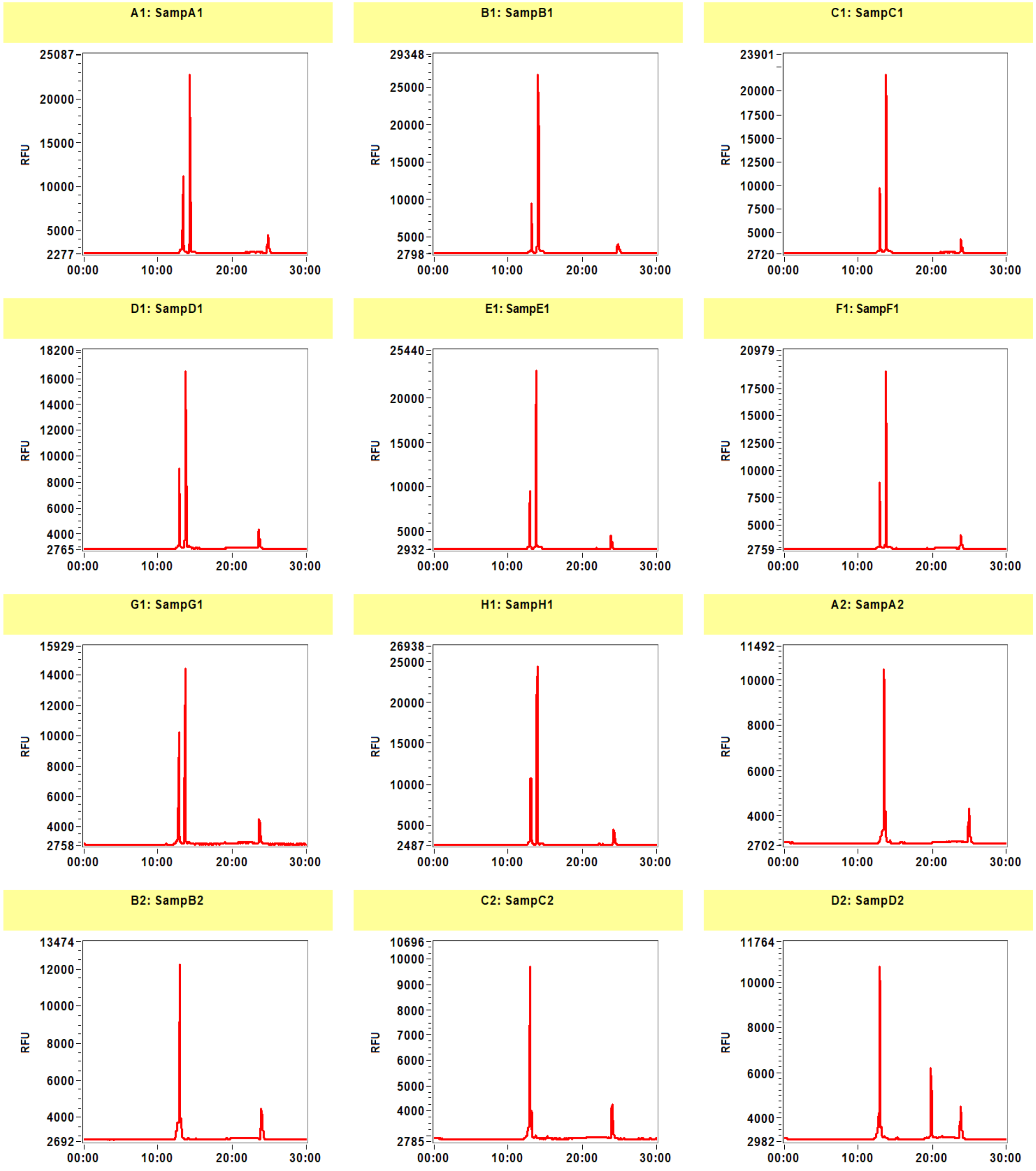

Filename and data path: C:\AATI\Data\2021 09 14\18-04-19\2021 09 14 18H 04M..raw

E2: SampE2

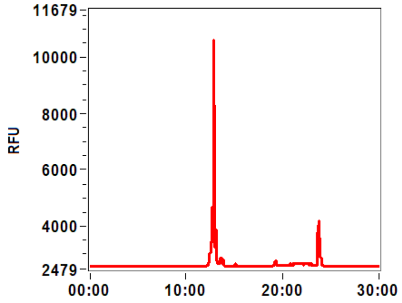

F2: SampF2

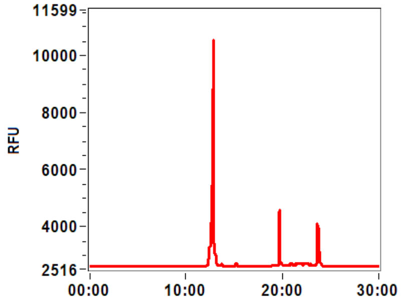

G2: SampG2

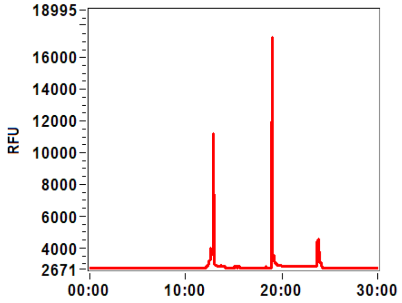

H2: SampH2

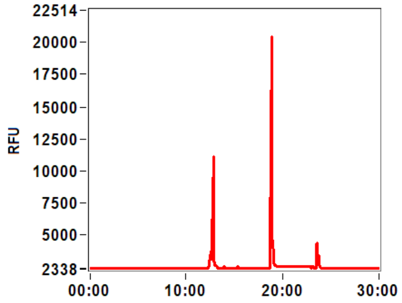

A3: SampA3

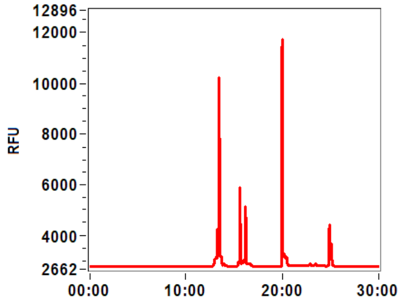

B3: SampB3

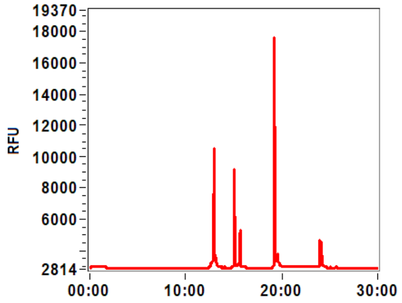

C3: SampC3

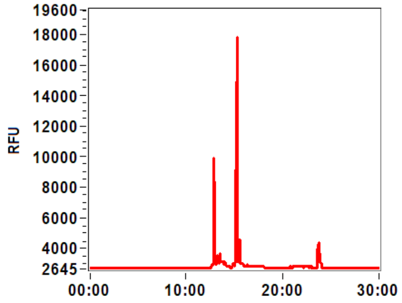

D3: SampD3

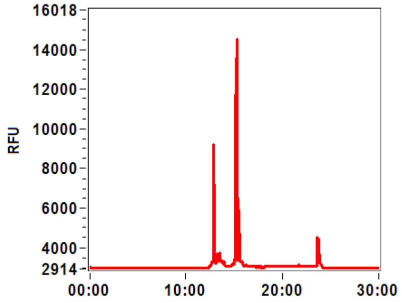

E3: SampE3

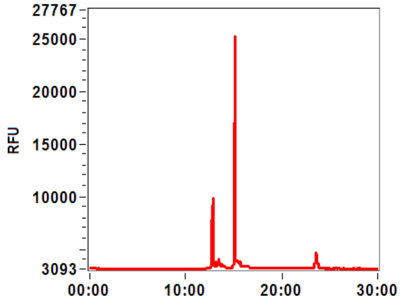

F3: SampF3

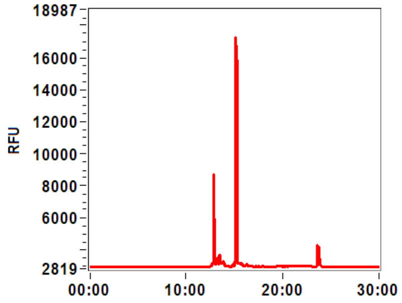

G3: SampG3

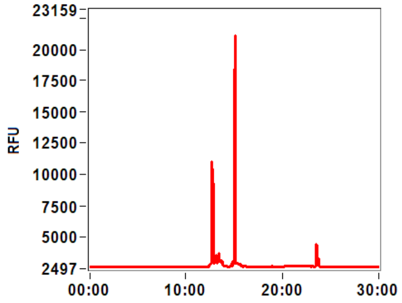

H3: SampH3

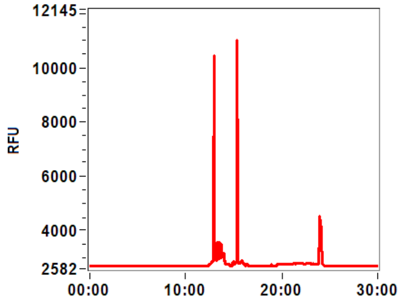

Filename and data path: C:\AATI\Data\2021 09 14\18-04-19\2021 09 14 18H 04M..raw

A4: SampA4

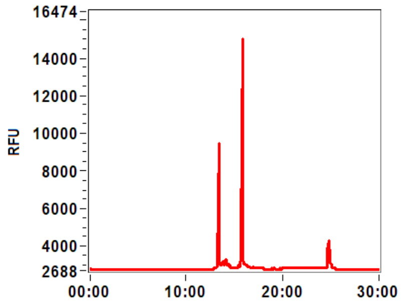

B4: SampB4

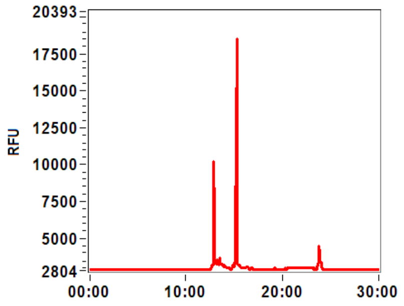

C4: SampC4

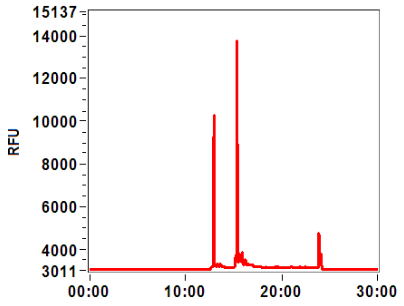

D4: SampD4

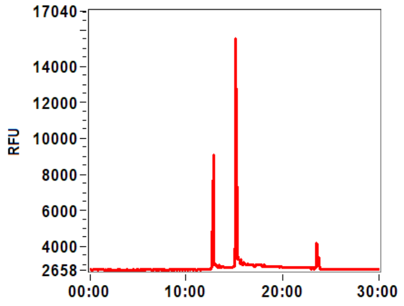

E4: SampE4

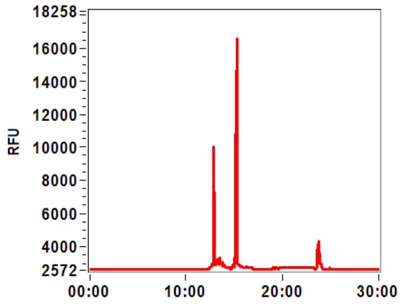

F4: SampF4

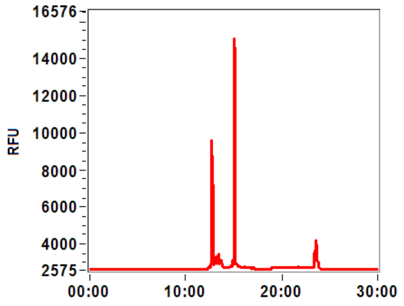

G4: SampG4

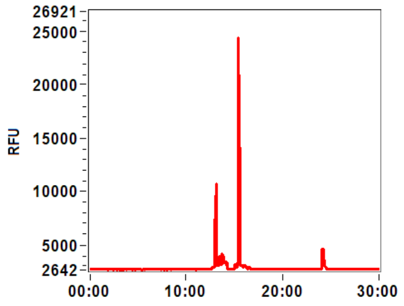

H4: SampH4

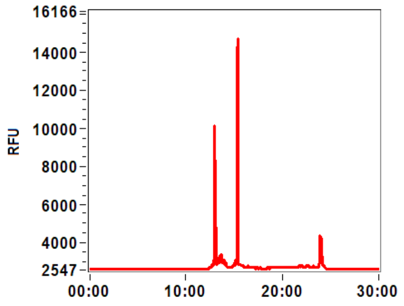

A5: SampA5

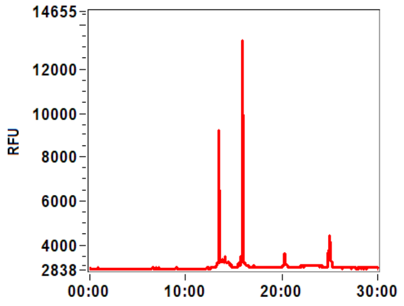

B5: SampB5

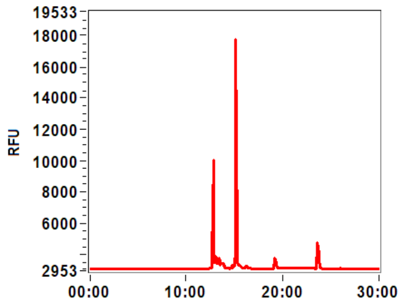

C5: SampC5

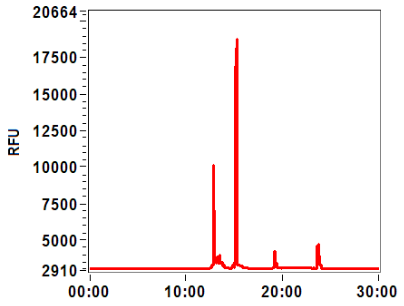

D5: SampD5

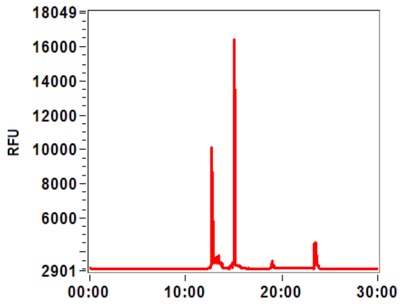

Filename and data path: C:\AATI\Data\2021 09 14\18-04-19\2021 09 14 18H 04M..raw

E5: SampE5

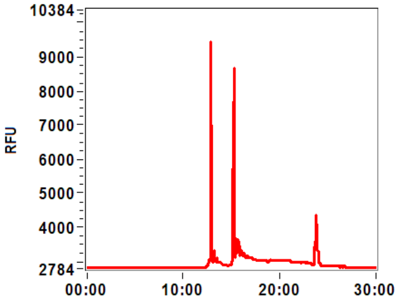

F5: SampF5

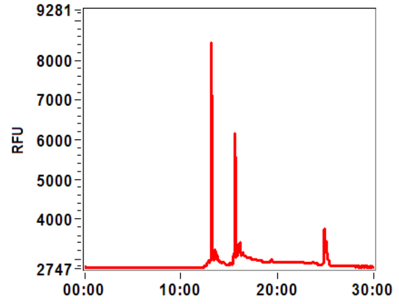

G5: SampG5

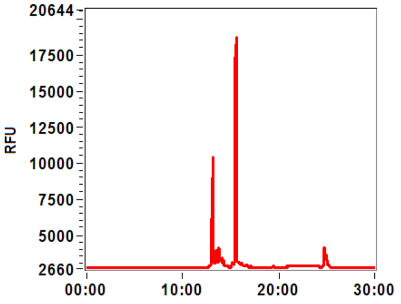

H5: SampH5

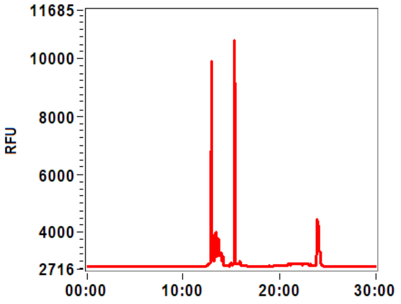

A6: SampA6

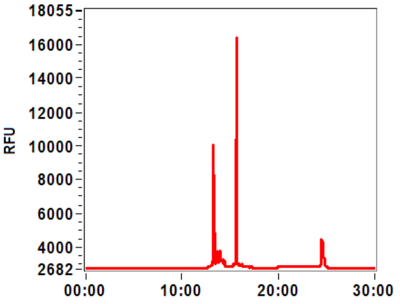

B6: SampB6

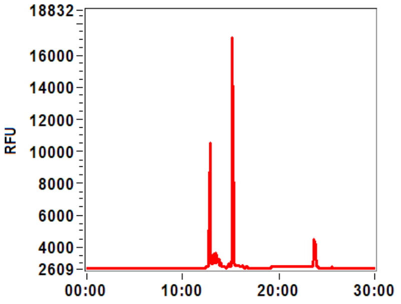

C6: SampC6

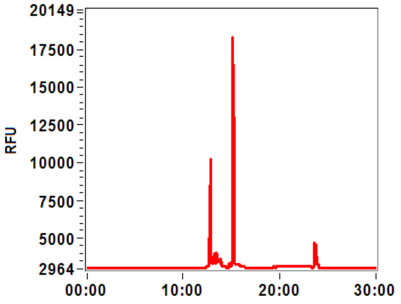

D6: SampD6

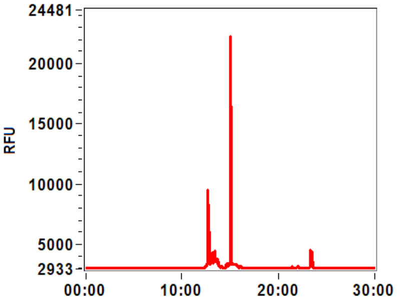

E6: SampE6

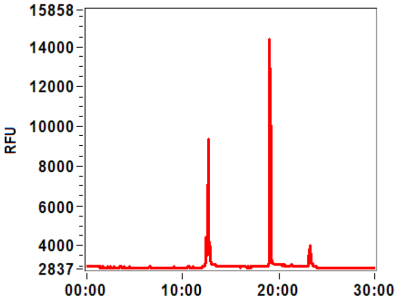

F6: SampF6

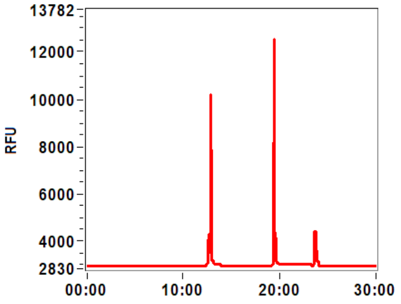

G6: SampG6

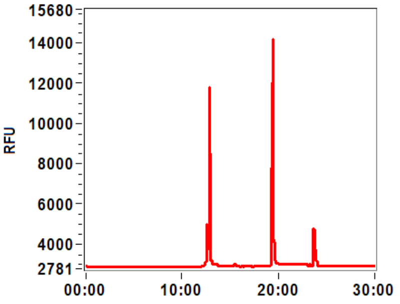

H6: SampH6

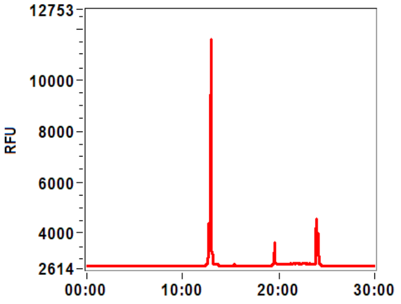

Filename and data path: C:\AATI\Data\2021 09 14\18-04-19\2021 09 14 18H 04M..raw

A7: SampA7

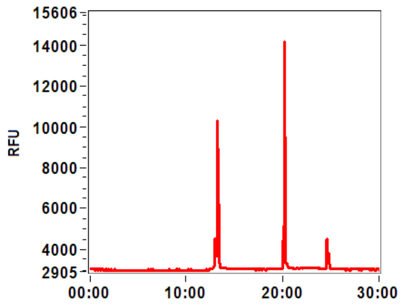

B7: SampB7

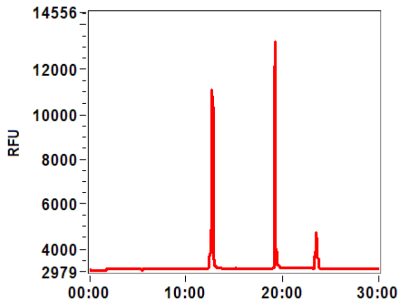

C7: SampC7

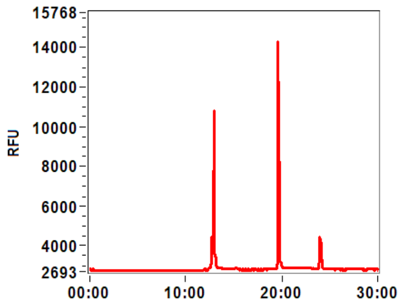

D7: SampD7

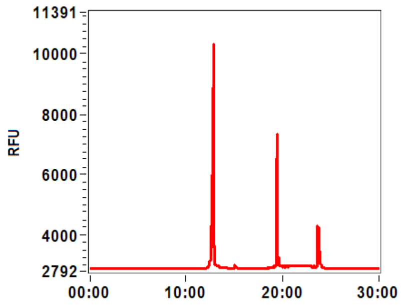

E7: SampE7

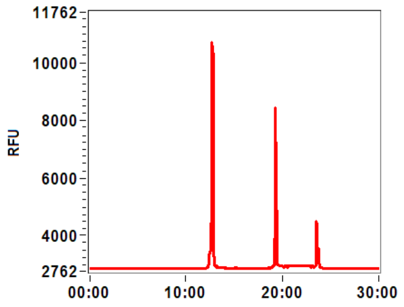

F7: SampF7

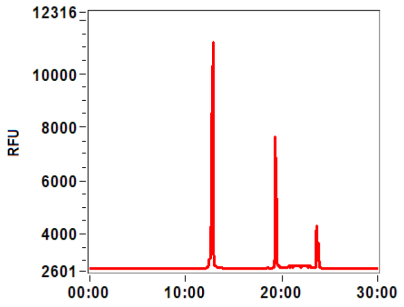

G7: SampG7

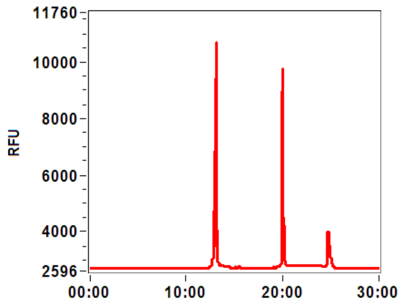

H7: SampH7

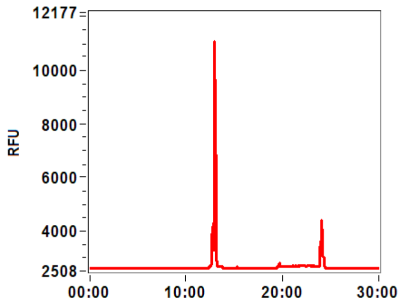

A8: SampA8

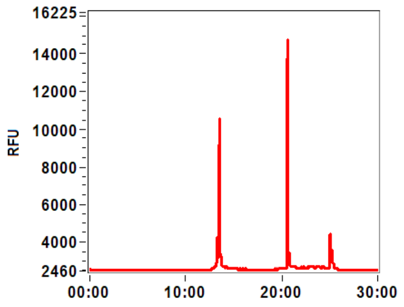

B8: SampB8

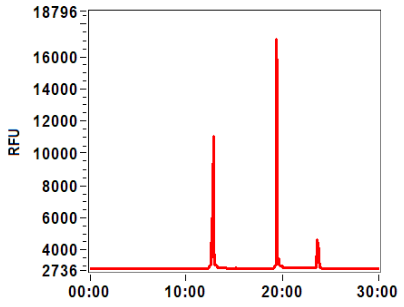

C8: SampC8

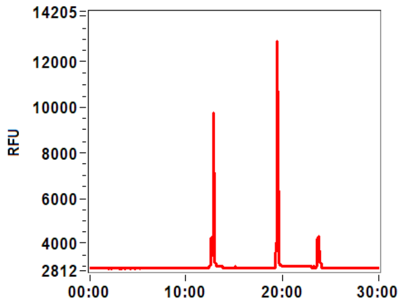

D8: SampD8

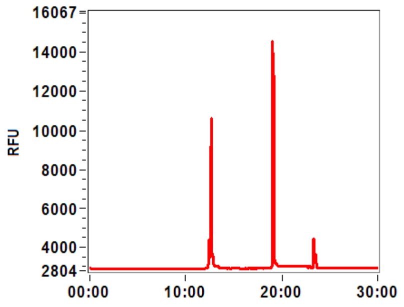

Filename and data path: C:\AATI\Data\2021 09 14\18-04-19\2021 09 14 18H 04M..raw

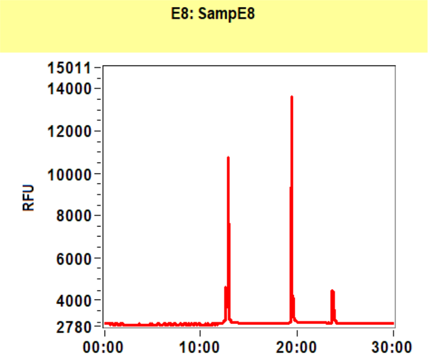

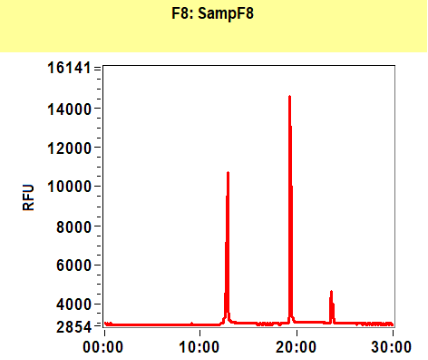

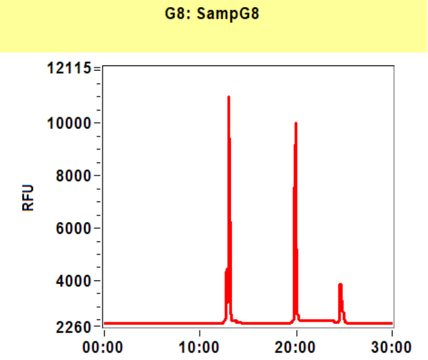

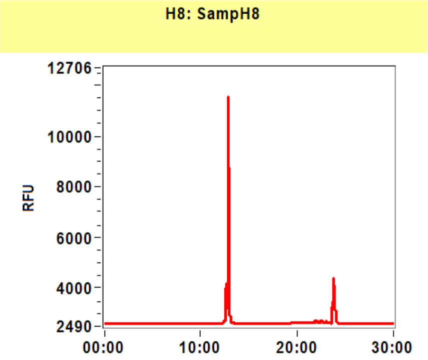

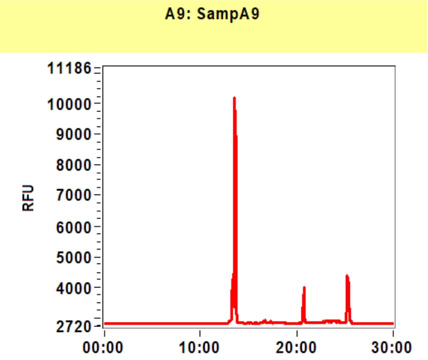

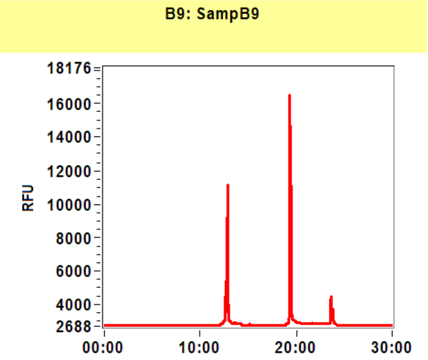

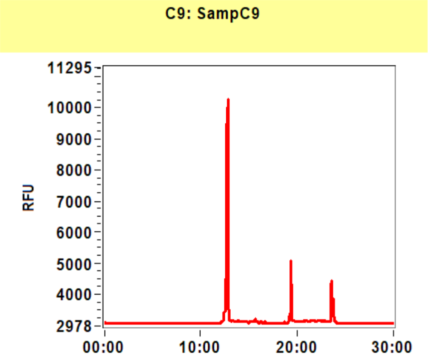

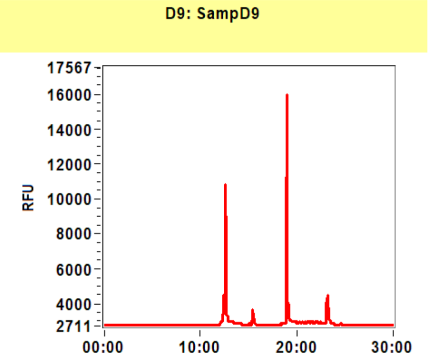

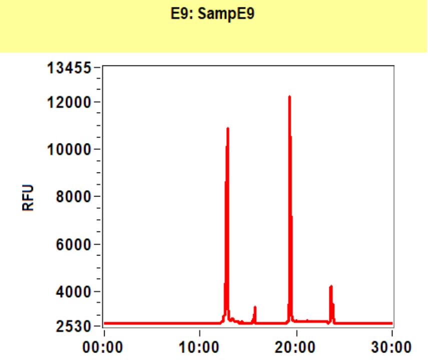

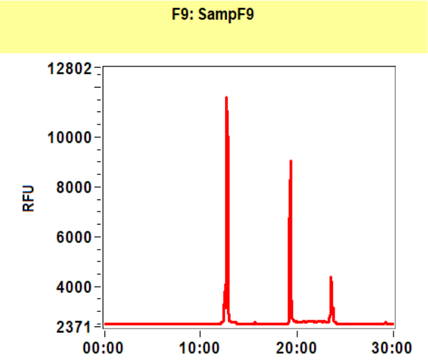

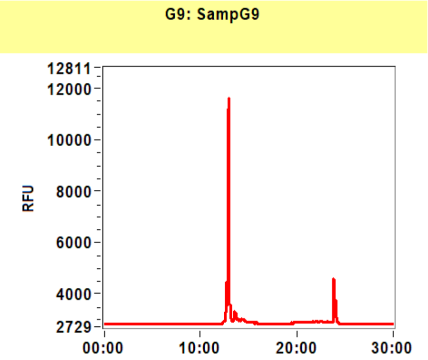

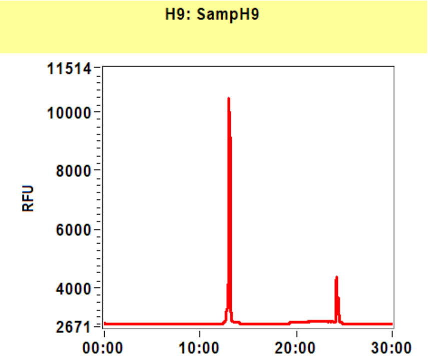

Filename and data path: C:\AATI\Data\2021 09 14\18-04-19\2021 09 14 18H 04M..raw

A10: SampA10

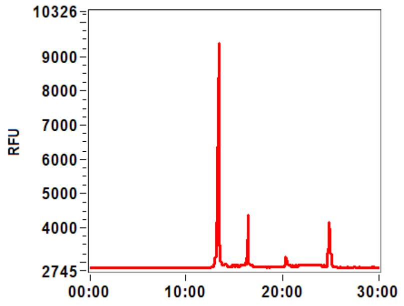

B10: SampB10

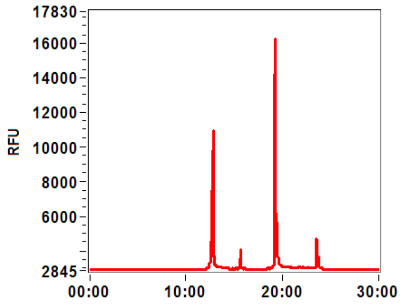

C10: SampC10

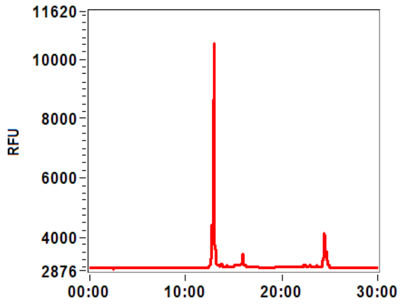

D10: SampD10

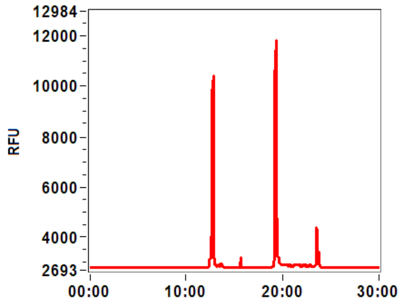

E10: SampE10

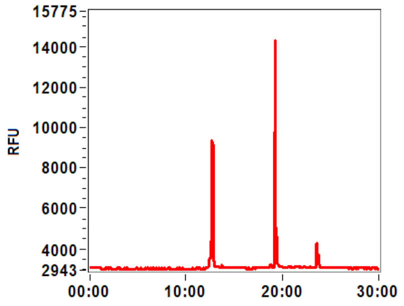

F10: SampF10

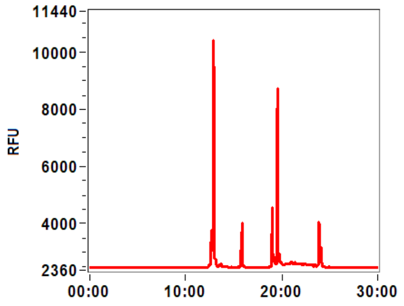

G10: SampG10

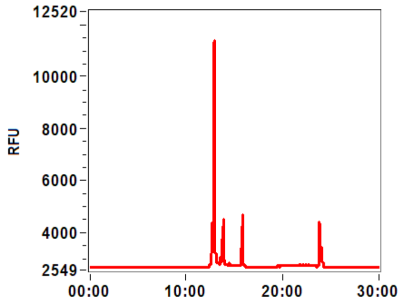

H10: SampH10

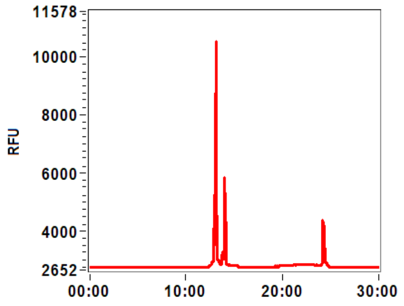

A11: SampA11

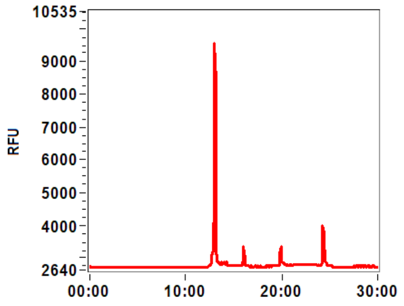

B11: SampB11

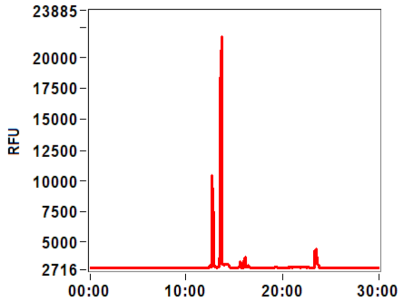

C11: SampC11

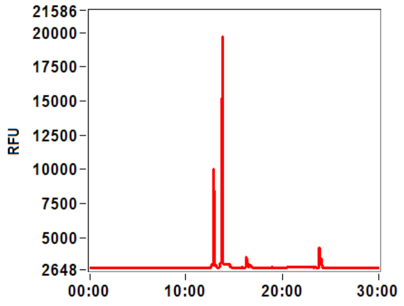

D11: SampD11

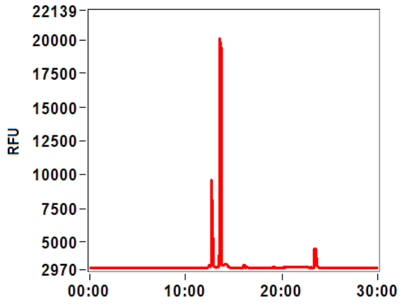

Filename and data path: C:\AATI\Data\2021 09 14\18-04-19\2021 09 14 18H 04M..raw

E11: SampE11

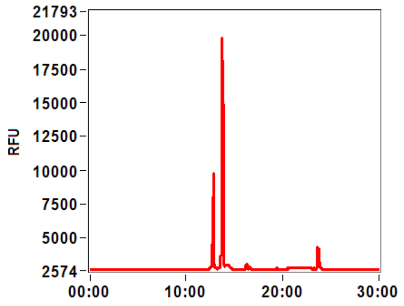

F11: SampF11

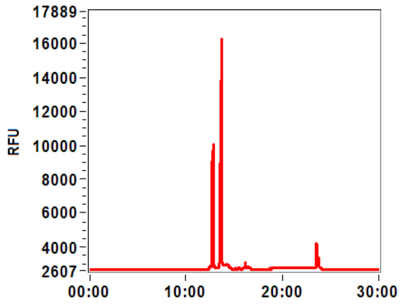

G11: SampG11

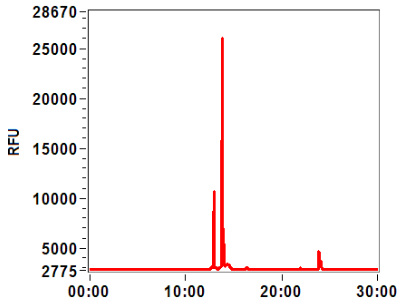

H11: SampH11

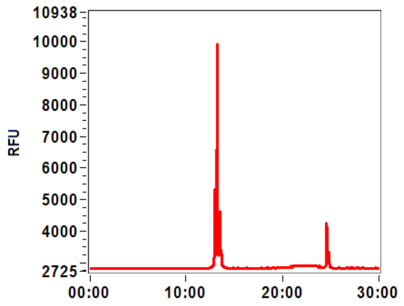

A12: SampA12

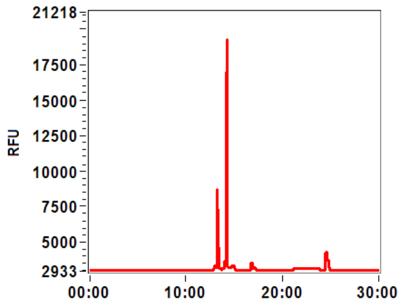

B12: SampB12

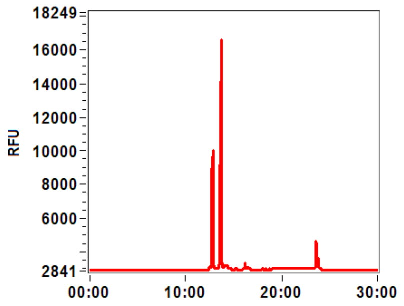

C12: SampC12

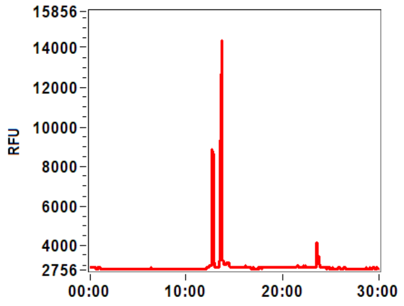

D12: SampD12

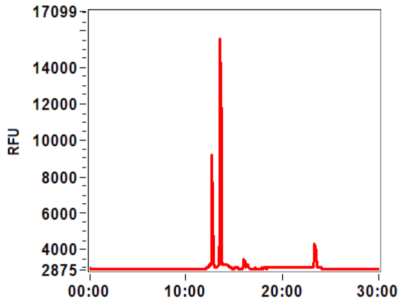

E12: SampE12

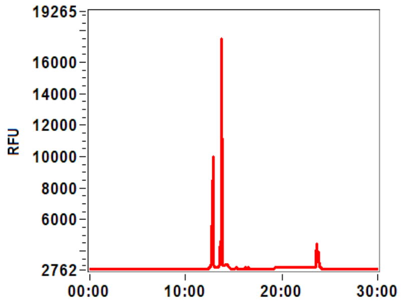

F12: SampF12

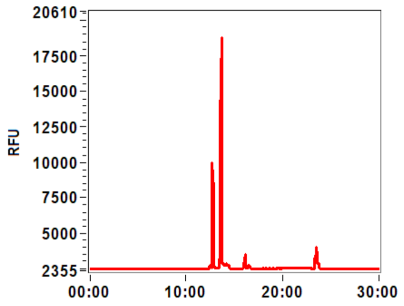

G12: SampG12

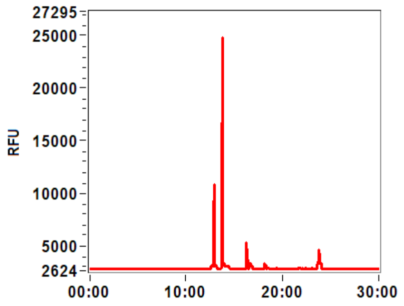

H12: SampH12

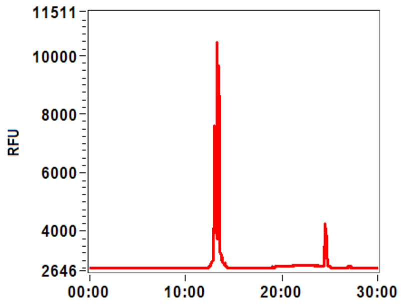

Sample: SampA1
Well location: A1
Created: Tuesday, September 14, 2021 6:14:00 PM

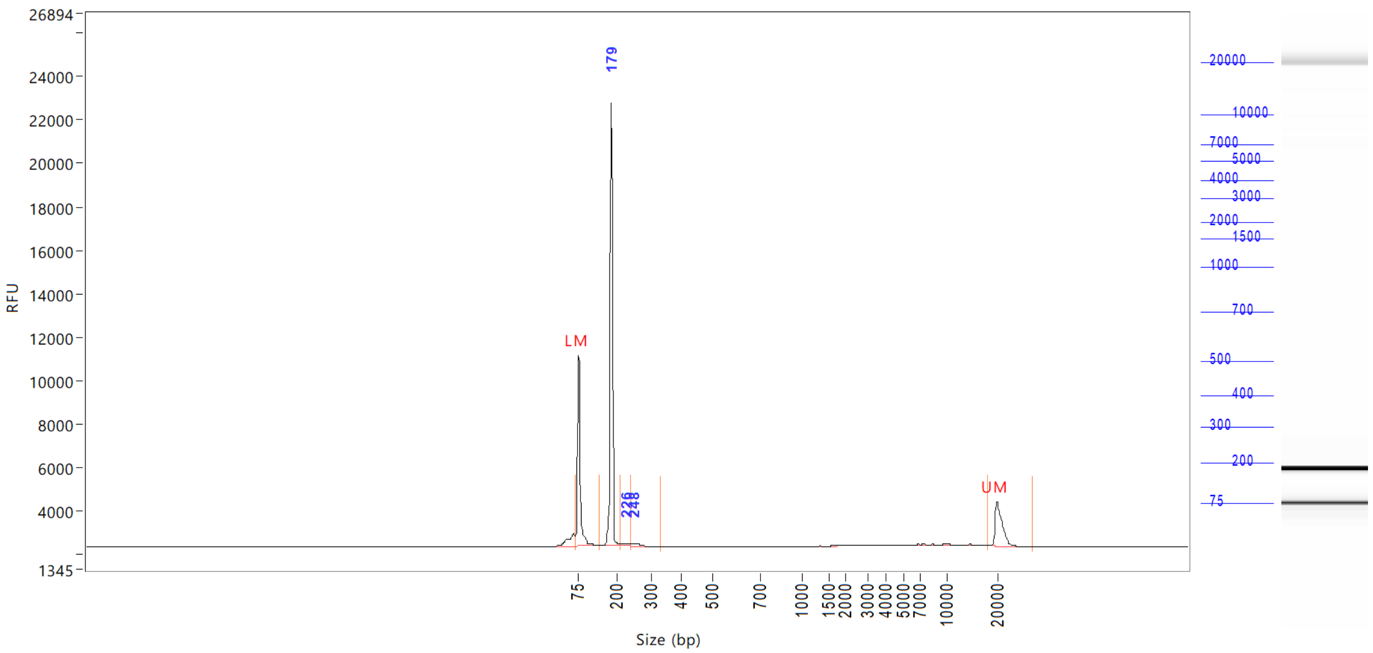

Peak	Size<br>(bp)	Concentration<br>(ng/uL)	From<br>(bp)	To<br>(bp)	Average size<br>(bp)	CV%	RFU	Corrected peak area
1	75 (LM)	0.5000	66	143	76	10.46	8766	56.978
2	179	11.1529	143	208	177	2.58	20385	105.911
3	226	0.1834	208	239	224	4.08	92	1.741
4	248	0.2058	239	333	255	5.13	108	1.954
5	20000 (UM)	0.1584	18171	27254	20483	3.99	2037	18.048

TIC:	11.5421	ng/uL
TIM:	106.2596	nmole/L
Total concentration:	11.8291	ng/uL

```
Sample peak width (sec): 5    Sample min peak height: 50    Sample baseline V to V?: Y    Sample baseline V to V points: 3
Sample filter: Binomial       Number of points for filter: 3  Sample start region (min): 0  Sample end region (min): 30
Marker peak width (sec): 5    Marker min peak height: 200   Marker baseline V to V?: Y    Marker baseline V to V points: 3
Lower marker selection: First peak > 500 RFU                Upper marker selection: Last peak > 200 RFU
Ladder size (bp)75, 200, 300, 400, 500, 700, 1000, 1500, 2000, 3000, 4000, 5000, 7000, 10000, 20000
Quantification using: Lower Marker          Final concentration (ng/uL): 0.5000          Dilution factor: 12.0
```

Sample: SampB1
Well location: B1
Created: Tuesday, September 14, 2021 6:14:00 PM

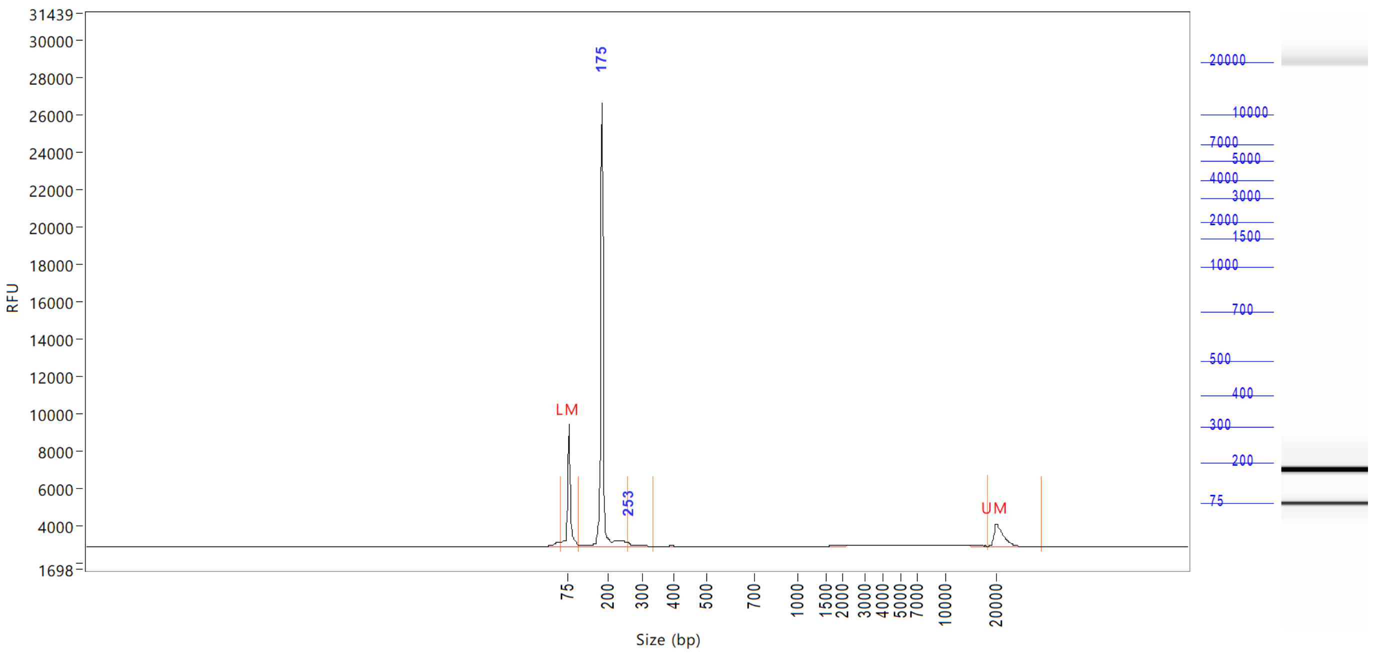

Peak	Size (bp)	Concentration (ng/uL)	From (bp)	To (bp)	Average size (bp)	CV%	RFU	Corrected peak area
1	75 (LM)	0.5000	48	107	73	10.68	6587	46.584
2	175	19.8523	107	253	176	8.90	23775	154.132
3	253	0.3149	253	329	273	6.71	218	2.445
4	20000 (UM)	0.1489	18293	28947	20733	5.49	1197	13.873

TIC:	20.1672	ng/uL
TIM:	187.3280	nmole/L
Total concentration:	20.7234	ng/uL

Sample peak width (sec): 5 Sample min peak height: 50 Sample baseline V to V?: Y Sample baseline V to V points: 3
Sample filter: Binomial Number of points for filter: 3 Sample start region (min): 0 Sample end region (min): 30
Marker peak width (sec): 5 Marker min peak height: 200 Marker baseline V to V?: Y Marker baseline V to V points: 3
Lower marker selection: First peak > 500 RFU Upper marker selection: Last peak > 200 RFU
Ladder size (bp)75, 200, 300, 400, 500, 700, 1000, 1500, 2000, 3000, 4000, 5000, 7000, 10000, 20000
Quantification using: Lower Marker Final concentration (ng/uL): 0.5000 Dilution factor: 12.0

Sample: SampC1
Well location: C1
Created: Tuesday, September 14, 2021 6:14:00 PM

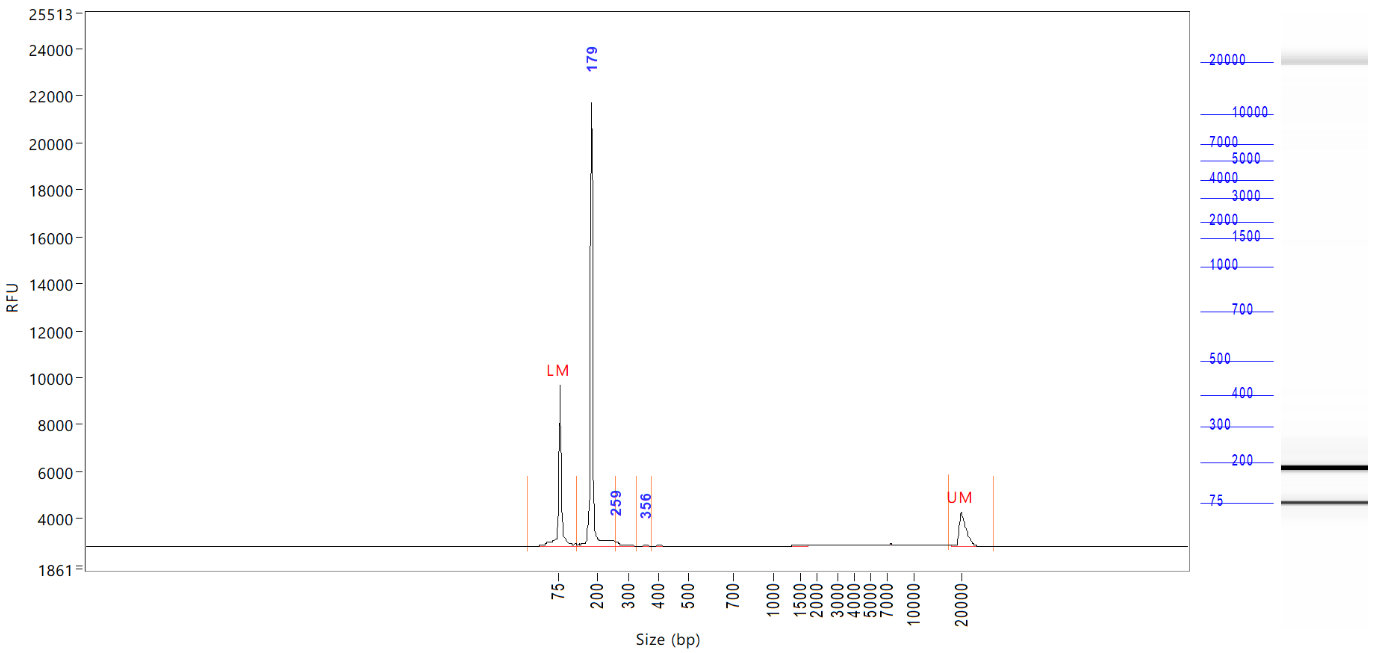

Peak	Size (bp)	Concentration (ng/uL)	From (bp)	To (bp)	Average size (bp)	CV%	RFU	Corrected peak area
1	75 (LM)	0.5000	0	131	72	22.38	6888	50.677
2	179	13.9590	131	259	181	8.21	18903	117.900
3	259	0.2343	259	325	277	6.13	186	1.979
4	356	0.0583	325	378	351	2.75	50	0.493
5	20000 (UM)	0.1282	17439	26649	20478	4.12	1412	12.998

TIC:	14.2517	ng/uL
TIM:	128.4528	nmole/L
Total concentration:	14.6830	ng/uL

```
Sample peak width (sec): 5    Sample min peak height: 50    Sample baseline V to V?: Y    Sample baseline V to V points: 3
Sample filter: Binomial       Number of points for filter: 3  Sample start region (min): 0  Sample end region (min): 30
Marker peak width (sec): 5    Marker min peak height: 200   Marker baseline V to V?: Y    Marker baseline V to V points: 3
Lower marker selection: First peak > 500 RFU                Upper marker selection: Last peak > 200 RFU
Ladder size (bp)75, 200, 300, 400, 500, 700, 1000, 1500, 2000, 3000, 4000, 5000, 7000, 10000, 20000
Quantification using: Lower Marker          Final concentration (ng/uL): 0.5000          Dilution factor: 12.0
```

Sample: SampD1
Well location: D1
Created: Tuesday, September 14, 2021 6:14:00 PM

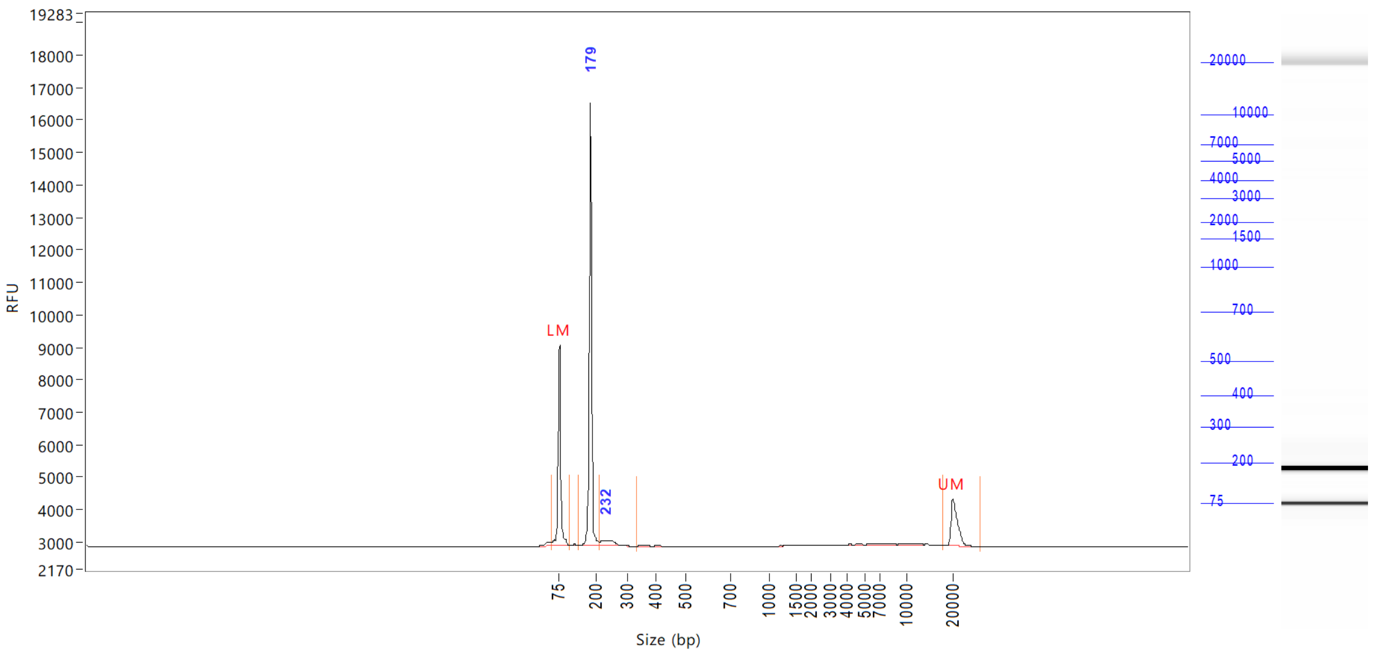

Peak	Size (bp)	Concentration (ng/uL)	From (bp)	To (bp)	Average size (bp)	CV%	RFU	Corrected peak area
1	75 (LM)	0.5000	48	108	73	8.57	6160	39.135
2	179	12.1056	143	208	177	3.06	13639	78.959
3	232	0.7466	208	329	236	7.55	160	4.870
4	20000 (UM)	0.1615	17927	25924	20421	3.77	1457	12.638

TIC:	12.8522	ng/uL
TIM:	117.6371	nmole/L
Total concentration:	13.3874	ng/uL

Sample peak width (sec): 5 Sample min peak height: 50 Sample baseline V to V?: Y Sample baseline V to V points: 3
Sample filter: Binomial Number of points for filter: 3 Sample start region (min): 0 Sample end region (min): 30
Marker peak width (sec): 5 Marker min peak height: 200 Marker baseline V to V?: Y Marker baseline V to V points: 3
Lower marker selection: First peak > 500 RFU Upper marker selection: Last peak > 200 RFU
Ladder size (bp)75, 200, 300, 400, 500, 700, 1000, 1500, 2000, 3000, 4000, 5000, 7000, 10000, 20000
Quantification using: Lower Marker Final concentration (ng/uL): 0.5000 Dilution factor: 12.0

Sample: SampE1
Well location: E1
Created: Tuesday, September 14, 2021 6:14:00 PM

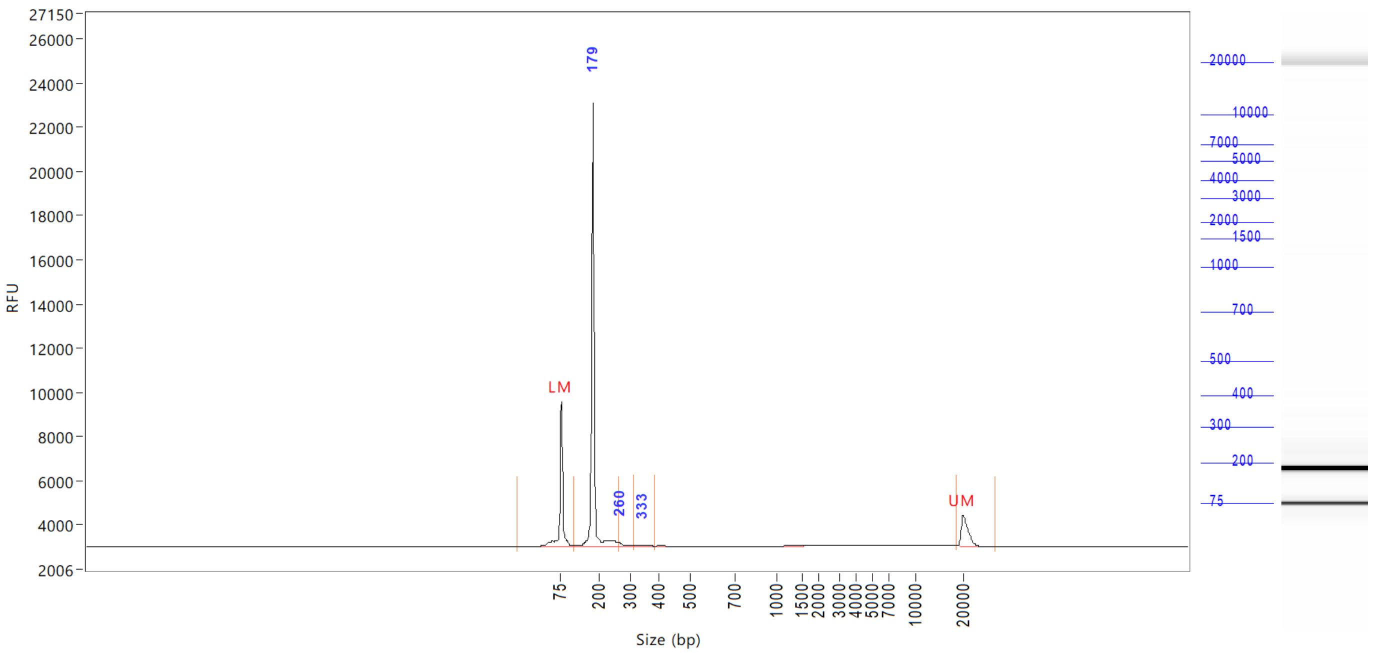

Peak	Size (bp)	Concentration (ng/uL)	From (bp)	To (bp)	Average size (bp)	CV%	RFU	Corrected peak area
1	75 (LM)	0.5000	0	118	70	23.63	6581	48.357
2	179	15.4981	118	260	180	8.77	20092	124.906
3	260	0.2525	260	311	278	5.26	204	2.035
4	333	0.1059	311	382	336	4.28	61	0.854
5	20000 (UM)	0.1311	18415	26649	20399	3.86	1409	12.680

TIC:	15.8565	ng/uL
TIM:	143.5603	nmole/L
Total concentration:	16.1960	ng/uL

Sample peak width (sec): 5   Sample min peak height: 50   Sample baseline V to V?: Y   Sample baseline V to V points: 3
Sample filter: Binomial   Number of points for filter: 3 Sample start region (min): 0 Sample end region (min): 30
Marker peak width (sec): 5   Marker min peak height: 200   Marker baseline V to V?: Y   Marker baseline V to V points: 3
Lower marker selection: First peak > 500 RFU   Upper marker selection: Last peak > 200 RFU
Ladder size (bp)75, 200, 300, 400, 500, 700, 1000, 1500, 2000, 3000, 4000, 5000, 7000, 10000, 20000
Quantification using: Lower Marker   Final concentration (ng/uL): 0.5000   Dilution factor: 12.0

Sample: SampF1
Well location: F1
Created: Tuesday, September 14, 2021 6:14:00 PM

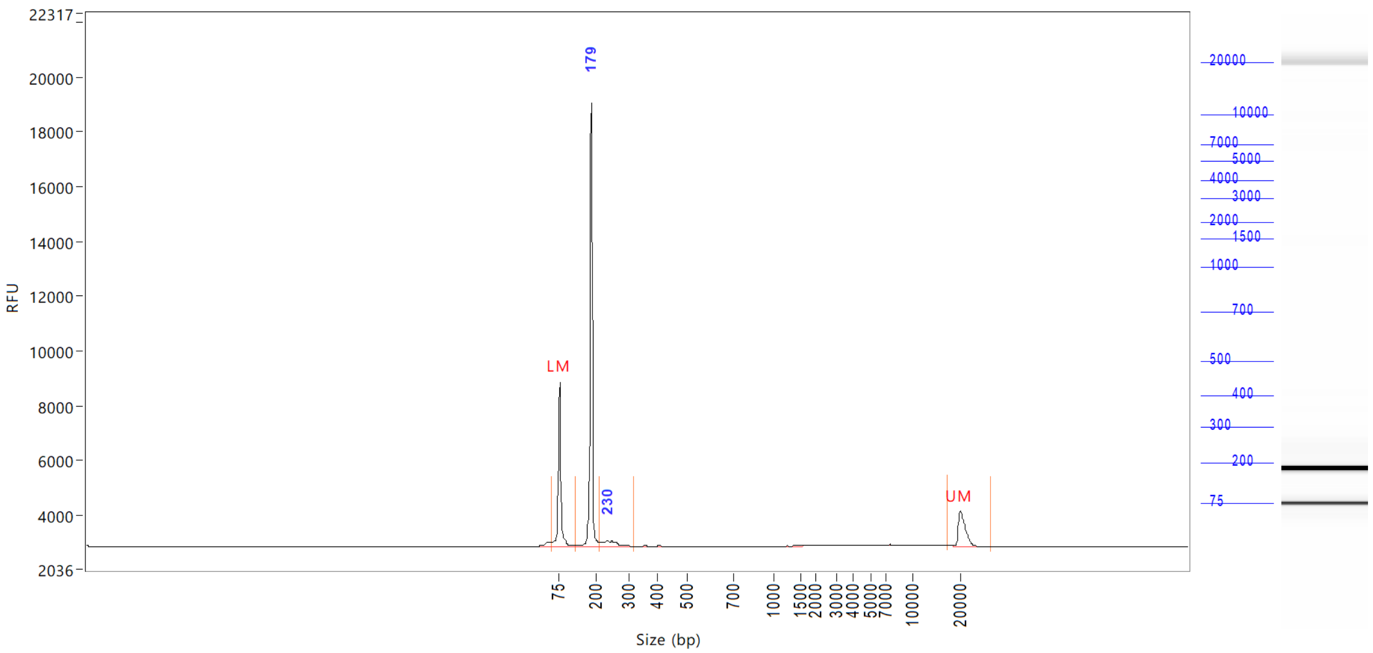

Peak	Size (bp)	Concentration (ng/uL)	From (bp)	To (bp)	Average size (bp)	CV%	RFU	Corrected peak area
1	75 (LM)	0.5000	48	131	74	13.08	6003	39.532
2	179	13.8898	131	206	177	3.47	16208	91.515
3	230	0.9494	206	317	238	8.48	187	6.256
4	20000 (UM)	0.1485	17439	26408	20475	4.08	1289	11.738

TIC:	14.8393	ng/uL
TIM:	135.5670	nmole/L
Total concentration:	15.3333	ng/uL

Sample peak width (sec): 5   Sample min peak height: 50   Sample baseline V to V?: Y   Sample baseline V to V points: 3
Sample filter: Binomial   Number of points for filter: 3 Sample start region (min): 0 Sample end region (min): 30
Marker peak width (sec): 5   Marker min peak height: 200   Marker baseline V to V?: Y   Marker baseline V to V points: 3
Lower marker selection: First peak > 500 RFU   Upper marker selection: Last peak > 200 RFU
Ladder size (bp)75, 200, 300, 400, 500, 700, 1000, 1500, 2000, 3000, 4000, 5000, 7000, 10000, 20000
Quantification using: Lower Marker   Final concentration (ng/uL): 0.5000   Dilution factor: 12.0

Sample: SampG1
Well location: G1
Created: Tuesday, September 14, 2021 6:14:00 PM

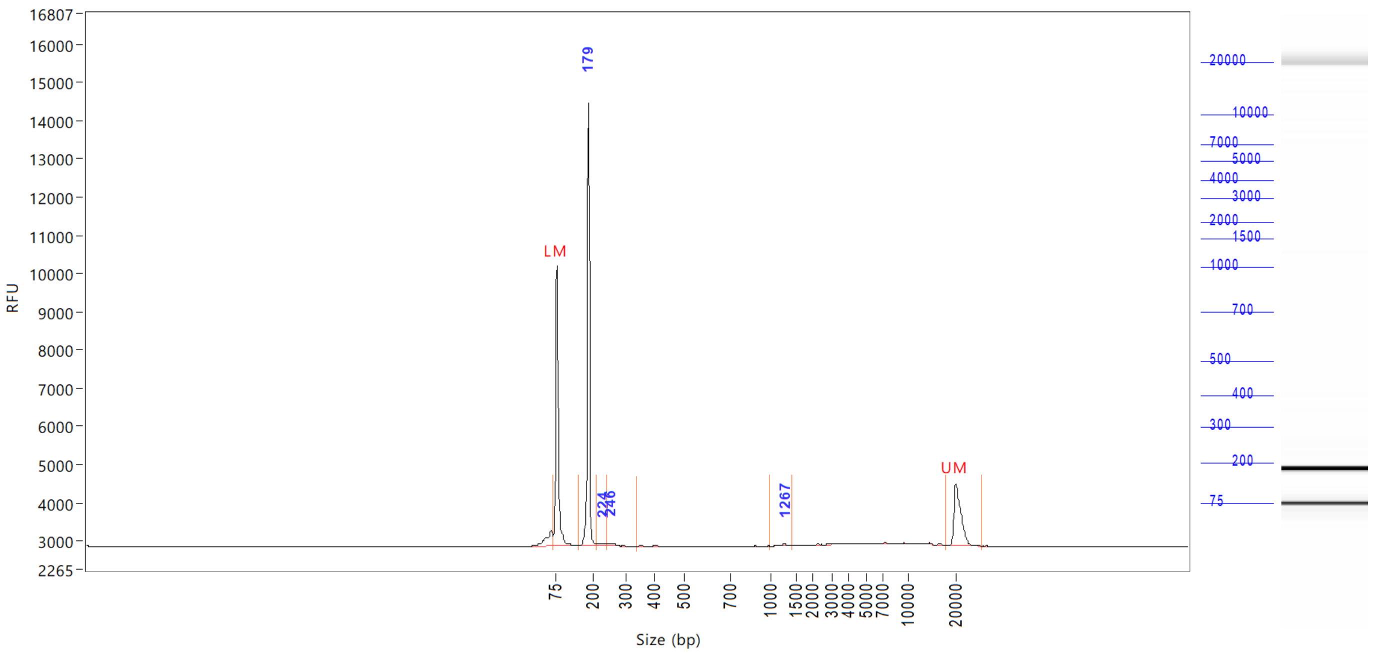

Peak	Size (bp)	Concentration (ng/uL)	From (bp)	To (bp)	Average size (bp)	CV%	RFU	Corrected peak area
1	75 (LM)	0.5000	64	147	74	9.85	7328	46.295
2	179	8.0912	147	208	177	2.78	11595	62.430
3	224	0.1390	208	239	224	4.03	62	1.072
4	246	0.1527	239	333	256	6.40	67	1.178
5	1267	0.0452	1000	1411	1250	4.28	58	0.348
6	20000 (UM)	0.1605	17805	25440	20452	3.97	1627	14.861

TIC:	8.4280	ng/uL
TIM:	77.2104	nmole/L
Total concentration:	8.8414	ng/uL

Sample peak width (sec): 5   Sample min peak height: 50   Sample baseline V to V?: Y   Sample baseline V to V points: 3
Sample filter: Binomial   Number of points for filter: 3 Sample start region (min): 0 Sample end region (min): 30
Marker peak width (sec): 5   Marker min peak height: 200   Marker baseline V to V?: Y   Marker baseline V to V points: 3
Lower marker selection: First peak > 500 RFU   Upper marker selection: Last peak > 200 RFU
Ladder size (bp)75, 200, 300, 400, 500, 700, 1000, 1500, 2000, 3000, 4000, 5000, 7000, 10000, 20000
Quantification using: Lower Marker   Final concentration (ng/uL): 0.5000   Dilution factor: 12.0

Sample: SampH1
Well location: H1
Created: Tuesday, September 14, 2021 6:14:00 PM

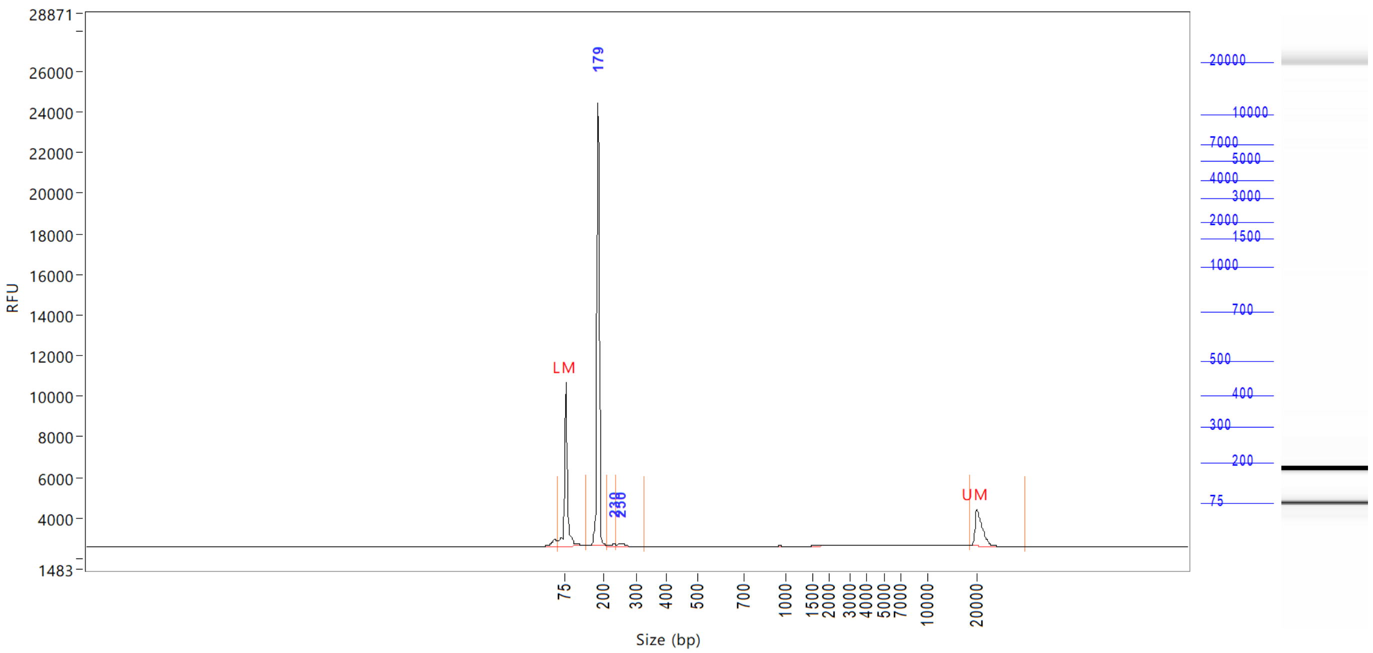

Peak	Size (bp)	Concentration (ng/uL)	From (bp)	To (bp)	Average size (bp)	CV%	RFU	Corrected peak area
1	75 (LM)	0.5000	52	143	75	11.89	8082	57.323
2	179	12.0762	143	208	177	2.52	21861	115.373
3	230	0.1494	208	237	223	3.84	81	1.427
4	250	0.1894	237	325	253	5.11	100	1.810
5	20000 (UM)	0.1461	18537	29793	20578	4.49	1770	16.755

TIC:	12.4149	ng/uL
TIM:	114.4942	nmole/L
Total concentration:	12.7580	ng/uL

Sample peak width (sec): 5 Sample min peak height: 50 Sample baseline V to V?: Y Sample baseline V to V points: 3
Sample filter: Binomial Number of points for filter: 3 Sample start region (min): 0 Sample end region (min): 30
Marker peak width (sec): 5 Marker min peak height: 200 Marker baseline V to V?: Y Marker baseline V to V points: 3
Lower marker selection: First peak > 500 RFU Upper marker selection: Last peak > 200 RFU
Ladder size (bp)75, 200, 300, 400, 500, 700, 1000, 1500, 2000, 3000, 4000, 5000, 7000, 10000, 20000
Quantification using: Lower Marker Final concentration (ng/uL): 0.5000 Dilution factor: 12.0

Sample: SampA2
Well location: A2
Created: Tuesday, September 14, 2021 6:14:00 PM

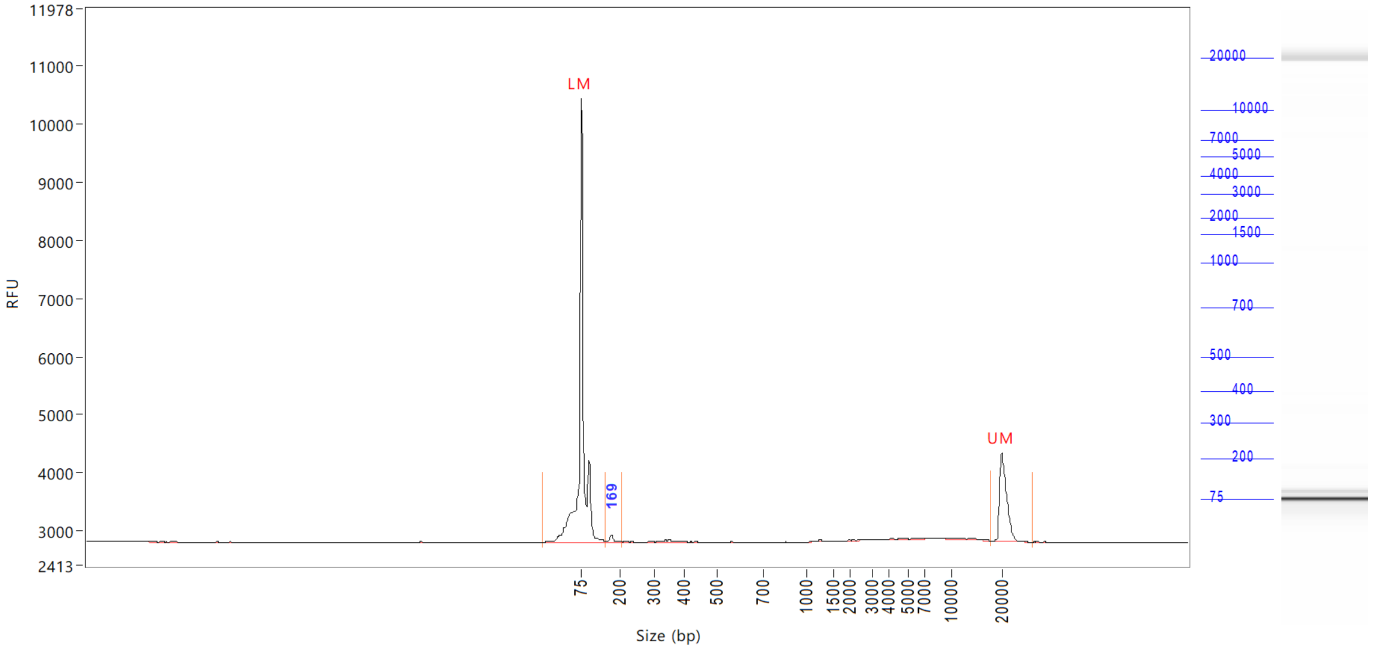

Peak	Size (bp)	Concentration (ng/uL)	From (bp)	To (bp)	Average size (bp)	CV%	RFU	Corrected peak area
1	75 (LM)	0.5000	0	150	70	32.34	7644	76.702
2	169	0.0867	150	203	169	5.93	130	1.108
3	20000 (UM)	0.0869	17927	26166	20358	3.75	1524	13.327

TIC:	0.0867	ng/uL
TIM:	0.8430	nmole/L
Total concentration:	0.4251	ng/uL

Sample peak width (sec): 5   Sample min peak height: 50   Sample baseline V to V?: Y   Sample baseline V to V points: 3
Sample filter: Binomial   Number of points for filter: 3   Sample start region (min): 0   Sample end region (min): 30
Marker peak width (sec): 5   Marker min peak height: 200   Marker baseline V to V?: Y   Marker baseline V to V points: 3
Lower marker selection: First peak > 500 RFU   Upper marker selection: Last peak > 200 RFU
Ladder size (bp)75, 200, 300, 400, 500, 700, 1000, 1500, 2000, 3000, 4000, 5000, 7000, 10000, 20000
Quantification using: Lower Marker   Final concentration (ng/uL): 0.5000   Dilution factor: 12.0

Sample: SampB2
Well location: B2
Created: Tuesday, September 14, 2021 6:14:00 PM

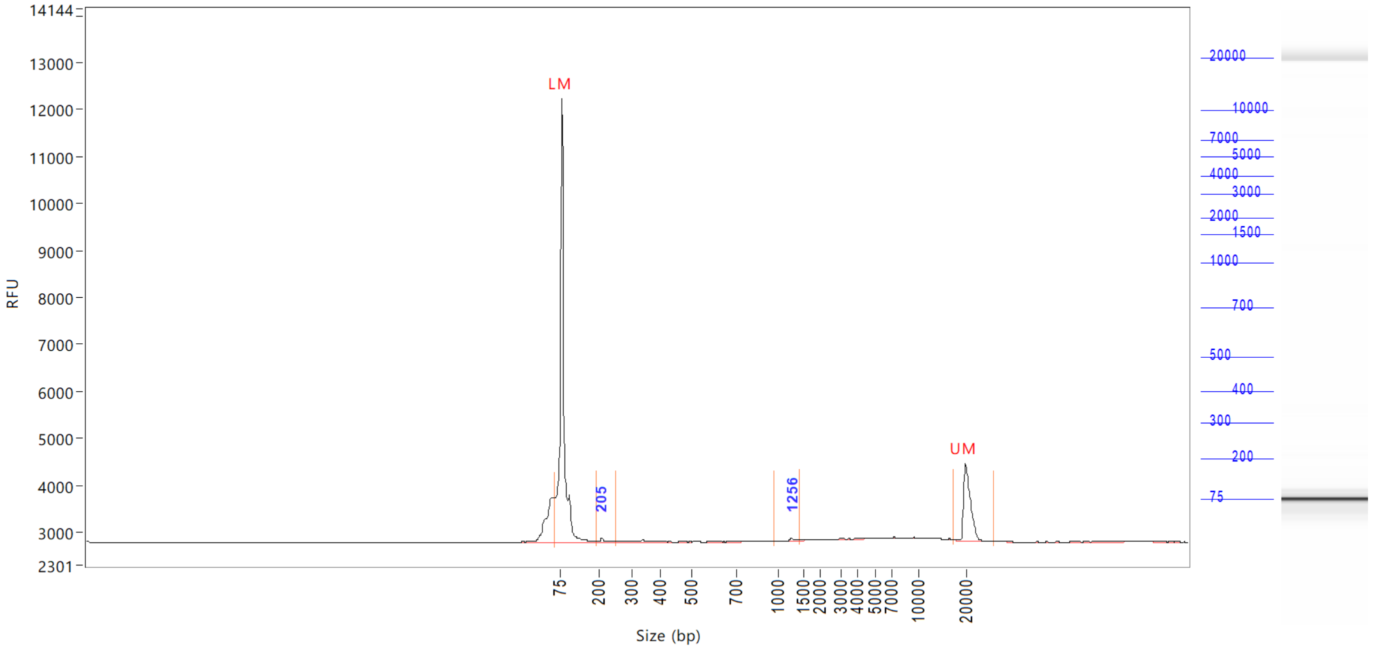

Peak	Size	Concentration	From	To	Average size	CV%	RFU	Corrected peak area
	(bp)	(ng/uL)	(bp)	(bp)	(bp)			
1	75 (LM)	0.5000	54	187	77	18.98	9459	86.507
2	205	0.0636	187	250	210	6.28	88	0.917
3	1256	0.0337	974	1422	1247	6.63	66	0.486
4	20000 (UM)	0.0869	17439	26045	20394	3.95	1645	15.027

TIC:	0.0973	ng/uL
TIM:	0.5427	nmole/L
Total concentration:	0.3293	ng/uL

```
Sample peak width (sec): 5    Sample min peak height: 50    Sample baseline V to V?: Y    Sample baseline V to V points: 3
Sample filter: Binomial       Number of points for filter: 3  Sample start region (min): 0  Sample end region (min): 30
Marker peak width (sec): 5    Marker min peak height: 200   Marker baseline V to V?: Y    Marker baseline V to V points: 3
Lower marker selection: First peak > 500 RFU                Upper marker selection: Last peak > 200 RFU
Ladder size (bp)75, 200, 300, 400, 500, 700, 1000, 1500, 2000, 3000, 4000, 5000, 7000, 10000, 20000
Quantification using: Lower Marker          Final concentration (ng/uL): 0.5000          Dilution factor: 12.0
```

Sample: SampC2
Well location: C2
Created: Tuesday, September 14, 2021 6:14:00 PM

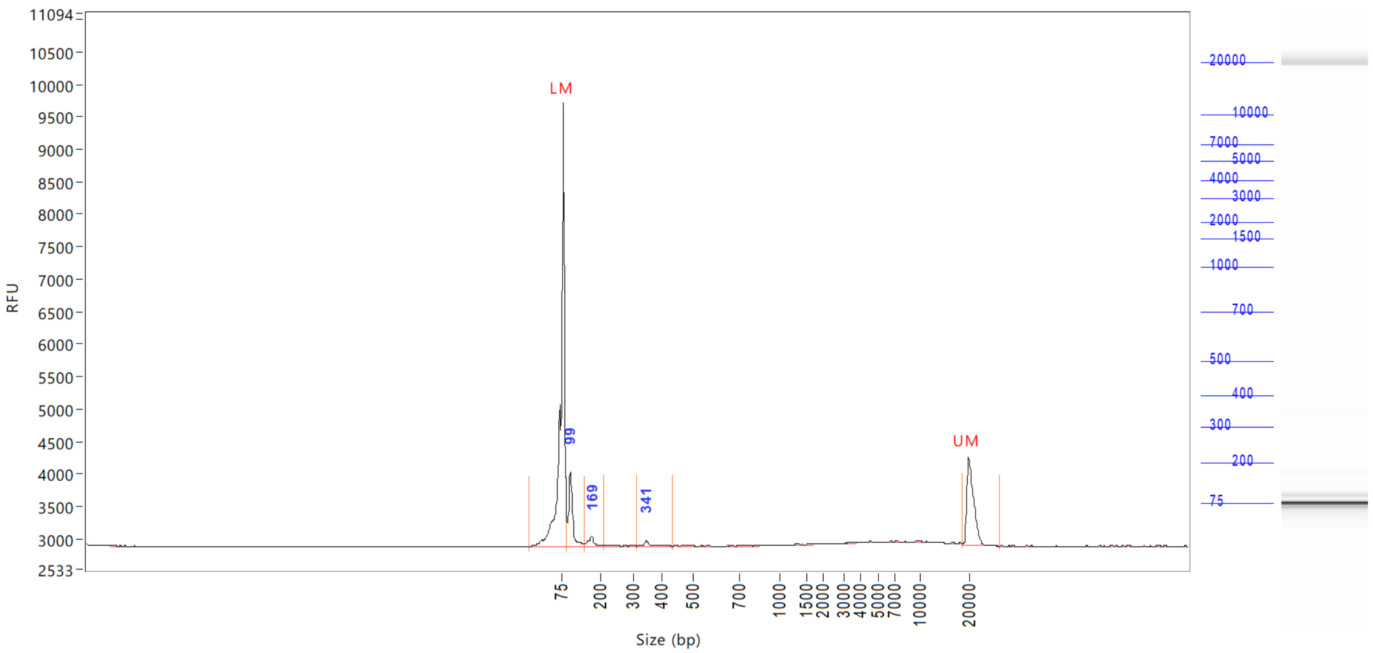

Peak	Size (bp)	Concentration (ng/uL)	From (bp)	To (bp)	Average size (bp)	CV%	RFU	Corrected peak area
1	75 (LM)	0.5000	0	87	64	26.16	6839	64.488
2	99	1.0037	87	145	100	11.09	1144	10.787
3	169	0.1866	145	210	166	7.96	150	2.006
4	341	0.0897	309	433	351	6.72	93	0.964
5	20000 (UM)	0.0931	18659	26408	20413	3.76	1350	12.013

TIC:	1.2800	ng/uL
TIM:	18.7497	nmole/L
Total concentration:	1.5296	ng/uL

Sample peak width (sec): 5   Sample min peak height: 50   Sample baseline V to V?: Y   Sample baseline V to V points: 3
Sample filter: Binomial   Number of points for filter: 3 Sample start region (min): 0 Sample end region (min): 30
Marker peak width (sec): 5   Marker min peak height: 200   Marker baseline V to V?: Y   Marker baseline V to V points: 3
Lower marker selection: First peak > 500 RFU   Upper marker selection: Last peak > 200 RFU
Ladder size (bp)75, 200, 300, 400, 500, 700, 1000, 1500, 2000, 3000, 4000, 5000, 7000, 10000, 20000
Quantification using: Lower Marker   Final concentration (ng/uL): 0.5000   Dilution factor: 12.0

Sample: SampD2
Well location: D2
Created: Tuesday, September 14, 2021 6:14:00 PM

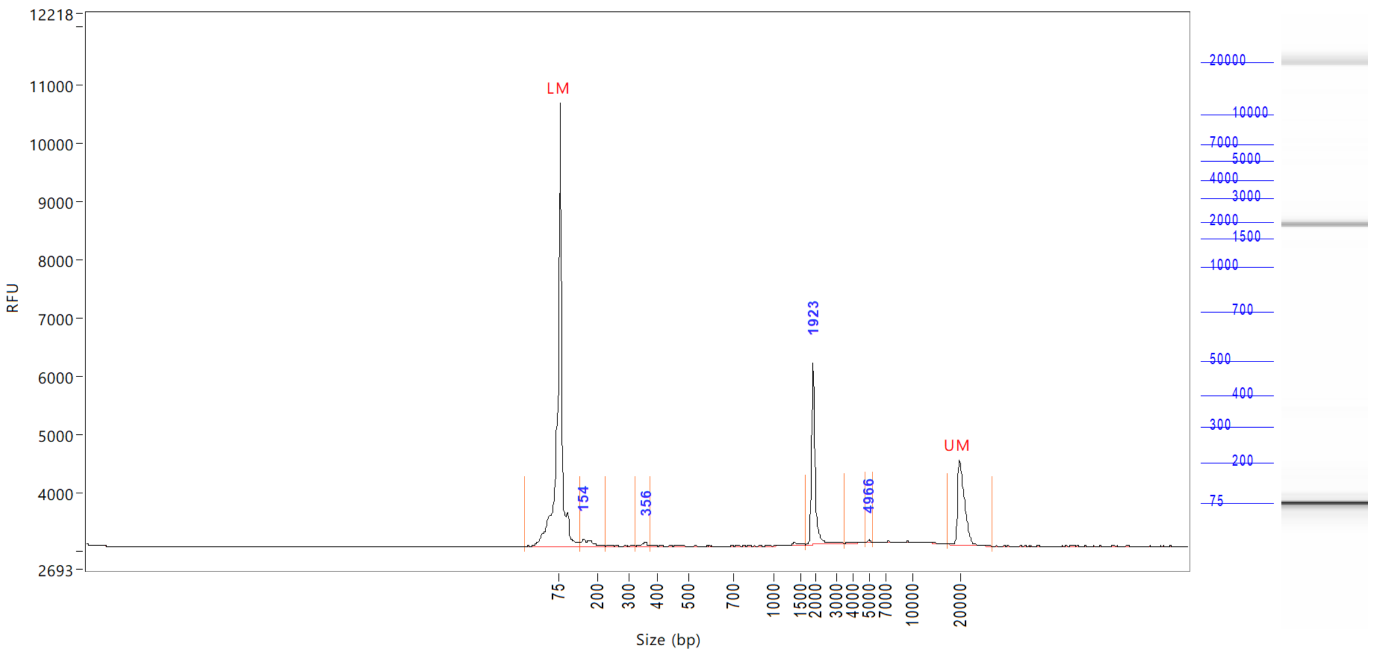

Peak	Size (bp)	Concentration (ng/uL)	From (bp)	To (bp)	Average size (bp)	CV%	RFU	Corrected peak area
1	75 (LM)	0.5000	0	141	67	30.93	7613	81.377
2	154	0.2080	141	223	167	10.72	132	2.821
3	356	0.0568	321	372	346	3.15	70	0.771
4	1923	1.3358	1655	3500	1952	9.42	3119	18.117
5	4966	0.0195	4724	5297	4941	2.44	51	0.265
6	20000 (UM)	0.0801	17561	26891	20463	4.31	1442	13.034

TIC:	1.6201	ng/uL
TIM:	3.4507	nmole/L
Total concentration:	1.8600	ng/uL

```
Sample peak width (sec): 5    Sample min peak height: 50    Sample baseline V to V?: Y    Sample baseline V to V points: 3
Sample filter: Binomial       Number of points for filter: 3  Sample start region (min): 0  Sample end region (min): 30
Marker peak width (sec): 5    Marker min peak height: 200   Marker baseline V to V?: Y    Marker baseline V to V points: 3
Lower marker selection: First peak > 500 RFU                Upper marker selection: Last peak > 200 RFU
Ladder size (bp)75, 200, 300, 400, 500, 700, 1000, 1500, 2000, 3000, 4000, 5000, 7000, 10000, 20000
Quantification using: Lower Marker          Final concentration (ng/uL): 0.5000          Dilution factor: 12.0
```

Sample: SampE2
Well location: E2
Created: Tuesday, September 14, 2021 6:14:00 PM

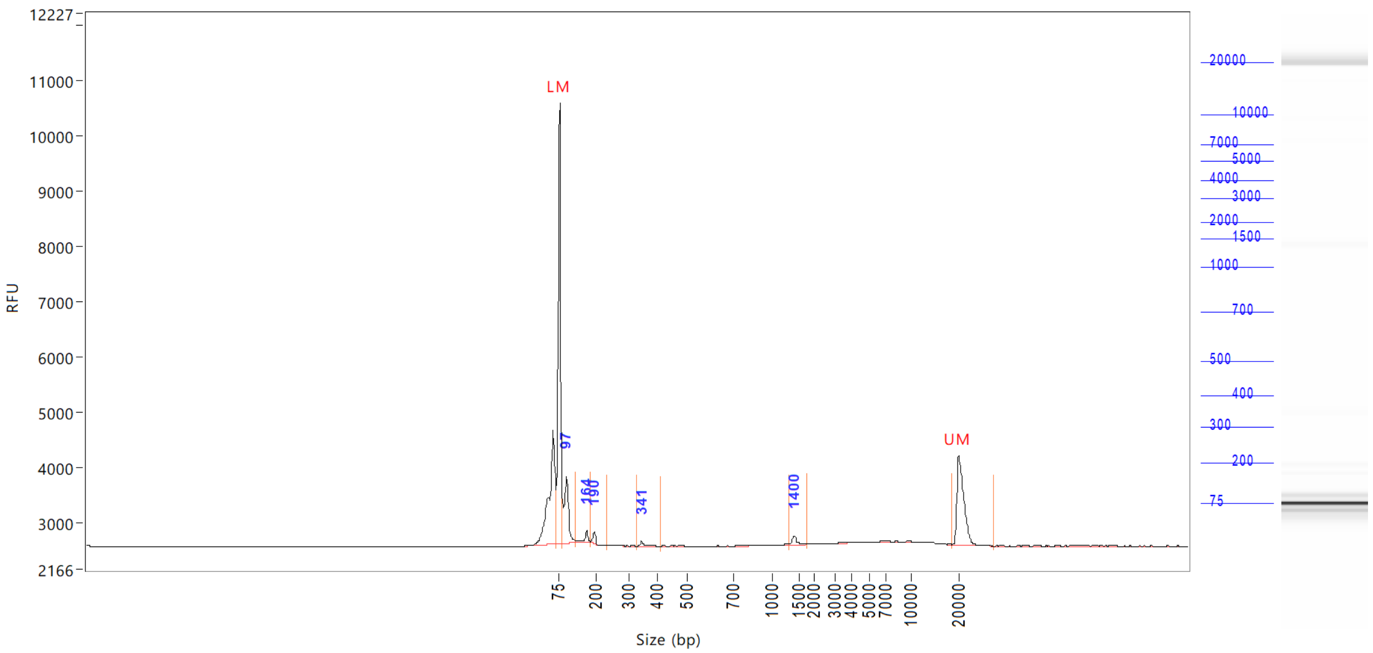

Peak	Size (bp)	Concentration (ng/uL)	From (bp)	To (bp)	Average size (bp)	CV%	RFU	Corrected peak area
1	75 (LM)	0.5000	66	86	73	5.41	7988	49.693
2	97	1.5444	86	131	97	8.19	1219	12.791
3	164	0.2073	131	179	158	6.60	220	1.717
4	190	0.1739	179	230	189	2.98	217	1.440
5	341	0.0933	325	409	349	4.28	89	0.773
6	1400	0.1483	1289	1750	1408	5.00	166	1.228
7	20000 (UM)	0.1463	18415	27496	20431	3.98	1608	14.541

TIC:	2.1671	ng/uL
TIM:	30.4245	nmole/L
Total concentration:	2.4552	ng/uL

```
Sample peak width (sec): 5    Sample min peak height: 50    Sample baseline V to V?: Y    Sample baseline V to V points: 3
Sample filter: Binomial       Number of points for filter: 3  Sample start region (min): 0  Sample end region (min): 30
Marker peak width (sec): 5    Marker min peak height: 200   Marker baseline V to V?: Y    Marker baseline V to V points: 3
Lower marker selection: First peak > 500 RFU                Upper marker selection: Last peak > 200 RFU
Ladder size (bp)75, 200, 300, 400, 500, 700, 1000, 1500, 2000, 3000, 4000, 5000, 7000, 10000, 20000
Quantification using: Lower Marker          Final concentration (ng/uL): 0.5000          Dilution factor: 12.0
```

Sample: SampF2
Well location: F2
Created: Tuesday, September 14, 2021 6:14:00 PM

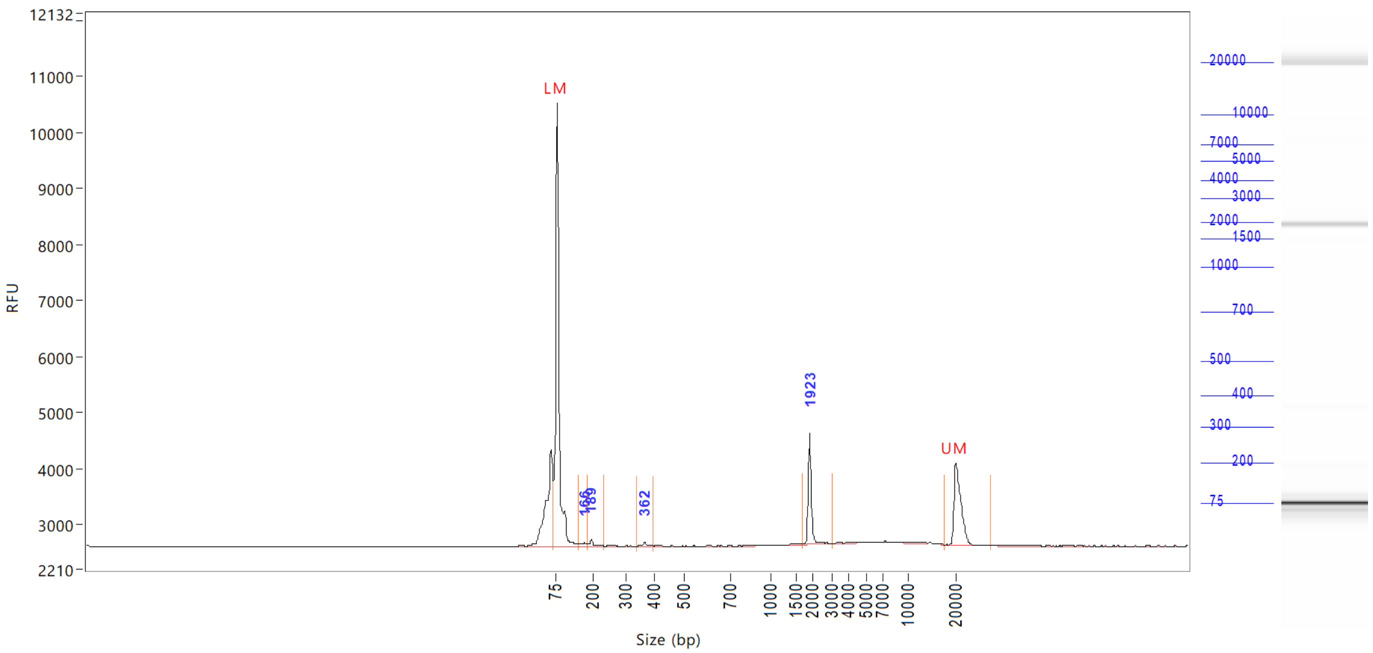

Peak	Size (bp)	Concentration (ng/uL)	From (bp)	To (bp)	Average size (bp)	CV%	RFU	Corrected peak area
1	75 (LM)	0.5000	64	147	77	14.56	7923	63.217
2	166	0.0848	147	177	161	5.53	58	0.893
3	189	0.0952	177	232	190	5.56	113	1.004
4	362	0.0605	333	394	360	3.19	76	0.637
5	1923	0.9681	1693	2971	1928	6.89	1974	10.200
6	20000 (UM)	0.1069	17683	27496	20475	4.25	1469	13.515

TIC:	1.2086	ng/uL
TIM:	2.7928	nmole/L
Total concentration:	1.4850	ng/uL

Sample peak width (sec): 5 Sample min peak height: 50 Sample baseline V to V?: Y Sample baseline V to V points: 3
Sample filter: Binomial Number of points for filter: 3 Sample start region (min): 0 Sample end region (min): 30
Marker peak width (sec): 5 Marker min peak height: 200 Marker baseline V to V?: Y Marker baseline V to V points: 3
Lower marker selection: First peak > 500 RFU Upper marker selection: Last peak > 200 RFU
Ladder size (bp)75, 200, 300, 400, 500, 700, 1000, 1500, 2000, 3000, 4000, 5000, 7000, 10000, 20000
Quantification using: Lower Marker Final concentration (ng/uL): 0.5000 Dilution factor: 12.0

Sample: SampG2
Well location: G2
Created: Tuesday, September 14, 2021 6:14:00 PM

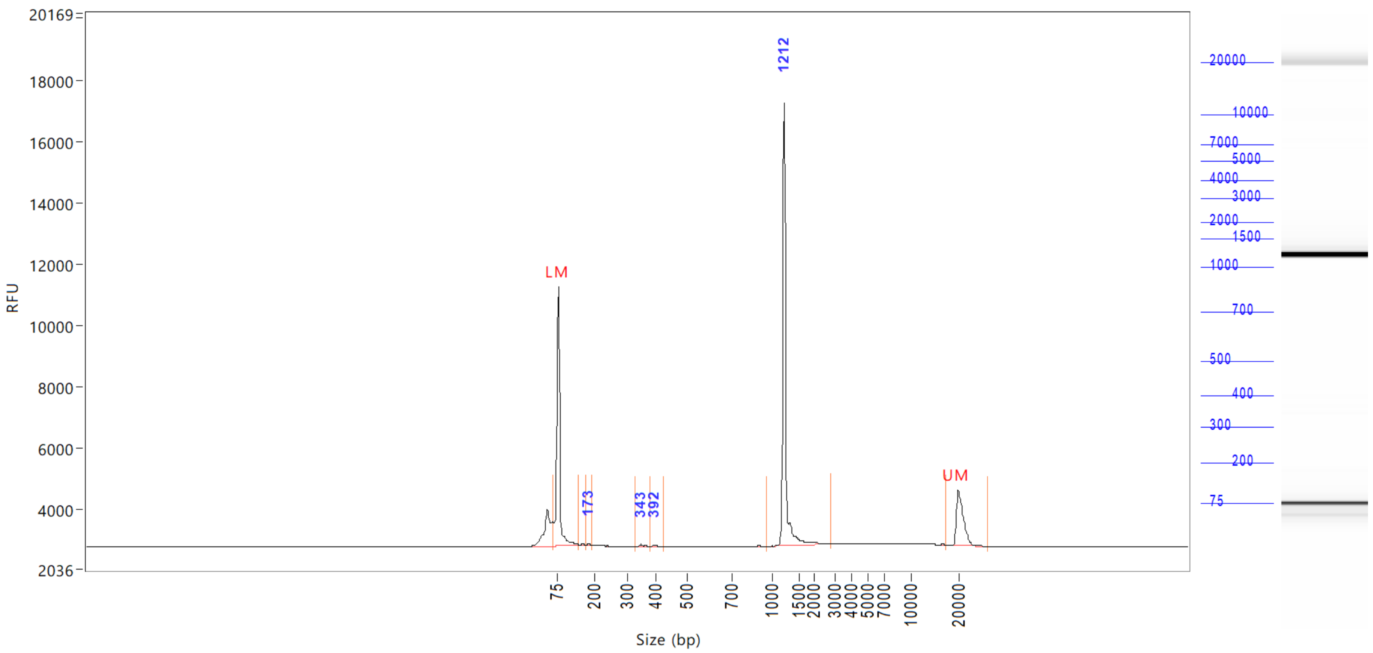

Peak	Size (bp)	Concentration (ng/uL)	From (bp)	To (bp)	Average size (bp)	CV%	RFU	Corrected peak area
1	75 (LM)	0.5000	62	145	75	14.22	8462	58.140
2	173	0.0606	166	190	174	2.72	81	0.587
3	343	0.0603	327	378	349	3.27	67	0.584
4	392	0.0495	378	424	393	2.12	65	0.479
5	1212	7.5937	952	2800	1241	10.19	14466	73.583
6	20000 (UM)	0.1419	17561	26166	20455	4.11	1803	16.499

TIC:	7.7640	ng/uL
TIM:	11.1354	nmole/L
Total concentration:	8.0821	ng/uL

Sample peak width (sec): 5   Sample min peak height: 50   Sample baseline V to V?: Y   Sample baseline V to V points: 3
Sample filter: Binomial   Number of points for filter: 3 Sample start region (min): 0 Sample end region (min): 30
Marker peak width (sec): 5   Marker min peak height: 200   Marker baseline V to V?: Y   Marker baseline V to V points: 3
Lower marker selection: First peak > 500 RFU   Upper marker selection: Last peak > 200 RFU
Ladder size (bp)75, 200, 300, 400, 500, 700, 1000, 1500, 2000, 3000, 4000, 5000, 7000, 10000, 20000
Quantification using: Lower Marker   Final concentration (ng/uL): 0.5000   Dilution factor: 12.0

Sample: SampH2
Well location: H2
Created: Tuesday, September 14, 2021 6:14:00 PM

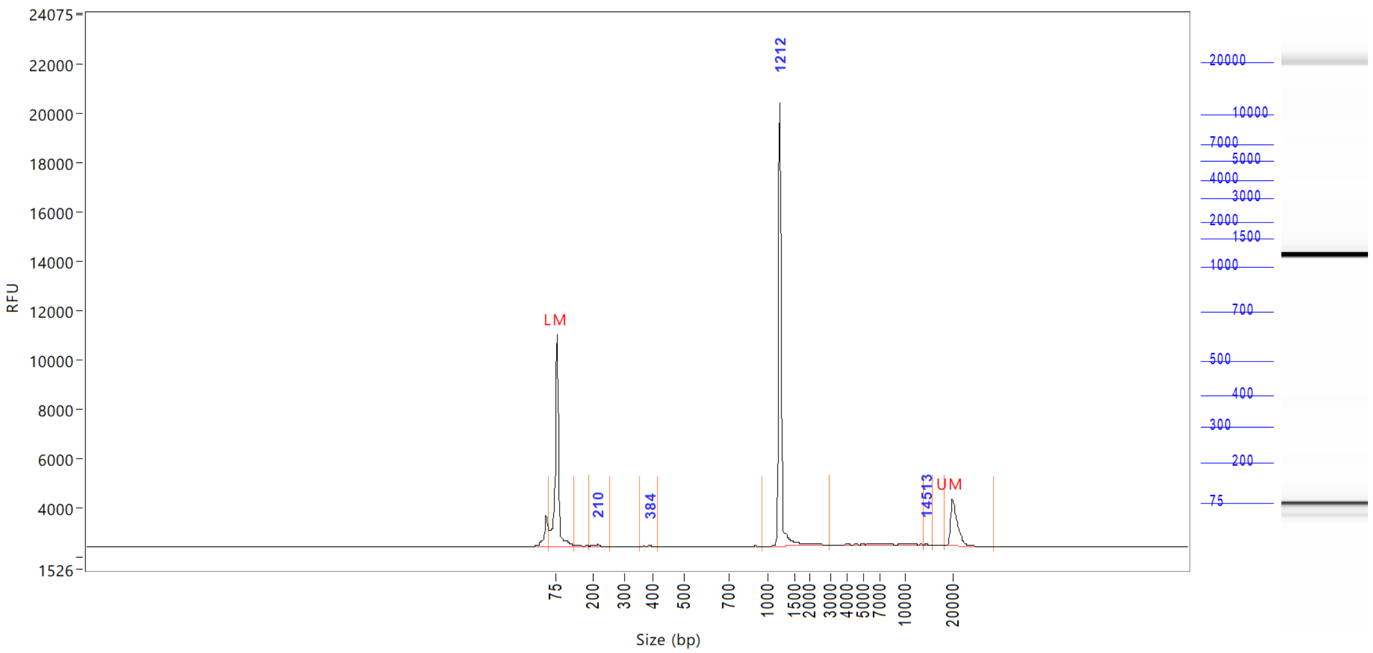

Peak	Size (bp)	Concentration (ng/uL)	From (bp)	To (bp)	Average size (bp)	CV%	RFU	Corrected peak area
1	75 (LM)	0.5000	48	133	73	14.95	8586	68.990
2	210	0.0850	181	248	204	4.55	78	0.977
3	384	0.0530	349	409	377	3.13	70	0.609
4	1212	7.3457	961	2971	1243	13.45	18006	84.463
5	14513	0.0279	13781	15610	14451	3.00	54	0.320
6	20000 (UM)	0.1262	18415	29068	20544	4.48	1909	17.412

TIC:	7.5115	ng/uL
TIM:	10.6466	nmole/L
Total concentration:	7.8049	ng/uL

```
Sample peak width (sec): 5    Sample min peak height: 50    Sample baseline V to V?: Y    Sample baseline V to V points: 3
Sample filter: Binomial       Number of points for filter: 3  Sample start region (min): 0  Sample end region (min): 30
Marker peak width (sec): 5    Marker min peak height: 200   Marker baseline V to V?: Y    Marker baseline V to V points: 3
Lower marker selection: First peak > 500 RFU                Upper marker selection: Last peak > 200 RFU
Ladder size (bp)75, 200, 300, 400, 500, 700, 1000, 1500, 2000, 3000, 4000, 5000, 7000, 10000, 20000
Quantification using: Lower Marker          Final concentration (ng/uL): 0.5000          Dilution factor: 12.0
```

Sample: SampA3
Well location: A3
Created: Tuesday, September 14, 2021 6:14:00 PM

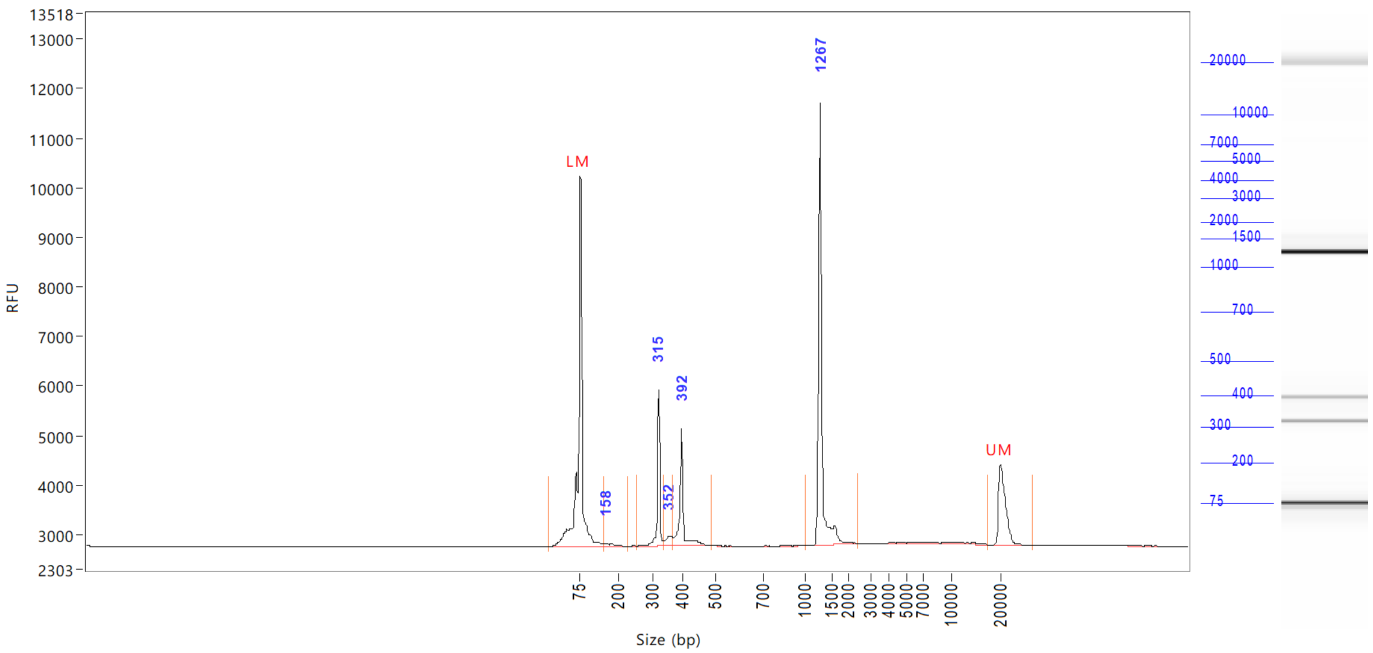

Peak	Size (bp)	Concentration (ng/uL)	From (bp)	To (bp)	Average size (bp)	CV%	RFU	Corrected peak area
1	75 (LM)	0.5000	0	152	69	30.15	7480	70.616
2	158	0.0888	152	223	171	8.46	65	1.045
3	315	1.3972	251	333	312	2.61	3145	16.444
4	352	0.2317	333	366	351	2.65	197	2.727
5	392	1.4098	366	487	395	4.64	2363	16.593
6	1267	3.9725	1001	2429	1292	8.29	8937	46.754
7	20000 (UM)	0.1031	17439	26408	20446	4.29	1638	14.566

TIC:	7.1001	ng/uL
TIM:	20.2396	nmole/L
Total concentration:	7.2880	ng/uL

```
Sample peak width (sec): 5    Sample min peak height: 50    Sample baseline V to V?: Y    Sample baseline V to V points: 3
Sample filter: Binomial       Number of points for filter: 3  Sample start region (min): 0  Sample end region (min): 30
Marker peak width (sec): 5    Marker min peak height: 200   Marker baseline V to V?: Y    Marker baseline V to V points: 3
Lower marker selection: First peak > 500 RFU                Upper marker selection: Last peak > 200 RFU
Ladder size (bp)75, 200, 300, 400, 500, 700, 1000, 1500, 2000, 3000, 4000, 5000, 7000, 10000, 20000
Quantification using: Lower Marker          Final concentration (ng/uL): 0.5000          Dilution factor: 12.0
```

Sample: SampB3
Well location: B3
Created: Tuesday, September 14, 2021 6:14:00 PM

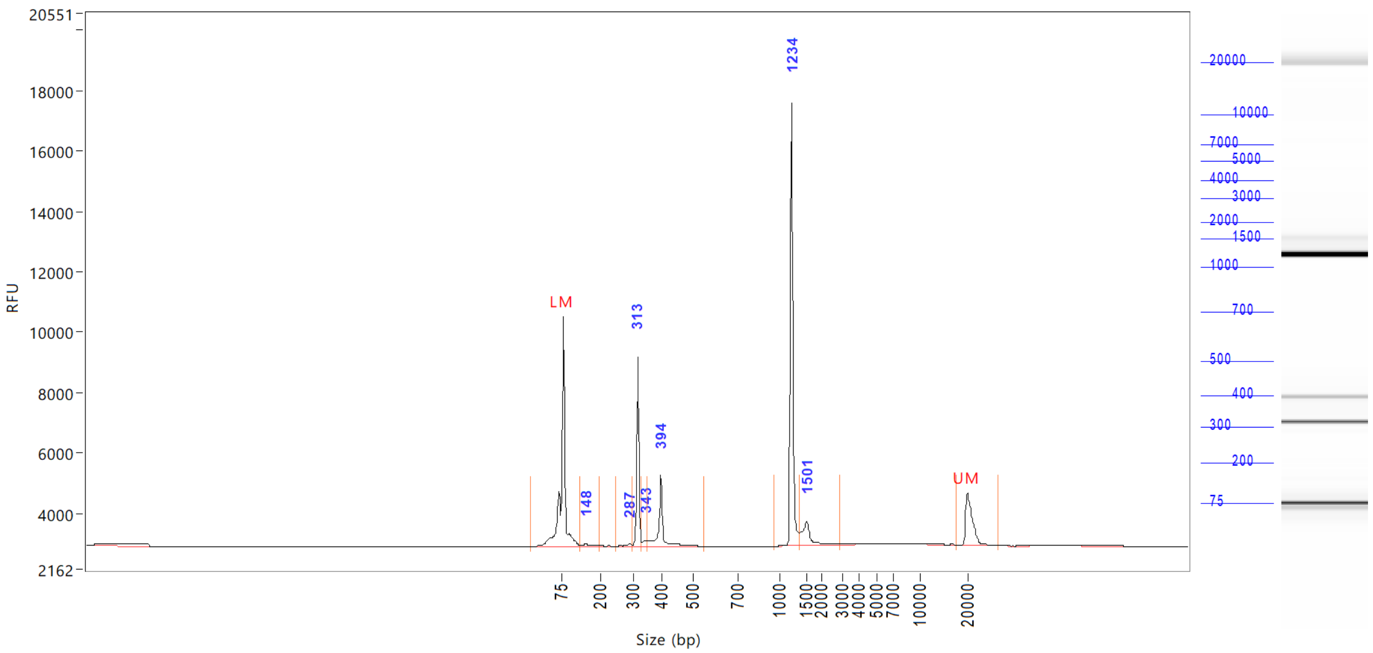

Peak	Size (bp)	Concentration (ng/uL)	From (bp)	To (bp)	Average size (bp)	CV%	RFU	Corrected peak area
1	75 (LM)	0.5000	0	133	69	26.69	7607	70.882
2	148	0.1322	133	192	155	10.09	108	1.562
3	287	0.0671	246	295	282	3.80	97	0.793
4	313	2.5329	295	327	311	1.31	6278	29.923
5	343	0.1790	327	349	339	1.99	200	2.115
6	394	1.5912	349	547	394	6.30	2349	18.798
7	1234	5.6473	965	1367	1219	2.79	14674	66.715
8	1501	0.9406	1367	2857	1595	17.57	818	11.112
9	20000 (UM)	0.1105	17561	26529	20486	4.33	1728	15.666

TIC:	11.0904	ng/uL
TIM:	31.3006	nmole/L
Total concentration:	11.2725	ng/uL

Sample peak width (sec): 5   Sample min peak height: 50   Sample baseline V to V?: Y   Sample baseline V to V points: 3
Sample filter: Binomial   Number of points for filter: 3   Sample start region (min): 0   Sample end region (min): 30
Marker peak width (sec): 5   Marker min peak height: 200   Marker baseline V to V?: Y   Marker baseline V to V points: 3
Lower marker selection: First peak > 500 RFU   Upper marker selection: Last peak > 200 RFU
Ladder size (bp)75, 200, 300, 400, 500, 700, 1000, 1500, 2000, 3000, 4000, 5000, 7000, 10000, 20000
Quantification using: Lower Marker   Final concentration (ng/uL): 0.5000   Dilution factor: 12.0

Sample: SampC3
Well location: C3
Created: Tuesday, September 14, 2021 6:14:00 PM

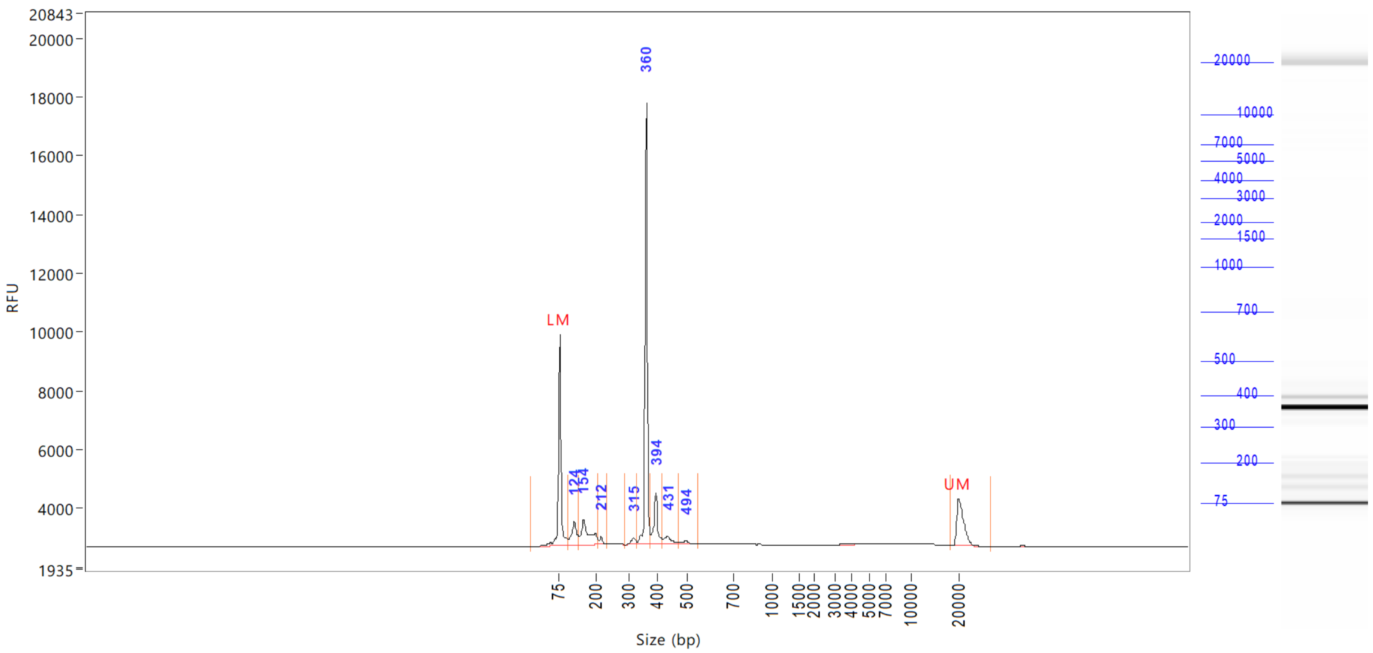

Peak	Size (bp)	Concentration (ng/uL)	From (bp)	To (bp)	Average size (bp)	CV%	RFU	Corrected peak area
1	75 (LM)	0.5000	0	103	73	14.16	7176	46.219
2	124	1.3548	103	139	121	7.45	813	10.436
3	154	2.0763	139	205	167	10.70	851	15.995
4	212	0.2313	205	233	210	1.91	284	1.781
5	315	0.2747	289	327	312	2.47	217	2.116
6	360	9.6699	327	374	357	1.52	15024	74.489
7	394	1.7328	374	416	391	2.26	1731	13.348
8	431	0.4410	416	471	433	2.75	232	3.397
9	494	0.1184	471	544	492	2.27	88	0.912
10	20000 (UM)	0.1563	18293	26891	20411	3.99	1588	14.445

TIC:	15.8992	ng/uL
TIM:	96.0159	nmole/L
Total concentration:	16.1875	ng/uL

Sample peak width (sec): 5 Sample min peak height: 50 Sample baseline V to V?: Y Sample baseline V to V points: 3
Sample filter: Binomial Number of points for filter: 3 Sample start region (min): 0 Sample end region (min): 30
Marker peak width (sec): 5 Marker min peak height: 200 Marker baseline V to V?: Y Marker baseline V to V points: 3
Lower marker selection: First peak > 500 RFU Upper marker selection: Last peak > 200 RFU
Ladder size (bp)75, 200, 300, 400, 500, 700, 1000, 1500, 2000, 3000, 4000, 5000, 7000, 10000, 20000
Quantification using: Lower Marker Final concentration (ng/uL): 0.5000 Dilution factor: 12.0

Sample: SampD3
Well location: D3
Created: Tuesday, September 14, 2021 6:14:00 PM

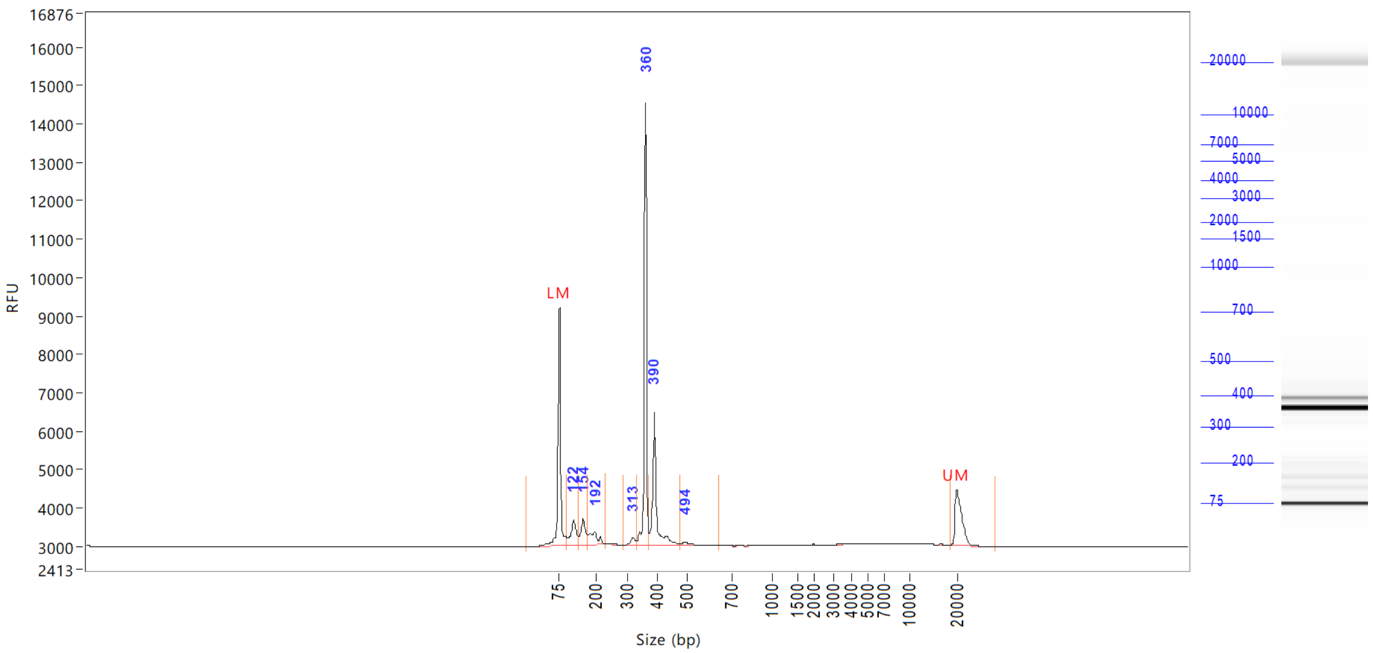

Peak	Size (bp)	Concentration (ng/uL)	From (bp)	To (bp)	Average size (bp)	CV%	RFU	Corrected peak area
1	75 (LM)	0.5000	0	101	71	15.56	6213	41.381
2	122	1.2944	101	139	120	7.96	675	8.927
3	154	1.0946	139	168	153	4.81	694	7.549
4	192	1.0010	168	226	188	6.94	316	6.904
5	313	0.2913	282	325	311	2.73	187	2.009
6	360	8.5928	325	372	355	1.66	11522	59.263
7	390	3.9112	372	475	395	4.81	3461	26.975
8	494	0.2439	475	639	514	7.29	94	1.682
9	20000 (UM)	0.1604	18415	28100	20421	4.23	1466	13.274

TIC:	16.4292	ng/uL
TIM:	96.6665	nmole/L
Total concentration:	16.6563	ng/uL

Sample peak width (sec): 5   Sample min peak height: 50   Sample baseline V to V?: Y   Sample baseline V to V points: 3
Sample filter: Binomial   Number of points for filter: 3 Sample start region (min): 0 Sample end region (min): 30
Marker peak width (sec): 5   Marker min peak height: 200   Marker baseline V to V?: Y   Marker baseline V to V points: 3
Lower marker selection: First peak > 500 RFU   Upper marker selection: Last peak > 200 RFU
Ladder size (bp)75, 200, 300, 400, 500, 700, 1000, 1500, 2000, 3000, 4000, 5000, 7000, 10000, 20000
Quantification using: Lower Marker   Final concentration (ng/uL): 0.5000   Dilution factor: 12.0

Sample: SampE3
Well location: E3
Created: Tuesday, September 14, 2021 6:14:00 PM

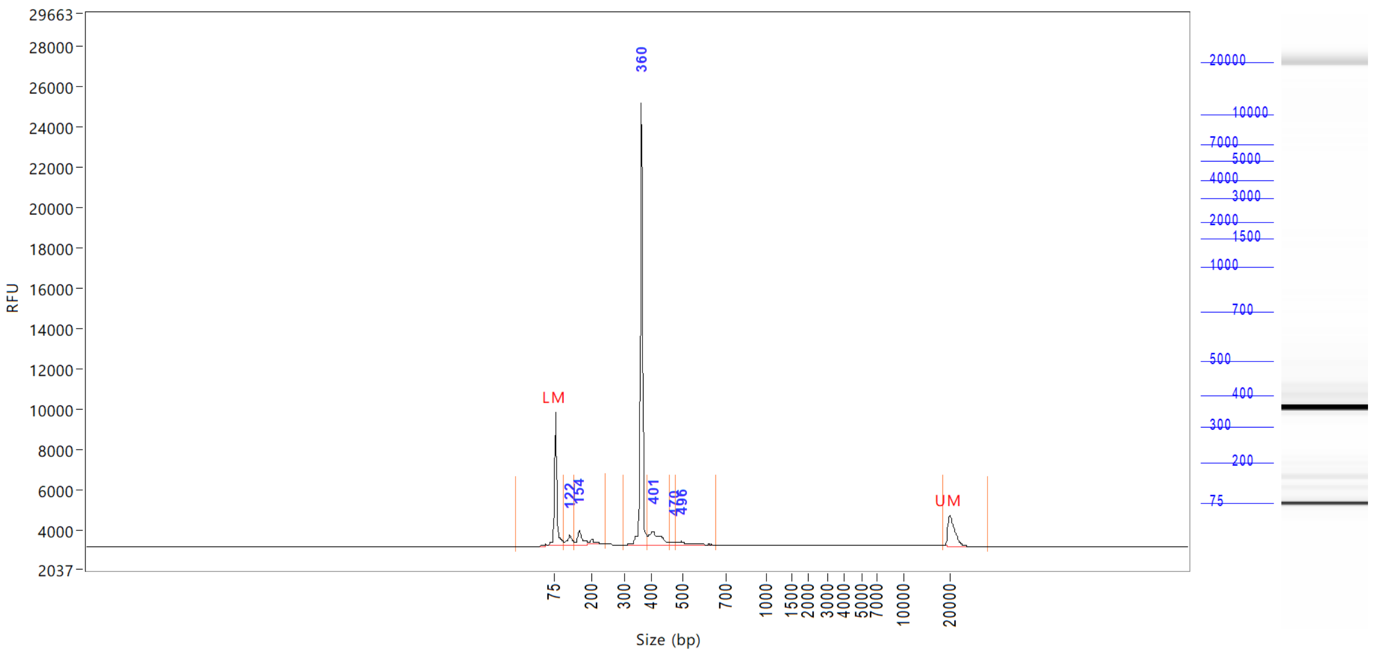

Peak	Size (bp)	Concentration (ng/uL)	From (bp)	To (bp)	Average size (bp)	CV%	RFU	Corrected peak area
1	75 (LM)	0.5000	0	103	73	14.01	6637	42.680
2	122	0.8577	103	139	120	7.61	480	6.101
3	154	1.6352	139	241	168	12.68	707	11.632
4	360	15.8605	295	382	357	1.97	22018	112.821
5	401	2.2665	382	456	410	4.54	696	16.123
6	470	0.2292	456	479	468	1.50	137	1.630
7	496	0.5926	479	652	522	7.53	176	4.216
8	20000 (UM)	0.1621	18537	28100	20495	4.19	1524	13.840

TIC:	21.4417	ng/uL
TIM:	112.6007	nmole/L
Total concentration:	21.8098	ng/uL

Sample peak width (sec): 5 Sample min peak height: 50 Sample baseline V to V?: Y Sample baseline V to V points: 3
Sample filter: Binomial Number of points for filter: 3 Sample start region (min): 0 Sample end region (min): 30
Marker peak width (sec): 5 Marker min peak height: 200 Marker baseline V to V?: Y Marker baseline V to V points: 3
Lower marker selection: First peak > 500 RFU Upper marker selection: Last peak > 200 RFU
Ladder size (bp)75, 200, 300, 400, 500, 700, 1000, 1500, 2000, 3000, 4000, 5000, 7000, 10000, 20000
Quantification using: Lower Marker Final concentration (ng/uL): 0.5000 Dilution factor: 12.0

Sample: SampF3
Well location: F3
Created: Tuesday, September 14, 2021 6:14:00 PM

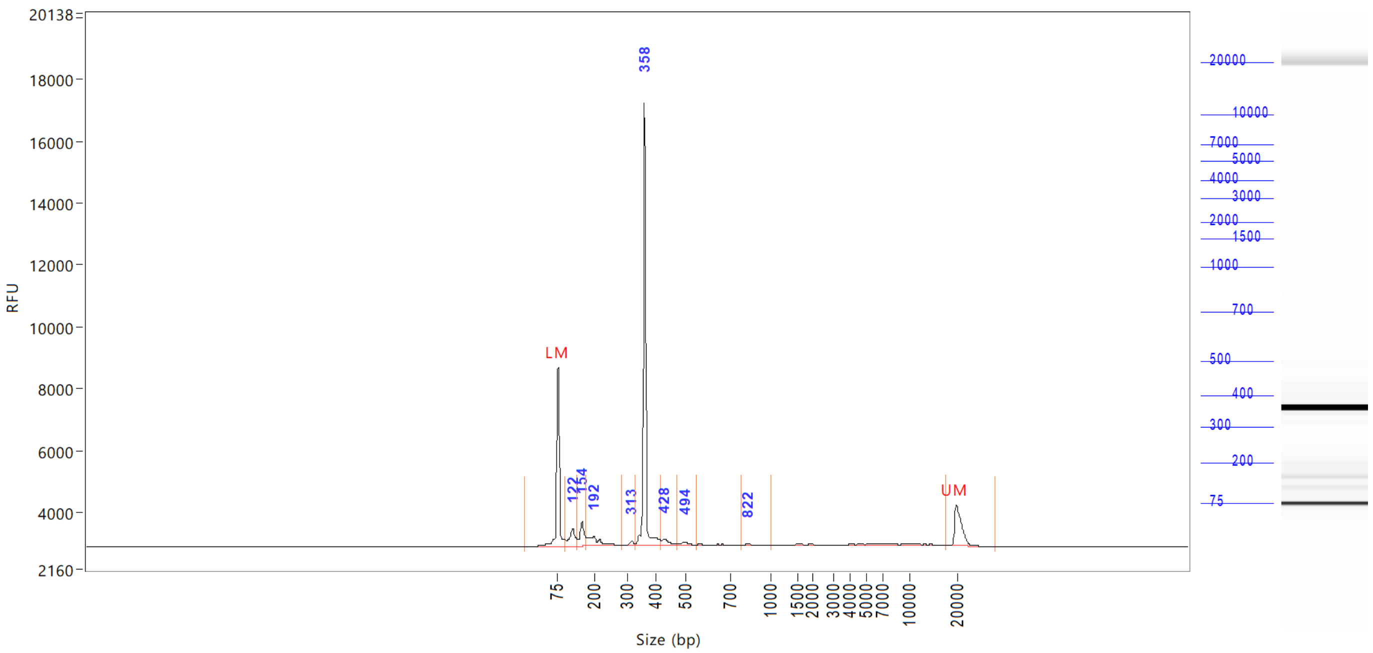

Peak	Size (bp)	Concentration (ng/uL)	From (bp)	To (bp)	Average size (bp)	CV%	RFU	Corrected peak area
1	75 (LM)	0.5000	0	101	71	17.34	5791	39.280
2	122	1.1925	101	139	120	8.08	567	7.807
3	154	1.2961	139	169	154	4.92	810	8.485
4	192	1.2696	169	284	199	11.55	312	8.312
5	313	0.1940	284	325	312	2.48	132	1.270
6	358	11.5895	325	416	358	2.95	14328	75.873
7	428	0.4280	416	471	435	3.37	186	2.802
8	494	0.2025	471	544	494	2.94	103	1.325
9	822	0.1031	775	1001	835	5.61	55	0.675
10	20000 (UM)	0.1566	17683	28221	20478	4.34	1338	12.303

TIC:	16.2752	ng/uL
TIM:	97.4262	nmole/L
Total concentration:	16.7899	ng/uL

```
Sample peak width (sec): 5    Sample min peak height: 50    Sample baseline V to V?: Y    Sample baseline V to V points: 3
Sample filter: Binomial       Number of points for filter: 3  Sample start region (min): 0  Sample end region (min): 30
Marker peak width (sec): 5    Marker min peak height: 200   Marker baseline V to V?: Y    Marker baseline V to V points: 3
Lower marker selection: First peak > 500 RFU                Upper marker selection: Last peak > 200 RFU
Ladder size (bp)75, 200, 300, 400, 500, 700, 1000, 1500, 2000, 3000, 4000, 5000, 7000, 10000, 20000
Quantification using: Lower Marker          Final concentration (ng/uL): 0.5000          Dilution factor: 12.0
```

Sample: SampG3
Well location: G3
Created: Tuesday, September 14, 2021 6:14:00 PM

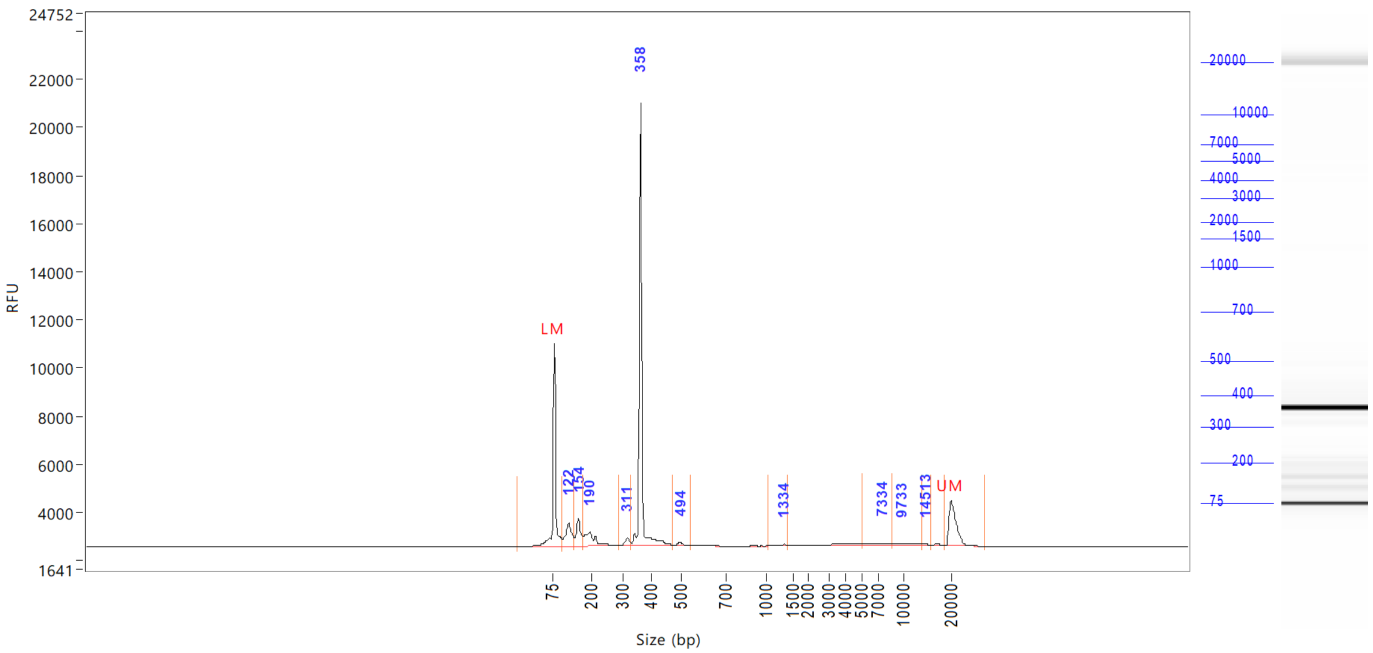

Peak	Size (bp)	Concentration (ng/uL)	From (bp)	To (bp)	Average size (bp)	CV%	RFU	Corrected peak area
1	75 (LM)	0.5000	0	101	70	19.66	8418	58.139
2	122	1.4811	101	141	120	8.50	990	14.352
3	154	1.2629	141	169	154	4.84	1133	12.237
4	190	1.4680	169	284	197	10.75	575	14.225
5	311	0.3399	284	327	310	2.73	313	3.294
6	358	10.8115	327	470	361	5.01	18437	104.761
7	494	0.1860	470	539	494	3.05	146	1.802
8	1334	0.0279	1034	1400	1316	3.18	52	0.271
9	7334	0.1501	5001	8734	6940	14.09	59	1.454
10	9733	0.1512	8734	13781	10869	13.30	52	1.465
11	14513	0.0398	13781	15732	14492	3.22	58	0.386
12	20000 (UM)	0.1510	18537	27254	20487	4.09	1904	17.562

TIC:	15.9184	ng/uL
TIM:	97.7993	nmole/L
Total concentration:	16.0637	ng/uL

```
Sample peak width (sec): 5    Sample min peak height: 50    Sample baseline V to V?: Y    Sample baseline V to V points: 3
Sample filter: Binomial       Number of points for filter: 3  Sample start region (min): 0  Sample end region (min): 30
Marker peak width (sec): 5    Marker min peak height: 200   Marker baseline V to V?: Y    Marker baseline V to V points: 3
Lower marker selection: First peak > 500 RFU                Upper marker selection: Last peak > 200 RFU
Ladder size (bp)75, 200, 300, 400, 500, 700, 1000, 1500, 2000, 3000, 4000, 5000, 7000, 10000, 20000
Quantification using: Lower Marker          Final concentration (ng/uL): 0.5000          Dilution factor: 12.0
```

Sample: SampH3
Well location: H3
Created: Tuesday, September 14, 2021 6:14:00 PM

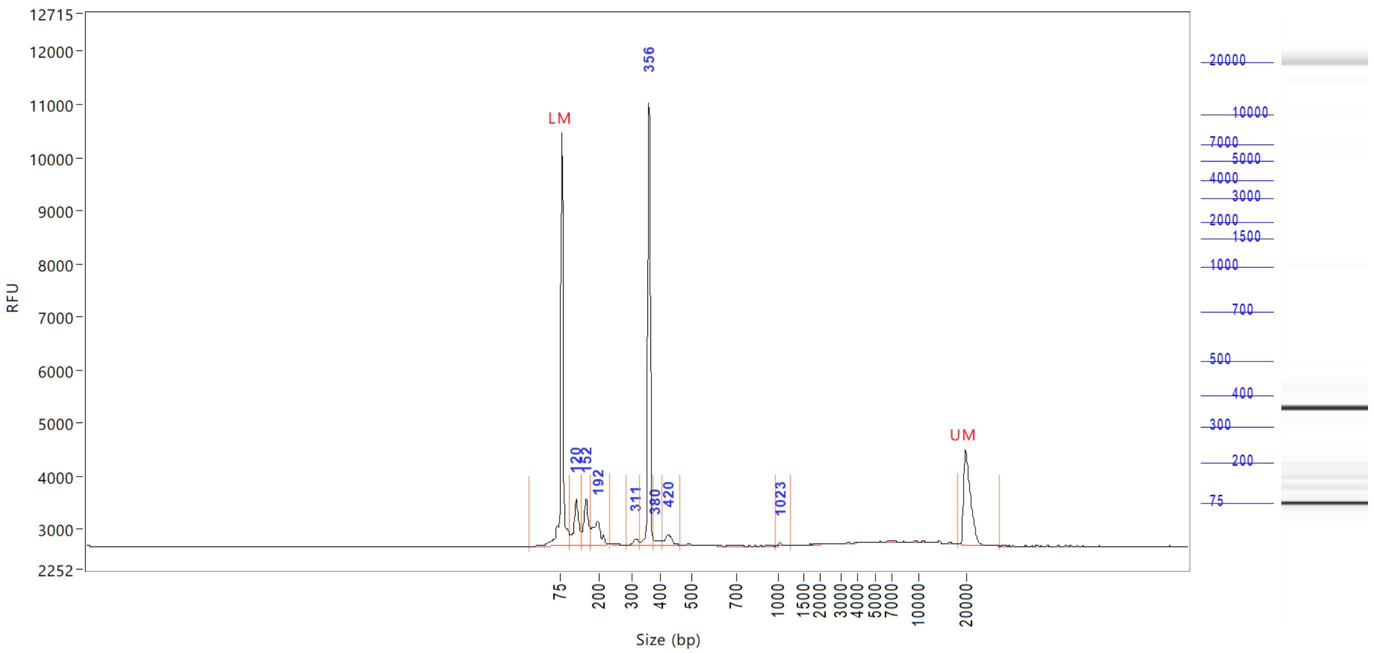

Peak	Size (bp)	Concentration (ng/uL)	From (bp)	To (bp)	Average size (bp)	CV%	RFU	Corrected peak area
1	75 (LM)	0.5000	0	103	71	16.56	7766	50.767
2	120	1.2123	103	139	121	7.15	874	10.258
3	152	1.1176	139	169	153	5.09	872	9.456
4	192	0.9725	169	232	188	6.25	436	8.229
5	311	0.1594	280	327	310	2.78	124	1.349
6	356	4.8641	327	376	355	1.41	8339	41.156
7	380	0.1660	376	405	390	2.29	92	1.404
8	420	0.3257	405	462	423	2.46	207	2.756
9	1023	0.0483	983	1245	1051	5.79	63	0.409
10	20000 (UM)	0.1616	18415	27133	20413	3.96	1791	16.408

TIC:	8.8660	ng/uL
TIM:	62.4007	nmole/L
Total concentration:	9.1386	ng/uL

```
Sample peak width (sec): 5    Sample min peak height: 50    Sample baseline V to V?: Y    Sample baseline V to V points: 3
Sample filter: Binomial       Number of points for filter: 3  Sample start region (min): 0  Sample end region (min): 30
Marker peak width (sec): 5    Marker min peak height: 200   Marker baseline V to V?: Y    Marker baseline V to V points: 3
Lower marker selection: First peak > 500 RFU                Upper marker selection: Last peak > 200 RFU
Ladder size (bp)75, 200, 300, 400, 500, 700, 1000, 1500, 2000, 3000, 4000, 5000, 7000, 10000, 20000
Quantification using: Lower Marker          Final concentration (ng/uL): 0.5000          Dilution factor: 12.0
```

Sample: SampA4
Well location: A4
Created: Tuesday, September 14, 2021 6:14:00 PM

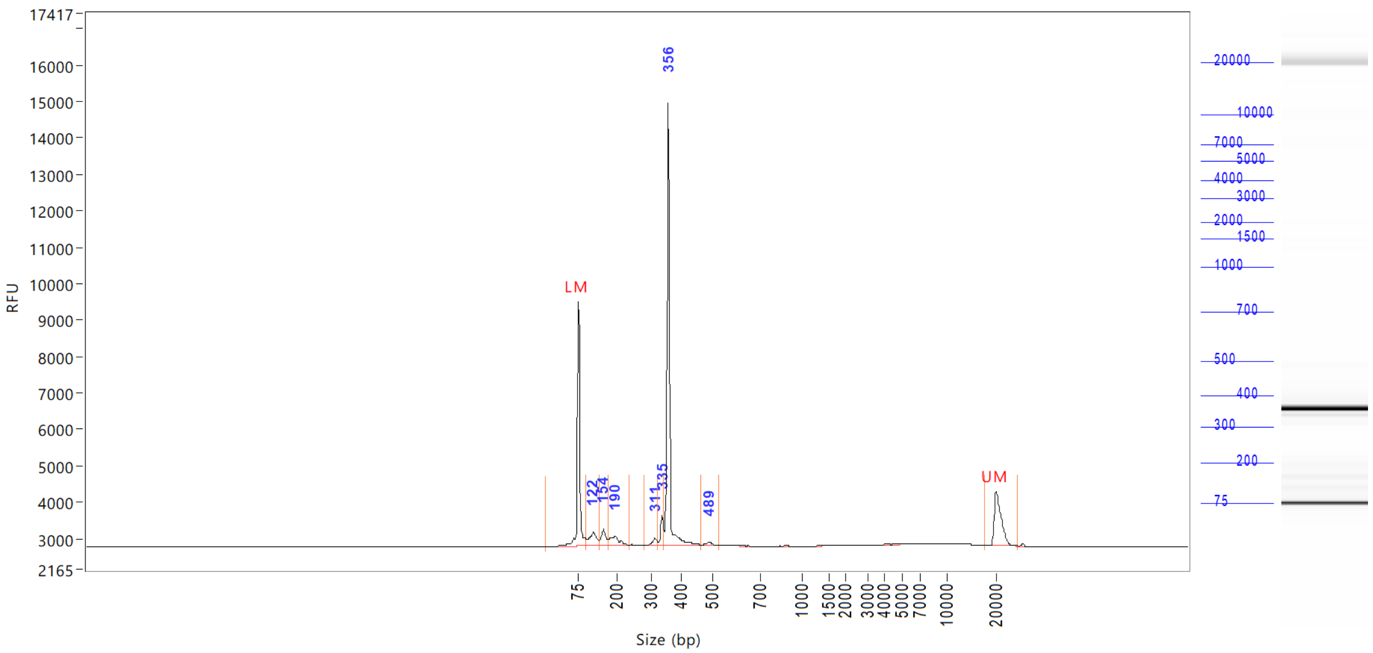

Peak	Size (bp)	Concentration (ng/uL)	From (bp)	To (bp)	Average size (bp)	CV%	RFU	Corrected peak area
1	75 (LM)	0.5000	0	101	72	14.81	6700	41.331
2	122	0.8725	101	141	120	8.89	366	6.010
3	154	0.7084	141	169	154	4.87	442	4.880
4	190	0.6870	169	233	189	6.91	228	4.732
5	311	0.3004	282	323	309	2.61	195	2.069
6	335	0.6632	323	341	334	1.35	825	4.569
7	356	9.4884	341	464	357	4.16	12162	65.360
8	489	0.1557	464	524	485	2.04	96	1.073
9	20000 (UM)	0.1599	17683	24473	20391	3.72	1503	13.221

TIC:	12.8755	ng/uL
TIM:	74.6002	nmole/L
Total concentration:	13.1742	ng/uL

Sample peak width (sec): 5   Sample min peak height: 50   Sample baseline V to V?: Y   Sample baseline V to V points: 3
Sample filter: Binomial   Number of points for filter: 3 Sample start region (min): 0 Sample end region (min): 30
Marker peak width (sec): 5   Marker min peak height: 200   Marker baseline V to V?: Y   Marker baseline V to V points: 3
Lower marker selection: First peak > 500 RFU   Upper marker selection: Last peak > 200 RFU
Ladder size (bp)75, 200, 300, 400, 500, 700, 1000, 1500, 2000, 3000, 4000, 5000, 7000, 10000, 20000
Quantification using: Lower Marker   Final concentration (ng/uL): 0.5000   Dilution factor: 12.0

Sample: SampB4
Well location: B4
Created: Tuesday, September 14, 2021 6:14:00 PM

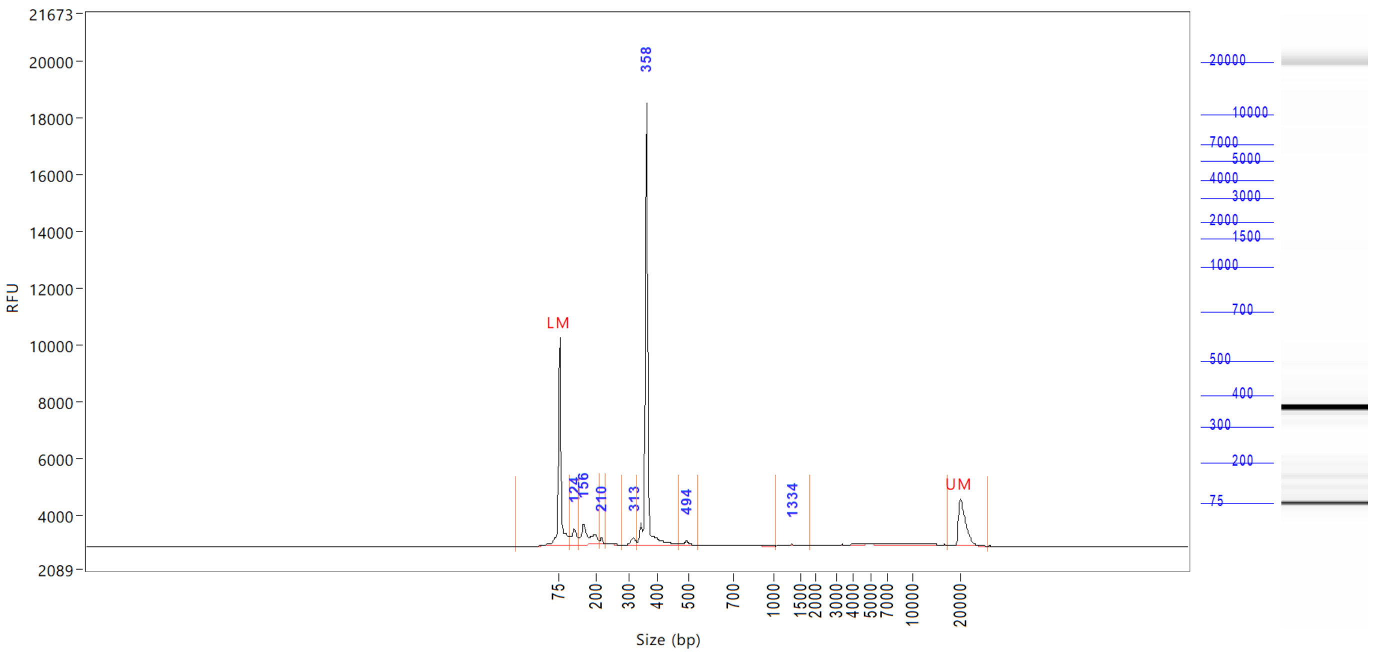

Peak	Size (bp)	Concentration (ng/uL)	From (bp)	To (bp)	Average size (bp)	CV%	RFU	Corrected peak area
1	75 (LM)	0.5000	0	110	75	16.20	7301	49.989
2	124	0.8537	110	139	123	6.30	571	7.113
3	156	1.6394	139	205	167	10.83	708	13.659
4	210	0.1727	205	226	210	1.76	237	1.438
5	313	0.3347	278	325	310	2.74	266	2.789
6	358	10.8415	325	470	360	4.32	15607	90.326
7	494	0.2325	470	544	495	3.32	147	1.937
8	1334	0.0769	1012	1808	1410	9.35	51	0.641
9	20000 (UM)	0.1518	17317	25682	20437	4.19	1620	15.177

TIC:	14.1515	ng/uL
TIM:	81.0760	nmole/L
Total concentration:	14.5235	ng/uL

```
Sample peak width (sec): 5    Sample min peak height: 50    Sample baseline V to V?: Y    Sample baseline V to V points: 3
Sample filter: Binomial       Number of points for filter: 3  Sample start region (min): 0  Sample end region (min): 30
Marker peak width (sec): 5    Marker min peak height: 200   Marker baseline V to V?: Y    Marker baseline V to V points: 3
Lower marker selection: First peak > 500 RFU                Upper marker selection: Last peak > 200 RFU
Ladder size (bp)75, 200, 300, 400, 500, 700, 1000, 1500, 2000, 3000, 4000, 5000, 7000, 10000, 20000
Quantification using: Lower Marker          Final concentration (ng/uL): 0.5000          Dilution factor: 12.0
```

Sample: SampC4
Well location: C4
Created: Tuesday, September 14, 2021 6:14:00 PM

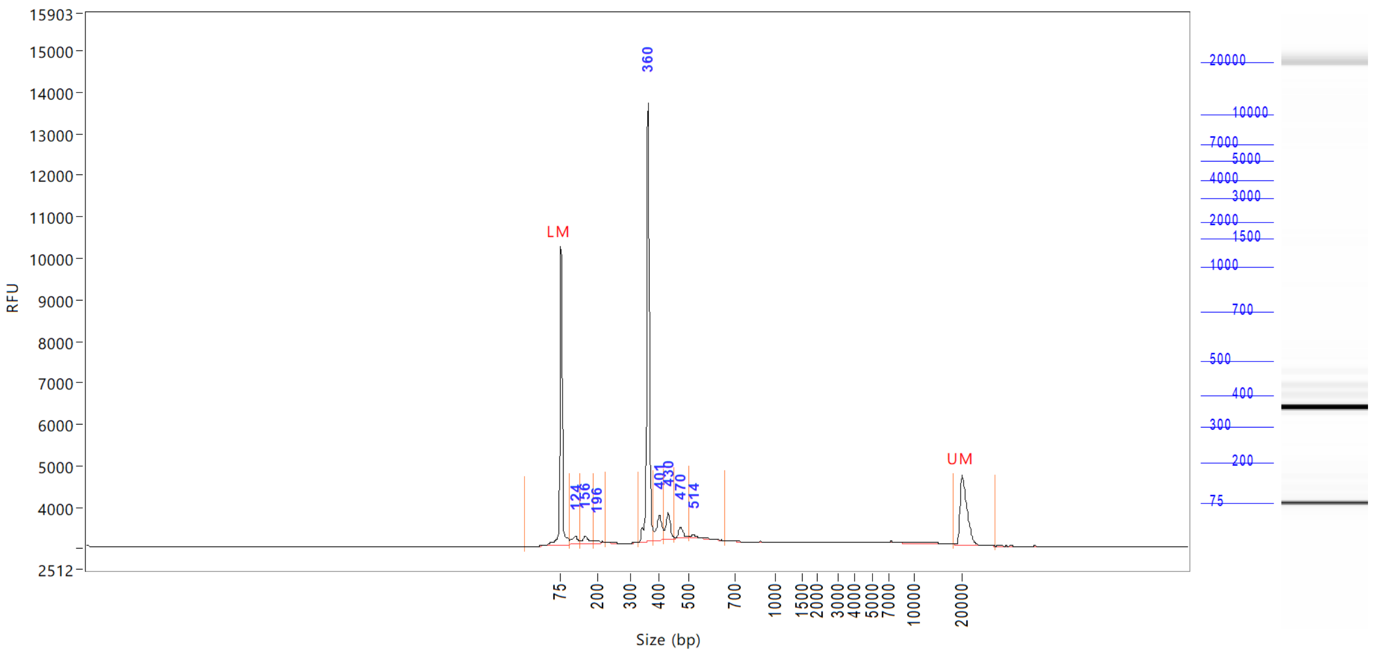

Peak	Size<br>(bp)	Concentration<br>(ng/uL)	From<br>(bp)	To<br>(bp)	Average size<br>(bp)	CV%	RFU	Corrected peak area
1	75 (LM)	0.5000	0	105	73	12.36	7183	44.257
2	124	0.4036	105	139	120	7.53	201	2.977
3	156	0.3936	139	187	159	7.76	197	2.903
4	196	0.1234	187	224	199	4.29	74	0.910
5	360	7.7193	325	382	358	1.74	10579	56.939
6	401	0.9145	382	414	398	2.07	591	6.745
7	430	0.8000	414	450	427	1.63	637	5.901
8	470	0.3250	450	500	468	1.66	266	2.397
9	514	0.1298	500	657	543	6.07	57	0.958
10	20000 (UM)	0.1744	18049	27133	20449	4.09	1693	15.438

TIC:	10.8092	ng/uL
TIM:	54.4863	nmole/L
Total concentration:	11.2596	ng/uL

Sample peak width (sec): 5 Sample min peak height: 50 Sample baseline V to V?: Y Sample baseline V to V points: 3
Sample filter: Binomial Number of points for filter: 3 Sample start region (min): 0 Sample end region (min): 30
Marker peak width (sec): 5 Marker min peak height: 200 Marker baseline V to V?: Y Marker baseline V to V points: 3
Lower marker selection: First peak > 500 RFU Upper marker selection: Last peak > 200 RFU
Ladder size (bp)75, 200, 300, 400, 500, 700, 1000, 1500, 2000, 3000, 4000, 5000, 7000, 10000, 20000
Quantification using: Lower Marker Final concentration (ng/uL): 0.5000 Dilution factor: 12.0

Sample: SampD4
Well location: D4
Created: Tuesday, September 14, 2021 6:14:00 PM

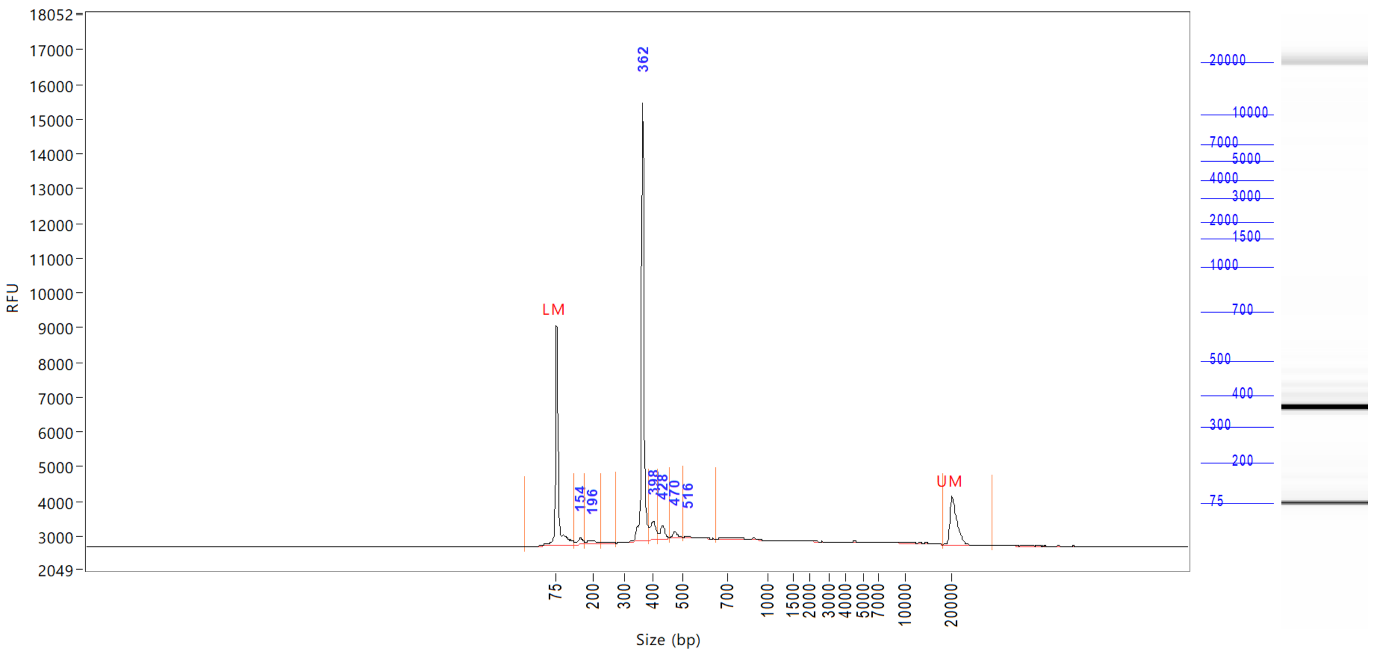

Peak	Size (bp)	Concentration (ng/uL)	From (bp)	To (bp)	Average size (bp)	CV%	RFU	Corrected peak area
1	75 (LM)	0.5000	0	135	77	18.39	6314	44.404
2	154	0.3286	135	171	153	6.22	179	2.432
3	196	0.3175	171	223	194	7.04	99	2.350
4	362	9.6016	271	384	359	2.03	12625	71.058
5	398	0.8792	384	416	398	2.15	532	6.506
6	428	0.5015	416	452	428	1.78	384	3.711
7	470	0.2127	452	498	470	1.74	167	1.574
8	516	0.1066	498	644	541	5.91	51	0.789
9	20000 (UM)	0.1479	18171	28584	20578	4.57	1417	13.135

TIC:	11.9477	ng/uL
TIM:	56.8526	nmole/L
Total concentration:	12.4614	ng/uL

Sample peak width (sec): 5 Sample min peak height: 50 Sample baseline V to V?: Y Sample baseline V to V points: 3
Sample filter: Binomial Number of points for filter: 3 Sample start region (min): 0 Sample end region (min): 30
Marker peak width (sec): 5 Marker min peak height: 200 Marker baseline V to V?: Y Marker baseline V to V points: 3
Lower marker selection: First peak > 500 RFU Upper marker selection: Last peak > 200 RFU
Ladder size (bp)75, 200, 300, 400, 500, 700, 1000, 1500, 2000, 3000, 4000, 5000, 7000, 10000, 20000
Quantification using: Lower Marker Final concentration (ng/uL): 0.5000 Dilution factor: 12.0

Sample: SampE4
Well location: E4
Created: Tuesday, September 14, 2021 6:14:00 PM

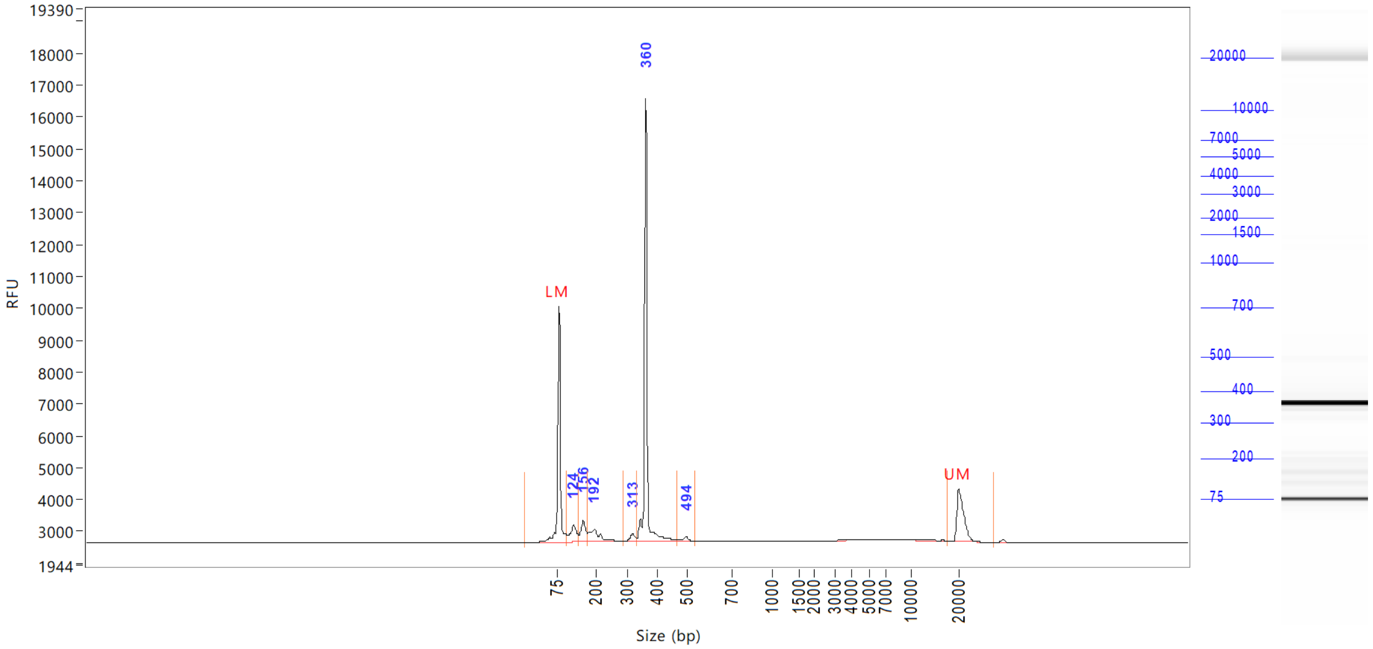

Peak	Size (bp)	Concentration (ng/uL)	From (bp)	To (bp)	Average size (bp)	CV%	RFU	Corrected peak area
1	75 (LM)	0.5000	0	103	72	15.67	7376	47.748
2	124	1.0052	103	139	121	7.86	551	7.999
3	156	1.0114	139	169	154	5.14	690	8.049
4	192	1.1498	169	282	196	10.45	373	9.150
5	313	0.3108	282	327	311	2.68	242	2.473
6	360	9.9040	327	468	361	4.75	13909	78.817
7	494	0.2284	468	532	493	2.81	138	1.818
8	20000 (UM)	0.1556	17561	27496	20420	4.07	1641	14.855

TIC:	13.6096	ng/uL
TIM:	81.6293	nmole/L
Total concentration:	13.9725	ng/uL

```
Sample peak width (sec): 5    Sample min peak height: 50    Sample baseline V to V?: Y    Sample baseline V to V points: 3
Sample filter: Binomial       Number of points for filter: 3  Sample start region (min): 0  Sample end region (min): 30
Marker peak width (sec): 5    Marker min peak height: 200   Marker baseline V to V?: Y    Marker baseline V to V points: 3
Lower marker selection: First peak > 500 RFU                Upper marker selection: Last peak > 200 RFU
Ladder size (bp)75, 200, 300, 400, 500, 700, 1000, 1500, 2000, 3000, 4000, 5000, 7000, 10000, 20000
Quantification using: Lower Marker          Final concentration (ng/uL): 0.5000          Dilution factor: 12.0
```

Sample: SampF4
Well location: F4
Created: Tuesday, September 14, 2021 6:14:00 PM

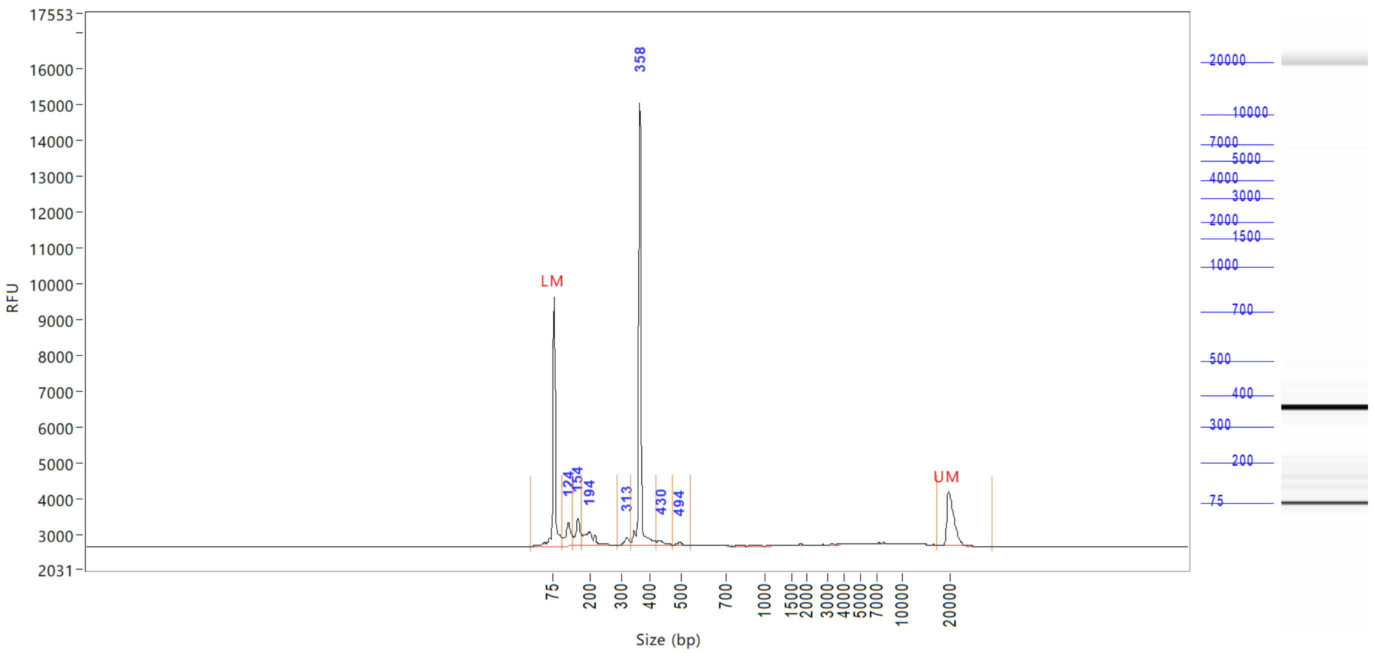

Peak	Size (bp)	Concentration (ng/uL)	From (bp)	To (bp)	Average size (bp)	CV%	RFU	Corrected peak area
1	75 (LM)	0.5000	0	103	73	14.14	6936	44.758
2	124	1.1883	103	141	122	8.04	636	8.864
3	154	1.1045	141	169	155	4.82	757	8.239
4	194	1.3075	169	284	196	10.29	397	9.754
5	313	0.2962	284	327	312	2.68	212	2.209
6	358	8.8755	327	418	358	2.83	12373	66.209
7	430	0.2580	418	471	435	2.92	127	1.924
8	494	0.1188	471	539	492	1.93	95	0.886
9	20000 (UM)	0.1589	17439	29189	20439	4.46	1519	14.220

TIC:	13.1488	ng/uL
TIM:	82.4038	nmole/L
Total concentration:	13.3885	ng/uL

Sample peak width (sec): 5 Sample min peak height: 50 Sample baseline V to V?: Y Sample baseline V to V points: 3
Sample filter: Binomial Number of points for filter: 3 Sample start region (min): 0 Sample end region (min): 30
Marker peak width (sec): 5 Marker min peak height: 200 Marker baseline V to V?: Y Marker baseline V to V points: 3
Lower marker selection: First peak > 500 RFU Upper marker selection: Last peak > 200 RFU
Ladder size (bp)75, 200, 300, 400, 500, 700, 1000, 1500, 2000, 3000, 4000, 5000, 7000, 10000, 20000
Quantification using: Lower Marker Final concentration (ng/uL): 0.5000 Dilution factor: 12.0

Sample: SampG4
Well location: G4
Created: Tuesday, September 14, 2021 6:14:00 PM

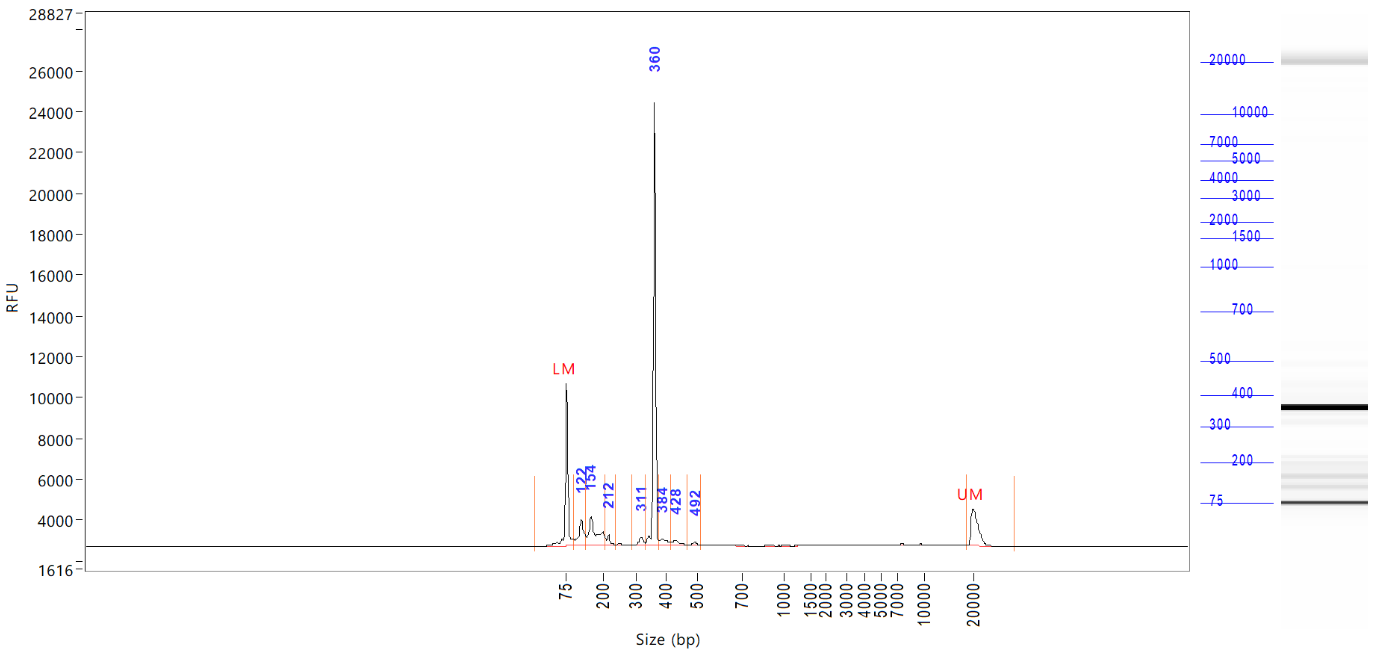

Peak	Size (bp)	Concentration (ng/uL)	From (bp)	To (bp)	Average size (bp)	CV%	RFU	Corrected peak area
1	75 (LM)	0.5000	0	101	72	17.09	7920	51.498
2	122	1.8378	101	141	122	8.08	1230	15.774
3	154	2.8843	141	203	167	10.48	1385	24.756
4	212	0.3589	203	233	210	2.29	489	3.081
5	311	0.4560	284	327	310	2.62	400	3.914
6	360	12.3321	327	374	355	1.46	21696	105.847
7	384	0.6013	374	416	393	3.11	278	5.161
8	428	0.3515	416	470	431	2.42	231	3.017
9	492	0.1371	470	516	489	1.23	148	1.177
10	20000 (UM)	0.1604	18659	28826	20541	4.24	1807	16.523

TIC:	18.9590	ng/uL
TIM:	119.8388	nmole/L
Total concentration:	19.2122	ng/uL

Sample peak width (sec): 5   Sample min peak height: 50   Sample baseline V to V?: Y   Sample baseline V to V points: 3
Sample filter: Binomial   Number of points for filter: 3   Sample start region (min): 0   Sample end region (min): 30
Marker peak width (sec): 5   Marker min peak height: 200   Marker baseline V to V?: Y   Marker baseline V to V points: 3
Lower marker selection: First peak > 500 RFU   Upper marker selection: Last peak > 200 RFU
Ladder size (bp)75, 200, 300, 400, 500, 700, 1000, 1500, 2000, 3000, 4000, 5000, 7000, 10000, 20000
Quantification using: Lower Marker   Final concentration (ng/uL): 0.5000   Dilution factor: 12.0

Sample: SampH4
Well location: H4
Created: Tuesday, September 14, 2021 6:14:00 PM

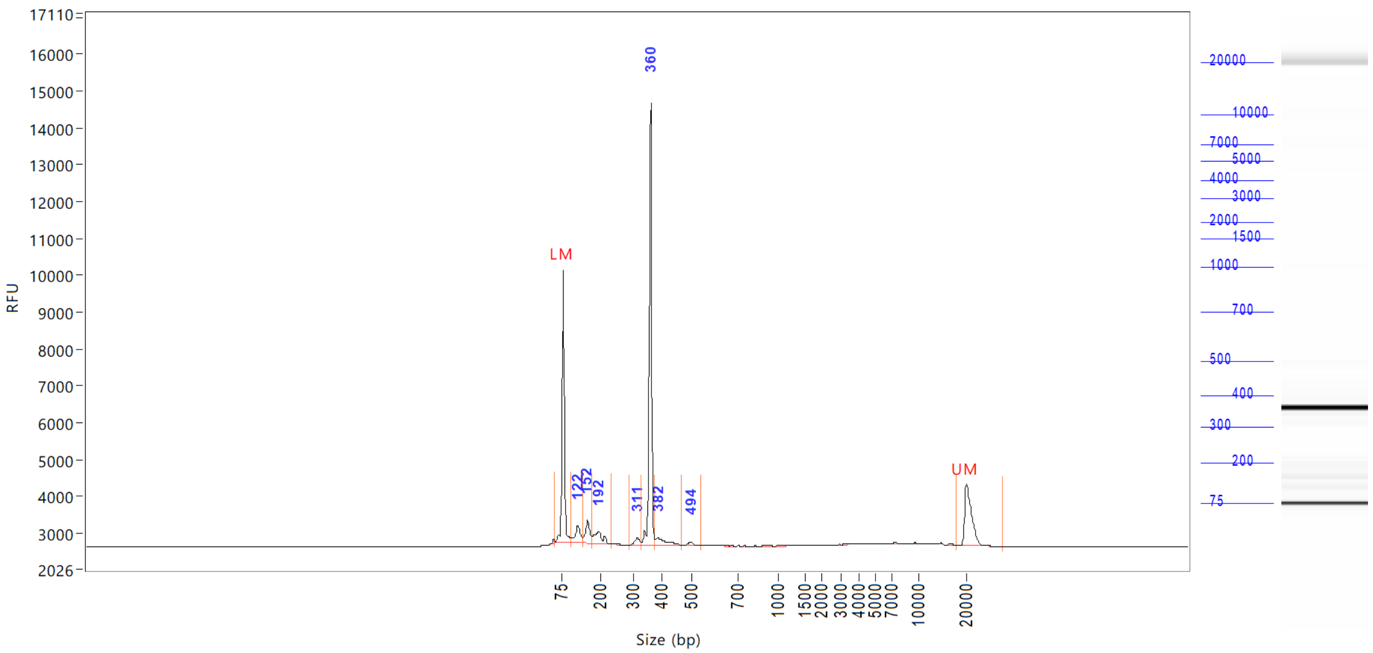

Peak	Size (bp)	Concentration (ng/uL)	From (bp)	To (bp)	Average size (bp)	CV%	RFU	Corrected peak area
1	75 (LM)	0.5000	50	103	73	8.13	7380	42.825
2	122	0.7958	103	139	121	7.18	464	5.680
3	152	0.9030	139	169	154	4.91	615	6.445
4	192	0.9085	169	232	190	6.74	318	6.484
5	311	0.3007	287	327	311	2.74	204	2.146
6	360	8.4492	327	374	356	1.62	12023	60.306
7	382	0.6640	374	466	404	5.52	194	4.739
8	494	0.1422	466	539	489	1.97	105	1.015
9	20000 (UM)	0.1769	17805	27738	20502	3.97	1669	15.150

TIC:	12.1634	ng/uL
TIM:	72.1221	nmole/L
Total concentration:	12.4840	ng/uL

Sample peak width (sec): 5   Sample min peak height: 50   Sample baseline V to V?: Y   Sample baseline V to V points: 3
Sample filter: Binomial   Number of points for filter: 3   Sample start region (min): 0   Sample end region (min): 30
Marker peak width (sec): 5   Marker min peak height: 200   Marker baseline V to V?: Y   Marker baseline V to V points: 3
Lower marker selection: First peak > 500 RFU   Upper marker selection: Last peak > 200 RFU
Ladder size (bp)75, 200, 300, 400, 500, 700, 1000, 1500, 2000, 3000, 4000, 5000, 7000, 10000, 20000
Quantification using: Lower Marker   Final concentration (ng/uL): 0.5000   Dilution factor: 12.0

Sample: SampA5
Well location: A5
Created: Tuesday, September 14, 2021 6:14:00 PM

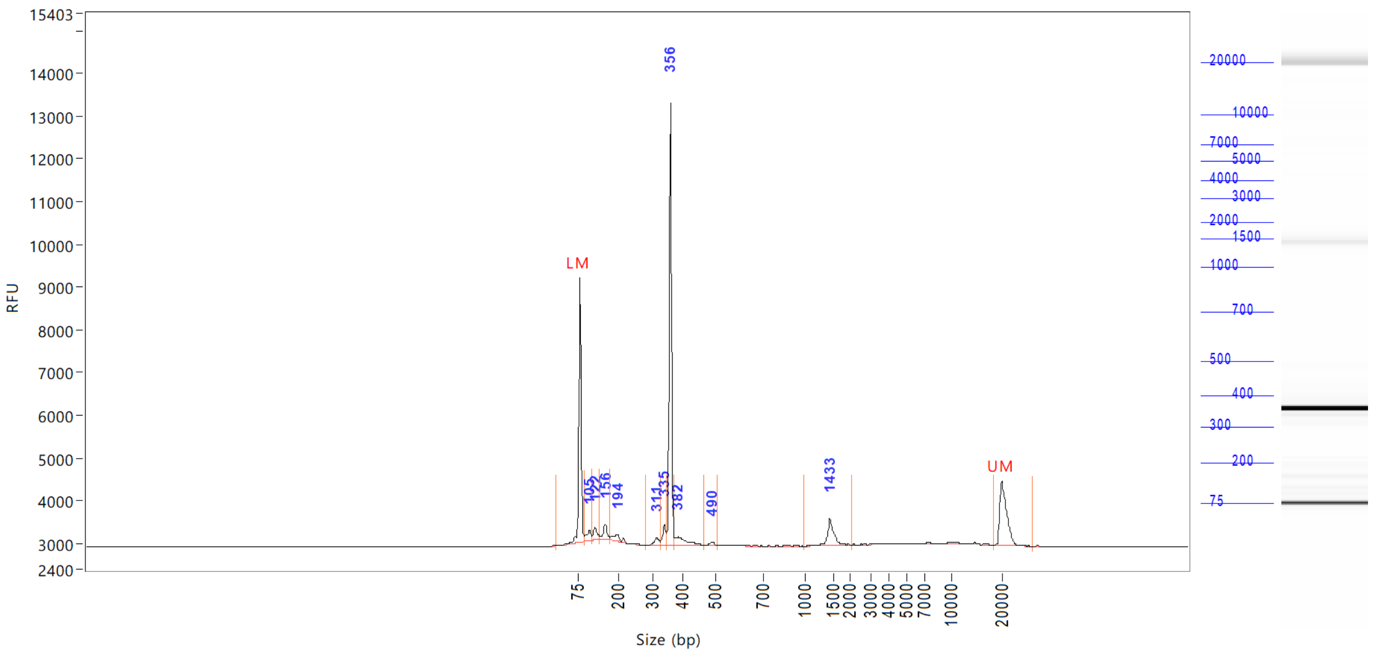

Peak	Size (bp)	Concentration (ng/uL)	From (bp)	To (bp)	Average size (bp)	CV%	RFU	Corrected peak area
1	75 (LM)	0.5000	5	89	73	8.06	6178	35.318
2	105	0.4381	89	112	100	6.50	239	2.579
3	122	0.4732	112	139	123	5.56	279	2.786
4	156	0.5076	139	169	154	4.42	344	2.988
5	194	0.4779	169	277	194	9.33	158	2.813
6	311	0.3093	277	323	308	2.85	167	1.821
7	335	0.5513	323	343	335	1.60	498	3.245
8	356	8.5745	343	372	355	1.14	10361	50.472
9	382	0.7184	372	466	399	5.26	190	4.229
10	490	0.1135	466	506	487	1.40	76	0.668
11	1433	1.0239	991	2087	1458	8.11	622	6.027
12	20000 (UM)	0.1826	18537	26166	20402	3.78	1485	12.900

TIC:	13.1877	ng/uL
TIM:	71.5793	nmole/L
Total concentration:	13.4442	ng/uL

```
Sample peak width (sec): 5    Sample min peak height: 50    Sample baseline V to V?: Y    Sample baseline V to V points: 3
Sample filter: Binomial       Number of points for filter: 3  Sample start region (min): 0  Sample end region (min): 30
Marker peak width (sec): 5    Marker min peak height: 200   Marker baseline V to V?: Y    Marker baseline V to V points: 3
Lower marker selection: First peak > 500 RFU                Upper marker selection: Last peak > 200 RFU
Ladder size (bp)75, 200, 300, 400, 500, 700, 1000, 1500, 2000, 3000, 4000, 5000, 7000, 10000, 20000
Quantification using: Lower Marker          Final concentration (ng/uL): 0.5000          Dilution factor: 12.0
```

Sample: SampB5
Well location: B5
Created: Tuesday, September 14, 2021 6:14:00 PM

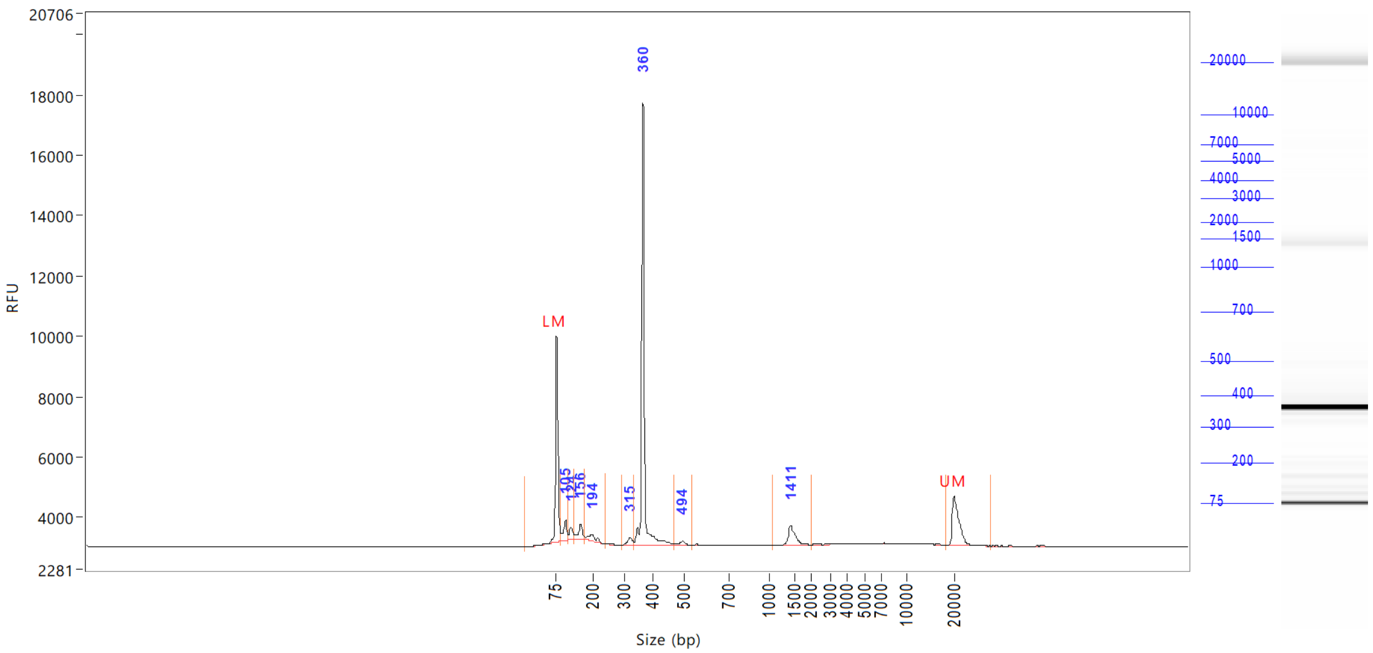

Peak	Size (bp)	Concentration (ng/uL)	From (bp)	To (bp)	Average size (bp)	CV%	RFU	Corrected peak area
1	75 (LM)	0.5000	0	91	74	8.38	6831	40.936
2	105	0.8548	91	112	101	5.25	689	5.832
3	124	0.5406	112	135	123	4.88	394	3.689
4	156	0.7206	135	171	152	5.40	494	4.917
5	194	0.5381	171	235	193	6.41	218	3.672
6	315	0.3922	287	329	313	2.81	251	2.676
7	360	12.6631	329	470	362	4.79	14680	86.396
8	494	0.2462	470	537	494	2.86	130	1.680
9	1411	1.1168	1068	2001	1444	7.47	653	7.620
10	20000 (UM)	0.1819	18415	27859	20510	4.35	1627	14.889

TIC:	17.0726	ng/uL
TIM:	95.2003	nmole/L
Total concentration:	17.4107	ng/uL

Sample peak width (sec): 5   Sample min peak height: 50   Sample baseline V to V?: Y   Sample baseline V to V points: 3
Sample filter: Binomial   Number of points for filter: 3 Sample start region (min): 0 Sample end region (min): 30
Marker peak width (sec): 5   Marker min peak height: 200   Marker baseline V to V?: Y   Marker baseline V to V points: 3
Lower marker selection: First peak > 500 RFU   Upper marker selection: Last peak > 200 RFU
Ladder size (bp)75, 200, 300, 400, 500, 700, 1000, 1500, 2000, 3000, 4000, 5000, 7000, 10000, 20000
Quantification using: Lower Marker   Final concentration (ng/uL): 0.5000   Dilution factor: 12.0

Sample: SampC5
Well location: C5
Created: Tuesday, September 14, 2021 6:14:00 PM

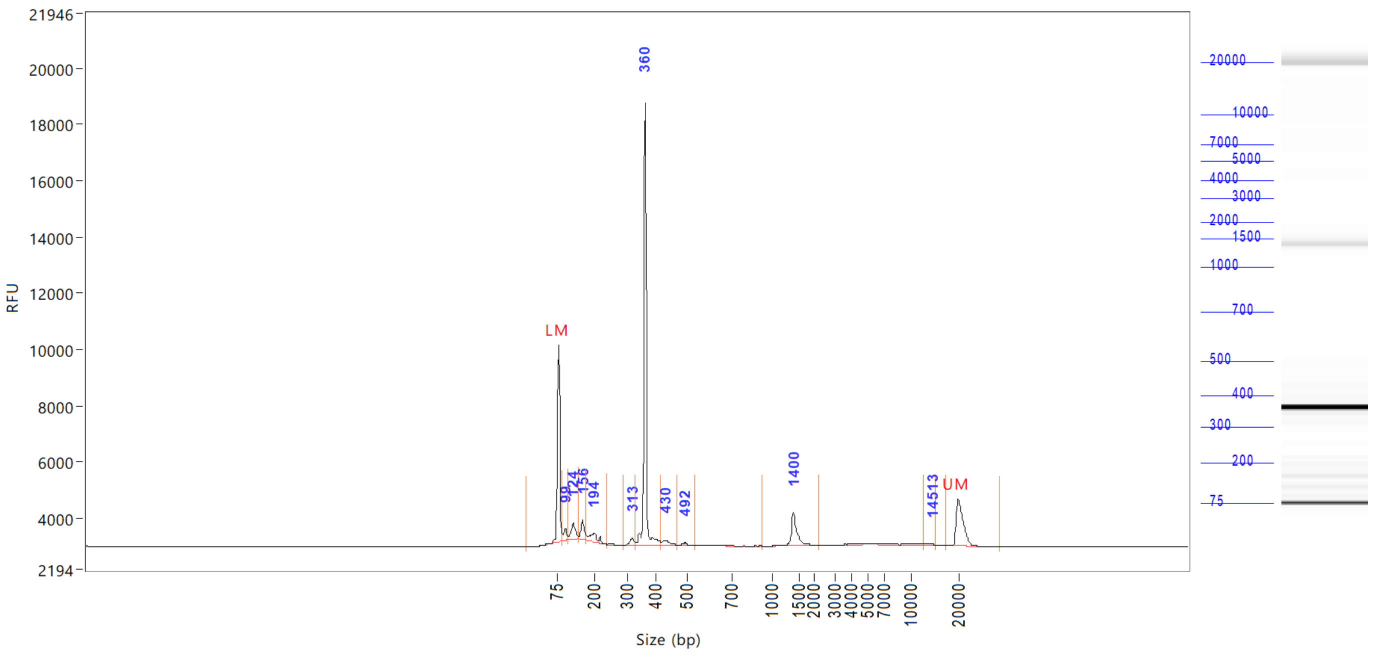

Peak	Size (bp)	Concentration (ng/uL)	From (bp)	To (bp)	Average size (bp)	CV%	RFU	Corrected peak area
1	75 (LM)	0.5000	0	91	74	8.61	6965	40.546
2	99	0.4345	91	107	97	4.19	406	2.937
3	124	0.9027	107	141	123	6.20	569	6.100
4	156	0.8551	141	169	155	4.14	679	5.778
5	194	0.8319	169	235	191	6.64	317	5.622
6	313	0.3823	284	327	311	2.70	247	2.583
7	360	12.1174	327	416	359	2.89	15745	81.885
8	430	0.4098	416	468	433	2.80	187	2.769
9	492	0.1333	468	537	489	2.08	90	0.901
10	1400	1.5161	931	2201	1427	7.87	1188	10.245
11	14513	0.0706	12684	15366	13867	4.91	50	0.477
12	20000 (UM)	0.1908	17561	28826	20424	4.21	1687	15.474

TIC:	17.6536	ng/uL
TIM:	96.9553	nmole/L
Total concentration:	17.9755	ng/uL

```
Sample peak width (sec): 5    Sample min peak height: 50    Sample baseline V to V?: Y    Sample baseline V to V points: 3
Sample filter: Binomial       Number of points for filter: 3  Sample start region (min): 0  Sample end region (min): 30
Marker peak width (sec): 5    Marker min peak height: 200   Marker baseline V to V?: Y    Marker baseline V to V points: 3
Lower marker selection: First peak > 500 RFU                Upper marker selection: Last peak > 200 RFU
Ladder size (bp)75, 200, 300, 400, 500, 700, 1000, 1500, 2000, 3000, 4000, 5000, 7000, 10000, 20000
Quantification using: Lower Marker          Final concentration (ng/uL): 0.5000          Dilution factor: 12.0
```

Sample: SampD5
Well location: D5
Created: Tuesday, September 14, 2021 6:14:00 PM

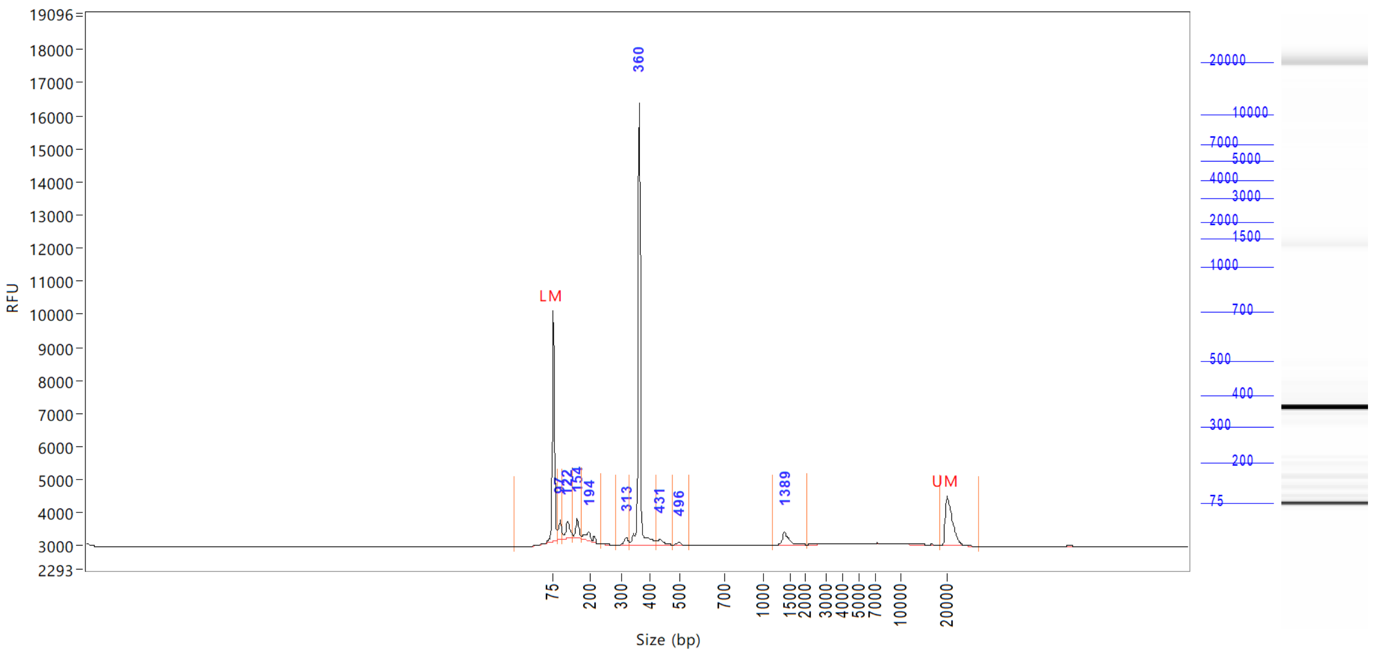

Peak	Size (bp)	Concentration (ng/uL)	From (bp)	To (bp)	Average size (bp)	CV%	RFU	Corrected peak area
1	75 (LM)	0.5000	0	89	73	9.19	6966	39.984
2	97	0.6756	89	107	96	4.58	586	4.502
3	122	0.8155	107	143	123	6.48	503	5.434
4	154	0.6897	143	169	154	3.75	586	4.596
5	194	0.6737	169	235	192	6.43	267	4.489
6	313	0.3356	284	329	312	2.80	211	2.236
7	360	10.7537	329	418	360	3.04	13389	71.662
8	431	0.3667	418	475	435	3.00	169	2.443
9	496	0.1119	475	539	494	1.89	77	0.746
10	1389	0.6429	1178	2030	1432	8.00	410	4.285
11	20000 (UM)	0.1757	18537	26770	20520	4.28	1511	14.052

TIC:	15.0653	ng/uL
TIM:	88.9897	nmole/L
Total concentration:	15.4536	ng/uL

Sample peak width (sec): 5   Sample min peak height: 50   Sample baseline V to V?: Y   Sample baseline V to V points: 3
Sample filter: Binomial   Number of points for filter: 3 Sample start region (min): 0 Sample end region (min): 30
Marker peak width (sec): 5   Marker min peak height: 200   Marker baseline V to V?: Y   Marker baseline V to V points: 3
Lower marker selection: First peak > 500 RFU   Upper marker selection: Last peak > 200 RFU
Ladder size (bp)75, 200, 300, 400, 500, 700, 1000, 1500, 2000, 3000, 4000, 5000, 7000, 10000, 20000
Quantification using: Lower Marker   Final concentration (ng/uL): 0.5000   Dilution factor: 12.0

Sample: SampE5
Well location: E5
Created: Tuesday, September 14, 2021 6:14:00 PM

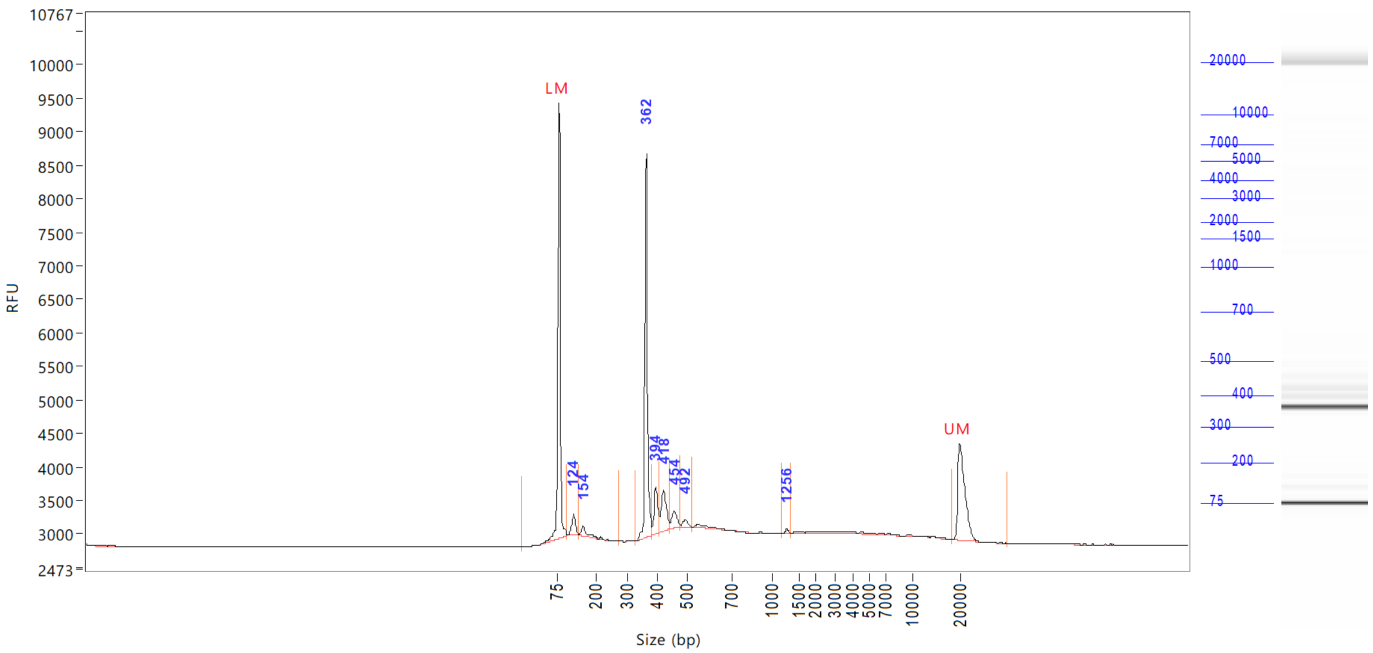

Peak	Size (bp)	Concentration (ng/uL)	From (bp)	To (bp)	Average size (bp)	CV%	RFU	Corrected peak area
1	75 (LM)	0.5000	0	103	73	10.79	6507	37.899
2	124	0.4510	103	141	121	5.20	326	2.849
3	154	0.3135	141	269	170	14.37	143	1.980
4	362	4.9254	323	380	359	1.67	5717	31.111
5	394	0.9379	380	407	394	1.70	693	5.924
6	418	1.0169	407	441	420	1.93	606	6.423
7	454	0.4250	441	475	454	1.71	258	2.685
8	492	0.1720	475	519	490	1.52	109	1.086
9	1256	0.0505	1167	1345	1246	2.40	60	0.319
10	20000 (UM)	0.1775	18293	29914	20548	4.47	1450	13.454

TIC:	8.2922	ng/uL
TIM:	41.8115	nmole/L
Total concentration:	8.8721	ng/uL

Sample peak width (sec): 5   Sample min peak height: 50   Sample baseline V to V?: Y   Sample baseline V to V points: 3
Sample filter: Binomial   Number of points for filter: 3 Sample start region (min): 0 Sample end region (min): 30
Marker peak width (sec): 5   Marker min peak height: 200   Marker baseline V to V?: Y   Marker baseline V to V points: 3
Lower marker selection: First peak > 500 RFU   Upper marker selection: Last peak > 200 RFU
Ladder size (bp)75, 200, 300, 400, 500, 700, 1000, 1500, 2000, 3000, 4000, 5000, 7000, 10000, 20000
Quantification using: Lower Marker   Final concentration (ng/uL): 0.5000   Dilution factor: 12.0

Sample: SampF5
Well location: F5
Created: Tuesday, September 14, 2021 6:14:00 PM

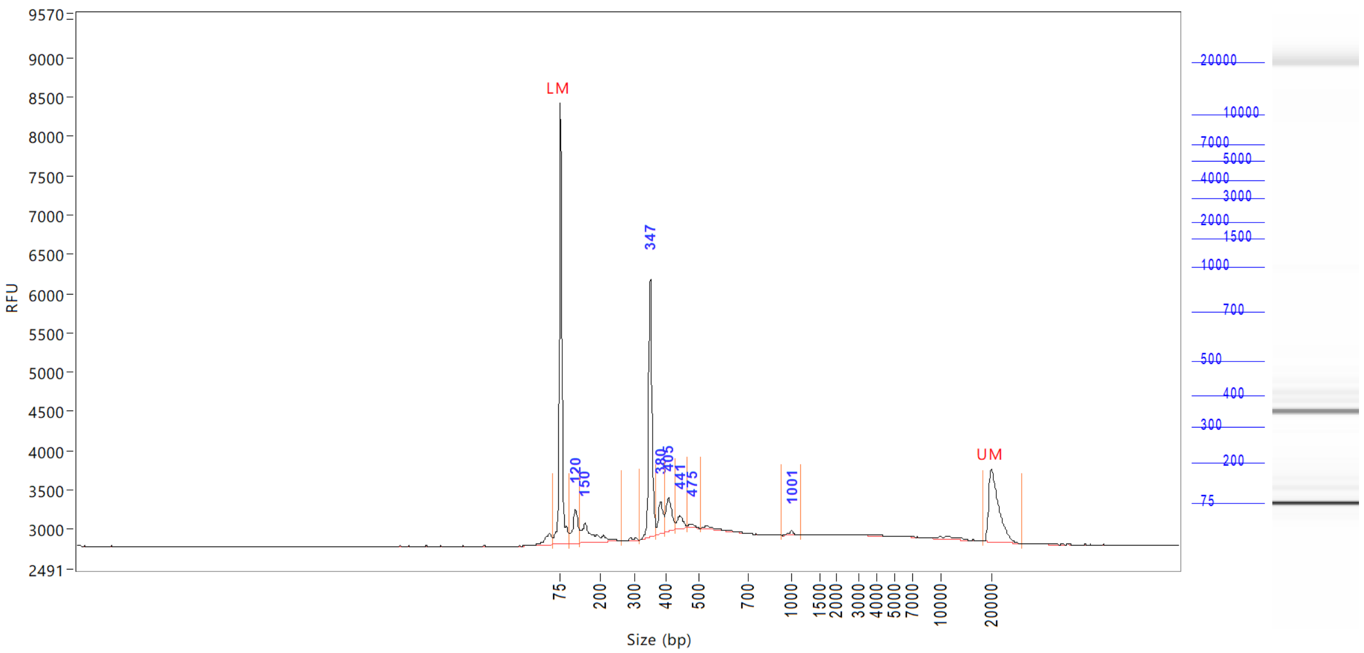

Peak	Size (bp)	Concentration (ng/uL)	From (bp)	To (bp)	Average size (bp)	CV%	RFU	Corrected peak area
1	75 (LM)	0.5000	50	103	74	8.99	5626	36.098
2	120	0.9234	103	137	120	7.21	429	5.555
3	150	1.0568	137	259	171	15.78	249	6.358
4	347	3.8986	311	366	345	1.81	3283	23.455
5	380	0.6882	366	394	380	1.95	409	4.140
6	405	0.8426	394	428	407	2.02	430	5.069
7	441	0.3270	428	464	442	2.00	160	1.968
8	475	0.0953	464	504	475	1.47	53	0.573
9	1001	0.0866	935	1167	999	3.31	63	0.521
10	20000 (UM)	0.1600	18415	26166	20710	5.45	919	11.549

TIC:	7.9185	ng/uL
TIM:	49.4670	nmole/L
Total concentration:	8.4202	ng/uL

```
Sample peak width (sec): 5     Sample min peak height: 50     Sample baseline V to V?: Y     Sample baseline V to V points: 3
Sample filter: Binomial        Number of points for filter: 3  Sample start region (min): 0  Sample end region (min): 30
Marker peak width (sec): 5     Marker min peak height: 200    Marker baseline V to V?: Y     Marker baseline V to V points: 3
Lower marker selection: First peak > 500 RFU                  Upper marker selection: Last peak > 200 RFU
Ladder size (bp)75, 200, 300, 400, 500, 700, 1000, 1500, 2000, 3000, 4000, 5000, 7000, 10000, 20000
Quantification using: Lower Marker          Final concentration (ng/uL): 0.5000          Dilution factor: 12.0
```

Sample: SampG5
Well location: G5
Created: Tuesday, September 14, 2021 6:14:00 PM

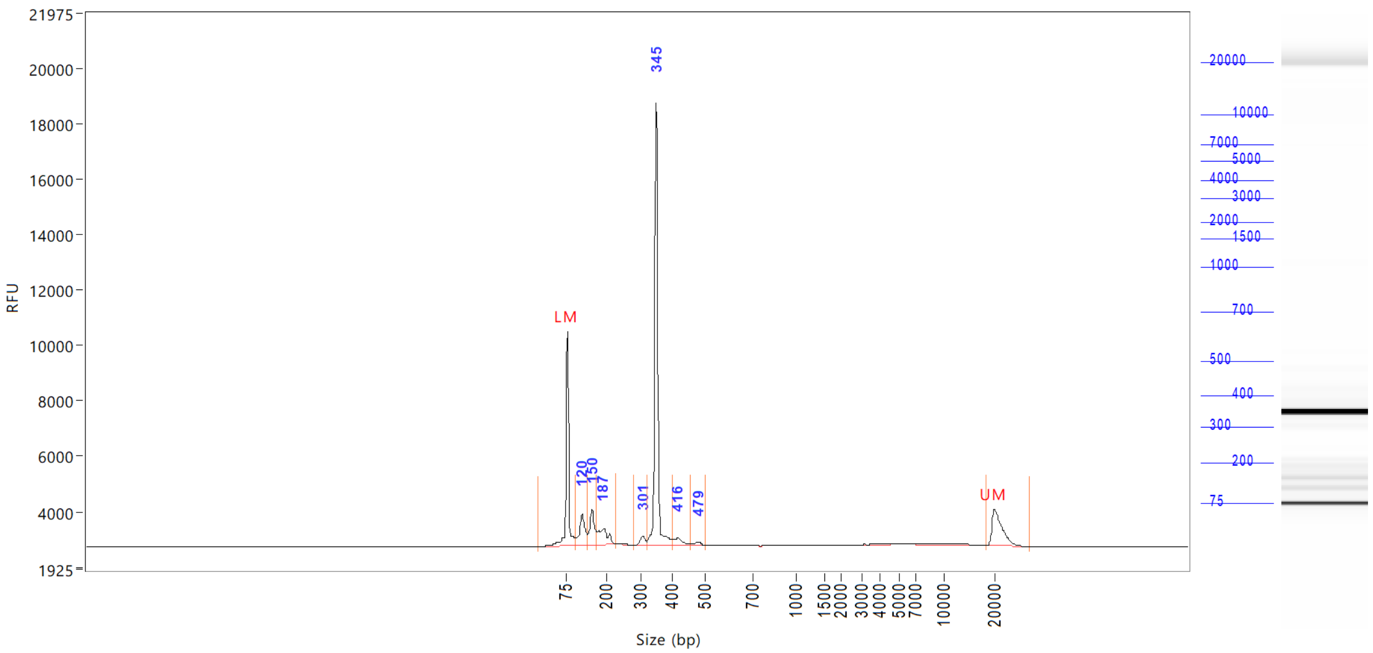

Peak	Size (bp)	Concentration (ng/uL)	From (bp)	To (bp)	Average size (bp)	CV%	RFU	Corrected peak area
1	75 (LM)	0.5000	0	99	72	15.38	7704	51.851
2	120	1.7399	99	137	119	7.83	1155	15.036
3	150	1.6669	137	166	151	4.91	1283	14.405
4	187	1.4894	166	226	186	6.89	588	12.871
5	301	0.4104	275	315	300	2.52	319	3.547
6	345	12.3684	315	401	345	3.00	15975	106.885
7	416	0.4672	401	456	419	3.02	251	4.038
8	479	0.1386	456	500	475	1.67	115	1.198
9	20000 (UM)	0.1619	18415	27012	20748	5.47	1327	16.788

TIC:	18.2808	ng/uL
TIM:	118.8701	nmole/L
Total concentration:	18.6472	ng/uL

Sample peak width (sec): 5 Sample min peak height: 50 Sample baseline V to V?: Y Sample baseline V to V points: 3
Sample filter: Binomial Number of points for filter: 3 Sample start region (min): 0 Sample end region (min): 30
Marker peak width (sec): 5 Marker min peak height: 200 Marker baseline V to V?: Y Marker baseline V to V points: 3
Lower marker selection: First peak > 500 RFU Upper marker selection: Last peak > 200 RFU
Ladder size (bp)75, 200, 300, 400, 500, 700, 1000, 1500, 2000, 3000, 4000, 5000, 7000, 10000, 20000
Quantification using: Lower Marker Final concentration (ng/uL): 0.5000 Dilution factor: 12.0

Sample: SampH5
Well location: H5
Created: Tuesday, September 14, 2021 6:14:00 PM

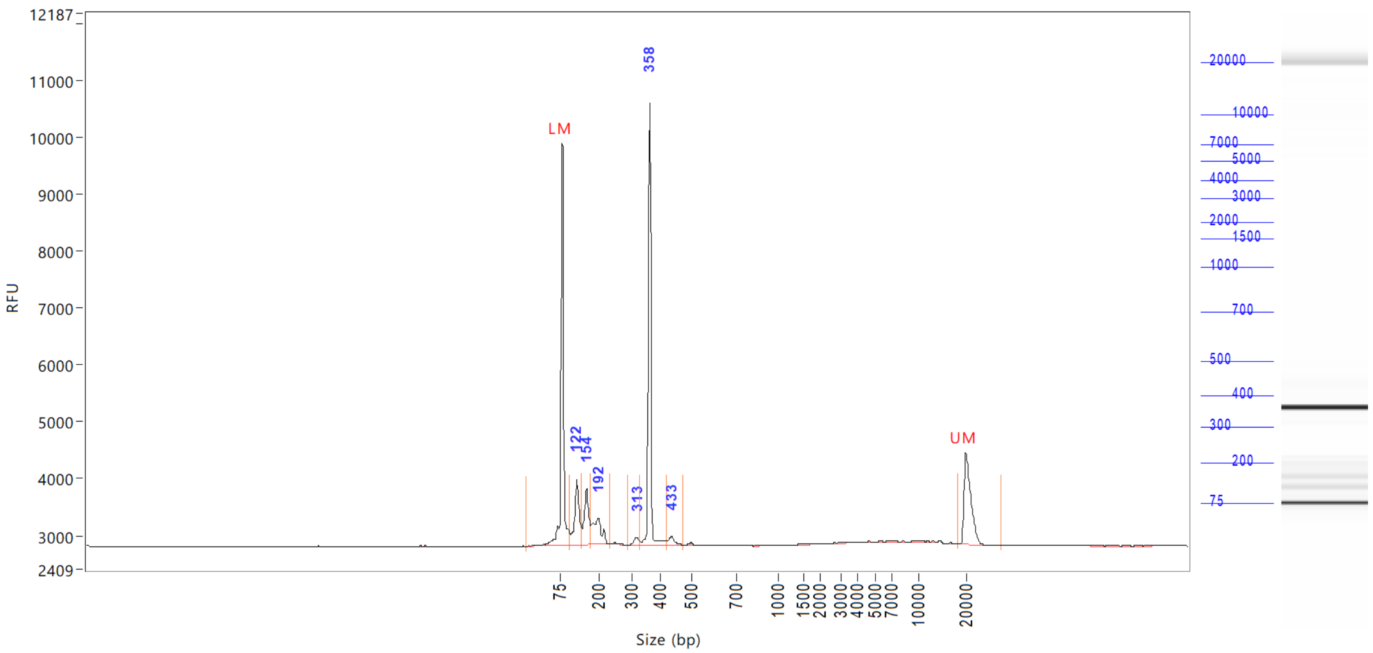

Peak	Size (bp)	Concentration (ng/uL)	From (bp)	To (bp)	Average size (bp)	CV%	RFU	Corrected peak area
1	75 (LM)	0.5000	0	103	73	15.14	7082	47.199
2	122	1.6457	103	141	122	7.06	1158	12.945
3	154	1.3138	141	169	154	4.75	986	10.335
4	192	1.1983	169	233	190	6.66	449	9.426
5	313	0.1937	287	329	312	2.81	138	1.524
6	358	5.1117	329	416	358	2.57	7794	40.211
7	433	0.2660	416	473	434	2.87	160	2.093
8	20000 (UM)	0.1559	18293	27375	20502	4.05	1602	14.721

TIC:	9.7292	ng/uL
TIM:	72.0715	nmole/L
Total concentration:	10.0833	ng/uL

Sample peak width (sec): 5 Sample min peak height: 50 Sample baseline V to V?: Y Sample baseline V to V points: 3
Sample filter: Binomial Number of points for filter: 3 Sample start region (min): 0 Sample end region (min): 30
Marker peak width (sec): 5 Marker min peak height: 200 Marker baseline V to V?: Y Marker baseline V to V points: 3
Lower marker selection: First peak > 500 RFU Upper marker selection: Last peak > 200 RFU
Ladder size (bp)75, 200, 300, 400, 500, 700, 1000, 1500, 2000, 3000, 4000, 5000, 7000, 10000, 20000
Quantification using: Lower Marker Final concentration (ng/uL): 0.5000 Dilution factor: 12.0

Sample: SampA6
Well location: A6
Created: Tuesday, September 14, 2021 6:14:00 PM

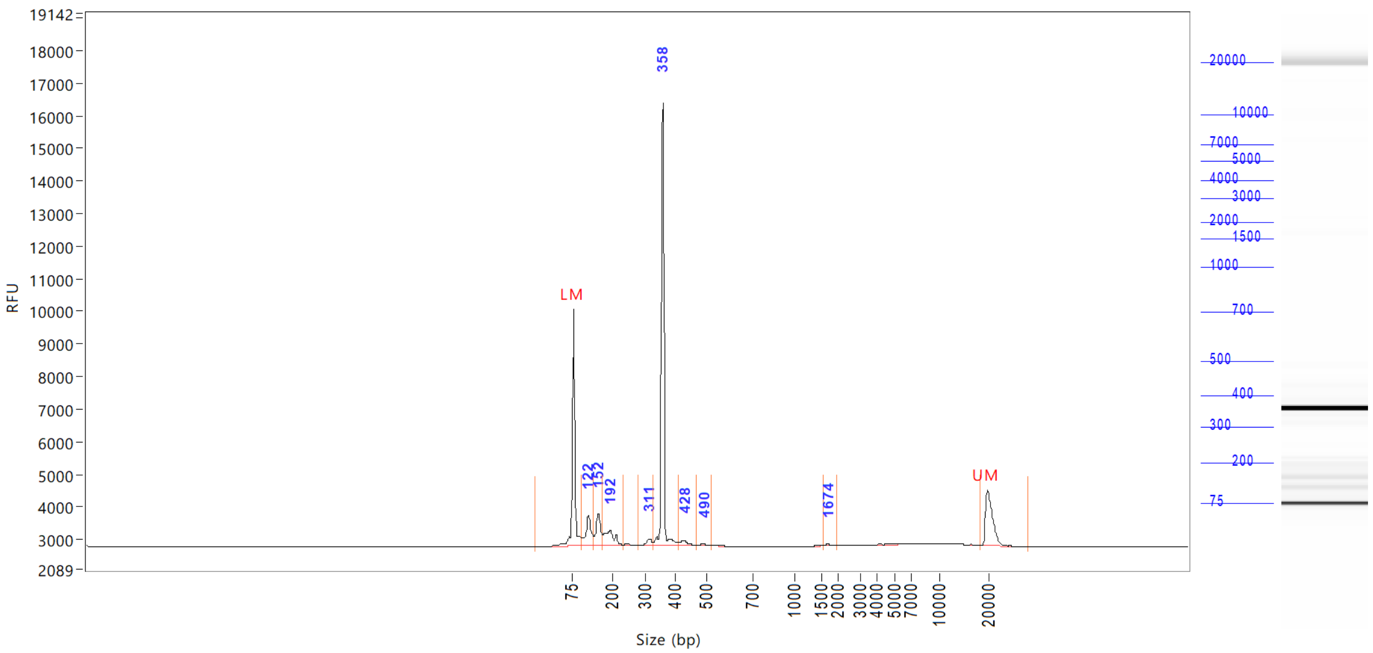

Peak	Size (bp)	Concentration (ng/uL)	From (bp)	To (bp)	Average size (bp)	CV%	RFU	Corrected peak area
1	75 (LM)	0.5000	0	101	72	15.37	7268	45.798
2	122	1.4991	101	139	121	7.67	934	11.443
3	152	1.3628	139	169	153	5.13	966	10.402
4	192	1.2518	169	232	190	6.74	457	9.555
5	311	0.3036	278	325	309	2.74	213	2.317
6	358	9.0476	325	412	356	2.66	13604	69.060
7	428	0.3153	412	470	429	2.72	174	2.407
8	490	0.0834	470	516	487	1.42	76	0.637
9	1674	0.0554	1520	1981	1680	5.81	59	0.423
10	20000 (UM)	0.1679	18537	28100	20427	4.29	1695	15.380

TIC:	13.9190	ng/uL
TIM:	90.7982	nmole/L
Total concentration:	14.1782	ng/uL

Sample peak width (sec): 5   Sample min peak height: 50   Sample baseline V to V?: Y   Sample baseline V to V points: 3
Sample filter: Binomial   Number of points for filter: 3   Sample start region (min): 0   Sample end region (min): 30
Marker peak width (sec): 5   Marker min peak height: 200   Marker baseline V to V?: Y   Marker baseline V to V points: 3
Lower marker selection: First peak > 500 RFU   Upper marker selection: Last peak > 200 RFU
Ladder size (bp)75, 200, 300, 400, 500, 700, 1000, 1500, 2000, 3000, 4000, 5000, 7000, 10000, 20000
Quantification using: Lower Marker   Final concentration (ng/uL): 0.5000   Dilution factor: 12.0

Sample: SampB6
Well location: B6
Created: Tuesday, September 14, 2021 6:14:00 PM

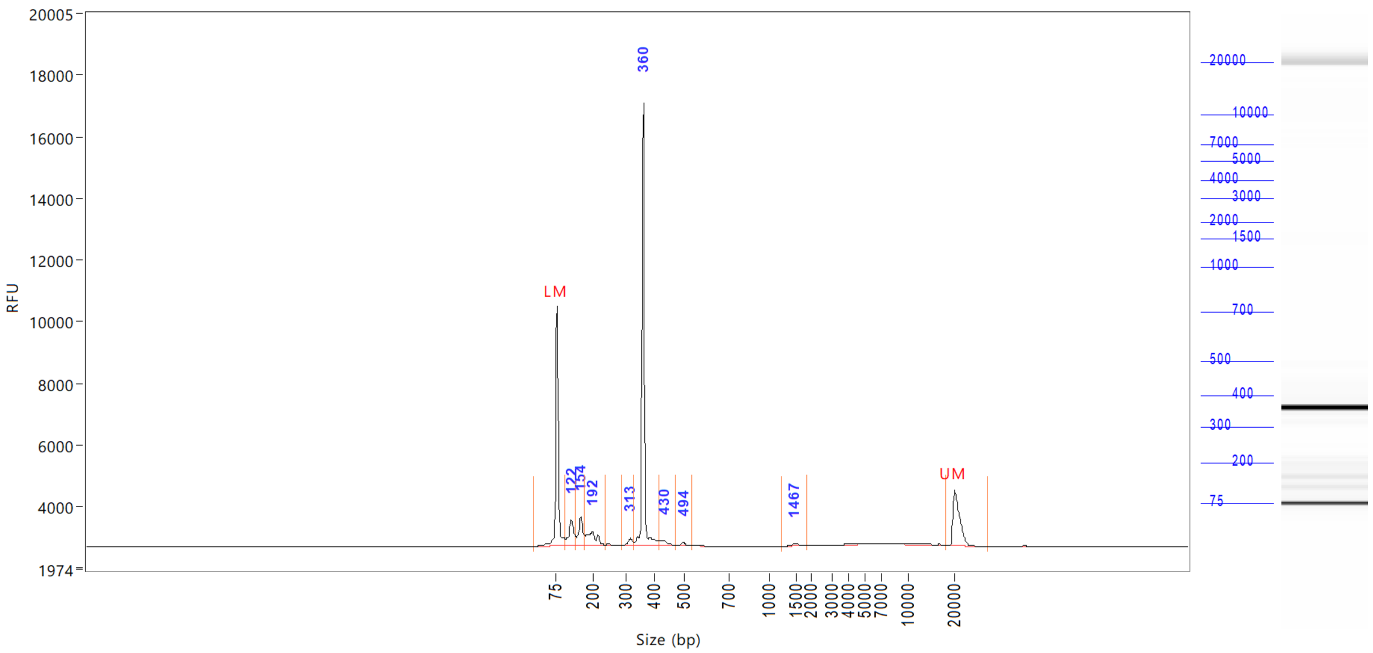

Peak	Size (bp)	Concentration (ng/uL)	From (bp)	To (bp)	Average size (bp)	CV%	RFU	Corrected peak area
1	75 (LM)	0.5000	0	101	73	12.48	7773	48.108
2	122	1.3167	101	139	121	7.85	818	10.557
3	154	1.2046	139	168	153	4.73	914	9.658
4	192	1.1520	168	233	189	7.20	420	9.237
5	313	0.3120	286	329	312	2.88	234	2.501
6	360	9.3644	329	416	358	2.92	14385	75.084
7	430	0.3231	416	471	433	2.86	176	2.590
8	494	0.1108	471	532	491	1.69	95	0.889
9	1467	0.0923	1223	1846	1498	7.34	59	0.740
10	20000 (UM)	0.1691	18049	26891	20503	4.09	1802	16.272

TIC:	13.8759	ng/uL
TIM:	87.2173	nmole/L
Total concentration:	14.1541	ng/uL

Sample peak width (sec): 5   Sample min peak height: 50   Sample baseline V to V?: Y   Sample baseline V to V points: 3
Sample filter: Binomial   Number of points for filter: 3   Sample start region (min): 0   Sample end region (min): 30
Marker peak width (sec): 5   Marker min peak height: 200   Marker baseline V to V?: Y   Marker baseline V to V points: 3
Lower marker selection: First peak > 500 RFU   Upper marker selection: Last peak > 200 RFU
Ladder size (bp)75, 200, 300, 400, 500, 700, 1000, 1500, 2000, 3000, 4000, 5000, 7000, 10000, 20000
Quantification using: Lower Marker   Final concentration (ng/uL): 0.5000   Dilution factor: 12.0

Sample: SampC6
Well location: C6
Created: Tuesday, September 14, 2021 6:14:00 PM

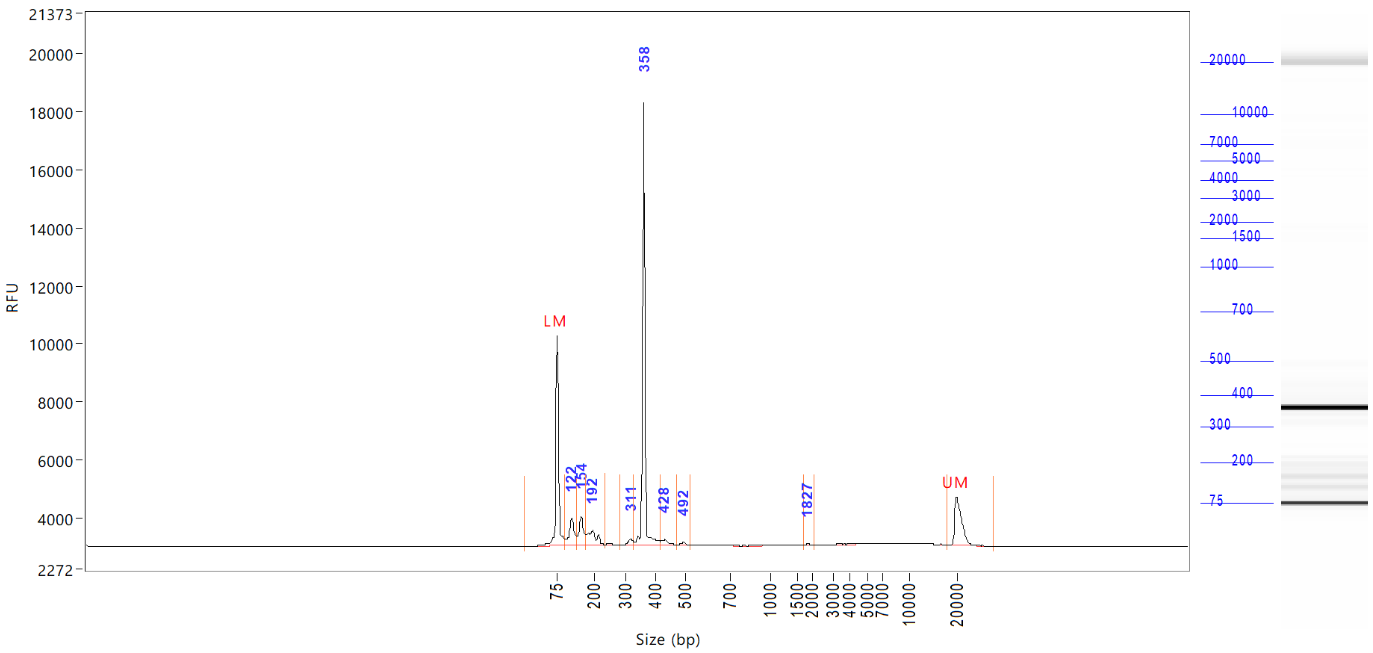

Peak	Size (bp)	Concentration (ng/uL)	From (bp)	To (bp)	Average size (bp)	CV%	RFU	Corrected peak area
1	75 (LM)	0.5000	0	101	72	14.12	7194	47.460
2	122	1.4430	101	139	121	7.64	906	11.414
3	154	1.3258	139	168	153	4.78	996	10.487
4	192	1.2735	168	230	188	7.05	477	10.073
5	311	0.3107	280	325	309	2.71	232	2.458
6	358	10.0246	325	414	357	2.89	15229	79.295
7	428	0.3581	414	470	432	2.93	190	2.832
8	492	0.1139	470	519	488	1.57	95	0.901
9	1827	0.0449	1712	2087	1848	4.65	56	0.355
10	20000 (UM)	0.1629	17927	27617	20420	4.16	1651	15.463

TIC:	14.8945	ng/uL
TIM:	94.6090	nmole/L
Total concentration:	15.1851	ng/uL

```
Sample peak width (sec): 5    Sample min peak height: 50    Sample baseline V to V?: Y    Sample baseline V to V points: 3
Sample filter: Binomial       Number of points for filter: 3  Sample start region (min): 0  Sample end region (min): 30
Marker peak width (sec): 5    Marker min peak height: 200   Marker baseline V to V?: Y    Marker baseline V to V points: 3
Lower marker selection: First peak > 500 RFU                Upper marker selection: Last peak > 200 RFU
Ladder size (bp)75, 200, 300, 400, 500, 700, 1000, 1500, 2000, 3000, 4000, 5000, 7000, 10000, 20000
Quantification using: Lower Marker          Final concentration (ng/uL): 0.5000          Dilution factor: 12.0
```

Sample: SampD6
Well location: D6
Created: Tuesday, September 14, 2021 6:14:00 PM

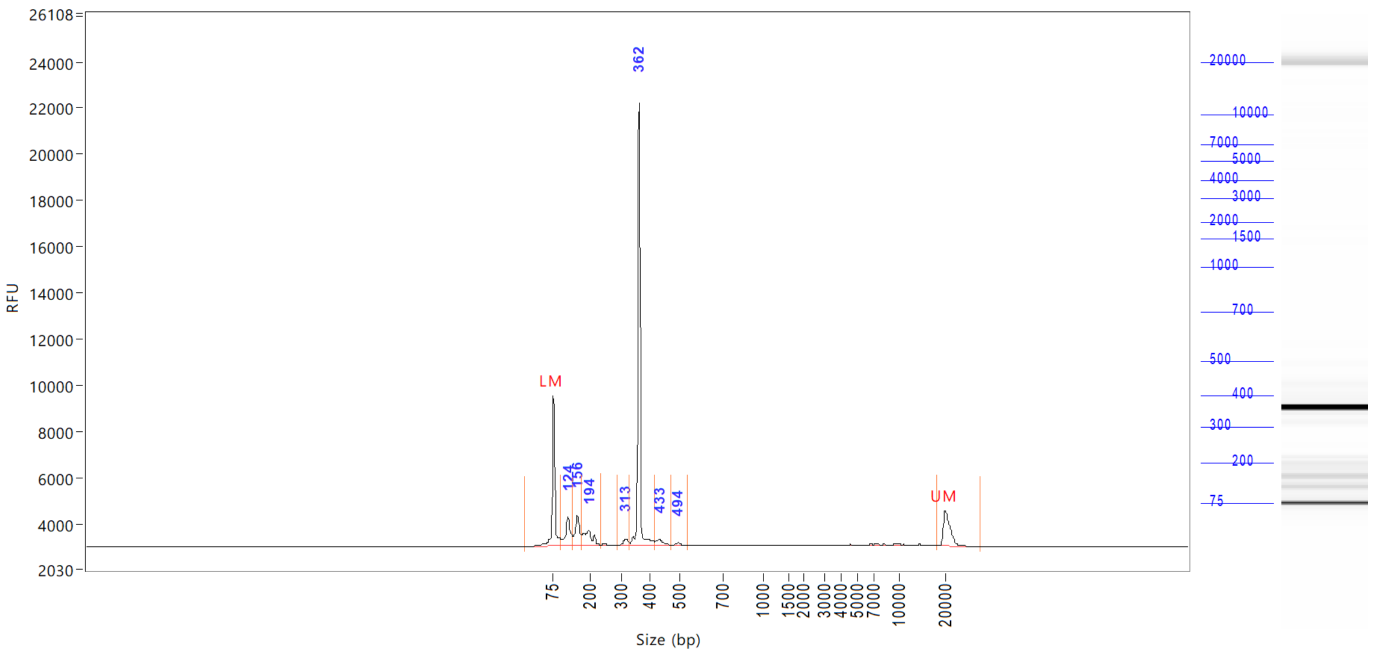

Peak	Size (bp)	Concentration (ng/uL)	From (bp)	To (bp)	Average size (bp)	CV%	RFU	Corrected peak area
1	75 (LM)	0.5000	0	101	73	15.41	6486	43.998
2	124	2.0243	101	141	122	7.83	1212	14.844
3	156	1.8797	141	169	155	4.63	1314	13.784
4	194	1.7658	169	233	191	6.90	615	12.948
5	313	0.4236	287	327	311	2.62	297	3.106
6	362	13.5815	327	416	359	2.68	19198	99.594
7	433	0.5118	416	475	434	2.97	262	3.753
8	494	0.1417	475	537	493	1.73	114	1.039
9	20000 (UM)	0.1587	18171	27617	20410	4.50	1549	13.965

TIC:	20.3284	ng/uL
TIM:	129.2846	nmole/L
Total concentration:	20.5957	ng/uL

Sample peak width (sec): 5 Sample min peak height: 50 Sample baseline V to V?: Y Sample baseline V to V points: 3
Sample filter: Binomial Number of points for filter: 3 Sample start region (min): 0 Sample end region (min): 30
Marker peak width (sec): 5 Marker min peak height: 200 Marker baseline V to V?: Y Marker baseline V to V points: 3
Lower marker selection: First peak > 500 RFU Upper marker selection: Last peak > 200 RFU
Ladder size (bp)75, 200, 300, 400, 500, 700, 1000, 1500, 2000, 3000, 4000, 5000, 7000, 10000, 20000
Quantification using: Lower Marker Final concentration (ng/uL): 0.5000 Dilution factor: 12.0

Sample: SampE6
Well location: E6
Created: Tuesday, September 14, 2021 6:14:00 PM

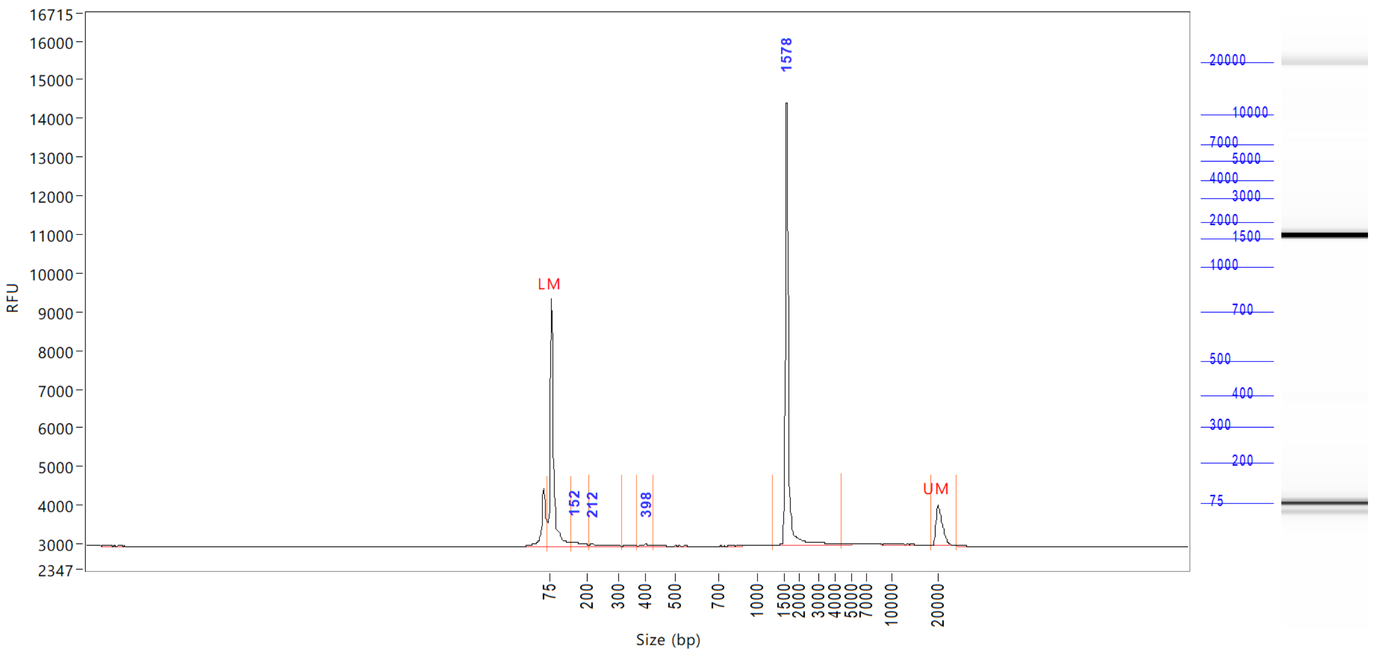

Peak	Size (bp)	Concentration (ng/uL)	From (bp)	To (bp)	Average size (bp)	CV%	RFU	Corrected peak area
1	75 (LM)	0.5000	62	143	78	16.57	6417	54.118
2	152	0.2769	143	201	167	10.04	109	2.498
3	212	0.1456	201	311	231	11.37	64	1.313
4	398	0.0723	368	424	392	2.90	65	0.652
5	1578	6.8984	1289	4345	1655	20.12	11457	62.222
6	20000 (UM)	0.0849	18537	23869	20379	3.84	1046	9.193

TIC:	7.3933	ng/uL
TIM:	10.9273	nmole/L
Total concentration:	7.6753	ng/uL

Sample peak width (sec): 5   Sample min peak height: 50   Sample baseline V to V?: Y   Sample baseline V to V points: 3
Sample filter: Binomial   Number of points for filter: 3   Sample start region (min): 0   Sample end region (min): 30
Marker peak width (sec): 5   Marker min peak height: 200   Marker baseline V to V?: Y   Marker baseline V to V points: 3
Lower marker selection: First peak > 500 RFU   Upper marker selection: Last peak > 200 RFU
Ladder size (bp)75, 200, 300, 400, 500, 700, 1000, 1500, 2000, 3000, 4000, 5000, 7000, 10000, 20000
Quantification using: Lower Marker   Final concentration (ng/uL): 0.5000   Dilution factor: 12.0

Sample: SampF6
Well location: F6
Created: Tuesday, September 14, 2021 6:14:00 PM

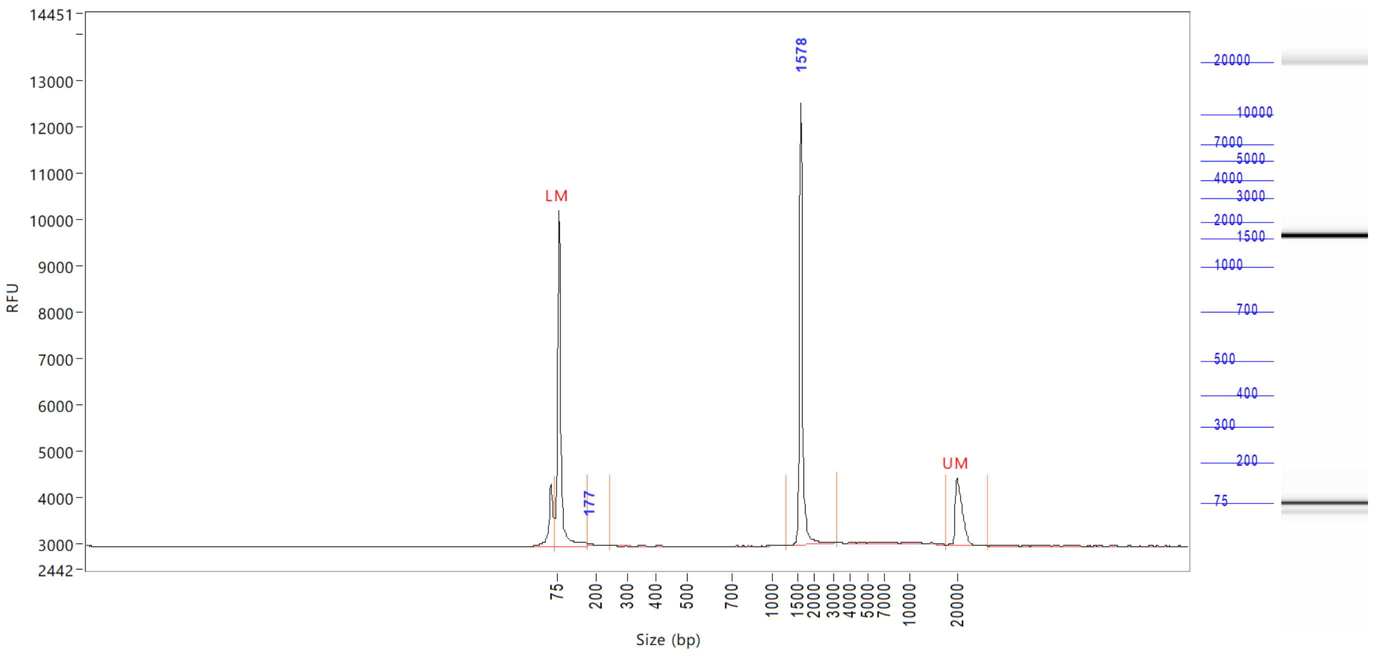

Peak	Size (bp)	Concentration (ng/uL)	From (bp)	To (bp)	Average size (bp)	CV%	RFU	Corrected peak area
1	75 (LM)	0.5000	64	169	79	20.69	7250	55.667
2	177	0.1173	169	241	189	8.09	62	1.089
3	1578	5.2941	1289	3108	1589	8.20	9560	49.118
4	20000 (UM)	0.1205	17805	26649	20493	4.16	1457	13.419

TIC:	5.4115	ng/uL
TIM:	6.5050	nmole/L
Total concentration:	5.7767	ng/uL

Sample peak width (sec): 5   Sample min peak height: 50   Sample baseline V to V?: Y   Sample baseline V to V points: 3
Sample filter: Binomial   Number of points for filter: 3 Sample start region (min): 0 Sample end region (min): 30
Marker peak width (sec): 5   Marker min peak height: 200   Marker baseline V to V?: Y   Marker baseline V to V points: 3
Lower marker selection: First peak > 500 RFU   Upper marker selection: Last peak > 200 RFU
Ladder size (bp)75, 200, 300, 400, 500, 700, 1000, 1500, 2000, 3000, 4000, 5000, 7000, 10000, 20000
Quantification using: Lower Marker   Final concentration (ng/uL): 0.5000   Dilution factor: 12.0

Sample: SampG6
Well location: G6
Created: Tuesday, September 14, 2021 6:14:00 PM

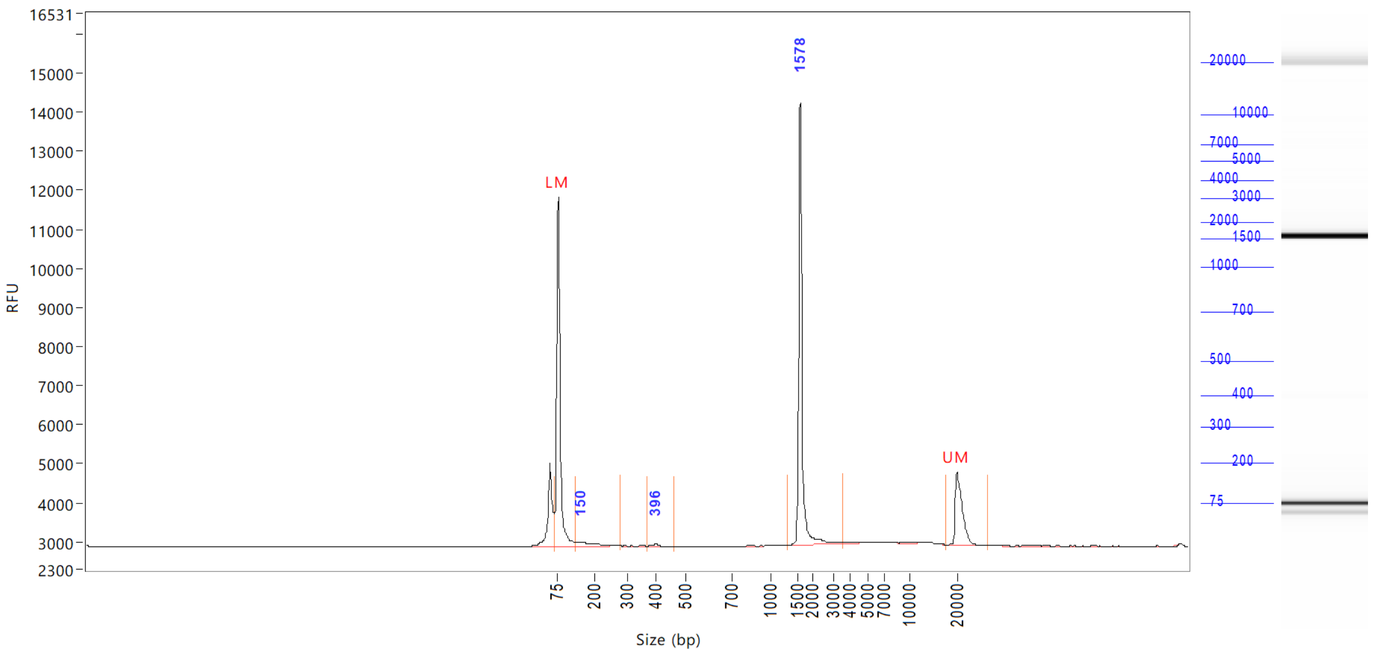

Peak	Size (bp)	Concentration (ng/uL)	From (bp)	To (bp)	Average size (bp)	CV%	RFU	Corrected peak area
1	75 (LM)	0.5000	64	131	76	13.31	8947	71.598
2	150	0.3555	131	277	172	18.07	109	4.242
3	396	0.0686	366	460	399	4.07	81	0.819
4	1578	5.2873	1300	3572	1600	13.00	11334	63.093
5	20000 (UM)	0.1214	17683	26408	20526	4.39	1850	17.381

TIC:	5.7114	ng/uL
TIM:	9.1202	nmole/L
Total concentration:	5.9499	ng/uL

```
Sample peak width (sec): 5    Sample min peak height: 50    Sample baseline V to V?: Y    Sample baseline V to V points: 3
Sample filter: Binomial       Number of points for filter: 3  Sample start region (min): 0  Sample end region (min): 30
Marker peak width (sec): 5    Marker min peak height: 200   Marker baseline V to V?: Y    Marker baseline V to V points: 3
Lower marker selection: First peak > 500 RFU                Upper marker selection: Last peak > 200 RFU
Ladder size (bp)75, 200, 300, 400, 500, 700, 1000, 1500, 2000, 3000, 4000, 5000, 7000, 10000, 20000
Quantification using: Lower Marker          Final concentration (ng/uL): 0.5000          Dilution factor: 12.0
```

Sample: SampH6
Well location: H6
Created: Tuesday, September 14, 2021 6:14:00 PM

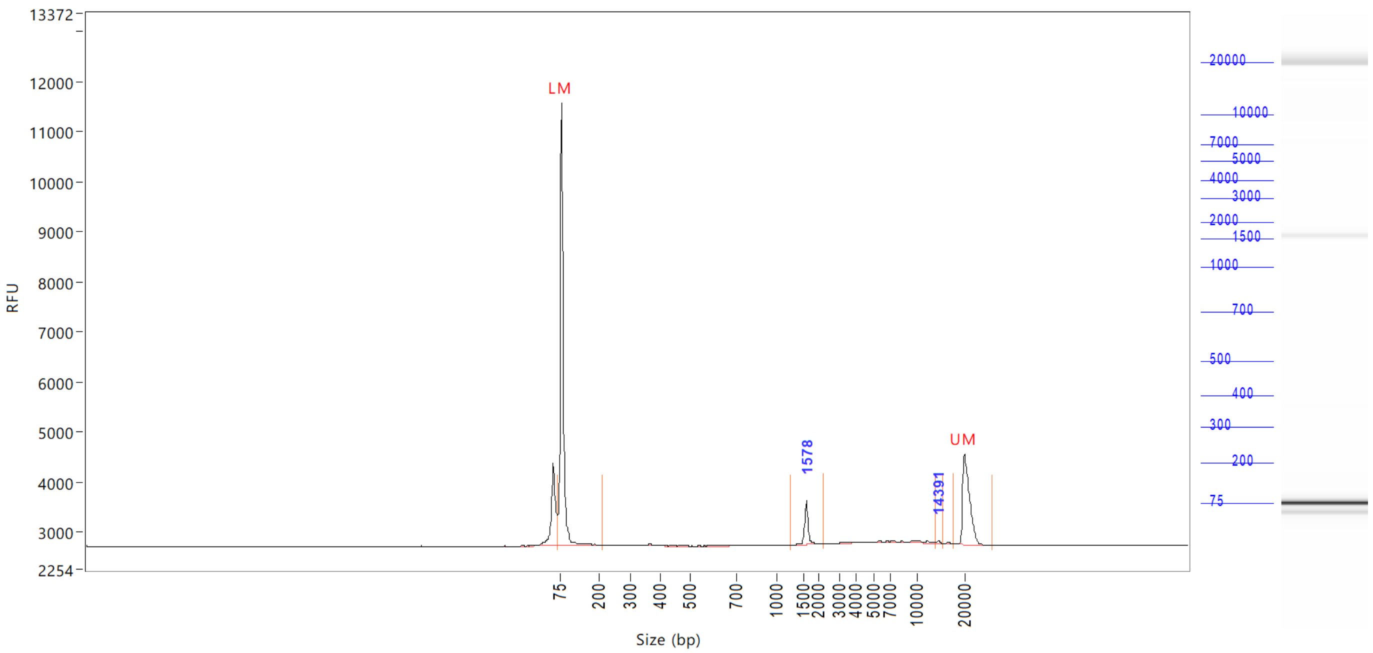

Peak	Size (bp)	Concentration (ng/uL)	From (bp)	To (bp)	Average size (bp)	CV%	RFU	Corrected peak area
1	75 (LM)	0.5000	64	208	77	16.72	8867	65.775
2	1578	0.4610	1267	2201	1581	6.80	886	5.054
3	14391	0.0226	13903	15488	14444	2.41	51	0.247
4	20000 (UM)	0.1285	17561	25924	20464	4.04	1814	16.910

TIC:	0.4836	ng/uL
TIM:	0.4826	nmole/L
Total concentration:	0.8238	ng/uL

Sample peak width (sec): 5   Sample min peak height: 50   Sample baseline V to V?: Y   Sample baseline V to V points: 3
Sample filter: Binomial   Number of points for filter: 3 Sample start region (min): 0 Sample end region (min): 30
Marker peak width (sec): 5   Marker min peak height: 200   Marker baseline V to V?: Y   Marker baseline V to V points: 3
Lower marker selection: First peak > 500 RFU   Upper marker selection: Last peak > 200 RFU
Ladder size (bp)75, 200, 300, 400, 500, 700, 1000, 1500, 2000, 3000, 4000, 5000, 7000, 10000, 20000
Quantification using: Lower Marker   Final concentration (ng/uL): 0.5000   Dilution factor: 12.0

Sample: SampA7
Well location: A7
Created: Tuesday, September 14, 2021 6:14:00 PM

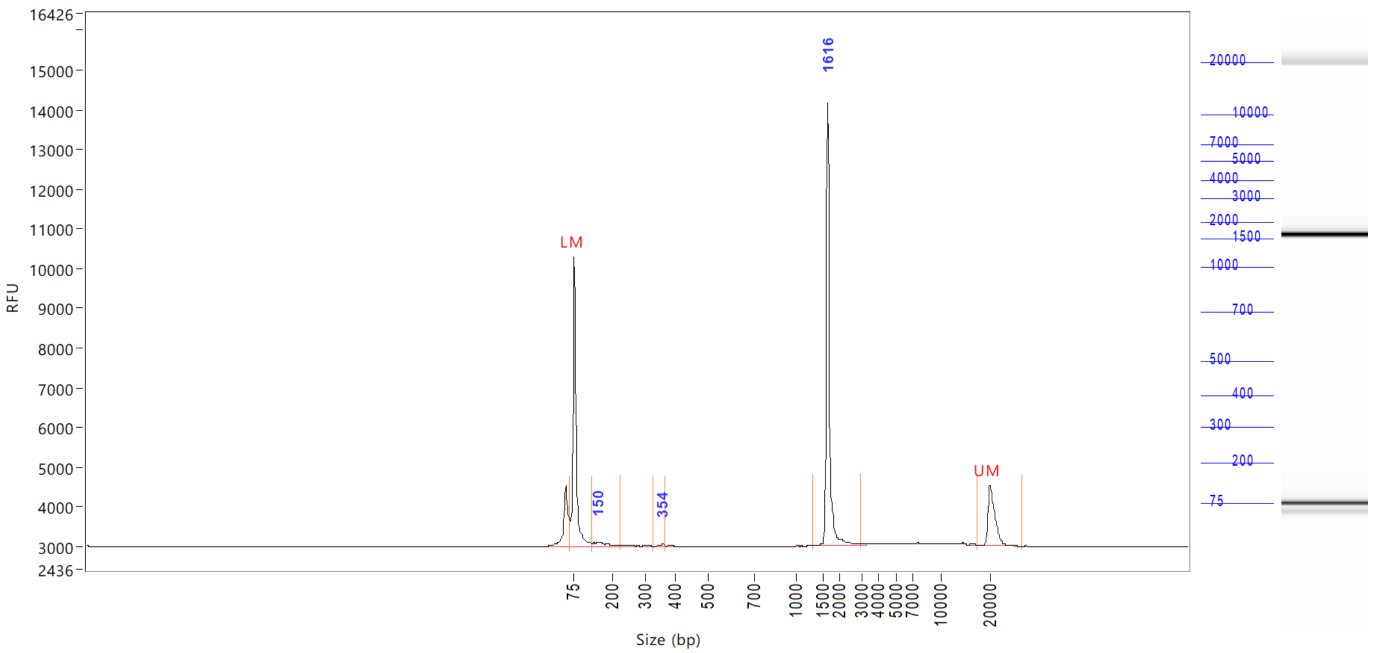

Peak	Size (bp)	Concentration (ng/uL)	From (bp)	To (bp)	Average size (bp)	CV%	RFU	Corrected peak area
1	75 (LM)	0.5000	64	131	77	13.48	7290	54.590
2	150	0.3380	131	223	164	14.40	91	3.075
3	354	0.0446	325	364	349	2.25	60	0.406
4	1616	6.2939	1289	2971	1632	9.22	11155	57.265
5	20000 (UM)	0.1233	17561	26649	20518	4.11	1509	13.460

TIC:	6.6766	ng/uL
TIM:	9.9462	nmole/L
Total concentration:	6.9888	ng/uL

```
Sample peak width (sec): 5    Sample min peak height: 50    Sample baseline V to V?: Y    Sample baseline V to V points: 3
Sample filter: Binomial       Number of points for filter: 3  Sample start region (min): 0  Sample end region (min): 30
Marker peak width (sec): 5    Marker min peak height: 200   Marker baseline V to V?: Y    Marker baseline V to V points: 3
Lower marker selection: First peak > 500 RFU                Upper marker selection: Last peak > 200 RFU
Ladder size (bp)75, 200, 300, 400, 500, 700, 1000, 1500, 2000, 3000, 4000, 5000, 7000, 10000, 20000
Quantification using: Lower Marker          Final concentration (ng/uL): 0.5000          Dilution factor: 12.0
```

Sample: SampB7
Well location: B7
Created: Tuesday, September 14, 2021 6:14:00 PM

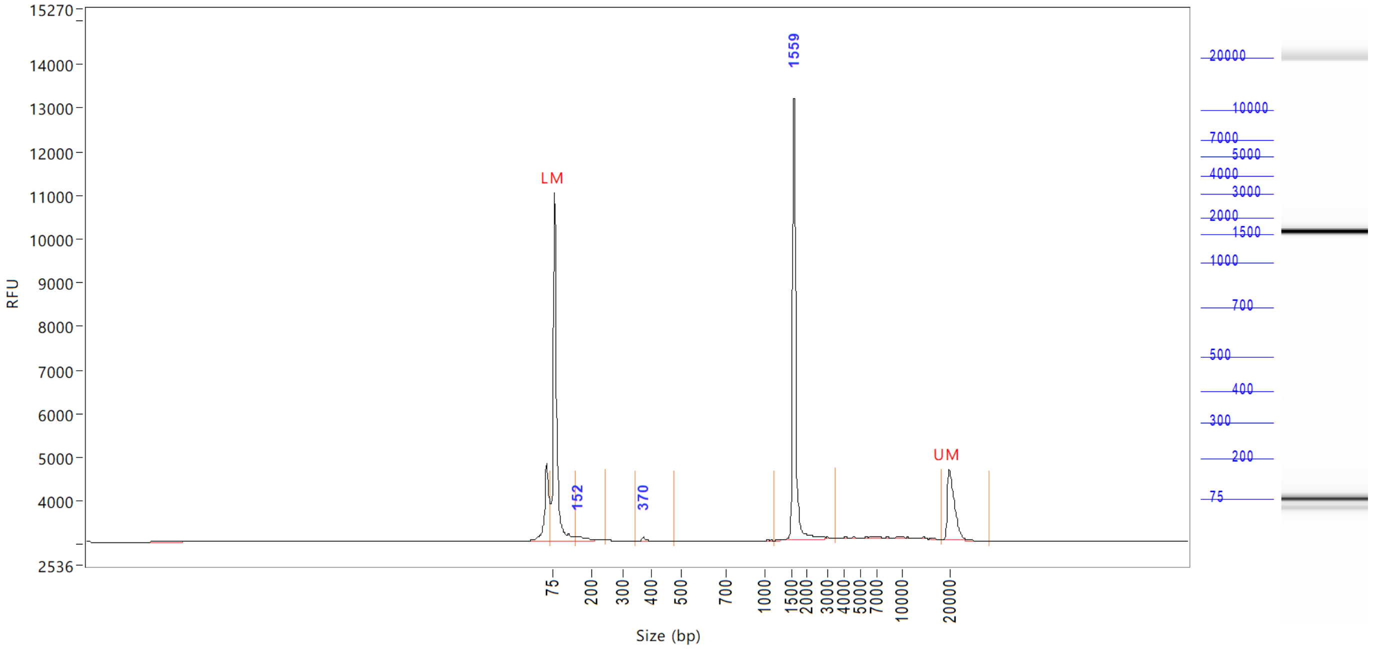

Peak	Size<br>(bp)	Concentration<br>(ng/uL)	From<br>(bp)	To<br>(bp)	Average size<br>(bp)	CV%	RFU	Corrected peak area
1	75 (LM)	0.5000	62	145	77	15.39	7980	66.198
2	152	0.2168	145	244	173	12.87	90	2.392
3	370	0.0627	343	473	379	6.24	83	0.692
4	1559	4.9134	1156	3536	1602	12.98	10122	54.209
5	20000 (UM)	0.1141	18415	28463	20574	4.52	1618	15.112

TIC:	5.1929	ng/uL
TIM:	7.3808	nmole/L
Total concentration:	5.4471	ng/uL

Sample peak width (sec): 5 Sample min peak height: 50 Sample baseline V to V?: Y Sample baseline V to V points: 3
Sample filter: Binomial Number of points for filter: 3 Sample start region (min): 0 Sample end region (min): 30
Marker peak width (sec): 5 Marker min peak height: 200 Marker baseline V to V?: Y Marker baseline V to V points: 3
Lower marker selection: First peak > 500 RFU Upper marker selection: Last peak > 200 RFU
Ladder size (bp)75, 200, 300, 400, 500, 700, 1000, 1500, 2000, 3000, 4000, 5000, 7000, 10000, 20000
Quantification using: Lower Marker Final concentration (ng/uL): 0.5000 Dilution factor: 12.0

Sample: SampC7
Well location: C7
Created: Tuesday, September 14, 2021 6:14:00 PM

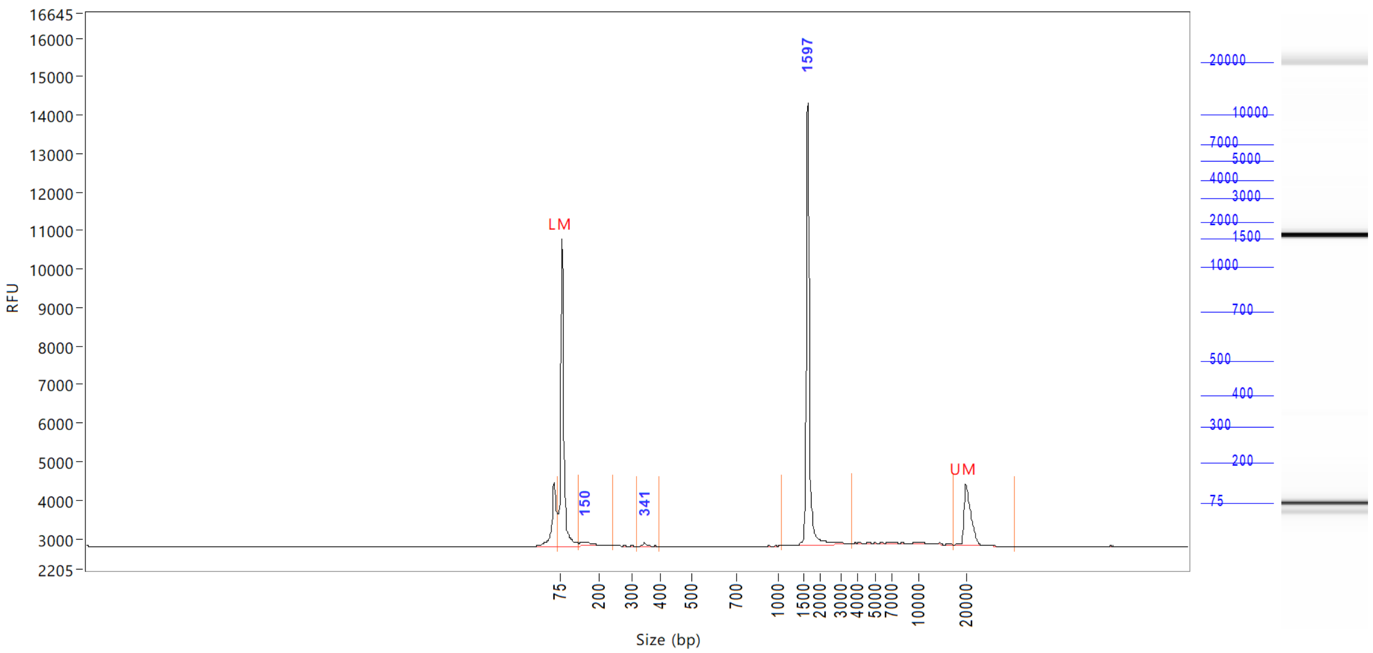

Peak	Size (bp)	Concentration (ng/uL)	From (bp)	To (bp)	Average size (bp)	CV%	RFU	Corrected peak area
1	75 (LM)	0.5000	64	131	76	12.84	7979	60.372
2	150	0.3001	131	242	163	14.26	93	3.020
3	341	0.0776	315	394	344	3.60	89	0.781
4	1597	6.0161	1068	3643	1621	13.13	11509	60.534
5	20000 (UM)	0.1191	17561	30277	20491	4.41	1595	14.386

TIC:	6.3938	ng/uL
TIM:	9.5068	nmole/L
Total concentration:	6.7045	ng/uL

Sample peak width (sec): 5 Sample min peak height: 50 Sample baseline V to V?: Y Sample baseline V to V points: 3
Sample filter: Binomial Number of points for filter: 3 Sample start region (min): 0 Sample end region (min): 30
Marker peak width (sec): 5 Marker min peak height: 200 Marker baseline V to V?: Y Marker baseline V to V points: 3
Lower marker selection: First peak > 500 RFU Upper marker selection: Last peak > 200 RFU
Ladder size (bp)75, 200, 300, 400, 500, 700, 1000, 1500, 2000, 3000, 4000, 5000, 7000, 10000, 20000
Quantification using: Lower Marker Final concentration (ng/uL): 0.5000 Dilution factor: 12.0

Sample: SampD7
Well location: D7
Created: Tuesday, September 14, 2021 6:14:00 PM

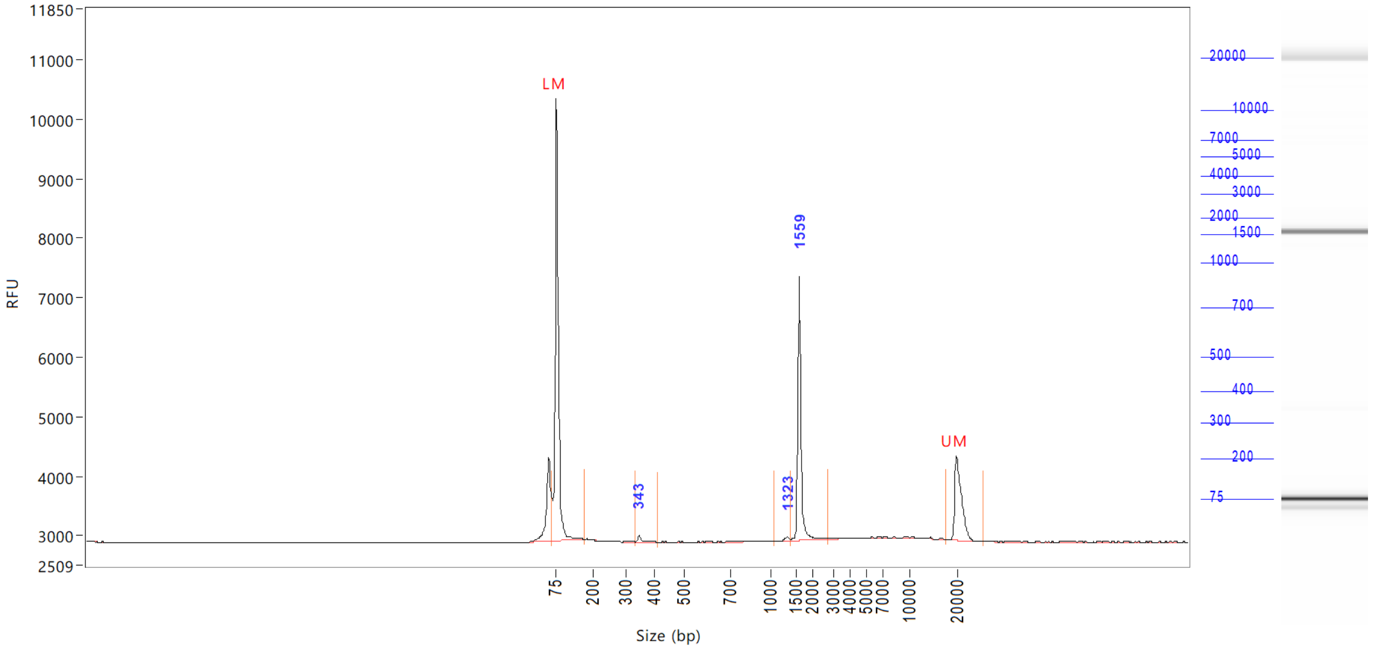

Peak	Size (bp)	Concentration (ng/uL)	From (bp)	To (bp)	Average size (bp)	CV%	RFU	Corrected peak area
1	75 (LM)	0.5000	64	169	76	12.25	7436	56.757
2	343	0.0987	331	409	352	4.68	110	0.933
3	1323	0.0549	1079	1378	1292	3.86	64	0.520
4	1559	2.4930	1378	2800	1580	8.21	4424	23.582
5	20000 (UM)	0.1127	17927	25803	20483	4.12	1406	12.793

TIC:	2.6466	ng/uL
TIM:	3.1285	nmole/L
Total concentration:	2.8861	ng/uL

Sample peak width (sec): 5 Sample min peak height: 50 Sample baseline V to V?: Y Sample baseline V to V points: 3
Sample filter: Binomial Number of points for filter: 3 Sample start region (min): 0 Sample end region (min): 30
Marker peak width (sec): 5 Marker min peak height: 200 Marker baseline V to V?: Y Marker baseline V to V points: 3
Lower marker selection: First peak > 500 RFU Upper marker selection: Last peak > 200 RFU
Ladder size (bp)75, 200, 300, 400, 500, 700, 1000, 1500, 2000, 3000, 4000, 5000, 7000, 10000, 20000
Quantification using: Lower Marker Final concentration (ng/uL): 0.5000 Dilution factor: 12.0

Sample: SampE7
Well location: E7
Created: Tuesday, September 14, 2021 6:14:00 PM

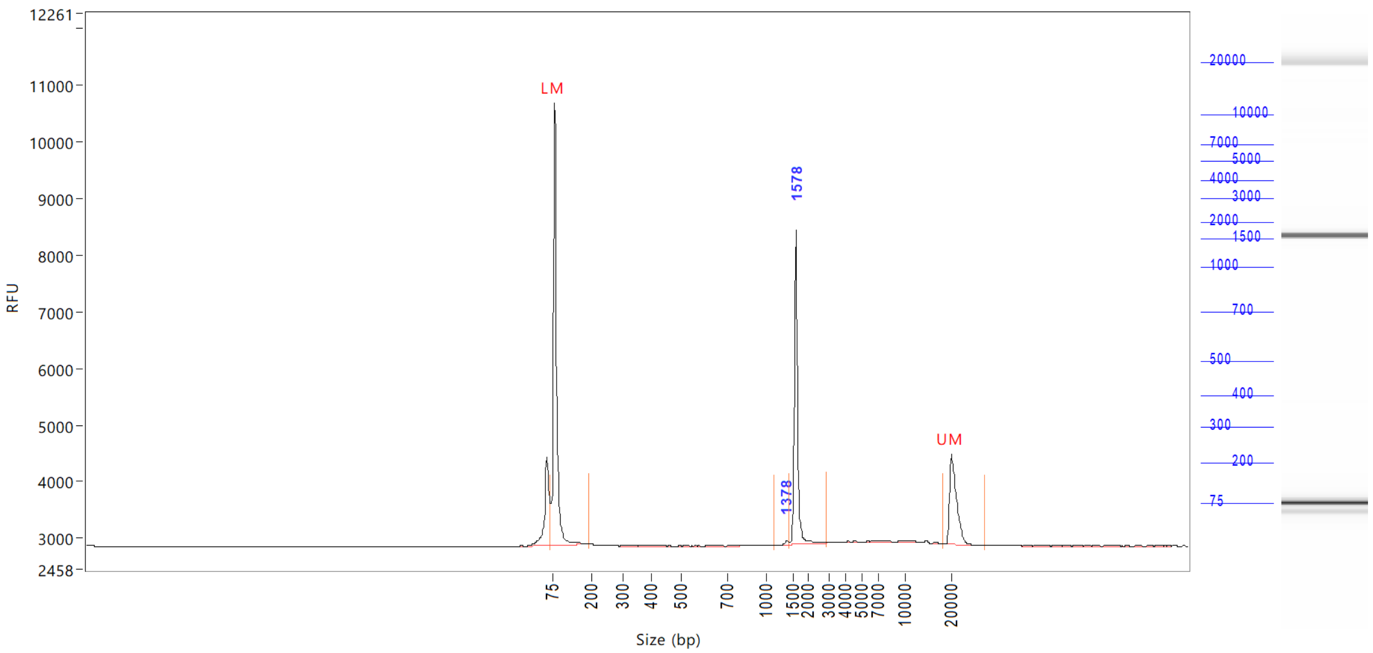

Peak	Size (bp)	Concentration (ng/uL)	From (bp)	To (bp)	Average size (bp)	CV%	RFU	Corrected peak area
1	75 (LM)	0.5000	64	189	77	15.29	7813	61.445
2	1378	0.0436	1145	1433	1364	4.00	63	0.446
3	1578	2.8477	1433	2857	1590	8.48	5556	29.163
4	20000 (UM)	0.1194	18171	27133	20483	4.23	1586	14.671

TIC:	2.8913	ng/uL
TIM:	3.0008	nmole/L
Total concentration:	3.1874	ng/uL

Sample peak width (sec): 5 Sample min peak height: 50 Sample baseline V to V?: Y Sample baseline V to V points: 3
Sample filter: Binomial Number of points for filter: 3 Sample start region (min): 0 Sample end region (min): 30
Marker peak width (sec): 5 Marker min peak height: 200 Marker baseline V to V?: Y Marker baseline V to V points: 3
Lower marker selection: First peak > 500 RFU Upper marker selection: Last peak > 200 RFU
Ladder size (bp)75, 200, 300, 400, 500, 700, 1000, 1500, 2000, 3000, 4000, 5000, 7000, 10000, 20000
Quantification using: Lower Marker Final concentration (ng/uL): 0.5000 Dilution factor: 12.0

Sample: SampF7
Well location: F7
Created: Tuesday, September 14, 2021 6:14:00 PM

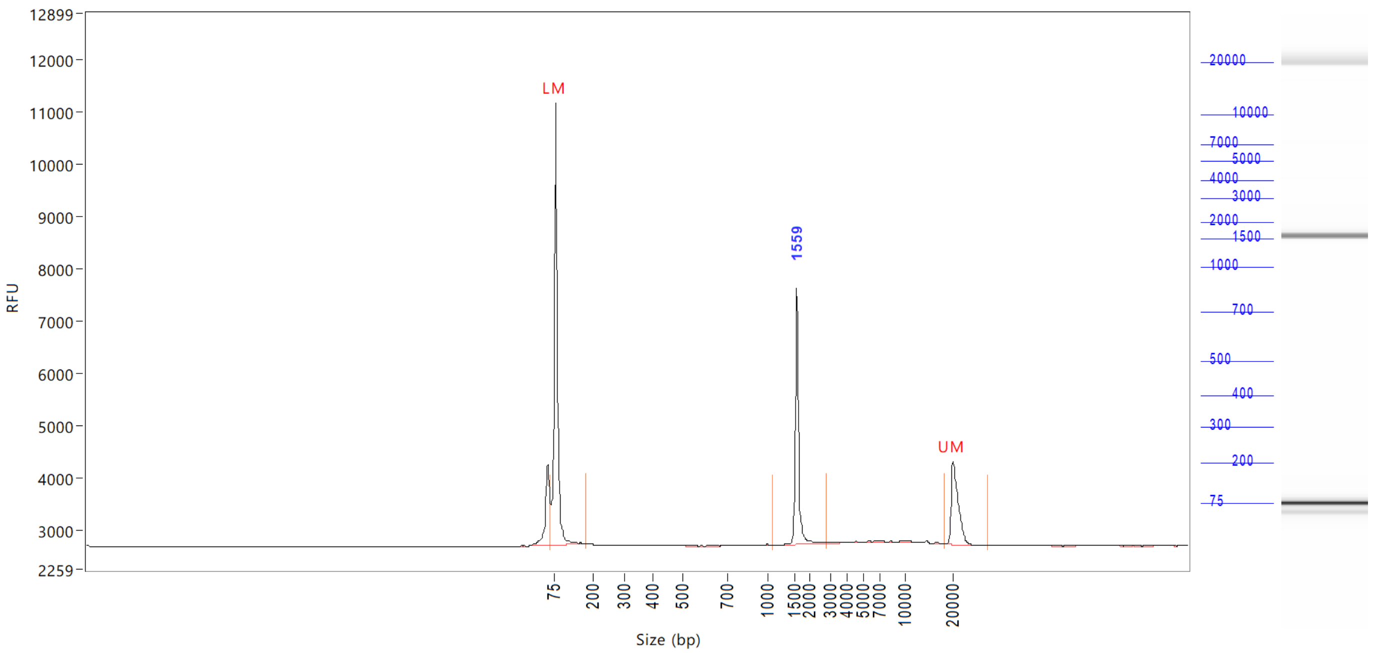

Peak	Size (bp)	Concentration (ng/uL)	From (bp)	To (bp)	Average size (bp)	CV%	RFU	Corrected peak area
1	75 (LM)	0.5000	58	177	75	13.94	8470	65.921
2	1559	2.4178	1090	2800	1587	9.21	4891	26.564
3	20000 (UM)	0.1140	18171	27496	20553	4.19	1598	15.025

TIC:	2.4178	ng/uL
TIM:	2.5079	nmole/L
Total concentration:	2.6792	ng/uL

```
Sample peak width (sec): 5    Sample min peak height: 50    Sample baseline V to V?: Y    Sample baseline V to V points: 3
Sample filter: Binomial       Number of points for filter: 3  Sample start region (min): 0  Sample end region (min): 30
Marker peak width (sec): 5    Marker min peak height: 200   Marker baseline V to V?: Y    Marker baseline V to V points: 3
Lower marker selection: First peak > 500 RFU                Upper marker selection: Last peak > 200 RFU
Ladder size (bp)75, 200, 300, 400, 500, 700, 1000, 1500, 2000, 3000, 4000, 5000, 7000, 10000, 20000
Quantification using: Lower Marker          Final concentration (ng/uL): 0.5000          Dilution factor: 12.0
```

Sample: SampG7
Well location: G7
Created: Tuesday, September 14, 2021 6:14:00 PM

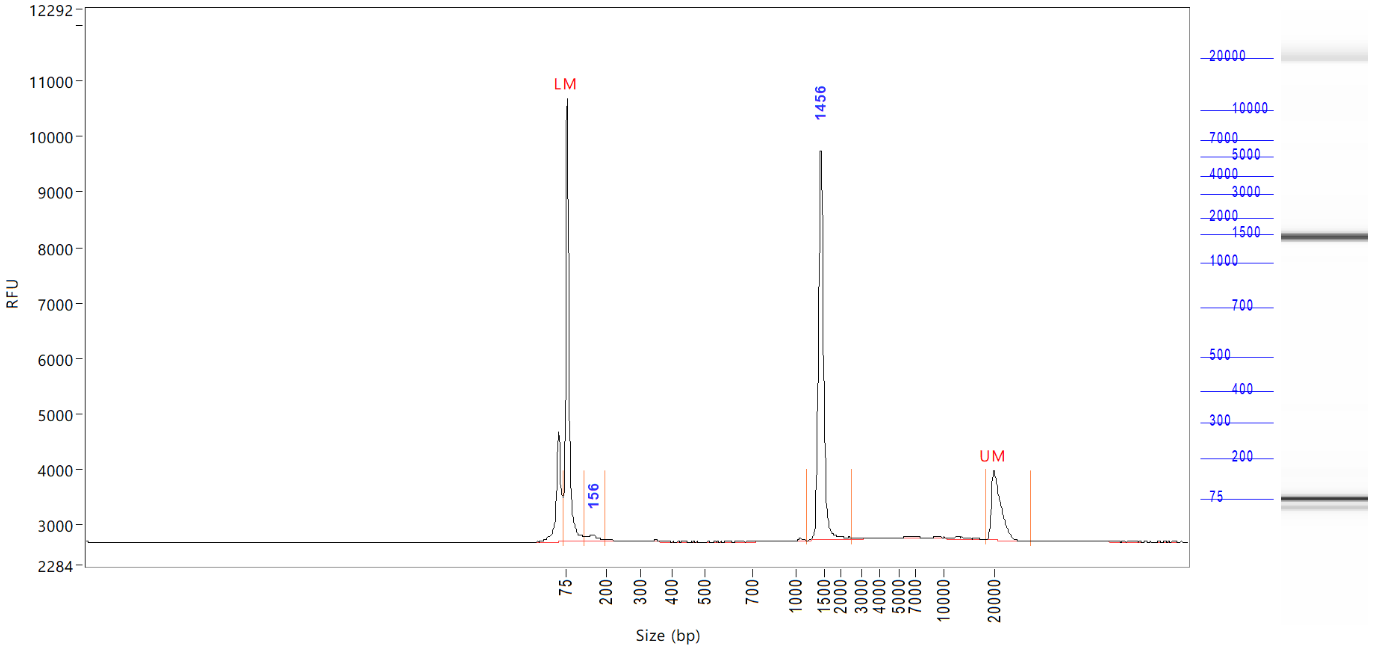

Peak	Size (bp)	Concentration (ng/uL)	From (bp)	To (bp)	Average size (bp)	CV%	RFU	Corrected peak area
1	75 (LM)	0.5000	64	128	76	12.26	7989	65.141
2	156	0.3053	128	194	153	11.16	109	3.315
3	1456	5.3800	1201	2515	1461	7.14	7011	58.410
4	20000 (UM)	0.1152	18415	27133	20598	5.15	1253	15.009

TIC:	5.6853	ng/uL
TIM:	9.3411	nmole/L
Total concentration:	6.1672	ng/uL

```
Sample peak width (sec): 5    Sample min peak height: 50    Sample baseline V to V?: Y    Sample baseline V to V points: 3
Sample filter: Binomial       Number of points for filter: 3  Sample start region (min): 0  Sample end region (min): 30
Marker peak width (sec): 5    Marker min peak height: 200   Marker baseline V to V?: Y    Marker baseline V to V points: 3
Lower marker selection: First peak > 500 RFU                Upper marker selection: Last peak > 200 RFU
Ladder size (bp)75, 200, 300, 400, 500, 700, 1000, 1500, 2000, 3000, 4000, 5000, 7000, 10000, 20000
Quantification using: Lower Marker          Final concentration (ng/uL): 0.5000          Dilution factor: 12.0
```

Sample: SampH7
Well location: H7
Created: Tuesday, September 14, 2021 6:14:00 PM

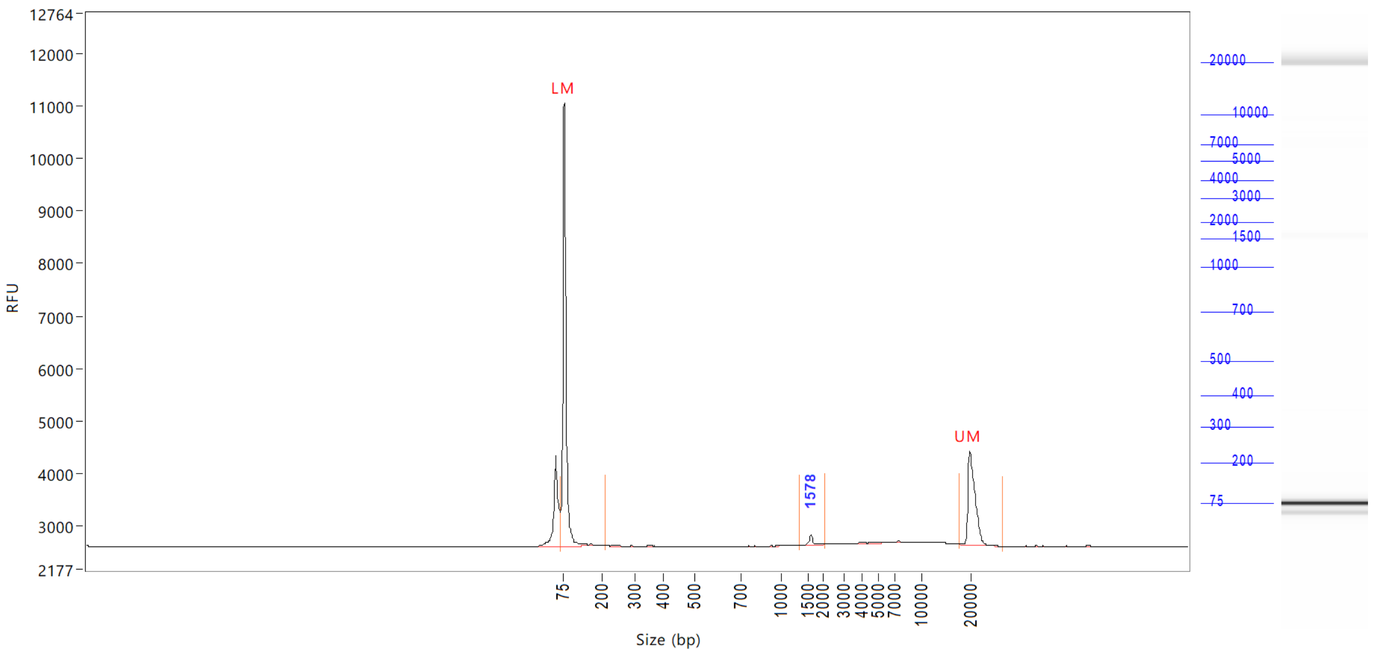

Peak	Size (bp)	Concentration (ng/uL)	From (bp)	To (bp)	Average size (bp)	CV%	RFU	Corrected peak area
1	75 (LM)	0.5000	64	208	76	16.56	8455	64.757
2	1578	0.1151	1345	2030	1582	6.77	190	1.243
3	20000 (UM)	0.1273	17683	26891	20464	4.09	1774	16.484

TIC:	0.1151	ng/uL
TIM:	0.1198	nmole/L
Total concentration:	0.3853	ng/uL

Sample peak width (sec): 5 Sample min peak height: 50 Sample baseline V to V?: Y Sample baseline V to V points: 3
Sample filter: Binomial Number of points for filter: 3 Sample start region (min): 0 Sample end region (min): 30
Marker peak width (sec): 5 Marker min peak height: 200 Marker baseline V to V?: Y Marker baseline V to V points: 3
Lower marker selection: First peak > 500 RFU Upper marker selection: Last peak > 200 RFU
Ladder size (bp)75, 200, 300, 400, 500, 700, 1000, 1500, 2000, 3000, 4000, 5000, 7000, 10000, 20000
Quantification using: Lower Marker Final concentration (ng/uL): 0.5000 Dilution factor: 12.0

Sample: SampA8
Well location: A8
Created: Tuesday, September 14, 2021 6:14:00 PM

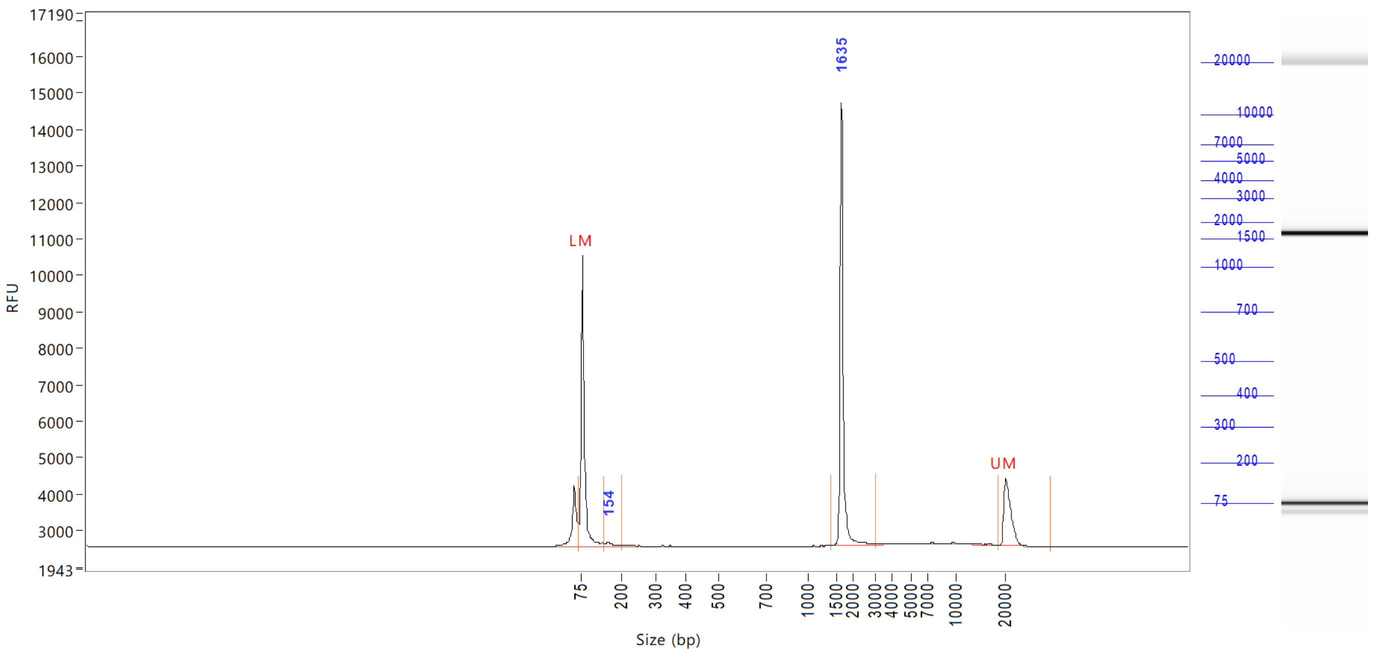

Peak	Size (bp)	Concentration (ng/uL)	From (bp)	To (bp)	Average size (bp)	CV%	RFU	Corrected peak area
1	75 (LM)	0.5000	64	141	76	14.86	7974	59.637
2	154	0.2013	141	200	162	9.34	97	2.000
3	1635	6.0188	1400	3037	1659	9.25	12160	59.823
4	20000 (UM)	0.1381	18659	29068	20482	4.13	1851	16.472

TIC:	6.2200	ng/uL
TIM:	8.0140	nmole/L
Total concentration:	6.6150	ng/uL

```
Sample peak width (sec): 5    Sample min peak height: 50    Sample baseline V to V?: Y    Sample baseline V to V points: 3
Sample filter: Binomial       Number of points for filter: 3  Sample start region (min): 0  Sample end region (min): 30
Marker peak width (sec): 5    Marker min peak height: 200   Marker baseline V to V?: Y    Marker baseline V to V points: 3
Lower marker selection: First peak > 500 RFU                Upper marker selection: Last peak > 200 RFU
Ladder size (bp)75, 200, 300, 400, 500, 700, 1000, 1500, 2000, 3000, 4000, 5000, 7000, 10000, 20000
Quantification using: Lower Marker          Final concentration (ng/uL): 0.5000          Dilution factor: 12.0
```

Sample: SampB8
Well location: B8
Created: Tuesday, September 14, 2021 6:14:00 PM

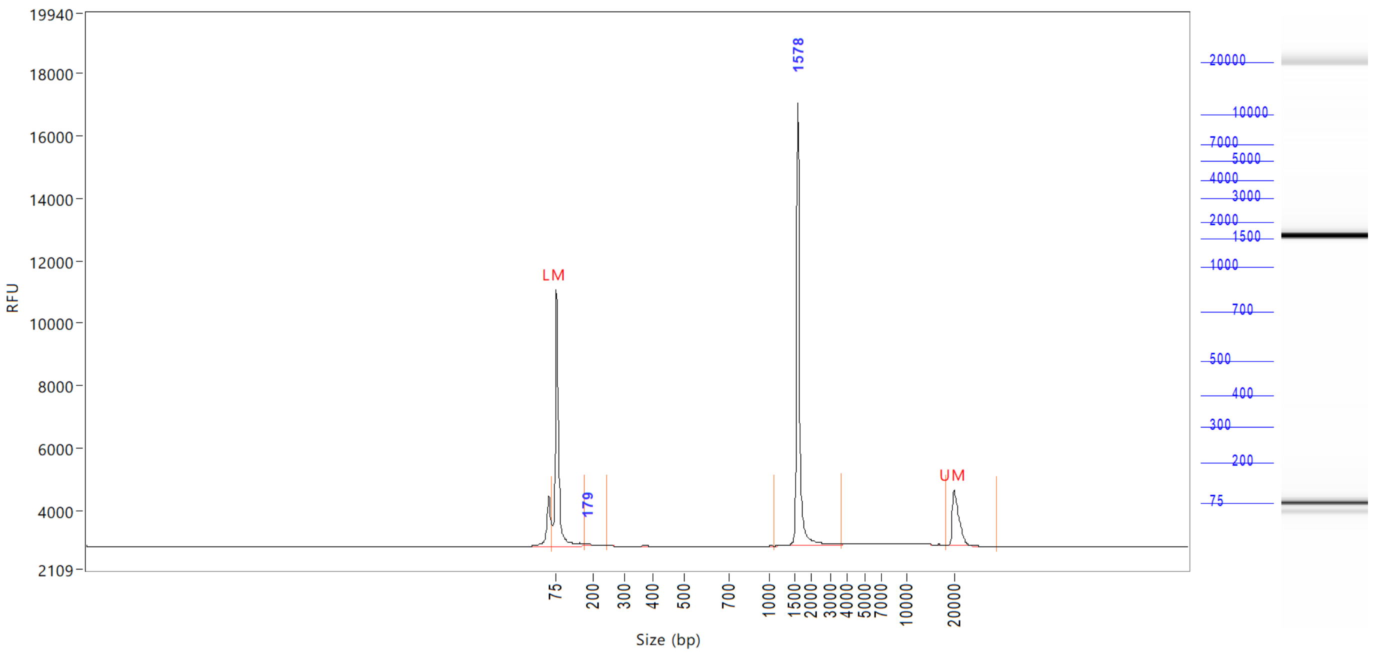

Peak	Size (bp)	Concentration (ng/uL)	From (bp)	To (bp)	Average size (bp)	CV%	RFU	Corrected peak area
1	75 (LM)	0.5000	64	171	80	20.44	8232	65.527
2	179	0.1152	171	242	191	8.17	73	1.258
3	1578	6.9057	1090	3572	1608	12.86	14220	75.419
4	20000 (UM)	0.1225	18171	28947	20495	4.29	1769	16.054

TIC:	7.0209	ng/uL
TIM:	8.0611	nmole/L
Total concentration:	7.3048	ng/uL

Sample peak width (sec): 5   Sample min peak height: 50   Sample baseline V to V?: Y   Sample baseline V to V points: 3
Sample filter: Binomial   Number of points for filter: 3 Sample start region (min): 0 Sample end region (min): 30
Marker peak width (sec): 5   Marker min peak height: 200   Marker baseline V to V?: Y   Marker baseline V to V points: 3
Lower marker selection: First peak > 500 RFU   Upper marker selection: Last peak > 200 RFU
Ladder size (bp)75, 200, 300, 400, 500, 700, 1000, 1500, 2000, 3000, 4000, 5000, 7000, 10000, 20000
Quantification using: Lower Marker   Final concentration (ng/uL): 0.5000   Dilution factor: 12.0

Sample: SampC8
Well location: C8
Created: Tuesday, September 14, 2021 6:14:00 PM

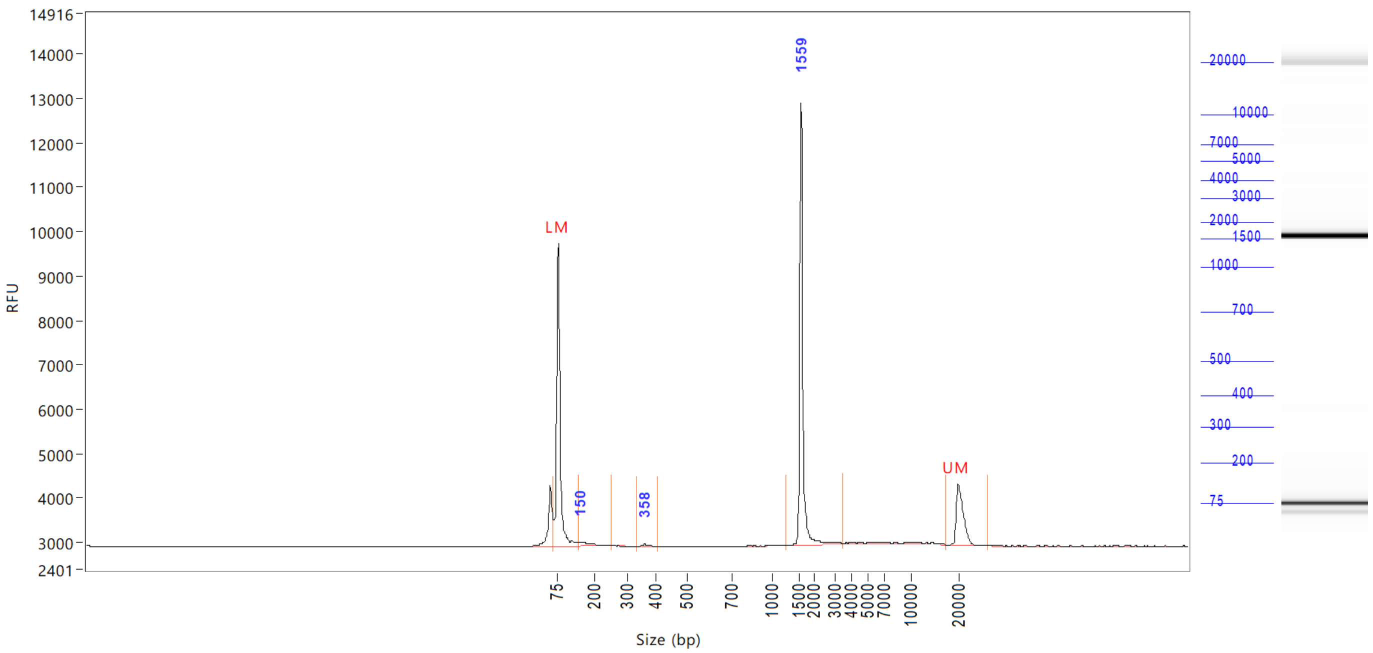

Peak	Size (bp)	Concentration (ng/uL)	From (bp)	To (bp)	Average size (bp)	CV%	RFU	Corrected peak area
1	75 (LM)	0.5000	62	141	77	15.49	6834	54.080
2	150	0.2936	141	250	171	13.27	93	2.646
3	358	0.0547	331	403	359	3.30	51	0.493
4	1559	5.7019	1267	3500	1601	12.32	9972	51.393
5	20000 (UM)	0.1183	17439	26166	20443	4.25	1390	12.794

TIC:	6.0502	ng/uL
TIM:	8.9357	nmole/L
Total concentration:	6.2976	ng/uL

```
Sample peak width (sec): 5    Sample min peak height: 50    Sample baseline V to V?: Y    Sample baseline V to V points: 3
Sample filter: Binomial       Number of points for filter: 3  Sample start region (min): 0  Sample end region (min): 30
Marker peak width (sec): 5    Marker min peak height: 200   Marker baseline V to V?: Y    Marker baseline V to V points: 3
Lower marker selection: First peak > 500 RFU                Upper marker selection: Last peak > 200 RFU
Ladder size (bp)75, 200, 300, 400, 500, 700, 1000, 1500, 2000, 3000, 4000, 5000, 7000, 10000, 20000
Quantification using: Lower Marker          Final concentration (ng/uL): 0.5000          Dilution factor: 12.0
```

Sample: SampD8
Well location: D8
Created: Tuesday, September 14, 2021 6:14:00 PM

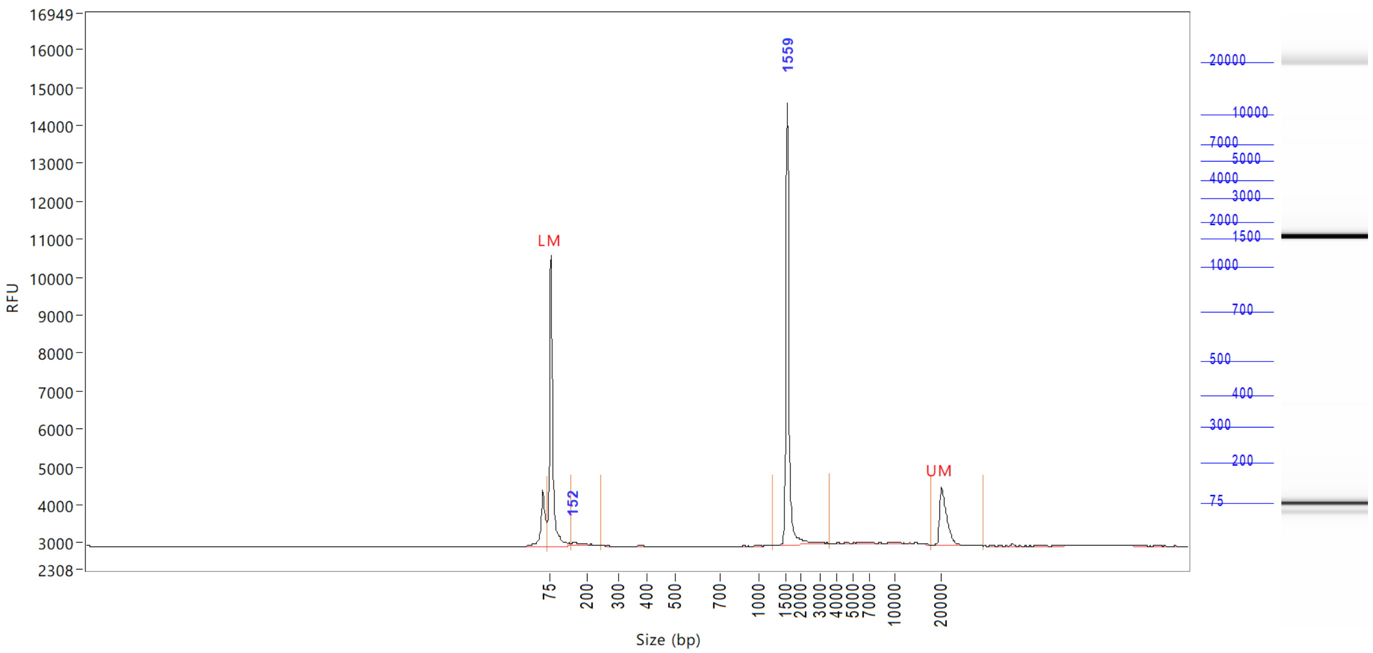

Peak	Size (bp)	Concentration (ng/uL)	From (bp)	To (bp)	Average size (bp)	CV%	RFU	Corrected peak area
1	75 (LM)	0.5000	62	143	77	15.40	7661	60.727
2	152	0.2467	143	242	174	13.56	92	2.497
3	1559	6.1958	1256	3536	1582	11.09	11663	62.708
4	20000 (UM)	0.1211	17683	29309	20635	4.84	1523	14.702

TIC:	6.4425	ng/uL
TIM:	8.7773	nmole/L
Total concentration:	6.7009	ng/uL

```
Sample peak width (sec): 5    Sample min peak height: 50    Sample baseline V to V?: Y    Sample baseline V to V points: 3
Sample filter: Binomial       Number of points for filter: 3  Sample start region (min): 0  Sample end region (min): 30
Marker peak width (sec): 5    Marker min peak height: 200   Marker baseline V to V?: Y    Marker baseline V to V points: 3
Lower marker selection: First peak > 500 RFU                Upper marker selection: Last peak > 200 RFU
Ladder size (bp)75, 200, 300, 400, 500, 700, 1000, 1500, 2000, 3000, 4000, 5000, 7000, 10000, 20000
Quantification using: Lower Marker          Final concentration (ng/uL): 0.5000          Dilution factor: 12.0
```

Sample: SampE8
Well location: E8
Created: Tuesday, September 14, 2021 6:14:00 PM

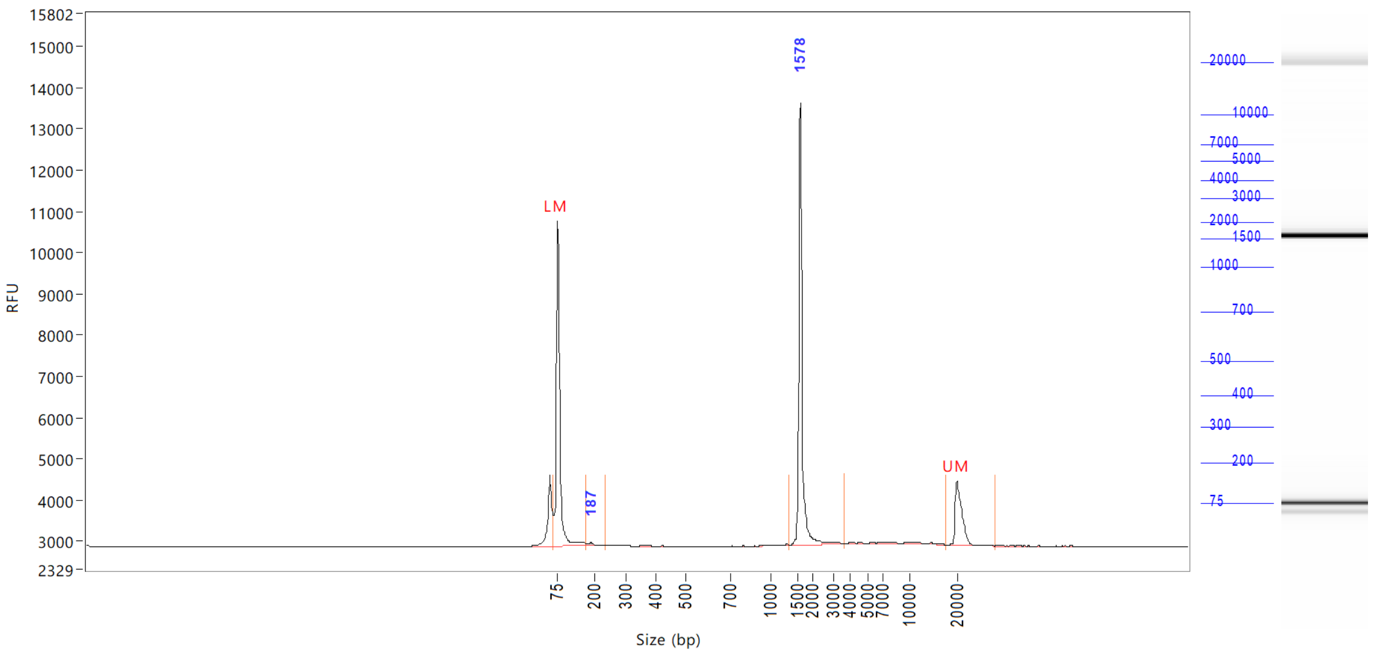

Peak	Size (bp)	Concentration (ng/uL)	From (bp)	To (bp)	Average size (bp)	CV%	RFU	Corrected peak area
1	75 (LM)	0.5000	64	171	79	20.17	7865	63.291
2	187	0.1048	171	233	189	7.09	67	1.105
3	1578	5.5447	1356	3608	1616	13.32	10733	58.488
4	20000 (UM)	0.1110	17683	27979	20436	4.21	1552	14.051

TIC:	5.6494	ng/uL
TIM:	6.5592	nmole/L
Total concentration:	5.9384	ng/uL

Sample peak width (sec): 5 Sample min peak height: 50 Sample baseline V to V?: Y Sample baseline V to V points: 3
Sample filter: Binomial Number of points for filter: 3 Sample start region (min): 0 Sample end region (min): 30
Marker peak width (sec): 5 Marker min peak height: 200 Marker baseline V to V?: Y Marker baseline V to V points: 3
Lower marker selection: First peak > 500 RFU Upper marker selection: Last peak > 200 RFU
Ladder size (bp)75, 200, 300, 400, 500, 700, 1000, 1500, 2000, 3000, 4000, 5000, 7000, 10000, 20000
Quantification using: Lower Marker Final concentration (ng/uL): 0.5000 Dilution factor: 12.0

Sample: SampF8
Well location: F8
Created: Tuesday, September 14, 2021 6:14:00 PM

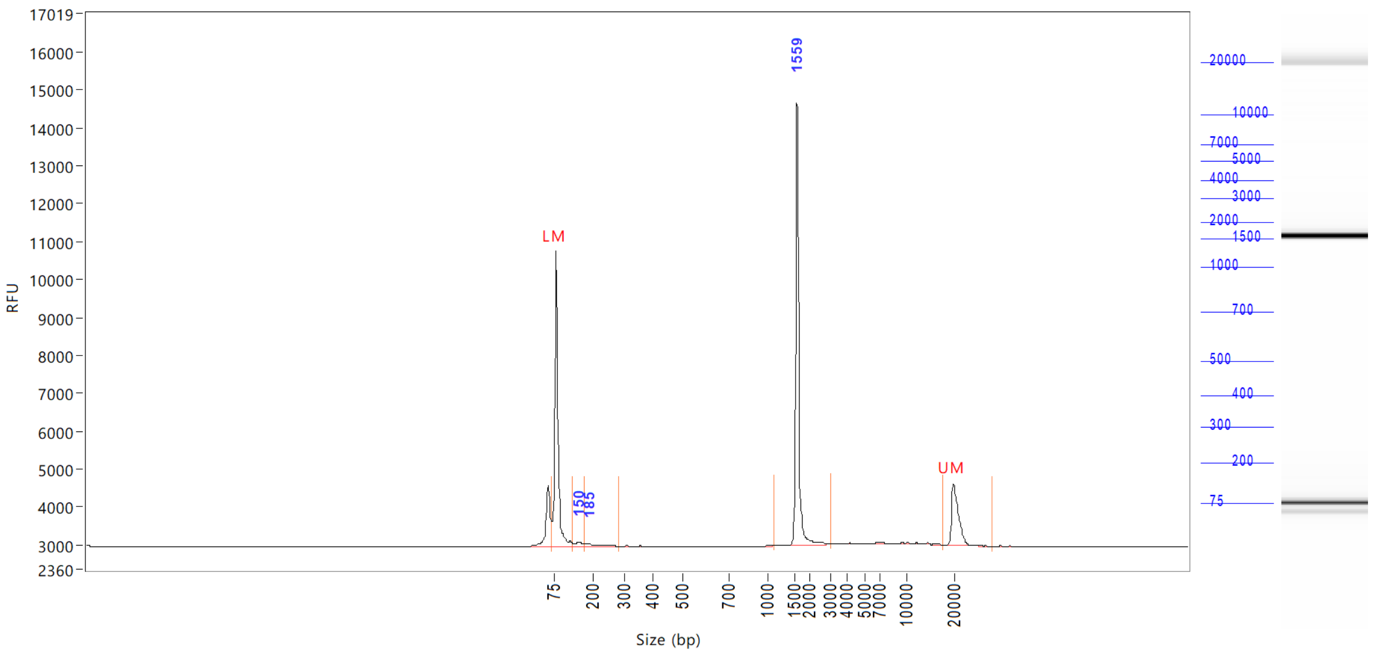

Peak	Size (bp)	Concentration (ng/uL)	From (bp)	To (bp)	Average size (bp)	CV%	RFU	Corrected peak area
1	75 (LM)	0.5000	64	133	78	13.38	7793	61.640
2	150	0.1974	133	171	151	7.17	105	2.028
3	185	0.1970	171	282	203	12.85	80	2.024
4	1559	5.8721	1101	3000	1586	9.84	11685	60.326
5	20000 (UM)	0.1230	17805	28221	20502	4.54	1638	15.165

TIC:	6.2665	ng/uL
TIM:	9.8388	nmole/L
Total concentration:	6.5471	ng/uL

```
Sample peak width (sec): 5    Sample min peak height: 50    Sample baseline V to V?: Y    Sample baseline V to V points: 3
Sample filter: Binomial       Number of points for filter: 3  Sample start region (min): 0  Sample end region (min): 30
Marker peak width (sec): 5    Marker min peak height: 200   Marker baseline V to V?: Y    Marker baseline V to V points: 3
Lower marker selection: First peak > 500 RFU                Upper marker selection: Last peak > 200 RFU
Ladder size (bp)75, 200, 300, 400, 500, 700, 1000, 1500, 2000, 3000, 4000, 5000, 7000, 10000, 20000
Quantification using: Lower Marker          Final concentration (ng/uL): 0.5000          Dilution factor: 12.0
```

Sample: SampG8
Well location: G8
Created: Tuesday, September 14, 2021 6:14:00 PM

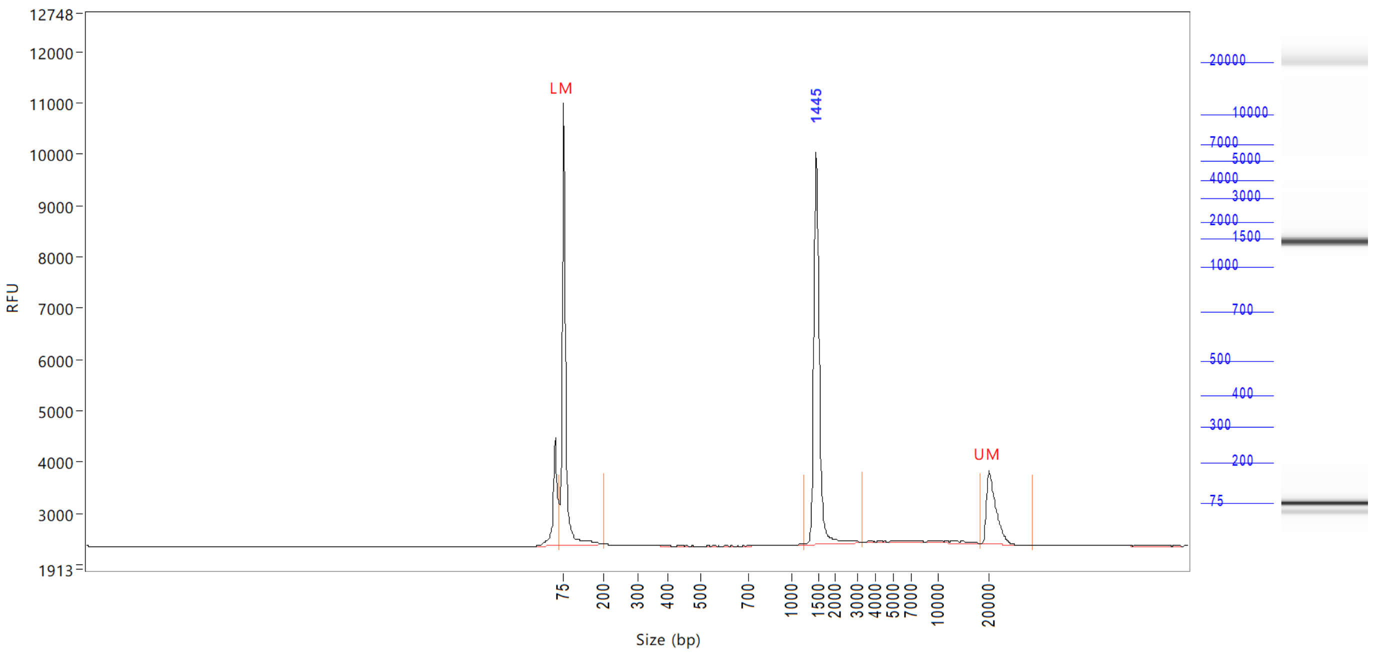

Peak	Size (bp)	Concentration (ng/uL)	From (bp)	To (bp)	Average size (bp)	CV%	RFU	Corrected peak area
1	75 (LM)	0.5000	62	201	78	21.09	8639	73.796
2	1445	5.4369	1212	3286	1475	12.54	7633	66.870
3	20000 (UM)	0.1166	18171	28584	20691	5.39	1431	17.209

TIC:	5.4369	ng/uL
TIM:	6.0674	nmole/L
Total concentration:	5.7898	ng/uL

Sample peak width (sec): 5 Sample min peak height: 50 Sample baseline V to V?: Y Sample baseline V to V points: 3
Sample filter: Binomial Number of points for filter: 3 Sample start region (min): 0 Sample end region (min): 30
Marker peak width (sec): 5 Marker min peak height: 200 Marker baseline V to V?: Y Marker baseline V to V points: 3
Lower marker selection: First peak > 500 RFU Upper marker selection: Last peak > 200 RFU
Ladder size (bp)75, 200, 300, 400, 500, 700, 1000, 1500, 2000, 3000, 4000, 5000, 7000, 10000, 20000
Quantification using: Lower Marker Final concentration (ng/uL): 0.5000 Dilution factor: 12.0

Sample: SampH8
Well location: H8
Created: Tuesday, September 14, 2021 6:14:00 PM

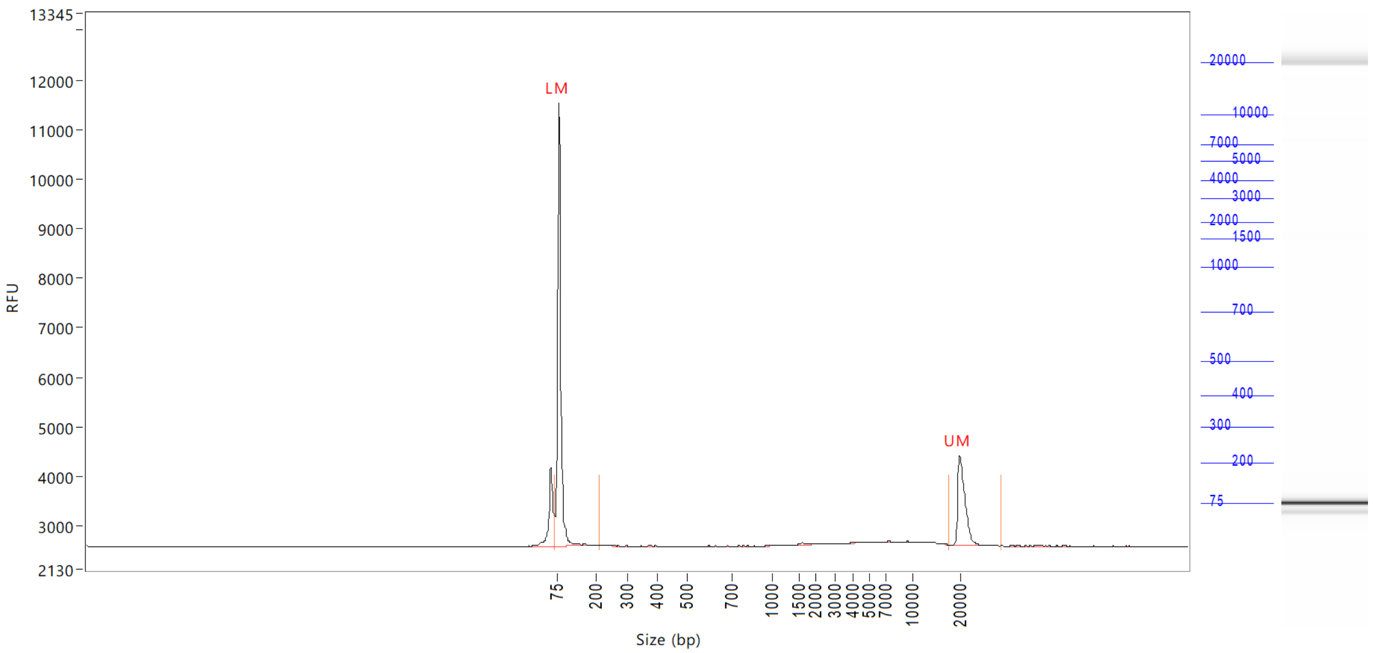

Peak	Size	Concentration	From	To	Average size	CV%	RFU	Corrected peak area
	(bp)	(ng/uL)	(bp)	(bp)	(bp)			
1	75 (LM)	0.5000	64	212	77	15.32	8955	66.148
2	20000 (UM)	0.1284	17683	28705	20544	4.27	1805	16.984

TIC:	0.0000	ng/uL
TIM:	0.0000	nmole/L
Total concentration:	0.2656	ng/uL

Sample peak width (sec): 5   Sample min peak height: 50   Sample baseline V to V?: Y   Sample baseline V to V points: 3
Sample filter: Binomial   Number of points for filter: 3 Sample start region (min): 0 Sample end region (min): 30
Marker peak width (sec): 5   Marker min peak height: 200   Marker baseline V to V?: Y   Marker baseline V to V points: 3
Lower marker selection: First peak > 500 RFU   Upper marker selection: Last peak > 200 RFU
Ladder size (bp)75, 200, 300, 400, 500, 700, 1000, 1500, 2000, 3000, 4000, 5000, 7000, 10000, 20000
Quantification using: Lower Marker   Final concentration (ng/uL): 0.5000   Dilution factor: 12.0

Sample: SampA9
Well location: A9
Created: Tuesday, September 14, 2021 6:14:00 PM

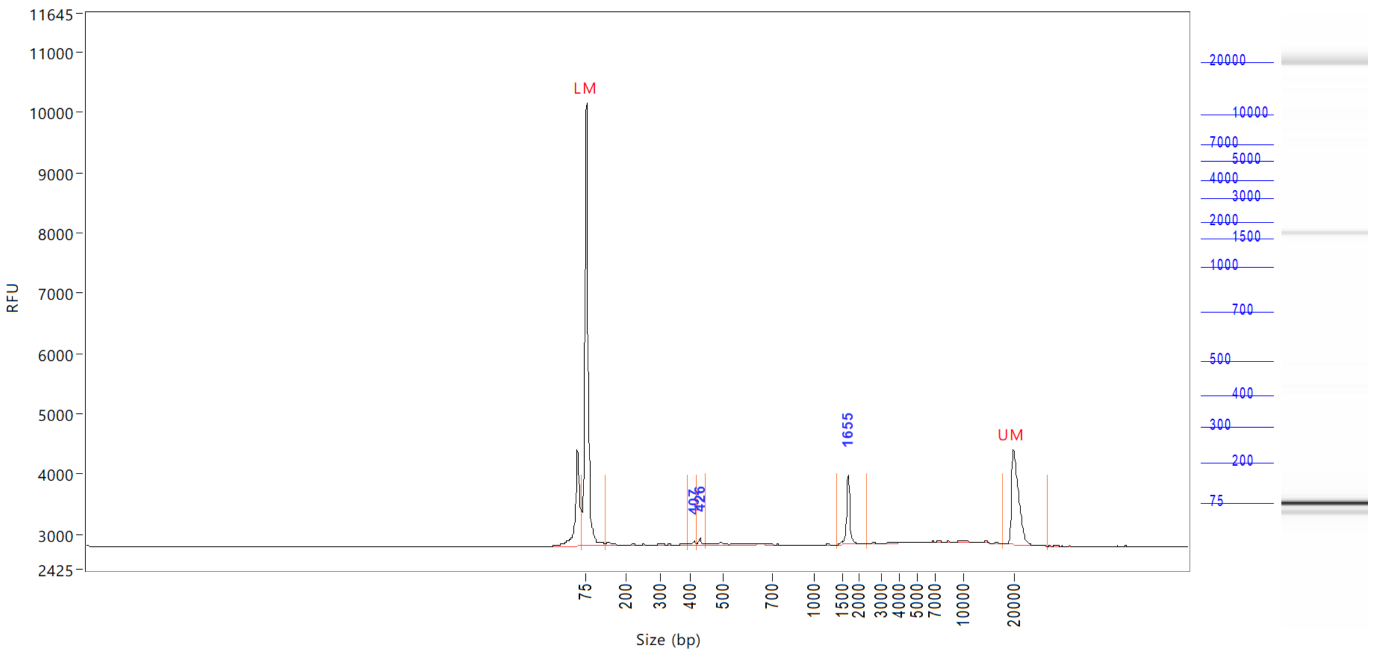

Peak	Size (bp)	Concentration (ng/uL)	From (bp)	To (bp)	Average size (bp)	CV%	RFU	Corrected peak area
1	75 (LM)	0.5000	62	133	75	11.18	7357	54.726
2	407	0.0392	386	416	403	1.64	59	0.357
3	426	0.0573	416	441	424	1.07	109	0.522
4	1655	0.6650	1389	2315	1641	5.27	1153	6.066
5	20000 (UM)	0.1252	17805	26770	20464	3.89	1563	13.701

TIC:	0.7615	ng/uL
TIM:	1.0493	nmole/L
Total concentration:	1.2632	ng/uL

Sample peak width (sec): 5   Sample min peak height: 50   Sample baseline V to V?: Y   Sample baseline V to V points: 3
Sample filter: Binomial   Number of points for filter: 3 Sample start region (min): 0 Sample end region (min): 30
Marker peak width (sec): 5   Marker min peak height: 200   Marker baseline V to V?: Y   Marker baseline V to V points: 3
Lower marker selection: First peak > 500 RFU   Upper marker selection: Last peak > 200 RFU
Ladder size (bp)75, 200, 300, 400, 500, 700, 1000, 1500, 2000, 3000, 4000, 5000, 7000, 10000, 20000
Quantification using: Lower Marker   Final concentration (ng/uL): 0.5000   Dilution factor: 12.0

Sample: SampB9
Well location: B9
Created: Tuesday, September 14, 2021 6:14:00 PM

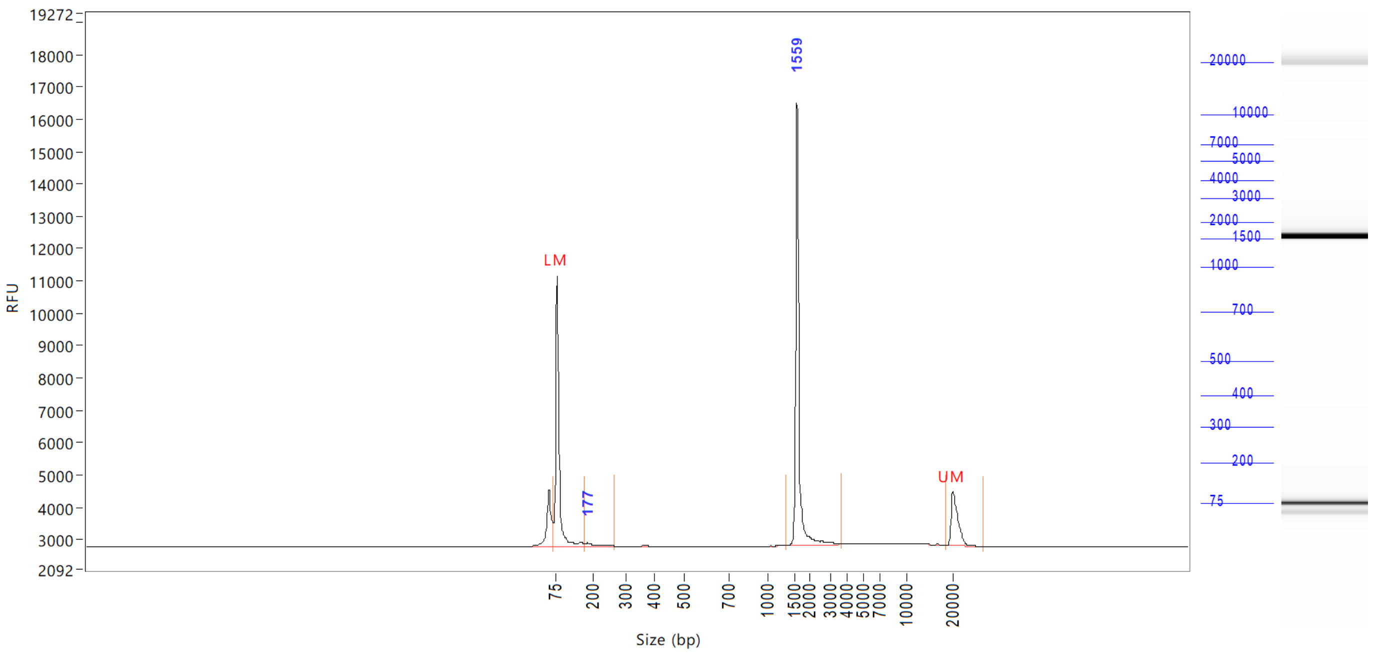

Peak	Size (bp)	Concentration (ng/uL)	From (bp)	To (bp)	Average size (bp)	CV%	RFU	Corrected peak area
1	75 (LM)	0.5000	64	169	79	21.70	8341	66.272
2	177	0.1807	169	266	195	10.89	100	1.996
3	1559	6.8153	1334	3679	1609	14.62	13701	75.277
4	20000 (UM)	0.1141	18415	26649	20485	4.09	1682	15.127

TIC:	6.9960	ng/uL
TIM:	8.4960	nmole/L
Total concentration:	7.2541	ng/uL

Sample peak width (sec): 5   Sample min peak height: 50   Sample baseline V to V?: Y   Sample baseline V to V points: 3
Sample filter: Binomial   Number of points for filter: 3 Sample start region (min): 0 Sample end region (min): 30
Marker peak width (sec): 5   Marker min peak height: 200   Marker baseline V to V?: Y   Marker baseline V to V points: 3
Lower marker selection: First peak > 500 RFU   Upper marker selection: Last peak > 200 RFU
Ladder size (bp)75, 200, 300, 400, 500, 700, 1000, 1500, 2000, 3000, 4000, 5000, 7000, 10000, 20000
Quantification using: Lower Marker   Final concentration (ng/uL): 0.5000   Dilution factor: 12.0

Sample: SampC9
Well location: C9
Created: Tuesday, September 14, 2021 6:14:00 PM

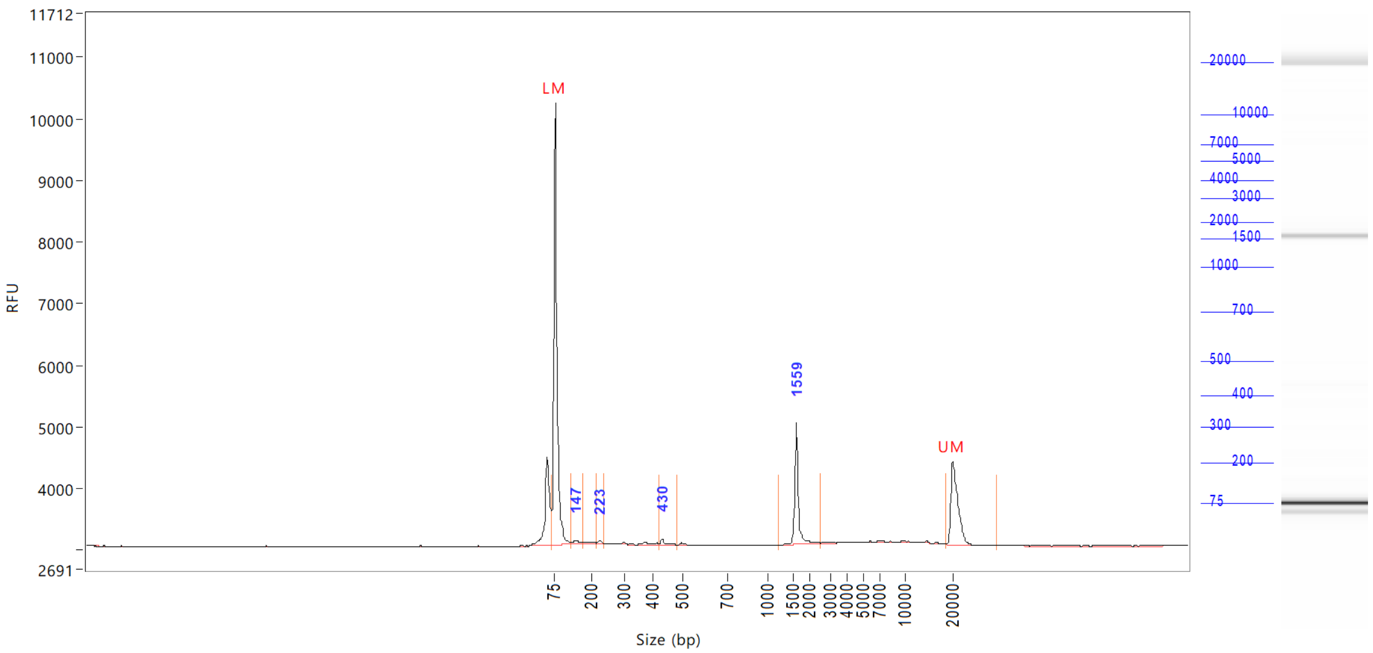

Peak	Size (bp)	Concentration (ng/uL)	From (bp)	To (bp)	Average size (bp)	CV%	RFU	Corrected peak area
1	75 (LM)	0.5000	64	129	76	10.53	7183	54.965
2	147	0.0931	129	168	145	6.68	62	0.853
3	223	0.0472	210	237	221	2.33	52	0.432
4	430	0.0745	420	479	436	3.34	100	0.683
5	1559	1.2370	1212	2543	1584	8.65	1965	11.332
6	20000 (UM)	0.1163	18415	29189	20485	4.42	1342	12.783

TIC:	1.4517	ng/uL
TIM:	2.9727	nmole/L
Total concentration:	1.8725	ng/uL

Sample peak width (sec): 5   Sample min peak height: 50   Sample baseline V to V?: Y   Sample baseline V to V points: 3
Sample filter: Binomial   Number of points for filter: 3   Sample start region (min): 0   Sample end region (min): 30
Marker peak width (sec): 5   Marker min peak height: 200   Marker baseline V to V?: Y   Marker baseline V to V points: 3
Lower marker selection: First peak > 500 RFU   Upper marker selection: Last peak > 200 RFU
Ladder size (bp)75, 200, 300, 400, 500, 700, 1000, 1500, 2000, 3000, 4000, 5000, 7000, 10000, 20000
Quantification using: Lower Marker   Final concentration (ng/uL): 0.5000   Dilution factor: 12.0

Sample: SampD9
Well location: D9
Created: Tuesday, September 14, 2021 6:14:00 PM

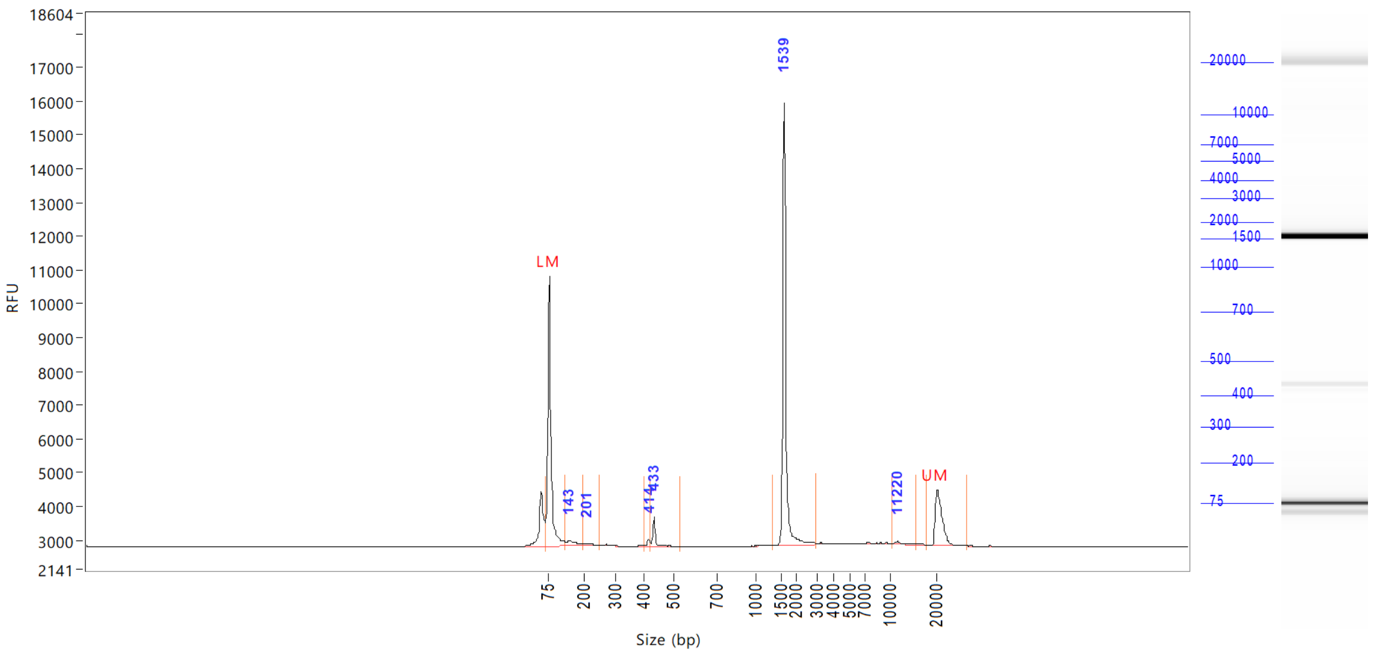

Peak	Size (bp)	Concentration (ng/uL)	From (bp)	To (bp)	Average size (bp)	CV%	RFU	Corrected peak area
1	75 (LM)	0.5000	62	133	77	14.76	7980	63.687
2	143	0.3254	133	194	157	10.71	140	3.454
3	201	0.0935	194	248	209	5.24	61	0.992
4	414	0.1000	401	420	412	1.03	218	1.061
5	433	0.4626	420	527	434	3.36	861	4.910
6	1539	6.5678	1334	2971	1584	10.62	13123	69.714
7	11220	0.0975	10245	15244	12317	11.68	85	1.035
8	20000 (UM)	0.1185	17561	26529	20537	4.23	1630	15.092

TIC:	7.6468	ng/uL
TIM:	13.1337	nmole/L
Total concentration:	7.9616	ng/uL

Sample peak width (sec): 5 Sample min peak height: 50 Sample baseline V to V?: Y Sample baseline V to V points: 3
Sample filter: Binomial Number of points for filter: 3 Sample start region (min): 0 Sample end region (min): 30
Marker peak width (sec): 5 Marker min peak height: 200 Marker baseline V to V?: Y Marker baseline V to V points: 3
Lower marker selection: First peak > 500 RFU Upper marker selection: Last peak > 200 RFU
Ladder size (bp)75, 200, 300, 400, 500, 700, 1000, 1500, 2000, 3000, 4000, 5000, 7000, 10000, 20000
Quantification using: Lower Marker Final concentration (ng/uL): 0.5000 Dilution factor: 12.0

Sample: SampE9
Well location: E9
Created: Tuesday, September 14, 2021 6:14:00 PM

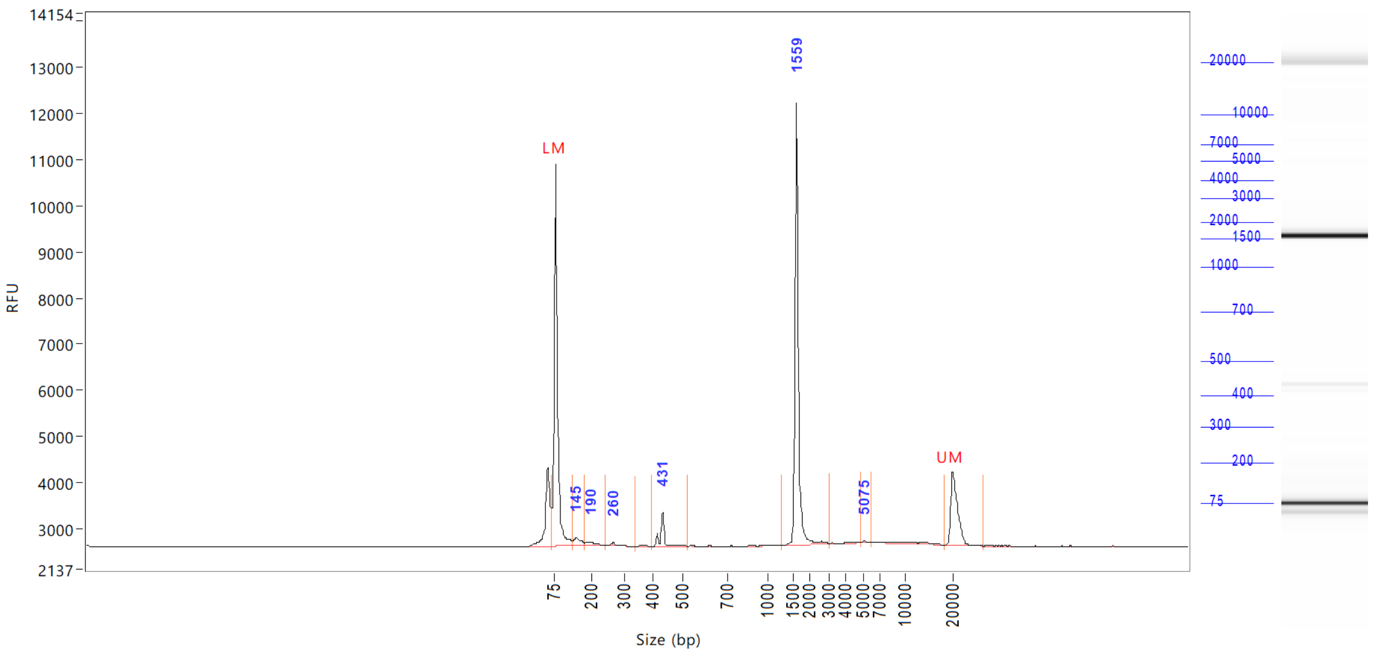

Peak	Size (bp)	Concentration (ng/uL)	From (bp)	To (bp)	Average size (bp)	CV%	RFU	Corrected peak area
1	75 (LM)	0.5000	60	129	76	13.58	8239	66.858
2	145	0.2753	129	171	147	7.68	176	3.068
3	190	0.1343	171	239	191	7.21	78	1.497
4	260	0.0386	239	335	263	5.34	56	0.430
5	431	0.4675	396	521	428	3.56	719	5.210
6	1559	4.7577	1256	3000	1591	9.51	9573	53.015
7	5075	0.0278	4828	6112	5197	6.35	54	0.310
8	20000 (UM)	0.1094	18415	26770	20534	4.05	1594	14.626

TIC:	5.7013	ng/uL
TIM:	11.2042	nmole/L
Total concentration:	5.9429	ng/uL

```
Sample peak width (sec): 5    Sample min peak height: 50    Sample baseline V to V?: Y    Sample baseline V to V points: 3
Sample filter: Binomial       Number of points for filter: 3  Sample start region (min): 0  Sample end region (min): 30
Marker peak width (sec): 5    Marker min peak height: 200   Marker baseline V to V?: Y    Marker baseline V to V points: 3
Lower marker selection: First peak > 500 RFU                Upper marker selection: Last peak > 200 RFU
Ladder size (bp)75, 200, 300, 400, 500, 700, 1000, 1500, 2000, 3000, 4000, 5000, 7000, 10000, 20000
Quantification using: Lower Marker          Final concentration (ng/uL): 0.5000          Dilution factor: 12.0
```

Sample: SampF9
Well location: F9
Created: Tuesday, September 14, 2021 6:14:00 PM

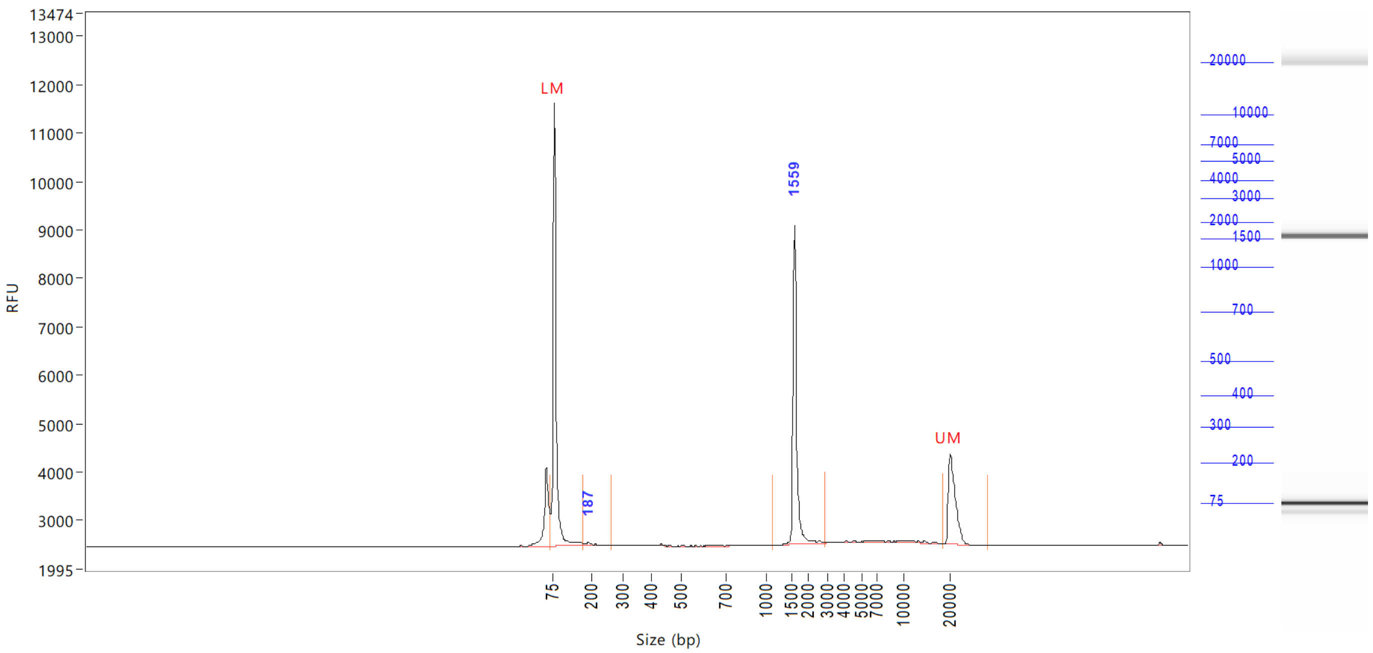

Peak	Size (bp)	Concentration (ng/uL)	From (bp)	To (bp)	Average size (bp)	CV%	RFU	Corrected peak area
1	75 (LM)	0.5000	64	171	77	19.13	9164	69.750
2	187	0.1086	171	260	195	9.50	65	1.263
3	1559	3.2572	1134	2857	1583	9.41	6579	37.865
4	20000 (UM)	0.1245	18415	27979	20491	4.28	1867	17.368

TIC:	3.3658	ng/uL
TIM:	4.3029	nmole/L
Total concentration:	3.6580	ng/uL

```
Sample peak width (sec): 5    Sample min peak height: 50    Sample baseline V to V?: Y    Sample baseline V to V points: 3
Sample filter: Binomial       Number of points for filter: 3  Sample start region (min): 0  Sample end region (min): 30
Marker peak width (sec): 5    Marker min peak height: 200   Marker baseline V to V?: Y    Marker baseline V to V points: 3
Lower marker selection: First peak > 500 RFU                Upper marker selection: Last peak > 200 RFU
Ladder size (bp)75, 200, 300, 400, 500, 700, 1000, 1500, 2000, 3000, 4000, 5000, 7000, 10000, 20000
Quantification using: Lower Marker          Final concentration (ng/uL): 0.5000          Dilution factor: 12.0
```

Sample: SampG9
Well location: G9
Created: Tuesday, September 14, 2021 6:14:00 PM

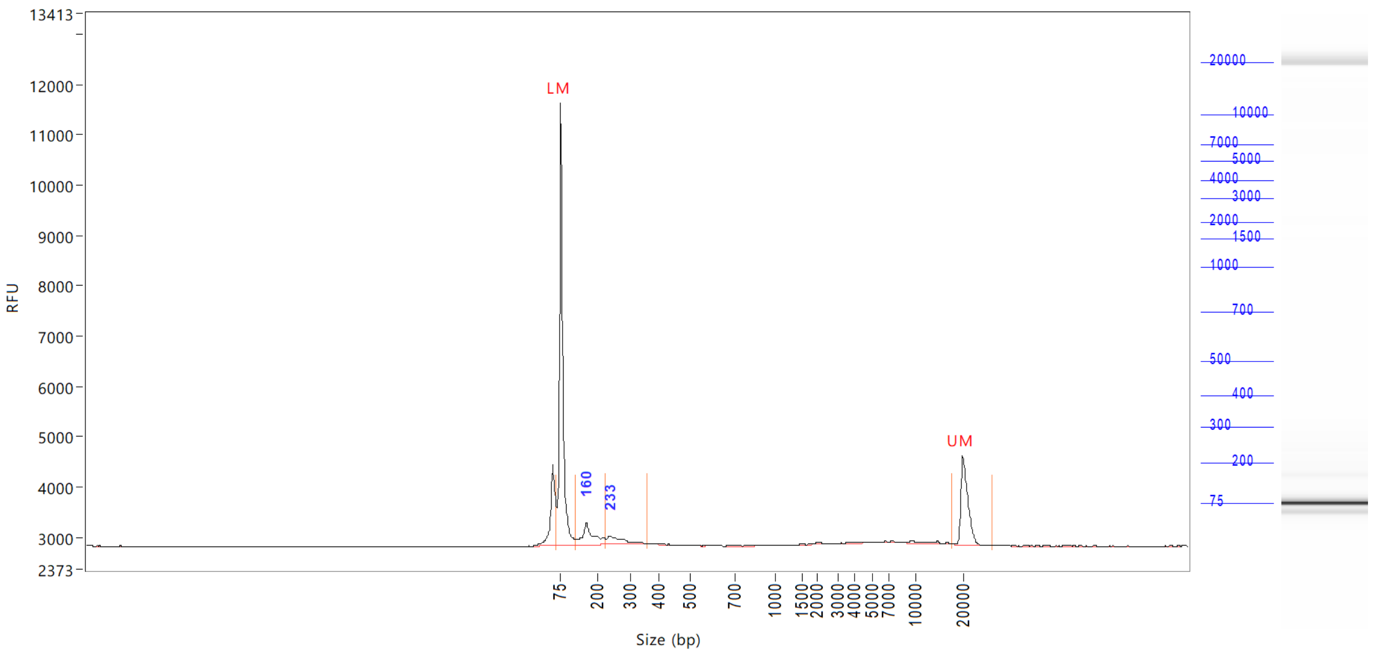

Peak	Size (bp)	Concentration (ng/uL)	From (bp)	To (bp)	Average size (bp)	CV%	RFU	Corrected peak area
1	75 (LM)	0.5000	64	126	77	12.39	8803	72.019
2	160	0.9242	126	224	171	14.88	444	11.093
3	233	0.4363	224	356	260	10.61	167	5.237
4	20000 (UM)	0.1136	17683	26166	20498	4.04	1771	16.359

TIC:	1.3605	ng/uL
TIM:	11.6446	nmole/L
Total concentration:	1.7212	ng/uL

Sample peak width (sec): 5 Sample min peak height: 50 Sample baseline V to V?: Y Sample baseline V to V points: 3
Sample filter: Binomial Number of points for filter: 3 Sample start region (min): 0 Sample end region (min): 30
Marker peak width (sec): 5 Marker min peak height: 200 Marker baseline V to V?: Y Marker baseline V to V points: 3
Lower marker selection: First peak > 500 RFU Upper marker selection: Last peak > 200 RFU
Ladder size (bp)75, 200, 300, 400, 500, 700, 1000, 1500, 2000, 3000, 4000, 5000, 7000, 10000, 20000
Quantification using: Lower Marker Final concentration (ng/uL): 0.5000 Dilution factor: 12.0

Sample: SampH9
Well location: H9
Created: Tuesday, September 14, 2021 6:14:00 PM

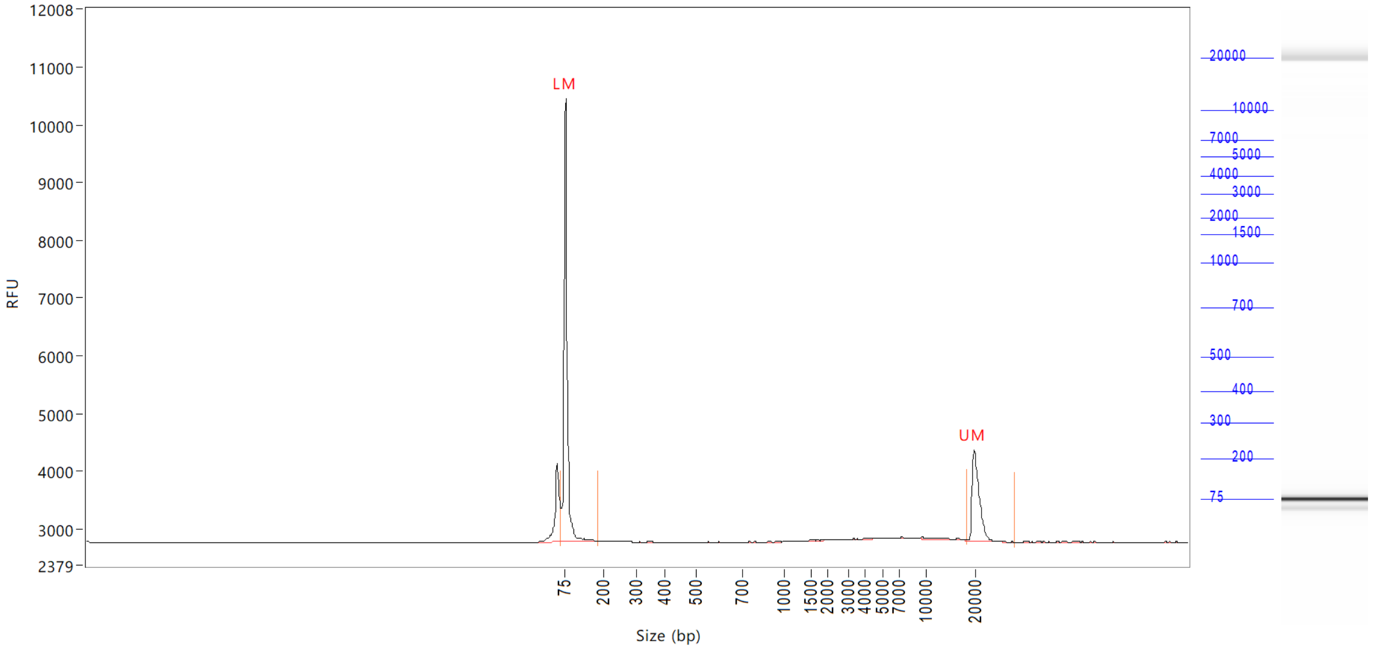

Peak	Size	Concentration	From	To	Average size	CV%	RFU	Corrected peak area
	(bp)	(ng/uL)	(bp)	(bp)	(bp)			
1	75 (LM)	0.5000	62	183	75	14.12	7682	58.247
2	20000 (UM)	0.1239	18537	28463	20478	4.52	1571	14.436

TIC:	0.0000	ng/uL
TIM:	0.0000	nmole/L
Total concentration:	0.3205	ng/uL

Sample peak width (sec): 5 Sample min peak height: 50 Sample baseline V to V?: Y Sample baseline V to V points: 3
Sample filter: Binomial Number of points for filter: 3 Sample start region (min): 0 Sample end region (min): 30
Marker peak width (sec): 5 Marker min peak height: 200 Marker baseline V to V?: Y Marker baseline V to V points: 3
Lower marker selection: First peak > 500 RFU Upper marker selection: Last peak > 200 RFU
Ladder size (bp)75, 200, 300, 400, 500, 700, 1000, 1500, 2000, 3000, 4000, 5000, 7000, 10000, 20000
Quantification using: Lower Marker Final concentration (ng/uL): 0.5000 Dilution factor: 12.0

Sample: SampA10
Well location: A10
Created: Tuesday, September 14, 2021 6:14:00 PM

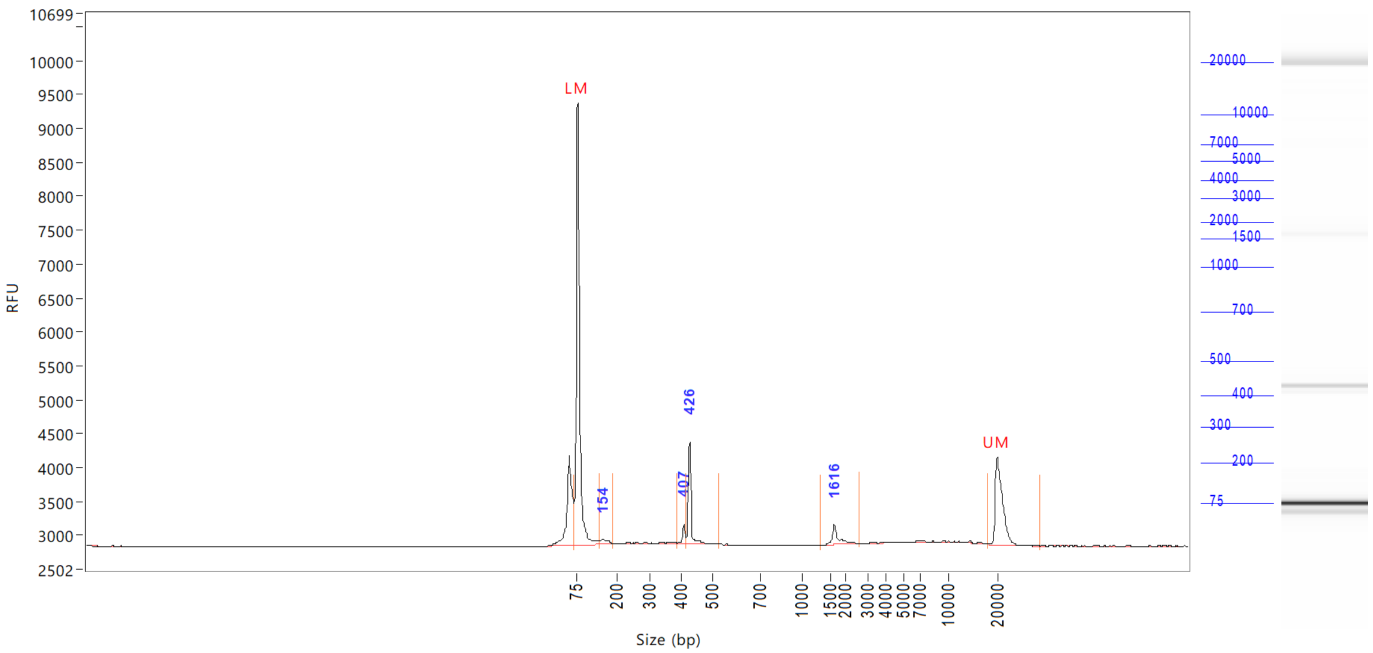

Peak	Size (bp)	Concentration (ng/uL)	From (bp)	To (bp)	Average size (bp)	CV%	RFU	Corrected peak area
1	75 (LM)	0.5000	64	143	76	13.78	6531	51.163
2	154	0.1530	143	189	159	6.75	74	1.304
3	407	0.1845	386	414	406	1.27	296	1.573
4	426	0.9637	414	524	428	2.84	1510	8.217
5	1616	0.3082	1323	2600	1696	10.94	298	2.628
6	20000 (UM)	0.1161	17927	28463	20450	4.48	1283	11.880

TIC:	1.6093	ng/uL
TIM:	6.3326	nmole/L
Total concentration:	1.8782	ng/uL

```
Sample peak width (sec): 5    Sample min peak height: 50    Sample baseline V to V?: Y    Sample baseline V to V points: 3
Sample filter: Binomial       Number of points for filter: 3  Sample start region (min): 0  Sample end region (min): 30
Marker peak width (sec): 5    Marker min peak height: 200   Marker baseline V to V?: Y    Marker baseline V to V points: 3
Lower marker selection: First peak > 500 RFU                Upper marker selection: Last peak > 200 RFU
Ladder size (bp)75, 200, 300, 400, 500, 700, 1000, 1500, 2000, 3000, 4000, 5000, 7000, 10000, 20000
Quantification using: Lower Marker          Final concentration (ng/uL): 0.5000          Dilution factor: 12.0
```

Sample: SampB10
Well location: B10
Created: Tuesday, September 14, 2021 6:14:00 PM

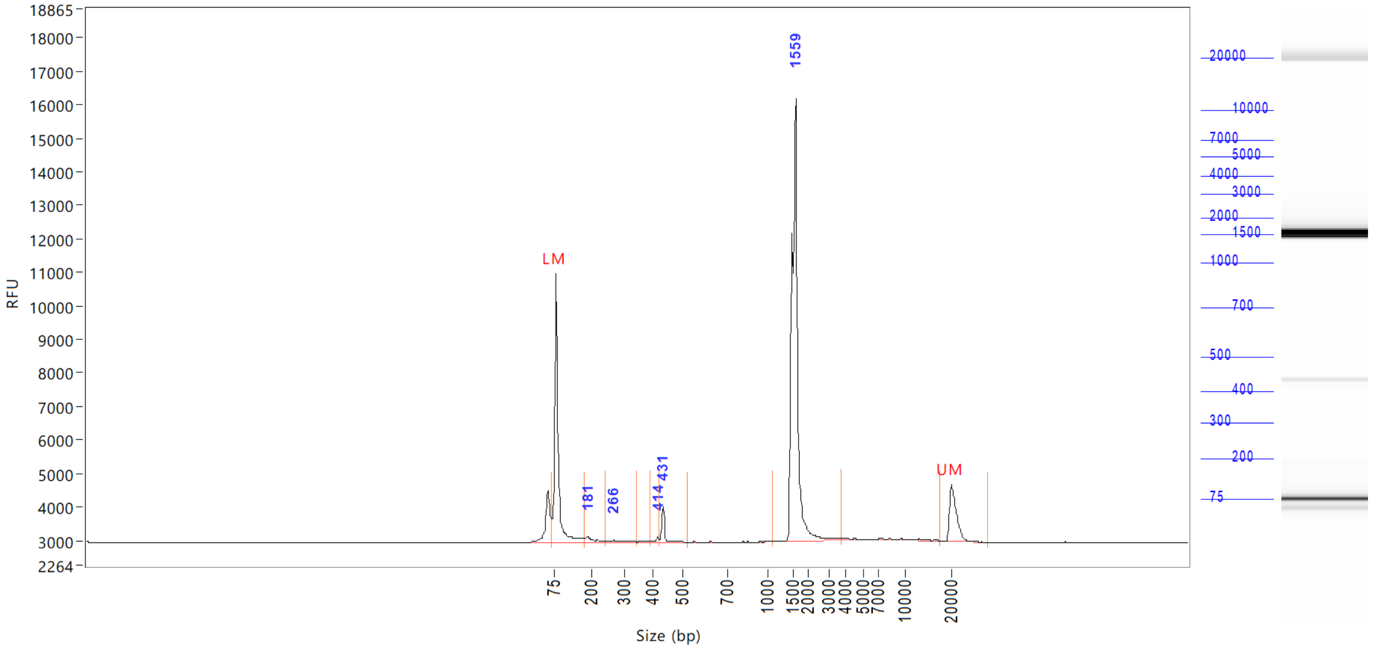

Peak	Size (bp)	Concentration (ng/uL)	From (bp)	To (bp)	Average size (bp)	CV%	RFU	Corrected peak area
1	75 (LM)	0.5000	60	171	81	23.75	8019	68.979
2	181	0.2735	171	239	195	9.43	174	3.145
3	266	0.0860	239	341	268	8.71	53	0.989
4	414	0.0711	390	422	413	1.33	156	0.817
5	431	0.4981	422	521	434	3.03	1058	5.726
6	1559	10.4379	1112	3750	1565	14.02	13230	119.999
7	20000 (UM)	0.1130	17683	27617	20459	4.41	1689	15.592

TIC:	11.3666	ng/uL
TIM:	15.9847	nmole/L
Total concentration:	11.6617	ng/uL

```
Sample peak width (sec): 5    Sample min peak height: 50    Sample baseline V to V?: Y    Sample baseline V to V points: 3
Sample filter: Binomial       Number of points for filter: 3  Sample start region (min): 0  Sample end region (min): 30
Marker peak width (sec): 5    Marker min peak height: 200   Marker baseline V to V?: Y    Marker baseline V to V points: 3
Lower marker selection: First peak > 500 RFU                Upper marker selection: Last peak > 200 RFU
Ladder size (bp)75, 200, 300, 400, 500, 700, 1000, 1500, 2000, 3000, 4000, 5000, 7000, 10000, 20000
Quantification using: Lower Marker          Final concentration (ng/uL): 0.5000          Dilution factor: 12.0
```

Sample: SampC10
Well location: C10
Created: Tuesday, September 14, 2021 6:14:00 PM

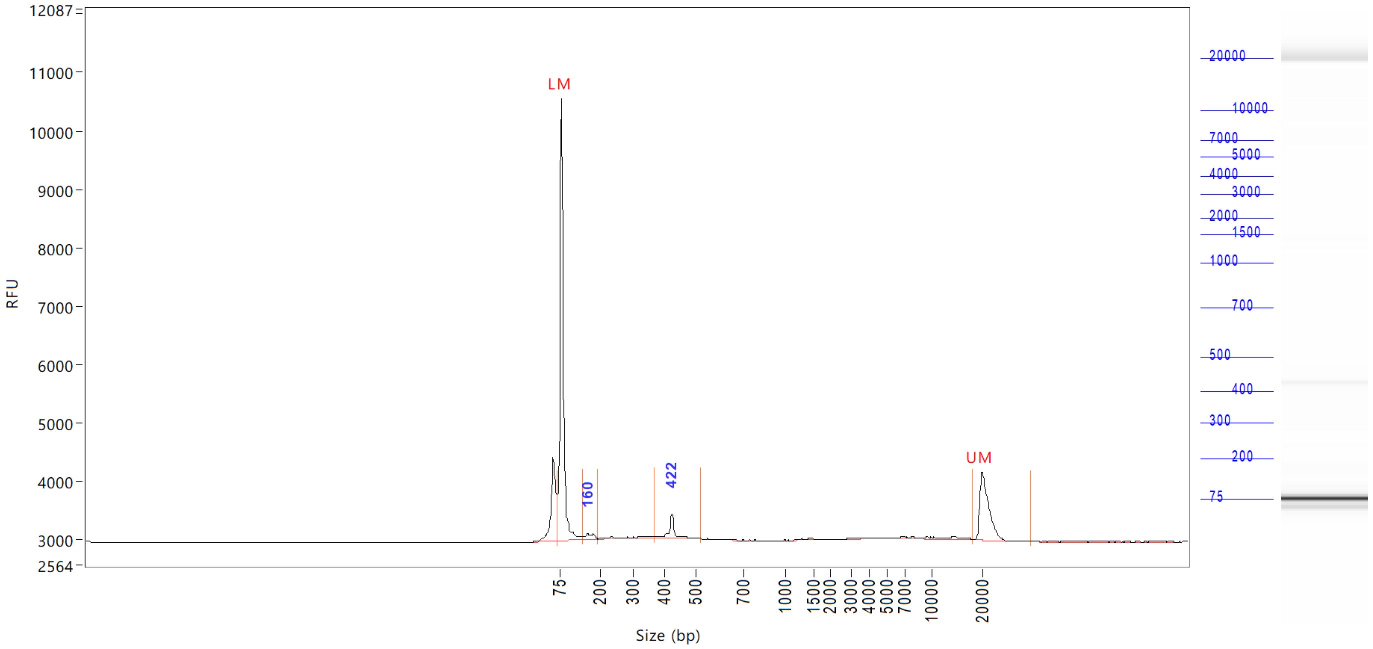

Peak	Size (bp)	Concentration (ng/uL)	From (bp)	To (bp)	Average size (bp)	CV%	RFU	Corrected peak area
1	75 (LM)	0.5000	64	145	77	13.25	7582	62.490
2	160	0.1696	145	190	164	6.51	91	1.766
3	422	0.4853	366	516	421	5.75	425	5.054
4	20000 (UM)	0.1099	18293	29793	20698	5.39	1162	13.736

TIC:	0.6549	ng/uL
TIM:	3.5964	nmole/L
Total concentration:	1.2424	ng/uL

Sample peak width (sec): 5 Sample min peak height: 50 Sample baseline V to V?: Y Sample baseline V to V points: 3
Sample filter: Binomial Number of points for filter: 3 Sample start region (min): 0 Sample end region (min): 30
Marker peak width (sec): 5 Marker min peak height: 200 Marker baseline V to V?: Y Marker baseline V to V points: 3
Lower marker selection: First peak > 500 RFU Upper marker selection: Last peak > 200 RFU
Ladder size (bp)75, 200, 300, 400, 500, 700, 1000, 1500, 2000, 3000, 4000, 5000, 7000, 10000, 20000
Quantification using: Lower Marker Final concentration (ng/uL): 0.5000 Dilution factor: 12.0

Sample: SampD10
Well location: D10
Created: Tuesday, September 14, 2021 6:14:00 PM

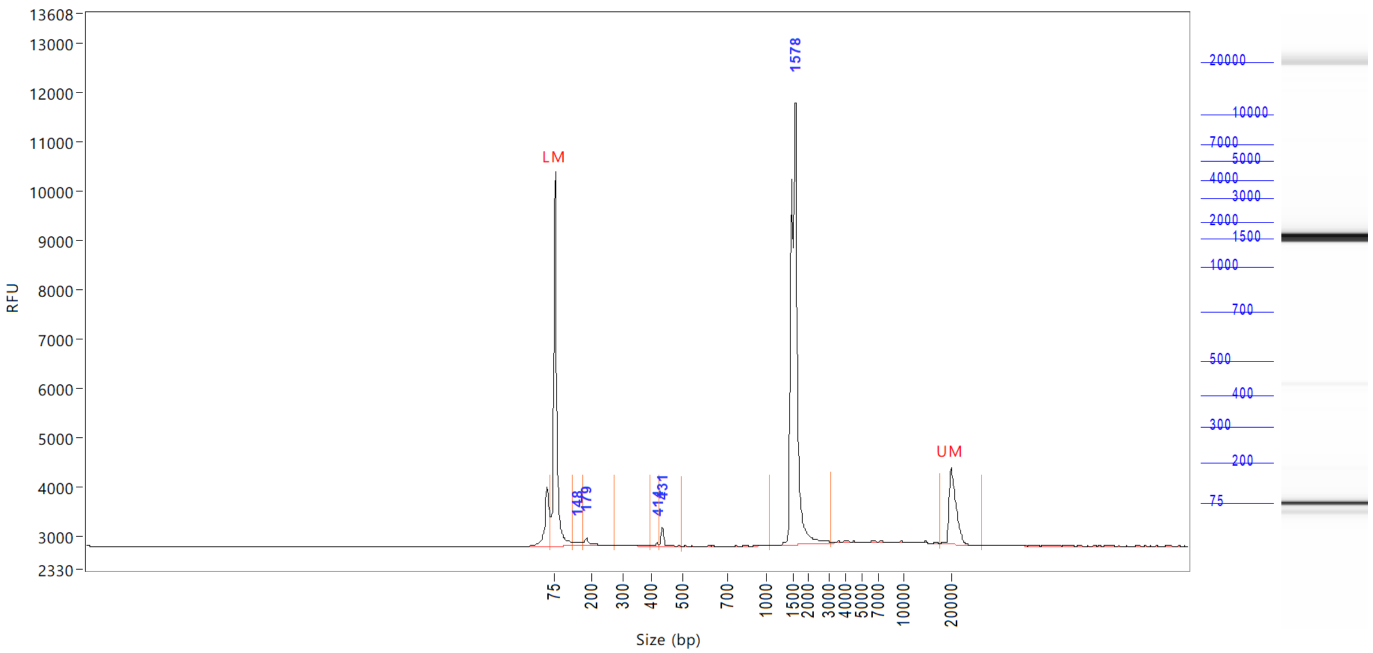

Peak	Size (bp)	Concentration (ng/uL)	From (bp)	To (bp)	Average size (bp)	CV%	RFU	Corrected peak area
1	75 (LM)	0.5000	60	131	75	13.17	7594	56.773
2	148	0.1839	131	168	149	7.20	84	1.740
3	179	0.2529	168	271	190	10.29	156	2.393
4	414	0.0441	390	422	411	1.81	64	0.418
5	431	0.2249	422	496	433	2.63	395	2.128
6	1578	9.3873	1045	3144	1556	11.08	8975	88.824
7	20000 (UM)	0.1255	17683	26529	20456	4.06	1558	14.247

TIC:	10.0931	ng/uL
TIM:	15.1787	nmole/L
Total concentration:	10.3394	ng/uL

```
Sample peak width (sec): 5     Sample min peak height: 50     Sample baseline V to V?: Y     Sample baseline V to V points: 3
Sample filter: Binomial        Number of points for filter: 3  Sample start region (min): 0  Sample end region (min): 30
Marker peak width (sec): 5     Marker min peak height: 200    Marker baseline V to V?: Y     Marker baseline V to V points: 3
Lower marker selection: First peak > 500 RFU                  Upper marker selection: Last peak > 200 RFU
Ladder size (bp)75, 200, 300, 400, 500, 700, 1000, 1500, 2000, 3000, 4000, 5000, 7000, 10000, 20000
Quantification using: Lower Marker          Final concentration (ng/uL): 0.5000          Dilution factor: 12.0
```

Sample: SampE10
Well location: E10
Created: Tuesday, September 14, 2021 6:14:00 PM

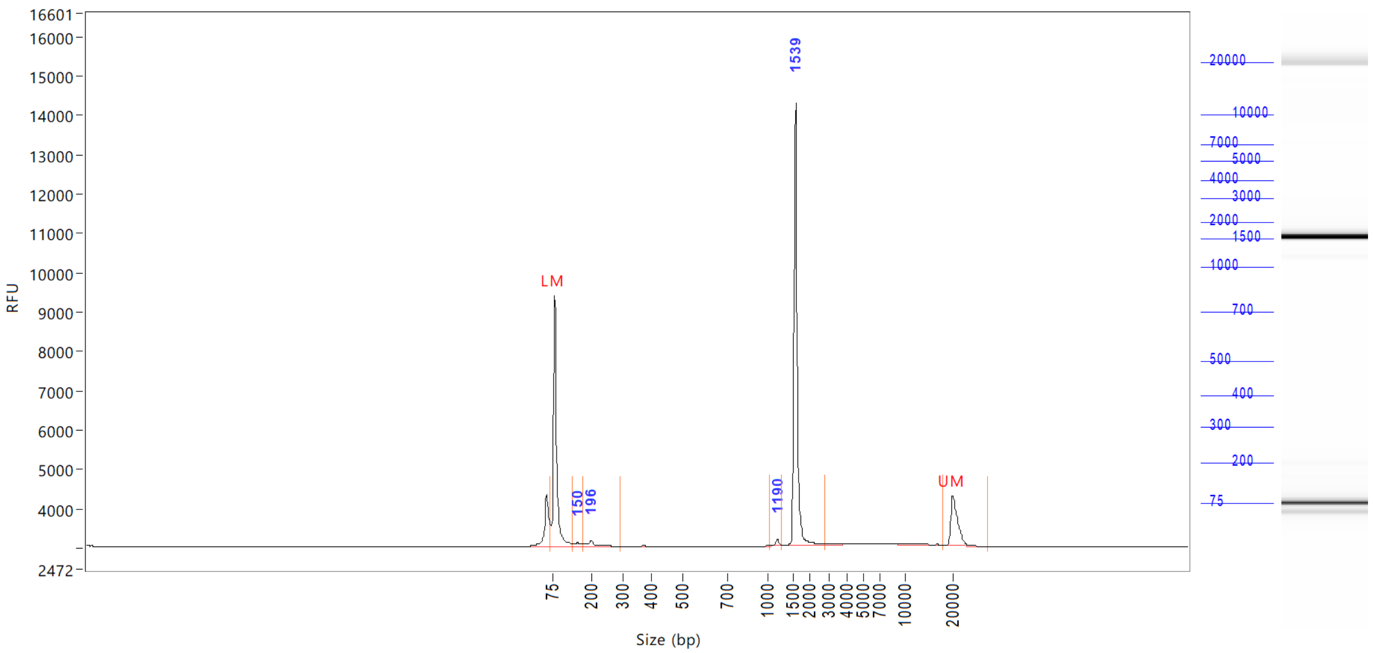

Peak	Size<br>(bp)	Concentration<br>(ng/uL)	From<br>(bp)	To<br>(bp)	Average size<br>(bp)	CV%	RFU	Corrected peak area
1	75 (LM)	0.5000	64	133	78	13.65	6367	50.673
2	150	0.2171	133	169	150	6.95	93	1.833
3	196	0.3219	169	289	202	11.93	156	2.718
4	1190	0.1213	1034	1267	1173	3.14	172	1.025
5	1539	6.9738	1267	2800	1556	8.05	11267	58.897
6	20000 (UM)	0.1160	17805	27254	20451	4.25	1279	11.753

TIC:	7.6340	ng/uL
TIM:	12.5462	nmole/L
Total concentration:	7.9740	ng/uL

```
Sample peak width (sec): 5    Sample min peak height: 50    Sample baseline V to V?: Y    Sample baseline V to V points: 3
Sample filter: Binomial       Number of points for filter: 3  Sample start region (min): 0  Sample end region (min): 30
Marker peak width (sec): 5    Marker min peak height: 200   Marker baseline V to V?: Y    Marker baseline V to V points: 3
Lower marker selection: First peak > 500 RFU                Upper marker selection: Last peak > 200 RFU
Ladder size (bp)75, 200, 300, 400, 500, 700, 1000, 1500, 2000, 3000, 4000, 5000, 7000, 10000, 20000
Quantification using: Lower Marker          Final concentration (ng/uL): 0.5000          Dilution factor: 12.0
```

Sample: SampF10
Well location: F10
Created: Tuesday, September 14, 2021 6:14:00 PM

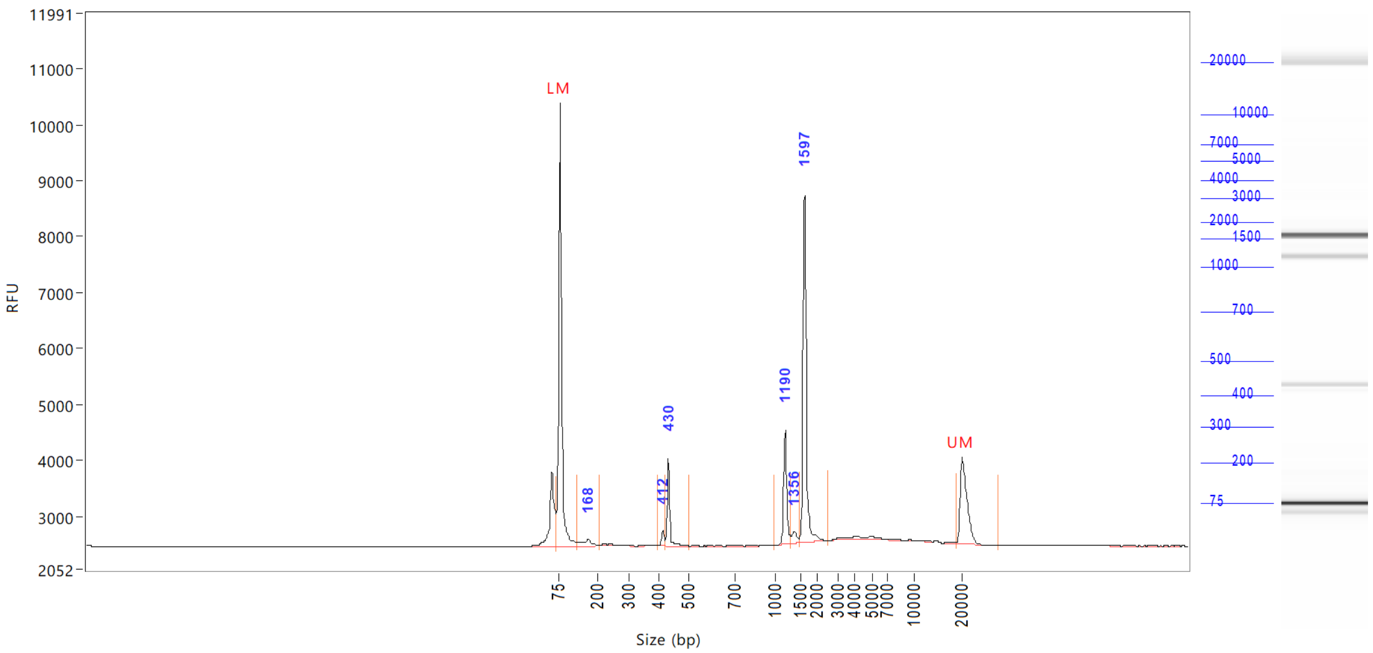

Peak	Size (bp)	Concentration (ng/uL)	From (bp)	To (bp)	Average size (bp)	CV%	RFU	Corrected peak area
1	75 (LM)	0.5000	64	131	75	12.28	7939	57.284
2	168	0.2825	131	205	161	11.03	122	2.697
3	412	0.1476	398	420	412	1.15	271	1.409
4	430	0.8595	420	501	431	2.31	1567	8.205
5	1190	1.1649	991	1300	1178	3.19	2030	11.121
6	1356	0.1760	1300	1456	1366	3.13	206	1.681
7	1597	3.3850	1456	2515	1585	5.81	6193	32.318
8	20000 (UM)	0.1215	18659	27617	20462	3.89	1558	13.925

TIC:	6.0155	ng/uL
TIM:	12.1099	nmole/L
Total concentration:	6.4745	ng/uL

```
Sample peak width (sec): 5    Sample min peak height: 50    Sample baseline V to V?: Y    Sample baseline V to V points: 3
Sample filter: Binomial       Number of points for filter: 3  Sample start region (min): 0  Sample end region (min): 30
Marker peak width (sec): 5    Marker min peak height: 200   Marker baseline V to V?: Y    Marker baseline V to V points: 3
Lower marker selection: First peak > 500 RFU                Upper marker selection: Last peak > 200 RFU
Ladder size (bp)75, 200, 300, 400, 500, 700, 1000, 1500, 2000, 3000, 4000, 5000, 7000, 10000, 20000
Quantification using: Lower Marker          Final concentration (ng/uL): 0.5000          Dilution factor: 12.0
```

Sample: SampG10
Well location: G10
Created: Tuesday, September 14, 2021 6:14:00 PM

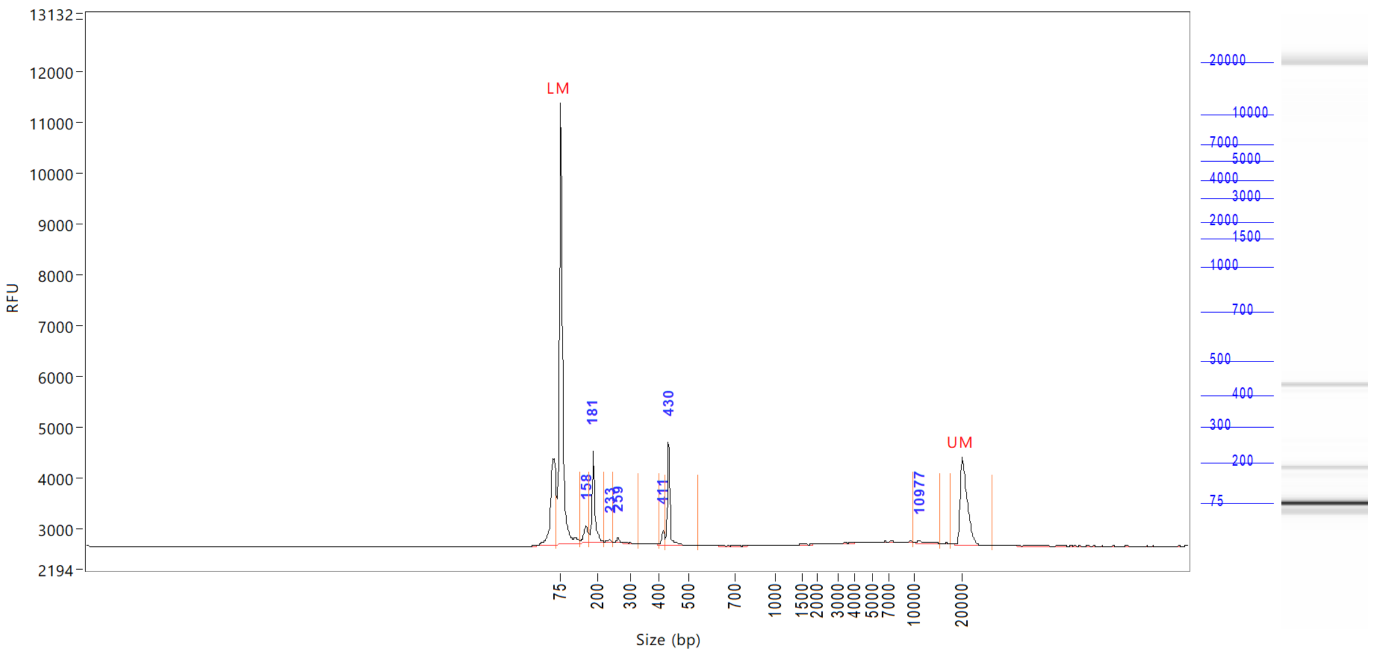

Peak	Size (bp)	Concentration (ng/uL)	From (bp)	To (bp)	Average size (bp)	CV%	RFU	Corrected peak area
1	75 (LM)	0.5000	64	139	76	13.40	8699	71.092
2	158	0.2947	139	169	157	4.77	332	3.492
3	181	1.0102	169	217	182	3.72	1785	11.970
4	233	0.0375	217	246	231	2.78	52	0.444
5	259	0.0685	246	327	268	6.74	84	0.811
6	411	0.1271	400	418	411	1.05	279	1.507
7	430	0.8964	418	537	430	2.31	2011	10.622
8	10977	0.0773	9933	15366	12238	12.73	56	0.916
9	20000 (UM)	0.1108	17683	26287	20472	3.89	1731	15.752

TIC:	2.5118	ng/uL
TIM:	16.8471	nmole/L
Total concentration:	2.6985	ng/uL

Sample peak width (sec): 5   Sample min peak height: 50   Sample baseline V to V?: Y   Sample baseline V to V points: 3
Sample filter: Binomial   Number of points for filter: 3   Sample start region (min): 0   Sample end region (min): 30
Marker peak width (sec): 5   Marker min peak height: 200   Marker baseline V to V?: Y   Marker baseline V to V points: 3
Lower marker selection: First peak > 500 RFU   Upper marker selection: Last peak > 200 RFU
Ladder size (bp)75, 200, 300, 400, 500, 700, 1000, 1500, 2000, 3000, 4000, 5000, 7000, 10000, 20000
Quantification using: Lower Marker   Final concentration (ng/uL): 0.5000   Dilution factor: 12.0

Sample: SampH10
Well location: H10
Created: Tuesday, September 14, 2021 6:14:00 PM

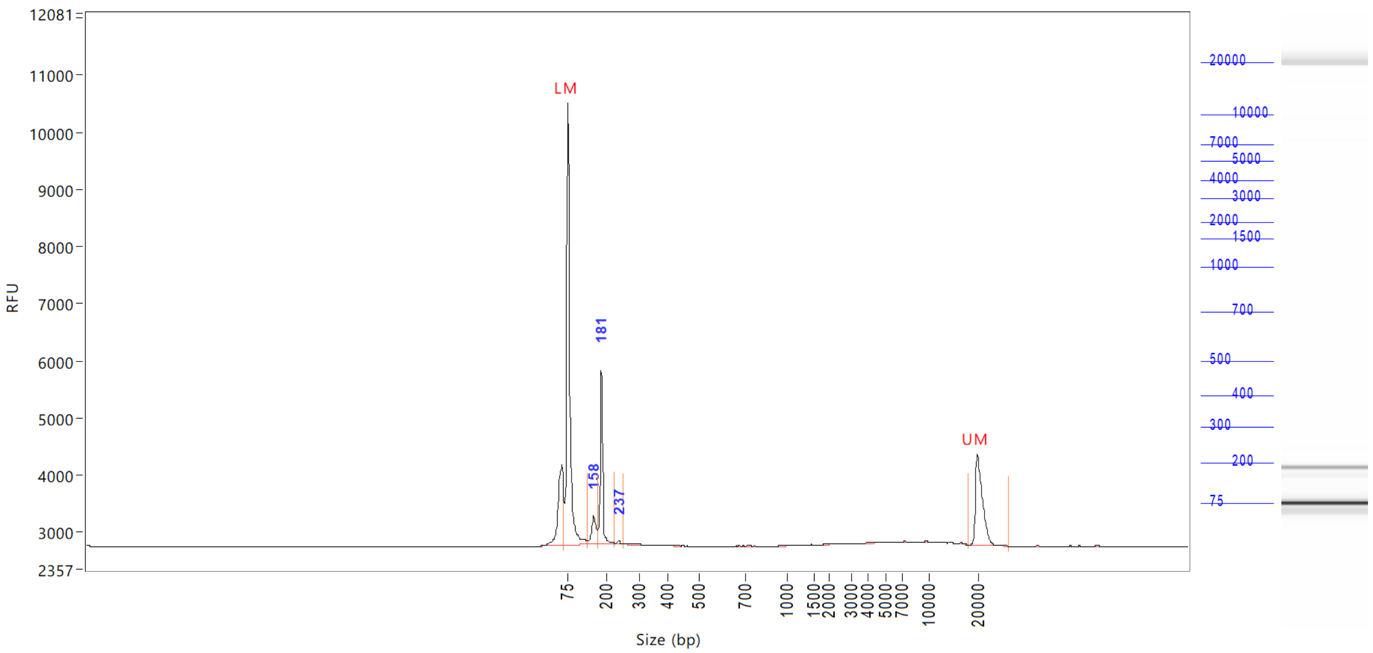

Peak	Size	Concentration	From	To	Average size	CV%	RFU	Corrected peak area
	(bp)	(ng/uL)	(bp)	(bp)	(bp)			
1	75 (LM)	0.5000	64	137	76	13.55	7745	59.376
2	158	0.5556	137	171	158	5.08	491	5.498
3	181	1.7581	171	224	181	2.99	3027	17.399
4	237	0.0308	224	251	235	1.60	55	0.305
5	20000 (UM)	0.1217	18049	26529	20474	4.06	1584	14.450

TIC:	2.3445	ng/uL
TIM:	21.9639	nmole/L
Total concentration:	2.6336	ng/uL

```
Sample peak width (sec): 5    Sample min peak height: 50    Sample baseline V to V?: Y    Sample baseline V to V points: 3
Sample filter: Binomial       Number of points for filter: 3  Sample start region (min): 0  Sample end region (min): 30
Marker peak width (sec): 5    Marker min peak height: 200   Marker baseline V to V?: Y    Marker baseline V to V points: 3
Lower marker selection: First peak > 500 RFU                Upper marker selection: Last peak > 200 RFU
Ladder size (bp)75, 200, 300, 400, 500, 700, 1000, 1500, 2000, 3000, 4000, 5000, 7000, 10000, 20000
Quantification using: Lower Marker          Final concentration (ng/uL): 0.5000          Dilution factor: 12.0
```

Sample: SampA11
Well location: A11
Created: Tuesday, September 14, 2021 6:14:00 PM

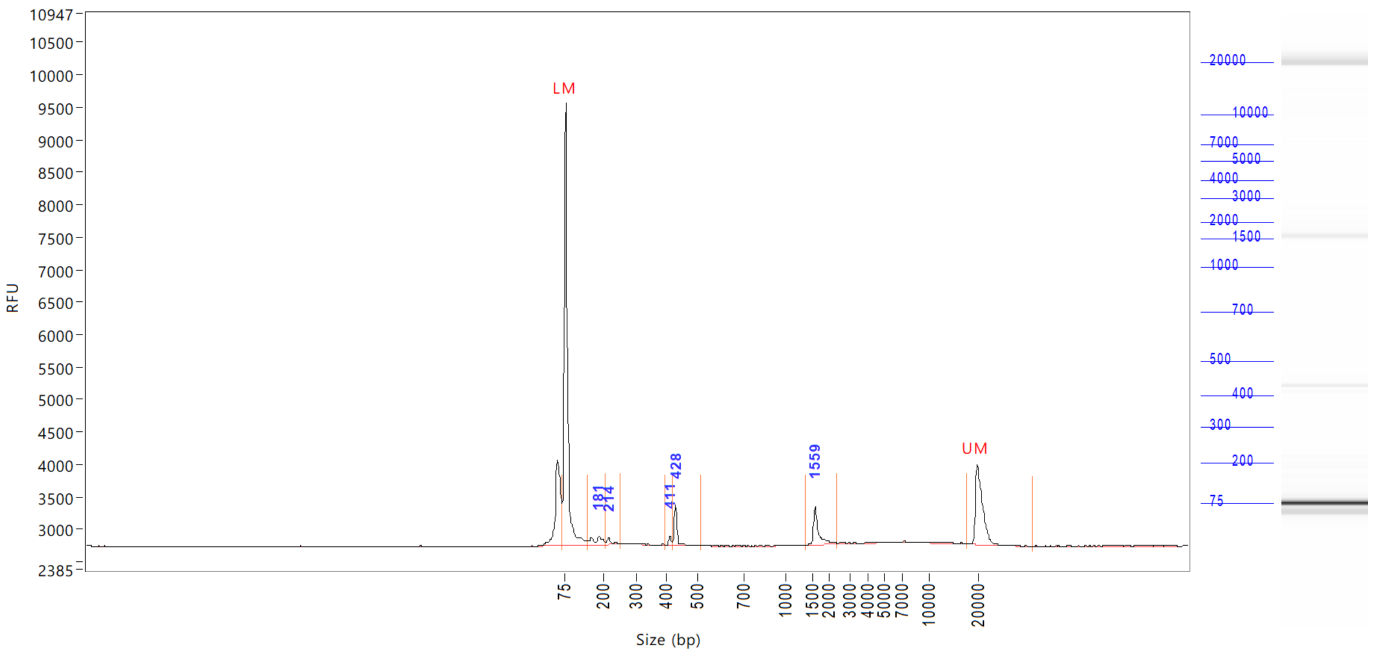

Peak	Size (bp)	Concentration (ng/uL)	From (bp)	To (bp)	Average size (bp)	CV%	RFU	Corrected peak area
1	75 (LM)	0.5000	64	147	77	15.88	6827	55.196
2	181	0.2857	147	203	173	9.21	133	2.628
3	214	0.1211	203	248	216	4.53	109	1.114
4	411	0.0867	396	418	409	1.27	145	0.797
5	428	0.3560	418	514	429	2.52	617	3.275
6	1559	0.5163	1356	2372	1643	10.80	601	4.749
7	20000 (UM)	0.1048	17927	31486	20570	5.55	1225	11.573

TIC:	1.3657	ng/uL
TIM:	5.8675	nmole/L
Total concentration:	1.6530	ng/uL

```
Sample peak width (sec): 5    Sample min peak height: 50    Sample baseline V to V?: Y    Sample baseline V to V points: 3
Sample filter: Binomial       Number of points for filter: 3  Sample start region (min): 0  Sample end region (min): 30
Marker peak width (sec): 5    Marker min peak height: 200   Marker baseline V to V?: Y    Marker baseline V to V points: 3
Lower marker selection: First peak > 500 RFU                Upper marker selection: Last peak > 200 RFU
Ladder size (bp)75, 200, 300, 400, 500, 700, 1000, 1500, 2000, 3000, 4000, 5000, 7000, 10000, 20000
Quantification using: Lower Marker          Final concentration (ng/uL): 0.5000          Dilution factor: 12.0
```

Sample: SampB11
Well location: B11
Created: Tuesday, September 14, 2021 6:14:00 PM

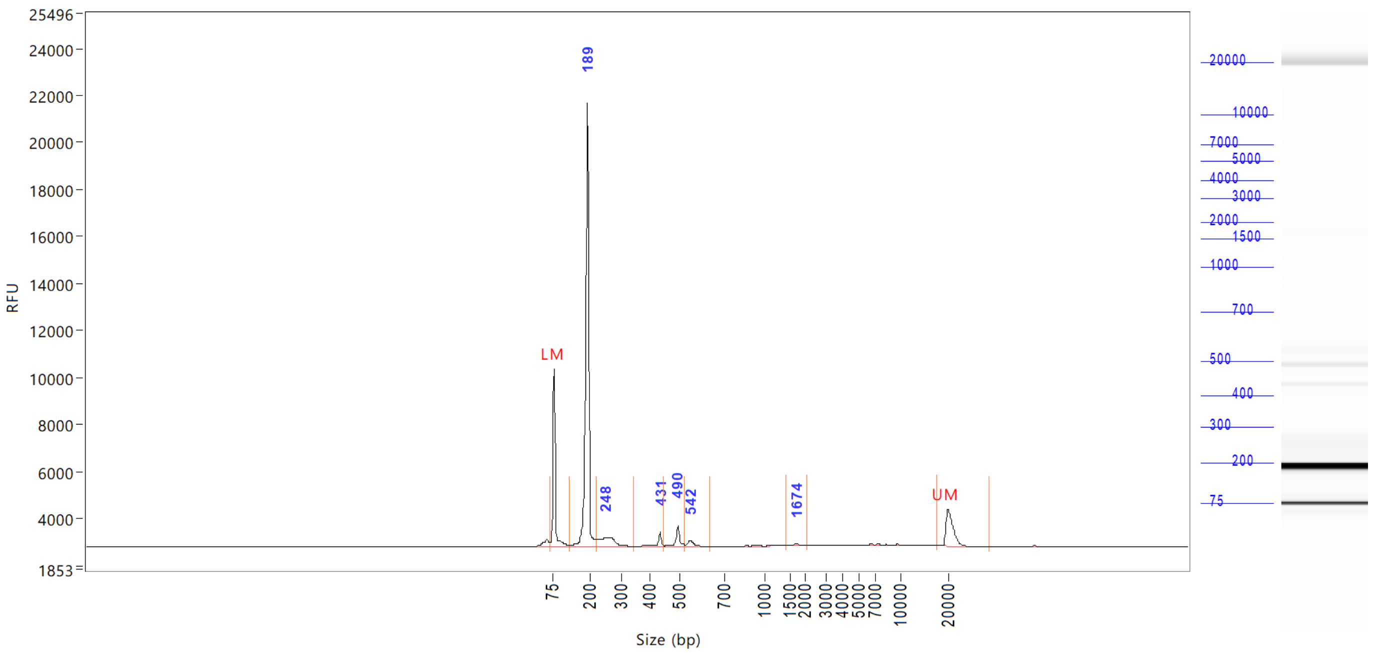

Peak	Size (bp)	Concentration (ng/uL)	From (bp)	To (bp)	Average size (bp)	CV%	RFU	Corrected peak area
1	75 (LM)	0.5000	62	128	76	11.95	7575	46.080
2	189	18.3068	128	219	184	4.71	18896	140.595
3	248	1.6599	219	341	252	8.28	373	12.748
4	431	0.5418	341	445	420	4.13	594	4.161
5	490	0.8444	445	519	488	2.39	894	6.485
6	542	0.4027	519	634	546	3.27	259	3.092
7	1674	0.1105	1400	2030	1639	6.85	78	0.849
8	20000 (UM)	0.1610	17561	28826	20528	4.61	1587	14.839

TIC:	21.8661	ng/uL
TIM:	180.6992	nmole/L
Total concentration:	22.2256	ng/uL

```
Sample peak width (sec): 5    Sample min peak height: 50    Sample baseline V to V?: Y    Sample baseline V to V points: 3
Sample filter: Binomial       Number of points for filter: 3  Sample start region (min): 0  Sample end region (min): 30
Marker peak width (sec): 5    Marker min peak height: 200   Marker baseline V to V?: Y    Marker baseline V to V points: 3
Lower marker selection: First peak > 500 RFU                Upper marker selection: Last peak > 200 RFU
Ladder size (bp)75, 200, 300, 400, 500, 700, 1000, 1500, 2000, 3000, 4000, 5000, 7000, 10000, 20000
Quantification using: Lower Marker          Final concentration (ng/uL): 0.5000          Dilution factor: 12.0
```

Sample: SampC11
Well location: C11
Created: Tuesday, September 14, 2021 6:14:00 PM

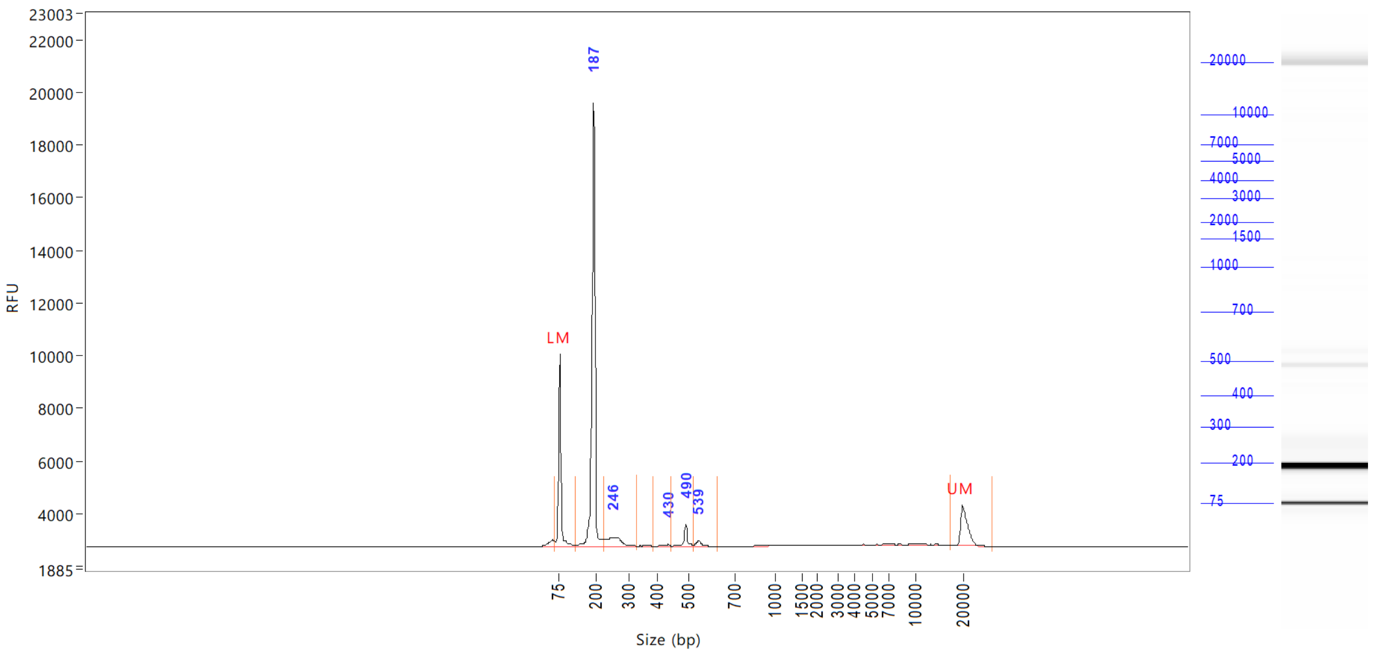

Peak	Size (bp)	Concentration (ng/uL)	From (bp)	To (bp)	Average size (bp)	CV%	RFU	Corrected peak area
1	75 (LM)	0.5000	64	128	75	10.84	7336	43.617
2	187	17.3504	128	219	184	4.61	16872	126.130
3	246	1.5398	219	327	249	7.63	344	11.194
4	430	0.1015	382	443	419	2.93	77	0.738
5	490	0.7699	443	516	487	2.23	817	5.597
6	539	0.3574	516	626	543	3.17	219	2.598
7	20000 (UM)	0.1624	17439	26287	20445	4.05	1522	14.165

TIC:	20.1190	ng/uL
TIM:	169.2795	nmole/L
Total concentration:	20.4720	ng/uL

Sample peak width (sec): 5 Sample min peak height: 50 Sample baseline V to V?: Y Sample baseline V to V points: 3
Sample filter: Binomial Number of points for filter: 3 Sample start region (min): 0 Sample end region (min): 30
Marker peak width (sec): 5 Marker min peak height: 200 Marker baseline V to V?: Y Marker baseline V to V points: 3
Lower marker selection: First peak > 500 RFU Upper marker selection: Last peak > 200 RFU
Ladder size (bp)75, 200, 300, 400, 500, 700, 1000, 1500, 2000, 3000, 4000, 5000, 7000, 10000, 20000
Quantification using: Lower Marker Final concentration (ng/uL): 0.5000 Dilution factor: 12.0

Sample: SampD11
Well location: D11
Created: Tuesday, September 14, 2021 6:14:00 PM

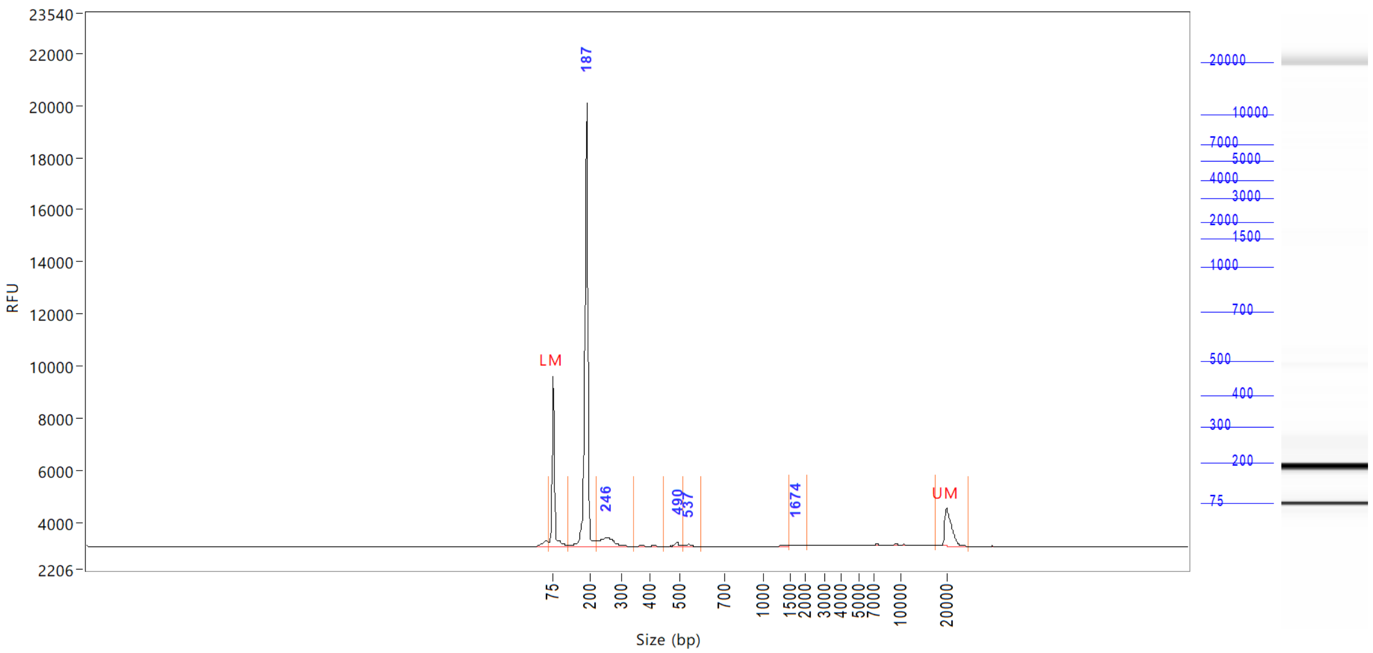

Peak	Size (bp)	Concentration (ng/uL)	From (bp)	To (bp)	Average size (bp)	CV%	RFU	Corrected peak area
1	75 (LM)	0.5000	60	128	75	11.83	6560	40.718
2	187	17.8969	128	217	183	4.53	17051	121.455
3	246	1.5842	217	345	251	8.32	312	10.751
4	490	0.2186	445	516	487	2.62	185	1.483
5	537	0.1446	516	598	541	2.75	82	0.982
6	1674	0.0897	1500	2058	1670	7.32	52	0.609
7	20000 (UM)	0.1637	17561	24715	20457	4.05	1450	13.334

TIC:	19.9340	ng/uL
TIM:	172.4281	nmole/L
Total concentration:	20.4210	ng/uL

Sample peak width (sec): 5 Sample min peak height: 50 Sample baseline V to V?: Y Sample baseline V to V points: 3
Sample filter: Binomial Number of points for filter: 3 Sample start region (min): 0 Sample end region (min): 30
Marker peak width (sec): 5 Marker min peak height: 200 Marker baseline V to V?: Y Marker baseline V to V points: 3
Lower marker selection: First peak > 500 RFU Upper marker selection: Last peak > 200 RFU
Ladder size (bp)75, 200, 300, 400, 500, 700, 1000, 1500, 2000, 3000, 4000, 5000, 7000, 10000, 20000
Quantification using: Lower Marker Final concentration (ng/uL): 0.5000 Dilution factor: 12.0

Sample: SampE11
Well location: E11
Created: Tuesday, September 14, 2021 6:14:00 PM

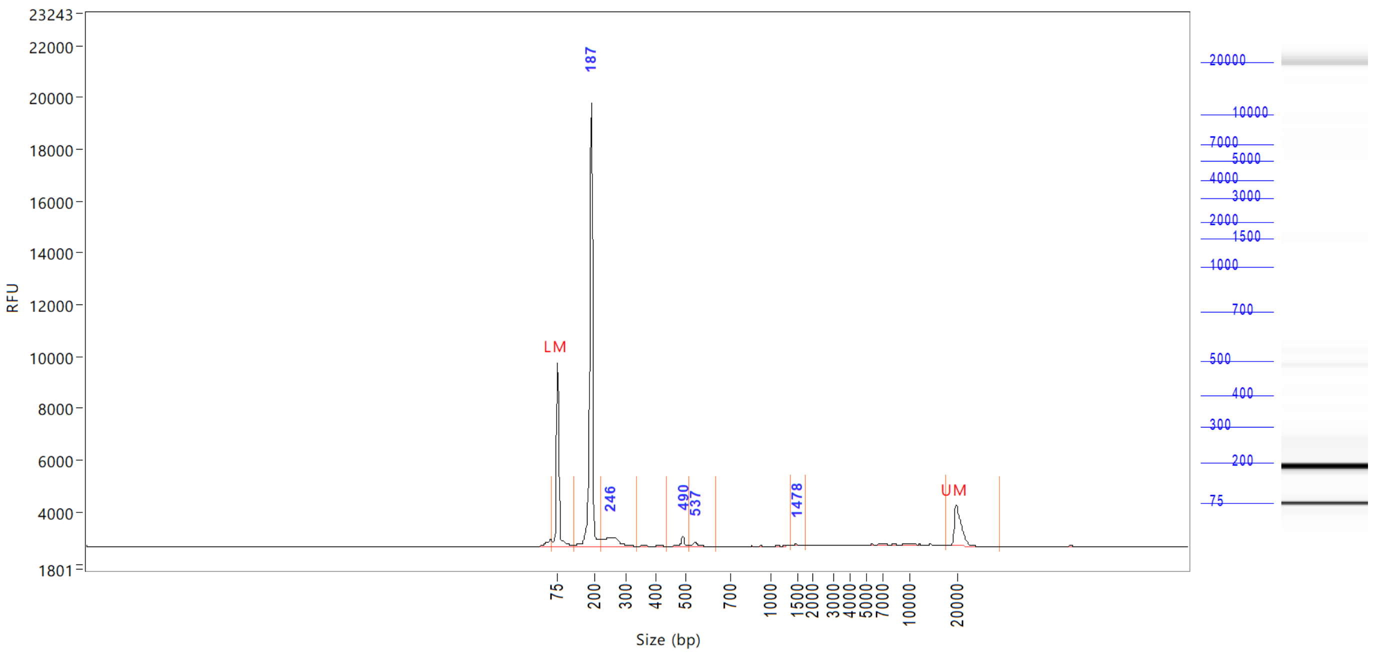

Peak	Size (bp)	Concentration (ng/uL)	From (bp)	To (bp)	Average size (bp)	CV%	RFU	Corrected peak area
1	75 (LM)	0.5000	58	129	75	12.08	7069	43.216
2	187	17.1268	129	219	183	4.71	17134	123.359
3	246	1.6132	219	335	251	8.01	349	11.619
4	490	0.4170	437	516	486	2.59	394	3.004
5	537	0.2650	516	634	545	3.85	157	1.909
6	1478	0.0725	1367	1770	1501	4.75	68	0.522
7	20000 (UM)	0.1669	17805	29068	20418	4.25	1575	14.423

TIC:	19.4944	ng/uL
TIM:	166.7243	nmole/L
Total concentration:	19.8204	ng/uL

Sample peak width (sec): 5   Sample min peak height: 50   Sample baseline V to V?: Y   Sample baseline V to V points: 3
Sample filter: Binomial   Number of points for filter: 3 Sample start region (min): 0 Sample end region (min): 30
Marker peak width (sec): 5   Marker min peak height: 200   Marker baseline V to V?: Y   Marker baseline V to V points: 3
Lower marker selection: First peak > 500 RFU   Upper marker selection: Last peak > 200 RFU
Ladder size (bp)75, 200, 300, 400, 500, 700, 1000, 1500, 2000, 3000, 4000, 5000, 7000, 10000, 20000
Quantification using: Lower Marker   Final concentration (ng/uL): 0.5000   Dilution factor: 12.0

Sample: SampF11
Well location: F11
Created: Tuesday, September 14, 2021 6:14:00 PM

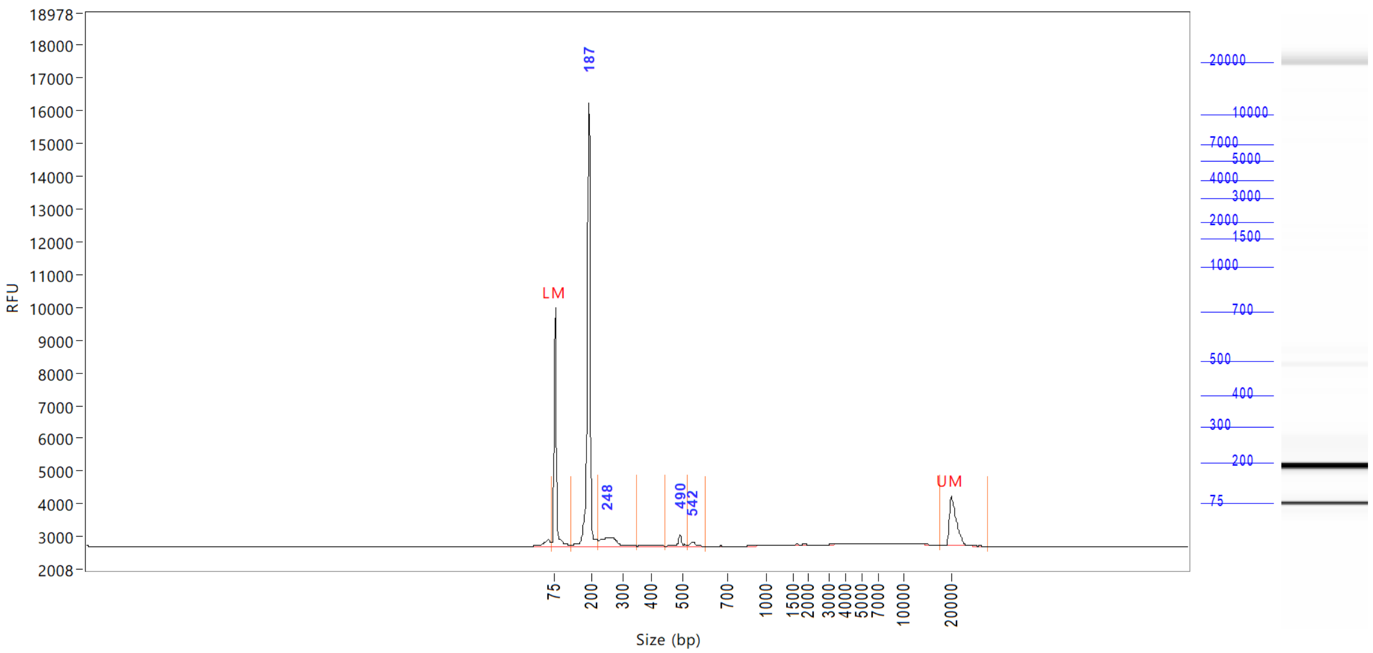

Peak	Size (bp)	Concentration (ng/uL)	From (bp)	To (bp)	Average size (bp)	CV%	RFU	Corrected peak area
1	75 (LM)	0.5000	64	129	75	10.66	7297	42.493
2	187	13.9913	129	219	184	4.45	13553	99.090
3	248	1.2949	219	343	252	8.44	274	9.171
4	490	0.3837	445	521	489	2.64	358	2.717
5	542	0.1937	521	600	545	2.53	122	1.372
6	20000 (UM)	0.1651	17805	27859	20572	4.33	1509	14.031

TIC:	15.8636	ng/uL
TIM:	135.3394	nmole/L
Total concentration:	16.2525	ng/uL

Sample peak width (sec): 5 Sample min peak height: 50 Sample baseline V to V?: Y Sample baseline V to V points: 3
Sample filter: Binomial Number of points for filter: 3 Sample start region (min): 0 Sample end region (min): 30
Marker peak width (sec): 5 Marker min peak height: 200 Marker baseline V to V?: Y Marker baseline V to V points: 3
Lower marker selection: First peak > 500 RFU Upper marker selection: Last peak > 200 RFU
Ladder size (bp)75, 200, 300, 400, 500, 700, 1000, 1500, 2000, 3000, 4000, 5000, 7000, 10000, 20000
Quantification using: Lower Marker Final concentration (ng/uL): 0.5000 Dilution factor: 12.0

Sample: SampG11
Well location: G11
Created: Tuesday, September 14, 2021 6:14:00 PM

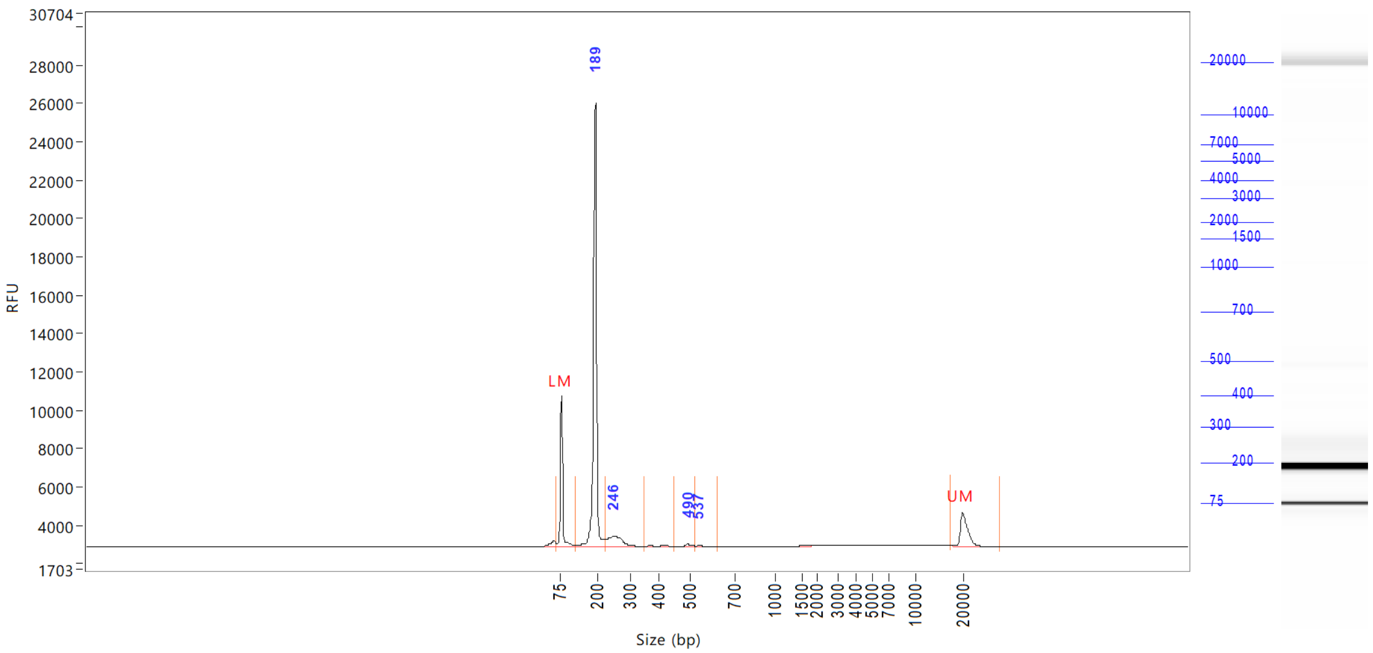

Peak	Size (bp)	Concentration (ng/uL)	From (bp)	To (bp)	Average size (bp)	CV%	RFU	Corrected peak area
1	75 (LM)	0.5000	62	122	75	10.24	7851	46.380
2	189	22.6241	122	219	184	4.79	23177	174.884
3	246	2.2273	219	345	250	8.06	521	17.217
4	490	0.2048	445	521	488	3.06	170	1.583
5	537	0.0926	521	621	546	3.61	60	0.716
6	20000 (UM)	0.1780	17561	27738	20427	4.17	1765	16.510

TIC:	25.1489	ng/uL
TIM:	217.7688	nmole/L
Total concentration:	25.6705	ng/uL

Sample peak width (sec): 5   Sample min peak height: 50   Sample baseline V to V?: Y   Sample baseline V to V points: 3
Sample filter: Binomial   Number of points for filter: 3 Sample start region (min): 0 Sample end region (min): 30
Marker peak width (sec): 5   Marker min peak height: 200   Marker baseline V to V?: Y   Marker baseline V to V points: 3
Lower marker selection: First peak > 500 RFU   Upper marker selection: Last peak > 200 RFU
Ladder size (bp)75, 200, 300, 400, 500, 700, 1000, 1500, 2000, 3000, 4000, 5000, 7000, 10000, 20000
Quantification using: Lower Marker   Final concentration (ng/uL): 0.5000   Dilution factor: 12.0

Sample: SampH11
Well location: H11
Created: Tuesday, September 14, 2021 6:14:00 PM

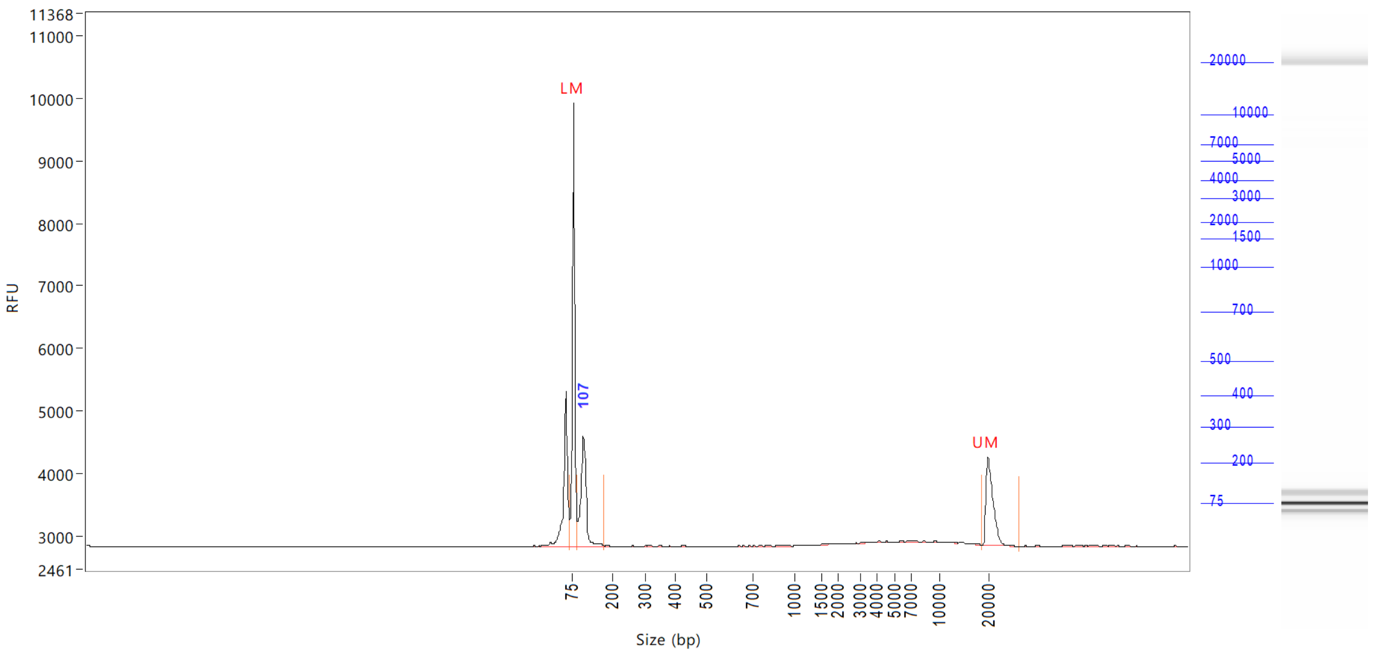

Peak	Size (bp)	Concentration (ng/uL)	From (bp)	To (bp)	Average size (bp)	CV%	RFU	Corrected peak area
1	75 (LM)	0.5000	64	86	73	5.20	7113	40.975
2	107	3.2235	86	175	105	11.22	1769	22.014
3	20000 (UM)	0.1534	18781	26408	20505	3.94	1400	12.574

TIC:	3.2235	ng/uL
TIM:	50.4183	nmole/L
Total concentration:	3.6404	ng/uL

Sample peak width (sec): 5 Sample min peak height: 50 Sample baseline V to V?: Y Sample baseline V to V points: 3
Sample filter: Binomial Number of points for filter: 3 Sample start region (min): 0 Sample end region (min): 30
Marker peak width (sec): 5 Marker min peak height: 200 Marker baseline V to V?: Y Marker baseline V to V points: 3
Lower marker selection: First peak > 500 RFU Upper marker selection: Last peak > 200 RFU
Ladder size (bp)75, 200, 300, 400, 500, 700, 1000, 1500, 2000, 3000, 4000, 5000, 7000, 10000, 20000
Quantification using: Lower Marker Final concentration (ng/uL): 0.5000 Dilution factor: 12.0

Sample: SampA12
Well location: A12
Created: Tuesday, September 14, 2021 6:14:00 PM

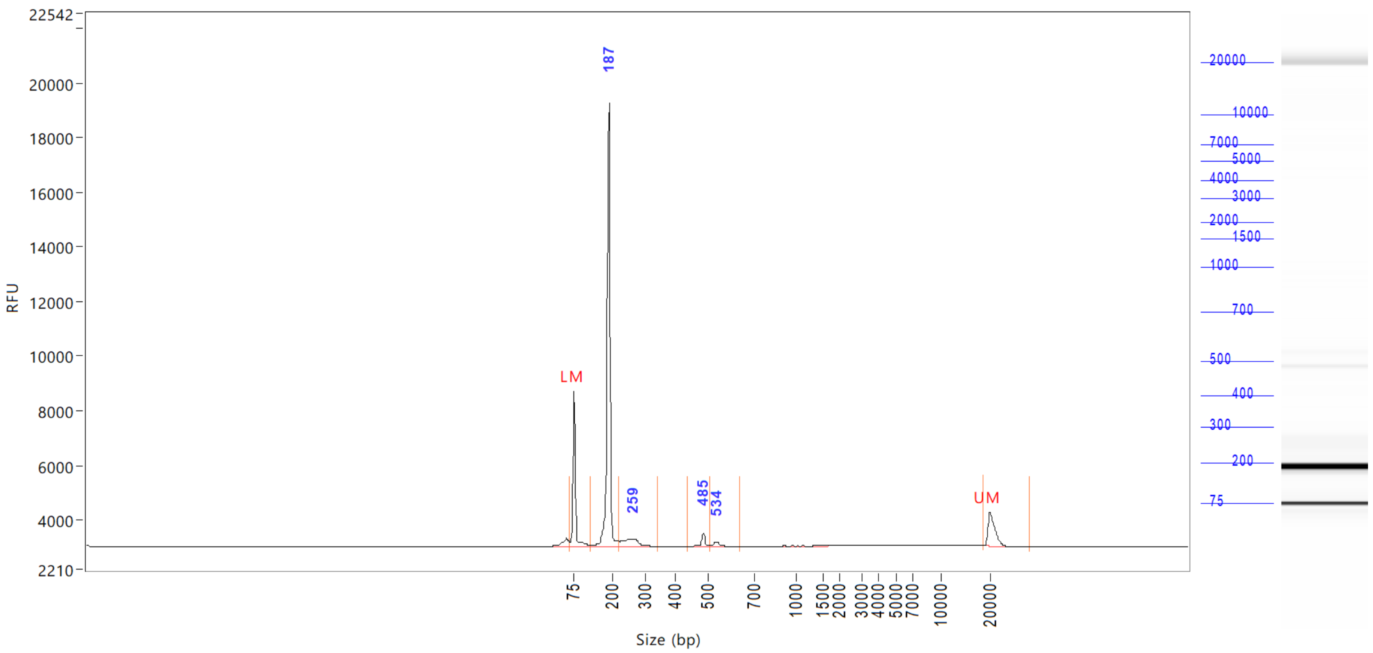

Peak	Size (bp)	Concentration (ng/uL)	From (bp)	To (bp)	Average size (bp)	CV%	RFU	Corrected peak area
1	75 (LM)	0.5000	64	128	75	12.43	5699	34.846
2	187	19.8093	128	217	183	4.54	16252	115.047
3	259	1.5995	217	339	251	8.14	276	9.290
4	485	0.6240	435	511	483	2.59	506	3.624
5	534	0.3693	511	639	541	4.06	174	2.145
6	20000 (UM)	0.1585	18903	28221	20519	4.31	1230	11.048

TIC:	22.4021	ng/uL
TIM:	191.6918	nmole/L
Total concentration:	22.9967	ng/uL

Sample peak width (sec): 5 Sample min peak height: 50 Sample baseline V to V?: Y Sample baseline V to V points: 3
Sample filter: Binomial Number of points for filter: 3 Sample start region (min): 0 Sample end region (min): 30
Marker peak width (sec): 5 Marker min peak height: 200 Marker baseline V to V?: Y Marker baseline V to V points: 3
Lower marker selection: First peak > 500 RFU Upper marker selection: Last peak > 200 RFU
Ladder size (bp)75, 200, 300, 400, 500, 700, 1000, 1500, 2000, 3000, 4000, 5000, 7000, 10000, 20000
Quantification using: Lower Marker Final concentration (ng/uL): 0.5000 Dilution factor: 12.0

Sample: SampB12
Well location: B12
Created: Tuesday, September 14, 2021 6:14:00 PM

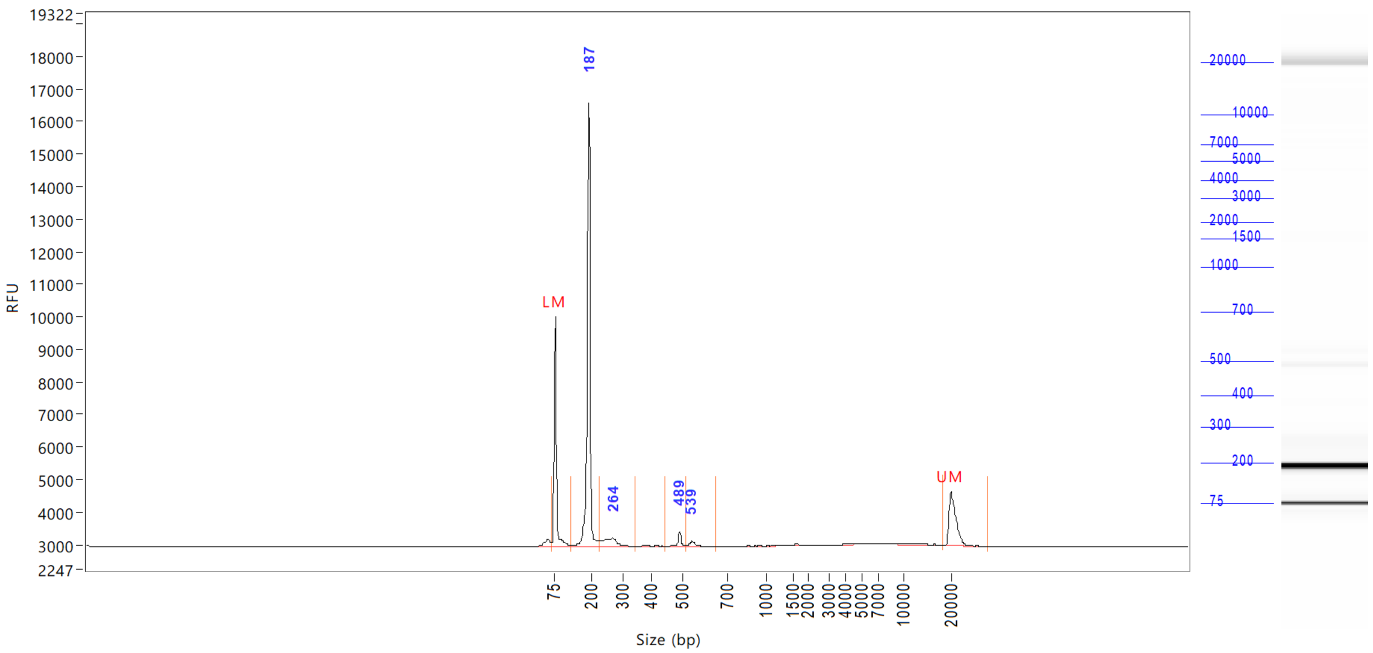

Peak	Size (bp)	Concentration (ng/uL)	From (bp)	To (bp)	Average size (bp)	CV%	RFU	Corrected peak area
1	75 (LM)	0.5000	64	129	76	11.54	7081	42.417
2	187	13.6915	129	223	184	4.61	13649	96.792
3	264	1.1485	223	341	254	7.75	247	8.119
4	489	0.4488	445	516	488	2.34	434	3.173
5	539	0.2853	516	652	548	4.17	160	2.017
6	20000 (UM)	0.1797	18171	27738	20506	4.46	1650	15.242

TIC:	15.5741	ng/uL
TIM:	132.1402	nmole/L
Total concentration:	16.1418	ng/uL

Sample peak width (sec): 5   Sample min peak height: 50   Sample baseline V to V?: Y   Sample baseline V to V points: 3
Sample filter: Binomial   Number of points for filter: 3   Sample start region (min): 0   Sample end region (min): 30
Marker peak width (sec): 5   Marker min peak height: 200   Marker baseline V to V?: Y   Marker baseline V to V points: 3
Lower marker selection: First peak > 500 RFU   Upper marker selection: Last peak > 200 RFU
Ladder size (bp)75, 200, 300, 400, 500, 700, 1000, 1500, 2000, 3000, 4000, 5000, 7000, 10000, 20000
Quantification using: Lower Marker   Final concentration (ng/uL): 0.5000   Dilution factor: 12.0

Sample: SampC12
Well location: C12
Created: Tuesday, September 14, 2021 6:14:00 PM

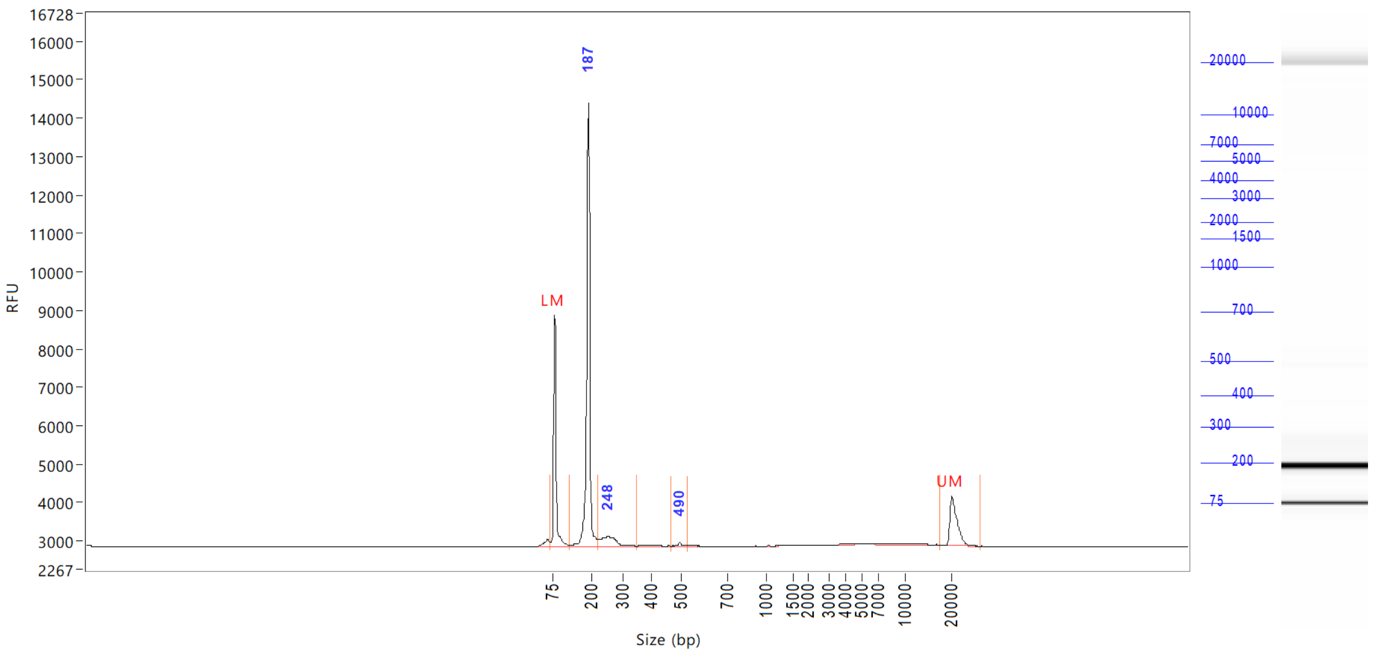

Peak	Size (bp)	Concentration (ng/uL)	From (bp)	To (bp)	Average size (bp)	CV%	RFU	Corrected peak area
1	75 (LM)	0.5000	64	128	76	10.34	5999	38.139
2	187	13.7606	128	217	185	4.64	11549	87.470
3	248	1.3351	217	343	251	8.58	245	8.487
4	490	0.1302	464	524	493	2.53	98	0.827
5	20000 (UM)	0.1644	17683	26166	20593	4.42	1313	12.539

TIC:	15.2260	ng/uL
TIM:	131.4705	nmole/L
Total concentration:	15.8304	ng/uL

Sample peak width (sec): 5 Sample min peak height: 50 Sample baseline V to V?: Y Sample baseline V to V points: 3
Sample filter: Binomial Number of points for filter: 3 Sample start region (min): 0 Sample end region (min): 30
Marker peak width (sec): 5 Marker min peak height: 200 Marker baseline V to V?: Y Marker baseline V to V points: 3
Lower marker selection: First peak > 500 RFU Upper marker selection: Last peak > 200 RFU
Ladder size (bp)75, 200, 300, 400, 500, 700, 1000, 1500, 2000, 3000, 4000, 5000, 7000, 10000, 20000
Quantification using: Lower Marker Final concentration (ng/uL): 0.5000 Dilution factor: 12.0

Sample: SampD12
Well location: D12
Created: Tuesday, September 14, 2021 6:14:00 PM

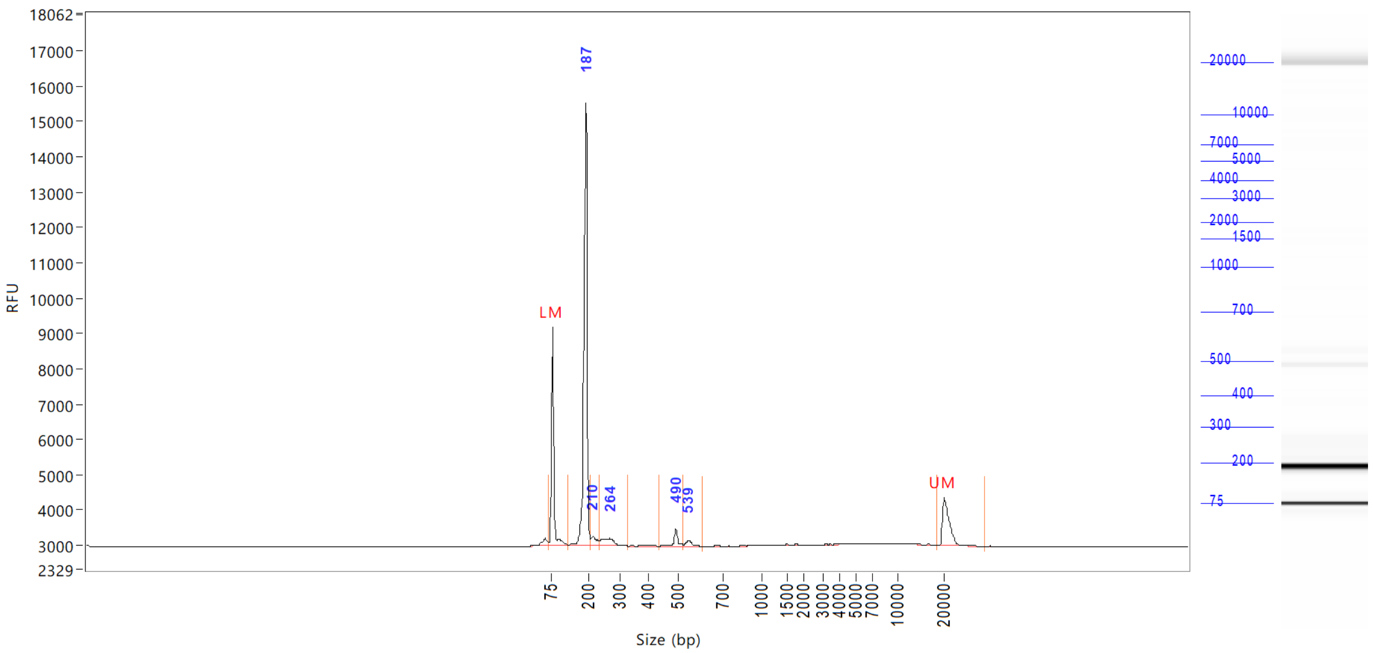

Peak	Size (bp)	Concentration (ng/uL)	From (bp)	To (bp)	Average size (bp)	CV%	RFU	Corrected peak area
1	75 (LM)	0.5000	60	129	74	9.86	6166	36.983
2	187	14.7293	129	203	183	3.65	12535	90.790
3	210	0.5452	203	230	215	3.84	264	3.360
4	264	0.9231	230	325	256	6.41	201	5.690
5	490	0.5191	433	519	489	2.34	470	3.200
6	539	0.3174	519	608	544	2.76	169	1.957
7	20000 (UM)	0.1686	18415	28826	20562	4.91	1348	12.473

TIC:	17.0342	ng/uL
TIM:	145.1318	nmole/L
Total concentration:	17.5323	ng/uL

Sample peak width (sec): 5 Sample min peak height: 50 Sample baseline V to V?: Y Sample baseline V to V points: 3
Sample filter: Binomial Number of points for filter: 3 Sample start region (min): 0 Sample end region (min): 30
Marker peak width (sec): 5 Marker min peak height: 200 Marker baseline V to V?: Y Marker baseline V to V points: 3
Lower marker selection: First peak > 500 RFU Upper marker selection: Last peak > 200 RFU
Ladder size (bp)75, 200, 300, 400, 500, 700, 1000, 1500, 2000, 3000, 4000, 5000, 7000, 10000, 20000
Quantification using: Lower Marker Final concentration (ng/uL): 0.5000 Dilution factor: 12.0

Sample: SampE12
Well location: E12
Created: Tuesday, September 14, 2021 6:14:00 PM

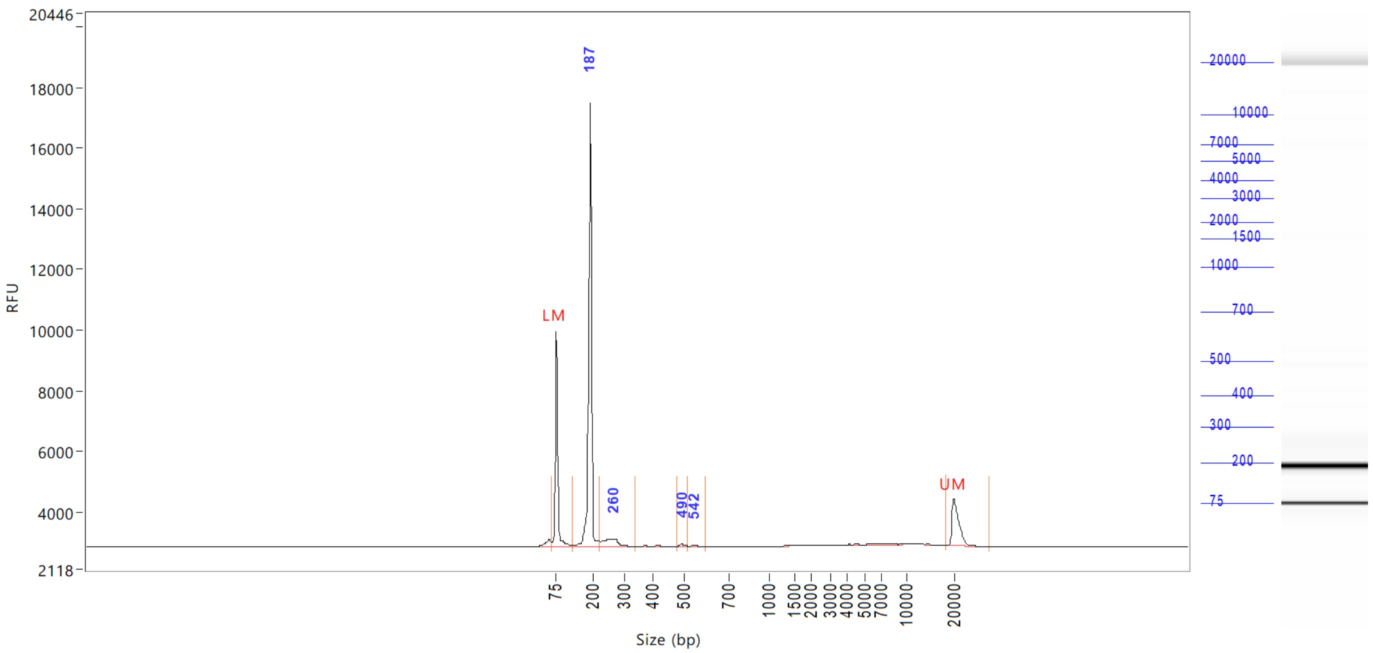

Peak	Size (bp)	Concentration (ng/uL)	From (bp)	To (bp)	Average size (bp)	CV%	RFU	Corrected peak area
1	75 (LM)	0.5000	60	129	75	11.39	7093	42.063
2	187	14.7472	129	219	184	4.55	14650	103.386
3	260	1.2195	219	335	252	8.08	256	8.550
4	490	0.0934	475	516	491	1.75	92	0.655
5	542	0.0831	516	590	543	2.64	53	0.583
6	20000 (UM)	0.1707	18415	27496	20442	4.22	1549	14.361

TIC:	16.1432	ng/uL
TIM:	140.2902	nmole/L
Total concentration:	16.5699	ng/uL

```
Sample peak width (sec): 5    Sample min peak height: 50    Sample baseline V to V?: Y    Sample baseline V to V points: 3
Sample filter: Binomial       Number of points for filter: 3  Sample start region (min): 0  Sample end region (min): 30
Marker peak width (sec): 5    Marker min peak height: 200   Marker baseline V to V?: Y    Marker baseline V to V points: 3
Lower marker selection: First peak > 500 RFU                Upper marker selection: Last peak > 200 RFU
Ladder size (bp)75, 200, 300, 400, 500, 700, 1000, 1500, 2000, 3000, 4000, 5000, 7000, 10000, 20000
Quantification using: Lower Marker          Final concentration (ng/uL): 0.5000          Dilution factor: 12.0
```

Sample: SampF12
Well location: F12
Created: Tuesday, September 14, 2021 6:14:00 PM

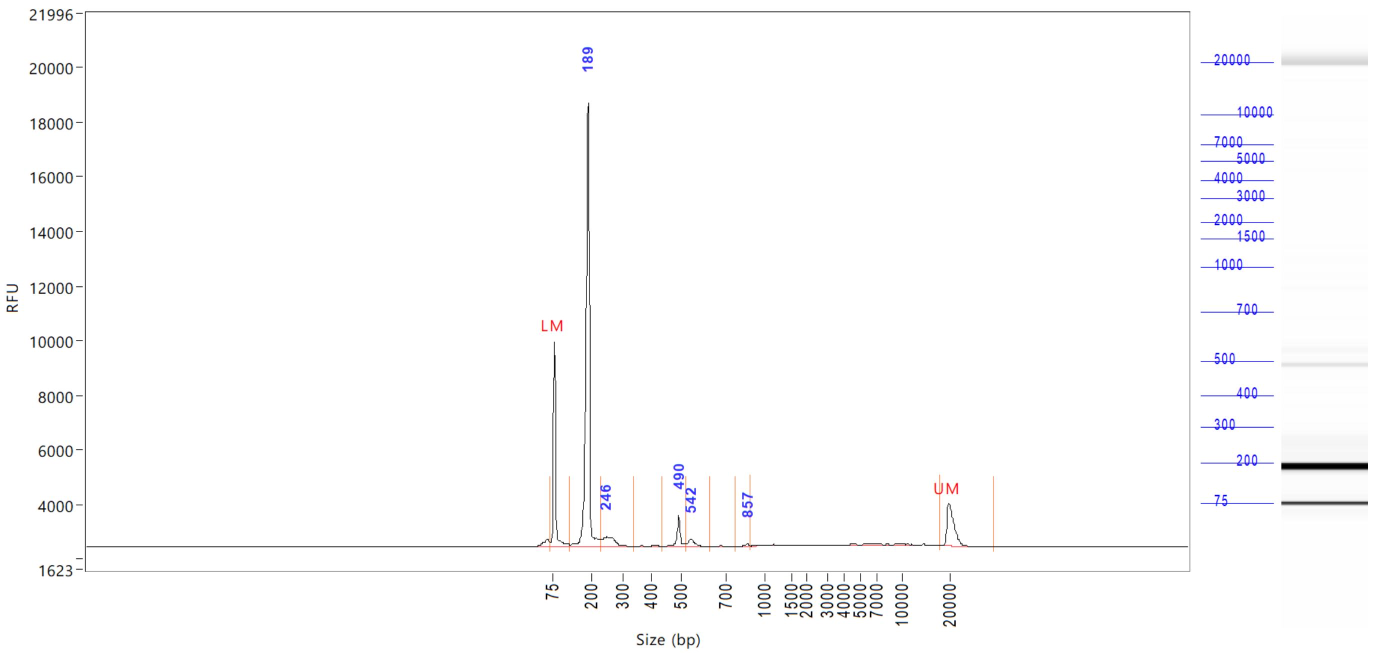

Peak	Size (bp)	Concentration (ng/uL)	From (bp)	To (bp)	Average size (bp)	CV%	RFU	Corrected peak area
1	75 (LM)	0.5000	62	128	75	11.88	7460	44.768
2	189	17.9916	128	228	184	5.32	16276	134.242
3	246	1.3099	228	337	254	7.00	344	9.774
4	490	1.0292	437	521	488	2.53	1120	7.679
5	542	0.4254	521	629	545	2.98	279	3.174
6	857	0.0563	766	887	849	2.09	78	0.420
7	20000 (UM)	0.1626	18171	29430	20465	4.48	1570	14.555

TIC:	20.8125	ng/uL
TIM:	174.1008	nmole/L
Total concentration:	21.2608	ng/uL

```
Sample peak width (sec): 5    Sample min peak height: 50    Sample baseline V to V?: Y    Sample baseline V to V points: 3
Sample filter: Binomial       Number of points for filter: 3  Sample start region (min): 0  Sample end region (min): 30
Marker peak width (sec): 5    Marker min peak height: 200   Marker baseline V to V?: Y    Marker baseline V to V points: 3
Lower marker selection: First peak > 500 RFU                Upper marker selection: Last peak > 200 RFU
Ladder size (bp)75, 200, 300, 400, 500, 700, 1000, 1500, 2000, 3000, 4000, 5000, 7000, 10000, 20000
Quantification using: Lower Marker          Final concentration (ng/uL): 0.5000          Dilution factor: 12.0
```

Sample: SampG12
Well location: G12
Created: Tuesday, September 14, 2021 6:14:00 PM

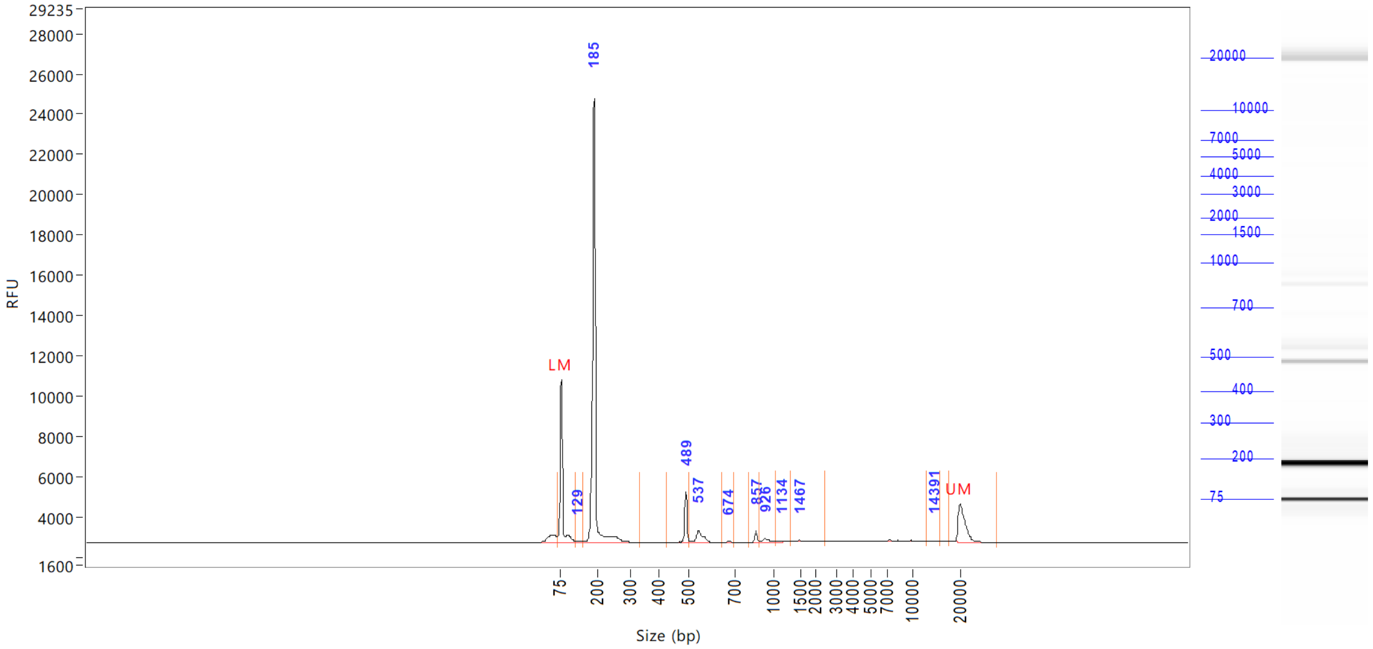

Peak	Size (bp)	Concentration (ng/uL)	From (bp)	To (bp)	Average size (bp)	CV%	RFU	Corrected peak area
1	75 (LM)	0.5000	64	122	75	12.13	8063	51.038
2	129	0.1225	122	147	133	5.71	74	1.042
3	185	18.0387	147	331	185	9.31	22092	153.442
4	489	1.4357	424	500	485	1.30	2512	12.213
5	537	0.9167	500	646	543	3.45	614	7.797
6	674	0.0521	646	697	671	1.16	70	0.443
7	857	0.2756	805	883	853	1.09	540	2.344
8	926	0.2283	883	1001	935	2.82	191	1.942
9	1134	0.0997	1001	1300	1137	6.89	66	0.848
10	1467	0.1578	1300	2429	1586	14.16	71	1.342
11	14391	0.0614	12684	15854	13983	5.70	53	0.522
12	20000 (UM)	0.1713	17561	27617	20469	4.34	1866	17.490

TIC:	21.3885	ng/uL
TIM:	170.8454	nmole/L
Total concentration:	21.7206	ng/uL

Sample peak width (sec): 5 Sample min peak height: 50 Sample baseline V to V?: Y Sample baseline V to V points: 3
Sample filter: Binomial Number of points for filter: 3 Sample start region (min): 0 Sample end region (min): 30
Marker peak width (sec): 5 Marker min peak height: 200 Marker baseline V to V?: Y Marker baseline V to V points: 3
Lower marker selection: First peak > 500 RFU Upper marker selection: Last peak > 200 RFU
Ladder size (bp)75, 200, 300, 400, 500, 700, 1000, 1500, 2000, 3000, 4000, 5000, 7000, 10000, 20000
Quantification using: Lower Marker Final concentration (ng/uL): 0.5000 Dilution factor: 12.0

Sample: SampH12
Well location: H12
Created: Tuesday, September 14, 2021 6:14:00 PM

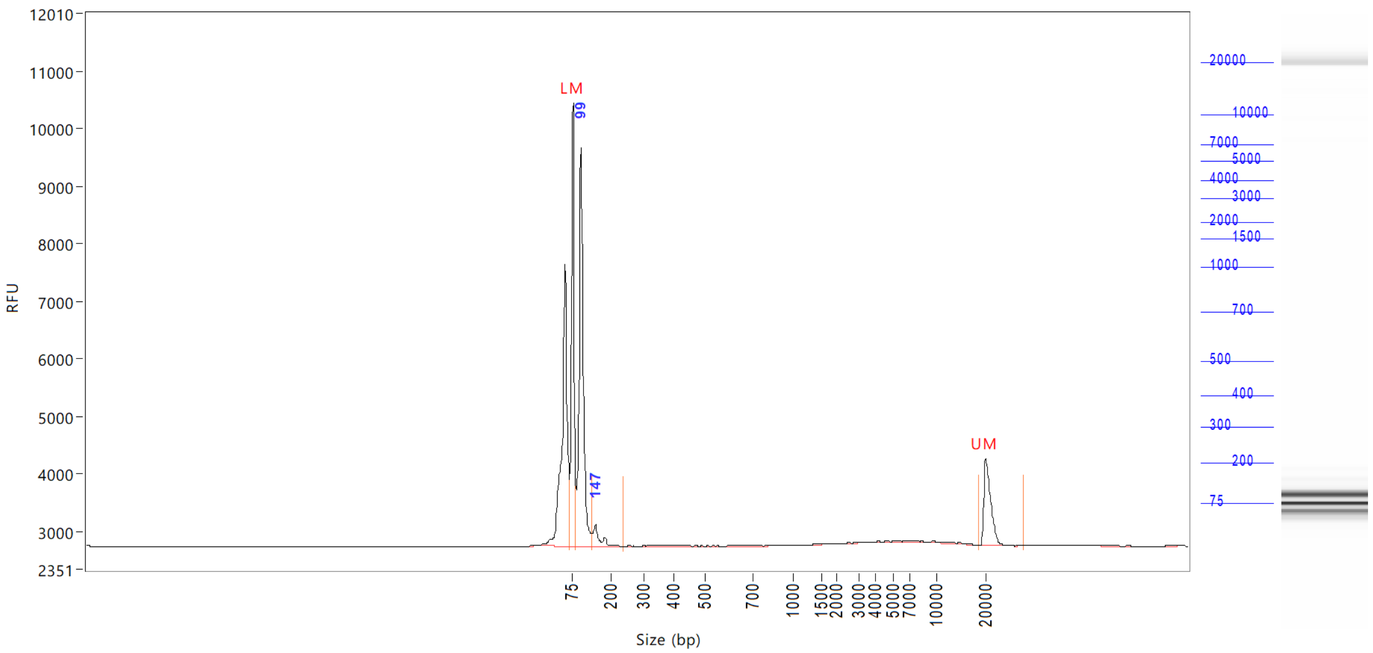

Peak	Size (bp)	Concentration (ng/uL)	From (bp)	To (bp)	Average size (bp)	CV%	RFU	Corrected peak area
1	75 (LM)	0.5000	66	84	73	5.40	7712	48.768
2	99	8.4311	84	137	100	8.87	6911	68.528
3	147	0.6302	137	233	156	10.74	372	5.122
4	20000 (UM)	0.1359	18537	27617	20460	4.23	1508	13.254

TIC:	9.0612	ng/uL
TIM:	145.0856	nmole/L
Total concentration:	9.4722	ng/uL

Sample peak width (sec): 5 Sample min peak height: 200 Sample baseline V to V?: Y Sample baseline V to V points: 3
Sample filter: Binomial Number of points for filter: 3 Sample start region (min): 0 Sample end region (min): 30
Marker peak width (sec): 5 Marker min peak height: 200 Marker baseline V to V?: Y Marker baseline V to V points: 3
Lower marker selection: First peak > 500 RFU Upper marker selection: Last peak > 200 RFU
Ladder size (bp)75, 200, 300, 400, 500, 700, 1000, 1500, 2000, 3000, 4000, 5000, 7000, 10000, 20000
Quantification using: Lower Marker Final concentration (ng/uL): 0.5000 Dilution factor: 12.0

Sample: DNA size ladder
Well location: A7
Created: Tuesday, September 14, 2021 6:14:00 PM
Import from: C:\Users\admin\Desktop\210810_alberto_ladder.SCAL
Fit type: Point to point

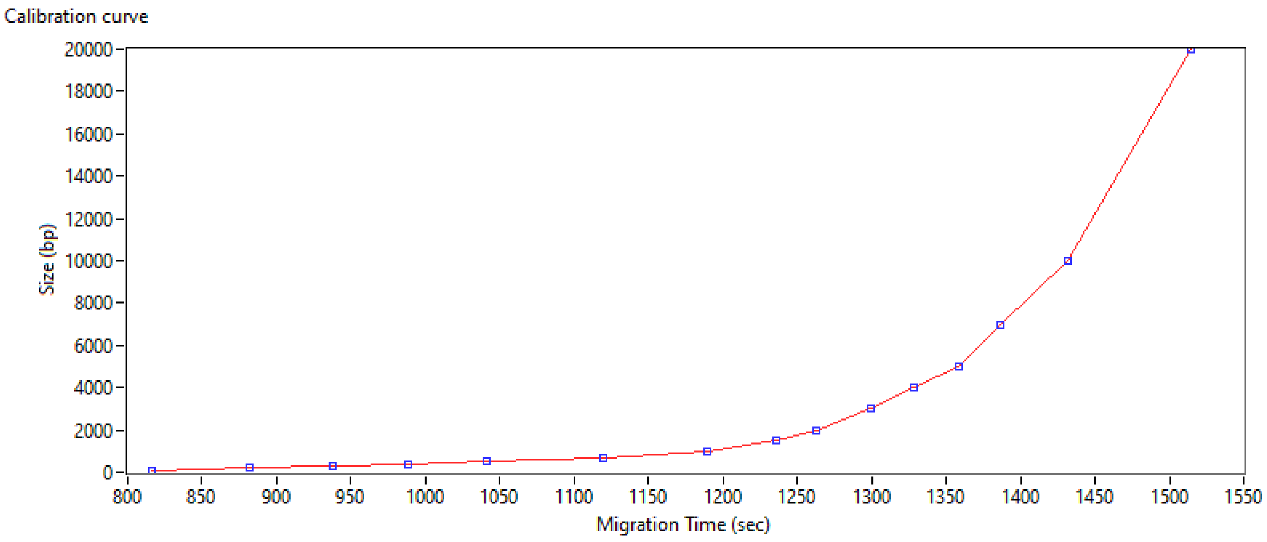

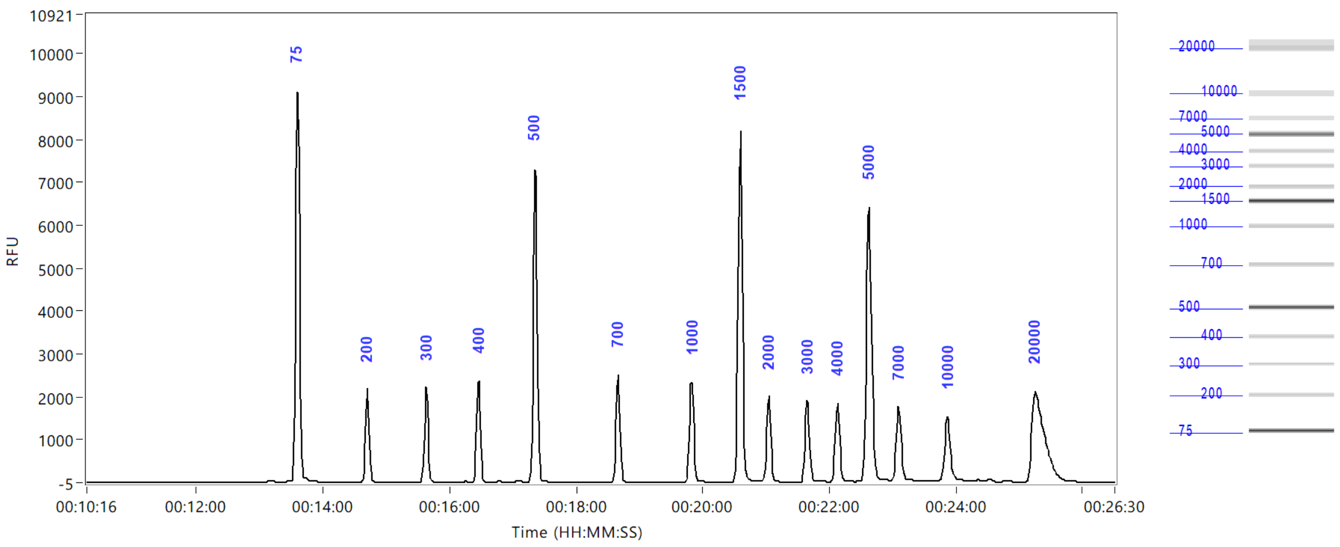

Supplement: Supplementary file 2 — sb3c00292_si_002.zip [file sb3c00292_si_002.zip › dnada_supplementary_material_pks_library_build/ZAG_trials/ZAG_Trial_5/18-04-19_plate3/plate3-t1.pdf]
