# Supplementary material for: Automated Platform for the Plasmid Construction Process
Source: ACS Synth Biol. 2023 Nov 10;12(12):3506–13. doi: 10.1021/acssynbio.3c00292 (PMC10729297; doi:10.1021/acssynbio.3c00292)

Instrument controller software run summary:

Filename and data path: C:\AATI\Data\2021 09 14\16-33-14\2021 09 14 16H 33M..raw
Created: Tuesday, September 14, 2021 4:54:05 PM
Number of capillaries: 96
Array serial number: 111920-09SFS
Effect length: 33 cm
Array usage count: 213
Instrument type: ZAG DNA Analyzer
Instrument controller software version: 1.0.1.9
Device serial number: Z7597

Method Information

Method name: ZAG130FC33 - DNA 75-20000bp - Full Conditioning.mthds
Gel prime: No
Full conditioning: Yes
Gel prime to buffer: No
Gel selection: Gel 1
Perform prerun: 8.0 kV, 30 sec.
Rinse: No
Marker 1: Row: A, 5.0 kV, 5 sec.
Rinse: No
Sample injection: 5.0 kV, 5 sec.
Separation: 8.0 kV, 30.0 min.
Tray name: Sample Tray-1

Analysis mode: DNA

Notes

Gel Image

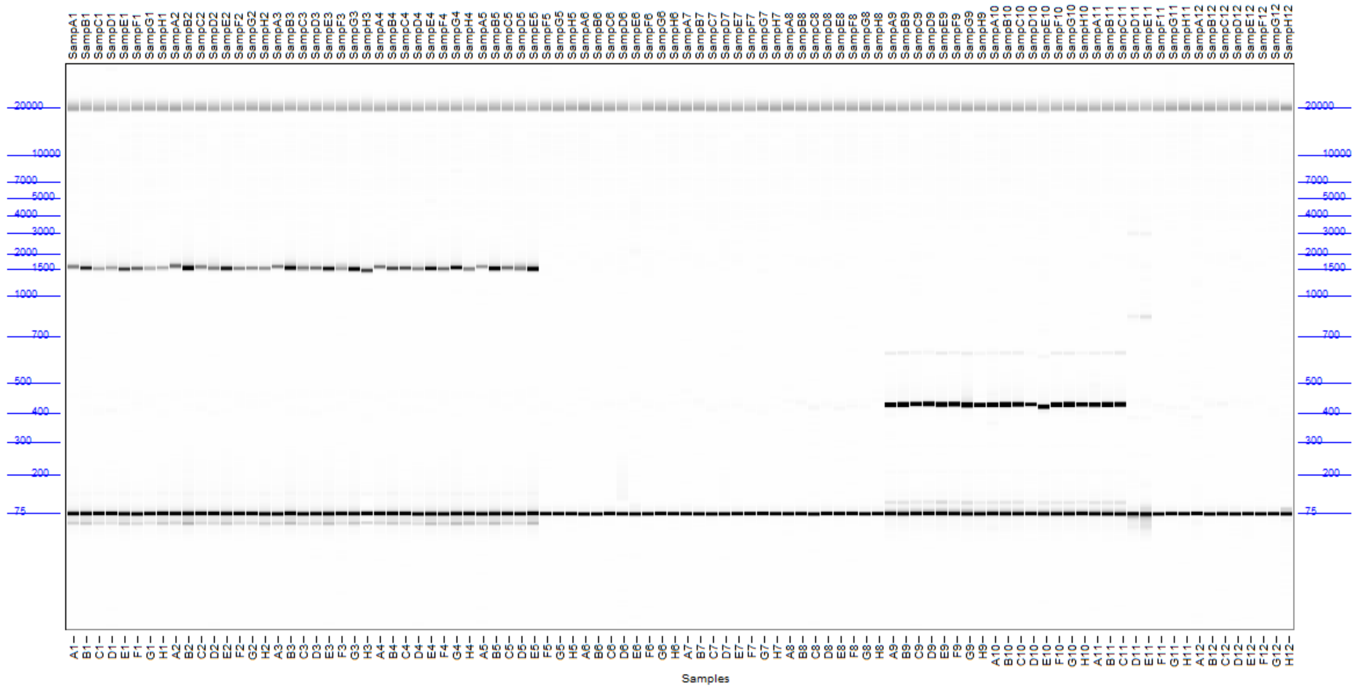

Filename and data path: C:\AATI\Data\2021 09 14\16-33-14\2021 09 14 16H 33M..raw

A1: SampA1

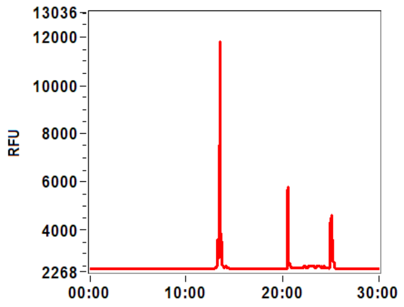

B1: SampB1

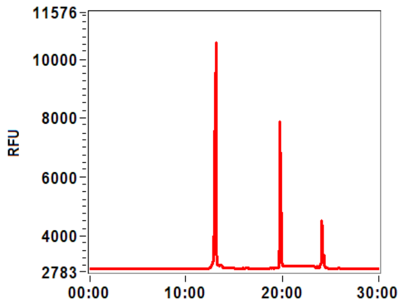

C1: SampC1

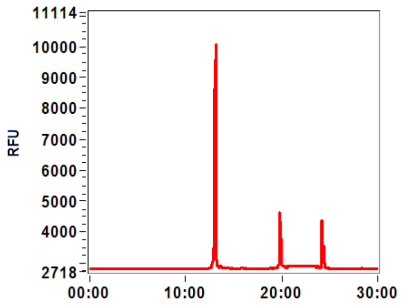

D1: SampD1

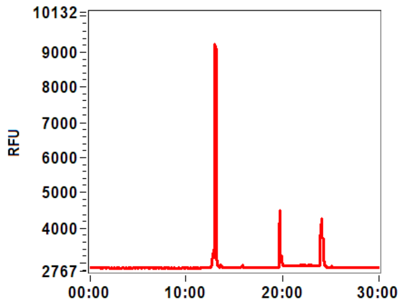

E1: SampE1

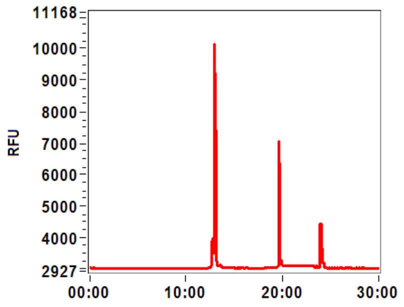

F1: SampF1

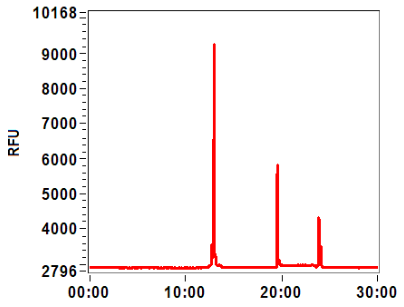

G1: SampG1

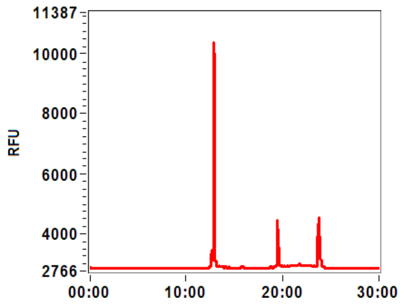

H1: SampH1

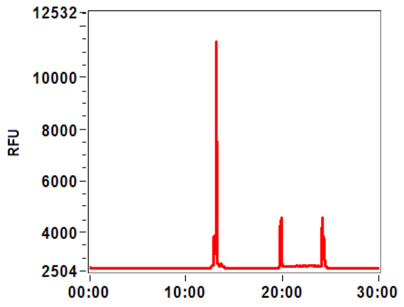

A2: SampA2

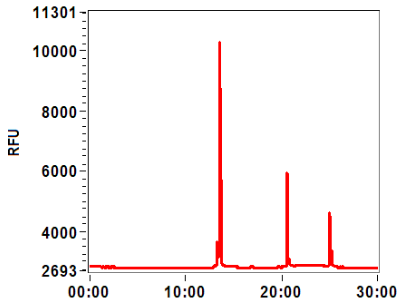

B2: SampB2

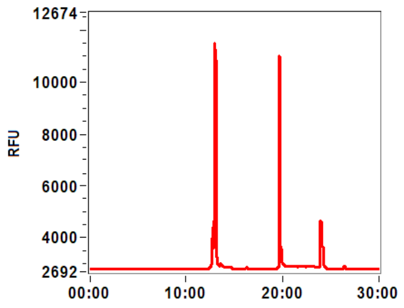

C2: SampC2

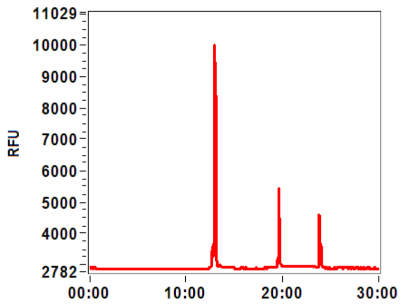

D2: SampD2

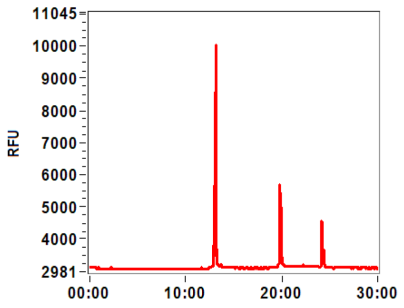

Filename and data path: C:\AATI\Data\2021 09 14\16-33-14\2021 09 14 16H 33M..raw

E2: SampE2

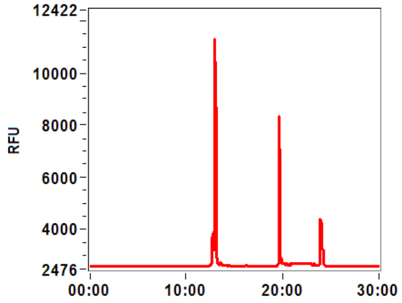

F2: SampF2

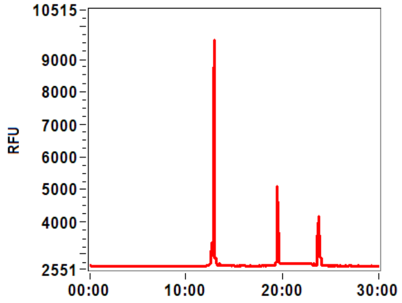

G2: SampG2

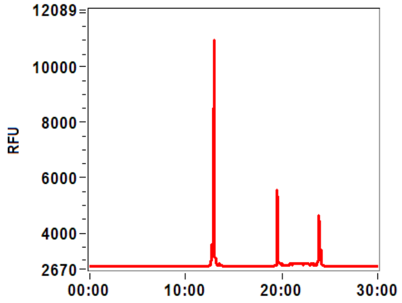

H2: SampH2

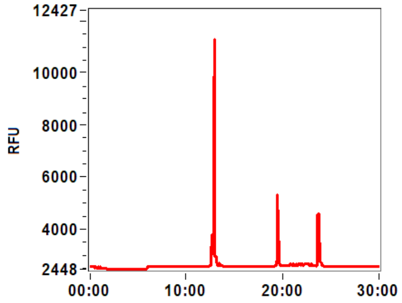

A3: SampA3

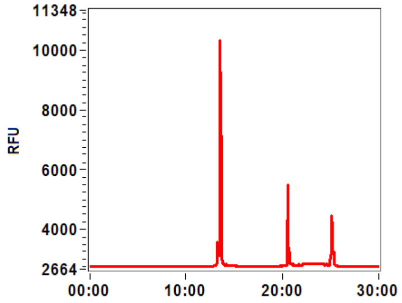

B3: SampB3

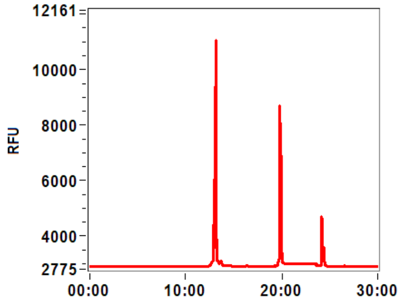

C3: SampC3

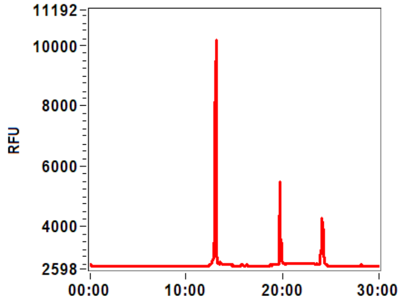

D3: SampD3

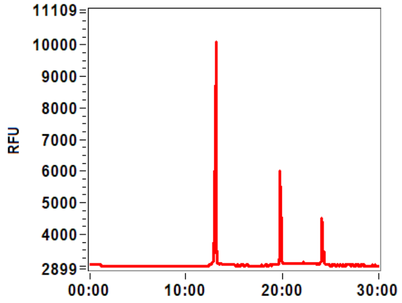

E3: SampE3

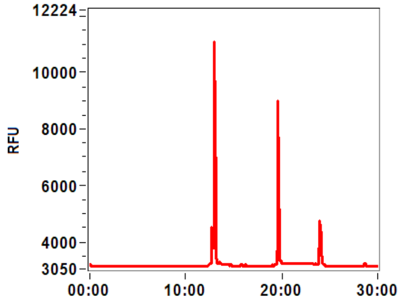

F3: SampF3

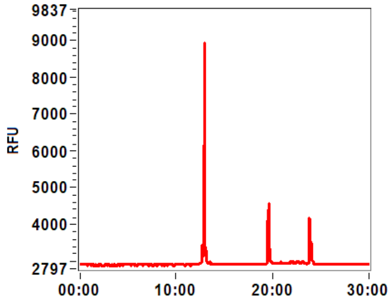

G3: SampG3

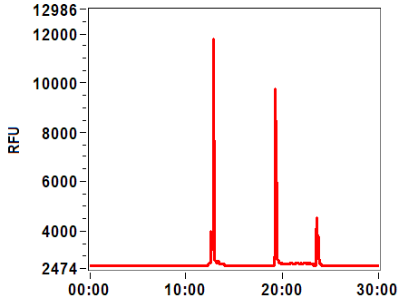

H3: SampH3

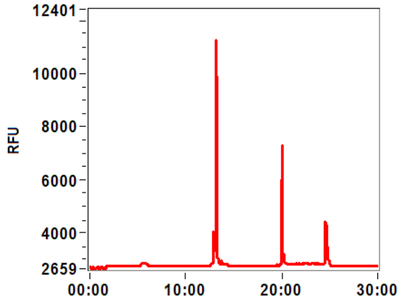

Filename and data path: C:\AATI\Data\2021 09 14\16-33-14\2021 09 14 16H 33M..raw

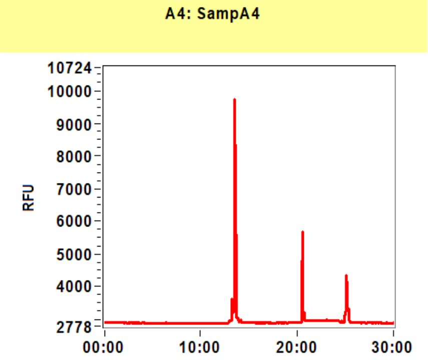

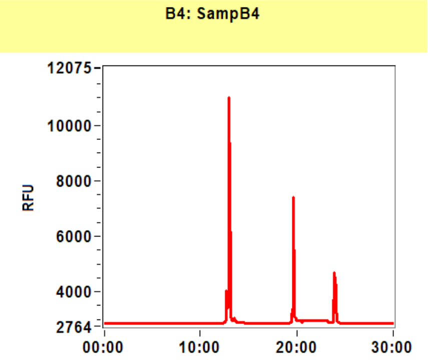

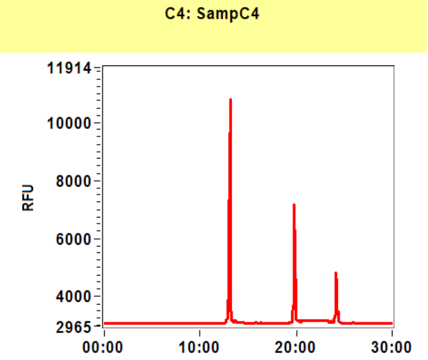

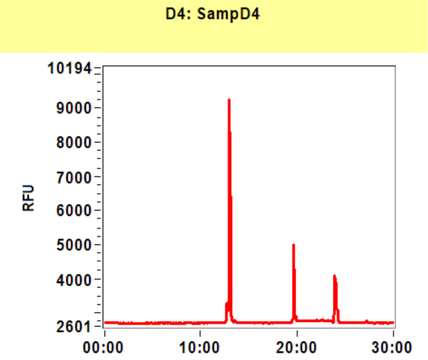

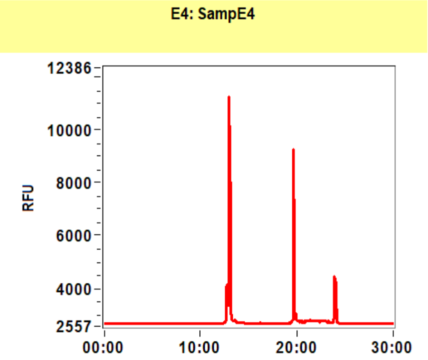

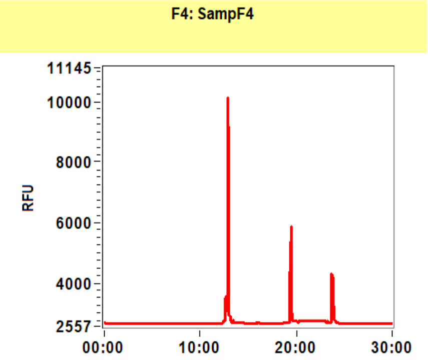

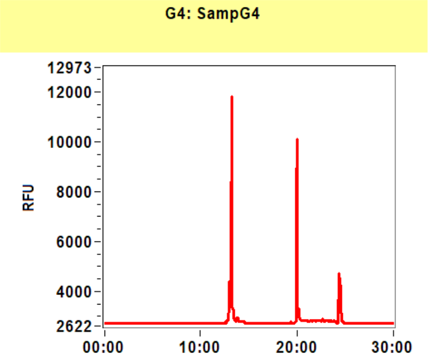

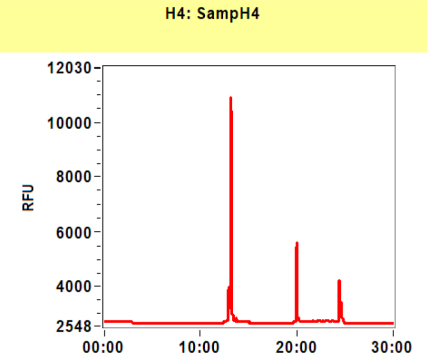

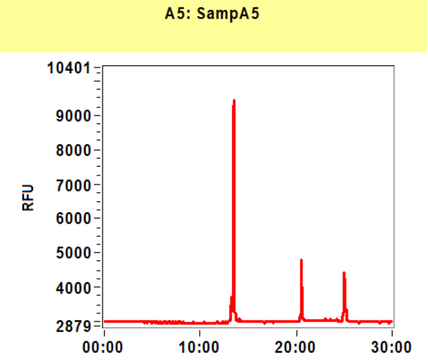

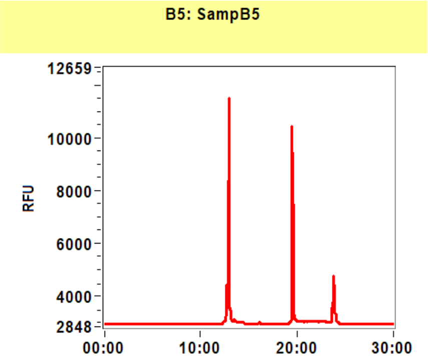

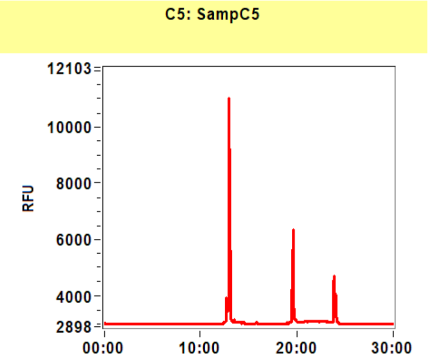

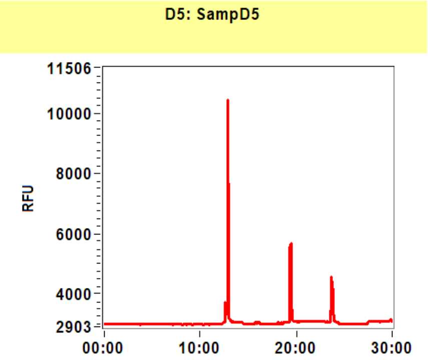

Filename and data path: C:\AATI\Data\2021 09 14\16-33-14\2021 09 14 16H 33M..raw

E5: SampE5

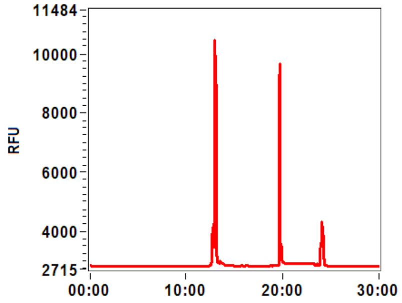

F5: SampF5

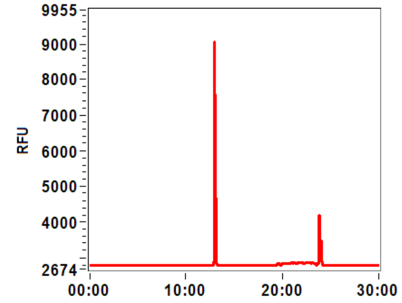

G5: SampG5

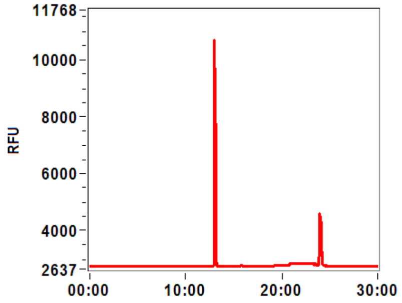

H5: SampH5

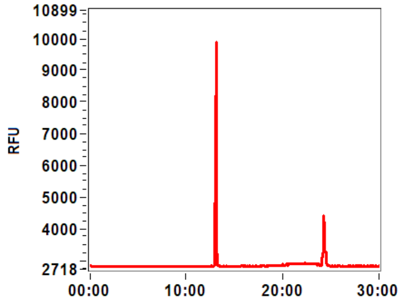

A6: SampA6

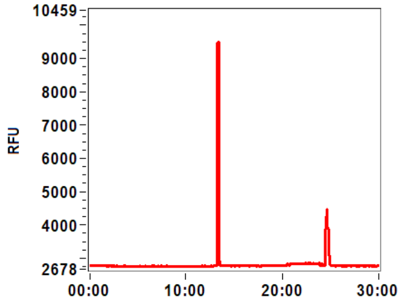

B6: SampB6

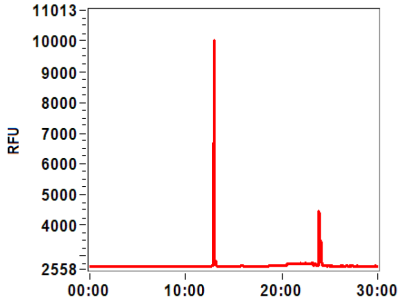

C6: SampC6

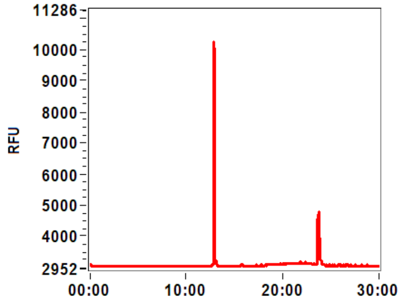

D6: SampD6

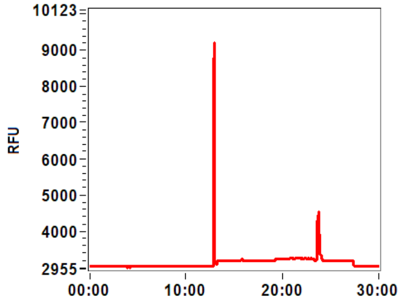

E6: SampE6

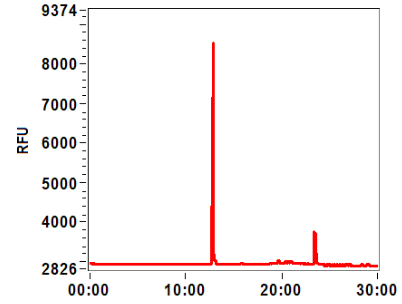

F6: SampF6

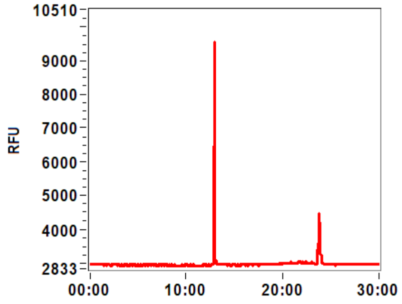

G6: SampG6

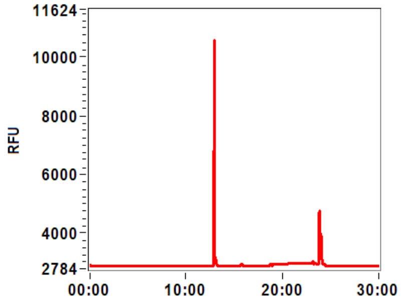

H6: SampH6

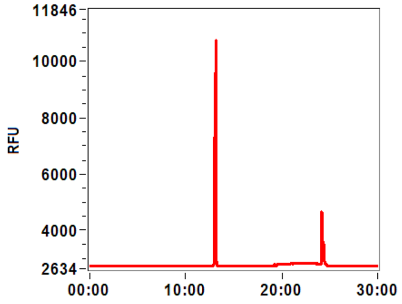

Filename and data path: C:\AATI\Data\2021 09 14\16-33-14\2021 09 14 16H 33M..raw

A7: SampA7

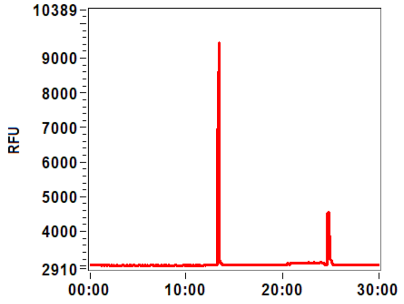

B7: SampB7

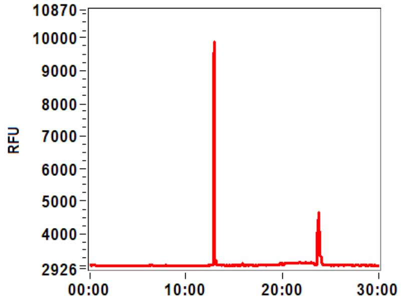

C7: SampC7

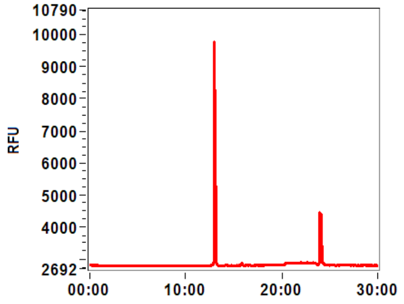

D7: SampD7

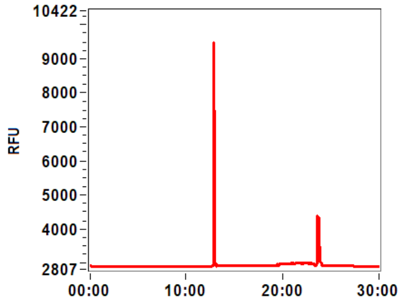

E7: SampE7

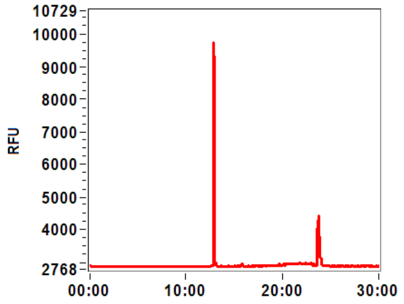

F7: SampF7

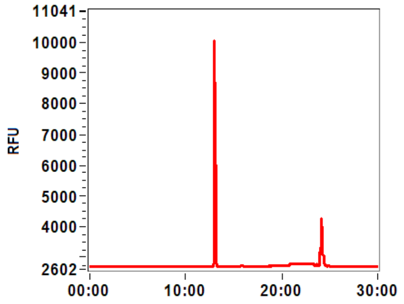

G7: SampG7

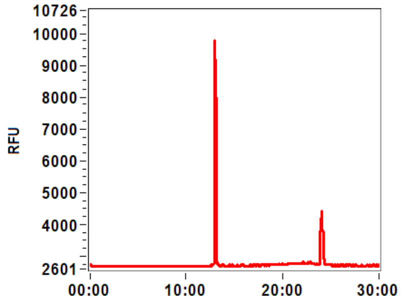

H7: SampH7

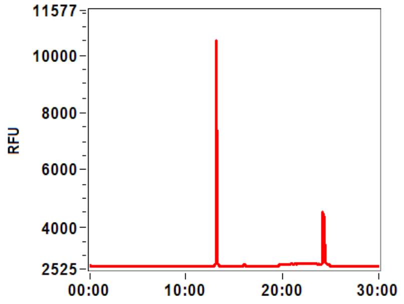

A8: SampA8

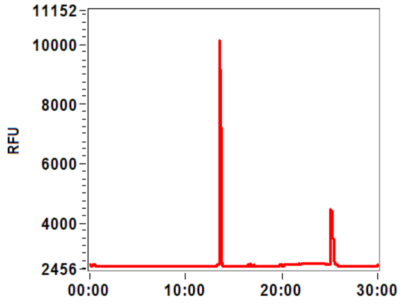

B8: SampB8

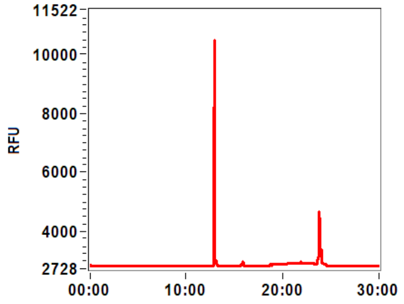

C8: SampC8

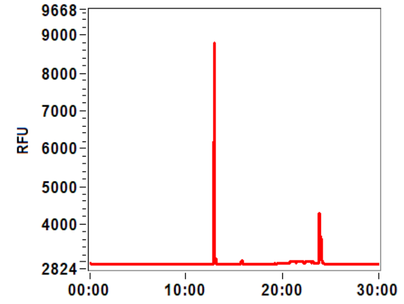

D8: SampD8

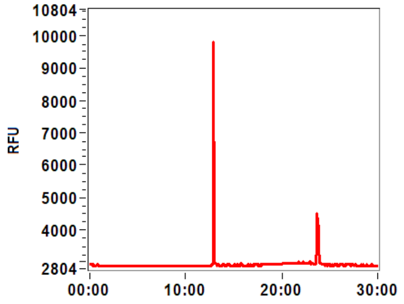

Filename and data path: C:\AATI\Data\2021 09 14\16-33-14\2021 09 14 16H 33M..raw

E8: SampE8

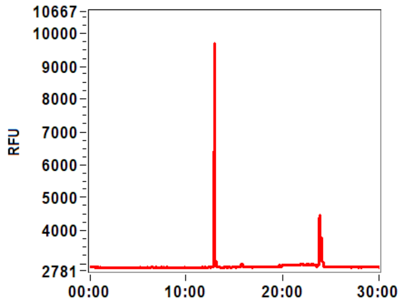

F8: SampF8

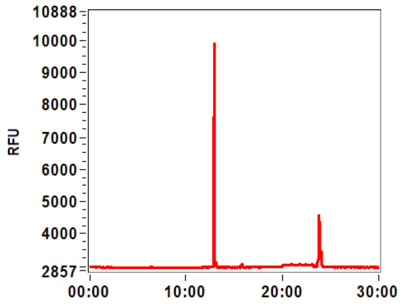

G8: SampG8

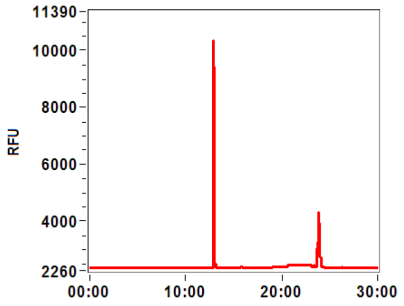

H8: SampH8

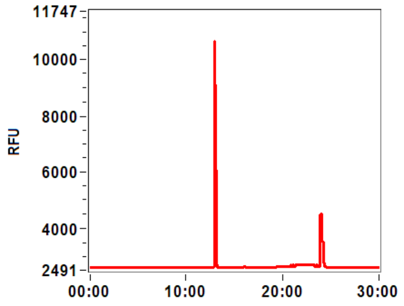

A9: SampA9

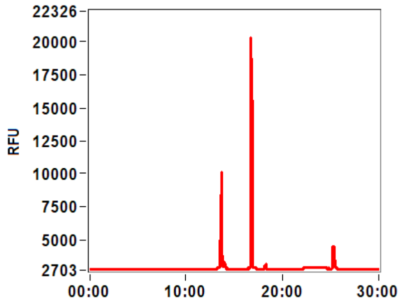

B9: SampB9

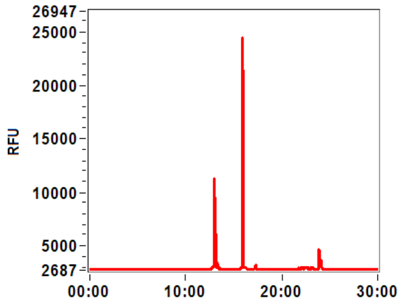

C9: SampC9

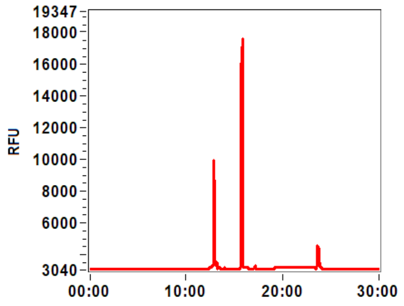

D9: SampD9

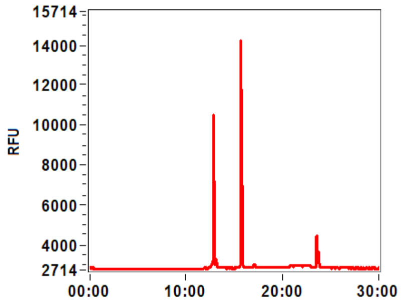

E9: SampE9

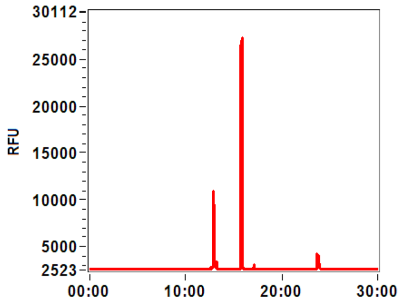

F9: SampF9

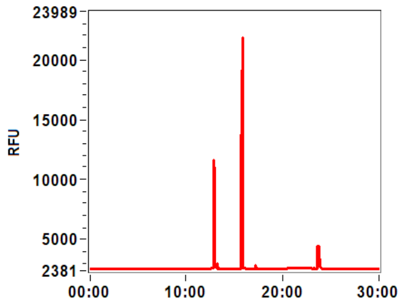

G9: SampG9

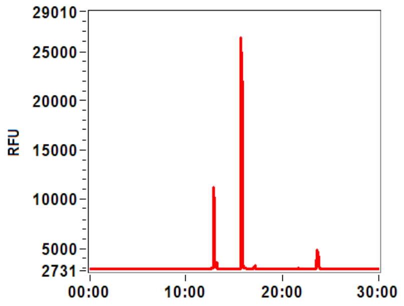

H9: SampH9

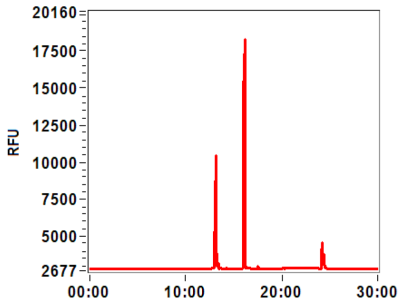

Filename and data path: C:\AATI\Data\2021 09 14\16-33-14\2021 09 14 16H 33M..raw

A10: SampA10

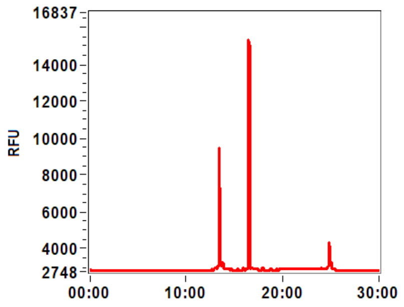

B10: SampB10

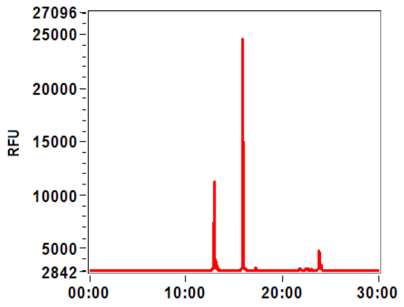

C10: SampC10

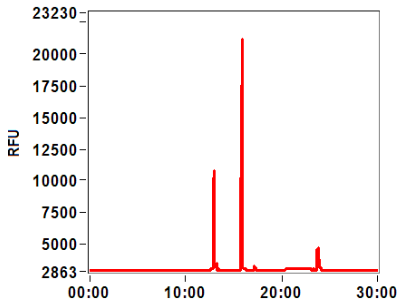

D10: SampD10

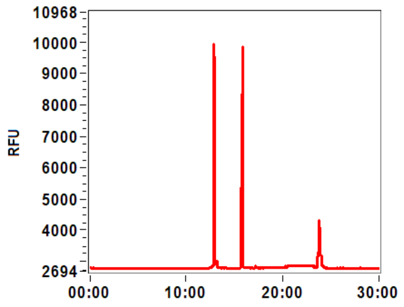

E10: SampE10

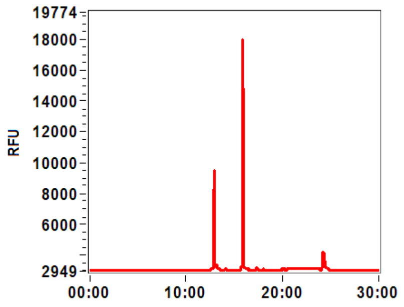

F10: SampF10

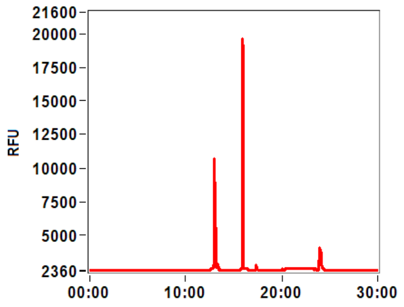

G10: SampG10

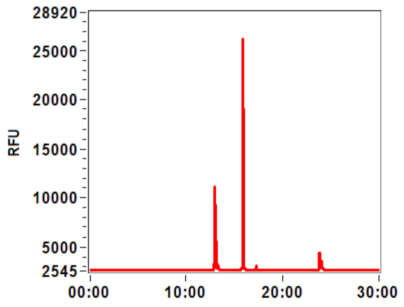

H10: SampH10

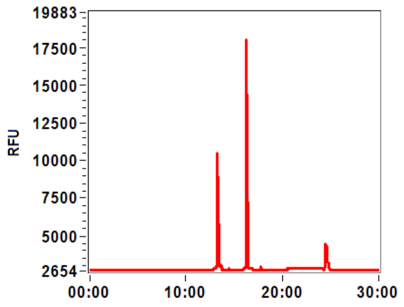

A11: SampA11

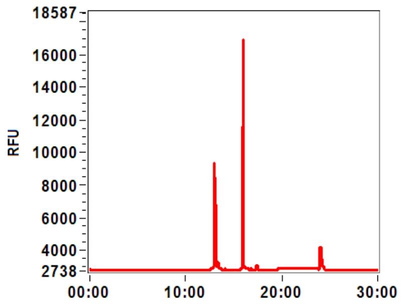

B11: SampB11

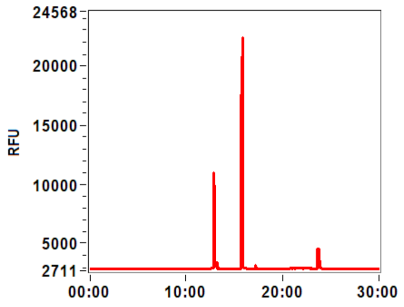

C11: SampC11

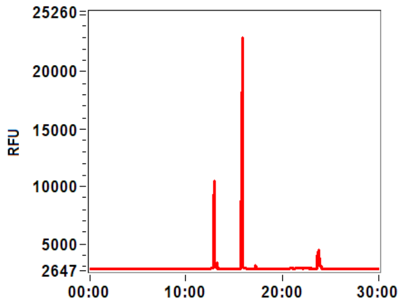

D11: SampD11

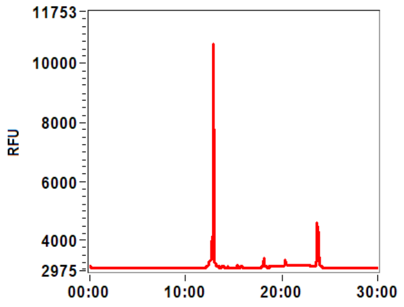

Filename and data path: C:\AATI\Data\2021 09 14\16-33-14\2021 09 14 16H 33M..raw

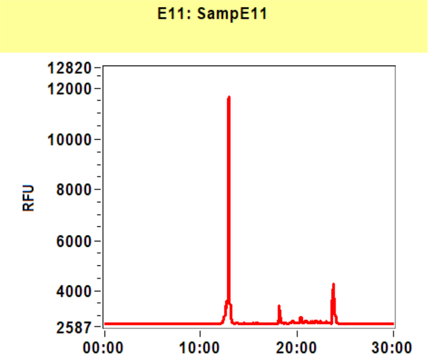

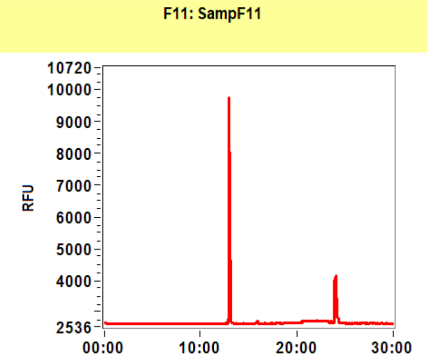

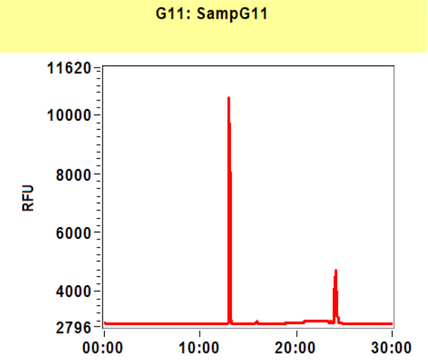

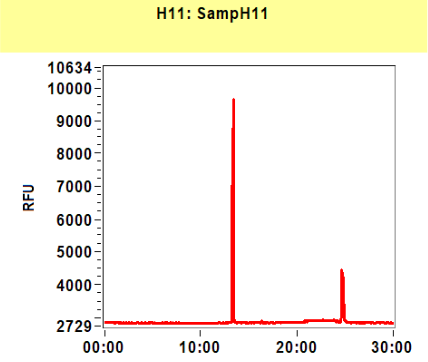

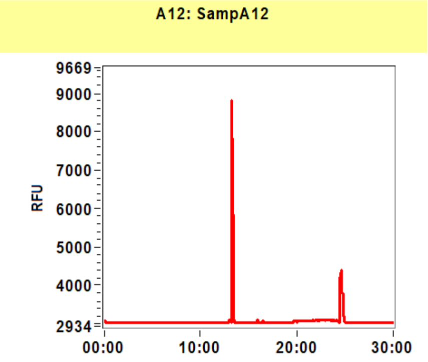

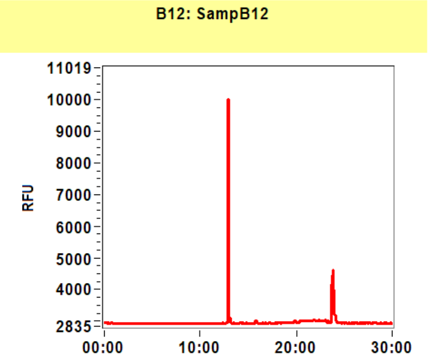

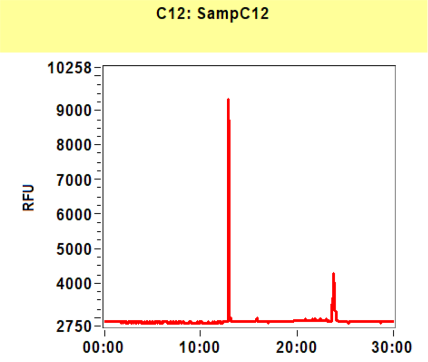

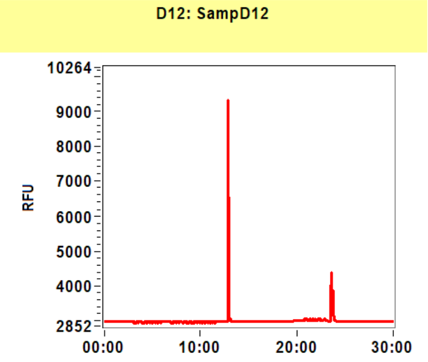

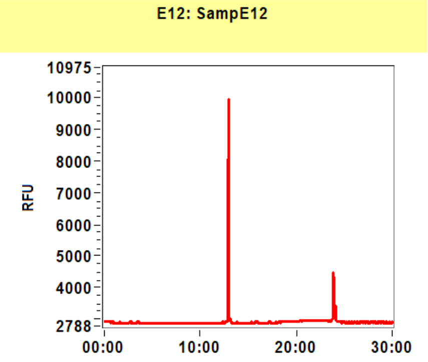

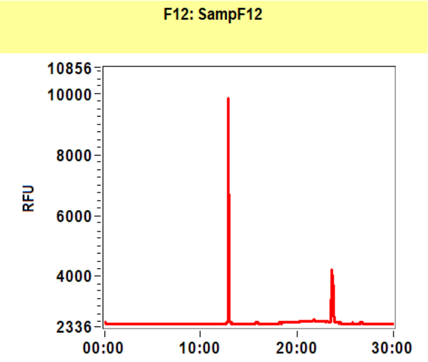

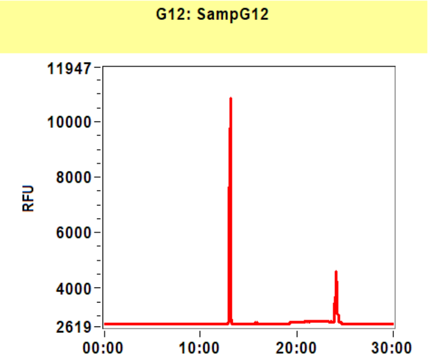

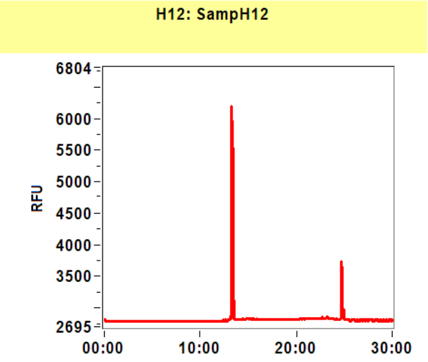

Sample: SampA1
Well location: A1
Created: Tuesday, September 14, 2021 4:54:05 PM

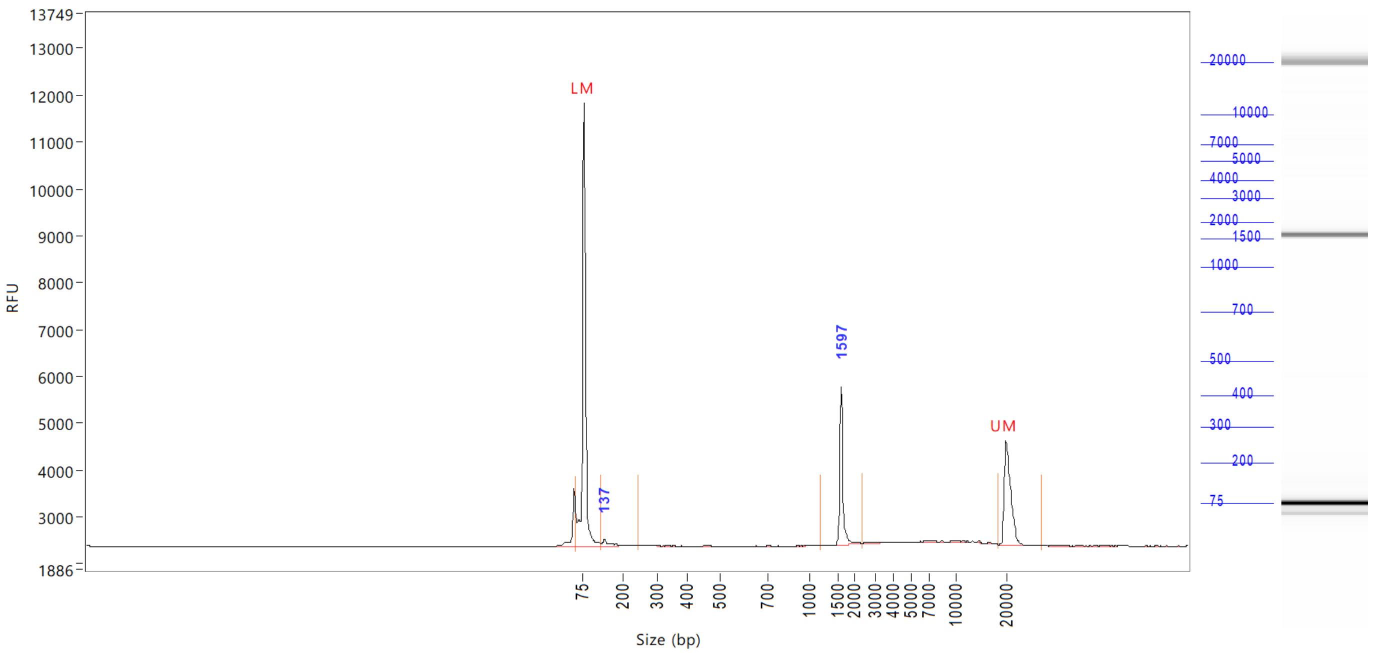

Peak	Size	Concentration	From	To	Average size	CV%	RFU	Corrected peak area
	(bp)	(ng/uL)	(bp)	(bp)	(bp)			
1	75 (LM)	0.5000	52	128	74	12.03	9478	65.146
2	137	0.2353	128	242	161	18.49	137	2.554
3	1597	1.5264	1190	2372	1608	6.49	3363	16.573
4	20000 (UM)	0.1419	18537	27012	20363	3.61	2226	18.486

TIC:	1.7617	ng/uL
TIM:	3.9644	nmole/L
Total concentration:	2.0589	ng/uL

Sample peak width (sec): 5 Sample min peak height: 50 Sample baseline V to V?: Y Sample baseline V to V points: 3
Sample filter: Binomial Number of points for filter: 3 Sample start region (min): 0 Sample end region (min): 30
Marker peak width (sec): 5 Marker min peak height: 200 Marker baseline V to V?: Y Marker baseline V to V points: 3
Lower marker selection: First peak > 500 RFU Upper marker selection: Last peak > 200 RFU
Ladder size (bp)75, 200, 300, 400, 500, 700, 1000, 1500, 2000, 3000, 4000, 5000, 7000, 10000, 20000
Quantification using: Lower Marker Final concentration (ng/uL): 0.5000 Dilution factor: 12.0

Sample: SampB1
Well location: B1
Created: Tuesday, September 14, 2021 4:54:05 PM

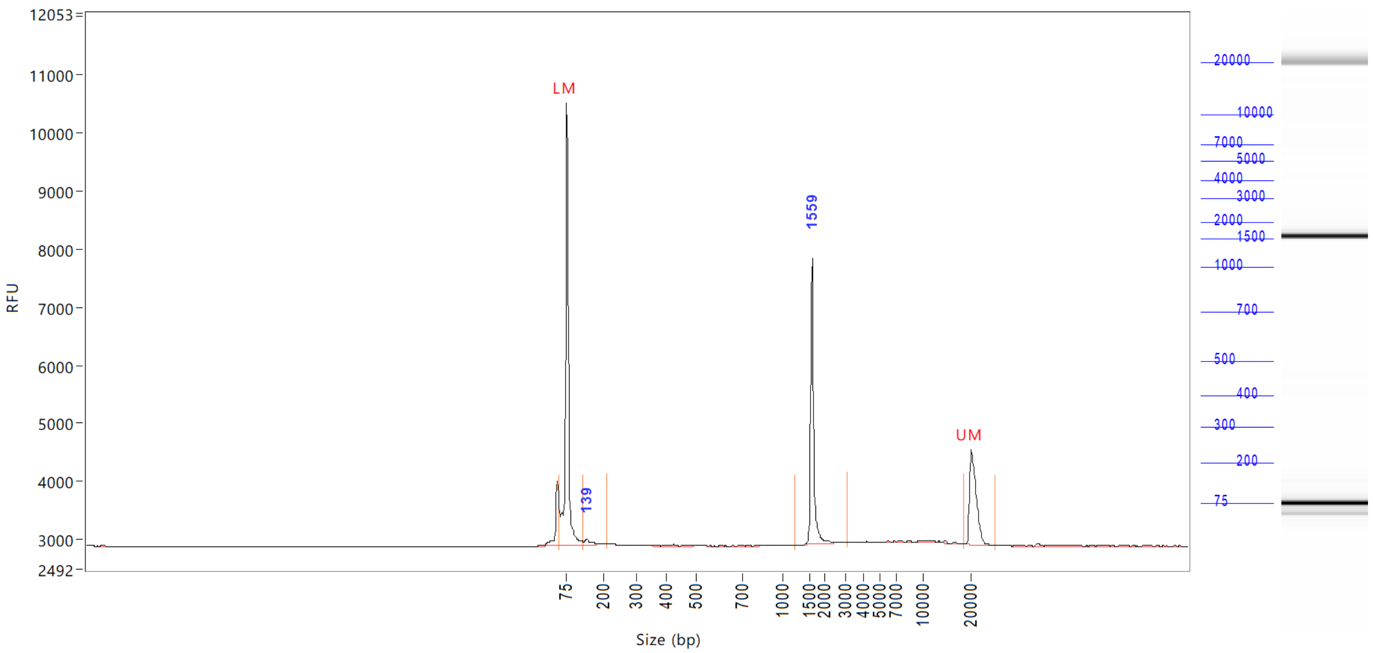

Peak	Size (bp)	Concentration (ng/uL)	From (bp)	To (bp)	Average size (bp)	CV%	RFU	Corrected peak area
1	75 (LM)	0.5000	52	129	74	12.93	7621	55.373
2	139	0.1710	129	208	149	10.21	103	1.578
3	1559	2.6836	1234	3072	1579	8.61	4919	24.767
4	20000 (UM)	0.1272	18537	25078	20478	3.79	1627	14.084

TIC:	2.8546	ng/uL
TIM:	4.6839	nmole/L
Total concentration:	3.1894	ng/uL

```
Sample peak width (sec): 5    Sample min peak height: 50    Sample baseline V to V?: Y    Sample baseline V to V points: 3
Sample filter: Binomial       Number of points for filter: 3  Sample start region (min): 0  Sample end region (min): 30
Marker peak width (sec): 5    Marker min peak height: 200   Marker baseline V to V?: Y    Marker baseline V to V points: 3
Lower marker selection: First peak > 500 RFU                Upper marker selection: Last peak > 200 RFU
Ladder size (bp)75, 200, 300, 400, 500, 700, 1000, 1500, 2000, 3000, 4000, 5000, 7000, 10000, 20000
Quantification using: Lower Marker          Final concentration (ng/uL): 0.5000          Dilution factor: 12.0
```

Sample: SampC1
Well location: C1
Created: Tuesday, September 14, 2021 4:54:05 PM

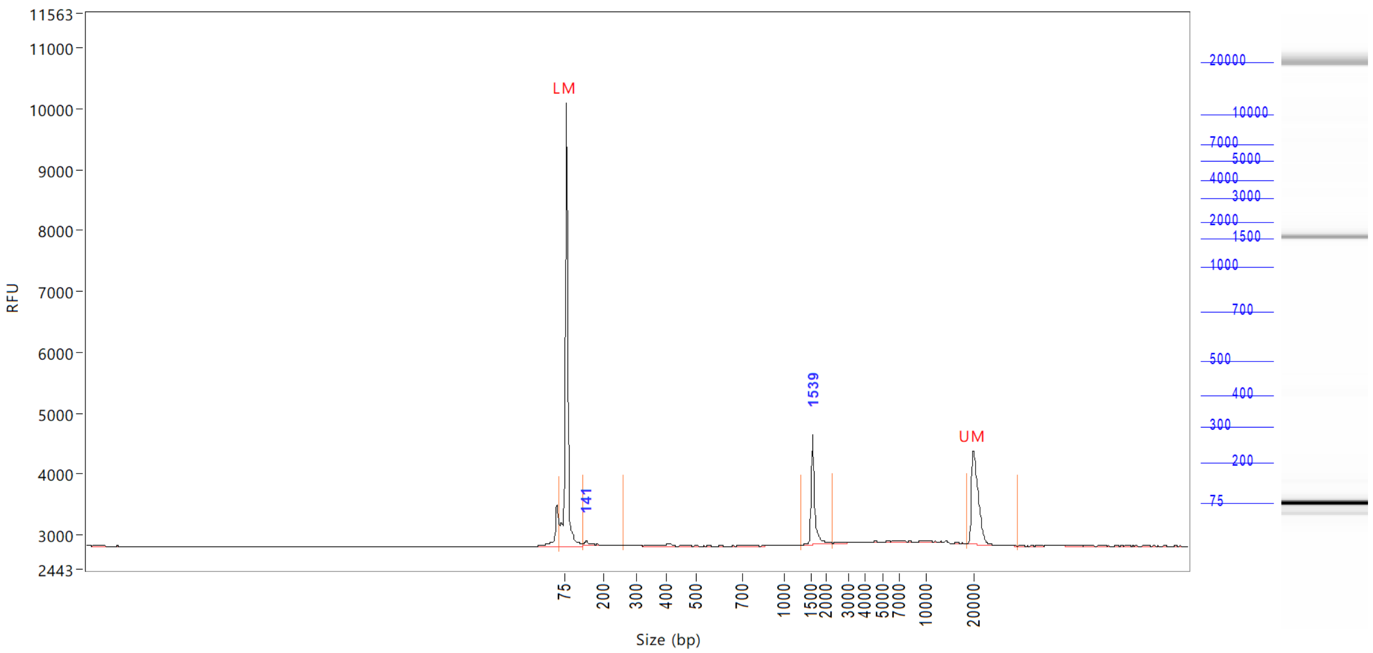

Peak	Size (bp)	Concentration (ng/uL)	From (bp)	To (bp)	Average size (bp)	CV%	RFU	Corrected peak area
1	75 (LM)	0.5000	52	128	74	11.30	7283	49.575
2	141	0.2189	128	260	165	19.58	94	1.809
3	1539	1.2226	1323	2286	1567	7.35	1813	10.102
4	20000 (UM)	0.1370	18415	29068	20409	4.24	1535	13.582

TIC:	1.4416	ng/uL
TIM:	3.4651	nmole/L
Total concentration:	1.8842	ng/uL

Sample peak width (sec): 5 Sample min peak height: 50 Sample baseline V to V?: Y Sample baseline V to V points: 3
Sample filter: Binomial Number of points for filter: 3 Sample start region (min): 0 Sample end region (min): 30
Marker peak width (sec): 5 Marker min peak height: 200 Marker baseline V to V?: Y Marker baseline V to V points: 3
Lower marker selection: First peak > 500 RFU Upper marker selection: Last peak > 200 RFU
Ladder size (bp)75, 200, 300, 400, 500, 700, 1000, 1500, 2000, 3000, 4000, 5000, 7000, 10000, 20000
Quantification using: Lower Marker Final concentration (ng/uL): 0.5000 Dilution factor: 12.0

Sample: SampD1
Well location: D1
Created: Tuesday, September 14, 2021 4:54:05 PM

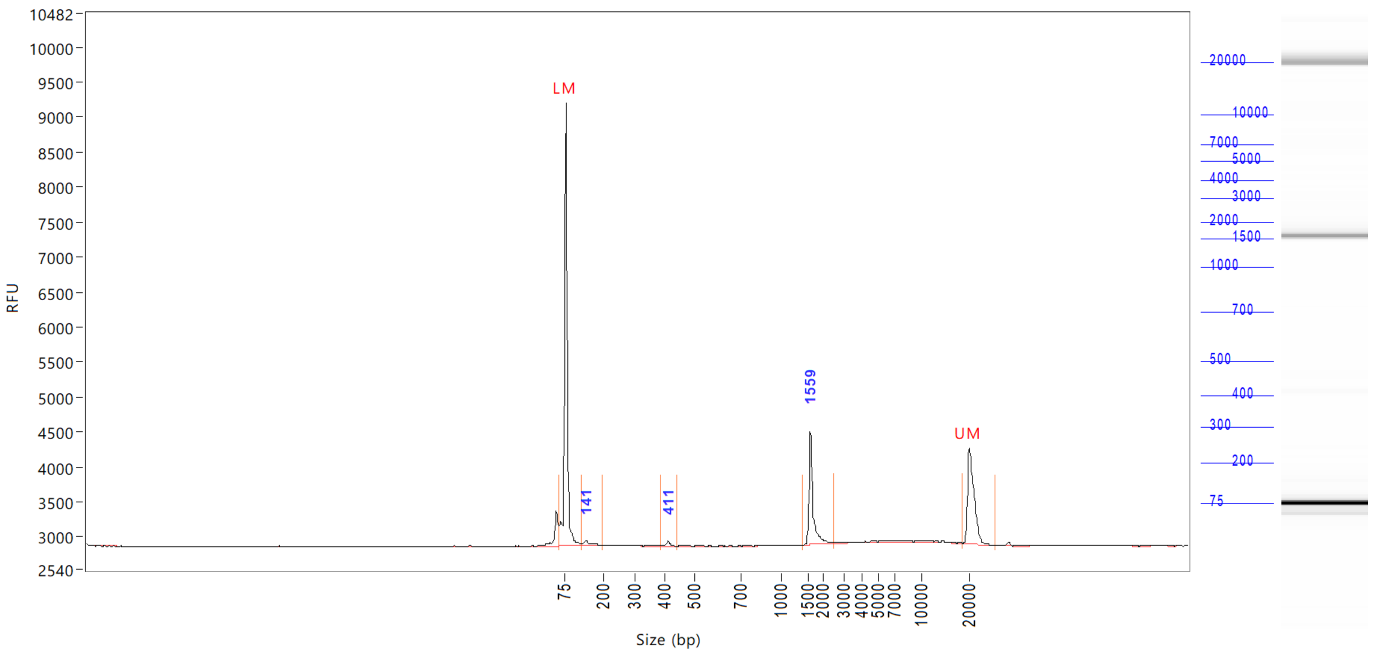

Peak	Size (bp)	Concentration (ng/uL)	From (bp)	To (bp)	Average size (bp)	CV%	RFU	Corrected peak area
1	75 (LM)	0.5000	52	129	74	11.40	6339	43.341
2	141	0.1625	129	198	152	11.60	76	1.174
3	411	0.0767	384	441	408	1.86	76	0.554
4	1559	1.4115	1400	2458	1610	9.22	1612	10.196
5	20000 (UM)	0.1344	18537	25561	20424	3.83	1360	11.653

TIC:	1.6507	ng/uL
TIM:	3.5094	nmole/L
Total concentration:	2.0088	ng/uL

Sample peak width (sec): 5 Sample min peak height: 50 Sample baseline V to V?: Y Sample baseline V to V points: 3
Sample filter: Binomial Number of points for filter: 3 Sample start region (min): 0 Sample end region (min): 30
Marker peak width (sec): 5 Marker min peak height: 200 Marker baseline V to V?: Y Marker baseline V to V points: 3
Lower marker selection: First peak > 500 RFU Upper marker selection: Last peak > 200 RFU
Ladder size (bp)75, 200, 300, 400, 500, 700, 1000, 1500, 2000, 3000, 4000, 5000, 7000, 10000, 20000
Quantification using: Lower Marker Final concentration (ng/uL): 0.5000 Dilution factor: 12.0

Sample: SampE1
Well location: E1
Created: Tuesday, September 14, 2021 4:54:05 PM

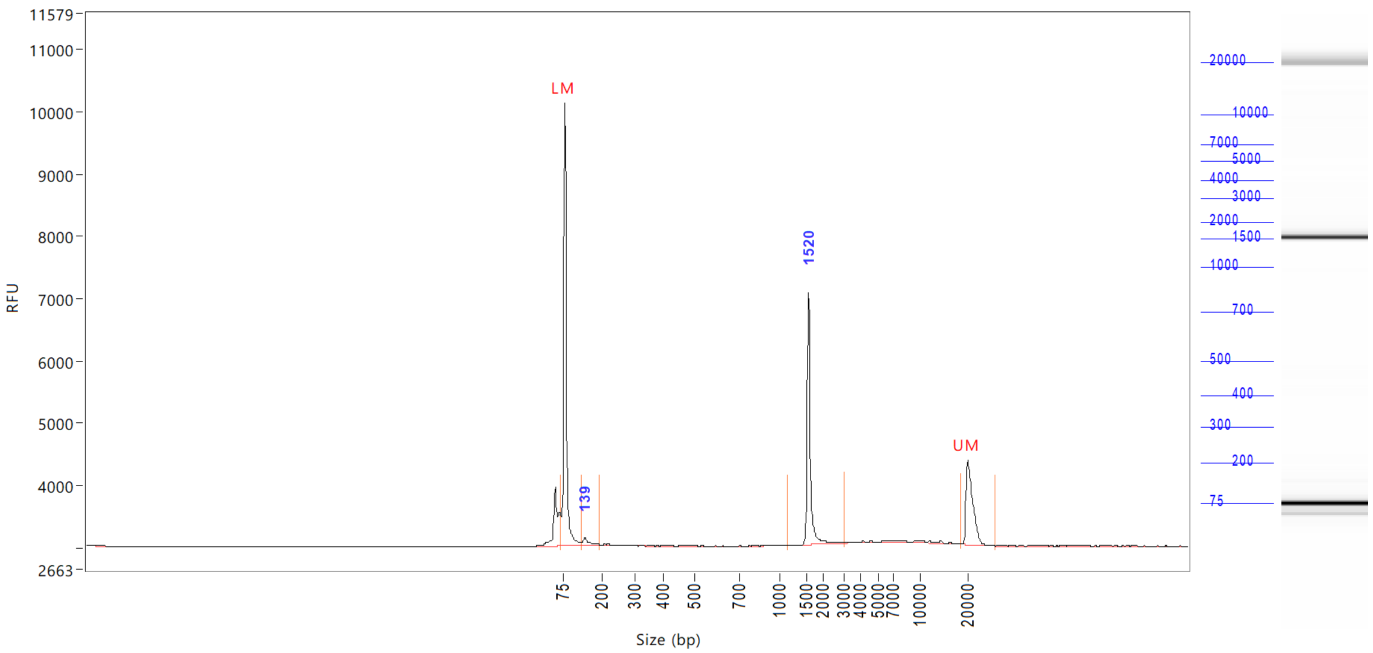

Peak	Size<br>(bp)	Concentration<br>(ng/uL)	From<br>(bp)	To<br>(bp)	Average size<br>(bp)	CV%	RFU	Corrected peak area
1	75 (LM)	0.5000	64	129	75	11.17	7116	48.166
2	139	0.2188	129	189	149	10.21	124	1.756
3	1520	2.6536	1123	3037	1568	11.49	4029	21.302
4	20000 (UM)	0.1261	18537	25803	20409	3.94	1357	12.143

TIC:	2.8724	ng/uL
TIM:	5.1990	nmole/L
Total concentration:	3.3415	ng/uL

```
Sample peak width (sec): 5    Sample min peak height: 50    Sample baseline V to V?: Y    Sample baseline V to V points: 3
Sample filter: Binomial       Number of points for filter: 3  Sample start region (min): 0  Sample end region (min): 30
Marker peak width (sec): 5    Marker min peak height: 200   Marker baseline V to V?: Y    Marker baseline V to V points: 3
Lower marker selection: First peak > 500 RFU                Upper marker selection: Last peak > 200 RFU
Ladder size (bp)75, 200, 300, 400, 500, 700, 1000, 1500, 2000, 3000, 4000, 5000, 7000, 10000, 20000
Quantification using: Lower Marker          Final concentration (ng/uL): 0.5000          Dilution factor: 12.0
```

Sample: SampF1
Well location: F1
Created: Tuesday, September 14, 2021 4:54:05 PM

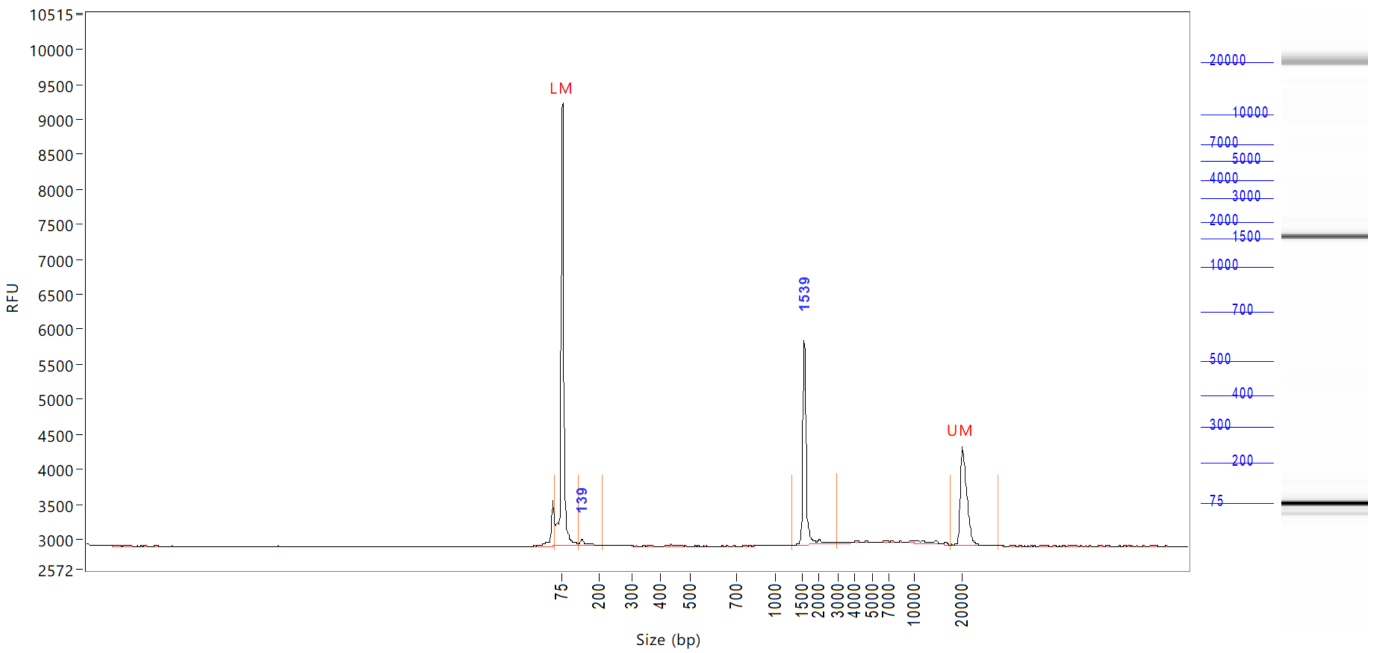

Peak	Size (bp)	Concentration (ng/uL)	From (bp)	To (bp)	Average size (bp)	CV%	RFU	Corrected peak area
1	75 (LM)	0.5000	50	128	73	11.57	6338	44.401
2	139	0.1518	128	208	148	11.22	83	1.123
3	1539	2.0850	1289	2971	1583	11.42	2926	15.429
4	20000 (UM)	0.1355	17439	27617	20421	3.93	1412	12.031

TIC:	2.2367	ng/uL
TIM:	3.8534	nmole/L
Total concentration:	2.7286	ng/uL

```
Sample peak width (sec): 5    Sample min peak height: 50    Sample baseline V to V?: Y    Sample baseline V to V points: 3
Sample filter: Binomial       Number of points for filter: 3  Sample start region (min): 0  Sample end region (min): 30
Marker peak width (sec): 5    Marker min peak height: 200   Marker baseline V to V?: Y    Marker baseline V to V points: 3
Lower marker selection: First peak > 500 RFU                Upper marker selection: Last peak > 200 RFU
Ladder size (bp)75, 200, 300, 400, 500, 700, 1000, 1500, 2000, 3000, 4000, 5000, 7000, 10000, 20000
Quantification using: Lower Marker          Final concentration (ng/uL): 0.5000          Dilution factor: 12.0
```

Sample: SampG1
Well location: G1
Created: Tuesday, September 14, 2021 4:54:05 PM

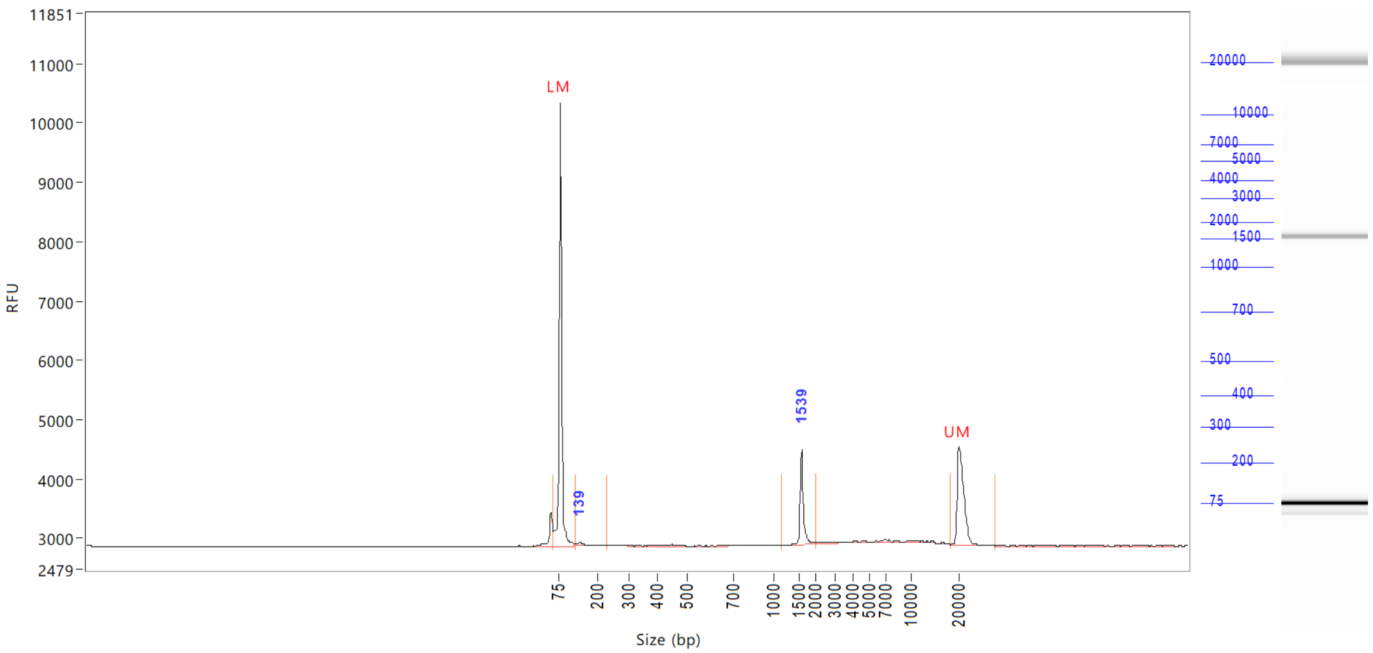

Peak	Size (bp)	Concentration (ng/uL)	From (bp)	To (bp)	Average size (bp)	CV%	RFU	Corrected peak area
1	75 (LM)	0.5000	52	129	74	11.06	7484	48.874
2	139	0.1461	129	232	158	15.74	67	1.190
3	1539	1.0540	1145	2001	1555	5.73	1595	8.585
4	20000 (UM)	0.1465	18171	27617	20451	4.02	1659	14.317

TIC:	1.2000	ng/uL
TIM:	2.6351	nmole/L
Total concentration:	1.6413	ng/uL

Sample peak width (sec): 5 Sample min peak height: 50 Sample baseline V to V?: Y Sample baseline V to V points: 3
Sample filter: Binomial Number of points for filter: 3 Sample start region (min): 0 Sample end region (min): 30
Marker peak width (sec): 5 Marker min peak height: 200 Marker baseline V to V?: Y Marker baseline V to V points: 3
Lower marker selection: First peak > 500 RFU Upper marker selection: Last peak > 200 RFU
Ladder size (bp)75, 200, 300, 400, 500, 700, 1000, 1500, 2000, 3000, 4000, 5000, 7000, 10000, 20000
Quantification using: Lower Marker Final concentration (ng/uL): 0.5000 Dilution factor: 12.0

Sample: SampH1
Well location: H1
Created: Tuesday, September 14, 2021 4:54:05 PM

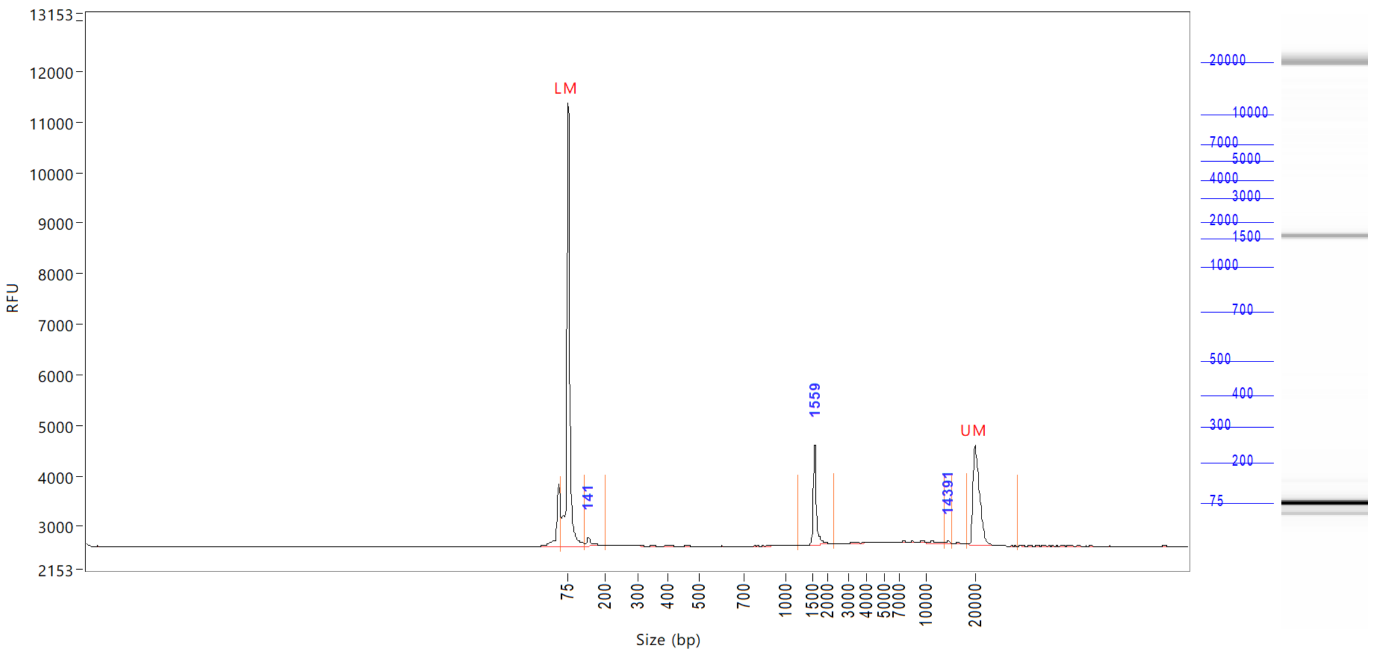

Peak	Size (bp)	Concentration (ng/uL)	From (bp)	To (bp)	Average size (bp)	CV%	RFU	Corrected peak area
1	75 (LM)	0.5000	52	128	74	12.26	8786	64.386
2	141	0.2104	128	200	150	11.90	175	2.257
3	1559	0.9386	1223	2258	1569	6.11	1976	10.072
4	14391	0.0255	13659	15244	14282	2.35	57	0.273
5	20000 (UM)	0.1300	18293	29068	20402	3.84	1975	16.737

TIC:	1.1744	ng/uL
TIM:	3.2926	nmole/L
Total concentration:	1.5188	ng/uL

```
Sample peak width (sec): 5    Sample min peak height: 50    Sample baseline V to V?: Y    Sample baseline V to V points: 3
Sample filter: Binomial       Number of points for filter: 3  Sample start region (min): 0  Sample end region (min): 30
Marker peak width (sec): 5    Marker min peak height: 200   Marker baseline V to V?: Y    Marker baseline V to V points: 3
Lower marker selection: First peak > 500 RFU                Upper marker selection: Last peak > 200 RFU
Ladder size (bp)75, 200, 300, 400, 500, 700, 1000, 1500, 2000, 3000, 4000, 5000, 7000, 10000, 20000
Quantification using: Lower Marker          Final concentration (ng/uL): 0.5000          Dilution factor: 12.0
```

Sample: SampA2
Well location: A2
Created: Tuesday, September 14, 2021 4:54:05 PM

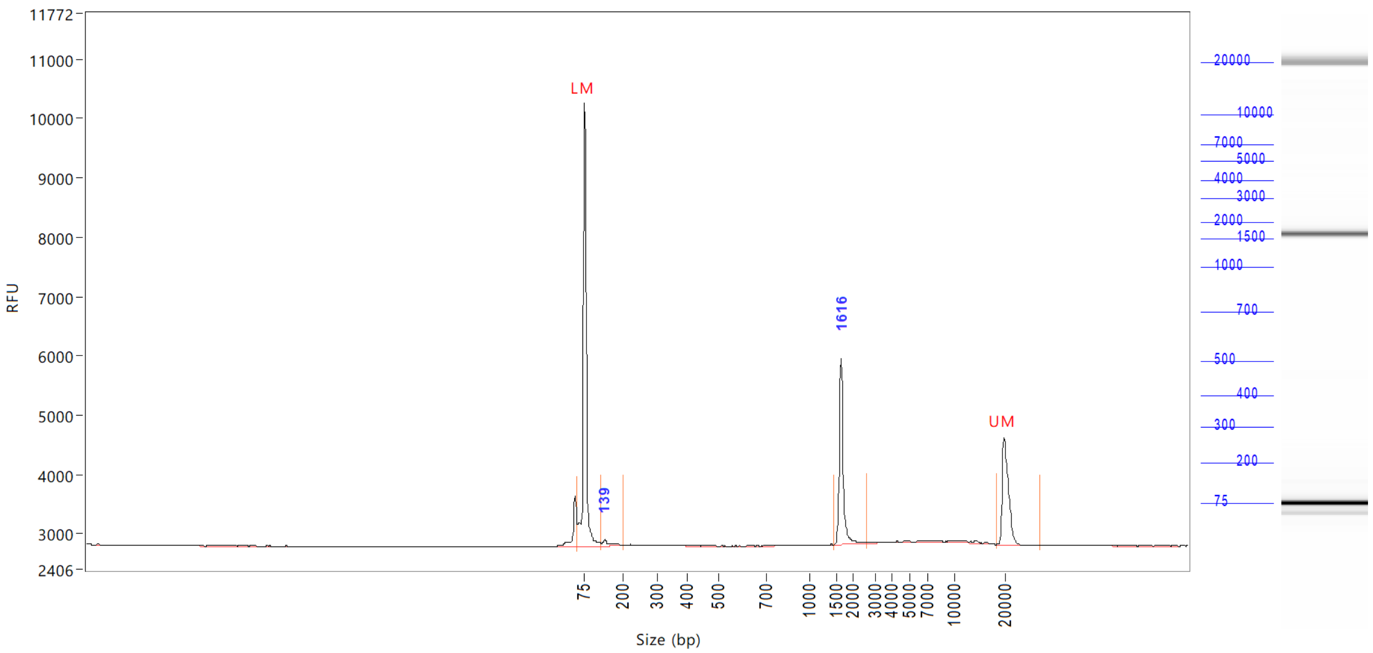

Peak	Size (bp)	Concentration (ng/uL)	From (bp)	To (bp)	Average size (bp)	CV%	RFU	Corrected peak area
1	75 (LM)	0.5000	54	129	75	11.85	7480	50.964
2	139	0.2199	129	200	155	12.64	102	1.868
3	1616	2.2525	1445	2657	1642	7.97	3130	19.133
4	20000 (UM)	0.1409	18415	27254	20270	3.32	1801	14.359

TIC:	2.4724	ng/uL
TIM:	4.5898	nmole/L
Total concentration:	2.9399	ng/uL

Sample peak width (sec): 5   Sample min peak height: 50   Sample baseline V to V?: Y   Sample baseline V to V points: 3
Sample filter: Binomial   Number of points for filter: 3   Sample start region (min): 0   Sample end region (min): 30
Marker peak width (sec): 5   Marker min peak height: 200   Marker baseline V to V?: Y   Marker baseline V to V points: 3
Lower marker selection: First peak > 500 RFU   Upper marker selection: Last peak > 200 RFU
Ladder size (bp)75, 200, 300, 400, 500, 700, 1000, 1500, 2000, 3000, 4000, 5000, 7000, 10000, 20000
Quantification using: Lower Marker   Final concentration (ng/uL): 0.5000   Dilution factor: 12.0

Sample: SampB2
Well location: B2
Created: Tuesday, September 14, 2021 4:54:05 PM

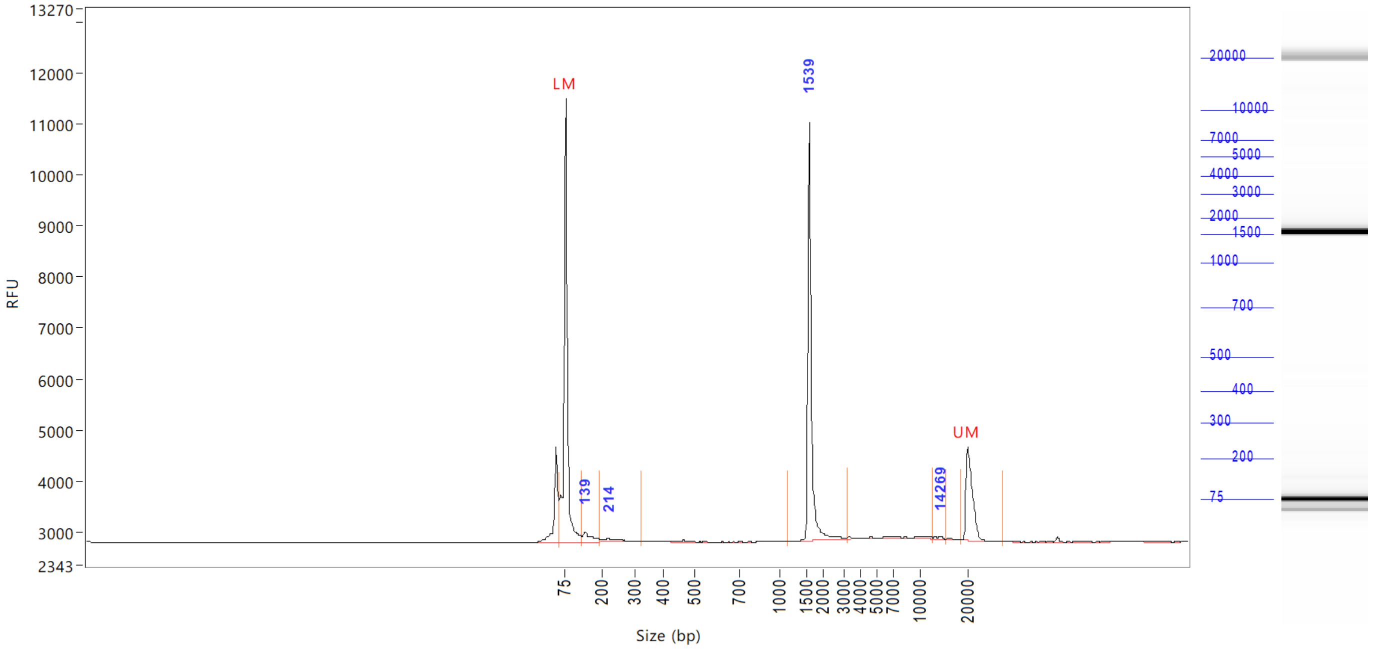

Peak	Size (bp)	Concentration (ng/uL)	From (bp)	To (bp)	Average size (bp)	CV%	RFU	Corrected peak area
1	75 (LM)	0.5000	52	129	75	15.07	8727	71.271
2	139	0.3391	129	190	154	11.31	207	4.028
3	214	0.2069	190	321	231	12.96	62	2.458
4	1539	3.9582	1134	3179	1601	12.19	8205	47.018
5	14269	0.0458	12684	15366	13914	4.83	61	0.544
6	20000 (UM)	0.1097	18537	27375	20465	3.77	1825	15.643

TIC:	4.5501	ng/uL
TIM:	9.1675	nmole/L
Total concentration:	4.8270	ng/uL

```
Sample peak width (sec): 5    Sample min peak height: 50    Sample baseline V to V?: Y    Sample baseline V to V points: 3
Sample filter: Binomial       Number of points for filter: 3  Sample start region (min): 0  Sample end region (min): 30
Marker peak width (sec): 5    Marker min peak height: 200   Marker baseline V to V?: Y    Marker baseline V to V points: 3
Lower marker selection: First peak > 500 RFU                Upper marker selection: Last peak > 200 RFU
Ladder size (bp)75, 200, 300, 400, 500, 700, 1000, 1500, 2000, 3000, 4000, 5000, 7000, 10000, 20000
Quantification using: Lower Marker          Final concentration (ng/uL): 0.5000          Dilution factor: 12.0
```

Sample: SampC2
Well location: C2
Created: Tuesday, September 14, 2021 4:54:05 PM

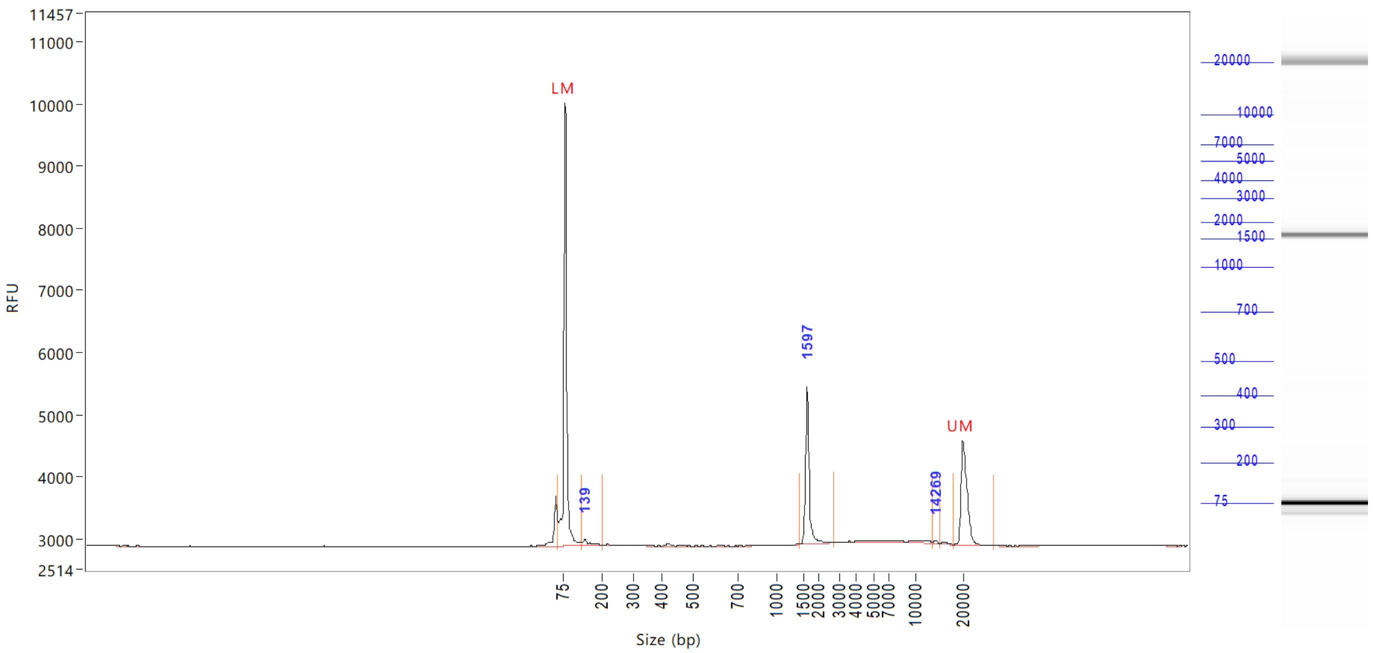

Peak	Size (bp)	Concentration (ng/uL)	From (bp)	To (bp)	Average size (bp)	CV%	RFU	Corrected peak area
1	75 (LM)	0.5000	52	129	75	12.36	7139	51.008
2	139	0.2137	129	200	154	12.21	99	1.817
3	1597	1.7636	1422	2715	1614	8.51	2521	14.993
4	14269	0.0335	13537	15122	14189	2.83	50	0.284
5	20000 (UM)	0.1341	18049	26770	20397	3.82	1689	13.678

TIC:	2.0107	ng/uL
TIM:	4.0831	nmole/L
Total concentration:	2.4548	ng/uL

Sample peak width (sec): 5 Sample min peak height: 50 Sample baseline V to V?: Y Sample baseline V to V points: 3
Sample filter: Binomial Number of points for filter: 3 Sample start region (min): 0 Sample end region (min): 30
Marker peak width (sec): 5 Marker min peak height: 200 Marker baseline V to V?: Y Marker baseline V to V points: 3
Lower marker selection: First peak > 500 RFU Upper marker selection: Last peak > 200 RFU
Ladder size (bp)75, 200, 300, 400, 500, 700, 1000, 1500, 2000, 3000, 4000, 5000, 7000, 10000, 20000
Quantification using: Lower Marker Final concentration (ng/uL): 0.5000 Dilution factor: 12.0

Sample: SampD2
Well location: D2
Created: Tuesday, September 14, 2021 4:54:05 PM

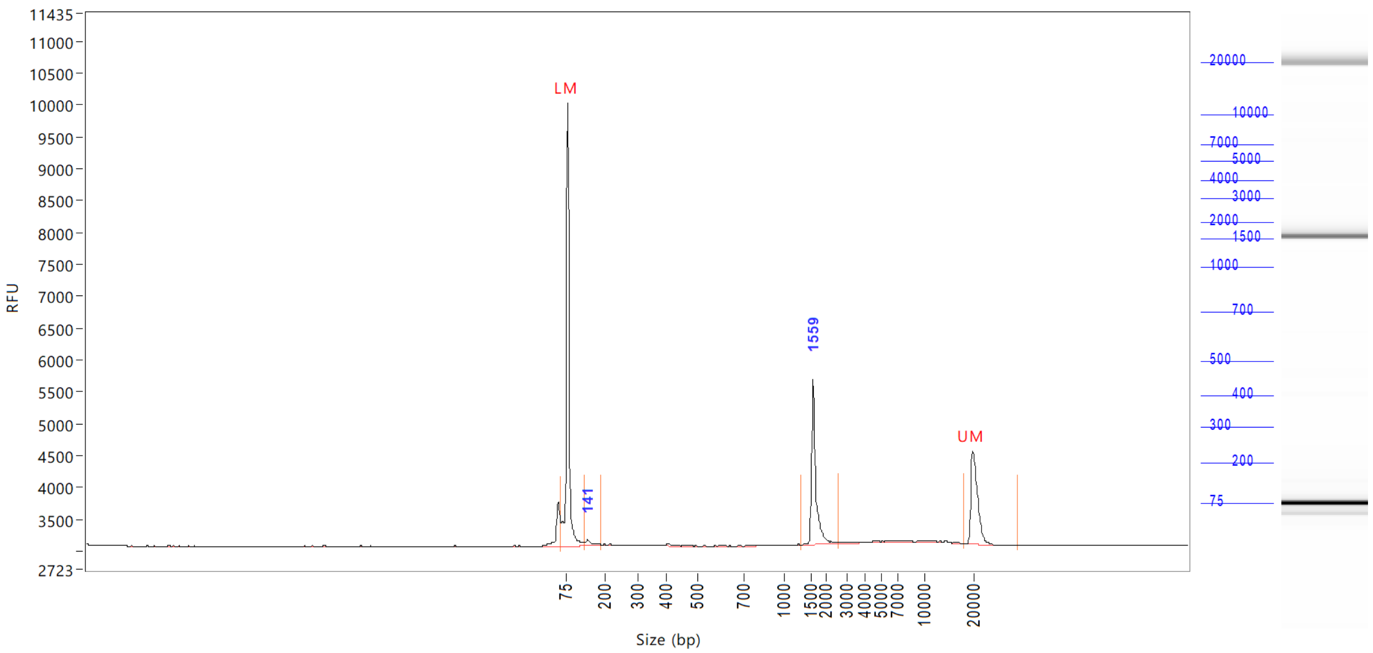

Peak	Size (bp)	Concentration (ng/uL)	From (bp)	To (bp)	Average size (bp)	CV%	RFU	Corrected peak area
1	75 (LM)	0.5000	52	131	75	12.72	6957	48.867
2	141	0.1969	131	187	152	10.14	96	1.604
3	1559	2.1601	1323	2572	1607	9.59	2598	17.593
4	20000 (UM)	0.1303	18171	29309	20411	4.34	1471	12.731

TIC:	2.3570	ng/uL
TIM:	4.3416	nmole/L
Total concentration:	2.8932	ng/uL

Sample peak width (sec): 5 Sample min peak height: 50 Sample baseline V to V?: Y Sample baseline V to V points: 3
Sample filter: Binomial Number of points for filter: 3 Sample start region (min): 0 Sample end region (min): 30
Marker peak width (sec): 5 Marker min peak height: 200 Marker baseline V to V?: Y Marker baseline V to V points: 3
Lower marker selection: First peak > 500 RFU Upper marker selection: Last peak > 200 RFU
Ladder size (bp)75, 200, 300, 400, 500, 700, 1000, 1500, 2000, 3000, 4000, 5000, 7000, 10000, 20000
Quantification using: Lower Marker Final concentration (ng/uL): 0.5000 Dilution factor: 12.0

Sample: SampE2
Well location: E2
Created: Tuesday, September 14, 2021 4:54:05 PM

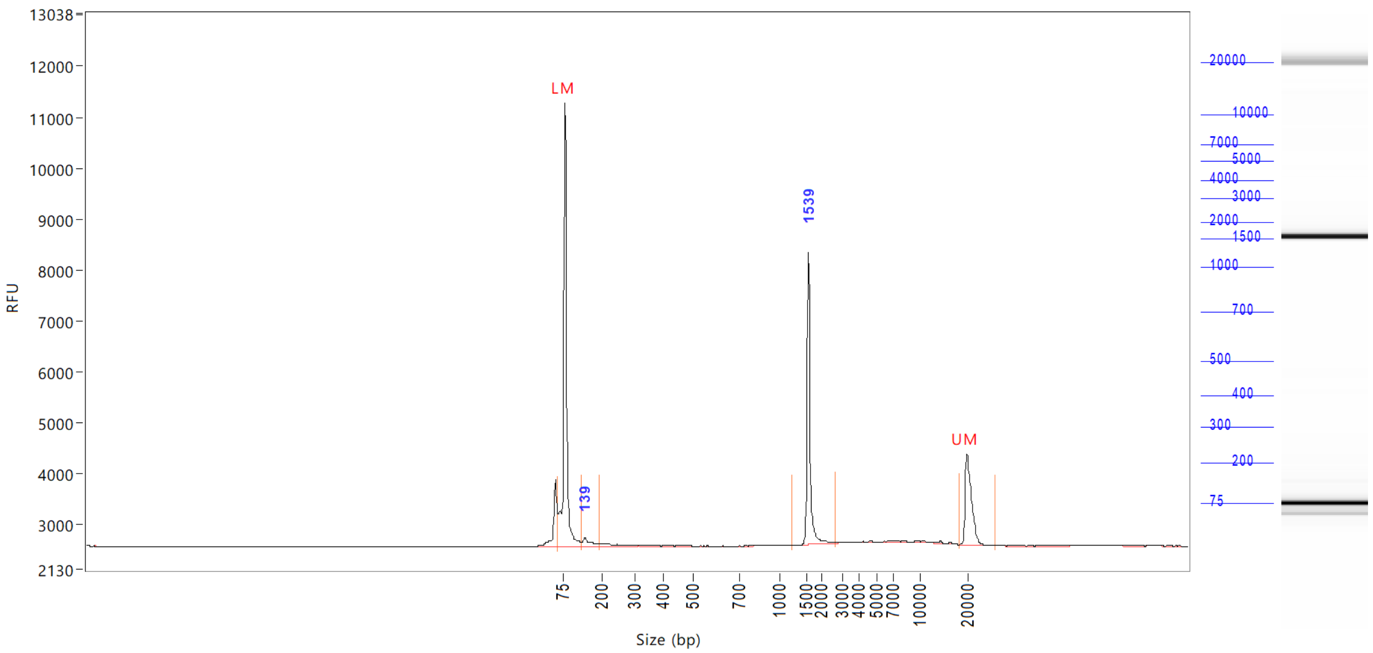

Peak	Size (bp)	Concentration (ng/uL)	From (bp)	To (bp)	Average size (bp)	CV%	RFU	Corrected peak area
1	75 (LM)	0.5000	52	129	74	13.68	8712	65.093
2	139	0.2653	129	189	152	10.88	161	2.879
3	1539	2.6559	1234	2629	1566	7.94	5733	28.814
4	20000 (UM)	0.1150	18293	25924	20412	3.70	1792	14.973

TIC:	2.9213	ng/uL
TIM:	5.6608	nmole/L
Total concentration:	3.3863	ng/uL

Sample peak width (sec): 5   Sample min peak height: 50   Sample baseline V to V?: Y   Sample baseline V to V points: 3
Sample filter: Binomial   Number of points for filter: 3 Sample start region (min): 0 Sample end region (min): 30
Marker peak width (sec): 5   Marker min peak height: 200   Marker baseline V to V?: Y   Marker baseline V to V points: 3
Lower marker selection: First peak > 500 RFU   Upper marker selection: Last peak > 200 RFU
Ladder size (bp)75, 200, 300, 400, 500, 700, 1000, 1500, 2000, 3000, 4000, 5000, 7000, 10000, 20000
Quantification using: Lower Marker   Final concentration (ng/uL): 0.5000   Dilution factor: 12.0

Sample: SampF2
Well location: F2
Created: Tuesday, September 14, 2021 4:54:05 PM

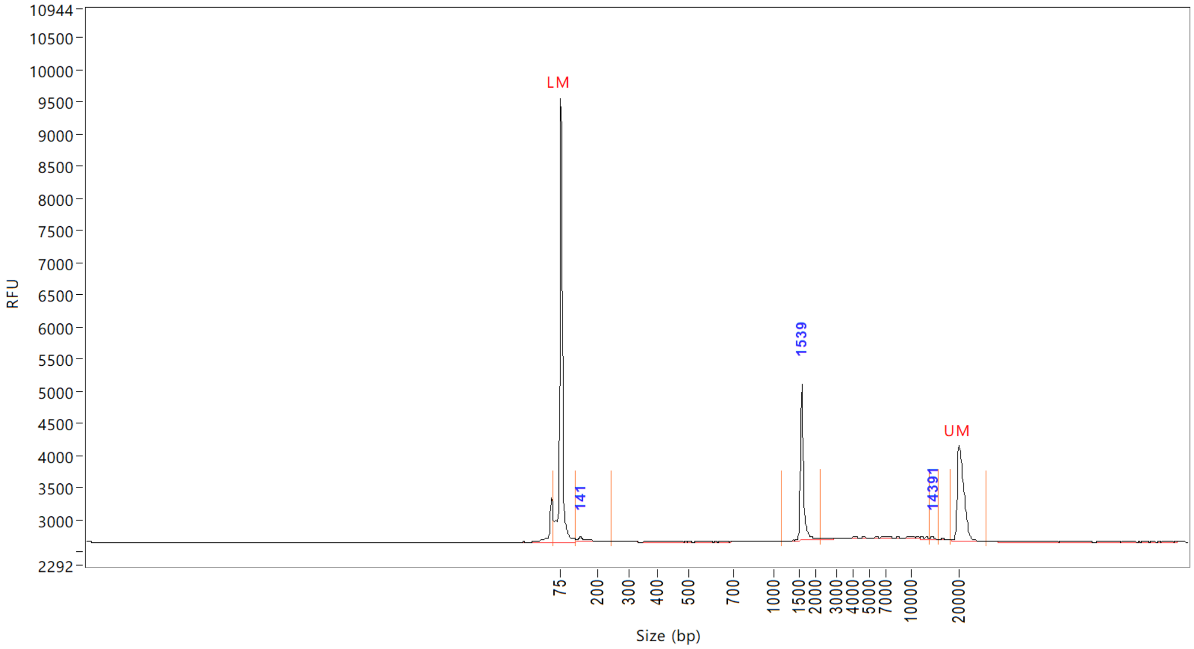

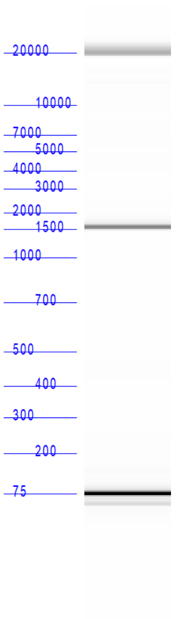

Peak	Size (bp)	Concentration (ng/uL)	From (bp)	To (bp)	Average size (bp)	CV%	RFU	Corrected peak area
1	75 (LM)	0.5000	52	128	75	11.62	6908	47.225
2	141	0.2382	128	242	165	18.85	79	1.875
3	1539	1.5790	1145	2229	1560	6.44	2422	12.428
4	14391	0.0348	13659	15732	14432	3.27	51	0.274
5	20000 (UM)	0.1338	18293	25682	20449	3.61	1477	12.636

TIC:	1.8519	ng/uL
TIM:	4.0431	nmole/L
Total concentration:	2.2546	ng/uL

```
Sample peak width (sec): 5    Sample min peak height: 50    Sample baseline V to V?: Y    Sample baseline V to V points: 3
Sample filter: Binomial       Number of points for filter: 3  Sample start region (min): 0  Sample end region (min): 30
Marker peak width (sec): 5    Marker min peak height: 200   Marker baseline V to V?: Y    Marker baseline V to V points: 3
Lower marker selection: First peak > 500 RFU                Upper marker selection: Last peak > 200 RFU
Ladder size (bp)75, 200, 300, 400, 500, 700, 1000, 1500, 2000, 3000, 4000, 5000, 7000, 10000, 20000
Quantification using: Lower Marker          Final concentration (ng/uL): 0.5000          Dilution factor: 12.0
```

Sample: SampG2
Well location: G2
Created: Tuesday, September 14, 2021 4:54:05 PM

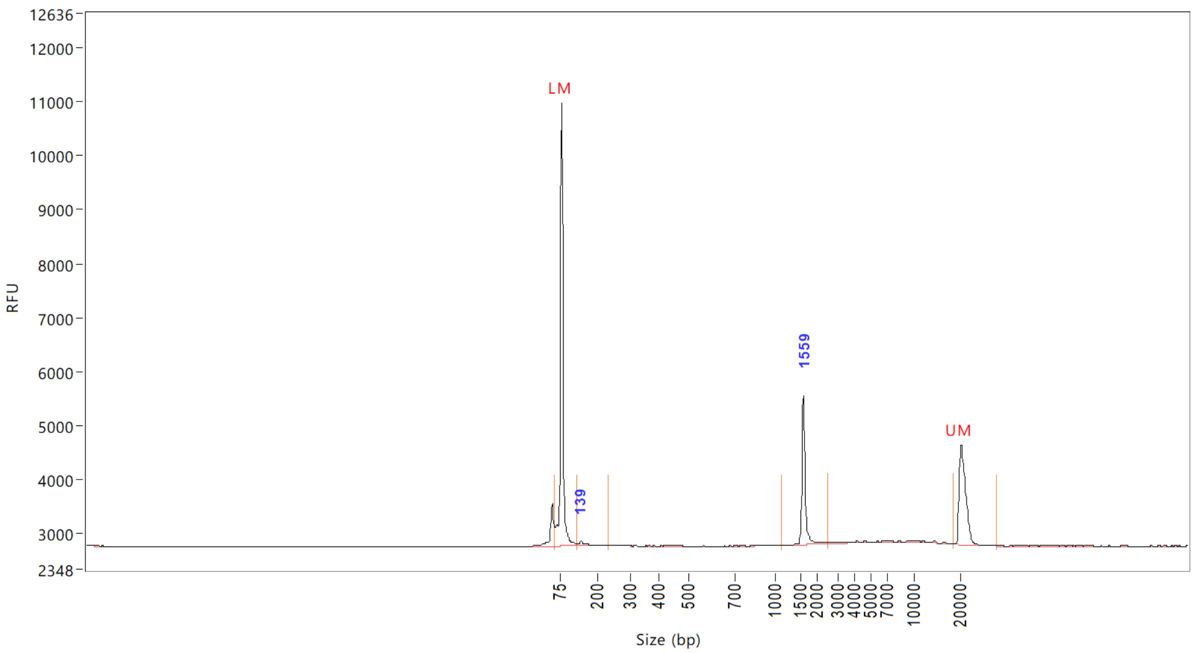

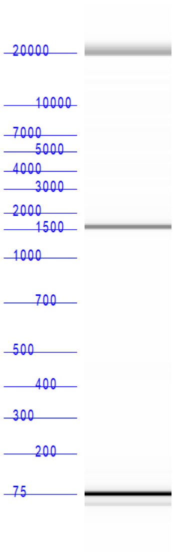

Peak	Size (bp)	Concentration (ng/uL)	From (bp)	To (bp)	Average size (bp)	CV%	RFU	Corrected peak area
1	75 (LM)	0.5000	52	129	74	11.39	8213	55.464
2	139	0.1454	129	228	155	14.29	83	1.344
3	1559	1.5340	1112	2572	1578	9.12	2754	14.181
4	20000 (UM)	0.1431	18293	27496	20450	3.81	1839	15.872

TIC:	1.6794	ng/uL
TIM:	3.1420	nmole/L
Total concentration:	2.0409	ng/uL

Sample peak width (sec): 5   Sample min peak height: 50   Sample baseline V to V?: Y   Sample baseline V to V points: 3
Sample filter: Binomial   Number of points for filter: 3 Sample start region (min): 0 Sample end region (min): 30
Marker peak width (sec): 5   Marker min peak height: 200   Marker baseline V to V?: Y   Marker baseline V to V points: 3
Lower marker selection: First peak > 500 RFU   Upper marker selection: Last peak > 200 RFU
Ladder size (bp)75, 200, 300, 400, 500, 700, 1000, 1500, 2000, 3000, 4000, 5000, 7000, 10000, 20000
Quantification using: Lower Marker   Final concentration (ng/uL): 0.5000   Dilution factor: 12.0

Sample: SampH2
Well location: H2
Created: Tuesday, September 14, 2021 4:54:05 PM

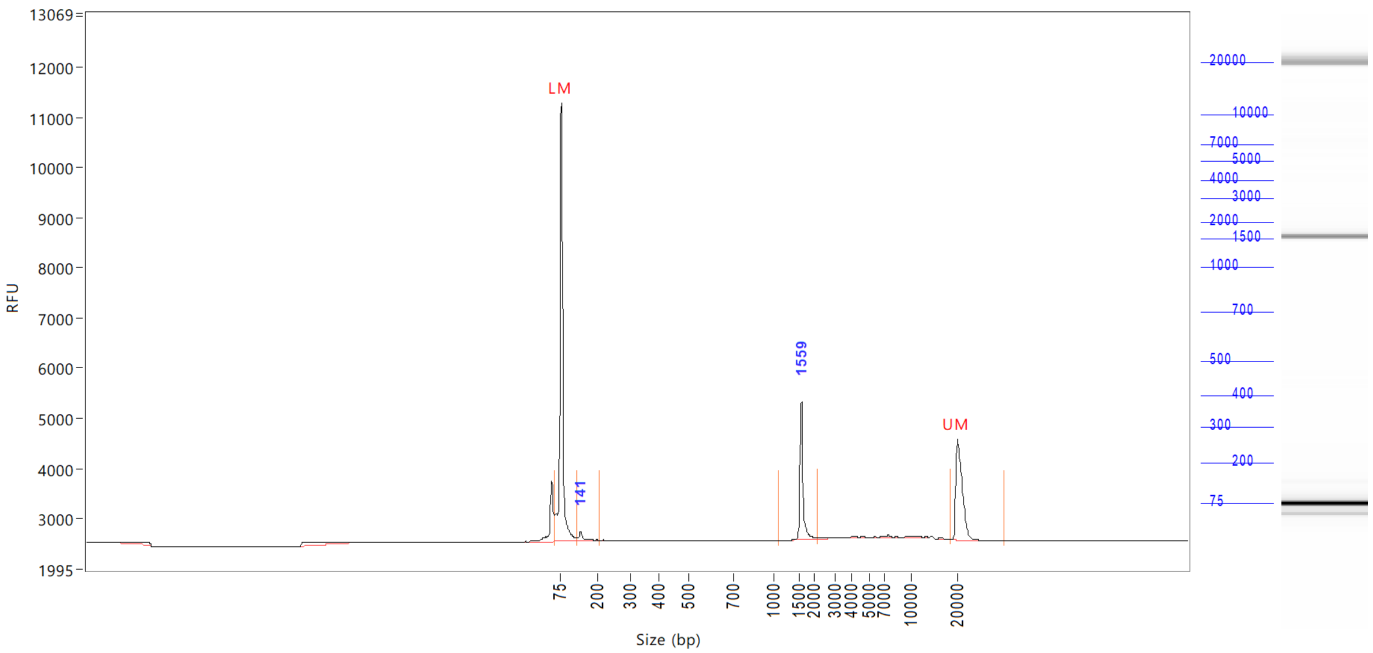

Peak	Size (bp)	Concentration (ng/uL)	From (bp)	To (bp)	Average size (bp)	CV%	RFU	Corrected peak area
1	75 (LM)	0.5000	52	128	74	12.91	8750	64.078
2	141	0.2293	128	201	150	12.68	177	2.449
3	1559	1.3222	1090	2087	1560	6.02	2732	14.121
4	20000 (UM)	0.1339	18293	29793	20420	4.16	2022	17.154

TIC:	1.5516	ng/uL
TIM:	3.9079	nmole/L
Total concentration:	1.8916	ng/uL

Sample peak width (sec): 5 Sample min peak height: 50 Sample baseline V to V?: Y Sample baseline V to V points: 3
Sample filter: Binomial Number of points for filter: 3 Sample start region (min): 0 Sample end region (min): 30
Marker peak width (sec): 5 Marker min peak height: 200 Marker baseline V to V?: Y Marker baseline V to V points: 3
Lower marker selection: First peak > 500 RFU Upper marker selection: Last peak > 200 RFU
Ladder size (bp)75, 200, 300, 400, 500, 700, 1000, 1500, 2000, 3000, 4000, 5000, 7000, 10000, 20000
Quantification using: Lower Marker Final concentration (ng/uL): 0.5000 Dilution factor: 12.0

Sample: SampA3
Well location: A3
Created: Tuesday, September 14, 2021 4:54:05 PM

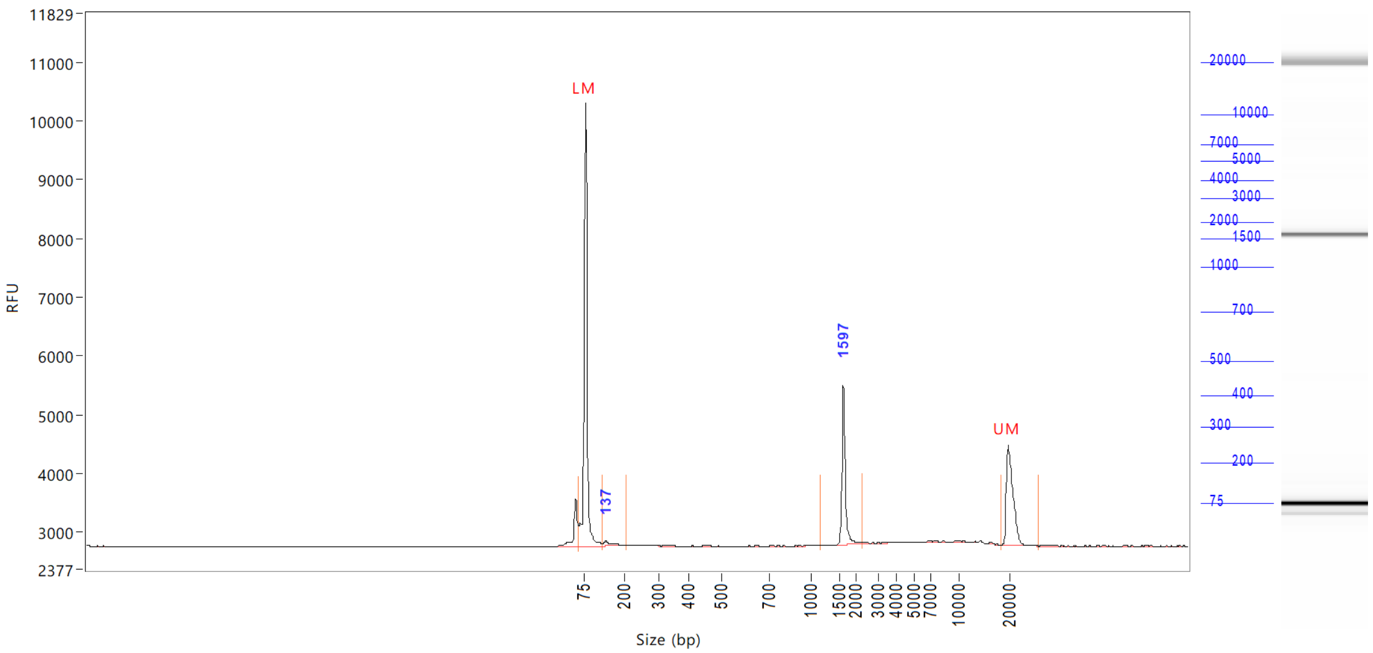

Peak	Size (bp)	Concentration (ng/uL)	From (bp)	To (bp)	Average size (bp)	CV%	RFU	Corrected peak area
1	75 (LM)	0.5000	52	126	73	11.27	7549	50.335
2	137	0.1964	126	205	153	13.73	89	1.647
3	1597	1.6163	1145	2258	1612	6.01	2697	13.559
4	20000 (UM)	0.1412	18415	26166	20316	3.67	1690	14.215

TIC:	1.8127	ng/uL
TIM:	3.7599	nmole/L
Total concentration:	2.1743	ng/uL

```
Sample peak width (sec): 5    Sample min peak height: 50    Sample baseline V to V?: Y    Sample baseline V to V points: 3
Sample filter: Binomial       Number of points for filter: 3  Sample start region (min): 0  Sample end region (min): 30
Marker peak width (sec): 5    Marker min peak height: 200   Marker baseline V to V?: Y    Marker baseline V to V points: 3
Lower marker selection: First peak > 500 RFU                Upper marker selection: Last peak > 200 RFU
Ladder size (bp)75, 200, 300, 400, 500, 700, 1000, 1500, 2000, 3000, 4000, 5000, 7000, 10000, 20000
Quantification using: Lower Marker          Final concentration (ng/uL): 0.5000          Dilution factor: 12.0
```

Sample: SampB3
Well location: B3
Created: Tuesday, September 14, 2021 4:54:05 PM

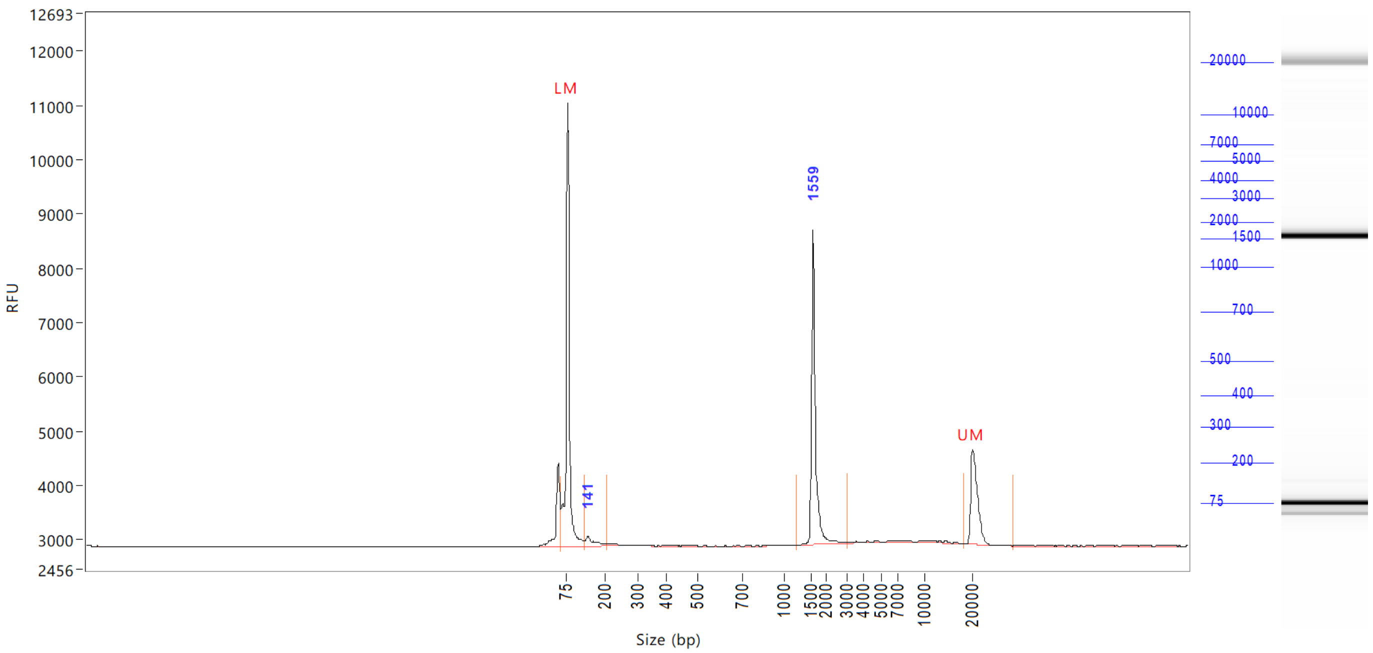

Peak	Size (bp)	Concentration (ng/uL)	From (bp)	To (bp)	Average size (bp)	CV%	RFU	Corrected peak area
1	75 (LM)	0.5000	52	131	75	14.62	8177	65.445
2	141	0.3367	131	206	159	13.40	191	3.673
3	1559	3.2784	1245	3000	1603	10.89	5802	35.759
4	20000 (UM)	0.1164	18293	28463	20489	4.03	1750	15.237

TIC:	3.6151	ng/uL
TIM:	6.8473	nmole/L
Total concentration:	4.1022	ng/uL

Sample peak width (sec): 5 Sample min peak height: 50 Sample baseline V to V?: Y Sample baseline V to V points: 3
Sample filter: Binomial Number of points for filter: 3 Sample start region (min): 0 Sample end region (min): 30
Marker peak width (sec): 5 Marker min peak height: 200 Marker baseline V to V?: Y Marker baseline V to V points: 3
Lower marker selection: First peak > 500 RFU Upper marker selection: Last peak > 200 RFU
Ladder size (bp)75, 200, 300, 400, 500, 700, 1000, 1500, 2000, 3000, 4000, 5000, 7000, 10000, 20000
Quantification using: Lower Marker Final concentration (ng/uL): 0.5000 Dilution factor: 12.0

Sample: SampC3
Well location: C3
Created: Tuesday, September 14, 2021 4:54:05 PM

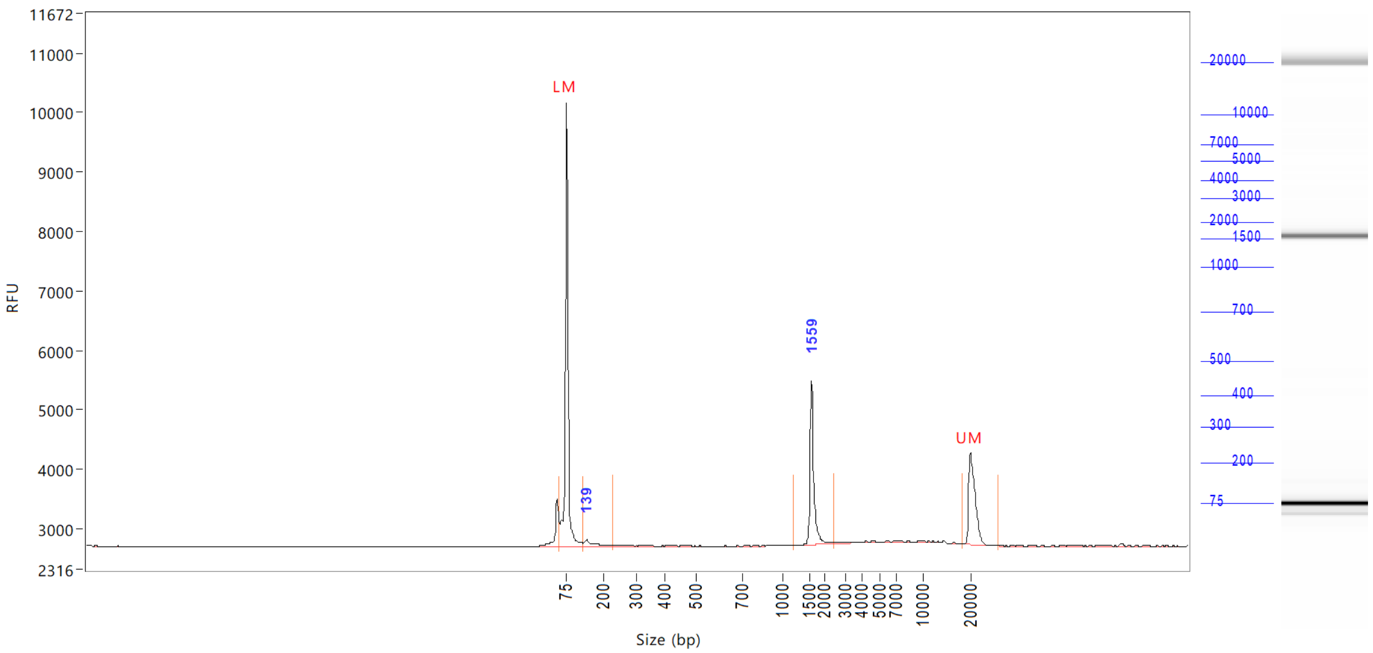

Peak	Size<br>(bp)	Concentration<br>(ng/uL)	From<br>(bp)	To<br>(bp)	Average size<br>(bp)	CV%	RFU	Corrected peak area
1	75 (LM)	0.5000	50	128	73	12.71	7475	52.219
2	139	0.2854	128	223	162	16.63	110	2.484
3	1559	1.9334	1212	2401	1580	7.33	2767	16.826
4	20000 (UM)	0.1254	18293	25803	20381	3.61	1556	13.097

TIC:	2.2188	ng/uL
TIM:	4.9100	nmole/L
Total concentration:	2.6183	ng/uL

Sample peak width (sec): 5 Sample min peak height: 50 Sample baseline V to V?: Y Sample baseline V to V points: 3
Sample filter: Binomial Number of points for filter: 3 Sample start region (min): 0 Sample end region (min): 30
Marker peak width (sec): 5 Marker min peak height: 200 Marker baseline V to V?: Y Marker baseline V to V points: 3
Lower marker selection: First peak > 500 RFU Upper marker selection: Last peak > 200 RFU
Ladder size (bp)75, 200, 300, 400, 500, 700, 1000, 1500, 2000, 3000, 4000, 5000, 7000, 10000, 20000
Quantification using: Lower Marker Final concentration (ng/uL): 0.5000 Dilution factor: 12.0

Sample: SampD3
Well location: D3
Created: Tuesday, September 14, 2021 4:54:05 PM

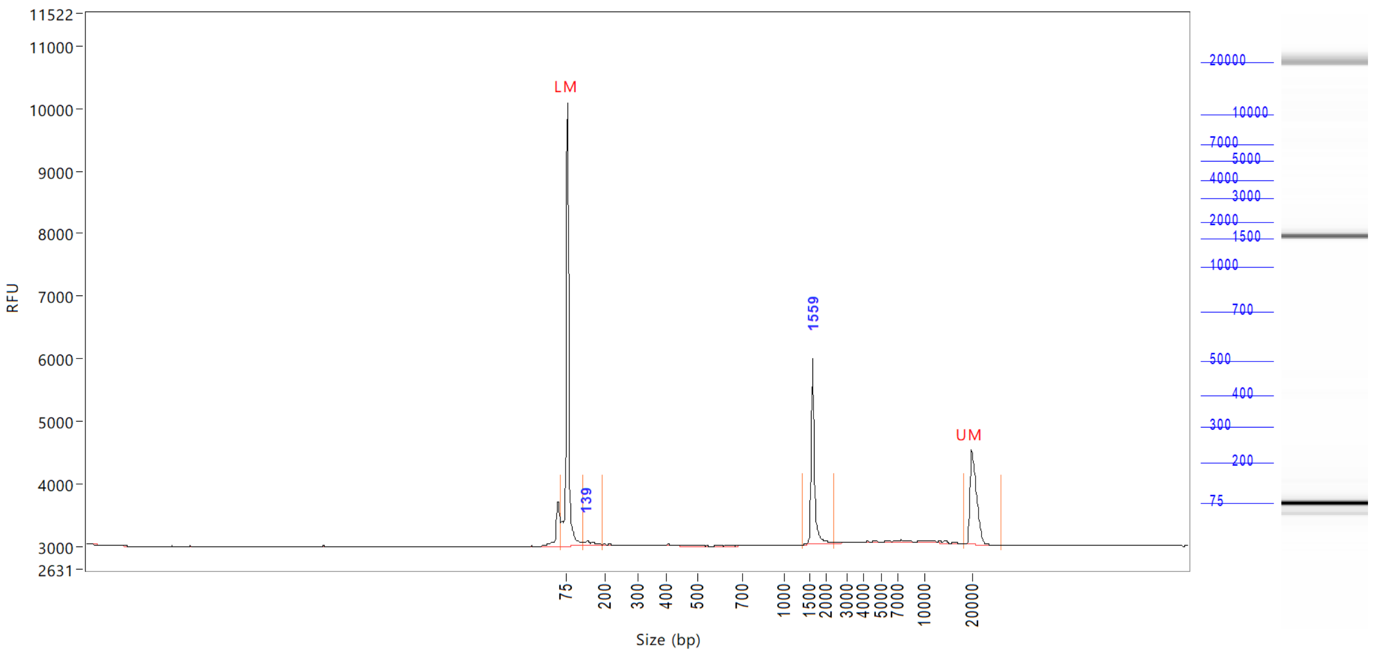

Peak	Size (bp)	Concentration (ng/uL)	From (bp)	To (bp)	Average size (bp)	CV%	RFU	Corrected peak area
1	75 (LM)	0.5000	52	129	74	12.43	7098	48.248
2	139	0.2169	129	189	153	10.68	91	1.744
3	1559	1.9589	1345	2401	1579	7.42	2970	15.752
4	20000 (UM)	0.1324	18293	26045	20415	3.66	1508	12.772

TIC:	2.1758	ng/uL
TIM:	4.3723	nmole/L
Total concentration:	2.6994	ng/uL

Sample peak width (sec): 5 Sample min peak height: 50 Sample baseline V to V?: Y Sample baseline V to V points: 3
Sample filter: Binomial Number of points for filter: 3 Sample start region (min): 0 Sample end region (min): 30
Marker peak width (sec): 5 Marker min peak height: 200 Marker baseline V to V?: Y Marker baseline V to V points: 3
Lower marker selection: First peak > 500 RFU Upper marker selection: Last peak > 200 RFU
Ladder size (bp)75, 200, 300, 400, 500, 700, 1000, 1500, 2000, 3000, 4000, 5000, 7000, 10000, 20000
Quantification using: Lower Marker Final concentration (ng/uL): 0.5000 Dilution factor: 12.0

Sample: SampE3
Well location: E3
Created: Tuesday, September 14, 2021 4:54:05 PM

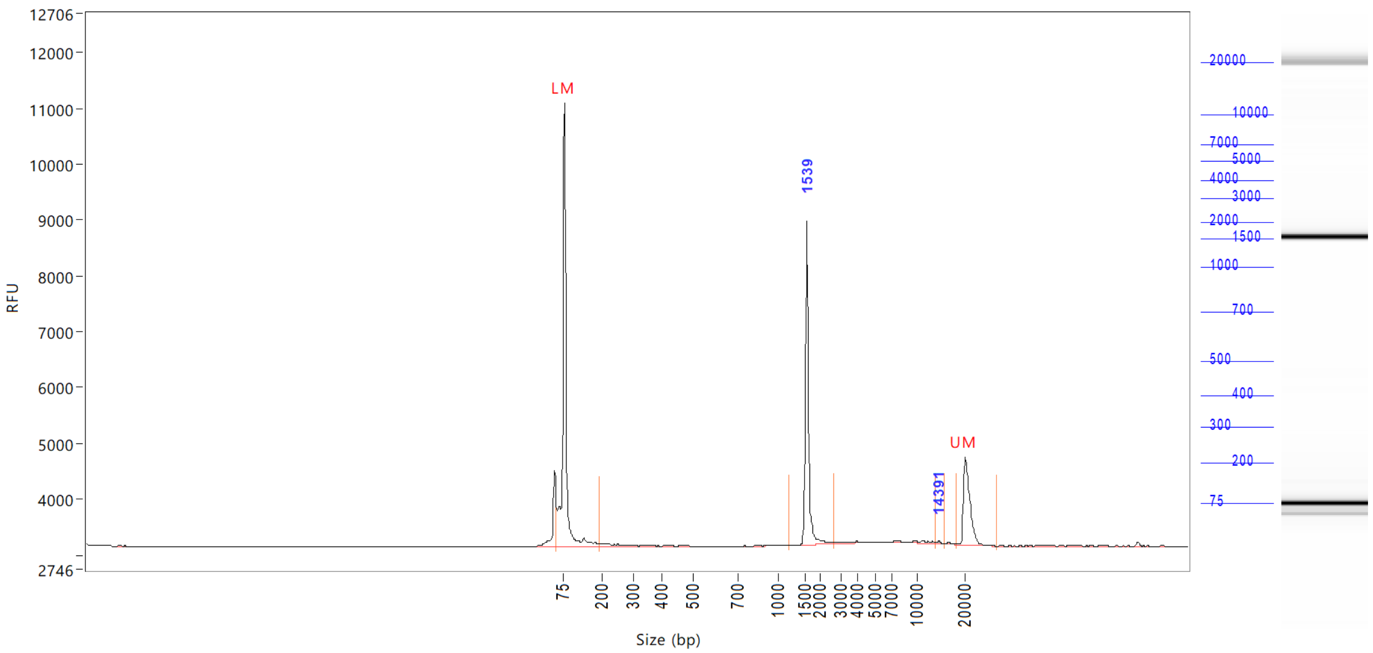

Peak	Size (bp)	Concentration (ng/uL)	From (bp)	To (bp)	Average size (bp)	CV%	RFU	Corrected peak area
1	75 (LM)	0.5000	50	192	77	25.84	7958	64.607
2	1539	2.7354	1201	2657	1565	9.07	5792	29.455
3	14391	0.0260	13781	15732	14435	3.45	51	0.280
4	20000 (UM)	0.1050	18171	26529	20461	3.93	1585	13.571

TIC:	2.7615	ng/uL
TIM:	2.8801	nmole/L
Total concentration:	3.2739	ng/uL

Sample peak width (sec): 5 Sample min peak height: 50 Sample baseline V to V?: Y Sample baseline V to V points: 3
Sample filter: Binomial Number of points for filter: 3 Sample start region (min): 0 Sample end region (min): 30
Marker peak width (sec): 5 Marker min peak height: 200 Marker baseline V to V?: Y Marker baseline V to V points: 3
Lower marker selection: First peak > 500 RFU Upper marker selection: Last peak > 200 RFU
Ladder size (bp)75, 200, 300, 400, 500, 700, 1000, 1500, 2000, 3000, 4000, 5000, 7000, 10000, 20000
Quantification using: Lower Marker Final concentration (ng/uL): 0.5000 Dilution factor: 12.0

Sample: SampF3
Well location: F3
Created: Tuesday, September 14, 2021 4:54:05 PM

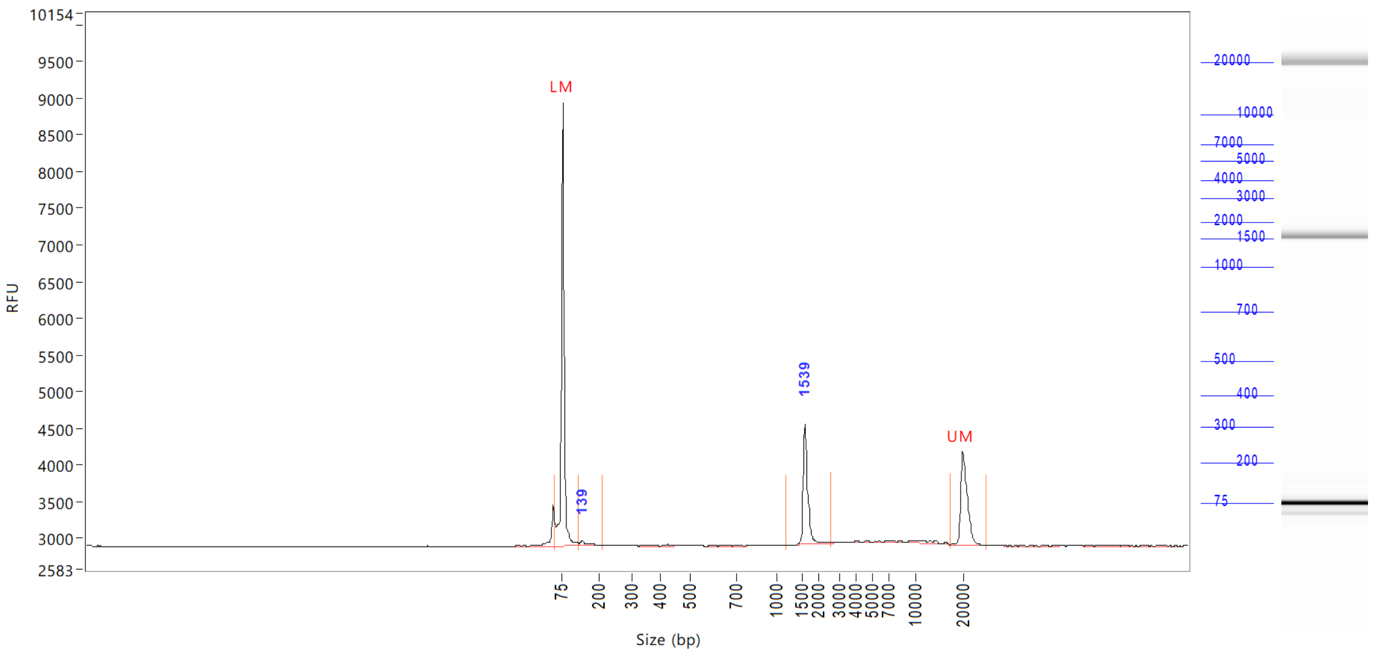

Peak	Size (bp)	Concentration (ng/uL)	From (bp)	To (bp)	Average size (bp)	CV%	RFU	Corrected peak area
1	75 (LM)	0.5000	52	129	74	11.67	6045	41.240
2	139	0.1747	129	205	155	13.02	70	1.201
3	1539	1.6534	1167	2572	1587	8.32	1645	11.364
4	20000 (UM)	0.1351	17439	25078	20378	3.85	1284	11.145

TIC:	1.8281	ng/uL
TIM:	3.5673	nmole/L
Total concentration:	2.3248	ng/uL

Sample peak width (sec): 5 Sample min peak height: 50 Sample baseline V to V?: Y Sample baseline V to V points: 3
Sample filter: Binomial Number of points for filter: 3 Sample start region (min): 0 Sample end region (min): 30
Marker peak width (sec): 5 Marker min peak height: 200 Marker baseline V to V?: Y Marker baseline V to V points: 3
Lower marker selection: First peak > 500 RFU Upper marker selection: Last peak > 200 RFU
Ladder size (bp)75, 200, 300, 400, 500, 700, 1000, 1500, 2000, 3000, 4000, 5000, 7000, 10000, 20000
Quantification using: Lower Marker Final concentration (ng/uL): 0.5000 Dilution factor: 12.0

Sample: SampG3
Well location: G3
Created: Tuesday, September 14, 2021 4:54:05 PM

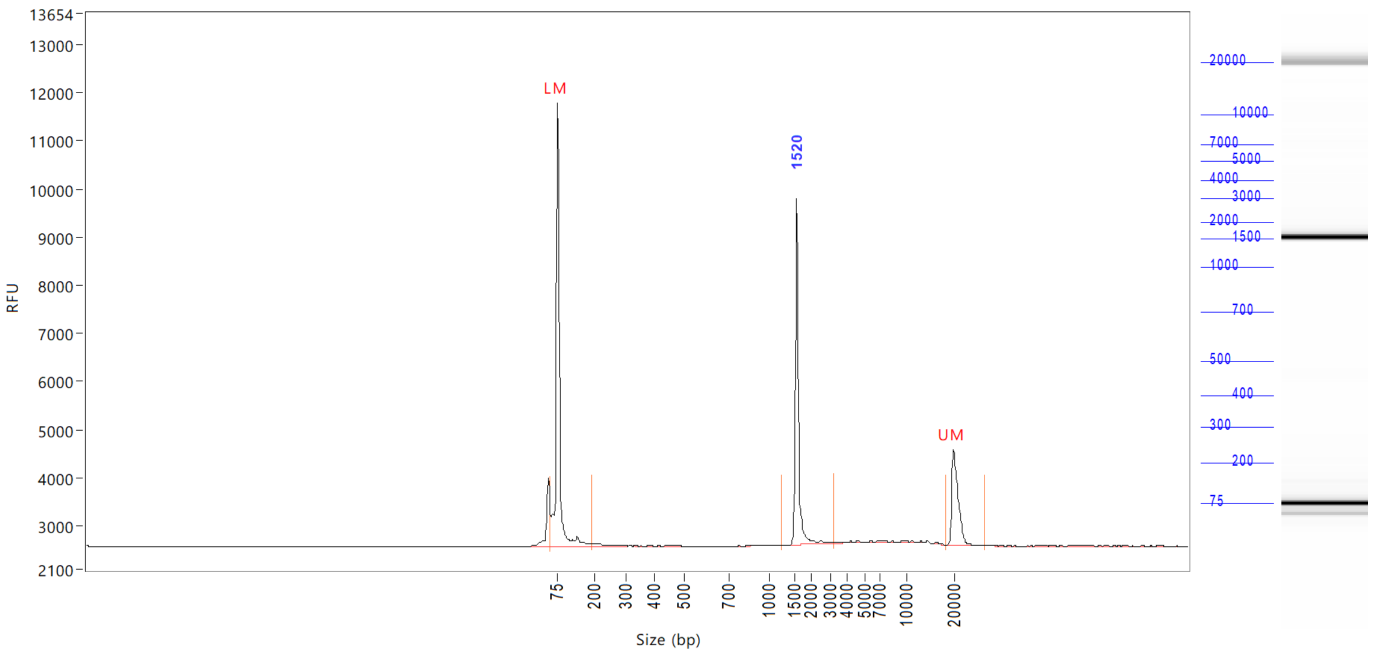

Peak	Size (bp)	Concentration (ng/uL)	From (bp)	To (bp)	Average size (bp)	CV%	RFU	Corrected peak area
1	75 (LM)	0.5000	52	190	78	24.92	9230	72.094
2	1520	3.0919	1223	3108	1565	11.51	7193	37.151
3	20000 (UM)	0.1179	18415	26649	20449	4.01	1970	16.997

TIC:	3.0919	ng/uL
TIM:	3.2521	nmole/L
Total concentration:	3.4762	ng/uL

```
Sample peak width (sec): 5    Sample min peak height: 50    Sample baseline V to V?: Y    Sample baseline V to V points: 3
Sample filter: Binomial       Number of points for filter: 3  Sample start region (min): 0  Sample end region (min): 30
Marker peak width (sec): 5    Marker min peak height: 200   Marker baseline V to V?: Y    Marker baseline V to V points: 3
Lower marker selection: First peak > 500 RFU                Upper marker selection: Last peak > 200 RFU
Ladder size (bp)75, 200, 300, 400, 500, 700, 1000, 1500, 2000, 3000, 4000, 5000, 7000, 10000, 20000
Quantification using: Lower Marker          Final concentration (ng/uL): 0.5000          Dilution factor: 12.0
```

Sample: SampH3
Well location: H3
Created: Tuesday, September 14, 2021 4:54:05 PM

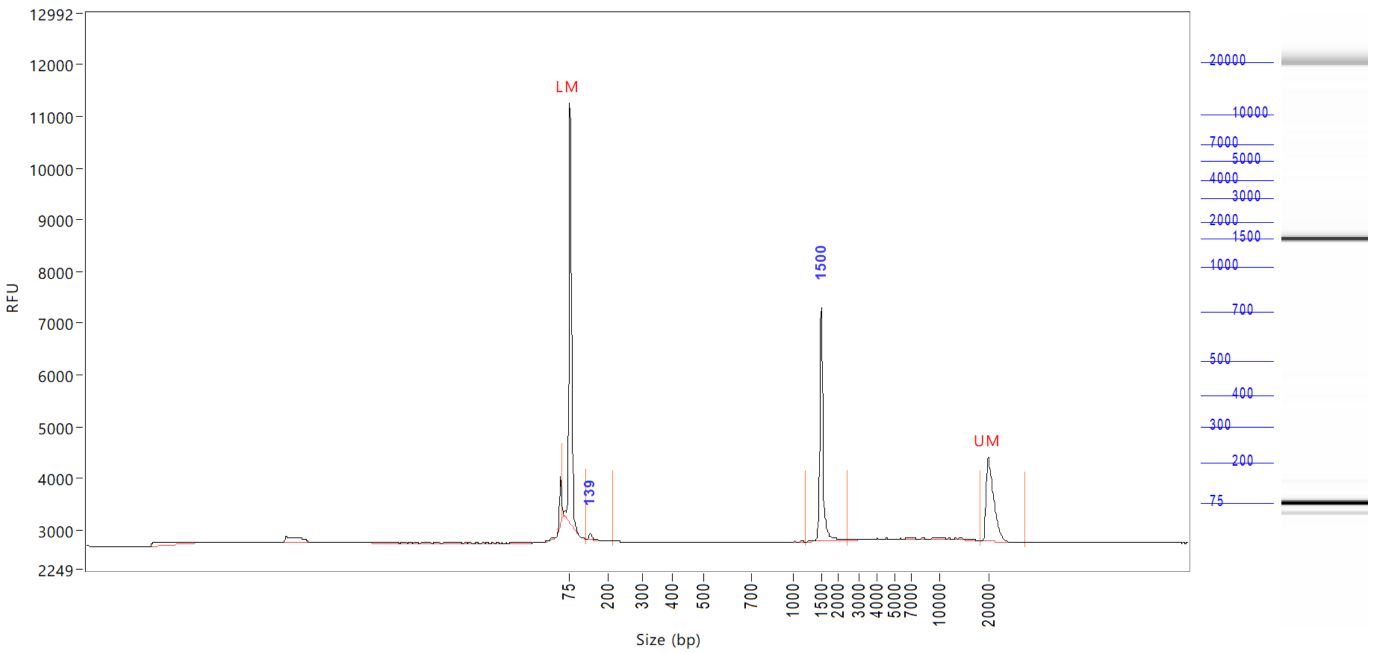

Peak	Size<br>(bp)	Concentration<br>(ng/uL)	From<br>(bp)	To<br>(bp)	Average size<br>(bp)	CV%	RFU	Corrected peak area
1	75 (LM)	0.5000	54	128	74	5.83	8133	49.631
2	139	0.1345	128	210	144	9.14	135	1.113
3	1500	2.8551	1212	2486	1518	7.80	4503	23.617
4	20000 (UM)	0.1578	18537	27496	20490	4.33	1623	15.667

TIC:	2.9896	ng/uL
TIM:	4.6310	nmole/L
Total concentration:	3.4325	ng/uL

```
Sample peak width (sec): 5    Sample min peak height: 50    Sample baseline V to V?: Y    Sample baseline V to V points: 3
Sample filter: Binomial       Number of points for filter: 3  Sample start region (min): 0  Sample end region (min): 30
Marker peak width (sec): 5    Marker min peak height: 200   Marker baseline V to V?: Y    Marker baseline V to V points: 3
Lower marker selection: First peak > 500 RFU                Upper marker selection: Last peak > 200 RFU
Ladder size (bp)75, 200, 300, 400, 500, 700, 1000, 1500, 2000, 3000, 4000, 5000, 7000, 10000, 20000
Quantification using: Lower Marker          Final concentration (ng/uL): 0.5000          Dilution factor: 12.0
```

Sample: SampA4
Well location: A4
Created: Tuesday, September 14, 2021 4:54:05 PM

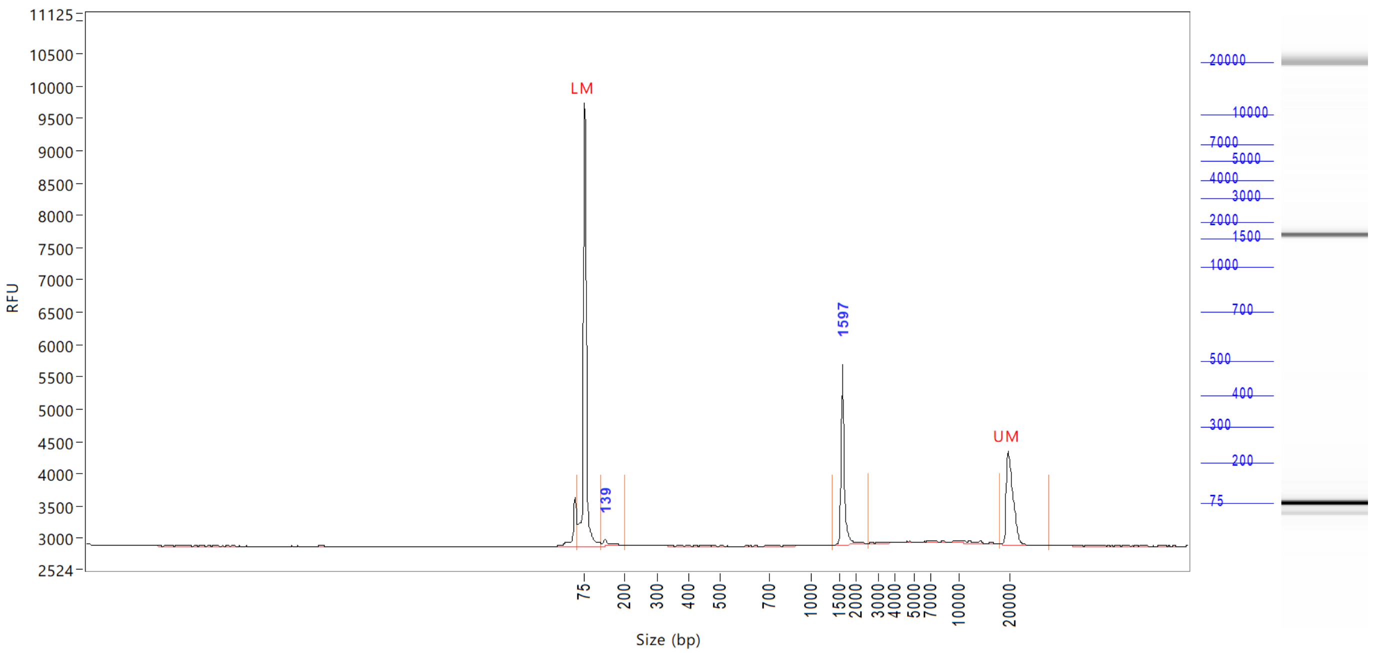

Peak	Size (bp)	Concentration (ng/uL)	From (bp)	To (bp)	Average size (bp)	CV%	RFU	Corrected peak area
1	75 (LM)	0.5000	54	128	75	11.32	6868	46.539
2	139	0.2213	128	201	153	12.98	101	1.716
3	1597	1.7795	1378	2572	1611	7.53	2784	13.803
4	20000 (UM)	0.1341	18171	28100	20333	3.99	1458	12.484

TIC:	2.0008	ng/uL
TIM:	4.1955	nmole/L
Total concentration:	2.5001	ng/uL

```
Sample peak width (sec): 5    Sample min peak height: 50    Sample baseline V to V?: Y    Sample baseline V to V points: 3
Sample filter: Binomial       Number of points for filter: 3  Sample start region (min): 0  Sample end region (min): 30
Marker peak width (sec): 5    Marker min peak height: 200   Marker baseline V to V?: Y    Marker baseline V to V points: 3
Lower marker selection: First peak > 500 RFU                Upper marker selection: Last peak > 200 RFU
Ladder size (bp)75, 200, 300, 400, 500, 700, 1000, 1500, 2000, 3000, 4000, 5000, 7000, 10000, 20000
Quantification using: Lower Marker          Final concentration (ng/uL): 0.5000          Dilution factor: 12.0
```

Sample: SampB4
Well location: B4
Created: Tuesday, September 14, 2021 4:54:05 PM

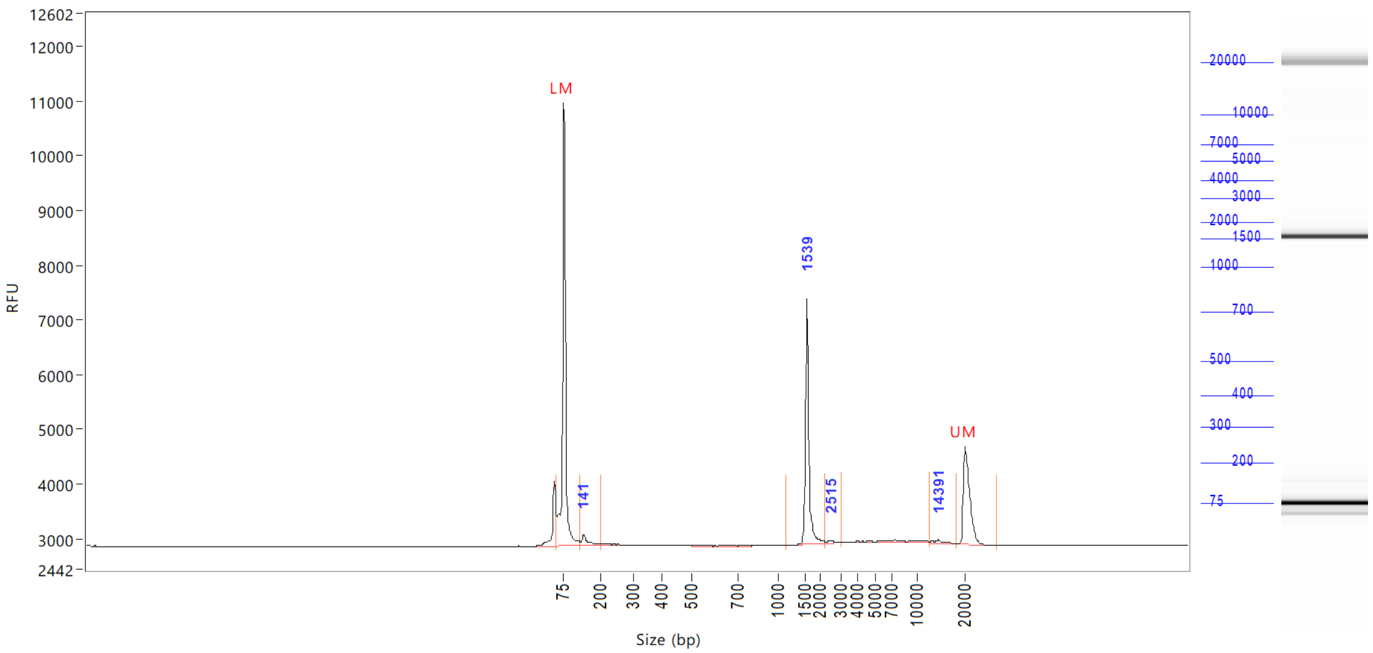

Peak	Size (bp)	Concentration (ng/uL)	From (bp)	To (bp)	Average size (bp)	CV%	RFU	Corrected peak area
1	75 (LM)	0.5000	52	129	75	13.46	8110	60.555
2	141	0.3098	129	200	153	12.19	204	3.126
3	1539	2.4161	1134	2229	1569	6.79	4478	24.385
4	2515	0.0622	2229	3000	2541	7.92	53	0.627
5	14391	0.0786	12440	18293	14524	9.11	61	0.793
6	20000 (UM)	0.1272	18293	26529	20438	4.04	1792	15.408

TIC:	2.8666	ng/uL
TIM:	5.9116	nmole/L
Total concentration:	3.2657	ng/uL

Sample peak width (sec): 5 Sample min peak height: 50 Sample baseline V to V?: Y Sample baseline V to V points: 3
Sample filter: Binomial Number of points for filter: 3 Sample start region (min): 0 Sample end region (min): 30
Marker peak width (sec): 5 Marker min peak height: 200 Marker baseline V to V?: Y Marker baseline V to V points: 3
Lower marker selection: First peak > 500 RFU Upper marker selection: Last peak > 200 RFU
Ladder size (bp)75, 200, 300, 400, 500, 700, 1000, 1500, 2000, 3000, 4000, 5000, 7000, 10000, 20000
Quantification using: Lower Marker Final concentration (ng/uL): 0.5000 Dilution factor: 12.0

Sample: SampC4
Well location: C4
Created: Tuesday, September 14, 2021 4:54:05 PM

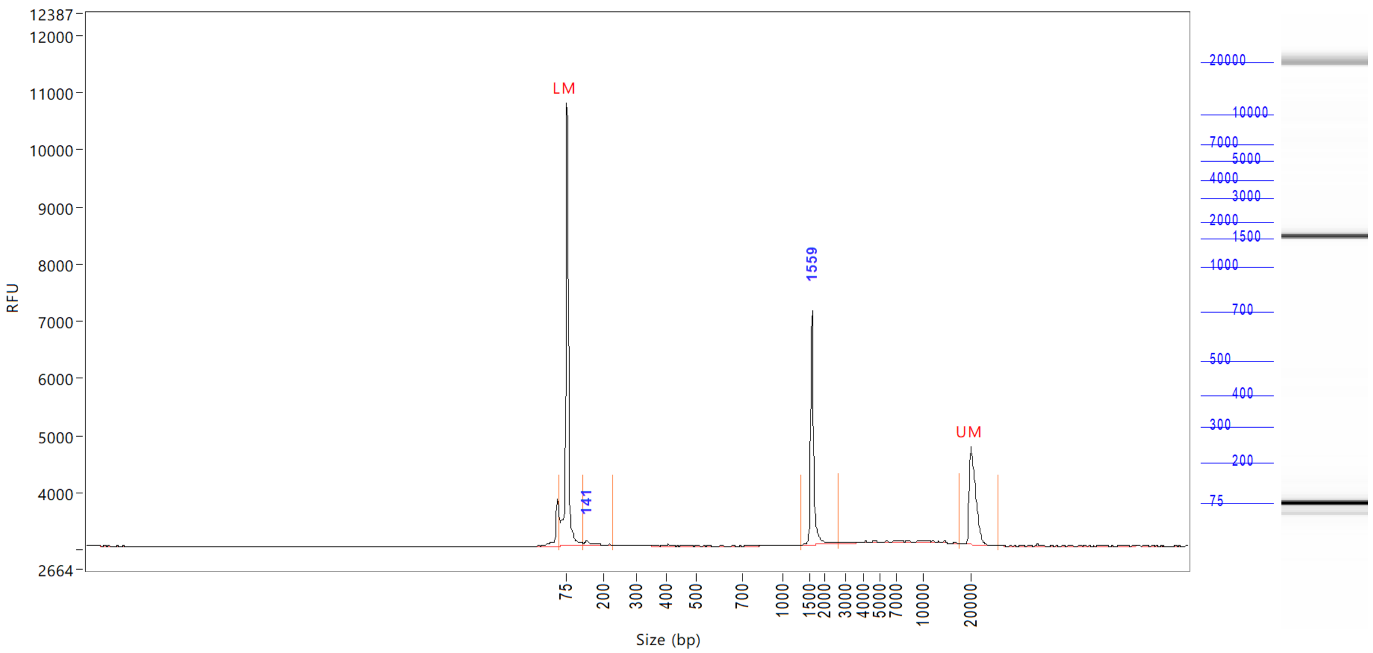

Peak	Size (bp)	Concentration (ng/uL)	From (bp)	To (bp)	Average size (bp)	CV%	RFU	Corrected peak area
1	75 (LM)	0.5000	52	129	74	11.84	7760	53.918
2	141	0.1683	129	223	155	13.70	85	1.513
3	1559	2.3001	1345	2629	1578	8.65	4099	20.669
4	20000 (UM)	0.1326	17561	25803	20367	3.62	1713	14.299

TIC:	2.4684	ng/uL
TIM:	4.1841	nmole/L
Total concentration:	2.8828	ng/uL

Sample peak width (sec): 5 Sample min peak height: 50 Sample baseline V to V?: Y Sample baseline V to V points: 3
Sample filter: Binomial Number of points for filter: 3 Sample start region (min): 0 Sample end region (min): 30
Marker peak width (sec): 5 Marker min peak height: 200 Marker baseline V to V?: Y Marker baseline V to V points: 3
Lower marker selection: First peak > 500 RFU Upper marker selection: Last peak > 200 RFU
Ladder size (bp)75, 200, 300, 400, 500, 700, 1000, 1500, 2000, 3000, 4000, 5000, 7000, 10000, 20000
Quantification using: Lower Marker Final concentration (ng/uL): 0.5000 Dilution factor: 12.0

Sample: SampD4
Well location: D4
Created: Tuesday, September 14, 2021 4:54:05 PM

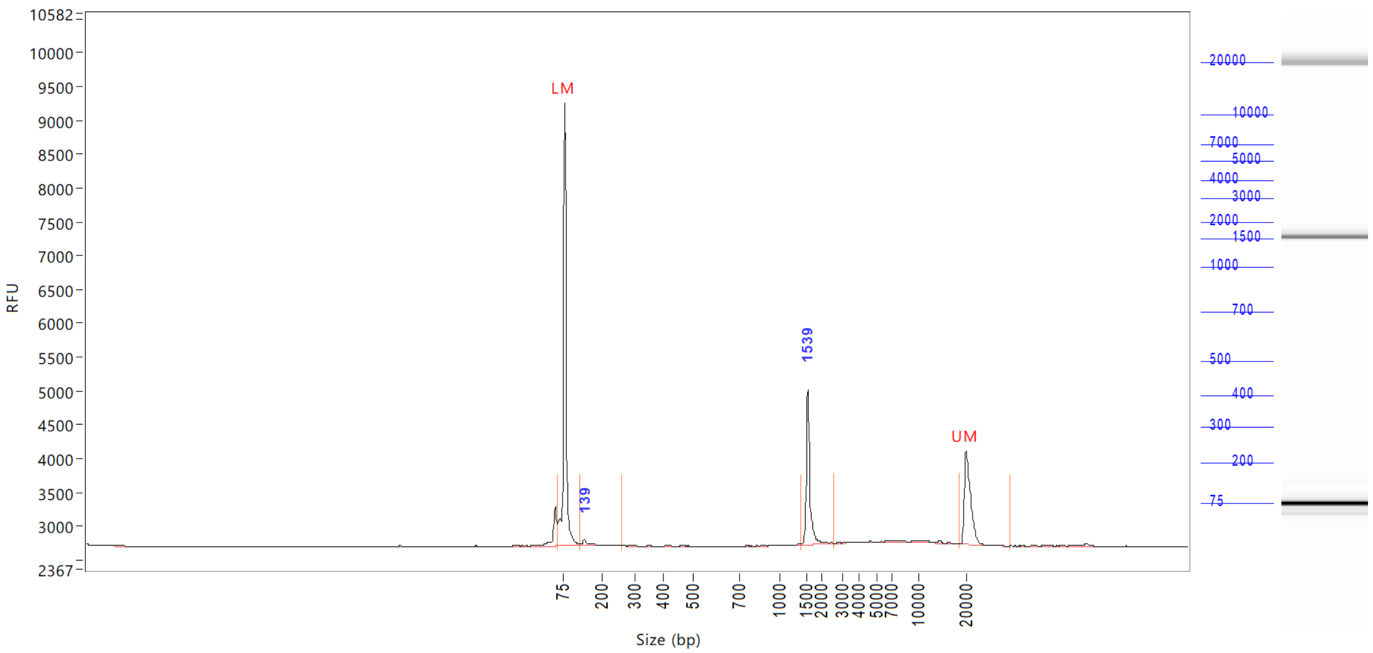

Peak	Size (bp)	Concentration (ng/uL)	From (bp)	To (bp)	Average size (bp)	CV%	RFU	Corrected peak area
1	75 (LM)	0.5000	50	128	73	11.99	6557	46.584
2	139	0.2100	128	260	163	19.77	91	1.631
3	1539	1.7174	1411	2600	1571	8.09	2286	13.334
4	20000 (UM)	0.1240	18537	29189	20380	4.11	1371	11.555

TIC:	1.9274	ng/uL
TIM:	3.9175	nmole/L
Total concentration:	2.2984	ng/uL

Sample peak width (sec): 5 Sample min peak height: 50 Sample baseline V to V?: Y Sample baseline V to V points: 3
Sample filter: Binomial Number of points for filter: 3 Sample start region (min): 0 Sample end region (min): 30
Marker peak width (sec): 5 Marker min peak height: 200 Marker baseline V to V?: Y Marker baseline V to V points: 3
Lower marker selection: First peak > 500 RFU Upper marker selection: Last peak > 200 RFU
Ladder size (bp)75, 200, 300, 400, 500, 700, 1000, 1500, 2000, 3000, 4000, 5000, 7000, 10000, 20000
Quantification using: Lower Marker Final concentration (ng/uL): 0.5000 Dilution factor: 12.0

Sample: SampE4
Well location: E4
Created: Tuesday, September 14, 2021 4:54:05 PM

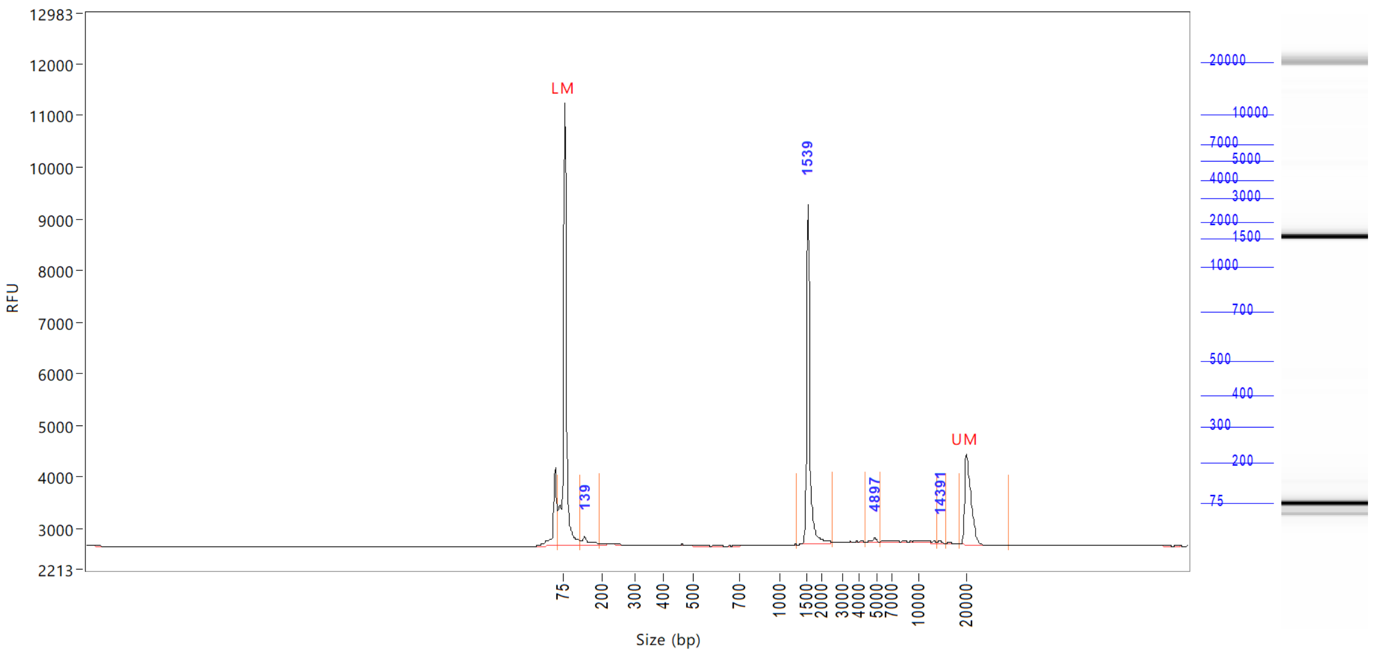

Peak	Size (bp)	Concentration (ng/uL)	From (bp)	To (bp)	Average size (bp)	CV%	RFU	Corrected peak area
1	75 (LM)	0.5000	50	128	73	14.14	8590	66.970
2	139	0.2374	128	189	150	10.99	157	2.650
3	1539	3.0777	1334	2543	1571	7.88	6583	34.353
4	4897	0.0409	4380	5445	4851	3.33	73	0.456
5	14391	0.0295	13781	15732	14454	3.24	61	0.329
6	20000 (UM)	0.1098	18415	28947	20443	3.99	1755	14.707

TIC:	3.3855	ng/uL
TIM:	5.8434	nmole/L
Total concentration:	3.7561	ng/uL

Sample peak width (sec): 5 Sample min peak height: 50 Sample baseline V to V?: Y Sample baseline V to V points: 3
Sample filter: Binomial Number of points for filter: 3 Sample start region (min): 0 Sample end region (min): 30
Marker peak width (sec): 5 Marker min peak height: 200 Marker baseline V to V?: Y Marker baseline V to V points: 3
Lower marker selection: First peak > 500 RFU Upper marker selection: Last peak > 200 RFU
Ladder size (bp)75, 200, 300, 400, 500, 700, 1000, 1500, 2000, 3000, 4000, 5000, 7000, 10000, 20000
Quantification using: Lower Marker Final concentration (ng/uL): 0.5000 Dilution factor: 12.0

Sample: SampF4
Well location: F4
Created: Tuesday, September 14, 2021 4:54:05 PM

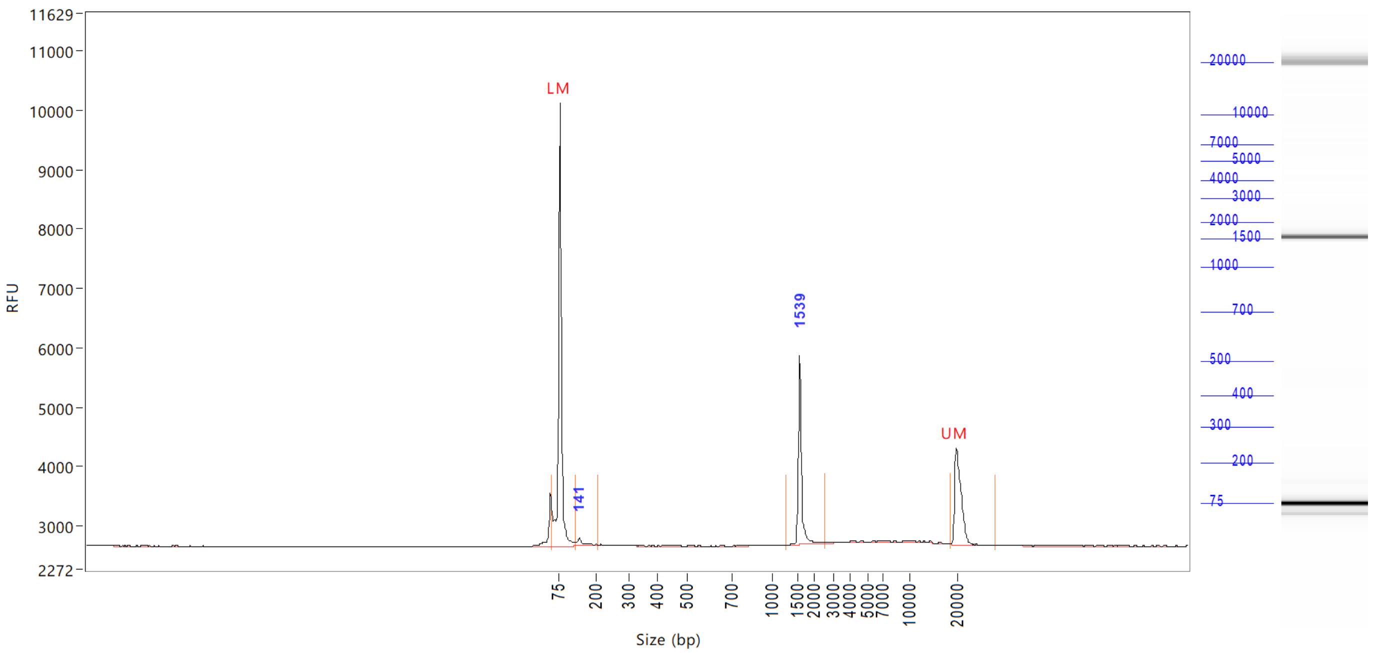

Peak	Size (bp)	Concentration (ng/uL)	From (bp)	To (bp)	Average size (bp)	CV%	RFU	Corrected peak area
1	75 (LM)	0.5000	50	128	73	12.73	7472	54.309
2	141	0.2324	128	201	153	13.29	126	2.103
3	1539	1.8064	1267	2572	1560	8.30	3171	16.350
4	20000 (UM)	0.1288	18537	28221	20422	4.05	1619	13.993

TIC:	2.0387	ng/uL
TIM:	4.4023	nmole/L
Total concentration:	2.4006	ng/uL

Sample peak width (sec): 5 Sample min peak height: 50 Sample baseline V to V?: Y Sample baseline V to V points: 3
Sample filter: Binomial Number of points for filter: 3 Sample start region (min): 0 Sample end region (min): 30
Marker peak width (sec): 5 Marker min peak height: 200 Marker baseline V to V?: Y Marker baseline V to V points: 3
Lower marker selection: First peak > 500 RFU Upper marker selection: Last peak > 200 RFU
Ladder size (bp)75, 200, 300, 400, 500, 700, 1000, 1500, 2000, 3000, 4000, 5000, 7000, 10000, 20000
Quantification using: Lower Marker Final concentration (ng/uL): 0.5000 Dilution factor: 12.0

Sample: SampG4
Well location: G4
Created: Tuesday, September 14, 2021 4:54:05 PM

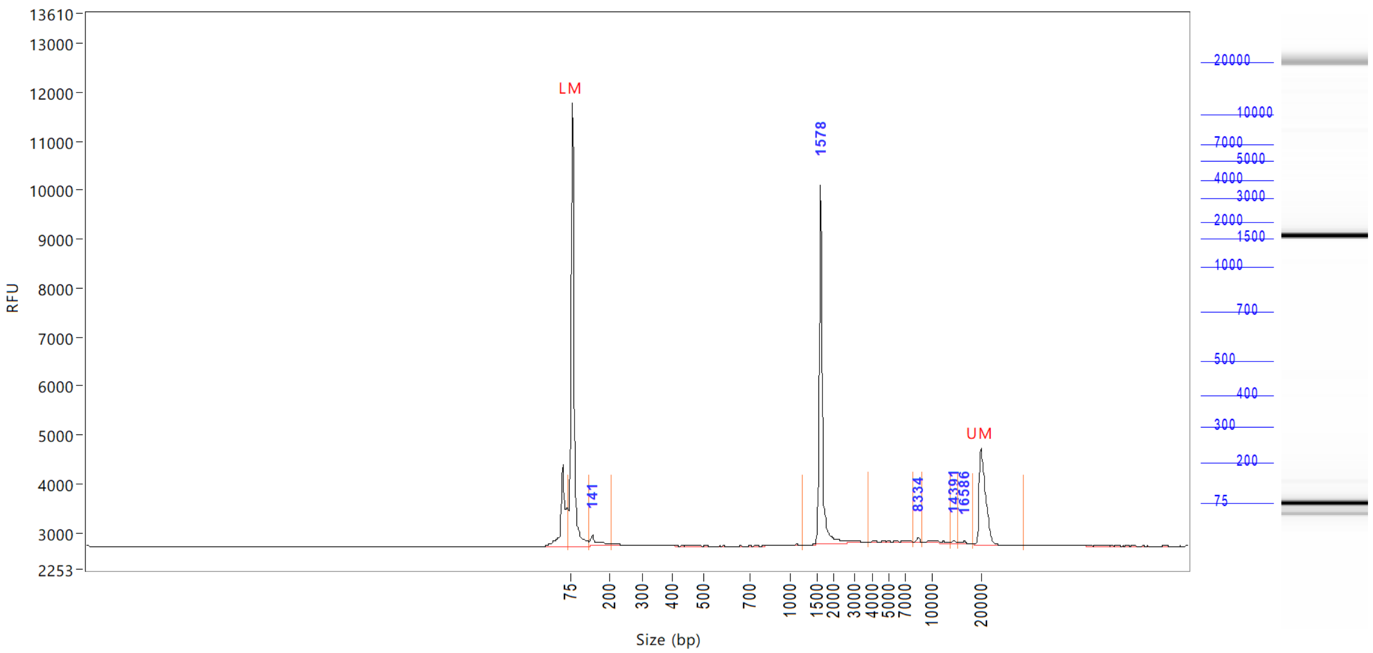

Peak	Size (bp)	Concentration (ng/uL)	From (bp)	To (bp)	Average size (bp)	CV%	RFU	Corrected peak area
1	75 (LM)	0.5000	64	128	76	12.06	9069	63.401
2	141	0.3627	128	203	154	13.51	216	3.833
3	1578	3.5748	1212	3786	1625	14.46	7345	37.774
4	8334	0.0383	7867	8867	8317	2.04	92	0.405
5	14391	0.0360	13781	15244	14411	2.61	72	0.381
6	16586	0.0395	15244	18293	16401	3.93	57	0.418
7	20000 (UM)	0.1314	18293	28826	20424	4.05	1960	16.662

TIC:	4.0514	ng/uL
TIM:	7.5081	nmole/L
Total concentration:	4.4351	ng/uL

Sample peak width (sec): 5   Sample min peak height: 50   Sample baseline V to V?: Y   Sample baseline V to V points: 3
Sample filter: Binomial   Number of points for filter: 3   Sample start region (min): 0   Sample end region (min): 30
Marker peak width (sec): 5   Marker min peak height: 200   Marker baseline V to V?: Y   Marker baseline V to V points: 3
Lower marker selection: First peak > 500 RFU   Upper marker selection: Last peak > 200 RFU
Ladder size (bp)75, 200, 300, 400, 500, 700, 1000, 1500, 2000, 3000, 4000, 5000, 7000, 10000, 20000
Quantification using: Lower Marker   Final concentration (ng/uL): 0.5000   Dilution factor: 12.0

Sample: SampH4
Well location: H4
Created: Tuesday, September 14, 2021 4:54:05 PM

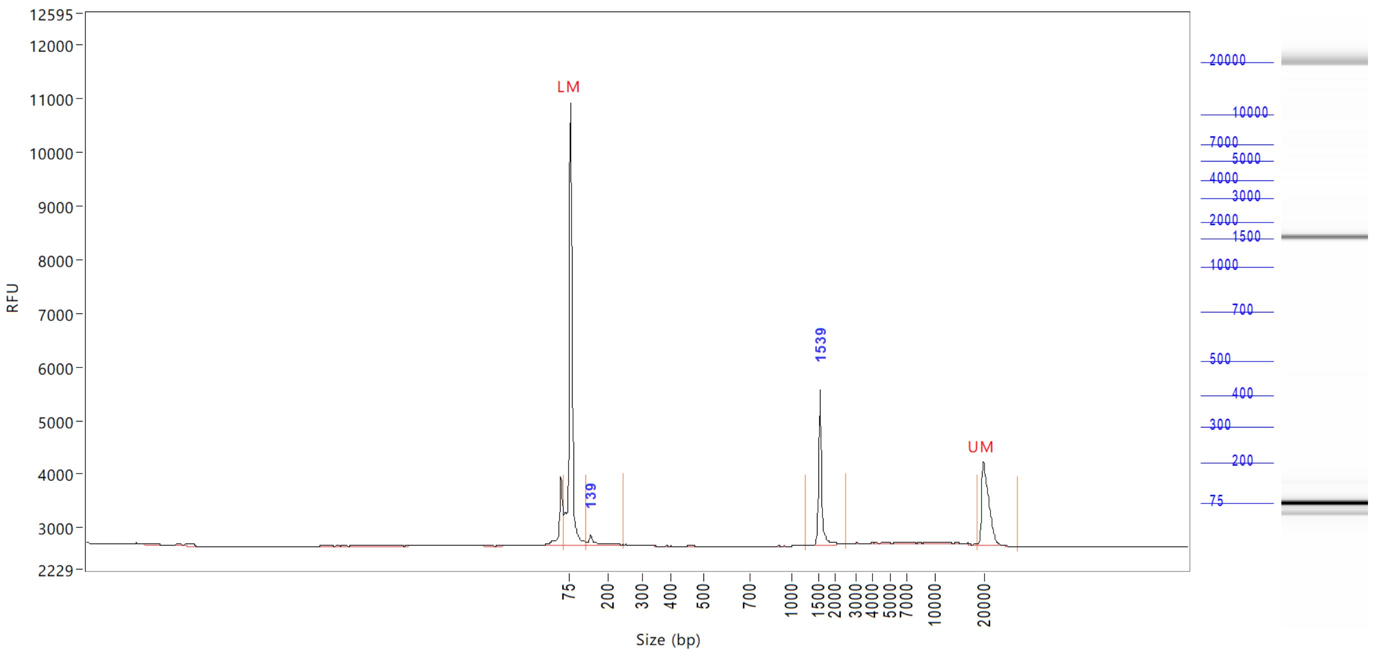

Peak	Size (bp)	Concentration (ng/uL)	From (bp)	To (bp)	Average size (bp)	CV%	RFU	Corrected peak area
1	75 (LM)	0.5000	54	128	74	11.95	8262	59.761
2	139	0.2349	128	241	152	15.69	184	2.339
3	1539	1.4074	1256	2543	1552	8.16	2897	14.018
4	20000 (UM)	0.1201	18781	27012	20526	4.12	1562	14.359

TIC:	1.6423	ng/uL
TIM:	4.0324	nmole/L
Total concentration:	1.9328	ng/uL

Sample peak width (sec): 5   Sample min peak height: 50   Sample baseline V to V?: Y   Sample baseline V to V points: 3
Sample filter: Binomial   Number of points for filter: 3 Sample start region (min): 0 Sample end region (min): 30
Marker peak width (sec): 5   Marker min peak height: 200   Marker baseline V to V?: Y   Marker baseline V to V points: 3
Lower marker selection: First peak > 500 RFU   Upper marker selection: Last peak > 200 RFU
Ladder size (bp)75, 200, 300, 400, 500, 700, 1000, 1500, 2000, 3000, 4000, 5000, 7000, 10000, 20000
Quantification using: Lower Marker   Final concentration (ng/uL): 0.5000   Dilution factor: 12.0

Sample: SampA5
Well location: A5
Created: Tuesday, September 14, 2021 4:54:05 PM

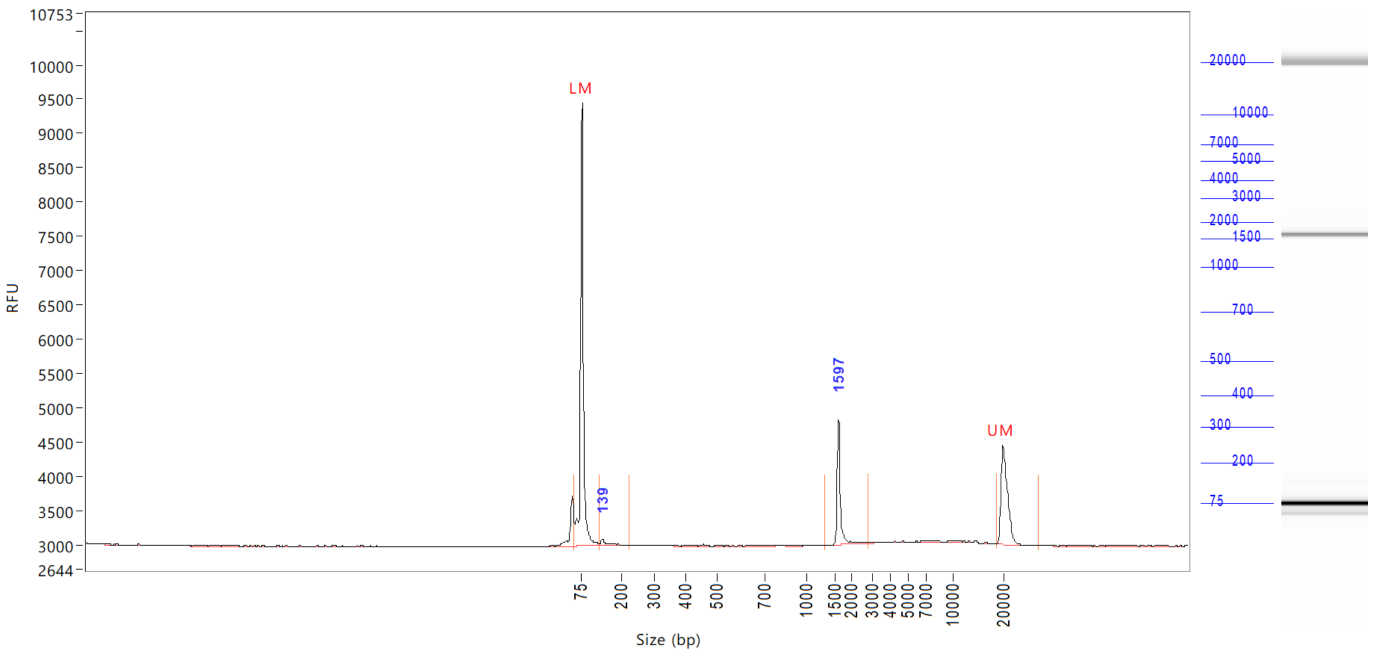

Peak	Size (bp)	Concentration (ng/uL)	From (bp)	To (bp)	Average size (bp)	CV%	RFU	Corrected peak area
1	75 (LM)	0.5000	52	128	73	11.83	6473	44.783
2	139	0.2183	128	221	156	15.60	93	1.629
3	1597	1.2740	1311	2800	1634	9.92	1829	9.509
4	20000 (UM)	0.1354	18659	27133	20343	3.84	1445	12.125

TIC:	1.4923	ng/uL
TIM:	3.5835	nmole/L
Total concentration:	1.8527	ng/uL

```
Sample peak width (sec): 5    Sample min peak height: 50    Sample baseline V to V?: Y    Sample baseline V to V points: 3
Sample filter: Binomial       Number of points for filter: 3  Sample start region (min): 0  Sample end region (min): 30
Marker peak width (sec): 5    Marker min peak height: 200   Marker baseline V to V?: Y    Marker baseline V to V points: 3
Lower marker selection: First peak > 500 RFU                Upper marker selection: Last peak > 200 RFU
Ladder size (bp)75, 200, 300, 400, 500, 700, 1000, 1500, 2000, 3000, 4000, 5000, 7000, 10000, 20000
Quantification using: Lower Marker          Final concentration (ng/uL): 0.5000          Dilution factor: 12.0
```

Sample: SampB5
Well location: B5
Created: Tuesday, September 14, 2021 4:54:05 PM

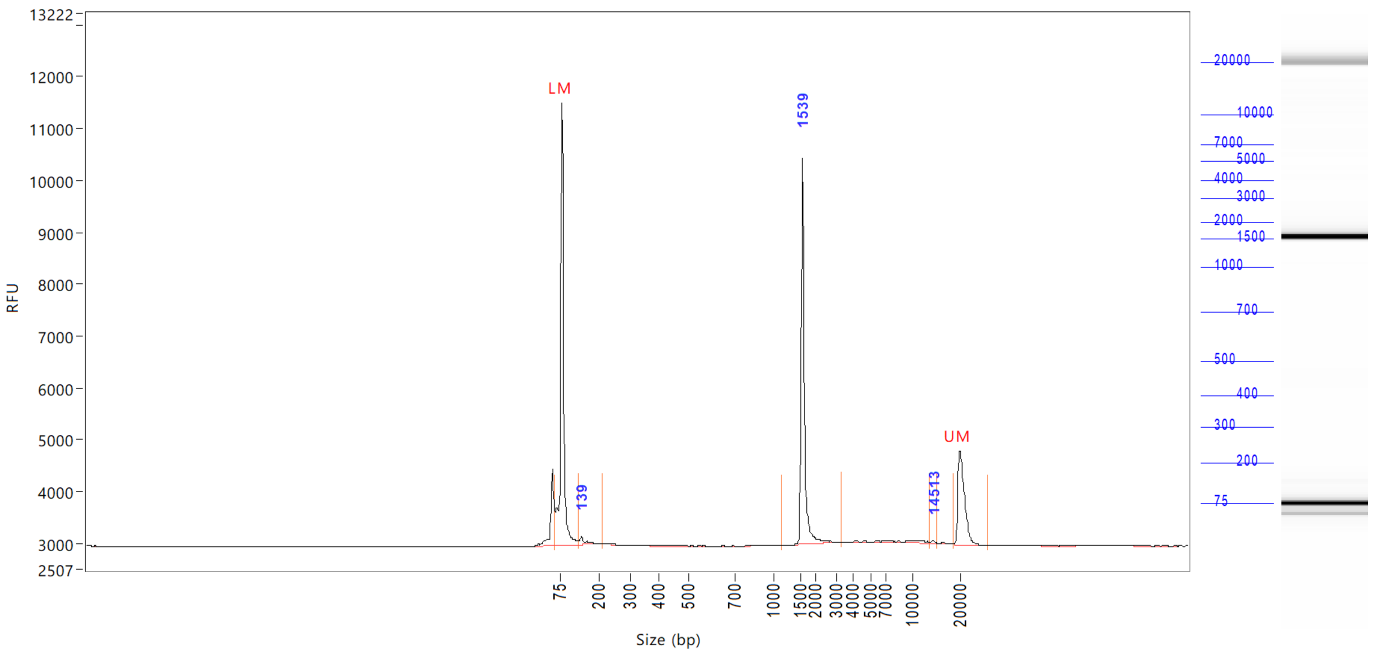

Peak	Size (bp)	Concentration (ng/uL)	From (bp)	To (bp)	Average size (bp)	CV%	RFU	Corrected peak area
1	75 (LM)	0.5000	52	129	74	13.58	8540	65.254
2	139	0.1914	129	208	149	10.60	147	2.081
3	1539	3.6162	1123	3322	1573	9.63	7451	39.328
4	14513	0.0285	13659	15244	14327	2.76	54	0.310
5	20000 (UM)	0.1200	18537	26166	20485	3.95	1812	15.663

TIC:	3.8361	ng/uL
TIM:	5.8981	nmole/L
Total concentration:	4.1764	ng/uL

Sample peak width (sec): 5   Sample min peak height: 50   Sample baseline V to V?: Y   Sample baseline V to V points: 3
Sample filter: Binomial   Number of points for filter: 3 Sample start region (min): 0 Sample end region (min): 30
Marker peak width (sec): 5   Marker min peak height: 200   Marker baseline V to V?: Y   Marker baseline V to V points: 3
Lower marker selection: First peak > 500 RFU   Upper marker selection: Last peak > 200 RFU
Ladder size (bp)75, 200, 300, 400, 500, 700, 1000, 1500, 2000, 3000, 4000, 5000, 7000, 10000, 20000
Quantification using: Lower Marker   Final concentration (ng/uL): 0.5000   Dilution factor: 12.0

Sample: SampC5
Well location: C5
Created: Tuesday, September 14, 2021 4:54:05 PM

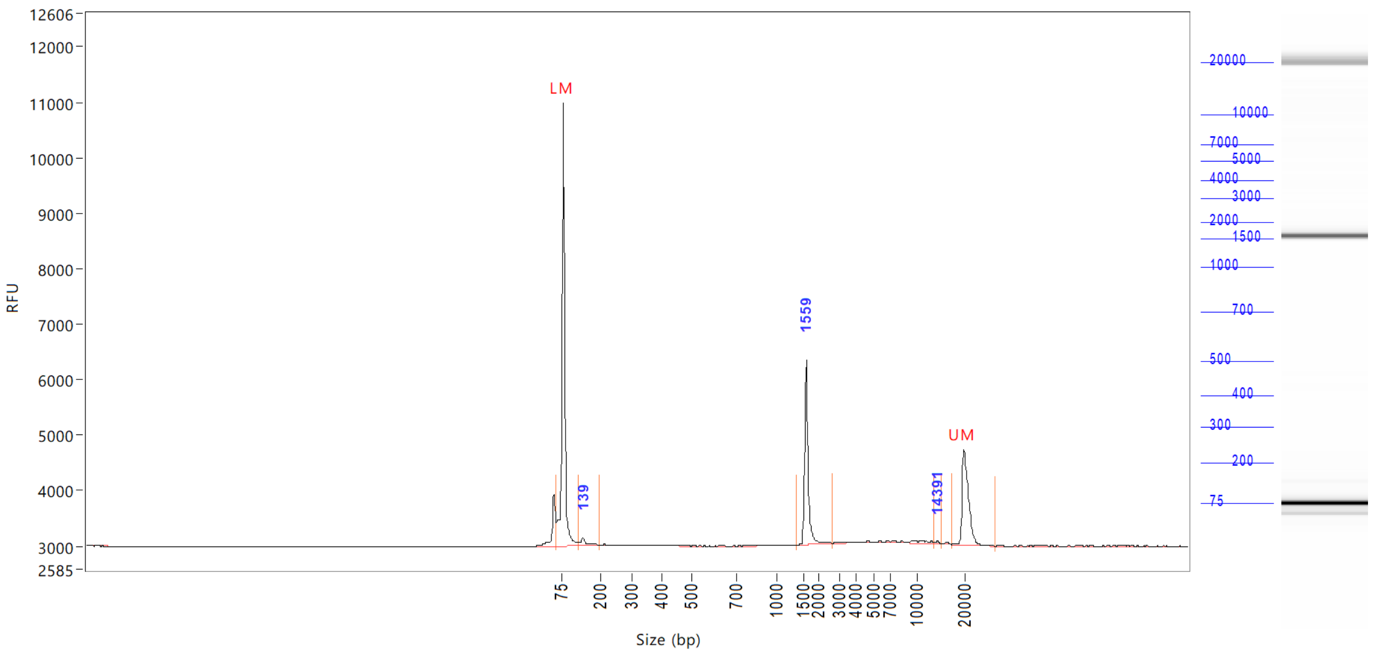

Peak	Size (bp)	Concentration (ng/uL)	From (bp)	To (bp)	Average size (bp)	CV%	RFU	Corrected peak area
1	75 (LM)	0.5000	52	129	74	12.66	8001	56.719
2	139	0.2337	129	196	151	11.60	156	2.209
3	1559	1.9443	1378	2629	1583	7.98	3325	18.380
4	14391	0.0331	13659	15244	14285	2.76	53	0.313
5	20000 (UM)	0.1293	17439	26649	20420	4.05	1721	14.669

TIC:	2.2110	ng/uL
TIM:	4.5691	nmole/L
Total concentration:	2.6566	ng/uL

Sample peak width (sec): 5 Sample min peak height: 50 Sample baseline V to V?: Y Sample baseline V to V points: 3
Sample filter: Binomial Number of points for filter: 3 Sample start region (min): 0 Sample end region (min): 30
Marker peak width (sec): 5 Marker min peak height: 200 Marker baseline V to V?: Y Marker baseline V to V points: 3
Lower marker selection: First peak > 500 RFU Upper marker selection: Last peak > 200 RFU
Ladder size (bp)75, 200, 300, 400, 500, 700, 1000, 1500, 2000, 3000, 4000, 5000, 7000, 10000, 20000
Quantification using: Lower Marker Final concentration (ng/uL): 0.5000 Dilution factor: 12.0

Sample: SampD5
Well location: D5
Created: Tuesday, September 14, 2021 4:54:05 PM

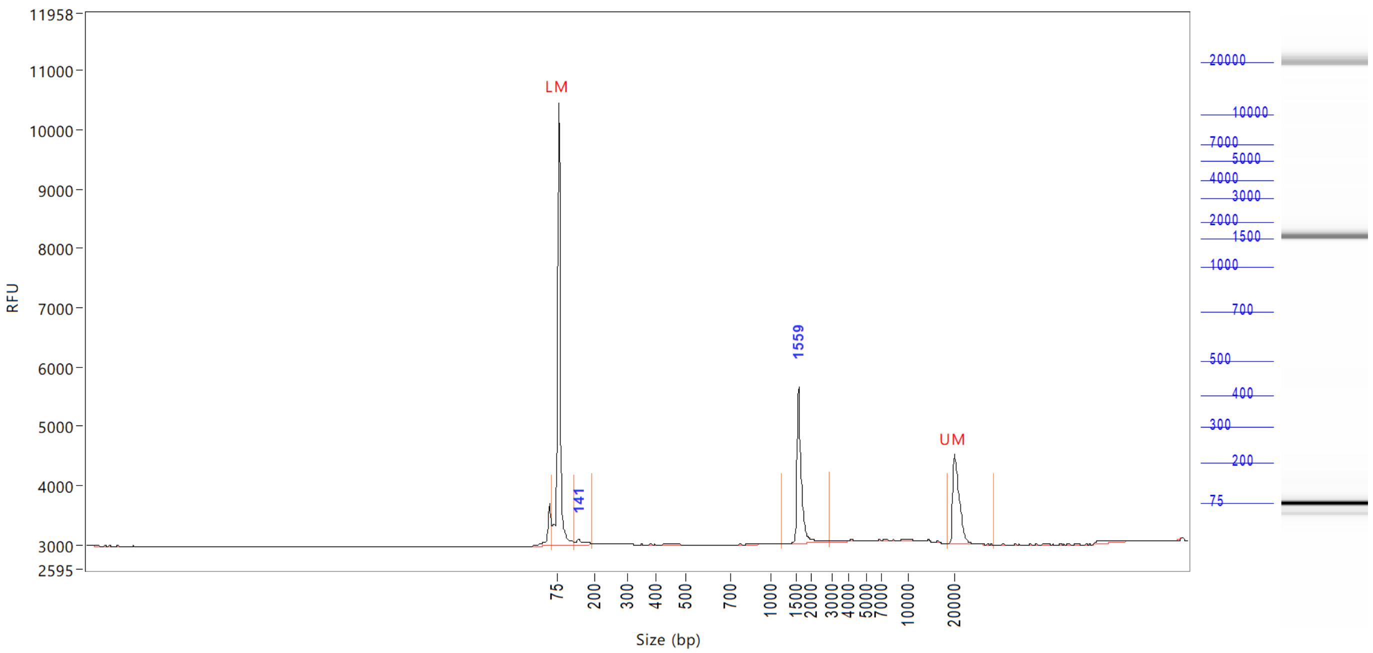

Peak	Size<br>(bp)	Concentration<br>(ng/uL)	From<br>(bp)	To<br>(bp)	Average size<br>(bp)	CV%	RFU	Corrected peak area
1	75 (LM)	0.5000	52	128	74	11.97	7466	50.715
2	141	0.1946	128	187	149	10.53	95	1.645
3	1559	2.2080	1201	2857	1595	10.29	2639	18.663
4	20000 (UM)	0.1311	18415	28221	20445	4.41	1518	13.297

TIC:	2.4026	ng/uL
TIM:	4.4249	nmole/L
Total concentration:	2.8275	ng/uL

Sample peak width (sec): 5 Sample min peak height: 50 Sample baseline V to V?: Y Sample baseline V to V points: 3
Sample filter: Binomial Number of points for filter: 3 Sample start region (min): 0 Sample end region (min): 30
Marker peak width (sec): 5 Marker min peak height: 200 Marker baseline V to V?: Y Marker baseline V to V points: 3
Lower marker selection: First peak > 500 RFU Upper marker selection: Last peak > 200 RFU
Ladder size (bp)75, 200, 300, 400, 500, 700, 1000, 1500, 2000, 3000, 4000, 5000, 7000, 10000, 20000
Quantification using: Lower Marker Final concentration (ng/uL): 0.5000 Dilution factor: 12.0

Sample: SampE5
Well location: E5
Created: Tuesday, September 14, 2021 4:54:05 PM

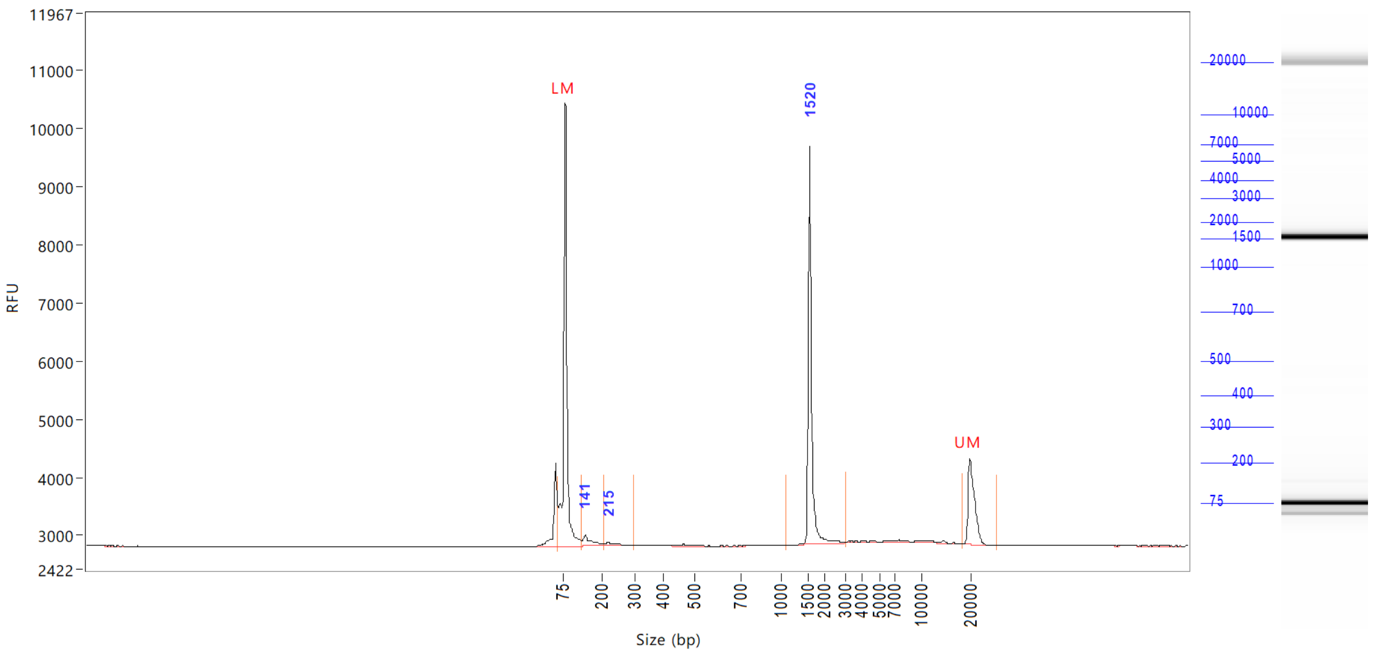

Peak	Size (bp)	Concentration (ng/uL)	From (bp)	To (bp)	Average size (bp)	CV%	RFU	Corrected peak area
1	75 (LM)	0.5000	52	131	75	14.96	7624	61.564
2	141	0.3610	131	203	158	12.91	187	3.704
3	215	0.1298	203	296	232	9.67	51	1.332
4	1520	3.5383	1090	3037	1569	11.16	6827	36.305
5	20000 (UM)	0.1009	18293	25440	20402	3.61	1471	12.424

TIC:	4.0290	ng/uL
TIM:	8.3875	nmole/L
Total concentration:	4.3654	ng/uL

```
Sample peak width (sec): 5    Sample min peak height: 50    Sample baseline V to V?: Y    Sample baseline V to V points: 3
Sample filter: Binomial       Number of points for filter: 3  Sample start region (min): 0  Sample end region (min): 30
Marker peak width (sec): 5    Marker min peak height: 200   Marker baseline V to V?: Y    Marker baseline V to V points: 3
Lower marker selection: First peak > 500 RFU                Upper marker selection: Last peak > 200 RFU
Ladder size (bp)75, 200, 300, 400, 500, 700, 1000, 1500, 2000, 3000, 4000, 5000, 7000, 10000, 20000
Quantification using: Lower Marker          Final concentration (ng/uL): 0.5000          Dilution factor: 12.0
```

Sample: SampF5
Well location: F5
Created: Tuesday, September 14, 2021 4:54:05 PM

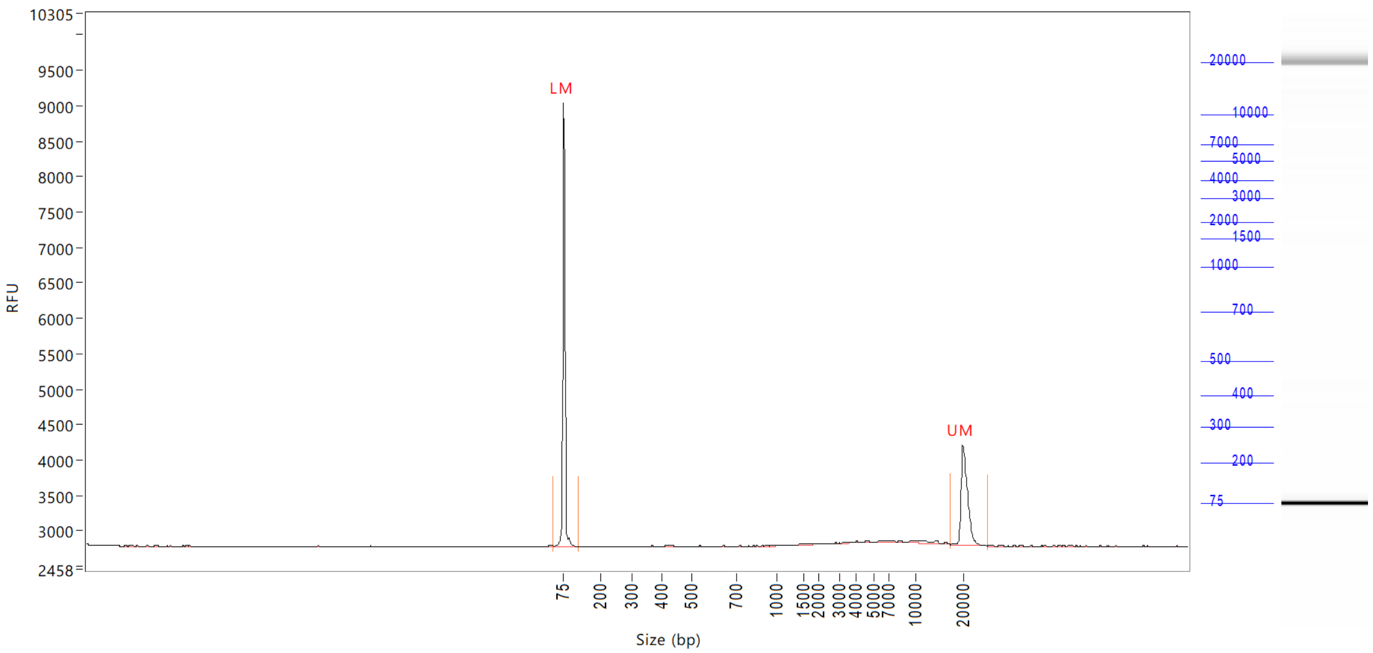

Peak	Size	Concentration	From	To	Average size	CV%	RFU	Corrected peak area
	(bp)	(ng/uL)	(bp)	(bp)	(bp)			
1	75 (LM)	0.5000	43	124	73	6.86	6273	34.987
2	20000 (UM)	0.1740	17439	25199	20447	3.82	1411	12.175

TIC:	0.0000	ng/uL
TIM:	0.0000	nmole/L
Total concentration:	0.7009	ng/uL

Sample peak width (sec): 5   Sample min peak height: 50   Sample baseline V to V?: Y   Sample baseline V to V points: 3
Sample filter: Binomial   Number of points for filter: 3   Sample start region (min): 0   Sample end region (min): 30
Marker peak width (sec): 5   Marker min peak height: 200   Marker baseline V to V?: Y   Marker baseline V to V points: 3
Lower marker selection: First peak > 500 RFU   Upper marker selection: Last peak > 200 RFU
Ladder size (bp)75, 200, 300, 400, 500, 700, 1000, 1500, 2000, 3000, 4000, 5000, 7000, 10000, 20000
Quantification using: Lower Marker   Final concentration (ng/uL): 0.5000   Dilution factor: 12.0

Sample: SampG5
Well location: G5
Created: Tuesday, September 14, 2021 4:54:05 PM

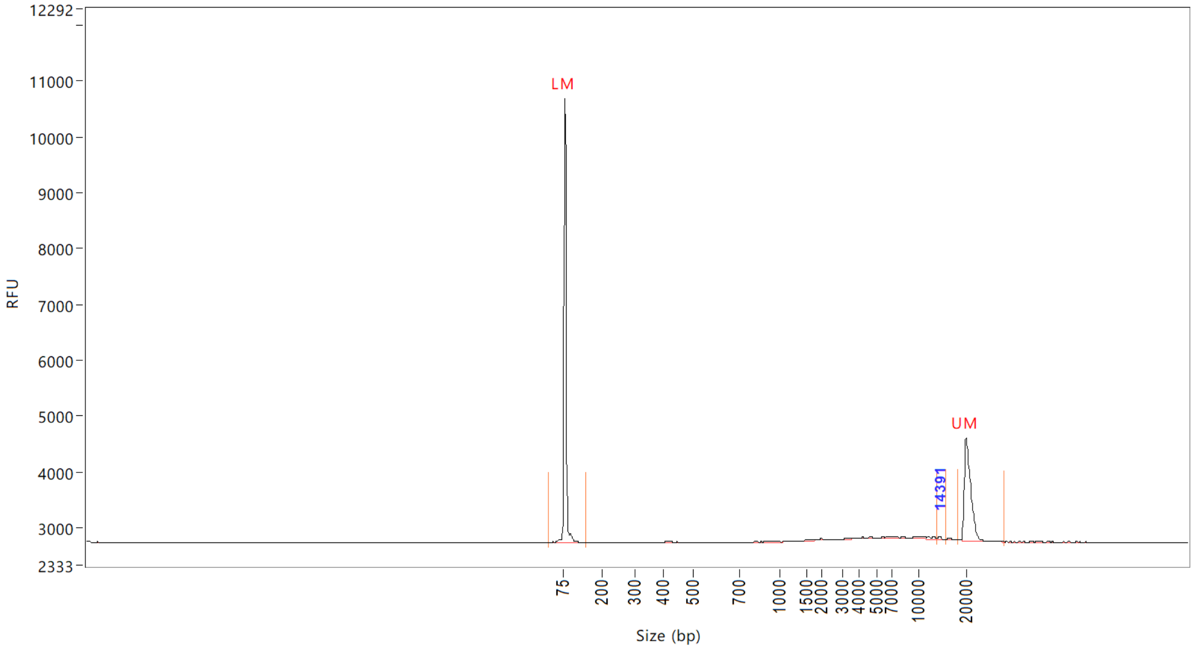

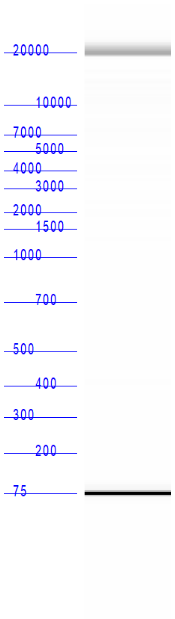

Peak	Size (bp)	Concentration (ng/uL)	From (bp)	To (bp)	Average size (bp)	CV%	RFU	Corrected peak area
1	75 (LM)	0.5000	24	143	74	8.42	7960	44.222
2	14391	0.0357	13781	15732	14373	3.00	56	0.263
3	20000 (UM)	0.1787	18293	28100	20376	3.97	1830	15.801

TIC:	0.0357	ng/uL
TIM:	0.0041	nmole/L
Total concentration:	0.6003	ng/uL

Sample peak width (sec): 5   Sample min peak height: 50   Sample baseline V to V?: Y   Sample baseline V to V points: 3
Sample filter: Binomial   Number of points for filter: 3   Sample start region (min): 0   Sample end region (min): 30
Marker peak width (sec): 5   Marker min peak height: 200   Marker baseline V to V?: Y   Marker baseline V to V points: 3
Lower marker selection: First peak > 500 RFU   Upper marker selection: Last peak > 200 RFU
Ladder size (bp)75, 200, 300, 400, 500, 700, 1000, 1500, 2000, 3000, 4000, 5000, 7000, 10000, 20000
Quantification using: Lower Marker   Final concentration (ng/uL): 0.5000   Dilution factor: 12.0

Sample: SampH5
Well location: H5
Created: Tuesday, September 14, 2021 4:54:05 PM

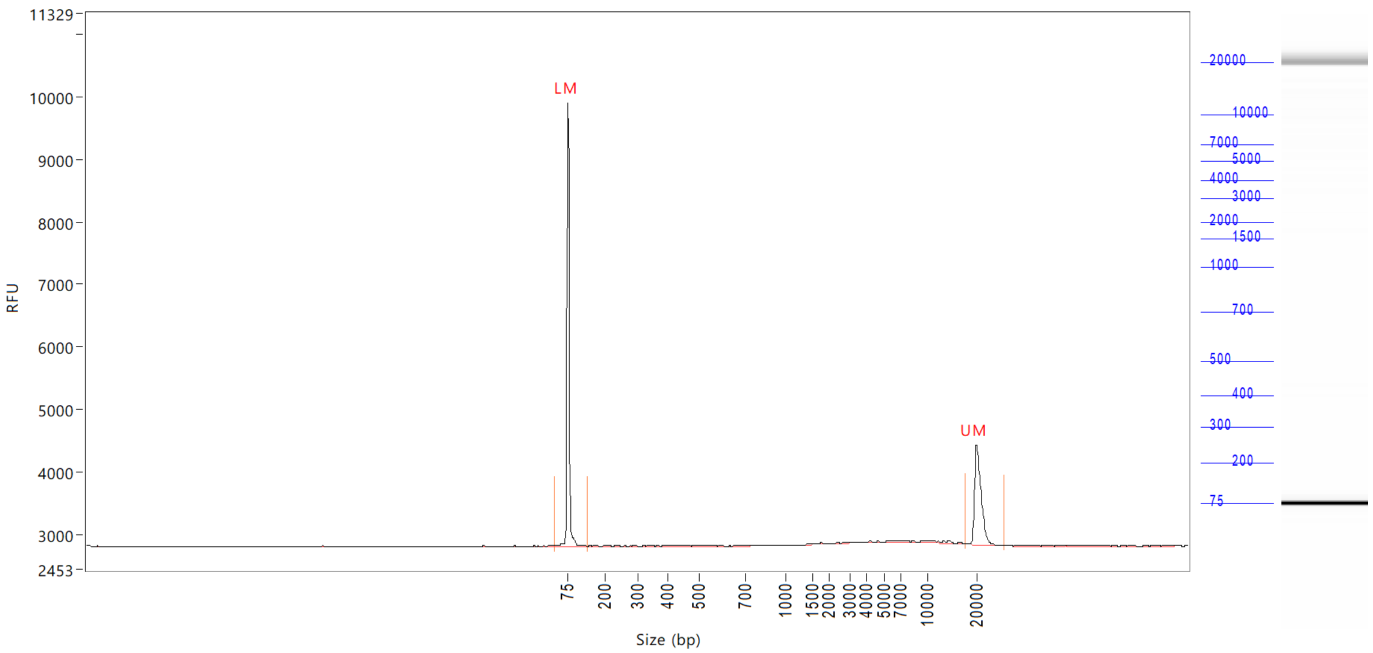

Peak	Size (bp)	Concentration (ng/uL)	From (bp)	To (bp)	Average size (bp)	CV%	RFU	Corrected peak area
1	75 (LM)	0.5000	33	139	74	7.71	7090	39.502
2	20000 (UM)	0.1761	17683	25924	20468	3.94	1599	13.911

TIC:	0.0000	ng/uL
TIM:	0.0000	nmole/L
Total concentration:	0.6037	ng/uL

Sample peak width (sec): 5   Sample min peak height: 50   Sample baseline V to V?: Y   Sample baseline V to V points: 3
Sample filter: Binomial   Number of points for filter: 3 Sample start region (min): 0 Sample end region (min): 30
Marker peak width (sec): 5   Marker min peak height: 200   Marker baseline V to V?: Y   Marker baseline V to V points: 3
Lower marker selection: First peak > 500 RFU   Upper marker selection: Last peak > 200 RFU
Ladder size (bp)75, 200, 300, 400, 500, 700, 1000, 1500, 2000, 3000, 4000, 5000, 7000, 10000, 20000
Quantification using: Lower Marker   Final concentration (ng/uL): 0.5000   Dilution factor: 12.0

Sample: SampA6
Well location: A6
Created: Tuesday, September 14, 2021 4:54:05 PM

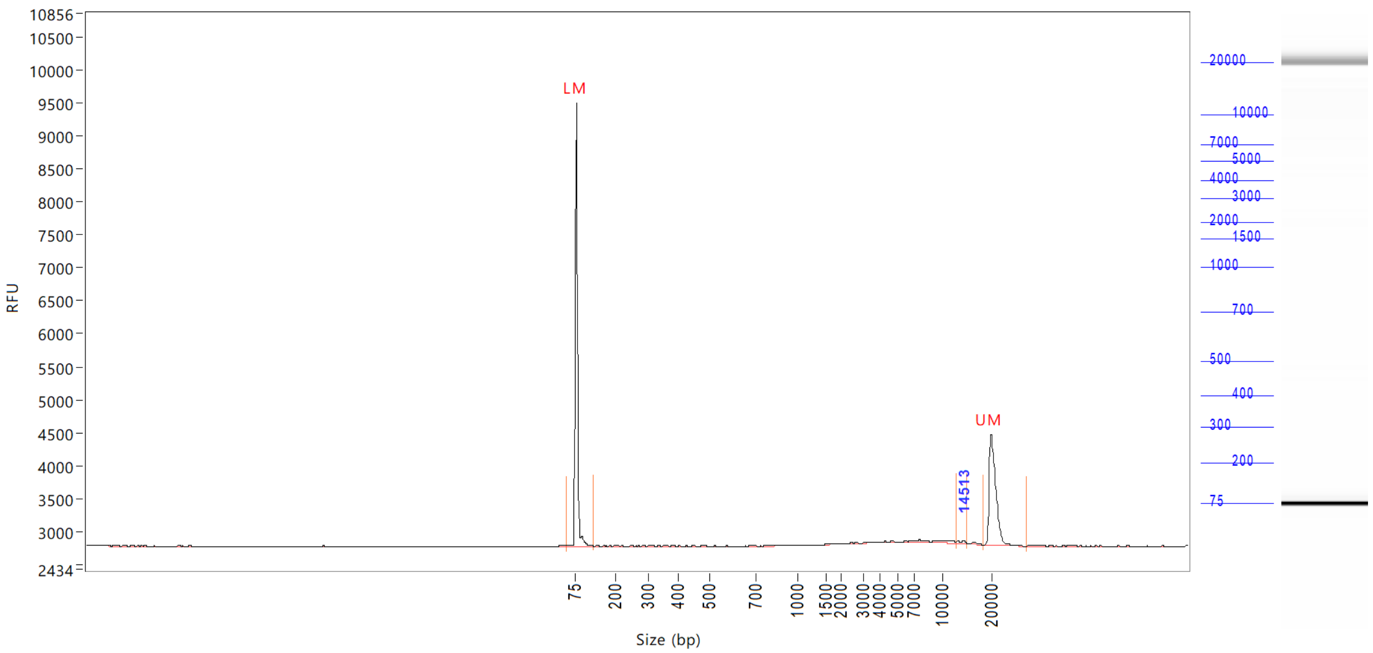

Peak	Size (bp)	Concentration (ng/uL)	From (bp)	To (bp)	Average size (bp)	CV%	RFU	Corrected peak area
1	75 (LM)	0.5000	47	131	73	7.70	6728	38.216
2	14513	0.0702	12806	15244	13959	4.57	55	0.447
3	20000 (UM)	0.1894	18537	27254	20457	4.07	1681	14.474

TIC:	0.0702	ng/uL
TIM:	0.0083	nmole/L
Total concentration:	0.6564	ng/uL

```
Sample peak width (sec): 5    Sample min peak height: 50    Sample baseline V to V?: Y    Sample baseline V to V points: 3
Sample filter: Binomial       Number of points for filter: 3  Sample start region (min): 0  Sample end region (min): 30
Marker peak width (sec): 5    Marker min peak height: 200   Marker baseline V to V?: Y    Marker baseline V to V points: 3
Lower marker selection: First peak > 500 RFU                Upper marker selection: Last peak > 200 RFU
Ladder size (bp)75, 200, 300, 400, 500, 700, 1000, 1500, 2000, 3000, 4000, 5000, 7000, 10000, 20000
Quantification using: Lower Marker          Final concentration (ng/uL): 0.5000          Dilution factor: 12.0
```

Sample: SampB6
Well location: B6
Created: Tuesday, September 14, 2021 4:54:05 PM

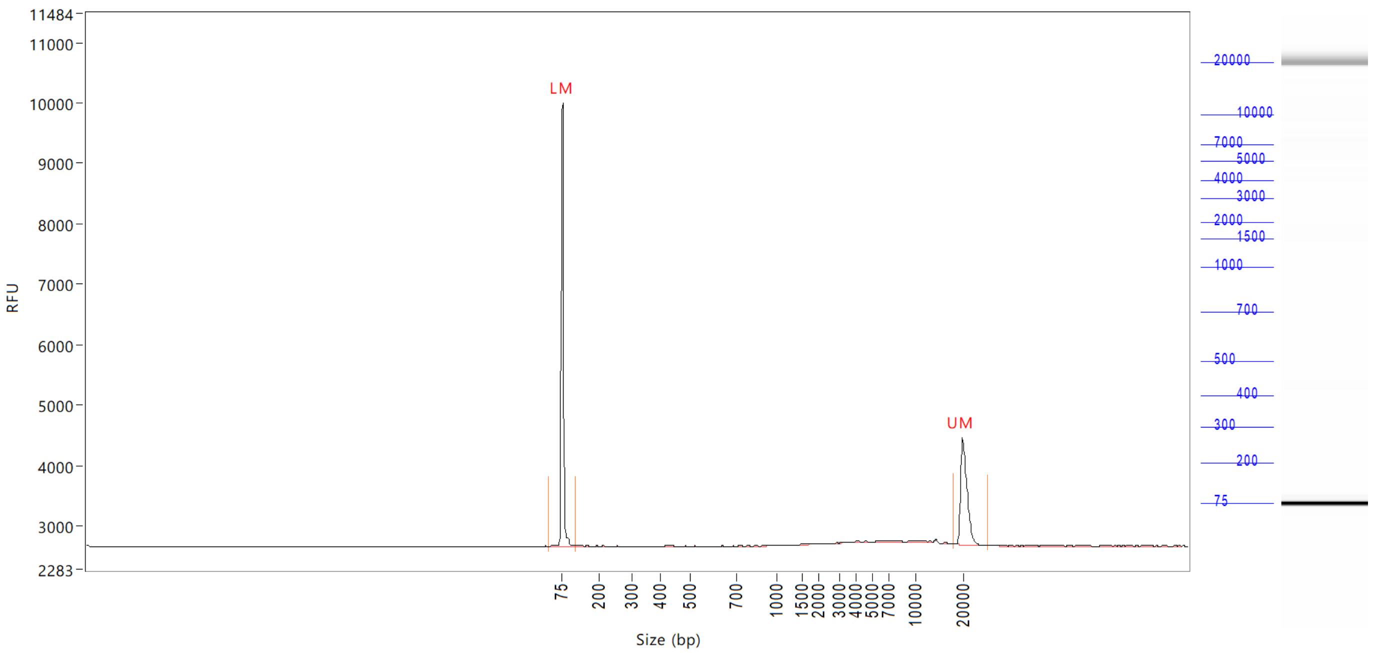

Peak	Size	Concentration	From	To	Average size	CV%	RFU	Corrected peak area
	(bp)	(ng/uL)	(bp)	(bp)	(bp)			
1	75 (LM)	0.5000	33	122	73	6.99	7351	42.198
2	20000 (UM)	0.1795	18171	25199	20344	3.71	1764	15.152

TIC:	0.0000	ng/uL
TIM:	0.0000	nmole/L
Total concentration:	0.5596	ng/uL

```
Sample peak width (sec): 5    Sample min peak height: 50    Sample baseline V to V?: Y    Sample baseline V to V points: 3
Sample filter: Binomial       Number of points for filter: 3  Sample start region (min): 0  Sample end region (min): 30
Marker peak width (sec): 5    Marker min peak height: 200   Marker baseline V to V?: Y    Marker baseline V to V points: 3
Lower marker selection: First peak > 500 RFU                Upper marker selection: Last peak > 200 RFU
Ladder size (bp)75, 200, 300, 400, 500, 700, 1000, 1500, 2000, 3000, 4000, 5000, 7000, 10000, 20000
Quantification using: Lower Marker          Final concentration (ng/uL): 0.5000          Dilution factor: 12.0
```

Sample: SampC6
Well location: C6
Created: Tuesday, September 14, 2021 4:54:05 PM

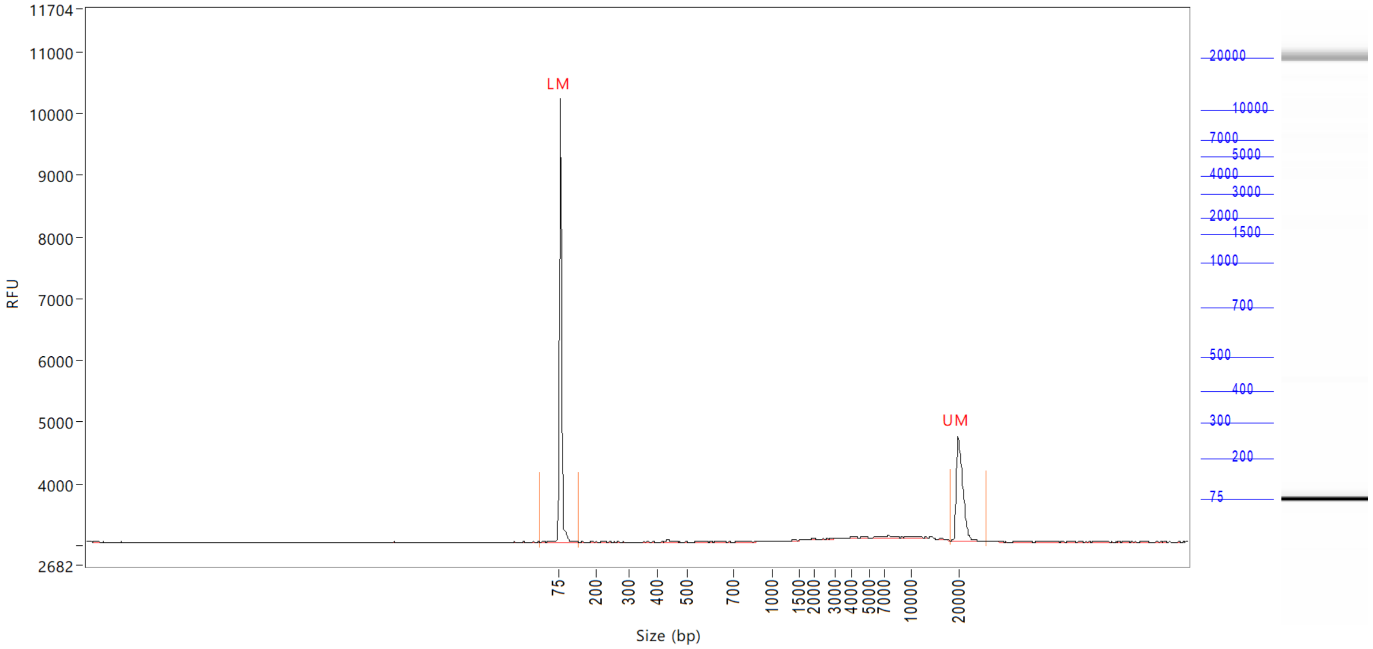

Peak	Size (bp)	Concentration (ng/uL)	From (bp)	To (bp)	Average size (bp)	CV%	RFU	Corrected peak area
1	75 (LM)	0.5000	11	135	74	8.85	7208	41.824
2	20000 (UM)	0.1721	18293	25803	20382	3.86	1690	14.395

TIC:	0.0000	ng/uL
TIM:	0.0000	nmole/L
Total concentration:	0.5443	ng/uL

Sample peak width (sec): 5   Sample min peak height: 50   Sample baseline V to V?: Y   Sample baseline V to V points: 3
Sample filter: Binomial   Number of points for filter: 3   Sample start region (min): 0   Sample end region (min): 30
Marker peak width (sec): 5   Marker min peak height: 200   Marker baseline V to V?: Y   Marker baseline V to V points: 3
Lower marker selection: First peak > 500 RFU   Upper marker selection: Last peak > 200 RFU
Ladder size (bp)75, 200, 300, 400, 500, 700, 1000, 1500, 2000, 3000, 4000, 5000, 7000, 10000, 20000
Quantification using: Lower Marker   Final concentration (ng/uL): 0.5000   Dilution factor: 12.0

Sample: SampD6
Well location: D6
Created: Tuesday, September 14, 2021 4:54:05 PM

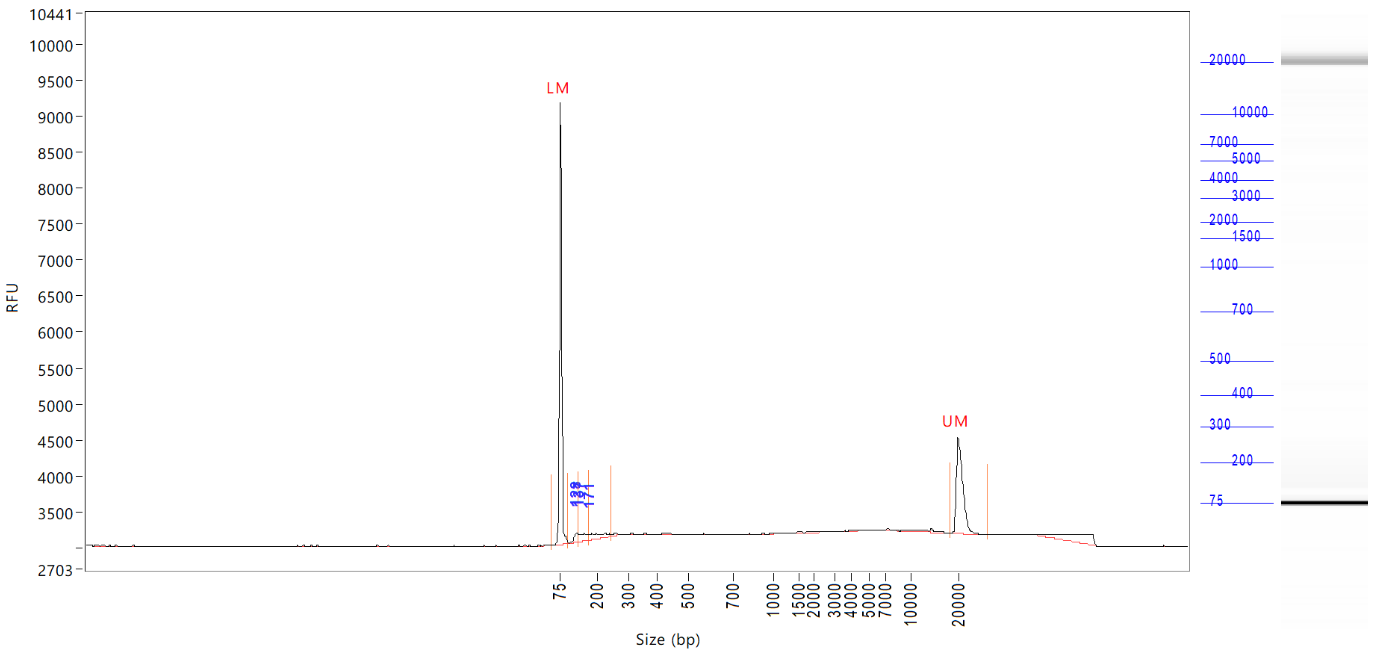

Peak	Size (bp)	Concentration (ng/uL)	From (bp)	To (bp)	Average size (bp)	CV%	RFU	Corrected peak area
1	75 (LM)	0.5000	45	103	73	5.66	6161	34.154
2	128	0.2765	103	137	126	6.31	122	1.574
3	137	0.3576	137	171	153	6.77	108	2.036
4	171	0.4676	171	242	201	9.85	83	2.662
5	20000 (UM)	0.1672	18415	26166	20453	4.05	1341	11.424

TIC:	1.1017	ng/uL
TIM:	11.2718	nmole/L
Total concentration:	1.7775	ng/uL

Sample peak width (sec): 5   Sample min peak height: 50   Sample baseline V to V?: Y   Sample baseline V to V points: 3
Sample filter: Binomial   Number of points for filter: 3 Sample start region (min): 0 Sample end region (min): 30
Marker peak width (sec): 5   Marker min peak height: 200   Marker baseline V to V?: Y   Marker baseline V to V points: 3
Lower marker selection: First peak > 500 RFU   Upper marker selection: Last peak > 200 RFU
Ladder size (bp)75, 200, 300, 400, 500, 700, 1000, 1500, 2000, 3000, 4000, 5000, 7000, 10000, 20000
Quantification using: Lower Marker   Final concentration (ng/uL): 0.5000   Dilution factor: 12.0

Sample: SampE6
Well location: E6
Created: Tuesday, September 14, 2021 4:54:05 PM

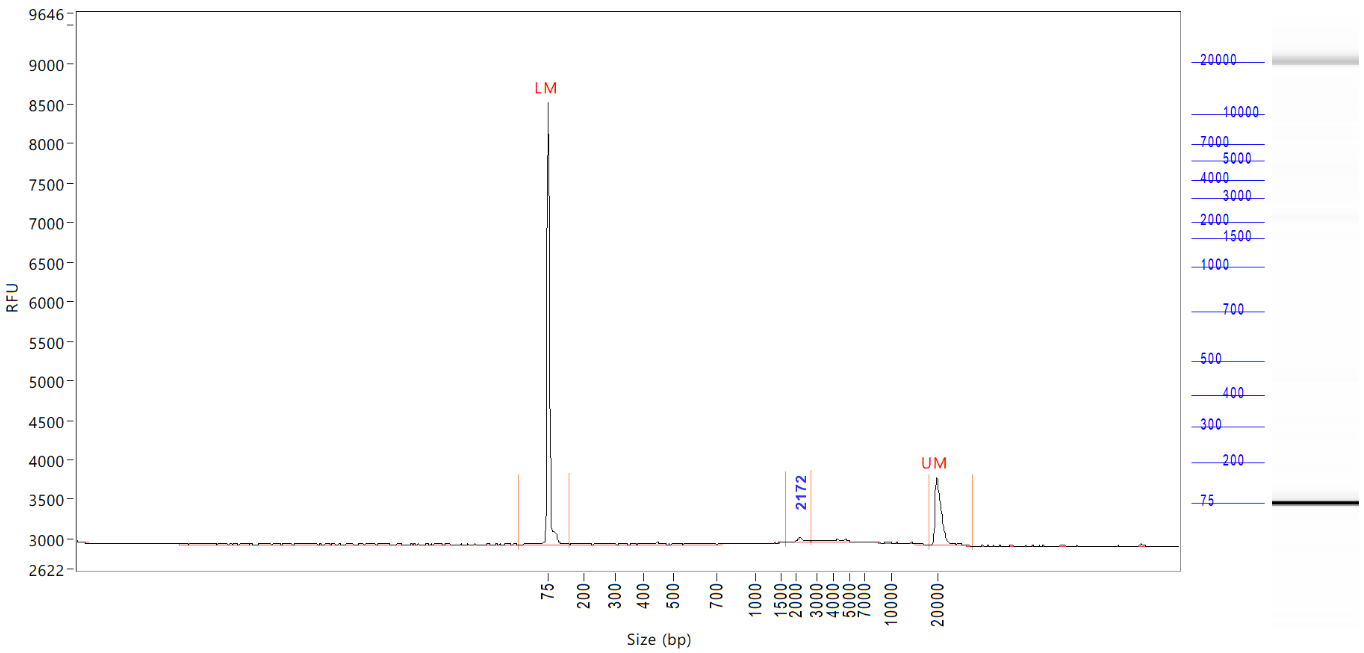

Peak	Size (bp)	Concentration (ng/uL)	From (bp)	To (bp)	Average size (bp)	CV%	RFU	Corrected peak area
1	75 (LM)	0.5000	0	148	73	12.63	5591	33.696
2	2172	0.1089	1616	2686	2122	9.75	63	0.611
3	20000 (UM)	0.1076	18415	27738	20557	5.57	853	7.254

TIC:	0.1089	ng/uL
TIM:	0.0844	nmole/L
Total concentration:	0.7078	ng/uL

```
Sample peak width (sec): 5    Sample min peak height: 50    Sample baseline V to V?: Y    Sample baseline V to V points: 3
Sample filter: Binomial       Number of points for filter: 3  Sample start region (min): 0  Sample end region (min): 30
Marker peak width (sec): 5    Marker min peak height: 200   Marker baseline V to V?: Y    Marker baseline V to V points: 3
Lower marker selection: First peak > 500 RFU                Upper marker selection: Last peak > 200 RFU
Ladder size (bp)75, 200, 300, 400, 500, 700, 1000, 1500, 2000, 3000, 4000, 5000, 7000, 10000, 20000
Quantification using: Lower Marker          Final concentration (ng/uL): 0.5000          Dilution factor: 12.0
```

Sample: SampF6
Well location: F6
Created: Tuesday, September 14, 2021 4:54:05 PM

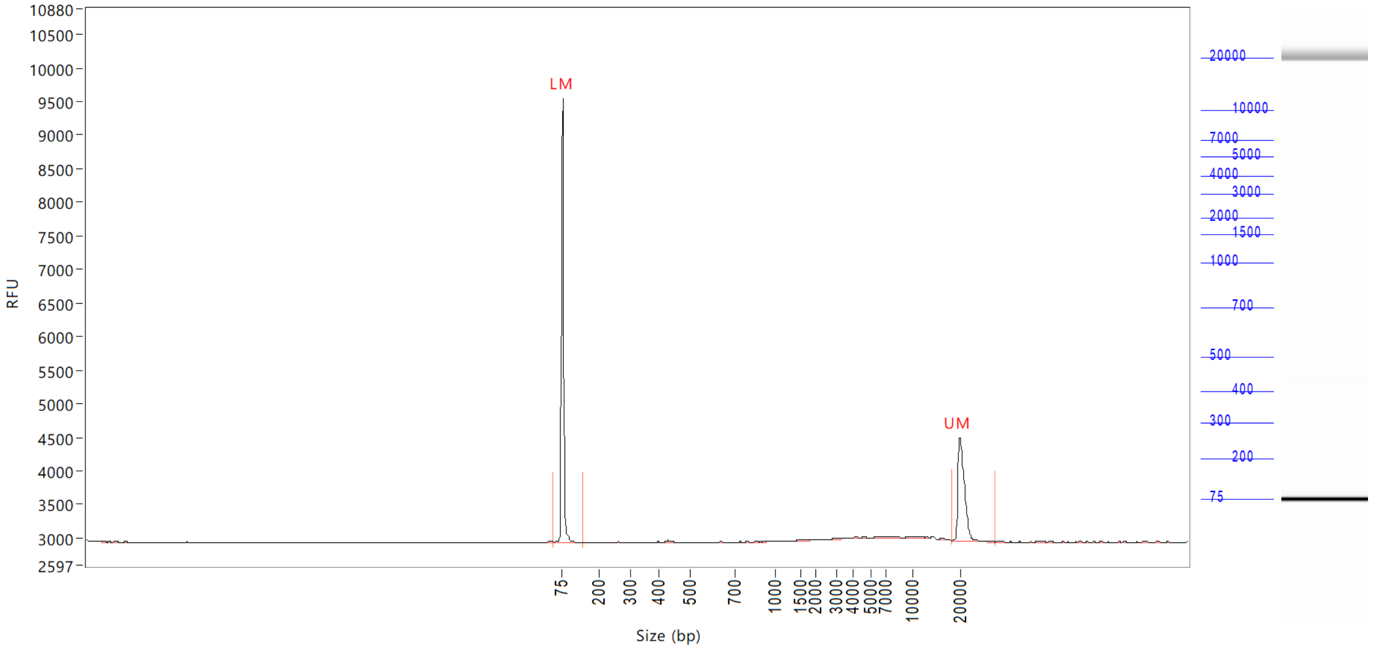

Peak	Size (bp)	Concentration (ng/uL)	From (bp)	To (bp)	Average size (bp)	CV%	RFU	Corrected peak area
1	75 (LM)	0.5000	47	147	73	6.89	6620	37.075
2	20000 (UM)	0.1776	18293	27738	20495	4.20	1527	13.172

TIC:	0.0000	ng/uL
TIM:	0.0000	nmole/L
Total concentration:	0.5831	ng/uL

Sample peak width (sec): 5 Sample min peak height: 50 Sample baseline V to V?: Y Sample baseline V to V points: 3
Sample filter: Binomial Number of points for filter: 3 Sample start region (min): 0 Sample end region (min): 30
Marker peak width (sec): 5 Marker min peak height: 200 Marker baseline V to V?: Y Marker baseline V to V points: 3
Lower marker selection: First peak > 500 RFU Upper marker selection: Last peak > 200 RFU
Ladder size (bp)75, 200, 300, 400, 500, 700, 1000, 1500, 2000, 3000, 4000, 5000, 7000, 10000, 20000
Quantification using: Lower Marker Final concentration (ng/uL): 0.5000 Dilution factor: 12.0

Sample: SampG6
Well location: G6
Created: Tuesday, September 14, 2021 4:54:05 PM

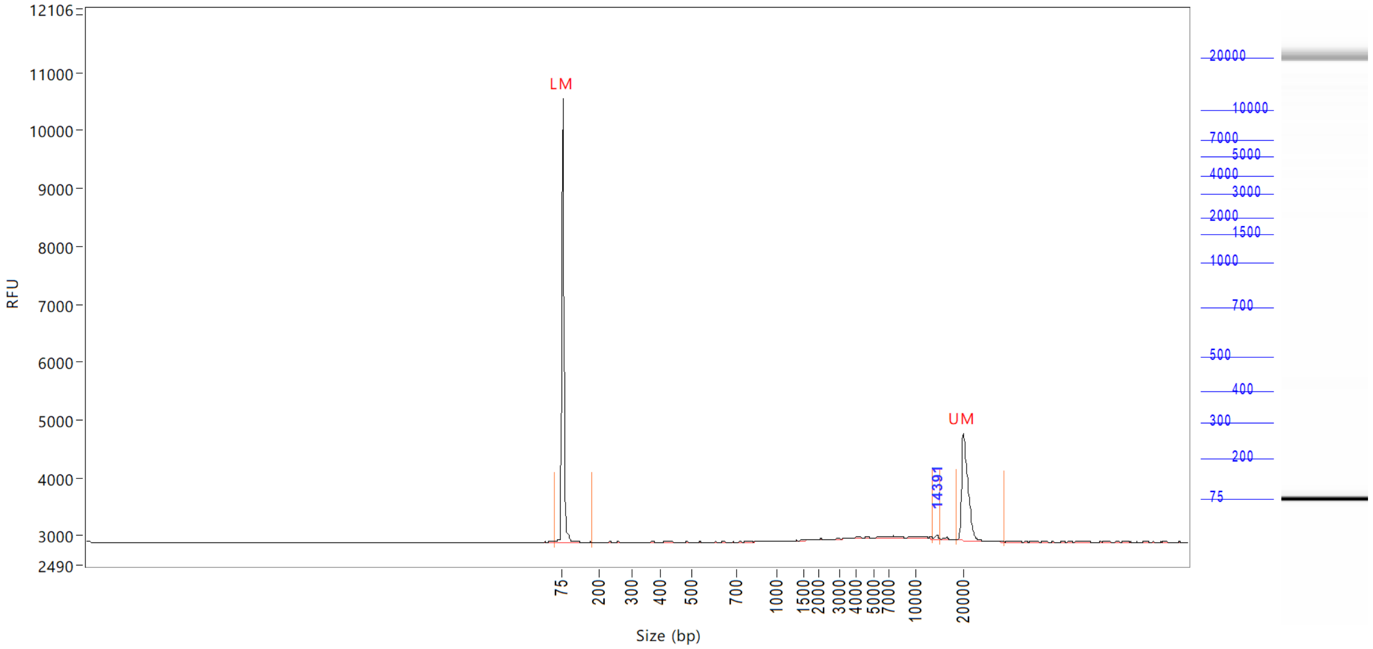

Peak	Size (bp)	Concentration (ng/uL)	From (bp)	To (bp)	Average size (bp)	CV%	RFU	Corrected peak area
1	75 (LM)	0.5000	50	173	75	8.54	7682	43.905
2	14391	0.0432	13659	15244	14268	2.43	64	0.316
3	20000 (UM)	0.1802	18537	28826	20458	4.15	1840	15.823

TIC:	0.0432	ng/uL
TIM:	0.0050	nmole/L
Total concentration:	0.5727	ng/uL

Sample peak width (sec): 5 Sample min peak height: 50 Sample baseline V to V?: Y Sample baseline V to V points: 3
Sample filter: Binomial Number of points for filter: 3 Sample start region (min): 0 Sample end region (min): 30
Marker peak width (sec): 5 Marker min peak height: 200 Marker baseline V to V?: Y Marker baseline V to V points: 3
Lower marker selection: First peak > 500 RFU Upper marker selection: Last peak > 200 RFU
Ladder size (bp)75, 200, 300, 400, 500, 700, 1000, 1500, 2000, 3000, 4000, 5000, 7000, 10000, 20000
Quantification using: Lower Marker Final concentration (ng/uL): 0.5000 Dilution factor: 12.0

Sample: SampH6
Well location: H6
Created: Tuesday, September 14, 2021 4:54:05 PM

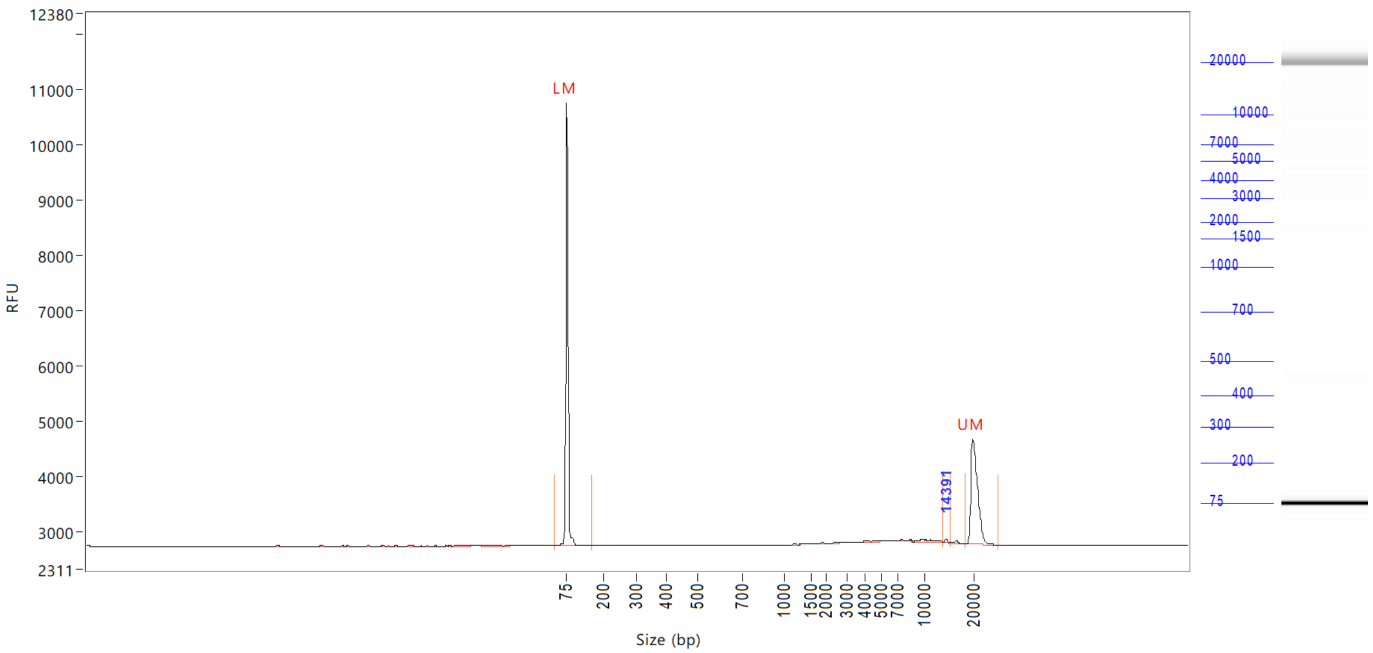

Peak	Size (bp)	Concentration (ng/uL)	From (bp)	To (bp)	Average size (bp)	CV%	RFU	Corrected peak area
1	75 (LM)	0.5000	35	158	74	8.09	8034	44.319
2	14391	0.0405	13659	15366	14325	2.67	59	0.299
3	20000 (UM)	0.1860	18293	25320	20364	3.67	1911	16.490

TIC:	0.0405	ng/uL
TIM:	0.0046	nmole/L
Total concentration:	0.5616	ng/uL

Sample peak width (sec): 5 Sample min peak height: 50 Sample baseline V to V?: Y Sample baseline V to V points: 3
Sample filter: Binomial Number of points for filter: 3 Sample start region (min): 0 Sample end region (min): 30
Marker peak width (sec): 5 Marker min peak height: 200 Marker baseline V to V?: Y Marker baseline V to V points: 3
Lower marker selection: First peak > 500 RFU Upper marker selection: Last peak > 200 RFU
Ladder size (bp)75, 200, 300, 400, 500, 700, 1000, 1500, 2000, 3000, 4000, 5000, 7000, 10000, 20000
Quantification using: Lower Marker Final concentration (ng/uL): 0.5000 Dilution factor: 12.0

Sample: SampA7
Well location: A7
Created: Tuesday, September 14, 2021 4:54:05 PM

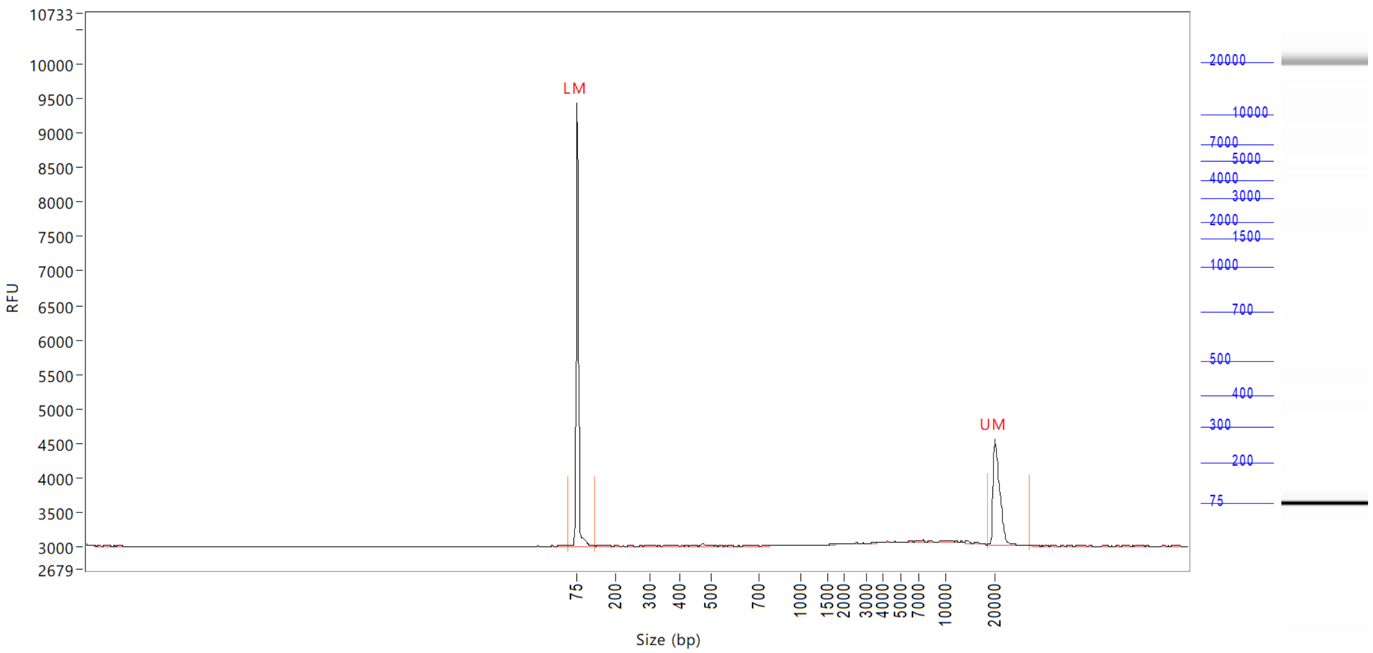

Peak	Size (bp)	Concentration (ng/uL)	From (bp)	To (bp)	Average size (bp)	CV%	RFU	Corrected peak area
1	75 (LM)	0.5000	48	133	74	8.12	6433	35.425
2	20000 (UM)	0.1845	18415	26891	20392	4.10	1535	13.069

TIC:	0.0000	ng/uL
TIM:	0.0000	nmole/L
Total concentration:	0.6353	ng/uL

Sample peak width (sec): 5 Sample min peak height: 50 Sample baseline V to V?: Y Sample baseline V to V points: 3
Sample filter: Binomial Number of points for filter: 3 Sample start region (min): 0 Sample end region (min): 30
Marker peak width (sec): 5 Marker min peak height: 200 Marker baseline V to V?: Y Marker baseline V to V points: 3
Lower marker selection: First peak > 500 RFU Upper marker selection: Last peak > 200 RFU
Ladder size (bp)75, 200, 300, 400, 500, 700, 1000, 1500, 2000, 3000, 4000, 5000, 7000, 10000, 20000
Quantification using: Lower Marker Final concentration (ng/uL): 0.5000 Dilution factor: 12.0

Sample: SampB7
Well location: B7
Created: Tuesday, September 14, 2021 4:54:05 PM

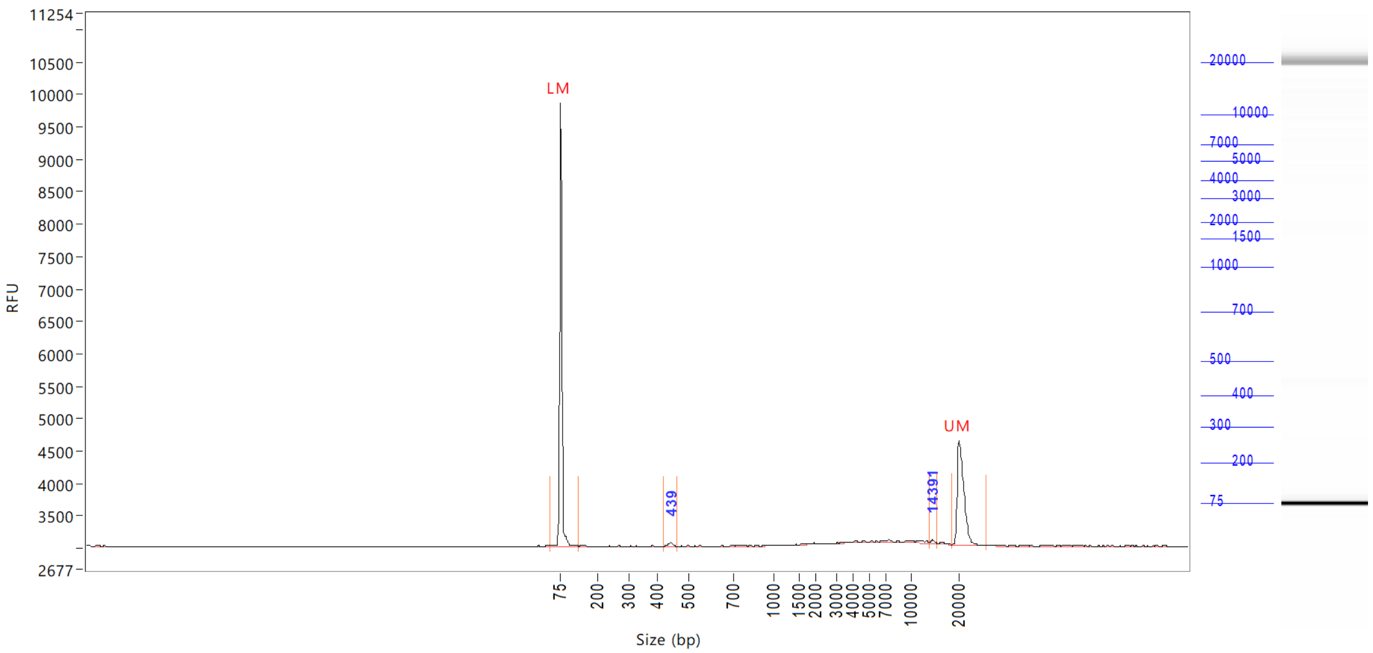

Peak	Size (bp)	Concentration (ng/uL)	From (bp)	To (bp)	Average size (bp)	CV%	RFU	Corrected peak area
1	75 (LM)	0.5000	41	133	73	7.73	6852	38.929
2	439	0.0827	414	462	436	1.92	58	0.537
3	14391	0.0532	13537	15366	14321	3.10	58	0.345
4	20000 (UM)	0.1792	18415	25682	20472	3.93	1603	13.956

TIC:	0.1359	ng/uL
TIM:	0.3184	nmole/L
Total concentration:	0.7060	ng/uL

Sample peak width (sec): 5   Sample min peak height: 50   Sample baseline V to V?: Y   Sample baseline V to V points: 3
Sample filter: Binomial   Number of points for filter: 3 Sample start region (min): 0 Sample end region (min): 30
Marker peak width (sec): 5   Marker min peak height: 200   Marker baseline V to V?: Y   Marker baseline V to V points: 3
Lower marker selection: First peak > 500 RFU   Upper marker selection: Last peak > 200 RFU
Ladder size (bp)75, 200, 300, 400, 500, 700, 1000, 1500, 2000, 3000, 4000, 5000, 7000, 10000, 20000
Quantification using: Lower Marker   Final concentration (ng/uL): 0.5000   Dilution factor: 12.0

Sample: SampC7
Well location: C7
Created: Tuesday, September 14, 2021 4:54:05 PM

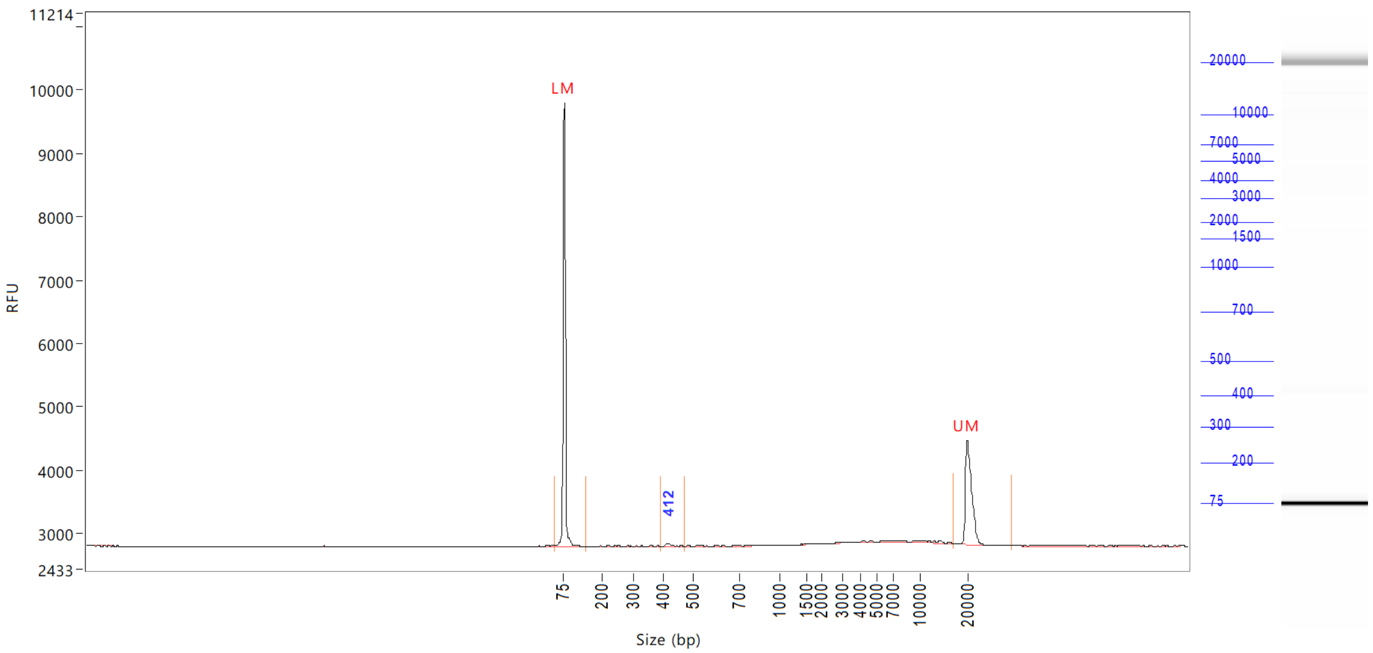

Peak	Size (bp)	Concentration (ng/uL)	From (bp)	To (bp)	Average size (bp)	CV%	RFU	Corrected peak area
1	75 (LM)	0.5000	47	148	73	7.10	7015	40.156
2	412	0.0862	390	468	418	3.84	52	0.577
3	20000 (UM)	0.1723	17195	29309	20324	4.12	1645	13.839

TIC:	0.0862	ng/uL
TIM:	0.3393	nmole/L
Total concentration:	0.6955	ng/uL

Sample peak width (sec): 5 Sample min peak height: 50 Sample baseline V to V?: Y Sample baseline V to V points: 3
Sample filter: Binomial Number of points for filter: 3 Sample start region (min): 0 Sample end region (min): 30
Marker peak width (sec): 5 Marker min peak height: 200 Marker baseline V to V?: Y Marker baseline V to V points: 3
Lower marker selection: First peak > 500 RFU Upper marker selection: Last peak > 200 RFU
Ladder size (bp)75, 200, 300, 400, 500, 700, 1000, 1500, 2000, 3000, 4000, 5000, 7000, 10000, 20000
Quantification using: Lower Marker Final concentration (ng/uL): 0.5000 Dilution factor: 12.0

Sample: SampD7
Well location: D7
Created: Tuesday, September 14, 2021 4:54:05 PM

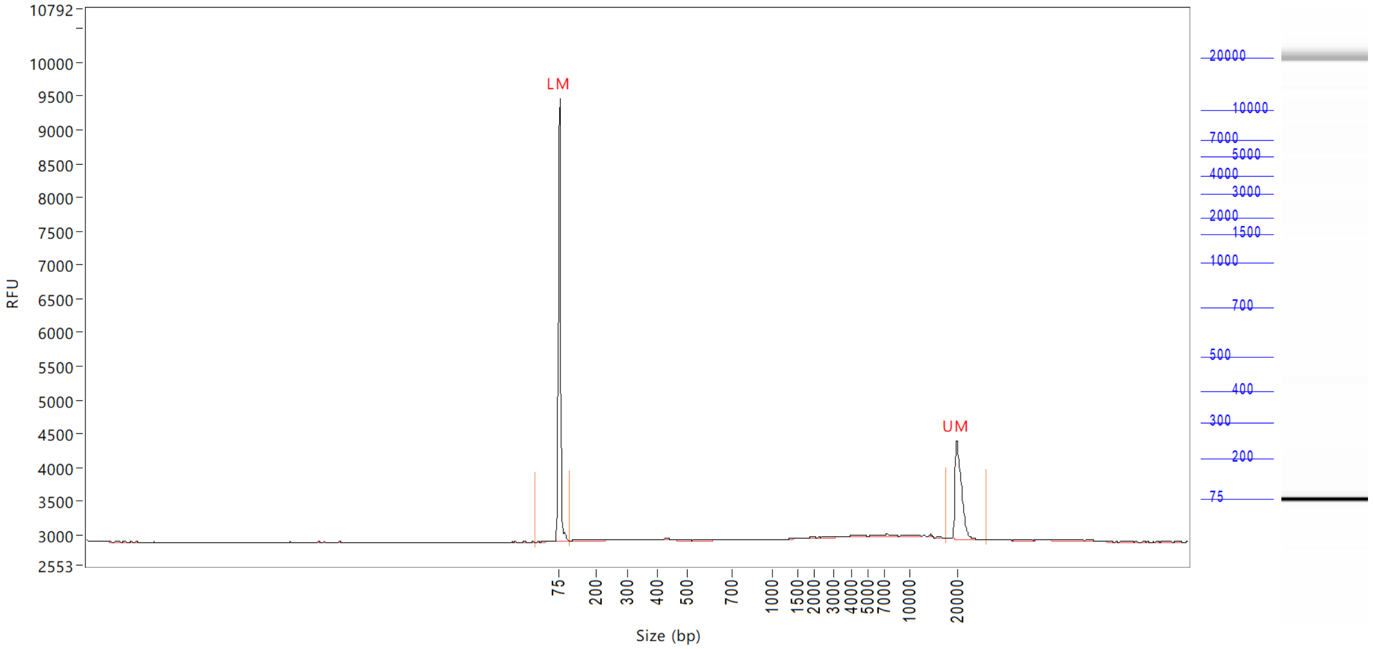

Peak	Size (bp)	Concentration (ng/uL)	From (bp)	To (bp)	Average size (bp)	CV%	RFU	Corrected peak area
1	75 (LM)	0.5000	0	110	73	7.14	6572	36.786
2	20000 (UM)	0.1694	17805	26287	20371	3.97	1452	12.463

TIC:	0.0000	ng/uL
TIM:	0.0000	nmole/L
Total concentration:	0.7475	ng/uL

Sample peak width (sec): 5 Sample min peak height: 50 Sample baseline V to V?: Y Sample baseline V to V points: 3
Sample filter: Binomial Number of points for filter: 3 Sample start region (min): 0 Sample end region (min): 30
Marker peak width (sec): 5 Marker min peak height: 200 Marker baseline V to V?: Y Marker baseline V to V points: 3
Lower marker selection: First peak > 500 RFU Upper marker selection: Last peak > 200 RFU
Ladder size (bp)75, 200, 300, 400, 500, 700, 1000, 1500, 2000, 3000, 4000, 5000, 7000, 10000, 20000
Quantification using: Lower Marker Final concentration (ng/uL): 0.5000 Dilution factor: 12.0

Sample: SampE7
Well location: E7
Created: Tuesday, September 14, 2021 4:54:05 PM

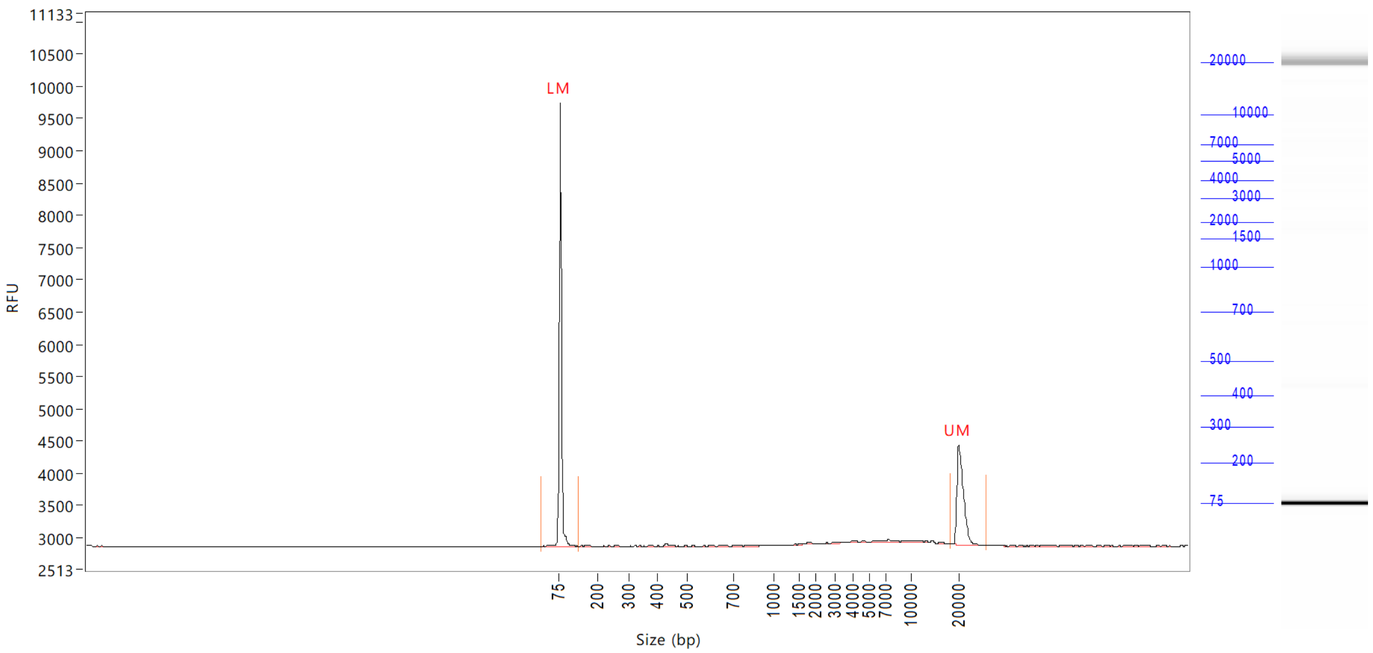

Peak	Size (bp)	Concentration (ng/uL)	From (bp)	To (bp)	Average size (bp)	CV%	RFU	Corrected peak area
1	75 (LM)	0.5000	14	137	74	8.67	6887	39.114
2	20000 (UM)	0.1702	18171	25682	20385	3.91	1536	13.311

TIC:	0.0000	ng/uL
TIM:	0.0000	nmole/L
Total concentration:	0.5885	ng/uL

```
Sample peak width (sec): 5    Sample min peak height: 50    Sample baseline V to V?: Y    Sample baseline V to V points: 3
Sample filter: Binomial       Number of points for filter: 3  Sample start region (min): 0  Sample end region (min): 30
Marker peak width (sec): 5    Marker min peak height: 200   Marker baseline V to V?: Y    Marker baseline V to V points: 3
Lower marker selection: First peak > 500 RFU                Upper marker selection: Last peak > 200 RFU
Ladder size (bp)75, 200, 300, 400, 500, 700, 1000, 1500, 2000, 3000, 4000, 5000, 7000, 10000, 20000
Quantification using: Lower Marker          Final concentration (ng/uL): 0.5000          Dilution factor: 12.0
```

Sample: SampF7
Well location: F7
Created: Tuesday, September 14, 2021 4:54:05 PM

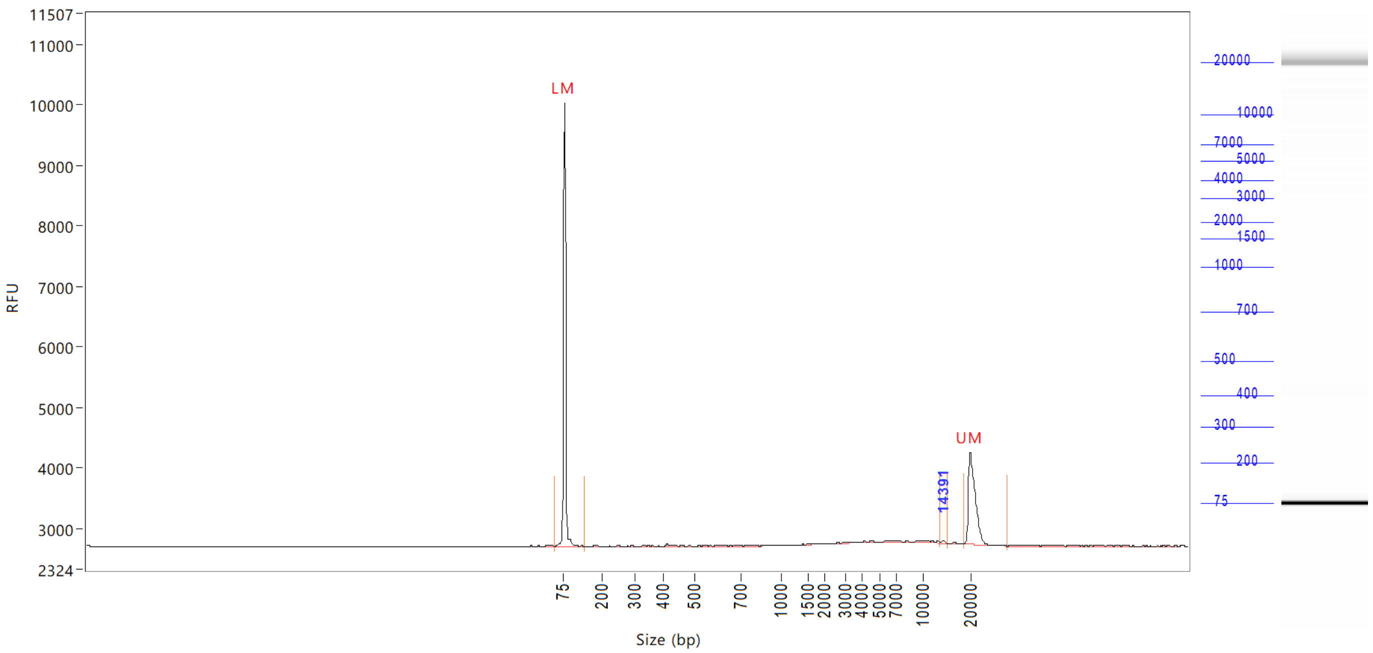

Peak	Size (bp)	Concentration (ng/uL)	From (bp)	To (bp)	Average size (bp)	CV%	RFU	Corrected peak area
1	75 (LM)	0.5000	43	143	74	6.89	7335	40.968
2	14391	0.0360	13659	15244	14244	2.20	53	0.246
3	20000 (UM)	0.1670	18537	27738	20432	4.34	1516	13.686

TIC:	0.0360	ng/uL
TIM:	0.0042	nmole/L
Total concentration:	0.6012	ng/uL

```
Sample peak width (sec): 5    Sample min peak height: 50    Sample baseline V to V?: Y    Sample baseline V to V points: 3
Sample filter: Binomial       Number of points for filter: 3  Sample start region (min): 0  Sample end region (min): 30
Marker peak width (sec): 5    Marker min peak height: 200   Marker baseline V to V?: Y    Marker baseline V to V points: 3
Lower marker selection: First peak > 500 RFU                Upper marker selection: Last peak > 200 RFU
Ladder size (bp)75, 200, 300, 400, 500, 700, 1000, 1500, 2000, 3000, 4000, 5000, 7000, 10000, 20000
Quantification using: Lower Marker          Final concentration (ng/uL): 0.5000          Dilution factor: 12.0
```

Sample: SampG7
Well location: G7
Created: Tuesday, September 14, 2021 4:54:05 PM

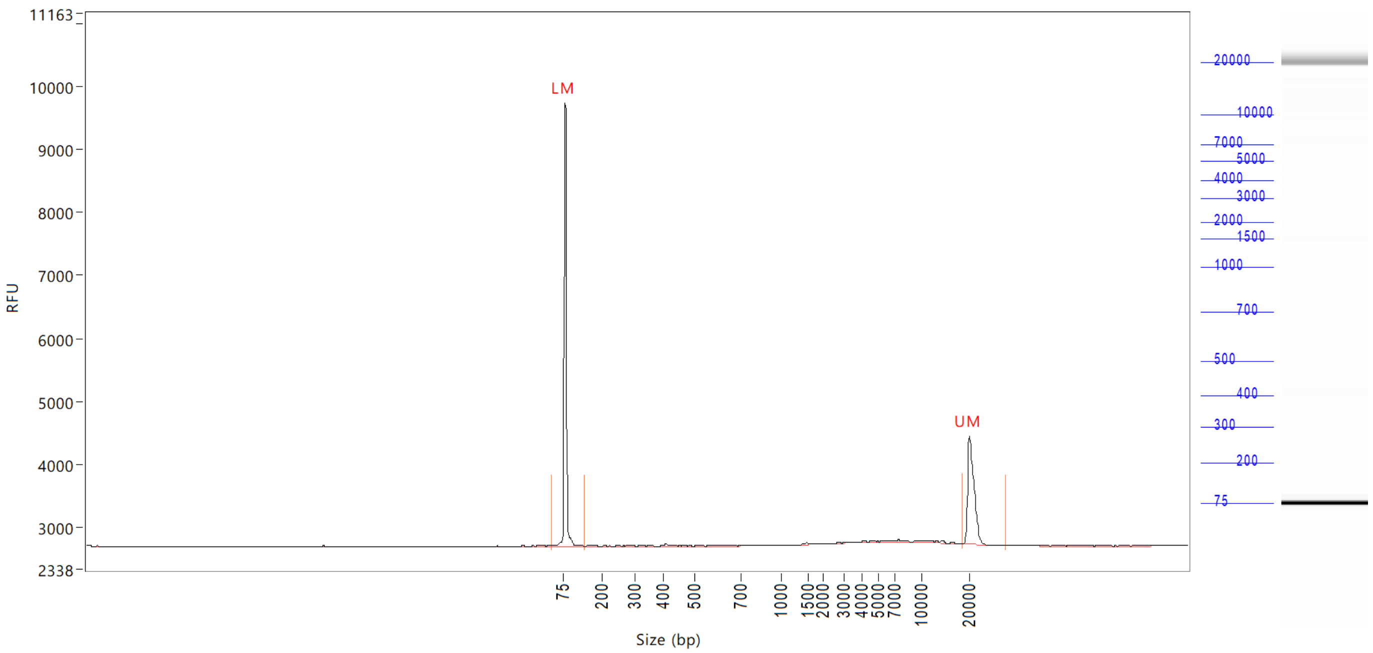

Peak	Size<br>(bp)	Concentration<br>(ng/uL)	From<br>(bp)	To<br>(bp)	Average size<br>(bp)	CV%	RFU	Corrected peak area
1	75 (LM)	0.5000	33	141	74	7.25	7048	40.446
2	20000 (UM)	0.1763	18537	27617	20411	3.63	1710	14.262

TIC:	0.0000	ng/uL
TIM:	0.0000	nmole/L
Total concentration:	0.6468	ng/uL

Sample peak width (sec): 5   Sample min peak height: 50   Sample baseline V to V?: Y   Sample baseline V to V points: 3
Sample filter: Binomial   Number of points for filter: 3 Sample start region (min): 0 Sample end region (min): 30
Marker peak width (sec): 5   Marker min peak height: 200   Marker baseline V to V?: Y   Marker baseline V to V points: 3
Lower marker selection: First peak > 500 RFU   Upper marker selection: Last peak > 200 RFU
Ladder size (bp)75, 200, 300, 400, 500, 700, 1000, 1500, 2000, 3000, 4000, 5000, 7000, 10000, 20000
Quantification using: Lower Marker   Final concentration (ng/uL): 0.5000   Dilution factor: 12.0

Sample: SampH7
Well location: H7
Created: Tuesday, September 14, 2021 4:54:05 PM

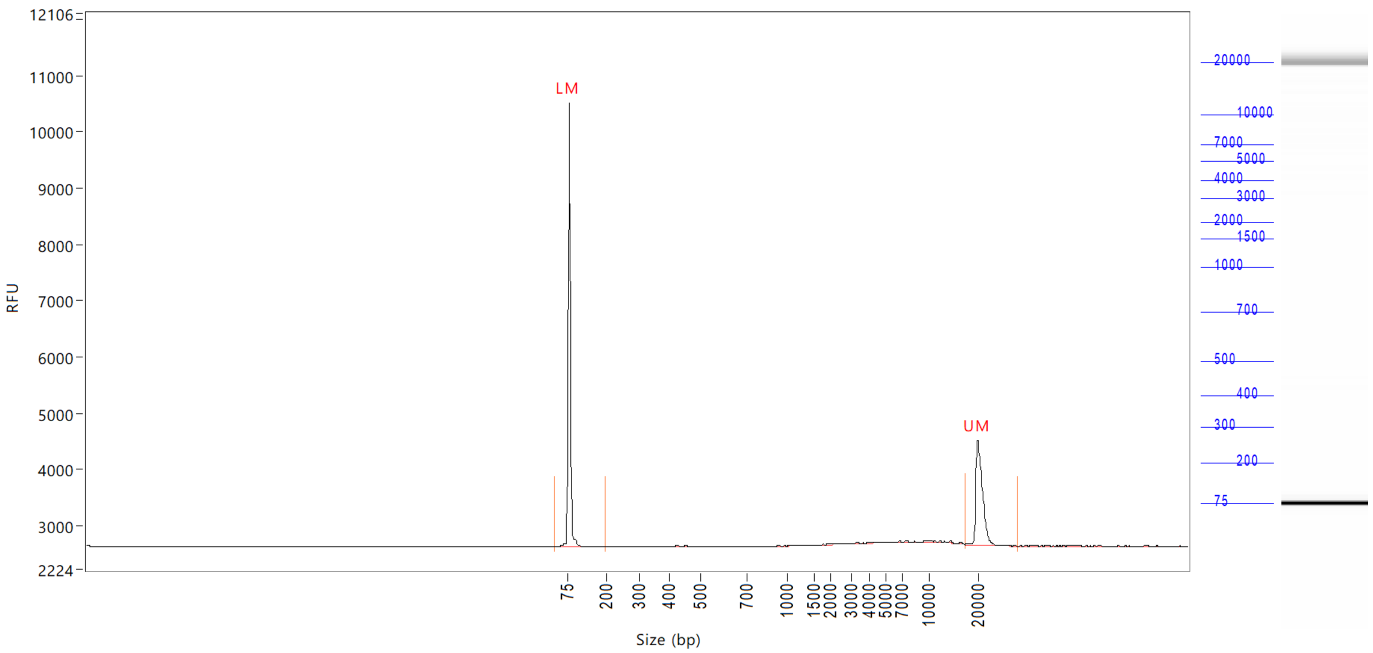

Peak	Size (bp)	Concentration (ng/uL)	From (bp)	To (bp)	Average size (bp)	CV%	RFU	Corrected peak area
1	75 (LM)	0.5000	31	194	74	11.23	7899	43.589
2	20000 (UM)	0.1867	17317	28342	20378	4.27	1869	16.272

TIC:	0.0000	ng/uL
TIM:	0.0000	nmole/L
Total concentration:	0.4494	ng/uL

Sample peak width (sec): 5 Sample min peak height: 50 Sample baseline V to V?: Y Sample baseline V to V points: 3
Sample filter: Binomial Number of points for filter: 3 Sample start region (min): 0 Sample end region (min): 30
Marker peak width (sec): 5 Marker min peak height: 200 Marker baseline V to V?: Y Marker baseline V to V points: 3
Lower marker selection: First peak > 500 RFU Upper marker selection: Last peak > 200 RFU
Ladder size (bp)75, 200, 300, 400, 500, 700, 1000, 1500, 2000, 3000, 4000, 5000, 7000, 10000, 20000
Quantification using: Lower Marker Final concentration (ng/uL): 0.5000 Dilution factor: 12.0

Sample: SampA8
Well location: A8
Created: Tuesday, September 14, 2021 4:54:05 PM

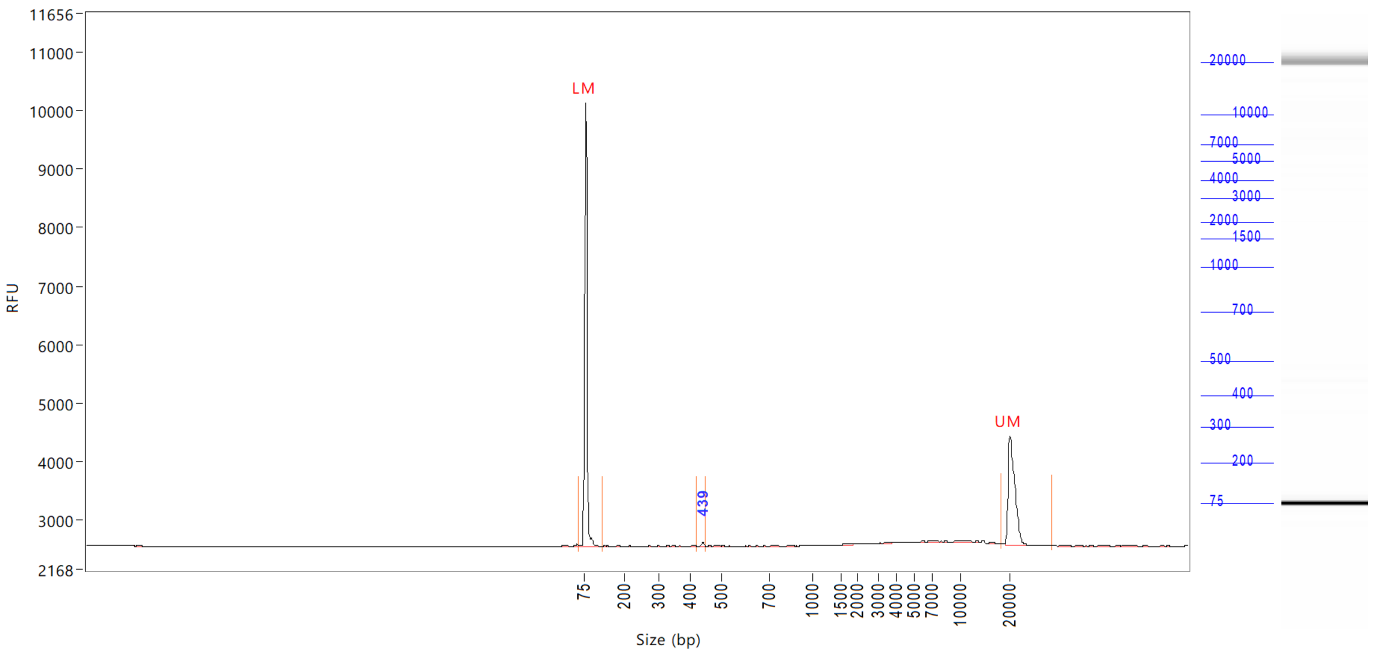

Peak	Size (bp)	Concentration (ng/uL)	From (bp)	To (bp)	Average size (bp)	CV%	RFU	Corrected peak area
1	75 (LM)	0.5000	54	126	74	6.19	7581	40.244
2	439	0.0471	422	450	438	0.99	63	0.316
3	20000 (UM)	0.1959	18293	28463	20438	3.75	1864	15.768

TIC:	0.0471	ng/uL
TIM:	0.1768	nmole/L
Total concentration:	0.5595	ng/uL

Sample peak width (sec): 5 Sample min peak height: 50 Sample baseline V to V?: Y Sample baseline V to V points: 3
Sample filter: Binomial Number of points for filter: 3 Sample start region (min): 0 Sample end region (min): 30
Marker peak width (sec): 5 Marker min peak height: 200 Marker baseline V to V?: Y Marker baseline V to V points: 3
Lower marker selection: First peak > 500 RFU Upper marker selection: Last peak > 200 RFU
Ladder size (bp)75, 200, 300, 400, 500, 700, 1000, 1500, 2000, 3000, 4000, 5000, 7000, 10000, 20000
Quantification using: Lower Marker Final concentration (ng/uL): 0.5000 Dilution factor: 12.0

Sample: SampB8
Well location: B8
Created: Tuesday, September 14, 2021 4:54:05 PM

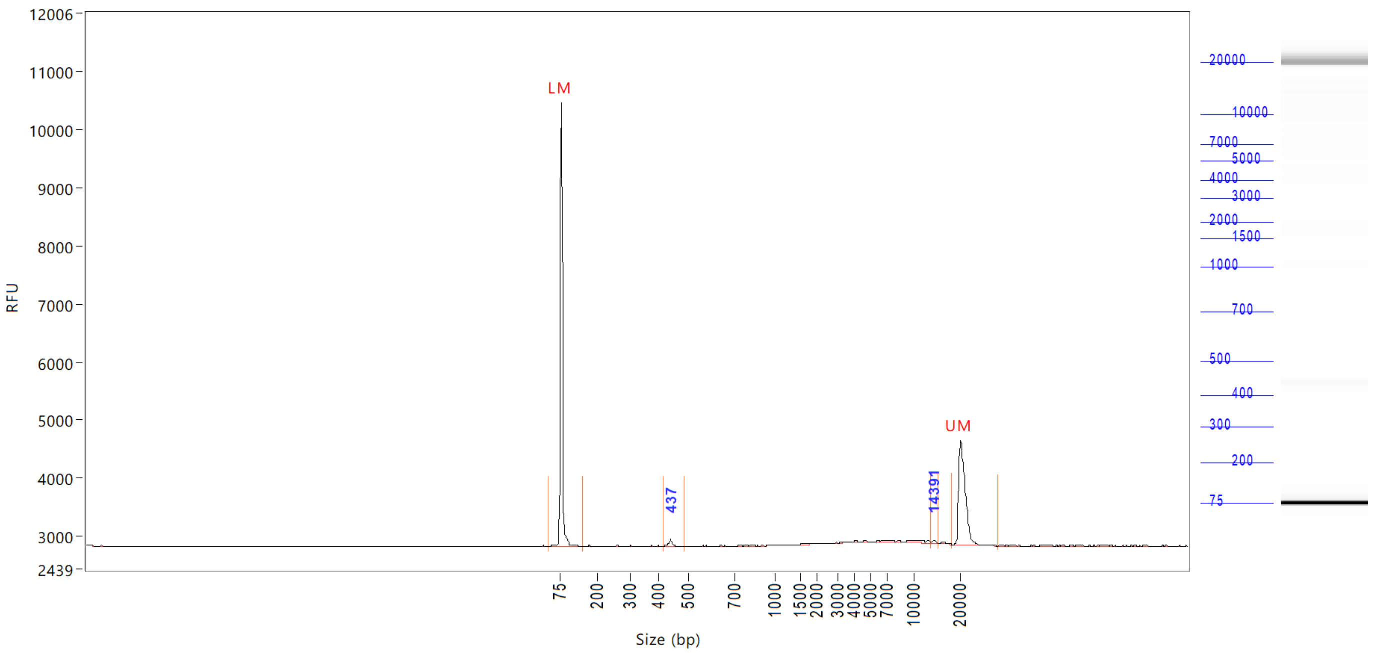

Peak	Size (bp)	Concentration (ng/uL)	From (bp)	To (bp)	Average size (bp)	CV%	RFU	Corrected peak area
1	75 (LM)	0.5000	37	150	74	8.05	7645	42.960
2	437	0.1292	412	483	436	2.61	112	0.925
3	14391	0.0425	13781	15122	14320	2.30	60	0.304
4	20000 (UM)	0.1816	18171	27859	20434	4.04	1804	15.604

TIC:	0.1717	ng/uL
TIM:	0.4924	nmole/L
Total concentration:	0.7150	ng/uL

Sample peak width (sec): 5   Sample min peak height: 50   Sample baseline V to V?: Y   Sample baseline V to V points: 3
Sample filter: Binomial   Number of points for filter: 3 Sample start region (min): 0 Sample end region (min): 30
Marker peak width (sec): 5   Marker min peak height: 200   Marker baseline V to V?: Y   Marker baseline V to V points: 3
Lower marker selection: First peak > 500 RFU   Upper marker selection: Last peak > 200 RFU
Ladder size (bp)75, 200, 300, 400, 500, 700, 1000, 1500, 2000, 3000, 4000, 5000, 7000, 10000, 20000
Quantification using: Lower Marker   Final concentration (ng/uL): 0.5000   Dilution factor: 12.0

Sample: SampC8
Well location: C8
Created: Tuesday, September 14, 2021 4:54:05 PM

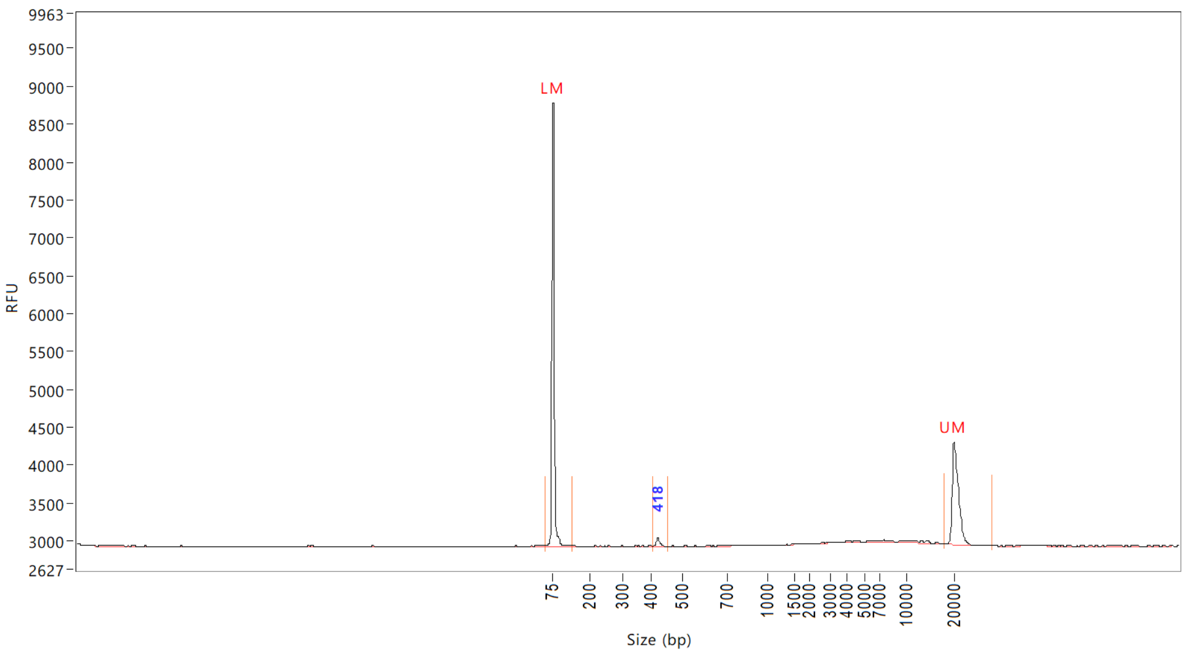

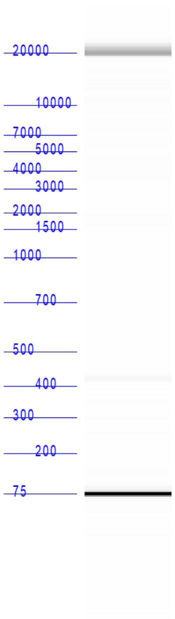

Peak	Size (bp)	Concentration (ng/uL)	From (bp)	To (bp)	Average size (bp)	CV%	RFU	Corrected peak area
1	75 (LM)	0.5000	48	139	73	8.28	5863	34.601
2	418	0.1443	403	452	418	1.59	112	0.832
3	20000 (UM)	0.1653	17927	27859	20370	4.01	1346	11.442

TIC:	0.1443	ng/uL
TIM:	0.5678	nmole/L
Total concentration:	0.7473	ng/uL

Sample peak width (sec): 5   Sample min peak height: 50   Sample baseline V to V?: Y   Sample baseline V to V points: 3
Sample filter: Binomial   Number of points for filter: 3 Sample start region (min): 0 Sample end region (min): 30
Marker peak width (sec): 5   Marker min peak height: 200   Marker baseline V to V?: Y   Marker baseline V to V points: 3
Lower marker selection: First peak > 500 RFU   Upper marker selection: Last peak > 200 RFU
Ladder size (bp)75, 200, 300, 400, 500, 700, 1000, 1500, 2000, 3000, 4000, 5000, 7000, 10000, 20000
Quantification using: Lower Marker   Final concentration (ng/uL): 0.5000   Dilution factor: 12.0

Sample: SampD8
Well location: D8
Created: Tuesday, September 14, 2021 4:54:05 PM

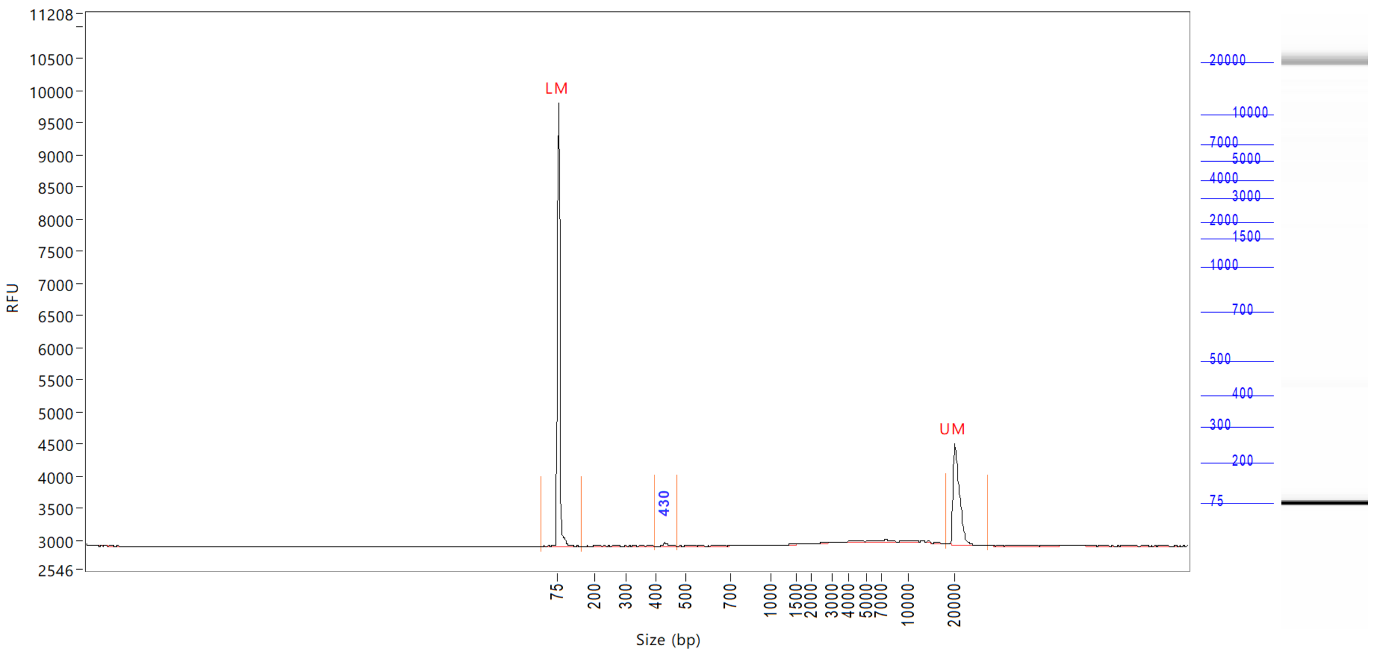

Peak	Size (bp)	Concentration (ng/uL)	From (bp)	To (bp)	Average size (bp)	CV%	RFU	Corrected peak area
1	75 (LM)	0.5000	18	152	75	8.70	6921	40.293
2	430	0.0769	396	473	432	2.69	53	0.517
3	20000 (UM)	0.1689	18049	26891	20480	4.18	1574	13.608

TIC:	0.0769	ng/uL
TIM:	0.2931	nmole/L
Total concentration:	0.5949	ng/uL

```
Sample peak width (sec): 5    Sample min peak height: 50    Sample baseline V to V?: Y    Sample baseline V to V points: 3
Sample filter: Binomial       Number of points for filter: 3  Sample start region (min): 0  Sample end region (min): 30
Marker peak width (sec): 5    Marker min peak height: 200   Marker baseline V to V?: Y    Marker baseline V to V points: 3
Lower marker selection: First peak > 500 RFU                Upper marker selection: Last peak > 200 RFU
Ladder size (bp)75, 200, 300, 400, 500, 700, 1000, 1500, 2000, 3000, 4000, 5000, 7000, 10000, 20000
Quantification using: Lower Marker          Final concentration (ng/uL): 0.5000          Dilution factor: 12.0
```

Sample: SampE8
Well location: E8
Created: Tuesday, September 14, 2021 4:54:05 PM

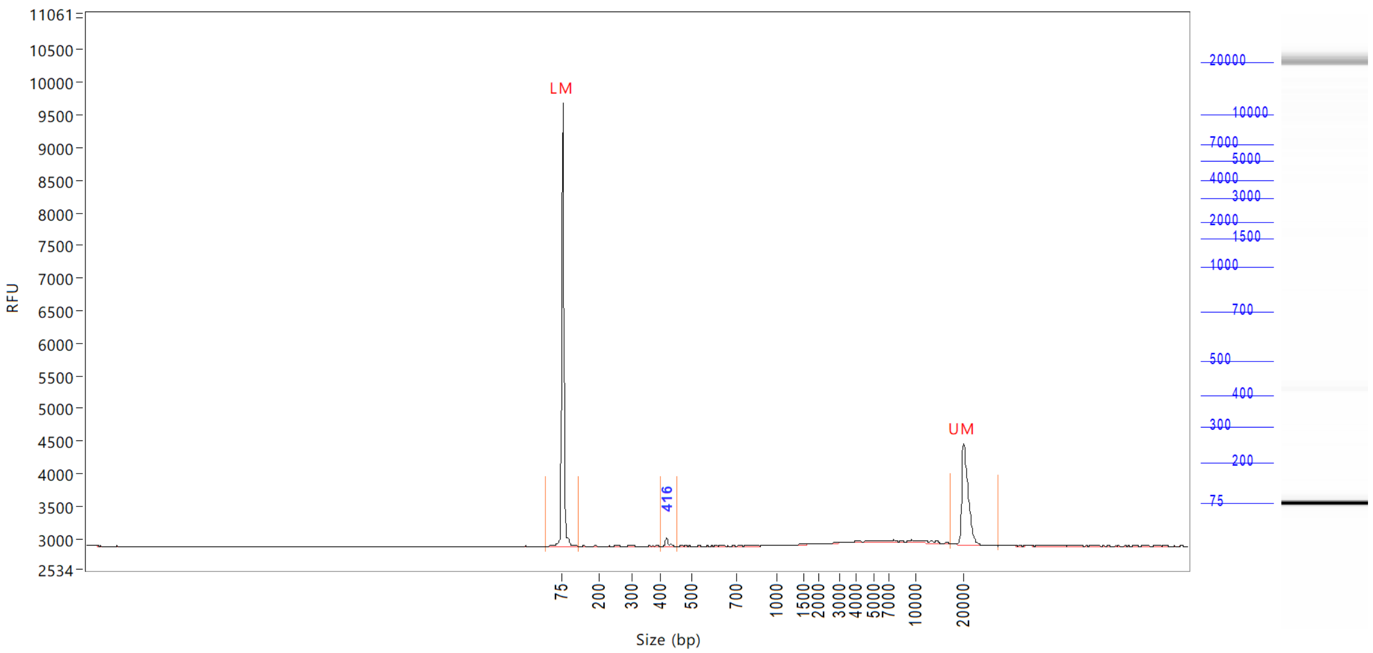

Peak	Size<br>(bp)	Concentration<br>(ng/uL)	From<br>(bp)	To<br>(bp)	Average size<br>(bp)	CV%	RFU	Corrected peak area
1	75 (LM)	0.5000	22	131	74	7.96	6815	39.184
2	416	0.1298	398	456	419	2.05	121	0.848
3	20000 (UM)	0.1723	17439	27375	20481	4.16	1559	13.504

TIC:	0.1298	ng/uL
TIM:	0.5098	nmole/L
Total concentration:	0.7681	ng/uL

```
Sample peak width (sec): 5    Sample min peak height: 50    Sample baseline V to V?: Y    Sample baseline V to V points: 3
Sample filter: Binomial       Number of points for filter: 3  Sample start region (min): 0  Sample end region (min): 30
Marker peak width (sec): 5    Marker min peak height: 200   Marker baseline V to V?: Y    Marker baseline V to V points: 3
Lower marker selection: First peak > 500 RFU                Upper marker selection: Last peak > 200 RFU
Ladder size (bp)75, 200, 300, 400, 500, 700, 1000, 1500, 2000, 3000, 4000, 5000, 7000, 10000, 20000
Quantification using: Lower Marker          Final concentration (ng/uL): 0.5000          Dilution factor: 12.0
```

Sample: SampF8
Well location: F8
Created: Tuesday, September 14, 2021 4:54:05 PM

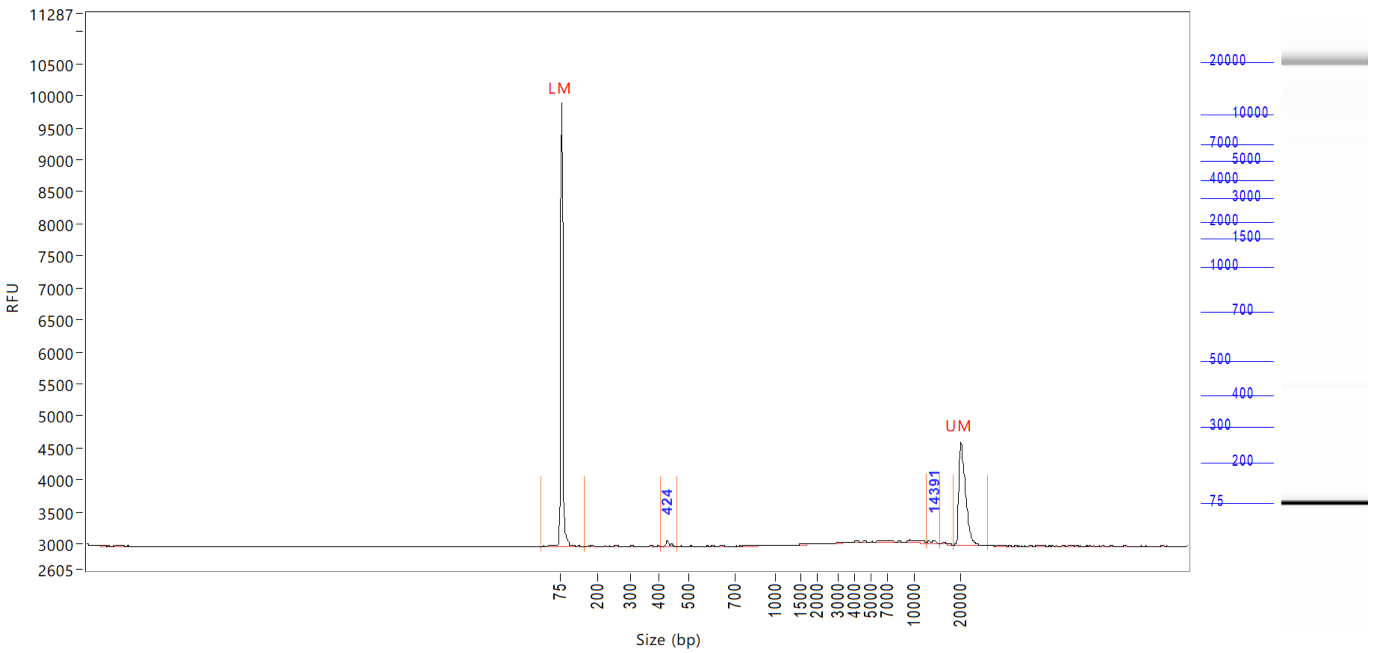

Peak	Size (bp)	Concentration (ng/uL)	From (bp)	To (bp)	Average size (bp)	CV%	RFU	Corrected peak area
1	75 (LM)	0.5000	9	152	74	8.88	6938	40.345
2	424	0.1073	403	460	427	2.19	94	0.721
3	14391	0.0804	12562	15732	13924	5.68	59	0.541
4	20000 (UM)	0.1734	18415	25682	20441	3.98	1603	13.993

TIC:	0.1876	ng/uL
TIM:	0.4228	nmole/L
Total concentration:	0.7650	ng/uL

Sample peak width (sec): 5 Sample min peak height: 50 Sample baseline V to V?: Y Sample baseline V to V points: 3
Sample filter: Binomial Number of points for filter: 3 Sample start region (min): 0 Sample end region (min): 30
Marker peak width (sec): 5 Marker min peak height: 200 Marker baseline V to V?: Y Marker baseline V to V points: 3
Lower marker selection: First peak > 500 RFU Upper marker selection: Last peak > 200 RFU
Ladder size (bp)75, 200, 300, 400, 500, 700, 1000, 1500, 2000, 3000, 4000, 5000, 7000, 10000, 20000
Quantification using: Lower Marker Final concentration (ng/uL): 0.5000 Dilution factor: 12.0

Sample: SampG8
Well location: G8
Created: Tuesday, September 14, 2021 4:54:05 PM

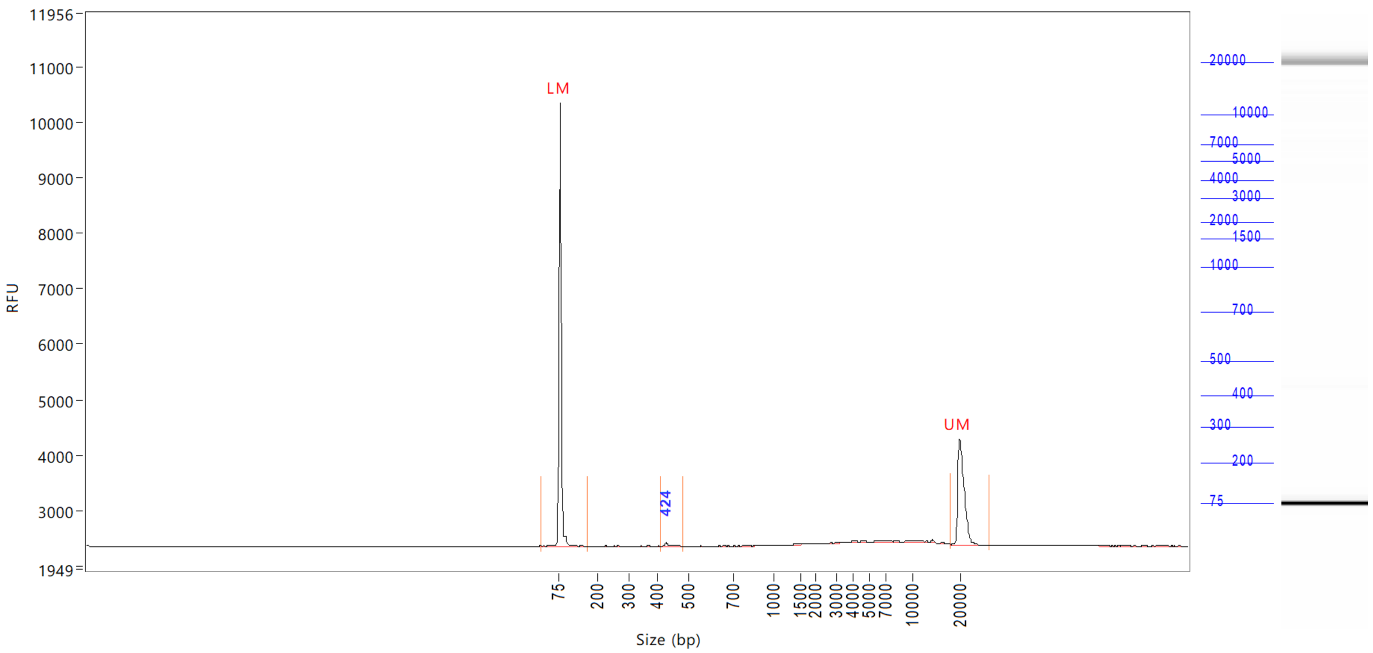

Peak	Size (bp)	Concentration (ng/uL)	From (bp)	To (bp)	Average size (bp)	CV%	RFU	Corrected peak area
1	75 (LM)	0.5000	16	168	74	10.06	7997	44.933
2	424	0.0868	407	483	432	3.50	62	0.650
3	20000 (UM)	0.1766	18171	26287	20412	3.73	1912	15.870

TIC:	0.0868	ng/uL
TIM:	0.3307	nmole/L
Total concentration:	0.4962	ng/uL

Sample peak width (sec): 5   Sample min peak height: 50   Sample baseline V to V?: Y   Sample baseline V to V points: 3
Sample filter: Binomial   Number of points for filter: 3 Sample start region (min): 0 Sample end region (min): 30
Marker peak width (sec): 5   Marker min peak height: 200   Marker baseline V to V?: Y   Marker baseline V to V points: 3
Lower marker selection: First peak > 500 RFU   Upper marker selection: Last peak > 200 RFU
Ladder size (bp)75, 200, 300, 400, 500, 700, 1000, 1500, 2000, 3000, 4000, 5000, 7000, 10000, 20000
Quantification using: Lower Marker   Final concentration (ng/uL): 0.5000   Dilution factor: 12.0

Sample: SampH8
Well location: H8
Created: Tuesday, September 14, 2021 4:54:05 PM

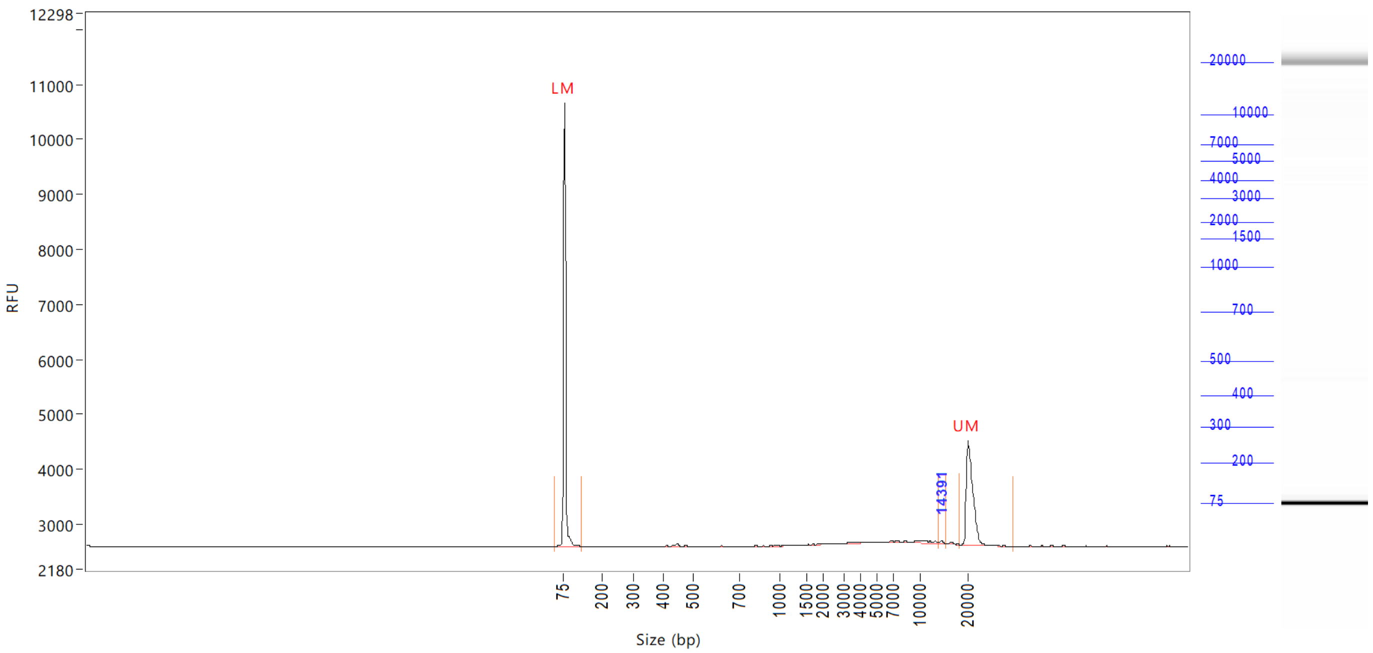

Peak	Size<br>(bp)	Concentration<br>(ng/uL)	From<br>(bp)	To<br>(bp)	Average size<br>(bp)	CV%	RFU	Corrected peak area
1	75 (LM)	0.5000	47	131	74	7.24	8088	44.885
2	14391	0.0323	13659	15488	14244	2.11	54	0.242
3	20000 (UM)	0.1823	18293	29309	20420	4.03	1901	16.369

TIC:	0.0323	ng/uL
TIM:	0.0037	nmole/L
Total concentration:	0.5996	ng/uL

Sample peak width (sec): 5   Sample min peak height: 50   Sample baseline V to V?: Y   Sample baseline V to V points: 3
Sample filter: Binomial   Number of points for filter: 3 Sample start region (min): 0 Sample end region (min): 30
Marker peak width (sec): 5   Marker min peak height: 200   Marker baseline V to V?: Y   Marker baseline V to V points: 3
Lower marker selection: First peak > 500 RFU   Upper marker selection: Last peak > 200 RFU
Ladder size (bp)75, 200, 300, 400, 500, 700, 1000, 1500, 2000, 3000, 4000, 5000, 7000, 10000, 20000
Quantification using: Lower Marker   Final concentration (ng/uL): 0.5000   Dilution factor: 12.0

Sample: SampA9
Well location: A9
Created: Tuesday, September 14, 2021 4:54:05 PM

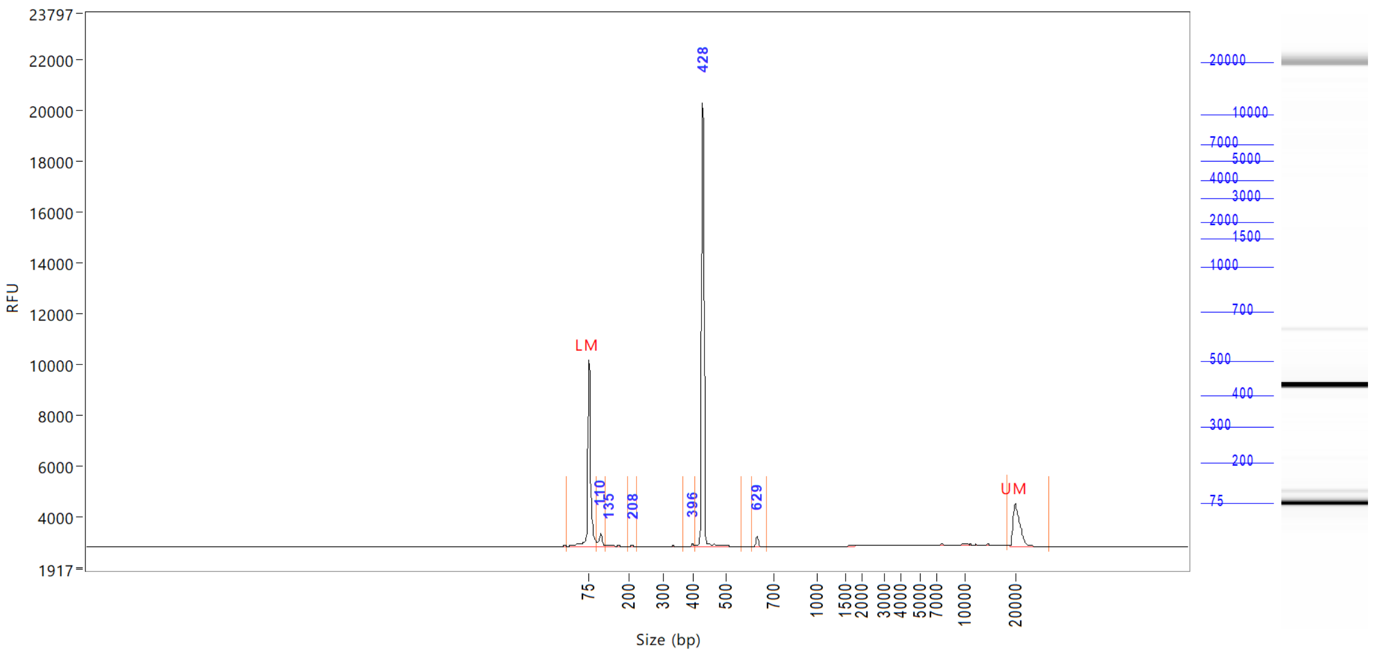

Peak	Size (bp)	Concentration (ng/uL)	From (bp)	To (bp)	Average size (bp)	CV%	RFU	Corrected peak area
1	75 (LM)	0.5000	9	101	73	13.88	7351	50.448
2	110	0.4788	101	128	110	4.89	551	4.026
3	135	0.1435	128	198	150	12.44	71	1.207
4	208	0.0368	198	223	207	2.02	52	0.309
5	396	0.0686	364	403	393	1.89	88	0.577
6	428	9.3502	403	562	428	2.22	17487	78.617
7	629	0.1824	611	667	626	0.81	381	1.533
8	20000 (UM)	0.1387	18415	26770	20357	3.78	1686	13.990

TIC:	10.2603	ng/uL
TIM:	45.7259	nmole/L
Total concentration:	10.5891	ng/uL

Sample peak width (sec): 5 Sample min peak height: 50 Sample baseline V to V?: Y Sample baseline V to V points: 3
Sample filter: Binomial Number of points for filter: 3 Sample start region (min): 0 Sample end region (min): 30
Marker peak width (sec): 5 Marker min peak height: 200 Marker baseline V to V?: Y Marker baseline V to V points: 3
Lower marker selection: First peak > 500 RFU Upper marker selection: Last peak > 200 RFU
Ladder size (bp)75, 200, 300, 400, 500, 700, 1000, 1500, 2000, 3000, 4000, 5000, 7000, 10000, 20000
Quantification using: Lower Marker Final concentration (ng/uL): 0.5000 Dilution factor: 12.0

Sample: SampB9
Well location: B9
Created: Tuesday, September 14, 2021 4:54:05 PM

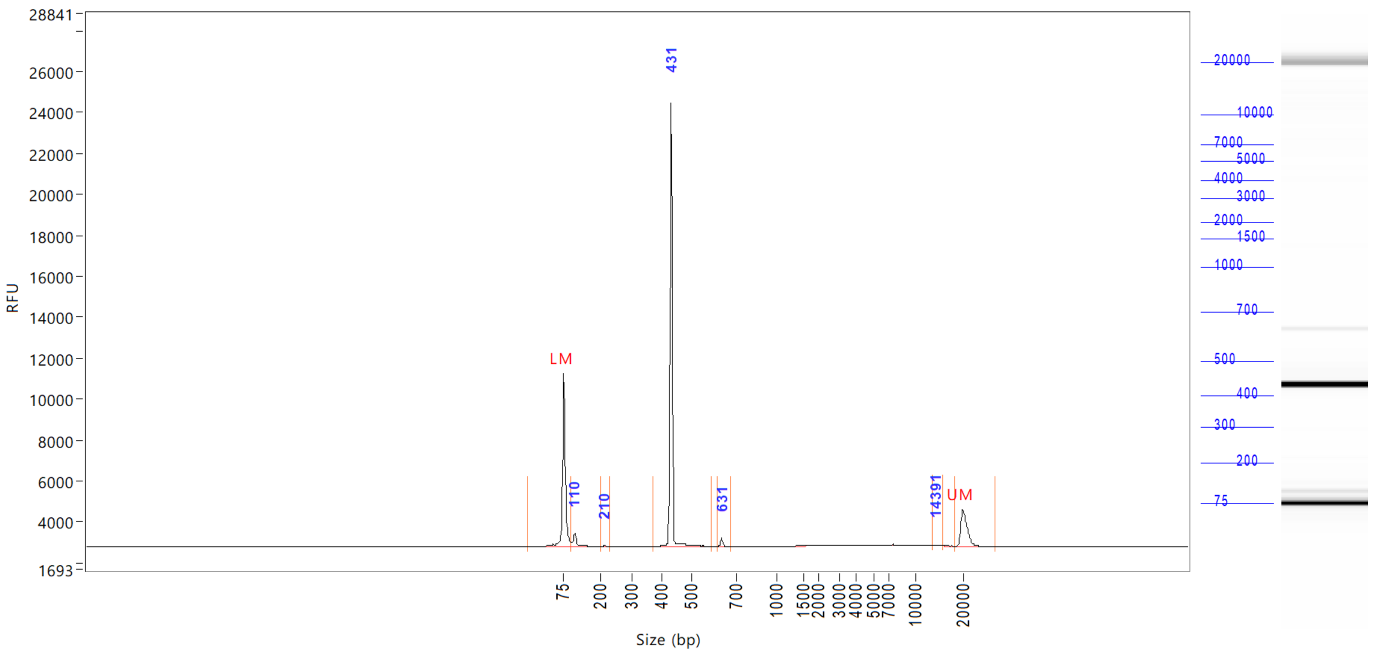

Peak	Size (bp)	Concentration (ng/uL)	From (bp)	To (bp)	Average size (bp)	CV%	RFU	Corrected peak area
1	75 (LM)	0.5000	0	101	72	16.53	8433	60.993
2	110	0.6310	101	200	119	16.74	666	6.415
3	210	0.0304	200	224	208	1.57	59	0.309
4	431	10.1526	374	585	429	2.39	21696	103.206
5	631	0.1768	613	672	628	1.00	423	1.798
6	14391	0.0360	13781	15732	14472	3.55	62	0.366
7	20000 (UM)	0.1268	18293	27012	20371	3.83	1819	15.473

TIC:	11.0269	ng/uL
TIM:	48.3583	nmole/L
Total concentration:	11.3457	ng/uL

Sample peak width (sec): 5 Sample min peak height: 50 Sample baseline V to V?: Y Sample baseline V to V points: 3
Sample filter: Binomial Number of points for filter: 3 Sample start region (min): 0 Sample end region (min): 30
Marker peak width (sec): 5 Marker min peak height: 200 Marker baseline V to V?: Y Marker baseline V to V points: 3
Lower marker selection: First peak > 500 RFU Upper marker selection: Last peak > 200 RFU
Ladder size (bp)75, 200, 300, 400, 500, 700, 1000, 1500, 2000, 3000, 4000, 5000, 7000, 10000, 20000
Quantification using: Lower Marker Final concentration (ng/uL): 0.5000 Dilution factor: 12.0

Sample: SampC9
Well location: C9
Created: Tuesday, September 14, 2021 4:54:05 PM

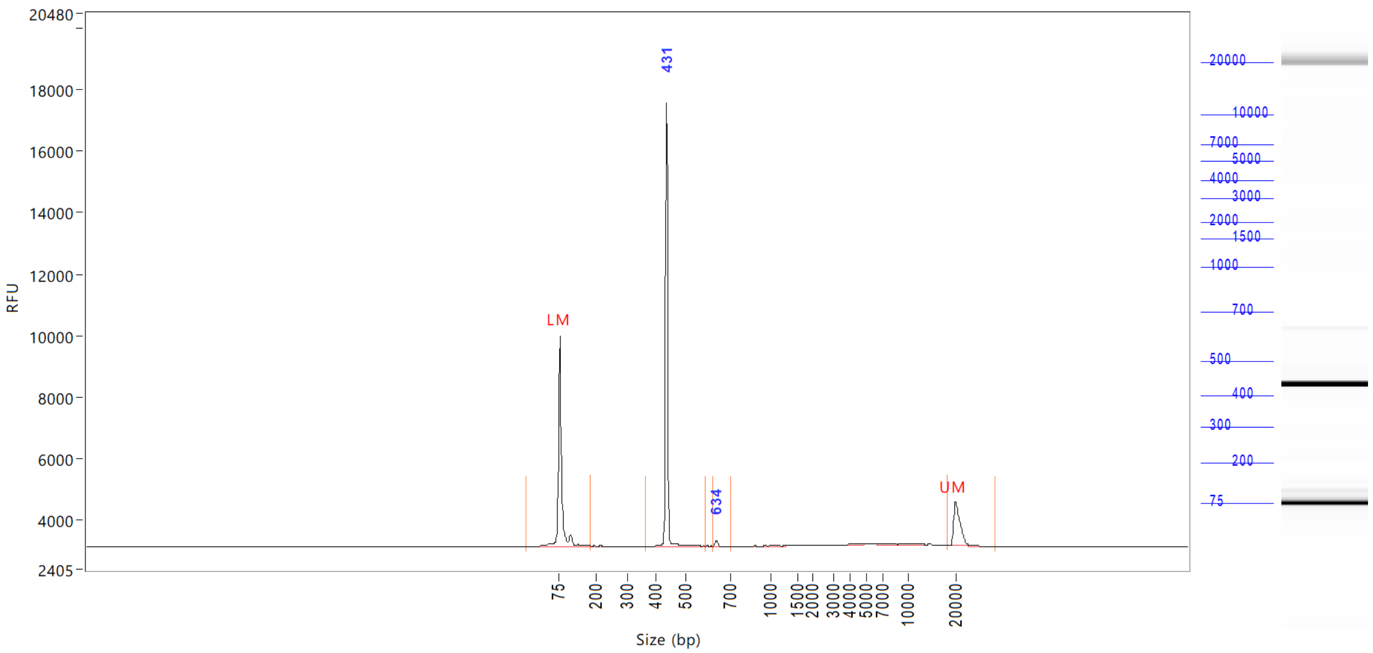

Peak	Size (bp)	Concentration (ng/uL)	From (bp)	To (bp)	Average size (bp)	CV%	RFU	Corrected peak area
1	75 (LM)	0.5000	0	181	76	20.41	6816	51.274
2	431	7.8865	360	580	431	2.82	14444	67.395
3	634	0.1138	616	697	633	1.97	196	0.972
4	20000 (UM)	0.1240	18415	28463	20523	4.80	1443	12.718

TIC:	8.0003	ng/uL
TIM:	30.4030	nmole/L
Total concentration:	8.3577	ng/uL

Sample peak width (sec): 5 Sample min peak height: 50 Sample baseline V to V?: Y Sample baseline V to V points: 3
Sample filter: Binomial Number of points for filter: 3 Sample start region (min): 0 Sample end region (min): 30
Marker peak width (sec): 5 Marker min peak height: 200 Marker baseline V to V?: Y Marker baseline V to V points: 3
Lower marker selection: First peak > 500 RFU Upper marker selection: Last peak > 200 RFU
Ladder size (bp)75, 200, 300, 400, 500, 700, 1000, 1500, 2000, 3000, 4000, 5000, 7000, 10000, 20000
Quantification using: Lower Marker Final concentration (ng/uL): 0.5000 Dilution factor: 12.0

Sample: SampD9
Well location: D9
Created: Tuesday, September 14, 2021 4:54:05 PM

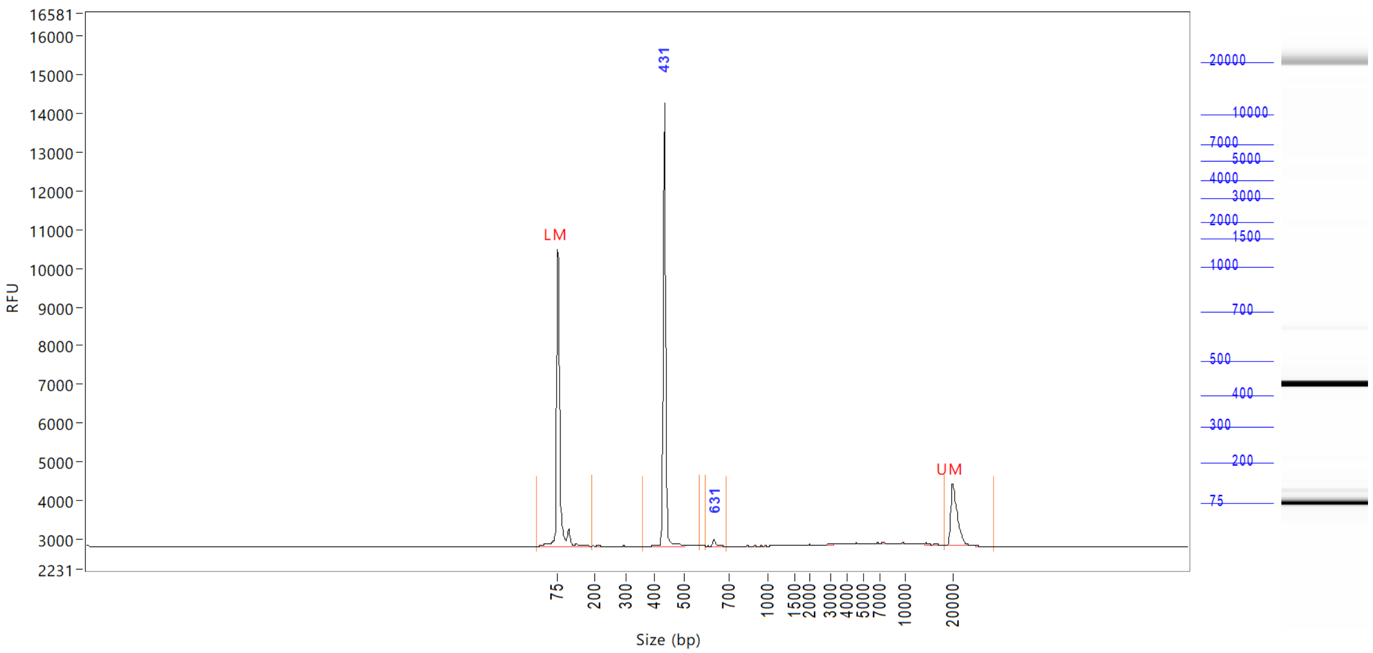

Peak	Size (bp)	Concentration (ng/uL)	From (bp)	To (bp)	Average size (bp)	CV%	RFU	Corrected peak area
1	75 (LM)	0.5000	5	192	77	19.47	7662	57.408
2	431	6.1146	358	570	431	2.13	11462	58.504
3	631	0.1018	593	687	633	1.69	178	0.974
4	20000 (UM)	0.1239	18415	28826	20554	4.36	1596	14.227

TIC:	6.2164	ng/uL
TIM:	23.6075	nmole/L
Total concentration:	6.5489	ng/uL

```
Sample peak width (sec): 5    Sample min peak height: 50    Sample baseline V to V?: Y    Sample baseline V to V points: 3
Sample filter: Binomial       Number of points for filter: 3  Sample start region (min): 0  Sample end region (min): 30
Marker peak width (sec): 5    Marker min peak height: 200   Marker baseline V to V?: Y    Marker baseline V to V points: 3
Lower marker selection: First peak > 500 RFU                Upper marker selection: Last peak > 200 RFU
Ladder size (bp)75, 200, 300, 400, 500, 700, 1000, 1500, 2000, 3000, 4000, 5000, 7000, 10000, 20000
Quantification using: Lower Marker          Final concentration (ng/uL): 0.5000          Dilution factor: 12.0
```

Sample: SampE9
Well location: E9
Created: Tuesday, September 14, 2021 4:54:05 PM

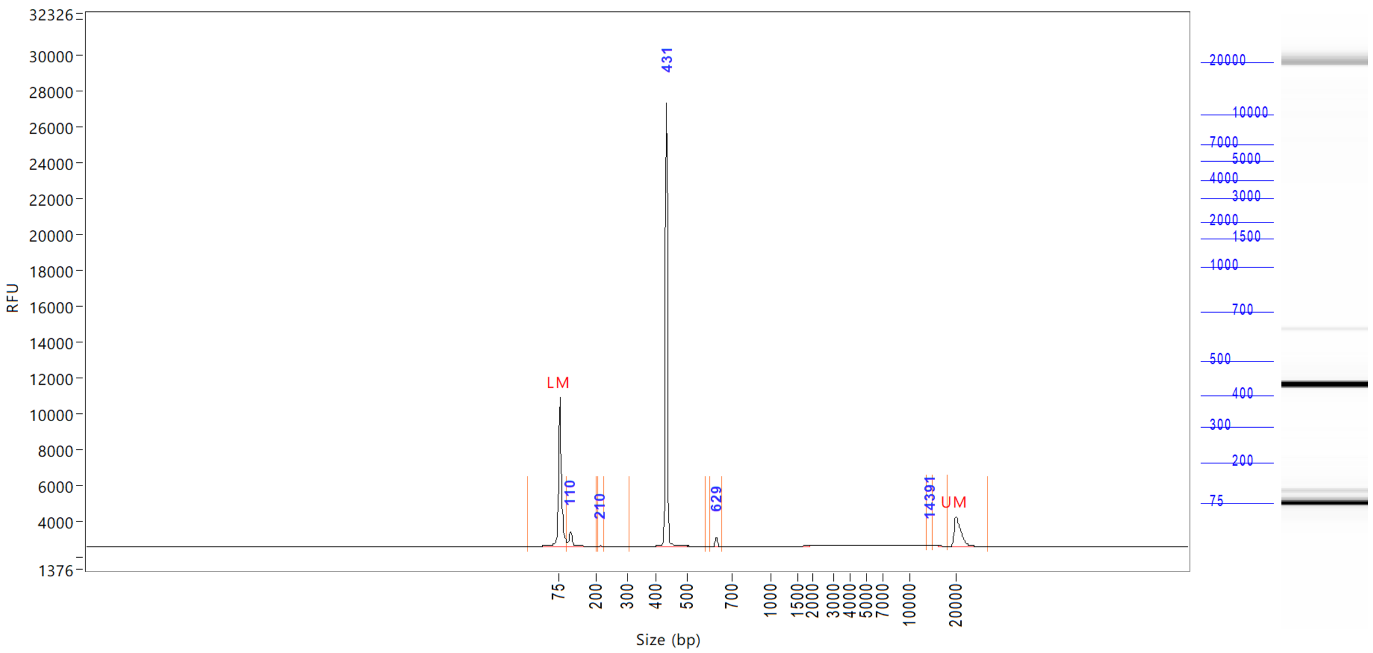

Peak	Size (bp)	Concentration (ng/uL)	From (bp)	To (bp)	Average size (bp)	CV%	RFU	Corrected peak area
1	75 (LM)	0.5000	0	101	73	16.36	8289	62.760
2	110	0.7503	101	201	118	16.01	841	7.848
3	210	0.0243	205	224	209	1.46	58	0.254
4	431	11.0312	301	580	429	2.08	24738	115.386
5	629	0.1861	603	654	627	0.75	464	1.947
6	14391	0.0248	13659	15122	14275	2.48	54	0.259
7	20000 (UM)	0.1179	18171	26770	20513	4.34	1668	14.801

TIC:	12.0166	ng/uL
TIM:	53.4354	nmole/L
Total concentration:	12.3486	ng/uL

Sample peak width (sec): 5   Sample min peak height: 50   Sample baseline V to V?: Y   Sample baseline V to V points: 3
Sample filter: Binomial   Number of points for filter: 3   Sample start region (min): 0   Sample end region (min): 30
Marker peak width (sec): 5   Marker min peak height: 200   Marker baseline V to V?: Y   Marker baseline V to V points: 3
Lower marker selection: First peak > 500 RFU   Upper marker selection: Last peak > 200 RFU
Ladder size (bp)75, 200, 300, 400, 500, 700, 1000, 1500, 2000, 3000, 4000, 5000, 7000, 10000, 20000
Quantification using: Lower Marker   Final concentration (ng/uL): 0.5000   Dilution factor: 12.0

Sample: SampF9
Well location: F9
Created: Tuesday, September 14, 2021 4:54:05 PM

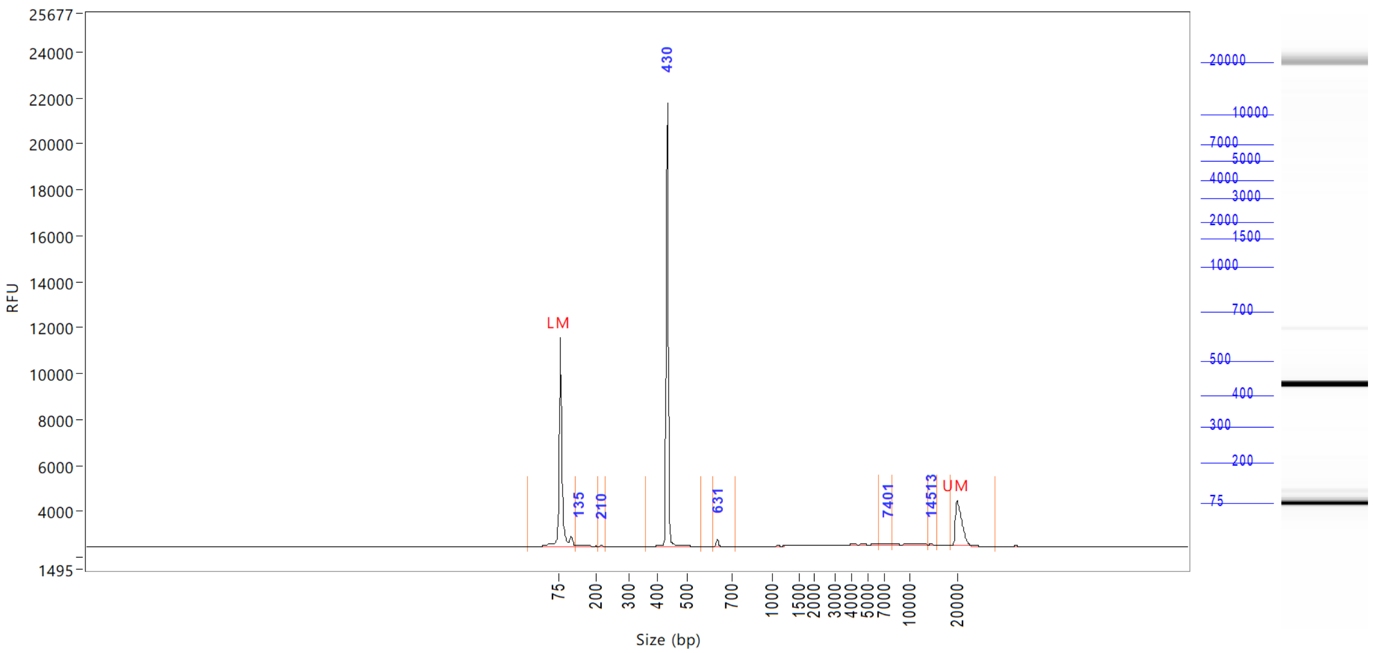

Peak	Size (bp)	Concentration (ng/uL)	From (bp)	To (bp)	Average size (bp)	CV%	RFU	Corrected peak area
1	75 (LM)	0.5000	0	128	75	17.85	9114	66.366
2	135	0.1367	128	203	153	13.51	80	1.512
3	210	0.0265	203	226	210	2.14	51	0.293
4	430	8.0009	358	557	430	2.05	19323	88.498
5	631	0.1248	613	714	632	1.96	309	1.380
6	7401	0.0471	6408	8001	7225	5.69	52	0.521
7	14513	0.0383	13659	15854	14529	3.94	66	0.424
8	20000 (UM)	0.1278	18537	28100	20469	3.93	1969	16.959

TIC:	8.3743	ng/uL
TIM:	32.6309	nmole/L
Total concentration:	8.7115	ng/uL

Sample peak width (sec): 5 Sample min peak height: 50 Sample baseline V to V?: Y Sample baseline V to V points: 3
Sample filter: Binomial Number of points for filter: 3 Sample start region (min): 0 Sample end region (min): 30
Marker peak width (sec): 5 Marker min peak height: 200 Marker baseline V to V?: Y Marker baseline V to V points: 3
Lower marker selection: First peak > 500 RFU Upper marker selection: Last peak > 200 RFU
Ladder size (bp)75, 200, 300, 400, 500, 700, 1000, 1500, 2000, 3000, 4000, 5000, 7000, 10000, 20000
Quantification using: Lower Marker Final concentration (ng/uL): 0.5000 Dilution factor: 12.0

Sample: SampG9
Well location: G9
Created: Tuesday, September 14, 2021 4:54:05 PM

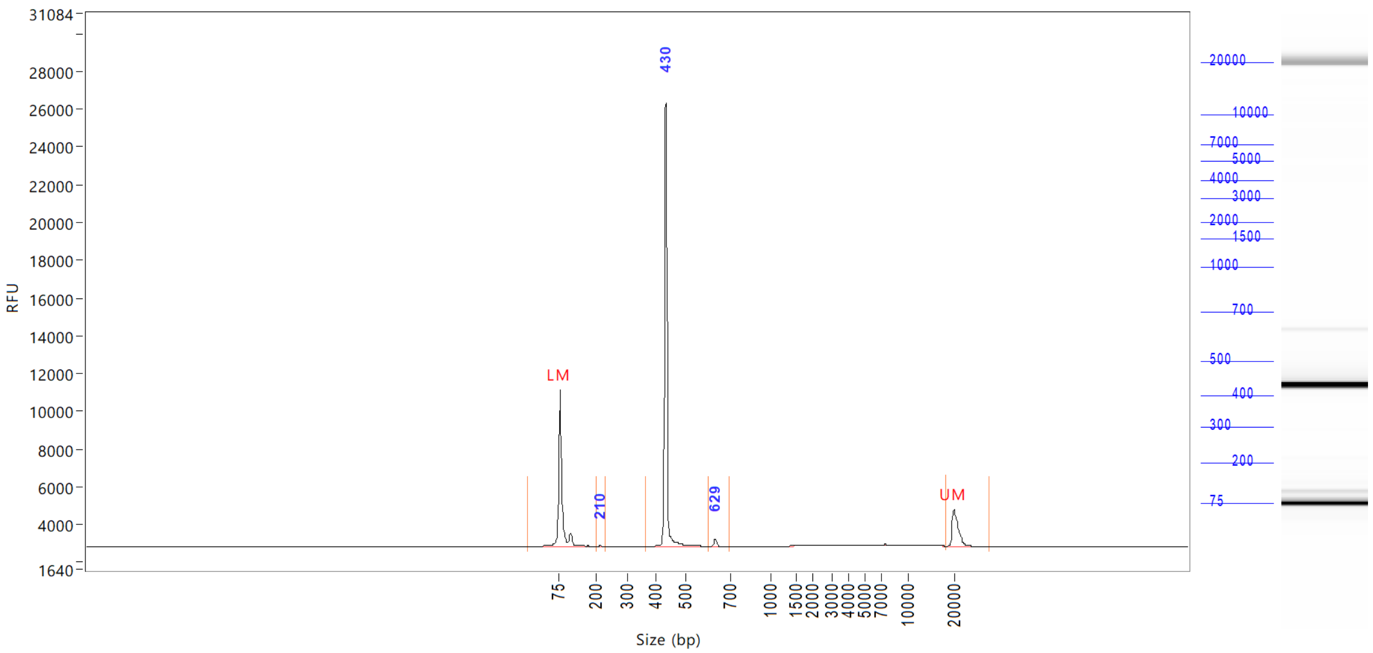

Peak	Size (bp)	Concentration (ng/uL)	From (bp)	To (bp)	Average size (bp)	CV%	RFU	Corrected peak area
1	75 (LM)	0.5000	0	200	77	25.71	8329	69.569
2	210	0.0330	200	224	207	1.94	65	0.382
3	430	11.0540	360	600	429	3.43	23537	128.170
4	629	0.1942	600	695	627	1.92	409	2.251
5	20000 (UM)	0.1153	18171	27496	20354	3.87	1941	16.046

TIC:	11.2811	ng/uL
TIM:	43.1675	nmole/L
Total concentration:	11.4889	ng/uL

Sample peak width (sec): 5 Sample min peak height: 50 Sample baseline V to V?: Y Sample baseline V to V points: 3
Sample filter: Binomial Number of points for filter: 3 Sample start region (min): 0 Sample end region (min): 30
Marker peak width (sec): 5 Marker min peak height: 200 Marker baseline V to V?: Y Marker baseline V to V points: 3
Lower marker selection: First peak > 500 RFU Upper marker selection: Last peak > 200 RFU
Ladder size (bp)75, 200, 300, 400, 500, 700, 1000, 1500, 2000, 3000, 4000, 5000, 7000, 10000, 20000
Quantification using: Lower Marker Final concentration (ng/uL): 0.5000 Dilution factor: 12.0

Sample: SampH9
Well location: H9
Created: Tuesday, September 14, 2021 4:54:05 PM

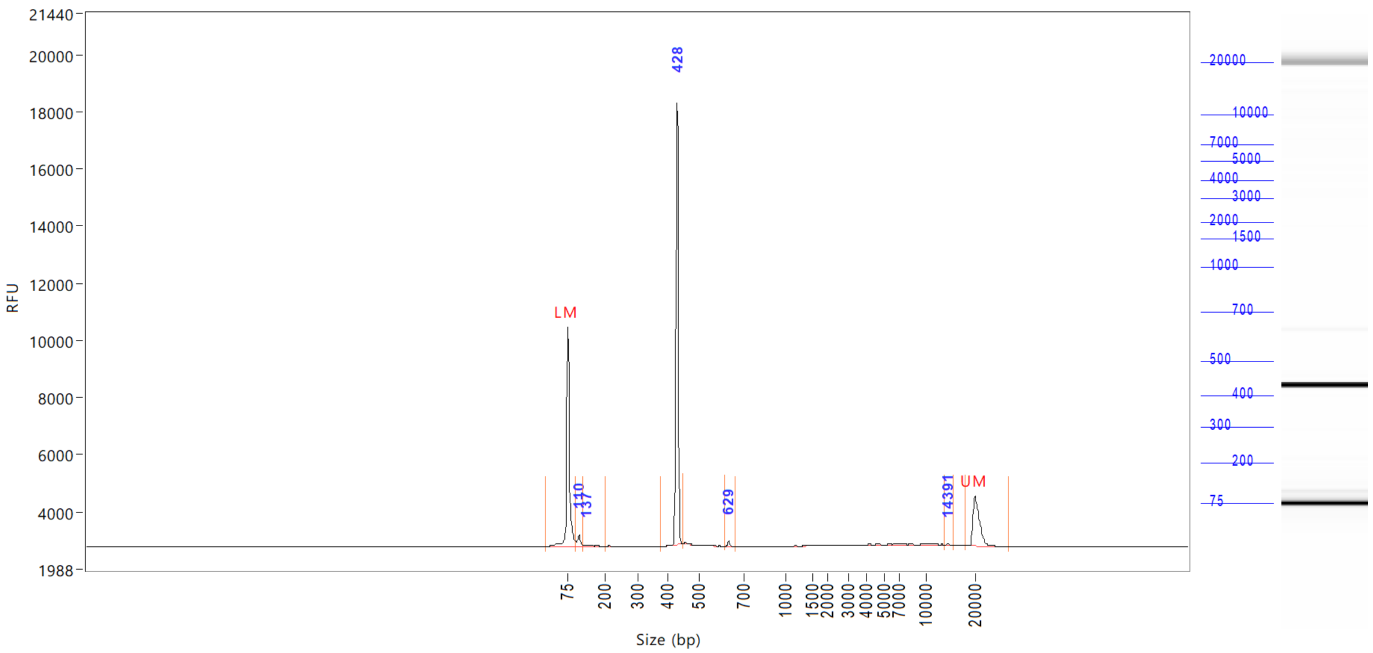

Peak	Size (bp)	Concentration (ng/uL)	From (bp)	To (bp)	Average size (bp)	CV%	RFU	Corrected peak area
1	75 (LM)	0.5000	5	101	72	13.74	7705	53.474
2	110	0.3580	101	126	109	4.92	395	3.191
3	137	0.1286	126	200	149	13.20	69	1.146
4	428	7.6521	374	449	426	0.80	15479	68.199
5	629	0.0798	611	659	626	0.86	170	0.712
6	14391	0.0290	13659	15366	14322	2.50	53	0.259
7	20000 (UM)	0.1365	17927	27012	20421	4.13	1740	14.595

TIC:	8.2476	ng/uL
TIM:	36.5815	nmole/L
Total concentration:	8.6500	ng/uL

Sample peak width (sec): 5   Sample min peak height: 50   Sample baseline V to V?: Y   Sample baseline V to V points: 3
Sample filter: Binomial   Number of points for filter: 3 Sample start region (min): 0 Sample end region (min): 30
Marker peak width (sec): 5   Marker min peak height: 200   Marker baseline V to V?: Y   Marker baseline V to V points: 3
Lower marker selection: First peak > 500 RFU   Upper marker selection: Last peak > 200 RFU
Ladder size (bp)75, 200, 300, 400, 500, 700, 1000, 1500, 2000, 3000, 4000, 5000, 7000, 10000, 20000
Quantification using: Lower Marker   Final concentration (ng/uL): 0.5000   Dilution factor: 12.0

Sample: SampA10
Well location: A10
Created: Tuesday, September 14, 2021 4:54:05 PM

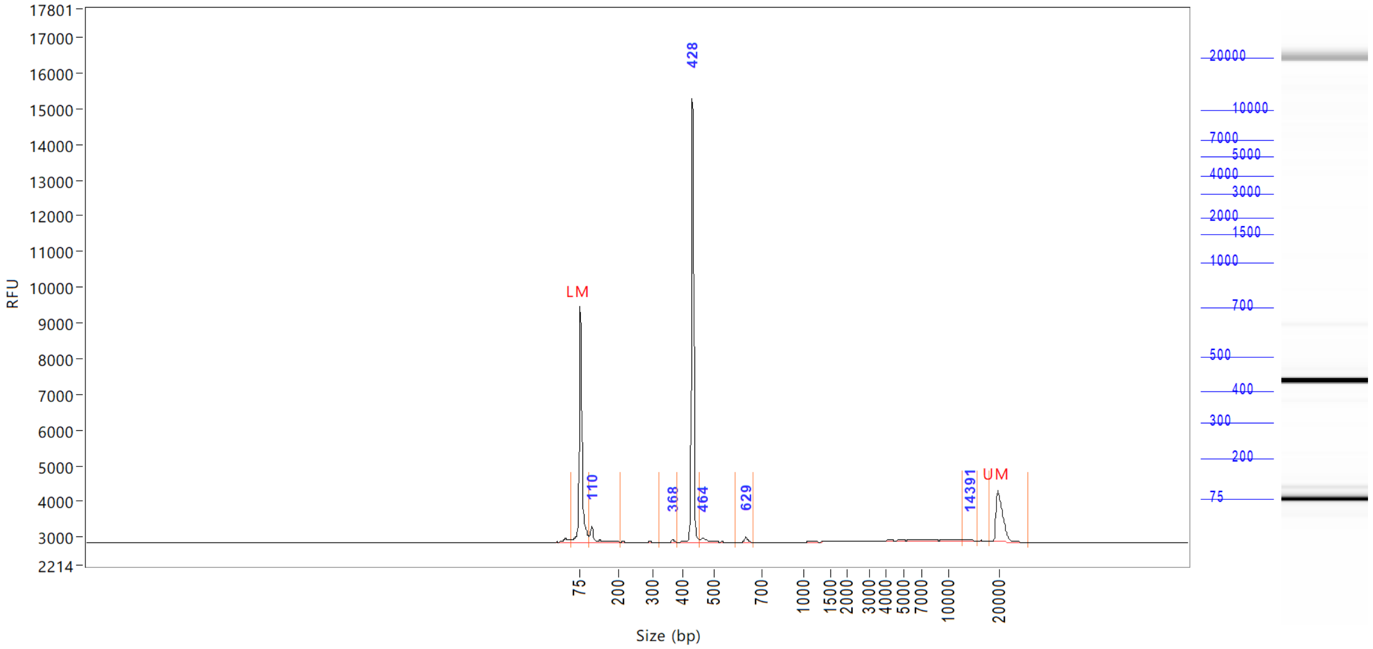

Peak	Size (bp)	Concentration (ng/uL)	From (bp)	To (bp)	Average size (bp)	CV%	RFU	Corrected peak area
1	75 (LM)	0.5000	48	101	75	8.92	6628	44.555
2	110	0.5832	101	201	119	16.86	444	4.331
3	368	0.0888	321	384	362	3.03	94	0.659
4	428	8.0012	384	454	427	1.15	12458	59.416
5	464	0.2696	454	585	485	6.52	128	2.002
6	629	0.1385	585	664	627	1.93	160	1.029
7	14391	0.0709	12806	15854	14171	5.39	59	0.527
8	20000 (UM)	0.1355	18293	26166	20361	3.82	1441	12.075

TIC:	9.1524	ng/uL
TIM:	40.5729	nmole/L
Total concentration:	9.5026	ng/uL

```
Sample peak width (sec): 5    Sample min peak height: 50    Sample baseline V to V?: Y    Sample baseline V to V points: 3
Sample filter: Binomial       Number of points for filter: 3  Sample start region (min): 0  Sample end region (min): 30
Marker peak width (sec): 5    Marker min peak height: 200   Marker baseline V to V?: Y    Marker baseline V to V points: 3
Lower marker selection: First peak > 500 RFU                Upper marker selection: Last peak > 200 RFU
Ladder size (bp)75, 200, 300, 400, 500, 700, 1000, 1500, 2000, 3000, 4000, 5000, 7000, 10000, 20000
Quantification using: Lower Marker          Final concentration (ng/uL): 0.5000          Dilution factor: 12.0
```

Sample: SampB10
Well location: B10
Created: Tuesday, September 14, 2021 4:54:05 PM

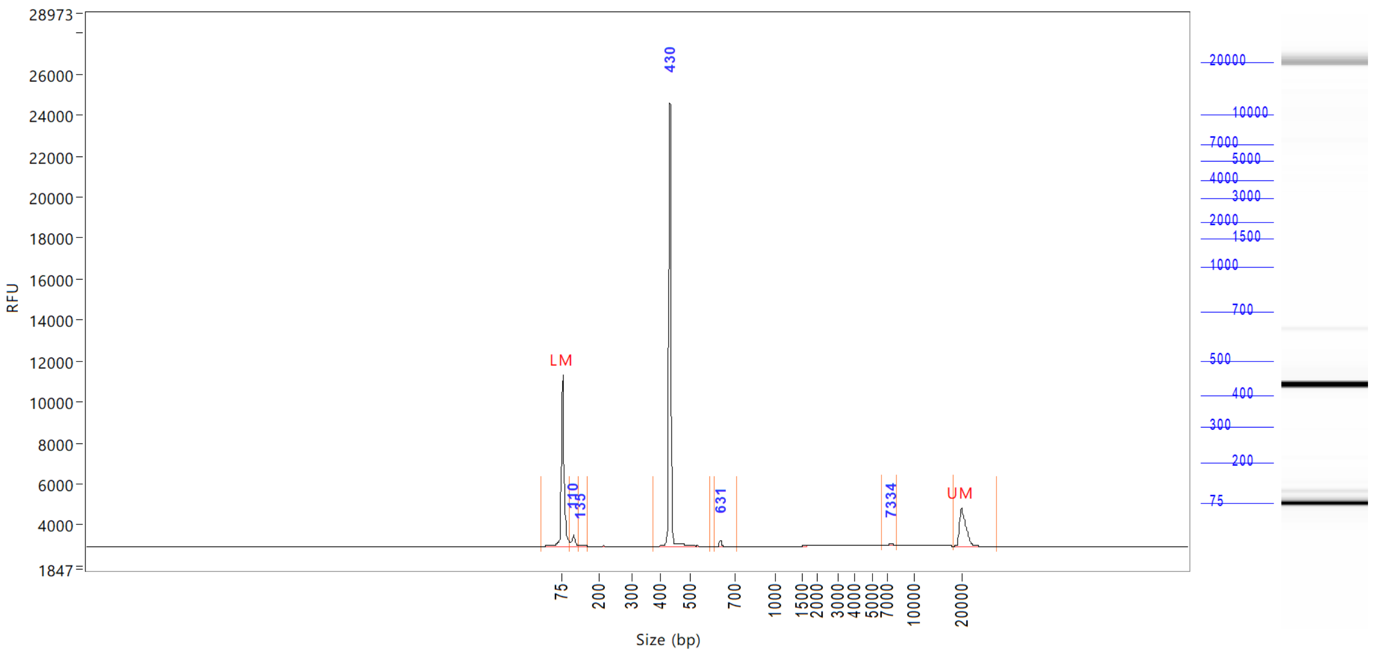

Peak	Size (bp)	Concentration (ng/uL)	From (bp)	To (bp)	Average size (bp)	CV%	RFU	Corrected peak area
1	75 (LM)	0.5000	7	101	73	13.60	8349	58.892
2	110	0.4433	101	128	110	5.00	587	4.351
3	135	0.0953	128	160	139	5.93	84	0.935
4	430	10.7982	374	583	429	2.21	21678	105.988
5	631	0.1365	603	705	629	1.42	303	1.340
6	7334	0.0686	6260	7934	7143	5.35	80	0.674
7	20000 (UM)	0.1332	18293	27375	20418	4.15	1836	15.691

TIC:	11.5419	ng/uL
TIM:	49.5336	nmole/L
Total concentration:	11.9072	ng/uL

Sample peak width (sec): 5 Sample min peak height: 50 Sample baseline V to V?: Y Sample baseline V to V points: 3
Sample filter: Binomial Number of points for filter: 3 Sample start region (min): 0 Sample end region (min): 30
Marker peak width (sec): 5 Marker min peak height: 200 Marker baseline V to V?: Y Marker baseline V to V points: 3
Lower marker selection: First peak > 500 RFU Upper marker selection: Last peak > 200 RFU
Ladder size (bp)75, 200, 300, 400, 500, 700, 1000, 1500, 2000, 3000, 4000, 5000, 7000, 10000, 20000
Quantification using: Lower Marker Final concentration (ng/uL): 0.5000 Dilution factor: 12.0

Sample: SampC10
Well location: C10
Created: Tuesday, September 14, 2021 4:54:05 PM

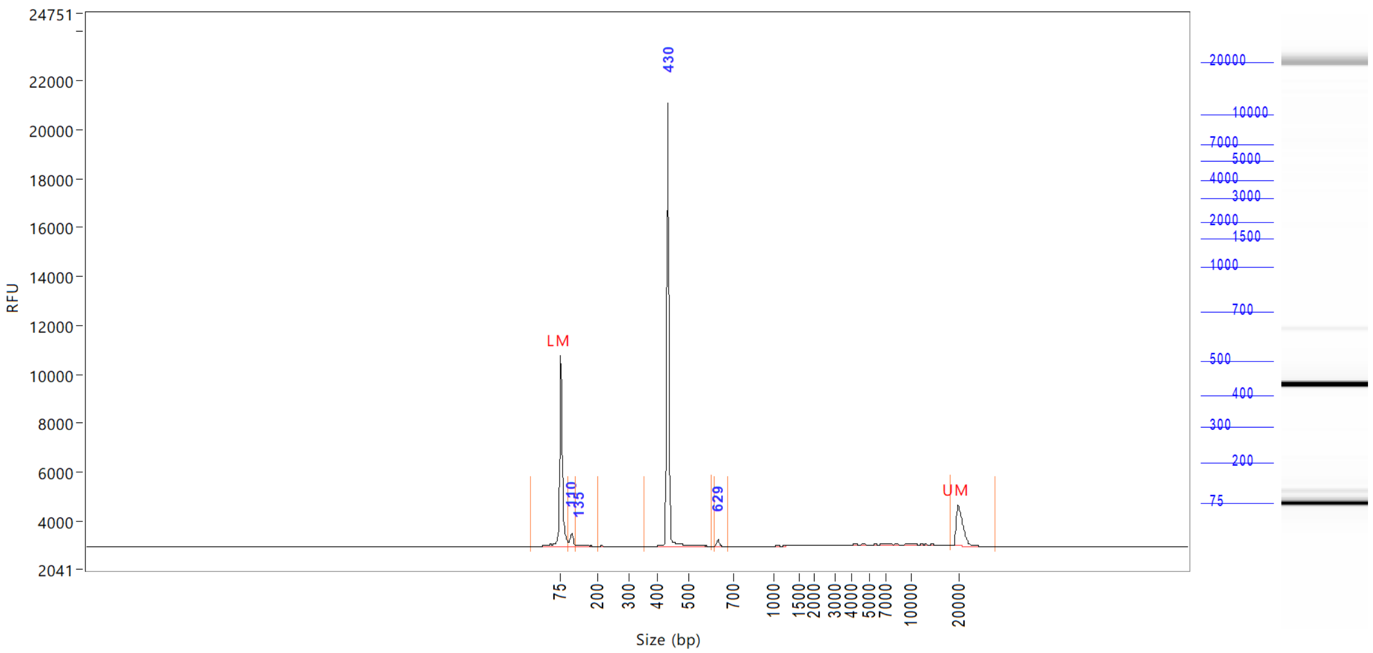

Peak	Size<br>(bp)	Concentration<br>(ng/uL)	From<br>(bp)	To<br>(bp)	Average size<br>(bp)	CV%	RFU	Corrected peak area
1	75 (LM)	0.5000	0	101	73	15.20	7838	54.814
2	110	0.4378	101	126	110	4.77	534	4.000
3	135	0.1712	126	201	152	13.57	82	1.564
4	430	9.3038	352	606	430	2.76	18146	84.997
5	629	0.1506	613	674	629	1.14	308	1.376
6	20000 (UM)	0.1333	18415	27979	20404	4.37	1693	14.609

TIC:	10.0634	ng/uL
TIM:	44.3827	nmole/L
Total concentration:	10.4357	ng/uL

Sample peak width (sec): 5 Sample min peak height: 50 Sample baseline V to V?: Y Sample baseline V to V points: 3
Sample filter: Binomial Number of points for filter: 3 Sample start region (min): 0 Sample end region (min): 30
Marker peak width (sec): 5 Marker min peak height: 200 Marker baseline V to V?: Y Marker baseline V to V points: 3
Lower marker selection: First peak > 500 RFU Upper marker selection: Last peak > 200 RFU
Ladder size (bp)75, 200, 300, 400, 500, 700, 1000, 1500, 2000, 3000, 4000, 5000, 7000, 10000, 20000
Quantification using: Lower Marker Final concentration (ng/uL): 0.5000 Dilution factor: 12.0

Sample: SampD10
Well location: D10
Created: Tuesday, September 14, 2021 4:54:05 PM

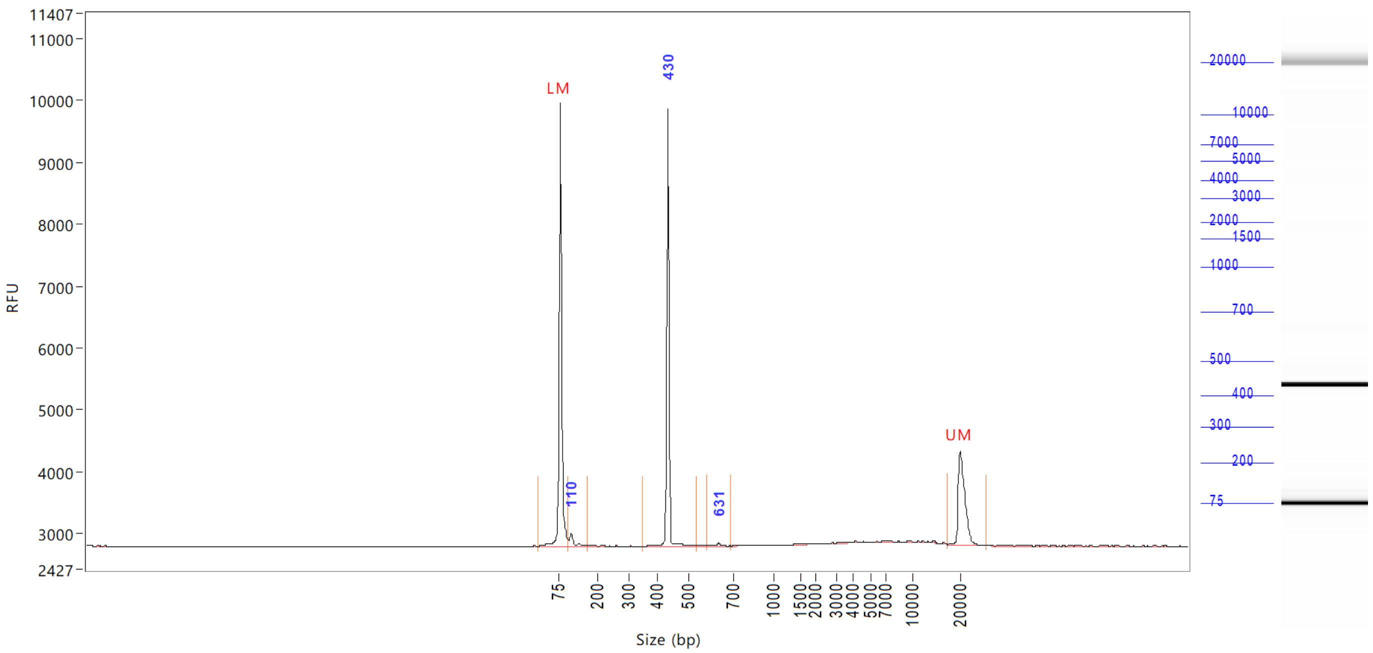

Peak	Size (bp)	Concentration (ng/uL)	From (bp)	To (bp)	Average size (bp)	CV%	RFU	Corrected peak area
1	75 (LM)	0.5000	3	103	73	12.03	7175	46.425
2	110	0.2796	103	168	118	12.92	203	2.164
3	430	4.2680	343	534	429	2.51	7059	33.024
4	631	0.0513	583	687	627	2.82	55	0.397
5	20000 (UM)	0.1417	17439	25440	20404	3.89	1507	13.159

TIC:	4.5989	ng/uL
TIM:	20.3965	nmole/L
Total concentration:	4.9909	ng/uL

Sample peak width (sec): 5   Sample min peak height: 50   Sample baseline V to V?: Y   Sample baseline V to V points: 3
Sample filter: Binomial   Number of points for filter: 3 Sample start region (min): 0 Sample end region (min): 30
Marker peak width (sec): 5   Marker min peak height: 200   Marker baseline V to V?: Y   Marker baseline V to V points: 3
Lower marker selection: First peak > 500 RFU   Upper marker selection: Last peak > 200 RFU
Ladder size (bp)75, 200, 300, 400, 500, 700, 1000, 1500, 2000, 3000, 4000, 5000, 7000, 10000, 20000
Quantification using: Lower Marker   Final concentration (ng/uL): 0.5000   Dilution factor: 12.0

Sample: SampE10
Well location: E10
Created: Tuesday, September 14, 2021 4:54:05 PM

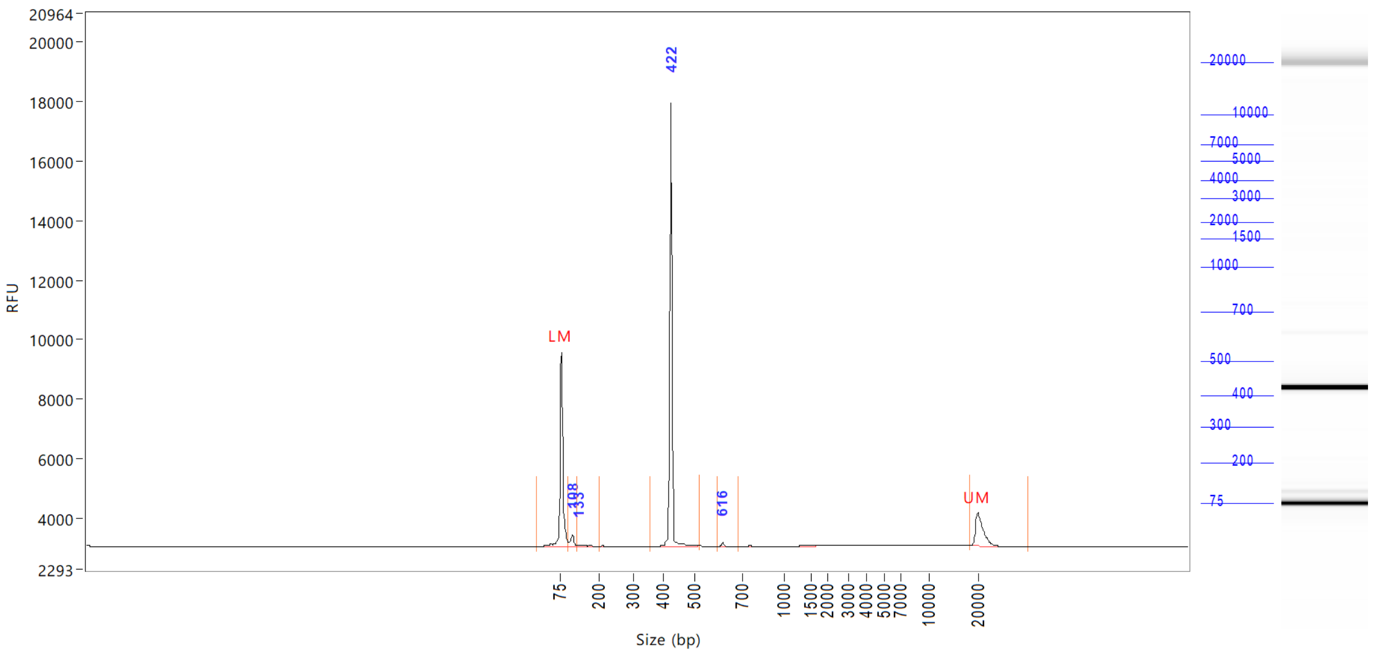

Peak	Size (bp)	Concentration (ng/uL)	From (bp)	To (bp)	Average size (bp)	CV%	RFU	Corrected peak area
1	75 (LM)	0.5000	0	99	72	13.56	6511	46.631
2	108	0.3967	99	126	108	5.31	388	3.083
3	133	0.1646	126	198	149	12.67	69	1.279
4	422	9.1560	358	519	421	1.83	14915	71.159
5	616	0.0808	595	682	616	1.94	122	0.628
6	20000 (UM)	0.1223	18293	30277	20477	5.20	1113	11.405

TIC:	9.7981	ng/uL
TIM:	43.8476	nmole/L
Total concentration:	10.2581	ng/uL

Sample peak width (sec): 5 Sample min peak height: 50 Sample baseline V to V?: Y Sample baseline V to V points: 3
Sample filter: Binomial Number of points for filter: 3 Sample start region (min): 0 Sample end region (min): 30
Marker peak width (sec): 5 Marker min peak height: 200 Marker baseline V to V?: Y Marker baseline V to V points: 3
Lower marker selection: First peak > 500 RFU Upper marker selection: Last peak > 200 RFU
Ladder size (bp)75, 200, 300, 400, 500, 700, 1000, 1500, 2000, 3000, 4000, 5000, 7000, 10000, 20000
Quantification using: Lower Marker Final concentration (ng/uL): 0.5000 Dilution factor: 12.0

Sample: SampF10
Well location: F10
Created: Tuesday, September 14, 2021 4:54:05 PM

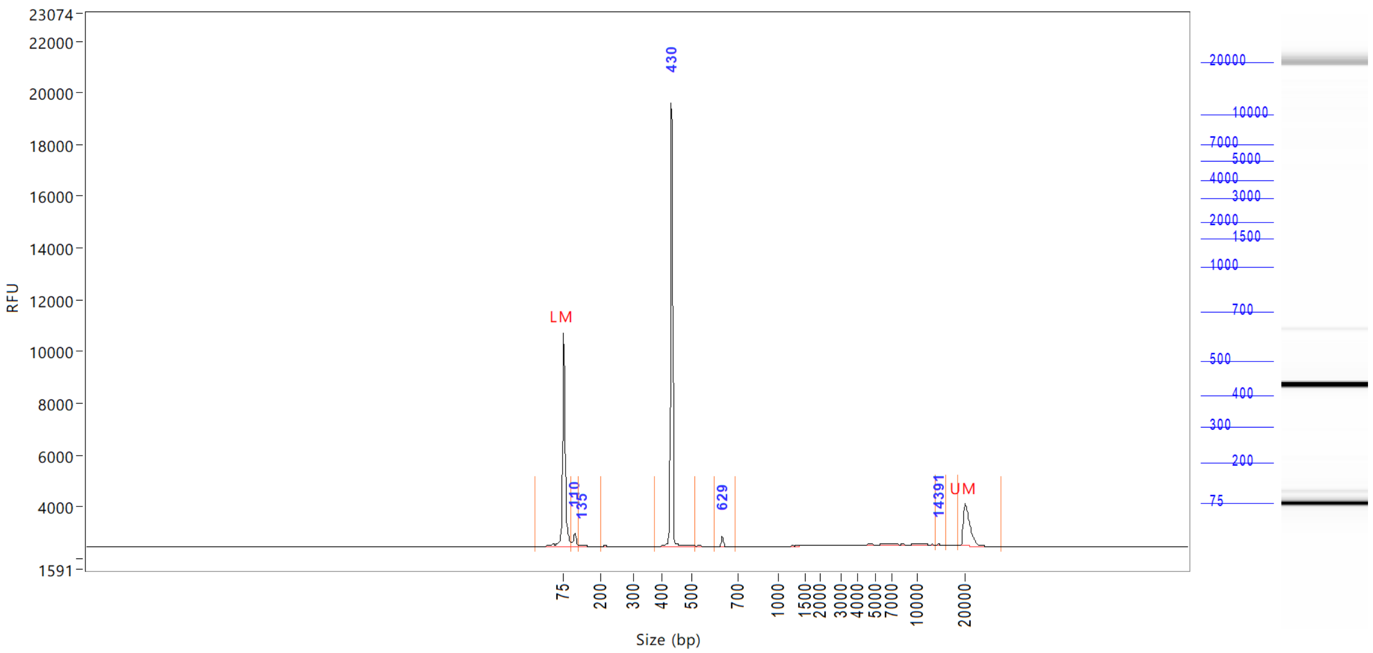

Peak	Size (bp)	Concentration (ng/uL)	From (bp)	To (bp)	Average size (bp)	CV%	RFU	Corrected peak area
1	75 (LM)	0.5000	0	101	72	15.03	8240	56.742
2	110	0.4008	101	128	110	5.18	505	3.790
3	135	0.1369	128	200	152	12.65	74	1.294
4	430	8.3945	374	511	428	1.85	17172	79.387
5	629	0.1744	595	685	628	1.57	366	1.649
6	14391	0.0299	13781	15854	14504	3.86	51	0.283
7	20000 (UM)	0.1252	18415	27738	20446	3.89	1650	14.212

TIC:	9.1364	ng/uL
TIM:	40.1955	nmole/L
Total concentration:	9.5040	ng/uL

Sample peak width (sec): 5 Sample min peak height: 50 Sample baseline V to V?: Y Sample baseline V to V points: 3
Sample filter: Binomial Number of points for filter: 3 Sample start region (min): 0 Sample end region (min): 30
Marker peak width (sec): 5 Marker min peak height: 200 Marker baseline V to V?: Y Marker baseline V to V points: 3
Lower marker selection: First peak > 500 RFU Upper marker selection: Last peak > 200 RFU
Ladder size (bp)75, 200, 300, 400, 500, 700, 1000, 1500, 2000, 3000, 4000, 5000, 7000, 10000, 20000
Quantification using: Lower Marker Final concentration (ng/uL): 0.5000 Dilution factor: 12.0

Sample: SampG10
Well location: G10
Created: Tuesday, September 14, 2021 4:54:05 PM

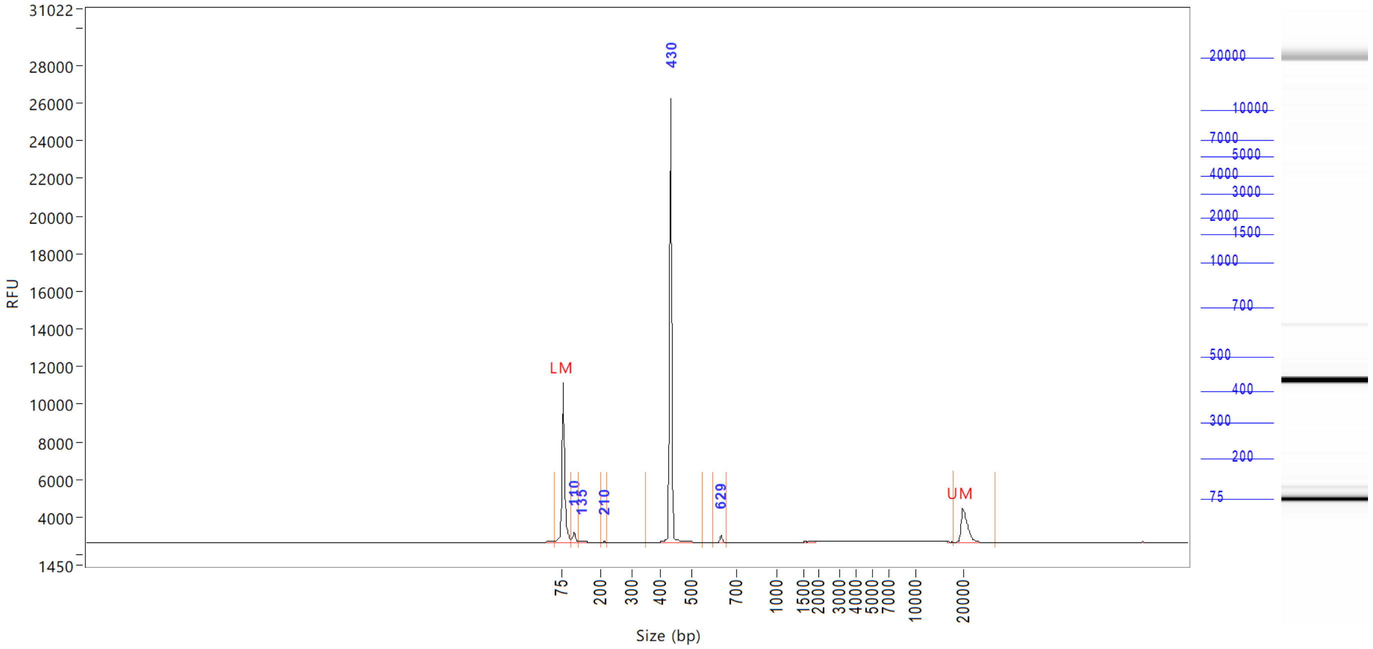

Peak	Size (bp)	Concentration (ng/uL)	From (bp)	To (bp)	Average size (bp)	CV%	RFU	Corrected peak area
1	75 (LM)	0.5000	47	101	74	9.07	8507	58.085
2	110	0.4281	101	128	109	5.13	555	4.145
3	135	0.1245	128	200	148	11.62	83	1.205
4	210	0.0279	200	221	208	1.53	56	0.270
5	430	11.2800	345	549	429	1.79	23634	109.200
6	629	0.1748	595	657	627	0.87	399	1.692
7	20000 (UM)	0.1315	18171	27012	20484	4.12	1760	15.278

TIC:	12.0353	ng/uL
TIM:	51.7761	nmole/L
Total concentration:	12.3508	ng/uL

Sample peak width (sec): 5 Sample min peak height: 50 Sample baseline V to V?: Y Sample baseline V to V points: 3
Sample filter: Binomial Number of points for filter: 3 Sample start region (min): 0 Sample end region (min): 30
Marker peak width (sec): 5 Marker min peak height: 200 Marker baseline V to V?: Y Marker baseline V to V points: 3
Lower marker selection: First peak > 500 RFU Upper marker selection: Last peak > 200 RFU
Ladder size (bp)75, 200, 300, 400, 500, 700, 1000, 1500, 2000, 3000, 4000, 5000, 7000, 10000, 20000
Quantification using: Lower Marker Final concentration (ng/uL): 0.5000 Dilution factor: 12.0

Sample: SampH10
Well location: H10
Created: Tuesday, September 14, 2021 4:54:05 PM

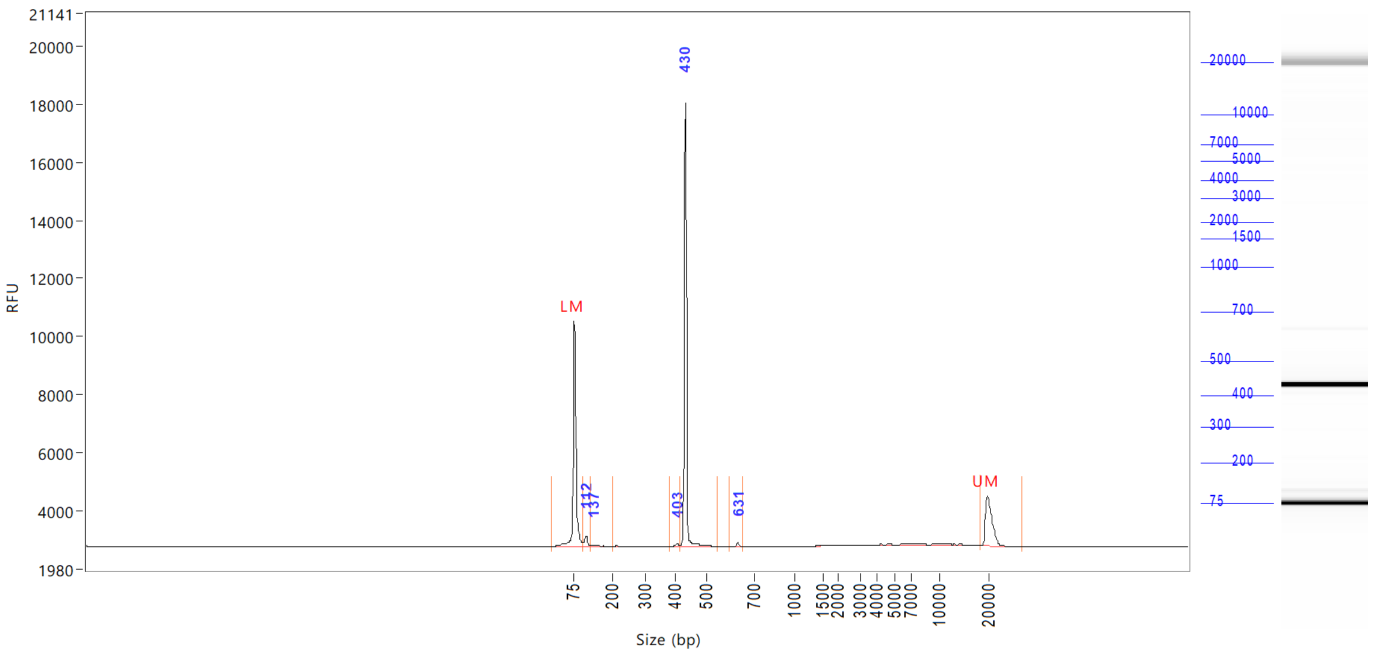

Peak	Size (bp)	Concentration (ng/uL)	From (bp)	To (bp)	Average size (bp)	CV%	RFU	Corrected peak area
1	75 (LM)	0.5000	7	101	73	13.27	7759	52.603
2	112	0.3191	101	128	110	5.15	372	2.797
3	137	0.1302	128	198	152	12.98	64	1.142
4	403	0.0904	378	414	403	1.93	98	0.792
5	430	7.8470	414	542	429	2.14	15316	68.796
6	631	0.0782	598	652	628	0.90	158	0.686
7	20000 (UM)	0.1383	18537	27133	20345	3.97	1707	14.545

TIC:	8.4649	ng/uL
TIM:	36.8424	nmole/L
Total concentration:	8.8092	ng/uL

Sample peak width (sec): 5 Sample min peak height: 50 Sample baseline V to V?: Y Sample baseline V to V points: 3
Sample filter: Binomial Number of points for filter: 3 Sample start region (min): 0 Sample end region (min): 30
Marker peak width (sec): 5 Marker min peak height: 200 Marker baseline V to V?: Y Marker baseline V to V points: 3
Lower marker selection: First peak > 500 RFU Upper marker selection: Last peak > 200 RFU
Ladder size (bp)75, 200, 300, 400, 500, 700, 1000, 1500, 2000, 3000, 4000, 5000, 7000, 10000, 20000
Quantification using: Lower Marker Final concentration (ng/uL): 0.5000 Dilution factor: 12.0

Sample: SampA11
Well location: A11
Created: Tuesday, September 14, 2021 4:54:05 PM

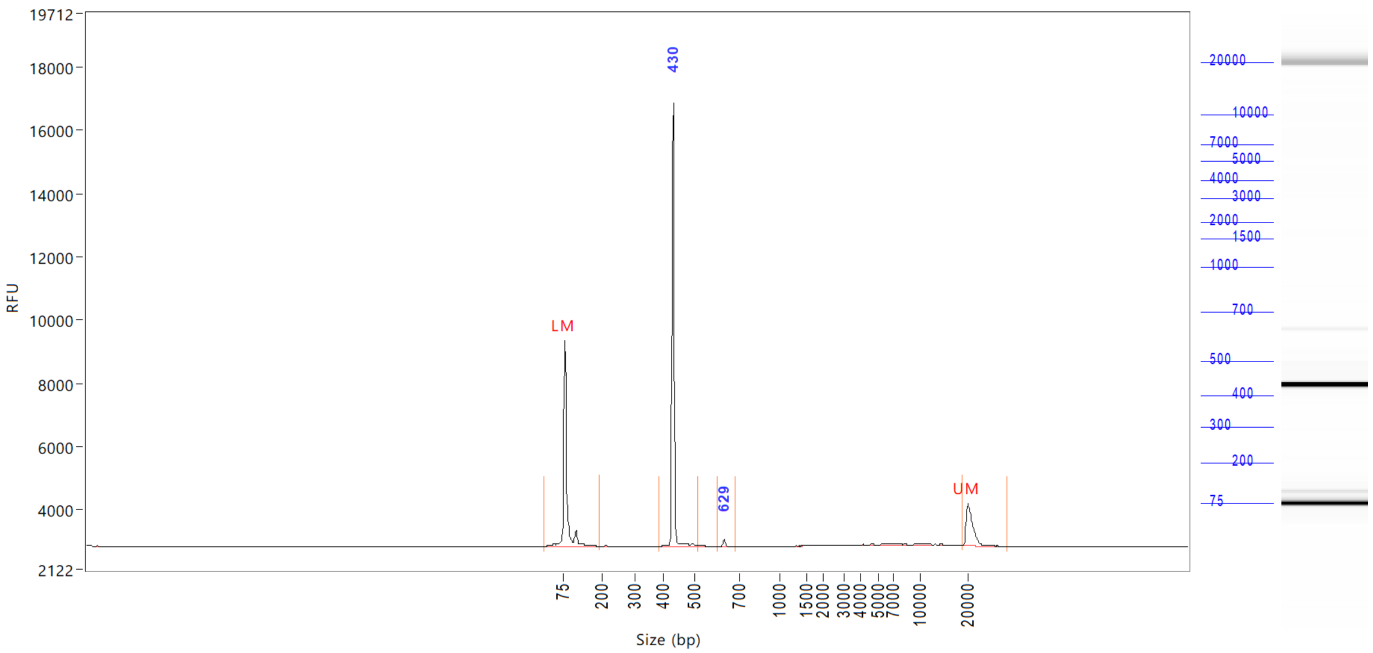

Peak	Size (bp)	Concentration (ng/uL)	From (bp)	To (bp)	Average size (bp)	CV%	RFU	Corrected peak area
1	75 (LM)	0.5000	7	189	77	21.11	6516	49.546
2	430	7.9414	384	516	429	2.41	14058	65.577
3	629	0.1403	600	682	628	1.37	252	1.158
4	20000 (UM)	0.1167	18659	28221	20474	4.89	1321	11.567

TIC:	8.0816	ng/uL
TIM:	30.8254	nmole/L
Total concentration:	8.4382	ng/uL

Sample peak width (sec): 5 Sample min peak height: 50 Sample baseline V to V?: Y Sample baseline V to V points: 3
Sample filter: Binomial Number of points for filter: 3 Sample start region (min): 0 Sample end region (min): 30
Marker peak width (sec): 5 Marker min peak height: 200 Marker baseline V to V?: Y Marker baseline V to V points: 3
Lower marker selection: First peak > 500 RFU Upper marker selection: Last peak > 200 RFU
Ladder size (bp)75, 200, 300, 400, 500, 700, 1000, 1500, 2000, 3000, 4000, 5000, 7000, 10000, 20000
Quantification using: Lower Marker Final concentration (ng/uL): 0.5000 Dilution factor: 12.0

Sample: SampB11
Well location: B11
Created: Tuesday, September 14, 2021 4:54:05 PM

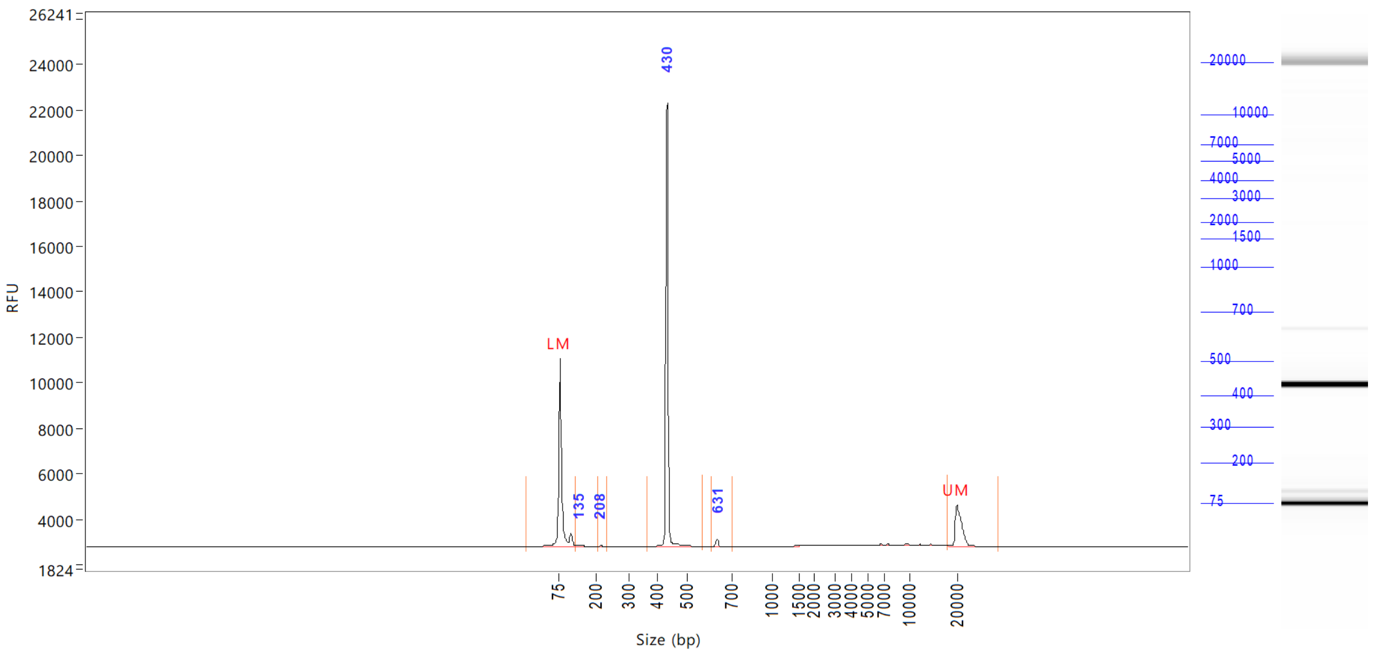

Peak	Size (bp)	Concentration (ng/uL)	From (bp)	To (bp)	Average size (bp)	CV%	RFU	Corrected peak area
1	75 (LM)	0.5000	0	126	75	19.85	8247	62.644
2	135	0.1595	126	201	151	13.62	88	1.665
3	208	0.0359	201	230	210	3.11	56	0.375
4	430	8.9328	362	567	429	1.93	19513	93.265
5	631	0.1354	608	697	629	1.29	319	1.413
6	20000 (UM)	0.1218	17927	28705	20429	4.06	1797	15.260

TIC:	9.2635	ng/uL
TIM:	36.6314	nmole/L
Total concentration:	9.5264	ng/uL

```
Sample peak width (sec): 5    Sample min peak height: 50    Sample baseline V to V?: Y    Sample baseline V to V points: 3
Sample filter: Binomial       Number of points for filter: 3  Sample start region (min): 0  Sample end region (min): 30
Marker peak width (sec): 5    Marker min peak height: 200   Marker baseline V to V?: Y    Marker baseline V to V points: 3
Lower marker selection: First peak > 500 RFU                Upper marker selection: Last peak > 200 RFU
Ladder size (bp)75, 200, 300, 400, 500, 700, 1000, 1500, 2000, 3000, 4000, 5000, 7000, 10000, 20000
Quantification using: Lower Marker          Final concentration (ng/uL): 0.5000          Dilution factor: 12.0
```

Sample: SampC11
Well location: C11
Created: Tuesday, September 14, 2021 4:54:05 PM

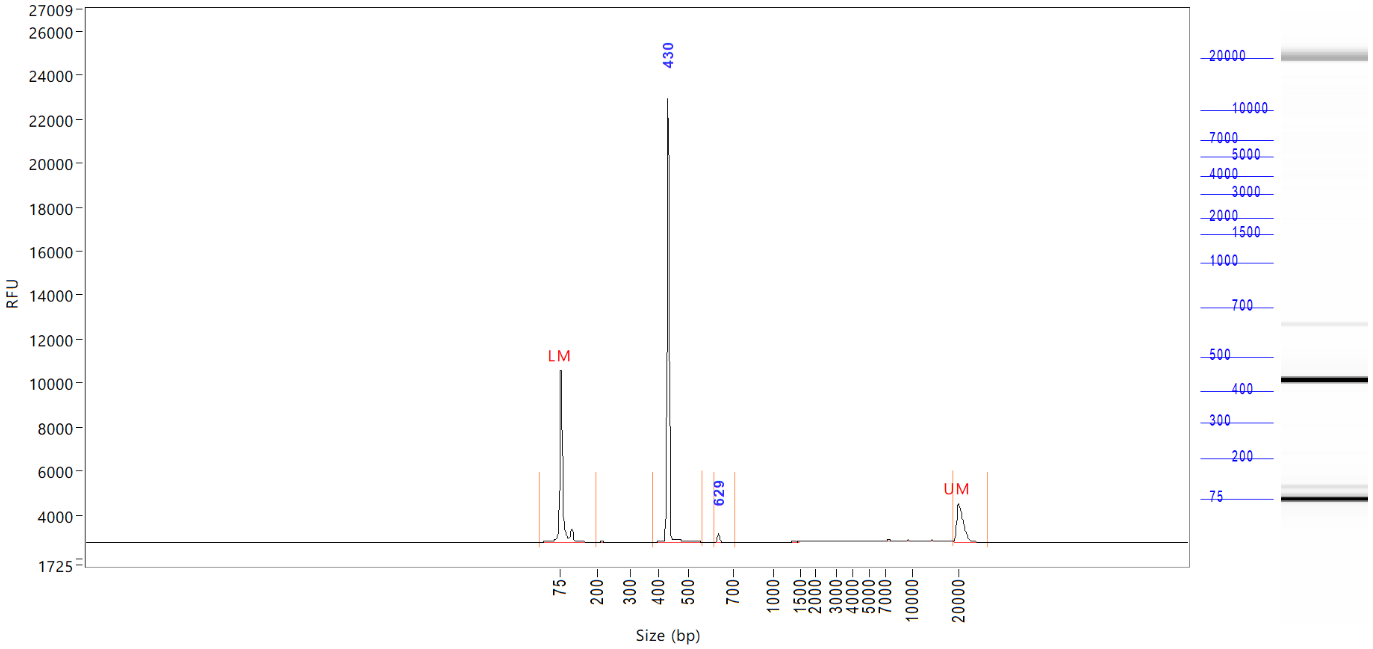

Peak	Size (bp)	Concentration (ng/uL)	From (bp)	To (bp)	Average size (bp)	CV%	RFU	Corrected peak area
1	75 (LM)	0.5000	5	194	76	21.42	7812	60.473
2	430	9.1899	382	557	429	1.99	20202	92.623
3	629	0.1843	611	714	629	1.29	426	1.857
4	20000 (UM)	0.1223	18659	25924	20403	3.78	1739	14.795

TIC:	9.3741	ng/uL
TIM:	35.7284	nmole/L
Total concentration:	9.6669	ng/uL

Sample peak width (sec): 5 Sample min peak height: 50 Sample baseline V to V?: Y Sample baseline V to V points: 3
Sample filter: Binomial Number of points for filter: 3 Sample start region (min): 0 Sample end region (min): 30
Marker peak width (sec): 5 Marker min peak height: 200 Marker baseline V to V?: Y Marker baseline V to V points: 3
Lower marker selection: First peak > 500 RFU Upper marker selection: Last peak > 200 RFU
Ladder size (bp)75, 200, 300, 400, 500, 700, 1000, 1500, 2000, 3000, 4000, 5000, 7000, 10000, 20000
Quantification using: Lower Marker Final concentration (ng/uL): 0.5000 Dilution factor: 12.0

Sample: SampD11
Well location: D11
Created: Tuesday, September 14, 2021 4:54:05 PM

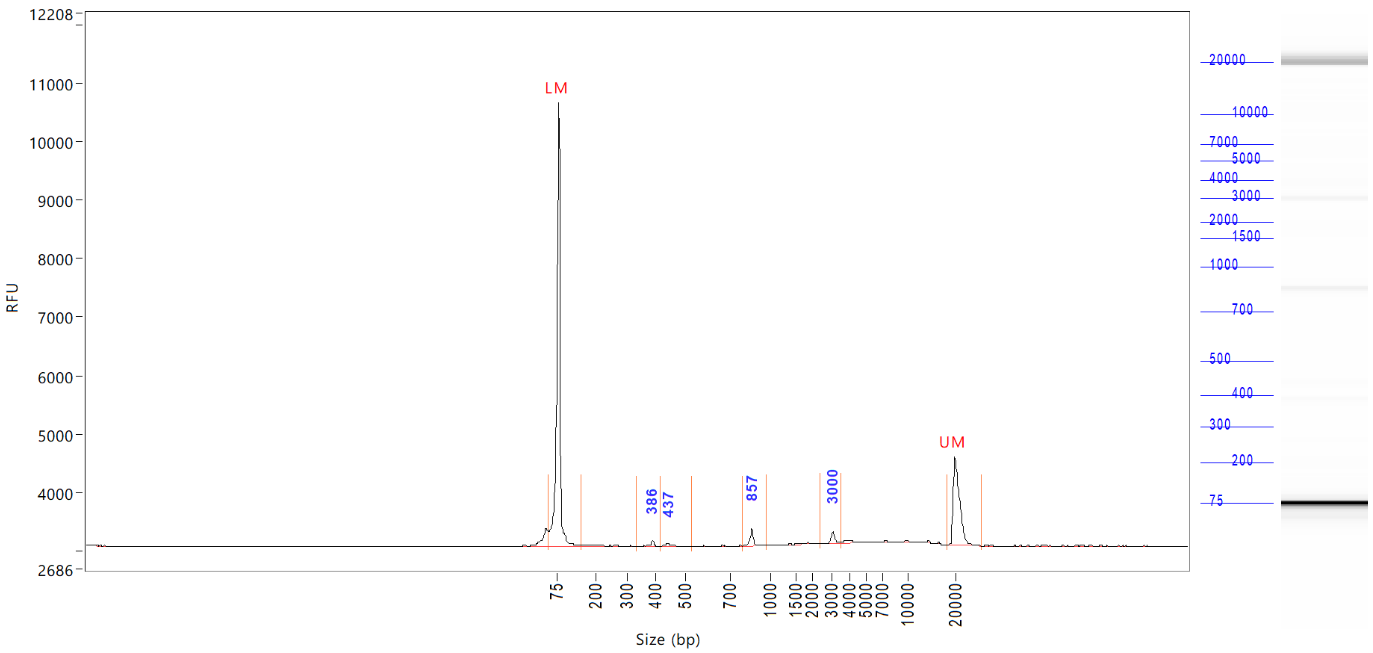

Peak	Size (bp)	Concentration (ng/uL)	From (bp)	To (bp)	Average size (bp)	CV%	RFU	Corrected peak area
1	75 (LM)	0.5000	43	152	71	14.22	7605	58.561
2	386	0.0679	333	414	380	3.48	91	0.662
3	437	0.0599	414	524	442	4.68	52	0.585
4	857	0.1891	788	970	854	3.06	302	1.846
5	3000	0.1204	2401	3465	2992	4.92	208	1.175
6	20000 (UM)	0.1110	18293	25561	20422	4.07	1500	13.005

TIC:	0.4373	ng/uL
TIM:	0.9476	nmole/L
Total concentration:	0.8103	ng/uL

Sample peak width (sec): 5 Sample min peak height: 50 Sample baseline V to V?: Y Sample baseline V to V points: 3
Sample filter: Binomial Number of points for filter: 3 Sample start region (min): 0 Sample end region (min): 30
Marker peak width (sec): 5 Marker min peak height: 200 Marker baseline V to V?: Y Marker baseline V to V points: 3
Lower marker selection: First peak > 500 RFU Upper marker selection: Last peak > 200 RFU
Ladder size (bp)75, 200, 300, 400, 500, 700, 1000, 1500, 2000, 3000, 4000, 5000, 7000, 10000, 20000
Quantification using: Lower Marker Final concentration (ng/uL): 0.5000 Dilution factor: 12.0

Sample: SampE11
Well location: E11
Created: Tuesday, September 14, 2021 4:54:05 PM

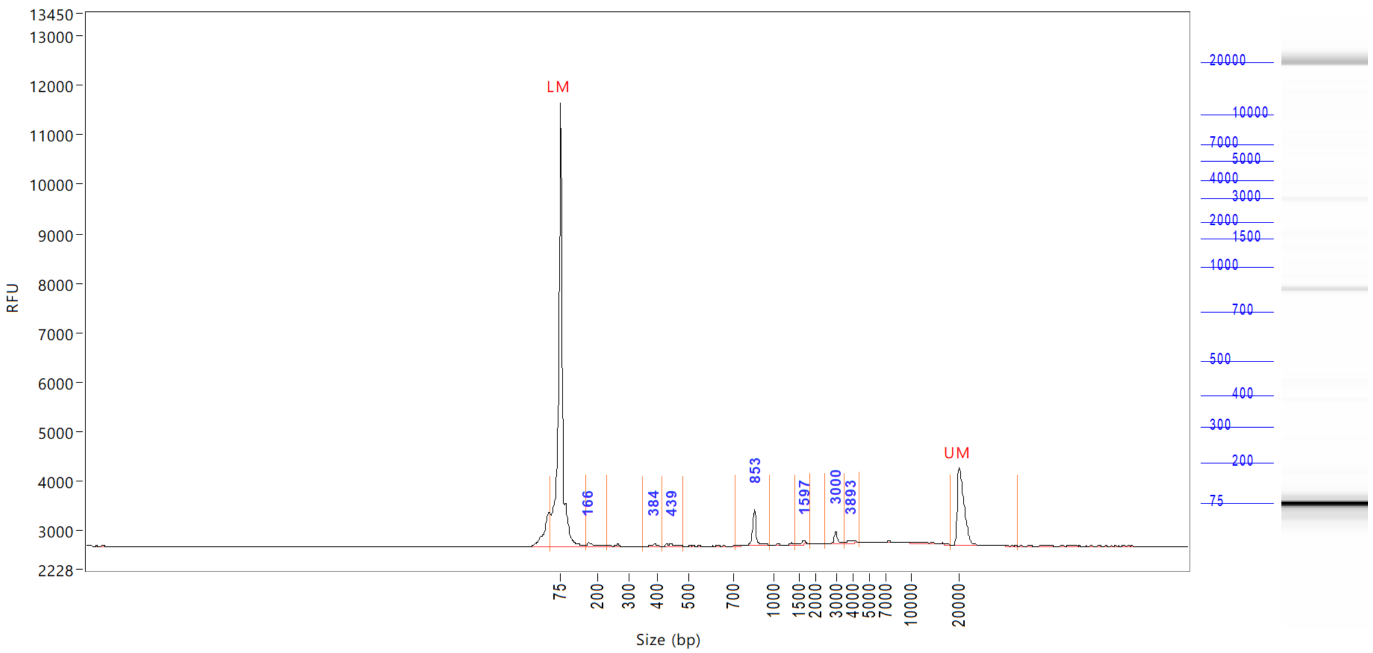

Peak	Size (bp)	Concentration (ng/uL)	From (bp)	To (bp)	Average size (bp)	CV%	RFU	Corrected peak area
1	75 (LM)	0.5000	43	158	72	18.17	8967	87.191
2	166	0.0668	158	230	177	9.75	77	0.971
3	384	0.0353	343	412	381	3.23	64	0.513
4	439	0.0537	412	483	440	3.41	66	0.781
5	853	0.3112	710	965	852	3.23	728	4.523
6	1597	0.0423	1411	1846	1593	5.52	74	0.615
7	3000	0.0948	2486	3465	2984	4.75	247	1.377
8	3893	0.0442	3465	4380	3911	5.60	50	0.642
9	20000 (UM)	0.0796	18293	32574	20559	5.12	1563	13.883

TIC:	0.6484	ng/uL
TIM:	1.6898	nmole/L
Total concentration:	0.8526	ng/uL

Sample peak width (sec): 5   Sample min peak height: 50   Sample baseline V to V?: Y   Sample baseline V to V points: 3
Sample filter: Binomial   Number of points for filter: 3 Sample start region (min): 0 Sample end region (min): 30
Marker peak width (sec): 5   Marker min peak height: 200   Marker baseline V to V?: Y   Marker baseline V to V points: 3
Lower marker selection: First peak > 500 RFU   Upper marker selection: Last peak > 200 RFU
Ladder size (bp)75, 200, 300, 400, 500, 700, 1000, 1500, 2000, 3000, 4000, 5000, 7000, 10000, 20000
Quantification using: Lower Marker   Final concentration (ng/uL): 0.5000   Dilution factor: 12.0

Sample: SampF11
Well location: F11
Created: Tuesday, September 14, 2021 4:54:05 PM

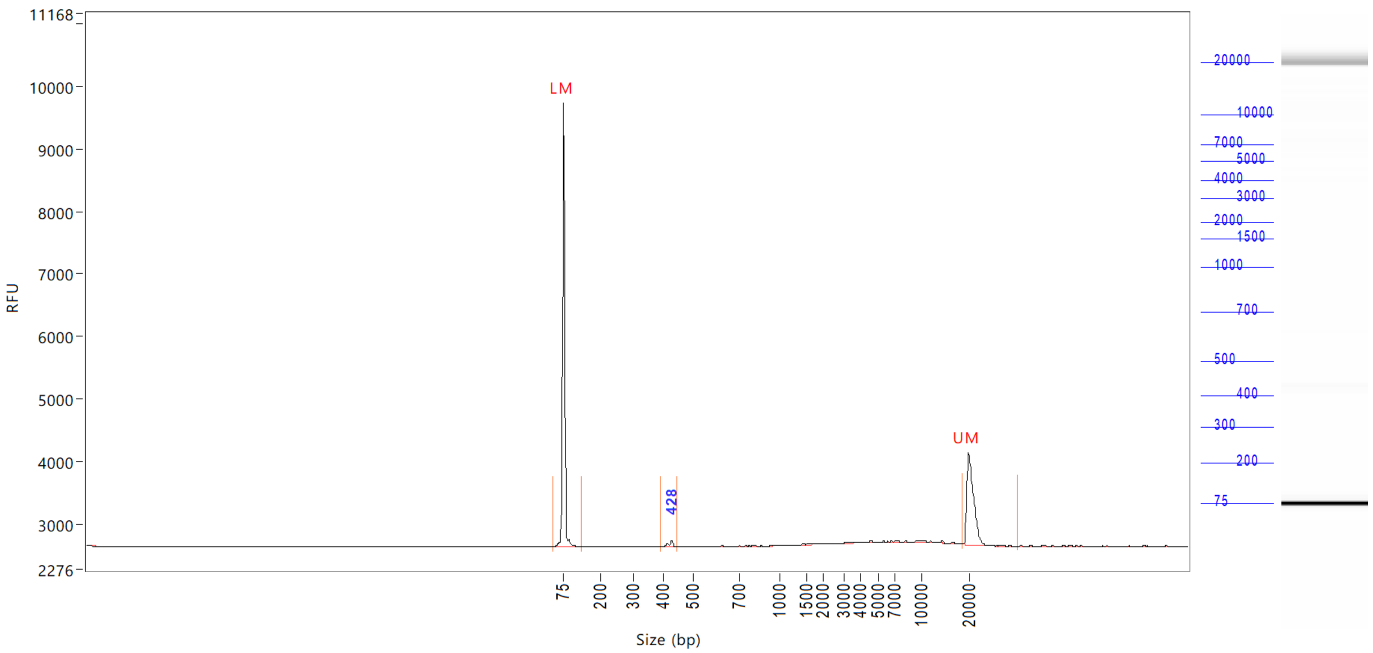

Peak	Size (bp)	Concentration (ng/uL)	From (bp)	To (bp)	Average size (bp)	CV%	RFU	Corrected peak area
1	75 (LM)	0.5000	41	135	73	7.13	7108	40.273
2	428	0.1029	392	447	420	2.17	84	0.691
3	20000 (UM)	0.1629	18537	30398	20444	4.35	1478	13.118

TIC:	0.1029	ng/uL
TIM:	0.4032	nmole/L
Total concentration:	0.5807	ng/uL

Sample peak width (sec): 5   Sample min peak height: 50   Sample baseline V to V?: Y   Sample baseline V to V points: 3
Sample filter: Binomial   Number of points for filter: 3 Sample start region (min): 0 Sample end region (min): 30
Marker peak width (sec): 5   Marker min peak height: 200   Marker baseline V to V?: Y   Marker baseline V to V points: 3
Lower marker selection: First peak > 500 RFU   Upper marker selection: Last peak > 200 RFU
Ladder size (bp)75, 200, 300, 400, 500, 700, 1000, 1500, 2000, 3000, 4000, 5000, 7000, 10000, 20000
Quantification using: Lower Marker   Final concentration (ng/uL): 0.5000   Dilution factor: 12.0

Sample: SampG11
Well location: G11
Created: Tuesday, September 14, 2021 4:54:05 PM

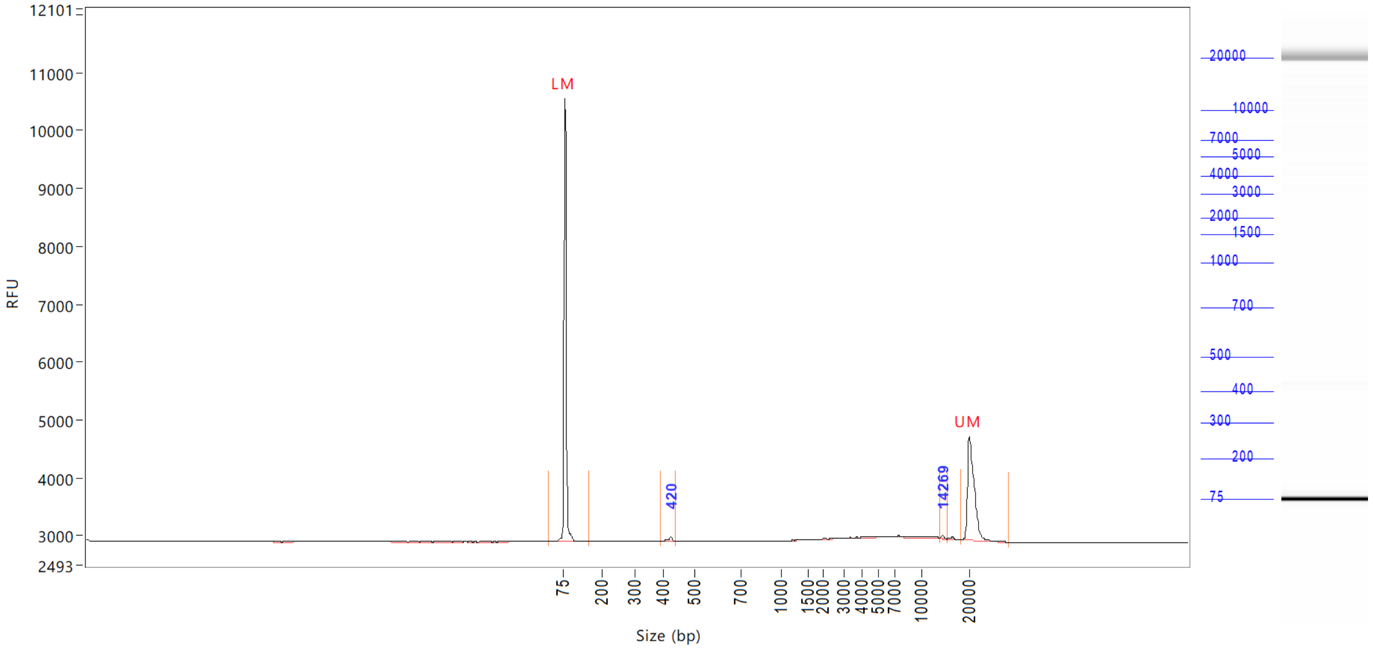

Peak	Size (bp)	Concentration (ng/uL)	From (bp)	To (bp)	Average size (bp)	CV%	RFU	Corrected peak area
1	75 (LM)	0.5000	26	154	74	8.19	7666	43.127
2	420	0.1102	386	435	412	2.28	95	0.792
3	14269	0.0381	13659	15366	14276	2.45	55	0.274
4	20000 (UM)	0.1834	18293	28342	20544	5.38	1787	15.823

TIC:	0.1483	ng/uL
TIM:	0.4446	nmole/L
Total concentration:	0.6324	ng/uL

Sample peak width (sec): 5   Sample min peak height: 50   Sample baseline V to V?: Y   Sample baseline V to V points: 3
Sample filter: Binomial   Number of points for filter: 3 Sample start region (min): 0 Sample end region (min): 30
Marker peak width (sec): 5   Marker min peak height: 200   Marker baseline V to V?: Y   Marker baseline V to V points: 3
Lower marker selection: First peak > 500 RFU   Upper marker selection: Last peak > 200 RFU
Ladder size (bp)75, 200, 300, 400, 500, 700, 1000, 1500, 2000, 3000, 4000, 5000, 7000, 10000, 20000
Quantification using: Lower Marker   Final concentration (ng/uL): 0.5000   Dilution factor: 12.0

Sample: SampH11
Well location: H11
Created: Tuesday, September 14, 2021 4:54:05 PM

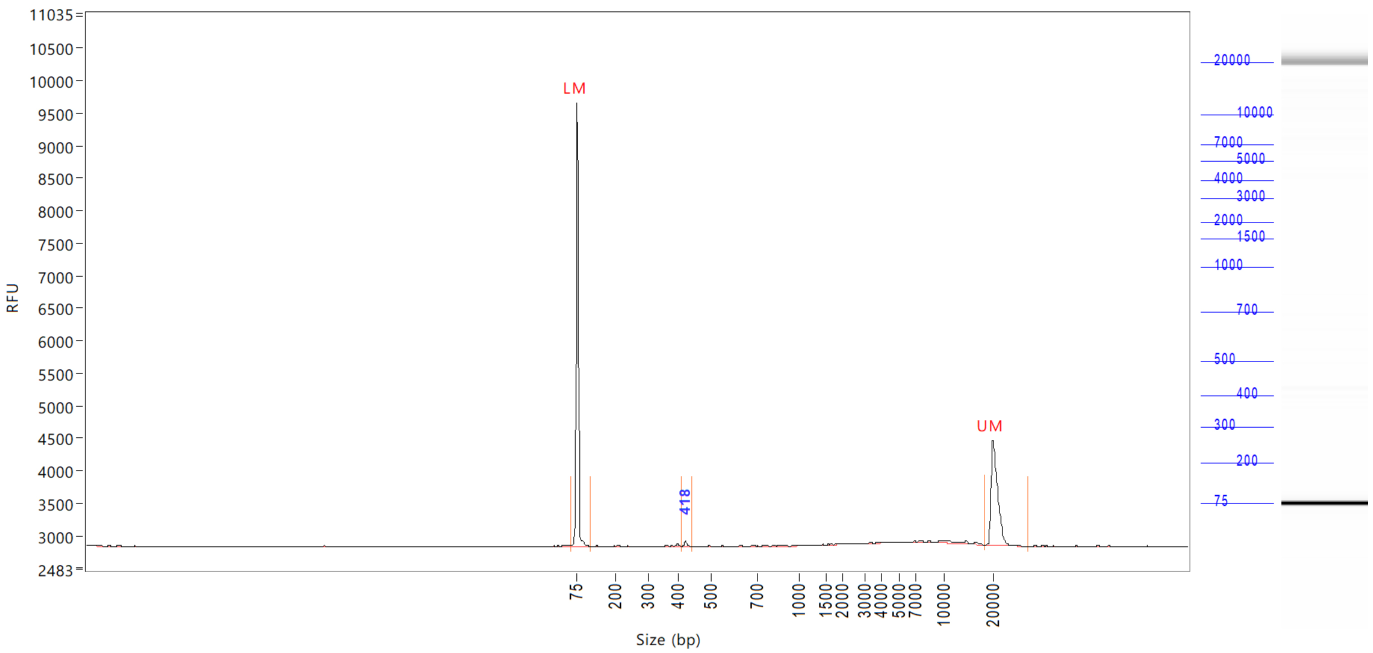

Peak	Size (bp)	Concentration (ng/uL)	From (bp)	To (bp)	Average size (bp)	CV%	RFU	Corrected peak area
1	75 (LM)	0.5000	58	118	73	5.56	6834	36.258
2	418	0.0747	405	443	418	1.33	82	0.452
3	20000 (UM)	0.1901	18537	27375	20450	3.81	1609	13.783

TIC:	0.0747	ng/uL
TIM:	0.2942	nmole/L
Total concentration:	0.7387	ng/uL

Sample peak width (sec): 5   Sample min peak height: 50   Sample baseline V to V?: Y   Sample baseline V to V points: 3
Sample filter: Binomial   Number of points for filter: 3 Sample start region (min): 0 Sample end region (min): 30
Marker peak width (sec): 5   Marker min peak height: 200   Marker baseline V to V?: Y   Marker baseline V to V points: 3
Lower marker selection: First peak > 500 RFU   Upper marker selection: Last peak > 200 RFU
Ladder size (bp)75, 200, 300, 400, 500, 700, 1000, 1500, 2000, 3000, 4000, 5000, 7000, 10000, 20000
Quantification using: Lower Marker   Final concentration (ng/uL): 0.5000   Dilution factor: 12.0

Sample: SampA12
Well location: A12
Created: Tuesday, September 14, 2021 4:54:05 PM

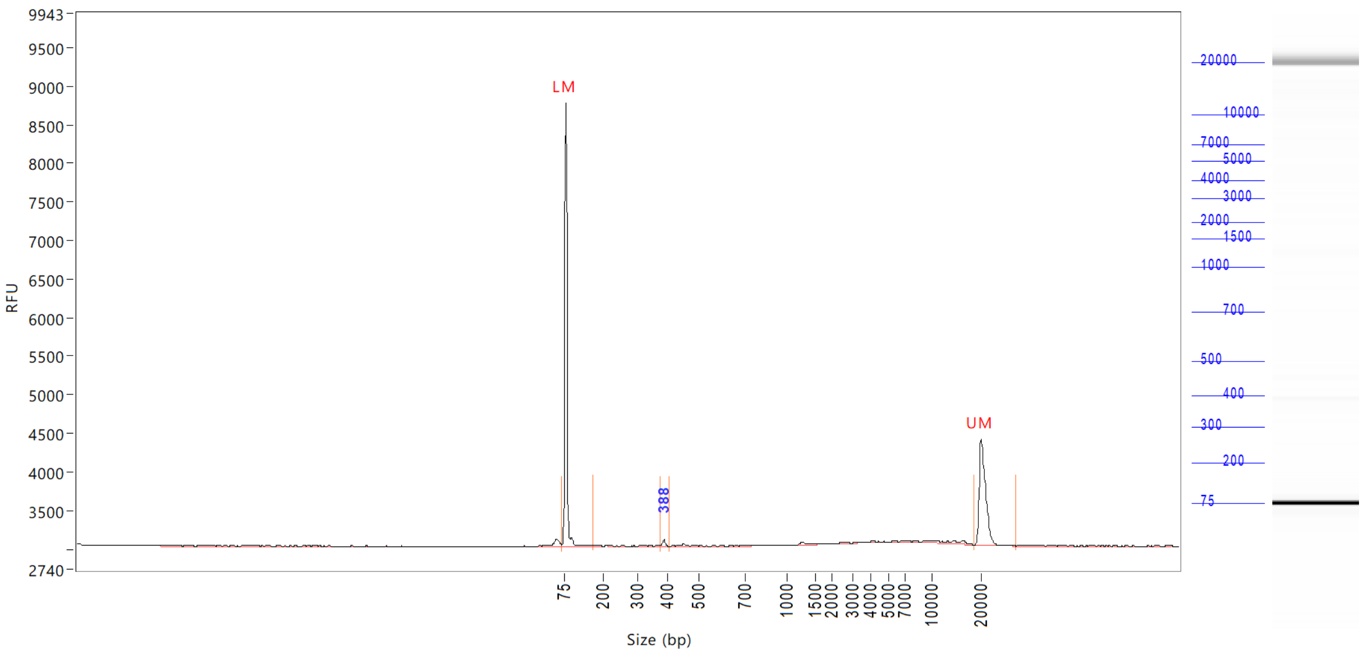

Peak	Size (bp)	Concentration (ng/uL)	From (bp)	To (bp)	Average size (bp)	CV%	RFU	Corrected peak area
1	75 (LM)	0.5000	64	162	75	9.51	5753	32.317
2	388	0.0945	376	405	386	1.18	89	0.509
3	20000 (UM)	0.1780	18659	27254	20385	3.98	1372	11.508

TIC:	0.0945	ng/uL
TIM:	0.4030	nmole/L
Total concentration:	0.9632	ng/uL

Sample peak width (sec): 5 Sample min peak height: 50 Sample baseline V to V?: Y Sample baseline V to V points: 3
Sample filter: Binomial Number of points for filter: 3 Sample start region (min): 0 Sample end region (min): 30
Marker peak width (sec): 5 Marker min peak height: 200 Marker baseline V to V?: Y Marker baseline V to V points: 3
Lower marker selection: First peak > 500 RFU Upper marker selection: Last peak > 200 RFU
Ladder size (bp)75, 200, 300, 400, 500, 700, 1000, 1500, 2000, 3000, 4000, 5000, 7000, 10000, 20000
Quantification using: Lower Marker Final concentration (ng/uL): 0.5000 Dilution factor: 12.0

Sample: SampB12
Well location: B12
Created: Tuesday, September 14, 2021 4:54:05 PM

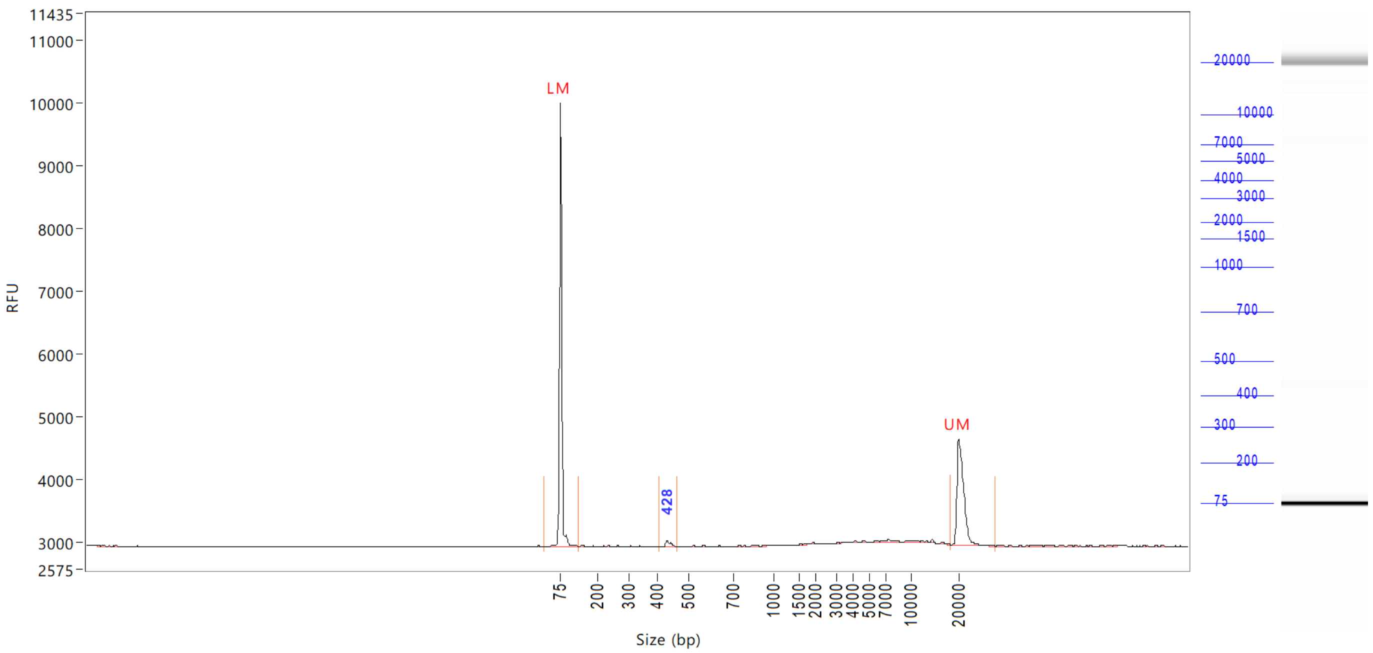

Peak	Size (bp)	Concentration (ng/uL)	From (bp)	To (bp)	Average size (bp)	CV%	RFU	Corrected peak area
1	75 (LM)	0.5000	22	135	73	8.32	7080	41.248
2	428	0.1249	403	460	432	2.15	86	0.859
3	20000 (UM)	0.1791	18293	27738	20449	4.45	1690	14.778

TIC:	0.1249	ng/uL
TIM:	0.4756	nmole/L
Total concentration:	0.6438	ng/uL

Sample peak width (sec): 5 Sample min peak height: 50 Sample baseline V to V?: Y Sample baseline V to V points: 3
Sample filter: Binomial Number of points for filter: 3 Sample start region (min): 0 Sample end region (min): 30
Marker peak width (sec): 5 Marker min peak height: 200 Marker baseline V to V?: Y Marker baseline V to V points: 3
Lower marker selection: First peak > 500 RFU Upper marker selection: Last peak > 200 RFU
Ladder size (bp)75, 200, 300, 400, 500, 700, 1000, 1500, 2000, 3000, 4000, 5000, 7000, 10000, 20000
Quantification using: Lower Marker Final concentration (ng/uL): 0.5000 Dilution factor: 12.0

Sample: SampC12
Well location: C12
Created: Tuesday, September 14, 2021 4:54:05 PM

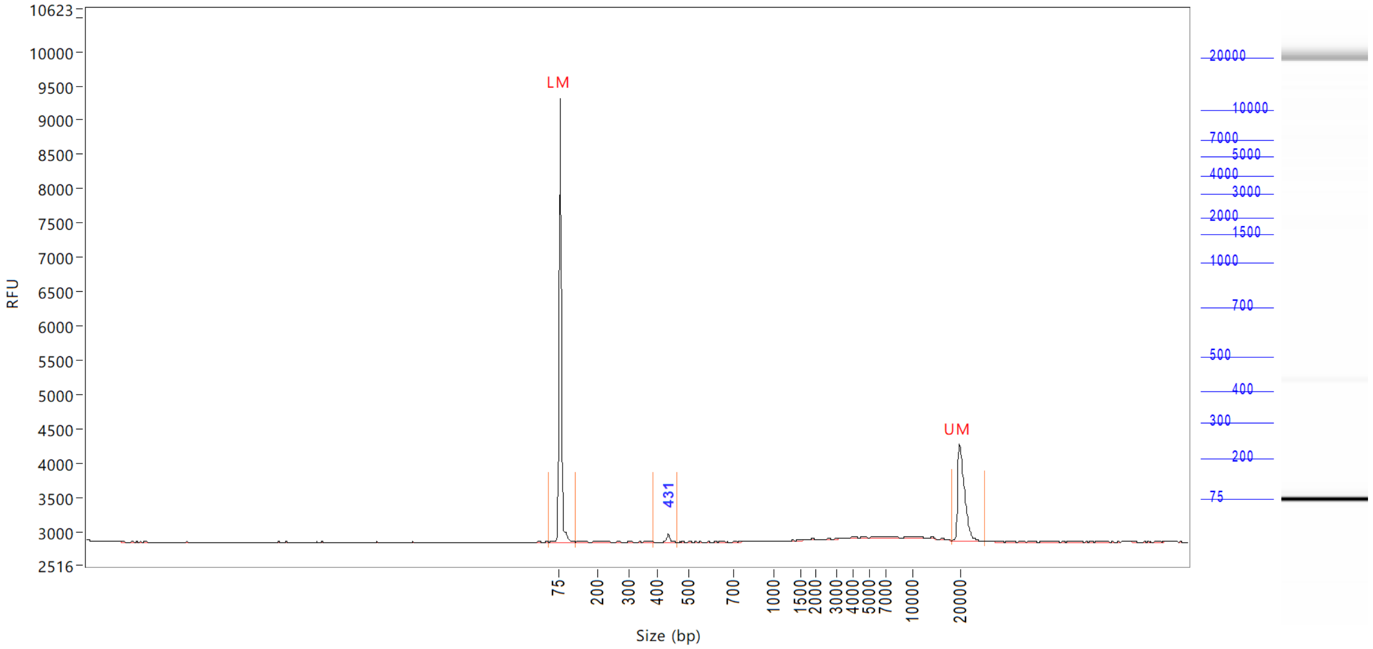

Peak	Size (bp)	Concentration (ng/uL)	From (bp)	To (bp)	Average size (bp)	CV%	RFU	Corrected peak area
1	75 (LM)	0.5000	39	126	74	7.69	6476	37.935
2	431	0.1344	386	466	430	2.08	126	0.849
3	20000 (UM)	0.1649	18293	25320	20486	4.08	1403	12.510

TIC:	0.1344	ng/uL
TIM:	0.5141	nmole/L
Total concentration:	0.6726	ng/uL

Sample peak width (sec): 5   Sample min peak height: 50   Sample baseline V to V?: Y   Sample baseline V to V points: 3
Sample filter: Binomial   Number of points for filter: 3   Sample start region (min): 0   Sample end region (min): 30
Marker peak width (sec): 5   Marker min peak height: 200   Marker baseline V to V?: Y   Marker baseline V to V points: 3
Lower marker selection: First peak > 500 RFU   Upper marker selection: Last peak > 200 RFU
Ladder size (bp)75, 200, 300, 400, 500, 700, 1000, 1500, 2000, 3000, 4000, 5000, 7000, 10000, 20000
Quantification using: Lower Marker   Final concentration (ng/uL): 0.5000   Dilution factor: 12.0

Sample: SampD12
Well location: D12
Created: Tuesday, September 14, 2021 4:54:05 PM

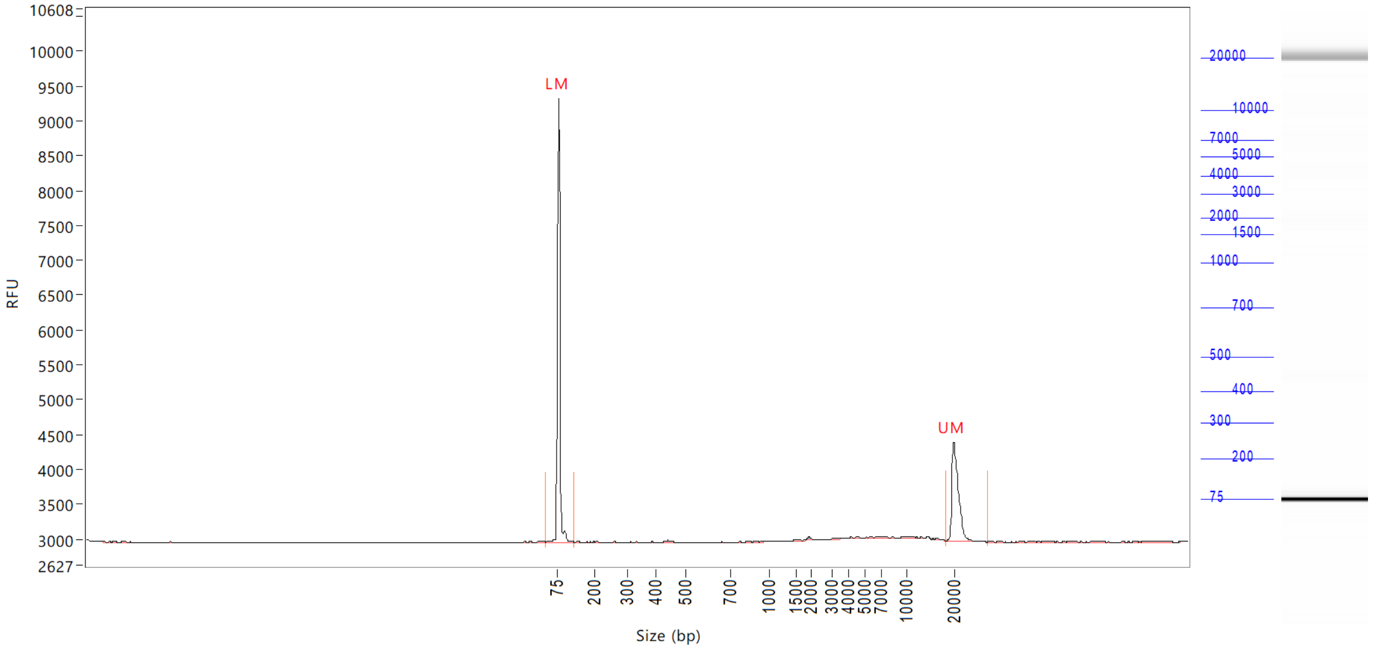

Peak	Size (bp)	Concentration (ng/uL)	From (bp)	To (bp)	Average size (bp)	CV%	RFU	Corrected peak area
1	75 (LM)	0.5000	31	128	73	8.15	6377	36.953
2	20000 (UM)	0.1686	18537	27254	20502	4.18	1420	12.462

TIC:	0.0000	ng/uL
TIM:	0.0000	nmole/L
Total concentration:	0.6261	ng/uL

Sample peak width (sec): 5   Sample min peak height: 50   Sample baseline V to V?: Y   Sample baseline V to V points: 3
Sample filter: Binomial   Number of points for filter: 3   Sample start region (min): 0   Sample end region (min): 30
Marker peak width (sec): 5   Marker min peak height: 200   Marker baseline V to V?: Y   Marker baseline V to V points: 3
Lower marker selection: First peak > 500 RFU   Upper marker selection: Last peak > 200 RFU
Ladder size (bp)75, 200, 300, 400, 500, 700, 1000, 1500, 2000, 3000, 4000, 5000, 7000, 10000, 20000
Quantification using: Lower Marker   Final concentration (ng/uL): 0.5000   Dilution factor: 12.0

Sample: SampE12
Well location: E12
Created: Tuesday, September 14, 2021 4:54:05 PM

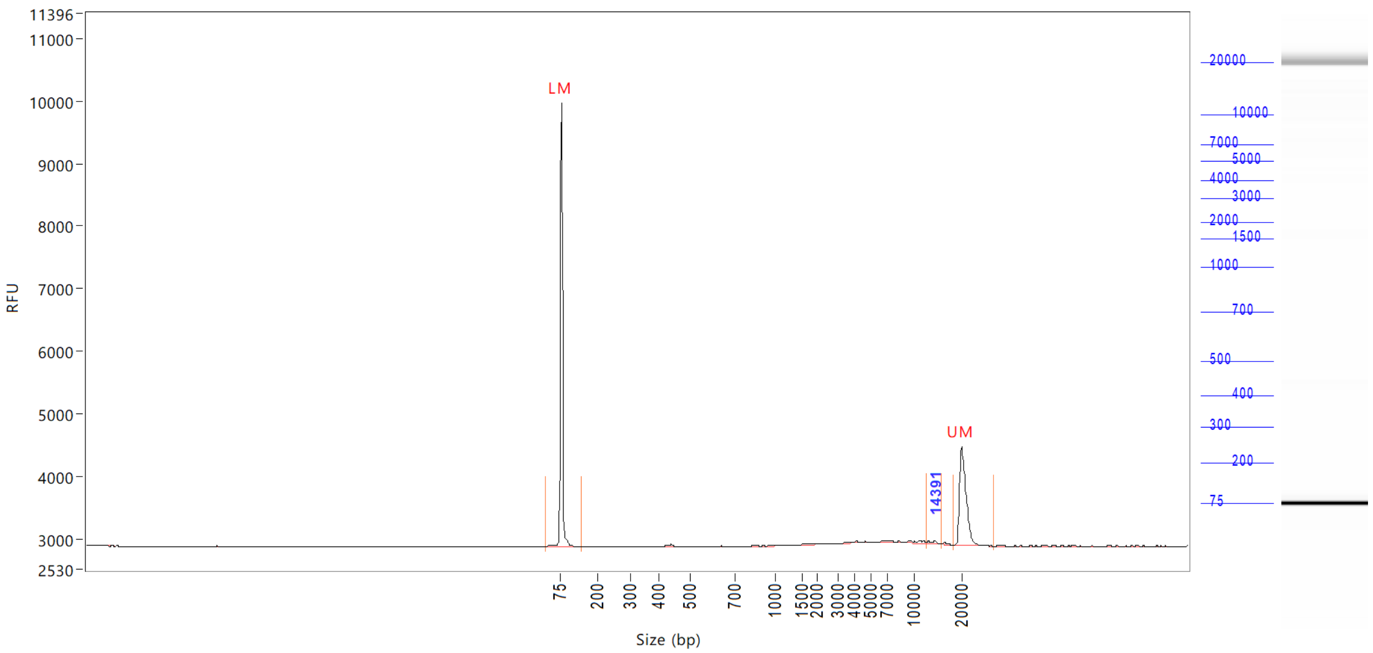

Peak	Size (bp)	Concentration (ng/uL)	From (bp)	To (bp)	Average size (bp)	CV%	RFU	Corrected peak area
1	75 (LM)	0.5000	26	143	73	8.28	7090	39.614
2	14391	0.0709	12562	15732	13860	5.73	52	0.468
3	20000 (UM)	0.1700	18171	26891	20409	3.86	1577	13.469

TIC:	0.0709	ng/uL
TIM:	0.0084	nmole/L
Total concentration:	0.6402	ng/uL

Sample peak width (sec): 5 Sample min peak height: 50 Sample baseline V to V?: Y Sample baseline V to V points: 3
Sample filter: Binomial Number of points for filter: 3 Sample start region (min): 0 Sample end region (min): 30
Marker peak width (sec): 5 Marker min peak height: 200 Marker baseline V to V?: Y Marker baseline V to V points: 3
Lower marker selection: First peak > 500 RFU Upper marker selection: Last peak > 200 RFU
Ladder size (bp)75, 200, 300, 400, 500, 700, 1000, 1500, 2000, 3000, 4000, 5000, 7000, 10000, 20000
Quantification using: Lower Marker Final concentration (ng/uL): 0.5000 Dilution factor: 12.0

Sample: SampF12
Well location: F12
Created: Tuesday, September 14, 2021 4:54:05 PM

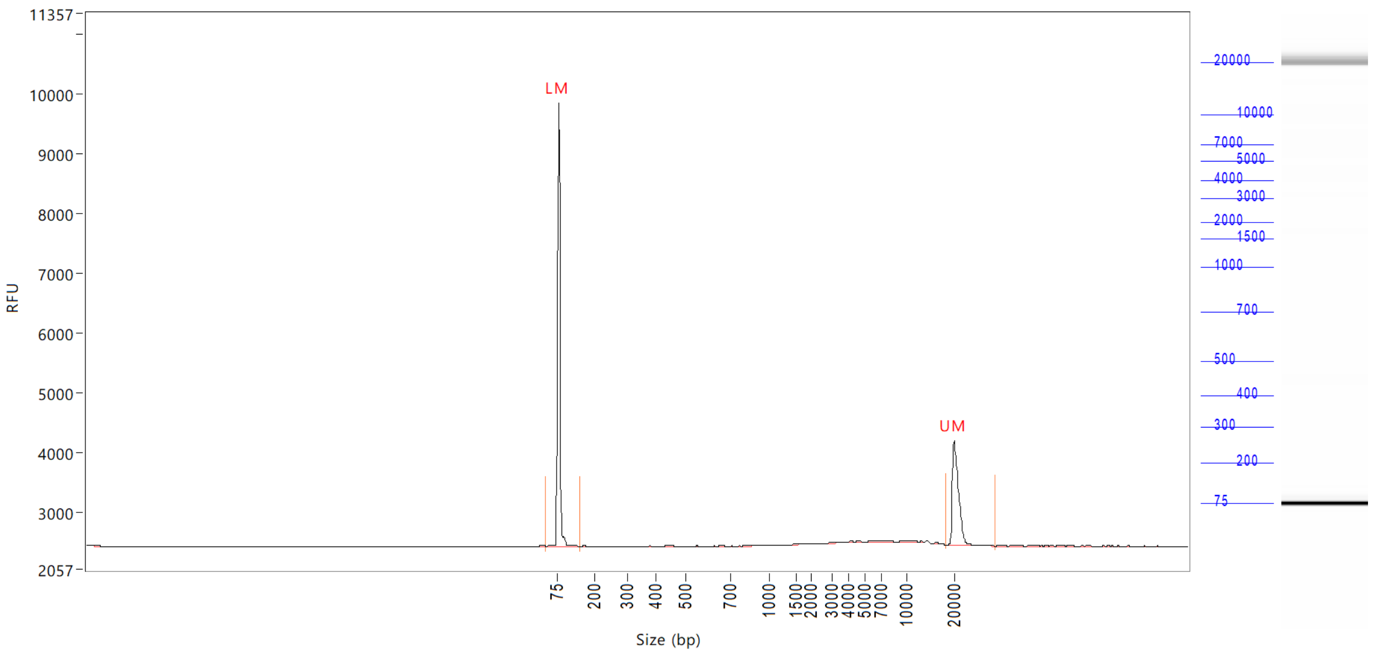

Peak	Size (bp)	Concentration (ng/uL)	From (bp)	To (bp)	Average size (bp)	CV%	RFU	Corrected peak area
1	75 (LM)	0.5000	33	148	73	8.10	7431	42.253
2	20000 (UM)	0.1725	18171	28705	20411	4.10	1753	14.577

TIC:	0.0000	ng/uL
TIM:	0.0000	nmole/L
Total concentration:	0.4863	ng/uL

Sample peak width (sec): 5   Sample min peak height: 50   Sample baseline V to V?: Y   Sample baseline V to V points: 3
Sample filter: Binomial   Number of points for filter: 3   Sample start region (min): 0   Sample end region (min): 30
Marker peak width (sec): 5   Marker min peak height: 200   Marker baseline V to V?: Y   Marker baseline V to V points: 3
Lower marker selection: First peak > 500 RFU   Upper marker selection: Last peak > 200 RFU
Ladder size (bp)75, 200, 300, 400, 500, 700, 1000, 1500, 2000, 3000, 4000, 5000, 7000, 10000, 20000
Quantification using: Lower Marker   Final concentration (ng/uL): 0.5000   Dilution factor: 12.0

Sample: SampG12
Well location: G12
Created: Tuesday, September 14, 2021 4:54:05 PM

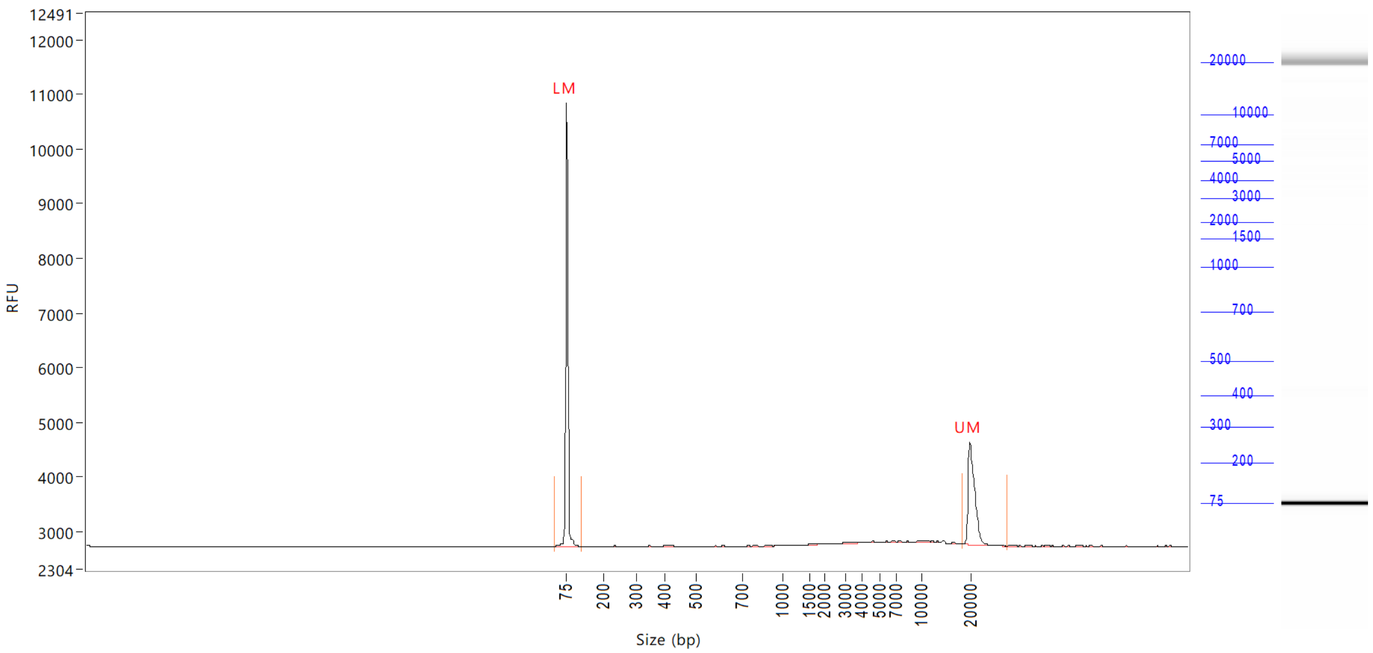

Peak	Size (bp)	Concentration (ng/uL)	From (bp)	To (bp)	Average size (bp)	CV%	RFU	Corrected peak area
1	75 (LM)	0.5000	37	126	73	6.66	8140	44.569
2	20000 (UM)	0.1808	18293	27859	20430	3.91	1876	16.114

TIC:	0.0000	ng/uL
TIM:	0.0000	nmole/L
Total concentration:	0.5161	ng/uL

Sample peak width (sec): 5 Sample min peak height: 50 Sample baseline V to V?: Y Sample baseline V to V points: 3
Sample filter: Binomial Number of points for filter: 3 Sample start region (min): 0 Sample end region (min): 30
Marker peak width (sec): 5 Marker min peak height: 200 Marker baseline V to V?: Y Marker baseline V to V points: 3
Lower marker selection: First peak > 500 RFU Upper marker selection: Last peak > 200 RFU
Ladder size (bp)75, 200, 300, 400, 500, 700, 1000, 1500, 2000, 3000, 4000, 5000, 7000, 10000, 20000
Quantification using: Lower Marker Final concentration (ng/uL): 0.5000 Dilution factor: 12.0

Sample: SampH12
Well location: H12
Created: Tuesday, September 14, 2021 4:54:05 PM

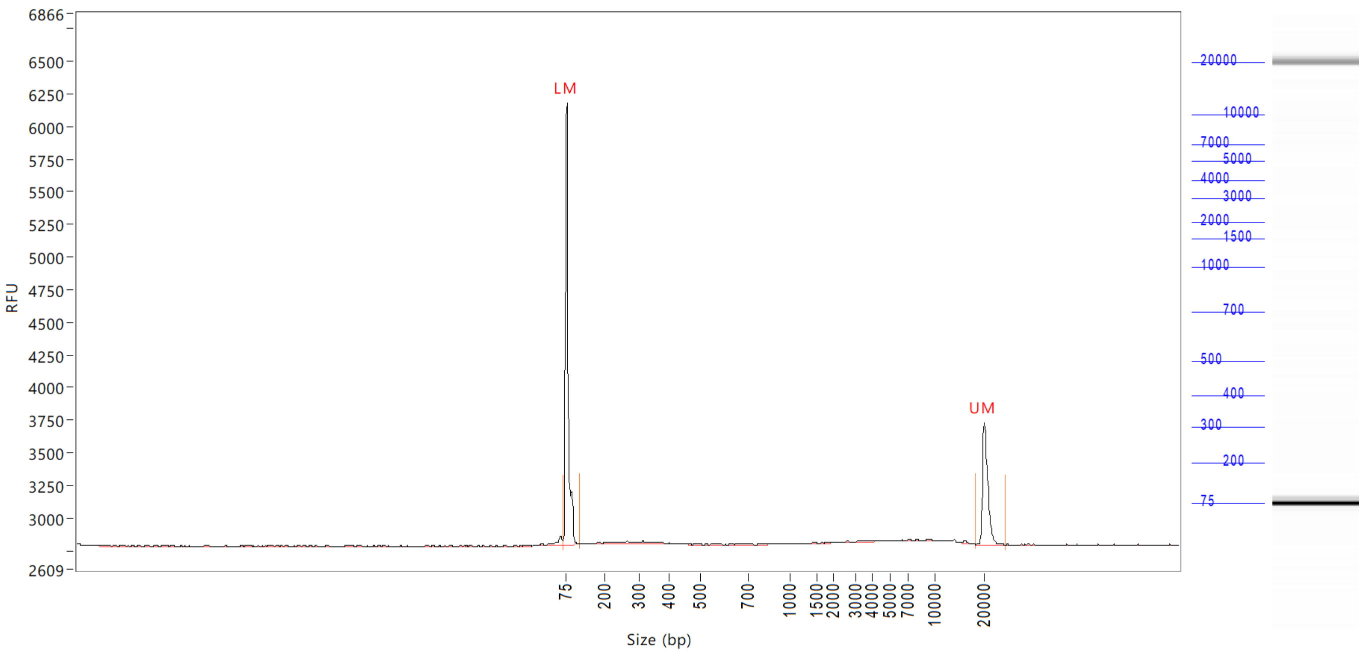

Peak	Size (bp)	Concentration (ng/uL)	From (bp)	To (bp)	Average size (bp)	CV%	RFU	Corrected peak area
1	75 (LM)	0.5000	64	120	75	8.47	3389	22.796
2	20000 (UM)	0.1401	18415	24231	20256	3.08	929	6.386

TIC:	0.0000	ng/uL
TIM:	0.0000	nmole/L
Total concentration:	0.8094	ng/uL

Sample peak width (sec): 5 Sample min peak height: 200 Sample baseline V to V?: Y Sample baseline V to V points: 3
Sample filter: Binomial Number of points for filter: 3 Sample start region (min): 0 Sample end region (min): 30
Marker peak width (sec): 5 Marker min peak height: 200 Marker baseline V to V?: Y Marker baseline V to V points: 3
Lower marker selection: First peak > 500 RFU Upper marker selection: Last peak > 200 RFU
Ladder size (bp)75, 200, 300, 400, 500, 700, 1000, 1500, 2000, 3000, 4000, 5000, 7000, 10000, 20000
Quantification using: Lower Marker Final concentration (ng/uL): 0.5000 Dilution factor: 12.0

Sample: DNA size ladder
Well location: A7
Created: Tuesday, September 14, 2021 4:54:05 PM
Import from: C:\Users\admin\Desktop\210810_alberto_ladder.SCAL
Fit type: Point to point

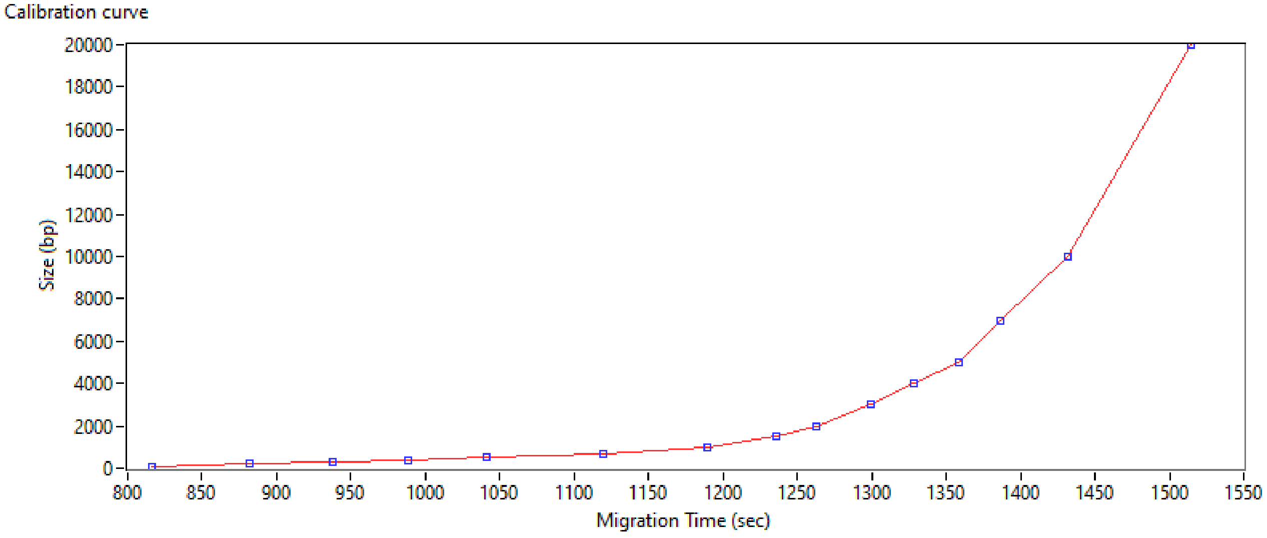

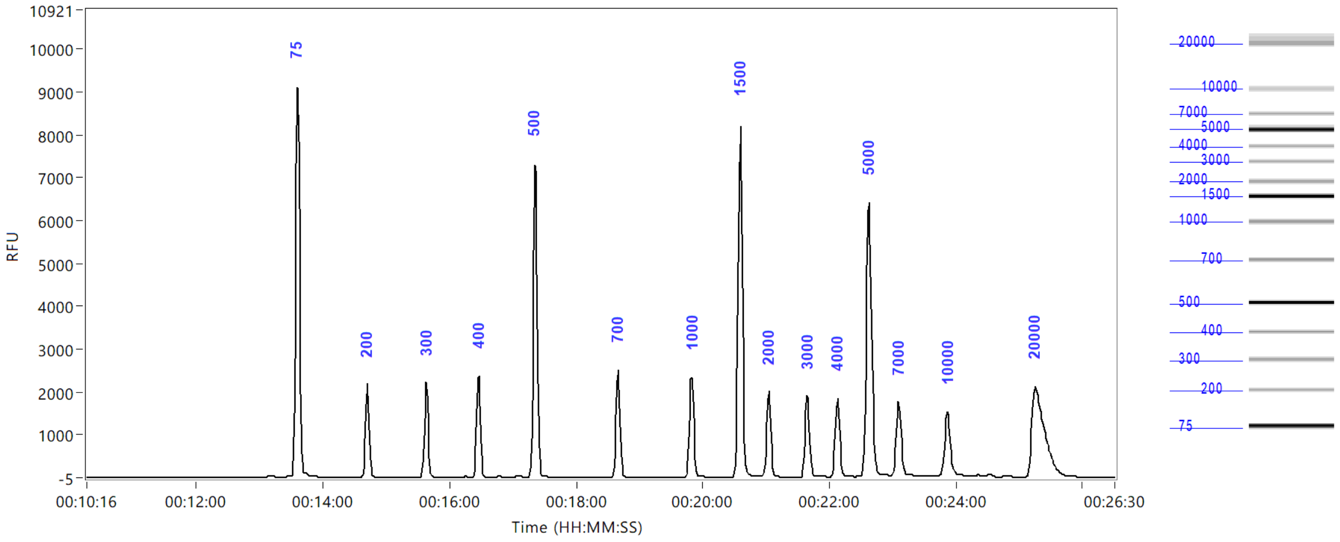

Supplement: Supplementary file 2 — sb3c00292_si_002.zip [file sb3c00292_si_002.zip › dnada_supplementary_material_pks_library_build/ZAG_trials/ZAG_Trial_5/16-33-14_plate1/plate1-t1.pdf]
