# Supplementary material for: Automated Platform for the Plasmid Construction Process
Source: ACS Synth Biol. 2023 Nov 10;12(12):3506–13. doi: 10.1021/acssynbio.3c00292 (PMC10729297; doi:10.1021/acssynbio.3c00292)

Instrument controller software run summary:

Filename and data path: C:\AATI\Data\2021 09 14\21-24-18\2021 09 14 21H 24M..raw
Created: Tuesday, September 14, 2021 9:33:53 PM
Number of capillaries: 96
Array serial number: 111920-09SFS
Effect length: 33 cm
Array usage count: 220
Instrument type: ZAG DNA Analyzer
Instrument controller software version: 1.0.1.9
Device serial number: Z7597

Method Information

Method name: ZAG130GP33 - DNA 75-20000bp - Gel Prime Only.mthds
Gel prime: Yes
Full conditioning: No
Gel prime to buffer: No
Gel selection: Gel 1
Perform prerun: 8.0 kV, 30 sec.
Rinse: No
Marker 1: Row: A, 5.0 kV, 5 sec.
Rinse: No
Sample injection: 5.0 kV, 5 sec.
Separation: 8.0 kV, 30.0 min.
Tray name: Sample Tray-8

Analysis mode: DNA

Notes

Gel Image

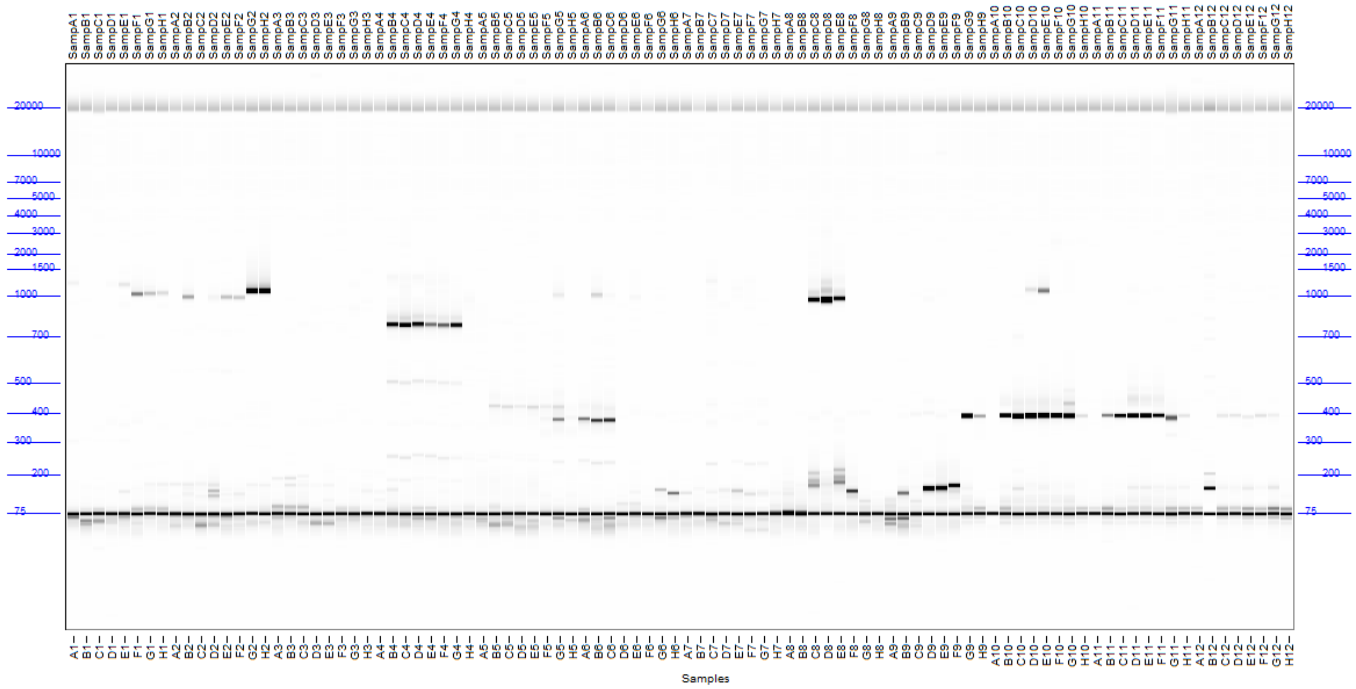
20000
10000
7000
5000
4000
3000
2000
1500
1000
700
500
400
300
200
75
Samples

Filename and data path: C:\AATI\Data\2021 09 14\21-24-18\2021 09 14 21H 24M..raw

A1: SampA1

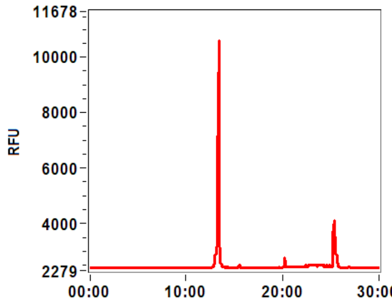

B1: SampB1

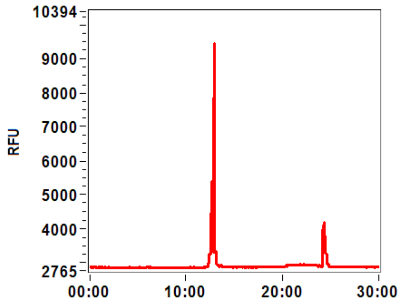

C1: SampC1

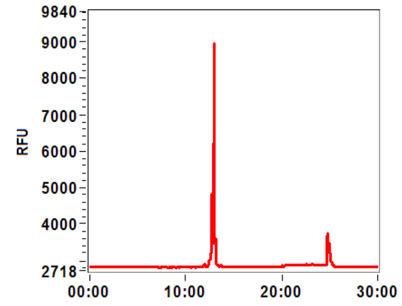

D1: SampD1

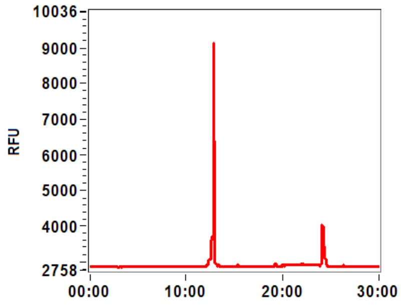

E1: SampE1

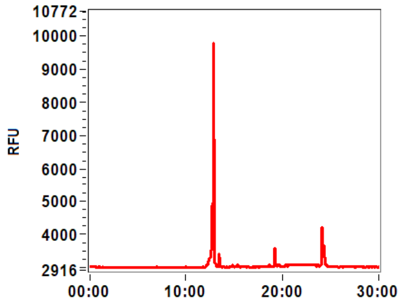

F1: SampF1

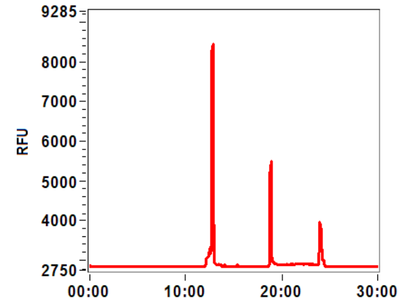

G1: SampG1

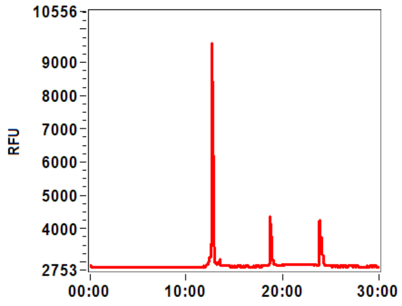

H1: SampH1

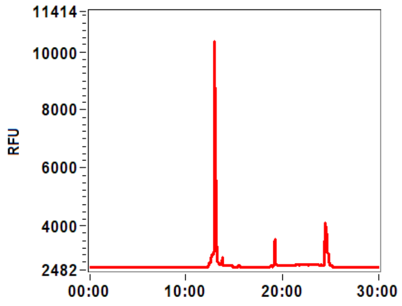

A2: SampA2

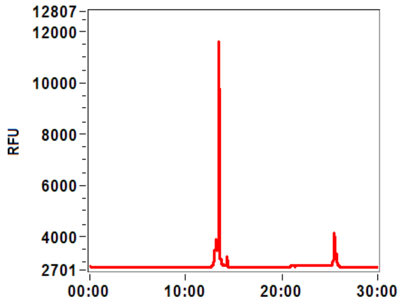

B2: SampB2

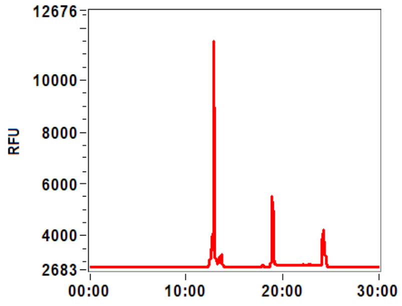

C2: SampC2

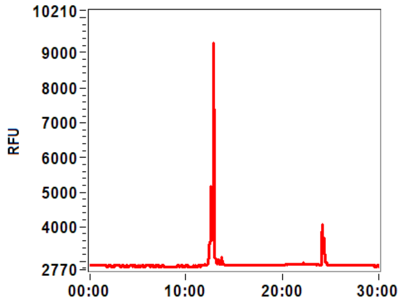

D2: SampD2

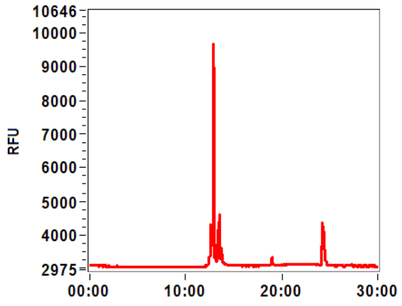

Filename and data path: C:\AATI\Data\2021 09 14\21-24-18\2021 09 14 21H 24M..raw

E2: SampE2

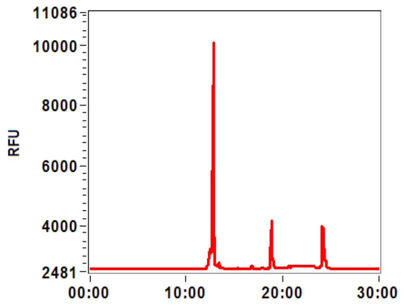

F2: SampF2

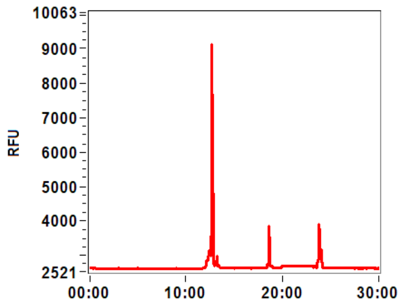

G2: SampG2

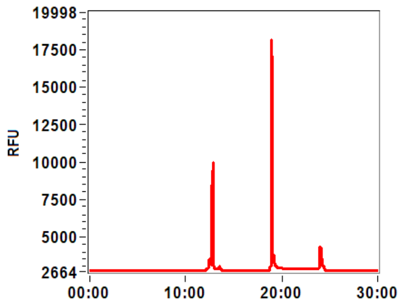

H2: SampH2

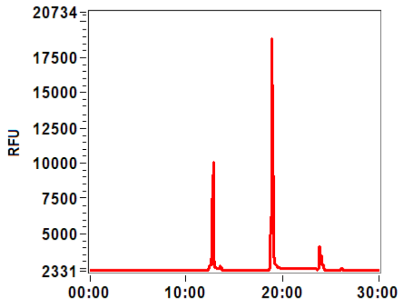

A3: SampA3

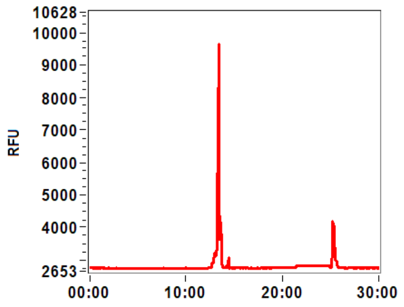

B3: SampB3

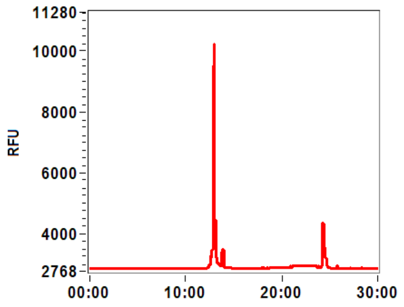

C3: SampC3

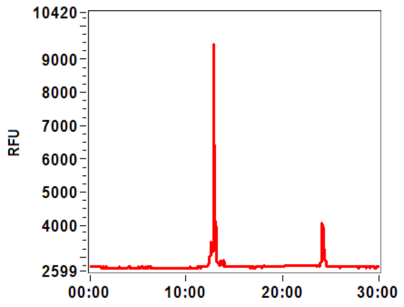

D3: SampD3

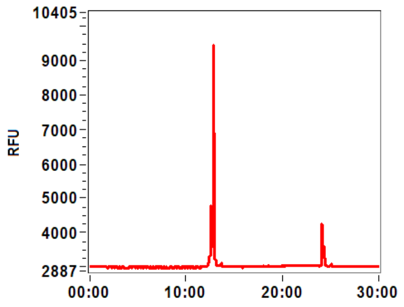

E3: SampE3

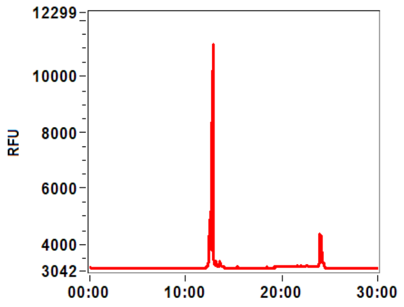

F3: SampF3

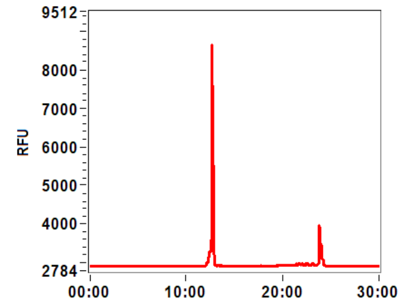

G3: SampG3

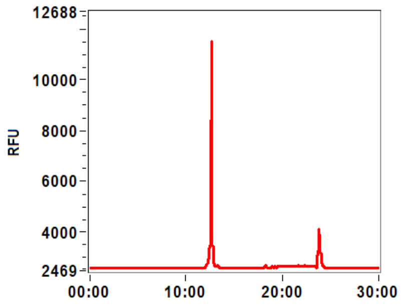

H3: SampH3

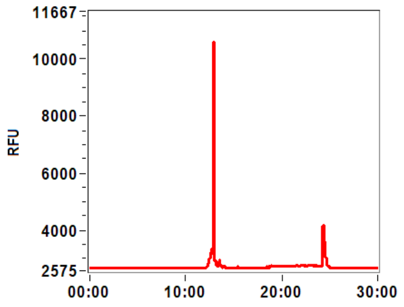

Filename and data path: C:\AATI\Data\2021 09 14\21-24-18\2021 09 14 21H 24M..raw

A4: SampA4

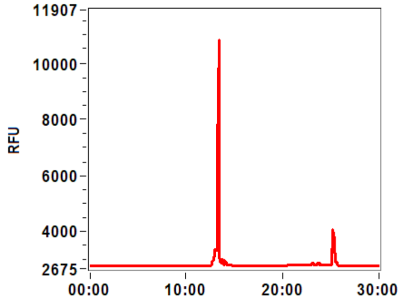

B4: SampB4

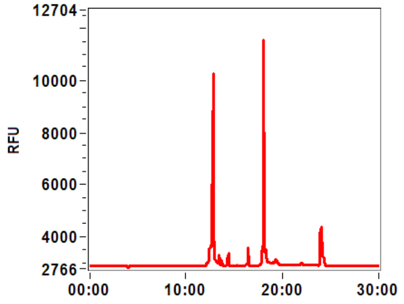

C4: SampC4

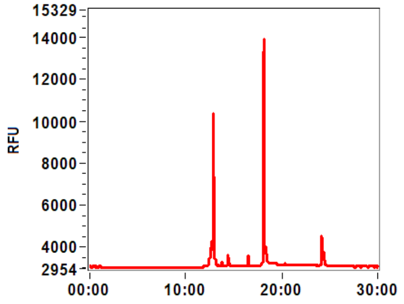

D4: SampD4

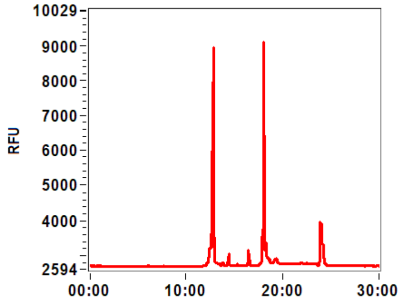

E4: SampE4

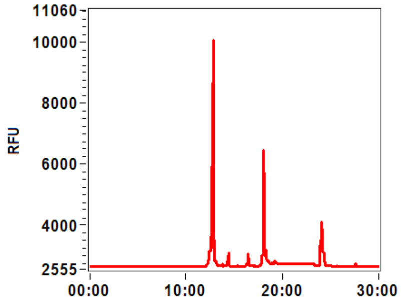

F4: SampF4

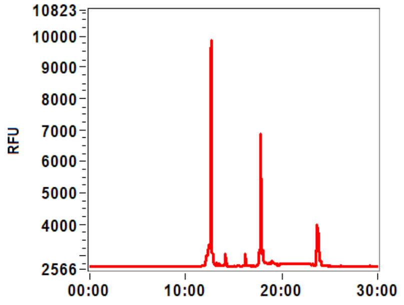

G4: SampG4

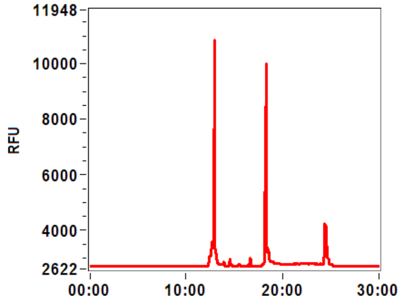

H4: SampH4

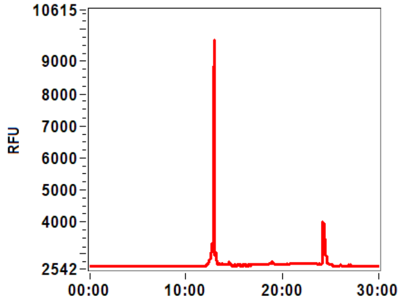

A5: SampA5

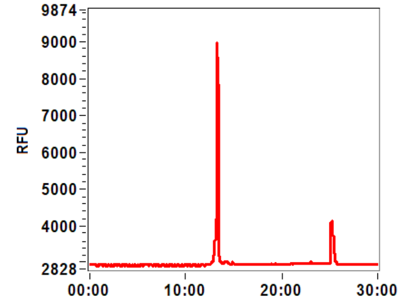

B5: SampB5

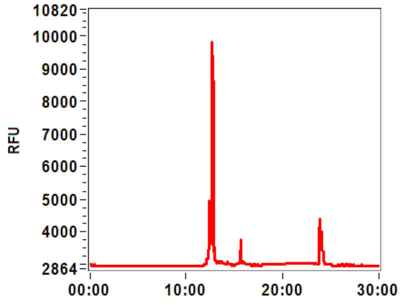

C5: SampC5

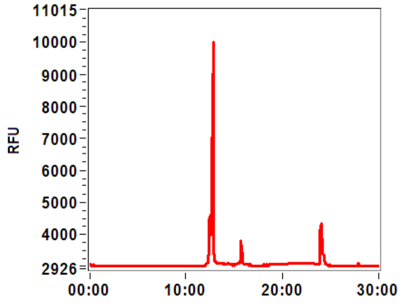

D5: SampD5

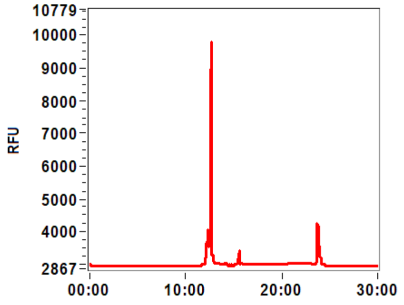

Filename and data path: C:\AATI\Data\2021 09 14\21-24-18\2021 09 14 21H 24M..raw

E5: SampE5

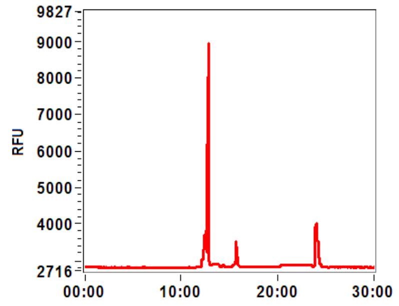

F5: SampF5

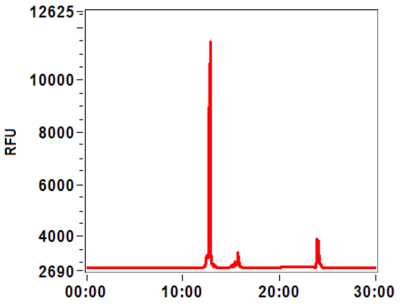

G5: SampG5

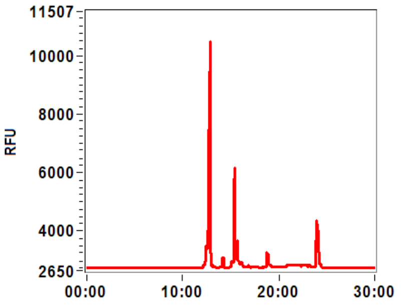

H5: SampH5

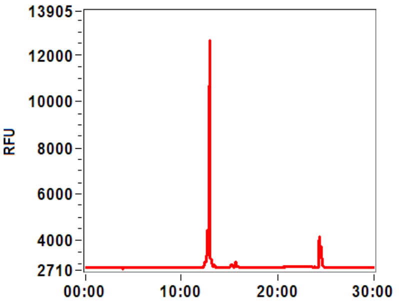

A6: SampA6

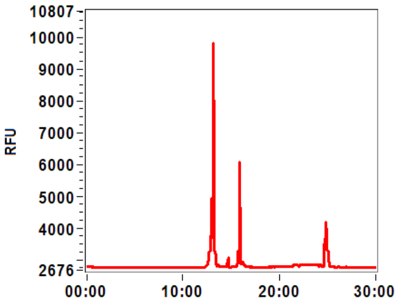

B6: SampB6

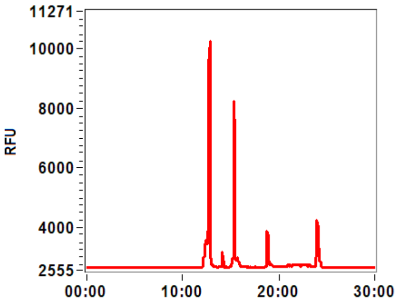

C6: SampC6

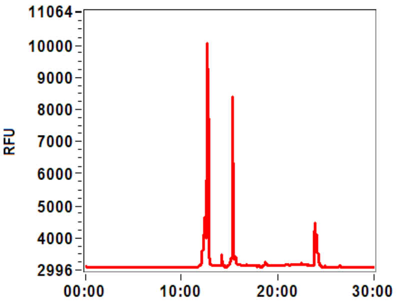

D6: SampD6

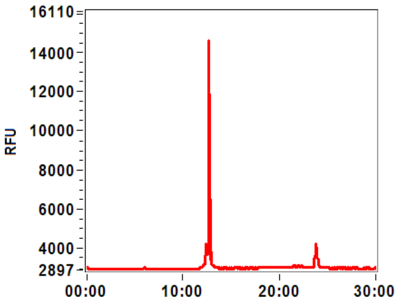

E6: SampE6

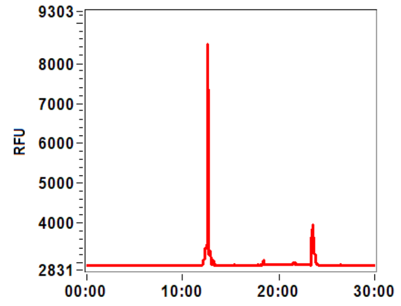

F6: SampF6

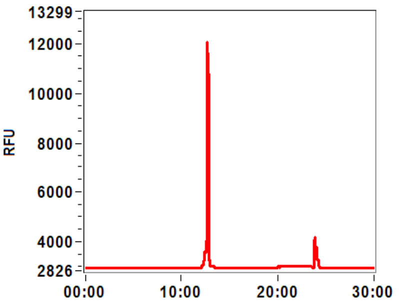

G6: SampG6

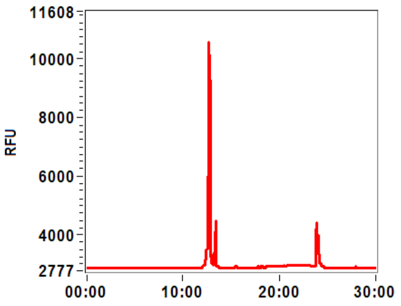

H6: SampH6

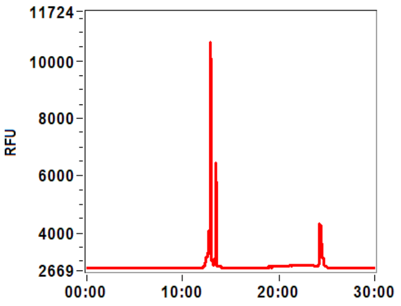

Filename and data path: C:\AATI\Data\2021 09 14\21-24-18\2021 09 14 21H 24M..raw

A7: SampA7

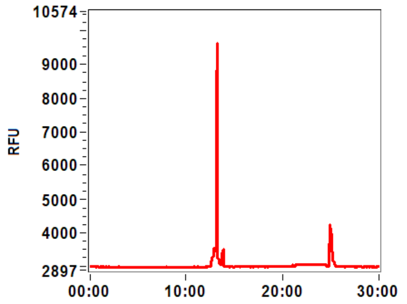

B7: SampB7

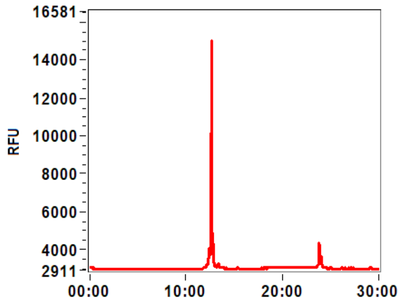

C7: SampC7

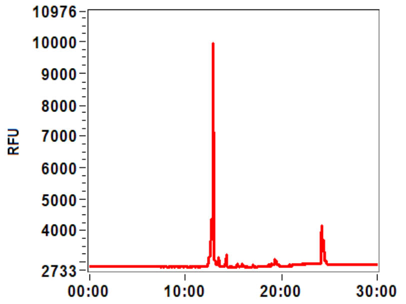

D7: SampD7

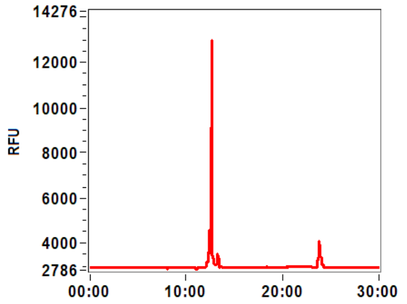

E7: SampE7

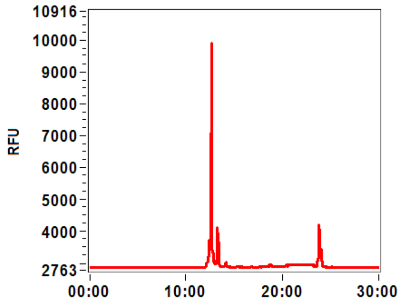

F7: SampF7

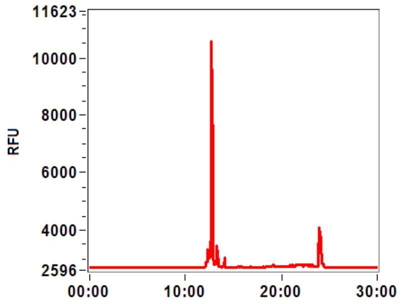

G7: SampG7

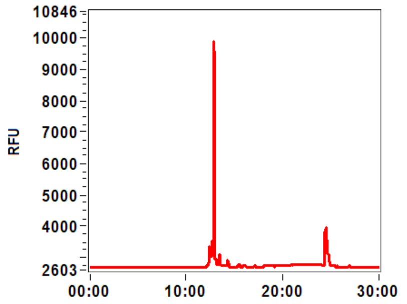

H7: SampH7

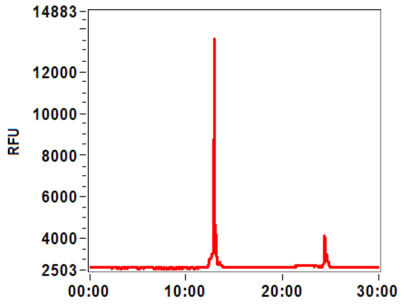

A8: SampA8

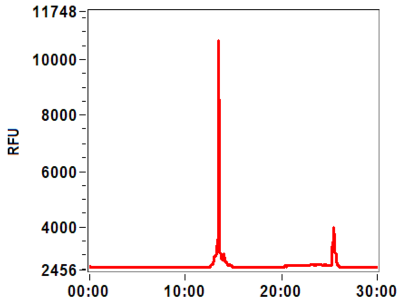

B8: SampB8

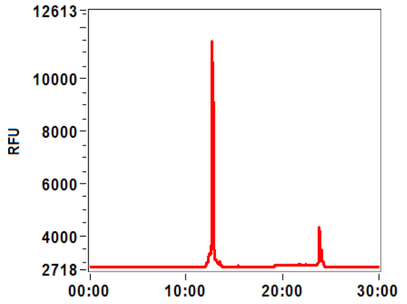

C8: SampC8

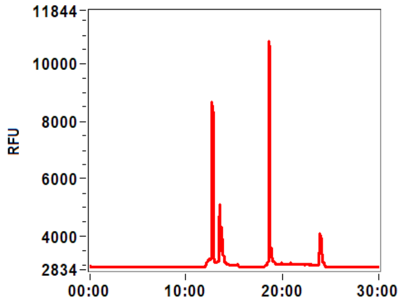

D8: SampD8

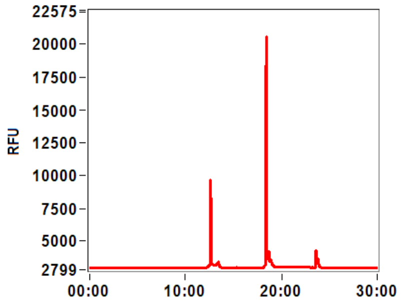

Filename and data path: C:\AATI\Data\2021 09 14\21-24-18\2021 09 14 21H 24M..raw

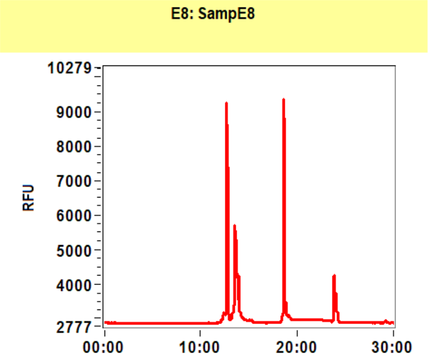

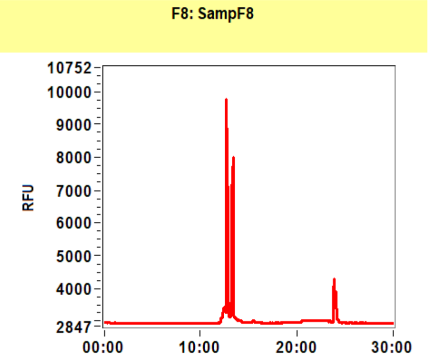

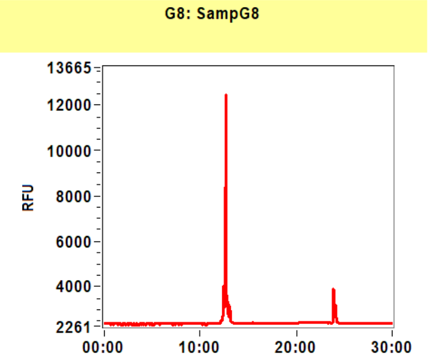

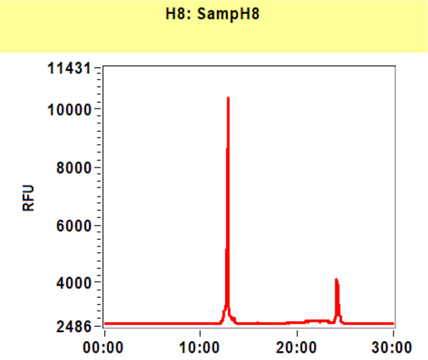

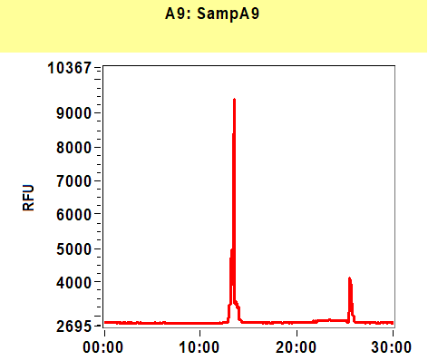

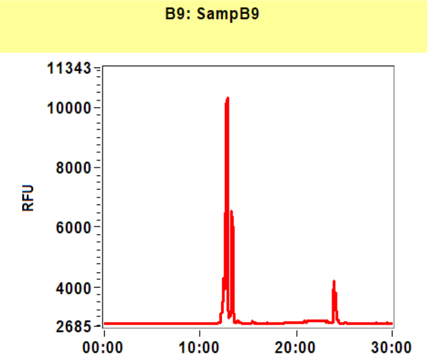

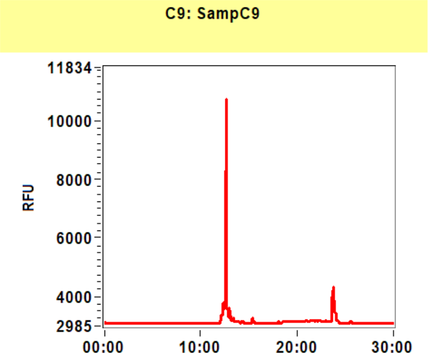

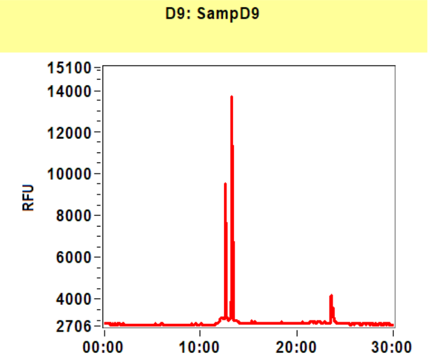

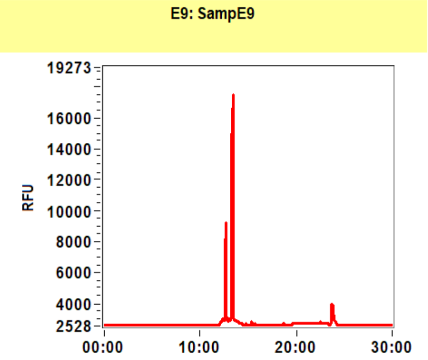

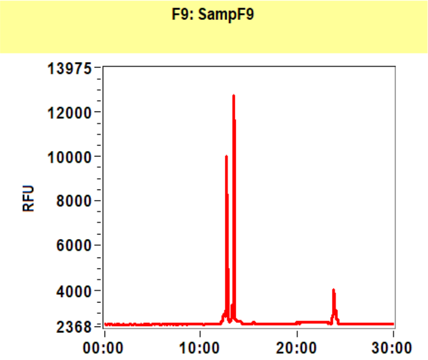

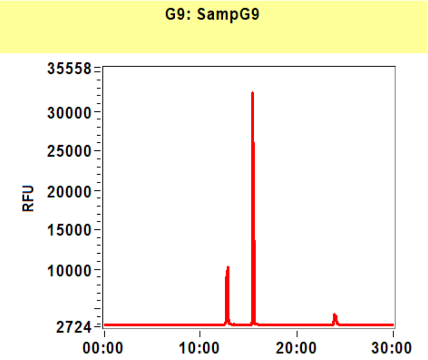

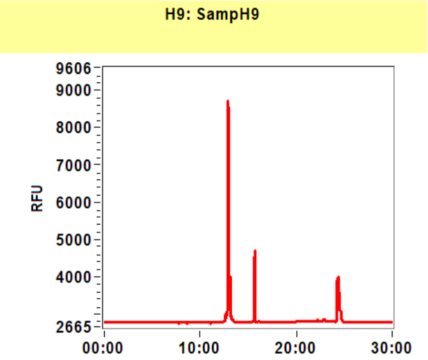

Filename and data path: C:\AATI\Data\2021 09 14\21-24-18\2021 09 14 21H 24M..raw

A10: SampA10

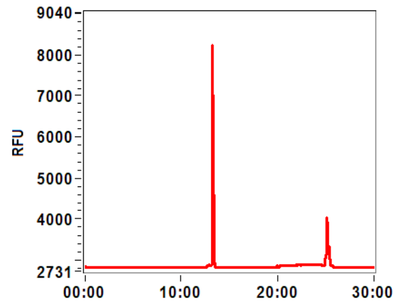

B10: SampB10

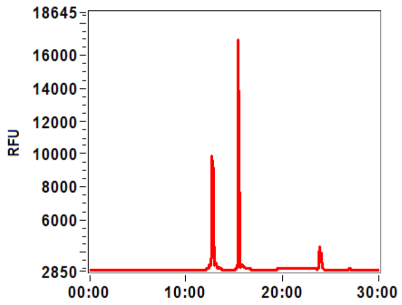

C10: SampC10

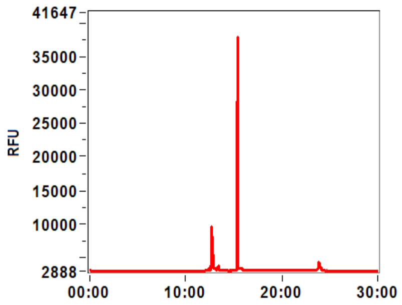

D10: SampD10

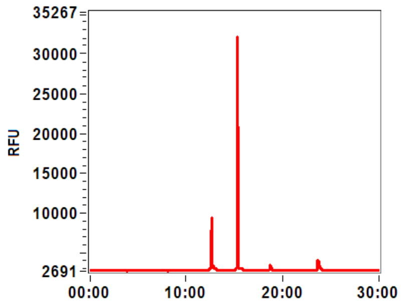

E10: SampE10

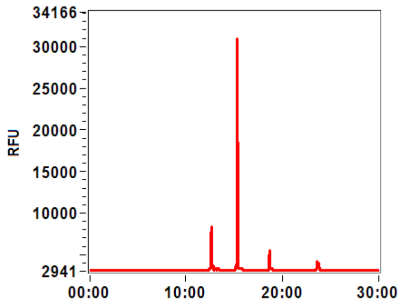

F10: SampF10

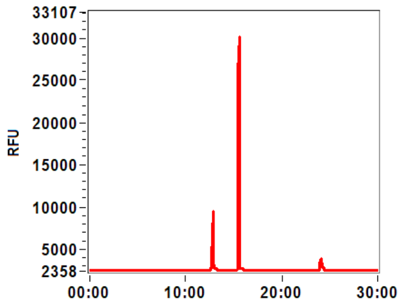

G10: SampG10

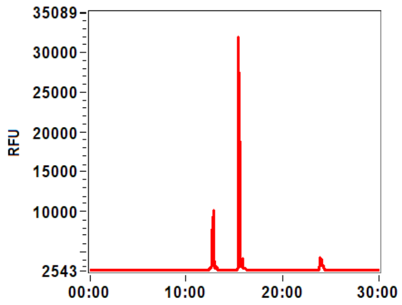

H10: SampH10

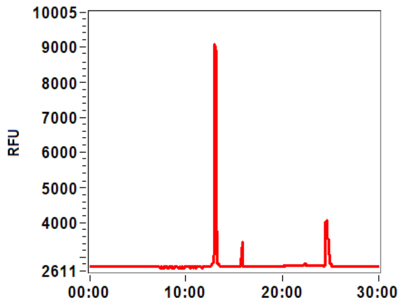

A11: SampA11

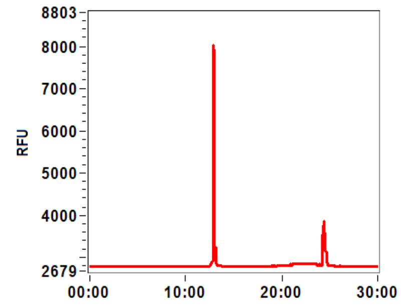

B11: SampB11

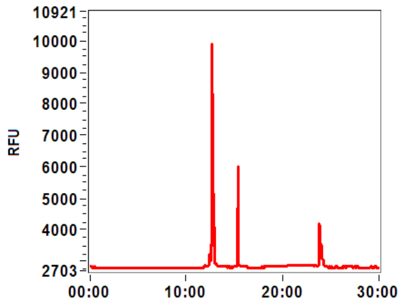

C11: SampC11

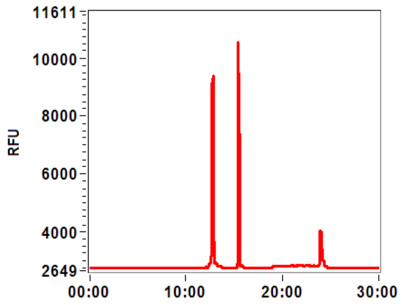

D11: SampD11

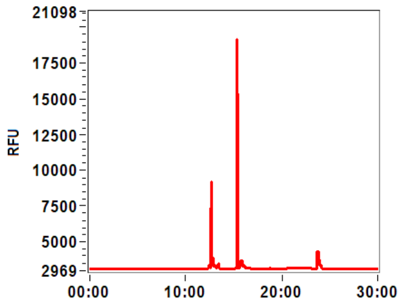

Filename and data path: C:\AATI\Data\2021 09 14\21-24-18\2021 09 14 21H 24M..raw

E11: SampE11

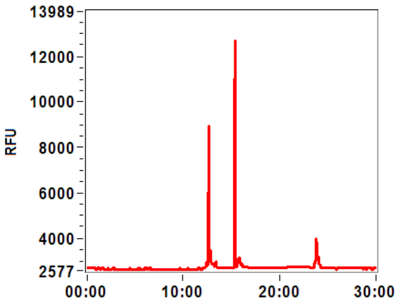

F11: SampF11

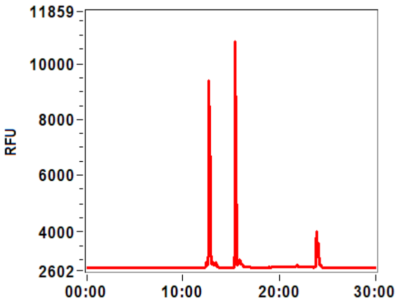

G11: SampG11

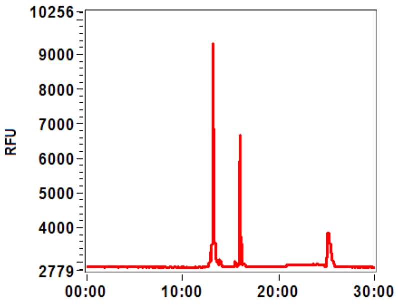

H11: SampH11

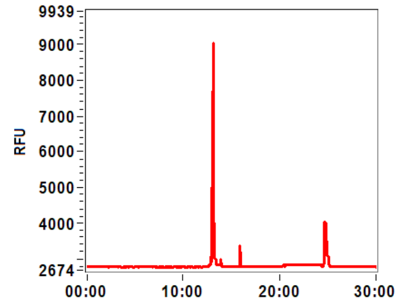

A12: SampA12

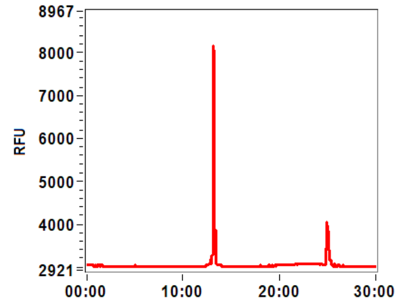

B12: SampB12

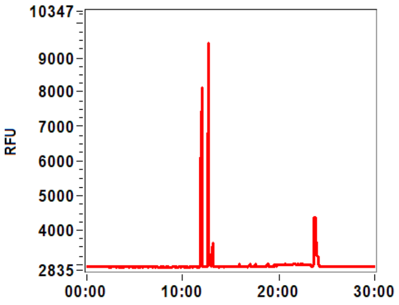

C12: SampC12

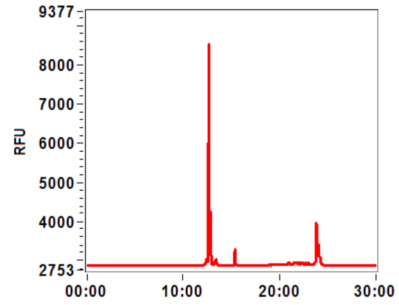

D12: SampD12

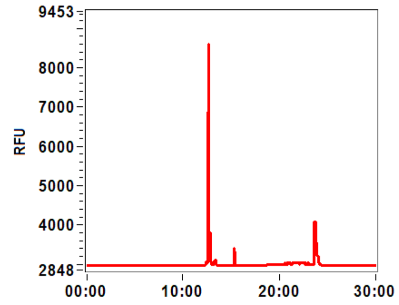

E12: SampE12

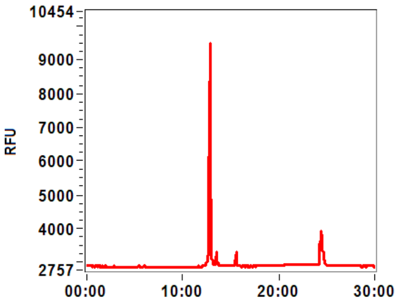

F12: SampF12

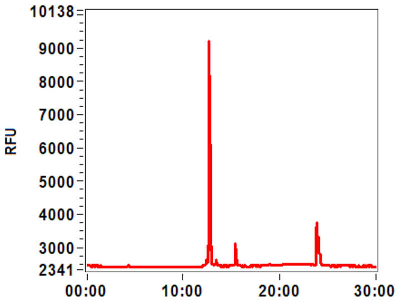

G12: SampG12

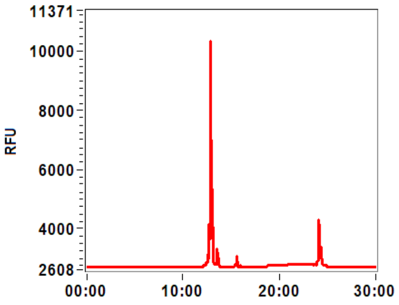

H12: SampH12

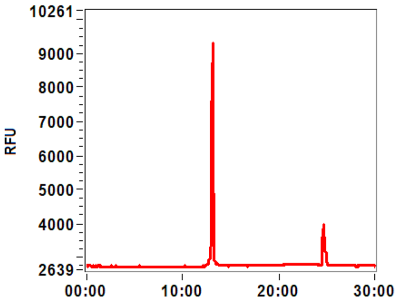

Sample: SampA1
Well location: A1
Created: Tuesday, September 14, 2021 9:33:53 PM

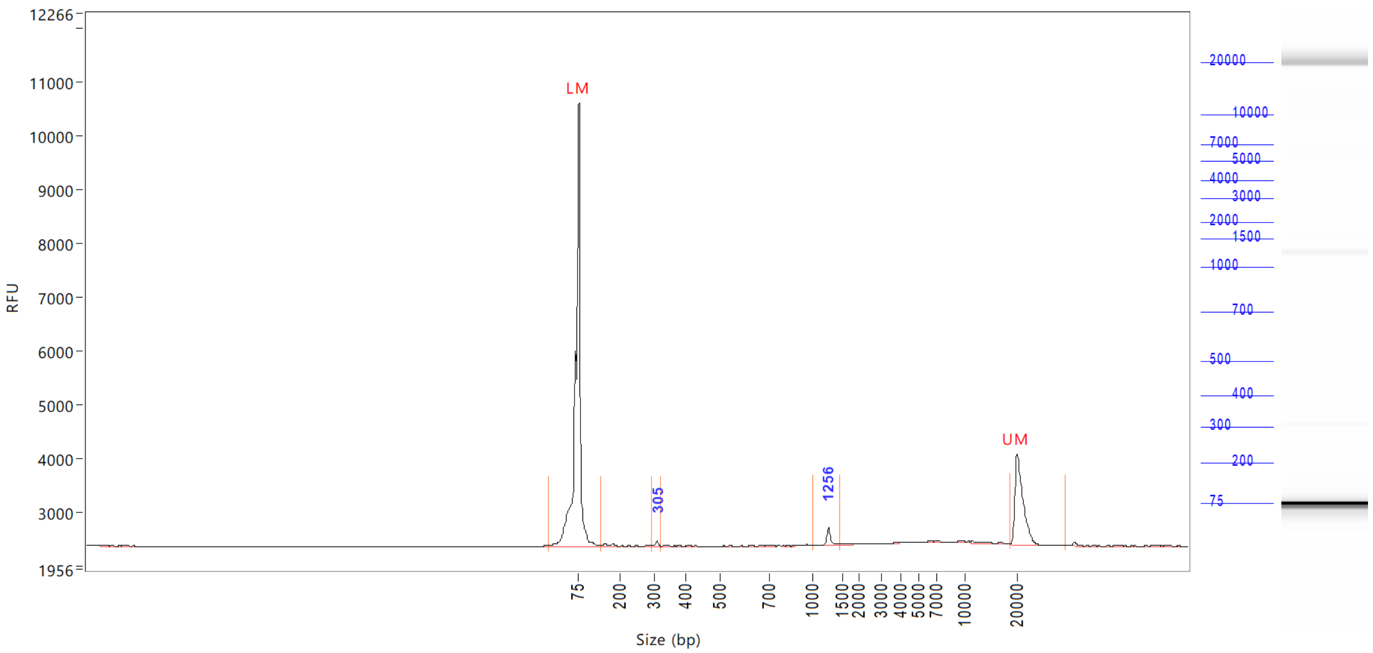

Peak	Size (bp)	Concentration (ng/uL)	From (bp)	To (bp)	Average size (bp)	CV%	RFU	Corrected peak area
1	75 (LM)	0.5000	0	141	65	24.34	8238	89.880
2	305	0.0398	289	317	302	1.40	111	0.596
3	1256	0.1300	1000	1467	1257	4.39	319	1.948
4	20000 (UM)	0.0960	18537	29430	20551	4.69	1688	17.255

TIC:	0.1698	ng/uL
TIM:	0.3870	nmole/L
Total concentration:	0.4860	ng/uL

```
Sample peak width (sec): 5    Sample min peak height: 50    Sample baseline V to V?: Y    Sample baseline V to V points: 3
Sample filter: Binomial       Number of points for filter: 3  Sample start region (min): 0  Sample end region (min): 30
Marker peak width (sec): 5    Marker min peak height: 200   Marker baseline V to V?: Y    Marker baseline V to V points: 3
Lower marker selection: First peak > 500 RFU                Upper marker selection: Last peak > 200 RFU
Ladder size (bp)75, 200, 300, 400, 500, 700, 1000, 1500, 2000, 3000, 4000, 5000, 7000, 10000, 20000
Quantification using: Lower Marker          Final concentration (ng/uL): 0.5000          Dilution factor: 12.0
```

Sample: SampB1
Well location: B1
Created: Tuesday, September 14, 2021 9:33:53 PM

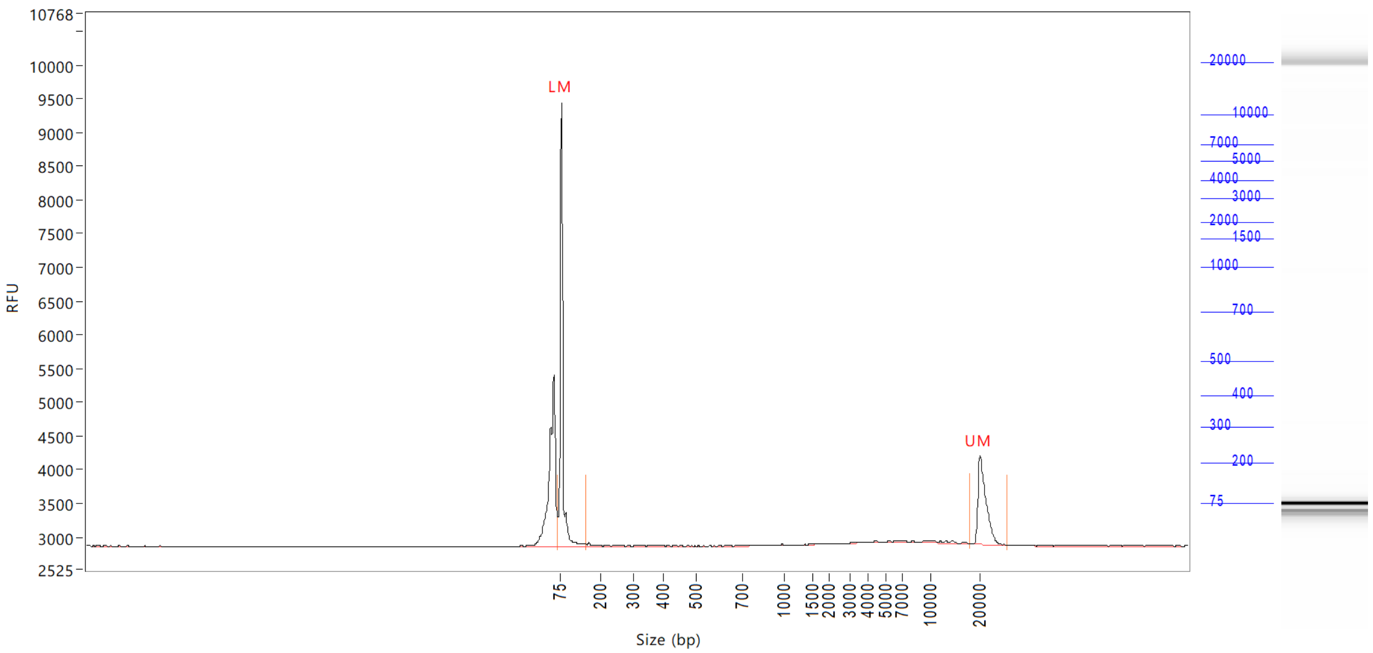

Peak	Size (bp)	Concentration (ng/uL)	From (bp)	To (bp)	Average size (bp)	CV%	RFU	Corrected peak area
1	75 (LM)	0.5000	66	154	76	14.23	6583	42.205
2	20000 (UM)	0.1630	18049	25561	20628	4.64	1308	13.760

TIC:	0.0000	ng/uL
TIM:	0.0000	nmole/L
Total concentration:	0.6240	ng/uL

Sample peak width (sec): 5   Sample min peak height: 50   Sample baseline V to V?: Y   Sample baseline V to V points: 3
Sample filter: Binomial   Number of points for filter: 3   Sample start region (min): 0   Sample end region (min): 30
Marker peak width (sec): 5   Marker min peak height: 200   Marker baseline V to V?: Y   Marker baseline V to V points: 3
Lower marker selection: First peak > 500 RFU   Upper marker selection: Last peak > 200 RFU
Ladder size (bp)75, 200, 300, 400, 500, 700, 1000, 1500, 2000, 3000, 4000, 5000, 7000, 10000, 20000
Quantification using: Lower Marker   Final concentration (ng/uL): 0.5000   Dilution factor: 12.0

Sample: SampC1
Well location: C1
Created: Tuesday, September 14, 2021 9:33:53 PM

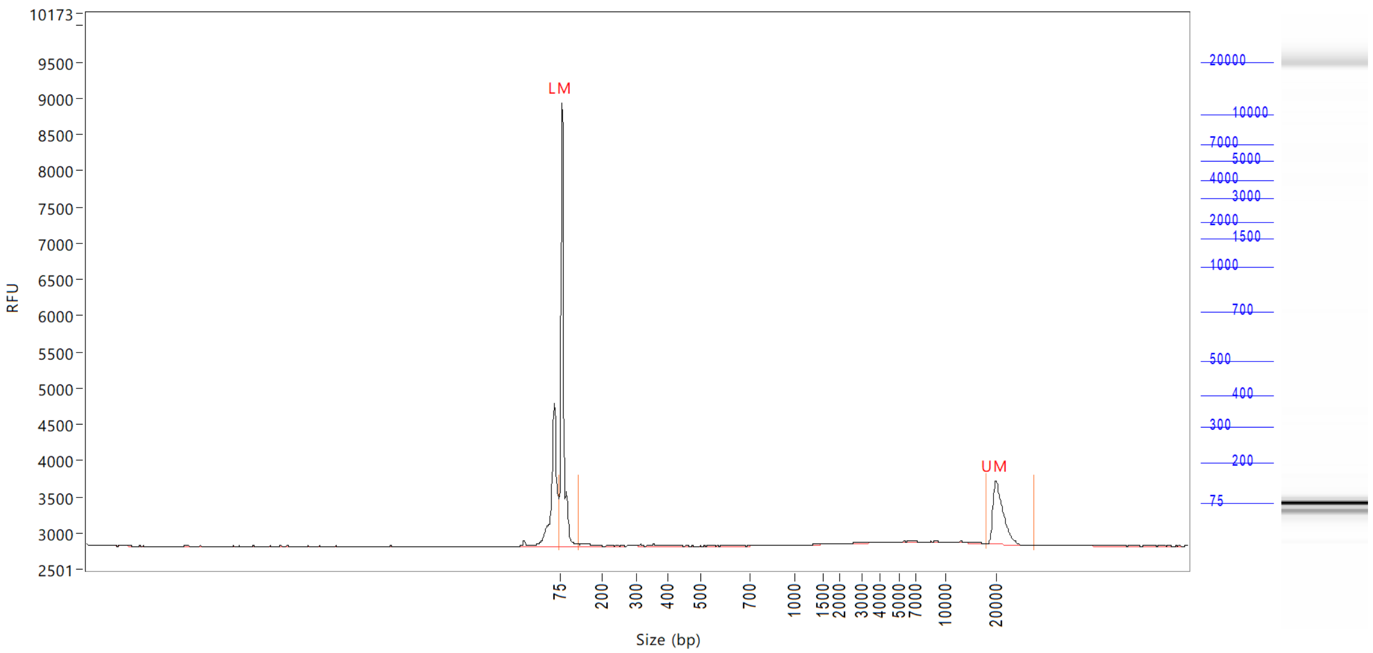

Peak	Size (bp)	Concentration (ng/uL)	From (bp)	To (bp)	Average size (bp)	CV%	RFU	Corrected peak area
1	75 (LM)	0.5000	66	129	76	10.19	6127	43.722
2	20000 (UM)	0.1314	18049	27375	20551	5.49	882	11.489

TIC:	0.0000	ng/uL
TIM:	0.0000	nmole/L
Total concentration:	0.5567	ng/uL

```
Sample peak width (sec): 5    Sample min peak height: 50    Sample baseline V to V?: Y    Sample baseline V to V points: 3
Sample filter: Binomial       Number of points for filter: 3  Sample start region (min): 0  Sample end region (min): 30
Marker peak width (sec): 5    Marker min peak height: 200   Marker baseline V to V?: Y    Marker baseline V to V points: 3
Lower marker selection: First peak > 500 RFU                Upper marker selection: Last peak > 200 RFU
Ladder size (bp)75, 200, 300, 400, 500, 700, 1000, 1500, 2000, 3000, 4000, 5000, 7000, 10000, 20000
Quantification using: Lower Marker          Final concentration (ng/uL): 0.5000         Dilution factor: 12.0
```

Sample: SampD1
Well location: D1
Created: Tuesday, September 14, 2021 9:33:53 PM

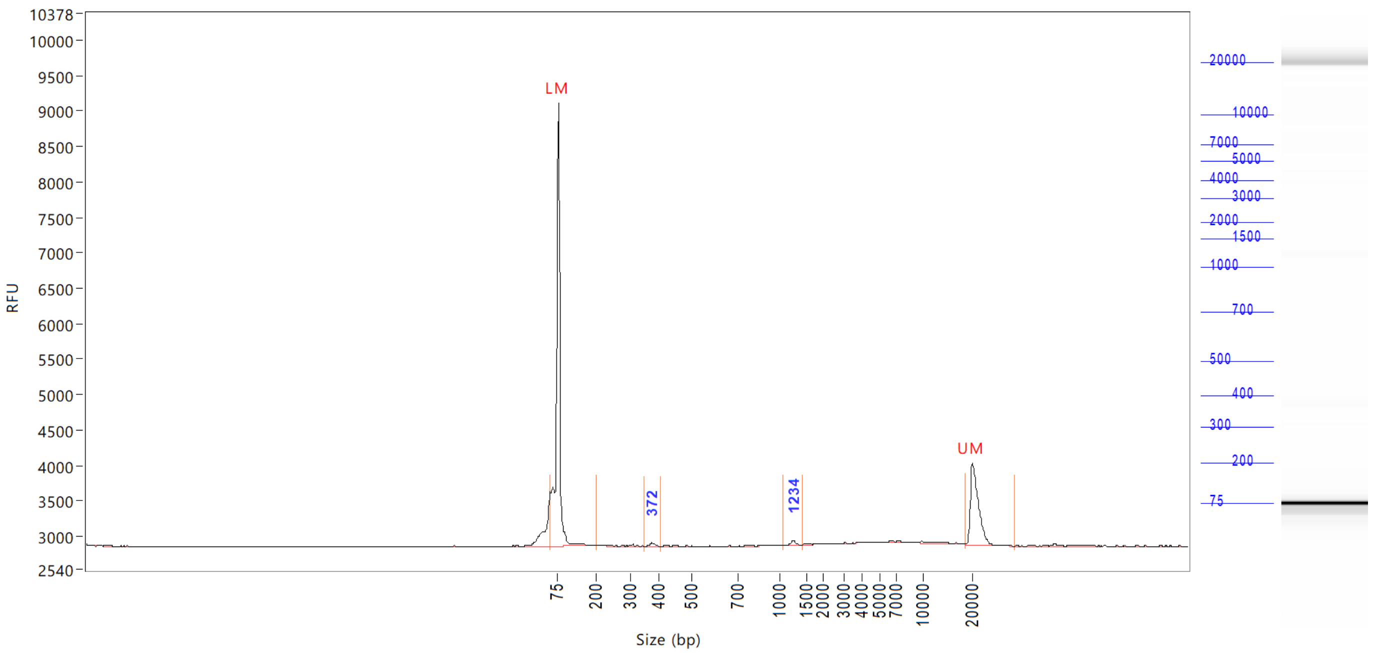

Peak	Size (bp)	Concentration (ng/uL)	From (bp)	To (bp)	Average size (bp)	CV%	RFU	Corrected peak area
1	75 (LM)	0.5000	52	198	73	17.33	6258	49.038
2	372	0.0664	347	400	372	2.39	52	0.543
3	1234	0.0592	1068	1411	1230	3.75	70	0.484
4	20000 (UM)	0.1211	18537	28463	20607	4.73	1144	11.872

TIC:	0.1257	ng/uL
TIM:	0.3730	nmole/L
Total concentration:	0.5155	ng/uL

Sample peak width (sec): 5   Sample min peak height: 50   Sample baseline V to V?: Y   Sample baseline V to V points: 3
Sample filter: Binomial   Number of points for filter: 3 Sample start region (min): 0 Sample end region (min): 30
Marker peak width (sec): 5   Marker min peak height: 200   Marker baseline V to V?: Y   Marker baseline V to V points: 3
Lower marker selection: First peak > 500 RFU   Upper marker selection: Last peak > 200 RFU
Ladder size (bp)75, 200, 300, 400, 500, 700, 1000, 1500, 2000, 3000, 4000, 5000, 7000, 10000, 20000
Quantification using: Lower Marker   Final concentration (ng/uL): 0.5000   Dilution factor: 12.0

Sample: SampE1
Well location: E1
Created: Tuesday, September 14, 2021 9:33:53 PM

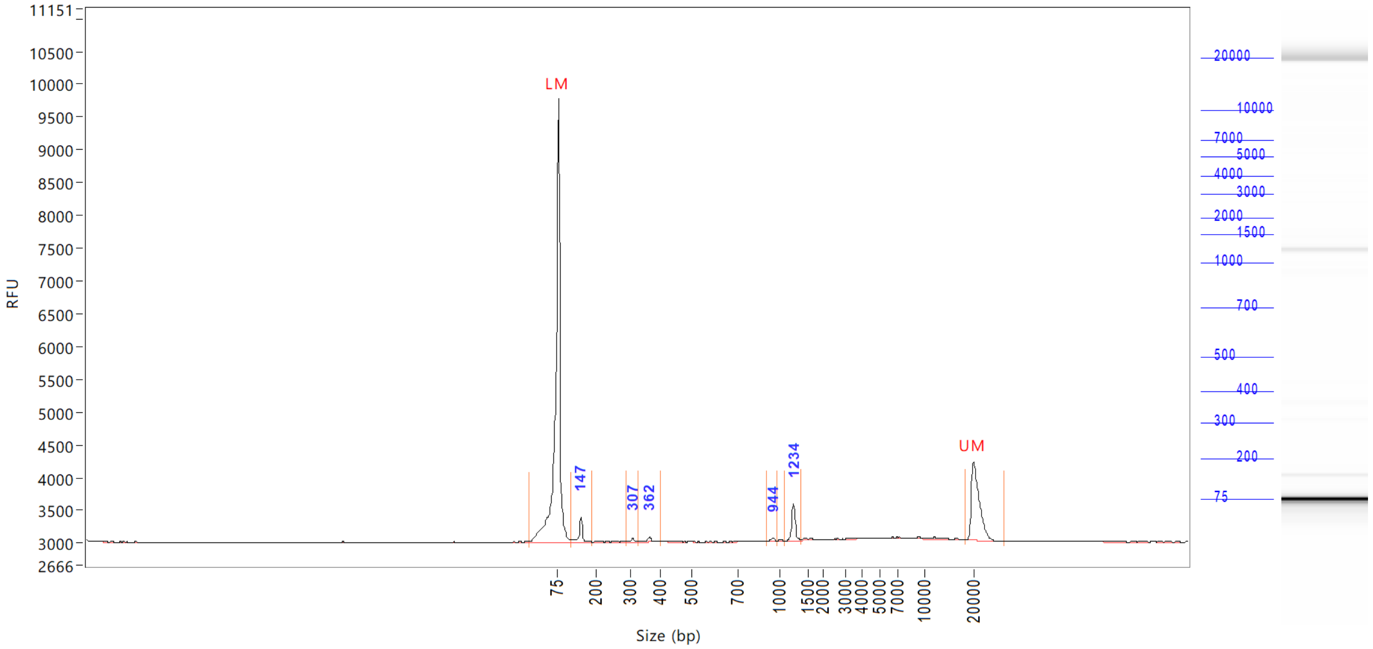

Peak	Size (bp)	Concentration (ng/uL)	From (bp)	To (bp)	Average size (bp)	CV%	RFU	Corrected peak area
1	75 (LM)	0.5000	0	116	65	27.41	6778	68.852
2	147	0.2723	116	185	143	7.44	383	3.124
3	307	0.0356	286	327	305	2.35	67	0.409
4	362	0.0491	327	400	361	2.96	69	0.564
5	944	0.0339	896	978	939	1.72	54	0.389
6	1234	0.2869	1068	1389	1223	3.33	563	3.293
7	20000 (UM)	0.0900	18293	26408	20533	4.62	1191	12.397

TIC:	0.6779	ng/uL
TIM:	3.9906	nmole/L
Total concentration:	0.9588	ng/uL

Sample peak width (sec): 5   Sample min peak height: 50   Sample baseline V to V?: Y   Sample baseline V to V points: 3
Sample filter: Binomial   Number of points for filter: 3 Sample start region (min): 0 Sample end region (min): 30
Marker peak width (sec): 5   Marker min peak height: 200   Marker baseline V to V?: Y   Marker baseline V to V points: 3
Lower marker selection: First peak > 500 RFU   Upper marker selection: Last peak > 200 RFU
Ladder size (bp)75, 200, 300, 400, 500, 700, 1000, 1500, 2000, 3000, 4000, 5000, 7000, 10000, 20000
Quantification using: Lower Marker   Final concentration (ng/uL): 0.5000   Dilution factor: 12.0

Sample: SampF1
Well location: F1
Created: Tuesday, September 14, 2021 9:33:53 PM

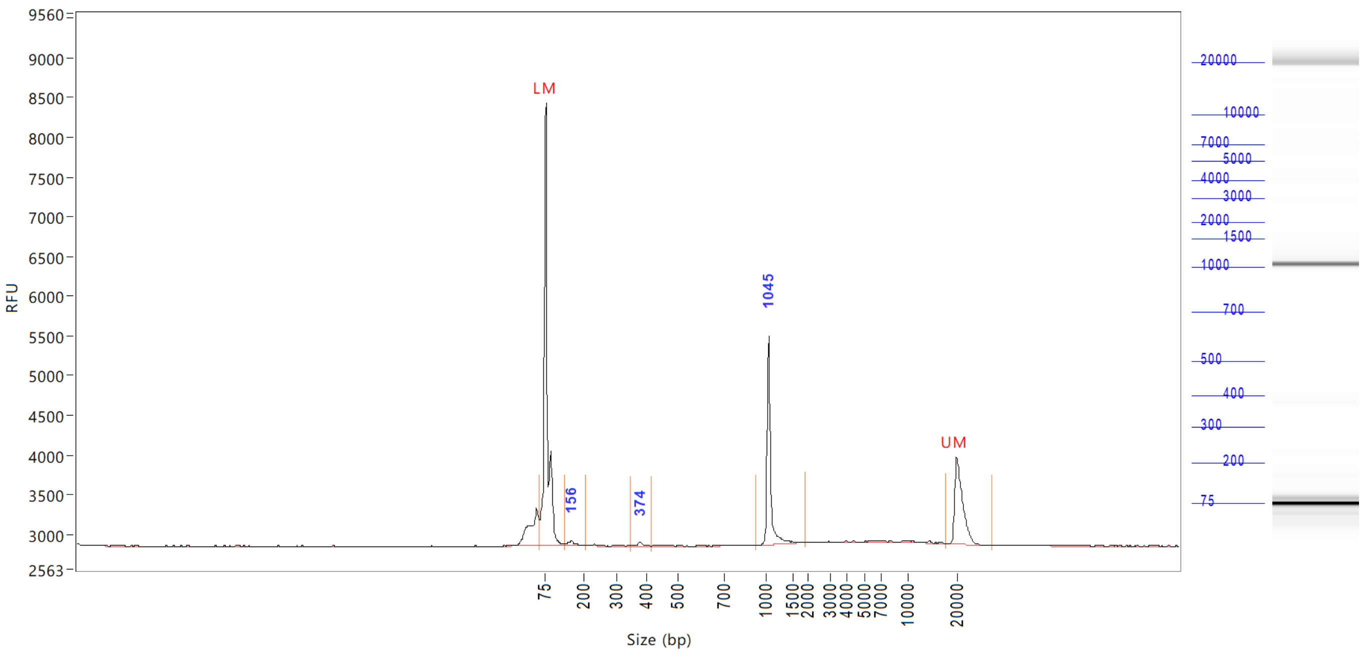

Peak	Size (bp)	Concentration (ng/uL)	From (bp)	To (bp)	Average size (bp)	CV%	RFU	Corrected peak area
1	75 (LM)	0.5000	54	135	76	14.10	5582	48.393
2	156	0.1267	135	203	155	8.26	71	1.022
3	374	0.0725	347	412	378	3.27	52	0.584
4	1045	1.9817	931	1904	1069	8.70	2626	15.983
5	20000 (UM)	0.1180	17683	27375	20628	4.75	1091	11.419

TIC:	2.1808	ng/uL
TIM:	4.7100	nmole/L
Total concentration:	2.5162	ng/uL

Sample peak width (sec): 5 Sample min peak height: 50 Sample baseline V to V?: Y Sample baseline V to V points: 3
Sample filter: Binomial Number of points for filter: 3 Sample start region (min): 0 Sample end region (min): 30
Marker peak width (sec): 5 Marker min peak height: 200 Marker baseline V to V?: Y Marker baseline V to V points: 3
Lower marker selection: First peak > 500 RFU Upper marker selection: Last peak > 200 RFU
Ladder size (bp)75, 200, 300, 400, 500, 700, 1000, 1500, 2000, 3000, 4000, 5000, 7000, 10000, 20000
Quantification using: Lower Marker Final concentration (ng/uL): 0.5000 Dilution factor: 12.0

Sample: SampG1
Well location: G1
Created: Tuesday, September 14, 2021 9:33:53 PM

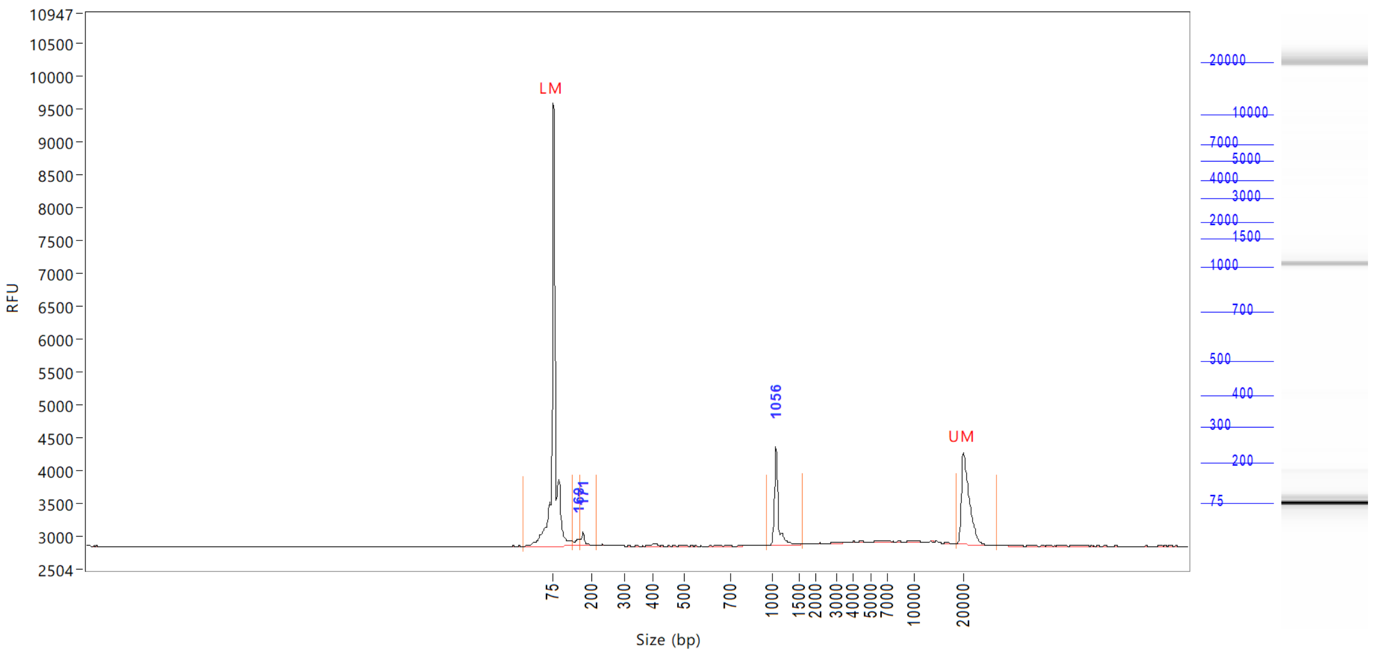

Peak	Size (bp)	Concentration (ng/uL)	From (bp)	To (bp)	Average size (bp)	CV%	RFU	Corrected peak area
1	75 (LM)	0.5000	0	139	73	24.12	6742	60.573
2	160	0.1370	139	164	153	4.86	103	1.383
3	171	0.1624	164	212	172	4.62	219	1.639
4	1056	0.9937	952	1597	1082	8.42	1506	10.031
5	20000 (UM)	0.1201	18659	27012	20624	4.66	1385	14.555

TIC:	1.2931	ng/uL
TIM:	4.5357	nmole/L
Total concentration:	1.5853	ng/uL

Sample peak width (sec): 5   Sample min peak height: 50   Sample baseline V to V?: Y   Sample baseline V to V points: 3
Sample filter: Binomial   Number of points for filter: 3 Sample start region (min): 0 Sample end region (min): 30
Marker peak width (sec): 5   Marker min peak height: 200   Marker baseline V to V?: Y   Marker baseline V to V points: 3
Lower marker selection: First peak > 500 RFU   Upper marker selection: Last peak > 200 RFU
Ladder size (bp)75, 200, 300, 400, 500, 700, 1000, 1500, 2000, 3000, 4000, 5000, 7000, 10000, 20000
Quantification using: Lower Marker   Final concentration (ng/uL): 0.5000   Dilution factor: 12.0

Sample: SampH1
Well location: H1
Created: Tuesday, September 14, 2021 9:33:53 PM

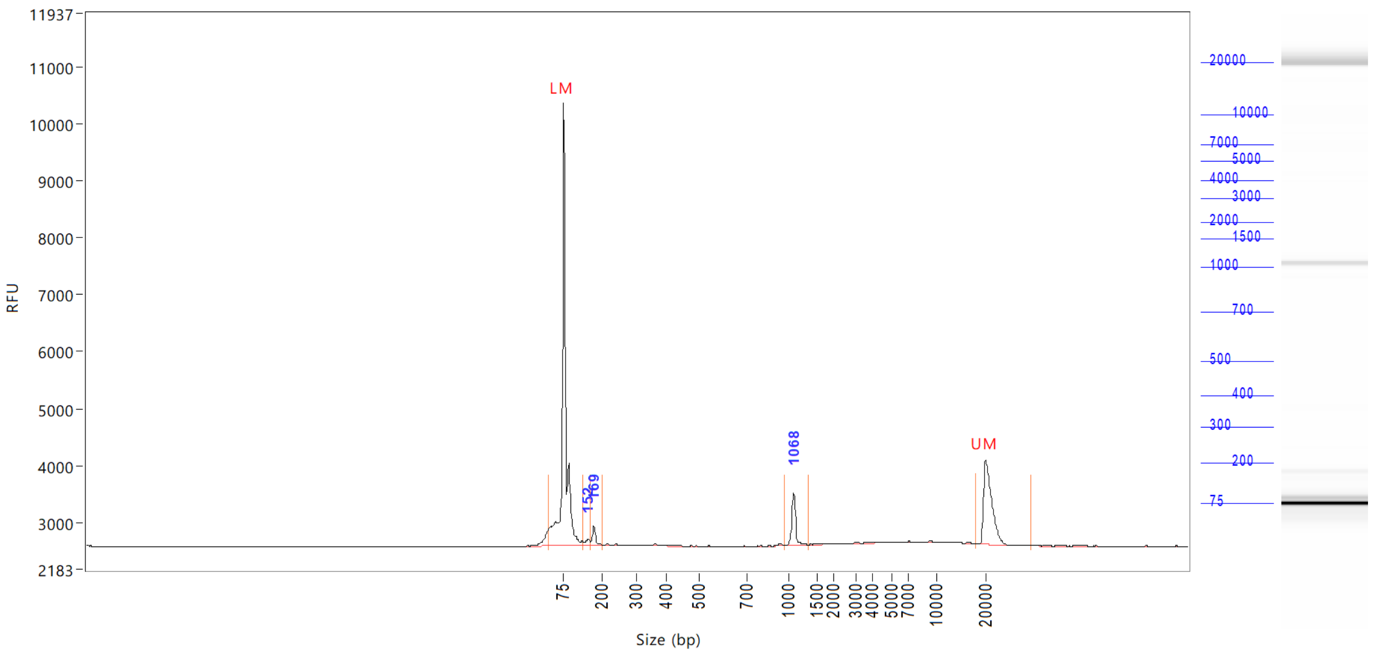

Peak	Size<br>(bp)	Concentration<br>(ng/uL)	From<br>(bp)	To<br>(bp)	Average size<br>(bp)	CV%	RFU	Corrected peak area
1	75 (LM)	0.5000	30	137	74	20.40	7780	69.195
2	152	0.0993	137	160	149	4.32	108	1.145
3	169	0.2160	160	198	169	3.32	326	2.491
4	1068	0.4916	965	1334	1064	4.31	928	5.669
5	20000 (UM)	0.1124	17927	29189	20552	4.83	1484	15.549

TIC:	0.8069	ng/uL
TIM:	3.9571	nmole/L
Total concentration:	1.0785	ng/uL

Sample peak width (sec): 5 Sample min peak height: 50 Sample baseline V to V?: Y Sample baseline V to V points: 3
Sample filter: Binomial Number of points for filter: 3 Sample start region (min): 0 Sample end region (min): 30
Marker peak width (sec): 5 Marker min peak height: 200 Marker baseline V to V?: Y Marker baseline V to V points: 3
Lower marker selection: First peak > 500 RFU Upper marker selection: Last peak > 200 RFU
Ladder size (bp)75, 200, 300, 400, 500, 700, 1000, 1500, 2000, 3000, 4000, 5000, 7000, 10000, 20000
Quantification using: Lower Marker Final concentration (ng/uL): 0.5000 Dilution factor: 12.0

Sample: SampA2
Well location: A2
Created: Tuesday, September 14, 2021 9:33:53 PM

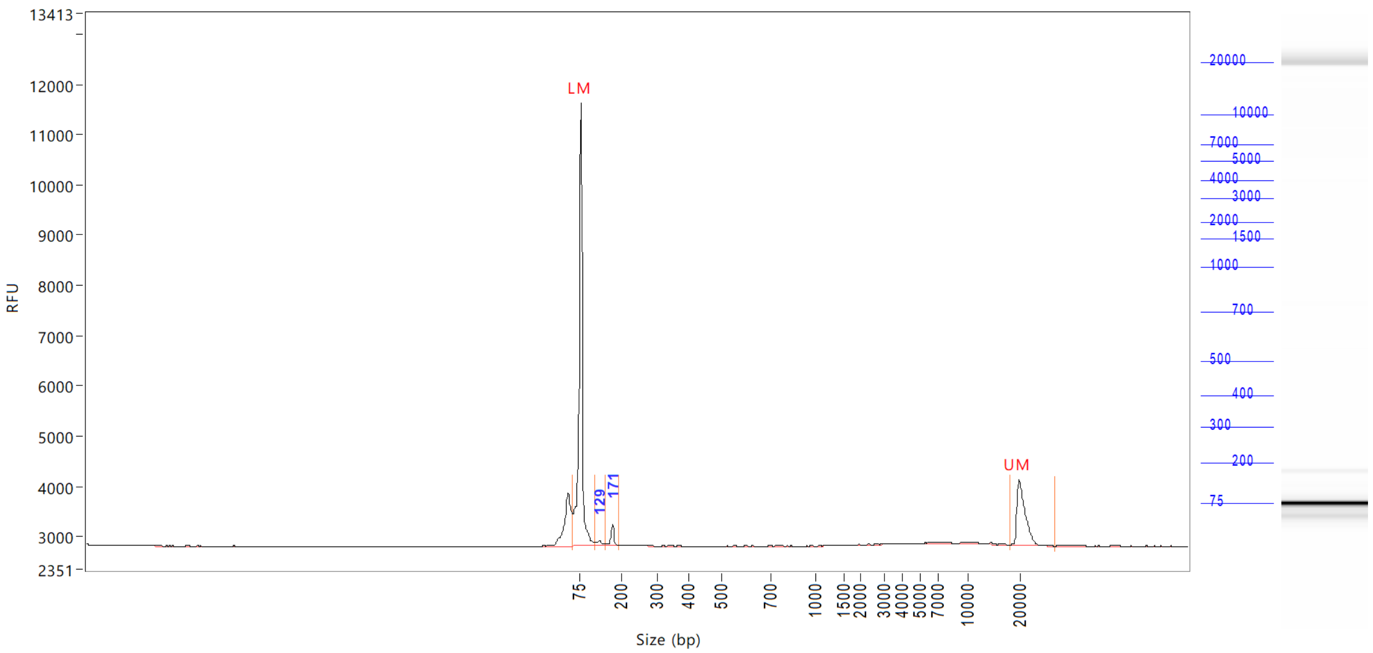

Peak	Size (bp)	Concentration (ng/uL)	From (bp)	To (bp)	Average size (bp)	CV%	RFU	Corrected peak area
1	75 (LM)	0.5000	48	118	72	12.52	8830	70.600
2	129	0.1111	118	150	132	7.18	91	1.307
3	171	0.2362	150	190	168	3.64	417	2.780
4	20000 (UM)	0.0959	18171	26891	20578	4.61	1315	13.542

TIC:	0.3474	ng/uL
TIM:	3.6947	nmole/L
Total concentration:	0.7179	ng/uL

```
Sample peak width (sec): 5    Sample min peak height: 50    Sample baseline V to V?: Y    Sample baseline V to V points: 3
Sample filter: Binomial       Number of points for filter: 3  Sample start region (min): 0  Sample end region (min): 30
Marker peak width (sec): 5    Marker min peak height: 200   Marker baseline V to V?: Y    Marker baseline V to V points: 3
Lower marker selection: First peak > 500 RFU                Upper marker selection: Last peak > 200 RFU
Ladder size (bp)75, 200, 300, 400, 500, 700, 1000, 1500, 2000, 3000, 4000, 5000, 7000, 10000, 20000
Quantification using: Lower Marker          Final concentration (ng/uL): 0.5000          Dilution factor: 12.0
```

Sample: SampB2
Well location: B2
Created: Tuesday, September 14, 2021 9:33:53 PM

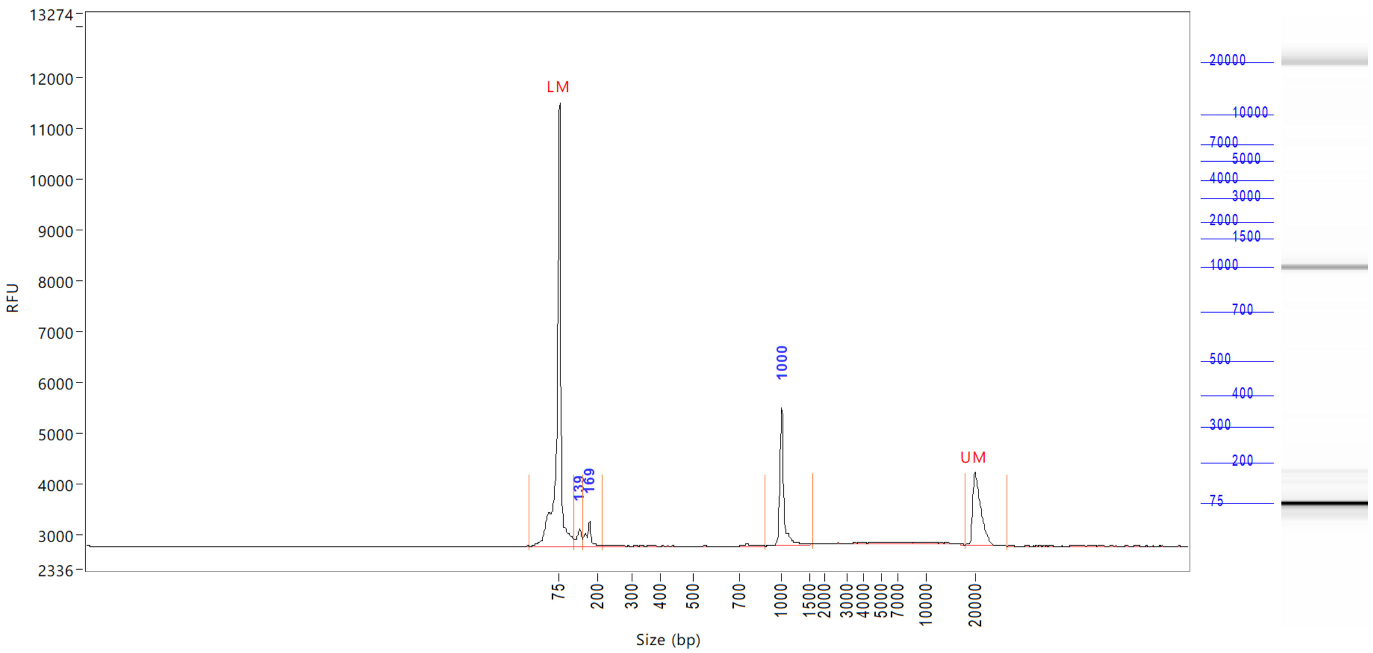

Peak	Size (bp)	Concentration (ng/uL)	From (bp)	To (bp)	Average size (bp)	CV%	RFU	Corrected peak area
1	75 (LM)	0.5000	0	124	67	26.36	8735	84.764
2	139	0.2678	124	148	136	4.83	350	3.783
3	169	0.3865	148	210	167	7.16	502	5.461
4	1000	1.2585	883	1597	1028	8.73	2708	17.779
5	20000 (UM)	0.0882	18049	26529	20577	4.60	1431	14.945

TIC:	1.9128	ng/uL
TIM:	9.0551	nmole/L
Total concentration:	2.2438	ng/uL

Sample peak width (sec): 5   Sample min peak height: 50   Sample baseline V to V?: Y   Sample baseline V to V points: 3
Sample filter: Binomial   Number of points for filter: 3 Sample start region (min): 0 Sample end region (min): 30
Marker peak width (sec): 5   Marker min peak height: 200   Marker baseline V to V?: Y   Marker baseline V to V points: 3
Lower marker selection: First peak > 500 RFU   Upper marker selection: Last peak > 200 RFU
Ladder size (bp)75, 200, 300, 400, 500, 700, 1000, 1500, 2000, 3000, 4000, 5000, 7000, 10000, 20000
Quantification using: Lower Marker   Final concentration (ng/uL): 0.5000   Dilution factor: 12.0

Sample: SampC2
Well location: C2
Created: Tuesday, September 14, 2021 9:33:53 PM

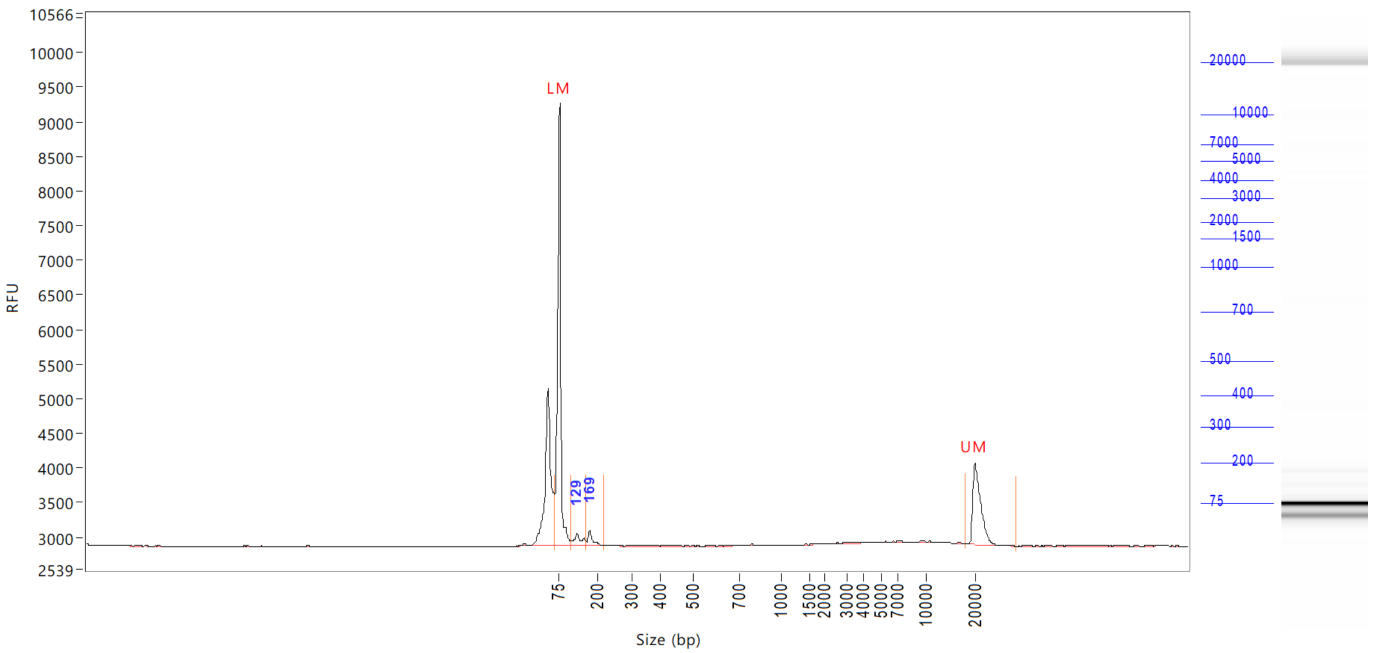

Peak	Size (bp)	Concentration (ng/uL)	From (bp)	To (bp)	Average size (bp)	CV%	RFU	Corrected peak area
1	75 (LM)	0.5000	60	112	73	10.08	6401	49.208
2	129	0.3609	112	160	134	9.73	182	2.960
3	169	0.2860	160	215	174	5.82	227	2.346
4	20000 (UM)	0.1230	18049	28463	20588	4.84	1186	12.102

TIC:	0.6470	ng/uL
TIM:	7.1283	nmole/L
Total concentration:	1.0134	ng/uL

Sample peak width (sec): 5 Sample min peak height: 50 Sample baseline V to V?: Y Sample baseline V to V points: 3
Sample filter: Binomial Number of points for filter: 3 Sample start region (min): 0 Sample end region (min): 30
Marker peak width (sec): 5 Marker min peak height: 200 Marker baseline V to V?: Y Marker baseline V to V points: 3
Lower marker selection: First peak > 500 RFU Upper marker selection: Last peak > 200 RFU
Ladder size (bp)75, 200, 300, 400, 500, 700, 1000, 1500, 2000, 3000, 4000, 5000, 7000, 10000, 20000
Quantification using: Lower Marker Final concentration (ng/uL): 0.5000 Dilution factor: 12.0

Sample: SampD2
Well location: D2
Created: Tuesday, September 14, 2021 9:33:53 PM

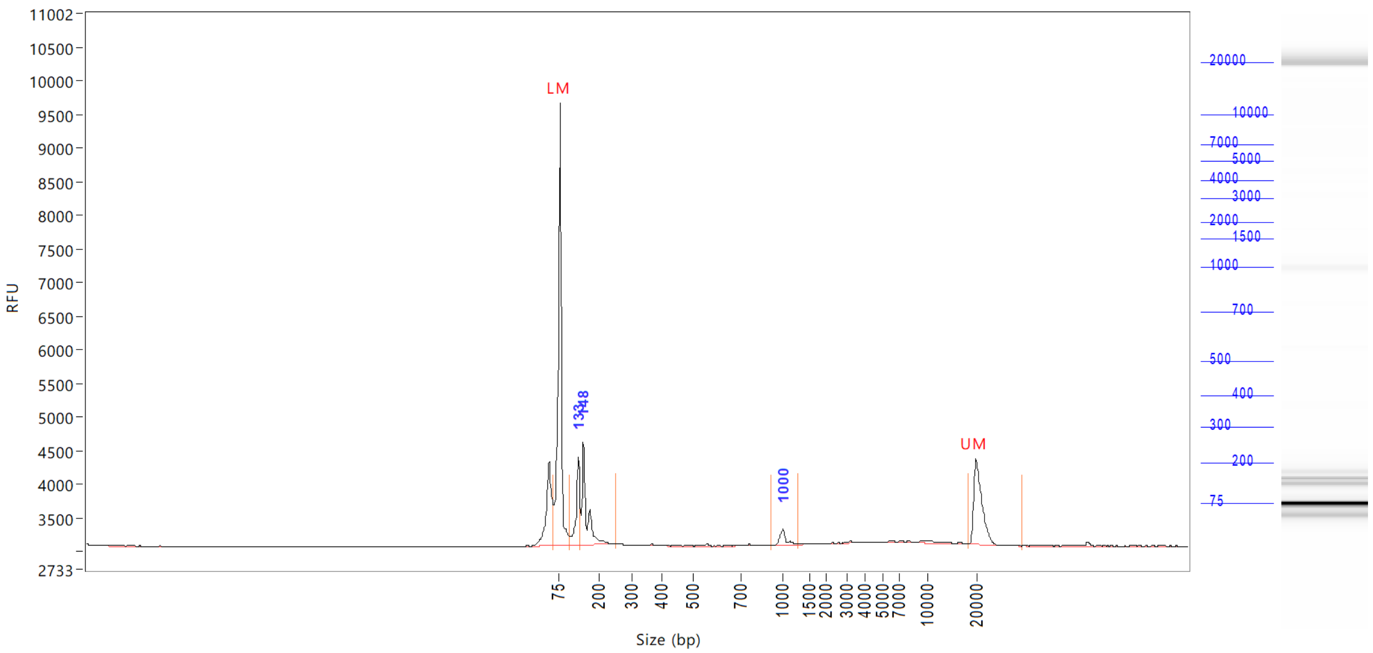

Peak	Size (bp)	Concentration (ng/uL)	From (bp)	To (bp)	Average size (bp)	CV%	RFU	Corrected peak area
1	75 (LM)	0.5000	56	105	72	10.03	6592	51.597
2	133	1.3492	105	141	129	6.36	1304	11.602
3	148	1.8907	141	253	159	11.37	1516	16.259
4	1000	0.2758	918	1278	1021	6.21	243	2.372
5	20000 (UM)	0.1302	18415	29430	20543	4.97	1265	13.441

TIC:	3.5157	ng/uL
TIM:	37.1742	nmole/L
Total concentration:	3.8885	ng/uL

Sample peak width (sec): 5   Sample min peak height: 50   Sample baseline V to V?: Y   Sample baseline V to V points: 3
Sample filter: Binomial   Number of points for filter: 3   Sample start region (min): 0   Sample end region (min): 30
Marker peak width (sec): 5   Marker min peak height: 200   Marker baseline V to V?: Y   Marker baseline V to V points: 3
Lower marker selection: First peak > 500 RFU   Upper marker selection: Last peak > 200 RFU
Ladder size (bp)75, 200, 300, 400, 500, 700, 1000, 1500, 2000, 3000, 4000, 5000, 7000, 10000, 20000
Quantification using: Lower Marker   Final concentration (ng/uL): 0.5000   Dilution factor: 12.0

Sample: SampE2
Well location: E2
Created: Tuesday, September 14, 2021 9:33:53 PM

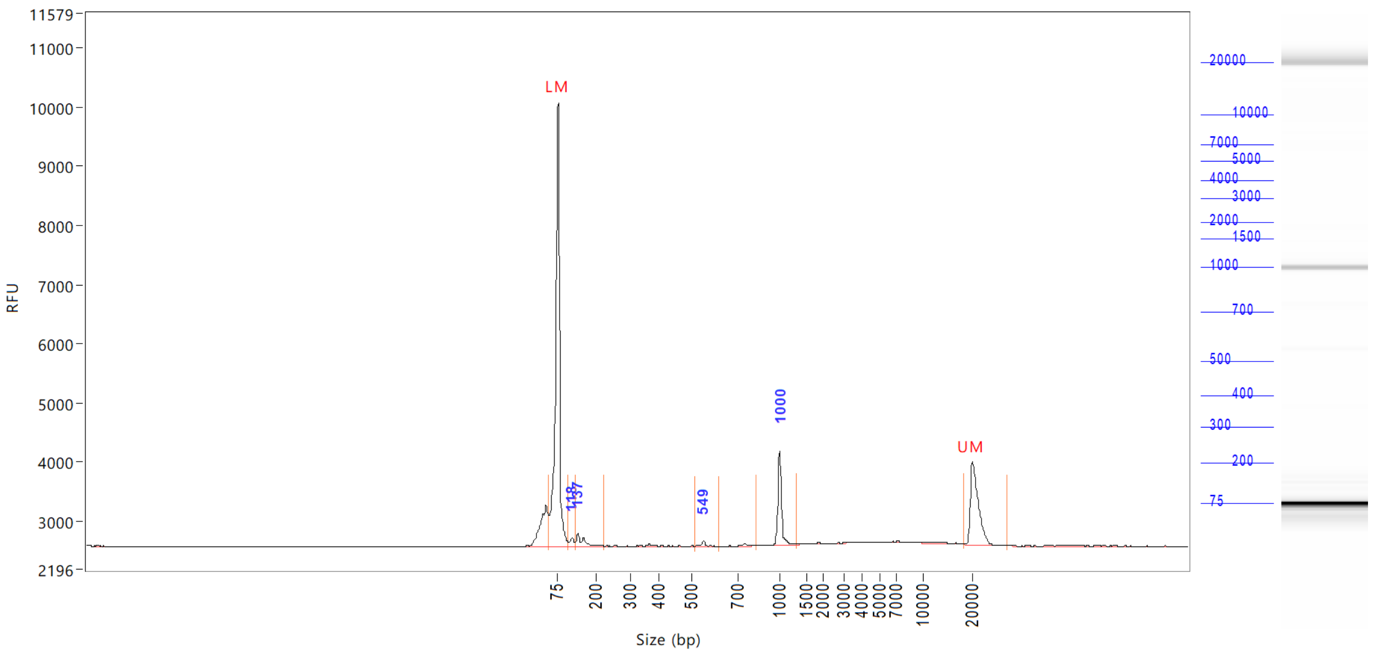

Peak	Size (bp)	Concentration (ng/uL)	From (bp)	To (bp)	Average size (bp)	CV%	RFU	Corrected peak area
1	75 (LM)	0.5000	48	110	70	12.12	7492	69.163
2	118	0.1138	110	129	119	4.68	142	1.311
3	137	0.2591	129	219	150	10.94	215	2.987
4	549	0.0540	511	618	550	3.06	93	0.622
5	1000	0.8101	835	1311	1006	4.21	1589	9.338
6	20000 (UM)	0.1041	18171	27133	20604	4.73	1392	14.406

TIC:	1.2369	ng/uL
TIM:	5.8966	nmole/L
Total concentration:	1.5140	ng/uL

Sample peak width (sec): 5   Sample min peak height: 50   Sample baseline V to V?: Y   Sample baseline V to V points: 3
Sample filter: Binomial   Number of points for filter: 3 Sample start region (min): 0 Sample end region (min): 30
Marker peak width (sec): 5   Marker min peak height: 200   Marker baseline V to V?: Y   Marker baseline V to V points: 3
Lower marker selection: First peak > 500 RFU   Upper marker selection: Last peak > 200 RFU
Ladder size (bp)75, 200, 300, 400, 500, 700, 1000, 1500, 2000, 3000, 4000, 5000, 7000, 10000, 20000
Quantification using: Lower Marker   Final concentration (ng/uL): 0.5000   Dilution factor: 12.0

Sample: SampF2
Well location: F2
Created: Tuesday, September 14, 2021 9:33:53 PM

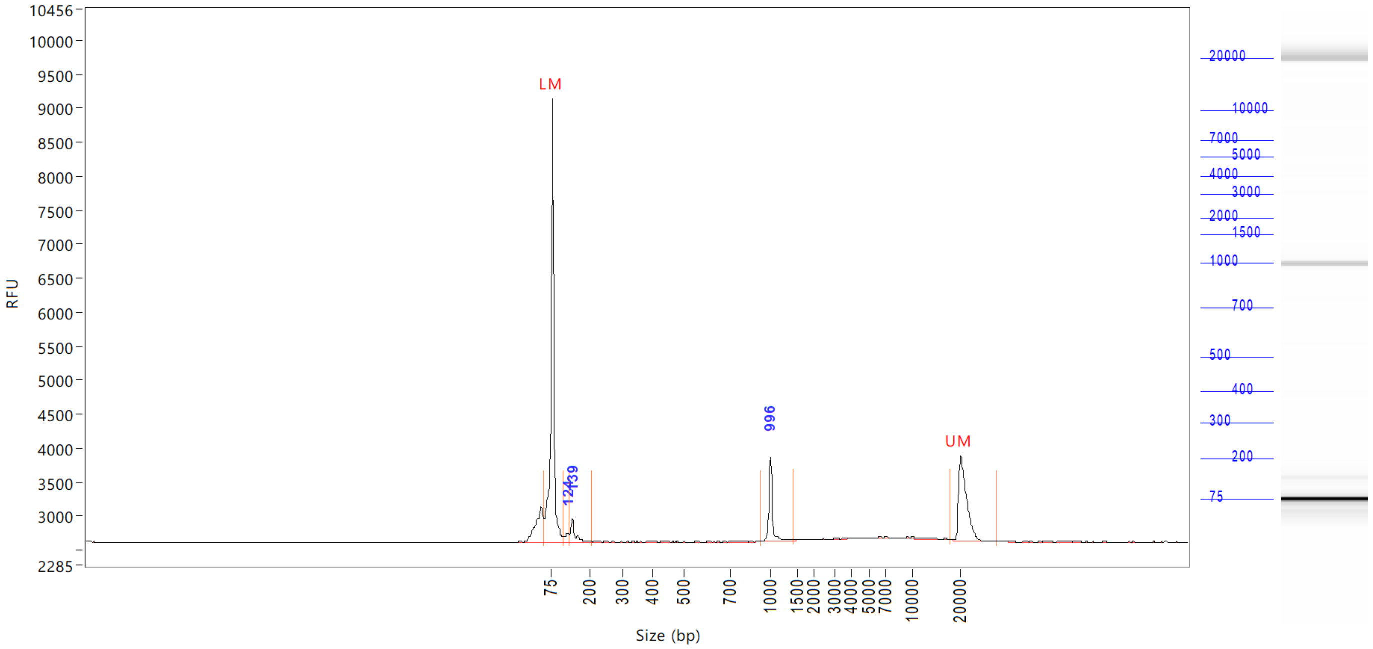

Peak	Size (bp)	Concentration (ng/uL)	From (bp)	To (bp)	Average size (bp)	CV%	RFU	Corrected peak area
1	75 (LM)	0.5000	48	112	72	12.30	6524	49.383
2	124	0.1571	112	129	121	4.28	130	1.293
3	139	0.4060	129	205	144	9.20	348	3.342
4	996	0.8956	926	1433	1011	6.15	1223	7.371
5	20000 (UM)	0.1311	17927	27617	20621	4.65	1235	12.945

TIC:	1.4587	ng/uL
TIM:	8.2244	nmole/L
Total concentration:	1.8666	ng/uL

```
Sample peak width (sec): 5    Sample min peak height: 50    Sample baseline V to V?: Y    Sample baseline V to V points: 3
Sample filter: Binomial       Number of points for filter: 3  Sample start region (min): 0  Sample end region (min): 30
Marker peak width (sec): 5    Marker min peak height: 200   Marker baseline V to V?: Y    Marker baseline V to V points: 3
Lower marker selection: First peak > 500 RFU                Upper marker selection: Last peak > 200 RFU
Ladder size (bp)75, 200, 300, 400, 500, 700, 1000, 1500, 2000, 3000, 4000, 5000, 7000, 10000, 20000
Quantification using: Lower Marker          Final concentration (ng/uL): 0.5000          Dilution factor: 12.0
```

Sample: SampG2
Well location: G2
Created: Tuesday, September 14, 2021 9:33:53 PM

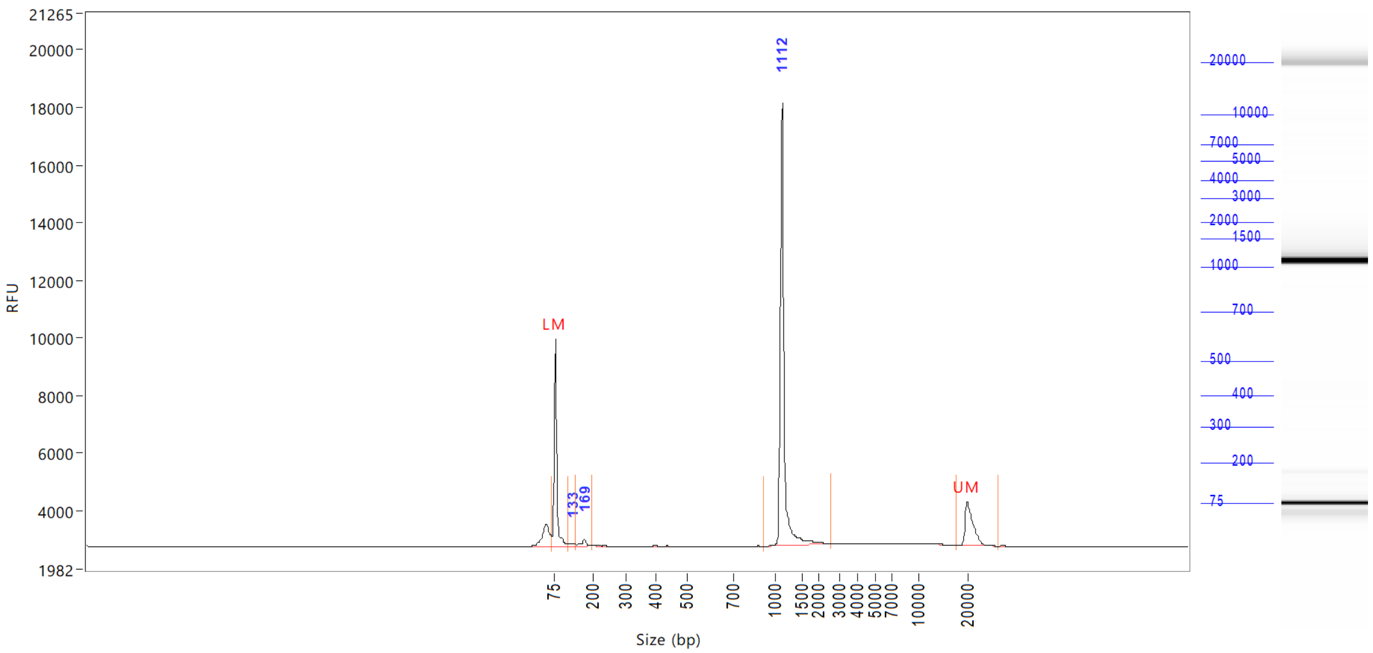

Peak	Size (bp)	Concentration (ng/uL)	From (bp)	To (bp)	Average size (bp)	CV%	RFU	Corrected peak area
1	75 (LM)	0.5000	66	120	76	9.91	7212	47.215
2	133	0.1434	120	143	131	5.16	83	1.129
3	169	0.2974	143	196	163	5.87	257	2.341
4	1112	11.9237	918	2600	1140	11.60	15379	93.828
5	20000 (UM)	0.1683	17683	26287	20503	4.59	1523	15.890

TIC:	12.3645	ng/uL
TIM:	22.0143	nmole/L
Total concentration:	12.7591	ng/uL

Sample peak width (sec): 5   Sample min peak height: 50   Sample baseline V to V?: Y   Sample baseline V to V points: 3
Sample filter: Binomial   Number of points for filter: 3 Sample start region (min): 0 Sample end region (min): 30
Marker peak width (sec): 5   Marker min peak height: 200   Marker baseline V to V?: Y   Marker baseline V to V points: 3
Lower marker selection: First peak > 500 RFU   Upper marker selection: Last peak > 200 RFU
Ladder size (bp)75, 200, 300, 400, 500, 700, 1000, 1500, 2000, 3000, 4000, 5000, 7000, 10000, 20000
Quantification using: Lower Marker   Final concentration (ng/uL): 0.5000   Dilution factor: 12.0

Sample: SampH2
Well location: H2
Created: Tuesday, September 14, 2021 9:33:53 PM

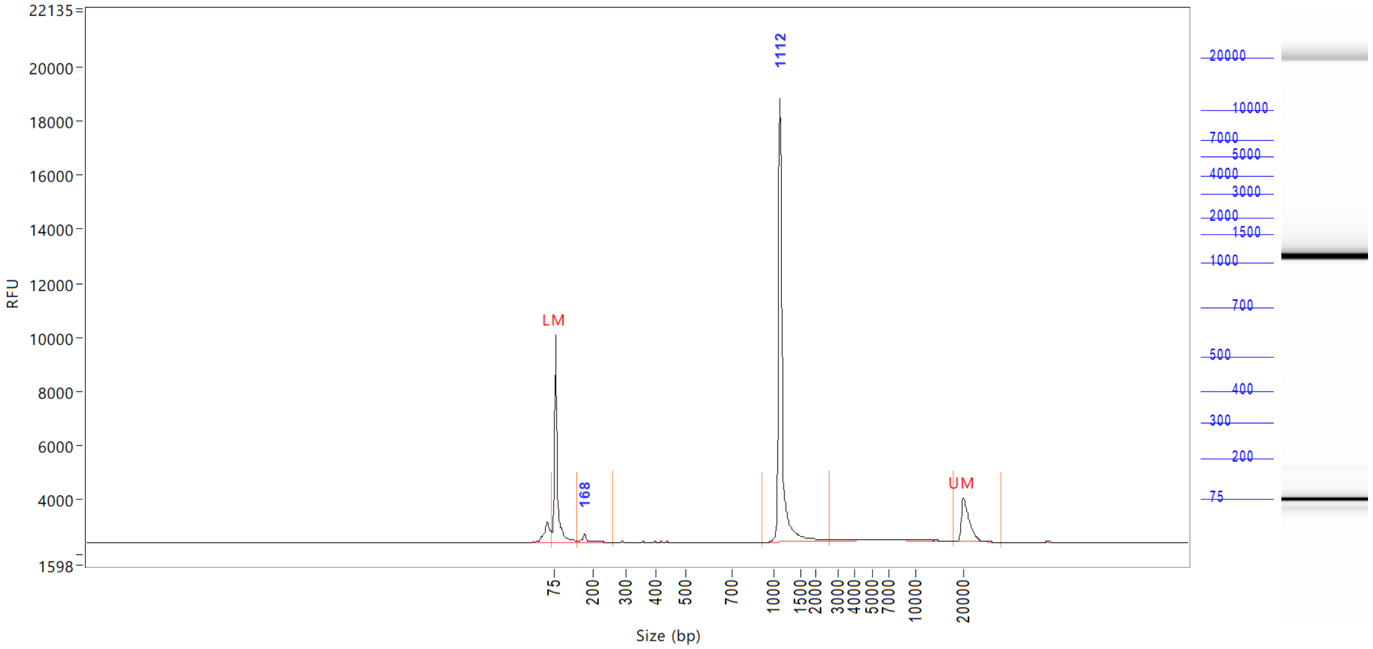

Peak	Size (bp)	Concentration (ng/uL)	From (bp)	To (bp)	Average size (bp)	CV%	RFU	Corrected peak area
1	75 (LM)	0.5000	64	145	78	16.20	7673	54.261
2	168	0.3701	145	259	173	11.65	296	3.347
3	1112	11.6029	913	2629	1150	14.06	16394	104.932
4	20000 (UM)	0.1547	18049	27979	20620	4.71	1625	16.784

TIC:	11.9730	ng/uL
TIM:	20.1239	nmole/L
Total concentration:	12.4635	ng/uL

```
Sample peak width (sec): 5    Sample min peak height: 50    Sample baseline V to V?: Y    Sample baseline V to V points: 3
Sample filter: Binomial       Number of points for filter: 3  Sample start region (min): 0  Sample end region (min): 30
Marker peak width (sec): 5    Marker min peak height: 200   Marker baseline V to V?: Y    Marker baseline V to V points: 3
Lower marker selection: First peak > 500 RFU                Upper marker selection: Last peak > 200 RFU
Ladder size (bp)75, 200, 300, 400, 500, 700, 1000, 1500, 2000, 3000, 4000, 5000, 7000, 10000, 20000
Quantification using: Lower Marker          Final concentration (ng/uL): 0.5000          Dilution factor: 12.0
```

Sample: SampA3
Well location: A3
Created: Tuesday, September 14, 2021 9:33:53 PM

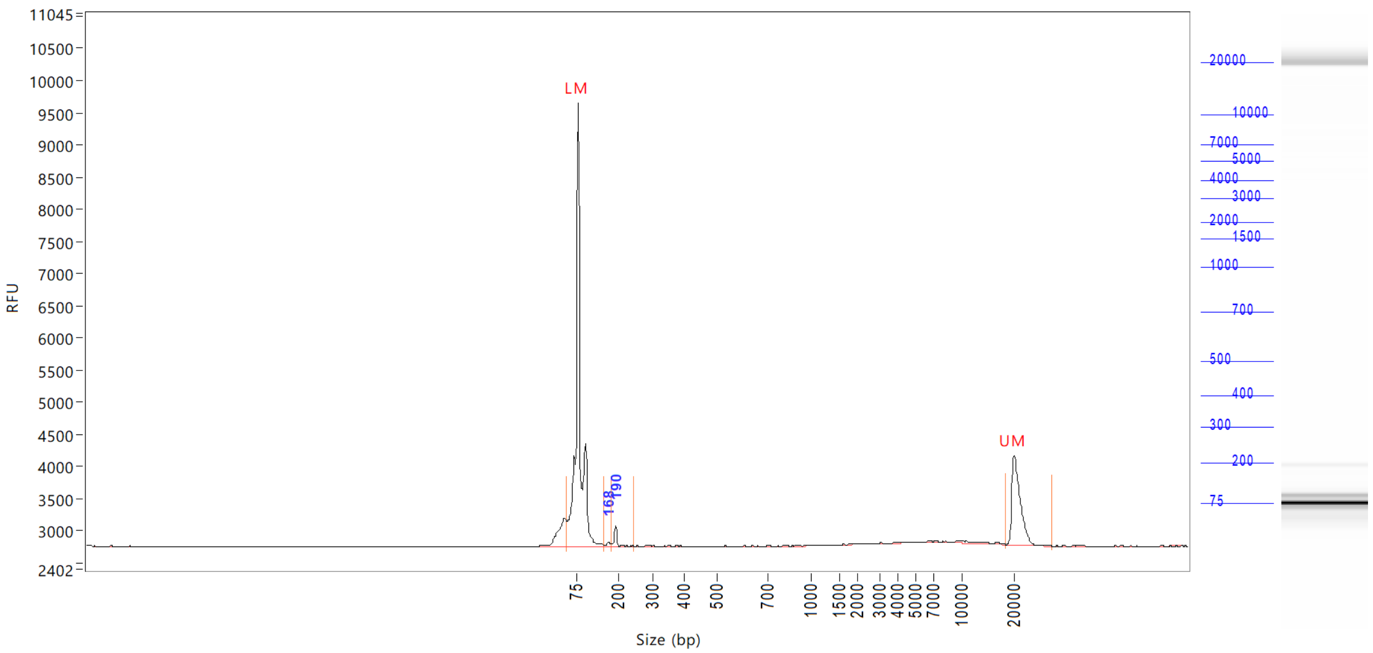

Peak	Size (bp)	Concentration (ng/uL)	From (bp)	To (bp)	Average size (bp)	CV%	RFU	Corrected peak area
1	75 (LM)	0.5000	41	156	76	19.90	6906	73.104
2	168	0.0434	156	177	167	3.48	59	0.529
3	190	0.1553	177	244	189	4.43	323	1.893
4	20000 (UM)	0.0994	18415	27496	20585	4.64	1398	14.535

TIC:	0.1988	ng/uL
TIM:	1.7789	nmole/L
Total concentration:	0.4798	ng/uL

Sample peak width (sec): 5   Sample min peak height: 50   Sample baseline V to V?: Y   Sample baseline V to V points: 3
Sample filter: Binomial   Number of points for filter: 3 Sample start region (min): 0 Sample end region (min): 30
Marker peak width (sec): 5   Marker min peak height: 200   Marker baseline V to V?: Y   Marker baseline V to V points: 3
Lower marker selection: First peak > 500 RFU   Upper marker selection: Last peak > 200 RFU
Ladder size (bp)75, 200, 300, 400, 500, 700, 1000, 1500, 2000, 3000, 4000, 5000, 7000, 10000, 20000
Quantification using: Lower Marker   Final concentration (ng/uL): 0.5000   Dilution factor: 12.0

Sample: SampB3
Well location: B3
Created: Tuesday, September 14, 2021 9:33:53 PM

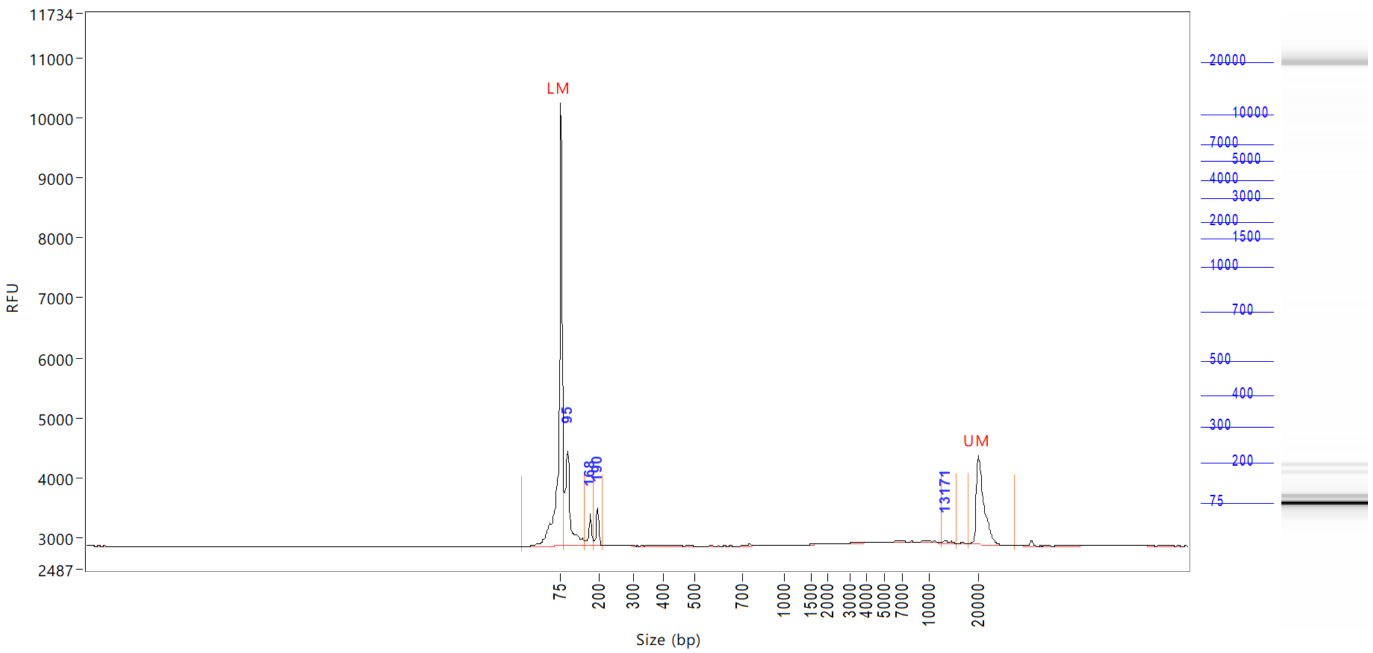

Peak	Size (bp)	Concentration (ng/uL)	From (bp)	To (bp)	Average size (bp)	CV%	RFU	Corrected peak area
1	75 (LM)	0.5000	0	86	67	23.20	7380	61.011
2	95	1.9113	86	150	101	14.92	1572	19.435
3	168	0.3652	150	179	165	3.63	532	3.714
4	190	0.3520	179	210	189	2.12	625	3.579
5	13171	0.0462	12562	15610	13650	4.95	51	0.470
6	20000 (UM)	0.1213	18049	27133	20515	4.90	1481	14.803

TIC:	2.6748	ng/uL
TIM:	37.7814	nmole/L
Total concentration:	3.0310	ng/uL

Sample peak width (sec): 5 Sample min peak height: 50 Sample baseline V to V?: Y Sample baseline V to V points: 3
Sample filter: Binomial Number of points for filter: 3 Sample start region (min): 0 Sample end region (min): 30
Marker peak width (sec): 5 Marker min peak height: 200 Marker baseline V to V?: Y Marker baseline V to V points: 3
Lower marker selection: First peak > 500 RFU Upper marker selection: Last peak > 200 RFU
Ladder size (bp)75, 200, 300, 400, 500, 700, 1000, 1500, 2000, 3000, 4000, 5000, 7000, 10000, 20000
Quantification using: Lower Marker Final concentration (ng/uL): 0.5000 Dilution factor: 12.0

Sample: SampC3
Well location: C3
Created: Tuesday, September 14, 2021 9:33:53 PM

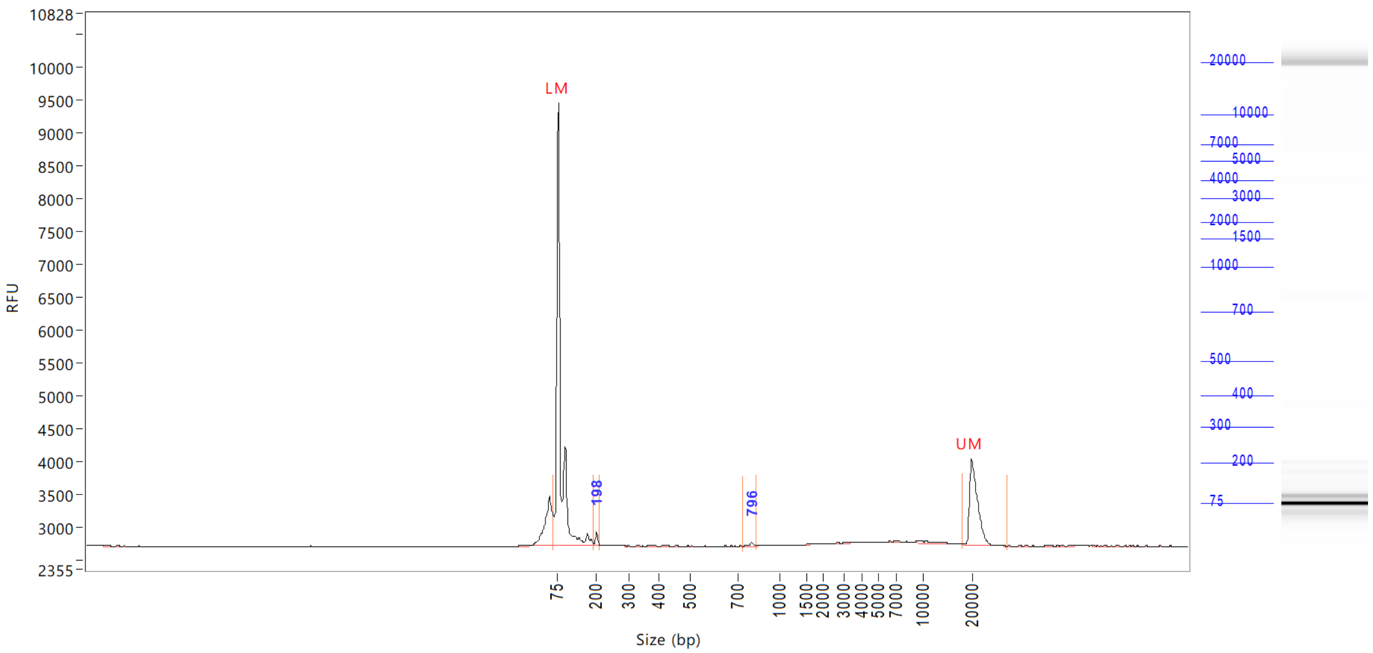

Peak	Size (bp)	Concentration (ng/uL)	From (bp)	To (bp)	Average size (bp)	CV%	RFU	Corrected peak area
1	75 (LM)	0.5000	62	189	84	26.45	6762	60.886
2	198	0.1144	189	208	195	1.62	216	1.161
3	796	0.0339	727	827	789	2.40	53	0.344
4	20000 (UM)	0.1110	17927	27133	20535	4.57	1321	13.521

TIC:	0.1483	ng/uL
TIM:	1.0356	nmole/L
Total concentration:	0.5006	ng/uL

Sample peak width (sec): 5   Sample min peak height: 50   Sample baseline V to V?: Y   Sample baseline V to V points: 3
Sample filter: Binomial   Number of points for filter: 3   Sample start region (min): 0   Sample end region (min): 30
Marker peak width (sec): 5   Marker min peak height: 200   Marker baseline V to V?: Y   Marker baseline V to V points: 3
Lower marker selection: First peak > 500 RFU   Upper marker selection: Last peak > 200 RFU
Ladder size (bp)75, 200, 300, 400, 500, 700, 1000, 1500, 2000, 3000, 4000, 5000, 7000, 10000, 20000
Quantification using: Lower Marker   Final concentration (ng/uL): 0.5000   Dilution factor: 12.0

Sample: SampD3
Well location: D3
Created: Tuesday, September 14, 2021 9:33:53 PM

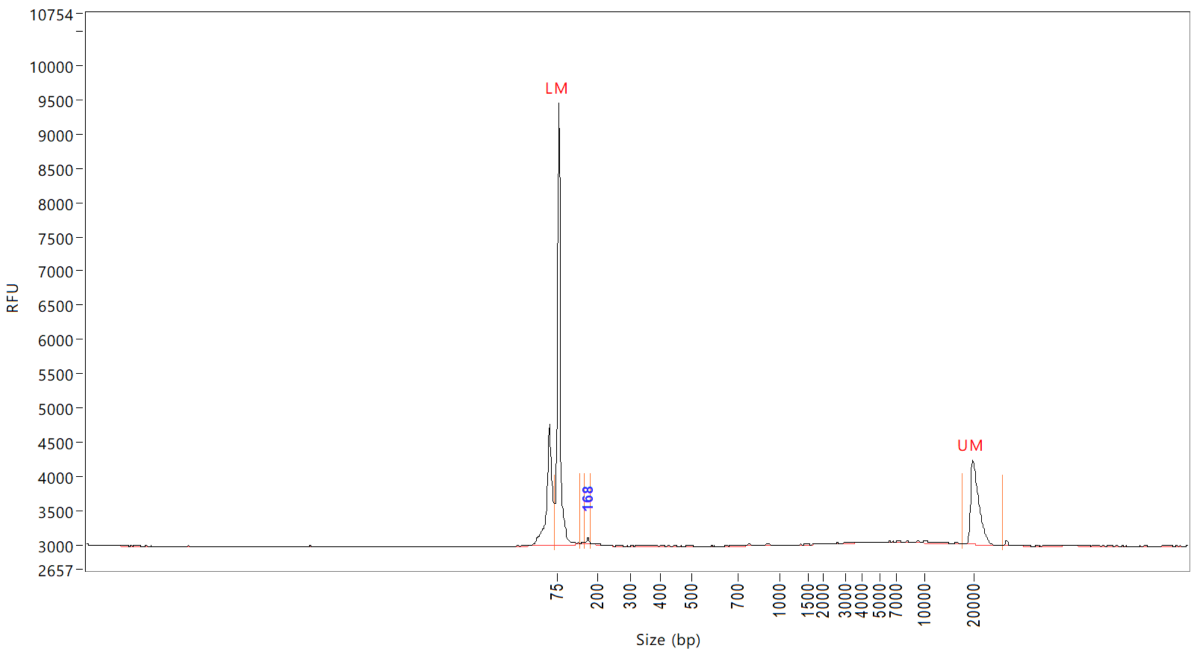

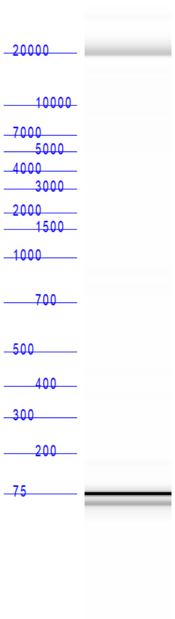

Peak	Size (bp)	Concentration (ng/uL)	From (bp)	To (bp)	Average size (bp)	CV%	RFU	Corrected peak area
1	75 (LM)	0.5000	62	143	75	11.54	6450	45.166
2	168	0.0728	158	179	165	2.49	93	0.548
3	20000 (UM)	0.1427	17683	26045	20516	4.51	1228	12.895

TIC:	0.0728	ng/uL
TIM:	0.7257	nmole/L
Total concentration:	0.7007	ng/uL

Sample peak width (sec): 5   Sample min peak height: 50   Sample baseline V to V?: Y   Sample baseline V to V points: 3
Sample filter: Binomial   Number of points for filter: 3   Sample start region (min): 0   Sample end region (min): 30
Marker peak width (sec): 5   Marker min peak height: 200   Marker baseline V to V?: Y   Marker baseline V to V points: 3
Lower marker selection: First peak > 500 RFU   Upper marker selection: Last peak > 200 RFU
Ladder size (bp)75, 200, 300, 400, 500, 700, 1000, 1500, 2000, 3000, 4000, 5000, 7000, 10000, 20000
Quantification using: Lower Marker   Final concentration (ng/uL): 0.5000   Dilution factor: 12.0

Sample: SampE3
Well location: E3
Created: Tuesday, September 14, 2021 9:33:53 PM

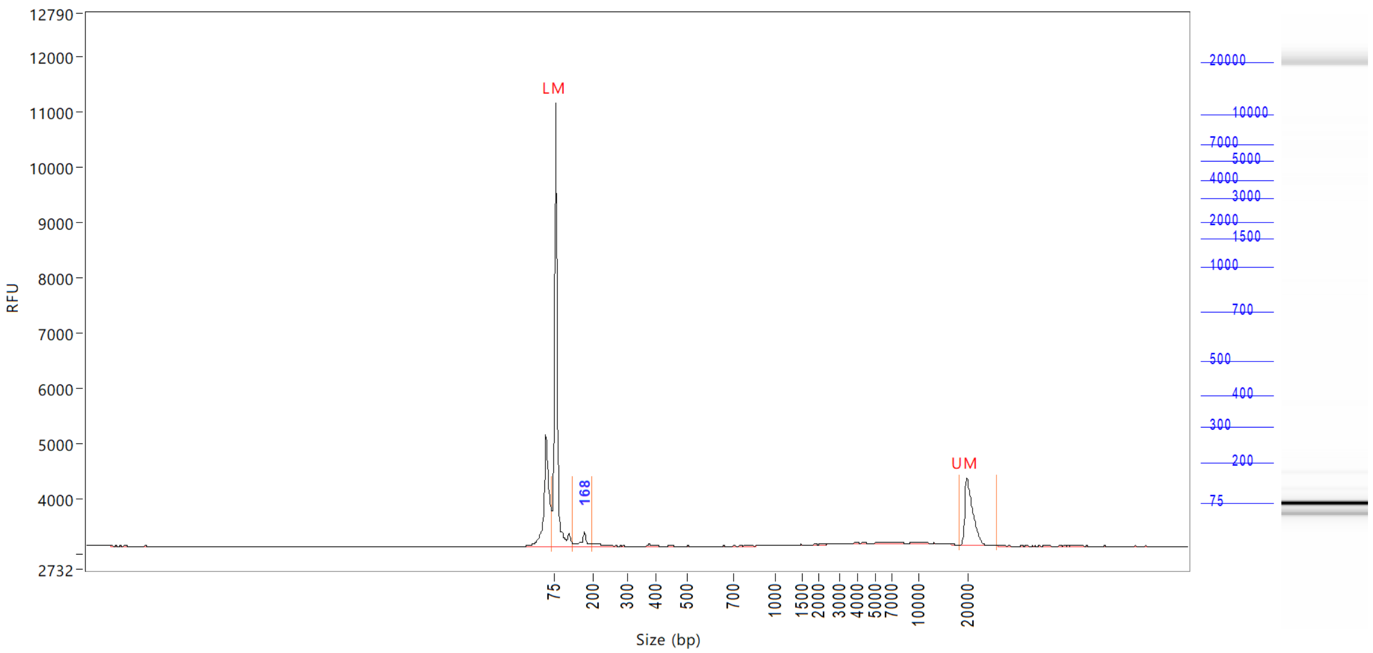

Peak	Size (bp)	Concentration (ng/uL)	From (bp)	To (bp)	Average size (bp)	CV%	RFU	Corrected peak area
1	75 (LM)	0.5000	64	129	76	13.05	8035	56.579
2	168	0.3172	129	192	161	9.40	252	2.991
3	20000 (UM)	0.1118	18537	26045	20565	4.43	1212	12.646

TIC:	0.3172	ng/uL
TIM:	3.2380	nmole/L
Total concentration:	0.7541	ng/uL

Sample peak width (sec): 5   Sample min peak height: 50   Sample baseline V to V?: Y   Sample baseline V to V points: 3
Sample filter: Binomial   Number of points for filter: 3   Sample start region (min): 0   Sample end region (min): 30
Marker peak width (sec): 5   Marker min peak height: 200   Marker baseline V to V?: Y   Marker baseline V to V points: 3
Lower marker selection: First peak > 500 RFU   Upper marker selection: Last peak > 200 RFU
Ladder size (bp)75, 200, 300, 400, 500, 700, 1000, 1500, 2000, 3000, 4000, 5000, 7000, 10000, 20000
Quantification using: Lower Marker   Final concentration (ng/uL): 0.5000   Dilution factor: 12.0

Sample: SampF3
Well location: F3
Created: Tuesday, September 14, 2021 9:33:53 PM

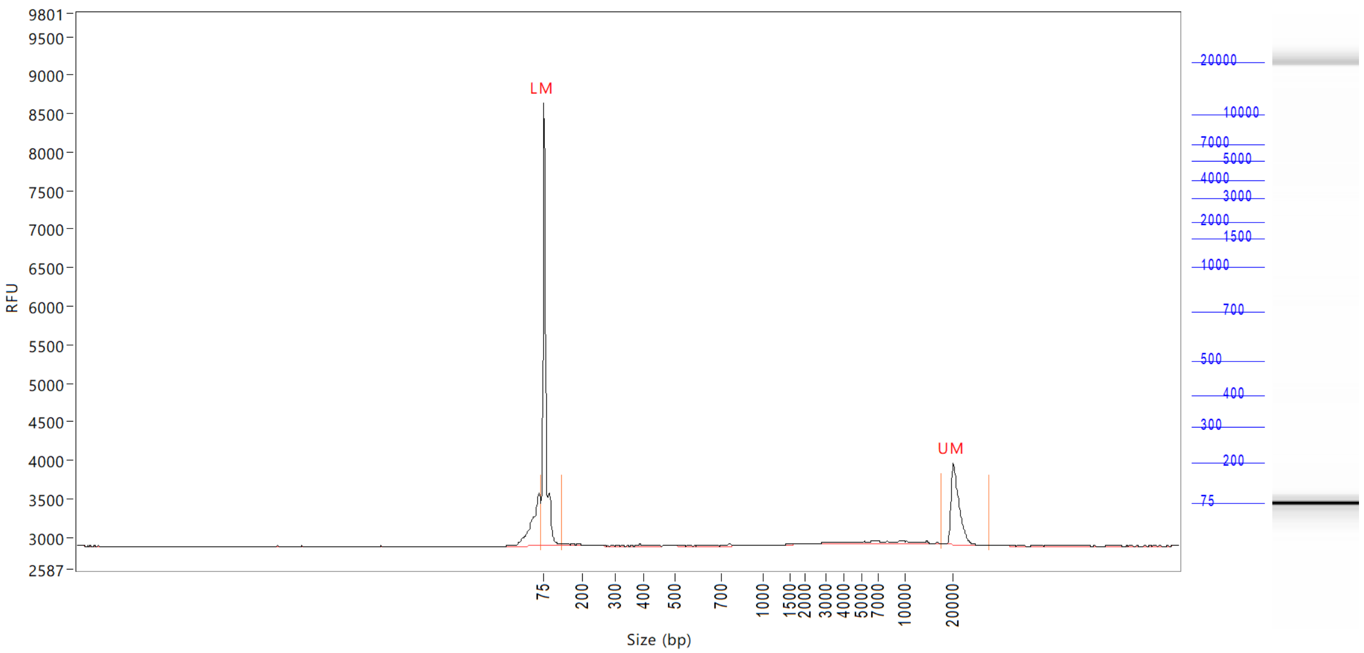

Peak	Size	Concentration	From	To	Average size	CV%	RFU	Corrected peak area
	(bp)	(ng/uL)	(bp)	(bp)	(bp)			
1	75 (LM)	0.5000	66	131	76	11.20	5755	41.496
2	20000 (UM)	0.1353	17683	27496	20638	4.78	1066	11.228

TIC:	0.0000	ng/uL
TIM:	0.0000	nmole/L
Total concentration:	0.7410	ng/uL

Sample peak width (sec): 5   Sample min peak height: 50   Sample baseline V to V?: Y   Sample baseline V to V points: 3
Sample filter: Binomial   Number of points for filter: 3   Sample start region (min): 0   Sample end region (min): 30
Marker peak width (sec): 5   Marker min peak height: 200   Marker baseline V to V?: Y   Marker baseline V to V points: 3
Lower marker selection: First peak > 500 RFU   Upper marker selection: Last peak > 200 RFU
Ladder size (bp)75, 200, 300, 400, 500, 700, 1000, 1500, 2000, 3000, 4000, 5000, 7000, 10000, 20000
Quantification using: Lower Marker   Final concentration (ng/uL): 0.5000   Dilution factor: 12.0

Sample: SampG3
Well location: G3
Created: Tuesday, September 14, 2021 9:33:53 PM

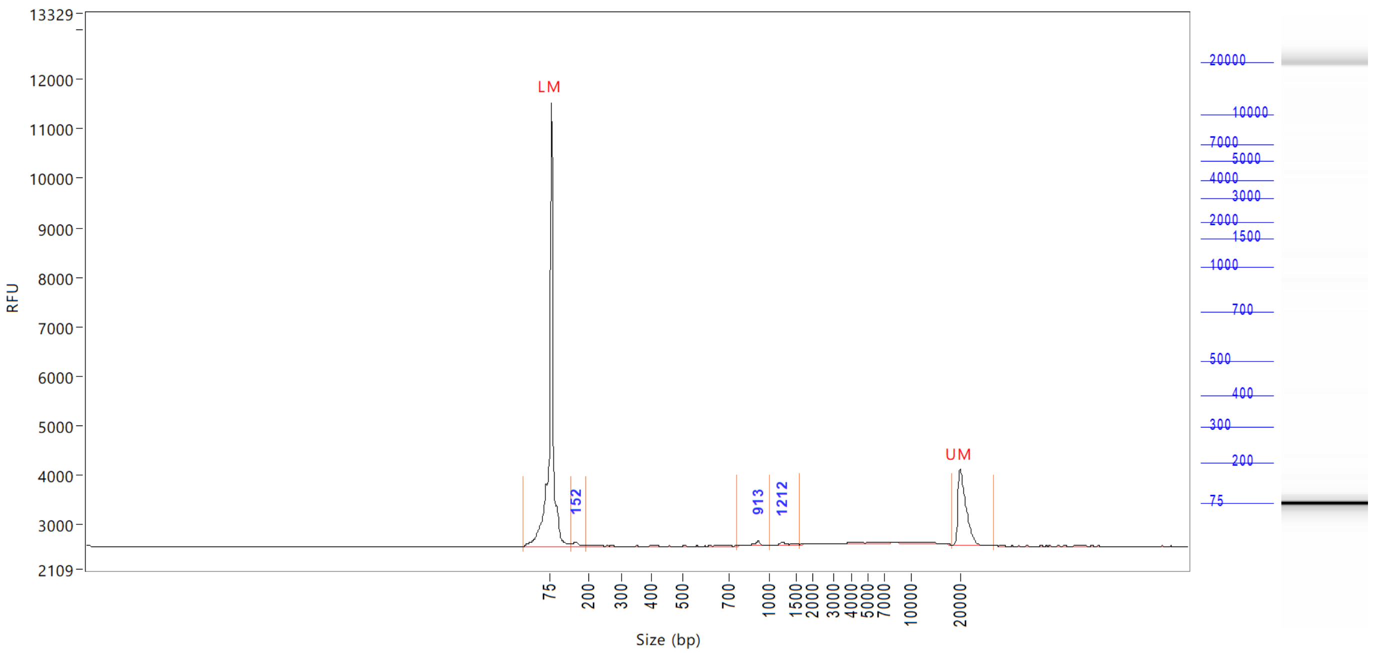

Peak	Size (bp)	Concentration (ng/uL)	From (bp)	To (bp)	Average size (bp)	CV%	RFU	Corrected peak area
1	75 (LM)	0.5000	0	137	68	26.29	8964	87.395
2	152	0.0988	137	189	155	7.80	94	1.439
3	913	0.0695	753	1000	894	4.42	105	1.012
4	1212	0.0442	1000	1559	1247	7.59	65	0.644
5	20000 (UM)	0.0925	18171	27012	20585	4.67	1531	16.165

TIC:	0.2125	ng/uL
TIM:	1.2338	nmole/L
Total concentration:	0.5018	ng/uL

Sample peak width (sec): 5   Sample min peak height: 50   Sample baseline V to V?: Y   Sample baseline V to V points: 3
Sample filter: Binomial   Number of points for filter: 3 Sample start region (min): 0 Sample end region (min): 30
Marker peak width (sec): 5   Marker min peak height: 200   Marker baseline V to V?: Y   Marker baseline V to V points: 3
Lower marker selection: First peak > 500 RFU   Upper marker selection: Last peak > 200 RFU
Ladder size (bp)75, 200, 300, 400, 500, 700, 1000, 1500, 2000, 3000, 4000, 5000, 7000, 10000, 20000
Quantification using: Lower Marker   Final concentration (ng/uL): 0.5000   Dilution factor: 12.0

Sample: SampH3
Well location: H3
Created: Tuesday, September 14, 2021 9:33:53 PM

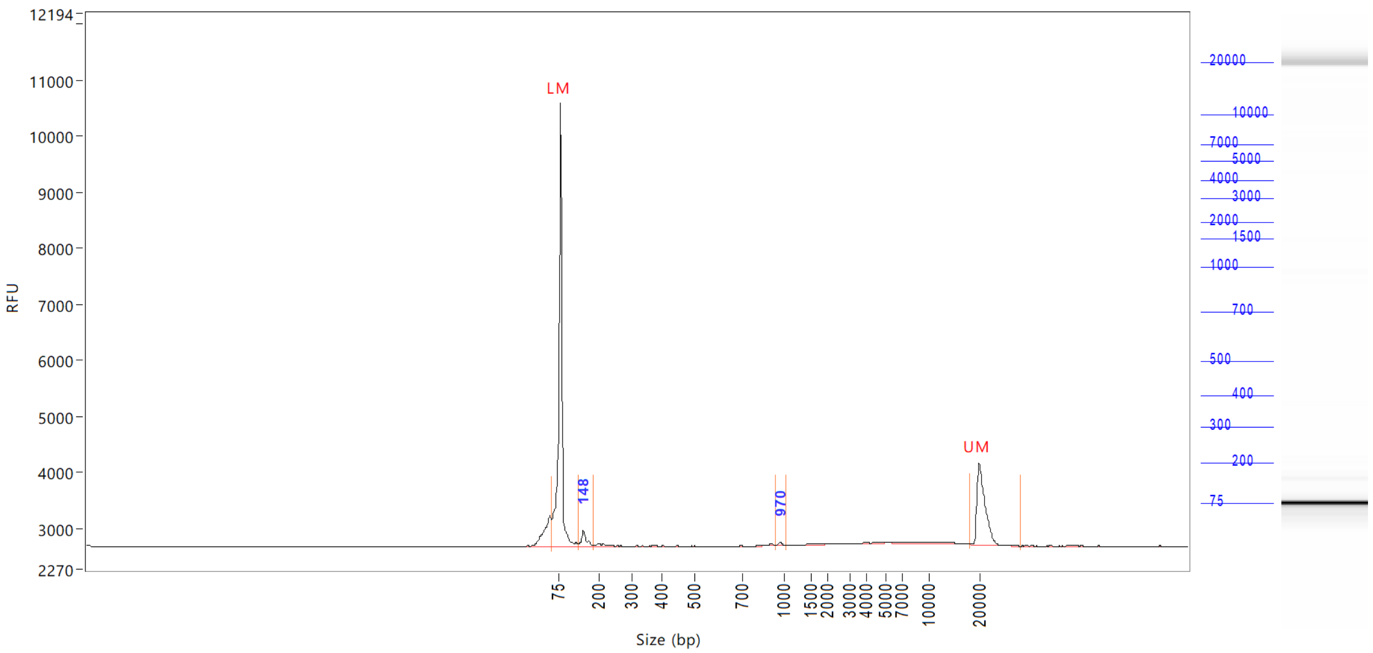

Peak	Size (bp)	Concentration (ng/uL)	From (bp)	To (bp)	Average size (bp)	CV%	RFU	Corrected peak area
1	75 (LM)	0.5000	48	135	73	14.04	7926	63.586
2	148	0.2865	135	183	151	6.20	276	3.037
3	970	0.0302	935	1023	966	1.51	53	0.320
4	20000 (UM)	0.1220	18293	28221	20540	4.73	1471	15.519

TIC:	0.3167	ng/uL
TIM:	3.1703	nmole/L
Total concentration:	0.7686	ng/uL

Sample peak width (sec): 5   Sample min peak height: 50   Sample baseline V to V?: Y   Sample baseline V to V points: 3
Sample filter: Binomial   Number of points for filter: 3 Sample start region (min): 0 Sample end region (min): 30
Marker peak width (sec): 5   Marker min peak height: 200   Marker baseline V to V?: Y   Marker baseline V to V points: 3
Lower marker selection: First peak > 500 RFU   Upper marker selection: Last peak > 200 RFU
Ladder size (bp)75, 200, 300, 400, 500, 700, 1000, 1500, 2000, 3000, 4000, 5000, 7000, 10000, 20000
Quantification using: Lower Marker   Final concentration (ng/uL): 0.5000   Dilution factor: 12.0

Sample: SampA4
Well location: A4
Created: Tuesday, September 14, 2021 9:33:53 PM

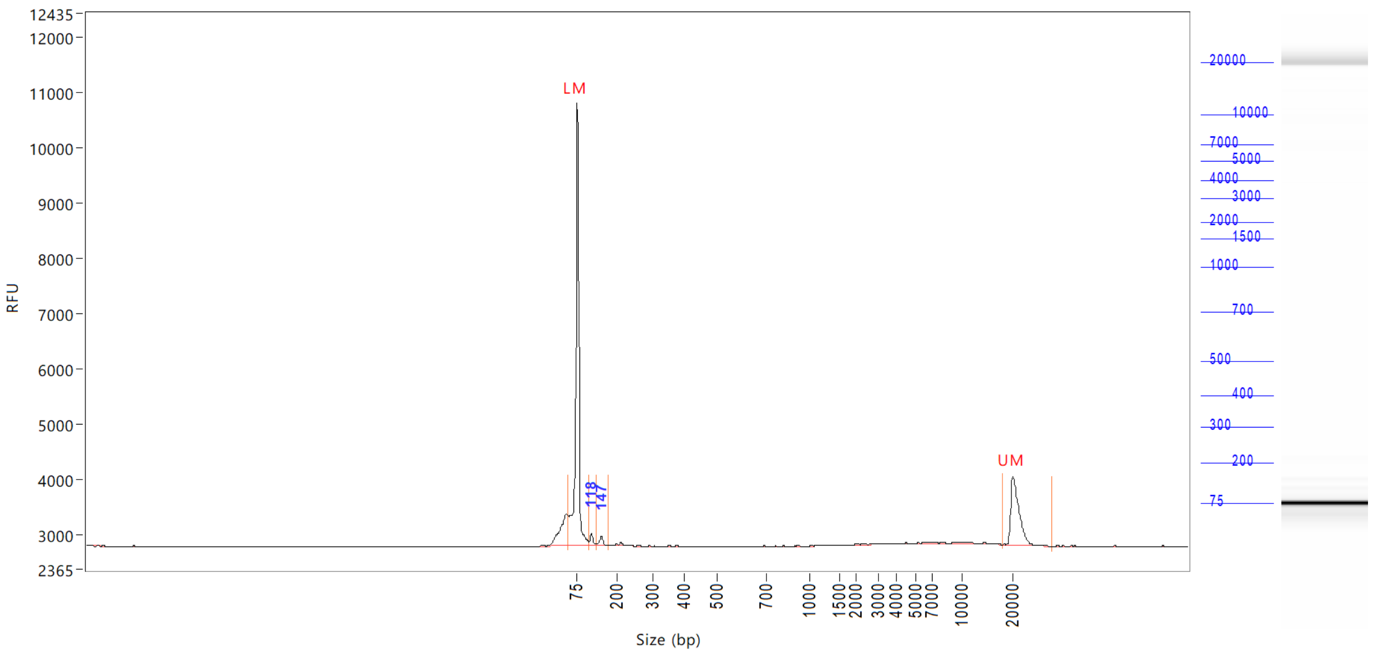

Peak	Size (bp)	Concentration (ng/uL)	From (bp)	To (bp)	Average size (bp)	CV%	RFU	Corrected peak area
1	75 (LM)	0.5000	48	110	72	11.23	8032	60.502
2	118	0.1500	110	133	118	4.33	209	1.512
3	147	0.1225	133	171	145	3.73	167	1.236
4	20000 (UM)	0.1044	17927	27496	20588	4.48	1238	12.632

TIC:	0.2725	ng/uL
TIM:	3.4769	nmole/L
Total concentration:	0.5583	ng/uL

```
Sample peak width (sec): 5    Sample min peak height: 50    Sample baseline V to V?: Y    Sample baseline V to V points: 3
Sample filter: Binomial       Number of points for filter: 3  Sample start region (min): 0  Sample end region (min): 30
Marker peak width (sec): 5    Marker min peak height: 200   Marker baseline V to V?: Y    Marker baseline V to V points: 3
Lower marker selection: First peak > 500 RFU                Upper marker selection: Last peak > 200 RFU
Ladder size (bp)75, 200, 300, 400, 500, 700, 1000, 1500, 2000, 3000, 4000, 5000, 7000, 10000, 20000
Quantification using: Lower Marker          Final concentration (ng/uL): 0.5000          Dilution factor: 12.0
```

Sample: SampB4
Well location: B4
Created: Tuesday, September 14, 2021 9:33:53 PM

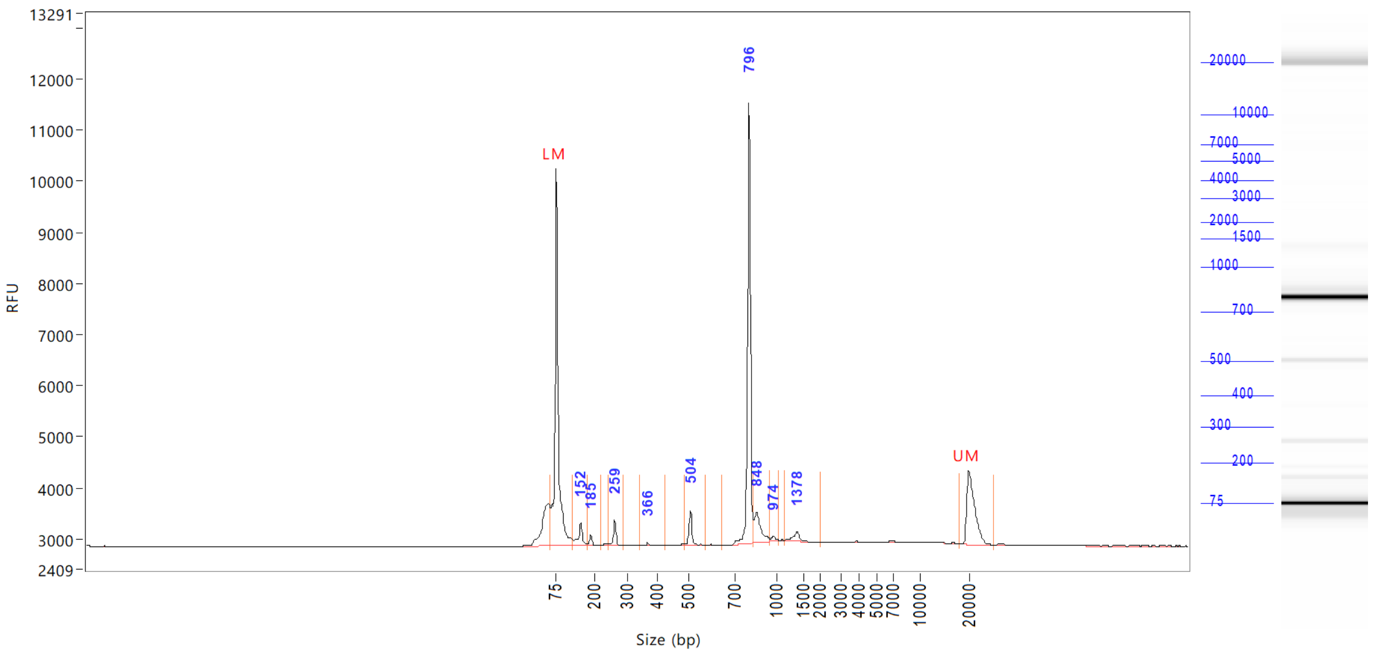

Peak	Size (bp)	Concentration (ng/uL)	From (bp)	To (bp)	Average size (bp)	CV%	RFU	Corrected peak area
1	75 (LM)	0.5000	56	129	76	15.32	7383	61.161
2	152	0.4199	129	175	149	6.26	436	4.280
3	185	0.1153	175	219	184	3.49	189	1.176
4	259	0.2899	239	287	256	2.08	497	2.955
5	366	0.0392	341	426	370	4.34	57	0.399
6	504	0.4148	489	570	506	2.67	692	4.229
7	796	4.5248	641	827	789	2.20	8636	46.123
8	848	0.7019	827	948	863	3.40	595	7.154
9	974	0.0712	948	1034	971	1.68	88	0.725
10	1378	0.1970	1134	2000	1336	6.82	189	2.008
11	20000 (UM)	0.1239	18049	25320	20584	4.55	1459	15.155

TIC:	6.7739	ng/uL
TIM:	20.1875	nmole/L
Total concentration:	6.9791	ng/uL

Sample peak width (sec): 5   Sample min peak height: 50   Sample baseline V to V?: Y   Sample baseline V to V points: 3
Sample filter: Binomial   Number of points for filter: 3   Sample start region (min): 0   Sample end region (min): 30
Marker peak width (sec): 5   Marker min peak height: 200   Marker baseline V to V?: Y   Marker baseline V to V points: 3
Lower marker selection: First peak > 500 RFU   Upper marker selection: Last peak > 200 RFU
Ladder size (bp)75, 200, 300, 400, 500, 700, 1000, 1500, 2000, 3000, 4000, 5000, 7000, 10000, 20000
Quantification using: Lower Marker   Final concentration (ng/uL): 0.5000   Dilution factor: 12.0

Sample: SampC4
Well location: C4
Created: Tuesday, September 14, 2021 9:33:53 PM

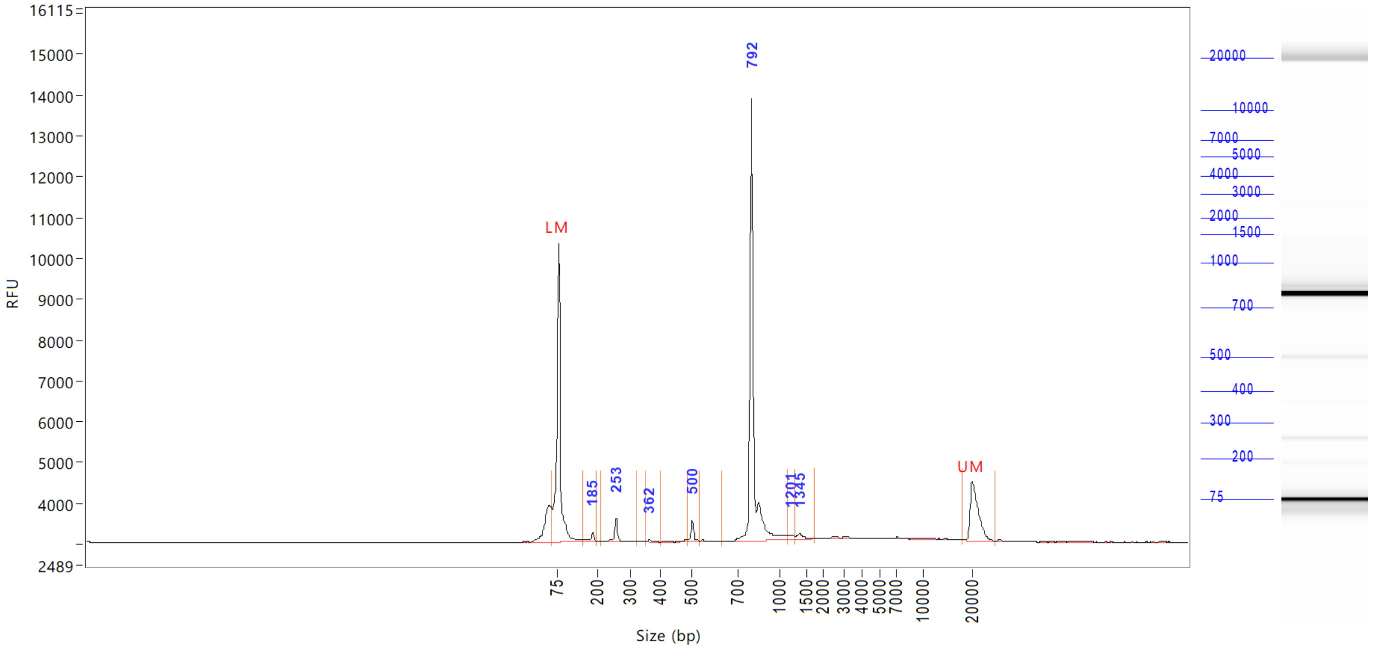

Peak	Size (bp)	Concentration (ng/uL)	From (bp)	To (bp)	Average size (bp)	CV%	RFU	Corrected peak area
1	75 (LM)	0.5000	56	154	75	17.09	7290	61.219
2	185	0.1693	154	196	177	5.25	229	1.727
3	253	0.3815	210	317	251	4.16	568	3.893
4	362	0.0429	349	400	365	2.74	62	0.438
5	500	0.3082	485	534	501	1.67	516	3.145
6	792	6.9686	629	1145	806	6.91	10854	71.102
7	1201	0.1055	1145	1267	1202	3.06	120	1.077
8	1345	0.1580	1267	1731	1379	6.36	127	1.612
9	20000 (UM)	0.1237	17927	24594	20560	4.55	1454	15.143

TIC:	8.1340	ng/uL
TIM:	19.8409	nmole/L
Total concentration:	8.5355	ng/uL

Sample peak width (sec): 5   Sample min peak height: 50   Sample baseline V to V?: Y   Sample baseline V to V points: 3
Sample filter: Binomial   Number of points for filter: 3 Sample start region (min): 0 Sample end region (min): 30
Marker peak width (sec): 5   Marker min peak height: 200   Marker baseline V to V?: Y   Marker baseline V to V points: 3
Lower marker selection: First peak > 500 RFU   Upper marker selection: Last peak > 200 RFU
Ladder size (bp)75, 200, 300, 400, 500, 700, 1000, 1500, 2000, 3000, 4000, 5000, 7000, 10000, 20000
Quantification using: Lower Marker   Final concentration (ng/uL): 0.5000   Dilution factor: 12.0

Sample: SampD4
Well location: D4
Created: Tuesday, September 14, 2021 9:33:53 PM

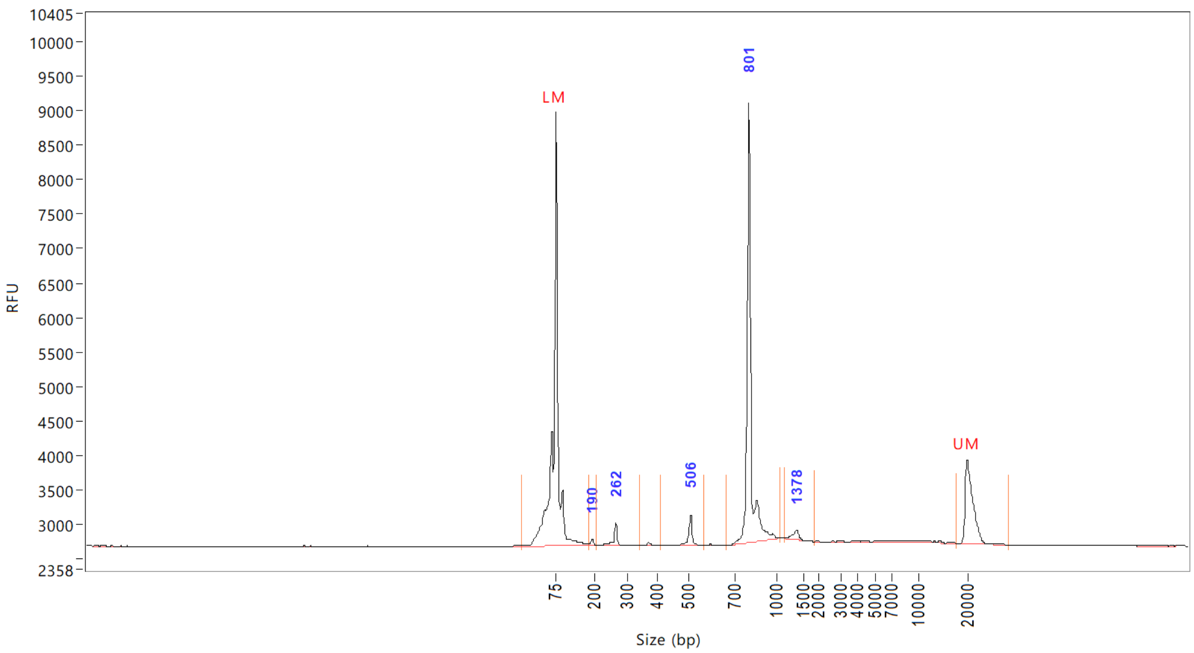

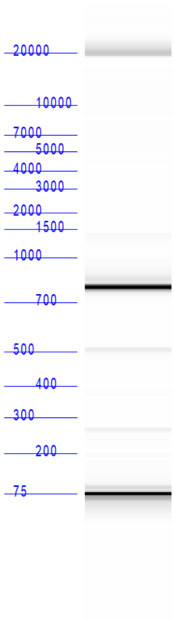

Peak	Size (bp)	Concentration (ng/uL)	From (bp)	To (bp)	Average size (bp)	CV%	RFU	Corrected peak area
1	75 (LM)	0.5000	0	181	67	35.68	6275	72.367
2	190	0.0550	181	203	189	2.62	96	0.664
3	262	0.2254	203	337	253	6.57	333	2.718
4	506	0.2619	409	567	500	3.72	441	3.158
5	801	3.6684	664	1068	804	4.77	6375	44.245
6	1378	0.1200	1145	1846	1339	6.55	142	1.447
7	20000 (UM)	0.0869	17805	28463	20568	5.46	1209	12.572

TIC:	4.3307	ng/uL
TIM:	10.4627	nmole/L
Total concentration:	4.5452	ng/uL

Sample peak width (sec): 5 Sample min peak height: 50 Sample baseline V to V?: Y Sample baseline V to V points: 3
Sample filter: Binomial Number of points for filter: 3 Sample start region (min): 0 Sample end region (min): 30
Marker peak width (sec): 5 Marker min peak height: 200 Marker baseline V to V?: Y Marker baseline V to V points: 3
Lower marker selection: First peak > 500 RFU Upper marker selection: Last peak > 200 RFU
Ladder size (bp)75, 200, 300, 400, 500, 700, 1000, 1500, 2000, 3000, 4000, 5000, 7000, 10000, 20000
Quantification using: Lower Marker Final concentration (ng/uL): 0.5000 Dilution factor: 12.0

Sample: SampE4
Well location: E4
Created: Tuesday, September 14, 2021 9:33:53 PM

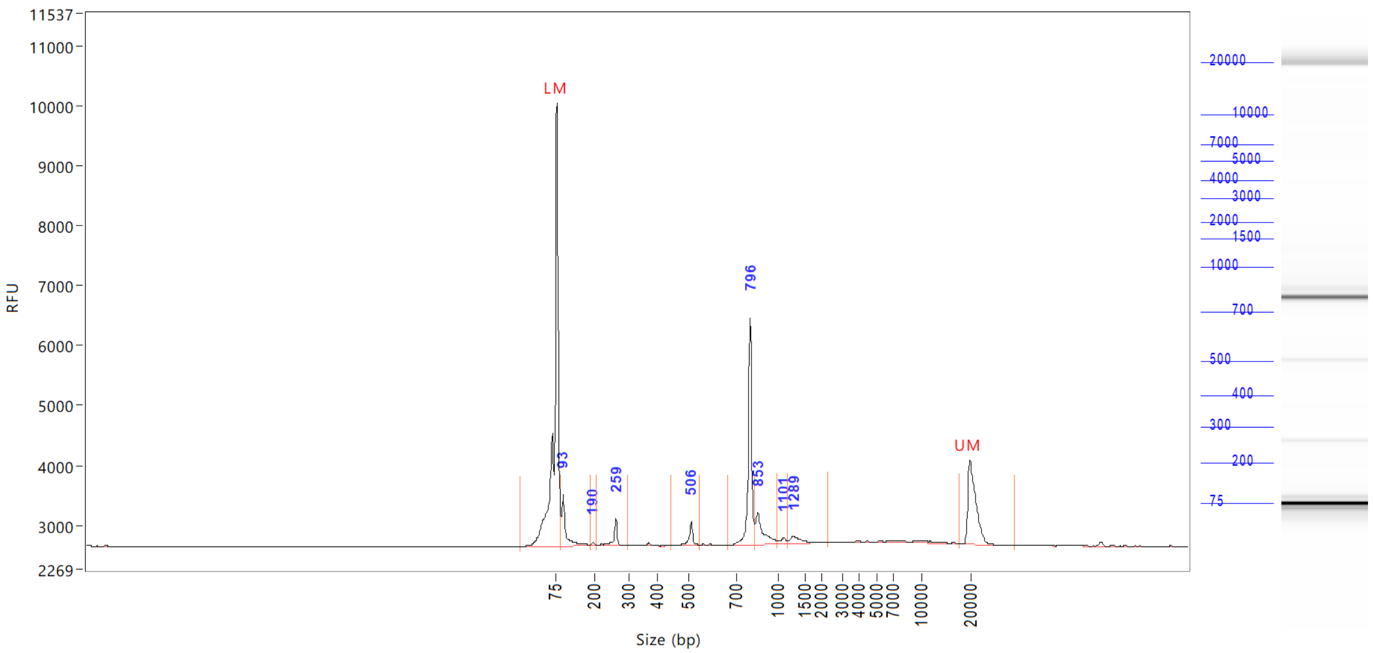

Peak	Size (bp)	Concentration (ng/uL)	From (bp)	To (bp)	Average size (bp)	CV%	RFU	Corrected peak area
1	75 (LM)	0.5000	0	87	62	28.23	7401	73.972
2	93	0.6990	87	183	105	20.89	860	8.618
3	190	0.0369	183	201	190	2.34	69	0.455
4	259	0.2901	201	298	250	6.01	446	3.576
5	506	0.2605	441	539	499	2.73	409	3.212
6	796	1.9234	667	827	787	2.65	3779	23.712
7	853	0.5816	827	996	879	4.96	527	7.170
8	1101	0.0897	996	1167	1075	4.79	94	1.105
9	1289	0.1822	1167	2344	1385	13.64	118	2.246
10	20000 (UM)	0.0995	17927	29068	20639	5.40	1404	14.718

TIC:	4.0633	ng/uL
TIM:	19.4851	nmole/L
Total concentration:	4.3136	ng/uL

```
Sample peak width (sec): 5    Sample min peak height: 50    Sample baseline V to V?: Y    Sample baseline V to V points: 3
Sample filter: Binomial       Number of points for filter: 3  Sample start region (min): 0  Sample end region (min): 30
Marker peak width (sec): 5    Marker min peak height: 200    Marker baseline V to V?: Y    Marker baseline V to V points: 3
Lower marker selection: First peak > 500 RFU                 Upper marker selection: Last peak > 200 RFU
Ladder size (bp)75, 200, 300, 400, 500, 700, 1000, 1500, 2000, 3000, 4000, 5000, 7000, 10000, 20000
Quantification using: Lower Marker          Final concentration (ng/uL): 0.5000          Dilution factor: 12.0
```

Sample: SampF4
Well location: F4
Created: Tuesday, September 14, 2021 9:33:53 PM

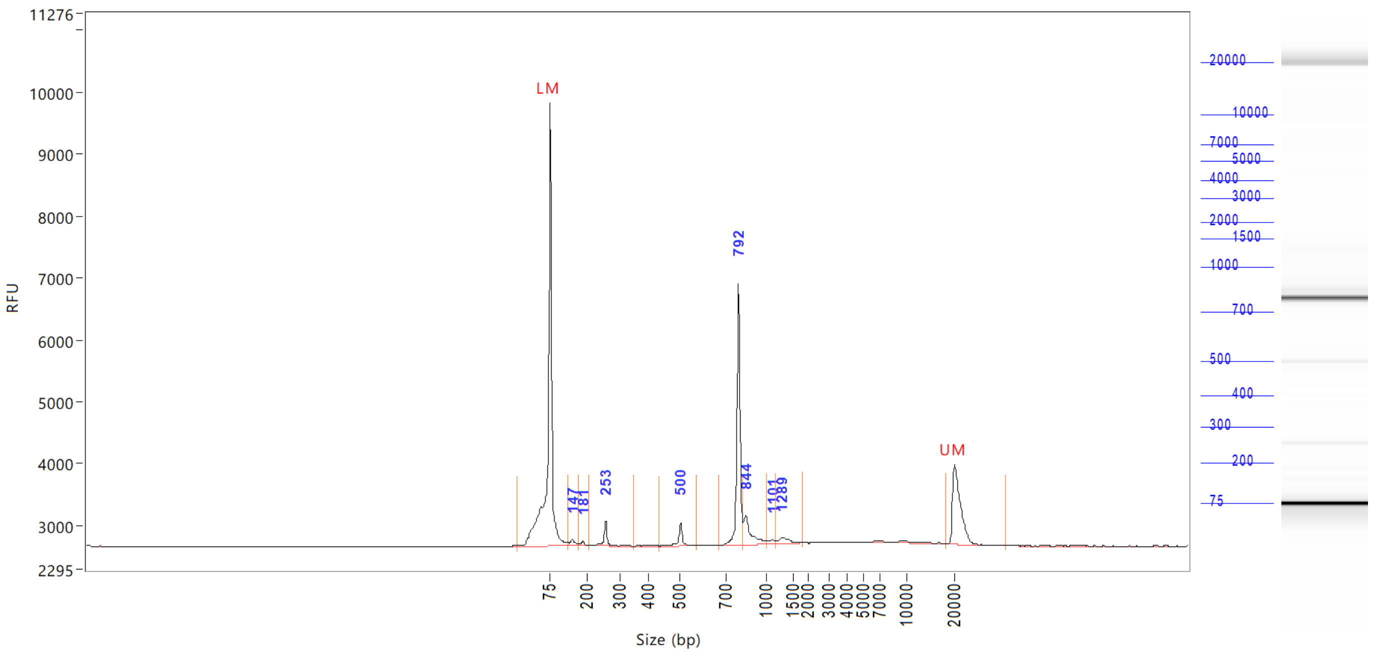

Peak	Size (bp)	Concentration (ng/uL)	From (bp)	To (bp)	Average size (bp)	CV%	RFU	Corrected peak area
1	75 (LM)	0.5000	0	137	64	34.46	7169	71.533
2	147	0.0992	137	168	149	5.56	100	1.183
3	181	0.0642	168	201	180	4.38	83	0.765
4	253	0.2709	201	343	248	6.89	400	3.229
5	500	0.2496	431	570	497	3.78	384	2.975
6	792	2.0644	667	822	783	2.43	4221	24.612
7	844	0.5364	822	1000	873	5.19	479	6.395
8	1101	0.0665	1000	1178	1082	4.89	68	0.793
9	1289	0.1453	1178	1808	1339	7.80	101	1.732
10	20000 (UM)	0.0971	18293	30639	20649	5.64	1301	13.896

TIC:	3.4964	ng/uL
TIM:	9.9333	nmole/L
Total concentration:	3.6804	ng/uL

```
Sample peak width (sec): 5    Sample min peak height: 50    Sample baseline V to V?: Y    Sample baseline V to V points: 3
Sample filter: Binomial       Number of points for filter: 3  Sample start region (min): 0  Sample end region (min): 30
Marker peak width (sec): 5    Marker min peak height: 200   Marker baseline V to V?: Y    Marker baseline V to V points: 3
Lower marker selection: First peak > 500 RFU                Upper marker selection: Last peak > 200 RFU
Ladder size (bp)75, 200, 300, 400, 500, 700, 1000, 1500, 2000, 3000, 4000, 5000, 7000, 10000, 20000
Quantification using: Lower Marker          Final concentration (ng/uL): 0.5000          Dilution factor: 12.0
```

Sample: SampG4
Well location: G4
Created: Tuesday, September 14, 2021 9:33:53 PM

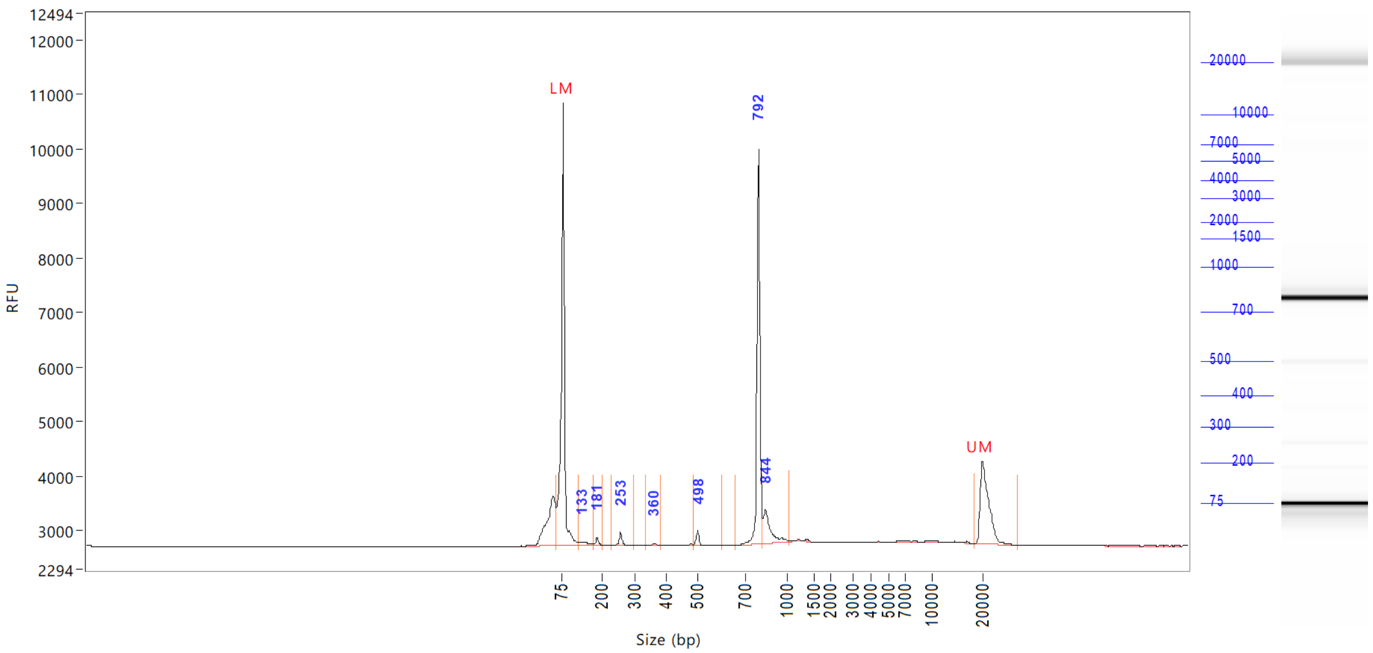

Peak	Size (bp)	Concentration (ng/uL)	From (bp)	To (bp)	Average size (bp)	CV%	RFU	Corrected peak area
1	75 (LM)	0.5000	54	126	72	12.39	8137	63.675
2	133	0.1331	126	173	145	9.40	69	1.412
3	181	0.0851	173	200	180	2.22	149	0.904
4	253	0.1491	226	293	251	2.69	238	1.582
5	360	0.0411	333	380	356	2.23	51	0.436
6	498	0.1718	485	598	503	3.73	288	1.823
7	792	3.6969	662	827	786	2.13	7238	39.233
8	844	0.7053	827	1034	872	5.01	619	7.485
9	20000 (UM)	0.1308	18415	27012	20645	4.93	1524	16.656

TIC:	4.9823	ng/uL
TIM:	13.0867	nmole/L
Total concentration:	5.2007	ng/uL

Sample peak width (sec): 5   Sample min peak height: 50   Sample baseline V to V?: Y   Sample baseline V to V points: 3
Sample filter: Binomial   Number of points for filter: 3   Sample start region (min): 0   Sample end region (min): 30
Marker peak width (sec): 5   Marker min peak height: 200   Marker baseline V to V?: Y   Marker baseline V to V points: 3
Lower marker selection: First peak > 500 RFU   Upper marker selection: Last peak > 200 RFU
Ladder size (bp)75, 200, 300, 400, 500, 700, 1000, 1500, 2000, 3000, 4000, 5000, 7000, 10000, 20000
Quantification using: Lower Marker   Final concentration (ng/uL): 0.5000   Dilution factor: 12.0

Sample: SampH4
Well location: H4
Created: Tuesday, September 14, 2021 9:33:53 PM

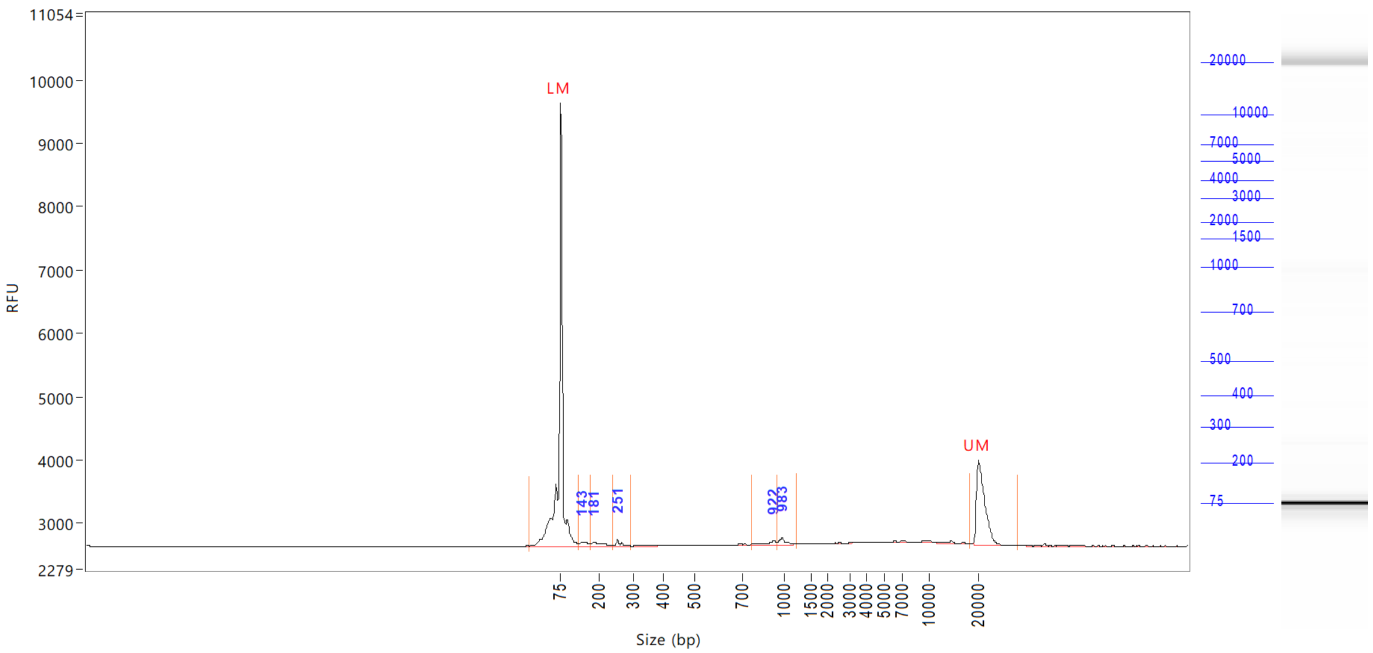

Peak	Size (bp)	Concentration (ng/uL)	From (bp)	To (bp)	Average size (bp)	CV%	RFU	Corrected peak area
1	75 (LM)	0.5000	0	131	67	28.24	7008	62.337
2	143	0.1191	131	169	150	7.44	55	1.238
3	181	0.1408	169	241	196	9.75	59	1.462
4	251	0.0905	241	295	256	4.06	100	0.940
5	922	0.0922	770	948	881	5.70	60	0.958
6	983	0.1074	948	1212	999	4.45	106	1.116
7	20000 (UM)	0.1168	18293	27738	20596	4.77	1327	14.564

TIC:	0.5500	ng/uL
TIM:	3.4164	nmole/L
Total concentration:	0.8848	ng/uL

```
Sample peak width (sec): 5    Sample min peak height: 50    Sample baseline V to V?: Y    Sample baseline V to V points: 3
Sample filter: Binomial       Number of points for filter: 3  Sample start region (min): 0  Sample end region (min): 30
Marker peak width (sec): 5    Marker min peak height: 200   Marker baseline V to V?: Y    Marker baseline V to V points: 3
Lower marker selection: First peak > 500 RFU                Upper marker selection: Last peak > 200 RFU
Ladder size (bp)75, 200, 300, 400, 500, 700, 1000, 1500, 2000, 3000, 4000, 5000, 7000, 10000, 20000
Quantification using: Lower Marker          Final concentration (ng/uL): 0.5000          Dilution factor: 12.0
```

Sample: SampA5
Well location: A5
Created: Tuesday, September 14, 2021 9:33:53 PM

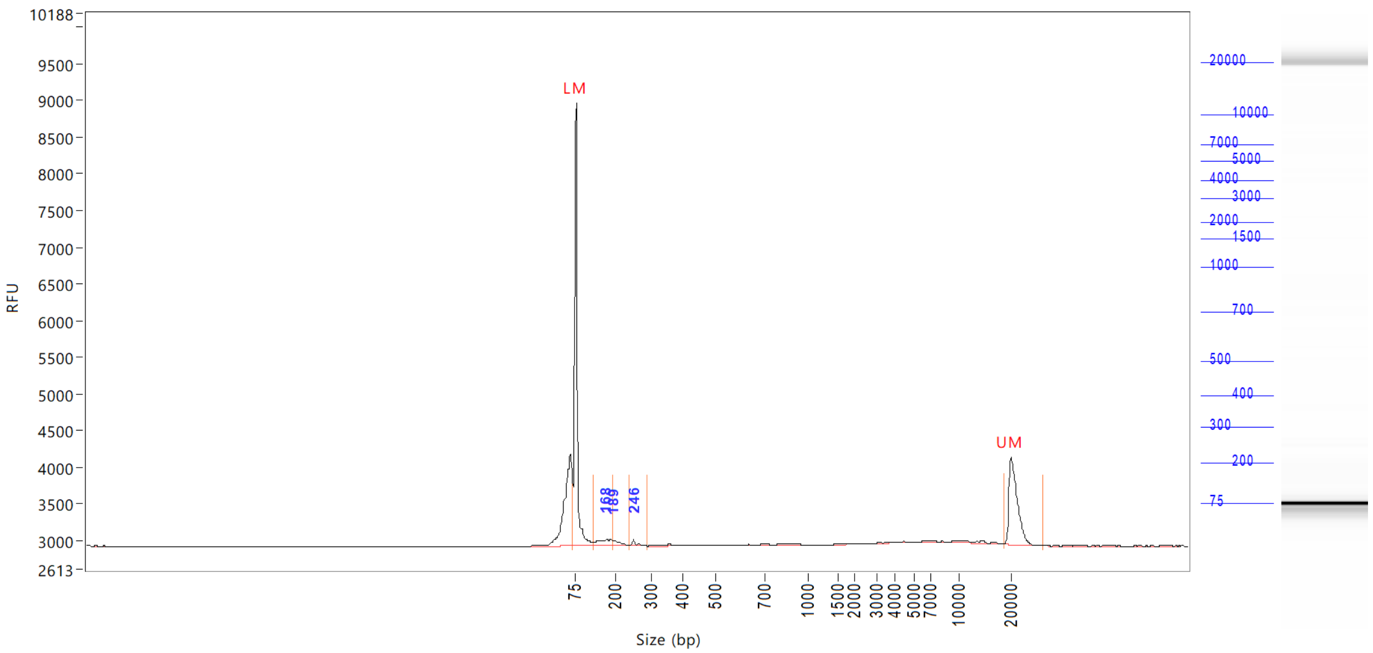

Peak	Size (bp)	Concentration (ng/uL)	From (bp)	To (bp)	Average size (bp)	CV%	RFU	Corrected peak area
1	75 (LM)	0.5000	66	129	75	12.05	6044	38.828
2	168	0.3936	129	189	160	10.66	75	2.547
3	189	0.1297	189	235	201	4.96	64	0.840
4	246	0.0867	235	287	251	3.88	73	0.561
5	20000 (UM)	0.1610	18537	26287	20596	4.52	1202	12.505

TIC:	0.6101	ng/uL
TIM:	5.6730	nmole/L
Total concentration:	1.2462	ng/uL

Sample peak width (sec): 5 Sample min peak height: 50 Sample baseline V to V?: Y Sample baseline V to V points: 3
Sample filter: Binomial Number of points for filter: 3 Sample start region (min): 0 Sample end region (min): 30
Marker peak width (sec): 5 Marker min peak height: 200 Marker baseline V to V?: Y Marker baseline V to V points: 3
Lower marker selection: First peak > 500 RFU Upper marker selection: Last peak > 200 RFU
Ladder size (bp)75, 200, 300, 400, 500, 700, 1000, 1500, 2000, 3000, 4000, 5000, 7000, 10000, 20000
Quantification using: Lower Marker Final concentration (ng/uL): 0.5000 Dilution factor: 12.0

Sample: SampB5
Well location: B5
Created: Tuesday, September 14, 2021 9:33:53 PM

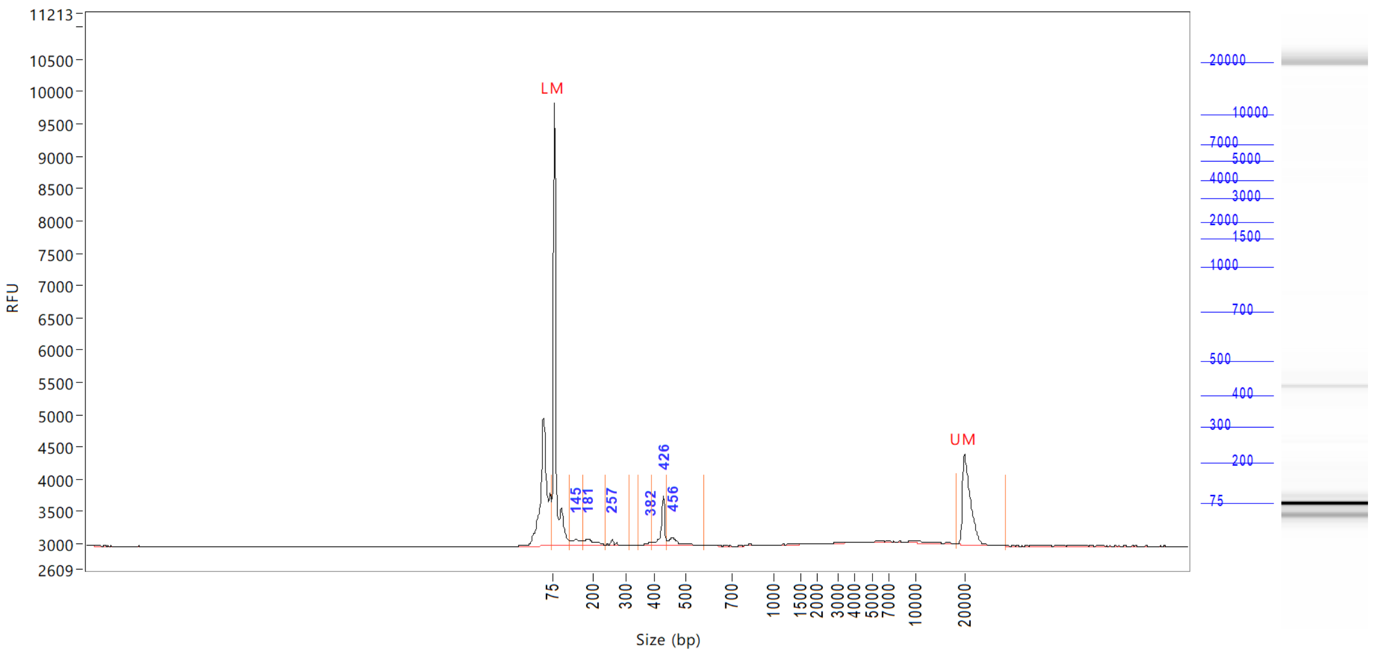

Peak	Size (bp)	Concentration (ng/uL)	From (bp)	To (bp)	Average size (bp)	CV%	RFU	Corrected peak area
1	75 (LM)	0.5000	66	124	77	13.45	6865	48.019
2	145	0.2747	124	168	145	8.86	89	2.199
3	181	0.2628	168	237	190	8.34	96	2.103
4	257	0.0927	237	309	261	4.41	90	0.742
5	382	0.0886	343	388	373	2.84	60	0.709
6	426	0.6884	388	437	419	2.38	770	5.510
7	456	0.3378	437	575	468	5.65	123	2.703
8	20000 (UM)	0.1547	18293	28584	20628	5.12	1408	14.859

TIC:	1.7451	ng/uL
TIM:	10.2540	nmole/L
Total concentration:	2.1271	ng/uL

Sample peak width (sec): 5 Sample min peak height: 50 Sample baseline V to V?: Y Sample baseline V to V points: 3
Sample filter: Binomial Number of points for filter: 3 Sample start region (min): 0 Sample end region (min): 30
Marker peak width (sec): 5 Marker min peak height: 200 Marker baseline V to V?: Y Marker baseline V to V points: 3
Lower marker selection: First peak > 500 RFU Upper marker selection: Last peak > 200 RFU
Ladder size (bp)75, 200, 300, 400, 500, 700, 1000, 1500, 2000, 3000, 4000, 5000, 7000, 10000, 20000
Quantification using: Lower Marker Final concentration (ng/uL): 0.5000 Dilution factor: 12.0

Sample: SampC5
Well location: C5
Created: Tuesday, September 14, 2021 9:33:53 PM

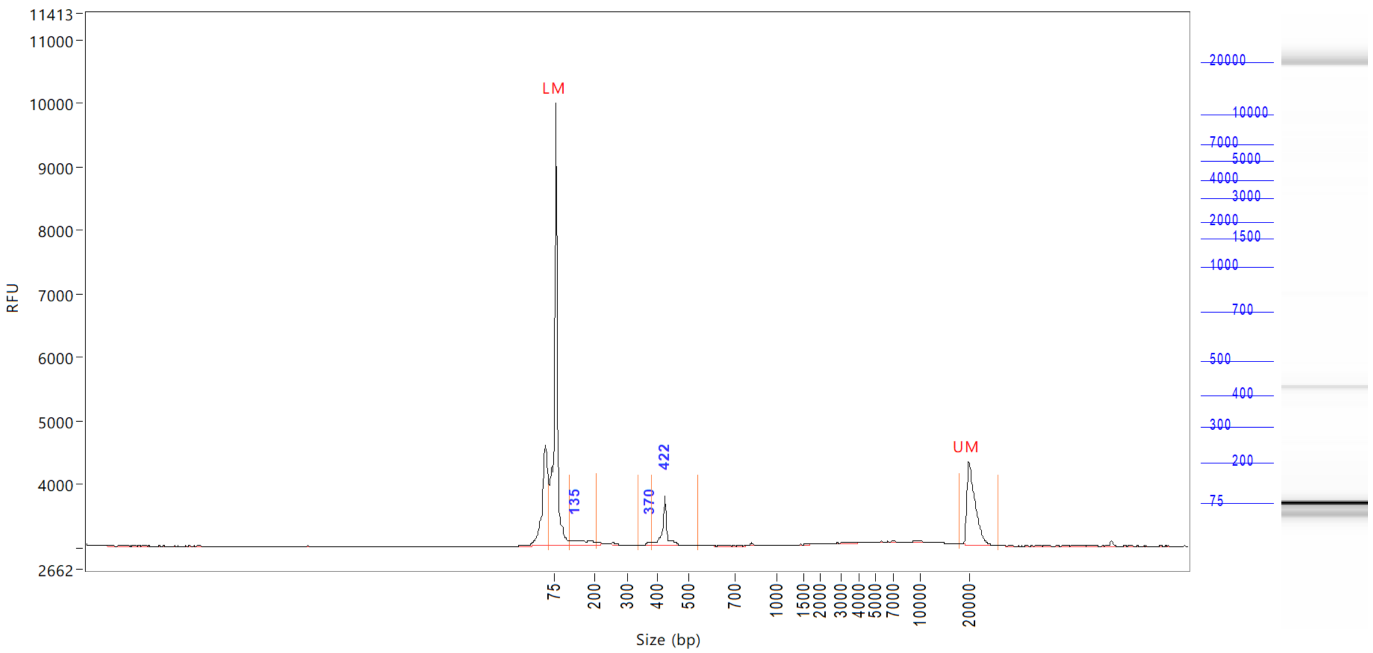

Peak	Size (bp)	Concentration (ng/uL)	From (bp)	To (bp)	Average size (bp)	CV%	RFU	Corrected peak area
1	75 (LM)	0.5000	54	122	73	13.12	6978	56.952
2	135	0.3270	122	205	159	14.84	72	3.104
3	370	0.0549	337	382	368	2.55	53	0.521
4	422	0.7109	382	537	423	4.44	770	6.748
5	20000 (UM)	0.1247	18049	26166	20680	4.85	1309	14.208

TIC:	1.0929	ng/uL
TIM:	6.3918	nmole/L
Total concentration:	1.4501	ng/uL

```
Sample peak width (sec): 5    Sample min peak height: 50    Sample baseline V to V?: Y    Sample baseline V to V points: 3
Sample filter: Binomial       Number of points for filter: 3  Sample start region (min): 0  Sample end region (min): 30
Marker peak width (sec): 5    Marker min peak height: 200   Marker baseline V to V?: Y    Marker baseline V to V points: 3
Lower marker selection: First peak > 500 RFU                Upper marker selection: Last peak > 200 RFU
Ladder size (bp)75, 200, 300, 400, 500, 700, 1000, 1500, 2000, 3000, 4000, 5000, 7000, 10000, 20000
Quantification using: Lower Marker          Final concentration (ng/uL): 0.5000          Dilution factor: 12.0
```

Sample: SampD5
Well location: D5
Created: Tuesday, September 14, 2021 9:33:53 PM

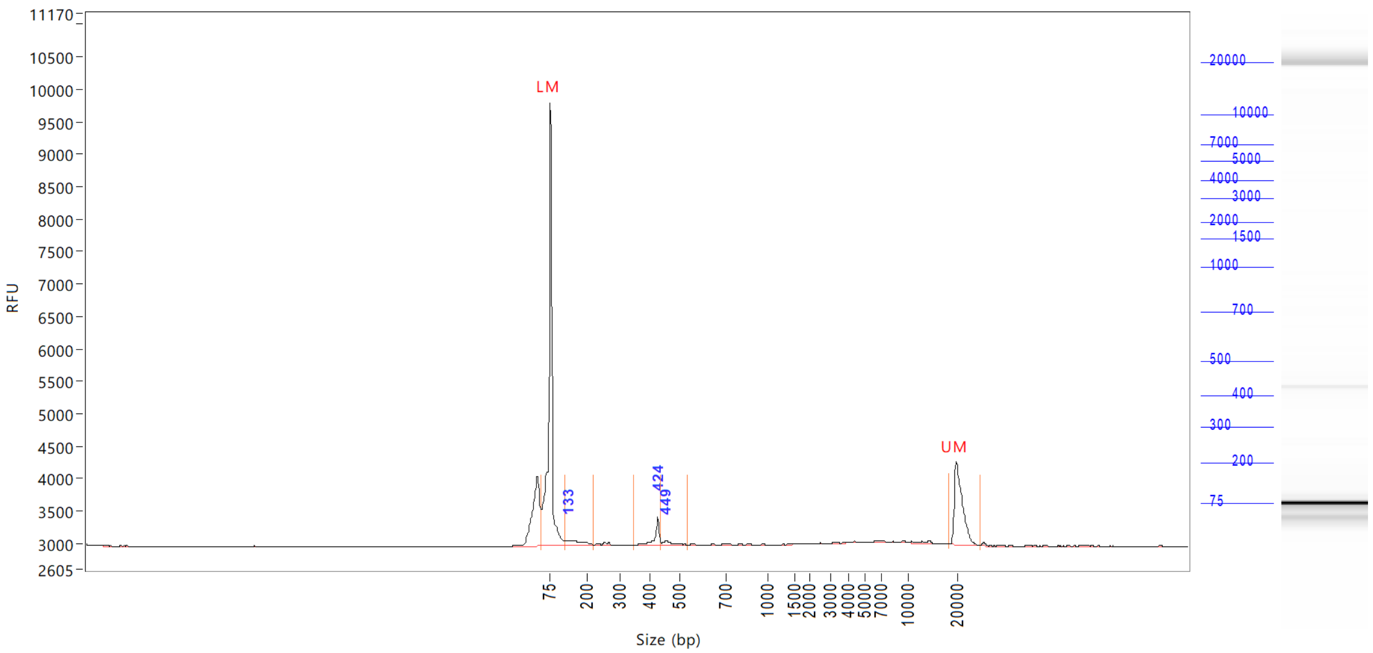

Peak	Size (bp)	Concentration (ng/uL)	From (bp)	To (bp)	Average size (bp)	CV%	RFU	Corrected peak area
1	75 (LM)	0.5000	45	124	72	16.10	6833	58.413
2	133	0.2760	124	215	157	14.31	70	2.687
3	424	0.3948	343	437	410	4.60	436	3.843
4	449	0.1403	437	529	466	4.92	65	1.366
5	20000 (UM)	0.1163	18415	24836	20579	4.57	1277	13.582

TIC:	0.8110	ng/uL
TIM:	4.9688	nmole/L
Total concentration:	1.2131	ng/uL

Sample peak width (sec): 5   Sample min peak height: 50   Sample baseline V to V?: Y   Sample baseline V to V points: 3
Sample filter: Binomial   Number of points for filter: 3   Sample start region (min): 0   Sample end region (min): 30
Marker peak width (sec): 5   Marker min peak height: 200   Marker baseline V to V?: Y   Marker baseline V to V points: 3
Lower marker selection: First peak > 500 RFU   Upper marker selection: Last peak > 200 RFU
Ladder size (bp)75, 200, 300, 400, 500, 700, 1000, 1500, 2000, 3000, 4000, 5000, 7000, 10000, 20000
Quantification using: Lower Marker   Final concentration (ng/uL): 0.5000   Dilution factor: 12.0

Sample: SampE5
Well location: E5
Created: Tuesday, September 14, 2021 9:33:53 PM

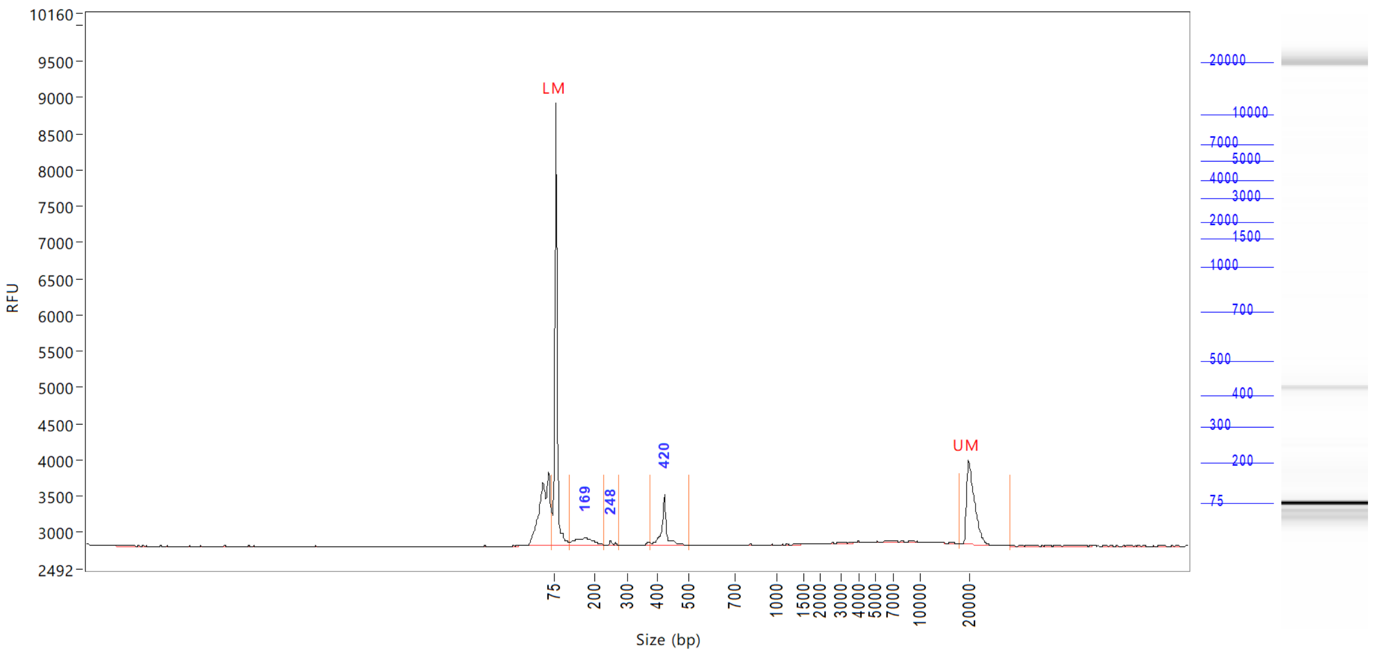

Peak	Size (bp)	Concentration (ng/uL)	From (bp)	To (bp)	Average size (bp)	CV%	RFU	Corrected peak area
1	75 (LM)	0.5000	66	122	75	9.78	6116	37.706
2	169	0.7081	122	230	166	15.15	101	4.450
3	248	0.0914	230	273	250	3.59	62	0.575
4	420	1.1008	380	501	421	4.50	704	6.918
5	20000 (UM)	0.1610	18049	28342	20569	4.87	1164	12.143

TIC:	1.9003	ng/uL
TIM:	11.9156	nmole/L
Total concentration:	2.3712	ng/uL

```
Sample peak width (sec): 5    Sample min peak height: 50    Sample baseline V to V?: Y    Sample baseline V to V points: 3
Sample filter: Binomial       Number of points for filter: 3  Sample start region (min): 0  Sample end region (min): 30
Marker peak width (sec): 5    Marker min peak height: 200   Marker baseline V to V?: Y    Marker baseline V to V points: 3
Lower marker selection: First peak > 500 RFU                Upper marker selection: Last peak > 200 RFU
Ladder size (bp)75, 200, 300, 400, 500, 700, 1000, 1500, 2000, 3000, 4000, 5000, 7000, 10000, 20000
Quantification using: Lower Marker          Final concentration (ng/uL): 0.5000          Dilution factor: 12.0
```

Sample: SampF5
Well location: F5
Created: Tuesday, September 14, 2021 9:33:53 PM

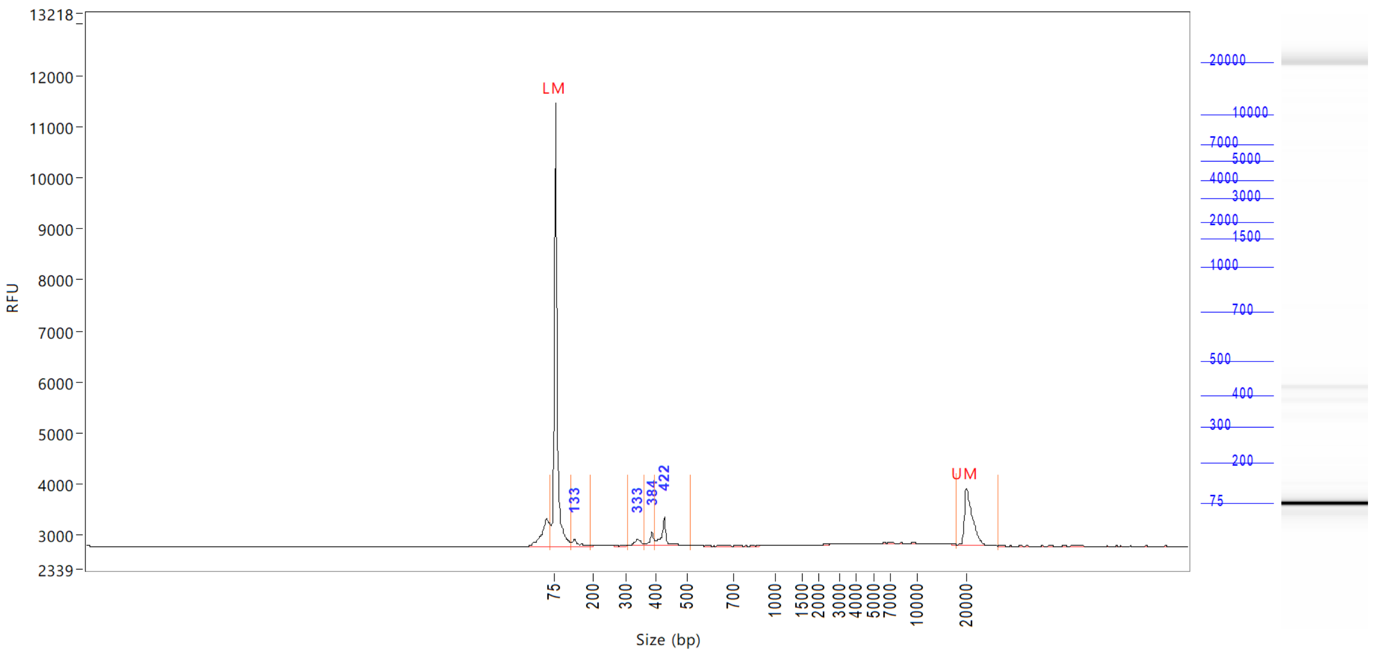

Peak	Size (bp)	Concentration (ng/uL)	From (bp)	To (bp)	Average size (bp)	CV%	RFU	Corrected peak area
1	75 (LM)	0.5000	60	126	75	12.08	8687	62.058
2	133	0.1773	126	187	144	11.45	142	1.834
3	333	0.1718	303	356	332	3.37	125	1.776
4	384	0.2186	356	394	379	2.31	278	2.261
5	422	0.5693	394	509	424	4.64	561	5.888
6	20000 (UM)	0.0936	18049	26529	20626	4.60	1111	11.621

TIC:	1.1369	ng/uL
TIM:	6.0328	nmole/L
Total concentration:	1.4082	ng/uL

Sample peak width (sec): 5 Sample min peak height: 50 Sample baseline V to V?: Y Sample baseline V to V points: 3
Sample filter: Binomial Number of points for filter: 3 Sample start region (min): 0 Sample end region (min): 30
Marker peak width (sec): 5 Marker min peak height: 200 Marker baseline V to V?: Y Marker baseline V to V points: 3
Lower marker selection: First peak > 500 RFU Upper marker selection: Last peak > 200 RFU
Ladder size (bp)75, 200, 300, 400, 500, 700, 1000, 1500, 2000, 3000, 4000, 5000, 7000, 10000, 20000
Quantification using: Lower Marker Final concentration (ng/uL): 0.5000 Dilution factor: 12.0

Sample: SampG5
Well location: G5
Created: Tuesday, September 14, 2021 9:33:53 PM

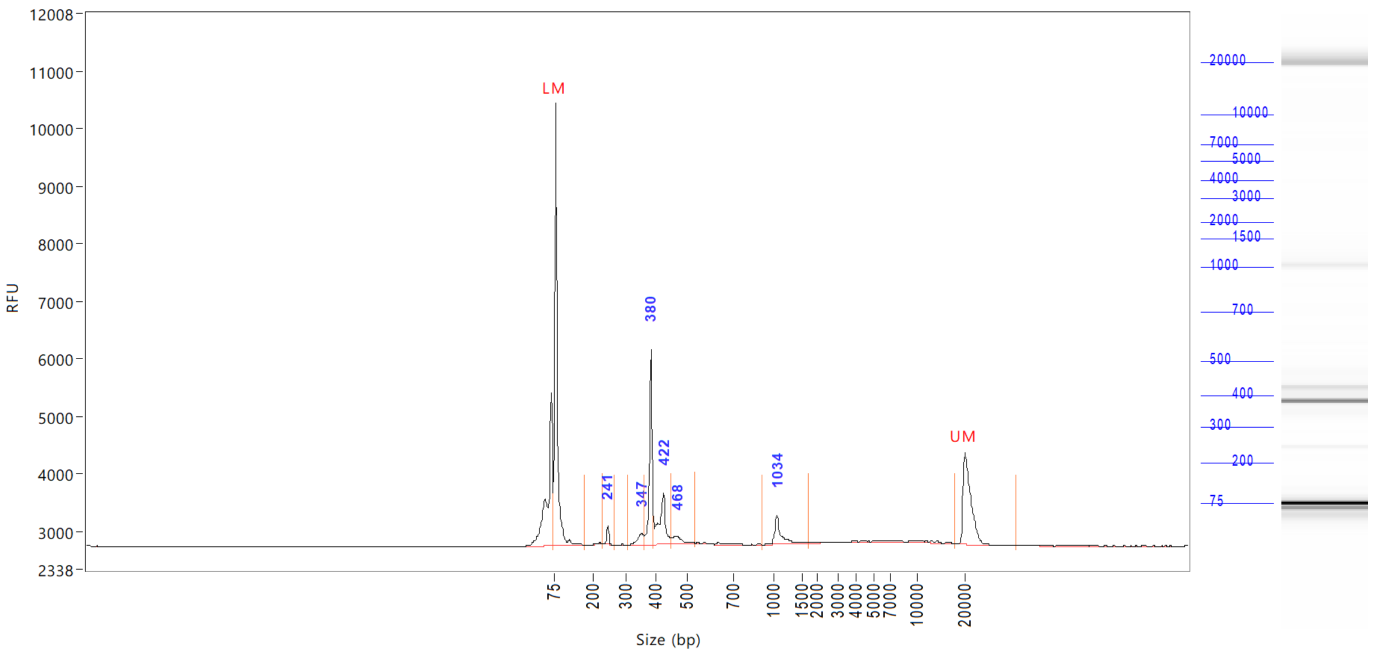

Peak	Size (bp)	Concentration (ng/uL)	From (bp)	To (bp)	Average size (bp)	CV%	RFU	Corrected peak area
1	75 (LM)	0.5000	67	168	77	13.71	7716	51.283
2	241	0.1847	226	260	239	1.39	314	1.579
3	347	0.3395	303	360	342	3.53	201	2.902
4	380	2.2967	360	392	378	1.47	3403	19.630
5	422	1.2908	392	447	415	3.06	904	11.033
6	468	0.2911	447	532	468	3.56	133	2.488
7	1034	0.6456	909	1731	1099	12.29	514	5.518
8	20000 (UM)	0.1645	17805	30519	20622	5.46	1603	16.870

TIC:	5.0484	ng/uL
TIM:	20.0082	nmole/L
Total concentration:	5.4264	ng/uL

Sample peak width (sec): 5 Sample min peak height: 50 Sample baseline V to V?: Y Sample baseline V to V points: 3
Sample filter: Binomial Number of points for filter: 3 Sample start region (min): 0 Sample end region (min): 30
Marker peak width (sec): 5 Marker min peak height: 200 Marker baseline V to V?: Y Marker baseline V to V points: 3
Lower marker selection: First peak > 500 RFU Upper marker selection: Last peak > 200 RFU
Ladder size (bp)75, 200, 300, 400, 500, 700, 1000, 1500, 2000, 3000, 4000, 5000, 7000, 10000, 20000
Quantification using: Lower Marker Final concentration (ng/uL): 0.5000 Dilution factor: 12.0

Sample: SampH5
Well location: H5
Created: Tuesday, September 14, 2021 9:33:53 PM

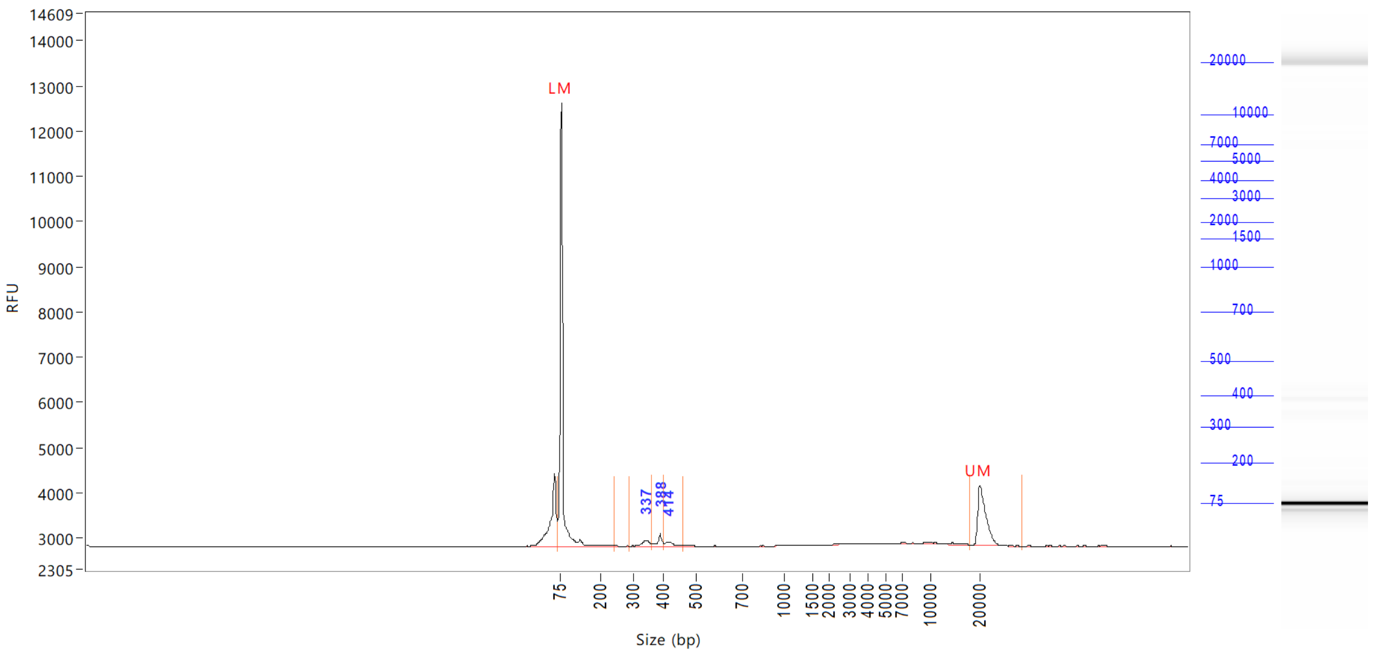

Peak	Size	Concentration	From	To	Average size	CV%	RFU	Corrected peak area
	(bp)	(ng/uL)	(bp)	(bp)	(bp)			
1	75 (LM)	0.5000	64	241	78	24.33	9830	66.013
2	337	0.1951	284	360	335	4.10	130	2.147
3	388	0.2009	360	400	383	2.44	278	2.211
4	414	0.1404	400	458	417	3.16	91	1.544
5	20000 (UM)	0.1074	17927	28705	20611	4.86	1337	14.177

TIC:	0.5365	ng/uL
TIM:	2.3753	nmole/L
Total concentration:	0.8385	ng/uL

```
Sample peak width (sec): 5     Sample min peak height: 50     Sample baseline V to V?: Y     Sample baseline V to V points: 3
Sample filter: Binomial        Number of points for filter: 3  Sample start region (min): 0  Sample end region (min): 30
Marker peak width (sec): 5     Marker min peak height: 200    Marker baseline V to V?: Y     Marker baseline V to V points: 3
Lower marker selection: First peak > 500 RFU                  Upper marker selection: Last peak > 200 RFU
Ladder size (bp)75, 200, 300, 400, 500, 700, 1000, 1500, 2000, 3000, 4000, 5000, 7000, 10000, 20000
Quantification using: Lower Marker          Final concentration (ng/uL): 0.5000          Dilution factor: 12.0
```

Sample: SampA6
Well location: A6
Created: Tuesday, September 14, 2021 9:33:53 PM

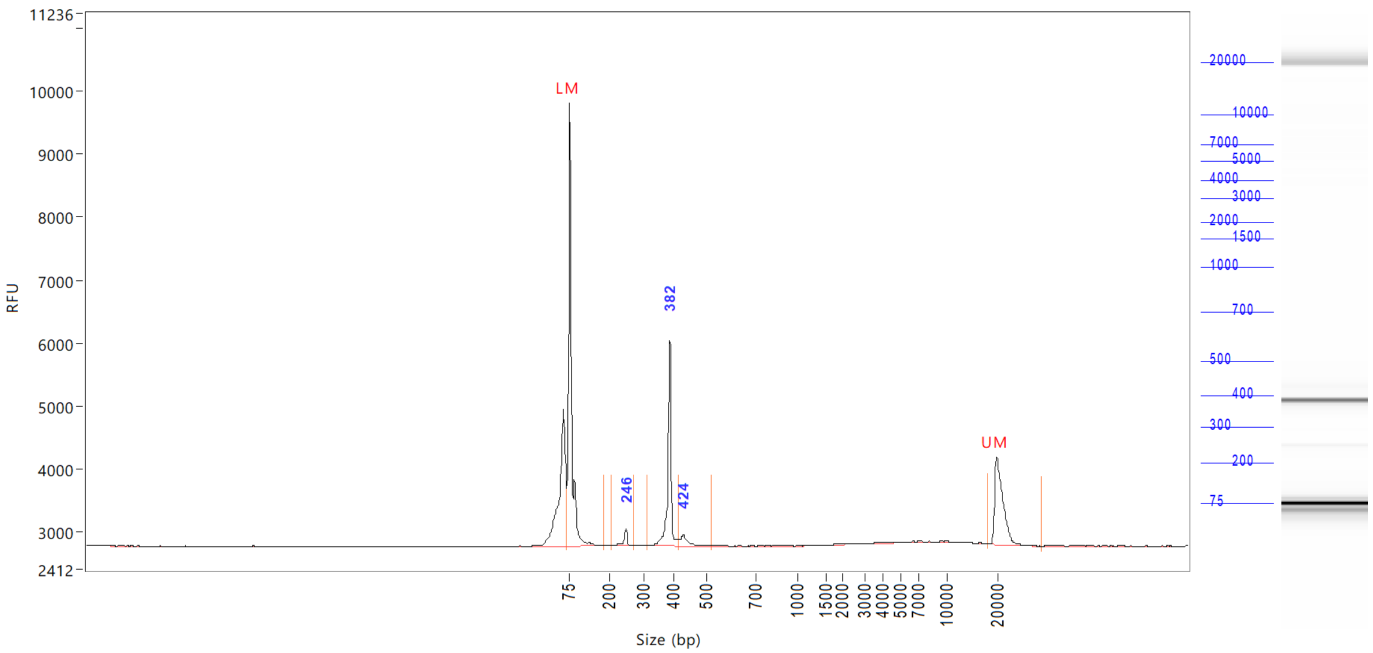

Peak	Size (bp)	Concentration (ng/uL)	From (bp)	To (bp)	Average size (bp)	CV%	RFU	Corrected peak area
1	75 (LM)	0.5000	66	181	77	16.10	7045	51.257
2	246	0.2273	205	268	238	4.34	267	1.942
3	382	2.5551	307	411	378	3.09	3273	21.828
4	424	0.3328	411	519	435	5.13	172	2.843
5	20000 (UM)	0.1425	18049	28826	20607	5.22	1397	14.612

TIC:	3.1151	ng/uL
TIM:	13.9502	nmole/L
Total concentration:	3.4603	ng/uL

```
Sample peak width (sec): 5    Sample min peak height: 50    Sample baseline V to V?: Y    Sample baseline V to V points: 3
Sample filter: Binomial       Number of points for filter: 3  Sample start region (min): 0  Sample end region (min): 30
Marker peak width (sec): 5    Marker min peak height: 200   Marker baseline V to V?: Y    Marker baseline V to V points: 3
Lower marker selection: First peak > 500 RFU                Upper marker selection: Last peak > 200 RFU
Ladder size (bp)75, 200, 300, 400, 500, 700, 1000, 1500, 2000, 3000, 4000, 5000, 7000, 10000, 20000
Quantification using: Lower Marker          Final concentration (ng/uL): 0.5000          Dilution factor: 12.0
```

Sample: SampB6
Well location: B6
Created: Tuesday, September 14, 2021 9:33:53 PM

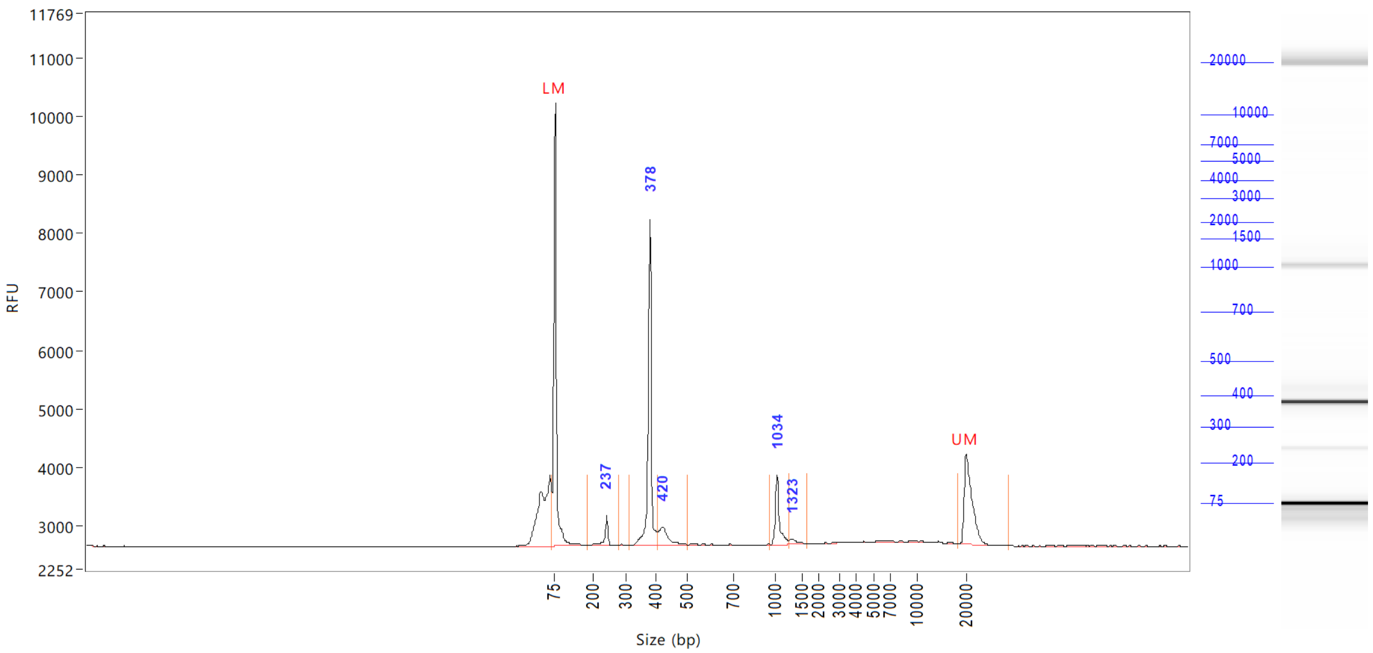

Peak	Size (bp)	Concentration (ng/uL)	From (bp)	To (bp)	Average size (bp)	CV%	RFU	Corrected peak area
1	75 (LM)	0.5000	66	179	76	15.49	7597	50.054
2	237	0.4520	179	275	230	5.99	510	3.771
3	378	4.3116	309	405	374	3.01	5569	35.968
4	420	0.7359	405	500	429	4.94	317	6.139
5	1034	0.9941	965	1245	1044	5.51	1185	8.293
6	1323	0.1425	1245	1674	1358	6.83	87	1.189
7	20000 (UM)	0.1592	18293	28826	20577	4.80	1531	15.935

TIC:	6.6362	ng/uL
TIM:	26.7610	nmole/L
Total concentration:	6.9430	ng/uL

Sample peak width (sec): 5   Sample min peak height: 50   Sample baseline V to V?: Y   Sample baseline V to V points: 3
Sample filter: Binomial   Number of points for filter: 3   Sample start region (min): 0   Sample end region (min): 30
Marker peak width (sec): 5   Marker min peak height: 200   Marker baseline V to V?: Y   Marker baseline V to V points: 3
Lower marker selection: First peak > 500 RFU   Upper marker selection: Last peak > 200 RFU
Ladder size (bp)75, 200, 300, 400, 500, 700, 1000, 1500, 2000, 3000, 4000, 5000, 7000, 10000, 20000
Quantification using: Lower Marker   Final concentration (ng/uL): 0.5000   Dilution factor: 12.0

Sample: SampC6
Well location: C6
Created: Tuesday, September 14, 2021 9:33:53 PM

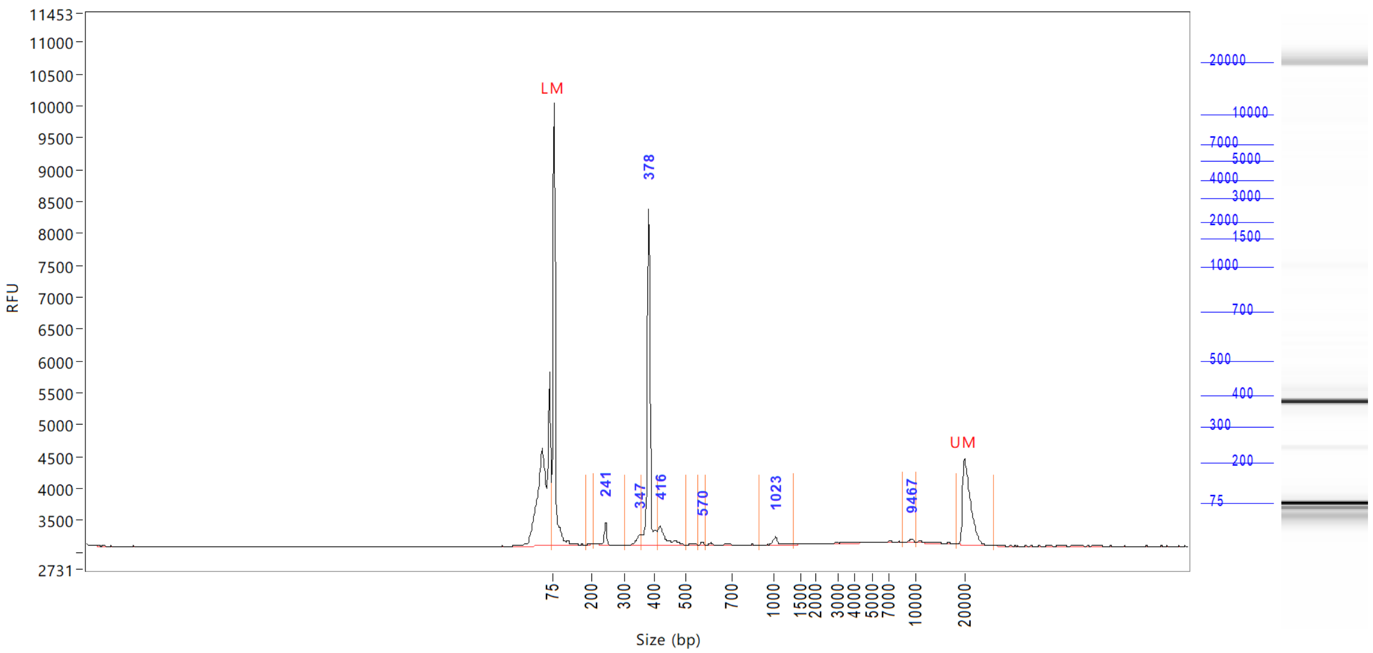

Peak	Size (bp)	Concentration (ng/uL)	From (bp)	To (bp)	Average size (bp)	CV%	RFU	Corrected peak area
1	75 (LM)	0.5000	67	177	76	13.33	6956	44.701
2	241	0.2881	203	300	237	4.64	341	2.146
3	347	0.2883	300	356	341	3.21	175	2.148
4	378	4.1563	356	407	377	2.06	5287	30.965
5	416	0.6607	407	501	431	5.09	311	4.923
6	570	0.0323	552	583	566	0.87	51	0.241
7	1023	0.2001	892	1378	1056	9.11	132	1.491
8	9467	0.0517	8534	10000	9406	3.29	54	0.385
9	20000 (UM)	0.1597	18293	25924	20614	4.61	1349	14.278

TIC:	5.6775	ng/uL
TIM:	24.4649	nmole/L
Total concentration:	6.0149	ng/uL

Sample peak width (sec): 5   Sample min peak height: 50   Sample baseline V to V?: Y   Sample baseline V to V points: 3
Sample filter: Binomial   Number of points for filter: 3 Sample start region (min): 0 Sample end region (min): 30
Marker peak width (sec): 5   Marker min peak height: 200   Marker baseline V to V?: Y   Marker baseline V to V points: 3
Lower marker selection: First peak > 500 RFU   Upper marker selection: Last peak > 200 RFU
Ladder size (bp)75, 200, 300, 400, 500, 700, 1000, 1500, 2000, 3000, 4000, 5000, 7000, 10000, 20000
Quantification using: Lower Marker   Final concentration (ng/uL): 0.5000   Dilution factor: 12.0

Sample: SampD6
Well location: D6
Created: Tuesday, September 14, 2021 9:33:53 PM

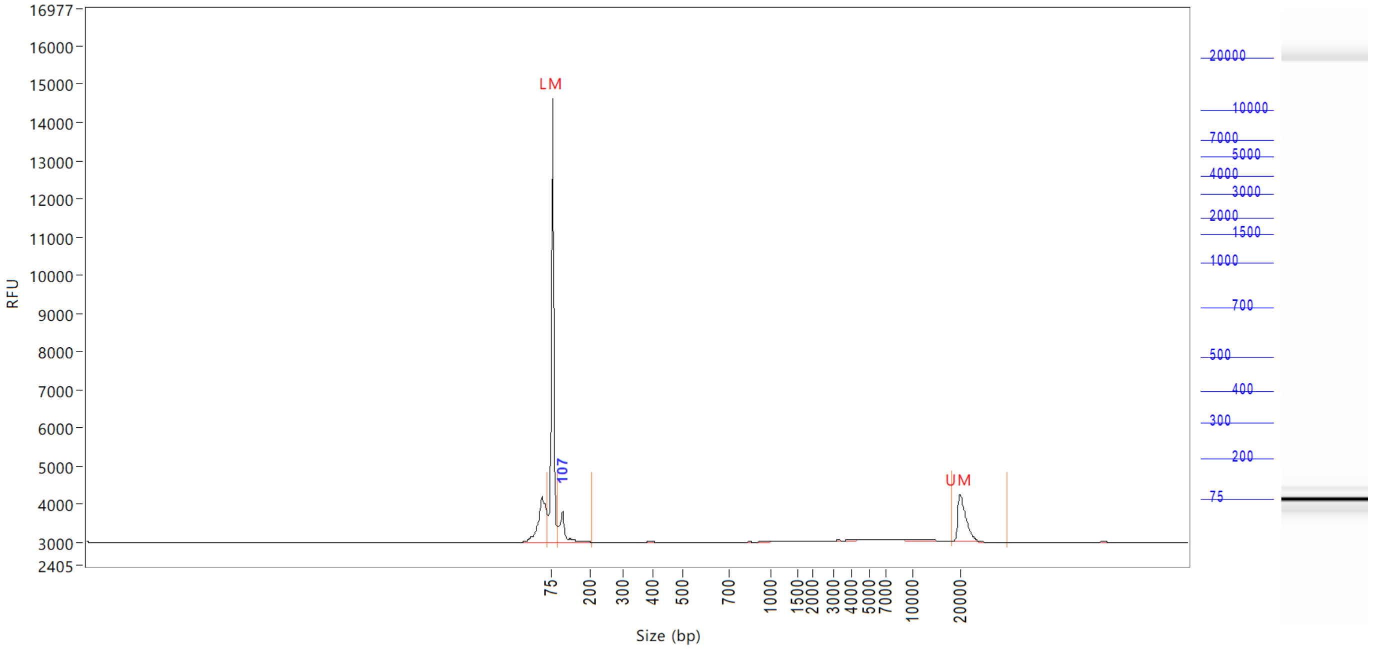

Peak	Size (bp)	Concentration (ng/uL)	From (bp)	To (bp)	Average size (bp)	CV%	RFU	Corrected peak area
1	75 (LM)	0.5000	60	89	73	6.26	11649	73.697
2	107	0.9092	89	201	109	17.40	826	11.167
3	20000 (UM)	0.0899	18171	29672	20588	5.14	1232	13.254

TIC:	0.9092	ng/uL
TIM:	13.6998	nmole/L
Total concentration:	1.2373	ng/uL

Sample peak width (sec): 5   Sample min peak height: 50   Sample baseline V to V?: Y   Sample baseline V to V points: 3
Sample filter: Binomial   Number of points for filter: 3 Sample start region (min): 0 Sample end region (min): 30
Marker peak width (sec): 5   Marker min peak height: 200   Marker baseline V to V?: Y   Marker baseline V to V points: 3
Lower marker selection: First peak > 500 RFU   Upper marker selection: Last peak > 200 RFU
Ladder size (bp)75, 200, 300, 400, 500, 700, 1000, 1500, 2000, 3000, 4000, 5000, 7000, 10000, 20000
Quantification using: Lower Marker   Final concentration (ng/uL): 0.5000   Dilution factor: 12.0

Sample: SampE6
Well location: E6
Created: Tuesday, September 14, 2021 9:33:53 PM

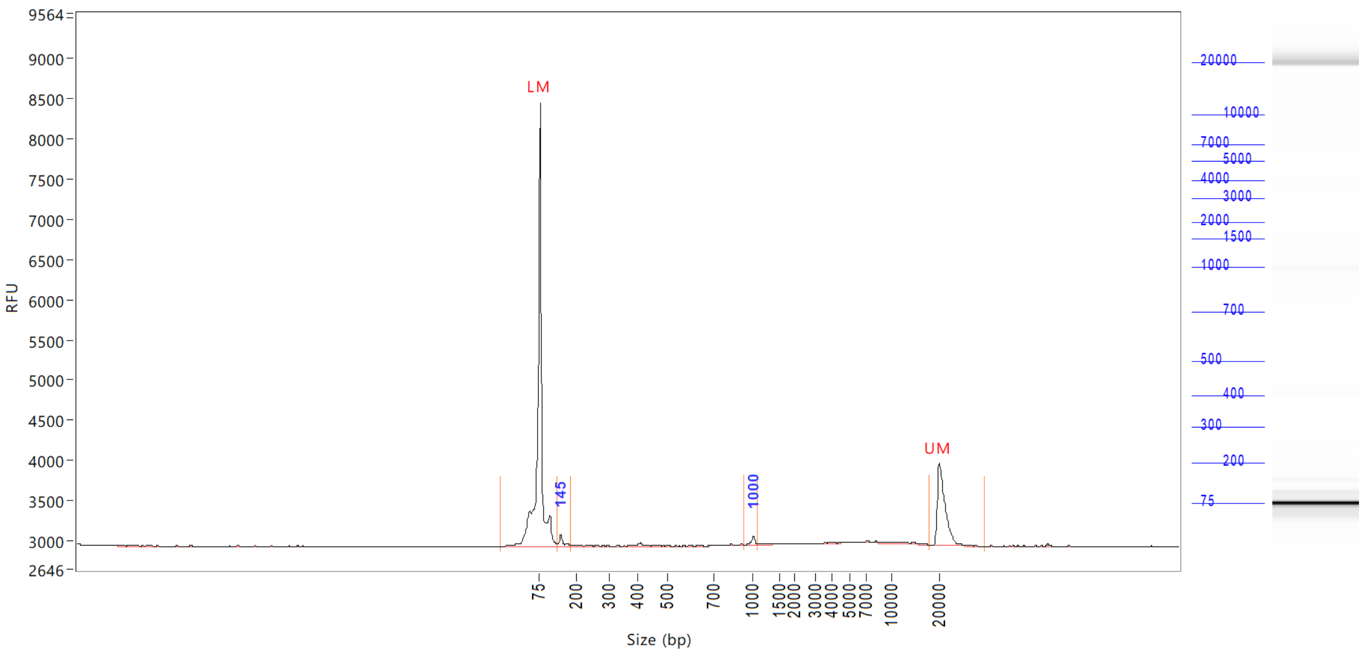

Peak	Size (bp)	Concentration (ng/uL)	From (bp)	To (bp)	Average size (bp)	CV%	RFU	Corrected peak area
1	75 (LM)	0.5000	0	135	69	30.27	5529	54.246
2	145	0.1476	135	179	149	7.11	152	1.334
3	1000	0.0842	935	1079	993	2.25	114	0.761
4	20000 (UM)	0.0976	17805	29551	20717	5.84	1024	10.588

TIC:	0.2318	ng/uL
TIM:	1.7671	nmole/L
Total concentration:	0.6439	ng/uL

Sample peak width (sec): 5 Sample min peak height: 50 Sample baseline V to V?: Y Sample baseline V to V points: 3
Sample filter: Binomial Number of points for filter: 3 Sample start region (min): 0 Sample end region (min): 30
Marker peak width (sec): 5 Marker min peak height: 200 Marker baseline V to V?: Y Marker baseline V to V points: 3
Lower marker selection: First peak > 500 RFU Upper marker selection: Last peak > 200 RFU
Ladder size (bp)75, 200, 300, 400, 500, 700, 1000, 1500, 2000, 3000, 4000, 5000, 7000, 10000, 20000
Quantification using: Lower Marker Final concentration (ng/uL): 0.5000 Dilution factor: 12.0

Sample: SampF6
Well location: F6
Created: Tuesday, September 14, 2021 9:33:53 PM

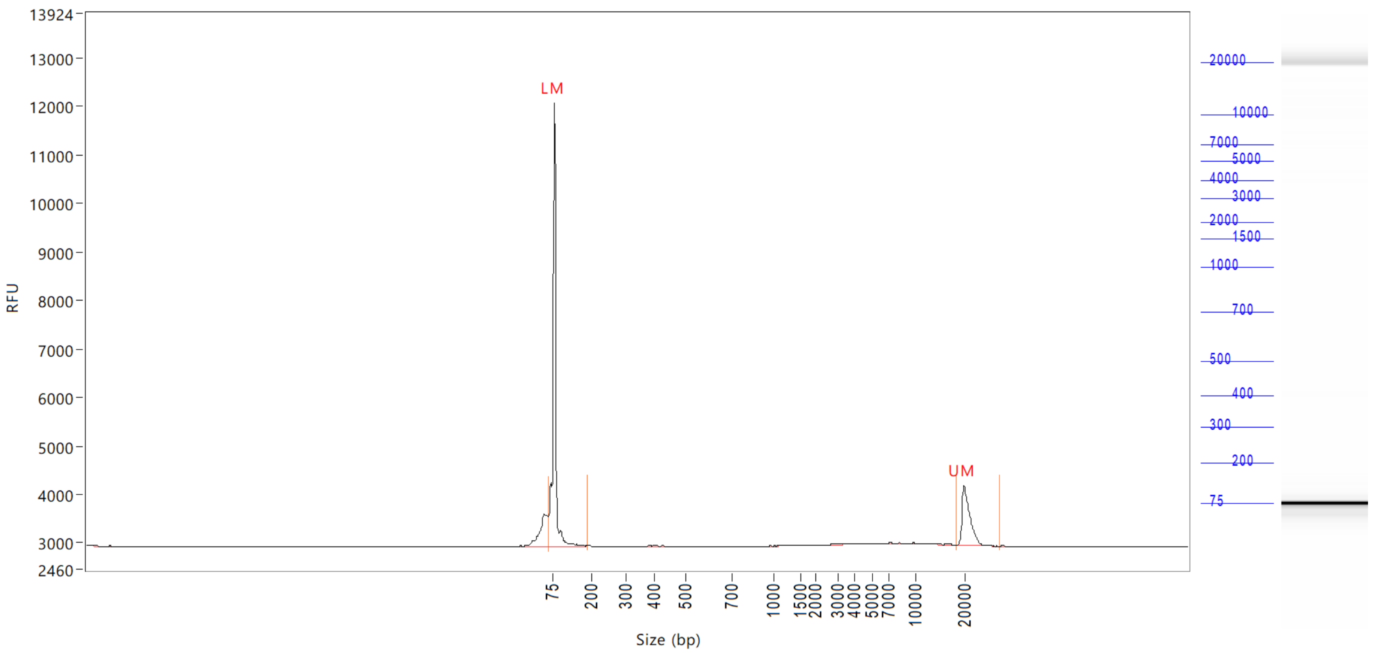

Peak	Size (bp)	Concentration (ng/uL)	From (bp)	To (bp)	Average size (bp)	CV%	RFU	Corrected peak area
1	75 (LM)	0.5000	56	183	74	15.19	9158	65.399
2	20000 (UM)	0.0968	18415	27375	20612	4.57	1219	12.663

TIC:	0.0000	ng/uL
TIM:	0.0000	nmole/L
Total concentration:	0.3189	ng/uL

```
Sample peak width (sec): 5    Sample min peak height: 50    Sample baseline V to V?: Y    Sample baseline V to V points: 3
Sample filter: Binomial       Number of points for filter: 3  Sample start region (min): 0  Sample end region (min): 30
Marker peak width (sec): 5    Marker min peak height: 200   Marker baseline V to V?: Y    Marker baseline V to V points: 3
Lower marker selection: First peak > 500 RFU                Upper marker selection: Last peak > 200 RFU
Ladder size (bp)75, 200, 300, 400, 500, 700, 1000, 1500, 2000, 3000, 4000, 5000, 7000, 10000, 20000
Quantification using: Lower Marker          Final concentration (ng/uL): 0.5000          Dilution factor: 12.0
```

Sample: SampG6
Well location: G6
Created: Tuesday, September 14, 2021 9:33:53 PM

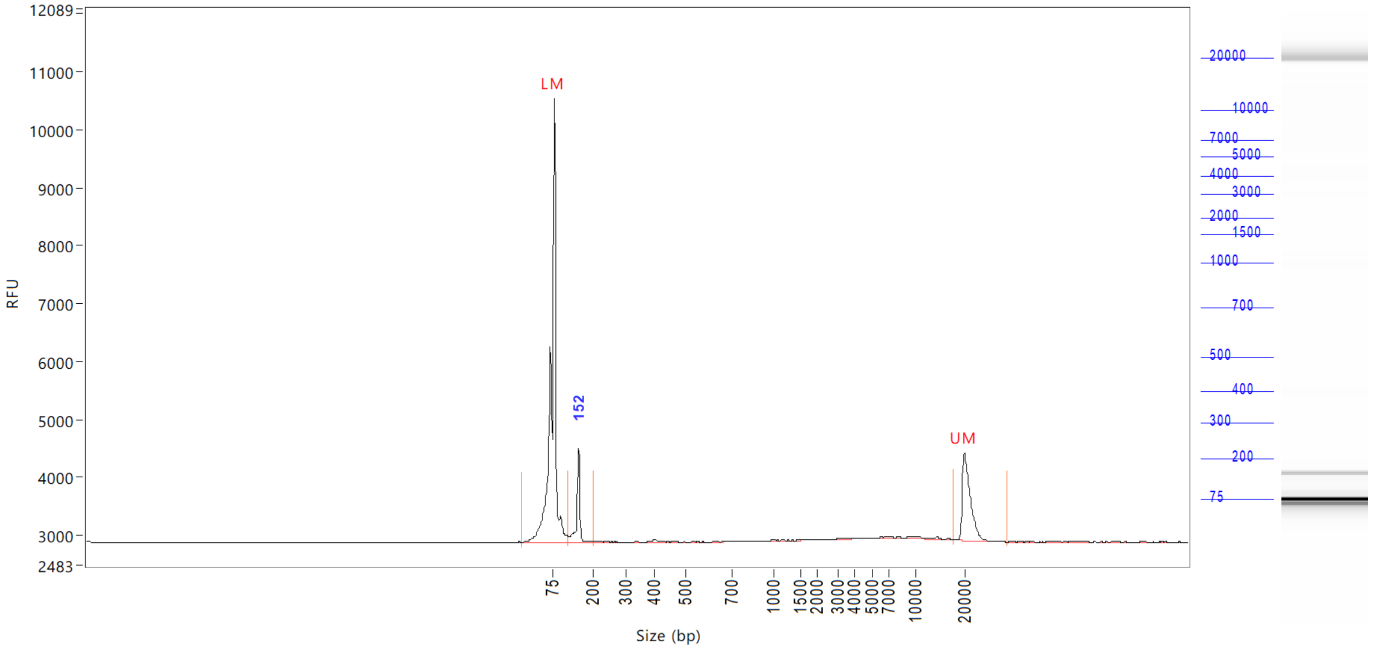

Peak	Size	Concentration	From	To	Average size	CV%	RFU	Corrected peak area
	(bp)	(ng/uL)	(bp)	(bp)	(bp)			
1	75 (LM)	0.5000	0	122	65	27.36	7673	86.840
2	152	0.7768	122	203	149	6.68	1615	11.243
3	20000 (UM)	0.0932	17683	28826	20595	5.08	1513	16.182

TIC:	0.7768	ng/uL
TIM:	8.5686	nmole/L
Total concentration:	1.0842	ng/uL

Sample peak width (sec): 5 Sample min peak height: 50 Sample baseline V to V?: Y Sample baseline V to V points: 3
Sample filter: Binomial Number of points for filter: 3 Sample start region (min): 0 Sample end region (min): 30
Marker peak width (sec): 5 Marker min peak height: 200 Marker baseline V to V?: Y Marker baseline V to V points: 3
Lower marker selection: First peak > 500 RFU Upper marker selection: Last peak > 200 RFU
Ladder size (bp)75, 200, 300, 400, 500, 700, 1000, 1500, 2000, 3000, 4000, 5000, 7000, 10000, 20000
Quantification using: Lower Marker Final concentration (ng/uL): 0.5000 Dilution factor: 12.0

Sample: SampH6
Well location: H6
Created: Tuesday, September 14, 2021 9:33:53 PM

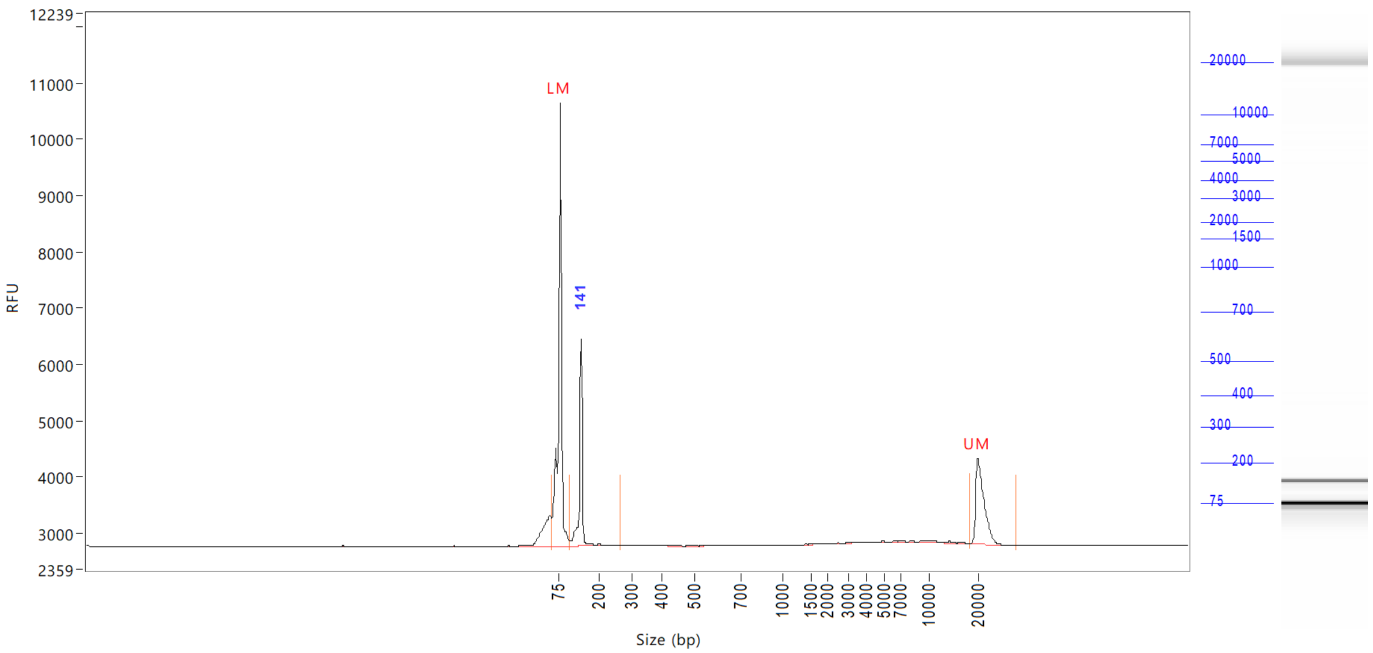

Peak	Size (bp)	Concentration (ng/uL)	From (bp)	To (bp)	Average size (bp)	CV%	RFU	Corrected peak area
1	75 (LM)	0.5000	48	105	71	12.10	7888	63.355
2	141	2.4252	105	260	139	10.35	3686	25.608
3	20000 (UM)	0.1315	18415	27738	20599	4.93	1535	16.668

TIC:	2.4252	ng/uL
TIM:	28.6715	nmole/L
Total concentration:	2.7755	ng/uL

```
Sample peak width (sec): 5    Sample min peak height: 50    Sample baseline V to V?: Y    Sample baseline V to V points: 3
Sample filter: Binomial       Number of points for filter: 3  Sample start region (min): 0  Sample end region (min): 30
Marker peak width (sec): 5    Marker min peak height: 200   Marker baseline V to V?: Y    Marker baseline V to V points: 3
Lower marker selection: First peak > 500 RFU                Upper marker selection: Last peak > 200 RFU
Ladder size (bp)75, 200, 300, 400, 500, 700, 1000, 1500, 2000, 3000, 4000, 5000, 7000, 10000, 20000
Quantification using: Lower Marker          Final concentration (ng/uL): 0.5000          Dilution factor: 12.0
```

Sample: SampA7
Well location: A7
Created: Tuesday, September 14, 2021 9:33:53 PM

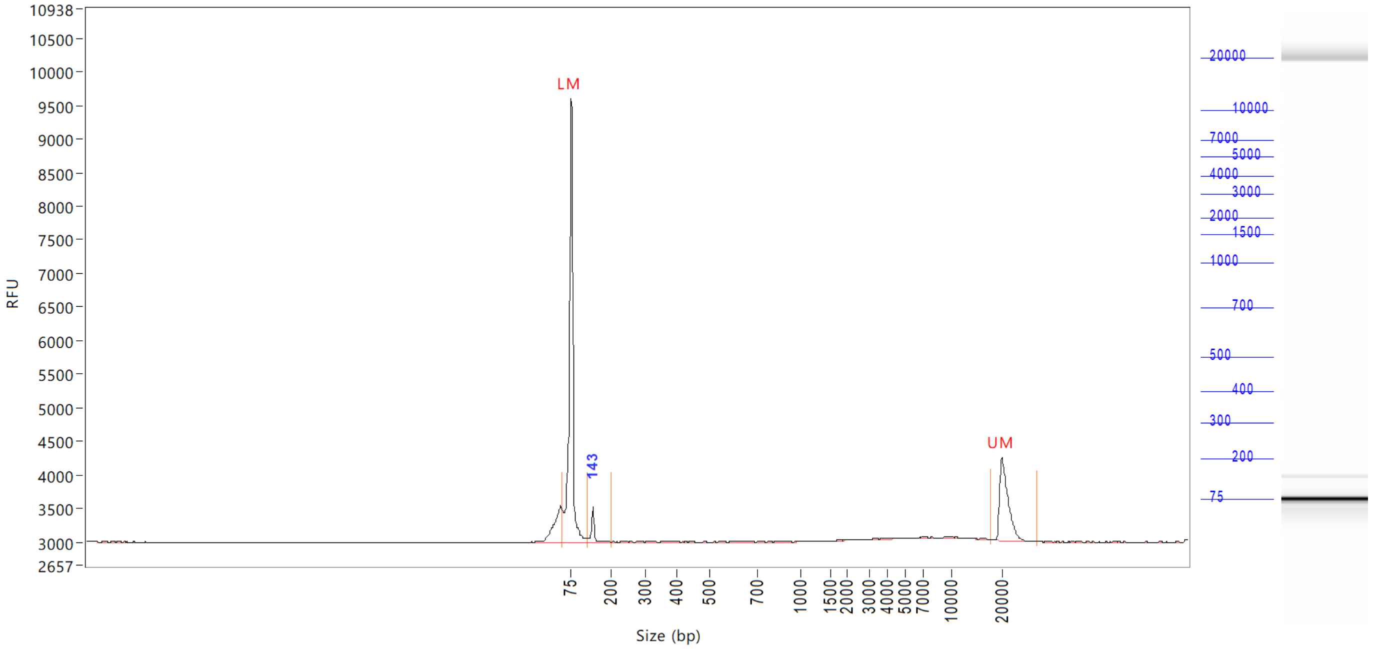

Peak	Size (bp)	Concentration (ng/uL)	From (bp)	To (bp)	Average size (bp)	CV%	RFU	Corrected peak area
1	75 (LM)	0.5000	50	126	73	13.00	6616	54.009
2	143	0.3905	126	200	142	7.66	532	3.515
3	20000 (UM)	0.1221	17927	27012	20626	4.83	1240	13.194

TIC:	0.3905	ng/uL
TIM:	4.5191	nmole/L
Total concentration:	0.7729	ng/uL

Sample peak width (sec): 5   Sample min peak height: 50   Sample baseline V to V?: Y   Sample baseline V to V points: 3
Sample filter: Binomial   Number of points for filter: 3 Sample start region (min): 0 Sample end region (min): 30
Marker peak width (sec): 5   Marker min peak height: 200   Marker baseline V to V?: Y   Marker baseline V to V points: 3
Lower marker selection: First peak > 500 RFU   Upper marker selection: Last peak > 200 RFU
Ladder size (bp)75, 200, 300, 400, 500, 700, 1000, 1500, 2000, 3000, 4000, 5000, 7000, 10000, 20000
Quantification using: Lower Marker   Final concentration (ng/uL): 0.5000   Dilution factor: 12.0

Sample: SampB7
Well location: B7
Created: Tuesday, September 14, 2021 9:33:53 PM

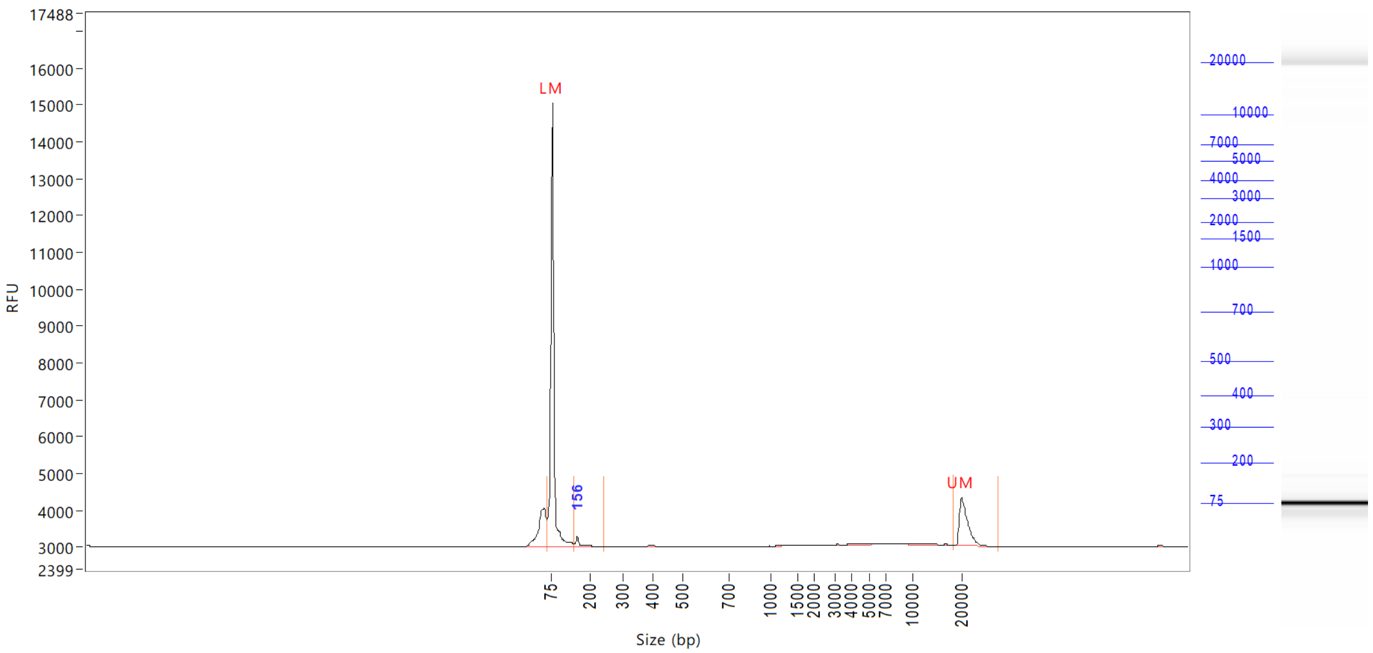

Peak	Size	Concentration	From	To	Average size	CV%	RFU	Corrected peak area
	(bp)	(ng/uL)	(bp)	(bp)	(bp)			
1	75 (LM)	0.5000	60	147	76	14.97	12062	95.715
2	156	0.1689	147	242	167	12.97	271	2.694
3	20000 (UM)	0.0745	18415	27617	20670	4.92	1324	14.253

TIC:	0.1689	ng/uL
TIM:	1.6624	nmole/L
Total concentration:	0.3976	ng/uL

```
Sample peak width (sec): 5    Sample min peak height: 50    Sample baseline V to V?: Y    Sample baseline V to V points: 3
Sample filter: Binomial       Number of points for filter: 3  Sample start region (min): 0  Sample end region (min): 30
Marker peak width (sec): 5    Marker min peak height: 200   Marker baseline V to V?: Y    Marker baseline V to V points: 3
Lower marker selection: First peak > 500 RFU                Upper marker selection: Last peak > 200 RFU
Ladder size (bp)75, 200, 300, 400, 500, 700, 1000, 1500, 2000, 3000, 4000, 5000, 7000, 10000, 20000
Quantification using: Lower Marker          Final concentration (ng/uL): 0.5000          Dilution factor: 12.0
```

Sample: SampC7
Well location: C7
Created: Tuesday, September 14, 2021 9:33:53 PM

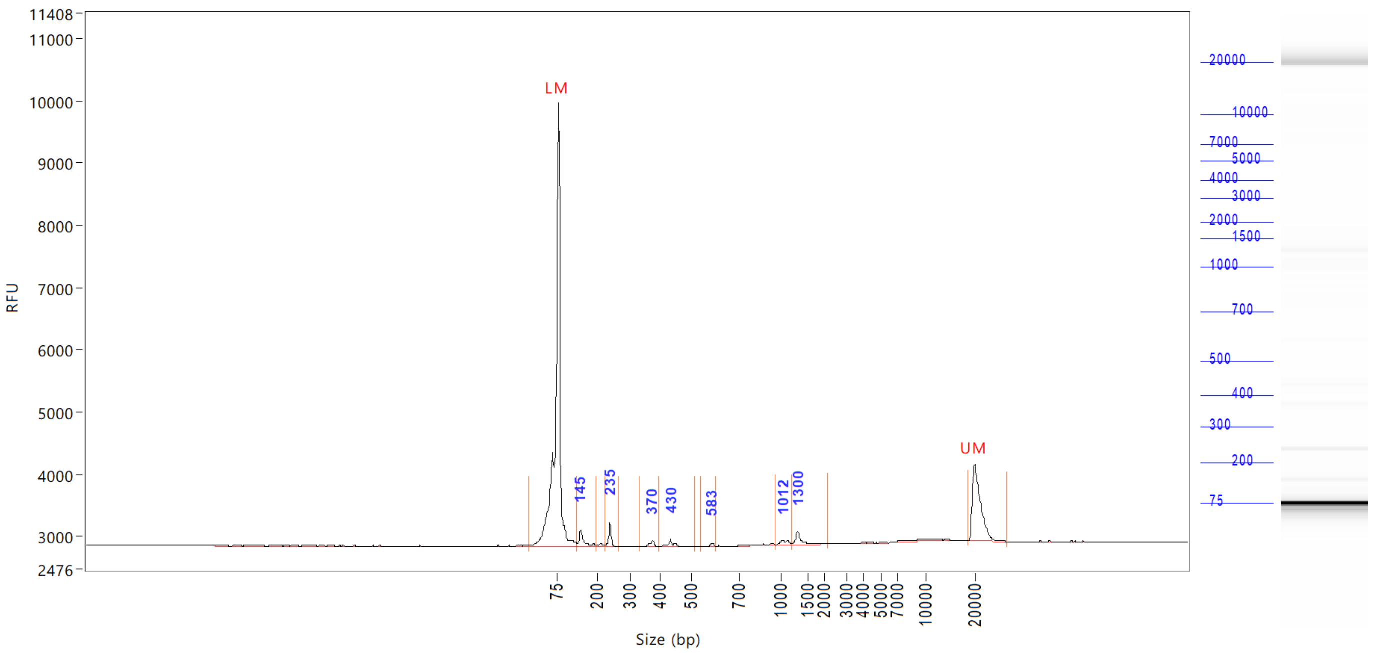

Peak	Size (bp)	Concentration (ng/uL)	From (bp)	To (bp)	Average size (bp)	CV%	RFU	Corrected peak area
1	75 (LM)	0.5000	0	133	65	27.06	7135	77.093
2	145	0.2171	133	196	152	10.20	267	2.790
3	235	0.1686	221	262	233	2.01	390	2.167
4	370	0.0757	331	394	365	3.03	104	0.973
5	430	0.1052	394	509	434	4.66	115	1.352
6	583	0.0286	539	600	578	1.82	56	0.367
7	1012	0.0803	965	1190	1056	5.97	78	1.032
8	1300	0.1629	1190	2144	1365	11.53	213	2.093
9	20000 (UM)	0.0850	18659	26408	20601	4.92	1231	13.100

TIC:	0.8384	ng/uL
TIM:	4.6810	nmole/L
Total concentration:	1.0877	ng/uL

Sample peak width (sec): 5 Sample min peak height: 50 Sample baseline V to V?: Y Sample baseline V to V points: 3
Sample filter: Binomial Number of points for filter: 3 Sample start region (min): 0 Sample end region (min): 30
Marker peak width (sec): 5 Marker min peak height: 200 Marker baseline V to V?: Y Marker baseline V to V points: 3
Lower marker selection: First peak > 500 RFU Upper marker selection: Last peak > 200 RFU
Ladder size (bp)75, 200, 300, 400, 500, 700, 1000, 1500, 2000, 3000, 4000, 5000, 7000, 10000, 20000
Quantification using: Lower Marker Final concentration (ng/uL): 0.5000 Dilution factor: 12.0

Sample: SampD7
Well location: D7
Created: Tuesday, September 14, 2021 9:33:53 PM

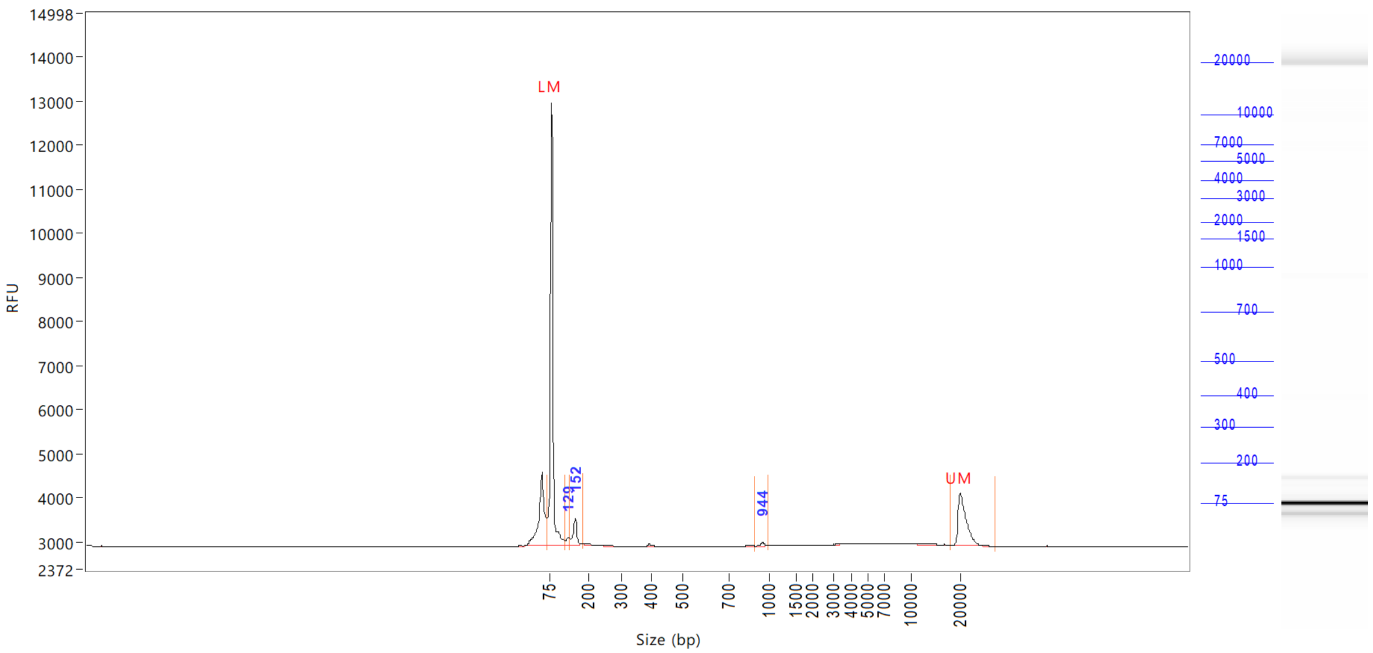

Peak	Size<br>(bp)	Concentration<br>(ng/uL)	From<br>(bp)	To<br>(bp)	Average size<br>(bp)	CV%	RFU	Corrected peak area
1	75 (LM)	0.5000	62	120	75	10.54	10063	67.695
2	129	0.1381	120	135	128	3.63	170	1.558
3	152	0.4512	135	177	148	4.19	584	5.091
4	944	0.0475	887	983	937	1.56	82	0.535
5	20000 (UM)	0.0924	17927	27375	20635	4.85	1177	12.504

TIC:	0.6368	ng/uL
TIM:	6.8663	nmole/L
Total concentration:	0.9364	ng/uL

Sample peak width (sec): 5   Sample min peak height: 50   Sample baseline V to V?: Y   Sample baseline V to V points: 3
Sample filter: Binomial   Number of points for filter: 3   Sample start region (min): 0   Sample end region (min): 30
Marker peak width (sec): 5   Marker min peak height: 200   Marker baseline V to V?: Y   Marker baseline V to V points: 3
Lower marker selection: First peak > 500 RFU   Upper marker selection: Last peak > 200 RFU
Ladder size (bp)75, 200, 300, 400, 500, 700, 1000, 1500, 2000, 3000, 4000, 5000, 7000, 10000, 20000
Quantification using: Lower Marker   Final concentration (ng/uL): 0.5000   Dilution factor: 12.0

Sample: SampE7
Well location: E7
Created: Tuesday, September 14, 2021 9:33:53 PM

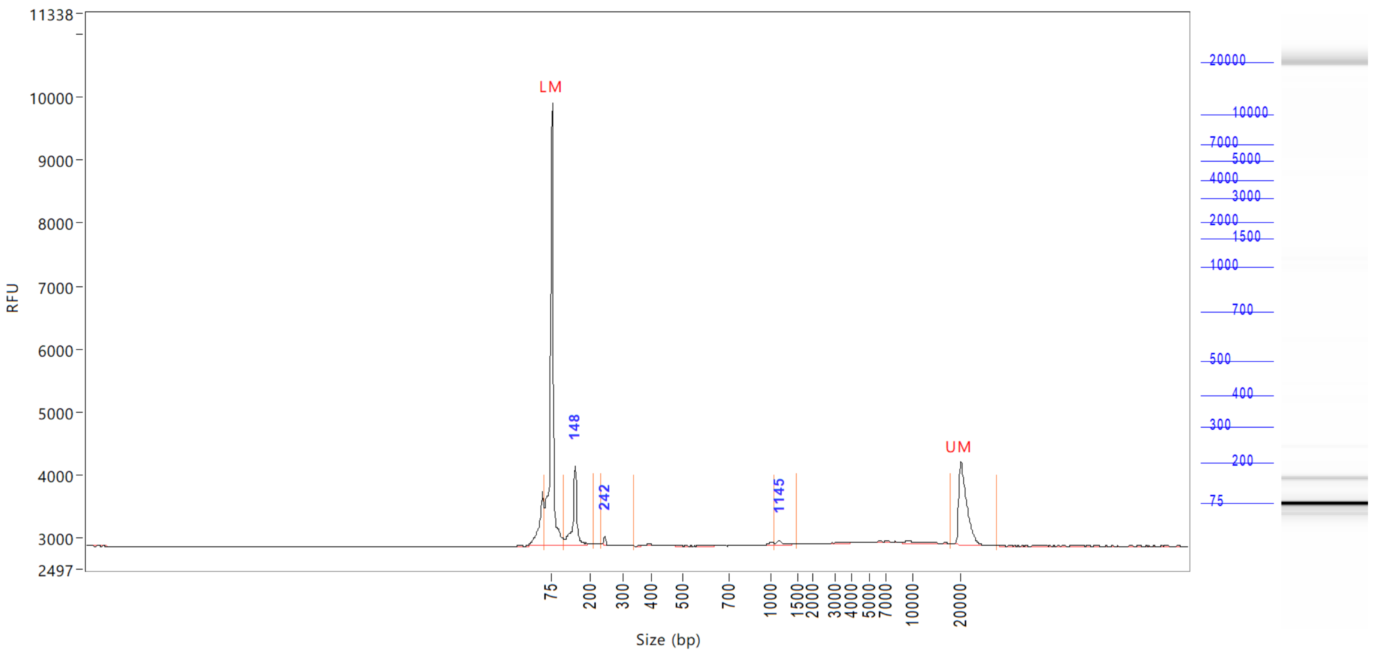

Peak	Size (bp)	Concentration (ng/uL)	From (bp)	To (bp)	Average size (bp)	CV%	RFU	Corrected peak area
1	75 (LM)	0.5000	48	114	72	13.53	7049	55.496
2	148	1.3363	114	210	145	8.88	1241	12.360
3	242	0.0824	233	333	249	8.08	132	0.762
4	1145	0.0838	1068	1478	1186	7.58	74	0.775
5	20000 (UM)	0.1237	17805	27375	20610	4.73	1328	13.735

TIC:	1.5025	ng/uL
TIM:	15.8061	nmole/L
Total concentration:	1.9187	ng/uL

Sample peak width (sec): 5   Sample min peak height: 50   Sample baseline V to V?: Y   Sample baseline V to V points: 3
Sample filter: Binomial   Number of points for filter: 3 Sample start region (min): 0 Sample end region (min): 30
Marker peak width (sec): 5   Marker min peak height: 200   Marker baseline V to V?: Y   Marker baseline V to V points: 3
Lower marker selection: First peak > 500 RFU   Upper marker selection: Last peak > 200 RFU
Ladder size (bp)75, 200, 300, 400, 500, 700, 1000, 1500, 2000, 3000, 4000, 5000, 7000, 10000, 20000
Quantification using: Lower Marker   Final concentration (ng/uL): 0.5000   Dilution factor: 12.0

Sample: SampF7
Well location: F7
Created: Tuesday, September 14, 2021 9:33:53 PM

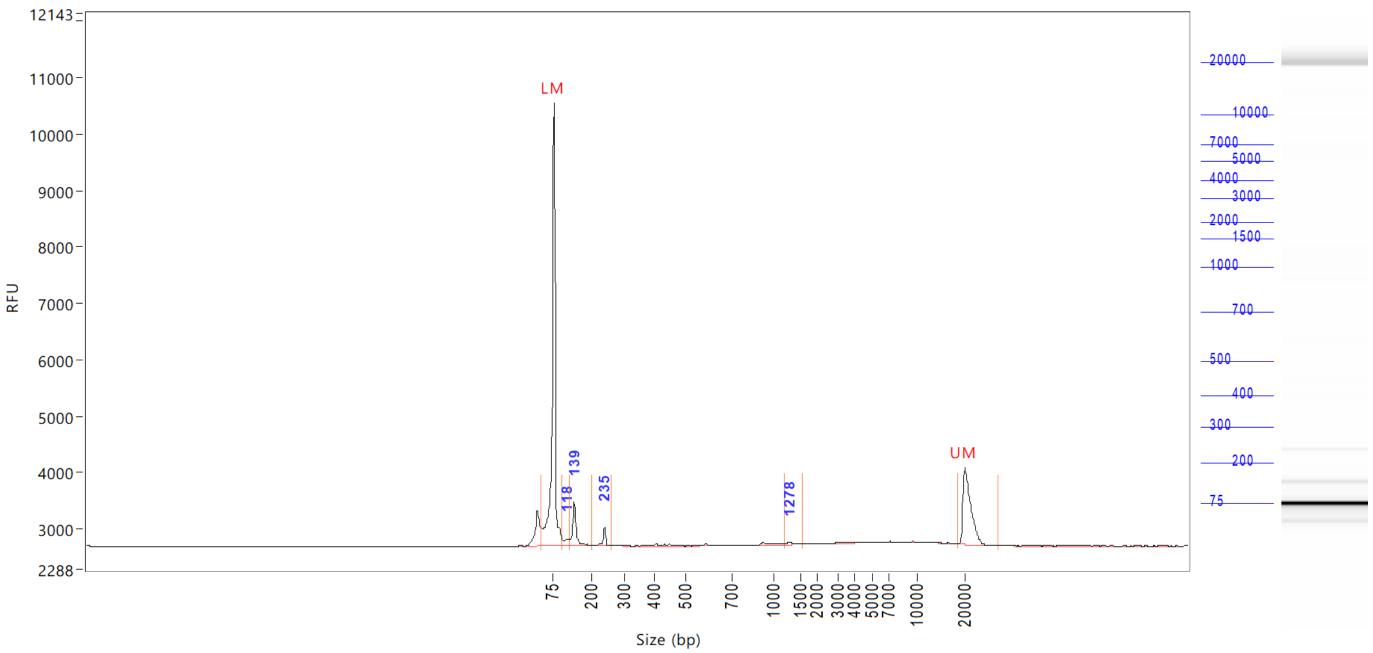

Peak	Size (bp)	Concentration (ng/uL)	From (bp)	To (bp)	Average size (bp)	CV%	RFU	Corrected peak area
1	75 (LM)	0.5000	37	105	70	13.80	7864	62.292
2	118	0.1731	105	128	117	5.90	125	1.797
3	139	0.6285	128	196	140	7.11	776	6.525
4	235	0.1842	196	259	231	3.05	317	1.912
5	1278	0.0513	1190	1520	1299	5.35	50	0.533
6	20000 (UM)	0.1147	18537	26891	20566	4.66	1360	14.290

TIC:	1.0371	ng/uL
TIM:	11.1837	nmole/L
Total concentration:	1.4196	ng/uL

```
Sample peak width (sec): 5    Sample min peak height: 50    Sample baseline V to V?: Y    Sample baseline V to V points: 3
Sample filter: Binomial       Number of points for filter: 3  Sample start region (min): 0  Sample end region (min): 30
Marker peak width (sec): 5    Marker min peak height: 200   Marker baseline V to V?: Y    Marker baseline V to V points: 3
Lower marker selection: First peak > 500 RFU                Upper marker selection: Last peak > 200 RFU
Ladder size (bp)75, 200, 300, 400, 500, 700, 1000, 1500, 2000, 3000, 4000, 5000, 7000, 10000, 20000
Quantification using: Lower Marker          Final concentration (ng/uL): 0.5000          Dilution factor: 12.0
```

Sample: SampG7
Well location: G7
Created: Tuesday, September 14, 2021 9:33:53 PM

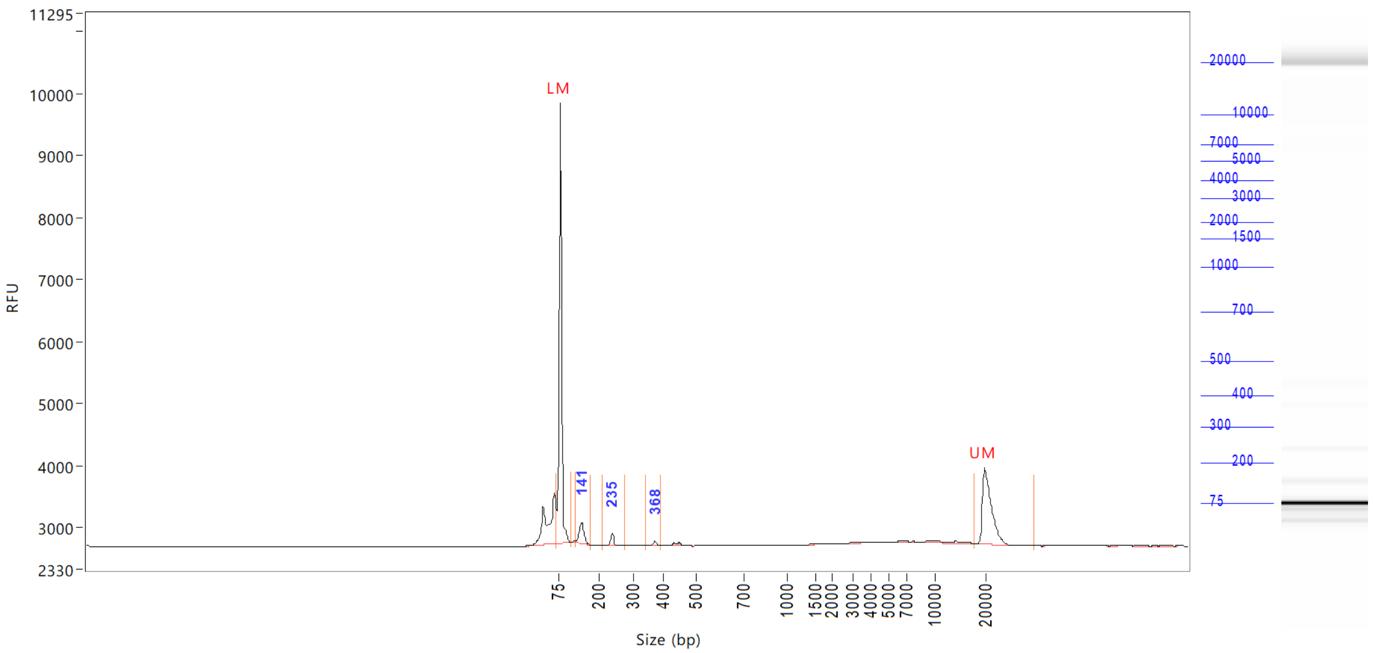

Peak	Size (bp)	Concentration (ng/uL)	From (bp)	To (bp)	Average size (bp)	CV%	RFU	Corrected peak area
1	75 (LM)	0.5000	66	108	74	6.22	7111	44.559
2	141	0.5214	126	171	140	5.01	343	3.872
3	235	0.1728	205	275	233	2.85	201	1.283
4	368	0.0788	337	386	365	1.89	74	0.586
5	20000 (UM)	0.1585	18049	29914	20675	5.24	1227	14.130

TIC:	0.7730	ng/uL
TIM:	7.6952	nmole/L
Total concentration:	1.3586	ng/uL

Sample peak width (sec): 5 Sample min peak height: 50 Sample baseline V to V?: Y Sample baseline V to V points: 3
Sample filter: Binomial Number of points for filter: 3 Sample start region (min): 0 Sample end region (min): 30
Marker peak width (sec): 5 Marker min peak height: 200 Marker baseline V to V?: Y Marker baseline V to V points: 3
Lower marker selection: First peak > 500 RFU Upper marker selection: Last peak > 200 RFU
Ladder size (bp)75, 200, 300, 400, 500, 700, 1000, 1500, 2000, 3000, 4000, 5000, 7000, 10000, 20000
Quantification using: Lower Marker Final concentration (ng/uL): 0.5000 Dilution factor: 12.0

Sample: SampH7
Well location: H7
Created: Tuesday, September 14, 2021 9:33:53 PM

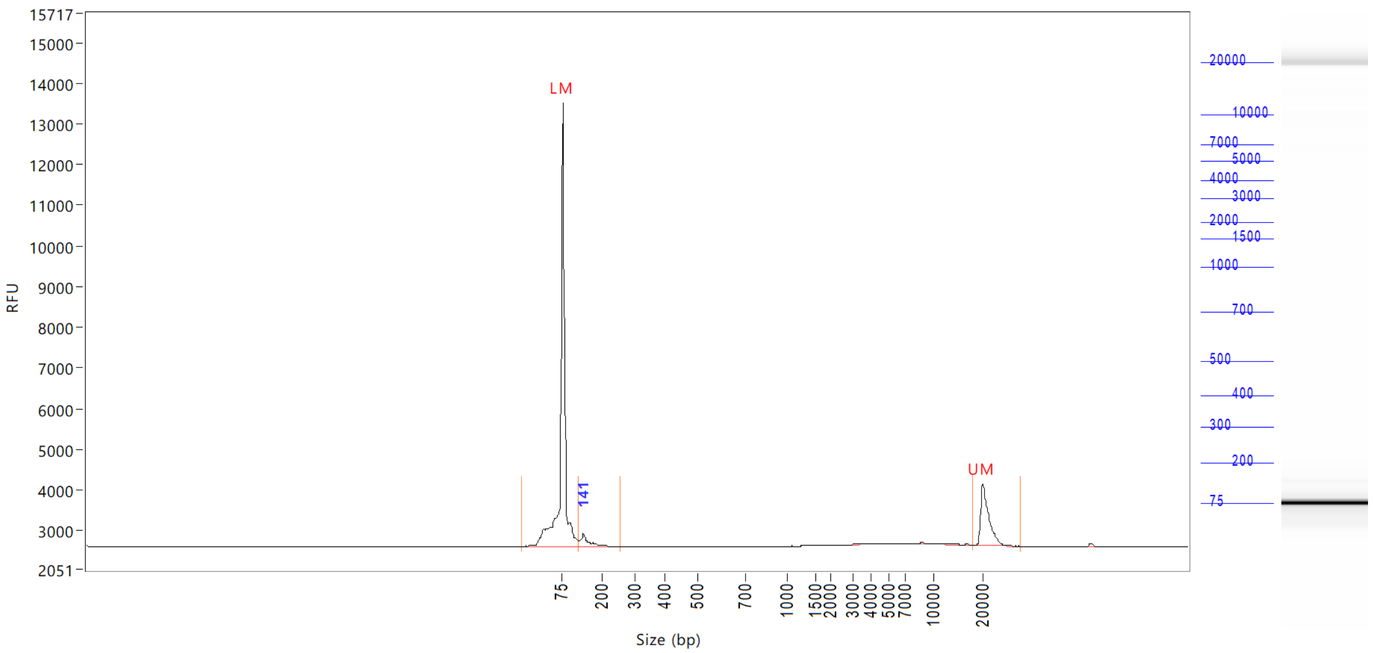

Peak	Size	Concentration	From	To	Average size	CV%	RFU	Corrected peak area
	(bp)	(ng/uL)	(bp)	(bp)	(bp)			
1	75 (LM)	0.5000	0	126	68	32.78	10924	109.465
2	141	0.3096	126	255	152	14.57	309	5.648
3	20000 (UM)	0.0718	18171	27617	20628	5.12	1505	15.729

TIC:	0.3096	ng/uL
TIM:	3.3475	nmole/L
Total concentration:	0.4645	ng/uL

Sample peak width (sec): 5   Sample min peak height: 50   Sample baseline V to V?: Y   Sample baseline V to V points: 3
Sample filter: Binomial   Number of points for filter: 3 Sample start region (min): 0 Sample end region (min): 30
Marker peak width (sec): 5   Marker min peak height: 200   Marker baseline V to V?: Y   Marker baseline V to V points: 3
Lower marker selection: First peak > 500 RFU   Upper marker selection: Last peak > 200 RFU
Ladder size (bp)75, 200, 300, 400, 500, 700, 1000, 1500, 2000, 3000, 4000, 5000, 7000, 10000, 20000
Quantification using: Lower Marker   Final concentration (ng/uL): 0.5000   Dilution factor: 12.0

Sample: SampA8
Well location: A8
Created: Tuesday, September 14, 2021 9:33:53 PM

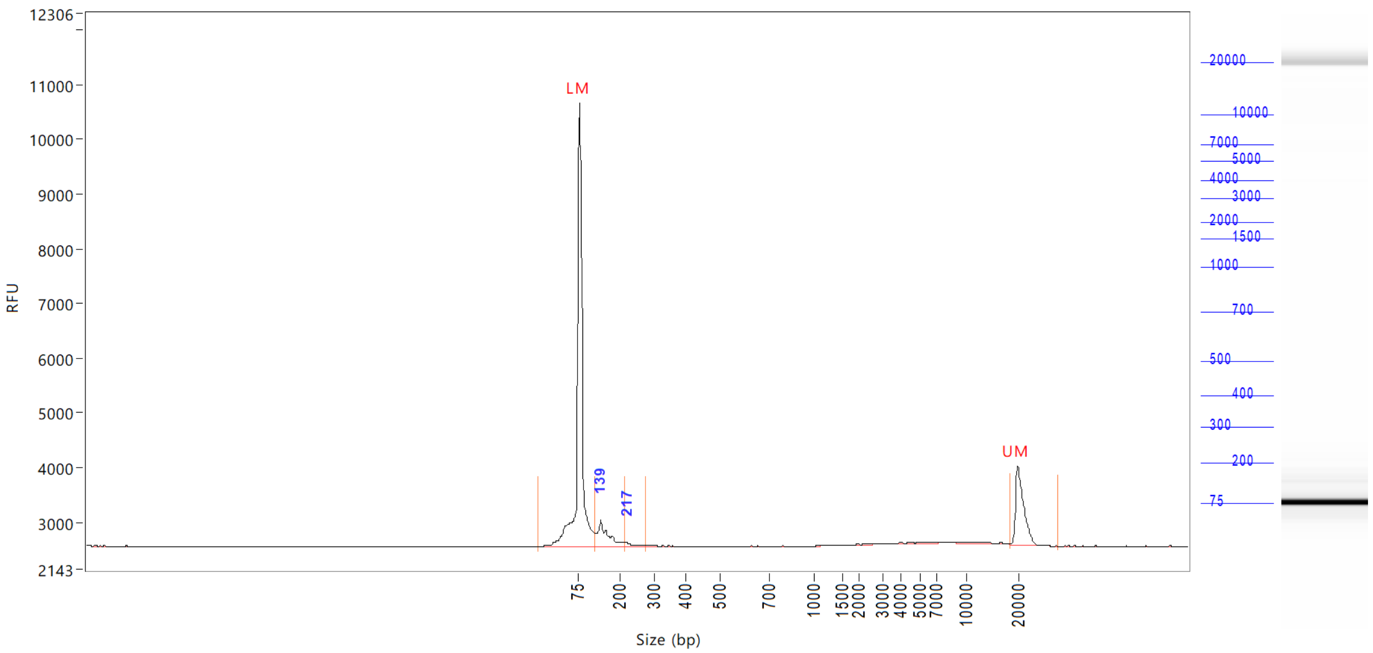

Peak	Size (bp)	Concentration (ng/uL)	From (bp)	To (bp)	Average size (bp)	CV%	RFU	Corrected peak area
1	75 (LM)	0.5000	0	124	72	26.51	8122	98.636
2	139	0.6616	124	212	154	14.35	473	10.877
3	217	0.0625	212	271	230	6.94	65	1.027
4	20000 (UM)	0.0736	18415	27859	20530	4.36	1431	14.513

TIC:	0.7241	ng/uL
TIM:	7.5078	nmole/L
Total concentration:	0.9242	ng/uL

Sample peak width (sec): 5   Sample min peak height: 50   Sample baseline V to V?: Y   Sample baseline V to V points: 3
Sample filter: Binomial   Number of points for filter: 3   Sample start region (min): 0   Sample end region (min): 30
Marker peak width (sec): 5   Marker min peak height: 200   Marker baseline V to V?: Y   Marker baseline V to V points: 3
Lower marker selection: First peak > 500 RFU   Upper marker selection: Last peak > 200 RFU
Ladder size (bp)75, 200, 300, 400, 500, 700, 1000, 1500, 2000, 3000, 4000, 5000, 7000, 10000, 20000
Quantification using: Lower Marker   Final concentration (ng/uL): 0.5000   Dilution factor: 12.0

Sample: SampB8
Well location: B8
Created: Tuesday, September 14, 2021 9:33:53 PM

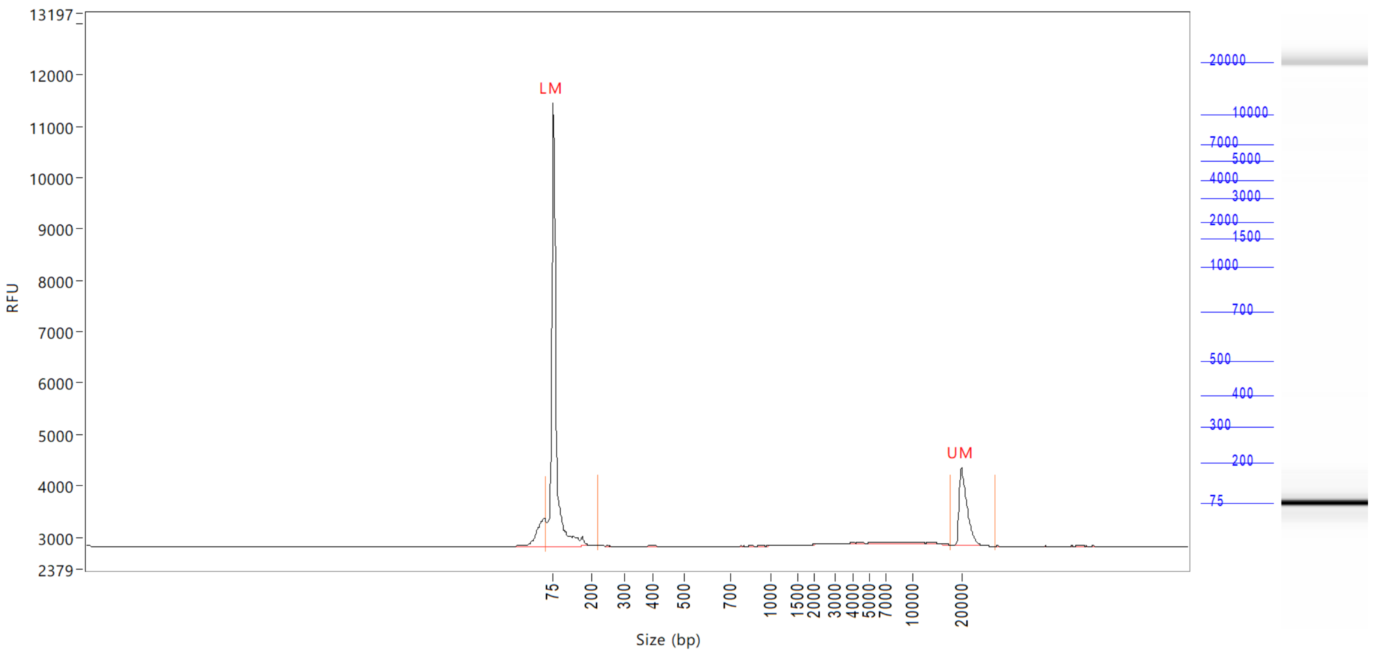

Peak	Size (bp)	Concentration (ng/uL)	From (bp)	To (bp)	Average size (bp)	CV%	RFU	Corrected peak area
1	75 (LM)	0.5000	52	221	81	25.32	8641	95.992
2	20000 (UM)	0.0807	17805	27012	20541	4.53	1512	15.498

TIC:	0.0000	ng/uL
TIM:	0.0000	nmole/L
Total concentration:	0.2426	ng/uL

```
Sample peak width (sec): 5    Sample min peak height: 50    Sample baseline V to V?: Y    Sample baseline V to V points: 3
Sample filter: Binomial       Number of points for filter: 3  Sample start region (min): 0  Sample end region (min): 30
Marker peak width (sec): 5    Marker min peak height: 200   Marker baseline V to V?: Y    Marker baseline V to V points: 3
Lower marker selection: First peak > 500 RFU                Upper marker selection: Last peak > 200 RFU
Ladder size (bp)75, 200, 300, 400, 500, 700, 1000, 1500, 2000, 3000, 4000, 5000, 7000, 10000, 20000
Quantification using: Lower Marker          Final concentration (ng/uL): 0.5000          Dilution factor: 12.0
```

Sample: SampC8
Well location: C8
Created: Tuesday, September 14, 2021 9:33:53 PM

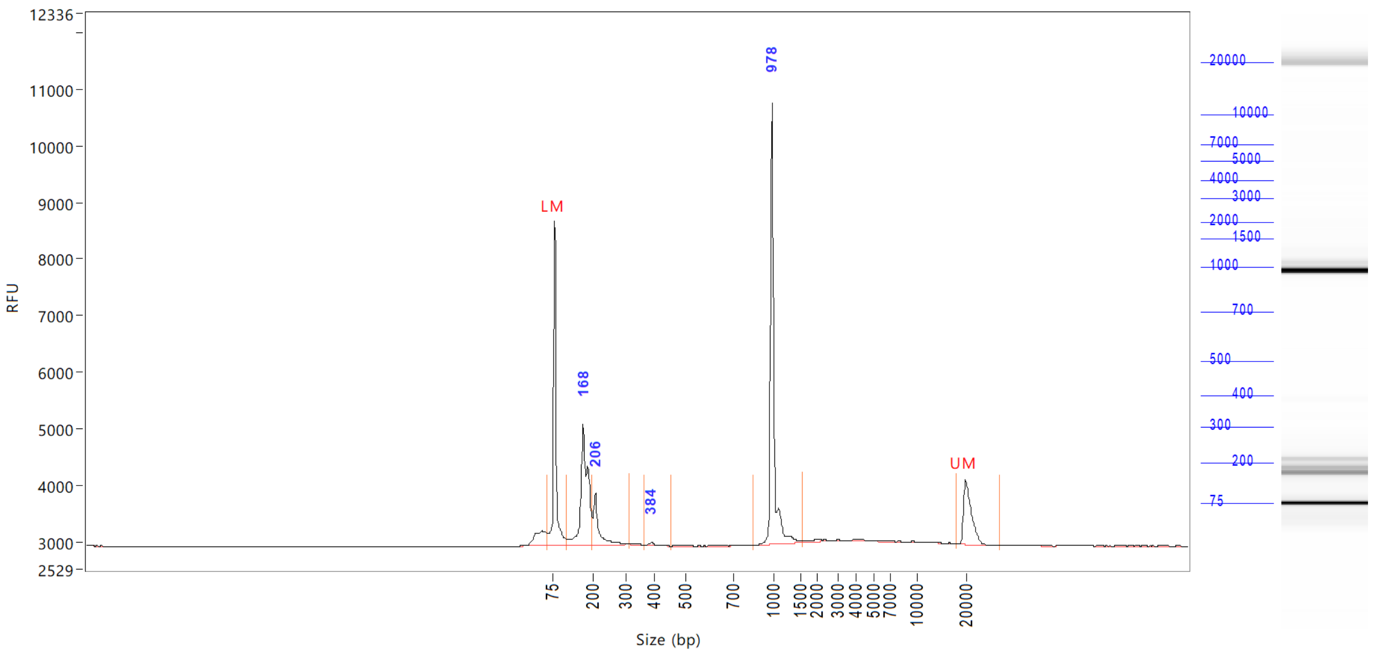

Peak	Size (bp)	Concentration (ng/uL)	From (bp)	To (bp)	Average size (bp)	CV%	RFU	Corrected peak area
1	75 (LM)	0.5000	54	114	75	11.54	5733	38.985
2	168	5.4868	114	196	169	9.25	2139	35.650
3	206	1.6956	196	309	215	9.57	915	11.017
4	384	0.0949	362	449	390	4.27	66	0.617
5	978	7.3467	848	1501	1000	7.43	7801	47.735
6	20000 (UM)	0.1530	18049	26891	20532	4.55	1142	11.927

TIC:	14.6239	ng/uL
TIM:	78.8293	nmole/L
Total concentration:	15.1288	ng/uL

Sample peak width (sec): 5   Sample min peak height: 50   Sample baseline V to V?: Y   Sample baseline V to V points: 3
Sample filter: Binomial   Number of points for filter: 3   Sample start region (min): 0   Sample end region (min): 30
Marker peak width (sec): 5   Marker min peak height: 200   Marker baseline V to V?: Y   Marker baseline V to V points: 3
Lower marker selection: First peak > 500 RFU   Upper marker selection: Last peak > 200 RFU
Ladder size (bp)75, 200, 300, 400, 500, 700, 1000, 1500, 2000, 3000, 4000, 5000, 7000, 10000, 20000
Quantification using: Lower Marker   Final concentration (ng/uL): 0.5000   Dilution factor: 12.0

Sample: SampD8
Well location: D8
Created: Tuesday, September 14, 2021 9:33:53 PM

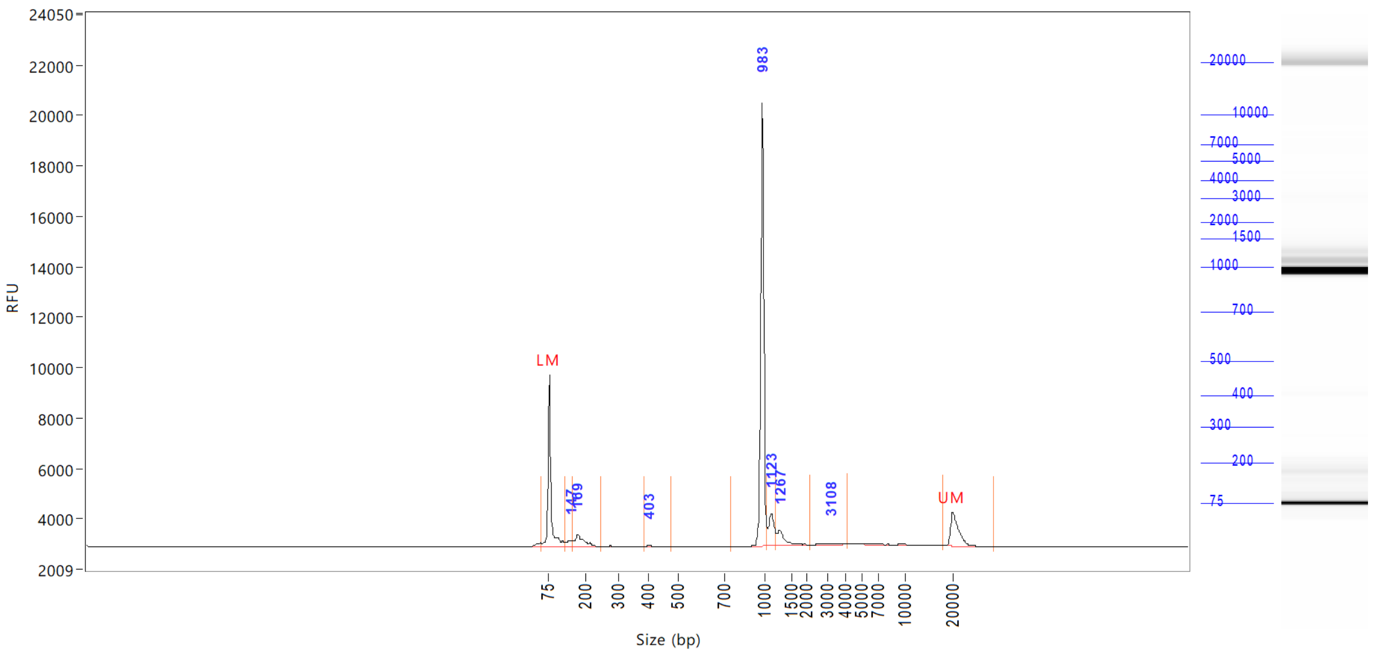

Peak	Size (bp)	Concentration (ng/uL)	From (bp)	To (bp)	Average size (bp)	CV%	RFU	Corrected peak area
1	75 (LM)	0.5000	52	128	78	15.96	6784	48.992
2	147	0.4314	128	152	140	5.32	230	3.522
3	169	1.1624	152	242	177	9.02	469	9.491
4	403	0.0848	388	477	412	4.64	74	0.693
5	983	12.1100	749	1034	975	1.52	17596	98.883
6	1123	1.5372	1034	1212	1113	4.27	1252	12.552
7	1267	0.9828	1212	2087	1354	11.78	597	8.025
8	3108	0.1886	2087	4139	3034	15.78	63	1.540
9	20000 (UM)	0.1459	17927	28463	20569	5.04	1347	14.298

TIC:	16.4971	ng/uL
TIM:	40.2116	nmole/L
Total concentration:	16.6510	ng/uL

```
Sample peak width (sec): 5     Sample min peak height: 50     Sample baseline V to V?: Y     Sample baseline V to V points: 3
Sample filter: Binomial        Number of points for filter: 3  Sample start region (min): 0  Sample end region (min): 30
Marker peak width (sec): 5     Marker min peak height: 200    Marker baseline V to V?: Y     Marker baseline V to V points: 3
Lower marker selection: First peak > 500 RFU                  Upper marker selection: Last peak > 200 RFU
Ladder size (bp)75, 200, 300, 400, 500, 700, 1000, 1500, 2000, 3000, 4000, 5000, 7000, 10000, 20000
Quantification using: Lower Marker          Final concentration (ng/uL): 0.5000          Dilution factor: 12.0
```

Sample: SampE8
Well location: E8
Created: Tuesday, September 14, 2021 9:33:53 PM

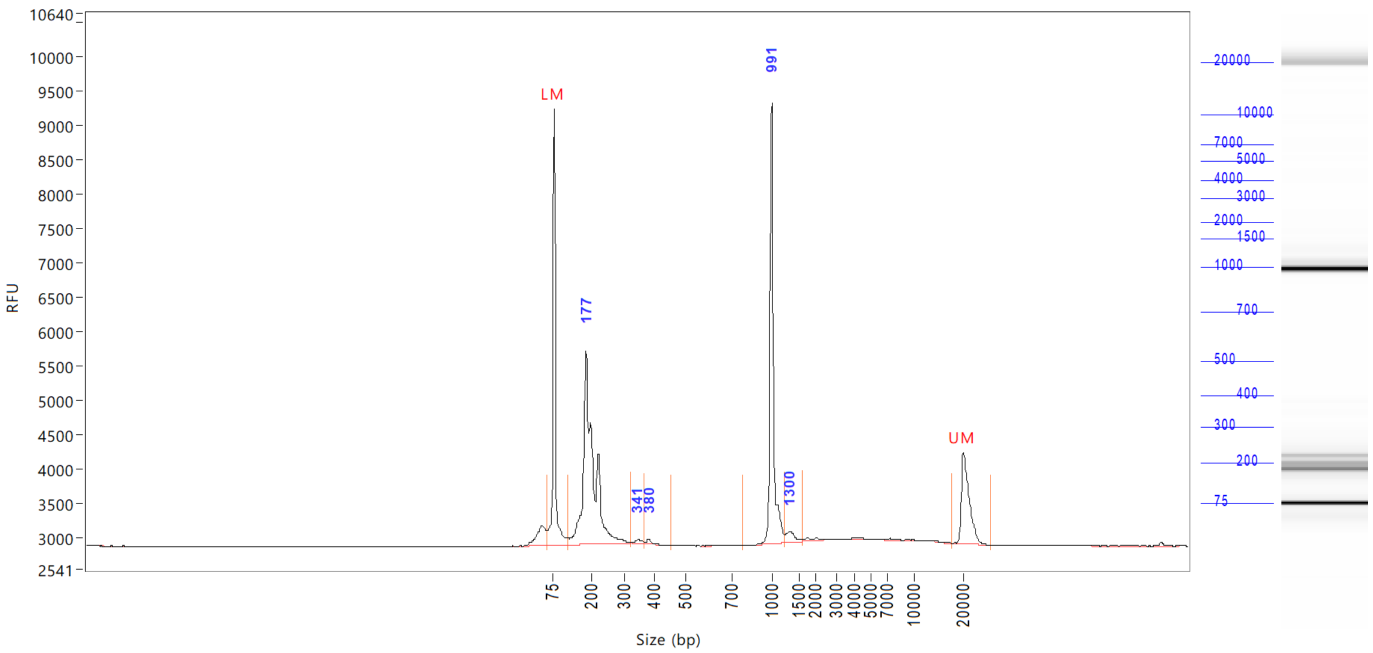

Peak	Size (bp)	Concentration (ng/uL)	From (bp)	To (bp)	Average size (bp)	CV%	RFU	Corrected peak area
1	75 (LM)	0.5000	54	124	75	12.85	6354	42.574
2	177	8.9701	124	319	191	14.27	2817	63.650
3	341	0.1047	319	368	345	3.23	51	0.743
4	380	0.0963	368	456	386	4.52	70	0.683
5	991	5.5925	775	1212	999	4.34	6433	39.683
6	1300	0.3481	1212	1559	1330	6.17	159	2.470
7	20000 (UM)	0.1621	17683	25561	20574	4.47	1340	13.807

TIC:	15.1117	ng/uL
TIM:	87.7693	nmole/L
Total concentration:	15.4606	ng/uL

Sample peak width (sec): 5 Sample min peak height: 50 Sample baseline V to V?: Y Sample baseline V to V points: 3
Sample filter: Binomial Number of points for filter: 3 Sample start region (min): 0 Sample end region (min): 30
Marker peak width (sec): 5 Marker min peak height: 200 Marker baseline V to V?: Y Marker baseline V to V points: 3
Lower marker selection: First peak > 500 RFU Upper marker selection: Last peak > 200 RFU
Ladder size (bp)75, 200, 300, 400, 500, 700, 1000, 1500, 2000, 3000, 4000, 5000, 7000, 10000, 20000
Quantification using: Lower Marker Final concentration (ng/uL): 0.5000 Dilution factor: 12.0

Sample: SampF8
Well location: F8
Created: Tuesday, September 14, 2021 9:33:53 PM

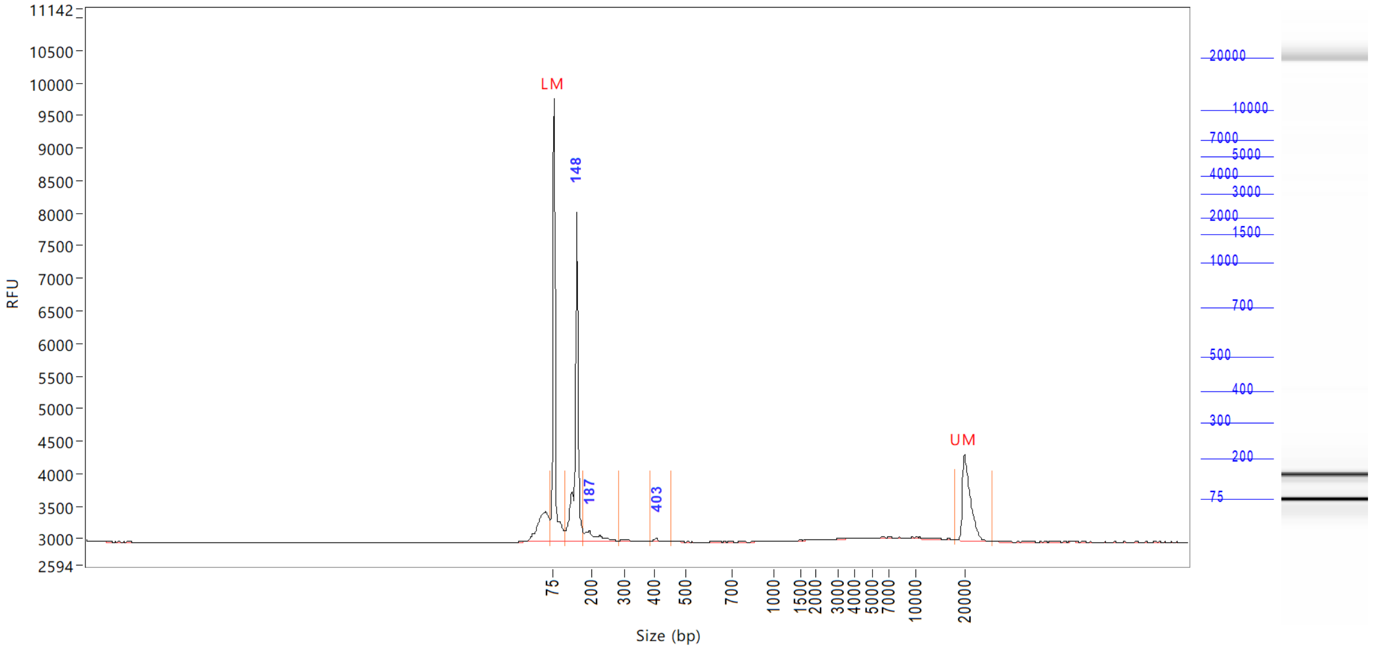

Peak	Size (bp)	Concentration (ng/uL)	From (bp)	To (bp)	Average size (bp)	CV%	RFU	Corrected peak area
1	75 (LM)	0.5000	64	110	75	10.54	6817	43.166
2	148	5.5527	110	171	143	7.02	5057	39.948
3	187	0.5965	171	278	203	11.74	153	4.291
4	403	0.0556	388	450	404	2.86	53	0.400
5	20000 (UM)	0.1624	17927	25803	20597	4.76	1323	14.017

TIC:	6.2048	ng/uL
TIM:	68.8700	nmole/L
Total concentration:	6.5984	ng/uL

Sample peak width (sec): 5 Sample min peak height: 50 Sample baseline V to V?: Y Sample baseline V to V points: 3
Sample filter: Binomial Number of points for filter: 3 Sample start region (min): 0 Sample end region (min): 30
Marker peak width (sec): 5 Marker min peak height: 200 Marker baseline V to V?: Y Marker baseline V to V points: 3
Lower marker selection: First peak > 500 RFU Upper marker selection: Last peak > 200 RFU
Ladder size (bp)75, 200, 300, 400, 500, 700, 1000, 1500, 2000, 3000, 4000, 5000, 7000, 10000, 20000
Quantification using: Lower Marker Final concentration (ng/uL): 0.5000 Dilution factor: 12.0

Sample: SampG8
Well location: G8
Created: Tuesday, September 14, 2021 9:33:53 PM

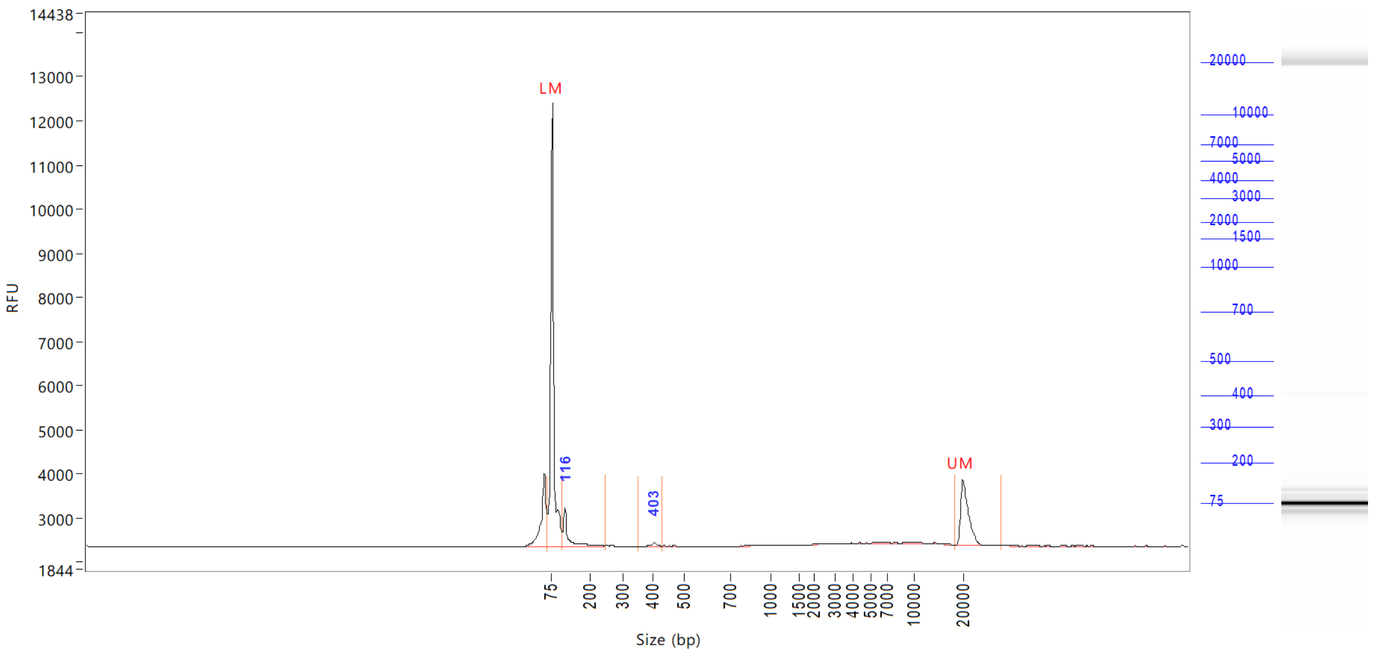

Peak	Size (bp)	Concentration (ng/uL)	From (bp)	To (bp)	Average size (bp)	CV%	RFU	Corrected peak area
1	75 (LM)	0.5000	60	108	75	11.61	10059	79.789
2	116	0.6876	108	244	129	19.70	861	9.144
3	403	0.0587	352	430	400	3.02	102	0.781
4	20000 (UM)	0.0953	18415	27979	20561	4.57	1499	15.201

TIC:	0.7463	ng/uL
TIM:	8.9993	nmole/L
Total concentration:	0.9659	ng/uL

Sample peak width (sec): 5   Sample min peak height: 50   Sample baseline V to V?: Y   Sample baseline V to V points: 3
Sample filter: Binomial   Number of points for filter: 3 Sample start region (min): 0 Sample end region (min): 30
Marker peak width (sec): 5   Marker min peak height: 200   Marker baseline V to V?: Y   Marker baseline V to V points: 3
Lower marker selection: First peak > 500 RFU   Upper marker selection: Last peak > 200 RFU
Ladder size (bp)75, 200, 300, 400, 500, 700, 1000, 1500, 2000, 3000, 4000, 5000, 7000, 10000, 20000
Quantification using: Lower Marker   Final concentration (ng/uL): 0.5000   Dilution factor: 12.0

Sample: SampH8
Well location: H8
Created: Tuesday, September 14, 2021 9:33:53 PM

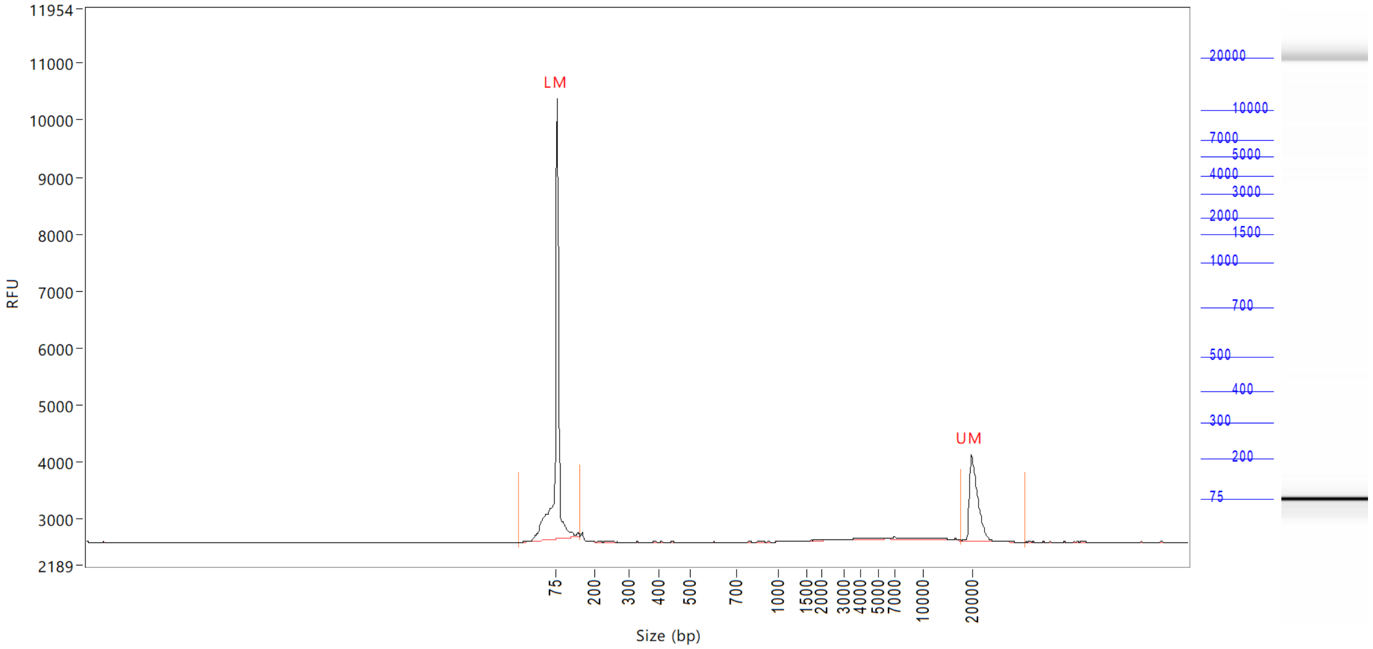

Peak	Size (bp)	Concentration (ng/uL)	From (bp)	To (bp)	Average size (bp)	CV%	RFU	Corrected peak area
1	75 (LM)	0.5000	0	150	67	30.55	7742	67.342
2	20000 (UM)	0.1208	17805	30760	20577	5.09	1511	16.266

TIC:	0.0000	ng/uL
TIM:	0.0000	nmole/L
Total concentration:	0.3868	ng/uL

Sample peak width (sec): 5 Sample min peak height: 50 Sample baseline V to V?: Y Sample baseline V to V points: 3
Sample filter: Binomial Number of points for filter: 3 Sample start region (min): 0 Sample end region (min): 30
Marker peak width (sec): 5 Marker min peak height: 200 Marker baseline V to V?: Y Marker baseline V to V points: 3
Lower marker selection: First peak > 500 RFU Upper marker selection: Last peak > 200 RFU
Ladder size (bp)75, 200, 300, 400, 500, 700, 1000, 1500, 2000, 3000, 4000, 5000, 7000, 10000, 20000
Quantification using: Lower Marker Final concentration (ng/uL): 0.5000 Dilution factor: 12.0

Sample: SampA9
Well location: A9
Created: Tuesday, September 14, 2021 9:33:53 PM

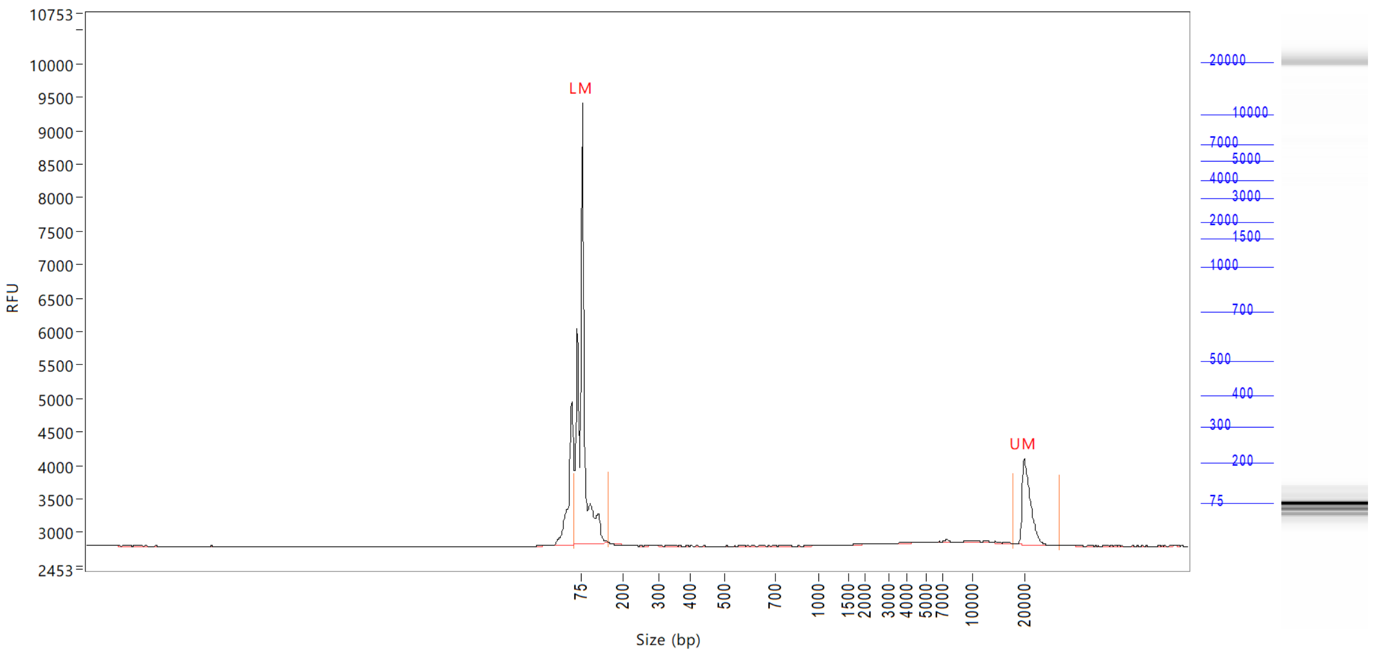

Peak	Size (bp)	Concentration (ng/uL)	From (bp)	To (bp)	Average size (bp)	CV%	RFU	Corrected peak area
1	75 (LM)	0.5000	48	154	73	23.52	6597	78.374
2	20000 (UM)	0.0838	17927	26770	20538	4.64	1293	13.142

TIC:	0.0000	ng/uL
TIM:	0.0000	nmole/L
Total concentration:	0.2288	ng/uL

Sample peak width (sec): 5 Sample min peak height: 50 Sample baseline V to V?: Y Sample baseline V to V points: 3
Sample filter: Binomial Number of points for filter: 3 Sample start region (min): 0 Sample end region (min): 30
Marker peak width (sec): 5 Marker min peak height: 200 Marker baseline V to V?: Y Marker baseline V to V points: 3
Lower marker selection: First peak > 500 RFU Upper marker selection: Last peak > 200 RFU
Ladder size (bp)75, 200, 300, 400, 500, 700, 1000, 1500, 2000, 3000, 4000, 5000, 7000, 10000, 20000
Quantification using: Lower Marker Final concentration (ng/uL): 0.5000 Dilution factor: 12.0

Sample: SampB9
Well location: B9
Created: Tuesday, September 14, 2021 9:33:53 PM

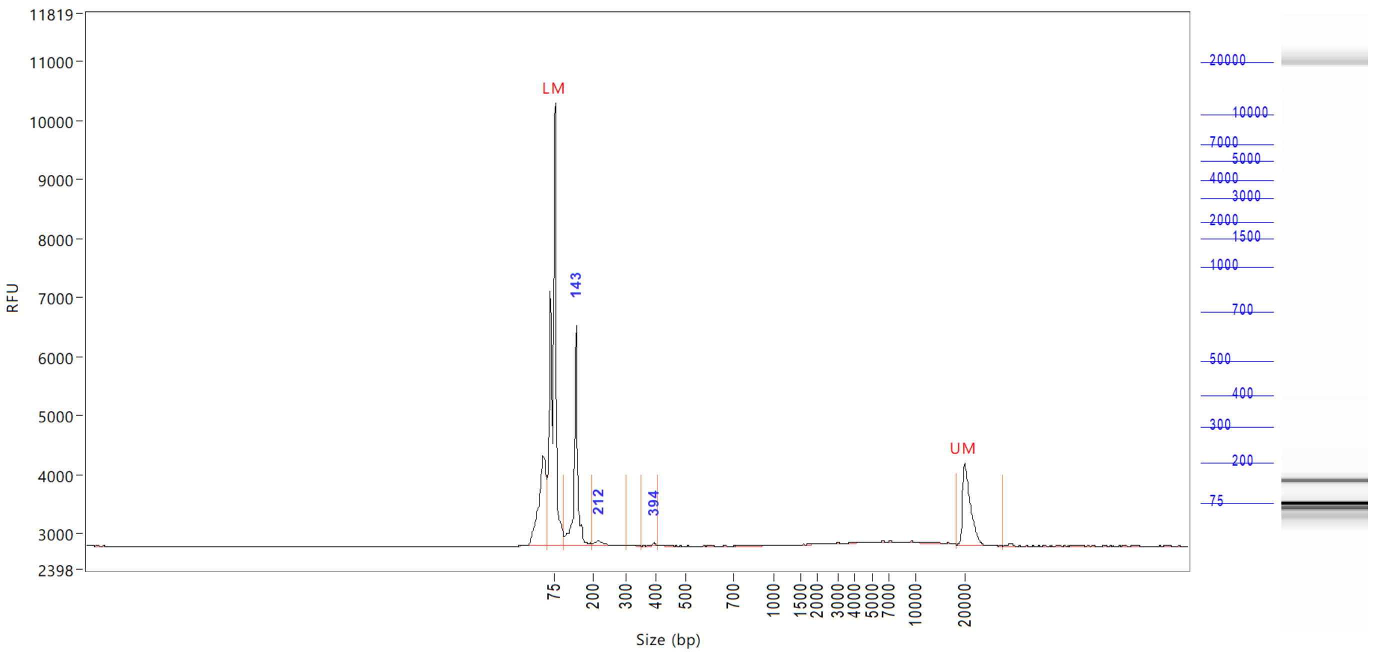

Peak	Size (bp)	Concentration (ng/uL)	From (bp)	To (bp)	Average size (bp)	CV%	RFU	Corrected peak area
1	75 (LM)	0.5000	48	105	68	14.87	7509	79.764
2	143	2.2865	105	196	140	8.60	3727	30.397
3	212	0.1208	196	300	219	7.97	75	1.607
4	394	0.0349	349	409	387	2.74	63	0.464
5	20000 (UM)	0.0905	18415	27859	20615	4.75	1384	14.433

TIC:	2.4422	ng/uL
TIM:	27.8947	nmole/L
Total concentration:	2.6655	ng/uL

Sample peak width (sec): 5 Sample min peak height: 50 Sample baseline V to V?: Y Sample baseline V to V points: 3
Sample filter: Binomial Number of points for filter: 3 Sample start region (min): 0 Sample end region (min): 30
Marker peak width (sec): 5 Marker min peak height: 200 Marker baseline V to V?: Y Marker baseline V to V points: 3
Lower marker selection: First peak > 500 RFU Upper marker selection: Last peak > 200 RFU
Ladder size (bp)75, 200, 300, 400, 500, 700, 1000, 1500, 2000, 3000, 4000, 5000, 7000, 10000, 20000
Quantification using: Lower Marker Final concentration (ng/uL): 0.5000 Dilution factor: 12.0

Sample: SampC9
Well location: C9
Created: Tuesday, September 14, 2021 9:33:53 PM

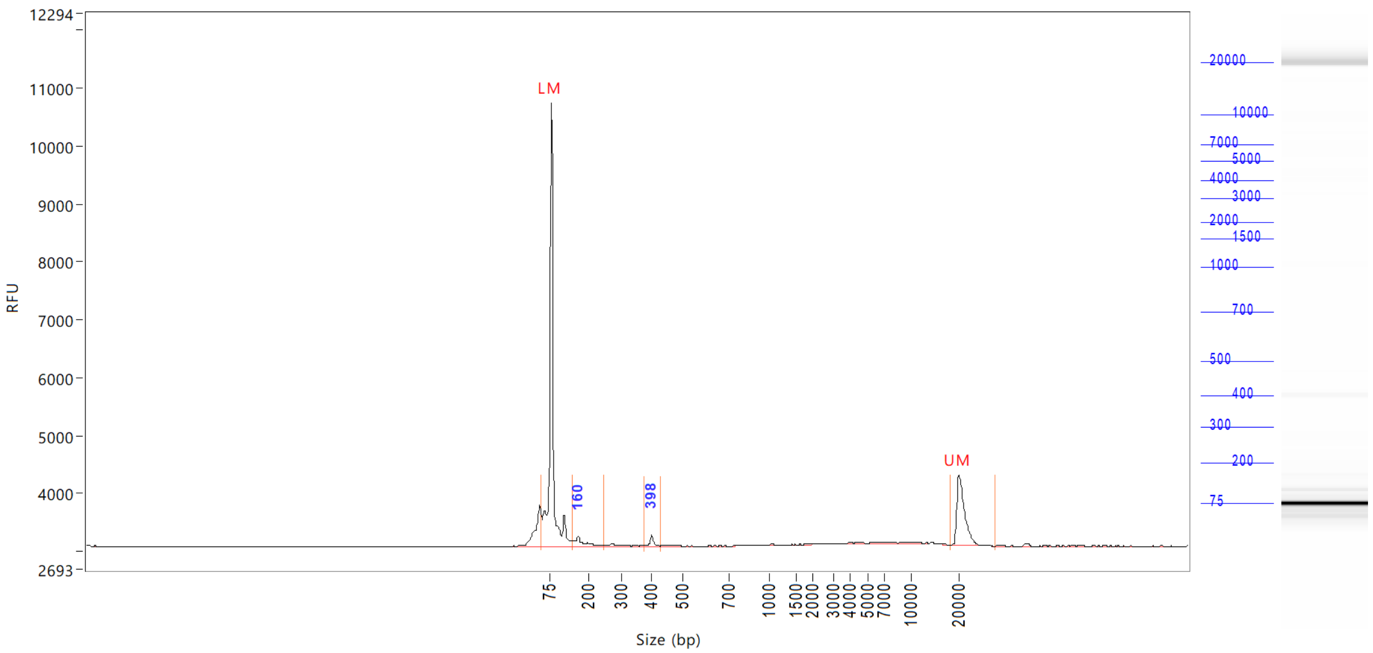

Peak	Size (bp)	Concentration (ng/uL)	From (bp)	To (bp)	Average size (bp)	CV%	RFU	Corrected peak area
1	75 (LM)	0.5000	45	143	75	20.79	7666	66.572
2	160	0.3205	143	242	174	14.18	175	3.556
3	398	0.1244	376	428	398	1.86	202	1.380
4	20000 (UM)	0.0920	18293	27496	20571	4.82	1205	12.249

TIC:	0.4449	ng/uL
TIM:	3.5421	nmole/L
Total concentration:	0.7920	ng/uL

Sample peak width (sec): 5 Sample min peak height: 50 Sample baseline V to V?: Y Sample baseline V to V points: 3
Sample filter: Binomial Number of points for filter: 3 Sample start region (min): 0 Sample end region (min): 30
Marker peak width (sec): 5 Marker min peak height: 200 Marker baseline V to V?: Y Marker baseline V to V points: 3
Lower marker selection: First peak > 500 RFU Upper marker selection: Last peak > 200 RFU
Ladder size (bp)75, 200, 300, 400, 500, 700, 1000, 1500, 2000, 3000, 4000, 5000, 7000, 10000, 20000
Quantification using: Lower Marker Final concentration (ng/uL): 0.5000 Dilution factor: 12.0

Sample: SampD9
Well location: D9
Created: Tuesday, September 14, 2021 9:33:53 PM

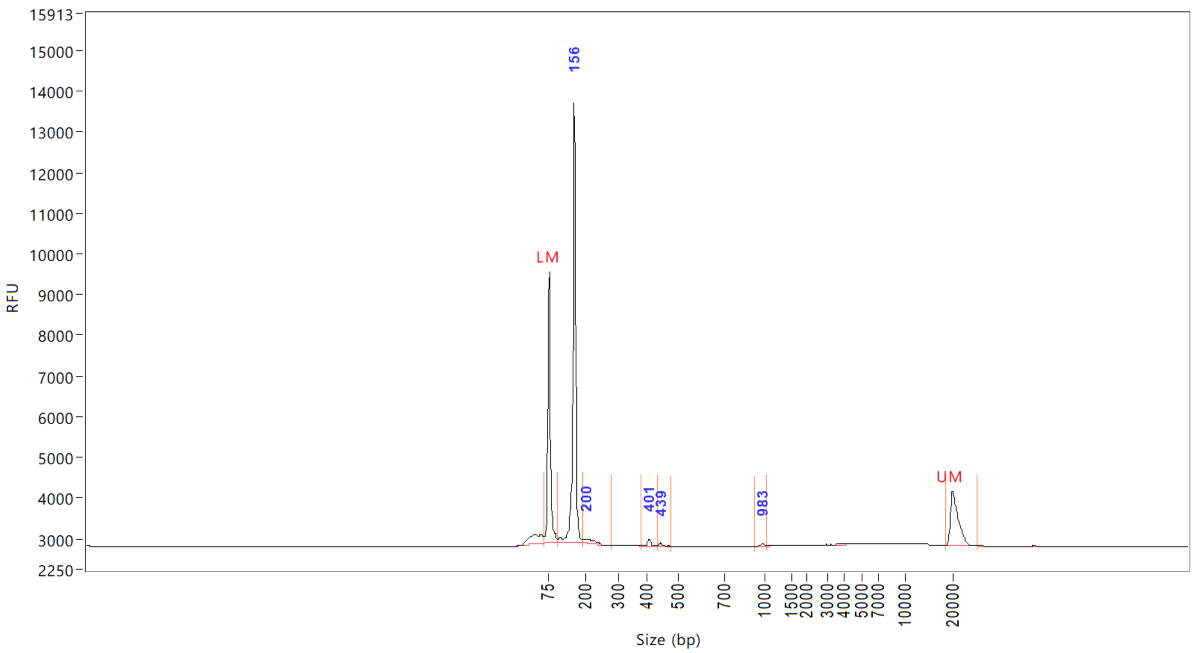

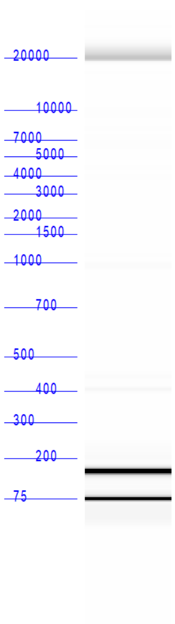

Peak	Size (bp)	Concentration (ng/uL)	From (bp)	To (bp)	Average size (bp)	CV%	RFU	Corrected peak area
1	75 (LM)	0.5000	62	103	74	7.58	6662	42.453
2	156	10.1973	103	187	154	5.36	10817	72.151
3	200	0.3693	187	277	209	7.18	108	2.613
4	401	0.1868	376	430	404	2.40	180	1.322
5	439	0.0892	430	475	442	2.02	79	0.631
6	983	0.0678	926	1045	979	1.73	74	0.479
7	20000 (UM)	0.1629	18537	25440	20623	4.41	1336	13.831

TIC:	10.9104	ng/uL
TIM:	112.9449	nmole/L
Total concentration:	11.2137	ng/uL

```
Sample peak width (sec): 5     Sample min peak height: 50     Sample baseline V to V?: Y     Sample baseline V to V points: 3
Sample filter: Binomial        Number of points for filter: 3  Sample start region (min): 0  Sample end region (min): 30
Marker peak width (sec): 5     Marker min peak height: 200    Marker baseline V to V?: Y     Marker baseline V to V points: 3
Lower marker selection: First peak > 500 RFU                  Upper marker selection: Last peak > 200 RFU
Ladder size (bp)75, 200, 300, 400, 500, 700, 1000, 1500, 2000, 3000, 4000, 5000, 7000, 10000, 20000
Quantification using: Lower Marker          Final concentration (ng/uL): 0.5000          Dilution factor: 12.0
```

Sample: SampE9
Well location: E9
Created: Tuesday, September 14, 2021 9:33:53 PM

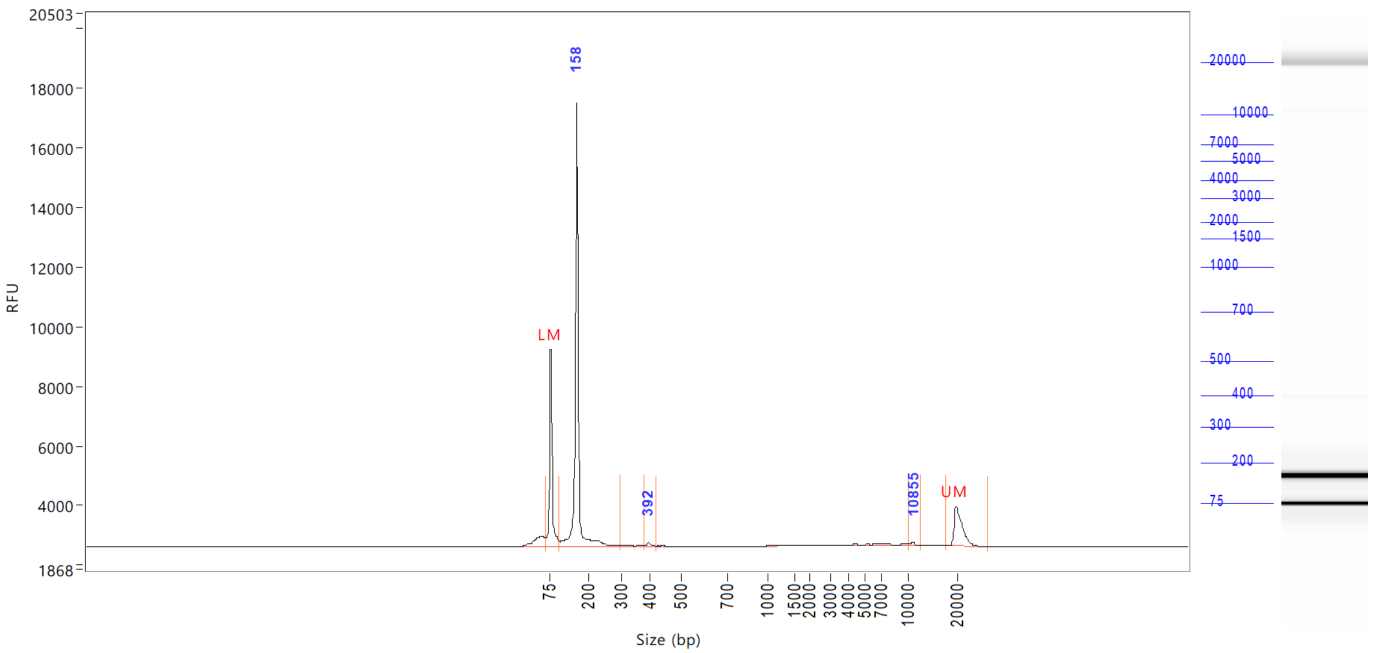

Peak	Size (bp)	Concentration (ng/uL)	From (bp)	To (bp)	Average size (bp)	CV%	RFU	Corrected peak area
1	75 (LM)	0.5000	58	103	74	9.38	6604	46.197
2	158	14.3378	103	298	160	12.69	14887	110.393
3	392	0.1142	376	416	393	1.86	122	0.879
4	10855	0.0617	10123	12562	10953	5.58	84	0.475
5	20000 (UM)	0.1522	17927	26649	20548	4.71	1338	14.058

TIC:	14.5137	ng/uL
TIM:	147.7804	nmole/L
Total concentration:	14.9408	ng/uL

```
Sample peak width (sec): 5    Sample min peak height: 50    Sample baseline V to V?: Y    Sample baseline V to V points: 3
Sample filter: Binomial       Number of points for filter: 3  Sample start region (min): 0  Sample end region (min): 30
Marker peak width (sec): 5    Marker min peak height: 200   Marker baseline V to V?: Y    Marker baseline V to V points: 3
Lower marker selection: First peak > 500 RFU                Upper marker selection: Last peak > 200 RFU
Ladder size (bp)75, 200, 300, 400, 500, 700, 1000, 1500, 2000, 3000, 4000, 5000, 7000, 10000, 20000
Quantification using: Lower Marker          Final concentration (ng/uL): 0.5000          Dilution factor: 12.0
```

Sample: SampF9
Well location: F9
Created: Tuesday, September 14, 2021 9:33:53 PM

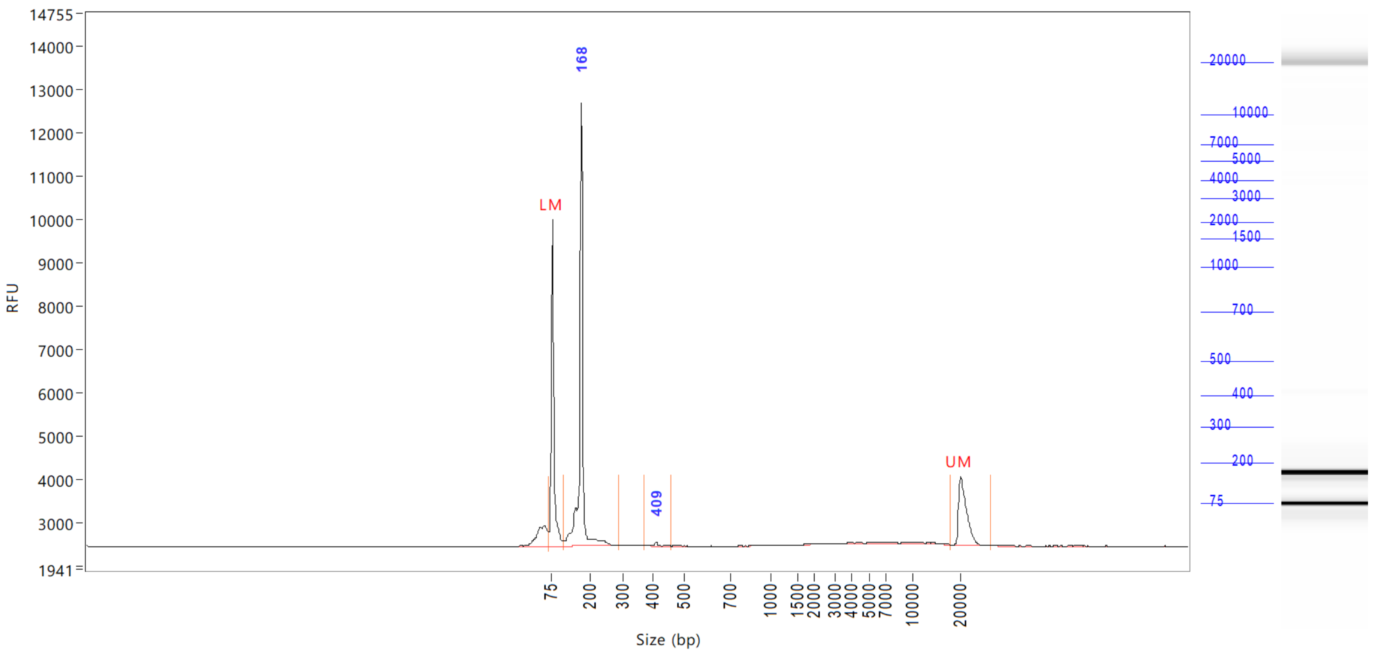

Peak	Size (bp)	Concentration (ng/uL)	From (bp)	To (bp)	Average size (bp)	CV%	RFU	Corrected peak area
1	75 (LM)	0.5000	62	112	74	9.96	7517	51.595
2	168	9.0597	112	284	165	11.43	10228	77.906
3	409	0.0850	368	458	407	3.50	93	0.731
4	20000 (UM)	0.1599	17927	26045	20592	4.70	1571	16.497

TIC:	9.1447	ng/uL
TIM:	90.5990	nmole/L
Total concentration:	9.4963	ng/uL

```
Sample peak width (sec): 5     Sample min peak height: 50     Sample baseline V to V?: Y     Sample baseline V to V points: 3
Sample filter: Binomial        Number of points for filter: 3  Sample start region (min): 0  Sample end region (min): 30
Marker peak width (sec): 5     Marker min peak height: 200    Marker baseline V to V?: Y     Marker baseline V to V points: 3
Lower marker selection: First peak > 500 RFU                  Upper marker selection: Last peak > 200 RFU
Ladder size (bp)75, 200, 300, 400, 500, 700, 1000, 1500, 2000, 3000, 4000, 5000, 7000, 10000, 20000
Quantification using: Lower Marker          Final concentration (ng/uL): 0.5000          Dilution factor: 12.0
```

Sample: SampG9
Well location: G9
Created: Tuesday, September 14, 2021 9:33:53 PM

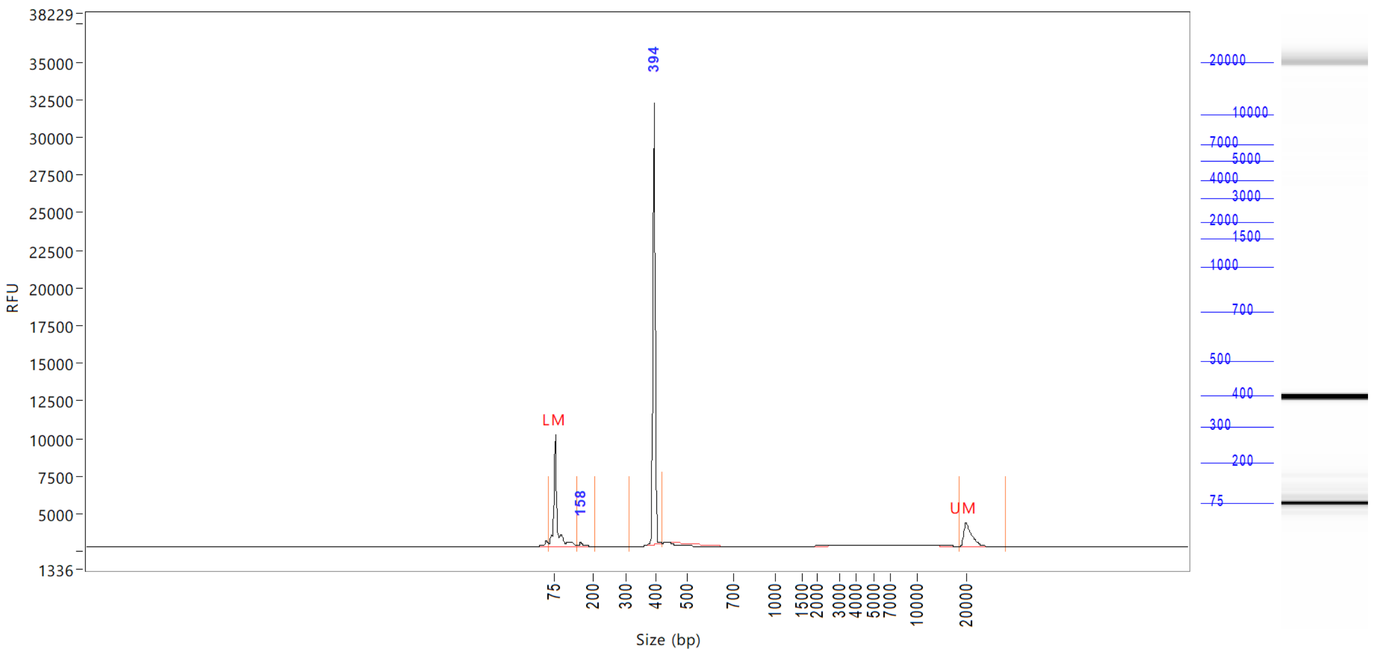

Peak	Size (bp)	Concentration (ng/uL)	From (bp)	To (bp)	Average size (bp)	CV%	RFU	Corrected peak area
1	75 (LM)	0.5000	54	148	80	21.02	7440	61.696
2	158	0.3237	148	203	164	7.49	261	3.328
3	394	14.0704	309	420	391	1.03	29348	144.682
4	20000 (UM)	0.1321	18659	28342	20655	5.03	1532	16.300

TIC:	14.3941	ng/uL
TIM:	62.4503	nmole/L
Total concentration:	14.7229	ng/uL

Sample peak width (sec): 5   Sample min peak height: 50   Sample baseline V to V?: Y   Sample baseline V to V points: 3
Sample filter: Binomial   Number of points for filter: 3 Sample start region (min): 0 Sample end region (min): 30
Marker peak width (sec): 5   Marker min peak height: 200   Marker baseline V to V?: Y   Marker baseline V to V points: 3
Lower marker selection: First peak > 500 RFU   Upper marker selection: Last peak > 200 RFU
Ladder size (bp)75, 200, 300, 400, 500, 700, 1000, 1500, 2000, 3000, 4000, 5000, 7000, 10000, 20000
Quantification using: Lower Marker   Final concentration (ng/uL): 0.5000   Dilution factor: 12.0

Sample: SampH9
Well location: H9
Created: Tuesday, September 14, 2021 9:33:53 PM

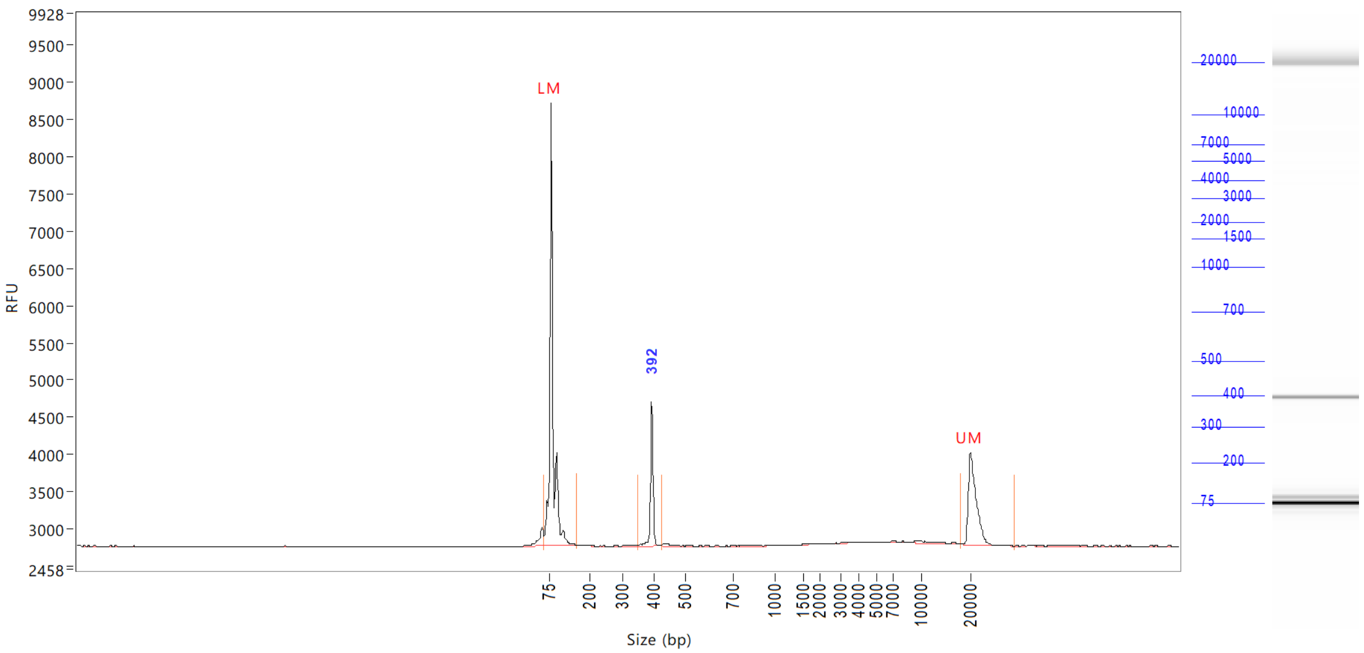

Peak	Size (bp)	Concentration (ng/uL)	From (bp)	To (bp)	Average size (bp)	CV%	RFU	Corrected peak area
1	75 (LM)	0.5000	54	160	78	15.27	5957	49.290
2	392	1.2229	347	422	389	1.94	1933	10.046
3	20000 (UM)	0.1297	18049	28705	20512	4.84	1230	12.789

TIC:	1.2229	ng/uL
TIM:	5.1722	nmole/L
Total concentration:	1.6796	ng/uL

Sample peak width (sec): 5 Sample min peak height: 50 Sample baseline V to V?: Y Sample baseline V to V points: 3
Sample filter: Binomial Number of points for filter: 3 Sample start region (min): 0 Sample end region (min): 30
Marker peak width (sec): 5 Marker min peak height: 200 Marker baseline V to V?: Y Marker baseline V to V points: 3
Lower marker selection: First peak > 500 RFU Upper marker selection: Last peak > 200 RFU
Ladder size (bp)75, 200, 300, 400, 500, 700, 1000, 1500, 2000, 3000, 4000, 5000, 7000, 10000, 20000
Quantification using: Lower Marker Final concentration (ng/uL): 0.5000 Dilution factor: 12.0

Sample: SampA10
Well location: A10
Created: Tuesday, September 14, 2021 9:33:53 PM

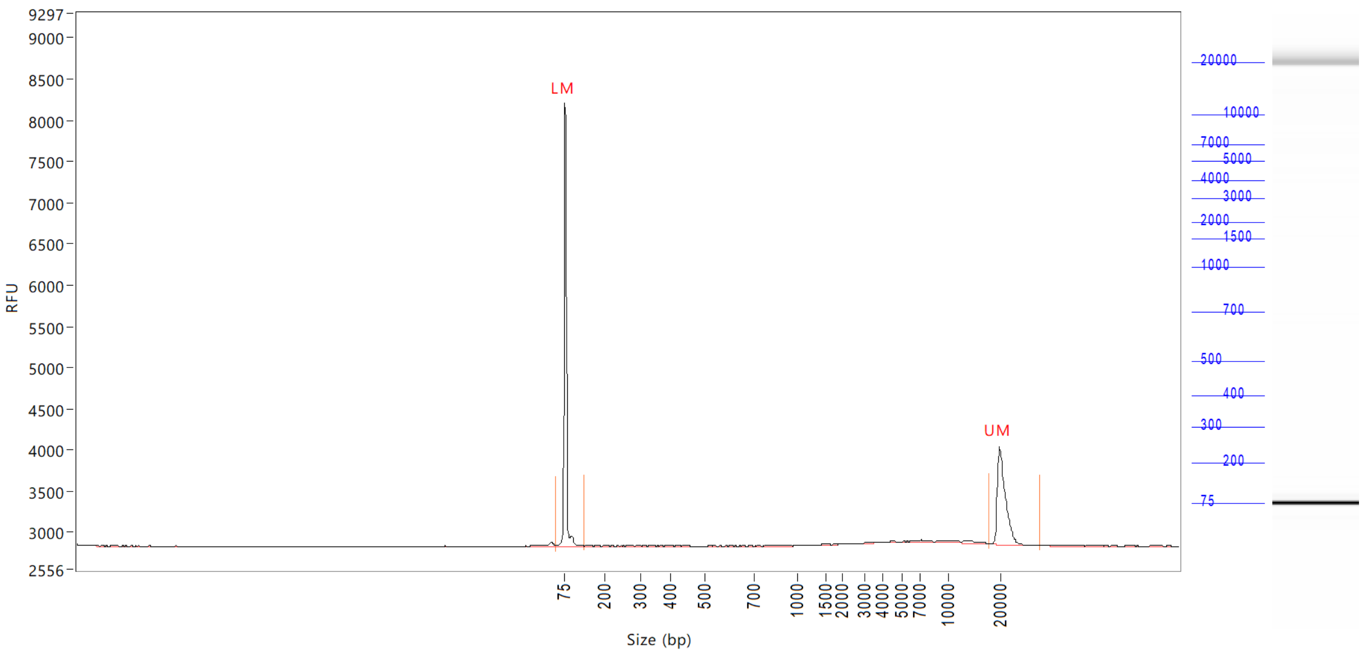

Peak	Size (bp)	Concentration (ng/uL)	From (bp)	To (bp)	Average size (bp)	CV%	RFU	Corrected peak area
1	75 (LM)	0.5000	47	135	75	8.18	5384	30.766
2	20000 (UM)	0.1967	18049	27738	20576	4.78	1180	12.103

TIC:	0.0000	ng/uL
TIM:	0.0000	nmole/L
Total concentration:	0.6729	ng/uL

Sample peak width (sec): 5 Sample min peak height: 50 Sample baseline V to V?: Y Sample baseline V to V points: 3
Sample filter: Binomial Number of points for filter: 3 Sample start region (min): 0 Sample end region (min): 30
Marker peak width (sec): 5 Marker min peak height: 200 Marker baseline V to V?: Y Marker baseline V to V points: 3
Lower marker selection: First peak > 500 RFU Upper marker selection: Last peak > 200 RFU
Ladder size (bp)75, 200, 300, 400, 500, 700, 1000, 1500, 2000, 3000, 4000, 5000, 7000, 10000, 20000
Quantification using: Lower Marker Final concentration (ng/uL): 0.5000 Dilution factor: 12.0

Sample: SampB10
Well location: B10
Created: Tuesday, September 14, 2021 9:33:53 PM

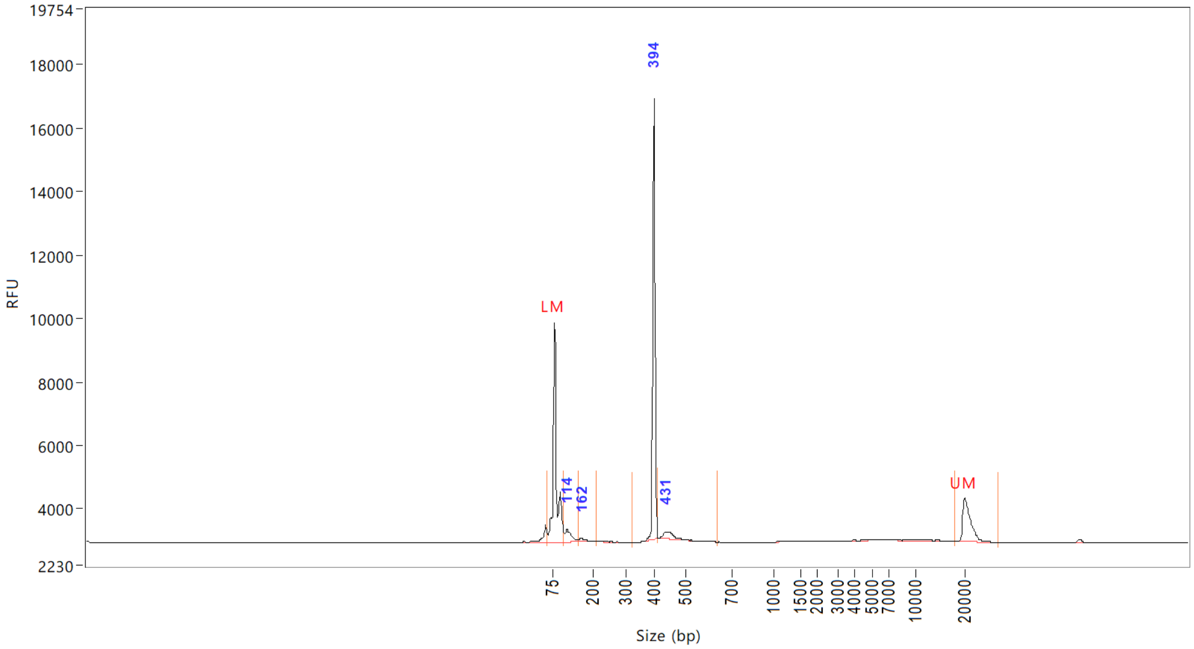

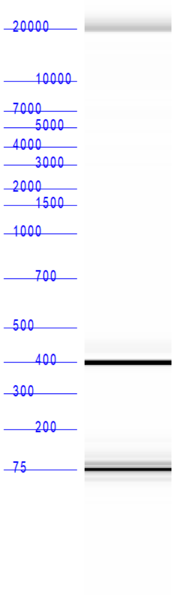

Peak	Size (bp)	Concentration (ng/uL)	From (bp)	To (bp)	Average size (bp)	CV%	RFU	Corrected peak area
1	75 (LM)	0.5000	54	107	77	12.94	6910	58.228
2	114	0.6309	107	152	121	9.50	409	6.123
3	162	0.1707	152	208	167	6.67	114	1.656
4	394	7.0284	323	409	392	1.05	13905	68.209
5	431	0.4630	409	636	445	5.50	224	4.494
6	20000 (UM)	0.1236	17927	27012	20633	4.75	1385	14.390

TIC:	8.2931	ng/uL
TIM:	41.4574	nmole/L
Total concentration:	8.5484	ng/uL

Sample peak width (sec): 5 Sample min peak height: 50 Sample baseline V to V?: Y Sample baseline V to V points: 3
Sample filter: Binomial Number of points for filter: 3 Sample start region (min): 0 Sample end region (min): 30
Marker peak width (sec): 5 Marker min peak height: 200 Marker baseline V to V?: Y Marker baseline V to V points: 3
Lower marker selection: First peak > 500 RFU Upper marker selection: Last peak > 200 RFU
Ladder size (bp)75, 200, 300, 400, 500, 700, 1000, 1500, 2000, 3000, 4000, 5000, 7000, 10000, 20000
Quantification using: Lower Marker Final concentration (ng/uL): 0.5000 Dilution factor: 12.0

Sample: SampC10
Well location: C10
Created: Tuesday, September 14, 2021 9:33:53 PM

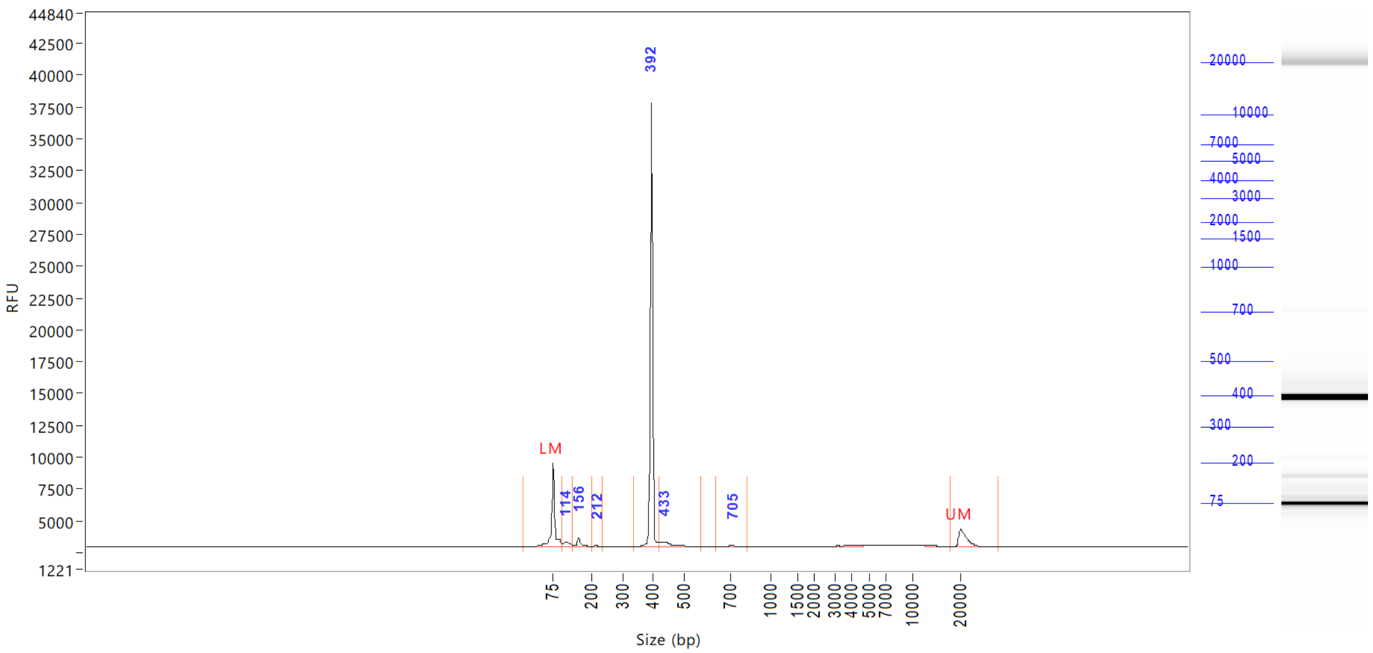

Peak	Size (bp)	Concentration (ng/uL)	From (bp)	To (bp)	Average size (bp)	CV%	RFU	Corrected peak area
1	75 (LM)	0.5000	0	105	71	20.06	6550	53.517
2	114	0.6398	105	141	120	8.30	335	5.707
3	156	0.7129	141	200	159	7.24	634	6.358
4	212	0.0761	200	233	211	2.78	86	0.679
5	392	19.6510	329	420	390	1.40	34866	175.277
6	433	0.8892	420	575	450	6.00	336	7.931
7	705	0.1255	639	827	707	4.71	124	1.119
8	20000 (UM)	0.1318	17927	27738	20628	4.96	1342	14.112

TIC:	22.0945	ng/uL
TIM:	103.1654	nmole/L
Total concentration:	22.3431	ng/uL

Sample peak width (sec): 5   Sample min peak height: 50   Sample baseline V to V?: Y   Sample baseline V to V points: 3
Sample filter: Binomial   Number of points for filter: 3 Sample start region (min): 0 Sample end region (min): 30
Marker peak width (sec): 5   Marker min peak height: 200   Marker baseline V to V?: Y   Marker baseline V to V points: 3
Lower marker selection: First peak > 500 RFU   Upper marker selection: Last peak > 200 RFU
Ladder size (bp)75, 200, 300, 400, 500, 700, 1000, 1500, 2000, 3000, 4000, 5000, 7000, 10000, 20000
Quantification using: Lower Marker   Final concentration (ng/uL): 0.5000   Dilution factor: 12.0

Sample: SampD10
Well location: D10
Created: Tuesday, September 14, 2021 9:33:53 PM

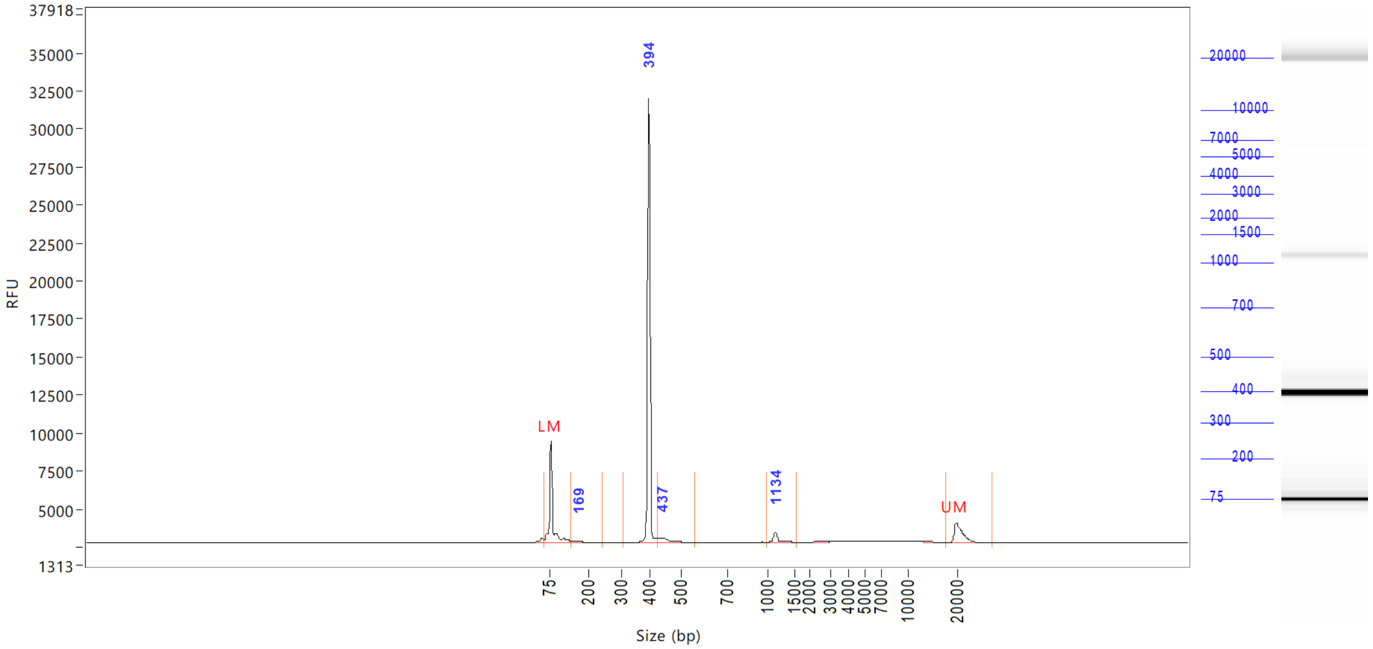

Peak	Size (bp)	Concentration (ng/uL)	From (bp)	To (bp)	Average size (bp)	CV%	RFU	Corrected peak area
1	75 (LM)	0.5000	52	143	79	20.53	6675	53.335
2	169	0.3396	143	244	167	11.44	136	3.019
3	394	16.3733	309	420	392	1.42	29260	145.546
4	437	0.7468	420	560	447	5.08	297	6.638
5	1134	0.6006	991	1559	1149	7.40	702	5.339
6	20000 (UM)	0.1274	17927	27496	20610	4.94	1276	13.590

TIC:	18.0603	ng/uL
TIM:	75.6724	nmole/L
Total concentration:	18.2920	ng/uL

Sample peak width (sec): 5   Sample min peak height: 50   Sample baseline V to V?: Y   Sample baseline V to V points: 3
Sample filter: Binomial   Number of points for filter: 3   Sample start region (min): 0   Sample end region (min): 30
Marker peak width (sec): 5   Marker min peak height: 200   Marker baseline V to V?: Y   Marker baseline V to V points: 3
Lower marker selection: First peak > 500 RFU   Upper marker selection: Last peak > 200 RFU
Ladder size (bp)75, 200, 300, 400, 500, 700, 1000, 1500, 2000, 3000, 4000, 5000, 7000, 10000, 20000
Quantification using: Lower Marker   Final concentration (ng/uL): 0.5000   Dilution factor: 12.0

Sample: SampE10
Well location: E10
Created: Tuesday, September 14, 2021 9:33:53 PM

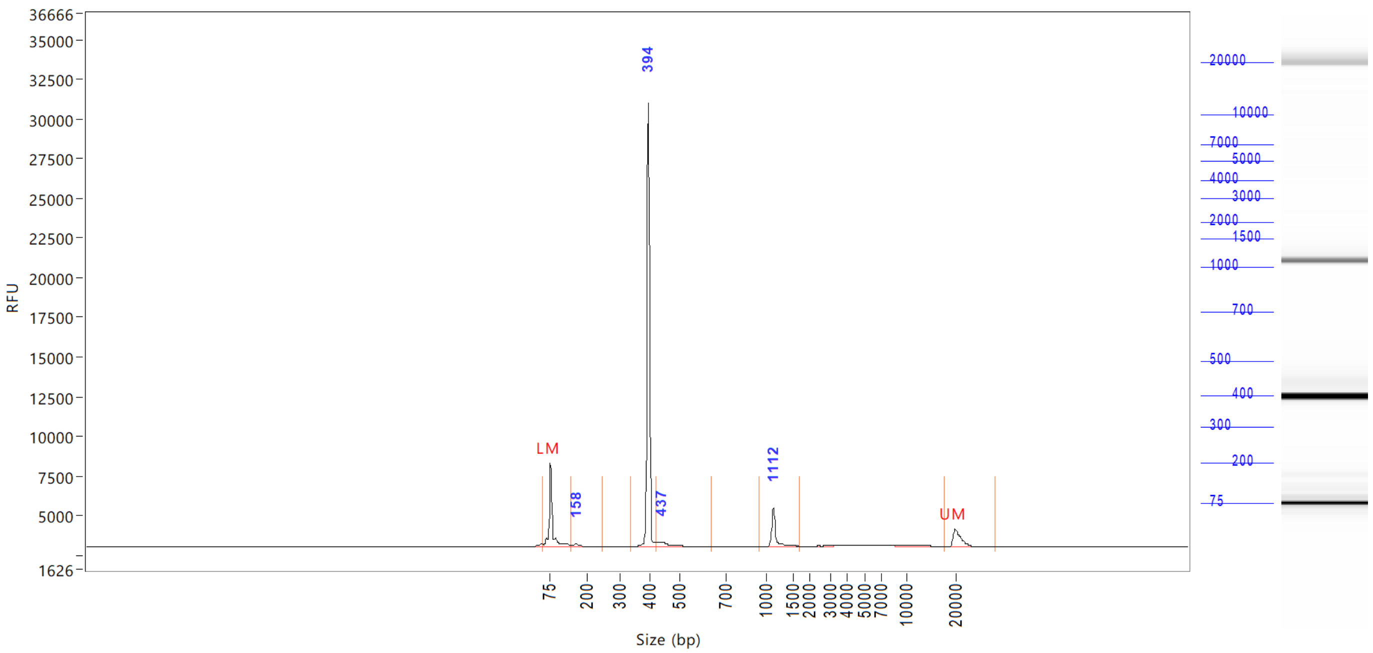

Peak	Size (bp)	Concentration (ng/uL)	From (bp)	To (bp)	Average size (bp)	CV%	RFU	Corrected peak area
1	75 (LM)	0.5000	52	143	79	20.11	5259	43.295
2	158	0.4558	143	244	170	13.29	192	3.289
3	394	20.0814	333	422	392	1.43	28013	144.905
4	437	1.0034	422	634	456	7.34	297	7.240
5	1112	2.2015	944	1693	1127	7.06	2411	15.886
6	20000 (UM)	0.1280	17683	28463	20574	4.89	1069	11.080

TIC:	23.7421	ng/uL
TIM:	95.5273	nmole/L
Total concentration:	24.0570	ng/uL

Sample peak width (sec): 5   Sample min peak height: 50   Sample baseline V to V?: Y   Sample baseline V to V points: 3
Sample filter: Binomial   Number of points for filter: 3 Sample start region (min): 0 Sample end region (min): 30
Marker peak width (sec): 5   Marker min peak height: 200   Marker baseline V to V?: Y   Marker baseline V to V points: 3
Lower marker selection: First peak > 500 RFU   Upper marker selection: Last peak > 200 RFU
Ladder size (bp)75, 200, 300, 400, 500, 700, 1000, 1500, 2000, 3000, 4000, 5000, 7000, 10000, 20000
Quantification using: Lower Marker   Final concentration (ng/uL): 0.5000   Dilution factor: 12.0

Sample: SampF10
Well location: F10
Created: Tuesday, September 14, 2021 9:33:53 PM

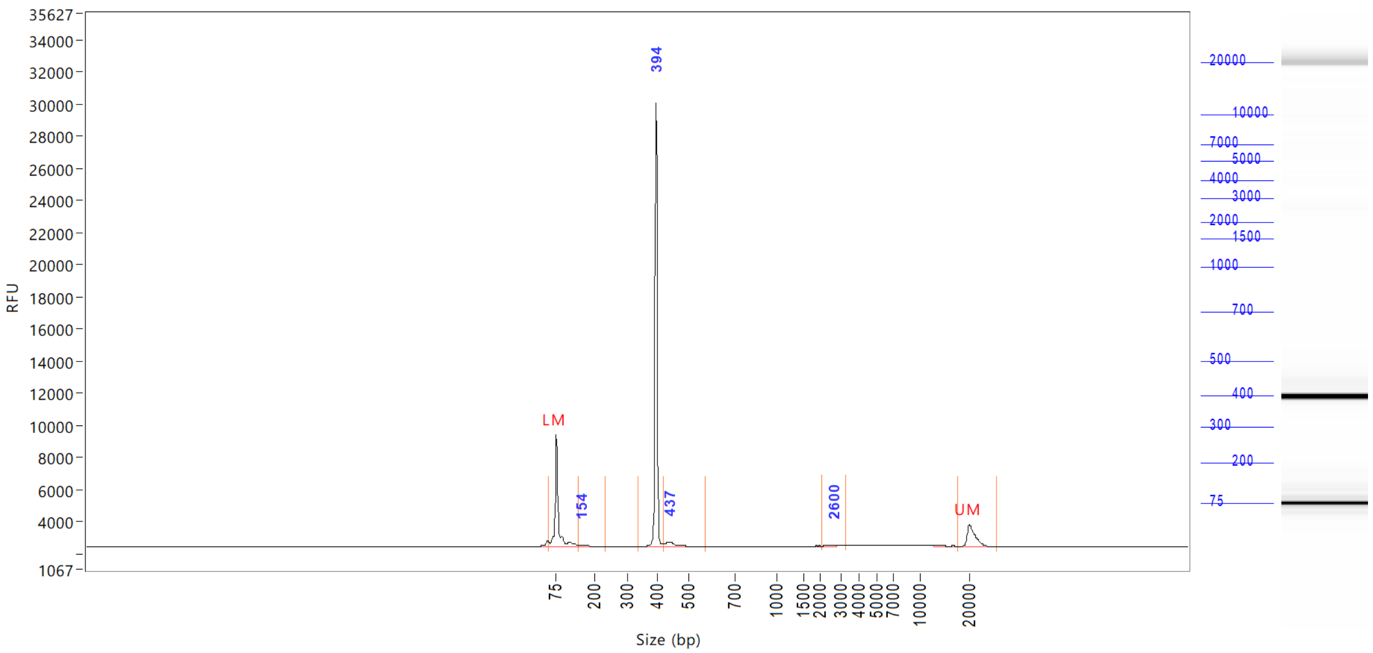

Peak	Size (bp)	Concentration (ng/uL)	From (bp)	To (bp)	Average size (bp)	CV%	RFU	Corrected peak area
1	75 (LM)	0.5000	54	148	79	20.96	6967	55.037
2	154	0.2444	148	233	169	9.87	106	2.242
3	394	14.7080	335	420	392	1.35	27635	134.915
4	437	0.6854	420	572	451	6.26	279	6.287
5	2600	0.0870	2030	3251	2619	8.92	72	0.798
6	20000 (UM)	0.1264	17683	25561	20549	4.56	1338	13.915

TIC:	15.7248	ng/uL
TIM:	66.6634	nmole/L
Total concentration:	16.0035	ng/uL

Sample peak width (sec): 5 Sample min peak height: 50 Sample baseline V to V?: Y Sample baseline V to V points: 3
Sample filter: Binomial Number of points for filter: 3 Sample start region (min): 0 Sample end region (min): 30
Marker peak width (sec): 5 Marker min peak height: 200 Marker baseline V to V?: Y Marker baseline V to V points: 3
Lower marker selection: First peak > 500 RFU Upper marker selection: Last peak > 200 RFU
Ladder size (bp)75, 200, 300, 400, 500, 700, 1000, 1500, 2000, 3000, 4000, 5000, 7000, 10000, 20000
Quantification using: Lower Marker Final concentration (ng/uL): 0.5000 Dilution factor: 12.0

Sample: SampG10
Well location: G10
Created: Tuesday, September 14, 2021 9:33:53 PM

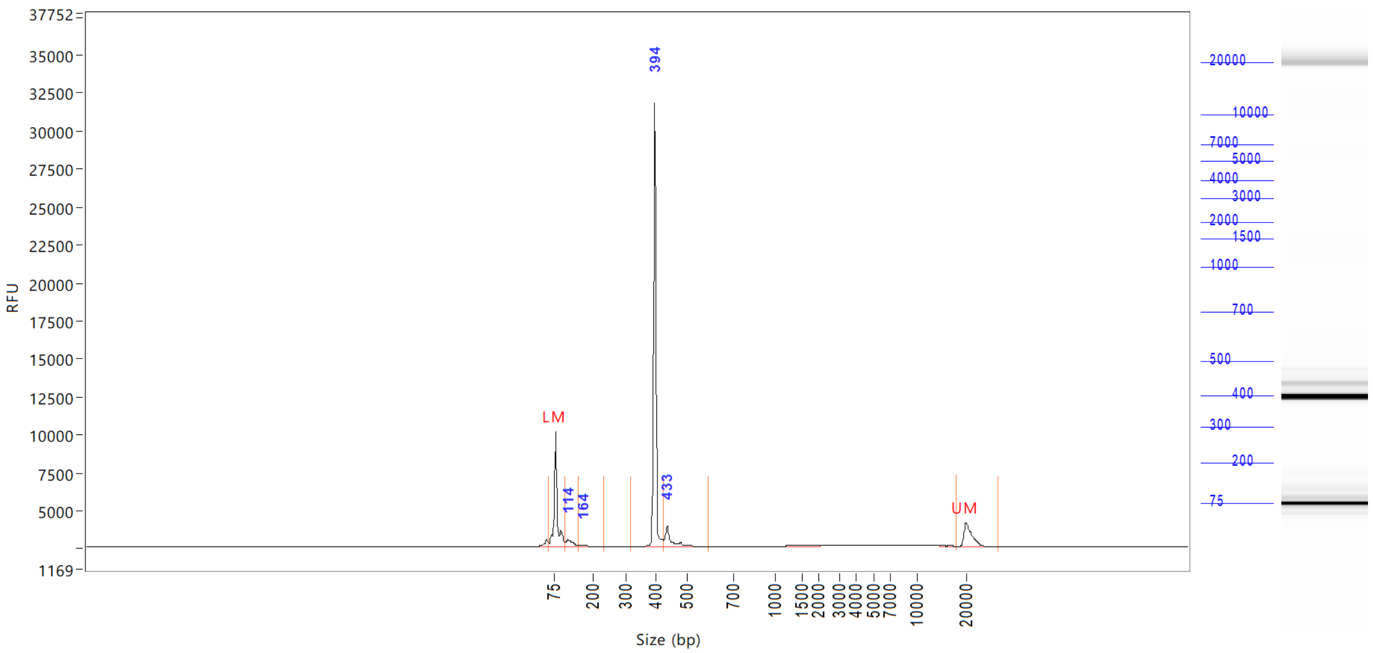

Peak	Size (bp)	Concentration (ng/uL)	From (bp)	To (bp)	Average size (bp)	CV%	RFU	Corrected peak area
1	75 (LM)	0.5000	54	107	75	12.64	7540	58.996
2	114	0.7535	107	152	122	9.45	447	7.409
3	164	0.2110	152	230	170	8.56	122	2.075
4	394	15.3707	311	420	391	1.56	29247	151.134
5	433	1.6070	420	585	447	6.20	1370	15.801
6	20000 (UM)	0.1363	17927	26649	20516	4.63	1542	16.083

TIC:	17.9422	ng/uL
TIM:	82.7795	nmole/L
Total concentration:	18.3090	ng/uL

Sample peak width (sec): 5 Sample min peak height: 50 Sample baseline V to V?: Y Sample baseline V to V points: 3
Sample filter: Binomial Number of points for filter: 3 Sample start region (min): 0 Sample end region (min): 30
Marker peak width (sec): 5 Marker min peak height: 200 Marker baseline V to V?: Y Marker baseline V to V points: 3
Lower marker selection: First peak > 500 RFU Upper marker selection: Last peak > 200 RFU
Ladder size (bp)75, 200, 300, 400, 500, 700, 1000, 1500, 2000, 3000, 4000, 5000, 7000, 10000, 20000
Quantification using: Lower Marker Final concentration (ng/uL): 0.5000 Dilution factor: 12.0

Sample: SampH10
Well location: H10
Created: Tuesday, September 14, 2021 9:33:53 PM

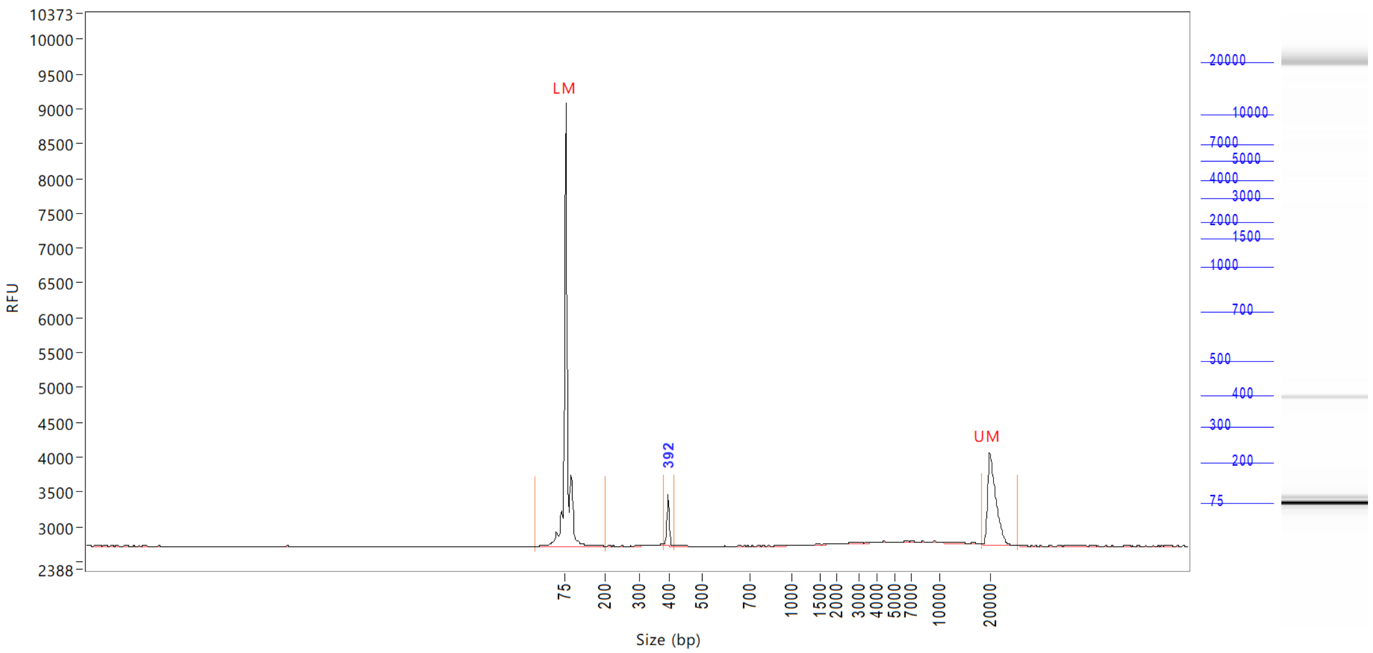

Peak	Size (bp)	Concentration (ng/uL)	From (bp)	To (bp)	Average size (bp)	CV%	RFU	Corrected peak area
1	75 (LM)	0.5000	0	200	75	22.48	6384	51.089
2	392	0.4314	380	414	391	1.03	737	3.673
3	20000 (UM)	0.1370	18537	25561	20596	4.49	1319	13.995

TIC:	0.4314	ng/uL
TIM:	1.8151	nmole/L
Total concentration:	0.8887	ng/uL

Sample peak width (sec): 5 Sample min peak height: 50 Sample baseline V to V?: Y Sample baseline V to V points: 3
Sample filter: Binomial Number of points for filter: 3 Sample start region (min): 0 Sample end region (min): 30
Marker peak width (sec): 5 Marker min peak height: 200 Marker baseline V to V?: Y Marker baseline V to V points: 3
Lower marker selection: First peak > 500 RFU Upper marker selection: Last peak > 200 RFU
Ladder size (bp)75, 200, 300, 400, 500, 700, 1000, 1500, 2000, 3000, 4000, 5000, 7000, 10000, 20000
Quantification using: Lower Marker Final concentration (ng/uL): 0.5000 Dilution factor: 12.0

Sample: SampA11
Well location: A11
Created: Tuesday, September 14, 2021 9:33:53 PM

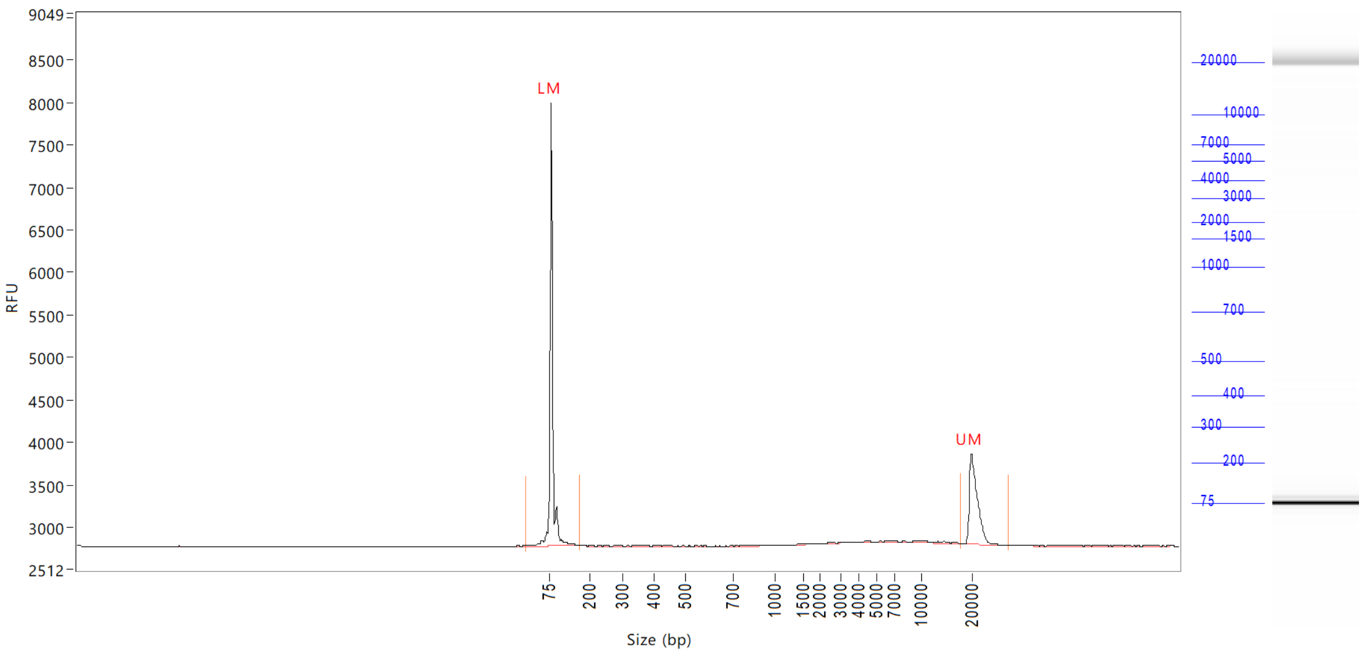

Peak	Size (bp)	Concentration (ng/uL)	From (bp)	To (bp)	Average size (bp)	CV%	RFU	Corrected peak area
1	75 (LM)	0.5000	0	168	76	16.67	5218	35.686
2	20000 (UM)	0.1562	17805	27375	20617	4.82	1066	11.152

TIC:	0.0000	ng/uL
TIM:	0.0000	nmole/L
Total concentration:	0.5324	ng/uL

Sample peak width (sec): 5 Sample min peak height: 50 Sample baseline V to V?: Y Sample baseline V to V points: 3
Sample filter: Binomial Number of points for filter: 3 Sample start region (min): 0 Sample end region (min): 30
Marker peak width (sec): 5 Marker min peak height: 200 Marker baseline V to V?: Y Marker baseline V to V points: 3
Lower marker selection: First peak > 500 RFU Upper marker selection: Last peak > 200 RFU
Ladder size (bp)75, 200, 300, 400, 500, 700, 1000, 1500, 2000, 3000, 4000, 5000, 7000, 10000, 20000
Quantification using: Lower Marker Final concentration (ng/uL): 0.5000 Dilution factor: 12.0

Sample: SampB11
Well location: B11
Created: Tuesday, September 14, 2021 9:33:53 PM

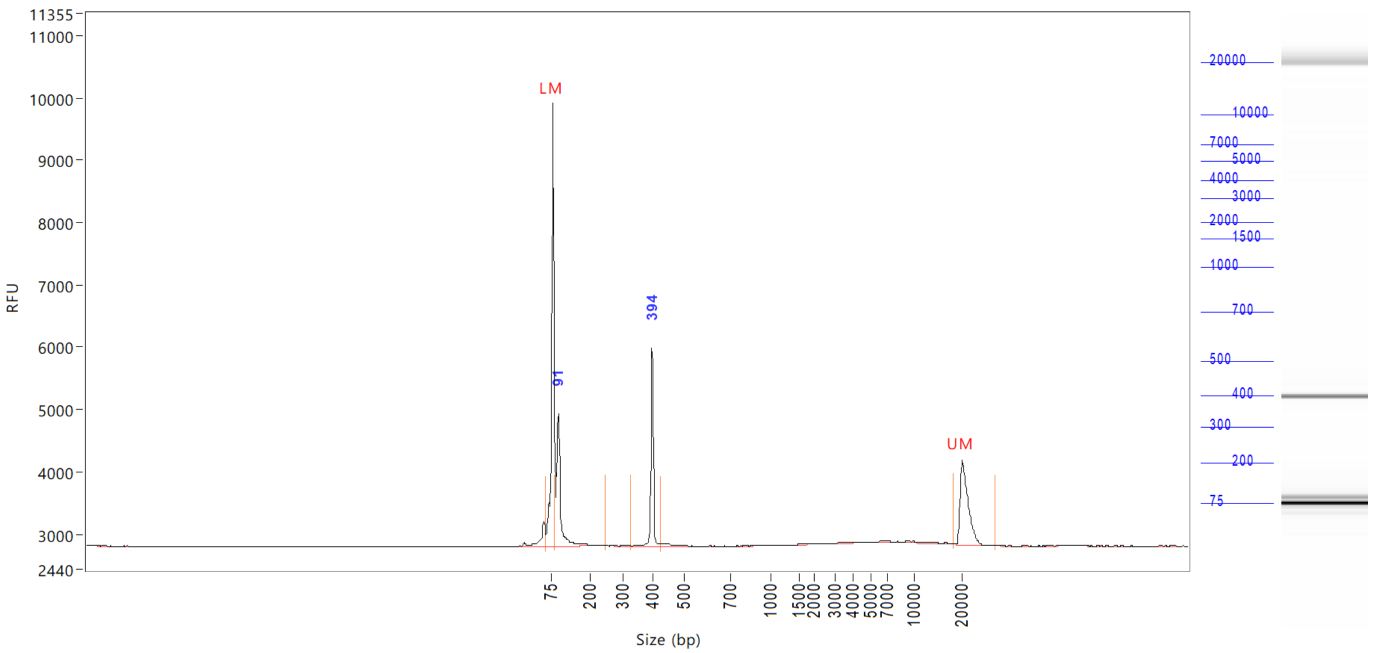

Peak	Size (bp)	Concentration (ng/uL)	From (bp)	To (bp)	Average size (bp)	CV%	RFU	Corrected peak area
1	75 (LM)	0.5000	54	84	73	7.28	7120	45.951
2	91	2.5670	84	242	99	22.06	2126	19.660
3	394	2.1769	325	422	392	1.94	3175	16.672
4	20000 (UM)	0.1540	18293	26891	20619	4.56	1356	14.152

TIC:	4.7439	ng/uL
TIM:	51.7139	nmole/L
Total concentration:	5.2771	ng/uL

Sample peak width (sec): 5 Sample min peak height: 50 Sample baseline V to V?: Y Sample baseline V to V points: 3
Sample filter: Binomial Number of points for filter: 3 Sample start region (min): 0 Sample end region (min): 30
Marker peak width (sec): 5 Marker min peak height: 200 Marker baseline V to V?: Y Marker baseline V to V points: 3
Lower marker selection: First peak > 500 RFU Upper marker selection: Last peak > 200 RFU
Ladder size (bp)75, 200, 300, 400, 500, 700, 1000, 1500, 2000, 3000, 4000, 5000, 7000, 10000, 20000
Quantification using: Lower Marker Final concentration (ng/uL): 0.5000 Dilution factor: 12.0

Sample: SampC11
Well location: C11
Created: Tuesday, September 14, 2021 9:33:53 PM

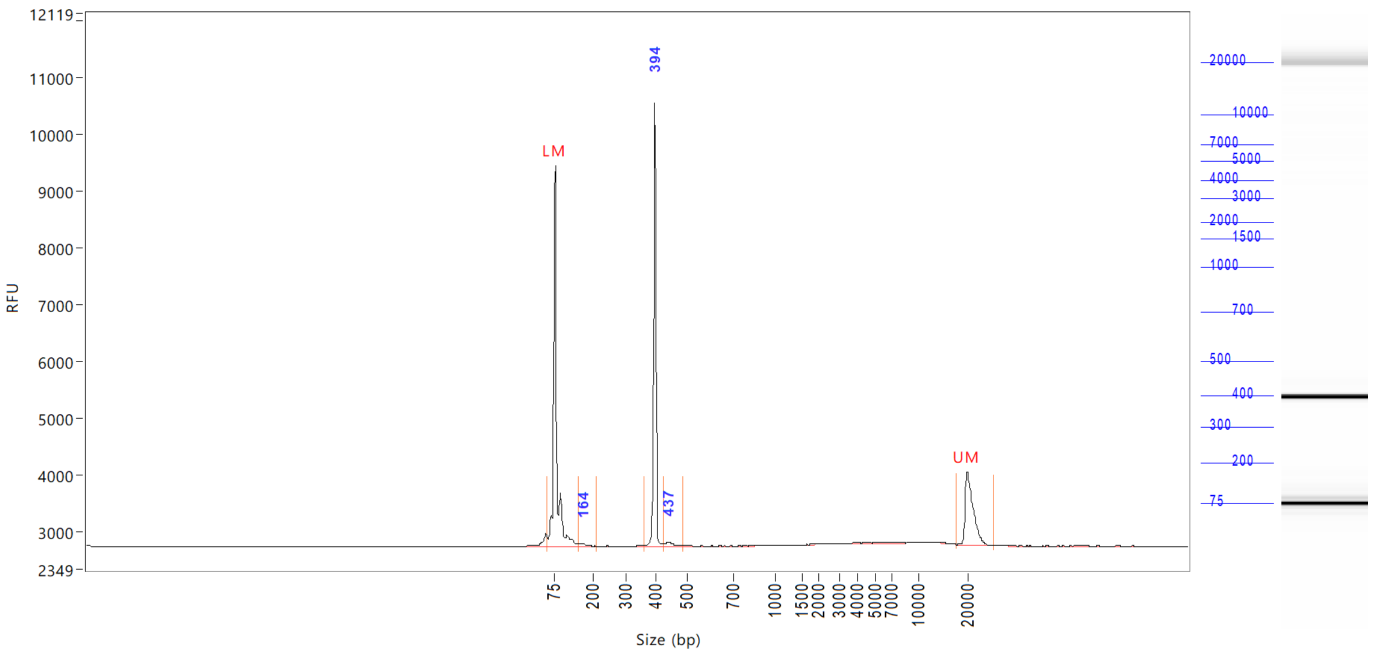

Peak	Size (bp)	Concentration (ng/uL)	From (bp)	To (bp)	Average size (bp)	CV%	RFU	Corrected peak area
1	75 (LM)	0.5000	52	150	77	17.94	6681	52.476
2	164	0.0935	150	210	167	7.39	50	0.818
3	394	4.4570	356	422	391	1.32	7805	38.981
4	437	0.1370	422	485	442	3.31	72	1.198
5	20000 (UM)	0.1297	17805	25440	20521	4.66	1284	13.615

TIC:	4.6876	ng/uL
TIM:	20.1851	nmole/L
Total concentration:	4.9890	ng/uL

```
Sample peak width (sec): 5     Sample min peak height: 50     Sample baseline V to V?: Y     Sample baseline V to V points: 3
Sample filter: Binomial        Number of points for filter: 3  Sample start region (min): 0  Sample end region (min): 30
Marker peak width (sec): 5     Marker min peak height: 200    Marker baseline V to V?: Y     Marker baseline V to V points: 3
Lower marker selection: First peak > 500 RFU                  Upper marker selection: Last peak > 200 RFU
Ladder size (bp)75, 200, 300, 400, 500, 700, 1000, 1500, 2000, 3000, 4000, 5000, 7000, 10000, 20000
Quantification using: Lower Marker          Final concentration (ng/uL): 0.5000          Dilution factor: 12.0
```

Sample: SampD11
Well location: D11
Created: Tuesday, September 14, 2021 9:33:53 PM

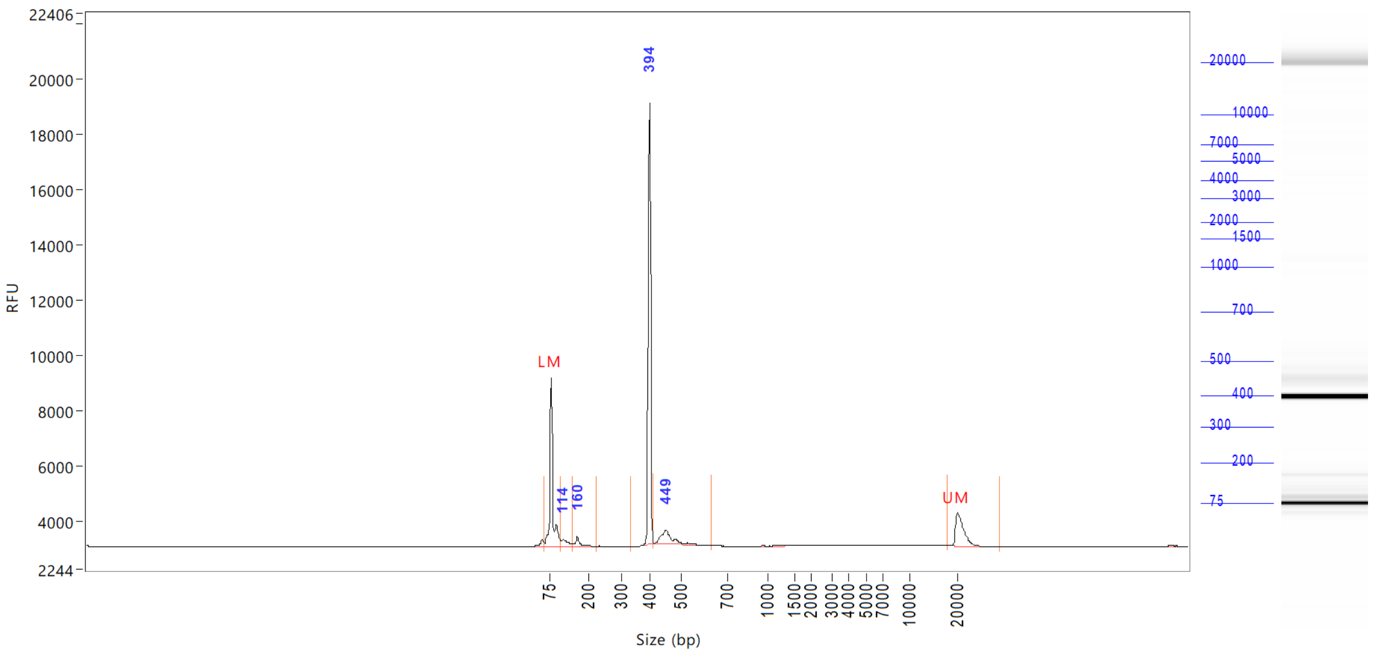

Peak	Size (bp)	Concentration (ng/uL)	From (bp)	To (bp)	Average size (bp)	CV%	RFU	Corrected peak area
1	75 (LM)	0.5000	54	107	76	11.70	6090	44.481
2	114	0.5930	107	147	123	9.25	259	4.396
3	160	0.4883	147	223	164	8.28	348	3.620
4	394	10.4271	333	411	392	0.98	16033	77.301
5	449	1.5014	411	626	459	6.97	495	11.131
6	20000 (UM)	0.1451	17805	28584	20609	5.06	1235	12.904

TIC:	13.0098	ng/uL
TIM:	61.9605	nmole/L
Total concentration:	13.3256	ng/uL

Sample peak width (sec): 5 Sample min peak height: 50 Sample baseline V to V?: Y Sample baseline V to V points: 3
Sample filter: Binomial Number of points for filter: 3 Sample start region (min): 0 Sample end region (min): 30
Marker peak width (sec): 5 Marker min peak height: 200 Marker baseline V to V?: Y Marker baseline V to V points: 3
Lower marker selection: First peak > 500 RFU Upper marker selection: Last peak > 200 RFU
Ladder size (bp)75, 200, 300, 400, 500, 700, 1000, 1500, 2000, 3000, 4000, 5000, 7000, 10000, 20000
Quantification using: Lower Marker Final concentration (ng/uL): 0.5000 Dilution factor: 12.0

Sample: SampE11
Well location: E11
Created: Tuesday, September 14, 2021 9:33:53 PM

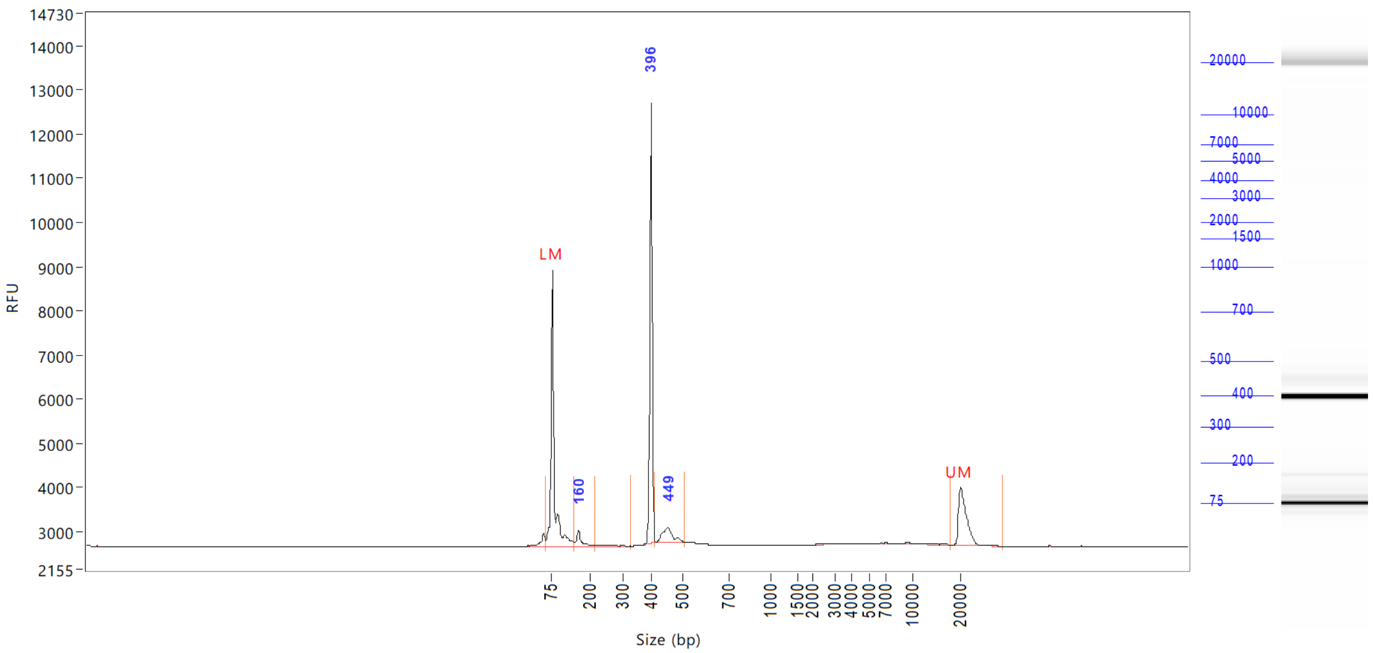

Peak	Size (bp)	Concentration (ng/uL)	From (bp)	To (bp)	Average size (bp)	CV%	RFU	Corrected peak area
1	75 (LM)	0.5000	56	148	80	20.25	6263	50.371
2	160	0.4637	148	214	166	8.46	358	3.893
3	396	5.8865	327	409	392	1.10	9973	49.418
4	449	0.7715	409	504	447	3.83	339	6.477
5	20000 (UM)	0.1353	17927	28584	20555	4.77	1313	13.629

TIC:	7.1217	ng/uL
TIM:	32.1377	nmole/L
Total concentration:	7.5442	ng/uL

Sample peak width (sec): 5 Sample min peak height: 50 Sample baseline V to V?: Y Sample baseline V to V points: 3
Sample filter: Binomial Number of points for filter: 3 Sample start region (min): 0 Sample end region (min): 30
Marker peak width (sec): 5 Marker min peak height: 200 Marker baseline V to V?: Y Marker baseline V to V points: 3
Lower marker selection: First peak > 500 RFU Upper marker selection: Last peak > 200 RFU
Ladder size (bp)75, 200, 300, 400, 500, 700, 1000, 1500, 2000, 3000, 4000, 5000, 7000, 10000, 20000
Quantification using: Lower Marker Final concentration (ng/uL): 0.5000 Dilution factor: 12.0

Sample: SampF11
Well location: F11
Created: Tuesday, September 14, 2021 9:33:53 PM

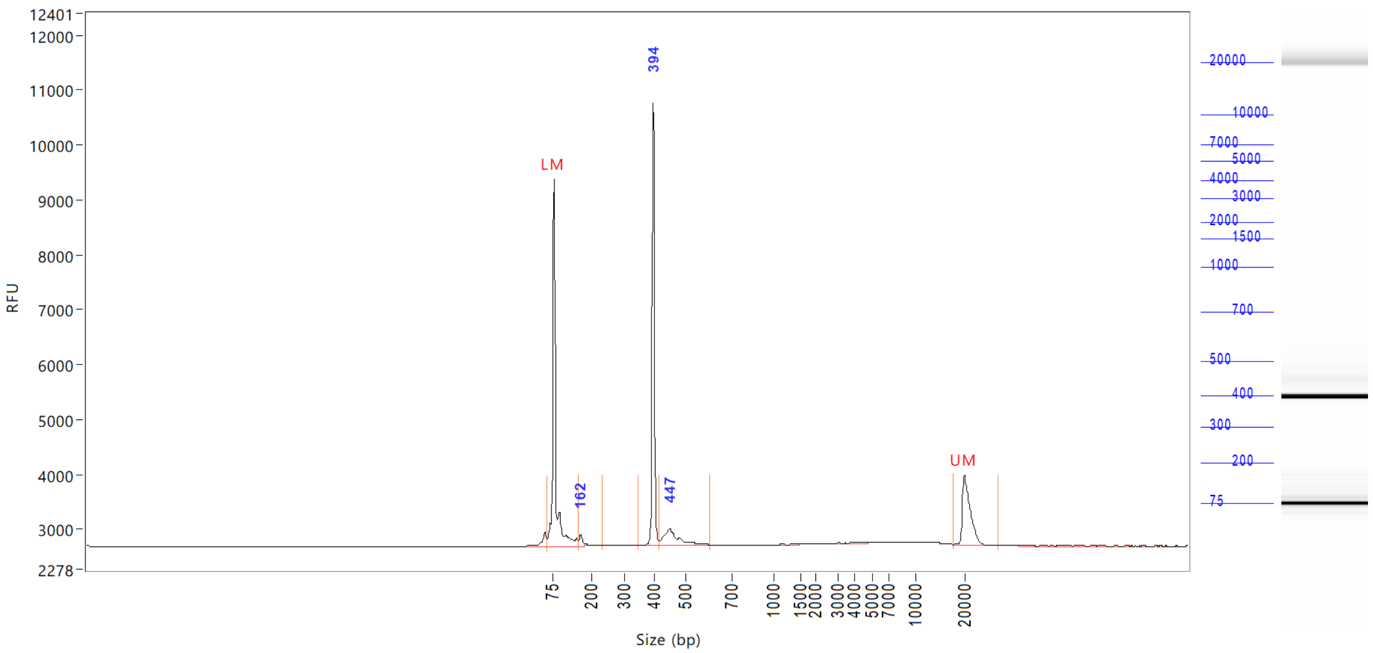

Peak	Size (bp)	Concentration (ng/uL)	From (bp)	To (bp)	Average size (bp)	CV%	RFU	Corrected peak area
1	75 (LM)	0.5000	54	154	79	21.95	6694	50.028
2	162	0.2657	154	228	170	9.78	211	2.215
3	394	4.8379	345	412	392	1.17	8073	40.339
4	447	1.0092	412	603	460	7.38	308	8.415
5	20000 (UM)	0.1326	17683	26891	20565	4.57	1263	13.269

TIC:	6.1128	ng/uL
TIM:	26.4843	nmole/L
Total concentration:	6.3908	ng/uL

Sample peak width (sec): 5 Sample min peak height: 50 Sample baseline V to V?: Y Sample baseline V to V points: 3
Sample filter: Binomial Number of points for filter: 3 Sample start region (min): 0 Sample end region (min): 30
Marker peak width (sec): 5 Marker min peak height: 200 Marker baseline V to V?: Y Marker baseline V to V points: 3
Lower marker selection: First peak > 500 RFU Upper marker selection: Last peak > 200 RFU
Ladder size (bp)75, 200, 300, 400, 500, 700, 1000, 1500, 2000, 3000, 4000, 5000, 7000, 10000, 20000
Quantification using: Lower Marker Final concentration (ng/uL): 0.5000 Dilution factor: 12.0

Sample: SampG11
Well location: G11
Created: Tuesday, September 14, 2021 9:33:53 PM

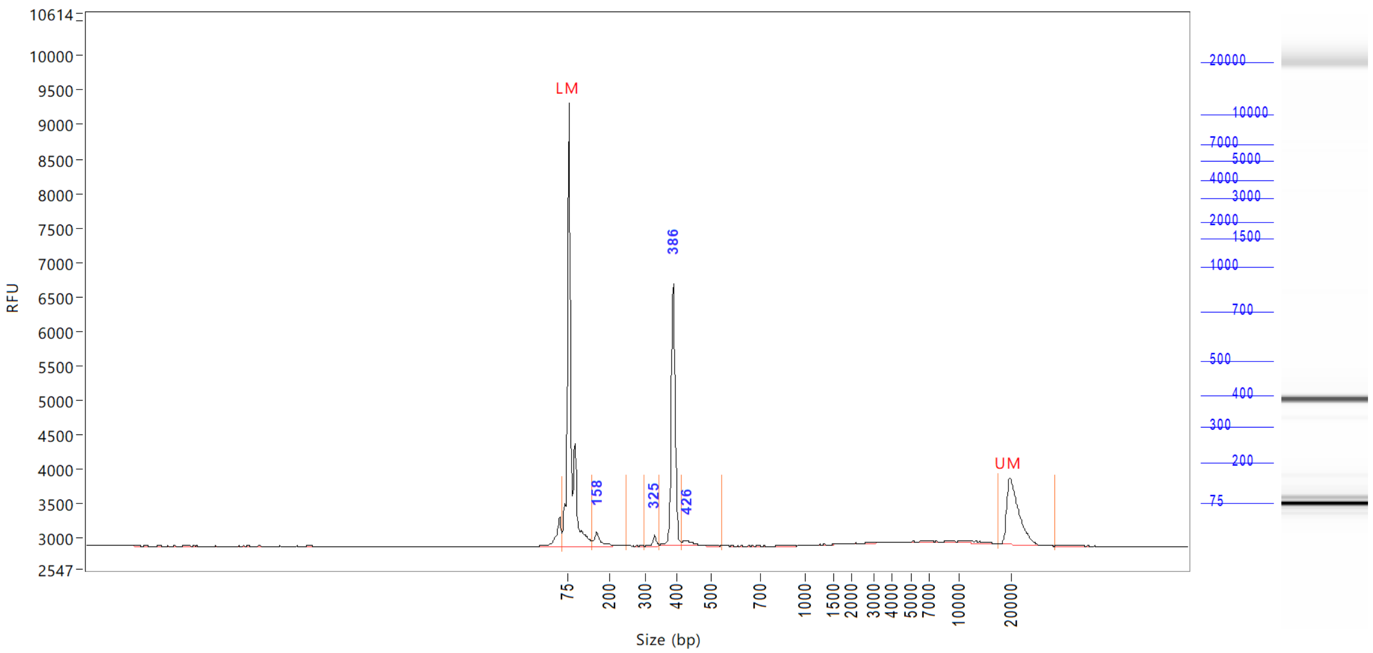

Peak	Size (bp)	Concentration (ng/uL)	From (bp)	To (bp)	Average size (bp)	CV%	RFU	Corrected peak area
1	75 (LM)	0.5000	52	147	78	18.83	6446	61.884
2	158	0.2793	147	246	166	10.48	205	2.881
3	325	0.1127	295	341	324	2.27	163	1.162
4	386	2.9964	341	409	384	1.94	3813	30.905
5	426	0.1794	409	539	437	5.50	79	1.850
6	20000 (UM)	0.1176	17683	28463	20725	6.41	965	14.551

TIC:	3.5678	ng/uL
TIM:	16.8514	nmole/L
Total concentration:	3.8074	ng/uL

Sample peak width (sec): 5 Sample min peak height: 50 Sample baseline V to V?: Y Sample baseline V to V points: 3
Sample filter: Binomial Number of points for filter: 3 Sample start region (min): 0 Sample end region (min): 30
Marker peak width (sec): 5 Marker min peak height: 200 Marker baseline V to V?: Y Marker baseline V to V points: 3
Lower marker selection: First peak > 500 RFU Upper marker selection: Last peak > 200 RFU
Ladder size (bp)75, 200, 300, 400, 500, 700, 1000, 1500, 2000, 3000, 4000, 5000, 7000, 10000, 20000
Quantification using: Lower Marker Final concentration (ng/uL): 0.5000 Dilution factor: 12.0

Sample: SampH11
Well location: H11
Created: Tuesday, September 14, 2021 9:33:53 PM

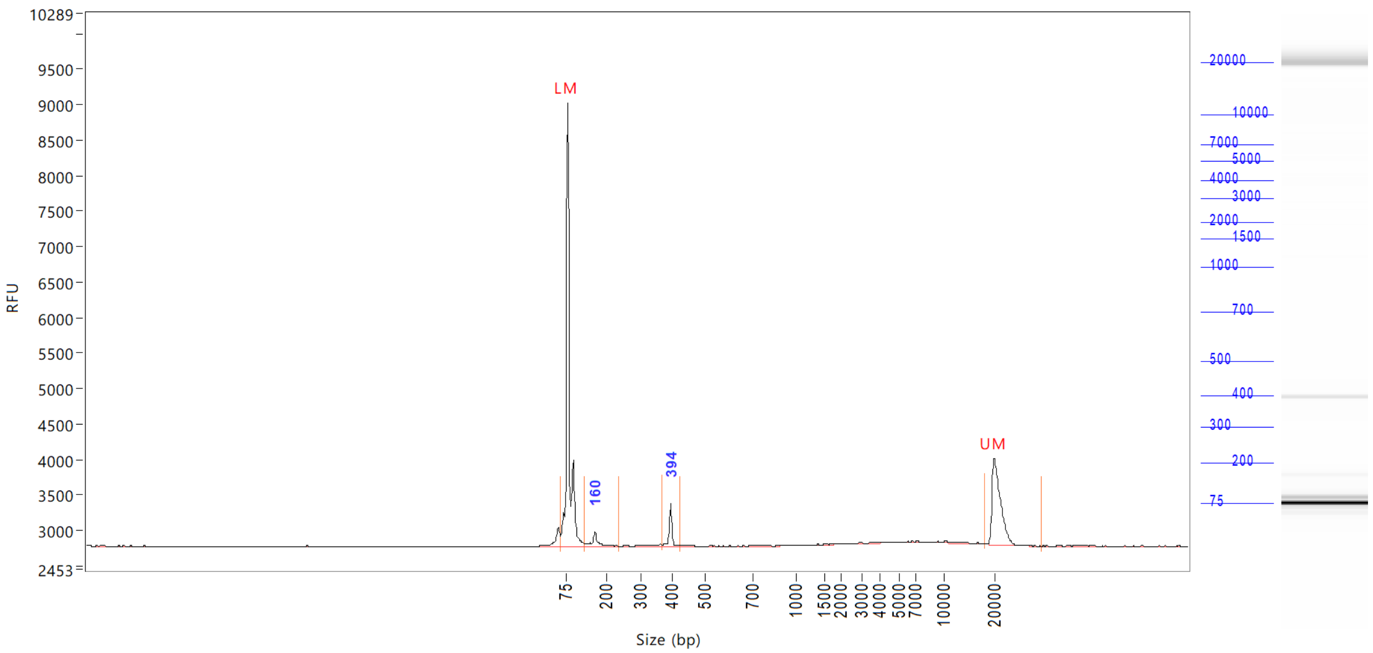

Peak	Size	Concentration	From	To	Average size	CV%	RFU	Corrected peak area
	(bp)	(ng/uL)	(bp)	(bp)	(bp)			
1	75 (LM)	0.5000	54	129	77	13.24	6260	49.228
2	160	0.3096	129	233	161	11.57	207	2.540
3	394	0.4051	366	424	391	1.97	592	3.323
4	20000 (UM)	0.1357	18171	29430	20557	5.00	1225	13.356

TIC:	0.7146	ng/uL
TIM:	4.8650	nmole/L
Total concentration:	1.1592	ng/uL

Sample peak width (sec): 5 Sample min peak height: 50 Sample baseline V to V?: Y Sample baseline V to V points: 3
Sample filter: Binomial Number of points for filter: 3 Sample start region (min): 0 Sample end region (min): 30
Marker peak width (sec): 5 Marker min peak height: 200 Marker baseline V to V?: Y Marker baseline V to V points: 3
Lower marker selection: First peak > 500 RFU Upper marker selection: Last peak > 200 RFU
Ladder size (bp)75, 200, 300, 400, 500, 700, 1000, 1500, 2000, 3000, 4000, 5000, 7000, 10000, 20000
Quantification using: Lower Marker Final concentration (ng/uL): 0.5000 Dilution factor: 12.0

Sample: SampA12
Well location: A12
Created: Tuesday, September 14, 2021 9:33:53 PM

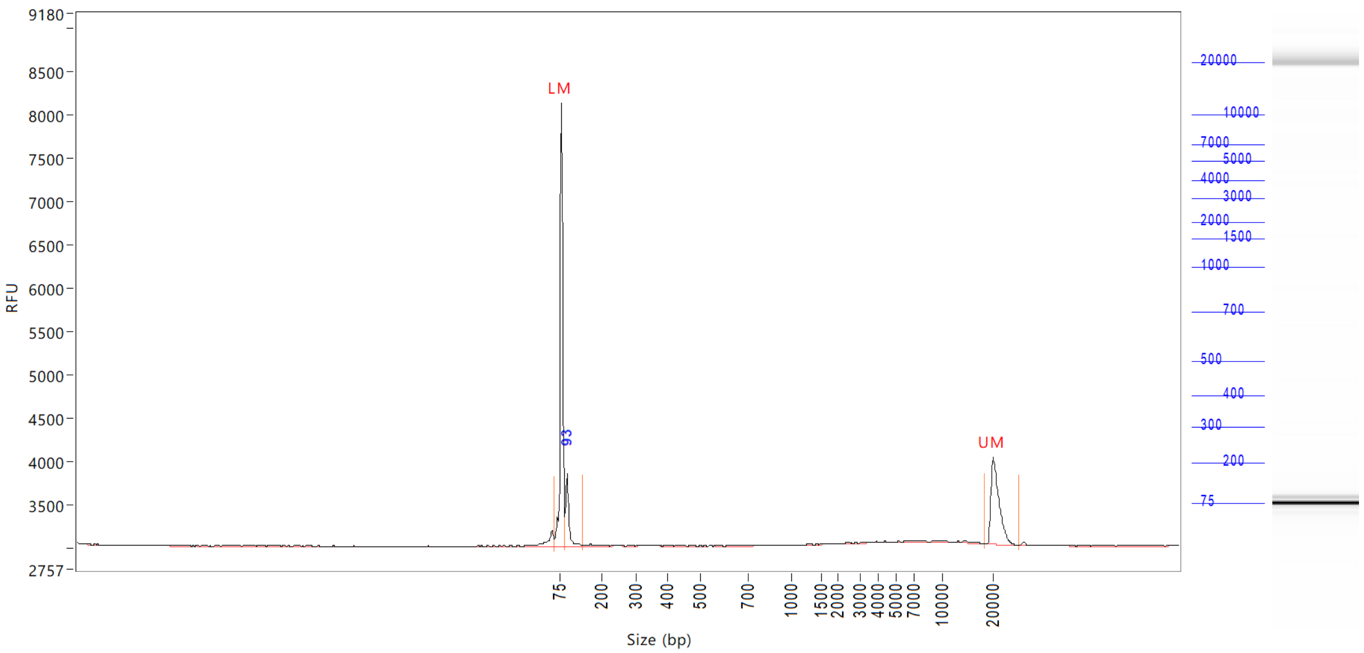

Peak	Size (bp)	Concentration (ng/uL)	From (bp)	To (bp)	Average size (bp)	CV%	RFU	Corrected peak area
1	75 (LM)	0.5000	54	86	73	6.62	5130	31.003
2	93	1.3076	86	145	94	10.74	839	6.756
3	20000 (UM)	0.1660	18171	25199	20523	4.48	1013	10.294

TIC:	1.3076	ng/uL
TIM:	22.8385	nmole/L
Total concentration:	1.9744	ng/uL

```
Sample peak width (sec): 5    Sample min peak height: 50    Sample baseline V to V?: Y    Sample baseline V to V points: 3
Sample filter: Binomial       Number of points for filter: 3  Sample start region (min): 0  Sample end region (min): 30
Marker peak width (sec): 5    Marker min peak height: 200   Marker baseline V to V?: Y    Marker baseline V to V points: 3
Lower marker selection: First peak > 500 RFU                Upper marker selection: Last peak > 200 RFU
Ladder size (bp)75, 200, 300, 400, 500, 700, 1000, 1500, 2000, 3000, 4000, 5000, 7000, 10000, 20000
Quantification using: Lower Marker          Final concentration (ng/uL): 0.5000          Dilution factor: 12.0
```

Sample: SampB12
Well location: B12
Created: Tuesday, September 14, 2021 9:33:53 PM

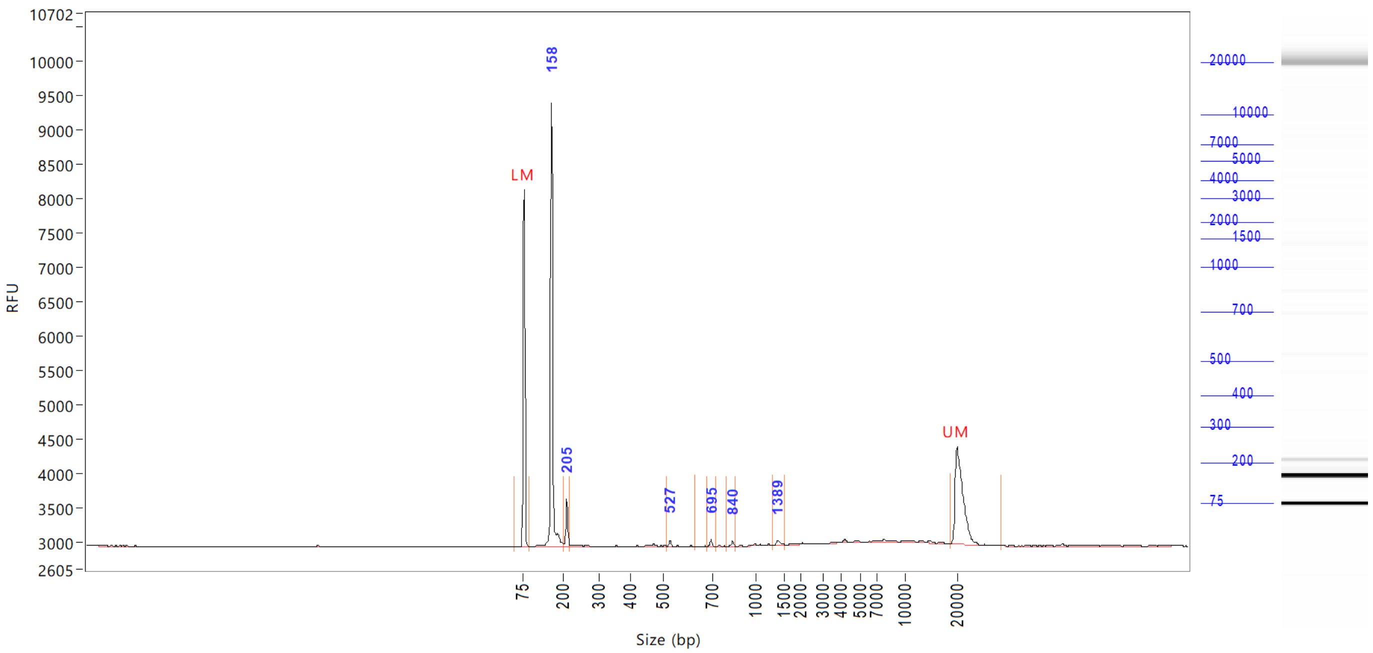

Peak	Size (bp)	Concentration (ng/uL)	From (bp)	To (bp)	Average size (bp)	CV%	RFU	Corrected peak area
1	75 (LM)	0.5000	47	93	73	3.68	5194	26.796
2	158	8.5288	93	196	157	4.67	6470	38.090
3	205	0.7419	196	215	203	1.32	683	3.314
4	527	0.1062	514	626	535	4.49	90	0.474
5	695	0.0884	680	727	693	0.85	99	0.395
6	840	0.0772	801	866	833	1.04	86	0.345
7	1389	0.1093	1311	1520	1401	3.00	70	0.488
8	20000 (UM)	0.2772	18659	28705	20572	4.80	1411	14.854

TIC:	9.6518	ng/uL
TIM:	96.1155	nmole/L
Total concentration:	10.5459	ng/uL

Sample peak width (sec): 5   Sample min peak height: 50   Sample baseline V to V?: Y   Sample baseline V to V points: 3
Sample filter: Binomial   Number of points for filter: 3   Sample start region (min): 0   Sample end region (min): 30
Marker peak width (sec): 5   Marker min peak height: 200   Marker baseline V to V?: Y   Marker baseline V to V points: 3
Lower marker selection: First peak > 500 RFU   Upper marker selection: Last peak > 200 RFU
Ladder size (bp)75, 200, 300, 400, 500, 700, 1000, 1500, 2000, 3000, 4000, 5000, 7000, 10000, 20000
Quantification using: Lower Marker   Final concentration (ng/uL): 0.5000   Dilution factor: 12.0

Sample: SampC12
Well location: C12
Created: Tuesday, September 14, 2021 9:33:53 PM

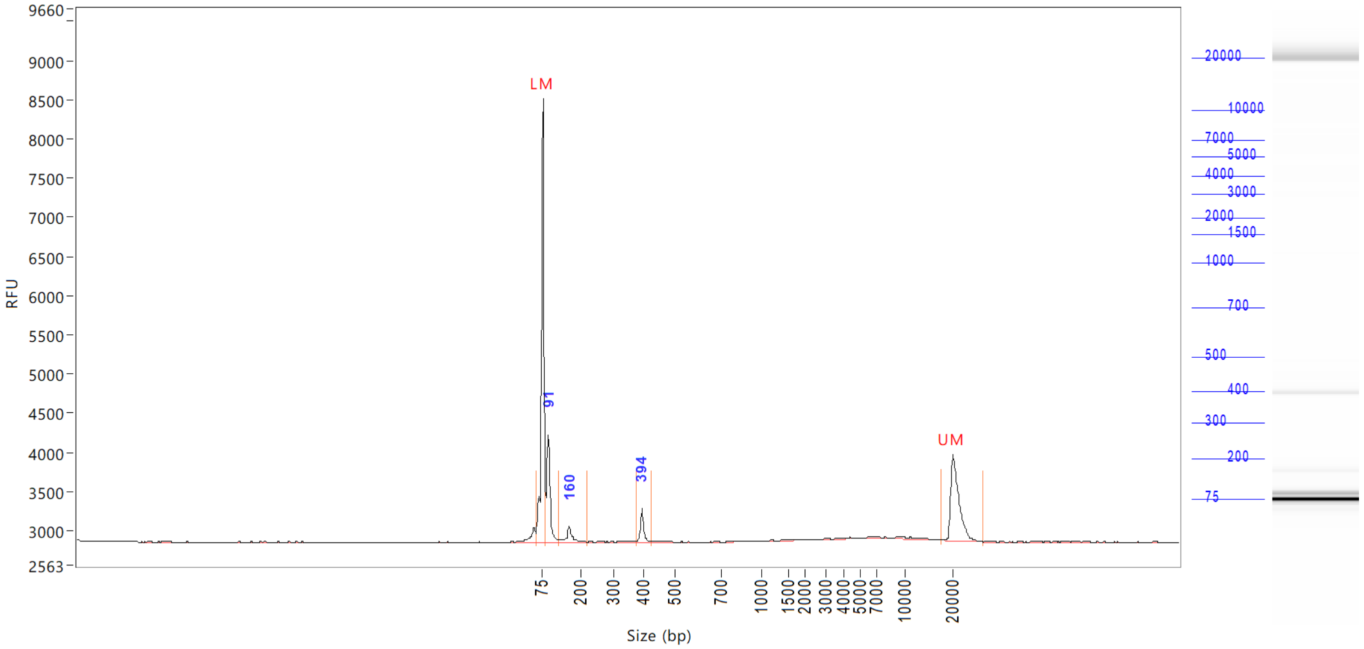

Peak	Size	Concentration	From	To	Average size	CV%	RFU	Corrected peak area
	(bp)	(ng/uL)	(bp)	(bp)	(bp)			
1	75 (LM)	0.5000	54	84	72	7.34	5668	37.374
2	91	1.8172	84	129	92	8.71	1367	11.319
3	160	0.4008	129	215	160	9.30	194	2.497
4	394	0.4411	376	426	393	1.76	424	2.748
5	20000 (UM)	0.1591	17561	26287	20649	5.21	1093	11.890

TIC:	2.6591	ng/uL
TIM:	38.3922	nmole/L
Total concentration:	3.1915	ng/uL

```
Sample peak width (sec): 5    Sample min peak height: 50    Sample baseline V to V?: Y    Sample baseline V to V points: 3
Sample filter: Binomial       Number of points for filter: 3  Sample start region (min): 0  Sample end region (min): 30
Marker peak width (sec): 5    Marker min peak height: 200   Marker baseline V to V?: Y    Marker baseline V to V points: 3
Lower marker selection: First peak > 500 RFU                Upper marker selection: Last peak > 200 RFU
Ladder size (bp)75, 200, 300, 400, 500, 700, 1000, 1500, 2000, 3000, 4000, 5000, 7000, 10000, 20000
Quantification using: Lower Marker          Final concentration (ng/uL): 0.5000          Dilution factor: 12.0
```

Sample: SampD12
Well location: D12
Created: Tuesday, September 14, 2021 9:33:53 PM

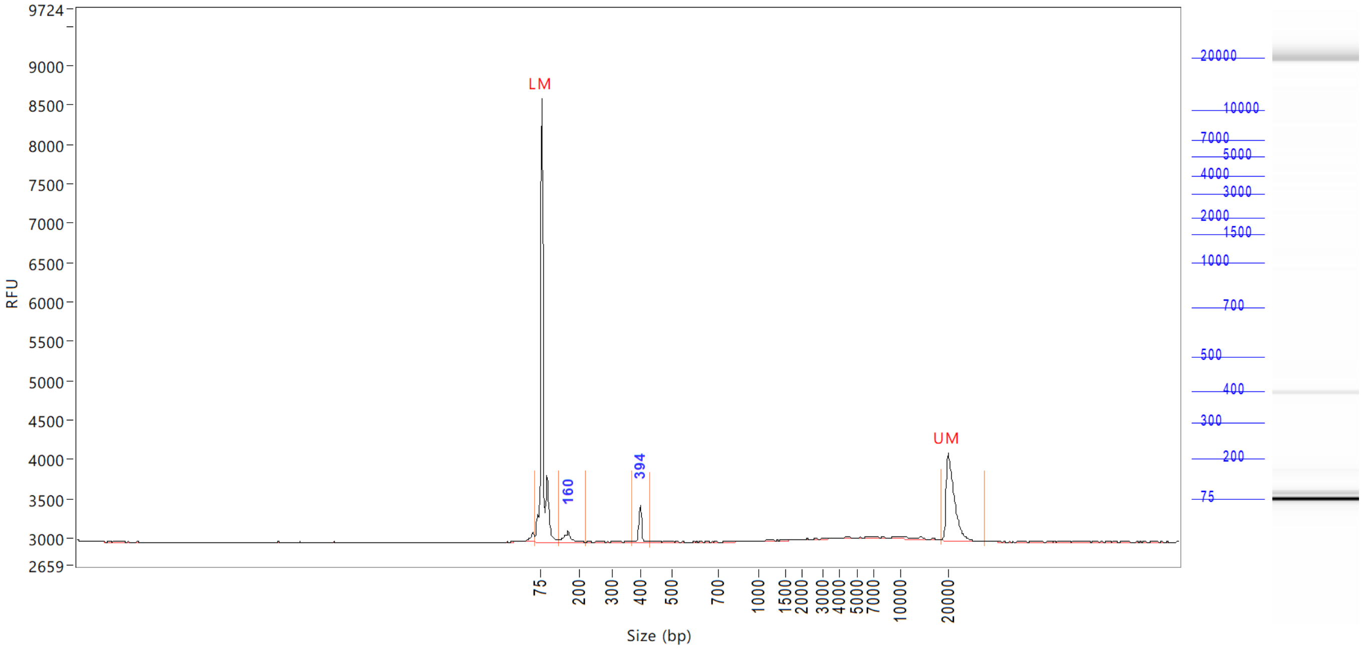

Peak	Size (bp)	Concentration (ng/uL)	From (bp)	To (bp)	Average size (bp)	CV%	RFU	Corrected peak area
1	75 (LM)	0.5000	52	129	77	12.85	5641	40.966
2	160	0.3122	129	217	158	10.15	137	2.132
3	394	0.3816	368	428	393	1.81	460	2.605
4	20000 (UM)	0.1456	18415	27375	20612	5.31	1105	11.932

TIC:	0.6938	ng/uL
TIM:	4.8451	nmole/L
Total concentration:	1.2682	ng/uL

```
Sample peak width (sec): 5    Sample min peak height: 50    Sample baseline V to V?: Y    Sample baseline V to V points: 3
Sample filter: Binomial       Number of points for filter: 3  Sample start region (min): 0  Sample end region (min): 30
Marker peak width (sec): 5    Marker min peak height: 200   Marker baseline V to V?: Y    Marker baseline V to V points: 3
Lower marker selection: First peak > 500 RFU                Upper marker selection: Last peak > 200 RFU
Ladder size (bp)75, 200, 300, 400, 500, 700, 1000, 1500, 2000, 3000, 4000, 5000, 7000, 10000, 20000
Quantification using: Lower Marker          Final concentration (ng/uL): 0.5000          Dilution factor: 12.0
```

Sample: SampE12
Well location: E12
Created: Tuesday, September 14, 2021 9:33:53 PM

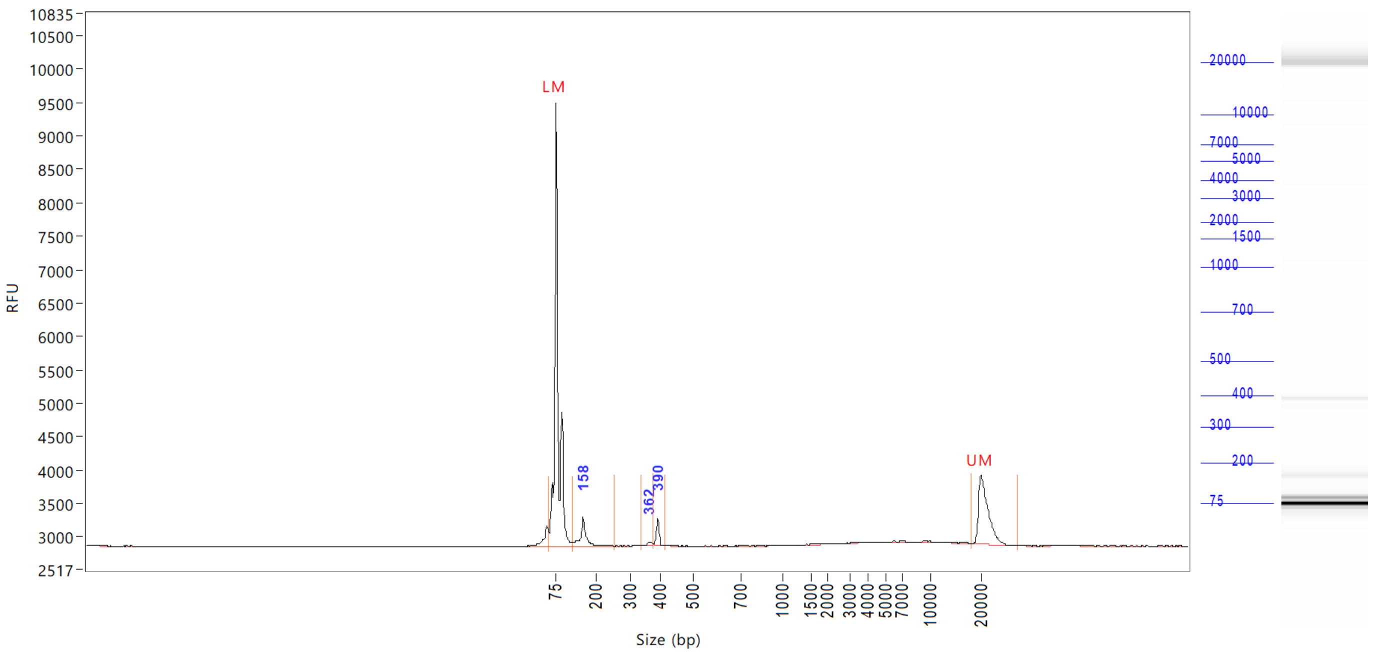

Peak	Size (bp)	Concentration (ng/uL)	From (bp)	To (bp)	Average size (bp)	CV%	RFU	Corrected peak area
1	75 (LM)	0.5000	52	126	76	14.49	6647	61.333
2	158	0.5404	126	253	159	12.03	432	5.524
3	362	0.0431	335	376	360	1.67	51	0.440
4	390	0.2205	376	412	387	1.09	405	2.254
5	20000 (UM)	0.1050	18049	27133	20685	5.48	1038	12.878

TIC:	0.8040	ng/uL
TIM:	6.7208	nmole/L
Total concentration:	1.0641	ng/uL

Sample peak width (sec): 5 Sample min peak height: 50 Sample baseline V to V?: Y Sample baseline V to V points: 3
Sample filter: Binomial Number of points for filter: 3 Sample start region (min): 0 Sample end region (min): 30
Marker peak width (sec): 5 Marker min peak height: 200 Marker baseline V to V?: Y Marker baseline V to V points: 3
Lower marker selection: First peak > 500 RFU Upper marker selection: Last peak > 200 RFU
Ladder size (bp)75, 200, 300, 400, 500, 700, 1000, 1500, 2000, 3000, 4000, 5000, 7000, 10000, 20000
Quantification using: Lower Marker Final concentration (ng/uL): 0.5000 Dilution factor: 12.0

Sample: SampF12
Well location: F12
Created: Tuesday, September 14, 2021 9:33:53 PM

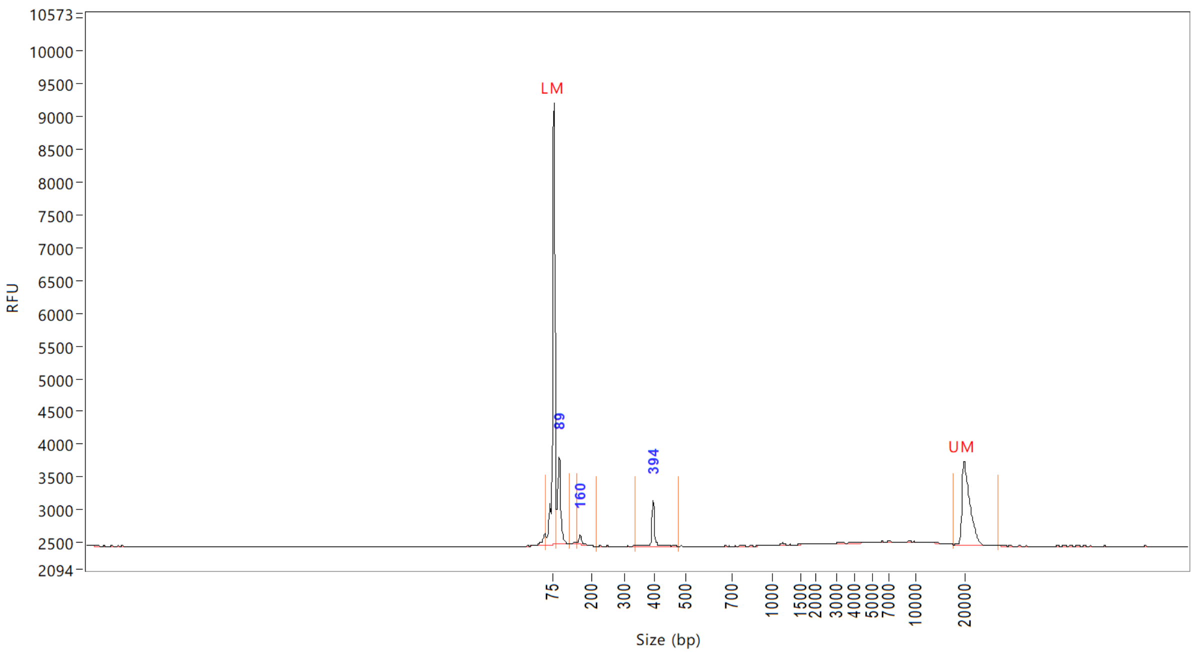

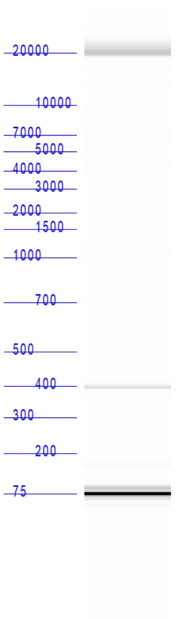

Peak	Size (bp)	Concentration (ng/uL)	From (bp)	To (bp)	Average size (bp)	CV%	RFU	Corrected peak area
1	75 (LM)	0.5000	50	84	72	7.47	6745	42.217
2	89	1.3880	84	128	90	6.18	1334	9.766
3	160	0.1481	150	212	161	5.07	142	1.042
4	394	0.5823	333	477	393	3.71	698	4.097
5	20000 (UM)	0.1578	17805	27012	20608	4.56	1278	13.322

TIC:	2.1184	ng/uL
TIM:	29.2680	nmole/L
Total concentration:	2.4934	ng/uL

Sample peak width (sec): 5 Sample min peak height: 50 Sample baseline V to V?: Y Sample baseline V to V points: 3
Sample filter: Binomial Number of points for filter: 3 Sample start region (min): 0 Sample end region (min): 30
Marker peak width (sec): 5 Marker min peak height: 200 Marker baseline V to V?: Y Marker baseline V to V points: 3
Lower marker selection: First peak > 500 RFU Upper marker selection: Last peak > 200 RFU
Ladder size (bp)75, 200, 300, 400, 500, 700, 1000, 1500, 2000, 3000, 4000, 5000, 7000, 10000, 20000
Quantification using: Lower Marker Final concentration (ng/uL): 0.5000 Dilution factor: 12.0

Sample: SampG12
Well location: G12
Created: Tuesday, September 14, 2021 9:33:53 PM

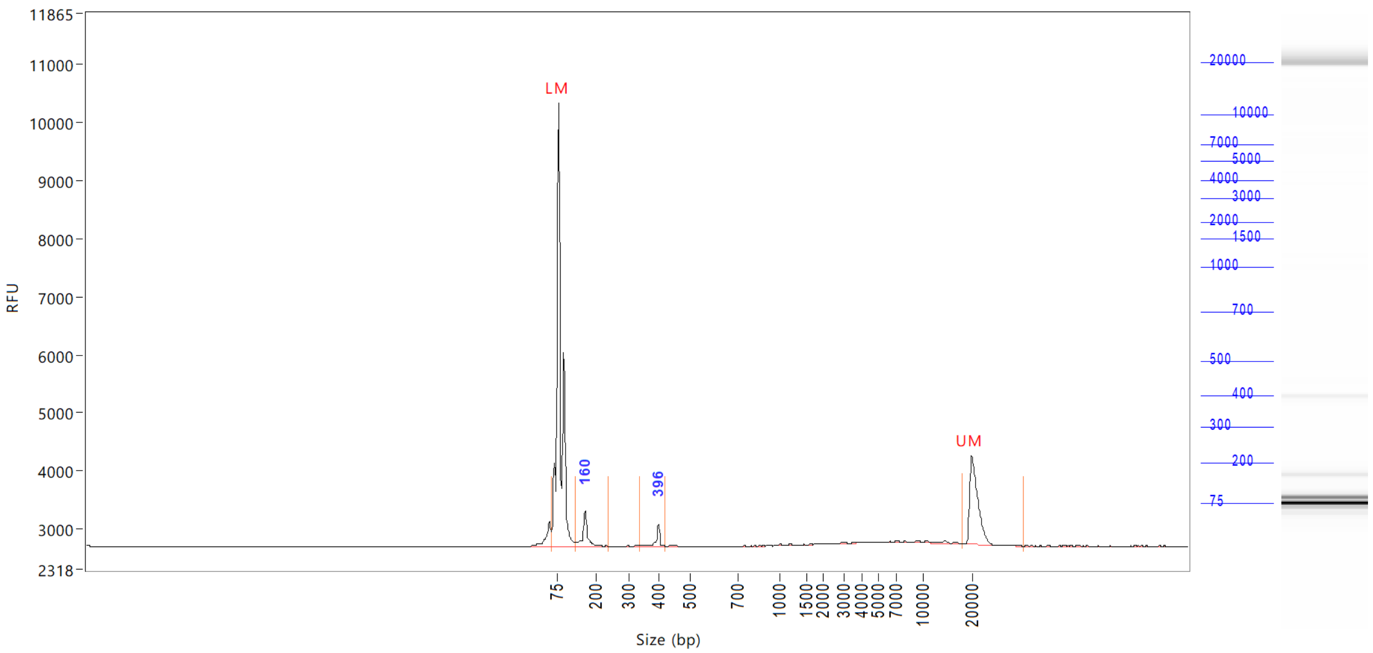

Peak	Size (bp)	Concentration (ng/uL)	From (bp)	To (bp)	Average size (bp)	CV%	RFU	Corrected peak area
1	75 (LM)	0.5000	54	133	78	15.54	7631	78.786
2	160	0.4658	133	235	160	8.67	603	6.116
3	396	0.1963	335	418	388	3.15	386	2.578
4	20000 (UM)	0.1024	17927	30519	20625	5.27	1531	16.141

TIC:	0.6621	ng/uL
TIM:	5.6173	nmole/L
Total concentration:	0.9302	ng/uL

Sample peak width (sec): 5 Sample min peak height: 50 Sample baseline V to V?: Y Sample baseline V to V points: 3
Sample filter: Binomial Number of points for filter: 3 Sample start region (min): 0 Sample end region (min): 30
Marker peak width (sec): 5 Marker min peak height: 200 Marker baseline V to V?: Y Marker baseline V to V points: 3
Lower marker selection: First peak > 500 RFU Upper marker selection: Last peak > 200 RFU
Ladder size (bp)75, 200, 300, 400, 500, 700, 1000, 1500, 2000, 3000, 4000, 5000, 7000, 10000, 20000
Quantification using: Lower Marker Final concentration (ng/uL): 0.5000 Dilution factor: 12.0

Sample: SampH12
Well location: H12
Created: Tuesday, September 14, 2021 9:33:53 PM

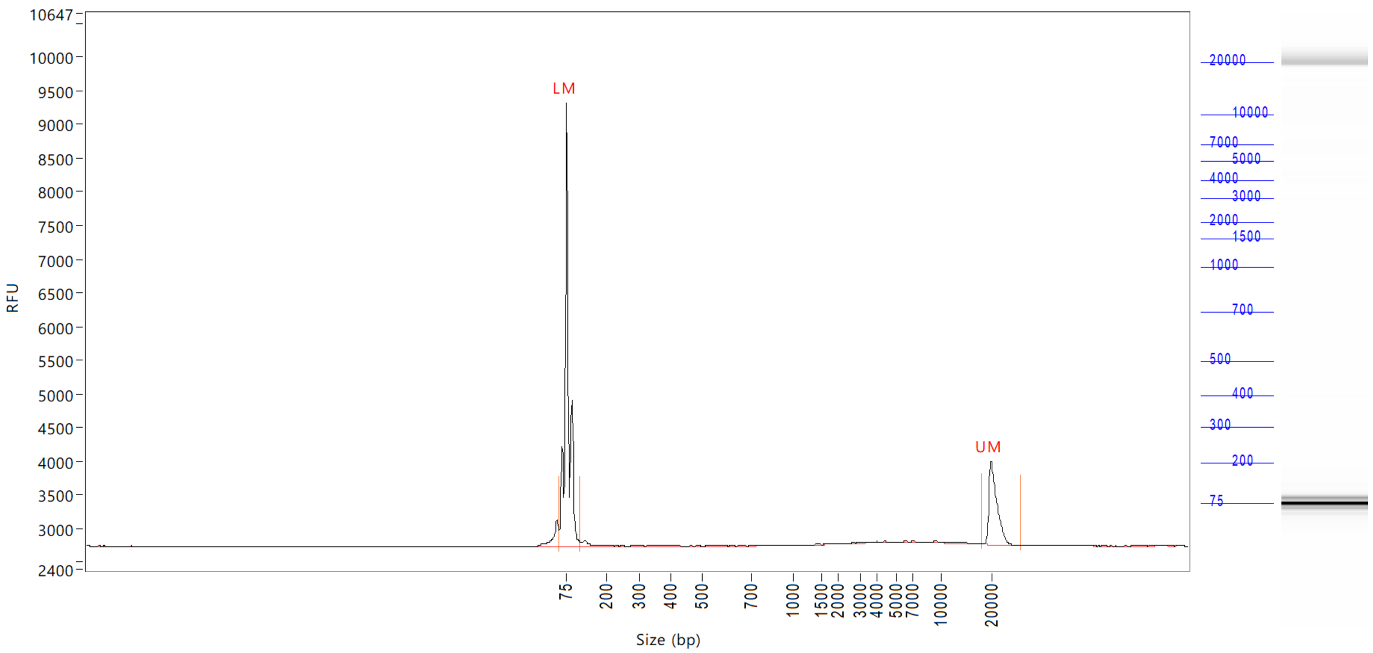

Peak	Size	Concentration	From	To	Average size	CV%	RFU	Corrected peak area
	(bp)	(ng/uL)	(bp)	(bp)	(bp)			
1	75 (LM)	0.5000	50	118	75	14.59	6589	63.081
2	20000 (UM)	0.1020	18171	25924	20601	4.54	1239	12.865

TIC:	0.0000	ng/uL
TIM:	0.0000	nmole/L
Total concentration:	0.4652	ng/uL

```
Sample peak width (sec): 5    Sample min peak height: 200   Sample baseline V to V?: Y    Sample baseline V to V points: 3
Sample filter: Binomial       Number of points for filter: 3  Sample start region (min): 0  Sample end region (min): 30
Marker peak width (sec): 5    Marker min peak height: 200   Marker baseline V to V?: Y    Marker baseline V to V points: 3
Lower marker selection: First peak > 500 RFU                Upper marker selection: Last peak > 200 RFU
Ladder size (bp)75, 200, 300, 400, 500, 700, 1000, 1500, 2000, 3000, 4000, 5000, 7000, 10000, 20000
Quantification using: Lower Marker          Final concentration (ng/uL): 0.5000          Dilution factor: 12.0
```

Sample: DNA size ladder
Well location: A7
Created: Tuesday, September 14, 2021 9:33:53 PM
Import from: C:\Users\admin\Desktop\210810_alberto_ladder.SCAL
Fit type: Point to point

Calibration curve

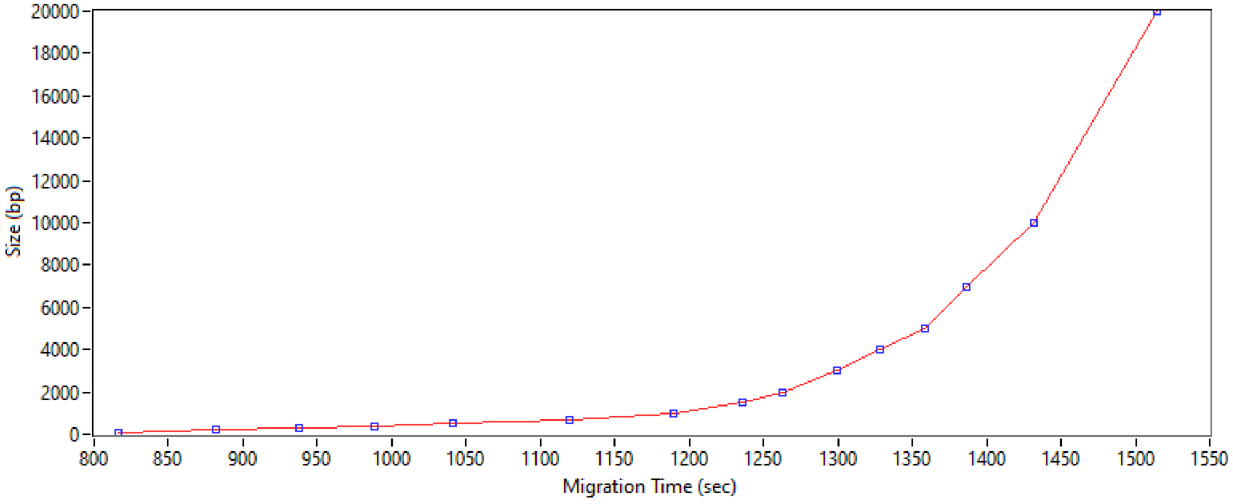

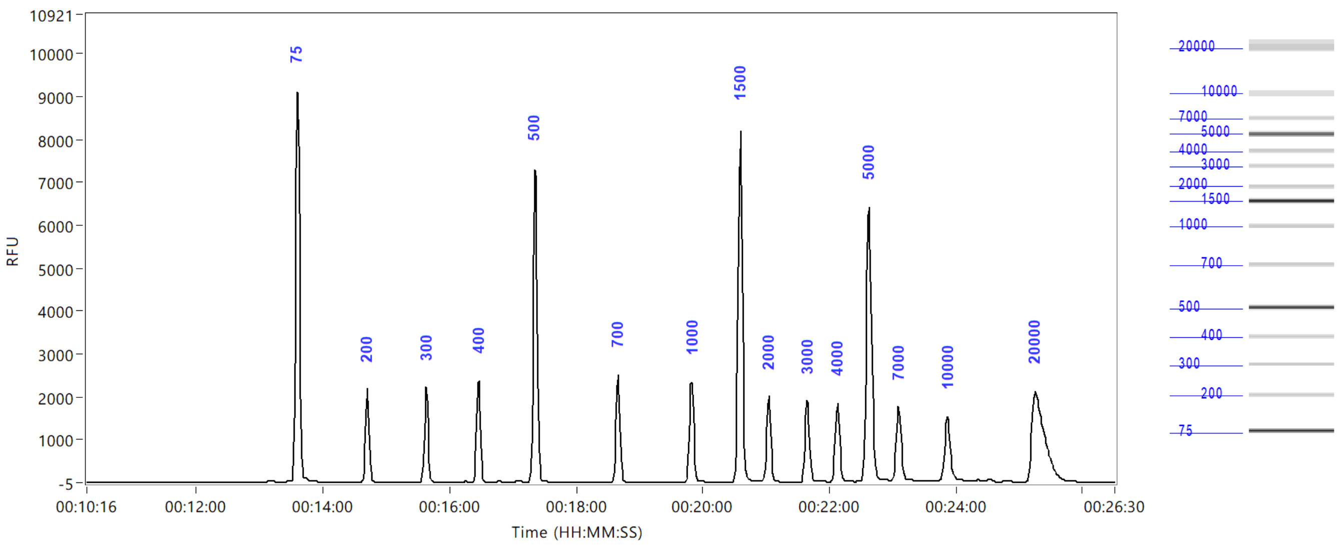

Supplement: Supplementary file 2 — sb3c00292_si_002.zip [file sb3c00292_si_002.zip › dnada_supplementary_material_pks_library_build/ZAG_trials/ZAG_Trial_5/21-24-18_plate8/plate8-t1.pdf]
