# Supplementary material for: Automated Platform for the Plasmid Construction Process
Source: ACS Synth Biol. 2023 Nov 10;12(12):3506–13. doi: 10.1021/acssynbio.3c00292 (PMC10729297; doi:10.1021/acssynbio.3c00292)

Instrument controller software run summary:

Filename and data path: C:\AATI\Data\2021 09 14\20-44-15\2021 09 14 20H 44M..raw
Created: Tuesday, September 14, 2021 8:54:07 PM
Number of capillaries: 96
Array serial number: 111920-09SFS
Effect length: 33 cm
Array usage count: 219
Instrument type: ZAG DNA Analyzer
Instrument controller software version: 1.0.1.9
Device serial number: Z7597

Method Information

Method name: ZAG130GP33 - DNA 75-20000bp - Gel Prime Only.mthds
Gel prime: Yes
Full conditioning: No
Gel prime to buffer: No
Gel selection: Gel 1
Perform prerun: 8.0 kV, 30 sec.
Rinse: No
Marker 1: Row: A, 5.0 kV, 5 sec.
Rinse: No
Sample injection: 5.0 kV, 5 sec.
Separation: 8.0 kV, 30.0 min.
Tray name: Sample Tray-7

Analysis mode: DNA

Notes

Gel Image

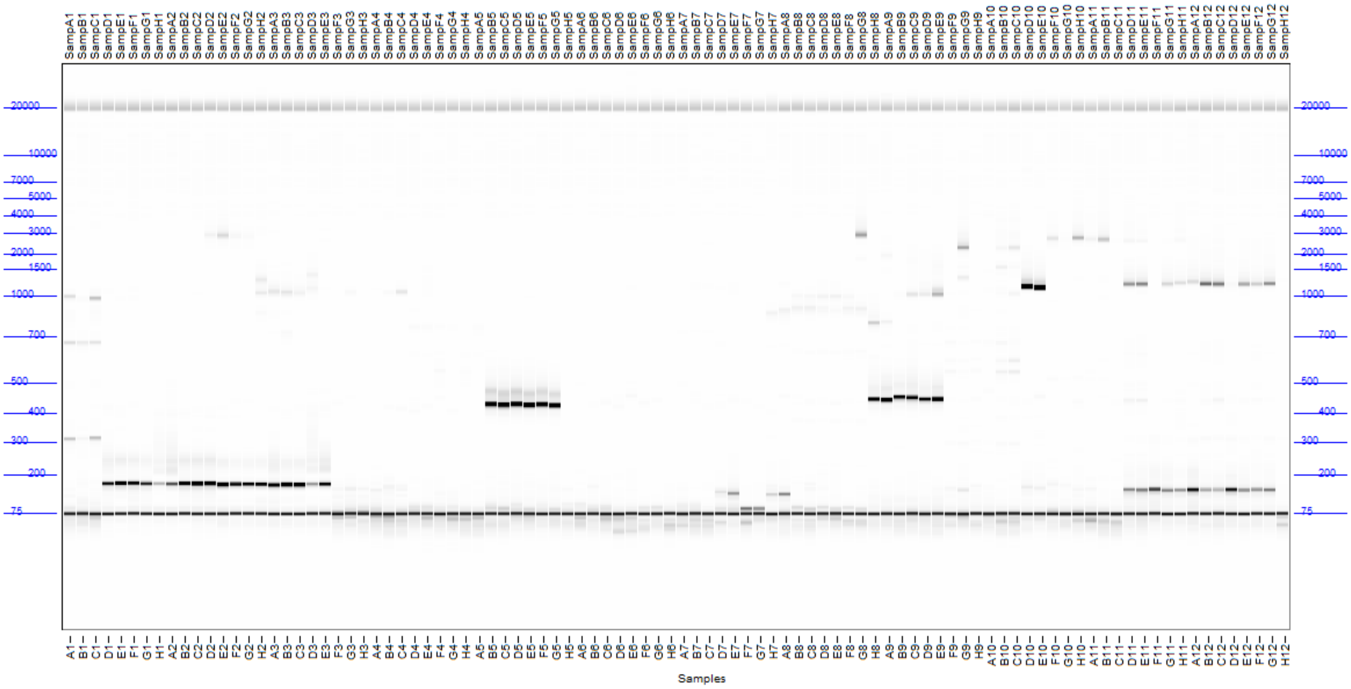
20000
10000
7000
5000
4000
3000
2000
1500
1000
700
500
400
300
200
75
Samples

Filename and data path: C:\AATI\Data\2021 09 14\20-44-15\2021 09 14 20H 44M..raw

A1: SampA1

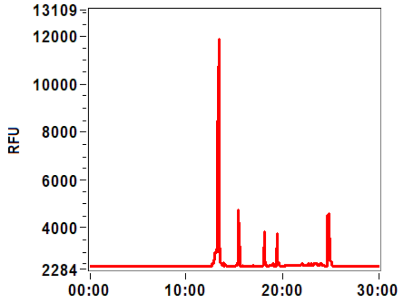

B1: SampB1

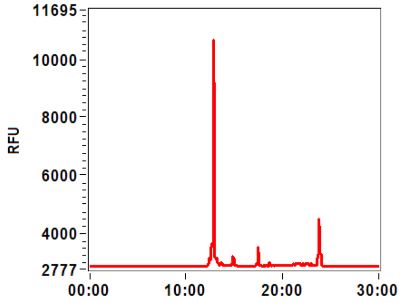

C1: SampC1

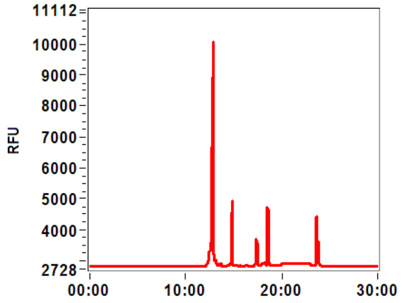

D1: SampD1

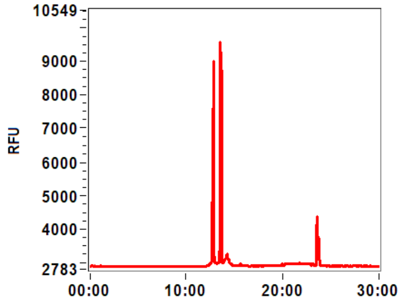

E1: SampE1

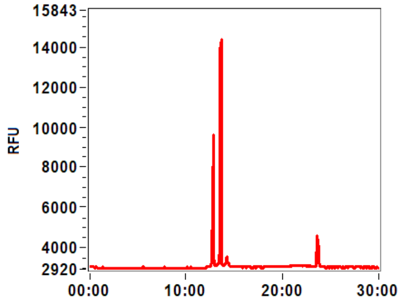

F1: SampF1

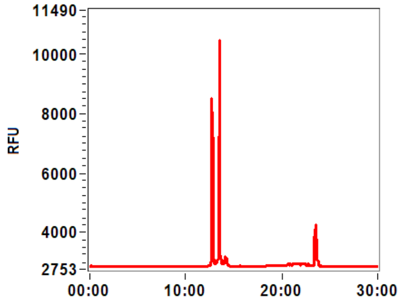

G1: SampG1

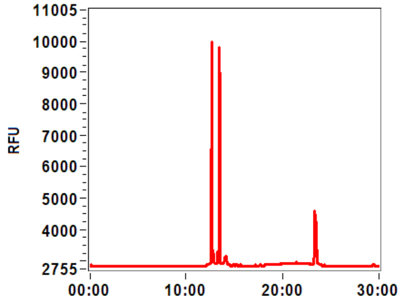

H1: SampH1

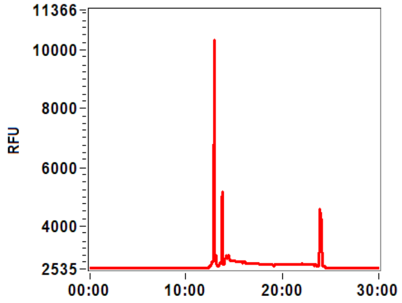

A2: SampA2

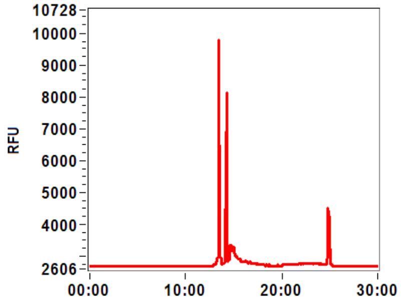

B2: SampB2

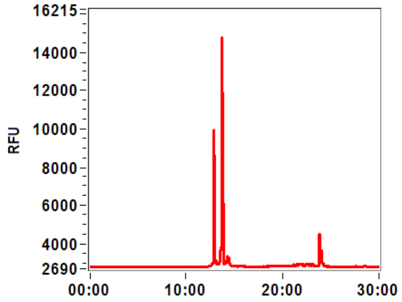

C2: SampC2

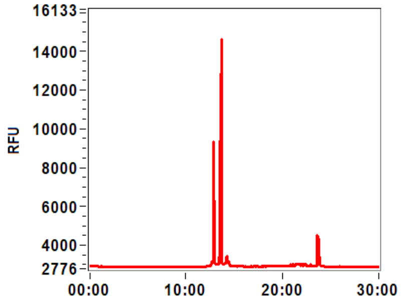

D2: SampD2

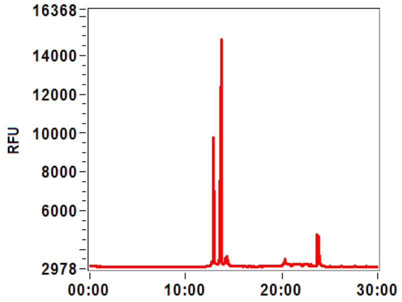

Filename and data path: C:\AATI\Data\2021 09 14\20-44-15\2021 09 14 20H 44M..raw

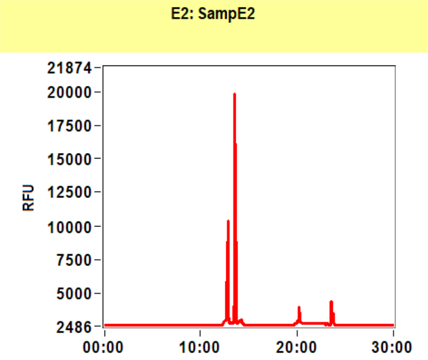

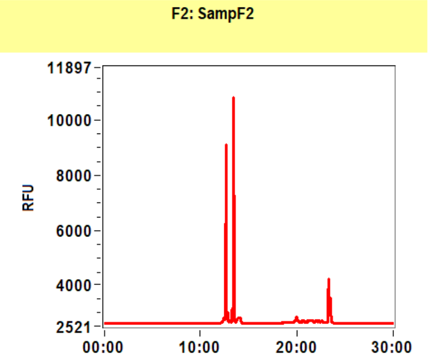

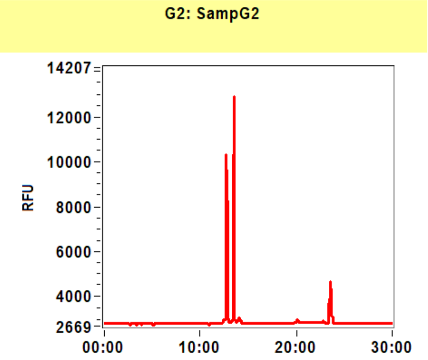

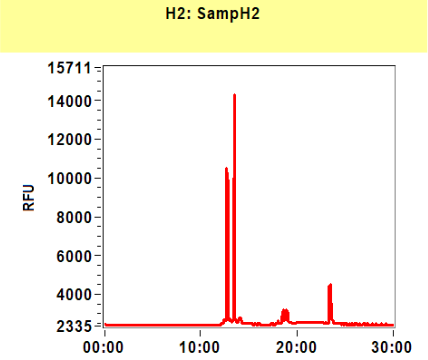

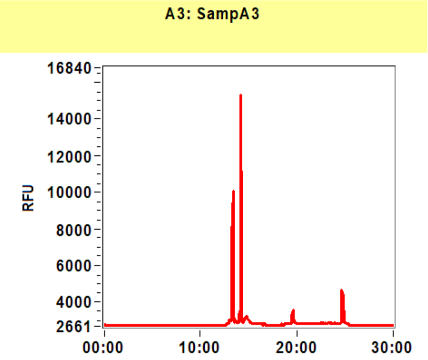

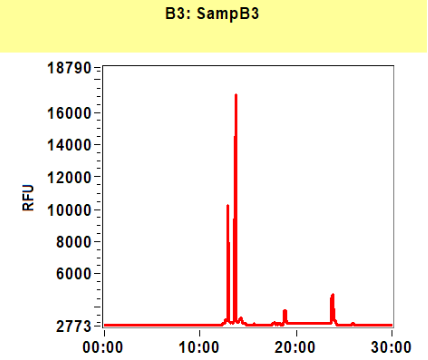

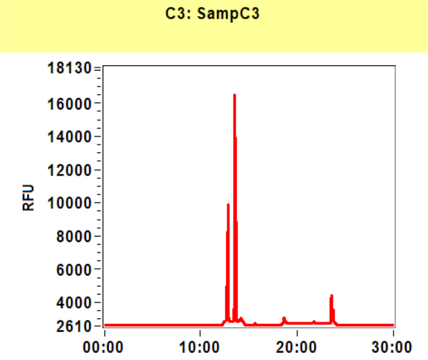

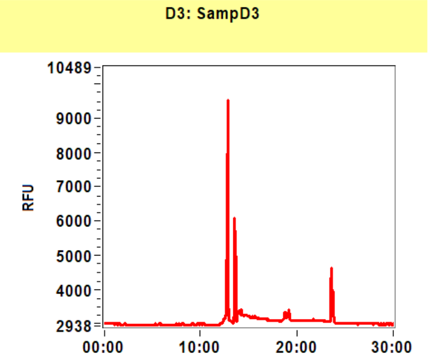

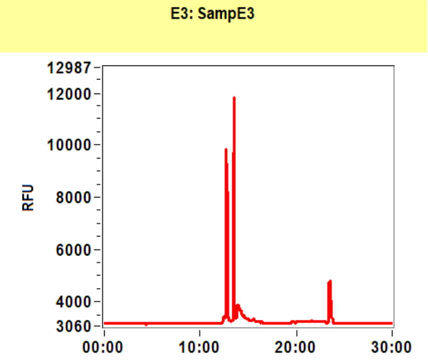

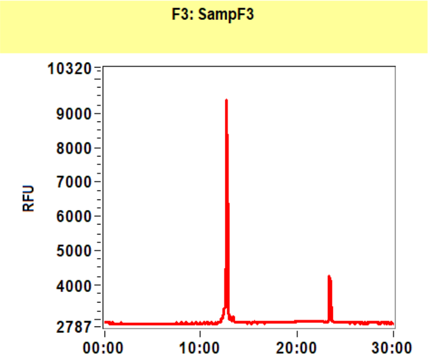

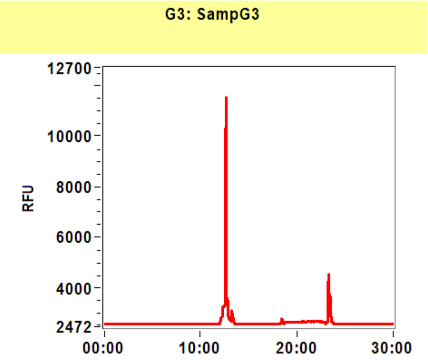

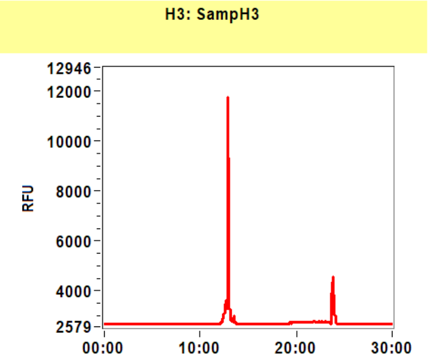

Filename and data path: C:\AATI\Data\2021 09 14\20-44-15\2021 09 14 20H 44M..raw

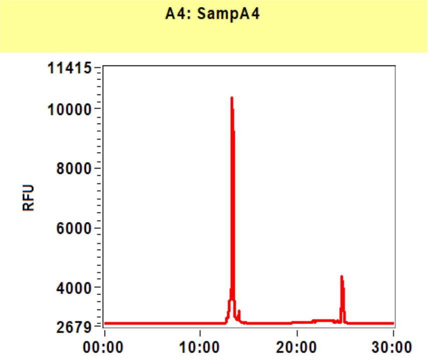

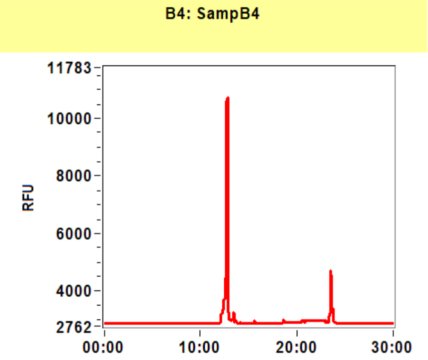

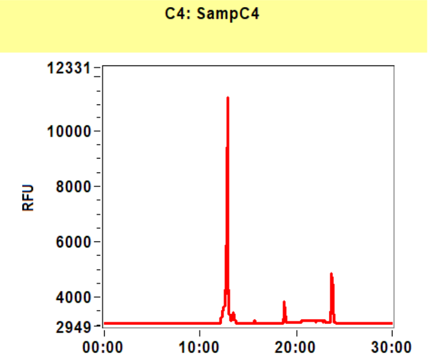

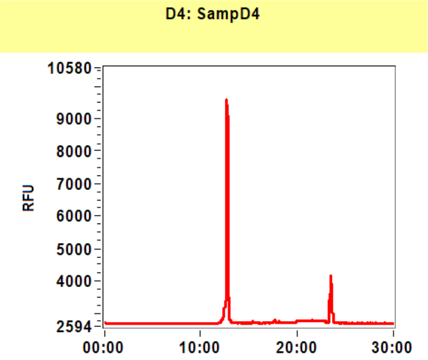

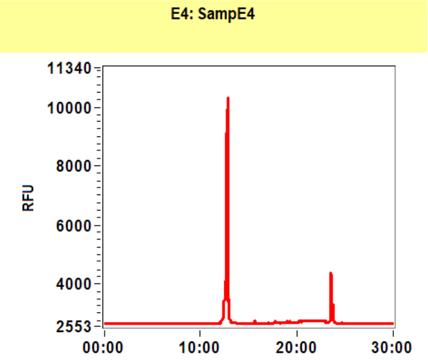

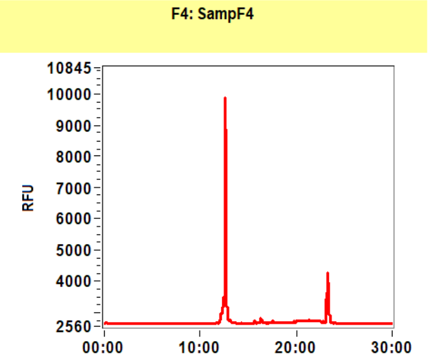

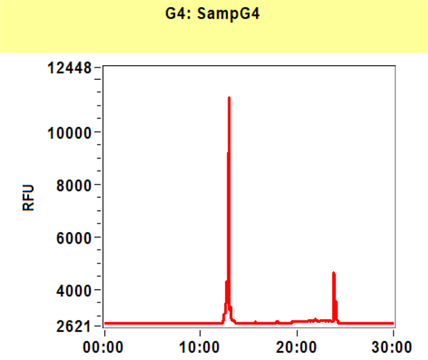

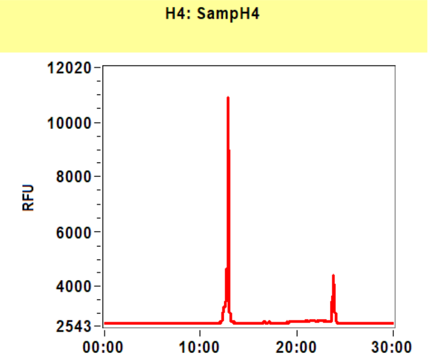

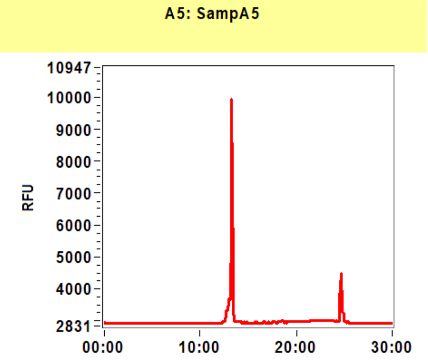

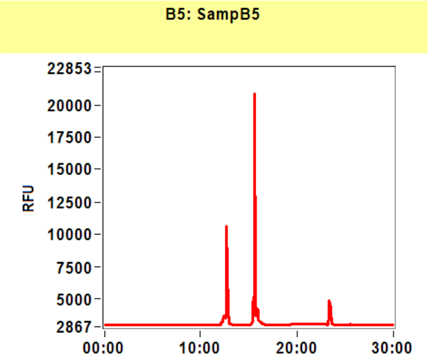

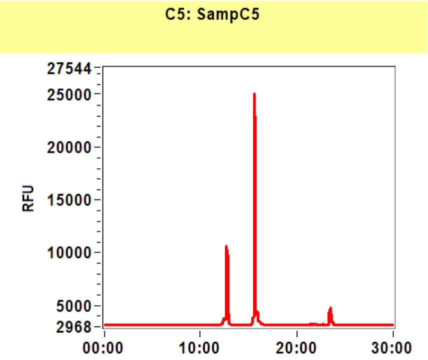

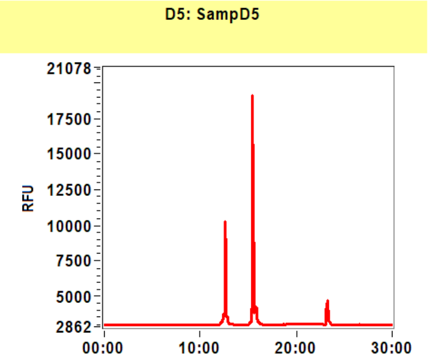

Filename and data path: C:\AATI\Data\2021 09 14\20-44-15\2021 09 14 20H 44M..raw

E5: SampE5

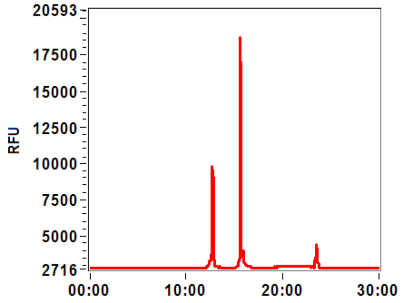

F5: SampF5

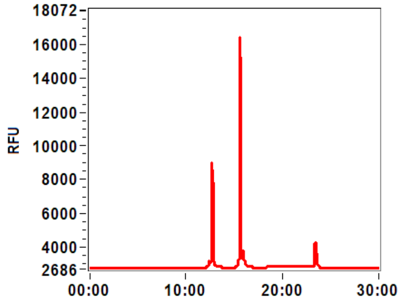

G5: SampG5

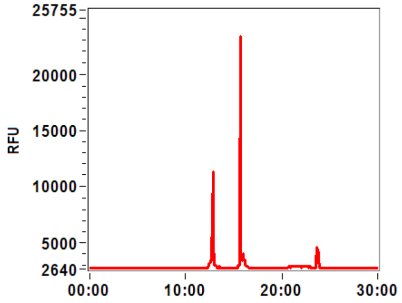

H5: SampH5

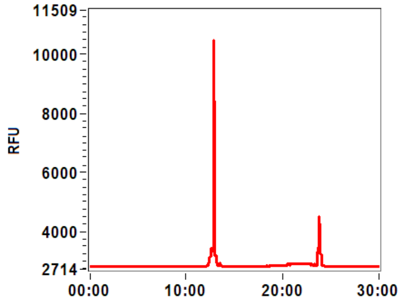

A6: SampA6

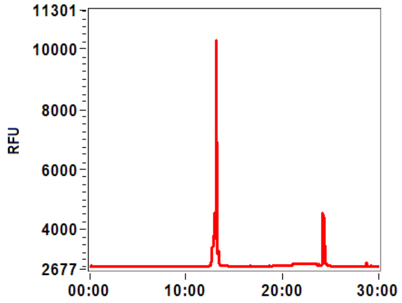

B6: SampB6

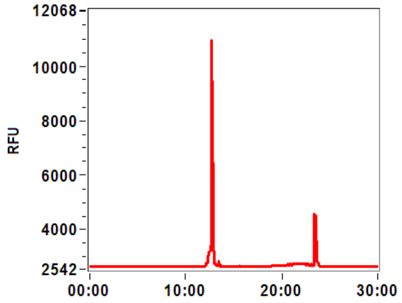

C6: SampC6

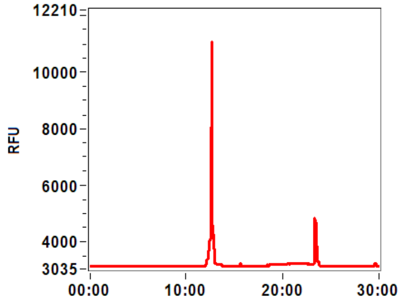

D6: SampD6

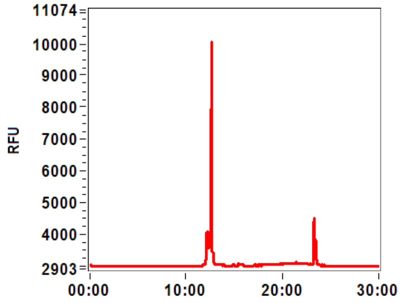

E6: SampE6

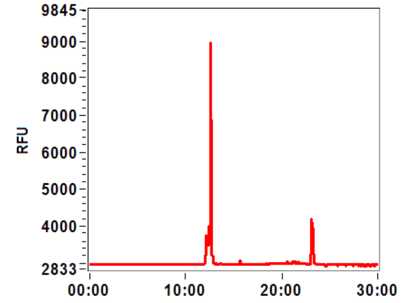

F6: SampF6

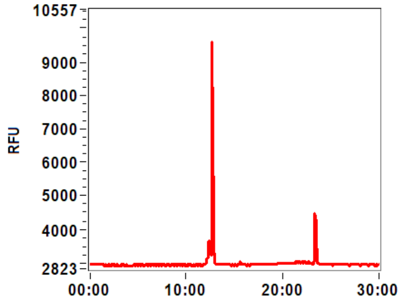

G6: SampG6

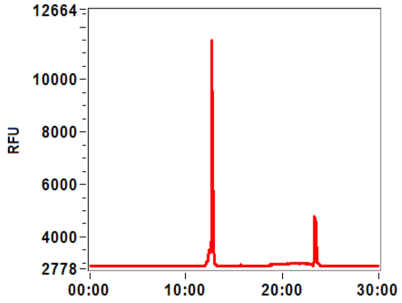

H6: SampH6

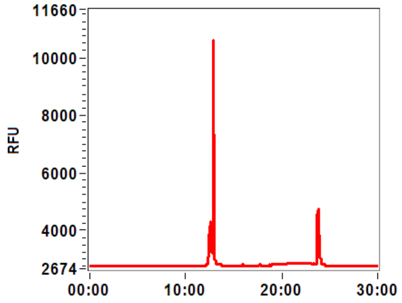

Filename and data path: C:\AATI\Data\2021 09 14\20-44-15\2021 09 14 20H 44M..raw

A7: SampA7

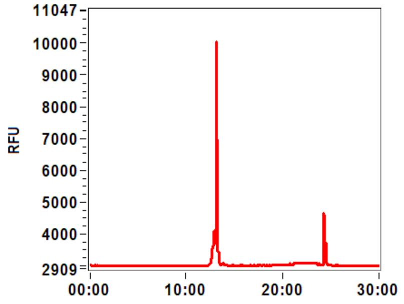

B7: SampB7

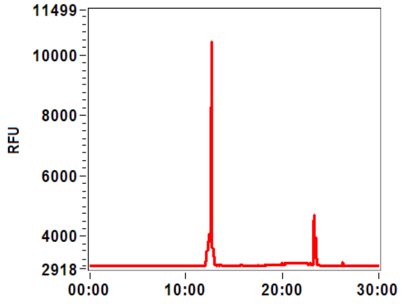

C7: SampC7

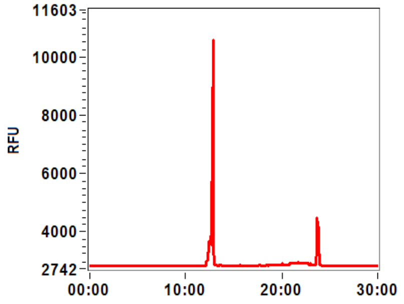

D7: SampD7

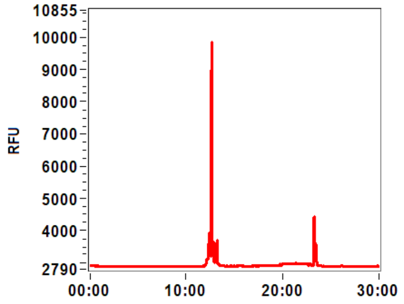

E7: SampE7

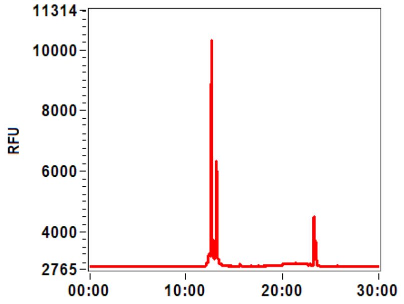

F7: SampF7

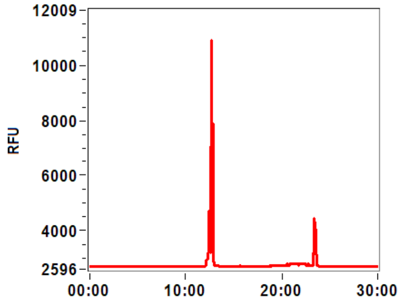

G7: SampG7

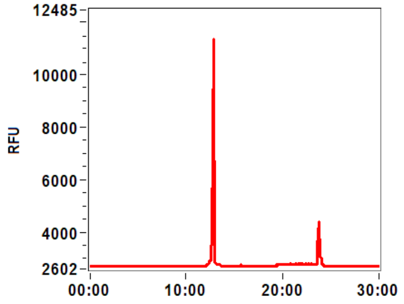

H7: SampH7

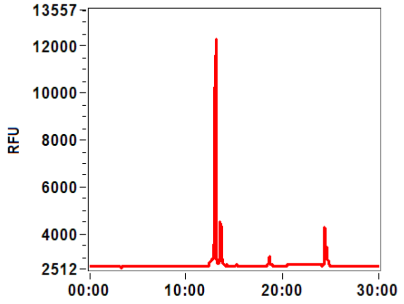

A8: SampA8

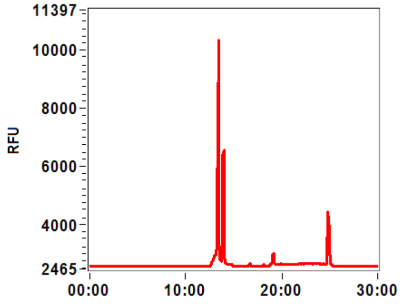

B8: SampB8

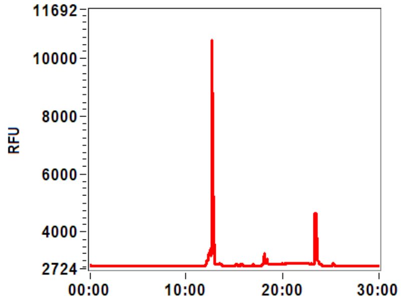

C8: SampC8

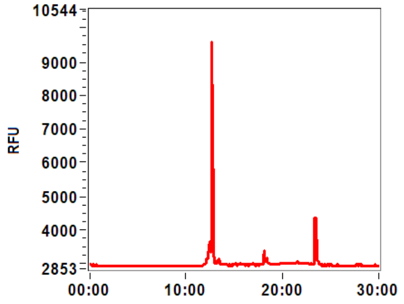

D8: SampD8

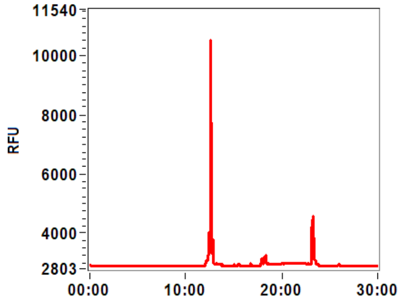

Filename and data path: C:\AATI\Data\2021 09 14\20-44-15\2021 09 14 20H 44M..raw

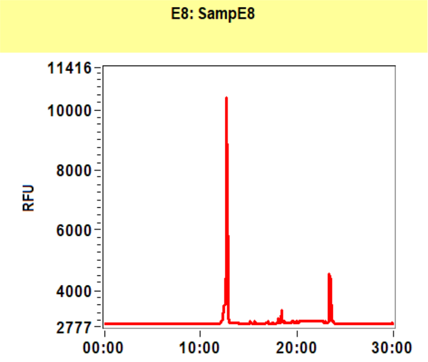

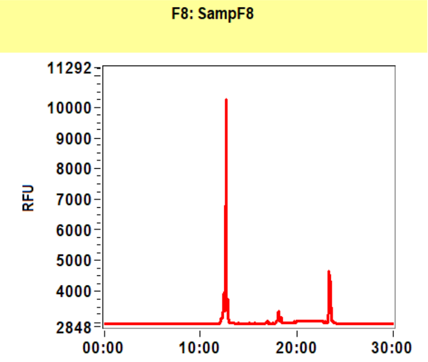

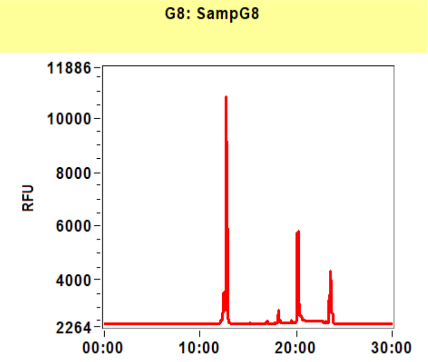

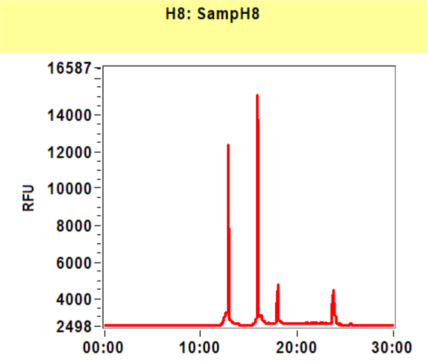

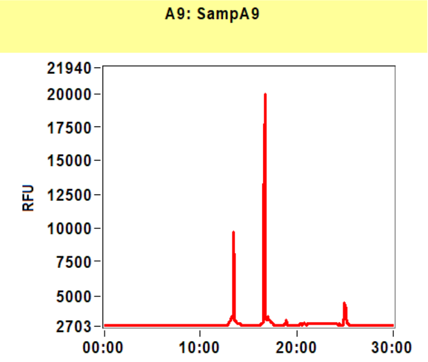

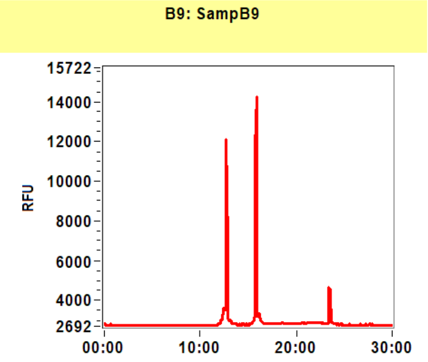

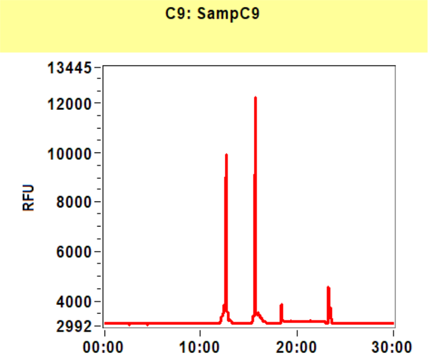

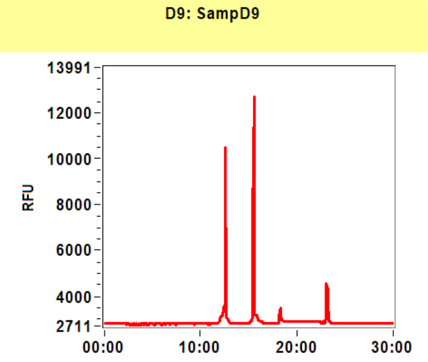

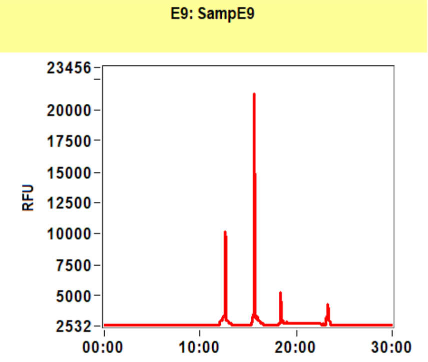

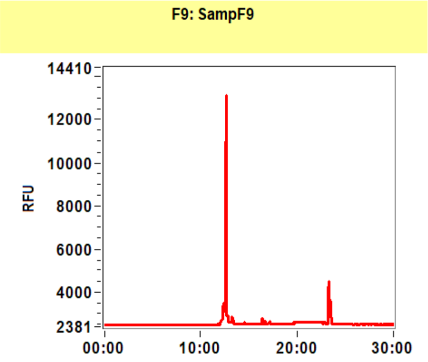

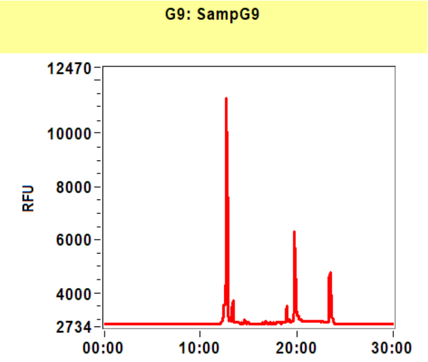

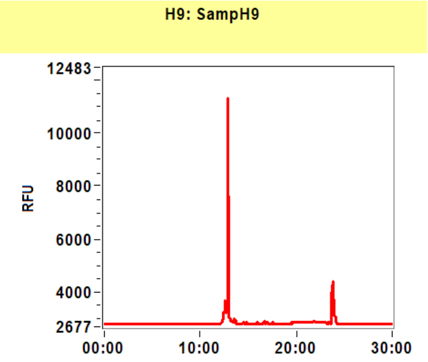

Filename and data path: C:\AATI\Data\2021 09 14\20-44-15\2021 09 14 20H 44M..raw

A10: SampA10

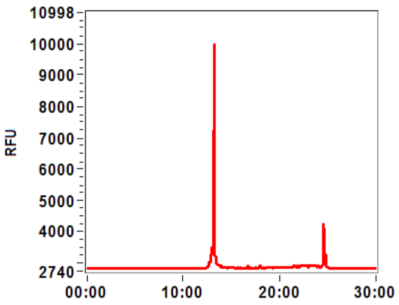

B10: SampB10

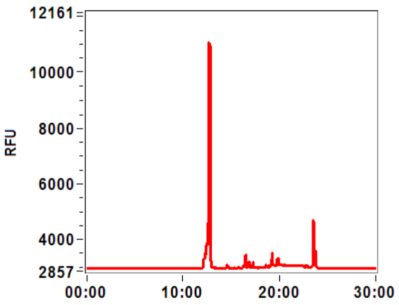

C10: SampC10

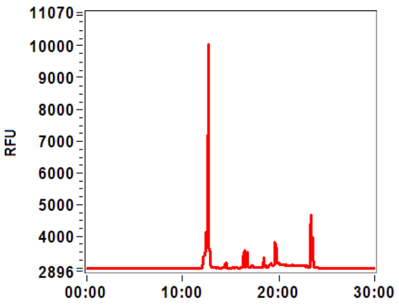

D10: SampD10

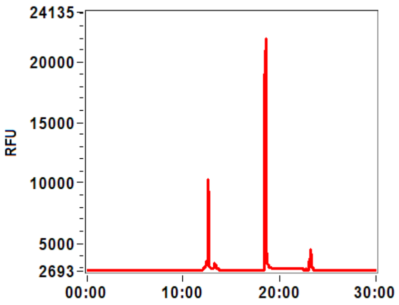

E10: SampE10

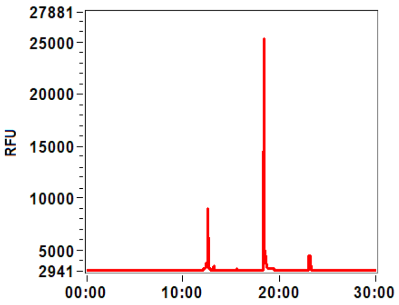

F10: SampF10

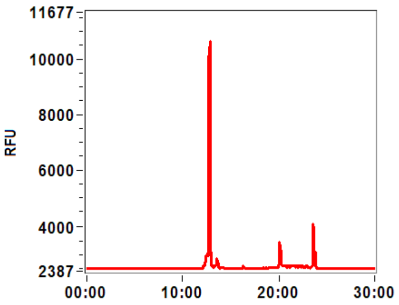

G10: SampG10

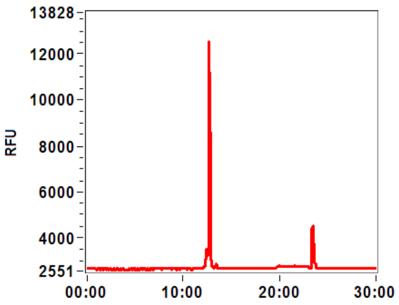

H10: SampH10

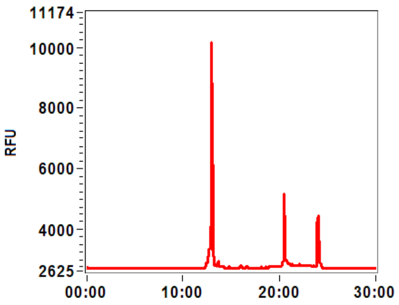

A11: SampA11

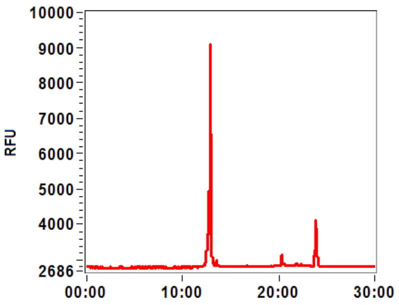

B11: SampB11

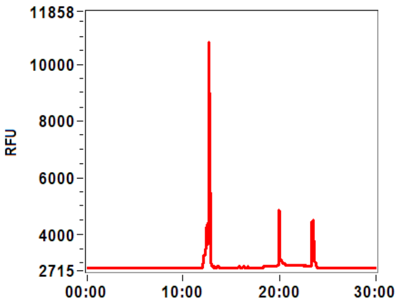

C11: SampC11

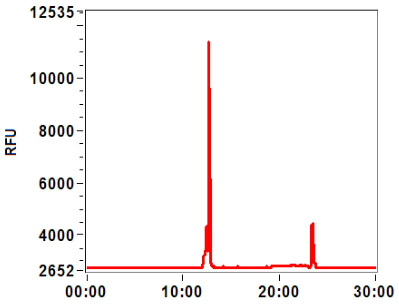

D11: SampD11

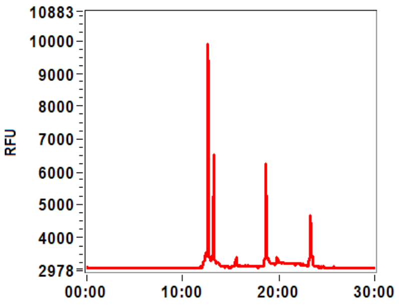

Filename and data path: C:\AATI\Data\2021 09 14\20-44-15\2021 09 14 20H 44M..raw

E11: SampE11

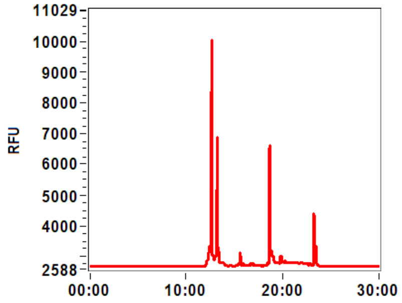

F11: SampF11

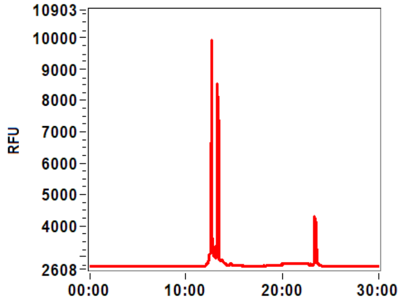

G11: SampG11

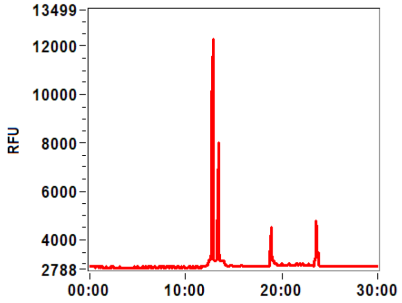

H11: SampH11

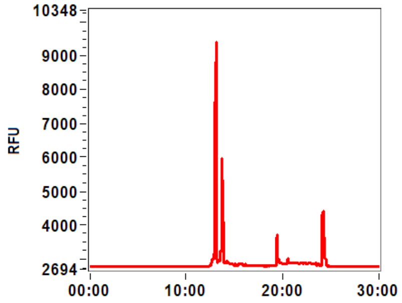

A12: SampA12

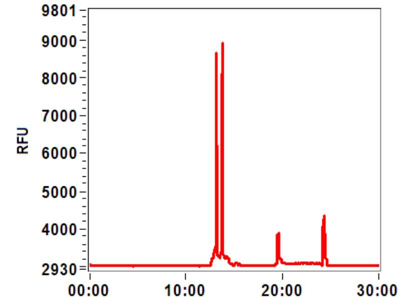

B12: SampB12

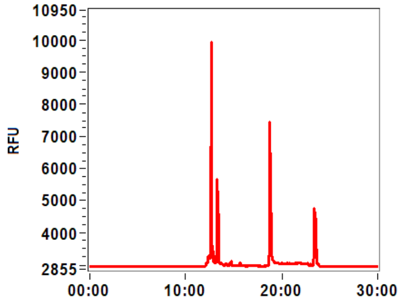

C12: SampC12

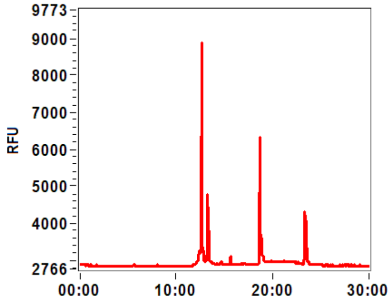

D12: SampD12

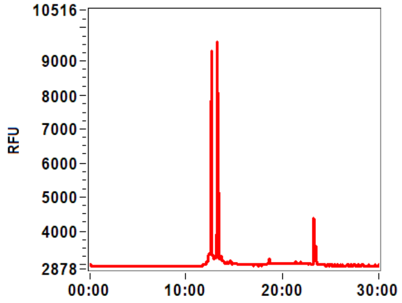

E12: SampE12

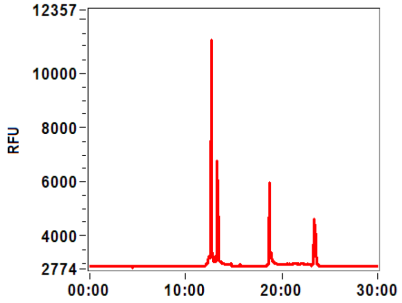

F12: SampF12

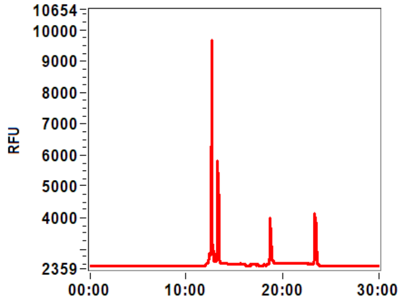

G12: SampG12

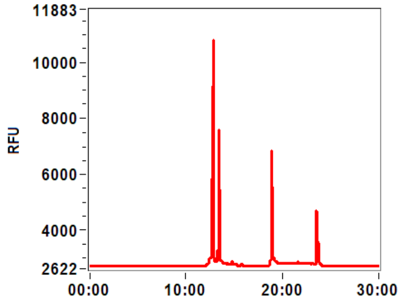

H12: SampH12

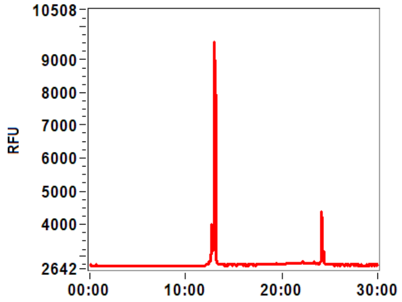

Sample: SampA1
Well location: A1
Created: Tuesday, September 14, 2021 8:54:07 PM

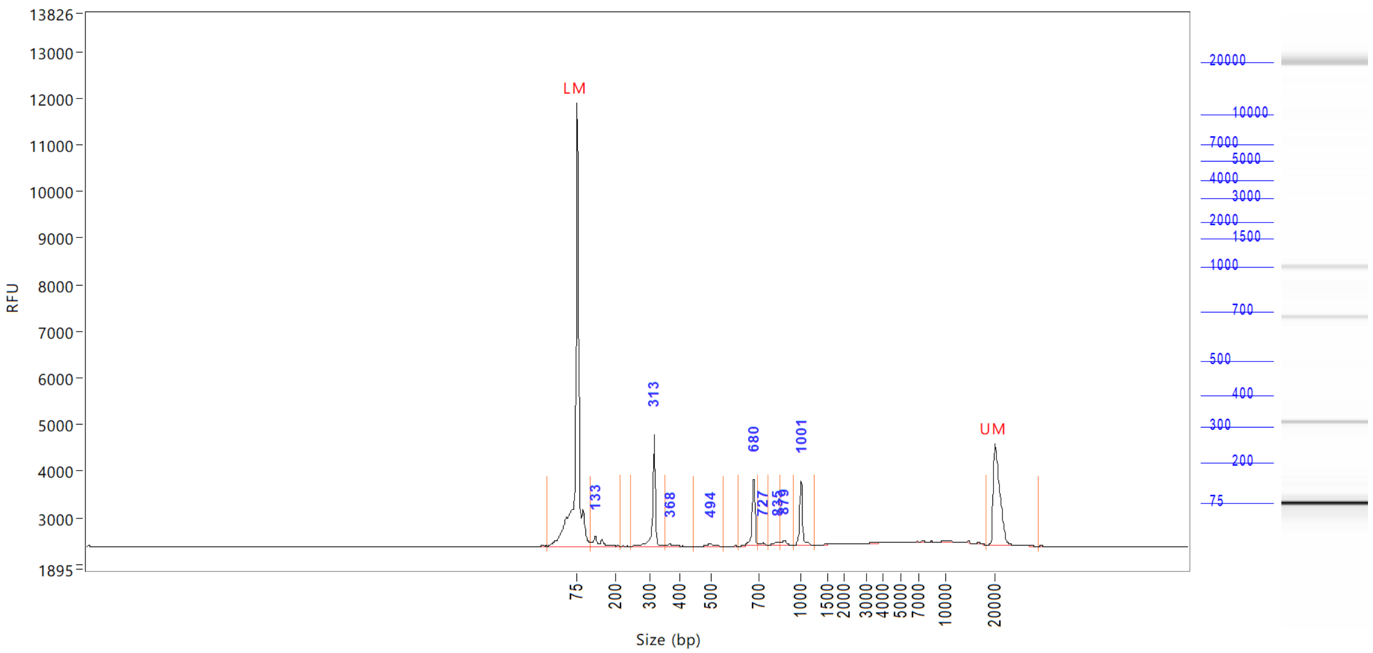

Peak	Size (bp)	Concentration (ng/uL)	From (bp)	To (bp)	Average size (bp)	CV%	RFU	Corrected peak area
1	75 (LM)	0.5000	0	120	67	28.77	9535	83.369
2	133	0.2431	120	214	144	12.50	217	3.378
3	313	0.9872	244	349	308	3.71	2403	13.718
4	368	0.0530	349	443	374	5.45	50	0.736
5	494	0.0534	443	552	492	3.40	55	0.742
6	680	0.5203	611	697	673	1.43	1431	7.229
7	727	0.0375	697	762	722	2.25	57	0.521
8	835	0.0621	762	853	819	2.85	78	0.862
9	879	0.0618	853	944	876	1.77	93	0.859
10	1001	0.4802	944	1256	1006	2.94	1379	6.673
11	20000 (UM)	0.1103	18293	28826	20391	3.78	2185	18.385

TIC:	2.4986	ng/uL
TIM:	10.8430	nmole/L
Total concentration:	2.6532	ng/uL

```
Sample peak width (sec): 5    Sample min peak height: 50    Sample baseline V to V?: Y    Sample baseline V to V points: 3
Sample filter: Binomial       Number of points for filter: 3  Sample start region (min): 0  Sample end region (min): 30
Marker peak width (sec): 5    Marker min peak height: 200   Marker baseline V to V?: Y    Marker baseline V to V points: 3
Lower marker selection: First peak > 500 RFU                Upper marker selection: Last peak > 200 RFU
Ladder size (bp)75, 200, 300, 400, 500, 700, 1000, 1500, 2000, 3000, 4000, 5000, 7000, 10000, 20000
Quantification using: Lower Marker          Final concentration (ng/uL): 0.5000          Dilution factor: 12.0
```

Sample: SampB1
Well location: B1
Created: Tuesday, September 14, 2021 8:54:07 PM

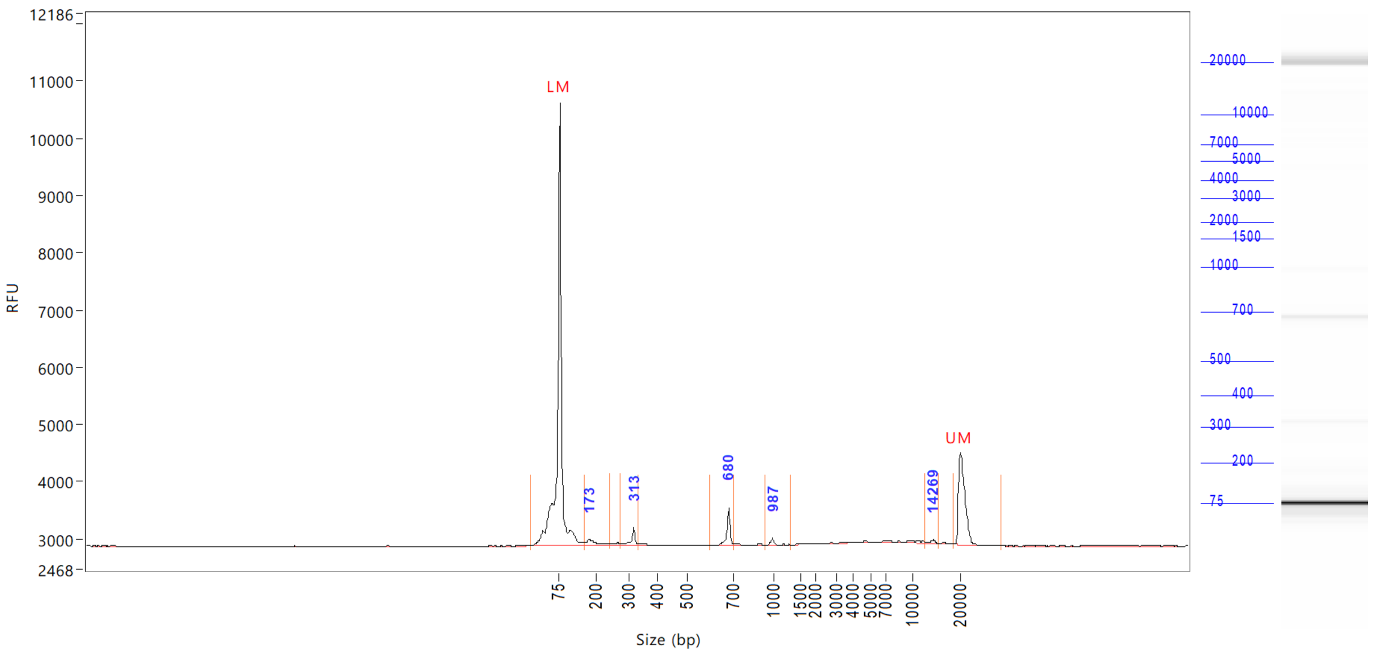

Peak	Size (bp)	Concentration (ng/uL)	From (bp)	To (bp)	Average size (bp)	CV%	RFU	Corrected peak area
1	75 (LM)	0.5000	0	160	69	31.59	7747	76.681
2	173	0.1400	160	241	184	10.12	100	1.789
3	313	0.1814	271	331	306	4.03	303	2.319
4	680	0.3112	598	700	671	1.91	664	3.977
5	987	0.0577	939	1323	1004	6.28	116	0.737
6	14269	0.0410	12562	15366	13827	4.62	68	0.524
7	20000 (UM)	0.0885	18659	28463	20434	3.79	1604	13.575

TIC:	0.7312	ng/uL
TIM:	3.0884	nmole/L
Total concentration:	1.0255	ng/uL

Sample peak width (sec): 5 Sample min peak height: 50 Sample baseline V to V?: Y Sample baseline V to V points: 3
Sample filter: Binomial Number of points for filter: 3 Sample start region (min): 0 Sample end region (min): 30
Marker peak width (sec): 5 Marker min peak height: 200 Marker baseline V to V?: Y Marker baseline V to V points: 3
Lower marker selection: First peak > 500 RFU Upper marker selection: Last peak > 200 RFU
Ladder size (bp)75, 200, 300, 400, 500, 700, 1000, 1500, 2000, 3000, 4000, 5000, 7000, 10000, 20000
Quantification using: Lower Marker Final concentration (ng/uL): 0.5000 Dilution factor: 12.0

Sample: SampC1
Well location: C1
Created: Tuesday, September 14, 2021 8:54:07 PM

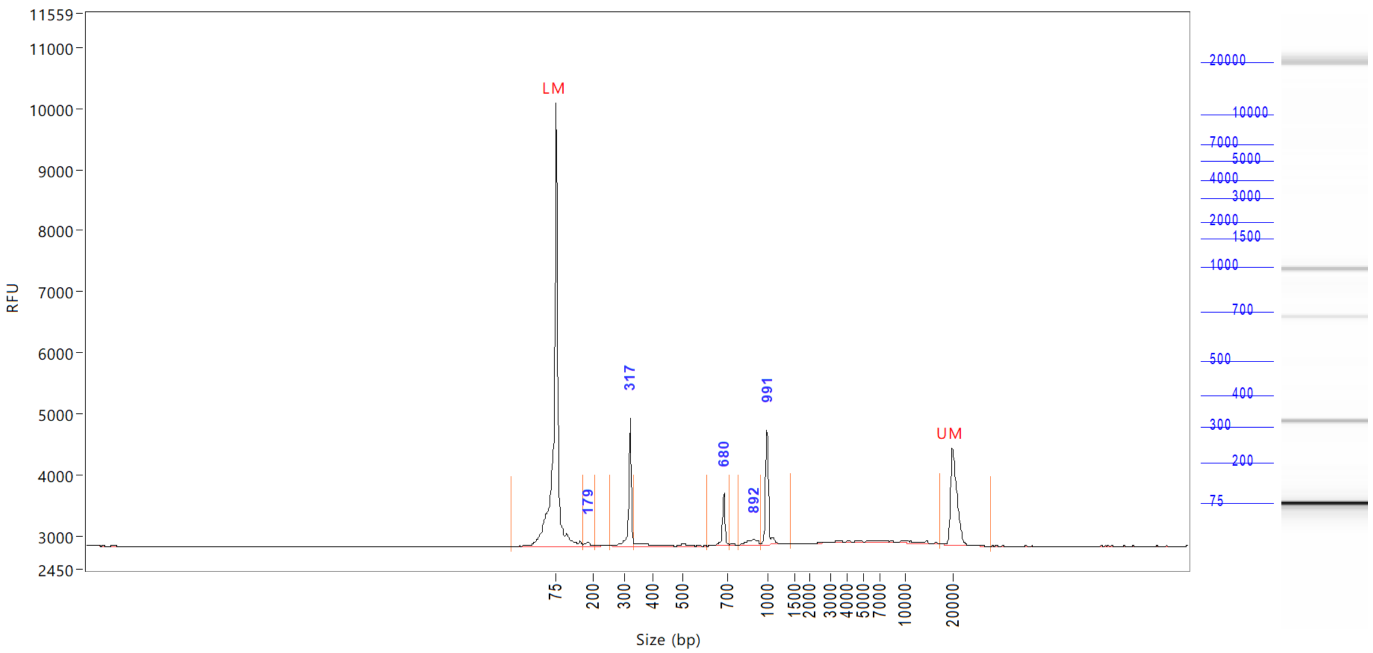

Peak	Size (bp)	Concentration (ng/uL)	From (bp)	To (bp)	Average size (bp)	CV%	RFU	Corrected peak area
1	75 (LM)	0.5000	0	162	67	33.20	7276	74.036
2	179	0.0638	162	203	177	4.80	72	0.787
3	317	0.9788	250	331	311	3.37	2097	12.078
4	680	0.3492	606	705	675	1.46	863	4.308
5	892	0.1349	770	944	868	4.57	87	1.665
6	991	0.7705	944	1445	998	4.63	1876	9.507
7	20000 (UM)	0.0925	17317	28100	20410	3.84	1592	13.699

TIC:	2.2972	ng/uL
TIM:	8.1476	nmole/L
Total concentration:	2.6859	ng/uL

```
Sample peak width (sec): 5     Sample min peak height: 50     Sample baseline V to V?: Y     Sample baseline V to V points: 3
Sample filter: Binomial        Number of points for filter: 3  Sample start region (min): 0  Sample end region (min): 30
Marker peak width (sec): 5     Marker min peak height: 200    Marker baseline V to V?: Y     Marker baseline V to V points: 3
Lower marker selection: First peak > 500 RFU                  Upper marker selection: Last peak > 200 RFU
Ladder size (bp)75, 200, 300, 400, 500, 700, 1000, 1500, 2000, 3000, 4000, 5000, 7000, 10000, 20000
Quantification using: Lower Marker          Final concentration (ng/uL): 0.5000          Dilution factor: 12.0
```

Sample: SampD1
Well location: D1
Created: Tuesday, September 14, 2021 8:54:07 PM

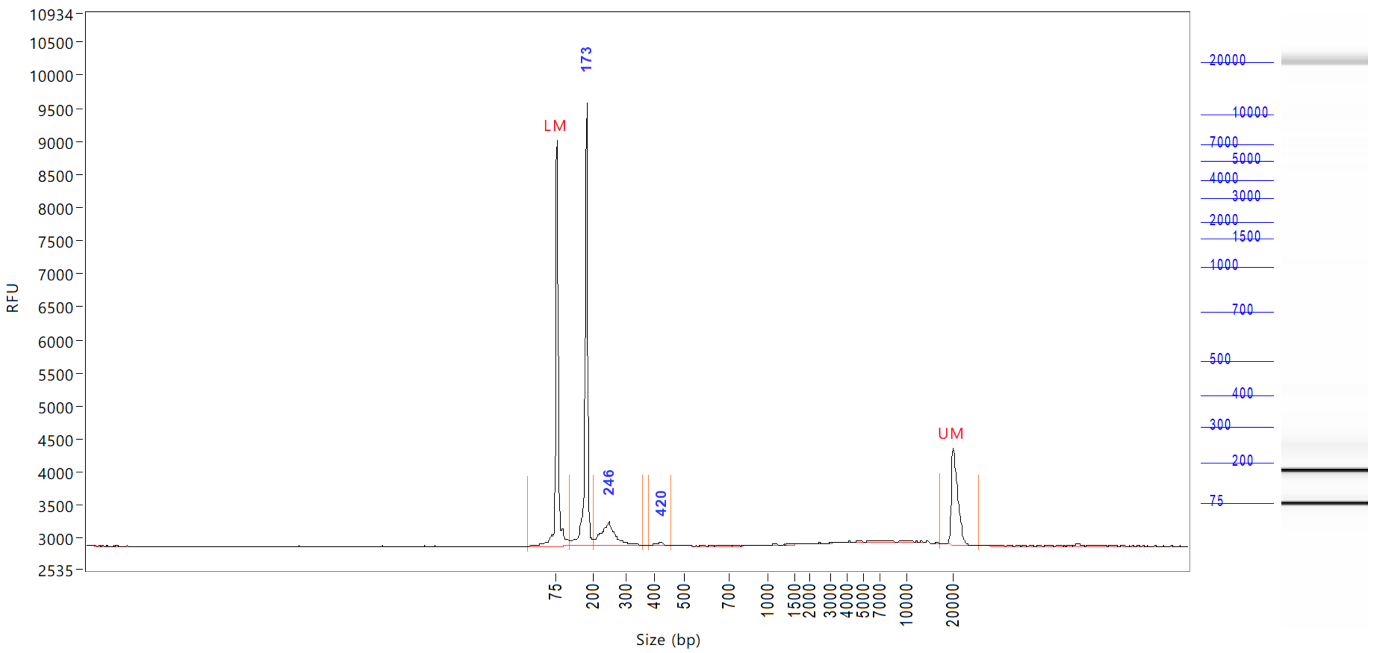

Peak	Size (bp)	Concentration (ng/uL)	From (bp)	To (bp)	Average size (bp)	CV%	RFU	Corrected peak area
1	75 (LM)	0.5000	0	118	72	18.13	6126	40.225
2	173	6.3888	118	200	169	6.08	6701	42.831
3	246	1.6819	200	360	246	11.67	356	11.276
4	420	0.1003	380	454	416	3.28	50	0.673
5	20000 (UM)	0.1568	17317	25440	20434	3.63	1448	12.616

TIC:	8.1710	ng/uL
TIM:	73.7835	nmole/L
Total concentration:	8.5220	ng/uL

Sample peak width (sec): 5 Sample min peak height: 50 Sample baseline V to V?: Y Sample baseline V to V points: 3
Sample filter: Binomial Number of points for filter: 3 Sample start region (min): 0 Sample end region (min): 30
Marker peak width (sec): 5 Marker min peak height: 200 Marker baseline V to V?: Y Marker baseline V to V points: 3
Lower marker selection: First peak > 500 RFU Upper marker selection: Last peak > 200 RFU
Ladder size (bp)75, 200, 300, 400, 500, 700, 1000, 1500, 2000, 3000, 4000, 5000, 7000, 10000, 20000
Quantification using: Lower Marker Final concentration (ng/uL): 0.5000 Dilution factor: 12.0

Sample: SampE1
Well location: E1
Created: Tuesday, September 14, 2021 8:54:07 PM

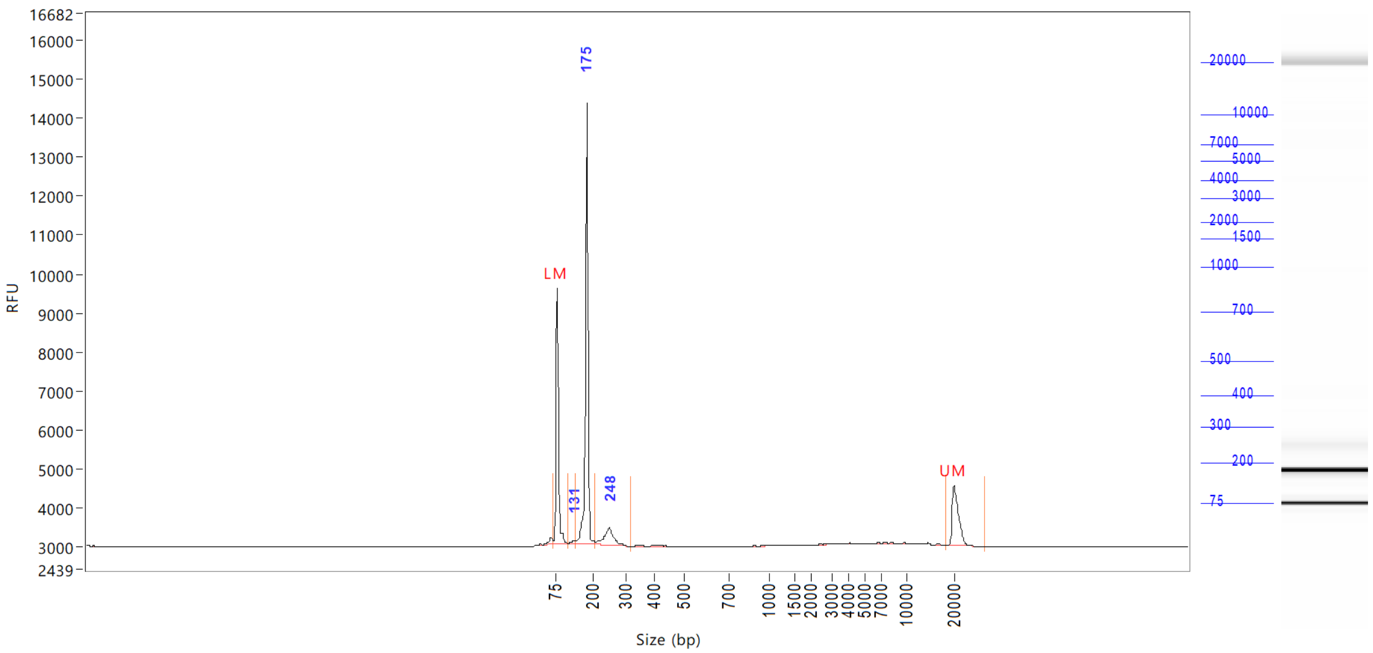

Peak	Size (bp)	Concentration (ng/uL)	From (bp)	To (bp)	Average size (bp)	CV%	RFU	Corrected peak area
1	75 (LM)	0.5000	64	110	74	6.93	6546	37.761
2	131	0.1327	110	137	128	4.93	85	0.835
3	175	10.8755	137	203	171	3.95	11317	68.445
4	248	1.4712	203	317	243	7.29	455	9.259
5	20000 (UM)	0.1722	18293	26529	20368	3.61	1535	13.002

TIC:	12.4794	ng/uL
TIM:	116.2090	nmole/L
Total concentration:	12.9766	ng/uL

Sample peak width (sec): 5   Sample min peak height: 50   Sample baseline V to V?: Y   Sample baseline V to V points: 3
Sample filter: Binomial   Number of points for filter: 3   Sample start region (min): 0   Sample end region (min): 30
Marker peak width (sec): 5   Marker min peak height: 200   Marker baseline V to V?: Y   Marker baseline V to V points: 3
Lower marker selection: First peak > 500 RFU   Upper marker selection: Last peak > 200 RFU
Ladder size (bp)75, 200, 300, 400, 500, 700, 1000, 1500, 2000, 3000, 4000, 5000, 7000, 10000, 20000
Quantification using: Lower Marker   Final concentration (ng/uL): 0.5000   Dilution factor: 12.0

Sample: SampF1
Well location: F1
Created: Tuesday, September 14, 2021 8:54:07 PM

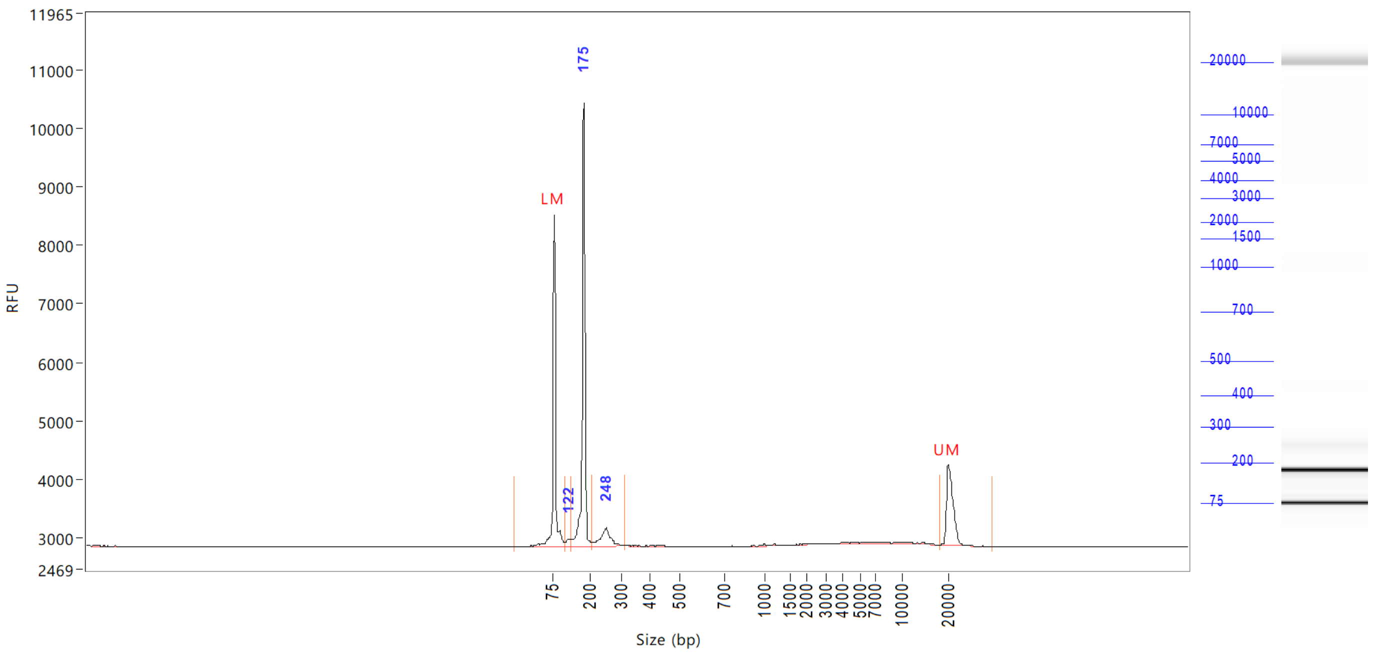

Peak	Size (bp)	Concentration (ng/uL)	From (bp)	To (bp)	Average size (bp)	CV%	RFU	Corrected peak area
1	75 (LM)	0.5000	0	112	73	18.47	5649	38.134
2	122	0.2422	112	133	123	5.13	115	1.540
3	175	7.5665	133	205	171	5.01	7585	48.090
4	248	1.0726	205	309	243	7.47	311	6.817
5	20000 (UM)	0.1571	18049	29430	20394	4.23	1385	11.984

TIC:	8.8813	ng/uL
TIM:	83.2334	nmole/L
Total concentration:	9.5319	ng/uL

Sample peak width (sec): 5   Sample min peak height: 50   Sample baseline V to V?: Y   Sample baseline V to V points: 3
Sample filter: Binomial   Number of points for filter: 3   Sample start region (min): 0   Sample end region (min): 30
Marker peak width (sec): 5   Marker min peak height: 200   Marker baseline V to V?: Y   Marker baseline V to V points: 3
Lower marker selection: First peak > 500 RFU   Upper marker selection: Last peak > 200 RFU
Ladder size (bp)75, 200, 300, 400, 500, 700, 1000, 1500, 2000, 3000, 4000, 5000, 7000, 10000, 20000
Quantification using: Lower Marker   Final concentration (ng/uL): 0.5000   Dilution factor: 12.0

Sample: SampG1
Well location: G1
Created: Tuesday, September 14, 2021 8:54:07 PM

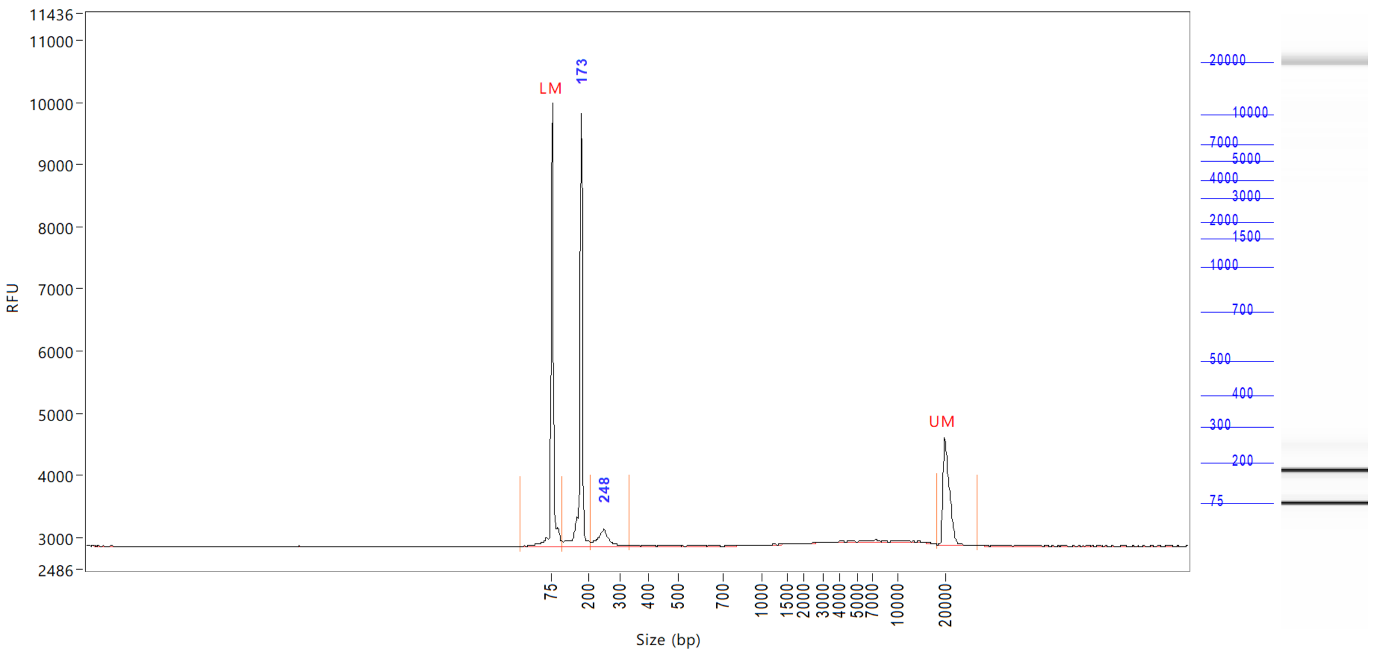

Peak	Size (bp)	Concentration (ng/uL)	From (bp)	To (bp)	Average size (bp)	CV%	RFU	Corrected peak area
1	75 (LM)	0.5000	0	112	73	16.42	7149	44.642
2	173	6.0450	112	205	169	6.65	6963	44.976
3	248	0.8858	205	333	243	8.61	284	6.591
4	20000 (UM)	0.1680	18415	27133	20396	3.95	1719	15.000

TIC:	6.9308	ng/uL
TIM:	64.7933	nmole/L
Total concentration:	7.3736	ng/uL

Sample peak width (sec): 5 Sample min peak height: 50 Sample baseline V to V?: Y Sample baseline V to V points: 3
Sample filter: Binomial Number of points for filter: 3 Sample start region (min): 0 Sample end region (min): 30
Marker peak width (sec): 5 Marker min peak height: 200 Marker baseline V to V?: Y Marker baseline V to V points: 3
Lower marker selection: First peak > 500 RFU Upper marker selection: Last peak > 200 RFU
Ladder size (bp)75, 200, 300, 400, 500, 700, 1000, 1500, 2000, 3000, 4000, 5000, 7000, 10000, 20000
Quantification using: Lower Marker Final concentration (ng/uL): 0.5000 Dilution factor: 12.0

Sample: SampH1
Well location: H1
Created: Tuesday, September 14, 2021 8:54:07 PM

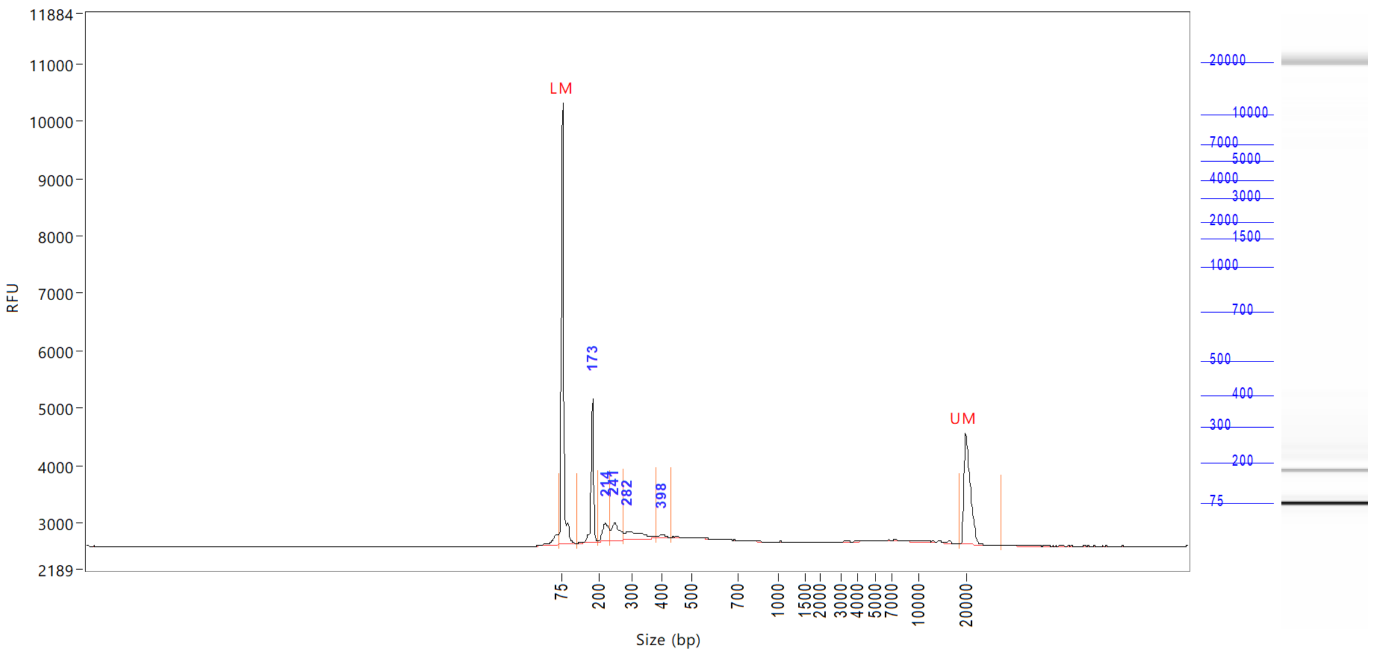

Peak	Size (bp)	Concentration (ng/uL)	From (bp)	To (bp)	Average size (bp)	CV%	RFU	Corrected peak area
1	75 (LM)	0.5000	64	122	74	7.99	7709	45.439
2	173	1.9241	122	192	170	3.93	2509	14.572
3	214	0.6212	192	232	215	4.24	306	4.705
4	241	0.7170	232	273	249	4.70	316	5.430
5	282	0.7000	273	382	312	9.23	141	5.301
6	398	0.1043	382	430	398	2.45	55	0.790
7	20000 (UM)	0.1802	18659	27496	20394	3.63	1943	16.378

TIC:	4.0666	ng/uL
TIM:	32.2143	nmole/L
Total concentration:	4.3431	ng/uL

Sample peak width (sec): 5   Sample min peak height: 50   Sample baseline V to V?: Y   Sample baseline V to V points: 3
Sample filter: Binomial   Number of points for filter: 3 Sample start region (min): 0 Sample end region (min): 30
Marker peak width (sec): 5   Marker min peak height: 200   Marker baseline V to V?: Y   Marker baseline V to V points: 3
Lower marker selection: First peak > 500 RFU   Upper marker selection: Last peak > 200 RFU
Ladder size (bp)75, 200, 300, 400, 500, 700, 1000, 1500, 2000, 3000, 4000, 5000, 7000, 10000, 20000
Quantification using: Lower Marker   Final concentration (ng/uL): 0.5000   Dilution factor: 12.0

Sample: SampA2
Well location: A2
Created: Tuesday, September 14, 2021 8:54:07 PM

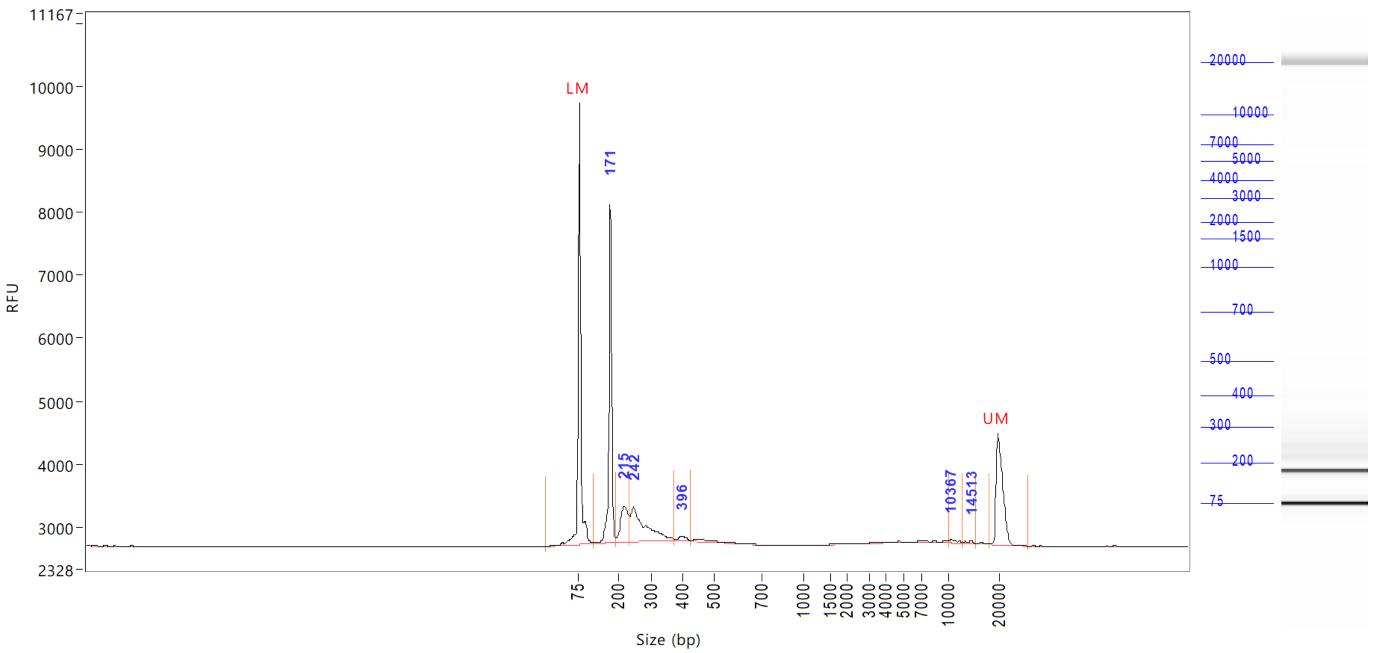

Peak	Size (bp)	Concentration (ng/uL)	From (bp)	To (bp)	Average size (bp)	CV%	RFU	Corrected peak area
1	75 (LM)	0.5000	0	122	72	15.95	7033	44.534
2	171	4.3587	122	189	169	4.15	5368	32.351
3	215	1.3369	189	232	214	4.86	573	9.923
4	242	2.2986	232	372	272	12.24	551	17.061
5	396	0.1821	372	426	397	3.14	78	1.352
6	10367	0.0770	10000	12806	11075	7.44	54	0.572
7	14513	0.0569	12806	15366	13987	4.59	53	0.423
8	20000 (UM)	0.1649	18293	26045	20332	3.46	1783	14.688

TIC:	8.3102	ng/uL
TIM:	67.3413	nmole/L
Total concentration:	8.7075	ng/uL

Sample peak width (sec): 5 Sample min peak height: 50 Sample baseline V to V?: Y Sample baseline V to V points: 3
Sample filter: Binomial Number of points for filter: 3 Sample start region (min): 0 Sample end region (min): 30
Marker peak width (sec): 5 Marker min peak height: 200 Marker baseline V to V?: Y Marker baseline V to V points: 3
Lower marker selection: First peak > 500 RFU Upper marker selection: Last peak > 200 RFU
Ladder size (bp)75, 200, 300, 400, 500, 700, 1000, 1500, 2000, 3000, 4000, 5000, 7000, 10000, 20000
Quantification using: Lower Marker Final concentration (ng/uL): 0.5000 Dilution factor: 12.0

Sample: SampB2
Well location: B2
Created: Tuesday, September 14, 2021 8:54:07 PM

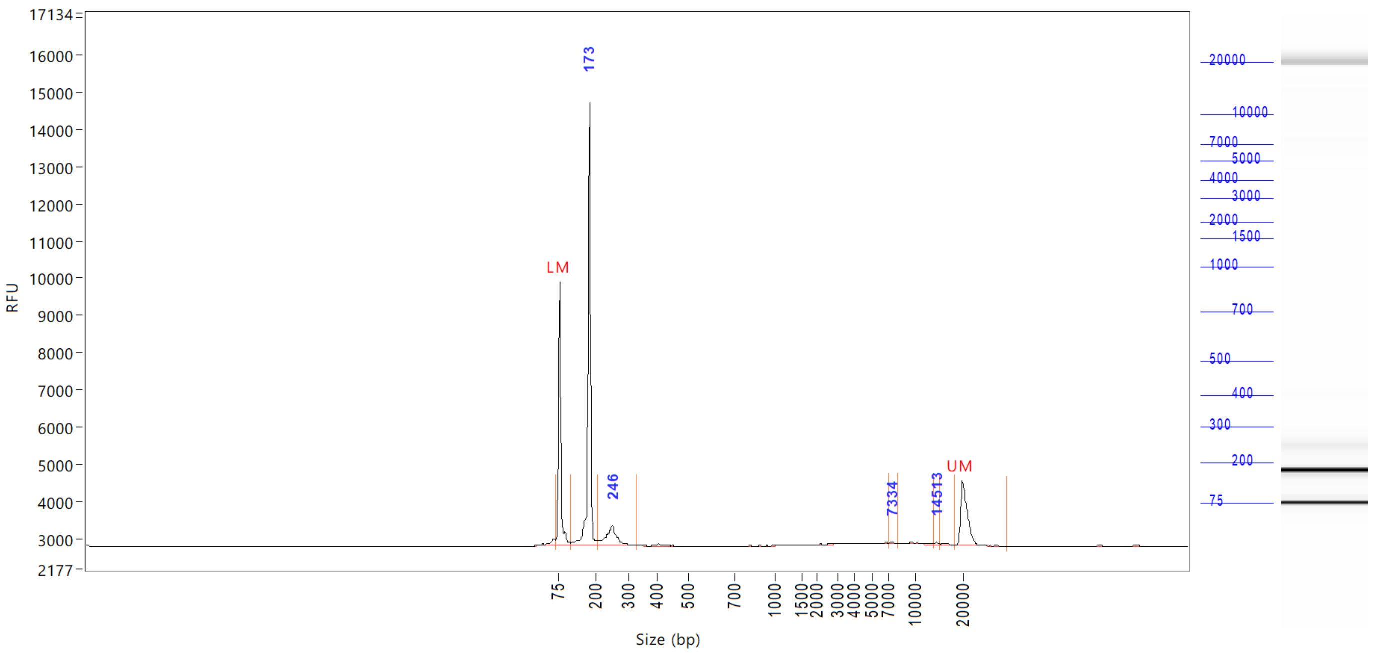

Peak	Size (bp)	Concentration (ng/uL)	From (bp)	To (bp)	Average size (bp)	CV%	RFU	Corrected peak area
1	75 (LM)	0.5000	66	112	75	8.62	7087	42.694
2	173	10.3976	112	201	170	5.73	11923	73.986
3	246	1.7060	201	325	241	8.17	522	12.139
4	7334	0.0492	7001	8134	7349	3.04	58	0.350
5	14513	0.0356	13903	15366	14472	2.42	51	0.253
6	20000 (UM)	0.1793	18415	29430	20493	4.44	1712	15.309

TIC:	12.1884	ng/uL
TIM:	112.1986	nmole/L
Total concentration:	12.7270	ng/uL

Sample peak width (sec): 5   Sample min peak height: 50   Sample baseline V to V?: Y   Sample baseline V to V points: 3
Sample filter: Binomial   Number of points for filter: 3 Sample start region (min): 0 Sample end region (min): 30
Marker peak width (sec): 5   Marker min peak height: 200   Marker baseline V to V?: Y   Marker baseline V to V points: 3
Lower marker selection: First peak > 500 RFU   Upper marker selection: Last peak > 200 RFU
Ladder size (bp)75, 200, 300, 400, 500, 700, 1000, 1500, 2000, 3000, 4000, 5000, 7000, 10000, 20000
Quantification using: Lower Marker   Final concentration (ng/uL): 0.5000   Dilution factor: 12.0

Sample: SampC2
Well location: C2
Created: Tuesday, September 14, 2021 8:54:07 PM

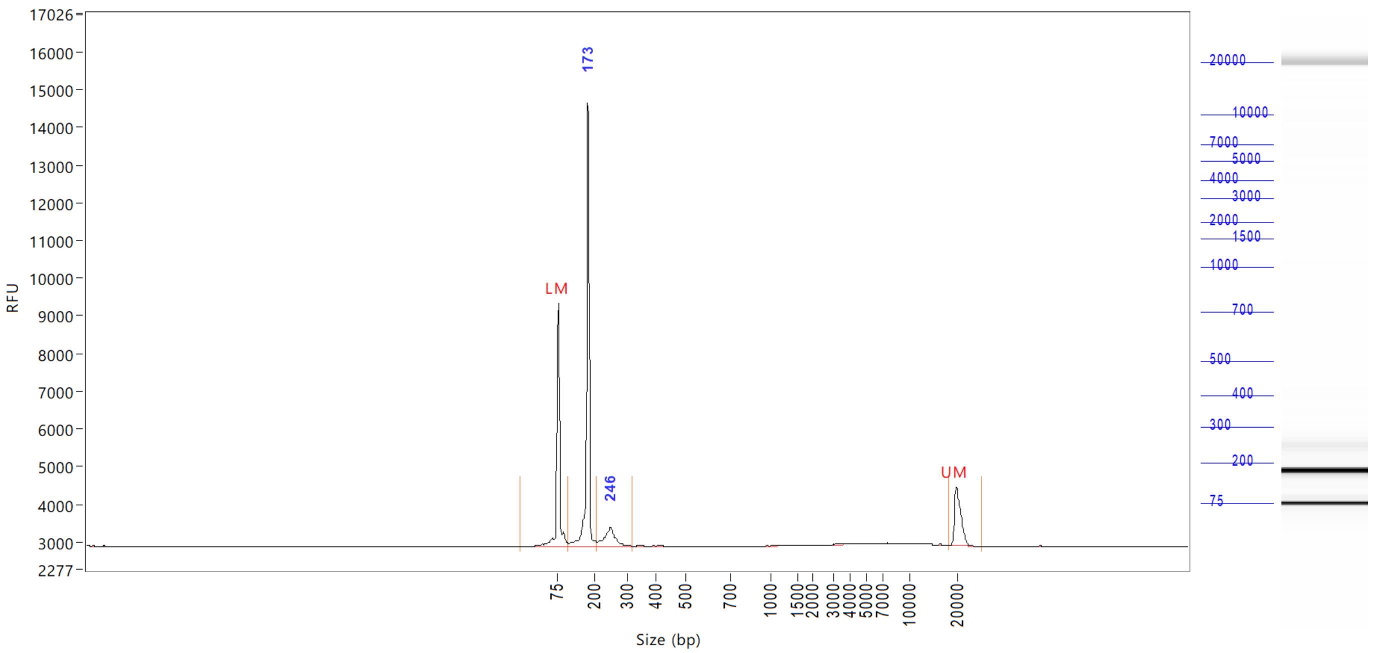

Peak	Size (bp)	Concentration (ng/uL)	From (bp)	To (bp)	Average size (bp)	CV%	RFU	Corrected peak area
1	75 (LM)	0.5000	0	110	71	21.59	6456	44.049
2	173	10.3901	110	203	169	6.17	11790	76.280
3	246	1.6244	203	317	242	8.27	516	11.926
4	20000 (UM)	0.1493	18415	25440	20336	3.47	1570	13.150

TIC:	12.0146	ng/uL
TIM:	112.1025	nmole/L
Total concentration:	12.4917	ng/uL

Sample peak width (sec): 5 Sample min peak height: 50 Sample baseline V to V?: Y Sample baseline V to V points: 3
Sample filter: Binomial Number of points for filter: 3 Sample start region (min): 0 Sample end region (min): 30
Marker peak width (sec): 5 Marker min peak height: 200 Marker baseline V to V?: Y Marker baseline V to V points: 3
Lower marker selection: First peak > 500 RFU Upper marker selection: Last peak > 200 RFU
Ladder size (bp)75, 200, 300, 400, 500, 700, 1000, 1500, 2000, 3000, 4000, 5000, 7000, 10000, 20000
Quantification using: Lower Marker Final concentration (ng/uL): 0.5000 Dilution factor: 12.0

Sample: SampD2
Well location: D2
Created: Tuesday, September 14, 2021 8:54:07 PM

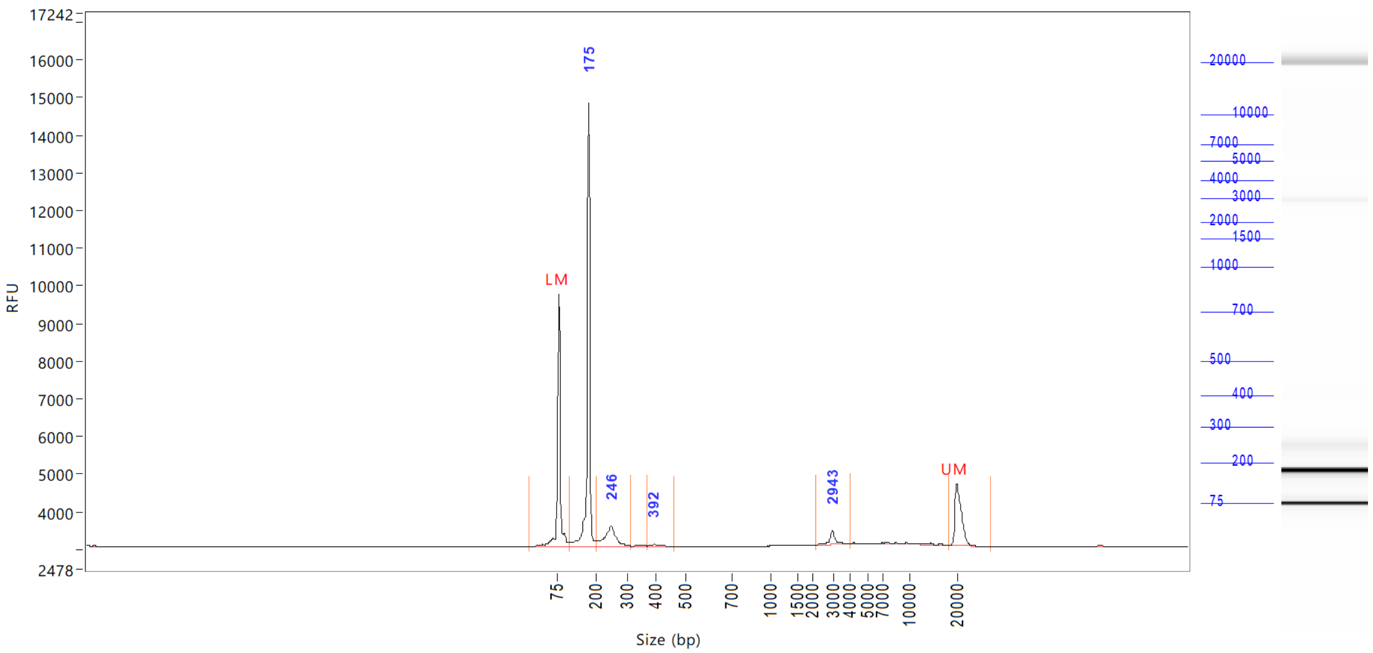

Peak	Size (bp)	Concentration (ng/uL)	From (bp)	To (bp)	Average size (bp)	CV%	RFU	Corrected peak area
1	75 (LM)	0.5000	0	110	72	18.55	6689	44.694
2	175	10.3290	110	203	169	6.34	11795	76.941
3	246	1.6692	203	311	241	7.80	540	12.434
4	392	0.0829	368	460	401	3.99	51	0.617
5	2943	0.4419	2115	4035	2970	10.97	360	3.292
6	20000 (UM)	0.1567	18293	27254	20449	3.76	1636	14.007

TIC:	12.5230	ng/uL
TIM:	112.4442	nmole/L
Total concentration:	12.9491	ng/uL

```
Sample peak width (sec): 5    Sample min peak height: 50    Sample baseline V to V?: Y    Sample baseline V to V points: 3
Sample filter: Binomial       Number of points for filter: 3  Sample start region (min): 0  Sample end region (min): 30
Marker peak width (sec): 5    Marker min peak height: 200   Marker baseline V to V?: Y    Marker baseline V to V points: 3
Lower marker selection: First peak > 500 RFU                Upper marker selection: Last peak > 200 RFU
Ladder size (bp)75, 200, 300, 400, 500, 700, 1000, 1500, 2000, 3000, 4000, 5000, 7000, 10000, 20000
Quantification using: Lower Marker          Final concentration (ng/uL): 0.5000          Dilution factor: 12.0
```

Sample: SampE2
Well location: E2
Created: Tuesday, September 14, 2021 8:54:07 PM

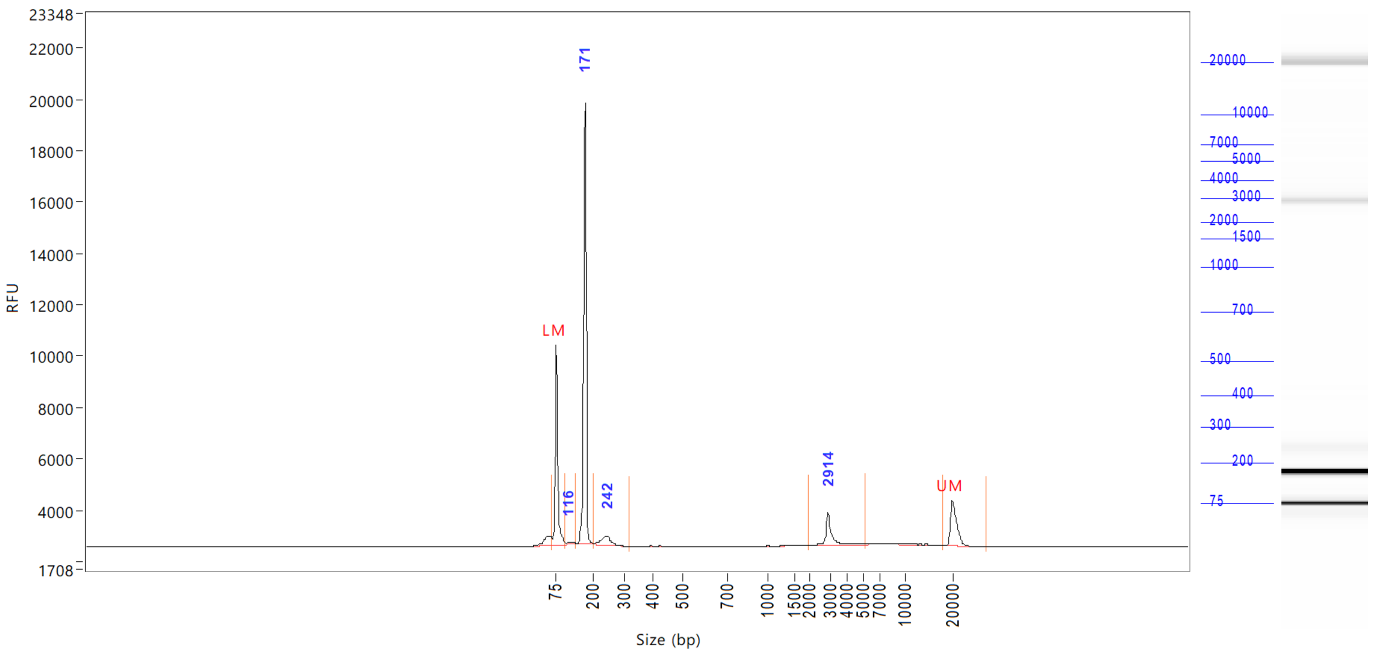

Peak	Size (bp)	Concentration (ng/uL)	From (bp)	To (bp)	Average size (bp)	CV%	RFU	Corrected peak area
1	75 (LM)	0.5000	64	107	74	7.94	7759	47.834
2	116	0.1671	107	143	122	8.10	91	1.332
3	171	12.1508	143	200	168	3.06	17206	96.870
4	242	0.9989	200	317	236	6.91	373	7.964
5	2914	1.4025	1962	5149	3054	14.81	1260	11.181
6	20000 (UM)	0.1565	18171	27133	20396	3.75	1772	14.972

TIC:	14.7192	ng/uL
TIM:	128.8577	nmole/L
Total concentration:	15.1147	ng/uL

Sample peak width (sec): 5 Sample min peak height: 50 Sample baseline V to V?: Y Sample baseline V to V points: 3
Sample filter: Binomial Number of points for filter: 3 Sample start region (min): 0 Sample end region (min): 30
Marker peak width (sec): 5 Marker min peak height: 200 Marker baseline V to V?: Y Marker baseline V to V points: 3
Lower marker selection: First peak > 500 RFU Upper marker selection: Last peak > 200 RFU
Ladder size (bp)75, 200, 300, 400, 500, 700, 1000, 1500, 2000, 3000, 4000, 5000, 7000, 10000, 20000
Quantification using: Lower Marker Final concentration (ng/uL): 0.5000 Dilution factor: 12.0

Sample: SampF2
Well location: F2
Created: Tuesday, September 14, 2021 8:54:07 PM

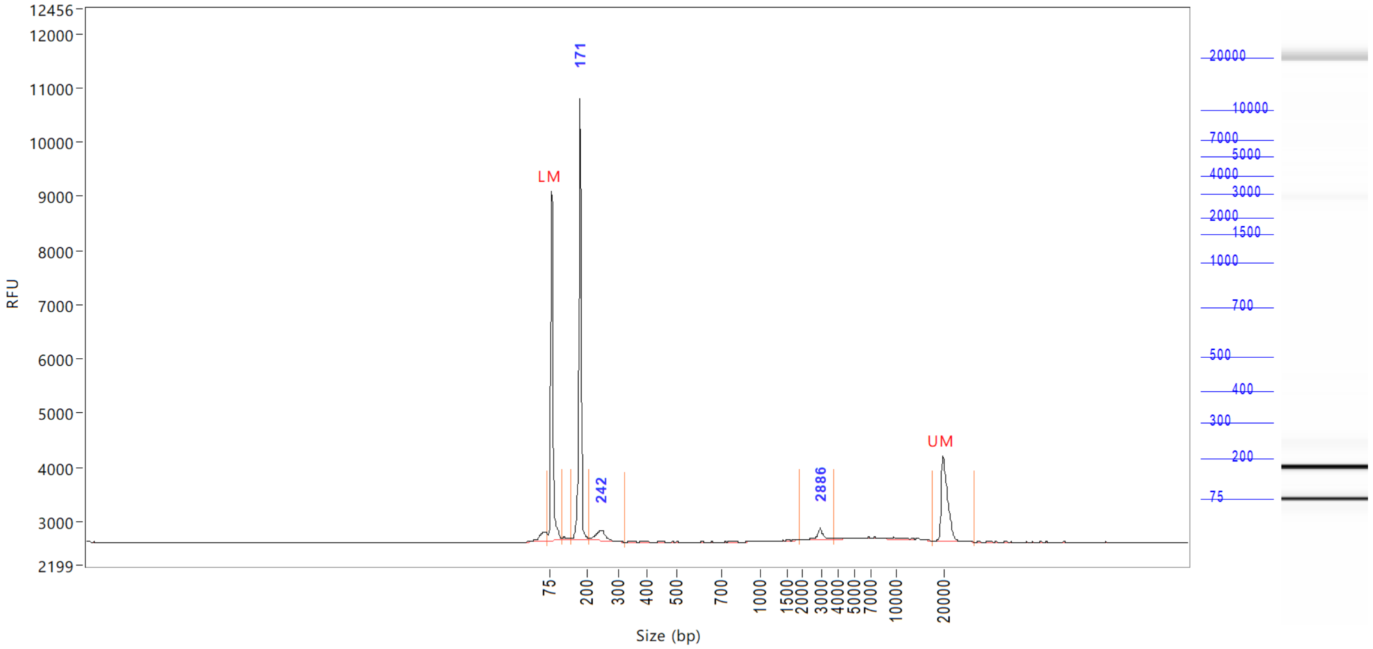

Peak	Size (bp)	Concentration (ng/uL)	From (bp)	To (bp)	Average size (bp)	CV%	RFU	Corrected peak area
1	75 (LM)	0.5000	62	110	75	7.48	6448	39.260
2	171	7.1774	143	201	169	3.17	8144	46.965
3	242	0.6544	201	317	237	6.91	194	4.282
4	2886	0.3105	1962	3786	2865	10.03	207	2.032
5	20000 (UM)	0.1740	17561	26770	20417	3.93	1560	13.659

TIC:	8.1424	ng/uL
TIM:	74.5330	nmole/L
Total concentration:	8.6750	ng/uL

Sample peak width (sec): 5   Sample min peak height: 50   Sample baseline V to V?: Y   Sample baseline V to V points: 3
Sample filter: Binomial   Number of points for filter: 3 Sample start region (min): 0 Sample end region (min): 30
Marker peak width (sec): 5   Marker min peak height: 200   Marker baseline V to V?: Y   Marker baseline V to V points: 3
Lower marker selection: First peak > 500 RFU   Upper marker selection: Last peak > 200 RFU
Ladder size (bp)75, 200, 300, 400, 500, 700, 1000, 1500, 2000, 3000, 4000, 5000, 7000, 10000, 20000
Quantification using: Lower Marker   Final concentration (ng/uL): 0.5000   Dilution factor: 12.0

Sample: SampG2
Well location: G2
Created: Tuesday, September 14, 2021 8:54:07 PM

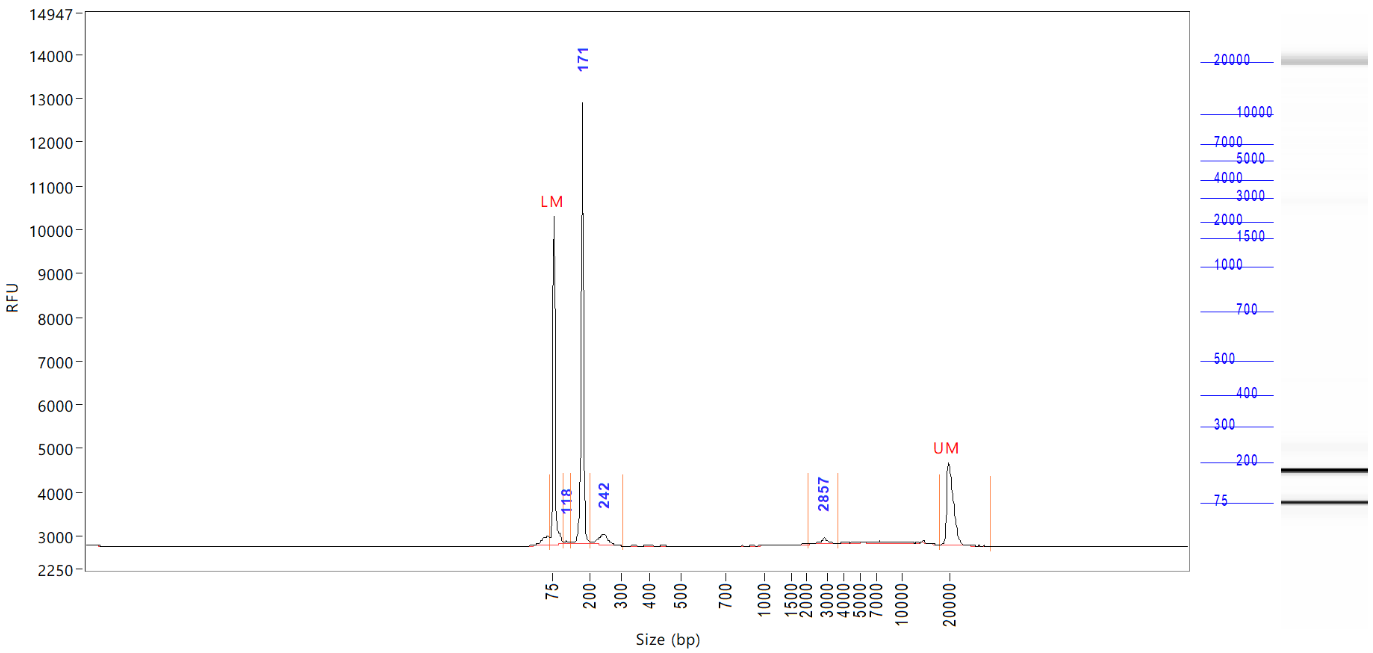

Peak	Size<br>(bp)	Concentration<br>(ng/uL)	From<br>(bp)	To<br>(bp)	Average size<br>(bp)	CV%	RFU	Corrected peak area
1	75 (LM)	0.5000	64	108	75	7.16	7508	45.083
2	118	0.0888	108	133	120	5.86	58	0.668
3	171	7.5758	133	200	169	3.35	10088	56.923
4	242	0.7228	200	303	236	7.11	238	5.431
5	2857	0.2124	2058	3679	2827	10.28	135	1.596
6	20000 (UM)	0.1829	18171	28947	20379	4.23	1861	16.493

TIC:	8.5999	ng/uL
TIM:	80.0657	nmole/L
Total concentration:	8.9841	ng/uL

Sample peak width (sec): 5   Sample min peak height: 50   Sample baseline V to V?: Y   Sample baseline V to V points: 3
Sample filter: Binomial   Number of points for filter: 3   Sample start region (min): 0   Sample end region (min): 30
Marker peak width (sec): 5   Marker min peak height: 200   Marker baseline V to V?: Y   Marker baseline V to V points: 3
Lower marker selection: First peak > 500 RFU   Upper marker selection: Last peak > 200 RFU
Ladder size (bp)75, 200, 300, 400, 500, 700, 1000, 1500, 2000, 3000, 4000, 5000, 7000, 10000, 20000
Quantification using: Lower Marker   Final concentration (ng/uL): 0.5000   Dilution factor: 12.0

Sample: SampH2
Well location: H2
Created: Tuesday, September 14, 2021 8:54:07 PM

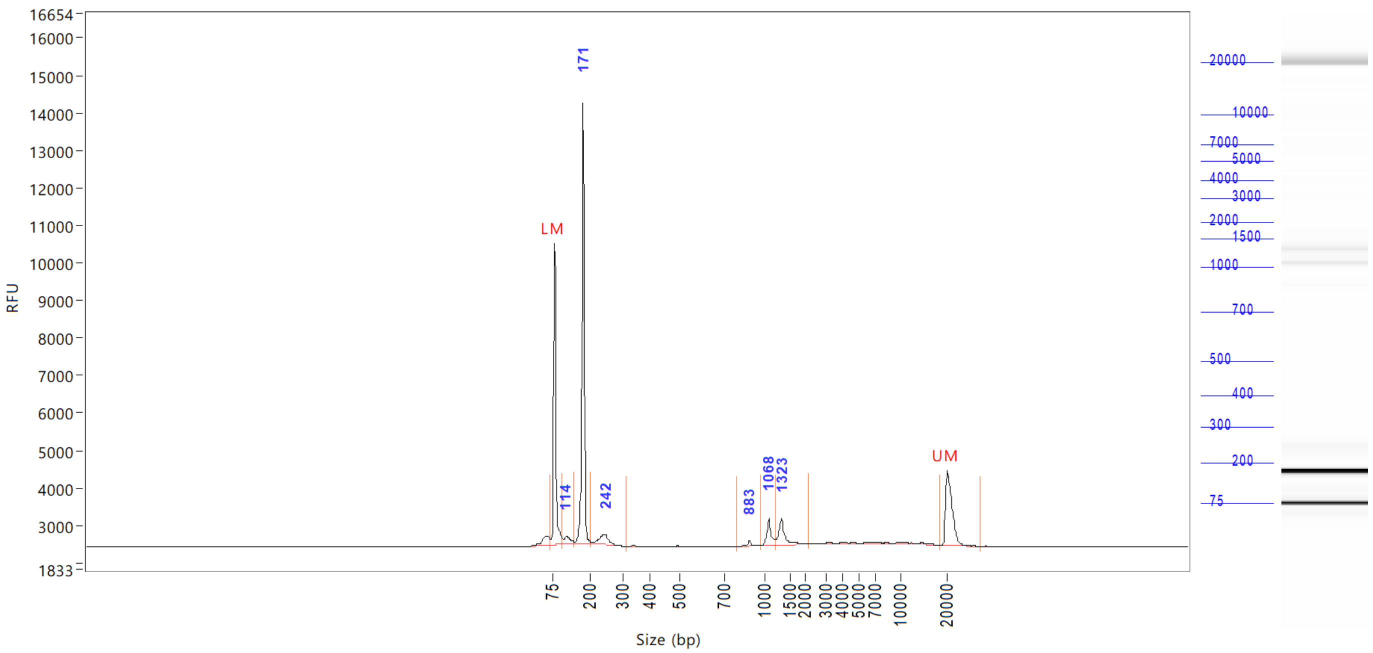

Peak	Size (bp)	Concentration (ng/uL)	From (bp)	To (bp)	Average size (bp)	CV%	RFU	Corrected peak area
1	75 (LM)	0.5000	62	103	75	7.51	8030	47.715
2	114	0.3679	103	143	118	8.53	195	2.925
3	171	8.3324	143	198	168	3.11	11757	66.264
4	242	0.7325	198	309	236	6.85	269	5.825
5	883	0.1430	783	974	881	3.64	139	1.137
6	1068	0.6444	974	1201	1077	4.92	730	5.125
7	1323	0.8941	1201	2115	1359	9.86	700	7.110
8	20000 (UM)	0.1828	18293	27012	20470	4.18	2008	17.447

TIC:	11.1142	ng/uL
TIM:	94.0898	nmole/L
Total concentration:	11.4957	ng/uL

Sample peak width (sec): 5 Sample min peak height: 50 Sample baseline V to V?: Y Sample baseline V to V points: 3
Sample filter: Binomial Number of points for filter: 3 Sample start region (min): 0 Sample end region (min): 30
Marker peak width (sec): 5 Marker min peak height: 200 Marker baseline V to V?: Y Marker baseline V to V points: 3
Lower marker selection: First peak > 500 RFU Upper marker selection: Last peak > 200 RFU
Ladder size (bp)75, 200, 300, 400, 500, 700, 1000, 1500, 2000, 3000, 4000, 5000, 7000, 10000, 20000
Quantification using: Lower Marker Final concentration (ng/uL): 0.5000 Dilution factor: 12.0

Sample: SampA3
Well location: A3
Created: Tuesday, September 14, 2021 8:54:07 PM

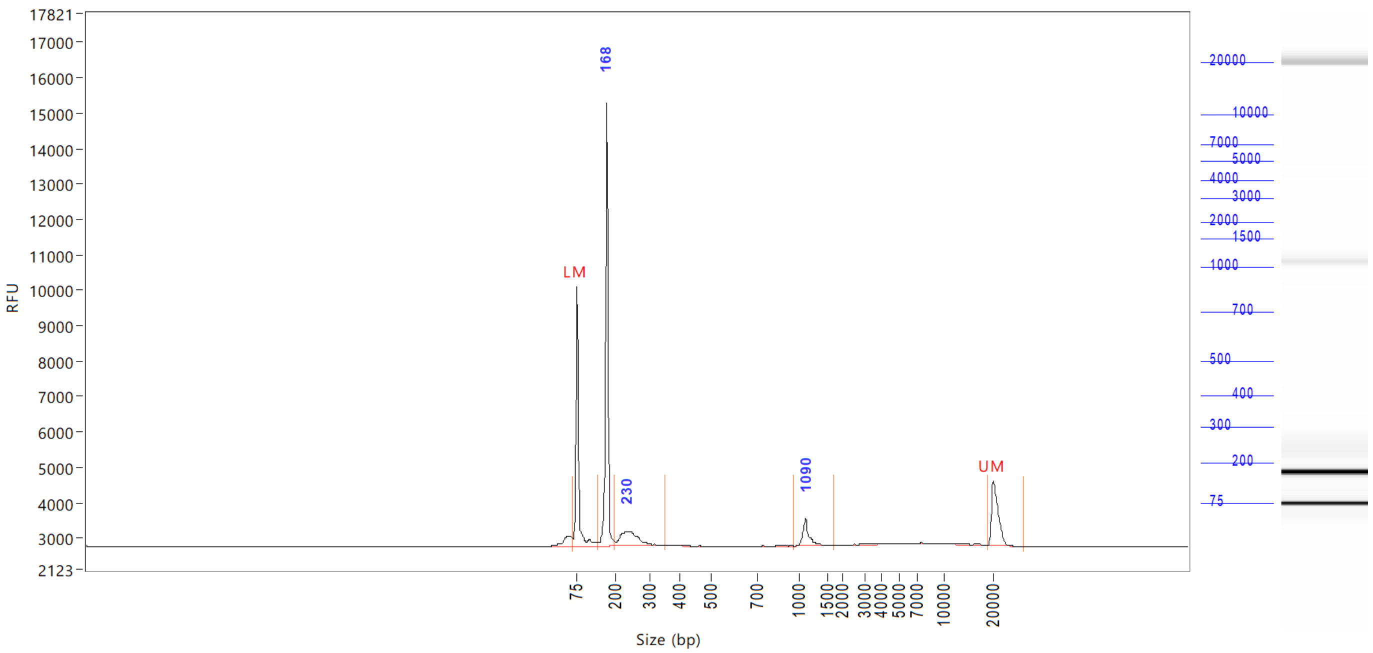

Peak	Size (bp)	Concentration (ng/uL)	From (bp)	To (bp)	Average size (bp)	CV%	RFU	Corrected peak area
1	75 (LM)	0.5000	64	141	77	16.95	7341	47.121
2	168	9.3446	141	196	166	3.59	12533	73.388
3	230	2.0351	196	352	241	11.33	409	15.983
4	1090	0.9316	961	1712	1126	9.36	765	7.317
5	20000 (UM)	0.1654	18781	26045	20424	3.59	1804	15.588

TIC:	12.3113	ng/uL
TIM:	107.7828	nmole/L
Total concentration:	12.7976	ng/uL

```
Sample peak width (sec): 5    Sample min peak height: 50    Sample baseline V to V?: Y    Sample baseline V to V points: 3
Sample filter: Binomial       Number of points for filter: 3  Sample start region (min): 0  Sample end region (min): 30
Marker peak width (sec): 5    Marker min peak height: 200   Marker baseline V to V?: Y    Marker baseline V to V points: 3
Lower marker selection: First peak > 500 RFU                Upper marker selection: Last peak > 200 RFU
Ladder size (bp)75, 200, 300, 400, 500, 700, 1000, 1500, 2000, 3000, 4000, 5000, 7000, 10000, 20000
Quantification using: Lower Marker          Final concentration (ng/uL): 0.5000          Dilution factor: 12.0
```

Sample: SampB3
Well location: B3
Created: Tuesday, September 14, 2021 8:54:07 PM

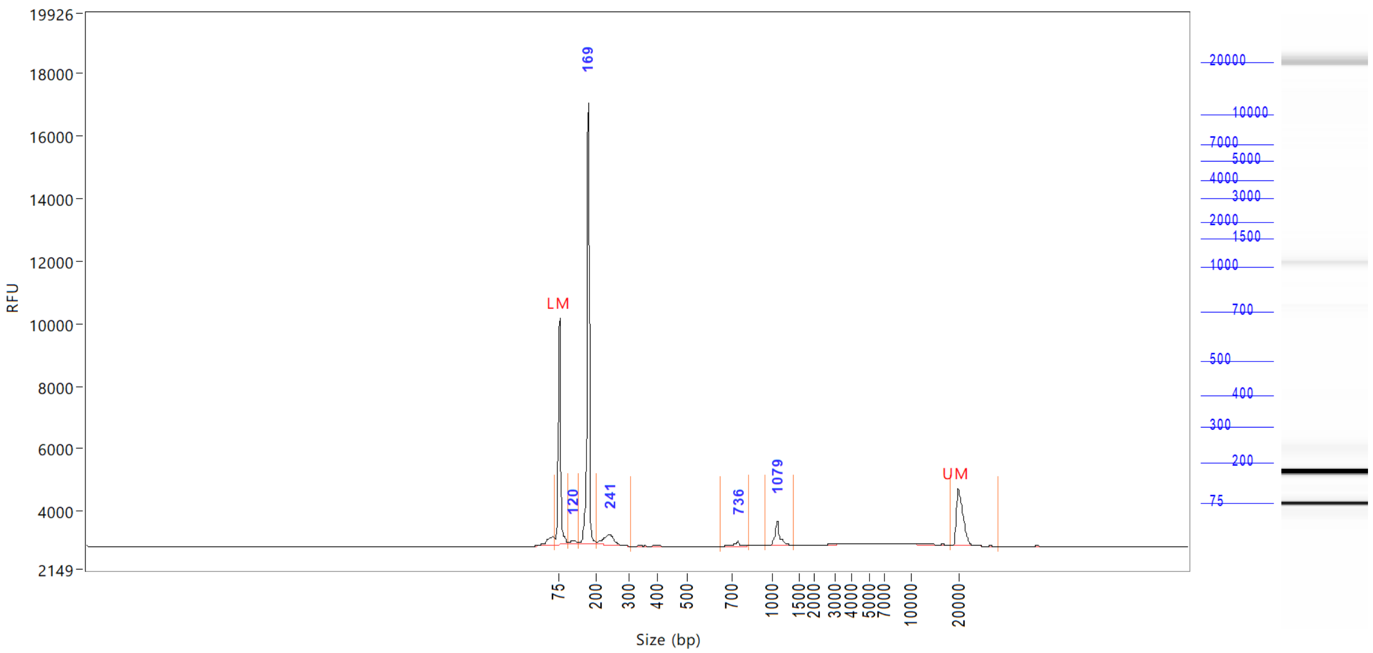

Peak	Size (bp)	Concentration (ng/uL)	From (bp)	To (bp)	Average size (bp)	CV%	RFU	Corrected peak area
1	75 (LM)	0.5000	64	105	73	6.84	7257	42.823
2	120	0.1850	105	141	119	7.00	103	1.321
3	169	11.1804	141	198	168	3.16	14120	79.797
4	241	1.1056	198	309	233	7.47	323	7.891
5	736	0.2149	644	818	719	4.22	153	1.534
6	1079	0.9188	944	1389	1089	6.81	792	6.557
7	20000 (UM)	0.1832	18293	28463	20396	3.99	1824	15.690

TIC:	13.6047	ng/uL
TIM:	121.6344	nmole/L
Total concentration:	14.1497	ng/uL

Sample peak width (sec): 5 Sample min peak height: 50 Sample baseline V to V?: Y Sample baseline V to V points: 3
Sample filter: Binomial Number of points for filter: 3 Sample start region (min): 0 Sample end region (min): 30
Marker peak width (sec): 5 Marker min peak height: 200 Marker baseline V to V?: Y Marker baseline V to V points: 3
Lower marker selection: First peak > 500 RFU Upper marker selection: Last peak > 200 RFU
Ladder size (bp)75, 200, 300, 400, 500, 700, 1000, 1500, 2000, 3000, 4000, 5000, 7000, 10000, 20000
Quantification using: Lower Marker Final concentration (ng/uL): 0.5000 Dilution factor: 12.0

Sample: SampC3
Well location: C3
Created: Tuesday, September 14, 2021 8:54:07 PM

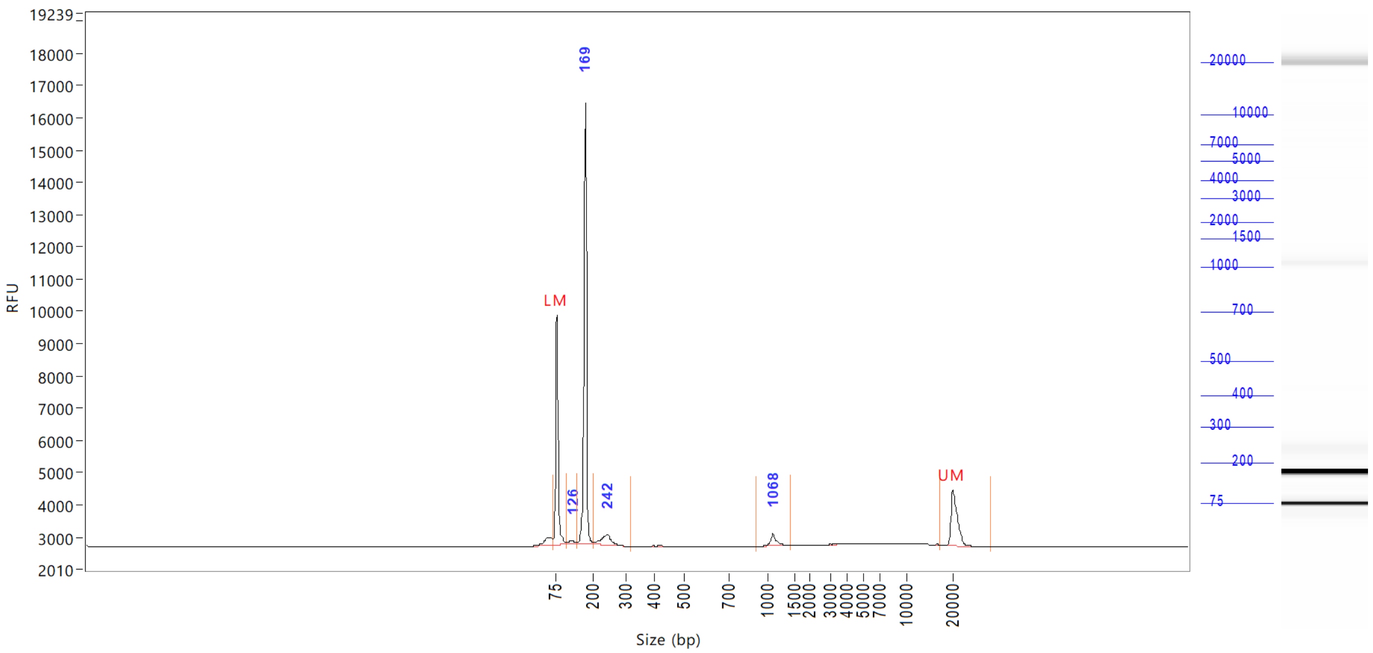

Peak	Size (bp)	Concentration (ng/uL)	From (bp)	To (bp)	Average size (bp)	CV%	RFU	Corrected peak area
1	75 (LM)	0.5000	64	107	73	7.32	7128	42.841
2	126	0.1983	107	143	123	7.75	91	1.416
3	169	10.8518	143	200	167	3.22	13685	77.484
4	242	1.0211	200	319	234	7.09	313	7.291
5	1068	0.5343	905	1433	1099	7.96	387	3.815
6	20000 (UM)	0.1746	17317	27979	20396	4.19	1718	14.956

TIC:	12.6056	ng/uL
TIM:	117.4413	nmole/L
Total concentration:	12.9841	ng/uL

```
Sample peak width (sec): 5     Sample min peak height: 50     Sample baseline V to V?: Y     Sample baseline V to V points: 3
Sample filter: Binomial        Number of points for filter: 3  Sample start region (min): 0  Sample end region (min): 30
Marker peak width (sec): 5     Marker min peak height: 200    Marker baseline V to V?: Y     Marker baseline V to V points: 3
Lower marker selection: First peak > 500 RFU                  Upper marker selection: Last peak > 200 RFU
Ladder size (bp)75, 200, 300, 400, 500, 700, 1000, 1500, 2000, 3000, 4000, 5000, 7000, 10000, 20000
Quantification using: Lower Marker          Final concentration (ng/uL): 0.5000          Dilution factor: 12.0
```

Sample: SampD3
Well location: D3
Created: Tuesday, September 14, 2021 8:54:07 PM

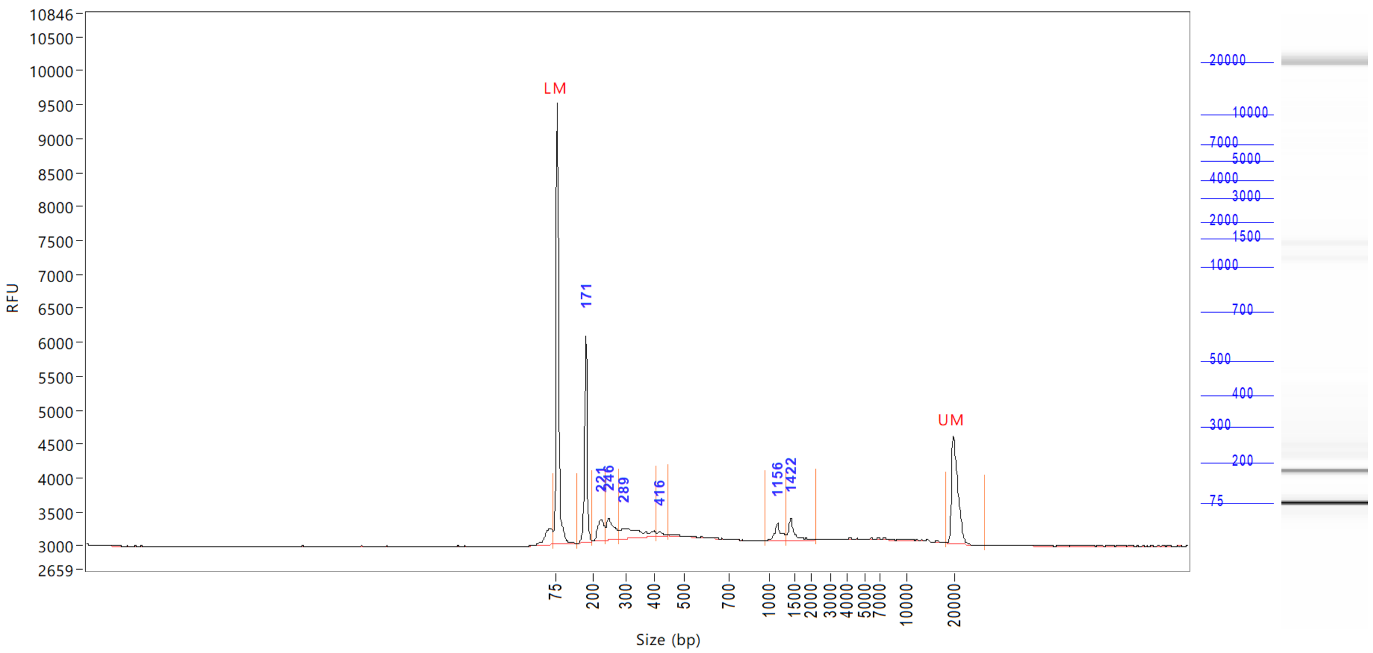

Peak	Size (bp)	Concentration (ng/uL)	From (bp)	To (bp)	Average size (bp)	CV%	RFU	Corrected peak area
1	75 (LM)	0.5000	64	145	75	9.30	6509	40.379
2	171	2.6057	145	194	169	3.02	3037	17.536
3	221	0.6882	194	233	218	4.00	314	4.631
4	246	0.8583	233	278	252	4.99	326	5.776
5	289	1.0348	278	407	330	11.10	148	6.964
6	416	0.1070	407	445	418	1.95	62	0.720
7	1156	0.4336	974	1311	1161	6.69	253	2.918
8	1422	0.4787	1311	2258	1476	11.31	327	3.221
9	20000 (UM)	0.1693	18293	26770	20353	3.68	1590	13.673

TIC:	6.2062	ng/uL
TIM:	42.8655	nmole/L
Total concentration:	6.4768	ng/uL

```
Sample peak width (sec): 5     Sample min peak height: 50     Sample baseline V to V?: Y     Sample baseline V to V points: 3
Sample filter: Binomial        Number of points for filter: 3  Sample start region (min): 0  Sample end region (min): 30
Marker peak width (sec): 5     Marker min peak height: 200    Marker baseline V to V?: Y     Marker baseline V to V points: 3
Lower marker selection: First peak > 500 RFU                  Upper marker selection: Last peak > 200 RFU
Ladder size (bp)75, 200, 300, 400, 500, 700, 1000, 1500, 2000, 3000, 4000, 5000, 7000, 10000, 20000
Quantification using: Lower Marker          Final concentration (ng/uL): 0.5000          Dilution factor: 12.0
```

Sample: SampE3
Well location: E3
Created: Tuesday, September 14, 2021 8:54:07 PM

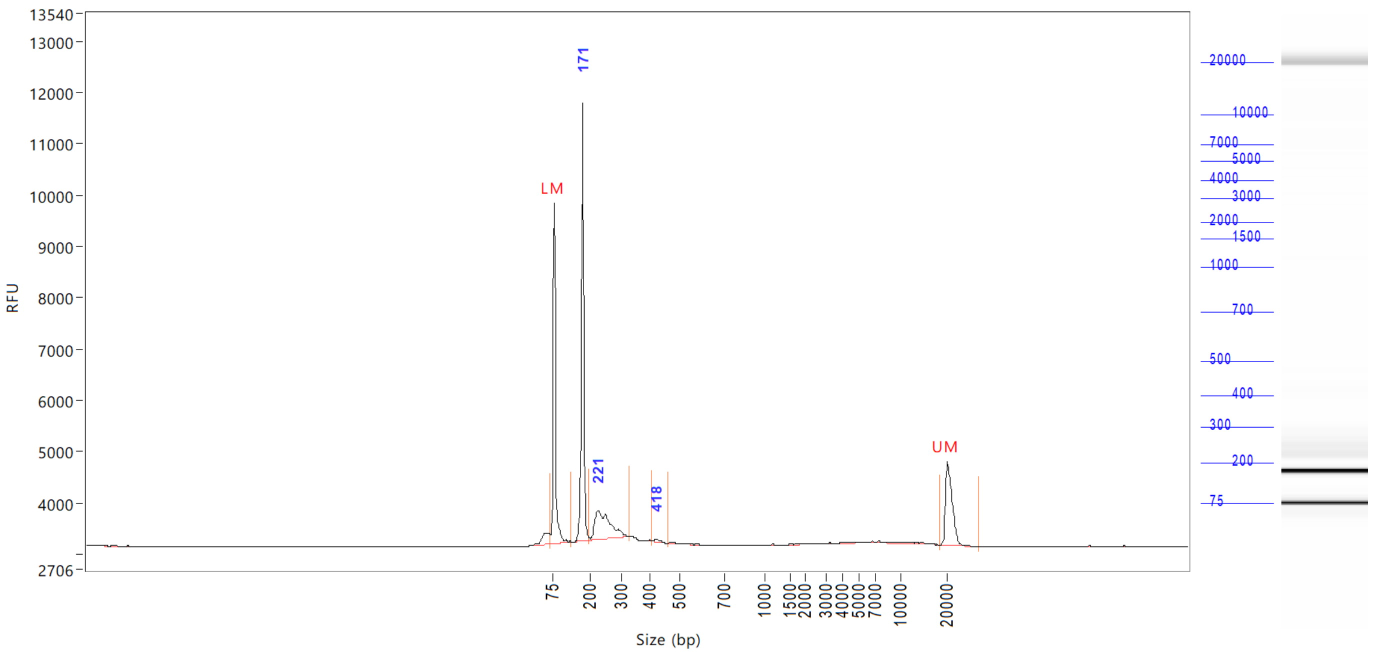

Peak	Size (bp)	Concentration (ng/uL)	From (bp)	To (bp)	Average size (bp)	CV%	RFU	Corrected peak area
1	75 (LM)	0.5000	66	135	76	9.08	6644	41.888
2	171	6.9579	135	196	169	3.04	8549	48.576
3	221	2.6908	196	325	241	10.43	562	18.786
4	418	0.0940	403	456	421	2.12	63	0.656
5	20000 (UM)	0.1733	18293	26891	20434	3.83	1639	14.519

TIC:	9.7427	ng/uL
TIM:	86.4076	nmole/L
Total concentration:	10.2134	ng/uL

Sample peak width (sec): 5 Sample min peak height: 50 Sample baseline V to V?: Y Sample baseline V to V points: 3
Sample filter: Binomial Number of points for filter: 3 Sample start region (min): 0 Sample end region (min): 30
Marker peak width (sec): 5 Marker min peak height: 200 Marker baseline V to V?: Y Marker baseline V to V points: 3
Lower marker selection: First peak > 500 RFU Upper marker selection: Last peak > 200 RFU
Ladder size (bp)75, 200, 300, 400, 500, 700, 1000, 1500, 2000, 3000, 4000, 5000, 7000, 10000, 20000
Quantification using: Lower Marker Final concentration (ng/uL): 0.5000 Dilution factor: 12.0

Sample: SampF3
Well location: F3
Created: Tuesday, September 14, 2021 8:54:07 PM

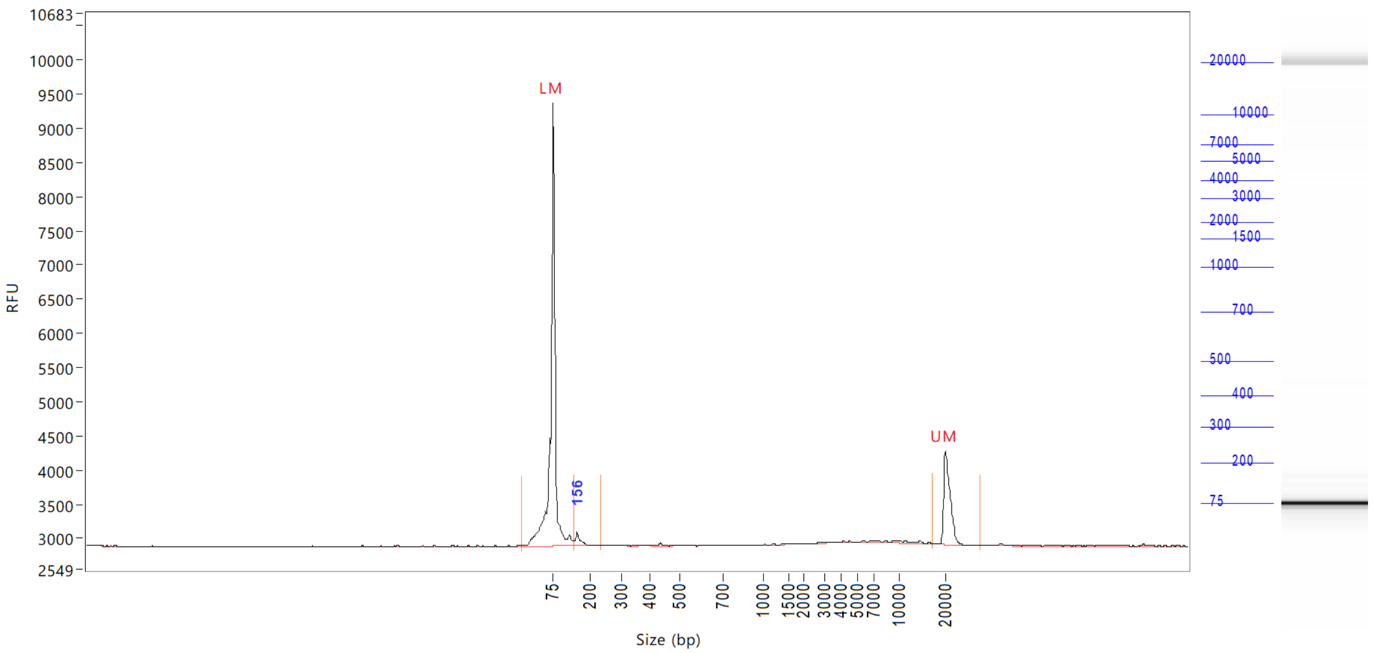

Peak	Size (bp)	Concentration (ng/uL)	From (bp)	To (bp)	Average size (bp)	CV%	RFU	Corrected peak area
1	75 (LM)	0.5000	0	145	68	31.88	6494	72.625
2	156	0.1923	145	235	163	10.25	195	2.328
3	20000 (UM)	0.0822	17195	27496	20424	4.08	1359	11.933

TIC:	0.1923	ng/uL
TIM:	1.9394	nmole/L
Total concentration:	0.4904	ng/uL

Sample peak width (sec): 5 Sample min peak height: 50 Sample baseline V to V?: Y Sample baseline V to V points: 3
Sample filter: Binomial Number of points for filter: 3 Sample start region (min): 0 Sample end region (min): 30
Marker peak width (sec): 5 Marker min peak height: 200 Marker baseline V to V?: Y Marker baseline V to V points: 3
Lower marker selection: First peak > 500 RFU Upper marker selection: Last peak > 200 RFU
Ladder size (bp)75, 200, 300, 400, 500, 700, 1000, 1500, 2000, 3000, 4000, 5000, 7000, 10000, 20000
Quantification using: Lower Marker Final concentration (ng/uL): 0.5000 Dilution factor: 12.0

Sample: SampG3
Well location: G3
Created: Tuesday, September 14, 2021 8:54:07 PM

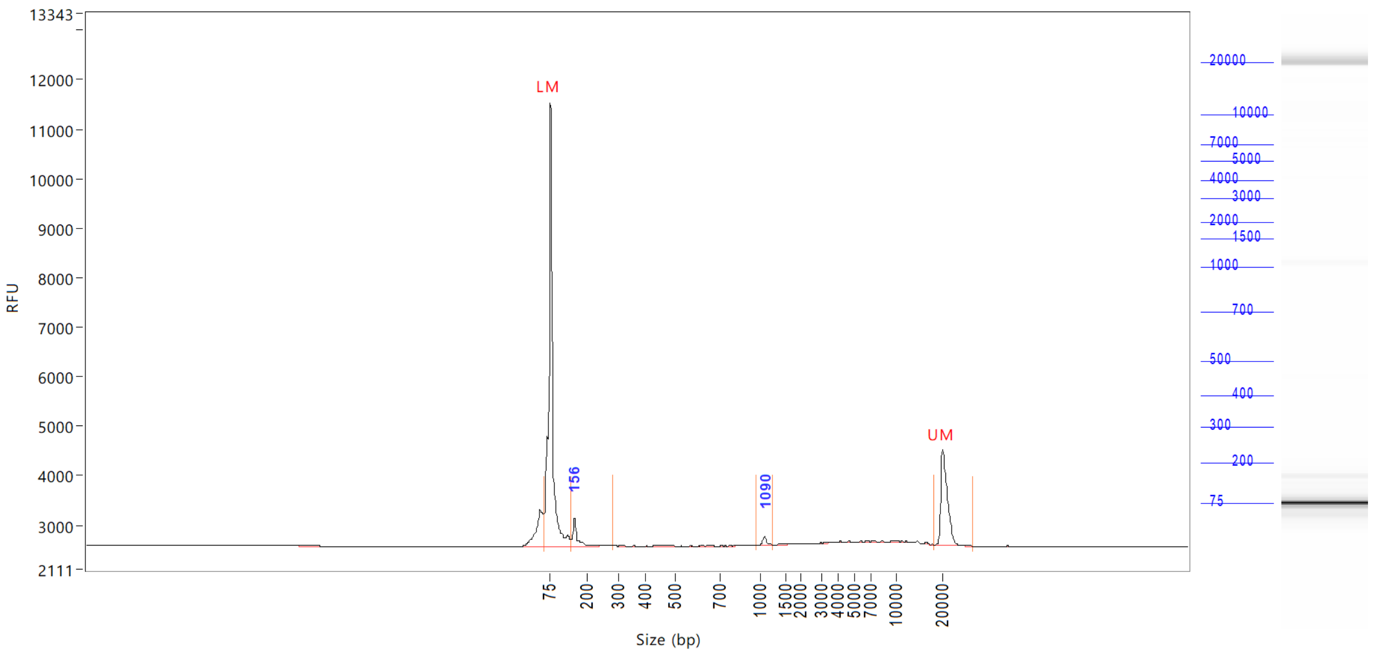

Peak	Size (bp)	Concentration (ng/uL)	From (bp)	To (bp)	Average size (bp)	CV%	RFU	Corrected peak area
1	75 (LM)	0.5000	54	145	77	19.40	8975	84.538
2	156	0.4235	145	282	165	13.05	568	5.967
3	1090	0.0913	970	1256	1075	4.60	199	1.286
4	20000 (UM)	0.1030	18171	26770	20499	4.15	1936	17.420

TIC:	0.5148	ng/uL
TIM:	4.3588	nmole/L
Total concentration:	0.7452	ng/uL

Sample peak width (sec): 5 Sample min peak height: 50 Sample baseline V to V?: Y Sample baseline V to V points: 3
Sample filter: Binomial Number of points for filter: 3 Sample start region (min): 0 Sample end region (min): 30
Marker peak width (sec): 5 Marker min peak height: 200 Marker baseline V to V?: Y Marker baseline V to V points: 3
Lower marker selection: First peak > 500 RFU Upper marker selection: Last peak > 200 RFU
Ladder size (bp)75, 200, 300, 400, 500, 700, 1000, 1500, 2000, 3000, 4000, 5000, 7000, 10000, 20000
Quantification using: Lower Marker Final concentration (ng/uL): 0.5000 Dilution factor: 12.0

Sample: SampH3
Well location: H3
Created: Tuesday, September 14, 2021 8:54:07 PM

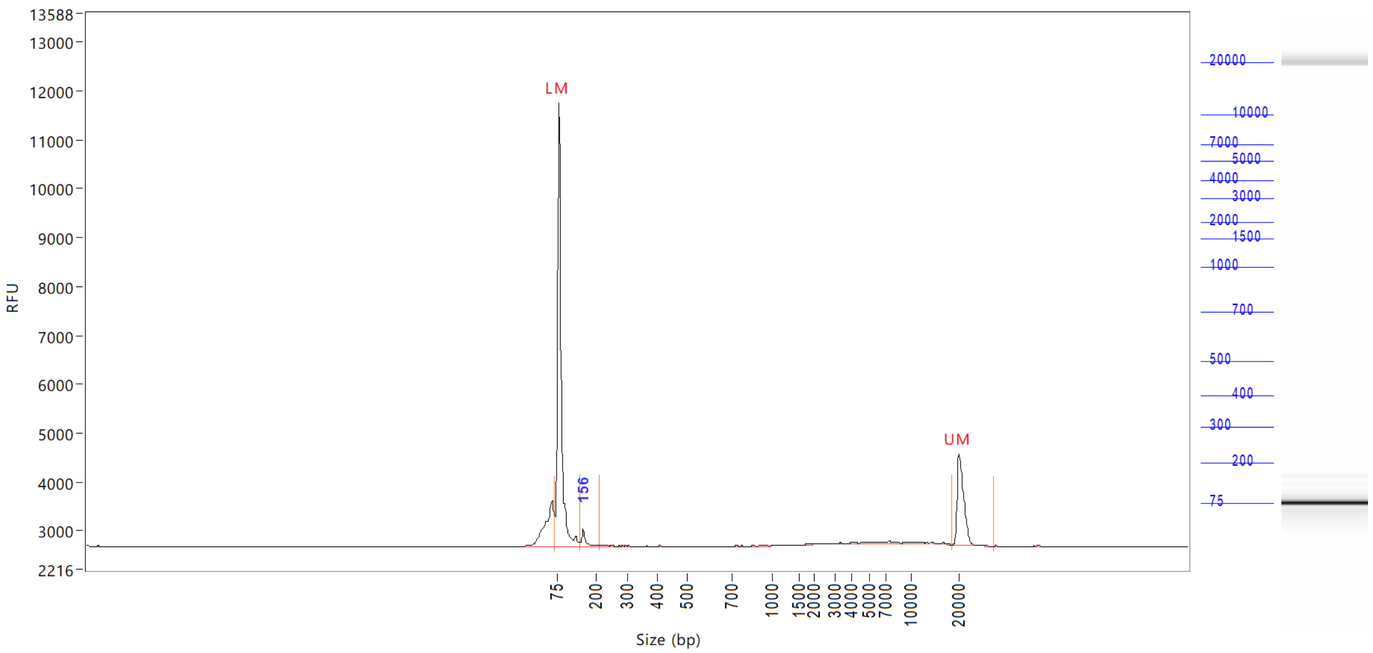

Peak	Size	Concentration	From	To	Average size	CV%	RFU	Corrected peak area
	(bp)	(ng/uL)	(bp)	(bp)	(bp)			
1	75 (LM)	0.5000	64	145	78	15.86	9085	77.141
2	156	0.2175	145	208	159	7.65	340	2.797
3	20000 (UM)	0.1039	18415	27254	20420	3.73	1865	16.029

TIC:	0.2175	ng/uL
TIM:	2.2485	nmole/L
Total concentration:	0.5069	ng/uL

```
Sample peak width (sec): 5    Sample min peak height: 50    Sample baseline V to V?: Y    Sample baseline V to V points: 3
Sample filter: Binomial       Number of points for filter: 3  Sample start region (min): 0  Sample end region (min): 30
Marker peak width (sec): 5    Marker min peak height: 200   Marker baseline V to V?: Y    Marker baseline V to V points: 3
Lower marker selection: First peak > 500 RFU                Upper marker selection: Last peak > 200 RFU
Ladder size (bp)75, 200, 300, 400, 500, 700, 1000, 1500, 2000, 3000, 4000, 5000, 7000, 10000, 20000
Quantification using: Lower Marker          Final concentration (ng/uL): 0.5000          Dilution factor: 12.0
```

Sample: SampA4
Well location: A4
Created: Tuesday, September 14, 2021 8:54:07 PM

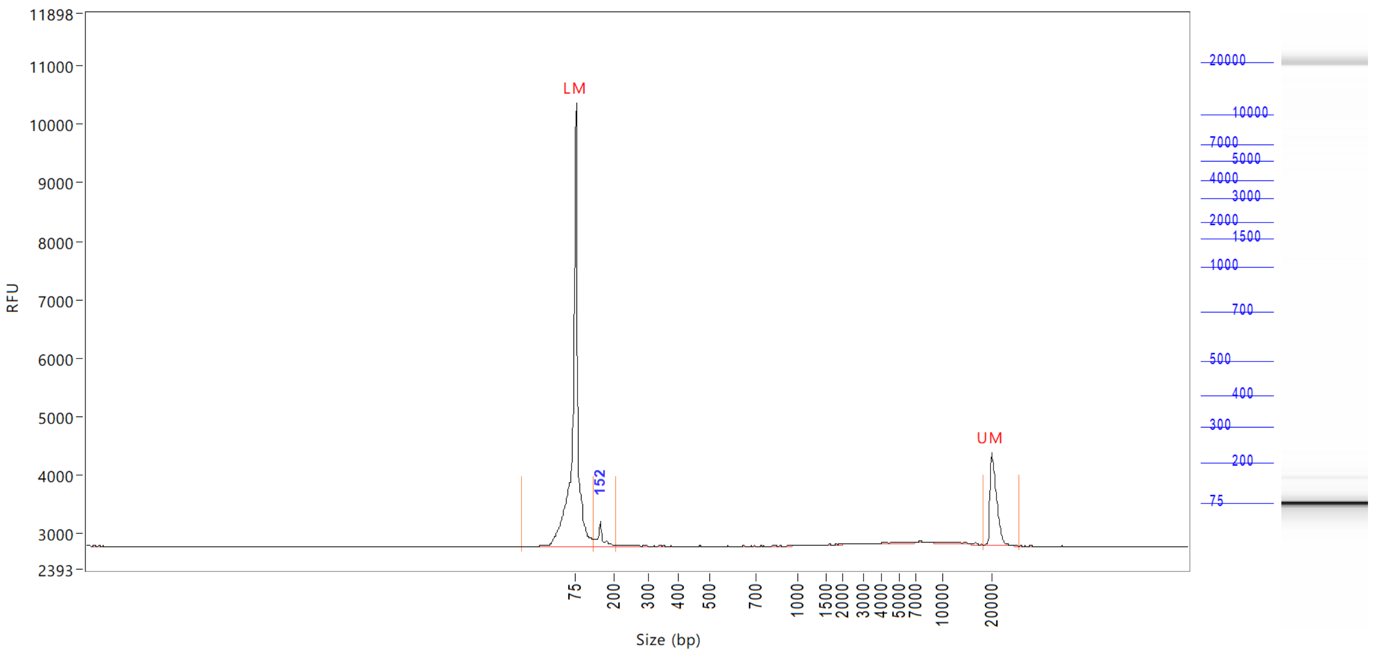

Peak	Size (bp)	Concentration (ng/uL)	From (bp)	To (bp)	Average size (bp)	CV%	RFU	Corrected peak area
1	75 (LM)	0.5000	0	133	65	35.36	7599	91.711
2	152	0.3028	133	201	155	9.31	420	4.628
3	20000 (UM)	0.0735	18415	25440	20358	3.78	1582	13.476

TIC:	0.3028	ng/uL
TIM:	3.2109	nmole/L
Total concentration:	0.5662	ng/uL

```
Sample peak width (sec): 5    Sample min peak height: 50    Sample baseline V to V?: Y    Sample baseline V to V points: 3
Sample filter: Binomial       Number of points for filter: 3  Sample start region (min): 0  Sample end region (min): 30
Marker peak width (sec): 5    Marker min peak height: 200   Marker baseline V to V?: Y    Marker baseline V to V points: 3
Lower marker selection: First peak > 500 RFU                Upper marker selection: Last peak > 200 RFU
Ladder size (bp)75, 200, 300, 400, 500, 700, 1000, 1500, 2000, 3000, 4000, 5000, 7000, 10000, 20000
Quantification using: Lower Marker          Final concentration (ng/uL): 0.5000          Dilution factor: 12.0
```

Sample: SampB4
Well location: B4
Created: Tuesday, September 14, 2021 8:54:07 PM

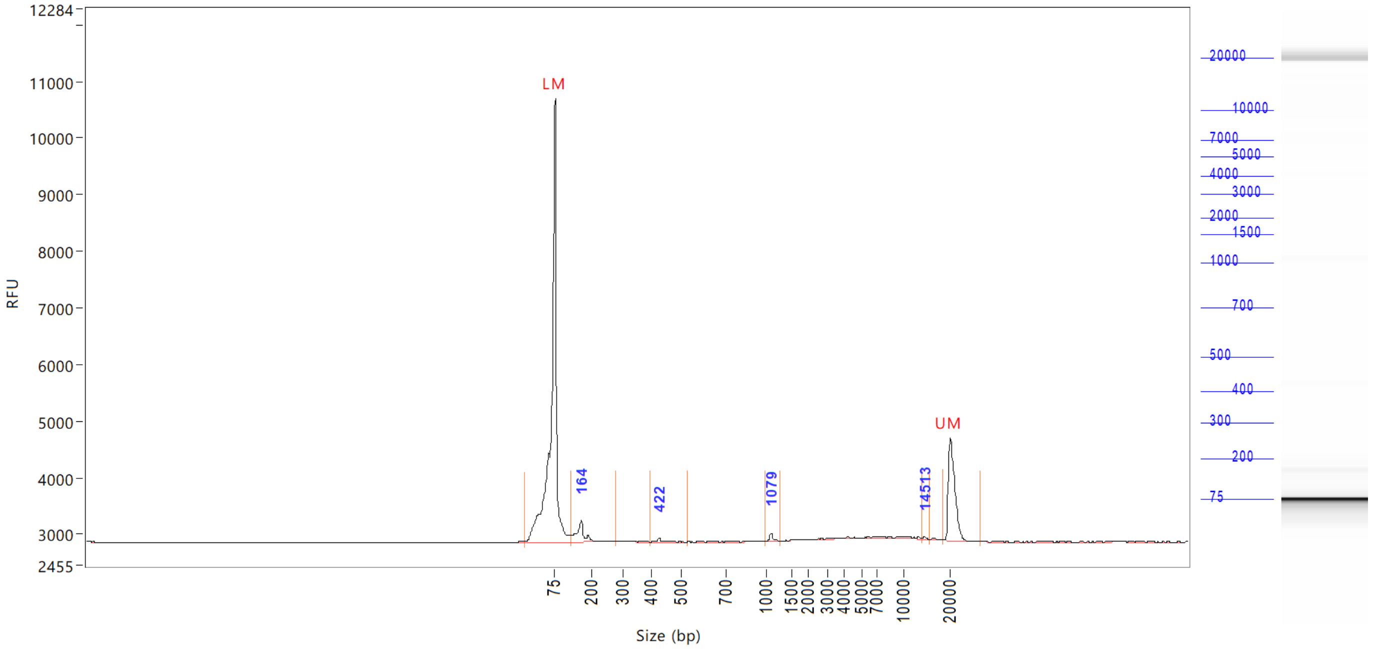

Peak	Size (bp)	Concentration (ng/uL)	From (bp)	To (bp)	Average size (bp)	CV%	RFU	Corrected peak area
1	75 (LM)	0.5000	0	129	60	38.24	7851	100.303
2	164	0.3972	129	277	163	14.34	381	6.641
3	422	0.0401	394	521	432	5.28	71	0.670
4	1079	0.0492	991	1256	1074	4.05	141	0.822
5	14513	0.0131	13781	15488	14318	2.08	51	0.219
6	20000 (UM)	0.0781	18537	26408	20368	3.71	1808	15.660

TIC:	0.4996	ng/uL
TIM:	4.2356	nmole/L
Total concentration:	0.6720	ng/uL

```
Sample peak width (sec): 5    Sample min peak height: 50    Sample baseline V to V?: Y    Sample baseline V to V points: 3
Sample filter: Binomial       Number of points for filter: 3  Sample start region (min): 0  Sample end region (min): 30
Marker peak width (sec): 5    Marker min peak height: 200   Marker baseline V to V?: Y    Marker baseline V to V points: 3
Lower marker selection: First peak > 500 RFU                Upper marker selection: Last peak > 200 RFU
Ladder size (bp)75, 200, 300, 400, 500, 700, 1000, 1500, 2000, 3000, 4000, 5000, 7000, 10000, 20000
Quantification using: Lower Marker          Final concentration (ng/uL): 0.5000          Dilution factor: 12.0
```

Sample: SampC4
Well location: C4
Created: Tuesday, September 14, 2021 8:54:07 PM

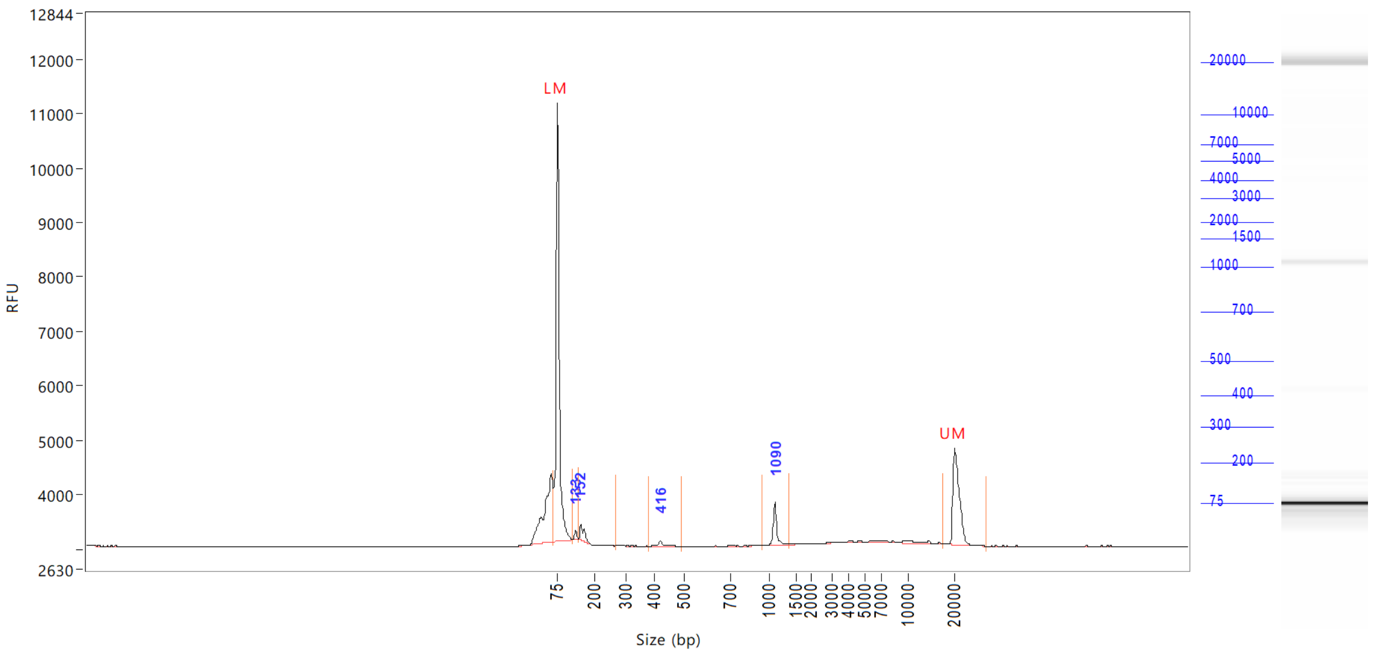

Peak	Size (bp)	Concentration (ng/uL)	From (bp)	To (bp)	Average size (bp)	CV%	RFU	Corrected peak area
1	75 (LM)	0.5000	60	124	74	10.99	8067	59.922
2	133	0.0843	124	145	133	3.16	163	0.842
3	152	0.2945	145	266	162	12.10	276	2.941
4	416	0.1021	376	487	421	4.44	115	1.020
5	1090	0.4798	948	1378	1089	5.31	798	4.792
6	20000 (UM)	0.1268	17561	26770	20395	3.74	1767	15.193

TIC:	0.9608	ng/uL
TIM:	5.1542	nmole/L
Total concentration:	1.3205	ng/uL

```
Sample peak width (sec): 5    Sample min peak height: 50    Sample baseline V to V?: Y    Sample baseline V to V points: 3
Sample filter: Binomial       Number of points for filter: 3  Sample start region (min): 0  Sample end region (min): 30
Marker peak width (sec): 5    Marker min peak height: 200   Marker baseline V to V?: Y    Marker baseline V to V points: 3
Lower marker selection: First peak > 500 RFU                Upper marker selection: Last peak > 200 RFU
Ladder size (bp)75, 200, 300, 400, 500, 700, 1000, 1500, 2000, 3000, 4000, 5000, 7000, 10000, 20000
Quantification using: Lower Marker          Final concentration (ng/uL): 0.5000          Dilution factor: 12.0
```

Sample: SampD4
Well location: D4
Created: Tuesday, September 14, 2021 8:54:07 PM

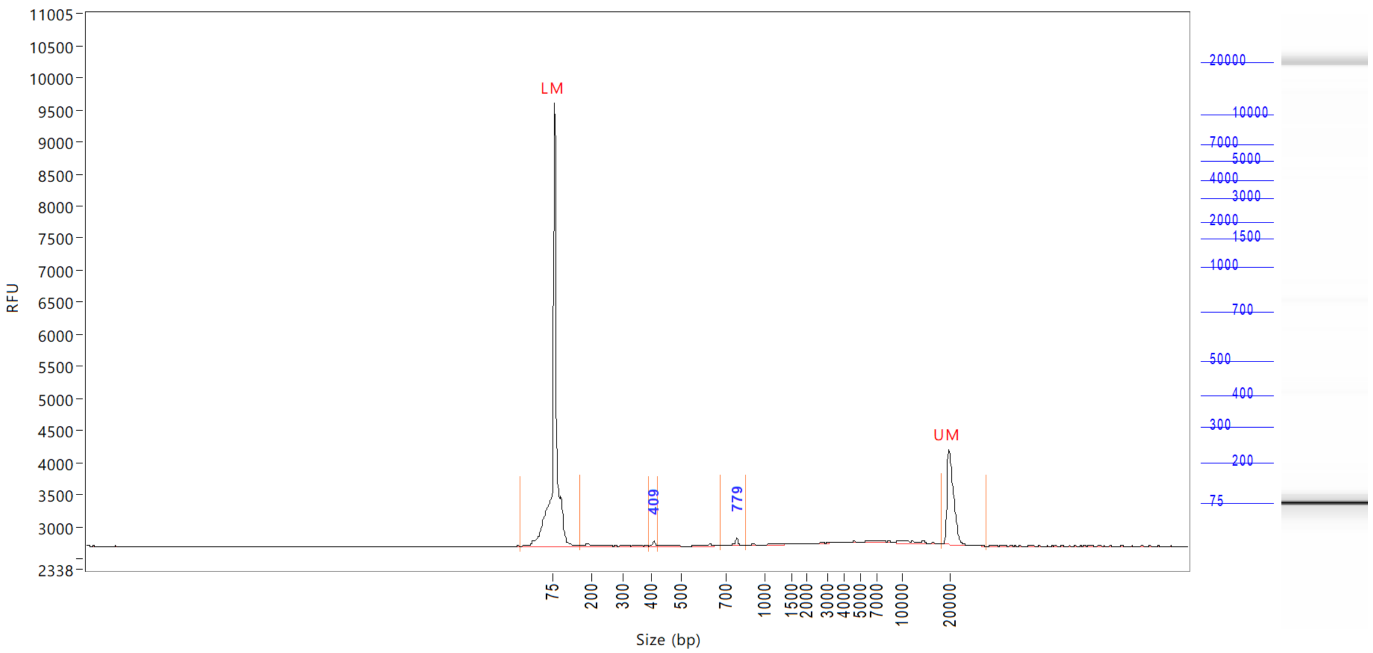

Peak	Size (bp)	Concentration (ng/uL)	From (bp)	To (bp)	Average size (bp)	CV%	RFU	Corrected peak area
1	75 (LM)	0.5000	0	164	69	30.17	6924	68.916
2	409	0.0466	390	418	405	1.38	84	0.535
3	779	0.0691	677	844	769	3.26	114	0.794
4	20000 (UM)	0.0925	18293	27979	20377	3.94	1472	12.752

TIC:	0.1157	ng/uL
TIM:	0.3373	nmole/L
Total concentration:	0.5638	ng/uL

Sample peak width (sec): 5 Sample min peak height: 50 Sample baseline V to V?: Y Sample baseline V to V points: 3
Sample filter: Binomial Number of points for filter: 3 Sample start region (min): 0 Sample end region (min): 30
Marker peak width (sec): 5 Marker min peak height: 200 Marker baseline V to V?: Y Marker baseline V to V points: 3
Lower marker selection: First peak > 500 RFU Upper marker selection: Last peak > 200 RFU
Ladder size (bp)75, 200, 300, 400, 500, 700, 1000, 1500, 2000, 3000, 4000, 5000, 7000, 10000, 20000
Quantification using: Lower Marker Final concentration (ng/uL): 0.5000 Dilution factor: 12.0

Sample: SampE4
Well location: E4
Created: Tuesday, September 14, 2021 8:54:07 PM

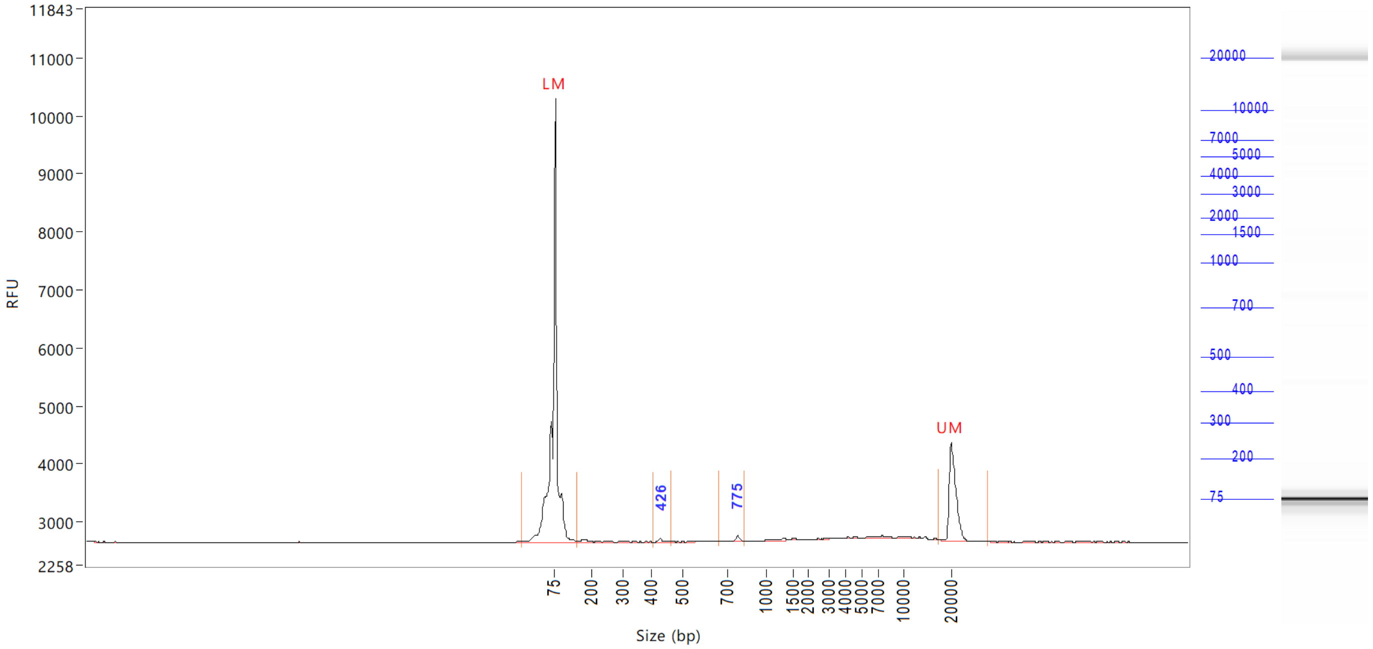

Peak	Size (bp)	Concentration (ng/uL)	From (bp)	To (bp)	Average size (bp)	CV%	RFU	Corrected peak area
1	75 (LM)	0.5000	0	148	66	31.23	7658	85.594
2	426	0.0407	403	460	426	2.45	78	0.580
3	775	0.0591	664	831	767	3.81	99	0.842
4	20000 (UM)	0.0874	17317	27979	20465	4.04	1701	14.967

TIC:	0.0997	ng/uL
TIM:	0.2838	nmole/L
Total concentration:	0.4511	ng/uL

Sample peak width (sec): 5   Sample min peak height: 50   Sample baseline V to V?: Y   Sample baseline V to V points: 3
Sample filter: Binomial   Number of points for filter: 3 Sample start region (min): 0 Sample end region (min): 30
Marker peak width (sec): 5   Marker min peak height: 200   Marker baseline V to V?: Y   Marker baseline V to V points: 3
Lower marker selection: First peak > 500 RFU   Upper marker selection: Last peak > 200 RFU
Ladder size (bp)75, 200, 300, 400, 500, 700, 1000, 1500, 2000, 3000, 4000, 5000, 7000, 10000, 20000
Quantification using: Lower Marker   Final concentration (ng/uL): 0.5000   Dilution factor: 12.0

Sample: SampF4
Well location: F4
Created: Tuesday, September 14, 2021 8:54:07 PM

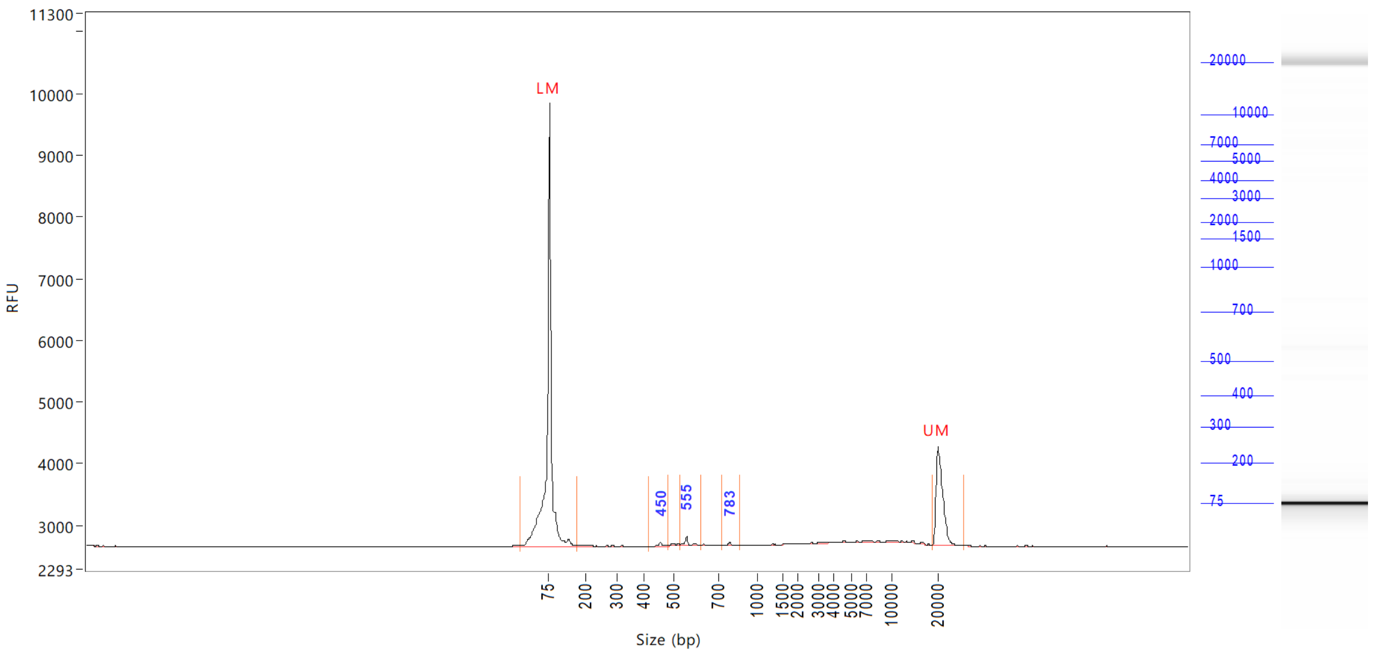

Peak	Size (bp)	Concentration (ng/uL)	From (bp)	To (bp)	Average size (bp)	CV%	RFU	Corrected peak area
1	75 (LM)	0.5000	0	169	67	34.30	7199	75.254
2	450	0.0386	411	475	448	2.30	68	0.484
3	555	0.1045	521	618	559	3.97	153	1.311
4	783	0.0316	731	861	783	3.46	54	0.397
5	20000 (UM)	0.0952	18659	25682	20383	3.92	1598	14.323

TIC:	0.1748	ng/uL
TIM:	0.5159	nmole/L
Total concentration:	0.4783	ng/uL

Sample peak width (sec): 5   Sample min peak height: 50   Sample baseline V to V?: Y   Sample baseline V to V points: 3
Sample filter: Binomial   Number of points for filter: 3 Sample start region (min): 0 Sample end region (min): 30
Marker peak width (sec): 5   Marker min peak height: 200   Marker baseline V to V?: Y   Marker baseline V to V points: 3
Lower marker selection: First peak > 500 RFU   Upper marker selection: Last peak > 200 RFU
Ladder size (bp)75, 200, 300, 400, 500, 700, 1000, 1500, 2000, 3000, 4000, 5000, 7000, 10000, 20000
Quantification using: Lower Marker   Final concentration (ng/uL): 0.5000   Dilution factor: 12.0

Sample: SampG4
Well location: G4
Created: Tuesday, September 14, 2021 8:54:07 PM

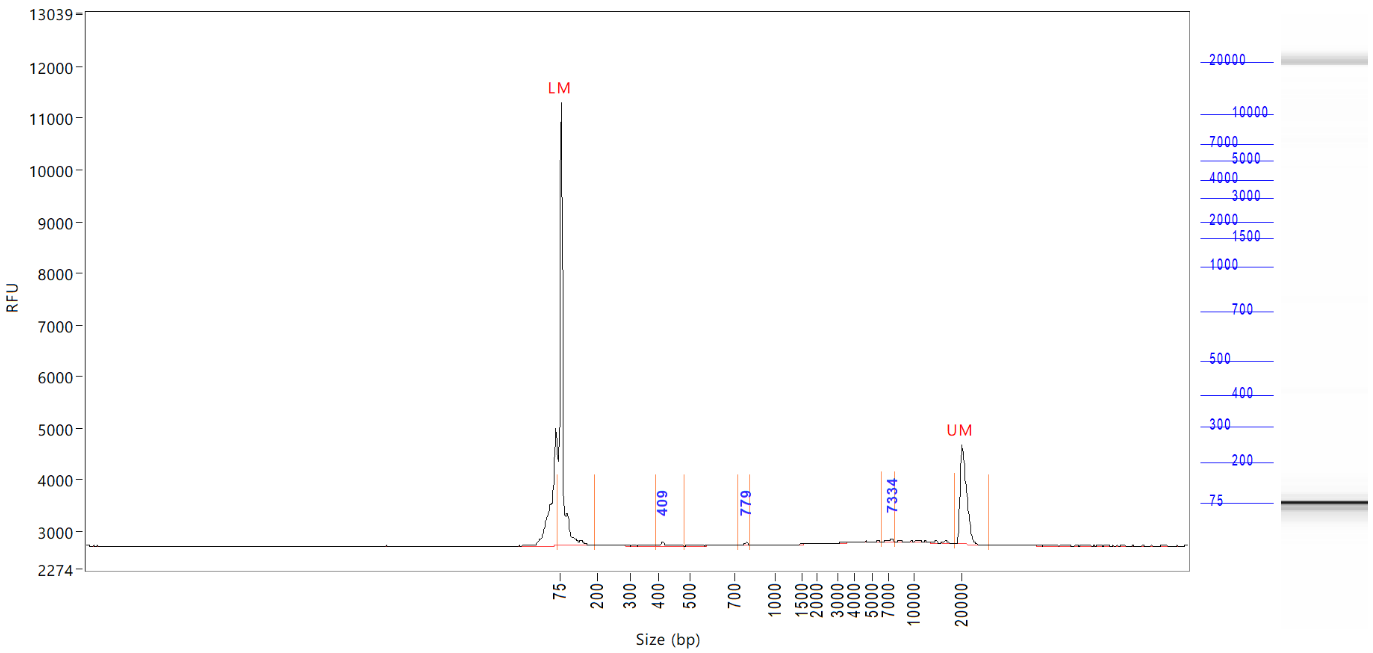

Peak	Size (bp)	Concentration (ng/uL)	From (bp)	To (bp)	Average size (bp)	CV%	RFU	Corrected peak area
1	75 (LM)	0.5000	66	187	78	18.14	8592	63.932
2	409	0.0677	386	483	418	4.73	72	0.722
3	779	0.0339	727	809	770	2.20	54	0.361
4	7334	0.0433	6260	7867	7134	5.07	62	0.462
5	20000 (UM)	0.1295	18537	25803	20432	3.59	1930	16.564

TIC:	0.1449	ng/uL
TIM:	0.3490	nmole/L
Total concentration:	0.4238	ng/uL

Sample peak width (sec): 5 Sample min peak height: 50 Sample baseline V to V?: Y Sample baseline V to V points: 3
Sample filter: Binomial Number of points for filter: 3 Sample start region (min): 0 Sample end region (min): 30
Marker peak width (sec): 5 Marker min peak height: 200 Marker baseline V to V?: Y Marker baseline V to V points: 3
Lower marker selection: First peak > 500 RFU Upper marker selection: Last peak > 200 RFU
Ladder size (bp)75, 200, 300, 400, 500, 700, 1000, 1500, 2000, 3000, 4000, 5000, 7000, 10000, 20000
Quantification using: Lower Marker Final concentration (ng/uL): 0.5000 Dilution factor: 12.0

Sample: SampH4
Well location: H4
Created: Tuesday, September 14, 2021 8:54:07 PM

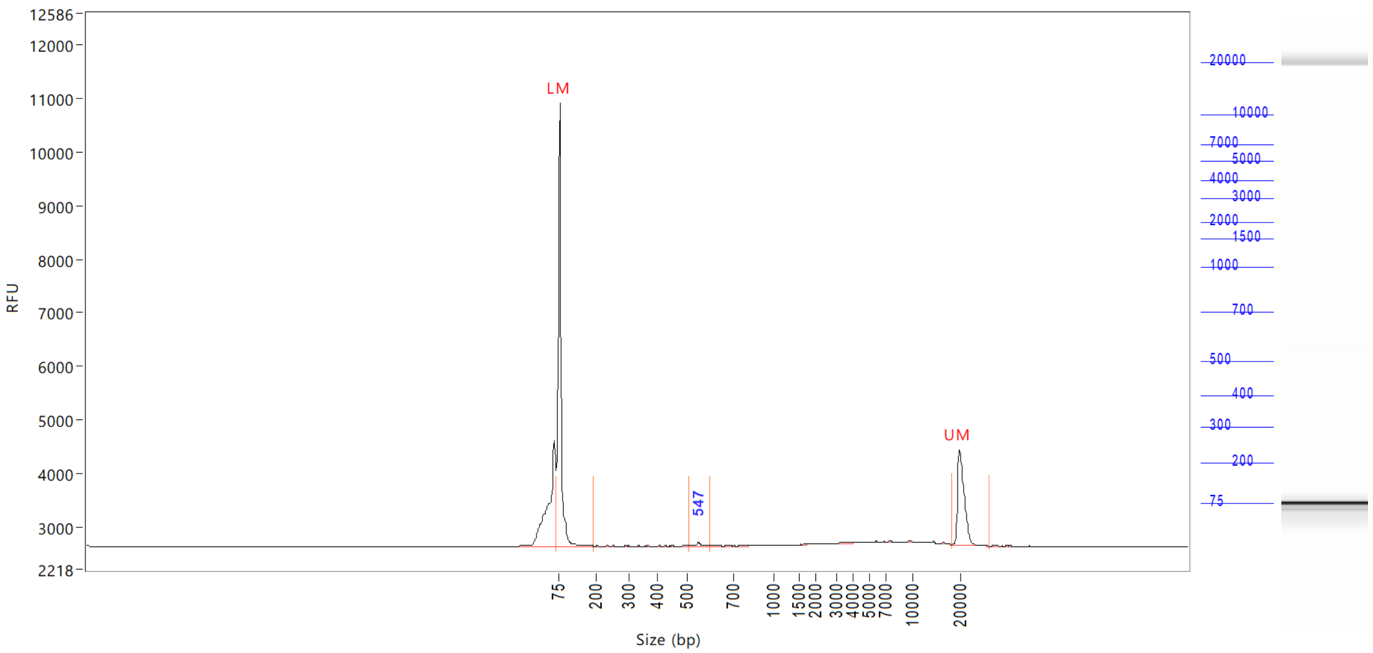

Peak	Size (bp)	Concentration (ng/uL)	From (bp)	To (bp)	Average size (bp)	CV%	RFU	Corrected peak area
1	75 (LM)	0.5000	66	190	76	15.29	8285	58.713
2	547	0.0865	504	595	546	4.18	70	0.847
3	20000 (UM)	0.1301	18293	26287	20396	3.80	1766	15.274

TIC:	0.0865	ng/uL
TIM:	0.2607	nmole/L
Total concentration:	0.4562	ng/uL

```
Sample peak width (sec): 5    Sample min peak height: 50    Sample baseline V to V?: Y    Sample baseline V to V points: 3
Sample filter: Binomial       Number of points for filter: 3  Sample start region (min): 0  Sample end region (min): 30
Marker peak width (sec): 5    Marker min peak height: 200   Marker baseline V to V?: Y    Marker baseline V to V points: 3
Lower marker selection: First peak > 500 RFU                Upper marker selection: Last peak > 200 RFU
Ladder size (bp)75, 200, 300, 400, 500, 700, 1000, 1500, 2000, 3000, 4000, 5000, 7000, 10000, 20000
Quantification using: Lower Marker          Final concentration (ng/uL): 0.5000          Dilution factor: 12.0
```

Sample: SampA5
Well location: A5
Created: Tuesday, September 14, 2021 8:54:07 PM

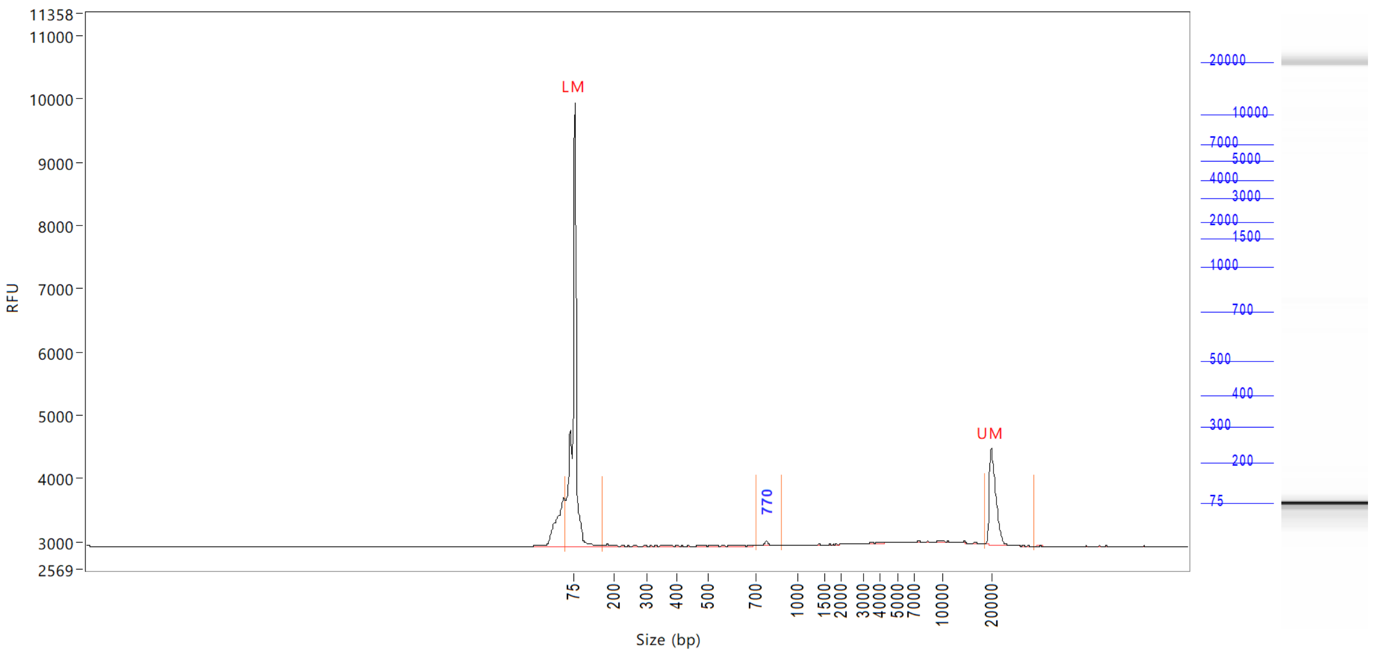

Peak	Size (bp)	Concentration (ng/uL)	From (bp)	To (bp)	Average size (bp)	CV%	RFU	Corrected peak area
1	75 (LM)	0.5000	47	166	71	17.17	7022	63.228
2	770	0.0602	705	883	768	3.88	71	0.635
3	20000 (UM)	0.1026	18781	28584	20329	3.86	1517	12.968

TIC:	0.0602	ng/uL
TIM:	0.1291	nmole/L
Total concentration:	0.3743	ng/uL

```
Sample peak width (sec): 5    Sample min peak height: 50    Sample baseline V to V?: Y    Sample baseline V to V points: 3
Sample filter: Binomial       Number of points for filter: 3  Sample start region (min): 0  Sample end region (min): 30
Marker peak width (sec): 5    Marker min peak height: 200   Marker baseline V to V?: Y    Marker baseline V to V points: 3
Lower marker selection: First peak > 500 RFU                Upper marker selection: Last peak > 200 RFU
Ladder size (bp)75, 200, 300, 400, 500, 700, 1000, 1500, 2000, 3000, 4000, 5000, 7000, 10000, 20000
Quantification using: Lower Marker          Final concentration (ng/uL): 0.5000          Dilution factor: 12.0
```

Sample: SampB5
Well location: B5
Created: Tuesday, September 14, 2021 8:54:07 PM

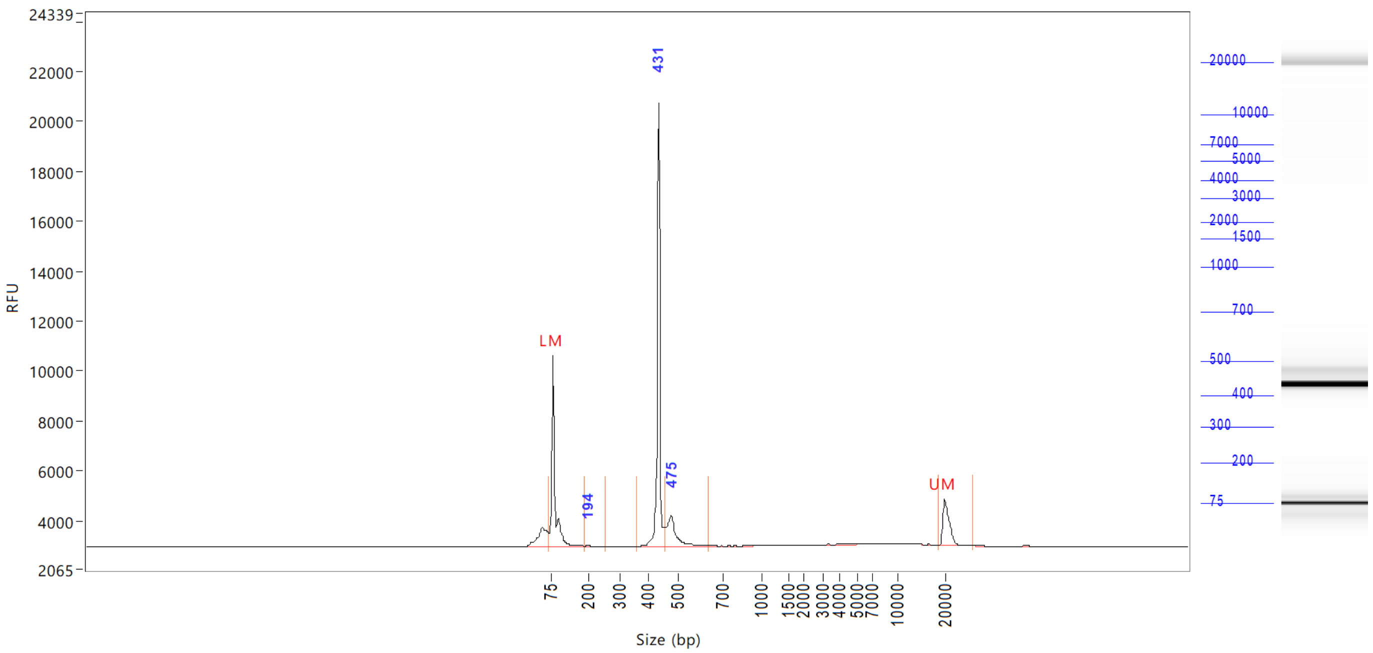

Peak	Size (bp)	Concentration (ng/uL)	From (bp)	To (bp)	Average size (bp)	CV%	RFU	Corrected peak area
1	75 (LM)	0.5000	66	183	82	20.98	7683	61.301
2	194	0.0795	183	251	204	8.50	53	0.812
3	431	9.8827	356	454	429	2.01	17800	100.970
4	475	2.2286	454	634	484	6.12	1268	22.769
5	20000 (UM)	0.1306	18537	26045	20380	3.95	1838	16.011

TIC:	12.1908	ng/uL
TIM:	46.1209	nmole/L
Total concentration:	12.4446	ng/uL

Sample peak width (sec): 5   Sample min peak height: 50   Sample baseline V to V?: Y   Sample baseline V to V points: 3
Sample filter: Binomial   Number of points for filter: 3 Sample start region (min): 0 Sample end region (min): 30
Marker peak width (sec): 5   Marker min peak height: 200   Marker baseline V to V?: Y   Marker baseline V to V points: 3
Lower marker selection: First peak > 500 RFU   Upper marker selection: Last peak > 200 RFU
Ladder size (bp)75, 200, 300, 400, 500, 700, 1000, 1500, 2000, 3000, 4000, 5000, 7000, 10000, 20000
Quantification using: Lower Marker   Final concentration (ng/uL): 0.5000   Dilution factor: 12.0

Sample: SampC5
Well location: C5
Created: Tuesday, September 14, 2021 8:54:07 PM

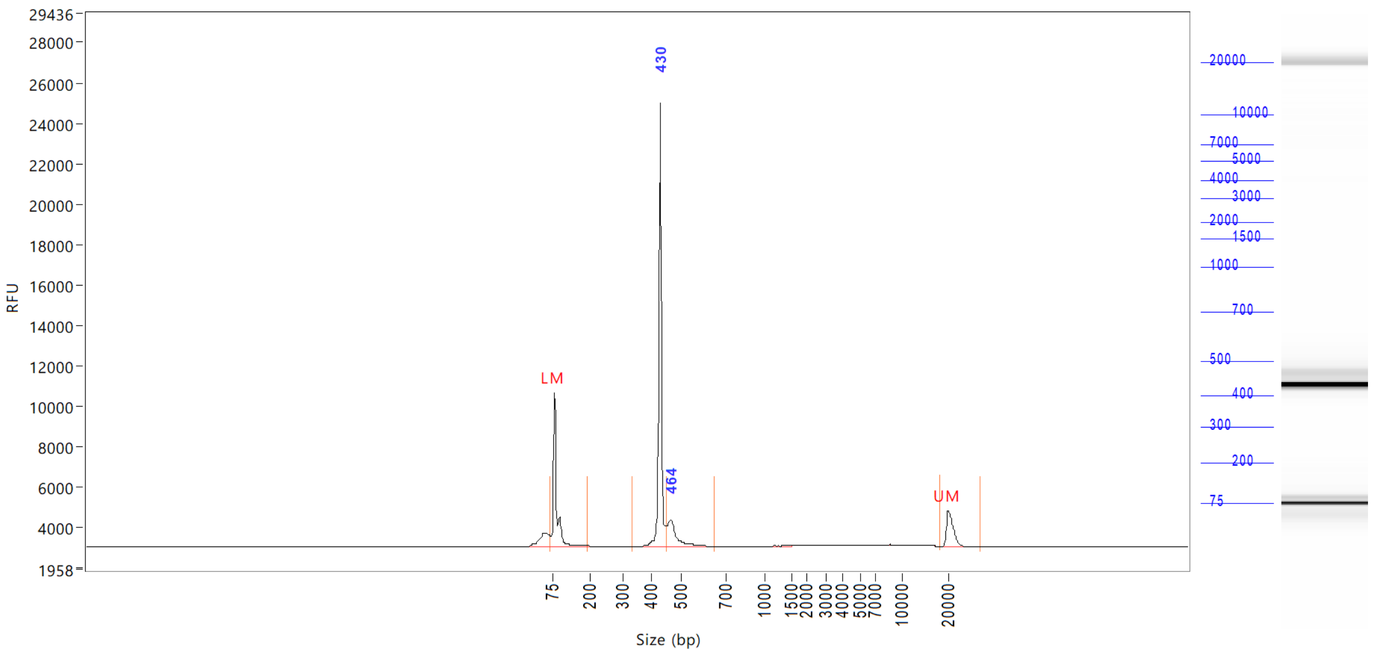

Peak	Size (bp)	Concentration (ng/uL)	From (bp)	To (bp)	Average size (bp)	CV%	RFU	Corrected peak area
1	75 (LM)	0.5000	62	187	81	21.89	7573	61.775
2	430	11.8568	333	450	427	2.07	21962	122.077
3	464	2.3881	450	652	478	6.13	1327	24.588
4	20000 (UM)	0.1253	18293	26649	20402	3.88	1770	15.479

TIC:	14.2450	ng/uL
TIM:	53.9089	nmole/L
Total concentration:	14.4689	ng/uL

```
Sample peak width (sec): 5     Sample min peak height: 50    Sample baseline V to V?: Y    Sample baseline V to V points: 3
Sample filter: Binomial        Number of points for filter: 3  Sample start region (min): 0  Sample end region (min): 30
Marker peak width (sec): 5     Marker min peak height: 200   Marker baseline V to V?: Y    Marker baseline V to V points: 3
Lower marker selection: First peak > 500 RFU                 Upper marker selection: Last peak > 200 RFU
Ladder size (bp)75, 200, 300, 400, 500, 700, 1000, 1500, 2000, 3000, 4000, 5000, 7000, 10000, 20000
Quantification using: Lower Marker          Final concentration (ng/uL): 0.5000          Dilution factor: 12.0
```

Sample: SampD5
Well location: D5
Created: Tuesday, September 14, 2021 8:54:07 PM

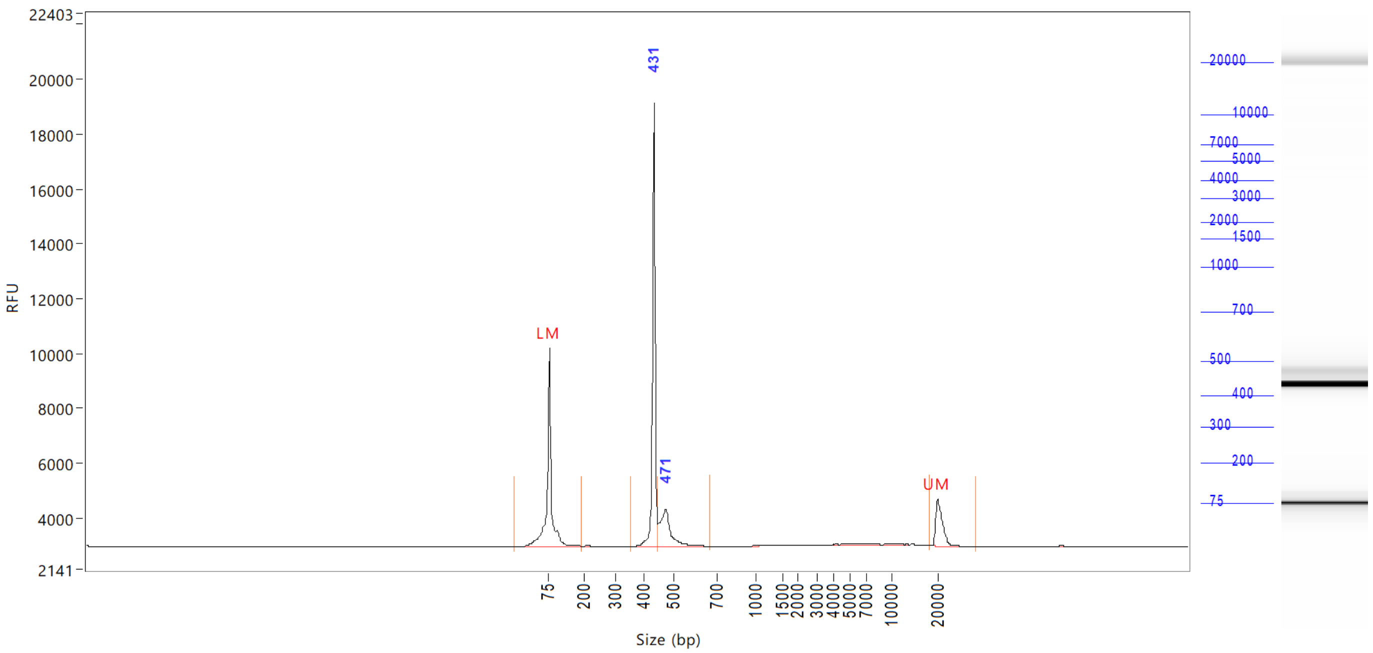

Peak	Size<br>(bp)	Concentration<br>(ng/uL)	From<br>(bp)	To<br>(bp)	Average size<br>(bp)	CV%	RFU	Corrected peak area
1	75 (LM)	0.5000	0	187	72	32.33	7266	75.072
2	431	7.4128	352	445	428	1.88	16192	92.750
3	471	2.2685	445	667	476	5.88	1356	28.384
4	20000 (UM)	0.1014	18415	28221	20487	4.30	1699	15.227

TIC:	9.6813	ng/uL
TIM:	36.3390	nmole/L
Total concentration:	9.8902	ng/uL

Sample peak width (sec): 5 Sample min peak height: 50 Sample baseline V to V?: Y Sample baseline V to V points: 3
Sample filter: Binomial Number of points for filter: 3 Sample start region (min): 0 Sample end region (min): 30
Marker peak width (sec): 5 Marker min peak height: 200 Marker baseline V to V?: Y Marker baseline V to V points: 3
Lower marker selection: First peak > 500 RFU Upper marker selection: Last peak > 200 RFU
Ladder size (bp)75, 200, 300, 400, 500, 700, 1000, 1500, 2000, 3000, 4000, 5000, 7000, 10000, 20000
Quantification using: Lower Marker Final concentration (ng/uL): 0.5000 Dilution factor: 12.0

Sample: SampE5
Well location: E5
Created: Tuesday, September 14, 2021 8:54:07 PM

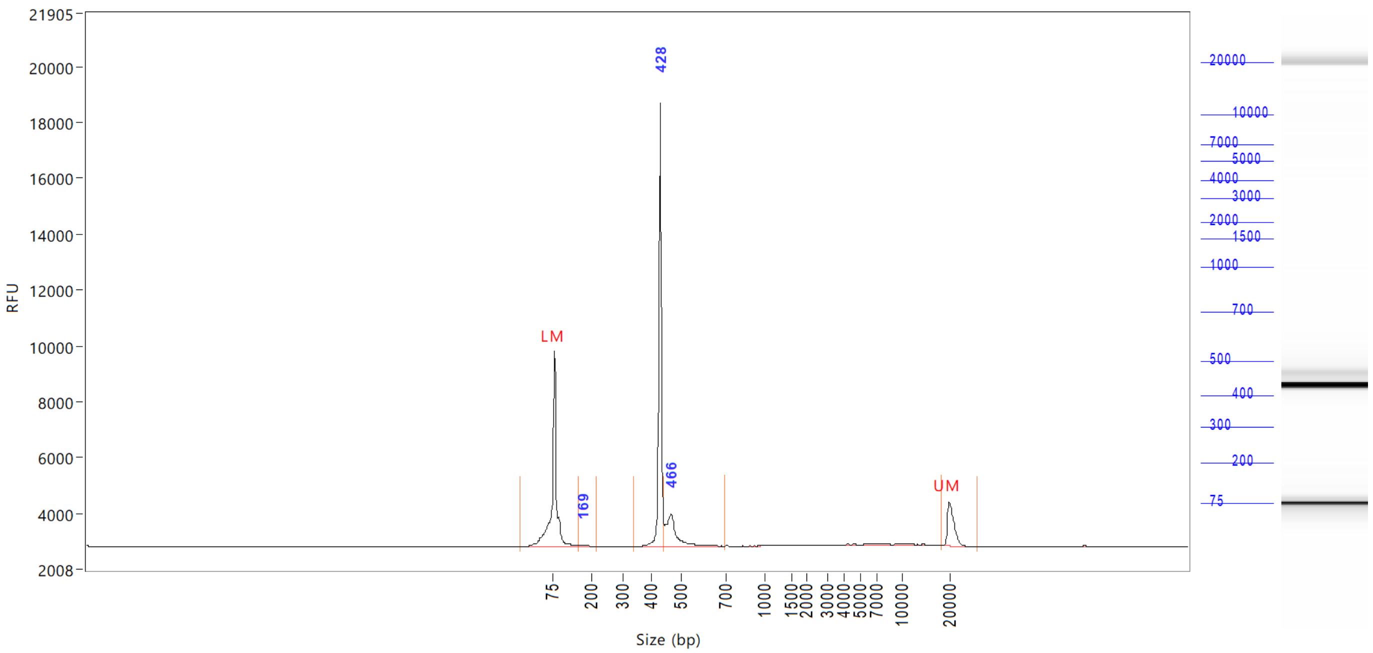

Peak	Size (bp)	Concentration (ng/uL)	From (bp)	To (bp)	Average size (bp)	CV%	RFU	Corrected peak area
1	75 (LM)	0.5000	0	154	68	30.87	7015	73.171
2	169	0.0952	154	215	176	8.33	55	1.161
3	428	7.1102	337	443	425	1.76	15898	86.711
4	466	1.8765	443	695	474	6.81	1166	22.884
5	20000 (UM)	0.0921	18293	25924	20447	3.67	1558	13.484

TIC:	9.0819	ng/uL
TIM:	34.9297	nmole/L
Total concentration:	9.2472	ng/uL

Sample peak width (sec): 5 Sample min peak height: 50 Sample baseline V to V?: Y Sample baseline V to V points: 3
Sample filter: Binomial Number of points for filter: 3 Sample start region (min): 0 Sample end region (min): 30
Marker peak width (sec): 5 Marker min peak height: 200 Marker baseline V to V?: Y Marker baseline V to V points: 3
Lower marker selection: First peak > 500 RFU Upper marker selection: Last peak > 200 RFU
Ladder size (bp)75, 200, 300, 400, 500, 700, 1000, 1500, 2000, 3000, 4000, 5000, 7000, 10000, 20000
Quantification using: Lower Marker Final concentration (ng/uL): 0.5000 Dilution factor: 12.0

Sample: SampF5
Well location: F5
Created: Tuesday, September 14, 2021 8:54:07 PM

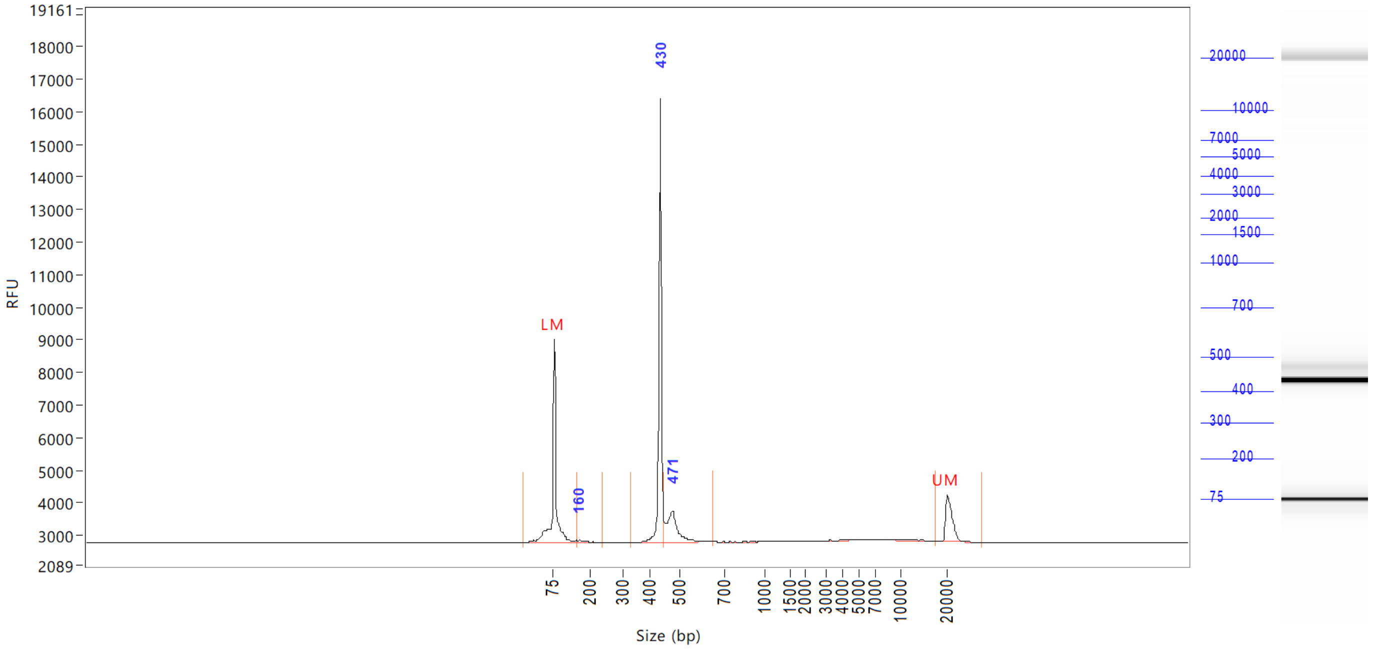

Peak	Size (bp)	Concentration (ng/uL)	From (bp)	To (bp)	Average size (bp)	CV%	RFU	Corrected peak area
1	75 (LM)	0.5000	0	152	70	29.86	6220	54.513
2	160	0.1074	152	237	172	10.20	62	0.976
3	430	7.9776	329	445	427	1.85	13636	72.481
4	471	2.2021	445	644	476	5.61	964	20.007
5	20000 (UM)	0.1160	17317	27375	20440	3.92	1449	12.649

TIC:	10.2871	ng/uL
TIM:	39.3788	nmole/L
Total concentration:	10.4966	ng/uL

Sample peak width (sec): 5 Sample min peak height: 50 Sample baseline V to V?: Y Sample baseline V to V points: 3
Sample filter: Binomial Number of points for filter: 3 Sample start region (min): 0 Sample end region (min): 30
Marker peak width (sec): 5 Marker min peak height: 200 Marker baseline V to V?: Y Marker baseline V to V points: 3
Lower marker selection: First peak > 500 RFU Upper marker selection: Last peak > 200 RFU
Ladder size (bp)75, 200, 300, 400, 500, 700, 1000, 1500, 2000, 3000, 4000, 5000, 7000, 10000, 20000
Quantification using: Lower Marker Final concentration (ng/uL): 0.5000 Dilution factor: 12.0

Sample: SampG5
Well location: G5
Created: Tuesday, September 14, 2021 8:54:07 PM

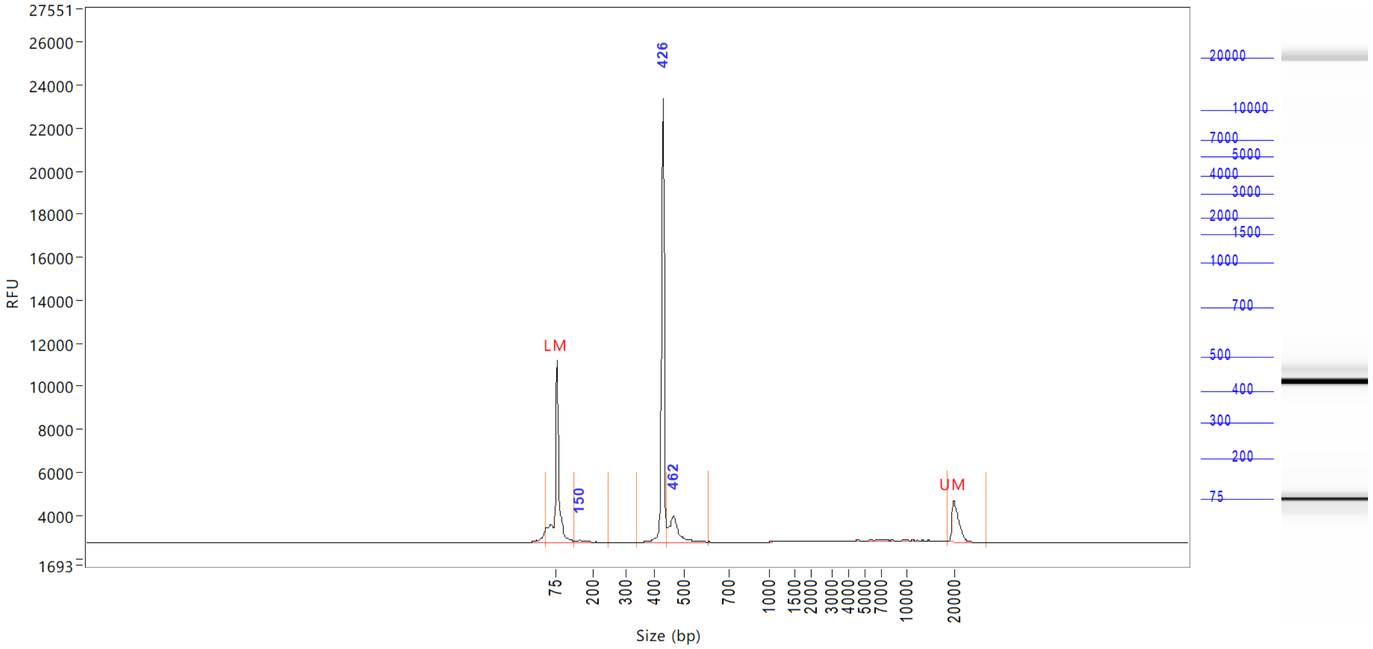

Peak	Size (bp)	Concentration (ng/uL)	From (bp)	To (bp)	Average size (bp)	CV%	RFU	Corrected peak area
1	75 (LM)	0.5000	39	131	73	19.50	8501	74.312
2	150	0.2471	131	246	164	15.63	110	3.060
3	426	8.5529	339	441	424	1.72	20661	105.931
4	462	1.7923	441	606	468	5.06	1241	22.198
5	20000 (UM)	0.1175	18659	26770	20426	4.11	1908	17.462

TIC:	10.5922	ng/uL
TIM:	41.9677	nmole/L
Total concentration:	10.7667	ng/uL

Sample peak width (sec): 5   Sample min peak height: 50   Sample baseline V to V?: Y   Sample baseline V to V points: 3
Sample filter: Binomial   Number of points for filter: 3 Sample start region (min): 0 Sample end region (min): 30
Marker peak width (sec): 5   Marker min peak height: 200   Marker baseline V to V?: Y   Marker baseline V to V points: 3
Lower marker selection: First peak > 500 RFU   Upper marker selection: Last peak > 200 RFU
Ladder size (bp)75, 200, 300, 400, 500, 700, 1000, 1500, 2000, 3000, 4000, 5000, 7000, 10000, 20000
Quantification using: Lower Marker   Final concentration (ng/uL): 0.5000   Dilution factor: 12.0

Sample: SampH5
Well location: H5
Created: Tuesday, September 14, 2021 8:54:07 PM

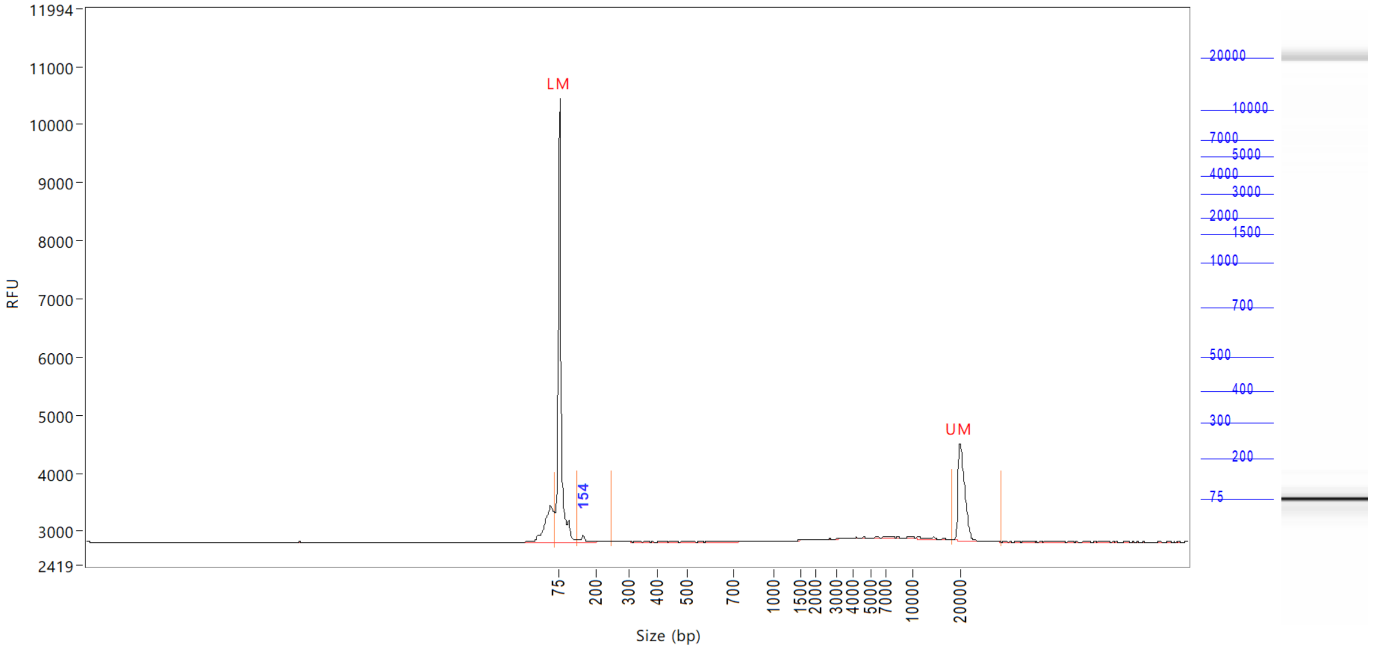

Peak	Size (bp)	Concentration (ng/uL)	From (bp)	To (bp)	Average size (bp)	CV%	RFU	Corrected peak area
1	75 (LM)	0.5000	62	135	76	13.02	7648	54.376
2	154	0.1365	135	246	160	14.43	121	1.237
3	20000 (UM)	0.1351	18293	28705	20384	4.11	1674	14.690

TIC:	0.1365	ng/uL
TIM:	1.4021	nmole/L
Total concentration:	0.5322	ng/uL

```
Sample peak width (sec): 5    Sample min peak height: 50    Sample baseline V to V?: Y    Sample baseline V to V points: 3
Sample filter: Binomial       Number of points for filter: 3  Sample start region (min): 0  Sample end region (min): 30
Marker peak width (sec): 5    Marker min peak height: 200   Marker baseline V to V?: Y    Marker baseline V to V points: 3
Lower marker selection: First peak > 500 RFU                Upper marker selection: Last peak > 200 RFU
Ladder size (bp)75, 200, 300, 400, 500, 700, 1000, 1500, 2000, 3000, 4000, 5000, 7000, 10000, 20000
Quantification using: Lower Marker          Final concentration (ng/uL): 0.5000          Dilution factor: 12.0
```

Sample: SampA6
Well location: A6
Created: Tuesday, September 14, 2021 8:54:07 PM

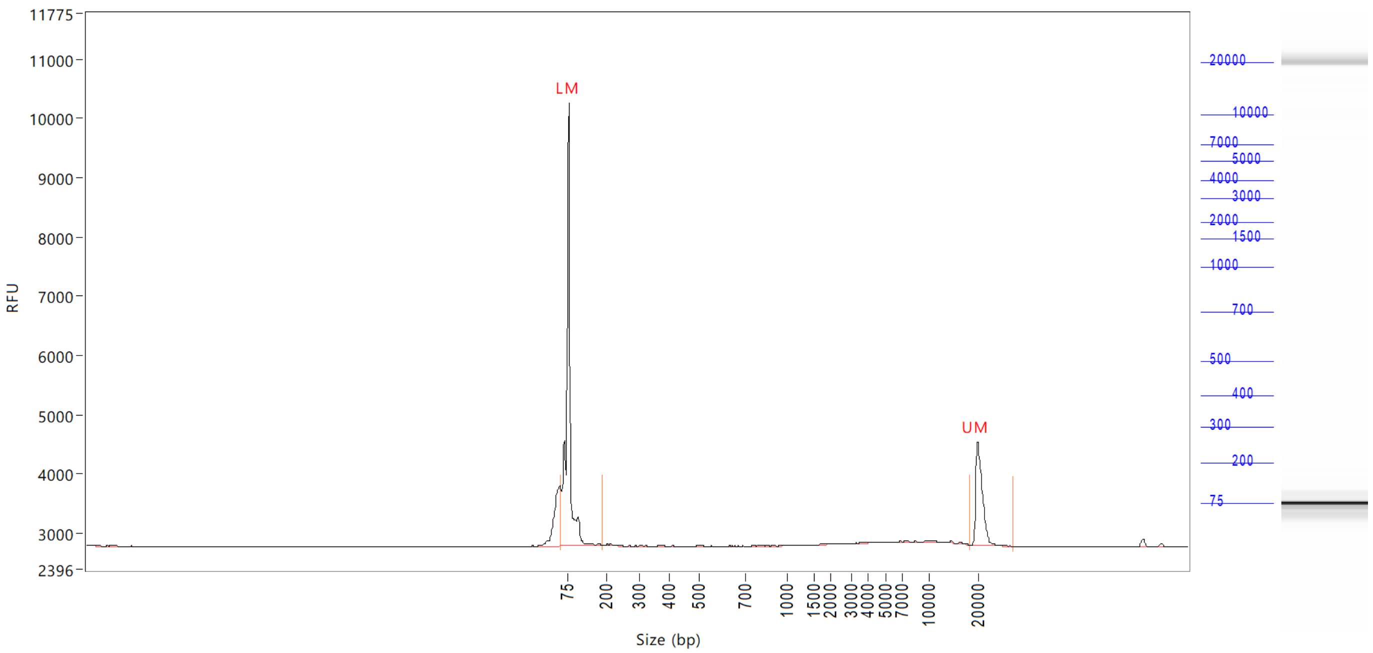

Peak	Size<br>(bp)	Concentration<br>(ng/uL)	From<br>(bp)	To<br>(bp)	Average size<br>(bp)	CV%	RFU	Corrected peak area
1	75 (LM)	0.5000	50	187	74	19.36	7484	64.996
2	20000 (UM)	0.1165	18537	27496	20501	4.21	1743	15.148

TIC:	0.0000	ng/uL
TIM:	0.0000	nmole/L
Total concentration:	0.3068	ng/uL

```
Sample peak width (sec): 5    Sample min peak height: 50    Sample baseline V to V?: Y    Sample baseline V to V points: 3
Sample filter: Binomial       Number of points for filter: 3  Sample start region (min): 0  Sample end region (min): 30
Marker peak width (sec): 5    Marker min peak height: 200   Marker baseline V to V?: Y    Marker baseline V to V points: 3
Lower marker selection: First peak > 500 RFU                Upper marker selection: Last peak > 200 RFU
Ladder size (bp)75, 200, 300, 400, 500, 700, 1000, 1500, 2000, 3000, 4000, 5000, 7000, 10000, 20000
Quantification using: Lower Marker          Final concentration (ng/uL): 0.5000          Dilution factor: 12.0
```

Sample: SampB6
Well location: B6
Created: Tuesday, September 14, 2021 8:54:07 PM

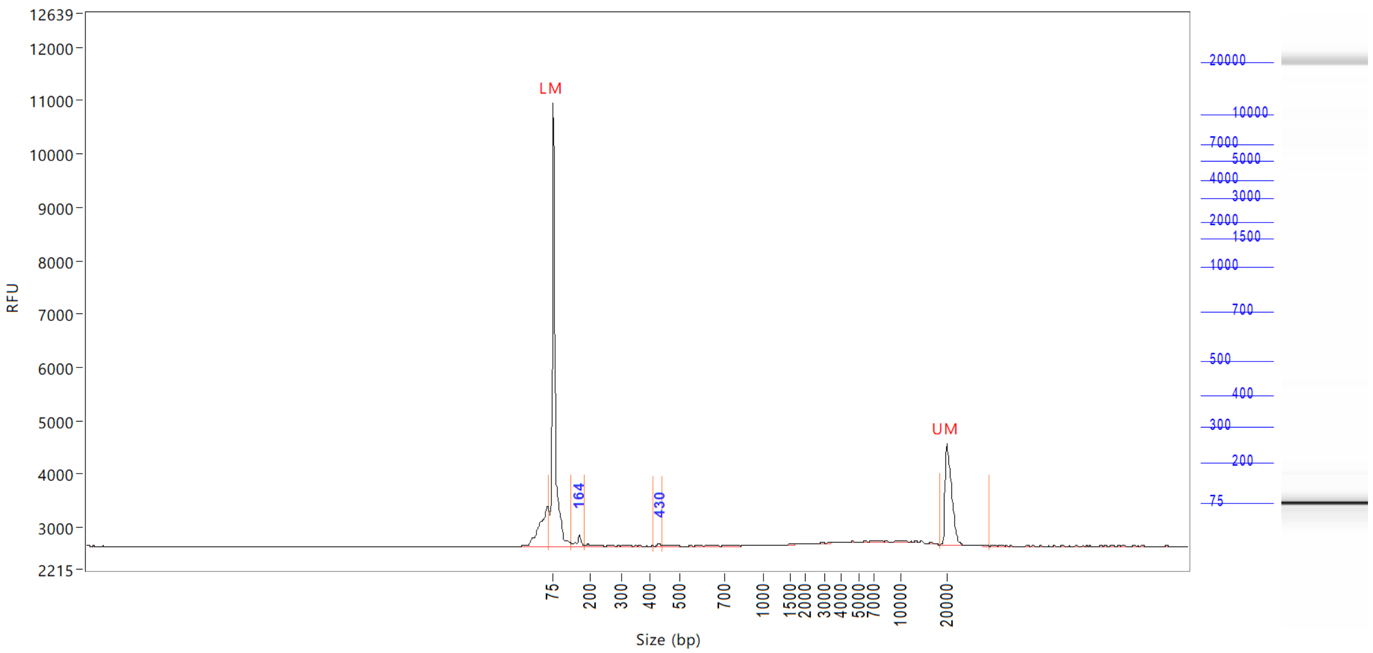

Peak	Size (bp)	Concentration (ng/uL)	From (bp)	To (bp)	Average size (bp)	CV%	RFU	Corrected peak area
1	75 (LM)	0.5000	62	137	77	13.78	8325	61.364
2	164	0.2085	137	183	158	6.44	221	2.133
3	430	0.0342	412	443	429	1.45	53	0.350
4	20000 (UM)	0.1349	18415	29189	20500	4.14	1888	16.562

TIC:	0.2427	ng/uL
TIM:	2.3005	nmole/L
Total concentration:	0.6330	ng/uL

Sample peak width (sec): 5   Sample min peak height: 50   Sample baseline V to V?: Y   Sample baseline V to V points: 3
Sample filter: Binomial   Number of points for filter: 3 Sample start region (min): 0 Sample end region (min): 30
Marker peak width (sec): 5   Marker min peak height: 200   Marker baseline V to V?: Y   Marker baseline V to V points: 3
Lower marker selection: First peak > 500 RFU   Upper marker selection: Last peak > 200 RFU
Ladder size (bp)75, 200, 300, 400, 500, 700, 1000, 1500, 2000, 3000, 4000, 5000, 7000, 10000, 20000
Quantification using: Lower Marker   Final concentration (ng/uL): 0.5000   Dilution factor: 12.0

Sample: SampC6
Well location: C6
Created: Tuesday, September 14, 2021 8:54:07 PM

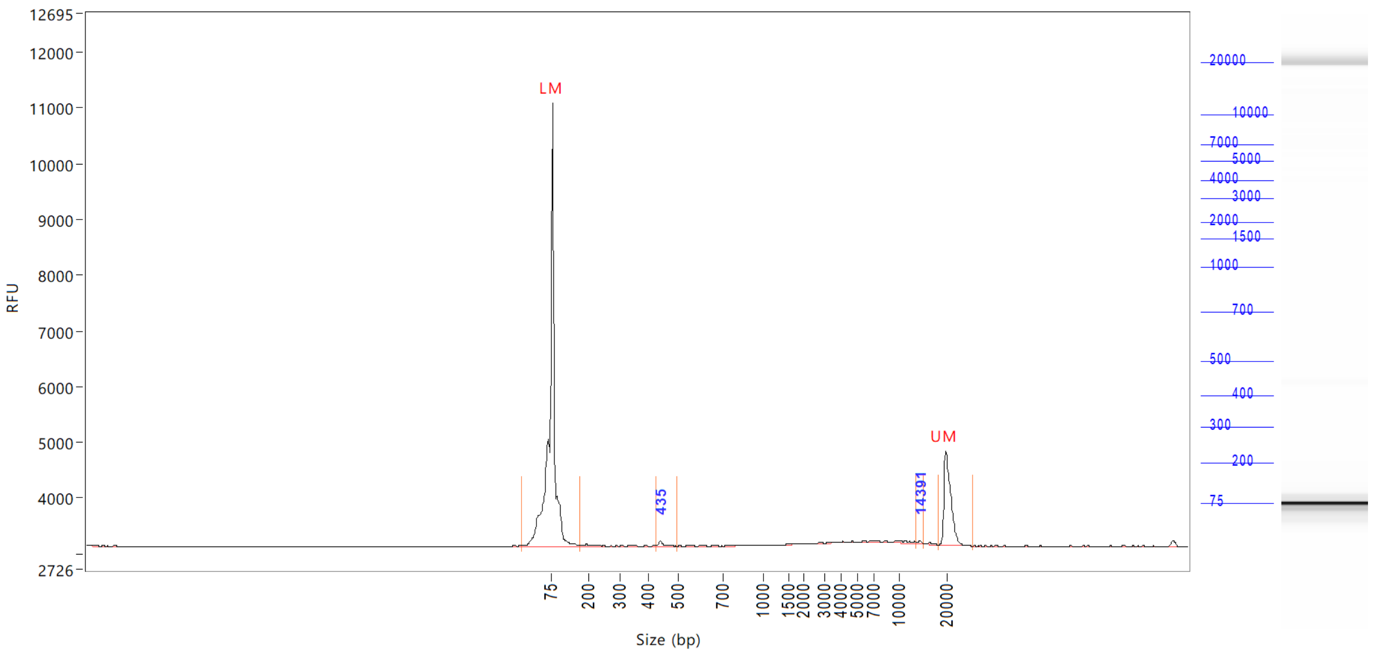

Peak	Size (bp)	Concentration (ng/uL)	From (bp)	To (bp)	Average size (bp)	CV%	RFU	Corrected peak area
1	75 (LM)	0.5000	0	168	65	34.20	7964	96.037
2	435	0.0585	422	492	442	3.34	105	0.936
3	14391	0.0205	13659	15244	14318	2.65	59	0.329
4	20000 (UM)	0.0798	18293	25682	20419	3.97	1684	15.332

TIC:	0.0790	ng/uL
TIM:	0.2200	nmole/L
Total concentration:	0.3615	ng/uL

Sample peak width (sec): 5 Sample min peak height: 50 Sample baseline V to V?: Y Sample baseline V to V points: 3
Sample filter: Binomial Number of points for filter: 3 Sample start region (min): 0 Sample end region (min): 30
Marker peak width (sec): 5 Marker min peak height: 200 Marker baseline V to V?: Y Marker baseline V to V points: 3
Lower marker selection: First peak > 500 RFU Upper marker selection: Last peak > 200 RFU
Ladder size (bp)75, 200, 300, 400, 500, 700, 1000, 1500, 2000, 3000, 4000, 5000, 7000, 10000, 20000
Quantification using: Lower Marker Final concentration (ng/uL): 0.5000 Dilution factor: 12.0

Sample: SampD6
Well location: D6
Created: Tuesday, September 14, 2021 8:54:07 PM

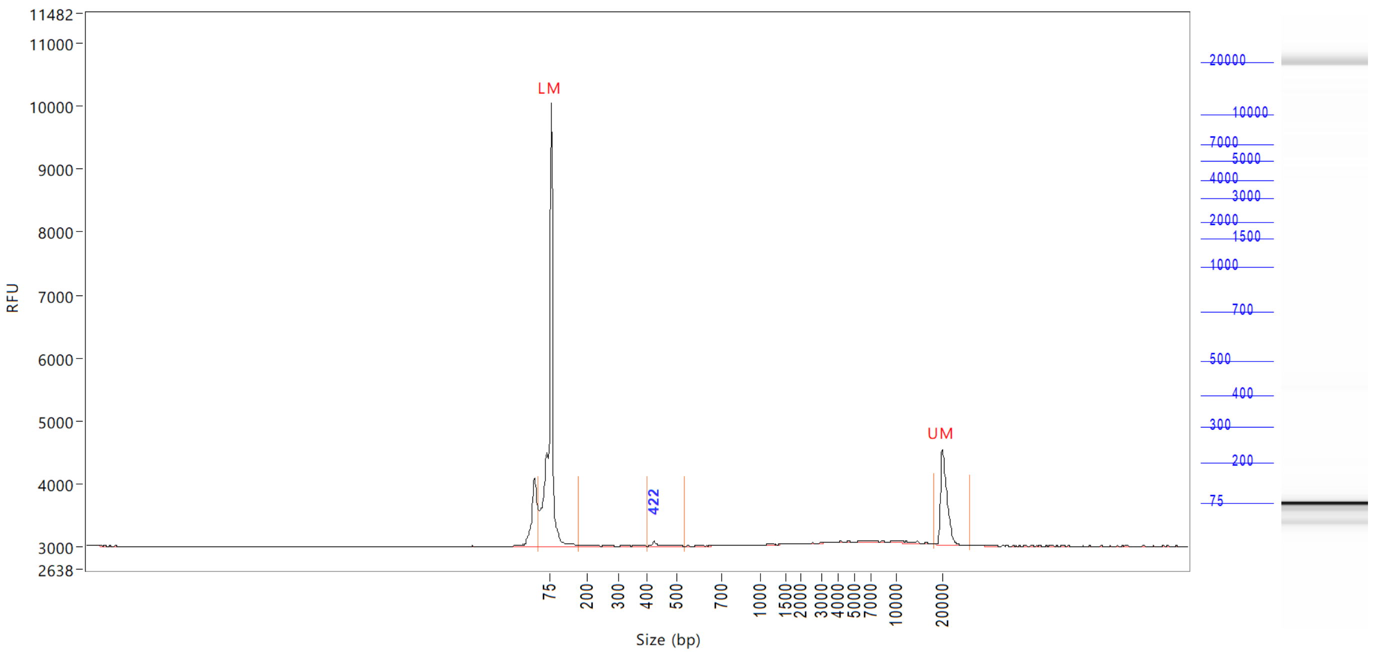

Peak	Size (bp)	Concentration (ng/uL)	From (bp)	To (bp)	Average size (bp)	CV%	RFU	Corrected peak area
1	75 (LM)	0.5000	33	166	68	22.41	7065	69.177
2	422	0.0922	400	532	440	6.35	85	1.063
3	20000 (UM)	0.0964	18171	25924	20415	3.85	1507	13.332

TIC:	0.0922	ng/uL
TIM:	0.3449	nmole/L
Total concentration:	0.4144	ng/uL

Sample peak width (sec): 5 Sample min peak height: 50 Sample baseline V to V?: Y Sample baseline V to V points: 3
Sample filter: Binomial Number of points for filter: 3 Sample start region (min): 0 Sample end region (min): 30
Marker peak width (sec): 5 Marker min peak height: 200 Marker baseline V to V?: Y Marker baseline V to V points: 3
Lower marker selection: First peak > 500 RFU Upper marker selection: Last peak > 200 RFU
Ladder size (bp)75, 200, 300, 400, 500, 700, 1000, 1500, 2000, 3000, 4000, 5000, 7000, 10000, 20000
Quantification using: Lower Marker Final concentration (ng/uL): 0.5000 Dilution factor: 12.0

Sample: SampE6
Well location: E6
Created: Tuesday, September 14, 2021 8:54:07 PM

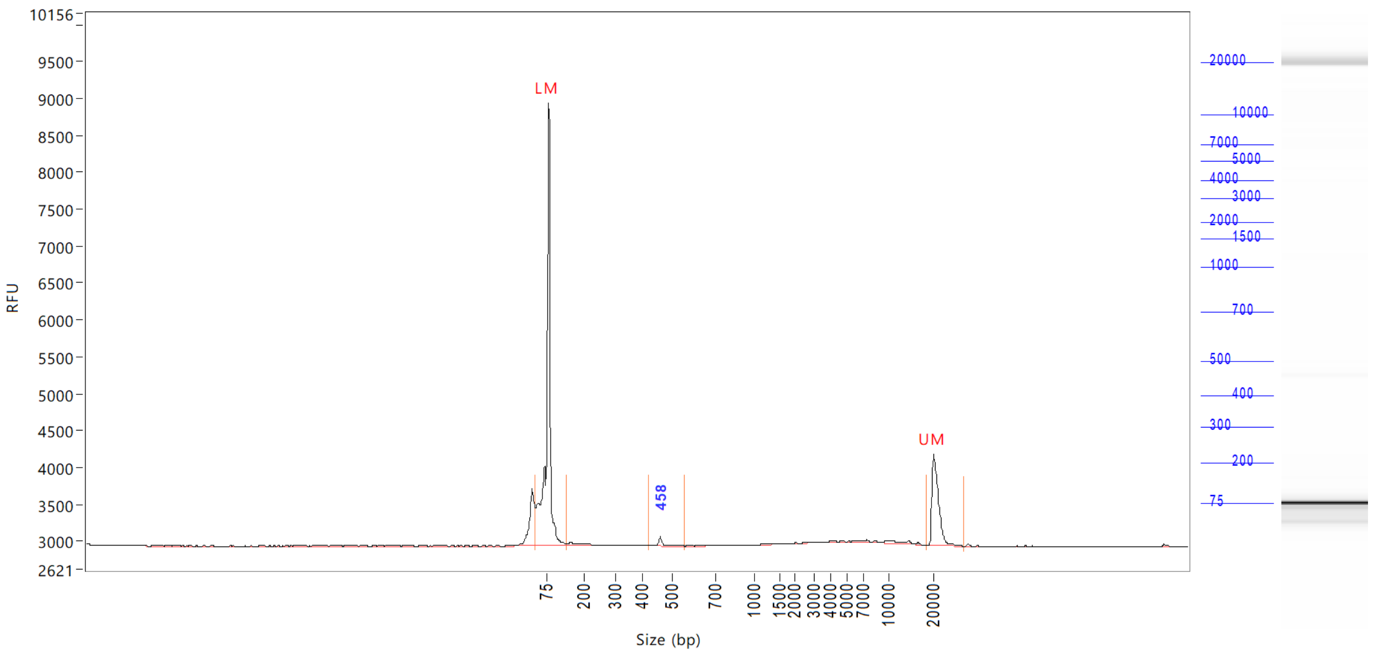

Peak	Size (bp)	Concentration (ng/uL)	From (bp)	To (bp)	Average size (bp)	CV%	RFU	Corrected peak area
1	75 (LM)	0.5000	30	139	68	23.11	6012	56.566
2	458	0.1211	418	555	466	4.75	130	1.142
3	20000 (UM)	0.0967	18537	26649	20472	4.60	1239	10.939

TIC:	0.1211	ng/uL
TIM:	0.4278	nmole/L
Total concentration:	0.6012	ng/uL

```
Sample peak width (sec): 5    Sample min peak height: 50    Sample baseline V to V?: Y    Sample baseline V to V points: 3
Sample filter: Binomial       Number of points for filter: 3  Sample start region (min): 0  Sample end region (min): 30
Marker peak width (sec): 5    Marker min peak height: 200   Marker baseline V to V?: Y    Marker baseline V to V points: 3
Lower marker selection: First peak > 500 RFU                Upper marker selection: Last peak > 200 RFU
Ladder size (bp)75, 200, 300, 400, 500, 700, 1000, 1500, 2000, 3000, 4000, 5000, 7000, 10000, 20000
Quantification using: Lower Marker          Final concentration (ng/uL): 0.5000          Dilution factor: 12.0
```

Sample: SampF6
Well location: F6
Created: Tuesday, September 14, 2021 8:54:07 PM

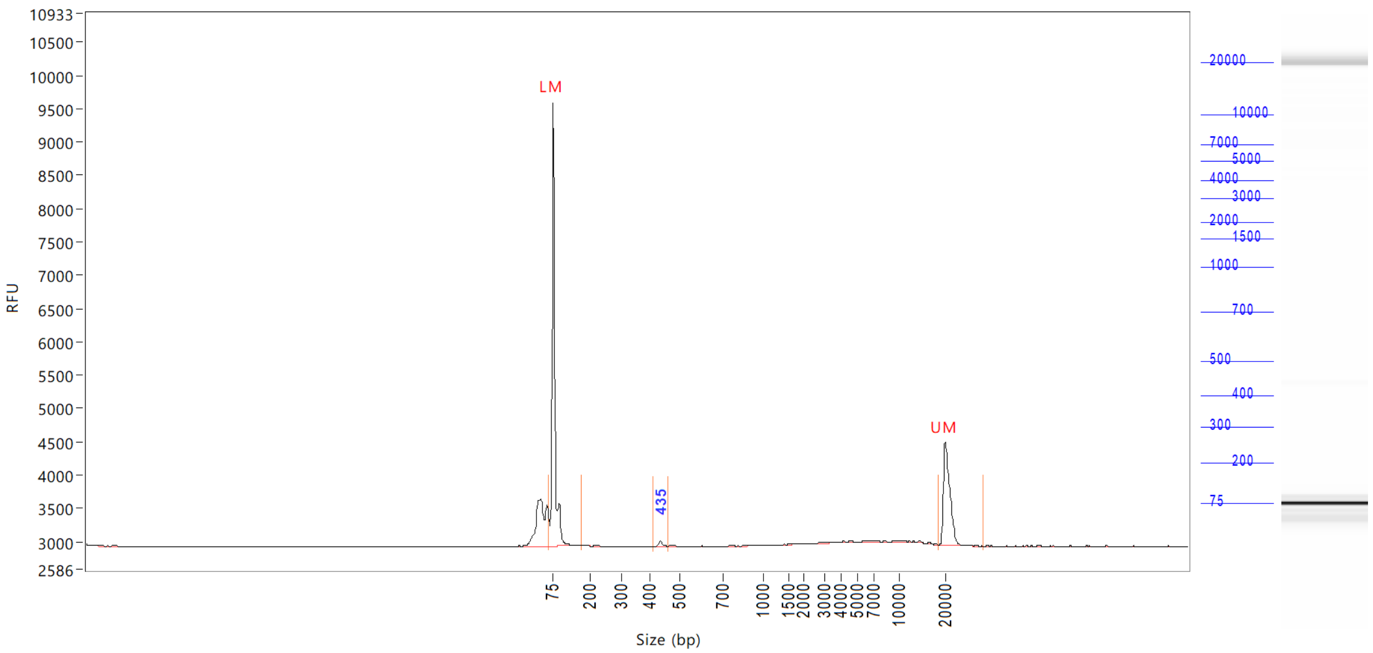

Peak	Size (bp)	Concentration (ng/uL)	From (bp)	To (bp)	Average size (bp)	CV%	RFU	Corrected peak area
1	75 (LM)	0.5000	62	171	76	12.08	6664	45.215
2	435	0.0885	414	460	435	1.99	93	0.667
3	20000 (UM)	0.1495	18537	28221	20361	3.96	1541	13.519

TIC:	0.0885	ng/uL
TIM:	0.3349	nmole/L
Total concentration:	0.5294	ng/uL

Sample peak width (sec): 5 Sample min peak height: 50 Sample baseline V to V?: Y Sample baseline V to V points: 3
Sample filter: Binomial Number of points for filter: 3 Sample start region (min): 0 Sample end region (min): 30
Marker peak width (sec): 5 Marker min peak height: 200 Marker baseline V to V?: Y Marker baseline V to V points: 3
Lower marker selection: First peak > 500 RFU Upper marker selection: Last peak > 200 RFU
Ladder size (bp)75, 200, 300, 400, 500, 700, 1000, 1500, 2000, 3000, 4000, 5000, 7000, 10000, 20000
Quantification using: Lower Marker Final concentration (ng/uL): 0.5000 Dilution factor: 12.0

Sample: SampG6
Well location: G6
Created: Tuesday, September 14, 2021 8:54:07 PM

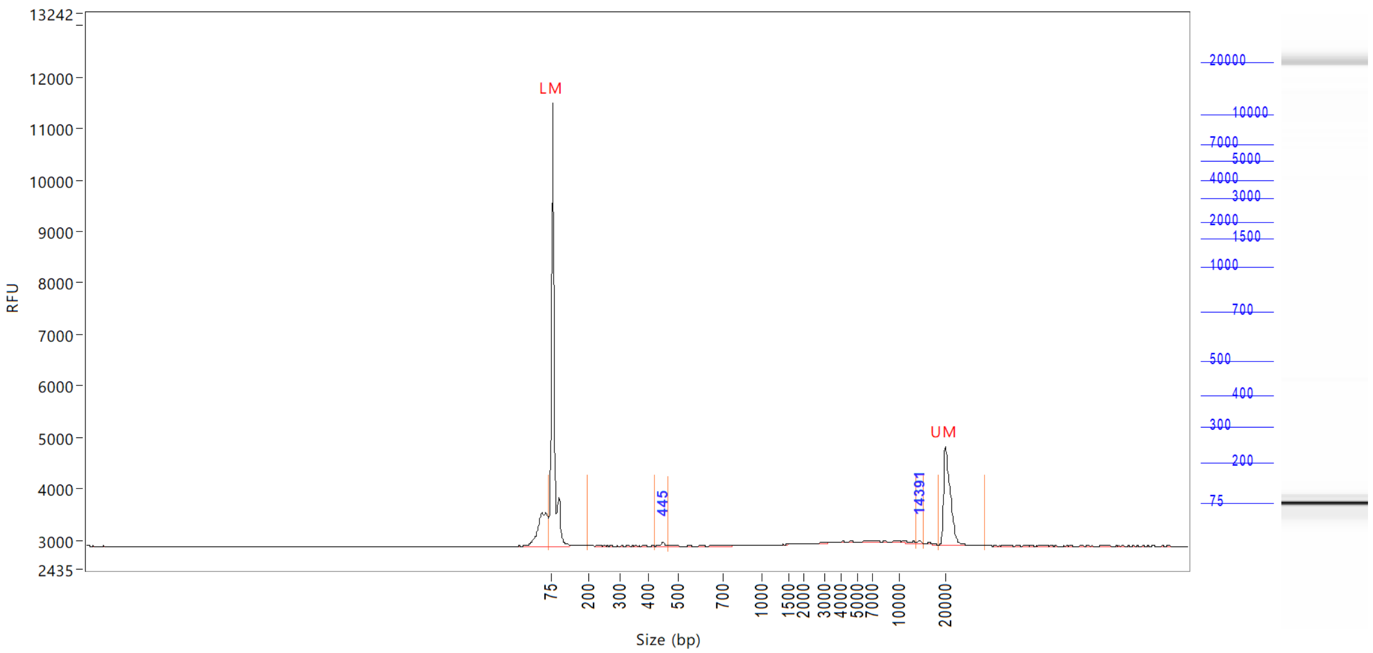

Peak	Size (bp)	Concentration (ng/uL)	From (bp)	To (bp)	Average size (bp)	CV%	RFU	Corrected peak area
1	75 (LM)	0.5000	60	192	76	14.77	8629	63.530
2	445	0.0480	420	466	443	1.71	80	0.509
3	14391	0.0303	13781	15244	14354	2.43	65	0.321
4	20000 (UM)	0.1357	18415	28584	20471	4.24	1903	17.237

TIC:	0.0784	ng/uL
TIM:	0.1819	nmole/L
Total concentration:	0.4211	ng/uL

```
Sample peak width (sec): 5    Sample min peak height: 50    Sample baseline V to V?: Y    Sample baseline V to V points: 3
Sample filter: Binomial       Number of points for filter: 3  Sample start region (min): 0  Sample end region (min): 30
Marker peak width (sec): 5    Marker min peak height: 200   Marker baseline V to V?: Y    Marker baseline V to V points: 3
Lower marker selection: First peak > 500 RFU                Upper marker selection: Last peak > 200 RFU
Ladder size (bp)75, 200, 300, 400, 500, 700, 1000, 1500, 2000, 3000, 4000, 5000, 7000, 10000, 20000
Quantification using: Lower Marker          Final concentration (ng/uL): 0.5000          Dilution factor: 12.0
```

Sample: SampH6
Well location: H6
Created: Tuesday, September 14, 2021 8:54:07 PM

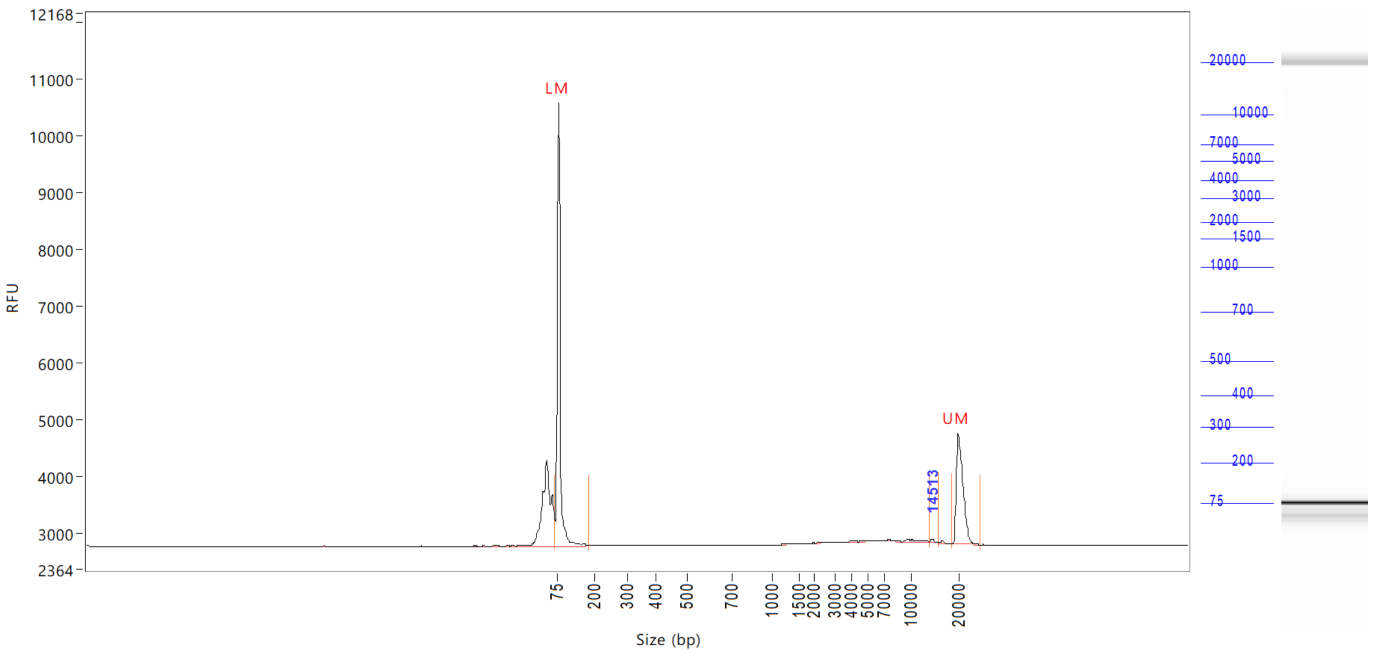

Peak	Size (bp)	Concentration (ng/uL)	From (bp)	To (bp)	Average size (bp)	CV%	RFU	Corrected peak area
1	75 (LM)	0.5000	66	181	78	16.66	7829	52.949
2	14513	0.0372	13781	15854	14511	3.48	59	0.328
3	20000 (UM)	0.1599	18537	24836	20372	3.56	1948	16.932

TIC:	0.0372	ng/uL
TIM:	0.0042	nmole/L
Total concentration:	0.4651	ng/uL

```
Sample peak width (sec): 5    Sample min peak height: 50    Sample baseline V to V?: Y    Sample baseline V to V points: 3
Sample filter: Binomial       Number of points for filter: 3  Sample start region (min): 0  Sample end region (min): 30
Marker peak width (sec): 5    Marker min peak height: 200   Marker baseline V to V?: Y    Marker baseline V to V points: 3
Lower marker selection: First peak > 500 RFU                Upper marker selection: Last peak > 200 RFU
Ladder size (bp)75, 200, 300, 400, 500, 700, 1000, 1500, 2000, 3000, 4000, 5000, 7000, 10000, 20000
Quantification using: Lower Marker          Final concentration (ng/uL): 0.5000          Dilution factor: 12.0
```

Sample: SampA7
Well location: A7
Created: Tuesday, September 14, 2021 8:54:07 PM

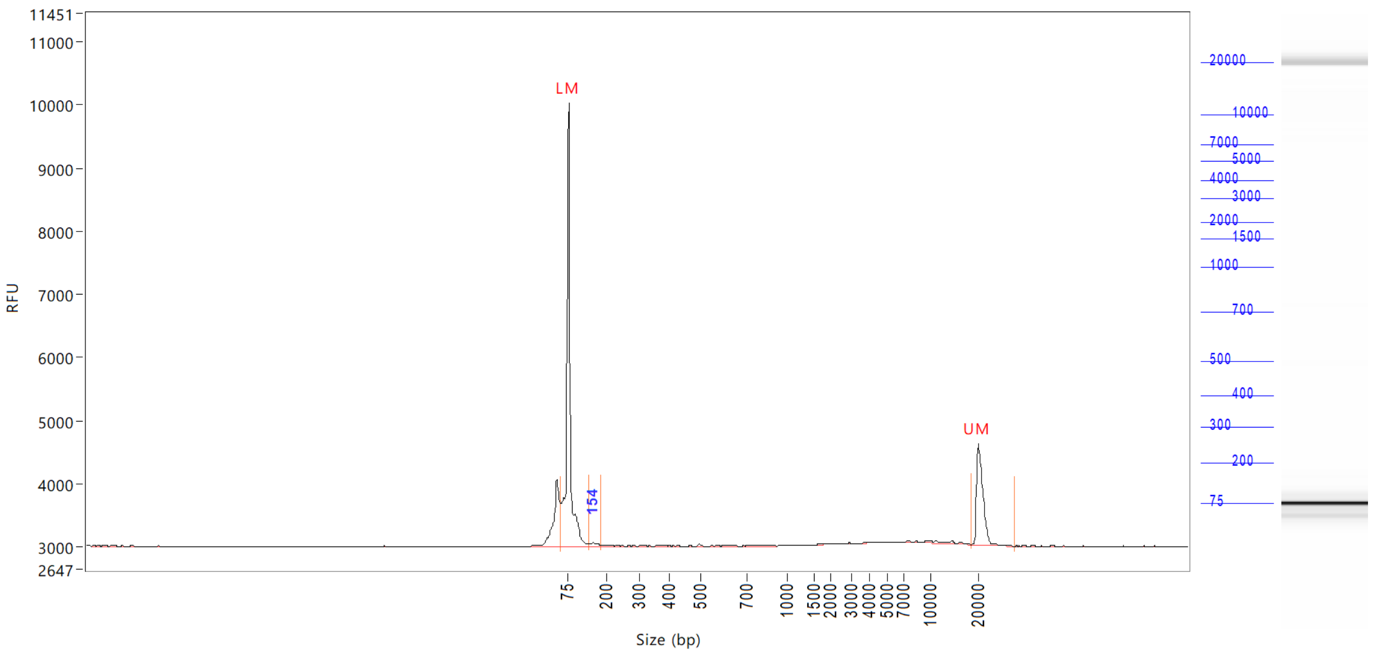

Peak	Size (bp)	Concentration (ng/uL)	From (bp)	To (bp)	Average size (bp)	CV%	RFU	Corrected peak area
1	75 (LM)	0.5000	48	141	74	17.20	7031	59.367
2	154	0.0795	141	179	156	5.72	68	0.787
3	20000 (UM)	0.1133	18415	27496	20387	4.40	1606	13.449

TIC:	0.0795	ng/uL
TIM:	0.8381	nmole/L
Total concentration:	0.5044	ng/uL

Sample peak width (sec): 5   Sample min peak height: 50   Sample baseline V to V?: Y   Sample baseline V to V points: 3
Sample filter: Binomial   Number of points for filter: 3 Sample start region (min): 0 Sample end region (min): 30
Marker peak width (sec): 5   Marker min peak height: 200   Marker baseline V to V?: Y   Marker baseline V to V points: 3
Lower marker selection: First peak > 500 RFU   Upper marker selection: Last peak > 200 RFU
Ladder size (bp)75, 200, 300, 400, 500, 700, 1000, 1500, 2000, 3000, 4000, 5000, 7000, 10000, 20000
Quantification using: Lower Marker   Final concentration (ng/uL): 0.5000   Dilution factor: 12.0

Sample: SampB7
Well location: B7
Created: Tuesday, September 14, 2021 8:54:07 PM

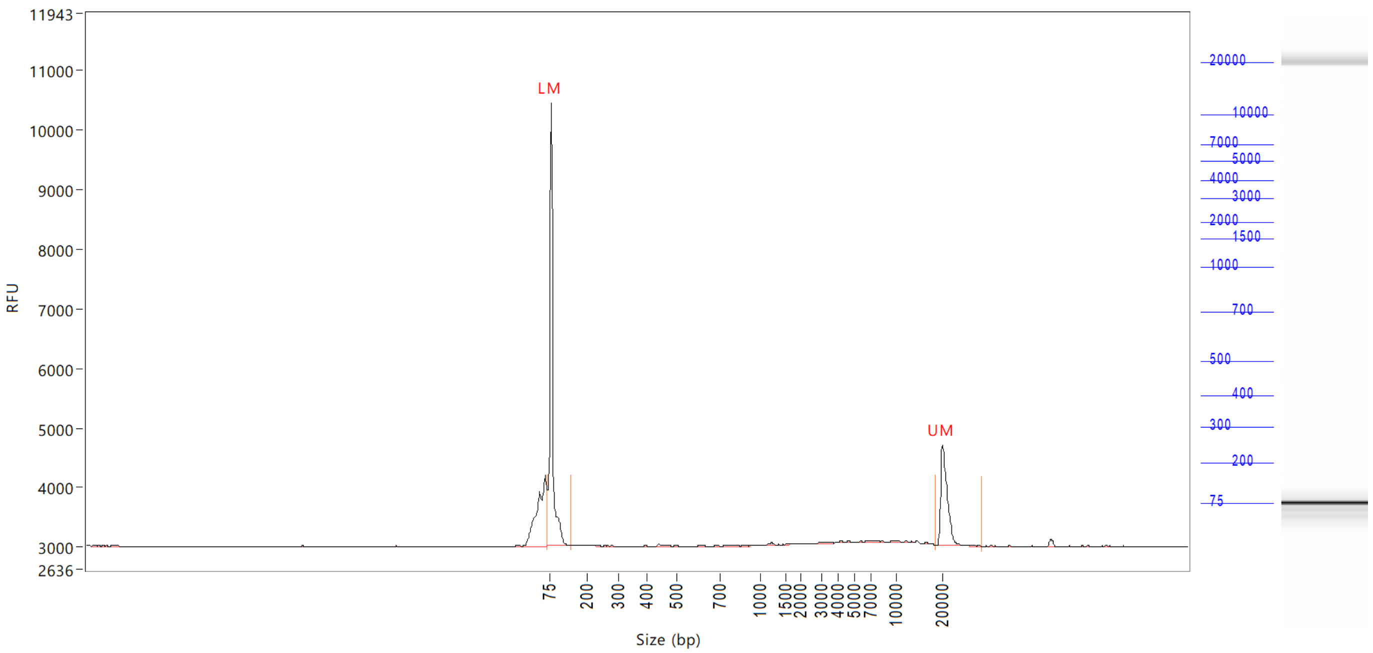

Peak	Size (bp)	Concentration (ng/uL)	From (bp)	To (bp)	Average size (bp)	CV%	RFU	Corrected peak area
1	75 (LM)	0.5000	62	145	77	12.89	7429	57.341
2	20000 (UM)	0.1295	18659	28584	20501	4.61	1670	14.856

TIC:	0.0000	ng/uL
TIM:	0.0000	nmole/L
Total concentration:	0.4118	ng/uL

Sample peak width (sec): 5 Sample min peak height: 50 Sample baseline V to V?: Y Sample baseline V to V points: 3
Sample filter: Binomial Number of points for filter: 3 Sample start region (min): 0 Sample end region (min): 30
Marker peak width (sec): 5 Marker min peak height: 200 Marker baseline V to V?: Y Marker baseline V to V points: 3
Lower marker selection: First peak > 500 RFU Upper marker selection: Last peak > 200 RFU
Ladder size (bp)75, 200, 300, 400, 500, 700, 1000, 1500, 2000, 3000, 4000, 5000, 7000, 10000, 20000
Quantification using: Lower Marker Final concentration (ng/uL): 0.5000 Dilution factor: 12.0

Sample: SampC7
Well location: C7
Created: Tuesday, September 14, 2021 8:54:07 PM

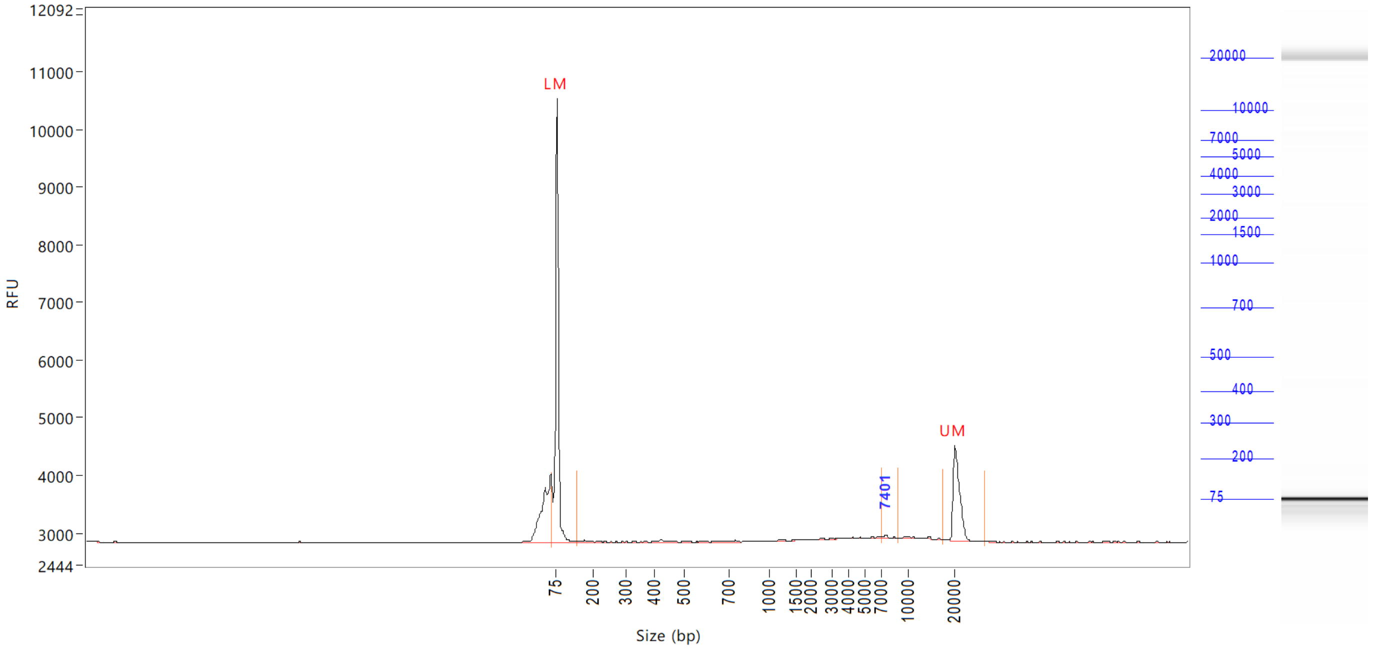

Peak	Size (bp)	Concentration (ng/uL)	From (bp)	To (bp)	Average size (bp)	CV%	RFU	Corrected peak area
1	75 (LM)	0.5000	60	145	74	10.46	7706	51.524
2	7401	0.0477	7000	8867	7598	6.19	53	0.409
3	20000 (UM)	0.1393	17561	26529	20444	3.74	1640	14.359

TIC:	0.0477	ng/uL
TIM:	0.0103	nmole/L
Total concentration:	0.5050	ng/uL

```
Sample peak width (sec): 5    Sample min peak height: 50    Sample baseline V to V?: Y    Sample baseline V to V points: 3
Sample filter: Binomial       Number of points for filter: 3  Sample start region (min): 0  Sample end region (min): 30
Marker peak width (sec): 5    Marker min peak height: 200   Marker baseline V to V?: Y    Marker baseline V to V points: 3
Lower marker selection: First peak > 500 RFU                Upper marker selection: Last peak > 200 RFU
Ladder size (bp)75, 200, 300, 400, 500, 700, 1000, 1500, 2000, 3000, 4000, 5000, 7000, 10000, 20000
Quantification using: Lower Marker          Final concentration (ng/uL): 0.5000          Dilution factor: 12.0
```

Sample: SampD7
Well location: D7
Created: Tuesday, September 14, 2021 8:54:07 PM

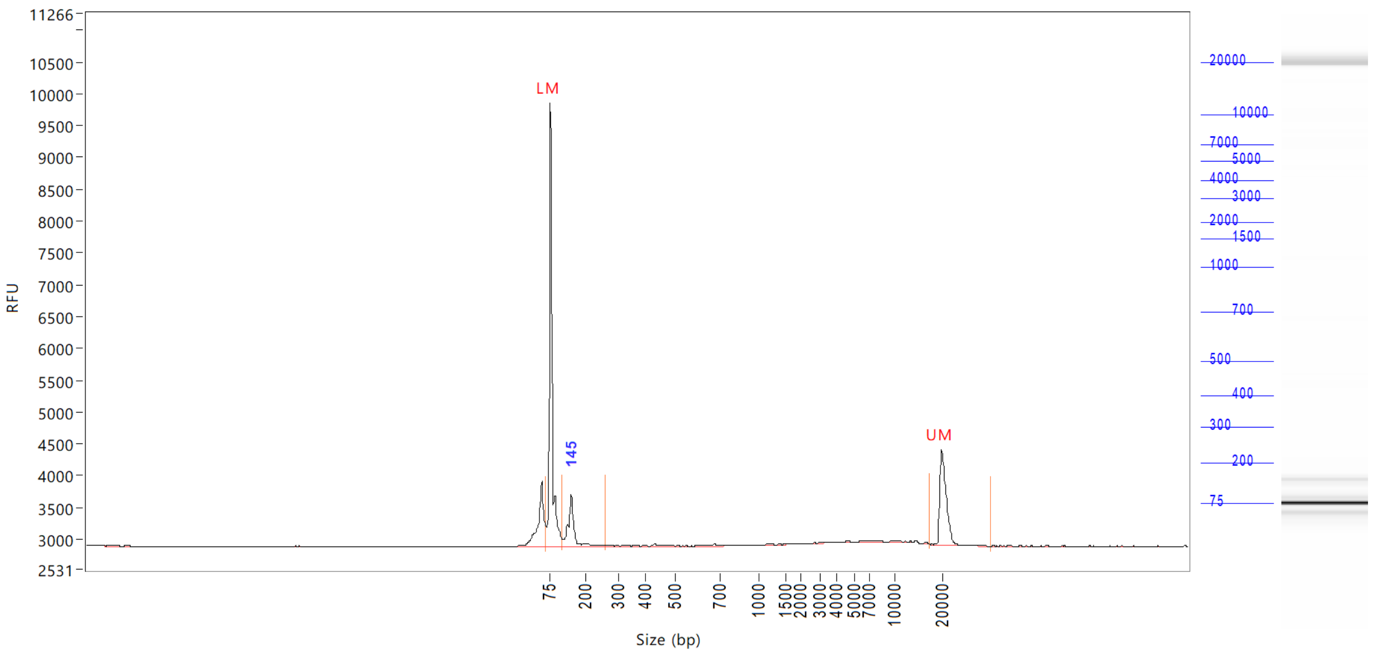

Peak	Size (bp)	Concentration (ng/uL)	From (bp)	To (bp)	Average size (bp)	CV%	RFU	Corrected peak area
1	75 (LM)	0.5000	62	116	77	11.53	6976	48.445
2	145	1.2599	116	259	147	14.72	824	10.172
3	20000 (UM)	0.1372	17439	30398	20379	4.64	1502	13.290

TIC:	1.2599	ng/uL
TIM:	14.0853	nmole/L
Total concentration:	1.7183	ng/uL

```
Sample peak width (sec): 5     Sample min peak height: 50     Sample baseline V to V?: Y     Sample baseline V to V points: 3
Sample filter: Binomial        Number of points for filter: 3  Sample start region (min): 0  Sample end region (min): 30
Marker peak width (sec): 5     Marker min peak height: 200    Marker baseline V to V?: Y     Marker baseline V to V points: 3
Lower marker selection: First peak > 500 RFU                  Upper marker selection: Last peak > 200 RFU
Ladder size (bp)75, 200, 300, 400, 500, 700, 1000, 1500, 2000, 3000, 4000, 5000, 7000, 10000, 20000
Quantification using: Lower Marker          Final concentration (ng/uL): 0.5000          Dilution factor: 12.0
```

Sample: SampE7
Well location: E7
Created: Tuesday, September 14, 2021 8:54:07 PM

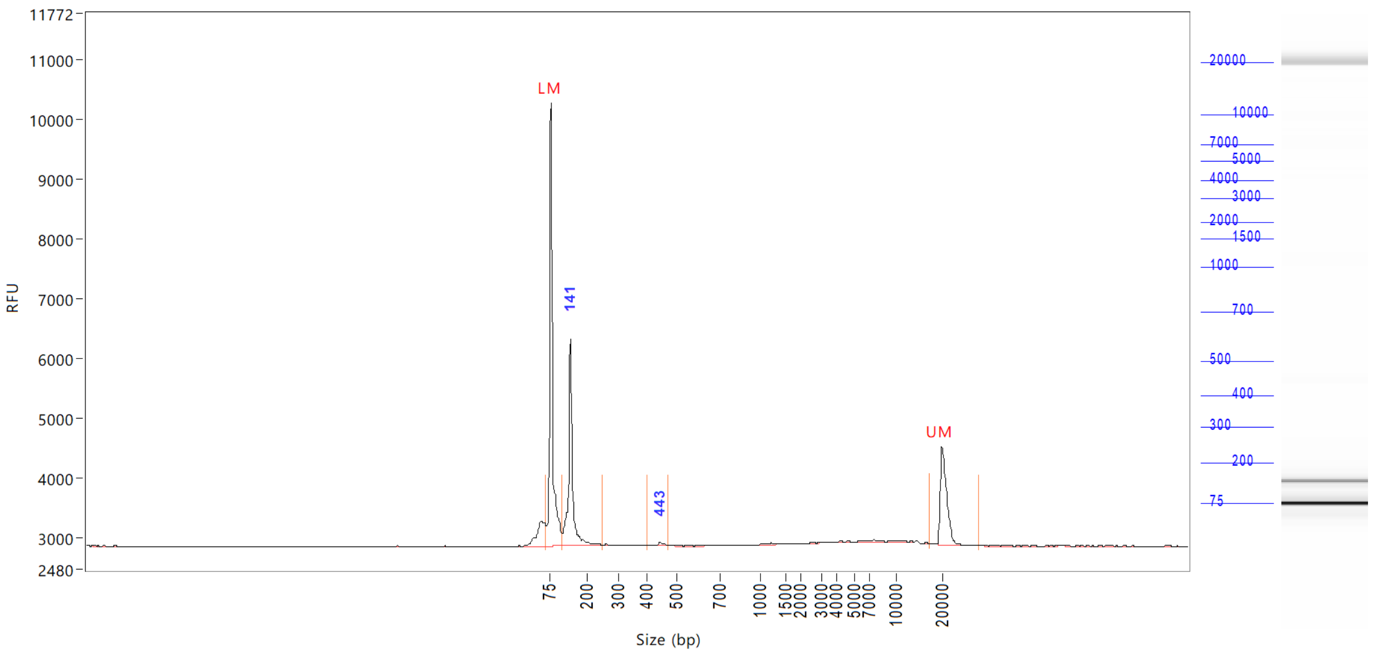

Peak	Size (bp)	Concentration (ng/uL)	From (bp)	To (bp)	Average size (bp)	CV%	RFU	Corrected peak area
1	75 (LM)	0.5000	60	112	76	12.45	7418	55.030
2	141	3.4887	112	250	143	13.53	3447	31.998
3	443	0.0736	401	471	443	2.95	61	0.675
4	20000 (UM)	0.1322	17439	28100	20493	4.22	1634	14.545

TIC:	3.5623	ng/uL
TIM:	40.3661	nmole/L
Total concentration:	3.9526	ng/uL

Sample peak width (sec): 5 Sample min peak height: 50 Sample baseline V to V?: Y Sample baseline V to V points: 3
Sample filter: Binomial Number of points for filter: 3 Sample start region (min): 0 Sample end region (min): 30
Marker peak width (sec): 5 Marker min peak height: 200 Marker baseline V to V?: Y Marker baseline V to V points: 3
Lower marker selection: First peak > 500 RFU Upper marker selection: Last peak > 200 RFU
Ladder size (bp)75, 200, 300, 400, 500, 700, 1000, 1500, 2000, 3000, 4000, 5000, 7000, 10000, 20000
Quantification using: Lower Marker Final concentration (ng/uL): 0.5000 Dilution factor: 12.0

Sample: SampF7
Well location: F7
Created: Tuesday, September 14, 2021 8:54:07 PM

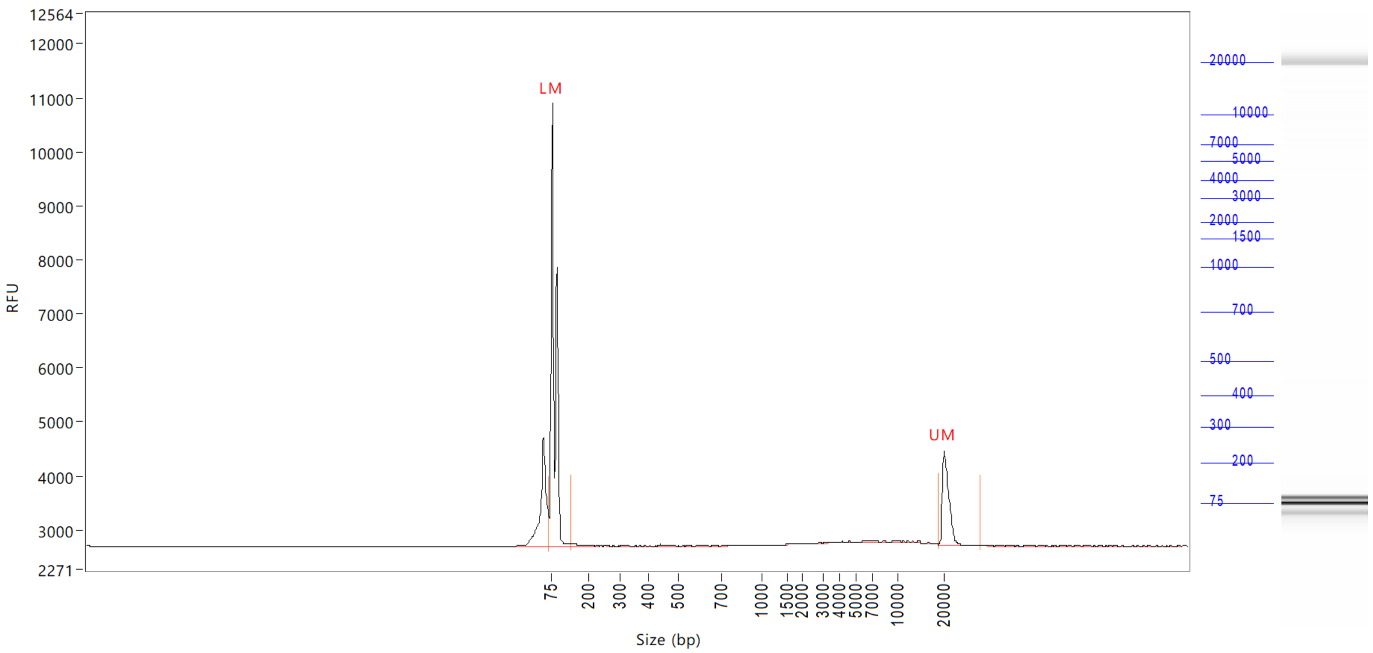

Peak	Size (bp)	Concentration (ng/uL)	From (bp)	To (bp)	Average size (bp)	CV%	RFU	Corrected peak area
1	75 (LM)	0.5000	66	139	80	11.96	8221	78.894
2	20000 (UM)	0.0965	18659	27738	20394	3.82	1739	15.225

TIC:	0.0000	ng/uL
TIM:	0.0000	nmole/L
Total concentration:	0.3006	ng/uL

```
Sample peak width (sec): 5    Sample min peak height: 50    Sample baseline V to V?: Y    Sample baseline V to V points: 3
Sample filter: Binomial       Number of points for filter: 3  Sample start region (min): 0  Sample end region (min): 30
Marker peak width (sec): 5    Marker min peak height: 200   Marker baseline V to V?: Y    Marker baseline V to V points: 3
Lower marker selection: First peak > 500 RFU                Upper marker selection: Last peak > 200 RFU
Ladder size (bp)75, 200, 300, 400, 500, 700, 1000, 1500, 2000, 3000, 4000, 5000, 7000, 10000, 20000
Quantification using: Lower Marker          Final concentration (ng/uL): 0.5000          Dilution factor: 12.0
```

Sample: SampG7
Well location: G7
Created: Tuesday, September 14, 2021 8:54:07 PM

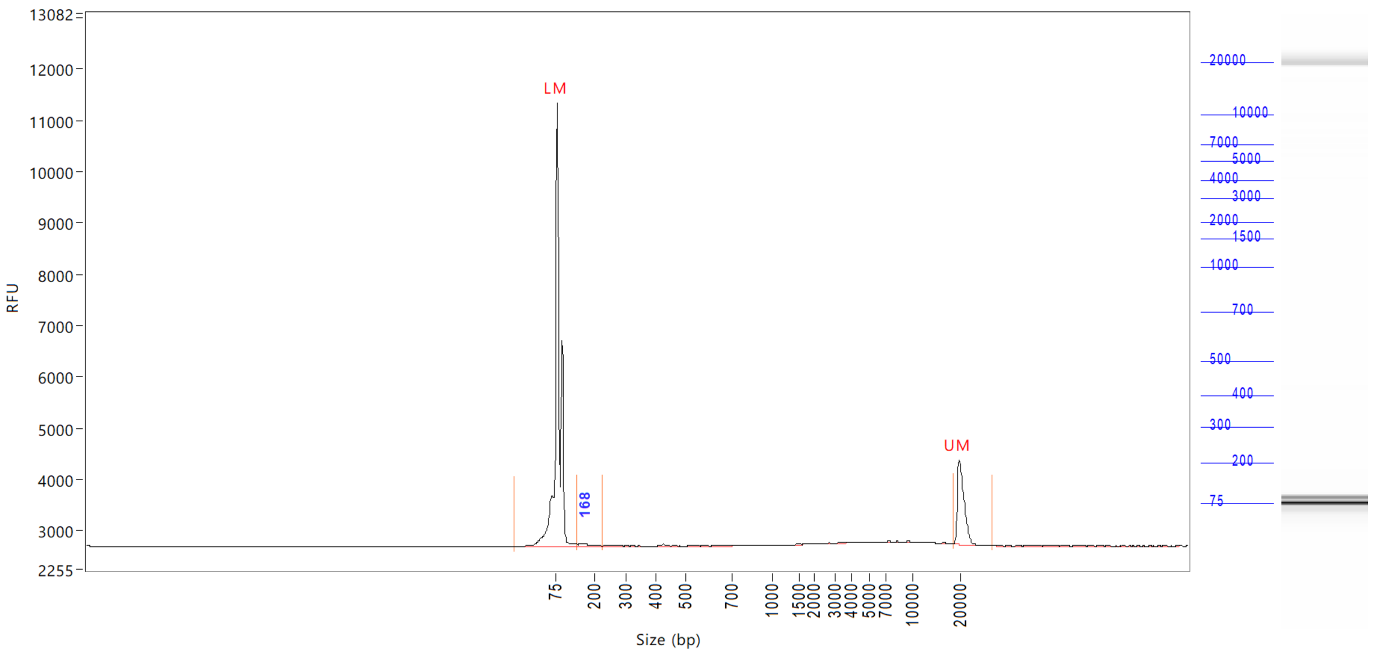

Peak	Size (bp)	Concentration (ng/uL)	From (bp)	To (bp)	Average size (bp)	CV%	RFU	Corrected peak area
1	75 (LM)	0.5000	0	143	73	24.59	8649	93.323
2	168	0.0653	143	223	165	10.18	54	1.016
3	20000 (UM)	0.0766	18537	26891	20358	3.70	1645	14.298

TIC:	0.0653	ng/uL
TIM:	0.6506	nmole/L
Total concentration:	0.2827	ng/uL

Sample peak width (sec): 5   Sample min peak height: 50   Sample baseline V to V?: Y   Sample baseline V to V points: 3
Sample filter: Binomial   Number of points for filter: 3   Sample start region (min): 0   Sample end region (min): 30
Marker peak width (sec): 5   Marker min peak height: 200   Marker baseline V to V?: Y   Marker baseline V to V points: 3
Lower marker selection: First peak > 500 RFU   Upper marker selection: Last peak > 200 RFU
Ladder size (bp)75, 200, 300, 400, 500, 700, 1000, 1500, 2000, 3000, 4000, 5000, 7000, 10000, 20000
Quantification using: Lower Marker   Final concentration (ng/uL): 0.5000   Dilution factor: 12.0

Sample: SampH7
Well location: H7
Created: Tuesday, September 14, 2021 8:54:07 PM

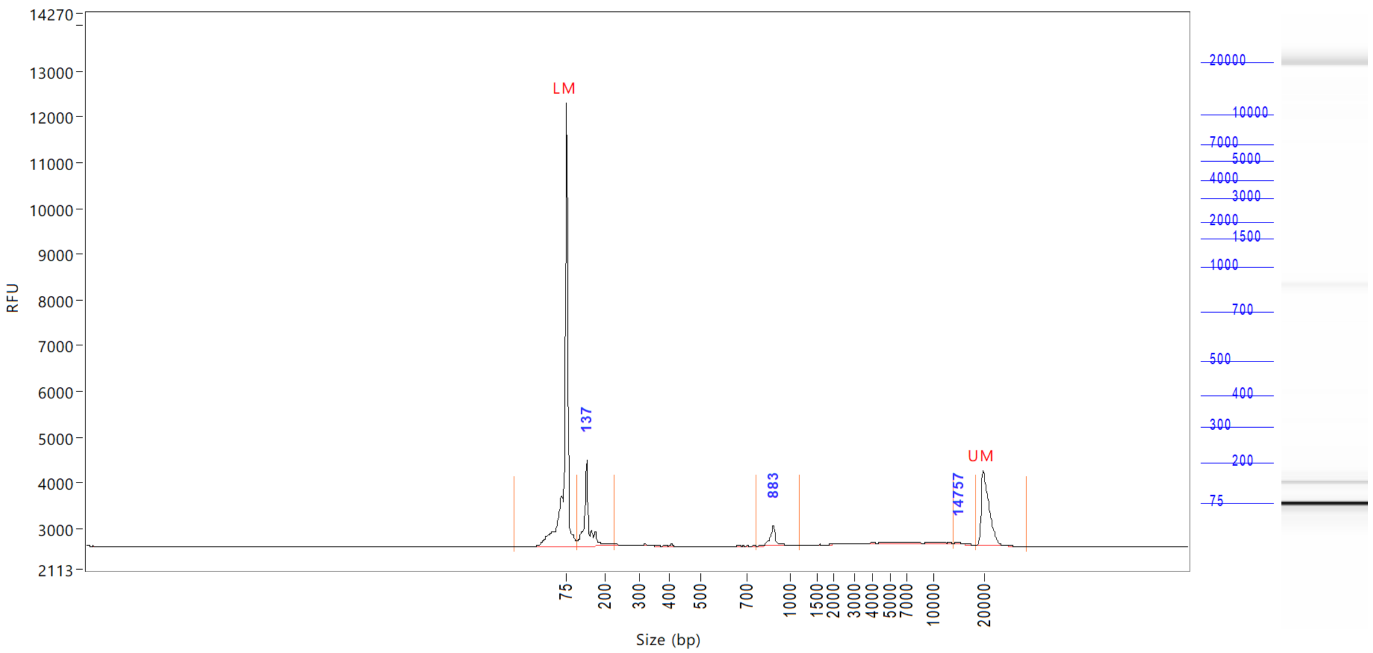

Peak	Size (bp)	Concentration (ng/uL)	From (bp)	To (bp)	Average size (bp)	CV%	RFU	Corrected peak area
1	75 (LM)	0.5000	0	107	64	30.52	9710	82.558
2	137	1.4372	107	224	142	14.53	1881	19.776
3	883	0.3090	757	1190	879	4.76	447	4.252
4	14757	0.0477	13903	18537	15465	6.57	55	0.656
5	20000 (UM)	0.0995	18537	28705	20557	4.74	1632	16.423

TIC:	1.7939	ng/uL
TIM:	17.2165	nmole/L
Total concentration:	2.0381	ng/uL

Sample peak width (sec): 5   Sample min peak height: 50   Sample baseline V to V?: Y   Sample baseline V to V points: 3
Sample filter: Binomial   Number of points for filter: 3 Sample start region (min): 0 Sample end region (min): 30
Marker peak width (sec): 5   Marker min peak height: 200   Marker baseline V to V?: Y   Marker baseline V to V points: 3
Lower marker selection: First peak > 500 RFU   Upper marker selection: Last peak > 200 RFU
Ladder size (bp)75, 200, 300, 400, 500, 700, 1000, 1500, 2000, 3000, 4000, 5000, 7000, 10000, 20000
Quantification using: Lower Marker   Final concentration (ng/uL): 0.5000   Dilution factor: 12.0

Sample: SampA8
Well location: A8
Created: Tuesday, September 14, 2021 8:54:07 PM

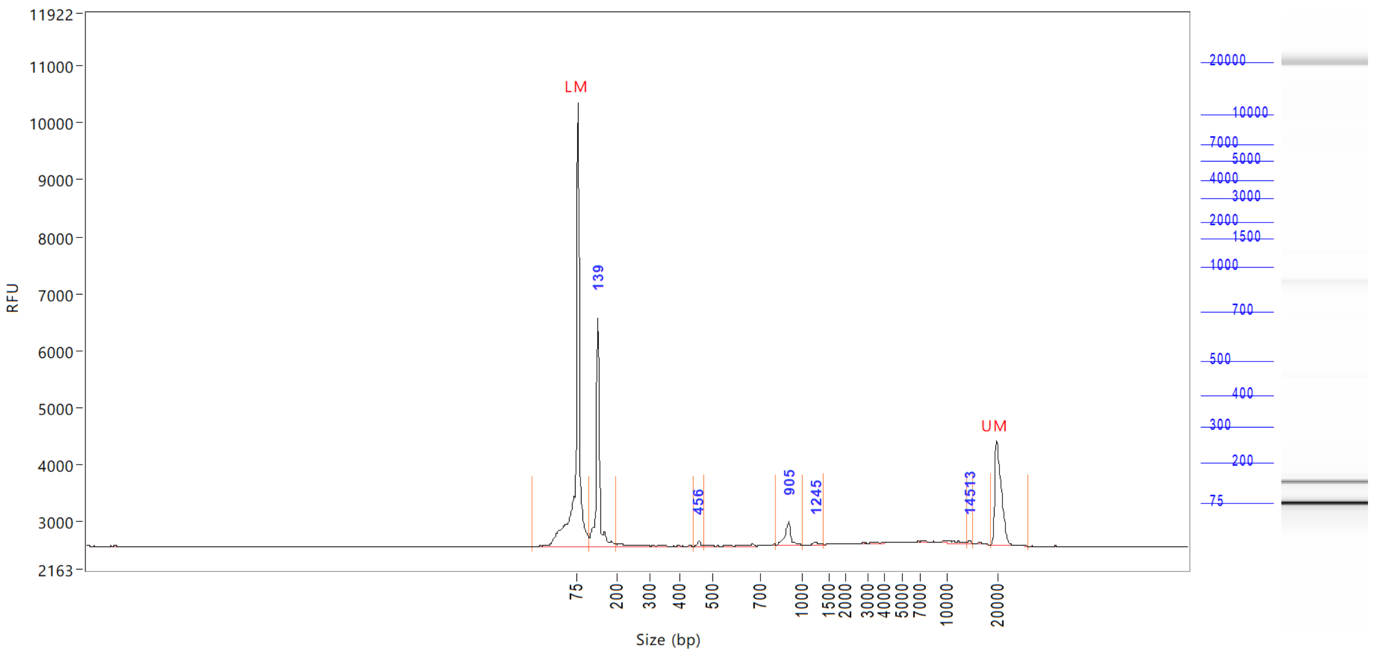

Peak	Size (bp)	Concentration (ng/uL)	From (bp)	To (bp)	Average size (bp)	CV%	RFU	Corrected peak area
1	75 (LM)	0.5000	0	108	64	34.42	7799	71.401
2	139	2.4586	108	198	137	9.11	3994	29.258
3	456	0.0432	439	471	454	1.13	88	0.514
4	905	0.3526	818	1012	892	3.28	427	4.196
5	1245	0.0362	1012	1400	1226	5.61	53	0.430
6	14513	0.0229	13781	15244	14389	2.48	54	0.273
7	20000 (UM)	0.1059	18659	26408	20312	3.48	1830	15.126

TIC:	2.9135	ng/uL
TIM:	30.3483	nmole/L
Total concentration:	3.2777	ng/uL

```
Sample peak width (sec): 5    Sample min peak height: 50    Sample baseline V to V?: Y    Sample baseline V to V points: 3
Sample filter: Binomial       Number of points for filter: 3  Sample start region (min): 0  Sample end region (min): 30
Marker peak width (sec): 5    Marker min peak height: 200   Marker baseline V to V?: Y    Marker baseline V to V points: 3
Lower marker selection: First peak > 500 RFU                Upper marker selection: Last peak > 200 RFU
Ladder size (bp)75, 200, 300, 400, 500, 700, 1000, 1500, 2000, 3000, 4000, 5000, 7000, 10000, 20000
Quantification using: Lower Marker          Final concentration (ng/uL): 0.5000          Dilution factor: 12.0
```

Sample: SampB8
Well location: B8
Created: Tuesday, September 14, 2021 8:54:07 PM

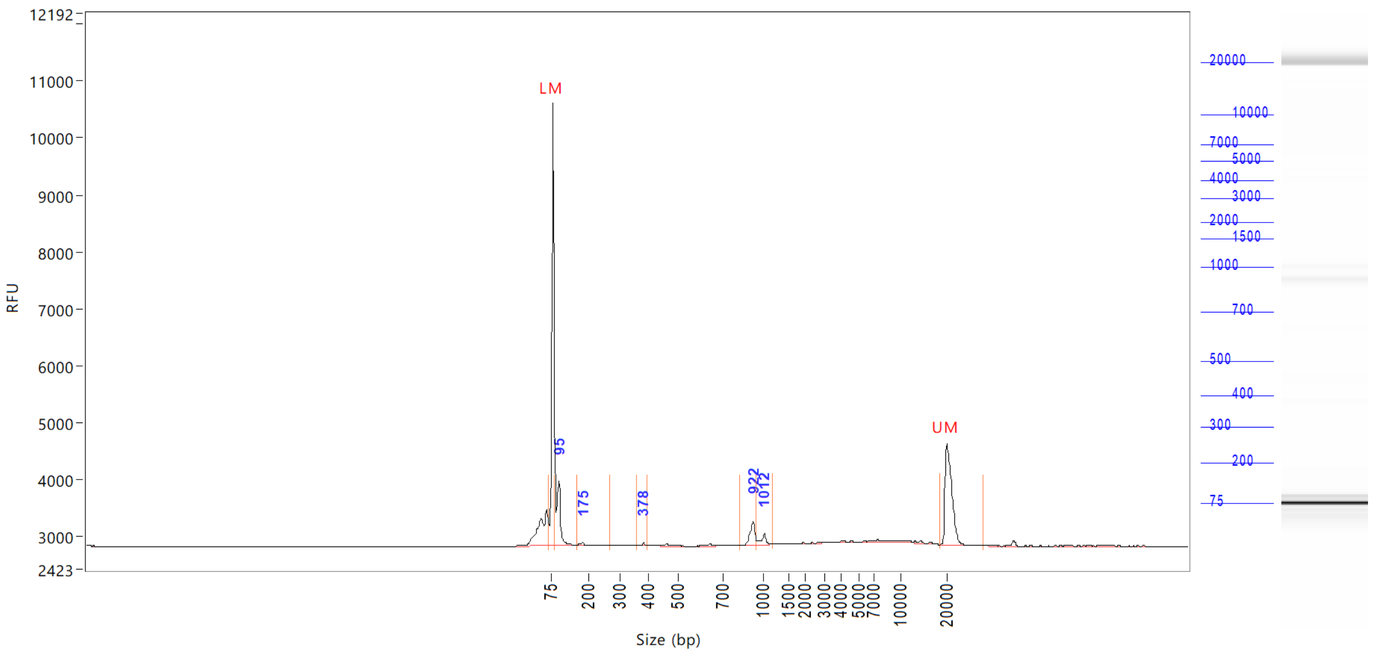

Peak	Size (bp)	Concentration (ng/uL)	From (bp)	To (bp)	Average size (bp)	CV%	RFU	Corrected peak area
1	75 (LM)	0.5000	66	84	74	4.56	7793	45.019
2	95	1.4341	84	156	95	12.44	1128	10.760
3	175	0.1130	156	262	181	11.10	59	0.847
4	378	0.0433	356	394	374	1.77	54	0.325
5	922	0.4653	818	952	909	2.30	427	3.491
6	1012	0.2350	952	1178	1004	4.15	190	1.764
7	20000 (UM)	0.1798	18415	27738	20488	3.86	1789	16.189

TIC:	2.2906	ng/uL
TIM:	27.2286	nmole/L
Total concentration:	2.8833	ng/uL

Sample peak width (sec): 5   Sample min peak height: 50   Sample baseline V to V?: Y   Sample baseline V to V points: 3
Sample filter: Binomial   Number of points for filter: 3 Sample start region (min): 0 Sample end region (min): 30
Marker peak width (sec): 5   Marker min peak height: 200   Marker baseline V to V?: Y   Marker baseline V to V points: 3
Lower marker selection: First peak > 500 RFU   Upper marker selection: Last peak > 200 RFU
Ladder size (bp)75, 200, 300, 400, 500, 700, 1000, 1500, 2000, 3000, 4000, 5000, 7000, 10000, 20000
Quantification using: Lower Marker   Final concentration (ng/uL): 0.5000   Dilution factor: 12.0

Sample: SampC8
Well location: C8
Created: Tuesday, September 14, 2021 8:54:07 PM

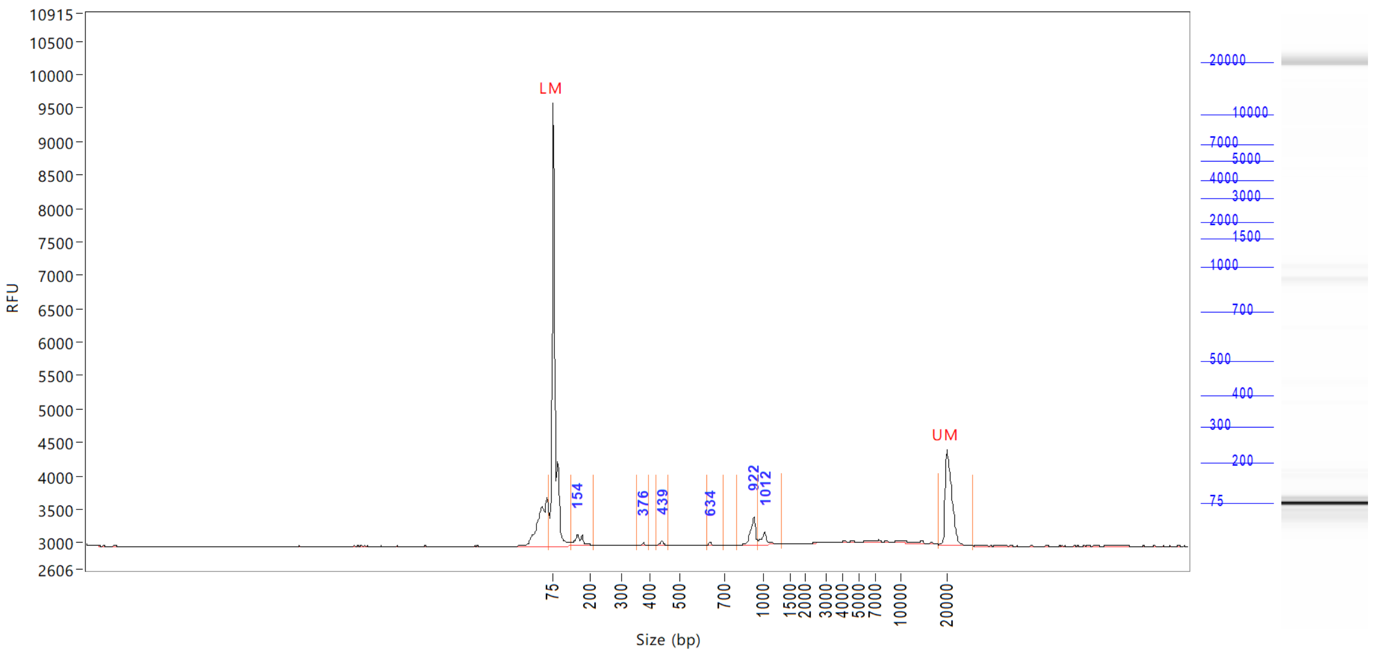

Peak	Size (bp)	Concentration (ng/uL)	From (bp)	To (bp)	Average size (bp)	CV%	RFU	Corrected peak area
1	75 (LM)	0.5000	62	137	77	13.59	6635	50.250
2	154	0.3689	137	212	162	9.26	170	3.089
3	376	0.0395	354	396	372	1.89	54	0.331
4	439	0.0674	418	460	436	1.72	67	0.565
5	634	0.0459	618	695	638	2.27	59	0.384
6	922	0.4224	796	957	906	2.79	412	3.538
7	1012	0.2270	957	1334	1025	7.28	196	1.902
8	20000 (UM)	0.1242	18171	25682	20377	3.86	1418	12.484

TIC:	1.1711	ng/uL
TIM:	5.4221	nmole/L
Total concentration:	1.5196	ng/uL

Sample peak width (sec): 5 Sample min peak height: 50 Sample baseline V to V?: Y Sample baseline V to V points: 3
Sample filter: Binomial Number of points for filter: 3 Sample start region (min): 0 Sample end region (min): 30
Marker peak width (sec): 5 Marker min peak height: 200 Marker baseline V to V?: Y Marker baseline V to V points: 3
Lower marker selection: First peak > 500 RFU Upper marker selection: Last peak > 200 RFU
Ladder size (bp)75, 200, 300, 400, 500, 700, 1000, 1500, 2000, 3000, 4000, 5000, 7000, 10000, 20000
Quantification using: Lower Marker Final concentration (ng/uL): 0.5000 Dilution factor: 12.0

Sample: SampD8
Well location: D8
Created: Tuesday, September 14, 2021 8:54:07 PM

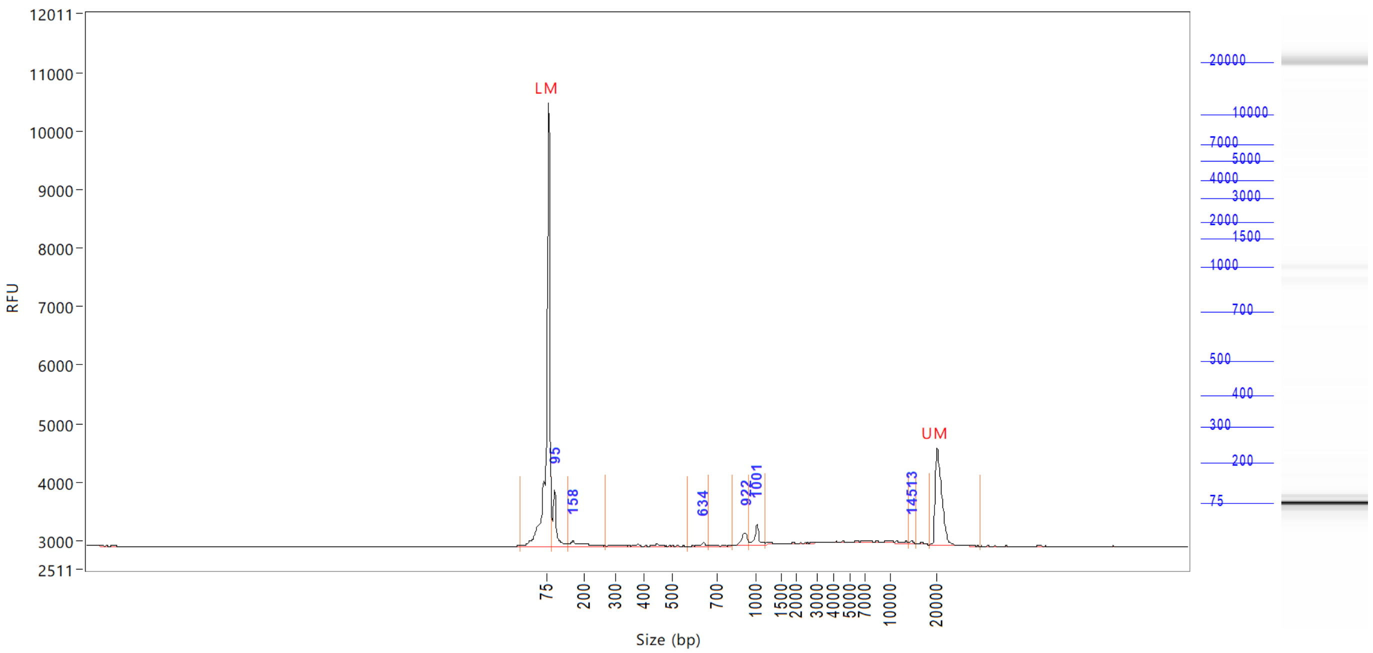

Peak	Size (bp)	Concentration (ng/uL)	From (bp)	To (bp)	Average size (bp)	CV%	RFU	Corrected peak area
1	75 (LM)	0.5000	0	87	66	23.20	7589	62.677
2	95	0.8295	87	145	99	12.85	964	8.665
3	158	0.2136	145	266	182	14.92	91	2.232
4	634	0.0423	562	657	625	2.64	62	0.442
5	922	0.2164	809	944	900	2.54	218	2.261
6	1001	0.2583	944	1178	1011	4.51	354	2.698
7	14513	0.0243	13781	15244	14367	2.37	50	0.254
8	20000 (UM)	0.1164	18293	29309	20499	4.38	1643	14.594

TIC:	1.5845	ng/uL
TIM:	16.6181	nmole/L
Total concentration:	1.9493	ng/uL

```
Sample peak width (sec): 5     Sample min peak height: 50     Sample baseline V to V?: Y     Sample baseline V to V points: 3
Sample filter: Binomial        Number of points for filter: 3  Sample start region (min): 0  Sample end region (min): 30
Marker peak width (sec): 5     Marker min peak height: 200    Marker baseline V to V?: Y     Marker baseline V to V points: 3
Lower marker selection: First peak > 500 RFU                  Upper marker selection: Last peak > 200 RFU
Ladder size (bp)75, 200, 300, 400, 500, 700, 1000, 1500, 2000, 3000, 4000, 5000, 7000, 10000, 20000
Quantification using: Lower Marker          Final concentration (ng/uL): 0.5000          Dilution factor: 12.0
```

Sample: SampE8
Well location: E8
Created: Tuesday, September 14, 2021 8:54:07 PM

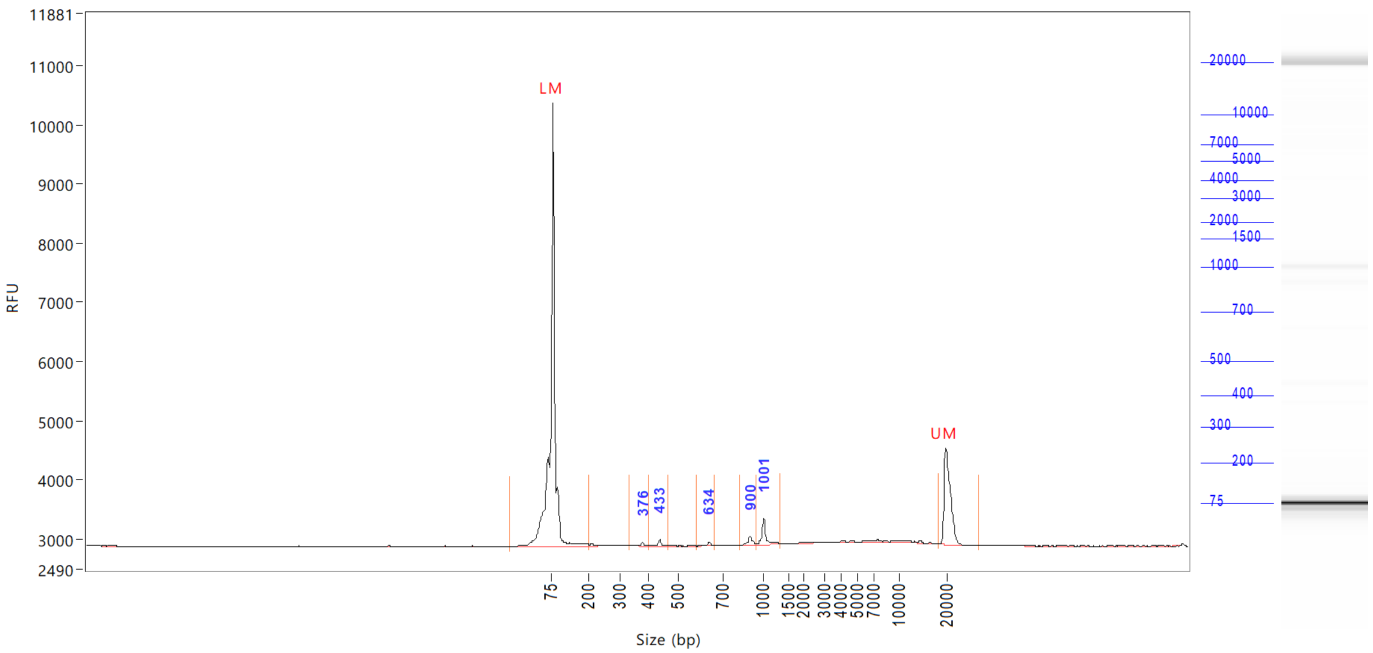

Peak	Size (bp)	Concentration (ng/uL)	From (bp)	To (bp)	Average size (bp)	CV%	RFU	Corrected peak area
1	75 (LM)	0.5000	0	198	68	33.87	7501	81.237
2	376	0.0330	329	400	368	3.84	60	0.447
3	433	0.0573	400	462	432	2.36	103	0.776
4	634	0.0370	580	659	625	2.46	71	0.501
5	900	0.0976	822	948	895	2.69	151	1.321
6	1001	0.2355	948	1334	1029	6.96	444	3.188
7	20000 (UM)	0.0859	18415	27012	20363	3.74	1629	13.958

TIC:	0.4604	ng/uL
TIM:	1.0194	nmole/L
Total concentration:	0.7287	ng/uL

Sample peak width (sec): 5   Sample min peak height: 50   Sample baseline V to V?: Y   Sample baseline V to V points: 3
Sample filter: Binomial   Number of points for filter: 3   Sample start region (min): 0   Sample end region (min): 30
Marker peak width (sec): 5   Marker min peak height: 200   Marker baseline V to V?: Y   Marker baseline V to V points: 3
Lower marker selection: First peak > 500 RFU   Upper marker selection: Last peak > 200 RFU
Ladder size (bp)75, 200, 300, 400, 500, 700, 1000, 1500, 2000, 3000, 4000, 5000, 7000, 10000, 20000
Quantification using: Lower Marker   Final concentration (ng/uL): 0.5000   Dilution factor: 12.0

Sample: SampF8
Well location: F8
Created: Tuesday, September 14, 2021 8:54:07 PM

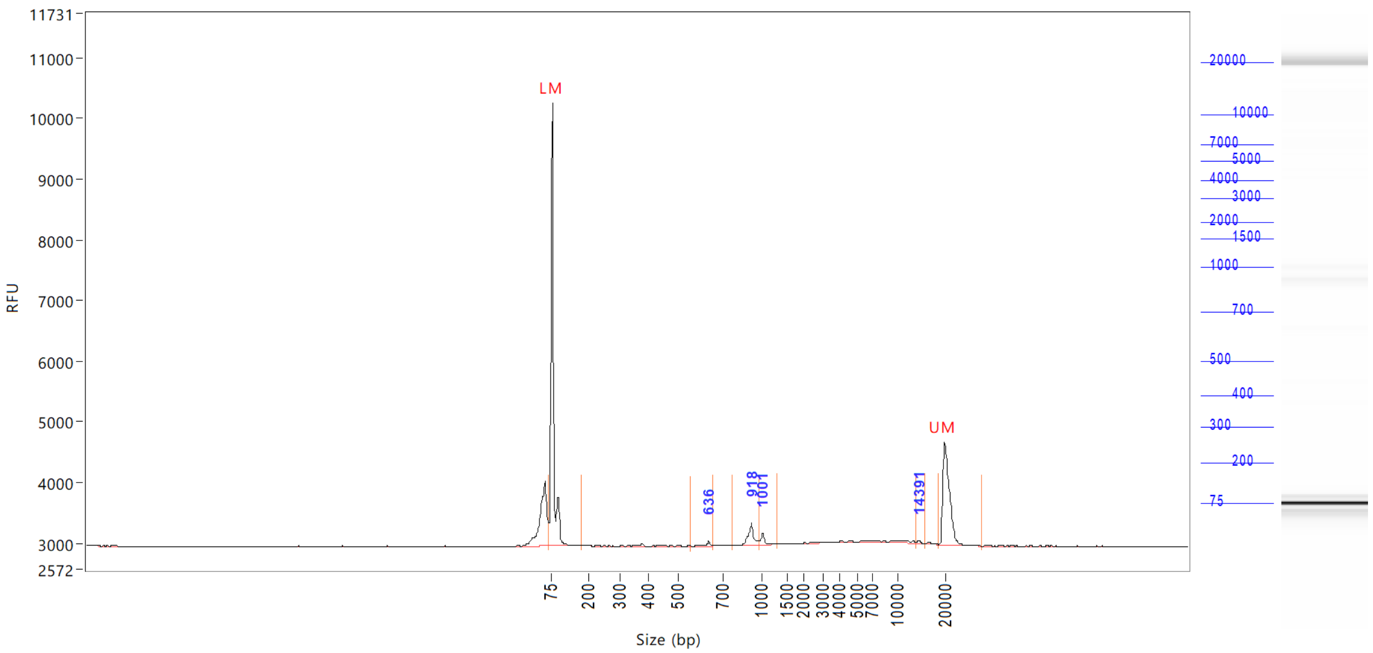

Peak	Size (bp)	Concentration (ng/uL)	From (bp)	To (bp)	Average size (bp)	CV%	RFU	Corrected peak area
1	75 (LM)	0.5000	62	173	76	12.62	7309	48.465
2	636	0.0769	557	657	621	3.09	78	0.621
3	918	0.4272	770	974	910	3.17	357	3.450
4	1001	0.1746	974	1300	1025	6.18	190	1.410
5	14391	0.0336	13781	15732	14444	3.12	52	0.272
6	20000 (UM)	0.1567	18659	27979	20400	4.17	1703	15.189

TIC:	0.7122	ng/uL
TIM:	1.2605	nmole/L
Total concentration:	1.1553	ng/uL

```
Sample peak width (sec): 5    Sample min peak height: 50    Sample baseline V to V?: Y    Sample baseline V to V points: 3
Sample filter: Binomial       Number of points for filter: 3  Sample start region (min): 0  Sample end region (min): 30
Marker peak width (sec): 5    Marker min peak height: 200   Marker baseline V to V?: Y    Marker baseline V to V points: 3
Lower marker selection: First peak > 500 RFU                Upper marker selection: Last peak > 200 RFU
Ladder size (bp)75, 200, 300, 400, 500, 700, 1000, 1500, 2000, 3000, 4000, 5000, 7000, 10000, 20000
Quantification using: Lower Marker          Final concentration (ng/uL): 0.5000          Dilution factor: 12.0
```

Sample: SampG8
Well location: G8
Created: Tuesday, September 14, 2021 8:54:07 PM

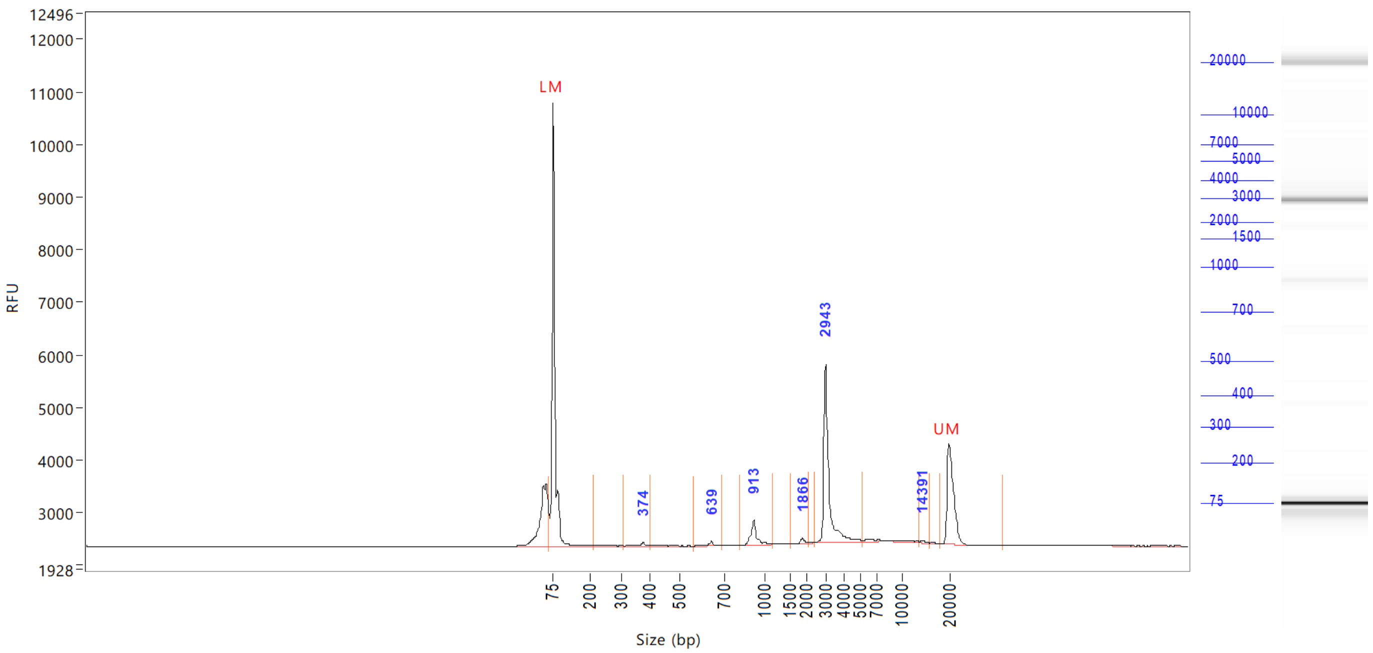

Peak	Size (bp)	Concentration (ng/uL)	From (bp)	To (bp)	Average size (bp)	CV%	RFU	Corrected peak area
1	75 (LM)	0.5000	64	210	77	16.36	8443	59.655
2	374	0.0731	305	398	362	5.33	72	0.727
3	639	0.0981	555	687	624	4.00	102	0.976
4	913	0.4641	809	1156	915	4.30	482	4.615
5	1866	0.0644	1500	2115	1848	4.73	103	0.640
6	2943	2.7486	2372	5297	3098	13.36	3387	27.328
7	14391	0.0321	13659	15732	14377	3.44	57	0.319
8	20000 (UM)	0.1378	18171	31365	20390	4.28	1926	16.443

TIC:	3.4803	ng/uL
TIM:	2.9474	nmole/L
Total concentration:	3.7729	ng/uL

```
Sample peak width (sec): 5    Sample min peak height: 50    Sample baseline V to V?: Y    Sample baseline V to V points: 3
Sample filter: Binomial       Number of points for filter: 3  Sample start region (min): 0  Sample end region (min): 30
Marker peak width (sec): 5    Marker min peak height: 200   Marker baseline V to V?: Y    Marker baseline V to V points: 3
Lower marker selection: First peak > 500 RFU                Upper marker selection: Last peak > 200 RFU
Ladder size (bp)75, 200, 300, 400, 500, 700, 1000, 1500, 2000, 3000, 4000, 5000, 7000, 10000, 20000
Quantification using: Lower Marker          Final concentration (ng/uL): 0.5000          Dilution factor: 12.0
```

Sample: SampH8
Well location: H8
Created: Tuesday, September 14, 2021 8:54:07 PM

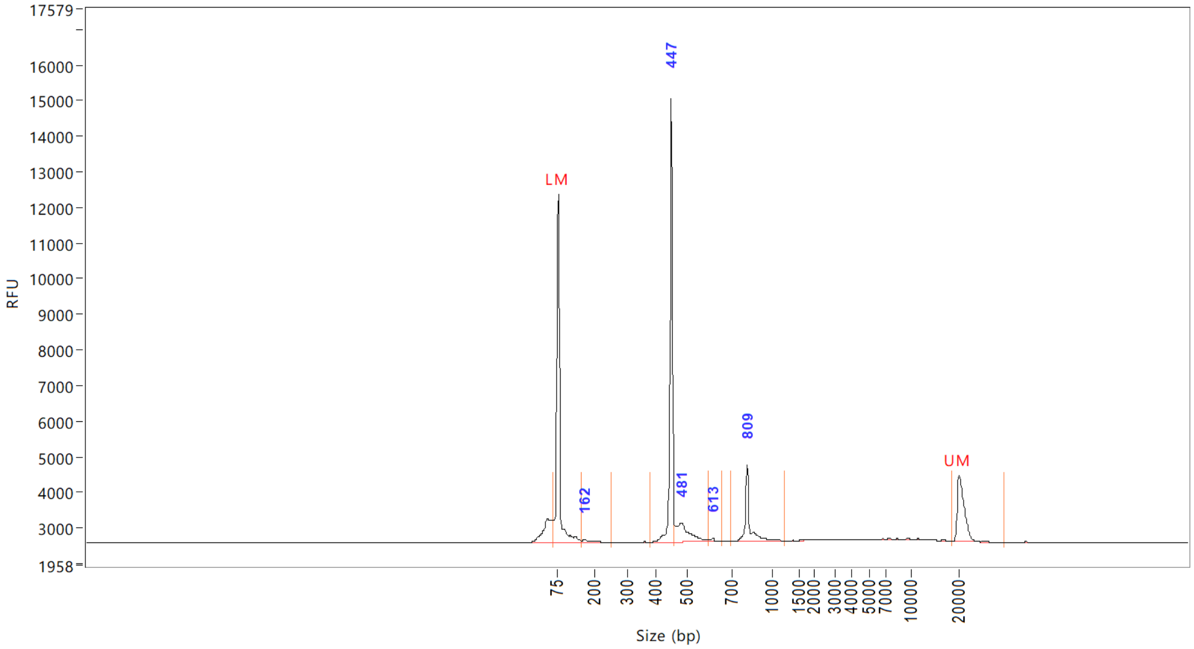

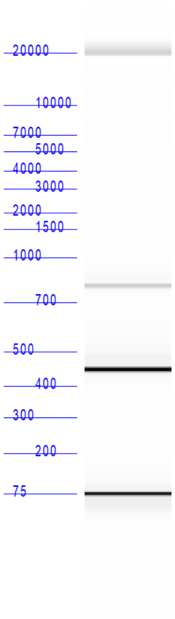

Peak	Size (bp)	Concentration (ng/uL)	From (bp)	To (bp)	Average size (bp)	CV%	RFU	Corrected peak area
1	75 (LM)	0.5000	58	152	76	17.93	9756	67.162
2	162	0.1012	152	250	173	11.00	73	1.133
3	447	5.6232	378	460	444	1.69	12473	62.944
4	481	1.2381	460	590	496	6.12	527	13.859
5	613	0.0775	590	657	610	2.38	93	0.868
6	809	1.4012	692	1234	824	6.71	2127	15.684
7	20000 (UM)	0.1247	18415	29551	20537	4.25	1853	16.747

TIC:	8.4412	ng/uL
TIM:	28.9155	nmole/L
Total concentration:	8.6567	ng/uL

Sample peak width (sec): 5 Sample min peak height: 50 Sample baseline V to V?: Y Sample baseline V to V points: 3
Sample filter: Binomial Number of points for filter: 3 Sample start region (min): 0 Sample end region (min): 30
Marker peak width (sec): 5 Marker min peak height: 200 Marker baseline V to V?: Y Marker baseline V to V points: 3
Lower marker selection: First peak > 500 RFU Upper marker selection: Last peak > 200 RFU
Ladder size (bp)75, 200, 300, 400, 500, 700, 1000, 1500, 2000, 3000, 4000, 5000, 7000, 10000, 20000
Quantification using: Lower Marker Final concentration (ng/uL): 0.5000 Dilution factor: 12.0

Sample: SampA9
Well location: A9
Created: Tuesday, September 14, 2021 8:54:07 PM

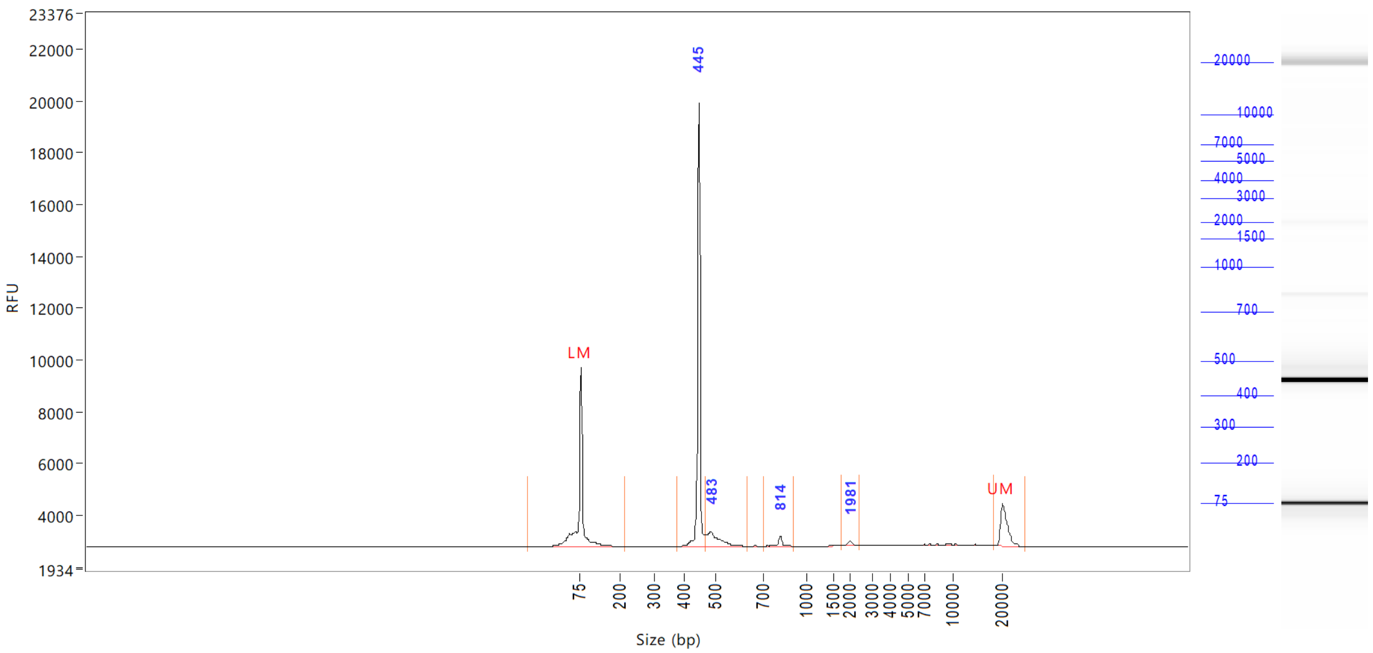

Peak	Size (bp)	Concentration (ng/uL)	From (bp)	To (bp)	Average size (bp)	CV%	RFU	Corrected peak area
1	75 (LM)	0.5000	0	212	69	37.00	6901	62.230
2	445	8.2338	374	468	443	1.70	17136	85.398
3	483	1.2331	468	631	501	5.97	585	12.789
4	814	0.2407	697	909	803	3.92	381	2.497
5	1981	0.1423	1712	2401	1975	5.87	202	1.476
6	20000 (UM)	0.1137	18293	24473	20424	3.78	1646	14.152

TIC:	9.8499	ng/uL
TIM:	35.2441	nmole/L
Total concentration:	10.1542	ng/uL

Sample peak width (sec): 5 Sample min peak height: 50 Sample baseline V to V?: Y Sample baseline V to V points: 3
Sample filter: Binomial Number of points for filter: 3 Sample start region (min): 0 Sample end region (min): 30
Marker peak width (sec): 5 Marker min peak height: 200 Marker baseline V to V?: Y Marker baseline V to V points: 3
Lower marker selection: First peak > 500 RFU Upper marker selection: Last peak > 200 RFU
Ladder size (bp)75, 200, 300, 400, 500, 700, 1000, 1500, 2000, 3000, 4000, 5000, 7000, 10000, 20000
Quantification using: Lower Marker Final concentration (ng/uL): 0.5000 Dilution factor: 12.0

Sample: SampB9
Well location: B9
Created: Tuesday, September 14, 2021 8:54:07 PM

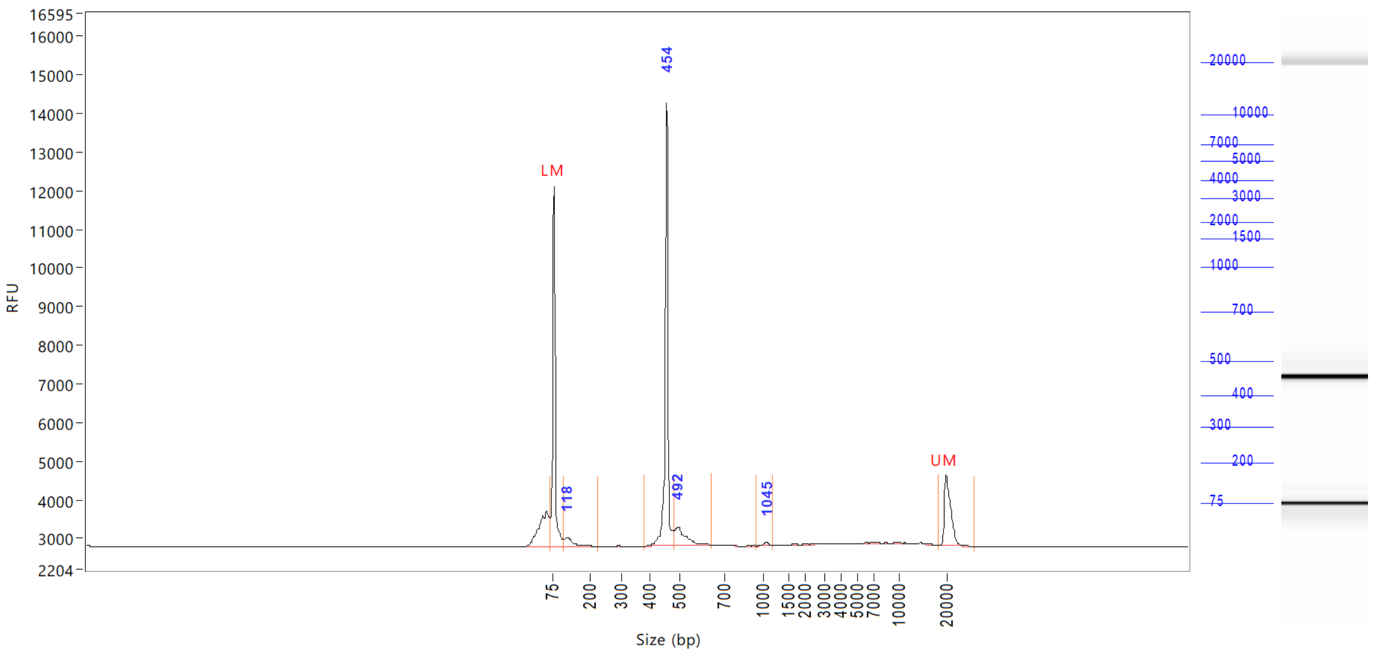

Peak	Size (bp)	Concentration (ng/uL)	From (bp)	To (bp)	Average size (bp)	CV%	RFU	Corrected peak area
1	75 (LM)	0.5000	62	107	74	9.15	9310	60.403
2	118	0.4991	107	224	132	17.85	227	5.024
3	454	6.5291	376	479	451	2.08	11479	65.730
4	492	0.9933	479	641	510	5.55	481	10.000
5	1045	0.0755	948	1178	1034	4.44	86	0.760
6	20000 (UM)	0.1283	18293	26045	20424	3.73	1821	15.501

TIC:	8.0970	ng/uL
TIM:	33.3580	nmole/L
Total concentration:	8.3206	ng/uL

```
Sample peak width (sec): 5    Sample min peak height: 50    Sample baseline V to V?: Y    Sample baseline V to V points: 3
Sample filter: Binomial       Number of points for filter: 3  Sample start region (min): 0  Sample end region (min): 30
Marker peak width (sec): 5    Marker min peak height: 200   Marker baseline V to V?: Y    Marker baseline V to V points: 3
Lower marker selection: First peak > 500 RFU                Upper marker selection: Last peak > 200 RFU
Ladder size (bp)75, 200, 300, 400, 500, 700, 1000, 1500, 2000, 3000, 4000, 5000, 7000, 10000, 20000
Quantification using: Lower Marker          Final concentration (ng/uL): 0.5000          Dilution factor: 12.0
```

Sample: SampC9
Well location: C9
Created: Tuesday, September 14, 2021 8:54:07 PM

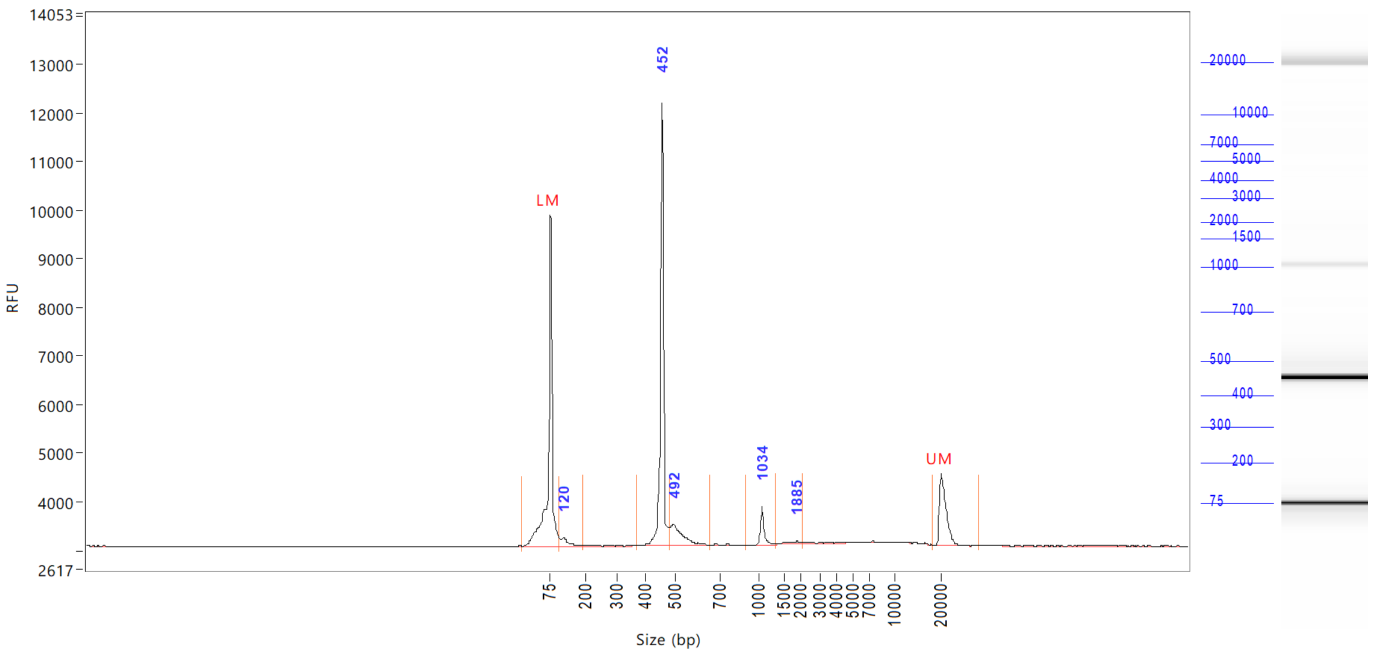

Peak	Size (bp)	Concentration (ng/uL)	From (bp)	To (bp)	Average size (bp)	CV%	RFU	Corrected peak area
1	75 (LM)	0.5000	0	108	66	29.87	6829	67.078
2	120	0.3124	108	185	131	14.77	176	3.492
3	452	4.9399	368	477	449	2.27	9125	55.226
4	492	0.9981	477	657	518	7.39	432	11.158
5	1034	0.4519	892	1334	1052	5.97	783	5.053
6	1885	0.1156	1334	2087	1683	11.97	51	1.293
7	20000 (UM)	0.1007	18293	28221	20532	4.58	1458	13.504

TIC:	6.8179	ng/uL
TIM:	26.0117	nmole/L
Total concentration:	7.0959	ng/uL

Sample peak width (sec): 5 Sample min peak height: 50 Sample baseline V to V?: Y Sample baseline V to V points: 3
Sample filter: Binomial Number of points for filter: 3 Sample start region (min): 0 Sample end region (min): 30
Marker peak width (sec): 5 Marker min peak height: 200 Marker baseline V to V?: Y Marker baseline V to V points: 3
Lower marker selection: First peak > 500 RFU Upper marker selection: Last peak > 200 RFU
Ladder size (bp)75, 200, 300, 400, 500, 700, 1000, 1500, 2000, 3000, 4000, 5000, 7000, 10000, 20000
Quantification using: Lower Marker Final concentration (ng/uL): 0.5000 Dilution factor: 12.0

Sample: SampD9
Well location: D9
Created: Tuesday, September 14, 2021 8:54:07 PM

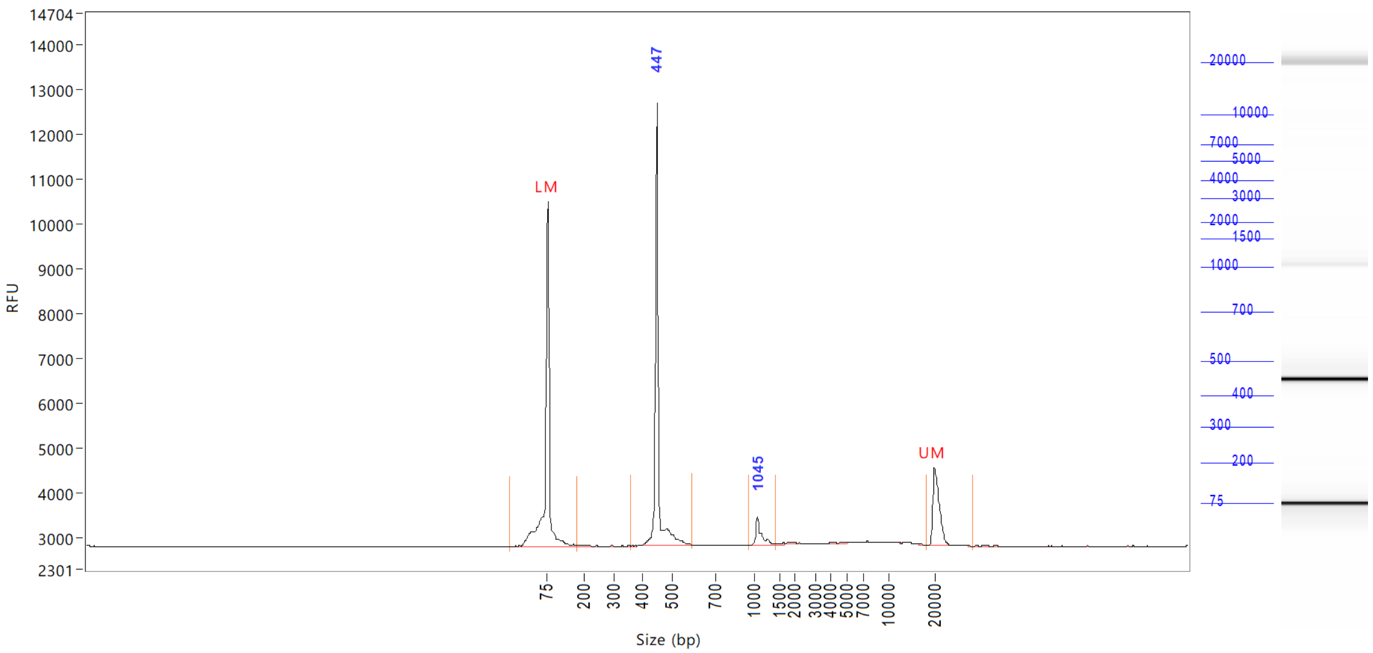

Peak	Size (bp)	Concentration (ng/uL)	From (bp)	To (bp)	Average size (bp)	CV%	RFU	Corrected peak area
1	75 (LM)	0.5000	0	177	66	38.88	7707	72.674
2	447	5.1353	356	593	451	4.66	9890	62.200
3	1045	0.5016	952	1400	1093	8.28	646	6.076
4	20000 (UM)	0.1028	18415	28463	20484	3.95	1715	14.941

TIC:	5.6369	ng/uL
TIM:	19.4909	nmole/L
Total concentration:	5.9519	ng/uL

Sample peak width (sec): 5 Sample min peak height: 50 Sample baseline V to V?: Y Sample baseline V to V points: 3
Sample filter: Binomial Number of points for filter: 3 Sample start region (min): 0 Sample end region (min): 30
Marker peak width (sec): 5 Marker min peak height: 200 Marker baseline V to V?: Y Marker baseline V to V points: 3
Lower marker selection: First peak > 500 RFU Upper marker selection: Last peak > 200 RFU
Ladder size (bp)75, 200, 300, 400, 500, 700, 1000, 1500, 2000, 3000, 4000, 5000, 7000, 10000, 20000
Quantification using: Lower Marker Final concentration (ng/uL): 0.5000 Dilution factor: 12.0

Sample: SampE9
Well location: E9
Created: Tuesday, September 14, 2021 8:54:07 PM

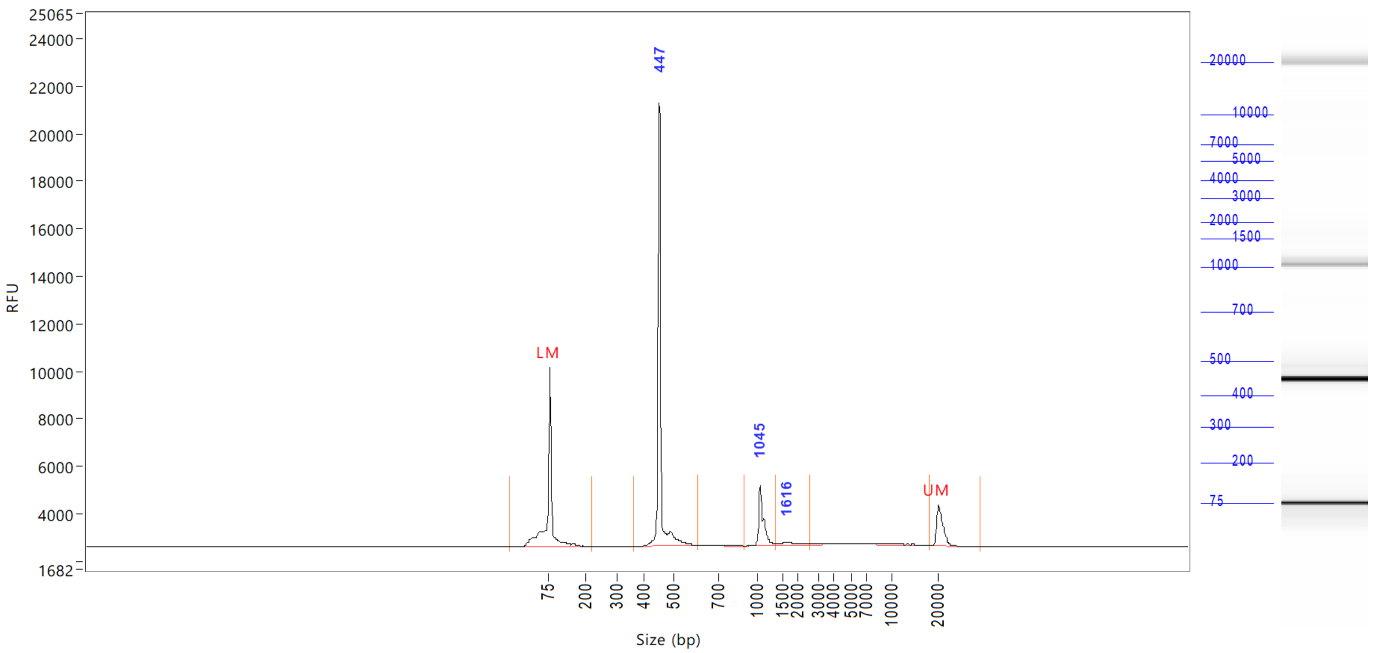

Peak	Size (bp)	Concentration (ng/uL)	From (bp)	To (bp)	Average size (bp)	CV%	RFU	Corrected peak area
1	75 (LM)	0.5000	0	221	67	44.62	7557	76.762
2	447	8.5821	362	606	451	4.41	18661	109.796
3	1045	1.5900	900	1367	1076	6.77	2542	20.342
4	1616	0.2521	1367	2572	1697	15.31	125	3.226
5	20000 (UM)	0.0960	18293	29309	20467	4.17	1711	14.744

TIC:	10.4242	ng/uL
TIM:	33.9873	nmole/L
Total concentration:	10.6559	ng/uL

Sample peak width (sec): 5   Sample min peak height: 50   Sample baseline V to V?: Y   Sample baseline V to V points: 3
Sample filter: Binomial   Number of points for filter: 3 Sample start region (min): 0 Sample end region (min): 30
Marker peak width (sec): 5   Marker min peak height: 200   Marker baseline V to V?: Y   Marker baseline V to V points: 3
Lower marker selection: First peak > 500 RFU   Upper marker selection: Last peak > 200 RFU
Ladder size (bp)75, 200, 300, 400, 500, 700, 1000, 1500, 2000, 3000, 4000, 5000, 7000, 10000, 20000
Quantification using: Lower Marker   Final concentration (ng/uL): 0.5000   Dilution factor: 12.0

Sample: SampF9
Well location: F9
Created: Tuesday, September 14, 2021 8:54:07 PM

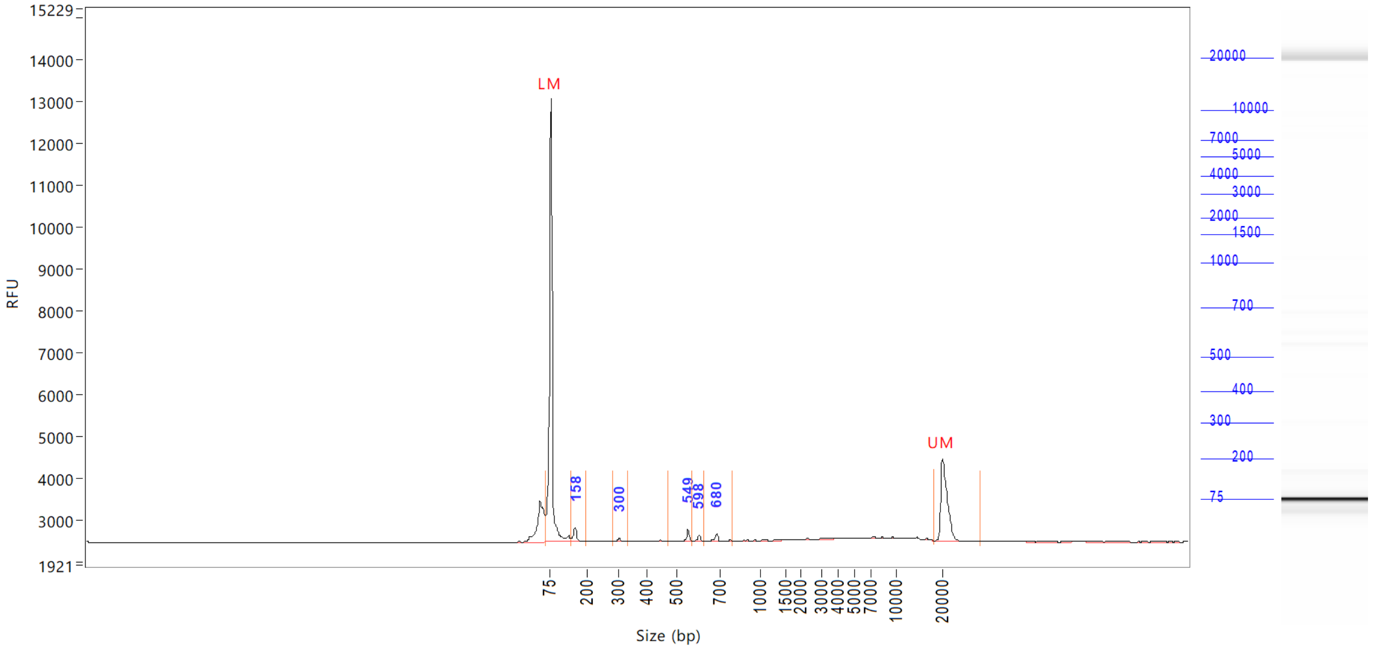

Peak	Size (bp)	Concentration (ng/uL)	From (bp)	To (bp)	Average size (bp)	CV%	RFU	Corrected peak area
1	75 (LM)	0.5000	56	143	74	15.43	10623	77.673
2	158	0.2405	143	192	156	6.12	345	3.113
3	300	0.0483	280	333	299	3.22	81	0.625
4	549	0.1376	470	570	540	3.37	277	1.782
5	598	0.0747	570	623	596	1.67	160	0.968
6	680	0.1030	623	788	682	4.59	175	1.333
7	20000 (UM)	0.1126	18293	28221	20470	4.03	1974	17.485

TIC:	0.6041	ng/uL
TIM:	3.6736	nmole/L
Total concentration:	0.9052	ng/uL

Sample peak width (sec): 5 Sample min peak height: 50 Sample baseline V to V?: Y Sample baseline V to V points: 3
Sample filter: Binomial Number of points for filter: 3 Sample start region (min): 0 Sample end region (min): 30
Marker peak width (sec): 5 Marker min peak height: 200 Marker baseline V to V?: Y Marker baseline V to V points: 3
Lower marker selection: First peak > 500 RFU Upper marker selection: Last peak > 200 RFU
Ladder size (bp)75, 200, 300, 400, 500, 700, 1000, 1500, 2000, 3000, 4000, 5000, 7000, 10000, 20000
Quantification using: Lower Marker Final concentration (ng/uL): 0.5000 Dilution factor: 12.0

Sample: SampG9
Well location: G9
Created: Tuesday, September 14, 2021 8:54:07 PM

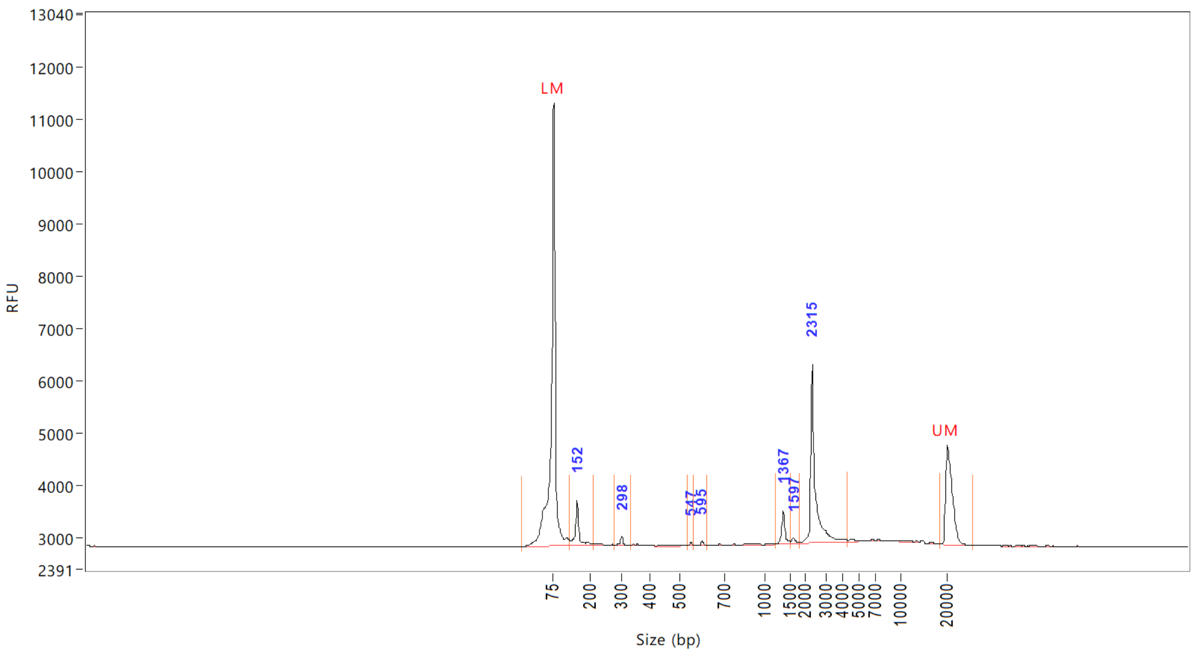

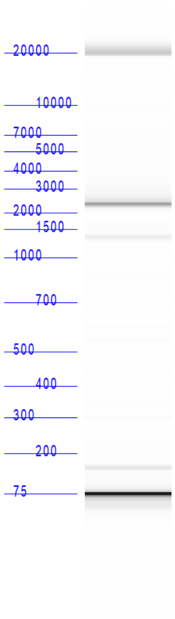

Peak	Size (bp)	Concentration (ng/uL)	From (bp)	To (bp)	Average size (bp)	CV%	RFU	Corrected peak area
1	75 (LM)	0.5000	0	129	66	27.25	8500	87.499
2	152	0.5332	129	210	155	10.14	873	7.776
3	298	0.0773	275	329	297	3.11	163	1.127
4	547	0.0234	529	560	543	1.08	69	0.341
5	595	0.0280	560	621	592	1.53	79	0.409
6	1367	0.2509	1234	1501	1359	3.43	644	3.659
7	1597	0.0441	1501	1808	1594	4.25	103	0.643
8	2315	1.7293	1808	4311	2488	15.62	3420	25.219
9	20000 (UM)	0.0951	18415	25682	20489	3.81	1896	16.635

TIC:	2.6863	ng/uL
TIM:	7.7247	nmole/L
Total concentration:	3.0083	ng/uL

```
Sample peak width (sec): 5    Sample min peak height: 50    Sample baseline V to V?: Y    Sample baseline V to V points: 3
Sample filter: Binomial       Number of points for filter: 3  Sample start region (min): 0  Sample end region (min): 30
Marker peak width (sec): 5    Marker min peak height: 200   Marker baseline V to V?: Y    Marker baseline V to V points: 3
Lower marker selection: First peak > 500 RFU                Upper marker selection: Last peak > 200 RFU
Ladder size (bp)75, 200, 300, 400, 500, 700, 1000, 1500, 2000, 3000, 4000, 5000, 7000, 10000, 20000
Quantification using: Lower Marker          Final concentration (ng/uL): 0.5000          Dilution factor: 12.0
```

Sample: SampH9
Well location: H9
Created: Tuesday, September 14, 2021 8:54:07 PM

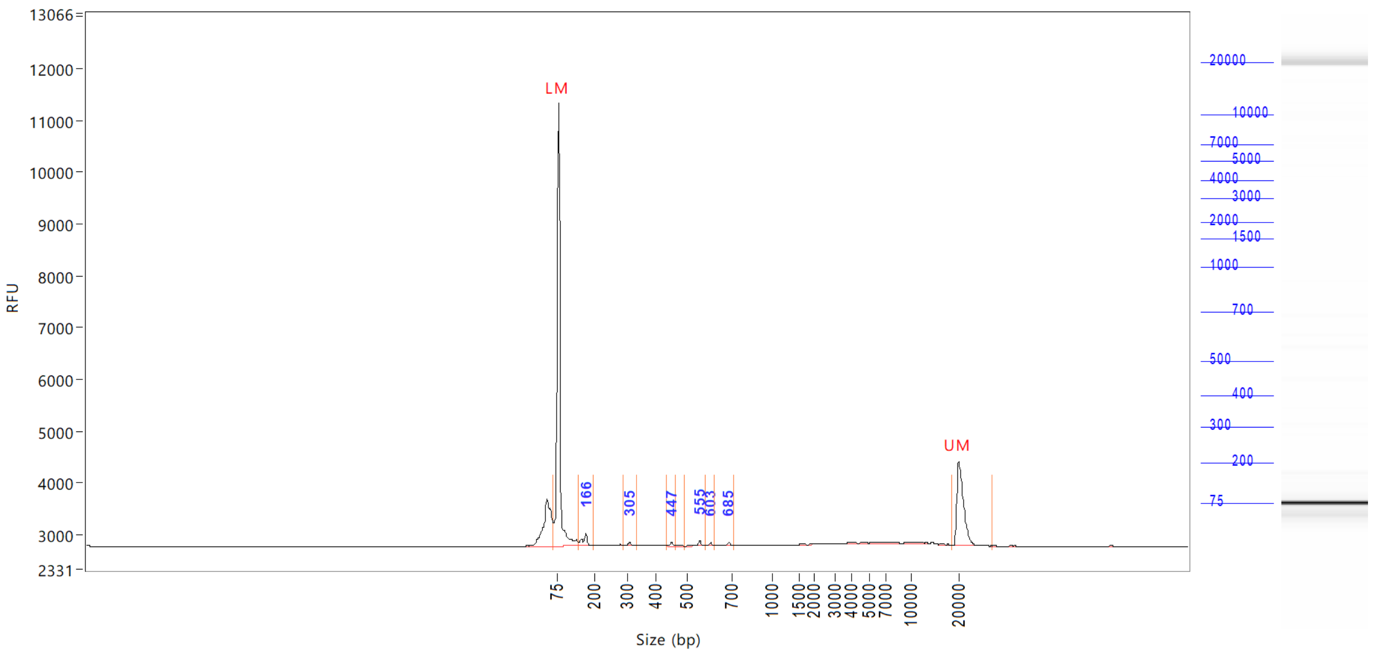

Peak	Size (bp)	Concentration (ng/uL)	From (bp)	To (bp)	Average size (bp)	CV%	RFU	Corrected peak area
1	75 (LM)	0.5000	58	141	76	15.17	8571	58.899
2	166	0.2449	141	194	158	6.09	230	2.404
3	305	0.0474	286	331	301	2.64	67	0.465
4	447	0.0453	433	464	446	1.21	80	0.445
5	555	0.0755	492	583	544	3.37	100	0.741
6	603	0.0334	583	621	600	1.17	59	0.327
7	685	0.0487	621	710	673	2.31	73	0.478
8	20000 (UM)	0.1197	18415	27012	20401	3.97	1614	14.099

TIC:	0.4951	ng/uL
TIM:	3.4128	nmole/L
Total concentration:	0.8429	ng/uL

```
Sample peak width (sec): 5    Sample min peak height: 50    Sample baseline V to V?: Y    Sample baseline V to V points: 3
Sample filter: Binomial       Number of points for filter: 3  Sample start region (min): 0  Sample end region (min): 30
Marker peak width (sec): 5    Marker min peak height: 200   Marker baseline V to V?: Y    Marker baseline V to V points: 3
Lower marker selection: First peak > 500 RFU                Upper marker selection: Last peak > 200 RFU
Ladder size (bp)75, 200, 300, 400, 500, 700, 1000, 1500, 2000, 3000, 4000, 5000, 7000, 10000, 20000
Quantification using: Lower Marker          Final concentration (ng/uL): 0.5000          Dilution factor: 12.0
```

Sample: SampA10
Well location: A10
Created: Tuesday, September 14, 2021 8:54:07 PM

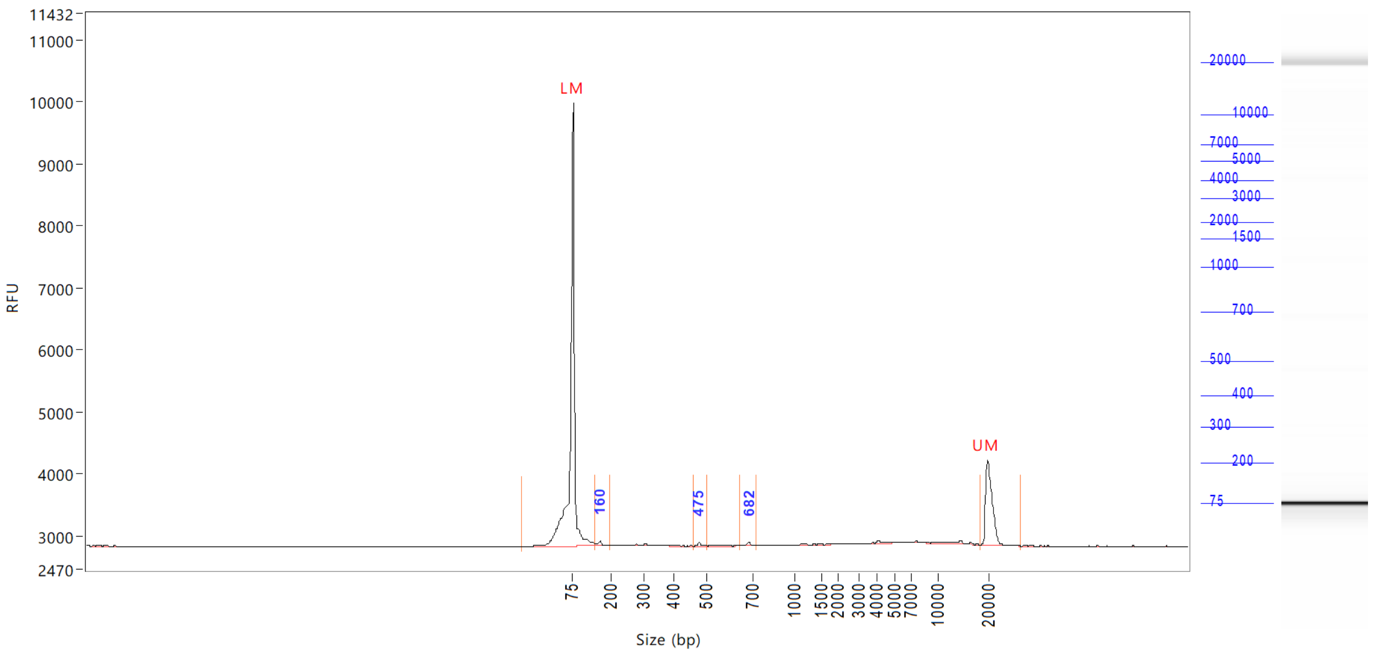

Peak	Size (bp)	Concentration (ng/uL)	From (bp)	To (bp)	Average size (bp)	CV%	RFU	Corrected peak area
1	75 (LM)	0.5000	0	145	65	33.90	7155	67.823
2	160	0.0759	145	192	160	7.10	76	0.858
3	475	0.0327	458	504	476	1.53	60	0.370
4	682	0.0420	641	714	675	1.88	67	0.475
5	20000 (UM)	0.0847	18415	26891	20332	3.68	1367	11.483

TIC:	0.1505	ng/uL
TIM:	0.9947	nmole/L
Total concentration:	0.5333	ng/uL

Sample peak width (sec): 5 Sample min peak height: 50 Sample baseline V to V?: Y Sample baseline V to V points: 3
Sample filter: Binomial Number of points for filter: 3 Sample start region (min): 0 Sample end region (min): 30
Marker peak width (sec): 5 Marker min peak height: 200 Marker baseline V to V?: Y Marker baseline V to V points: 3
Lower marker selection: First peak > 500 RFU Upper marker selection: Last peak > 200 RFU
Ladder size (bp)75, 200, 300, 400, 500, 700, 1000, 1500, 2000, 3000, 4000, 5000, 7000, 10000, 20000
Quantification using: Lower Marker Final concentration (ng/uL): 0.5000 Dilution factor: 12.0

Sample: SampB10
Well location: B10
Created: Tuesday, September 14, 2021 8:54:07 PM

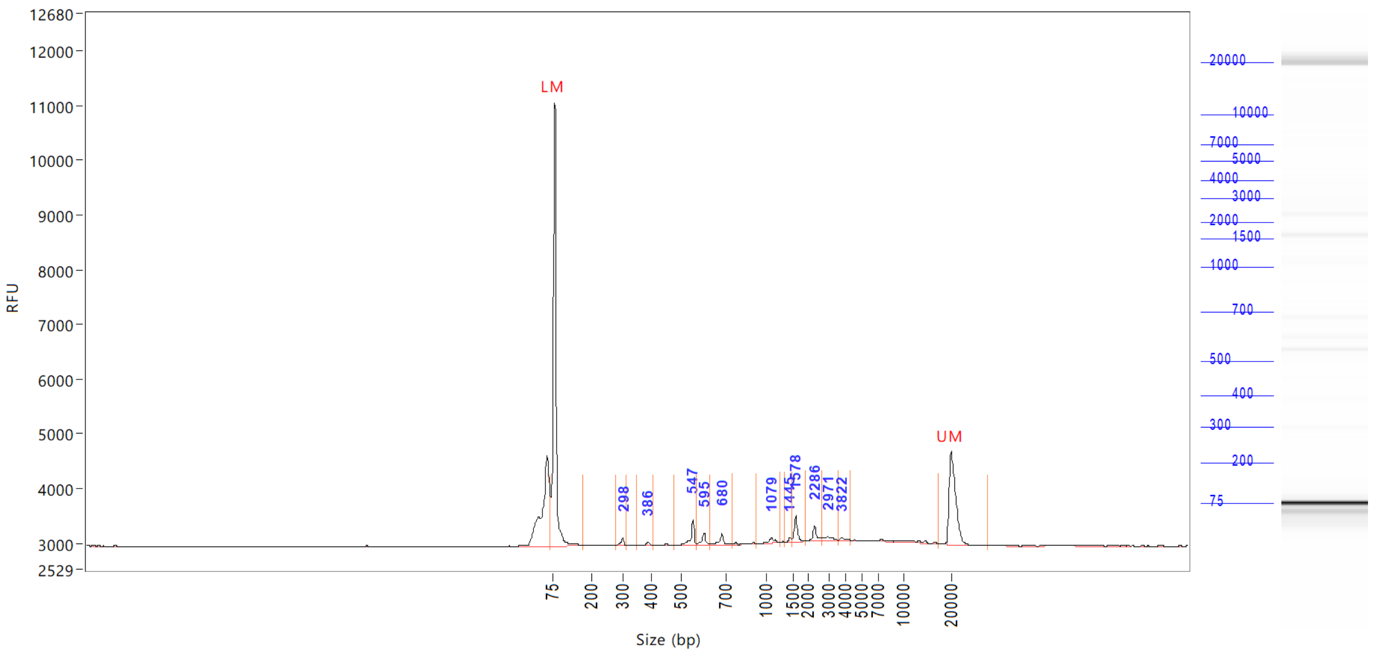

Peak	Size (bp)	Concentration (ng/uL)	From (bp)	To (bp)	Average size (bp)	CV%	RFU	Corrected peak area
1	75 (LM)	0.5000	64	173	76	14.03	8106	60.051
2	298	0.1046	275	309	292	2.20	145	1.047
3	386	0.0598	347	405	375	3.40	58	0.599
4	547	0.3051	471	562	537	2.87	469	3.054
5	595	0.1589	562	623	591	2.11	216	1.590
6	680	0.1727	623	744	673	3.01	221	1.728
7	1079	0.0956	922	1245	1076	6.13	90	0.957
8	1445	0.0377	1356	1489	1439	1.95	82	0.378
9	1578	0.2613	1489	1923	1588	4.82	480	2.616
10	2286	0.1857	1923	2657	2310	6.48	271	1.859
11	2971	0.1168	2657	3608	3009	7.74	71	1.169
12	3822	0.0539	3608	4345	3902	4.86	63	0.539
13	20000 (UM)	0.1243	17317	27859	20424	4.08	1713	14.925

TIC:	1.5522	ng/uL
TIM:	3.3308	nmole/L
Total concentration:	1.8885	ng/uL

```
Sample peak width (sec): 5    Sample min peak height: 50    Sample baseline V to V?: Y    Sample baseline V to V points: 3
Sample filter: Binomial       Number of points for filter: 3  Sample start region (min): 0  Sample end region (min): 30
Marker peak width (sec): 5    Marker min peak height: 200   Marker baseline V to V?: Y    Marker baseline V to V points: 3
Lower marker selection: First peak > 500 RFU                Upper marker selection: Last peak > 200 RFU
Ladder size (bp)75, 200, 300, 400, 500, 700, 1000, 1500, 2000, 3000, 4000, 5000, 7000, 10000, 20000
Quantification using: Lower Marker          Final concentration (ng/uL): 0.5000          Dilution factor: 12.0
```

Sample: SampC10
Well location: C10
Created: Tuesday, September 14, 2021 8:54:07 PM

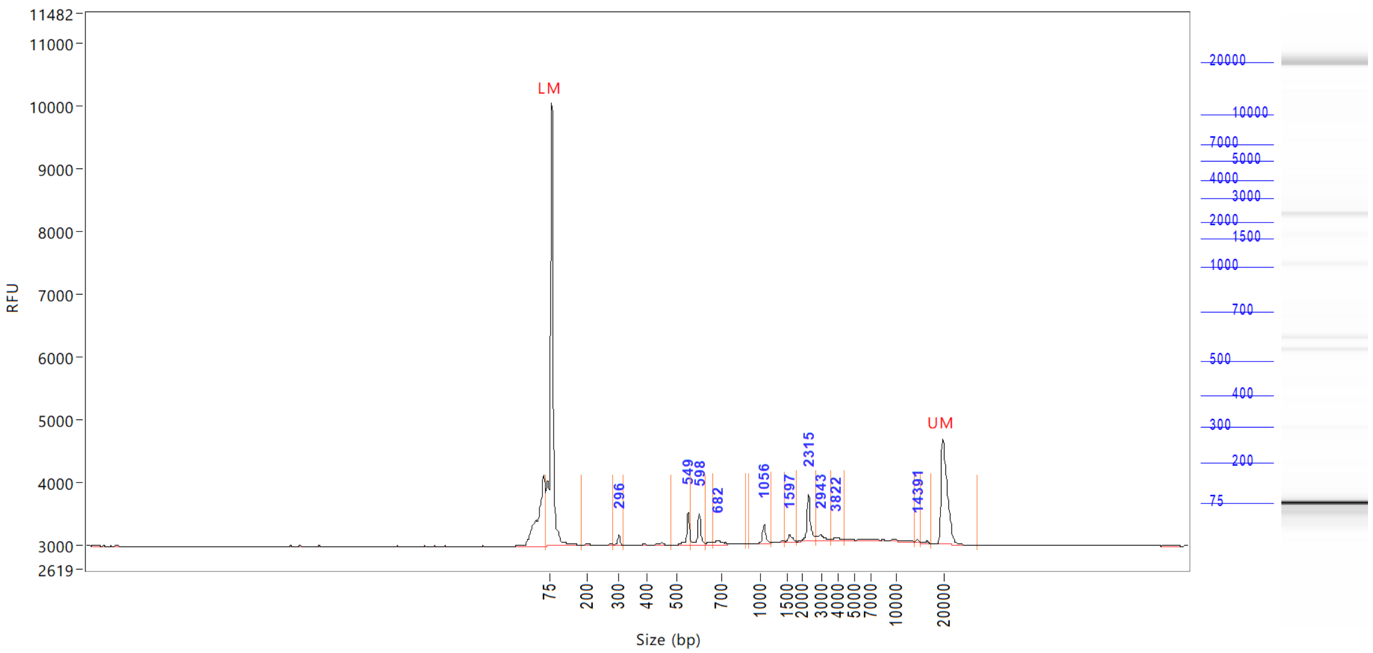

Peak	Size (bp)	Concentration (ng/uL)	From (bp)	To (bp)	Average size (bp)	CV%	RFU	Corrected peak area
1	75 (LM)	0.5000	56	177	75	16.04	7075	54.421
2	296	0.1060	280	313	294	1.71	161	0.962
3	549	0.3296	479	562	541	2.33	531	2.989
4	598	0.3522	562	623	594	1.82	505	3.194
5	682	0.1331	659	879	706	5.94	73	1.207
6	1056	0.2084	909	1201	1045	4.29	292	1.890
7	1597	0.0875	1467	1808	1594	4.33	115	0.794
8	2315	0.4951	1808	2686	2309	5.87	759	4.491
9	2943	0.1383	2686	3536	2979	7.36	90	1.254
10	3822	0.0678	3536	4345	3891	5.25	54	0.615
11	14391	0.0265	13781	15244	14375	2.38	51	0.240
12	20000 (UM)	0.1328	17317	27254	20355	3.88	1676	14.456

TIC:	1.9445	ng/uL
TIM:	3.7613	nmole/L
Total concentration:	2.3282	ng/uL

```
Sample peak width (sec): 5    Sample min peak height: 50    Sample baseline V to V?: Y    Sample baseline V to V points: 3
Sample filter: Binomial       Number of points for filter: 3  Sample start region (min): 0  Sample end region (min): 30
Marker peak width (sec): 5    Marker min peak height: 200   Marker baseline V to V?: Y    Marker baseline V to V points: 3
Lower marker selection: First peak > 500 RFU                Upper marker selection: Last peak > 200 RFU
Ladder size (bp)75, 200, 300, 400, 500, 700, 1000, 1500, 2000, 3000, 4000, 5000, 7000, 10000, 20000
Quantification using: Lower Marker          Final concentration (ng/uL): 0.5000          Dilution factor: 12.0
```

Sample: SampD10
Well location: D10
Created: Tuesday, September 14, 2021 8:54:07 PM

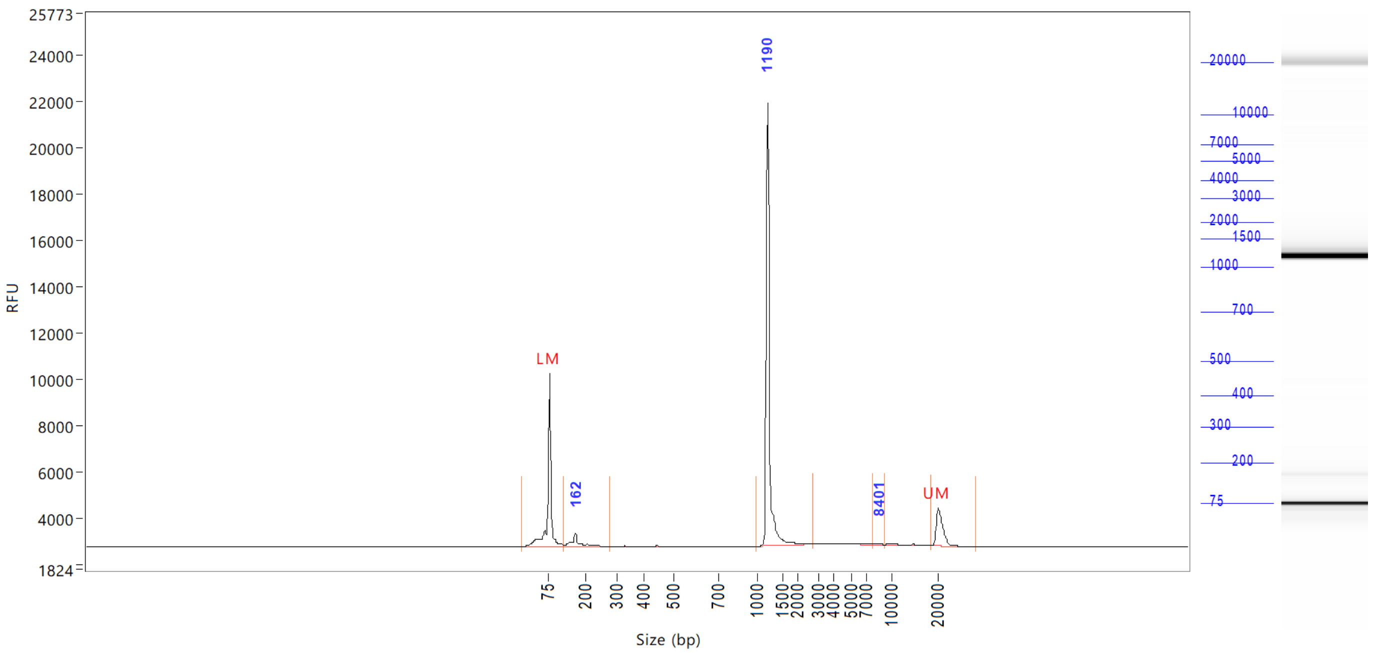

Peak	Size (bp)	Concentration (ng/uL)	From (bp)	To (bp)	Average size (bp)	CV%	RFU	Corrected peak area
1	75 (LM)	0.5000	0	124	67	28.71	7485	61.329
2	162	0.8760	124	278	165	14.83	572	8.954
3	1190	9.9933	987	2715	1218	9.73	19113	102.147
4	8401	0.0336	7734	9067	8332	3.01	61	0.343
5	20000 (UM)	0.1168	18415	28221	20408	4.12	1659	14.332

TIC:	10.9029	ng/uL
TIM:	22.2386	nmole/L
Total concentration:	11.1870	ng/uL

Sample peak width (sec): 5   Sample min peak height: 50   Sample baseline V to V?: Y   Sample baseline V to V points: 3
Sample filter: Binomial   Number of points for filter: 3 Sample start region (min): 0 Sample end region (min): 30
Marker peak width (sec): 5   Marker min peak height: 200   Marker baseline V to V?: Y   Marker baseline V to V points: 3
Lower marker selection: First peak > 500 RFU   Upper marker selection: Last peak > 200 RFU
Ladder size (bp)75, 200, 300, 400, 500, 700, 1000, 1500, 2000, 3000, 4000, 5000, 7000, 10000, 20000
Quantification using: Lower Marker   Final concentration (ng/uL): 0.5000   Dilution factor: 12.0

Sample: SampE10
Well location: E10
Created: Tuesday, September 14, 2021 8:54:07 PM

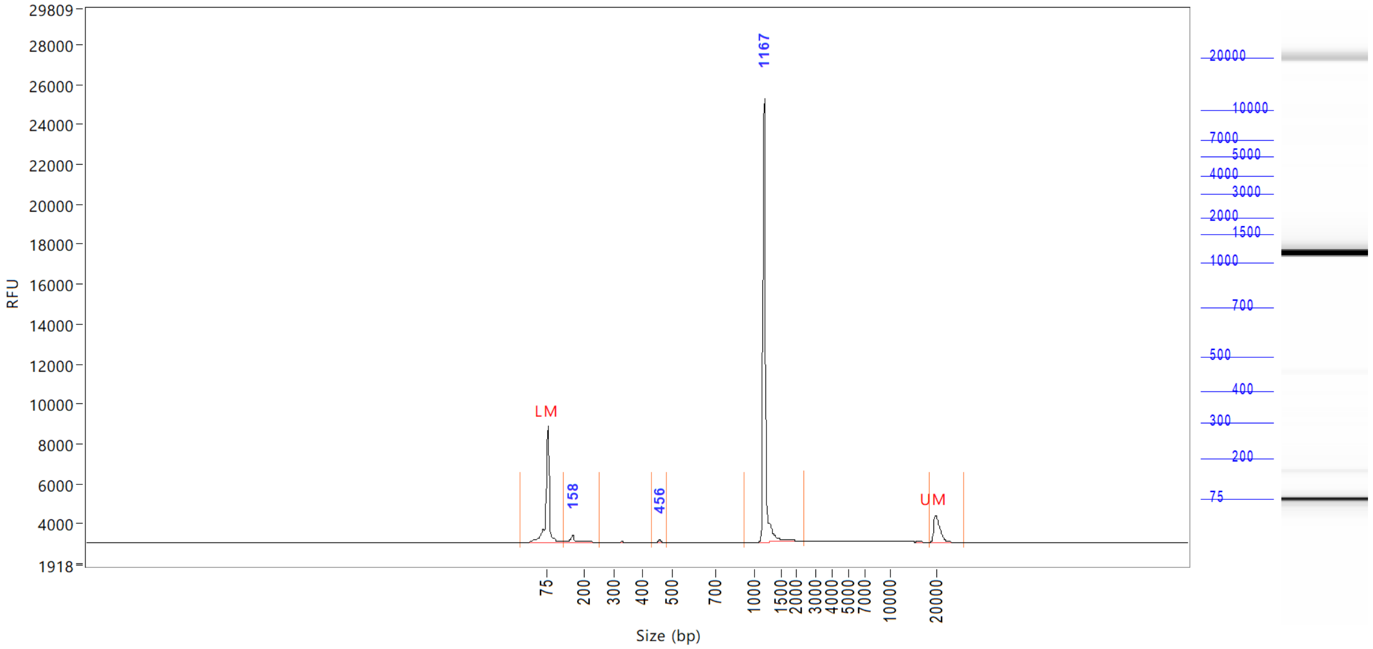

Peak	Size (bp)	Concentration (ng/uL)	From (bp)	To (bp)	Average size (bp)	CV%	RFU	Corrected peak area
1	75 (LM)	0.5000	0	129	69	22.05	5842	47.537
2	158	0.5812	129	251	167	14.10	356	4.605
3	456	0.0975	428	479	454	1.54	122	0.772
4	1167	13.3427	918	2372	1178	7.51	22274	105.712
5	20000 (UM)	0.1194	18659	26166	20379	3.94	1355	11.356

TIC:	14.0214	ng/uL
TIM:	24.7175	nmole/L
Total concentration:	14.3212	ng/uL

Sample peak width (sec): 5 Sample min peak height: 50 Sample baseline V to V?: Y Sample baseline V to V points: 3
Sample filter: Binomial Number of points for filter: 3 Sample start region (min): 0 Sample end region (min): 30
Marker peak width (sec): 5 Marker min peak height: 200 Marker baseline V to V?: Y Marker baseline V to V points: 3
Lower marker selection: First peak > 500 RFU Upper marker selection: Last peak > 200 RFU
Ladder size (bp)75, 200, 300, 400, 500, 700, 1000, 1500, 2000, 3000, 4000, 5000, 7000, 10000, 20000
Quantification using: Lower Marker Final concentration (ng/uL): 0.5000 Dilution factor: 12.0

Sample: SampF10
Well location: F10
Created: Tuesday, September 14, 2021 8:54:07 PM

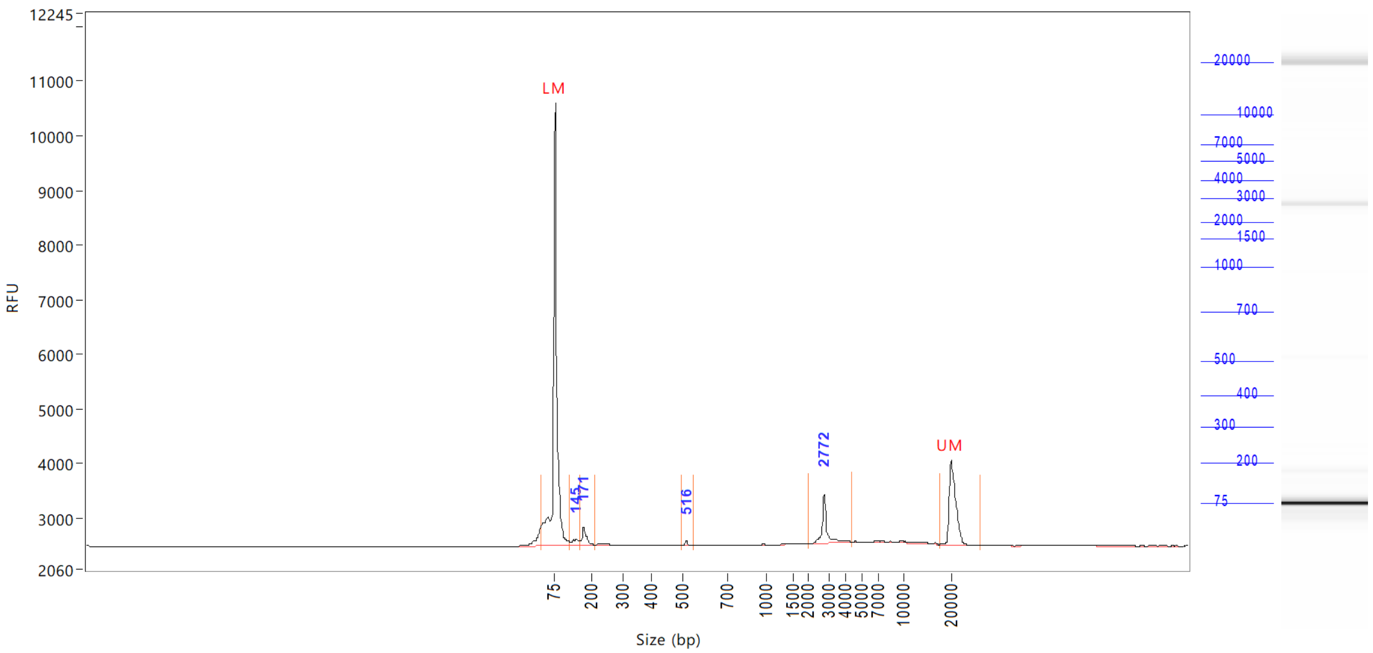

Peak	Size (bp)	Concentration (ng/uL)	From (bp)	To (bp)	Average size (bp)	CV%	RFU	Corrected peak area
1	75 (LM)	0.5000	30	126	71	19.42	8123	69.659
2	145	0.1627	126	160	143	6.86	107	1.888
3	171	0.2498	160	206	172	4.41	328	2.900
4	516	0.0477	496	547	515	1.64	100	0.554
5	2772	0.5664	2000	4380	2856	12.25	900	6.575
6	20000 (UM)	0.0970	17439	26287	20419	3.87	1551	13.513

TIC:	1.0265	ng/uL
TIM:	4.7356	nmole/L
Total concentration:	1.3194	ng/uL

```
Sample peak width (sec): 5    Sample min peak height: 50    Sample baseline V to V?: Y    Sample baseline V to V points: 3
Sample filter: Binomial       Number of points for filter: 3  Sample start region (min): 0  Sample end region (min): 30
Marker peak width (sec): 5    Marker min peak height: 200   Marker baseline V to V?: Y    Marker baseline V to V points: 3
Lower marker selection: First peak > 500 RFU                Upper marker selection: Last peak > 200 RFU
Ladder size (bp)75, 200, 300, 400, 500, 700, 1000, 1500, 2000, 3000, 4000, 5000, 7000, 10000, 20000
Quantification using: Lower Marker          Final concentration (ng/uL): 0.5000          Dilution factor: 12.0
```

Sample: SampG10
Well location: G10
Created: Tuesday, September 14, 2021 8:54:07 PM

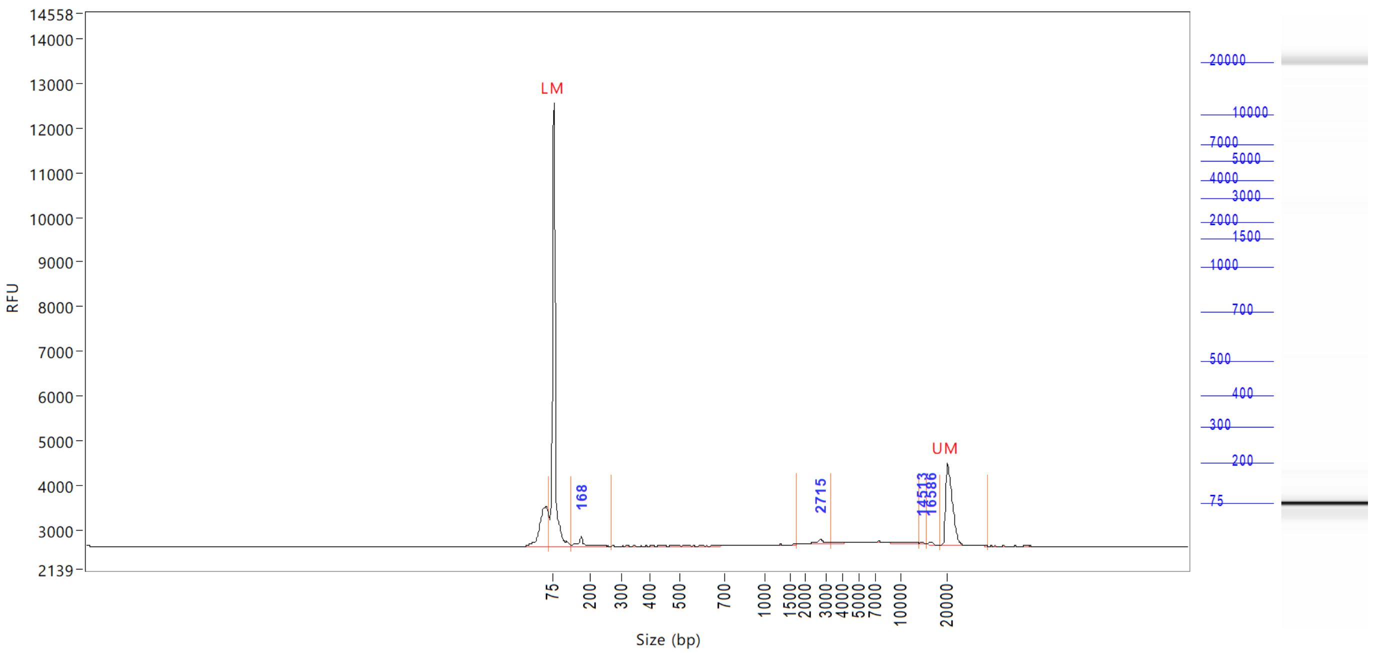

Peak	Size (bp)	Concentration (ng/uL)	From (bp)	To (bp)	Average size (bp)	CV%	RFU	Corrected peak area
1	75 (LM)	0.5000	60	131	75	11.85	9924	71.570
2	168	0.2412	131	264	168	14.03	207	2.877
3	2715	0.1091	1731	3286	2646	10.63	109	1.301
4	14513	0.0234	13903	15366	14497	2.52	54	0.279
5	16586	0.0301	15366	18293	16348	3.03	58	0.360
6	20000 (UM)	0.1136	18293	28705	20503	4.05	1844	16.266

TIC:	0.4038	ng/uL
TIM:	2.4338	nmole/L
Total concentration:	0.6639	ng/uL

Sample peak width (sec): 5 Sample min peak height: 50 Sample baseline V to V?: Y Sample baseline V to V points: 3
Sample filter: Binomial Number of points for filter: 3 Sample start region (min): 0 Sample end region (min): 30
Marker peak width (sec): 5 Marker min peak height: 200 Marker baseline V to V?: Y Marker baseline V to V points: 3
Lower marker selection: First peak > 500 RFU Upper marker selection: Last peak > 200 RFU
Ladder size (bp)75, 200, 300, 400, 500, 700, 1000, 1500, 2000, 3000, 4000, 5000, 7000, 10000, 20000
Quantification using: Lower Marker Final concentration (ng/uL): 0.5000 Dilution factor: 12.0

Sample: SampH10
Well location: H10
Created: Tuesday, September 14, 2021 8:54:07 PM

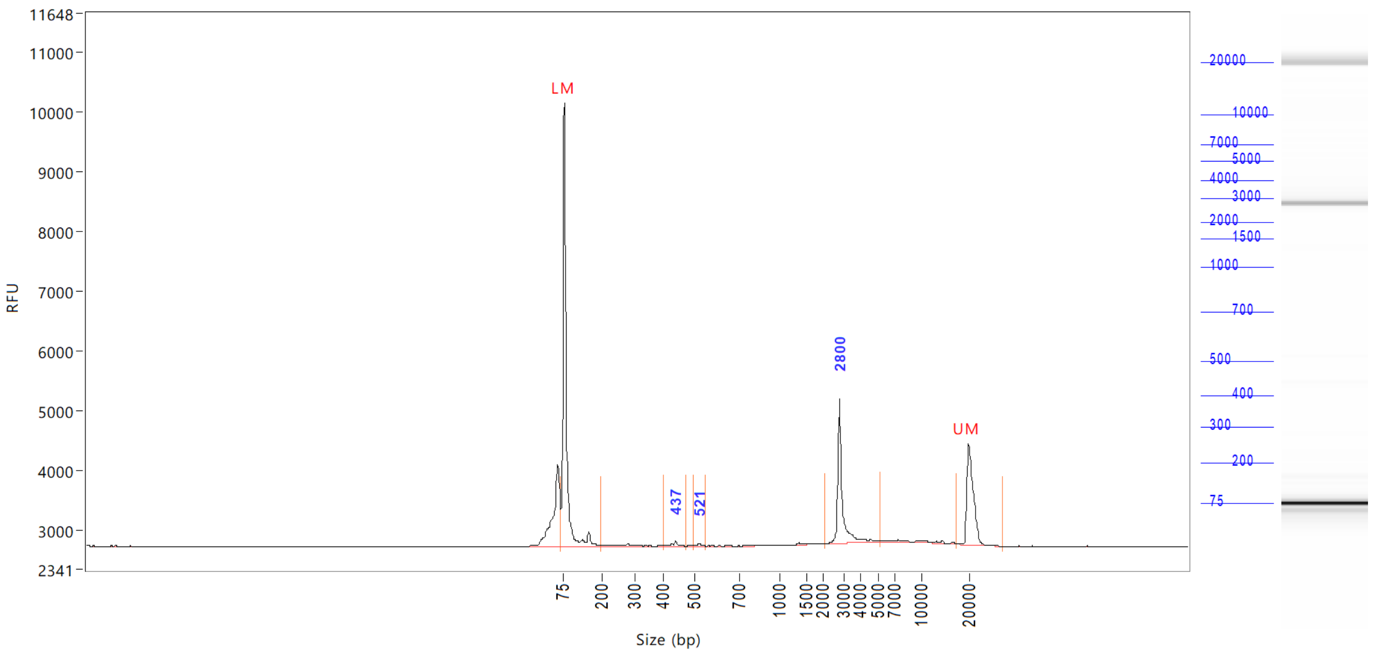

Peak	Size (bp)	Concentration (ng/uL)	From (bp)	To (bp)	Average size (bp)	CV%	RFU	Corrected peak area
1	75 (LM)	0.5000	64	198	80	25.17	7437	58.756
2	437	0.0777	400	471	432	2.82	83	0.761
3	521	0.0315	496	547	517	1.75	51	0.309
4	2800	1.7028	2058	5297	2959	14.88	2421	16.675
5	20000 (UM)	0.1226	17439	27012	20370	3.80	1696	14.404

TIC:	1.8120	ng/uL
TIM:	1.3435	nmole/L
Total concentration:	2.2037	ng/uL

Sample peak width (sec): 5   Sample min peak height: 50   Sample baseline V to V?: Y   Sample baseline V to V points: 3
Sample filter: Binomial   Number of points for filter: 3 Sample start region (min): 0 Sample end region (min): 30
Marker peak width (sec): 5   Marker min peak height: 200   Marker baseline V to V?: Y   Marker baseline V to V points: 3
Lower marker selection: First peak > 500 RFU   Upper marker selection: Last peak > 200 RFU
Ladder size (bp)75, 200, 300, 400, 500, 700, 1000, 1500, 2000, 3000, 4000, 5000, 7000, 10000, 20000
Quantification using: Lower Marker   Final concentration (ng/uL): 0.5000   Dilution factor: 12.0

Sample: SampA11
Well location: A11
Created: Tuesday, September 14, 2021 8:54:07 PM

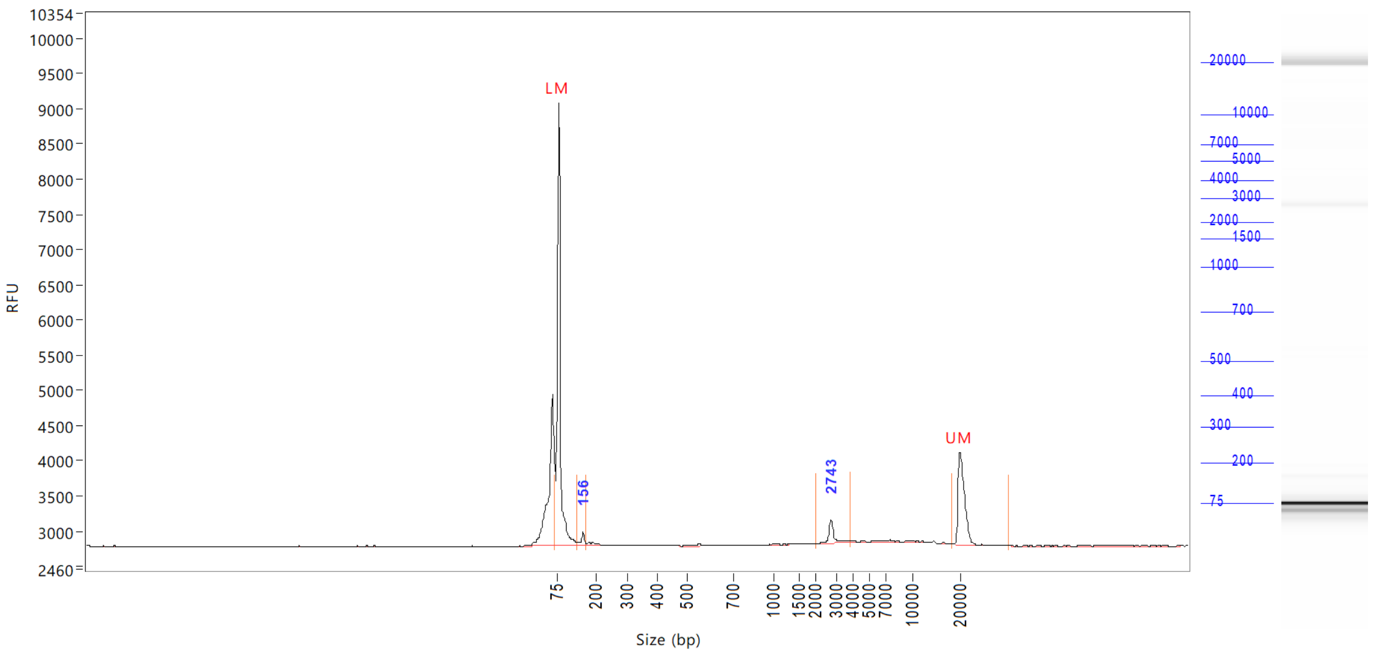

Peak	Size (bp)	Concentration (ng/uL)	From (bp)	To (bp)	Average size (bp)	CV%	RFU	Corrected peak area
1	75 (LM)	0.5000	66	135	76	13.98	6296	44.367
2	156	0.2040	135	166	151	4.63	191	1.509
3	2743	0.3556	2001	3857	2825	10.86	331	2.630
4	20000 (UM)	0.1265	18171	30156	20401	4.68	1315	11.227

TIC:	0.5596	ng/uL
TIM:	2.4277	nmole/L
Total concentration:	1.0629	ng/uL

```
Sample peak width (sec): 5    Sample min peak height: 50    Sample baseline V to V?: Y    Sample baseline V to V points: 3
Sample filter: Binomial       Number of points for filter: 3  Sample start region (min): 0  Sample end region (min): 30
Marker peak width (sec): 5    Marker min peak height: 200   Marker baseline V to V?: Y    Marker baseline V to V points: 3
Lower marker selection: First peak > 500 RFU                Upper marker selection: Last peak > 200 RFU
Ladder size (bp)75, 200, 300, 400, 500, 700, 1000, 1500, 2000, 3000, 4000, 5000, 7000, 10000, 20000
Quantification using: Lower Marker          Final concentration (ng/uL): 0.5000          Dilution factor: 12.0
```

Sample: SampB11
Well location: B11
Created: Tuesday, September 14, 2021 8:54:07 PM

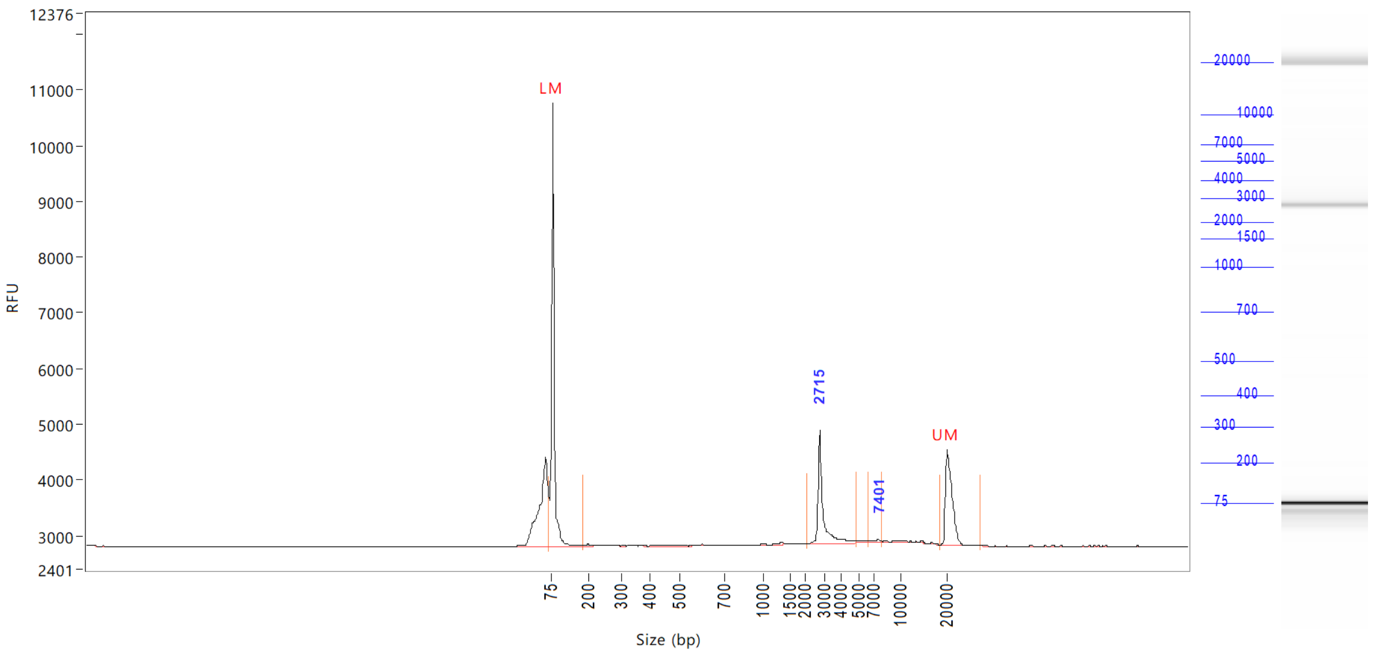

Peak	Size (bp)	Concentration (ng/uL)	From (bp)	To (bp)	Average size (bp)	CV%	RFU	Corrected peak area
1	75 (LM)	0.5000	66	175	76	14.02	7967	52.637
2	2715	1.7392	2115	4897	2928	16.27	2021	15.257
3	7401	0.0739	6260	7867	7040	6.23	53	0.648
4	20000 (UM)	0.1426	18415	27133	20448	3.86	1699	15.017

TIC:	1.8130	ng/uL
TIM:	0.9951	nmole/L
Total concentration:	2.3151	ng/uL

```
Sample peak width (sec): 5    Sample min peak height: 50    Sample baseline V to V?: Y    Sample baseline V to V points: 3
Sample filter: Binomial       Number of points for filter: 3  Sample start region (min): 0  Sample end region (min): 30
Marker peak width (sec): 5    Marker min peak height: 200   Marker baseline V to V?: Y    Marker baseline V to V points: 3
Lower marker selection: First peak > 500 RFU                Upper marker selection: Last peak > 200 RFU
Ladder size (bp)75, 200, 300, 400, 500, 700, 1000, 1500, 2000, 3000, 4000, 5000, 7000, 10000, 20000
Quantification using: Lower Marker          Final concentration (ng/uL): 0.5000          Dilution factor: 12.0
```

Sample: SampC11
Well location: C11
Created: Tuesday, September 14, 2021 8:54:07 PM

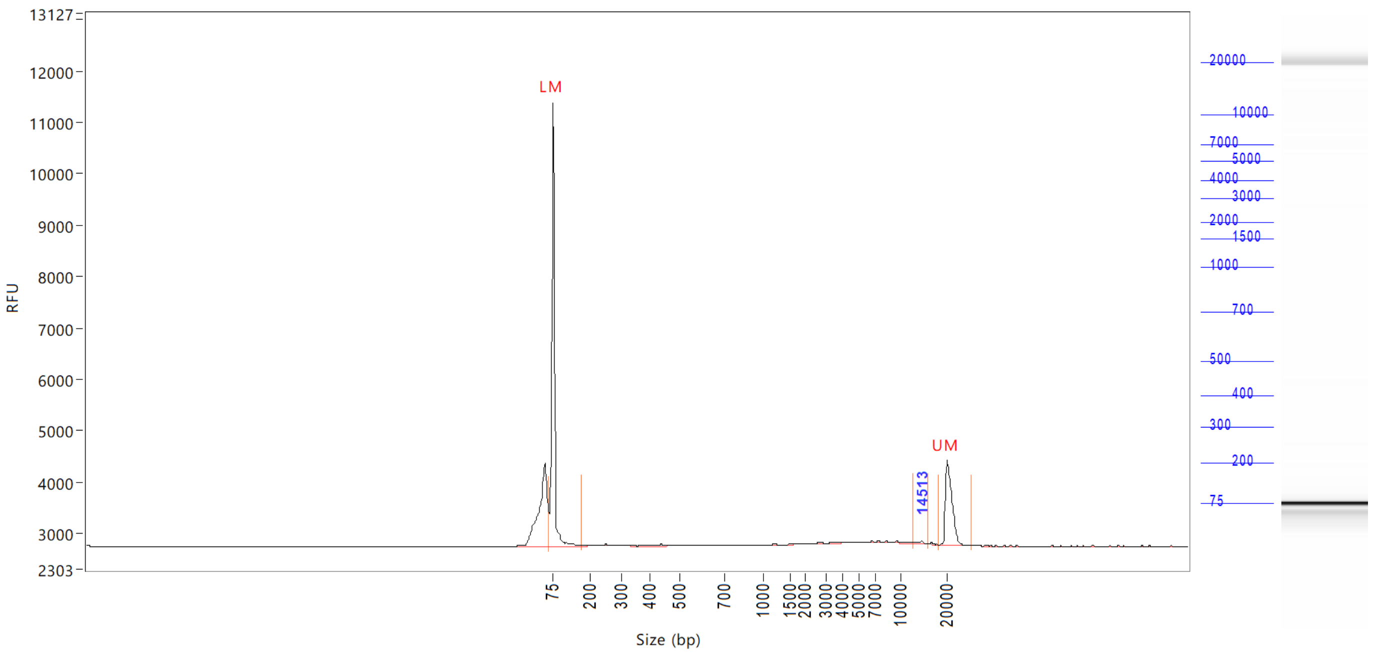

Peak	Size	Concentration	From	To	Average size	CV%	RFU	Corrected peak area
	(bp)	(ng/uL)	(bp)	(bp)	(bp)			
1	75 (LM)	0.5000	60	171	74	14.40	8645	60.898
2	14513	0.0438	12684	15854	14087	5.40	57	0.444
3	20000 (UM)	0.1181	18171	25199	20406	3.66	1663	14.384

TIC:	0.0438	ng/uL
TIM:	0.0051	nmole/L
Total concentration:	0.4818	ng/uL

Sample peak width (sec): 5 Sample min peak height: 50 Sample baseline V to V?: Y Sample baseline V to V points: 3
Sample filter: Binomial Number of points for filter: 3 Sample start region (min): 0 Sample end region (min): 30
Marker peak width (sec): 5 Marker min peak height: 200 Marker baseline V to V?: Y Marker baseline V to V points: 3
Lower marker selection: First peak > 500 RFU Upper marker selection: Last peak > 200 RFU
Ladder size (bp)75, 200, 300, 400, 500, 700, 1000, 1500, 2000, 3000, 4000, 5000, 7000, 10000, 20000
Quantification using: Lower Marker Final concentration (ng/uL): 0.5000 Dilution factor: 12.0

Sample: SampD11
Well location: D11
Created: Tuesday, September 14, 2021 8:54:07 PM

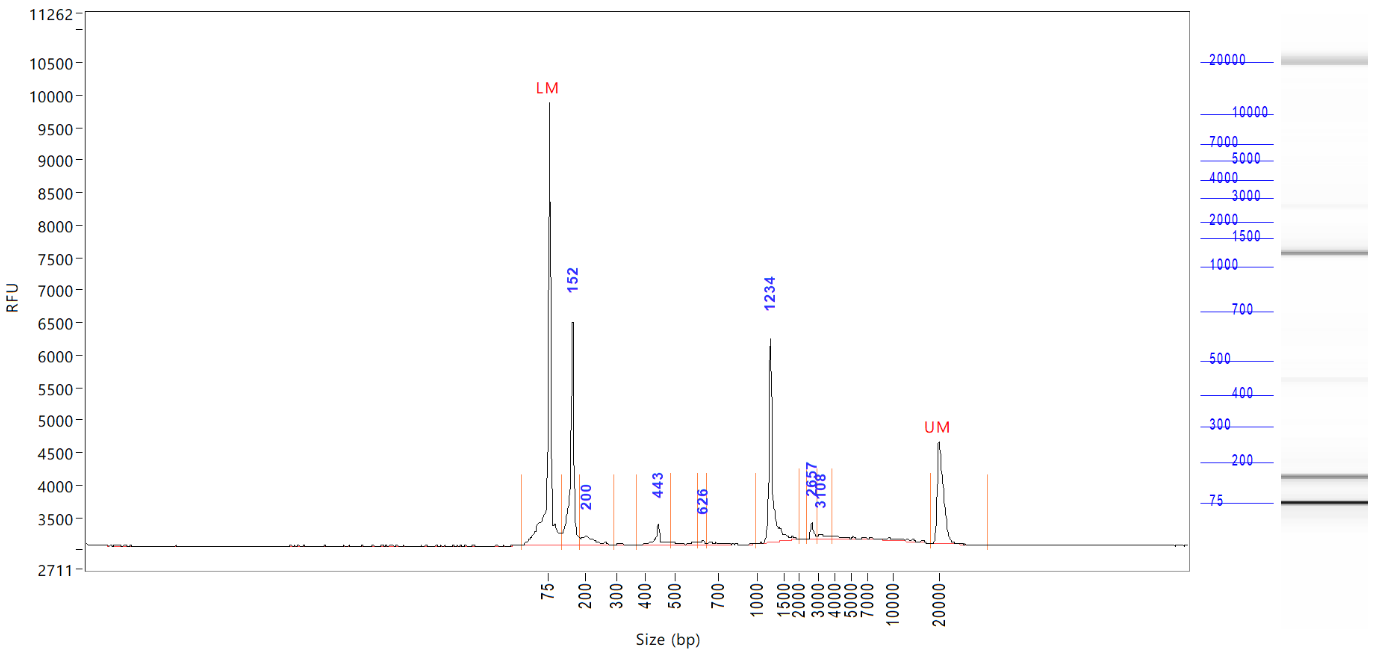

Peak	Size (bp)	Concentration (ng/uL)	From (bp)	To (bp)	Average size (bp)	CV%	RFU	Corrected peak area
1	75 (LM)	0.5000	0	116	68	29.11	6818	57.098
2	152	3.1316	116	181	148	7.40	3436	29.802
3	200	0.4436	181	291	213	11.35	139	4.222
4	443	0.3320	370	485	440	4.54	311	3.160
5	626	0.0550	603	644	622	1.65	61	0.523
6	1234	2.1258	983	2030	1272	8.71	3130	20.230
7	2657	0.1438	2429	2914	2665	3.85	253	1.369
8	3108	0.1194	2914	3822	3303	8.15	66	1.136
9	20000 (UM)	0.1181	18293	30639	20433	5.05	1556	13.489

TIC:	6.3512	ng/uL
TIM:	42.4862	nmole/L
Total concentration:	6.7745	ng/uL

```
Sample peak width (sec): 5    Sample min peak height: 50    Sample baseline V to V?: Y    Sample baseline V to V points: 3
Sample filter: Binomial       Number of points for filter: 3  Sample start region (min): 0  Sample end region (min): 30
Marker peak width (sec): 5    Marker min peak height: 200   Marker baseline V to V?: Y    Marker baseline V to V points: 3
Lower marker selection: First peak > 500 RFU                Upper marker selection: Last peak > 200 RFU
Ladder size (bp)75, 200, 300, 400, 500, 700, 1000, 1500, 2000, 3000, 4000, 5000, 7000, 10000, 20000
Quantification using: Lower Marker          Final concentration (ng/uL): 0.5000          Dilution factor: 12.0
```

Sample: SampE11
Well location: E11
Created: Tuesday, September 14, 2021 8:54:07 PM

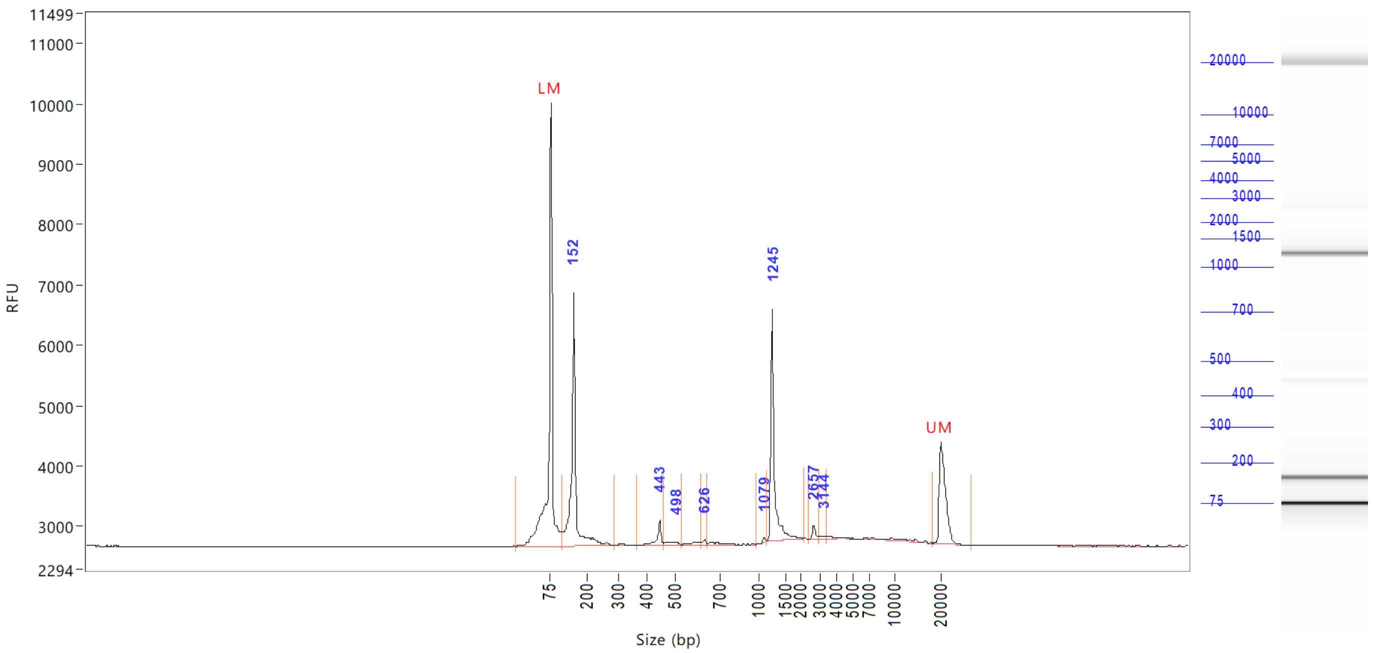

Peak	Size (bp)	Concentration (ng/uL)	From (bp)	To (bp)	Average size (bp)	CV%	RFU	Corrected peak area
1	75 (LM)	0.5000	0	116	65	34.21	7353	67.988
2	152	3.5219	116	286	155	15.97	4187	39.908
3	443	0.2745	368	456	433	3.53	423	3.111
4	498	0.0943	456	519	482	2.89	51	1.069
5	626	0.0444	616	639	625	0.96	83	0.503
6	1079	0.0243	970	1134	1071	2.14	69	0.275
7	1245	2.0442	1134	2115	1270	8.33	3836	23.164
8	2657	0.1043	2372	2914	2667	3.75	220	1.182
9	3144	0.0434	2914	3322	3092	3.88	54	0.491
10	20000 (UM)	0.1067	18293	26649	20382	3.83	1670	14.510

TIC:	6.1514	ng/uL
TIM:	41.6027	nmole/L
Total concentration:	6.5449	ng/uL

```
Sample peak width (sec): 5    Sample min peak height: 50    Sample baseline V to V?: Y    Sample baseline V to V points: 3
Sample filter: Binomial       Number of points for filter: 3  Sample start region (min): 0  Sample end region (min): 30
Marker peak width (sec): 5    Marker min peak height: 200   Marker baseline V to V?: Y    Marker baseline V to V points: 3
Lower marker selection: First peak > 500 RFU                Upper marker selection: Last peak > 200 RFU
Ladder size (bp)75, 200, 300, 400, 500, 700, 1000, 1500, 2000, 3000, 4000, 5000, 7000, 10000, 20000
Quantification using: Lower Marker          Final concentration (ng/uL): 0.5000          Dilution factor: 12.0
```

Sample: SampF11
Well location: F11
Created: Tuesday, September 14, 2021 8:54:07 PM

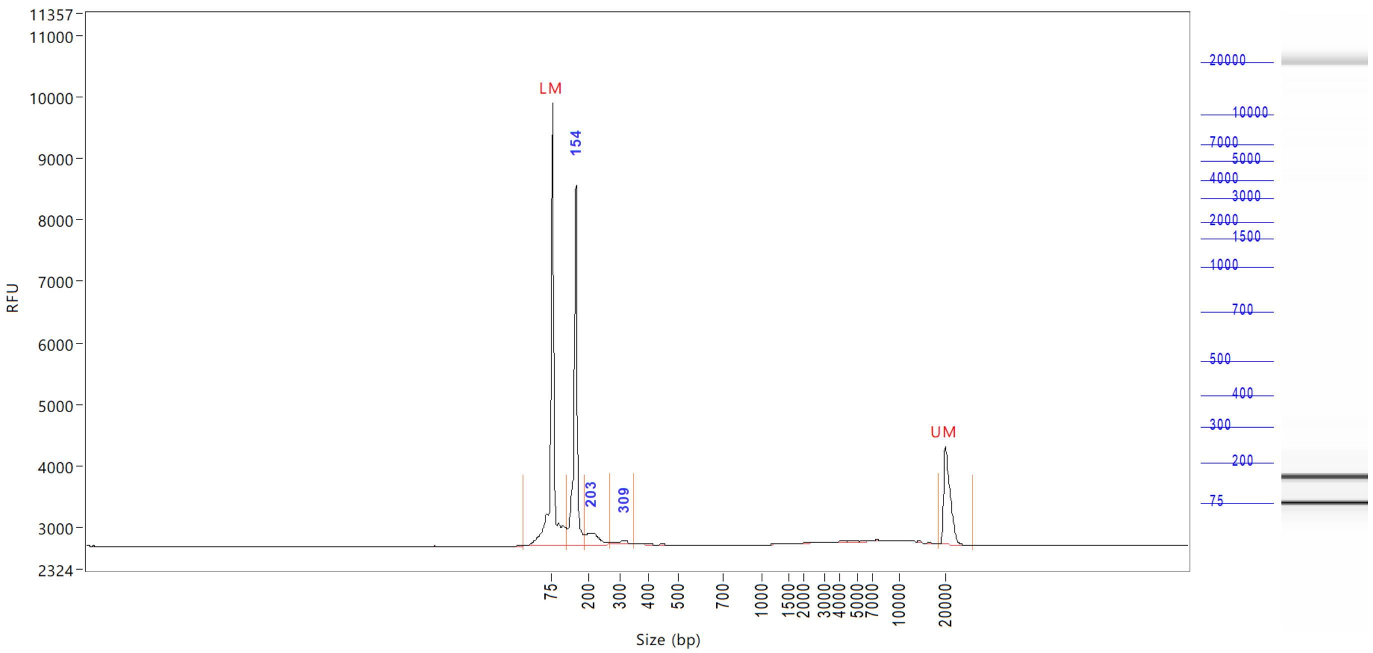

Peak	Size<br>(bp)	Concentration<br>(ng/uL)	From<br>(bp)	To<br>(bp)	Average size<br>(bp)	CV%	RFU	Corrected peak area
1	75 (LM)	0.5000	0	126	73	25.88	7201	61.484
2	154	4.4254	126	185	151	5.82	5844	45.349
3	203	0.5781	185	264	212	8.86	184	5.924
4	309	0.1495	264	345	297	5.84	69	1.532
5	20000 (UM)	0.1126	18537	26045	20439	3.81	1578	13.847

TIC:	5.1530	ng/uL
TIM:	53.4795	nmole/L
Total concentration:	5.4281	ng/uL

Sample peak width (sec): 5 Sample min peak height: 50 Sample baseline V to V?: Y Sample baseline V to V points: 3
Sample filter: Binomial Number of points for filter: 3 Sample start region (min): 0 Sample end region (min): 30
Marker peak width (sec): 5 Marker min peak height: 200 Marker baseline V to V?: Y Marker baseline V to V points: 3
Lower marker selection: First peak > 500 RFU Upper marker selection: Last peak > 200 RFU
Ladder size (bp)75, 200, 300, 400, 500, 700, 1000, 1500, 2000, 3000, 4000, 5000, 7000, 10000, 20000
Quantification using: Lower Marker Final concentration (ng/uL): 0.5000 Dilution factor: 12.0

Sample: SampG11
Well location: G11
Created: Tuesday, September 14, 2021 8:54:07 PM

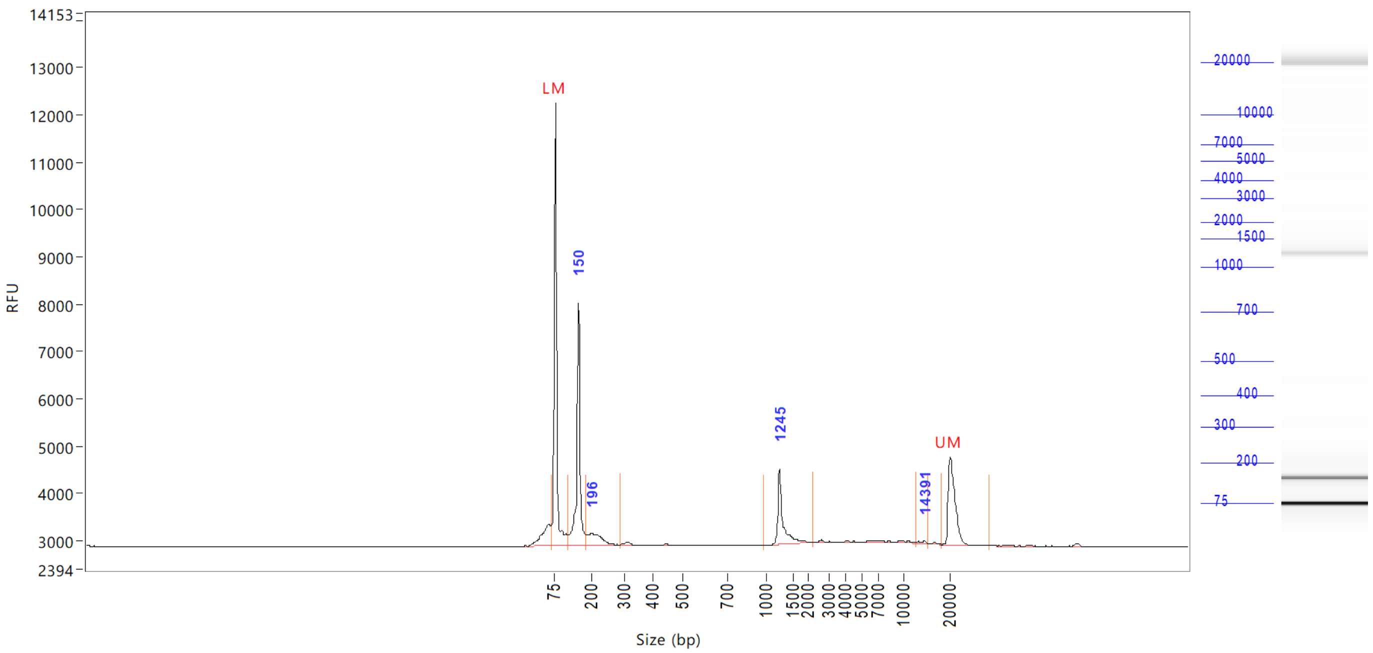

Peak	Size (bp)	Concentration (ng/uL)	From (bp)	To (bp)	Average size (bp)	CV%	RFU	Corrected peak area
1	75 (LM)	0.5000	60	116	74	12.22	9380	61.999
2	150	3.8742	116	175	147	6.75	5109	40.033
3	196	0.7809	175	286	205	9.66	252	8.069
4	1245	1.2204	974	2229	1302	10.88	1598	12.610
5	14391	0.0448	12562	15244	13805	4.73	57	0.463
6	20000 (UM)	0.1360	18171	28221	20427	4.22	1864	16.860

TIC:	5.9203	ng/uL
TIM:	51.1248	nmole/L
Total concentration:	6.2544	ng/uL

```
Sample peak width (sec): 5    Sample min peak height: 50    Sample baseline V to V?: Y    Sample baseline V to V points: 3
Sample filter: Binomial       Number of points for filter: 3  Sample start region (min): 0  Sample end region (min): 30
Marker peak width (sec): 5    Marker min peak height: 200   Marker baseline V to V?: Y    Marker baseline V to V points: 3
Lower marker selection: First peak > 500 RFU                Upper marker selection: Last peak > 200 RFU
Ladder size (bp)75, 200, 300, 400, 500, 700, 1000, 1500, 2000, 3000, 4000, 5000, 7000, 10000, 20000
Quantification using: Lower Marker          Final concentration (ng/uL): 0.5000          Dilution factor: 12.0
```

Sample: SampH11
Well location: H11
Created: Tuesday, September 14, 2021 8:54:07 PM

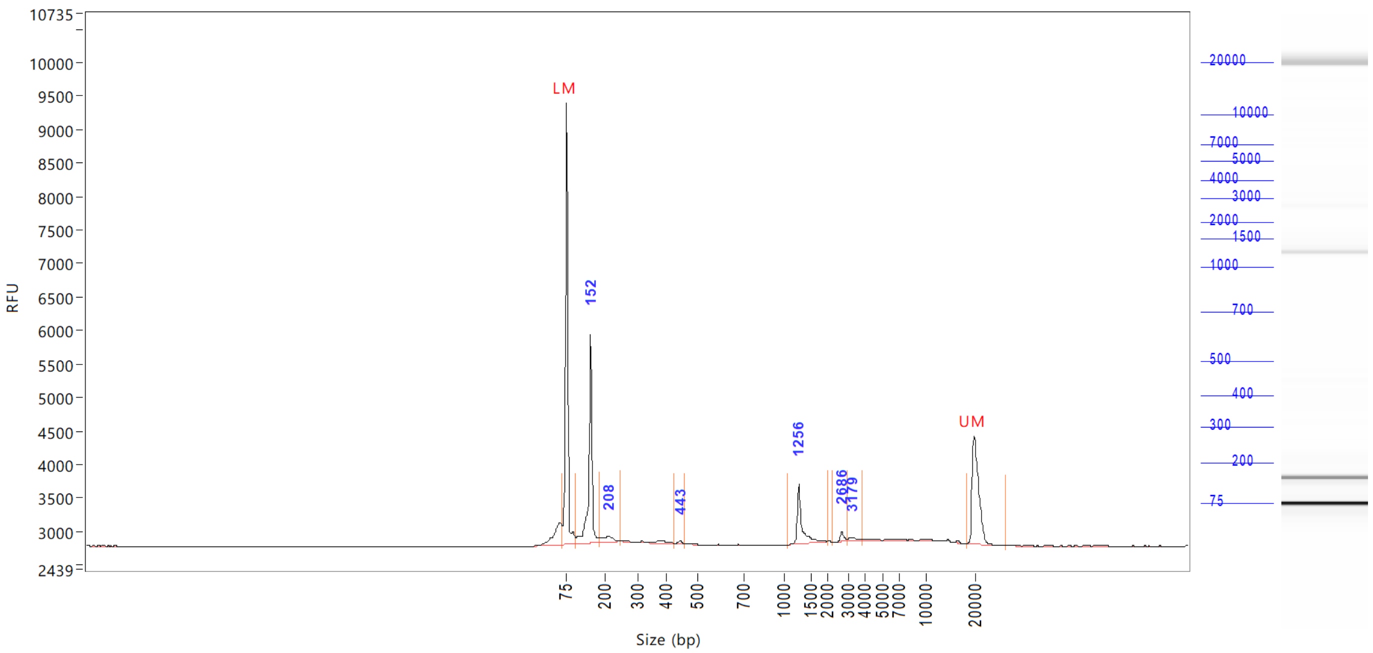

Peak	Size (bp)	Concentration (ng/uL)	From (bp)	To (bp)	Average size (bp)	CV%	RFU	Corrected peak area
1	75 (LM)	0.5000	62	103	73	7.70	6598	39.745
2	152	3.6292	103	183	146	8.36	3119	24.040
3	208	0.3120	183	244	204	6.39	79	2.067
4	443	0.0611	424	460	441	1.82	52	0.404
5	1256	1.0012	1045	2001	1314	9.33	903	6.632
6	2686	0.1285	2258	2943	2699	3.95	151	0.851
7	3179	0.1163	2943	3822	3311	7.81	51	0.771
8	20000 (UM)	0.1724	18293	26649	20331	3.63	1610	13.702

TIC:	5.2483	ng/uL
TIM:	44.9845	nmole/L
Total concentration:	5.7965	ng/uL

Sample peak width (sec): 5 Sample min peak height: 50 Sample baseline V to V?: Y Sample baseline V to V points: 3
Sample filter: Binomial Number of points for filter: 3 Sample start region (min): 0 Sample end region (min): 30
Marker peak width (sec): 5 Marker min peak height: 200 Marker baseline V to V?: Y Marker baseline V to V points: 3
Lower marker selection: First peak > 500 RFU Upper marker selection: Last peak > 200 RFU
Ladder size (bp)75, 200, 300, 400, 500, 700, 1000, 1500, 2000, 3000, 4000, 5000, 7000, 10000, 20000
Quantification using: Lower Marker Final concentration (ng/uL): 0.5000 Dilution factor: 12.0

Sample: SampA12
Well location: A12
Created: Tuesday, September 14, 2021 8:54:07 PM

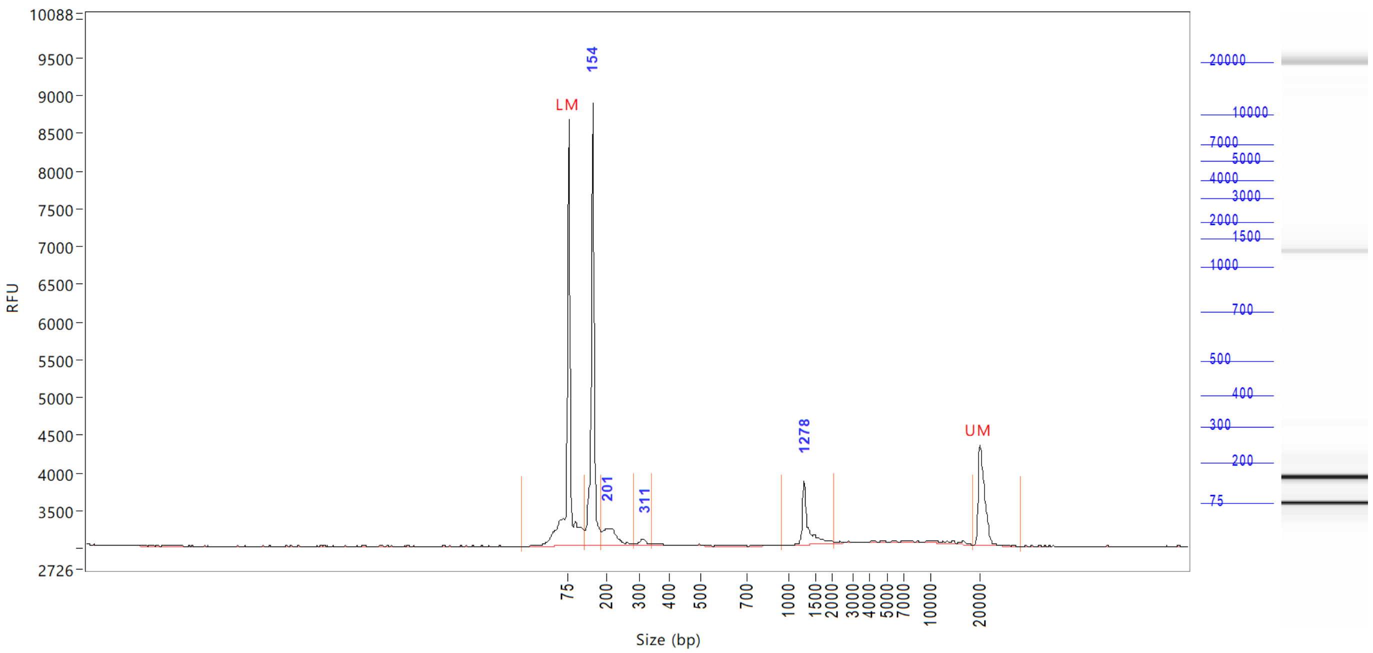

Peak	Size (bp)	Concentration (ng/uL)	From (bp)	To (bp)	Average size (bp)	CV%	RFU	Corrected peak area
1	75 (LM)	0.5000	0	126	70	32.63	5643	49.550
2	154	5.2234	126	181	150	5.58	5868	43.137
3	201	0.8195	181	280	208	9.22	220	6.768
4	311	0.1134	280	337	306	3.56	66	0.937
5	1278	0.9223	948	2115	1364	13.09	847	7.617
6	20000 (UM)	0.1136	18659	28463	20504	4.75	1318	11.261

TIC:	7.0787	ng/uL
TIM:	65.4330	nmole/L
Total concentration:	7.4926	ng/uL

Sample peak width (sec): 5   Sample min peak height: 50   Sample baseline V to V?: Y   Sample baseline V to V points: 3
Sample filter: Binomial   Number of points for filter: 3 Sample start region (min): 0 Sample end region (min): 30
Marker peak width (sec): 5   Marker min peak height: 200   Marker baseline V to V?: Y   Marker baseline V to V points: 3
Lower marker selection: First peak > 500 RFU   Upper marker selection: Last peak > 200 RFU
Ladder size (bp)75, 200, 300, 400, 500, 700, 1000, 1500, 2000, 3000, 4000, 5000, 7000, 10000, 20000
Quantification using: Lower Marker   Final concentration (ng/uL): 0.5000   Dilution factor: 12.0

Sample: SampB12
Well location: B12
Created: Tuesday, September 14, 2021 8:54:07 PM

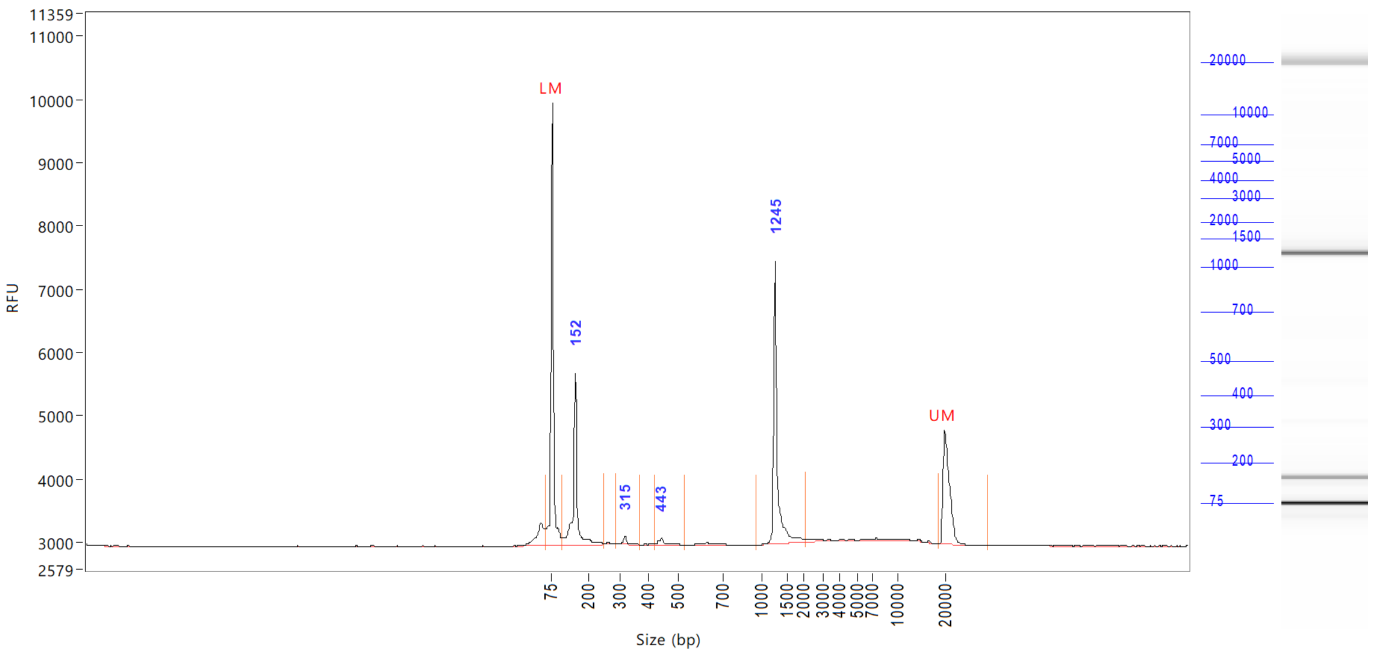

Peak	Size (bp)	Concentration (ng/uL)	From (bp)	To (bp)	Average size (bp)	CV%	RFU	Corrected peak area
1	75 (LM)	0.5000	52	110	74	10.57	7001	45.237
2	152	3.2192	110	248	153	13.20	2703	24.271
3	315	0.1209	284	372	314	4.23	125	0.912
4	443	0.1813	422	524	450	4.70	102	1.367
5	1245	3.6608	957	2087	1288	10.91	4450	27.601
6	20000 (UM)	0.1753	18537	29068	20442	4.38	1792	15.858

TIC:	7.1822	ng/uL
TIM:	40.5563	nmole/L
Total concentration:	7.7997	ng/uL

```
Sample peak width (sec): 5    Sample min peak height: 50    Sample baseline V to V?: Y    Sample baseline V to V points: 3
Sample filter: Binomial       Number of points for filter: 3  Sample start region (min): 0  Sample end region (min): 30
Marker peak width (sec): 5    Marker min peak height: 200   Marker baseline V to V?: Y    Marker baseline V to V points: 3
Lower marker selection: First peak > 500 RFU               Upper marker selection: Last peak > 200 RFU
Ladder size (bp)75, 200, 300, 400, 500, 700, 1000, 1500, 2000, 3000, 4000, 5000, 7000, 10000, 20000
Quantification using: Lower Marker          Final concentration (ng/uL): 0.5000          Dilution factor: 12.0
```

Sample: SampC12
Well location: C12
Created: Tuesday, September 14, 2021 8:54:07 PM

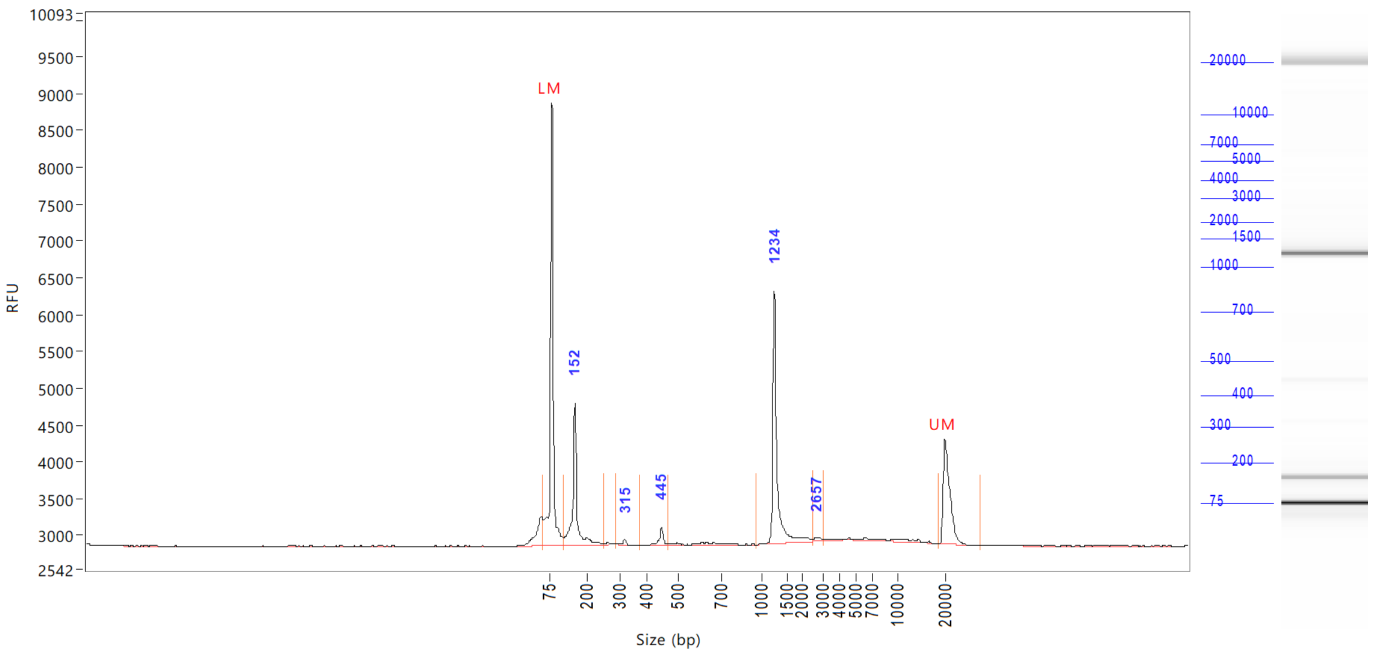

Peak	Size (bp)	Concentration (ng/uL)	From (bp)	To (bp)	Average size (bp)	CV%	RFU	Corrected peak area
1	75 (LM)	0.5000	48	116	74	13.10	6023	45.040
2	152	2.5615	116	251	155	13.19	1927	19.229
3	315	0.0785	286	374	315	4.36	75	0.590
4	445	0.2726	374	468	438	3.14	247	2.046
5	1234	3.2372	957	2458	1303	14.99	3421	24.300
6	2657	0.0802	2458	3001	2715	5.86	53	0.602
7	20000 (UM)	0.1425	18537	27617	20532	4.28	1426	12.840

TIC:	6.2300	ng/uL
TIM:	32.7345	nmole/L
Total concentration:	6.8481	ng/uL

Sample peak width (sec): 5 Sample min peak height: 50 Sample baseline V to V?: Y Sample baseline V to V points: 3
Sample filter: Binomial Number of points for filter: 3 Sample start region (min): 0 Sample end region (min): 30
Marker peak width (sec): 5 Marker min peak height: 200 Marker baseline V to V?: Y Marker baseline V to V points: 3
Lower marker selection: First peak > 500 RFU Upper marker selection: Last peak > 200 RFU
Ladder size (bp)75, 200, 300, 400, 500, 700, 1000, 1500, 2000, 3000, 4000, 5000, 7000, 10000, 20000
Quantification using: Lower Marker Final concentration (ng/uL): 0.5000 Dilution factor: 12.0

Sample: SampD12
Well location: D12
Created: Tuesday, September 14, 2021 8:54:07 PM

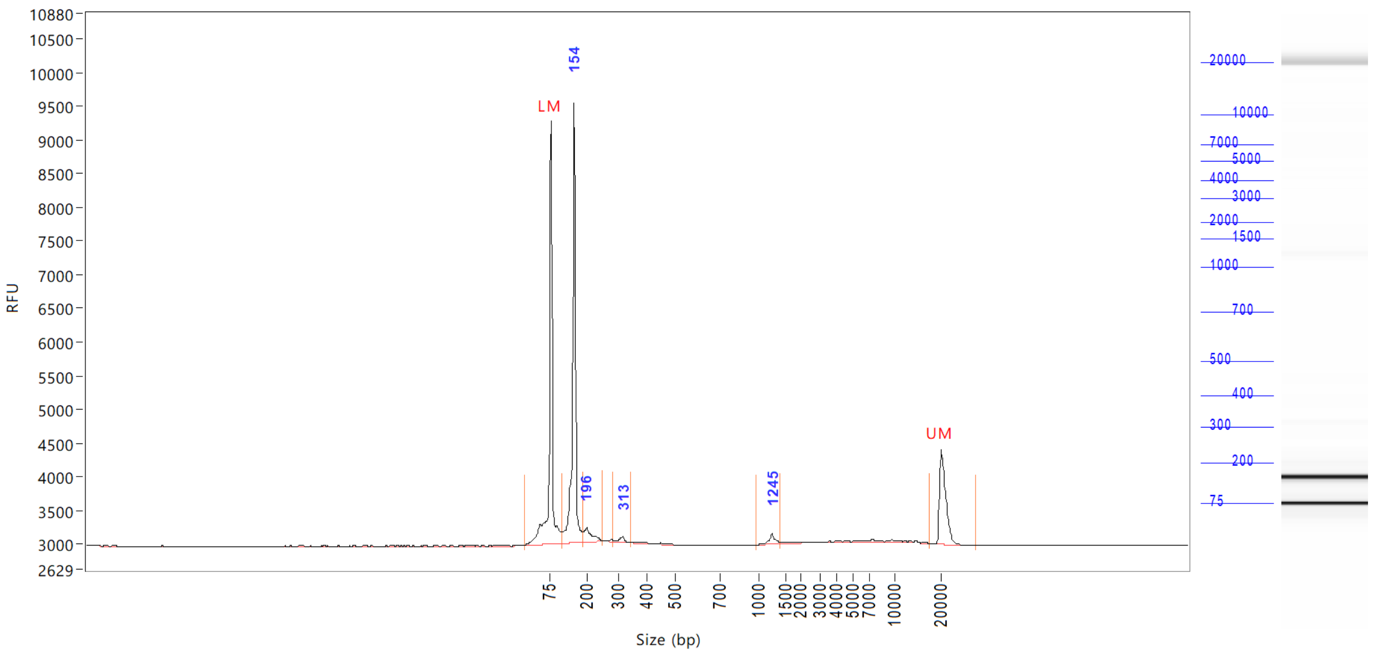

Peak	Size (bp)	Concentration (ng/uL)	From (bp)	To (bp)	Average size (bp)	CV%	RFU	Corrected peak area
1	75 (LM)	0.5000	0	112	69	25.26	6277	48.575
2	154	6.0003	112	185	150	6.79	6539	48.578
3	196	0.4788	185	248	204	7.03	212	3.877
4	313	0.1295	284	343	306	3.37	97	1.048
5	1245	0.2012	974	1400	1232	6.13	170	1.629
6	20000 (UM)	0.1254	17439	27375	20446	4.03	1401	12.185

TIC:	6.8098	ng/uL
TIM:	70.5679	nmole/L
Total concentration:	7.2867	ng/uL

Sample peak width (sec): 5 Sample min peak height: 50 Sample baseline V to V?: Y Sample baseline V to V points: 3
Sample filter: Binomial Number of points for filter: 3 Sample start region (min): 0 Sample end region (min): 30
Marker peak width (sec): 5 Marker min peak height: 200 Marker baseline V to V?: Y Marker baseline V to V points: 3
Lower marker selection: First peak > 500 RFU Upper marker selection: Last peak > 200 RFU
Ladder size (bp)75, 200, 300, 400, 500, 700, 1000, 1500, 2000, 3000, 4000, 5000, 7000, 10000, 20000
Quantification using: Lower Marker Final concentration (ng/uL): 0.5000 Dilution factor: 12.0

Sample: SampE12
Well location: E12
Created: Tuesday, September 14, 2021 8:54:07 PM

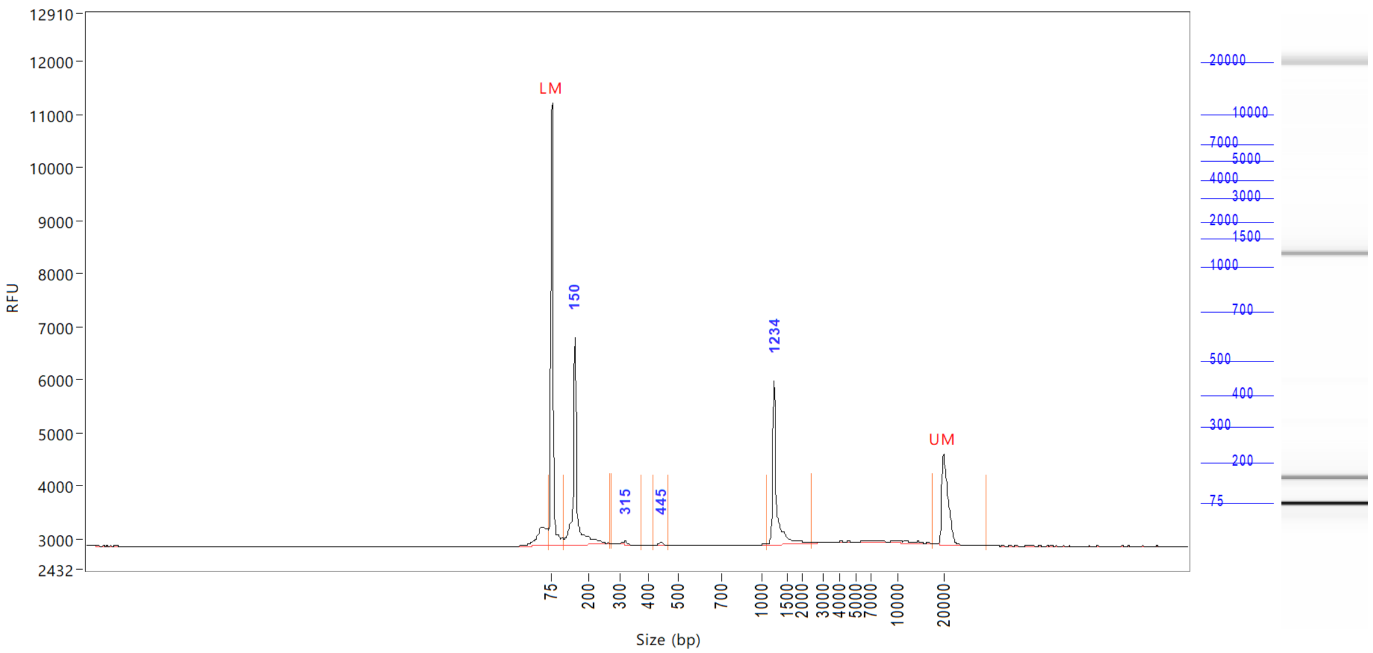

Peak	Size (bp)	Concentration (ng/uL)	From (bp)	To (bp)	Average size (bp)	CV%	RFU	Corrected peak area
1	75 (LM)	0.5000	62	114	74	9.93	8355	51.846
2	150	4.0347	114	268	156	14.23	3894	34.863
3	315	0.0743	273	378	312	4.44	66	0.642
4	445	0.0674	418	464	440	1.82	73	0.583
5	1234	2.3895	1090	2458	1301	14.08	3086	20.647
6	20000 (UM)	0.1431	17439	29068	20396	4.11	1706	14.834

TIC:	6.5658	ng/uL
TIM:	46.1761	nmole/L
Total concentration:	7.0268	ng/uL

Sample peak width (sec): 5   Sample min peak height: 50   Sample baseline V to V?: Y   Sample baseline V to V points: 3
Sample filter: Binomial   Number of points for filter: 3 Sample start region (min): 0 Sample end region (min): 30
Marker peak width (sec): 5   Marker min peak height: 200   Marker baseline V to V?: Y   Marker baseline V to V points: 3
Lower marker selection: First peak > 500 RFU   Upper marker selection: Last peak > 200 RFU
Ladder size (bp)75, 200, 300, 400, 500, 700, 1000, 1500, 2000, 3000, 4000, 5000, 7000, 10000, 20000
Quantification using: Lower Marker   Final concentration (ng/uL): 0.5000   Dilution factor: 12.0

Sample: SampF12
Well location: F12
Created: Tuesday, September 14, 2021 8:54:07 PM

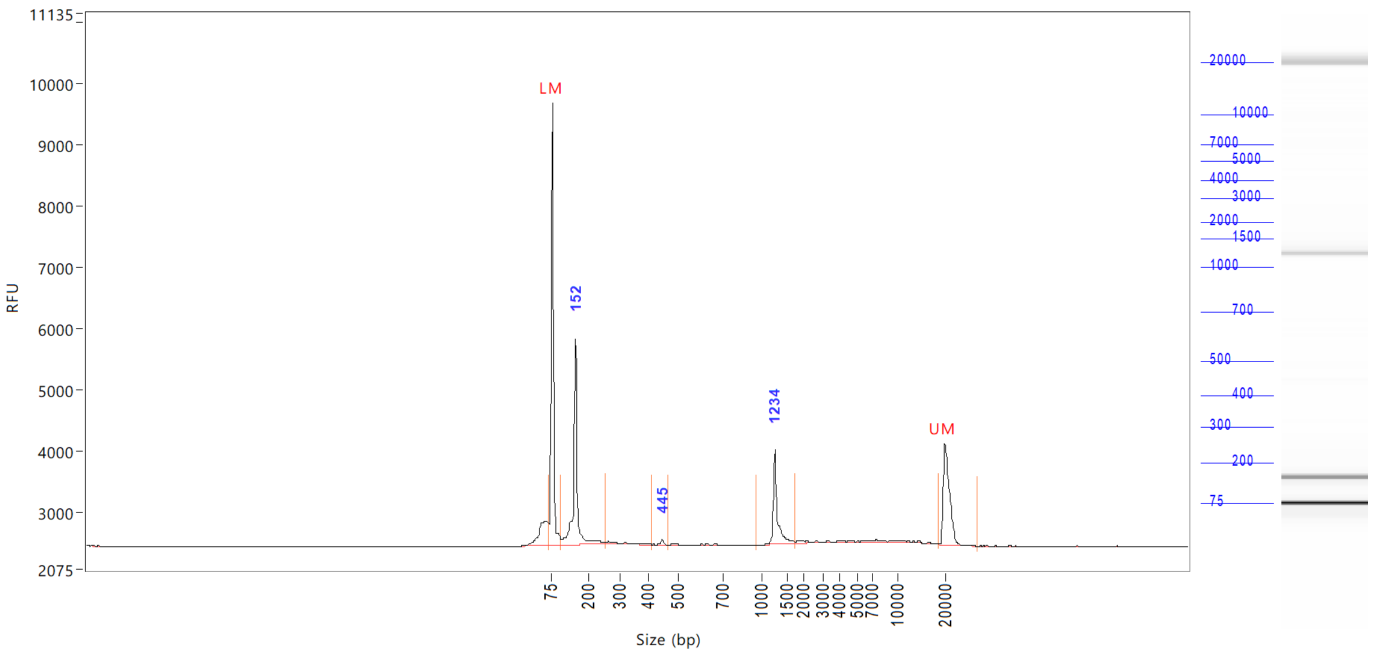

Peak	Size (bp)	Concentration (ng/uL)	From (bp)	To (bp)	Average size (bp)	CV%	RFU	Corrected peak area
1	75 (LM)	0.5000	66	105	75	7.33	7220	43.543
2	152	3.9078	105	251	153	13.50	3348	28.360
3	445	0.0784	409	464	441	1.99	88	0.569
4	1234	1.4371	961	1712	1273	7.67	1532	10.430
5	20000 (UM)	0.1609	18537	27012	20425	3.75	1651	14.012

TIC:	5.4233	ng/uL
TIM:	44.1293	nmole/L
Total concentration:	6.1031	ng/uL

Sample peak width (sec): 5 Sample min peak height: 50 Sample baseline V to V?: Y Sample baseline V to V points: 3
Sample filter: Binomial Number of points for filter: 3 Sample start region (min): 0 Sample end region (min): 30
Marker peak width (sec): 5 Marker min peak height: 200 Marker baseline V to V?: Y Marker baseline V to V points: 3
Lower marker selection: First peak > 500 RFU Upper marker selection: Last peak > 200 RFU
Ladder size (bp)75, 200, 300, 400, 500, 700, 1000, 1500, 2000, 3000, 4000, 5000, 7000, 10000, 20000
Quantification using: Lower Marker Final concentration (ng/uL): 0.5000 Dilution factor: 12.0

Sample: SampG12
Well location: G12
Created: Tuesday, September 14, 2021 8:54:07 PM

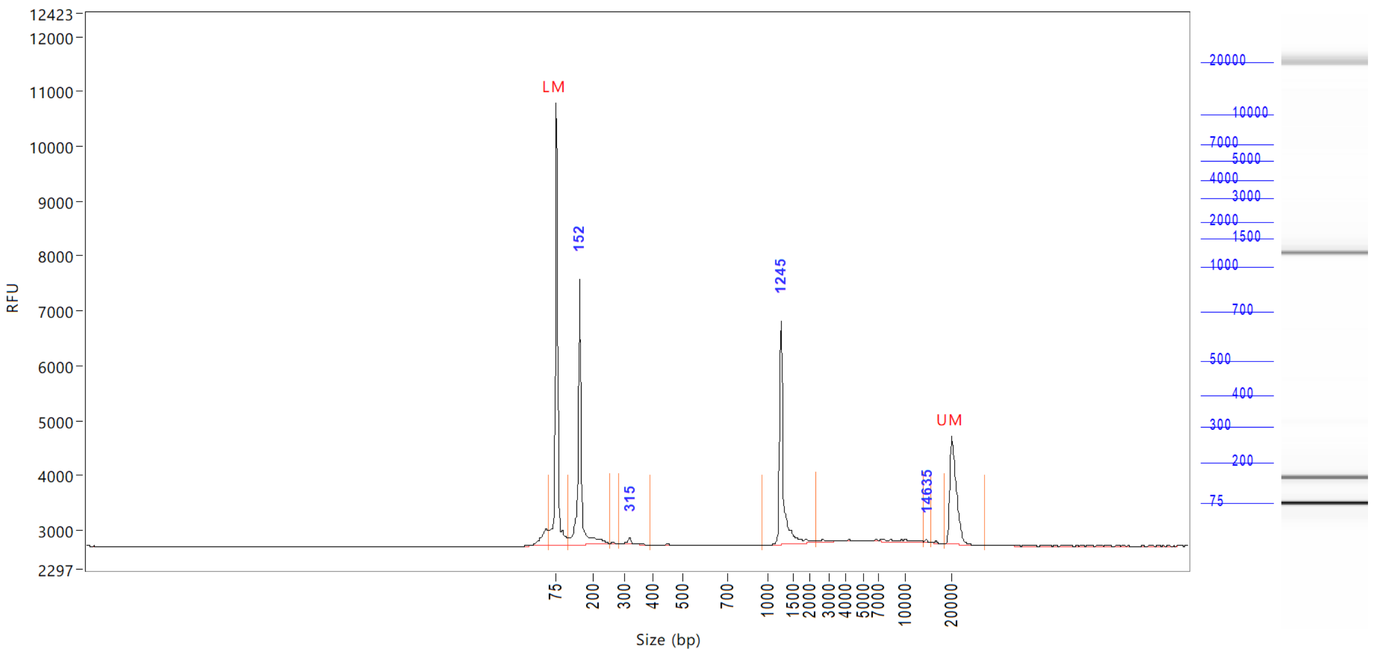

Peak	Size (bp)	Concentration (ng/uL)	From (bp)	To (bp)	Average size (bp)	CV%	RFU	Corrected peak area
1	75 (LM)	0.5000	50	114	74	11.58	8070	51.515
2	152	4.4189	114	250	154	12.67	4821	37.940
3	315	0.1278	280	388	314	5.11	123	1.097
4	1245	2.9315	957	2344	1306	12.76	4065	25.170
5	14635	0.0272	13903	15610	14400	1.94	54	0.233
6	20000 (UM)	0.1657	18415	27012	20428	3.85	1963	17.074

TIC:	7.5055	ng/uL
TIM:	51.5293	nmole/L
Total concentration:	8.0339	ng/uL

```
Sample peak width (sec): 5    Sample min peak height: 50    Sample baseline V to V?: Y    Sample baseline V to V points: 3
Sample filter: Binomial       Number of points for filter: 3  Sample start region (min): 0  Sample end region (min): 30
Marker peak width (sec): 5    Marker min peak height: 200   Marker baseline V to V?: Y    Marker baseline V to V points: 3
Lower marker selection: First peak > 500 RFU                Upper marker selection: Last peak > 200 RFU
Ladder size (bp)75, 200, 300, 400, 500, 700, 1000, 1500, 2000, 3000, 4000, 5000, 7000, 10000, 20000
Quantification using: Lower Marker          Final concentration (ng/uL): 0.5000         Dilution factor: 12.0
```

Sample: SampH12
Well location: H12
Created: Tuesday, September 14, 2021 8:54:07 PM

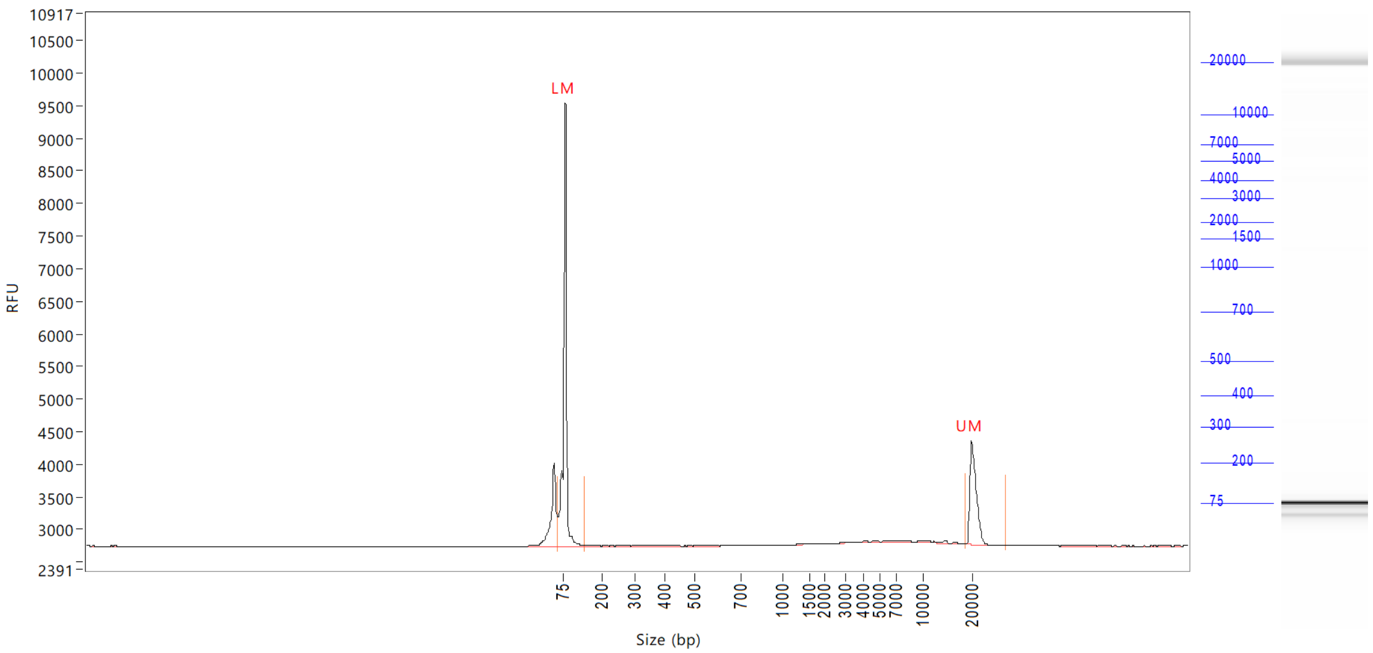

Peak	Size (bp)	Concentration (ng/uL)	From (bp)	To (bp)	Average size (bp)	CV%	RFU	Corrected peak area
1	75 (LM)	0.5000	52	141	73	12.82	6813	49.630
2	20000 (UM)	0.1322	18659	27012	20359	3.58	1588	13.117

TIC:	0.0000	ng/uL
TIM:	0.0000	nmole/L
Total concentration:	0.5042	ng/uL

Sample peak width (sec): 5 Sample min peak height: 200 Sample baseline V to V?: Y Sample baseline V to V points: 3
Sample filter: Binomial Number of points for filter: 3 Sample start region (min): 0 Sample end region (min): 30
Marker peak width (sec): 5 Marker min peak height: 200 Marker baseline V to V?: Y Marker baseline V to V points: 3
Lower marker selection: First peak > 500 RFU Upper marker selection: Last peak > 200 RFU
Ladder size (bp)75, 200, 300, 400, 500, 700, 1000, 1500, 2000, 3000, 4000, 5000, 7000, 10000, 20000
Quantification using: Lower Marker Final concentration (ng/uL): 0.5000 Dilution factor: 12.0

Sample: DNA size ladder
Well location: A7
Created: Tuesday, September 14, 2021 8:54:07 PM
Import from: C:\Users\admin\Desktop\210810_alberto_ladder.SCAL
Fit type: Point to point

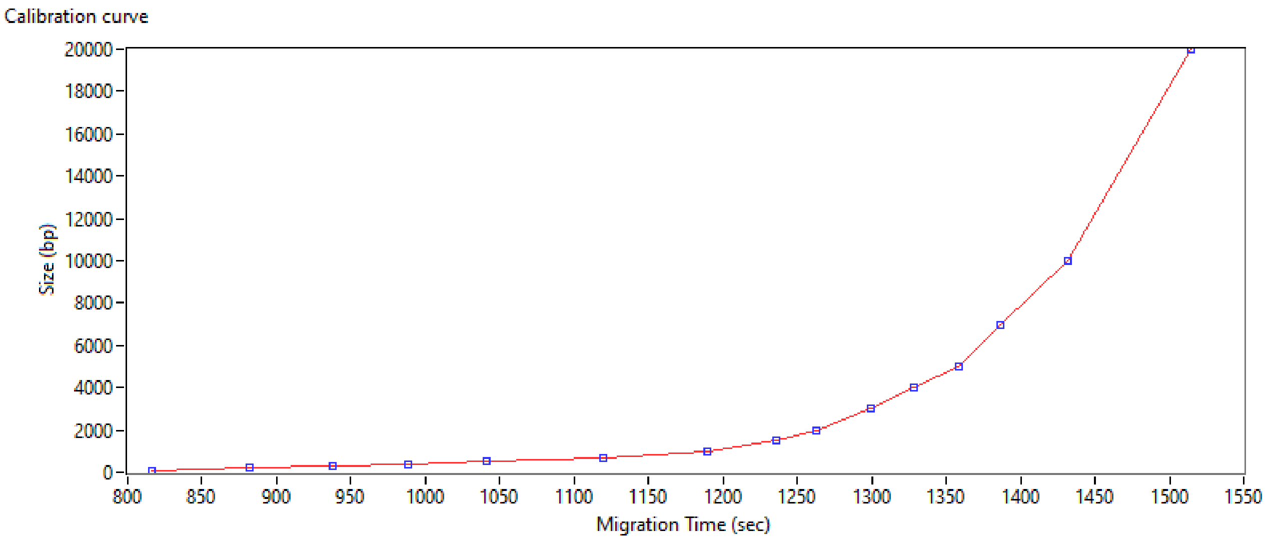

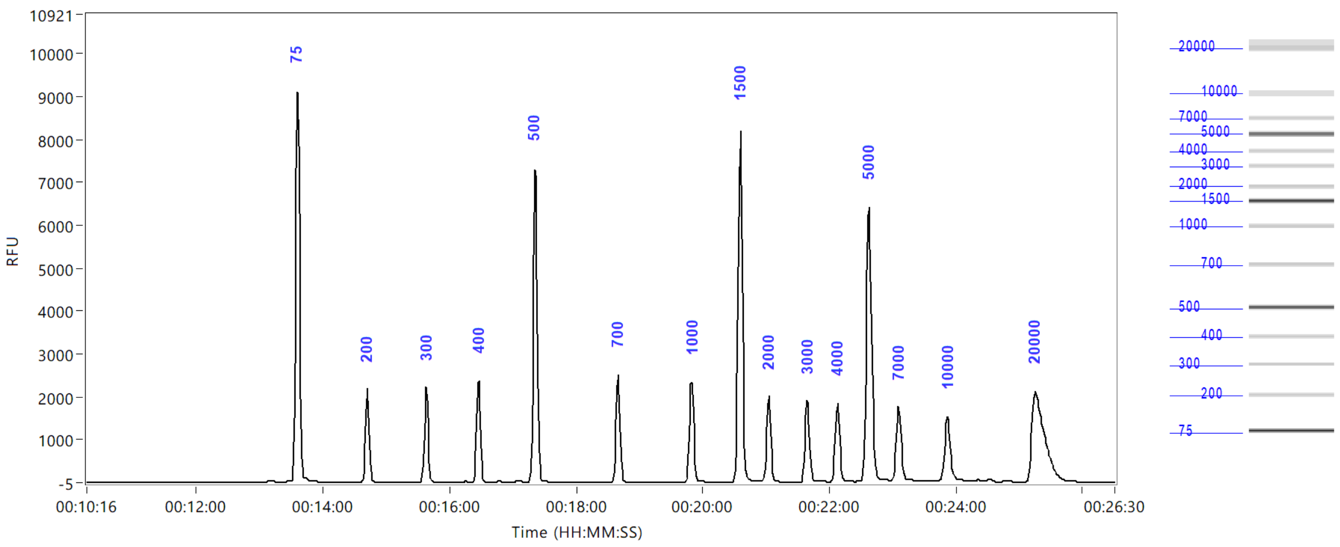

Supplement: Supplementary file 2 — sb3c00292_si_002.zip [file sb3c00292_si_002.zip › dnada_supplementary_material_pks_library_build/ZAG_trials/ZAG_Trial_5/20-44-15_plate7/plate7-t1.pdf]
