# Supplementary material for: Automated Platform for the Plasmid Construction Process
Source: ACS Synth Biol. 2023 Nov 10;12(12):3506–13. doi: 10.1021/acssynbio.3c00292 (PMC10729297; doi:10.1021/acssynbio.3c00292)

# Instrument controller software run summary:

**Filename and data path:** C:\AATI\Data\2021 09 14\18-44-09\2021 09 14 18H 44M..raw
**Created:** Tuesday, September 14, 2021 6:54:01 PM
**Number of capillaries:** 96
**Array serial number:** 111920-09SFS
**Effect length:** 33 cm
**Array usage count:** 216
**Instrument type:** ZAG DNA Analyzer
**Instrument controller software version:** 1.0.1.9
**Device serial number:** Z7597

## Method Information

**Method name:** ZAG130GP33 - DNA 75-20000bp - Gel Prime Only.mthds
**Gel prime:** Yes
**Full conditioning:** No
**Gel prime to buffer:** No
**Gel selection:** Gel 1
**Perform prerun:** 8.0 kV, 30 sec.
**Rinse:** No
**Marker 1:** Row: A, 5.0 kV, 5 sec.
**Rinse:** No
**Sample injection:** 5.0 kV, 5 sec.
**Separation:** 8.0 kV, 30.0 min.
**Tray name:** Sample Tray-4

**Analysis mode:** DNA

**Notes**

# Gel Image

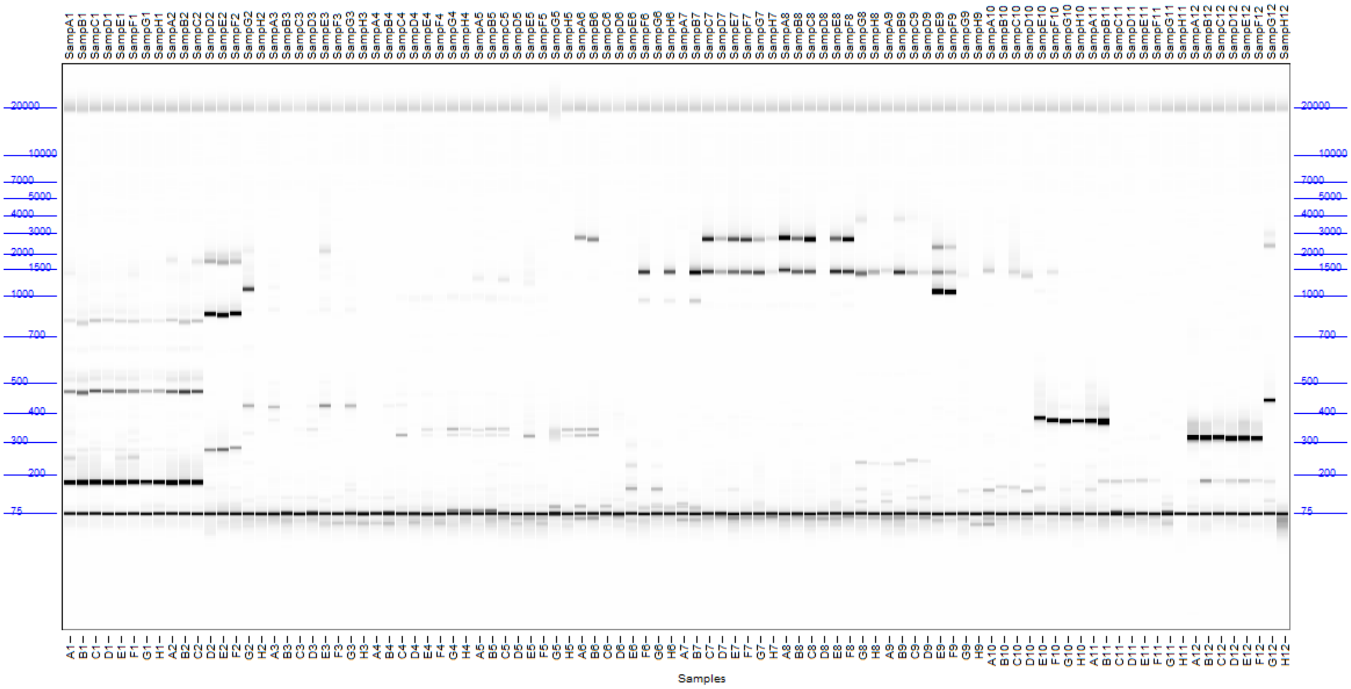
20000
10000
7000
5000
4000
3000
2000
1500
1000
700
500
400
300
200
75
Samples

**Filename and data path:** C:\AATI\Data\2021 09 14\18-44-09\2021 09 14 18H 44M..raw

A1: SampA1

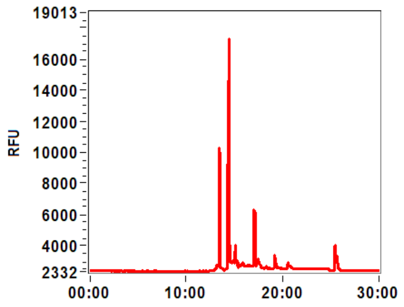


B1: SampB1

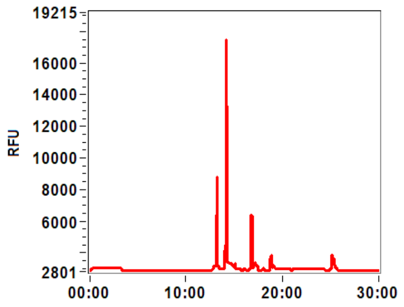


C1: SampC1

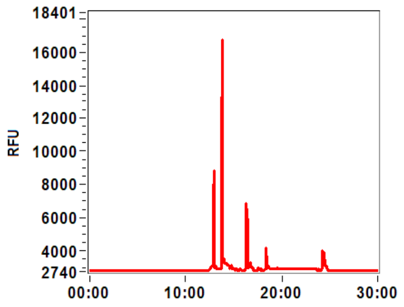


D1: SampD1

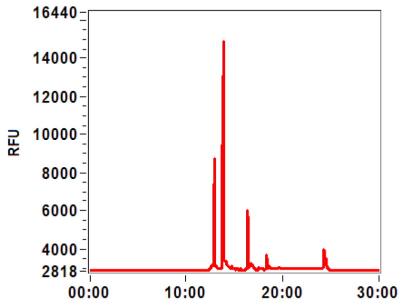


E1: SampE1

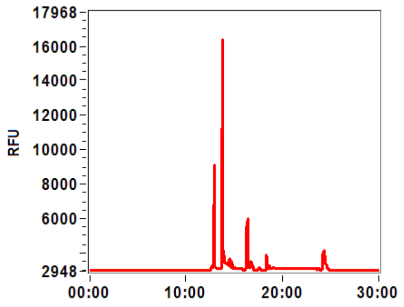


F1: SampF1

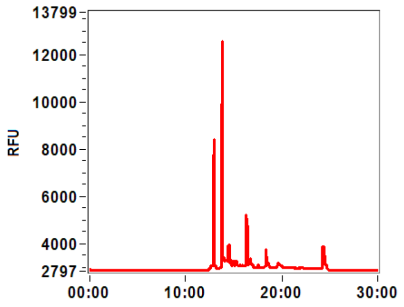


G1: SampG1

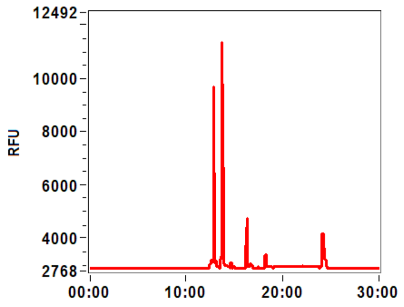


H1: SampH1

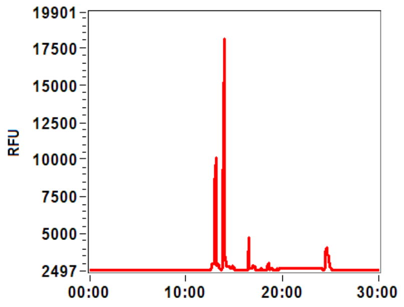


A2: SampA2

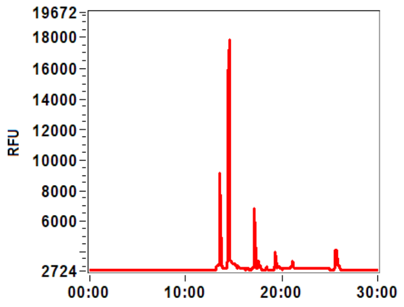


B2: SampB2

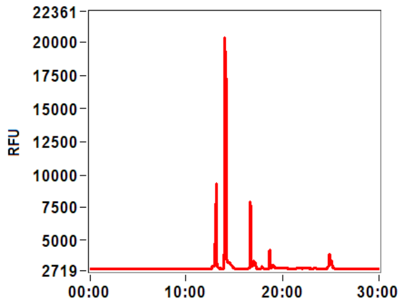


C2: SampC2

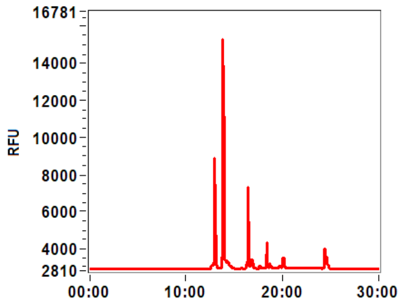


D2: SampD2

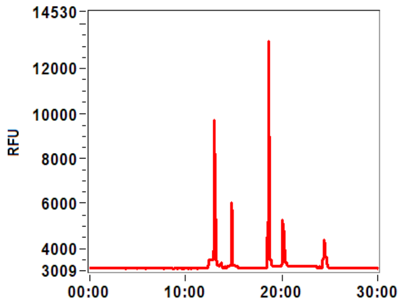

**Filename and data path:** C:\AATI\Data\2021 09 14\18-44-09\2021 09 14 18H 44M..raw

E2: SampE2

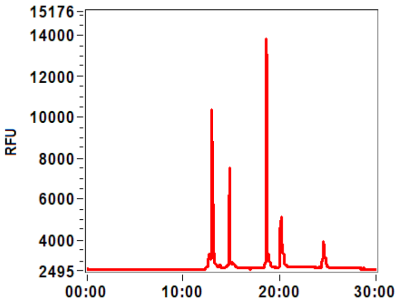


F2: SampF2

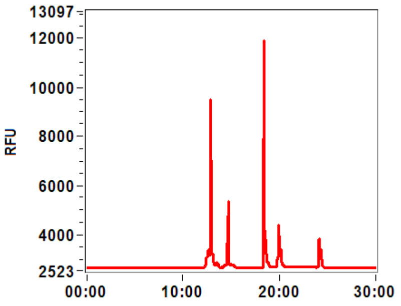


G2: SampG2

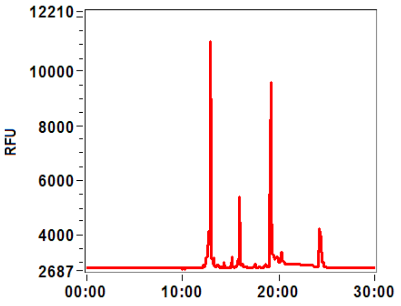


H2: SampH2

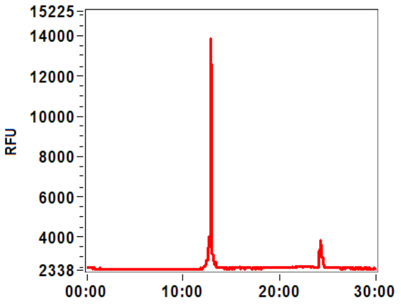


A3: SampA3

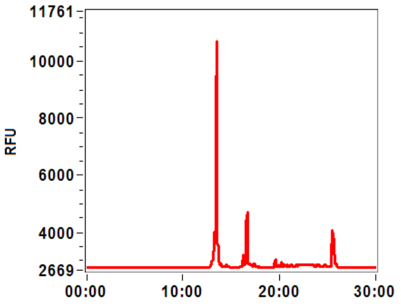


B3: SampB3

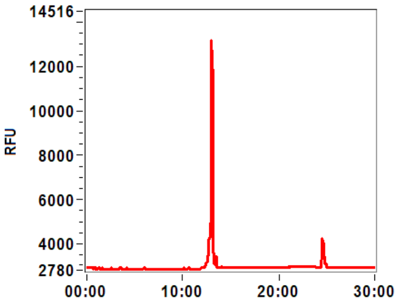


C3: SampC3

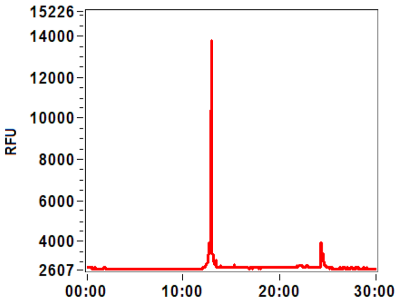


D3: SampD3

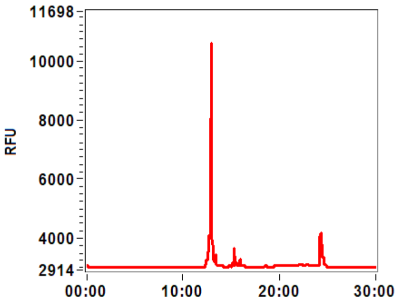


E3: SampE3

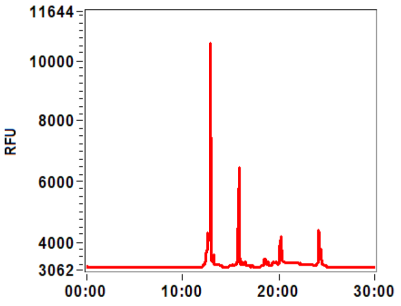


F3: SampF3

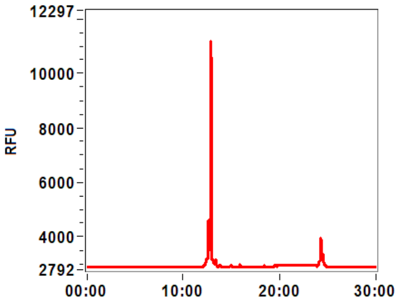


G3: SampG3

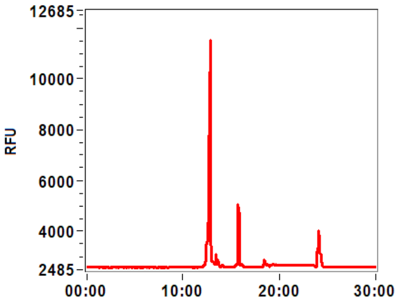


H3: SampH3

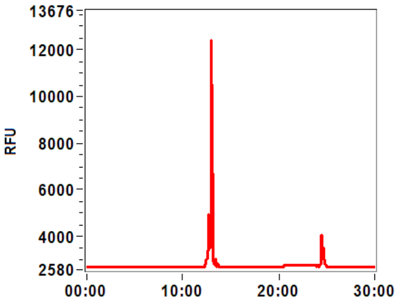

**Filename and data path:** C:\AATI\Data\2021 09 14\18-44-09\2021 09 14 18H 44M..raw

**A4: SampA4**

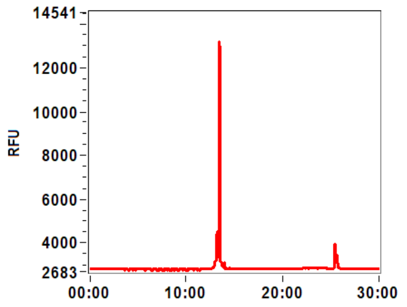


**B4: SampB4**

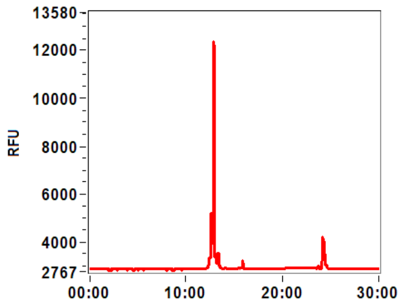


**C4: SampC4**

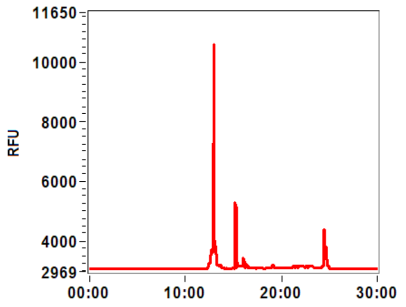


**D4: SampD4**

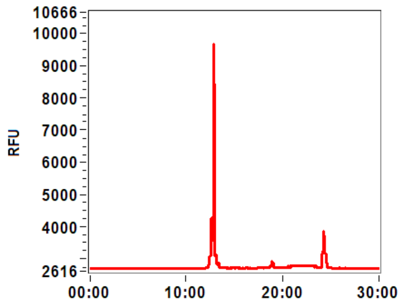


**E4: SampE4**

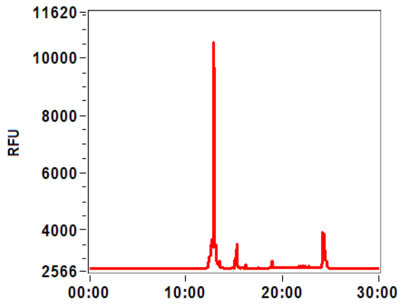


**F4: SampF4**

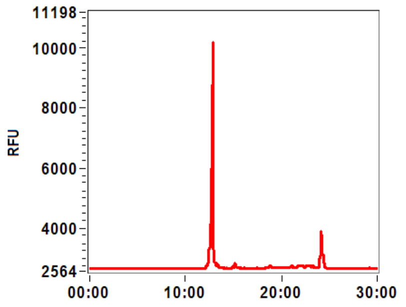


**G4: SampG4**

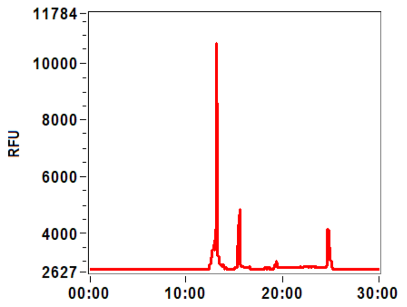


**H4: SampH4**

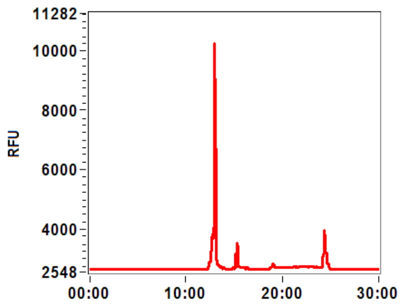


**A5: SampA5**

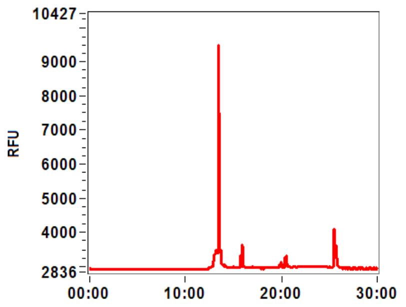


**B5: SampB5**

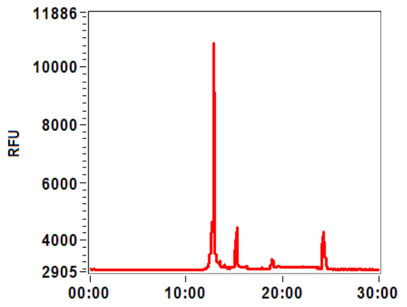


**C5: SampC5**

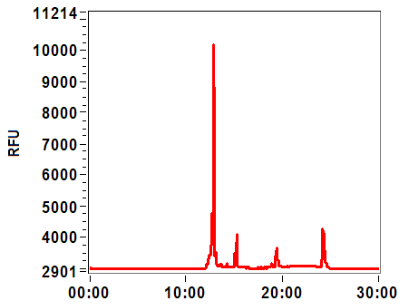


**D5: SampD5**

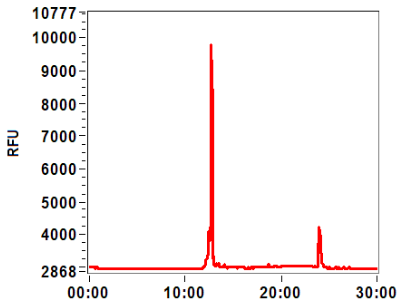

**Filename and data path:** C:\AATI\Data\2021 09 14\18-44-09\2021 09 14 18H 44M..raw

E5: SampE5

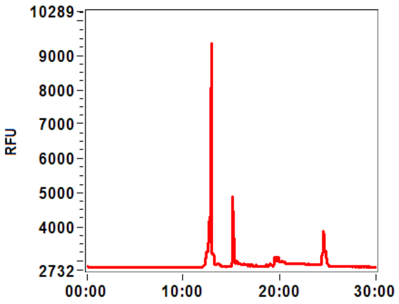


F5: SampF5

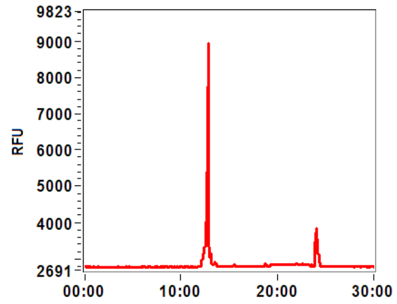


G5: SampG5

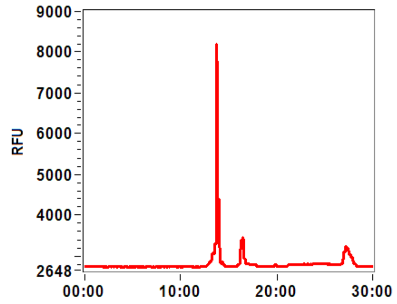


H5: SampH5

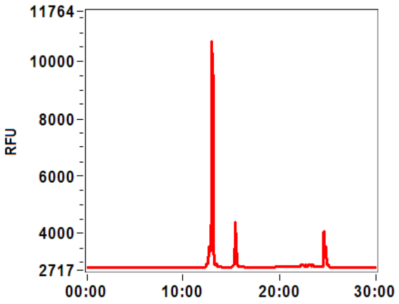


A6: SampA6

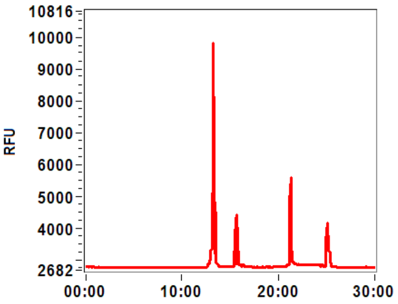


B6: SampB6

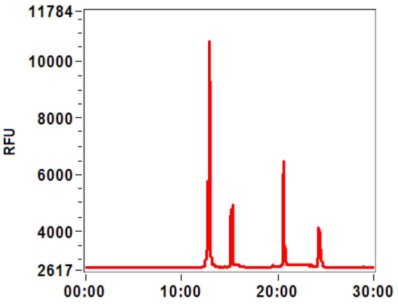


C6: SampC6

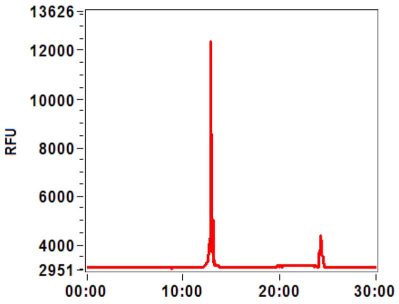


D6: SampD6

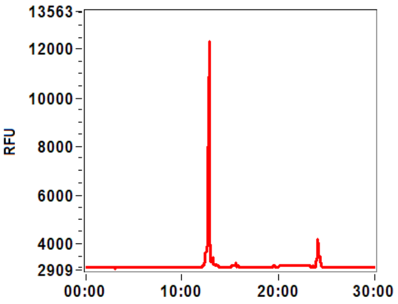


E6: SampE6

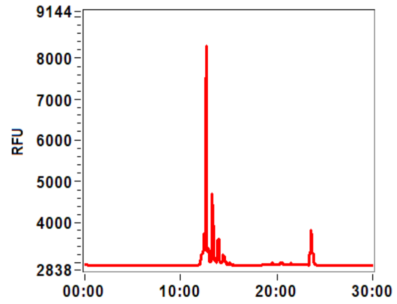


F6: SampF6

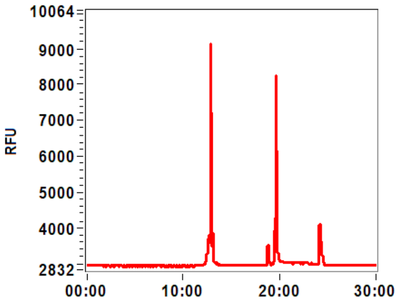


G6: SampG6

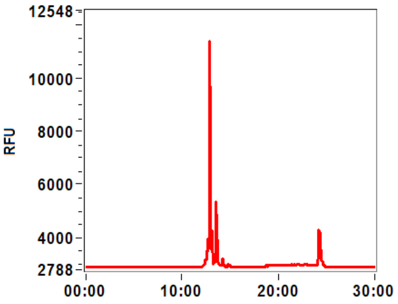


H6: SampH6

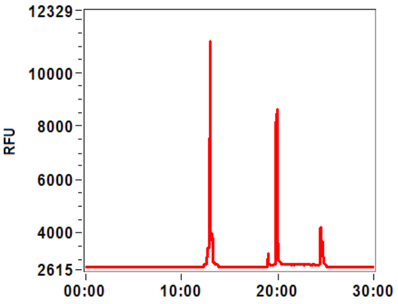

**Filename and data path:** C:\AATI\Data\2021 09 14\18-44-09\2021 09 14 18H 44M..raw

A7: SampA7

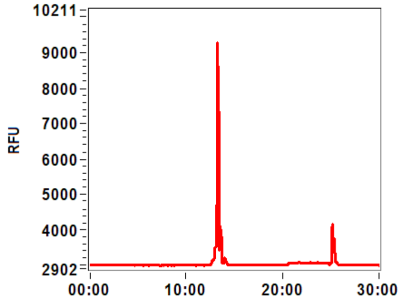


B7: SampB7

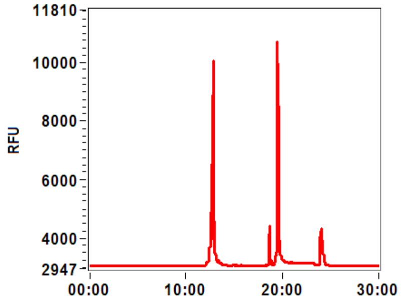


C7: SampC7

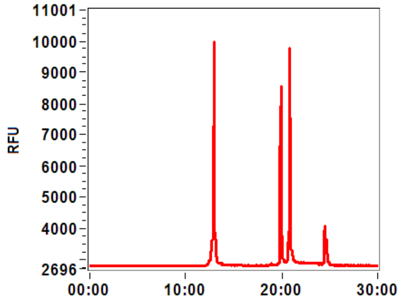


D7: SampD7

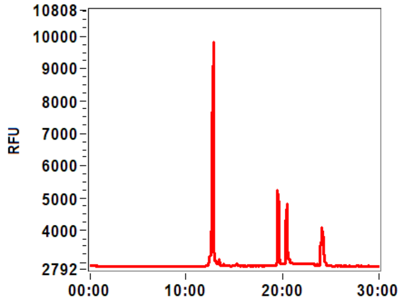


E7: SampE7

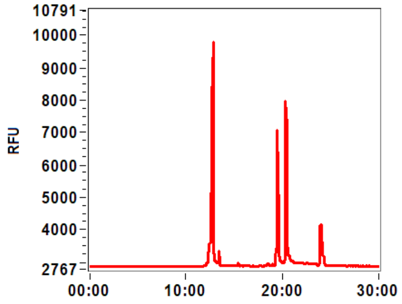


F7: SampF7

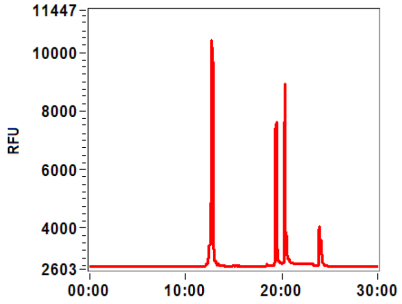


G7: SampG7

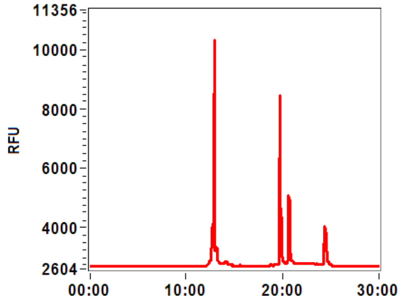


H7: SampH7

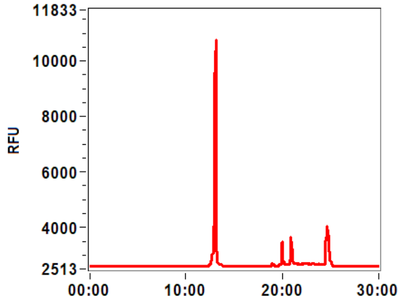


A8: SampA8

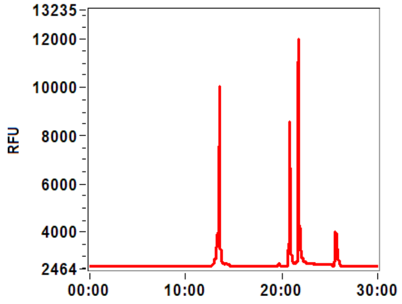


B8: SampB8

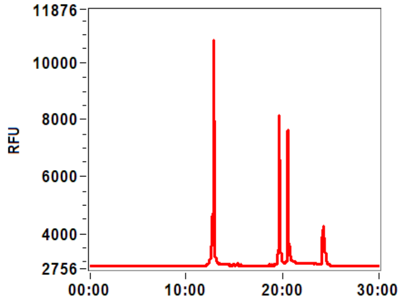


C8: SampC8

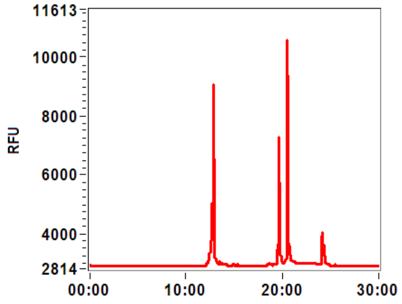


D8: SampD8

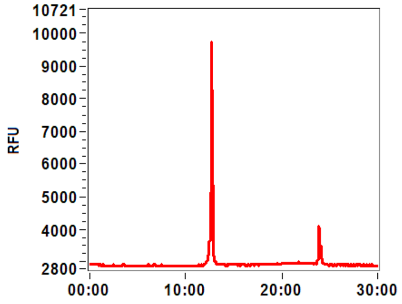

**Filename and data path:** C:\AATI\Data\2021 09 14\18-44-09\2021 09 14 18H 44M..raw

E8: SampE8

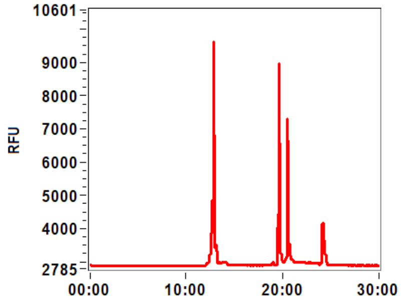


F8: SampF8

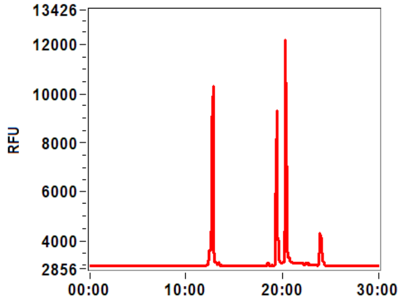


G8: SampG8

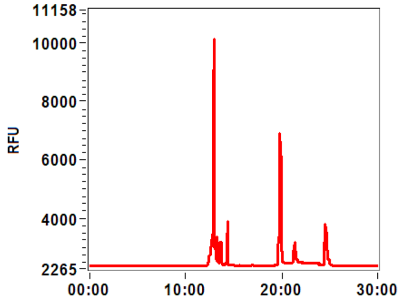


H8: SampH8

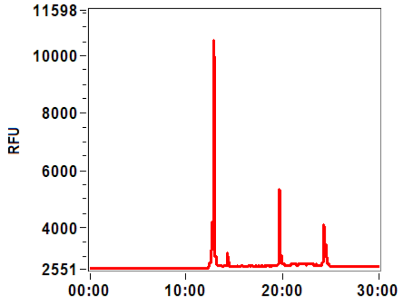


A9: SampA9

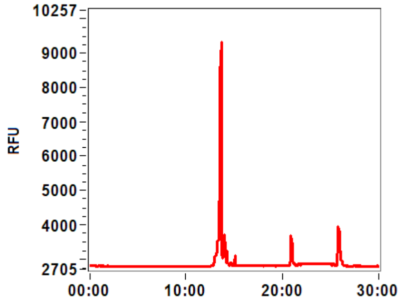


B9: SampB9

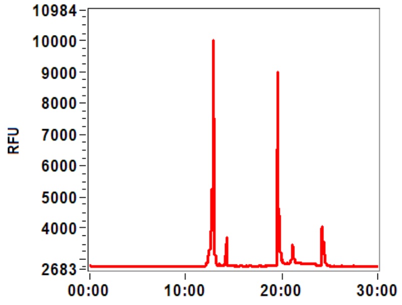


C9: SampC9

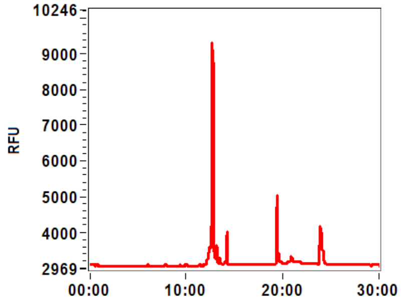


D9: SampD9

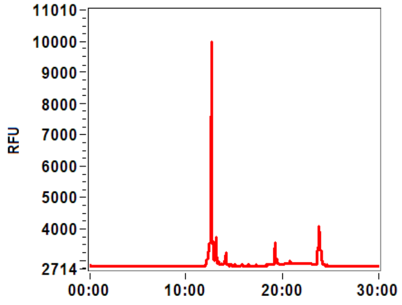


E9: SampE9

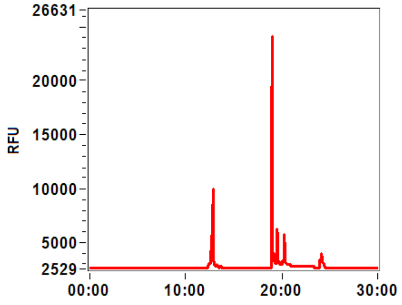


F9: SampF9

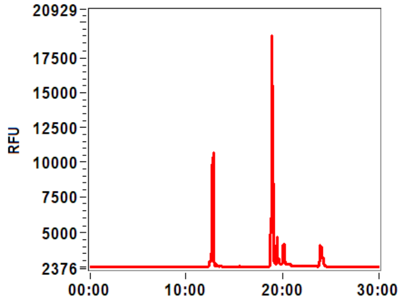


G9: SampG9

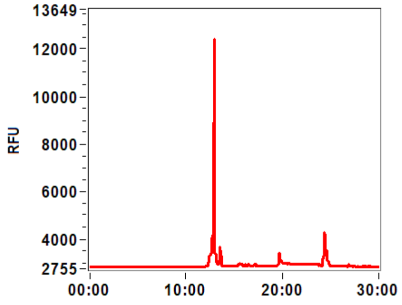


H9: SampH9

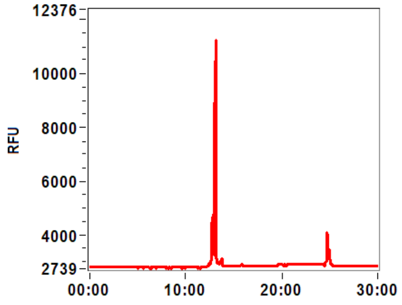

**Filename and data path:** C:\AATI\Data\2021 09 14\18-44-09\2021 09 14 18H 44M..raw

A10: SampA10

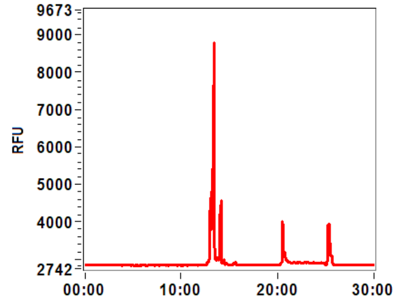


B10: SampB10

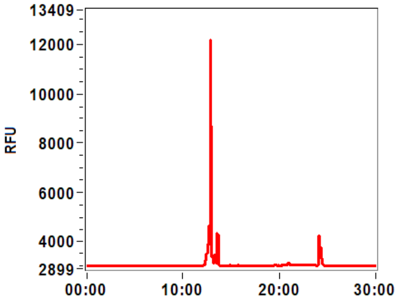


C10: SampC10

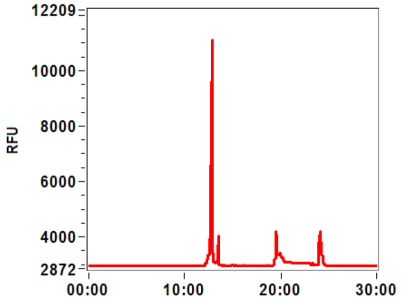


D10: SampD10

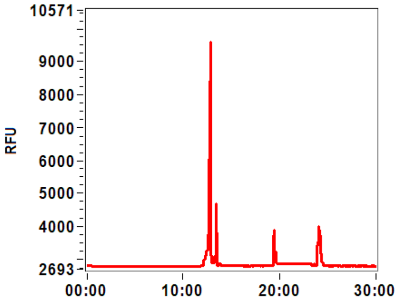


E10: SampE10

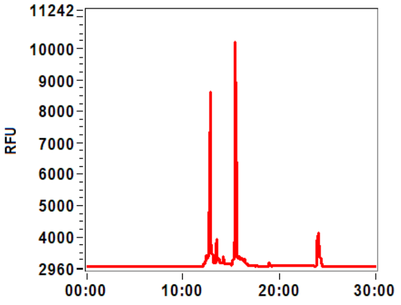


F10: SampF10

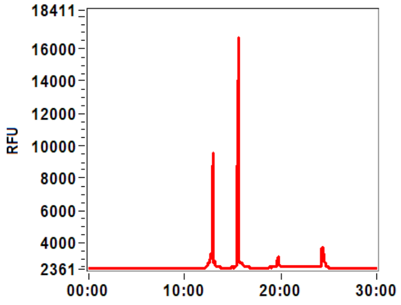


G10: SampG10

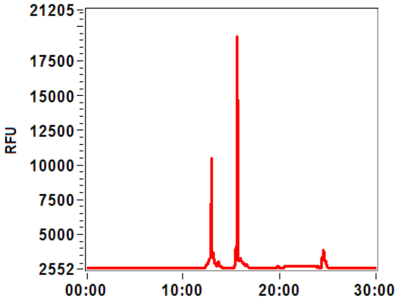


H10: SampH10

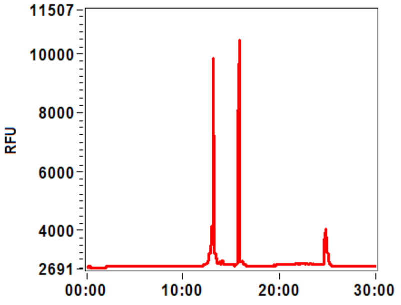


A11: SampA11

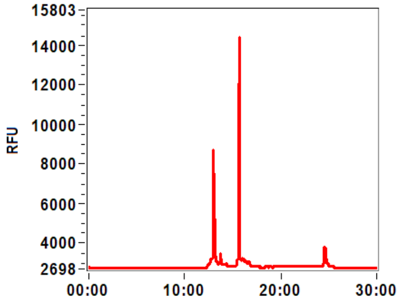


B11: SampB11

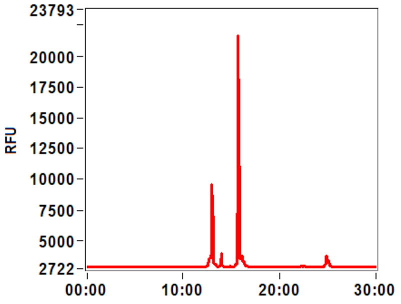


C11: SampC11

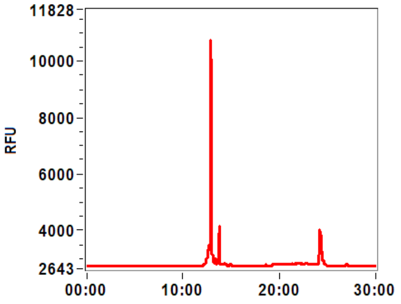


D11: SampD11

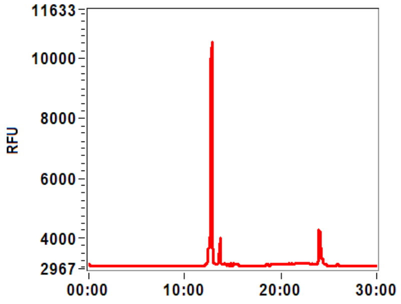

**Filename and data path:** C:\AATI\Data\2021 09 14\18-44-09\2021 09 14 18H 44M..raw

**E11: SampE11**

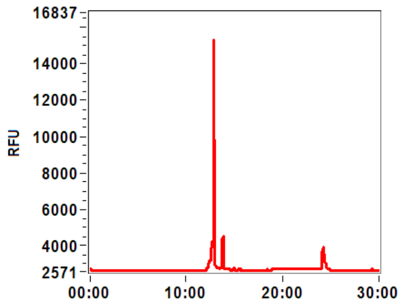


**F11: SampF11**

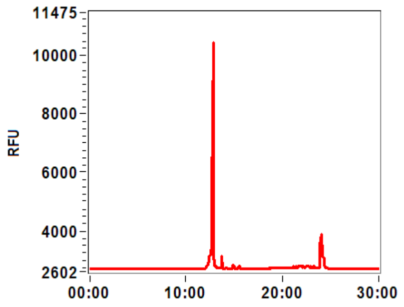


**G11: SampG11**

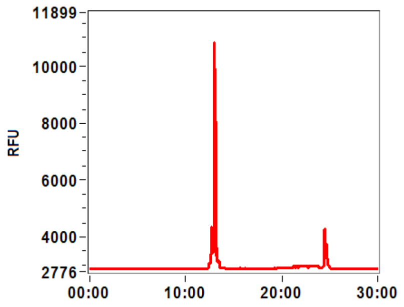


**H11: SampH11**

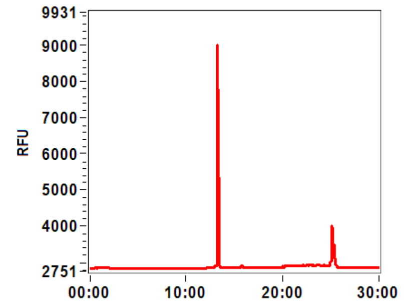


**A12: SampA12**

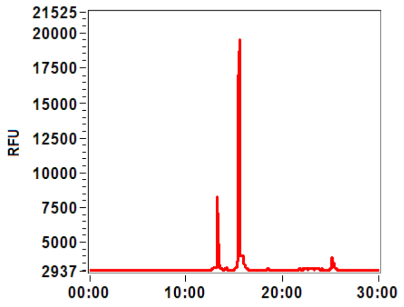


**B12: SampB12**

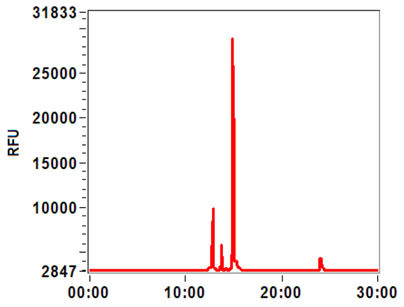


**C12: SampC12**

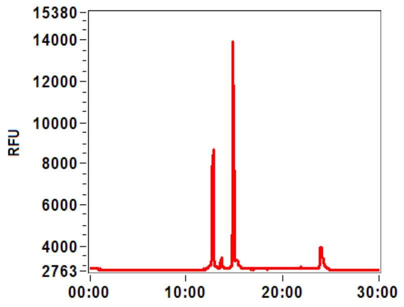


**D12: SampD12**

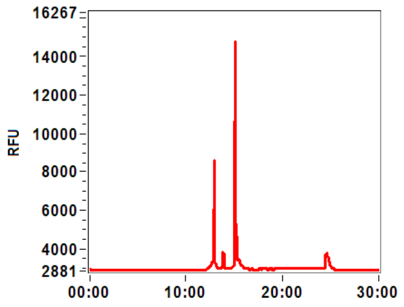


**E12: SampE12**

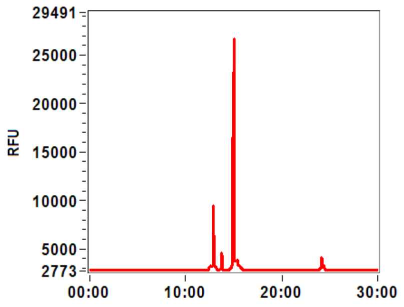


**F12: SampF12**

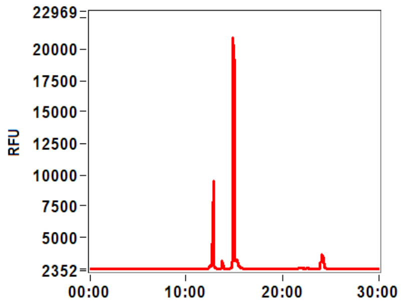


**G12: SampG12**

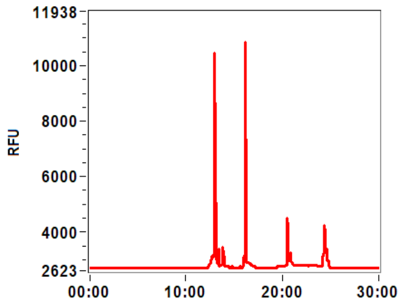


**H12: SampH12**

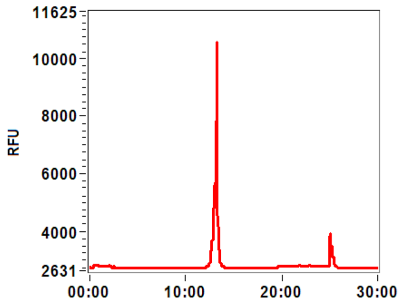

**Sample:** SampA1
**Well location:** A1
**Created:** Tuesday, September 14, 2021 6:54:01 PM

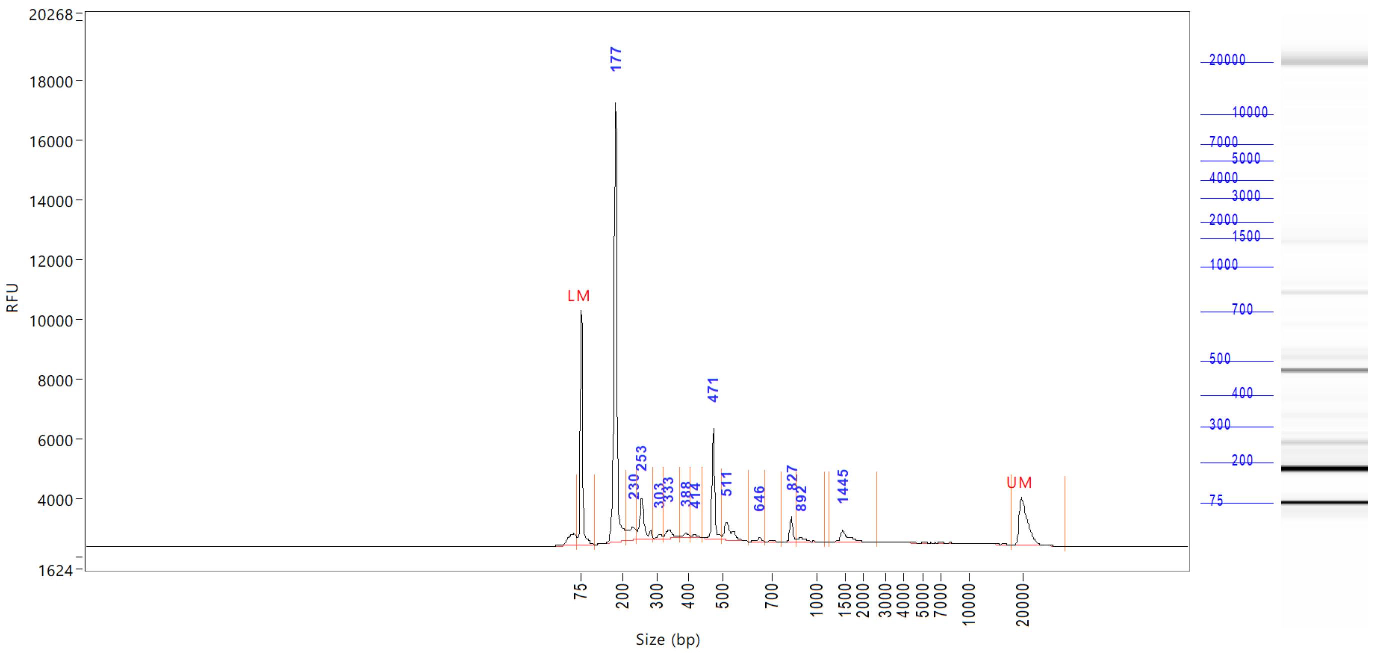

| Peak | Size (bp) | Concentration (ng/uL) | From (bp) | To (bp) | Average size (bp) | CV% | RFU | Corrected peak area |
|---|---|---|---|---|---|---|---|---|
| 1 | 75 (LM) | 0.5000 | 64 | 116 | 74 | 6.83 | 7857 | 45.061 |
| 2 | 177 | 13.7398 | 116 | 208 | 176 | 4.10 | 14761 | 103.189 |
| 3 | 230 | 1.0185 | 208 | 239 | 223 | 4.01 | 449 | 7.649 |
| 4 | 253 | 1.8581 | 239 | 289 | 254 | 3.93 | 1362 | 13.955 |
| 5 | 303 | 0.1760 | 289 | 315 | 303 | 2.11 | 141 | 1.322 |
| 6 | 333 | 0.5683 | 315 | 370 | 336 | 3.77 | 300 | 4.268 |
| 7 | 388 | 0.2566 | 370 | 405 | 387 | 2.34 | 156 | 1.927 |
| 8 | 414 | 0.1655 | 405 | 439 | 417 | 2.03 | 110 | 1.243 |
| 9 | 471 | 2.6569 | 439 | 496 | 470 | 1.42 | 3690 | 19.954 |
| 10 | 511 | 1.0460 | 496 | 606 | 523 | 3.45 | 574 | 7.856 |
| 11 | 646 | 0.1216 | 606 | 669 | 642 | 1.66 | 120 | 0.913 |
| 12 | 827 | 0.5701 | 757 | 861 | 822 | 1.59 | 824 | 4.281 |
| 13 | 892 | 0.2491 | 861 | 1134 | 909 | 4.29 | 157 | 1.871 |
| 14 | 1445 | 0.7086 | 1201 | 2629 | 1566 | 13.42 | 398 | 5.322 |
| 15 | 20000 (UM) | 0.1861 | 17927 | 28463 | 20485 | 4.79 | 1590 | 16.767 |

| | | |
|---|---|---|
| TIC: | 23.1351 | ng/uL |
| TIM: | 168.6029 | nmole/L |
| Total concentration: | 23.2768 | ng/uL |

Sample peak width (sec): 5    Sample min peak height: 50    Sample baseline V to V?: Y    Sample baseline V to V points: 3
Sample filter: Binomial    Number of points for filter: 3    Sample start region (min): 0    Sample end region (min): 30
Marker peak width (sec): 5    Marker min peak height: 200    Marker baseline V to V?: Y    Marker baseline V to V points: 3
Lower marker selection: First peak > 500 RFU    Upper marker selection: Last peak > 200 RFU
Ladder size (bp)75, 200, 300, 400, 500, 700, 1000, 1500, 2000, 3000, 4000, 5000, 7000, 10000, 20000
Quantification using: Lower Marker    Final concentration (ng/uL): 0.5000    Dilution factor: 12.0

**Sample:** SampB1
**Well location:** B1
**Created:** Tuesday, September 14, 2021 6:54:01 PM

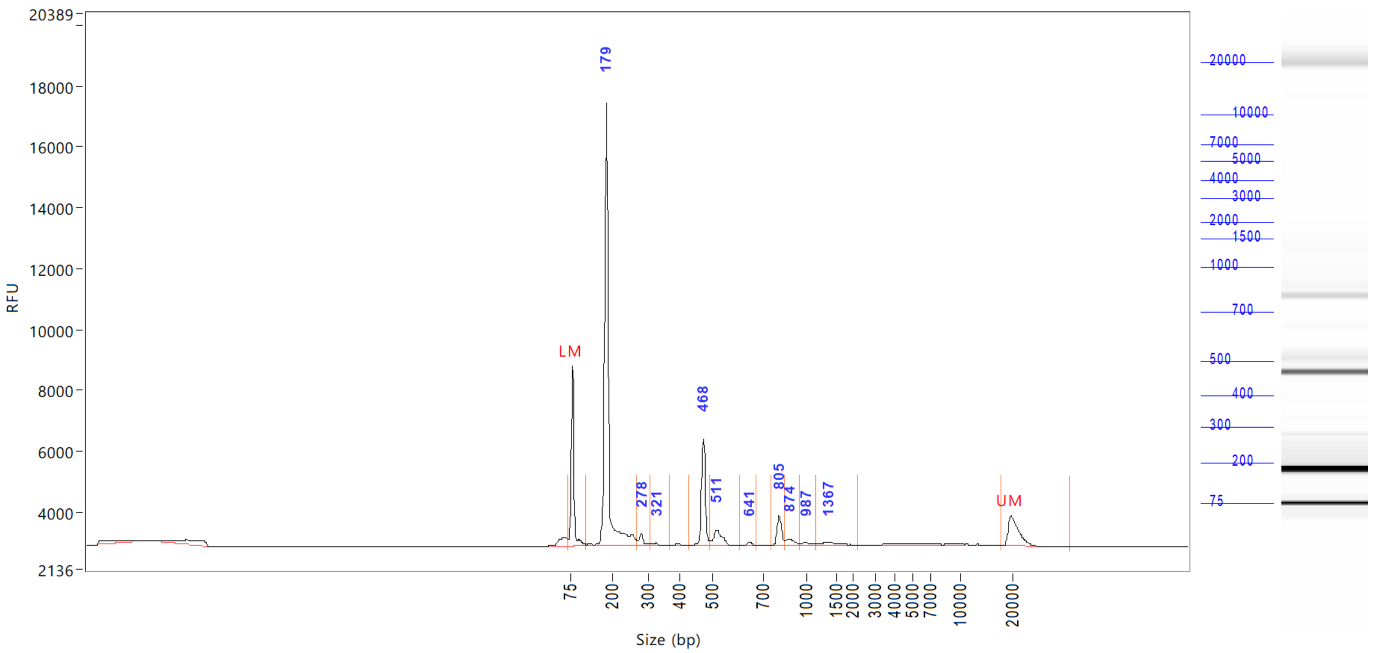


| Peak | Size (bp) | Concentration (ng/uL) | From (bp) | To (bp) | Average size (bp) | CV% | RFU | Corrected peak area |
|---|---|---|---|---|---|---|---|---|
| 1 | 75 (LM) | 0.5000 | 64 | 116 | 75 | 9.28 | 5891 | 37.013 |
| 2 | 179 | 21.5841 | 116 | 268 | 182 | 11.05 | 14555 | 133.150 |
| 3 | 278 | 0.5399 | 268 | 307 | 278 | 2.88 | 399 | 3.330 |
| 4 | 321 | 0.1287 | 307 | 368 | 325 | 3.68 | 74 | 0.794 |
| 5 | 468 | 4.0647 | 428 | 490 | 467 | 1.43 | 3511 | 25.075 |
| 6 | 511 | 1.3156 | 490 | 608 | 517 | 3.15 | 521 | 8.116 |
| 7 | 641 | 0.1592 | 608 | 669 | 640 | 1.41 | 120 | 0.982 |
| 8 | 805 | 1.2313 | 753 | 848 | 805 | 2.08 | 970 | 7.596 |
| 9 | 874 | 0.5045 | 848 | 952 | 888 | 3.11 | 218 | 3.112 |
| 10 | 987 | 0.2239 | 952 | 1134 | 1012 | 5.07 | 92 | 1.381 |
| 11 | 1367 | 0.4155 | 1134 | 2172 | 1406 | 13.10 | 89 | 2.563 |
| 12 | 20000 (UM) | 0.1707 | 18049 | 31244 | 20601 | 6.03 | 980 | 12.637 |

| | | |
|---|---|---|
| TIC: | 30.1673 | ng/uL |
| TIM: | 222.0374 | nmole/L |
| Total concentration: | 30.4745 | ng/uL |

```
Sample peak width (sec): 5     Sample min peak height: 50     Sample baseline V to V?: Y     Sample baseline V to V points: 3
Sample filter: Binomial        Number of points for filter: 3  Sample start region (min): 0  Sample end region (min): 30
Marker peak width (sec): 5     Marker min peak height: 200    Marker baseline V to V?: Y     Marker baseline V to V points: 3
Lower marker selection: First peak > 500 RFU                  Upper marker selection: Last peak > 200 RFU
Ladder size (bp)75, 200, 300, 400, 500, 700, 1000, 1500, 2000, 3000, 4000, 5000, 7000, 10000, 20000
Quantification using: Lower Marker          Final concentration (ng/uL): 0.5000          Dilution factor: 12.0
```

**Sample:** SampC1
**Well location:** C1
**Created:** Tuesday, September 14, 2021 6:54:01 PM

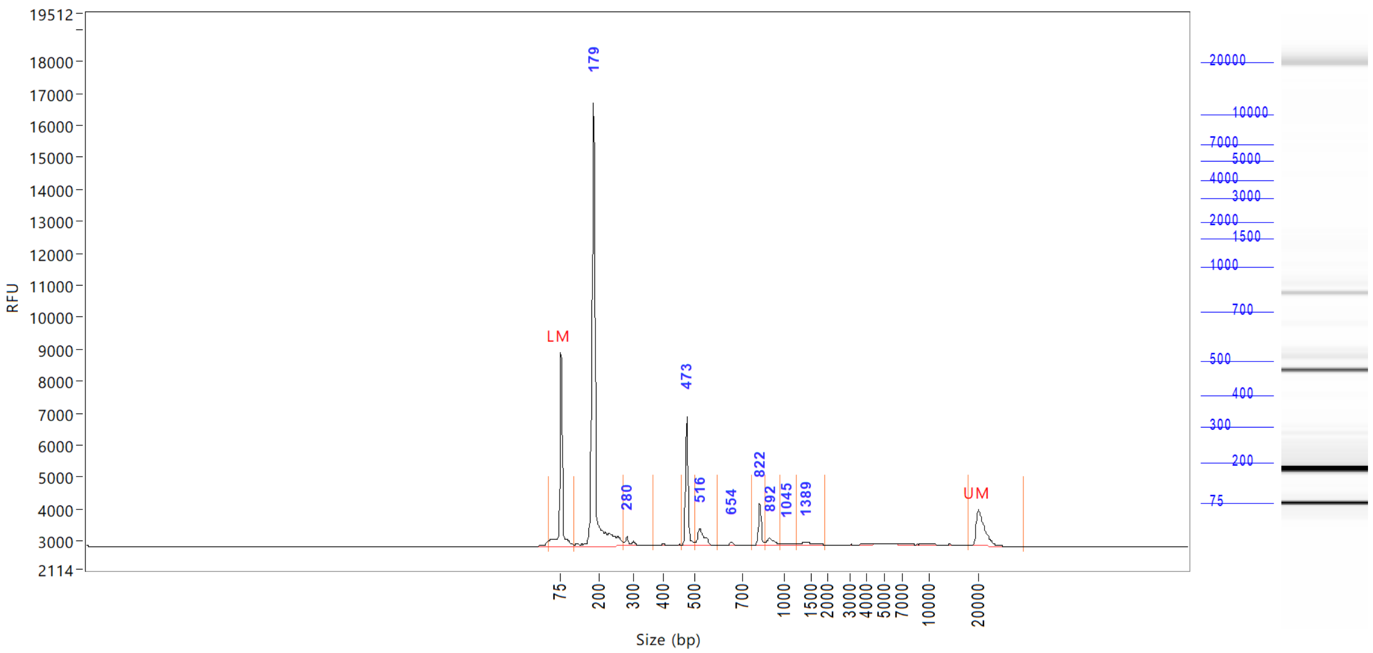


| Peak | Size (bp) | Concentration (ng/uL) | From (bp) | To (bp) | Average size (bp) | CV% | RFU | Corrected peak area |
|---|---|---|---|---|---|---|---|---|
| 1 | 75 (LM) | 0.5000 | 37 | 118 | 73 | 14.51 | 6050 | 41.936 |
| 2 | 179 | 16.8371 | 118 | 269 | 183 | 10.83 | 13895 | 117.681 |
| 3 | 280 | 0.5303 | 269 | 370 | 290 | 6.36 | 305 | 3.707 |
| 4 | 473 | 3.0778 | 458 | 498 | 473 | 1.20 | 4041 | 21.512 |
| 5 | 516 | 1.0250 | 498 | 595 | 526 | 3.24 | 516 | 7.164 |
| 6 | 654 | 0.1999 | 595 | 770 | 663 | 3.78 | 134 | 1.397 |
| 7 | 822 | 0.9837 | 770 | 861 | 822 | 1.52 | 1311 | 6.876 |
| 8 | 892 | 0.4207 | 861 | 974 | 904 | 3.02 | 236 | 2.941 |
| 9 | 1045 | 0.1300 | 974 | 1223 | 1066 | 6.77 | 64 | 0.908 |
| 10 | 1389 | 0.2516 | 1223 | 1923 | 1446 | 10.03 | 86 | 1.758 |
| 11 | 20000 (UM) | 0.1468 | 18049 | 29189 | 20580 | 5.24 | 1128 | 12.311 |

| | | |
|---|---|---|
| TIC: | 23.4560 | ng/uL |
| TIM: | 171.9004 | nmole/L |
| Total concentration: | 23.7670 | ng/uL |

```
Sample peak width (sec): 5    Sample min peak height: 50    Sample baseline V to V?: Y    Sample baseline V to V points: 3
Sample filter: Binomial       Number of points for filter: 3  Sample start region (min): 0  Sample end region (min): 30
Marker peak width (sec): 5    Marker min peak height: 200   Marker baseline V to V?: Y    Marker baseline V to V points: 3
Lower marker selection: First peak > 500 RFU                Upper marker selection: Last peak > 200 RFU
Ladder size (bp)75, 200, 300, 400, 500, 700, 1000, 1500, 2000, 3000, 4000, 5000, 7000, 10000, 20000
Quantification using: Lower Marker          Final concentration (ng/uL): 0.5000         Dilution factor: 12.0
```

**Sample:** SampD1
**Well location:** D1
**Created:** Tuesday, September 14, 2021 6:54:01 PM

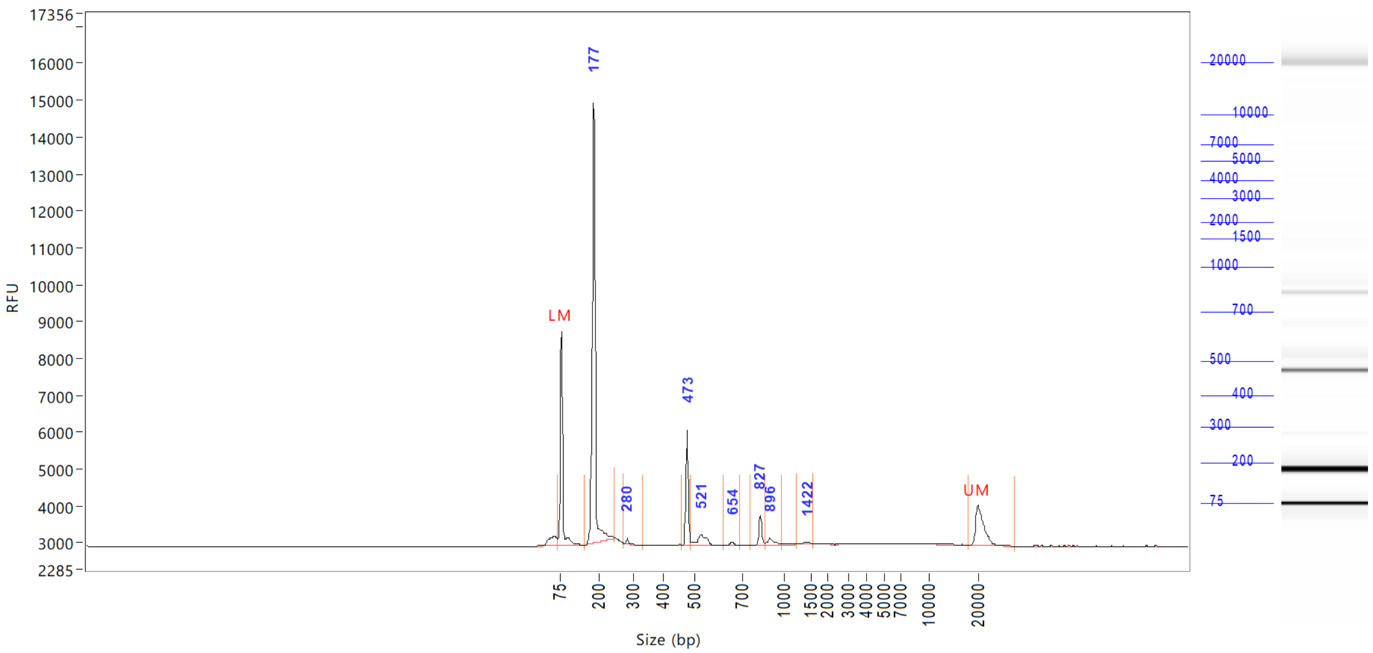


| Peak | Size (bp) | Concentration (ng/uL) | From (bp) | To (bp) | Average size (bp) | CV% | RFU | Corrected peak area |
|---|---|---|---|---|---|---|---|---|
| 1 | 75 (LM) | 0.5000 | 64 | 150 | 75 | 11.44 | 5797 | 35.252 |
| 2 | 177 | 14.5447 | 150 | 242 | 177 | 5.31 | 11950 | 85.454 |
| 3 | 280 | 0.1692 | 268 | 329 | 282 | 2.91 | 155 | 0.994 |
| 4 | 473 | 2.6324 | 456 | 489 | 472 | 0.97 | 3130 | 15.466 |
| 5 | 521 | 0.9216 | 489 | 618 | 526 | 3.78 | 310 | 5.415 |
| 6 | 654 | 0.1417 | 618 | 690 | 652 | 1.71 | 111 | 0.832 |
| 7 | 827 | 0.7152 | 753 | 861 | 824 | 1.50 | 796 | 4.202 |
| 8 | 896 | 0.3685 | 861 | 987 | 908 | 3.22 | 187 | 2.165 |
| 9 | 1422 | 0.1321 | 1223 | 1559 | 1398 | 5.25 | 52 | 0.776 |
| 10 | 20000 (UM) | 0.1634 | 18171 | 27496 | 20547 | 4.90 | 1091 | 11.524 |

| | | |
|---|---|---|
| TIC: | 19.6254 | ng/uL |
| TIM: | 150.7451 | nmole/L |
| Total concentration: | 20.1549 | ng/uL |

```
Sample peak width (sec): 5     Sample min peak height: 50    Sample baseline V to V?: Y    Sample baseline V to V points: 3
Sample filter: Binomial        Number of points for filter: 3  Sample start region (min): 0  Sample end region (min): 30
Marker peak width (sec): 5     Marker min peak height: 200   Marker baseline V to V?: Y    Marker baseline V to V points: 3
Lower marker selection: First peak > 500 RFU                  Upper marker selection: Last peak > 200 RFU
Ladder size (bp)75, 200, 300, 400, 500, 700, 1000, 1500, 2000, 3000, 4000, 5000, 7000, 10000, 20000
Quantification using: Lower Marker          Final concentration (ng/uL): 0.5000          Dilution factor: 12.0
```

**Sample:** SampE1
**Well location:** E1
**Created:** Tuesday, September 14, 2021 6:54:01 PM

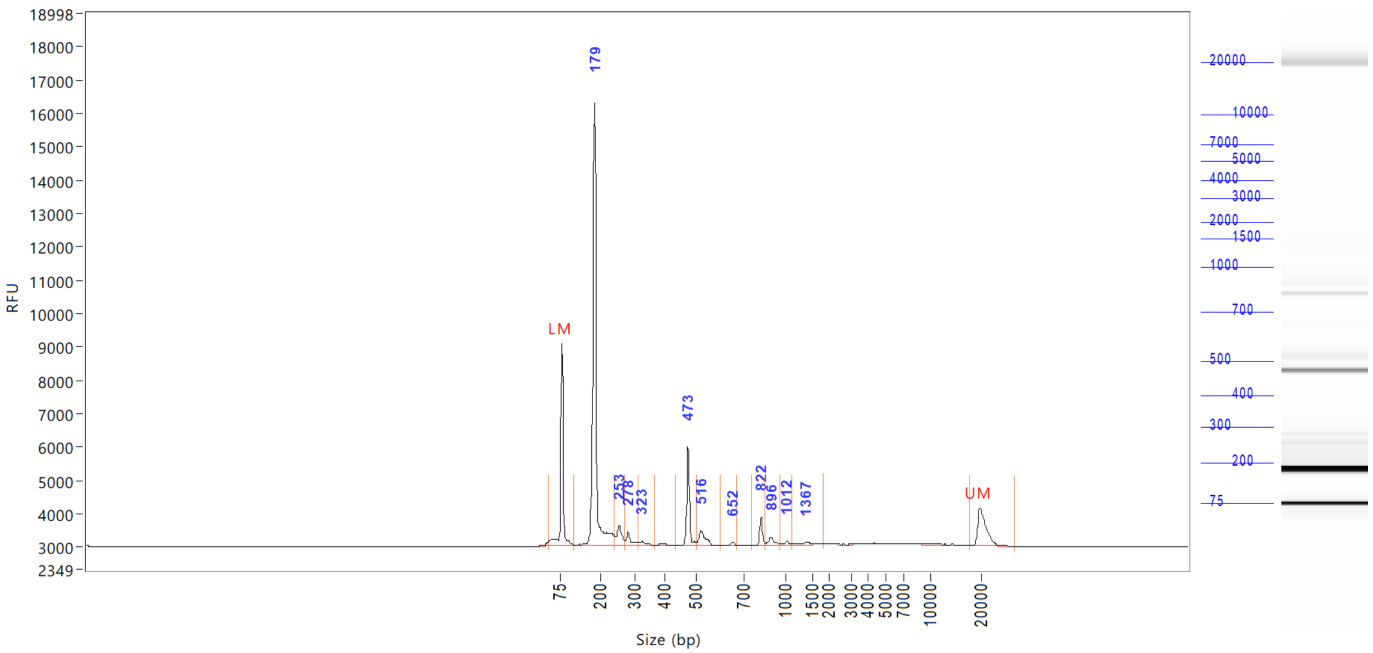

| Peak | Size (bp) | Concentration (ng/uL) | From (bp) | To (bp) | Average size (bp) | CV% | RFU | Corrected peak area |
|---|---|---|---|---|---|---|---|---|
| 1 | 75 (LM) | 0.5000 | 37 | 114 | 71 | 12.15 | 6061 | 38.619 |
| 2 | 179 | 16.7451 | 114 | 241 | 179 | 7.88 | 13262 | 107.781 |
| 3 | 253 | 1.0052 | 241 | 269 | 253 | 2.86 | 572 | 6.470 |
| 4 | 278 | 0.5831 | 269 | 313 | 284 | 4.18 | 397 | 3.753 |
| 5 | 323 | 0.1884 | 313 | 368 | 326 | 3.16 | 104 | 1.213 |
| 6 | 473 | 2.4667 | 435 | 500 | 472 | 1.44 | 2944 | 15.877 |
| 7 | 516 | 0.8783 | 500 | 603 | 525 | 3.10 | 438 | 5.653 |
| 8 | 652 | 0.1241 | 603 | 672 | 648 | 1.46 | 116 | 0.799 |
| 9 | 822 | 0.6705 | 753 | 853 | 818 | 1.42 | 848 | 4.316 |
| 10 | 896 | 0.4121 | 853 | 965 | 898 | 2.91 | 244 | 2.653 |
| 11 | 1012 | 0.1507 | 965 | 1112 | 1013 | 3.41 | 104 | 0.970 |
| 12 | 1367 | 0.2190 | 1112 | 1827 | 1396 | 10.98 | 59 | 1.409 |
| 13 | 20000 (UM) | 0.1579 | 17927 | 26770 | 20521 | 5.06 | 1128 | 12.197 |

| | | |
|---|---|---|
| TIC: | 23.4432 | ng/uL |
| TIM: | 178.9279 | nmole/L |
| Total concentration: | 23.7507 | ng/uL |

Sample peak width (sec): 5  Sample min peak height: 50  Sample baseline V to V?: Y  Sample baseline V to V points: 3
Sample filter: Binomial  Number of points for filter: 3  Sample start region (min): 0  Sample end region (min): 30
Marker peak width (sec): 5  Marker min peak height: 200  Marker baseline V to V?: Y  Marker baseline V to V points: 3
Lower marker selection: First peak > 500 RFU  Upper marker selection: Last peak > 200 RFU
Ladder size (bp)75, 200, 300, 400, 500, 700, 1000, 1500, 2000, 3000, 4000, 5000, 7000, 10000, 20000
Quantification using: Lower Marker  Final concentration (ng/uL): 0.5000  Dilution factor: 12.0

**Sample:** SampF1
**Well location:** F1
**Created:** Tuesday, September 14, 2021 6:54:01 PM

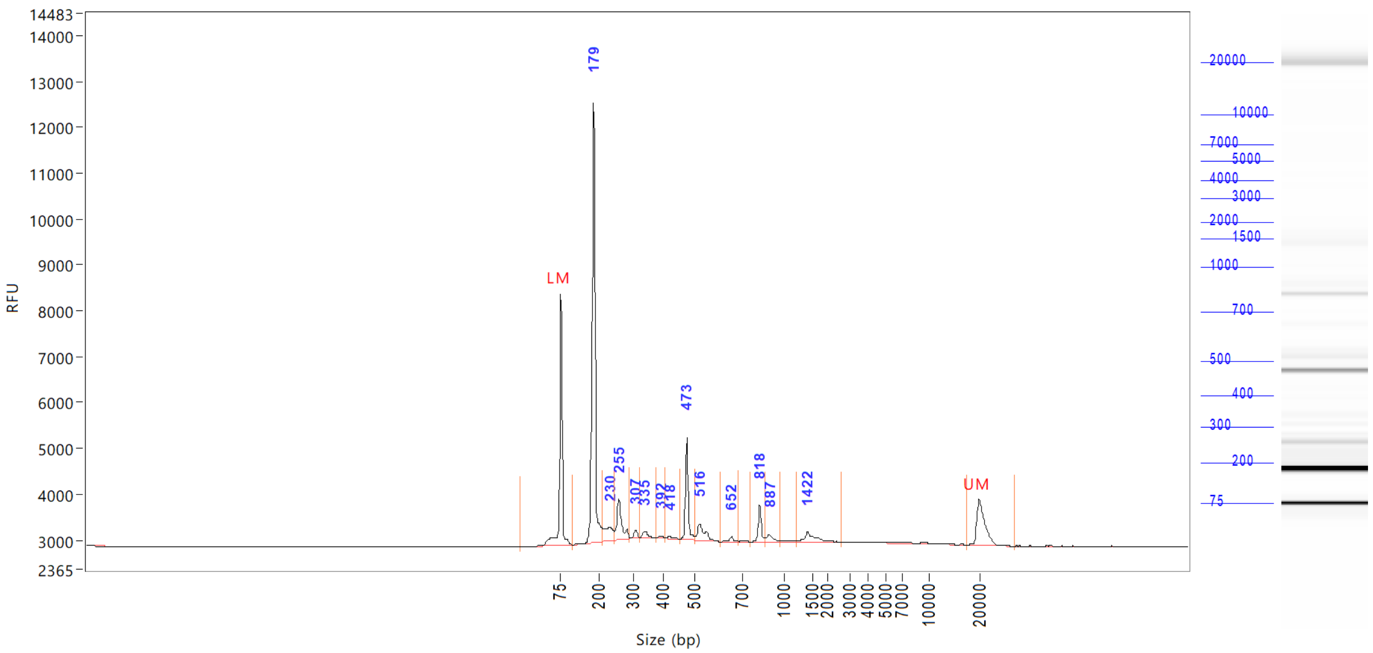


| Peak | Size (bp) | Concentration (ng/uL) | From (bp) | To (bp) | Average size (bp) | CV% | RFU | Corrected peak area |
|---|---|---|---|---|---|---|---|---|
| 1 | 75 (LM) | 0.5000 | 0 | 112 | 70 | 18.53 | 5482 | 36.196 |
| 2 | 179 | 11.7892 | 112 | 210 | 178 | 4.37 | 9601 | 71.121 |
| 3 | 230 | 0.9675 | 210 | 242 | 225 | 4.20 | 299 | 5.836 |
| 4 | 255 | 1.6286 | 242 | 289 | 257 | 3.75 | 887 | 9.825 |
| 5 | 307 | 0.2100 | 289 | 319 | 304 | 1.81 | 183 | 1.267 |
| 6 | 335 | 0.3180 | 319 | 374 | 339 | 3.45 | 151 | 1.918 |
| 7 | 392 | 0.0990 | 374 | 407 | 389 | 2.05 | 58 | 0.597 |
| 8 | 418 | 0.1049 | 407 | 452 | 424 | 2.89 | 54 | 0.633 |
| 9 | 473 | 2.0392 | 452 | 500 | 472 | 1.40 | 2219 | 12.302 |
| 10 | 516 | 0.8226 | 500 | 608 | 527 | 3.51 | 362 | 4.962 |
| 11 | 652 | 0.1298 | 608 | 685 | 646 | 1.66 | 102 | 0.783 |
| 12 | 818 | 0.7396 | 753 | 861 | 818 | 1.84 | 801 | 4.462 |
| 13 | 887 | 0.2912 | 861 | 965 | 897 | 2.80 | 166 | 1.757 |
| 14 | 1422 | 0.5926 | 1212 | 2600 | 1527 | 13.33 | 207 | 3.575 |
| 15 | 20000 (UM) | 0.1468 | 17439 | 27133 | 20487 | 4.90 | 997 | 10.630 |

| | | |
|---|---|---|
| TIC: | 19.7321 | ng/uL |
| TIM: | 142.5500 | nmole/L |
| Total concentration: | 19.9747 | ng/uL |

```
Sample peak width (sec): 5     Sample min peak height: 50    Sample baseline V to V?: Y    Sample baseline V to V points: 3
Sample filter: Binomial        Number of points for filter: 3  Sample start region (min): 0  Sample end region (min): 30
Marker peak width (sec): 5     Marker min peak height: 200   Marker baseline V to V?: Y    Marker baseline V to V points: 3
Lower marker selection: First peak > 500 RFU                 Upper marker selection: Last peak > 200 RFU
Ladder size (bp)75, 200, 300, 400, 500, 700, 1000, 1500, 2000, 3000, 4000, 5000, 7000, 10000, 20000
Quantification using: Lower Marker          Final concentration (ng/uL): 0.5000         Dilution factor: 12.0
```

**Sample:** SampG1
**Well location:** G1
**Created:** Tuesday, September 14, 2021 6:54:01 PM

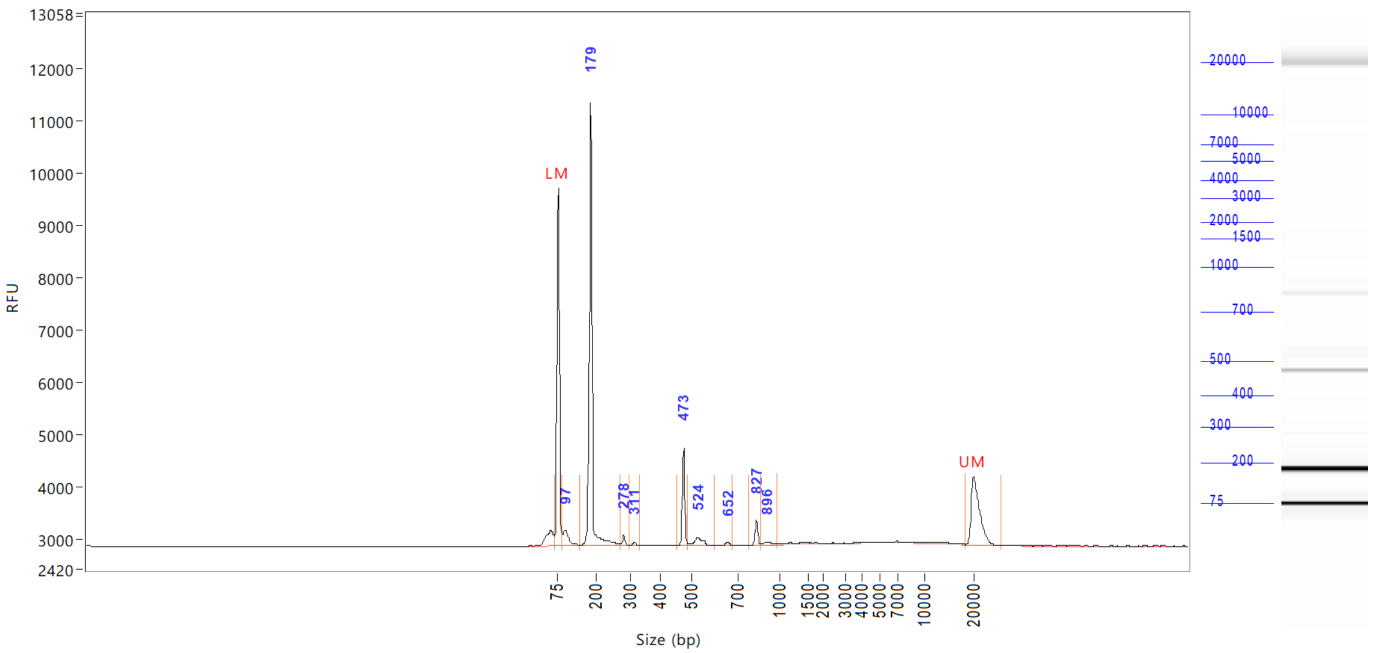


| Peak | Size (bp) | Concentration (ng/uL) | From (bp) | To (bp) | Average size (bp) | CV% | RFU | Corrected peak area |
|---|---|---|---|---|---|---|---|---|
| 1 | 75 (LM) | 0.5000 | 64 | 87 | 73 | 5.02 | 6833 | 38.879 |
| 2 | 97 | 0.6575 | 87 | 147 | 101 | 10.97 | 295 | 4.260 |
| 3 | 179 | 8.8623 | 147 | 269 | 179 | 7.63 | 8479 | 57.427 |
| 4 | 278 | 0.1782 | 269 | 296 | 278 | 1.69 | 203 | 1.155 |
| 5 | 311 | 0.0814 | 296 | 329 | 310 | 2.02 | 78 | 0.528 |
| 6 | 473 | 1.4062 | 456 | 487 | 471 | 0.85 | 1879 | 9.112 |
| 7 | 524 | 0.4198 | 487 | 595 | 525 | 3.64 | 164 | 2.720 |
| 8 | 652 | 0.0692 | 595 | 674 | 647 | 1.72 | 65 | 0.448 |
| 9 | 827 | 0.3816 | 775 | 857 | 820 | 1.41 | 496 | 2.473 |
| 10 | 896 | 0.1367 | 857 | 978 | 905 | 3.16 | 65 | 0.885 |
| 11 | 20000 (UM) | 0.1822 | 18293 | 25440 | 20541 | 4.67 | 1295 | 14.168 |

| | | |
|---|---|---|
| TIC: | 12.1928 | ng/uL |
| TIM: | 100.9882 | nmole/L |
| Total concentration: | 12.7471 | ng/uL |

```
Sample peak width (sec): 5     Sample min peak height: 50     Sample baseline V to V?: Y     Sample baseline V to V points: 3
Sample filter: Binomial        Number of points for filter: 3  Sample start region (min): 0  Sample end region (min): 30
Marker peak width (sec): 5     Marker min peak height: 200    Marker baseline V to V?: Y     Marker baseline V to V points: 3
Lower marker selection: First peak > 500 RFU                  Upper marker selection: Last peak > 200 RFU
Ladder size (bp)75, 200, 300, 400, 500, 700, 1000, 1500, 2000, 3000, 4000, 5000, 7000, 10000, 20000
Quantification using: Lower Marker          Final concentration (ng/uL): 0.5000          Dilution factor: 12.0
```

**Sample:** SampH1
**Well location:** H1
**Created:** Tuesday, September 14, 2021 6:54:01 PM

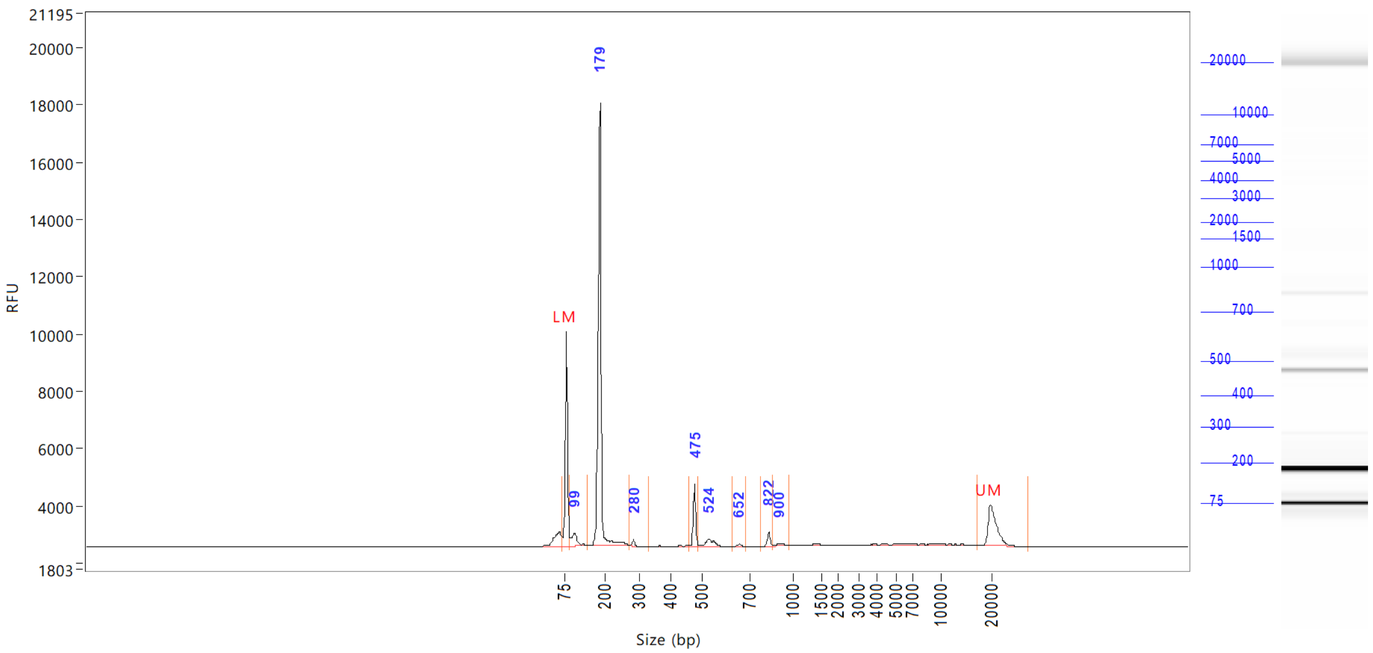


| Peak | Size (bp) | Concentration (ng/uL) | From (bp) | To (bp) | Average size (bp) | CV% | RFU | Corrected peak area |
|---|---|---|---|---|---|---|---|---|
| 1 | 75 (LM) | 0.5000 | 66 | 87 | 74 | 4.80 | 7488 | 42.282 |
| 2 | 99 | 0.8849 | 87 | 143 | 101 | 10.82 | 452 | 6.236 |
| 3 | 179 | 14.3816 | 143 | 271 | 179 | 7.60 | 15475 | 101.348 |
| 4 | 280 | 0.1722 | 271 | 327 | 279 | 2.39 | 228 | 1.214 |
| 5 | 475 | 1.4927 | 456 | 489 | 472 | 0.94 | 2156 | 10.519 |
| 6 | 524 | 0.5716 | 489 | 623 | 528 | 3.55 | 257 | 4.028 |
| 7 | 652 | 0.0701 | 623 | 680 | 650 | 1.19 | 72 | 0.494 |
| 8 | 822 | 0.3344 | 766 | 853 | 820 | 1.27 | 478 | 2.356 |
| 9 | 900 | 0.1427 | 853 | 978 | 905 | 3.11 | 74 | 1.005 |
| 10 | 20000 (UM) | 0.1824 | 17439 | 27254 | 20466 | 4.69 | 1431 | 15.424 |

| | | |
|---|---|---|
| TIC: | 18.0502 | ng/uL |
| TIM: | 155.5799 | nmole/L |
| Total concentration: | 18.5791 | ng/uL |

```
Sample peak width (sec): 5    Sample min peak height: 50    Sample baseline V to V?: Y    Sample baseline V to V points: 3
Sample filter: Binomial       Number of points for filter: 3  Sample start region (min): 0  Sample end region (min): 30
Marker peak width (sec): 5    Marker min peak height: 200   Marker baseline V to V?: Y    Marker baseline V to V points: 3
Lower marker selection: First peak > 500 RFU                Upper marker selection: Last peak > 200 RFU
Ladder size (bp)75, 200, 300, 400, 500, 700, 1000, 1500, 2000, 3000, 4000, 5000, 7000, 10000, 20000
Quantification using: Lower Marker          Final concentration (ng/uL): 0.5000          Dilution factor: 12.0
```

**Sample:** SampA2
**Well location:** A2
**Created:** Tuesday, September 14, 2021 6:54:01 PM

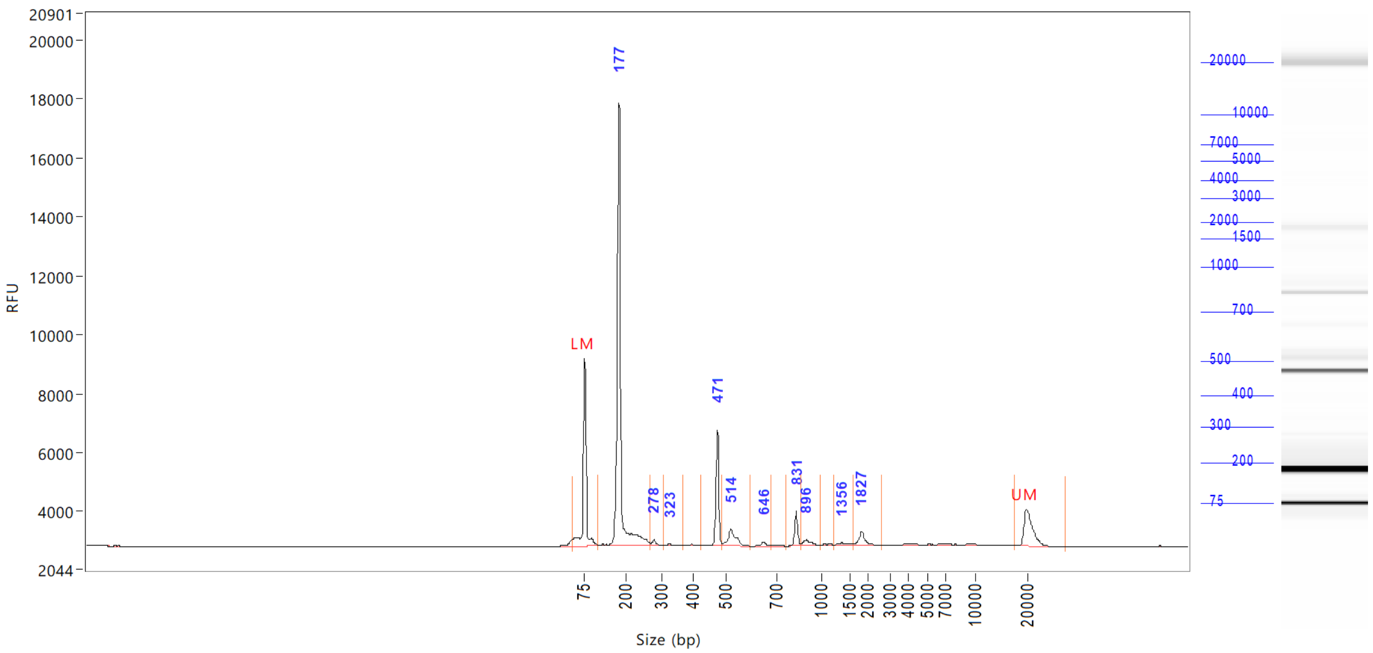


| Peak | Size (bp) | Concentration (ng/uL) | From (bp) | To (bp) | Average size (bp) | CV% | RFU | Corrected peak area |
|---|---|---|---|---|---|---|---|---|
| 1 | 75 (LM) | 0.5000 | 39 | 116 | 73 | 14.05 | 6338 | 42.696 |
| 2 | 177 | 16.7892 | 116 | 269 | 180 | 9.95 | 15040 | 119.472 |
| 3 | 278 | 0.2244 | 269 | 305 | 280 | 3.06 | 213 | 1.597 |
| 4 | 323 | 0.1037 | 305 | 370 | 328 | 4.88 | 62 | 0.738 |
| 5 | 471 | 2.7455 | 426 | 487 | 470 | 1.08 | 3922 | 19.537 |
| 6 | 514 | 1.1155 | 487 | 595 | 520 | 3.40 | 558 | 7.938 |
| 7 | 646 | 0.1624 | 595 | 680 | 645 | 1.88 | 150 | 1.155 |
| 8 | 831 | 0.8220 | 766 | 866 | 826 | 1.52 | 1170 | 5.849 |
| 9 | 896 | 0.3274 | 866 | 1000 | 906 | 2.90 | 193 | 2.330 |
| 10 | 1356 | 0.1615 | 1212 | 1559 | 1372 | 6.12 | 74 | 1.150 |
| 11 | 1827 | 0.5733 | 1559 | 2629 | 1831 | 7.08 | 452 | 4.079 |
| 12 | 20000 (UM) | 0.1495 | 17683 | 27375 | 20417 | 4.61 | 1224 | 12.765 |

| | | |
|---|---|---|
| TIC: | 23.0249 | ng/uL |
| TIM: | 171.6764 | nmole/L |
| Total concentration: | 23.4084 | ng/uL |

```
Sample peak width (sec): 5     Sample min peak height: 50     Sample baseline V to V?: Y     Sample baseline V to V points: 3
Sample filter: Binomial        Number of points for filter: 3  Sample start region (min): 0  Sample end region (min): 30
Marker peak width (sec): 5     Marker min peak height: 200    Marker baseline V to V?: Y     Marker baseline V to V points: 3
Lower marker selection: First peak > 500 RFU                  Upper marker selection: Last peak > 200 RFU
Ladder size (bp)75, 200, 300, 400, 500, 700, 1000, 1500, 2000, 3000, 4000, 5000, 7000, 10000, 20000
Quantification using: Lower Marker          Final concentration (ng/uL): 0.5000          Dilution factor: 12.0
```

**Sample:** SampB2
**Well location:** B2
**Created:** Tuesday, September 14, 2021 6:54:01 PM

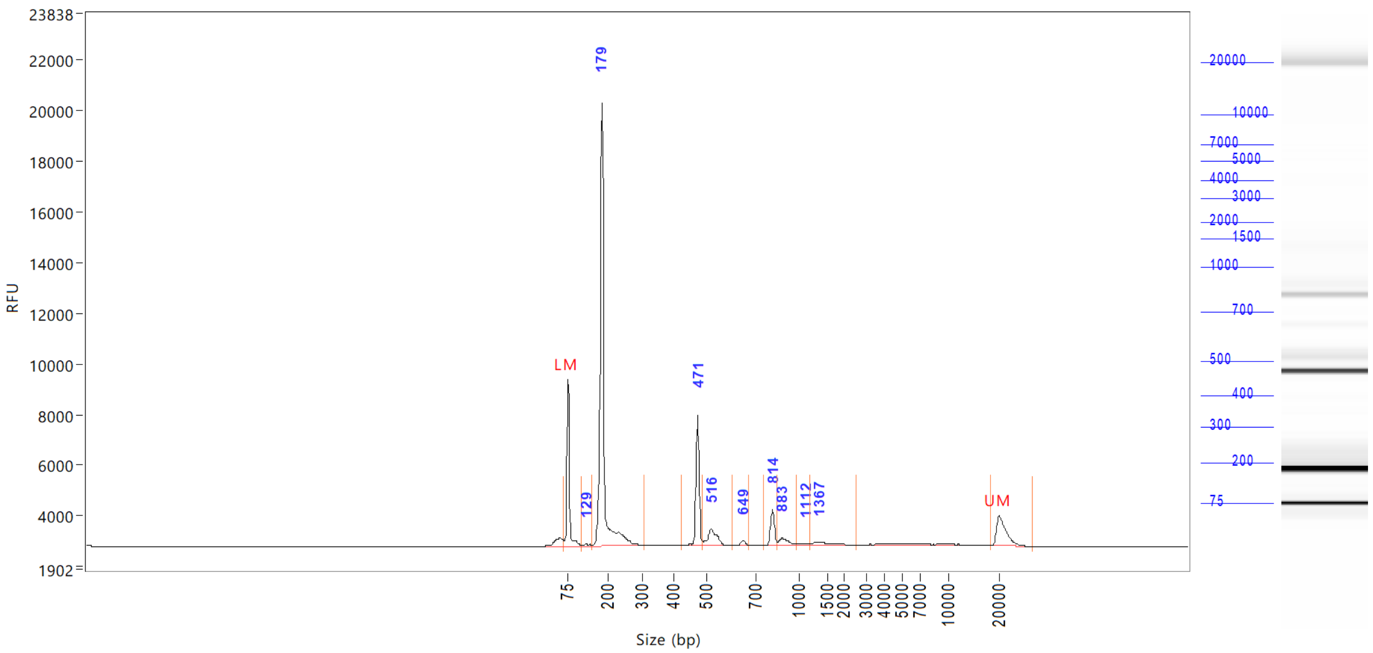


| Peak | Size (bp) | Concentration (ng/uL) | From (bp) | To (bp) | Average size (bp) | CV% | RFU | Corrected peak area |
|---|---|---|---|---|---|---|---|---|
| 1 | 75 (LM) | 0.5000 | 64 | 118 | 75 | 10.02 | 6570 | 40.847 |
| 2 | 129 | 0.2089 | 118 | 152 | 135 | 7.58 | 71 | 1.422 |
| 3 | 179 | 22.3537 | 152 | 301 | 183 | 10.30 | 17512 | 152.182 |
| 4 | 471 | 4.8182 | 424 | 489 | 469 | 1.30 | 5162 | 32.802 |
| 5 | 516 | 1.6337 | 489 | 603 | 522 | 3.53 | 675 | 11.122 |
| 6 | 649 | 0.2388 | 603 | 674 | 646 | 1.32 | 208 | 1.625 |
| 7 | 814 | 1.4394 | 757 | 853 | 812 | 1.81 | 1419 | 9.799 |
| 8 | 883 | 0.6322 | 853 | 991 | 901 | 3.71 | 278 | 4.304 |
| 9 | 1112 | 0.1606 | 991 | 1201 | 1090 | 5.94 | 66 | 1.093 |
| 10 | 1367 | 0.4657 | 1201 | 2543 | 1461 | 14.41 | 121 | 3.171 |
| 11 | 20000 (UM) | 0.1716 | 18293 | 26649 | 20609 | 5.27 | 1173 | 14.020 |

| | | |
|---|---|---|
| TIC: | 31.9510 | ng/uL |
| TIM: | 230.8642 | nmole/L |
| Total concentration: | 32.3802 | ng/uL |

Sample peak width (sec): 5 Sample min peak height: 50 Sample baseline V to V?: Y Sample baseline V to V points: 3
Sample filter: Binomial Number of points for filter: 3 Sample start region (min): 0 Sample end region (min): 30
Marker peak width (sec): 5 Marker min peak height: 200 Marker baseline V to V?: Y Marker baseline V to V points: 3
Lower marker selection: First peak > 500 RFU Upper marker selection: Last peak > 200 RFU
Ladder size (bp)75, 200, 300, 400, 500, 700, 1000, 1500, 2000, 3000, 4000, 5000, 7000, 10000, 20000
Quantification using: Lower Marker Final concentration (ng/uL): 0.5000 Dilution factor: 12.0

**Sample:** SampC2
**Well location:** C2
**Created:** Tuesday, September 14, 2021 6:54:01 PM

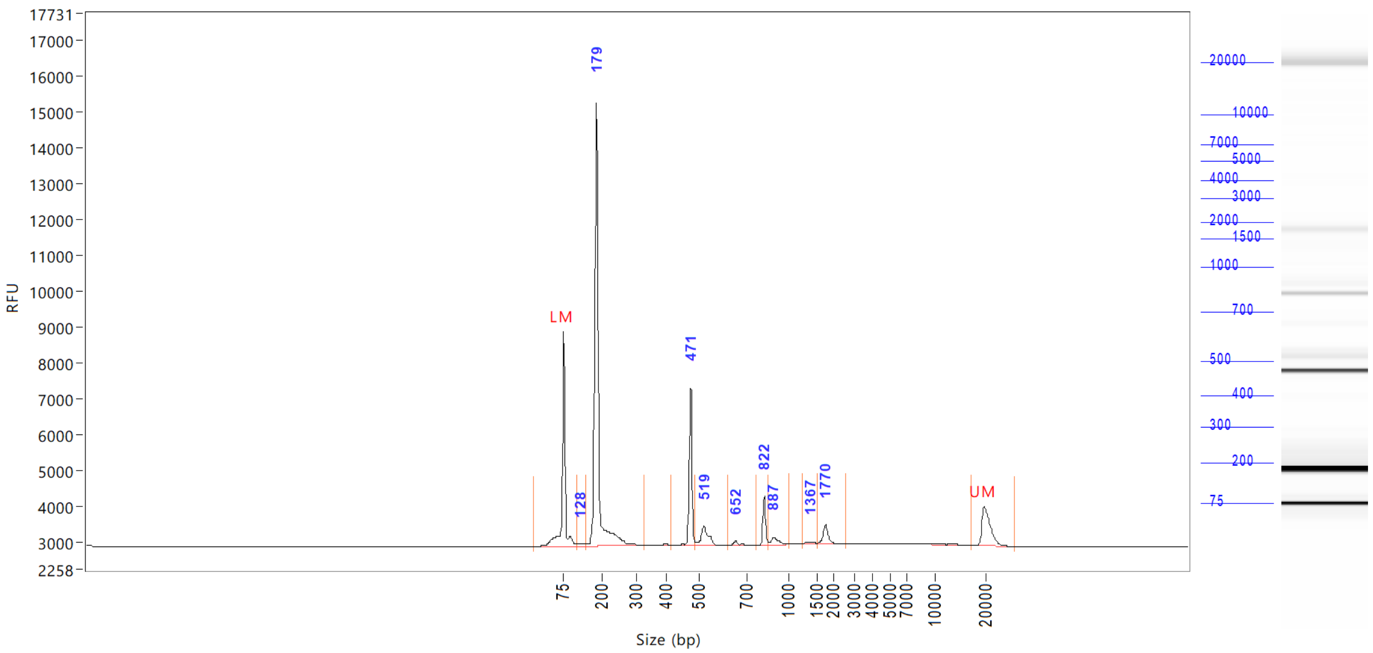


| Peak | Size (bp) | Concentration (ng/uL) | From (bp) | To (bp) | Average size (bp) | CV% | RFU | Corrected peak area |
|---|---|---|---|---|---|---|---|---|
| 1 | 75 (LM) | 0.5000 | 0 | 116 | 70 | 21.04 | 5996 | 43.114 |
| 2 | 128 | 0.1426 | 116 | 147 | 131 | 7.06 | 58 | 1.025 |
| 3 | 179 | 14.8444 | 147 | 323 | 183 | 11.00 | 12353 | 106.667 |
| 4 | 471 | 3.2064 | 414 | 487 | 470 | 1.19 | 4372 | 23.040 |
| 5 | 519 | 1.0828 | 487 | 623 | 523 | 3.65 | 529 | 7.781 |
| 6 | 652 | 0.1706 | 623 | 762 | 661 | 3.35 | 128 | 1.226 |
| 7 | 822 | 0.9146 | 762 | 853 | 818 | 1.26 | 1367 | 6.572 |
| 8 | 887 | 0.3545 | 853 | 1001 | 900 | 3.15 | 212 | 2.547 |
| 9 | 1367 | 0.1560 | 1234 | 1501 | 1376 | 5.18 | 74 | 1.121 |
| 10 | 1770 | 0.6844 | 1501 | 2572 | 1777 | 7.48 | 554 | 4.918 |
| 11 | 20000 (UM) | 0.1365 | 17439 | 26166 | 20442 | 4.68 | 1108 | 11.771 |

| | | |
|---|---|---|
| TIC: | 21.5564 | ng/uL |
| TIM: | 153.5139 | nmole/L |
| Total concentration: | 21.8553 | ng/uL |

Sample peak width (sec): 5 Sample min peak height: 50 Sample baseline V to V?: Y Sample baseline V to V points: 3
Sample filter: Binomial Number of points for filter: 3 Sample start region (min): 0 Sample end region (min): 30
Marker peak width (sec): 5 Marker min peak height: 200 Marker baseline V to V?: Y Marker baseline V to V points: 3
Lower marker selection: First peak > 500 RFU Upper marker selection: Last peak > 200 RFU
Ladder size (bp)75, 200, 300, 400, 500, 700, 1000, 1500, 2000, 3000, 4000, 5000, 7000, 10000, 20000
Quantification using: Lower Marker Final concentration (ng/uL): 0.5000 Dilution factor: 12.0

**Sample:** SampD2
**Well location:** D2
**Created:** Tuesday, September 14, 2021 6:54:01 PM

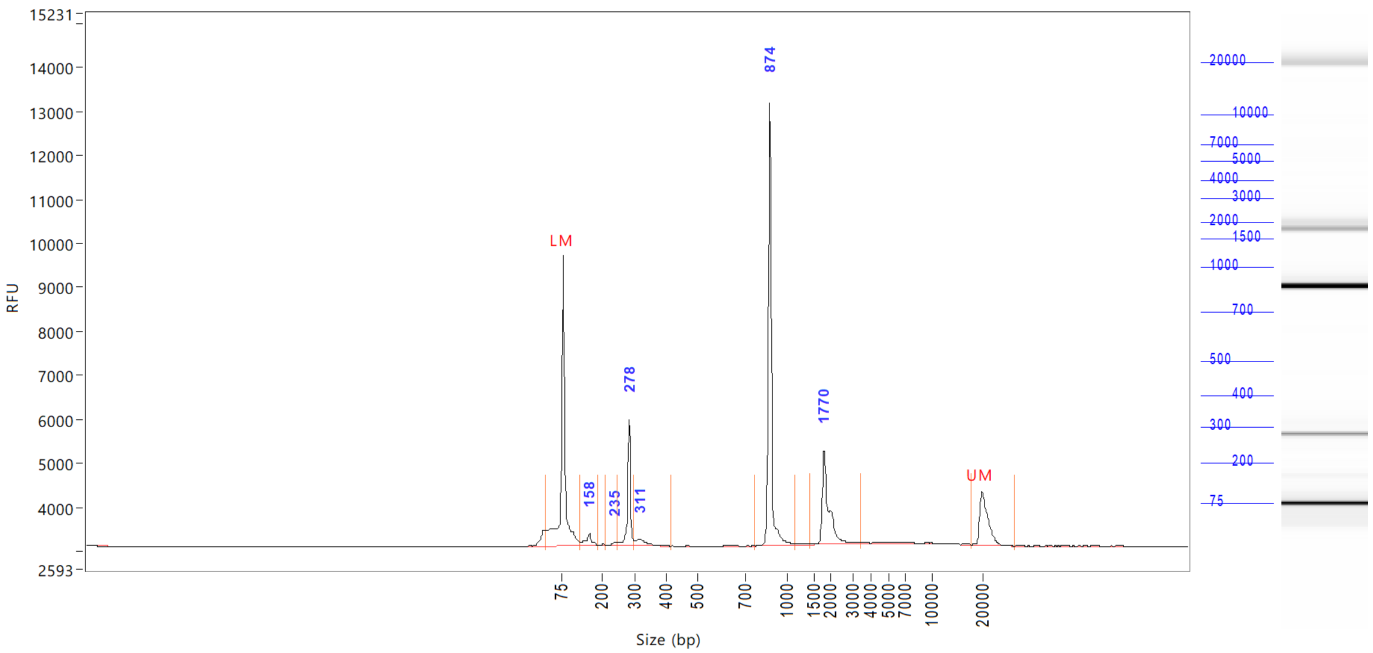


| Peak | Size (bp) | Concentration (ng/uL) | From (bp) | To (bp) | Average size (bp) | CV% | RFU | Corrected peak area |
|---|---|---|---|---|---|---|---|---|
| 1 | 75 (LM) | 0.5000 | 18 | 129 | 70 | 26.78 | 6612 | 61.639 |
| 2 | 158 | 0.3707 | 129 | 187 | 153 | 7.93 | 273 | 3.808 |
| 3 | 235 | 0.0762 | 210 | 244 | 234 | 2.85 | 70 | 0.783 |
| 4 | 278 | 1.7382 | 244 | 295 | 275 | 2.60 | 2879 | 17.857 |
| 5 | 311 | 0.3382 | 295 | 416 | 321 | 7.05 | 152 | 3.475 |
| 6 | 874 | 5.4680 | 766 | 1145 | 876 | 3.23 | 10081 | 56.173 |
| 7 | 1770 | 2.0962 | 1422 | 3429 | 1889 | 12.35 | 2119 | 21.534 |
| 8 | 20000 (UM) | 0.1051 | 17927 | 26408 | 20494 | 4.77 | 1211 | 12.958 |

| | | |
|---|---|---|
| TIC: | 10.0875 | ng/uL |
| TIM: | 28.7479 | nmole/L |
| Total concentration: | 10.3383 | ng/uL |

Sample peak width (sec): 5 Sample min peak height: 50 Sample baseline V to V?: Y Sample baseline V to V points: 3
Sample filter: Binomial Number of points for filter: 3 Sample start region (min): 0 Sample end region (min): 30
Marker peak width (sec): 5 Marker min peak height: 200 Marker baseline V to V?: Y Marker baseline V to V points: 3
Lower marker selection: First peak > 500 RFU Upper marker selection: Last peak > 200 RFU
Ladder size (bp)75, 200, 300, 400, 500, 700, 1000, 1500, 2000, 3000, 4000, 5000, 7000, 10000, 20000
Quantification using: Lower Marker Final concentration (ng/uL): 0.5000 Dilution factor: 12.0

**Sample:** SampE2
**Well location:** E2
**Created:** Tuesday, September 14, 2021 6:54:01 PM

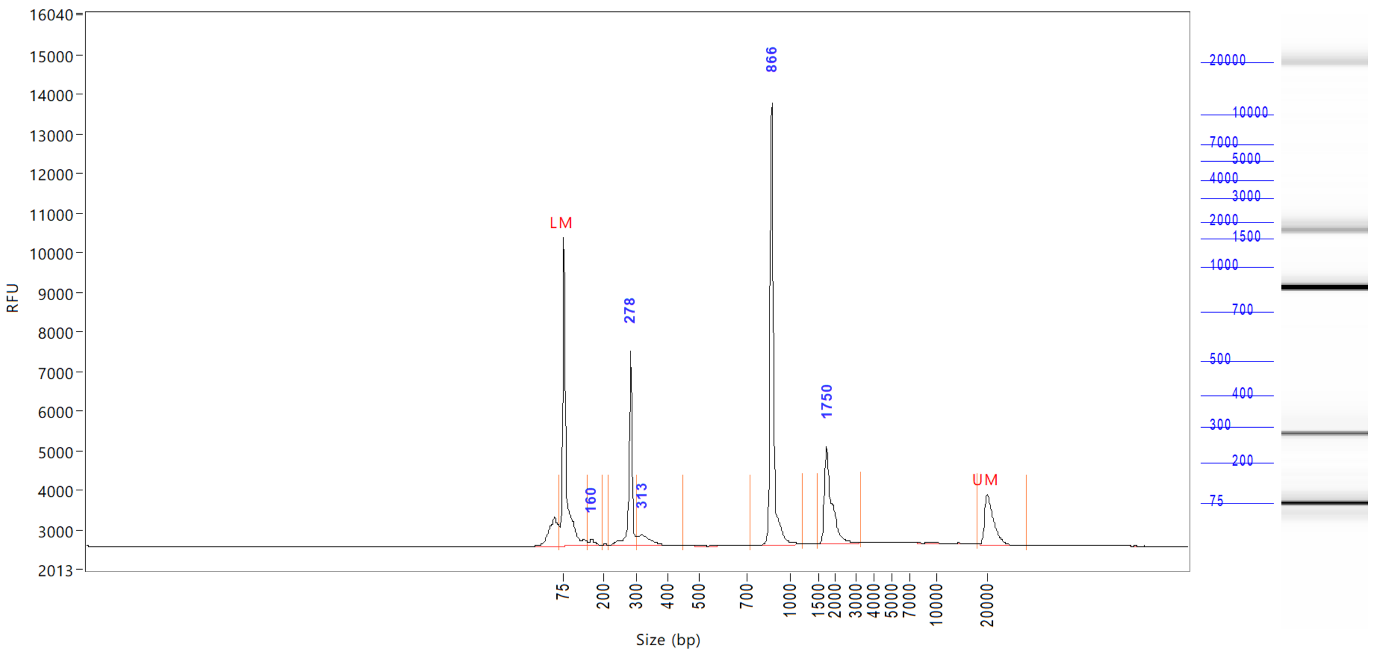


| Peak | Size (bp) | Concentration (ng/uL) | From (bp) | To (bp) | Average size (bp) | CV% | RFU | Corrected peak area |
|---|---|---|---|---|---|---|---|---|
| 1 | 75 (LM) | 0.5000 | 64 | 150 | 81 | 19.17 | 7797 | 66.523 |
| 2 | 160 | 0.1912 | 150 | 196 | 164 | 5.77 | 169 | 2.120 |
| 3 | 278 | 3.0601 | 215 | 298 | 275 | 3.95 | 4913 | 33.928 |
| 4 | 313 | 0.6927 | 298 | 449 | 331 | 8.50 | 263 | 7.681 |
| 5 | 866 | 6.3838 | 718 | 1212 | 869 | 3.74 | 11179 | 70.778 |
| 6 | 1750 | 2.5272 | 1489 | 3251 | 1852 | 11.82 | 2466 | 28.019 |
| 7 | 20000 (UM) | 0.1091 | 18049 | 27859 | 20593 | 5.14 | 1266 | 14.510 |

| | | |
|---|---|---|
| TIC: | 12.8550 | ng/uL |
| TIM: | 37.9992 | nmole/L |
| Total concentration: | 13.1042 | ng/uL |

```
Sample peak width (sec): 5    Sample min peak height: 50    Sample baseline V to V?: Y    Sample baseline V to V points: 3
Sample filter: Binomial       Number of points for filter: 3  Sample start region (min): 0  Sample end region (min): 30
Marker peak width (sec): 5    Marker min peak height: 200   Marker baseline V to V?: Y    Marker baseline V to V points: 3
Lower marker selection: First peak > 500 RFU                Upper marker selection: Last peak > 200 RFU
Ladder size (bp)75, 200, 300, 400, 500, 700, 1000, 1500, 2000, 3000, 4000, 5000, 7000, 10000, 20000
Quantification using: Lower Marker          Final concentration (ng/uL): 0.5000          Dilution factor: 12.0
```

**Sample:** SampF2
**Well location:** F2
**Created:** Tuesday, September 14, 2021 6:54:01 PM

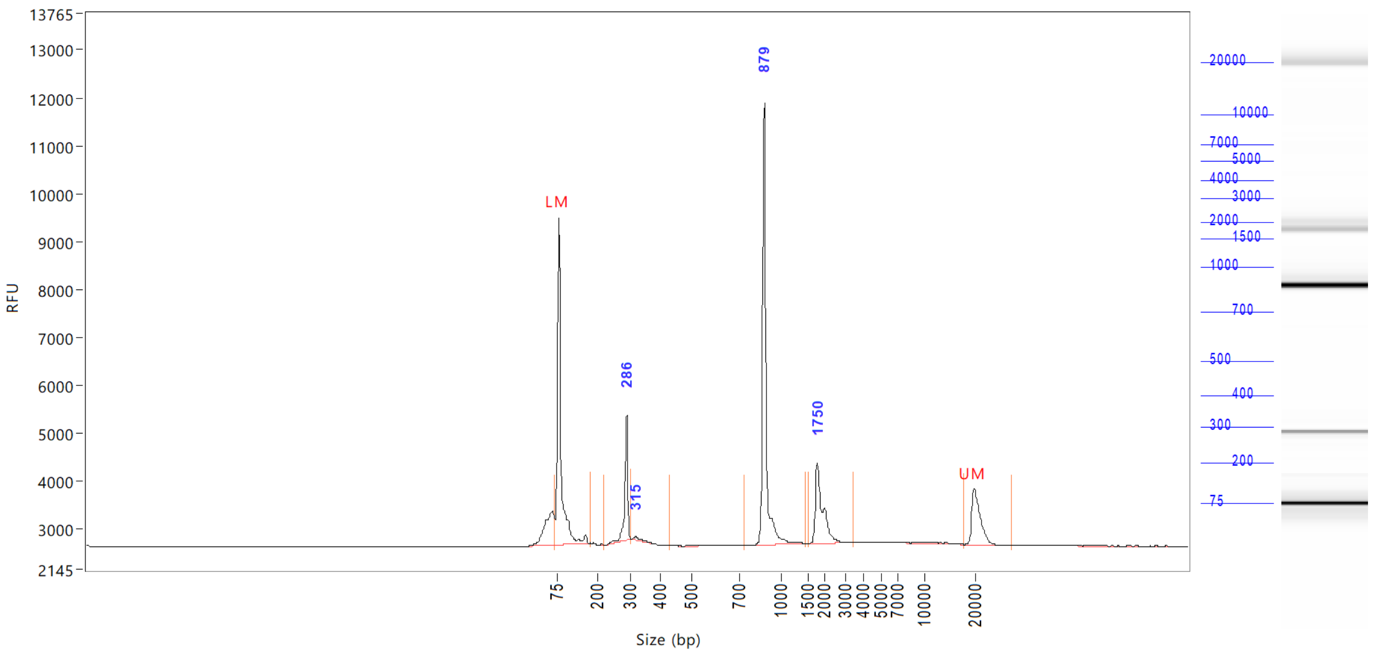


| Peak | Size (bp) | Concentration (ng/uL) | From (bp) | To (bp) | Average size (bp) | CV% | RFU | Corrected peak area |
|---|---|---|---|---|---|---|---|---|
| 1 | 75 (LM) | 0.5000 | 64 | 179 | 81 | 21.91 | 6837 | 57.913 |
| 2 | 286 | 1.6765 | 217 | 298 | 280 | 3.26 | 2611 | 16.181 |
| 3 | 315 | 0.1058 | 298 | 426 | 327 | 6.05 | 68 | 1.021 |
| 4 | 879 | 5.9069 | 736 | 1445 | 885 | 4.76 | 9256 | 57.014 |
| 5 | 1750 | 1.9604 | 1501 | 3429 | 1891 | 12.11 | 1684 | 18.922 |
| 6 | 20000 (UM) | 0.1119 | 18049 | 27496 | 20557 | 4.98 | 1191 | 12.957 |

| | | |
|---|---|---|
| TIC: | 9.6496 | ng/uL |
| TIM: | 23.0722 | nmole/L |
| Total concentration: | 9.9189 | ng/uL |

Sample peak width (sec): 5 Sample min peak height: 50 Sample baseline V to V?: Y Sample baseline V to V points: 3
Sample filter: Binomial Number of points for filter: 3 Sample start region (min): 0 Sample end region (min): 30
Marker peak width (sec): 5 Marker min peak height: 200 Marker baseline V to V?: Y Marker baseline V to V points: 3
Lower marker selection: First peak > 500 RFU Upper marker selection: Last peak > 200 RFU
Ladder size (bp)75, 200, 300, 400, 500, 700, 1000, 1500, 2000, 3000, 4000, 5000, 7000, 10000, 20000
Quantification using: Lower Marker Final concentration (ng/uL): 0.5000 Dilution factor: 12.0

**Sample:** SampG2
**Well location:** G2
**Created:** Tuesday, September 14, 2021 6:54:01 PM

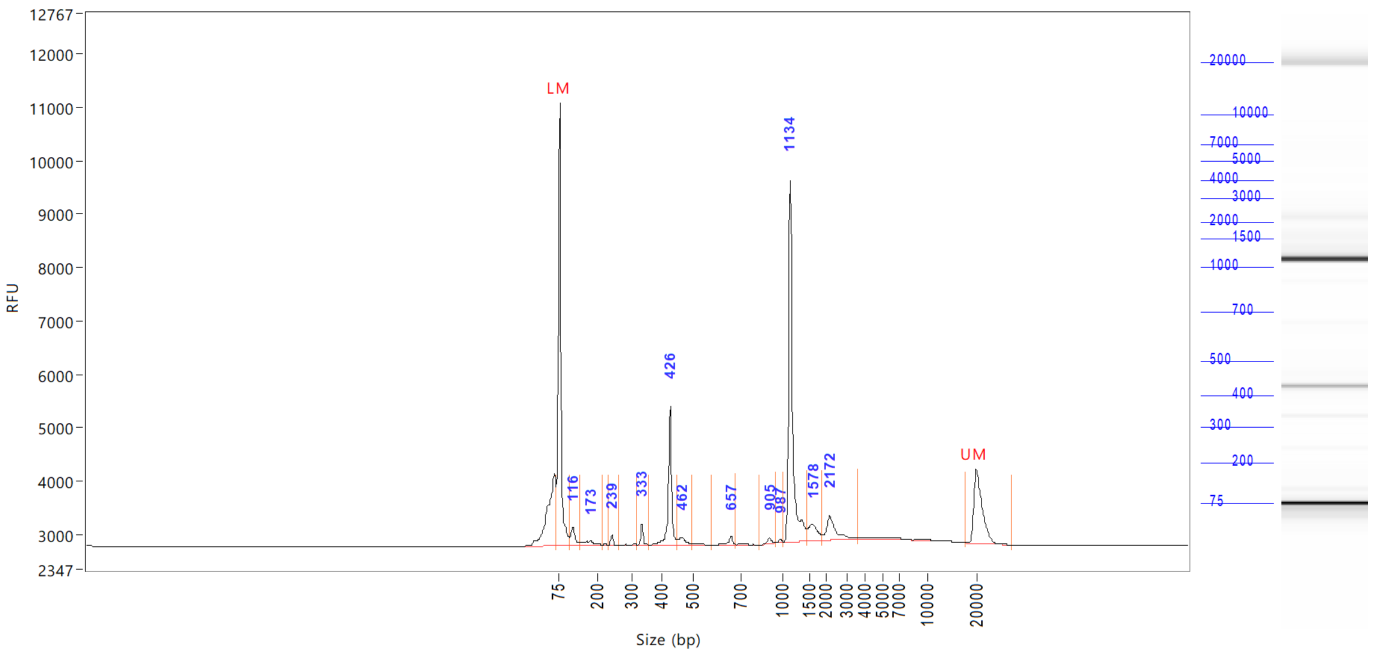


| Peak | Size (bp) | Concentration (ng/uL) | From (bp) | To (bp) | Average size (bp) | CV% | RFU | Corrected peak area |
|---|---|---|---|---|---|---|---|---|
| 1 | 75 (LM) | 0.5000 | 66 | 107 | 75 | 8.78 | 8313 | 55.272 |
| 2 | 116 | 0.3851 | 107 | 141 | 118 | 6.97 | 345 | 3.547 |
| 3 | 173 | 0.2064 | 141 | 210 | 165 | 9.15 | 80 | 1.902 |
| 4 | 239 | 0.1073 | 228 | 262 | 237 | 1.89 | 182 | 0.988 |
| 5 | 333 | 0.2231 | 315 | 356 | 330 | 1.59 | 399 | 2.056 |
| 6 | 426 | 1.9047 | 356 | 447 | 421 | 2.61 | 2612 | 17.546 |
| 7 | 462 | 0.2469 | 447 | 498 | 465 | 2.76 | 150 | 2.274 |
| 8 | 657 | 0.1310 | 578 | 682 | 646 | 2.52 | 150 | 1.207 |
| 9 | 905 | 0.0836 | 840 | 952 | 900 | 1.63 | 124 | 0.770 |
| 10 | 987 | 0.0314 | 952 | 1012 | 982 | 1.11 | 59 | 0.289 |
| 11 | 1134 | 4.9438 | 1012 | 1445 | 1159 | 6.50 | 6781 | 45.542 |
| 12 | 1578 | 0.5325 | 1445 | 1885 | 1599 | 7.49 | 300 | 4.905 |
| 13 | 2172 | 0.7017 | 1885 | 3572 | 2314 | 14.37 | 469 | 6.464 |
| 14 | 20000 (UM) | 0.1372 | 17805 | 27254 | 20560 | 4.86 | 1401 | 15.166 |

| | | |
|---|---|---|
| TIC: | 9.4975 | ng/uL |
| TIM: | 26.1994 | nmole/L |
| Total concentration: | 9.7729 | ng/uL |

```
Sample peak width (sec): 5    Sample min peak height: 50    Sample baseline V to V?: Y    Sample baseline V to V points: 3
Sample filter: Binomial       Number of points for filter: 3  Sample start region (min): 0  Sample end region (min): 30
Marker peak width (sec): 5    Marker min peak height: 200   Marker baseline V to V?: Y    Marker baseline V to V points: 3
Lower marker selection: First peak > 500 RFU                Upper marker selection: Last peak > 200 RFU
Ladder size (bp)75, 200, 300, 400, 500, 700, 1000, 1500, 2000, 3000, 4000, 5000, 7000, 10000, 20000
Quantification using: Lower Marker          Final concentration (ng/uL): 0.5000          Dilution factor: 12.0
```

**Sample:** SampH2
**Well location:** H2
**Created:** Tuesday, September 14, 2021 6:54:01 PM

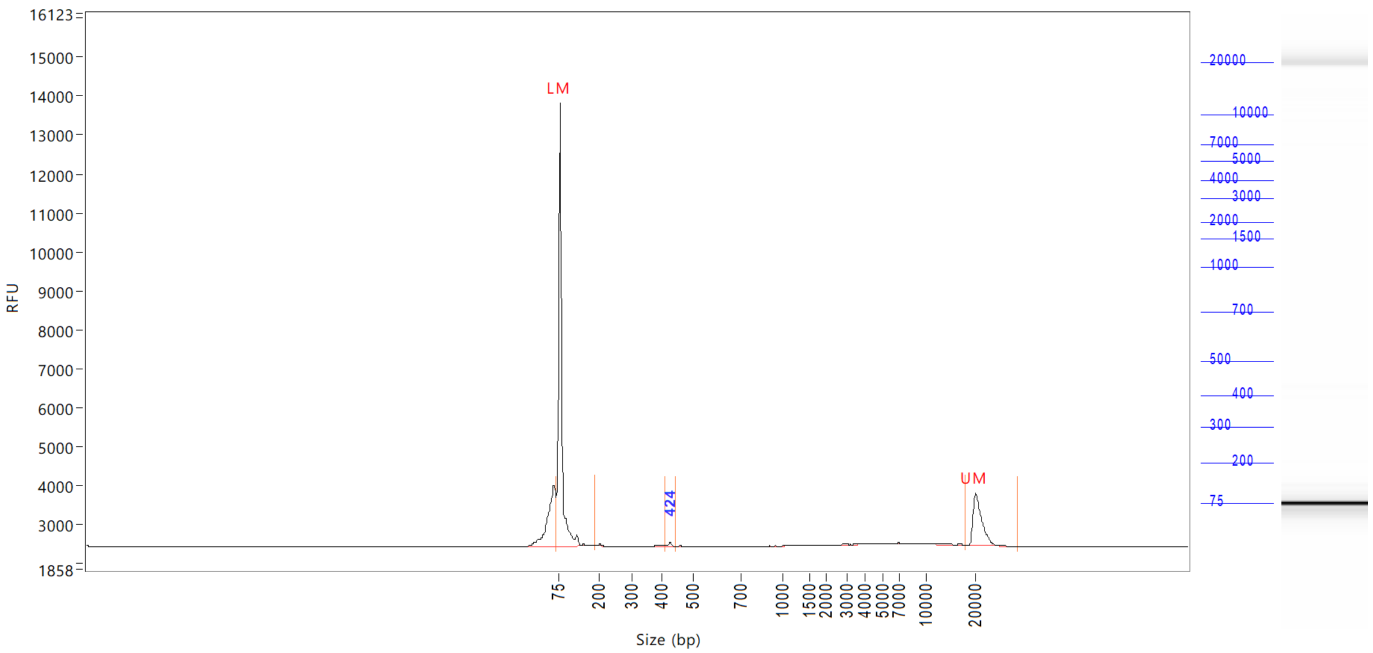


| Peak | Size (bp) | Concentration (ng/uL) | From (bp) | To (bp) | Average size (bp) | CV% | RFU | Corrected peak area |
|---|---|---|---|---|---|---|---|---|
| 1 | 75 (LM) | 0.5000 | 64 | 187 | 77 | 16.99 | 11394 | 89.114 |
| 2 | 424 | 0.0405 | 411 | 443 | 421 | 1.26 | 96 | 0.601 |
| 3 | 20000 (UM) | 0.0862 | 17805 | 28705 | 20610 | 5.17 | 1367 | 15.370 |

| | | |
|---|---|---|
| TIC: | 0.0405 | ng/uL |
| TIM: | 0.1581 | nmole/L |
| Total concentration: | 0.3202 | ng/uL |

Sample peak width (sec): 5 Sample min peak height: 50 Sample baseline V to V?: Y Sample baseline V to V points: 3
Sample filter: Binomial Number of points for filter: 3 Sample start region (min): 0 Sample end region (min): 30
Marker peak width (sec): 5 Marker min peak height: 200 Marker baseline V to V?: Y Marker baseline V to V points: 3
Lower marker selection: First peak > 500 RFU Upper marker selection: Last peak > 200 RFU
Ladder size (bp)75, 200, 300, 400, 500, 700, 1000, 1500, 2000, 3000, 4000, 5000, 7000, 10000, 20000
Quantification using: Lower Marker Final concentration (ng/uL): 0.5000 Dilution factor: 12.0

**Sample:** SampA3
**Well location:** A3
**Created:** Tuesday, September 14, 2021 6:54:01 PM

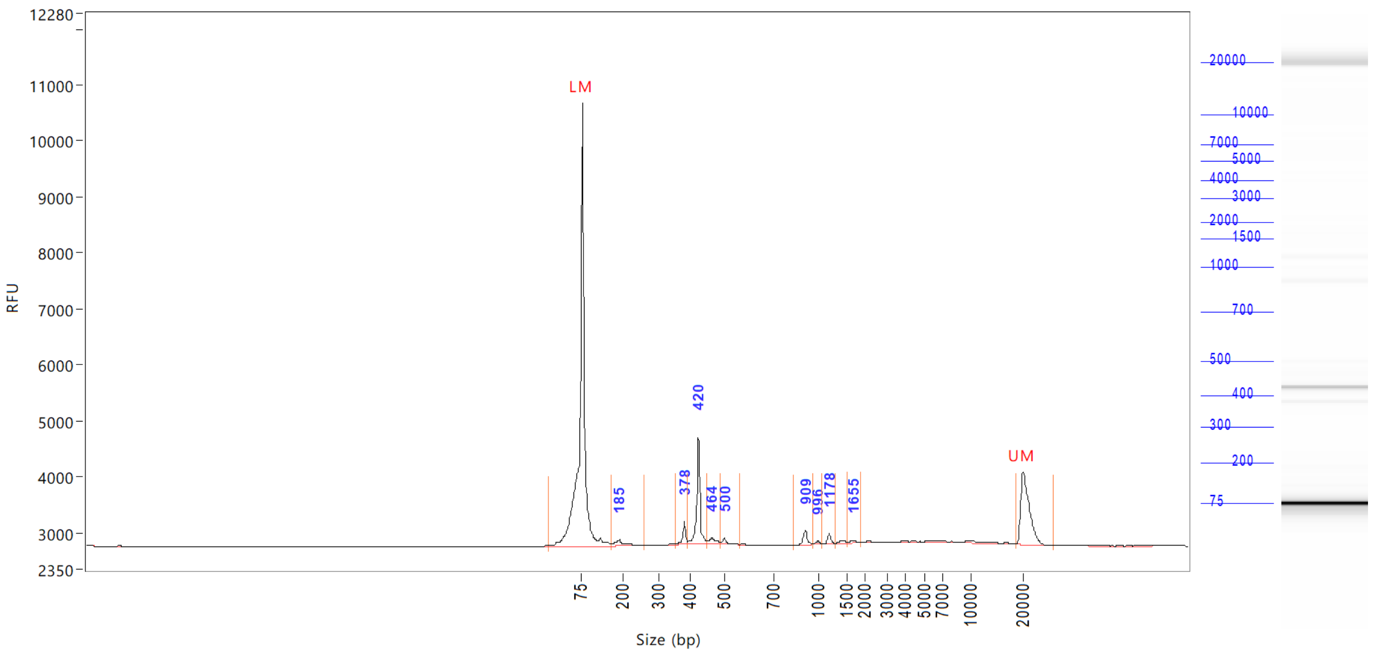


| Peak | Size (bp) | Concentration (ng/uL) | From (bp) | To (bp) | Average size (bp) | CV% | RFU | Corrected peak area |
|---|---|---|---|---|---|---|---|---|
| 1 | 75 (LM) | 0.5000 | 0 | 162 | 68 | 30.83 | 7932 | 87.825 |
| 2 | 185 | 0.1552 | 162 | 259 | 192 | 11.29 | 107 | 2.272 |
| 3 | 378 | 0.1613 | 354 | 390 | 375 | 1.87 | 401 | 2.361 |
| 4 | 420 | 0.8475 | 390 | 449 | 419 | 2.04 | 1909 | 12.405 |
| 5 | 464 | 0.1021 | 449 | 489 | 466 | 2.33 | 102 | 1.495 |
| 6 | 500 | 0.0614 | 489 | 562 | 503 | 2.74 | 119 | 0.898 |
| 7 | 909 | 0.1161 | 831 | 961 | 906 | 2.02 | 255 | 1.699 |
| 8 | 996 | 0.0243 | 961 | 1056 | 988 | 1.42 | 54 | 0.355 |
| 9 | 1178 | 0.0678 | 1056 | 1289 | 1169 | 3.06 | 179 | 0.992 |
| 10 | 1655 | 0.0322 | 1501 | 1923 | 1672 | 6.22 | 52 | 0.471 |
| 11 | 20000 (UM) | 0.0788 | 18537 | 25924 | 20497 | 4.53 | 1309 | 13.833 |

| | | |
|---|---|---|
| TIC: | 1.5678 | ng/uL |
| TIM: | 6.3045 | nmole/L |
| Total concentration: | 1.8359 | ng/uL |

```
Sample peak width (sec): 5     Sample min peak height: 50    Sample baseline V to V?: Y    Sample baseline V to V points: 3
Sample filter: Binomial        Number of points for filter: 3  Sample start region (min): 0  Sample end region (min): 30
Marker peak width (sec): 5     Marker min peak height: 200   Marker baseline V to V?: Y    Marker baseline V to V points: 3
Lower marker selection: First peak > 500 RFU                 Upper marker selection: Last peak > 200 RFU
Ladder size (bp)75, 200, 300, 400, 500, 700, 1000, 1500, 2000, 3000, 4000, 5000, 7000, 10000, 20000
Quantification using: Lower Marker          Final concentration (ng/uL): 0.5000         Dilution factor: 12.0
```

**Sample:** SampB3
**Well location:** B3
**Created:** Tuesday, September 14, 2021 6:54:01 PM

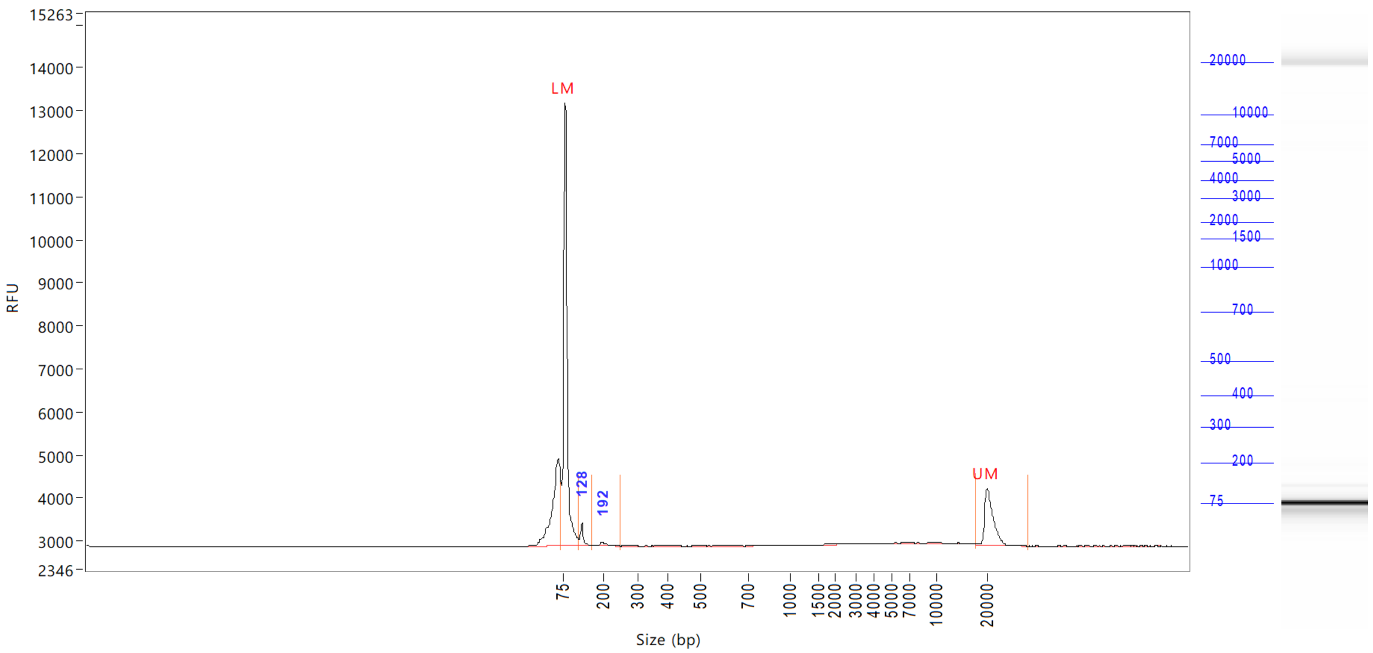


| Peak | Size<br>(bp) | Concentration<br>(ng/uL) | From<br>(bp) | To<br>(bp) | Average size<br>(bp) | CV% | RFU | Corrected peak area |
|---|---|---|---|---|---|---|---|---|
| 1 | 75 (LM) | 0.5000 | 64 | 118 | 76 | 10.89 | 10306 | 91.971 |
| 2 | 128 | 0.2534 | 118 | 162 | 128 | 6.74 | 517 | 3.884 |
| 3 | 192 | 0.0833 | 162 | 248 | 191 | 7.59 | 82 | 1.276 |
| 4 | 20000 (UM) | 0.0754 | 17683 | 28100 | 20531 | 4.77 | 1308 | 13.871 |

| | | |
|---|---|---|
| TIC: | 0.3367 | ng/uL |
| TIM: | 3.9694 | nmole/L |
| Total concentration: | 0.5632 | ng/uL |

Sample peak width (sec): 5    Sample min peak height: 50    Sample baseline V to V?: Y    Sample baseline V to V points: 3
Sample filter: Binomial    Number of points for filter: 3    Sample start region (min): 0    Sample end region (min): 30
Marker peak width (sec): 5    Marker min peak height: 200    Marker baseline V to V?: Y    Marker baseline V to V points: 3
Lower marker selection: First peak > 500 RFU    Upper marker selection: Last peak > 200 RFU
Ladder size (bp)75, 200, 300, 400, 500, 700, 1000, 1500, 2000, 3000, 4000, 5000, 7000, 10000, 20000
Quantification using: Lower Marker    Final concentration (ng/uL): 0.5000    Dilution factor: 12.0

**Sample:** SampC3
**Well location:** C3
**Created:** Tuesday, September 14, 2021 6:54:01 PM

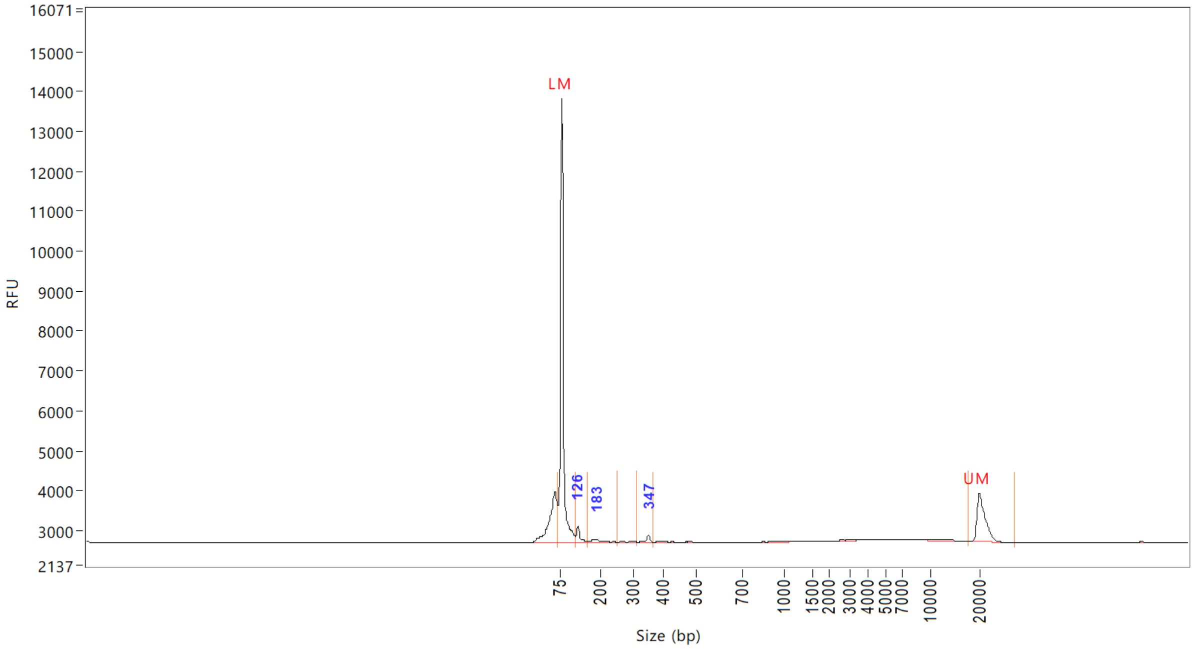


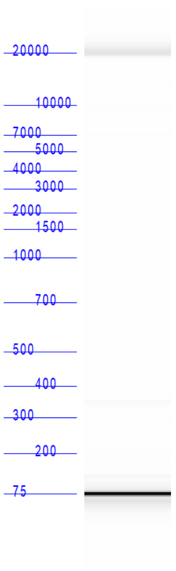


| Peak | Size (bp) | Concentration (ng/uL) | From (bp) | To (bp) | Average size (bp) | CV% | RFU | Corrected peak area |
|---|---|---|---|---|---|---|---|---|
| 1 | 75 (LM) | 0.5000 | 62 | 118 | 75 | 11.47 | 11135 | 82.409 |
| 2 | 126 | 0.2484 | 118 | 156 | 128 | 7.10 | 396 | 3.411 |
| 3 | 183 | 0.1221 | 156 | 248 | 183 | 9.97 | 77 | 1.677 |
| 4 | 347 | 0.0876 | 307 | 362 | 341 | 2.48 | 184 | 1.203 |
| 5 | 20000 (UM) | 0.0782 | 17927 | 27133 | 20569 | 4.78 | 1203 | 12.882 |

| | | |
|---|---|---|
| TIC: | 0.4581 | ng/uL |
| TIM: | 4.7079 | nmole/L |
| Total concentration: | 0.7395 | ng/uL |

Sample peak width (sec): 5    Sample min peak height: 50    Sample baseline V to V?: Y    Sample baseline V to V points: 3
Sample filter: Binomial    Number of points for filter: 3    Sample start region (min): 0    Sample end region (min): 30
Marker peak width (sec): 5    Marker min peak height: 200    Marker baseline V to V?: Y    Marker baseline V to V points: 3
Lower marker selection: First peak > 500 RFU    Upper marker selection: Last peak > 200 RFU
Ladder size (bp)75, 200, 300, 400, 500, 700, 1000, 1500, 2000, 3000, 4000, 5000, 7000, 10000, 20000
Quantification using: Lower Marker    Final concentration (ng/uL): 0.5000    Dilution factor: 12.0

**Sample:** SampD3
**Well location:** D3
**Created:** Tuesday, September 14, 2021 6:54:01 PM

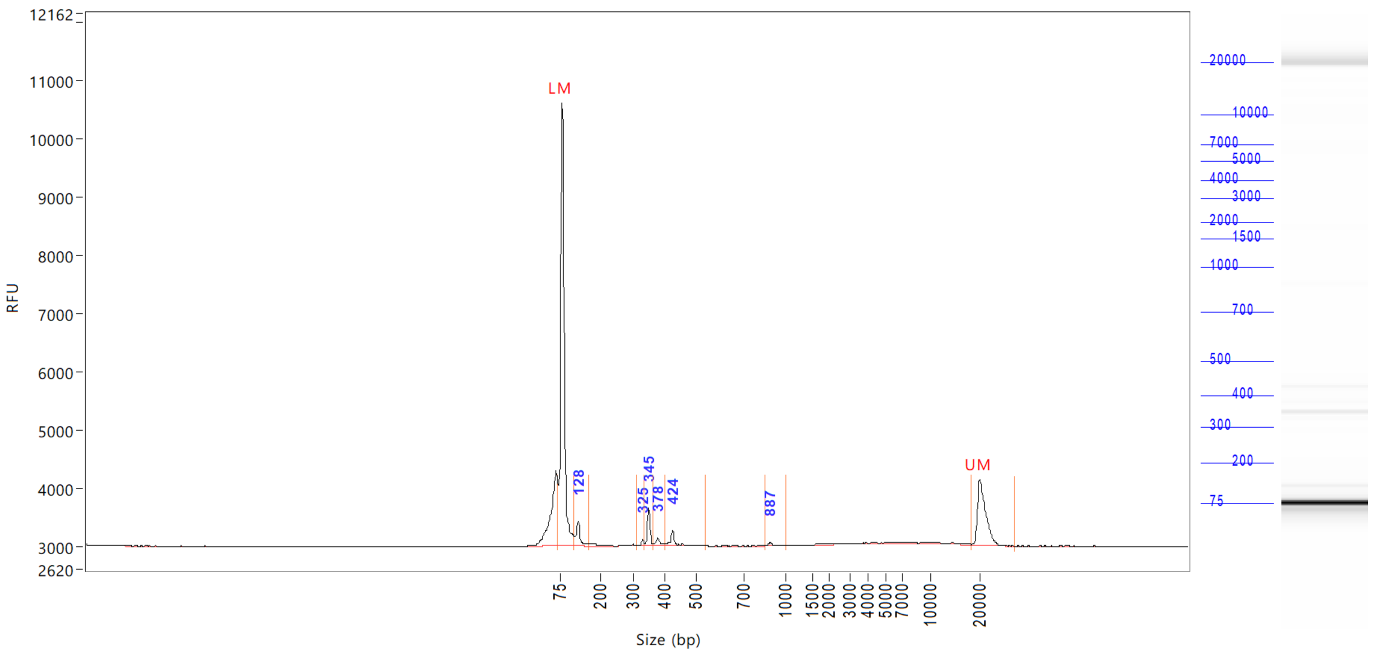


| Peak | Size (bp) | Concentration (ng/uL) | From (bp) | To (bp) | Average size (bp) | CV% | RFU | Corrected peak area |
|---|---|---|---|---|---|---|---|---|
| 1 | 75 (LM) | 0.5000 | 64 | 114 | 76 | 10.39 | 7616 | 68.872 |
| 2 | 128 | 0.3510 | 114 | 160 | 128 | 7.90 | 420 | 4.029 |
| 3 | 325 | 0.0515 | 309 | 333 | 324 | 1.47 | 106 | 0.592 |
| 4 | 345 | 0.2878 | 333 | 360 | 344 | 1.34 | 635 | 3.303 |
| 5 | 378 | 0.1087 | 360 | 401 | 379 | 2.86 | 129 | 1.248 |
| 6 | 424 | 0.2048 | 401 | 539 | 429 | 5.04 | 268 | 2.350 |
| 7 | 887 | 0.0376 | 853 | 1012 | 896 | 3.27 | 56 | 0.431 |
| 8 | 20000 (UM) | 0.0883 | 18415 | 27133 | 20558 | 4.84 | 1131 | 12.164 |

| | | |
|---|---|---|
| TIC: | 1.0414 | ng/uL |
| TIM: | 7.4701 | nmole/L |
| Total concentration: | 1.3820 | ng/uL |

Sample peak width (sec): 5    Sample min peak height: 50    Sample baseline V to V?: Y    Sample baseline V to V points: 3
Sample filter: Binomial    Number of points for filter: 3  Sample start region (min): 0  Sample end region (min): 30
Marker peak width (sec): 5    Marker min peak height: 200    Marker baseline V to V?: Y    Marker baseline V to V points: 3
Lower marker selection: First peak > 500 RFU    Upper marker selection: Last peak > 200 RFU
Ladder size (bp)75, 200, 300, 400, 500, 700, 1000, 1500, 2000, 3000, 4000, 5000, 7000, 10000, 20000
Quantification using: Lower Marker    Final concentration (ng/uL): 0.5000    Dilution factor: 12.0

**Sample:** SampE3
**Well location:** E3
**Created:** Tuesday, September 14, 2021 6:54:01 PM

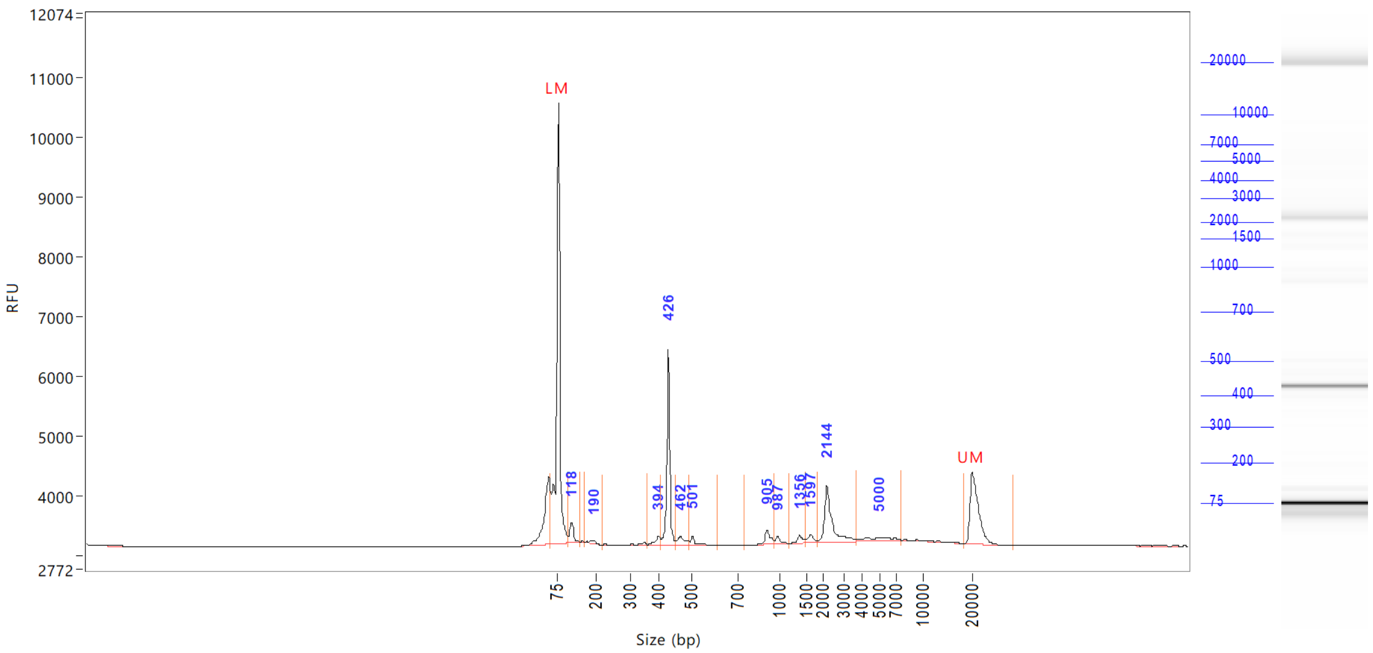


| Peak | Size (bp) | Concentration (ng/uL) | From (bp) | To (bp) | Average size (bp) | CV% | RFU | Corrected peak area |
|---|---|---|---|---|---|---|---|---|
| 1 | 75 (LM) | 0.5000 | 52 | 107 | 72 | 11.60 | 7385 | 57.832 |
| 2 | 118 | 0.3362 | 107 | 145 | 117 | 5.44 | 336 | 3.241 |
| 3 | 190 | 0.0830 | 158 | 214 | 182 | 5.90 | 57 | 0.800 |
| 4 | 394 | 0.1628 | 360 | 403 | 390 | 2.54 | 142 | 1.569 |
| 5 | 426 | 2.0268 | 403 | 449 | 424 | 1.52 | 3278 | 19.536 |
| 6 | 462 | 0.2205 | 449 | 494 | 469 | 2.71 | 139 | 2.125 |
| 7 | 501 | 0.1346 | 494 | 608 | 515 | 4.87 | 156 | 1.298 |
| 8 | 905 | 0.2179 | 740 | 957 | 907 | 3.06 | 240 | 2.100 |
| 9 | 987 | 0.1052 | 957 | 1178 | 993 | 3.45 | 132 | 1.014 |
| 10 | 1356 | 0.1249 | 1178 | 1467 | 1369 | 3.96 | 134 | 1.204 |
| 11 | 1597 | 0.1411 | 1467 | 1808 | 1596 | 5.56 | 143 | 1.360 |
| 12 | 2144 | 1.1560 | 1808 | 3679 | 2334 | 15.75 | 958 | 11.142 |
| 13 | 5000 | 0.2570 | 3679 | 7534 | 5110 | 19.74 | 57 | 2.477 |
| 14 | 20000 (UM) | 0.1133 | 18293 | 28342 | 20595 | 5.18 | 1196 | 13.102 |

| | | |
|---|---|---|
| TIC: | 4.9661 | ng/uL |
| TIM: | 16.9906 | nmole/L |
| Total concentration: | 5.0912 | ng/uL |

Sample peak width (sec): 5 Sample min peak height: 50 Sample baseline V to V?: Y Sample baseline V to V points: 3
Sample filter: Binomial Number of points for filter: 3 Sample start region (min): 0 Sample end region (min): 30
Marker peak width (sec): 5 Marker min peak height: 200 Marker baseline V to V?: Y Marker baseline V to V points: 3
Lower marker selection: First peak > 500 RFU Upper marker selection: Last peak > 200 RFU
Ladder size (bp)75, 200, 300, 400, 500, 700, 1000, 1500, 2000, 3000, 4000, 5000, 7000, 10000, 20000
Quantification using: Lower Marker Final concentration (ng/uL): 0.5000 Dilution factor: 12.0

**Sample:** SampF3
**Well location:** F3
**Created:** Tuesday, September 14, 2021 6:54:01 PM

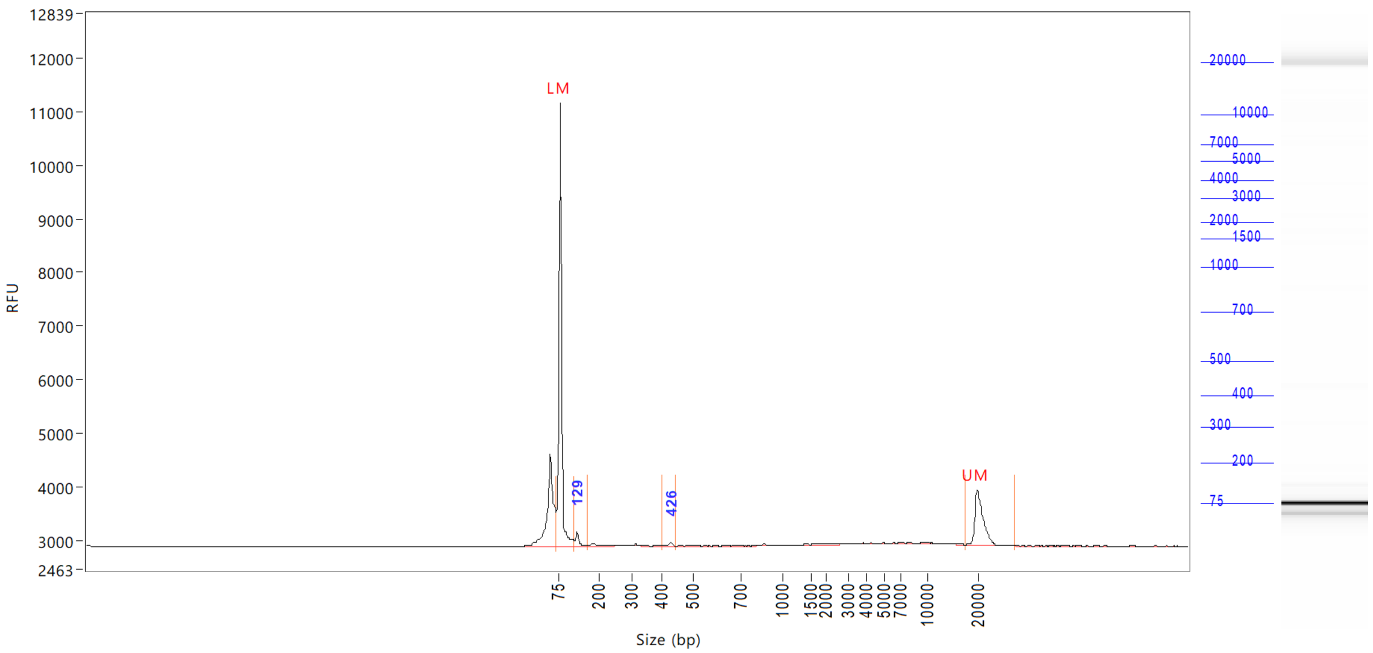


| Peak | Size | Concentration | From | To | Average size | CV% | RFU | Corrected peak area |
|---|---|---|---|---|---|---|---|---|
| | (bp) | (ng/uL) | (bp) | (bp) | (bp) | | | |
| 1 | 75 (LM) | 0.5000 | 64 | 120 | 75 | 10.93 | 8290 | 56.277 |
| 2 | 129 | 0.2515 | 120 | 164 | 131 | 7.27 | 263 | 2.358 |
| 3 | 426 | 0.0599 | 400 | 441 | 420 | 1.84 | 78 | 0.562 |
| 4 | 20000 (UM) | 0.0994 | 17561 | 27375 | 20526 | 4.99 | 1031 | 11.183 |

| | | |
|---|---|---|
| TIC: | 0.3113 | ng/uL |
| TIM: | 3.3885 | nmole/L |
| Total concentration: | 0.8438 | ng/uL |

```
Sample peak width (sec): 5    Sample min peak height: 50    Sample baseline V to V?: Y    Sample baseline V to V points: 3
Sample filter: Binomial       Number of points for filter: 3  Sample start region (min): 0  Sample end region (min): 30
Marker peak width (sec): 5    Marker min peak height: 200   Marker baseline V to V?: Y    Marker baseline V to V points: 3
Lower marker selection: First peak > 500 RFU                Upper marker selection: Last peak > 200 RFU
Ladder size (bp)75, 200, 300, 400, 500, 700, 1000, 1500, 2000, 3000, 4000, 5000, 7000, 10000, 20000
Quantification using: Lower Marker          Final concentration (ng/uL): 0.5000          Dilution factor: 12.0
```

**Sample:** SampG3
**Well location:** G3
**Created:** Tuesday, September 14, 2021 6:54:01 PM

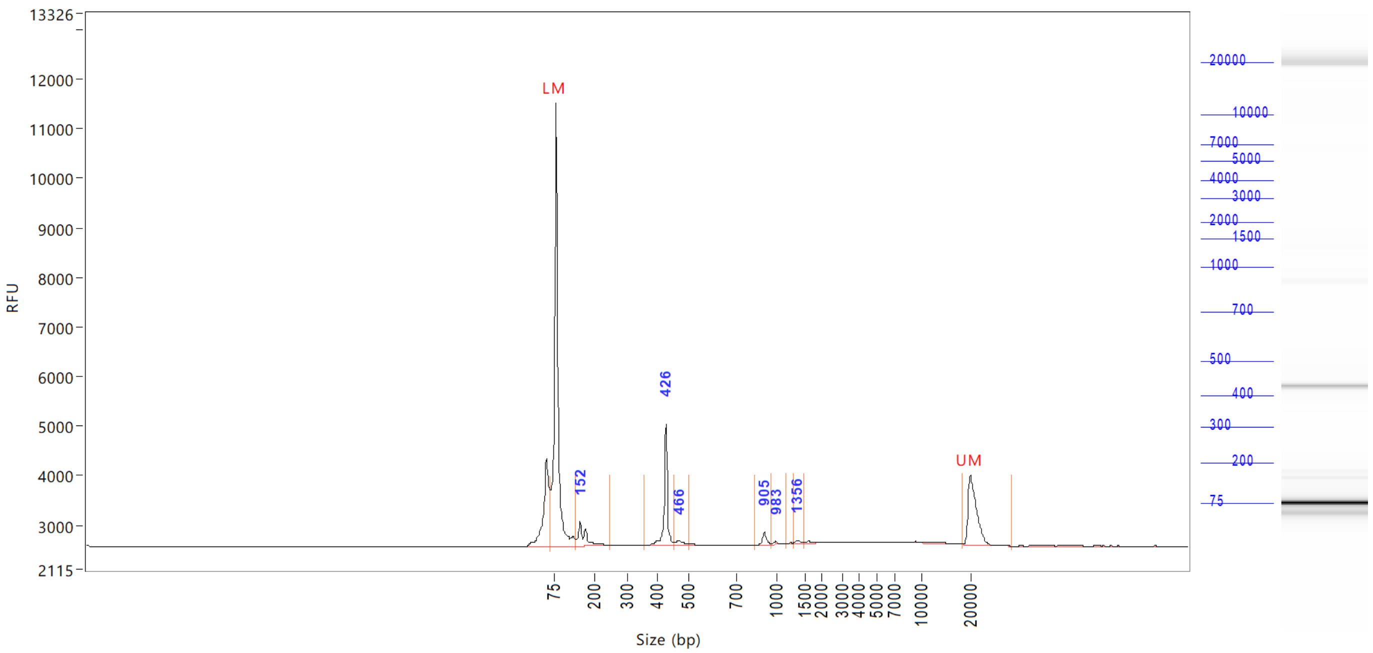


| Peak | Size (bp) | Concentration (ng/uL) | From (bp) | To (bp) | Average size (bp) | CV% | RFU | Corrected peak area |
|---|---|---|---|---|---|---|---|---|
| 1 | 75 (LM) | 0.5000 | 60 | 139 | 77 | 16.42 | 8956 | 79.219 |
| 2 | 152 | 0.5869 | 139 | 248 | 165 | 12.61 | 494 | 7.749 |
| 3 | 426 | 1.2412 | 354 | 452 | 421 | 2.70 | 2459 | 16.387 |
| 4 | 466 | 0.1281 | 452 | 500 | 471 | 2.67 | 105 | 1.691 |
| 5 | 905 | 0.1355 | 835 | 957 | 903 | 2.13 | 258 | 1.789 |
| 6 | 983 | 0.0383 | 957 | 1145 | 990 | 3.21 | 64 | 0.506 |
| 7 | 1356 | 0.0533 | 1278 | 1500 | 1376 | 4.26 | 71 | 0.703 |
| 8 | 20000 (UM) | 0.0980 | 18415 | 28342 | 20549 | 5.02 | 1405 | 15.522 |

| | | |
|---|---|---|
| TIC: | 2.1832 | ng/uL |
| TIM: | 11.5195 | nmole/L |
| Total concentration: | 2.4609 | ng/uL |

Sample peak width (sec): 5    Sample min peak height: 50    Sample baseline V to V?: Y    Sample baseline V to V points: 3
Sample filter: Binomial    Number of points for filter: 3  Sample start region (min): 0  Sample end region (min): 30
Marker peak width (sec): 5    Marker min peak height: 200    Marker baseline V to V?: Y    Marker baseline V to V points: 3
Lower marker selection: First peak > 500 RFU    Upper marker selection: Last peak > 200 RFU
Ladder size (bp)75, 200, 300, 400, 500, 700, 1000, 1500, 2000, 3000, 4000, 5000, 7000, 10000, 20000
Quantification using: Lower Marker    Final concentration (ng/uL): 0.5000    Dilution factor: 12.0

**Sample:** SampH3
**Well location:** H3
**Created:** Tuesday, September 14, 2021 6:54:01 PM

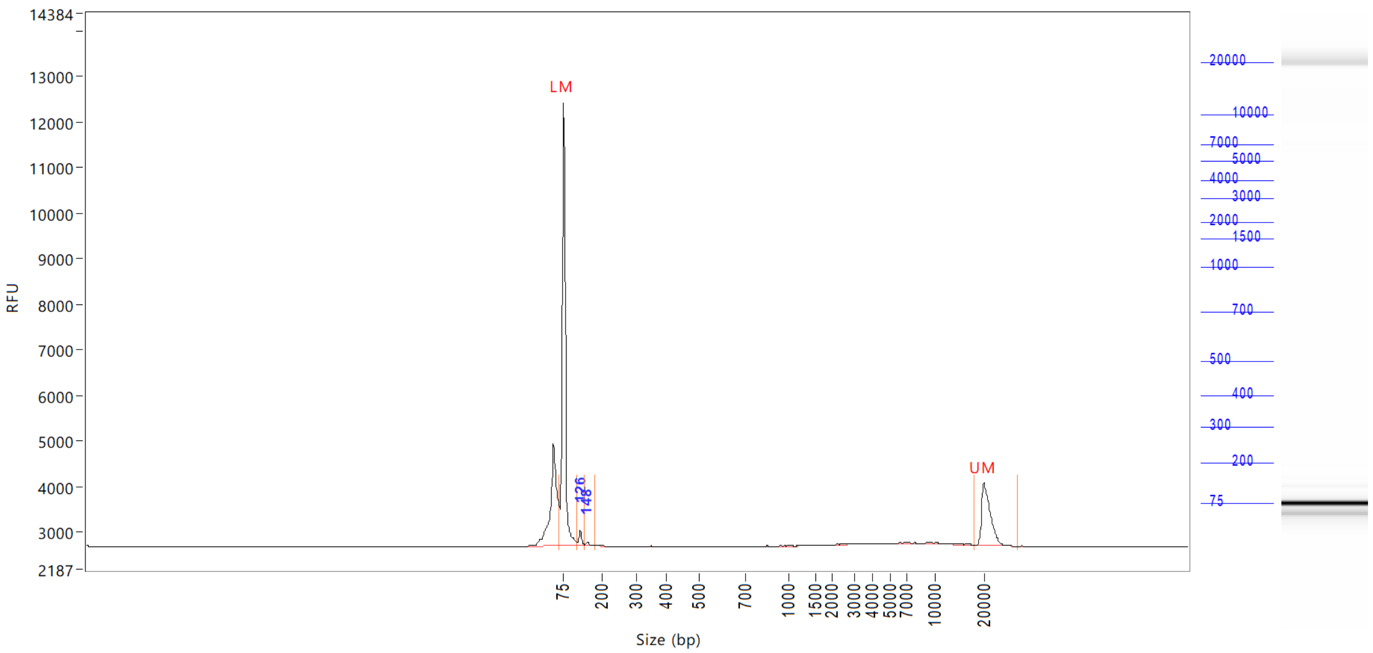


| Peak | Size (bp) | Concentration (ng/uL) | From (bp) | To (bp) | Average size (bp) | CV% | RFU | Corrected peak area |
|---|---|---|---|---|---|---|---|---|
| 1 | 75 (LM) | 0.5000 | 64 | 116 | 74 | 9.18 | 9731 | 78.636 |
| 2 | 126 | 0.1802 | 116 | 141 | 125 | 4.17 | 354 | 2.362 |
| 3 | 148 | 0.0495 | 141 | 175 | 149 | 3.80 | 91 | 0.648 |
| 4 | 20000 (UM) | 0.0968 | 18049 | 26770 | 20543 | 4.61 | 1372 | 15.219 |

| | | |
|---|---|---|
| TIC: | 0.2297 | ng/uL |
| TIM: | 2.9141 | nmole/L |
| Total concentration: | 0.5403 | ng/uL |

Sample peak width (sec): 5 Sample min peak height: 50 Sample baseline V to V?: Y Sample baseline V to V points: 3
Sample filter: Binomial Number of points for filter: 3 Sample start region (min): 0 Sample end region (min): 30
Marker peak width (sec): 5 Marker min peak height: 200 Marker baseline V to V?: Y Marker baseline V to V points: 3
Lower marker selection: First peak > 500 RFU Upper marker selection: Last peak > 200 RFU
Ladder size (bp)75, 200, 300, 400, 500, 700, 1000, 1500, 2000, 3000, 4000, 5000, 7000, 10000, 20000
Quantification using: Lower Marker Final concentration (ng/uL): 0.5000 Dilution factor: 12.0

**Sample:** SampA4
**Well location:** A4
**Created:** Tuesday, September 14, 2021 6:54:01 PM

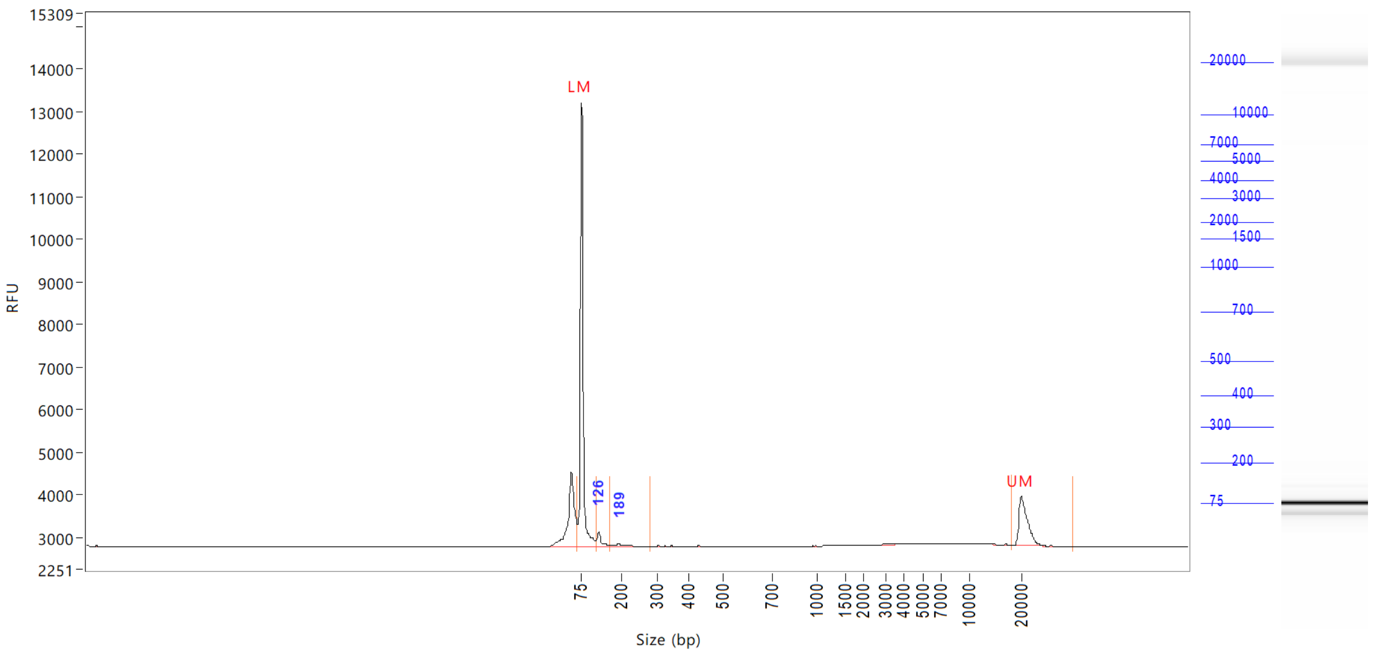


| Peak | Size (bp) | Concentration (ng/uL) | From (bp) | To (bp) | Average size (bp) | CV% | RFU | Corrected peak area |
|---|---|---|---|---|---|---|---|---|
| 1 | 75 (LM) | 0.5000 | 64 | 118 | 75 | 10.05 | 10431 | 70.139 |
| 2 | 126 | 0.2558 | 118 | 162 | 130 | 8.12 | 346 | 2.991 |
| 3 | 189 | 0.0964 | 162 | 280 | 194 | 12.50 | 55 | 1.126 |
| 4 | 20000 (UM) | 0.0878 | 18049 | 29914 | 20545 | 5.21 | 1160 | 12.319 |

| | | |
|---|---|---|
| TIC: | 0.3522 | ng/uL |
| TIM: | 4.0501 | nmole/L |
| Total concentration: | 0.5748 | ng/uL |

```
Sample peak width (sec): 5    Sample min peak height: 50    Sample baseline V to V?: Y    Sample baseline V to V points: 3
Sample filter: Binomial       Number of points for filter: 3  Sample start region (min): 0  Sample end region (min): 30
Marker peak width (sec): 5    Marker min peak height: 200   Marker baseline V to V?: Y    Marker baseline V to V points: 3
Lower marker selection: First peak > 500 RFU                Upper marker selection: Last peak > 200 RFU
Ladder size (bp)75, 200, 300, 400, 500, 700, 1000, 1500, 2000, 3000, 4000, 5000, 7000, 10000, 20000
Quantification using: Lower Marker          Final concentration (ng/uL): 0.5000          Dilution factor: 12.0
```

**Sample:** SampB4
**Well location:** B4
**Created:** Tuesday, September 14, 2021 6:54:01 PM

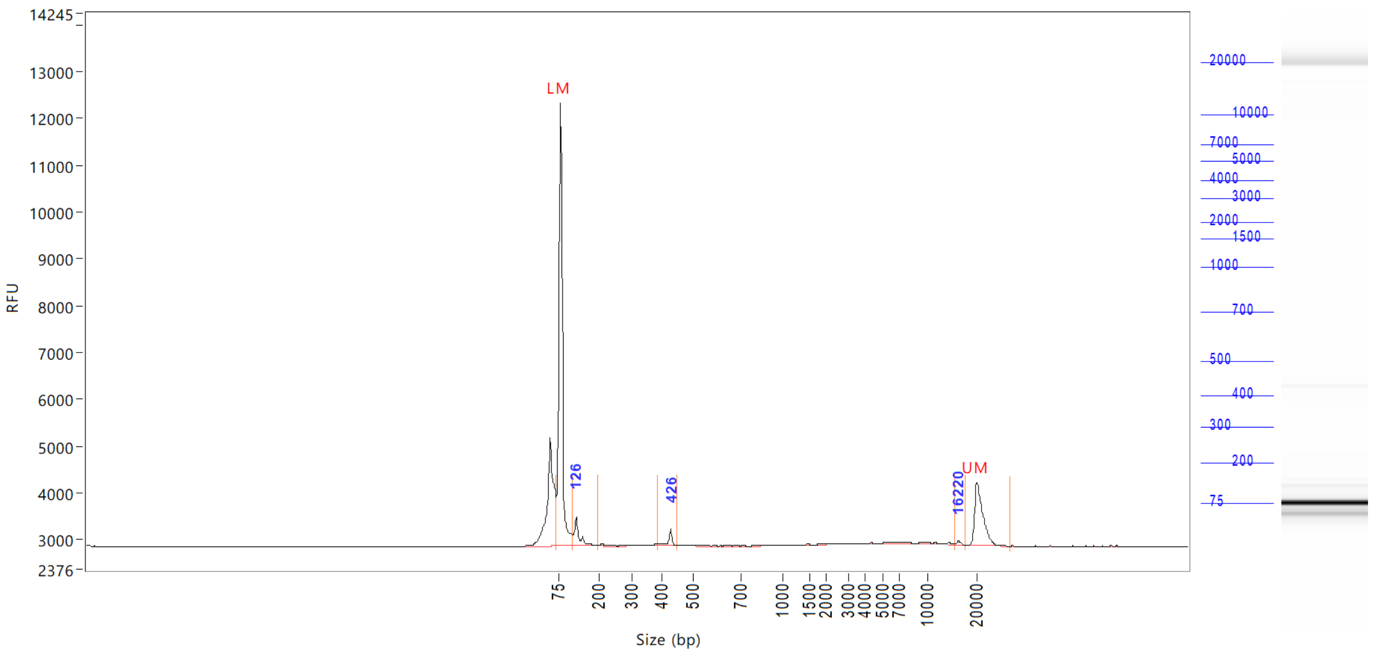


| Peak | Size (bp) | Concentration (ng/uL) | From (bp) | To (bp) | Average size (bp) | CV% | RFU | Corrected peak area |
|---|---|---|---|---|---|---|---|---|
| 1 | 75 (LM) | 0.5000 | 64 | 116 | 76 | 10.87 | 9473 | 86.211 |
| 2 | 126 | 0.5168 | 116 | 196 | 134 | 12.65 | 632 | 7.426 |
| 3 | 426 | 0.1903 | 386 | 445 | 418 | 2.90 | 356 | 2.735 |
| 4 | 16220 | 0.0275 | 15610 | 17805 | 16278 | 2.15 | 75 | 0.396 |
| 5 | 20000 (UM) | 0.0840 | 17805 | 26770 | 20598 | 4.84 | 1340 | 14.490 |

| | | |
|---|---|---|
| TIC: | 0.7347 | ng/uL |
| TIM: | 7.0896 | nmole/L |
| Total concentration: | 1.0603 | ng/uL |

```
Sample peak width (sec): 5    Sample min peak height: 50    Sample baseline V to V?: Y    Sample baseline V to V points: 3
Sample filter: Binomial       Number of points for filter: 3  Sample start region (min): 0  Sample end region (min): 30
Marker peak width (sec): 5    Marker min peak height: 200   Marker baseline V to V?: Y    Marker baseline V to V points: 3
Lower marker selection: First peak > 500 RFU                Upper marker selection: Last peak > 200 RFU
Ladder size (bp)75, 200, 300, 400, 500, 700, 1000, 1500, 2000, 3000, 4000, 5000, 7000, 10000, 20000
Quantification using: Lower Marker          Final concentration (ng/uL): 0.5000          Dilution factor: 12.0
```

**Sample:** SampC4
**Well location:** C4
**Created:** Tuesday, September 14, 2021 6:54:01 PM

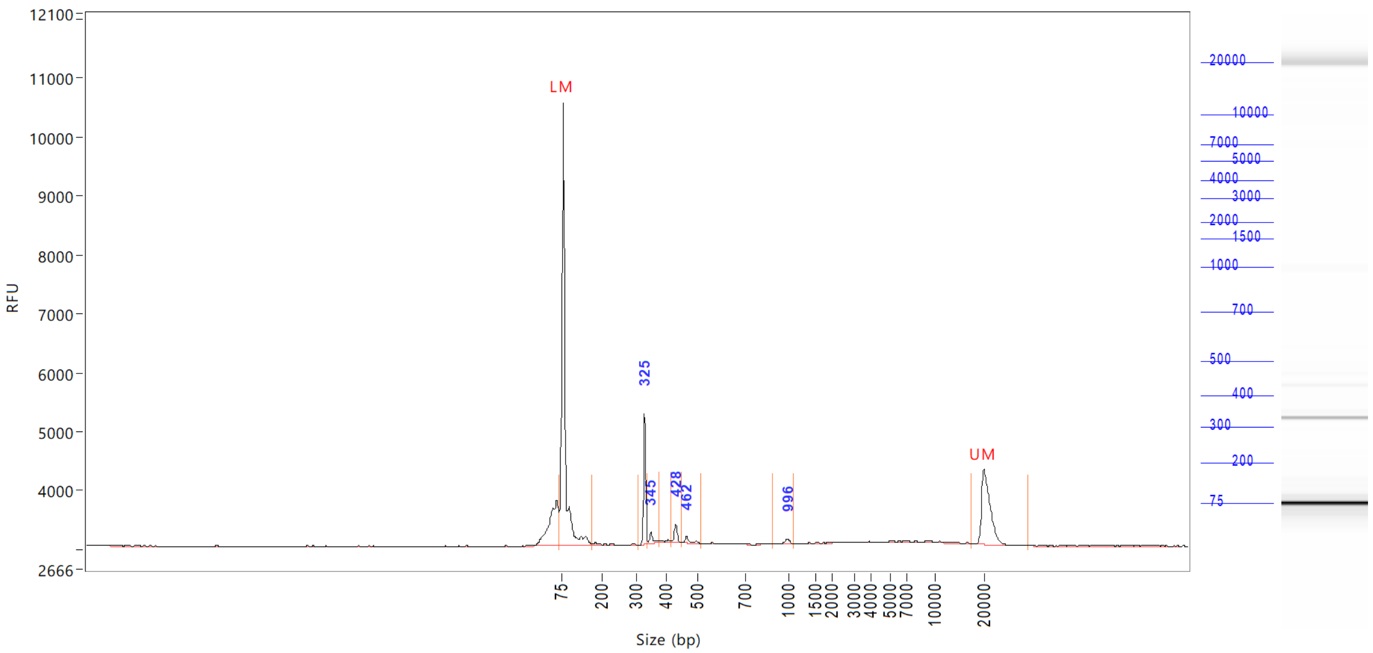


| Peak | Size (bp) | Concentration (ng/uL) | From (bp) | To (bp) | Average size (bp) | CV% | RFU | Corrected peak area |
|---|---|---|---|---|---|---|---|---|
| 1 | 75 (LM) | 0.5000 | 62 | 166 | 79 | 21.02 | 7525 | 61.504 |
| 2 | 325 | 1.0055 | 305 | 337 | 324 | 1.11 | 2217 | 10.307 |
| 3 | 345 | 0.1356 | 337 | 376 | 349 | 2.58 | 195 | 1.389 |
| 4 | 428 | 0.1669 | 416 | 447 | 426 | 1.11 | 315 | 1.710 |
| 5 | 462 | 0.1034 | 447 | 514 | 470 | 3.11 | 121 | 1.060 |
| 6 | 996 | 0.0771 | 896 | 1090 | 988 | 2.08 | 95 | 0.790 |
| 7 | 20000 (UM) | 0.1138 | 17561 | 28705 | 20518 | 4.74 | 1292 | 13.995 |

| | | |
|---|---|---|
| TIC: | 1.4884 | ng/uL |
| TIM: | 6.8792 | nmole/L |
| Total concentration: | 1.9523 | ng/uL |

Sample peak width (sec): 5 Sample min peak height: 50 Sample baseline V to V?: Y Sample baseline V to V points: 3
Sample filter: Binomial Number of points for filter: 3 Sample start region (min): 0 Sample end region (min): 30
Marker peak width (sec): 5 Marker min peak height: 200 Marker baseline V to V?: Y Marker baseline V to V points: 3
Lower marker selection: First peak > 500 RFU Upper marker selection: Last peak > 200 RFU
Ladder size (bp)75, 200, 300, 400, 500, 700, 1000, 1500, 2000, 3000, 4000, 5000, 7000, 10000, 20000
Quantification using: Lower Marker Final concentration (ng/uL): 0.5000 Dilution factor: 12.0

**Sample:** SampD4
**Well location:** D4
**Created:** Tuesday, September 14, 2021 6:54:01 PM

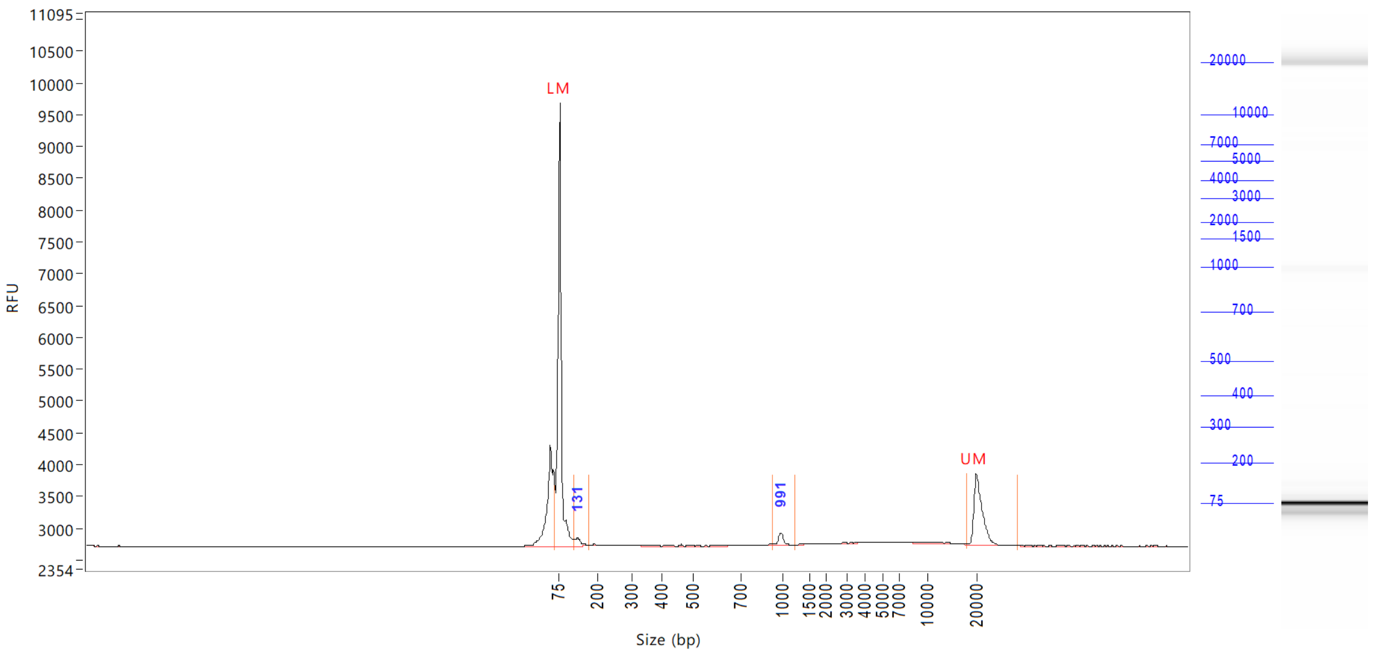


| Peak | Size (bp) | Concentration (ng/uL) | From (bp) | To (bp) | Average size (bp) | CV% | RFU | Corrected peak area |
|---|---|---|---|---|---|---|---|---|
| 1 | 75 (LM) | 0.5000 | 64 | 124 | 76 | 12.43 | 6980 | 55.400 |
| 2 | 131 | 0.1966 | 124 | 168 | 136 | 7.79 | 137 | 1.815 |
| 3 | 991 | 0.1727 | 926 | 1223 | 988 | 3.26 | 181 | 1.595 |
| 4 | 20000 (UM) | 0.1085 | 18049 | 28342 | 20598 | 5.01 | 1110 | 12.027 |

| | | |
|---|---|---|
| TIC: | 0.3693 | ng/uL |
| TIM: | 2.6630 | nmole/L |
| Total concentration: | 0.8355 | ng/uL |

Sample peak width (sec): 5    Sample min peak height: 50    Sample baseline V to V?: Y    Sample baseline V to V points: 3
Sample filter: Binomial    Number of points for filter: 3  Sample start region (min): 0  Sample end region (min): 30
Marker peak width (sec): 5    Marker min peak height: 200   Marker baseline V to V?: Y    Marker baseline V to V points: 3
Lower marker selection: First peak > 500 RFU    Upper marker selection: Last peak > 200 RFU
Ladder size (bp)75, 200, 300, 400, 500, 700, 1000, 1500, 2000, 3000, 4000, 5000, 7000, 10000, 20000
Quantification using: Lower Marker    Final concentration (ng/uL): 0.5000    Dilution factor: 12.0

**Sample:** SampE4
**Well location:** E4
**Created:** Tuesday, September 14, 2021 6:54:01 PM

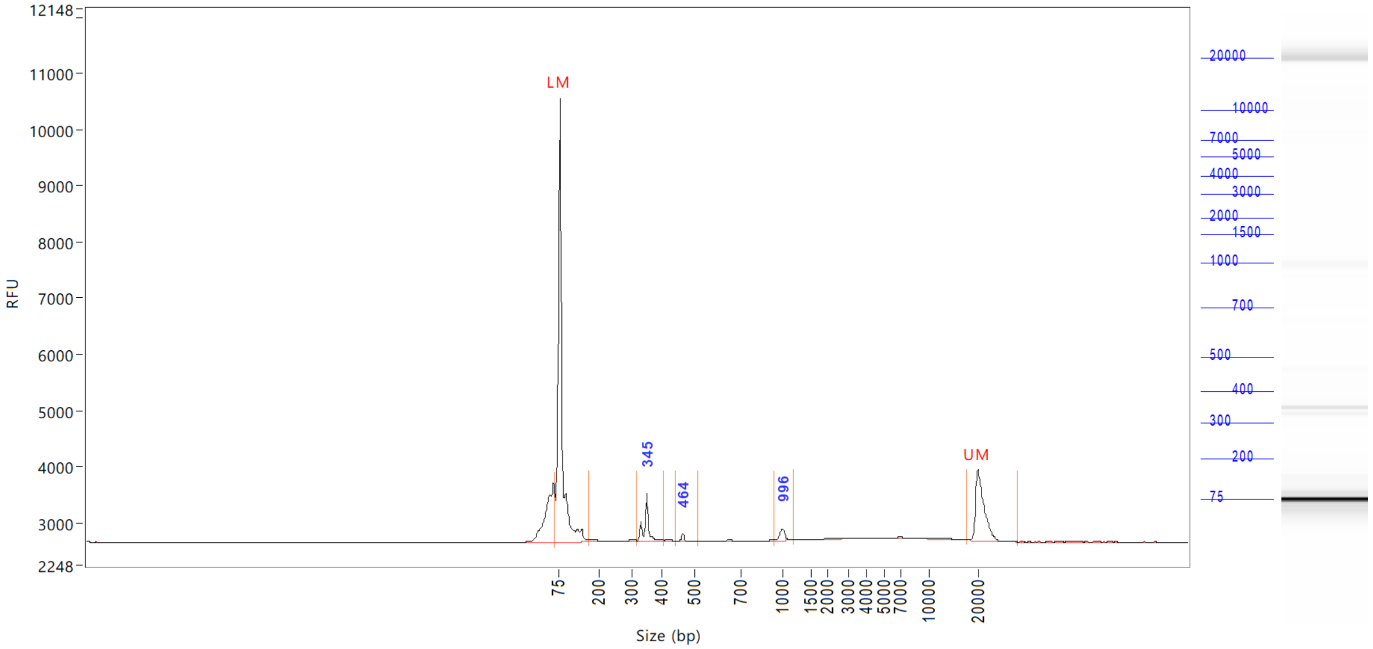


| Peak | Size (bp) | Concentration (ng/uL) | From (bp) | To (bp) | Average size (bp) | CV% | RFU | Corrected peak area |
|---|---|---|---|---|---|---|---|---|
| 1 | 75 (LM) | 0.5000 | 62 | 168 | 79 | 22.21 | 7908 | 72.646 |
| 2 | 345 | 0.6144 | 313 | 403 | 342 | 4.73 | 859 | 7.438 |
| 3 | 464 | 0.0775 | 441 | 511 | 463 | 2.27 | 138 | 0.938 |
| 4 | 996 | 0.1543 | 931 | 1178 | 991 | 2.91 | 214 | 1.868 |
| 5 | 20000 (UM) | 0.0957 | 17927 | 28221 | 20574 | 5.00 | 1259 | 13.911 |

| | | |
|---|---|---|
| TIC: | 0.8461 | ng/uL |
| TIM: | 3.4868 | nmole/L |
| Total concentration: | 1.2560 | ng/uL |

```
Sample peak width (sec): 5    Sample min peak height: 50    Sample baseline V to V?: Y    Sample baseline V to V points: 3
Sample filter: Binomial       Number of points for filter: 3  Sample start region (min): 0  Sample end region (min): 30
Marker peak width (sec): 5    Marker min peak height: 200   Marker baseline V to V?: Y    Marker baseline V to V points: 3
Lower marker selection: First peak > 500 RFU                Upper marker selection: Last peak > 200 RFU
Ladder size (bp)75, 200, 300, 400, 500, 700, 1000, 1500, 2000, 3000, 4000, 5000, 7000, 10000, 20000
Quantification using: Lower Marker          Final concentration (ng/uL): 0.5000          Dilution factor: 12.0
```

**Sample:** SampF4
**Well location:** F4
**Created:** Tuesday, September 14, 2021 6:54:01 PM

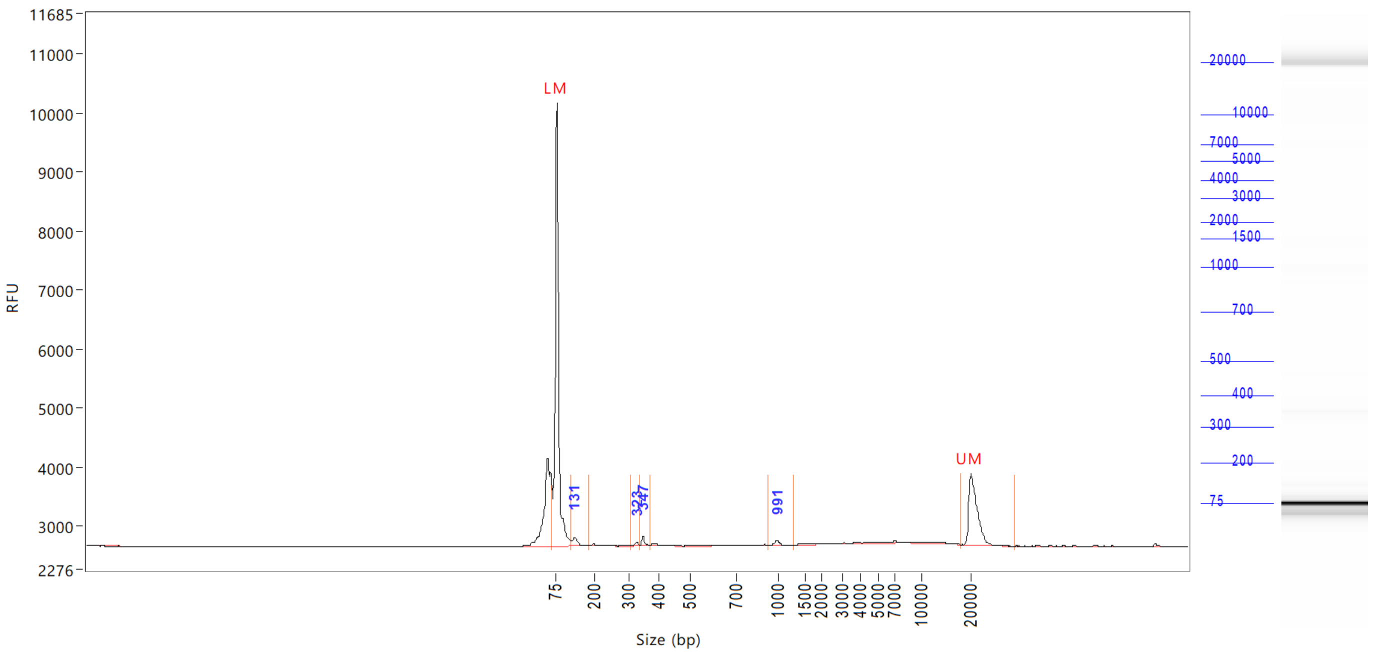


| Peak | Size (bp) | Concentration (ng/uL) | From (bp) | To (bp) | Average size (bp) | CV% | RFU | Corrected peak area |
|---|---|---|---|---|---|---|---|---|
| 1 | 75 (LM) | 0.5000 | 62 | 122 | 75 | 12.27 | 7514 | 59.432 |
| 2 | 131 | 0.2027 | 122 | 179 | 136 | 10.00 | 150 | 2.008 |
| 3 | 323 | 0.0434 | 305 | 333 | 321 | 1.97 | 59 | 0.429 |
| 4 | 347 | 0.1089 | 333 | 370 | 346 | 2.18 | 169 | 1.079 |
| 5 | 991 | 0.0750 | 926 | 1278 | 1001 | 5.63 | 73 | 0.743 |
| 6 | 20000 (UM) | 0.1107 | 17805 | 28705 | 20561 | 4.93 | 1204 | 13.153 |

| | | |
|---|---|---|
| TIC: | 0.4300 | ng/uL |
| TIM: | 3.3129 | nmole/L |
| Total concentration: | 0.8731 | ng/uL |

```
Sample peak width (sec): 5    Sample min peak height: 50    Sample baseline V to V?: Y    Sample baseline V to V points: 3
Sample filter: Binomial       Number of points for filter: 3  Sample start region (min): 0  Sample end region (min): 30
Marker peak width (sec): 5    Marker min peak height: 200   Marker baseline V to V?: Y    Marker baseline V to V points: 3
Lower marker selection: First peak > 500 RFU               Upper marker selection: Last peak > 200 RFU
Ladder size (bp)75, 200, 300, 400, 500, 700, 1000, 1500, 2000, 3000, 4000, 5000, 7000, 10000, 20000
Quantification using: Lower Marker          Final concentration (ng/uL): 0.5000          Dilution factor: 12.0
```

**Sample:** SampG4
**Well location:** G4
**Created:** Tuesday, September 14, 2021 6:54:01 PM

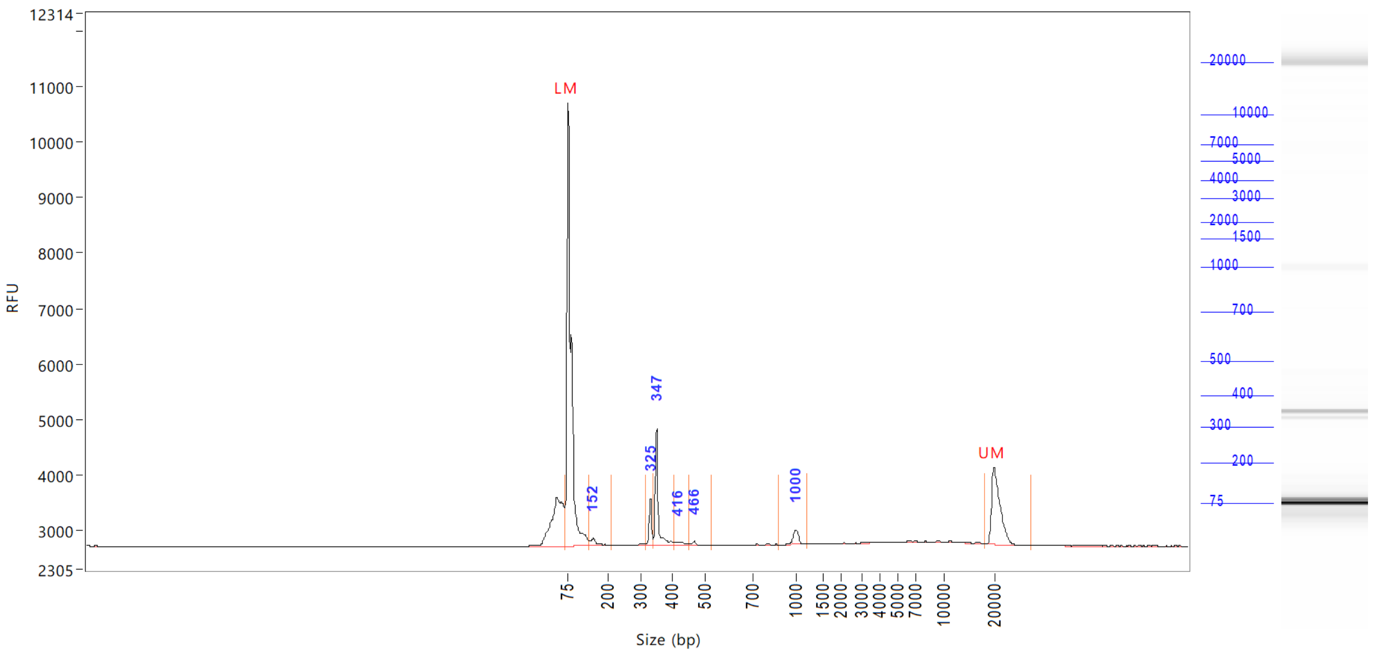


| Peak | Size (bp) | Concentration (ng/uL) | From (bp) | To (bp) | Average size (bp) | CV% | RFU | Corrected peak area |
|---|---|---|---|---|---|---|---|---|
| 1 | 75 (LM) | 0.5000 | 66 | 139 | 80 | 15.07 | 7991 | 77.348 |
| 2 | 152 | 0.1628 | 139 | 208 | 156 | 9.27 | 132 | 2.099 |
| 3 | 325 | 0.3220 | 313 | 335 | 325 | 1.29 | 832 | 4.151 |
| 4 | 347 | 0.9954 | 335 | 407 | 350 | 3.88 | 2099 | 12.832 |
| 5 | 416 | 0.0697 | 407 | 452 | 425 | 3.00 | 54 | 0.899 |
| 6 | 466 | 0.0441 | 452 | 527 | 469 | 2.97 | 76 | 0.568 |
| 7 | 1000 | 0.1807 | 874 | 1190 | 1003 | 3.24 | 264 | 2.330 |
| 8 | 20000 (UM) | 0.0983 | 18171 | 27496 | 20563 | 4.74 | 1394 | 15.199 |

| | | |
|---|---|---|
| TIC: | 1.7747 | ng/uL |
| TIM: | 8.7450 | nmole/L |
| Total concentration: | 1.9952 | ng/uL |

Sample peak width (sec): 5 Sample min peak height: 50 Sample baseline V to V?: Y Sample baseline V to V points: 3
Sample filter: Binomial Number of points for filter: 3 Sample start region (min): 0 Sample end region (min): 30
Marker peak width (sec): 5 Marker min peak height: 200 Marker baseline V to V?: Y Marker baseline V to V points: 3
Lower marker selection: First peak > 500 RFU Upper marker selection: Last peak > 200 RFU
Ladder size (bp)75, 200, 300, 400, 500, 700, 1000, 1500, 2000, 3000, 4000, 5000, 7000, 10000, 20000
Quantification using: Lower Marker Final concentration (ng/uL): 0.5000 Dilution factor: 12.0

**Sample:** SampH4
**Well location:** H4
**Created:** Tuesday, September 14, 2021 6:54:01 PM

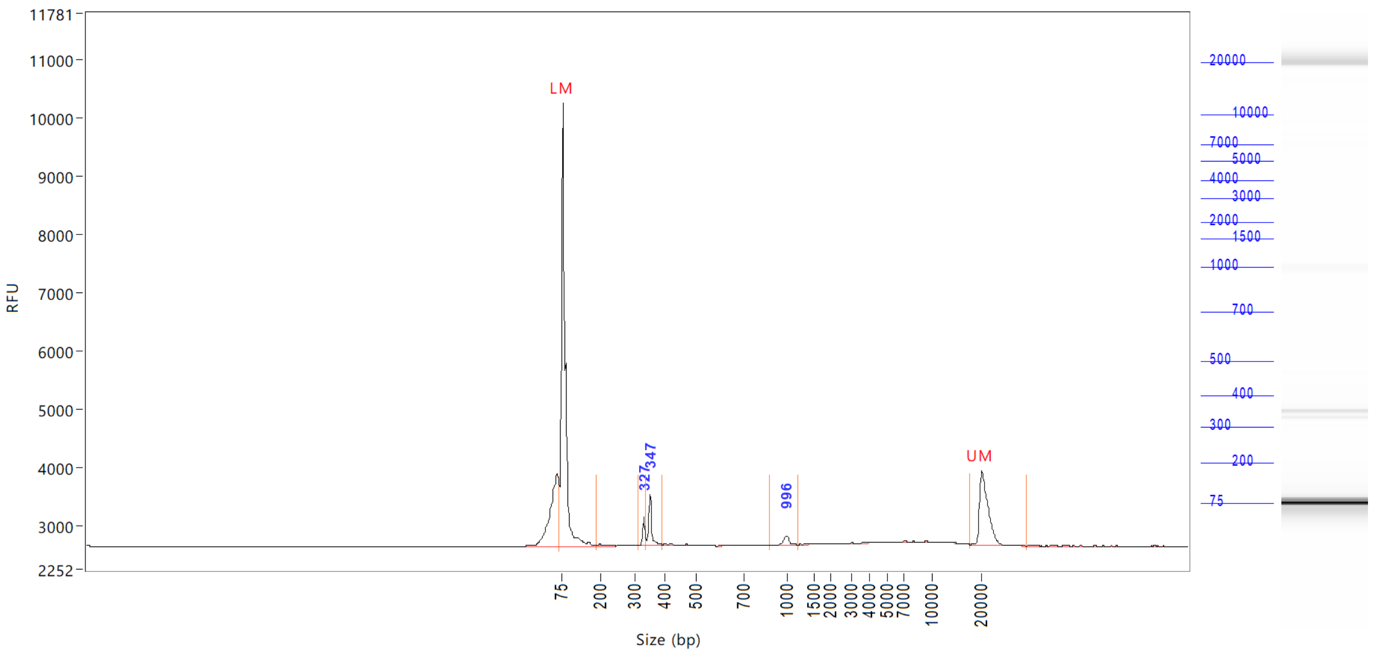


| Peak | Size (bp) | Concentration (ng/uL) | From (bp) | To (bp) | Average size (bp) | CV% | RFU | Corrected peak area |
|---|---|---|---|---|---|---|---|---|
| 1 | 75 (LM) | 0.5000 | 66 | 181 | 80 | 18.17 | 7613 | 70.550 |
| 2 | 327 | 0.2041 | 311 | 335 | 325 | 1.27 | 481 | 2.400 |
| 3 | 347 | 0.4561 | 335 | 390 | 349 | 2.77 | 868 | 5.363 |
| 4 | 996 | 0.1317 | 879 | 1201 | 997 | 3.63 | 161 | 1.549 |
| 5 | 20000 (UM) | 0.0988 | 17683 | 29189 | 20619 | 4.84 | 1272 | 13.939 |

| | | |
|---|---|---|
| TIC: | 0.7920 | ng/uL |
| TIM: | 3.4006 | nmole/L |
| Total concentration: | 1.0896 | ng/uL |

Sample peak width (sec): 5    Sample min peak height: 50    Sample baseline V to V?: Y    Sample baseline V to V points: 3
Sample filter: Binomial    Number of points for filter: 3  Sample start region (min): 0  Sample end region (min): 30
Marker peak width (sec): 5    Marker min peak height: 200    Marker baseline V to V?: Y    Marker baseline V to V points: 3
Lower marker selection: First peak > 500 RFU    Upper marker selection: Last peak > 200 RFU
Ladder size (bp)75, 200, 300, 400, 500, 700, 1000, 1500, 2000, 3000, 4000, 5000, 7000, 10000, 20000
Quantification using: Lower Marker    Final concentration (ng/uL): 0.5000    Dilution factor: 12.0

**Sample:** SampA5
**Well location:** A5
**Created:** Tuesday, September 14, 2021 6:54:01 PM

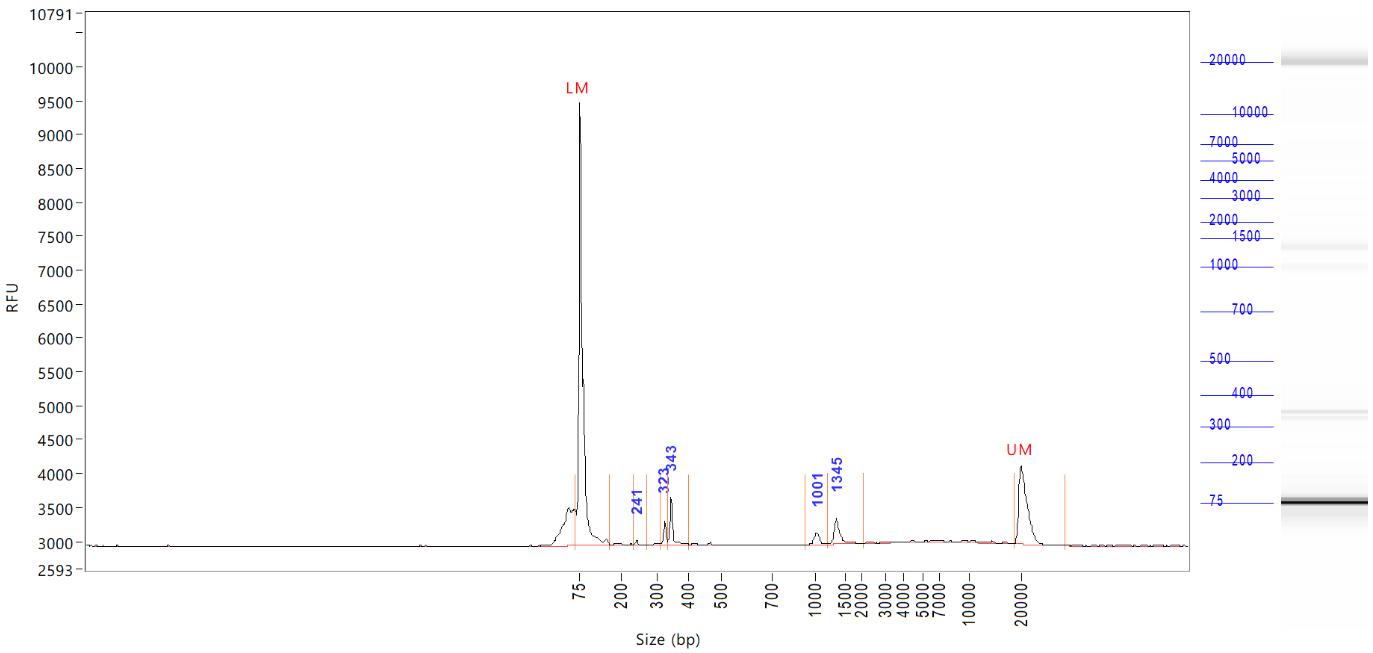


| Peak | Size (bp) | Concentration (ng/uL) | From (bp) | To (bp) | Average size (bp) | CV% | RFU | Corrected peak area |
|---|---|---|---|---|---|---|---|---|
| 1 | 75 (LM) | 0.5000 | 64 | 166 | 80 | 17.89 | 6542 | 58.250 |
| 2 | 241 | 0.0365 | 232 | 275 | 243 | 3.79 | 63 | 0.354 |
| 3 | 323 | 0.1900 | 311 | 333 | 323 | 1.36 | 361 | 1.844 |
| 4 | 343 | 0.4437 | 333 | 403 | 348 | 3.88 | 704 | 4.307 |
| 5 | 1001 | 0.1612 | 931 | 1201 | 1012 | 4.25 | 172 | 1.565 |
| 6 | 1345 | 0.3722 | 1201 | 2058 | 1390 | 9.41 | 384 | 3.613 |
| 7 | 20000 (UM) | 0.1045 | 18537 | 28584 | 20522 | 4.94 | 1149 | 12.176 |

| | | |
|---|---|---|
| TIC: | 1.2035 | ng/uL |
| TIM: | 4.0148 | nmole/L |
| Total concentration: | 1.6251 | ng/uL |

Sample peak width (sec): 5 Sample min peak height: 50 Sample baseline V to V?: Y Sample baseline V to V points: 3
Sample filter: Binomial Number of points for filter: 3 Sample start region (min): 0 Sample end region (min): 30
Marker peak width (sec): 5 Marker min peak height: 200 Marker baseline V to V?: Y Marker baseline V to V points: 3
Lower marker selection: First peak > 500 RFU Upper marker selection: Last peak > 200 RFU
Ladder size (bp)75, 200, 300, 400, 500, 700, 1000, 1500, 2000, 3000, 4000, 5000, 7000, 10000, 20000
Quantification using: Lower Marker Final concentration (ng/uL): 0.5000 Dilution factor: 12.0

**Sample:** SampB5
**Well location:** B5
**Created:** Tuesday, September 14, 2021 6:54:01 PM

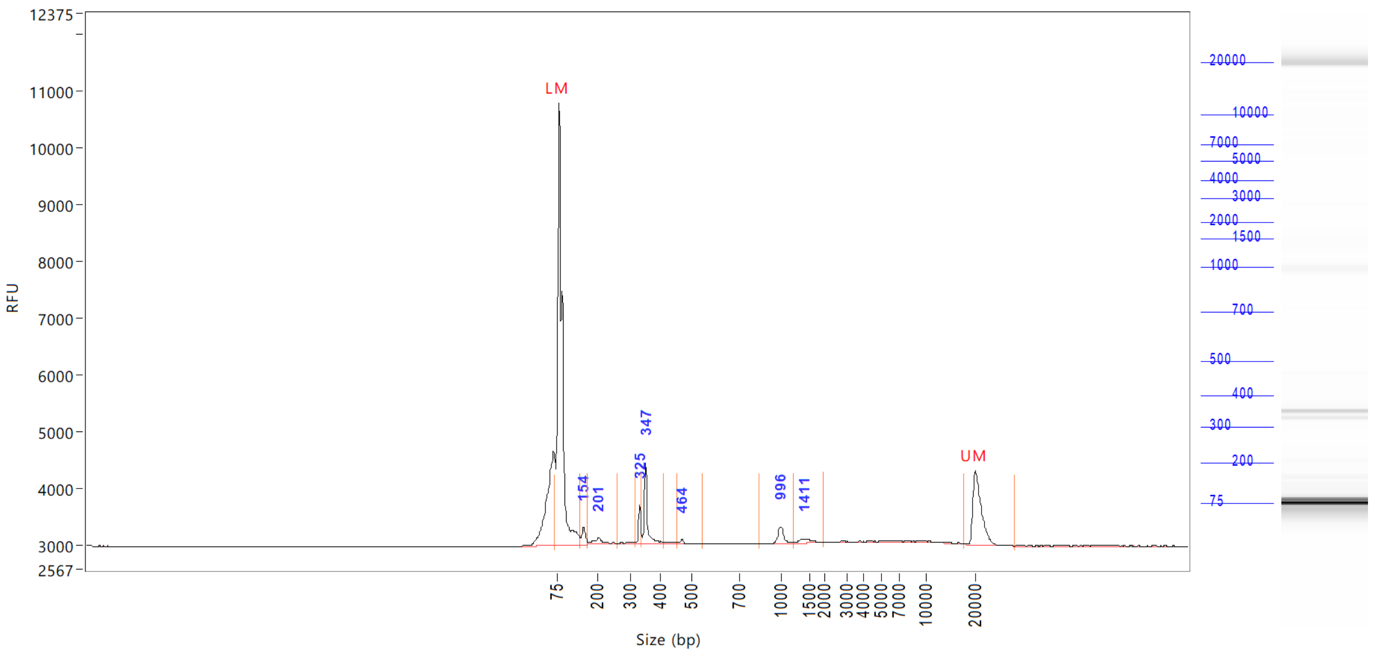


| Peak | Size (bp) | Concentration (ng/uL) | From (bp) | To (bp) | Average size (bp) | CV% | RFU | Corrected peak area |
|---|---|---|---|---|---|---|---|---|
| 1 | 75 (LM) | 0.5000 | 66 | 145 | 81 | 16.91 | 7818 | 84.355 |
| 2 | 154 | 0.1867 | 145 | 168 | 153 | 3.65 | 296 | 2.625 |
| 3 | 201 | 0.1689 | 168 | 260 | 199 | 9.66 | 121 | 2.374 |
| 4 | 325 | 0.2335 | 313 | 335 | 325 | 1.36 | 664 | 3.282 |
| 5 | 347 | 0.6350 | 335 | 409 | 350 | 3.71 | 1413 | 8.927 |
| 6 | 464 | 0.0411 | 450 | 542 | 468 | 3.73 | 80 | 0.578 |
| 7 | 996 | 0.2125 | 844 | 1201 | 996 | 4.38 | 299 | 2.987 |
| 8 | 1411 | 0.1131 | 1201 | 1962 | 1457 | 10.74 | 76 | 1.591 |
| 9 | 20000 (UM) | 0.0852 | 17561 | 27859 | 20541 | 5.24 | 1319 | 14.372 |

| | | |
|---|---|---|
| TIC: | 1.5908 | ng/uL |
| TIM: | 8.1910 | nmole/L |
| Total concentration: | 1.8174 | ng/uL |

Sample peak width (sec): 5 Sample min peak height: 50 Sample baseline V to V?: Y Sample baseline V to V points: 3
Sample filter: Binomial Number of points for filter: 3 Sample start region (min): 0 Sample end region (min): 30
Marker peak width (sec): 5 Marker min peak height: 200 Marker baseline V to V?: Y Marker baseline V to V points: 3
Lower marker selection: First peak > 500 RFU Upper marker selection: Last peak > 200 RFU
Ladder size (bp)75, 200, 300, 400, 500, 700, 1000, 1500, 2000, 3000, 4000, 5000, 7000, 10000, 20000
Quantification using: Lower Marker Final concentration (ng/uL): 0.5000 Dilution factor: 12.0

**Sample:** SampC5
**Well location:** C5
**Created:** Tuesday, September 14, 2021 6:54:01 PM

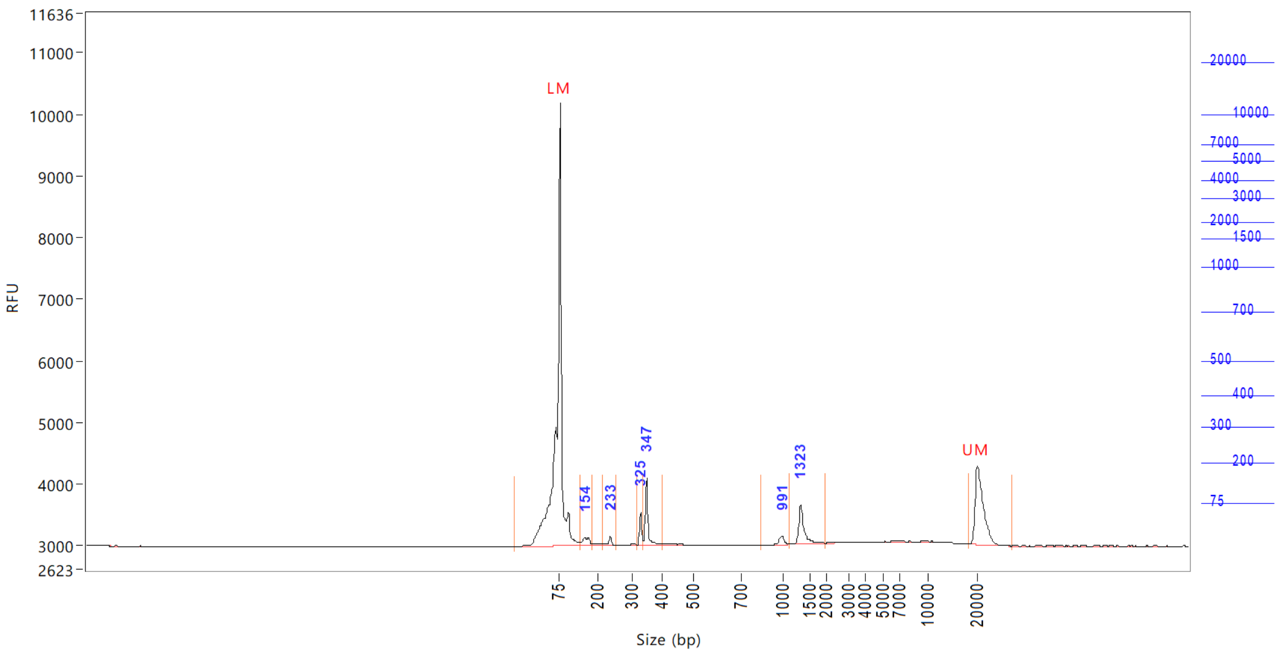




| Peak | Size (bp) | Concentration (ng/uL) | From (bp) | To (bp) | Average size (bp) | CV% | RFU | Corrected peak area |
|---|---|---|---|---|---|---|---|---|
| 1 | 75 (LM) | 0.5000 | 0 | 141 | 63 | 38.33 | 7194 | 87.668 |
| 2 | 154 | 0.1390 | 141 | 177 | 157 | 5.75 | 128 | 2.031 |
| 3 | 233 | 0.0618 | 210 | 250 | 229 | 2.81 | 140 | 0.903 |
| 4 | 325 | 0.1734 | 313 | 335 | 324 | 1.33 | 528 | 2.533 |
| 5 | 347 | 0.4183 | 335 | 401 | 348 | 3.00 | 1077 | 6.111 |
| 6 | 991 | 0.0852 | 840 | 1123 | 980 | 3.32 | 134 | 1.245 |
| 7 | 1323 | 0.3763 | 1123 | 1962 | 1354 | 7.60 | 637 | 5.499 |
| 8 | 20000 (UM) | 0.0780 | 18293 | 26891 | 20603 | 4.72 | 1270 | 13.676 |

| | | |
|---|---|---|
| TIC: | 1.2540 | ng/uL |
| TIM: | 5.3574 | nmole/L |
| Total concentration: | 1.4817 | ng/uL |

```
Sample peak width (sec): 5    Sample min peak height: 50    Sample baseline V to V?: Y    Sample baseline V to V points: 3
Sample filter: Binomial       Number of points for filter: 3  Sample start region (min): 0  Sample end region (min): 30
Marker peak width (sec): 5    Marker min peak height: 200   Marker baseline V to V?: Y    Marker baseline V to V points: 3
Lower marker selection: First peak > 500 RFU                Upper marker selection: Last peak > 200 RFU
Ladder size (bp)75, 200, 300, 400, 500, 700, 1000, 1500, 2000, 3000, 4000, 5000, 7000, 10000, 20000
Quantification using: Lower Marker          Final concentration (ng/uL): 0.5000         Dilution factor: 12.0
```

**Sample:** SampD5
**Well location:** D5
**Created:** Tuesday, September 14, 2021 6:54:01 PM

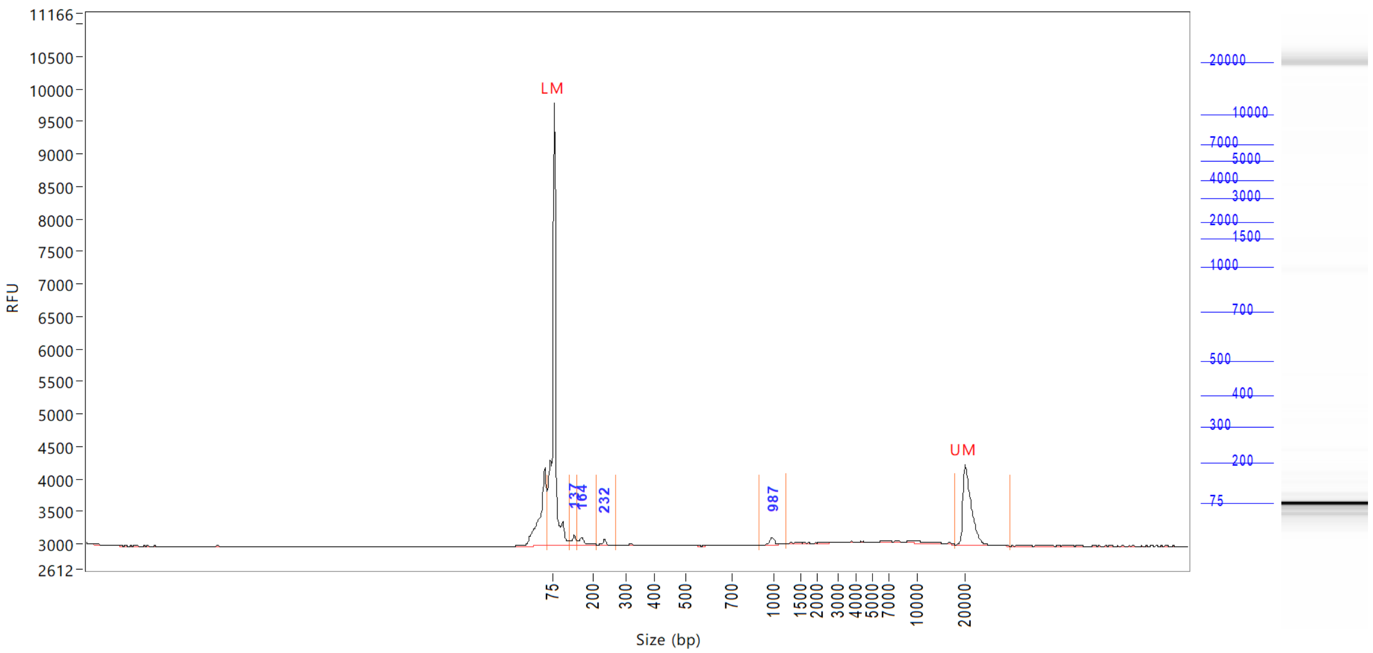


| Peak | Size (bp) | Concentration (ng/uL) | From (bp) | To (bp) | Average size (bp) | CV% | RFU | Corrected peak area |
|---|---|---|---|---|---|---|---|---|
| 1 | 75 (LM) | 0.5000 | 50 | 124 | 72 | 15.63 | 6823 | 58.506 |
| 2 | 137 | 0.1578 | 124 | 148 | 136 | 5.01 | 149 | 1.539 |
| 3 | 164 | 0.1769 | 148 | 212 | 165 | 8.31 | 119 | 1.725 |
| 4 | 232 | 0.0635 | 212 | 271 | 232 | 3.58 | 91 | 0.619 |
| 5 | 987 | 0.1033 | 892 | 1212 | 988 | 4.03 | 107 | 1.007 |
| 6 | 20000 (UM) | 0.1177 | 17927 | 29309 | 20646 | 5.48 | 1239 | 13.770 |

| | | |
|---|---|---|
| TIC: | 0.5015 | ng/uL |
| TIM: | 4.2911 | nmole/L |
| Total concentration: | 0.9433 | ng/uL |

```
Sample peak width (sec): 5    Sample min peak height: 50    Sample baseline V to V?: Y    Sample baseline V to V points: 3
Sample filter: Binomial       Number of points for filter: 3  Sample start region (min): 0  Sample end region (min): 30
Marker peak width (sec): 5    Marker min peak height: 200   Marker baseline V to V?: Y    Marker baseline V to V points: 3
Lower marker selection: First peak > 500 RFU                Upper marker selection: Last peak > 200 RFU
Ladder size (bp)75, 200, 300, 400, 500, 700, 1000, 1500, 2000, 3000, 4000, 5000, 7000, 10000, 20000
Quantification using: Lower Marker          Final concentration (ng/uL): 0.5000          Dilution factor: 12.0
```

**Sample:** SampE5
**Well location:** E5
**Created:** Tuesday, September 14, 2021 6:54:01 PM

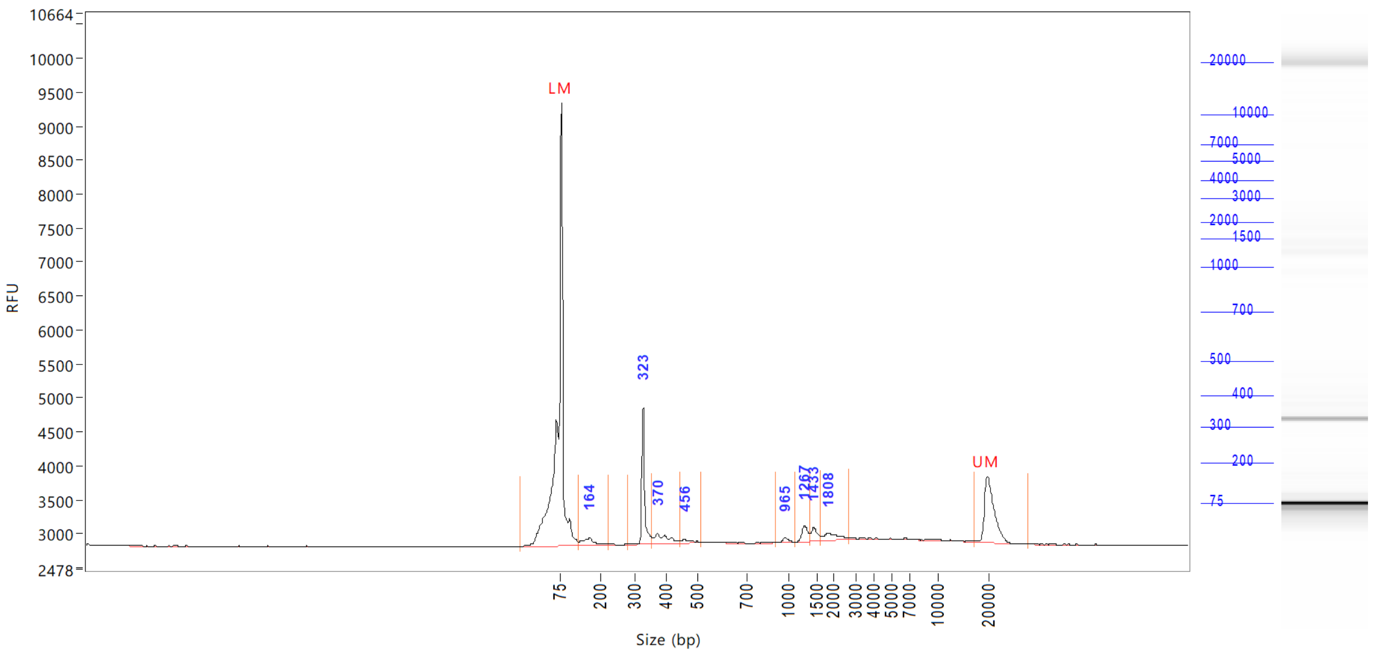


| Peak | Size (bp) | Concentration (ng/uL) | From (bp) | To (bp) | Average size (bp) | CV% | RFU | Corrected peak area |
|---|---|---|---|---|---|---|---|---|
| 1 | 75 (LM) | 0.5000 | 0 | 128 | 61 | 38.97 | 6534 | 83.653 |
| 2 | 164 | 0.2331 | 128 | 221 | 162 | 13.92 | 125 | 3.251 |
| 3 | 323 | 0.9223 | 277 | 352 | 322 | 2.82 | 2016 | 12.858 |
| 4 | 370 | 0.2994 | 352 | 445 | 389 | 6.28 | 165 | 4.174 |
| 5 | 456 | 0.0733 | 445 | 511 | 468 | 3.63 | 65 | 1.022 |
| 6 | 965 | 0.0567 | 900 | 1101 | 968 | 2.83 | 73 | 0.790 |
| 7 | 1267 | 0.1628 | 1101 | 1356 | 1262 | 4.02 | 234 | 2.270 |
| 8 | 1433 | 0.1583 | 1356 | 1597 | 1444 | 4.10 | 200 | 2.207 |
| 9 | 1808 | 0.1711 | 1597 | 2657 | 1951 | 12.89 | 104 | 2.385 |
| 10 | 20000 (UM) | 0.0727 | 17439 | 27859 | 20530 | 5.54 | 977 | 12.170 |

| | | |
|---|---|---|
| TIC: | 2.0770 | ng/uL |
| TIM: | 9.2347 | nmole/L |
| Total concentration: | 2.2268 | ng/uL |

```
Sample peak width (sec): 5    Sample min peak height: 50    Sample baseline V to V?: Y    Sample baseline V to V points: 3
Sample filter: Binomial       Number of points for filter: 3  Sample start region (min): 0  Sample end region (min): 30
Marker peak width (sec): 5    Marker min peak height: 200   Marker baseline V to V?: Y    Marker baseline V to V points: 3
Lower marker selection: First peak > 500 RFU                Upper marker selection: Last peak > 200 RFU
Ladder size (bp)75, 200, 300, 400, 500, 700, 1000, 1500, 2000, 3000, 4000, 5000, 7000, 10000, 20000
Quantification using: Lower Marker          Final concentration (ng/uL): 0.5000          Dilution factor: 12.0
```

**Sample:** SampF5
**Well location:** F5
**Created:** Tuesday, September 14, 2021 6:54:01 PM

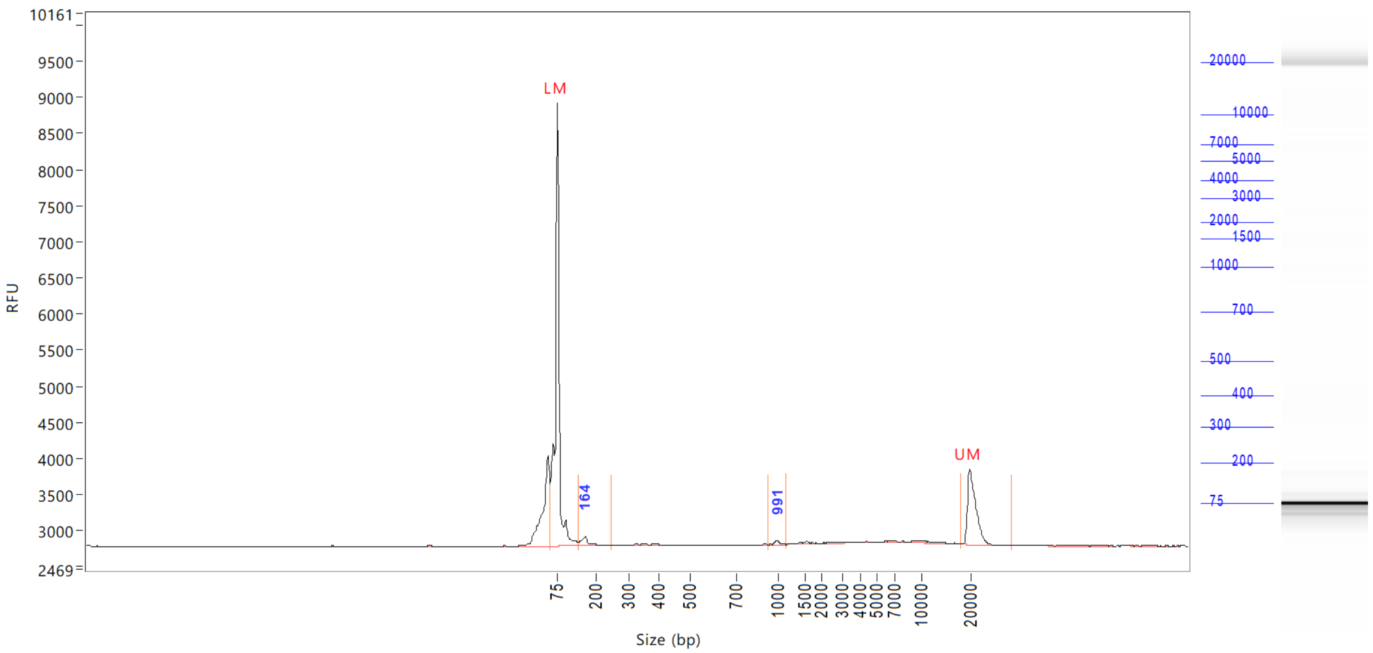


| Peak | Size (bp) | Concentration (ng/uL) | From (bp) | To (bp) | Average size (bp) | CV% | RFU | Corrected peak area |
|---|---|---|---|---|---|---|---|---|
| 1 | 75 (LM) | 0.5000 | 52 | 141 | 73 | 18.14 | 6141 | 56.443 |
| 2 | 164 | 0.2365 | 141 | 246 | 166 | 11.56 | 126 | 2.225 |
| 3 | 991 | 0.0620 | 931 | 1134 | 991 | 3.20 | 60 | 0.583 |
| 4 | 20000 (UM) | 0.1011 | 18049 | 28342 | 20560 | 4.99 | 1045 | 11.411 |

| | | |
|---|---|---|
| TIC: | 0.2985 | ng/uL |
| TIM: | 2.4452 | nmole/L |
| Total concentration: | 0.8235 | ng/uL |

```
Sample peak width (sec): 5    Sample min peak height: 50    Sample baseline V to V?: Y    Sample baseline V to V points: 3
Sample filter: Binomial       Number of points for filter: 3  Sample start region (min): 0  Sample end region (min): 30
Marker peak width (sec): 5    Marker min peak height: 200   Marker baseline V to V?: Y    Marker baseline V to V points: 3
Lower marker selection: First peak > 500 RFU                Upper marker selection: Last peak > 200 RFU
Ladder size (bp)75, 200, 300, 400, 500, 700, 1000, 1500, 2000, 3000, 4000, 5000, 7000, 10000, 20000
Quantification using: Lower Marker          Final concentration (ng/uL): 0.5000          Dilution factor: 12.0
```

**Sample:** SampG5
**Well location:** G5
**Created:** Tuesday, September 14, 2021 6:54:01 PM

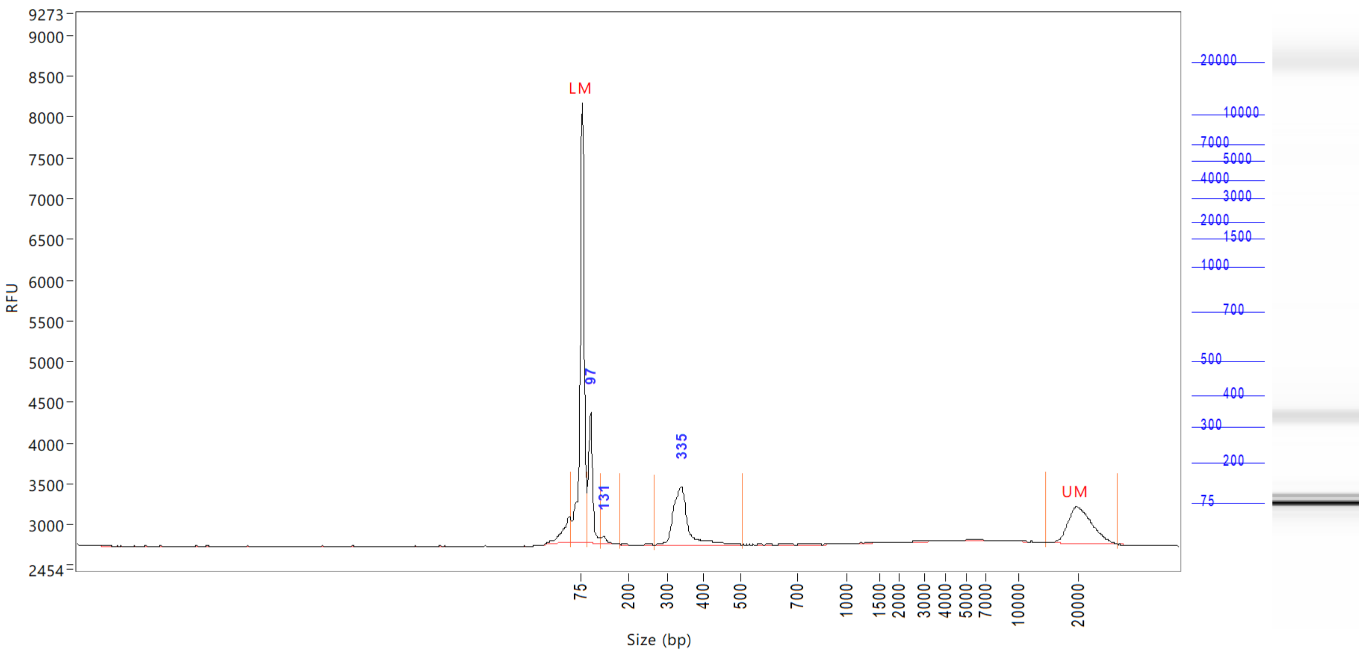


| Peak | Size (bp) | Concentration (ng/uL) | From (bp) | To (bp) | Average size (bp) | CV% | RFU | Corrected peak area |
|---|---|---|---|---|---|---|---|---|
| 1 | 75 (LM) | 0.5000 | 47 | 87 | 72 | 10.08 | 5400 | 53.658 |
| 2 | 97 | 1.7382 | 87 | 124 | 96 | 6.85 | 1607 | 15.545 |
| 3 | 131 | 0.1305 | 124 | 177 | 135 | 7.42 | 92 | 1.167 |
| 4 | 335 | 2.2265 | 266 | 506 | 339 | 9.79 | 715 | 19.911 |
| 5 | 20000 (UM) | 0.1162 | 14635 | 26891 | 20618 | 9.51 | 448 | 12.468 |

| | | |
|---|---|---|
| TIC: | 4.0952 | ng/uL |
| TIM: | 42.1220 | nmole/L |
| Total concentration: | 4.3745 | ng/uL |

```
Sample peak width (sec): 5    Sample min peak height: 50    Sample baseline V to V?: Y    Sample baseline V to V points: 3
Sample filter: Binomial       Number of points for filter: 3  Sample start region (min): 0  Sample end region (min): 30
Marker peak width (sec): 5    Marker min peak height: 200   Marker baseline V to V?: Y    Marker baseline V to V points: 3
Lower marker selection: First peak > 500 RFU                Upper marker selection: Last peak > 200 RFU
Ladder size (bp)75, 200, 300, 400, 500, 700, 1000, 1500, 2000, 3000, 4000, 5000, 7000, 10000, 20000
Quantification using: Lower Marker          Final concentration (ng/uL): 0.5000          Dilution factor: 12.0
```

**Sample:** SampH5
**Well location:** H5
**Created:** Tuesday, September 14, 2021 6:54:01 PM

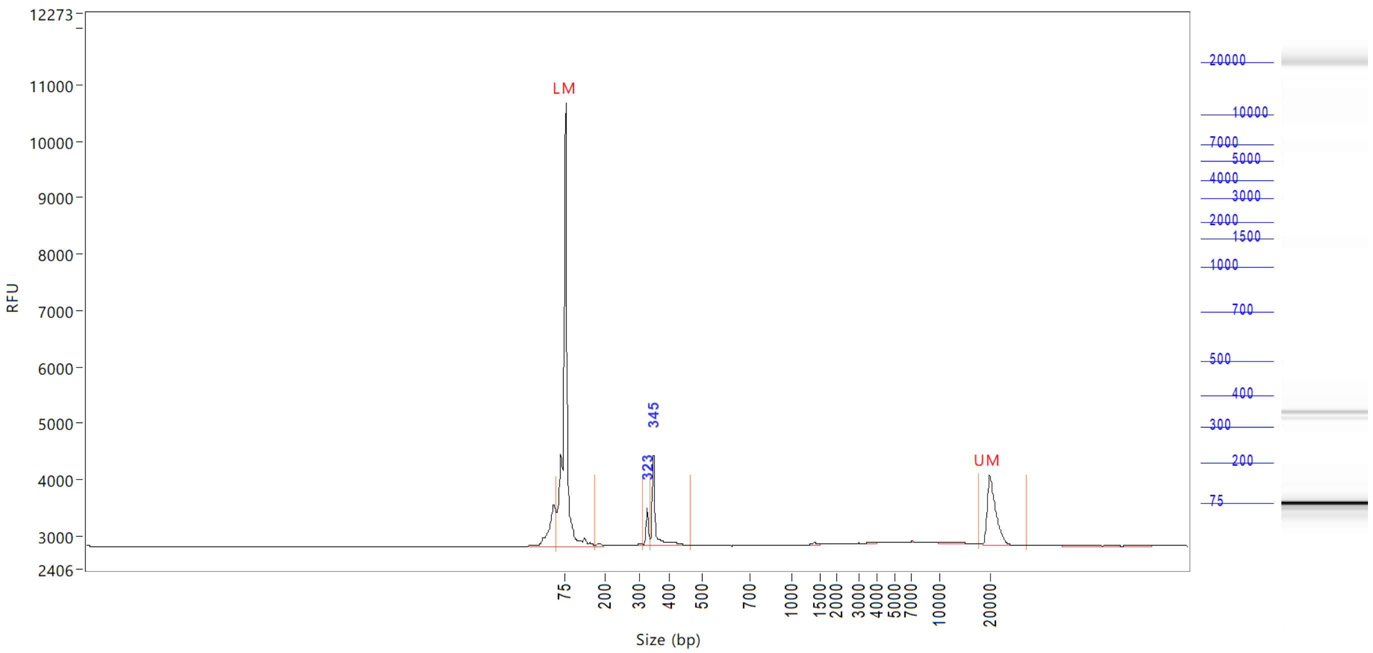


| Peak | Size<br>(bp) | Concentration<br>(ng/uL) | From<br>(bp) | To<br>(bp) | Average size<br>(bp) | CV% | RFU | Corrected peak area |
|---|---|---|---|---|---|---|---|---|
| 1 | 75 (LM) | 0.5000 | 48 | 166 | 73 | 20.23 | 7881 | 71.775 |
| 2 | 323 | 0.2939 | 311 | 333 | 323 | 1.39 | 674 | 3.516 |
| 3 | 345 | 0.9303 | 333 | 468 | 353 | 6.43 | 1604 | 11.128 |
| 4 | 20000 (UM) | 0.0933 | 17805 | 27375 | 20546 | 4.81 | 1224 | 13.390 |

| | | |
|---|---|---|
| TIC: | 1.2242 | ng/uL |
| TIM: | 5.8325 | nmole/L |
| Total concentration: | 1.5592 | ng/uL |

Sample peak width (sec): 5 Sample min peak height: 50 Sample baseline V to V?: Y Sample baseline V to V points: 3
Sample filter: Binomial Number of points for filter: 3 Sample start region (min): 0 Sample end region (min): 30
Marker peak width (sec): 5 Marker min peak height: 200 Marker baseline V to V?: Y Marker baseline V to V points: 3
Lower marker selection: First peak > 500 RFU Upper marker selection: Last peak > 200 RFU
Ladder size (bp)75, 200, 300, 400, 500, 700, 1000, 1500, 2000, 3000, 4000, 5000, 7000, 10000, 20000
Quantification using: Lower Marker Final concentration (ng/uL): 0.5000 Dilution factor: 12.0

**Sample:** SampA6
**Well location:** A6
**Created:** Tuesday, September 14, 2021 6:54:01 PM

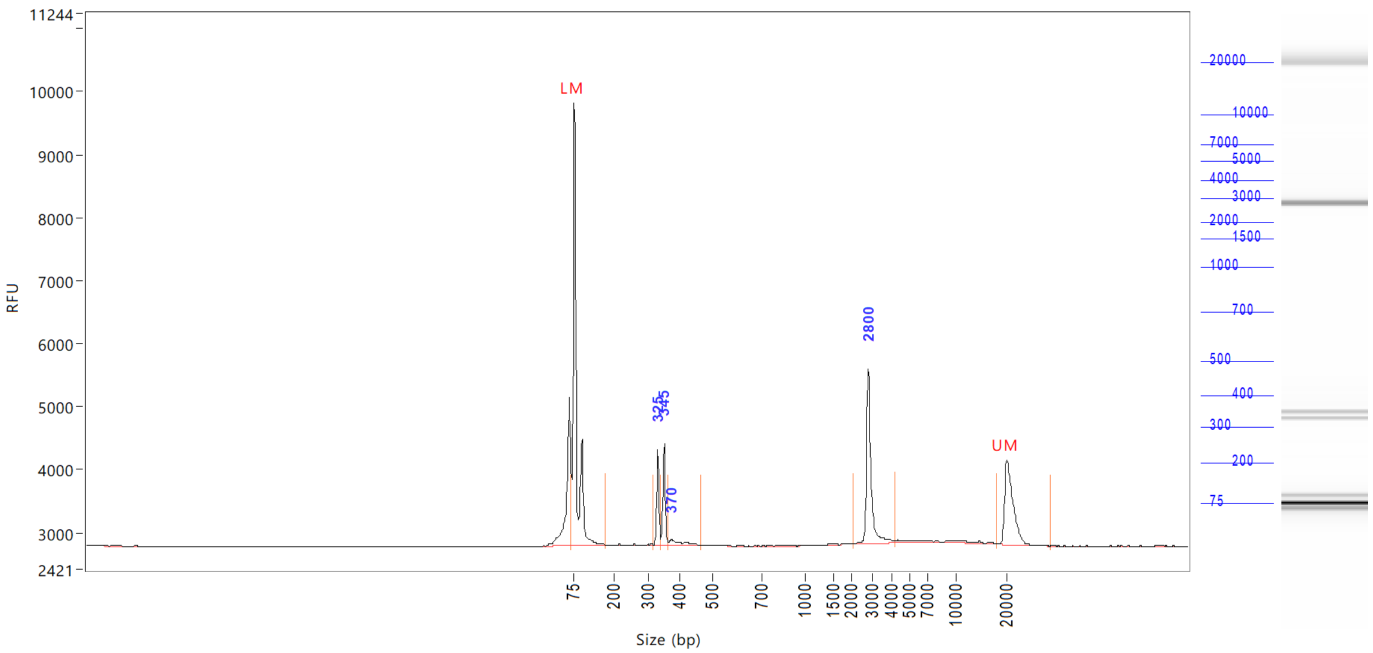


| Peak | Size (bp) | Concentration (ng/uL) | From (bp) | To (bp) | Average size (bp) | CV% | RFU | Corrected peak area |
|---|---|---|---|---|---|---|---|---|
| 1 | 75 (LM) | 0.5000 | 67 | 169 | 80 | 16.11 | 7035 | 56.133 |
| 2 | 325 | 0.7760 | 313 | 335 | 323 | 1.28 | 1518 | 7.260 |
| 3 | 345 | 0.8424 | 335 | 360 | 344 | 1.20 | 1617 | 7.881 |
| 4 | 370 | 0.2540 | 360 | 466 | 398 | 6.79 | 84 | 2.377 |
| 5 | 2800 | 2.0290 | 2115 | 4173 | 2857 | 8.95 | 2771 | 18.983 |
| 6 | 20000 (UM) | 0.1280 | 17927 | 28463 | 20623 | 4.94 | 1341 | 14.370 |

| | | |
|---|---|---|
| TIC: | 3.9014 | ng/uL |
| TIM: | 10.1999 | nmole/L |
| Total concentration: | 4.3177 | ng/uL |

```
Sample peak width (sec): 5    Sample min peak height: 50    Sample baseline V to V?: Y    Sample baseline V to V points: 3
Sample filter: Binomial       Number of points for filter: 3  Sample start region (min): 0  Sample end region (min): 30
Marker peak width (sec): 5    Marker min peak height: 200   Marker baseline V to V?: Y    Marker baseline V to V points: 3
Lower marker selection: First peak > 500 RFU                Upper marker selection: Last peak > 200 RFU
Ladder size (bp)75, 200, 300, 400, 500, 700, 1000, 1500, 2000, 3000, 4000, 5000, 7000, 10000, 20000
Quantification using: Lower Marker          Final concentration (ng/uL): 0.5000          Dilution factor: 12.0
```

**Sample:** SampB6
**Well location:** B6
**Created:** Tuesday, September 14, 2021 6:54:01 PM

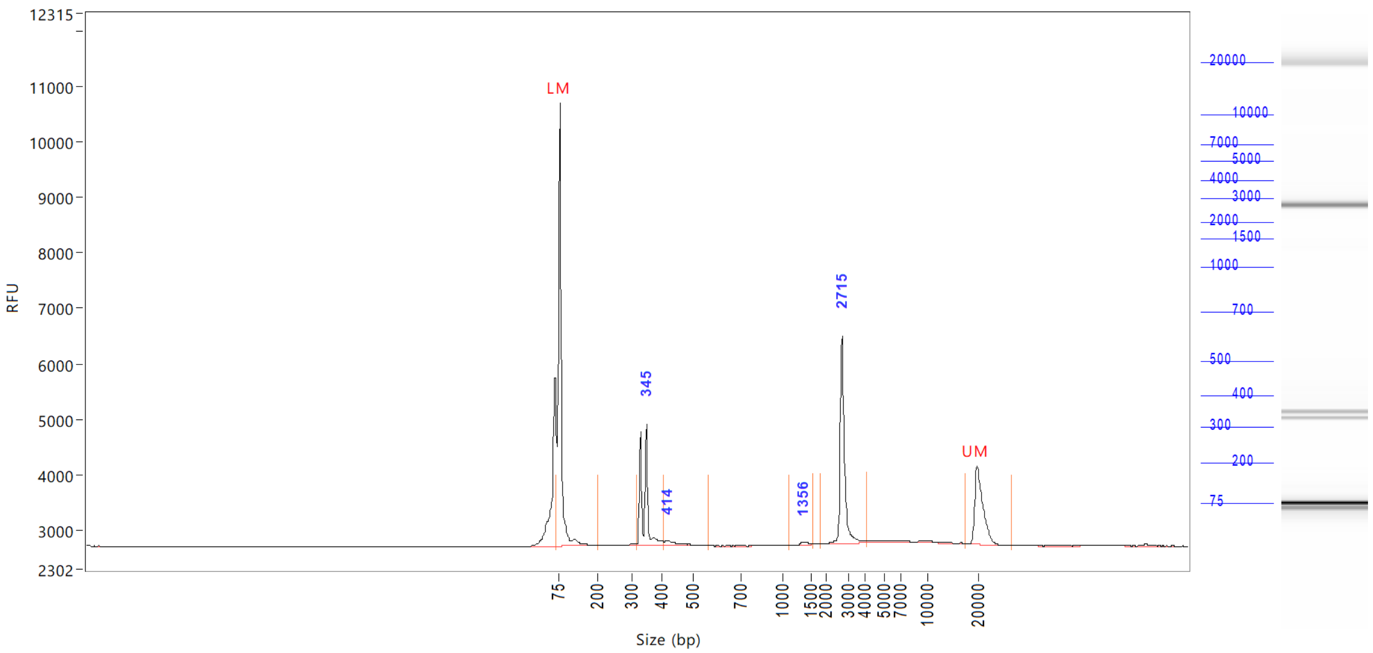


| Peak | Size (bp) | Concentration (ng/uL) | From (bp) | To (bp) | Average size (bp) | CV% | RFU | Corrected peak area |
|---|---|---|---|---|---|---|---|---|
| 1 | 75 (LM) | 0.5000 | 67 | 196 | 78 | 18.12 | 7995 | 58.266 |
| 2 | 345 | 2.4027 | 313 | 403 | 339 | 5.24 | 2191 | 23.332 |
| 3 | 414 | 0.2633 | 403 | 560 | 440 | 6.90 | 96 | 2.557 |
| 4 | 1356 | 0.0640 | 1123 | 1597 | 1372 | 6.13 | 52 | 0.621 |
| 5 | 2715 | 2.5762 | 1808 | 4035 | 2748 | 8.29 | 3743 | 25.018 |
| 6 | 20000 (UM) | 0.1299 | 17561 | 27012 | 20475 | 4.76 | 1401 | 15.133 |

| | | |
|---|---|---|
| TIC: | 5.3062 | ng/uL |
| TIM: | 14.2645 | nmole/L |
| Total concentration: | 5.6834 | ng/uL |

```
Sample peak width (sec): 5    Sample min peak height: 50    Sample baseline V to V?: Y    Sample baseline V to V points: 3
Sample filter: Binomial       Number of points for filter: 3  Sample start region (min): 0  Sample end region (min): 30
Marker peak width (sec): 5    Marker min peak height: 200   Marker baseline V to V?: Y    Marker baseline V to V points: 3
Lower marker selection: First peak > 500 RFU                Upper marker selection: Last peak > 200 RFU
Ladder size (bp)75, 200, 300, 400, 500, 700, 1000, 1500, 2000, 3000, 4000, 5000, 7000, 10000, 20000
Quantification using: Lower Marker          Final concentration (ng/uL): 0.5000          Dilution factor: 12.0
```

**Sample:** SampC6
**Well location:** C6
**Created:** Tuesday, September 14, 2021 6:54:01 PM

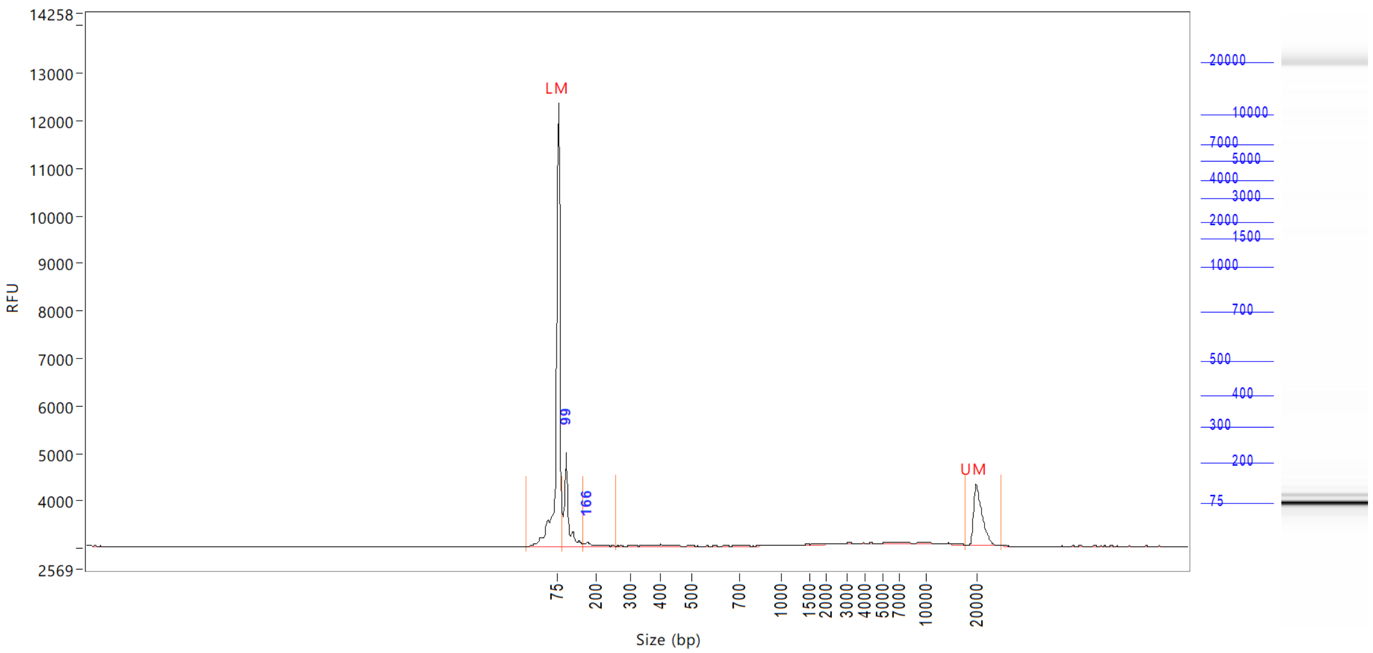


| Peak | Size (bp) | Concentration (ng/uL) | From (bp) | To (bp) | Average size (bp) | CV% | RFU | Corrected peak area |
|---|---|---|---|---|---|---|---|---|
| 1 | 75 (LM) | 0.5000 | 0 | 89 | 68 | 22.35 | 9339 | 84.030 |
| 2 | 99 | 1.3417 | 89 | 154 | 104 | 13.14 | 1972 | 18.791 |
| 3 | 166 | 0.1226 | 154 | 253 | 181 | 12.31 | 80 | 1.717 |
| 4 | 20000 (UM) | 0.0840 | 17805 | 25078 | 20511 | 4.61 | 1292 | 14.116 |

| | | |
|---|---|---|
| TIC: | 1.4643 | ng/uL |
| TIM: | 22.3000 | nmole/L |
| Total concentration: | 1.8121 | ng/uL |

```
Sample peak width (sec): 5    Sample min peak height: 50    Sample baseline V to V?: Y    Sample baseline V to V points: 3
Sample filter: Binomial       Number of points for filter: 3  Sample start region (min): 0  Sample end region (min): 30
Marker peak width (sec): 5    Marker min peak height: 200   Marker baseline V to V?: Y    Marker baseline V to V points: 3
Lower marker selection: First peak > 500 RFU                Upper marker selection: Last peak > 200 RFU
Ladder size (bp)75, 200, 300, 400, 500, 700, 1000, 1500, 2000, 3000, 4000, 5000, 7000, 10000, 20000
Quantification using: Lower Marker          Final concentration (ng/uL): 0.5000          Dilution factor: 12.0
```

**Sample:** SampD6
**Well location:** D6
**Created:** Tuesday, September 14, 2021 6:54:01 PM

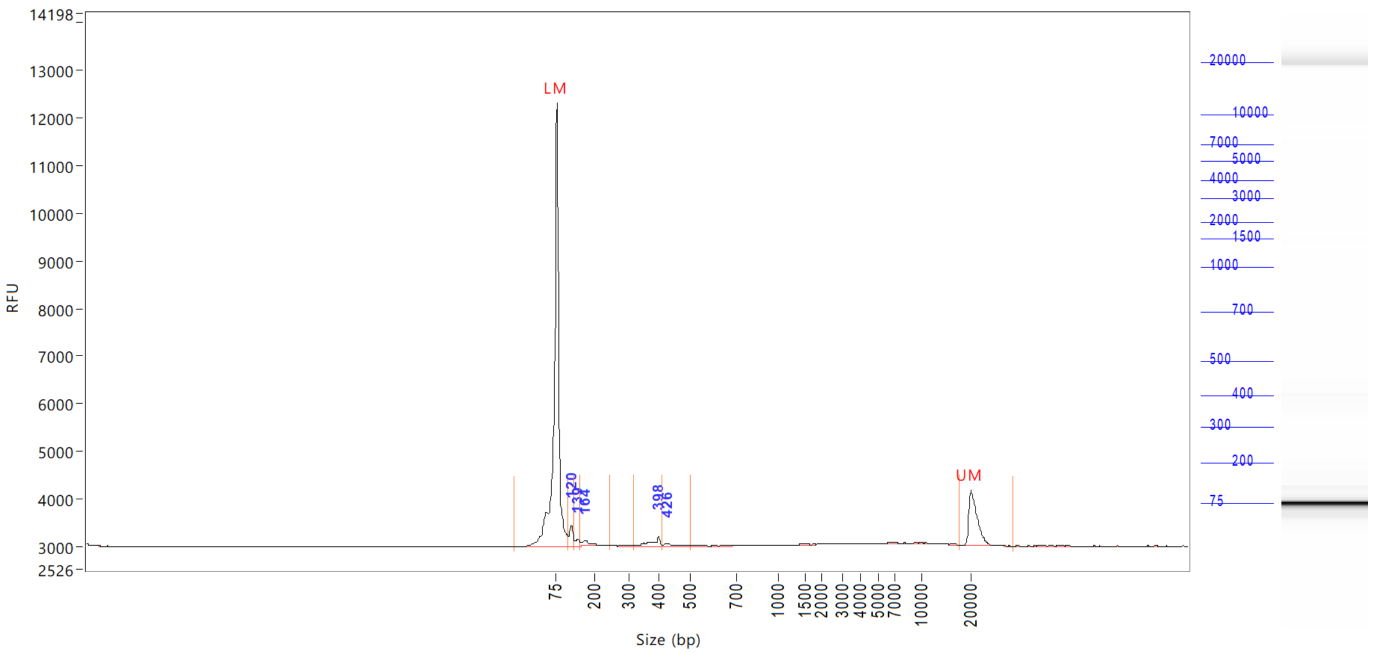


| Peak | Size (bp) | Concentration (ng/uL) | From (bp) | To (bp) | Average size (bp) | CV% | RFU | Corrected peak area |
|---|---|---|---|---|---|---|---|---|
| 1 | 75 (LM) | 0.5000 | 0 | 112 | 67 | 25.92 | 9322 | 103.855 |
| 2 | 120 | 0.2124 | 112 | 131 | 120 | 4.23 | 434 | 3.676 |
| 3 | 139 | 0.0908 | 131 | 150 | 140 | 3.98 | 152 | 1.572 |
| 4 | 164 | 0.1266 | 150 | 244 | 173 | 10.25 | 105 | 2.191 |
| 5 | 398 | 0.1946 | 315 | 411 | 374 | 6.16 | 194 | 3.369 |
| 6 | 426 | 0.0576 | 411 | 500 | 437 | 4.92 | 54 | 0.997 |
| 7 | 20000 (UM) | 0.0608 | 17683 | 28584 | 20593 | 5.15 | 1147 | 12.637 |

| | | |
|---|---|---|
| TIC: | 0.6820 | ng/uL |
| TIM: | 6.2493 | nmole/L |
| Total concentration: | 0.8150 | ng/uL |

```
Sample peak width (sec): 5    Sample min peak height: 50    Sample baseline V to V?: Y    Sample baseline V to V points: 3
Sample filter: Binomial       Number of points for filter: 3  Sample start region (min): 0  Sample end region (min): 30
Marker peak width (sec): 5    Marker min peak height: 200   Marker baseline V to V?: Y    Marker baseline V to V points: 3
Lower marker selection: First peak > 500 RFU                Upper marker selection: Last peak > 200 RFU
Ladder size (bp)75, 200, 300, 400, 500, 700, 1000, 1500, 2000, 3000, 4000, 5000, 7000, 10000, 20000
Quantification using: Lower Marker          Final concentration (ng/uL): 0.5000          Dilution factor: 12.0
```

**Sample:** SampE6
**Well location:** E6
**Created:** Tuesday, September 14, 2021 6:54:01 PM

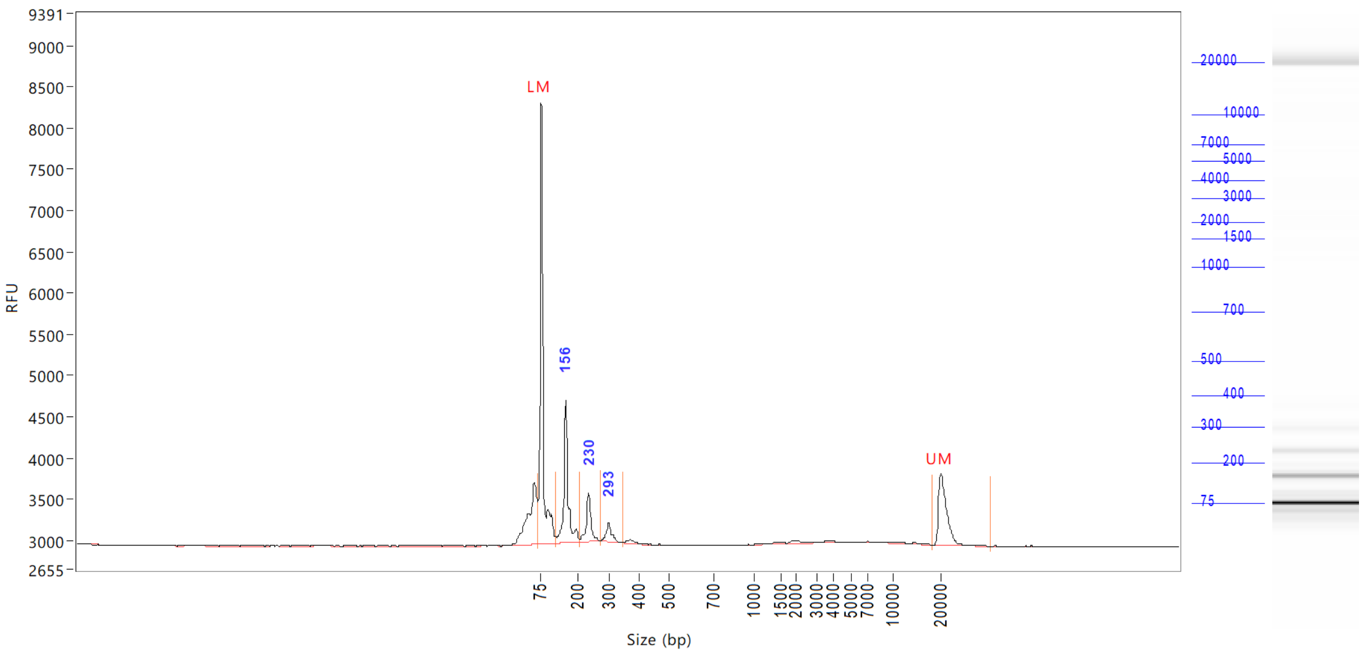


| Peak | Size (bp) | Concentration (ng/uL) | From (bp) | To (bp) | Average size (bp) | CV% | RFU | Corrected peak area |
|---|---|---|---|---|---|---|---|---|
| 1 | 75 (LM) | 0.5000 | 66 | 126 | 79 | 14.39 | 5353 | 40.783 |
| 2 | 156 | 2.5021 | 126 | 201 | 157 | 8.28 | 1724 | 17.007 |
| 3 | 230 | 0.8908 | 201 | 268 | 228 | 4.36 | 593 | 6.055 |
| 4 | 293 | 0.4023 | 268 | 345 | 295 | 4.12 | 223 | 2.735 |
| 5 | 20000 (UM) | 0.1157 | 18171 | 30639 | 20760 | 6.78 | 866 | 9.441 |

| | | |
|---|---|---|
| TIC: | 3.7952 | ng/uL |
| TIM: | 34.8628 | nmole/L |
| Total concentration: | 4.3593 | ng/uL |

```
Sample peak width (sec): 5    Sample min peak height: 50    Sample baseline V to V?: Y    Sample baseline V to V points: 3
Sample filter: Binomial       Number of points for filter: 3  Sample start region (min): 0  Sample end region (min): 30
Marker peak width (sec): 5    Marker min peak height: 200   Marker baseline V to V?: Y    Marker baseline V to V points: 3
Lower marker selection: First peak > 500 RFU                Upper marker selection: Last peak > 200 RFU
Ladder size (bp)75, 200, 300, 400, 500, 700, 1000, 1500, 2000, 3000, 4000, 5000, 7000, 10000, 20000
Quantification using: Lower Marker          Final concentration (ng/uL): 0.5000          Dilution factor: 12.0
```

**Sample:** SampF6
**Well location:** F6
**Created:** Tuesday, September 14, 2021 6:54:01 PM

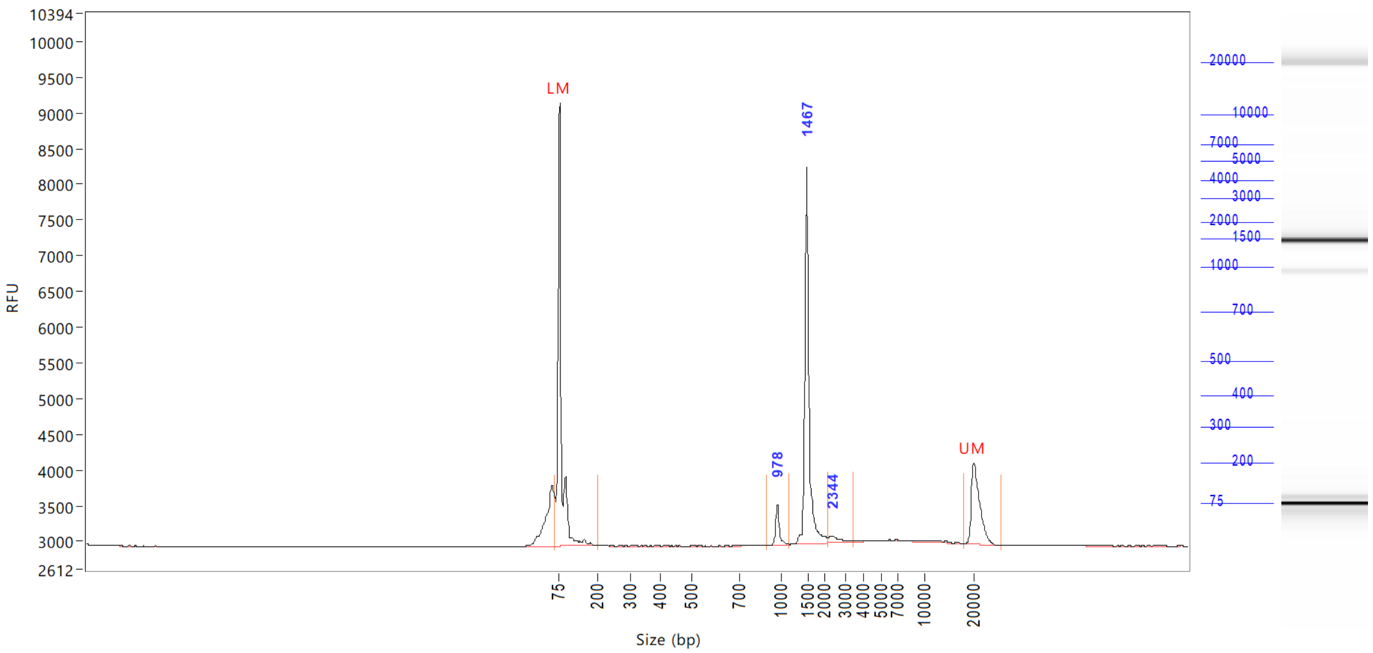


| Peak | Size (bp) | Concentration (ng/uL) | From (bp) | To (bp) | Average size (bp) | CV% | RFU | Corrected peak area |
|---|---|---|---|---|---|---|---|---|
| 1 | 75 (LM) | 0.5000 | 64 | 198 | 80 | 21.24 | 6212 | 50.497 |
| 2 | 978 | 0.4215 | 896 | 1156 | 975 | 2.35 | 564 | 3.547 |
| 3 | 1467 | 4.1998 | 1156 | 2144 | 1493 | 7.79 | 5283 | 35.346 |
| 4 | 2344 | 0.1962 | 2144 | 3429 | 2558 | 12.77 | 100 | 1.651 |
| 5 | 20000 (UM) | 0.1223 | 18171 | 25561 | 20568 | 4.70 | 1140 | 12.356 |

| | | |
|---|---|---|
| TIC: | 4.8174 | ng/uL |
| TIM: | 5.4681 | nmole/L |
| Total concentration: | 5.1541 | ng/uL |

```
Sample peak width (sec): 5     Sample min peak height: 50     Sample baseline V to V?: Y     Sample baseline V to V points: 3
Sample filter: Binomial        Number of points for filter: 3  Sample start region (min): 0  Sample end region (min): 30
Marker peak width (sec): 5     Marker min peak height: 200    Marker baseline V to V?: Y     Marker baseline V to V points: 3
Lower marker selection: First peak > 500 RFU                  Upper marker selection: Last peak > 200 RFU
Ladder size (bp)75, 200, 300, 400, 500, 700, 1000, 1500, 2000, 3000, 4000, 5000, 7000, 10000, 20000
Quantification using: Lower Marker          Final concentration (ng/uL): 0.5000          Dilution factor: 12.0
```

**Sample:** SampG6
**Well location:** G6
**Created:** Tuesday, September 14, 2021 6:54:01 PM

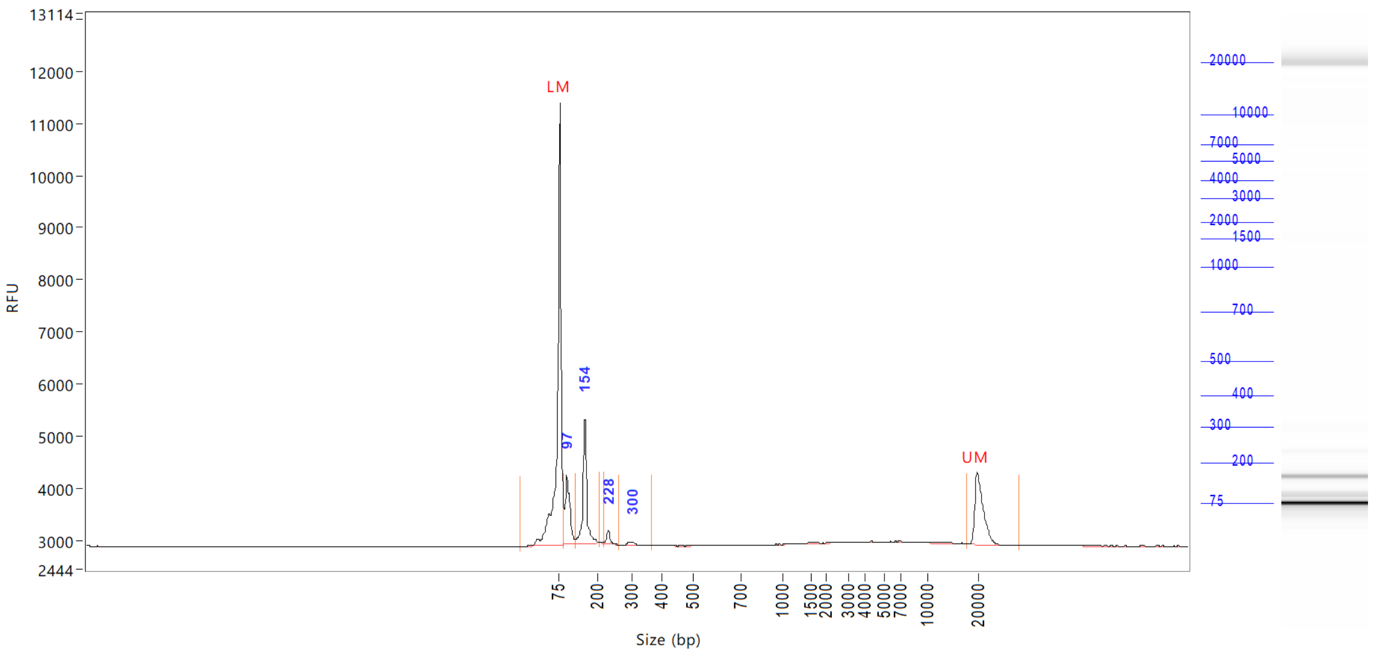


| Peak | Size (bp) | Concentration (ng/uL) | From (bp) | To (bp) | Average size (bp) | CV% | RFU | Corrected peak area |
|---|---|---|---|---|---|---|---|---|
| 1 | 75 (LM) | 0.5000 | 0 | 89 | 66 | 25.84 | 8493 | 79.027 |
| 2 | 97 | 1.1473 | 89 | 126 | 100 | 7.77 | 1317 | 15.111 |
| 3 | 154 | 1.5585 | 126 | 201 | 154 | 6.57 | 2389 | 20.527 |
| 4 | 228 | 0.1419 | 215 | 259 | 227 | 2.39 | 251 | 1.869 |
| 5 | 300 | 0.0811 | 259 | 368 | 297 | 5.32 | 68 | 1.068 |
| 6 | 20000 (UM) | 0.0977 | 17927 | 28463 | 20533 | 4.98 | 1385 | 15.448 |

| | | |
|---|---|---|
| TIC: | 2.9288 | ng/uL |
| TIM: | 36.9501 | nmole/L |
| Total concentration: | 3.2080 | ng/uL |

```
Sample peak width (sec): 5    Sample min peak height: 50    Sample baseline V to V?: Y    Sample baseline V to V points: 3
Sample filter: Binomial       Number of points for filter: 3  Sample start region (min): 0  Sample end region (min): 30
Marker peak width (sec): 5    Marker min peak height: 200   Marker baseline V to V?: Y    Marker baseline V to V points: 3
Lower marker selection: First peak > 500 RFU                Upper marker selection: Last peak > 200 RFU
Ladder size (bp)75, 200, 300, 400, 500, 700, 1000, 1500, 2000, 3000, 4000, 5000, 7000, 10000, 20000
Quantification using: Lower Marker          Final concentration (ng/uL): 0.5000          Dilution factor: 12.0
```

**Sample:** SampH6
**Well location:** H6
**Created:** Tuesday, September 14, 2021 6:54:01 PM

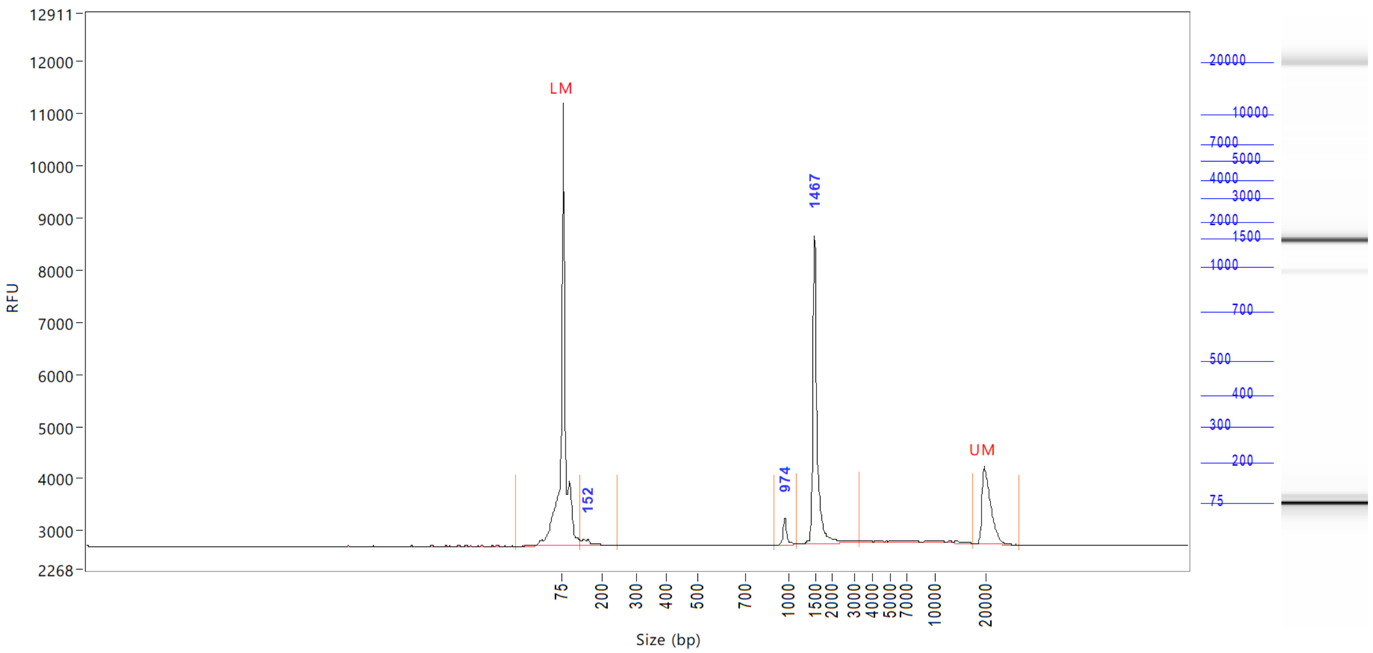


| Peak | Size (bp) | Concentration (ng/uL) | From (bp) | To (bp) | Average size (bp) | CV% | RFU | Corrected peak area |
|---|---|---|---|---|---|---|---|---|
| 1 | 75 (LM) | 0.5000 | 0 | 129 | 70 | 28.80 | 8492 | 89.306 |
| 2 | 152 | 0.1918 | 129 | 242 | 155 | 14.42 | 121 | 2.854 |
| 3 | 974 | 0.2207 | 900 | 1145 | 973 | 2.53 | 512 | 3.285 |
| 4 | 1467 | 2.7567 | 1145 | 3251 | 1521 | 13.79 | 5884 | 41.032 |
| 5 | 20000 (UM) | 0.0917 | 17683 | 27012 | 20521 | 4.91 | 1485 | 16.370 |

| | | |
|---|---|---|
| TIC: | 3.1692 | ng/uL |
| TIM: | 5.3903 | nmole/L |
| Total concentration: | 3.4172 | ng/uL |

Sample peak width (sec): 5    Sample min peak height: 50    Sample baseline V to V?: Y    Sample baseline V to V points: 3
Sample filter: Binomial    Number of points for filter: 3    Sample start region (min): 0    Sample end region (min): 30
Marker peak width (sec): 5    Marker min peak height: 200    Marker baseline V to V?: Y    Marker baseline V to V points: 3
Lower marker selection: First peak > 500 RFU    Upper marker selection: Last peak > 200 RFU
Ladder size (bp)75, 200, 300, 400, 500, 700, 1000, 1500, 2000, 3000, 4000, 5000, 7000, 10000, 20000
Quantification using: Lower Marker    Final concentration (ng/uL): 0.5000    Dilution factor: 12.0

**Sample:** SampA7
**Well location:** A7
**Created:** Tuesday, September 14, 2021 6:54:01 PM

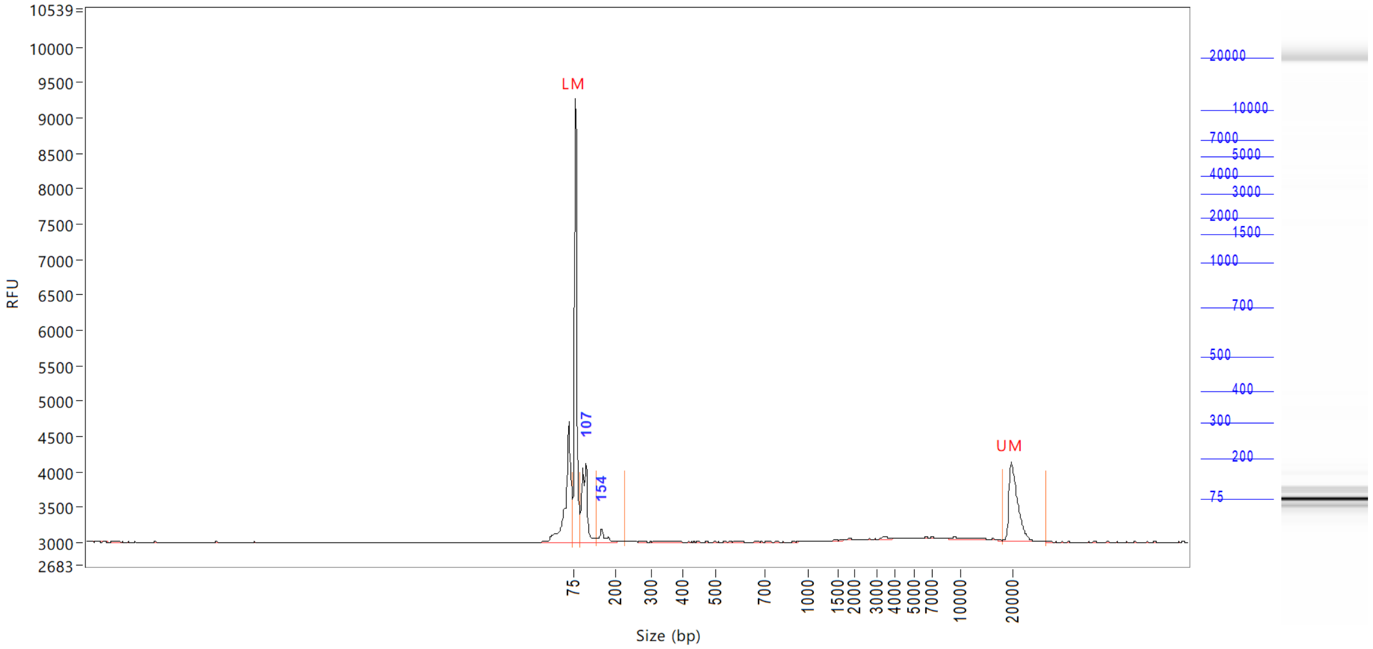


| Peak | Size (bp) | Concentration (ng/uL) | From (bp) | To (bp) | Average size (bp) | CV% | RFU | Corrected peak area |
|---|---|---|---|---|---|---|---|---|
| 1 | 75 (LM) | 0.5000 | 66 | 89 | 74 | 5.97 | 6277 | 38.260 |
| 2 | 107 | 2.1793 | 89 | 137 | 102 | 8.50 | 1109 | 13.897 |
| 3 | 154 | 0.3796 | 137 | 223 | 160 | 10.05 | 191 | 2.421 |
| 4 | 20000 (UM) | 0.1572 | 18171 | 26649 | 20526 | 4.81 | 1117 | 12.028 |

| | | |
|---|---|---|
| TIC: | 2.5590 | ng/uL |
| TIM: | 38.9868 | nmole/L |
| Total concentration: | 3.2704 | ng/uL |

Sample peak width (sec): 5    Sample min peak height: 50    Sample baseline V to V?: Y    Sample baseline V to V points: 3
Sample filter: Binomial    Number of points for filter: 3  Sample start region (min): 0  Sample end region (min): 30
Marker peak width (sec): 5    Marker min peak height: 200    Marker baseline V to V?: Y    Marker baseline V to V points: 3
Lower marker selection: First peak > 500 RFU    Upper marker selection: Last peak > 200 RFU
Ladder size (bp)75, 200, 300, 400, 500, 700, 1000, 1500, 2000, 3000, 4000, 5000, 7000, 10000, 20000
Quantification using: Lower Marker    Final concentration (ng/uL): 0.5000    Dilution factor: 12.0

**Sample:** SampB7
**Well location:** B7
**Created:** Tuesday, September 14, 2021 6:54:01 PM

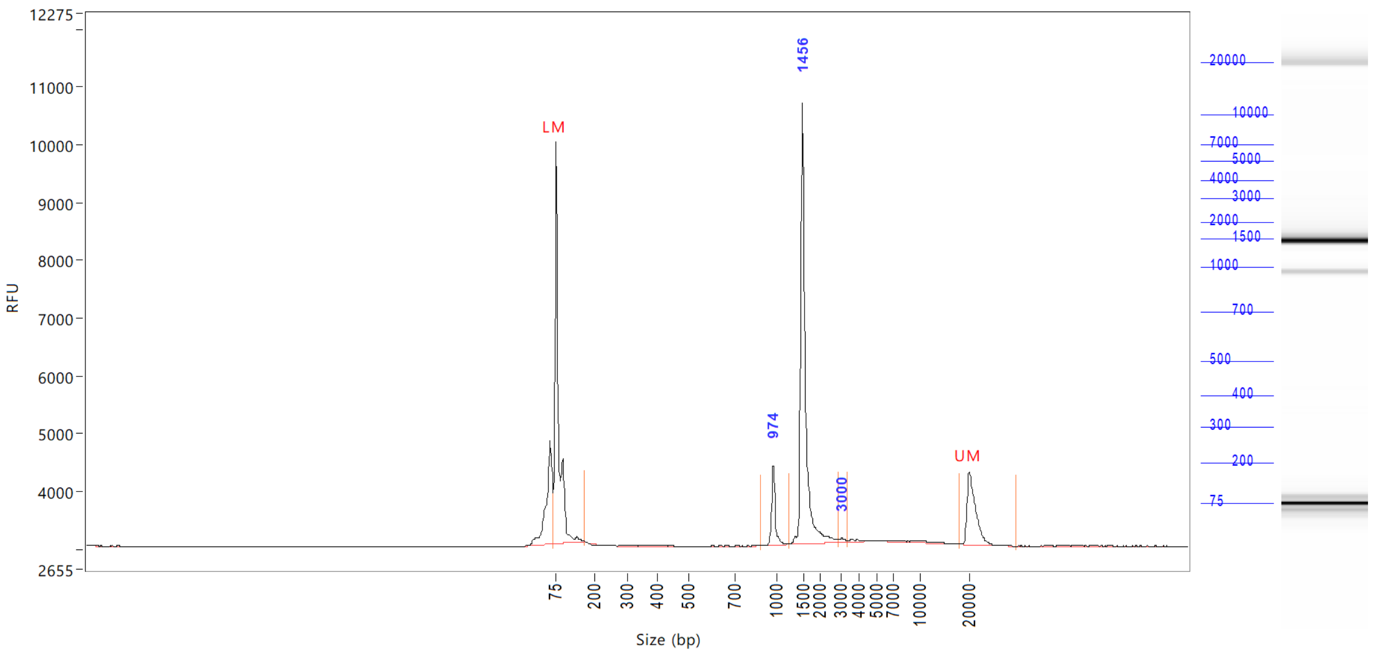


| Peak | Size (bp) | Concentration (ng/uL) | From (bp) | To (bp) | Average size (bp) | CV% | RFU | Corrected peak area |
|---|---|---|---|---|---|---|---|---|
| 1 | 75 (LM) | 0.5000 | 66 | 166 | 80 | 16.85 | 6958 | 61.870 |
| 2 | 974 | 0.8374 | 879 | 1223 | 972 | 2.64 | 1369 | 8.635 |
| 3 | 1456 | 5.6694 | 1223 | 2829 | 1522 | 12.88 | 7643 | 58.461 |
| 4 | 3000 | 0.0660 | 2829 | 3358 | 3036 | 4.71 | 71 | 0.680 |
| 5 | 20000 (UM) | 0.1146 | 17805 | 29551 | 20657 | 5.41 | 1260 | 14.178 |

| | | |
|---|---|---|
| TIC: | 6.5728 | ng/uL |
| TIM: | 7.5854 | nmole/L |
| Total concentration: | 6.9603 | ng/uL |

Sample peak width (sec): 5 Sample min peak height: 50 Sample baseline V to V?: Y Sample baseline V to V points: 3
Sample filter: Binomial Number of points for filter: 3 Sample start region (min): 0 Sample end region (min): 30
Marker peak width (sec): 5 Marker min peak height: 200 Marker baseline V to V?: Y Marker baseline V to V points: 3
Lower marker selection: First peak > 500 RFU Upper marker selection: Last peak > 200 RFU
Ladder size (bp)75, 200, 300, 400, 500, 700, 1000, 1500, 2000, 3000, 4000, 5000, 7000, 10000, 20000
Quantification using: Lower Marker Final concentration (ng/uL): 0.5000 Dilution factor: 12.0

**Sample:** SampC7
**Well location:** C7
**Created:** Tuesday, September 14, 2021 6:54:01 PM

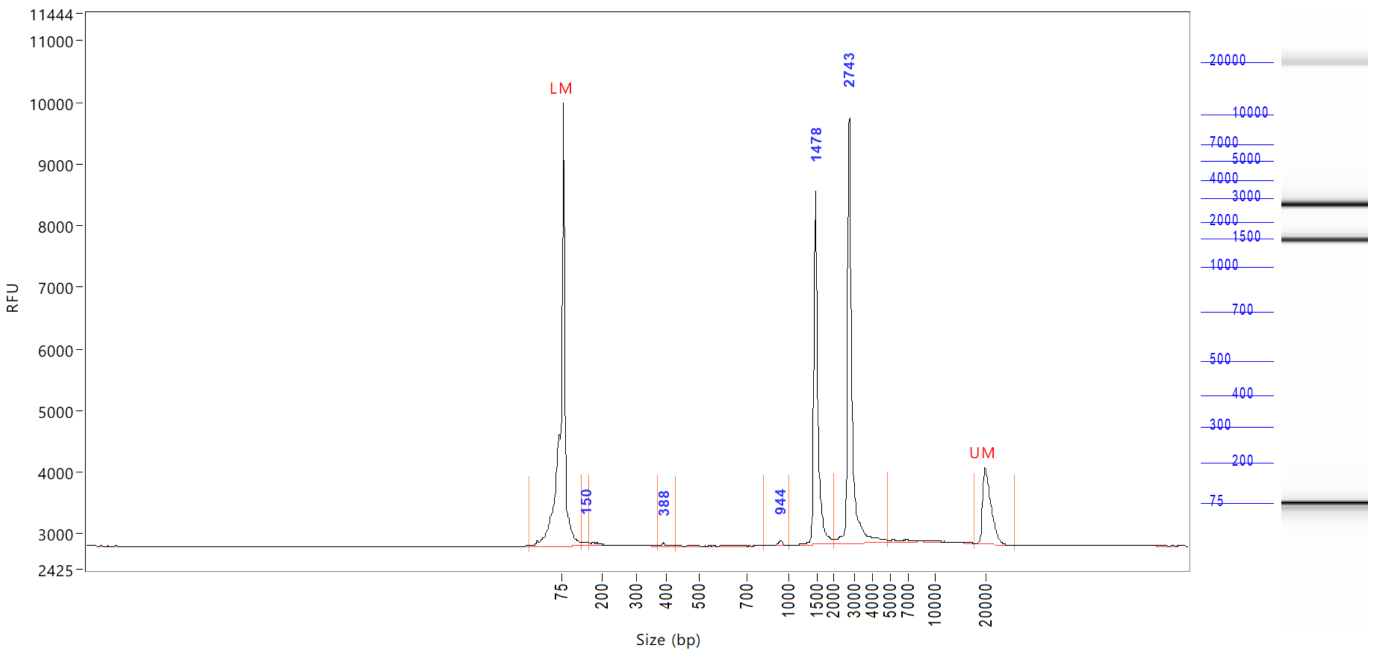


| Peak | Size (bp) | Concentration (ng/uL) | From (bp) | To (bp) | Average size (bp) | CV% | RFU | Corrected peak area |
|---|---|---|---|---|---|---|---|---|
| 1 | 75 (LM) | 0.5000 | 0 | 131 | 65 | 29.80 | 7202 | 80.945 |
| 2 | 150 | 0.0782 | 131 | 160 | 145 | 5.71 | 67 | 1.056 |
| 3 | 388 | 0.0365 | 372 | 428 | 391 | 3.30 | 51 | 0.492 |
| 4 | 944 | 0.0421 | 818 | 1001 | 928 | 2.87 | 72 | 0.568 |
| 5 | 1478 | 2.7641 | 1001 | 2058 | 1496 | 6.60 | 5731 | 37.290 |
| 6 | 2743 | 3.6034 | 2058 | 4862 | 2817 | 11.88 | 6916 | 48.613 |
| 7 | 20000 (UM) | 0.0825 | 17805 | 25924 | 20455 | 4.53 | 1243 | 13.354 |

| | | |
|---|---|---|
| TIC: | 6.5243 | ng/uL |
| TIM: | 6.2620 | nmole/L |
| Total concentration: | 6.8397 | ng/uL |

Sample peak width (sec): 5 Sample min peak height: 50 Sample baseline V to V?: Y Sample baseline V to V points: 3
Sample filter: Binomial Number of points for filter: 3 Sample start region (min): 0 Sample end region (min): 30
Marker peak width (sec): 5 Marker min peak height: 200 Marker baseline V to V?: Y Marker baseline V to V points: 3
Lower marker selection: First peak > 500 RFU Upper marker selection: Last peak > 200 RFU
Ladder size (bp)75, 200, 300, 400, 500, 700, 1000, 1500, 2000, 3000, 4000, 5000, 7000, 10000, 20000
Quantification using: Lower Marker Final concentration (ng/uL): 0.5000 Dilution factor: 12.0

**Sample:** SampD7
**Well location:** D7
**Created:** Tuesday, September 14, 2021 6:54:01 PM

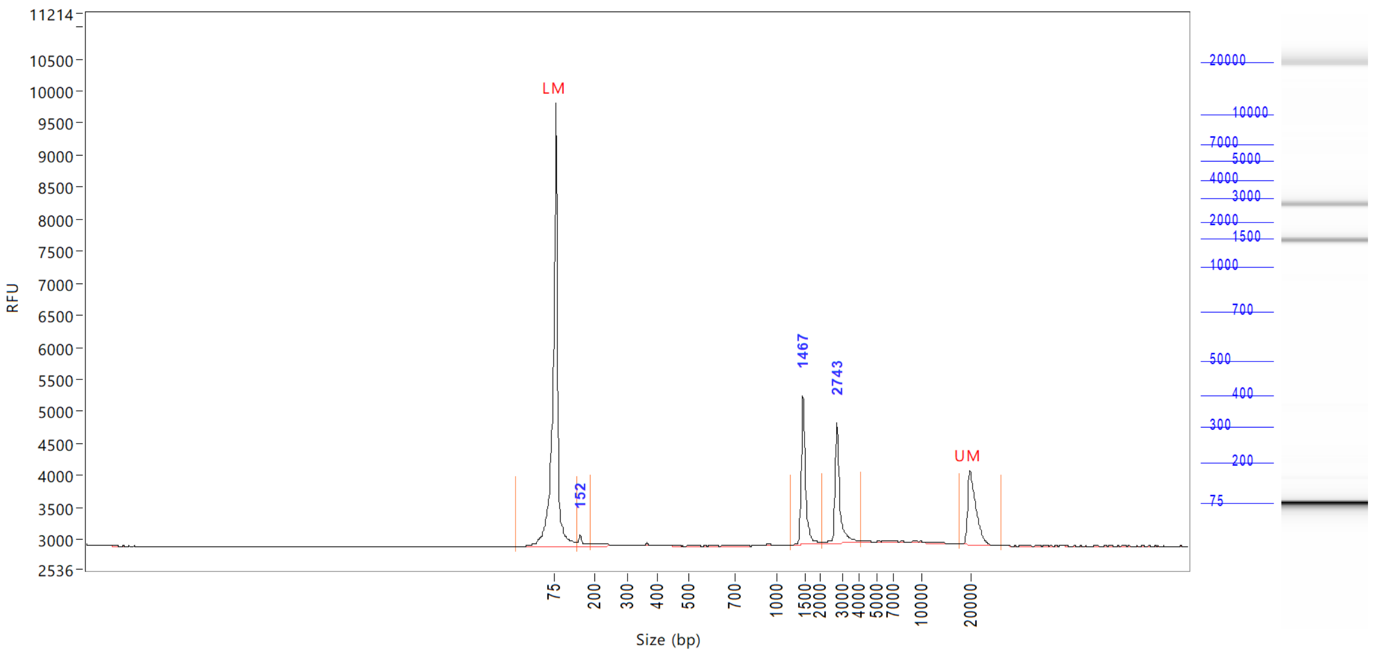


| Peak | Size (bp) | Concentration (ng/uL) | From (bp) | To (bp) | Average size (bp) | CV% | RFU | Corrected peak area |
|---|---|---|---|---|---|---|---|---|
| 1 | 75 (LM) | 0.5000 | 0 | 143 | 69 | 25.27 | 6933 | 79.385 |
| 2 | 152 | 0.1457 | 143 | 187 | 158 | 7.86 | 179 | 1.928 |
| 3 | 1467 | 1.1787 | 1234 | 2058 | 1488 | 6.23 | 2316 | 15.596 |
| 4 | 2743 | 1.0755 | 2058 | 4104 | 2820 | 9.45 | 1885 | 14.229 |
| 5 | 20000 (UM) | 0.0778 | 17927 | 26408 | 20556 | 4.73 | 1145 | 12.351 |

| | | |
|---|---|---|
| TIC: | 2.3999 | ng/uL |
| TIM: | 3.4477 | nmole/L |
| Total concentration: | 2.7738 | ng/uL |

```
Sample peak width (sec): 5    Sample min peak height: 50    Sample baseline V to V?: Y    Sample baseline V to V points: 3
Sample filter: Binomial       Number of points for filter: 3  Sample start region (min): 0  Sample end region (min): 30
Marker peak width (sec): 5    Marker min peak height: 200   Marker baseline V to V?: Y    Marker baseline V to V points: 3
Lower marker selection: First peak > 500 RFU                Upper marker selection: Last peak > 200 RFU
Ladder size (bp)75, 200, 300, 400, 500, 700, 1000, 1500, 2000, 3000, 4000, 5000, 7000, 10000, 20000
Quantification using: Lower Marker          Final concentration (ng/uL): 0.5000          Dilution factor: 12.0
```

**Sample:** SampE7
**Well location:** E7
**Created:** Tuesday, September 14, 2021 6:54:01 PM

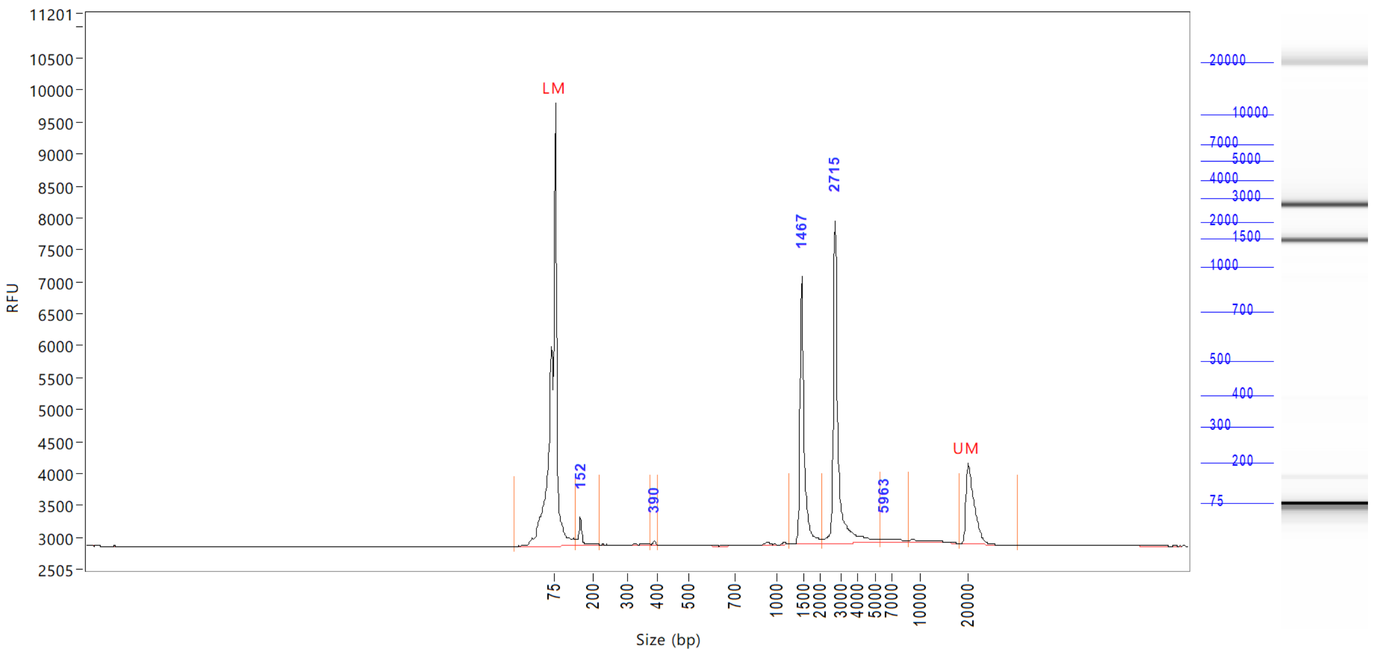


| Peak | Size (bp) | Concentration (ng/uL) | From (bp) | To (bp) | Average size (bp) | CV% | RFU | Corrected peak area |
|---|---|---|---|---|---|---|---|---|
| 1 | 75 (LM) | 0.5000 | 0 | 141 | 62 | 35.28 | 6943 | 95.901 |
| 2 | 152 | 0.2585 | 141 | 217 | 159 | 10.42 | 458 | 4.132 |
| 3 | 390 | 0.0305 | 378 | 403 | 387 | 1.14 | 86 | 0.487 |
| 4 | 1467 | 1.7834 | 1234 | 2087 | 1496 | 7.18 | 4180 | 28.505 |
| 5 | 2715 | 2.4500 | 2087 | 5519 | 2894 | 16.70 | 5055 | 39.160 |
| 6 | 5963 | 0.0915 | 5519 | 8800 | 6943 | 12.94 | 56 | 1.463 |
| 7 | 20000 (UM) | 0.0710 | 18415 | 30156 | 20628 | 5.20 | 1257 | 13.620 |

| | | |
|---|---|---|
| TIC: | 4.6140 | ng/uL |
| TIM: | 6.1798 | nmole/L |
| Total concentration: | 4.8422 | ng/uL |

Sample peak width (sec): 5 Sample min peak height: 50 Sample baseline V to V?: Y Sample baseline V to V points: 3
Sample filter: Binomial Number of points for filter: 3 Sample start region (min): 0 Sample end region (min): 30
Marker peak width (sec): 5 Marker min peak height: 200 Marker baseline V to V?: Y Marker baseline V to V points: 3
Lower marker selection: First peak > 500 RFU Upper marker selection: Last peak > 200 RFU
Ladder size (bp)75, 200, 300, 400, 500, 700, 1000, 1500, 2000, 3000, 4000, 5000, 7000, 10000, 20000
Quantification using: Lower Marker Final concentration (ng/uL): 0.5000 Dilution factor: 12.0

**Sample:** SampF7
**Well location:** F7
**Created:** Tuesday, September 14, 2021 6:54:01 PM

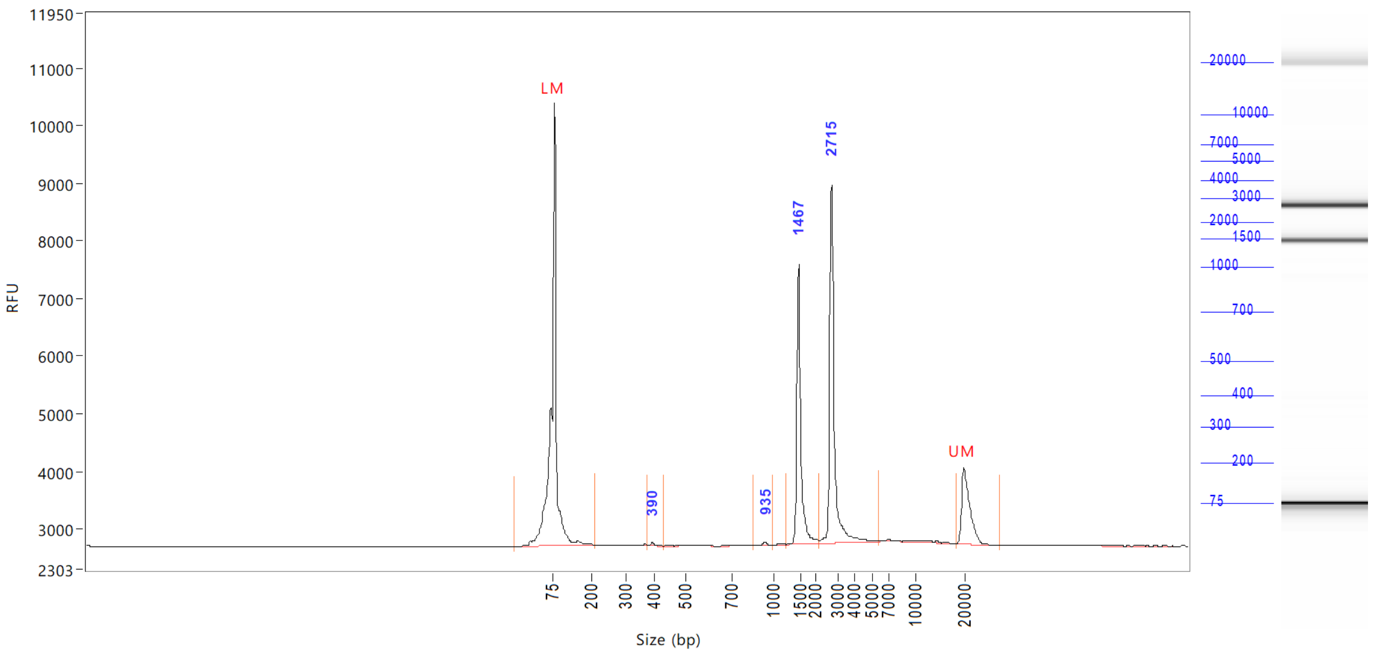


| Peak | Size (bp) | Concentration (ng/uL) | From (bp) | To (bp) | Average size (bp) | CV% | RFU | Corrected peak area |
|---|---|---|---|---|---|---|---|---|
| 1 | 75 (LM) | 0.5000 | 0 | 205 | 67 | 33.05 | 7694 | 90.978 |
| 2 | 390 | 0.0326 | 374 | 426 | 393 | 2.72 | 57 | 0.494 |
| 3 | 935 | 0.0313 | 853 | 991 | 927 | 2.29 | 64 | 0.475 |
| 4 | 1467 | 2.1137 | 1234 | 2115 | 1489 | 6.99 | 4860 | 32.050 |
| 5 | 2715 | 2.8738 | 2115 | 5815 | 2816 | 13.84 | 6200 | 43.576 |
| 6 | 20000 (UM) | 0.0798 | 18415 | 27496 | 20616 | 5.23 | 1322 | 14.518 |

| | | |
|---|---|---|
| TIC: | 5.0514 | ng/uL |
| TIM: | 4.2086 | nmole/L |
| Total concentration: | 5.2378 | ng/uL |

```
Sample peak width (sec): 5    Sample min peak height: 50    Sample baseline V to V?: Y    Sample baseline V to V points: 3
Sample filter: Binomial       Number of points for filter: 3  Sample start region (min): 0  Sample end region (min): 30
Marker peak width (sec): 5    Marker min peak height: 200   Marker baseline V to V?: Y    Marker baseline V to V points: 3
Lower marker selection: First peak > 500 RFU                Upper marker selection: Last peak > 200 RFU
Ladder size (bp)75, 200, 300, 400, 500, 700, 1000, 1500, 2000, 3000, 4000, 5000, 7000, 10000, 20000
Quantification using: Lower Marker          Final concentration (ng/uL): 0.5000          Dilution factor: 12.0
```

**Sample:** SampG7
**Well location:** G7
**Created:** Tuesday, September 14, 2021 6:54:01 PM

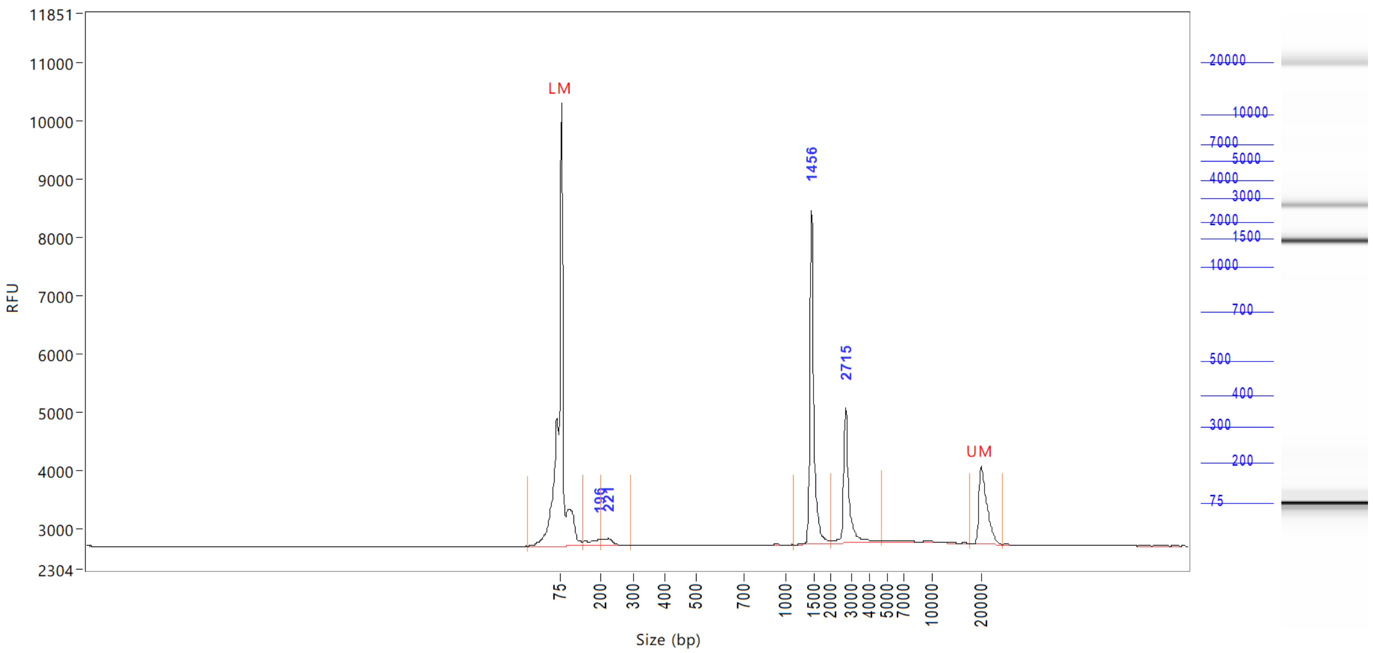


| Peak | Size (bp) | Concentration (ng/uL) | From (bp) | To (bp) | Average size (bp) | CV% | RFU | Corrected peak area |
|---|---|---|---|---|---|---|---|---|
| 1 | 75 (LM) | 0.5000 | 0 | 143 | 69 | 30.83 | 7623 | 92.056 |
| 2 | 196 | 0.2118 | 143 | 201 | 174 | 10.32 | 110 | 3.249 |
| 3 | 221 | 0.2001 | 201 | 291 | 225 | 8.76 | 124 | 3.070 |
| 4 | 1456 | 2.4294 | 1145 | 2001 | 1471 | 5.60 | 5726 | 37.273 |
| 5 | 2715 | 1.2263 | 2001 | 4724 | 2793 | 11.69 | 2325 | 18.815 |
| 6 | 20000 (UM) | 0.0754 | 17805 | 24231 | 20396 | 4.32 | 1332 | 13.889 |

| | | |
|---|---|---|
| TIC: | 4.0676 | ng/uL |
| TIM: | 6.9048 | nmole/L |
| Total concentration: | 4.2403 | ng/uL |

```
Sample peak width (sec): 5    Sample min peak height: 50    Sample baseline V to V?: Y    Sample baseline V to V points: 3
Sample filter: Binomial       Number of points for filter: 3  Sample start region (min): 0  Sample end region (min): 30
Marker peak width (sec): 5    Marker min peak height: 200   Marker baseline V to V?: Y    Marker baseline V to V points: 3
Lower marker selection: First peak > 500 RFU                Upper marker selection: Last peak > 200 RFU
Ladder size (bp)75, 200, 300, 400, 500, 700, 1000, 1500, 2000, 3000, 4000, 5000, 7000, 10000, 20000
Quantification using: Lower Marker          Final concentration (ng/uL): 0.5000          Dilution factor: 12.0
```

**Sample:** SampH7
**Well location:** H7
**Created:** Tuesday, September 14, 2021 6:54:01 PM

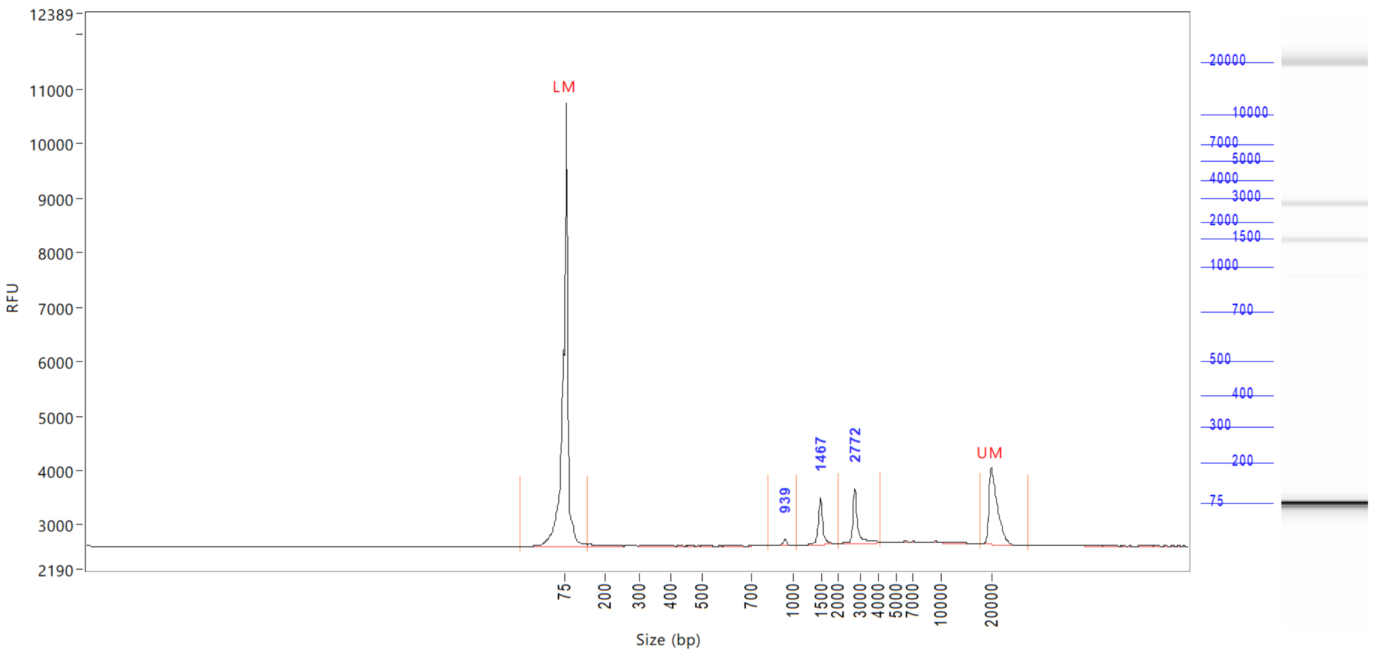


| Peak | Size (bp) | Concentration (ng/uL) | From (bp) | To (bp) | Average size (bp) | CV% | RFU | Corrected peak area |
|---|---|---|---|---|---|---|---|---|
| 1 | 75 (LM) | 0.5000 | 0 | 141 | 67 | 26.08 | 8150 | 93.724 |
| 2 | 939 | 0.0514 | 818 | 1056 | 932 | 2.71 | 110 | 0.802 |
| 3 | 1467 | 0.4049 | 1056 | 2001 | 1478 | 6.72 | 869 | 6.325 |
| 4 | 2772 | 0.4959 | 2001 | 4104 | 2840 | 10.69 | 1000 | 7.746 |
| 5 | 20000 (UM) | 0.0823 | 17927 | 27375 | 20532 | 4.86 | 1399 | 15.436 |

| | | |
|---|---|---|
| TIC: | 0.9521 | ng/uL |
| TIM: | 0.8291 | nmole/L |
| Total concentration: | 1.1917 | ng/uL |

Sample peak width (sec): 5    Sample min peak height: 50    Sample baseline V to V?: Y    Sample baseline V to V points: 3
Sample filter: Binomial    Number of points for filter: 3  Sample start region (min): 0  Sample end region (min): 30
Marker peak width (sec): 5    Marker min peak height: 200    Marker baseline V to V?: Y    Marker baseline V to V points: 3
Lower marker selection: First peak > 500 RFU    Upper marker selection: Last peak > 200 RFU
Ladder size (bp)75, 200, 300, 400, 500, 700, 1000, 1500, 2000, 3000, 4000, 5000, 7000, 10000, 20000
Quantification using: Lower Marker    Final concentration (ng/uL): 0.5000    Dilution factor: 12.0

**Sample:** SampA8
**Well location:** A8
**Created:** Tuesday, September 14, 2021 6:54:01 PM

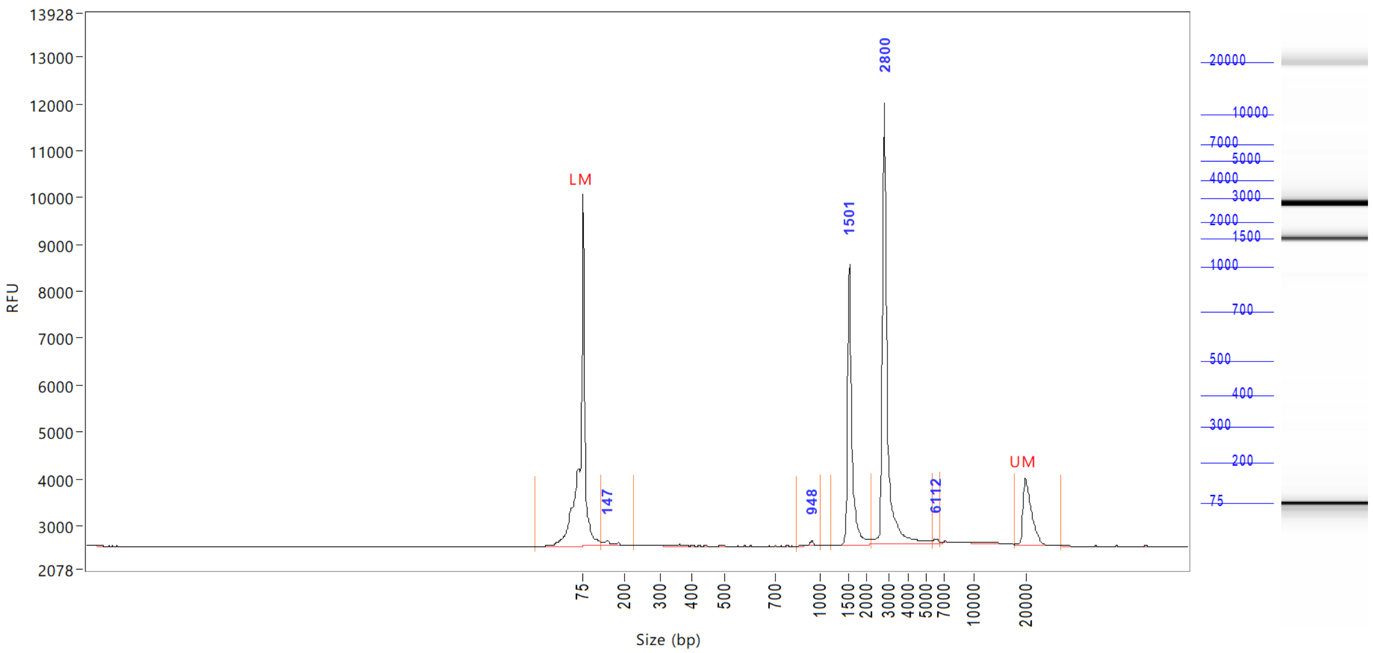


| Peak | Size (bp) | Concentration (ng/uL) | From (bp) | To (bp) | Average size (bp) | CV% | RFU | Corrected peak area |
|---|---|---|---|---|---|---|---|---|
| 1 | 75 (LM) | 0.5000 | 0 | 129 | 63 | 33.39 | 7503 | 85.691 |
| 2 | 147 | 0.1646 | 129 | 224 | 155 | 11.97 | 115 | 2.350 |
| 3 | 948 | 0.0494 | 835 | 1001 | 932 | 2.66 | 95 | 0.706 |
| 4 | 1501 | 2.8416 | 1178 | 2201 | 1544 | 7.98 | 5998 | 40.583 |
| 5 | 2800 | 4.7349 | 2201 | 5593 | 2899 | 12.88 | 9423 | 67.623 |
| 6 | 6112 | 0.0407 | 5593 | 6630 | 6055 | 4.46 | 84 | 0.581 |
| 7 | 20000 (UM) | 0.0859 | 17805 | 26649 | 20464 | 4.53 | 1417 | 14.725 |

| | | |
|---|---|---|
| TIC: | 7.8312 | ng/uL |
| TIM: | 7.5616 | nmole/L |
| Total concentration: | 8.0393 | ng/uL |

Sample peak width (sec): 5 Sample min peak height: 50 Sample baseline V to V?: Y Sample baseline V to V points: 3
Sample filter: Binomial Number of points for filter: 3 Sample start region (min): 0 Sample end region (min): 30
Marker peak width (sec): 5 Marker min peak height: 200 Marker baseline V to V?: Y Marker baseline V to V points: 3
Lower marker selection: First peak > 500 RFU Upper marker selection: Last peak > 200 RFU
Ladder size (bp)75, 200, 300, 400, 500, 700, 1000, 1500, 2000, 3000, 4000, 5000, 7000, 10000, 20000
Quantification using: Lower Marker Final concentration (ng/uL): 0.5000 Dilution factor: 12.0

**Sample:** SampB8
**Well location:** B8
**Created:** Tuesday, September 14, 2021 6:54:01 PM

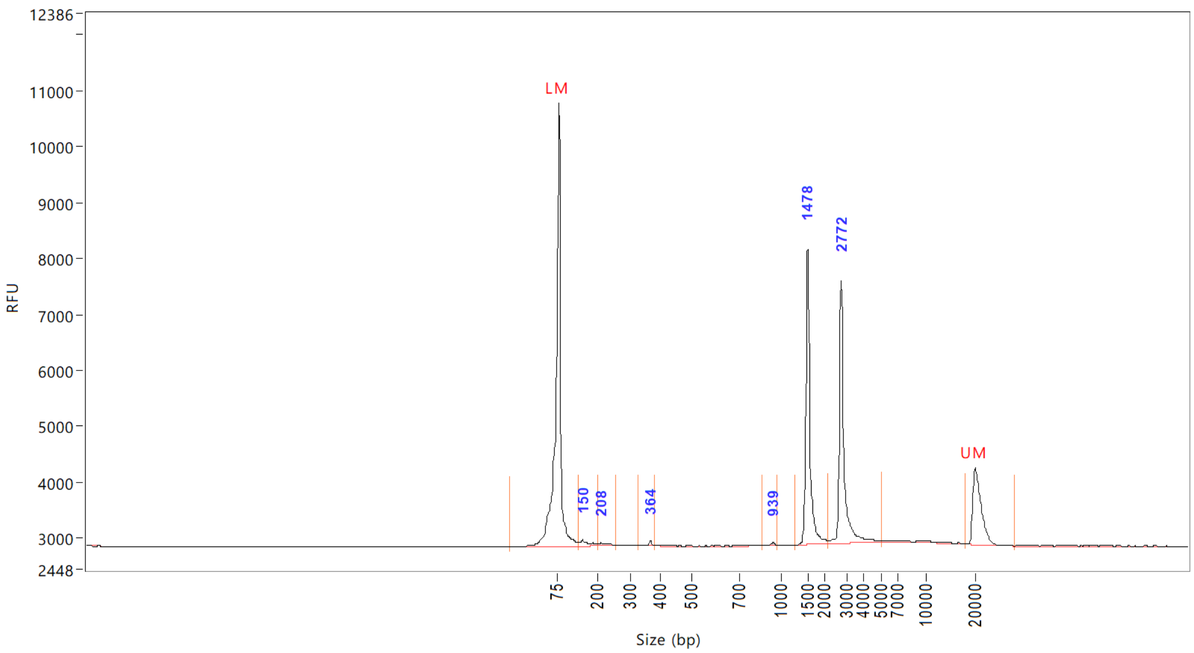


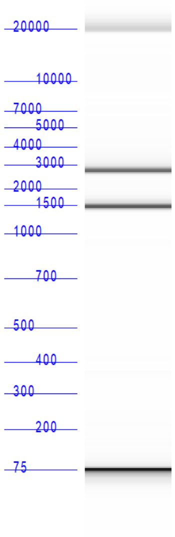


| Peak | Size (bp) | Concentration (ng/uL) | From (bp) | To (bp) | Average size (bp) | CV% | RFU | Corrected peak area |
|---|---|---|---|---|---|---|---|---|
| 1 | 75 (LM) | 0.5000 | 0 | 141 | 67 | 27.60 | 7939 | 87.401 |
| 2 | 150 | 0.1488 | 141 | 200 | 166 | 10.54 | 109 | 2.167 |
| 3 | 208 | 0.0798 | 200 | 257 | 220 | 6.40 | 53 | 1.163 |
| 4 | 364 | 0.0446 | 323 | 380 | 357 | 2.89 | 90 | 0.650 |
| 5 | 939 | 0.0287 | 866 | 974 | 929 | 2.16 | 55 | 0.419 |
| 6 | 1478 | 2.4068 | 1245 | 2087 | 1497 | 6.69 | 5279 | 35.059 |
| 7 | 2772 | 2.3489 | 2087 | 5001 | 2861 | 12.72 | 4700 | 34.216 |
| 8 | 20000 (UM) | 0.0859 | 18049 | 28100 | 20625 | 4.97 | 1390 | 15.020 |

| | | |
|---|---|---|
| TIC: | 5.0576 | ng/uL |
| TIM: | 6.3243 | nmole/L |
| Total concentration: | 5.2417 | ng/uL |

Sample peak width (sec): 5 Sample min peak height: 50 Sample baseline V to V?: Y Sample baseline V to V points: 3
Sample filter: Binomial Number of points for filter: 3 Sample start region (min): 0 Sample end region (min): 30
Marker peak width (sec): 5 Marker min peak height: 200 Marker baseline V to V?: Y Marker baseline V to V points: 3
Lower marker selection: First peak > 500 RFU Upper marker selection: Last peak > 200 RFU
Ladder size (bp)75, 200, 300, 400, 500, 700, 1000, 1500, 2000, 3000, 4000, 5000, 7000, 10000, 20000
Quantification using: Lower Marker Final concentration (ng/uL): 0.5000 Dilution factor: 12.0

**Sample:** SampC8
**Well location:** C8
**Created:** Tuesday, September 14, 2021 6:54:01 PM

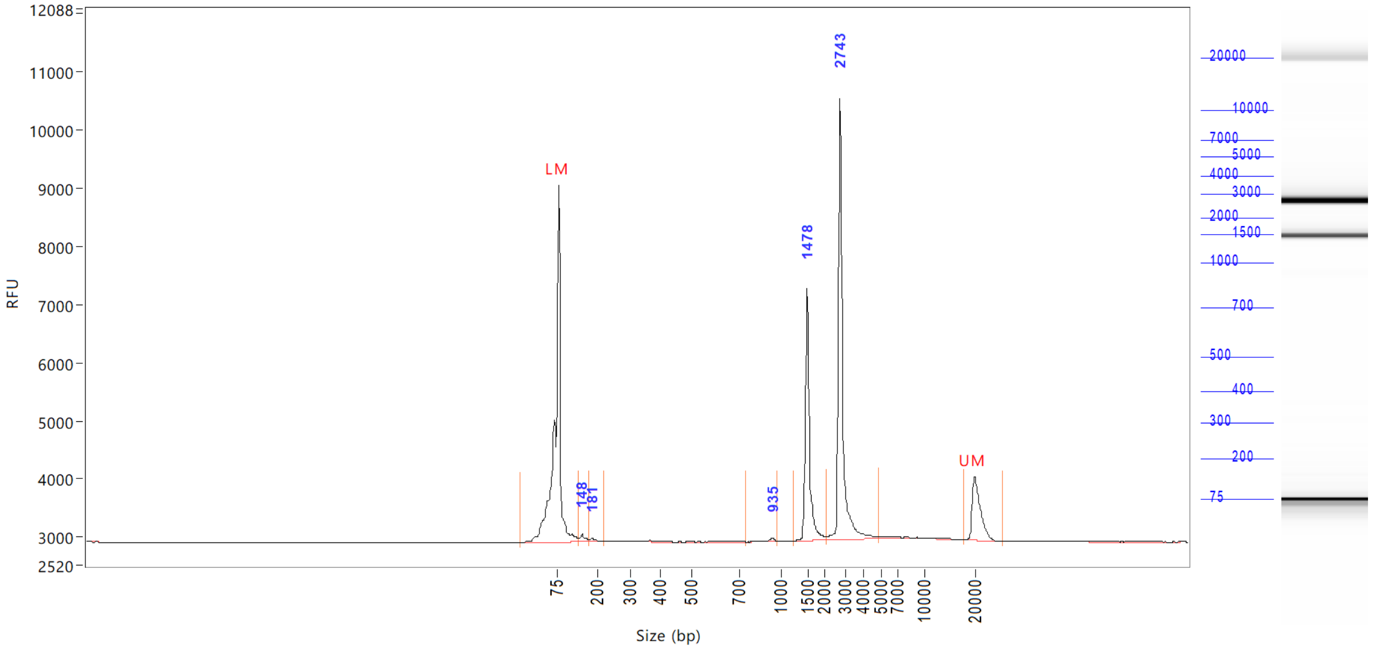


| Peak | Size (bp) | Concentration (ng/uL) | From (bp) | To (bp) | Average size (bp) | CV% | RFU | Corrected peak area |
|---|---|---|---|---|---|---|---|---|
| 1 | 75 (LM) | 0.5000 | 0 | 139 | 62 | 35.16 | 6141 | 77.050 |
| 2 | 148 | 0.1142 | 139 | 171 | 152 | 5.84 | 128 | 1.467 |
| 3 | 181 | 0.0652 | 171 | 219 | 189 | 6.68 | 60 | 0.838 |
| 4 | 935 | 0.0406 | 744 | 974 | 911 | 4.66 | 58 | 0.522 |
| 5 | 1478 | 2.2522 | 1212 | 2058 | 1498 | 6.76 | 4337 | 28.922 |
| 6 | 2743 | 4.0011 | 2058 | 4897 | 2793 | 11.16 | 7599 | 51.381 |
| 7 | 20000 (UM) | 0.0754 | 17805 | 25682 | 20597 | 4.63 | 1088 | 11.621 |

| | | |
|---|---|---|
| TIC: | 6.4734 | ng/uL |
| TIM: | 6.7091 | nmole/L |
| Total concentration: | 6.7095 | ng/uL |

Sample peak width (sec): 5    Sample min peak height: 50    Sample baseline V to V?: Y    Sample baseline V to V points: 3
Sample filter: Binomial    Number of points for filter: 3    Sample start region (min): 0    Sample end region (min): 30
Marker peak width (sec): 5    Marker min peak height: 200    Marker baseline V to V?: Y    Marker baseline V to V points: 3
Lower marker selection: First peak > 500 RFU    Upper marker selection: Last peak > 200 RFU
Ladder size (bp)75, 200, 300, 400, 500, 700, 1000, 1500, 2000, 3000, 4000, 5000, 7000, 10000, 20000
Quantification using: Lower Marker    Final concentration (ng/uL): 0.5000    Dilution factor: 12.0

**Sample:** SampD8
**Well location:** D8
**Created:** Tuesday, September 14, 2021 6:54:01 PM

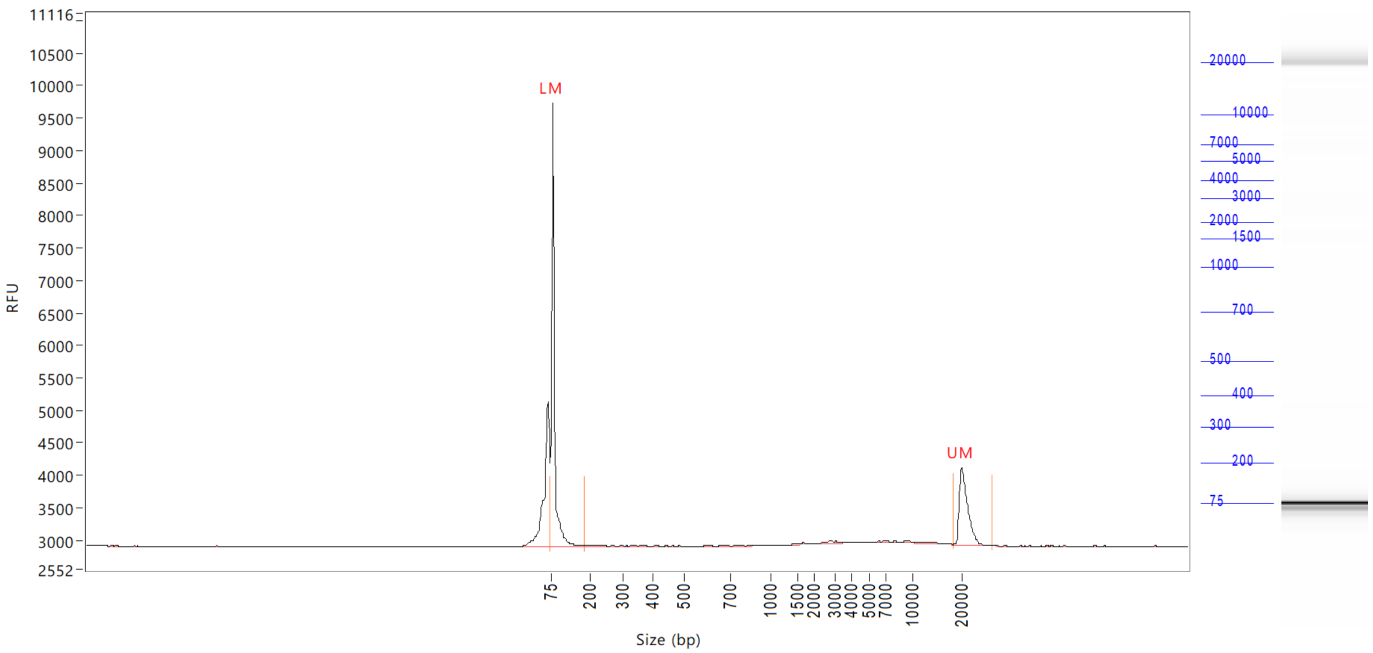


| Peak | Size (bp) | Concentration (ng/uL) | From (bp) | To (bp) | Average size (bp) | CV% | RFU | Corrected peak area |
|---|---|---|---|---|---|---|---|---|
| 1 | 75 (LM) | 0.5000 | 67 | 179 | 78 | 17.06 | 6844 | 49.477 |
| 2 | 20000 (UM) | 0.1331 | 18171 | 26287 | 20635 | 4.85 | 1202 | 13.173 |

| | | |
|---|---|---|
| TIC: | 0.0000 | ng/uL |
| TIM: | 0.0000 | nmole/L |
| Total concentration: | 0.6040 | ng/uL |

```
Sample peak width (sec): 5    Sample min peak height: 50    Sample baseline V to V?: Y    Sample baseline V to V points: 3
Sample filter: Binomial       Number of points for filter: 3  Sample start region (min): 0  Sample end region (min): 30
Marker peak width (sec): 5    Marker min peak height: 200   Marker baseline V to V?: Y    Marker baseline V to V points: 3
Lower marker selection: First peak > 500 RFU                Upper marker selection: Last peak > 200 RFU
Ladder size (bp)75, 200, 300, 400, 500, 700, 1000, 1500, 2000, 3000, 4000, 5000, 7000, 10000, 20000
Quantification using: Lower Marker          Final concentration (ng/uL): 0.5000          Dilution factor: 12.0
```

**Sample:** SampE8
**Well location:** E8
**Created:** Tuesday, September 14, 2021 6:54:01 PM

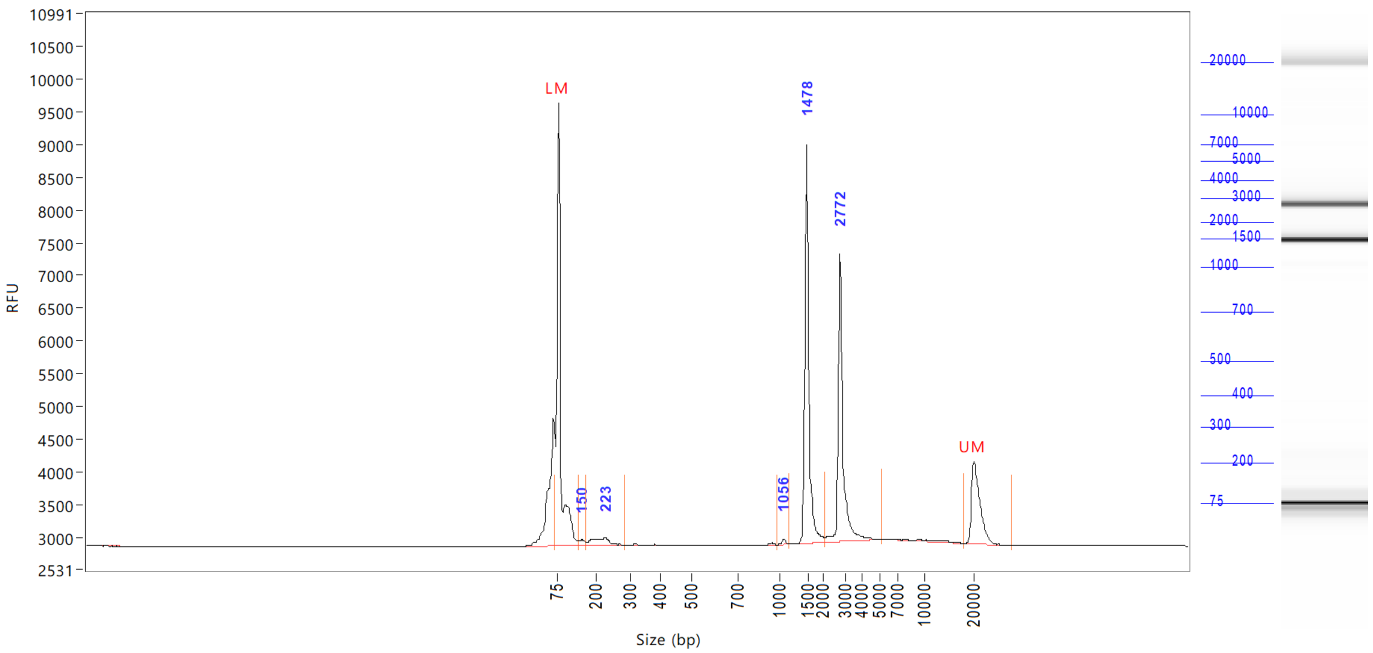


| Peak | Size (bp) | Concentration (ng/uL) | From (bp) | To (bp) | Average size (bp) | CV% | RFU | Corrected peak area |
|---|---|---|---|---|---|---|---|---|
| 1 | 75 (LM) | 0.5000 | 67 | 141 | 81 | 17.66 | 6751 | 56.687 |
| 2 | 150 | 0.1318 | 141 | 166 | 152 | 4.77 | 100 | 1.245 |
| 3 | 223 | 0.4674 | 166 | 282 | 206 | 11.22 | 115 | 4.416 |
| 4 | 1056 | 0.0598 | 978 | 1167 | 1043 | 3.16 | 89 | 0.565 |
| 5 | 1478 | 4.1024 | 1167 | 2058 | 1497 | 6.40 | 6063 | 38.759 |
| 6 | 2772 | 3.4030 | 2058 | 5149 | 2820 | 10.73 | 4367 | 32.151 |
| 7 | 20000 (UM) | 0.1224 | 17805 | 27617 | 20635 | 5.19 | 1256 | 13.879 |

| | | |
|---|---|---|
| TIC: | 8.1644 | ng/uL |
| TIM: | 11.7476 | nmole/L |
| Total concentration: | 8.4214 | ng/uL |

Sample peak width (sec): 5 Sample min peak height: 50 Sample baseline V to V?: Y Sample baseline V to V points: 3
Sample filter: Binomial Number of points for filter: 3 Sample start region (min): 0 Sample end region (min): 30
Marker peak width (sec): 5 Marker min peak height: 200 Marker baseline V to V?: Y Marker baseline V to V points: 3
Lower marker selection: First peak > 500 RFU Upper marker selection: Last peak > 200 RFU
Ladder size (bp)75, 200, 300, 400, 500, 700, 1000, 1500, 2000, 3000, 4000, 5000, 7000, 10000, 20000
Quantification using: Lower Marker Final concentration (ng/uL): 0.5000 Dilution factor: 12.0

**Sample:** SampF8
**Well location:** F8
**Created:** Tuesday, September 14, 2021 6:54:01 PM

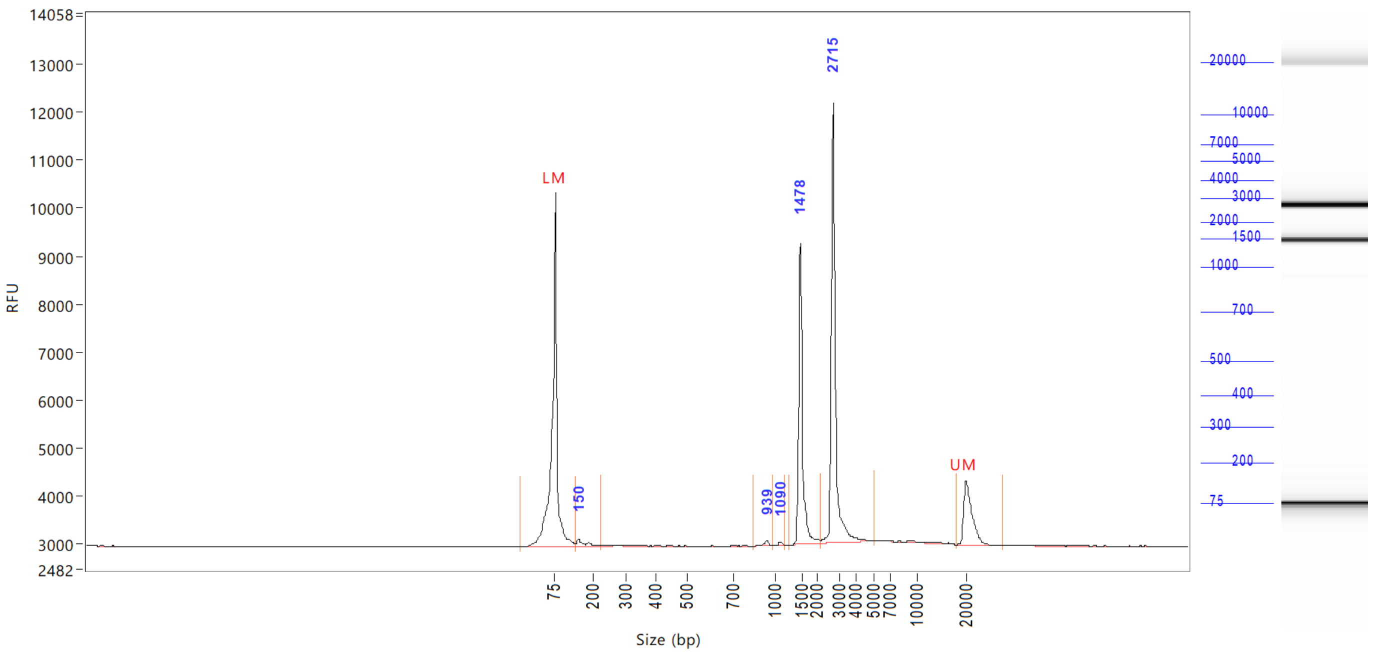


| Peak | Size (bp) | Concentration (ng/uL) | From (bp) | To (bp) | Average size (bp) | CV% | RFU | Corrected peak area |
|---|---|---|---|---|---|---|---|---|
| 1 | 75 (LM) | 0.5000 | 0 | 139 | 67 | 28.59 | 7375 | 87.387 |
| 2 | 150 | 0.1865 | 139 | 221 | 166 | 12.59 | 157 | 2.716 |
| 3 | 939 | 0.0509 | 848 | 991 | 931 | 2.18 | 109 | 0.741 |
| 4 | 1090 | 0.0243 | 991 | 1190 | 1081 | 3.04 | 66 | 0.354 |
| 5 | 1478 | 2.6833 | 1267 | 2087 | 1493 | 6.33 | 6272 | 39.080 |
| 6 | 2715 | 4.0252 | 2087 | 5001 | 2783 | 9.99 | 9173 | 58.624 |
| 7 | 20000 (UM) | 0.0855 | 18049 | 27617 | 20681 | 5.29 | 1327 | 14.943 |

| | | |
|---|---|---|
| TIC: | 6.9701 | ng/uL |
| TIM: | 7.3126 | nmole/L |
| Total concentration: | 7.1818 | ng/uL |

Sample peak width (sec): 5 Sample min peak height: 50 Sample baseline V to V?: Y Sample baseline V to V points: 3
Sample filter: Binomial Number of points for filter: 3 Sample start region (min): 0 Sample end region (min): 30
Marker peak width (sec): 5 Marker min peak height: 200 Marker baseline V to V?: Y Marker baseline V to V points: 3
Lower marker selection: First peak > 500 RFU Upper marker selection: Last peak > 200 RFU
Ladder size (bp)75, 200, 300, 400, 500, 700, 1000, 1500, 2000, 3000, 4000, 5000, 7000, 10000, 20000
Quantification using: Lower Marker Final concentration (ng/uL): 0.5000 Dilution factor: 12.0

**Sample:** SampG8
**Well location:** G8
**Created:** Tuesday, September 14, 2021 6:54:01 PM

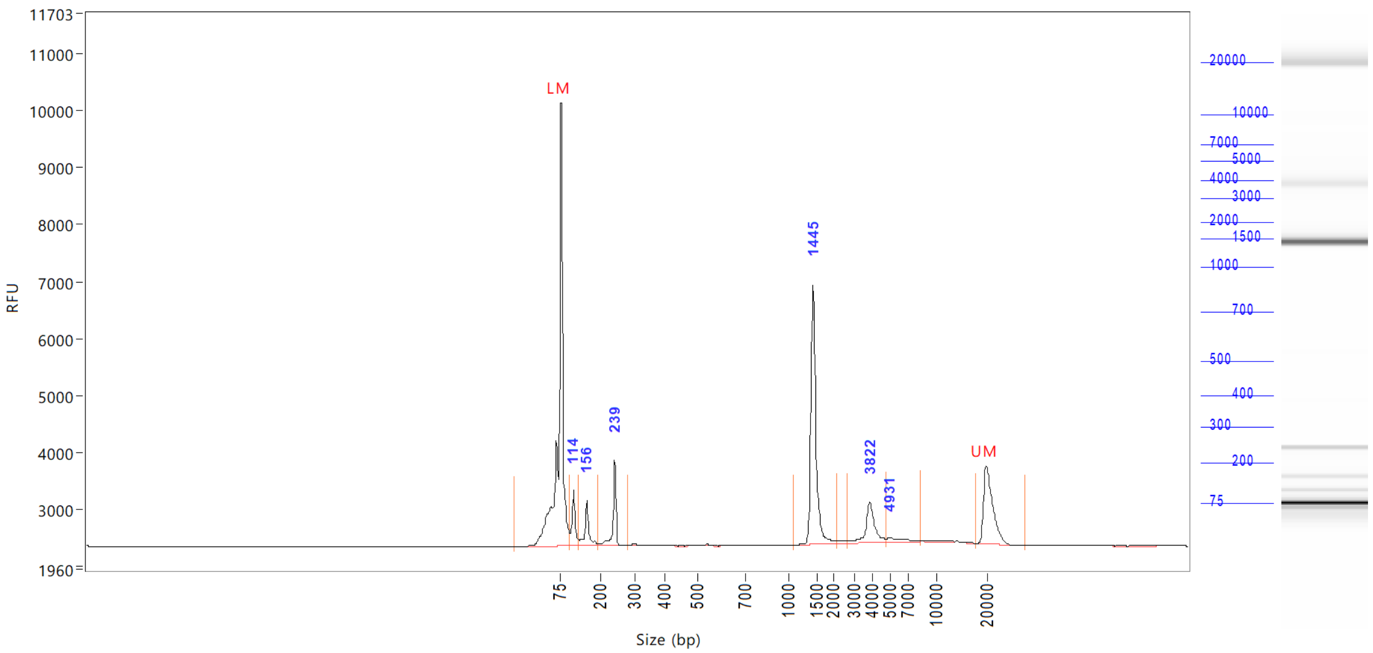


| Peak | Size (bp) | Concentration (ng/uL) | From (bp) | To (bp) | Average size (bp) | CV% | RFU | Corrected peak area |
|---|---|---|---|---|---|---|---|---|
| 1 | 75 (LM) | 0.5000 | 0 | 103 | 65 | 29.67 | 7777 | 83.566 |
| 2 | 114 | 0.5527 | 103 | 131 | 113 | 5.47 | 971 | 7.698 |
| 3 | 156 | 0.5210 | 131 | 192 | 155 | 7.22 | 804 | 7.257 |
| 4 | 239 | 0.7008 | 192 | 280 | 234 | 4.34 | 1492 | 9.761 |
| 5 | 1445 | 2.7117 | 1090 | 2172 | 1457 | 6.87 | 4548 | 37.767 |
| 6 | 3822 | 0.6719 | 2686 | 4724 | 3805 | 9.81 | 725 | 9.357 |
| 7 | 4931 | 0.1189 | 4724 | 8334 | 5877 | 16.89 | 72 | 1.656 |
| 8 | 20000 (UM) | 0.0963 | 17805 | 27617 | 20526 | 5.12 | 1371 | 16.093 |

| | | |
|---|---|---|
| TIC: | 5.2770 | ng/uL |
| TIM: | 21.8718 | nmole/L |
| Total concentration: | 5.4338 | ng/uL |

Sample peak width (sec): 5 Sample min peak height: 50 Sample baseline V to V?: Y Sample baseline V to V points: 3
Sample filter: Binomial Number of points for filter: 3 Sample start region (min): 0 Sample end region (min): 30
Marker peak width (sec): 5 Marker min peak height: 200 Marker baseline V to V?: Y Marker baseline V to V points: 3
Lower marker selection: First peak > 500 RFU Upper marker selection: Last peak > 200 RFU
Ladder size (bp)75, 200, 300, 400, 500, 700, 1000, 1500, 2000, 3000, 4000, 5000, 7000, 10000, 20000
Quantification using: Lower Marker Final concentration (ng/uL): 0.5000 Dilution factor: 12.0

**Sample:** SampH8
**Well location:** H8
**Created:** Tuesday, September 14, 2021 6:54:01 PM

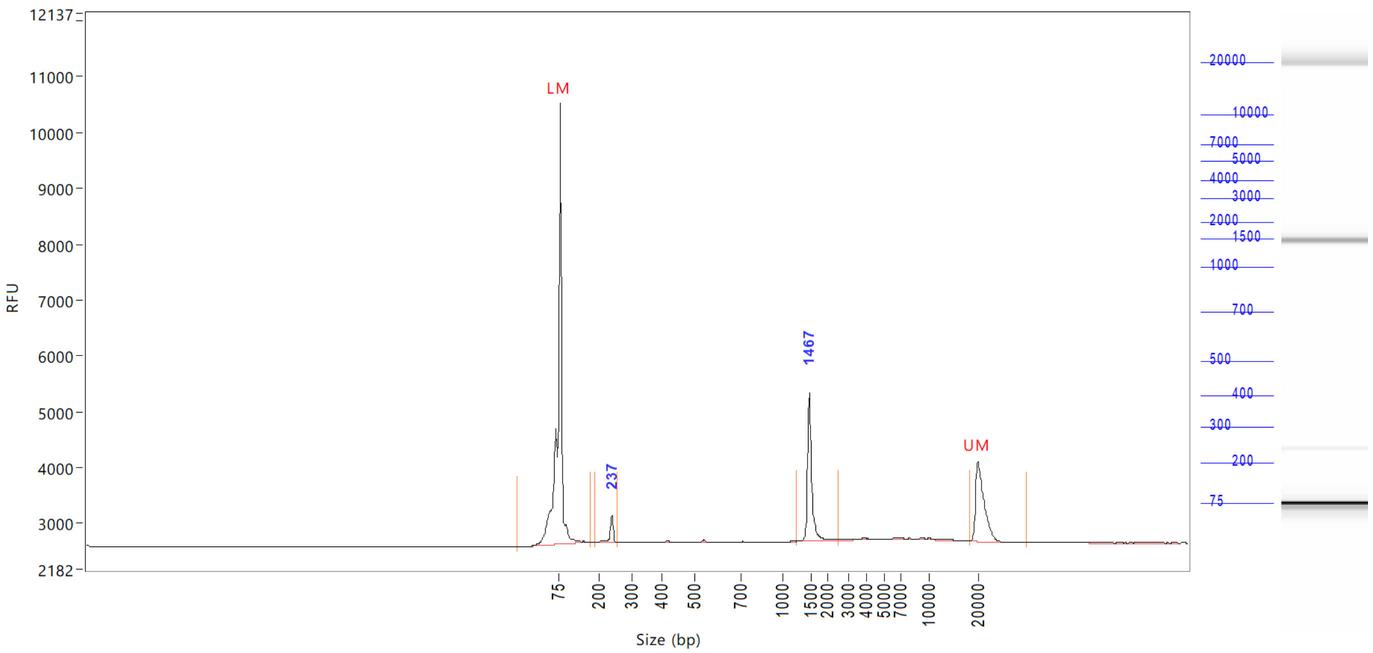


| Peak | Size (bp) | Concentration (ng/uL) | From (bp) | To (bp) | Average size (bp) | CV% | RFU | Corrected peak area |
|---|---|---|---|---|---|---|---|---|
| 1 | 75 (LM) | 0.5000 | 0 | 173 | 66 | 25.67 | 7913 | 77.154 |
| 2 | 237 | 0.2235 | 187 | 253 | 231 | 3.76 | 471 | 2.874 |
| 3 | 1467 | 1.4177 | 1223 | 2543 | 1488 | 7.96 | 2650 | 18.231 |
| 4 | 20000 (UM) | 0.1043 | 18293 | 29914 | 20611 | 5.41 | 1441 | 16.095 |

| | | |
|---|---|---|
| TIC: | 1.6413 | ng/uL |
| TIM: | 3.1596 | nmole/L |
| Total concentration: | 1.9695 | ng/uL |

Sample peak width (sec): 5    Sample min peak height: 50    Sample baseline V to V?: Y    Sample baseline V to V points: 3
Sample filter: Binomial    Number of points for filter: 3    Sample start region (min): 0    Sample end region (min): 30
Marker peak width (sec): 5    Marker min peak height: 200    Marker baseline V to V?: Y    Marker baseline V to V points: 3
Lower marker selection: First peak > 500 RFU    Upper marker selection: Last peak > 200 RFU
Ladder size (bp)75, 200, 300, 400, 500, 700, 1000, 1500, 2000, 3000, 4000, 5000, 7000, 10000, 20000
Quantification using: Lower Marker    Final concentration (ng/uL): 0.5000    Dilution factor: 12.0

**Sample:** SampA9
**Well location:** A9
**Created:** Tuesday, September 14, 2021 6:54:01 PM

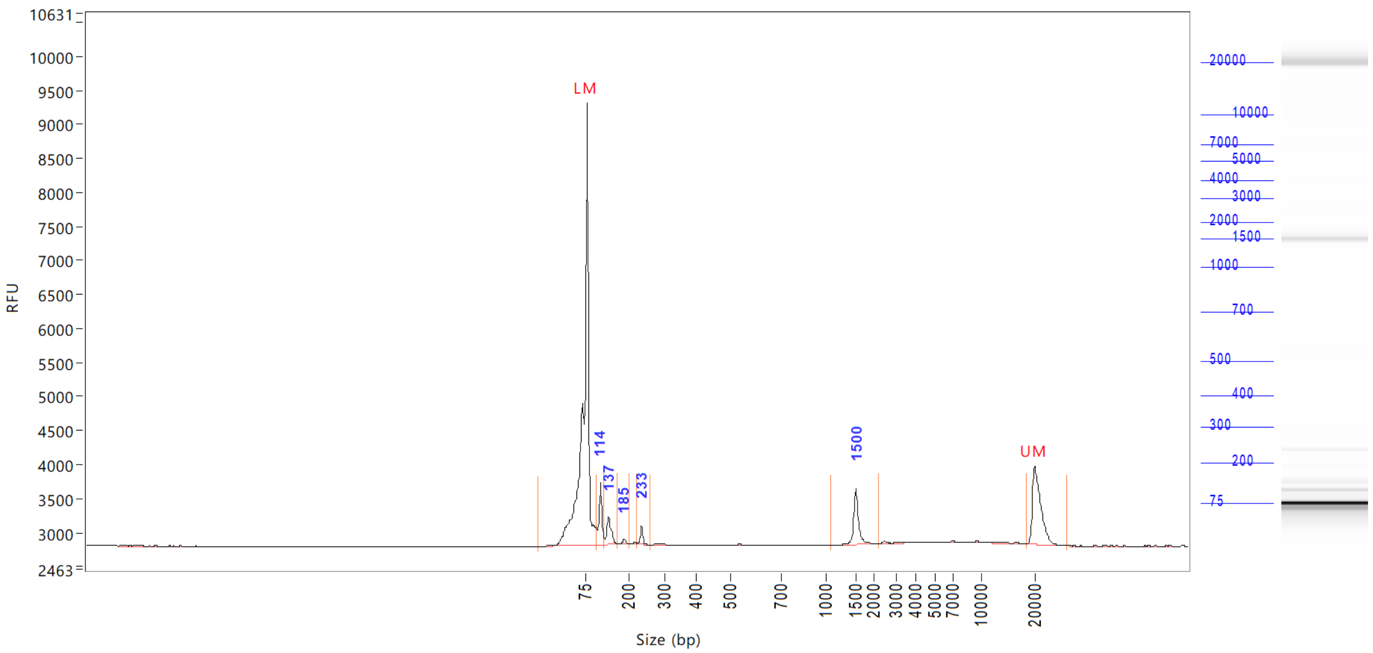


| Peak | Size (bp) | Concentration (ng/uL) | From (bp) | To (bp) | Average size (bp) | CV% | RFU | Corrected peak area |
|---|---|---|---|---|---|---|---|---|
| 1 | 75 (LM) | 0.5000 | 0 | 105 | 61 | 33.65 | 6507 | 77.300 |
| 2 | 114 | 0.4632 | 105 | 128 | 113 | 4.36 | 907 | 5.968 |
| 3 | 137 | 0.3053 | 128 | 168 | 139 | 5.10 | 401 | 3.933 |
| 4 | 185 | 0.0390 | 168 | 200 | 181 | 3.10 | 70 | 0.502 |
| 5 | 233 | 0.1121 | 221 | 259 | 232 | 1.65 | 282 | 1.444 |
| 6 | 1500 | 0.4733 | 1068 | 2229 | 1506 | 6.95 | 835 | 6.097 |
| 7 | 20000 (UM) | 0.0752 | 18415 | 26166 | 20484 | 4.54 | 1141 | 11.627 |

| | | |
|---|---|---|
| TIC: | 1.3928 | ng/uL |
| TIM: | 12.0084 | nmole/L |
| Total concentration: | 1.6520 | ng/uL |

```
Sample peak width (sec): 5    Sample min peak height: 50    Sample baseline V to V?: Y    Sample baseline V to V points: 3
Sample filter: Binomial       Number of points for filter: 3  Sample start region (min): 0  Sample end region (min): 30
Marker peak width (sec): 5    Marker min peak height: 200   Marker baseline V to V?: Y    Marker baseline V to V points: 3
Lower marker selection: First peak > 500 RFU                Upper marker selection: Last peak > 200 RFU
Ladder size (bp)75, 200, 300, 400, 500, 700, 1000, 1500, 2000, 3000, 4000, 5000, 7000, 10000, 20000
Quantification using: Lower Marker          Final concentration (ng/uL): 0.5000          Dilution factor: 12.0
```

**Sample:** SampB9
**Well location:** B9
**Created:** Tuesday, September 14, 2021 6:54:01 PM

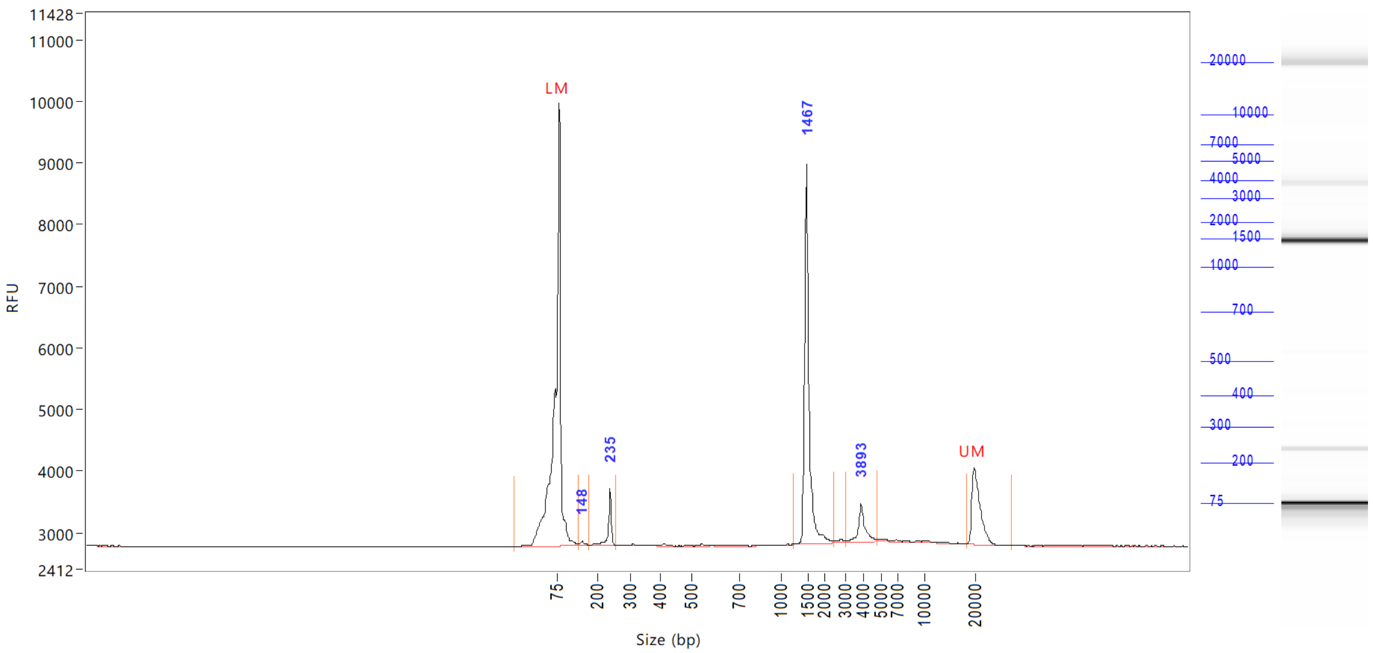


| Peak | Size (bp) | Concentration (ng/uL) | From (bp) | To (bp) | Average size (bp) | CV% | RFU | Corrected peak area |
|---|---|---|---|---|---|---|---|---|
| 1 | 75 (LM) | 0.5000 | 0 | 141 | 61 | 36.73 | 7199 | 96.555 |
| 2 | 148 | 0.0445 | 141 | 169 | 151 | 4.99 | 77 | 0.716 |
| 3 | 235 | 0.3812 | 169 | 255 | 229 | 5.80 | 913 | 6.135 |
| 4 | 1467 | 2.6792 | 1234 | 2458 | 1497 | 8.66 | 6156 | 43.116 |
| 5 | 3893 | 0.3813 | 3001 | 4793 | 3899 | 7.79 | 618 | 6.136 |
| 6 | 20000 (UM) | 0.0695 | 18415 | 27496 | 20556 | 4.85 | 1242 | 13.418 |

| | | |
|---|---|---|
| TIC: | 3.4863 | ng/uL |
| TIM: | 6.3291 | nmole/L |
| Total concentration: | 3.7488 | ng/uL |

Sample peak width (sec): 5 Sample min peak height: 50 Sample baseline V to V?: Y Sample baseline V to V points: 3
Sample filter: Binomial Number of points for filter: 3 Sample start region (min): 0 Sample end region (min): 30
Marker peak width (sec): 5 Marker min peak height: 200 Marker baseline V to V?: Y Marker baseline V to V points: 3
Lower marker selection: First peak > 500 RFU Upper marker selection: Last peak > 200 RFU
Ladder size (bp)75, 200, 300, 400, 500, 700, 1000, 1500, 2000, 3000, 4000, 5000, 7000, 10000, 20000
Quantification using: Lower Marker Final concentration (ng/uL): 0.5000 Dilution factor: 12.0

**Sample:** SampC9
**Well location:** C9
**Created:** Tuesday, September 14, 2021 6:54:01 PM

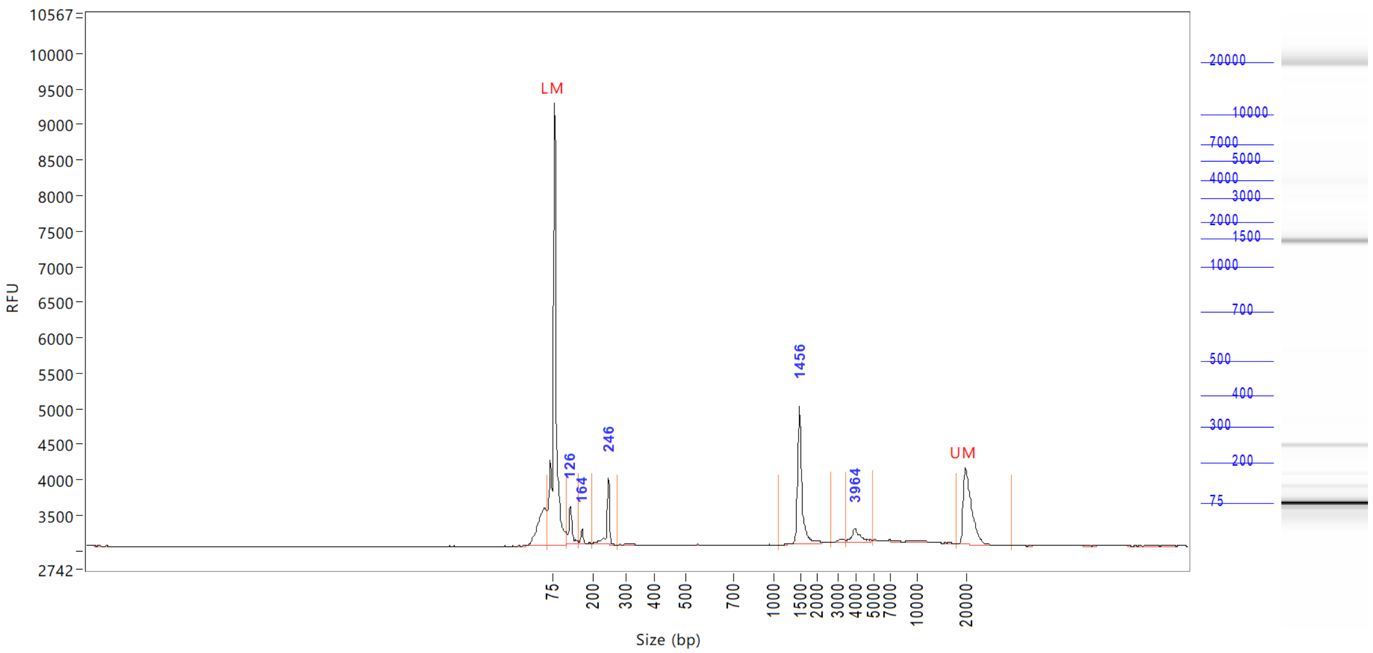


| Peak | Size (bp) | Concentration (ng/uL) | From (bp) | To (bp) | Average size (bp) | CV% | RFU | Corrected peak area |
|---|---|---|---|---|---|---|---|---|
| 1 | 75 (LM) | 0.5000 | 52 | 116 | 74 | 14.42 | 6231 | 53.411 |
| 2 | 126 | 0.4706 | 116 | 154 | 126 | 6.45 | 543 | 4.189 |
| 3 | 164 | 0.1385 | 154 | 194 | 164 | 4.37 | 203 | 1.233 |
| 4 | 246 | 0.6549 | 194 | 273 | 240 | 4.09 | 933 | 5.829 |
| 5 | 1456 | 1.5689 | 1079 | 2629 | 1495 | 9.87 | 1947 | 13.966 |
| 6 | 3964 | 0.2647 | 3393 | 4966 | 4030 | 8.09 | 188 | 2.357 |
| 7 | 20000 (UM) | 0.1125 | 18049 | 29309 | 20574 | 5.39 | 1088 | 12.021 |

| | | |
|---|---|---|
| TIC: | 3.0975 | ng/uL |
| TIM: | 13.8471 | nmole/L |
| Total concentration: | 3.4173 | ng/uL |

```
Sample peak width (sec): 5    Sample min peak height: 50    Sample baseline V to V?: Y    Sample baseline V to V points: 3
Sample filter: Binomial       Number of points for filter: 3  Sample start region (min): 0  Sample end region (min): 30
Marker peak width (sec): 5    Marker min peak height: 200   Marker baseline V to V?: Y    Marker baseline V to V points: 3
Lower marker selection: First peak > 500 RFU                Upper marker selection: Last peak > 200 RFU
Ladder size (bp)75, 200, 300, 400, 500, 700, 1000, 1500, 2000, 3000, 4000, 5000, 7000, 10000, 20000
Quantification using: Lower Marker          Final concentration (ng/uL): 0.5000          Dilution factor: 12.0
```

**Sample:** SampD9
**Well location:** D9
**Created:** Tuesday, September 14, 2021 6:54:01 PM

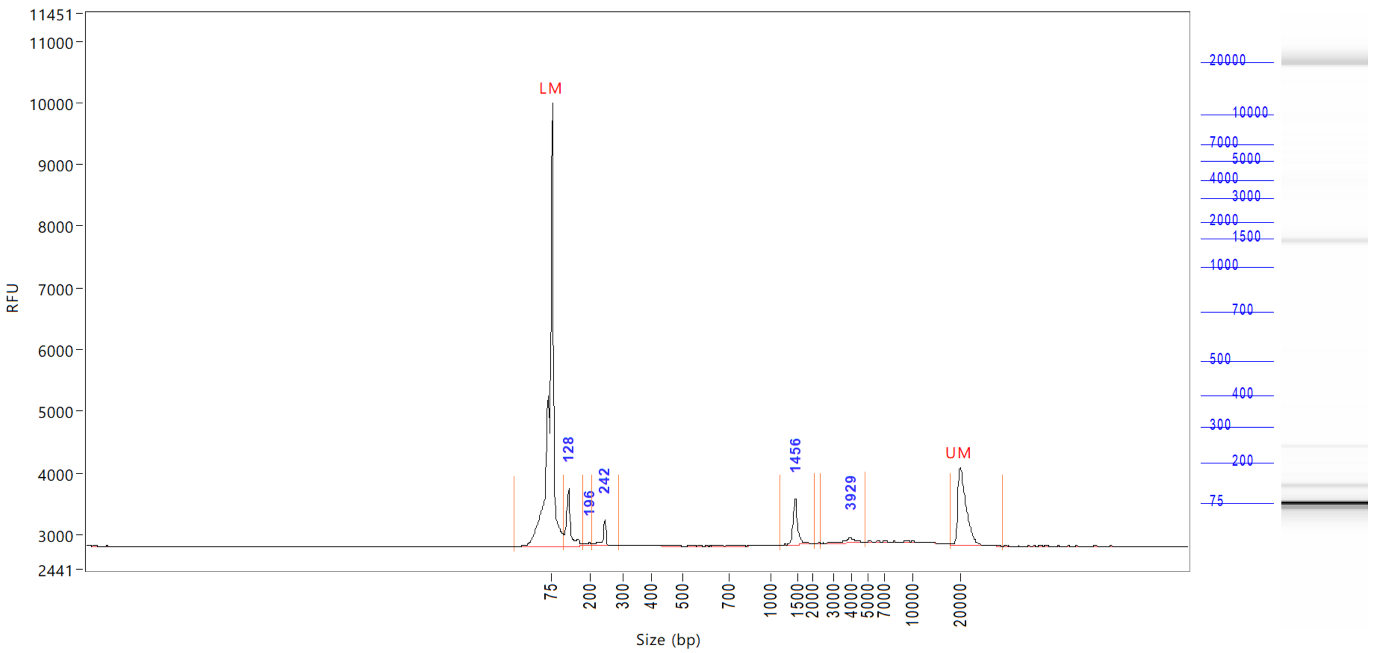


| Peak | Size (bp) | Concentration (ng/uL) | From (bp) | To (bp) | Average size (bp) | CV% | RFU | Corrected peak area |
|---|---|---|---|---|---|---|---|---|
| 1 | 75 (LM) | 0.5000 | 0 | 112 | 64 | 28.91 | 7197 | 80.758 |
| 2 | 128 | 0.7538 | 112 | 177 | 130 | 10.19 | 931 | 10.145 |
| 3 | 196 | 0.0464 | 177 | 203 | 192 | 3.95 | 54 | 0.624 |
| 4 | 242 | 0.2425 | 203 | 286 | 235 | 5.48 | 412 | 3.264 |
| 5 | 1456 | 0.4183 | 1178 | 2087 | 1479 | 7.13 | 740 | 5.630 |
| 6 | 3929 | 0.1230 | 2372 | 4793 | 3746 | 14.08 | 95 | 1.656 |
| 7 | 20000 (UM) | 0.0846 | 17805 | 28705 | 20607 | 5.16 | 1256 | 13.658 |

| | | |
|---|---|---|
| TIC: | 1.5840 | ng/uL |
| TIM: | 12.1409 | nmole/L |
| Total concentration: | 1.7541 | ng/uL |

```
Sample peak width (sec): 5    Sample min peak height: 50    Sample baseline V to V?: Y    Sample baseline V to V points: 3
Sample filter: Binomial       Number of points for filter: 3  Sample start region (min): 0  Sample end region (min): 30
Marker peak width (sec): 5    Marker min peak height: 200   Marker baseline V to V?: Y    Marker baseline V to V points: 3
Lower marker selection: First peak > 500 RFU                Upper marker selection: Last peak > 200 RFU
Ladder size (bp)75, 200, 300, 400, 500, 700, 1000, 1500, 2000, 3000, 4000, 5000, 7000, 10000, 20000
Quantification using: Lower Marker          Final concentration (ng/uL): 0.5000          Dilution factor: 12.0
```

**Sample:** SampE9
**Well location:** E9
**Created:** Tuesday, September 14, 2021 6:54:01 PM

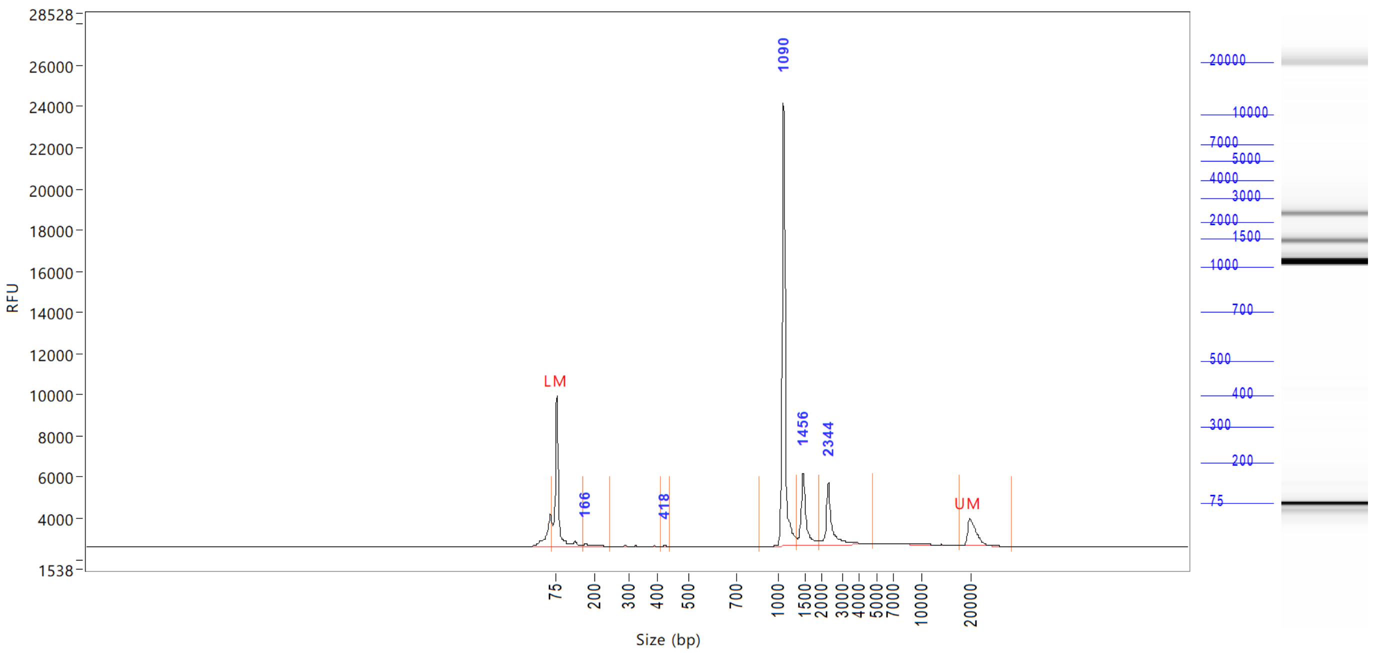


| Peak | Size (bp) | Concentration (ng/uL) | From (bp) | To (bp) | Average size (bp) | CV% | RFU | Corrected peak area |
|---|---|---|---|---|---|---|---|---|
| 1 | 75 (LM) | 0.5000 | 62 | 156 | 78 | 21.36 | 7321 | 58.376 |
| 2 | 166 | 0.2524 | 156 | 241 | 184 | 11.39 | 100 | 2.455 |
| 3 | 418 | 0.0473 | 409 | 437 | 418 | 1.33 | 84 | 0.460 |
| 4 | 1090 | 12.3576 | 870 | 1356 | 1098 | 4.87 | 21546 | 120.232 |
| 5 | 1456 | 2.9565 | 1356 | 1942 | 1500 | 7.88 | 3532 | 28.765 |
| 6 | 2344 | 2.8595 | 1942 | 4724 | 2509 | 17.88 | 3017 | 27.822 |
| 7 | 20000 (UM) | 0.1238 | 17805 | 28342 | 20627 | 5.20 | 1302 | 14.449 |

| | | |
|---|---|---|
| TIC: | 18.4733 | ng/uL |
| TIM: | 26.0864 | nmole/L |
| Total concentration: | 18.7178 | ng/uL |

```
Sample peak width (sec): 5    Sample min peak height: 50    Sample baseline V to V?: Y    Sample baseline V to V points: 3
Sample filter: Binomial       Number of points for filter: 3  Sample start region (min): 0  Sample end region (min): 30
Marker peak width (sec): 5    Marker min peak height: 200   Marker baseline V to V?: Y    Marker baseline V to V points: 3
Lower marker selection: First peak > 500 RFU                Upper marker selection: Last peak > 200 RFU
Ladder size (bp)75, 200, 300, 400, 500, 700, 1000, 1500, 2000, 3000, 4000, 5000, 7000, 10000, 20000
Quantification using: Lower Marker          Final concentration (ng/uL): 0.5000          Dilution factor: 12.0
```

**Sample:** SampF9
**Well location:** F9
**Created:** Tuesday, September 14, 2021 6:54:01 PM

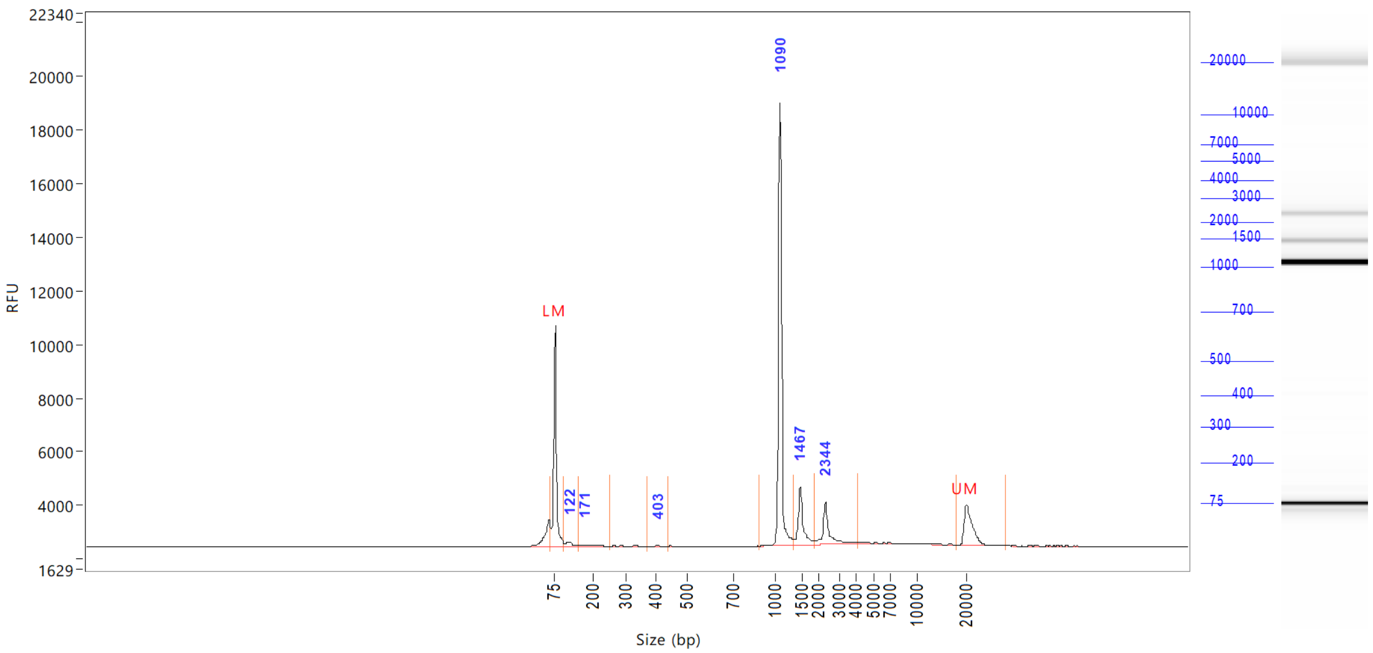


| Peak | Size (bp) | Concentration (ng/uL) | From (bp) | To (bp) | Average size (bp) | CV% | RFU | Corrected peak area |
|---|---|---|---|---|---|---|---|---|
| 1 | 75 (LM) | 0.5000 | 62 | 105 | 74 | 8.32 | 8241 | 55.492 |
| 2 | 122 | 0.3236 | 105 | 152 | 121 | 9.64 | 176 | 2.993 |
| 3 | 171 | 0.1702 | 152 | 251 | 183 | 11.22 | 60 | 1.574 |
| 4 | 403 | 0.0465 | 370 | 435 | 404 | 2.94 | 54 | 0.430 |
| 5 | 1090 | 9.4981 | 883 | 1356 | 1084 | 4.56 | 16518 | 87.845 |
| 6 | 1467 | 1.9159 | 1356 | 1904 | 1500 | 7.45 | 2143 | 17.720 |
| 7 | 2344 | 1.6316 | 1904 | 4035 | 2465 | 16.04 | 1563 | 15.090 |
| 8 | 20000 (UM) | 0.1494 | 17927 | 28221 | 20599 | 4.93 | 1518 | 16.577 |

| | | |
|---|---|---|
| TIC: | 13.5860 | ng/uL |
| TIM: | 23.7267 | nmole/L |
| Total concentration: | 13.8648 | ng/uL |

Sample peak width (sec): 5    Sample min peak height: 50    Sample baseline V to V?: Y    Sample baseline V to V points: 3
Sample filter: Binomial    Number of points for filter: 3  Sample start region (min): 0  Sample end region (min): 30
Marker peak width (sec): 5    Marker min peak height: 200    Marker baseline V to V?: Y    Marker baseline V to V points: 3
Lower marker selection: First peak > 500 RFU    Upper marker selection: Last peak > 200 RFU
Ladder size (bp)75, 200, 300, 400, 500, 700, 1000, 1500, 2000, 3000, 4000, 5000, 7000, 10000, 20000
Quantification using: Lower Marker    Final concentration (ng/uL): 0.5000    Dilution factor: 12.0

**Sample:** SampG9
**Well location:** G9
**Created:** Tuesday, September 14, 2021 6:54:01 PM

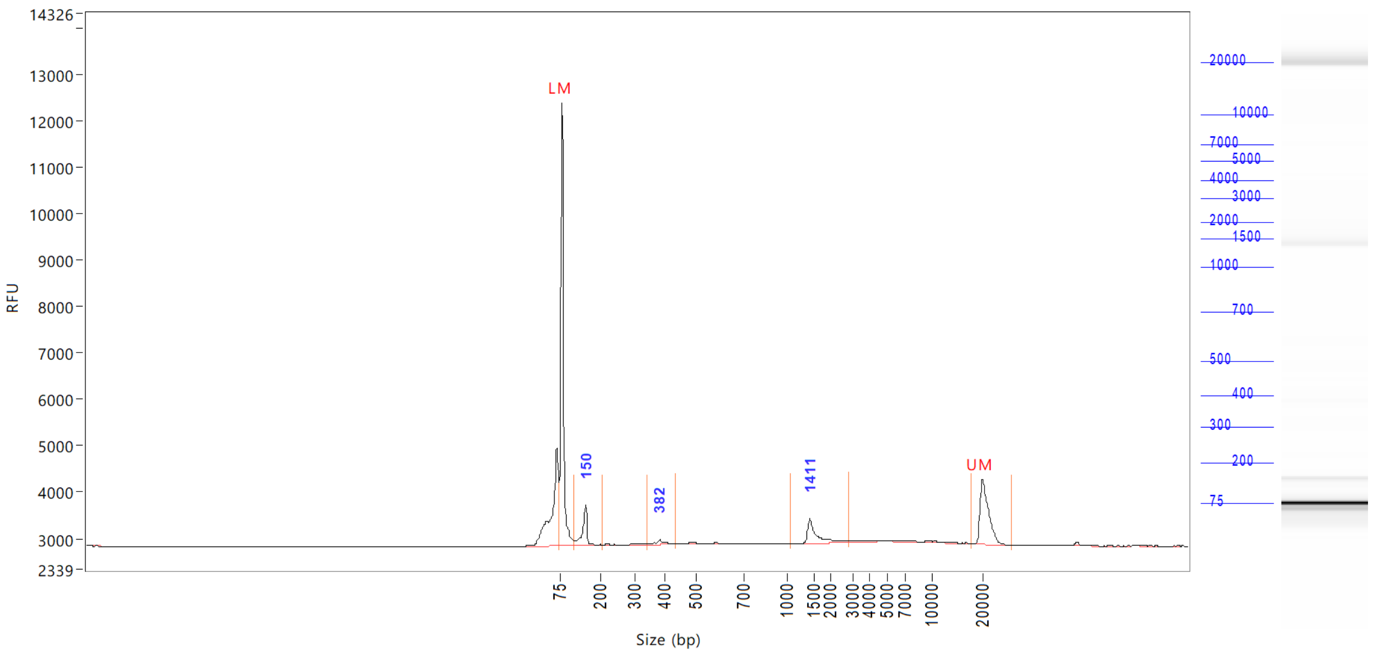


| Peak | Size<br>(bp) | Concentration<br>(ng/uL) | From<br>(bp) | To<br>(bp) | Average size<br>(bp) | CV% | RFU | Corrected peak area |
|---|---|---|---|---|---|---|---|---|
| 1 | 75 (LM) | 0.5000 | 66 | 114 | 75 | 9.17 | 9563 | 65.703 |
| 2 | 150 | 0.8097 | 114 | 201 | 144 | 9.38 | 859 | 8.866 |
| 3 | 382 | 0.1458 | 339 | 433 | 385 | 5.01 | 97 | 1.597 |
| 4 | 1411 | 0.7388 | 1068 | 2829 | 1638 | 21.89 | 532 | 8.090 |
| 5 | 20000 (UM) | 0.1186 | 17805 | 26045 | 20590 | 4.80 | 1405 | 15.587 |

| | | |
|---|---|---|
| TIC: | 1.6943 | ng/uL |
| TIM: | 10.6062 | nmole/L |
| Total concentration: | 2.2723 | ng/uL |

```
Sample peak width (sec): 5    Sample min peak height: 50    Sample baseline V to V?: Y    Sample baseline V to V points: 3
Sample filter: Binomial       Number of points for filter: 3  Sample start region (min): 0  Sample end region (min): 30
Marker peak width (sec): 5    Marker min peak height: 200   Marker baseline V to V?: Y    Marker baseline V to V points: 3
Lower marker selection: First peak > 500 RFU                Upper marker selection: Last peak > 200 RFU
Ladder size (bp)75, 200, 300, 400, 500, 700, 1000, 1500, 2000, 3000, 4000, 5000, 7000, 10000, 20000
Quantification using: Lower Marker          Final concentration (ng/uL): 0.5000          Dilution factor: 12.0
```

**Sample:** SampH9
**Well location:** H9
**Created:** Tuesday, September 14, 2021 6:54:01 PM

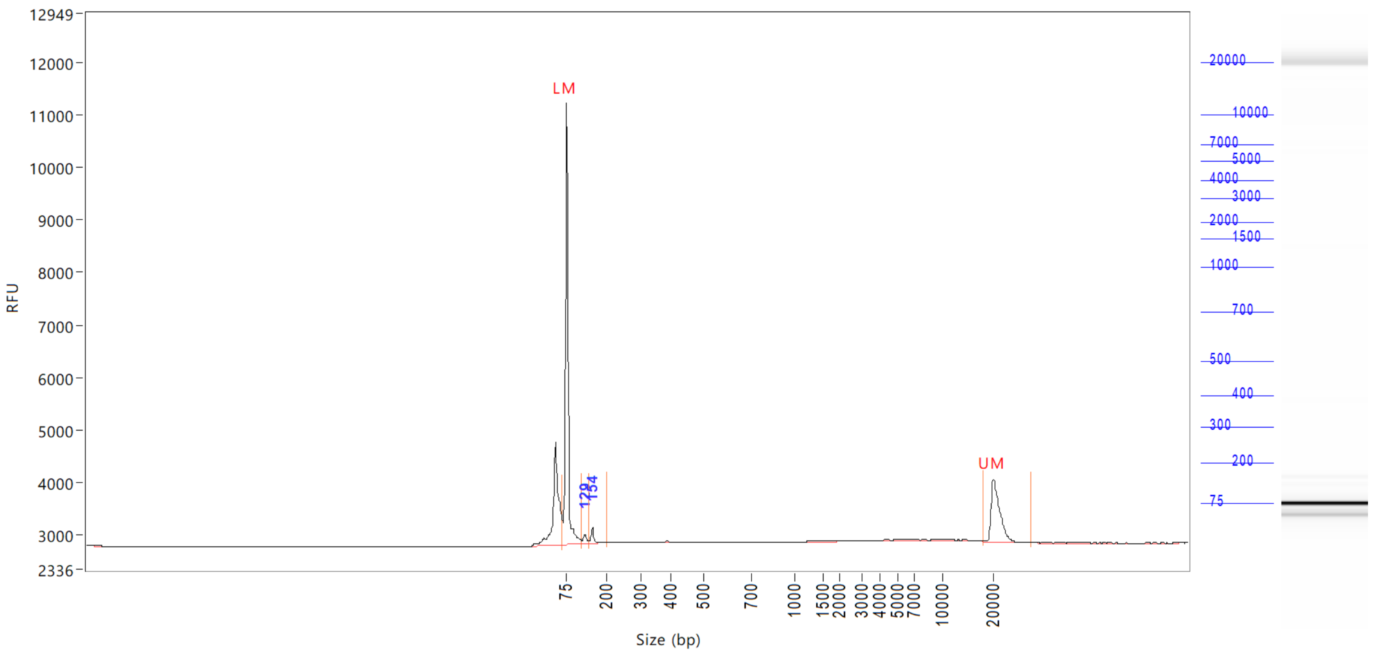


| Peak | Size (bp) | Concentration (ng/uL) | From (bp) | To (bp) | Average size (bp) | CV% | RFU | Corrected peak area |
|---|---|---|---|---|---|---|---|---|
| 1 | 75 (LM) | 0.5000 | 64 | 122 | 75 | 10.62 | 8444 | 55.872 |
| 2 | 129 | 0.1519 | 122 | 143 | 130 | 4.30 | 168 | 1.414 |
| 3 | 154 | 0.2192 | 143 | 200 | 155 | 5.85 | 290 | 2.041 |
| 4 | 20000 (UM) | 0.1182 | 18171 | 27496 | 20613 | 4.89 | 1202 | 13.213 |

| | | |
|---|---|---|
| TIC: | 0.3711 | ng/uL |
| TIM: | 4.2441 | nmole/L |
| Total concentration: | 0.8155 | ng/uL |

Sample peak width (sec): 5    Sample min peak height: 50    Sample baseline V to V?: Y    Sample baseline V to V points: 3
Sample filter: Binomial    Number of points for filter: 3  Sample start region (min): 0  Sample end region (min): 30
Marker peak width (sec): 5    Marker min peak height: 200    Marker baseline V to V?: Y    Marker baseline V to V points: 3
Lower marker selection: First peak > 500 RFU    Upper marker selection: Last peak > 200 RFU
Ladder size (bp)75, 200, 300, 400, 500, 700, 1000, 1500, 2000, 3000, 4000, 5000, 7000, 10000, 20000
Quantification using: Lower Marker    Final concentration (ng/uL): 0.5000    Dilution factor: 12.0

**Sample:** SampA10
**Well location:** A10
**Created:** Tuesday, September 14, 2021 6:54:01 PM

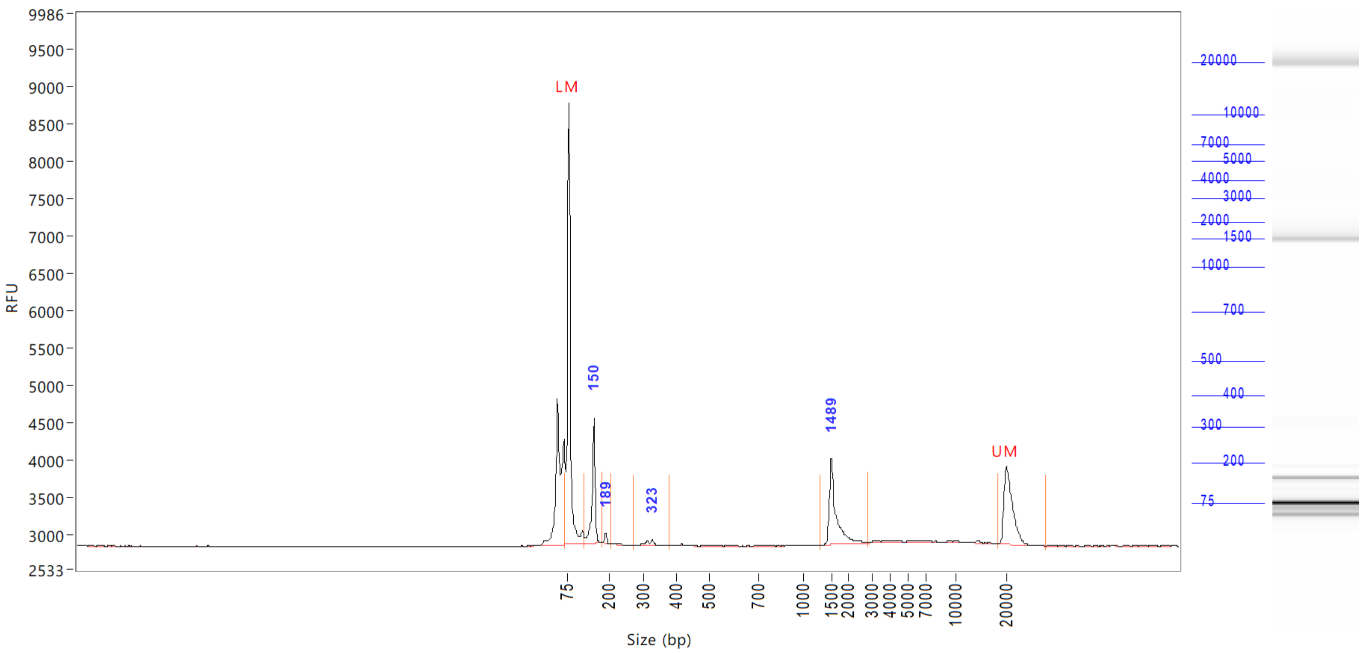


| Peak | Size (bp) | Concentration (ng/uL) | From (bp) | To (bp) | Average size (bp) | CV% | RFU | Corrected peak area |
|---|---|---|---|---|---|---|---|---|
| 1 | 75 (LM) | 0.5000 | 66 | 124 | 76 | 12.22 | 5928 | 42.620 |
| 2 | 150 | 1.6107 | 124 | 177 | 147 | 4.68 | 1670 | 11.441 |
| 3 | 189 | 0.1012 | 177 | 203 | 185 | 1.61 | 139 | 0.719 |
| 4 | 323 | 0.1580 | 268 | 378 | 313 | 5.76 | 74 | 1.122 |
| 5 | 1489 | 1.6585 | 1300 | 2857 | 1612 | 14.29 | 1144 | 11.781 |
| 6 | 20000 (UM) | 0.1304 | 18293 | 27496 | 20473 | 4.87 | 1046 | 11.114 |

| | | |
|---|---|---|
| TIC: | 3.5284 | ng/uL |
| TIM: | 21.4309 | nmole/L |
| Total concentration: | 3.9478 | ng/uL |

```
Sample peak width (sec): 5    Sample min peak height: 50    Sample baseline V to V?: Y    Sample baseline V to V points: 3
Sample filter: Binomial       Number of points for filter: 3  Sample start region (min): 0  Sample end region (min): 30
Marker peak width (sec): 5    Marker min peak height: 200   Marker baseline V to V?: Y    Marker baseline V to V points: 3
Lower marker selection: First peak > 500 RFU                Upper marker selection: Last peak > 200 RFU
Ladder size (bp)75, 200, 300, 400, 500, 700, 1000, 1500, 2000, 3000, 4000, 5000, 7000, 10000, 20000
Quantification using: Lower Marker          Final concentration (ng/uL): 0.5000          Dilution factor: 12.0
```

**Sample:** SampB10
**Well location:** B10
**Created:** Tuesday, September 14, 2021 6:54:01 PM

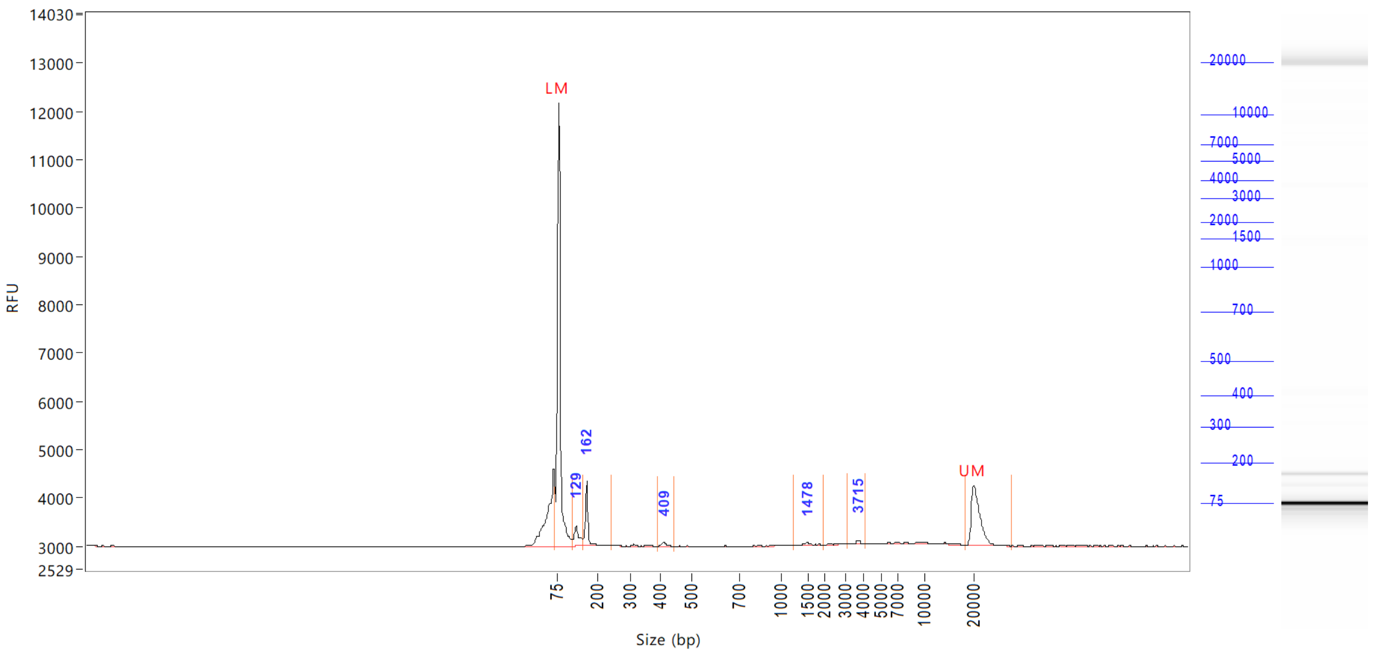


| Peak | Size (bp) | Concentration (ng/uL) | From (bp) | To (bp) | Average size (bp) | CV% | RFU | Corrected peak area |
|---|---|---|---|---|---|---|---|---|
| 1 | 75 (LM) | 0.5000 | 66 | 118 | 75 | 11.20 | 9185 | 65.200 |
| 2 | 129 | 0.4718 | 118 | 152 | 133 | 7.10 | 426 | 5.127 |
| 3 | 162 | 0.7698 | 152 | 242 | 164 | 6.88 | 1342 | 8.365 |
| 4 | 409 | 0.0771 | 388 | 441 | 409 | 2.32 | 88 | 0.838 |
| 5 | 1478 | 0.0672 | 1234 | 1962 | 1499 | 9.44 | 59 | 0.730 |
| 6 | 3715 | 0.0449 | 3144 | 4070 | 3668 | 4.14 | 81 | 0.488 |
| 7 | 20000 (UM) | 0.1046 | 18415 | 27617 | 20607 | 5.09 | 1249 | 13.635 |

| | | |
|---|---|---|
| TIC: | 1.4308 | ng/uL |
| TIM: | 13.9491 | nmole/L |
| Total concentration: | 1.7192 | ng/uL |

Sample peak width (sec): 5 Sample min peak height: 50 Sample baseline V to V?: Y Sample baseline V to V points: 3
Sample filter: Binomial Number of points for filter: 3 Sample start region (min): 0 Sample end region (min): 30
Marker peak width (sec): 5 Marker min peak height: 200 Marker baseline V to V?: Y Marker baseline V to V points: 3
Lower marker selection: First peak > 500 RFU Upper marker selection: Last peak > 200 RFU
Ladder size (bp)75, 200, 300, 400, 500, 700, 1000, 1500, 2000, 3000, 4000, 5000, 7000, 10000, 20000
Quantification using: Lower Marker Final concentration (ng/uL): 0.5000 Dilution factor: 12.0

**Sample:** SampC10
**Well location:** C10
**Created:** Tuesday, September 14, 2021 6:54:01 PM

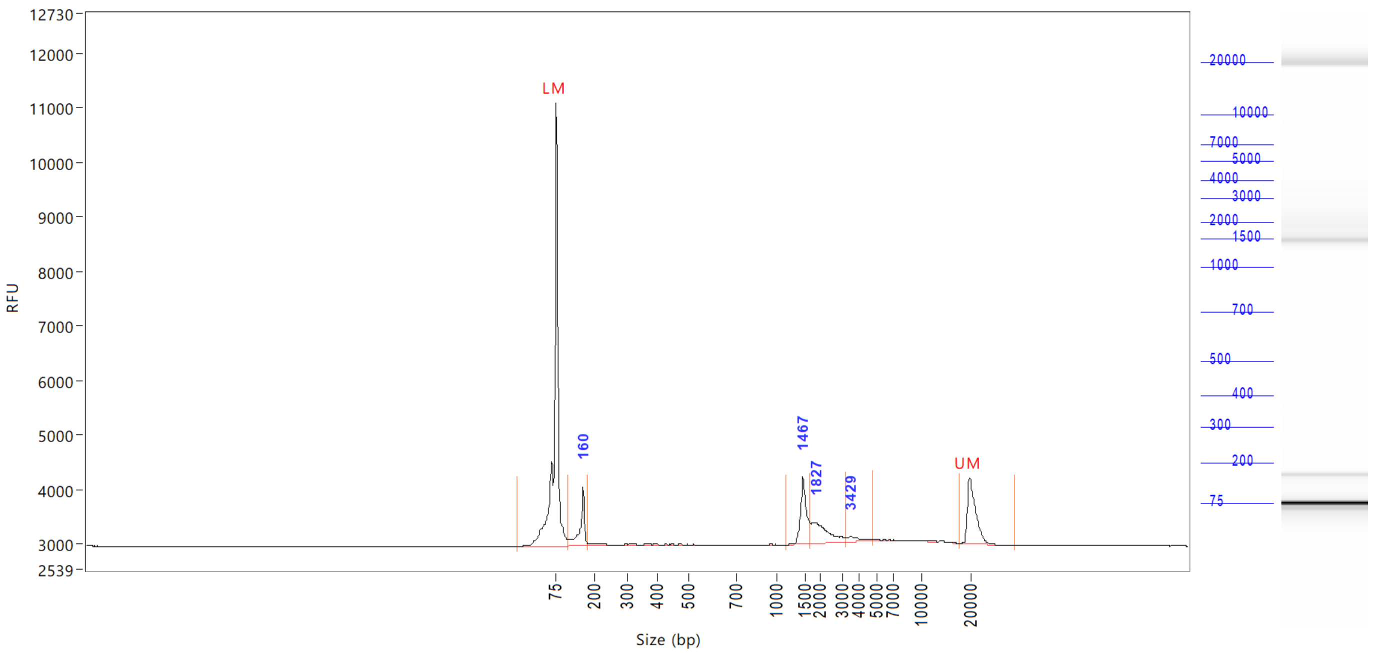


| Peak | Size (bp) | Concentration (ng/uL) | From (bp) | To (bp) | Average size (bp) | CV% | RFU | Corrected peak area |
|---|---|---|---|---|---|---|---|---|
| 1 | 75 (LM) | 0.5000 | 0 | 112 | 66 | 28.05 | 8138 | 78.488 |
| 2 | 160 | 0.8337 | 112 | 177 | 150 | 9.42 | 1085 | 10.906 |
| 3 | 1467 | 0.9151 | 1156 | 1674 | 1482 | 5.36 | 1231 | 11.971 |
| 4 | 1827 | 0.7929 | 1674 | 3179 | 2127 | 18.29 | 388 | 10.373 |
| 5 | 3429 | 0.1443 | 3179 | 4759 | 3749 | 10.74 | 94 | 1.888 |
| 6 | 20000 (UM) | 0.0842 | 17927 | 28947 | 20532 | 5.09 | 1215 | 13.224 |

| | | |
|---|---|---|
| TIC: | 2.6861 | ng/uL |
| TIM: | 10.8285 | nmole/L |
| Total concentration: | 2.9953 | ng/uL |

```
Sample peak width (sec): 5    Sample min peak height: 50    Sample baseline V to V?: Y    Sample baseline V to V points: 3
Sample filter: Binomial       Number of points for filter: 3  Sample start region (min): 0  Sample end region (min): 30
Marker peak width (sec): 5    Marker min peak height: 200   Marker baseline V to V?: Y    Marker baseline V to V points: 3
Lower marker selection: First peak > 500 RFU                Upper marker selection: Last peak > 200 RFU
Ladder size (bp)75, 200, 300, 400, 500, 700, 1000, 1500, 2000, 3000, 4000, 5000, 7000, 10000, 20000
Quantification using: Lower Marker          Final concentration (ng/uL): 0.5000          Dilution factor: 12.0
```

**Sample:** SampD10
**Well location:** D10
**Created:** Tuesday, September 14, 2021 6:54:01 PM

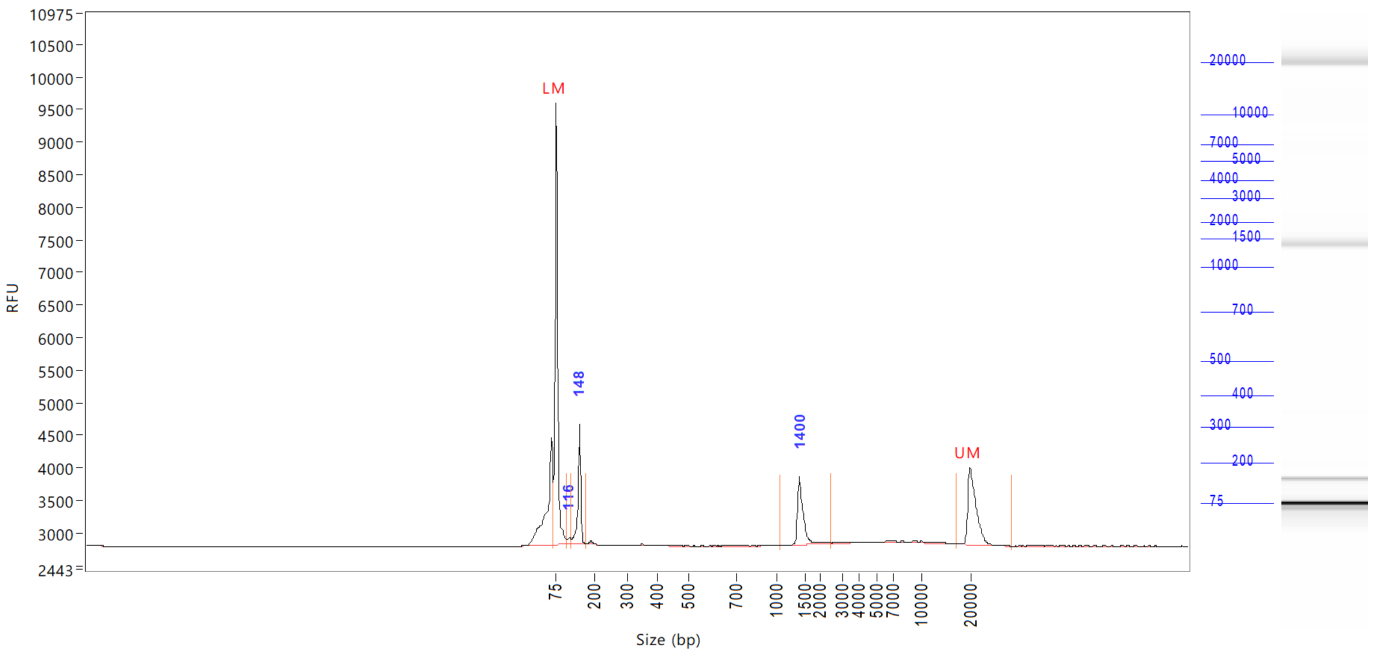


| Peak | Size (bp) | Concentration (ng/uL) | From (bp) | To (bp) | Average size (bp) | CV% | RFU | Corrected peak area |
|---|---|---|---|---|---|---|---|---|
| 1 | 75 (LM) | 0.5000 | 66 | 108 | 74 | 8.20 | 6789 | 44.411 |
| 2 | 116 | 0.1342 | 108 | 124 | 116 | 4.07 | 107 | 0.993 |
| 3 | 148 | 1.6311 | 124 | 169 | 144 | 4.19 | 1838 | 12.074 |
| 4 | 1400 | 1.3065 | 1045 | 2486 | 1443 | 10.27 | 1049 | 9.671 |
| 5 | 20000 (UM) | 0.1438 | 17317 | 28342 | 20608 | 5.02 | 1180 | 12.771 |

| | | |
|---|---|---|
| TIC: | 3.0718 | ng/uL |
| TIM: | 22.0057 | nmole/L |
| Total concentration: | 3.5405 | ng/uL |

```
Sample peak width (sec): 5     Sample min peak height: 50     Sample baseline V to V?: Y     Sample baseline V to V points: 3
Sample filter: Binomial        Number of points for filter: 3  Sample start region (min): 0  Sample end region (min): 30
Marker peak width (sec): 5     Marker min peak height: 200    Marker baseline V to V?: Y     Marker baseline V to V points: 3
Lower marker selection: First peak > 500 RFU                  Upper marker selection: Last peak > 200 RFU
Ladder size (bp)75, 200, 300, 400, 500, 700, 1000, 1500, 2000, 3000, 4000, 5000, 7000, 10000, 20000
Quantification using: Lower Marker          Final concentration (ng/uL): 0.5000          Dilution factor: 12.0
```

**Sample:** SampE10
**Well location:** E10
**Created:** Tuesday, September 14, 2021 6:54:01 PM

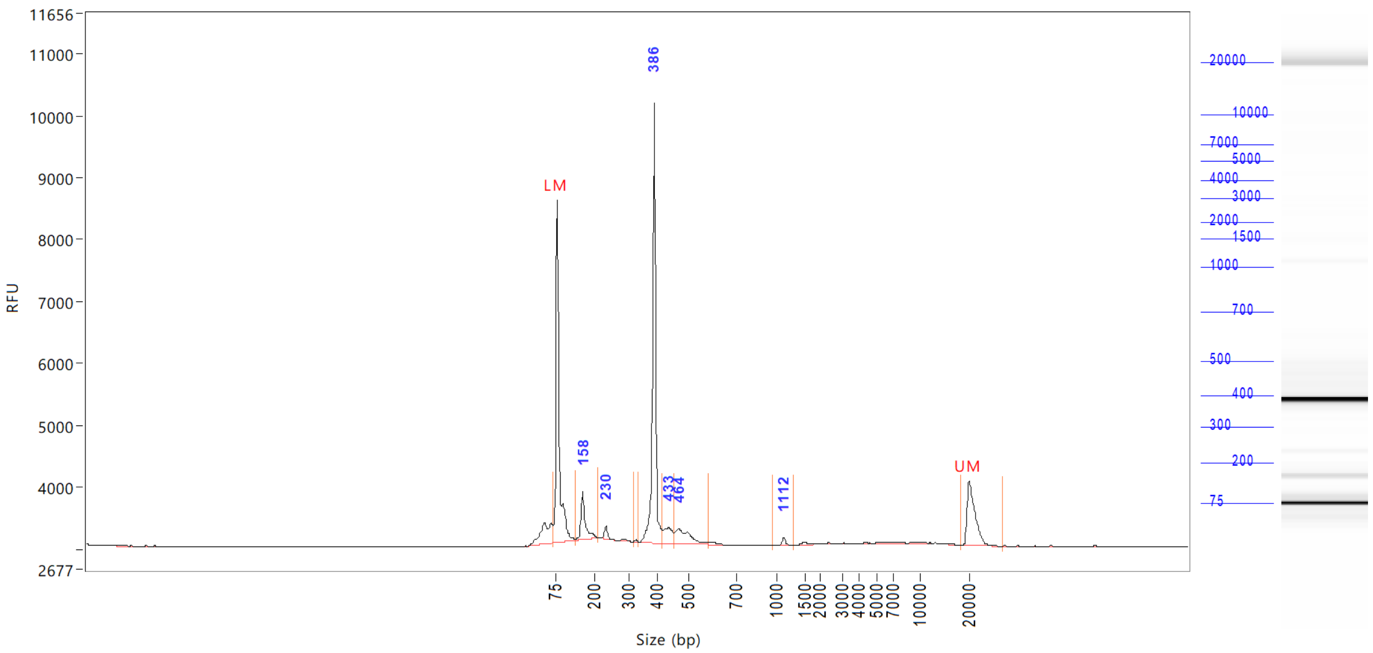


| Peak | Size<br>(bp) | Concentration<br>(ng/uL) | From<br>(bp) | To<br>(bp) | Average size<br>(bp) | CV% | RFU | Corrected peak area |
|---|---|---|---|---|---|---|---|---|
| 1 | 75 (LM) | 0.5000 | 64 | 133 | 78 | 13.43 | 5522 | 41.773 |
| 2 | 158 | 1.1105 | 133 | 208 | 160 | 7.38 | 778 | 7.732 |
| 3 | 230 | 0.2608 | 208 | 315 | 238 | 9.55 | 208 | 1.816 |
| 4 | 386 | 6.7745 | 333 | 412 | 382 | 2.51 | 7121 | 47.165 |
| 5 | 433 | 0.6446 | 412 | 449 | 430 | 2.45 | 252 | 4.487 |
| 6 | 464 | 1.0022 | 449 | 578 | 485 | 5.89 | 238 | 6.978 |
| 7 | 1112 | 0.1074 | 970 | 1289 | 1100 | 3.93 | 127 | 0.748 |
| 8 | 20000 (UM) | 0.1304 | 18171 | 26770 | 20559 | 4.86 | 1033 | 10.893 |

| | | |
|---|---|---|
| TIC: | 9.9000 | ng/uL |
| TIM: | 48.4152 | nmole/L |
| Total concentration: | 10.2687 | ng/uL |

```
Sample peak width (sec): 5    Sample min peak height: 50    Sample baseline V to V?: Y    Sample baseline V to V points: 3
Sample filter: Binomial       Number of points for filter: 3  Sample start region (min): 0  Sample end region (min): 30
Marker peak width (sec): 5    Marker min peak height: 200   Marker baseline V to V?: Y    Marker baseline V to V points: 3
Lower marker selection: First peak > 500 RFU                Upper marker selection: Last peak > 200 RFU
Ladder size (bp)75, 200, 300, 400, 500, 700, 1000, 1500, 2000, 3000, 4000, 5000, 7000, 10000, 20000
Quantification using: Lower Marker          Final concentration (ng/uL): 0.5000          Dilution factor: 12.0
```

**Sample:** SampF10
**Well location:** F10
**Created:** Tuesday, September 14, 2021 6:54:01 PM

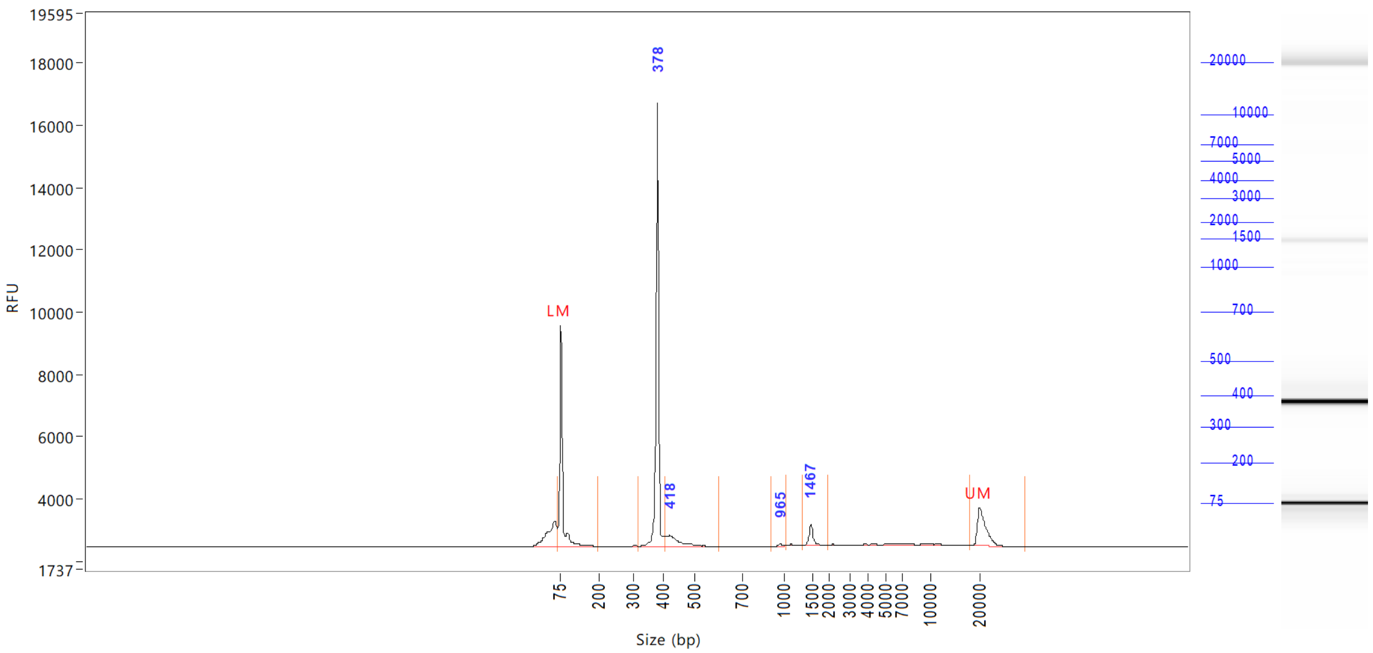


| Peak | Size (bp) | Concentration (ng/uL) | From (bp) | To (bp) | Average size (bp) | CV% | RFU | Corrected peak area |
|---|---|---|---|---|---|---|---|---|
| 1 | 75 (LM) | 0.5000 | 66 | 192 | 79 | 19.32 | 7108 | 48.685 |
| 2 | 378 | 9.7058 | 317 | 403 | 375 | 2.02 | 14271 | 78.755 |
| 3 | 418 | 1.1575 | 403 | 600 | 437 | 6.84 | 349 | 9.392 |
| 4 | 965 | 0.0430 | 905 | 1012 | 962 | 1.47 | 61 | 0.349 |
| 5 | 1467 | 0.5656 | 1323 | 1962 | 1481 | 5.69 | 656 | 4.589 |
| 6 | 20000 (UM) | 0.1401 | 17927 | 29189 | 20576 | 5.34 | 1235 | 13.644 |

| | | |
|---|---|---|
| TIC: | 11.4718 | ng/uL |
| TIM: | 47.6421 | nmole/L |
| Total concentration: | 11.7880 | ng/uL |

Sample peak width (sec): 5    Sample min peak height: 50    Sample baseline V to V?: Y    Sample baseline V to V points: 3
Sample filter: Binomial    Number of points for filter: 3  Sample start region (min): 0  Sample end region (min): 30
Marker peak width (sec): 5    Marker min peak height: 200    Marker baseline V to V?: Y    Marker baseline V to V points: 3
Lower marker selection: First peak > 500 RFU    Upper marker selection: Last peak > 200 RFU
Ladder size (bp)75, 200, 300, 400, 500, 700, 1000, 1500, 2000, 3000, 4000, 5000, 7000, 10000, 20000
Quantification using: Lower Marker    Final concentration (ng/uL): 0.5000    Dilution factor: 12.0

**Sample:** SampG10
**Well location:** G10
**Created:** Tuesday, September 14, 2021 6:54:01 PM

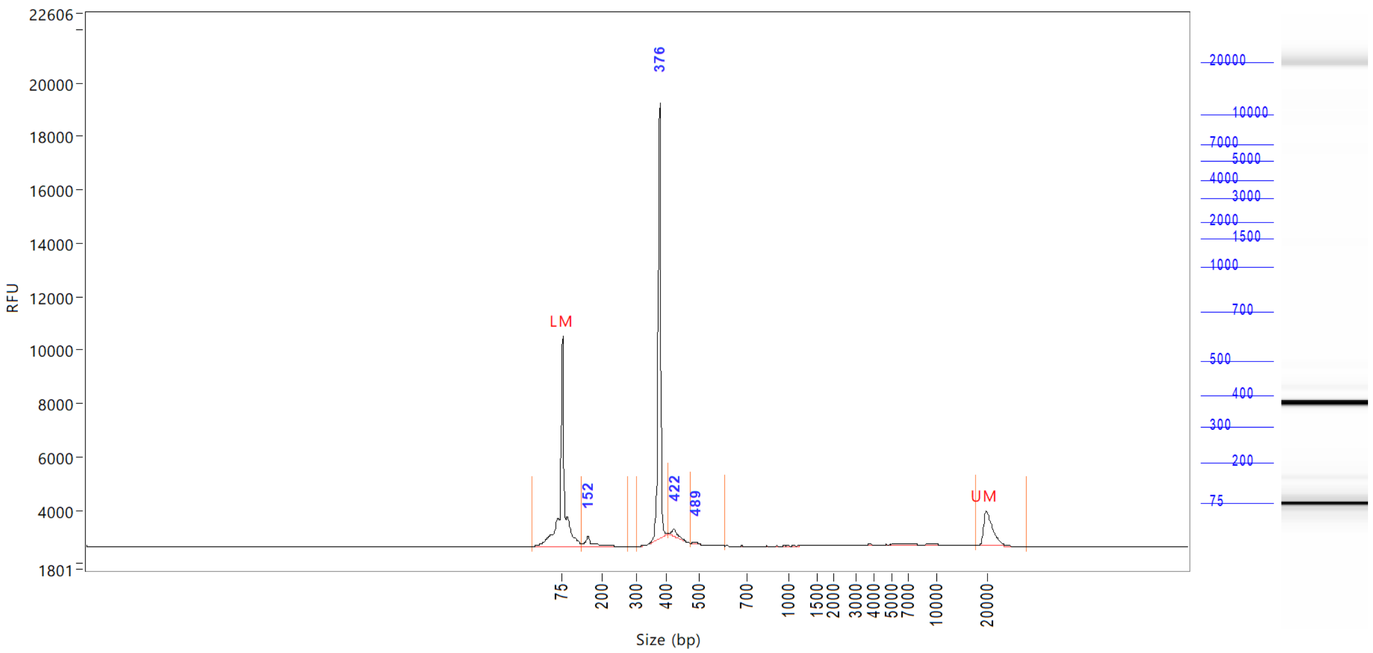


| Peak | Size (bp) | Concentration (ng/uL) | From (bp) | To (bp) | Average size (bp) | CV% | RFU | Corrected peak area |
|---|---|---|---|---|---|---|---|---|
| 1 | 75 (LM) | 0.5000 | 0 | 135 | 70 | 30.22 | 7854 | 84.900 |
| 2 | 152 | 0.4655 | 135 | 277 | 167 | 15.71 | 410 | 6.587 |
| 3 | 376 | 6.4051 | 300 | 403 | 372 | 1.46 | 16322 | 90.631 |
| 4 | 422 | 0.2220 | 403 | 473 | 425 | 2.55 | 300 | 3.141 |
| 5 | 489 | 0.0524 | 473 | 606 | 494 | 3.98 | 72 | 0.741 |
| 6 | 20000 (UM) | 0.0872 | 17805 | 28100 | 20521 | 5.14 | 1281 | 14.806 |

| | | |
|---|---|---|
| TIC: | 7.1449 | ng/uL |
| TIM: | 33.9430 | nmole/L |
| Total concentration: | 7.3532 | ng/uL |

Sample peak width (sec): 5 Sample min peak height: 50 Sample baseline V to V?: Y Sample baseline V to V points: 3
Sample filter: Binomial Number of points for filter: 3 Sample start region (min): 0 Sample end region (min): 30
Marker peak width (sec): 5 Marker min peak height: 200 Marker baseline V to V?: Y Marker baseline V to V points: 3
Lower marker selection: First peak > 500 RFU Upper marker selection: Last peak > 200 RFU
Ladder size (bp)75, 200, 300, 400, 500, 700, 1000, 1500, 2000, 3000, 4000, 5000, 7000, 10000, 20000
Quantification using: Lower Marker Final concentration (ng/uL): 0.5000 Dilution factor: 12.0

**Sample:** SampH10
**Well location:** H10
**Created:** Tuesday, September 14, 2021 6:54:01 PM

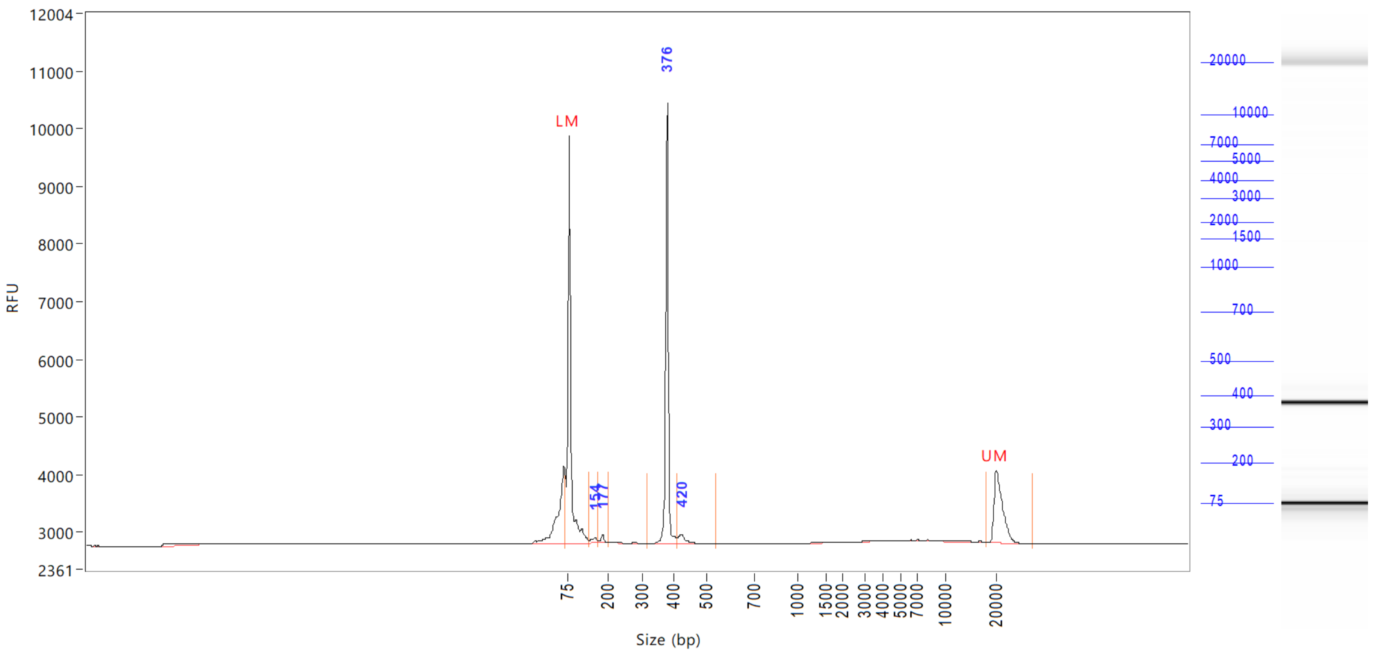


| Peak | Size (bp) | Concentration (ng/uL) | From (bp) | To (bp) | Average size (bp) | CV% | RFU | Corrected peak area |
|---|---|---|---|---|---|---|---|---|
| 1 | 75 (LM) | 0.5000 | 66 | 135 | 78 | 15.34 | 7058 | 50.476 |
| 2 | 154 | 0.1530 | 135 | 166 | 150 | 5.45 | 101 | 1.287 |
| 3 | 177 | 0.1210 | 166 | 198 | 176 | 3.05 | 143 | 1.018 |
| 4 | 376 | 4.9411 | 313 | 407 | 373 | 1.89 | 7659 | 41.568 |
| 5 | 420 | 0.3093 | 407 | 539 | 429 | 5.04 | 169 | 2.602 |
| 6 | 20000 (UM) | 0.1354 | 18049 | 27254 | 20583 | 4.77 | 1258 | 13.667 |

| | | |
|---|---|---|
| TIC: | 5.5244 | ng/uL |
| TIM: | 25.7872 | nmole/L |
| Total concentration: | 5.9067 | ng/uL |

Sample peak width (sec): 5    Sample min peak height: 50    Sample baseline V to V?: Y    Sample baseline V to V points: 3
Sample filter: Binomial    Number of points for filter: 3  Sample start region (min): 0  Sample end region (min): 30
Marker peak width (sec): 5    Marker min peak height: 200    Marker baseline V to V?: Y    Marker baseline V to V points: 3
Lower marker selection: First peak > 500 RFU    Upper marker selection: Last peak > 200 RFU
Ladder size (bp)75, 200, 300, 400, 500, 700, 1000, 1500, 2000, 3000, 4000, 5000, 7000, 10000, 20000
Quantification using: Lower Marker    Final concentration (ng/uL): 0.5000    Dilution factor: 12.0

**Sample:** SampA11
**Well location:** A11
**Created:** Tuesday, September 14, 2021 6:54:01 PM

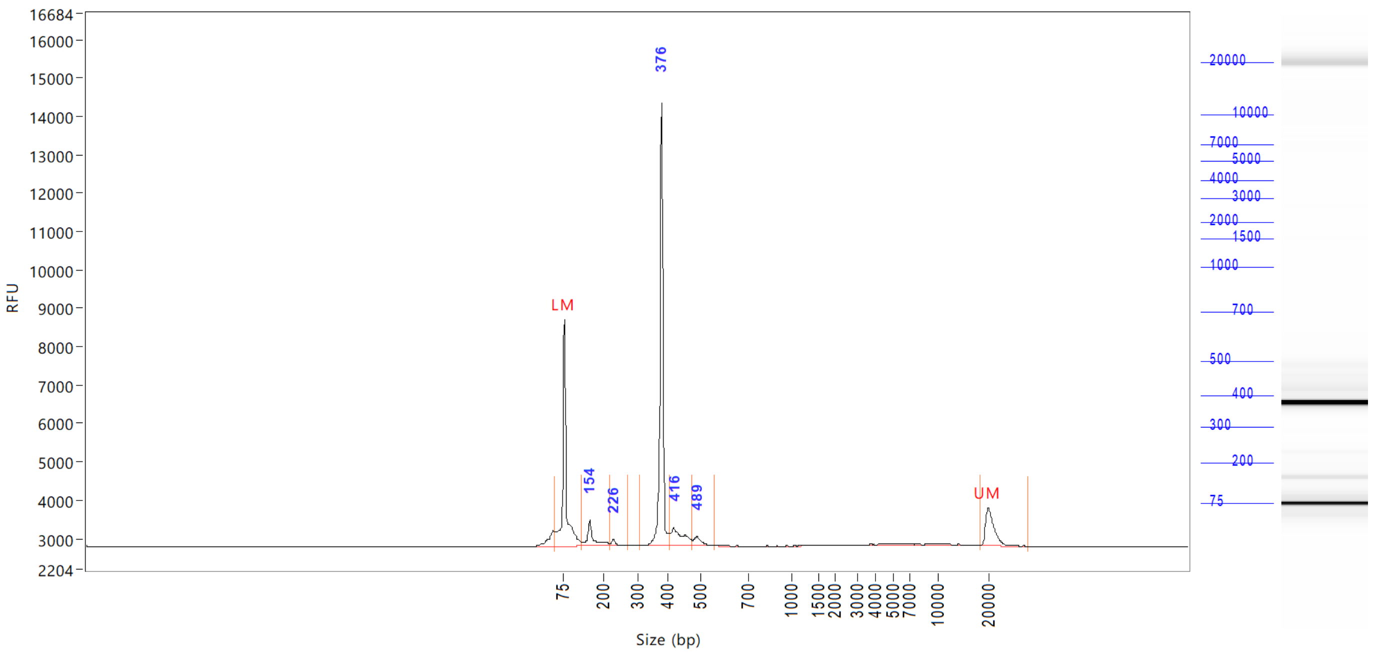


| Peak | Size (bp) | Concentration (ng/uL) | From (bp) | To (bp) | Average size (bp) | CV% | RFU | Corrected peak area |
|---|---|---|---|---|---|---|---|---|
| 1 | 75 (LM) | 0.5000 | 47 | 129 | 76 | 18.20 | 5888 | 50.107 |
| 2 | 154 | 1.0973 | 129 | 215 | 162 | 12.84 | 674 | 9.163 |
| 3 | 226 | 0.1900 | 215 | 269 | 228 | 3.95 | 164 | 1.587 |
| 4 | 376 | 8.2804 | 305 | 403 | 373 | 2.25 | 11550 | 69.150 |
| 5 | 416 | 1.3095 | 403 | 473 | 432 | 4.58 | 476 | 10.936 |
| 6 | 489 | 0.4555 | 473 | 557 | 495 | 3.67 | 240 | 3.804 |
| 7 | 20000 (UM) | 0.1064 | 18293 | 27979 | 20518 | 5.46 | 1000 | 10.663 |

| | | |
|---|---|---|
| TIC: | 11.3326 | ng/uL |
| TIM: | 55.5285 | nmole/L |
| Total concentration: | 11.6021 | ng/uL |

Sample peak width (sec): 5 Sample min peak height: 50 Sample baseline V to V?: Y Sample baseline V to V points: 3
Sample filter: Binomial Number of points for filter: 3 Sample start region (min): 0 Sample end region (min): 30
Marker peak width (sec): 5 Marker min peak height: 200 Marker baseline V to V?: Y Marker baseline V to V points: 3
Lower marker selection: First peak > 500 RFU Upper marker selection: Last peak > 200 RFU
Ladder size (bp)75, 200, 300, 400, 500, 700, 1000, 1500, 2000, 3000, 4000, 5000, 7000, 10000, 20000
Quantification using: Lower Marker Final concentration (ng/uL): 0.5000 Dilution factor: 12.0

**Sample:** SampB11
**Well location:** B11
**Created:** Tuesday, September 14, 2021 6:54:01 PM

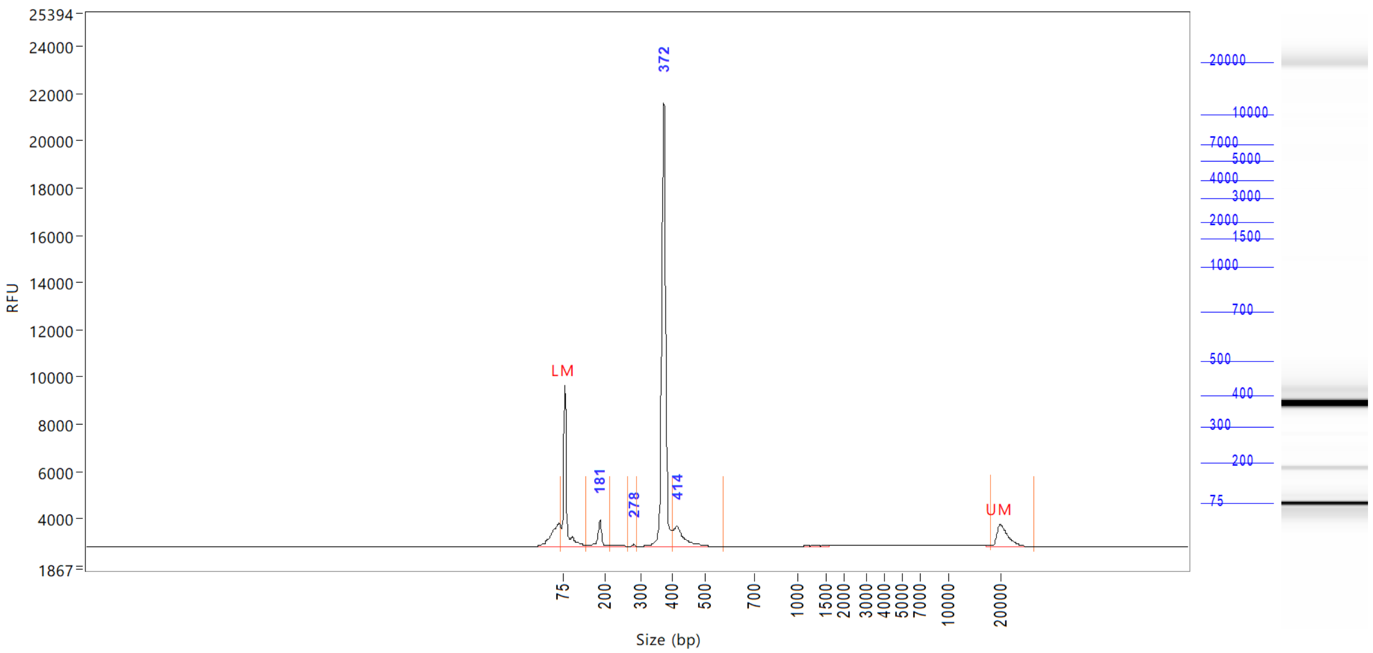


| Peak | Size (bp) | Concentration (ng/uL) | From (bp) | To (bp) | Average size (bp) | CV% | RFU | Corrected peak area |
|---|---|---|---|---|---|---|---|---|
| 1 | 75 (LM) | 0.5000 | 64 | 139 | 77 | 15.85 | 6801 | 51.147 |
| 2 | 181 | 1.1879 | 139 | 212 | 176 | 6.52 | 1133 | 10.126 |
| 3 | 278 | 0.0559 | 262 | 289 | 277 | 1.39 | 86 | 0.476 |
| 4 | 372 | 17.7857 | 289 | 403 | 371 | 2.48 | 18801 | 151.614 |
| 5 | 414 | 2.0679 | 403 | 575 | 430 | 5.98 | 857 | 17.628 |
| 6 | 20000 (UM) | 0.1221 | 17927 | 26408 | 20572 | 5.77 | 908 | 12.490 |

| | | |
|---|---|---|
| TIC: | 21.0973 | ng/uL |
| TIM: | 98.2108 | nmole/L |
| Total concentration: | 21.4717 | ng/uL |

Sample peak width (sec): 5 Sample min peak height: 50 Sample baseline V to V?: Y Sample baseline V to V points: 3
Sample filter: Binomial Number of points for filter: 3 Sample start region (min): 0 Sample end region (min): 30
Marker peak width (sec): 5 Marker min peak height: 200 Marker baseline V to V?: Y Marker baseline V to V points: 3
Lower marker selection: First peak > 500 RFU Upper marker selection: Last peak > 200 RFU
Ladder size (bp)75, 200, 300, 400, 500, 700, 1000, 1500, 2000, 3000, 4000, 5000, 7000, 10000, 20000
Quantification using: Lower Marker Final concentration (ng/uL): 0.5000 Dilution factor: 12.0

**Sample:** SampC11
**Well location:** C11
**Created:** Tuesday, September 14, 2021 6:54:01 PM

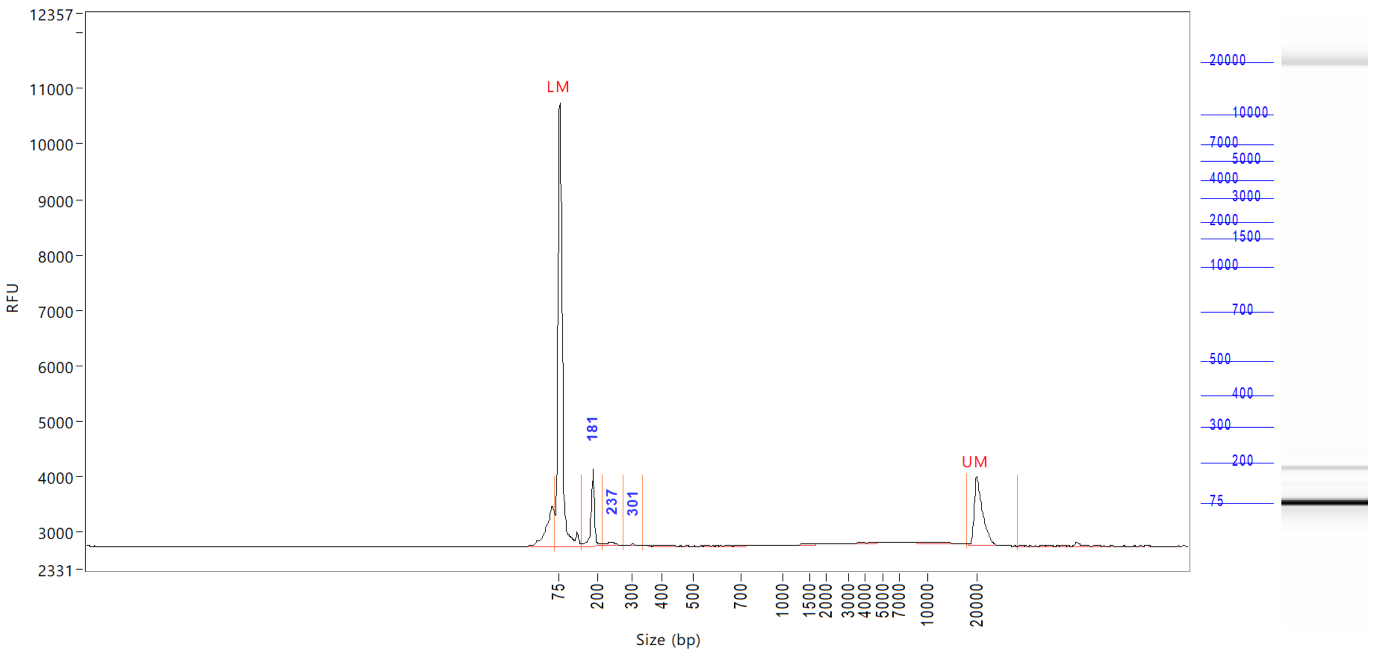


| Peak | Size<br>(bp) | Concentration<br>(ng/uL) | From<br>(bp) | To<br>(bp) | Average size<br>(bp) | CV% | RFU | Corrected peak area |
|---|---|---|---|---|---|---|---|---|
| 1 | 75 (LM) | 0.5000 | 64 | 145 | 77 | 14.91 | 8009 | 82.475 |
| 2 | 181 | 0.8086 | 145 | 210 | 177 | 5.38 | 1400 | 11.115 |
| 3 | 237 | 0.1206 | 210 | 277 | 234 | 6.02 | 75 | 1.658 |
| 4 | 301 | 0.0264 | 277 | 333 | 299 | 2.50 | 51 | 0.363 |
| 5 | 20000 (UM) | 0.0805 | 17927 | 28221 | 20524 | 4.96 | 1230 | 13.278 |

| | | |
|---|---|---|
| TIC: | 0.9556 | ng/uL |
| TIM: | 8.5030 | nmole/L |
| Total concentration: | 1.1968 | ng/uL |

Sample peak width (sec): 5 Sample min peak height: 50 Sample baseline V to V?: Y Sample baseline V to V points: 3
Sample filter: Binomial Number of points for filter: 3 Sample start region (min): 0 Sample end region (min): 30
Marker peak width (sec): 5 Marker min peak height: 200 Marker baseline V to V?: Y Marker baseline V to V points: 3
Lower marker selection: First peak > 500 RFU Upper marker selection: Last peak > 200 RFU
Ladder size (bp)75, 200, 300, 400, 500, 700, 1000, 1500, 2000, 3000, 4000, 5000, 7000, 10000, 20000
Quantification using: Lower Marker Final concentration (ng/uL): 0.5000 Dilution factor: 12.0

**Sample:** SampD11
**Well location:** D11
**Created:** Tuesday, September 14, 2021 6:54:01 PM

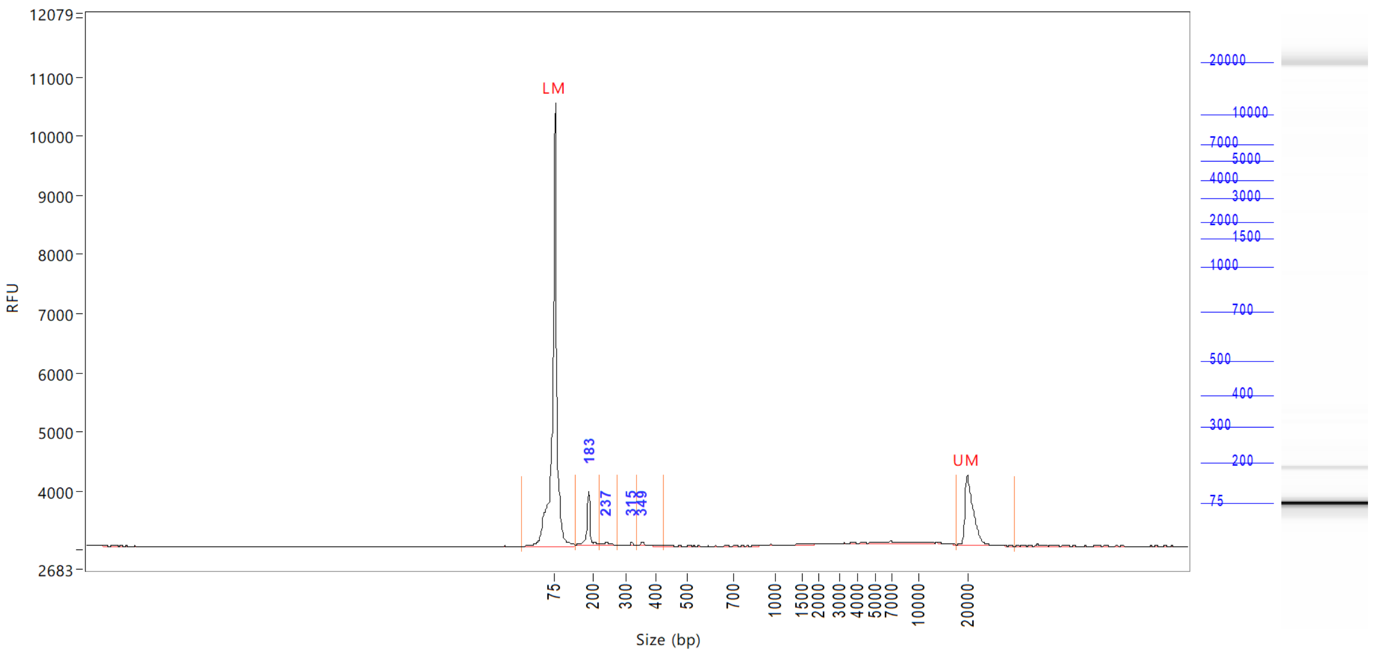


| Peak | Size (bp) | Concentration (ng/uL) | From (bp) | To (bp) | Average size (bp) | CV% | RFU | Corrected peak area |
|---|---|---|---|---|---|---|---|---|
| 1 | 75 (LM) | 0.5000 | 0 | 141 | 68 | 24.47 | 7504 | 77.700 |
| 2 | 183 | 0.5929 | 141 | 217 | 179 | 6.77 | 929 | 7.678 |
| 3 | 237 | 0.0851 | 217 | 271 | 237 | 5.26 | 55 | 1.102 |
| 4 | 315 | 0.0339 | 271 | 331 | 306 | 4.25 | 56 | 0.439 |
| 5 | 349 | 0.0670 | 331 | 424 | 364 | 6.30 | 68 | 0.867 |
| 6 | 20000 (UM) | 0.0836 | 17683 | 29672 | 20576 | 5.70 | 1175 | 12.996 |

| | | |
|---|---|---|
| TIC: | 0.7789 | ng/uL |
| TIM: | 6.5206 | nmole/L |
| Total concentration: | 1.0182 | ng/uL |

Sample peak width (sec): 5 Sample min peak height: 50 Sample baseline V to V?: Y Sample baseline V to V points: 3
Sample filter: Binomial Number of points for filter: 3 Sample start region (min): 0 Sample end region (min): 30
Marker peak width (sec): 5 Marker min peak height: 200 Marker baseline V to V?: Y Marker baseline V to V points: 3
Lower marker selection: First peak > 500 RFU Upper marker selection: Last peak > 200 RFU
Ladder size (bp)75, 200, 300, 400, 500, 700, 1000, 1500, 2000, 3000, 4000, 5000, 7000, 10000, 20000
Quantification using: Lower Marker Final concentration (ng/uL): 0.5000 Dilution factor: 12.0

**Sample:** SampE11
**Well location:** E11
**Created:** Tuesday, September 14, 2021 6:54:01 PM

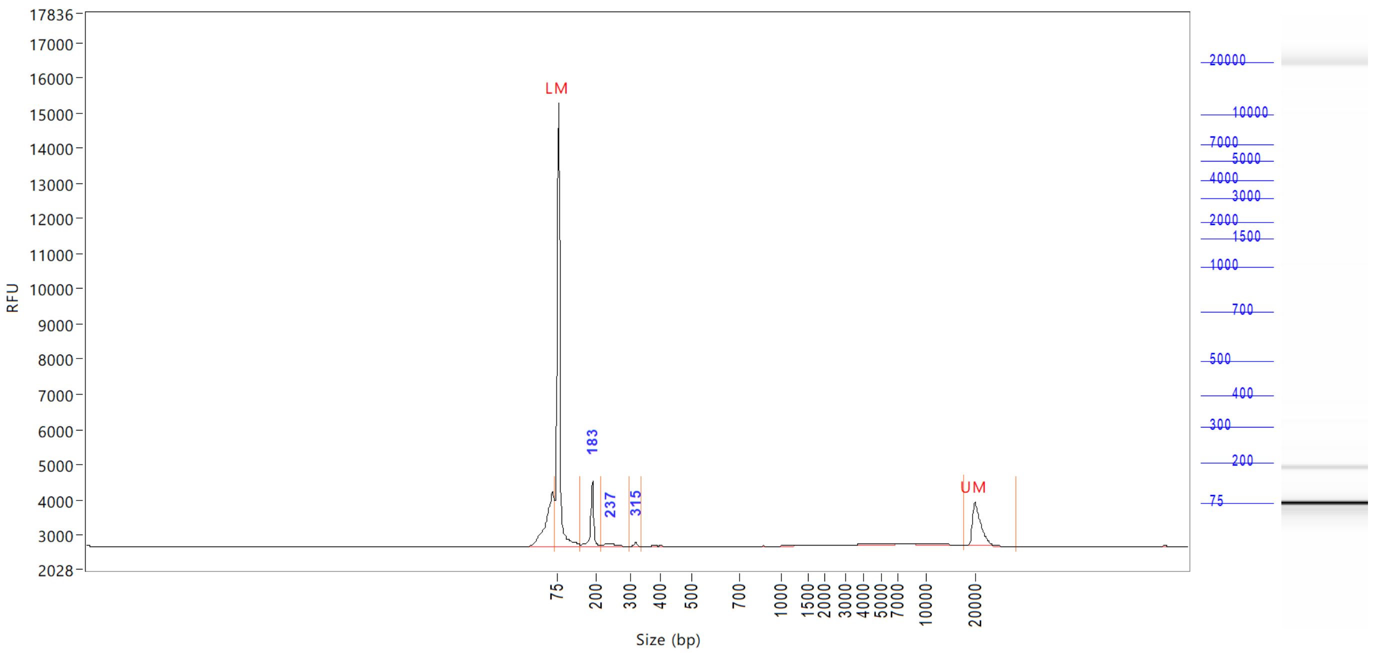


| Peak | Size (bp) | Concentration (ng/uL) | From (bp) | To (bp) | Average size (bp) | CV% | RFU | Corrected peak area |
|---|---|---|---|---|---|---|---|---|
| 1 | 75 (LM) | 0.5000 | 64 | 147 | 76 | 13.51 | 12627 | 86.218 |
| 2 | 183 | 0.9303 | 147 | 212 | 180 | 4.84 | 1844 | 13.367 |
| 3 | 237 | 0.1310 | 212 | 296 | 237 | 6.61 | 83 | 1.882 |
| 4 | 315 | 0.0445 | 296 | 335 | 312 | 1.78 | 102 | 0.639 |
| 5 | 20000 (UM) | 0.0778 | 17927 | 28463 | 20563 | 4.93 | 1238 | 13.424 |

| | | |
|---|---|---|
| TIC: | 1.1057 | ng/uL |
| TIM: | 9.6395 | nmole/L |
| Total concentration: | 1.3721 | ng/uL |

Sample peak width (sec): 5    Sample min peak height: 50    Sample baseline V to V?: Y    Sample baseline V to V points: 3
Sample filter: Binomial    Number of points for filter: 3  Sample start region (min): 0  Sample end region (min): 30
Marker peak width (sec): 5    Marker min peak height: 200    Marker baseline V to V?: Y    Marker baseline V to V points: 3
Lower marker selection: First peak > 500 RFU    Upper marker selection: Last peak > 200 RFU
Ladder size (bp)75, 200, 300, 400, 500, 700, 1000, 1500, 2000, 3000, 4000, 5000, 7000, 10000, 20000
Quantification using: Lower Marker    Final concentration (ng/uL): 0.5000    Dilution factor: 12.0

**Sample:** SampF11
**Well location:** F11
**Created:** Tuesday, September 14, 2021 6:54:01 PM

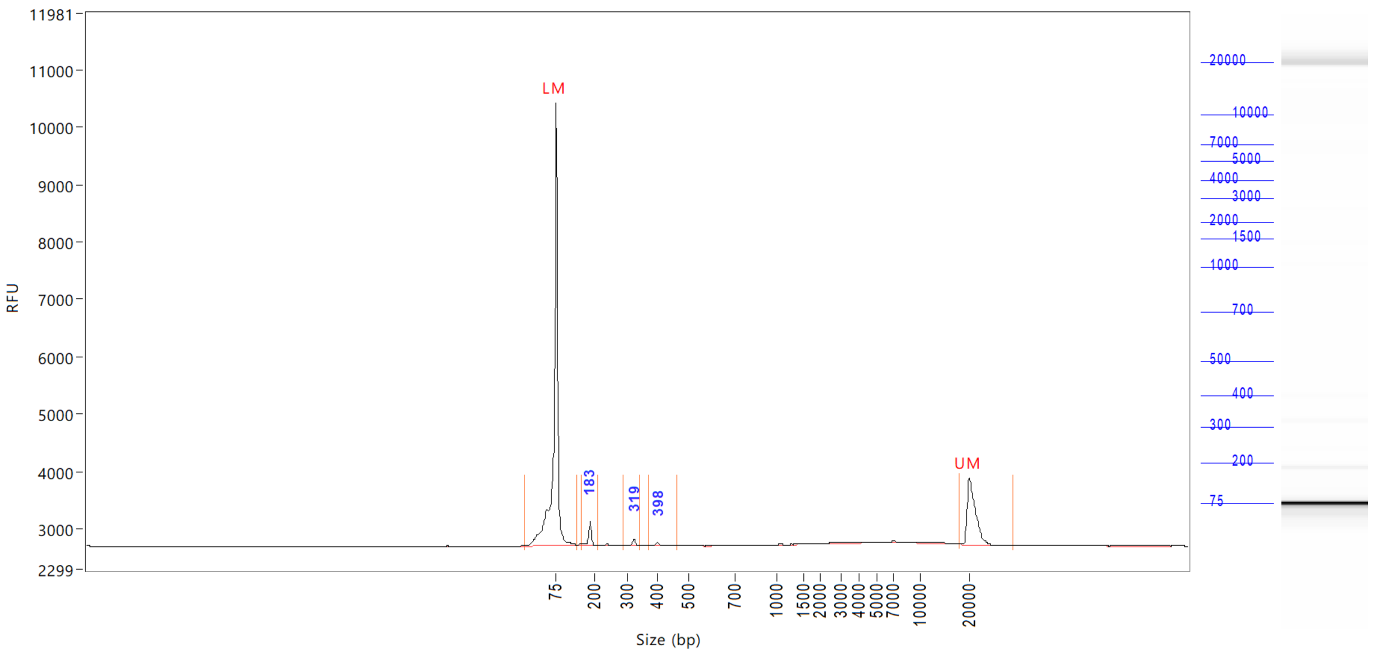


| Peak | Size (bp) | Concentration (ng/uL) | From (bp) | To (bp) | Average size (bp) | CV% | RFU | Corrected peak area |
|---|---|---|---|---|---|---|---|---|
| 1 | 75 (LM) | 0.5000 | 0 | 141 | 64 | 29.85 | 7727 | 71.321 |
| 2 | 183 | 0.2541 | 156 | 208 | 180 | 4.46 | 418 | 3.020 |
| 3 | 319 | 0.0695 | 287 | 339 | 315 | 2.33 | 121 | 0.826 |
| 4 | 398 | 0.0539 | 368 | 462 | 403 | 4.56 | 65 | 0.640 |
| 5 | 20000 (UM) | 0.0906 | 18049 | 29068 | 20658 | 5.51 | 1165 | 12.921 |

| | | |
|---|---|---|
| TIC: | 0.3774 | ng/uL |
| TIM: | 2.9033 | nmole/L |
| Total concentration: | 0.7328 | ng/uL |

Sample peak width (sec): 5    Sample min peak height: 50    Sample baseline V to V?: Y    Sample baseline V to V points: 3
Sample filter: Binomial    Number of points for filter: 3  Sample start region (min): 0  Sample end region (min): 30
Marker peak width (sec): 5    Marker min peak height: 200    Marker baseline V to V?: Y    Marker baseline V to V points: 3
Lower marker selection: First peak > 500 RFU    Upper marker selection: Last peak > 200 RFU
Ladder size (bp)75, 200, 300, 400, 500, 700, 1000, 1500, 2000, 3000, 4000, 5000, 7000, 10000, 20000
Quantification using: Lower Marker    Final concentration (ng/uL): 0.5000    Dilution factor: 12.0

**Sample:** SampG11
**Well location:** G11
**Created:** Tuesday, September 14, 2021 6:54:01 PM

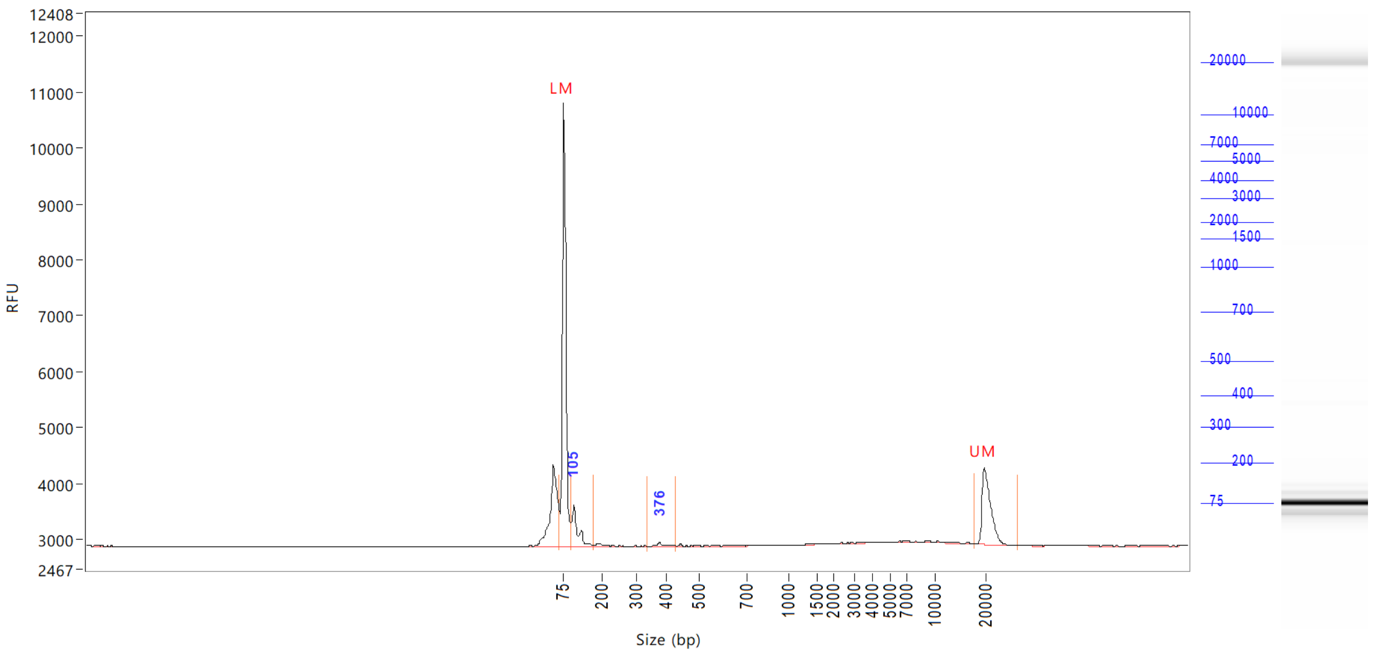


| Peak | Size (bp) | Concentration (ng/uL) | From (bp) | To (bp) | Average size (bp) | CV% | RFU | Corrected peak area |
|---|---|---|---|---|---|---|---|---|
| 1 | 75 (LM) | 0.5000 | 64 | 97 | 75 | 7.89 | 7938 | 72.417 |
| 2 | 105 | 0.8320 | 97 | 171 | 114 | 13.72 | 741 | 10.042 |
| 3 | 376 | 0.0523 | 333 | 428 | 376 | 4.65 | 68 | 0.631 |
| 4 | 20000 (UM) | 0.1046 | 18049 | 26529 | 20510 | 4.72 | 1381 | 15.144 |

| | | |
|---|---|---|
| TIC: | 0.8843 | ng/uL |
| TIM: | 12.2172 | nmole/L |
| Total concentration: | 1.2444 | ng/uL |

Sample peak width (sec): 5 Sample min peak height: 50 Sample baseline V to V?: Y Sample baseline V to V points: 3
Sample filter: Binomial Number of points for filter: 3 Sample start region (min): 0 Sample end region (min): 30
Marker peak width (sec): 5 Marker min peak height: 200 Marker baseline V to V?: Y Marker baseline V to V points: 3
Lower marker selection: First peak > 500 RFU Upper marker selection: Last peak > 200 RFU
Ladder size (bp)75, 200, 300, 400, 500, 700, 1000, 1500, 2000, 3000, 4000, 5000, 7000, 10000, 20000
Quantification using: Lower Marker Final concentration (ng/uL): 0.5000 Dilution factor: 12.0

**Sample:** SampH11
**Well location:** H11
**Created:** Tuesday, September 14, 2021 6:54:01 PM

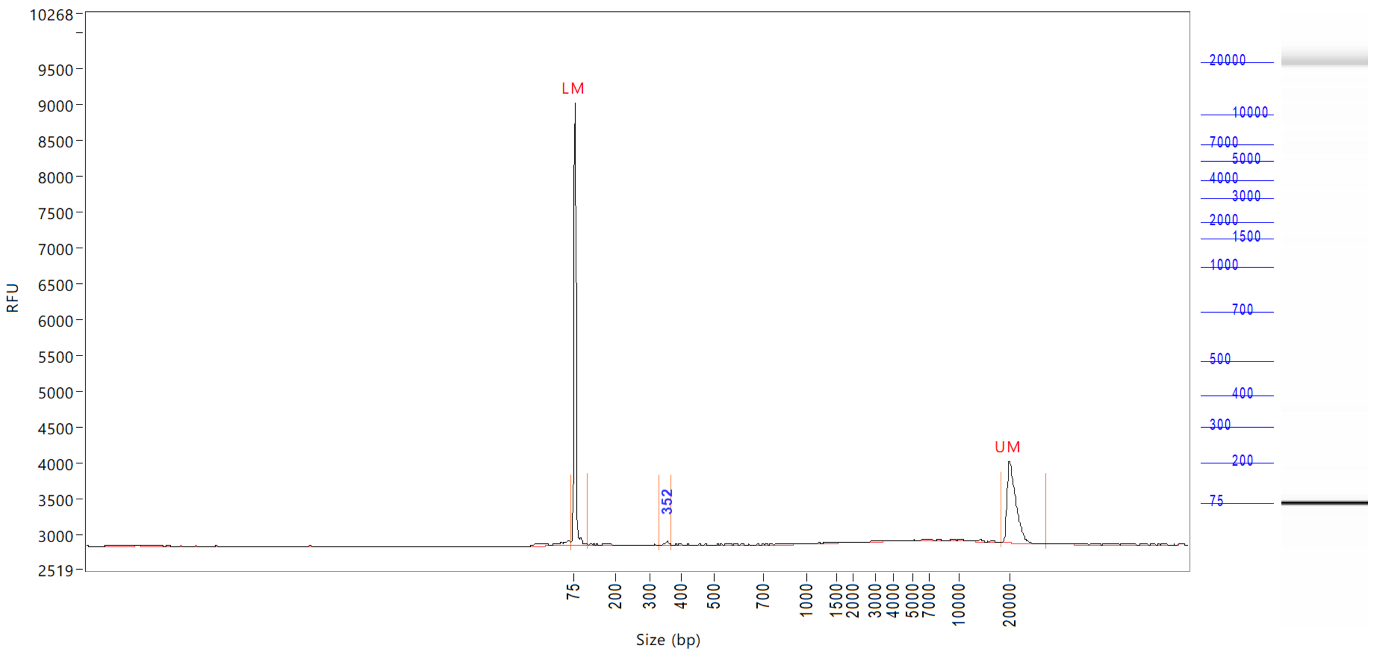

| Peak | Size (bp) | Concentration (ng/uL) | From (bp) | To (bp) | Average size (bp) | CV% | RFU | Corrected peak area |
|---|---|---|---|---|---|---|---|---|
| 1 | 75 (LM) | 0.5000 | 64 | 116 | 74 | 5.58 | 6172 | 32.897 |
| 2 | 352 | 0.0753 | 327 | 366 | 348 | 2.25 | 54 | 0.413 |
| 3 | 20000 (UM) | 0.1917 | 18171 | 27133 | 20512 | 4.77 | 1141 | 12.614 |

| | | |
|---|---|---|
| TIC: | 0.0753 | ng/uL |
| TIM: | 0.3561 | nmole/L |
| Total concentration: | 0.7770 | ng/uL |

Sample peak width (sec): 5 Sample min peak height: 50 Sample baseline V to V?: Y Sample baseline V to V points: 3
Sample filter: Binomial Number of points for filter: 3 Sample start region (min): 0 Sample end region (min): 30
Marker peak width (sec): 5 Marker min peak height: 200 Marker baseline V to V?: Y Marker baseline V to V points: 3
Lower marker selection: First peak > 500 RFU Upper marker selection: Last peak > 200 RFU
Ladder size (bp)75, 200, 300, 400, 500, 700, 1000, 1500, 2000, 3000, 4000, 5000, 7000, 10000, 20000
Quantification using: Lower Marker Final concentration (ng/uL): 0.5000 Dilution factor: 12.0

**Sample:** SampA12
**Well location:** A12
**Created:** Tuesday, September 14, 2021 6:54:01 PM

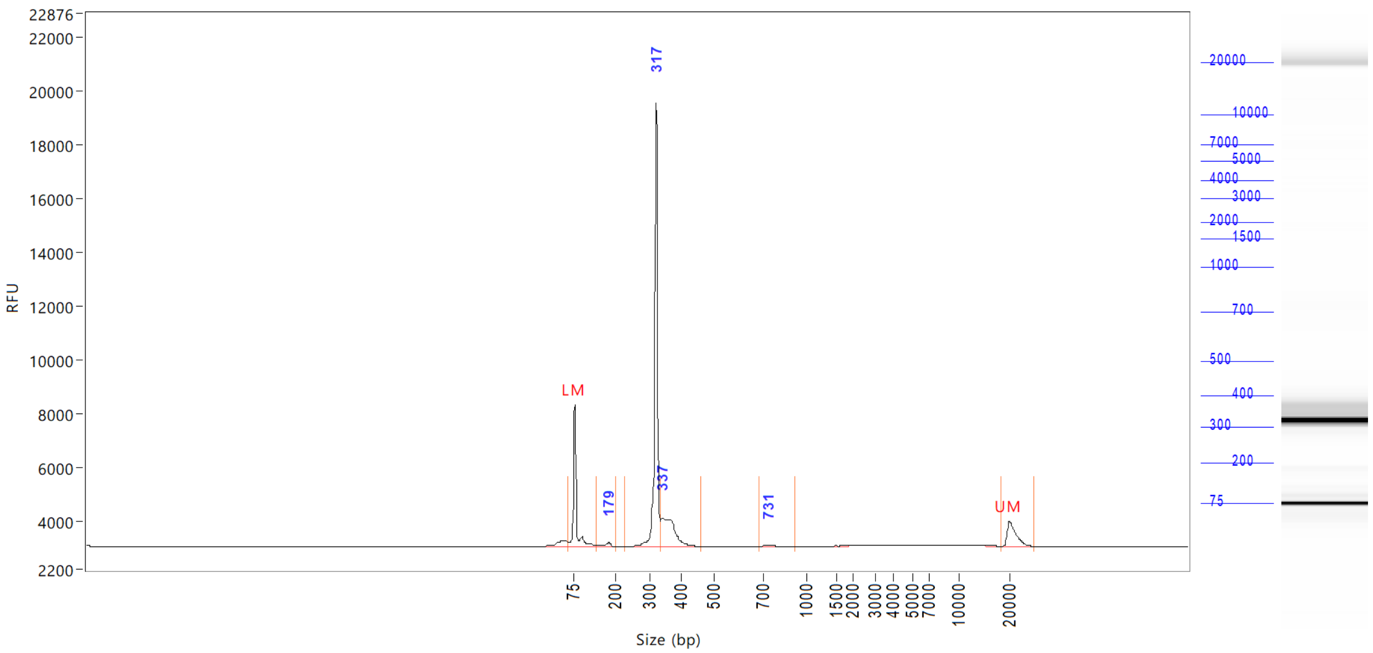


| Peak | Size (bp) | Concentration (ng/uL) | From (bp) | To (bp) | Average size (bp) | CV% | RFU | Corrected peak area |
|---|---|---|---|---|---|---|---|---|
| 1 | 75 (LM) | 0.5000 | 58 | 143 | 77 | 16.54 | 5264 | 36.027 |
| 2 | 179 | 0.3460 | 143 | 201 | 169 | 6.65 | 145 | 2.078 |
| 3 | 317 | 16.6050 | 226 | 331 | 314 | 2.89 | 16527 | 99.704 |
| 4 | 337 | 4.9867 | 331 | 458 | 360 | 6.07 | 1064 | 29.942 |
| 5 | 731 | 0.1837 | 685 | 918 | 748 | 6.19 | 53 | 1.103 |
| 6 | 20000 (UM) | 0.1413 | 18171 | 24594 | 20580 | 4.86 | 921 | 10.182 |

| | | |
|---|---|---|
| TIC: | 22.1213 | ng/uL |
| TIM: | 113.5494 | nmole/L |
| Total concentration: | 22.4601 | ng/uL |

Sample peak width (sec): 5 Sample min peak height: 50 Sample baseline V to V?: Y Sample baseline V to V points: 3
Sample filter: Binomial Number of points for filter: 3 Sample start region (min): 0 Sample end region (min): 30
Marker peak width (sec): 5 Marker min peak height: 200 Marker baseline V to V?: Y Marker baseline V to V points: 3
Lower marker selection: First peak > 500 RFU Upper marker selection: Last peak > 200 RFU
Ladder size (bp)75, 200, 300, 400, 500, 700, 1000, 1500, 2000, 3000, 4000, 5000, 7000, 10000, 20000
Quantification using: Lower Marker Final concentration (ng/uL): 0.5000 Dilution factor: 12.0

**Sample:** SampB12
**Well location:** B12
**Created:** Tuesday, September 14, 2021 6:54:01 PM

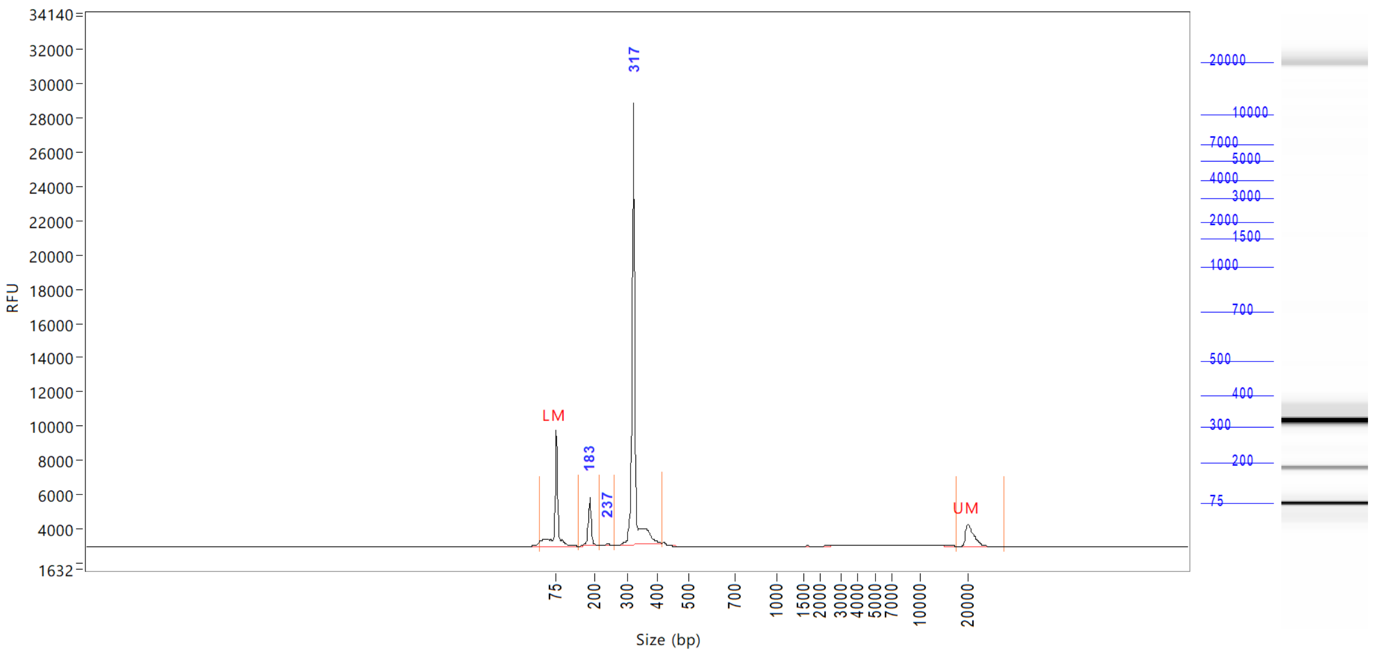


| Peak | Size (bp) | Concentration (ng/uL) | From (bp) | To (bp) | Average size (bp) | CV% | RFU | Corrected peak area |
|---|---|---|---|---|---|---|---|---|
| 1 | 75 (LM) | 0.5000 | 24 | 145 | 69 | 23.28 | 6828 | 53.913 |
| 2 | 183 | 2.2942 | 145 | 212 | 180 | 3.83 | 2837 | 20.615 |
| 3 | 237 | 0.1413 | 212 | 260 | 231 | 4.28 | 84 | 1.270 |
| 4 | 317 | 18.5490 | 260 | 414 | 320 | 5.11 | 25868 | 166.673 |
| 5 | 20000 (UM) | 0.1315 | 17683 | 27375 | 20516 | 4.88 | 1310 | 14.184 |

| | | |
|---|---|---|
| TIC: | 20.9845 | ng/uL |
| TIM: | 117.3143 | nmole/L |
| Total concentration: | 21.3437 | ng/uL |

Sample peak width (sec): 5    Sample min peak height: 50    Sample baseline V to V?: Y    Sample baseline V to V points: 3
Sample filter: Binomial    Number of points for filter: 3  Sample start region (min): 0  Sample end region (min): 30
Marker peak width (sec): 5    Marker min peak height: 200    Marker baseline V to V?: Y    Marker baseline V to V points: 3
Lower marker selection: First peak > 500 RFU    Upper marker selection: Last peak > 200 RFU
Ladder size (bp)75, 200, 300, 400, 500, 700, 1000, 1500, 2000, 3000, 4000, 5000, 7000, 10000, 20000
Quantification using: Lower Marker    Final concentration (ng/uL): 0.5000    Dilution factor: 12.0

**Sample:** SampC12
**Well location:** C12
**Created:** Tuesday, September 14, 2021 6:54:01 PM

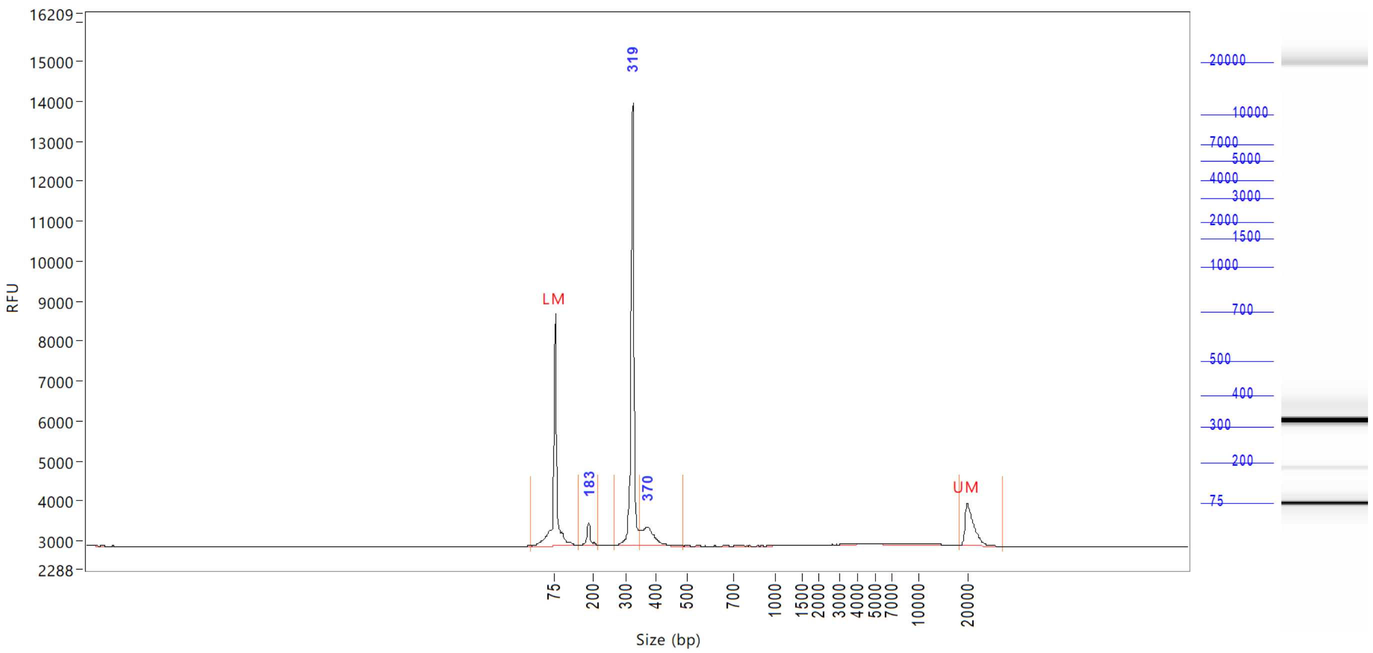

| Peak | Size (bp) | Concentration (ng/uL) | From (bp) | To (bp) | Average size (bp) | CV% | RFU | Corrected peak area |
|---|---|---|---|---|---|---|---|---|
| 1 | 75 (LM) | 0.5000 | 1 | 152 | 72 | 22.89 | 5805 | 48.237 |
| 2 | 183 | 0.6109 | 152 | 212 | 181 | 4.46 | 578 | 4.912 |
| 3 | 319 | 9.2789 | 264 | 341 | 315 | 2.59 | 11110 | 74.598 |
| 4 | 370 | 1.6339 | 341 | 487 | 374 | 6.45 | 465 | 13.136 |
| 5 | 20000 (UM) | 0.1262 | 18415 | 27254 | 20643 | 5.39 | 1067 | 12.178 |

| | | |
|---|---|---|
| TIC: | 11.5237 | ng/uL |
| TIM: | 61.1931 | nmole/L |
| Total concentration: | 11.9442 | ng/uL |

Sample peak width (sec): 5    Sample min peak height: 50    Sample baseline V to V?: Y    Sample baseline V to V points: 3
Sample filter: Binomial    Number of points for filter: 3  Sample start region (min): 0  Sample end region (min): 30
Marker peak width (sec): 5    Marker min peak height: 200    Marker baseline V to V?: Y    Marker baseline V to V points: 3
Lower marker selection: First peak > 500 RFU    Upper marker selection: Last peak > 200 RFU
Ladder size (bp)75, 200, 300, 400, 500, 700, 1000, 1500, 2000, 3000, 4000, 5000, 7000, 10000, 20000
Quantification using: Lower Marker    Final concentration (ng/uL): 0.5000    Dilution factor: 12.0

**Sample:** SampD12
**Well location:** D12
**Created:** Tuesday, September 14, 2021 6:54:01 PM

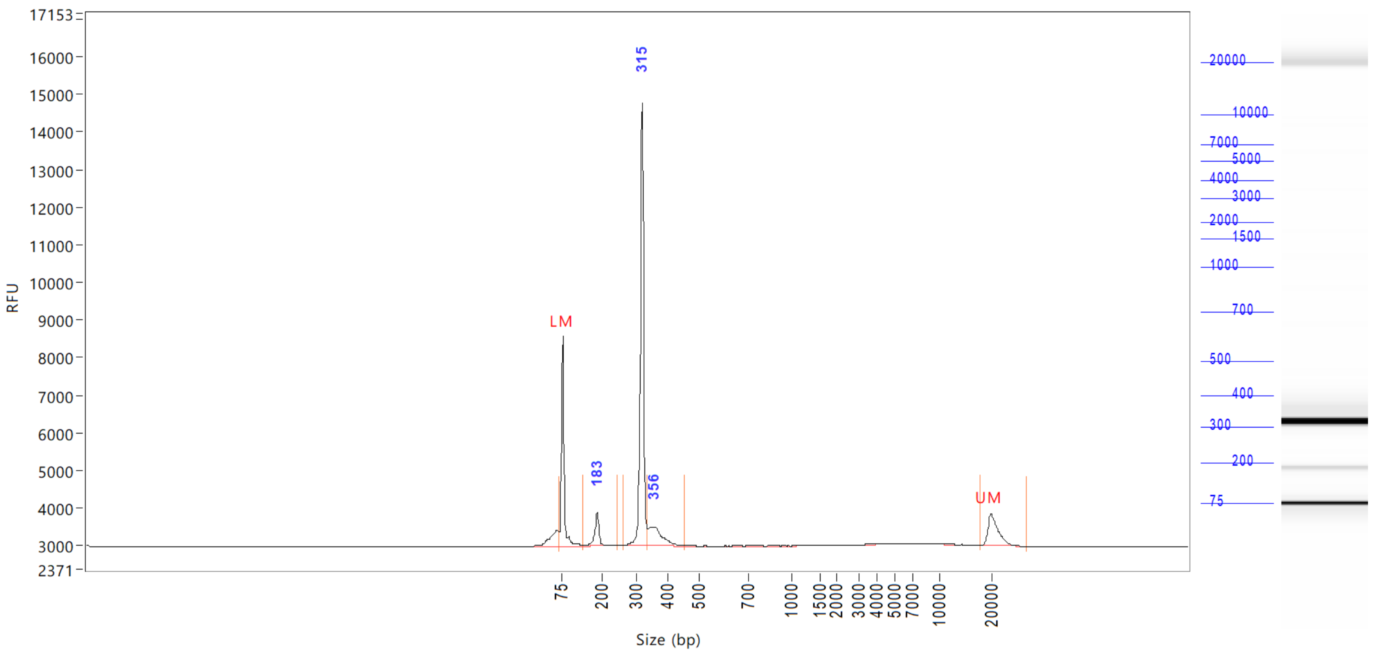


| Peak | Size (bp) | Concentration (ng/uL) | From (bp) | To (bp) | Average size (bp) | CV% | RFU | Corrected peak area |
|---|---|---|---|---|---|---|---|---|
| 1 | 75 (LM) | 0.5000 | 64 | 141 | 76 | 12.86 | 5593 | 37.455 |
| 2 | 183 | 1.3597 | 141 | 244 | 178 | 6.45 | 881 | 8.488 |
| 3 | 315 | 13.6062 | 262 | 333 | 312 | 2.34 | 11797 | 84.936 |
| 4 | 356 | 2.3667 | 333 | 454 | 364 | 6.52 | 511 | 14.774 |
| 5 | 20000 (UM) | 0.1420 | 18049 | 27133 | 20656 | 5.44 | 831 | 10.636 |

| | | |
|---|---|---|
| TIC: | 17.3325 | ng/uL |
| TIM: | 94.9919 | nmole/L |
| Total concentration: | 17.6666 | ng/uL |

Sample peak width (sec): 5 Sample min peak height: 50 Sample baseline V to V?: Y Sample baseline V to V points: 3
Sample filter: Binomial Number of points for filter: 3 Sample start region (min): 0 Sample end region (min): 30
Marker peak width (sec): 5 Marker min peak height: 200 Marker baseline V to V?: Y Marker baseline V to V points: 3
Lower marker selection: First peak > 500 RFU Upper marker selection: Last peak > 200 RFU
Ladder size (bp)75, 200, 300, 400, 500, 700, 1000, 1500, 2000, 3000, 4000, 5000, 7000, 10000, 20000
Quantification using: Lower Marker Final concentration (ng/uL): 0.5000 Dilution factor: 12.0

**Sample:** SampE12
**Well location:** E12
**Created:** Tuesday, September 14, 2021 6:54:01 PM

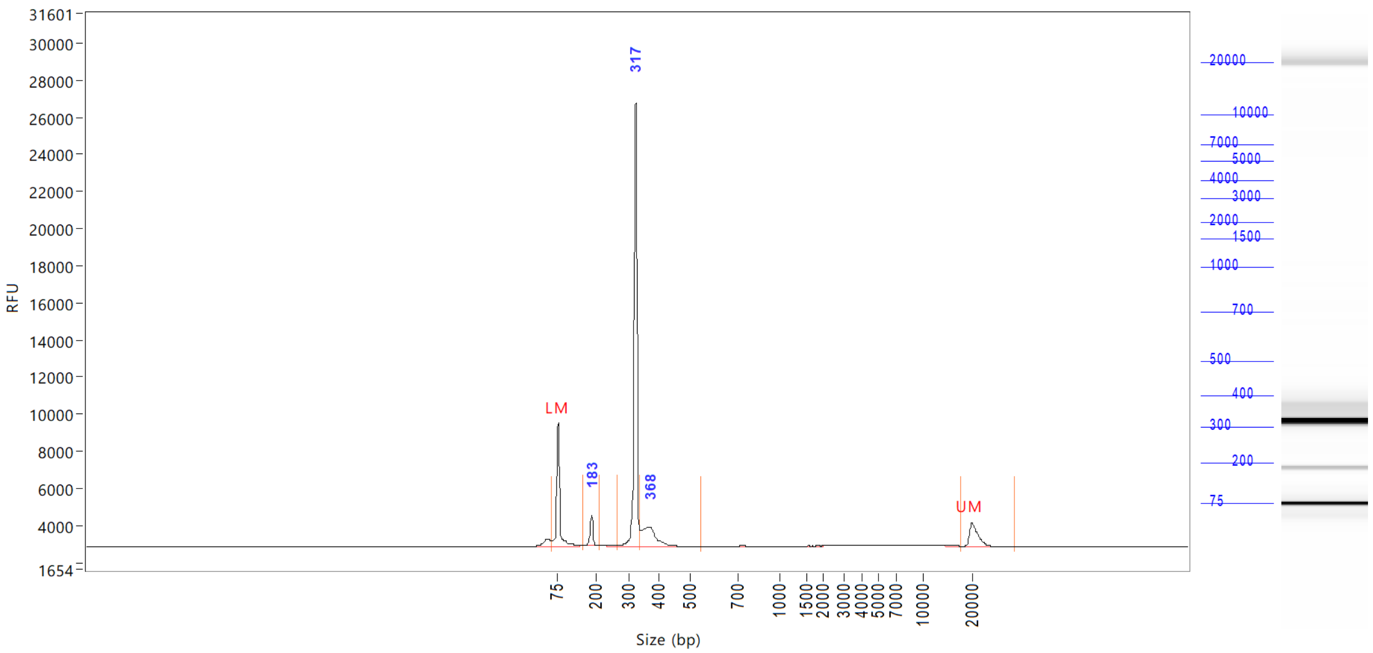


| Peak | Size (bp) | Concentration (ng/uL) | From (bp) | To (bp) | Average size (bp) | CV% | RFU | Corrected peak area |
|---|---|---|---|---|---|---|---|---|
| 1 | 75 (LM) | 0.5000 | 56 | 154 | 75 | 14.77 | 6649 | 47.037 |
| 2 | 183 | 1.5223 | 154 | 205 | 179 | 3.18 | 1668 | 11.934 |
| 3 | 317 | 18.3384 | 262 | 335 | 313 | 2.29 | 23914 | 143.765 |
| 4 | 368 | 3.8085 | 335 | 544 | 368 | 7.24 | 1046 | 29.857 |
| 5 | 20000 (UM) | 0.1447 | 17805 | 28705 | 20585 | 4.89 | 1250 | 13.610 |

| | | |
|---|---|---|
| TIC: | 23.6693 | ng/uL |
| TIM: | 127.3867 | nmole/L |
| Total concentration: | 24.2126 | ng/uL |

Sample peak width (sec): 5    Sample min peak height: 50    Sample baseline V to V?: Y    Sample baseline V to V points: 3
Sample filter: Binomial    Number of points for filter: 3  Sample start region (min): 0  Sample end region (min): 30
Marker peak width (sec): 5    Marker min peak height: 200    Marker baseline V to V?: Y    Marker baseline V to V points: 3
Lower marker selection: First peak > 500 RFU    Upper marker selection: Last peak > 200 RFU
Ladder size (bp)75, 200, 300, 400, 500, 700, 1000, 1500, 2000, 3000, 4000, 5000, 7000, 10000, 20000
Quantification using: Lower Marker    Final concentration (ng/uL): 0.5000    Dilution factor: 12.0

**Sample:** SampF12
**Well location:** F12
**Created:** Tuesday, September 14, 2021 6:54:01 PM

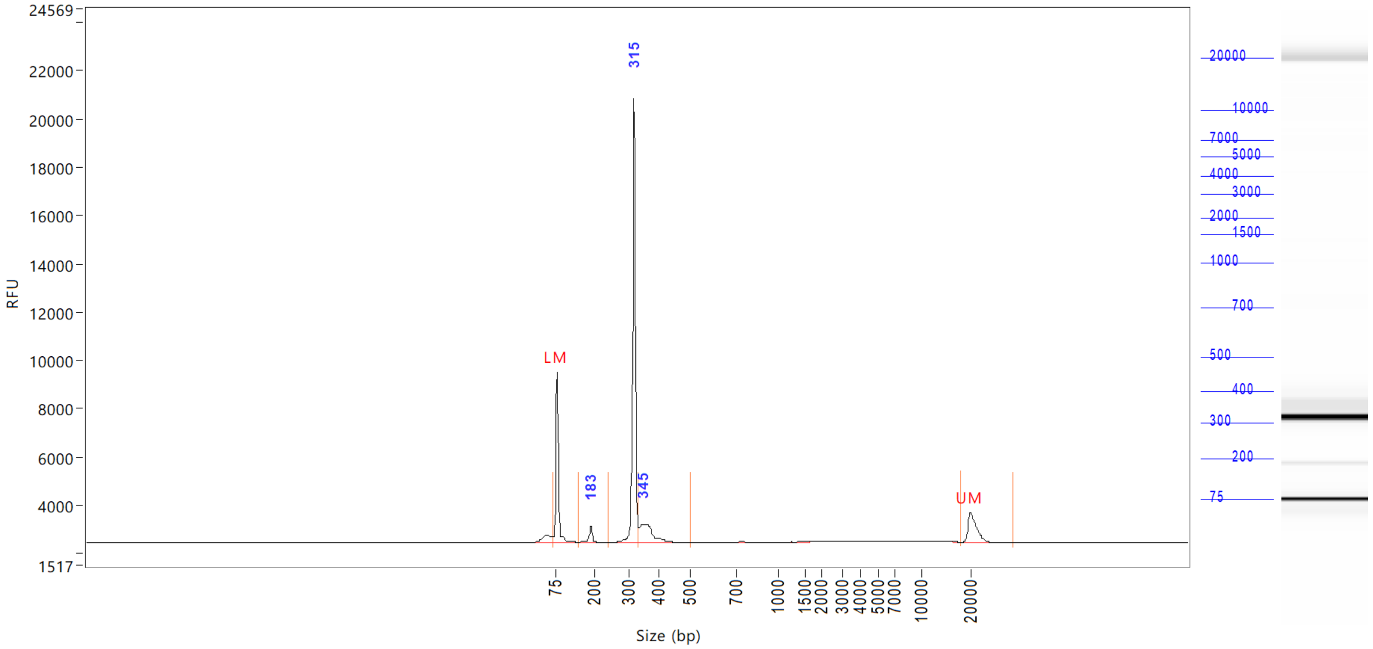


| Peak | Size (bp) | Concentration (ng/uL) | From (bp) | To (bp) | Average size (bp) | CV% | RFU | Corrected peak area |
|---|---|---|---|---|---|---|---|---|
| 1 | 75 (LM) | 0.5000 | 64 | 143 | 75 | 11.67 | 7091 | 43.224 |
| 2 | 183 | 0.7499 | 143 | 239 | 179 | 6.38 | 684 | 5.403 |
| 3 | 315 | 14.7221 | 239 | 333 | 312 | 2.23 | 18417 | 106.058 |
| 4 | 345 | 2.8341 | 333 | 501 | 364 | 6.74 | 726 | 20.417 |
| 5 | 20000 (UM) | 0.1558 | 17927 | 28705 | 20560 | 5.09 | 1219 | 13.472 |

| | | |
|---|---|---|
| TIC: | 18.3061 | ng/uL |
| TIM: | 97.3177 | nmole/L |
| Total concentration: | 18.7095 | ng/uL |

Sample peak width (sec): 5    Sample min peak height: 50    Sample baseline V to V?: Y    Sample baseline V to V points: 3
Sample filter: Binomial    Number of points for filter: 3    Sample start region (min): 0    Sample end region (min): 30
Marker peak width (sec): 5    Marker min peak height: 200    Marker baseline V to V?: Y    Marker baseline V to V points: 3
Lower marker selection: First peak > 500 RFU    Upper marker selection: Last peak > 200 RFU
Ladder size (bp)75, 200, 300, 400, 500, 700, 1000, 1500, 2000, 3000, 4000, 5000, 7000, 10000, 20000
Quantification using: Lower Marker    Final concentration (ng/uL): 0.5000    Dilution factor: 12.0

**Sample:** SampG12
**Well location:** G12
**Created:** Tuesday, September 14, 2021 6:54:01 PM

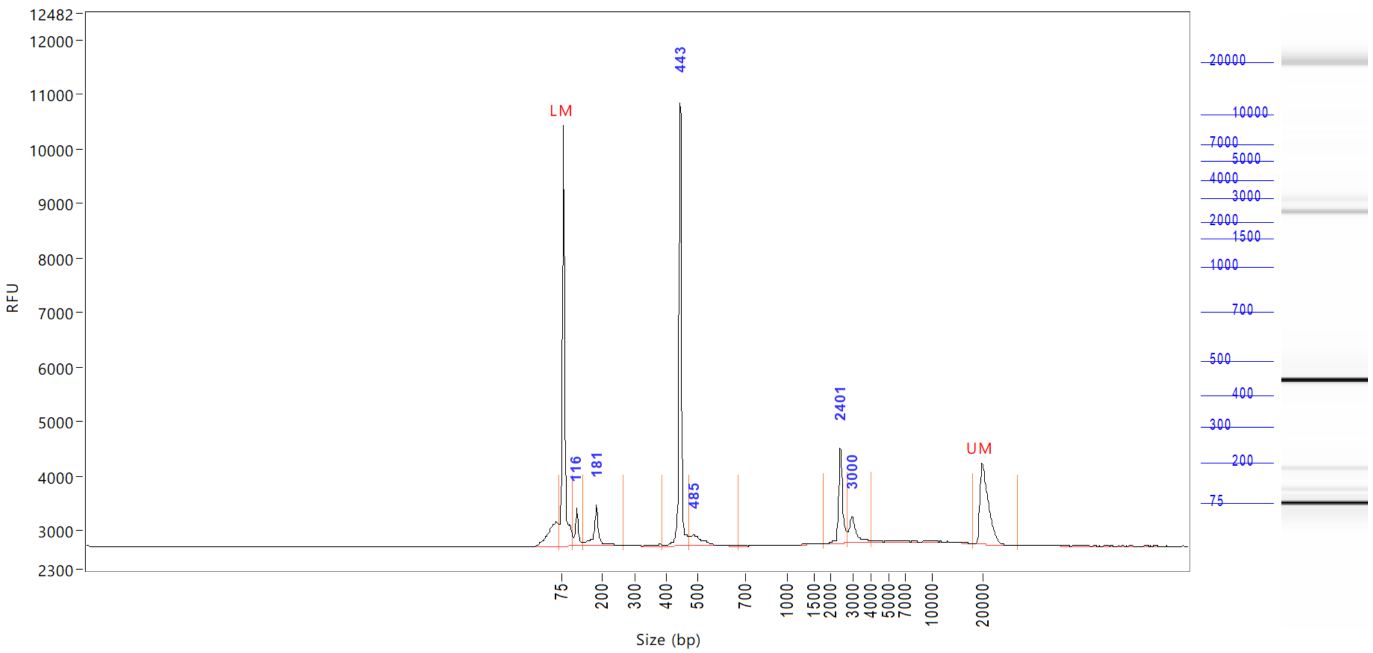


| Peak | Size (bp) | Concentration (ng/uL) | From (bp) | To (bp) | Average size (bp) | CV% | RFU | Corrected peak area |
|---|---|---|---|---|---|---|---|---|
| 1 | 75 (LM) | 0.5000 | 64 | 107 | 75 | 9.25 | 7704 | 49.735 |
| 2 | 116 | 0.6317 | 107 | 139 | 117 | 5.90 | 692 | 5.236 |
| 3 | 181 | 1.0043 | 139 | 264 | 178 | 10.52 | 759 | 8.325 |
| 4 | 443 | 5.3215 | 388 | 473 | 442 | 1.76 | 8128 | 44.110 |
| 5 | 485 | 0.6180 | 473 | 672 | 513 | 7.75 | 197 | 5.123 |
| 6 | 2401 | 1.3450 | 1789 | 2715 | 2402 | 5.12 | 1748 | 11.148 |
| 7 | 3000 | 0.6430 | 2715 | 4070 | 3060 | 9.04 | 484 | 5.330 |
| 8 | 20000 (UM) | 0.1662 | 18293 | 27012 | 20590 | 4.93 | 1491 | 16.528 |

| | | |
|---|---|---|
| TIC: | 9.5634 | ng/uL |
| TIM: | 41.2047 | nmole/L |
| Total concentration: | 9.7822 | ng/uL |

```
Sample peak width (sec): 5    Sample min peak height: 50    Sample baseline V to V?: Y    Sample baseline V to V points: 3
Sample filter: Binomial       Number of points for filter: 3  Sample start region (min): 0  Sample end region (min): 30
Marker peak width (sec): 5    Marker min peak height: 200   Marker baseline V to V?: Y    Marker baseline V to V points: 3
Lower marker selection: First peak > 500 RFU                Upper marker selection: Last peak > 200 RFU
Ladder size (bp)75, 200, 300, 400, 500, 700, 1000, 1500, 2000, 3000, 4000, 5000, 7000, 10000, 20000
Quantification using: Lower Marker          Final concentration (ng/uL): 0.5000          Dilution factor: 12.0
```

**Sample:** SampH12
**Well location:** H12
**Created:** Tuesday, September 14, 2021 6:54:01 PM

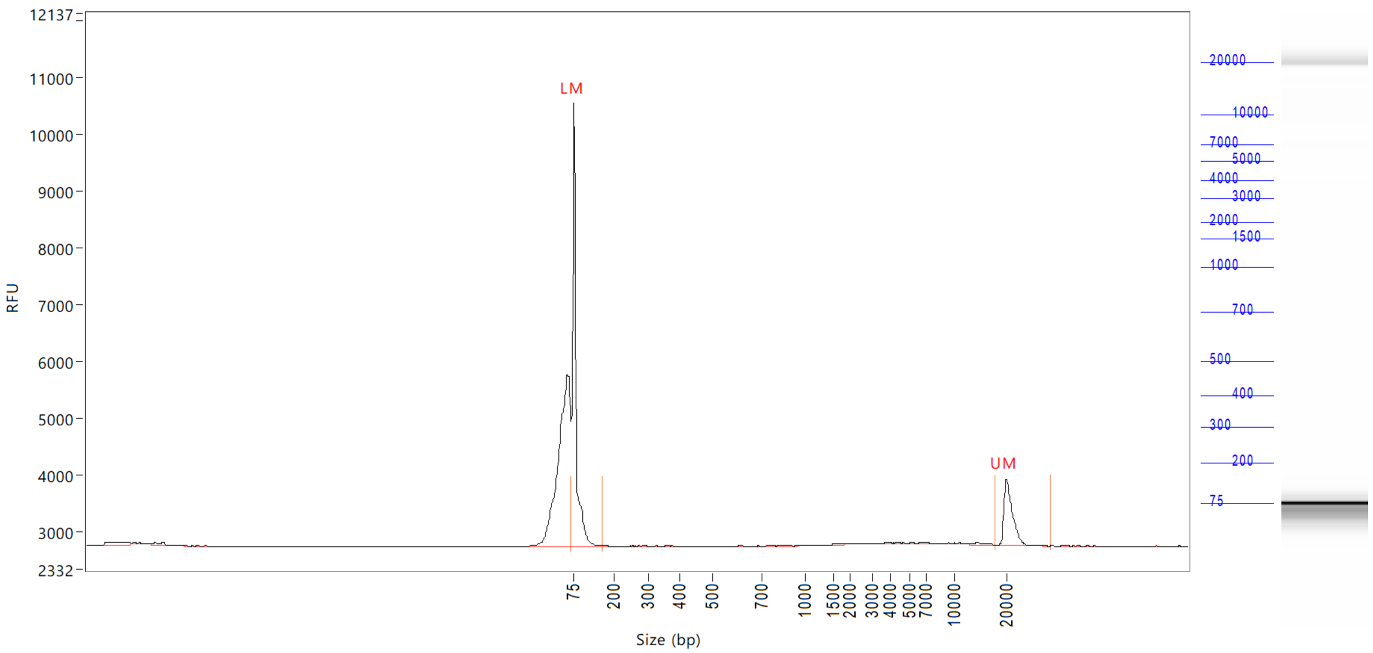


| Peak | Size (bp) | Concentration (ng/uL) | From (bp) | To (bp) | Average size (bp) | CV% | RFU | Corrected peak area |
|---|---|---|---|---|---|---|---|---|
| 1 | 75 (LM) | 0.5000 | 66 | 162 | 77 | 15.52 | 7839 | 65.098 |
| 2 | 20000 (UM) | 0.0976 | 17927 | 28705 | 20611 | 5.07 | 1172 | 12.705 |

| | | |
|---|---|---|
| TIC: | 0.0000 | ng/uL |
| TIM: | 0.0000 | nmole/L |
| Total concentration: | 0.3927 | ng/uL |

```
Sample peak width (sec): 5    Sample min peak height: 200   Sample baseline V to V?: Y    Sample baseline V to V points: 3
Sample filter: Binomial       Number of points for filter: 3  Sample start region (min): 0  Sample end region (min): 30
Marker peak width (sec): 5    Marker min peak height: 200   Marker baseline V to V?: Y    Marker baseline V to V points: 3
Lower marker selection: First peak > 500 RFU                Upper marker selection: Last peak > 200 RFU
Ladder size (bp)75, 200, 300, 400, 500, 700, 1000, 1500, 2000, 3000, 4000, 5000, 7000, 10000, 20000
Quantification using: Lower Marker          Final concentration (ng/uL): 0.5000          Dilution factor: 12.0
```

**Sample:** DNA size ladder
**Well location:** A7
**Created:** Tuesday, September 14, 2021 6:54:01 PM
**Import from:** C:\Users\admin\Desktop\210810_alberto_ladder.SCAL
**Fit type:** Point to point

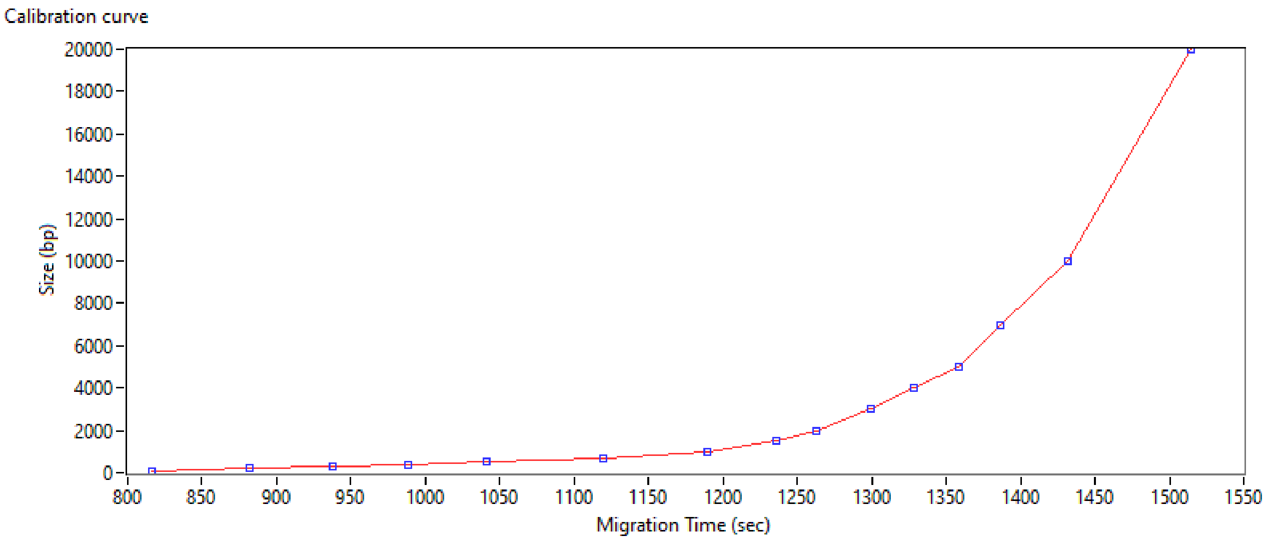


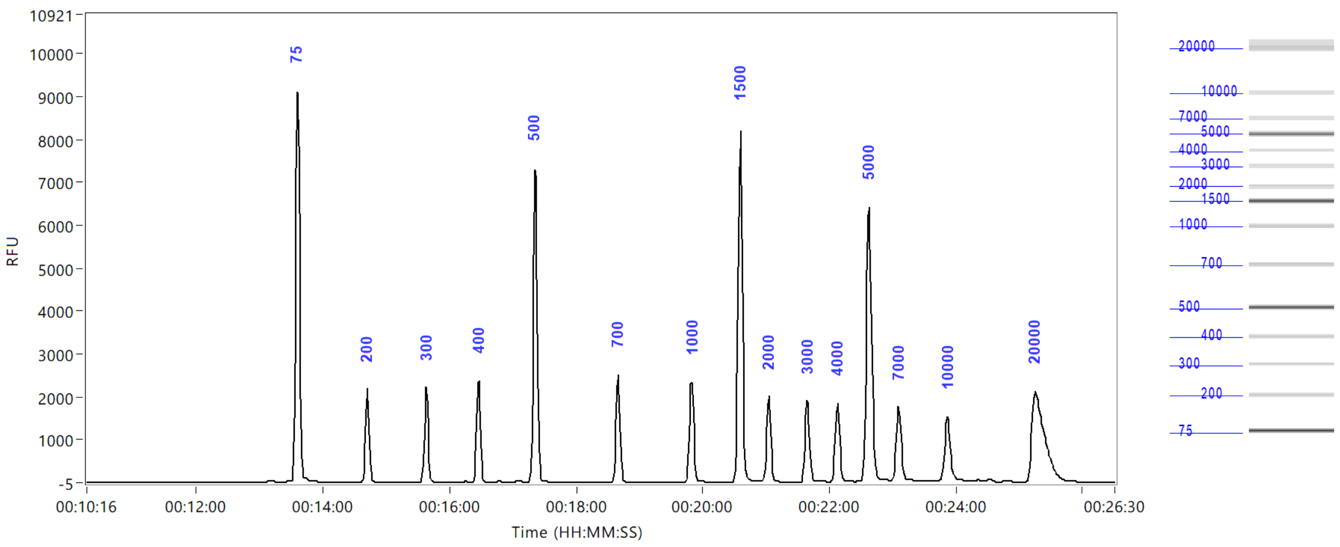

Supplement: Supplementary file 2 — sb3c00292_si_002.zip [file sb3c00292_si_002.zip › dnada_supplementary_material_pks_library_build/ZAG_trials/ZAG_Trial_5/18-44-09_plate4/plate4-t1.pdf]
