# Supplementary material for: Automated Platform for the Plasmid Construction Process
Source: ACS Synth Biol. 2023 Nov 10;12(12):3506–13. doi: 10.1021/acssynbio.3c00292 (PMC10729297; doi:10.1021/acssynbio.3c00292)

Instrument controller software run summary:

Filename and data path: C:\AATI\Data\2021 08 11\18-04-43\2021 08 11 18H 04M..raw
Created: Wednesday, August 11, 2021 6:14:43 PM
Number of capillaries: 96
Array serial number: 111920-09SFS
Effect length: 33 cm
Array usage count: 177
Instrument type: ZAG DNA Analyzer
Instrument controller software version: 1.0.1.9
Device serial number: Z7597

Method Information

Method name: ZAG130GP33 - DNA 75-20000bp - Gel Prime Only.mthds
Gel prime: Yes
Full conditioning: No
Gel prime to buffer: No
Gel selection: Gel 1
Perform prerun: 8.0 kV, 30 sec.
Rinse: No
Marker 1: Row: A, 5.0 kV, 5 sec.
Rinse: No
Sample injection: 5.0 kV, 5 sec.
Separation: 8.0 kV, 30.0 min.
Tray name: Sample Tray-6

Analysis mode: DNA

Notes

Gel Image

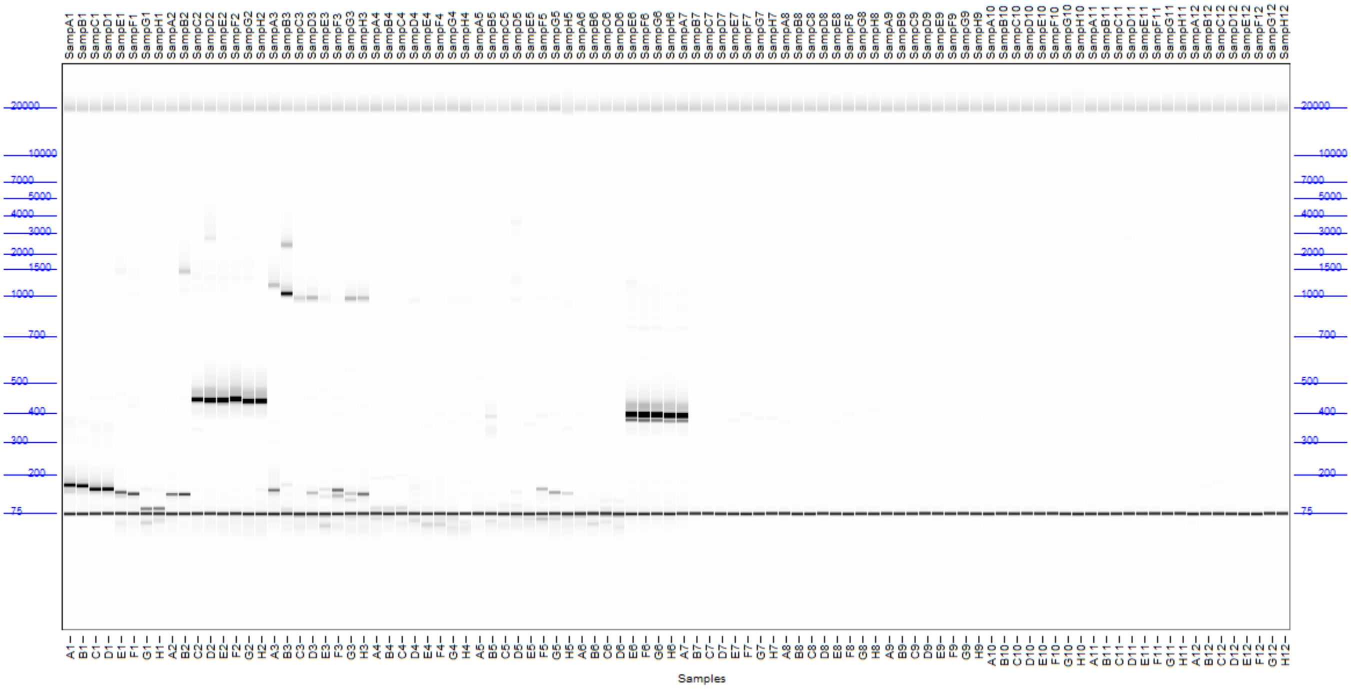

Filename and data path: C:\AATI\Data\2021 08 11\18-04-43\2021 08 11 18H 04M..raw

A1: SampA1

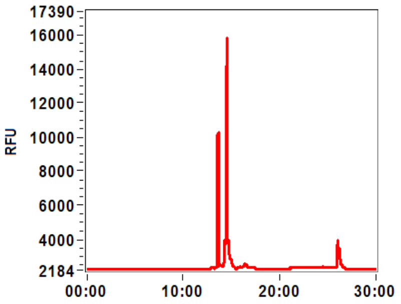

B1: SampB1

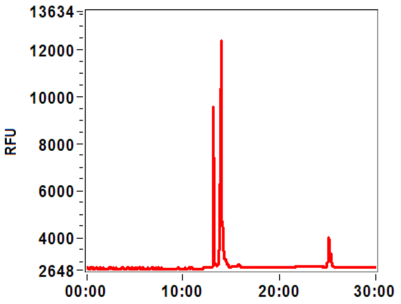

C1: SampC1

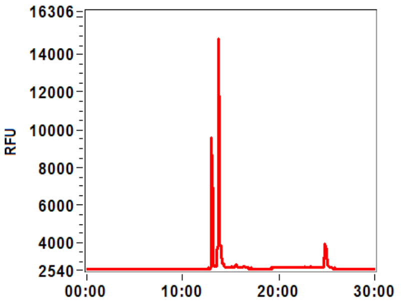

D1: SampD1

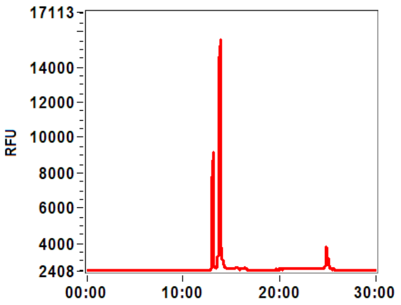

E1: SampE1

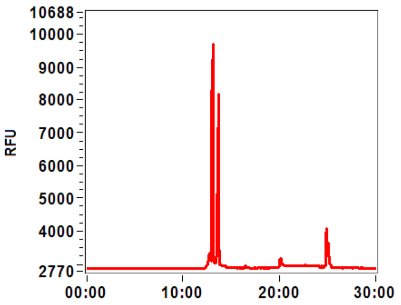

F1: SampF1

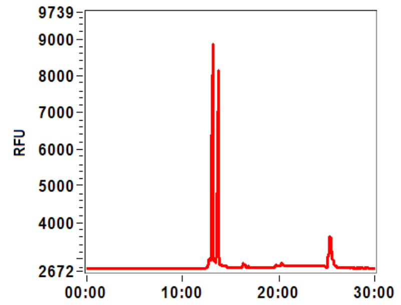

G1: SampG1

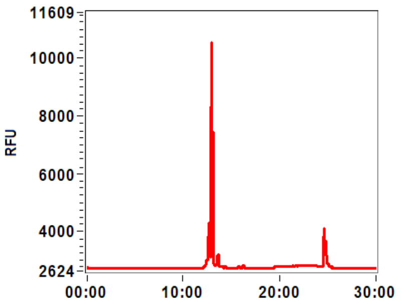

H1: SampH1

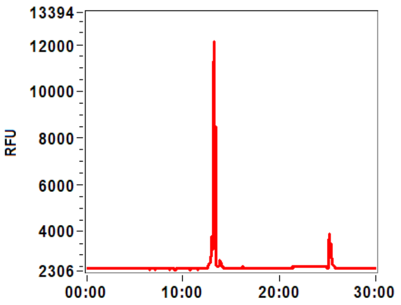

A2: SampA2

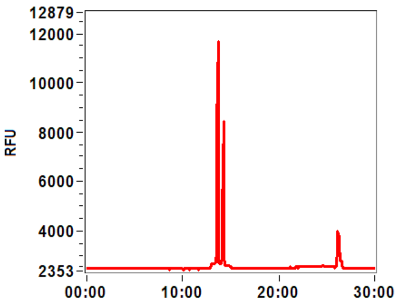

B2: SampB2

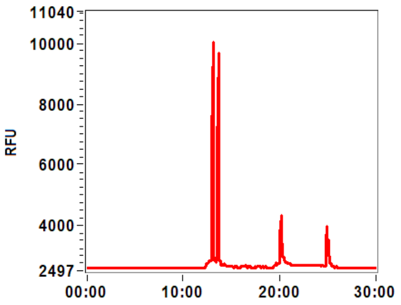

C2: SampC2

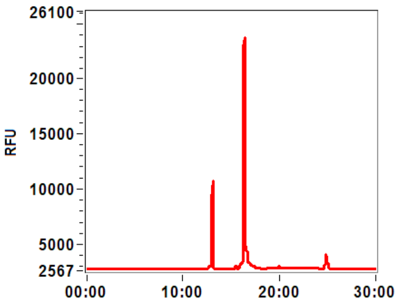

D2: SampD2

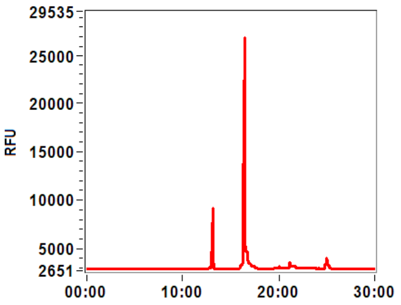

Filename and data path: C:\AATI\Data\2021 08 11\18-04-43\2021 08 11 18H 04M..raw

E2: SampE2

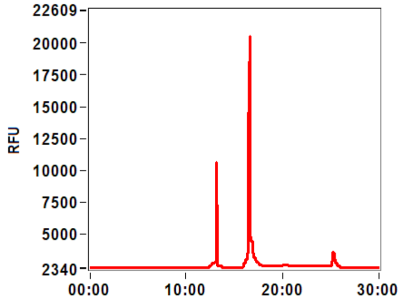

F2: SampF2

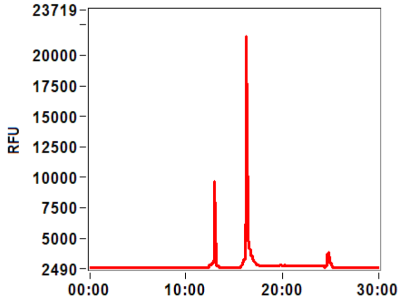

G2: SampG2

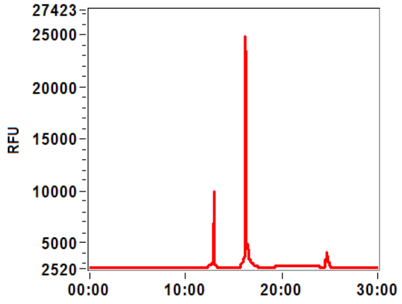

H2: SampH2

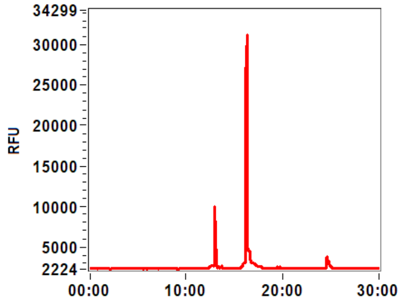

A3: SampA3

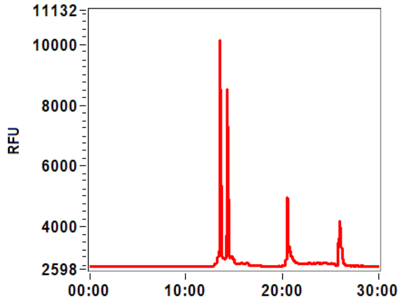

B3: SampB3

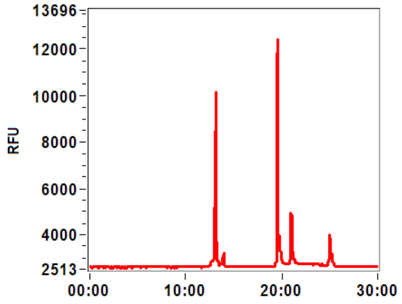

C3: SampC3

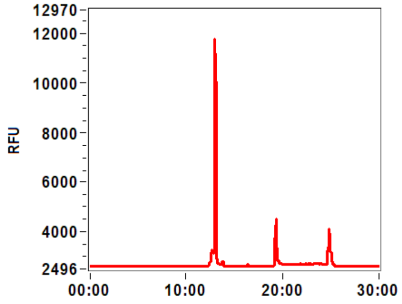

D3: SampD3

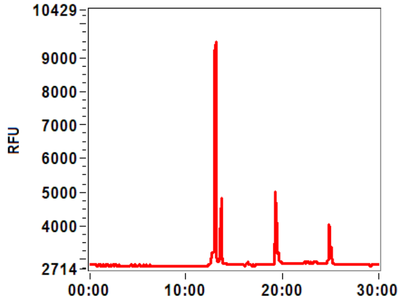

E3: SampE3

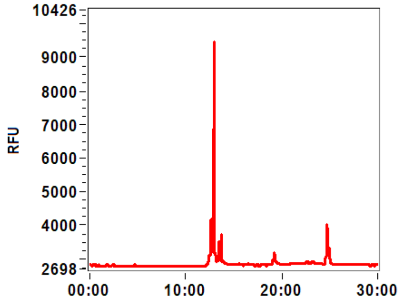

F3: SampF3

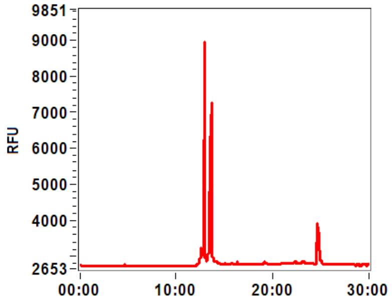

G3: SampG3

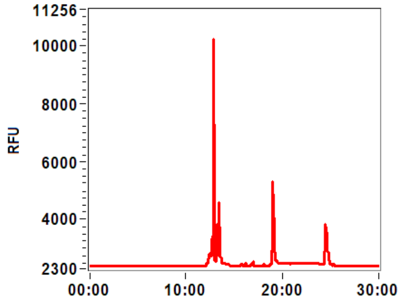

H3: SampH3

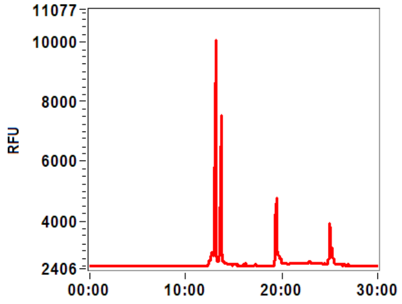

Filename and data path: C:\AATI\Data\2021 08 11\18-04-43\2021 08 11 18H 04M..raw

A4: SampA4

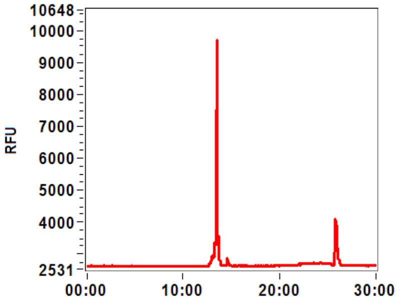

B4: SampB4

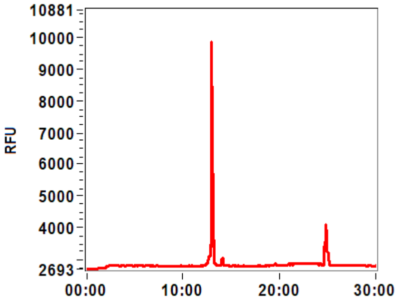

C4: SampC4

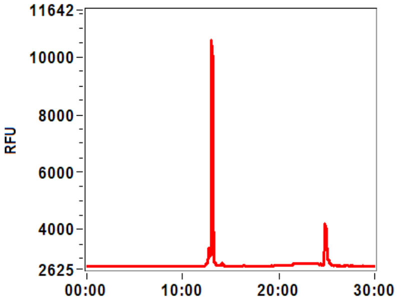

D4: SampD4

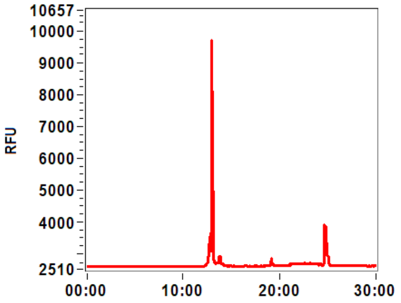

E4: SampE4

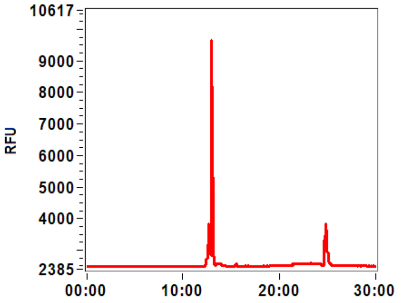

F4: SampF4

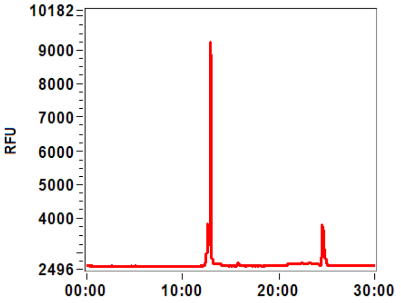

G4: SampG4

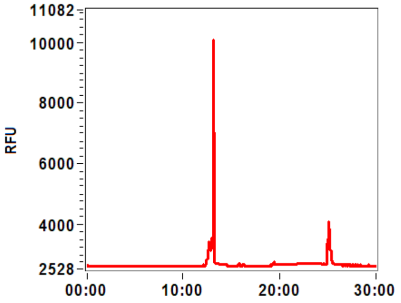

H4: SampH4

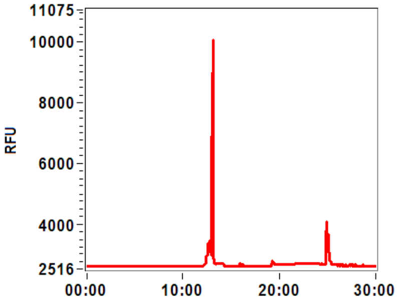

A5: SampA5

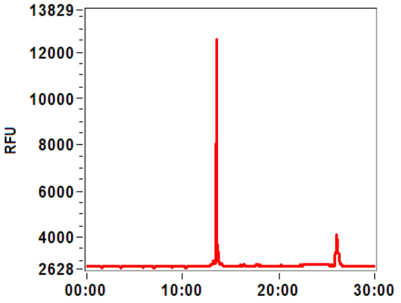

B5: SampB5

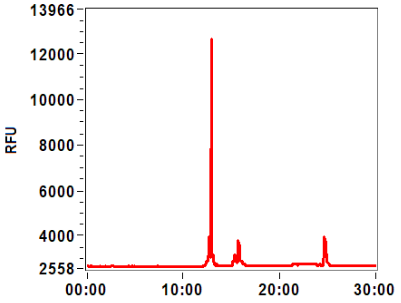

C5: SampC5

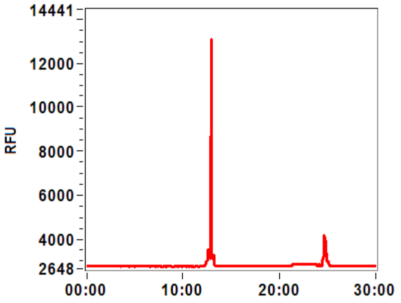

D5: SampD5

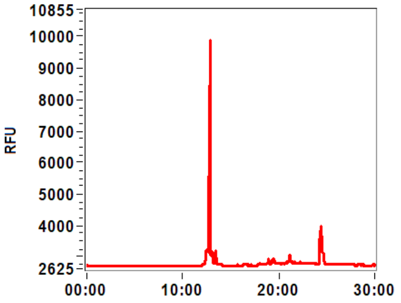

Filename and data path: C:\AATI\Data\2021 08 11\18-04-43\2021 08 11 18H 04M..raw

E5: SampE5

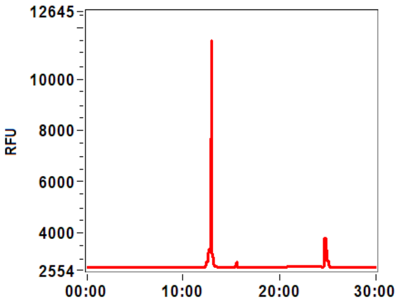

F5: SampF5

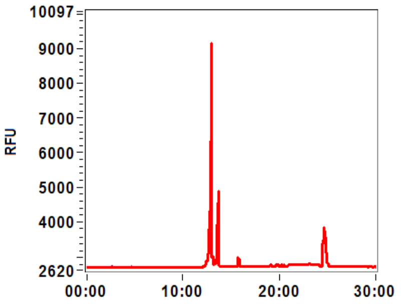

G5: SampG5

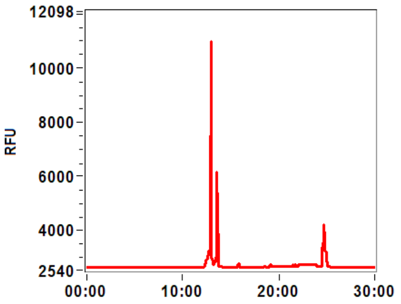

H5: SampH5

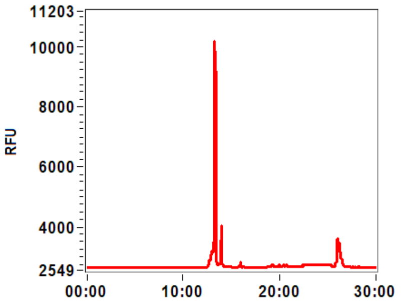

A6: SampA6

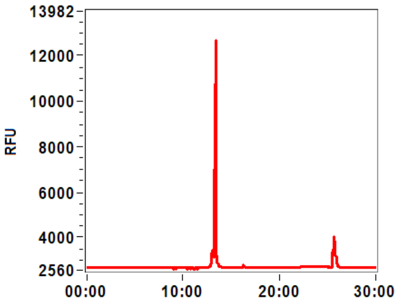

B6: SampB6

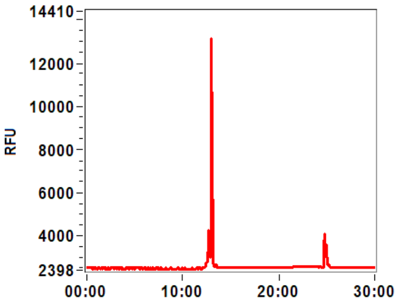

C6: SampC6

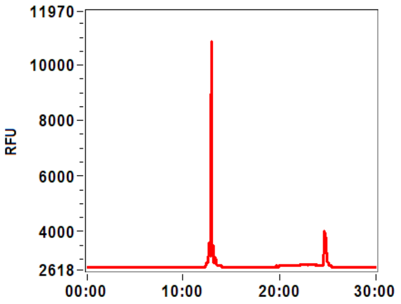

D6: SampD6

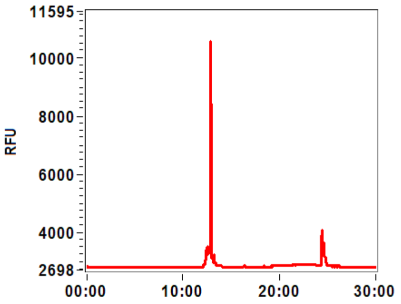

E6: SampE6

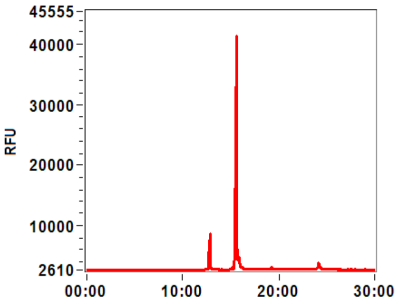

F6: SampF6

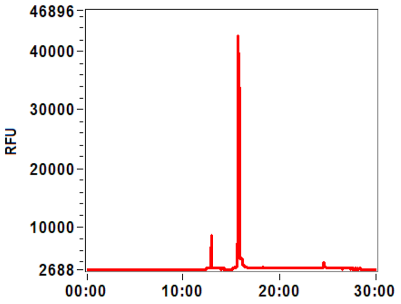

G6: SampG6

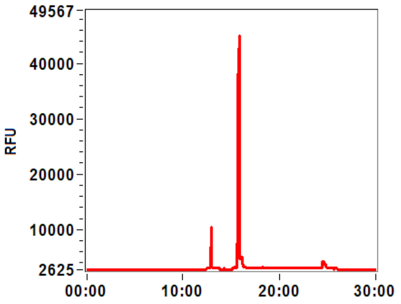

H6: SampH6

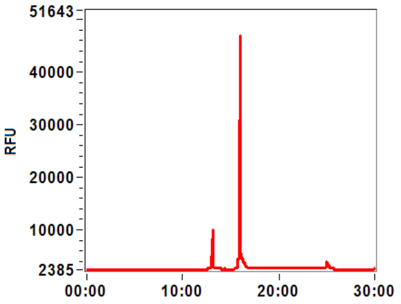

Filename and data path: C:\AATI\Data\2021 08 11\18-04-43\2021 08 11 18H 04M..raw

A7: SampA7

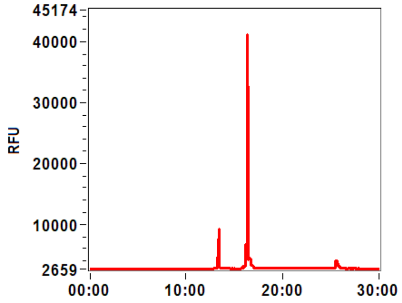

B7: SampB7

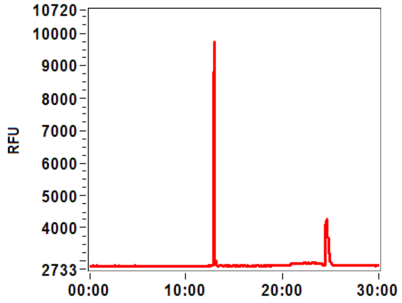

C7: SampC7

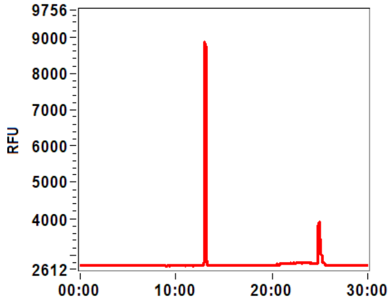

D7: SampD7

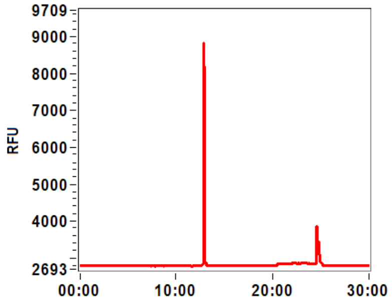

E7: SampE7

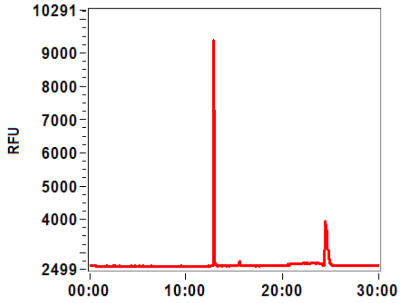

F7: SampF7

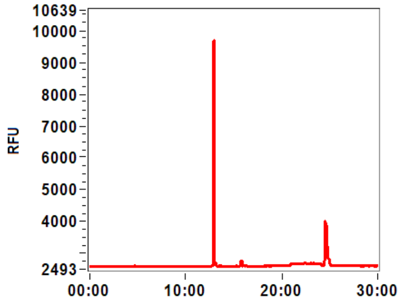

G7: SampG7

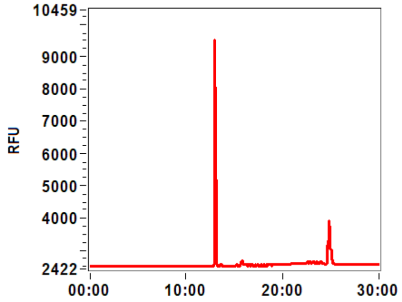

H7: SampH7

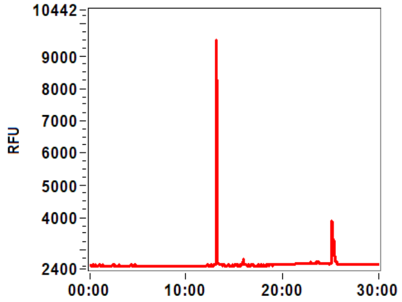

A8: SampA8

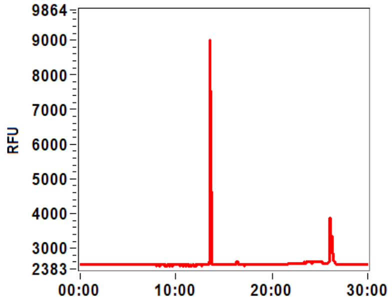

B8: SampB8

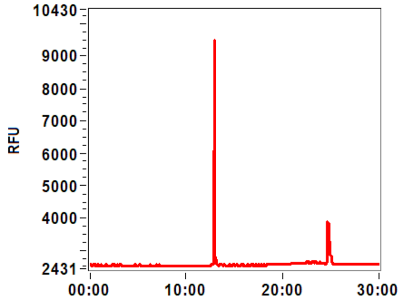

C8: SampC8

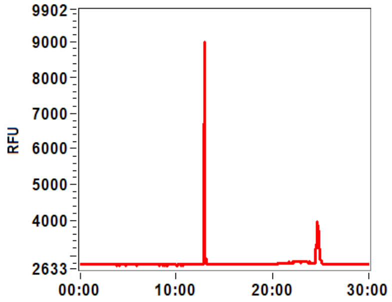

D8: SampD8

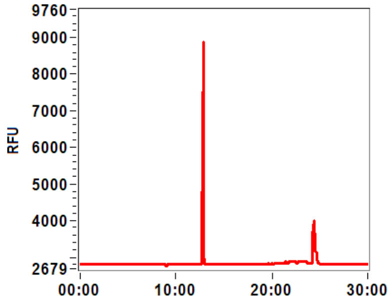

Filename and data path: C:\AATI\Data\2021 08 11\18-04-43\2021 08 11 18H 04M..raw

E8: SampE8

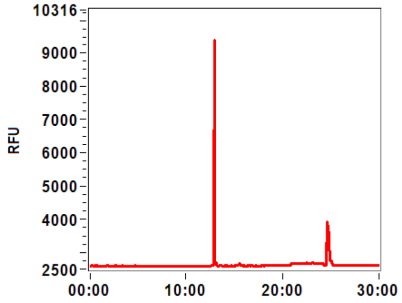

F8: SampF8

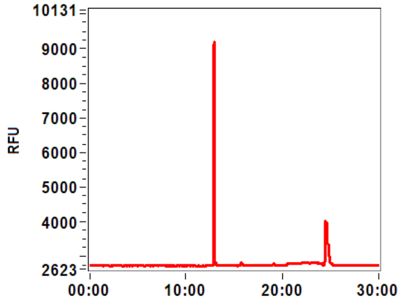

G8: SampG8

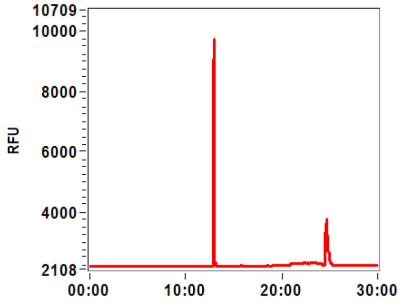

H8: SampH8

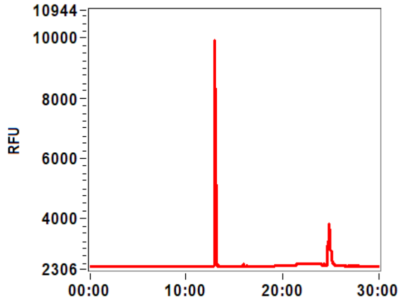

A9: SampA9

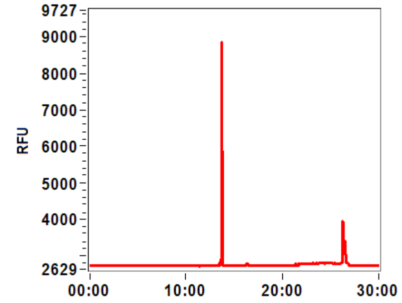

B9: SampB9

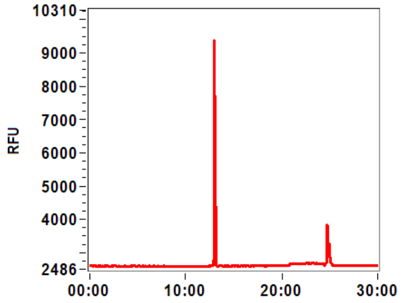

C9: SampC9

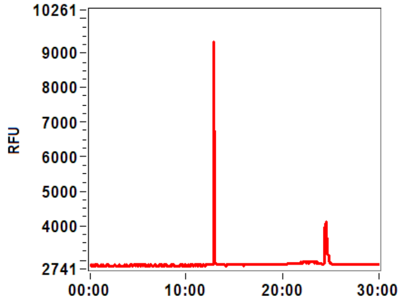

D9: SampD9

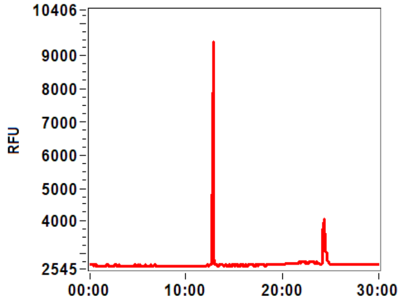

E9: SampE9

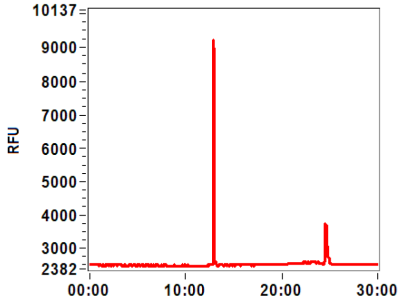

F9: SampF9

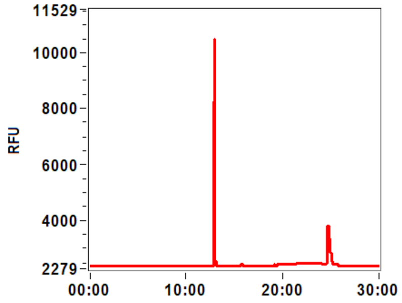

G9: SampG9

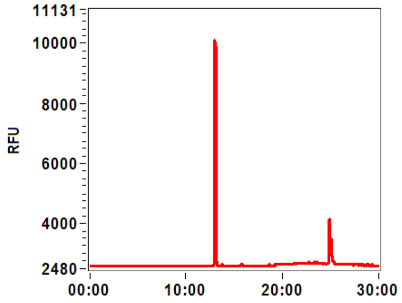

H9: SampH9

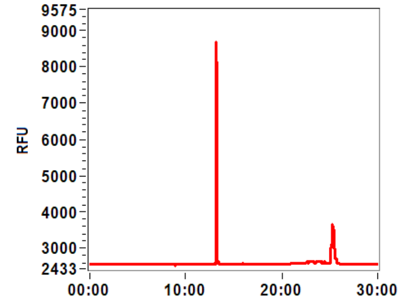

Filename and data path: C:\AATI\Data\2021 08 11\18-04-43\2021 08 11 18H 04M..raw

A10: SampA10

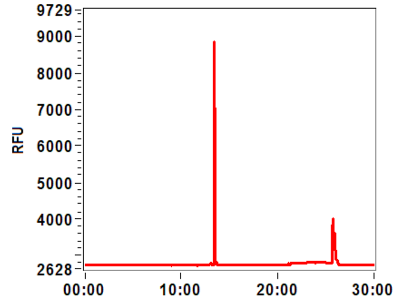

B10: SampB10

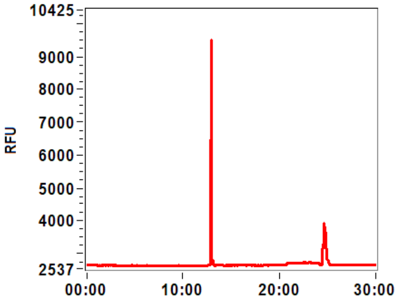

C10: SampC10

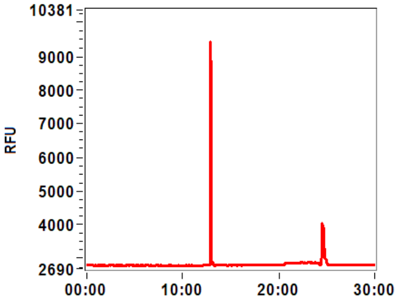

D10: SampD10

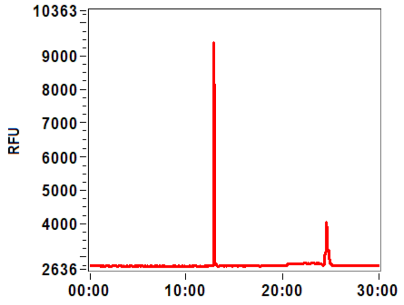

E10: SampE10

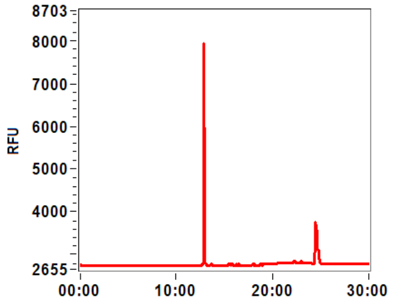

F10: SampF10

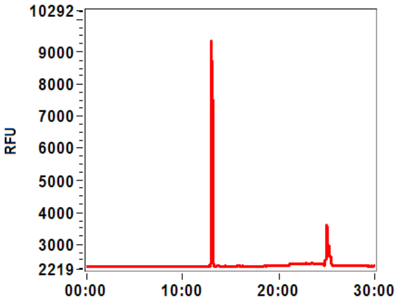

G10: SampG10

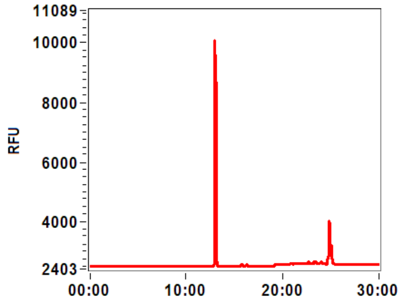

H10: SampH10

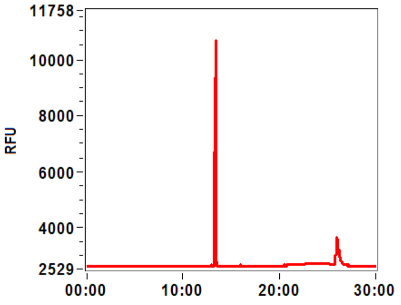

A11: SampA11

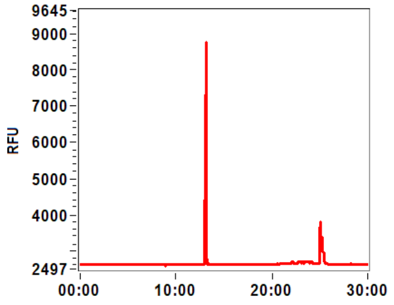

B11: SampB11

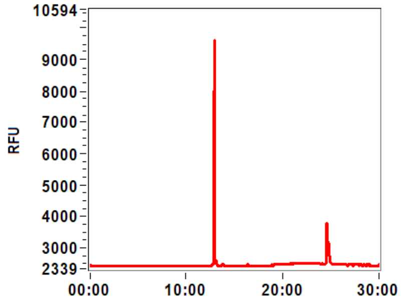

C11: SampC11

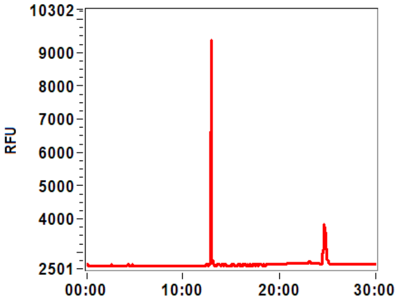

D11: SampD11

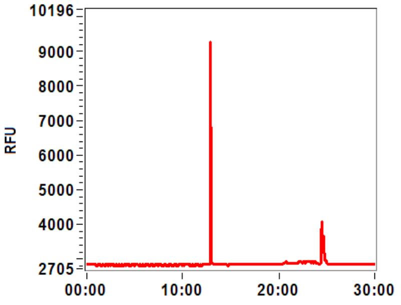

Filename and data path: C:\AATI\Data\2021 08 11\18-04-43\2021 08 11 18H 04M..raw

E11: SampE11

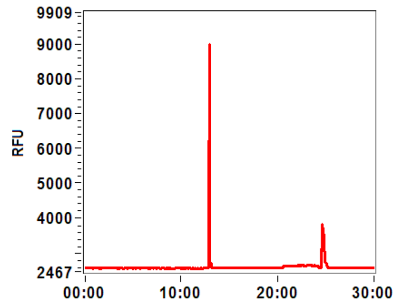

F11: SampF11

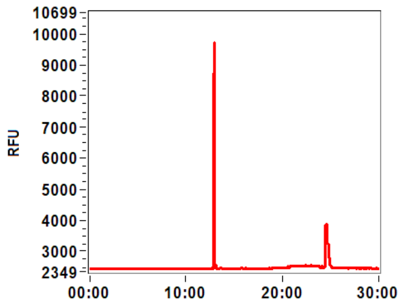

G11: SampG11

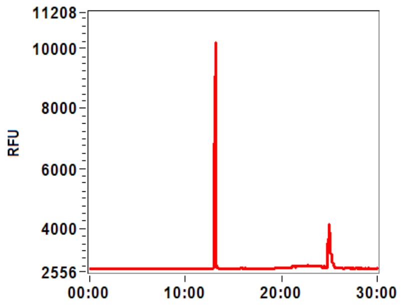

H11: SampH11

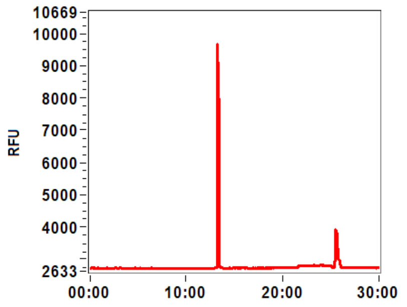

A12: SampA12

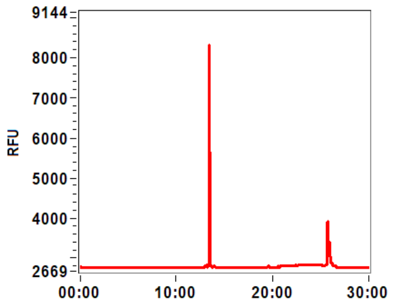

B12: SampB12

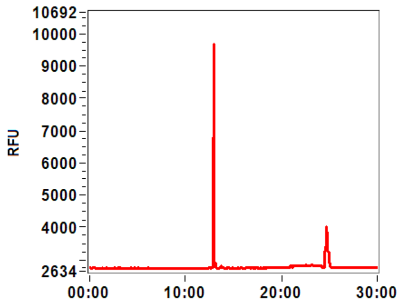

C12: SampC12

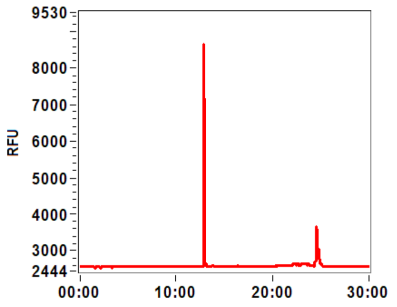

D12: SampD12

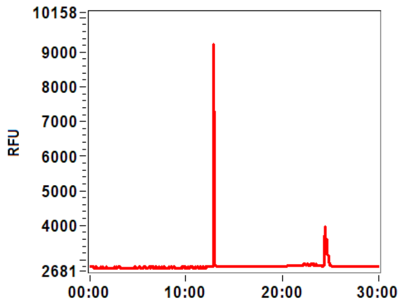

E12: SampE12

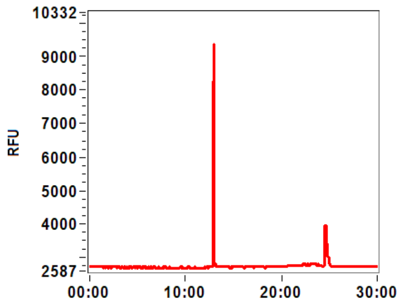

F12: SampF12

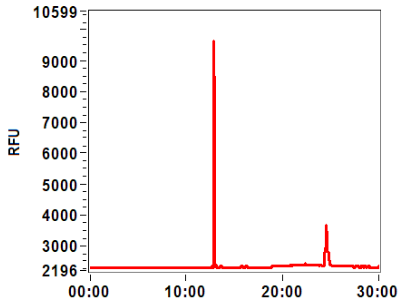

G12: SampG12

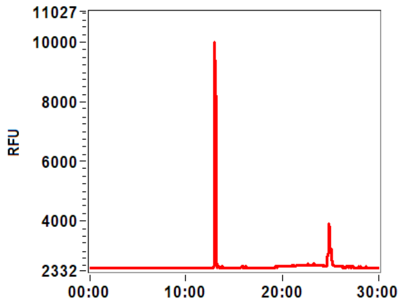

H12: SampH12

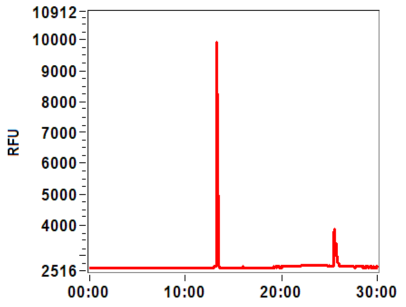

Sample: SampA1
Well location: A1
Created: Wednesday, August 11, 2021 6:14:43 PM

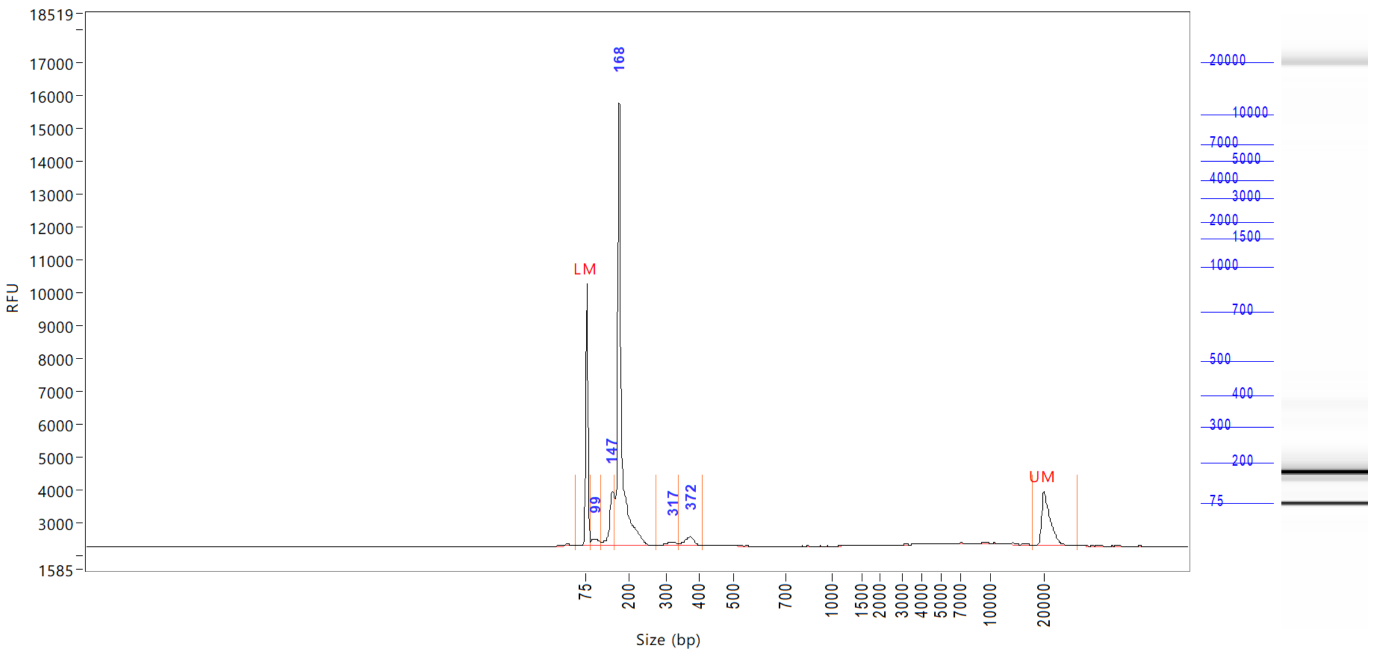

Peak	Size (bp)	Concentration (ng/uL)	From (bp)	To (bp)	Average size (bp)	CV%	RFU	Corrected peak area
1	75 (LM)	0.5000	43	86	72	4.57	7995	42.473
2	99	0.4991	86	116	99	8.87	205	3.533
3	147	2.7418	116	156	145	5.81	1660	19.409
4	168	16.6289	156	273	174	9.29	13506	117.713
5	317	0.3444	273	335	311	4.45	117	2.438
6	372	0.7447	335	411	366	4.06	268	5.272
7	20000 (UM)	0.1965	17805	26166	20522	4.60	1630	16.689

TIC:	20.9590	ng/uL
TIM:	201.6286	nmole/L
Total concentration:	21.2228	ng/uL

```
Sample peak width (sec): 5    Sample min peak height: 50    Sample baseline V to V?: Y    Sample baseline V to V points: 3
Sample filter: Binomial       Number of points for filter: 3  Sample start region (min): 0  Sample end region (min): 30
Marker peak width (sec): 5    Marker min peak height: 200   Marker baseline V to V?: Y    Marker baseline V to V points: 3
Lower marker selection: First peak > 500 RFU                Upper marker selection: Last peak > 200 RFU
Ladder size (bp)75, 200, 300, 400, 500, 700, 1000, 1500, 2000, 3000, 4000, 5000, 7000, 10000, 20000
Quantification using: Lower Marker          Final concentration (ng/uL): 0.5000          Dilution factor: 12.0
```

Sample: SampB1
Well location: B1
Created: Wednesday, August 11, 2021 6:14:43 PM

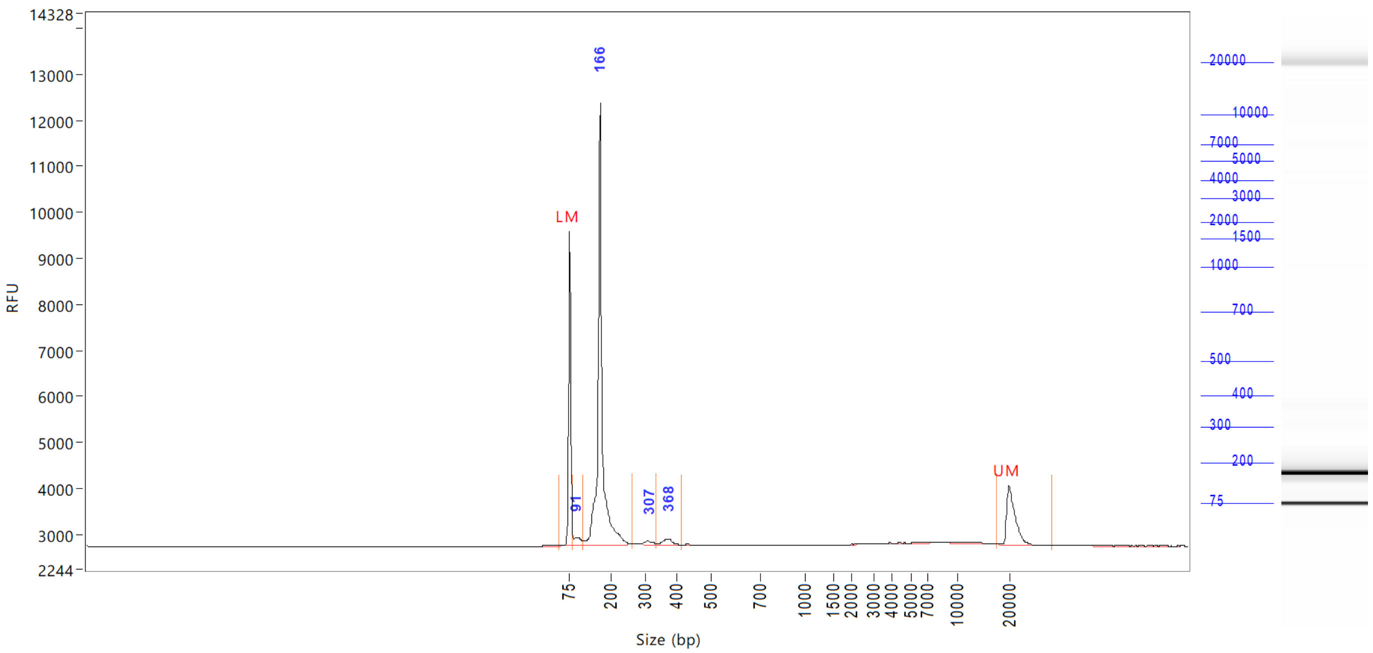

Peak	Size (bp)	Concentration (ng/uL)	From (bp)	To (bp)	Average size (bp)	CV%	RFU	Corrected peak area
1	75 (LM)	0.5000	41	86	72	5.33	6845	37.098
2	91	0.4899	86	114	98	8.46	174	3.029
3	166	14.6914	114	259	167	10.62	9628	90.837
4	307	0.2406	259	331	303	4.90	68	1.488
5	368	0.4004	331	411	364	4.05	132	2.476
6	20000 (UM)	0.1848	17561	28342	20535	5.07	1300	13.711

TIC:	15.8224	ng/uL
TIM:	155.9345	nmole/L
Total concentration:	16.3296	ng/uL

Sample peak width (sec): 5 Sample min peak height: 50 Sample baseline V to V?: Y Sample baseline V to V points: 3
Sample filter: Binomial Number of points for filter: 3 Sample start region (min): 0 Sample end region (min): 30
Marker peak width (sec): 5 Marker min peak height: 200 Marker baseline V to V?: Y Marker baseline V to V points: 3
Lower marker selection: First peak > 500 RFU Upper marker selection: Last peak > 200 RFU
Ladder size (bp)75, 200, 300, 400, 500, 700, 1000, 1500, 2000, 3000, 4000, 5000, 7000, 10000, 20000
Quantification using: Lower Marker Final concentration (ng/uL): 0.5000 Dilution factor: 12.0

Sample: SampC1
Well location: C1
Created: Wednesday, August 11, 2021 6:14:43 PM

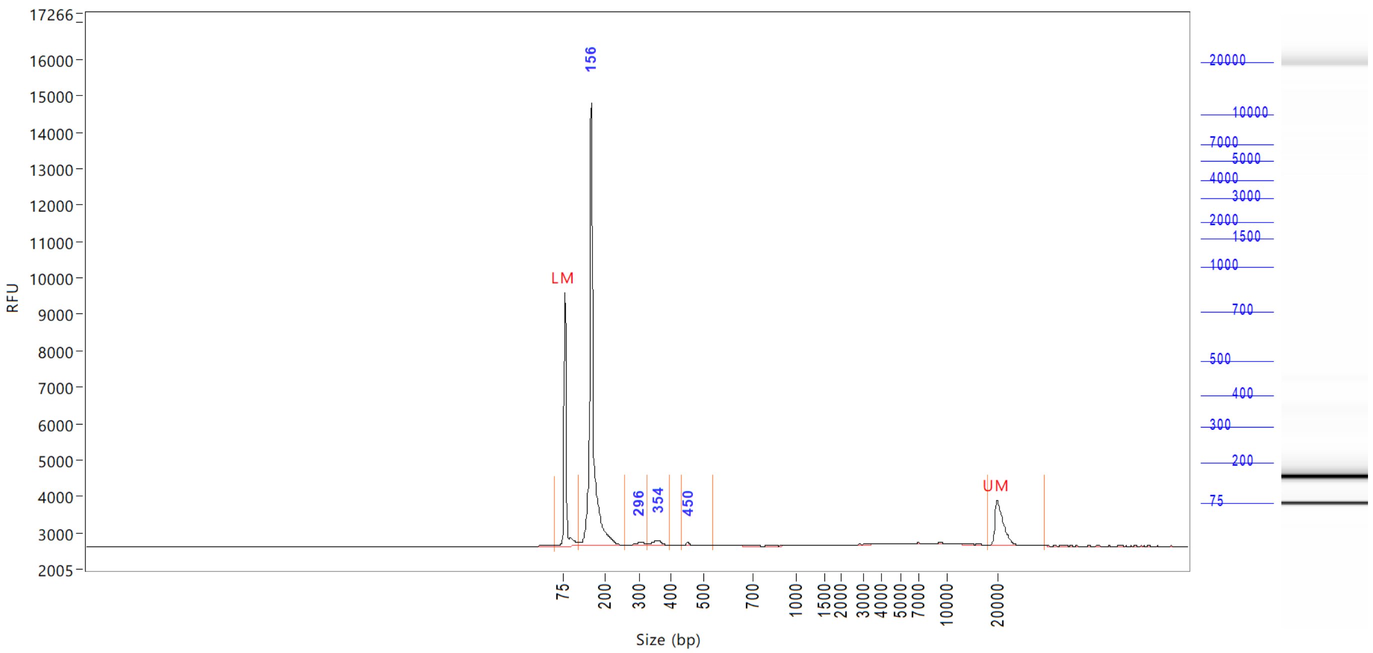

Peak	Size (bp)	Concentration (ng/uL)	From (bp)	To (bp)	Average size (bp)	CV%	RFU	Corrected peak area
1	75 (LM)	0.5000	47	120	75	11.30	6949	40.832
2	156	16.0690	120	255	159	10.28	12177	109.355
3	296	0.2839	255	321	295	5.12	80	1.932
4	354	0.4228	321	396	350	4.23	149	2.877
5	450	0.1424	431	537	456	3.96	108	0.969
6	20000 (UM)	0.1601	18049	29309	20515	5.00	1245	13.074

TIC:	16.9181	ng/uL
TIM:	170.1985	nmole/L
Total concentration:	17.4311	ng/uL

```
Sample peak width (sec): 5    Sample min peak height: 50    Sample baseline V to V?: Y    Sample baseline V to V points: 3
Sample filter: Binomial       Number of points for filter: 3  Sample start region (min): 0  Sample end region (min): 30
Marker peak width (sec): 5    Marker min peak height: 200   Marker baseline V to V?: Y    Marker baseline V to V points: 3
Lower marker selection: First peak > 500 RFU                Upper marker selection: Last peak > 200 RFU
Ladder size (bp)75, 200, 300, 400, 500, 700, 1000, 1500, 2000, 3000, 4000, 5000, 7000, 10000, 20000
Quantification using: Lower Marker          Final concentration (ng/uL): 0.5000          Dilution factor: 12.0
```

Sample: SampD1
Well location: D1
Created: Wednesday, August 11, 2021 6:14:43 PM

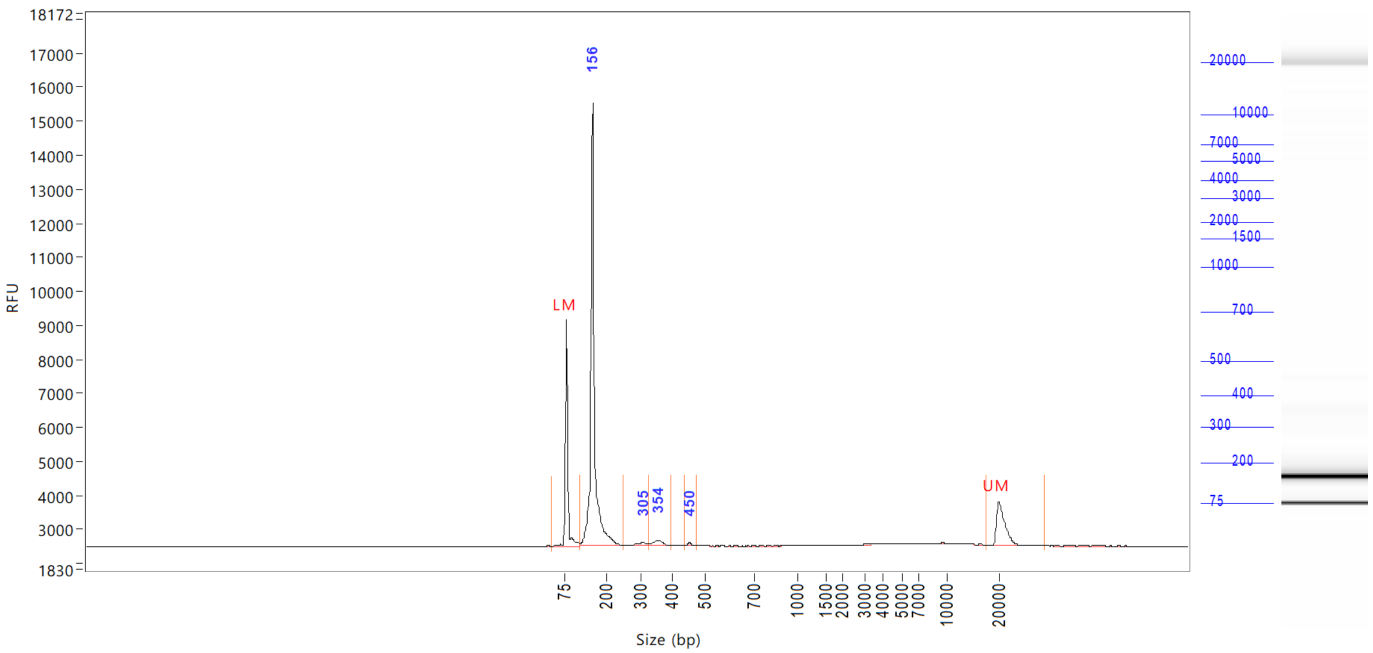

Peak	Size (bp)	Concentration (ng/uL)	From (bp)	To (bp)	Average size (bp)	CV%	RFU	Corrected peak area
1	75 (LM)	0.5000	30	118	75	10.92	6680	40.045
2	156	16.5906	118	250	158	10.15	13044	110.729
3	305	0.3034	250	321	295	5.73	79	2.025
4	354	0.4577	321	394	349	4.06	158	3.055
5	450	0.0937	435	475	451	1.61	88	0.625
6	20000 (UM)	0.1698	17561	29068	20607	5.12	1298	13.597

TIC:	17.4454	ng/uL
TIM:	176.7814	nmole/L
Total concentration:	17.8213	ng/uL

```
Sample peak width (sec): 5    Sample min peak height: 50    Sample baseline V to V?: Y    Sample baseline V to V points: 3
Sample filter: Binomial       Number of points for filter: 3  Sample start region (min): 0  Sample end region (min): 30
Marker peak width (sec): 5    Marker min peak height: 200   Marker baseline V to V?: Y    Marker baseline V to V points: 3
Lower marker selection: First peak > 500 RFU                Upper marker selection: Last peak > 200 RFU
Ladder size (bp)75, 200, 300, 400, 500, 700, 1000, 1500, 2000, 3000, 4000, 5000, 7000, 10000, 20000
Quantification using: Lower Marker          Final concentration (ng/uL): 0.5000          Dilution factor: 12.0
```

Sample: SampE1
Well location: E1
Created: Wednesday, August 11, 2021 6:14:43 PM

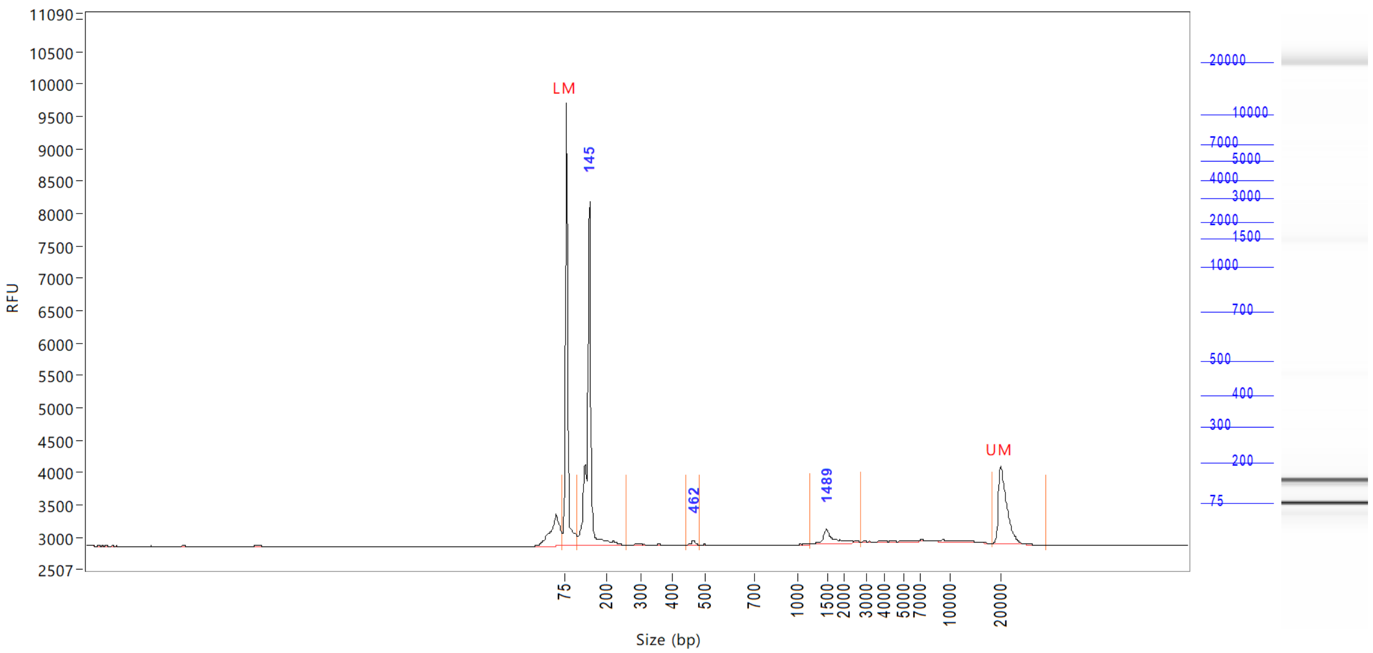

Peak	Size (bp)	Concentration (ng/uL)	From (bp)	To (bp)	Average size (bp)	CV%	RFU	Corrected peak area
1	75 (LM)	0.5000	64	110	76	9.61	6849	42.007
2	145	6.6030	110	257	143	11.52	5301	46.229
3	462	0.1153	441	481	460	1.79	86	0.807
4	1489	0.5556	1190	2772	1666	19.48	234	3.890
5	20000 (UM)	0.1505	18415	28826	20634	5.07	1187	12.641

TIC:	7.2739	ng/uL
TIM:	76.8437	nmole/L
Total concentration:	7.7962	ng/uL

```
Sample peak width (sec): 5    Sample min peak height: 50    Sample baseline V to V?: Y    Sample baseline V to V points: 3
Sample filter: Binomial       Number of points for filter: 3  Sample start region (min): 0  Sample end region (min): 30
Marker peak width (sec): 5    Marker min peak height: 200   Marker baseline V to V?: Y    Marker baseline V to V points: 3
Lower marker selection: First peak > 500 RFU                Upper marker selection: Last peak > 200 RFU
Ladder size (bp)75, 200, 300, 400, 500, 700, 1000, 1500, 2000, 3000, 4000, 5000, 7000, 10000, 20000
Quantification using: Lower Marker          Final concentration (ng/uL): 0.5000          Dilution factor: 12.0
```

Sample: SampF1
Well location: F1
Created: Wednesday, August 11, 2021 6:14:43 PM

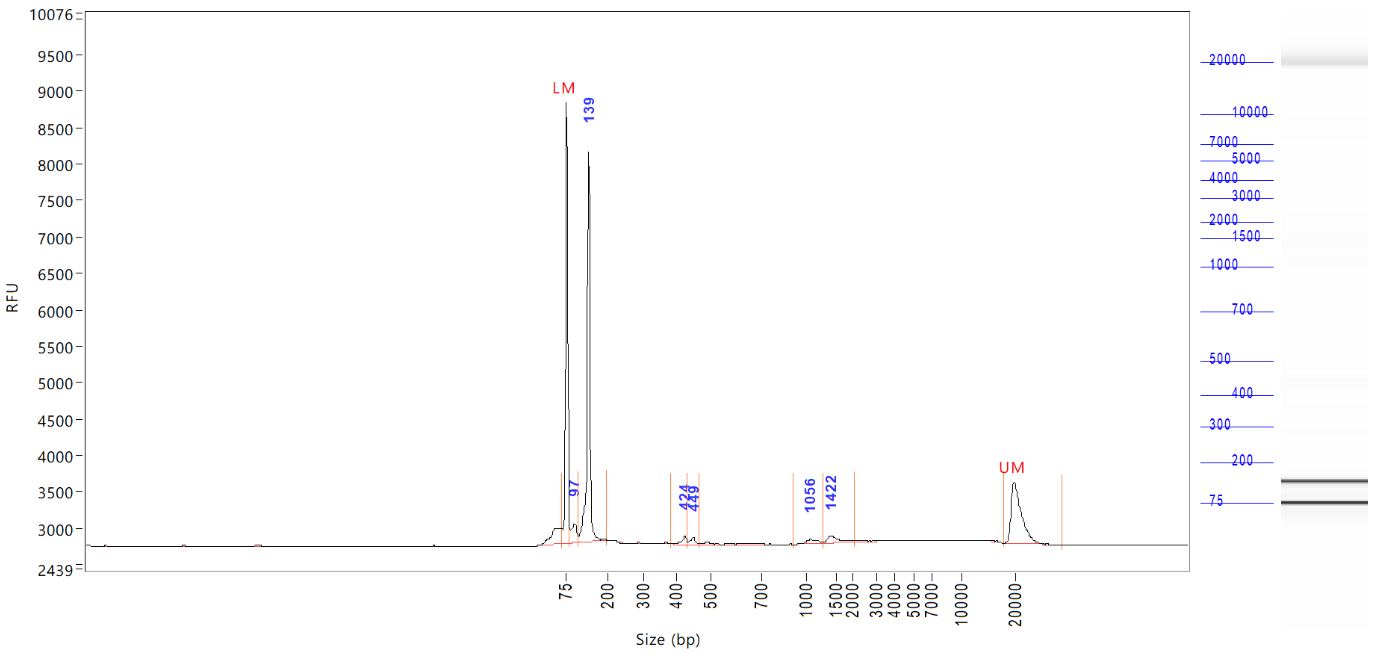

Peak	Size (bp)	Concentration (ng/uL)	From (bp)	To (bp)	Average size (bp)	CV%	RFU	Corrected peak area
1	75 (LM)	0.5000	64	86	73	4.58	6061	33.603
2	97	0.6263	86	110	96	6.99	263	3.508
3	139	6.5644	110	196	136	5.48	5346	36.764
4	424	0.2020	380	433	416	2.74	112	1.131
5	449	0.1668	433	466	446	1.65	97	0.934
6	1056	0.1830	913	1278	1089	7.78	62	1.025
7	1422	0.3199	1278	2030	1524	11.79	99	1.792
8	20000 (UM)	0.1597	17927	29068	20596	5.76	837	10.730

TIC:	8.0624	ng/uL
TIM:	92.0626	nmole/L
Total concentration:	8.5553	ng/uL

```
Sample peak width (sec): 5     Sample min peak height: 50    Sample baseline V to V?: Y    Sample baseline V to V points: 3
Sample filter: Binomial        Number of points for filter: 3  Sample start region (min): 0  Sample end region (min): 30
Marker peak width (sec): 5     Marker min peak height: 200   Marker baseline V to V?: Y    Marker baseline V to V points: 3
Lower marker selection: First peak > 500 RFU                 Upper marker selection: Last peak > 200 RFU
Ladder size (bp)75, 200, 300, 400, 500, 700, 1000, 1500, 2000, 3000, 4000, 5000, 7000, 10000, 20000
Quantification using: Lower Marker          Final concentration (ng/uL): 0.5000          Dilution factor: 12.0
```

Sample: SampG1
Well location: G1
Created: Wednesday, August 11, 2021 6:14:43 PM

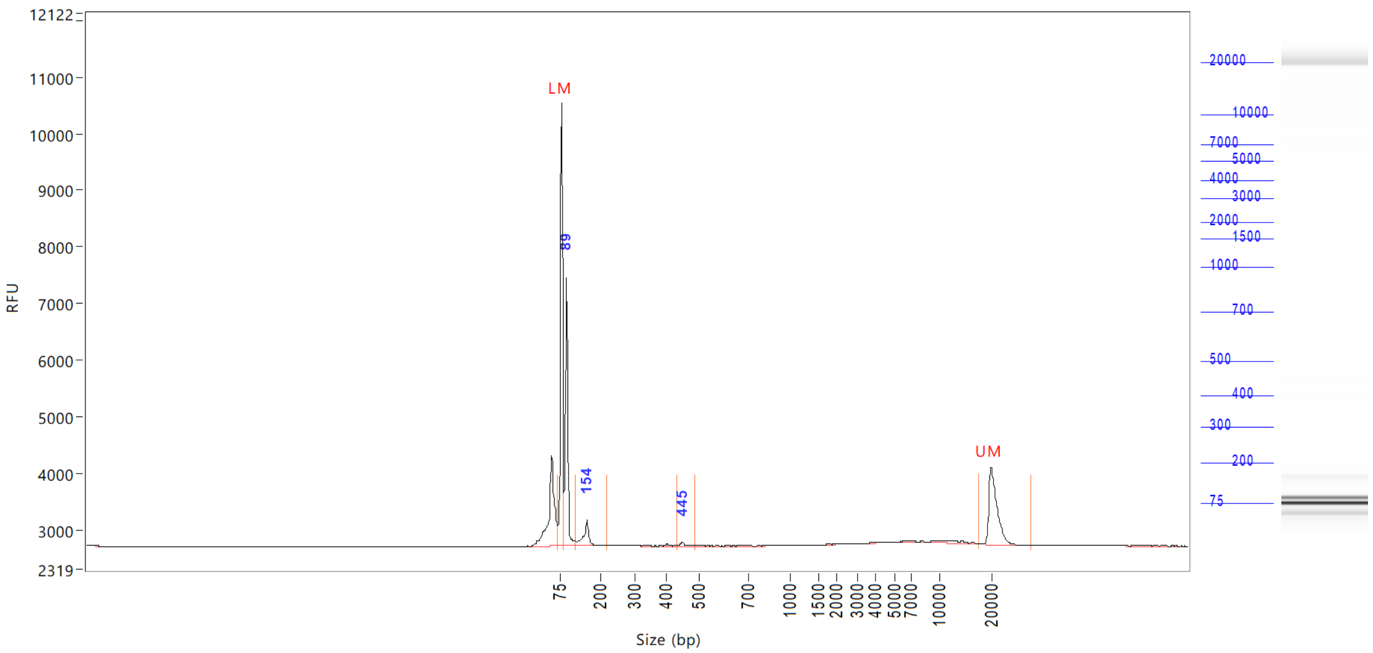

Peak	Size (bp)	Concentration (ng/uL)	From (bp)	To (bp)	Average size (bp)	CV%	RFU	Corrected peak area
1	75 (LM)	0.5000	64	82	73	4.63	7824	44.499
2	89	4.1122	82	122	89	6.47	4717	30.498
3	154	0.7053	122	215	149	9.38	445	5.231
4	445	0.0814	430	487	449	2.66	72	0.603
5	20000 (UM)	0.1609	17683	27859	20609	4.79	1356	14.318

TIC:	4.8988	ng/uL
TIM:	83.9252	nmole/L
Total concentration:	5.4571	ng/uL

Sample peak width (sec): 5   Sample min peak height: 50   Sample baseline V to V?: Y   Sample baseline V to V points: 3
Sample filter: Binomial   Number of points for filter: 3 Sample start region (min): 0 Sample end region (min): 30
Marker peak width (sec): 5   Marker min peak height: 200   Marker baseline V to V?: Y   Marker baseline V to V points: 3
Lower marker selection: First peak > 500 RFU   Upper marker selection: Last peak > 200 RFU
Ladder size (bp)75, 200, 300, 400, 500, 700, 1000, 1500, 2000, 3000, 4000, 5000, 7000, 10000, 20000
Quantification using: Lower Marker   Final concentration (ng/uL): 0.5000   Dilution factor: 12.0

Sample: SampH1
Well location: H1
Created: Wednesday, August 11, 2021 6:14:43 PM

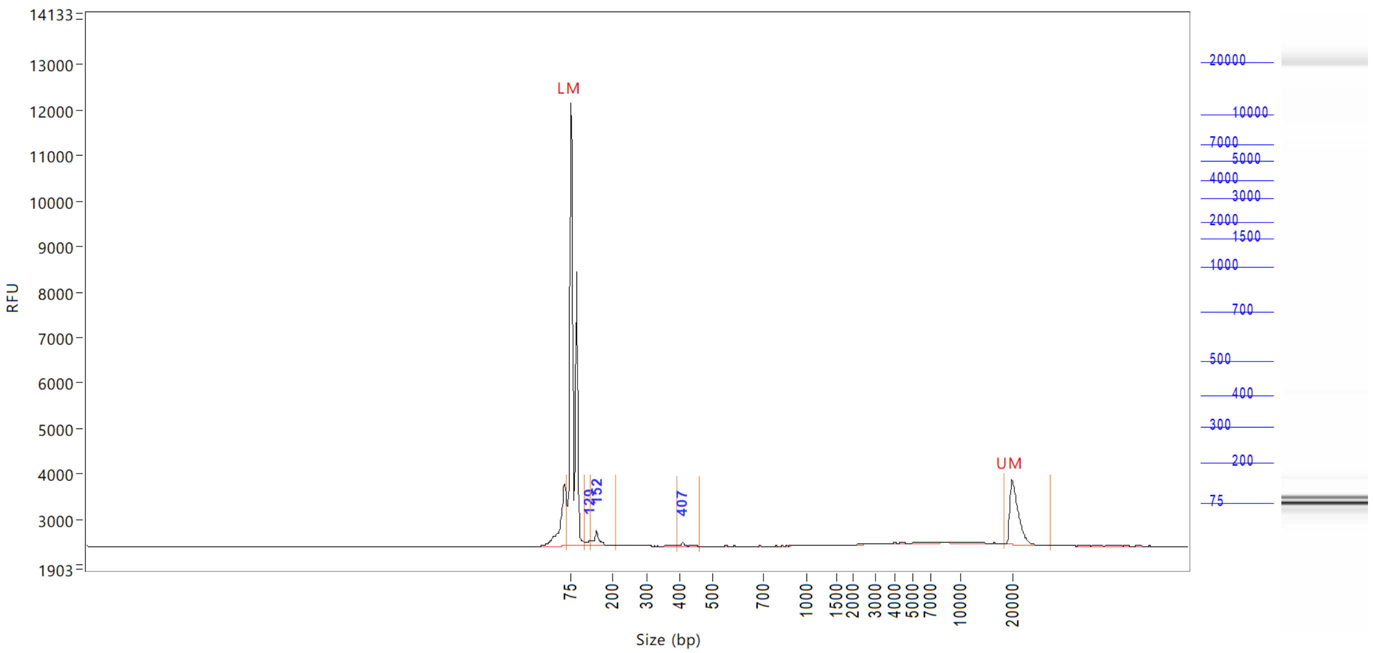

Peak	Size (bp)	Concentration (ng/uL)	From (bp)	To (bp)	Average size (bp)	CV%	RFU	Corrected peak area
1	75 (LM)	0.5000	64	116	79	11.53	9761	94.573
2	129	0.0670	116	133	125	4.34	93	1.056
3	152	0.2279	133	208	151	6.94	326	3.592
4	407	0.0379	394	462	413	3.53	74	0.597
5	20000 (UM)	0.0796	18415	27375	20552	4.83	1439	15.054

TIC:	0.3328	ng/uL
TIM:	3.5119	nmole/L
Total concentration:	0.5543	ng/uL

Sample peak width (sec): 5 Sample min peak height: 50 Sample baseline V to V?: Y Sample baseline V to V points: 3
Sample filter: Binomial Number of points for filter: 3 Sample start region (min): 0 Sample end region (min): 30
Marker peak width (sec): 5 Marker min peak height: 200 Marker baseline V to V?: Y Marker baseline V to V points: 3
Lower marker selection: First peak > 500 RFU Upper marker selection: Last peak > 200 RFU
Ladder size (bp)75, 200, 300, 400, 500, 700, 1000, 1500, 2000, 3000, 4000, 5000, 7000, 10000, 20000
Quantification using: Lower Marker Final concentration (ng/uL): 0.5000 Dilution factor: 12.0

Sample: SampA2
Well location: A2
Created: Wednesday, August 11, 2021 6:14:43 PM

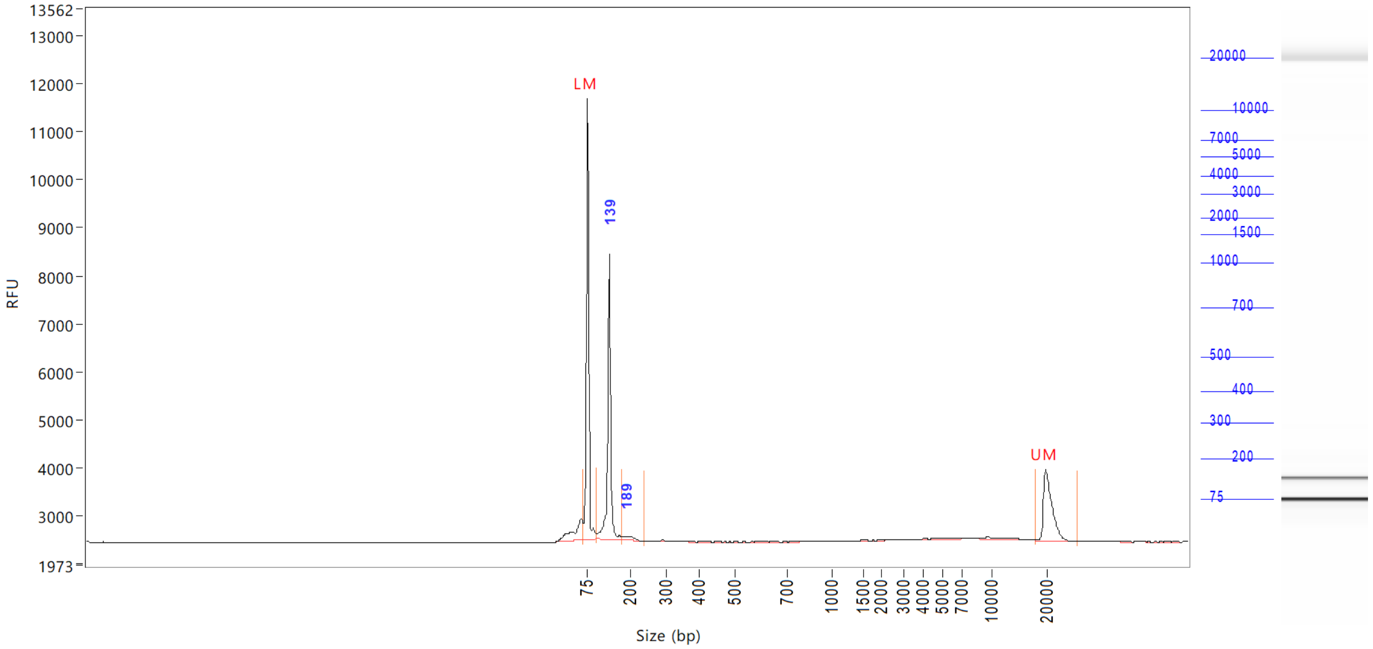

Peak	Size (bp)	Concentration (ng/uL)	From (bp)	To (bp)	Average size (bp)	CV%	RFU	Corrected peak area
1	75 (LM)	0.5000	62	99	73	6.43	9201	54.003
2	139	4.4677	99	173	134	6.73	5937	40.212
3	189	0.1999	173	239	193	6.28	78	1.799
4	20000 (UM)	0.1421	18049	25803	20496	4.60	1498	15.349

TIC:	4.6676	ng/uL
TIM:	56.4880	nmole/L
Total concentration:	5.1965	ng/uL

Sample peak width (sec): 5   Sample min peak height: 50   Sample baseline V to V?: Y   Sample baseline V to V points: 3
Sample filter: Binomial   Number of points for filter: 3 Sample start region (min): 0 Sample end region (min): 30
Marker peak width (sec): 5   Marker min peak height: 200   Marker baseline V to V?: Y   Marker baseline V to V points: 3
Lower marker selection: First peak > 500 RFU   Upper marker selection: Last peak > 200 RFU
Ladder size (bp)75, 200, 300, 400, 500, 700, 1000, 1500, 2000, 3000, 4000, 5000, 7000, 10000, 20000
Quantification using: Lower Marker   Final concentration (ng/uL): 0.5000   Dilution factor: 12.0

Sample: SampB2
Well location: B2
Created: Wednesday, August 11, 2021 6:14:43 PM

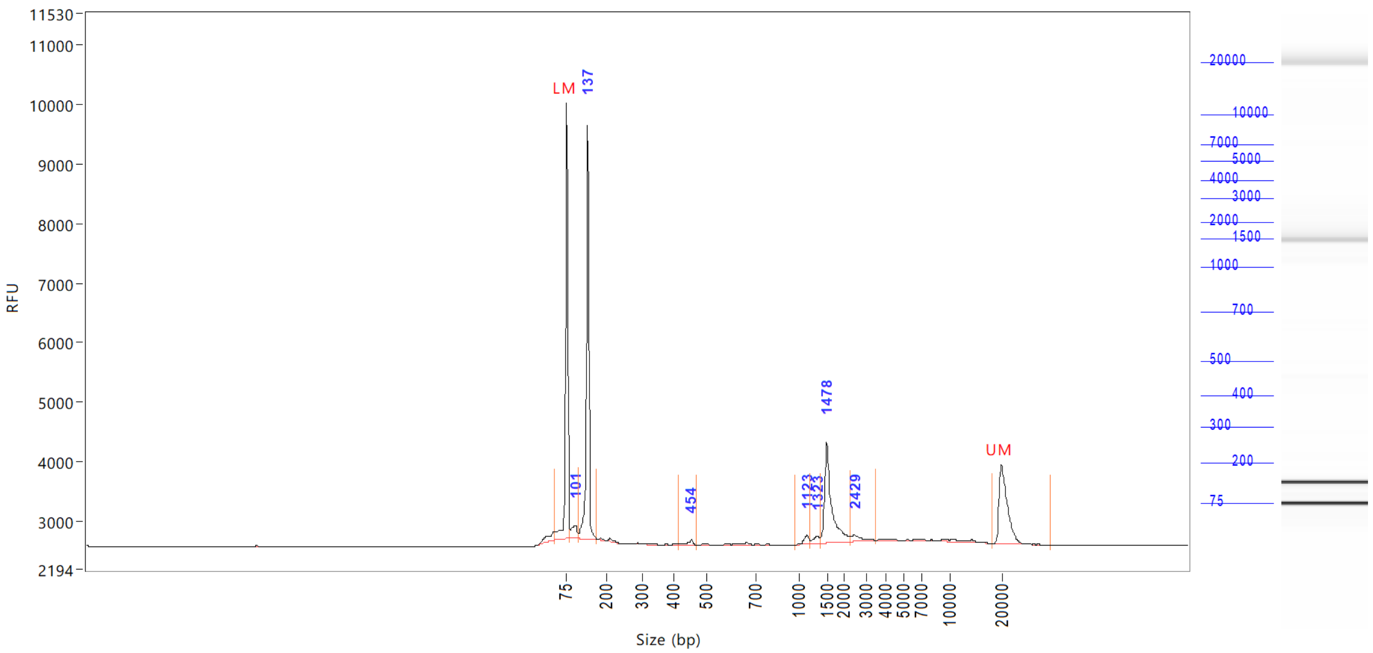

Peak	Size (bp)	Concentration (ng/uL)	From (bp)	To (bp)	Average size (bp)	CV%	RFU	Corrected peak area
1	75 (LM)	0.5000	41	86	72	8.18	7325	42.700
2	101	0.4097	86	110	97	7.07	221	2.915
3	137	6.0069	110	168	135	4.25	6955	42.750
4	454	0.1345	414	471	444	2.77	97	0.957
5	1123	0.1848	974	1201	1111	4.44	147	1.315
6	1323	0.1740	1201	1367	1290	3.72	122	1.239
7	1478	2.5931	1367	2229	1571	11.13	1690	18.454
8	2429	0.2301	2229	3465	2567	10.11	101	1.637
9	20000 (UM)	0.1601	18293	29551	20613	5.03	1320	13.670

TIC:	9.7330	ng/uL
TIM:	83.9085	nmole/L
Total concentration:	10.3923	ng/uL

```
Sample peak width (sec): 5    Sample min peak height: 50    Sample baseline V to V?: Y    Sample baseline V to V points: 3
Sample filter: Binomial       Number of points for filter: 3  Sample start region (min): 0  Sample end region (min): 30
Marker peak width (sec): 5    Marker min peak height: 200   Marker baseline V to V?: Y    Marker baseline V to V points: 3
Lower marker selection: First peak > 500 RFU                Upper marker selection: Last peak > 200 RFU
Ladder size (bp)75, 200, 300, 400, 500, 700, 1000, 1500, 2000, 3000, 4000, 5000, 7000, 10000, 20000
Quantification using: Lower Marker          Final concentration (ng/uL): 0.5000          Dilution factor: 12.0
```

Sample: SampC2
Well location: C2
Created: Wednesday, August 11, 2021 6:14:43 PM

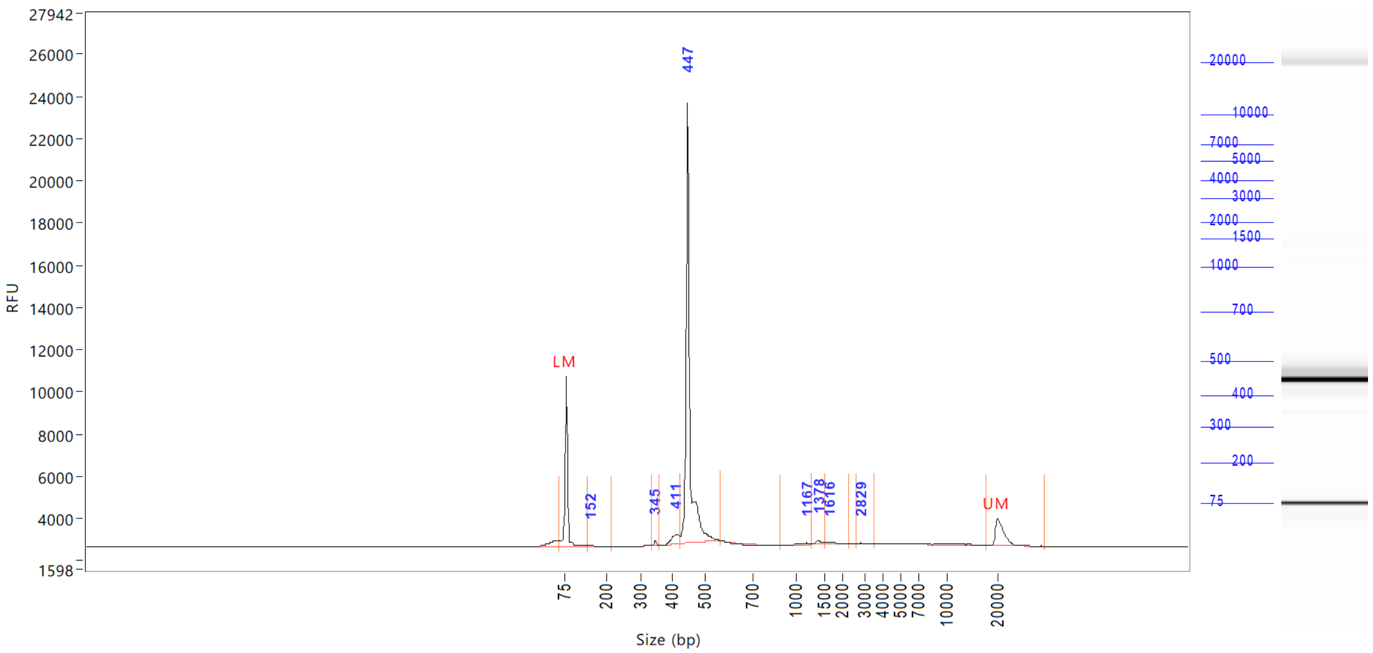

Peak	Size (bp)	Concentration (ng/uL)	From (bp)	To (bp)	Average size (bp)	CV%	RFU	Corrected peak area
1	75 (LM)	0.5000	54	143	75	13.12	8084	51.909
2	152	0.1275	143	212	163	10.73	72	1.103
3	345	0.1442	333	358	343	1.09	243	1.247
4	411	0.8018	358	422	406	2.54	427	6.937
5	447	17.2077	422	565	451	3.70	20881	148.873
6	1167	0.1554	879	1256	1098	9.25	76	1.344
7	1378	0.2256	1256	1489	1377	4.16	202	1.952
8	1616	0.1680	1489	2315	1679	9.18	89	1.453
9	2829	0.0802	2657	3608	3036	8.29	52	0.694
10	20000 (UM)	0.1312	17805	29068	20628	5.18	1314	13.623

TIC:	18.9104	ng/uL
TIM:	68.7177	nmole/L
Total concentration:	19.2093	ng/uL

```
Sample peak width (sec): 5    Sample min peak height: 50    Sample baseline V to V?: Y    Sample baseline V to V points: 3
Sample filter: Binomial       Number of points for filter: 3  Sample start region (min): 0  Sample end region (min): 30
Marker peak width (sec): 5    Marker min peak height: 200   Marker baseline V to V?: Y    Marker baseline V to V points: 3
Lower marker selection: First peak > 500 RFU                Upper marker selection: Last peak > 200 RFU
Ladder size (bp)75, 200, 300, 400, 500, 700, 1000, 1500, 2000, 3000, 4000, 5000, 7000, 10000, 20000
Quantification using: Lower Marker          Final concentration (ng/uL): 0.5000          Dilution factor: 12.0
```

Sample: SampD2
Well location: D2
Created: Wednesday, August 11, 2021 6:14:43 PM

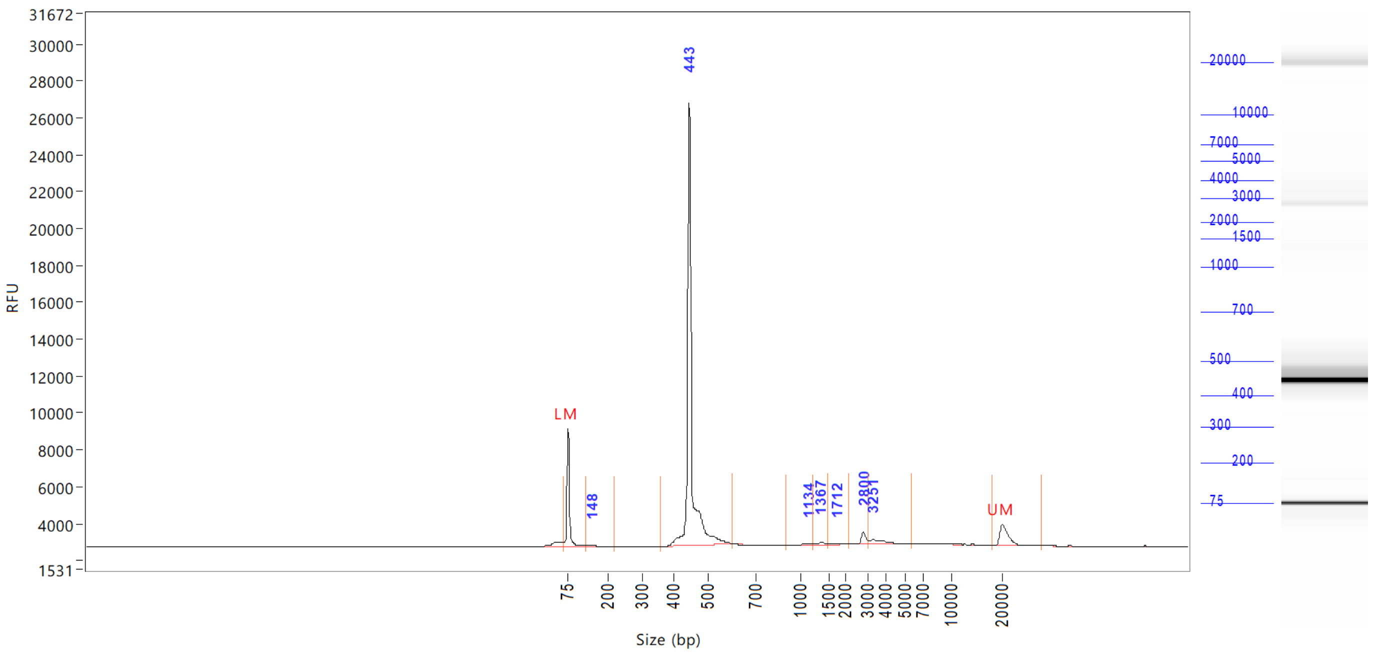

Peak	Size (bp)	Concentration (ng/uL)	From (bp)	To (bp)	Average size (bp)	CV%	RFU	Corrected peak area
1	75 (LM)	0.5000	64	131	76	11.60	6365	39.475
2	148	0.2410	131	215	154	12.04	83	1.586
3	443	28.2080	358	600	450	5.53	24031	185.587
4	1134	0.1764	905	1223	1075	7.72	62	1.160
5	1367	0.2658	1223	1489	1362	4.79	170	1.749
6	1712	0.1672	1489	2144	1675	8.33	61	1.100
7	2800	0.7969	2144	3001	2772	5.05	691	5.243
8	3251	0.9575	3001	5741	3743	15.35	262	6.300
9	20000 (UM)	0.1504	17927	27496	20501	4.75	1156	11.874

TIC:	30.8127	ng/uL
TIM:	107.3640	nmole/L
Total concentration:	31.0545	ng/uL

Sample peak width (sec): 5 Sample min peak height: 50 Sample baseline V to V?: Y Sample baseline V to V points: 3
Sample filter: Binomial Number of points for filter: 3 Sample start region (min): 0 Sample end region (min): 30
Marker peak width (sec): 5 Marker min peak height: 200 Marker baseline V to V?: Y Marker baseline V to V points: 3
Lower marker selection: First peak > 500 RFU Upper marker selection: Last peak > 200 RFU
Ladder size (bp)75, 200, 300, 400, 500, 700, 1000, 1500, 2000, 3000, 4000, 5000, 7000, 10000, 20000
Quantification using: Lower Marker Final concentration (ng/uL): 0.5000 Dilution factor: 12.0

Sample: SampE2
Well location: E2
Created: Wednesday, August 11, 2021 6:14:43 PM

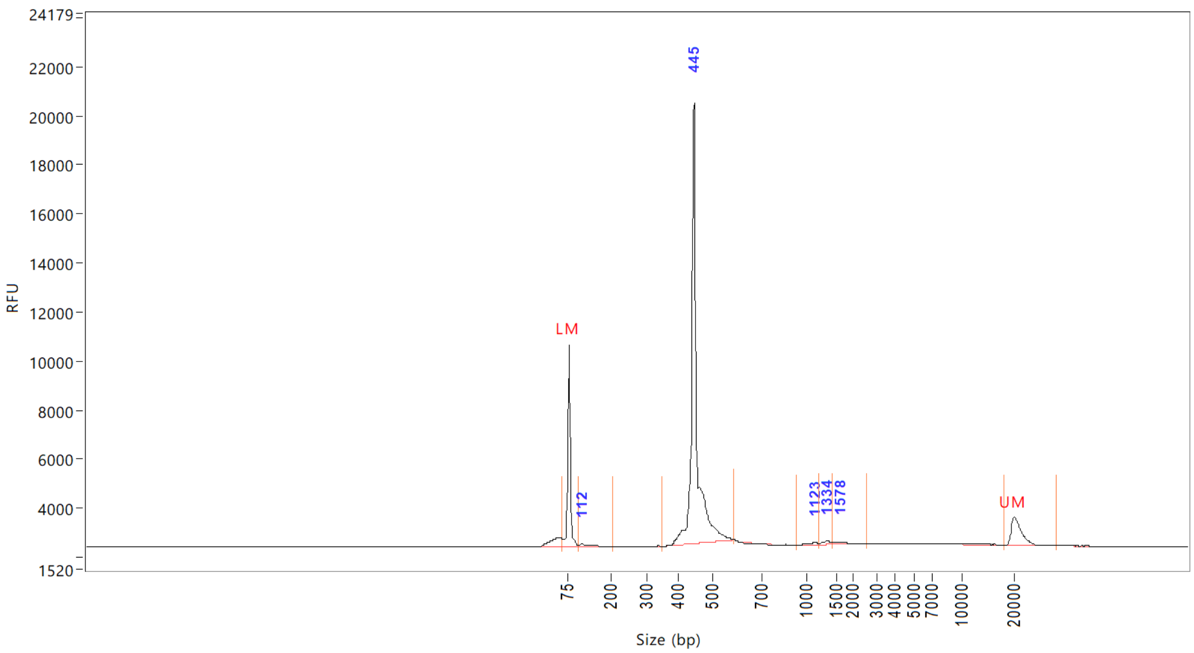

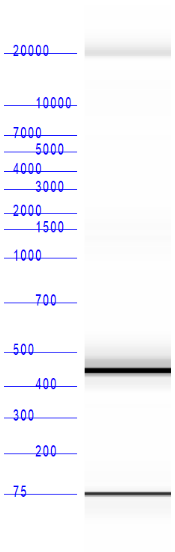

Peak	Size (bp)	Concentration (ng/uL)	From (bp)	To (bp)	Average size (bp)	CV%	RFU	Corrected peak area
1	75 (LM)	0.5000	54	103	73	8.03	8209	52.889
2	112	0.3206	103	203	132	17.71	101	2.826
3	445	20.3924	347	585	449	5.34	17989	179.756
4	1123	0.1290	935	1190	1076	6.34	66	1.137
5	1334	0.2109	1190	1422	1316	4.75	148	1.859
6	1578	0.2818	1422	2572	1696	15.41	110	2.484
7	20000 (UM)	0.1341	18293	28100	20651	5.47	1156	14.185

TIC:	21.3348	ng/uL
TIM:	79.4562	nmole/L
Total concentration:	21.6352	ng/uL

```
Sample peak width (sec): 5    Sample min peak height: 50    Sample baseline V to V?: Y    Sample baseline V to V points: 3
Sample filter: Binomial       Number of points for filter: 3  Sample start region (min): 0  Sample end region (min): 30
Marker peak width (sec): 5    Marker min peak height: 200   Marker baseline V to V?: Y    Marker baseline V to V points: 3
Lower marker selection: First peak > 500 RFU                Upper marker selection: Last peak > 200 RFU
Ladder size (bp)75, 200, 300, 400, 500, 700, 1000, 1500, 2000, 3000, 4000, 5000, 7000, 10000, 20000
Quantification using: Lower Marker          Final concentration (ng/uL): 0.5000          Dilution factor: 12.0
```

Sample: SampF2
Well location: F2
Created: Wednesday, August 11, 2021 6:14:43 PM

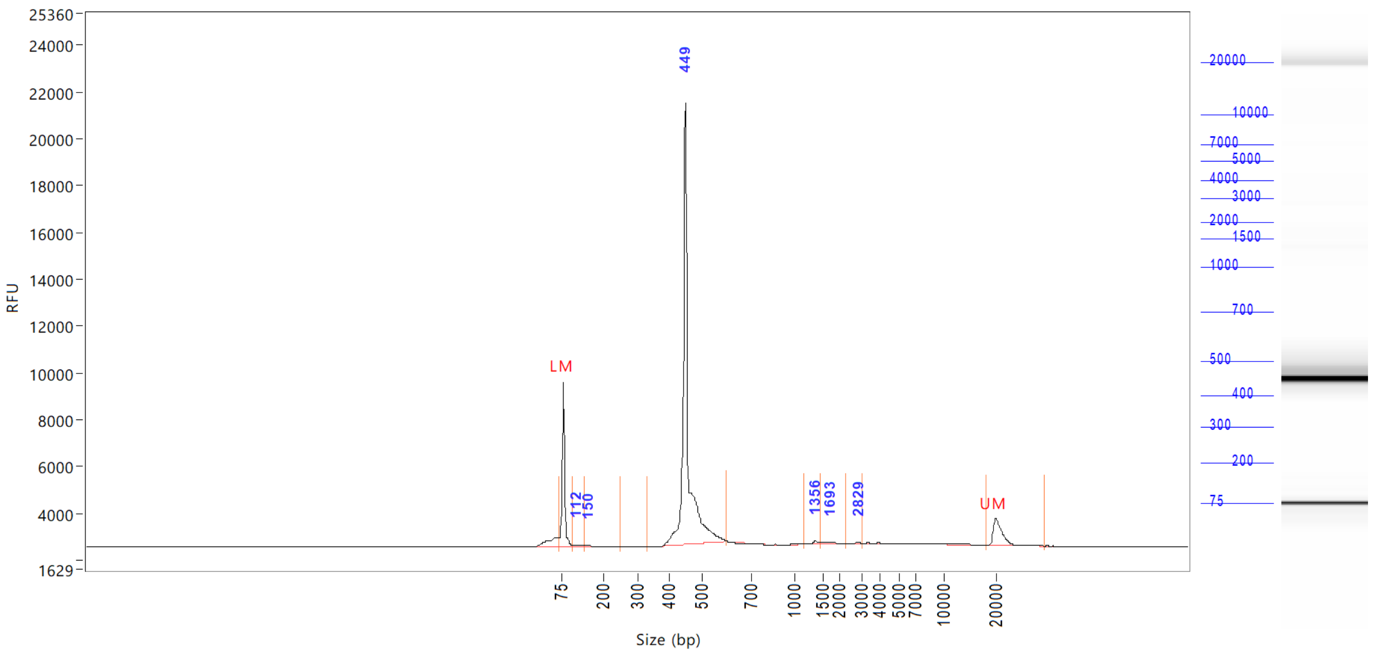

Peak	Size (bp)	Concentration (ng/uL)	From (bp)	To (bp)	Average size (bp)	CV%	RFU	Corrected peak area
1	75 (LM)	0.5000	62	103	74	7.34	6997	44.516
2	112	0.2383	103	143	119	9.56	90	1.768
3	150	0.1503	143	248	170	15.09	57	1.115
4	449	24.3827	327	600	455	5.98	18875	180.905
5	1356	0.1875	1178	1456	1338	4.97	136	1.391
6	1693	0.2069	1456	2258	1668	8.50	85	1.535
7	2829	0.0673	2258	3000	2789	4.68	61	0.499
8	20000 (UM)	0.1444	18049	29551	20612	5.63	1164	12.860

TIC:	25.2330	ng/uL
TIM:	93.3932	nmole/L
Total concentration:	25.6476	ng/uL

```
Sample peak width (sec): 5    Sample min peak height: 50    Sample baseline V to V?: Y    Sample baseline V to V points: 3
Sample filter: Binomial       Number of points for filter: 3  Sample start region (min): 0  Sample end region (min): 30
Marker peak width (sec): 5    Marker min peak height: 200   Marker baseline V to V?: Y    Marker baseline V to V points: 3
Lower marker selection: First peak > 500 RFU                Upper marker selection: Last peak > 200 RFU
Ladder size (bp)75, 200, 300, 400, 500, 700, 1000, 1500, 2000, 3000, 4000, 5000, 7000, 10000, 20000
Quantification using: Lower Marker          Final concentration (ng/uL): 0.5000          Dilution factor: 12.0
```

Sample: SampG2
Well location: G2
Created: Wednesday, August 11, 2021 6:14:43 PM

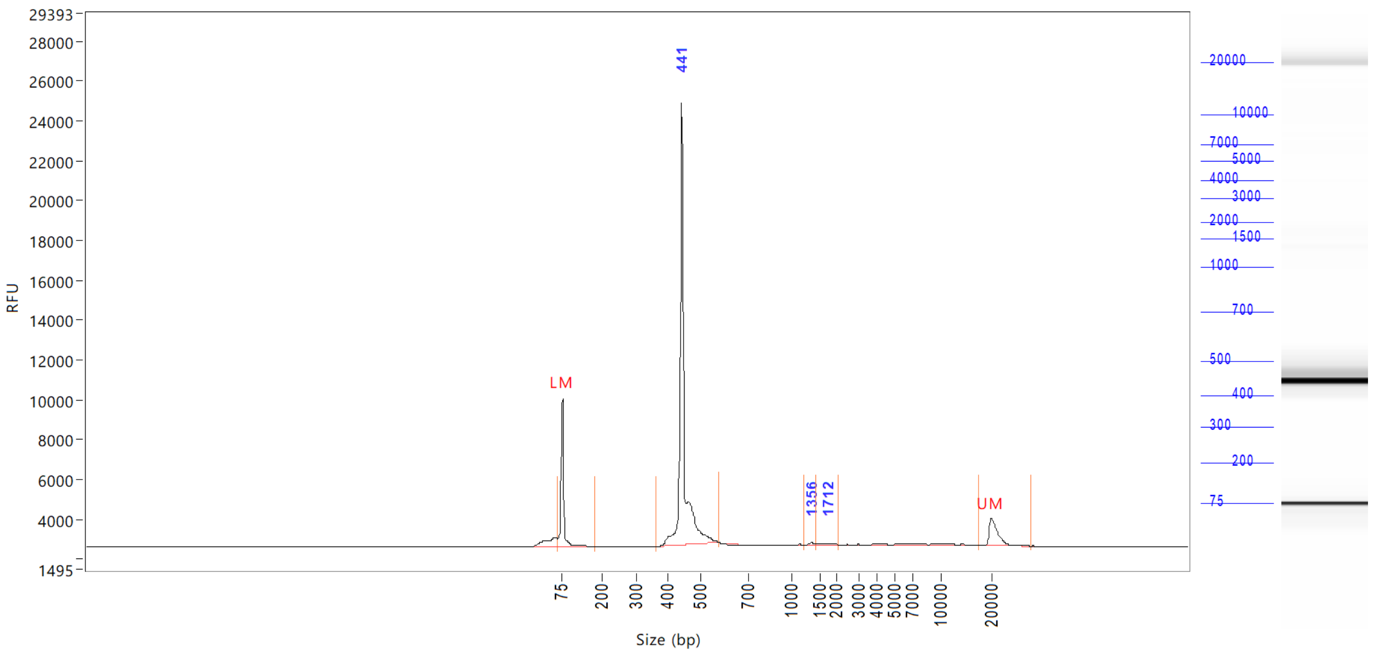

Peak	Size (bp)	Concentration (ng/uL)	From (bp)	To (bp)	Average size (bp)	CV%	RFU	Corrected peak area
1	75 (LM)	0.5000	60	175	74	14.54	7378	49.534
2	441	21.7097	364	572	446	5.08	22210	179.228
3	1356	0.1372	1223	1433	1340	3.81	129	1.132
4	1712	0.2360	1433	2087	1671	9.36	96	1.948
5	20000 (UM)	0.1498	17439	27859	20610	5.14	1384	14.836

TIC:	22.0829	ng/uL
TIM:	80.4935	nmole/L
Total concentration:	22.5410	ng/uL

```
Sample peak width (sec): 5    Sample min peak height: 50    Sample baseline V to V?: Y    Sample baseline V to V points: 3
Sample filter: Binomial       Number of points for filter: 3  Sample start region (min): 0  Sample end region (min): 30
Marker peak width (sec): 5    Marker min peak height: 200   Marker baseline V to V?: Y    Marker baseline V to V points: 3
Lower marker selection: First peak > 500 RFU                Upper marker selection: Last peak > 200 RFU
Ladder size (bp)75, 200, 300, 400, 500, 700, 1000, 1500, 2000, 3000, 4000, 5000, 7000, 10000, 20000
Quantification using: Lower Marker          Final concentration (ng/uL): 0.5000          Dilution factor: 12.0
```

Sample: SampH2
Well location: H2
Created: Wednesday, August 11, 2021 6:14:43 PM

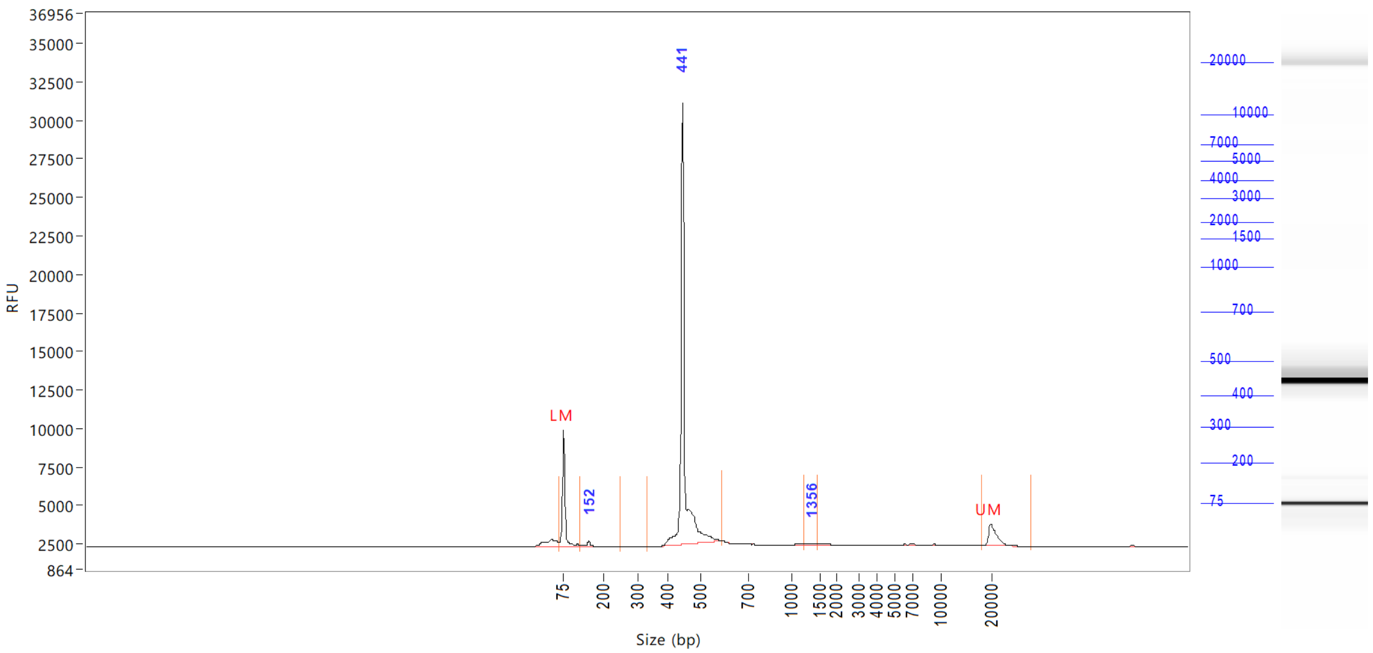

Peak	Size (bp)	Concentration (ng/uL)	From (bp)	To (bp)	Average size (bp)	CV%	RFU	Corrected peak area
1	75 (LM)	0.5000	60	128	75	13.34	7545	51.045
2	152	0.4679	128	250	151	11.06	339	3.980
3	441	25.0412	331	583	447	5.14	28708	213.040
4	1356	0.0897	1212	1467	1337	5.43	52	0.763
5	20000 (UM)	0.1437	18293	27859	20562	4.76	1405	14.675

TIC:	25.5987	ng/uL
TIM:	97.3791	nmole/L
Total concentration:	26.0770	ng/uL

```
Sample peak width (sec): 5    Sample min peak height: 50    Sample baseline V to V?: Y    Sample baseline V to V points: 3
Sample filter: Binomial       Number of points for filter: 3  Sample start region (min): 0  Sample end region (min): 30
Marker peak width (sec): 5    Marker min peak height: 200   Marker baseline V to V?: Y    Marker baseline V to V points: 3
Lower marker selection: First peak > 500 RFU                Upper marker selection: Last peak > 200 RFU
Ladder size (bp)75, 200, 300, 400, 500, 700, 1000, 1500, 2000, 3000, 4000, 5000, 7000, 10000, 20000
Quantification using: Lower Marker          Final concentration (ng/uL): 0.5000          Dilution factor: 12.0
```

Sample: SampA3
Well location: A3
Created: Wednesday, August 11, 2021 6:14:43 PM

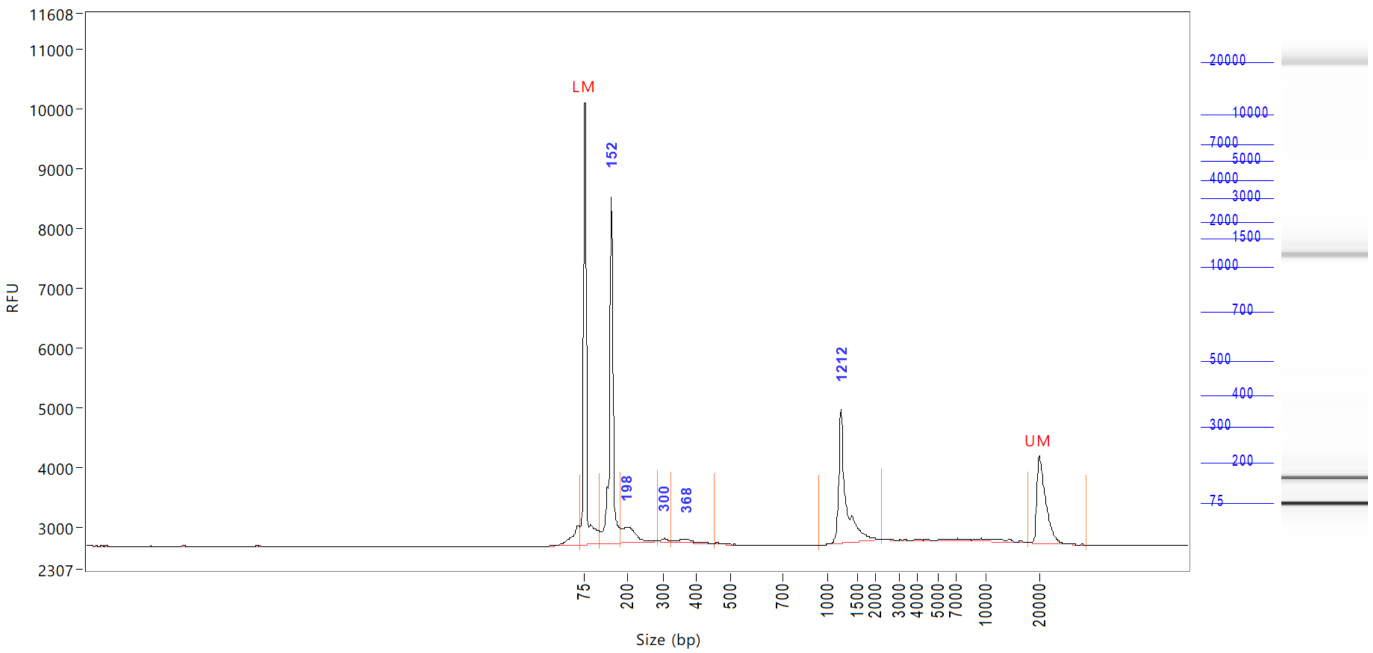

Peak	Size (bp)	Concentration (ng/uL)	From (bp)	To (bp)	Average size (bp)	CV%	RFU	Corrected peak area
1	75 (LM)	0.5000	60	118	75	13.67	7402	49.709
2	152	5.5363	118	181	149	7.06	5795	45.867
3	198	0.9186	181	280	205	8.93	258	7.611
4	300	0.0897	280	323	301	3.13	55	0.743
5	368	0.2062	323	450	372	8.06	65	1.708
6	1212	2.9892	939	2229	1287	11.94	2238	24.765
7	20000 (UM)	0.1565	18049	28826	20606	5.05	1474	15.562

TIC:	9.7399	ng/uL
TIM:	73.6591	nmole/L
Total concentration:	10.1543	ng/uL

Sample peak width (sec): 5 Sample min peak height: 50 Sample baseline V to V?: Y Sample baseline V to V points: 3
Sample filter: Binomial Number of points for filter: 3 Sample start region (min): 0 Sample end region (min): 30
Marker peak width (sec): 5 Marker min peak height: 200 Marker baseline V to V?: Y Marker baseline V to V points: 3
Lower marker selection: First peak > 500 RFU Upper marker selection: Last peak > 200 RFU
Ladder size (bp)75, 200, 300, 400, 500, 700, 1000, 1500, 2000, 3000, 4000, 5000, 7000, 10000, 20000
Quantification using: Lower Marker Final concentration (ng/uL): 0.5000 Dilution factor: 12.0

Sample: SampB3
Well location: B3
Created: Wednesday, August 11, 2021 6:14:43 PM

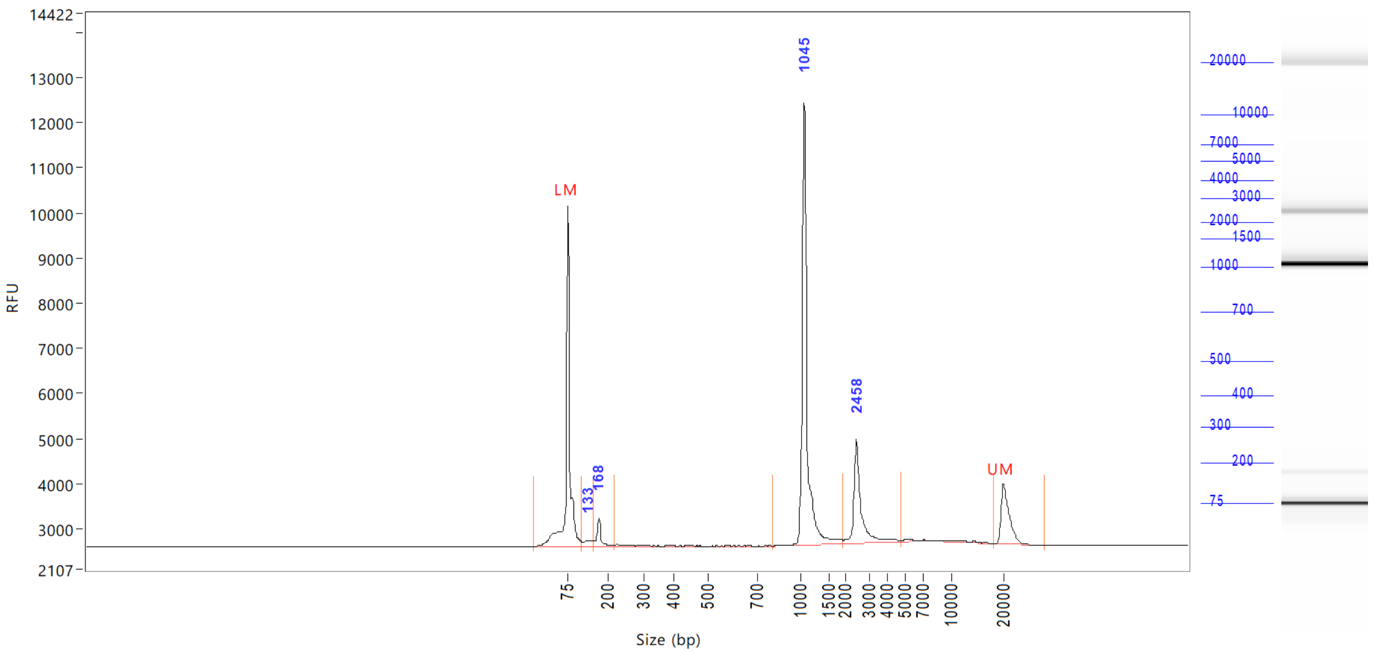

Peak	Size (bp)	Concentration (ng/uL)	From (bp)	To (bp)	Average size (bp)	CV%	RFU	Corrected peak area
1	75 (LM)	0.5000	0	118	69	27.84	7529	66.093
2	133	0.2675	118	154	136	7.85	133	2.946
3	168	0.4905	154	215	171	7.22	634	5.403
4	1045	6.7274	805	1904	1095	12.03	9812	74.105
5	2458	2.2188	1904	4793	2626	18.01	2292	24.442
6	20000 (UM)	0.1035	18171	27979	20598	4.74	1331	13.680

TIC:	9.7043	ng/uL
TIM:	19.4507	nmole/L
Total concentration:	10.0327	ng/uL

Sample peak width (sec): 5 Sample min peak height: 50 Sample baseline V to V?: Y Sample baseline V to V points: 3
Sample filter: Binomial Number of points for filter: 3 Sample start region (min): 0 Sample end region (min): 30
Marker peak width (sec): 5 Marker min peak height: 200 Marker baseline V to V?: Y Marker baseline V to V points: 3
Lower marker selection: First peak > 500 RFU Upper marker selection: Last peak > 200 RFU
Ladder size (bp)75, 200, 300, 400, 500, 700, 1000, 1500, 2000, 3000, 4000, 5000, 7000, 10000, 20000
Quantification using: Lower Marker Final concentration (ng/uL): 0.5000 Dilution factor: 12.0

Sample: SampC3
Well location: C3
Created: Wednesday, August 11, 2021 6:14:43 PM

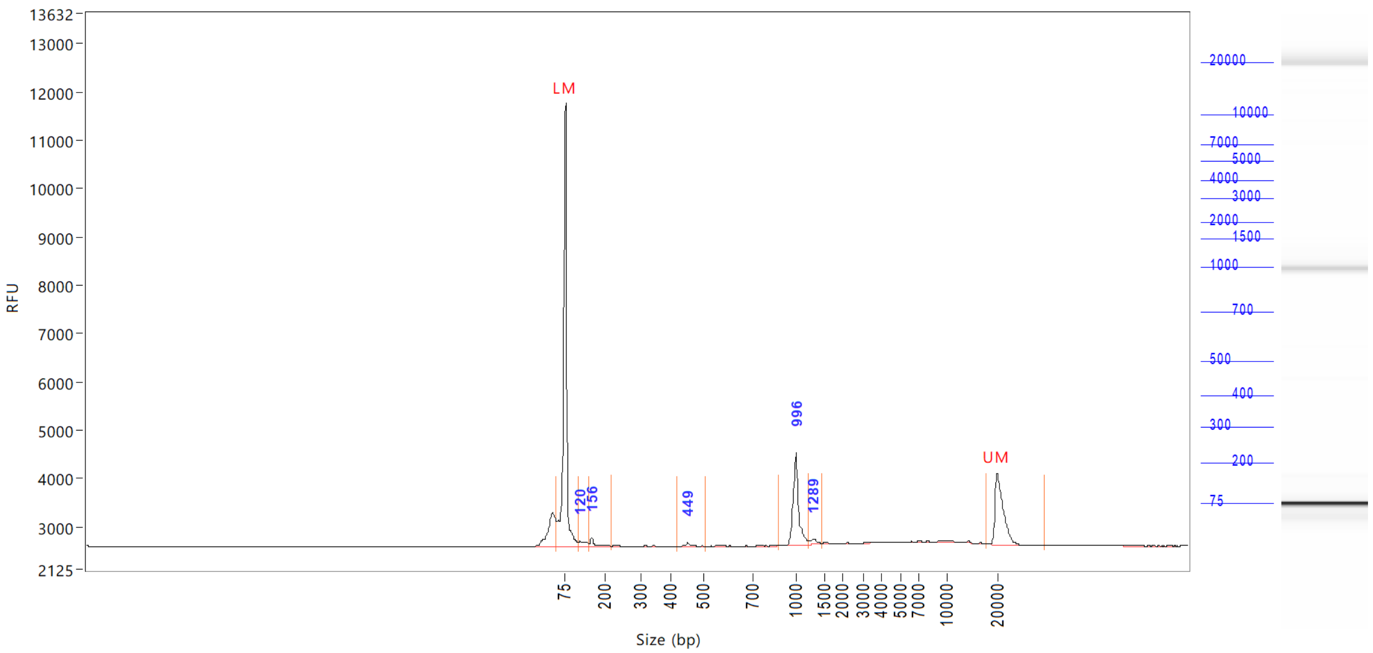

Peak	Size (bp)	Concentration (ng/uL)	From (bp)	To (bp)	Average size (bp)	CV%	RFU	Corrected peak area
1	75 (LM)	0.5000	47	114	71	12.17	9194	73.229
2	120	0.1485	114	147	129	7.62	102	1.812
3	156	0.1349	147	214	160	8.06	184	1.646
4	449	0.0520	418	501	452	3.55	71	0.635
5	996	1.3659	879	1212	1003	4.70	1904	16.671
6	1289	0.0971	1212	1445	1295	4.37	109	1.185
7	20000 (UM)	0.1050	17805	29309	20523	4.88	1472	15.382

TIC:	1.7984	ng/uL
TIM:	5.8307	nmole/L
Total concentration:	2.0235	ng/uL

Sample peak width (sec): 5 Sample min peak height: 50 Sample baseline V to V?: Y Sample baseline V to V points: 3
Sample filter: Binomial Number of points for filter: 3 Sample start region (min): 0 Sample end region (min): 30
Marker peak width (sec): 5 Marker min peak height: 200 Marker baseline V to V?: Y Marker baseline V to V points: 3
Lower marker selection: First peak > 500 RFU Upper marker selection: Last peak > 200 RFU
Ladder size (bp)75, 200, 300, 400, 500, 700, 1000, 1500, 2000, 3000, 4000, 5000, 7000, 10000, 20000
Quantification using: Lower Marker Final concentration (ng/uL): 0.5000 Dilution factor: 12.0

Sample: SampD3
Well location: D3
Created: Wednesday, August 11, 2021 6:14:43 PM

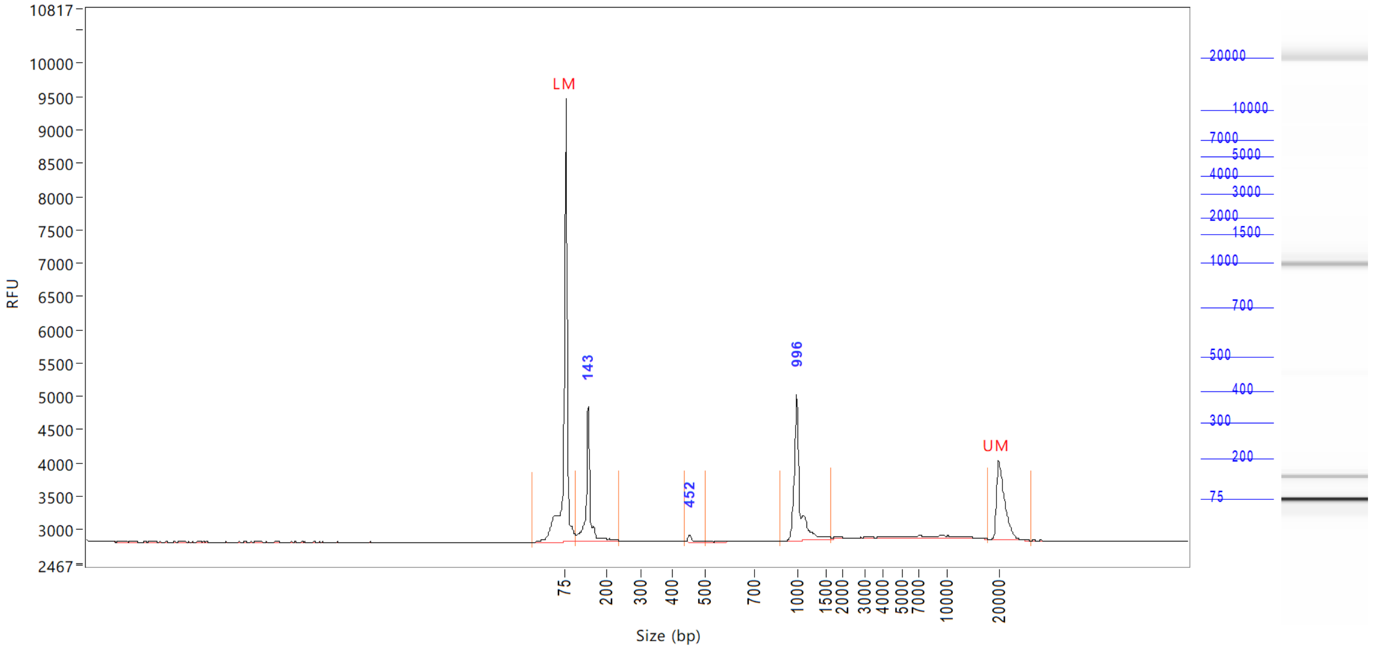

Peak	Size (bp)	Concentration (ng/uL)	From (bp)	To (bp)	Average size (bp)	CV%	RFU	Corrected peak area
1	75 (LM)	0.5000	0	107	66	25.58	6665	56.459
2	143	1.9370	107	235	142	11.70	2013	18.227
3	452	0.0907	435	498	455	2.60	111	0.853
4	996	2.1045	879	1655	1036	10.51	2198	19.803
5	20000 (UM)	0.1062	17927	26287	20562	4.64	1177	11.996

TIC:	4.1321	ng/uL
TIM:	26.0880	nmole/L
Total concentration:	4.5565	ng/uL

Sample peak width (sec): 5 Sample min peak height: 50 Sample baseline V to V?: Y Sample baseline V to V points: 3
Sample filter: Binomial Number of points for filter: 3 Sample start region (min): 0 Sample end region (min): 30
Marker peak width (sec): 5 Marker min peak height: 200 Marker baseline V to V?: Y Marker baseline V to V points: 3
Lower marker selection: First peak > 500 RFU Upper marker selection: Last peak > 200 RFU
Ladder size (bp)75, 200, 300, 400, 500, 700, 1000, 1500, 2000, 3000, 4000, 5000, 7000, 10000, 20000
Quantification using: Lower Marker Final concentration (ng/uL): 0.5000 Dilution factor: 12.0

Sample: SampE3
Well location: E3
Created: Wednesday, August 11, 2021 6:14:43 PM

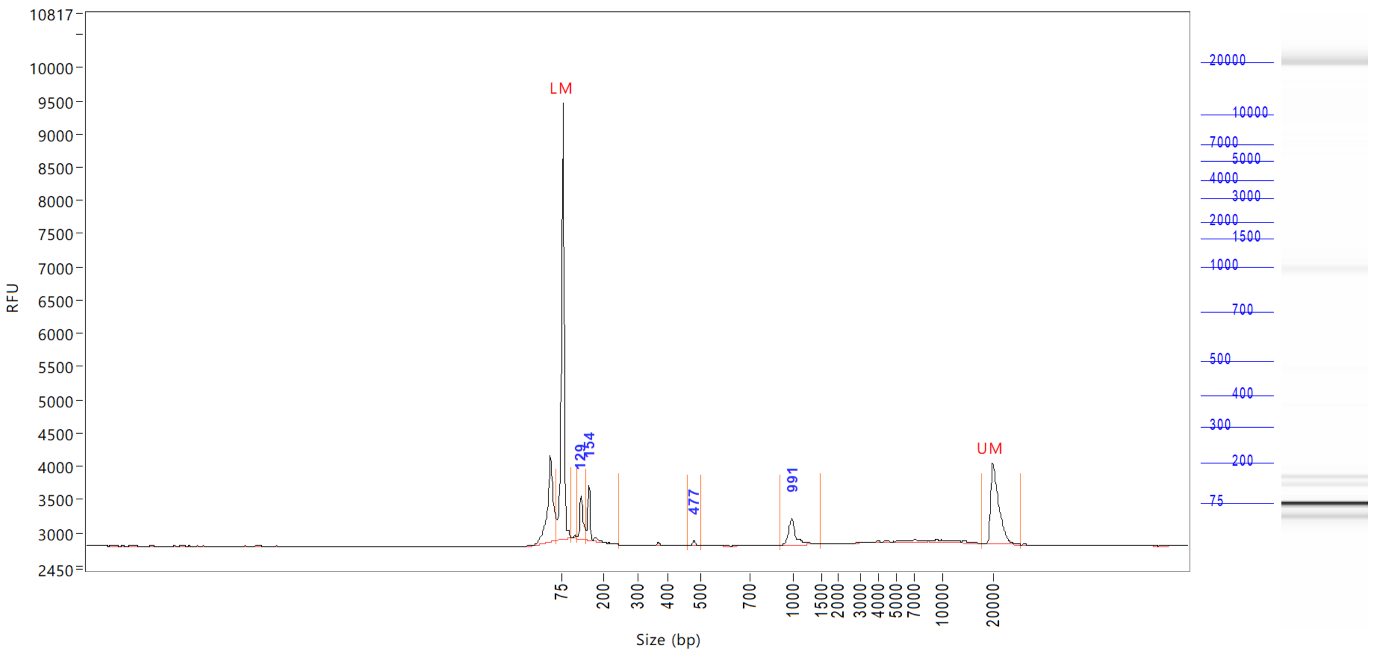

Peak	Size (bp)	Concentration (ng/uL)	From (bp)	To (bp)	Average size (bp)	CV%	RFU	Corrected peak area
1	75 (LM)	0.5000	56	101	71	7.22	6578	45.405
2	129	0.6892	118	145	131	4.60	644	5.215
3	154	0.7389	145	244	156	7.47	830	5.592
4	477	0.0759	458	498	476	1.57	78	0.574
5	991	0.6327	909	1478	1020	8.18	394	4.788
6	20000 (UM)	0.1401	17927	25682	20598	4.73	1210	12.727

TIC:	2.1367	ng/uL
TIM:	17.7128	nmole/L
Total concentration:	2.6198	ng/uL

Sample peak width (sec): 5 Sample min peak height: 50 Sample baseline V to V?: Y Sample baseline V to V points: 3
Sample filter: Binomial Number of points for filter: 3 Sample start region (min): 0 Sample end region (min): 30
Marker peak width (sec): 5 Marker min peak height: 200 Marker baseline V to V?: Y Marker baseline V to V points: 3
Lower marker selection: First peak > 500 RFU Upper marker selection: Last peak > 200 RFU
Ladder size (bp)75, 200, 300, 400, 500, 700, 1000, 1500, 2000, 3000, 4000, 5000, 7000, 10000, 20000
Quantification using: Lower Marker Final concentration (ng/uL): 0.5000 Dilution factor: 12.0

Sample: SampF3
Well location: F3
Created: Wednesday, August 11, 2021 6:14:43 PM

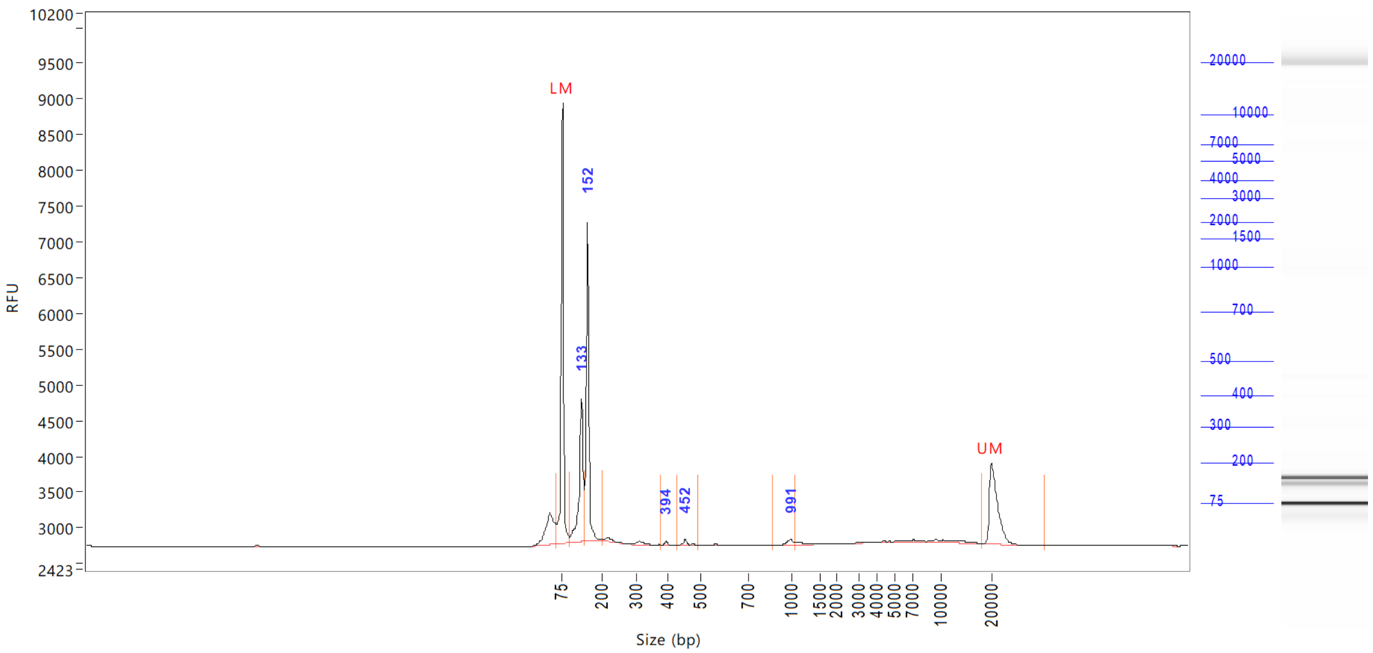

Peak	Size (bp)	Concentration (ng/uL)	From (bp)	To (bp)	Average size (bp)	CV%	RFU	Corrected peak area
1	75 (LM)	0.5000	56	97	72	7.10	6170	37.869
2	133	3.1076	97	143	129	7.50	2006	19.614
3	152	4.5514	143	200	151	4.37	4456	28.726
4	394	0.0571	380	426	394	2.02	55	0.361
5	452	0.1072	426	490	454	2.72	81	0.676
6	991	0.1425	866	1045	976	3.22	76	0.899
7	20000 (UM)	0.1614	18049	30639	20674	6.11	1137	12.228

TIC:	7.9657	ng/uL
TIM:	89.9869	nmole/L
Total concentration:	8.6541	ng/uL

Sample peak width (sec): 5 Sample min peak height: 50 Sample baseline V to V?: Y Sample baseline V to V points: 3
Sample filter: Binomial Number of points for filter: 3 Sample start region (min): 0 Sample end region (min): 30
Marker peak width (sec): 5 Marker min peak height: 200 Marker baseline V to V?: Y Marker baseline V to V points: 3
Lower marker selection: First peak > 500 RFU Upper marker selection: Last peak > 200 RFU
Ladder size (bp)75, 200, 300, 400, 500, 700, 1000, 1500, 2000, 3000, 4000, 5000, 7000, 10000, 20000
Quantification using: Lower Marker Final concentration (ng/uL): 0.5000 Dilution factor: 12.0

Sample: SampG3
Well location: G3
Created: Wednesday, August 11, 2021 6:14:43 PM

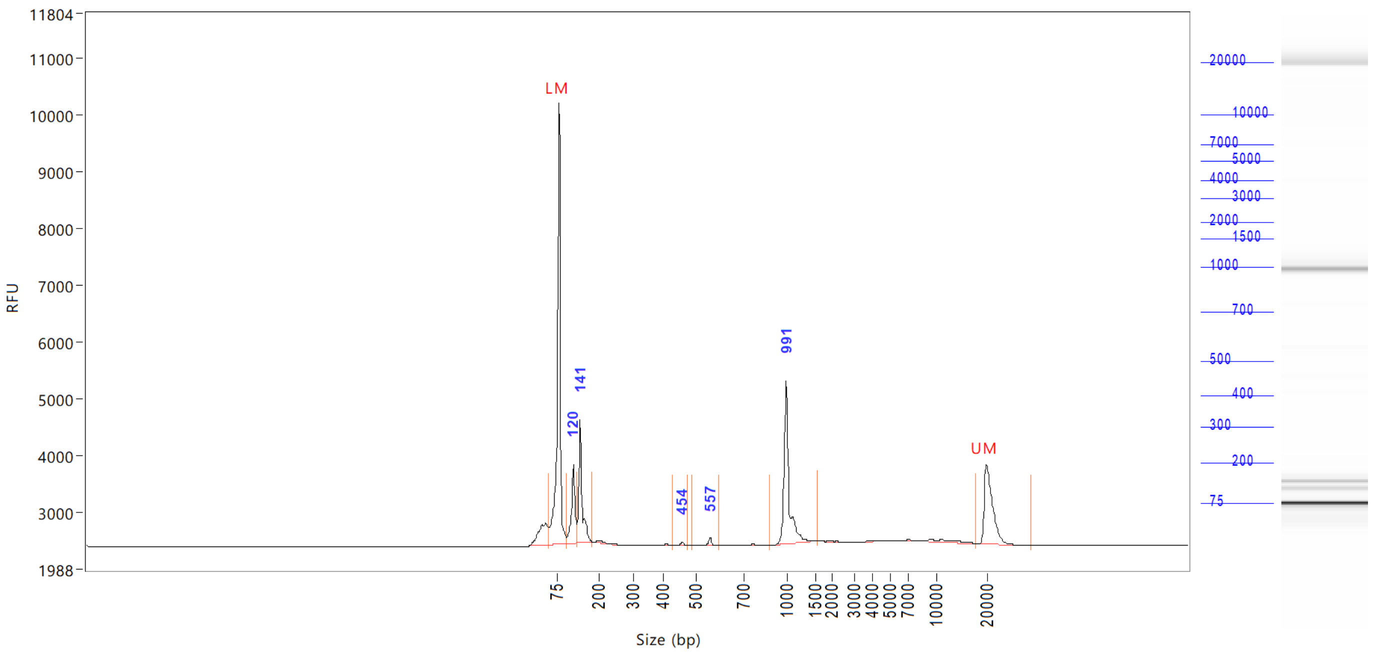

Peak	Size (bp)	Concentration (ng/uL)	From (bp)	To (bp)	Average size (bp)	CV%	RFU	Corrected peak area
1	75 (LM)	0.5000	47	99	71	10.75	7803	56.500
2	120	1.3072	99	131	117	6.08	1396	12.310
3	141	1.7967	131	181	142	5.74	2166	16.919
4	454	0.0553	428	473	452	1.75	73	0.521
5	557	0.1115	487	595	542	3.73	130	1.050
6	991	2.4932	870	1520	1014	7.09	2873	23.478
7	20000 (UM)	0.1315	17805	28947	20620	5.32	1404	14.856

TIC:	5.7639	ng/uL
TIM:	43.7332	nmole/L
Total concentration:	6.1988	ng/uL

Sample peak width (sec): 5 Sample min peak height: 50 Sample baseline V to V?: Y Sample baseline V to V points: 3
Sample filter: Binomial Number of points for filter: 3 Sample start region (min): 0 Sample end region (min): 30
Marker peak width (sec): 5 Marker min peak height: 200 Marker baseline V to V?: Y Marker baseline V to V points: 3
Lower marker selection: First peak > 500 RFU Upper marker selection: Last peak > 200 RFU
Ladder size (bp)75, 200, 300, 400, 500, 700, 1000, 1500, 2000, 3000, 4000, 5000, 7000, 10000, 20000
Quantification using: Lower Marker Final concentration (ng/uL): 0.5000 Dilution factor: 12.0

Sample: SampH3
Well location: H3
Created: Wednesday, August 11, 2021 6:14:43 PM

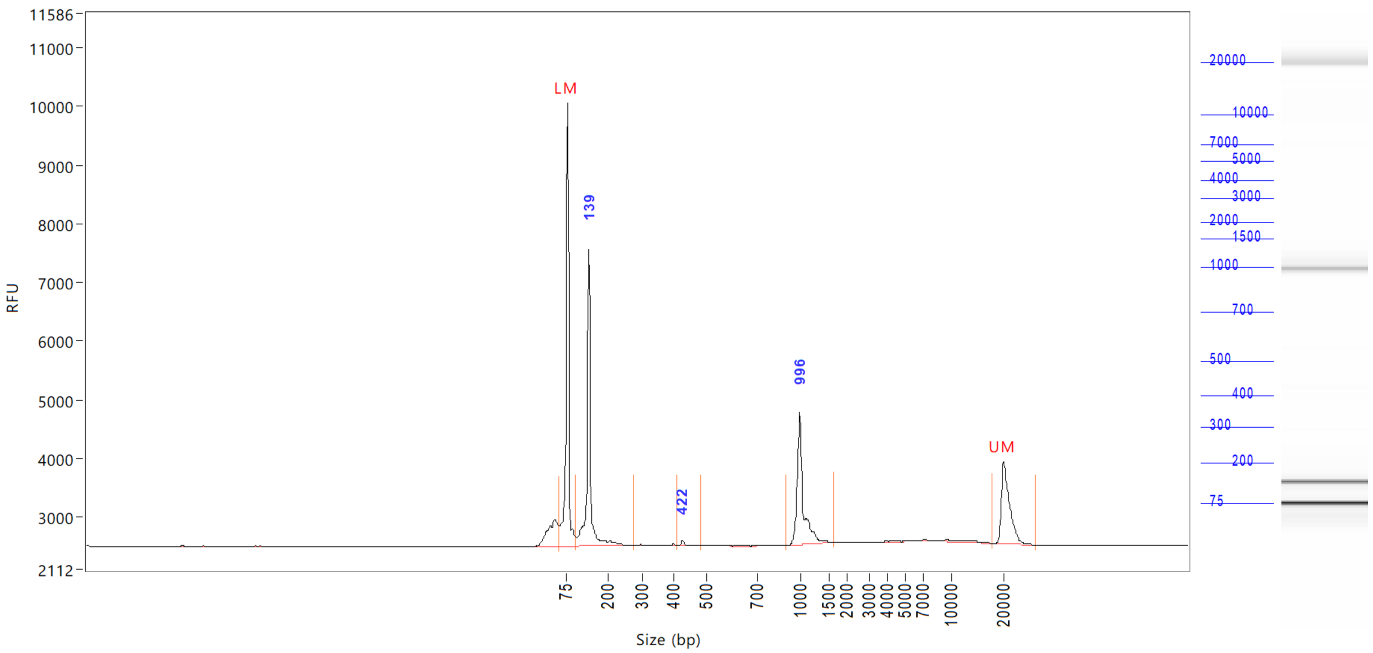

Peak	Size (bp)	Concentration (ng/uL)	From (bp)	To (bp)	Average size (bp)	CV%	RFU	Corrected peak area
1	75 (LM)	0.5000	48	101	72	10.24	7566	52.330
2	139	4.7821	101	273	140	15.17	5055	41.707
3	422	0.0786	407	479	426	3.04	90	0.685
4	996	2.5290	905	1616	1031	8.89	2254	22.057
5	20000 (UM)	0.1385	17805	26287	20542	4.79	1391	14.498

TIC:	7.3897	ng/uL
TIM:	60.4727	nmole/L
Total concentration:	7.7846	ng/uL

```
Sample peak width (sec): 5    Sample min peak height: 50    Sample baseline V to V?: Y    Sample baseline V to V points: 3
Sample filter: Binomial       Number of points for filter: 3  Sample start region (min): 0  Sample end region (min): 30
Marker peak width (sec): 5    Marker min peak height: 200   Marker baseline V to V?: Y    Marker baseline V to V points: 3
Lower marker selection: First peak > 500 RFU                Upper marker selection: Last peak > 200 RFU
Ladder size (bp)75, 200, 300, 400, 500, 700, 1000, 1500, 2000, 3000, 4000, 5000, 7000, 10000, 20000
Quantification using: Lower Marker          Final concentration (ng/uL): 0.5000          Dilution factor: 12.0
```

Sample: SampA4
Well location: A4
Created: Wednesday, August 11, 2021 6:14:43 PM

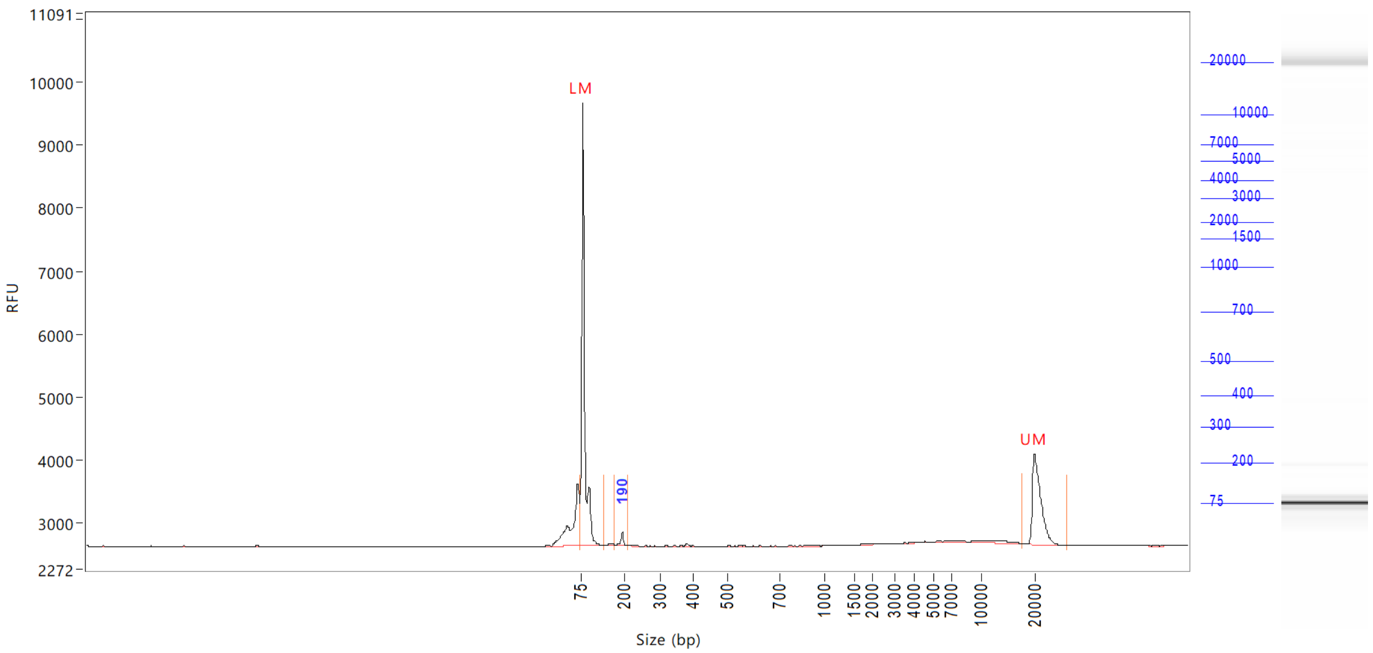

Peak	Size (bp)	Concentration (ng/uL)	From (bp)	To (bp)	Average size (bp)	CV%	RFU	Corrected peak area
1	75 (LM)	0.5000	67	137	77	10.13	7034	49.031
2	190	0.1565	169	208	188	2.78	230	1.279
3	20000 (UM)	0.1509	17561	26045	20484	4.56	1445	14.796

TIC:	0.1565	ng/uL
TIM:	1.3685	nmole/L
Total concentration:	0.6848	ng/uL

Sample peak width (sec): 5 Sample min peak height: 50 Sample baseline V to V?: Y Sample baseline V to V points: 3
Sample filter: Binomial Number of points for filter: 3 Sample start region (min): 0 Sample end region (min): 30
Marker peak width (sec): 5 Marker min peak height: 200 Marker baseline V to V?: Y Marker baseline V to V points: 3
Lower marker selection: First peak > 500 RFU Upper marker selection: Last peak > 200 RFU
Ladder size (bp)75, 200, 300, 400, 500, 700, 1000, 1500, 2000, 3000, 4000, 5000, 7000, 10000, 20000
Quantification using: Lower Marker Final concentration (ng/uL): 0.5000 Dilution factor: 12.0

Sample: SampB4
Well location: B4
Created: Wednesday, August 11, 2021 6:14:43 PM

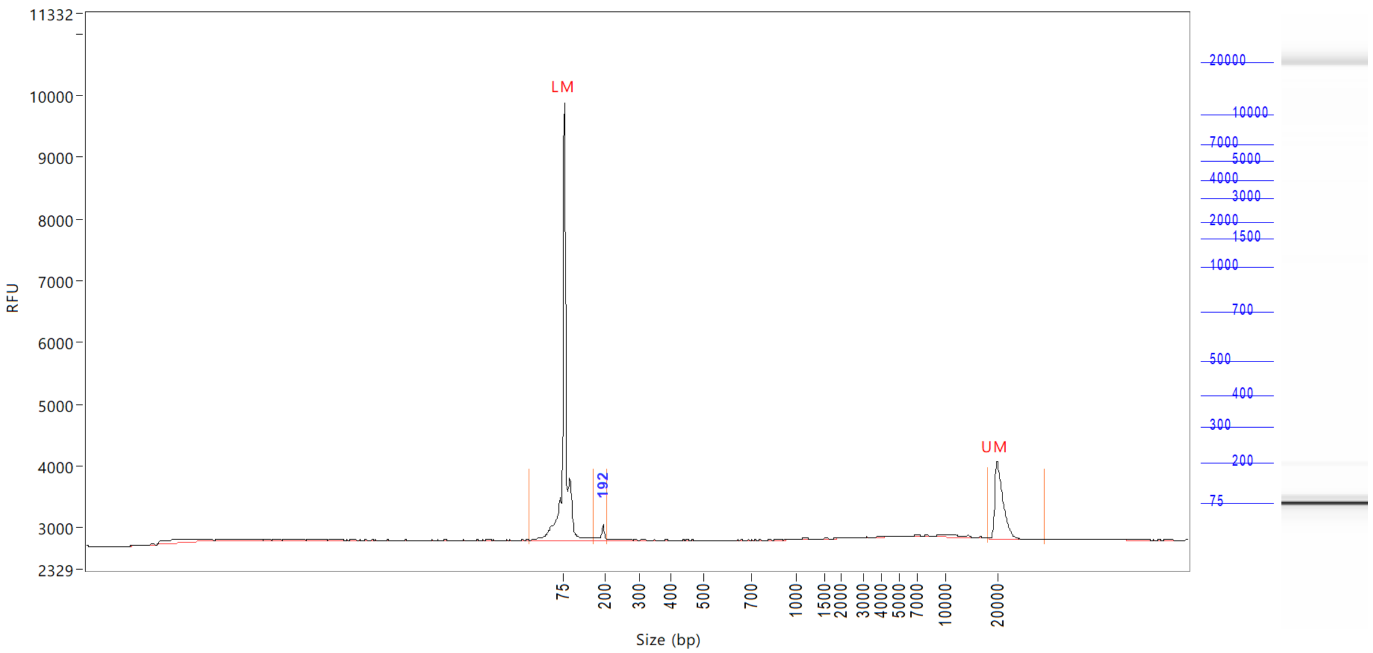

Peak	Size (bp)	Concentration (ng/uL)	From (bp)	To (bp)	Average size (bp)	CV%	RFU	Corrected peak area
1	75 (LM)	0.5000	0	166	72	26.70	7093	63.790
2	192	0.1779	166	206	188	4.41	246	1.891
3	20000 (UM)	0.1018	18171	29309	20601	5.02	1251	12.984

TIC:	0.1779	ng/uL
TIM:	1.5557	nmole/L
Total concentration:	0.5868	ng/uL

```
Sample peak width (sec): 5    Sample min peak height: 50    Sample baseline V to V?: Y    Sample baseline V to V points: 3
Sample filter: Binomial       Number of points for filter: 3  Sample start region (min): 0  Sample end region (min): 30
Marker peak width (sec): 5    Marker min peak height: 200   Marker baseline V to V?: Y    Marker baseline V to V points: 3
Lower marker selection: First peak > 500 RFU                Upper marker selection: Last peak > 200 RFU
Ladder size (bp)75, 200, 300, 400, 500, 700, 1000, 1500, 2000, 3000, 4000, 5000, 7000, 10000, 20000
Quantification using: Lower Marker          Final concentration (ng/uL): 0.5000          Dilution factor: 12.0
```

Sample: SampC4
Well location: C4
Created: Wednesday, August 11, 2021 6:14:43 PM

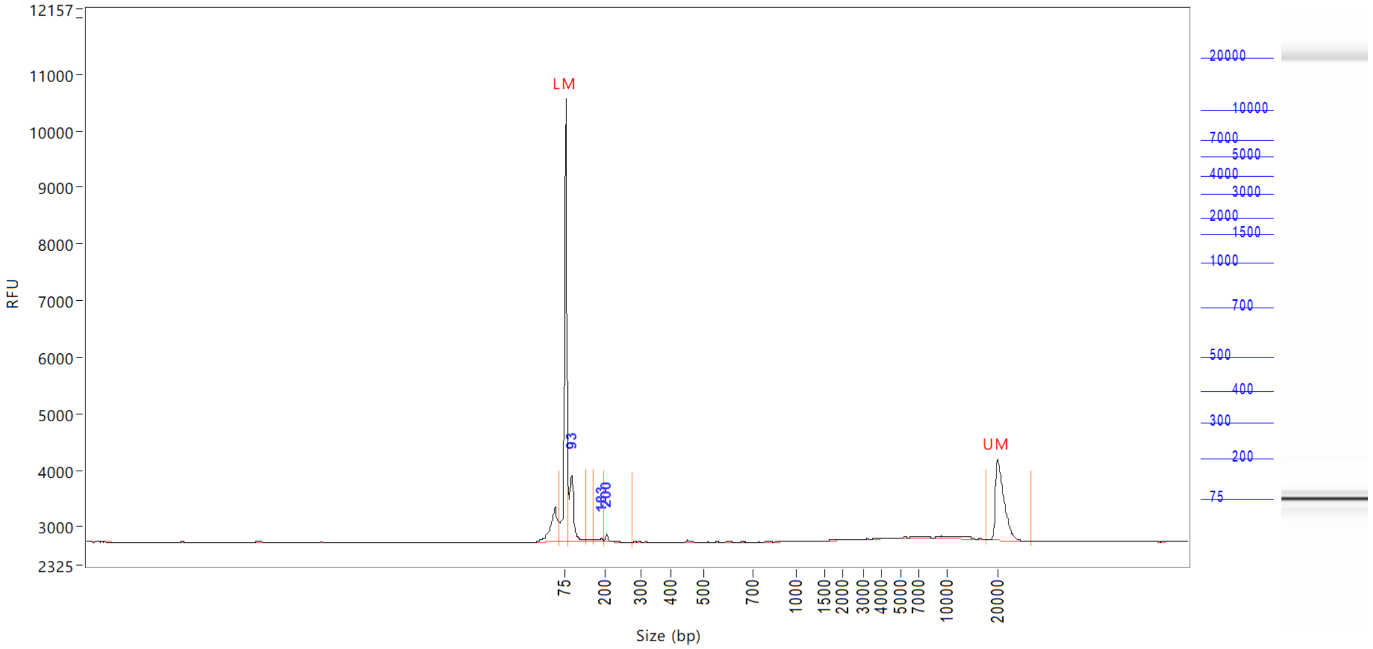

Peak	Size (bp)	Concentration (ng/uL)	From (bp)	To (bp)	Average size (bp)	CV%	RFU	Corrected peak area
1	75 (LM)	0.5000	56	86	73	6.58	7837	48.040
2	93	1.3490	86	141	94	8.16	1155	10.801
3	183	0.0557	164	192	180	3.72	58	0.446
4	200	0.1014	192	275	204	8.08	142	0.812
5	20000 (UM)	0.1611	17805	26529	20645	4.92	1451	15.481

TIC:	1.5061	ng/uL
TIM:	24.8876	nmole/L
Total concentration:	2.0533	ng/uL

```
Sample peak width (sec): 5    Sample min peak height: 50    Sample baseline V to V?: Y    Sample baseline V to V points: 3
Sample filter: Binomial       Number of points for filter: 3  Sample start region (min): 0  Sample end region (min): 30
Marker peak width (sec): 5    Marker min peak height: 200   Marker baseline V to V?: Y    Marker baseline V to V points: 3
Lower marker selection: First peak > 500 RFU                Upper marker selection: Last peak > 200 RFU
Ladder size (bp)75, 200, 300, 400, 500, 700, 1000, 1500, 2000, 3000, 4000, 5000, 7000, 10000, 20000
Quantification using: Lower Marker          Final concentration (ng/uL): 0.5000          Dilution factor: 12.0
```

Sample: SampD4
Well location: D4
Created: Wednesday, August 11, 2021 6:14:43 PM

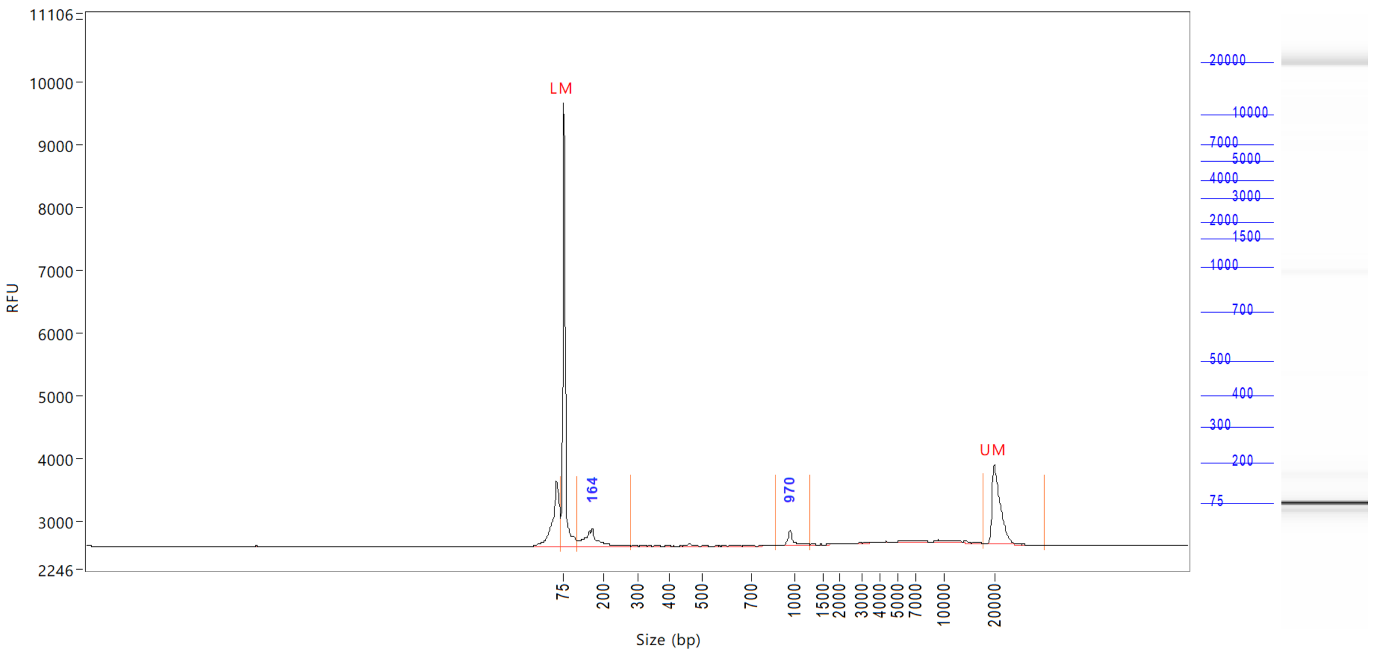

Peak	Size (bp)	Concentration (ng/uL)	From (bp)	To (bp)	Average size (bp)	CV%	RFU	Corrected peak area
1	75 (LM)	0.5000	66	116	75	10.20	7077	44.016
2	164	1.0216	116	277	160	16.95	283	7.495
3	970	0.2948	870	1267	990	6.59	251	2.162
4	20000 (UM)	0.1519	17683	29672	20563	5.24	1261	13.370

TIC:	1.3164	ng/uL
TIM:	10.9853	nmole/L
Total concentration:	1.8450	ng/uL

```
Sample peak width (sec): 5    Sample min peak height: 50    Sample baseline V to V?: Y    Sample baseline V to V points: 3
Sample filter: Binomial       Number of points for filter: 3  Sample start region (min): 0  Sample end region (min): 30
Marker peak width (sec): 5    Marker min peak height: 200   Marker baseline V to V?: Y    Marker baseline V to V points: 3
Lower marker selection: First peak > 500 RFU                Upper marker selection: Last peak > 200 RFU
Ladder size (bp)75, 200, 300, 400, 500, 700, 1000, 1500, 2000, 3000, 4000, 5000, 7000, 10000, 20000
Quantification using: Lower Marker          Final concentration (ng/uL): 0.5000          Dilution factor: 12.0
```

Sample: SampE4
Well location: E4
Created: Wednesday, August 11, 2021 6:14:43 PM

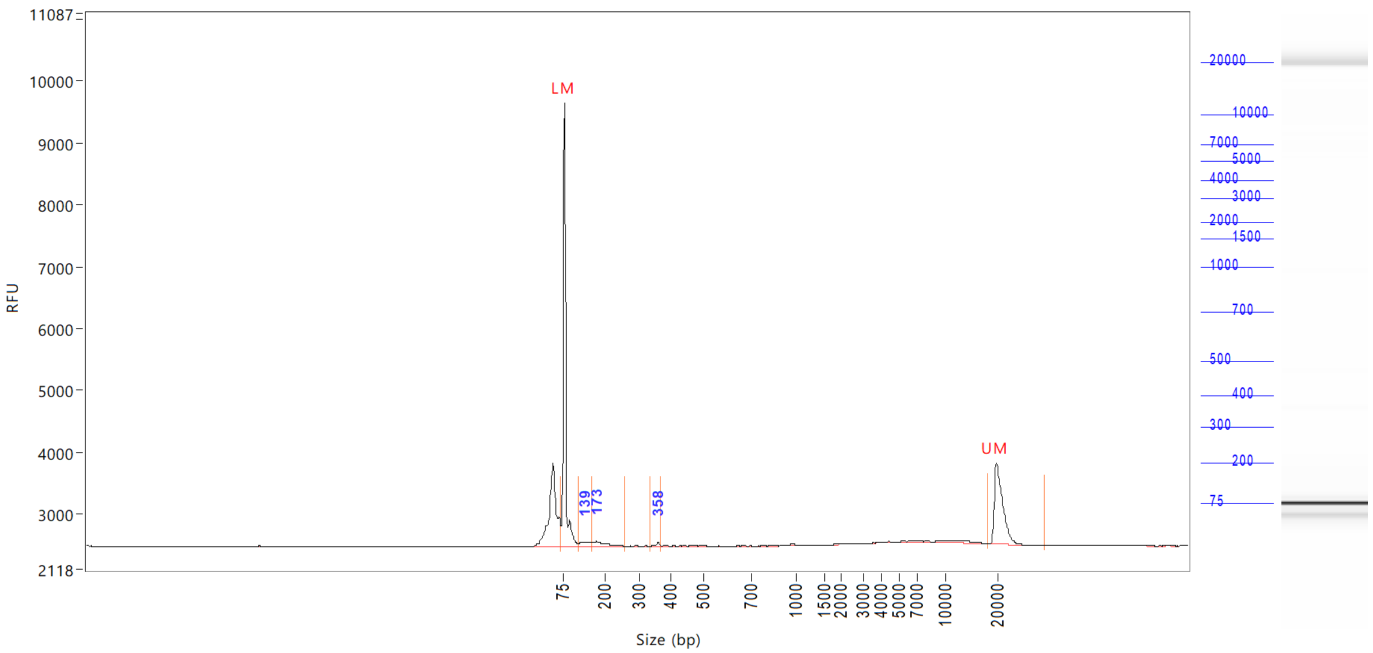

Peak	Size (bp)	Concentration (ng/uL)	From (bp)	To (bp)	Average size (bp)	CV%	RFU	Corrected peak area
1	75 (LM)	0.5000	66	120	75	10.17	7166	43.889
2	139	0.2361	120	160	140	8.49	65	1.727
3	173	0.2779	160	257	187	11.13	78	2.032
4	358	0.0623	331	370	354	1.99	70	0.456
5	20000 (UM)	0.1569	18171	29189	20527	4.98	1311	13.769

TIC:	0.5763	ng/uL
TIM:	5.5041	nmole/L
Total concentration:	1.1093	ng/uL

```
Sample peak width (sec): 5    Sample min peak height: 50    Sample baseline V to V?: Y    Sample baseline V to V points: 3
Sample filter: Binomial       Number of points for filter: 3  Sample start region (min): 0  Sample end region (min): 30
Marker peak width (sec): 5    Marker min peak height: 200   Marker baseline V to V?: Y    Marker baseline V to V points: 3
Lower marker selection: First peak > 500 RFU                Upper marker selection: Last peak > 200 RFU
Ladder size (bp)75, 200, 300, 400, 500, 700, 1000, 1500, 2000, 3000, 4000, 5000, 7000, 10000, 20000
Quantification using: Lower Marker          Final concentration (ng/uL): 0.5000          Dilution factor: 12.0
```

Sample: SampF4
Well location: F4
Created: Wednesday, August 11, 2021 6:14:43 PM

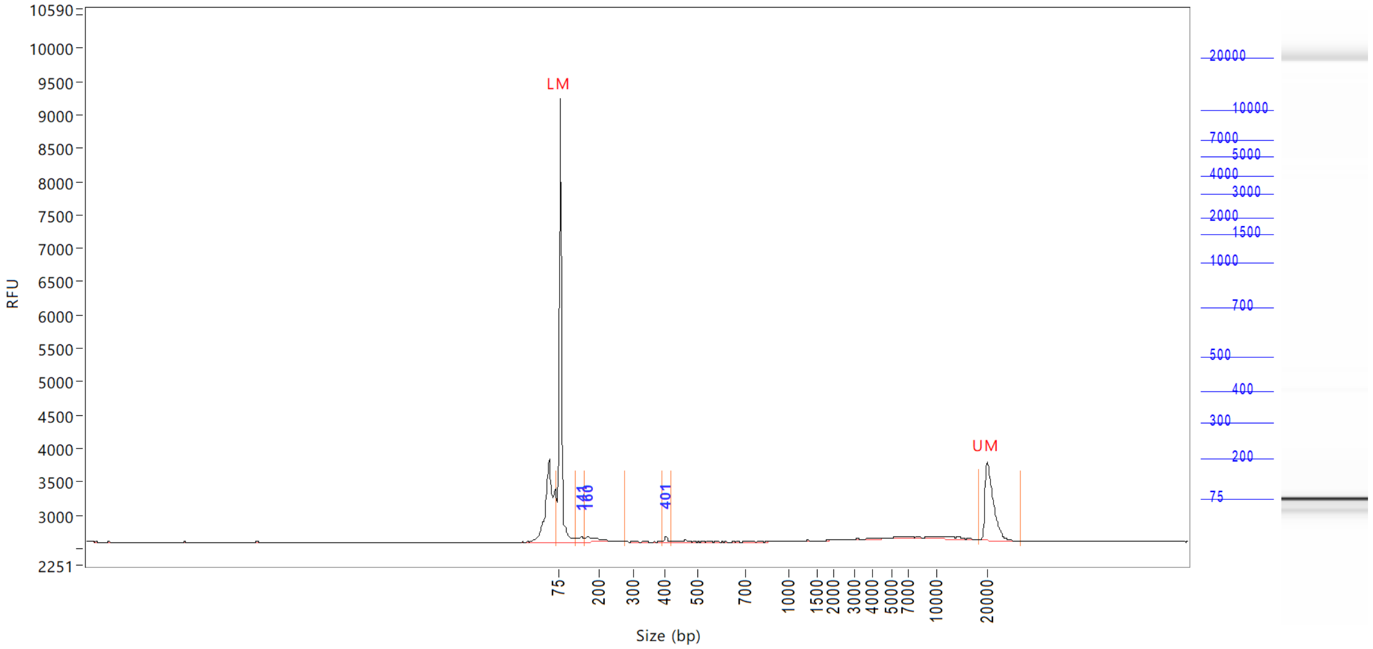

Peak	Size (bp)	Concentration (ng/uL)	From (bp)	To (bp)	Average size (bp)	CV%	RFU	Corrected peak area
1	75 (LM)	0.5000	66	124	75	10.17	6659	41.465
2	141	0.1698	124	150	137	5.89	70	1.174
3	160	0.3569	150	273	184	13.70	77	2.466
4	401	0.0774	390	416	400	1.18	90	0.535
5	20000 (UM)	0.1506	18293	26770	20627	4.91	1171	12.492

TIC:	0.6041	ng/uL
TIM:	5.5437	nmole/L
Total concentration:	1.2155	ng/uL

```
Sample peak width (sec): 5    Sample min peak height: 50    Sample baseline V to V?: Y    Sample baseline V to V points: 3
Sample filter: Binomial       Number of points for filter: 3  Sample start region (min): 0  Sample end region (min): 30
Marker peak width (sec): 5    Marker min peak height: 200   Marker baseline V to V?: Y    Marker baseline V to V points: 3
Lower marker selection: First peak > 500 RFU                Upper marker selection: Last peak > 200 RFU
Ladder size (bp)75, 200, 300, 400, 500, 700, 1000, 1500, 2000, 3000, 4000, 5000, 7000, 10000, 20000
Quantification using: Lower Marker          Final concentration (ng/uL): 0.5000          Dilution factor: 12.0
```

Sample: SampG4
Well location: G4
Created: Wednesday, August 11, 2021 6:14:43 PM

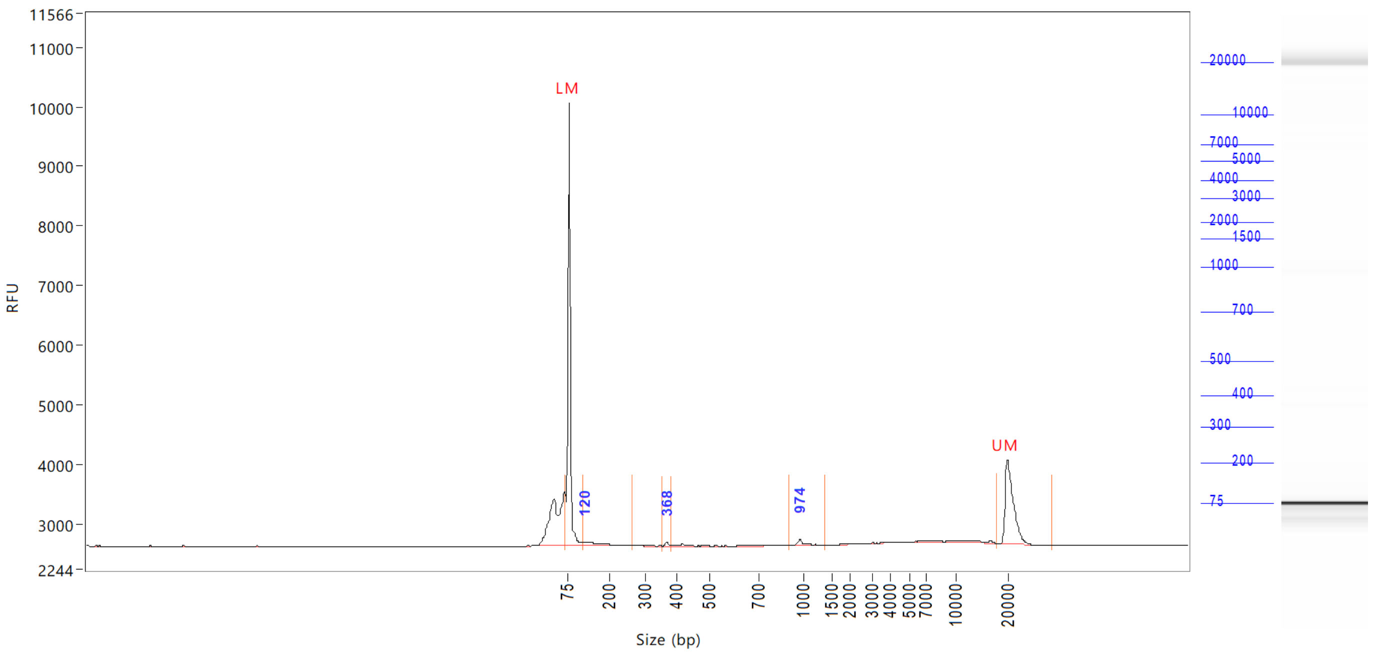

Peak	Size (bp)	Concentration (ng/uL)	From (bp)	To (bp)	Average size (bp)	CV%	RFU	Corrected peak area
1	75 (LM)	0.5000	66	114	74	8.11	7436	44.218
2	120	0.3434	114	264	158	20.82	59	2.530
3	368	0.0640	354	382	366	1.54	72	0.472
4	974	0.1586	900	1367	1012	8.52	96	1.169
5	20000 (UM)	0.1661	17927	28584	20571	4.75	1416	14.690

TIC:	0.5660	ng/uL
TIM:	4.1176	nmole/L
Total concentration:	1.0136	ng/uL

Sample peak width (sec): 5 Sample min peak height: 50 Sample baseline V to V?: Y Sample baseline V to V points: 3
Sample filter: Binomial Number of points for filter: 3 Sample start region (min): 0 Sample end region (min): 30
Marker peak width (sec): 5 Marker min peak height: 200 Marker baseline V to V?: Y Marker baseline V to V points: 3
Lower marker selection: First peak > 500 RFU Upper marker selection: Last peak > 200 RFU
Ladder size (bp)75, 200, 300, 400, 500, 700, 1000, 1500, 2000, 3000, 4000, 5000, 7000, 10000, 20000
Quantification using: Lower Marker Final concentration (ng/uL): 0.5000 Dilution factor: 12.0

Sample: SampH4
Well location: H4
Created: Wednesday, August 11, 2021 6:14:43 PM

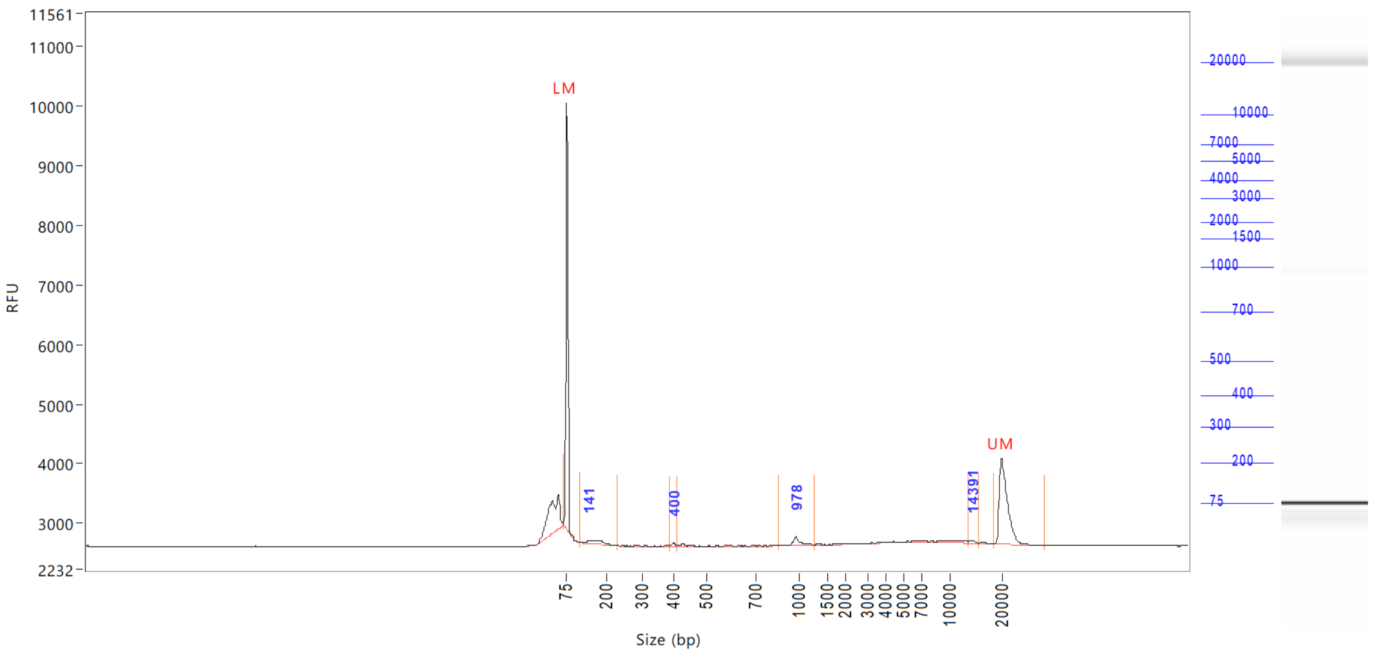

Peak	Size (bp)	Concentration (ng/uL)	From (bp)	To (bp)	Average size (bp)	CV%	RFU	Corrected peak area
1	75 (LM)	0.5000	66	116	73	4.26	7169	36.991
2	141	0.3340	116	230	162	13.09	55	2.059
3	400	0.0670	386	411	397	1.25	65	0.413
4	978	0.2701	857	1256	1003	6.77	160	1.665
5	14391	0.0554	13537	15488	14246	3.16	52	0.342
6	20000 (UM)	0.2009	18415	28463	20516	4.92	1439	14.865

TIC:	0.7265	ng/uL
TIM:	4.1162	nmole/L
Total concentration:	1.3294	ng/uL

```
Sample peak width (sec): 5    Sample min peak height: 50    Sample baseline V to V?: Y    Sample baseline V to V points: 3
Sample filter: Binomial       Number of points for filter: 3  Sample start region (min): 0  Sample end region (min): 30
Marker peak width (sec): 5    Marker min peak height: 200   Marker baseline V to V?: Y    Marker baseline V to V points: 3
Lower marker selection: First peak > 500 RFU                Upper marker selection: Last peak > 200 RFU
Ladder size (bp)75, 200, 300, 400, 500, 700, 1000, 1500, 2000, 3000, 4000, 5000, 7000, 10000, 20000
Quantification using: Lower Marker          Final concentration (ng/uL): 0.5000         Dilution factor: 12.0
```

Sample: SampA5
Well location: A5
Created: Wednesday, August 11, 2021 6:14:43 PM

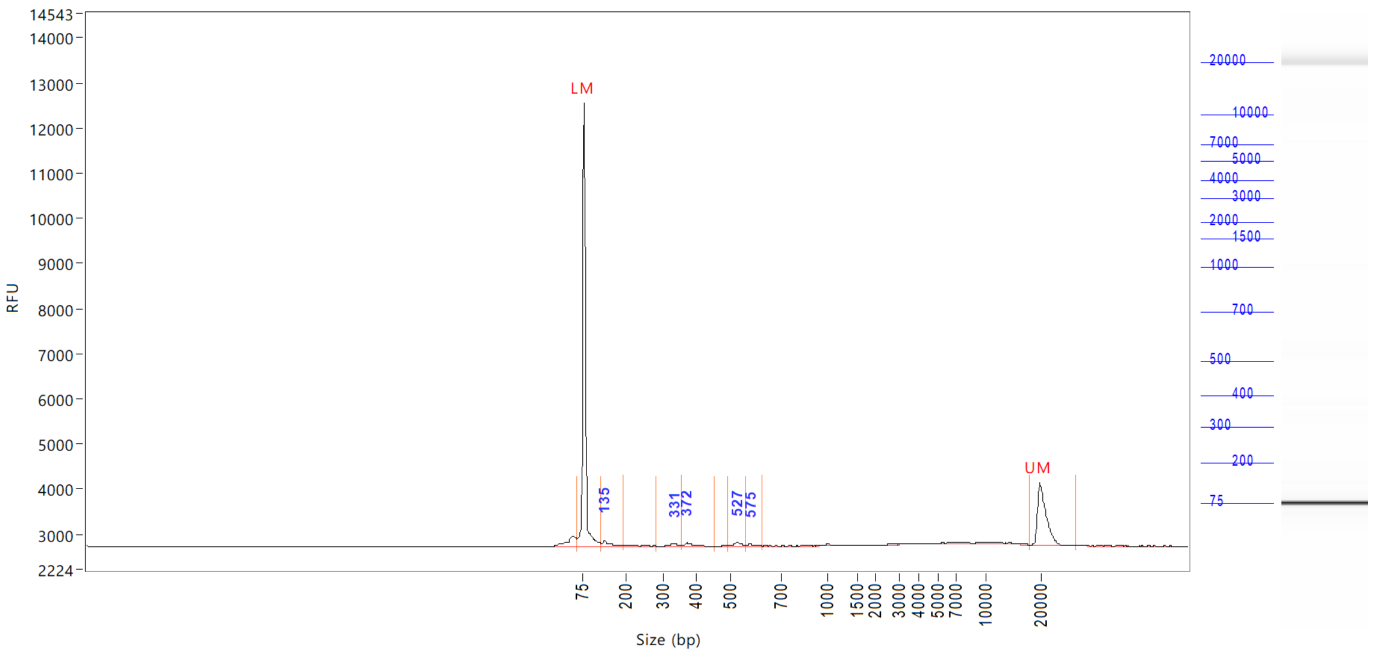

Peak	Size (bp)	Concentration (ng/uL)	From (bp)	To (bp)	Average size (bp)	CV%	RFU	Corrected peak area
1	75 (LM)	0.5000	56	126	75	10.94	9843	61.820
2	135	0.2103	126	192	149	12.89	136	2.166
3	331	0.0926	282	358	328	4.32	52	0.954
4	372	0.0973	358	452	382	4.86	75	1.002
5	527	0.1145	490	562	525	3.14	81	1.180
6	575	0.0500	562	623	581	2.41	52	0.515
7	20000 (UM)	0.1150	18171	26649	20543	4.56	1377	14.220

TIC:	0.5646	ng/uL
TIM:	3.7032	nmole/L
Total concentration:	0.9507	ng/uL

```
Sample peak width (sec): 5    Sample min peak height: 50    Sample baseline V to V?: Y    Sample baseline V to V points: 3
Sample filter: Binomial       Number of points for filter: 3  Sample start region (min): 0  Sample end region (min): 30
Marker peak width (sec): 5    Marker min peak height: 200   Marker baseline V to V?: Y    Marker baseline V to V points: 3
Lower marker selection: First peak > 500 RFU                Upper marker selection: Last peak > 200 RFU
Ladder size (bp)75, 200, 300, 400, 500, 700, 1000, 1500, 2000, 3000, 4000, 5000, 7000, 10000, 20000
Quantification using: Lower Marker          Final concentration (ng/uL): 0.5000          Dilution factor: 12.0
```

Sample: SampB5
Well location: B5
Created: Wednesday, August 11, 2021 6:14:43 PM

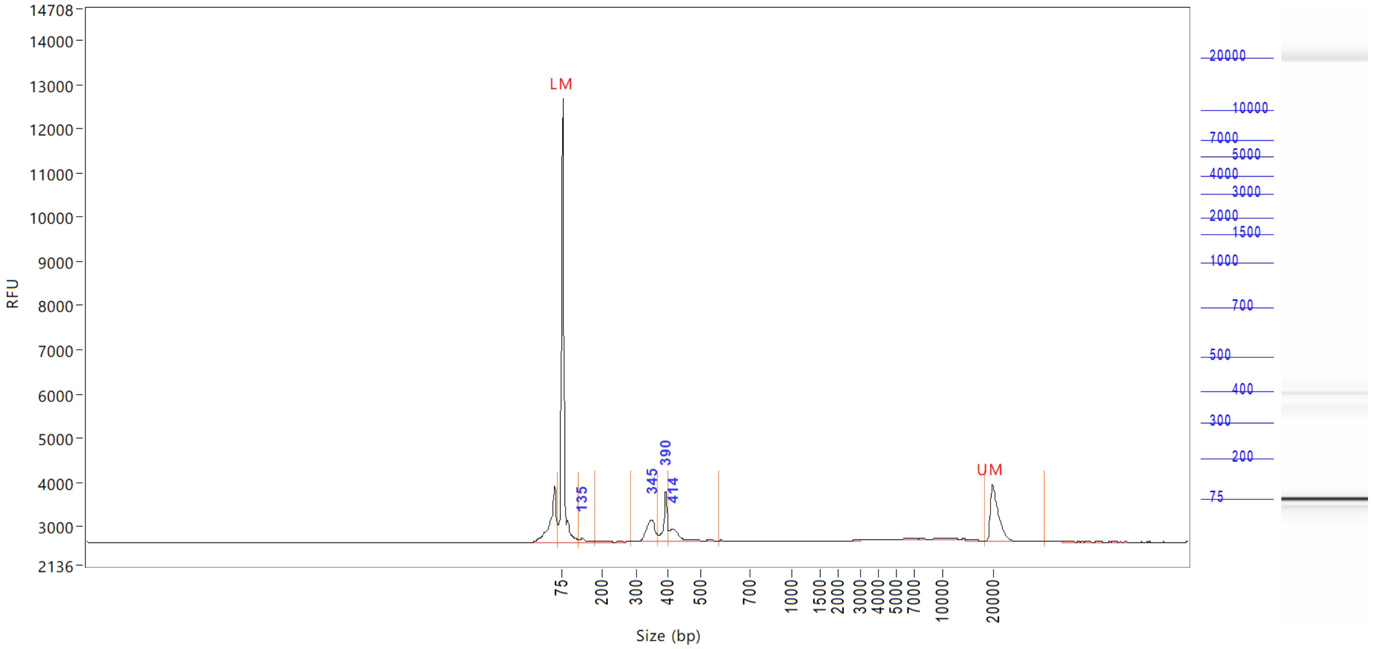

Peak	Size (bp)	Concentration (ng/uL)	From (bp)	To (bp)	Average size (bp)	CV%	RFU	Corrected peak area
1	75 (LM)	0.5000	62	128	75	10.76	10044	64.463
2	135	0.1224	128	175	143	8.75	97	1.316
3	345	0.8148	282	366	339	4.12	480	8.754
4	390	0.7747	366	401	387	2.07	1124	8.323
5	414	0.5978	401	572	438	9.26	284	6.423
6	20000 (UM)	0.1034	18537	30277	20560	5.14	1286	13.327

TIC:	2.3097	ng/uL
TIM:	10.9004	nmole/L
Total concentration:	2.6418	ng/uL

```
Sample peak width (sec): 5    Sample min peak height: 50    Sample baseline V to V?: Y    Sample baseline V to V points: 3
Sample filter: Binomial       Number of points for filter: 3  Sample start region (min): 0  Sample end region (min): 30
Marker peak width (sec): 5    Marker min peak height: 200   Marker baseline V to V?: Y    Marker baseline V to V points: 3
Lower marker selection: First peak > 500 RFU                Upper marker selection: Last peak > 200 RFU
Ladder size (bp)75, 200, 300, 400, 500, 700, 1000, 1500, 2000, 3000, 4000, 5000, 7000, 10000, 20000
Quantification using: Lower Marker          Final concentration (ng/uL): 0.5000          Dilution factor: 12.0
```

Sample: SampC5
Well location: C5
Created: Wednesday, August 11, 2021 6:14:43 PM

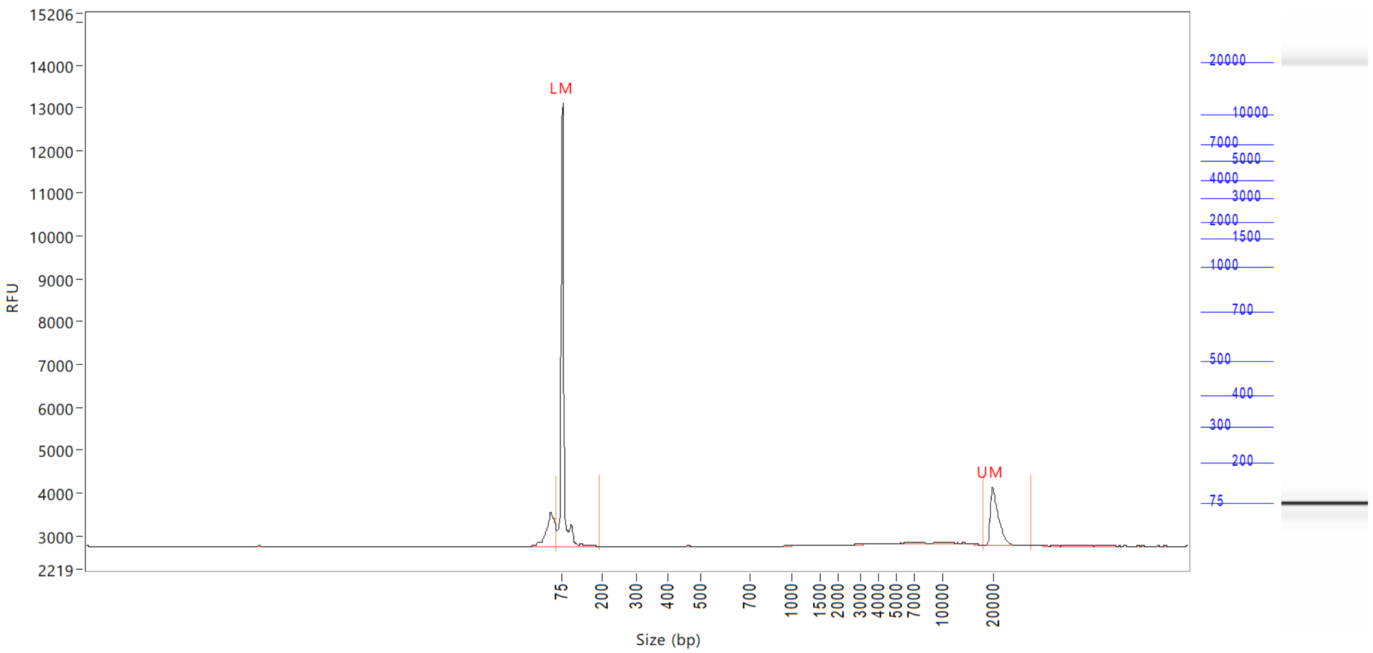

Peak	Size (bp)	Concentration (ng/uL)	From (bp)	To (bp)	Average size (bp)	CV%	RFU	Corrected peak area
1	75 (LM)	0.5000	58	189	75	14.21	10378	70.929
2	20000 (UM)	0.1004	18171	27617	20552	4.93	1362	14.239

TIC:	0.0000	ng/uL
TIM:	0.0000	nmole/L
Total concentration:	0.2632	ng/uL

```
Sample peak width (sec): 5    Sample min peak height: 50    Sample baseline V to V?: Y    Sample baseline V to V points: 3
Sample filter: Binomial       Number of points for filter: 3  Sample start region (min): 0  Sample end region (min): 30
Marker peak width (sec): 5    Marker min peak height: 200   Marker baseline V to V?: Y    Marker baseline V to V points: 3
Lower marker selection: First peak > 500 RFU                Upper marker selection: Last peak > 200 RFU
Ladder size (bp)75, 200, 300, 400, 500, 700, 1000, 1500, 2000, 3000, 4000, 5000, 7000, 10000, 20000
Quantification using: Lower Marker          Final concentration (ng/uL): 0.5000          Dilution factor: 12.0
```

Sample: SampD5
Well location: D5
Created: Wednesday, August 11, 2021 6:14:43 PM

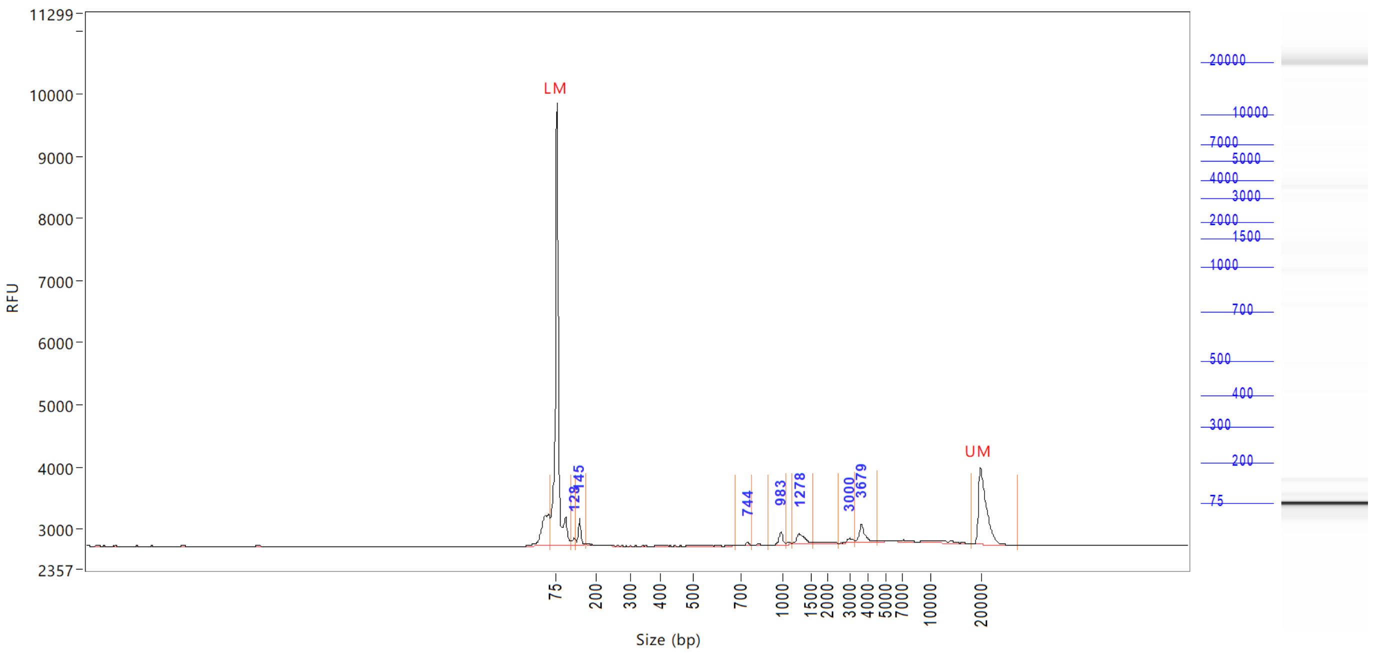

Peak	Size (bp)	Concentration (ng/uL)	From (bp)	To (bp)	Average size (bp)	CV%	RFU	Corrected peak area
1	75 (LM)	0.5000	54	118	74	13.22	7127	54.910
2	128	0.1046	118	135	126	4.02	105	0.957
3	145	0.2766	135	164	143	2.63	431	2.532
4	744	0.0500	674	775	732	2.76	62	0.458
5	983	0.1479	896	1034	978	1.84	205	1.353
6	1278	0.2475	1145	1539	1308	6.01	168	2.265
7	3000	0.0783	2515	3322	3020	5.03	73	0.717
8	3679	0.2827	3322	4518	3732	6.34	280	2.587
9	20000 (UM)	0.1156	18293	27496	20613	4.91	1225	12.696

TIC:	1.1876	ng/uL
TIM:	5.3834	nmole/L
Total concentration:	1.6256	ng/uL

Sample peak width (sec): 5   Sample min peak height: 50   Sample baseline V to V?: Y   Sample baseline V to V points: 3
Sample filter: Binomial   Number of points for filter: 3   Sample start region (min): 0   Sample end region (min): 30
Marker peak width (sec): 5   Marker min peak height: 200   Marker baseline V to V?: Y   Marker baseline V to V points: 3
Lower marker selection: First peak > 500 RFU   Upper marker selection: Last peak > 200 RFU
Ladder size (bp)75, 200, 300, 400, 500, 700, 1000, 1500, 2000, 3000, 4000, 5000, 7000, 10000, 20000
Quantification using: Lower Marker   Final concentration (ng/uL): 0.5000   Dilution factor: 12.0

Sample: SampE5
Well location: E5
Created: Wednesday, August 11, 2021 6:14:43 PM

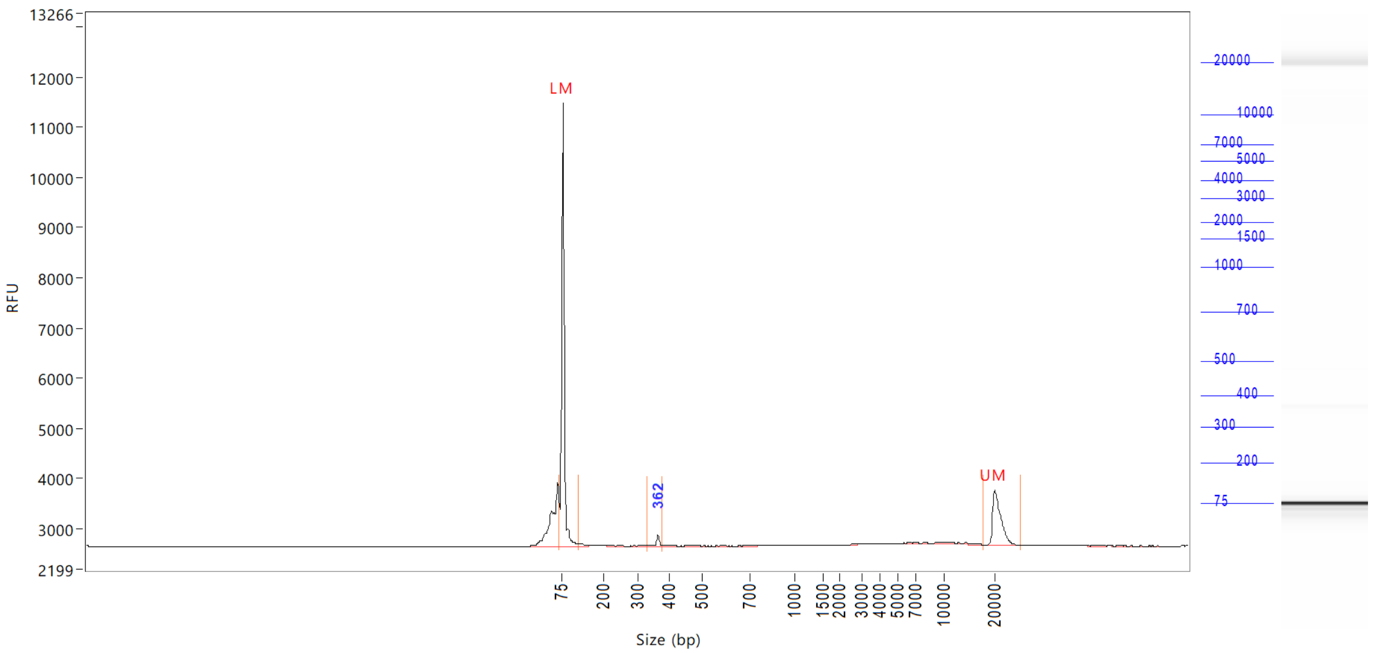

Peak	Size (bp)	Concentration (ng/uL)	From (bp)	To (bp)	Average size (bp)	CV%	RFU	Corrected peak area
1	75 (LM)	0.5000	64	124	74	9.22	8837	57.503
2	362	0.1520	331	376	358	2.11	231	1.456
3	20000 (UM)	0.0998	17561	25078	20498	4.59	1099	11.472

TIC:	0.1520	ng/uL
TIM:	0.6983	nmole/L
Total concentration:	0.6186	ng/uL

```
Sample peak width (sec): 5    Sample min peak height: 50    Sample baseline V to V?: Y    Sample baseline V to V points: 3
Sample filter: Binomial       Number of points for filter: 3  Sample start region (min): 0  Sample end region (min): 30
Marker peak width (sec): 5    Marker min peak height: 200   Marker baseline V to V?: Y    Marker baseline V to V points: 3
Lower marker selection: First peak > 500 RFU                Upper marker selection: Last peak > 200 RFU
Ladder size (bp)75, 200, 300, 400, 500, 700, 1000, 1500, 2000, 3000, 4000, 5000, 7000, 10000, 20000
Quantification using: Lower Marker          Final concentration (ng/uL): 0.5000          Dilution factor: 12.0
```

Sample: SampF5
Well location: F5
Created: Wednesday, August 11, 2021 6:14:43 PM

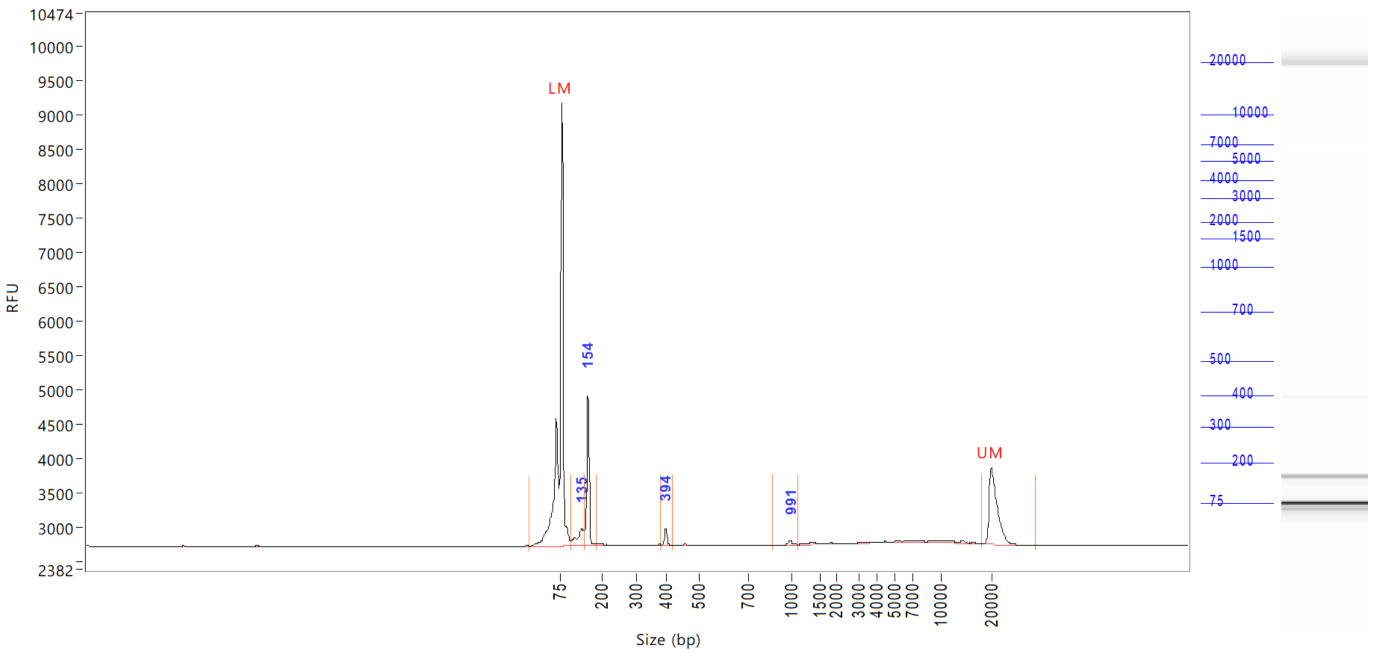

Peak	Size (bp)	Concentration (ng/uL)	From (bp)	To (bp)	Average size (bp)	CV%	RFU	Corrected peak area
1	75 (LM)	0.5000	0	103	64	26.18	6461	62.127
2	135	0.4138	103	145	128	9.45	239	4.285
3	154	1.2561	145	181	153	3.02	2195	13.007
4	394	0.1460	380	416	392	1.56	253	1.511
5	991	0.0496	866	1112	984	3.60	62	0.513
6	20000 (UM)	0.0945	18171	28584	20539	4.95	1127	11.741

TIC:	1.8655	ng/uL
TIM:	19.5012	nmole/L
Total concentration:	2.2798	ng/uL

```
Sample peak width (sec): 5    Sample min peak height: 50    Sample baseline V to V?: Y    Sample baseline V to V points: 3
Sample filter: Binomial       Number of points for filter: 3  Sample start region (min): 0  Sample end region (min): 30
Marker peak width (sec): 5    Marker min peak height: 200   Marker baseline V to V?: Y    Marker baseline V to V points: 3
Lower marker selection: First peak > 500 RFU                Upper marker selection: Last peak > 200 RFU
Ladder size (bp)75, 200, 300, 400, 500, 700, 1000, 1500, 2000, 3000, 4000, 5000, 7000, 10000, 20000
Quantification using: Lower Marker          Final concentration (ng/uL): 0.5000          Dilution factor: 12.0
```

Sample: SampG5
Well location: G5
Created: Wednesday, August 11, 2021 6:14:43 PM

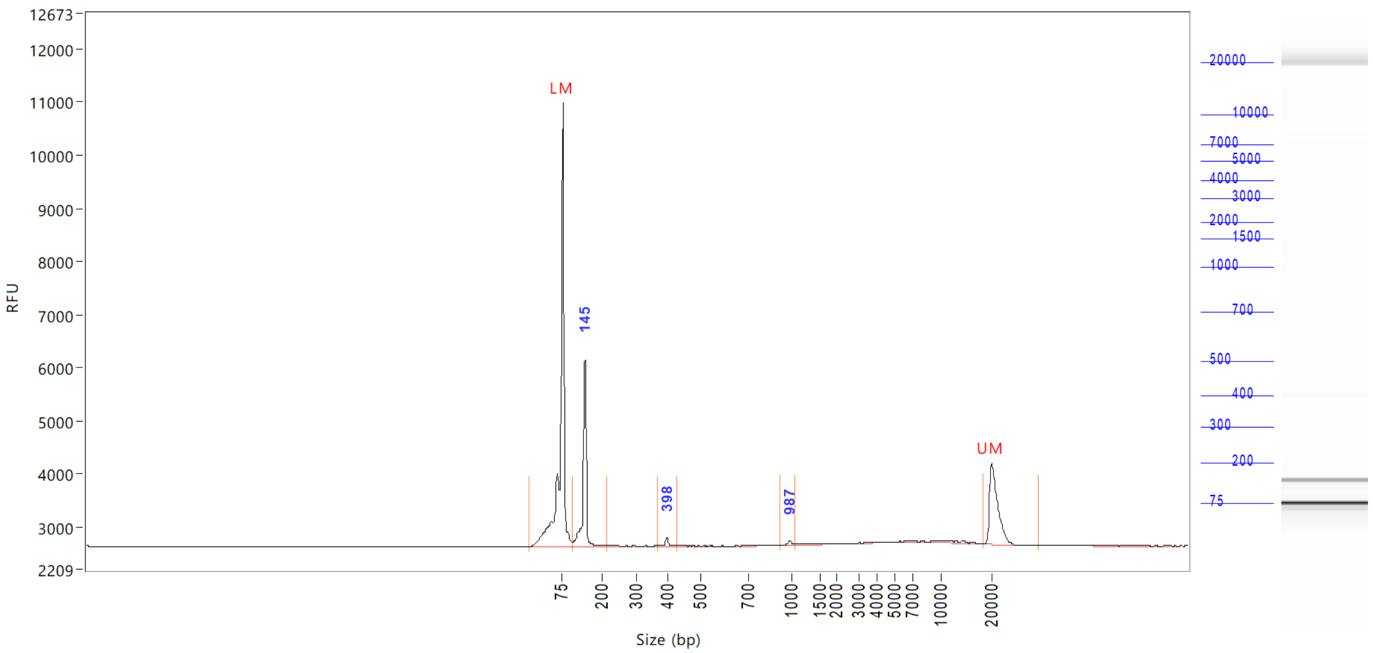

Peak	Size	Concentration	From	To	Average size	CV%	RFU	Corrected peak area
	(bp)	(ng/uL)	(bp)	(bp)	(bp)			
1	75 (LM)	0.5000	0	105	63	31.15	8363	75.603
2	145	2.0483	105	212	140	9.18	3515	25.810
3	398	0.0939	366	430	396	2.63	160	1.183
4	987	0.0539	918	1045	980	2.40	82	0.679
5	20000 (UM)	0.1087	18293	29189	20656	5.09	1534	16.440

TIC:	2.1961	ng/uL
TIM:	24.5237	nmole/L
Total concentration:	2.6067	ng/uL

```
Sample peak width (sec): 5    Sample min peak height: 50    Sample baseline V to V?: Y    Sample baseline V to V points: 3
Sample filter: Binomial       Number of points for filter: 3  Sample start region (min): 0  Sample end region (min): 30
Marker peak width (sec): 5    Marker min peak height: 200   Marker baseline V to V?: Y    Marker baseline V to V points: 3
Lower marker selection: First peak > 500 RFU                Upper marker selection: Last peak > 200 RFU
Ladder size (bp)75, 200, 300, 400, 500, 700, 1000, 1500, 2000, 3000, 4000, 5000, 7000, 10000, 20000
Quantification using: Lower Marker          Final concentration (ng/uL): 0.5000          Dilution factor: 12.0
```

Sample: SampH5
Well location: H5
Created: Wednesday, August 11, 2021 6:14:43 PM

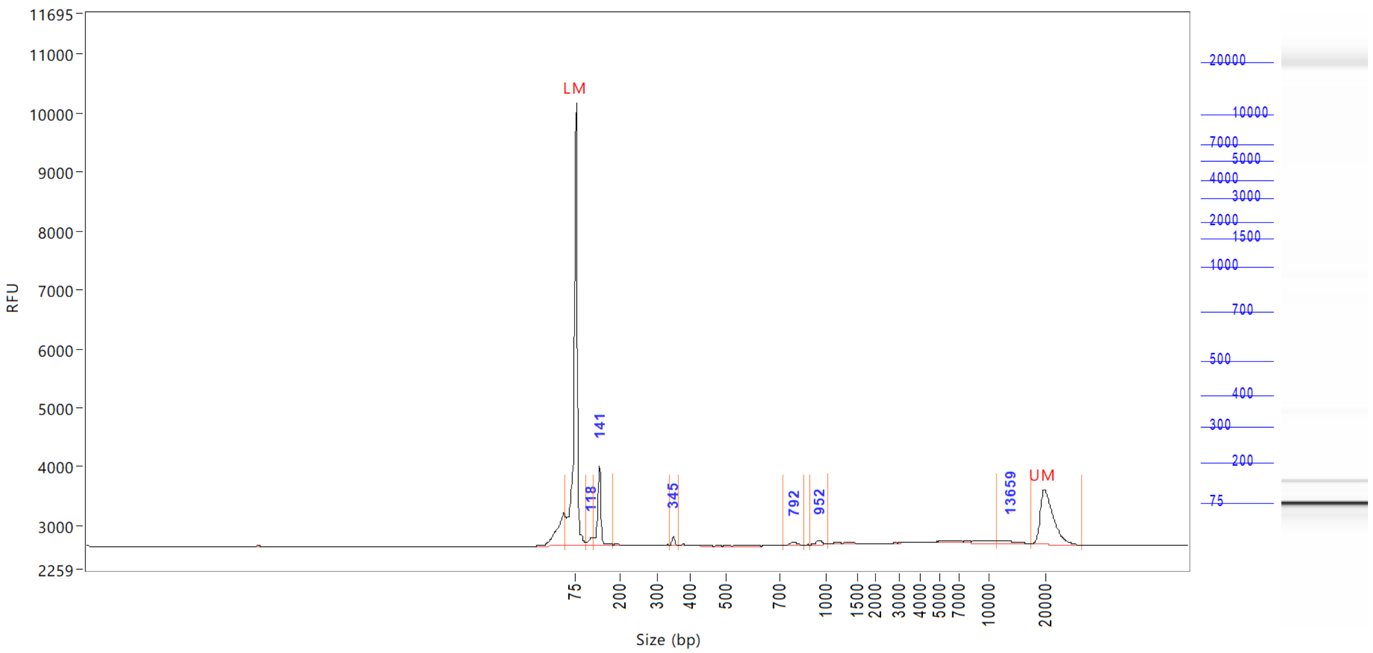

Peak	Size (bp)	Concentration (ng/uL)	From (bp)	To (bp)	Average size (bp)	CV%	RFU	Corrected peak area
1	75 (LM)	0.5000	47	105	70	10.75	7522	59.611
2	118	0.1494	105	124	115	4.84	128	1.484
3	141	0.9308	124	179	138	4.25	1337	9.248
4	345	0.1013	335	362	345	1.39	171	1.006
5	792	0.0646	718	848	779	3.14	54	0.641
6	952	0.0723	892	1012	950	2.34	64	0.718
7	13659	0.1204	11220	17561	13619	11.85	52	1.196
8	20000 (UM)	0.1238	17561	26891	20603	6.36	932	14.762

TIC:	1.4387	ng/uL
TIM:	13.9768	nmole/L
Total concentration:	1.9163	ng/uL

```
Sample peak width (sec): 5    Sample min peak height: 50    Sample baseline V to V?: Y    Sample baseline V to V points: 3
Sample filter: Binomial       Number of points for filter: 3  Sample start region (min): 0  Sample end region (min): 30
Marker peak width (sec): 5    Marker min peak height: 200   Marker baseline V to V?: Y    Marker baseline V to V points: 3
Lower marker selection: First peak > 500 RFU                Upper marker selection: Last peak > 200 RFU
Ladder size (bp)75, 200, 300, 400, 500, 700, 1000, 1500, 2000, 3000, 4000, 5000, 7000, 10000, 20000
Quantification using: Lower Marker          Final concentration (ng/uL): 0.5000          Dilution factor: 12.0
```

Sample: SampA6
Well location: A6
Created: Wednesday, August 11, 2021 6:14:43 PM

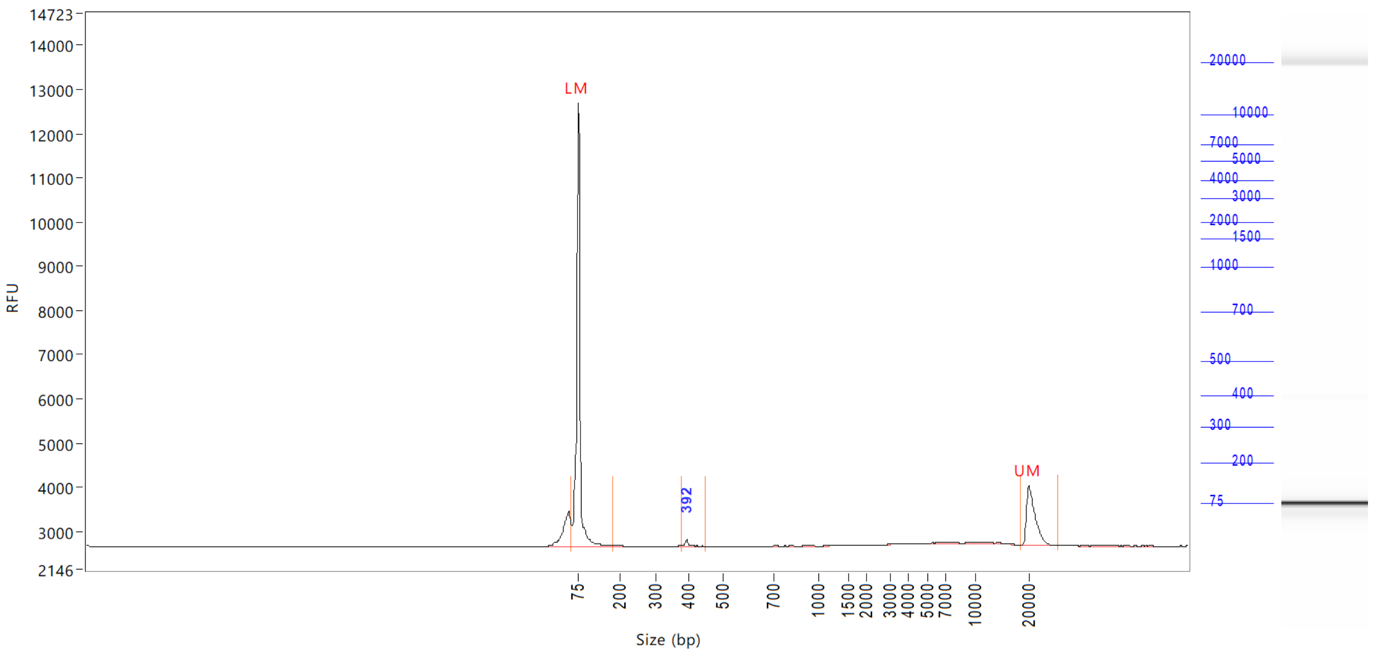

Peak	Size (bp)	Concentration (ng/uL)	From (bp)	To (bp)	Average size (bp)	CV%	RFU	Corrected peak area
1	75 (LM)	0.5000	56	177	74	15.91	10053	80.278
2	392	0.0813	378	447	395	3.18	155	1.088
3	20000 (UM)	0.0874	18537	25561	20573	4.55	1359	14.027

TIC:	0.0813	ng/uL
TIM:	0.3388	nmole/L
Total concentration:	0.3050	ng/uL

Sample peak width (sec): 5 Sample min peak height: 50 Sample baseline V to V?: Y Sample baseline V to V points: 3
Sample filter: Binomial Number of points for filter: 3 Sample start region (min): 0 Sample end region (min): 30
Marker peak width (sec): 5 Marker min peak height: 200 Marker baseline V to V?: Y Marker baseline V to V points: 3
Lower marker selection: First peak > 500 RFU Upper marker selection: Last peak > 200 RFU
Ladder size (bp)75, 200, 300, 400, 500, 700, 1000, 1500, 2000, 3000, 4000, 5000, 7000, 10000, 20000
Quantification using: Lower Marker Final concentration (ng/uL): 0.5000 Dilution factor: 12.0

Sample: SampB6
Well location: B6
Created: Wednesday, August 11, 2021 6:14:43 PM

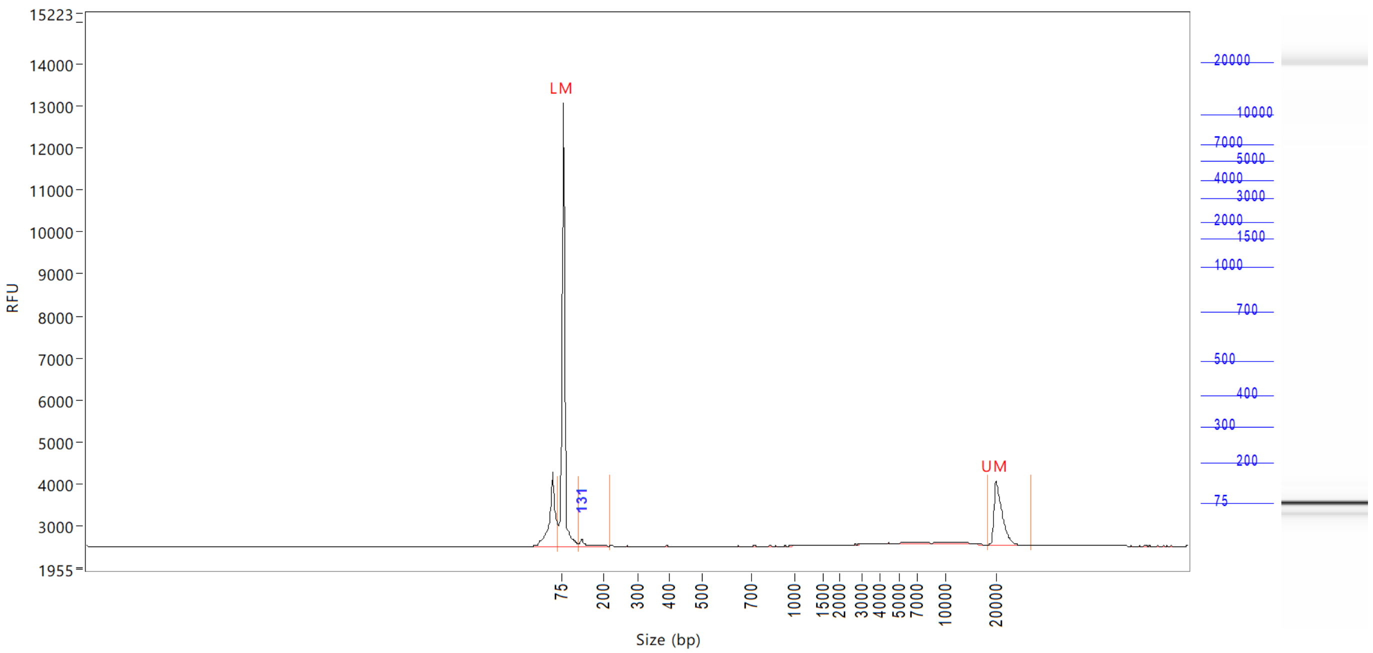

Peak	Size (bp)	Concentration (ng/uL)	From (bp)	To (bp)	Average size (bp)	CV%	RFU	Corrected peak area
1	75 (LM)	0.5000	60	122	75	9.99	10601	75.469
2	131	0.1679	122	217	142	14.03	168	2.112
3	20000 (UM)	0.1041	18171	26891	20562	4.86	1511	15.720

TIC:	0.1679	ng/uL
TIM:	1.9433	nmole/L
Total concentration:	0.4752	ng/uL

```
Sample peak width (sec): 5    Sample min peak height: 50    Sample baseline V to V?: Y    Sample baseline V to V points: 3
Sample filter: Binomial       Number of points for filter: 3  Sample start region (min): 0  Sample end region (min): 30
Marker peak width (sec): 5    Marker min peak height: 200   Marker baseline V to V?: Y    Marker baseline V to V points: 3
Lower marker selection: First peak > 500 RFU                Upper marker selection: Last peak > 200 RFU
Ladder size (bp)75, 200, 300, 400, 500, 700, 1000, 1500, 2000, 3000, 4000, 5000, 7000, 10000, 20000
Quantification using: Lower Marker          Final concentration (ng/uL): 0.5000          Dilution factor: 12.0
```

Sample: SampC6
Well location: C6
Created: Wednesday, August 11, 2021 6:14:43 PM

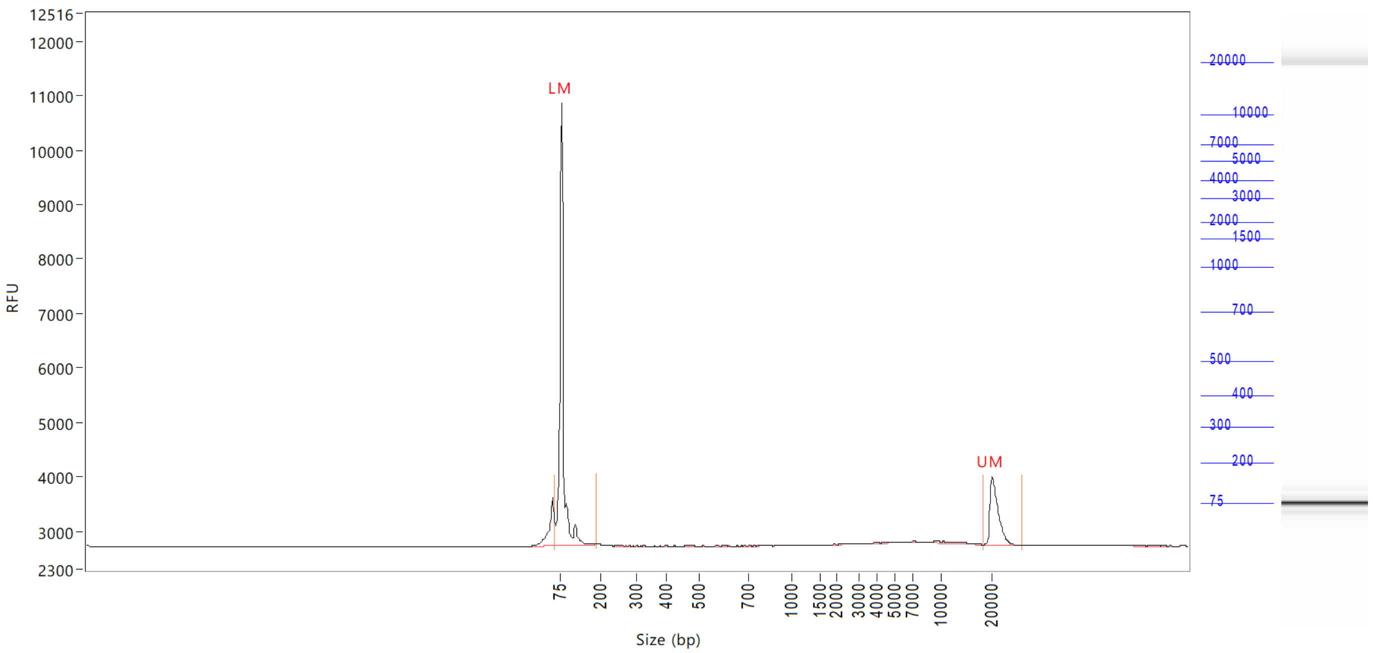

Peak	Size	Concentration	From	To	Average size	CV%	RFU	Corrected peak area
	(bp)	(ng/uL)	(bp)	(bp)	(bp)			
1	75 (LM)	0.5000	58	187	77	17.86	8146	70.291
2	20000 (UM)	0.0958	18171	26045	20589	4.72	1272	13.470

TIC:	0.0000	ng/uL
TIM:	0.0000	nmole/L
Total concentration:	0.3465	ng/uL

```
Sample peak width (sec): 5    Sample min peak height: 50    Sample baseline V to V?: Y    Sample baseline V to V points: 3
Sample filter: Binomial       Number of points for filter: 3  Sample start region (min): 0  Sample end region (min): 30
Marker peak width (sec): 5    Marker min peak height: 200   Marker baseline V to V?: Y    Marker baseline V to V points: 3
Lower marker selection: First peak > 500 RFU                Upper marker selection: Last peak > 200 RFU
Ladder size (bp)75, 200, 300, 400, 500, 700, 1000, 1500, 2000, 3000, 4000, 5000, 7000, 10000, 20000
Quantification using: Lower Marker          Final concentration (ng/uL): 0.5000          Dilution factor: 12.0
```

Sample: SampD6
Well location: D6
Created: Wednesday, August 11, 2021 6:14:43 PM

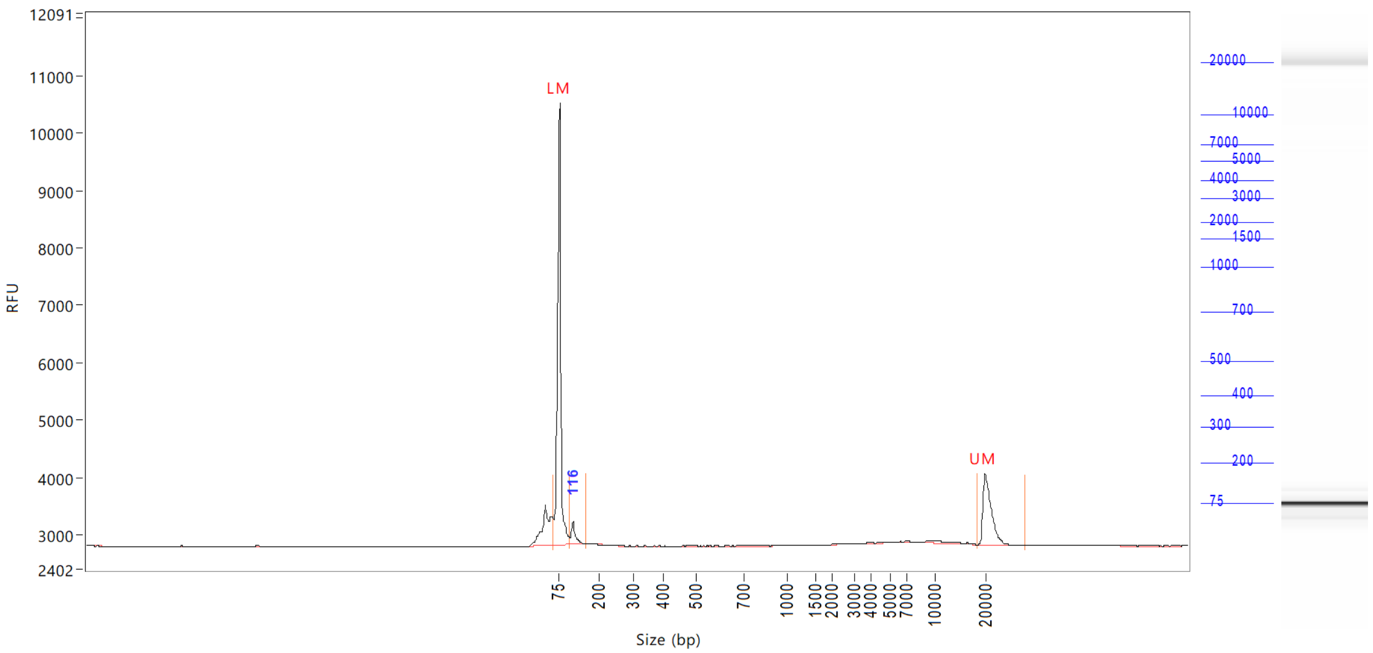

Peak	Size (bp)	Concentration (ng/uL)	From (bp)	To (bp)	Average size (bp)	CV%	RFU	Corrected peak area
1	75 (LM)	0.5000	58	107	73	9.60	7714	60.133
2	116	0.3589	107	156	117	6.69	384	3.597
3	20000 (UM)	0.1095	18293	27979	20570	4.90	1251	13.173

TIC:	0.3589	ng/uL
TIM:	5.0390	nmole/L
Total concentration:	0.7647	ng/uL

Sample peak width (sec): 5   Sample min peak height: 50   Sample baseline V to V?: Y   Sample baseline V to V points: 3
Sample filter: Binomial   Number of points for filter: 3 Sample start region (min): 0 Sample end region (min): 30
Marker peak width (sec): 5   Marker min peak height: 200   Marker baseline V to V?: Y   Marker baseline V to V points: 3
Lower marker selection: First peak > 500 RFU   Upper marker selection: Last peak > 200 RFU
Ladder size (bp)75, 200, 300, 400, 500, 700, 1000, 1500, 2000, 3000, 4000, 5000, 7000, 10000, 20000
Quantification using: Lower Marker   Final concentration (ng/uL): 0.5000   Dilution factor: 12.0

Sample: SampE6
Well location: E6
Created: Wednesday, August 11, 2021 6:14:43 PM

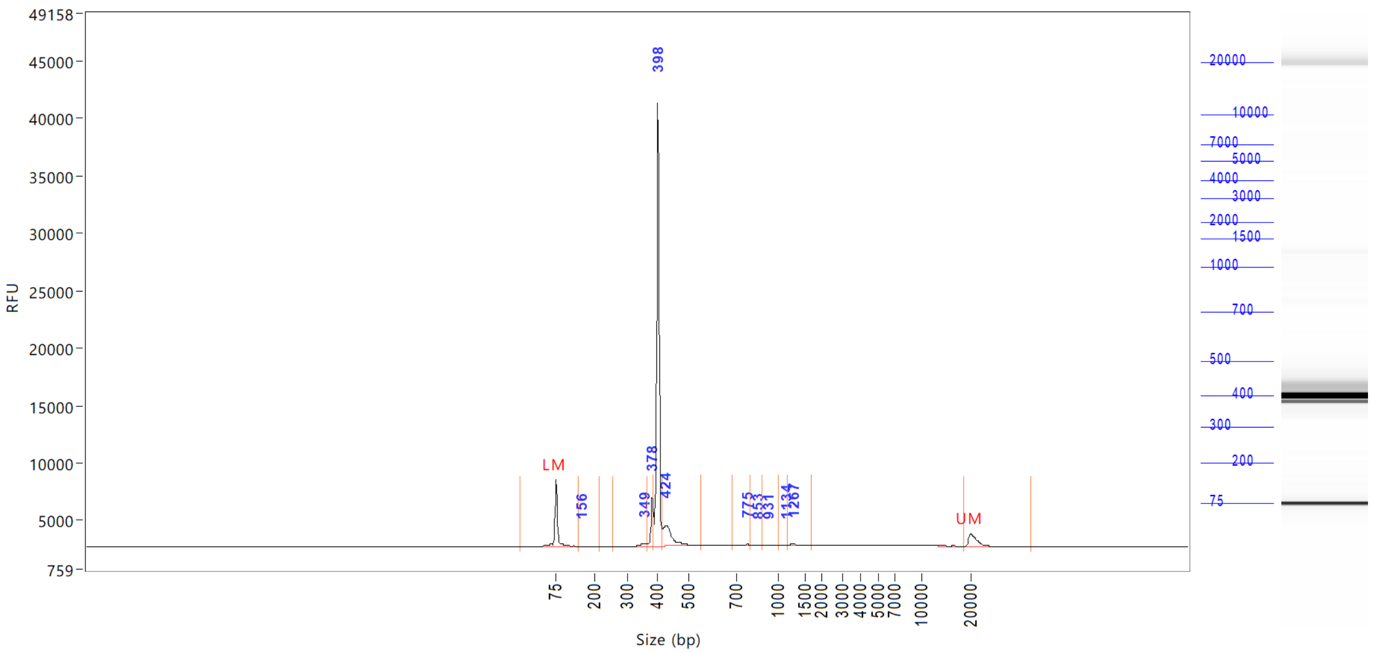

Peak	Size (bp)	Concentration (ng/uL)	From (bp)	To (bp)	Average size (bp)	CV%	RFU	Corrected peak area
1	75 (LM)	0.5000	0	145	72	22.64	5901	42.501
2	156	0.1801	145	210	168	9.58	59	1.275
3	349	0.5310	255	362	343	4.16	250	3.761
4	378	3.5236	362	386	378	1.35	4199	24.959
5	398	28.7647	386	411	396	1.07	38647	203.753
6	424	4.9189	411	547	434	4.98	1797	34.843
7	775	0.1283	682	801	760	2.64	143	0.909
8	853	0.0714	801	879	848	2.16	58	0.506
9	931	0.1941	879	1001	942	3.69	66	1.375
10	1134	0.1271	1001	1178	1096	4.79	76	0.900
11	1267	0.2774	1178	1693	1275	5.90	217	1.965
12	20000 (UM)	0.1352	18415	32332	20647	6.67	1107	11.491

TIC:	38.7165	ng/uL
TIM:	159.1080	nmole/L
Total concentration:	38.5522	ng/uL

Sample peak width (sec): 5   Sample min peak height: 50   Sample baseline V to V?: Y   Sample baseline V to V points: 3
Sample filter: Binomial   Number of points for filter: 3 Sample start region (min): 0 Sample end region (min): 30
Marker peak width (sec): 5   Marker min peak height: 200   Marker baseline V to V?: Y   Marker baseline V to V points: 3
Lower marker selection: First peak > 500 RFU   Upper marker selection: Last peak > 200 RFU
Ladder size (bp)75, 200, 300, 400, 500, 700, 1000, 1500, 2000, 3000, 4000, 5000, 7000, 10000, 20000
Quantification using: Lower Marker   Final concentration (ng/uL): 0.5000   Dilution factor: 12.0

Sample: SampF6
Well location: F6
Created: Wednesday, August 11, 2021 6:14:43 PM

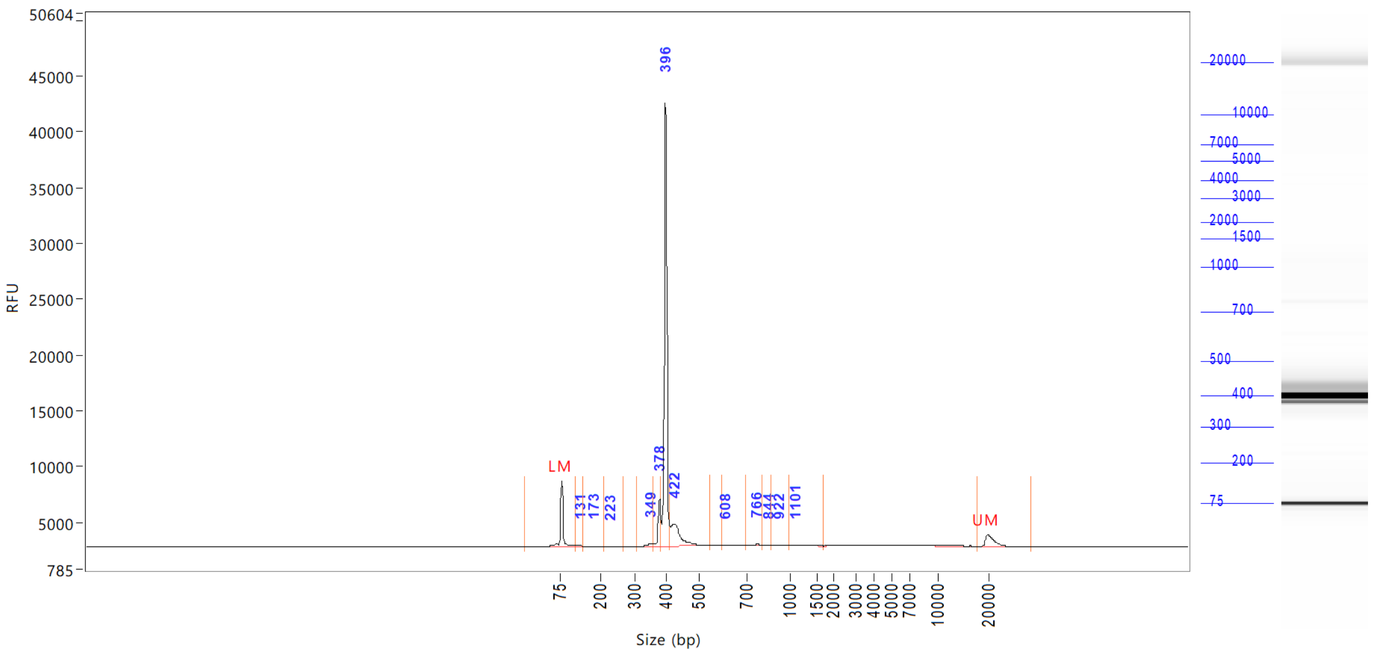

Peak	Size (bp)	Concentration (ng/uL)	From (bp)	To (bp)	Average size (bp)	CV%	RFU	Corrected peak area
1	75 (LM)	0.5000	0	120	71	19.29	5850	40.466
2	131	0.1752	120	143	130	5.01	95	1.182
3	173	0.2284	143	206	167	9.73	70	1.541
4	223	0.0788	206	264	226	5.48	52	0.532
5	349	0.5726	305	360	343	3.22	259	3.862
6	378	3.6790	360	384	376	1.34	4312	24.813
7	396	32.2773	384	411	395	1.15	39794	217.689
8	422	5.8154	411	544	434	4.87	1980	39.221
9	608	0.0511	593	695	616	3.07	54	0.344
10	766	0.2101	695	801	757	2.44	223	1.417
11	844	0.0876	801	870	839	2.15	59	0.591
12	922	0.2206	870	996	935	3.80	70	1.488
13	1101	0.2647	996	1674	1157	10.97	77	1.785
14	20000 (UM)	0.1410	18049	28584	20625	5.05	1064	11.410

TIC:	43.6610	ng/uL
TIM:	181.9024	nmole/L
Total concentration:	43.5300	ng/uL

Sample peak width (sec): 5 Sample min peak height: 50 Sample baseline V to V?: Y Sample baseline V to V points: 3
Sample filter: Binomial Number of points for filter: 3 Sample start region (min): 0 Sample end region (min): 30
Marker peak width (sec): 5 Marker min peak height: 200 Marker baseline V to V?: Y Marker baseline V to V points: 3
Lower marker selection: First peak > 500 RFU Upper marker selection: Last peak > 200 RFU
Ladder size (bp)75, 200, 300, 400, 500, 700, 1000, 1500, 2000, 3000, 4000, 5000, 7000, 10000, 20000
Quantification using: Lower Marker Final concentration (ng/uL): 0.5000 Dilution factor: 12.0

Sample: SampG6
Well location: G6
Created: Wednesday, August 11, 2021 6:14:43 PM

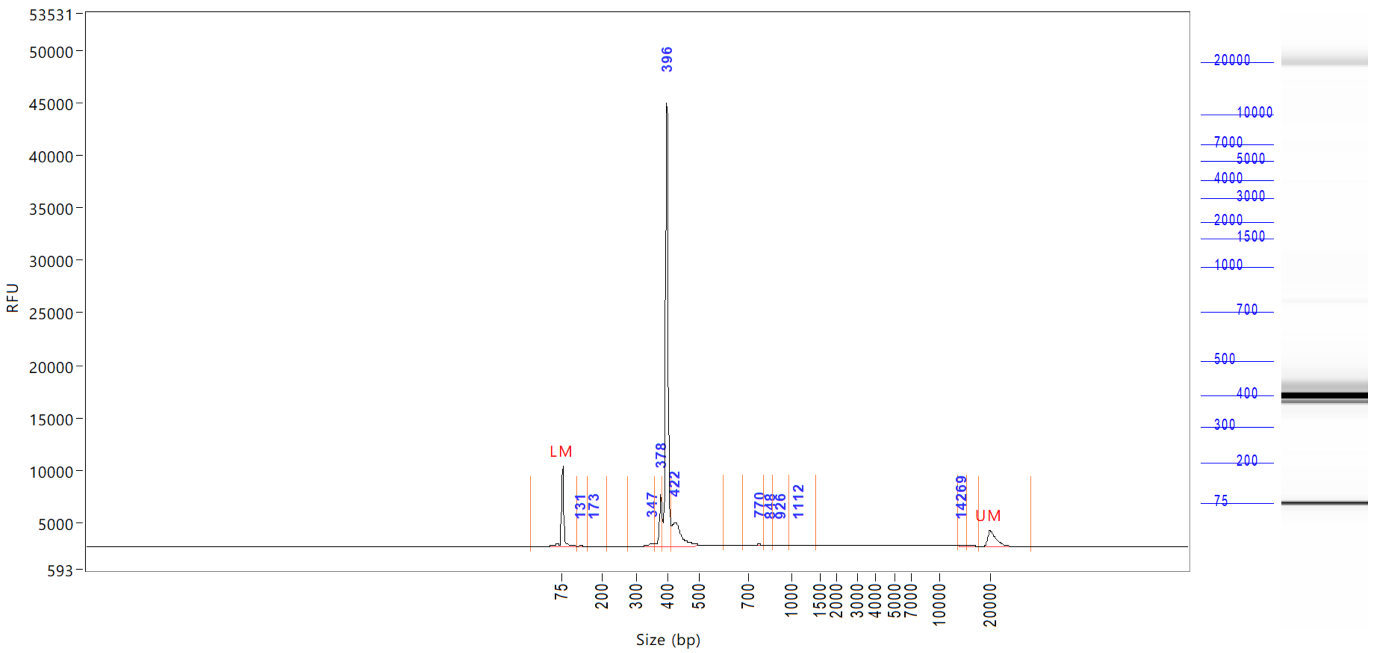

Peak	Size (bp)	Concentration (ng/uL)	From (bp)	To (bp)	Average size (bp)	CV%	RFU	Corrected peak area
1	75 (LM)	0.5000	0	122	72	17.24	7650	49.537
2	131	0.1693	122	154	135	7.01	91	1.398
3	173	0.1291	154	212	173	7.61	71	1.066
4	347	0.5414	275	360	342	3.52	295	4.470
5	378	3.4482	360	384	376	1.34	4941	28.469
6	396	28.4124	384	411	395	1.17	42294	234.579
7	422	5.4843	411	598	437	6.20	2233	45.279
8	770	0.1969	680	809	759	2.71	248	1.625
9	848	0.0784	809	874	845	2.01	71	0.647
10	926	0.1804	874	983	929	3.30	79	1.489
11	1112	0.2247	983	1433	1115	8.99	84	1.855
12	14269	0.0505	13537	15366	14260	3.34	53	0.417
13	20000 (UM)	0.1588	17683	28100	20529	4.96	1467	15.737

TIC:	38.9155	ng/uL
TIM:	161.2111	nmole/L
Total concentration:	38.7209	ng/uL

```
Sample peak width (sec): 5     Sample min peak height: 50    Sample baseline V to V?: Y    Sample baseline V to V points: 3
Sample filter: Binomial        Number of points for filter: 3  Sample start region (min): 0  Sample end region (min): 30
Marker peak width (sec): 5     Marker min peak height: 200   Marker baseline V to V?: Y    Marker baseline V to V points: 3
Lower marker selection: First peak > 500 RFU                 Upper marker selection: Last peak > 200 RFU
Ladder size (bp)75, 200, 300, 400, 500, 700, 1000, 1500, 2000, 3000, 4000, 5000, 7000, 10000, 20000
Quantification using: Lower Marker          Final concentration (ng/uL): 0.5000          Dilution factor: 12.0
```

Sample: SampH6
Well location: H6
Created: Wednesday, August 11, 2021 6:14:43 PM

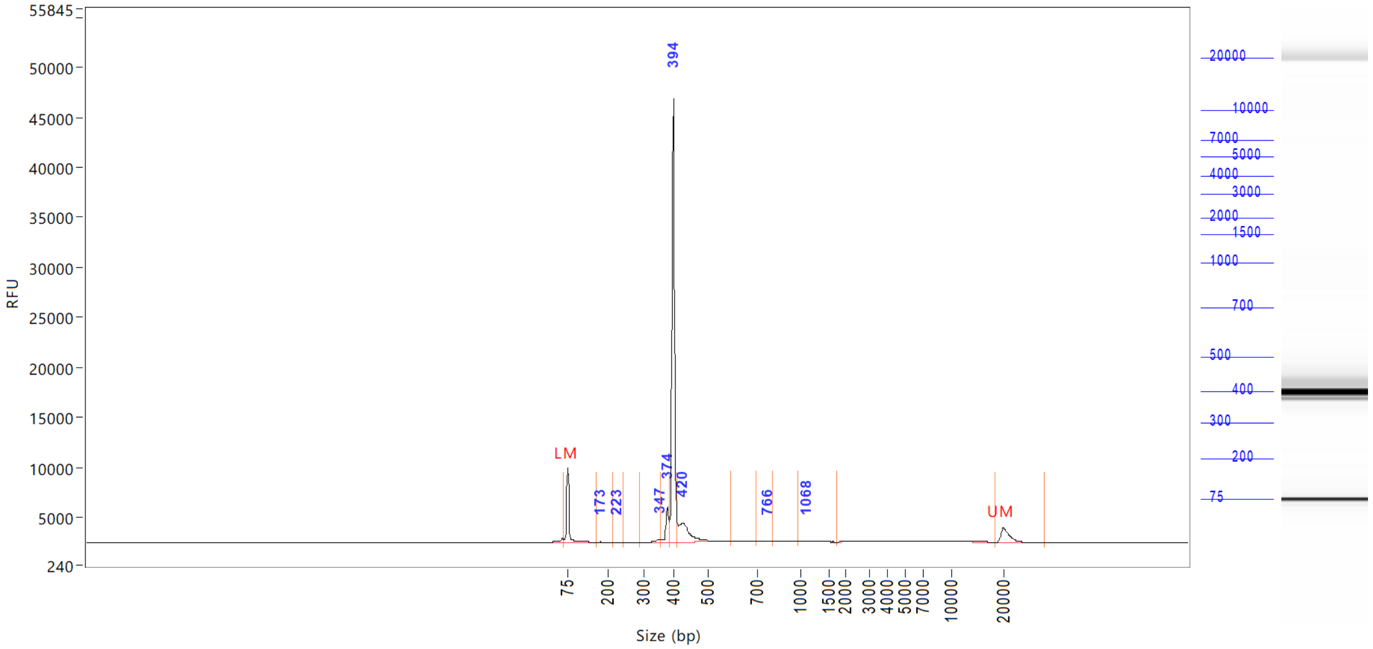

Peak	Size (bp)	Concentration (ng/uL)	From (bp)	To (bp)	Average size (bp)	CV%	RFU	Corrected peak area
1	75 (LM)	0.5000	64	164	77	19.10	7472	48.330
2	173	0.1037	164	210	177	6.55	75	0.835
3	223	0.0420	210	239	220	1.77	59	0.338
4	347	0.4346	291	356	341	2.97	253	3.501
5	374	2.8067	356	382	373	1.51	3620	22.608
6	394	29.6409	382	409	393	1.17	44417	238.759
7	420	5.3953	409	593	437	6.42	1863	43.459
8	766	0.0829	697	801	755	2.39	103	0.668
9	1068	0.1428	983	1712	1115	9.68	58	1.151
10	20000 (UM)	0.1548	18537	28221	20617	4.79	1396	14.963

TIC:	38.6488	ng/uL
TIM:	160.5483	nmole/L
Total concentration:	38.5874	ng/uL

```
Sample peak width (sec): 5    Sample min peak height: 50    Sample baseline V to V?: Y    Sample baseline V to V points: 3
Sample filter: Binomial       Number of points for filter: 3  Sample start region (min): 0  Sample end region (min): 30
Marker peak width (sec): 5    Marker min peak height: 200   Marker baseline V to V?: Y    Marker baseline V to V points: 3
Lower marker selection: First peak > 500 RFU                Upper marker selection: Last peak > 200 RFU
Ladder size (bp)75, 200, 300, 400, 500, 700, 1000, 1500, 2000, 3000, 4000, 5000, 7000, 10000, 20000
Quantification using: Lower Marker          Final concentration (ng/uL): 0.5000          Dilution factor: 12.0
```

Sample: SampA7
Well location: A7
Created: Wednesday, August 11, 2021 6:14:43 PM

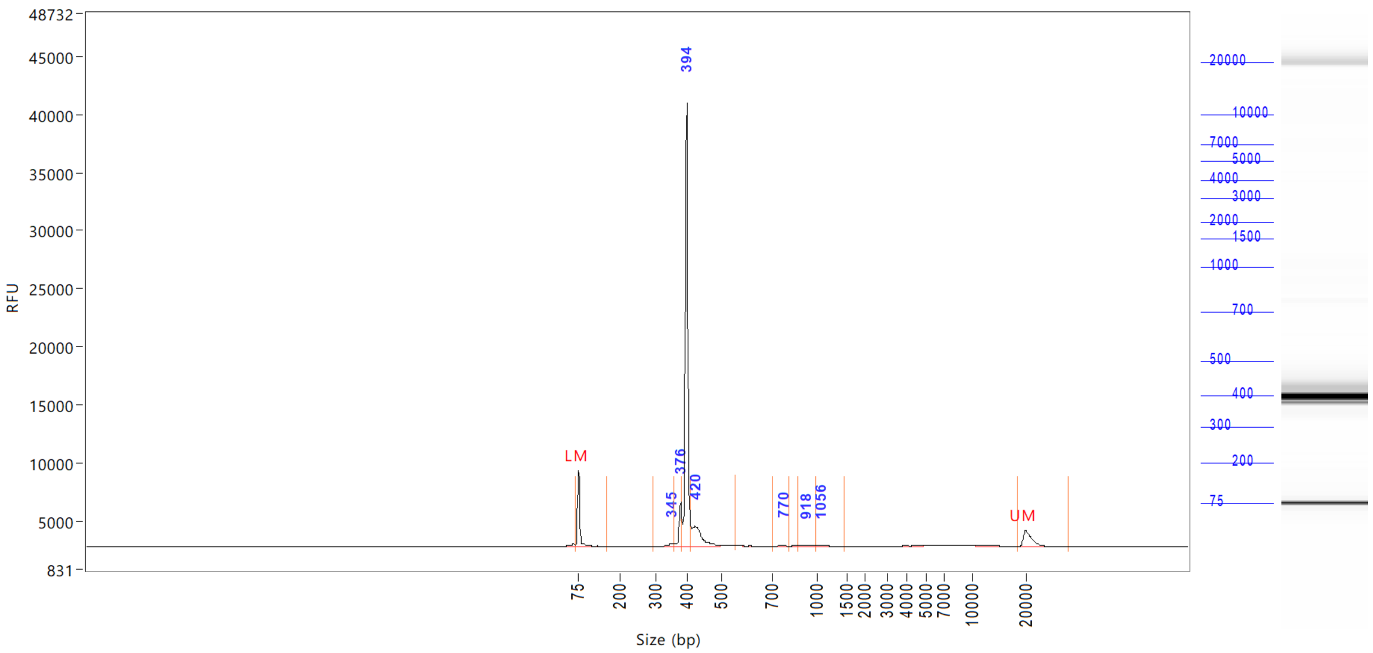

Peak	Size (bp)	Concentration (ng/uL)	From (bp)	To (bp)	Average size (bp)	CV%	RFU	Corrected peak area
1	75 (LM)	0.5000	66	160	77	16.06	6541	39.199
2	345	0.5380	291	358	341	3.23	231	3.515
3	376	3.2770	358	382	374	1.36	3895	21.409
4	394	30.7508	382	409	392	1.19	38274	200.902
5	420	5.1686	409	552	431	4.88	1723	33.767
6	770	0.1537	701	805	761	2.38	152	1.004
7	918	0.1962	866	987	926	3.71	64	1.282
8	1056	0.1761	987	1445	1096	8.90	64	1.150
9	20000 (UM)	0.1768	18537	28221	20611	5.12	1339	13.861

TIC:	40.2603	ng/uL
TIM:	166.7526	nmole/L
Total concentration:	40.3341	ng/uL

```
Sample peak width (sec): 5    Sample min peak height: 50    Sample baseline V to V?: Y    Sample baseline V to V points: 3
Sample filter: Binomial       Number of points for filter: 3  Sample start region (min): 0  Sample end region (min): 30
Marker peak width (sec): 5    Marker min peak height: 200   Marker baseline V to V?: Y    Marker baseline V to V points: 3
Lower marker selection: First peak > 500 RFU                Upper marker selection: Last peak > 200 RFU
Ladder size (bp)75, 200, 300, 400, 500, 700, 1000, 1500, 2000, 3000, 4000, 5000, 7000, 10000, 20000
Quantification using: Lower Marker          Final concentration (ng/uL): 0.5000          Dilution factor: 12.0
```

Sample: SampB7
Well location: B7
Created: Wednesday, August 11, 2021 6:14:43 PM

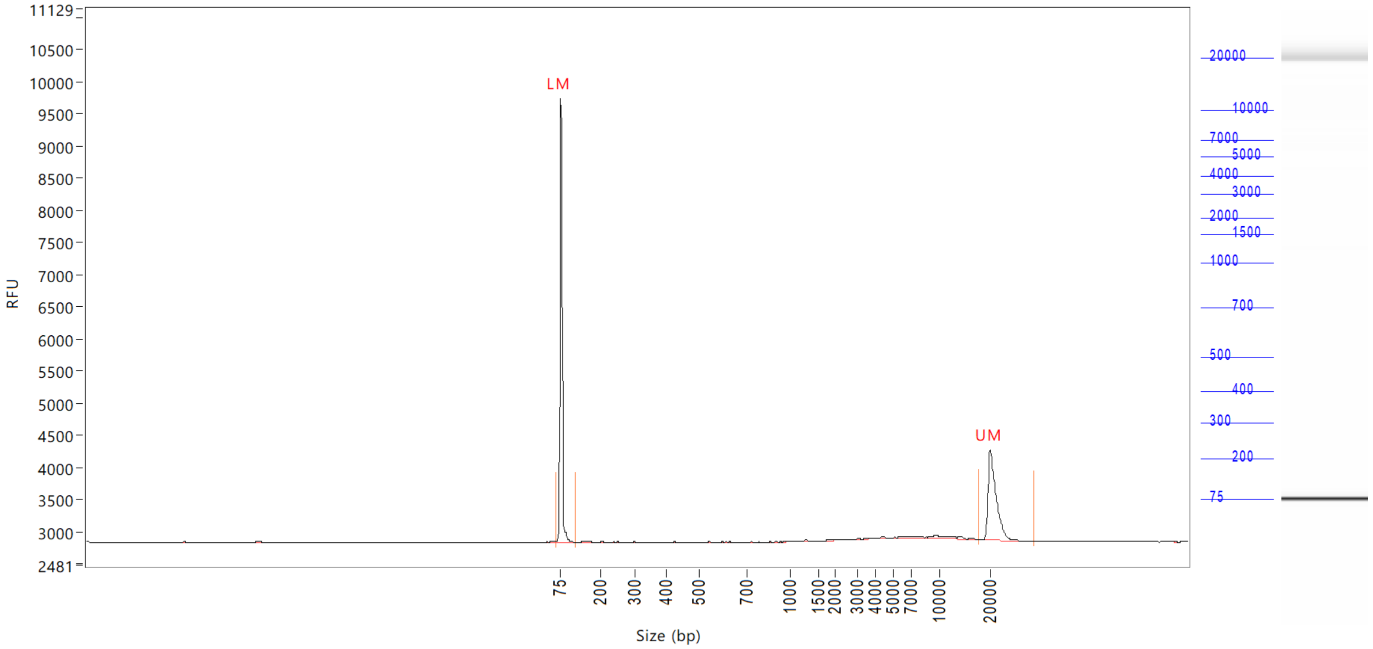

Peak	Size (bp)	Concentration (ng/uL)	From (bp)	To (bp)	Average size (bp)	CV%	RFU	Corrected peak area
1	75 (LM)	0.5000	64	122	74	5.74	6912	38.902
2	20000 (UM)	0.1879	17805	28705	20589	5.01	1400	14.617

TIC:	0.0000	ng/uL
TIM:	0.0000	nmole/L
Total concentration:	0.6679	ng/uL

```
Sample peak width (sec): 5    Sample min peak height: 50    Sample baseline V to V?: Y    Sample baseline V to V points: 3
Sample filter: Binomial       Number of points for filter: 3  Sample start region (min): 0  Sample end region (min): 30
Marker peak width (sec): 5    Marker min peak height: 200   Marker baseline V to V?: Y    Marker baseline V to V points: 3
Lower marker selection: First peak > 500 RFU                Upper marker selection: Last peak > 200 RFU
Ladder size (bp)75, 200, 300, 400, 500, 700, 1000, 1500, 2000, 3000, 4000, 5000, 7000, 10000, 20000
Quantification using: Lower Marker          Final concentration (ng/uL): 0.5000          Dilution factor: 12.0
```

Sample: SampC7
Well location: C7
Created: Wednesday, August 11, 2021 6:14:43 PM

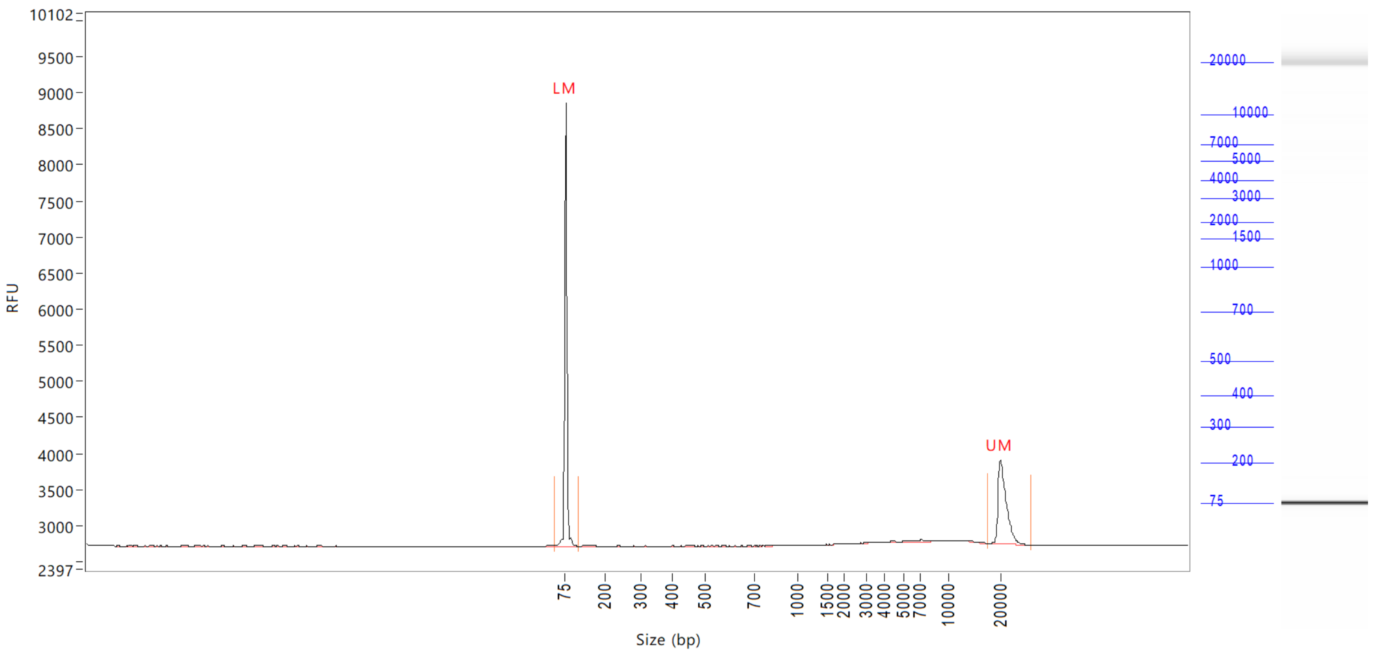

Peak	Size (bp)	Concentration (ng/uL)	From (bp)	To (bp)	Average size (bp)	CV%	RFU	Corrected peak area
1	75 (LM)	0.5000	45	118	74	6.35	6158	35.031
2	20000 (UM)	0.1714	17439	25924	20489	4.61	1168	12.011

TIC:	0.0000	ng/uL
TIM:	0.0000	nmole/L
Total concentration:	0.5232	ng/uL

```
Sample peak width (sec): 5    Sample min peak height: 50    Sample baseline V to V?: Y    Sample baseline V to V points: 3
Sample filter: Binomial       Number of points for filter: 3  Sample start region (min): 0  Sample end region (min): 30
Marker peak width (sec): 5    Marker min peak height: 200   Marker baseline V to V?: Y    Marker baseline V to V points: 3
Lower marker selection: First peak > 500 RFU                Upper marker selection: Last peak > 200 RFU
Ladder size (bp)75, 200, 300, 400, 500, 700, 1000, 1500, 2000, 3000, 4000, 5000, 7000, 10000, 20000
Quantification using: Lower Marker          Final concentration (ng/uL): 0.5000          Dilution factor: 12.0
```

Sample: SampD7
Well location: D7
Created: Wednesday, August 11, 2021 6:14:43 PM

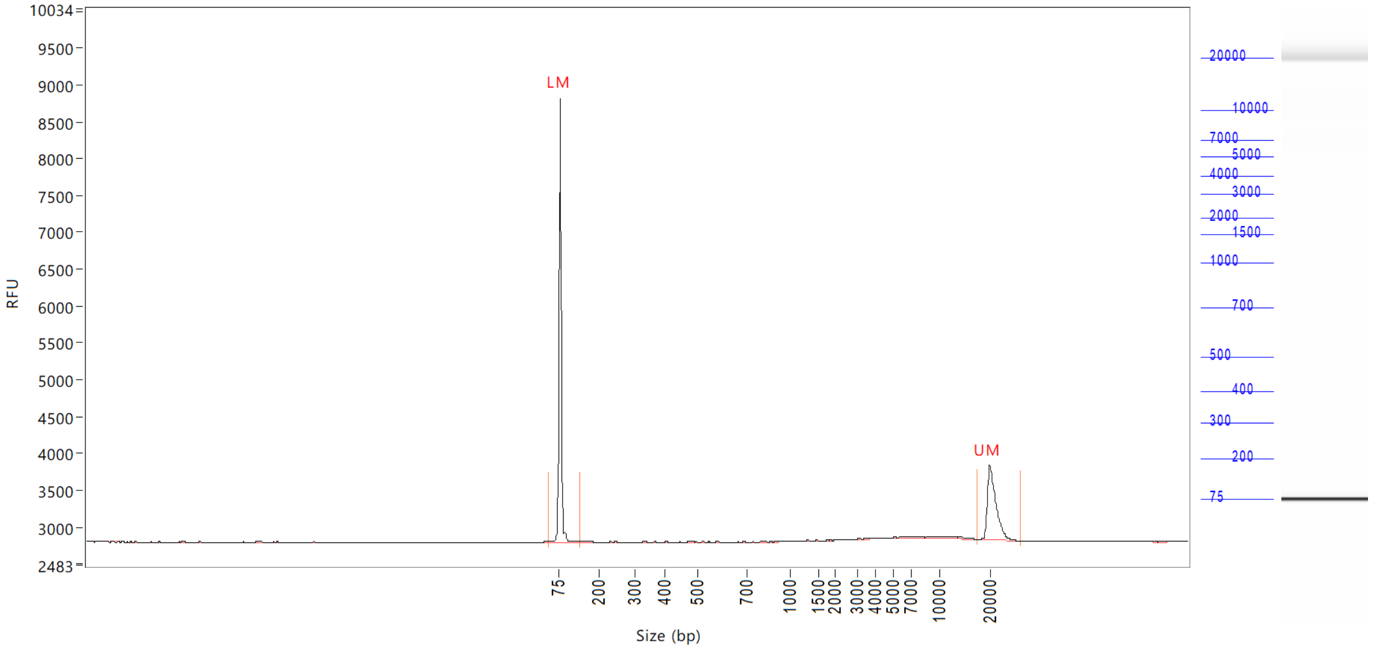

Peak	Size (bp)	Concentration (ng/uL)	From (bp)	To (bp)	Average size (bp)	CV%	RFU	Corrected peak area
1	75 (LM)	0.5000	39	137	73	8.00	6036	34.032
2	20000 (UM)	0.1637	17439	26166	20588	4.81	1020	11.141

TIC:	0.0000	ng/uL
TIM:	0.0000	nmole/L
Total concentration:	0.6667	ng/uL

Sample peak width (sec): 5   Sample min peak height: 50   Sample baseline V to V?: Y   Sample baseline V to V points: 3
Sample filter: Binomial   Number of points for filter: 3 Sample start region (min): 0 Sample end region (min): 30
Marker peak width (sec): 5   Marker min peak height: 200   Marker baseline V to V?: Y   Marker baseline V to V points: 3
Lower marker selection: First peak > 500 RFU   Upper marker selection: Last peak > 200 RFU
Ladder size (bp)75, 200, 300, 400, 500, 700, 1000, 1500, 2000, 3000, 4000, 5000, 7000, 10000, 20000
Quantification using: Lower Marker   Final concentration (ng/uL): 0.5000   Dilution factor: 12.0

Sample: SampE7
Well location: E7
Created: Wednesday, August 11, 2021 6:14:43 PM

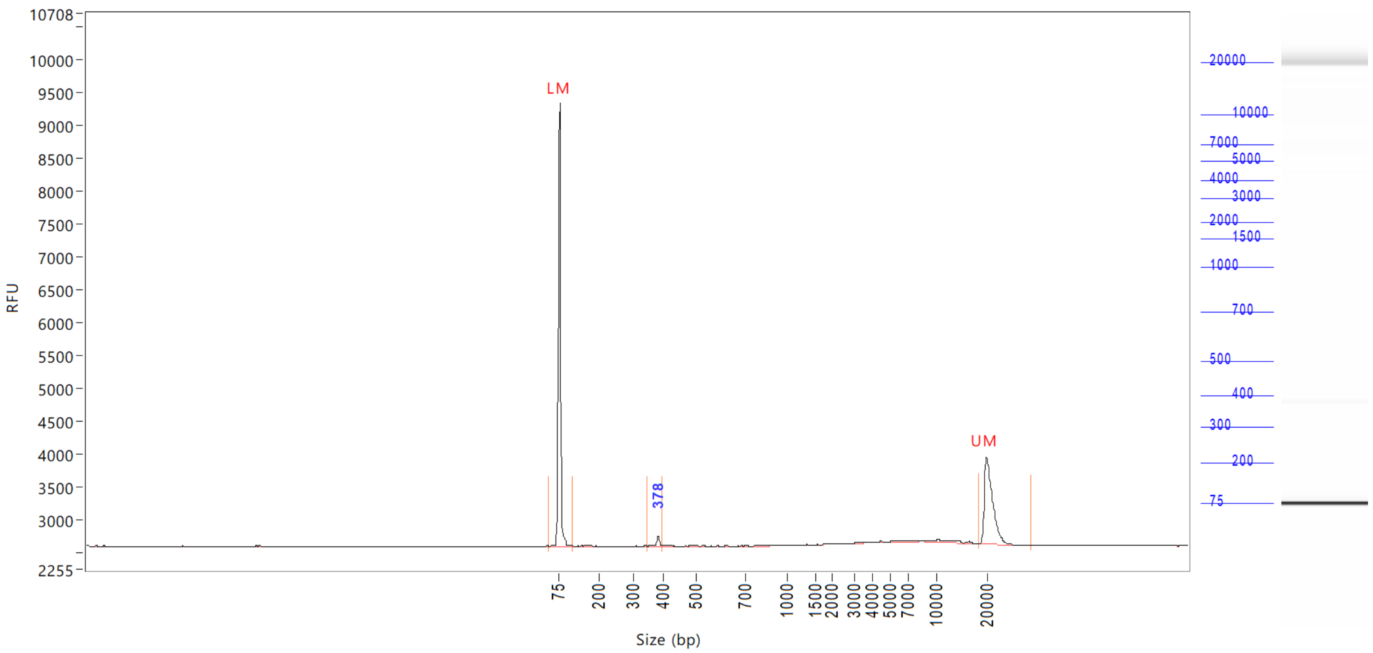

Peak	Size (bp)	Concentration (ng/uL)	From (bp)	To (bp)	Average size (bp)	CV%	RFU	Corrected peak area
1	75 (LM)	0.5000	43	118	73	6.40	6757	37.817
2	378	0.1559	343	392	374	2.08	158	0.982
3	20000 (UM)	0.1846	18415	28826	20619	5.00	1320	13.958

TIC:	0.1559	ng/uL
TIM:	0.6857	nmole/L
Total concentration:	0.8221	ng/uL

```
Sample peak width (sec): 5    Sample min peak height: 50    Sample baseline V to V?: Y    Sample baseline V to V points: 3
Sample filter: Binomial       Number of points for filter: 3  Sample start region (min): 0  Sample end region (min): 30
Marker peak width (sec): 5    Marker min peak height: 200   Marker baseline V to V?: Y    Marker baseline V to V points: 3
Lower marker selection: First peak > 500 RFU                Upper marker selection: Last peak > 200 RFU
Ladder size (bp)75, 200, 300, 400, 500, 700, 1000, 1500, 2000, 3000, 4000, 5000, 7000, 10000, 20000
Quantification using: Lower Marker          Final concentration (ng/uL): 0.5000          Dilution factor: 12.0
```

Sample: SampF7
Well location: F7
Created: Wednesday, August 11, 2021 6:14:43 PM

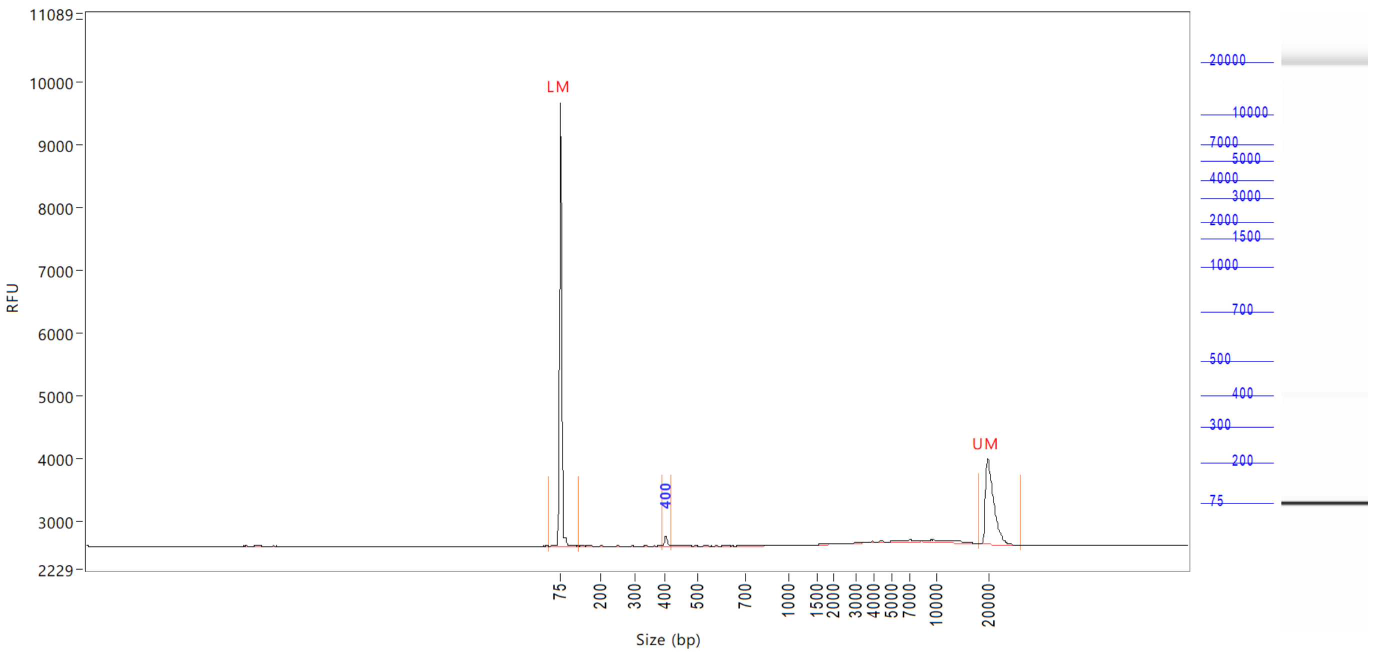

Peak	Size (bp)	Concentration (ng/uL)	From (bp)	To (bp)	Average size (bp)	CV%	RFU	Corrected peak area
1	75 (LM)	0.5000	39	131	73	6.24	7078	40.175
2	400	0.1443	388	414	398	1.14	165	0.966
3	20000 (UM)	0.1789	18293	26649	20570	4.78	1373	14.373

TIC:	0.1443	ng/uL
TIM:	0.5967	nmole/L
Total concentration:	0.7555	ng/uL

```
Sample peak width (sec): 5    Sample min peak height: 50    Sample baseline V to V?: Y    Sample baseline V to V points: 3
Sample filter: Binomial       Number of points for filter: 3  Sample start region (min): 0  Sample end region (min): 30
Marker peak width (sec): 5    Marker min peak height: 200   Marker baseline V to V?: Y    Marker baseline V to V points: 3
Lower marker selection: First peak > 500 RFU                Upper marker selection: Last peak > 200 RFU
Ladder size (bp)75, 200, 300, 400, 500, 700, 1000, 1500, 2000, 3000, 4000, 5000, 7000, 10000, 20000
Quantification using: Lower Marker          Final concentration (ng/uL): 0.5000          Dilution factor: 12.0
```

Sample: SampG7
Well location: G7
Created: Wednesday, August 11, 2021 6:14:43 PM

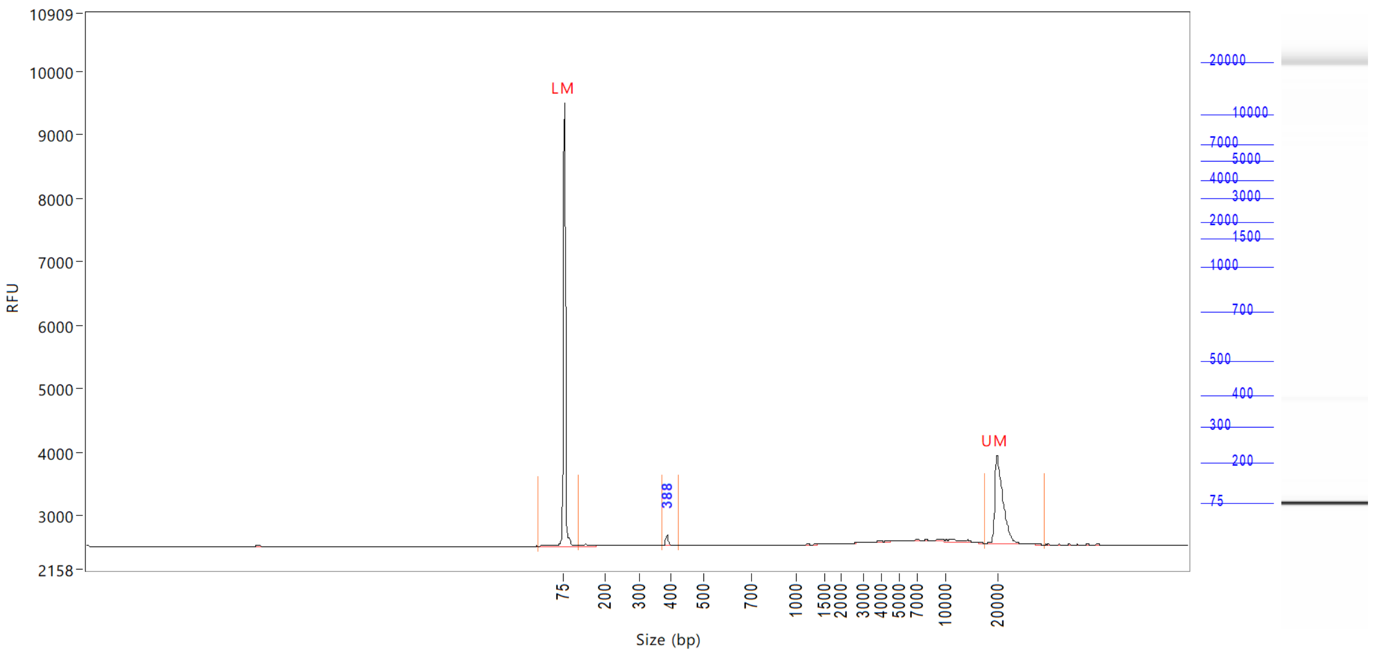

Peak	Size (bp)	Concentration (ng/uL)	From (bp)	To (bp)	Average size (bp)	CV%	RFU	Corrected peak area
1	75 (LM)	0.5000	0	122	73	8.51	6989	38.475
2	388	0.1701	374	424	388	2.52	164	1.091
3	20000 (UM)	0.1854	17561	29189	20596	4.92	1380	14.264

TIC:	0.1701	ng/uL
TIM:	0.7212	nmole/L
Total concentration:	0.7715	ng/uL

```
Sample peak width (sec): 5    Sample min peak height: 50    Sample baseline V to V?: Y    Sample baseline V to V points: 3
Sample filter: Binomial       Number of points for filter: 3  Sample start region (min): 0  Sample end region (min): 30
Marker peak width (sec): 5    Marker min peak height: 200   Marker baseline V to V?: Y    Marker baseline V to V points: 3
Lower marker selection: First peak > 500 RFU                Upper marker selection: Last peak > 200 RFU
Ladder size (bp)75, 200, 300, 400, 500, 700, 1000, 1500, 2000, 3000, 4000, 5000, 7000, 10000, 20000
Quantification using: Lower Marker          Final concentration (ng/uL): 0.5000          Dilution factor: 12.0
```

Sample: SampH7
Well location: H7
Created: Wednesday, August 11, 2021 6:14:43 PM

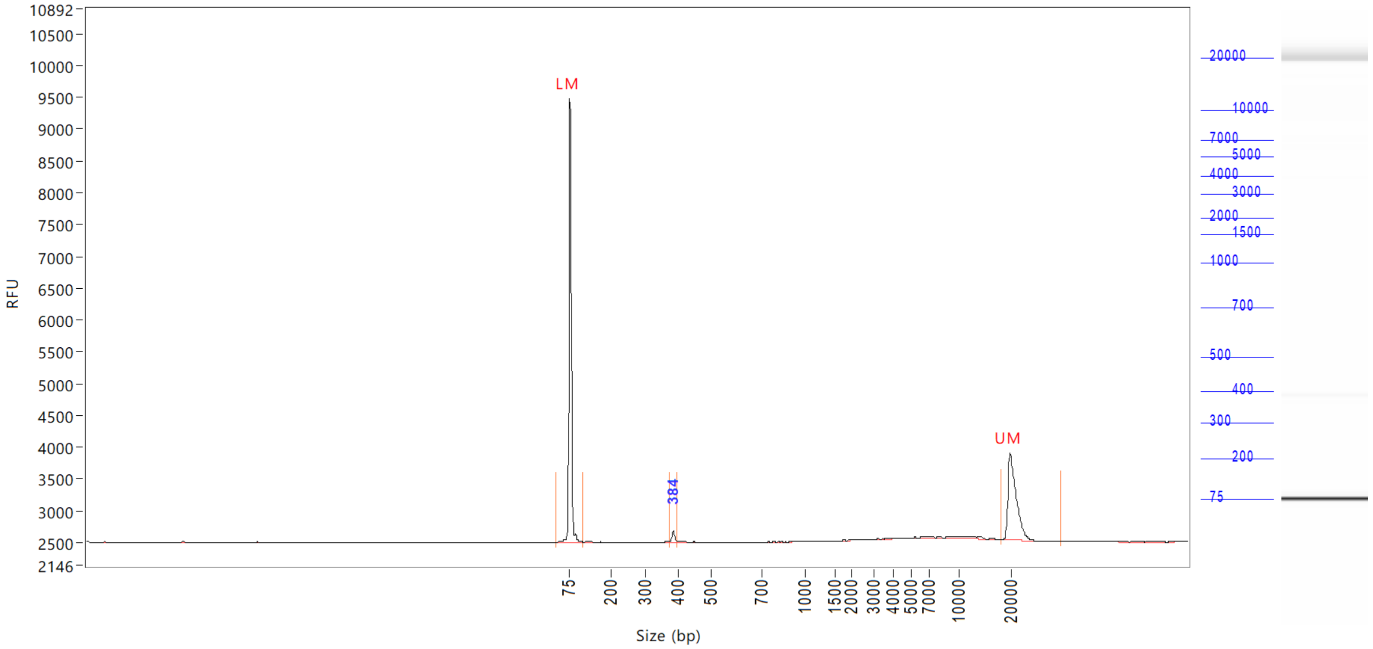

Peak	Size (bp)	Concentration (ng/uL)	From (bp)	To (bp)	Average size (bp)	CV%	RFU	Corrected peak area
1	75 (LM)	0.5000	35	114	74	6.38	6991	39.127
2	384	0.1649	372	398	383	1.24	189	1.075
3	20000 (UM)	0.1801	18293	29672	20541	4.92	1365	14.093

TIC:	0.1649	ng/uL
TIM:	0.7084	nmole/L
Total concentration:	0.7925	ng/uL

Sample peak width (sec): 5 Sample min peak height: 50 Sample baseline V to V?: Y Sample baseline V to V points: 3
Sample filter: Binomial Number of points for filter: 3 Sample start region (min): 0 Sample end region (min): 30
Marker peak width (sec): 5 Marker min peak height: 200 Marker baseline V to V?: Y Marker baseline V to V points: 3
Lower marker selection: First peak > 500 RFU Upper marker selection: Last peak > 200 RFU
Ladder size (bp)75, 200, 300, 400, 500, 700, 1000, 1500, 2000, 3000, 4000, 5000, 7000, 10000, 20000
Quantification using: Lower Marker Final concentration (ng/uL): 0.5000 Dilution factor: 12.0

Sample: SampA8
Well location: A8
Created: Wednesday, August 11, 2021 6:14:43 PM

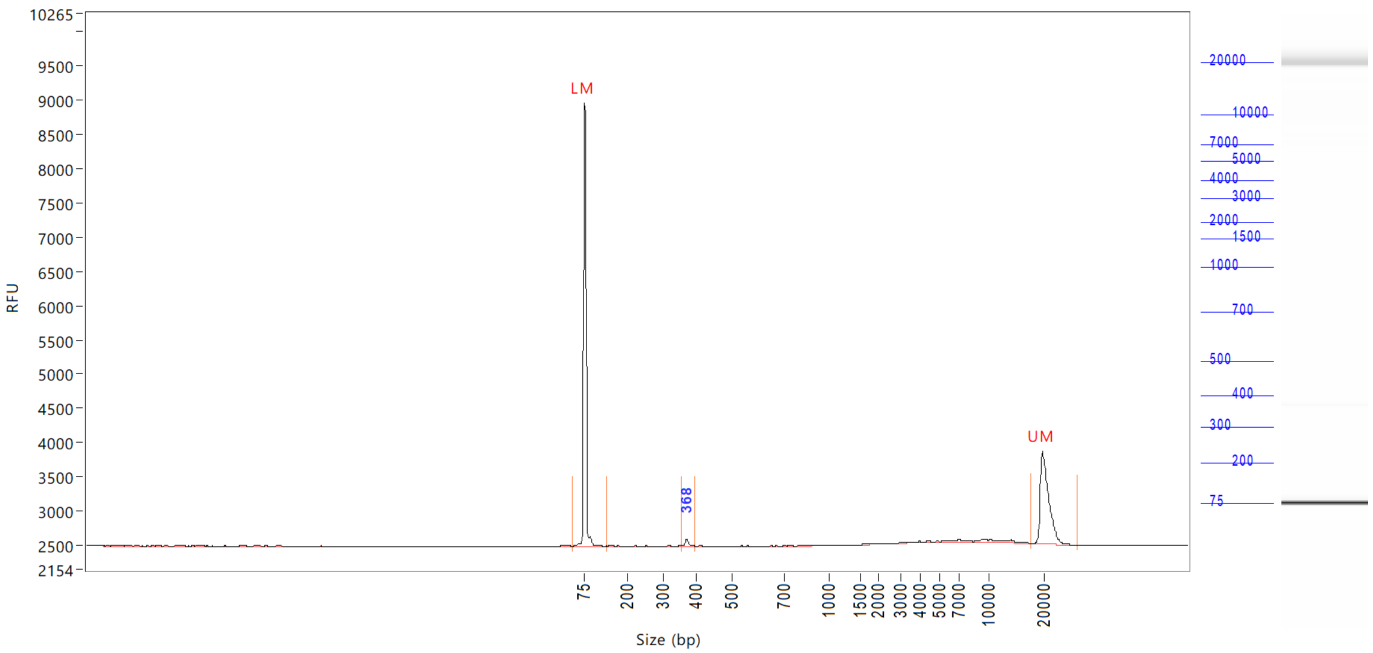

Peak	Size (bp)	Concentration (ng/uL)	From (bp)	To (bp)	Average size (bp)	CV%	RFU	Corrected peak area
1	75 (LM)	0.5000	41	141	74	7.08	6482	35.953
2	368	0.1300	354	396	368	2.19	113	0.779
3	20000 (UM)	0.1948	17683	26287	20456	4.59	1349	14.005

TIC:	0.1300	ng/uL
TIM:	0.5812	nmole/L
Total concentration:	0.7777	ng/uL

Sample peak width (sec): 5   Sample min peak height: 50   Sample baseline V to V?: Y   Sample baseline V to V points: 3
Sample filter: Binomial   Number of points for filter: 3   Sample start region (min): 0   Sample end region (min): 30
Marker peak width (sec): 5   Marker min peak height: 200   Marker baseline V to V?: Y   Marker baseline V to V points: 3
Lower marker selection: First peak > 500 RFU   Upper marker selection: Last peak > 200 RFU
Ladder size (bp)75, 200, 300, 400, 500, 700, 1000, 1500, 2000, 3000, 4000, 5000, 7000, 10000, 20000
Quantification using: Lower Marker   Final concentration (ng/uL): 0.5000   Dilution factor: 12.0

Sample: SampB8
Well location: B8
Created: Wednesday, August 11, 2021 6:14:43 PM

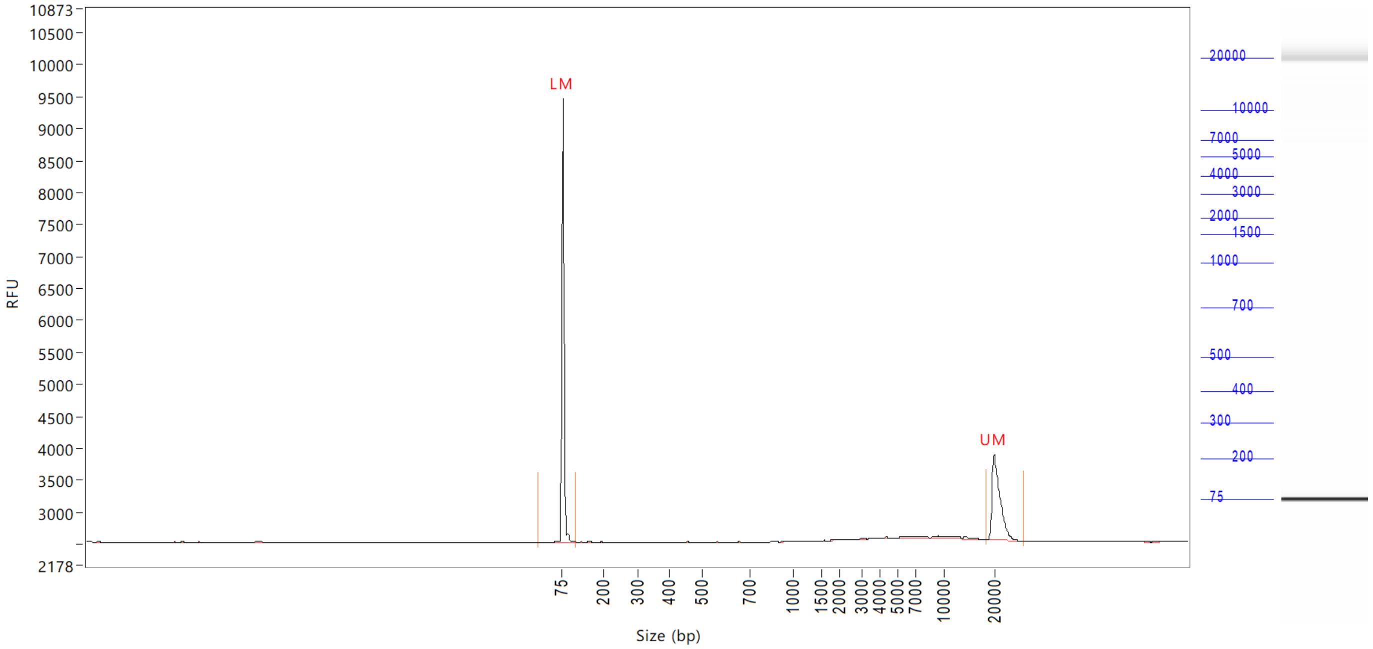

Peak	Size (bp)	Concentration (ng/uL)	From (bp)	To (bp)	Average size (bp)	CV%	RFU	Corrected peak area
1	75 (LM)	0.5000	0	116	72	8.09	6950	39.196
2	20000 (UM)	0.1748	18415	25803	20486	4.49	1333	13.706

TIC:	0.0000	ng/uL
TIM:	0.0000	nmole/L
Total concentration:	0.6146	ng/uL

```
Sample peak width (sec): 5    Sample min peak height: 50    Sample baseline V to V?: Y    Sample baseline V to V points: 3
Sample filter: Binomial       Number of points for filter: 3  Sample start region (min): 0  Sample end region (min): 30
Marker peak width (sec): 5    Marker min peak height: 200   Marker baseline V to V?: Y    Marker baseline V to V points: 3
Lower marker selection: First peak > 500 RFU                Upper marker selection: Last peak > 200 RFU
Ladder size (bp)75, 200, 300, 400, 500, 700, 1000, 1500, 2000, 3000, 4000, 5000, 7000, 10000, 20000
Quantification using: Lower Marker          Final concentration (ng/uL): 0.5000          Dilution factor: 12.0
```

Sample: SampC8
Well location: C8
Created: Wednesday, August 11, 2021 6:14:43 PM

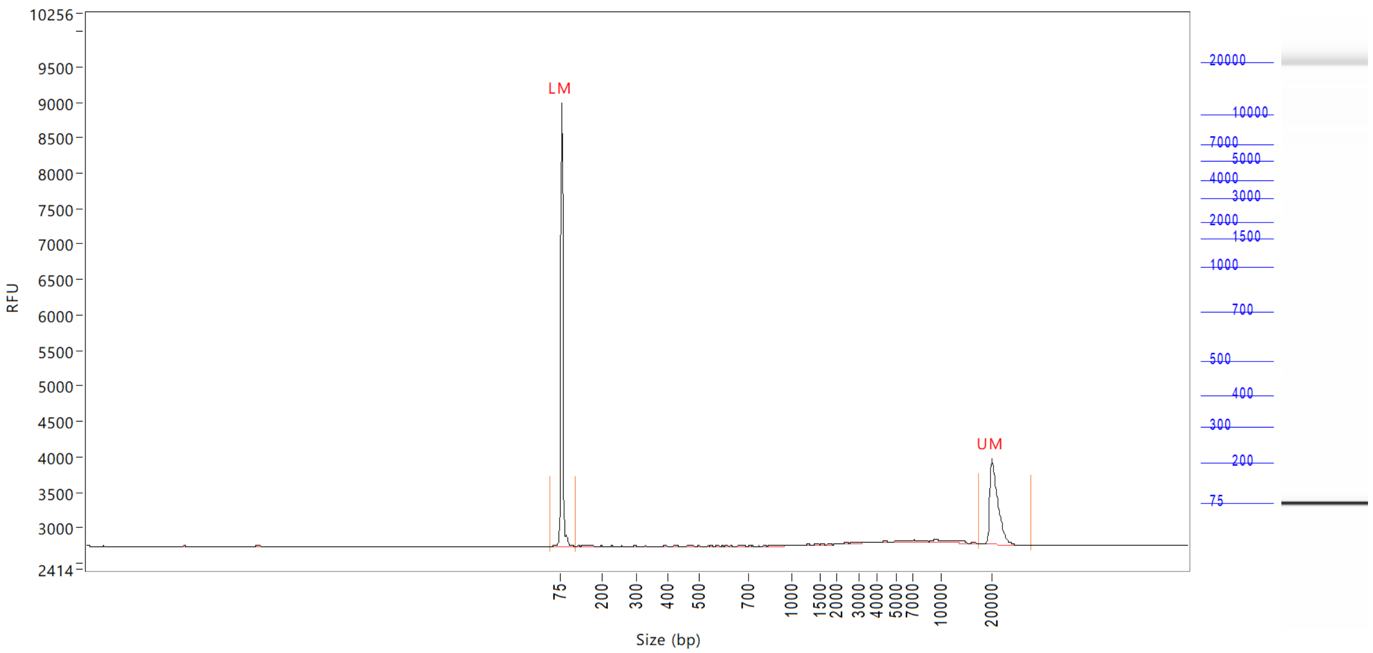

Peak	Size (bp)	Concentration (ng/uL)	From (bp)	To (bp)	Average size (bp)	CV%	RFU	Corrected peak area
1	75 (LM)	0.5000	41	118	73	6.05	6266	35.261
2	20000 (UM)	0.1785	17439	27617	20556	5.04	1202	12.585

TIC:	0.0000	ng/uL
TIM:	0.0000	nmole/L
Total concentration:	0.7649	ng/uL

Sample peak width (sec): 5 Sample min peak height: 50 Sample baseline V to V?: Y Sample baseline V to V points: 3
Sample filter: Binomial Number of points for filter: 3 Sample start region (min): 0 Sample end region (min): 30
Marker peak width (sec): 5 Marker min peak height: 200 Marker baseline V to V?: Y Marker baseline V to V points: 3
Lower marker selection: First peak > 500 RFU Upper marker selection: Last peak > 200 RFU
Ladder size (bp)75, 200, 300, 400, 500, 700, 1000, 1500, 2000, 3000, 4000, 5000, 7000, 10000, 20000
Quantification using: Lower Marker Final concentration (ng/uL): 0.5000 Dilution factor: 12.0

Sample: SampD8
Well location: D8
Created: Wednesday, August 11, 2021 6:14:43 PM

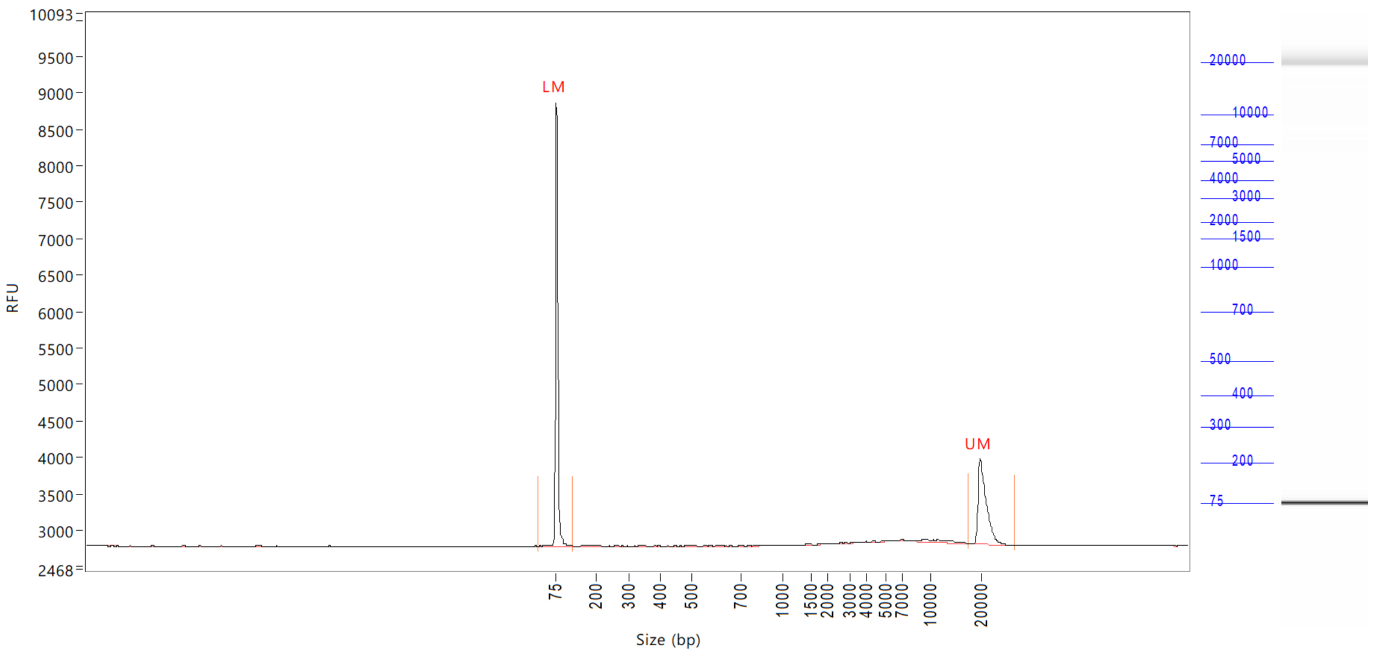

Peak	Size<br>(bp)	Concentration<br>(ng/uL)	From<br>(bp)	To<br>(bp)	Average size<br>(bp)	CV%	RFU	Corrected peak area
1	75 (LM)	0.5000	18	128	74	7.95	6094	35.065
2	20000 (UM)	0.1783	17561	26891	20564	4.92	1177	12.501

TIC:	0.0000	ng/uL
TIM:	0.0000	nmole/L
Total concentration:	0.6990	ng/uL

Sample peak width (sec): 5 Sample min peak height: 50 Sample baseline V to V?: Y Sample baseline V to V points: 3
Sample filter: Binomial Number of points for filter: 3 Sample start region (min): 0 Sample end region (min): 30
Marker peak width (sec): 5 Marker min peak height: 200 Marker baseline V to V?: Y Marker baseline V to V points: 3
Lower marker selection: First peak > 500 RFU Upper marker selection: Last peak > 200 RFU
Ladder size (bp)75, 200, 300, 400, 500, 700, 1000, 1500, 2000, 3000, 4000, 5000, 7000, 10000, 20000
Quantification using: Lower Marker Final concentration (ng/uL): 0.5000 Dilution factor: 12.0

Sample: SampE8
Well location: E8
Created: Wednesday, August 11, 2021 6:14:43 PM

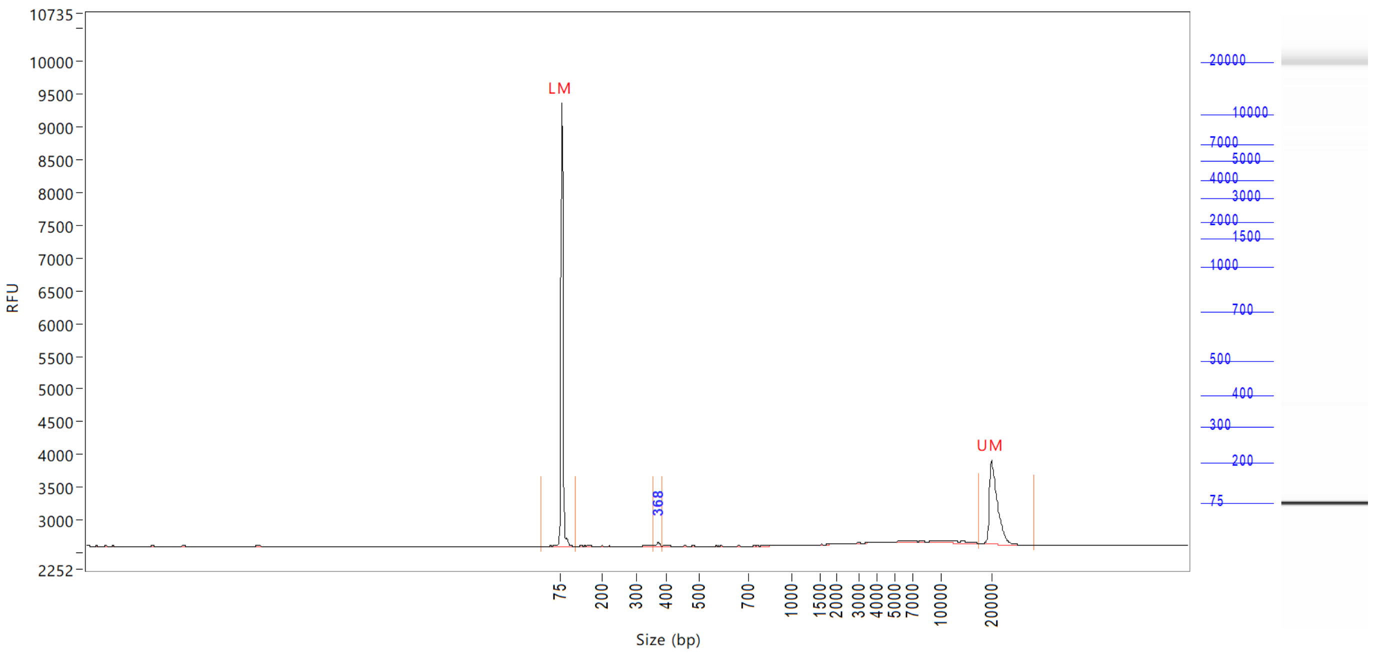

Peak	Size (bp)	Concentration (ng/uL)	From (bp)	To (bp)	Average size (bp)	CV%	RFU	Corrected peak area
1	75 (LM)	0.5000	14	120	73	7.01	6780	37.278
2	368	0.0652	354	382	366	1.45	66	0.405
3	20000 (UM)	0.1797	17561	28342	20588	4.87	1279	13.397

TIC:	0.0652	ng/uL
TIM:	0.2930	nmole/L
Total concentration:	0.6841	ng/uL

Sample peak width (sec): 5 Sample min peak height: 50 Sample baseline V to V?: Y Sample baseline V to V points: 3
Sample filter: Binomial Number of points for filter: 3 Sample start region (min): 0 Sample end region (min): 30
Marker peak width (sec): 5 Marker min peak height: 200 Marker baseline V to V?: Y Marker baseline V to V points: 3
Lower marker selection: First peak > 500 RFU Upper marker selection: Last peak > 200 RFU
Ladder size (bp)75, 200, 300, 400, 500, 700, 1000, 1500, 2000, 3000, 4000, 5000, 7000, 10000, 20000
Quantification using: Lower Marker Final concentration (ng/uL): 0.5000 Dilution factor: 12.0

Sample: SampF8
Well location: F8
Created: Wednesday, August 11, 2021 6:14:43 PM

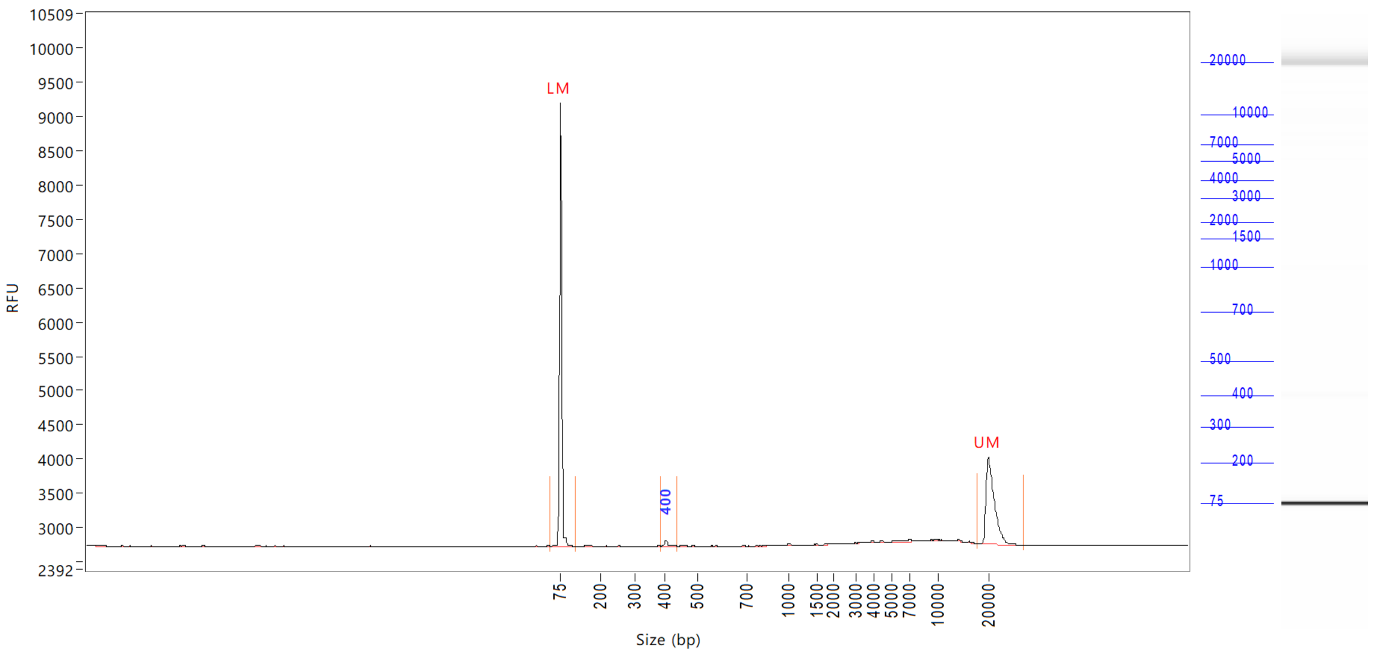

Peak	Size (bp)	Concentration (ng/uL)	From (bp)	To (bp)	Average size (bp)	CV%	RFU	Corrected peak area
1	75 (LM)	0.5000	45	122	73	5.94	6488	36.728
2	400	0.1060	386	435	401	2.55	85	0.649
3	20000 (UM)	0.1837	17927	26891	20575	4.89	1270	13.492

TIC:	0.1060	ng/uL
TIM:	0.4347	nmole/L
Total concentration:	0.5986	ng/uL

```
Sample peak width (sec): 5    Sample min peak height: 50    Sample baseline V to V?: Y    Sample baseline V to V points: 3
Sample filter: Binomial       Number of points for filter: 3  Sample start region (min): 0  Sample end region (min): 30
Marker peak width (sec): 5    Marker min peak height: 200   Marker baseline V to V?: Y    Marker baseline V to V points: 3
Lower marker selection: First peak > 500 RFU                Upper marker selection: Last peak > 200 RFU
Ladder size (bp)75, 200, 300, 400, 500, 700, 1000, 1500, 2000, 3000, 4000, 5000, 7000, 10000, 20000
Quantification using: Lower Marker          Final concentration (ng/uL): 0.5000          Dilution factor: 12.0
```

Sample: SampG8
Well location: G8
Created: Wednesday, August 11, 2021 6:14:43 PM

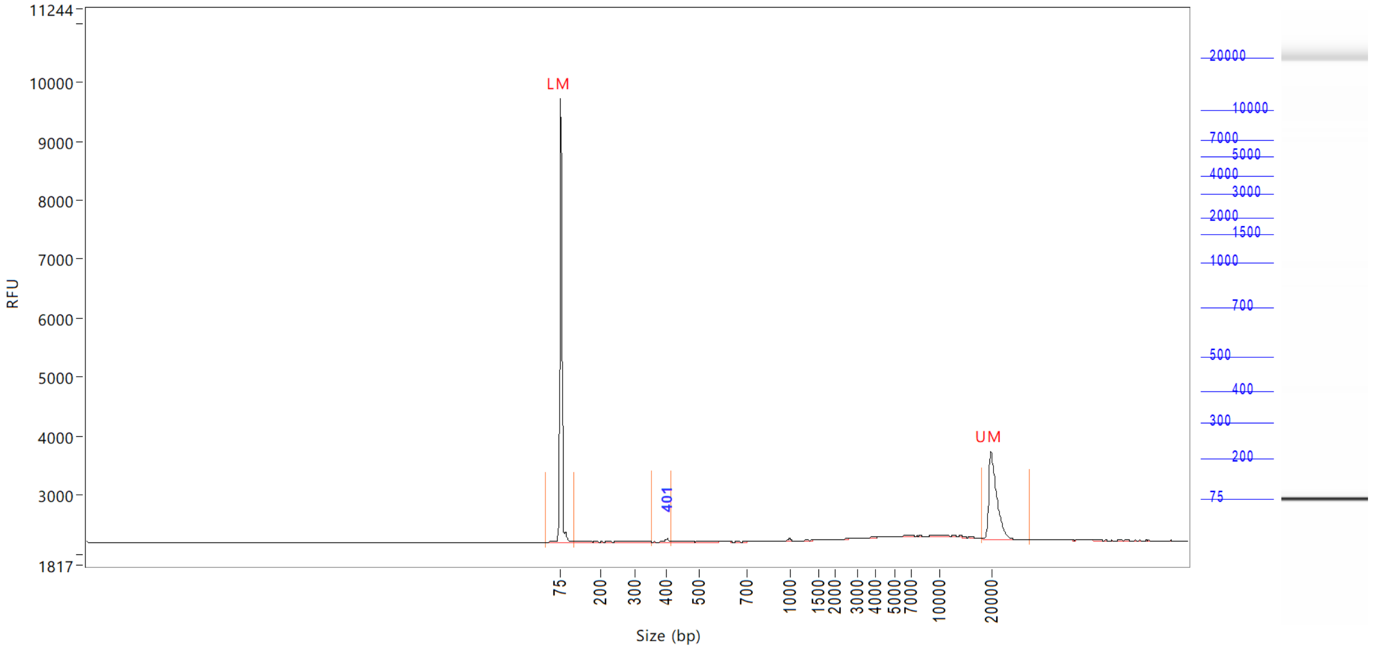

Peak	Size (bp)	Concentration (ng/uL)	From (bp)	To (bp)	Average size (bp)	CV%	RFU	Corrected peak area
1	75 (LM)	0.5000	28	118	73	6.57	7531	41.240
2	401	0.0555	352	414	397	2.30	53	0.381
3	20000 (UM)	0.1853	18293	27738	20575	4.70	1487	15.287

TIC:	0.0555	ng/uL
TIM:	0.2300	nmole/L
Total concentration:	0.6274	ng/uL

```
Sample peak width (sec): 5    Sample min peak height: 50    Sample baseline V to V?: Y    Sample baseline V to V points: 3
Sample filter: Binomial       Number of points for filter: 3  Sample start region (min): 0  Sample end region (min): 30
Marker peak width (sec): 5    Marker min peak height: 200   Marker baseline V to V?: Y    Marker baseline V to V points: 3
Lower marker selection: First peak > 500 RFU                Upper marker selection: Last peak > 200 RFU
Ladder size (bp)75, 200, 300, 400, 500, 700, 1000, 1500, 2000, 3000, 4000, 5000, 7000, 10000, 20000
Quantification using: Lower Marker          Final concentration (ng/uL): 0.5000          Dilution factor: 12.0
```

Sample: SampH8
Well location: H8
Created: Wednesday, August 11, 2021 6:14:43 PM

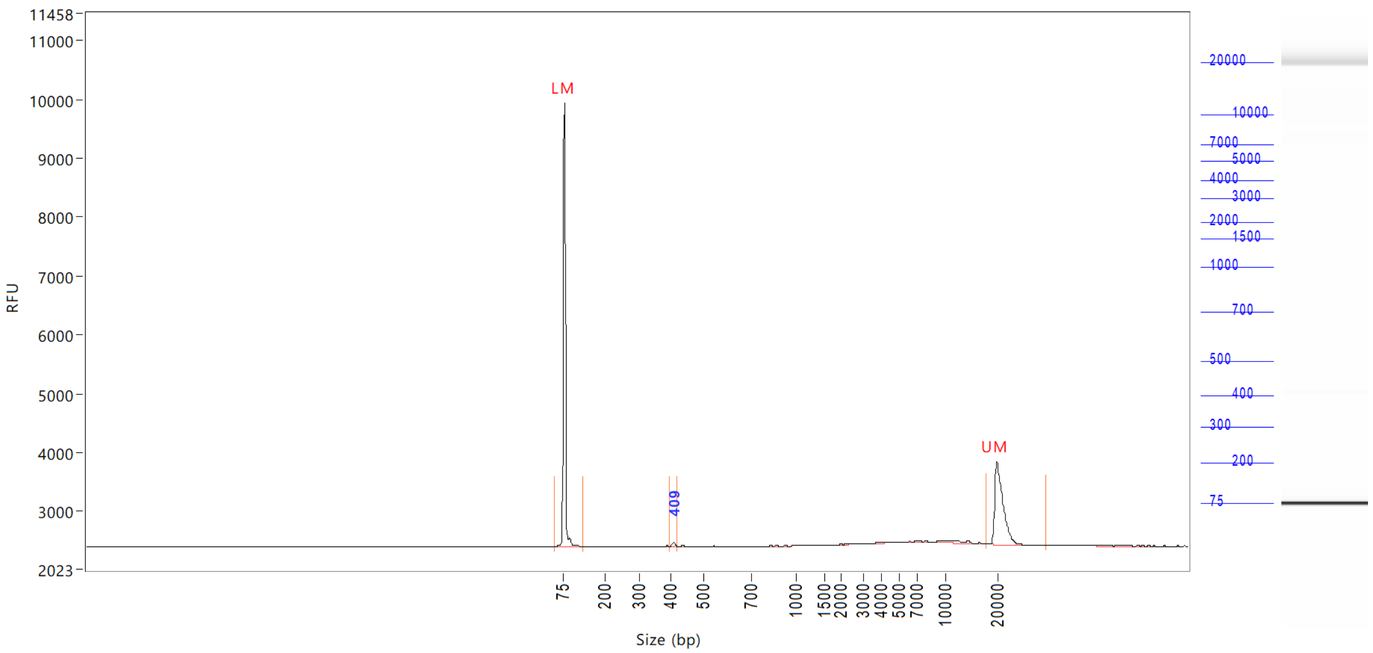

Peak	Size<br>(bp)	Concentration<br>(ng/uL)	From<br>(bp)	To<br>(bp)	Average size<br>(bp)	CV%	RFU	Corrected peak area
1	75 (LM)	0.5000	48	135	73	7.16	7543	41.337
2	409	0.0646	396	420	406	1.10	76	0.445
3	20000 (UM)	0.1789	17805	29672	20634	5.25	1409	14.789

TIC:	0.0646	ng/uL
TIM:	0.2617	nmole/L
Total concentration:	0.6482	ng/uL

```
Sample peak width (sec): 5    Sample min peak height: 50    Sample baseline V to V?: Y    Sample baseline V to V points: 3
Sample filter: Binomial       Number of points for filter: 3  Sample start region (min): 0  Sample end region (min): 30
Marker peak width (sec): 5    Marker min peak height: 200   Marker baseline V to V?: Y    Marker baseline V to V points: 3
Lower marker selection: First peak > 500 RFU                Upper marker selection: Last peak > 200 RFU
Ladder size (bp)75, 200, 300, 400, 500, 700, 1000, 1500, 2000, 3000, 4000, 5000, 7000, 10000, 20000
Quantification using: Lower Marker          Final concentration (ng/uL): 0.5000          Dilution factor: 12.0
```

Sample: SampA9
Well location: A9
Created: Wednesday, August 11, 2021 6:14:43 PM

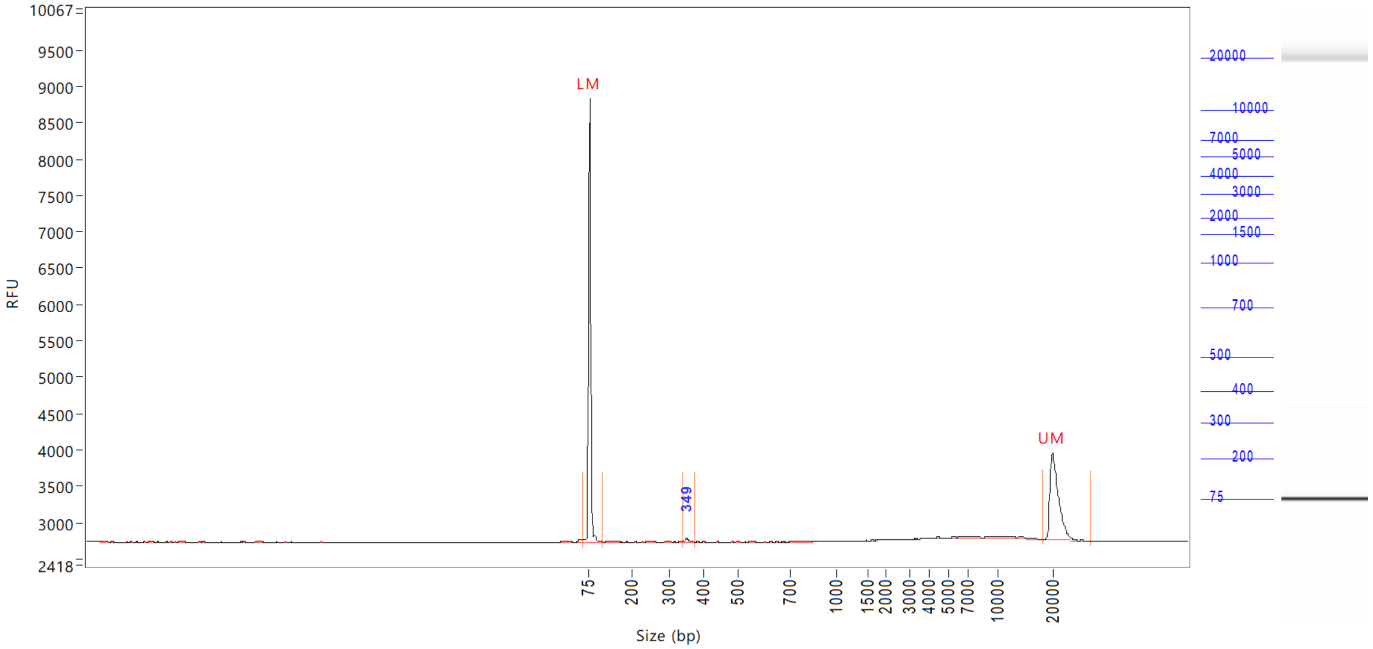

Peak	Size (bp)	Concentration (ng/uL)	From (bp)	To (bp)	Average size (bp)	CV%	RFU	Corrected peak area
1	75 (LM)	0.5000	56	114	74	5.36	6111	32.336
2	349	0.0755	337	376	351	2.26	58	0.407
3	20000 (UM)	0.1880	18293	26891	20468	4.58	1189	12.155

TIC:	0.0755	ng/uL
TIM:	0.3539	nmole/L
Total concentration:	0.7918	ng/uL

Sample peak width (sec): 5 Sample min peak height: 50 Sample baseline V to V?: Y Sample baseline V to V points: 3
Sample filter: Binomial Number of points for filter: 3 Sample start region (min): 0 Sample end region (min): 30
Marker peak width (sec): 5 Marker min peak height: 200 Marker baseline V to V?: Y Marker baseline V to V points: 3
Lower marker selection: First peak > 500 RFU Upper marker selection: Last peak > 200 RFU
Ladder size (bp)75, 200, 300, 400, 500, 700, 1000, 1500, 2000, 3000, 4000, 5000, 7000, 10000, 20000
Quantification using: Lower Marker Final concentration (ng/uL): 0.5000 Dilution factor: 12.0

Sample: SampB9
Well location: B9
Created: Wednesday, August 11, 2021 6:14:43 PM

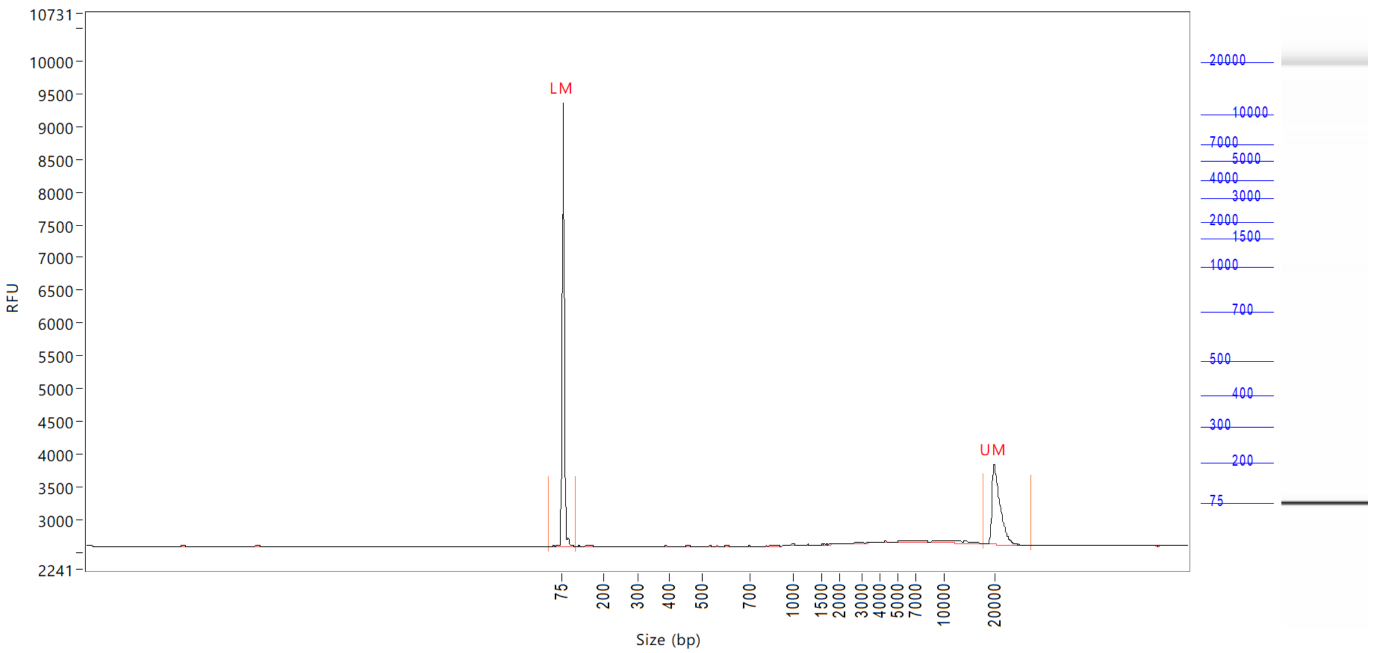

Peak	Size (bp)	Concentration (ng/uL)	From (bp)	To (bp)	Average size (bp)	CV%	RFU	Corrected peak area
1	75 (LM)	0.5000	30	114	73	6.10	6787	37.097
2	20000 (UM)	0.1714	17927	27133	20519	4.94	1224	12.715

TIC:	0.0000	ng/uL
TIM:	0.0000	nmole/L
Total concentration:	0.7406	ng/uL

```
Sample peak width (sec): 5    Sample min peak height: 50    Sample baseline V to V?: Y    Sample baseline V to V points: 3
Sample filter: Binomial       Number of points for filter: 3  Sample start region (min): 0  Sample end region (min): 30
Marker peak width (sec): 5    Marker min peak height: 200   Marker baseline V to V?: Y    Marker baseline V to V points: 3
Lower marker selection: First peak > 500 RFU                Upper marker selection: Last peak > 200 RFU
Ladder size (bp)75, 200, 300, 400, 500, 700, 1000, 1500, 2000, 3000, 4000, 5000, 7000, 10000, 20000
Quantification using: Lower Marker          Final concentration (ng/uL): 0.5000          Dilution factor: 12.0
```

Sample: SampC9
Well location: C9
Created: Wednesday, August 11, 2021 6:14:43 PM

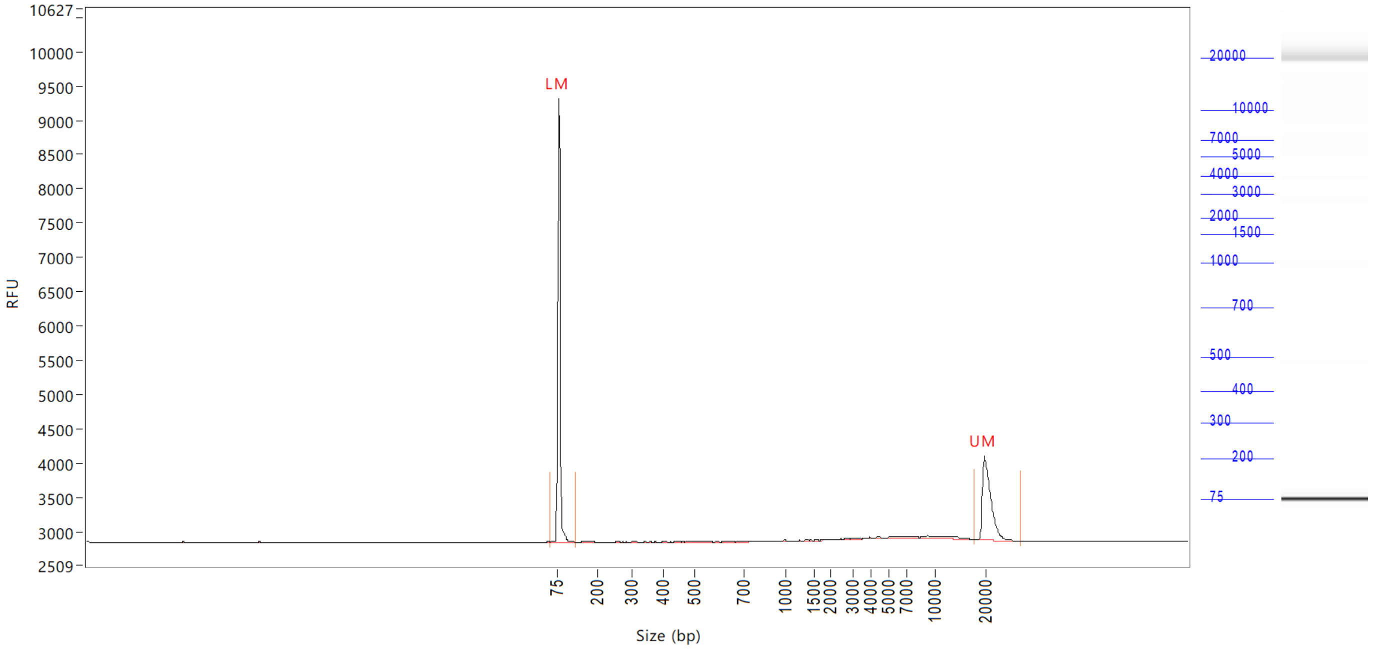

Peak	Size (bp)	Concentration (ng/uL)	From (bp)	To (bp)	Average size (bp)	CV%	RFU	Corrected peak area
1	75 (LM)	0.5000	48	128	74	6.60	6488	36.096
2	20000 (UM)	0.1812	18049	27133	20612	5.04	1219	13.083

TIC:	0.0000	ng/uL
TIM:	0.0000	nmole/L
Total concentration:	0.7992	ng/uL

```
Sample peak width (sec): 5    Sample min peak height: 50    Sample baseline V to V?: Y    Sample baseline V to V points: 3
Sample filter: Binomial       Number of points for filter: 3  Sample start region (min): 0  Sample end region (min): 30
Marker peak width (sec): 5    Marker min peak height: 200   Marker baseline V to V?: Y    Marker baseline V to V points: 3
Lower marker selection: First peak > 500 RFU                Upper marker selection: Last peak > 200 RFU
Ladder size (bp)75, 200, 300, 400, 500, 700, 1000, 1500, 2000, 3000, 4000, 5000, 7000, 10000, 20000
Quantification using: Lower Marker          Final concentration (ng/uL): 0.5000          Dilution factor: 12.0
```

Sample: SampD9
Well location: D9
Created: Wednesday, August 11, 2021 6:14:43 PM

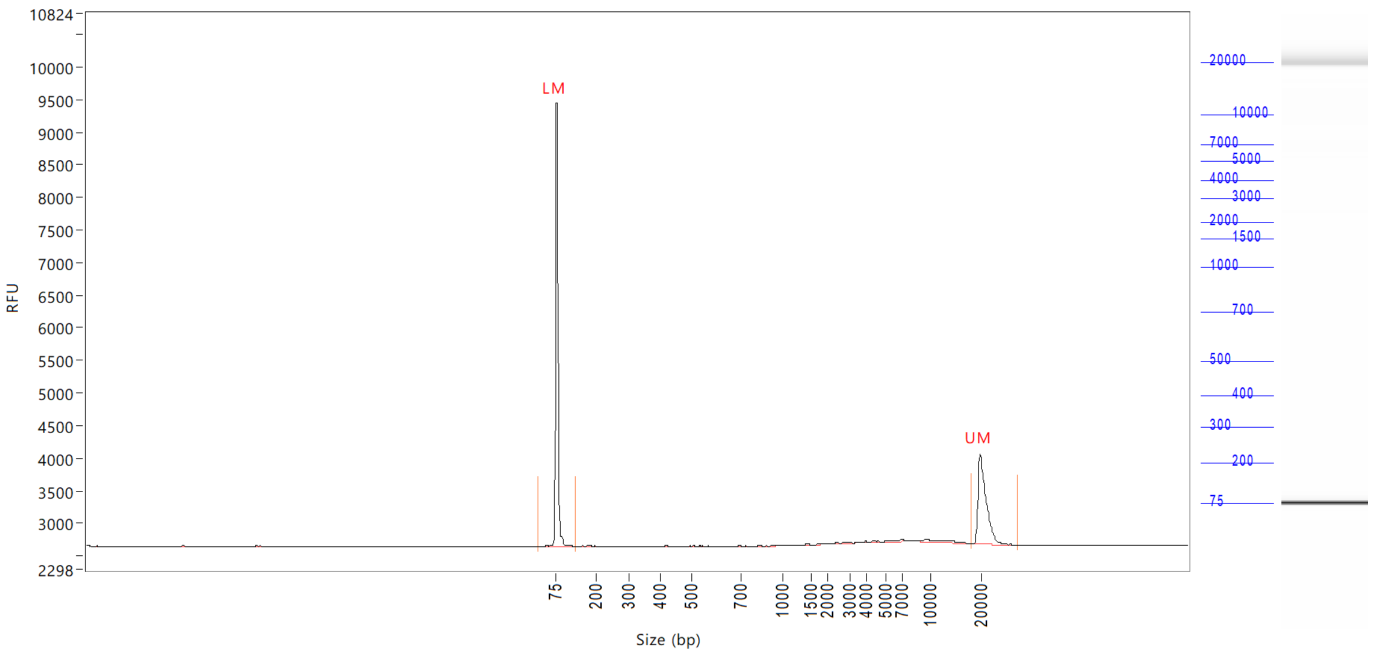

Peak	Size (bp)	Concentration (ng/uL)	From (bp)	To (bp)	Average size (bp)	CV%	RFU	Corrected peak area
1	75 (LM)	0.5000	18	137	74	7.53	6816	39.682
2	20000 (UM)	0.1835	18293	27375	20544	4.95	1386	14.566

TIC:	0.0000	ng/uL
TIM:	0.0000	nmole/L
Total concentration:	0.6351	ng/uL

```
Sample peak width (sec): 5    Sample min peak height: 50    Sample baseline V to V?: Y    Sample baseline V to V points: 3
Sample filter: Binomial       Number of points for filter: 3  Sample start region (min): 0  Sample end region (min): 30
Marker peak width (sec): 5    Marker min peak height: 200   Marker baseline V to V?: Y    Marker baseline V to V points: 3
Lower marker selection: First peak > 500 RFU                Upper marker selection: Last peak > 200 RFU
Ladder size (bp)75, 200, 300, 400, 500, 700, 1000, 1500, 2000, 3000, 4000, 5000, 7000, 10000, 20000
Quantification using: Lower Marker          Final concentration (ng/uL): 0.5000          Dilution factor: 12.0
```

Sample: SampE9
Well location: E9
Created: Wednesday, August 11, 2021 6:14:43 PM

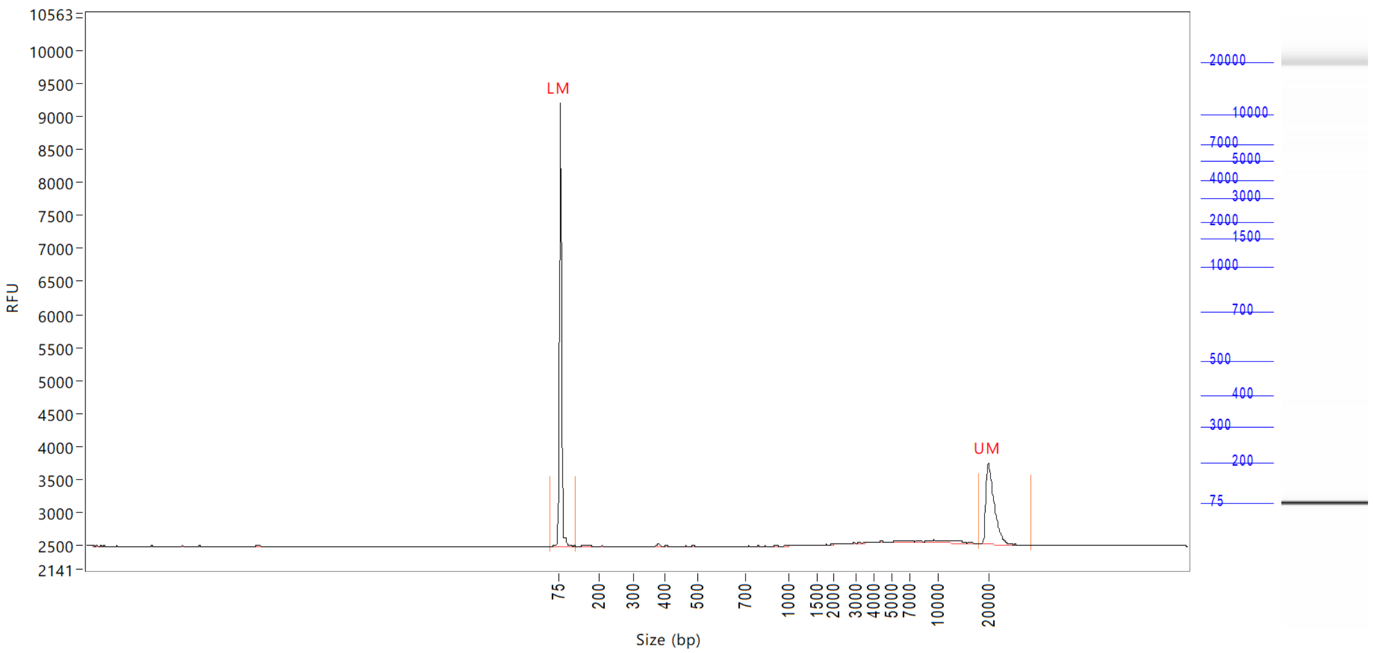

Peak	Size (bp)	Concentration (ng/uL)	From (bp)	To (bp)	Average size (bp)	CV%	RFU	Corrected peak area
1	75 (LM)	0.5000	47	126	74	6.13	6733	37.638
2	20000 (UM)	0.1752	18049	28463	20560	5.11	1242	13.189

TIC:	0.0000	ng/uL
TIM:	0.0000	nmole/L
Total concentration:	0.7604	ng/uL

```
Sample peak width (sec): 5    Sample min peak height: 50    Sample baseline V to V?: Y    Sample baseline V to V points: 3
Sample filter: Binomial       Number of points for filter: 3  Sample start region (min): 0  Sample end region (min): 30
Marker peak width (sec): 5    Marker min peak height: 200   Marker baseline V to V?: Y    Marker baseline V to V points: 3
Lower marker selection: First peak > 500 RFU                Upper marker selection: Last peak > 200 RFU
Ladder size (bp)75, 200, 300, 400, 500, 700, 1000, 1500, 2000, 3000, 4000, 5000, 7000, 10000, 20000
Quantification using: Lower Marker          Final concentration (ng/uL): 0.5000          Dilution factor: 12.0
```

Sample: SampF9
Well location: F9
Created: Wednesday, August 11, 2021 6:14:43 PM

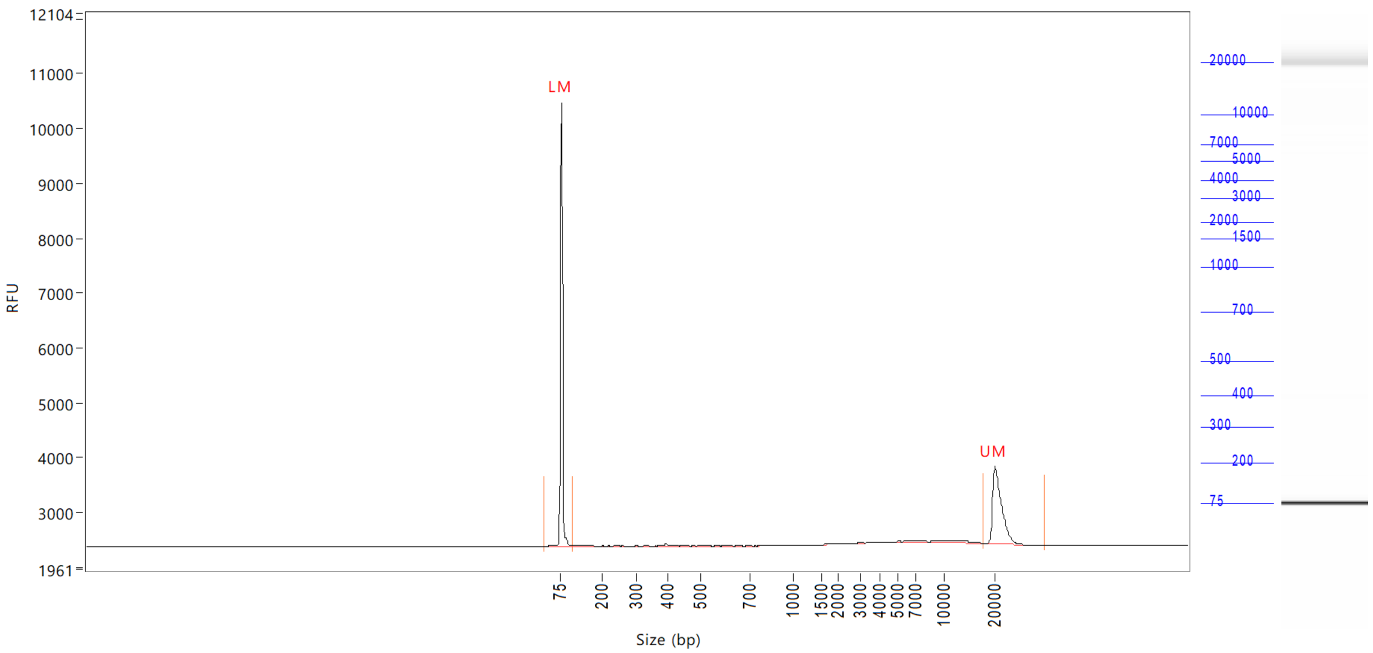

Peak	Size (bp)	Concentration (ng/uL)	From (bp)	To (bp)	Average size (bp)	CV%	RFU	Corrected peak area
1	75 (LM)	0.5000	24	112	73	5.89	8102	44.369
2	20000 (UM)	0.1834	17683	29672	20617	5.21	1411	16.277

TIC:	0.0000	ng/uL
TIM:	0.0000	nmole/L
Total concentration:	0.5813	ng/uL

```
Sample peak width (sec): 5    Sample min peak height: 50    Sample baseline V to V?: Y    Sample baseline V to V points: 3
Sample filter: Binomial       Number of points for filter: 3  Sample start region (min): 0  Sample end region (min): 30
Marker peak width (sec): 5    Marker min peak height: 200   Marker baseline V to V?: Y    Marker baseline V to V points: 3
Lower marker selection: First peak > 500 RFU                Upper marker selection: Last peak > 200 RFU
Ladder size (bp)75, 200, 300, 400, 500, 700, 1000, 1500, 2000, 3000, 4000, 5000, 7000, 10000, 20000
Quantification using: Lower Marker          Final concentration (ng/uL): 0.5000          Dilution factor: 12.0
```

Sample: SampG9
Well location: G9
Created: Wednesday, August 11, 2021 6:14:43 PM

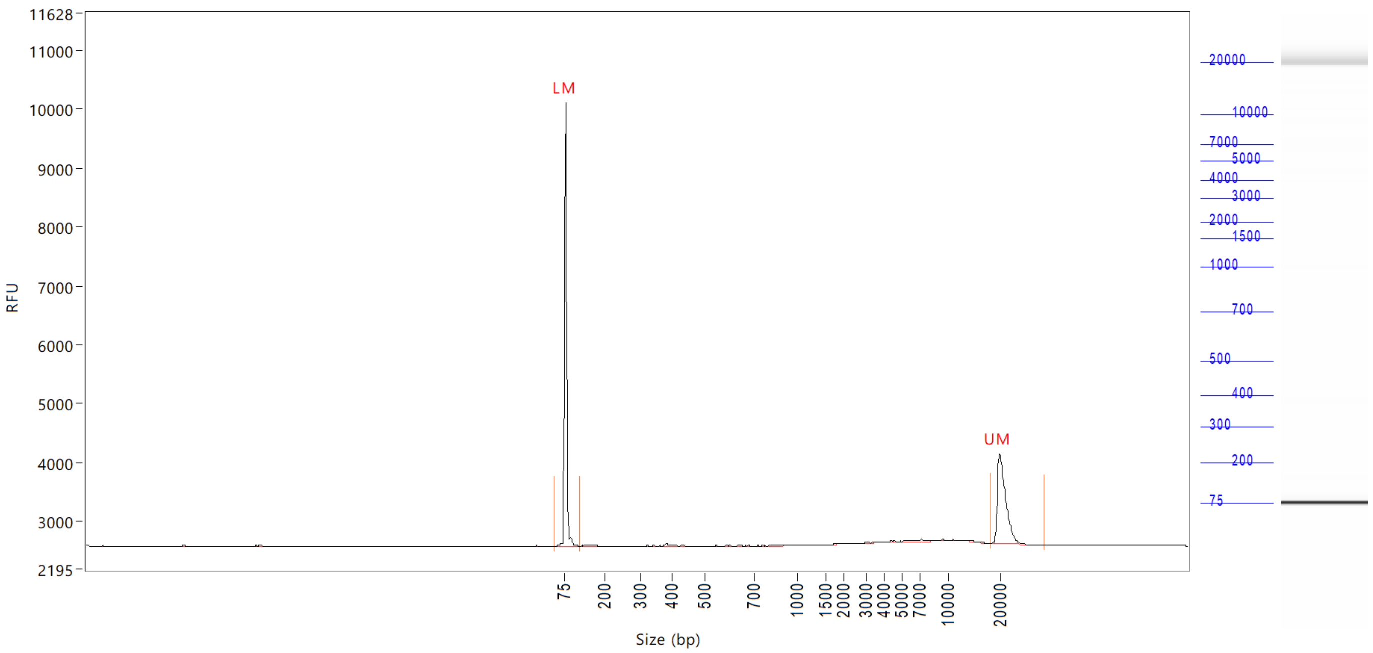

Peak	Size (bp)	Concentration (ng/uL)	From (bp)	To (bp)	Average size (bp)	CV%	RFU	Corrected peak area
1	75 (LM)	0.5000	45	120	74	6.27	7542	42.205
2	20000 (UM)	0.1874	18171	28826	20573	4.91	1531	15.819

TIC:	0.0000	ng/uL
TIM:	0.0000	nmole/L
Total concentration:	0.6071	ng/uL

Sample peak width (sec): 5 Sample min peak height: 50 Sample baseline V to V?: Y Sample baseline V to V points: 3
Sample filter: Binomial Number of points for filter: 3 Sample start region (min): 0 Sample end region (min): 30
Marker peak width (sec): 5 Marker min peak height: 200 Marker baseline V to V?: Y Marker baseline V to V points: 3
Lower marker selection: First peak > 500 RFU Upper marker selection: Last peak > 200 RFU
Ladder size (bp)75, 200, 300, 400, 500, 700, 1000, 1500, 2000, 3000, 4000, 5000, 7000, 10000, 20000
Quantification using: Lower Marker Final concentration (ng/uL): 0.5000 Dilution factor: 12.0

Sample: SampH9
Well location: H9
Created: Wednesday, August 11, 2021 6:14:43 PM

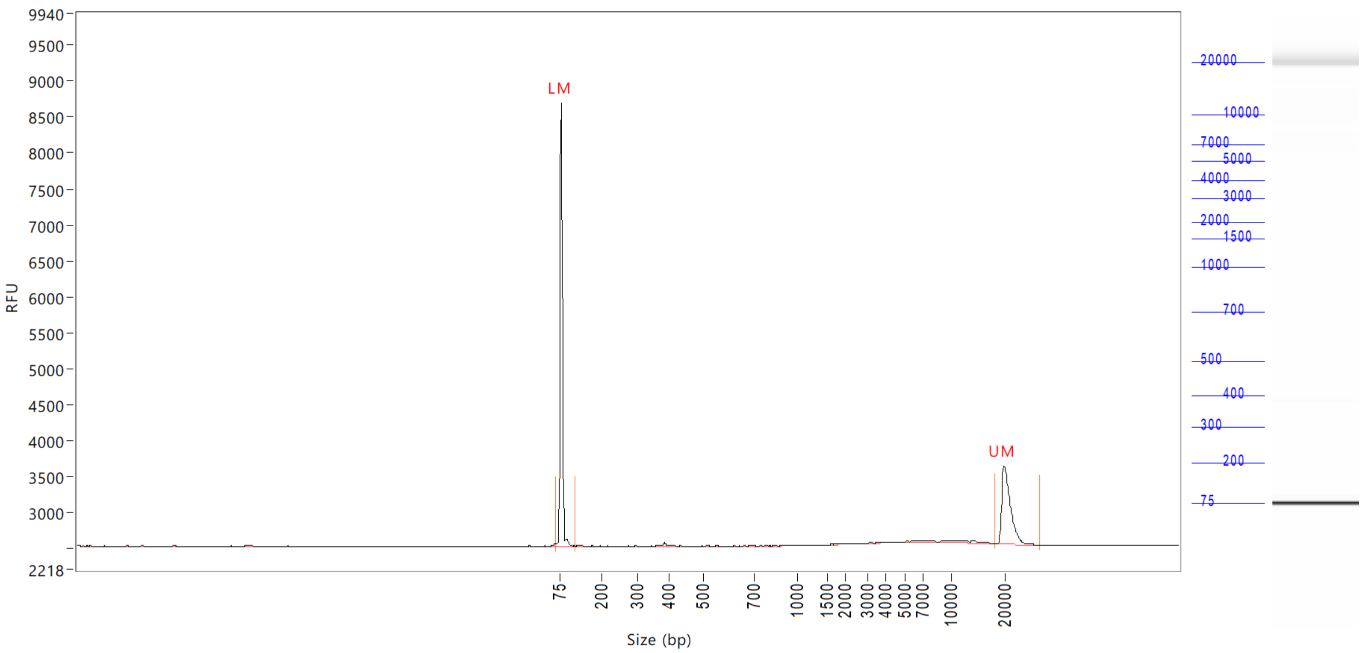

Peak	Size (bp)	Concentration (ng/uL)	From (bp)	To (bp)	Average size (bp)	CV%	RFU	Corrected peak area
1	75 (LM)	0.5000	62	118	73	5.73	6169	33.144
2	20000 (UM)	0.1766	18293	26891	20543	4.92	1096	11.709

TIC:	0.0000	ng/uL
TIM:	0.0000	nmole/L
Total concentration:	0.7339	ng/uL

Sample peak width (sec): 5 Sample min peak height: 50 Sample baseline V to V?: Y Sample baseline V to V points: 3
Sample filter: Binomial Number of points for filter: 3 Sample start region (min): 0 Sample end region (min): 30
Marker peak width (sec): 5 Marker min peak height: 200 Marker baseline V to V?: Y Marker baseline V to V points: 3
Lower marker selection: First peak > 500 RFU Upper marker selection: Last peak > 200 RFU
Ladder size (bp)75, 200, 300, 400, 500, 700, 1000, 1500, 2000, 3000, 4000, 5000, 7000, 10000, 20000
Quantification using: Lower Marker Final concentration (ng/uL): 0.5000 Dilution factor: 12.0

Sample: SampA10
Well location: A10
Created: Wednesday, August 11, 2021 6:14:43 PM

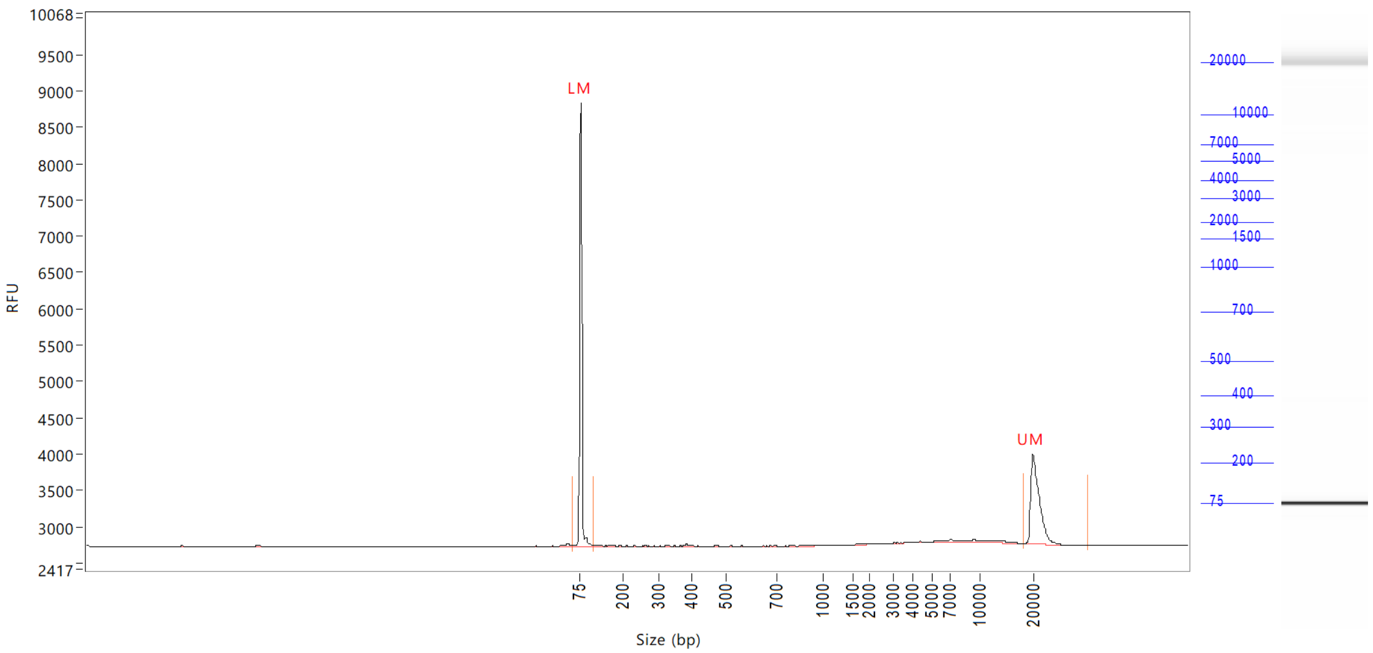

Peak	Size (bp)	Concentration (ng/uL)	From (bp)	To (bp)	Average size (bp)	CV%	RFU	Corrected peak area
1	75 (LM)	0.5000	52	112	73	6.03	6114	32.980
2	20000 (UM)	0.1996	18171	30277	20573	5.25	1248	13.162

TIC:	0.0000	ng/uL
TIM:	0.0000	nmole/L
Total concentration:	0.7859	ng/uL

```
Sample peak width (sec): 5    Sample min peak height: 50    Sample baseline V to V?: Y    Sample baseline V to V points: 3
Sample filter: Binomial       Number of points for filter: 3  Sample start region (min): 0  Sample end region (min): 30
Marker peak width (sec): 5    Marker min peak height: 200   Marker baseline V to V?: Y    Marker baseline V to V points: 3
Lower marker selection: First peak > 500 RFU                Upper marker selection: Last peak > 200 RFU
Ladder size (bp)75, 200, 300, 400, 500, 700, 1000, 1500, 2000, 3000, 4000, 5000, 7000, 10000, 20000
Quantification using: Lower Marker          Final concentration (ng/uL): 0.5000          Dilution factor: 12.0
```

Sample: SampB10
Well location: B10
Created: Wednesday, August 11, 2021 6:14:43 PM

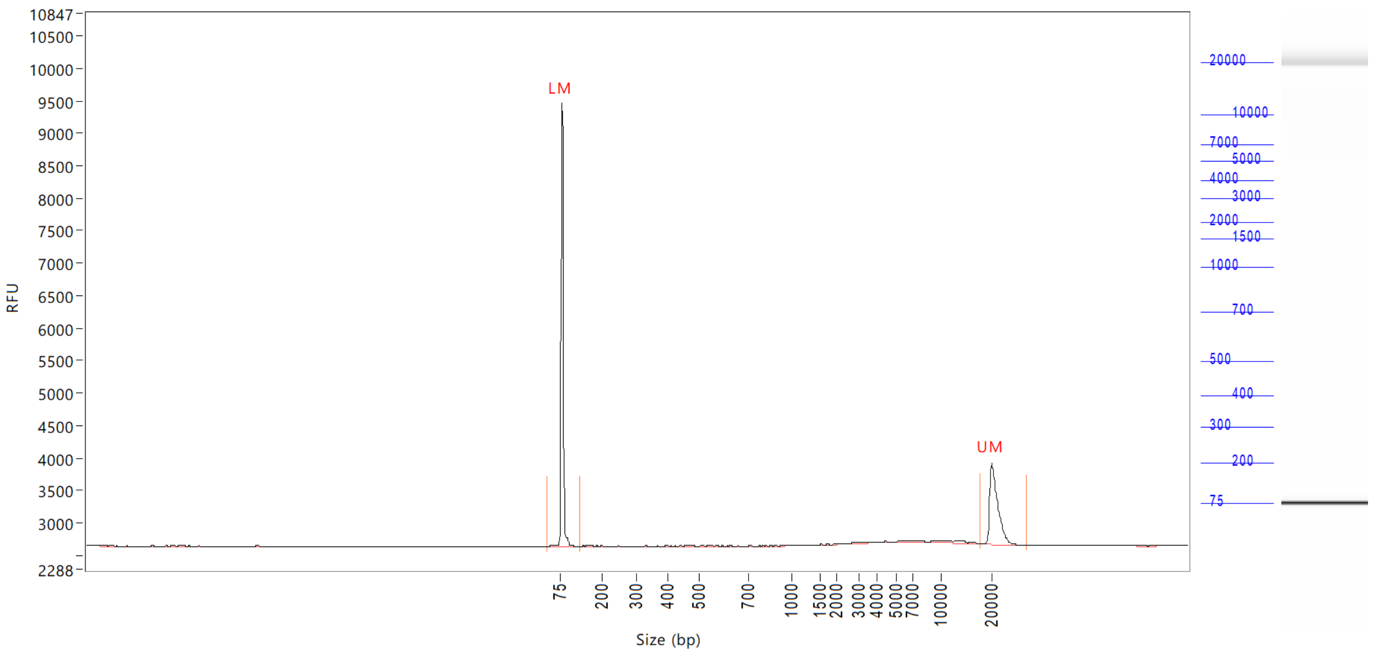

Peak	Size (bp)	Concentration (ng/uL)	From (bp)	To (bp)	Average size (bp)	CV%	RFU	Corrected peak area
1	75 (LM)	0.5000	31	131	74	6.45	6840	38.047
2	20000 (UM)	0.1728	17561	26891	20531	4.73	1258	13.146

TIC:	0.0000	ng/uL
TIM:	0.0000	nmole/L
Total concentration:	0.7422	ng/uL

Sample peak width (sec): 5 Sample min peak height: 50 Sample baseline V to V?: Y Sample baseline V to V points: 3
Sample filter: Binomial Number of points for filter: 3 Sample start region (min): 0 Sample end region (min): 30
Marker peak width (sec): 5 Marker min peak height: 200 Marker baseline V to V?: Y Marker baseline V to V points: 3
Lower marker selection: First peak > 500 RFU Upper marker selection: Last peak > 200 RFU
Ladder size (bp)75, 200, 300, 400, 500, 700, 1000, 1500, 2000, 3000, 4000, 5000, 7000, 10000, 20000
Quantification using: Lower Marker Final concentration (ng/uL): 0.5000 Dilution factor: 12.0

Sample: SampC10
Well location: C10
Created: Wednesday, August 11, 2021 6:14:43 PM

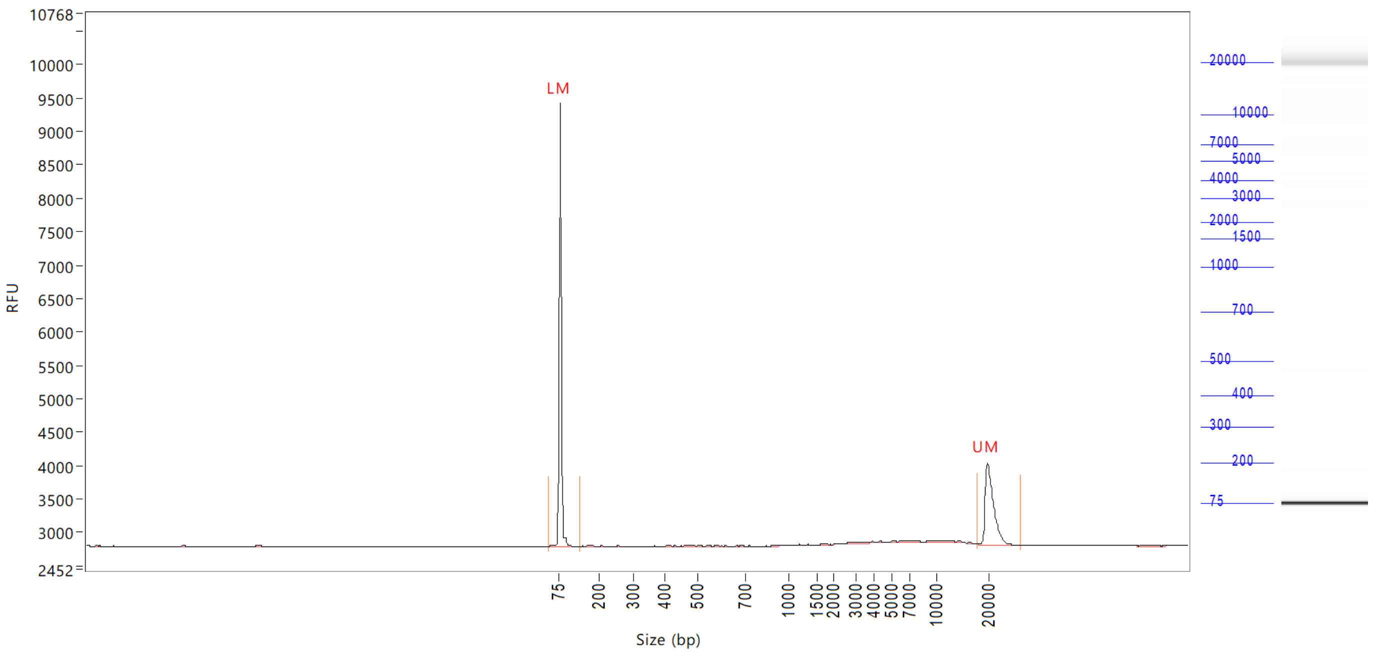

Peak	Size (bp)	Concentration (ng/uL)	From (bp)	To (bp)	Average size (bp)	CV%	RFU	Corrected peak area
1	75 (LM)	0.5000	39	137	73	6.80	6647	36.639
2	20000 (UM)	0.1768	17927	26529	20522	4.76	1229	12.952

TIC:	0.0000	ng/uL
TIM:	0.0000	nmole/L
Total concentration:	0.7151	ng/uL

Sample peak width (sec): 5 Sample min peak height: 50 Sample baseline V to V?: Y Sample baseline V to V points: 3
Sample filter: Binomial Number of points for filter: 3 Sample start region (min): 0 Sample end region (min): 30
Marker peak width (sec): 5 Marker min peak height: 200 Marker baseline V to V?: Y Marker baseline V to V points: 3
Lower marker selection: First peak > 500 RFU Upper marker selection: Last peak > 200 RFU
Ladder size (bp)75, 200, 300, 400, 500, 700, 1000, 1500, 2000, 3000, 4000, 5000, 7000, 10000, 20000
Quantification using: Lower Marker Final concentration (ng/uL): 0.5000 Dilution factor: 12.0

Sample: SampD10
Well location: D10
Created: Wednesday, August 11, 2021 6:14:43 PM

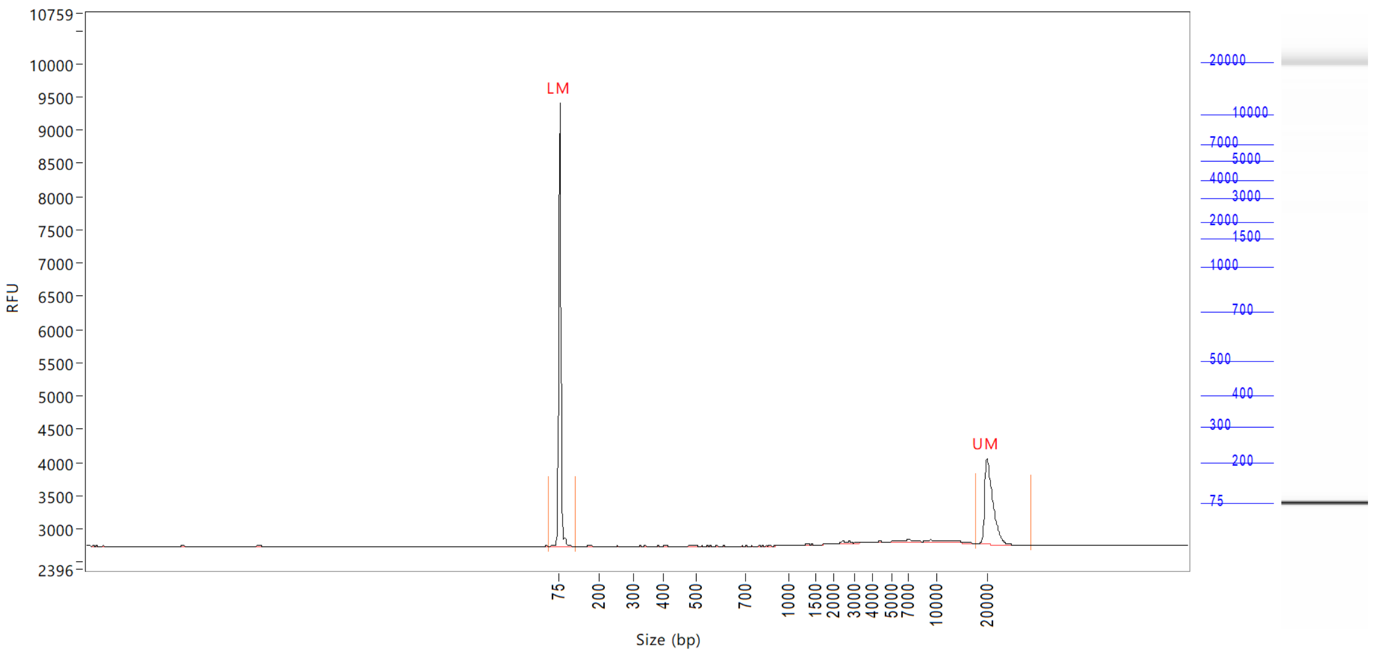

Peak	Size (bp)	Concentration (ng/uL)	From (bp)	To (bp)	Average size (bp)	CV%	RFU	Corrected peak area
1	75 (LM)	0.5000	43	128	74	6.22	6685	37.724
2	20000 (UM)	0.1794	17683	28705	20539	4.92	1287	13.538

TIC:	0.0000	ng/uL
TIM:	0.0000	nmole/L
Total concentration:	0.7290	ng/uL

Sample peak width (sec): 5   Sample min peak height: 50   Sample baseline V to V?: Y   Sample baseline V to V points: 3
Sample filter: Binomial   Number of points for filter: 3 Sample start region (min): 0 Sample end region (min): 30
Marker peak width (sec): 5   Marker min peak height: 200   Marker baseline V to V?: Y   Marker baseline V to V points: 3
Lower marker selection: First peak > 500 RFU   Upper marker selection: Last peak > 200 RFU
Ladder size (bp)75, 200, 300, 400, 500, 700, 1000, 1500, 2000, 3000, 4000, 5000, 7000, 10000, 20000
Quantification using: Lower Marker   Final concentration (ng/uL): 0.5000   Dilution factor: 12.0

Sample: SampE10
Well location: E10
Created: Wednesday, August 11, 2021 6:14:43 PM

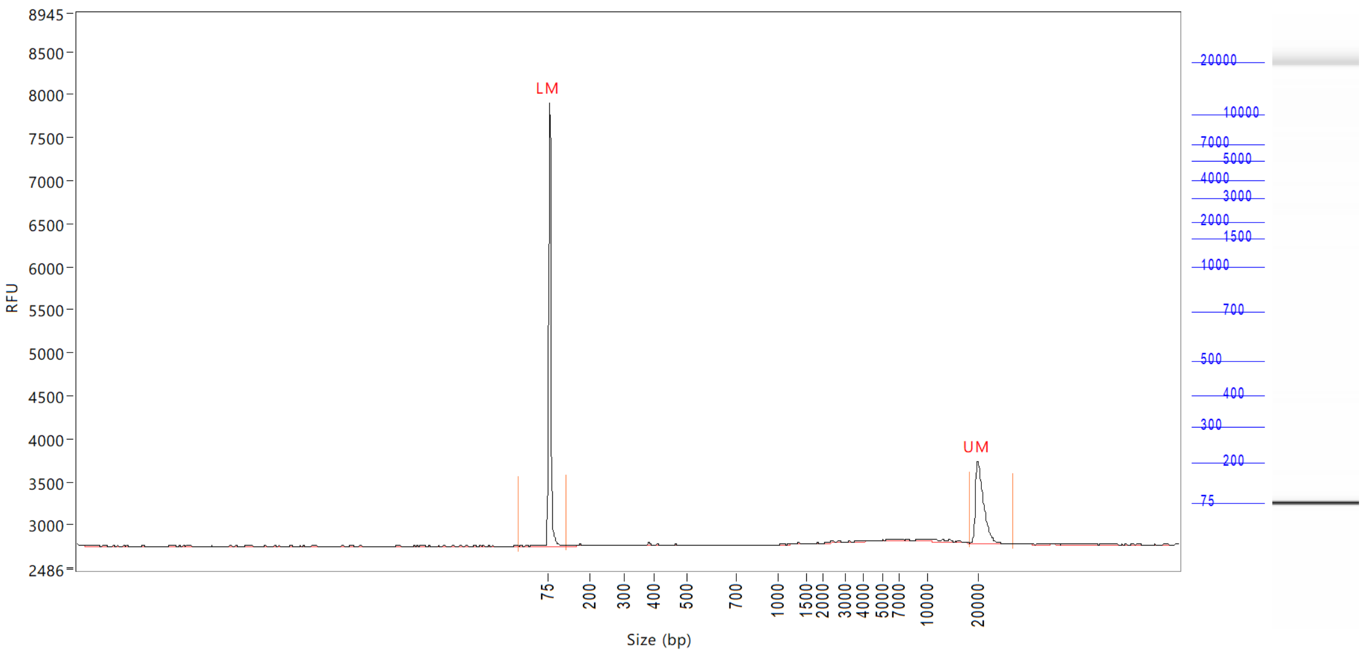

Peak	Size (bp)	Concentration (ng/uL)	From (bp)	To (bp)	Average size (bp)	CV%	RFU	Corrected peak area
1	75 (LM)	0.5000	0	128	73	10.59	5158	29.373
2	20000 (UM)	0.1682	18293	27012	20514	4.83	957	9.881

TIC:	0.0000	ng/uL
TIM:	0.0000	nmole/L
Total concentration:	0.9042	ng/uL

Sample peak width (sec): 5   Sample min peak height: 50   Sample baseline V to V?: Y   Sample baseline V to V points: 3
Sample filter: Binomial   Number of points for filter: 3 Sample start region (min): 0 Sample end region (min): 30
Marker peak width (sec): 5   Marker min peak height: 200   Marker baseline V to V?: Y   Marker baseline V to V points: 3
Lower marker selection: First peak > 500 RFU   Upper marker selection: Last peak > 200 RFU
Ladder size (bp)75, 200, 300, 400, 500, 700, 1000, 1500, 2000, 3000, 4000, 5000, 7000, 10000, 20000
Quantification using: Lower Marker   Final concentration (ng/uL): 0.5000   Dilution factor: 12.0

Sample: SampF10
Well location: F10
Created: Wednesday, August 11, 2021 6:14:43 PM

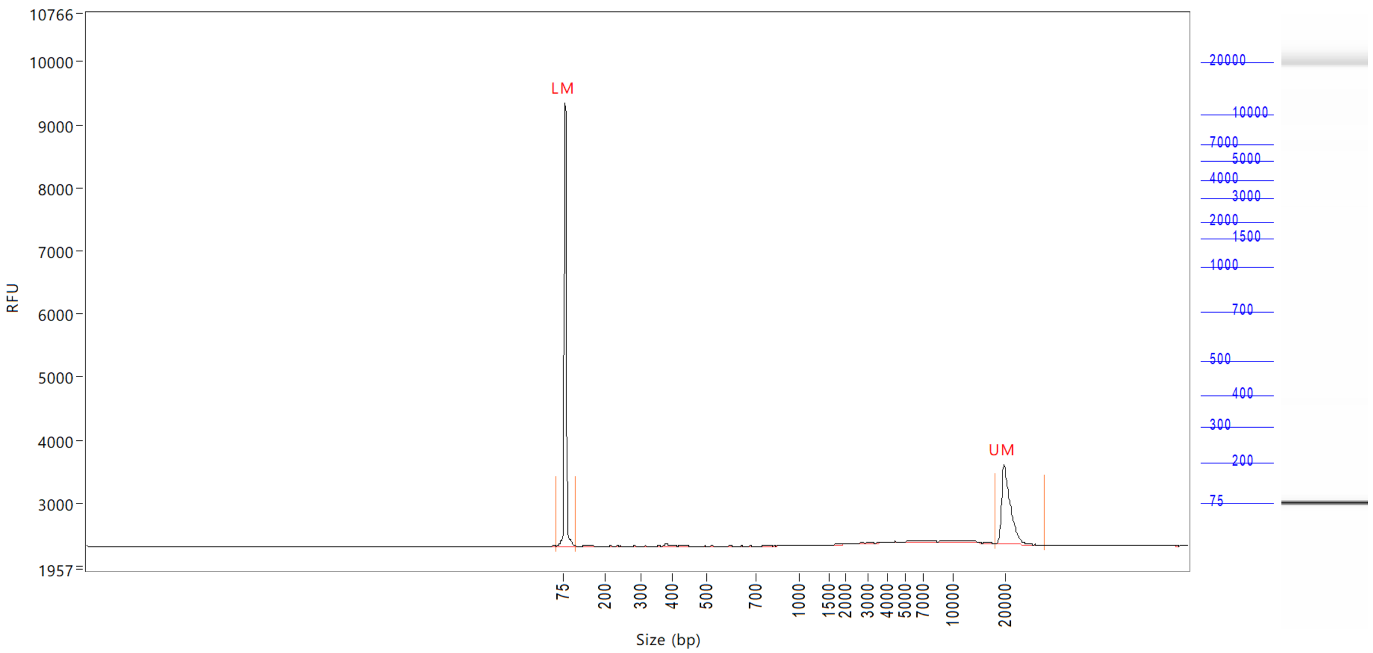

Peak	Size (bp)	Concentration (ng/uL)	From (bp)	To (bp)	Average size (bp)	CV%	RFU	Corrected peak area
1	75 (LM)	0.5000	48	108	74	5.56	7038	39.558
2	20000 (UM)	0.1760	18293	27859	20546	5.20	1255	13.928

TIC:	0.0000	ng/uL
TIM:	0.0000	nmole/L
Total concentration:	0.6497	ng/uL

Sample peak width (sec): 5   Sample min peak height: 50   Sample baseline V to V?: Y   Sample baseline V to V points: 3
Sample filter: Binomial   Number of points for filter: 3 Sample start region (min): 0 Sample end region (min): 30
Marker peak width (sec): 5   Marker min peak height: 200   Marker baseline V to V?: Y   Marker baseline V to V points: 3
Lower marker selection: First peak > 500 RFU   Upper marker selection: Last peak > 200 RFU
Ladder size (bp)75, 200, 300, 400, 500, 700, 1000, 1500, 2000, 3000, 4000, 5000, 7000, 10000, 20000
Quantification using: Lower Marker   Final concentration (ng/uL): 0.5000   Dilution factor: 12.0

Sample: SampG10
Well location: G10
Created: Wednesday, August 11, 2021 6:14:43 PM

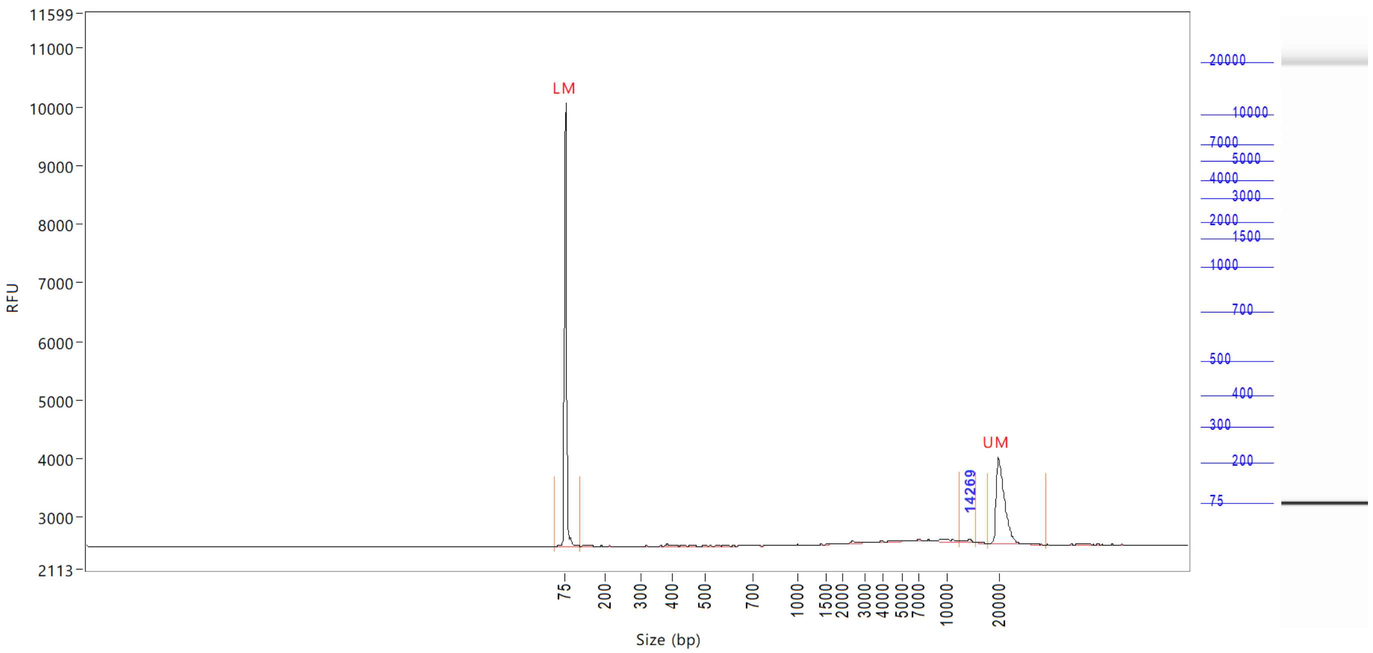

Peak	Size (bp)	Concentration (ng/uL)	From (bp)	To (bp)	Average size (bp)	CV%	RFU	Corrected peak area
1	75 (LM)	0.5000	45	120	73	6.07	7581	41.576
2	14269	0.0808	12440	15366	13821	5.54	50	0.560
3	20000 (UM)	0.1875	17805	29309	20531	5.16	1486	15.590

TIC:	0.0808	ng/uL
TIM:	0.0096	nmole/L
Total concentration:	0.6847	ng/uL

Sample peak width (sec): 5 Sample min peak height: 50 Sample baseline V to V?: Y Sample baseline V to V points: 3
Sample filter: Binomial Number of points for filter: 3 Sample start region (min): 0 Sample end region (min): 30
Marker peak width (sec): 5 Marker min peak height: 200 Marker baseline V to V?: Y Marker baseline V to V points: 3
Lower marker selection: First peak > 500 RFU Upper marker selection: Last peak > 200 RFU
Ladder size (bp)75, 200, 300, 400, 500, 700, 1000, 1500, 2000, 3000, 4000, 5000, 7000, 10000, 20000
Quantification using: Lower Marker Final concentration (ng/uL): 0.5000 Dilution factor: 12.0

Sample: SampH10
Well location: H10
Created: Wednesday, August 11, 2021 6:14:43 PM

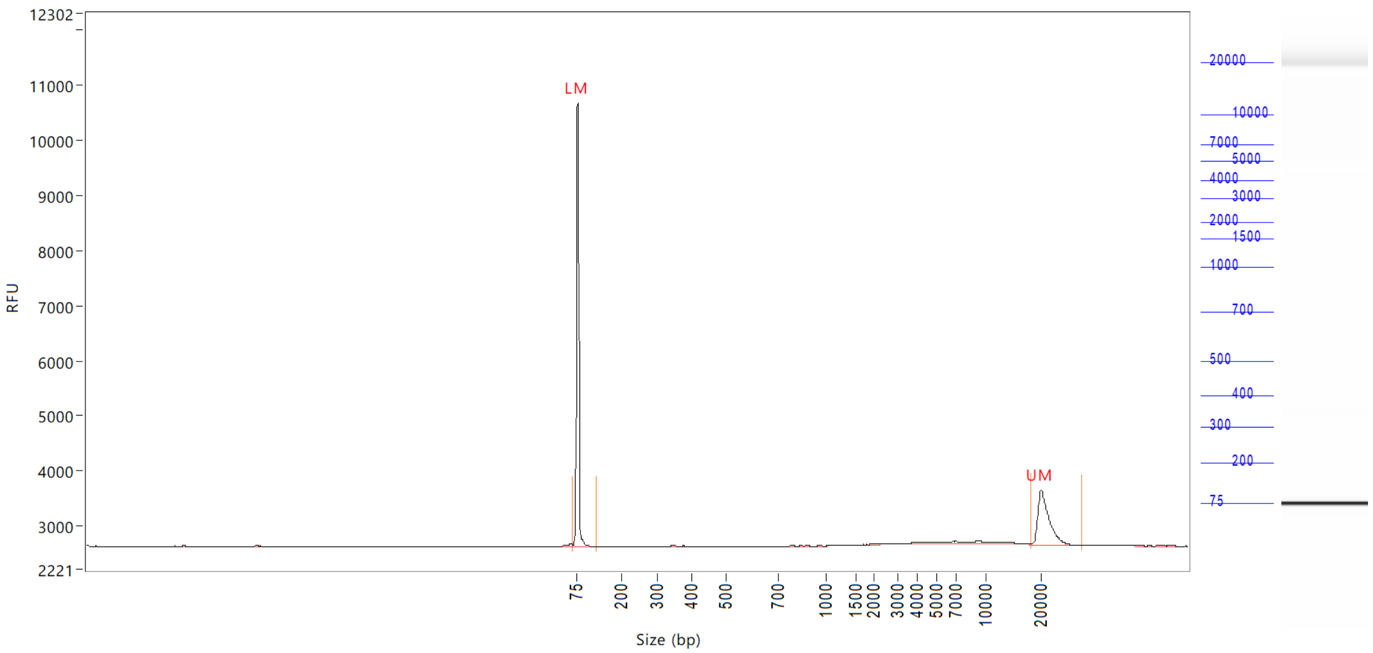

Peak	Size (bp)	Concentration (ng/uL)	From (bp)	To (bp)	Average size (bp)	CV%	RFU	Corrected peak area
1	75 (LM)	0.5000	62	129	73	5.81	8060	46.120
2	20000 (UM)	0.1453	18049	27496	20713	6.03	999	13.405

TIC:	0.0000	ng/uL
TIM:	0.0000	nmole/L
Total concentration:	0.7874	ng/uL

```
Sample peak width (sec): 5    Sample min peak height: 50    Sample baseline V to V?: Y    Sample baseline V to V points: 3
Sample filter: Binomial       Number of points for filter: 3  Sample start region (min): 0  Sample end region (min): 30
Marker peak width (sec): 5    Marker min peak height: 200   Marker baseline V to V?: Y    Marker baseline V to V points: 3
Lower marker selection: First peak > 500 RFU                Upper marker selection: Last peak > 200 RFU
Ladder size (bp)75, 200, 300, 400, 500, 700, 1000, 1500, 2000, 3000, 4000, 5000, 7000, 10000, 20000
Quantification using: Lower Marker          Final concentration (ng/uL): 0.5000          Dilution factor: 12.0
```

Sample: SampA11
Well location: A11
Created: Wednesday, August 11, 2021 6:14:43 PM

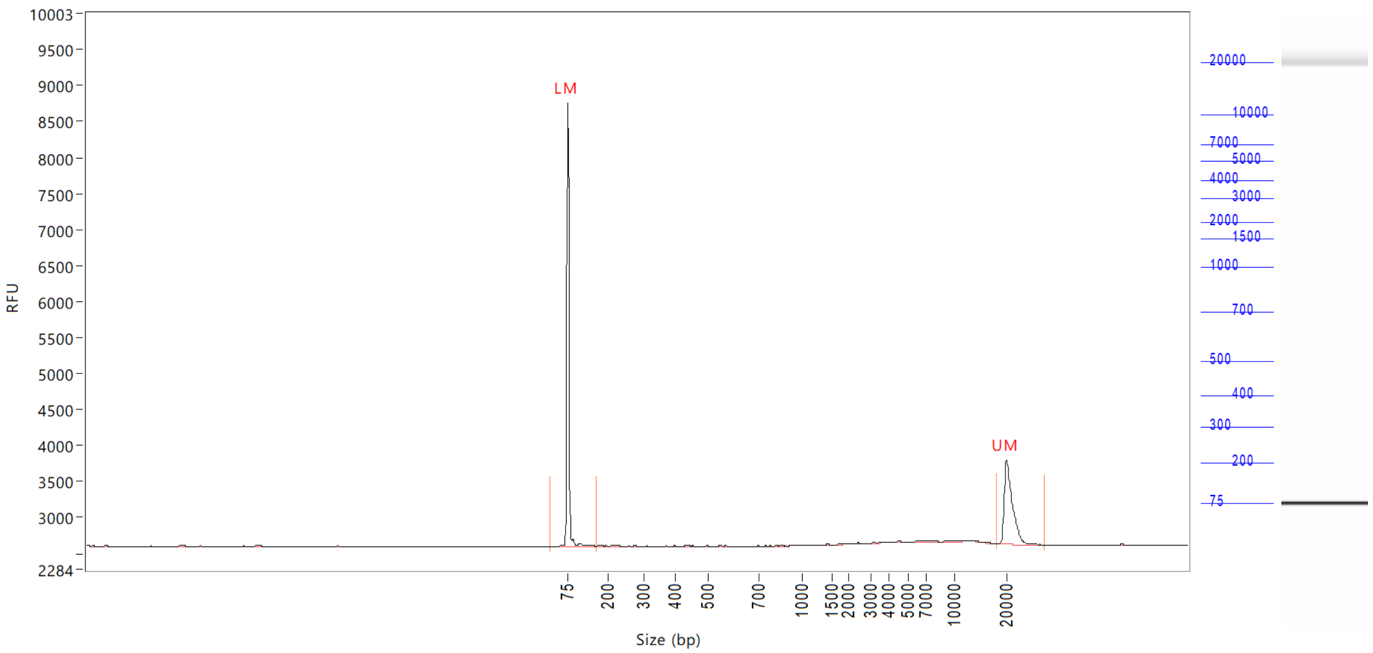

Peak	Size (bp)	Concentration (ng/uL)	From (bp)	To (bp)	Average size (bp)	CV%	RFU	Corrected peak area
1	75 (LM)	0.5000	24	160	74	11.08	6169	34.141
2	20000 (UM)	0.1793	17927	27254	20540	5.25	1171	12.243

TIC:	0.0000	ng/uL
TIM:	0.0000	nmole/L
Total concentration:	0.5803	ng/uL

Sample peak width (sec): 5 Sample min peak height: 50 Sample baseline V to V?: Y Sample baseline V to V points: 3
Sample filter: Binomial Number of points for filter: 3 Sample start region (min): 0 Sample end region (min): 30
Marker peak width (sec): 5 Marker min peak height: 200 Marker baseline V to V?: Y Marker baseline V to V points: 3
Lower marker selection: First peak > 500 RFU Upper marker selection: Last peak > 200 RFU
Ladder size (bp)75, 200, 300, 400, 500, 700, 1000, 1500, 2000, 3000, 4000, 5000, 7000, 10000, 20000
Quantification using: Lower Marker Final concentration (ng/uL): 0.5000 Dilution factor: 12.0

Sample: SampB11
Well location: B11
Created: Wednesday, August 11, 2021 6:14:43 PM

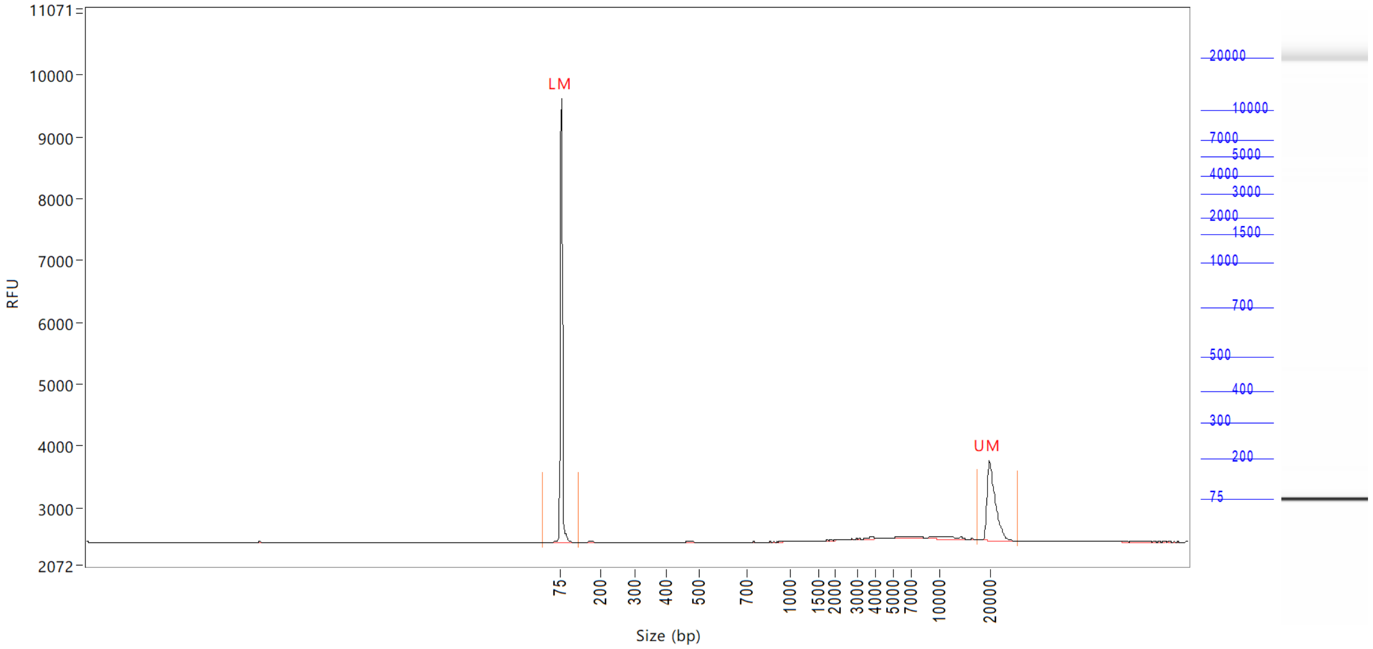

Peak	Size (bp)	Concentration (ng/uL)	From (bp)	To (bp)	Average size (bp)	CV%	RFU	Corrected peak area
1	75 (LM)	0.5000	18	131	73	6.83	7193	39.559
2	20000 (UM)	0.1721	17683	25440	20488	4.61	1306	13.619

TIC:	0.0000	ng/uL
TIM:	0.0000	nmole/L
Total concentration:	0.7414	ng/uL

Sample peak width (sec): 5 Sample min peak height: 50 Sample baseline V to V?: Y Sample baseline V to V points: 3
Sample filter: Binomial Number of points for filter: 3 Sample start region (min): 0 Sample end region (min): 30
Marker peak width (sec): 5 Marker min peak height: 200 Marker baseline V to V?: Y Marker baseline V to V points: 3
Lower marker selection: First peak > 500 RFU Upper marker selection: Last peak > 200 RFU
Ladder size (bp)75, 200, 300, 400, 500, 700, 1000, 1500, 2000, 3000, 4000, 5000, 7000, 10000, 20000
Quantification using: Lower Marker Final concentration (ng/uL): 0.5000 Dilution factor: 12.0

Sample: SampC11
Well location: C11
Created: Wednesday, August 11, 2021 6:14:43 PM

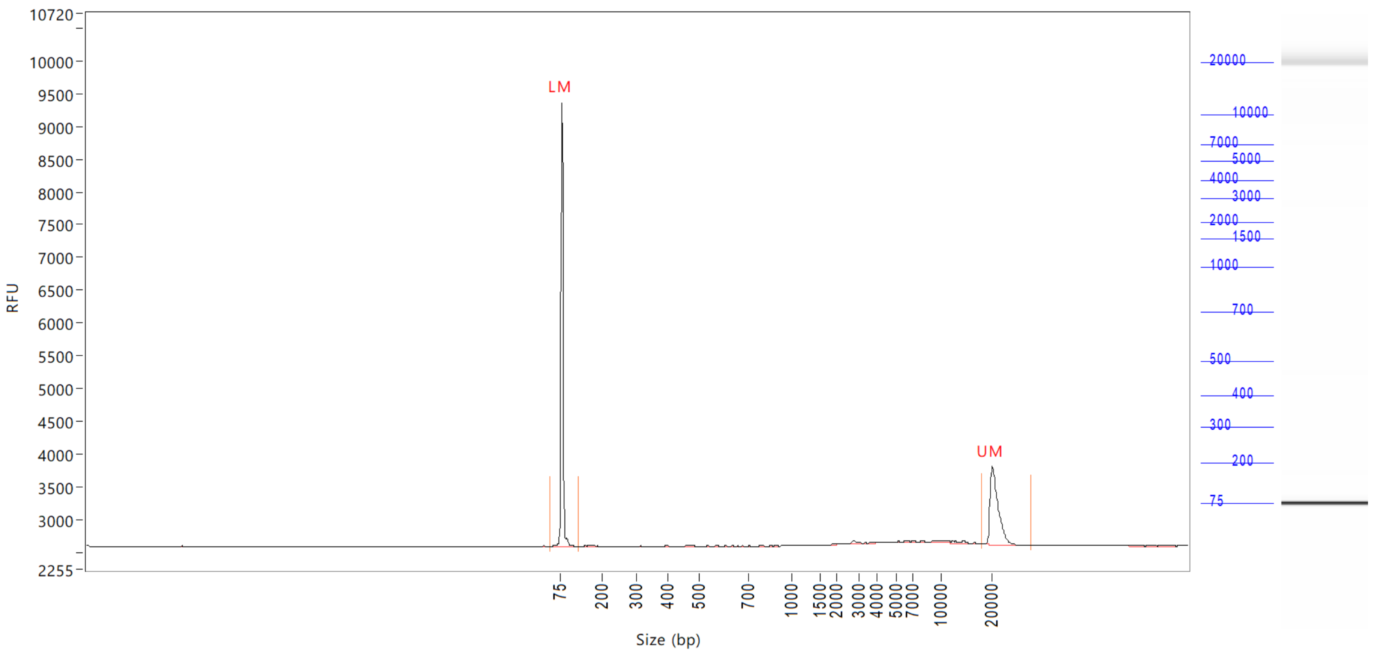

Peak	Size (bp)	Concentration (ng/uL)	From (bp)	To (bp)	Average size (bp)	CV%	RFU	Corrected peak area
1	75 (LM)	0.5000	41	129	73	6.25	6765	37.096
2	20000 (UM)	0.1705	18049	27617	20606	4.95	1204	12.648

TIC:	0.0000	ng/uL
TIM:	0.0000	nmole/L
Total concentration:	0.6582	ng/uL

Sample peak width (sec): 5   Sample min peak height: 50   Sample baseline V to V?: Y   Sample baseline V to V points: 3
Sample filter: Binomial   Number of points for filter: 3 Sample start region (min): 0 Sample end region (min): 30
Marker peak width (sec): 5   Marker min peak height: 200   Marker baseline V to V?: Y   Marker baseline V to V points: 3
Lower marker selection: First peak > 500 RFU   Upper marker selection: Last peak > 200 RFU
Ladder size (bp)75, 200, 300, 400, 500, 700, 1000, 1500, 2000, 3000, 4000, 5000, 7000, 10000, 20000
Quantification using: Lower Marker   Final concentration (ng/uL): 0.5000   Dilution factor: 12.0

Sample: SampD11
Well location: D11
Created: Wednesday, August 11, 2021 6:14:43 PM

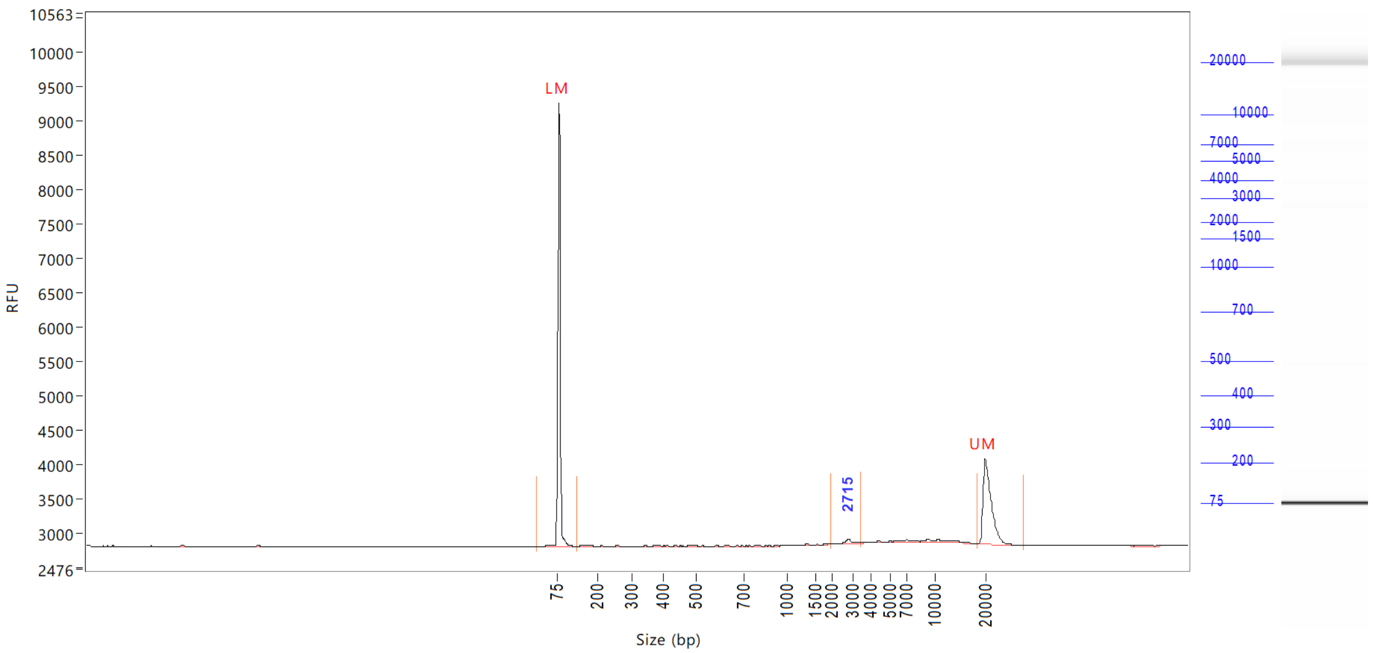

Peak	Size	Concentration	From	To	Average size	CV%	RFU	Corrected peak area
	(bp)	(ng/uL)	(bp)	(bp)	(bp)			
1	75 (LM)	0.5000	7	131	74	8.62	6465	36.221
2	2715	0.1219	1981	3358	2703	9.76	72	0.736
3	20000 (UM)	0.1799	18537	27617	20581	4.87	1244	13.034

TIC:	0.1219	ng/uL
TIM:	0.0743	nmole/L
Total concentration:	0.7896	ng/uL

Sample peak width (sec): 5   Sample min peak height: 50   Sample baseline V to V?: Y   Sample baseline V to V points: 3
Sample filter: Binomial   Number of points for filter: 3 Sample start region (min): 0 Sample end region (min): 30
Marker peak width (sec): 5   Marker min peak height: 200   Marker baseline V to V?: Y   Marker baseline V to V points: 3
Lower marker selection: First peak > 500 RFU   Upper marker selection: Last peak > 200 RFU
Ladder size (bp)75, 200, 300, 400, 500, 700, 1000, 1500, 2000, 3000, 4000, 5000, 7000, 10000, 20000
Quantification using: Lower Marker   Final concentration (ng/uL): 0.5000   Dilution factor: 12.0

Sample: SampE11
Well location: E11
Created: Wednesday, August 11, 2021 6:14:43 PM

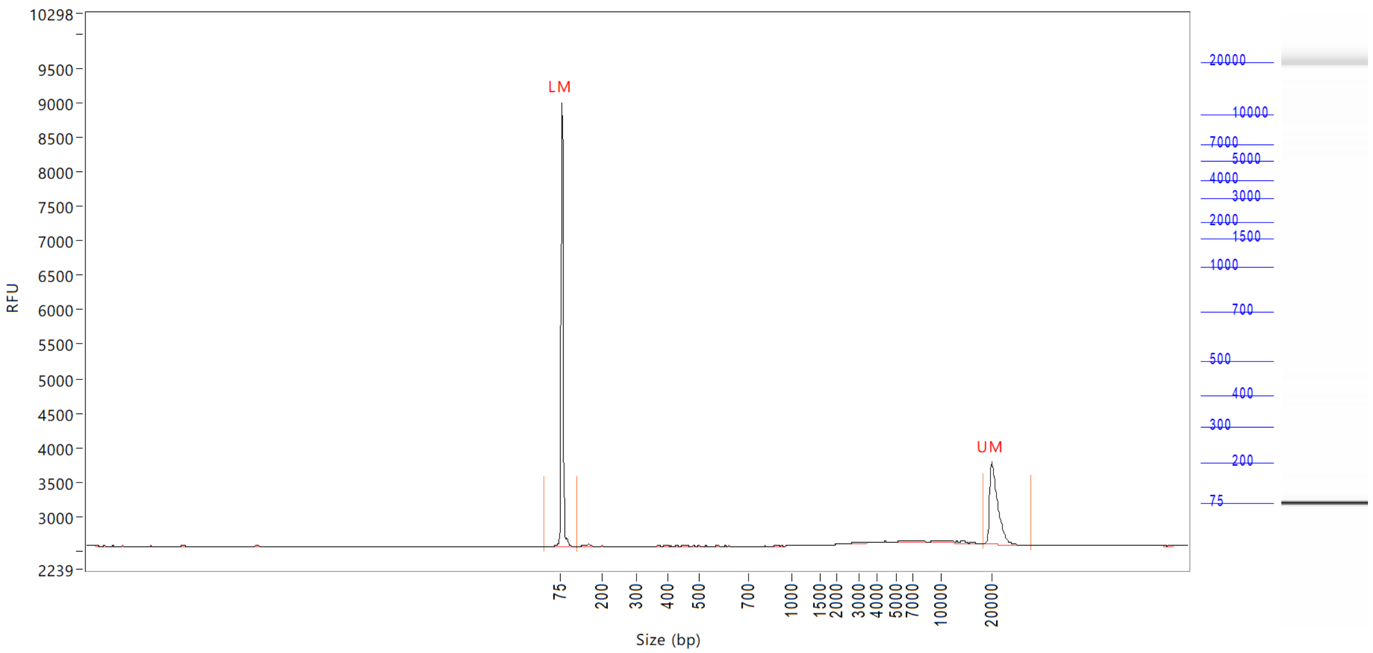

Peak	Size (bp)	Concentration (ng/uL)	From (bp)	To (bp)	Average size (bp)	CV%	RFU	Corrected peak area
1	75 (LM)	0.5000	22	122	73	6.81	6443	35.515
2	20000 (UM)	0.1765	18171	27859	20523	4.86	1204	12.537

TIC:	0.0000	ng/uL
TIM:	0.0000	nmole/L
Total concentration:	0.7588	ng/uL

Sample peak width (sec): 5 Sample min peak height: 50 Sample baseline V to V?: Y Sample baseline V to V points: 3
Sample filter: Binomial Number of points for filter: 3 Sample start region (min): 0 Sample end region (min): 30
Marker peak width (sec): 5 Marker min peak height: 200 Marker baseline V to V?: Y Marker baseline V to V points: 3
Lower marker selection: First peak > 500 RFU Upper marker selection: Last peak > 200 RFU
Ladder size (bp)75, 200, 300, 400, 500, 700, 1000, 1500, 2000, 3000, 4000, 5000, 7000, 10000, 20000
Quantification using: Lower Marker Final concentration (ng/uL): 0.5000 Dilution factor: 12.0

Sample: SampF11
Well location: F11
Created: Wednesday, August 11, 2021 6:14:43 PM

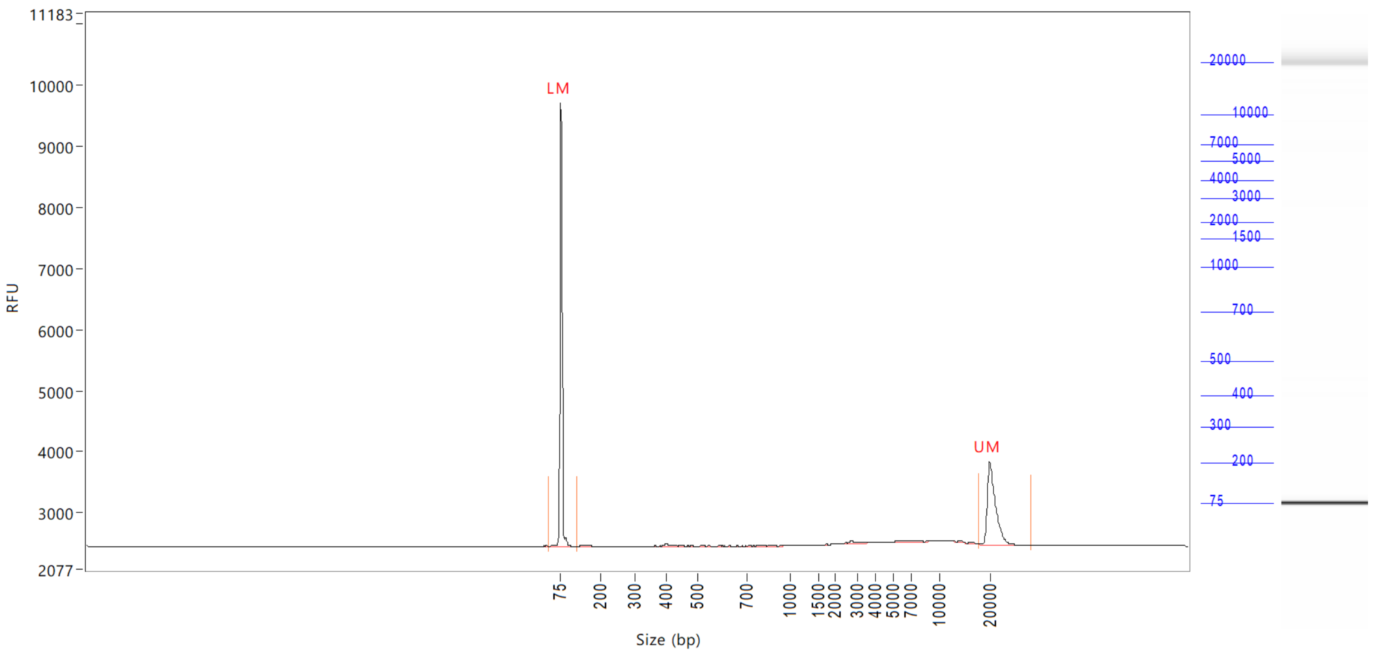

Peak	Size	Concentration	From	To	Average size	CV%	RFU	Corrected peak area
	(bp)	(ng/uL)	(bp)	(bp)	(bp)			
1	75 (LM)	0.5000	39	126	74	6.00	7279	40.417
2	20000 (UM)	0.1780	17805	28221	20588	5.19	1370	14.386

TIC:	0.0000	ng/uL
TIM:	0.0000	nmole/L
Total concentration:	0.5832	ng/uL

```
Sample peak width (sec): 5    Sample min peak height: 50    Sample baseline V to V?: Y    Sample baseline V to V points: 3
Sample filter: Binomial       Number of points for filter: 3  Sample start region (min): 0  Sample end region (min): 30
Marker peak width (sec): 5    Marker min peak height: 200   Marker baseline V to V?: Y    Marker baseline V to V points: 3
Lower marker selection: First peak > 500 RFU                Upper marker selection: Last peak > 200 RFU
Ladder size (bp)75, 200, 300, 400, 500, 700, 1000, 1500, 2000, 3000, 4000, 5000, 7000, 10000, 20000
Quantification using: Lower Marker          Final concentration (ng/uL): 0.5000          Dilution factor: 12.0
```

Sample: SampG11
Well location: G11
Created: Wednesday, August 11, 2021 6:14:43 PM

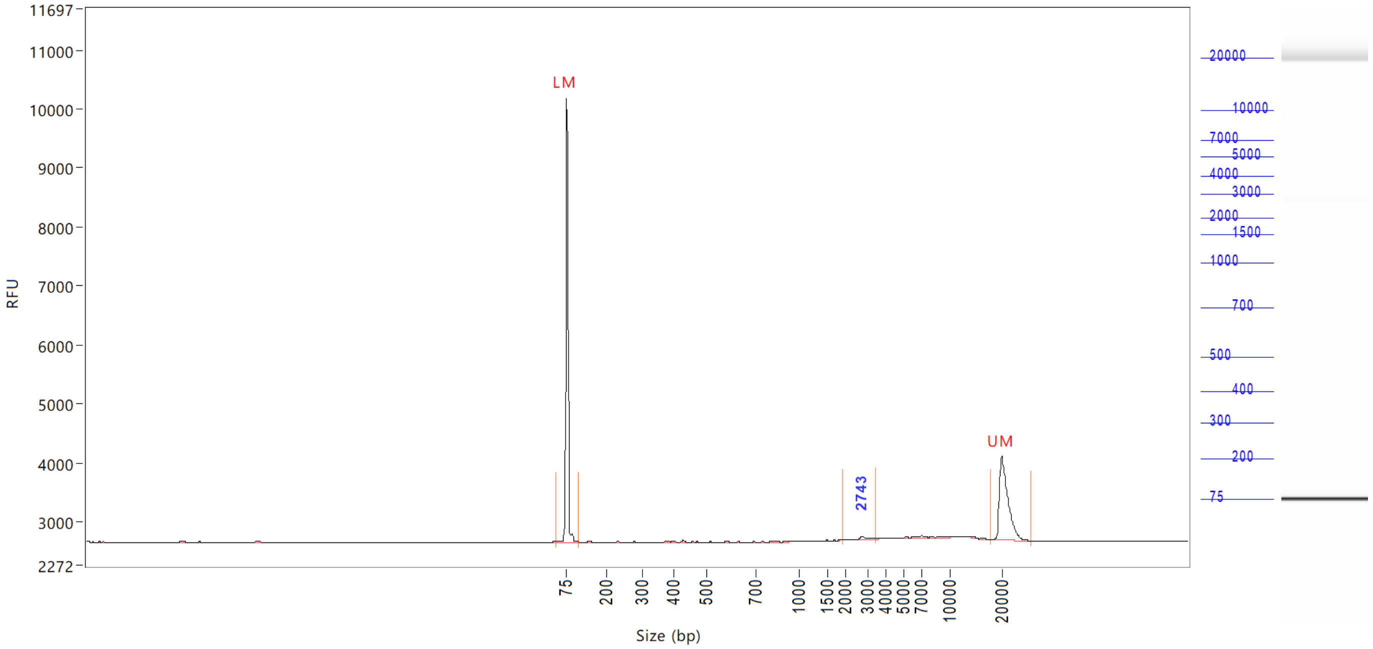

Peak	Size (bp)	Concentration (ng/uL)	From (bp)	To (bp)	Average size (bp)	CV%	RFU	Corrected peak area
1	75 (LM)	0.5000	43	114	74	5.82	7532	41.058
2	2743	0.0896	1923	3465	2735	10.31	55	0.613
3	20000 (UM)	0.1847	17927	25682	20579	4.80	1416	15.166

TIC:	0.0896	ng/uL
TIM:	0.0539	nmole/L
Total concentration:	0.6388	ng/uL

```
Sample peak width (sec): 5    Sample min peak height: 50    Sample baseline V to V?: Y    Sample baseline V to V points: 3
Sample filter: Binomial       Number of points for filter: 3  Sample start region (min): 0  Sample end region (min): 30
Marker peak width (sec): 5    Marker min peak height: 200   Marker baseline V to V?: Y    Marker baseline V to V points: 3
Lower marker selection: First peak > 500 RFU                Upper marker selection: Last peak > 200 RFU
Ladder size (bp)75, 200, 300, 400, 500, 700, 1000, 1500, 2000, 3000, 4000, 5000, 7000, 10000, 20000
Quantification using: Lower Marker          Final concentration (ng/uL): 0.5000          Dilution factor: 12.0
```

Sample: SampH11
Well location: H11
Created: Wednesday, August 11, 2021 6:14:43 PM

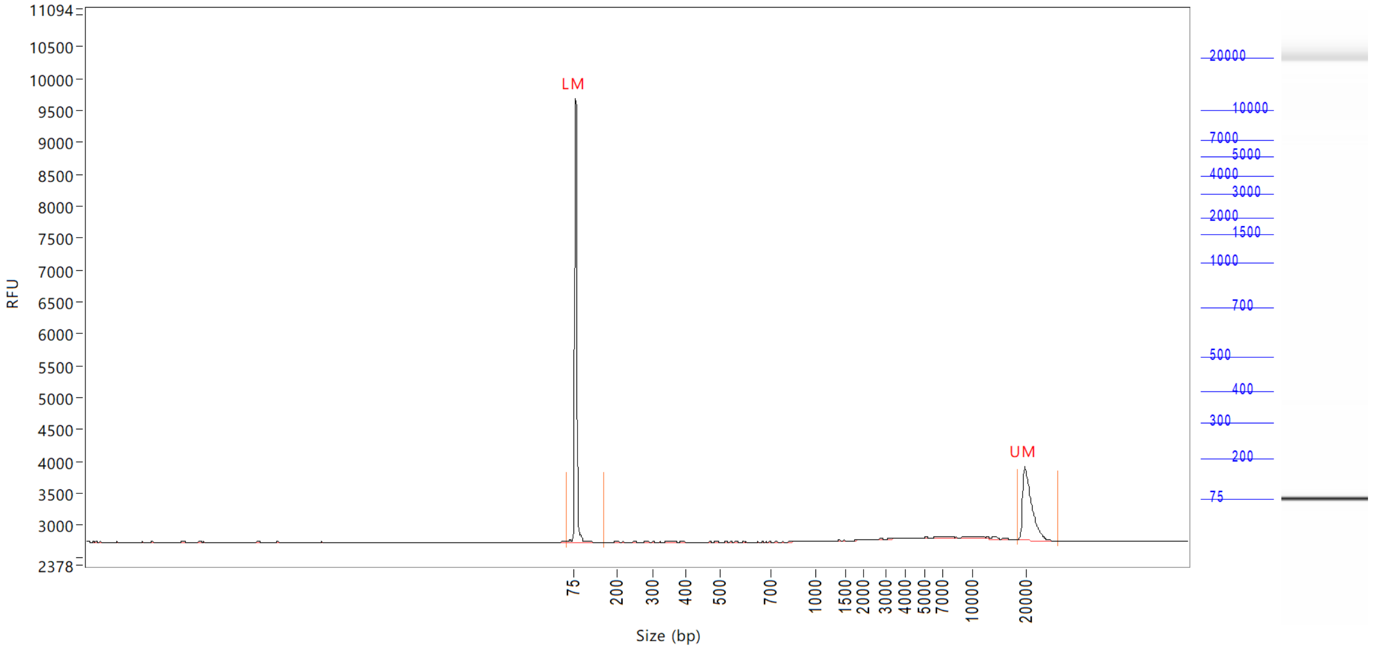

Peak	Size (bp)	Concentration (ng/uL)	From (bp)	To (bp)	Average size (bp)	CV%	RFU	Corrected peak area
1	75 (LM)	0.5000	52	160	74	7.43	6968	39.291
2	20000 (UM)	0.1626	18537	26166	20605	4.95	1170	12.776

TIC:	0.0000	ng/uL
TIM:	0.0000	nmole/L
Total concentration:	0.6395	ng/uL

```
Sample peak width (sec): 5    Sample min peak height: 50    Sample baseline V to V?: Y    Sample baseline V to V points: 3
Sample filter: Binomial       Number of points for filter: 3  Sample start region (min): 0  Sample end region (min): 30
Marker peak width (sec): 5    Marker min peak height: 200   Marker baseline V to V?: Y    Marker baseline V to V points: 3
Lower marker selection: First peak > 500 RFU                Upper marker selection: Last peak > 200 RFU
Ladder size (bp)75, 200, 300, 400, 500, 700, 1000, 1500, 2000, 3000, 4000, 5000, 7000, 10000, 20000
Quantification using: Lower Marker          Final concentration (ng/uL): 0.5000          Dilution factor: 12.0
```

Sample: SampA12
Well location: A12
Created: Wednesday, August 11, 2021 6:14:43 PM

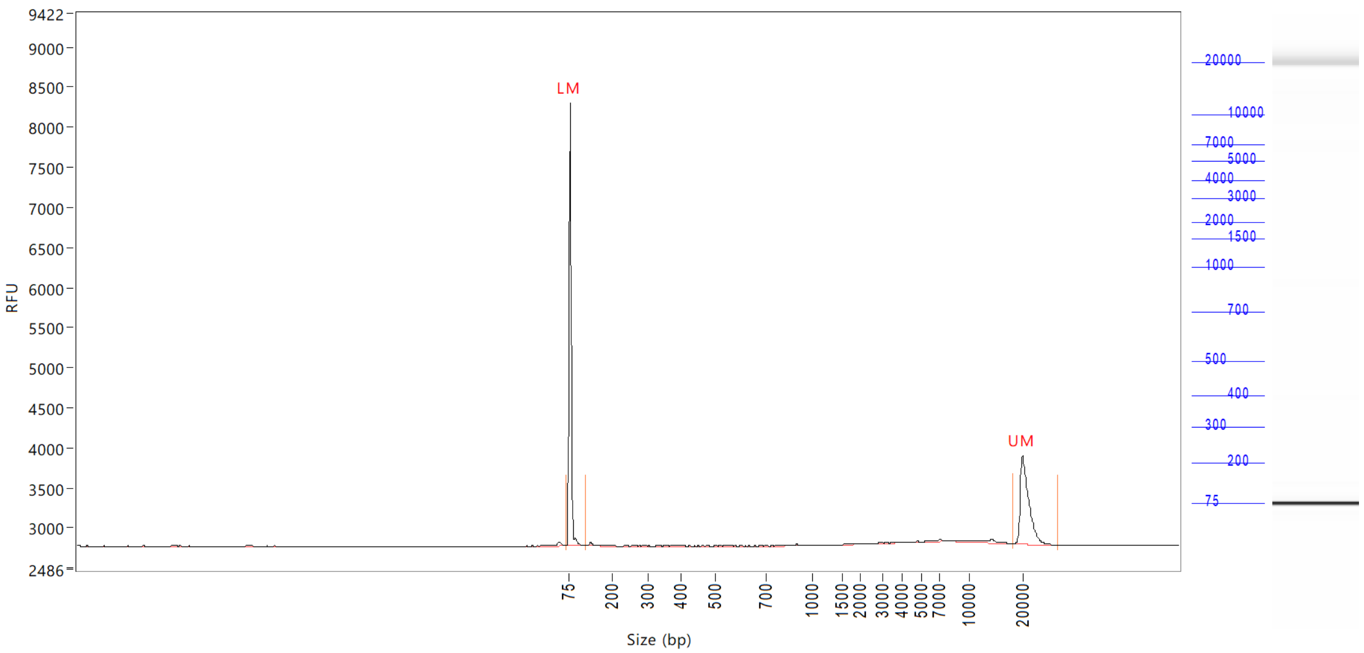

Peak	Size	Concentration	From	To	Average size	CV%	RFU	Corrected peak area
	(bp)	(ng/uL)	(bp)	(bp)	(bp)			
1	75 (LM)	0.5000	62	120	73	5.95	5537	30.277
2	20000 (UM)	0.1916	18293	26649	20526	4.88	1104	11.602

TIC:	0.0000	ng/uL
TIM:	0.0000	nmole/L
Total concentration:	0.8935	ng/uL

```
Sample peak width (sec): 5    Sample min peak height: 50    Sample baseline V to V?: Y    Sample baseline V to V points: 3
Sample filter: Binomial       Number of points for filter: 3  Sample start region (min): 0  Sample end region (min): 30
Marker peak width (sec): 5    Marker min peak height: 200   Marker baseline V to V?: Y    Marker baseline V to V points: 3
Lower marker selection: First peak > 500 RFU                Upper marker selection: Last peak > 200 RFU
Ladder size (bp)75, 200, 300, 400, 500, 700, 1000, 1500, 2000, 3000, 4000, 5000, 7000, 10000, 20000
Quantification using: Lower Marker          Final concentration (ng/uL): 0.5000          Dilution factor: 12.0
```

Sample: SampB12
Well location: B12
Created: Wednesday, August 11, 2021 6:14:43 PM

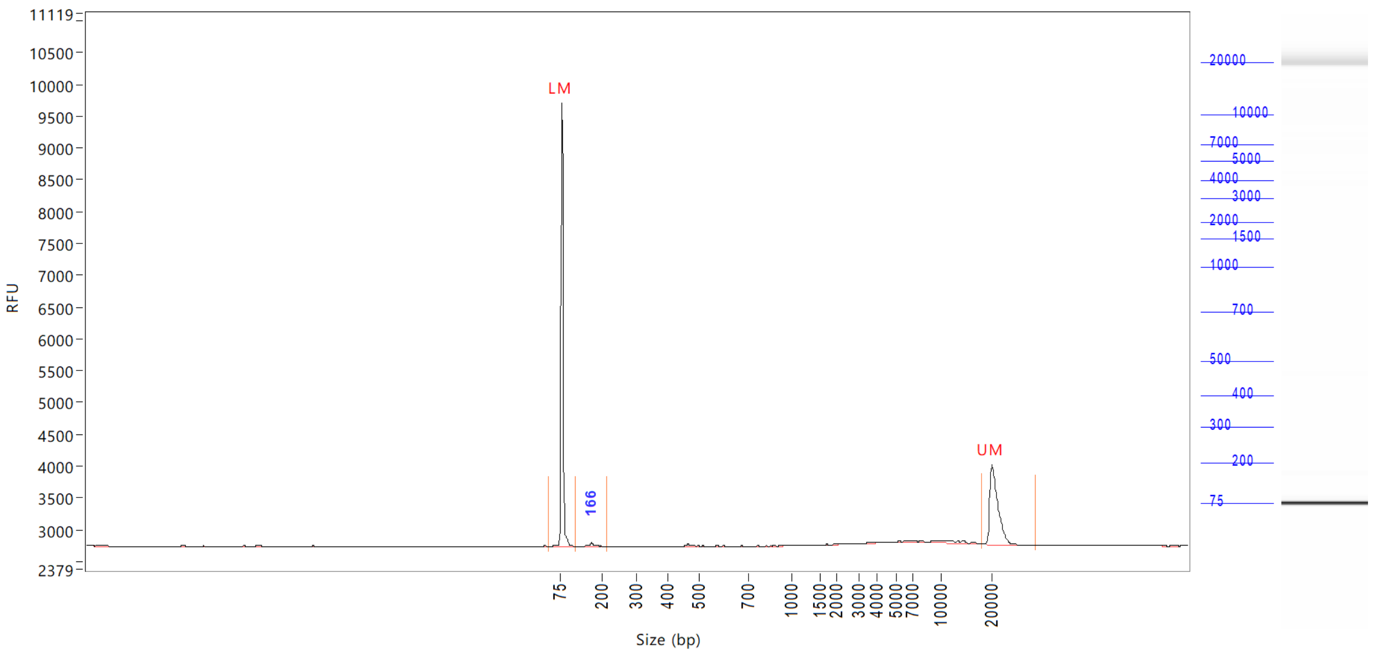

Peak	Size	Concentration	From	To	Average size	CV%	RFU	Corrected peak area
	(bp)	(ng/uL)	(bp)	(bp)	(bp)			
1	75 (LM)	0.5000	35	120	73	6.06	6985	38.214
2	166	0.1107	120	214	165	10.29	55	0.705
3	20000 (UM)	0.1754	18049	28705	20564	5.14	1263	13.403

TIC:	0.1107	ng/uL
TIM:	1.1025	nmole/L
Total concentration:	0.8246	ng/uL

```
Sample peak width (sec): 5    Sample min peak height: 50    Sample baseline V to V?: Y    Sample baseline V to V points: 3
Sample filter: Binomial       Number of points for filter: 3  Sample start region (min): 0  Sample end region (min): 30
Marker peak width (sec): 5    Marker min peak height: 200   Marker baseline V to V?: Y    Marker baseline V to V points: 3
Lower marker selection: First peak > 500 RFU                Upper marker selection: Last peak > 200 RFU
Ladder size (bp)75, 200, 300, 400, 500, 700, 1000, 1500, 2000, 3000, 4000, 5000, 7000, 10000, 20000
Quantification using: Lower Marker          Final concentration (ng/uL): 0.5000          Dilution factor: 12.0
```

Sample: SampC12
Well location: C12
Created: Wednesday, August 11, 2021 6:14:43 PM

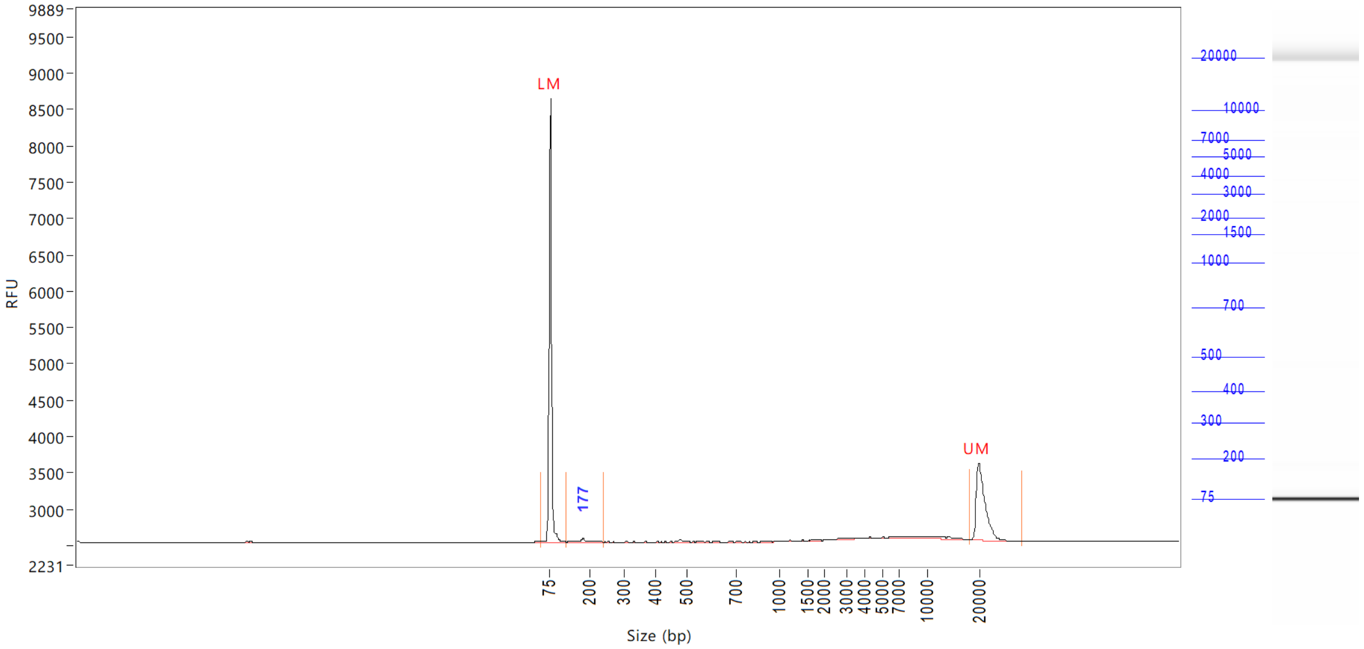

Peak	Size (bp)	Concentration (ng/uL)	From (bp)	To (bp)	Average size (bp)	CV%	RFU	Corrected peak area
1	75 (LM)	0.5000	48	126	74	6.92	6119	35.996
2	177	0.1482	126	237	178	12.85	62	0.889
3	20000 (UM)	0.1659	18293	28705	20663	5.46	1069	11.944

TIC:	0.1482	ng/uL
TIM:	1.3692	nmole/L
Total concentration:	0.8302	ng/uL

Sample peak width (sec): 5 Sample min peak height: 50 Sample baseline V to V?: Y Sample baseline V to V points: 3
Sample filter: Binomial Number of points for filter: 3 Sample start region (min): 0 Sample end region (min): 30
Marker peak width (sec): 5 Marker min peak height: 200 Marker baseline V to V?: Y Marker baseline V to V points: 3
Lower marker selection: First peak > 500 RFU Upper marker selection: Last peak > 200 RFU
Ladder size (bp)75, 200, 300, 400, 500, 700, 1000, 1500, 2000, 3000, 4000, 5000, 7000, 10000, 20000
Quantification using: Lower Marker Final concentration (ng/uL): 0.5000 Dilution factor: 12.0

Sample: SampD12
Well location: D12
Created: Wednesday, August 11, 2021 6:14:43 PM

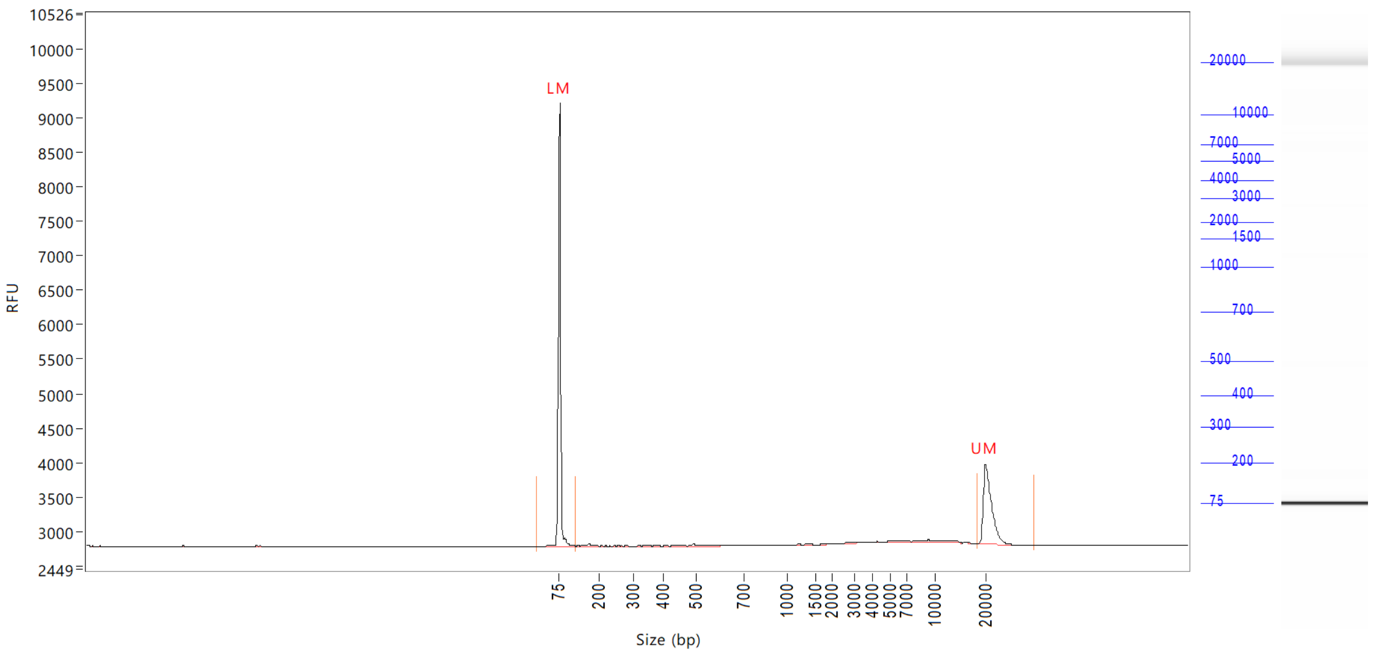

Peak	Size (bp)	Concentration (ng/uL)	From (bp)	To (bp)	Average size (bp)	CV%	RFU	Corrected peak area
1	75 (LM)	0.5000	7	126	73	8.55	6455	36.203
2	20000 (UM)	0.1692	18415	29551	20558	5.38	1157	12.253

TIC:	0.0000	ng/uL
TIM:	0.0000	nmole/L
Total concentration:	0.7485	ng/uL

```
Sample peak width (sec): 5    Sample min peak height: 50    Sample baseline V to V?: Y    Sample baseline V to V points: 3
Sample filter: Binomial       Number of points for filter: 3  Sample start region (min): 0  Sample end region (min): 30
Marker peak width (sec): 5    Marker min peak height: 200   Marker baseline V to V?: Y    Marker baseline V to V points: 3
Lower marker selection: First peak > 500 RFU                Upper marker selection: Last peak > 200 RFU
Ladder size (bp)75, 200, 300, 400, 500, 700, 1000, 1500, 2000, 3000, 4000, 5000, 7000, 10000, 20000
Quantification using: Lower Marker          Final concentration (ng/uL): 0.5000          Dilution factor: 12.0
```

Sample: SampE12
Well location: E12
Created: Wednesday, August 11, 2021 6:14:43 PM

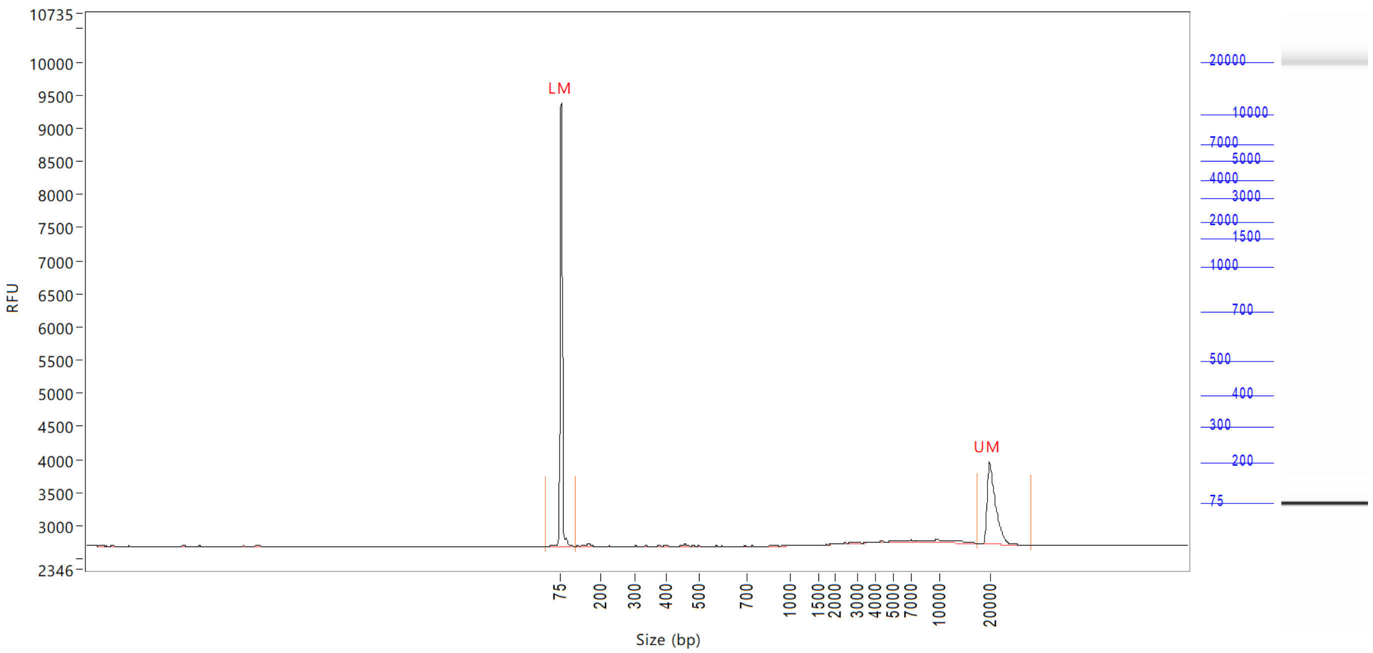

Peak	Size	Concentration	From	To	Average size	CV%	RFU	Corrected peak area
	(bp)	(ng/uL)	(bp)	(bp)	(bp)			
1	75 (LM)	0.5000	26	120	72	6.60	6704	38.185
2	20000 (UM)	0.1750	17683	28221	20564	5.10	1249	13.364

TIC:	0.0000	ng/uL
TIM:	0.0000	nmole/L
Total concentration:	0.7995	ng/uL

```
Sample peak width (sec): 5    Sample min peak height: 50    Sample baseline V to V?: Y    Sample baseline V to V points: 3
Sample filter: Binomial       Number of points for filter: 3  Sample start region (min): 0  Sample end region (min): 30
Marker peak width (sec): 5    Marker min peak height: 200   Marker baseline V to V?: Y    Marker baseline V to V points: 3
Lower marker selection: First peak > 500 RFU                Upper marker selection: Last peak > 200 RFU
Ladder size (bp)75, 200, 300, 400, 500, 700, 1000, 1500, 2000, 3000, 4000, 5000, 7000, 10000, 20000
Quantification using: Lower Marker          Final concentration (ng/uL): 0.5000          Dilution factor: 12.0
```

Sample: SampF12
Well location: F12
Created: Wednesday, August 11, 2021 6:14:43 PM

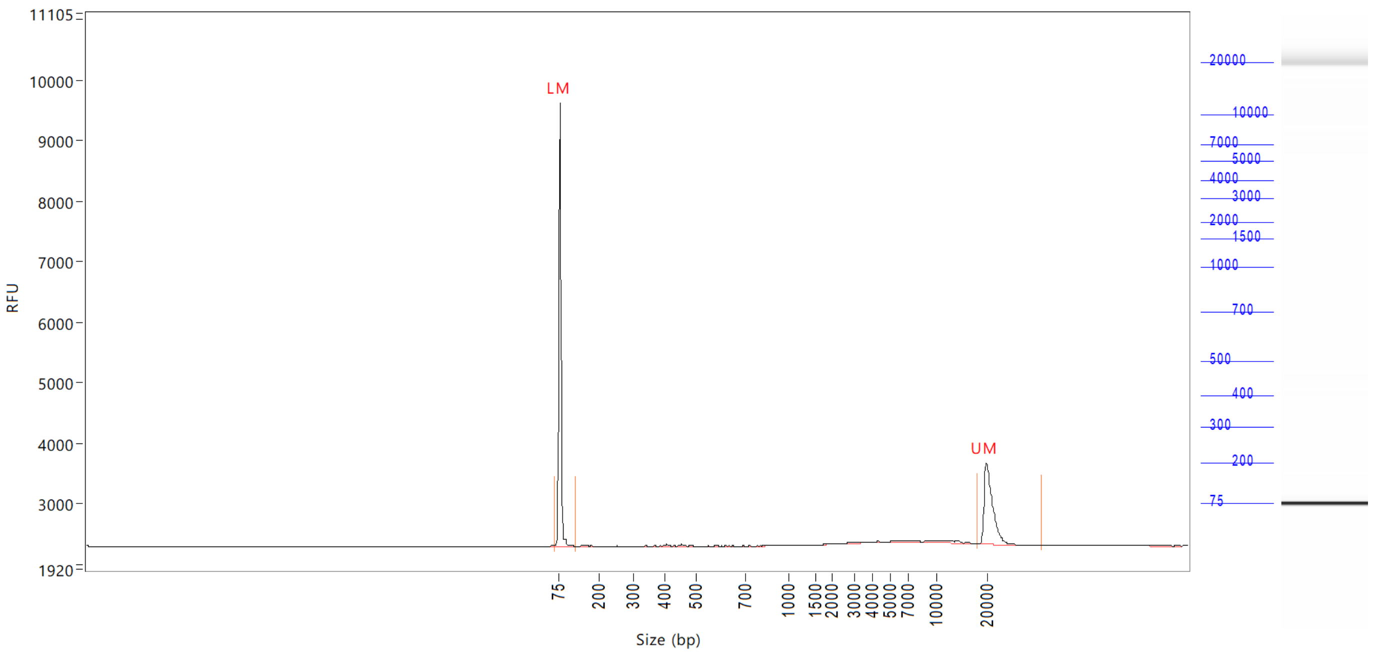

Peak	Size (bp)	Concentration (ng/uL)	From (bp)	To (bp)	Average size (bp)	CV%	RFU	Corrected peak area
1	75 (LM)	0.5000	62	124	73	5.89	7342	41.663
2	20000 (UM)	0.1747	18049	30881	20633	5.57	1348	14.556

TIC:	0.0000	ng/uL
TIM:	0.0000	nmole/L
Total concentration:	0.6122	ng/uL

Sample peak width (sec): 5   Sample min peak height: 50   Sample baseline V to V?: Y   Sample baseline V to V points: 3
Sample filter: Binomial   Number of points for filter: 3 Sample start region (min): 0 Sample end region (min): 30
Marker peak width (sec): 5   Marker min peak height: 200   Marker baseline V to V?: Y   Marker baseline V to V points: 3
Lower marker selection: First peak > 500 RFU   Upper marker selection: Last peak > 200 RFU
Ladder size (bp)75, 200, 300, 400, 500, 700, 1000, 1500, 2000, 3000, 4000, 5000, 7000, 10000, 20000
Quantification using: Lower Marker   Final concentration (ng/uL): 0.5000   Dilution factor: 12.0

Sample: SampG12
Well location: G12
Created: Wednesday, August 11, 2021 6:14:43 PM

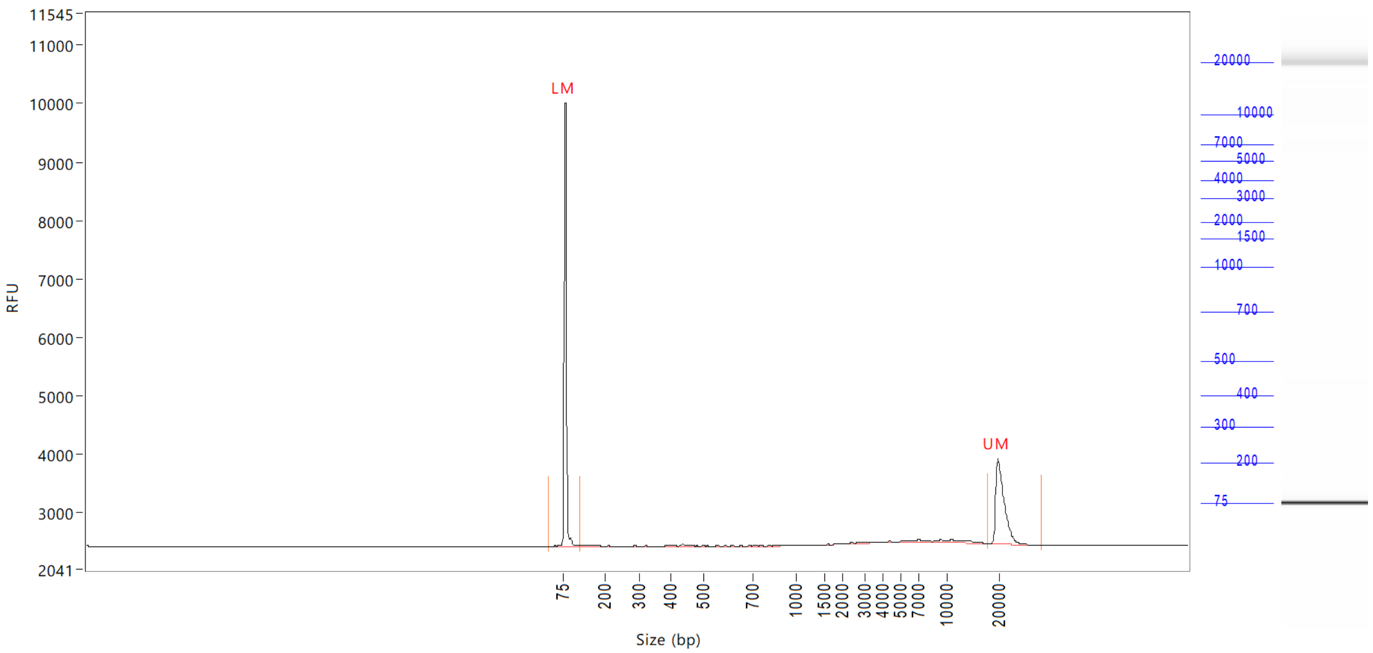

Peak	Size	Concentration	From	To	Average size	CV%	RFU	Corrected peak area
	(bp)	(ng/uL)	(bp)	(bp)	(bp)			
1	75 (LM)	0.5000	28	122	74	6.62	7595	43.093
2	20000 (UM)	0.1819	17927	28463	20578	4.97	1466	15.675

TIC:	0.0000	ng/uL
TIM:	0.0000	nmole/L
Total concentration:	0.6985	ng/uL

```
Sample peak width (sec): 5    Sample min peak height: 50    Sample baseline V to V?: Y    Sample baseline V to V points: 3
Sample filter: Binomial       Number of points for filter: 3  Sample start region (min): 0  Sample end region (min): 30
Marker peak width (sec): 5    Marker min peak height: 200   Marker baseline V to V?: Y    Marker baseline V to V points: 3
Lower marker selection: First peak > 500 RFU                Upper marker selection: Last peak > 200 RFU
Ladder size (bp)75, 200, 300, 400, 500, 700, 1000, 1500, 2000, 3000, 4000, 5000, 7000, 10000, 20000
Quantification using: Lower Marker          Final concentration (ng/uL): 0.5000          Dilution factor: 12.0
```

Sample: SampH12
Well location: H12
Created: Wednesday, August 11, 2021 6:14:43 PM

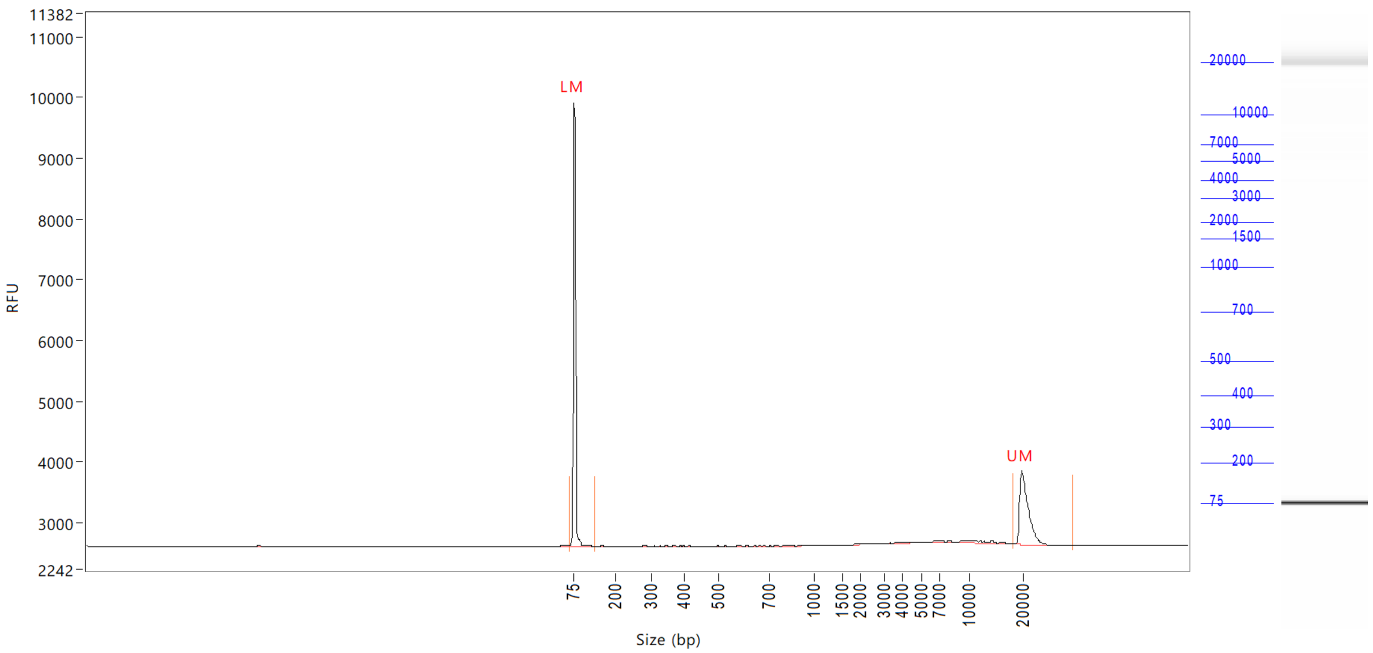

Peak	Size (bp)	Concentration (ng/uL)	From (bp)	To (bp)	Average size (bp)	CV%	RFU	Corrected peak area
1	75 (LM)	0.5000	64	137	74	5.87	7303	40.045
2	20000 (UM)	0.1624	18415	29672	20593	5.27	1212	13.004

TIC:	0.0000	ng/uL
TIM:	0.0000	nmole/L
Total concentration:	0.6563	ng/uL

```
Sample peak width (sec): 5    Sample min peak height: 200   Sample baseline V to V?: Y    Sample baseline V to V points: 3
Sample filter: Binomial       Number of points for filter: 3  Sample start region (min): 0  Sample end region (min): 30
Marker peak width (sec): 5    Marker min peak height: 200   Marker baseline V to V?: Y    Marker baseline V to V points: 3
Lower marker selection: First peak > 500 RFU                Upper marker selection: Last peak > 200 RFU
Ladder size (bp)75, 200, 300, 400, 500, 700, 1000, 1500, 2000, 3000, 4000, 5000, 7000, 10000, 20000
Quantification using: Lower Marker          Final concentration (ng/uL): 0.5000          Dilution factor: 12.0
```

Sample: DNA size ladder
Well location: A7
Created: Wednesday, August 11, 2021 6:14:43 PM
Import from: C:\Users\admin\Desktop\210811_alberto_ladder.SCAL
Fit type: Point to point

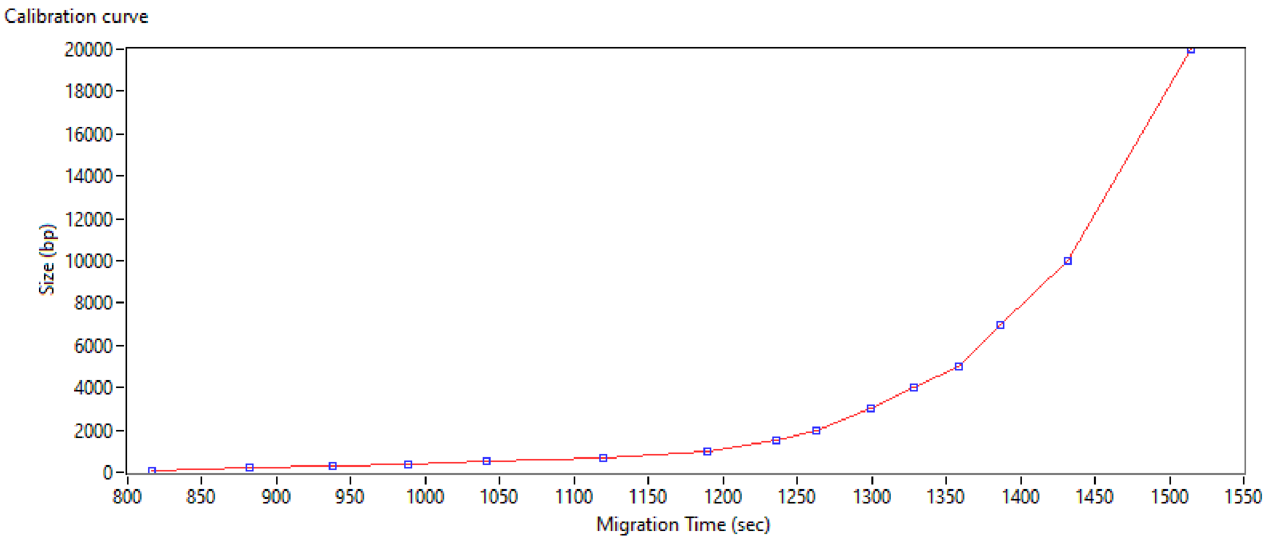

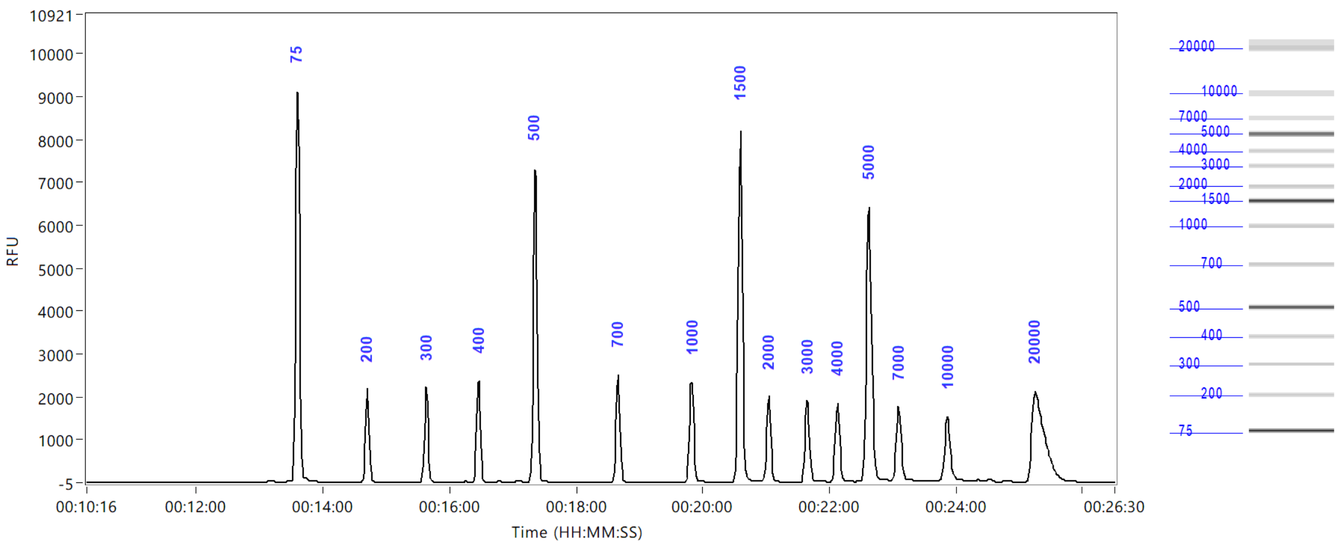

Supplement: Supplementary file 2 — sb3c00292_si_002.zip [file sb3c00292_si_002.zip › dnada_supplementary_material_pks_library_build/ZAG_trials/ZAG_Trial_2/18-04-43_redoplate2/redoplate2_t2.pdf]
