# Supplementary material for: Automated Platform for the Plasmid Construction Process
Source: ACS Synth Biol. 2023 Nov 10;12(12):3506–13. doi: 10.1021/acssynbio.3c00292 (PMC10729297; doi:10.1021/acssynbio.3c00292)

Instrument controller software run summary:

Filename and data path: C:\AATI\Data\2021 08 11\17-13-47\2021 08 11 17H 13M..raw
Created: Wednesday, August 11, 2021 5:34:32 PM
Number of capillaries: 96
Array serial number: 111920-09SFS
Effect length: 33 cm
Array usage count: 176
Instrument type: ZAG DNA Analyzer
Instrument controller software version: 1.0.1.9
Device serial number: Z7597

Method Information

Method name: ZAG130FC33 - DNA 75-20000bp - Full Conditioning.mthds
Gel prime: No
Full conditioning: Yes
Gel prime to buffer: No
Gel selection: Gel 1
Perform prerun: 8.0 kV, 30 sec.
Rinse: No
Marker 1: Row: A, 5.0 kV, 5 sec.
Rinse: No
Sample injection: 5.0 kV, 5 sec.
Separation: 8.0 kV, 30.0 min.
Tray name: Sample Tray-5

Analysis mode: DNA

Notes

Gel Image

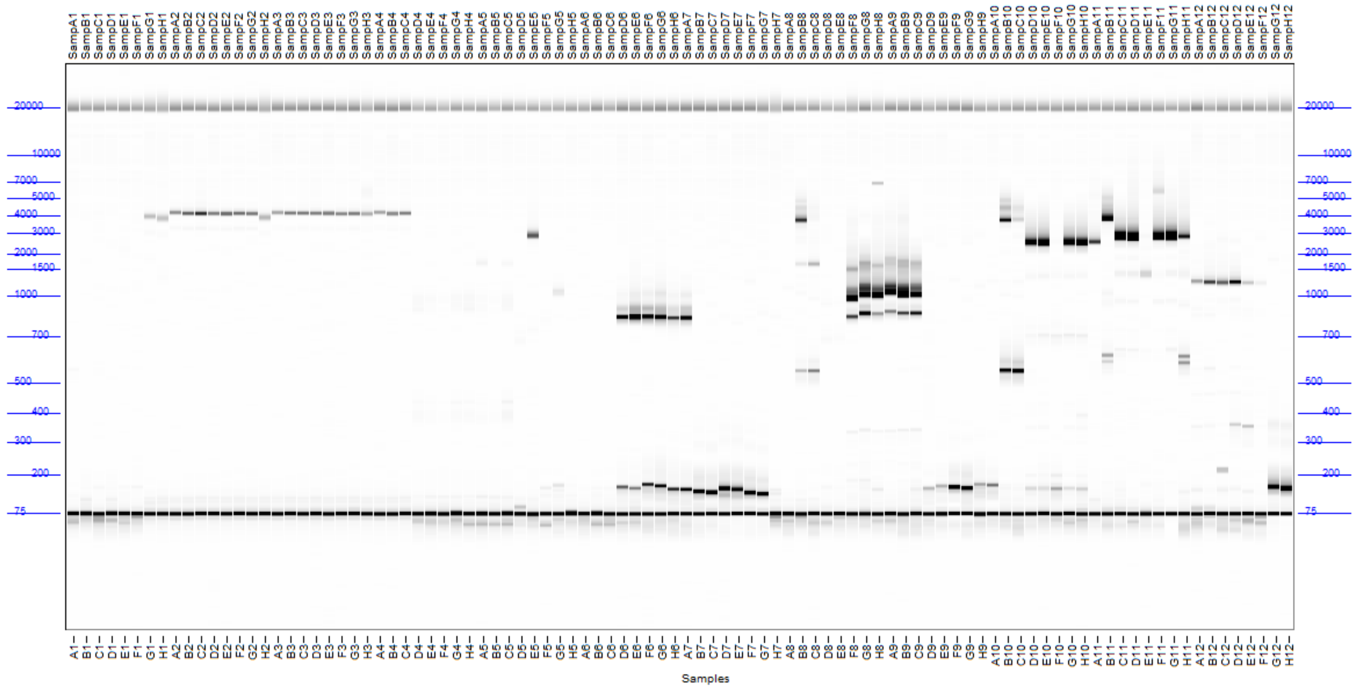
20000
10000
7000
5000
4000
3000
2000
1500
1000
700
500
400
300
200
75
Samples

Filename and data path: C:\AATI\Data\2021 08 11\17-13-47\2021 08 11 17H 13M..raw

A1: SampA1

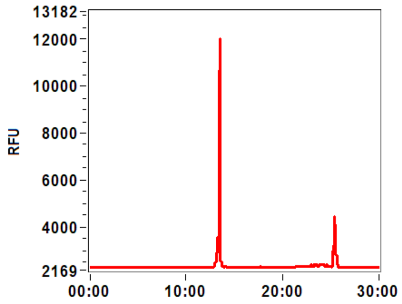

B1: SampB1

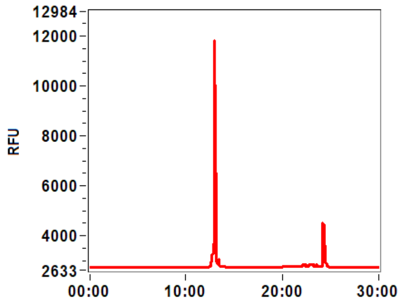

C1: SampC1

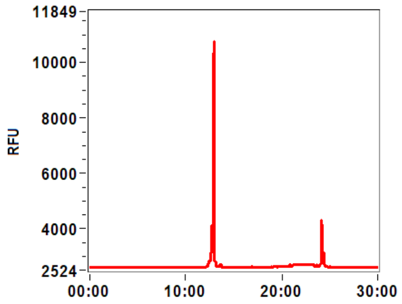

D1: SampD1

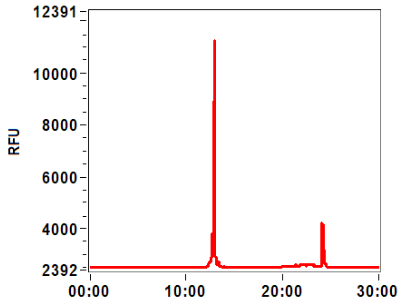

E1: SampE1

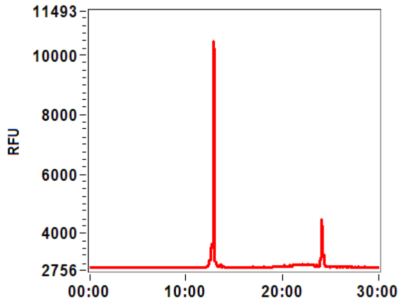

F1: SampF1

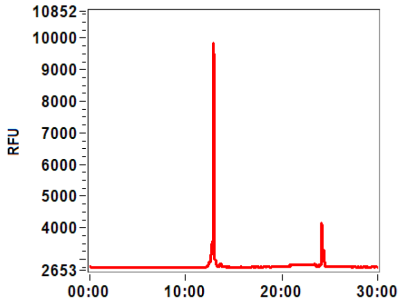

G1: SampG1

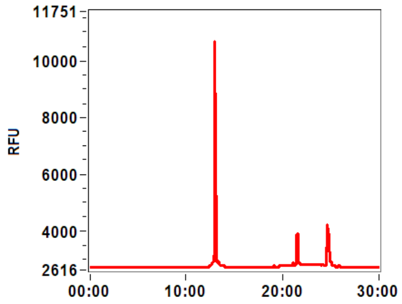

H1: SampH1

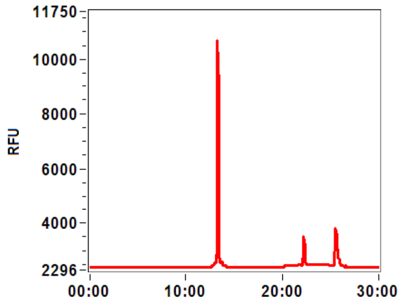

A2: SampA2

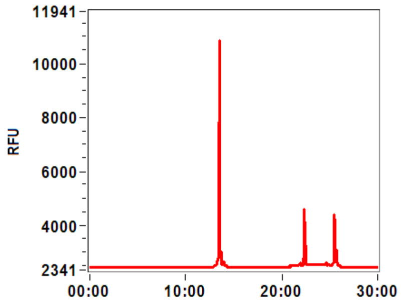

B2: SampB2

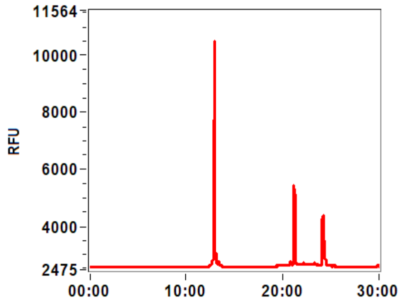

C2: SampC2

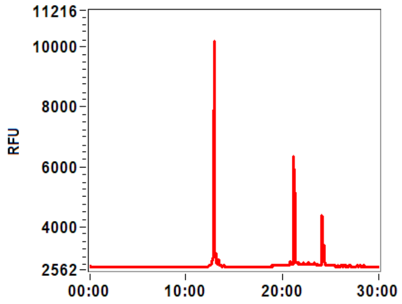

D2: SampD2

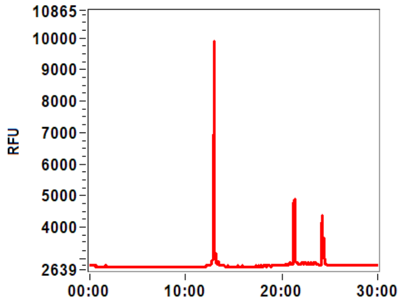

Filename and data path: C:\AATI\Data\2021 08 11\17-13-47\2021 08 11 17H 13M..raw

E2: SampE2

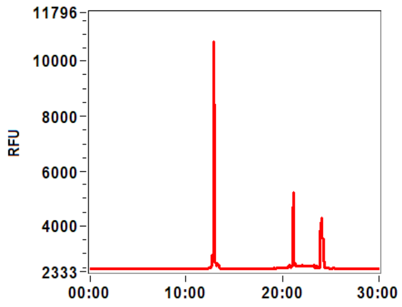

F2: SampF2

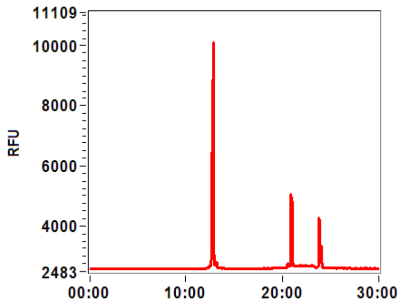

G2: SampG2

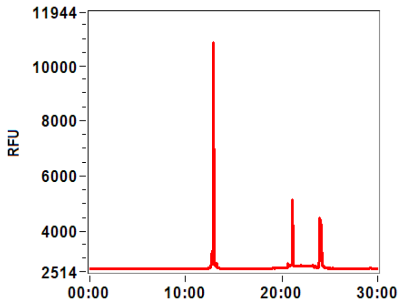

H2: SampH2

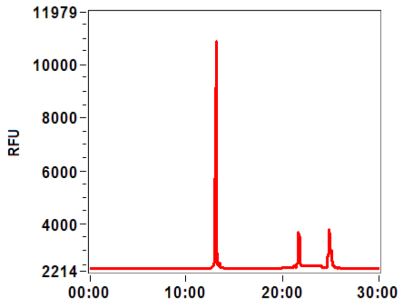

A3: SampA3

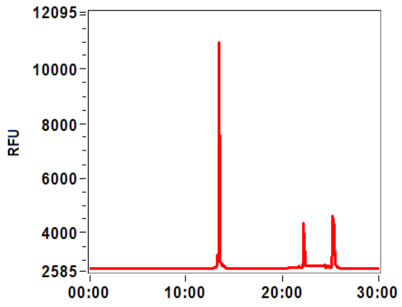

B3: SampB3

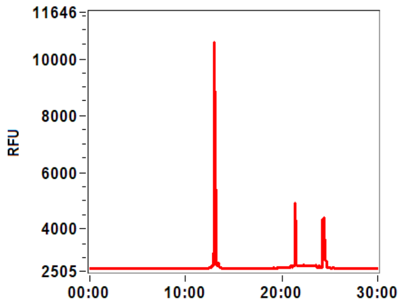

C3: SampC3

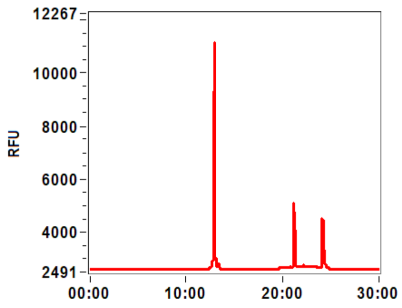

D3: SampD3

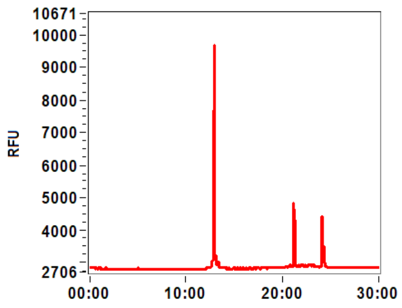

E3: SampE3

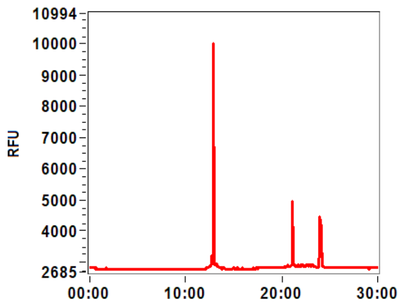

F3: SampF3

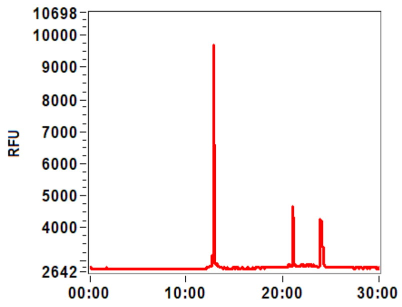

G3: SampG3

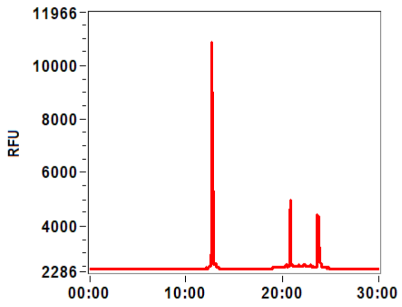

H3: SampH3

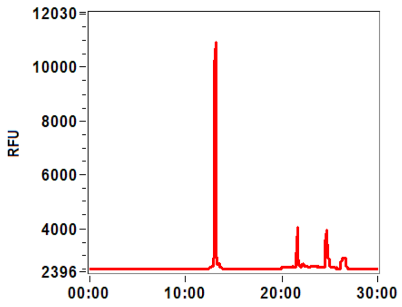

Filename and data path: C:\AATI\Data\2021 08 11\17-13-47\2021 08 11 17H 13M..raw

A4: SampA4

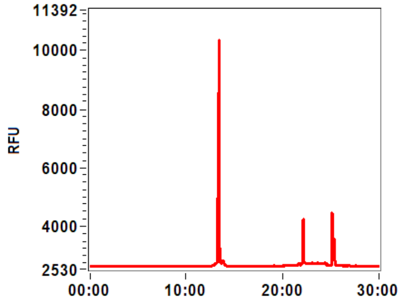

B4: SampB4

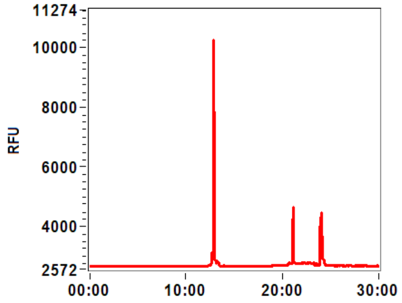

C4: SampC4

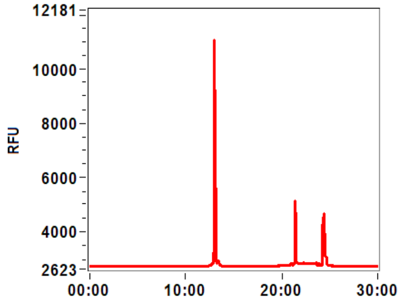

D4: SampD4

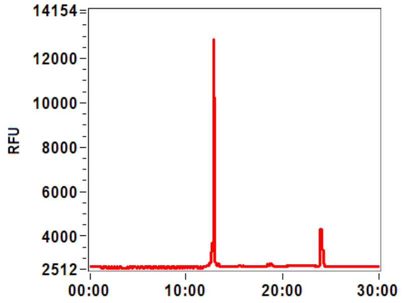

E4: SampE4

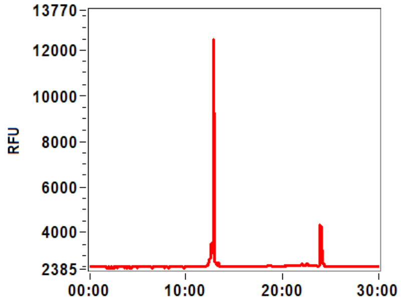

F4: SampF4

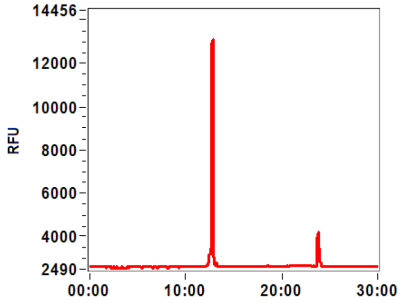

G4: SampG4

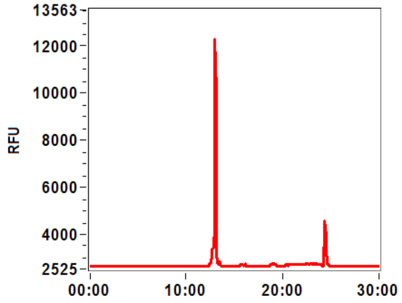

H4: SampH4

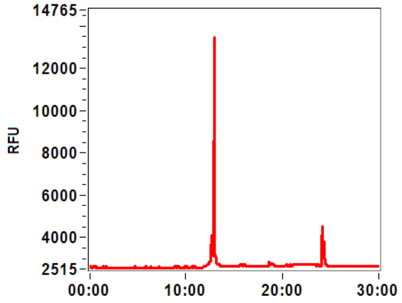

A5: SampA5

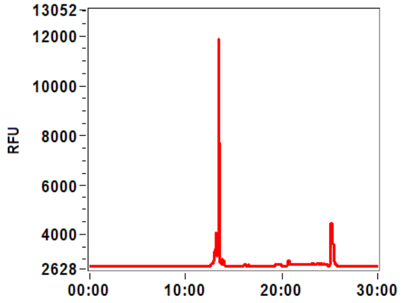

B5: SampB5

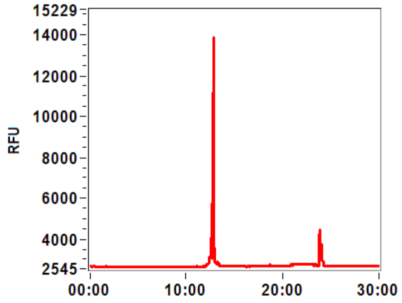

C5: SampC5

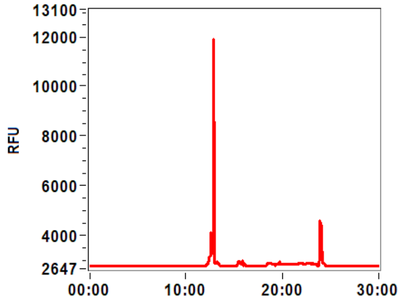

D5: SampD5

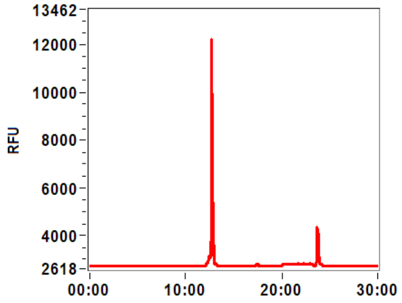

Filename and data path: C:\AATI\Data\2021 08 11\17-13-47\2021 08 11 17H 13M..raw

E5: SampE5

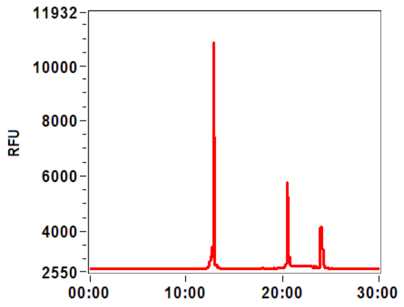

F5: SampF5

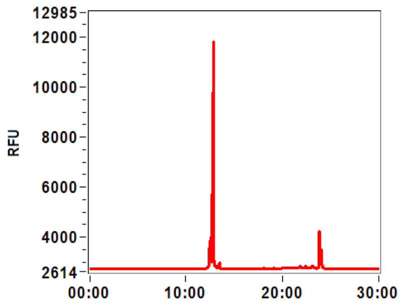

G5: SampG5

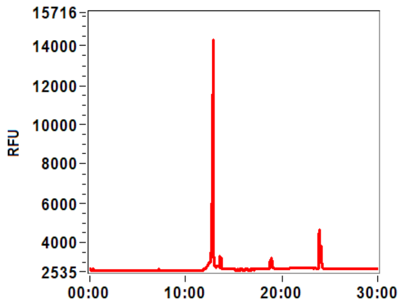

H5: SampH5

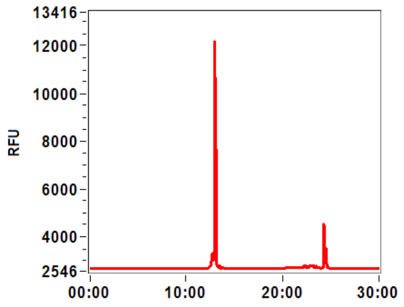

A6: SampA6

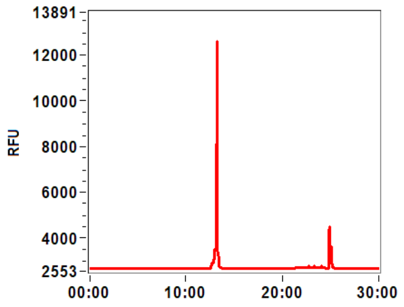

B6: SampB6

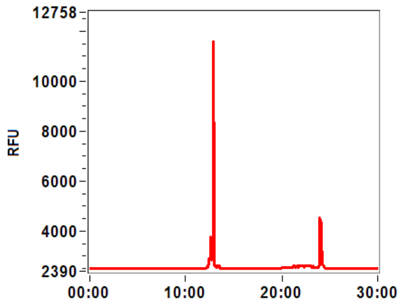

C6: SampC6

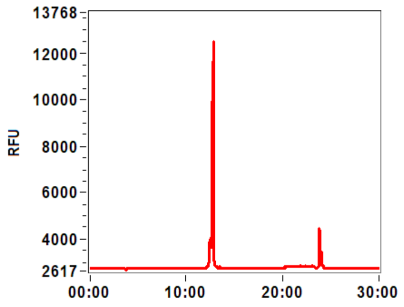

D6: SampD6

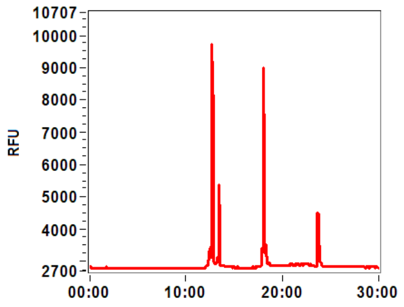

E6: SampE6

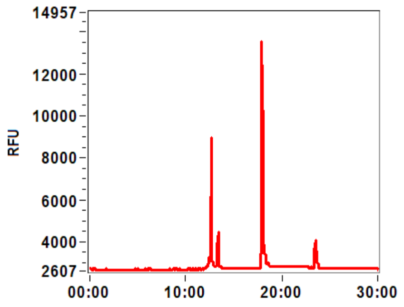

F6: SampF6

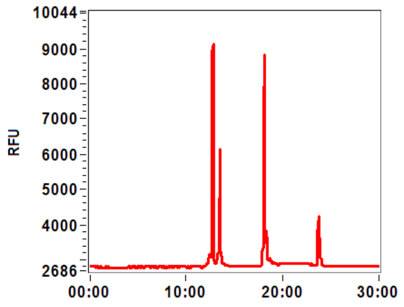

G6: SampG6

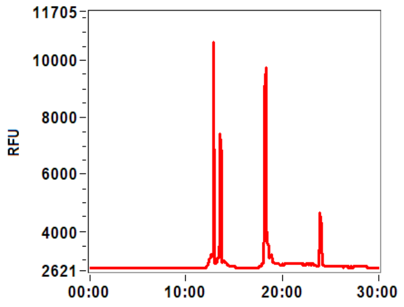

H6: SampH6

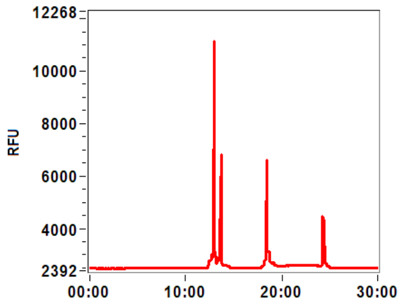

Filename and data path: C:\AATI\Data\2021 08 11\17-13-47\2021 08 11 17H 13M..raw

A7: SampA7

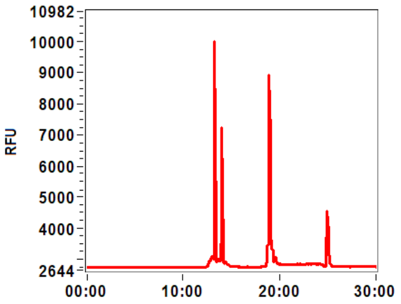

B7: SampB7

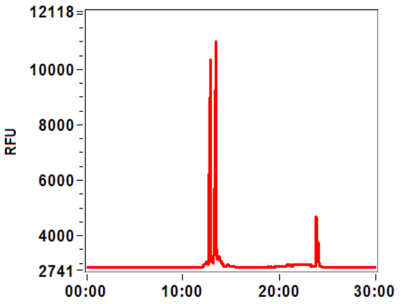

C7: SampC7

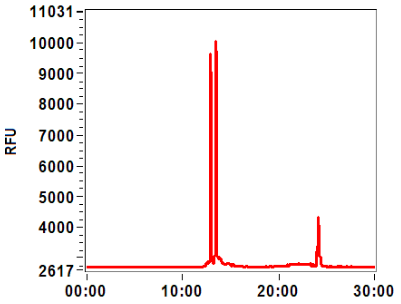

D7: SampD7

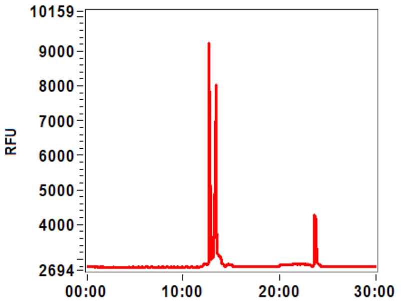

E7: SampE7

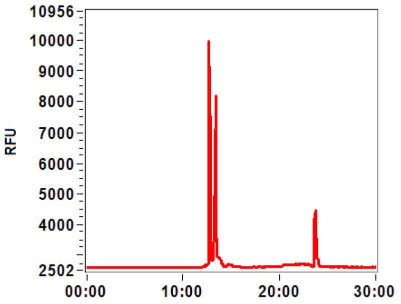

F7: SampF7

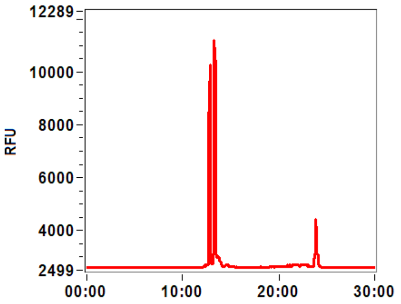

G7: SampG7

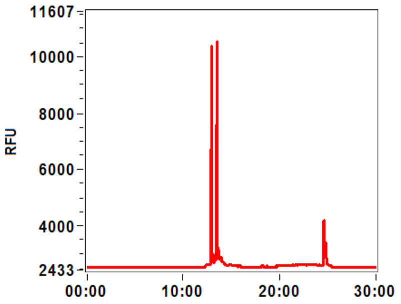

H7: SampH7

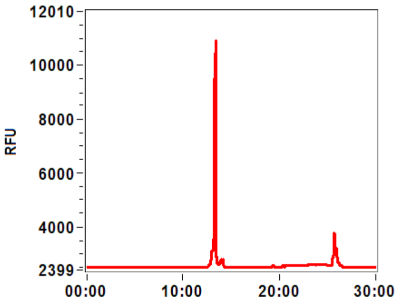

A8: SampA8

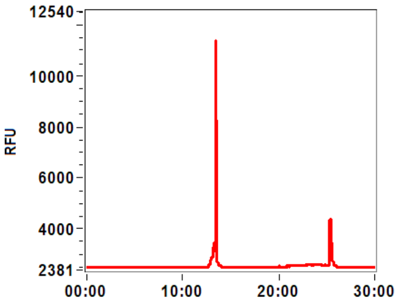

B8: SampB8

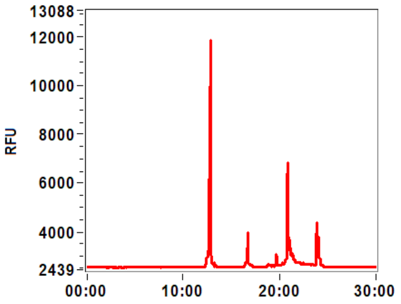

C8: SampC8

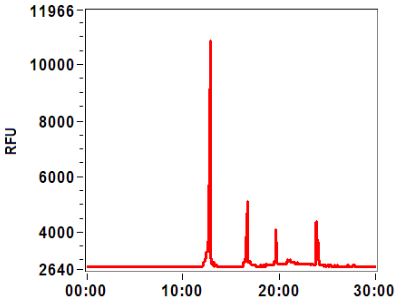

D8: SampD8

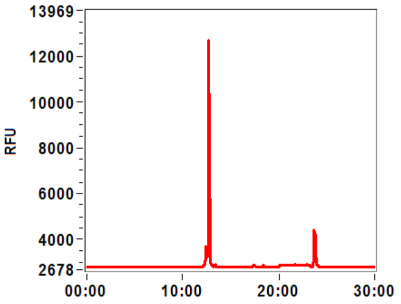

Filename and data path: C:\AATI\Data\2021 08 11\17-13-47\2021 08 11 17H 13M..raw

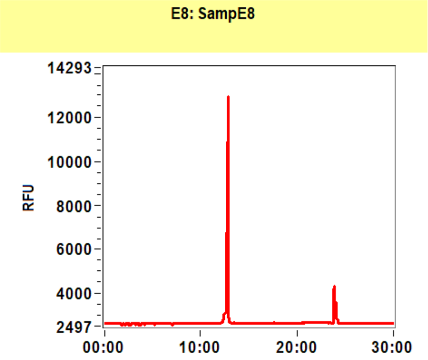

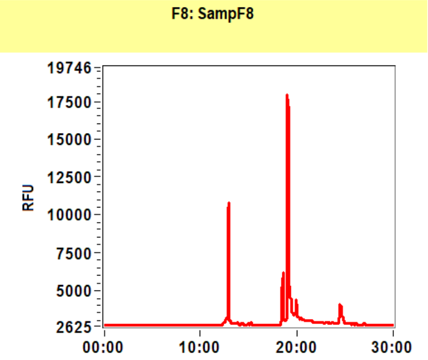

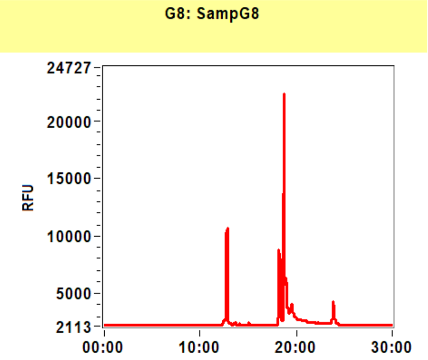

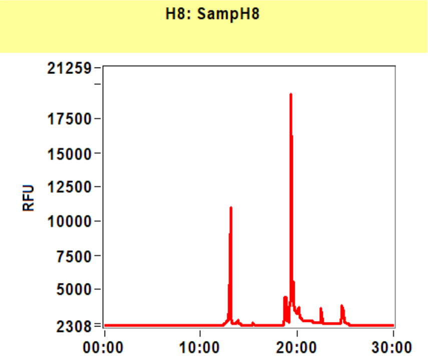

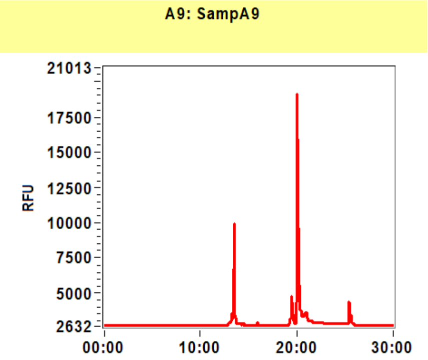

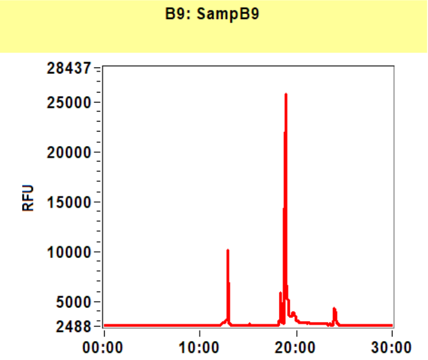

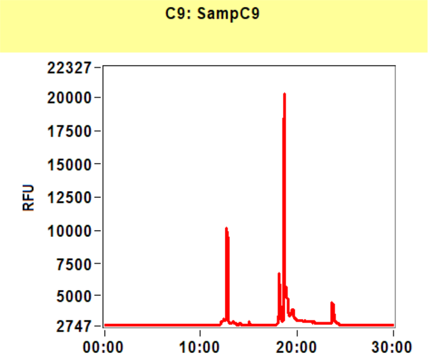

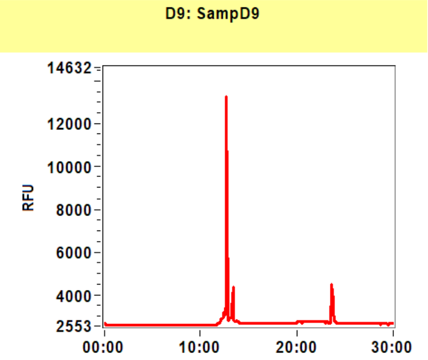

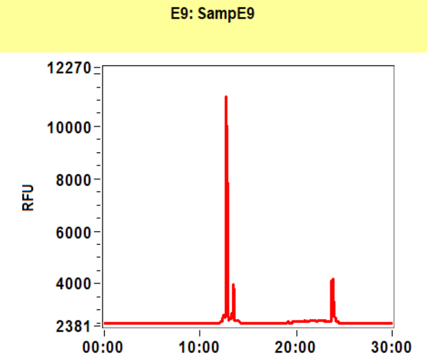

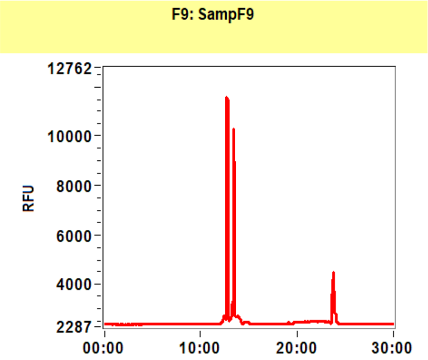

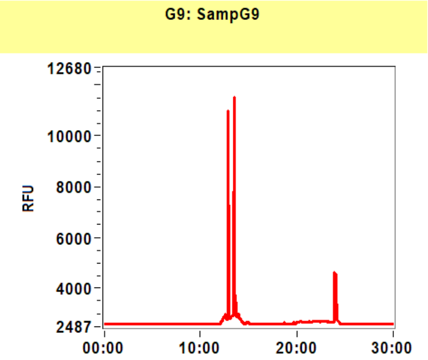

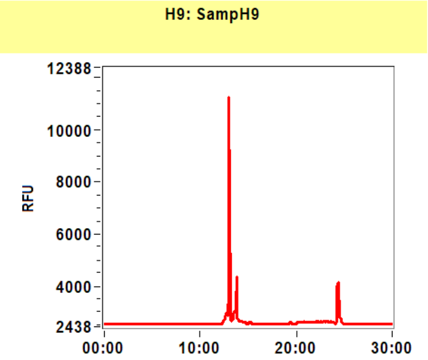

Filename and data path: C:\AATI\Data\2021 08 11\17-13-47\2021 08 11 17H 13M..raw

A10: SampA10

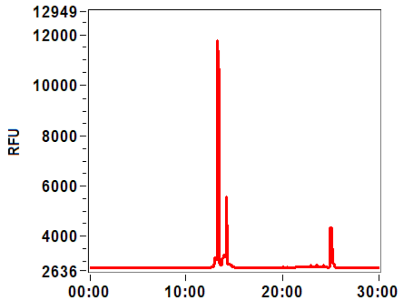

B10: SampB10

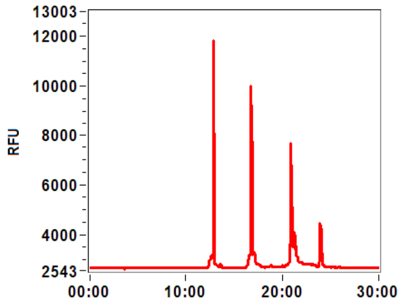

C10: SampC10

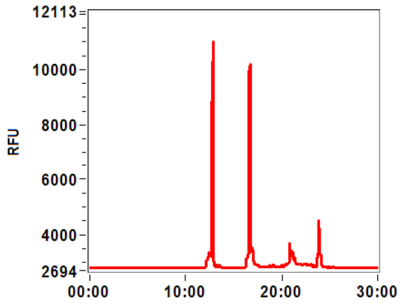

D10: SampD10

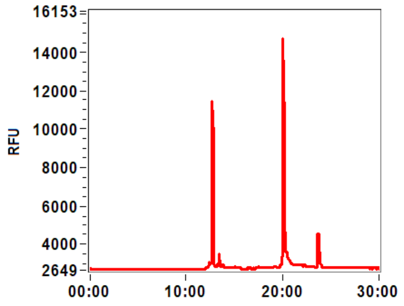

E10: SampE10

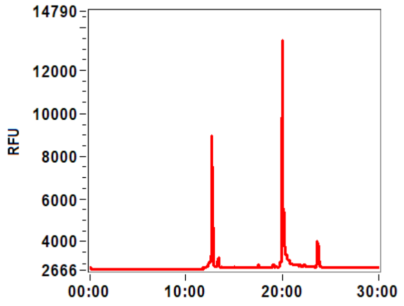

F10: SampF10

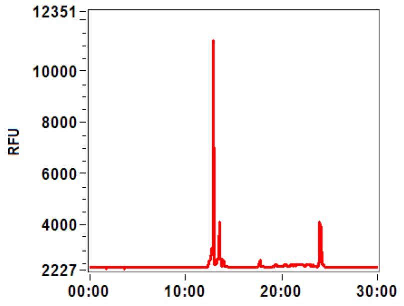

G10: SampG10

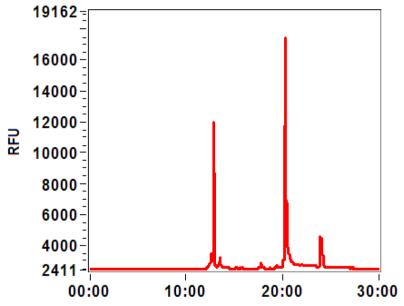

H10: SampH10

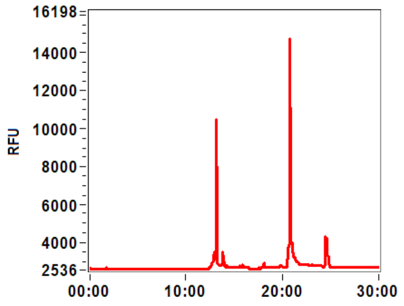

A11: SampA11

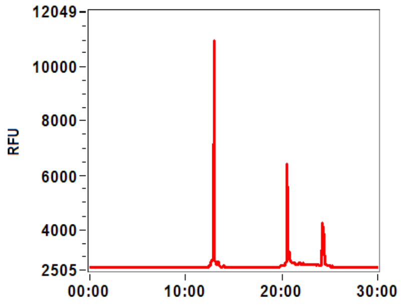

B11: SampB11

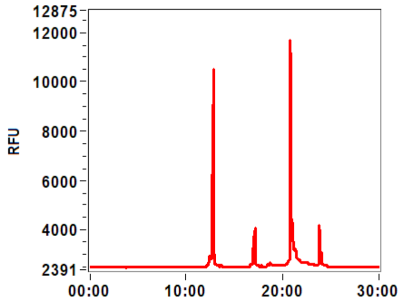

C11: SampC11

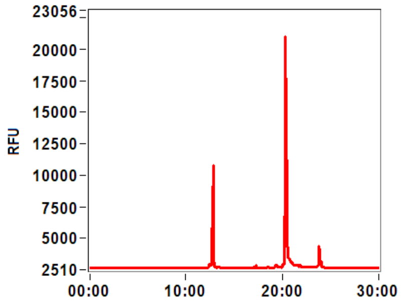

D11: SampD11

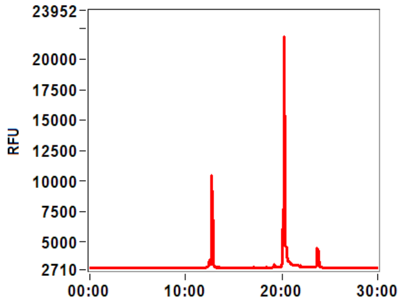

Filename and data path: C:\AATI\Data\2021 08 11\17-13-47\2021 08 11 17H 13M..raw

E11: SampE11

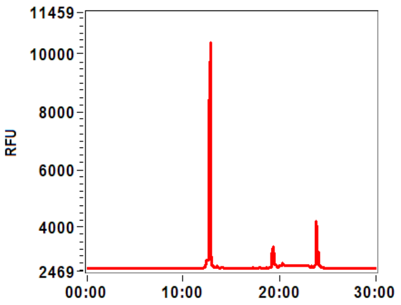

F11: SampF11

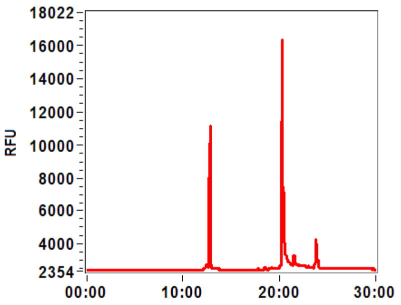

G11: SampG11

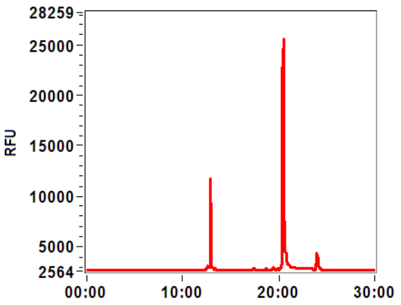

H11: SampH11

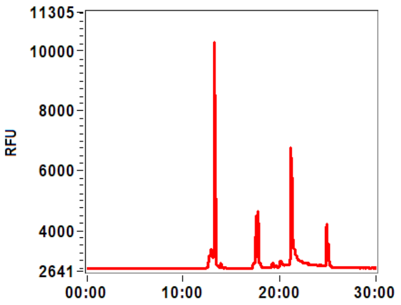

A12: SampA12

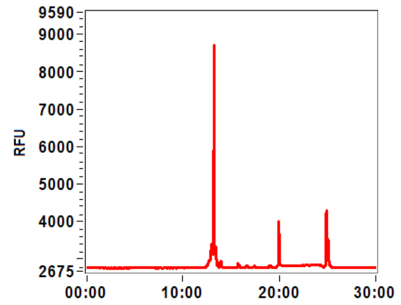

B12: SampB12

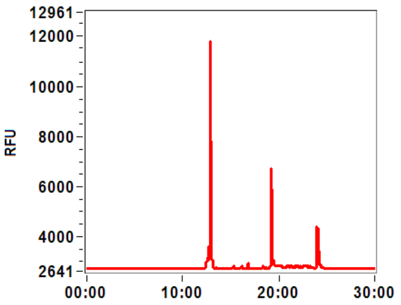

C12: SampC12

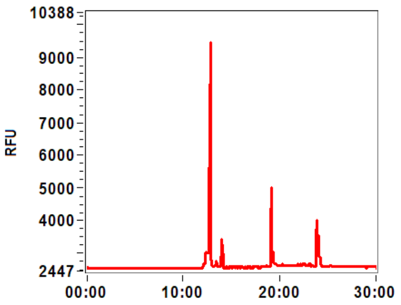

D12: SampD12

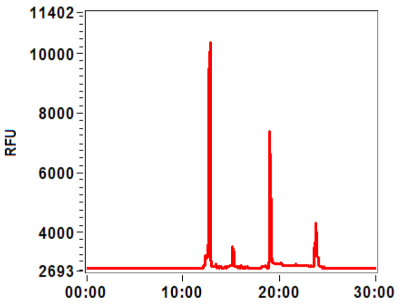

E12: SampE12

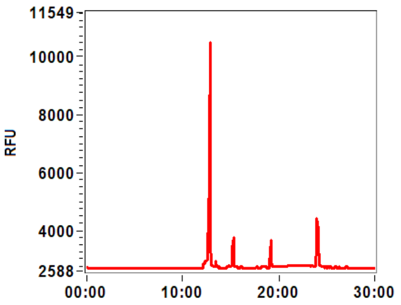

F12: SampF12

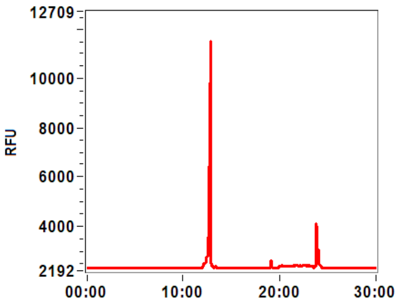

G12: SampG12

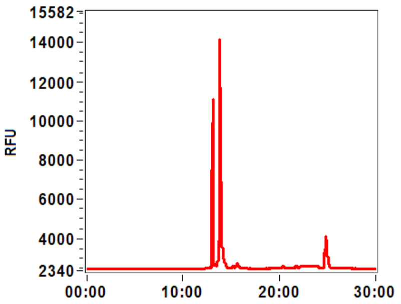

H12: SampH12

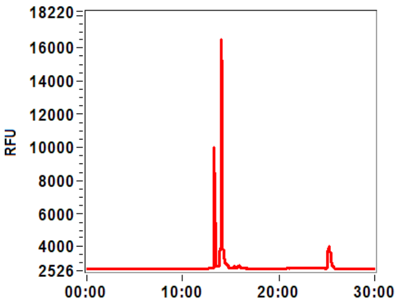

Sample: SampA1
Well location: A1
Created: Wednesday, August 11, 2021 5:34:32 PM

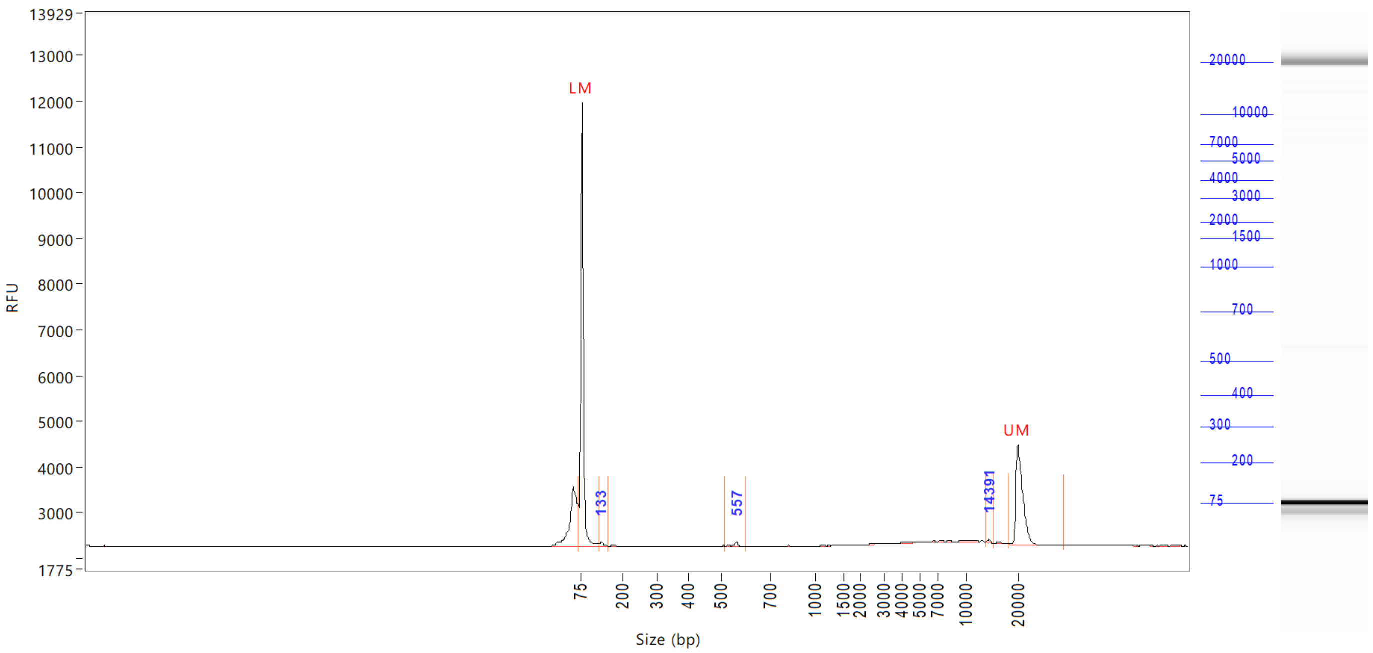

Peak	Size (bp)	Concentration (ng/uL)	From (bp)	To (bp)	Average size (bp)	CV%	RFU	Corrected peak area
1	75 (LM)	0.5000	67	126	75	8.51	9711	59.214
2	133	0.0745	126	156	134	4.79	90	0.735
3	557	0.0626	511	593	550	2.74	101	0.618
4	14391	0.0220	13659	15122	14212	1.77	60	0.217
5	20000 (UM)	0.1589	18049	28947	20373	4.13	2166	18.820

TIC:	0.1591	ng/uL
TIM:	1.1030	nmole/L
Total concentration:	0.5015	ng/uL

```
Sample peak width (sec): 5    Sample min peak height: 50    Sample baseline V to V?: Y    Sample baseline V to V points: 3
Sample filter: Binomial       Number of points for filter: 3  Sample start region (min): 0  Sample end region (min): 30
Marker peak width (sec): 5    Marker min peak height: 200   Marker baseline V to V?: Y    Marker baseline V to V points: 3
Lower marker selection: First peak > 500 RFU                Upper marker selection: Last peak > 200 RFU
Ladder size (bp)75, 200, 300, 400, 500, 700, 1000, 1500, 2000, 3000, 4000, 5000, 7000, 10000, 20000
Quantification using: Lower Marker          Final concentration (ng/uL): 0.5000          Dilution factor: 12.0
```

Sample: SampB1
Well location: B1
Created: Wednesday, August 11, 2021 5:34:32 PM

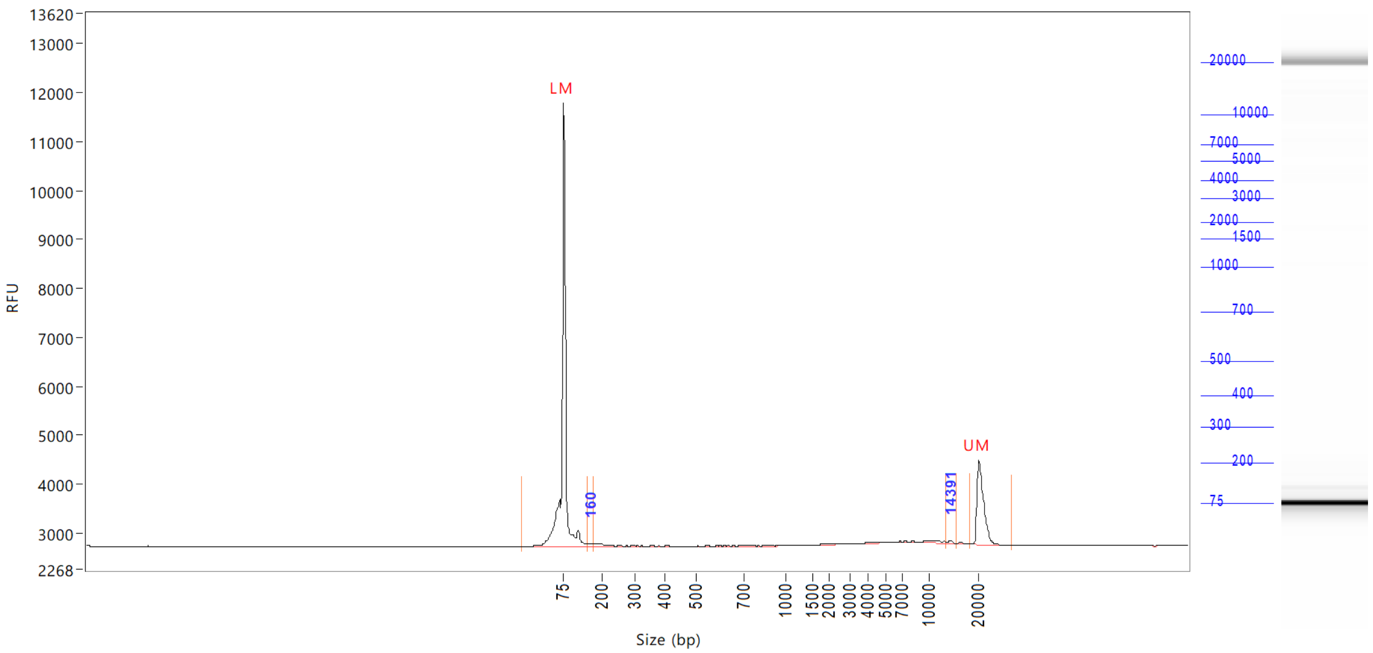

Peak	Size (bp)	Concentration (ng/uL)	From (bp)	To (bp)	Average size (bp)	CV%	RFU	Corrected peak area
1	75 (LM)	0.5000	0	150	71	26.82	9069	83.150
2	160	0.0411	150	171	160	3.94	54	0.570
3	14391	0.0271	13415	15610	14230	3.50	65	0.376
4	20000 (UM)	0.0911	18415	26770	20481	4.11	1723	15.144

TIC:	0.0683	ng/uL
TIM:	0.4258	nmole/L
Total concentration:	0.4055	ng/uL

Sample peak width (sec): 5   Sample min peak height: 50   Sample baseline V to V?: Y   Sample baseline V to V points: 3
Sample filter: Binomial   Number of points for filter: 3 Sample start region (min): 0 Sample end region (min): 30
Marker peak width (sec): 5   Marker min peak height: 200   Marker baseline V to V?: Y   Marker baseline V to V points: 3
Lower marker selection: First peak > 500 RFU   Upper marker selection: Last peak > 200 RFU
Ladder size (bp)75, 200, 300, 400, 500, 700, 1000, 1500, 2000, 3000, 4000, 5000, 7000, 10000, 20000
Quantification using: Lower Marker   Final concentration (ng/uL): 0.5000   Dilution factor: 12.0

Sample: SampC1
Well location: C1
Created: Wednesday, August 11, 2021 5:34:32 PM

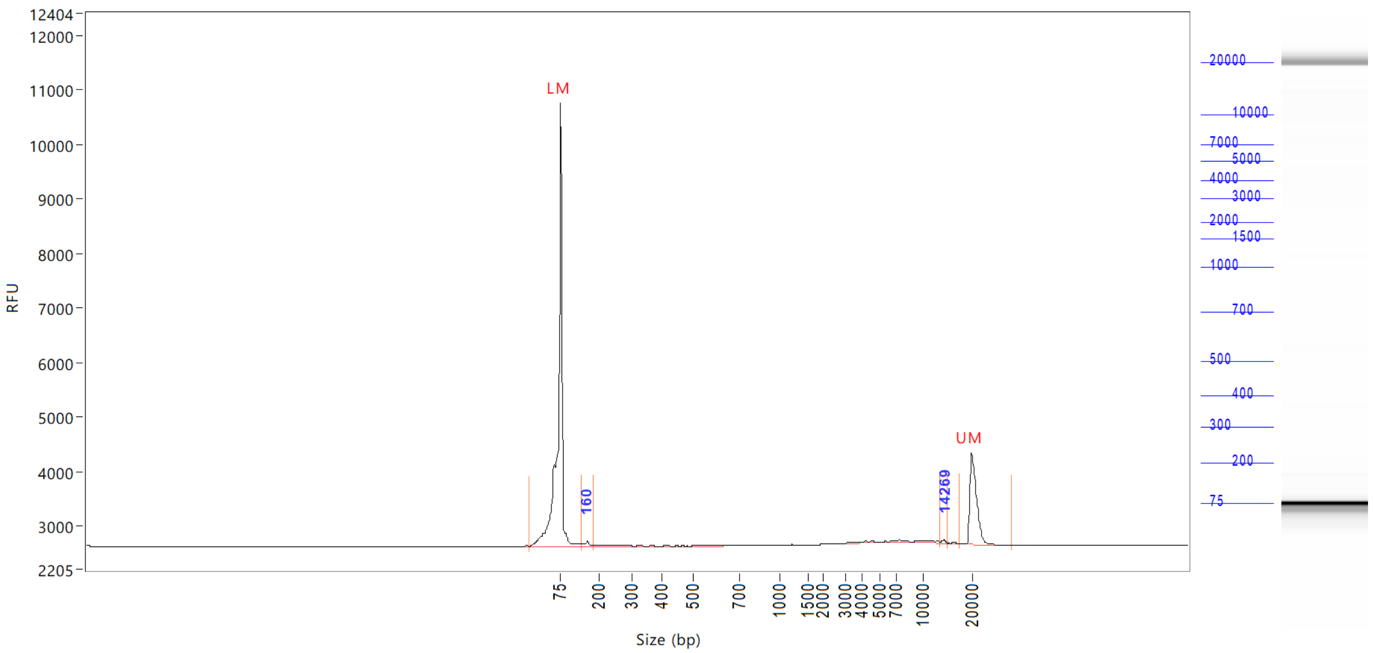

Peak	Size (bp)	Concentration (ng/uL)	From (bp)	To (bp)	Average size (bp)	CV%	RFU	Corrected peak area
1	75 (LM)	0.5000	0	141	63	30.71	8146	79.704
2	160	0.0727	141	183	157	5.63	100	0.966
3	14269	0.0251	13537	15000	14118	2.42	65	0.334
4	20000 (UM)	0.0920	17317	28221	20423	4.31	1679	14.668

TIC:	0.0979	ng/uL
TIM:	0.7644	nmole/L
Total concentration:	0.4486	ng/uL

Sample peak width (sec): 5   Sample min peak height: 50   Sample baseline V to V?: Y   Sample baseline V to V points: 3
Sample filter: Binomial   Number of points for filter: 3   Sample start region (min): 0   Sample end region (min): 30
Marker peak width (sec): 5   Marker min peak height: 200   Marker baseline V to V?: Y   Marker baseline V to V points: 3
Lower marker selection: First peak > 500 RFU   Upper marker selection: Last peak > 200 RFU
Ladder size (bp)75, 200, 300, 400, 500, 700, 1000, 1500, 2000, 3000, 4000, 5000, 7000, 10000, 20000
Quantification using: Lower Marker   Final concentration (ng/uL): 0.5000   Dilution factor: 12.0

Sample: SampD1
Well location: D1
Created: Wednesday, August 11, 2021 5:34:32 PM

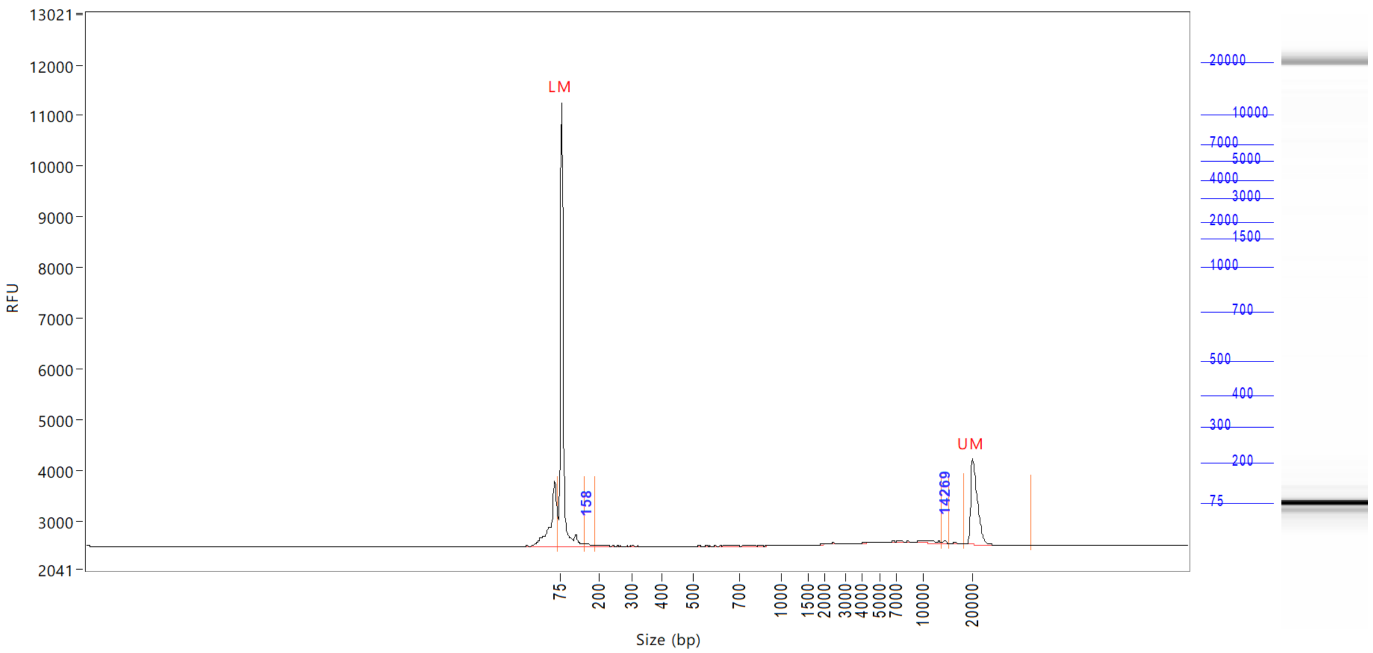

Peak	Size (bp)	Concentration (ng/uL)	From (bp)	To (bp)	Average size (bp)	CV%	RFU	Corrected peak area
1	75 (LM)	0.5000	66	150	77	15.18	8770	64.240
2	158	0.0784	150	185	165	6.27	60	0.839
3	14269	0.0247	13659	15122	14174	2.01	57	0.264
4	20000 (UM)	0.1143	18049	32090	20541	5.38	1682	14.680

TIC:	0.1031	ng/uL
TIM:	0.7836	nmole/L
Total concentration:	0.5053	ng/uL

Sample peak width (sec): 5 Sample min peak height: 50 Sample baseline V to V?: Y Sample baseline V to V points: 3
Sample filter: Binomial Number of points for filter: 3 Sample start region (min): 0 Sample end region (min): 30
Marker peak width (sec): 5 Marker min peak height: 200 Marker baseline V to V?: Y Marker baseline V to V points: 3
Lower marker selection: First peak > 500 RFU Upper marker selection: Last peak > 200 RFU
Ladder size (bp)75, 200, 300, 400, 500, 700, 1000, 1500, 2000, 3000, 4000, 5000, 7000, 10000, 20000
Quantification using: Lower Marker Final concentration (ng/uL): 0.5000 Dilution factor: 12.0

Sample: SampE1
Well location: E1
Created: Wednesday, August 11, 2021 5:34:32 PM

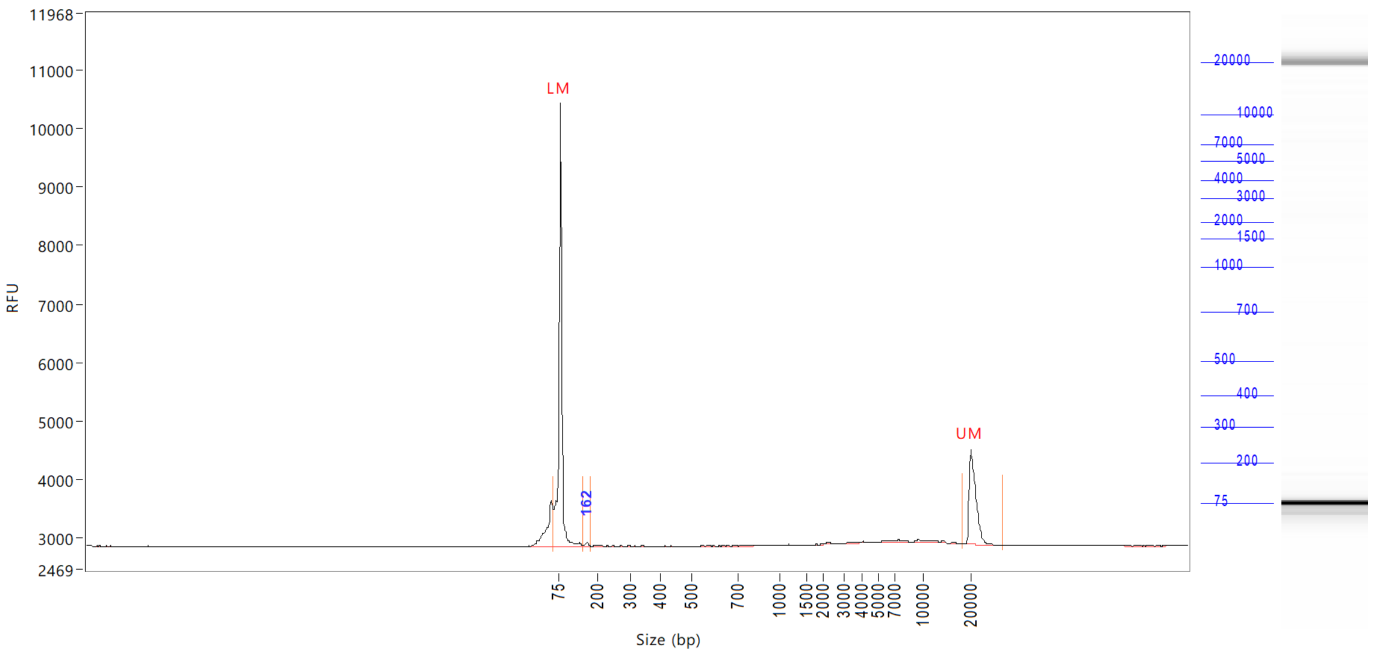

Peak	Size (bp)	Concentration (ng/uL)	From (bp)	To (bp)	Average size (bp)	CV%	RFU	Corrected peak area
1	75 (LM)	0.5000	54	152	74	14.60	7590	56.008
2	162	0.0514	152	173	160	2.64	71	0.480
3	20000 (UM)	0.1265	18049	26529	20427	4.25	1612	14.173

TIC:	0.0514	ng/uL
TIM:	0.5285	nmole/L
Total concentration:	0.4948	ng/uL

Sample peak width (sec): 5   Sample min peak height: 50   Sample baseline V to V?: Y   Sample baseline V to V points: 3
Sample filter: Binomial   Number of points for filter: 3 Sample start region (min): 0 Sample end region (min): 30
Marker peak width (sec): 5   Marker min peak height: 200   Marker baseline V to V?: Y   Marker baseline V to V points: 3
Lower marker selection: First peak > 500 RFU   Upper marker selection: Last peak > 200 RFU
Ladder size (bp)75, 200, 300, 400, 500, 700, 1000, 1500, 2000, 3000, 4000, 5000, 7000, 10000, 20000
Quantification using: Lower Marker   Final concentration (ng/uL): 0.5000   Dilution factor: 12.0

Sample: SampF1
Well location: F1
Created: Wednesday, August 11, 2021 5:34:32 PM

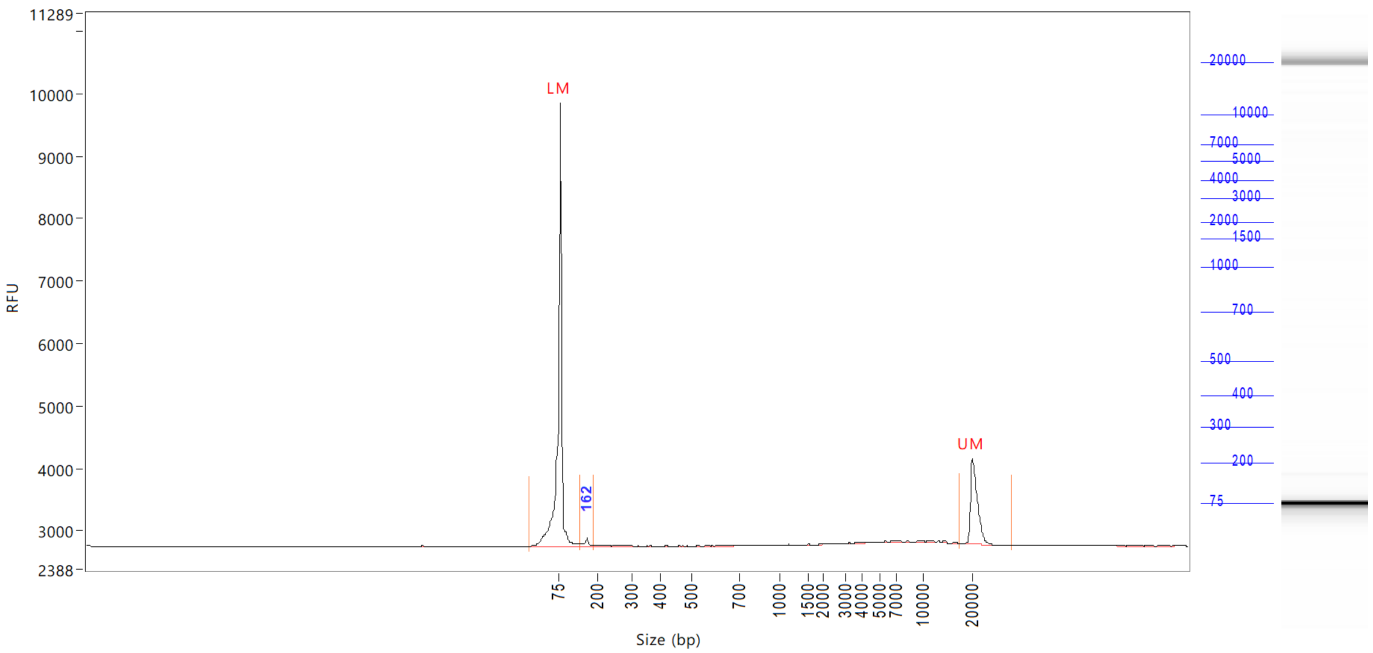

Peak	Size (bp)	Concentration (ng/uL)	From (bp)	To (bp)	Average size (bp)	CV%	RFU	Corrected peak area
1	75 (LM)	0.5000	0	139	67	25.83	7110	62.388
2	162	0.1110	139	185	157	6.17	123	1.154
3	20000 (UM)	0.0985	17317	27979	20454	4.32	1362	12.285

TIC:	0.1110	ng/uL
TIM:	1.1619	nmole/L
Total concentration:	0.4584	ng/uL

```
Sample peak width (sec): 5    Sample min peak height: 50    Sample baseline V to V?: Y    Sample baseline V to V points: 3
Sample filter: Binomial       Number of points for filter: 3  Sample start region (min): 0  Sample end region (min): 30
Marker peak width (sec): 5    Marker min peak height: 200   Marker baseline V to V?: Y    Marker baseline V to V points: 3
Lower marker selection: First peak > 500 RFU                Upper marker selection: Last peak > 200 RFU
Ladder size (bp)75, 200, 300, 400, 500, 700, 1000, 1500, 2000, 3000, 4000, 5000, 7000, 10000, 20000
Quantification using: Lower Marker          Final concentration (ng/uL): 0.5000          Dilution factor: 12.0
```

Sample: SampG1
Well location: G1
Created: Wednesday, August 11, 2021 5:34:32 PM

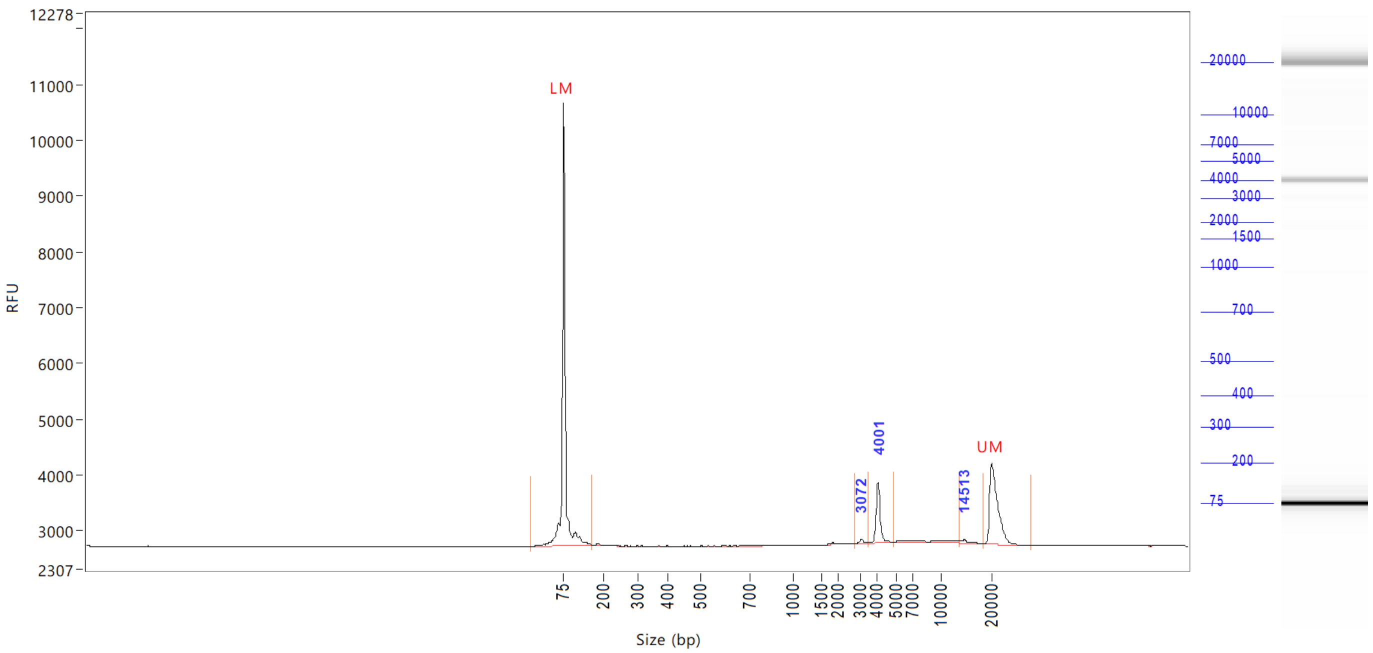

Peak	Size (bp)	Concentration (ng/uL)	From (bp)	To (bp)	Average size (bp)	CV%	RFU	Corrected peak area
1	75 (LM)	0.5000	0	164	74	22.15	7955	58.561
2	3072	0.0500	2772	3429	3059	3.88	76	0.488
3	4001	0.6994	3429	4862	4009	3.93	1093	6.826
4	14513	0.0734	13415	18171	14920	7.08	58	0.717
5	20000 (UM)	0.1317	18171	27738	20537	4.97	1464	15.419

TIC:	0.8228	ng/uL
TIM:	0.3222	nmole/L
Total concentration:	1.1880	ng/uL

Sample peak width (sec): 5   Sample min peak height: 50   Sample baseline V to V?: Y   Sample baseline V to V points: 3
Sample filter: Binomial   Number of points for filter: 3 Sample start region (min): 0 Sample end region (min): 30
Marker peak width (sec): 5   Marker min peak height: 200   Marker baseline V to V?: Y   Marker baseline V to V points: 3
Lower marker selection: First peak > 500 RFU   Upper marker selection: Last peak > 200 RFU
Ladder size (bp)75, 200, 300, 400, 500, 700, 1000, 1500, 2000, 3000, 4000, 5000, 7000, 10000, 20000
Quantification using: Lower Marker   Final concentration (ng/uL): 0.5000   Dilution factor: 12.0

Sample: SampH1
Well location: H1
Created: Wednesday, August 11, 2021 5:34:32 PM

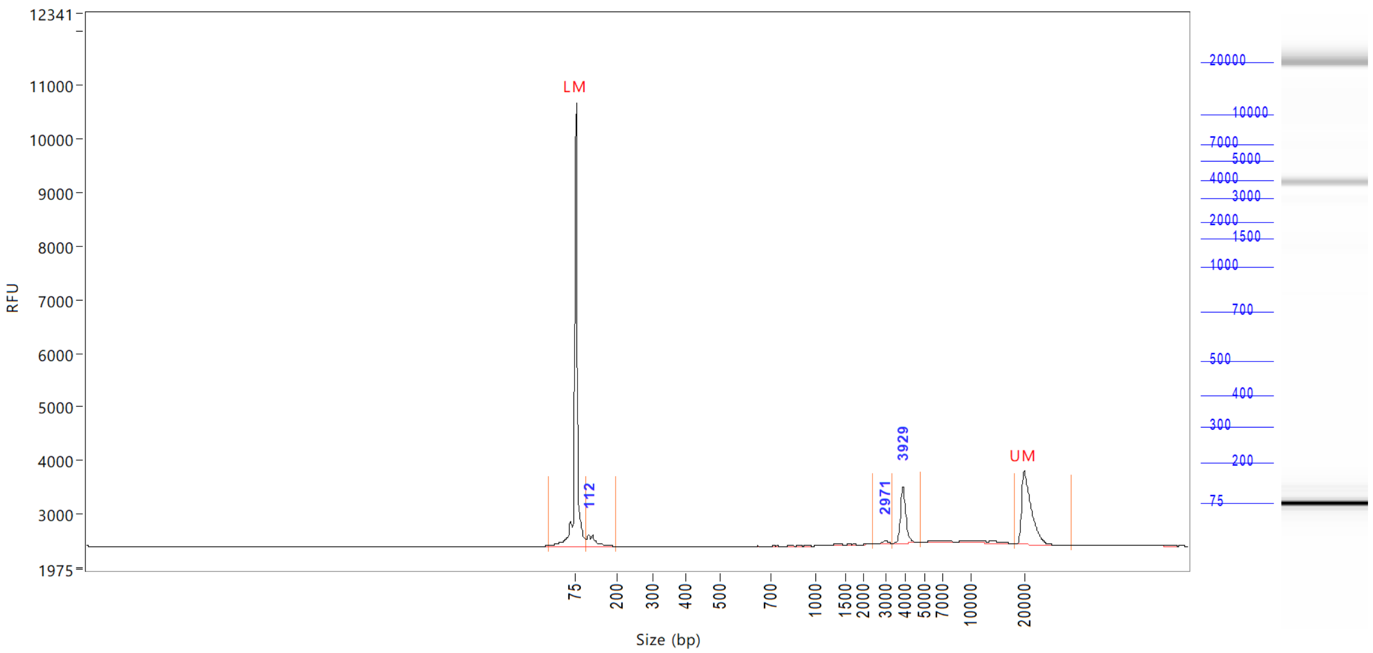

Peak	Size (bp)	Concentration (ng/uL)	From (bp)	To (bp)	Average size (bp)	CV%	RFU	Corrected peak area
1	75 (LM)	0.5000	0	103	71	16.19	8286	57.469
2	112	0.4624	103	192	123	13.58	213	4.429
3	2971	0.0534	2429	3322	2936	4.70	57	0.511
4	3929	0.8625	3322	4862	3920	4.59	1045	8.261
5	20000 (UM)	0.1407	18049	28705	20623	5.44	1369	16.171

TIC:	1.3783	ng/uL
TIM:	6.5688	nmole/L
Total concentration:	1.8111	ng/uL

```
Sample peak width (sec): 5    Sample min peak height: 50    Sample baseline V to V?: Y    Sample baseline V to V points: 3
Sample filter: Binomial       Number of points for filter: 3  Sample start region (min): 0  Sample end region (min): 30
Marker peak width (sec): 5    Marker min peak height: 200   Marker baseline V to V?: Y    Marker baseline V to V points: 3
Lower marker selection: First peak > 500 RFU                Upper marker selection: Last peak > 200 RFU
Ladder size (bp)75, 200, 300, 400, 500, 700, 1000, 1500, 2000, 3000, 4000, 5000, 7000, 10000, 20000
Quantification using: Lower Marker          Final concentration (ng/uL): 0.5000          Dilution factor: 12.0
```

Sample: SampA2
Well location: A2
Created: Wednesday, August 11, 2021 5:34:32 PM

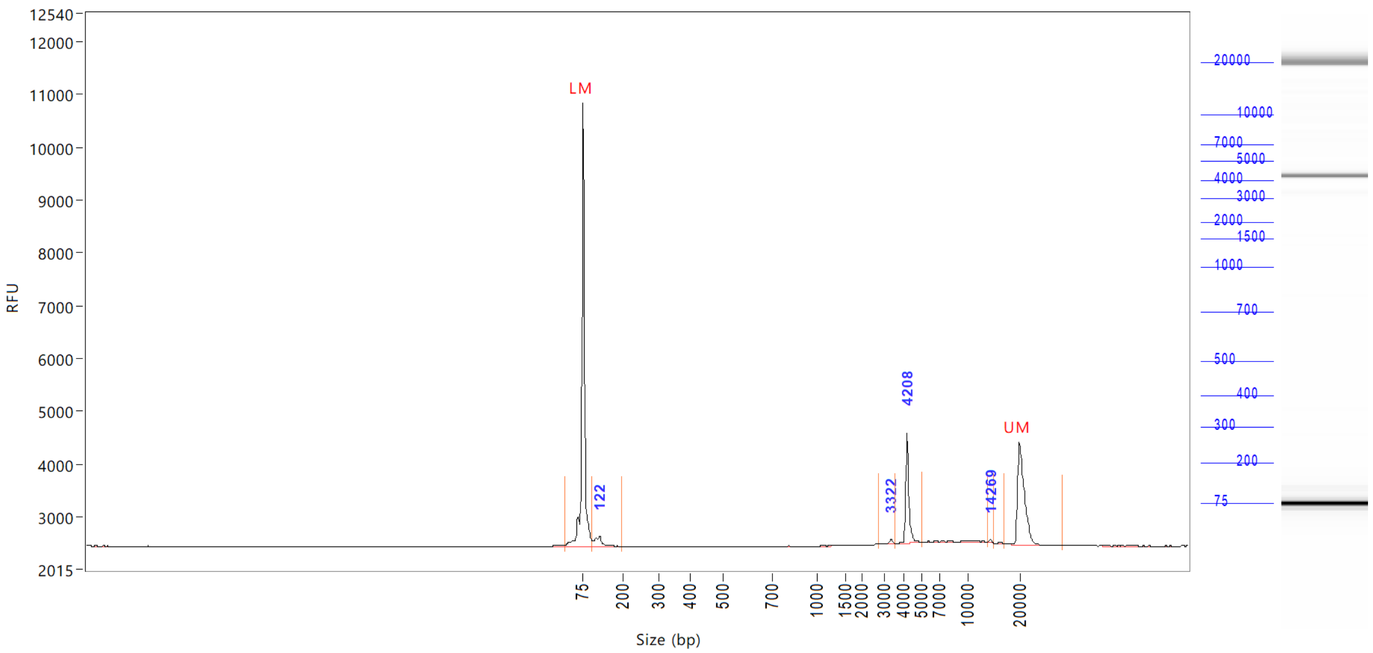

Peak	Size (bp)	Concentration (ng/uL)	From (bp)	To (bp)	Average size (bp)	CV%	RFU	Corrected peak area
1	75 (LM)	0.5000	20	105	71	13.43	8414	56.013
2	122	0.3682	105	194	125	13.78	190	3.437
3	3322	0.0436	2743	3608	3290	3.59	88	0.407
4	4208	0.9975	3608	5075	4218	3.12	2081	9.313
5	14269	0.0188	13781	15122	14219	1.62	53	0.176
6	20000 (UM)	0.1484	17073	28463	20398	3.99	1936	16.622

TIC:	1.4282	ng/uL
TIM:	5.2529	nmole/L
Total concentration:	1.7310	ng/uL

Sample peak width (sec): 5 Sample min peak height: 50 Sample baseline V to V?: Y Sample baseline V to V points: 3
Sample filter: Binomial Number of points for filter: 3 Sample start region (min): 0 Sample end region (min): 30
Marker peak width (sec): 5 Marker min peak height: 200 Marker baseline V to V?: Y Marker baseline V to V points: 3
Lower marker selection: First peak > 500 RFU Upper marker selection: Last peak > 200 RFU
Ladder size (bp)75, 200, 300, 400, 500, 700, 1000, 1500, 2000, 3000, 4000, 5000, 7000, 10000, 20000
Quantification using: Lower Marker Final concentration (ng/uL): 0.5000 Dilution factor: 12.0

Sample: SampB2
Well location: B2
Created: Wednesday, August 11, 2021 5:34:32 PM

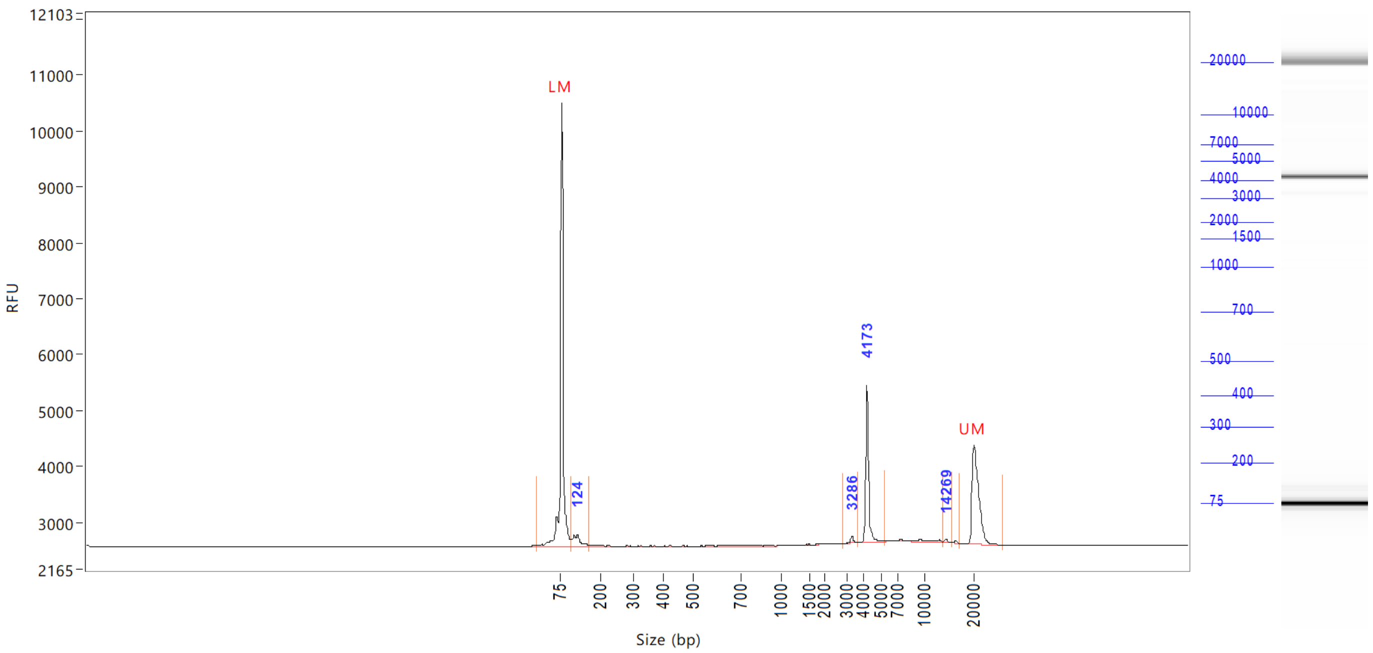

Peak	Size (bp)	Concentration (ng/uL)	From (bp)	To (bp)	Average size (bp)	CV%	RFU	Corrected peak area
1	75 (LM)	0.5000	0	105	71	16.19	7940	55.848
2	124	0.4050	105	166	123	11.08	215	3.770
3	3286	0.0594	2829	3715	3270	3.53	115	0.553
4	4173	1.3054	3715	5297	4176	3.40	2793	12.151
5	14269	0.0271	13537	15488	14105	2.09	54	0.252
6	20000 (UM)	0.1383	17195	26045	20475	3.91	1763	15.446

TIC:	1.7968	ng/uL
TIM:	5.9569	nmole/L
Total concentration:	2.1591	ng/uL

```
Sample peak width (sec): 5    Sample min peak height: 50    Sample baseline V to V?: Y    Sample baseline V to V points: 3
Sample filter: Binomial       Number of points for filter: 3  Sample start region (min): 0  Sample end region (min): 30
Marker peak width (sec): 5    Marker min peak height: 200   Marker baseline V to V?: Y    Marker baseline V to V points: 3
Lower marker selection: First peak > 500 RFU                Upper marker selection: Last peak > 200 RFU
Ladder size (bp)75, 200, 300, 400, 500, 700, 1000, 1500, 2000, 3000, 4000, 5000, 7000, 10000, 20000
Quantification using: Lower Marker          Final concentration (ng/uL): 0.5000          Dilution factor: 12.0
```

Sample: SampC2
Well location: C2
Created: Wednesday, August 11, 2021 5:34:32 PM

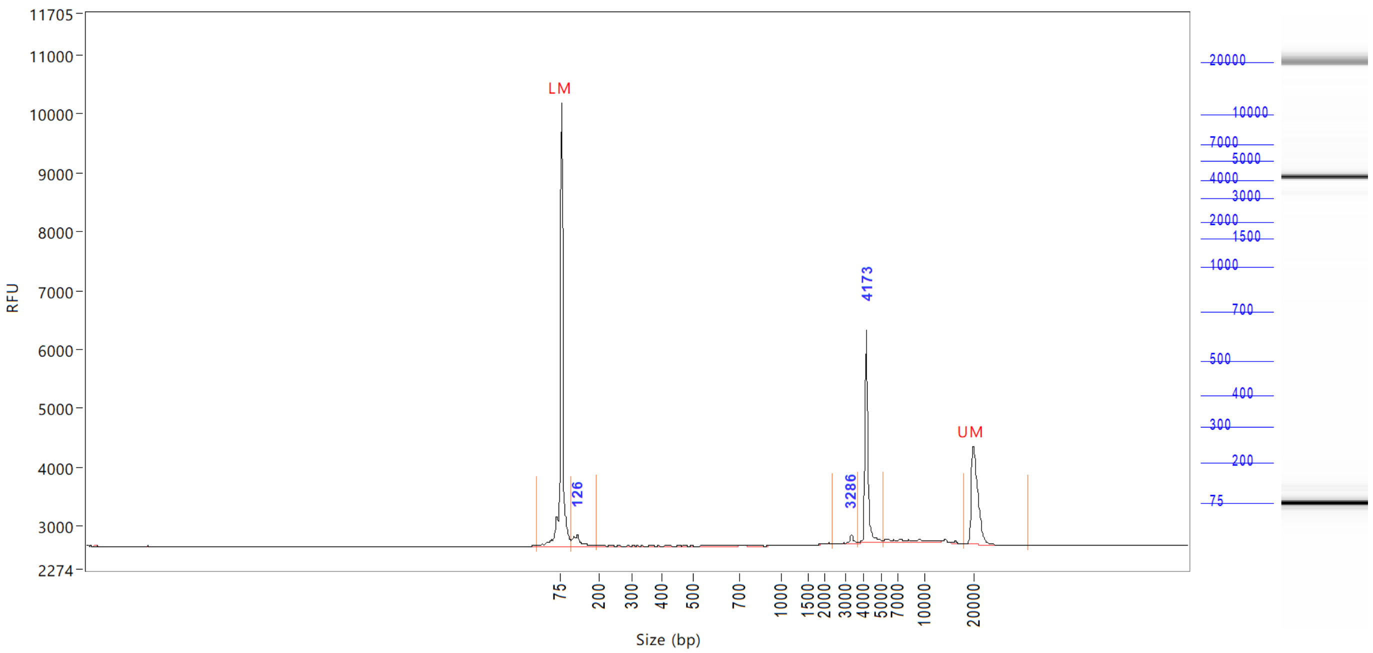

Peak	Size (bp)	Concentration (ng/uL)	From (bp)	To (bp)	Average size (bp)	CV%	RFU	Corrected peak area
1	75 (LM)	0.5000	0	107	72	15.80	7534	53.177
2	126	0.3884	107	187	126	12.76	205	3.442
3	3286	0.0991	2372	3715	3255	7.44	135	0.878
4	4173	1.8743	3715	5223	4182	4.14	3621	16.611
5	20000 (UM)	0.1342	18049	31244	20479	4.91	1664	14.275

TIC:	2.3617	ng/uL
TIM:	5.8520	nmole/L
Total concentration:	2.7409	ng/uL

Sample peak width (sec): 5   Sample min peak height: 50   Sample baseline V to V?: Y   Sample baseline V to V points: 3
Sample filter: Binomial   Number of points for filter: 3 Sample start region (min): 0 Sample end region (min): 30
Marker peak width (sec): 5   Marker min peak height: 200   Marker baseline V to V?: Y   Marker baseline V to V points: 3
Lower marker selection: First peak > 500 RFU   Upper marker selection: Last peak > 200 RFU
Ladder size (bp)75, 200, 300, 400, 500, 700, 1000, 1500, 2000, 3000, 4000, 5000, 7000, 10000, 20000
Quantification using: Lower Marker   Final concentration (ng/uL): 0.5000   Dilution factor: 12.0

Sample: SampD2
Well location: D2
Created: Wednesday, August 11, 2021 5:34:32 PM

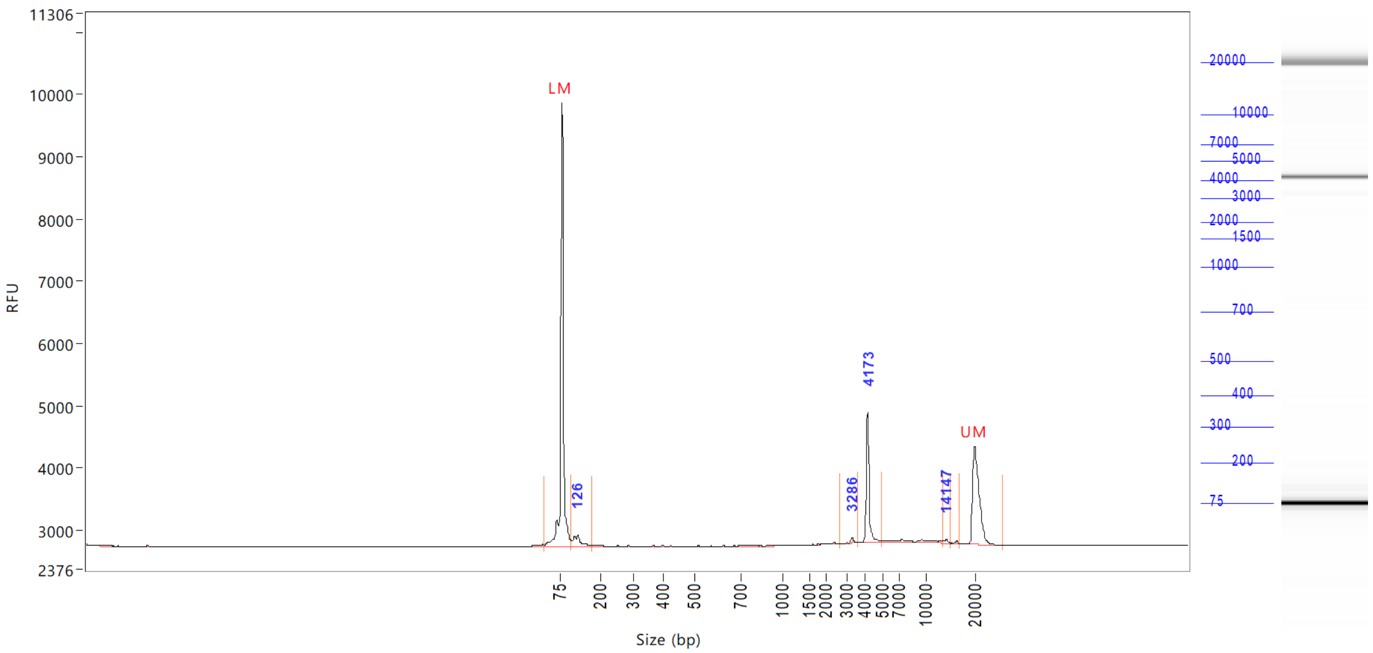

Peak	Size (bp)	Concentration (ng/uL)	From (bp)	To (bp)	Average size (bp)	CV%	RFU	Corrected peak area
1	75 (LM)	0.5000	18	107	72	13.65	7132	48.223
2	126	0.3653	107	173	125	11.33	172	2.936
3	3286	0.0480	2629	3643	3210	5.16	76	0.385
4	4173	1.1311	3643	4966	4162	3.07	2079	9.091
5	14147	0.0410	13415	15000	14022	2.63	63	0.329
6	20000 (UM)	0.1400	16952	25682	20313	3.95	1573	13.501

TIC:	1.5854	ng/uL
TIM:	5.2784	nmole/L
Total concentration:	2.0148	ng/uL

```
Sample peak width (sec): 5    Sample min peak height: 50    Sample baseline V to V?: Y    Sample baseline V to V points: 3
Sample filter: Binomial       Number of points for filter: 3  Sample start region (min): 0  Sample end region (min): 30
Marker peak width (sec): 5    Marker min peak height: 200   Marker baseline V to V?: Y    Marker baseline V to V points: 3
Lower marker selection: First peak > 500 RFU                Upper marker selection: Last peak > 200 RFU
Ladder size (bp)75, 200, 300, 400, 500, 700, 1000, 1500, 2000, 3000, 4000, 5000, 7000, 10000, 20000
Quantification using: Lower Marker          Final concentration (ng/uL): 0.5000          Dilution factor: 12.0
```

Sample: SampE2
Well location: E2
Created: Wednesday, August 11, 2021 5:34:32 PM

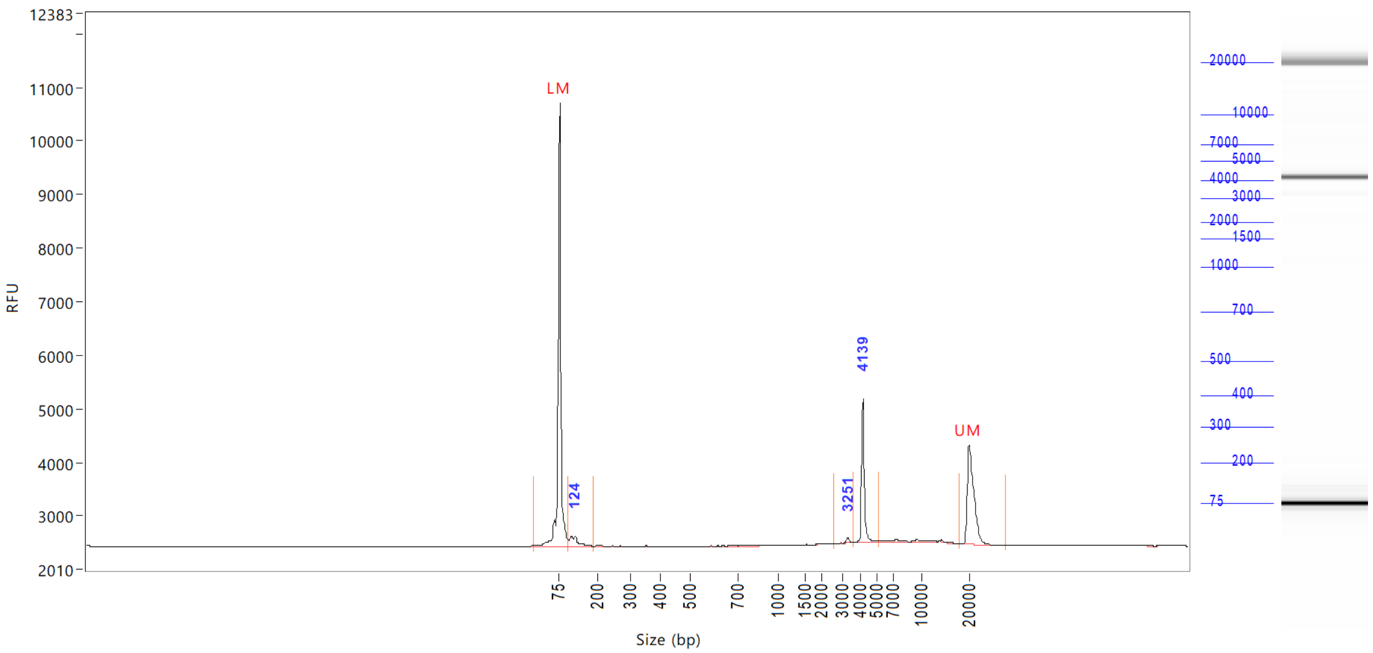

Peak	Size	Concentration	From	To	Average size	CV%	RFU	Corrected peak area
	(bp)	(ng/uL)	(bp)	(bp)	(bp)			
1	75 (LM)	0.5000	0	107	71	16.11	8291	56.709
2	124	0.3893	107	187	126	13.12	200	3.680
3	3251	0.0517	2543	3608	3220	4.86	103	0.488
4	4139	1.2049	3608	5075	4143	2.96	2689	11.388
5	20000 (UM)	0.1411	17927	27496	20378	3.99	1846	16.002

TIC:	1.6459	ng/uL
TIM:	5.5818	nmole/L
Total concentration:	2.0179	ng/uL

```
Sample peak width (sec): 5    Sample min peak height: 50    Sample baseline V to V?: Y    Sample baseline V to V points: 3
Sample filter: Binomial       Number of points for filter: 3  Sample start region (min): 0  Sample end region (min): 30
Marker peak width (sec): 5    Marker min peak height: 200   Marker baseline V to V?: Y    Marker baseline V to V points: 3
Lower marker selection: First peak > 500 RFU                Upper marker selection: Last peak > 200 RFU
Ladder size (bp)75, 200, 300, 400, 500, 700, 1000, 1500, 2000, 3000, 4000, 5000, 7000, 10000, 20000
Quantification using: Lower Marker          Final concentration (ng/uL): 0.5000          Dilution factor: 12.0
```

Sample: SampF2
Well location: F2
Created: Wednesday, August 11, 2021 5:34:32 PM

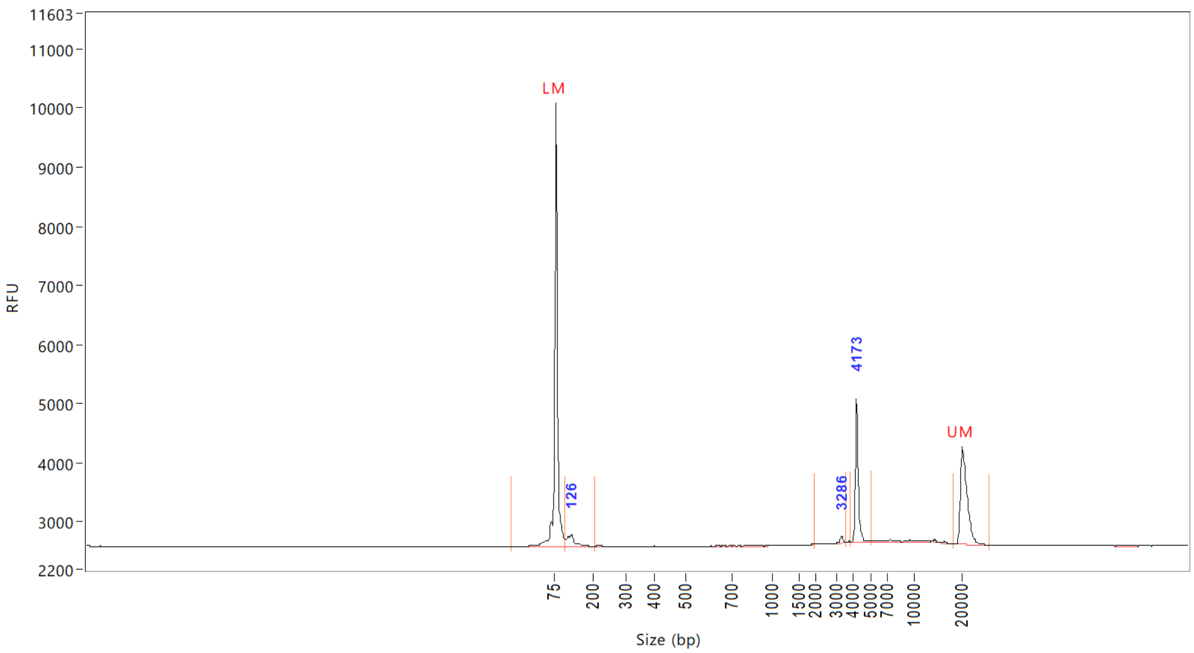

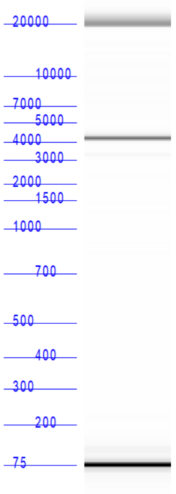

Peak	Size (bp)	Concentration (ng/uL)	From (bp)	To (bp)	Average size (bp)	CV%	RFU	Corrected peak area
1	75 (LM)	0.5000	0	107	72	17.39	7515	53.025
2	126	0.4132	107	201	128	14.37	202	3.652
3	3286	0.0733	1981	3536	3122	10.40	119	0.648
4	4173	1.1776	3857	5001	4168	2.81	2417	10.407
5	20000 (UM)	0.1379	18049	25682	20487	3.98	1645	14.628

TIC:	1.6640	ng/uL
TIM:	5.8075	nmole/L
Total concentration:	2.0058	ng/uL

```
Sample peak width (sec): 5    Sample min peak height: 50    Sample baseline V to V?: Y    Sample baseline V to V points: 3
Sample filter: Binomial       Number of points for filter: 3  Sample start region (min): 0  Sample end region (min): 30
Marker peak width (sec): 5    Marker min peak height: 200   Marker baseline V to V?: Y    Marker baseline V to V points: 3
Lower marker selection: First peak > 500 RFU                Upper marker selection: Last peak > 200 RFU
Ladder size (bp)75, 200, 300, 400, 500, 700, 1000, 1500, 2000, 3000, 4000, 5000, 7000, 10000, 20000
Quantification using: Lower Marker          Final concentration (ng/uL): 0.5000          Dilution factor: 12.0
```

Sample: SampG2
Well location: G2
Created: Wednesday, August 11, 2021 5:34:32 PM

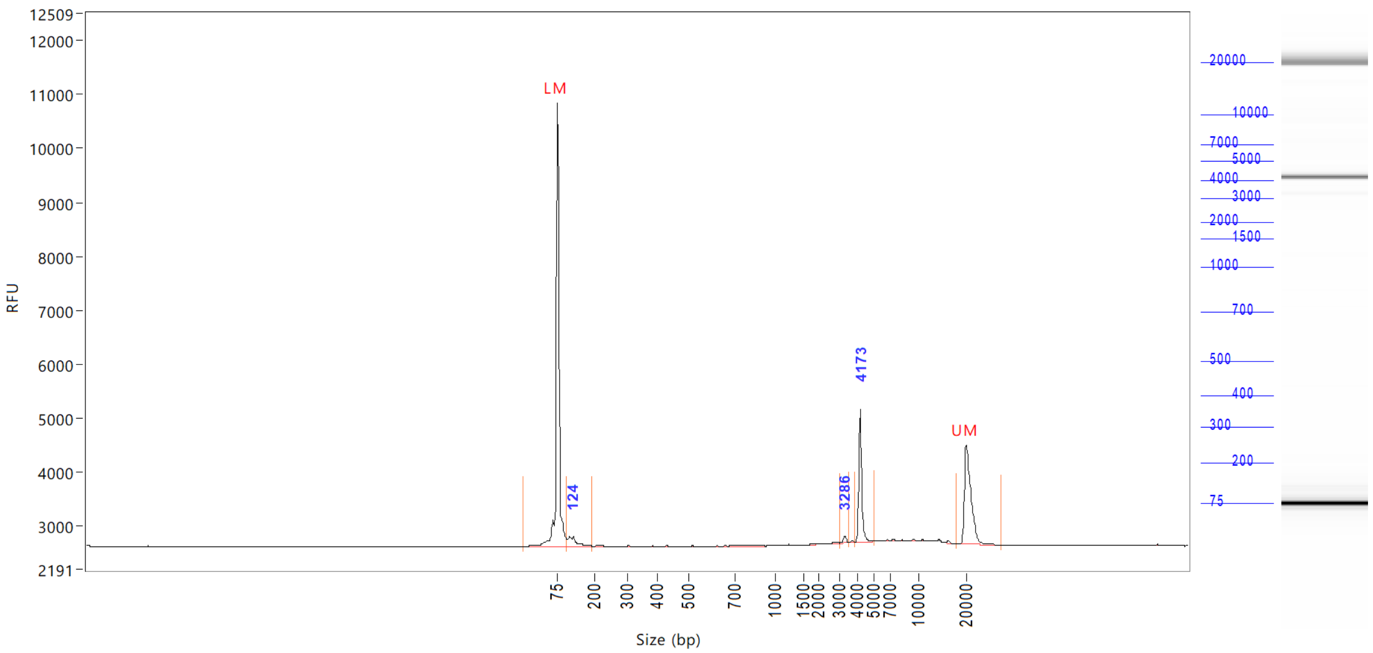

Peak	Size (bp)	Concentration (ng/uL)	From (bp)	To (bp)	Average size (bp)	CV%	RFU	Corrected peak area
1	75 (LM)	0.5000	0	107	71	17.68	8246	56.468
2	124	0.3991	107	189	128	14.18	193	3.756
3	3286	0.0580	2971	3500	3243	2.96	121	0.546
4	4173	1.1632	3893	5001	4152	2.80	2463	10.947
5	20000 (UM)	0.1414	18049	27375	20433	4.20	1844	15.972

TIC:	1.6202	ng/uL
TIM:	5.6130	nmole/L
Total concentration:	1.9948	ng/uL

```
Sample peak width (sec): 5     Sample min peak height: 50    Sample baseline V to V?: Y    Sample baseline V to V points: 3
Sample filter: Binomial        Number of points for filter: 3  Sample start region (min): 0  Sample end region (min): 30
Marker peak width (sec): 5     Marker min peak height: 200   Marker baseline V to V?: Y    Marker baseline V to V points: 3
Lower marker selection: First peak > 500 RFU                 Upper marker selection: Last peak > 200 RFU
Ladder size (bp)75, 200, 300, 400, 500, 700, 1000, 1500, 2000, 3000, 4000, 5000, 7000, 10000, 20000
Quantification using: Lower Marker          Final concentration (ng/uL): 0.5000          Dilution factor: 12.0
```

Sample: SampH2
Well location: H2
Created: Wednesday, August 11, 2021 5:34:32 PM

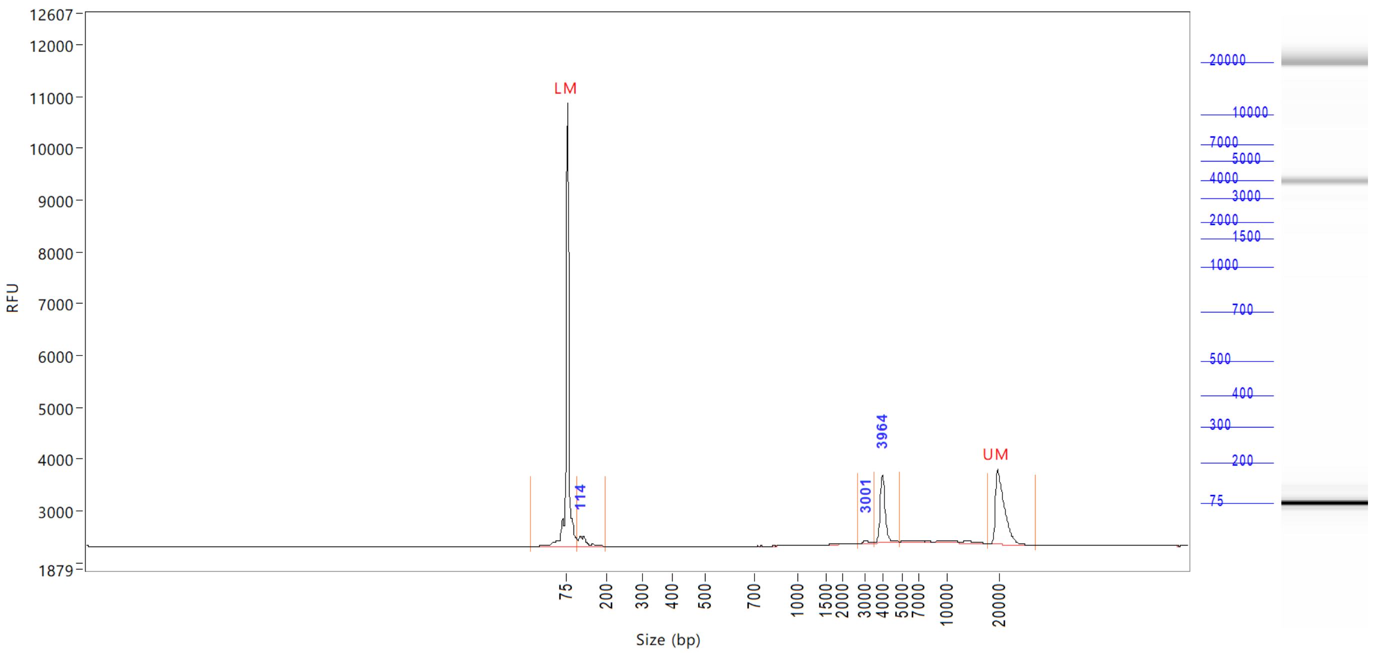

Peak	Size (bp)	Concentration (ng/uL)	From (bp)	To (bp)	Average size (bp)	CV%	RFU	Corrected peak area
1	75 (LM)	0.5000	0	107	72	17.04	8577	59.903
2	114	0.4076	107	196	128	15.07	208	4.070
3	3001	0.0493	2715	3465	3042	4.79	62	0.492
4	3964	0.9535	3465	4897	3961	4.39	1307	9.520
5	20000 (UM)	0.1358	17927	27254	20583	5.12	1448	16.273

TIC:	1.4104	ng/uL
TIM:	5.6554	nmole/L
Total concentration:	1.7970	ng/uL

```
Sample peak width (sec): 5    Sample min peak height: 50    Sample baseline V to V?: Y    Sample baseline V to V points: 3
Sample filter: Binomial       Number of points for filter: 3  Sample start region (min): 0  Sample end region (min): 30
Marker peak width (sec): 5    Marker min peak height: 200   Marker baseline V to V?: Y    Marker baseline V to V points: 3
Lower marker selection: First peak > 500 RFU                Upper marker selection: Last peak > 200 RFU
Ladder size (bp)75, 200, 300, 400, 500, 700, 1000, 1500, 2000, 3000, 4000, 5000, 7000, 10000, 20000
Quantification using: Lower Marker          Final concentration (ng/uL): 0.5000          Dilution factor: 12.0
```

Sample: SampA3
Well location: A3
Created: Wednesday, August 11, 2021 5:34:32 PM

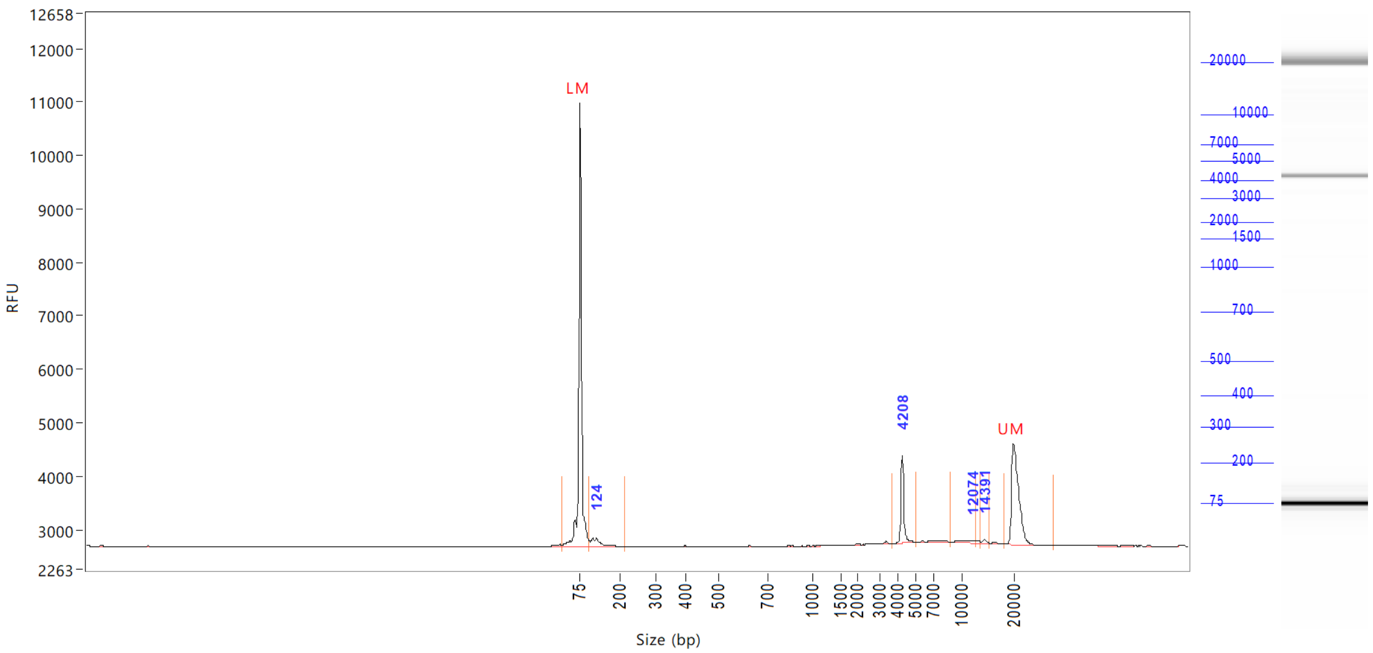

Peak	Size (bp)	Concentration (ng/uL)	From (bp)	To (bp)	Average size (bp)	CV%	RFU	Corrected peak area
1	75 (LM)	0.5000	18	105	72	13.35	8310	55.008
2	124	0.3711	105	210	128	16.10	175	3.402
3	4208	0.7749	3750	4966	4217	3.02	1629	7.104
4	12074	0.1106	8734	12562	10608	9.80	51	1.014
5	14391	0.0445	13659	15244	14304	2.63	74	0.408
6	20000 (UM)	0.1521	18171	27859	20496	4.21	1884	16.729

TIC:	1.3011	ng/uL
TIM:	5.0879	nmole/L
Total concentration:	1.6269	ng/uL

Sample peak width (sec): 5   Sample min peak height: 50   Sample baseline V to V?: Y   Sample baseline V to V points: 3
Sample filter: Binomial   Number of points for filter: 3 Sample start region (min): 0 Sample end region (min): 30
Marker peak width (sec): 5   Marker min peak height: 200   Marker baseline V to V?: Y   Marker baseline V to V points: 3
Lower marker selection: First peak > 500 RFU   Upper marker selection: Last peak > 200 RFU
Ladder size (bp)75, 200, 300, 400, 500, 700, 1000, 1500, 2000, 3000, 4000, 5000, 7000, 10000, 20000
Quantification using: Lower Marker   Final concentration (ng/uL): 0.5000   Dilution factor: 12.0

Sample: SampB3
Well location: B3
Created: Wednesday, August 11, 2021 5:34:32 PM

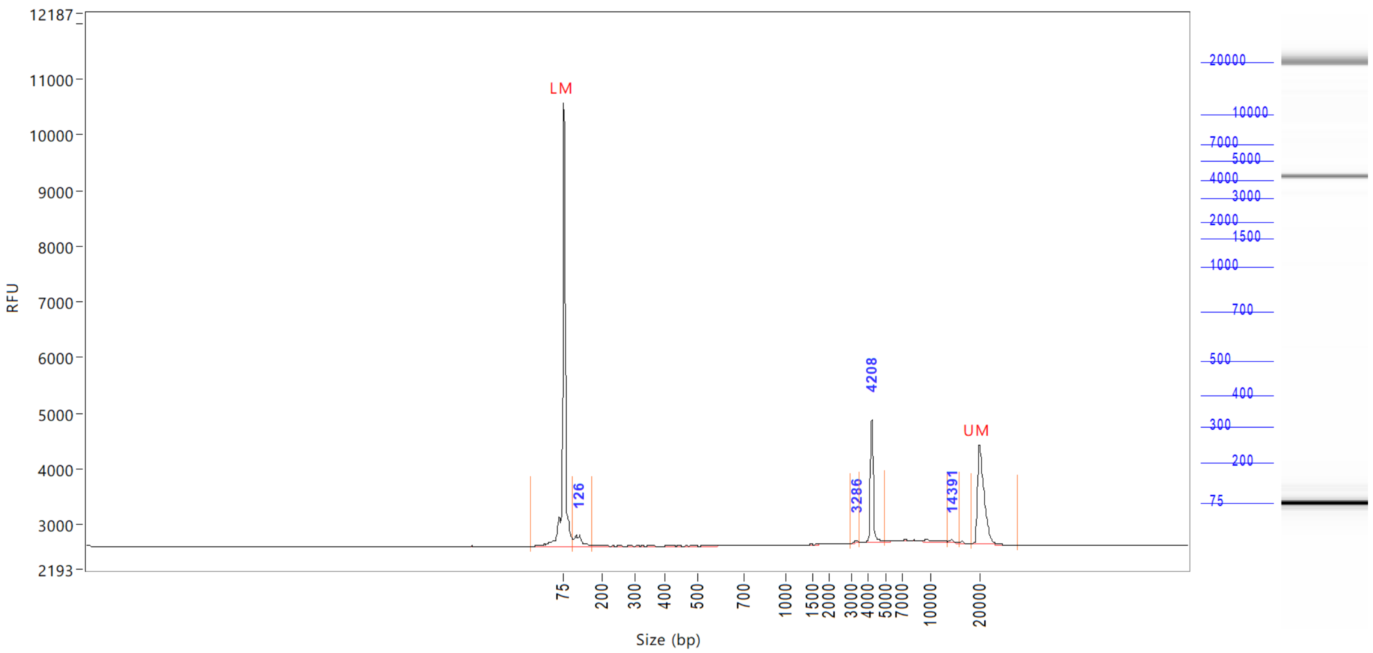

Peak	Size (bp)	Concentration (ng/uL)	From (bp)	To (bp)	Average size (bp)	CV%	RFU	Corrected peak area
1	75 (LM)	0.5000	0	105	72	17.44	7985	55.879
2	126	0.3816	105	166	123	10.77	209	3.554
3	3286	0.0262	3001	3500	3266	2.90	52	0.244
4	4208	1.0023	3500	4966	4174	2.85	2205	9.335
5	14391	0.0378	13537	15854	14386	3.96	63	0.352
6	20000 (UM)	0.1380	18415	27979	20456	4.16	1786	15.417

TIC:	1.4479	ng/uL
TIM:	5.5095	nmole/L
Total concentration:	1.8364	ng/uL

Sample peak width (sec): 5   Sample min peak height: 50   Sample baseline V to V?: Y   Sample baseline V to V points: 3
Sample filter: Binomial   Number of points for filter: 3 Sample start region (min): 0 Sample end region (min): 30
Marker peak width (sec): 5   Marker min peak height: 200   Marker baseline V to V?: Y   Marker baseline V to V points: 3
Lower marker selection: First peak > 500 RFU   Upper marker selection: Last peak > 200 RFU
Ladder size (bp)75, 200, 300, 400, 500, 700, 1000, 1500, 2000, 3000, 4000, 5000, 7000, 10000, 20000
Quantification using: Lower Marker   Final concentration (ng/uL): 0.5000   Dilution factor: 12.0

Sample: SampC3
Well location: C3
Created: Wednesday, August 11, 2021 5:34:32 PM

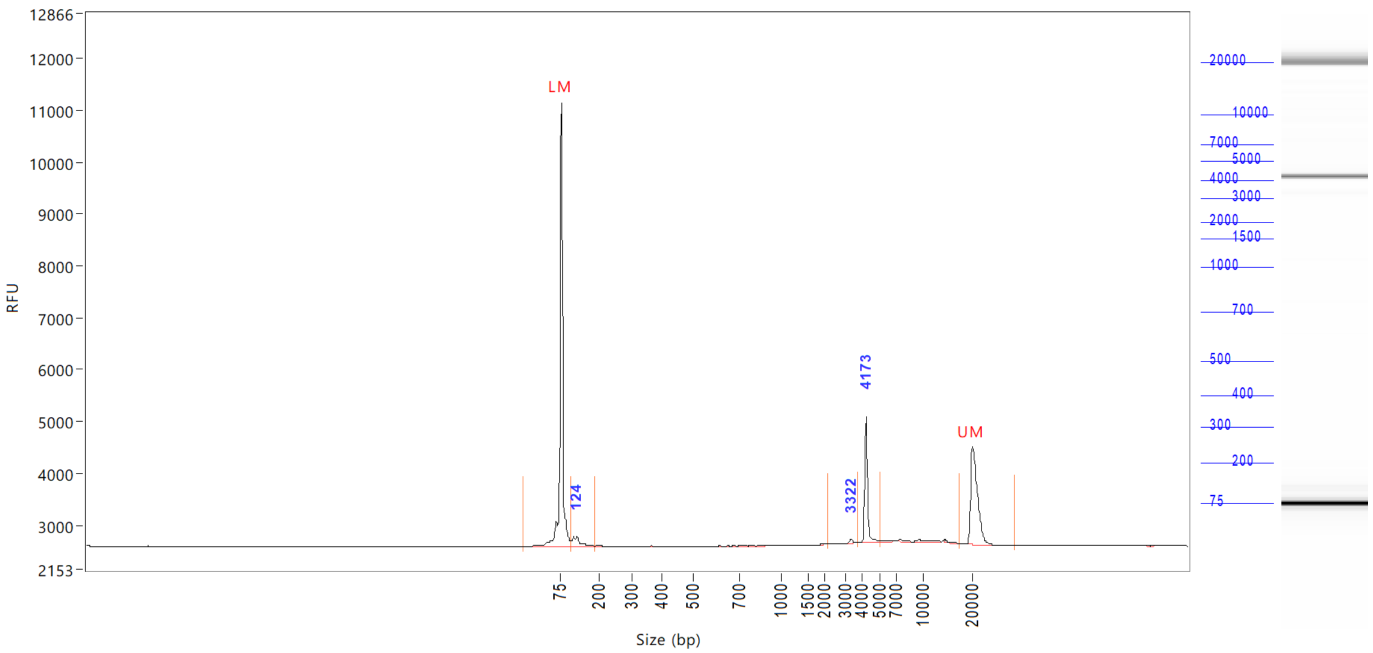

Peak	Size (bp)	Concentration (ng/uL)	From (bp)	To (bp)	Average size (bp)	CV%	RFU	Corrected peak area
1	75 (LM)	0.5000	0	107	71	17.38	8562	58.511
2	124	0.3562	107	183	126	12.69	198	3.474
3	3322	0.0495	2115	3786	3168	10.63	68	0.483
4	4173	1.0663	3786	5001	4183	3.22	2436	10.399
5	20000 (UM)	0.1429	17195	28826	20478	4.31	1893	16.728

TIC:	1.4720	ng/uL
TIM:	5.0903	nmole/L
Total concentration:	1.7894	ng/uL

```
Sample peak width (sec): 5    Sample min peak height: 50    Sample baseline V to V?: Y    Sample baseline V to V points: 3
Sample filter: Binomial       Number of points for filter: 3  Sample start region (min): 0  Sample end region (min): 30
Marker peak width (sec): 5    Marker min peak height: 200   Marker baseline V to V?: Y    Marker baseline V to V points: 3
Lower marker selection: First peak > 500 RFU                Upper marker selection: Last peak > 200 RFU
Ladder size (bp)75, 200, 300, 400, 500, 700, 1000, 1500, 2000, 3000, 4000, 5000, 7000, 10000, 20000
Quantification using: Lower Marker          Final concentration (ng/uL): 0.5000          Dilution factor: 12.0
```

Sample: SampD3
Well location: D3
Created: Wednesday, August 11, 2021 5:34:32 PM

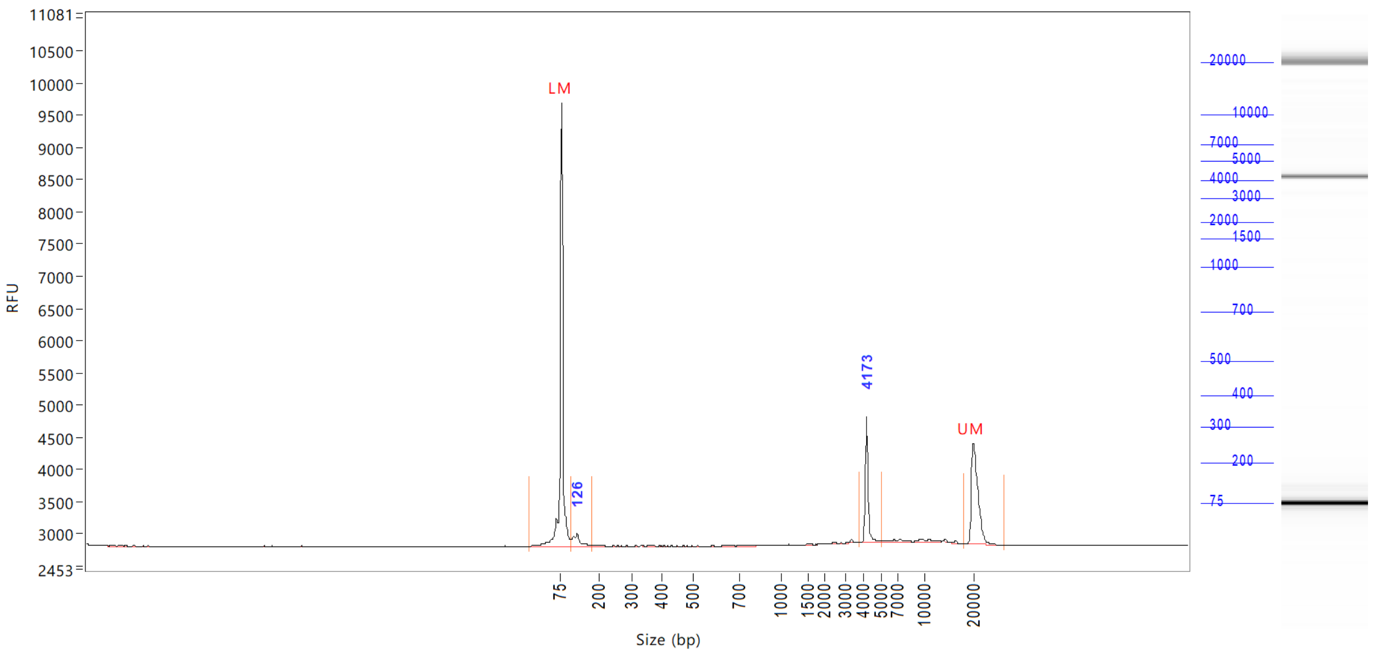

Peak	Size (bp)	Concentration (ng/uL)	From (bp)	To (bp)	Average size (bp)	CV%	RFU	Corrected peak area
1	75 (LM)	0.5000	0	107	71	19.19	6897	48.929
2	126	0.4197	107	177	126	12.21	203	3.422
3	4173	1.0199	3786	5001	4185	3.50	1948	8.317
4	20000 (UM)	0.1369	18171	26287	20467	3.92	1553	13.401

TIC:	1.4396	ng/uL
TIM:	5.8737	nmole/L
Total concentration:	1.9441	ng/uL

Sample peak width (sec): 5 Sample min peak height: 50 Sample baseline V to V?: Y Sample baseline V to V points: 3
Sample filter: Binomial Number of points for filter: 3 Sample start region (min): 0 Sample end region (min): 30
Marker peak width (sec): 5 Marker min peak height: 200 Marker baseline V to V?: Y Marker baseline V to V points: 3
Lower marker selection: First peak > 500 RFU Upper marker selection: Last peak > 200 RFU
Ladder size (bp)75, 200, 300, 400, 500, 700, 1000, 1500, 2000, 3000, 4000, 5000, 7000, 10000, 20000
Quantification using: Lower Marker Final concentration (ng/uL): 0.5000 Dilution factor: 12.0

Sample: SampE3
Well location: E3
Created: Wednesday, August 11, 2021 5:34:32 PM

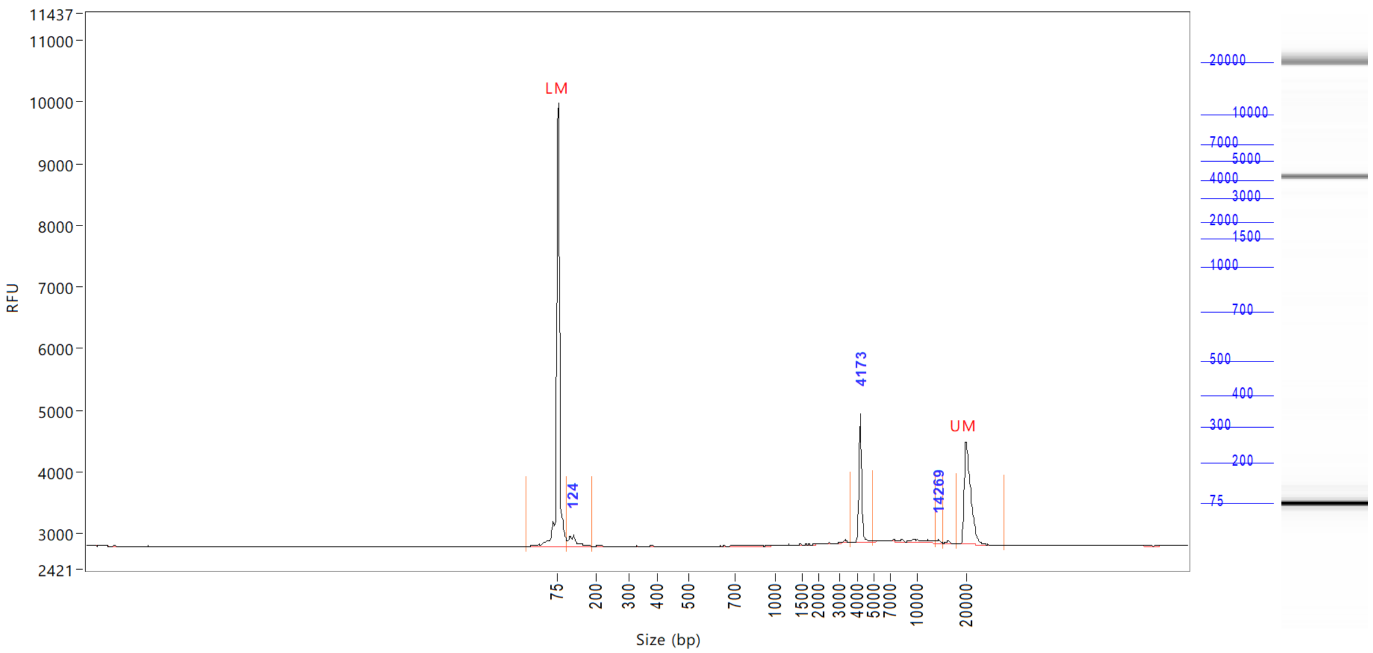

Peak	Size (bp)	Concentration (ng/uL)	From (bp)	To (bp)	Average size (bp)	CV%	RFU	Corrected peak area
1	75 (LM)	0.5000	0	105	71	17.10	7205	50.324
2	124	0.3853	105	189	125	13.44	178	3.232
3	4173	1.0358	3643	4966	4161	2.64	2088	8.687
4	14269	0.0398	13659	15244	14245	2.65	57	0.334
5	20000 (UM)	0.1441	18171	27979	20501	4.17	1653	14.502

TIC:	1.4610	ng/uL
TIM:	5.4792	nmole/L
Total concentration:	1.9706	ng/uL

Sample peak width (sec): 5 Sample min peak height: 50 Sample baseline V to V?: Y Sample baseline V to V points: 3
Sample filter: Binomial Number of points for filter: 3 Sample start region (min): 0 Sample end region (min): 30
Marker peak width (sec): 5 Marker min peak height: 200 Marker baseline V to V?: Y Marker baseline V to V points: 3
Lower marker selection: First peak > 500 RFU Upper marker selection: Last peak > 200 RFU
Ladder size (bp)75, 200, 300, 400, 500, 700, 1000, 1500, 2000, 3000, 4000, 5000, 7000, 10000, 20000
Quantification using: Lower Marker Final concentration (ng/uL): 0.5000 Dilution factor: 12.0

Sample: SampF3
Well location: F3
Created: Wednesday, August 11, 2021 5:34:32 PM

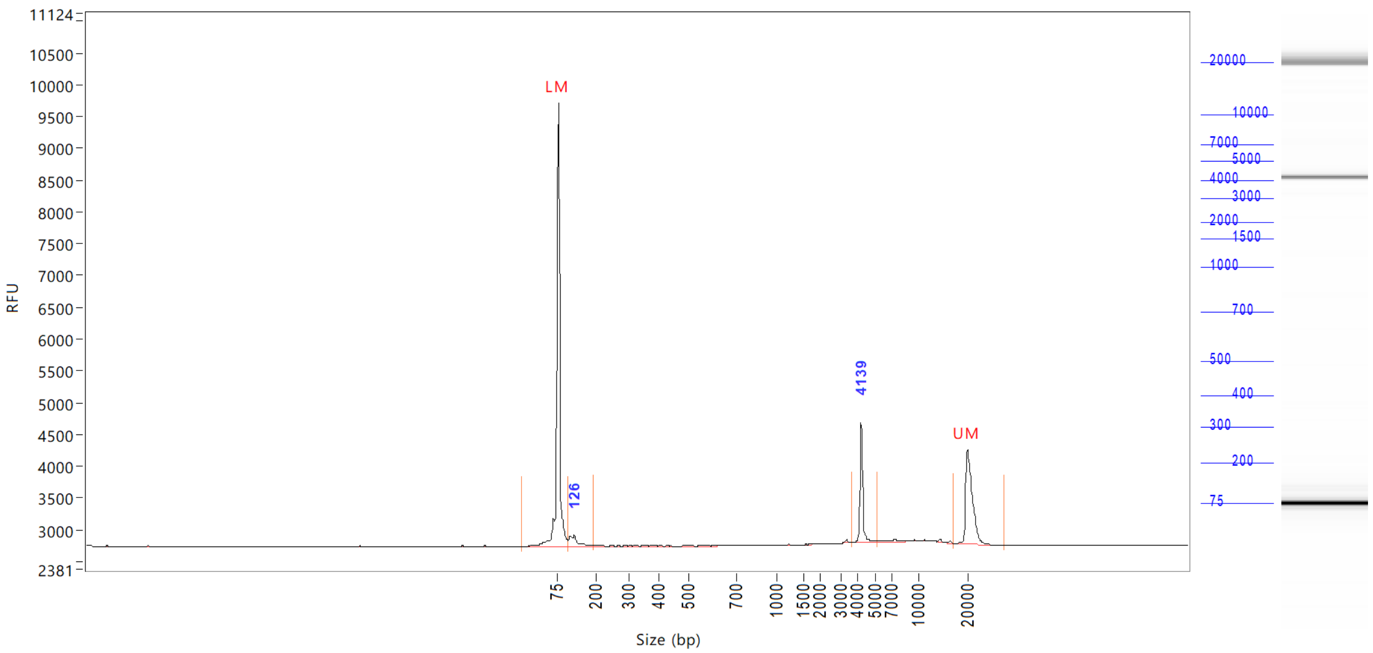

Peak	Size (bp)	Concentration (ng/uL)	From (bp)	To (bp)	Average size (bp)	CV%	RFU	Corrected peak area
1	75 (LM)	0.5000	0	107	71	17.58	6985	48.274
2	126	0.4088	107	190	128	14.12	175	3.289
3	4139	1.0076	3643	5149	4153	3.64	1879	8.107
4	20000 (UM)	0.1383	17195	27617	20425	4.31	1499	13.348

TIC:	1.4164	ng/uL
TIM:	5.6471	nmole/L
Total concentration:	1.8021	ng/uL

Sample peak width (sec): 5   Sample min peak height: 50   Sample baseline V to V?: Y   Sample baseline V to V points: 3
Sample filter: Binomial   Number of points for filter: 3 Sample start region (min): 0 Sample end region (min): 30
Marker peak width (sec): 5   Marker min peak height: 200   Marker baseline V to V?: Y   Marker baseline V to V points: 3
Lower marker selection: First peak > 500 RFU   Upper marker selection: Last peak > 200 RFU
Ladder size (bp)75, 200, 300, 400, 500, 700, 1000, 1500, 2000, 3000, 4000, 5000, 7000, 10000, 20000
Quantification using: Lower Marker   Final concentration (ng/uL): 0.5000   Dilution factor: 12.0

Sample: SampG3
Well location: G3
Created: Wednesday, August 11, 2021 5:34:32 PM

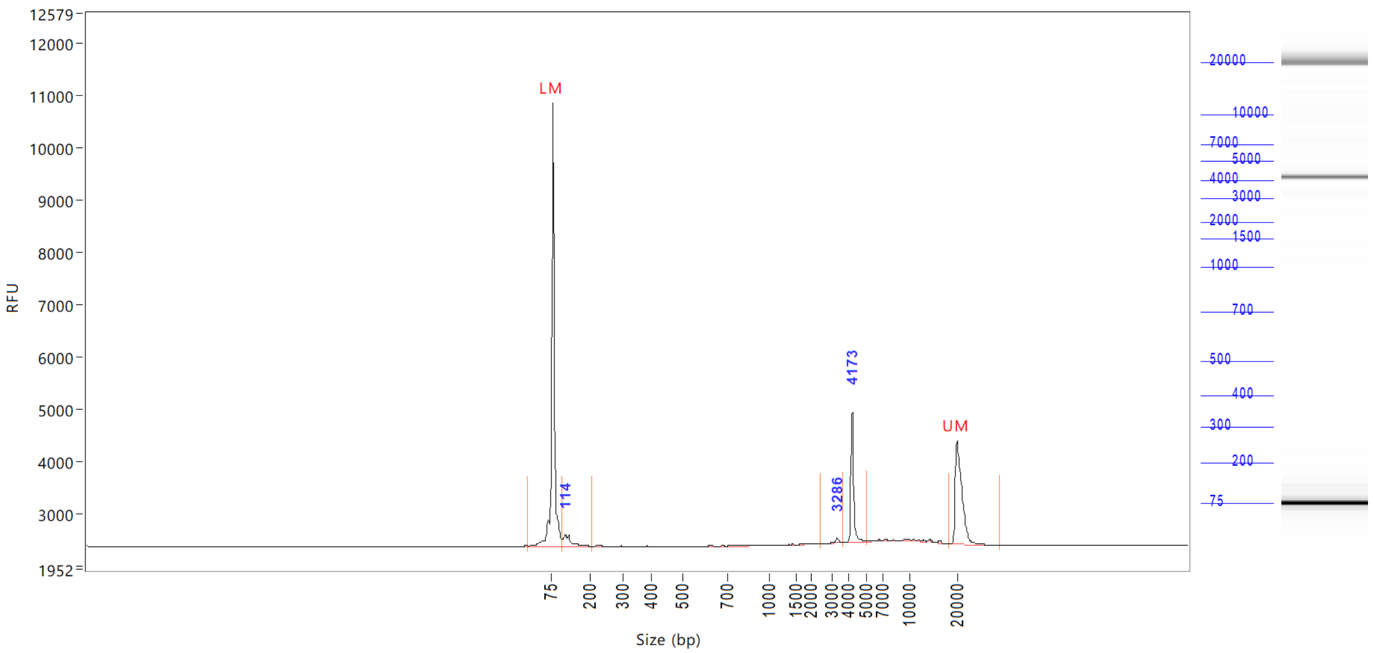

Peak	Size (bp)	Concentration (ng/uL)	From (bp)	To (bp)	Average size (bp)	CV%	RFU	Corrected peak area
1	75 (LM)	0.5000	0	107	73	15.29	8492	59.497
2	114	0.4297	107	205	128	15.07	220	4.260
3	3286	0.0464	2429	3608	3186	5.92	84	0.460
4	4173	1.1060	3608	5075	4158	3.16	2492	10.967
5	20000 (UM)	0.1480	18293	28826	20493	4.69	1980	17.608

TIC:	1.5820	ng/uL
TIM:	5.9769	nmole/L
Total concentration:	1.9349	ng/uL

Sample peak width (sec): 5   Sample min peak height: 50   Sample baseline V to V?: Y   Sample baseline V to V points: 3
Sample filter: Binomial   Number of points for filter: 3   Sample start region (min): 0   Sample end region (min): 30
Marker peak width (sec): 5   Marker min peak height: 200   Marker baseline V to V?: Y   Marker baseline V to V points: 3
Lower marker selection: First peak > 500 RFU   Upper marker selection: Last peak > 200 RFU
Ladder size (bp)75, 200, 300, 400, 500, 700, 1000, 1500, 2000, 3000, 4000, 5000, 7000, 10000, 20000
Quantification using: Lower Marker   Final concentration (ng/uL): 0.5000   Dilution factor: 12.0

Sample: SampH3
Well location: H3
Created: Wednesday, August 11, 2021 5:34:32 PM

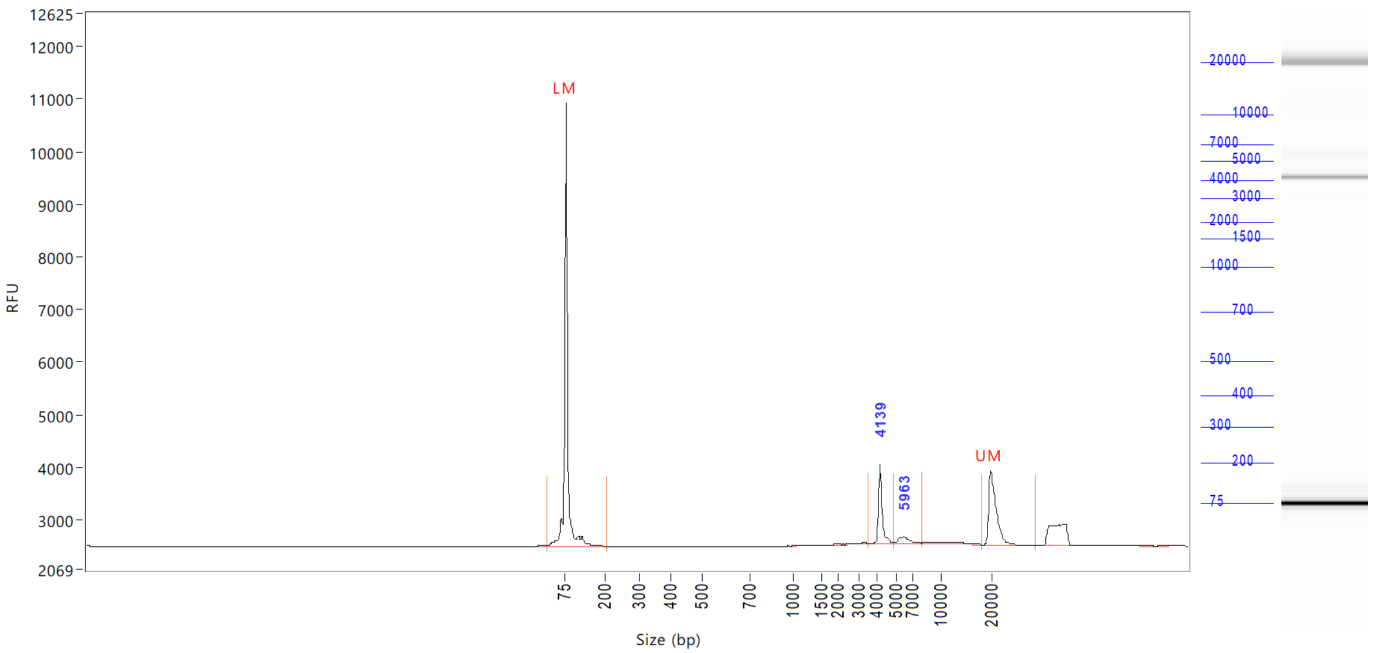

Peak	Size (bp)	Concentration (ng/uL)	From (bp)	To (bp)	Average size (bp)	CV%	RFU	Corrected peak area
1	75 (LM)	0.5000	18	201	75	22.39	8439	61.457
2	4139	0.7966	3465	4931	4159	4.09	1508	8.160
3	5963	0.1902	4931	7934	5980	10.23	122	1.948
4	20000 (UM)	0.1118	18293	28826	20553	5.20	1410	13.743

TIC:	0.9868	ng/uL
TIM:	0.3677	nmole/L
Total concentration:	1.3006	ng/uL

```
Sample peak width (sec): 5    Sample min peak height: 50    Sample baseline V to V?: Y    Sample baseline V to V points: 3
Sample filter: Binomial       Number of points for filter: 3  Sample start region (min): 0  Sample end region (min): 30
Marker peak width (sec): 5    Marker min peak height: 200   Marker baseline V to V?: Y    Marker baseline V to V points: 3
Lower marker selection: First peak > 500 RFU                Upper marker selection: Last peak > 200 RFU
Ladder size (bp)75, 200, 300, 400, 500, 700, 1000, 1500, 2000, 3000, 4000, 5000, 7000, 10000, 20000
Quantification using: Lower Marker          Final concentration (ng/uL): 0.5000          Dilution factor: 12.0
```

Sample: SampA4
Well location: A4
Created: Wednesday, August 11, 2021 5:34:32 PM

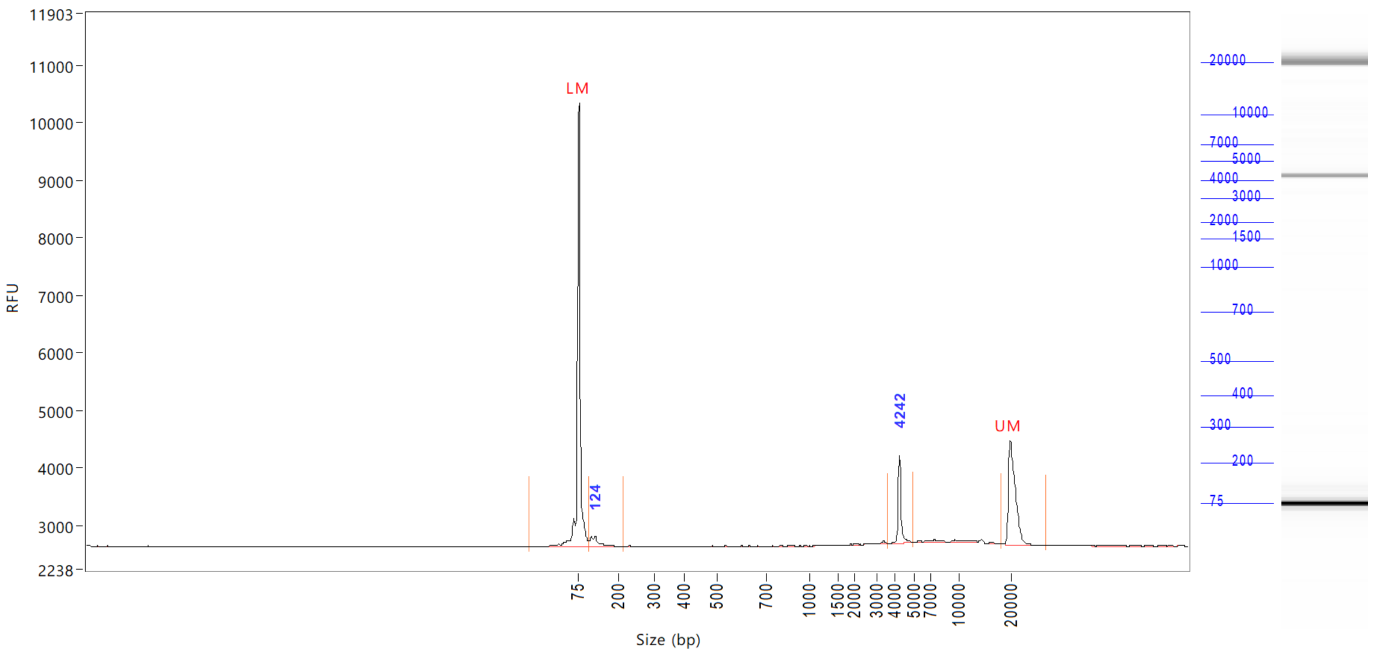

Peak	Size (bp)	Concentration (ng/uL)	From (bp)	To (bp)	Average size (bp)	CV%	RFU	Corrected peak area
1	75 (LM)	0.5000	0	105	70	20.37	7725	53.672
2	124	0.3608	105	212	126	14.34	173	3.228
3	4242	0.7481	3608	5000	4232	3.36	1525	6.692
4	20000 (UM)	0.1464	18049	27012	20434	3.95	1813	15.712

TIC:	1.1089	ng/uL
TIM:	4.9957	nmole/L
Total concentration:	1.4911	ng/uL

Sample peak width (sec): 5   Sample min peak height: 50   Sample baseline V to V?: Y   Sample baseline V to V points: 3
Sample filter: Binomial   Number of points for filter: 3 Sample start region (min): 0 Sample end region (min): 30
Marker peak width (sec): 5   Marker min peak height: 200   Marker baseline V to V?: Y   Marker baseline V to V points: 3
Lower marker selection: First peak > 500 RFU   Upper marker selection: Last peak > 200 RFU
Ladder size (bp)75, 200, 300, 400, 500, 700, 1000, 1500, 2000, 3000, 4000, 5000, 7000, 10000, 20000
Quantification using: Lower Marker   Final concentration (ng/uL): 0.5000   Dilution factor: 12.0

Sample: SampB4
Well location: B4
Created: Wednesday, August 11, 2021 5:34:32 PM

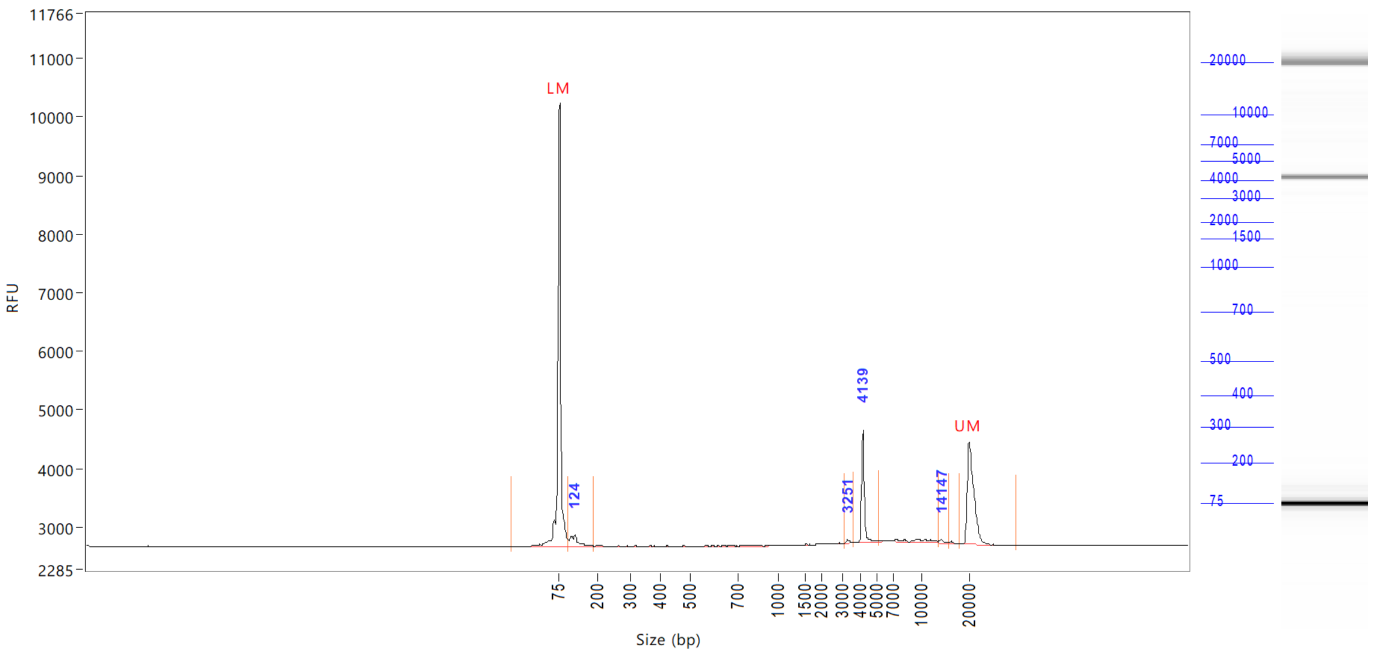

Peak	Size (bp)	Concentration (ng/uL)	From (bp)	To (bp)	Average size (bp)	CV%	RFU	Corrected peak area
1	75 (LM)	0.5000	0	105	70	20.75	7578	53.949
2	124	0.4010	105	185	125	13.03	194	3.606
3	3251	0.0329	3037	3643	3288	4.11	54	0.296
4	4139	0.8989	3643	5149	4146	3.16	1918	8.082
5	14147	0.0405	13537	15732	14276	3.84	63	0.364
6	20000 (UM)	0.1416	17927	29672	20412	4.71	1749	15.283

TIC:	1.3732	ng/uL
TIM:	5.6492	nmole/L
Total concentration:	1.7705	ng/uL

```
Sample peak width (sec): 5    Sample min peak height: 50    Sample baseline V to V?: Y    Sample baseline V to V points: 3
Sample filter: Binomial       Number of points for filter: 3  Sample start region (min): 0  Sample end region (min): 30
Marker peak width (sec): 5    Marker min peak height: 200   Marker baseline V to V?: Y    Marker baseline V to V points: 3
Lower marker selection: First peak > 500 RFU                Upper marker selection: Last peak > 200 RFU
Ladder size (bp)75, 200, 300, 400, 500, 700, 1000, 1500, 2000, 3000, 4000, 5000, 7000, 10000, 20000
Quantification using: Lower Marker          Final concentration (ng/uL): 0.5000          Dilution factor: 12.0
```

Sample: SampC4
Well location: C4
Created: Wednesday, August 11, 2021 5:34:32 PM

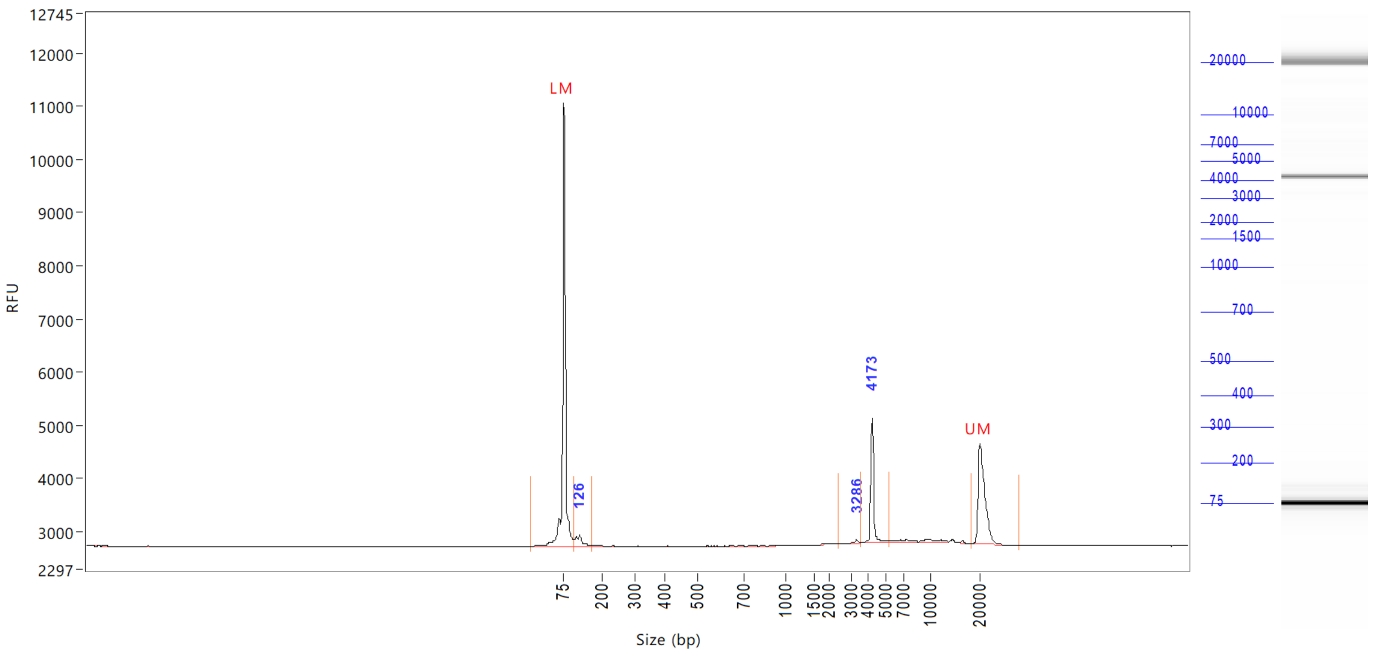

Peak	Size (bp)	Concentration (ng/uL)	From (bp)	To (bp)	Average size (bp)	CV%	RFU	Corrected peak area
1	75 (LM)	0.5000	0	107	72	16.82	8349	58.187
2	126	0.3533	107	166	124	10.09	215	3.427
3	3286	0.0389	2429	3608	3181	6.71	60	0.377
4	4173	1.0244	3608	5371	4184	4.13	2348	9.935
5	20000 (UM)	0.1420	18293	27979	20472	4.09	1884	16.525

TIC:	1.4166	ng/uL
TIM:	5.1046	nmole/L
Total concentration:	1.7998	ng/uL

Sample peak width (sec): 5   Sample min peak height: 50   Sample baseline V to V?: Y   Sample baseline V to V points: 3
Sample filter: Binomial   Number of points for filter: 3 Sample start region (min): 0 Sample end region (min): 30
Marker peak width (sec): 5   Marker min peak height: 200   Marker baseline V to V?: Y   Marker baseline V to V points: 3
Lower marker selection: First peak > 500 RFU   Upper marker selection: Last peak > 200 RFU
Ladder size (bp)75, 200, 300, 400, 500, 700, 1000, 1500, 2000, 3000, 4000, 5000, 7000, 10000, 20000
Quantification using: Lower Marker   Final concentration (ng/uL): 0.5000   Dilution factor: 12.0

Sample: SampD4
Well location: D4
Created: Wednesday, August 11, 2021 5:34:32 PM

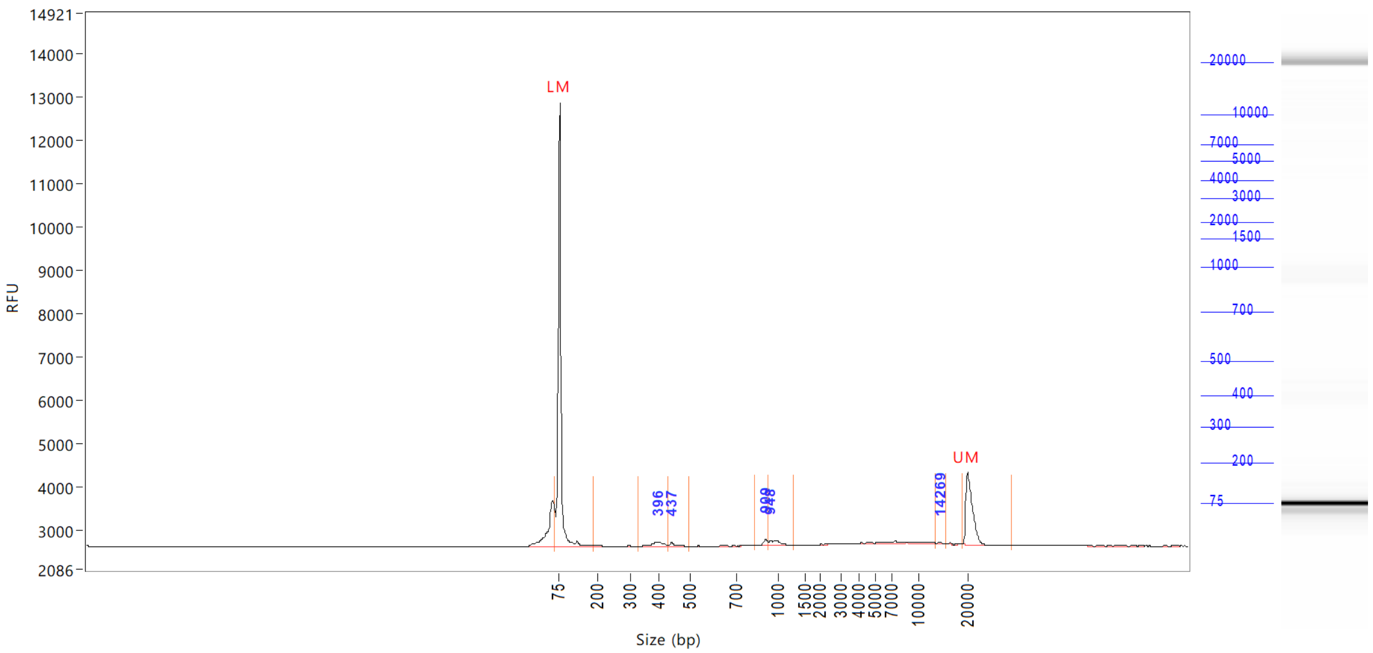

Peak	Size (bp)	Concentration (ng/uL)	From (bp)	To (bp)	Average size (bp)	CV%	RFU	Corrected peak area
1	75 (LM)	0.5000	64	189	76	16.51	10259	68.403
2	396	0.1905	327	428	391	5.03	90	2.172
3	437	0.0861	428	494	446	3.35	95	0.981
4	909	0.0930	831	931	905	1.82	137	1.060
5	948	0.1828	931	1289	994	6.22	117	2.084
6	14269	0.0256	13537	15488	14256	3.14	53	0.292
7	20000 (UM)	0.1067	18903	28947	20465	3.99	1667	14.604

TIC:	0.5780	ng/uL
TIM:	1.5939	nmole/L
Total concentration:	0.8570	ng/uL

```
Sample peak width (sec): 5    Sample min peak height: 50    Sample baseline V to V?: Y    Sample baseline V to V points: 3
Sample filter: Binomial       Number of points for filter: 3  Sample start region (min): 0  Sample end region (min): 30
Marker peak width (sec): 5    Marker min peak height: 200   Marker baseline V to V?: Y    Marker baseline V to V points: 3
Lower marker selection: First peak > 500 RFU                Upper marker selection: Last peak > 200 RFU
Ladder size (bp)75, 200, 300, 400, 500, 700, 1000, 1500, 2000, 3000, 4000, 5000, 7000, 10000, 20000
Quantification using: Lower Marker          Final concentration (ng/uL): 0.5000          Dilution factor: 12.0
```

Sample: SampE4
Well location: E4
Created: Wednesday, August 11, 2021 5:34:32 PM

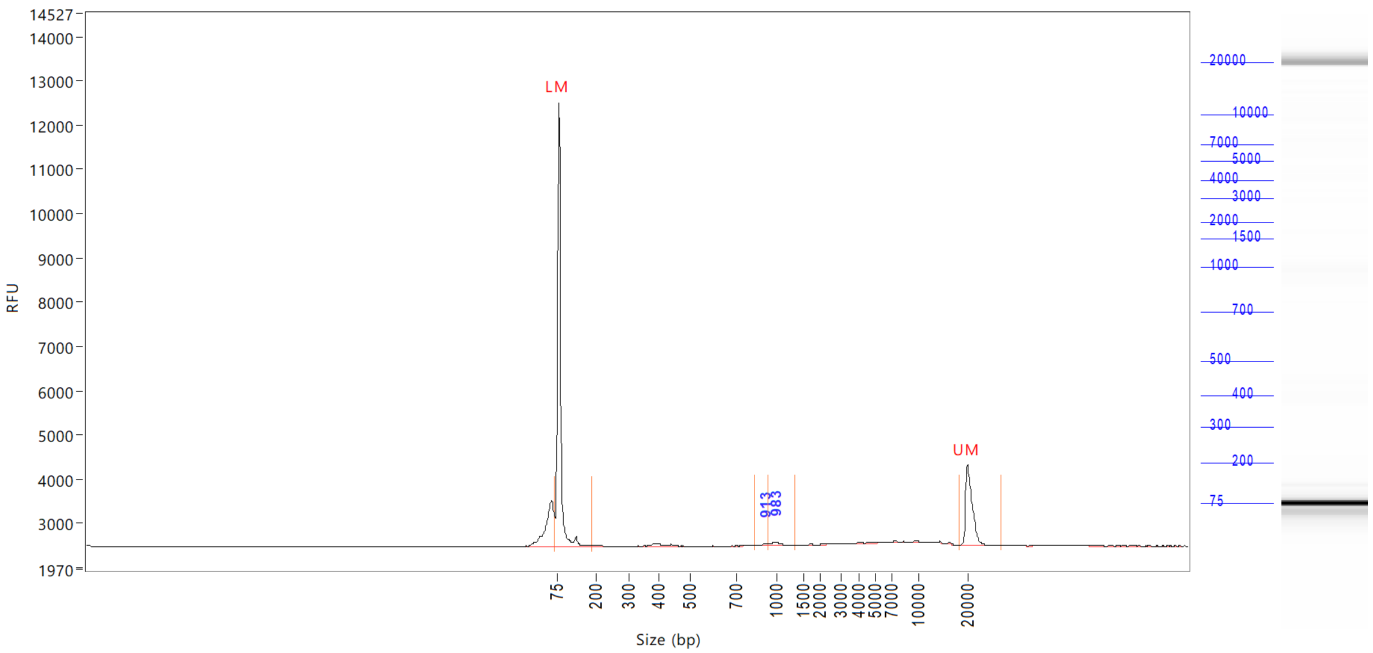

Peak	Size (bp)	Concentration (ng/uL)	From (bp)	To (bp)	Average size (bp)	CV%	RFU	Corrected peak area
1	75 (LM)	0.5000	64	183	77	17.60	10037	75.117
2	913	0.0396	831	935	907	2.06	54	0.496
3	983	0.0995	935	1334	1005	6.57	78	1.246
4	20000 (UM)	0.1057	18293	27012	20421	4.12	1815	15.880

TIC:	0.1391	ng/uL
TIM:	0.2348	nmole/L
Total concentration:	0.5415	ng/uL

```
Sample peak width (sec): 5    Sample min peak height: 50    Sample baseline V to V?: Y    Sample baseline V to V points: 3
Sample filter: Binomial       Number of points for filter: 3  Sample start region (min): 0  Sample end region (min): 30
Marker peak width (sec): 5    Marker min peak height: 200   Marker baseline V to V?: Y    Marker baseline V to V points: 3
Lower marker selection: First peak > 500 RFU                Upper marker selection: Last peak > 200 RFU
Ladder size (bp)75, 200, 300, 400, 500, 700, 1000, 1500, 2000, 3000, 4000, 5000, 7000, 10000, 20000
Quantification using: Lower Marker          Final concentration (ng/uL): 0.5000          Dilution factor: 12.0
```

Sample: SampF4
Well location: F4
Created: Wednesday, August 11, 2021 5:34:32 PM

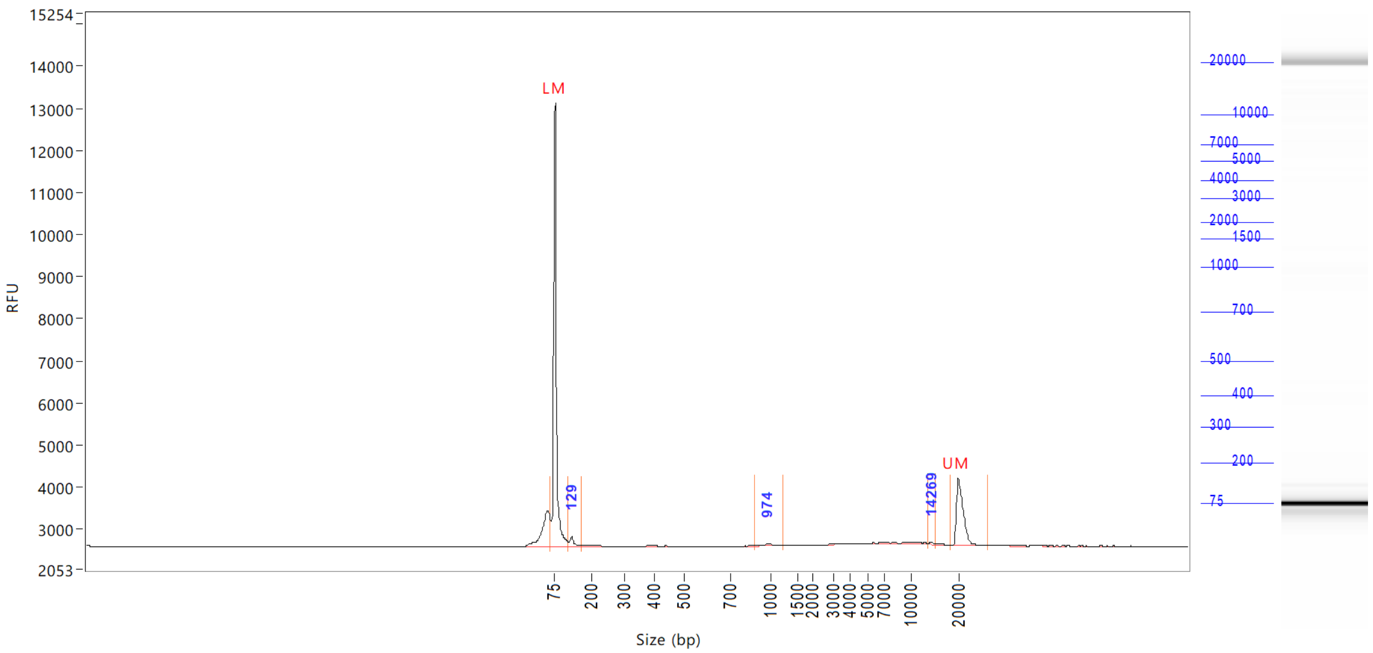

Peak	Size (bp)	Concentration (ng/uL)	From (bp)	To (bp)	Average size (bp)	CV%	RFU	Corrected peak area
1	75 (LM)	0.5000	62	120	74	10.85	10547	73.180
2	129	0.1723	120	164	133	8.35	228	2.101
3	974	0.0573	883	1245	979	5.67	51	0.699
4	14269	0.0289	13537	15366	14220	3.05	62	0.353
5	20000 (UM)	0.0963	18171	26287	20461	4.17	1599	14.093

TIC:	0.2585	ng/uL
TIM:	2.2282	nmole/L
Total concentration:	0.6427	ng/uL

```
Sample peak width (sec): 5    Sample min peak height: 50    Sample baseline V to V?: Y    Sample baseline V to V points: 3
Sample filter: Binomial       Number of points for filter: 3  Sample start region (min): 0  Sample end region (min): 30
Marker peak width (sec): 5    Marker min peak height: 200   Marker baseline V to V?: Y    Marker baseline V to V points: 3
Lower marker selection: First peak > 500 RFU                Upper marker selection: Last peak > 200 RFU
Ladder size (bp)75, 200, 300, 400, 500, 700, 1000, 1500, 2000, 3000, 4000, 5000, 7000, 10000, 20000
Quantification using: Lower Marker          Final concentration (ng/uL): 0.5000          Dilution factor: 12.0
```

Sample: SampG4
Well location: G4
Created: Wednesday, August 11, 2021 5:34:32 PM

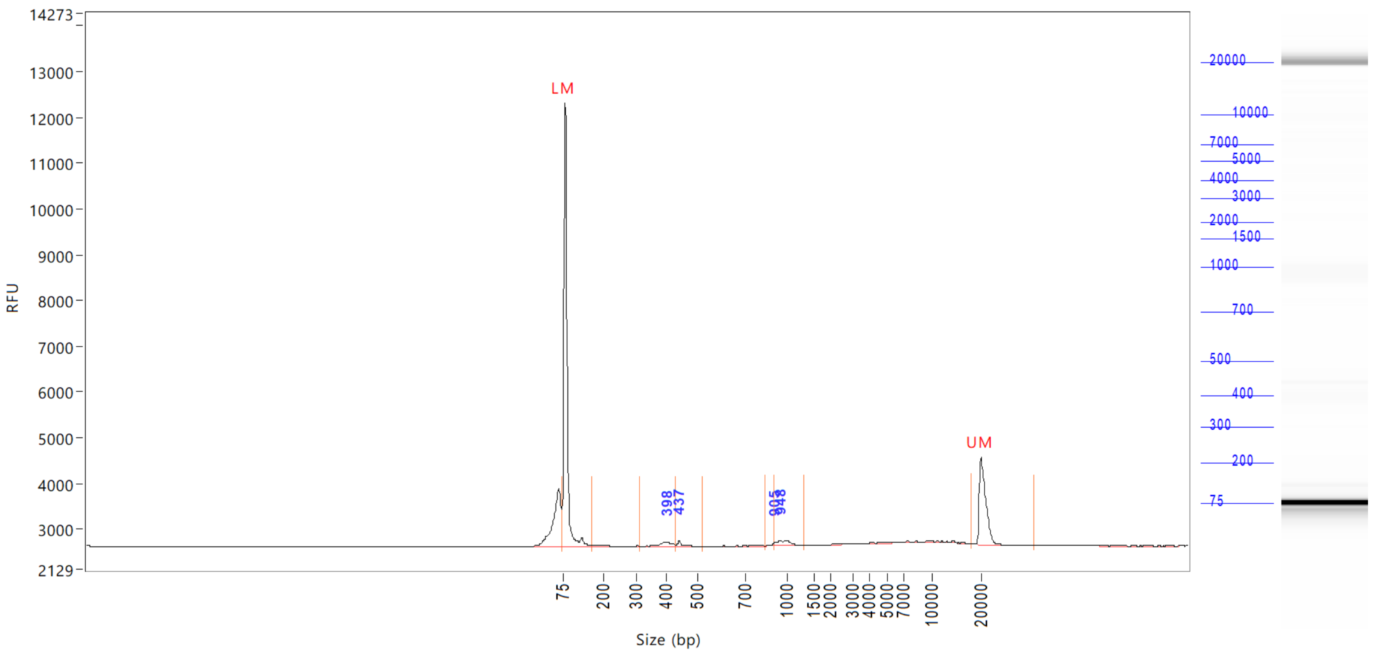

Peak	Size (bp)	Concentration (ng/uL)	From (bp)	To (bp)	Average size (bp)	CV%	RFU	Corrected peak area
1	75 (LM)	0.5000	66	160	77	15.10	9707	77.619
2	398	0.1745	307	426	388	6.28	87	2.258
3	437	0.0968	426	514	447	4.26	127	1.252
4	905	0.0245	835	909	895	1.52	73	0.317
5	948	0.1900	909	1289	981	6.22	117	2.458
6	20000 (UM)	0.1050	18171	30639	20458	4.43	1896	16.293

TIC:	0.4859	ng/uL
TIM:	1.4603	nmole/L
Total concentration:	0.8000	ng/uL

```
Sample peak width (sec): 5    Sample min peak height: 50    Sample baseline V to V?: Y    Sample baseline V to V points: 3
Sample filter: Binomial       Number of points for filter: 3  Sample start region (min): 0  Sample end region (min): 30
Marker peak width (sec): 5    Marker min peak height: 200   Marker baseline V to V?: Y    Marker baseline V to V points: 3
Lower marker selection: First peak > 500 RFU                Upper marker selection: Last peak > 200 RFU
Ladder size (bp)75, 200, 300, 400, 500, 700, 1000, 1500, 2000, 3000, 4000, 5000, 7000, 10000, 20000
Quantification using: Lower Marker          Final concentration (ng/uL): 0.5000          Dilution factor: 12.0
```

Sample: SampH4
Well location: H4
Created: Wednesday, August 11, 2021 5:34:32 PM

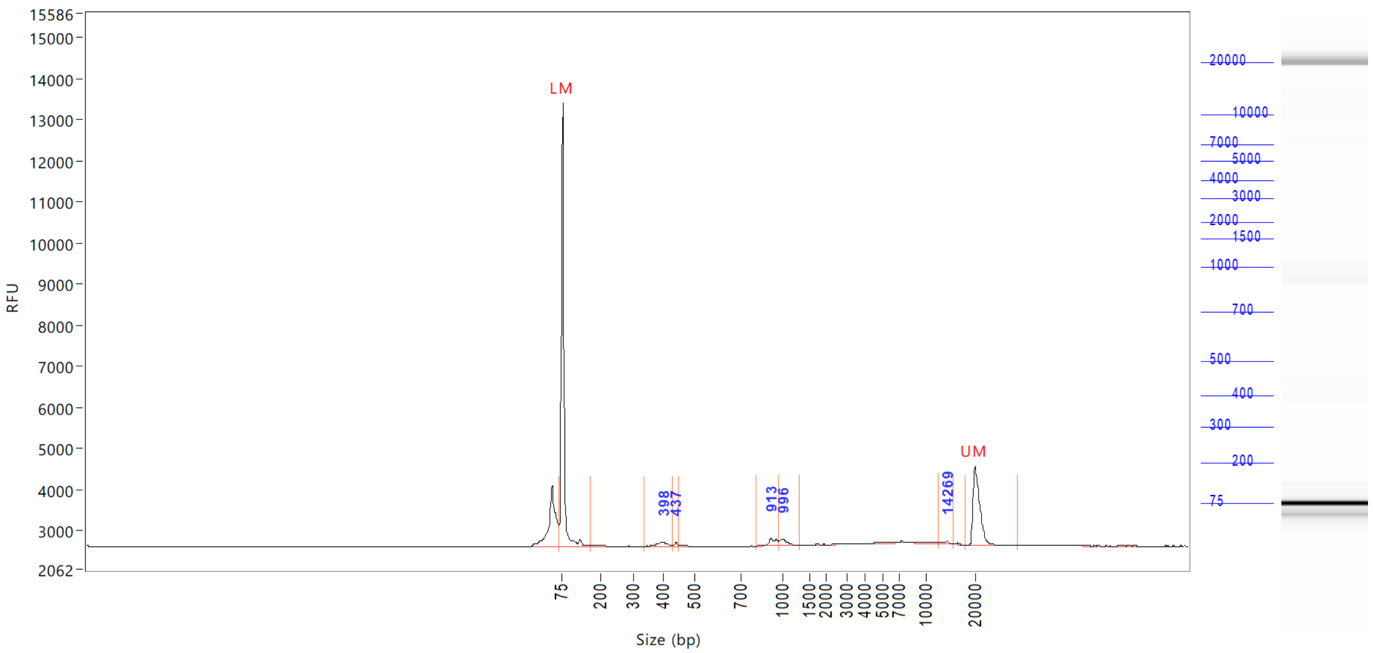

Peak	Size (bp)	Concentration (ng/uL)	From (bp)	To (bp)	Average size (bp)	CV%	RFU	Corrected peak area
1	75 (LM)	0.5000	64	168	76	16.04	10808	70.210
2	398	0.1759	331	430	392	4.71	87	2.058
3	437	0.0437	430	449	437	1.09	84	0.511
4	913	0.1927	801	965	919	3.14	183	2.255
5	996	0.1751	965	1300	1030	6.78	153	2.048
6	14269	0.0535	12440	15732	13801	5.83	70	0.626
7	20000 (UM)	0.1182	18049	28826	20472	4.40	1914	16.595

TIC:	0.6408	ng/uL
TIM:	1.5340	nmole/L
Total concentration:	0.9744	ng/uL

Sample peak width (sec): 5   Sample min peak height: 50   Sample baseline V to V?: Y   Sample baseline V to V points: 3
Sample filter: Binomial   Number of points for filter: 3   Sample start region (min): 0   Sample end region (min): 30
Marker peak width (sec): 5   Marker min peak height: 200   Marker baseline V to V?: Y   Marker baseline V to V points: 3
Lower marker selection: First peak > 500 RFU   Upper marker selection: Last peak > 200 RFU
Ladder size (bp)75, 200, 300, 400, 500, 700, 1000, 1500, 2000, 3000, 4000, 5000, 7000, 10000, 20000
Quantification using: Lower Marker   Final concentration (ng/uL): 0.5000   Dilution factor: 12.0

Sample: SampA5
Well location: A5
Created: Wednesday, August 11, 2021 5:34:32 PM

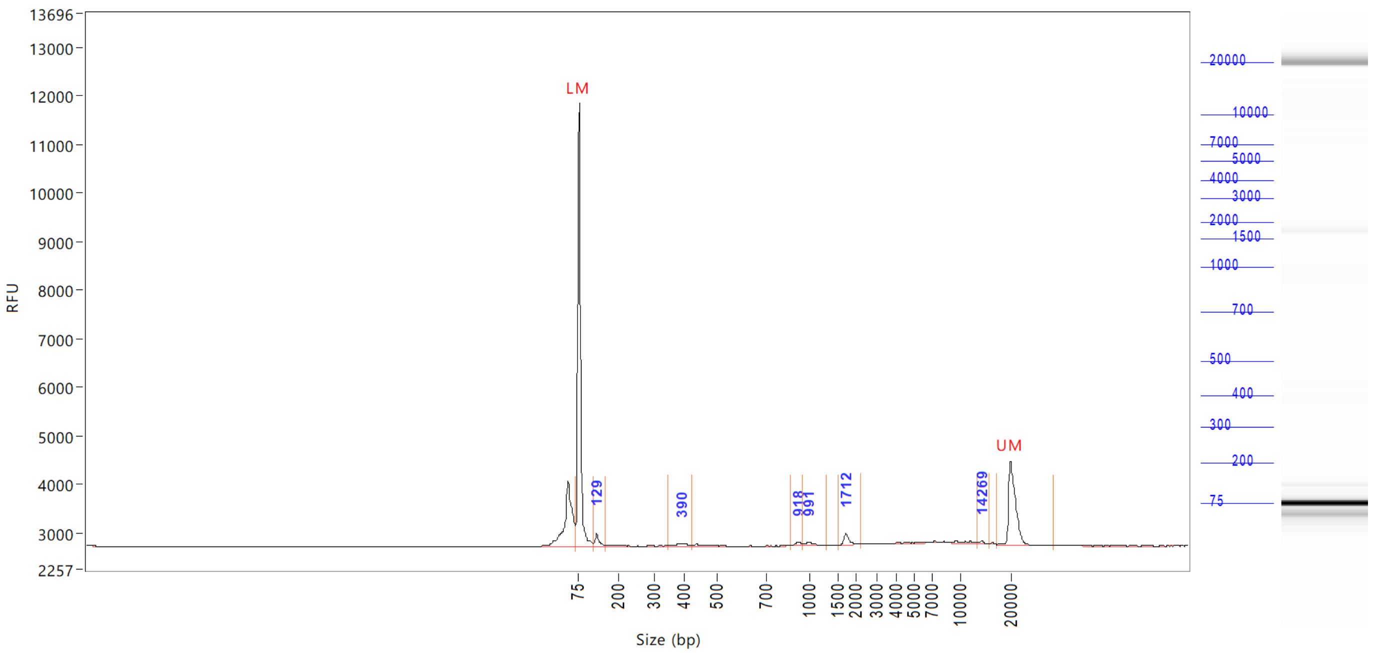

Peak	Size (bp)	Concentration (ng/uL)	From (bp)	To (bp)	Average size (bp)	CV%	RFU	Corrected peak area
1	75 (LM)	0.5000	64	120	75	9.46	9140	63.196
2	129	0.2272	120	158	132	6.51	279	2.393
3	390	0.1043	347	422	387	4.08	53	1.098
4	918	0.0708	866	948	915	2.15	67	0.746
5	991	0.0928	948	1289	1015	6.76	63	0.977
6	1712	0.1709	1501	2258	1755	6.53	223	1.800
7	14269	0.0377	13537	15610	14263	3.47	65	0.397
8	20000 (UM)	0.1170	17195	28221	20360	4.19	1721	14.783

TIC:	0.7037	ng/uL
TIM:	3.7136	nmole/L
Total concentration:	1.1028	ng/uL

```
Sample peak width (sec): 5    Sample min peak height: 50    Sample baseline V to V?: Y    Sample baseline V to V points: 3
Sample filter: Binomial       Number of points for filter: 3  Sample start region (min): 0  Sample end region (min): 30
Marker peak width (sec): 5    Marker min peak height: 200   Marker baseline V to V?: Y    Marker baseline V to V points: 3
Lower marker selection: First peak > 500 RFU                Upper marker selection: Last peak > 200 RFU
Ladder size (bp)75, 200, 300, 400, 500, 700, 1000, 1500, 2000, 3000, 4000, 5000, 7000, 10000, 20000
Quantification using: Lower Marker          Final concentration (ng/uL): 0.5000          Dilution factor: 12.0
```

Sample: SampB5
Well location: B5
Created: Wednesday, August 11, 2021 5:34:32 PM

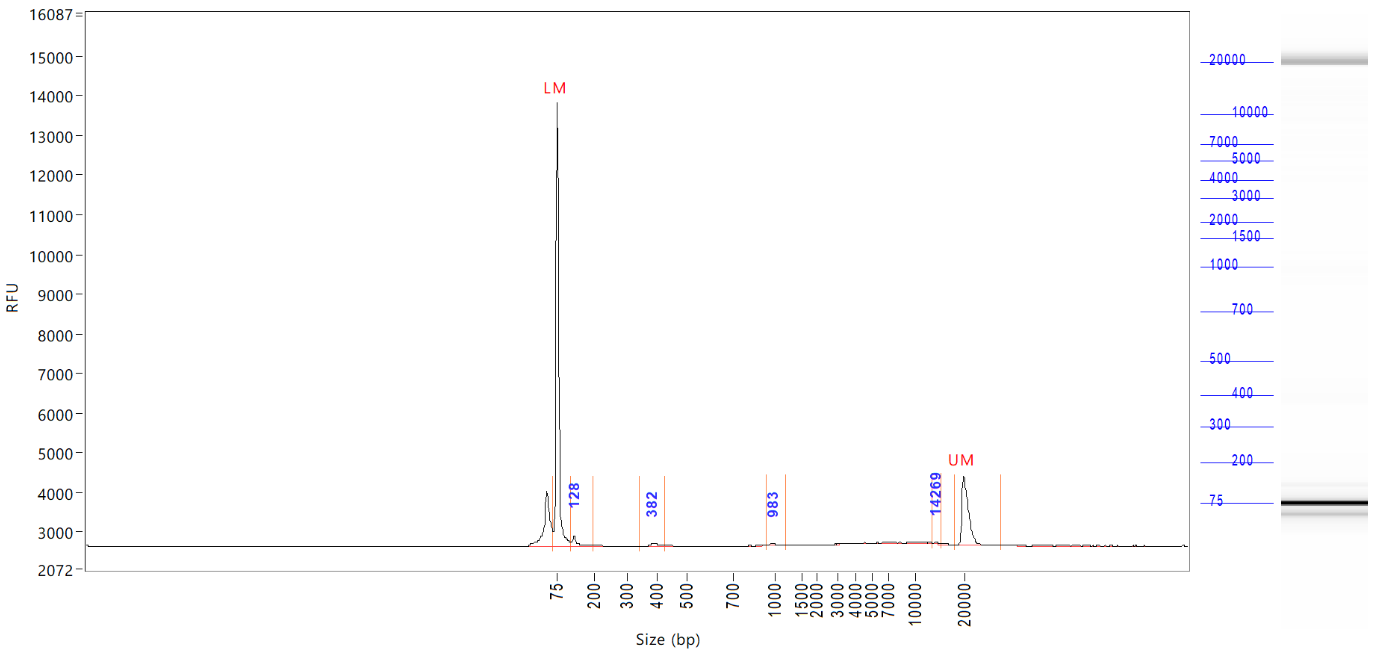

Peak	Size (bp)	Concentration (ng/uL)	From (bp)	To (bp)	Average size (bp)	CV%	RFU	Corrected peak area
1	75 (LM)	0.5000	62	118	74	10.10	11198	72.632
2	128	0.2402	118	194	137	13.56	269	2.908
3	382	0.0883	337	424	387	4.33	57	1.068
4	983	0.0584	935	1201	1000	4.89	56	0.707
5	14269	0.0259	13537	15244	14191	2.90	59	0.314
6	20000 (UM)	0.1058	18171	27617	20480	4.22	1747	15.375

TIC:	0.4128	ng/uL
TIM:	3.3553	nmole/L
Total concentration:	0.7140	ng/uL

Sample peak width (sec): 5   Sample min peak height: 50   Sample baseline V to V?: Y   Sample baseline V to V points: 3
Sample filter: Binomial   Number of points for filter: 3 Sample start region (min): 0 Sample end region (min): 30
Marker peak width (sec): 5   Marker min peak height: 200   Marker baseline V to V?: Y   Marker baseline V to V points: 3
Lower marker selection: First peak > 500 RFU   Upper marker selection: Last peak > 200 RFU
Ladder size (bp)75, 200, 300, 400, 500, 700, 1000, 1500, 2000, 3000, 4000, 5000, 7000, 10000, 20000
Quantification using: Lower Marker   Final concentration (ng/uL): 0.5000   Dilution factor: 12.0

Sample: SampC5
Well location: C5
Created: Wednesday, August 11, 2021 5:34:32 PM

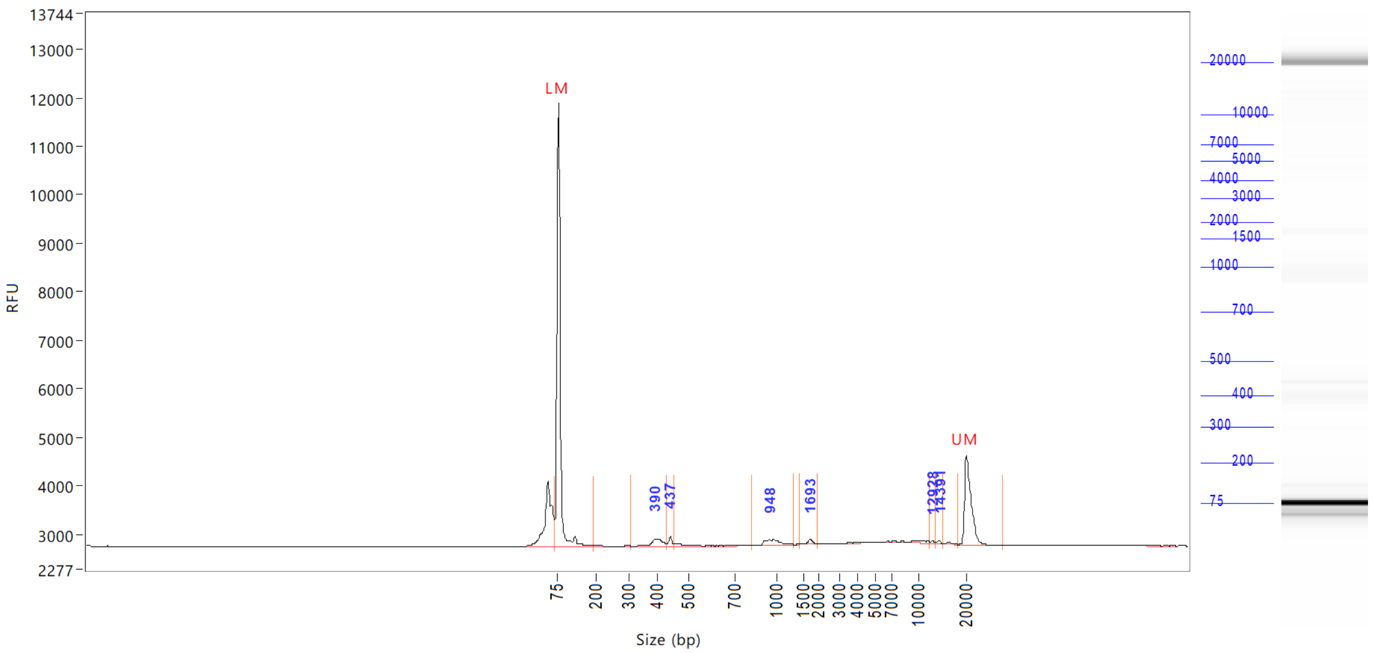

Peak	Size (bp)	Concentration (ng/uL)	From (bp)	To (bp)	Average size (bp)	CV%	RFU	Corrected peak area
1	75 (LM)	0.5000	64	190	77	17.80	9164	70.748
2	390	0.2981	307	428	393	4.80	157	3.515
3	437	0.1136	428	449	437	1.10	218	1.339
4	948	0.2487	822	1334	975	7.44	128	2.932
5	1693	0.0738	1422	1962	1690	5.53	111	0.871
6	12928	0.0223	12440	13659	12981	2.78	51	0.263
7	14391	0.0311	13659	15000	14223	2.31	74	0.367
8	20000 (UM)	0.1139	18293	27617	20474	4.27	1833	16.123

TIC:	0.7876	ng/uL
TIM:	2.1738	nmole/L
Total concentration:	1.1161	ng/uL

Sample peak width (sec): 5   Sample min peak height: 50   Sample baseline V to V?: Y   Sample baseline V to V points: 3
Sample filter: Binomial   Number of points for filter: 3   Sample start region (min): 0   Sample end region (min): 30
Marker peak width (sec): 5   Marker min peak height: 200   Marker baseline V to V?: Y   Marker baseline V to V points: 3
Lower marker selection: First peak > 500 RFU   Upper marker selection: Last peak > 200 RFU
Ladder size (bp)75, 200, 300, 400, 500, 700, 1000, 1500, 2000, 3000, 4000, 5000, 7000, 10000, 20000
Quantification using: Lower Marker   Final concentration (ng/uL): 0.5000   Dilution factor: 12.0

Sample: SampD5
Well location: D5
Created: Wednesday, August 11, 2021 5:34:32 PM

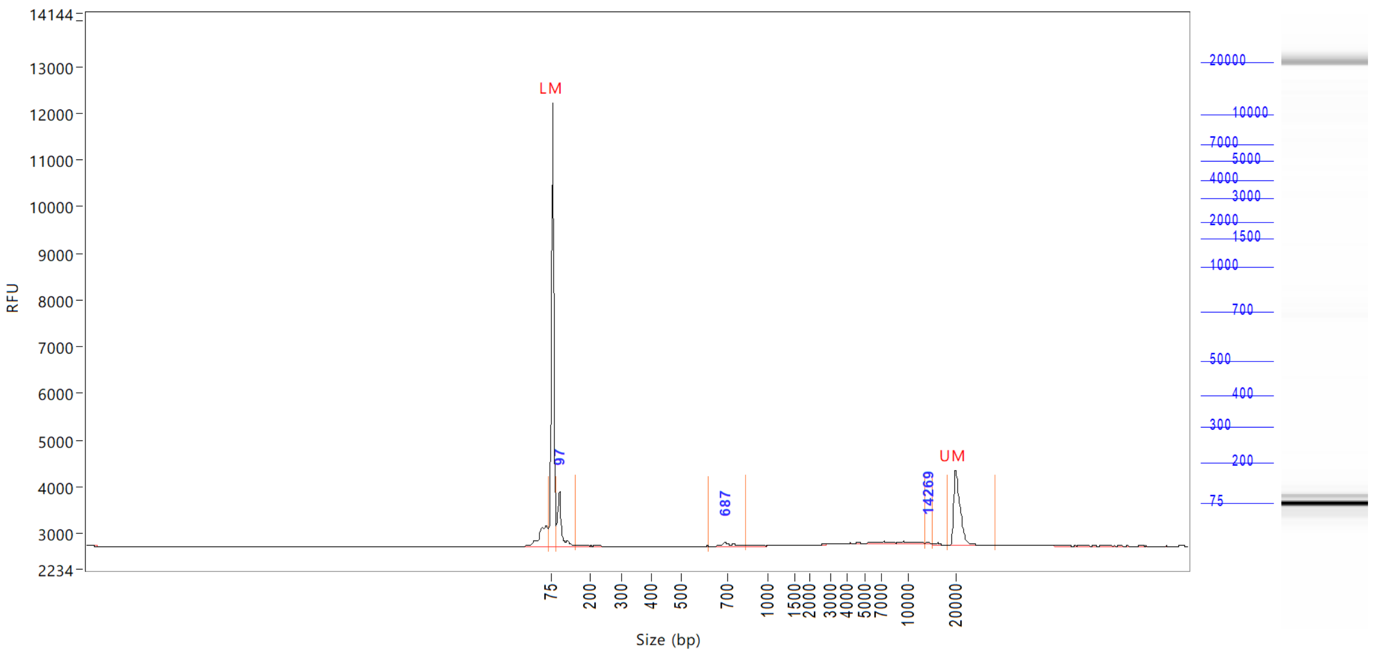

Peak	Size (bp)	Concentration (ng/uL)	From (bp)	To (bp)	Average size (bp)	CV%	RFU	Corrected peak area
1	75 (LM)	0.5000	62	86	73	5.40	9514	59.981
2	97	1.1257	86	148	98	12.11	1169	11.253
3	687	0.1318	618	835	705	5.79	75	1.317
4	14269	0.0311	13537	15122	14162	2.70	59	0.310
5	20000 (UM)	0.1162	18293	28463	20509	4.56	1614	13.941

TIC:	1.2885	ng/uL
TIM:	19.1719	nmole/L
Total concentration:	1.7076	ng/uL

Sample peak width (sec): 5 Sample min peak height: 50 Sample baseline V to V?: Y Sample baseline V to V points: 3
Sample filter: Binomial Number of points for filter: 3 Sample start region (min): 0 Sample end region (min): 30
Marker peak width (sec): 5 Marker min peak height: 200 Marker baseline V to V?: Y Marker baseline V to V points: 3
Lower marker selection: First peak > 500 RFU Upper marker selection: Last peak > 200 RFU
Ladder size (bp)75, 200, 300, 400, 500, 700, 1000, 1500, 2000, 3000, 4000, 5000, 7000, 10000, 20000
Quantification using: Lower Marker Final concentration (ng/uL): 0.5000 Dilution factor: 12.0

Sample: SampE5
Well location: E5
Created: Wednesday, August 11, 2021 5:34:32 PM

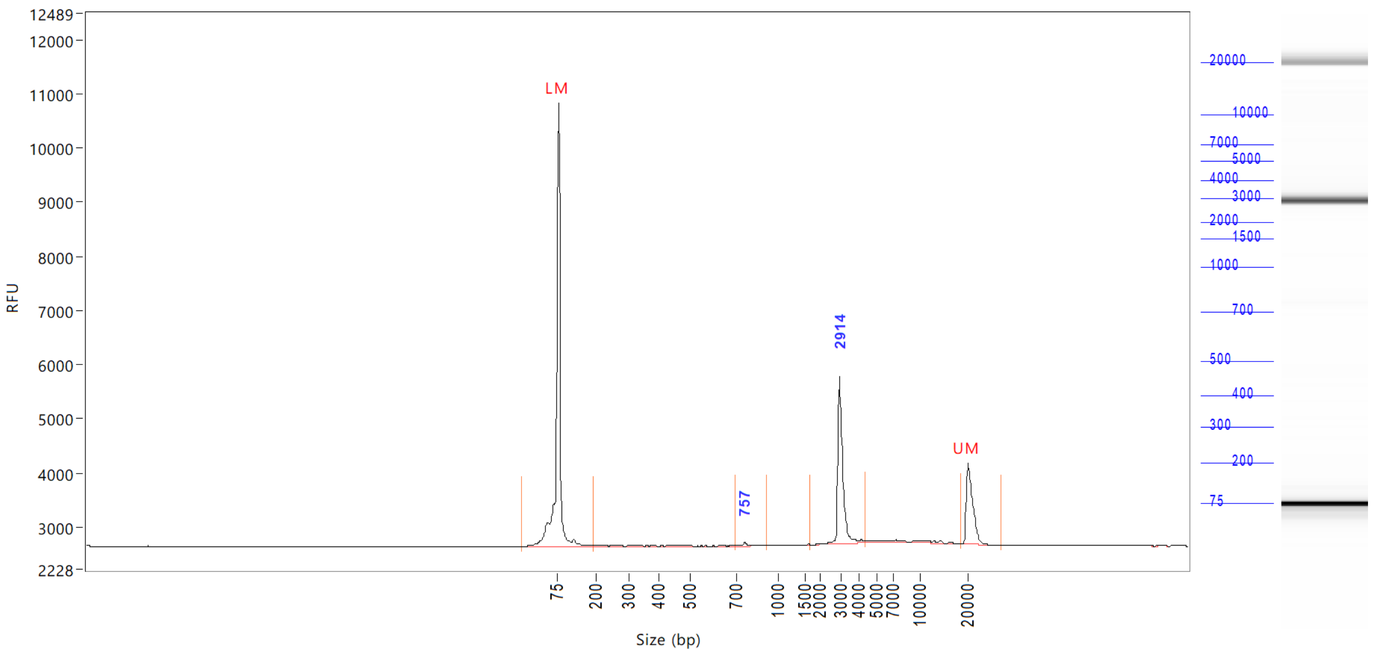

Peak	Size (bp)	Concentration (ng/uL)	From (bp)	To (bp)	Average size (bp)	CV%	RFU	Corrected peak area
1	75 (LM)	0.5000	0	190	68	30.14	8199	73.175
2	757	0.0625	697	918	780	6.44	66	0.763
3	2914	1.9546	1655	4345	2957	8.58	3076	23.838
4	20000 (UM)	0.0865	18659	26770	20412	3.91	1491	12.657

TIC:	2.0172	ng/uL
TIM:	1.2201	nmole/L
Total concentration:	2.3761	ng/uL

```
Sample peak width (sec): 5    Sample min peak height: 50    Sample baseline V to V?: Y    Sample baseline V to V points: 3
Sample filter: Binomial       Number of points for filter: 3  Sample start region (min): 0  Sample end region (min): 30
Marker peak width (sec): 5    Marker min peak height: 200   Marker baseline V to V?: Y    Marker baseline V to V points: 3
Lower marker selection: First peak > 500 RFU                Upper marker selection: Last peak > 200 RFU
Ladder size (bp)75, 200, 300, 400, 500, 700, 1000, 1500, 2000, 3000, 4000, 5000, 7000, 10000, 20000
Quantification using: Lower Marker          Final concentration (ng/uL): 0.5000          Dilution factor: 12.0
```

Sample: SampF5
Well location: F5
Created: Wednesday, August 11, 2021 5:34:32 PM

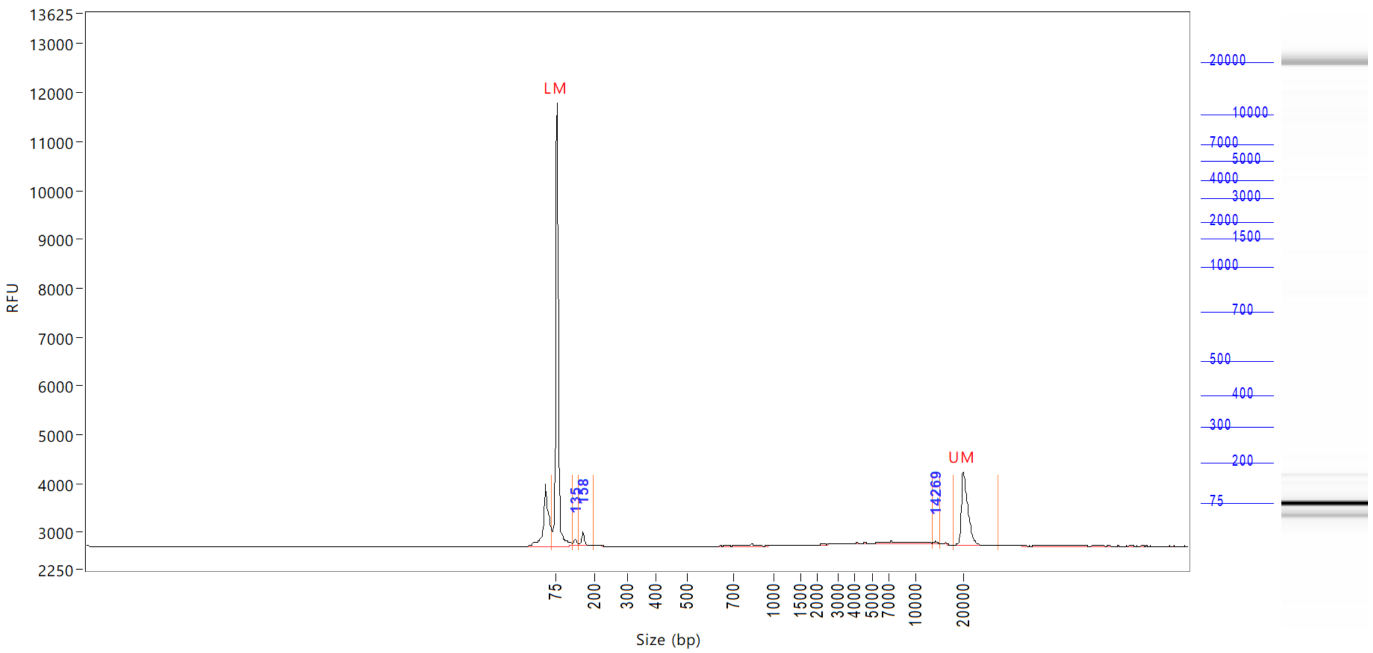

Peak	Size (bp)	Concentration (ng/uL)	From (bp)	To (bp)	Average size (bp)	CV%	RFU	Corrected peak area
1	75 (LM)	0.5000	62	124	74	10.02	9083	56.838
2	135	0.1313	124	147	134	4.67	133	1.244
3	158	0.2212	147	194	157	3.71	301	2.096
4	14269	0.0240	13415	15244	14045	2.21	50	0.228
5	20000 (UM)	0.1154	17927	27254	20414	4.24	1507	13.115

TIC:	0.3766	ng/uL
TIM:	3.9297	nmole/L
Total concentration:	0.7528	ng/uL

```
Sample peak width (sec): 5    Sample min peak height: 50    Sample baseline V to V?: Y    Sample baseline V to V points: 3
Sample filter: Binomial       Number of points for filter: 3  Sample start region (min): 0  Sample end region (min): 30
Marker peak width (sec): 5    Marker min peak height: 200   Marker baseline V to V?: Y    Marker baseline V to V points: 3
Lower marker selection: First peak > 500 RFU                Upper marker selection: Last peak > 200 RFU
Ladder size (bp)75, 200, 300, 400, 500, 700, 1000, 1500, 2000, 3000, 4000, 5000, 7000, 10000, 20000
Quantification using: Lower Marker          Final concentration (ng/uL): 0.5000          Dilution factor: 12.0
```

Sample: SampG5
Well location: G5
Created: Wednesday, August 11, 2021 5:34:32 PM

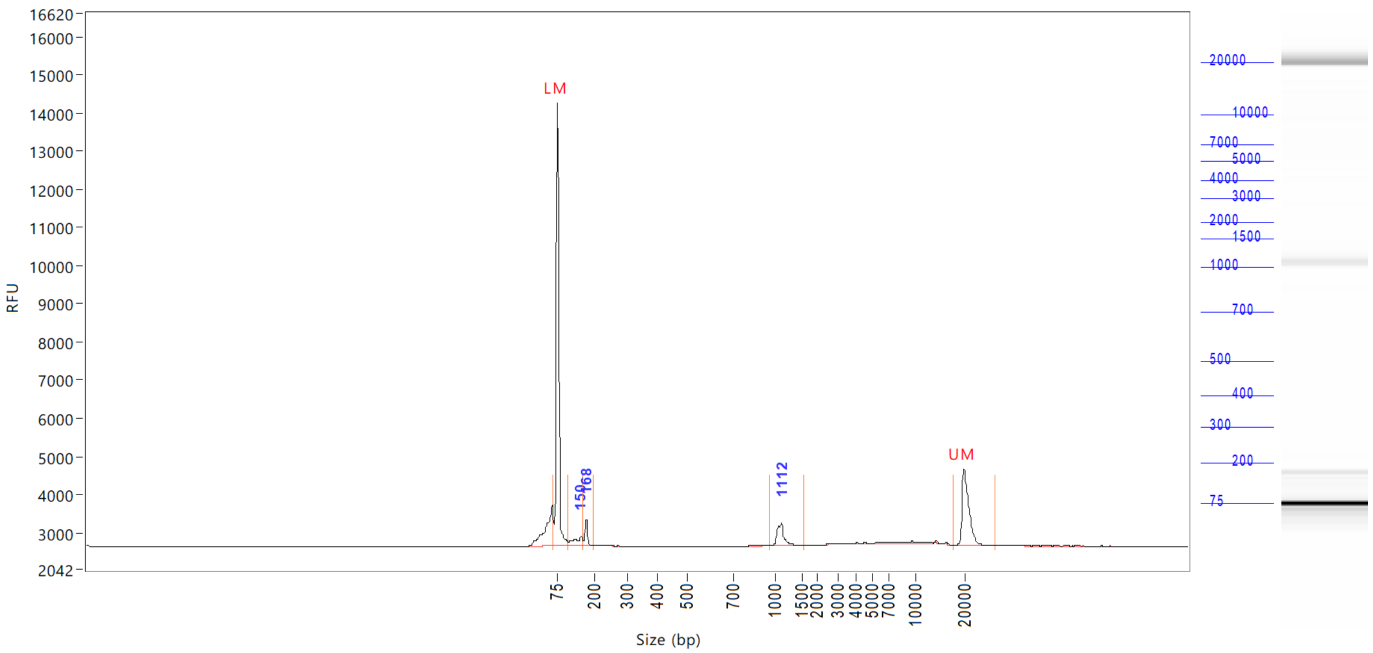

Peak	Size (bp)	Concentration (ng/uL)	From (bp)	To (bp)	Average size (bp)	CV%	RFU	Corrected peak area
1	75 (LM)	0.5000	64	110	74	8.05	11632	71.593
2	150	0.3923	110	158	136	10.17	249	4.681
3	168	0.3375	158	192	166	2.53	673	4.027
4	1112	0.5490	961	1559	1103	7.69	587	6.550
5	20000 (UM)	0.1262	17927	26529	20477	4.29	2002	18.072

TIC:	1.2787	ng/uL
TIM:	8.9008	nmole/L
Total concentration:	1.5903	ng/uL

Sample peak width (sec): 5 Sample min peak height: 50 Sample baseline V to V?: Y Sample baseline V to V points: 3
Sample filter: Binomial Number of points for filter: 3 Sample start region (min): 0 Sample end region (min): 30
Marker peak width (sec): 5 Marker min peak height: 200 Marker baseline V to V?: Y Marker baseline V to V points: 3
Lower marker selection: First peak > 500 RFU Upper marker selection: Last peak > 200 RFU
Ladder size (bp)75, 200, 300, 400, 500, 700, 1000, 1500, 2000, 3000, 4000, 5000, 7000, 10000, 20000
Quantification using: Lower Marker Final concentration (ng/uL): 0.5000 Dilution factor: 12.0

Sample: SampH5
Well location: H5
Created: Wednesday, August 11, 2021 5:34:32 PM

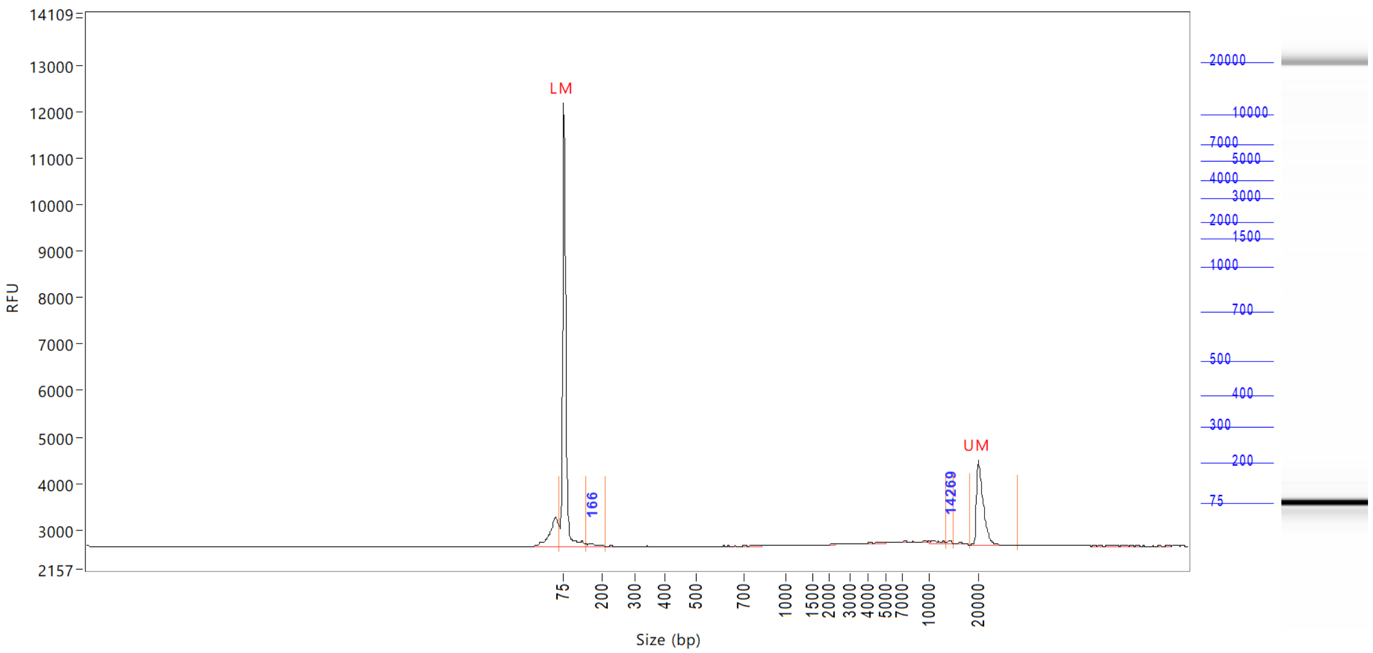

Peak	Size (bp)	Concentration (ng/uL)	From (bp)	To (bp)	Average size (bp)	CV%	RFU	Corrected peak area
1	75 (LM)	0.5000	64	148	76	13.47	9551	75.683
2	166	0.0833	148	208	169	8.52	53	1.050
3	14269	0.0306	13293	15000	14016	2.89	69	0.386
4	20000 (UM)	0.1050	18415	28100	20425	4.49	1818	15.889

TIC:	0.1139	ng/uL
TIM:	0.8134	nmole/L
Total concentration:	0.4157	ng/uL

Sample peak width (sec): 5   Sample min peak height: 50   Sample baseline V to V?: Y   Sample baseline V to V points: 3
Sample filter: Binomial   Number of points for filter: 3 Sample start region (min): 0 Sample end region (min): 30
Marker peak width (sec): 5   Marker min peak height: 200   Marker baseline V to V?: Y   Marker baseline V to V points: 3
Lower marker selection: First peak > 500 RFU   Upper marker selection: Last peak > 200 RFU
Ladder size (bp)75, 200, 300, 400, 500, 700, 1000, 1500, 2000, 3000, 4000, 5000, 7000, 10000, 20000
Quantification using: Lower Marker   Final concentration (ng/uL): 0.5000   Dilution factor: 12.0

Sample: SampA6
Well location: A6
Created: Wednesday, August 11, 2021 5:34:32 PM

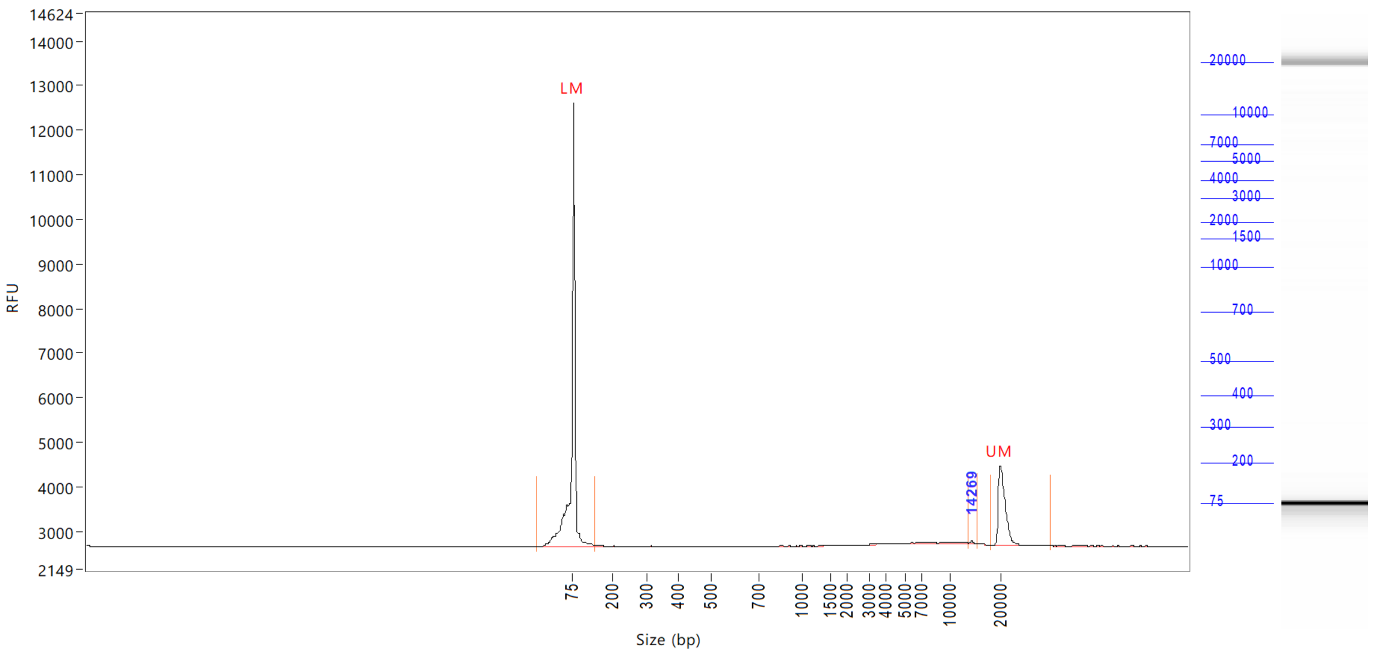

Peak	Size (bp)	Concentration (ng/uL)	From (bp)	To (bp)	Average size (bp)	CV%	RFU	Corrected peak area
1	75 (LM)	0.5000	0	141	65	31.16	9971	83.141
2	14269	0.0176	13537	15488	14135	2.17	55	0.243
3	20000 (UM)	0.0937	18171	29914	20403	4.49	1787	15.586

TIC:	0.0176	ng/uL
TIM:	0.0020	nmole/L
Total concentration:	0.3263	ng/uL

```
Sample peak width (sec): 5    Sample min peak height: 50    Sample baseline V to V?: Y    Sample baseline V to V points: 3
Sample filter: Binomial       Number of points for filter: 3  Sample start region (min): 0  Sample end region (min): 30
Marker peak width (sec): 5    Marker min peak height: 200   Marker baseline V to V?: Y    Marker baseline V to V points: 3
Lower marker selection: First peak > 500 RFU                Upper marker selection: Last peak > 200 RFU
Ladder size (bp)75, 200, 300, 400, 500, 700, 1000, 1500, 2000, 3000, 4000, 5000, 7000, 10000, 20000
Quantification using: Lower Marker          Final concentration (ng/uL): 0.5000          Dilution factor: 12.0
```

Sample: SampB6
Well location: B6
Created: Wednesday, August 11, 2021 5:34:32 PM

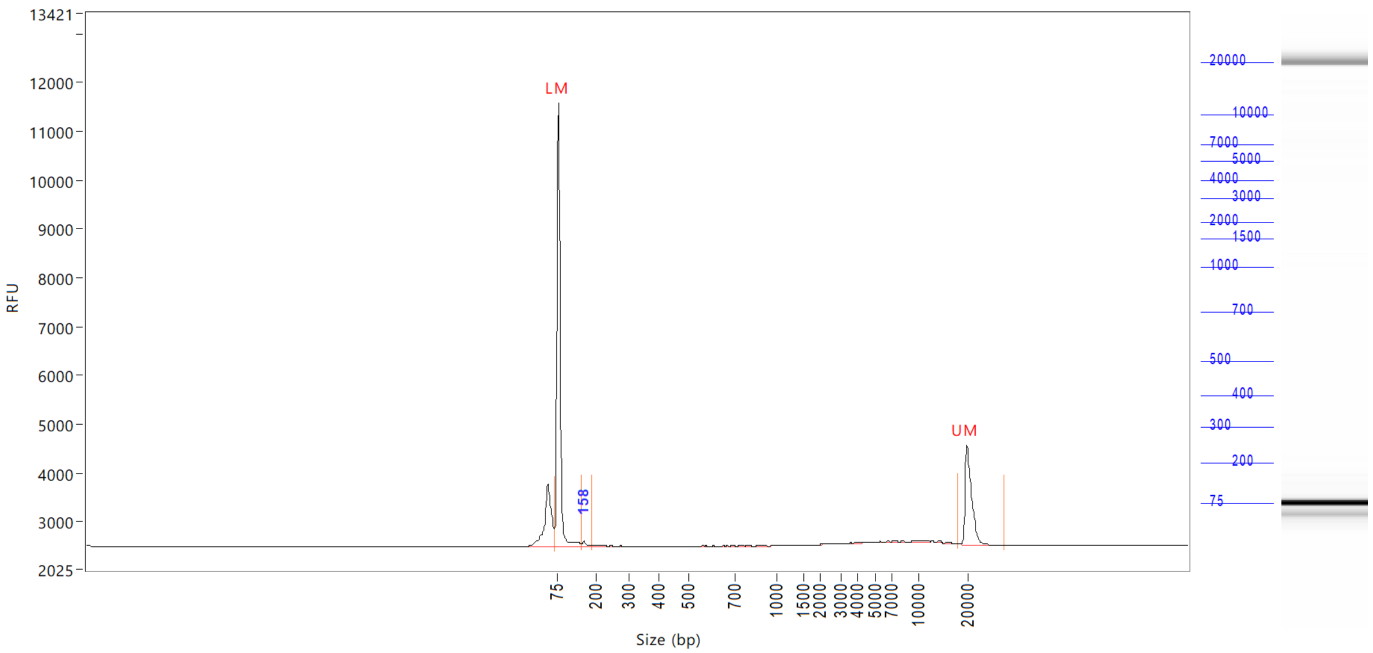

Peak	Size (bp)	Concentration (ng/uL)	From (bp)	To (bp)	Average size (bp)	CV%	RFU	Corrected peak area
1	75 (LM)	0.5000	64	150	76	14.18	9102	67.605
2	158	0.0704	150	187	160	5.24	98	0.794
3	20000 (UM)	0.1292	18171	27738	20446	4.17	2028	17.468

TIC:	0.0704	ng/uL
TIM:	0.7235	nmole/L
Total concentration:	0.3792	ng/uL

Sample peak width (sec): 5 Sample min peak height: 50 Sample baseline V to V?: Y Sample baseline V to V points: 3
Sample filter: Binomial Number of points for filter: 3 Sample start region (min): 0 Sample end region (min): 30
Marker peak width (sec): 5 Marker min peak height: 200 Marker baseline V to V?: Y Marker baseline V to V points: 3
Lower marker selection: First peak > 500 RFU Upper marker selection: Last peak > 200 RFU
Ladder size (bp)75, 200, 300, 400, 500, 700, 1000, 1500, 2000, 3000, 4000, 5000, 7000, 10000, 20000
Quantification using: Lower Marker Final concentration (ng/uL): 0.5000 Dilution factor: 12.0

Sample: SampC6
Well location: C6
Created: Wednesday, August 11, 2021 5:34:32 PM

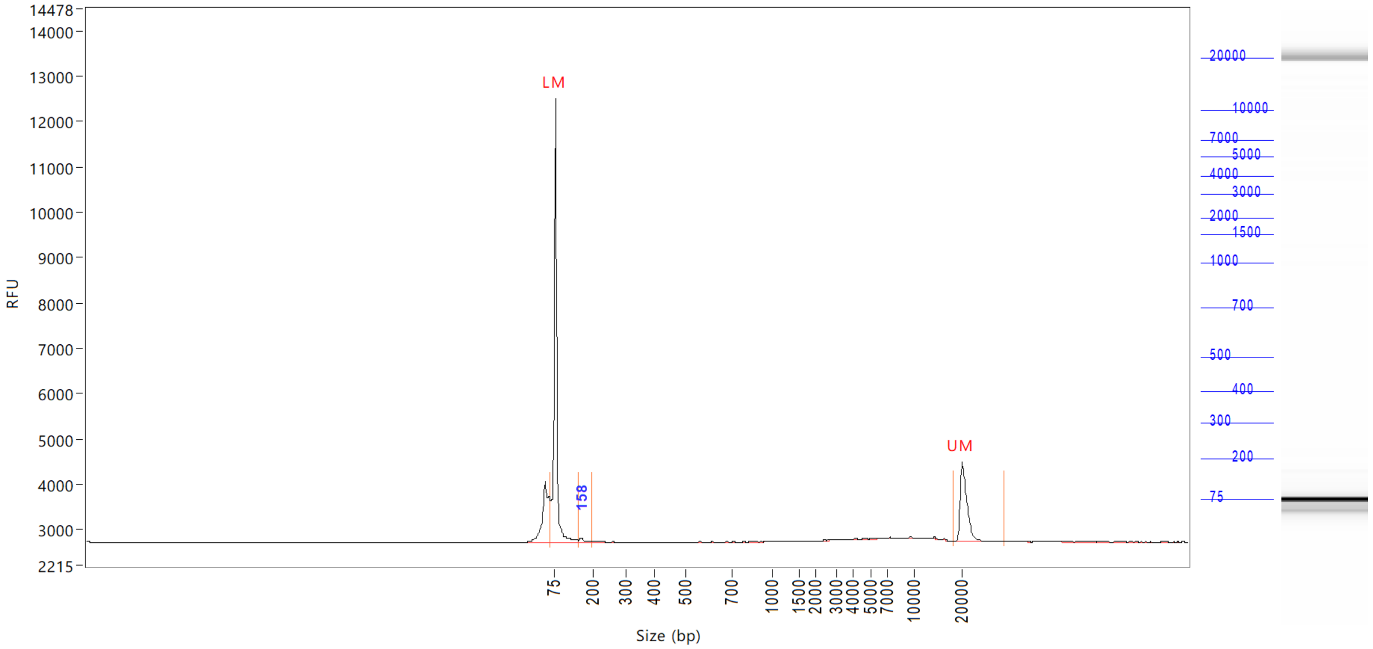

Peak	Size (bp)	Concentration (ng/uL)	From (bp)	To (bp)	Average size (bp)	CV%	RFU	Corrected peak area
1	75 (LM)	0.5000	58	148	74	14.43	9797	67.693
2	158	0.0872	148	192	160	6.06	102	0.983
3	20000 (UM)	0.1137	18293	28947	20491	4.62	1744	15.398

TIC:	0.0872	ng/uL
TIM:	0.8955	nmole/L
Total concentration:	0.4104	ng/uL

Sample peak width (sec): 5 Sample min peak height: 50 Sample baseline V to V?: Y Sample baseline V to V points: 3
Sample filter: Binomial Number of points for filter: 3 Sample start region (min): 0 Sample end region (min): 30
Marker peak width (sec): 5 Marker min peak height: 200 Marker baseline V to V?: Y Marker baseline V to V points: 3
Lower marker selection: First peak > 500 RFU Upper marker selection: Last peak > 200 RFU
Ladder size (bp)75, 200, 300, 400, 500, 700, 1000, 1500, 2000, 3000, 4000, 5000, 7000, 10000, 20000
Quantification using: Lower Marker Final concentration (ng/uL): 0.5000 Dilution factor: 12.0

Sample: SampD6
Well location: D6
Created: Wednesday, August 11, 2021 5:34:32 PM

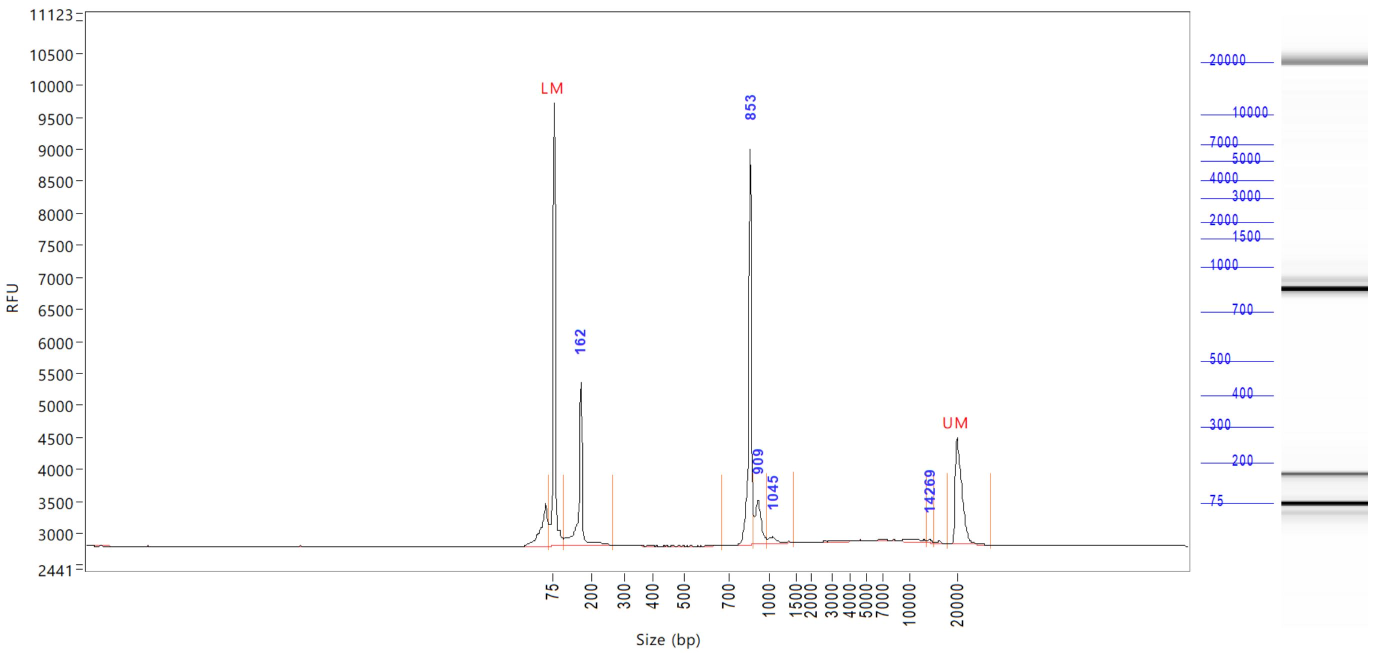

Peak	Size (bp)	Concentration (ng/uL)	From (bp)	To (bp)	Average size (bp)	CV%	RFU	Corrected peak area
1	75 (LM)	0.5000	56	105	73	8.87	6930	44.416
2	162	2.9919	105	266	157	13.40	2550	22.148
3	853	4.3896	664	879	843	2.02	6167	32.495
4	909	1.0115	879	983	913	2.62	685	7.488
5	1045	0.2360	983	1456	1094	10.36	110	1.747
6	14269	0.0464	13537	15122	14149	2.68	65	0.343
7	20000 (UM)	0.1638	18049	26891	20448	4.07	1658	14.555

TIC:	8.6754	ng/uL
TIM:	42.0763	nmole/L
Total concentration:	9.0456	ng/uL

Sample peak width (sec): 5 Sample min peak height: 50 Sample baseline V to V?: Y Sample baseline V to V points: 3
Sample filter: Binomial Number of points for filter: 3 Sample start region (min): 0 Sample end region (min): 30
Marker peak width (sec): 5 Marker min peak height: 200 Marker baseline V to V?: Y Marker baseline V to V points: 3
Lower marker selection: First peak > 500 RFU Upper marker selection: Last peak > 200 RFU
Ladder size (bp)75, 200, 300, 400, 500, 700, 1000, 1500, 2000, 3000, 4000, 5000, 7000, 10000, 20000
Quantification using: Lower Marker Final concentration (ng/uL): 0.5000 Dilution factor: 12.0

Sample: SampE6
Well location: E6
Created: Wednesday, August 11, 2021 5:34:32 PM

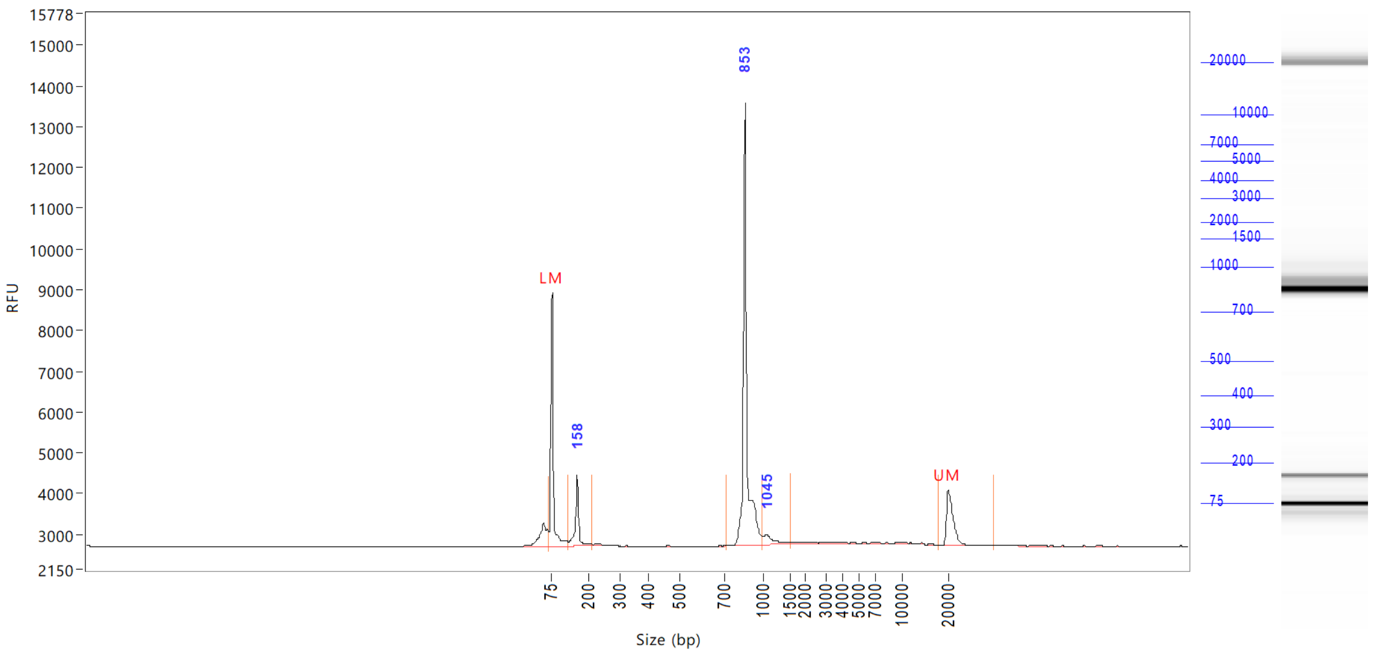

Peak	Size (bp)	Concentration (ng/uL)	From (bp)	To (bp)	Average size (bp)	CV%	RFU	Corrected peak area
1	75 (LM)	0.5000	66	129	76	15.04	6215	40.550
2	158	2.0573	129	210	156	8.36	1737	13.904
3	853	10.3732	718	987	861	3.98	10864	70.105
4	1045	0.6587	987	1501	1136	12.58	234	4.452
5	20000 (UM)	0.1493	17927	29793	20510	5.11	1360	12.110

TIC:	13.0893	ng/uL
TIM:	42.4599	nmole/L
Total concentration:	13.6835	ng/uL

```
Sample peak width (sec): 5    Sample min peak height: 50    Sample baseline V to V?: Y    Sample baseline V to V points: 3
Sample filter: Binomial       Number of points for filter: 3  Sample start region (min): 0  Sample end region (min): 30
Marker peak width (sec): 5    Marker min peak height: 200   Marker baseline V to V?: Y    Marker baseline V to V points: 3
Lower marker selection: First peak > 500 RFU                Upper marker selection: Last peak > 200 RFU
Ladder size (bp)75, 200, 300, 400, 500, 700, 1000, 1500, 2000, 3000, 4000, 5000, 7000, 10000, 20000
Quantification using: Lower Marker          Final concentration (ng/uL): 0.5000          Dilution factor: 12.0
```

Sample: SampF6
Well location: F6
Created: Wednesday, August 11, 2021 5:34:32 PM

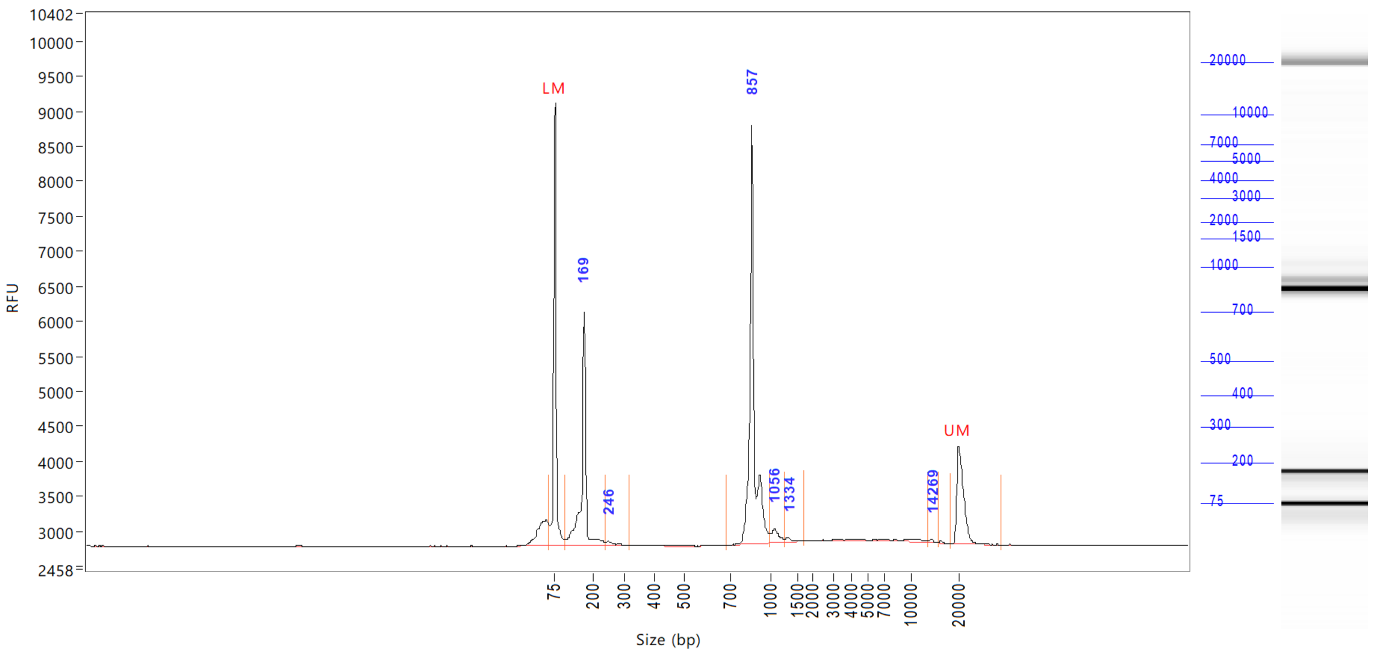

Peak	Size (bp)	Concentration (ng/uL)	From (bp)	To (bp)	Average size (bp)	CV%	RFU	Corrected peak area
1	75 (LM)	0.5000	56	110	73	9.53	6336	40.242
2	169	4.6694	110	239	164	12.45	3343	31.318
3	246	0.1485	239	315	257	6.12	56	0.996
4	857	6.9076	682	987	862	4.33	5986	46.330
5	1056	0.4069	987	1245	1071	6.45	194	2.729
6	1334	0.1089	1245	1693	1356	7.06	61	0.731
7	14269	0.0474	13415	15610	14214	3.59	54	0.318
8	20000 (UM)	0.1537	18171	28705	20429	4.56	1396	12.372

TIC:	12.2887	ng/uL
TIM:	61.7033	nmole/L
Total concentration:	12.6674	ng/uL

```
Sample peak width (sec): 5    Sample min peak height: 50    Sample baseline V to V?: Y    Sample baseline V to V points: 3
Sample filter: Binomial       Number of points for filter: 3  Sample start region (min): 0  Sample end region (min): 30
Marker peak width (sec): 5    Marker min peak height: 200   Marker baseline V to V?: Y    Marker baseline V to V points: 3
Lower marker selection: First peak > 500 RFU                Upper marker selection: Last peak > 200 RFU
Ladder size (bp)75, 200, 300, 400, 500, 700, 1000, 1500, 2000, 3000, 4000, 5000, 7000, 10000, 20000
Quantification using: Lower Marker          Final concentration (ng/uL): 0.5000          Dilution factor: 12.0
```

Sample: SampG6
Well location: G6
Created: Wednesday, August 11, 2021 5:34:32 PM

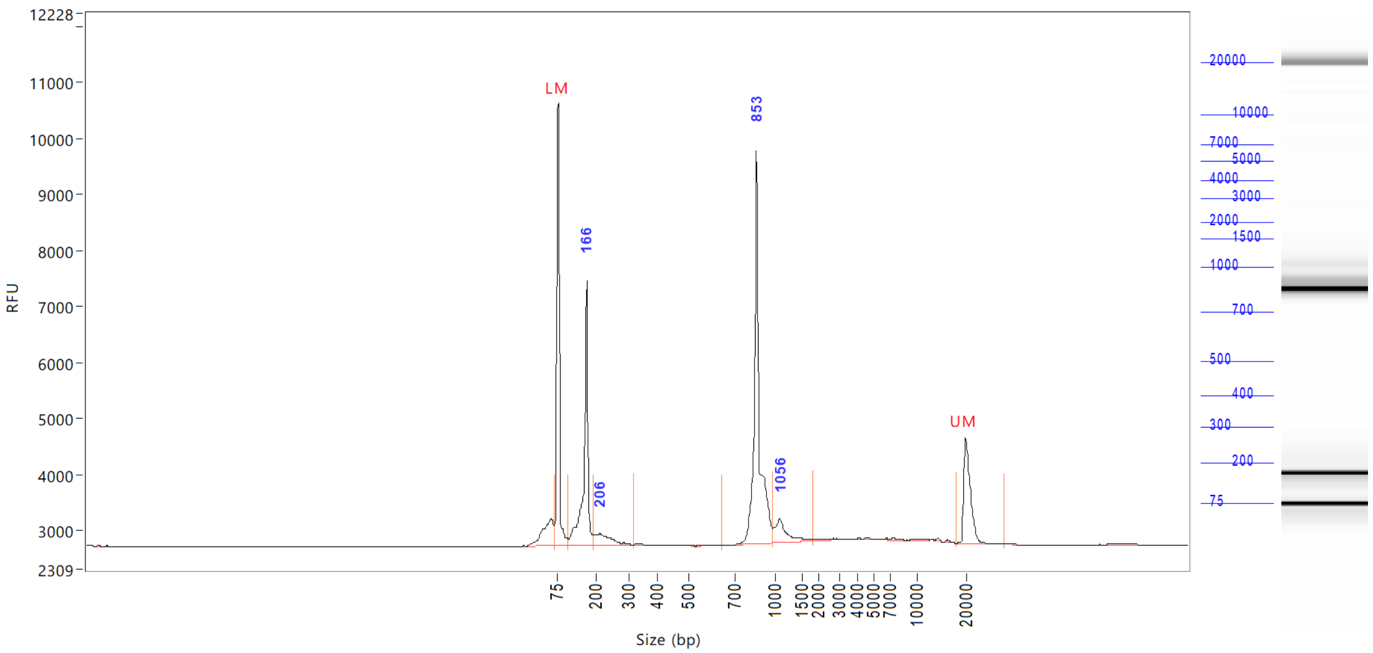

Peak	Size (bp)	Concentration (ng/uL)	From (bp)	To (bp)	Average size (bp)	CV%	RFU	Corrected peak area
1	75 (LM)	0.5000	64	107	73	8.33	7912	48.512
2	166	5.3073	107	192	157	10.11	4717	42.911
3	206	0.8638	192	313	225	10.54	199	6.984
4	853	7.4152	639	974	860	4.58	7006	59.954
5	1056	1.0259	974	1808	1139	15.04	421	8.295
6	20000 (UM)	0.1729	17927	27859	20419	4.29	1896	16.772

TIC:	14.6123	ng/uL
TIM:	77.5500	nmole/L
Total concentration:	14.9658	ng/uL

```
Sample peak width (sec): 5    Sample min peak height: 50    Sample baseline V to V?: Y    Sample baseline V to V points: 3
Sample filter: Binomial       Number of points for filter: 3  Sample start region (min): 0  Sample end region (min): 30
Marker peak width (sec): 5    Marker min peak height: 200   Marker baseline V to V?: Y    Marker baseline V to V points: 3
Lower marker selection: First peak > 500 RFU                Upper marker selection: Last peak > 200 RFU
Ladder size (bp)75, 200, 300, 400, 500, 700, 1000, 1500, 2000, 3000, 4000, 5000, 7000, 10000, 20000
Quantification using: Lower Marker          Final concentration (ng/uL): 0.5000          Dilution factor: 12.0
```

Sample: SampH6
Well location: H6
Created: Wednesday, August 11, 2021 5:34:32 PM

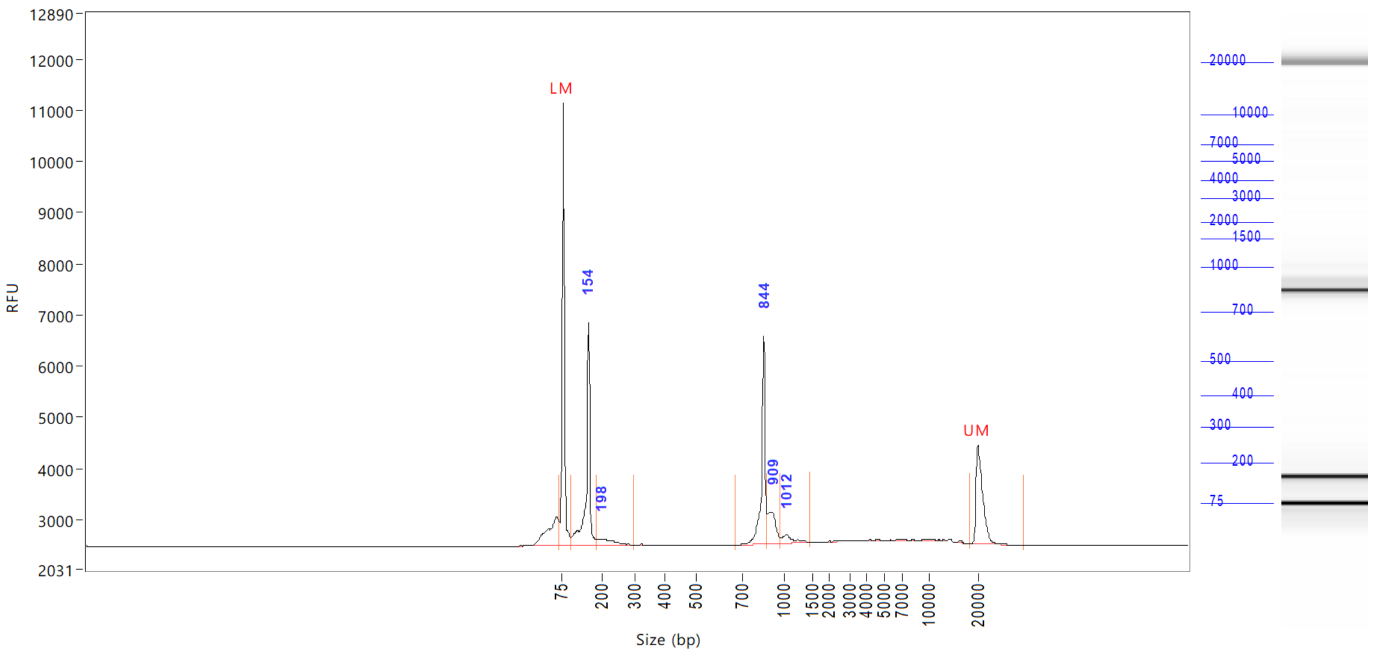

Peak	Size (bp)	Concentration (ng/uL)	From (bp)	To (bp)	Average size (bp)	CV%	RFU	Corrected peak area
1	75 (LM)	0.5000	64	99	74	6.78	8662	50.690
2	154	4.4351	99	185	147	10.56	4348	37.470
3	198	0.5242	185	295	216	10.63	130	4.429
4	844	2.9896	667	870	831	3.18	4070	25.258
5	909	1.0349	870	970	906	2.73	628	8.743
6	1012	0.3525	970	1467	1085	10.82	168	2.978
7	20000 (UM)	0.1673	18293	29309	20436	4.52	1939	16.963

TIC:	9.3364	ng/uL
TIM:	61.9116	nmole/L
Total concentration:	9.5984	ng/uL

```
Sample peak width (sec): 5    Sample min peak height: 50    Sample baseline V to V?: Y    Sample baseline V to V points: 3
Sample filter: Binomial       Number of points for filter: 3  Sample start region (min): 0  Sample end region (min): 30
Marker peak width (sec): 5    Marker min peak height: 200   Marker baseline V to V?: Y    Marker baseline V to V points: 3
Lower marker selection: First peak > 500 RFU                Upper marker selection: Last peak > 200 RFU
Ladder size (bp)75, 200, 300, 400, 500, 700, 1000, 1500, 2000, 3000, 4000, 5000, 7000, 10000, 20000
Quantification using: Lower Marker          Final concentration (ng/uL): 0.5000          Dilution factor: 12.0
```

Sample: SampA7
Well location: A7
Created: Wednesday, August 11, 2021 5:34:32 PM

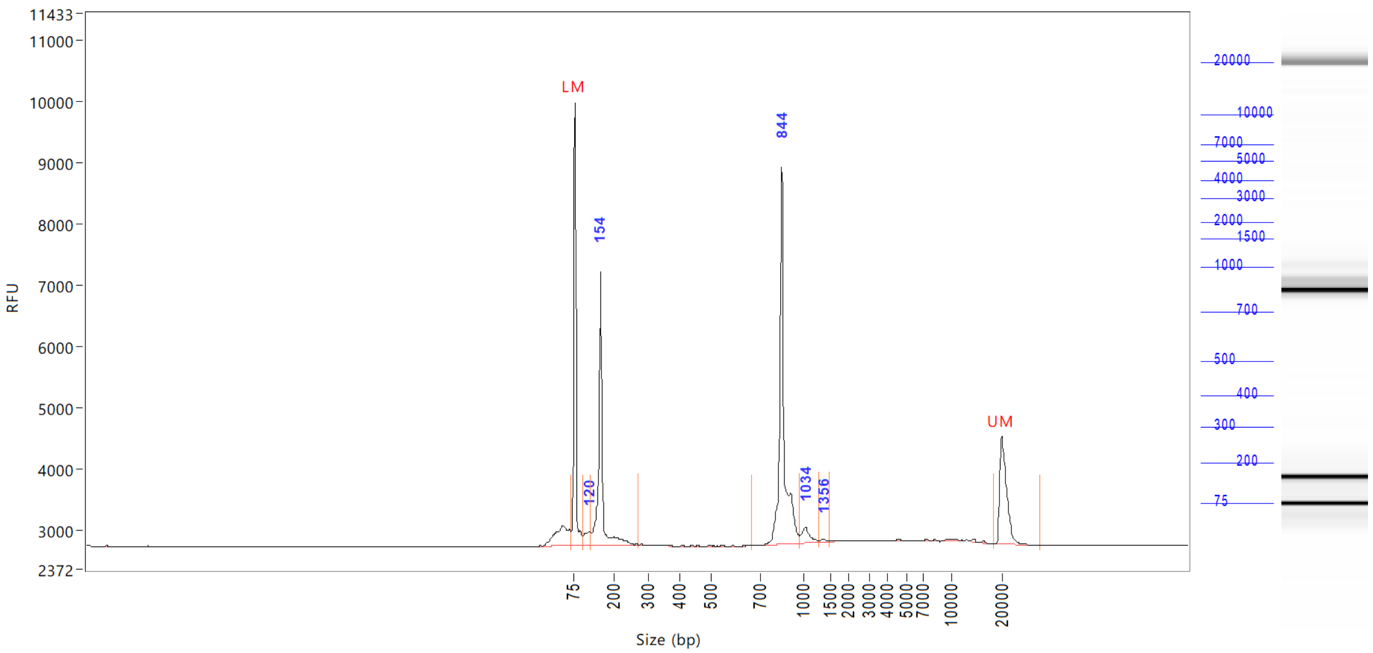

Peak	Size (bp)	Concentration (ng/uL)	From (bp)	To (bp)	Average size (bp)	CV%	RFU	Corrected peak area
1	75 (LM)	0.5000	64	99	74	6.95	7228	40.504
2	120	0.5431	99	126	113	7.10	225	3.666
3	154	5.2724	126	269	157	12.66	4468	35.593
4	844	6.7368	662	978	854	4.46	6147	45.478
5	1034	0.4935	978	1289	1061	7.18	253	3.332
6	1356	0.0599	1289	1478	1356	3.28	50	0.405
7	20000 (UM)	0.1890	18537	27617	20432	4.38	1766	15.313

TIC:	13.1058	ng/uL
TIM:	76.9140	nmole/L
Total concentration:	13.4766	ng/uL

```
Sample peak width (sec): 5    Sample min peak height: 50    Sample baseline V to V?: Y    Sample baseline V to V points: 3
Sample filter: Binomial       Number of points for filter: 3  Sample start region (min): 0  Sample end region (min): 30
Marker peak width (sec): 5    Marker min peak height: 200   Marker baseline V to V?: Y    Marker baseline V to V points: 3
Lower marker selection: First peak > 500 RFU                Upper marker selection: Last peak > 200 RFU
Ladder size (bp)75, 200, 300, 400, 500, 700, 1000, 1500, 2000, 3000, 4000, 5000, 7000, 10000, 20000
Quantification using: Lower Marker          Final concentration (ng/uL): 0.5000          Dilution factor: 12.0
```

Sample: SampB7
Well location: B7
Created: Wednesday, August 11, 2021 5:34:32 PM

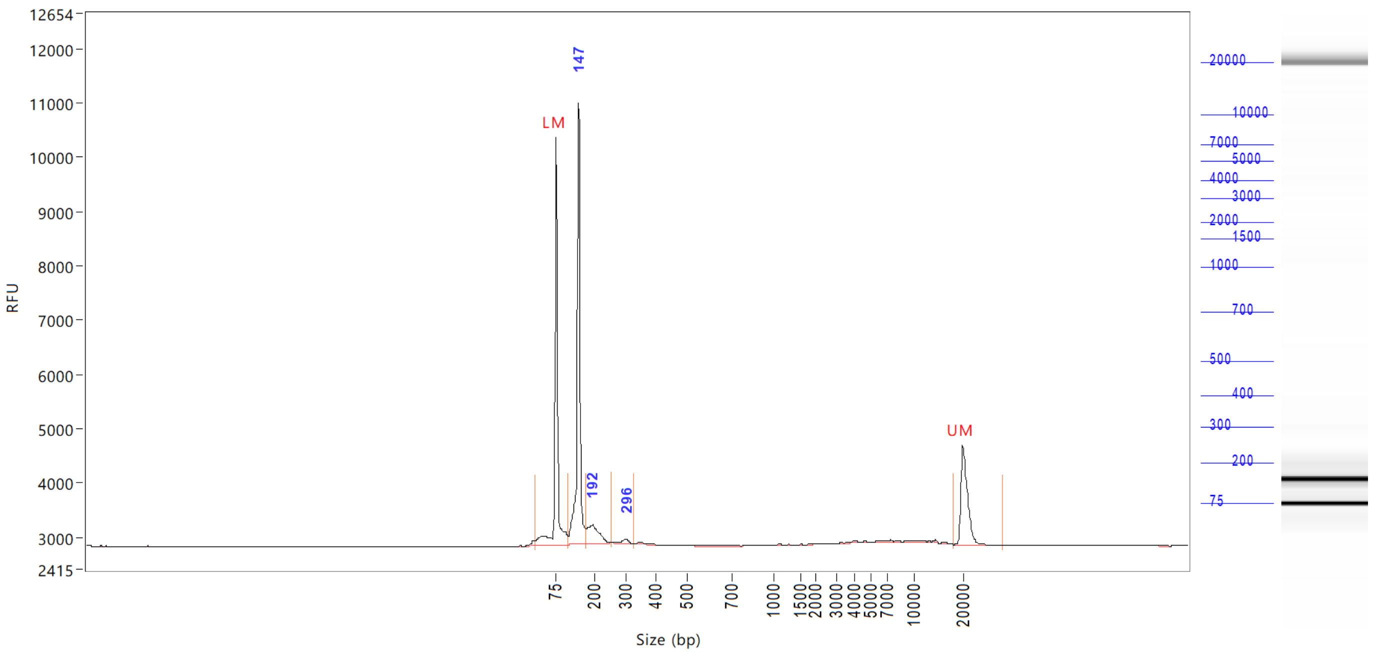

Peak	Size (bp)	Concentration (ng/uL)	From (bp)	To (bp)	Average size (bp)	CV%	RFU	Corrected peak area
1	75 (LM)	0.5000	5	114	70	23.49	7504	51.769
2	147	6.9218	114	173	144	6.28	8141	59.723
3	192	1.0504	173	251	197	7.98	344	9.063
4	296	0.1370	251	325	290	4.53	73	1.182
5	20000 (UM)	0.1564	18171	28342	20473	4.41	1840	16.189

TIC:	8.1091	ng/uL
TIM:	88.5377	nmole/L
Total concentration:	8.5135	ng/uL

Sample peak width (sec): 5   Sample min peak height: 50   Sample baseline V to V?: Y   Sample baseline V to V points: 3
Sample filter: Binomial   Number of points for filter: 3 Sample start region (min): 0 Sample end region (min): 30
Marker peak width (sec): 5   Marker min peak height: 200   Marker baseline V to V?: Y   Marker baseline V to V points: 3
Lower marker selection: First peak > 500 RFU   Upper marker selection: Last peak > 200 RFU
Ladder size (bp)75, 200, 300, 400, 500, 700, 1000, 1500, 2000, 3000, 4000, 5000, 7000, 10000, 20000
Quantification using: Lower Marker   Final concentration (ng/uL): 0.5000   Dilution factor: 12.0

Sample: SampC7
Well location: C7
Created: Wednesday, August 11, 2021 5:34:32 PM

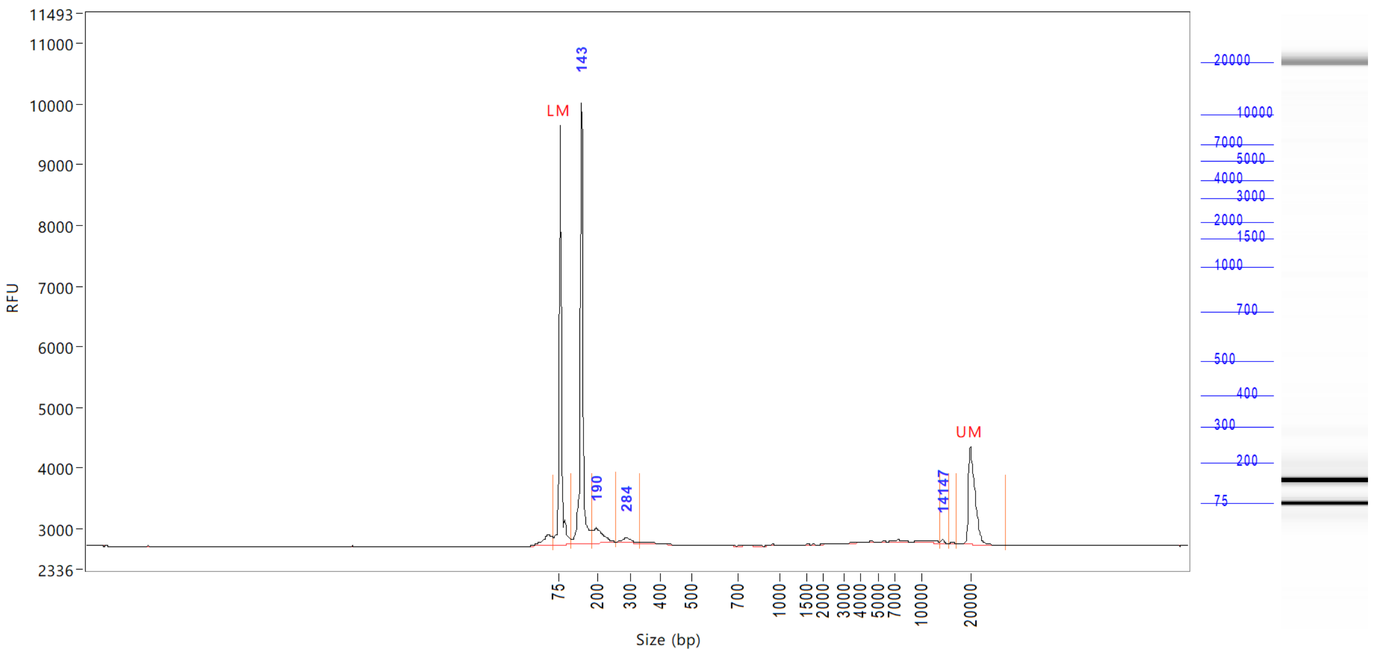

Peak	Size (bp)	Concentration (ng/uL)	From (bp)	To (bp)	Average size (bp)	CV%	RFU	Corrected peak area
1	75 (LM)	0.5000	54	112	74	9.72	6917	41.606
2	143	7.2719	112	181	143	6.35	7274	50.426
3	190	0.7801	181	251	201	7.41	250	5.410
4	284	0.2237	251	325	284	4.88	74	1.551
5	14147	0.0482	13537	15610	14210	3.43	60	0.334
6	20000 (UM)	0.1670	17195	27254	20347	4.11	1599	13.897

TIC:	8.3239	ng/uL
TIM:	91.2521	nmole/L
Total concentration:	8.8586	ng/uL

```
Sample peak width (sec): 5    Sample min peak height: 50    Sample baseline V to V?: Y    Sample baseline V to V points: 3
Sample filter: Binomial       Number of points for filter: 3  Sample start region (min): 0  Sample end region (min): 30
Marker peak width (sec): 5    Marker min peak height: 200   Marker baseline V to V?: Y    Marker baseline V to V points: 3
Lower marker selection: First peak > 500 RFU                Upper marker selection: Last peak > 200 RFU
Ladder size (bp)75, 200, 300, 400, 500, 700, 1000, 1500, 2000, 3000, 4000, 5000, 7000, 10000, 20000
Quantification using: Lower Marker          Final concentration (ng/uL): 0.5000          Dilution factor: 12.0
```

Sample: SampD7
Well location: D7
Created: Wednesday, August 11, 2021 5:34:32 PM

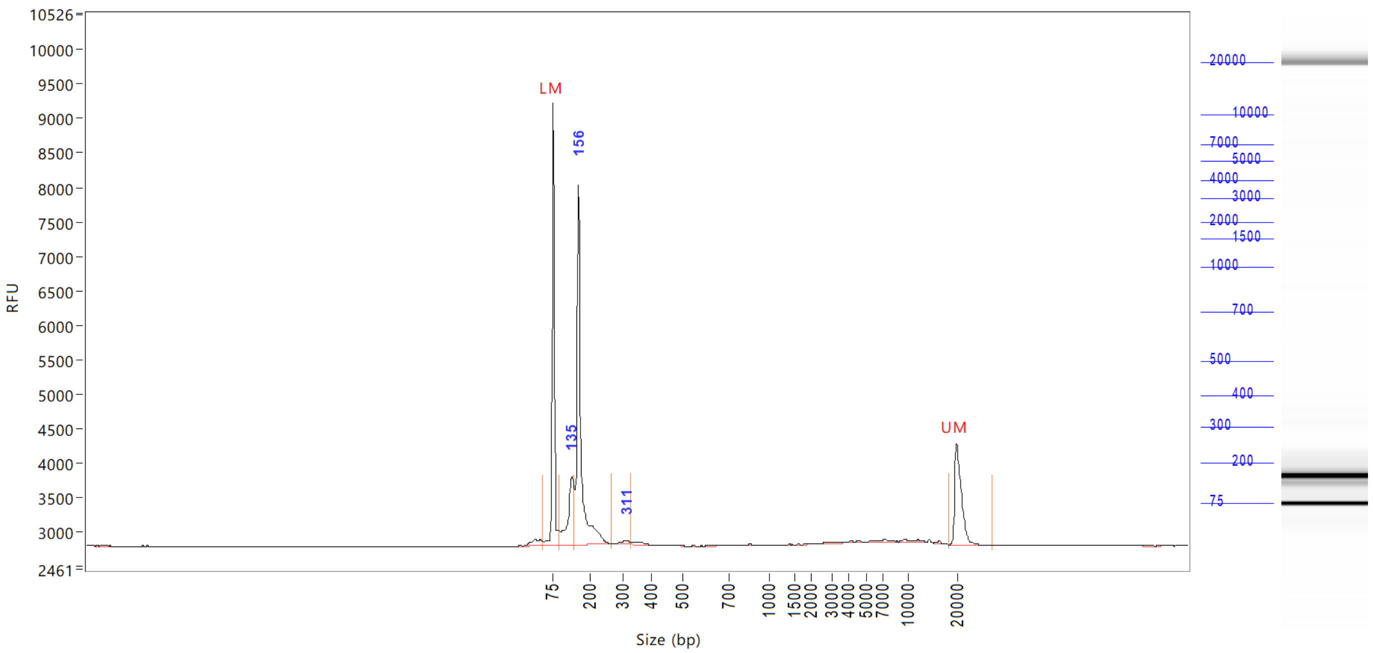

Peak	Size (bp)	Concentration (ng/uL)	From (bp)	To (bp)	Average size (bp)	CV%	RFU	Corrected peak area
1	75 (LM)	0.5000	43	97	73	8.42	6430	37.987
2	135	2.4213	97	145	128	9.71	990	15.330
3	156	7.7552	145	264	164	11.51	5219	49.100
4	311	0.1533	264	327	303	4.54	54	0.971
5	20000 (UM)	0.1703	18415	27617	20466	4.23	1476	12.935

TIC:	10.3298	ng/uL
TIM:	109.6420	nmole/L
Total concentration:	10.8253	ng/uL

Sample peak width (sec): 5   Sample min peak height: 50   Sample baseline V to V?: Y   Sample baseline V to V points: 3
Sample filter: Binomial   Number of points for filter: 3 Sample start region (min): 0 Sample end region (min): 30
Marker peak width (sec): 5   Marker min peak height: 200   Marker baseline V to V?: Y   Marker baseline V to V points: 3
Lower marker selection: First peak > 500 RFU   Upper marker selection: Last peak > 200 RFU
Ladder size (bp)75, 200, 300, 400, 500, 700, 1000, 1500, 2000, 3000, 4000, 5000, 7000, 10000, 20000
Quantification using: Lower Marker   Final concentration (ng/uL): 0.5000   Dilution factor: 12.0

Sample: SampE7
Well location: E7
Created: Wednesday, August 11, 2021 5:34:32 PM

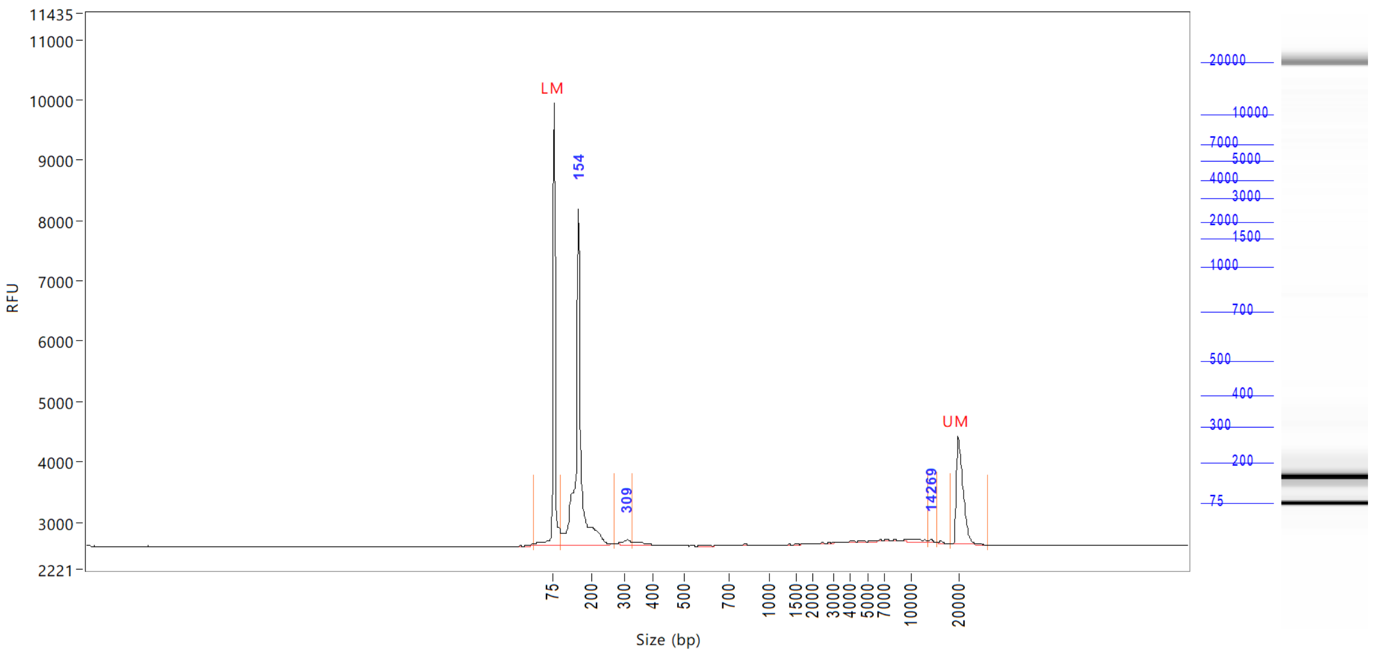

Peak	Size (bp)	Concentration (ng/uL)	From (bp)	To (bp)	Average size (bp)	CV%	RFU	Corrected peak area
1	75 (LM)	0.5000	9	99	73	14.11	7346	44.933
2	154	8.8219	99	271	155	15.42	5576	66.066
3	309	0.1930	271	325	302	4.43	81	1.445
4	14269	0.0478	13537	15610	14248	3.18	66	0.358
5	20000 (UM)	0.1740	18171	26166	20422	3.99	1789	15.635

TIC:	9.0627	ng/uL
TIM:	94.6034	nmole/L
Total concentration:	9.6964	ng/uL

Sample peak width (sec): 5 Sample min peak height: 50 Sample baseline V to V?: Y Sample baseline V to V points: 3
Sample filter: Binomial Number of points for filter: 3 Sample start region (min): 0 Sample end region (min): 30
Marker peak width (sec): 5 Marker min peak height: 200 Marker baseline V to V?: Y Marker baseline V to V points: 3
Lower marker selection: First peak > 500 RFU Upper marker selection: Last peak > 200 RFU
Ladder size (bp)75, 200, 300, 400, 500, 700, 1000, 1500, 2000, 3000, 4000, 5000, 7000, 10000, 20000
Quantification using: Lower Marker Final concentration (ng/uL): 0.5000 Dilution factor: 12.0

Sample: SampF7
Well location: F7
Created: Wednesday, August 11, 2021 5:34:32 PM

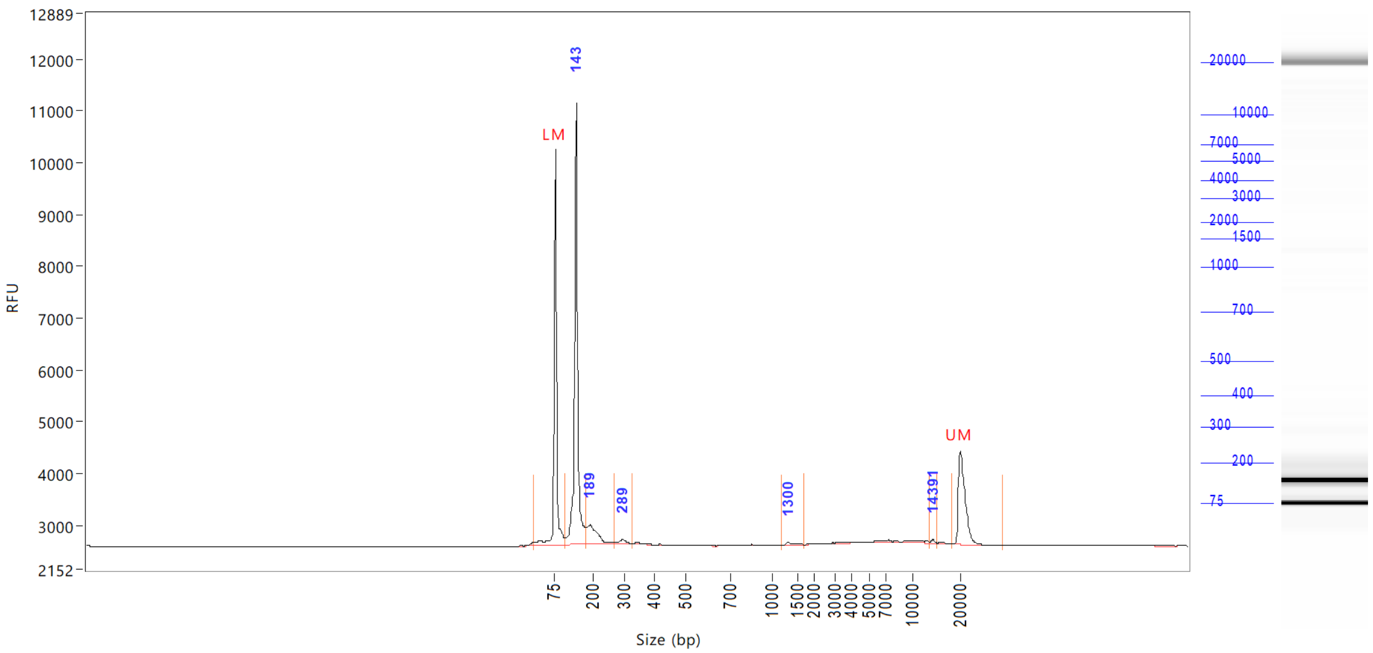

Peak	Size (bp)	Concentration (ng/uL)	From (bp)	To (bp)	Average size (bp)	CV%	RFU	Corrected peak area
1	75 (LM)	0.5000	5	107	72	17.63	7633	50.299
2	143	7.7124	107	175	142	7.00	8539	64.655
3	189	1.0756	175	266	198	8.56	372	9.017
4	289	0.1291	266	321	288	3.46	74	1.082
5	1300	0.0655	1178	1674	1337	6.90	59	0.549
6	14391	0.0479	13537	15244	14201	2.80	71	0.401
7	20000 (UM)	0.1595	18293	28947	20513	4.70	1807	16.047

TIC:	9.0304	ng/uL
TIM:	99.0101	nmole/L
Total concentration:	9.5094	ng/uL

Sample peak width (sec): 5   Sample min peak height: 50   Sample baseline V to V?: Y   Sample baseline V to V points: 3
Sample filter: Binomial   Number of points for filter: 3 Sample start region (min): 0 Sample end region (min): 30
Marker peak width (sec): 5   Marker min peak height: 200   Marker baseline V to V?: Y   Marker baseline V to V points: 3
Lower marker selection: First peak > 500 RFU   Upper marker selection: Last peak > 200 RFU
Ladder size (bp)75, 200, 300, 400, 500, 700, 1000, 1500, 2000, 3000, 4000, 5000, 7000, 10000, 20000
Quantification using: Lower Marker   Final concentration (ng/uL): 0.5000   Dilution factor: 12.0

Sample: SampG7
Well location: G7
Created: Wednesday, August 11, 2021 5:34:32 PM

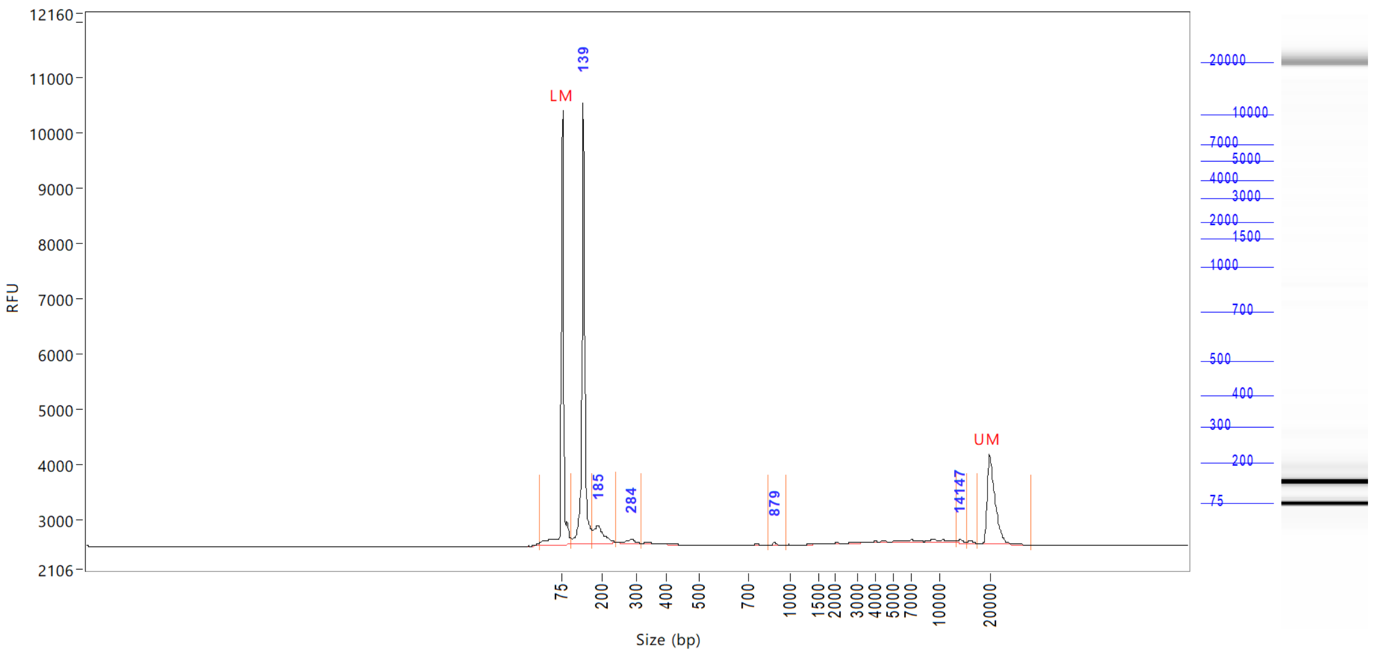

Peak	Size (bp)	Concentration (ng/uL)	From (bp)	To (bp)	Average size (bp)	CV%	RFU	Corrected peak area
1	75 (LM)	0.5000	7	103	71	17.19	7855	49.076
2	139	6.7875	103	169	137	6.48	7986	55.517
3	185	0.8579	169	239	191	8.13	322	7.017
4	284	0.1691	239	315	274	5.58	69	1.383
5	879	0.0477	835	974	892	3.09	51	0.390
6	14147	0.0656	13293	15488	14280	4.31	59	0.536
7	20000 (UM)	0.1664	17683	28221	20489	4.77	1618	16.334

TIC:	7.9278	ng/uL
TIM:	89.9081	nmole/L
Total concentration:	8.4380	ng/uL

```
Sample peak width (sec): 5    Sample min peak height: 50    Sample baseline V to V?: Y    Sample baseline V to V points: 3
Sample filter: Binomial       Number of points for filter: 3  Sample start region (min): 0  Sample end region (min): 30
Marker peak width (sec): 5    Marker min peak height: 200   Marker baseline V to V?: Y    Marker baseline V to V points: 3
Lower marker selection: First peak > 500 RFU                Upper marker selection: Last peak > 200 RFU
Ladder size (bp)75, 200, 300, 400, 500, 700, 1000, 1500, 2000, 3000, 4000, 5000, 7000, 10000, 20000
Quantification using: Lower Marker          Final concentration (ng/uL): 0.5000          Dilution factor: 12.0
```

Sample: SampH7
Well location: H7
Created: Wednesday, August 11, 2021 5:34:32 PM

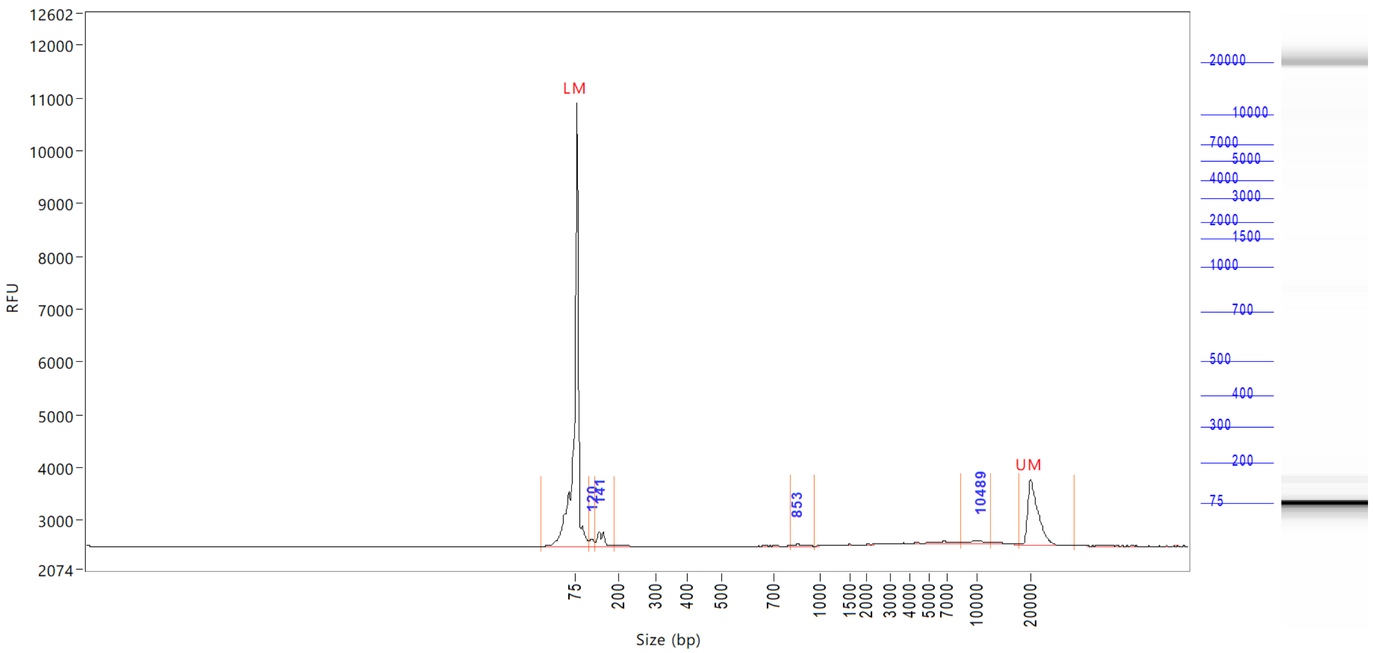

Peak	Size (bp)	Concentration (ng/uL)	From (bp)	To (bp)	Average size (bp)	CV%	RFU	Corrected peak area
1	75 (LM)	0.5000	0	110	66	24.07	8413	85.239
2	120	0.1078	110	128	118	4.37	147	1.532
3	141	0.2984	128	187	145	7.53	286	4.240
4	853	0.0329	814	970	865	3.55	52	0.468
5	10489	0.1017	8401	12440	10161	10.77	58	1.445
6	20000 (UM)	0.0919	17805	28100	20647	5.46	1241	15.669

TIC:	0.5409	ng/uL
TIM:	4.9629	nmole/L
Total concentration:	0.8324	ng/uL

```
Sample peak width (sec): 5    Sample min peak height: 50    Sample baseline V to V?: Y    Sample baseline V to V points: 3
Sample filter: Binomial       Number of points for filter: 3  Sample start region (min): 0  Sample end region (min): 30
Marker peak width (sec): 5    Marker min peak height: 200   Marker baseline V to V?: Y    Marker baseline V to V points: 3
Lower marker selection: First peak > 500 RFU                Upper marker selection: Last peak > 200 RFU
Ladder size (bp)75, 200, 300, 400, 500, 700, 1000, 1500, 2000, 3000, 4000, 5000, 7000, 10000, 20000
Quantification using: Lower Marker          Final concentration (ng/uL): 0.5000          Dilution factor: 12.0
```

Sample: SampA8
Well location: A8
Created: Wednesday, August 11, 2021 5:34:32 PM

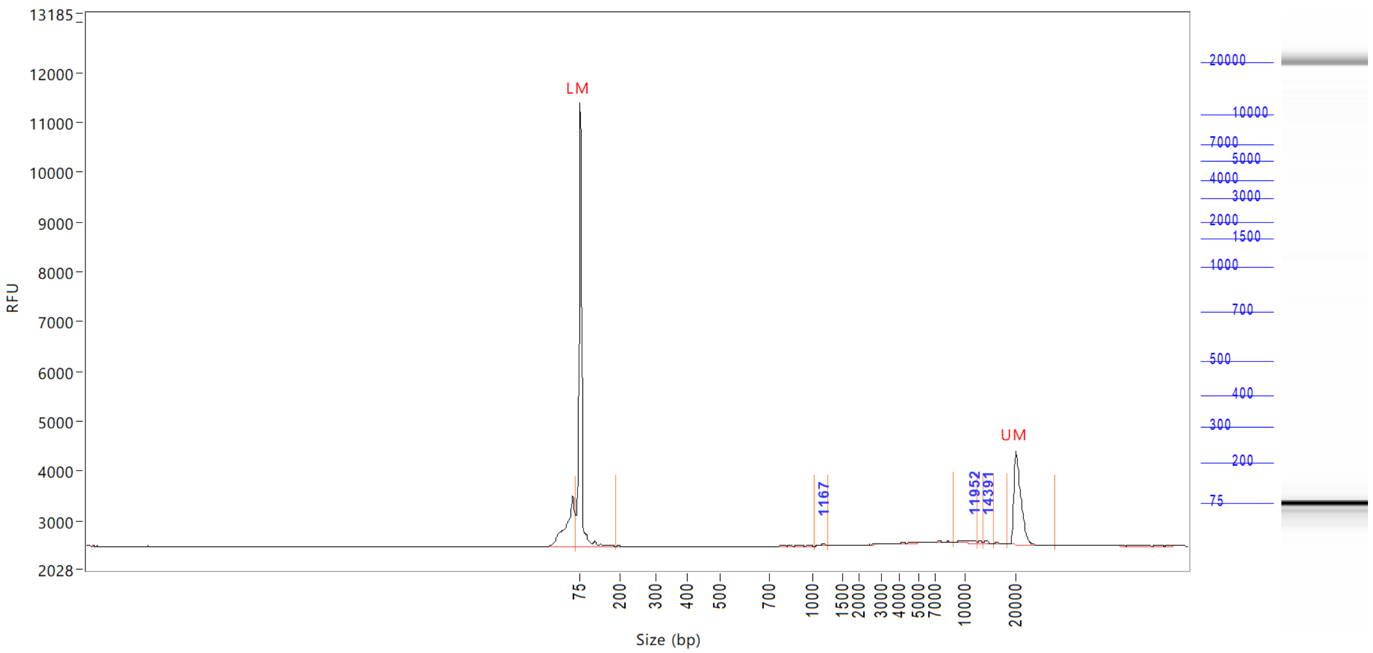

Peak	Size (bp)	Concentration (ng/uL)	From (bp)	To (bp)	Average size (bp)	CV%	RFU	Corrected peak area
1	75 (LM)	0.5000	60	183	75	13.20	8914	59.798
2	1167	0.0283	1023	1245	1162	2.87	52	0.282
3	11952	0.0919	8867	12562	10659	9.51	51	0.916
4	14391	0.0359	13537	15732	14265	3.36	65	0.358
5	20000 (UM)	0.1362	18293	27738	20428	3.99	1885	16.291

TIC:	0.1561	ng/uL
TIM:	0.0584	nmole/L
Total concentration:	0.4541	ng/uL

Sample peak width (sec): 5   Sample min peak height: 50   Sample baseline V to V?: Y   Sample baseline V to V points: 3
Sample filter: Binomial   Number of points for filter: 3   Sample start region (min): 0   Sample end region (min): 30
Marker peak width (sec): 5   Marker min peak height: 200   Marker baseline V to V?: Y   Marker baseline V to V points: 3
Lower marker selection: First peak > 500 RFU   Upper marker selection: Last peak > 200 RFU
Ladder size (bp)75, 200, 300, 400, 500, 700, 1000, 1500, 2000, 3000, 4000, 5000, 7000, 10000, 20000
Quantification using: Lower Marker   Final concentration (ng/uL): 0.5000   Dilution factor: 12.0

Sample: SampB8
Well location: B8
Created: Wednesday, August 11, 2021 5:34:32 PM

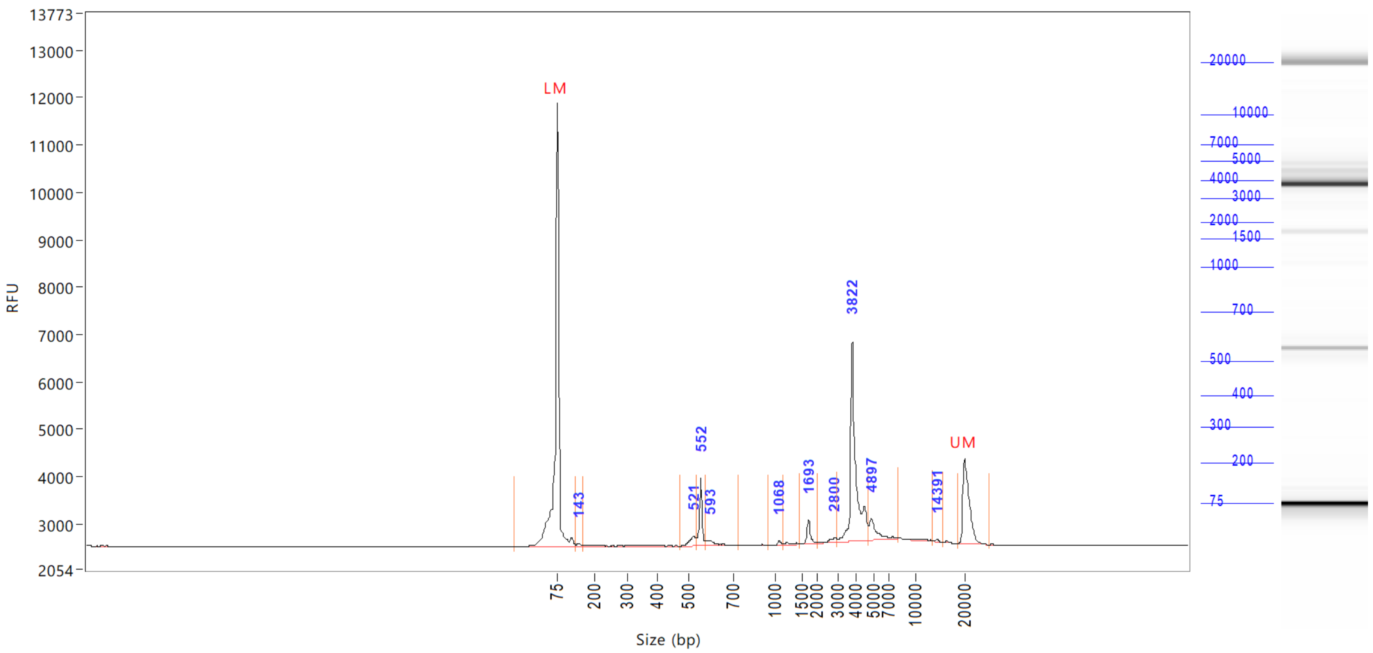

Peak	Size (bp)	Concentration (ng/uL)	From (bp)	To (bp)	Average size (bp)	CV%	RFU	Corrected peak area
1	75 (LM)	0.5000	0	131	67	26.70	9365	82.023
2	143	0.0515	131	156	143	4.80	62	0.703
3	521	0.1891	473	534	514	2.55	201	2.585
4	552	0.5451	534	570	550	1.21	1422	7.452
5	593	0.1713	570	723	611	5.91	118	2.341
6	1068	0.0343	952	1145	1061	3.52	80	0.469
7	1693	0.2138	1456	2001	1694	4.54	499	2.923
8	2800	0.0845	2001	2943	2630	8.30	98	1.156
9	3822	2.5938	2943	4759	3913	8.13	4197	35.459
10	4897	0.3389	4759	8067	5340	12.69	449	4.633
11	14391	0.0184	13415	15366	14147	2.41	54	0.251
12	20000 (UM)	0.0939	18659	25199	20446	3.75	1788	15.402

TIC:	4.2407	ng/uL
TIM:	4.8008	nmole/L
Total concentration:	4.4255	ng/uL

Sample peak width (sec): 5   Sample min peak height: 50   Sample baseline V to V?: Y   Sample baseline V to V points: 3
Sample filter: Binomial   Number of points for filter: 3   Sample start region (min): 0   Sample end region (min): 30
Marker peak width (sec): 5   Marker min peak height: 200   Marker baseline V to V?: Y   Marker baseline V to V points: 3
Lower marker selection: First peak > 500 RFU   Upper marker selection: Last peak > 200 RFU
Ladder size (bp)75, 200, 300, 400, 500, 700, 1000, 1500, 2000, 3000, 4000, 5000, 7000, 10000, 20000
Quantification using: Lower Marker   Final concentration (ng/uL): 0.5000   Dilution factor: 12.0

Sample: SampC8
Well location: C8
Created: Wednesday, August 11, 2021 5:34:32 PM

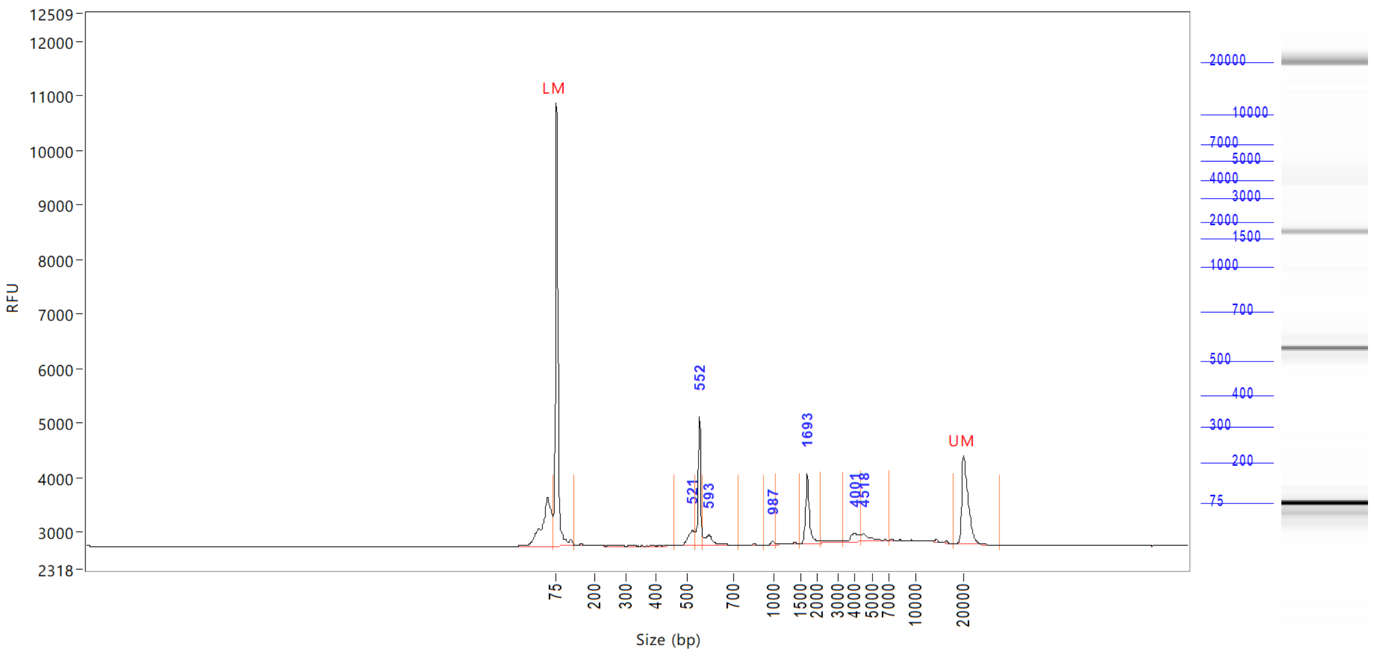

Peak	Size (bp)	Concentration (ng/uL)	From (bp)	To (bp)	Average size (bp)	CV%	RFU	Corrected peak area
1	75 (LM)	0.5000	66	135	76	11.27	8137	51.929
2	521	0.4390	456	534	513	2.76	276	3.799
3	552	1.3753	534	567	549	1.12	2352	11.903
4	593	0.4128	567	731	603	5.15	205	3.572
5	987	0.0438	922	1012	984	1.23	76	0.379
6	1693	0.8984	1478	2144	1705	5.76	1275	7.775
7	4001	0.2403	3286	4311	3960	5.86	155	2.080
8	4518	0.2501	4311	7001	4954	13.56	144	2.165
9	20000 (UM)	0.1355	18049	27617	20473	4.05	1625	14.068

TIC:	3.6597	ng/uL
TIM:	7.7806	nmole/L
Total concentration:	4.0601	ng/uL

```
Sample peak width (sec): 5    Sample min peak height: 50    Sample baseline V to V?: Y    Sample baseline V to V points: 3
Sample filter: Binomial       Number of points for filter: 3  Sample start region (min): 0  Sample end region (min): 30
Marker peak width (sec): 5    Marker min peak height: 200   Marker baseline V to V?: Y    Marker baseline V to V points: 3
Lower marker selection: First peak > 500 RFU                Upper marker selection: Last peak > 200 RFU
Ladder size (bp)75, 200, 300, 400, 500, 700, 1000, 1500, 2000, 3000, 4000, 5000, 7000, 10000, 20000
Quantification using: Lower Marker          Final concentration (ng/uL): 0.5000          Dilution factor: 12.0
```

Sample: SampD8
Well location: D8
Created: Wednesday, August 11, 2021 5:34:32 PM

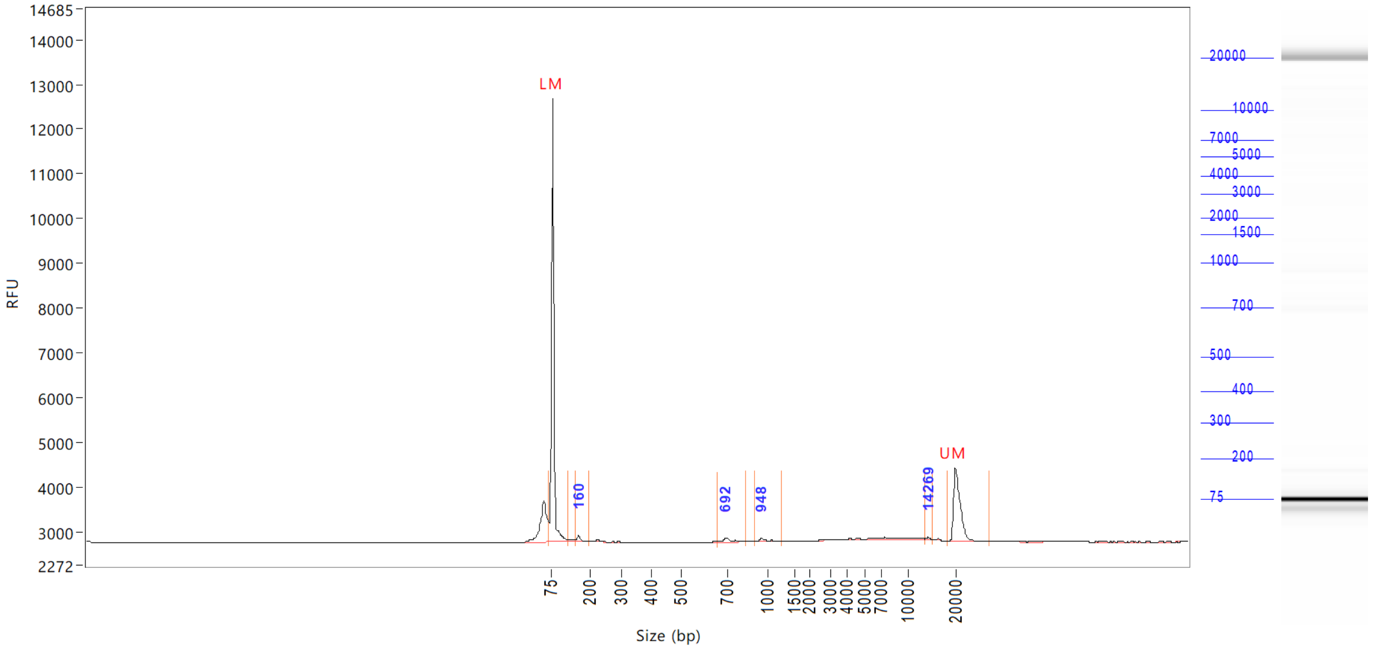

Peak	Size (bp)	Concentration (ng/uL)	From (bp)	To (bp)	Average size (bp)	CV%	RFU	Corrected peak area
1	75 (LM)	0.5000	64	128	74	9.70	9907	60.805
2	160	0.1031	148	194	160	4.81	125	1.045
3	692	0.1255	657	831	710	5.02	98	1.272
4	948	0.0852	905	1245	991	6.54	77	0.864
5	14269	0.0248	13537	15366	14134	2.16	55	0.251
6	20000 (UM)	0.1185	18415	27012	20479	4.20	1646	14.412

TIC:	0.3387	ng/uL
TIM:	1.4951	nmole/L
Total concentration:	0.7809	ng/uL

Sample peak width (sec): 5 Sample min peak height: 50 Sample baseline V to V?: Y Sample baseline V to V points: 3
Sample filter: Binomial Number of points for filter: 3 Sample start region (min): 0 Sample end region (min): 30
Marker peak width (sec): 5 Marker min peak height: 200 Marker baseline V to V?: Y Marker baseline V to V points: 3
Lower marker selection: First peak > 500 RFU Upper marker selection: Last peak > 200 RFU
Ladder size (bp)75, 200, 300, 400, 500, 700, 1000, 1500, 2000, 3000, 4000, 5000, 7000, 10000, 20000
Quantification using: Lower Marker Final concentration (ng/uL): 0.5000 Dilution factor: 12.0

Sample: SampE8
Well location: E8
Created: Wednesday, August 11, 2021 5:34:32 PM

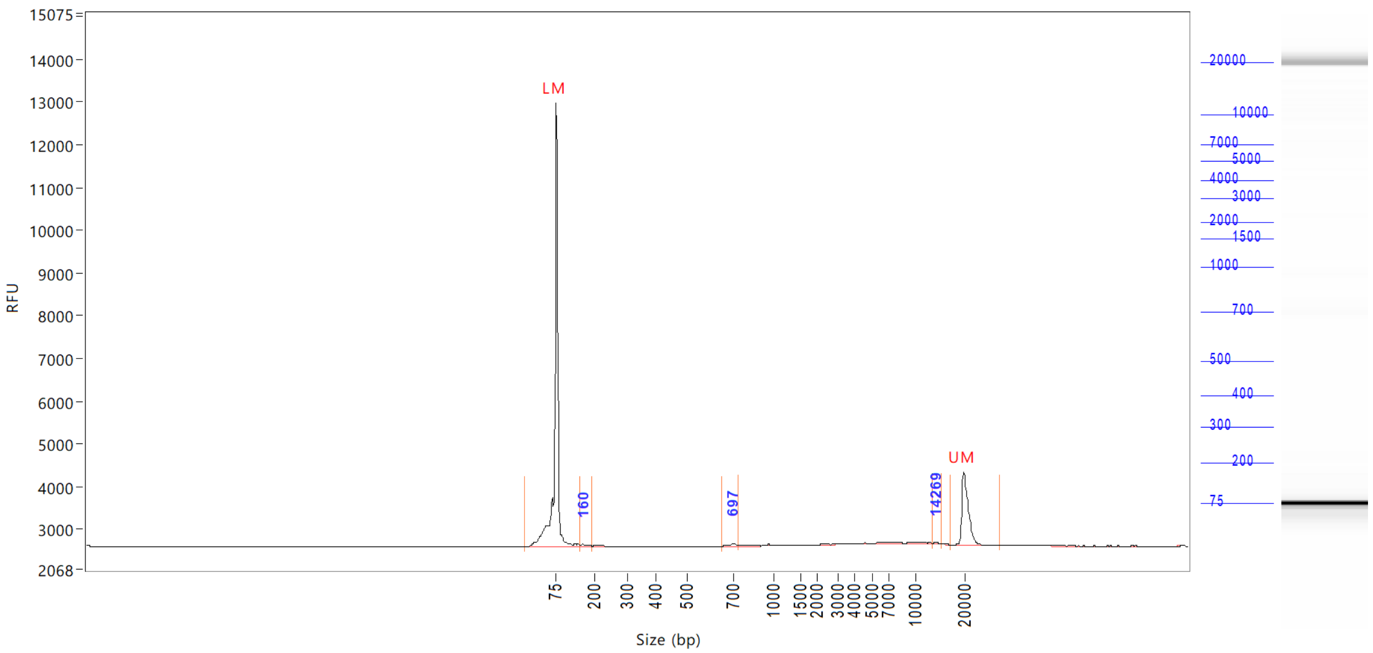

Peak	Size (bp)	Concentration (ng/uL)	From (bp)	To (bp)	Average size (bp)	CV%	RFU	Corrected peak area
1	75 (LM)	0.5000	0	152	67	27.92	10396	82.005
2	160	0.0491	152	192	165	6.47	58	0.672
3	697	0.0573	646	740	695	2.68	71	0.784
4	14269	0.0178	13415	15244	14043	2.17	53	0.243
5	20000 (UM)	0.0914	17195	27496	20388	4.35	1709	14.987

TIC:	0.1243	ng/uL
TIM:	0.6274	nmole/L
Total concentration:	0.4369	ng/uL

```
Sample peak width (sec): 5    Sample min peak height: 50    Sample baseline V to V?: Y    Sample baseline V to V points: 3
Sample filter: Binomial       Number of points for filter: 3  Sample start region (min): 0  Sample end region (min): 30
Marker peak width (sec): 5    Marker min peak height: 200   Marker baseline V to V?: Y    Marker baseline V to V points: 3
Lower marker selection: First peak > 500 RFU                Upper marker selection: Last peak > 200 RFU
Ladder size (bp)75, 200, 300, 400, 500, 700, 1000, 1500, 2000, 3000, 4000, 5000, 7000, 10000, 20000
Quantification using: Lower Marker          Final concentration (ng/uL): 0.5000          Dilution factor: 12.0
```

Sample: SampF8
Well location: F8
Created: Wednesday, August 11, 2021 5:34:32 PM

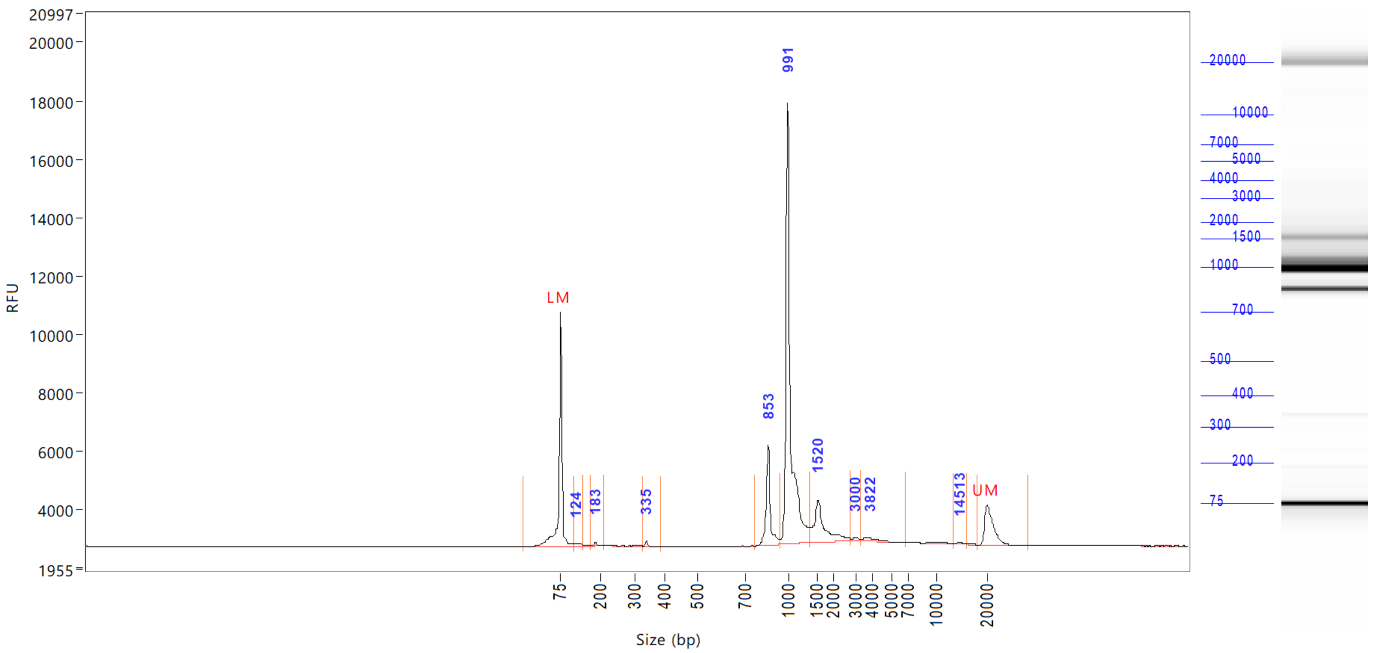

Peak	Size (bp)	Concentration (ng/uL)	From (bp)	To (bp)	Average size (bp)	CV%	RFU	Corrected peak area
1	75 (LM)	0.5000	0	116	67	24.04	8002	61.526
2	124	0.1361	116	145	128	6.33	93	1.396
3	183	0.1118	169	210	182	4.12	147	1.147
4	335	0.1061	325	384	336	2.36	212	1.088
5	853	2.2564	757	939	854	2.97	3408	23.138
6	991	11.6179	939	1378	1033	8.49	15128	119.133
7	1520	2.2858	1378	2800	1698	18.81	1446	23.439
8	3000	0.1256	2800	3322	3041	5.02	101	1.288
9	3822	0.2260	3322	6630	3990	15.14	100	2.318
10	14513	0.0522	13415	15854	14527	4.42	55	0.535
11	20000 (UM)	0.1227	18049	28221	20615	5.32	1350	15.104

TIC:	16.9180	ng/uL
TIM:	28.5203	nmole/L
Total concentration:	17.1852	ng/uL

```
Sample peak width (sec): 5    Sample min peak height: 50    Sample baseline V to V?: Y    Sample baseline V to V points: 3
Sample filter: Binomial       Number of points for filter: 3  Sample start region (min): 0  Sample end region (min): 30
Marker peak width (sec): 5    Marker min peak height: 200   Marker baseline V to V?: Y    Marker baseline V to V points: 3
Lower marker selection: First peak > 500 RFU                Upper marker selection: Last peak > 200 RFU
Ladder size (bp)75, 200, 300, 400, 500, 700, 1000, 1500, 2000, 3000, 4000, 5000, 7000, 10000, 20000
Quantification using: Lower Marker          Final concentration (ng/uL): 0.5000          Dilution factor: 12.0
```

Sample: SampG8
Well location: G8
Created: Wednesday, August 11, 2021 5:34:32 PM

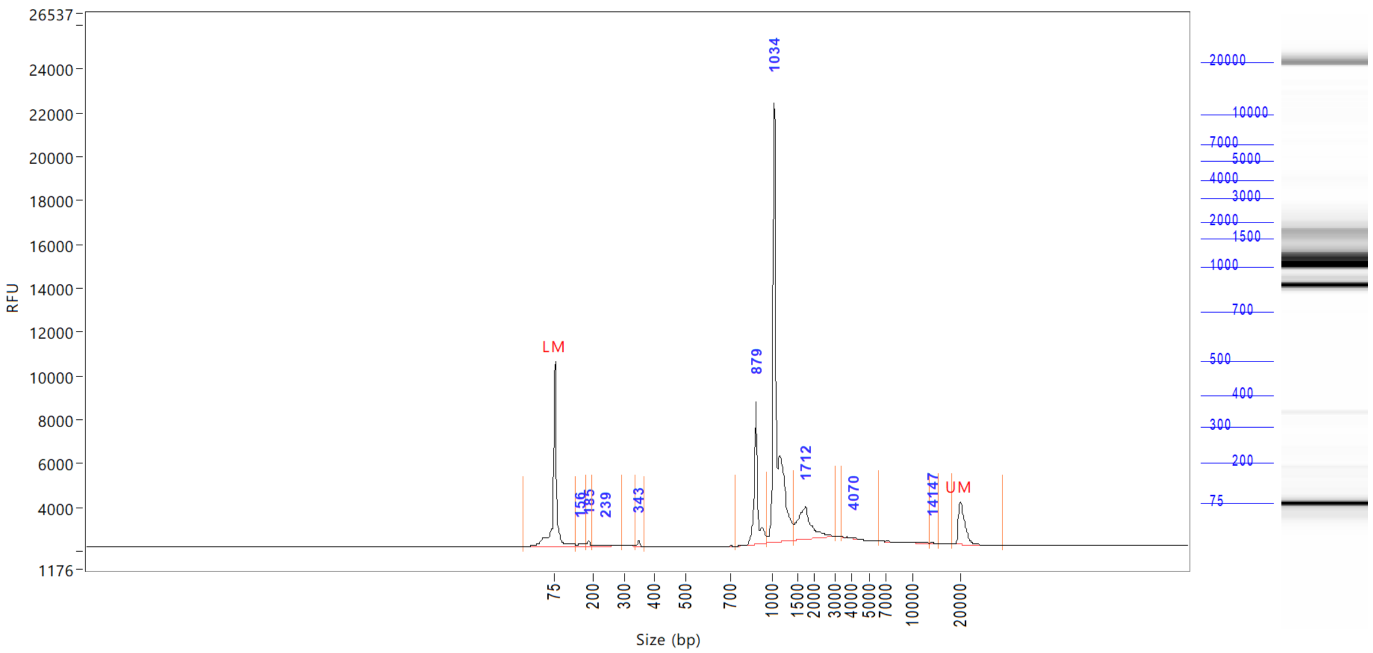

Peak	Size (bp)	Concentration (ng/uL)	From (bp)	To (bp)	Average size (bp)	CV%	RFU	Corrected peak area
1	75 (LM)	0.5000	0	145	69	27.27	8469	70.824
2	156	0.1852	145	177	161	5.84	136	2.186
3	185	0.1280	177	198	183	2.65	217	1.511
4	239	0.1372	198	289	229	8.28	68	1.620
5	343	0.1133	331	366	341	1.22	277	1.338
6	879	3.3836	723	965	882	2.93	6512	39.940
7	1034	12.5059	965	1411	1090	9.01	20075	147.621
8	1712	2.7388	1411	3037	1731	15.93	1490	32.329
9	4070	0.0686	3322	6112	4045	9.25	66	0.810
10	14147	0.0247	13415	15488	14049	2.63	62	0.292
11	20000 (UM)	0.1162	18293	28947	20454	4.38	1968	16.460

TIC:	19.2854	ng/uL
TIM:	32.4074	nmole/L
Total concentration:	19.3997	ng/uL

```
Sample peak width (sec): 5    Sample min peak height: 50    Sample baseline V to V?: Y    Sample baseline V to V points: 3
Sample filter: Binomial       Number of points for filter: 3  Sample start region (min): 0  Sample end region (min): 30
Marker peak width (sec): 5    Marker min peak height: 200   Marker baseline V to V?: Y    Marker baseline V to V points: 3
Lower marker selection: First peak > 500 RFU                Upper marker selection: Last peak > 200 RFU
Ladder size (bp)75, 200, 300, 400, 500, 700, 1000, 1500, 2000, 3000, 4000, 5000, 7000, 10000, 20000
Quantification using: Lower Marker          Final concentration (ng/uL): 0.5000          Dilution factor: 12.0
```

Sample: SampH8
Well location: H8
Created: Wednesday, August 11, 2021 5:34:32 PM

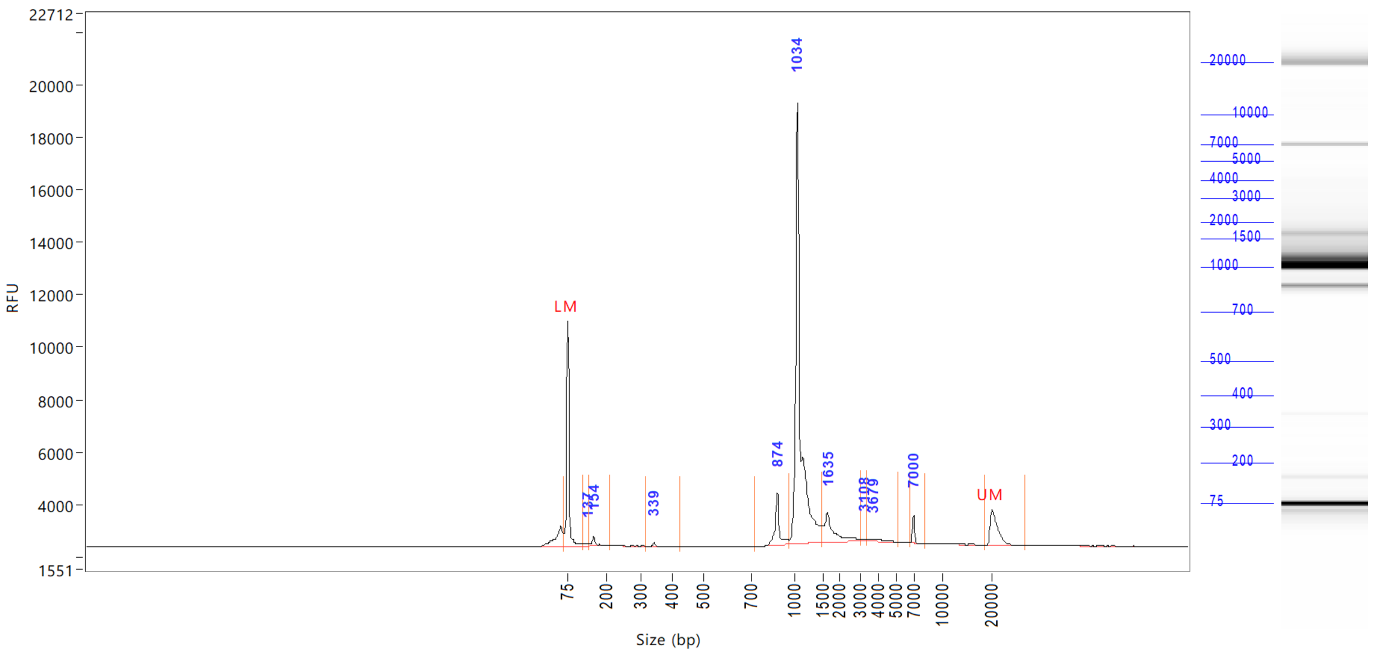

Peak	Size (bp)	Concentration (ng/uL)	From (bp)	To (bp)	Average size (bp)	CV%	RFU	Corrected peak area
1	75 (LM)	0.5000	64	122	74	10.02	8577	53.220
2	137	0.1228	122	143	133	4.83	81	1.090
3	154	0.3606	143	205	158	8.00	358	3.198
4	339	0.0979	311	424	340	5.25	147	0.869
5	874	1.6527	718	961	875	3.45	1988	14.660
6	1034	14.9424	961	1489	1089	10.44	16805	132.539
7	1635	1.9494	1489	2971	1840	19.55	1103	17.291
8	3108	0.0940	2971	3322	3127	3.58	95	0.834
9	3679	0.2371	3322	5075	3875	9.21	94	2.103
10	7000	0.3741	6556	8201	6903	1.71	1077	3.318
11	20000 (UM)	0.1256	18659	26408	20596	4.83	1365	13.370

TIC:	19.8311	ng/uL
TIM:	33.4193	nmole/L
Total concentration:	19.9939	ng/uL

Sample peak width (sec): 5   Sample min peak height: 50   Sample baseline V to V?: Y   Sample baseline V to V points: 3
Sample filter: Binomial   Number of points for filter: 3   Sample start region (min): 0   Sample end region (min): 30
Marker peak width (sec): 5   Marker min peak height: 200   Marker baseline V to V?: Y   Marker baseline V to V points: 3
Lower marker selection: First peak > 500 RFU   Upper marker selection: Last peak > 200 RFU
Ladder size (bp)75, 200, 300, 400, 500, 700, 1000, 1500, 2000, 3000, 4000, 5000, 7000, 10000, 20000
Quantification using: Lower Marker   Final concentration (ng/uL): 0.5000   Dilution factor: 12.0

Sample: SampA9
Well location: A9
Created: Wednesday, August 11, 2021 5:34:32 PM

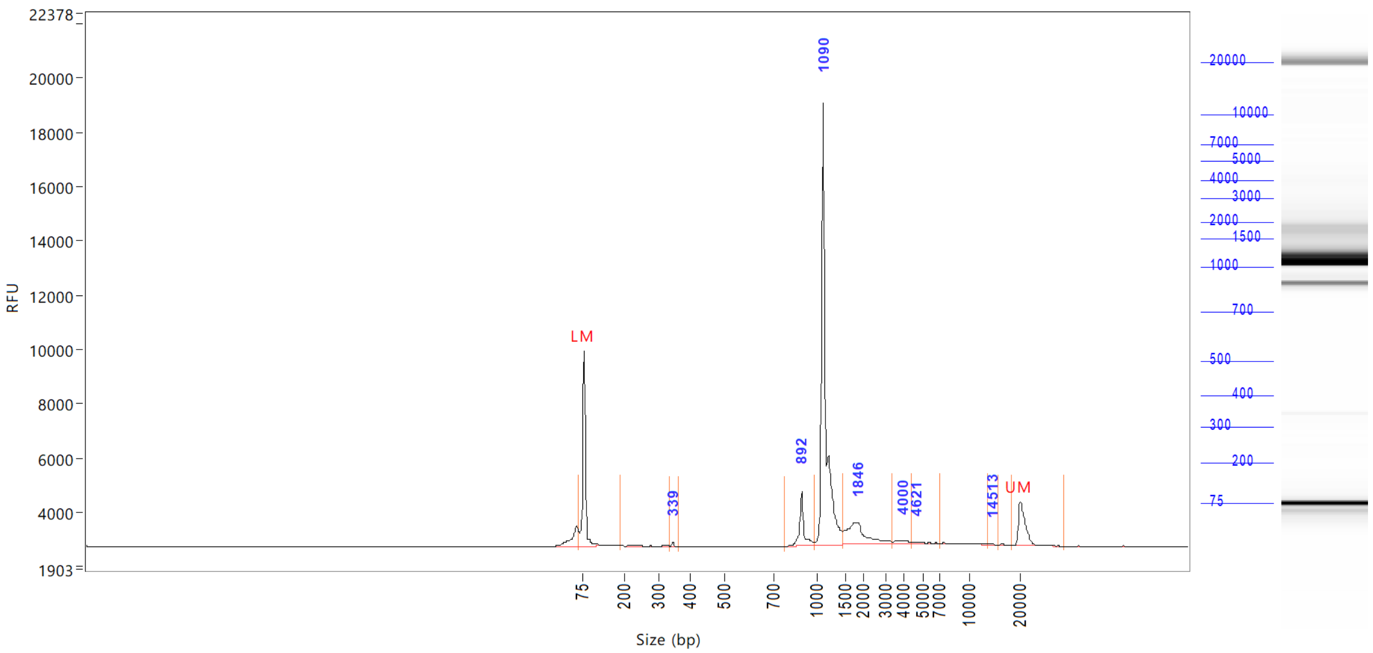

Peak	Size (bp)	Concentration (ng/uL)	From (bp)	To (bp)	Average size (bp)	CV%	RFU	Corrected peak area
1	75 (LM)	0.5000	62	187	76	16.41	7173	45.628
2	339	0.0936	331	362	338	1.06	152	0.712
3	892	1.8133	779	987	893	3.38	2009	13.790
4	1090	14.7046	987	1445	1125	7.86	16314	111.823
5	1846	2.9373	1445	3393	1895	21.94	788	22.337
6	4000	0.2194	3393	4380	3880	6.87	94	1.669
7	4621	0.0984	4380	7068	5003	12.99	51	0.749
8	14513	0.0360	13659	15732	14364	2.94	54	0.274
9	20000 (UM)	0.1500	18415	28463	20462	4.05	1616	13.693

TIC:	19.9027	ng/uL
TIM:	27.9932	nmole/L
Total concentration:	20.1782	ng/uL

Sample peak width (sec): 5   Sample min peak height: 50   Sample baseline V to V?: Y   Sample baseline V to V points: 3
Sample filter: Binomial   Number of points for filter: 3   Sample start region (min): 0   Sample end region (min): 30
Marker peak width (sec): 5   Marker min peak height: 200   Marker baseline V to V?: Y   Marker baseline V to V points: 3
Lower marker selection: First peak > 500 RFU   Upper marker selection: Last peak > 200 RFU
Ladder size (bp)75, 200, 300, 400, 500, 700, 1000, 1500, 2000, 3000, 4000, 5000, 7000, 10000, 20000
Quantification using: Lower Marker   Final concentration (ng/uL): 0.5000   Dilution factor: 12.0

Sample: SampB9
Well location: B9
Created: Wednesday, August 11, 2021 5:34:32 PM

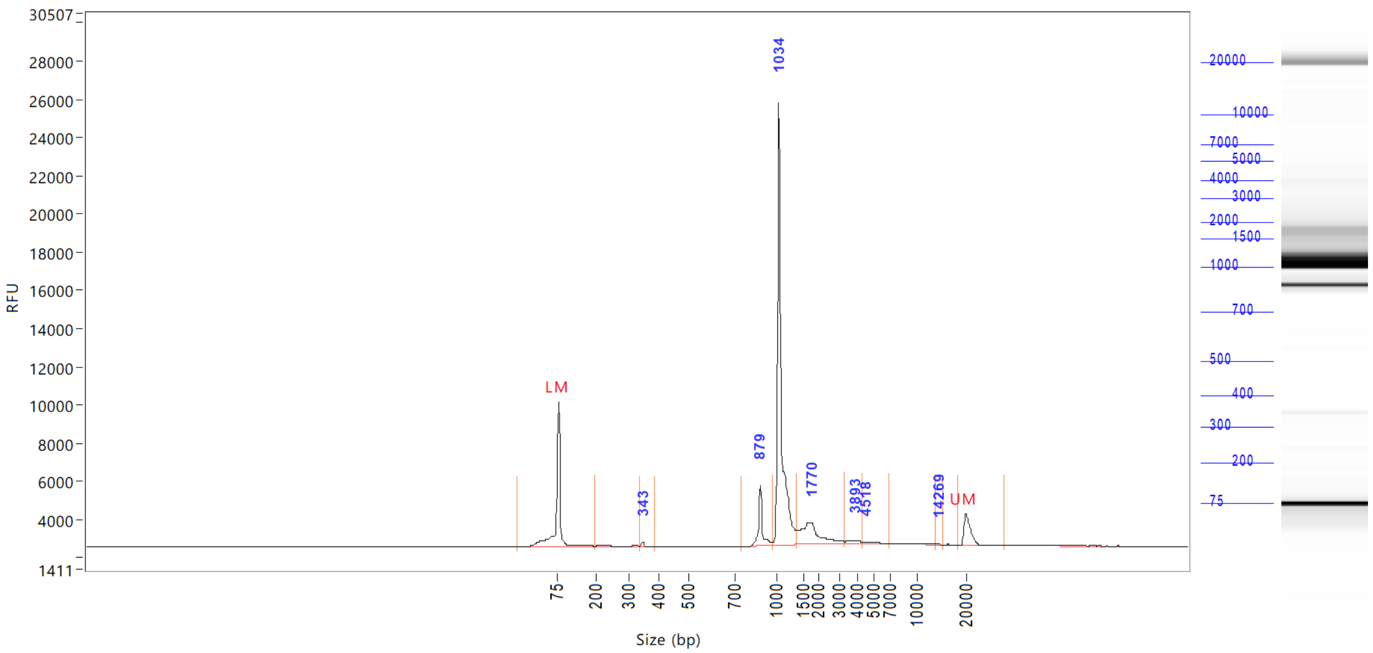

Peak	Size (bp)	Concentration (ng/uL)	From (bp)	To (bp)	Average size (bp)	CV%	RFU	Corrected peak area
1	75 (LM)	0.5000	0	194	65	41.21	7576	68.765
2	343	0.0946	333	388	341	1.49	232	1.084
3	879	1.7812	740	970	881	3.13	3134	20.414
4	1034	13.3461	970	1389	1074	7.94	23181	152.958
5	1770	2.8197	1389	3251	1815	22.21	1128	32.317
6	3893	0.2558	3251	4311	3783	7.40	163	2.931
7	4518	0.1112	4311	6926	4845	11.02	80	1.274
8	14269	0.0205	13537	15366	14136	2.24	53	0.235
9	20000 (UM)	0.1062	18415	27979	20477	4.24	1661	14.612

TIC:	18.4291	ng/uL
TIM:	26.9465	nmole/L
Total concentration:	18.6049	ng/uL

Sample peak width (sec): 5 Sample min peak height: 50 Sample baseline V to V?: Y Sample baseline V to V points: 3
Sample filter: Binomial Number of points for filter: 3 Sample start region (min): 0 Sample end region (min): 30
Marker peak width (sec): 5 Marker min peak height: 200 Marker baseline V to V?: Y Marker baseline V to V points: 3
Lower marker selection: First peak > 500 RFU Upper marker selection: Last peak > 200 RFU
Ladder size (bp)75, 200, 300, 400, 500, 700, 1000, 1500, 2000, 3000, 4000, 5000, 7000, 10000, 20000
Quantification using: Lower Marker Final concentration (ng/uL): 0.5000 Dilution factor: 12.0

Sample: SampC9
Well location: C9
Created: Wednesday, August 11, 2021 5:34:32 PM

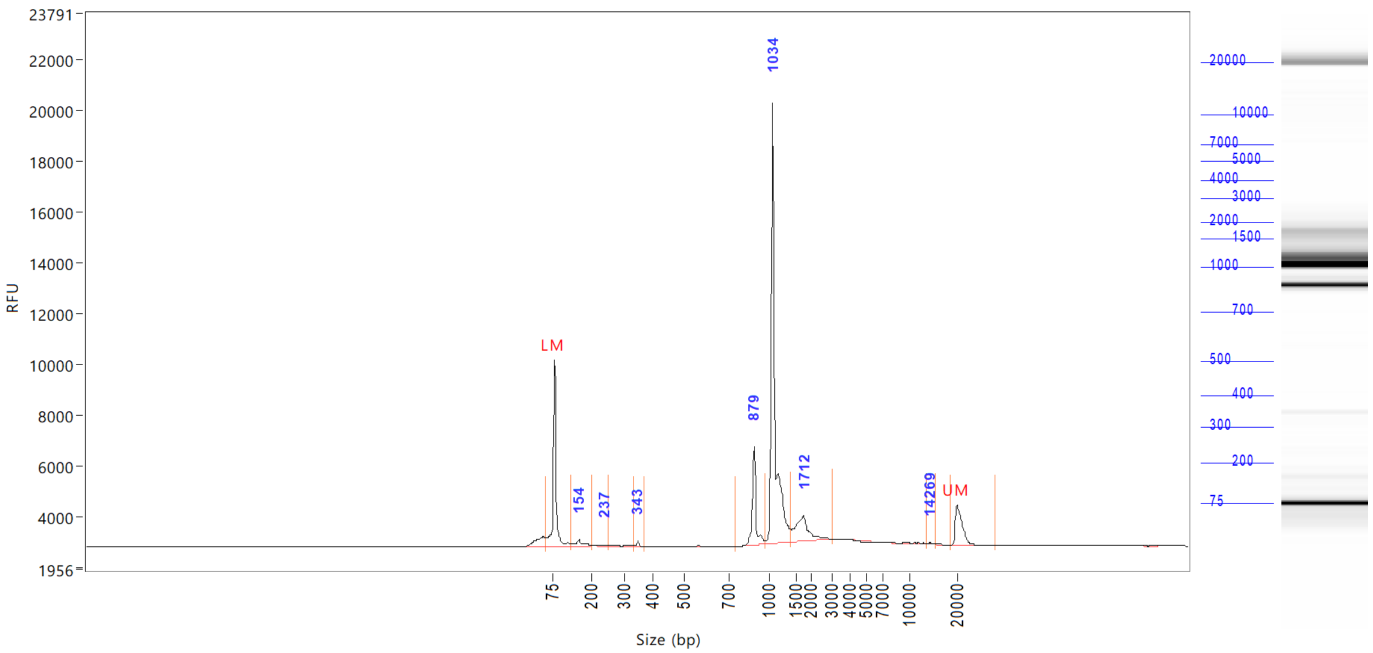

Peak	Size (bp)	Concentration (ng/uL)	From (bp)	To (bp)	Average size (bp)	CV%	RFU	Corrected peak area
1	75 (LM)	0.5000	47	129	74	15.30	7330	52.763
2	154	0.4566	129	200	155	10.01	242	4.016
3	237	0.0990	200	248	225	4.92	52	0.870
4	343	0.1244	331	368	342	1.31	223	1.094
5	879	2.5922	736	965	881	2.71	3845	22.795
6	1034	13.1638	965	1400	1080	8.39	17330	115.760
7	1712	2.3324	1400	2971	1704	14.96	992	20.511
8	14269	0.0377	13537	15610	14265	3.38	59	0.332
9	20000 (UM)	0.1386	18415	27979	20545	4.68	1604	14.628

TIC:	18.8062	ng/uL
TIM:	33.3266	nmole/L
Total concentration:	19.0650	ng/uL

```
Sample peak width (sec): 5    Sample min peak height: 50    Sample baseline V to V?: Y    Sample baseline V to V points: 3
Sample filter: Binomial       Number of points for filter: 3  Sample start region (min): 0  Sample end region (min): 30
Marker peak width (sec): 5    Marker min peak height: 200   Marker baseline V to V?: Y    Marker baseline V to V points: 3
Lower marker selection: First peak > 500 RFU                Upper marker selection: Last peak > 200 RFU
Ladder size (bp)75, 200, 300, 400, 500, 700, 1000, 1500, 2000, 3000, 4000, 5000, 7000, 10000, 20000
Quantification using: Lower Marker          Final concentration (ng/uL): 0.5000          Dilution factor: 12.0
```

Sample: SampD9
Well location: D9
Created: Wednesday, August 11, 2021 5:34:32 PM

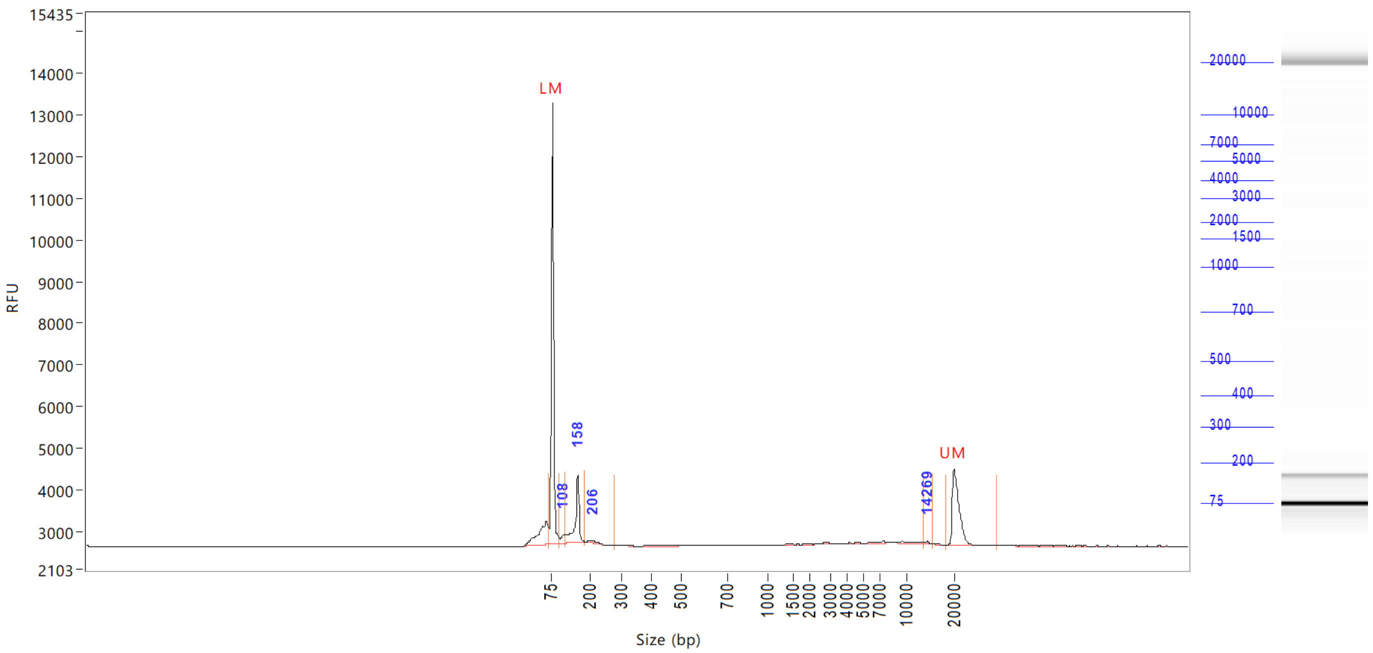

Peak	Size (bp)	Concentration (ng/uL)	From (bp)	To (bp)	Average size (bp)	CV%	RFU	Corrected peak area
1	75 (LM)	0.5000	64	97	73	5.85	10596	62.625
2	108	0.2275	97	116	107	5.24	213	2.374
3	158	1.4459	116	181	148	8.33	1614	15.092
4	206	0.1091	181	277	209	7.68	66	1.138
5	14269	0.0362	13537	15610	14252	3.28	66	0.378
6	20000 (UM)	0.1341	18293	28947	20527	4.65	1826	16.798

TIC:	1.8187	ng/uL
TIM:	20.4103	nmole/L
Total concentration:	2.4101	ng/uL

```
Sample peak width (sec): 5    Sample min peak height: 50    Sample baseline V to V?: Y    Sample baseline V to V points: 3
Sample filter: Binomial       Number of points for filter: 3  Sample start region (min): 0  Sample end region (min): 30
Marker peak width (sec): 5    Marker min peak height: 200   Marker baseline V to V?: Y    Marker baseline V to V points: 3
Lower marker selection: First peak > 500 RFU                Upper marker selection: Last peak > 200 RFU
Ladder size (bp)75, 200, 300, 400, 500, 700, 1000, 1500, 2000, 3000, 4000, 5000, 7000, 10000, 20000
Quantification using: Lower Marker          Final concentration (ng/uL): 0.5000          Dilution factor: 12.0
```

Sample: SampE9
Well location: E9
Created: Wednesday, August 11, 2021 5:34:32 PM

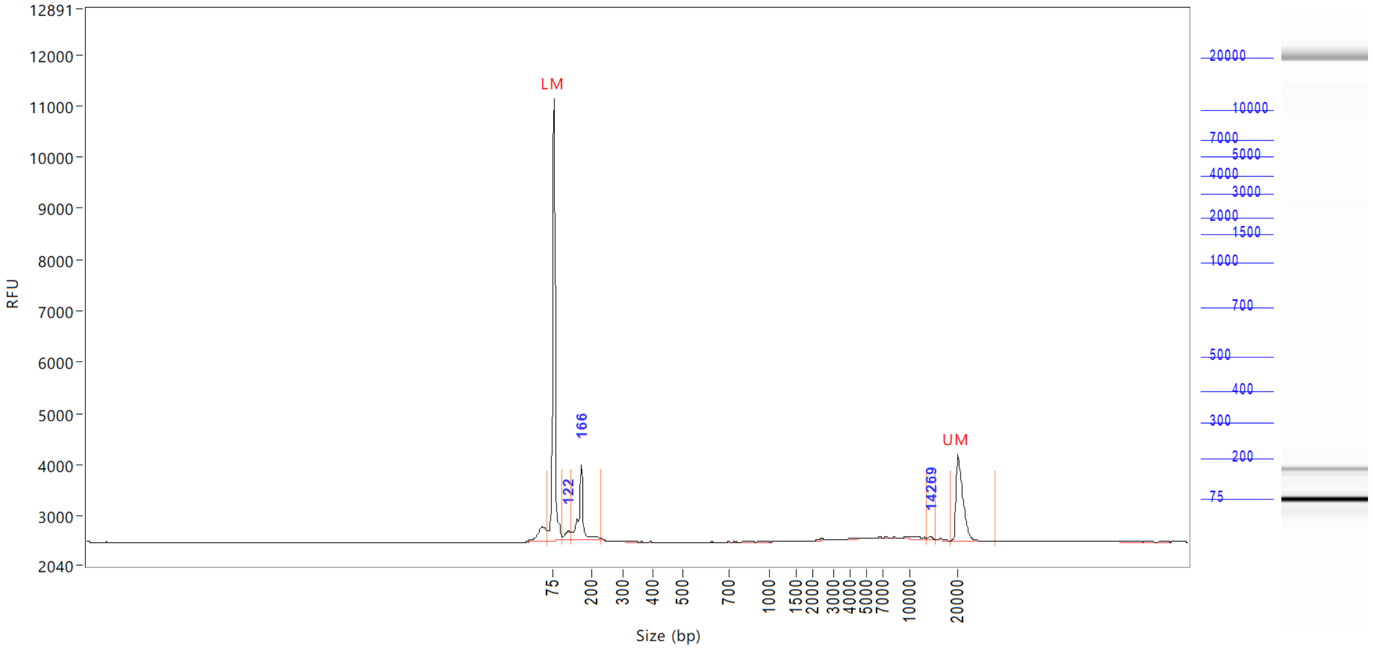

Peak	Size (bp)	Concentration (ng/uL)	From (bp)	To (bp)	Average size (bp)	CV%	RFU	Corrected peak area
1	75 (LM)	0.5000	54	103	73	7.78	8635	57.348
2	122	0.2499	103	131	119	6.63	179	2.389
3	166	1.5786	131	228	163	10.79	1446	15.088
4	14269	0.0439	13537	15244	14230	2.99	67	0.420
5	20000 (UM)	0.1317	18293	27859	20533	4.35	1677	15.106

TIC:	1.8724	ng/uL
TIM:	19.3743	nmole/L
Total concentration:	2.3406	ng/uL

Sample peak width (sec): 5   Sample min peak height: 50   Sample baseline V to V?: Y   Sample baseline V to V points: 3
Sample filter: Binomial   Number of points for filter: 3 Sample start region (min): 0 Sample end region (min): 30
Marker peak width (sec): 5   Marker min peak height: 200   Marker baseline V to V?: Y   Marker baseline V to V points: 3
Lower marker selection: First peak > 500 RFU   Upper marker selection: Last peak > 200 RFU
Ladder size (bp)75, 200, 300, 400, 500, 700, 1000, 1500, 2000, 3000, 4000, 5000, 7000, 10000, 20000
Quantification using: Lower Marker   Final concentration (ng/uL): 0.5000   Dilution factor: 12.0

Sample: SampF9
Well location: F9
Created: Wednesday, August 11, 2021 5:34:32 PM

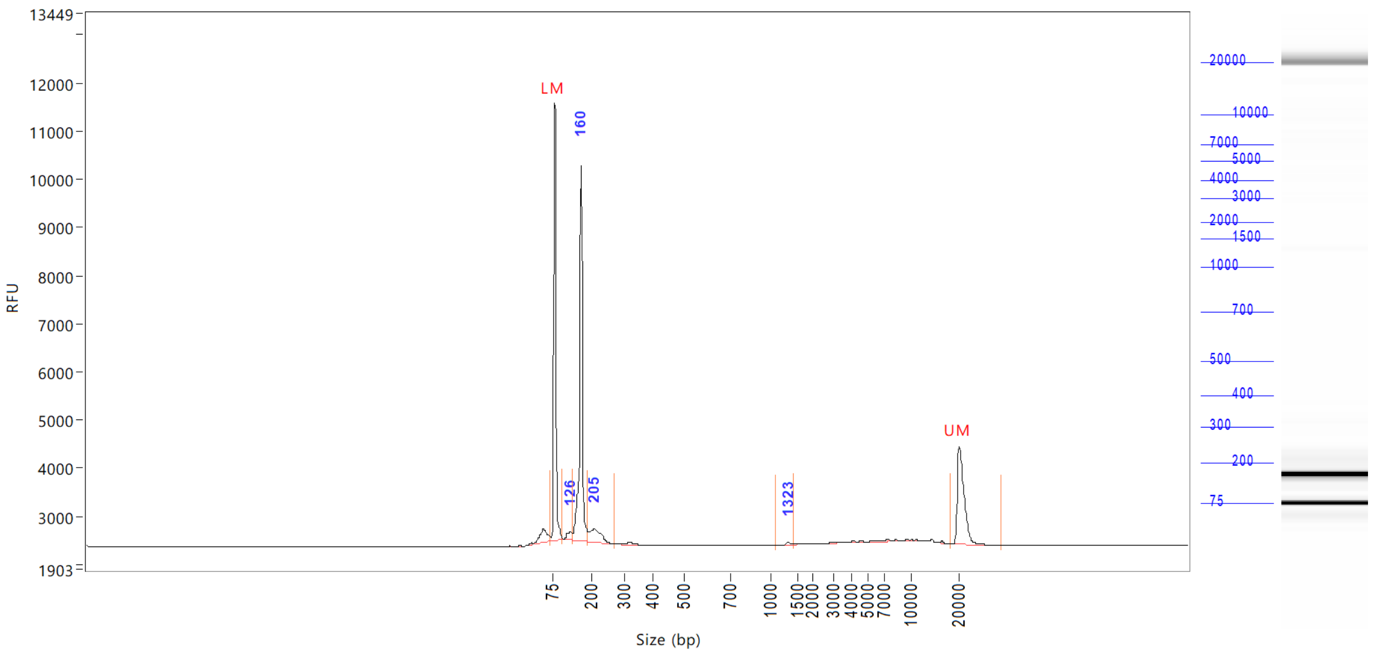

Peak	Size (bp)	Concentration (ng/uL)	From (bp)	To (bp)	Average size (bp)	CV%	RFU	Corrected peak area
1	75 (LM)	0.5000	64	103	74	5.30	9102	52.367
2	126	0.2411	103	133	122	6.00	164	2.105
3	160	6.2708	133	185	159	4.59	7798	54.731
4	205	0.8315	185	268	209	7.44	273	7.257
5	1323	0.0410	1101	1422	1311	3.25	59	0.358
6	20000 (UM)	0.1724	18171	28947	20533	4.60	2027	18.056

TIC:	7.3844	ng/uL
TIM:	74.6649	nmole/L
Total concentration:	7.8273	ng/uL

Sample peak width (sec): 5 Sample min peak height: 50 Sample baseline V to V?: Y Sample baseline V to V points: 3
Sample filter: Binomial Number of points for filter: 3 Sample start region (min): 0 Sample end region (min): 30
Marker peak width (sec): 5 Marker min peak height: 200 Marker baseline V to V?: Y Marker baseline V to V points: 3
Lower marker selection: First peak > 500 RFU Upper marker selection: Last peak > 200 RFU
Ladder size (bp)75, 200, 300, 400, 500, 700, 1000, 1500, 2000, 3000, 4000, 5000, 7000, 10000, 20000
Quantification using: Lower Marker Final concentration (ng/uL): 0.5000 Dilution factor: 12.0

Sample: SampG9
Well location: G9
Created: Wednesday, August 11, 2021 5:34:32 PM

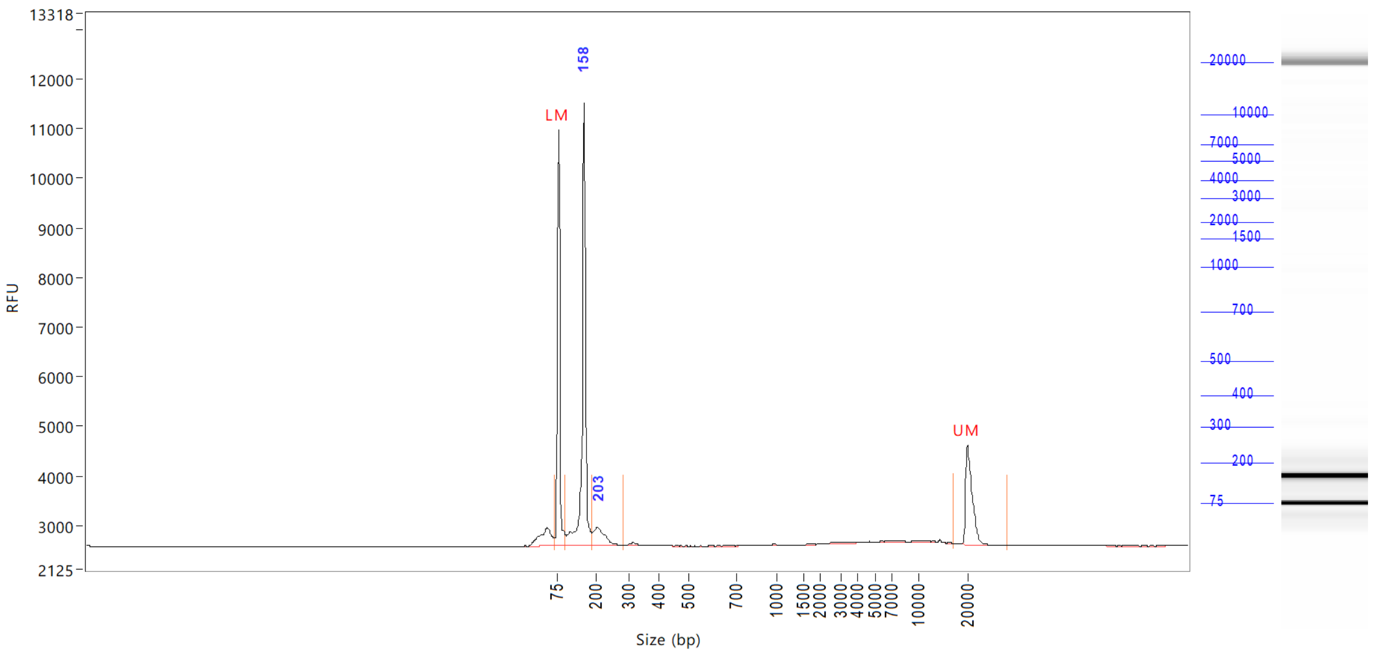

Peak	Size	Concentration	From	To	Average size	CV%	RFU	Corrected peak area
	(bp)	(ng/uL)	(bp)	(bp)	(bp)			
1	75 (LM)	0.5000	64	101	75	7.13	8377	50.096
2	158	8.1372	101	183	153	8.63	8924	67.941
3	203	1.1054	183	282	208	7.97	350	9.229
4	20000 (UM)	0.1748	17195	28342	20417	4.15	2009	17.509

TIC:	9.2426	ng/uL
TIM:	96.1509	nmole/L
Total concentration:	9.7535	ng/uL

Sample peak width (sec): 5   Sample min peak height: 50   Sample baseline V to V?: Y   Sample baseline V to V points: 3
Sample filter: Binomial   Number of points for filter: 3 Sample start region (min): 0 Sample end region (min): 30
Marker peak width (sec): 5   Marker min peak height: 200   Marker baseline V to V?: Y   Marker baseline V to V points: 3
Lower marker selection: First peak > 500 RFU   Upper marker selection: Last peak > 200 RFU
Ladder size (bp)75, 200, 300, 400, 500, 700, 1000, 1500, 2000, 3000, 4000, 5000, 7000, 10000, 20000
Quantification using: Lower Marker   Final concentration (ng/uL): 0.5000   Dilution factor: 12.0

Sample: SampH9
Well location: H9
Created: Wednesday, August 11, 2021 5:34:32 PM

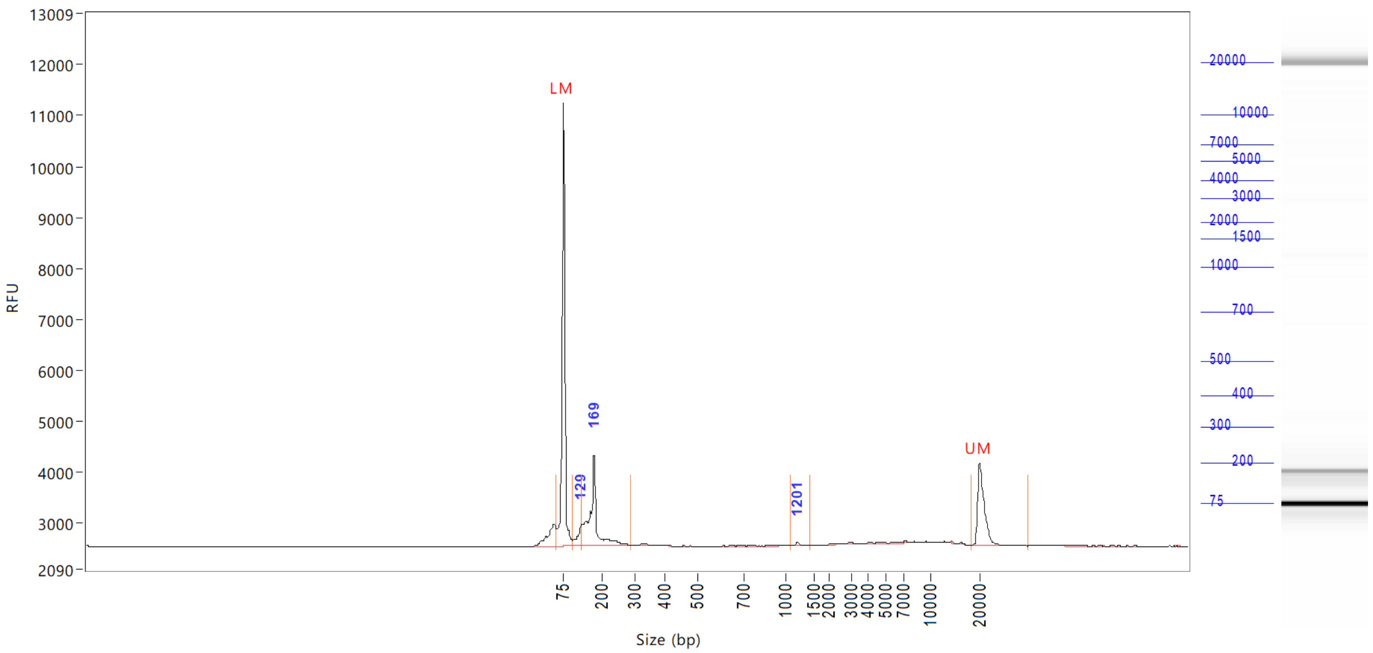

Peak	Size (bp)	Concentration (ng/uL)	From (bp)	To (bp)	Average size (bp)	CV%	RFU	Corrected peak area
1	75 (LM)	0.5000	54	101	72	8.21	8717	61.698
2	129	0.3972	101	131	120	7.55	382	4.084
3	169	2.5528	131	286	169	15.51	1781	26.250
4	1201	0.0503	1068	1445	1217	5.87	68	0.518
5	20000 (UM)	0.1144	18293	29793	20430	5.04	1599	14.112

TIC:	3.0003	ng/uL
TIM:	30.3355	nmole/L
Total concentration:	3.2993	ng/uL

Sample peak width (sec): 5   Sample min peak height: 50   Sample baseline V to V?: Y   Sample baseline V to V points: 3
Sample filter: Binomial   Number of points for filter: 3   Sample start region (min): 0   Sample end region (min): 30
Marker peak width (sec): 5   Marker min peak height: 200   Marker baseline V to V?: Y   Marker baseline V to V points: 3
Lower marker selection: First peak > 500 RFU   Upper marker selection: Last peak > 200 RFU
Ladder size (bp)75, 200, 300, 400, 500, 700, 1000, 1500, 2000, 3000, 4000, 5000, 7000, 10000, 20000
Quantification using: Lower Marker   Final concentration (ng/uL): 0.5000   Dilution factor: 12.0

Sample: SampA10
Well location: A10
Created: Wednesday, August 11, 2021 5:34:32 PM

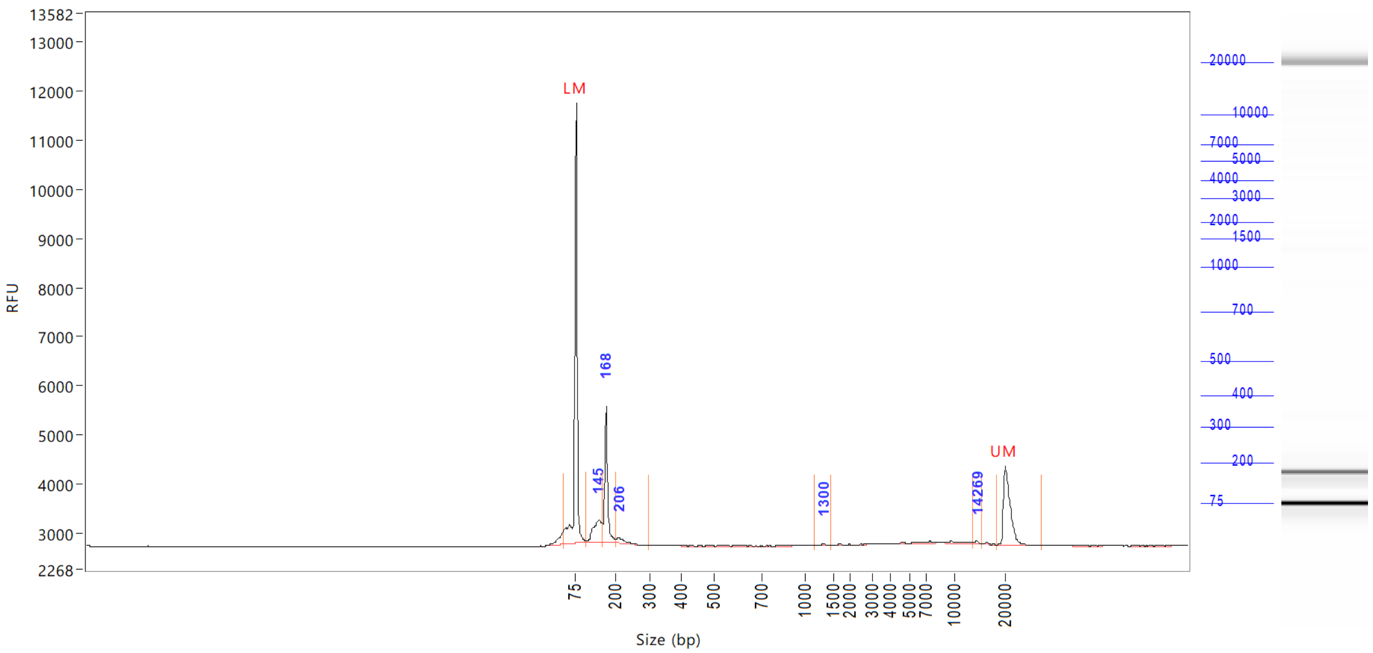

Peak	Size (bp)	Concentration (ng/uL)	From (bp)	To (bp)	Average size (bp)	CV%	RFU	Corrected peak area
1	75 (LM)	0.5000	37	108	71	12.33	8970	55.868
2	145	0.9674	108	158	139	8.61	449	9.008
3	168	1.9542	158	198	168	4.11	2766	18.196
4	206	0.2070	198	295	217	7.66	98	1.928
5	1300	0.0403	1145	1456	1301	4.40	53	0.375
6	14269	0.0444	13415	15244	14162	3.20	65	0.414
7	20000 (UM)	0.1238	18171	27012	20420	4.31	1612	13.832

TIC:	3.2133	ng/uL
TIM:	32.1823	nmole/L
Total concentration:	3.5423	ng/uL

Sample peak width (sec): 5 Sample min peak height: 50 Sample baseline V to V?: Y Sample baseline V to V points: 3
Sample filter: Binomial Number of points for filter: 3 Sample start region (min): 0 Sample end region (min): 30
Marker peak width (sec): 5 Marker min peak height: 200 Marker baseline V to V?: Y Marker baseline V to V points: 3
Lower marker selection: First peak > 500 RFU Upper marker selection: Last peak > 200 RFU
Ladder size (bp)75, 200, 300, 400, 500, 700, 1000, 1500, 2000, 3000, 4000, 5000, 7000, 10000, 20000
Quantification using: Lower Marker Final concentration (ng/uL): 0.5000 Dilution factor: 12.0

Sample: SampB10
Well location: B10
Created: Wednesday, August 11, 2021 5:34:32 PM

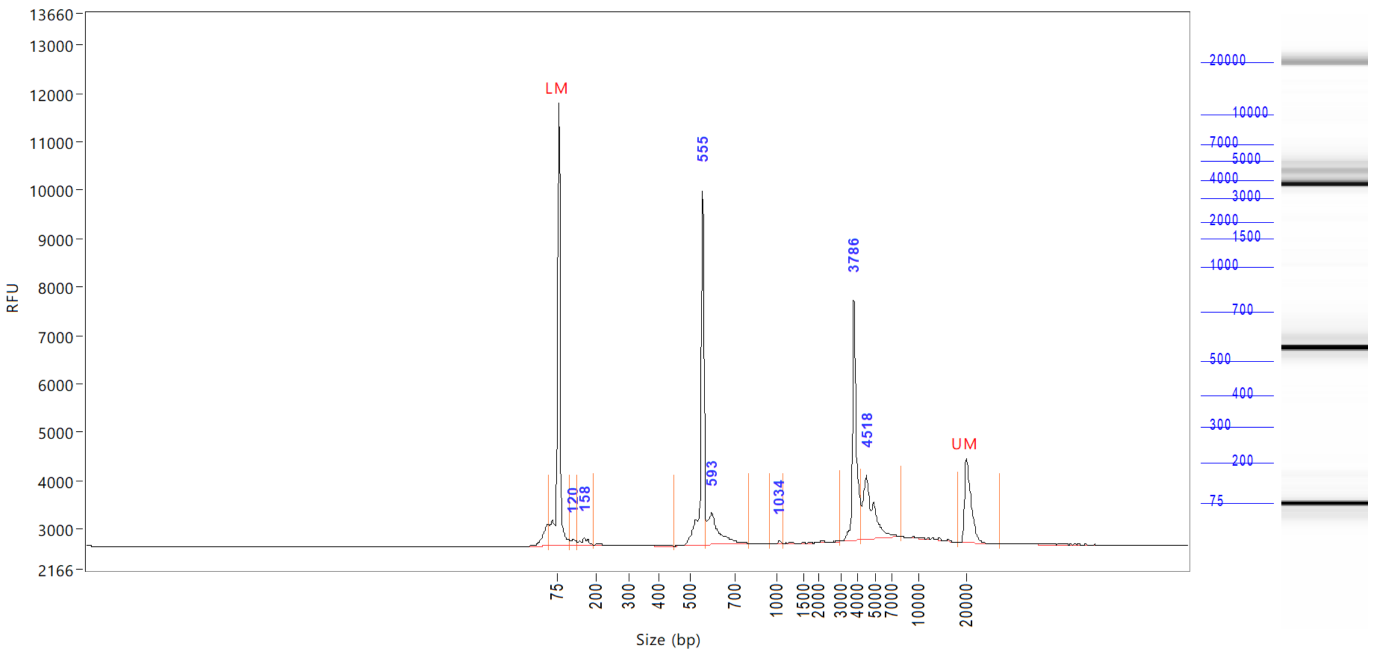

Peak	Size (bp)	Concentration (ng/uL)	From (bp)	To (bp)	Average size (bp)	CV%	RFU	Corrected peak area
1	75 (LM)	0.5000	45	112	72	12.32	9167	61.610
2	120	0.1419	112	133	122	5.00	126	1.457
3	158	0.2352	133	189	154	7.38	153	2.415
4	555	4.1057	447	570	546	2.86	7300	42.158
5	593	1.3533	570	788	613	6.07	658	13.897
6	1034	0.0380	952	1112	1032	3.11	73	0.391
7	3786	3.0129	3000	4242	3830	4.54	4953	30.937
8	4518	1.9208	4242	8067	4819	11.97	1331	19.723
9	20000 (UM)	0.1223	18171	27012	20435	4.05	1745	15.066

TIC:	10.8077	ng/uL
TIM:	22.4393	nmole/L
Total concentration:	10.9533	ng/uL

Sample peak width (sec): 5 Sample min peak height: 50 Sample baseline V to V?: Y Sample baseline V to V points: 3
Sample filter: Binomial Number of points for filter: 3 Sample start region (min): 0 Sample end region (min): 30
Marker peak width (sec): 5 Marker min peak height: 200 Marker baseline V to V?: Y Marker baseline V to V points: 3
Lower marker selection: First peak > 500 RFU Upper marker selection: Last peak > 200 RFU
Ladder size (bp)75, 200, 300, 400, 500, 700, 1000, 1500, 2000, 3000, 4000, 5000, 7000, 10000, 20000
Quantification using: Lower Marker Final concentration (ng/uL): 0.5000 Dilution factor: 12.0

Sample: SampC10
Well location: C10
Created: Wednesday, August 11, 2021 5:34:32 PM

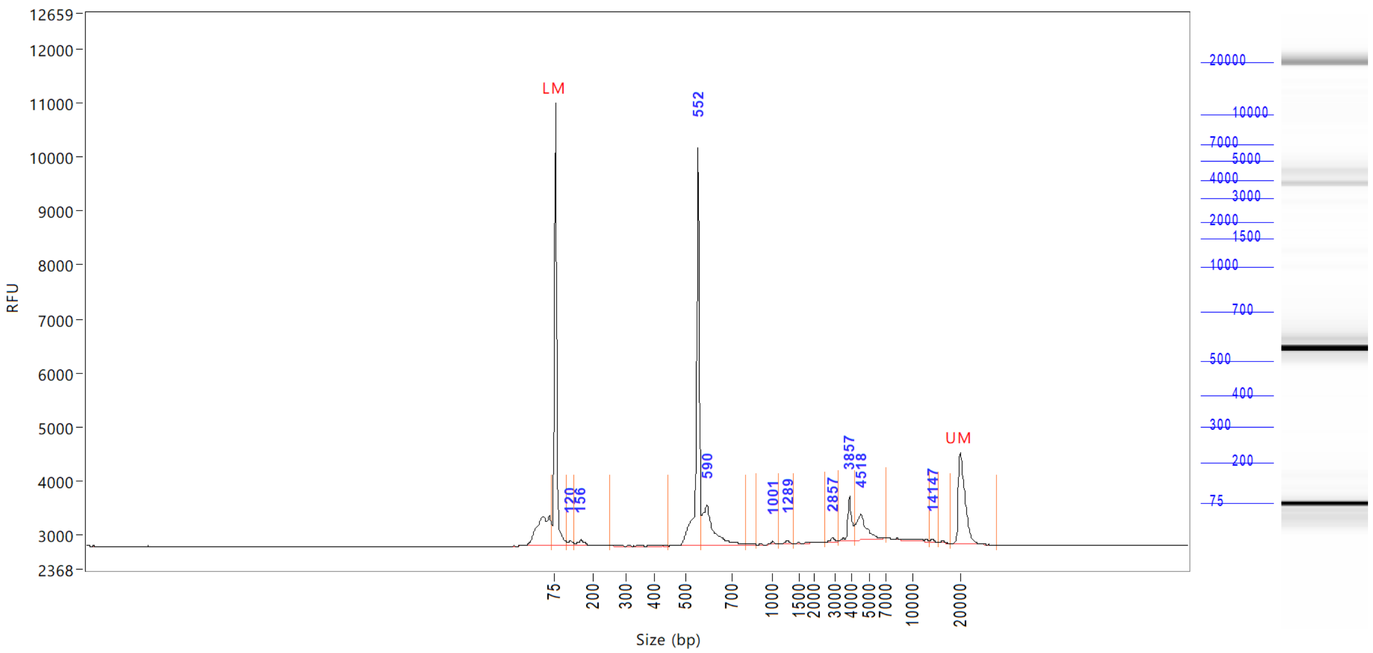

Peak	Size (bp)	Concentration (ng/uL)	From (bp)	To (bp)	Average size (bp)	CV%	RFU	Corrected peak area
1	75 (LM)	0.5000	64	112	74	8.53	8213	50.645
2	120	0.1485	112	137	123	5.80	108	1.253
3	156	0.2638	137	250	167	13.87	112	2.227
4	552	5.1647	445	567	544	2.87	7375	43.595
5	590	1.8248	567	809	612	7.03	747	15.403
6	1001	0.0431	887	1123	1006	4.62	53	0.364
7	1289	0.0570	1123	1400	1264	4.17	66	0.481
8	2857	0.0626	2486	3179	2848	4.79	73	0.529
9	3857	0.6154	3179	4104	3832	4.20	820	5.195
10	4518	0.8575	4104	7068	4610	9.12	479	7.238
11	14147	0.0385	13537	15488	14206	3.00	62	0.325
12	20000 (UM)	0.1462	17927	27617	20464	4.17	1684	14.804

TIC:	9.0761	ng/uL
TIM:	25.8666	nmole/L
Total concentration:	9.2207	ng/uL

```
Sample peak width (sec): 5    Sample min peak height: 50    Sample baseline V to V?: Y    Sample baseline V to V points: 3
Sample filter: Binomial       Number of points for filter: 3  Sample start region (min): 0  Sample end region (min): 30
Marker peak width (sec): 5    Marker min peak height: 200   Marker baseline V to V?: Y    Marker baseline V to V points: 3
Lower marker selection: First peak > 500 RFU                Upper marker selection: Last peak > 200 RFU
Ladder size (bp)75, 200, 300, 400, 500, 700, 1000, 1500, 2000, 3000, 4000, 5000, 7000, 10000, 20000
Quantification using: Lower Marker          Final concentration (ng/uL): 0.5000          Dilution factor: 12.0
```

Sample: SampD10
Well location: D10
Created: Wednesday, August 11, 2021 5:34:32 PM

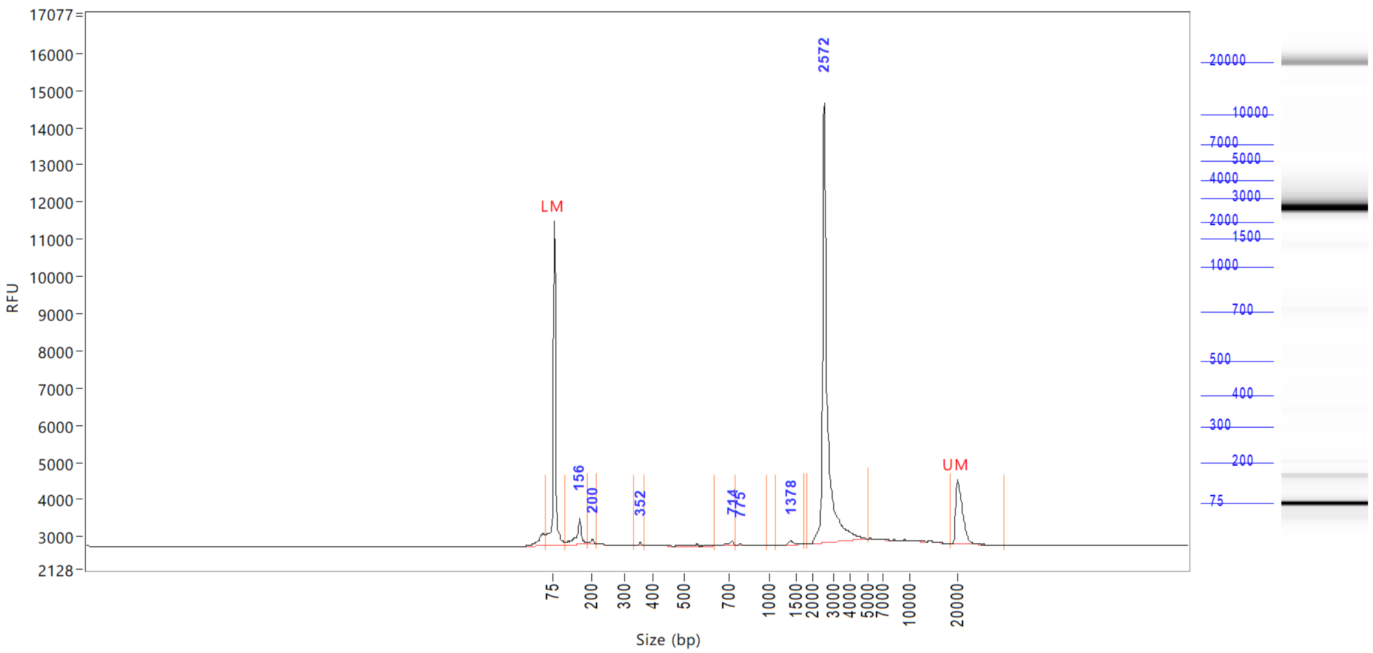

Peak	Size (bp)	Concentration (ng/uL)	From (bp)	To (bp)	Average size (bp)	CV%	RFU	Corrected peak area
1	75 (LM)	0.5000	45	112	72	11.15	8719	56.651
2	156	0.8914	112	185	149	9.56	716	8.416
3	200	0.1015	185	210	197	2.61	133	0.958
4	352	0.0494	329	368	349	1.87	80	0.466
5	714	0.1623	634	740	696	3.01	141	1.532
6	775	0.0767	740	974	799	6.22	57	0.724
7	1378	0.1189	1090	1750	1405	6.16	106	1.123
8	2572	9.5418	1846	5149	2708	14.10	11847	90.092
9	20000 (UM)	0.1361	18293	29793	20447	4.41	1750	15.425

TIC:	10.9419	ng/uL
TIM:	17.3935	nmole/L
Total concentration:	11.2050	ng/uL

Sample peak width (sec): 5 Sample min peak height: 50 Sample baseline V to V?: Y Sample baseline V to V points: 3
Sample filter: Binomial Number of points for filter: 3 Sample start region (min): 0 Sample end region (min): 30
Marker peak width (sec): 5 Marker min peak height: 200 Marker baseline V to V?: Y Marker baseline V to V points: 3
Lower marker selection: First peak > 500 RFU Upper marker selection: Last peak > 200 RFU
Ladder size (bp)75, 200, 300, 400, 500, 700, 1000, 1500, 2000, 3000, 4000, 5000, 7000, 10000, 20000
Quantification using: Lower Marker Final concentration (ng/uL): 0.5000 Dilution factor: 12.0

Sample: SampE10
Well location: E10
Created: Wednesday, August 11, 2021 5:34:32 PM

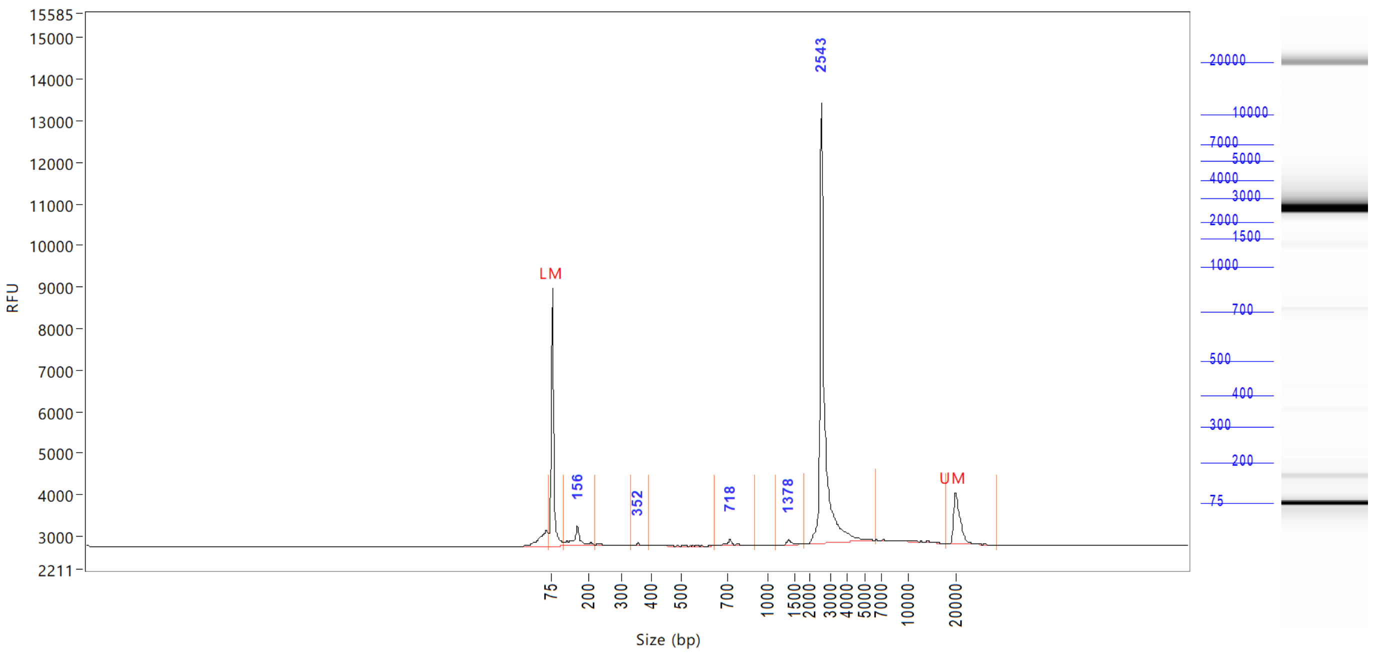

Peak	Size (bp)	Concentration (ng/uL)	From (bp)	To (bp)	Average size (bp)	CV%	RFU	Corrected peak area
1	75 (LM)	0.5000	66	114	76	9.38	6206	40.560
2	156	1.1496	114	214	155	14.07	465	7.771
3	352	0.0700	331	392	355	3.16	71	0.473
4	718	0.2979	646	905	724	6.27	171	2.014
5	1378	0.1764	1134	1827	1426	6.93	127	1.192
6	2543	11.8000	1827	6334	2711	15.75	10610	79.768
7	20000 (UM)	0.1317	18049	28705	20494	4.51	1237	10.683

TIC:	13.4937	ng/uL
TIM:	20.5600	nmole/L
Total concentration:	13.8396	ng/uL

```
Sample peak width (sec): 5    Sample min peak height: 50    Sample baseline V to V?: Y    Sample baseline V to V points: 3
Sample filter: Binomial       Number of points for filter: 3  Sample start region (min): 0  Sample end region (min): 30
Marker peak width (sec): 5    Marker min peak height: 200   Marker baseline V to V?: Y    Marker baseline V to V points: 3
Lower marker selection: First peak > 500 RFU                Upper marker selection: Last peak > 200 RFU
Ladder size (bp)75, 200, 300, 400, 500, 700, 1000, 1500, 2000, 3000, 4000, 5000, 7000, 10000, 20000
Quantification using: Lower Marker          Final concentration (ng/uL): 0.5000          Dilution factor: 12.0
```

Sample: SampF10
Well location: F10
Created: Wednesday, August 11, 2021 5:34:32 PM

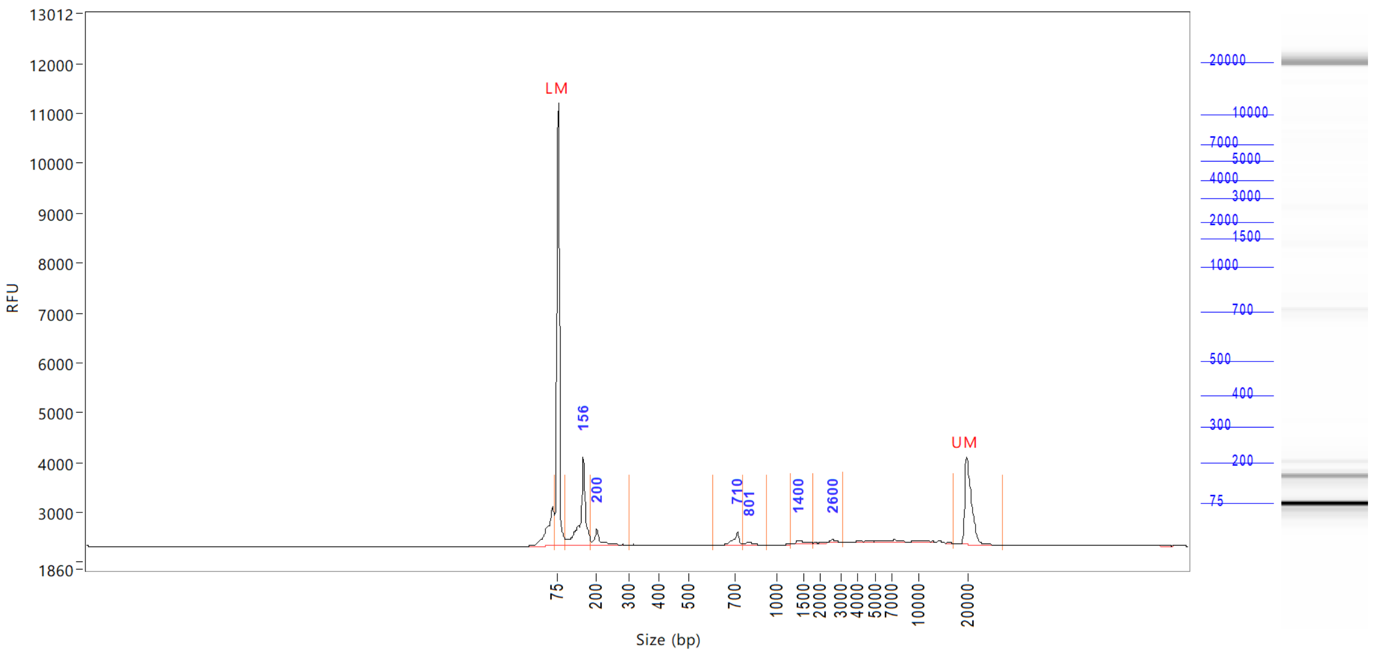

Peak	Size (bp)	Concentration (ng/uL)	From (bp)	To (bp)	Average size (bp)	CV%	RFU	Corrected peak area
1	75 (LM)	0.5000	64	101	73	6.89	8899	54.183
2	156	2.3988	101	183	147	11.40	1759	21.662
3	200	0.4575	183	298	208	9.63	335	4.131
4	710	0.3307	603	749	694	3.38	289	2.987
5	801	0.1142	749	922	806	5.25	60	1.031
6	1400	0.1217	1267	1789	1463	7.38	79	1.099
7	2600	0.0987	1789	3108	2536	12.15	74	0.891
8	20000 (UM)	0.1449	17195	27375	20387	4.36	1764	15.707

TIC:	3.5215	ng/uL
TIM:	31.6531	nmole/L
Total concentration:	3.7986	ng/uL

Sample peak width (sec): 5   Sample min peak height: 50   Sample baseline V to V?: Y   Sample baseline V to V points: 3
Sample filter: Binomial   Number of points for filter: 3 Sample start region (min): 0 Sample end region (min): 30
Marker peak width (sec): 5   Marker min peak height: 200   Marker baseline V to V?: Y   Marker baseline V to V points: 3
Lower marker selection: First peak > 500 RFU   Upper marker selection: Last peak > 200 RFU
Ladder size (bp)75, 200, 300, 400, 500, 700, 1000, 1500, 2000, 3000, 4000, 5000, 7000, 10000, 20000
Quantification using: Lower Marker   Final concentration (ng/uL): 0.5000   Dilution factor: 12.0

Sample: SampG10
Well location: G10
Created: Wednesday, August 11, 2021 5:34:32 PM

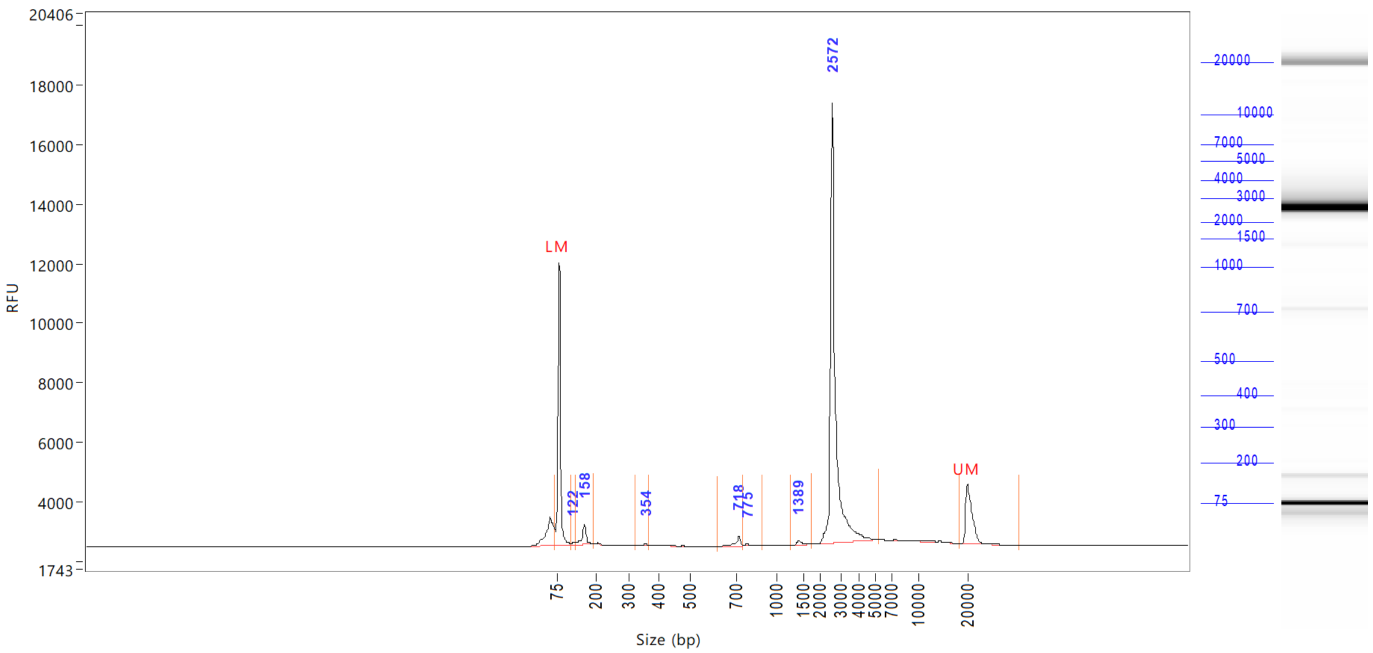

Peak	Size (bp)	Concentration (ng/uL)	From (bp)	To (bp)	Average size (bp)	CV%	RFU	Corrected peak area
1	75 (LM)	0.5000	66	114	75	7.64	9463	57.864
2	122	0.0745	114	128	121	3.41	89	0.719
3	158	0.6418	128	190	153	6.44	657	6.190
4	354	0.0574	317	366	348	2.44	92	0.554
5	718	0.2760	618	744	700	3.06	328	2.662
6	775	0.0914	744	887	792	4.38	72	0.882
7	1389	0.1322	1256	1731	1417	5.17	151	1.275
8	2572	11.5906	1731	5371	2705	13.97	14801	111.780
9	20000 (UM)	0.1499	18415	30639	20455	4.95	1988	17.351

TIC:	12.8640	ng/uL
TIM:	16.2240	nmole/L
Total concentration:	13.1247	ng/uL

```
Sample peak width (sec): 5    Sample min peak height: 50    Sample baseline V to V?: Y    Sample baseline V to V points: 3
Sample filter: Binomial       Number of points for filter: 3  Sample start region (min): 0  Sample end region (min): 30
Marker peak width (sec): 5    Marker min peak height: 200   Marker baseline V to V?: Y    Marker baseline V to V points: 3
Lower marker selection: First peak > 500 RFU                Upper marker selection: Last peak > 200 RFU
Ladder size (bp)75, 200, 300, 400, 500, 700, 1000, 1500, 2000, 3000, 4000, 5000, 7000, 10000, 20000
Quantification using: Lower Marker          Final concentration (ng/uL): 0.5000          Dilution factor: 12.0
```

Sample: SampH10
Well location: H10
Created: Wednesday, August 11, 2021 5:34:32 PM

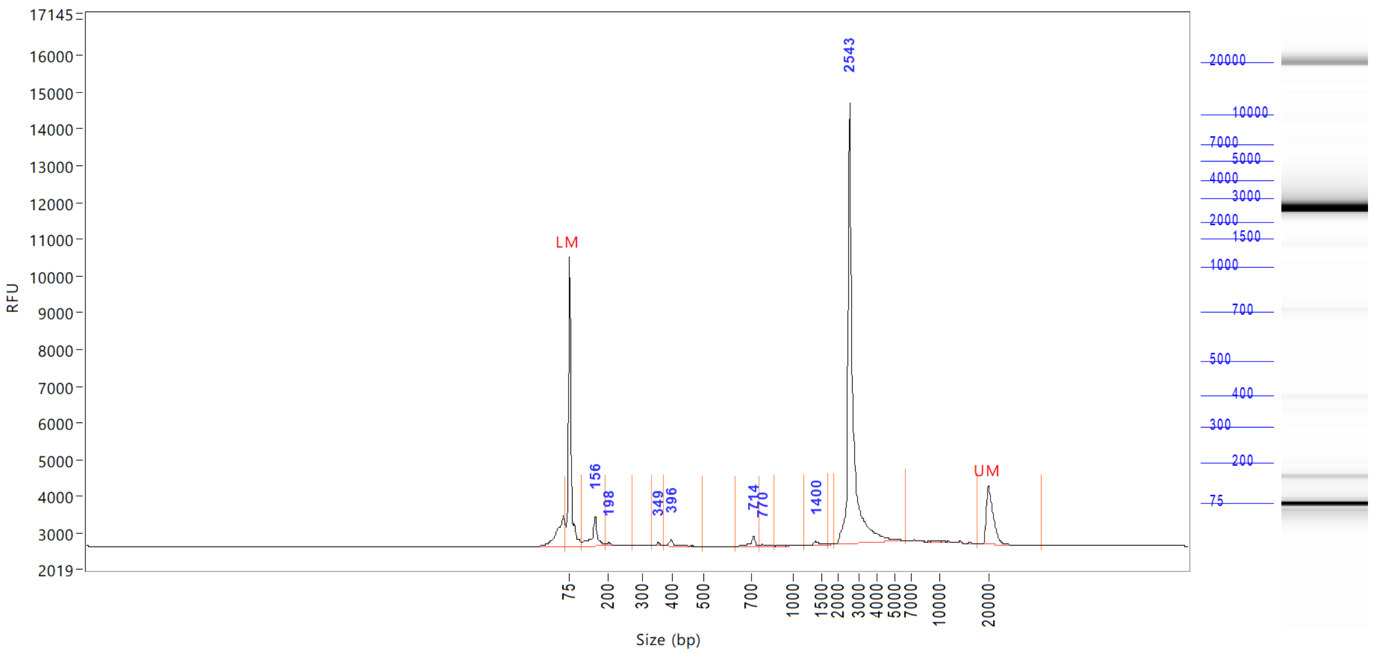

Peak	Size (bp)	Concentration (ng/uL)	From (bp)	To (bp)	Average size (bp)	CV%	RFU	Corrected peak area
1	75 (LM)	0.5000	64	112	74	10.04	7868	51.570
2	156	1.2004	112	189	149	10.60	807	10.317
3	198	0.1629	189	269	210	8.88	96	1.400
4	349	0.0842	331	368	350	2.37	88	0.724
5	396	0.2164	368	496	400	5.65	173	1.860
6	714	0.2513	634	749	699	2.94	257	2.160
7	770	0.0626	749	861	783	3.16	54	0.538
8	1400	0.1185	1167	1712	1431	5.85	106	1.018
9	2543	10.7543	1904	6260	2709	15.88	12008	92.432
10	20000 (UM)	0.1364	17683	30639	20465	5.09	1589	14.071

TIC:	12.8506	ng/uL
TIM:	23.1966	nmole/L
Total concentration:	13.0587	ng/uL

```
Sample peak width (sec): 5     Sample min peak height: 50     Sample baseline V to V?: Y     Sample baseline V to V points: 3
Sample filter: Binomial        Number of points for filter: 3  Sample start region (min): 0  Sample end region (min): 30
Marker peak width (sec): 5     Marker min peak height: 200    Marker baseline V to V?: Y     Marker baseline V to V points: 3
Lower marker selection: First peak > 500 RFU                  Upper marker selection: Last peak > 200 RFU
Ladder size (bp)75, 200, 300, 400, 500, 700, 1000, 1500, 2000, 3000, 4000, 5000, 7000, 10000, 20000
Quantification using: Lower Marker          Final concentration (ng/uL): 0.5000          Dilution factor: 12.0
```

Sample: SampA11
Well location: A11
Created: Wednesday, August 11, 2021 5:34:32 PM

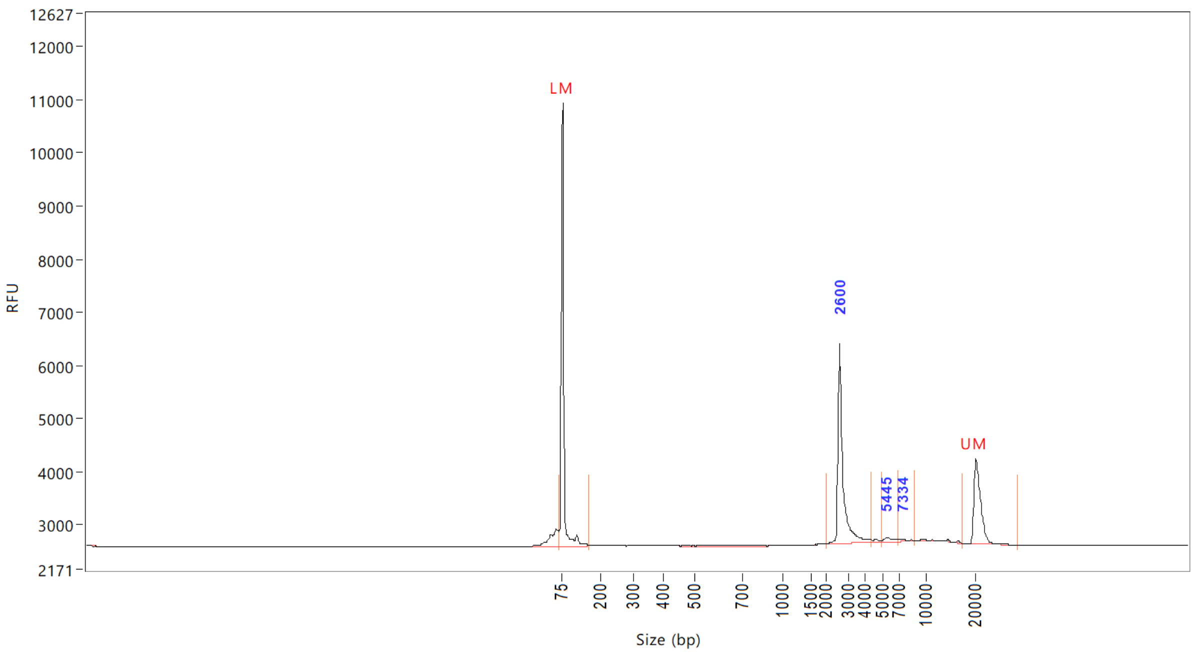

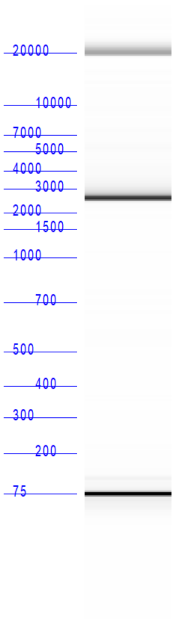

Peak	Size (bp)	Concentration (ng/uL)	From (bp)	To (bp)	Average size (bp)	CV%	RFU	Corrected peak area
1	75 (LM)	0.5000	64	164	76	16.44	8351	51.957
2	2600	3.2997	1981	4380	2740	12.57	3770	28.574
3	5445	0.1289	4931	7000	5757	10.18	82	1.116
4	7334	0.0589	7000	8667	7540	5.63	53	0.510
5	20000 (UM)	0.1362	17317	28705	20508	4.64	1606	14.152

TIC:	3.4875	ng/uL
TIM:	2.0322	nmole/L
Total concentration:	3.9142	ng/uL

Sample peak width (sec): 5   Sample min peak height: 50   Sample baseline V to V?: Y   Sample baseline V to V points: 3
Sample filter: Binomial   Number of points for filter: 3 Sample start region (min): 0 Sample end region (min): 30
Marker peak width (sec): 5   Marker min peak height: 200   Marker baseline V to V?: Y   Marker baseline V to V points: 3
Lower marker selection: First peak > 500 RFU   Upper marker selection: Last peak > 200 RFU
Ladder size (bp)75, 200, 300, 400, 500, 700, 1000, 1500, 2000, 3000, 4000, 5000, 7000, 10000, 20000
Quantification using: Lower Marker   Final concentration (ng/uL): 0.5000   Dilution factor: 12.0

Sample: SampB11
Well location: B11
Created: Wednesday, August 11, 2021 5:34:32 PM

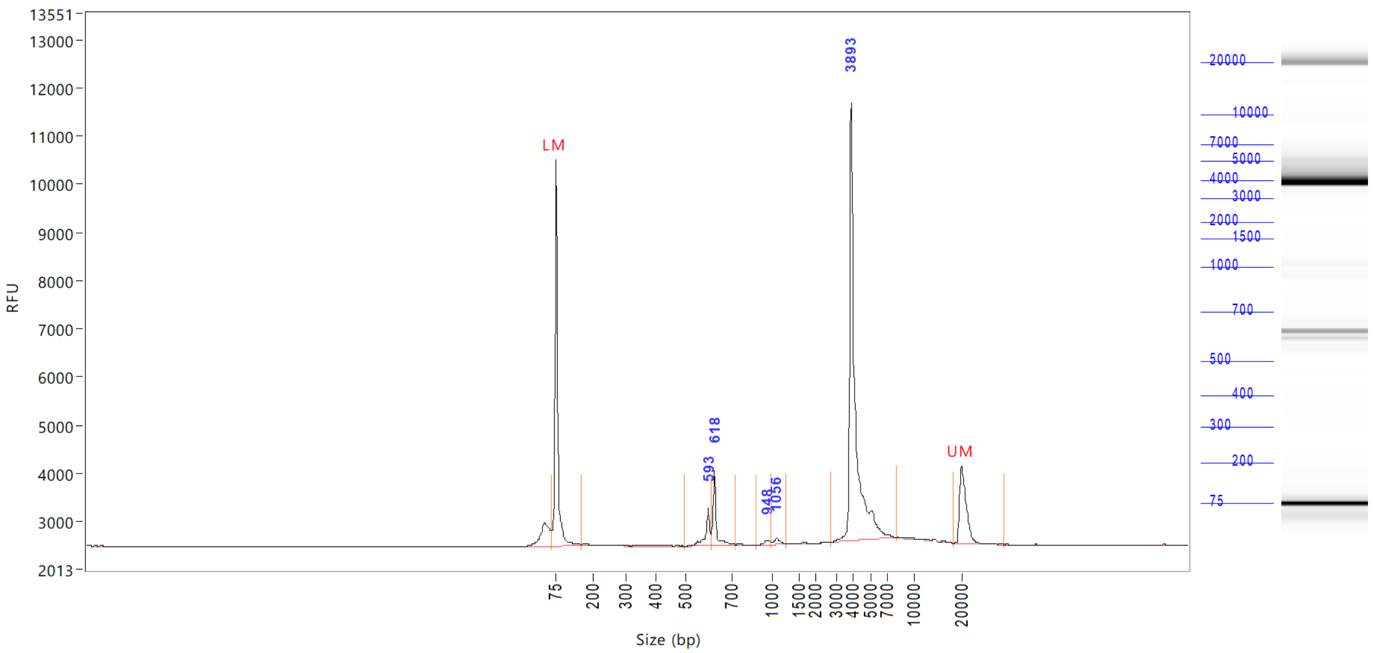

Peak	Size (bp)	Concentration (ng/uL)	From (bp)	To (bp)	Average size (bp)	CV%	RFU	Corrected peak area
1	75 (LM)	0.5000	62	156	76	15.26	8004	54.023
2	593	0.6835	492	606	581	3.34	772	6.155
3	618	1.0571	606	718	625	3.02	1563	9.518
4	948	0.1029	874	991	953	2.30	94	0.927
5	1056	0.1515	991	1256	1073	5.66	139	1.364
6	3893	8.4689	2715	8067	4151	13.30	9099	76.253
7	20000 (UM)	0.1298	18293	28947	20453	4.15	1610	14.027

TIC:	10.4640	ng/uL
TIM:	8.4884	nmole/L
Total concentration:	10.8051	ng/uL

Sample peak width (sec): 5   Sample min peak height: 50   Sample baseline V to V?: Y   Sample baseline V to V points: 3
Sample filter: Binomial   Number of points for filter: 3 Sample start region (min): 0 Sample end region (min): 30
Marker peak width (sec): 5   Marker min peak height: 200   Marker baseline V to V?: Y   Marker baseline V to V points: 3
Lower marker selection: First peak > 500 RFU   Upper marker selection: Last peak > 200 RFU
Ladder size (bp)75, 200, 300, 400, 500, 700, 1000, 1500, 2000, 3000, 4000, 5000, 7000, 10000, 20000
Quantification using: Lower Marker   Final concentration (ng/uL): 0.5000   Dilution factor: 12.0

Sample: SampC11
Well location: C11
Created: Wednesday, August 11, 2021 5:34:32 PM

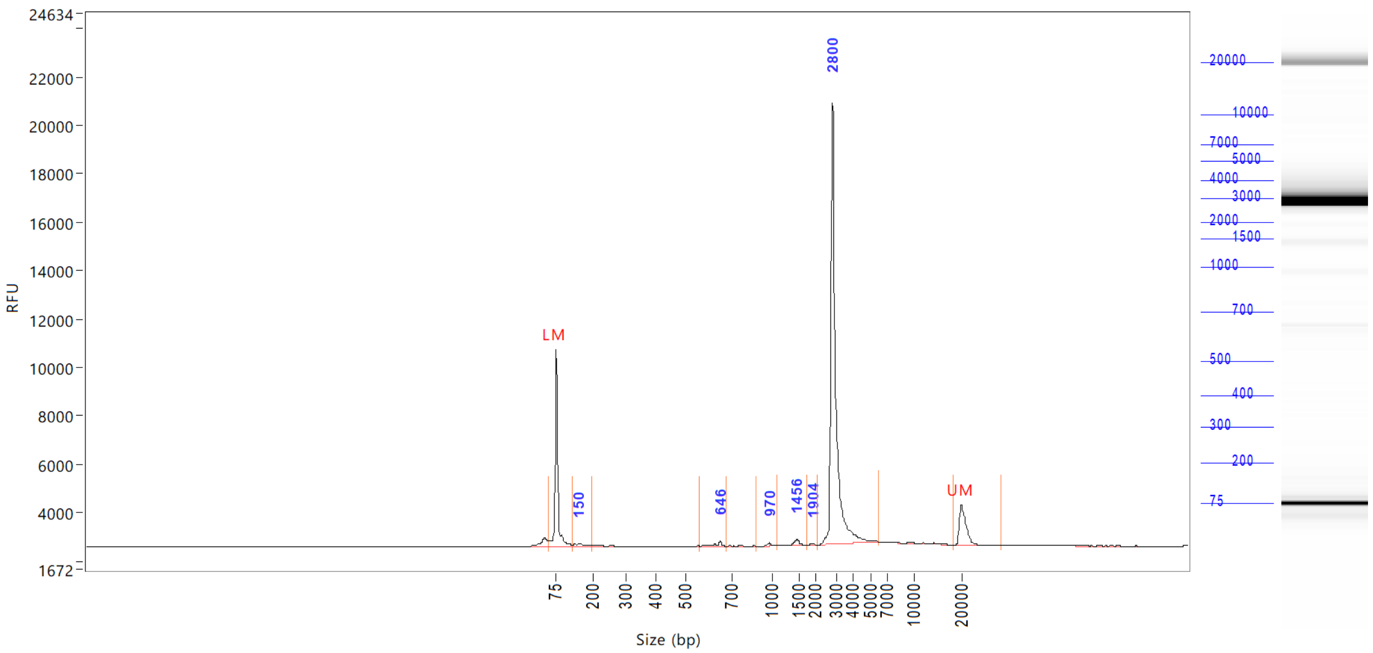

Peak	Size (bp)	Concentration (ng/uL)	From (bp)	To (bp)	Average size (bp)	CV%	RFU	Corrected peak area
1	75 (LM)	0.5000	52	126	75	12.57	8139	52.606
2	150	0.2859	126	192	152	10.90	105	2.506
3	646	0.2274	557	674	624	3.99	200	1.994
4	970	0.1003	874	1068	968	1.96	132	0.880
5	1456	0.2446	1068	1731	1436	5.68	227	2.145
6	1904	0.0471	1731	2087	1875	3.27	66	0.413
7	2800	15.3377	2087	5963	2948	11.97	18254	134.477
8	20000 (UM)	0.1392	18293	28463	20435	4.29	1669	14.641

TIC:	16.2431	ng/uL
TIM:	12.7479	nmole/L
Total concentration:	16.5613	ng/uL

Sample peak width (sec): 5   Sample min peak height: 50   Sample baseline V to V?: Y   Sample baseline V to V points: 3
Sample filter: Binomial   Number of points for filter: 3 Sample start region (min): 0 Sample end region (min): 30
Marker peak width (sec): 5   Marker min peak height: 200   Marker baseline V to V?: Y   Marker baseline V to V points: 3
Lower marker selection: First peak > 500 RFU   Upper marker selection: Last peak > 200 RFU
Ladder size (bp)75, 200, 300, 400, 500, 700, 1000, 1500, 2000, 3000, 4000, 5000, 7000, 10000, 20000
Quantification using: Lower Marker   Final concentration (ng/uL): 0.5000   Dilution factor: 12.0

Sample: SampD11
Well location: D11
Created: Wednesday, August 11, 2021 5:34:32 PM

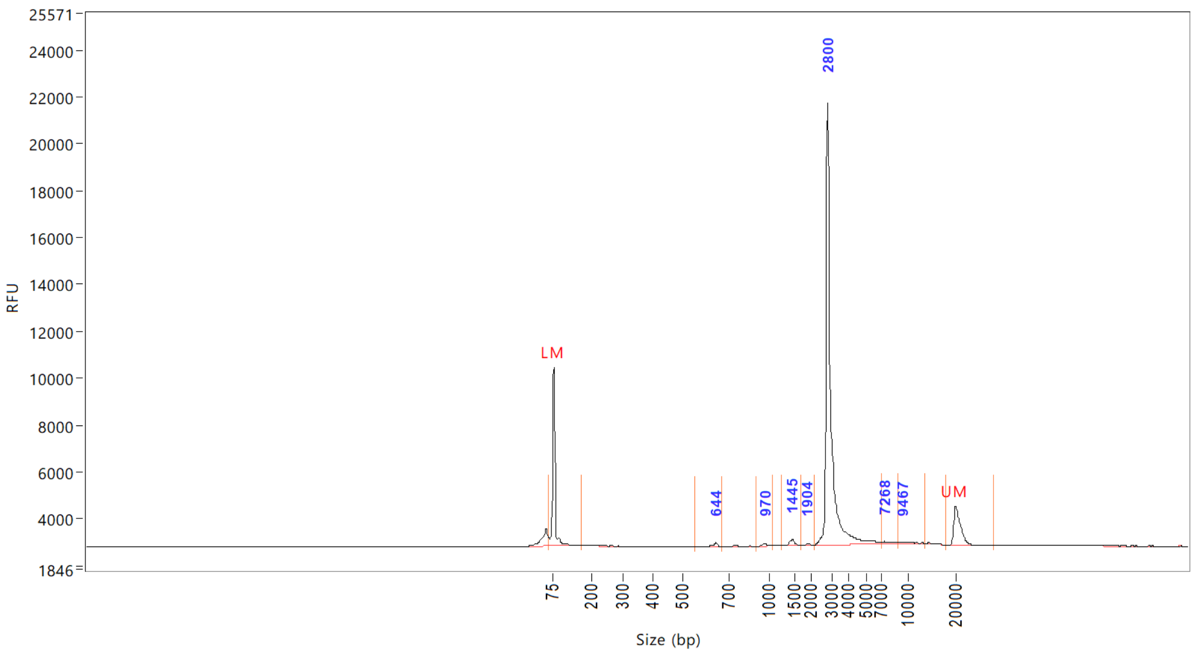

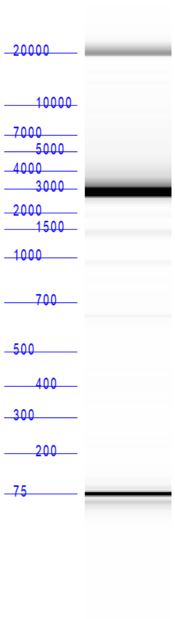

Peak	Size (bp)	Concentration (ng/uL)	From (bp)	To (bp)	Average size (bp)	CV%	RFU	Corrected peak area
1	75 (LM)	0.5000	62	168	75	12.66	7638	48.490
2	644	0.1421	549	672	631	3.24	164	1.149
3	970	0.0960	905	1056	963	1.76	127	0.776
4	1445	0.2895	1245	1693	1435	4.71	255	2.339
5	1904	0.0756	1693	2144	1881	4.54	84	0.611
6	2800	17.3869	2144	7000	2989	18.03	18890	140.514
7	7268	0.1851	7000	8867	7726	7.04	108	1.496
8	9467	0.1457	8867	13537	10264	11.12	64	1.178
9	20000 (UM)	0.1546	18049	27859	20440	4.43	1670	14.997

TIC:	18.3208	ng/uL
TIM:	10.5716	nmole/L
Total concentration:	18.5393	ng/uL

```
Sample peak width (sec): 5     Sample min peak height: 50     Sample baseline V to V?: Y     Sample baseline V to V points: 3
Sample filter: Binomial        Number of points for filter: 3  Sample start region (min): 0  Sample end region (min): 30
Marker peak width (sec): 5     Marker min peak height: 200    Marker baseline V to V?: Y     Marker baseline V to V points: 3
Lower marker selection: First peak > 500 RFU                  Upper marker selection: Last peak > 200 RFU
Ladder size (bp)75, 200, 300, 400, 500, 700, 1000, 1500, 2000, 3000, 4000, 5000, 7000, 10000, 20000
Quantification using: Lower Marker          Final concentration (ng/uL): 0.5000          Dilution factor: 12.0
```

Sample: SampE11
Well location: E11
Created: Wednesday, August 11, 2021 5:34:32 PM

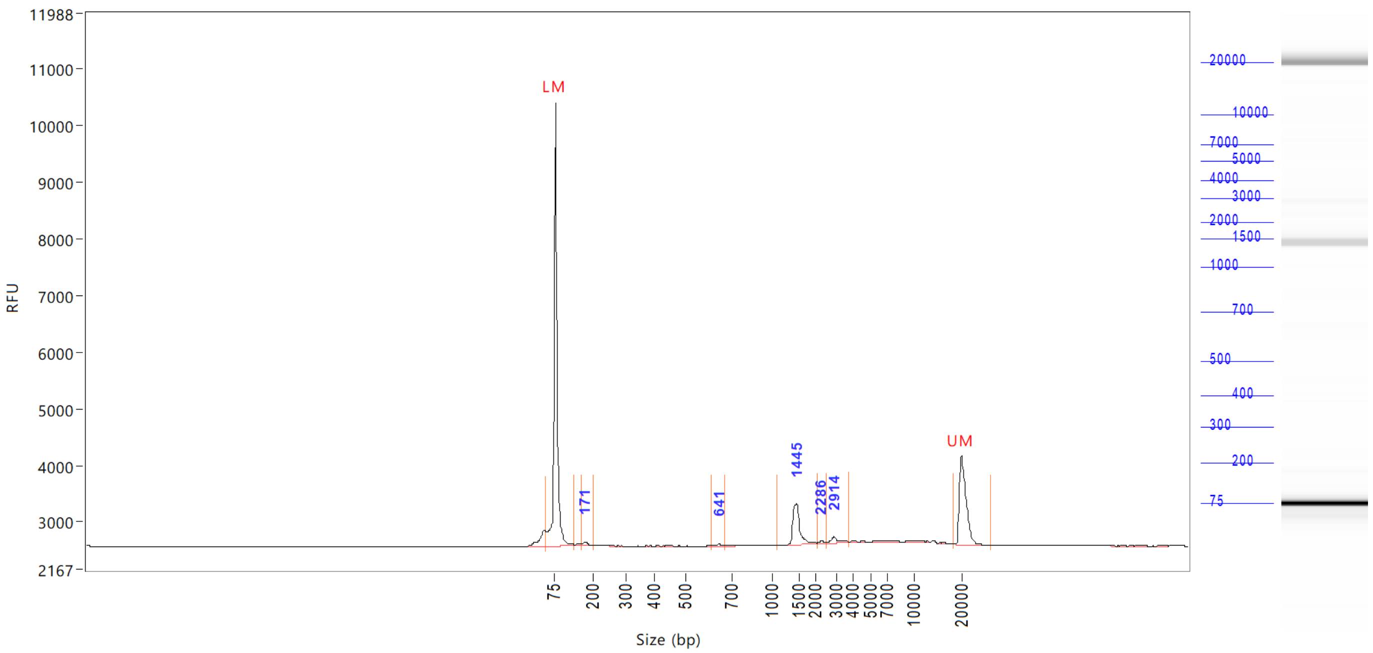

Peak	Size (bp)	Concentration (ng/uL)	From (bp)	To (bp)	Average size (bp)	CV%	RFU	Corrected peak area
1	75 (LM)	0.5000	45	137	73	12.66	7842	57.676
2	171	0.0710	158	200	170	4.18	71	0.682
3	641	0.0381	611	669	637	1.84	54	0.366
4	1445	0.8563	1079	2058	1461	8.20	730	8.231
5	2286	0.0453	2058	2515	2293	5.16	55	0.436
6	2914	0.1481	2515	3786	3004	10.02	120	1.424
7	20000 (UM)	0.1206	18171	26045	20407	4.00	1585	13.915

TIC:	1.1587	ng/uL
TIM:	1.8630	nmole/L
Total concentration:	1.5015	ng/uL

Sample peak width (sec): 5 Sample min peak height: 50 Sample baseline V to V?: Y Sample baseline V to V points: 3
Sample filter: Binomial Number of points for filter: 3 Sample start region (min): 0 Sample end region (min): 30
Marker peak width (sec): 5 Marker min peak height: 200 Marker baseline V to V?: Y Marker baseline V to V points: 3
Lower marker selection: First peak > 500 RFU Upper marker selection: Last peak > 200 RFU
Ladder size (bp)75, 200, 300, 400, 500, 700, 1000, 1500, 2000, 3000, 4000, 5000, 7000, 10000, 20000
Quantification using: Lower Marker Final concentration (ng/uL): 0.5000 Dilution factor: 12.0

Sample: SampF11
Well location: F11
Created: Wednesday, August 11, 2021 5:34:32 PM

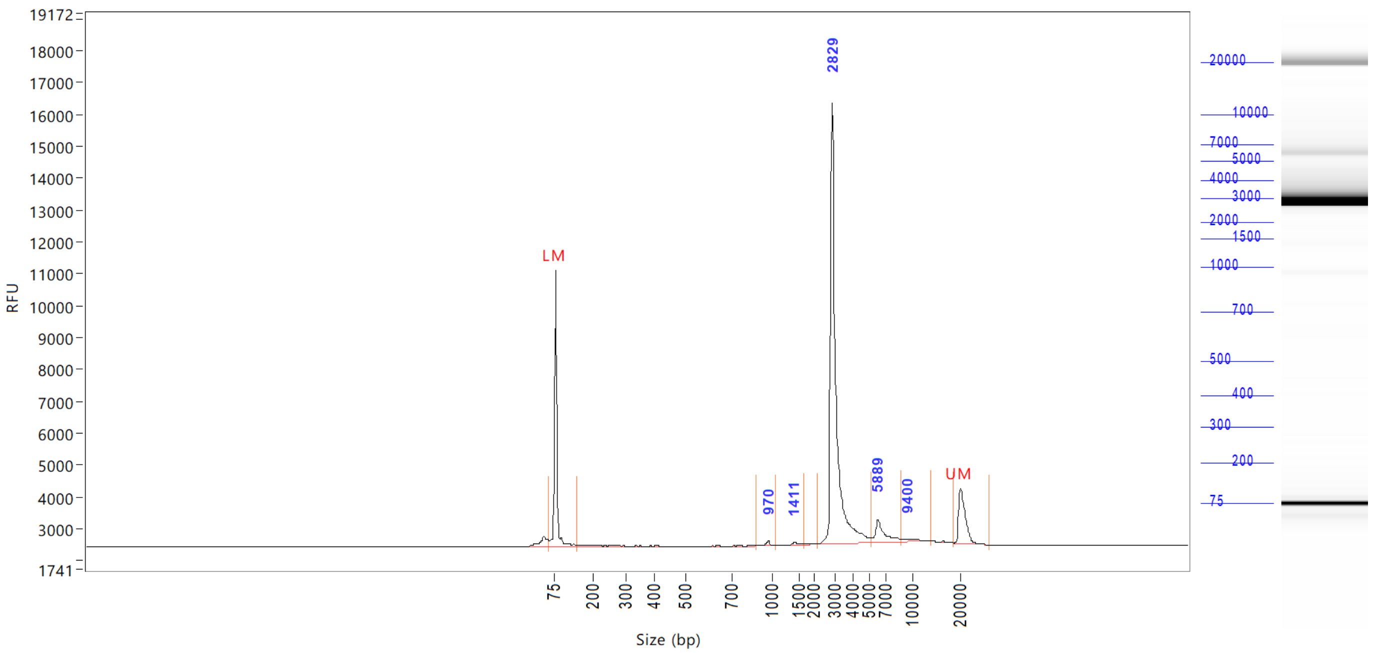

Peak	Size (bp)	Concentration (ng/uL)	From (bp)	To (bp)	Average size (bp)	CV%	RFU	Corrected peak area
1	75 (LM)	0.5000	52	147	75	14.06	8657	52.990
2	970	0.0981	879	1056	962	1.80	136	0.866
3	1411	0.0945	1056	1655	1410	6.35	71	0.834
4	2829	13.3137	2087	5223	3026	14.57	13849	117.582
5	5889	1.0559	5223	8667	6484	12.67	719	9.325
6	9400	0.2030	8667	13781	9950	10.62	86	1.793
7	20000 (UM)	0.1408	18537	25924	20364	3.70	1725	14.918

TIC:	14.7651	ng/uL
TIM:	7.8228	nmole/L
Total concentration:	15.0709	ng/uL

Sample peak width (sec): 5 Sample min peak height: 50 Sample baseline V to V?: Y Sample baseline V to V points: 3
Sample filter: Binomial Number of points for filter: 3 Sample start region (min): 0 Sample end region (min): 30
Marker peak width (sec): 5 Marker min peak height: 200 Marker baseline V to V?: Y Marker baseline V to V points: 3
Lower marker selection: First peak > 500 RFU Upper marker selection: Last peak > 200 RFU
Ladder size (bp)75, 200, 300, 400, 500, 700, 1000, 1500, 2000, 3000, 4000, 5000, 7000, 10000, 20000
Quantification using: Lower Marker Final concentration (ng/uL): 0.5000 Dilution factor: 12.0

Sample: SampG11
Well location: G11
Created: Wednesday, August 11, 2021 5:34:32 PM

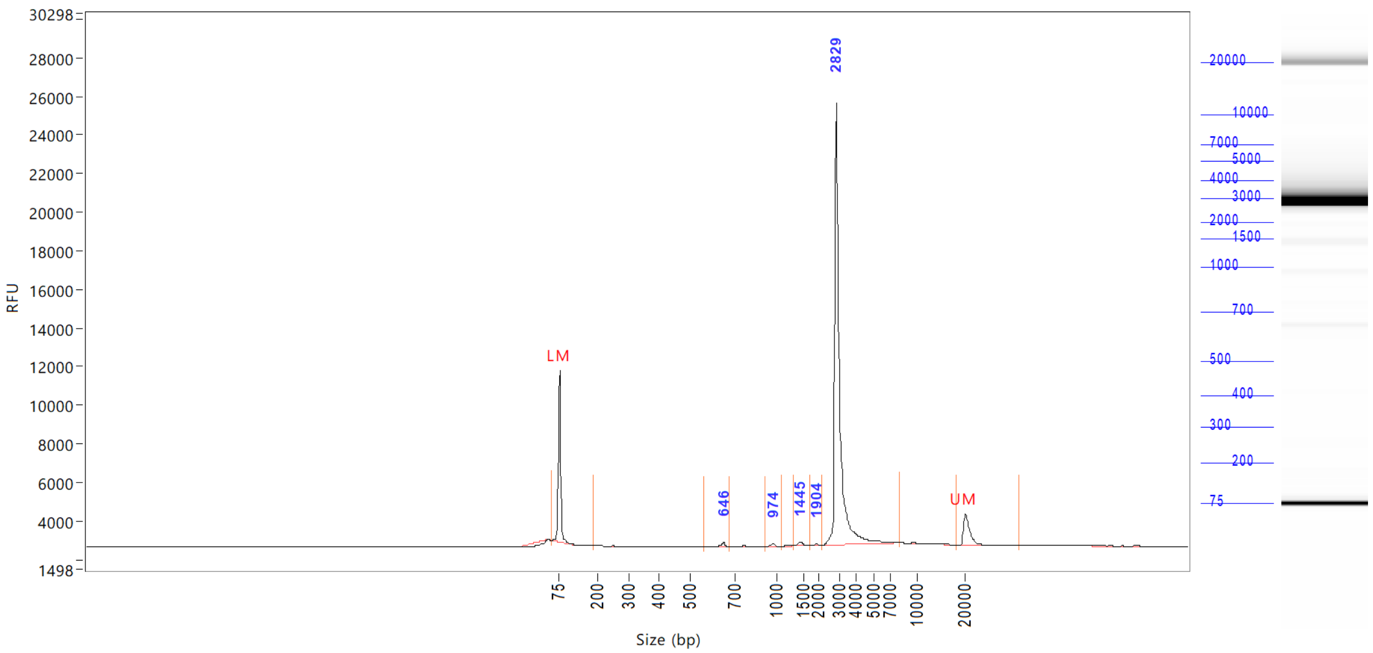

Peak	Size (bp)	Concentration (ng/uL)	From (bp)	To (bp)	Average size (bp)	CV%	RFU	Corrected peak area
1	75 (LM)	0.5000	48	187	74	14.47	8896	52.291
2	646	0.1685	560	672	636	2.78	212	1.469
3	974	0.1045	913	1090	968	1.72	139	0.910
4	1445	0.2110	1323	1731	1452	4.52	204	1.839
5	1904	0.0473	1731	2115	1887	3.39	64	0.413
6	2829	20.0143	2115	8001	2987	15.75	22929	174.429
7	20000 (UM)	0.1318	18293	31244	20580	6.02	1635	13.783

TIC:	20.5456	ng/uL
TIM:	11.9247	nmole/L
Total concentration:	20.7967	ng/uL

```
Sample peak width (sec): 5    Sample min peak height: 50    Sample baseline V to V?: Y    Sample baseline V to V points: 3
Sample filter: Binomial       Number of points for filter: 3  Sample start region (min): 0  Sample end region (min): 30
Marker peak width (sec): 5    Marker min peak height: 200   Marker baseline V to V?: Y    Marker baseline V to V points: 3
Lower marker selection: First peak > 500 RFU                Upper marker selection: Last peak > 200 RFU
Ladder size (bp)75, 200, 300, 400, 500, 700, 1000, 1500, 2000, 3000, 4000, 5000, 7000, 10000, 20000
Quantification using: Lower Marker          Final concentration (ng/uL): 0.5000         Dilution factor: 12.0
```

Sample: SampH11
Well location: H11
Created: Wednesday, August 11, 2021 5:34:32 PM

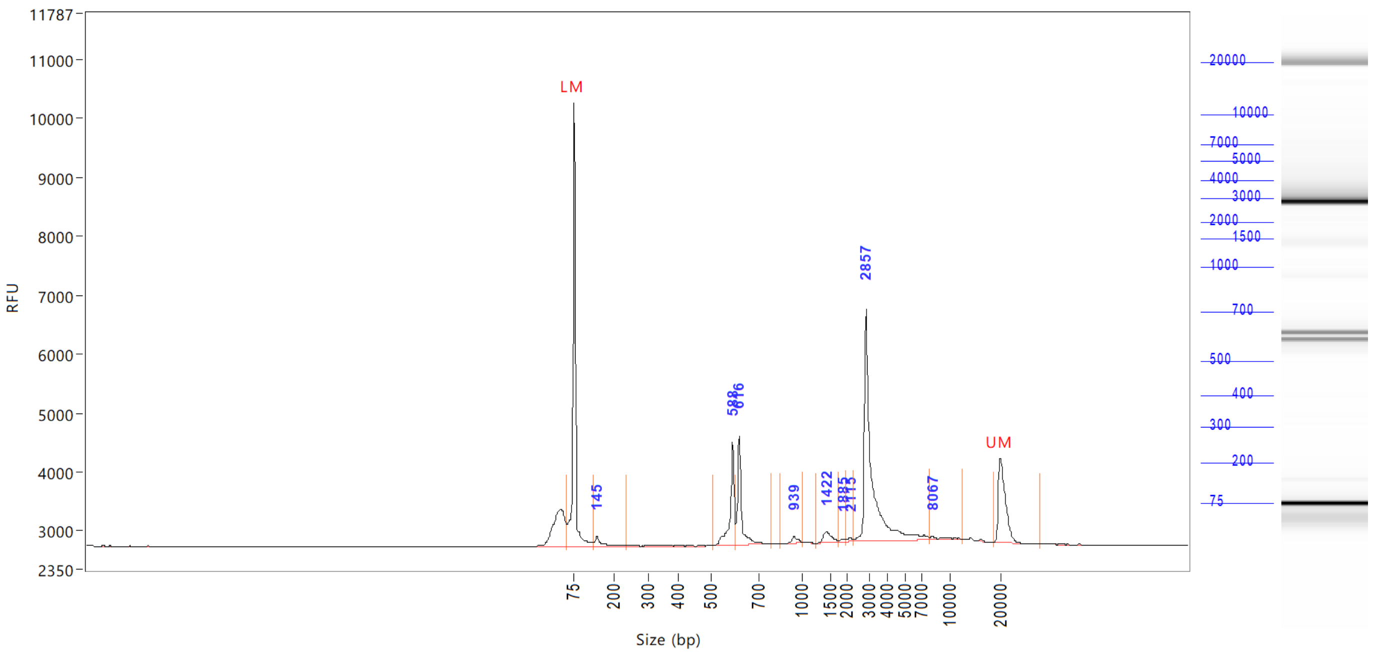

Peak	Size (bp)	Concentration (ng/uL)	From (bp)	To (bp)	Average size (bp)	CV%	RFU	Corrected peak area
1	75 (LM)	0.5000	52	135	74	13.86	7535	49.064
2	145	0.2677	135	233	157	13.37	167	2.189
3	588	1.5703	506	600	577	2.99	1739	12.841
4	616	1.5319	600	783	622	3.49	1852	12.527
5	939	0.1244	840	1001	941	2.65	107	1.017
6	1422	0.3029	1234	1750	1458	6.42	178	2.477
7	1885	0.0500	1750	1981	1867	3.52	51	0.409
8	2115	0.0569	1981	2258	2099	3.92	69	0.465
9	2857	5.1561	2258	7867	3333	26.87	3944	42.163
10	8067	0.1316	7867	12440	9455	12.83	51	1.076
11	20000 (UM)	0.1295	18537	27859	20395	3.97	1433	12.712

TIC:	9.1916	ng/uL
TIM:	14.5515	nmole/L
Total concentration:	9.2800	ng/uL

```
Sample peak width (sec): 5    Sample min peak height: 50    Sample baseline V to V?: Y    Sample baseline V to V points: 3
Sample filter: Binomial       Number of points for filter: 3  Sample start region (min): 0  Sample end region (min): 30
Marker peak width (sec): 5    Marker min peak height: 200   Marker baseline V to V?: Y    Marker baseline V to V points: 3
Lower marker selection: First peak > 500 RFU               Upper marker selection: Last peak > 200 RFU
Ladder size (bp)75, 200, 300, 400, 500, 700, 1000, 1500, 2000, 3000, 4000, 5000, 7000, 10000, 20000
Quantification using: Lower Marker          Final concentration (ng/uL): 0.5000          Dilution factor: 12.0
```

Sample: SampA12
Well location: A12
Created: Wednesday, August 11, 2021 5:34:32 PM

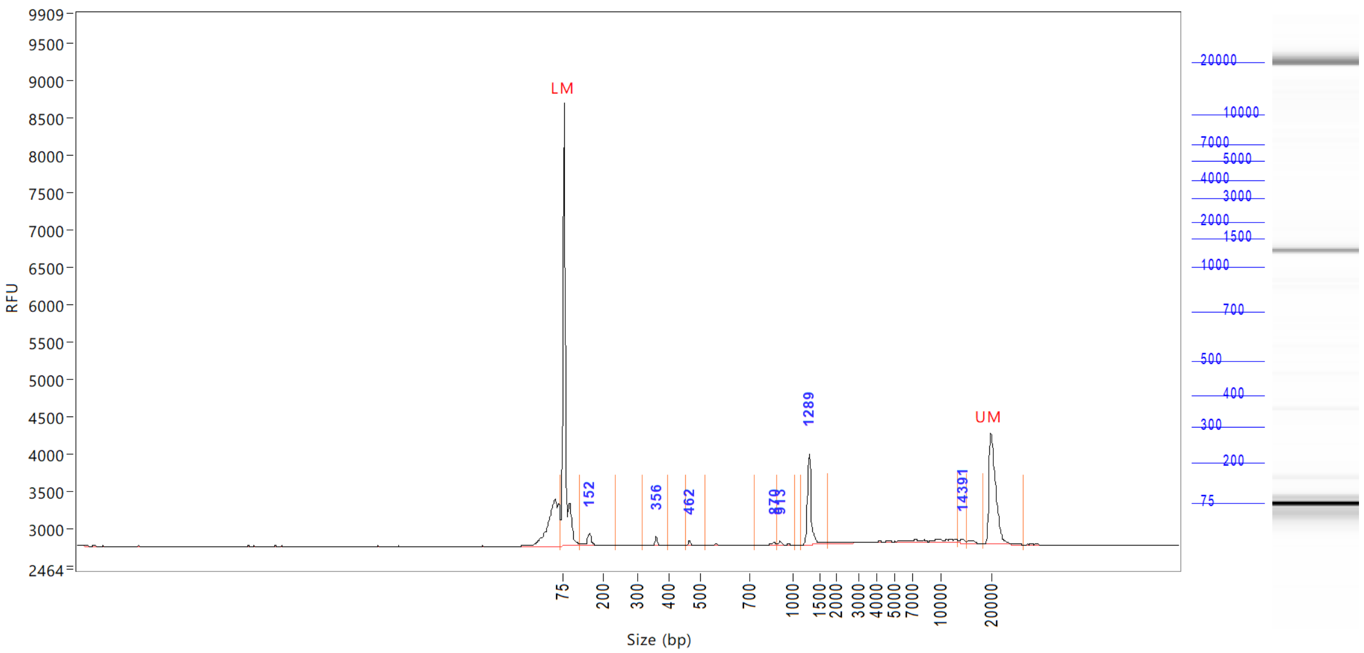

Peak	Size<br>(bp)	Concentration<br>(ng/uL)	From<br>(bp)	To<br>(bp)	Average size<br>(bp)	CV%	RFU	Corrected peak area
1	75 (LM)	0.5000	64	124	75	11.13	5943	39.772
2	152	0.2884	124	233	154	11.92	162	1.912
3	356	0.1140	313	398	351	3.90	119	0.755
4	462	0.0551	452	519	469	3.28	58	0.365
5	870	0.0578	731	887	848	3.79	51	0.383
6	913	0.0687	887	1023	926	3.26	57	0.455
7	1289	1.0726	1145	1693	1300	4.96	1218	7.110
8	14391	0.0581	13537	15122	14243	3.01	60	0.385
9	20000 (UM)	0.1581	18415	26408	20434	4.03	1487	12.573

TIC:	1.7146	ng/uL
TIM:	5.4043	nmole/L
Total concentration:	2.2319	ng/uL

```
Sample peak width (sec): 5    Sample min peak height: 50    Sample baseline V to V?: Y    Sample baseline V to V points: 3
Sample filter: Binomial       Number of points for filter: 3  Sample start region (min): 0  Sample end region (min): 30
Marker peak width (sec): 5    Marker min peak height: 200   Marker baseline V to V?: Y    Marker baseline V to V points: 3
Lower marker selection: First peak > 500 RFU                Upper marker selection: Last peak > 200 RFU
Ladder size (bp)75, 200, 300, 400, 500, 700, 1000, 1500, 2000, 3000, 4000, 5000, 7000, 10000, 20000
Quantification using: Lower Marker          Final concentration (ng/uL): 0.5000          Dilution factor: 12.0
```

Sample: SampB12
Well location: B12
Created: Wednesday, August 11, 2021 5:34:32 PM

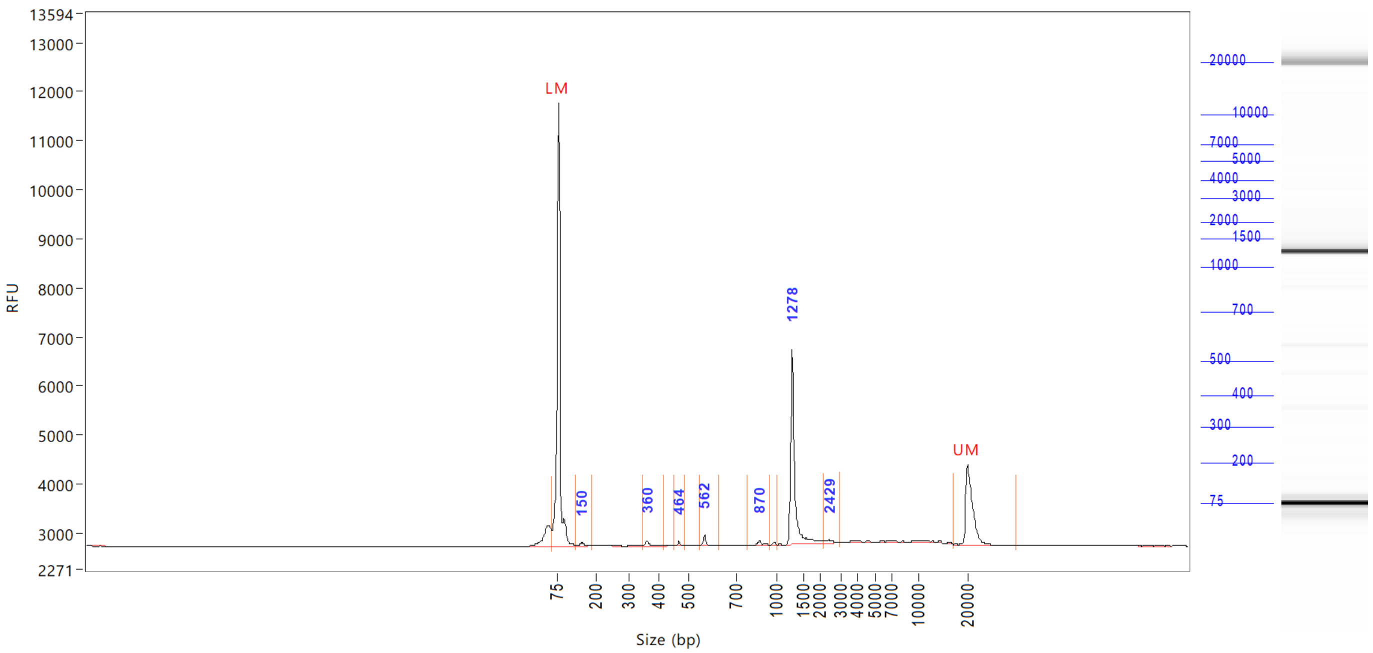

Peak	Size (bp)	Concentration (ng/uL)	From (bp)	To (bp)	Average size (bp)	CV%	RFU	Corrected peak area
1	75 (LM)	0.5000	56	129	75	11.06	9044	63.375
2	150	0.0853	129	189	149	7.50	66	0.901
3	360	0.0826	345	418	363	3.94	119	0.873
4	464	0.0426	447	485	462	0.99	91	0.450
5	562	0.1289	539	623	565	2.84	218	1.362
6	870	0.0828	783	948	870	3.29	106	0.874
7	1278	2.4163	1000	2144	1320	10.99	3969	25.523
8	2429	0.0806	2144	2943	2460	8.27	68	0.852
9	20000 (UM)	0.1171	17073	30035	20523	4.83	1637	14.840

TIC:	2.9191	ng/uL
TIM:	5.0657	nmole/L
Total concentration:	3.2809	ng/uL

Sample peak width (sec): 5 Sample min peak height: 50 Sample baseline V to V?: Y Sample baseline V to V points: 3
Sample filter: Binomial Number of points for filter: 3 Sample start region (min): 0 Sample end region (min): 30
Marker peak width (sec): 5 Marker min peak height: 200 Marker baseline V to V?: Y Marker baseline V to V points: 3
Lower marker selection: First peak > 500 RFU Upper marker selection: Last peak > 200 RFU
Ladder size (bp)75, 200, 300, 400, 500, 700, 1000, 1500, 2000, 3000, 4000, 5000, 7000, 10000, 20000
Quantification using: Lower Marker Final concentration (ng/uL): 0.5000 Dilution factor: 12.0

Sample: SampC12
Well location: C12
Created: Wednesday, August 11, 2021 5:34:32 PM

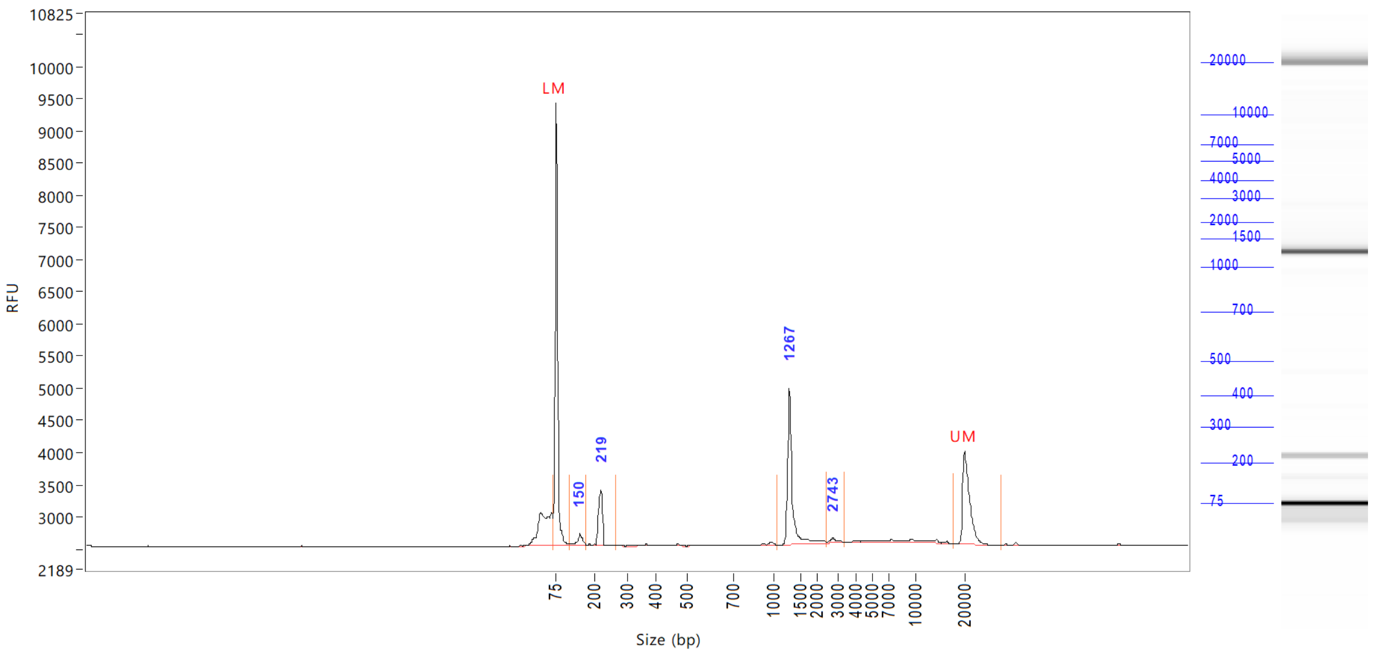

Peak	Size (bp)	Concentration (ng/uL)	From (bp)	To (bp)	Average size (bp)	CV%	RFU	Corrected peak area
1	75 (LM)	0.5000	66	120	74	8.21	6889	42.578
2	150	0.3189	120	173	147	7.49	180	2.263
3	219	1.1201	173	264	214	3.36	875	7.949
4	1267	2.6032	1045	2429	1325	13.53	2438	18.473
5	2743	0.1120	2429	3393	2785	7.70	76	0.795
6	20000 (UM)	0.1586	17805	27617	20517	4.58	1451	13.509

TIC:	4.1542	ng/uL
TIM:	15.4718	nmole/L
Total concentration:	4.8093	ng/uL

```
Sample peak width (sec): 5    Sample min peak height: 50    Sample baseline V to V?: Y    Sample baseline V to V points: 3
Sample filter: Binomial       Number of points for filter: 3  Sample start region (min): 0  Sample end region (min): 30
Marker peak width (sec): 5    Marker min peak height: 200   Marker baseline V to V?: Y    Marker baseline V to V points: 3
Lower marker selection: First peak > 500 RFU                Upper marker selection: Last peak > 200 RFU
Ladder size (bp)75, 200, 300, 400, 500, 700, 1000, 1500, 2000, 3000, 4000, 5000, 7000, 10000, 20000
Quantification using: Lower Marker          Final concentration (ng/uL): 0.5000          Dilution factor: 12.0
```

Sample: SampD12
Well location: D12
Created: Wednesday, August 11, 2021 5:34:32 PM

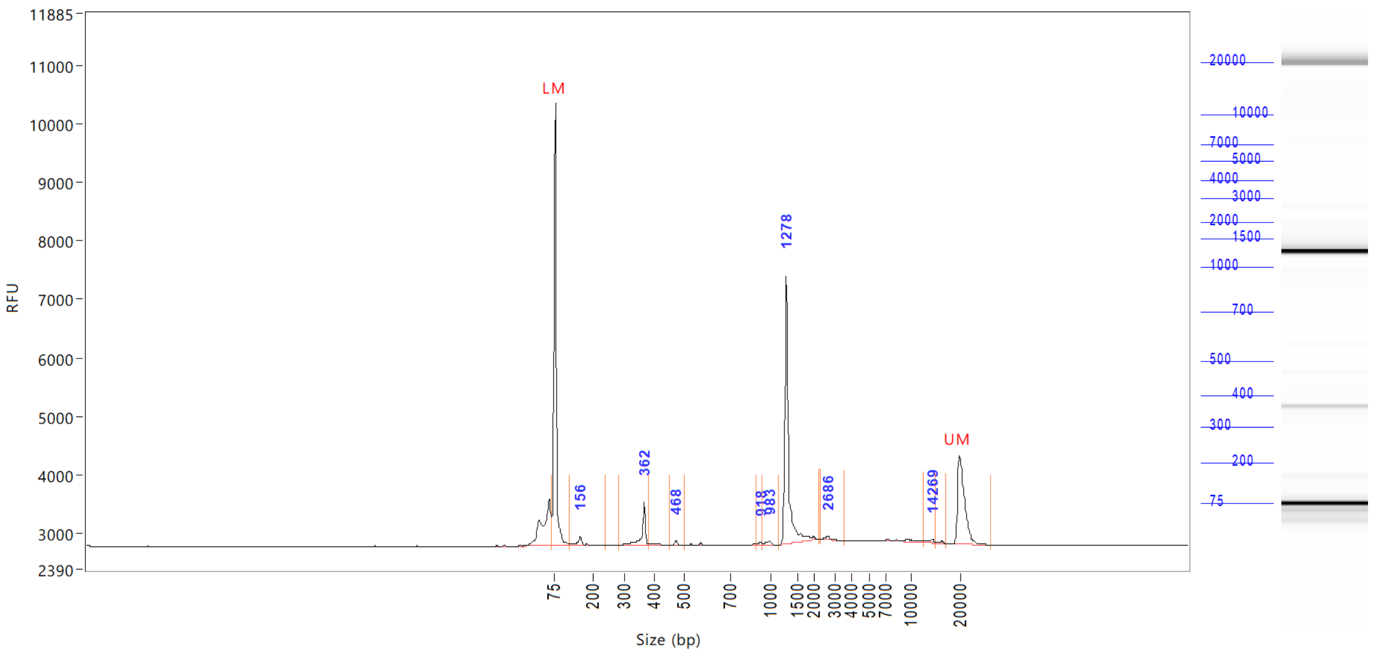

Peak	Size (bp)	Concentration (ng/uL)	From (bp)	To (bp)	Average size (bp)	CV%	RFU	Corrected peak area
1	75 (LM)	0.5000	64	124	74	8.98	7576	46.504
2	156	0.2613	124	239	158	13.94	149	2.025
3	362	0.7376	278	380	350	5.63	740	5.717
4	468	0.0617	449	500	467	2.02	77	0.478
5	918	0.0448	892	939	913	1.36	54	0.347
6	983	0.0839	939	1134	977	2.62	80	0.650
7	1278	3.6002	1134	2229	1310	9.35	4568	27.904
8	2686	0.0666	2286	3536	2698	7.80	60	0.516
9	14269	0.0657	12440	15122	13737	5.01	63	0.509
10	20000 (UM)	0.1493	17195	26408	20478	4.36	1516	13.883

TIC:	4.9217	ng/uL
TIM:	11.1970	nmole/L
Total concentration:	5.3799	ng/uL

```
Sample peak width (sec): 5    Sample min peak height: 50    Sample baseline V to V?: Y    Sample baseline V to V points: 3
Sample filter: Binomial       Number of points for filter: 3  Sample start region (min): 0  Sample end region (min): 30
Marker peak width (sec): 5    Marker min peak height: 200   Marker baseline V to V?: Y    Marker baseline V to V points: 3
Lower marker selection: First peak > 500 RFU                Upper marker selection: Last peak > 200 RFU
Ladder size (bp)75, 200, 300, 400, 500, 700, 1000, 1500, 2000, 3000, 4000, 5000, 7000, 10000, 20000
Quantification using: Lower Marker          Final concentration (ng/uL): 0.5000          Dilution factor: 12.0
```

Sample: SampE12
Well location: E12
Created: Wednesday, August 11, 2021 5:34:32 PM

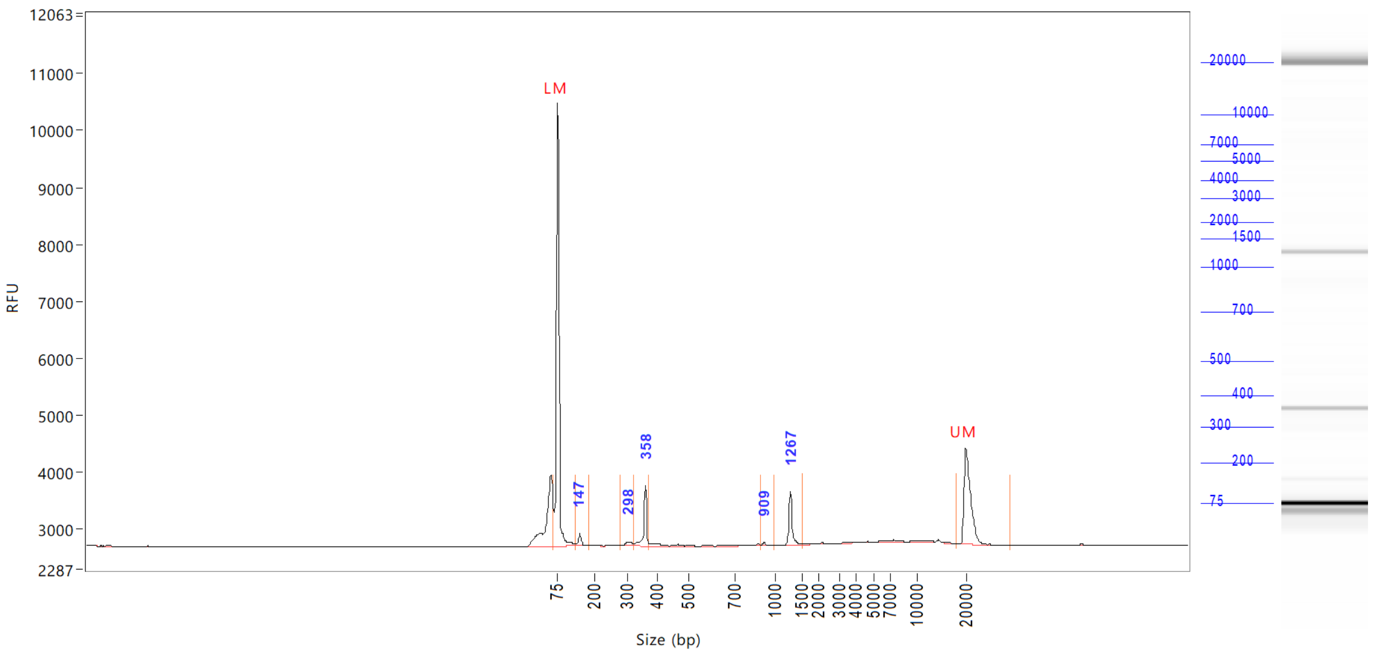

Peak	Size (bp)	Concentration (ng/uL)	From (bp)	To (bp)	Average size (bp)	CV%	RFU	Corrected peak area
1	75 (LM)	0.5000	64	133	75	10.83	7805	48.696
2	147	0.2127	133	181	147	5.27	211	1.726
3	298	0.1324	273	315	298	2.99	76	1.074
4	358	0.8014	315	370	351	2.91	1063	6.504
5	909	0.0480	887	991	917	2.52	50	0.390
6	1267	0.6279	991	1489	1264	3.55	935	5.096
7	20000 (UM)	0.1544	18049	29068	20489	4.38	1693	15.038

TIC:	1.8225	ng/uL
TIM:	7.7691	nmole/L
Total concentration:	2.3712	ng/uL

```
Sample peak width (sec): 5    Sample min peak height: 50    Sample baseline V to V?: Y    Sample baseline V to V points: 3
Sample filter: Binomial       Number of points for filter: 3  Sample start region (min): 0  Sample end region (min): 30
Marker peak width (sec): 5    Marker min peak height: 200   Marker baseline V to V?: Y    Marker baseline V to V points: 3
Lower marker selection: First peak > 500 RFU                Upper marker selection: Last peak > 200 RFU
Ladder size (bp)75, 200, 300, 400, 500, 700, 1000, 1500, 2000, 3000, 4000, 5000, 7000, 10000, 20000
Quantification using: Lower Marker          Final concentration (ng/uL): 0.5000          Dilution factor: 12.0
```

Sample: SampF12
Well location: F12
Created: Wednesday, August 11, 2021 5:34:32 PM

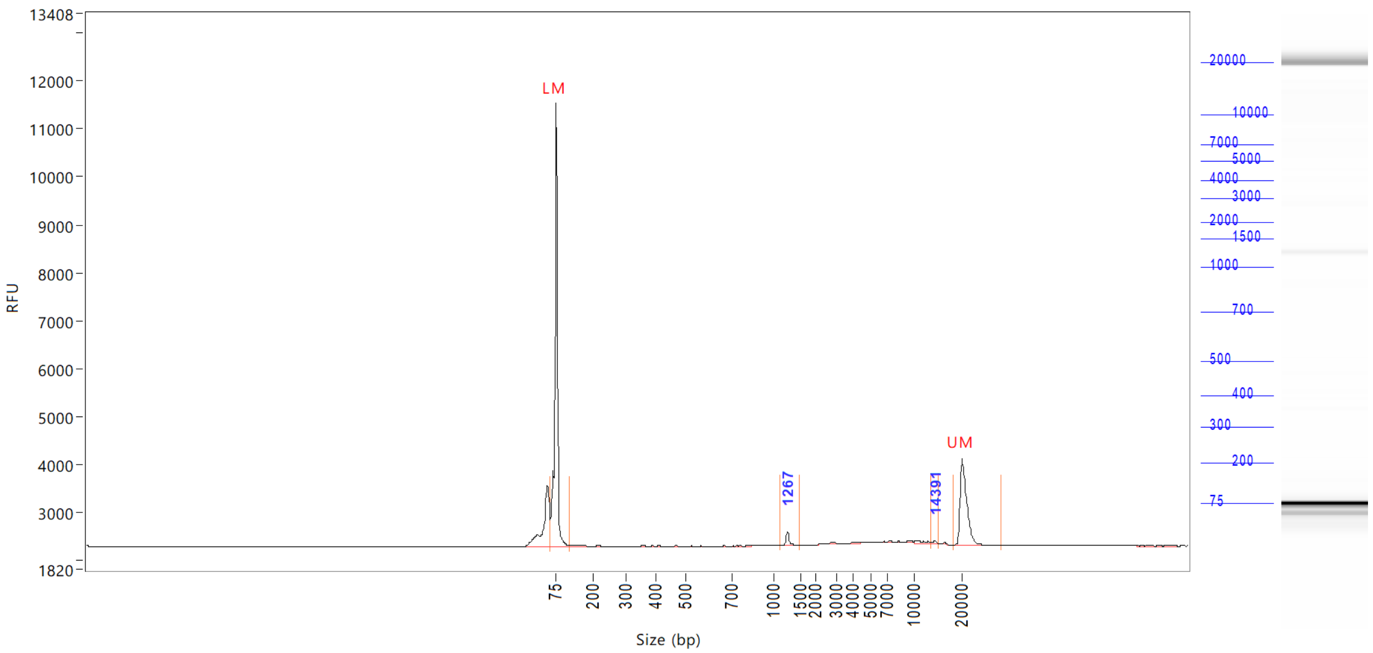

Peak	Size (bp)	Concentration (ng/uL)	From (bp)	To (bp)	Average size (bp)	CV%	RFU	Corrected peak area
1	75 (LM)	0.5000	54	118	72	9.56	9261	62.045
2	1267	0.1495	1134	1500	1258	3.46	288	1.546
3	14391	0.0324	13415	15000	14103	2.72	61	0.335
4	20000 (UM)	0.1264	18293	28342	20455	4.42	1787	15.685

TIC:	0.1819	ng/uL
TIM:	0.1994	nmole/L
Total concentration:	0.6579	ng/uL

```
Sample peak width (sec): 5    Sample min peak height: 50    Sample baseline V to V?: Y    Sample baseline V to V points: 3
Sample filter: Binomial       Number of points for filter: 3  Sample start region (min): 0  Sample end region (min): 30
Marker peak width (sec): 5    Marker min peak height: 200   Marker baseline V to V?: Y    Marker baseline V to V points: 3
Lower marker selection: First peak > 500 RFU                Upper marker selection: Last peak > 200 RFU
Ladder size (bp)75, 200, 300, 400, 500, 700, 1000, 1500, 2000, 3000, 4000, 5000, 7000, 10000, 20000
Quantification using: Lower Marker          Final concentration (ng/uL): 0.5000          Dilution factor: 12.0
```

Sample: SampG12
Well location: G12
Created: Wednesday, August 11, 2021 5:34:32 PM

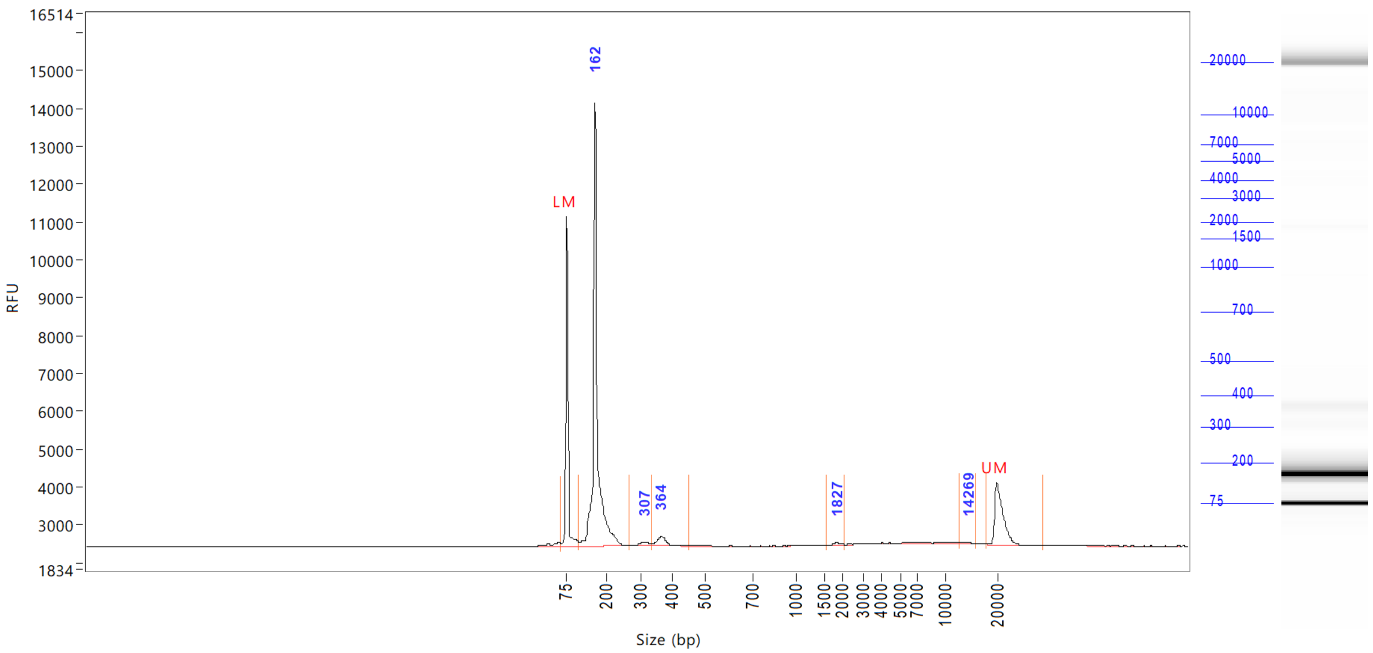

Peak	Size<br>(bp)	Concentration<br>(ng/uL)	From<br>(bp)	To<br>(bp)	Average size<br>(bp)	CV%	RFU	Corrected peak area
1	75 (LM)	0.5000	58	114	75	9.61	8716	49.968
2	162	13.2563	114	264	165	10.50	11722	110.397
3	307	0.2626	264	331	305	4.66	103	2.187
4	364	0.6007	331	450	368	6.74	240	5.003
5	1827	0.0727	1539	2115	1831	5.02	75	0.606
6	14269	0.0767	12562	15854	13997	5.83	55	0.639
7	20000 (UM)	0.1735	17805	29189	20631	5.17	1633	17.337

TIC:	14.2691	ng/uL
TIM:	136.2387	nmole/L
Total concentration:	14.6004	ng/uL

```
Sample peak width (sec): 5    Sample min peak height: 50    Sample baseline V to V?: Y    Sample baseline V to V points: 3
Sample filter: Binomial       Number of points for filter: 3  Sample start region (min): 0  Sample end region (min): 30
Marker peak width (sec): 5    Marker min peak height: 200   Marker baseline V to V?: Y    Marker baseline V to V points: 3
Lower marker selection: First peak > 500 RFU                Upper marker selection: Last peak > 200 RFU
Ladder size (bp)75, 200, 300, 400, 500, 700, 1000, 1500, 2000, 3000, 4000, 5000, 7000, 10000, 20000
Quantification using: Lower Marker          Final concentration (ng/uL): 0.5000          Dilution factor: 12.0
```

Sample: SampH12
Well location: H12
Created: Wednesday, August 11, 2021 5:34:32 PM

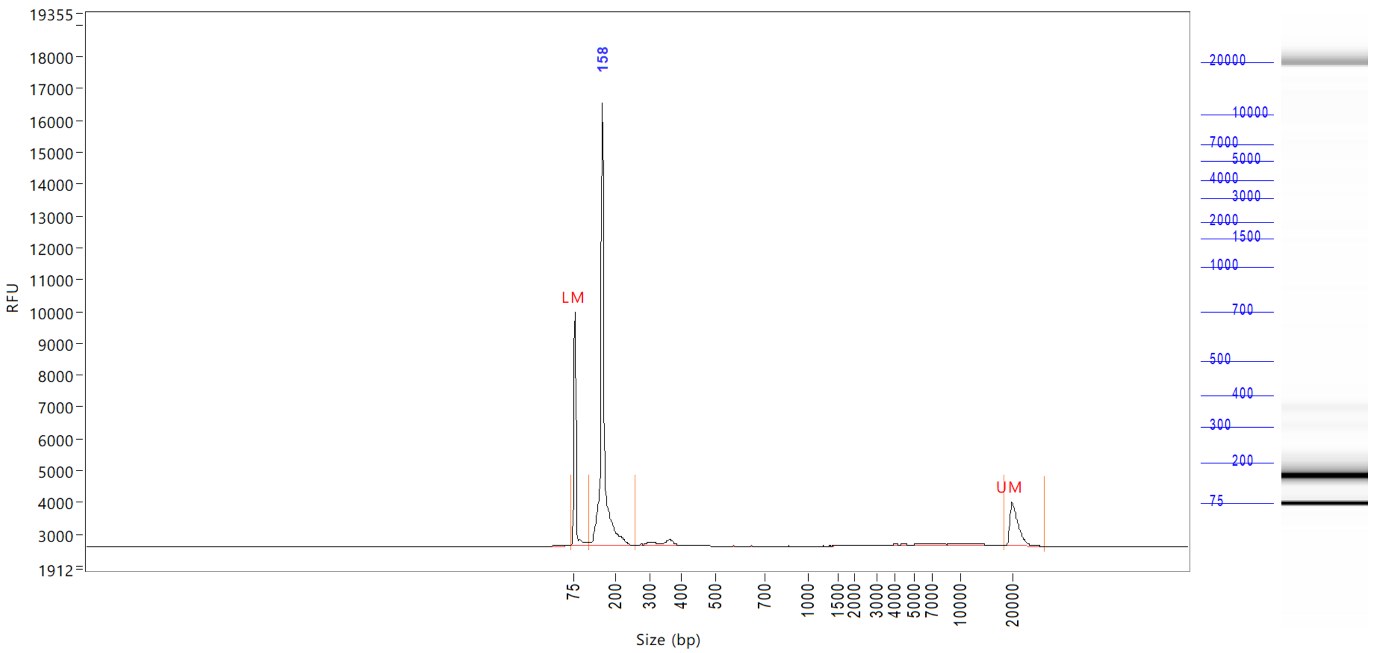

Peak	Size (bp)	Concentration (ng/uL)	From (bp)	To (bp)	Average size (bp)	CV%	RFU	Corrected peak area
1	75 (LM)	0.5000	64	118	75	10.65	7313	40.850
2	158	17.2500	118	255	160	10.18	13916	117.445
3	20000 (UM)	0.1710	18293	26408	20538	4.63	1370	13.968

TIC:	17.2500	ng/uL
TIM:	177.2107	nmole/L
Total concentration:	18.6778	ng/uL

```
Sample peak width (sec): 5    Sample min peak height: 200   Sample baseline V to V?: Y    Sample baseline V to V points: 3
Sample filter: Binomial       Number of points for filter: 3  Sample start region (min): 0  Sample end region (min): 30
Marker peak width (sec): 5    Marker min peak height: 200   Marker baseline V to V?: Y    Marker baseline V to V points: 3
Lower marker selection: First peak > 500 RFU                Upper marker selection: Last peak > 200 RFU
Ladder size (bp)75, 200, 300, 400, 500, 700, 1000, 1500, 2000, 3000, 4000, 5000, 7000, 10000, 20000
Quantification using: Lower Marker          Final concentration (ng/uL): 0.5000          Dilution factor: 12.0
```

Sample: DNA size ladder
Well location: A7
Created: Wednesday, August 11, 2021 5:34:32 PM
Import from: C:\Users\admin\Desktop\210811_alberto_ladder.SCAL
Fit type: Point to point

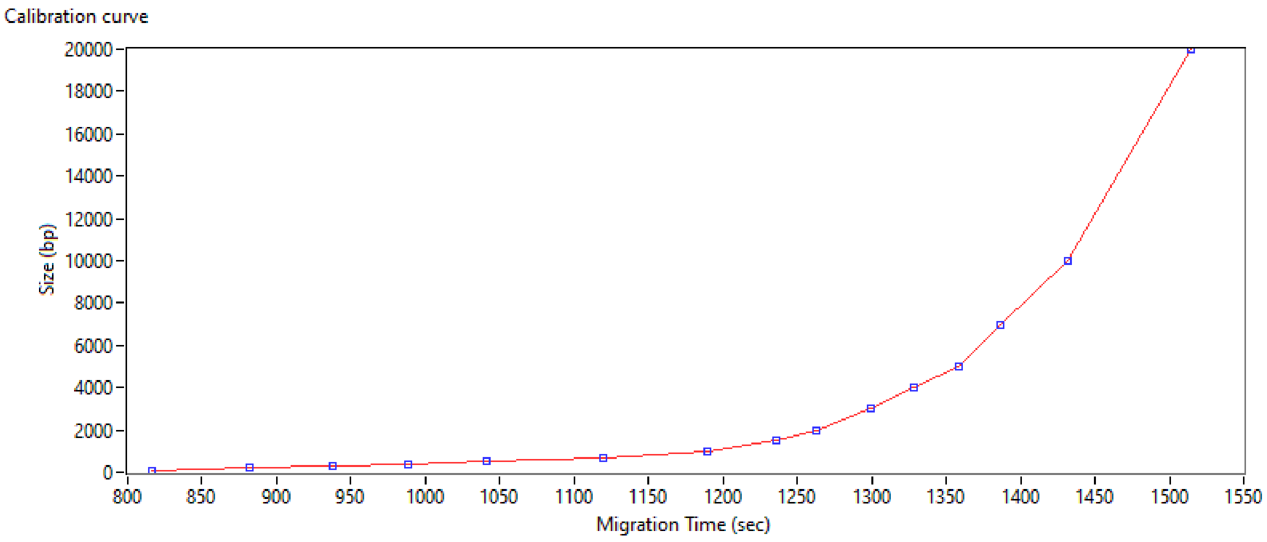

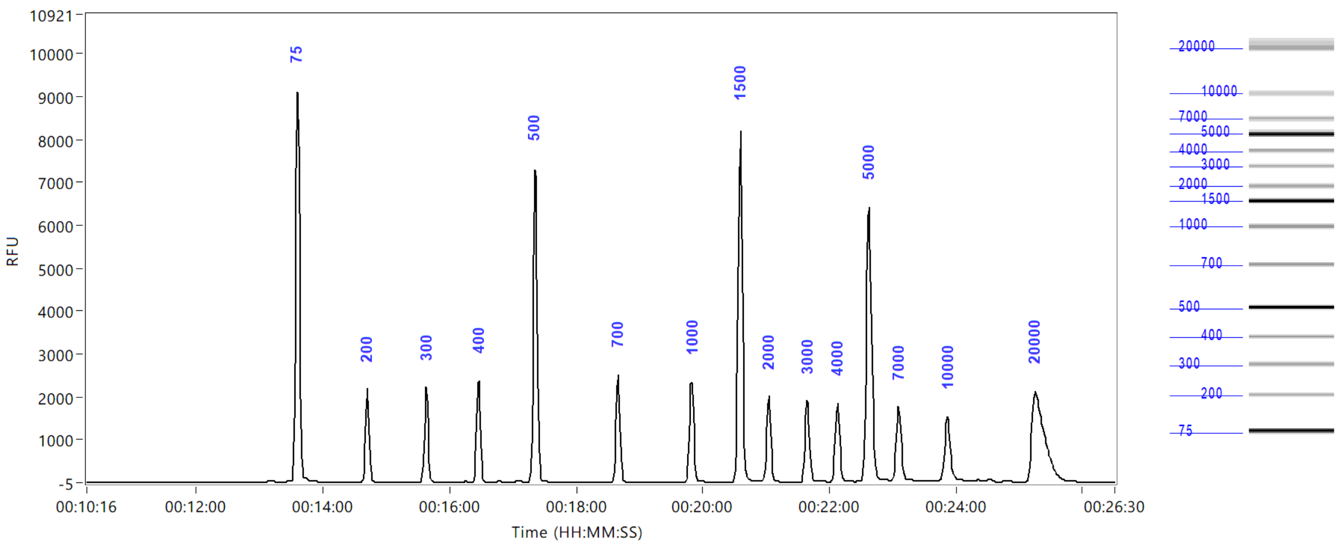

Supplement: Supplementary file 2 — sb3c00292_si_002.zip [file sb3c00292_si_002.zip › dnada_supplementary_material_pks_library_build/ZAG_trials/ZAG_Trial_2/17-13-47_redoplate1/redoplate1_t2.pdf]
