# Supplementary material for: Automated Platform for the Plasmid Construction Process
Source: ACS Synth Biol. 2023 Nov 10;12(12):3506–13. doi: 10.1021/acssynbio.3c00292 (PMC10729297; doi:10.1021/acssynbio.3c00292)

Instrument controller software run summary:

Filename and data path: C:\AATI\Data\2021 08 12\18-36-28\2021 08 12 18H 36M..raw
Created: Thursday, August 12, 2021 6:46:05 PM
Number of capillaries: 96
Array serial number: 111920-09SFS
Effect length: 33 cm
Array usage count: 188
Instrument type: ZAG DNA Analyzer
Instrument controller software version: 1.0.1.9
Device serial number: Z7597

Method Information

Method name: ZAG130GP33 - DNA 75-20000bp - Gel Prime Only.mthds
Gel prime: Yes
Full conditioning: No
Gel prime to buffer: No
Gel selection: Gel 1
Perform prerun: 8.0 kV, 30 sec.
Rinse: No
Marker 1: Row: A, 5.0 kV, 5 sec.
Rinse: No
Sample injection: 5.0 kV, 5 sec.
Separation: 8.0 kV, 30.0 min.
Tray name: Sample Tray-2

Analysis mode: DNA

Notes

Gel Image

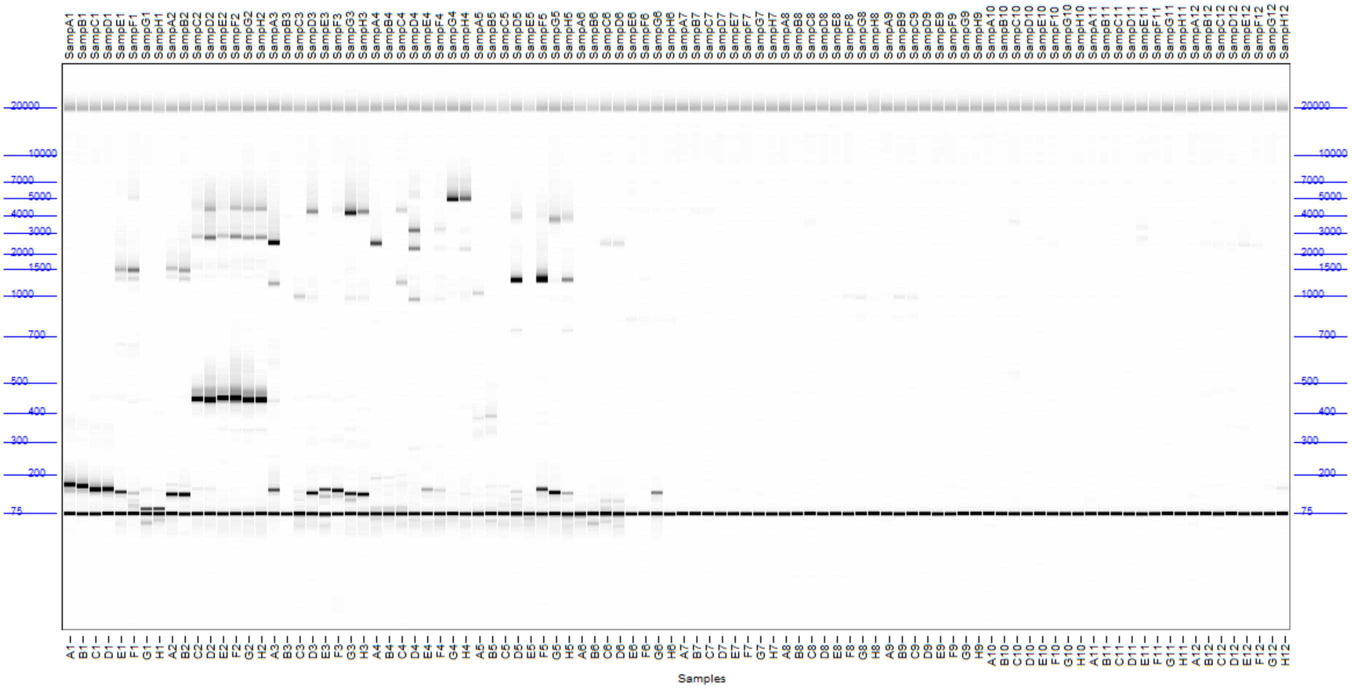

Filename and data path: C:\AATI\Data\2021 08 12\18-36-28\2021 08 12 18H 36M..raw

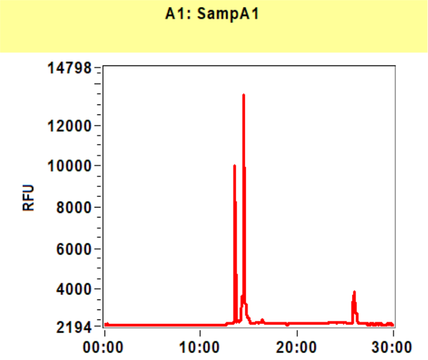

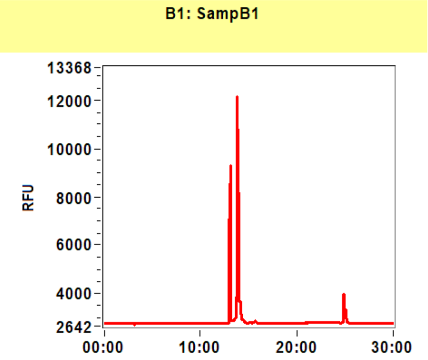

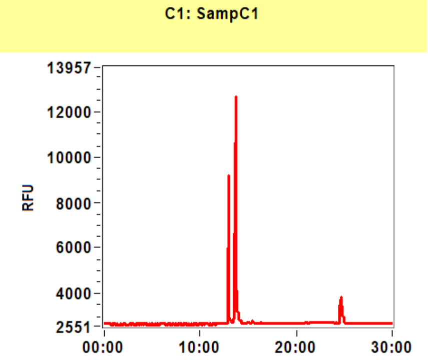

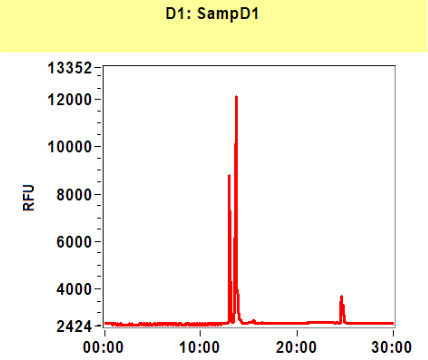

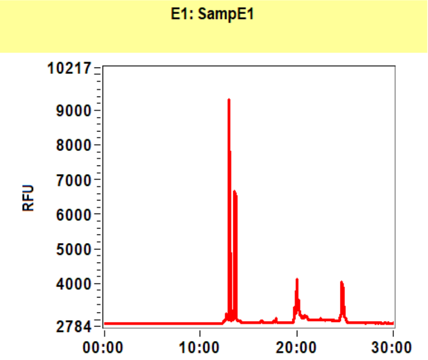

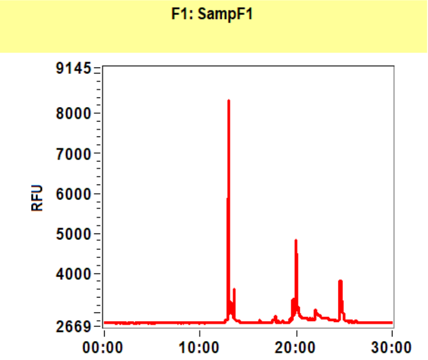

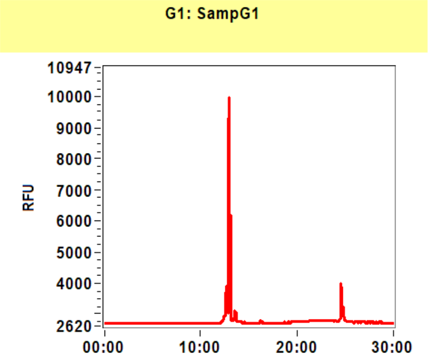

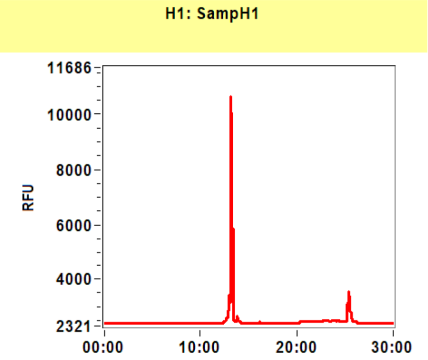

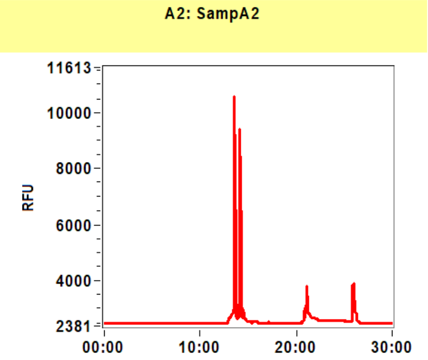

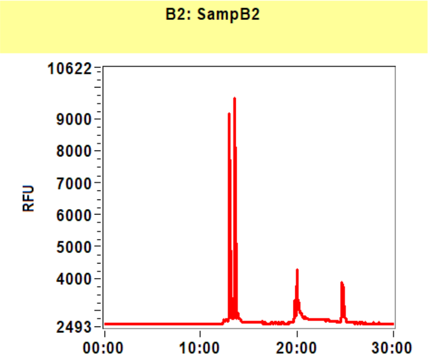

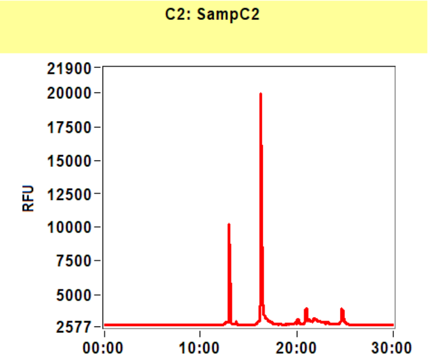

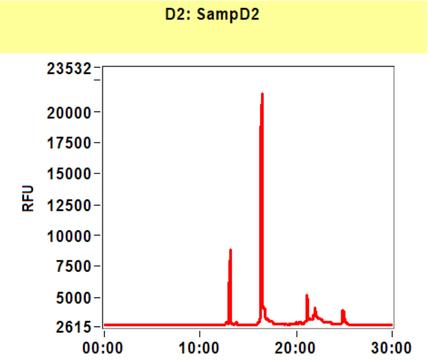

Filename and data path: C:\AATI\Data\2021 08 12\18-36-28\2021 08 12 18H 36M..raw

E2: SampE2

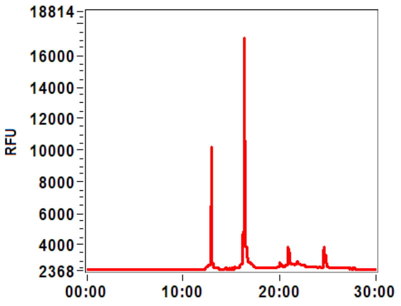

F2: SampF2

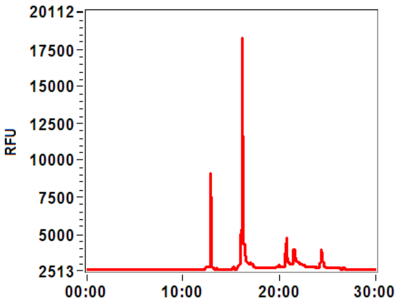

G2: SampG2

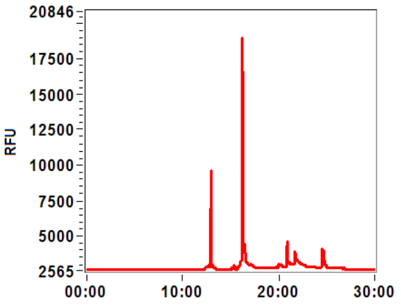

H2: SampH2

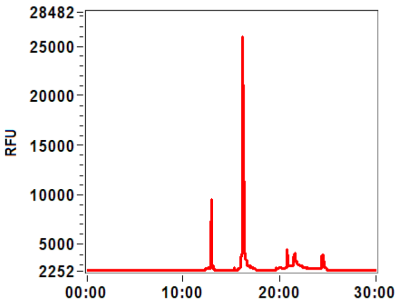

A3: SampA3

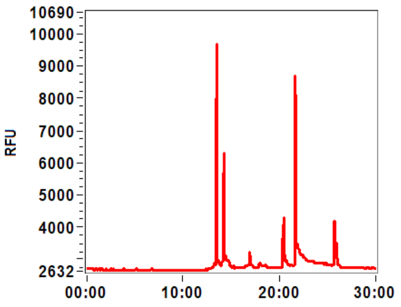

B3: SampB3

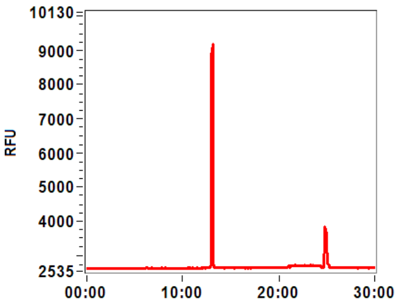

C3: SampC3

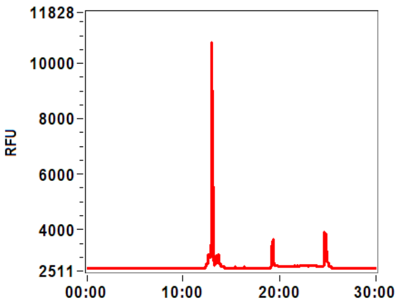

D3: SampD3

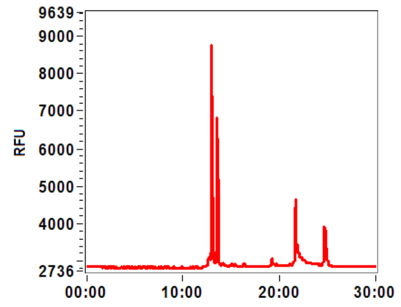

E3: SampE3

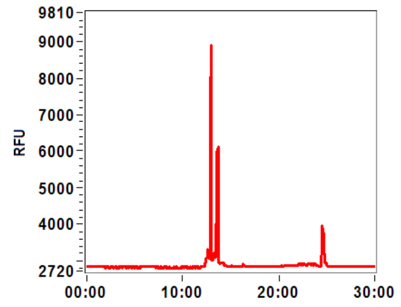

F3: SampF3

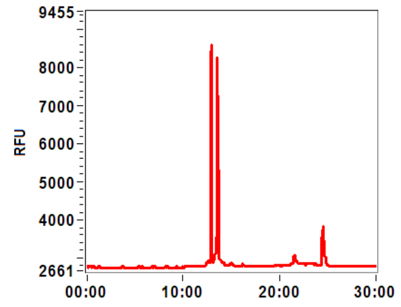

G3: SampG3

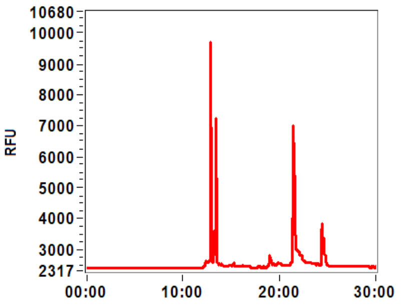

H3: SampH3

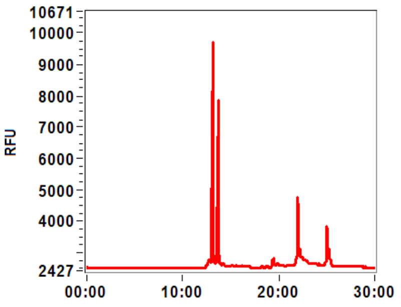

Filename and data path: C:\AATI\Data\2021 08 12\18-36-28\2021 08 12 18H 36M..raw

A4: SampA4

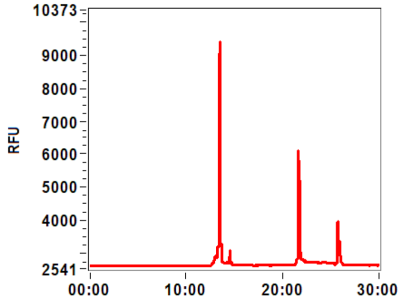

B4: SampB4

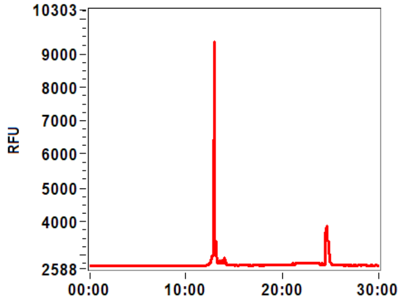

C4: SampC4

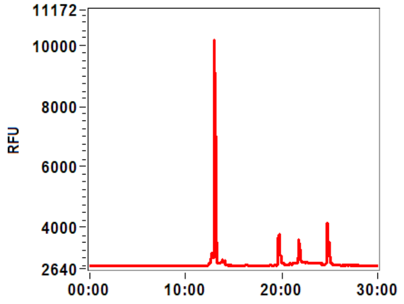

D4: SampD4

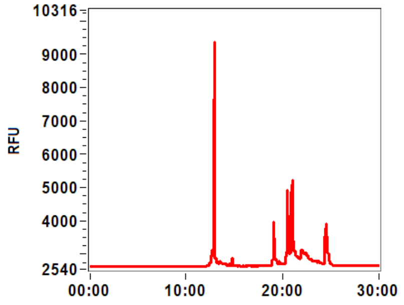

E4: SampE4

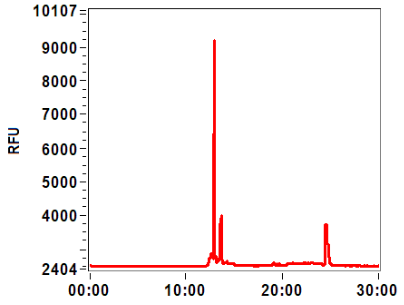

F4: SampF4

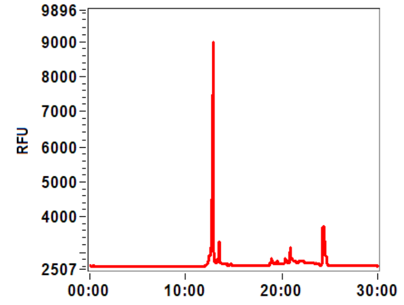

G4: SampG4

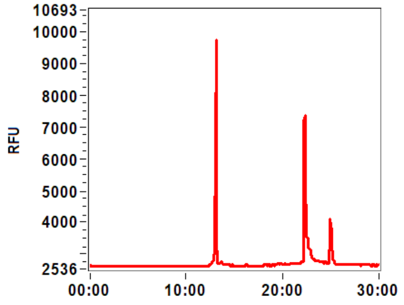

H4: SampH4

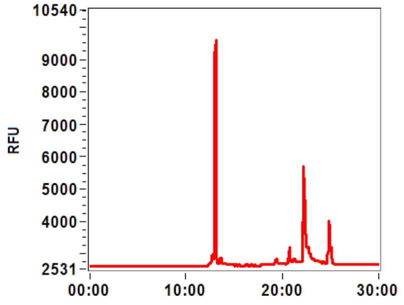

A5: SampA5

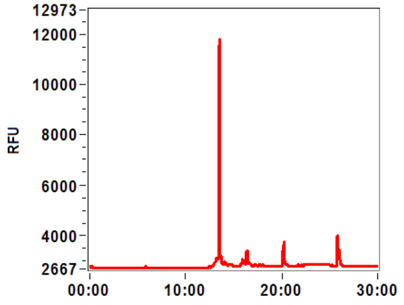

B5: SampB5

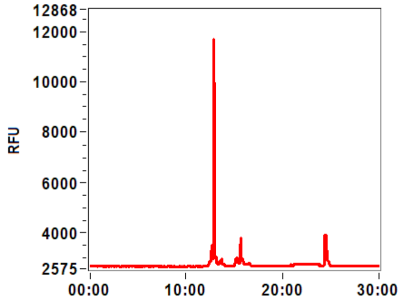

C5: SampC5

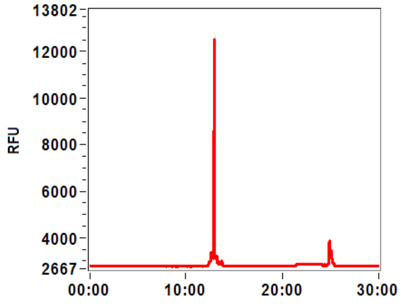

D5: SampD5

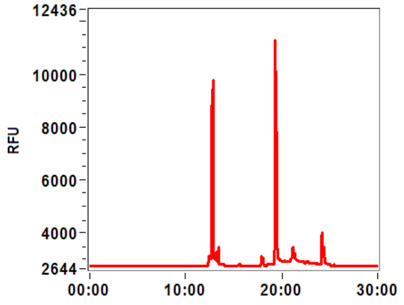

Filename and data path: C:\AATI\Data\2021 08 12\18-36-28\2021 08 12 18H 36M..raw

E5: SampE5

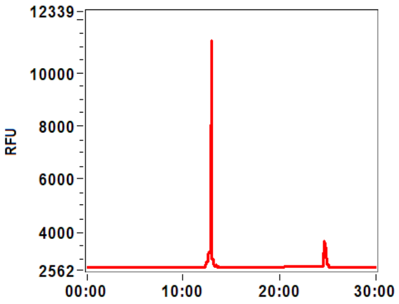

F5: SampF5

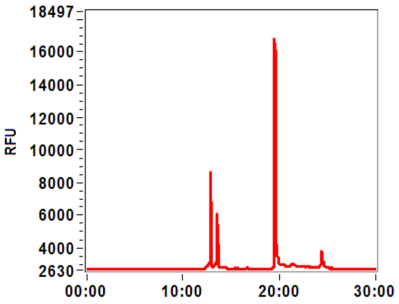

G5: SampG5

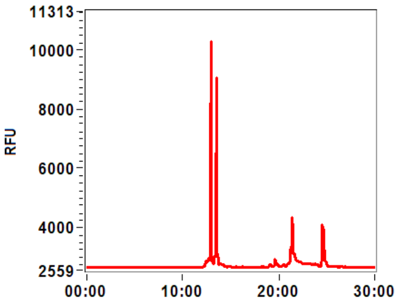

H5: SampH5

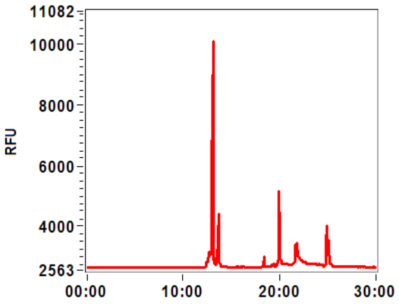

A6: SampA6

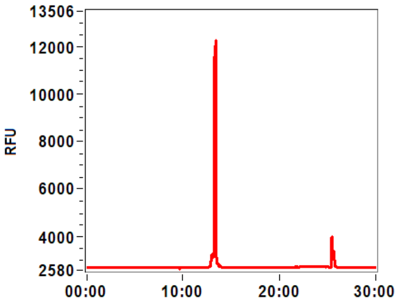

B6: SampB6

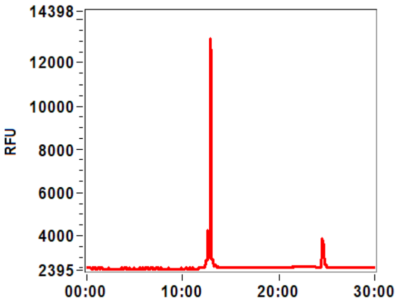

C6: SampC6

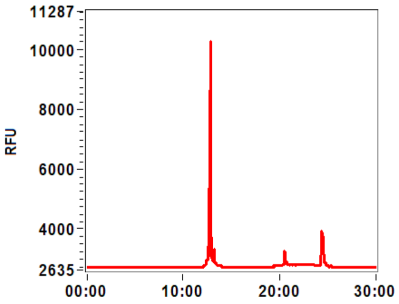

D6: SampD6

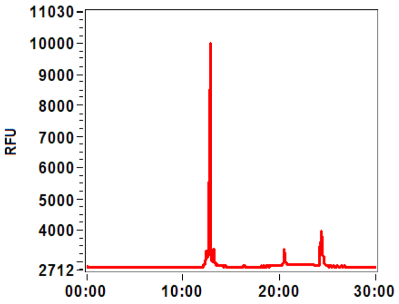

E6: SampE6

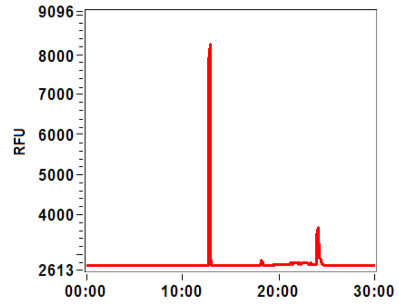

F6: SampF6

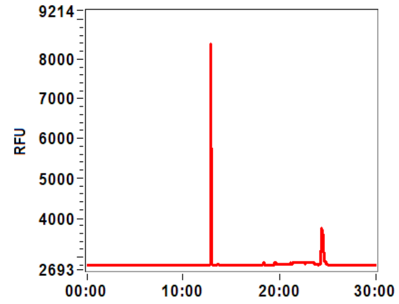

G6: SampG6

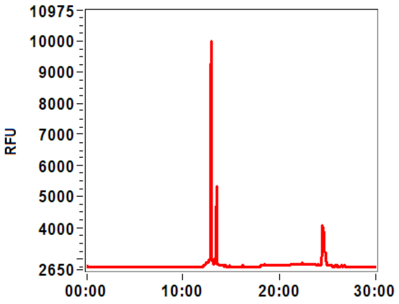

H6: SampH6

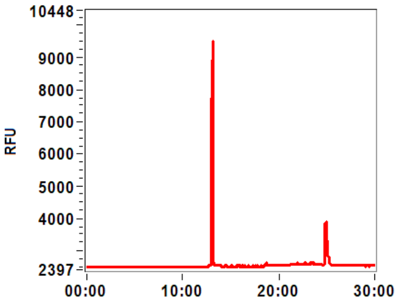

Filename and data path: C:\AATI\Data\2021 08 12\18-36-28\2021 08 12 18H 36M..raw

A7: SampA7

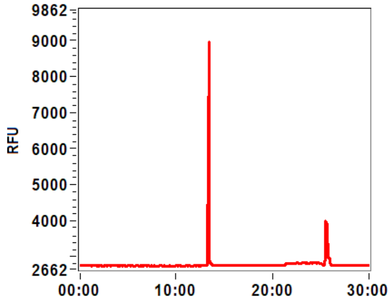

B7: SampB7

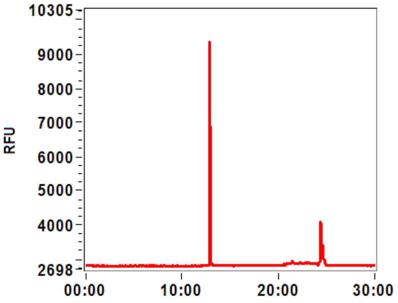

C7: SampC7

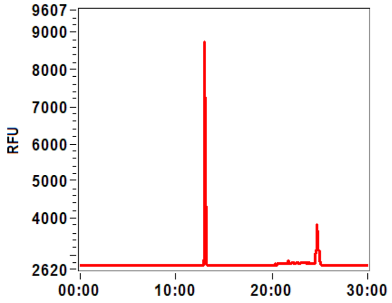

D7: SampD7

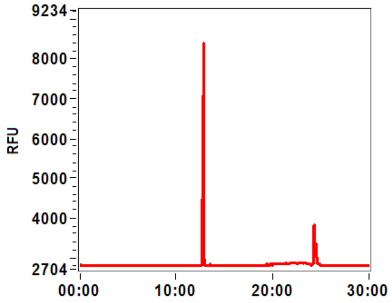

E7: SampE7

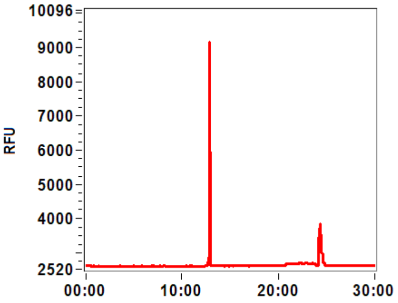

F7: SampF7

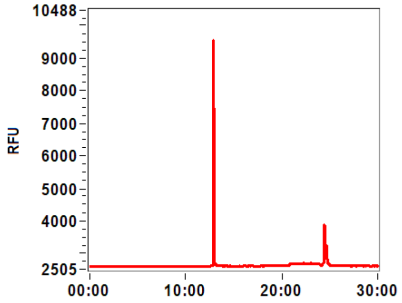

G7: SampG7

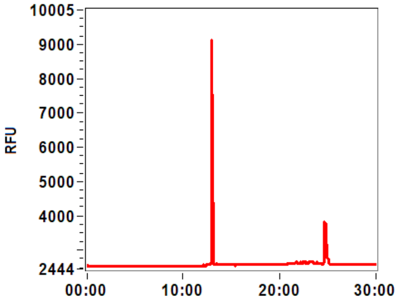

H7: SampH7

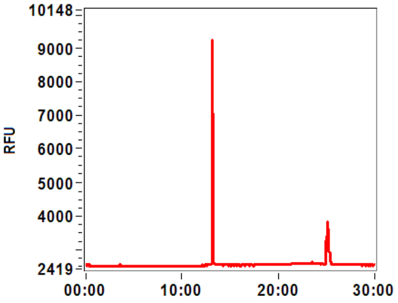

A8: SampA8

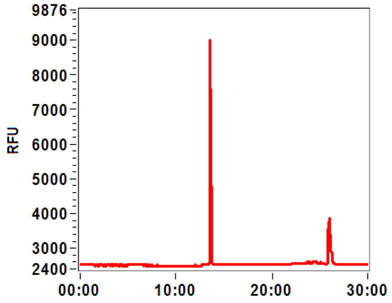

B8: SampB8

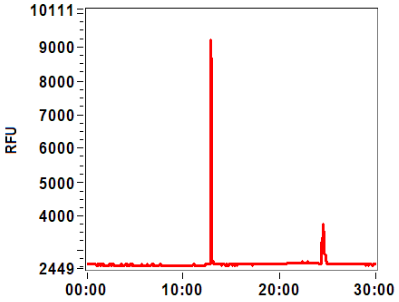

C8: SampC8

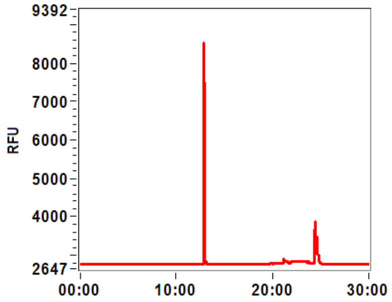

D8: SampD8

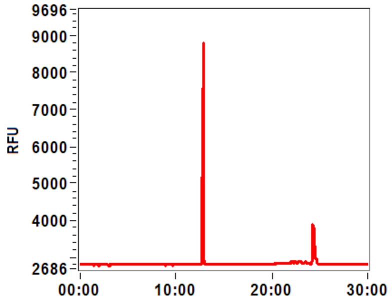

Filename and data path: C:\AATI\Data\2021 08 12\18-36-28\2021 08 12 18H 36M..raw

E8: SampE8

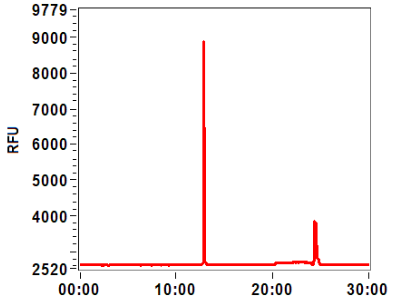

F8: SampF8

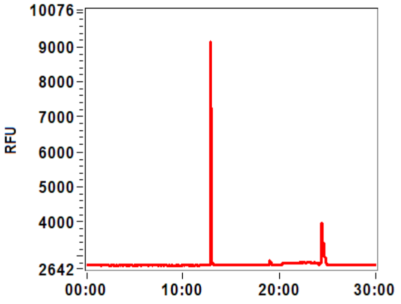

G8: SampG8

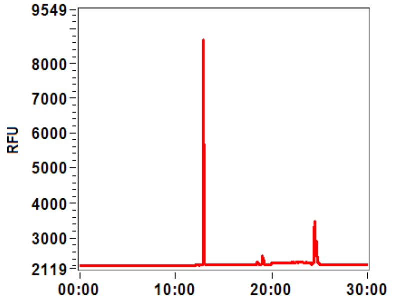

H8: SampH8

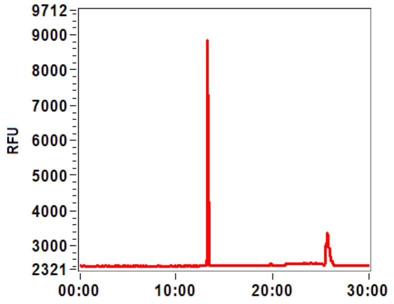

A9: SampA9

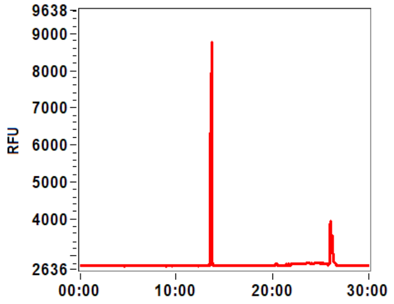

B9: SampB9

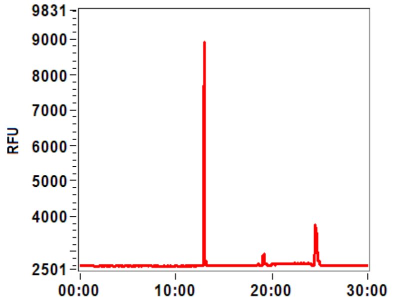

C9: SampC9

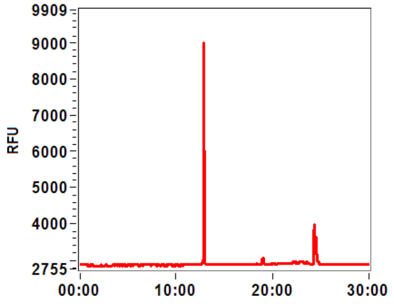

D9: SampD9

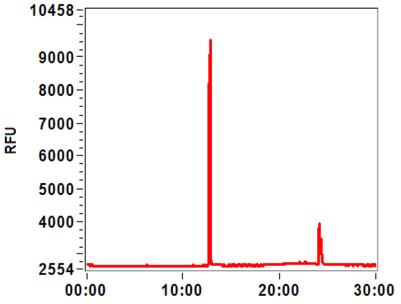

E9: SampE9

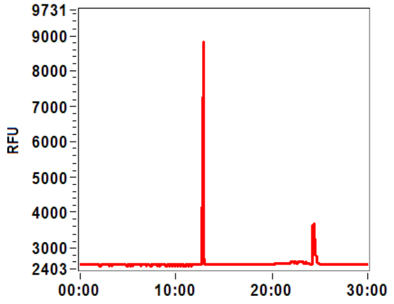

F9: SampF9

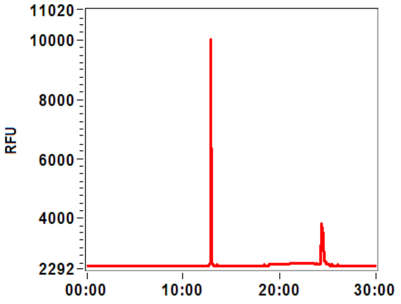

G9: SampG9

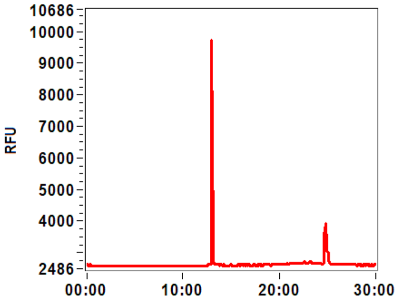

H9: SampH9

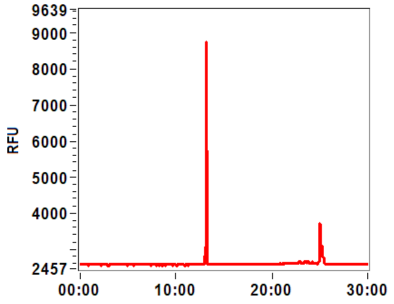

Filename and data path: C:\AATI\Data\2021 08 12\18-36-28\2021 08 12 18H 36M..raw

A10: SampA10

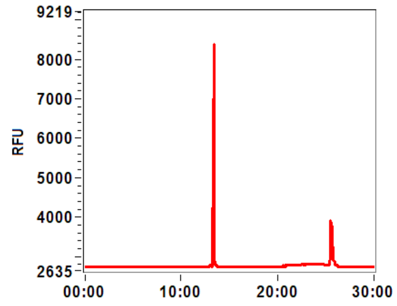

B10: SampB10

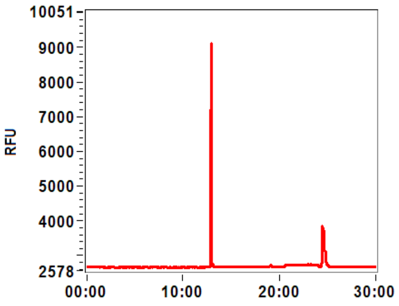

C10: SampC10

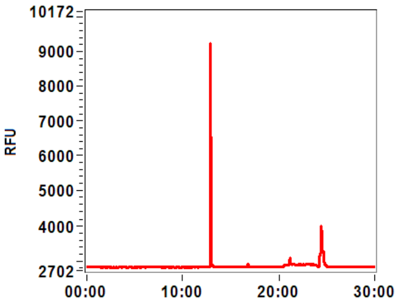

D10: SampD10

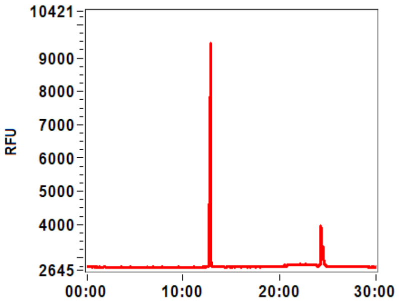

E10: SampE10

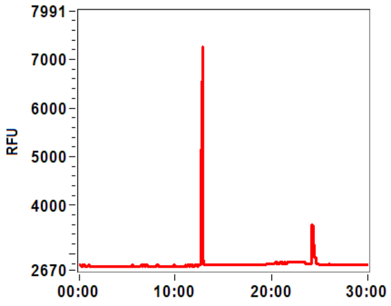

F10: SampF10

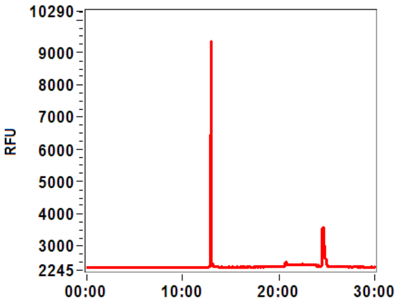

G10: SampG10

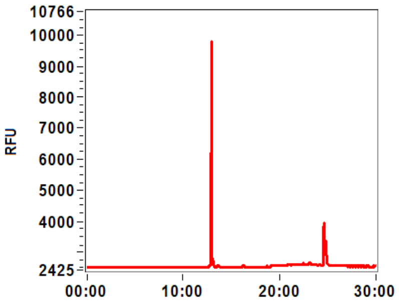

H10: SampH10

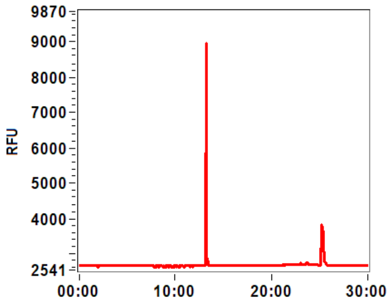

A11: SampA11

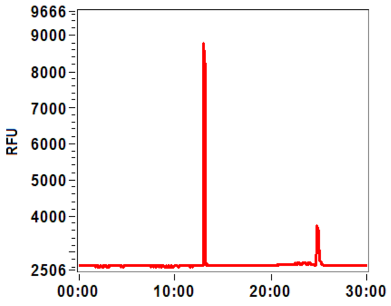

B11: SampB11

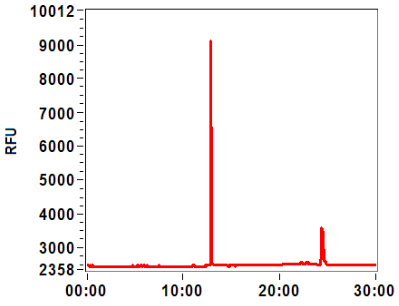

C11: SampC11

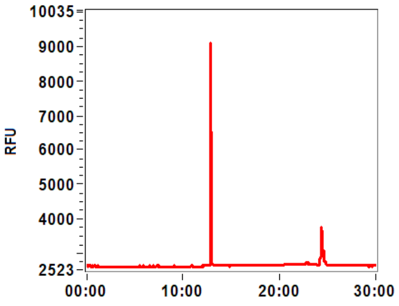

D11: SampD11

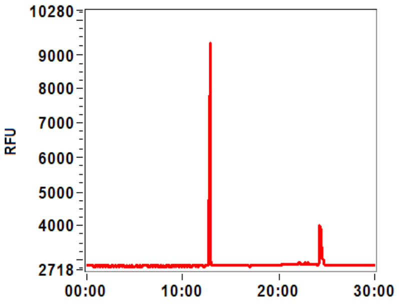

Filename and data path: C:\AATI\Data\2021 08 12\18-36-28\2021 08 12 18H 36M..raw

E11: SampE11

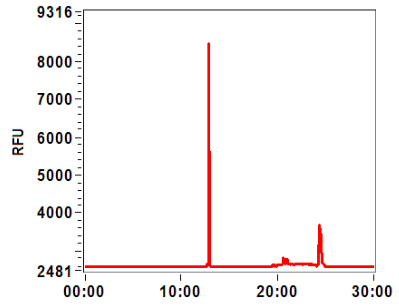

F11: SampF11

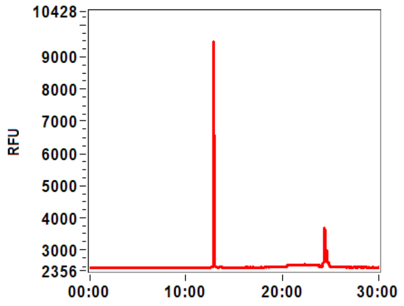

G11: SampG11

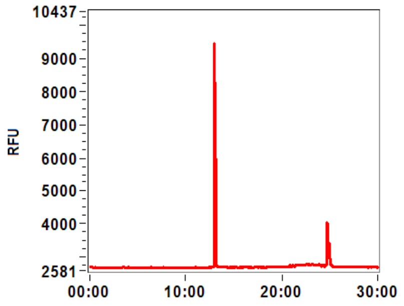

H11: SampH11

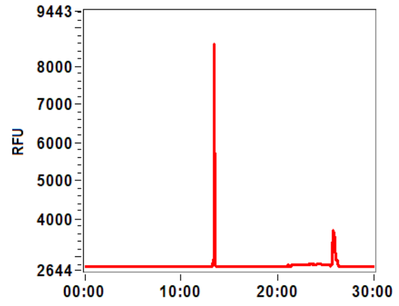

A12: SampA12

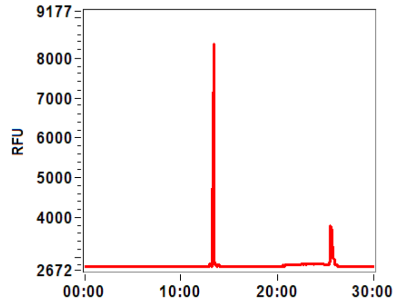

B12: SampB12

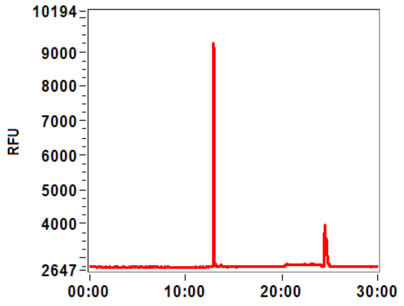

C12: SampC12

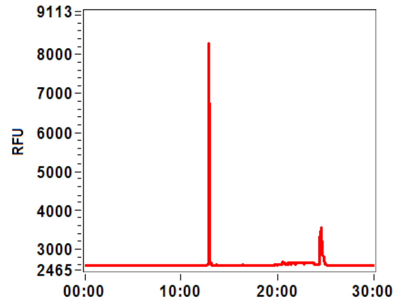

D12: SampD12

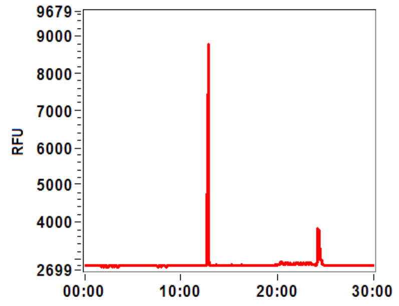

E12: SampE12

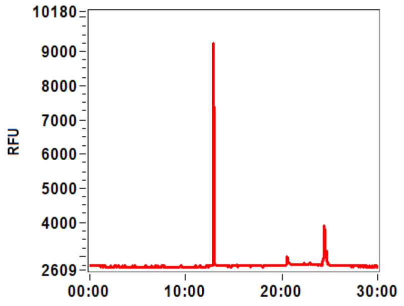

F12: SampF12

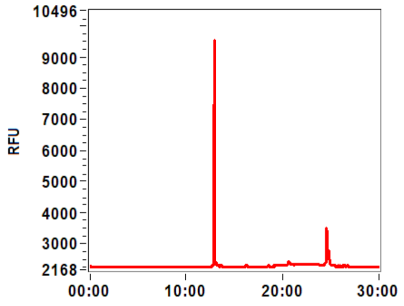

G12: SampG12

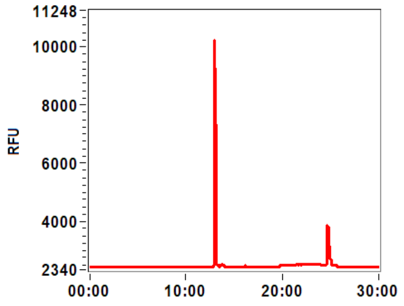

H12: SampH12

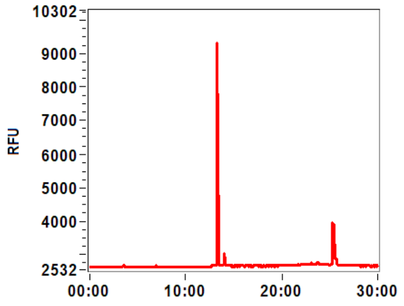

Sample: SampA1
Well location: A1
Created: Thursday, August 12, 2021 6:46:05 PM

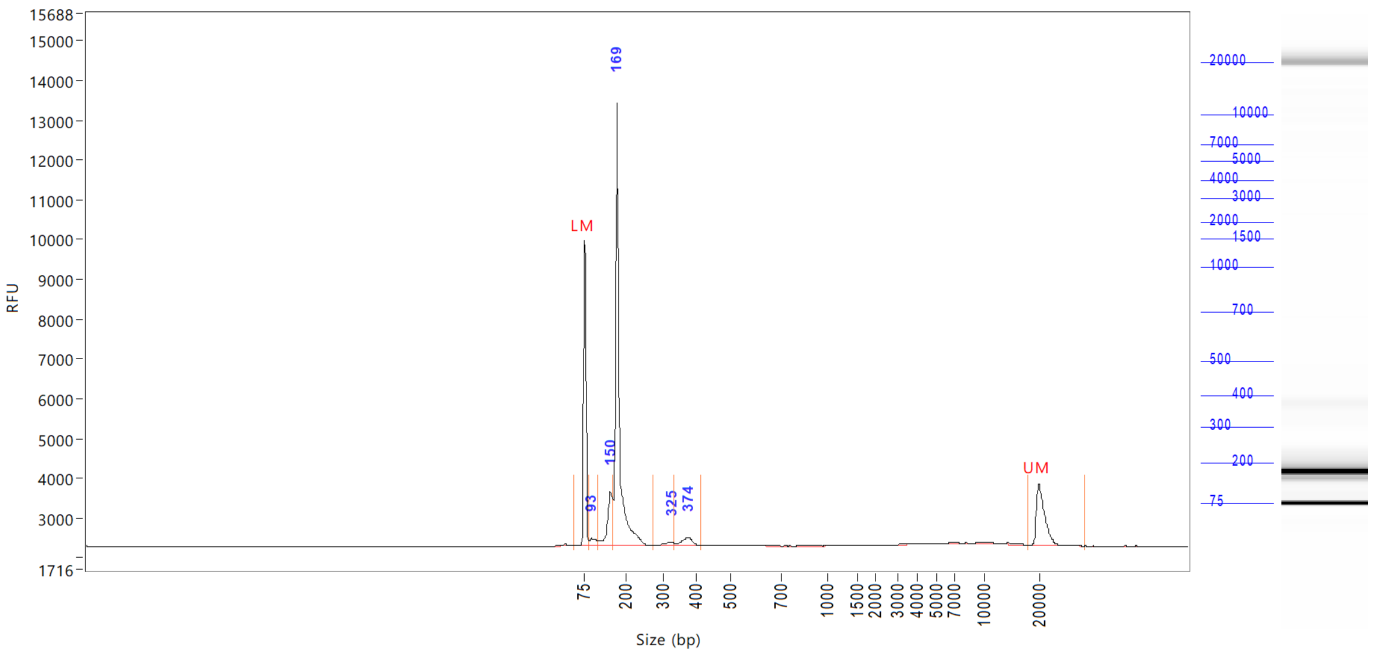

Peak	Size (bp)	Concentration (ng/uL)	From (bp)	To (bp)	Average size (bp)	CV%	RFU	Corrected peak area
1	75 (LM)	0.5000	45	87	74	4.33	7671	40.253
2	93	0.4295	87	116	100	8.42	169	2.881
3	150	2.4626	116	158	146	6.32	1341	16.522
4	169	13.4590	158	273	175	8.64	11136	90.295
5	325	0.2105	273	333	312	4.19	68	1.412
6	374	0.6097	333	414	369	4.42	202	4.091
7	20000 (UM)	0.1894	17927	28584	20523	4.47	1540	15.246

TIC:	17.1713	ng/uL
TIM:	165.0322	nmole/L
Total concentration:	17.5093	ng/uL

Sample peak width (sec): 5   Sample min peak height: 50   Sample baseline V to V?: Y   Sample baseline V to V points: 3
Sample filter: Binomial   Number of points for filter: 3 Sample start region (min): 0 Sample end region (min): 30
Marker peak width (sec): 5   Marker min peak height: 200   Marker baseline V to V?: Y   Marker baseline V to V points: 3
Lower marker selection: First peak > 500 RFU   Upper marker selection: Last peak > 200 RFU
Ladder size (bp)75, 200, 300, 400, 500, 700, 1000, 1500, 2000, 3000, 4000, 5000, 7000, 10000, 20000
Quantification using: Lower Marker   Final concentration (ng/uL): 0.5000   Dilution factor: 12.0

Sample: SampB1
Well location: B1
Created: Thursday, August 12, 2021 6:46:05 PM

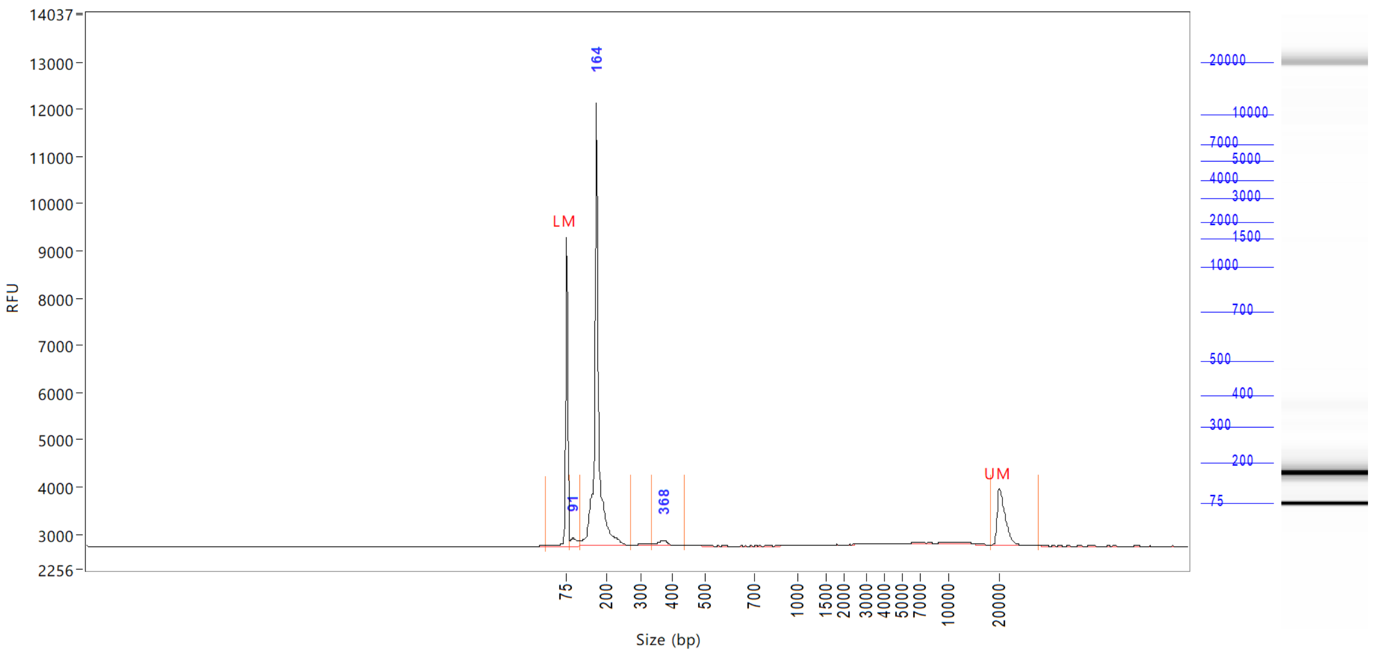

Peak	Size (bp)	Concentration (ng/uL)	From (bp)	To (bp)	Average size (bp)	CV%	RFU	Corrected peak area
1	75 (LM)	0.5000	12	86	72	9.15	6539	35.731
2	91	0.4937	86	118	101	9.73	163	2.940
3	164	14.7416	118	269	167	10.43	9401	87.790
4	368	0.3862	329	433	365	4.71	113	2.300
5	20000 (UM)	0.1714	18171	27617	20585	5.08	1210	12.250

TIC:	15.6215	ng/uL
TIM:	154.8714	nmole/L
Total concentration:	16.2398	ng/uL

Sample peak width (sec): 5   Sample min peak height: 50   Sample baseline V to V?: Y   Sample baseline V to V points: 3
Sample filter: Binomial   Number of points for filter: 3 Sample start region (min): 0 Sample end region (min): 30
Marker peak width (sec): 5   Marker min peak height: 200   Marker baseline V to V?: Y   Marker baseline V to V points: 3
Lower marker selection: First peak > 500 RFU   Upper marker selection: Last peak > 200 RFU
Ladder size (bp)75, 200, 300, 400, 500, 700, 1000, 1500, 2000, 3000, 4000, 5000, 7000, 10000, 20000
Quantification using: Lower Marker   Final concentration (ng/uL): 0.5000   Dilution factor: 12.0

Sample: SampC1
Well location: C1
Created: Thursday, August 12, 2021 6:46:05 PM

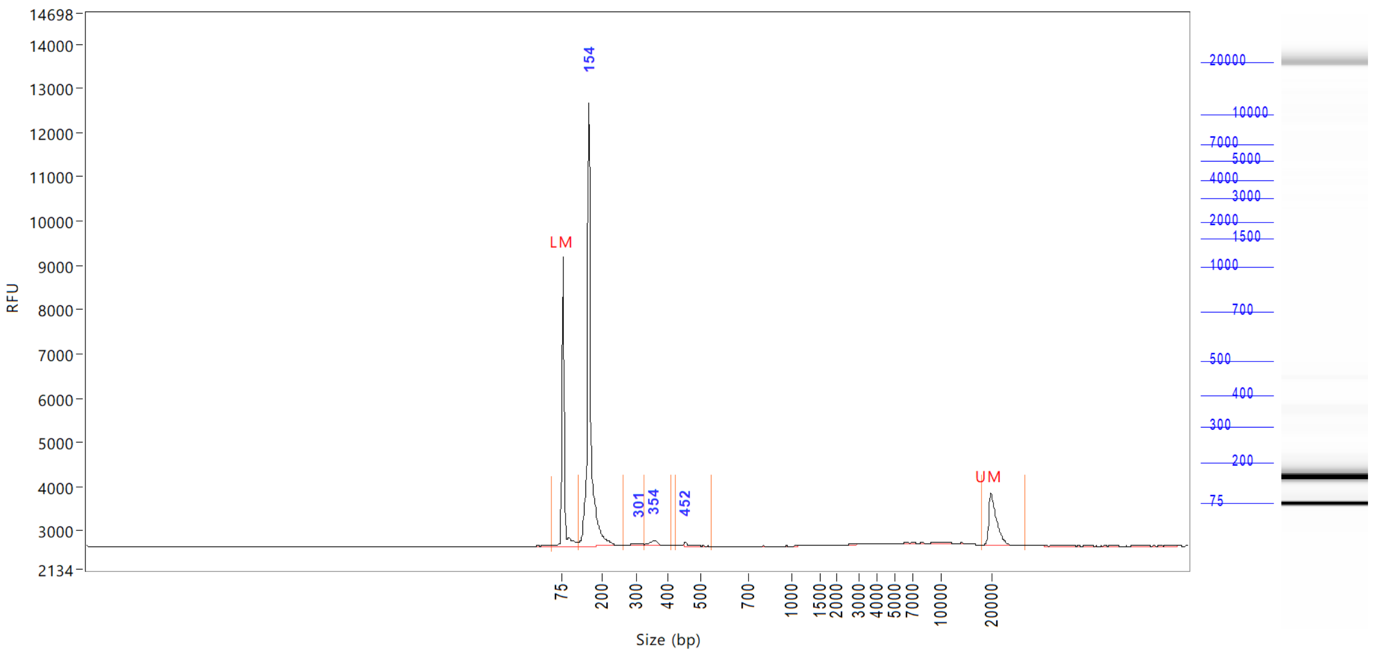

Peak	Size (bp)	Concentration (ng/uL)	From (bp)	To (bp)	Average size (bp)	CV%	RFU	Corrected peak area
1	75 (LM)	0.5000	41	122	75	12.09	6539	39.384
2	154	13.2180	122	259	156	9.33	10031	86.764
3	301	0.1934	259	321	295	4.78	56	1.270
4	354	0.3245	321	411	350	4.28	111	2.130
5	452	0.1286	424	544	460	4.65	89	0.844
6	20000 (UM)	0.1491	18171	26649	20548	4.58	1175	11.744

TIC:	13.8645	ng/uL
TIM:	142.3296	nmole/L
Total concentration:	14.2626	ng/uL

Sample peak width (sec): 5   Sample min peak height: 50   Sample baseline V to V?: Y   Sample baseline V to V points: 3
Sample filter: Binomial   Number of points for filter: 3 Sample start region (min): 0 Sample end region (min): 30
Marker peak width (sec): 5   Marker min peak height: 200   Marker baseline V to V?: Y   Marker baseline V to V points: 3
Lower marker selection: First peak > 500 RFU   Upper marker selection: Last peak > 200 RFU
Ladder size (bp)75, 200, 300, 400, 500, 700, 1000, 1500, 2000, 3000, 4000, 5000, 7000, 10000, 20000
Quantification using: Lower Marker   Final concentration (ng/uL): 0.5000   Dilution factor: 12.0

Sample: SampD1
Well location: D1
Created: Thursday, August 12, 2021 6:46:05 PM

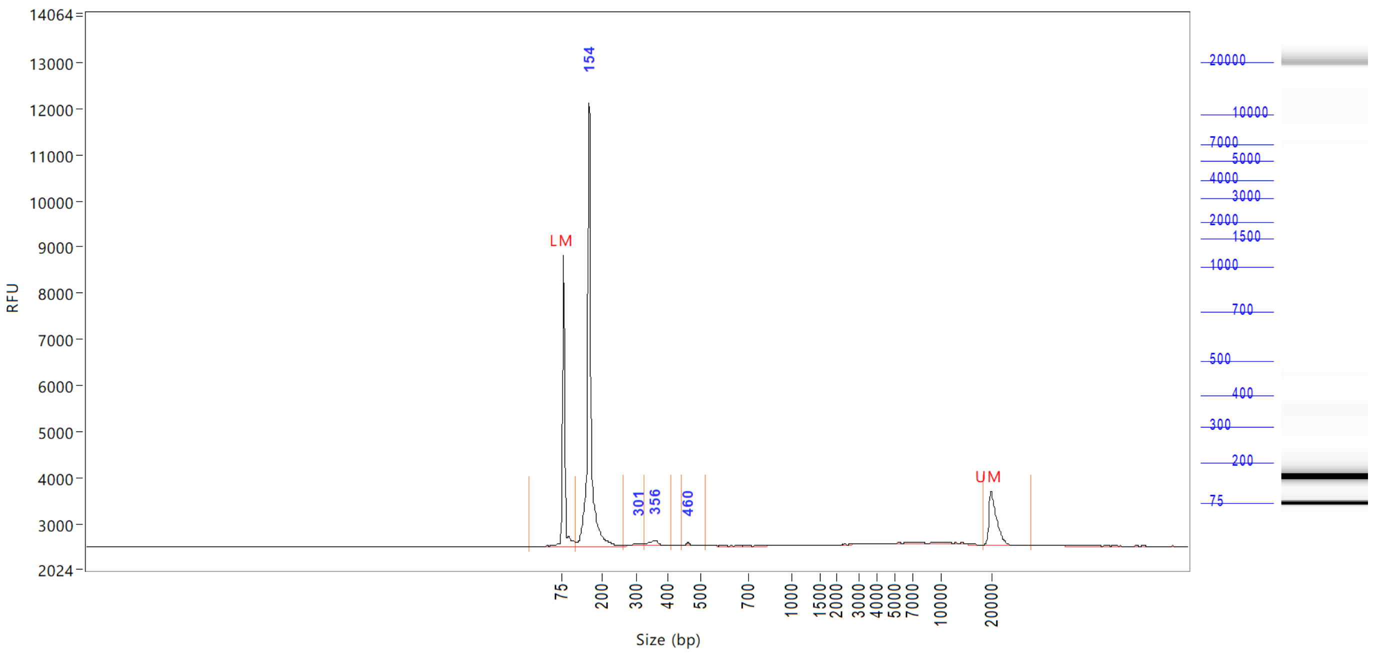

Peak	Size (bp)	Concentration (ng/uL)	From (bp)	To (bp)	Average size (bp)	CV%	RFU	Corrected peak area
1	75 (LM)	0.5000	0	116	75	14.20	6293	39.031
2	154	12.9898	116	259	156	9.65	9617	84.501
3	301	0.2266	259	321	296	5.39	61	1.474
4	356	0.3504	321	409	348	4.32	106	2.280
5	460	0.1065	443	516	466	3.48	71	0.693
6	20000 (UM)	0.1477	18415	27979	20562	4.53	1166	11.529

TIC:	13.6733	ng/uL
TIM:	140.1528	nmole/L
Total concentration:	14.1271	ng/uL

Sample peak width (sec): 5   Sample min peak height: 50   Sample baseline V to V?: Y   Sample baseline V to V points: 3
Sample filter: Binomial   Number of points for filter: 3 Sample start region (min): 0 Sample end region (min): 30
Marker peak width (sec): 5   Marker min peak height: 200   Marker baseline V to V?: Y   Marker baseline V to V points: 3
Lower marker selection: First peak > 500 RFU   Upper marker selection: Last peak > 200 RFU
Ladder size (bp)75, 200, 300, 400, 500, 700, 1000, 1500, 2000, 3000, 4000, 5000, 7000, 10000, 20000
Quantification using: Lower Marker   Final concentration (ng/uL): 0.5000   Dilution factor: 12.0

Sample: SampE1
Well location: E1
Created: Thursday, August 12, 2021 6:46:05 PM

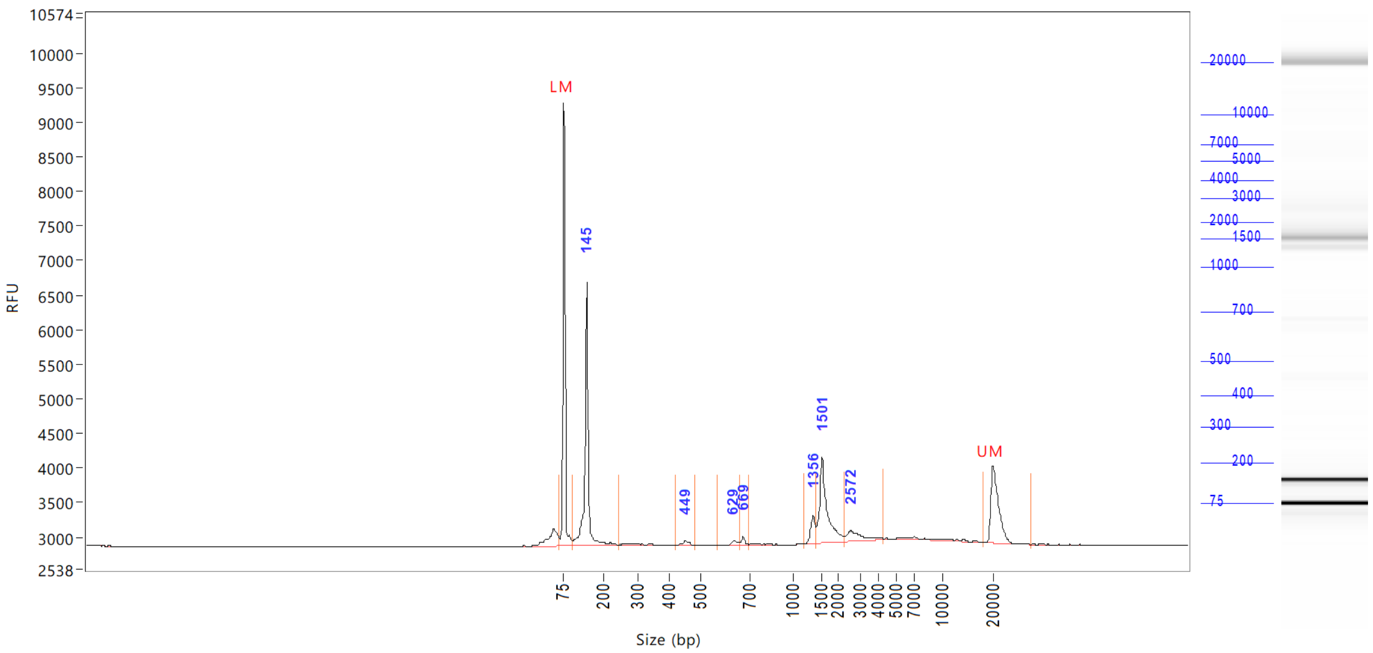

Peak	Size (bp)	Concentration (ng/uL)	From (bp)	To (bp)	Average size (bp)	CV%	RFU	Corrected peak area
1	75 (LM)	0.5000	64	101	74	6.14	6414	36.333
2	145	4.8447	101	242	144	10.59	3809	29.337
3	449	0.1270	420	481	447	2.36	69	0.769
4	629	0.1282	565	657	631	2.71	54	0.776
5	669	0.1250	657	695	669	1.20	118	0.757
6	1356	0.4812	1201	1400	1337	2.89	415	2.914
7	1501	2.4614	1400	2258	1612	11.32	1235	14.905
8	2572	0.5811	2258	4311	2885	16.74	152	3.519
9	20000 (UM)	0.1525	18049	27617	20554	4.43	1121	11.083

TIC:	8.7485	ng/uL
TIM:	59.8366	nmole/L
Total concentration:	9.2507	ng/uL

Sample peak width (sec): 5   Sample min peak height: 50   Sample baseline V to V?: Y   Sample baseline V to V points: 3
Sample filter: Binomial   Number of points for filter: 3 Sample start region (min): 0 Sample end region (min): 30
Marker peak width (sec): 5   Marker min peak height: 200   Marker baseline V to V?: Y   Marker baseline V to V points: 3
Lower marker selection: First peak > 500 RFU   Upper marker selection: Last peak > 200 RFU
Ladder size (bp)75, 200, 300, 400, 500, 700, 1000, 1500, 2000, 3000, 4000, 5000, 7000, 10000, 20000
Quantification using: Lower Marker   Final concentration (ng/uL): 0.5000   Dilution factor: 12.0

Sample: SampF1
Well location: F1
Created: Thursday, August 12, 2021 6:46:05 PM

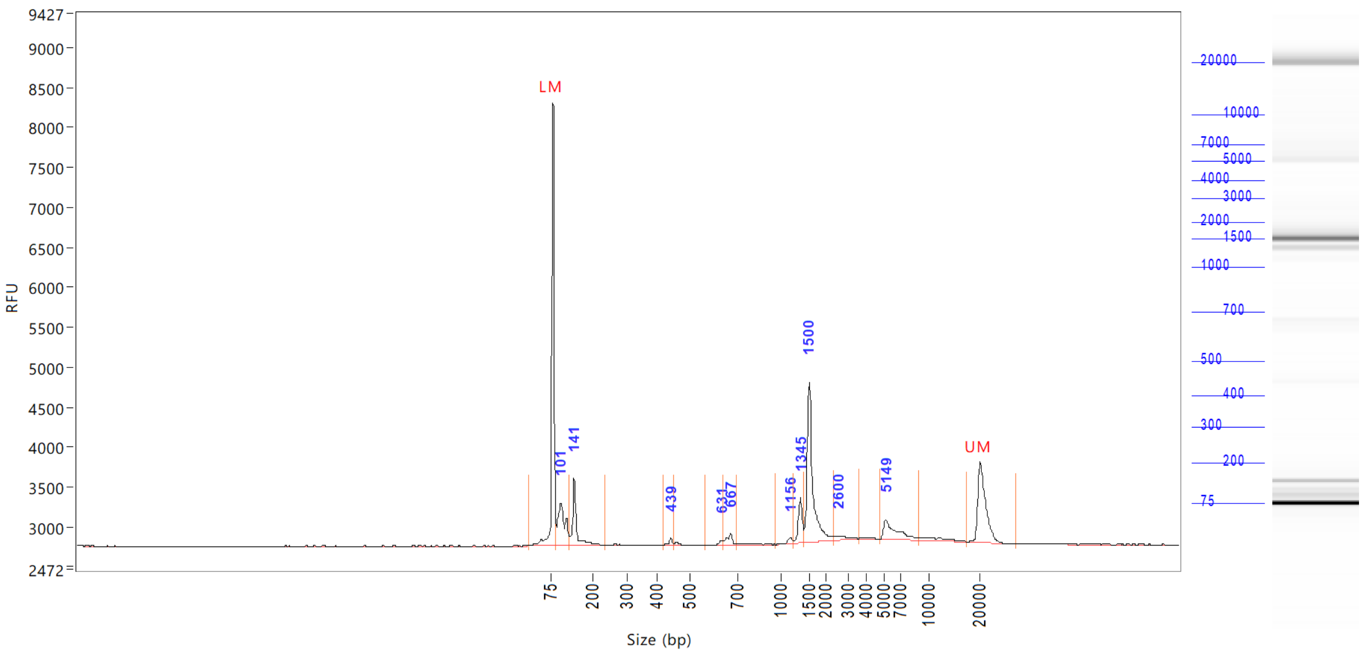

Peak	Size (bp)	Concentration (ng/uL)	From (bp)	To (bp)	Average size (bp)	CV%	RFU	Corrected peak area
1	75 (LM)	0.5000	5	86	72	12.30	5549	33.867
2	101	1.6428	86	128	103	10.72	535	9.273
3	141	1.2999	128	237	147	12.50	838	7.337
4	439	0.1039	418	450	437	1.57	84	0.586
5	631	0.0982	562	639	624	2.12	63	0.554
6	667	0.2406	639	695	659	1.79	145	1.358
7	1156	0.1063	965	1223	1141	4.21	78	0.600
8	1345	0.6650	1223	1411	1333	2.90	566	3.754
9	1500	3.1303	1411	2372	1576	10.64	1996	17.669
10	2600	0.1834	2372	3608	2756	10.13	56	1.035
11	5149	0.7913	4759	8934	6216	17.36	247	4.466
12	20000 (UM)	0.1512	17439	27133	20542	4.78	1013	10.241

TIC:	8.2617	ng/uL
TIM:	46.5388	nmole/L
Total concentration:	8.7194	ng/uL

```
Sample peak width (sec): 5    Sample min peak height: 50    Sample baseline V to V?: Y    Sample baseline V to V points: 3
Sample filter: Binomial       Number of points for filter: 3  Sample start region (min): 0  Sample end region (min): 30
Marker peak width (sec): 5    Marker min peak height: 200   Marker baseline V to V?: Y    Marker baseline V to V points: 3
Lower marker selection: First peak > 500 RFU                Upper marker selection: Last peak > 200 RFU
Ladder size (bp)75, 200, 300, 400, 500, 700, 1000, 1500, 2000, 3000, 4000, 5000, 7000, 10000, 20000
Quantification using: Lower Marker          Final concentration (ng/uL): 0.5000          Dilution factor: 12.0
```

Sample: SampG1
Well location: G1
Created: Thursday, August 12, 2021 6:46:05 PM

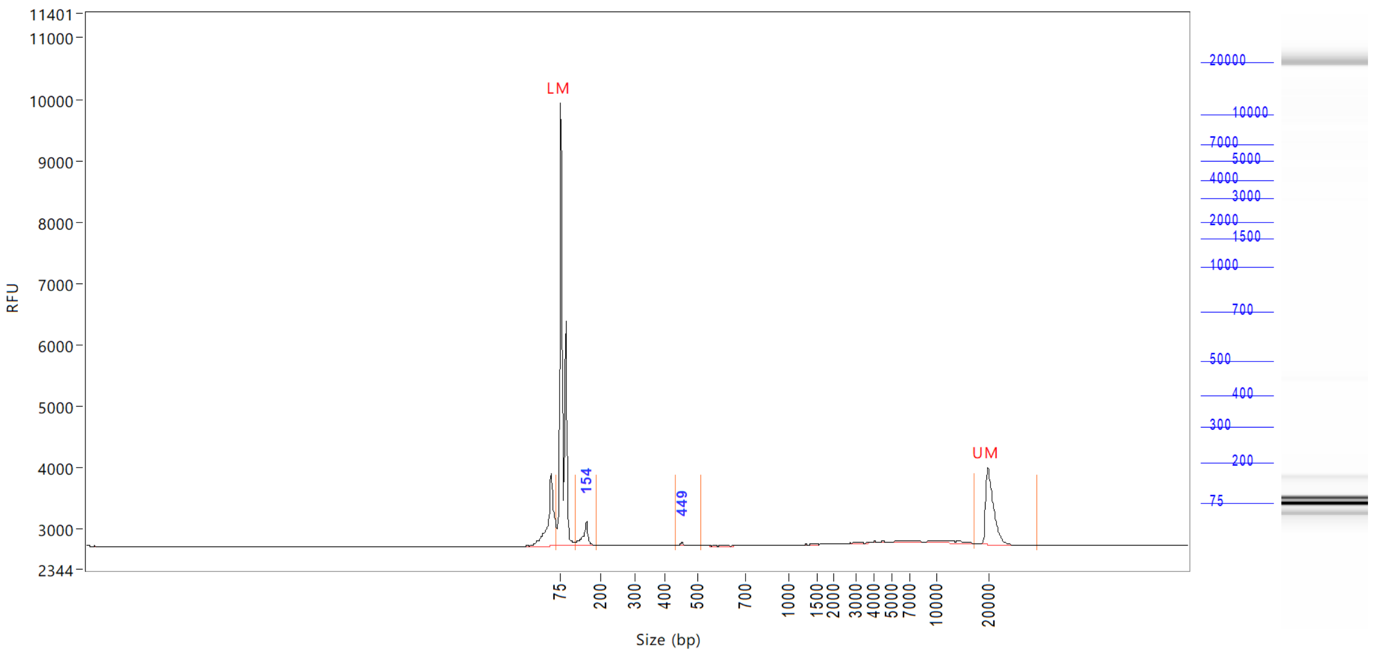

Peak	Size (bp)	Concentration (ng/uL)	From (bp)	To (bp)	Average size (bp)	CV%	RFU	Corrected peak area
1	75 (LM)	0.5000	64	120	79	11.27	7229	62.753
2	154	0.4682	120	189	147	8.58	406	4.897
3	449	0.0418	431	514	453	3.04	58	0.437
4	20000 (UM)	0.1006	17439	29672	20573	5.09	1255	12.621

TIC:	0.5100	ng/uL
TIM:	5.3861	nmole/L
Total concentration:	0.8779	ng/uL

Sample peak width (sec): 5 Sample min peak height: 50 Sample baseline V to V?: Y Sample baseline V to V points: 3
Sample filter: Binomial Number of points for filter: 3 Sample start region (min): 0 Sample end region (min): 30
Marker peak width (sec): 5 Marker min peak height: 200 Marker baseline V to V?: Y Marker baseline V to V points: 3
Lower marker selection: First peak > 500 RFU Upper marker selection: Last peak > 200 RFU
Ladder size (bp)75, 200, 300, 400, 500, 700, 1000, 1500, 2000, 3000, 4000, 5000, 7000, 10000, 20000
Quantification using: Lower Marker Final concentration (ng/uL): 0.5000 Dilution factor: 12.0

Sample: SampH1
Well location: H1
Created: Thursday, August 12, 2021 6:46:05 PM

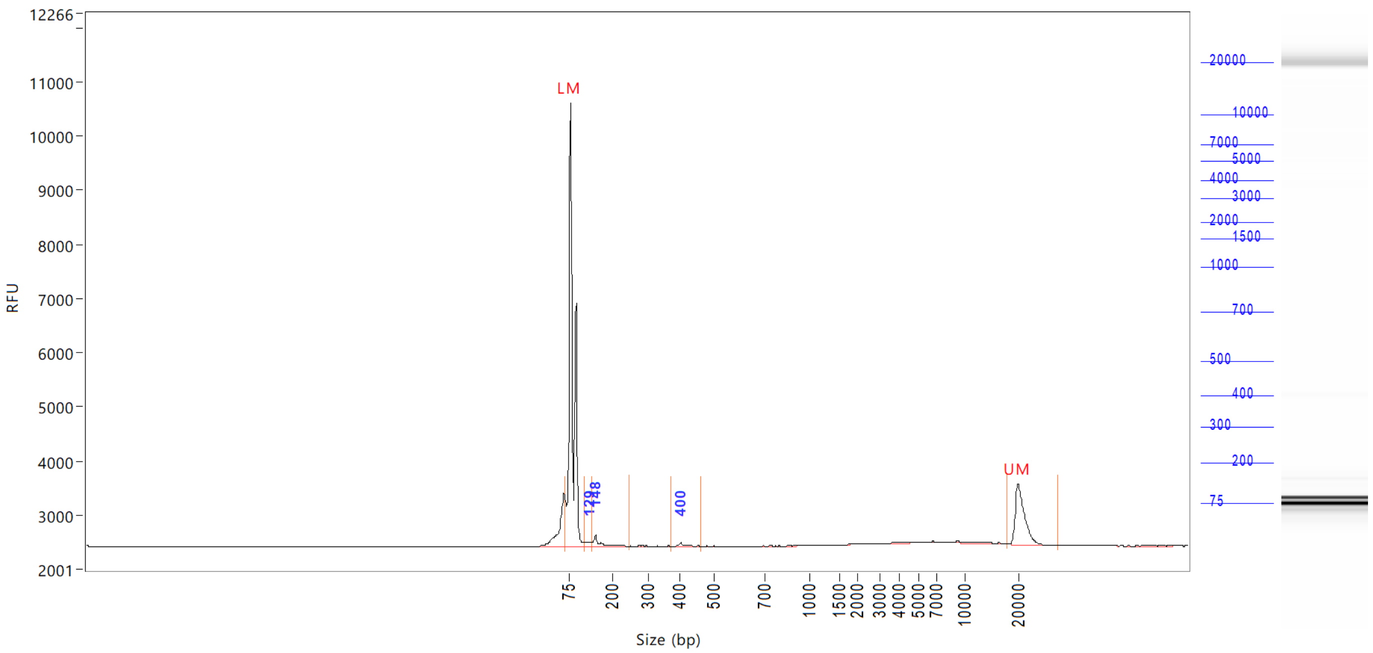

Peak	Size (bp)	Concentration (ng/uL)	From (bp)	To (bp)	Average size (bp)	CV%	RFU	Corrected peak area
1	75 (LM)	0.5000	62	116	78	11.35	8204	77.080
2	129	0.0978	116	139	128	5.51	79	1.257
3	148	0.1944	139	246	159	12.66	224	2.498
4	400	0.0479	372	462	406	4.65	62	0.615
5	20000 (UM)	0.0844	18049	27375	20490	5.22	1122	13.004

TIC:	0.3402	ng/uL
TIM:	3.4598	nmole/L
Total concentration:	0.5649	ng/uL

Sample peak width (sec): 5   Sample min peak height: 50   Sample baseline V to V?: Y   Sample baseline V to V points: 3
Sample filter: Binomial   Number of points for filter: 3 Sample start region (min): 0 Sample end region (min): 30
Marker peak width (sec): 5   Marker min peak height: 200   Marker baseline V to V?: Y   Marker baseline V to V points: 3
Lower marker selection: First peak > 500 RFU   Upper marker selection: Last peak > 200 RFU
Ladder size (bp)75, 200, 300, 400, 500, 700, 1000, 1500, 2000, 3000, 4000, 5000, 7000, 10000, 20000
Quantification using: Lower Marker   Final concentration (ng/uL): 0.5000   Dilution factor: 12.0

Sample: SampA2
Well location: A2
Created: Thursday, August 12, 2021 6:46:05 PM

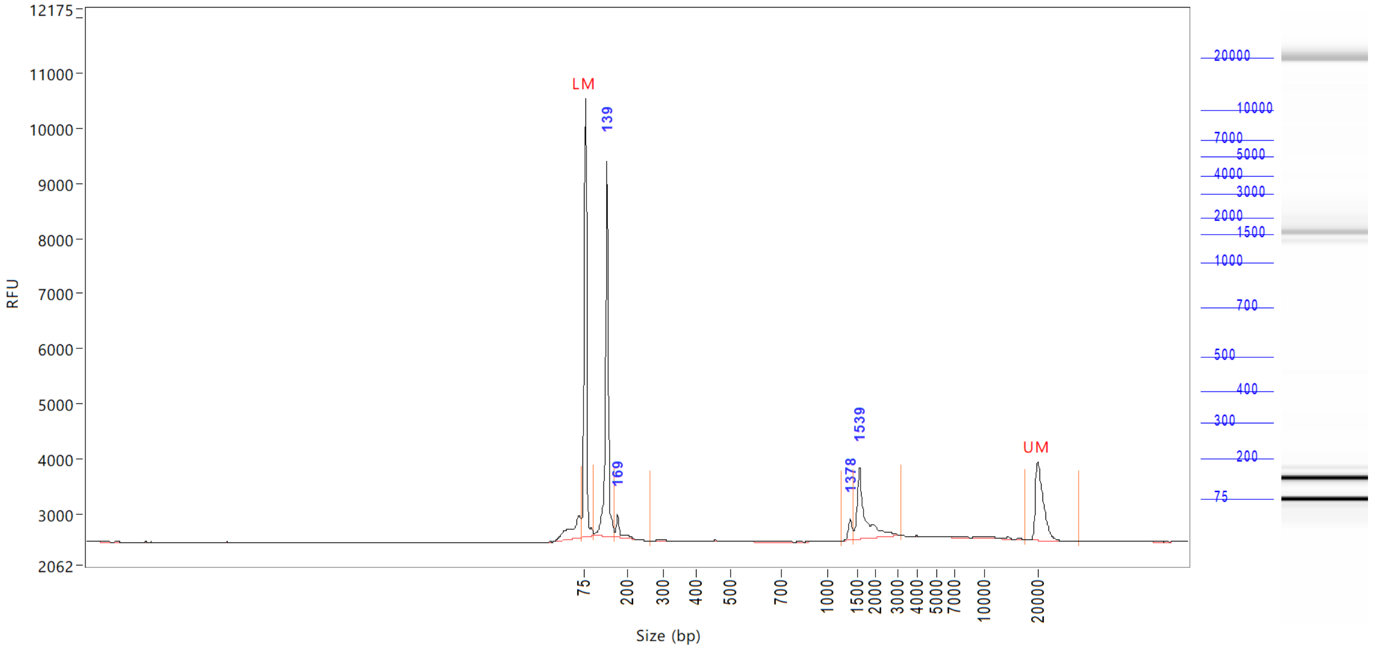

Peak	Size (bp)	Concentration (ng/uL)	From (bp)	To (bp)	Average size (bp)	CV%	RFU	Corrected peak area
1	75 (LM)	0.5000	64	101	74	5.64	7983	46.231
2	139	5.9649	101	160	135	5.17	6816	45.961
3	169	0.4624	160	262	176	8.37	427	3.563
4	1378	0.3108	1234	1445	1375	2.65	390	2.395
5	1539	1.9621	1445	3108	1739	17.95	1286	15.118
6	20000 (UM)	0.1471	17683	27617	20404	4.39	1420	13.605

TIC:	8.7002	ng/uL
TIM:	79.1521	nmole/L
Total concentration:	9.1248	ng/uL

Sample peak width (sec): 5 Sample min peak height: 50 Sample baseline V to V?: Y Sample baseline V to V points: 3
Sample filter: Binomial Number of points for filter: 3 Sample start region (min): 0 Sample end region (min): 30
Marker peak width (sec): 5 Marker min peak height: 200 Marker baseline V to V?: Y Marker baseline V to V points: 3
Lower marker selection: First peak > 500 RFU Upper marker selection: Last peak > 200 RFU
Ladder size (bp)75, 200, 300, 400, 500, 700, 1000, 1500, 2000, 3000, 4000, 5000, 7000, 10000, 20000
Quantification using: Lower Marker Final concentration (ng/uL): 0.5000 Dilution factor: 12.0

Sample: SampB2
Well location: B2
Created: Thursday, August 12, 2021 6:46:05 PM

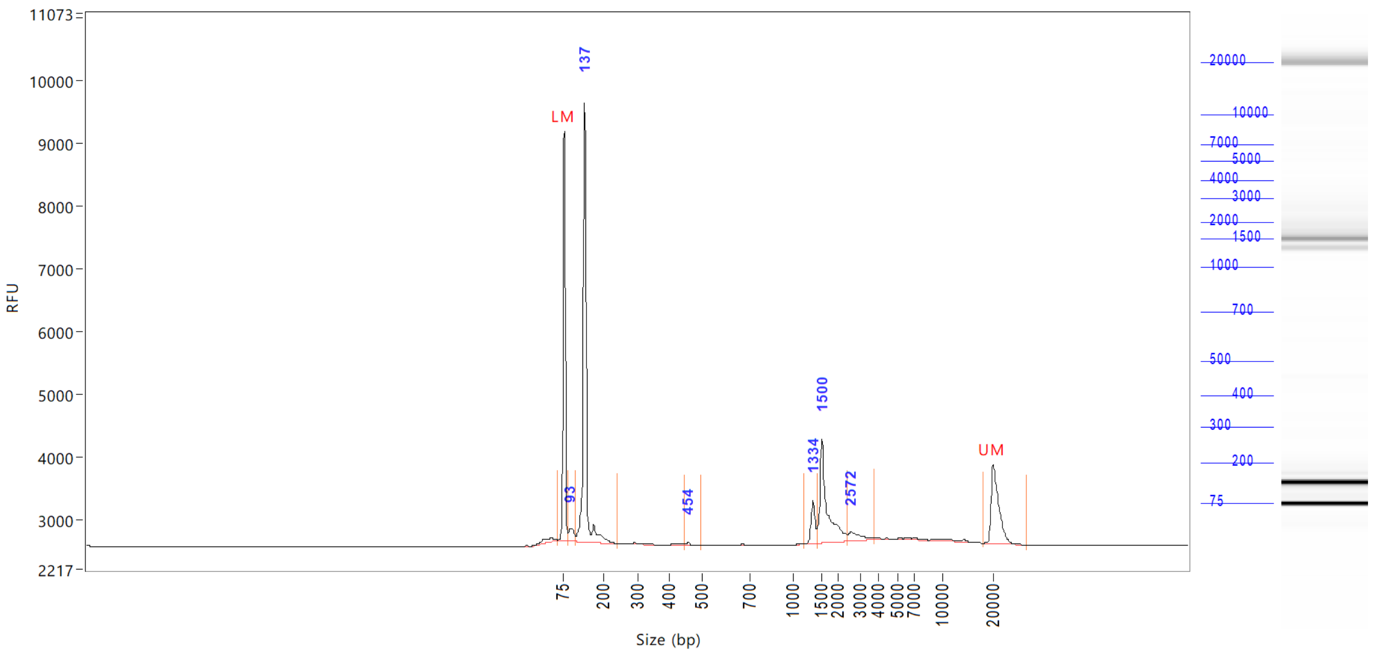

Peak	Size (bp)	Concentration (ng/uL)	From (bp)	To (bp)	Average size (bp)	CV%	RFU	Corrected peak area
1	75 (LM)	0.5000	54	86	72	4.68	6516	36.695
2	93	0.4290	86	108	96	6.78	196	2.624
3	137	8.2438	108	239	139	10.81	7007	50.418
4	454	0.0622	445	498	458	2.57	56	0.380
5	1334	0.7538	1178	1411	1334	2.90	694	4.610
6	1500	3.0987	1411	2401	1646	13.81	1653	18.951
7	2572	0.4447	2401	3822	2854	12.41	138	2.720
8	20000 (UM)	0.1671	17927	26649	20504	4.50	1250	12.265

TIC:	13.0321	ng/uL
TIM:	109.3058	nmole/L
Total concentration:	13.6001	ng/uL

Sample peak width (sec): 5   Sample min peak height: 50   Sample baseline V to V?: Y   Sample baseline V to V points: 3
Sample filter: Binomial   Number of points for filter: 3 Sample start region (min): 0 Sample end region (min): 30
Marker peak width (sec): 5   Marker min peak height: 200   Marker baseline V to V?: Y   Marker baseline V to V points: 3
Lower marker selection: First peak > 500 RFU   Upper marker selection: Last peak > 200 RFU
Ladder size (bp)75, 200, 300, 400, 500, 700, 1000, 1500, 2000, 3000, 4000, 5000, 7000, 10000, 20000
Quantification using: Lower Marker   Final concentration (ng/uL): 0.5000   Dilution factor: 12.0

Sample: SampC2
Well location: C2
Created: Thursday, August 12, 2021 6:46:05 PM

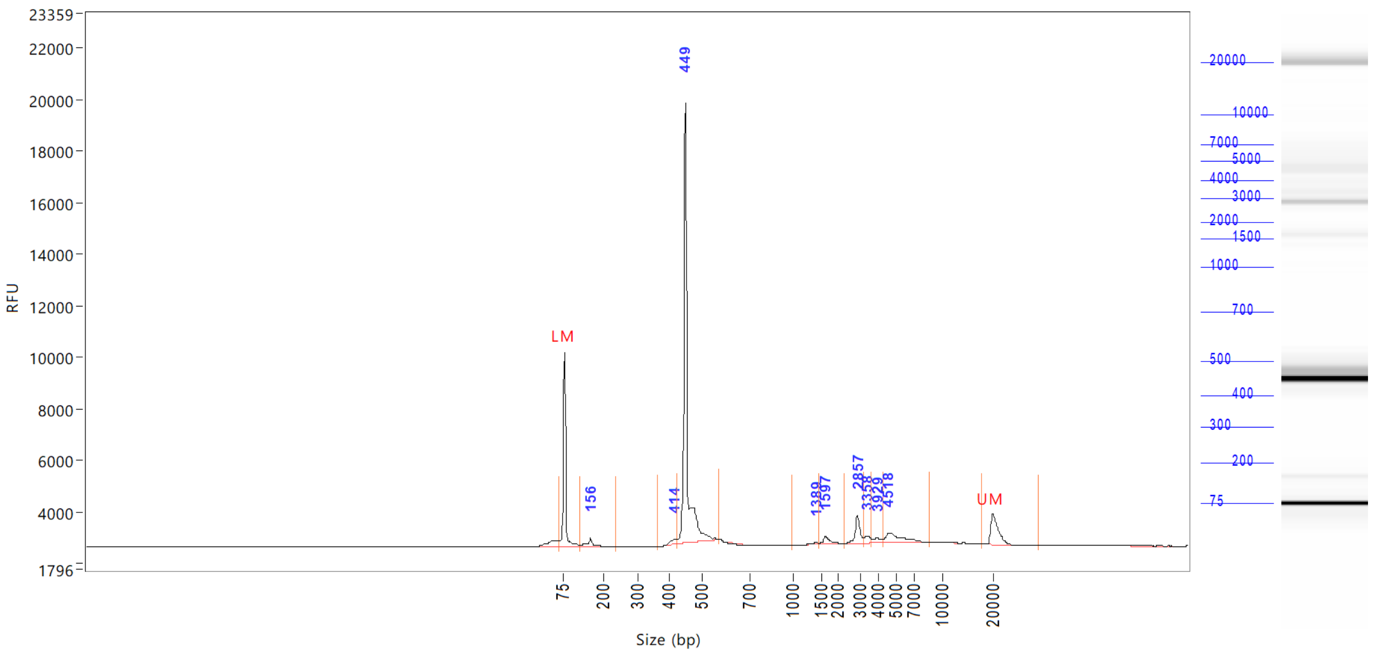

Peak	Size (bp)	Concentration (ng/uL)	From (bp)	To (bp)	Average size (bp)	CV%	RFU	Corrected peak area
1	75 (LM)	0.5000	58	126	75	11.11	7533	46.627
2	156	0.5505	126	232	154	11.83	312	4.278
3	414	0.3581	360	424	409	2.41	196	2.783
4	449	14.4751	424	567	453	3.74	17088	112.487
5	1389	0.0887	987	1445	1310	10.28	86	0.689
6	1597	0.3722	1445	2258	1636	7.66	293	2.892
7	2857	0.9545	2258	3144	2844	4.92	1068	7.417
8	3358	0.3439	3144	3643	3376	4.28	279	2.673
9	3929	0.2970	3643	4242	3928	4.40	176	2.308
10	4518	1.1593	4242	8734	5322	18.12	361	9.009
11	20000 (UM)	0.1242	17805	29068	20511	4.49	1211	11.579

TIC:	18.5992	ng/uL
TIM:	61.5822	nmole/L
Total concentration:	18.6368	ng/uL

```
Sample peak width (sec): 5    Sample min peak height: 50    Sample baseline V to V?: Y    Sample baseline V to V points: 3
Sample filter: Binomial       Number of points for filter: 3  Sample start region (min): 0  Sample end region (min): 30
Marker peak width (sec): 5    Marker min peak height: 200   Marker baseline V to V?: Y    Marker baseline V to V points: 3
Lower marker selection: First peak > 500 RFU                Upper marker selection: Last peak > 200 RFU
Ladder size (bp)75, 200, 300, 400, 500, 700, 1000, 1500, 2000, 3000, 4000, 5000, 7000, 10000, 20000
Quantification using: Lower Marker          Final concentration (ng/uL): 0.5000          Dilution factor: 12.0
```

Sample: SampD2
Well location: D2
Created: Thursday, August 12, 2021 6:46:05 PM

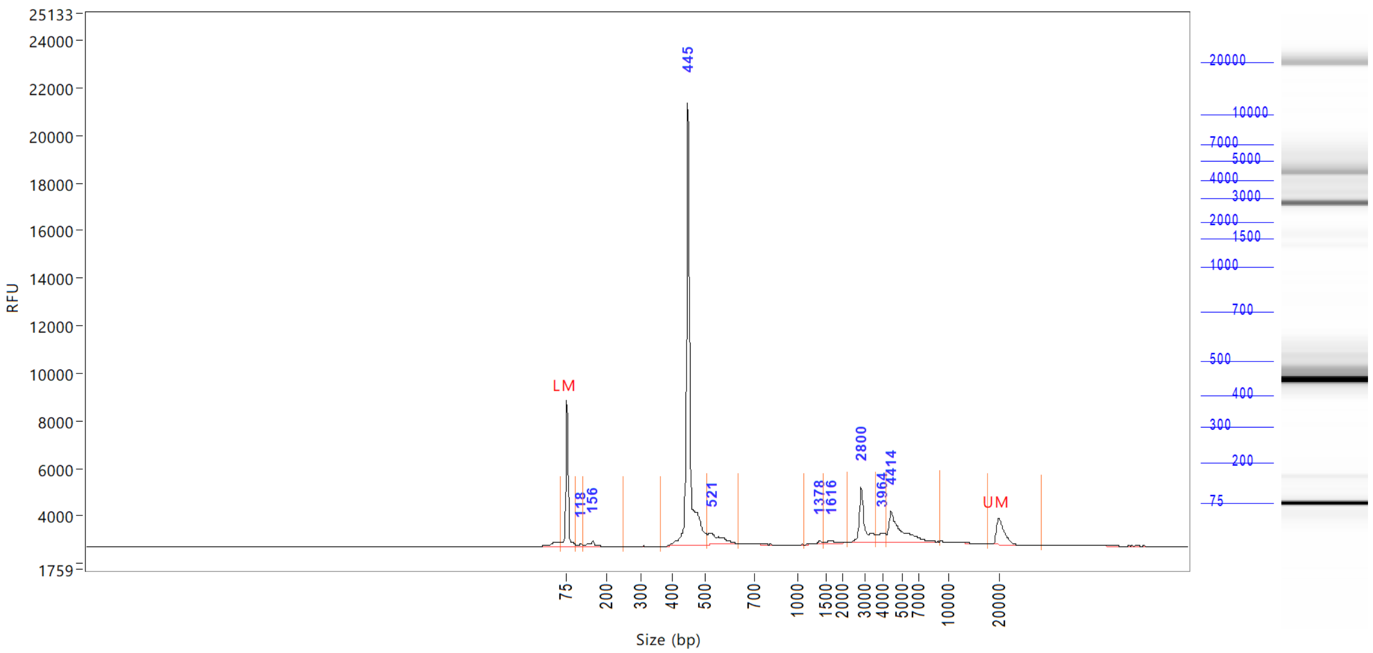

Peak	Size (bp)	Concentration (ng/uL)	From (bp)	To (bp)	Average size (bp)	CV%	RFU	Corrected peak area
1	75 (LM)	0.5000	58	105	74	7.72	6178	37.238
2	118	0.1981	105	126	115	5.56	93	1.230
3	156	0.5857	126	244	153	12.67	263	3.635
4	445	21.4658	360	506	448	3.64	18631	133.223
5	521	1.8239	506	634	543	5.09	486	11.320
6	1378	0.1356	1112	1456	1367	3.94	124	0.842
7	1616	0.2650	1456	2144	1639	7.66	133	1.645
8	2800	3.1273	2144	3572	2900	8.79	2321	19.409
9	3964	0.7978	3572	4139	3870	4.35	396	4.951
10	4414	3.5937	4139	9134	5049	17.71	1301	22.303
11	20000 (UM)	0.1470	17927	28221	20460	4.46	1111	10.951

TIC:	31.9929	ng/uL
TIM:	97.2040	nmole/L
Total concentration:	31.9847	ng/uL

Sample peak width (sec): 5   Sample min peak height: 50   Sample baseline V to V?: Y   Sample baseline V to V points: 3
Sample filter: Binomial   Number of points for filter: 3   Sample start region (min): 0   Sample end region (min): 30
Marker peak width (sec): 5   Marker min peak height: 200   Marker baseline V to V?: Y   Marker baseline V to V points: 3
Lower marker selection: First peak > 500 RFU   Upper marker selection: Last peak > 200 RFU
Ladder size (bp)75, 200, 300, 400, 500, 700, 1000, 1500, 2000, 3000, 4000, 5000, 7000, 10000, 20000
Quantification using: Lower Marker   Final concentration (ng/uL): 0.5000   Dilution factor: 12.0

Sample: SampE2
Well location: E2
Created: Thursday, August 12, 2021 6:46:05 PM

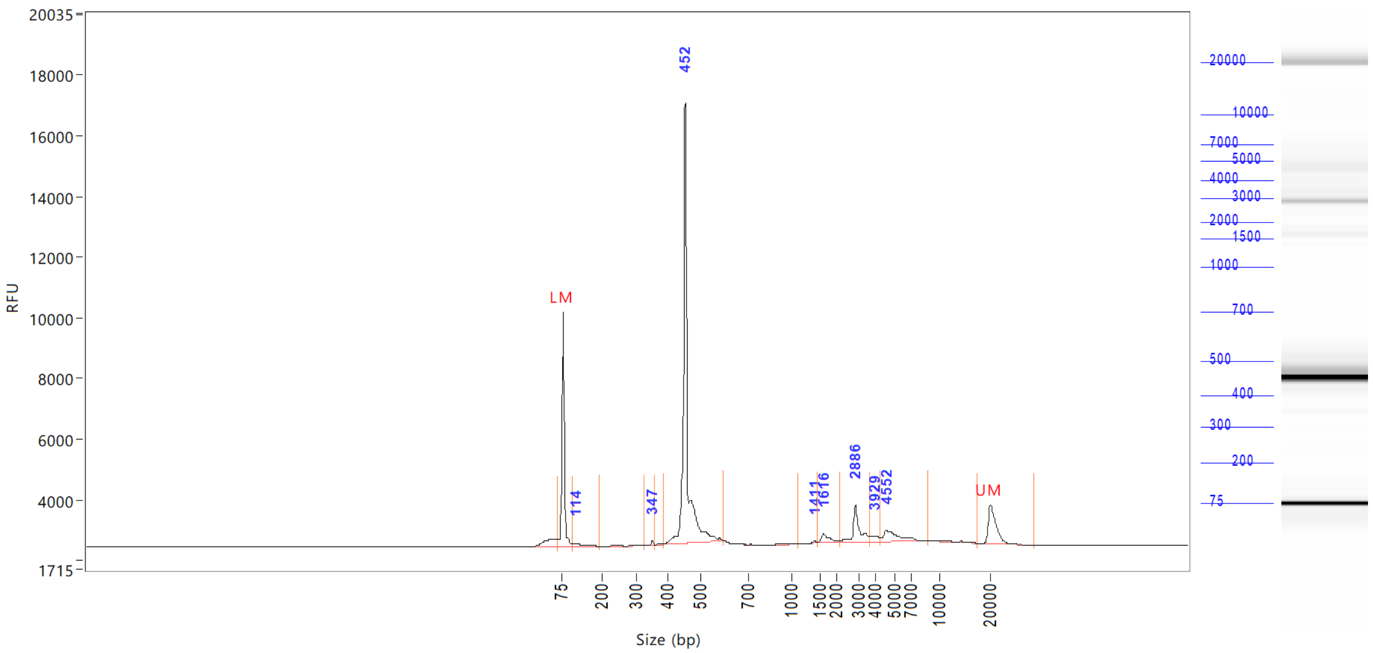

Peak	Size (bp)	Concentration (ng/uL)	From (bp)	To (bp)	Average size (bp)	CV%	RFU	Corrected peak area
1	75 (LM)	0.5000	58	103	73	7.38	7708	46.996
2	114	0.3158	103	190	127	14.33	106	2.474
3	347	0.1025	321	358	345	1.47	143	0.803
4	452	14.3311	386	593	456	5.12	14530	112.251
5	1411	0.0440	1112	1467	1380	4.62	62	0.345
6	1616	0.4125	1467	2144	1699	8.87	286	3.231
7	2886	1.4749	2144	3679	2966	9.95	1219	11.553
8	3929	0.2895	3679	4242	3949	4.11	179	2.268
9	4552	1.0789	4242	8734	5304	18.09	360	8.450
10	20000 (UM)	0.1348	17439	28584	20525	4.52	1282	12.668

TIC:	18.0493	ng/uL
TIM:	58.0135	nmole/L
Total concentration:	18.3457	ng/uL

```
Sample peak width (sec): 5    Sample min peak height: 50    Sample baseline V to V?: Y    Sample baseline V to V points: 3
Sample filter: Binomial       Number of points for filter: 3  Sample start region (min): 0  Sample end region (min): 30
Marker peak width (sec): 5    Marker min peak height: 200   Marker baseline V to V?: Y    Marker baseline V to V points: 3
Lower marker selection: First peak > 500 RFU                Upper marker selection: Last peak > 200 RFU
Ladder size (bp)75, 200, 300, 400, 500, 700, 1000, 1500, 2000, 3000, 4000, 5000, 7000, 10000, 20000
Quantification using: Lower Marker          Final concentration (ng/uL): 0.5000          Dilution factor: 12.0
```

Sample: SampF2
Well location: F2
Created: Thursday, August 12, 2021 6:46:05 PM

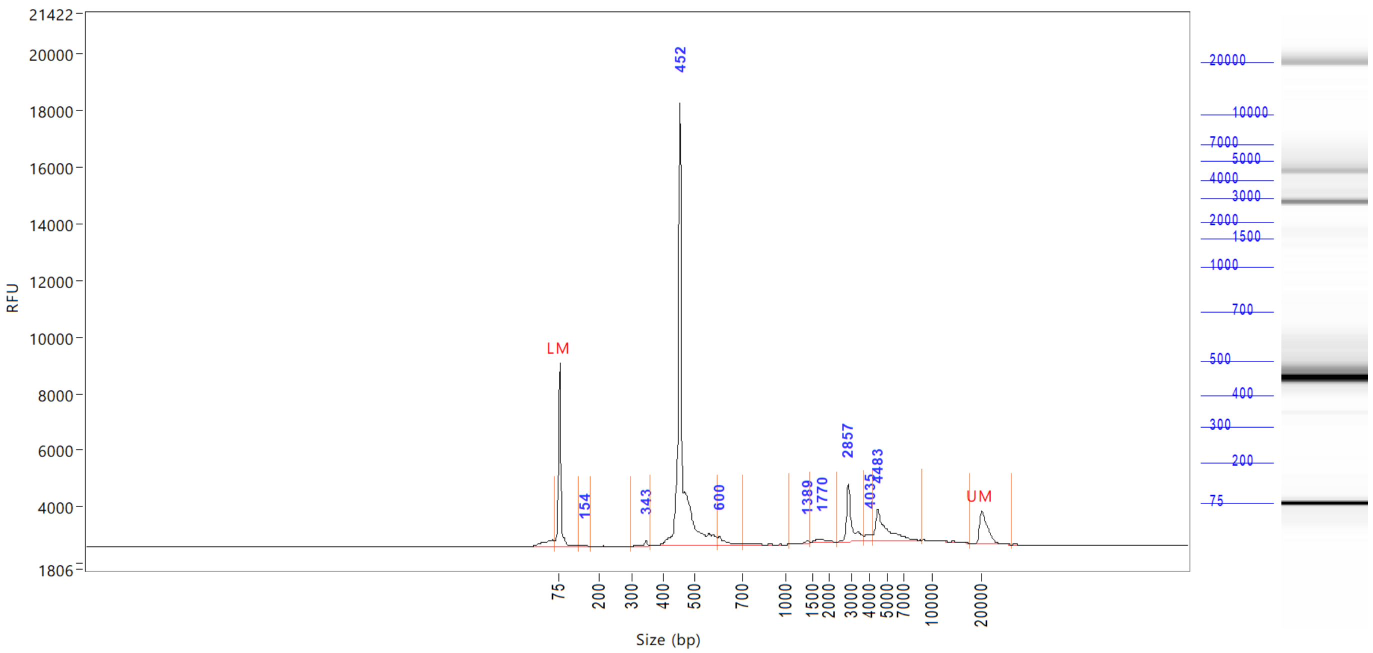

Peak	Size<br>(bp)	Concentration<br>(ng/uL)	From<br>(bp)	To<br>(bp)	Average size<br>(bp)	CV%	RFU	Corrected peak area
1	75 (LM)	0.5000	62	137	75	11.49	6479	39.747
2	154	0.0886	137	175	150	5.02	52	0.587
3	343	0.2237	296	360	334	3.96	184	1.482
4	452	20.8823	360	593	462	6.90	15647	138.333
5	600	0.6391	593	700	624	4.50	299	4.234
6	1389	0.1060	1068	1445	1341	6.78	89	0.703
7	1770	0.3395	1445	2315	1698	9.26	137	2.249
8	2857	2.4273	2315	3679	2965	8.33	2017	16.080
9	4035	0.4082	3679	4208	3953	3.94	233	2.704
10	4483	2.6187	4208	9000	5099	17.10	1113	17.347
11	20000 (UM)	0.1488	17805	25924	20539	4.51	1177	11.825

TIC:	27.7335	ng/uL
TIM:	80.9542	nmole/L
Total concentration:	27.9088	ng/uL

```
Sample peak width (sec): 5    Sample min peak height: 50    Sample baseline V to V?: Y    Sample baseline V to V points: 3
Sample filter: Binomial       Number of points for filter: 3  Sample start region (min): 0  Sample end region (min): 30
Marker peak width (sec): 5    Marker min peak height: 200   Marker baseline V to V?: Y    Marker baseline V to V points: 3
Lower marker selection: First peak > 500 RFU                Upper marker selection: Last peak > 200 RFU
Ladder size (bp)75, 200, 300, 400, 500, 700, 1000, 1500, 2000, 3000, 4000, 5000, 7000, 10000, 20000
Quantification using: Lower Marker          Final concentration (ng/uL): 0.5000          Dilution factor: 12.0
```

Sample: SampG2
Well location: G2
Created: Thursday, August 12, 2021 6:46:05 PM

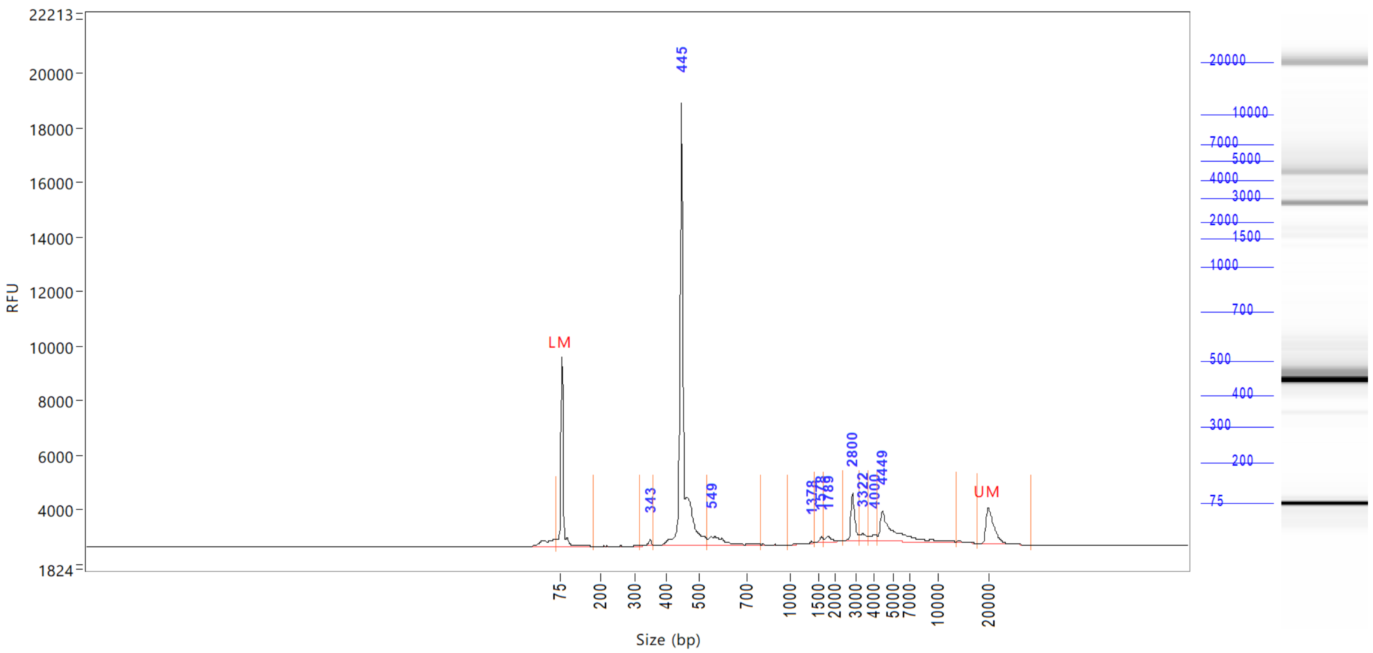

Peak	Size (bp)	Concentration (ng/uL)	From (bp)	To (bp)	Average size (bp)	CV%	RFU	Corrected peak area
1	75 (LM)	0.5000	60	175	75	13.91	6935	43.481
2	343	0.2543	311	358	336	3.06	241	1.843
3	445	17.5712	358	534	450	4.49	16260	127.337
4	549	1.3051	534	796	589	8.70	348	9.458
5	1378	0.0617	987	1433	1297	9.20	73	0.447
6	1578	0.1863	1433	1674	1570	3.60	208	1.350
7	1789	0.2813	1674	2372	1830	6.67	192	2.039
8	2800	1.5257	2372	3108	2811	4.00	1766	11.056
9	3322	0.3790	3108	3608	3340	4.38	279	2.747
10	4000	0.3745	3608	4139	3891	4.03	236	2.714
11	4449	2.9410	4139	13537	5628	30.83	1091	21.313
12	20000 (UM)	0.1520	17683	28342	20476	4.53	1332	13.221

TIC:	24.8802	ng/uL
TIM:	71.7659	nmole/L
Total concentration:	24.9114	ng/uL

```
Sample peak width (sec): 5     Sample min peak height: 50    Sample baseline V to V?: Y    Sample baseline V to V points: 3
Sample filter: Binomial        Number of points for filter: 3  Sample start region (min): 0  Sample end region (min): 30
Marker peak width (sec): 5     Marker min peak height: 200   Marker baseline V to V?: Y    Marker baseline V to V points: 3
Lower marker selection: First peak > 500 RFU                 Upper marker selection: Last peak > 200 RFU
Ladder size (bp)75, 200, 300, 400, 500, 700, 1000, 1500, 2000, 3000, 4000, 5000, 7000, 10000, 20000
Quantification using: Lower Marker          Final concentration (ng/uL): 0.5000          Dilution factor: 12.0
```

Sample: SampH2
Well location: H2
Created: Thursday, August 12, 2021 6:46:05 PM

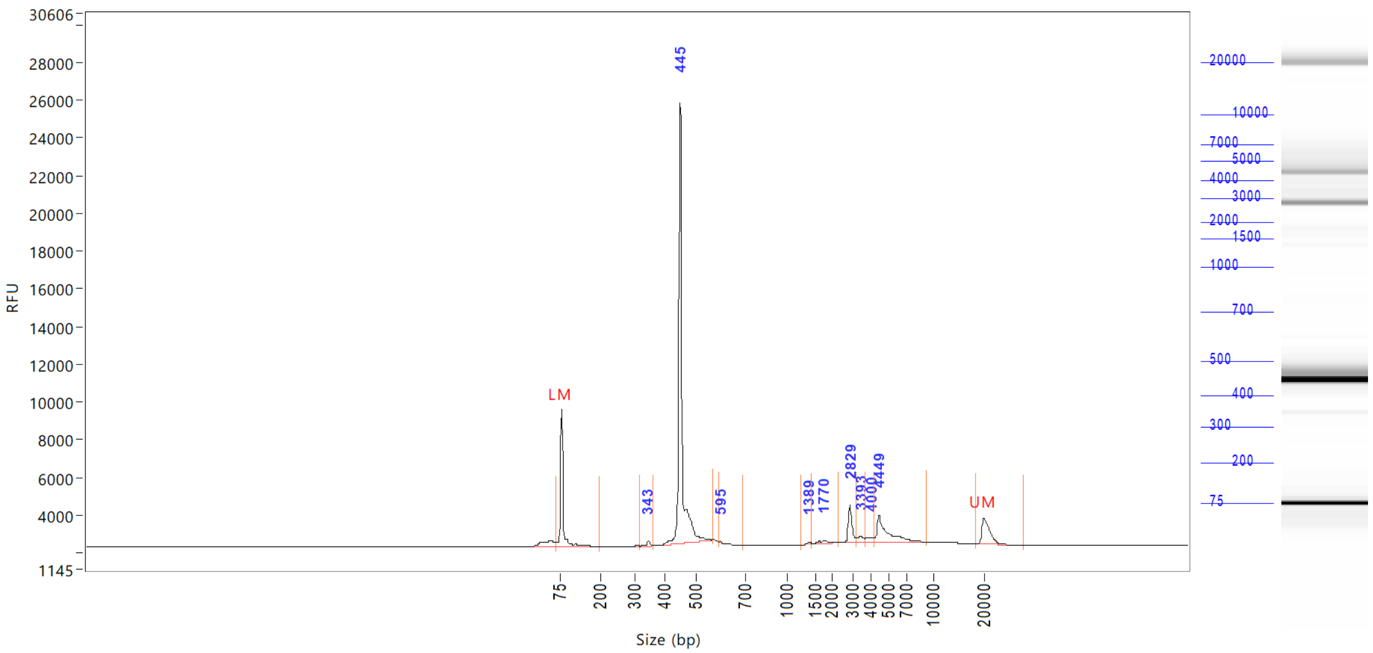

Peak	Size (bp)	Concentration (ng/uL)	From (bp)	To (bp)	Average size (bp)	CV%	RFU	Corrected peak area
1	75 (LM)	0.5000	62	194	78	18.29	7271	47.953
2	343	0.1892	313	362	340	1.77	261	1.512
3	445	19.5100	362	565	448	3.65	23387	155.928
4	595	0.0386	588	692	603	3.68	70	0.308
5	1389	0.0848	1245	1445	1377	2.07	137	0.678
6	1770	0.2897	1445	2286	1719	8.79	167	2.315
7	2829	1.5051	2286	3179	2832	4.33	1987	12.029
8	3393	0.3625	3179	3643	3400	4.00	316	2.897
9	4000	0.3483	3643	4139	3898	3.78	242	2.784
10	4449	2.6554	4139	9134	5074	17.99	1444	21.223
11	20000 (UM)	0.1431	18415	27979	20545	4.77	1405	13.728

TIC:	24.9835	ng/uL
TIM:	75.1142	nmole/L
Total concentration:	25.0654	ng/uL

Sample peak width (sec): 5 Sample min peak height: 50 Sample baseline V to V?: Y Sample baseline V to V points: 3
Sample filter: Binomial Number of points for filter: 3 Sample start region (min): 0 Sample end region (min): 30
Marker peak width (sec): 5 Marker min peak height: 200 Marker baseline V to V?: Y Marker baseline V to V points: 3
Lower marker selection: First peak > 500 RFU Upper marker selection: Last peak > 200 RFU
Ladder size (bp)75, 200, 300, 400, 500, 700, 1000, 1500, 2000, 3000, 4000, 5000, 7000, 10000, 20000
Quantification using: Lower Marker Final concentration (ng/uL): 0.5000 Dilution factor: 12.0

Sample: SampA3
Well location: A3
Created: Thursday, August 12, 2021 6:46:05 PM

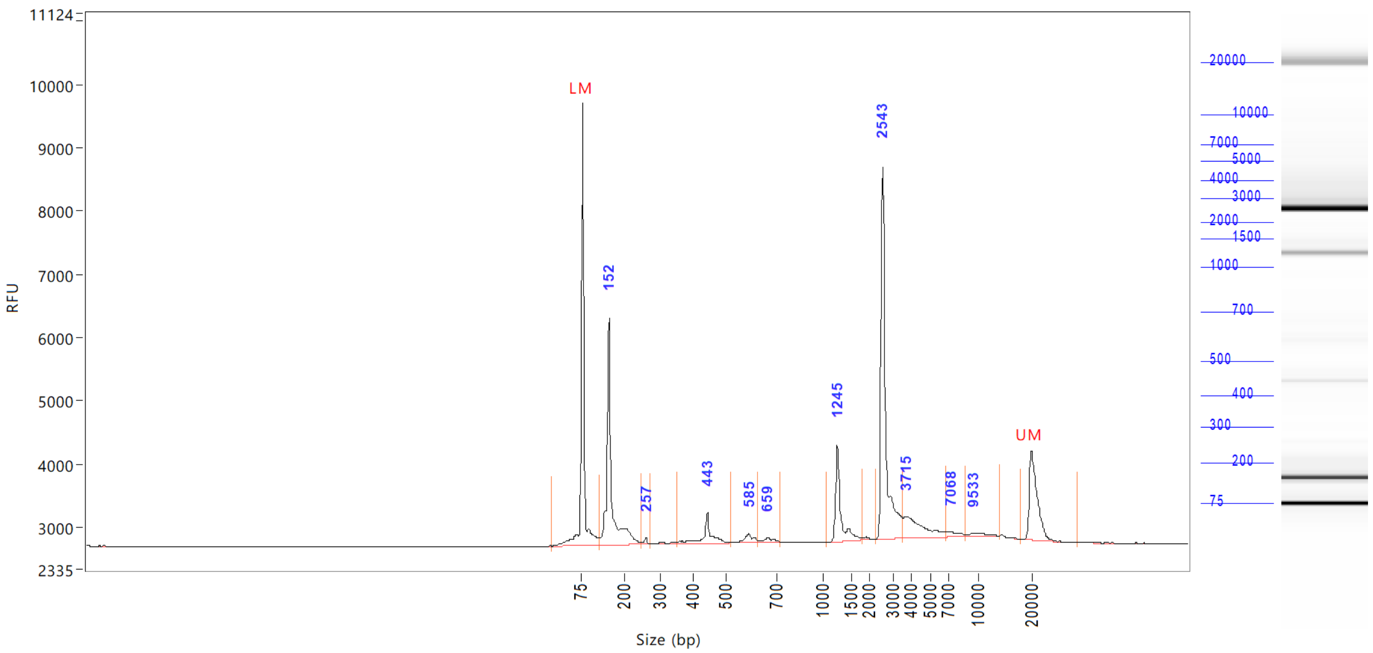

Peak	Size (bp)	Concentration (ng/uL)	From (bp)	To (bp)	Average size (bp)	CV%	RFU	Corrected peak area
1	75 (LM)	0.5000	0	126	73	21.38	7010	46.355
2	152	4.6922	126	246	160	14.04	3585	36.251
3	257	0.0791	246	269	254	1.61	95	0.611
4	443	0.8000	352	516	436	6.91	481	6.180
5	585	0.2994	516	623	583	3.51	156	2.313
6	659	0.2029	623	718	664	3.46	78	1.567
7	1245	1.5726	1056	1808	1291	8.64	1529	12.150
8	2543	5.2837	2258	3572	2669	9.87	5882	40.821
9	3715	1.2402	3572	6778	4464	17.76	342	9.582
10	7068	0.1759	6778	8667	7559	7.16	85	1.359
11	9533	0.1406	8667	14025	10113	11.02	50	1.086
12	20000 (UM)	0.1503	18171	28705	20586	4.97	1397	13.930

TIC:	14.4865	ng/uL
TIM:	58.8646	nmole/L
Total concentration:	14.6266	ng/uL

```
Sample peak width (sec): 5    Sample min peak height: 50    Sample baseline V to V?: Y    Sample baseline V to V points: 3
Sample filter: Binomial       Number of points for filter: 3  Sample start region (min): 0  Sample end region (min): 30
Marker peak width (sec): 5    Marker min peak height: 200   Marker baseline V to V?: Y    Marker baseline V to V points: 3
Lower marker selection: First peak > 500 RFU                Upper marker selection: Last peak > 200 RFU
Ladder size (bp)75, 200, 300, 400, 500, 700, 1000, 1500, 2000, 3000, 4000, 5000, 7000, 10000, 20000
Quantification using: Lower Marker          Final concentration (ng/uL): 0.5000          Dilution factor: 12.0
```

Sample: SampB3
Well location: B3
Created: Thursday, August 12, 2021 6:46:05 PM

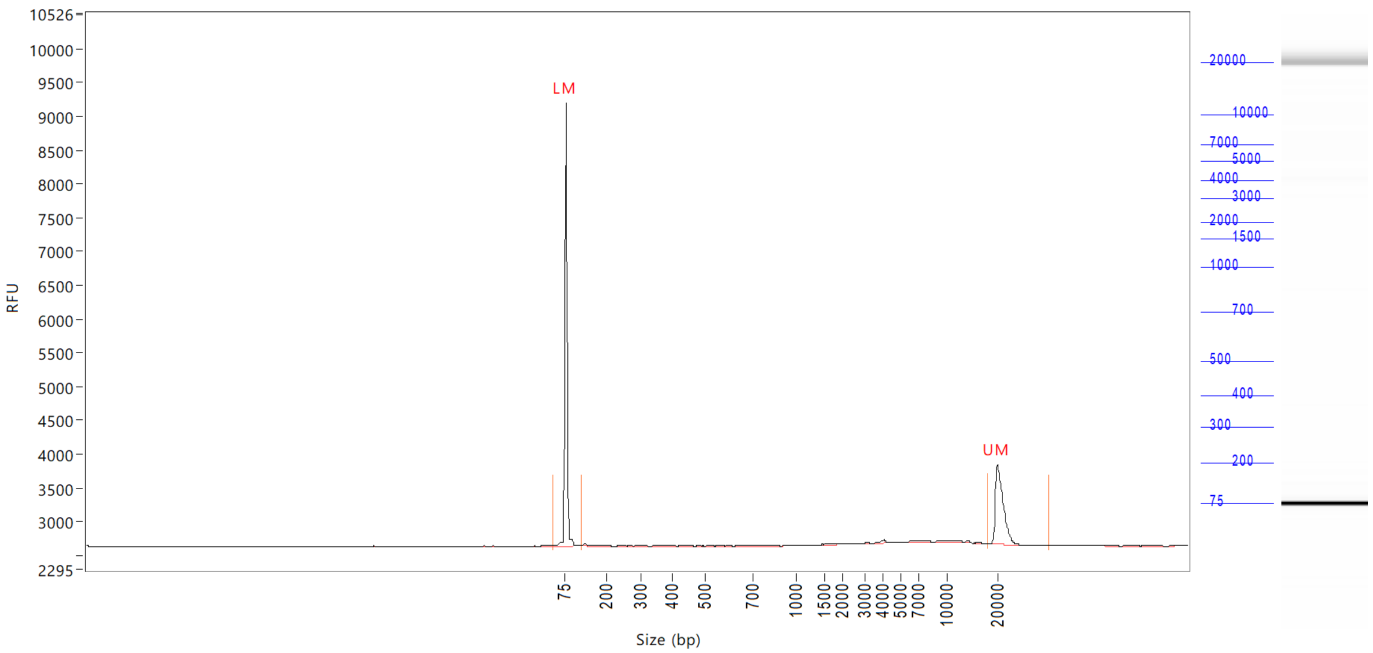

Peak	Size (bp)	Concentration (ng/uL)	From (bp)	To (bp)	Average size (bp)	CV%	RFU	Corrected peak area
1	75 (LM)	0.5000	35	122	73	7.36	6572	36.760
2	20000 (UM)	0.1601	18171	30156	20557	5.30	1187	11.773

TIC:	0.0000	ng/uL
TIM:	0.0000	nmole/L
Total concentration:	0.6533	ng/uL

```
Sample peak width (sec): 5    Sample min peak height: 50    Sample baseline V to V?: Y    Sample baseline V to V points: 3
Sample filter: Binomial       Number of points for filter: 3  Sample start region (min): 0  Sample end region (min): 30
Marker peak width (sec): 5    Marker min peak height: 200   Marker baseline V to V?: Y    Marker baseline V to V points: 3
Lower marker selection: First peak > 500 RFU                Upper marker selection: Last peak > 200 RFU
Ladder size (bp)75, 200, 300, 400, 500, 700, 1000, 1500, 2000, 3000, 4000, 5000, 7000, 10000, 20000
Quantification using: Lower Marker          Final concentration (ng/uL): 0.5000          Dilution factor: 12.0
```

Sample: SampC3
Well location: C3
Created: Thursday, August 12, 2021 6:46:05 PM

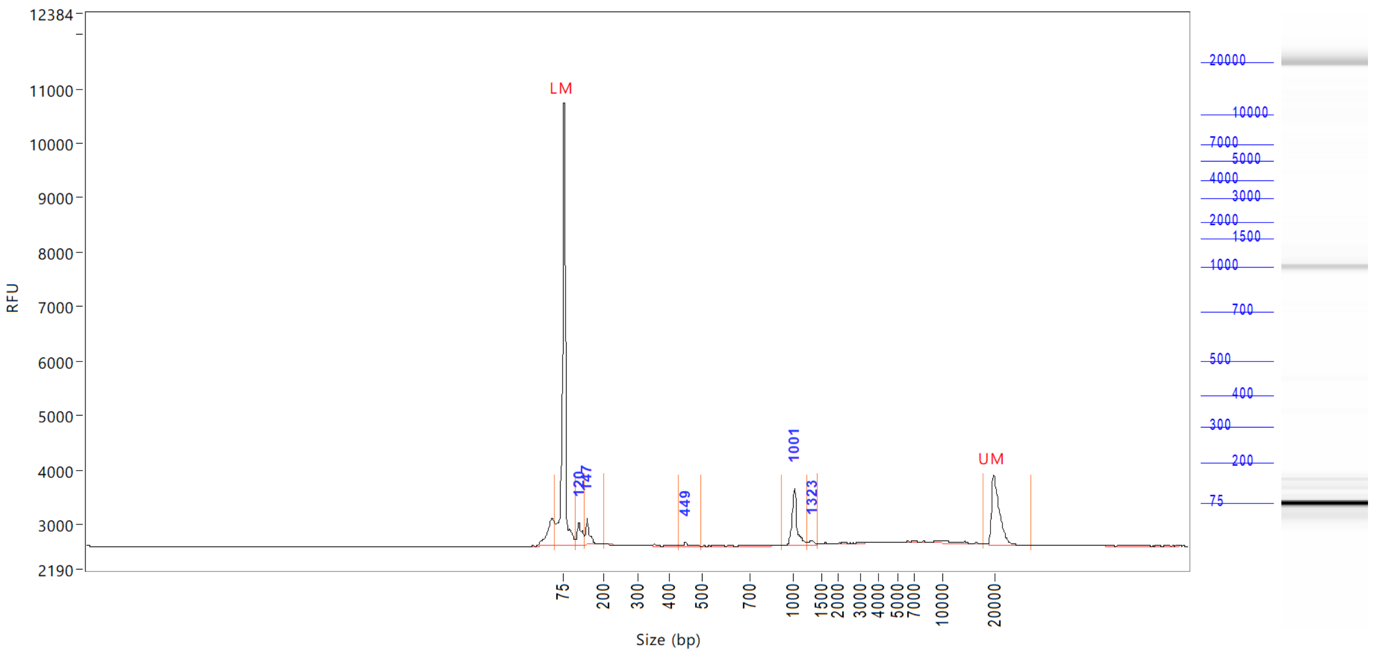

Peak	Size (bp)	Concentration (ng/uL)	From (bp)	To (bp)	Average size (bp)	CV%	RFU	Corrected peak area
1	75 (LM)	0.5000	50	110	73	10.76	8124	62.123
2	120	0.4494	110	139	124	6.27	400	4.653
3	147	0.4103	139	200	150	5.98	491	4.249
4	449	0.0544	428	498	452	3.11	70	0.563
5	1001	0.7828	918	1223	1021	5.15	1034	8.105
6	1323	0.0745	1223	1422	1301	3.86	69	0.772
7	20000 (UM)	0.1028	17927	27254	20492	4.60	1281	12.771

TIC:	1.7714	ng/uL
TIM:	12.0040	nmole/L
Total concentration:	2.1426	ng/uL

Sample peak width (sec): 5   Sample min peak height: 50   Sample baseline V to V?: Y   Sample baseline V to V points: 3
Sample filter: Binomial   Number of points for filter: 3 Sample start region (min): 0 Sample end region (min): 30
Marker peak width (sec): 5   Marker min peak height: 200   Marker baseline V to V?: Y   Marker baseline V to V points: 3
Lower marker selection: First peak > 500 RFU   Upper marker selection: Last peak > 200 RFU
Ladder size (bp)75, 200, 300, 400, 500, 700, 1000, 1500, 2000, 3000, 4000, 5000, 7000, 10000, 20000
Quantification using: Lower Marker   Final concentration (ng/uL): 0.5000   Dilution factor: 12.0

Sample: SampD3
Well location: D3
Created: Thursday, August 12, 2021 6:46:05 PM

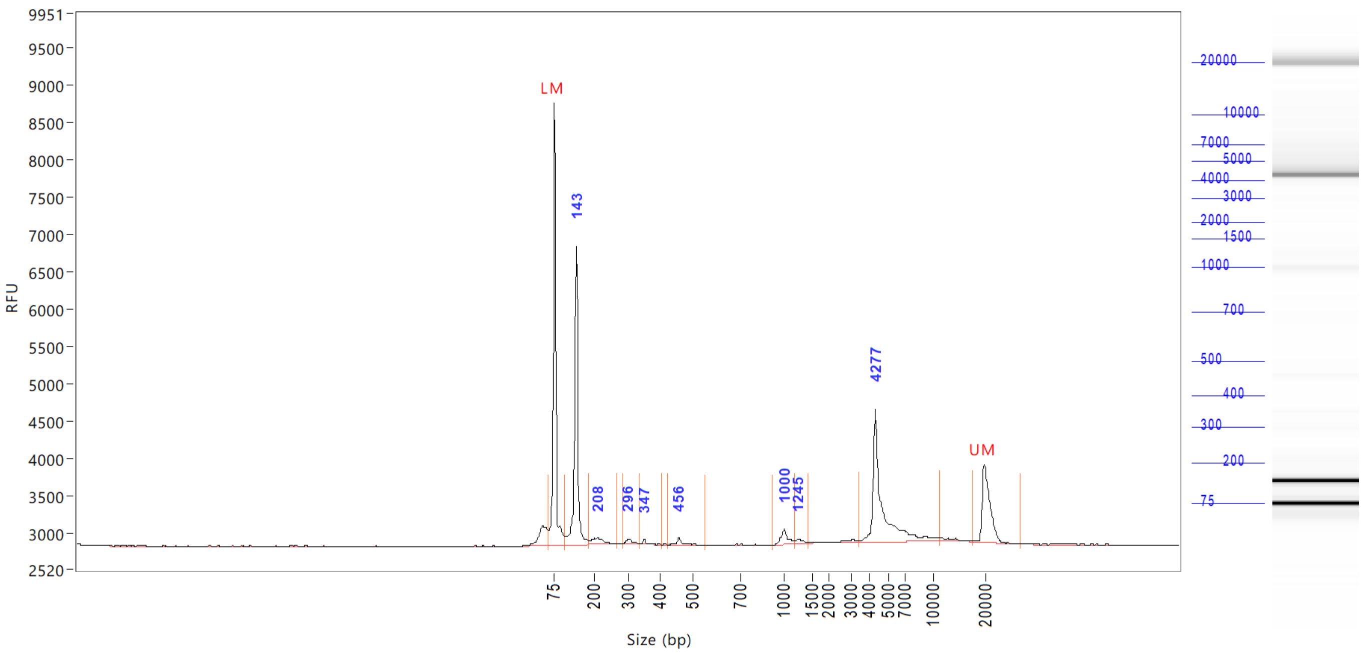

Peak	Size (bp)	Concentration (ng/uL)	From (bp)	To (bp)	Average size (bp)	CV%	RFU	Corrected peak area
1	75 (LM)	0.5000	56	107	74	9.72	5924	38.526
2	143	4.6578	107	183	140	7.06	3981	29.907
3	208	0.3432	183	264	207	7.39	89	2.204
4	296	0.1528	280	333	301	3.93	70	0.981
5	347	0.0752	333	405	351	4.23	63	0.483
6	456	0.1366	422	555	464	4.46	85	0.877
7	1000	0.3534	918	1178	1018	5.79	199	2.269
8	1245	0.1157	1178	1411	1267	4.97	69	0.743
9	4277	3.4747	3429	11098	5123	28.64	1777	22.311
10	20000 (UM)	0.1340	17683	27012	20443	4.66	1038	10.328

TIC:	9.3094	ng/uL
TIM:	60.9091	nmole/L
Total concentration:	9.6298	ng/uL

Sample peak width (sec): 5 Sample min peak height: 50 Sample baseline V to V?: Y Sample baseline V to V points: 3
Sample filter: Binomial Number of points for filter: 3 Sample start region (min): 0 Sample end region (min): 30
Marker peak width (sec): 5 Marker min peak height: 200 Marker baseline V to V?: Y Marker baseline V to V points: 3
Lower marker selection: First peak > 500 RFU Upper marker selection: Last peak > 200 RFU
Ladder size (bp)75, 200, 300, 400, 500, 700, 1000, 1500, 2000, 3000, 4000, 5000, 7000, 10000, 20000
Quantification using: Lower Marker Final concentration (ng/uL): 0.5000 Dilution factor: 12.0

Sample: SampE3
Well location: E3
Created: Thursday, August 12, 2021 6:46:05 PM

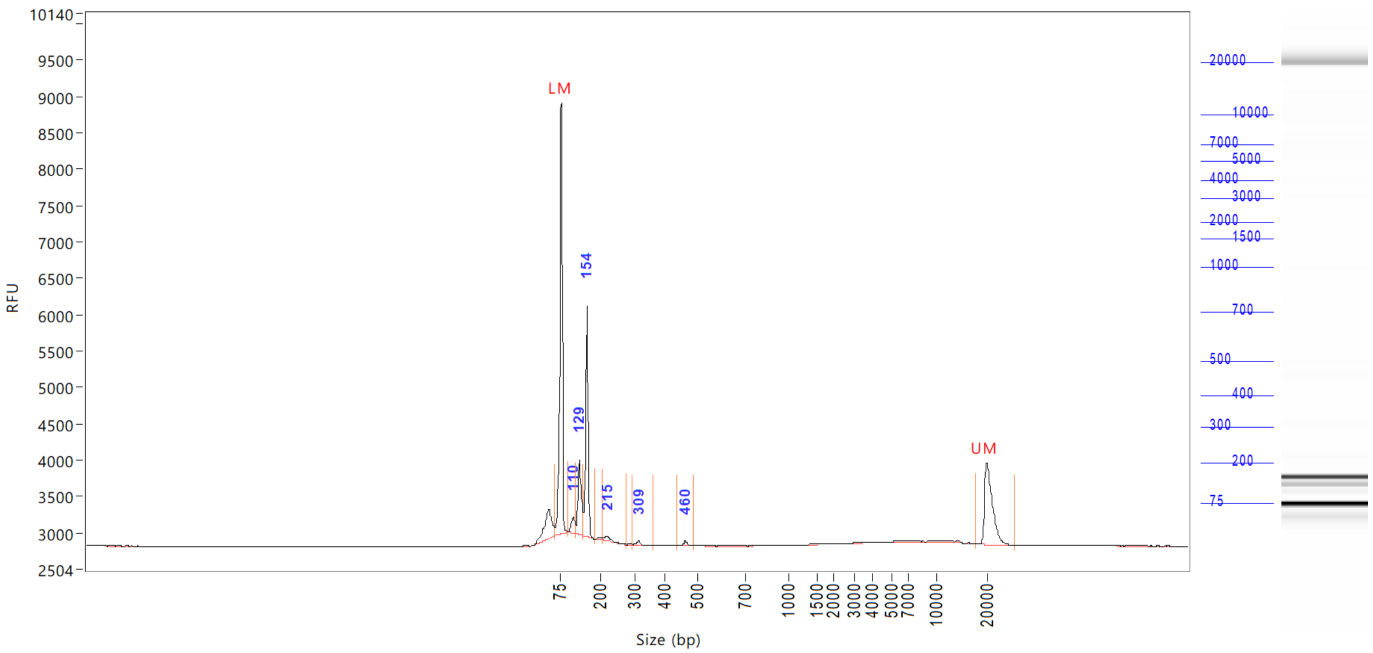

Peak	Size (bp)	Concentration (ng/uL)	From (bp)	To (bp)	Average size (bp)	CV%	RFU	Corrected peak area
1	75 (LM)	0.5000	58	95	71	5.64	5928	37.638
2	110	0.3253	95	120	110	5.12	220	2.040
3	129	1.4414	120	145	132	4.88	1019	9.042
4	154	2.8538	145	181	152	2.29	3167	17.902
5	215	0.1348	203	275	218	5.53	56	0.845
6	309	0.0729	293	358	309	3.11	51	0.458
7	460	0.0896	437	487	459	2.12	70	0.562
8	20000 (UM)	0.1478	17805	25440	20550	4.33	1120	11.125

TIC:	4.9177	ng/uL
TIM:	55.3825	nmole/L
Total concentration:	5.2874	ng/uL

Sample peak width (sec): 5   Sample min peak height: 50   Sample baseline V to V?: Y   Sample baseline V to V points: 3
Sample filter: Binomial   Number of points for filter: 3   Sample start region (min): 0   Sample end region (min): 30
Marker peak width (sec): 5   Marker min peak height: 200   Marker baseline V to V?: Y   Marker baseline V to V points: 3
Lower marker selection: First peak > 500 RFU   Upper marker selection: Last peak > 200 RFU
Ladder size (bp)75, 200, 300, 400, 500, 700, 1000, 1500, 2000, 3000, 4000, 5000, 7000, 10000, 20000
Quantification using: Lower Marker   Final concentration (ng/uL): 0.5000   Dilution factor: 12.0

Sample: SampF3
Well location: F3
Created: Thursday, August 12, 2021 6:46:05 PM

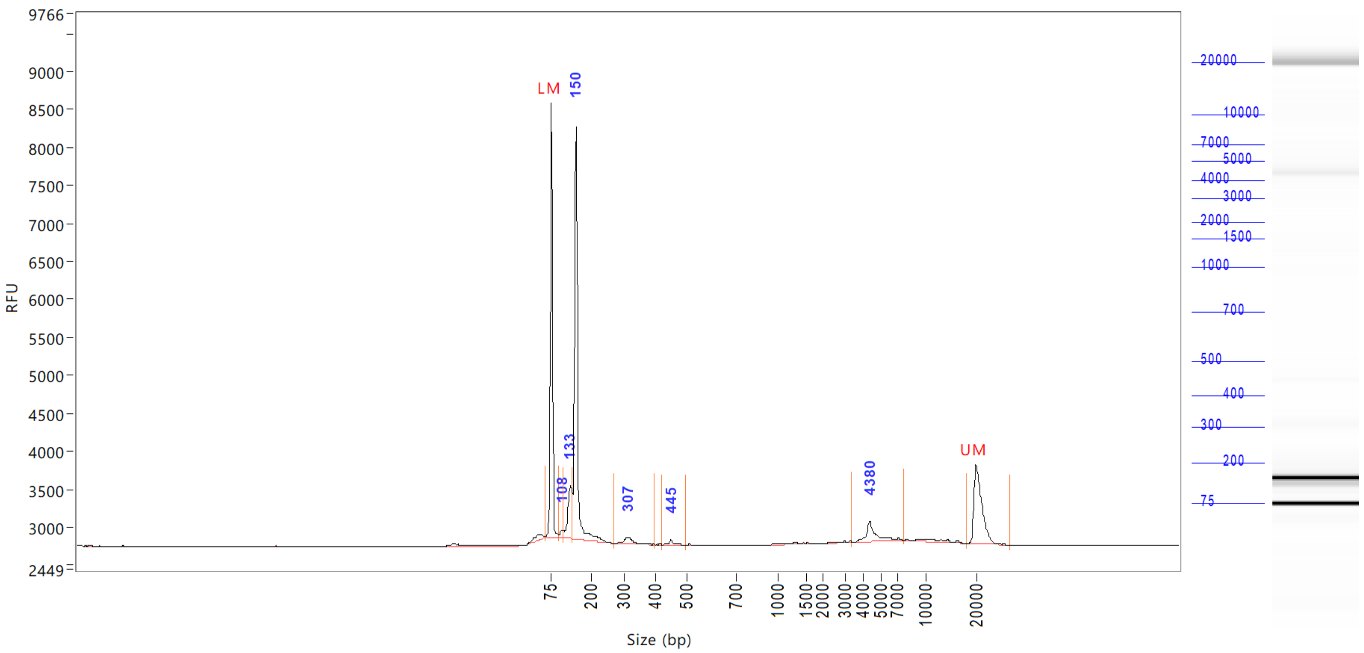

Peak	Size (bp)	Concentration (ng/uL)	From (bp)	To (bp)	Average size (bp)	CV%	RFU	Corrected peak area
1	75 (LM)	0.5000	58	97	73	5.05	5725	31.549
2	108	0.1787	97	114	107	4.63	102	0.940
3	133	1.4807	114	141	131	5.09	697	7.786
4	150	6.8521	141	268	153	10.06	5423	36.029
5	307	0.3044	268	398	311	6.60	83	1.601
6	445	0.1140	420	494	449	3.07	66	0.599
7	4380	0.6970	3429	7601	4696	15.87	262	3.665
8	20000 (UM)	0.1672	18171	26649	20528	4.58	1050	10.551

TIC:	9.6269	ng/uL
TIM:	97.1946	nmole/L
Total concentration:	9.9930	ng/uL

Sample peak width (sec): 5 Sample min peak height: 50 Sample baseline V to V?: Y Sample baseline V to V points: 3
Sample filter: Binomial Number of points for filter: 3 Sample start region (min): 0 Sample end region (min): 30
Marker peak width (sec): 5 Marker min peak height: 200 Marker baseline V to V?: Y Marker baseline V to V points: 3
Lower marker selection: First peak > 500 RFU Upper marker selection: Last peak > 200 RFU
Ladder size (bp)75, 200, 300, 400, 500, 700, 1000, 1500, 2000, 3000, 4000, 5000, 7000, 10000, 20000
Quantification using: Lower Marker Final concentration (ng/uL): 0.5000 Dilution factor: 12.0

Sample: SampG3
Well location: G3
Created: Thursday, August 12, 2021 6:46:05 PM

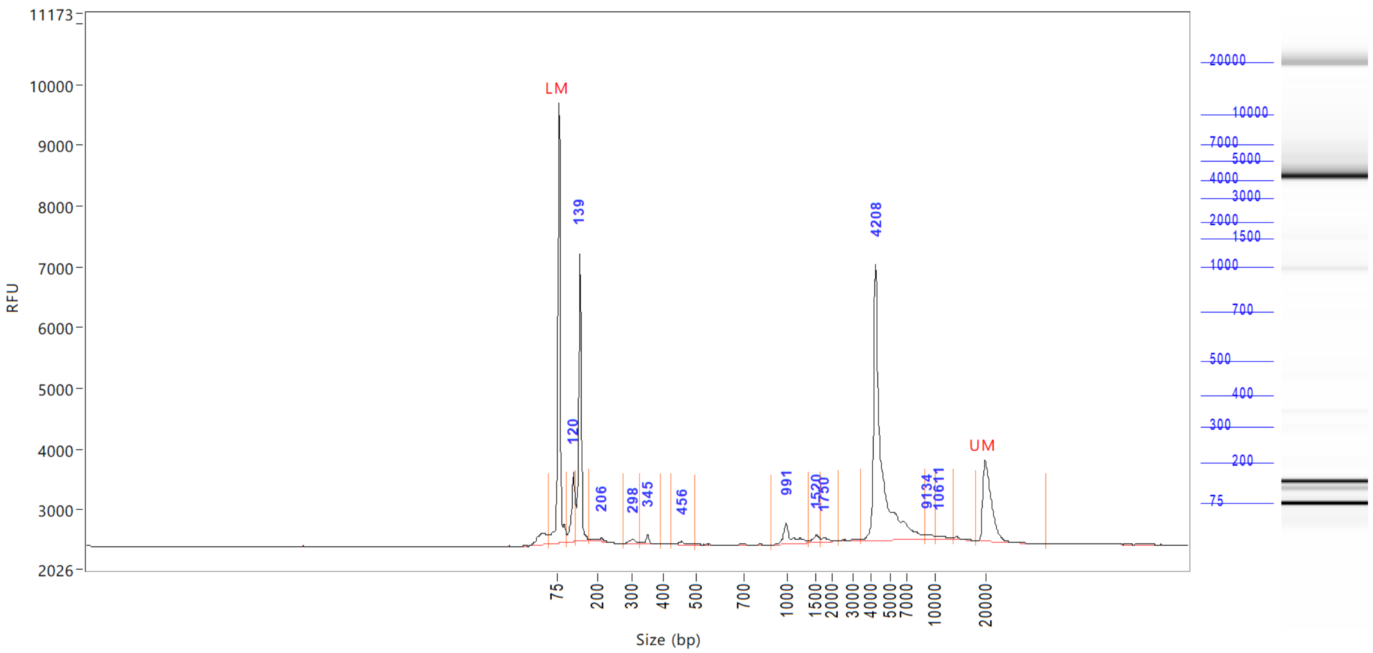

Peak	Size (bp)	Concentration (ng/uL)	From (bp)	To (bp)	Average size (bp)	CV%	RFU	Corrected peak area
1	75 (LM)	0.5000	47	99	73	8.89	7252	44.877
2	120	1.4933	99	129	118	6.08	1145	11.169
3	139	4.0571	129	169	138	3.27	4725	30.345
4	206	0.1315	169	269	199	8.08	54	0.983
5	298	0.1638	269	323	297	3.98	76	1.225
6	345	0.1670	323	388	344	3.08	164	1.249
7	456	0.1001	426	496	459	3.49	61	0.749
8	991	0.6139	892	1378	1062	10.75	360	4.591
9	1520	0.1607	1378	1655	1508	4.60	119	1.202
10	1750	0.1108	1655	2286	1827	7.55	67	0.829
11	4208	6.7493	3393	8934	4730	20.78	4527	50.481
12	9134	0.1517	8934	10245	9516	3.97	89	1.134
13	10611	0.1405	10245	13537	11674	8.14	65	1.051
14	20000 (UM)	0.1625	18171	32090	20605	5.82	1342	14.587

TIC:	14.0395	ng/uL
TIM:	75.8724	nmole/L
Total concentration:	14.1423	ng/uL

```
Sample peak width (sec): 5    Sample min peak height: 50    Sample baseline V to V?: Y    Sample baseline V to V points: 3
Sample filter: Binomial       Number of points for filter: 3  Sample start region (min): 0  Sample end region (min): 30
Marker peak width (sec): 5    Marker min peak height: 200   Marker baseline V to V?: Y    Marker baseline V to V points: 3
Lower marker selection: First peak > 500 RFU                Upper marker selection: Last peak > 200 RFU
Ladder size (bp)75, 200, 300, 400, 500, 700, 1000, 1500, 2000, 3000, 4000, 5000, 7000, 10000, 20000
Quantification using: Lower Marker          Final concentration (ng/uL): 0.5000          Dilution factor: 12.0
```

Sample: SampH3
Well location: H3
Created: Thursday, August 12, 2021 6:46:05 PM

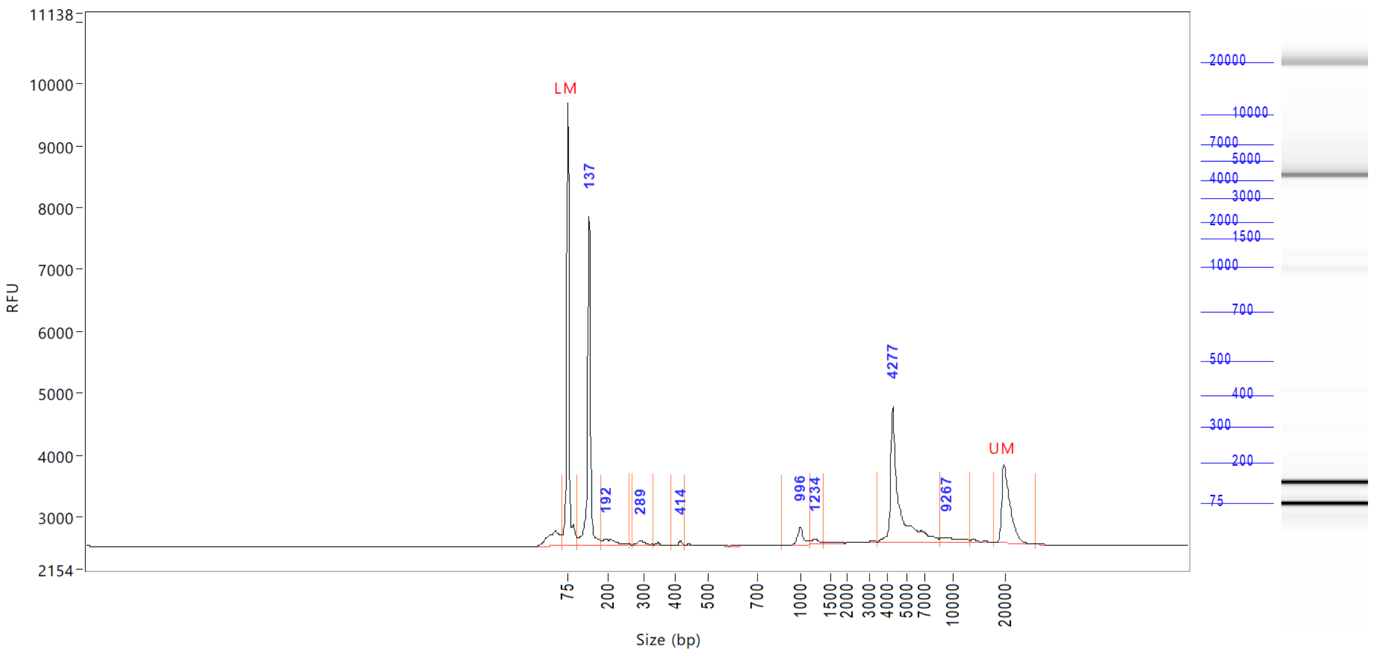

Peak	Size (bp)	Concentration (ng/uL)	From (bp)	To (bp)	Average size (bp)	CV%	RFU	Corrected peak area
1	75 (LM)	0.5000	56	105	73	8.94	7167	45.596
2	137	5.0748	105	177	135	6.60	5303	38.566
3	192	0.3192	177	257	200	7.62	84	2.425
4	289	0.1223	266	329	290	3.32	69	0.929
5	414	0.0712	390	430	413	1.63	80	0.541
6	996	0.3850	861	1134	1003	4.47	290	2.926
7	1234	0.1429	1134	1378	1234	5.15	90	1.086
8	4277	3.4468	3393	8600	4835	21.07	2191	26.194
9	9267	0.2613	8600	13415	10340	13.04	75	1.986
10	20000 (UM)	0.1470	17805	26045	20513	4.75	1276	13.401

TIC:	9.8234	ng/uL
TIM:	67.4085	nmole/L
Total concentration:	10.1334	ng/uL

Sample peak width (sec): 5   Sample min peak height: 50   Sample baseline V to V?: Y   Sample baseline V to V points: 3
Sample filter: Binomial   Number of points for filter: 3   Sample start region (min): 0   Sample end region (min): 30
Marker peak width (sec): 5   Marker min peak height: 200   Marker baseline V to V?: Y   Marker baseline V to V points: 3
Lower marker selection: First peak > 500 RFU   Upper marker selection: Last peak > 200 RFU
Ladder size (bp)75, 200, 300, 400, 500, 700, 1000, 1500, 2000, 3000, 4000, 5000, 7000, 10000, 20000
Quantification using: Lower Marker   Final concentration (ng/uL): 0.5000   Dilution factor: 12.0

Sample: SampA4
Well location: A4
Created: Thursday, August 12, 2021 6:46:05 PM

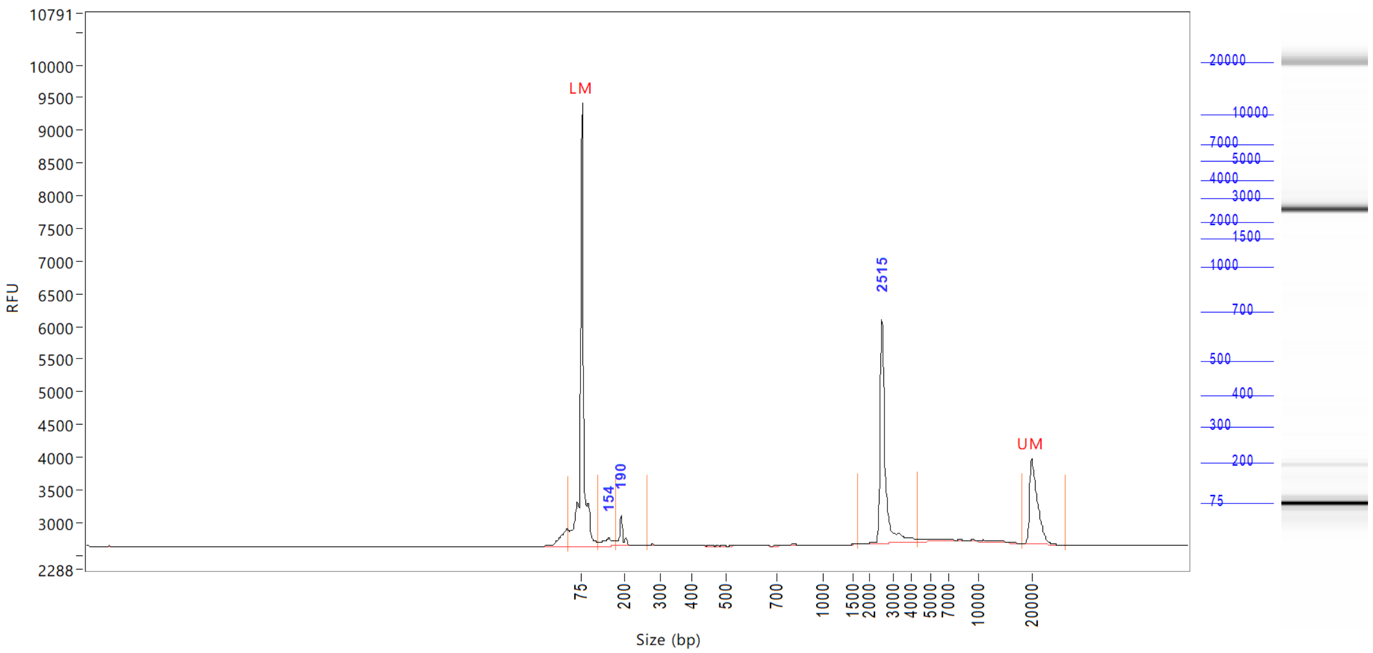

Peak	Size (bp)	Concentration (ng/uL)	From (bp)	To (bp)	Average size (bp)	CV%	RFU	Corrected peak area
1	75 (LM)	0.5000	37	124	73	16.18	6791	54.341
2	154	0.3138	124	173	149	9.17	127	2.842
3	190	0.4217	173	266	192	6.78	459	3.820
4	2515	2.7765	1655	4277	2634	12.76	3424	25.147
5	20000 (UM)	0.1161	18293	26166	20474	4.30	1294	12.619

TIC:	3.5121	ng/uL
TIM:	8.8079	nmole/L
Total concentration:	3.9784	ng/uL

Sample peak width (sec): 5 Sample min peak height: 50 Sample baseline V to V?: Y Sample baseline V to V points: 3
Sample filter: Binomial Number of points for filter: 3 Sample start region (min): 0 Sample end region (min): 30
Marker peak width (sec): 5 Marker min peak height: 200 Marker baseline V to V?: Y Marker baseline V to V points: 3
Lower marker selection: First peak > 500 RFU Upper marker selection: Last peak > 200 RFU
Ladder size (bp)75, 200, 300, 400, 500, 700, 1000, 1500, 2000, 3000, 4000, 5000, 7000, 10000, 20000
Quantification using: Lower Marker Final concentration (ng/uL): 0.5000 Dilution factor: 12.0

Sample: SampB4
Well location: B4
Created: Thursday, August 12, 2021 6:46:05 PM

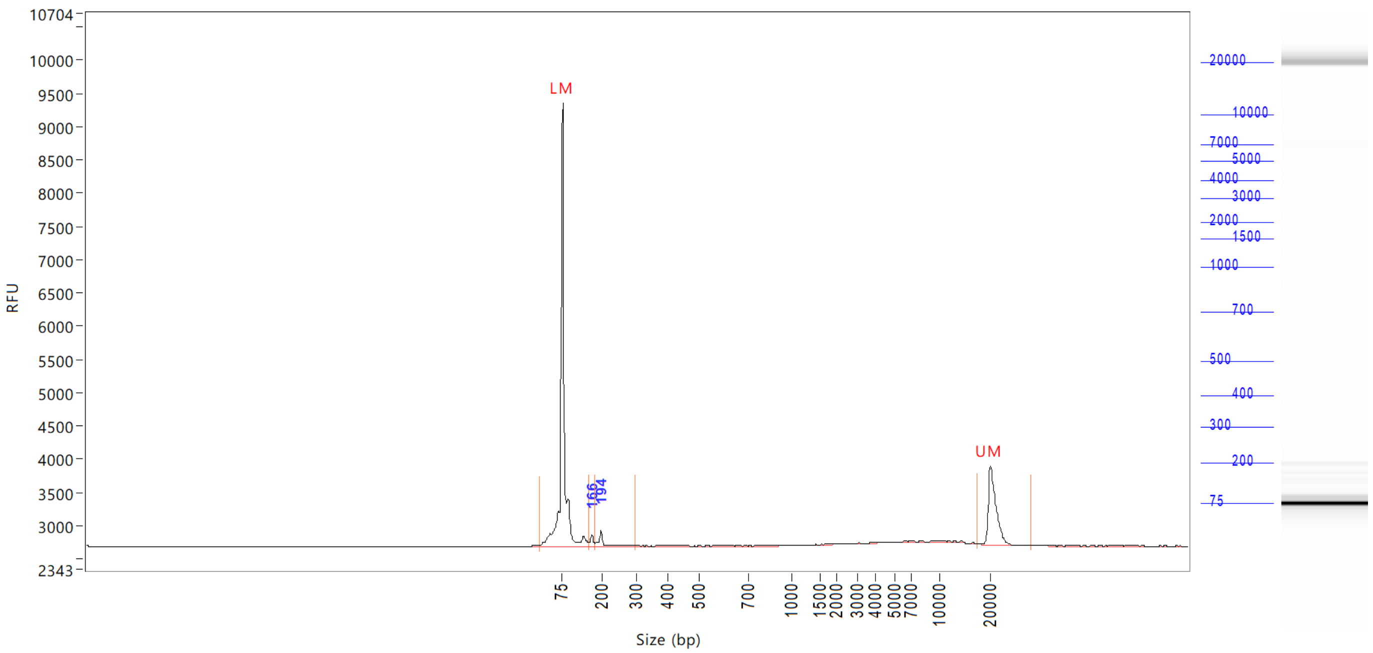

Peak	Size (bp)	Concentration (ng/uL)	From (bp)	To (bp)	Average size (bp)	CV%	RFU	Corrected peak area
1	75 (LM)	0.5000	5	156	74	25.61	6679	56.967
2	166	0.1250	156	175	165	2.99	162	1.186
3	194	0.2337	175	296	200	12.11	243	2.219
4	20000 (UM)	0.1035	17561	28221	20589	4.81	1176	11.797

TIC:	0.3587	ng/uL
TIM:	3.1661	nmole/L
Total concentration:	0.6894	ng/uL

Sample peak width (sec): 5 Sample min peak height: 50 Sample baseline V to V?: Y Sample baseline V to V points: 3
Sample filter: Binomial Number of points for filter: 3 Sample start region (min): 0 Sample end region (min): 30
Marker peak width (sec): 5 Marker min peak height: 200 Marker baseline V to V?: Y Marker baseline V to V points: 3
Lower marker selection: First peak > 500 RFU Upper marker selection: Last peak > 200 RFU
Ladder size (bp)75, 200, 300, 400, 500, 700, 1000, 1500, 2000, 3000, 4000, 5000, 7000, 10000, 20000
Quantification using: Lower Marker Final concentration (ng/uL): 0.5000 Dilution factor: 12.0

Sample: SampC4
Well location: C4
Created: Thursday, August 12, 2021 6:46:05 PM

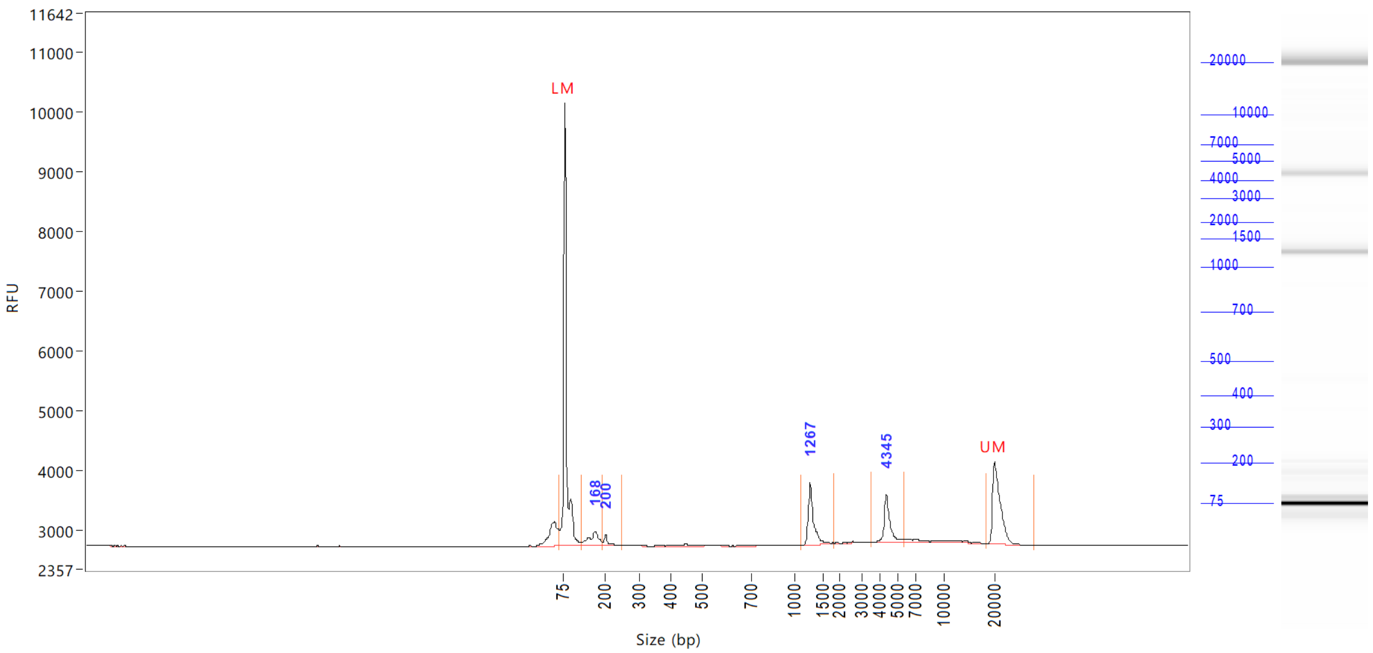

Peak	Size (bp)	Concentration (ng/uL)	From (bp)	To (bp)	Average size (bp)	CV%	RFU	Corrected peak area
1	75 (LM)	0.5000	58	128	76	12.82	7412	52.310
2	168	0.5938	128	192	161	10.12	229	5.177
3	200	0.1808	192	250	204	4.85	185	1.577
4	1267	1.0200	1101	1827	1296	7.12	1032	8.892
5	4345	0.7783	3572	5741	4442	6.69	787	6.786
6	20000 (UM)	0.1296	18293	27738	20566	4.54	1369	13.563

TIC:	2.5730	ng/uL
TIM:	9.1043	nmole/L
Total concentration:	3.0416	ng/uL

```
Sample peak width (sec): 5    Sample min peak height: 50    Sample baseline V to V?: Y    Sample baseline V to V points: 3
Sample filter: Binomial       Number of points for filter: 3  Sample start region (min): 0  Sample end region (min): 30
Marker peak width (sec): 5    Marker min peak height: 200   Marker baseline V to V?: Y    Marker baseline V to V points: 3
Lower marker selection: First peak > 500 RFU                Upper marker selection: Last peak > 200 RFU
Ladder size (bp)75, 200, 300, 400, 500, 700, 1000, 1500, 2000, 3000, 4000, 5000, 7000, 10000, 20000
Quantification using: Lower Marker          Final concentration (ng/uL): 0.5000          Dilution factor: 12.0
```

Sample: SampD4
Well location: D4
Created: Thursday, August 12, 2021 6:46:05 PM

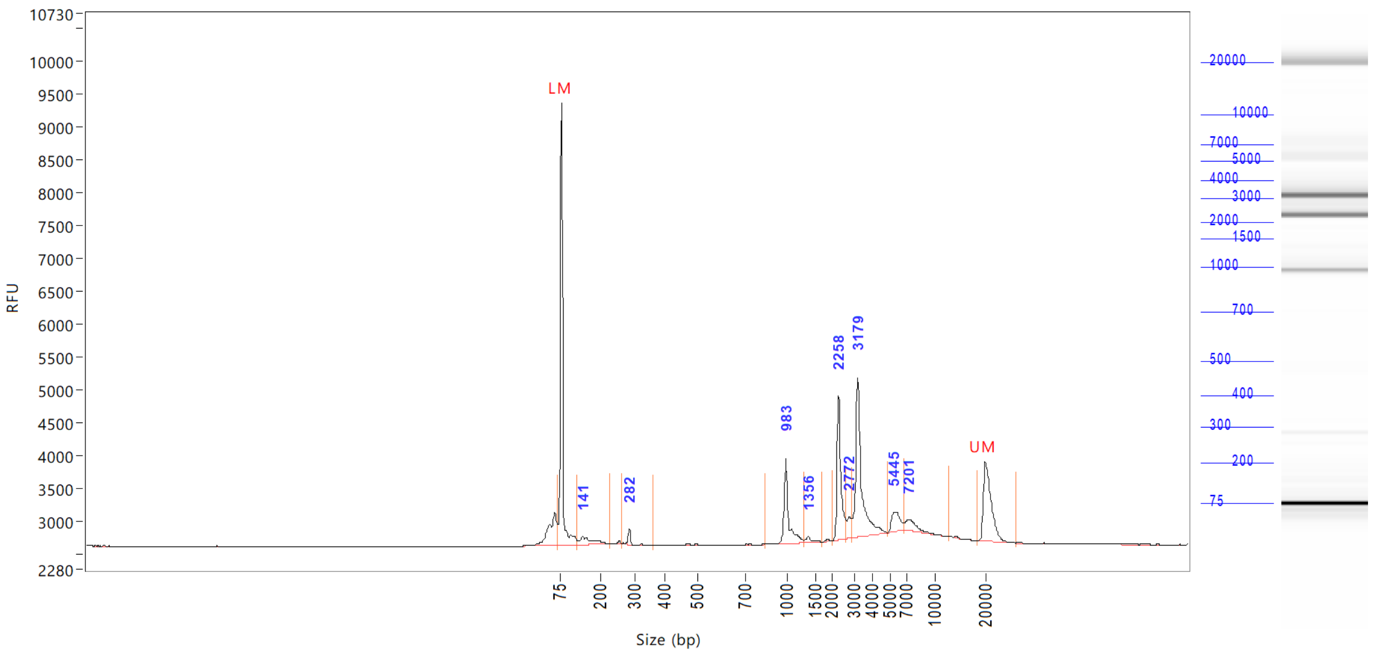

Peak	Size (bp)	Concentration (ng/uL)	From (bp)	To (bp)	Average size (bp)	CV%	RFU	Corrected peak area
1	75 (LM)	0.5000	66	126	76	12.36	6733	40.754
2	141	0.5297	126	224	161	14.70	130	3.598
3	282	0.2209	260	360	282	5.07	232	1.501
4	983	1.4241	835	1278	1010	6.76	1296	9.673
5	1356	0.1357	1278	1693	1383	5.81	92	0.922
6	2258	2.0296	2000	2629	2294	5.33	2180	13.786
7	2772	0.3565	2629	2886	2758	2.91	328	2.422
8	3179	3.0868	2886	4897	3324	10.68	2421	20.967
9	5445	0.5342	4897	6630	5668	7.85	291	3.628
10	7201	0.4027	6630	12684	7612	11.48	165	2.735
11	20000 (UM)	0.1473	18415	26045	20563	4.41	1197	12.004

TIC:	8.7202	ng/uL
TIM:	12.6187	nmole/L
Total concentration:	8.8636	ng/uL

```
Sample peak width (sec): 5    Sample min peak height: 50    Sample baseline V to V?: Y    Sample baseline V to V points: 3
Sample filter: Binomial       Number of points for filter: 3  Sample start region (min): 0  Sample end region (min): 30
Marker peak width (sec): 5    Marker min peak height: 200    Marker baseline V to V?: Y    Marker baseline V to V points: 3
Lower marker selection: First peak > 500 RFU              Upper marker selection: Last peak > 200 RFU
Ladder size (bp)75, 200, 300, 400, 500, 700, 1000, 1500, 2000, 3000, 4000, 5000, 7000, 10000, 20000
Quantification using: Lower Marker          Final concentration (ng/uL): 0.5000          Dilution factor: 12.0
```

Sample: SampE4
Well location: E4
Created: Thursday, August 12, 2021 6:46:05 PM

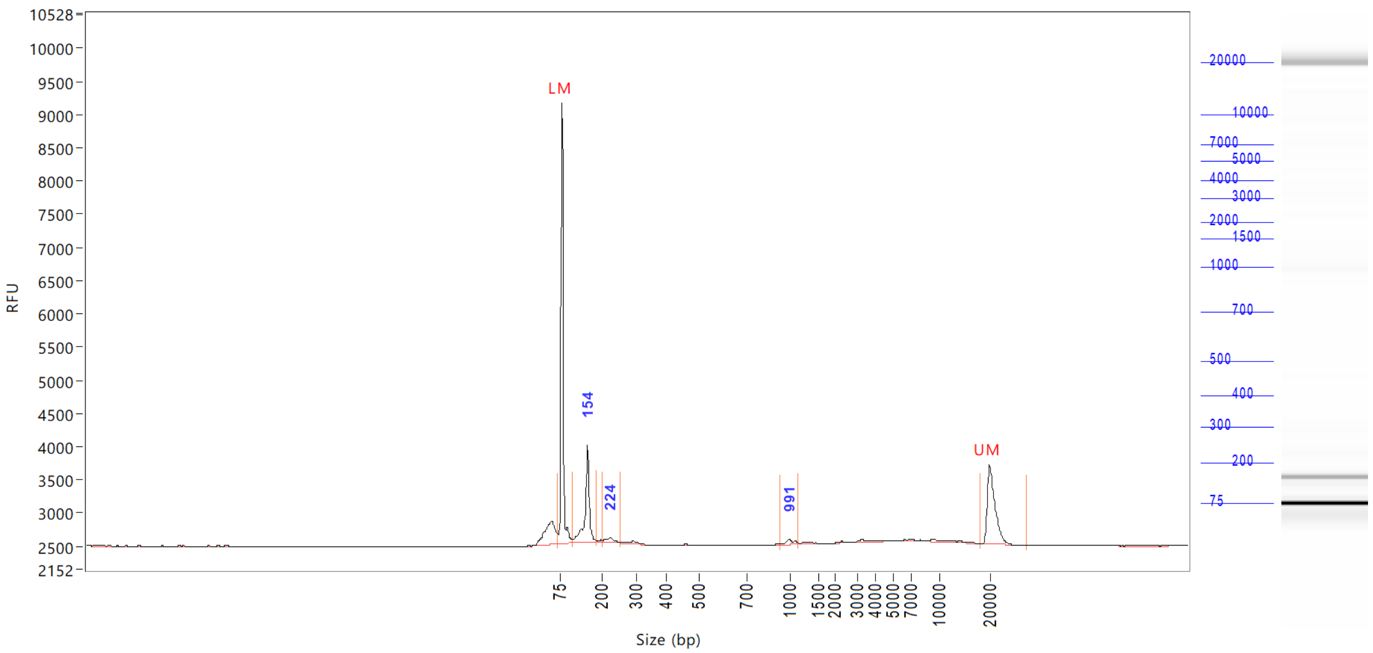

Peak	Size (bp)	Concentration (ng/uL)	From (bp)	To (bp)	Average size (bp)	CV%	RFU	Corrected peak area
1	75 (LM)	0.5000	64	107	74	7.12	6649	38.052
2	154	2.2076	107	185	147	8.54	1464	14.001
3	224	0.2073	200	255	222	5.62	79	1.315
4	991	0.1614	922	1134	1007	4.68	90	1.024
5	20000 (UM)	0.1552	18049	27375	20471	4.47	1204	11.813

TIC:	2.5763	ng/uL
TIM:	26.4806	nmole/L
Total concentration:	3.3755	ng/uL

```
Sample peak width (sec): 5    Sample min peak height: 50    Sample baseline V to V?: Y    Sample baseline V to V points: 3
Sample filter: Binomial       Number of points for filter: 3  Sample start region (min): 0  Sample end region (min): 30
Marker peak width (sec): 5    Marker min peak height: 200   Marker baseline V to V?: Y    Marker baseline V to V points: 3
Lower marker selection: First peak > 500 RFU                Upper marker selection: Last peak > 200 RFU
Ladder size (bp)75, 200, 300, 400, 500, 700, 1000, 1500, 2000, 3000, 4000, 5000, 7000, 10000, 20000
Quantification using: Lower Marker          Final concentration (ng/uL): 0.5000          Dilution factor: 12.0
```

Sample: SampF4
Well location: F4
Created: Thursday, August 12, 2021 6:46:05 PM

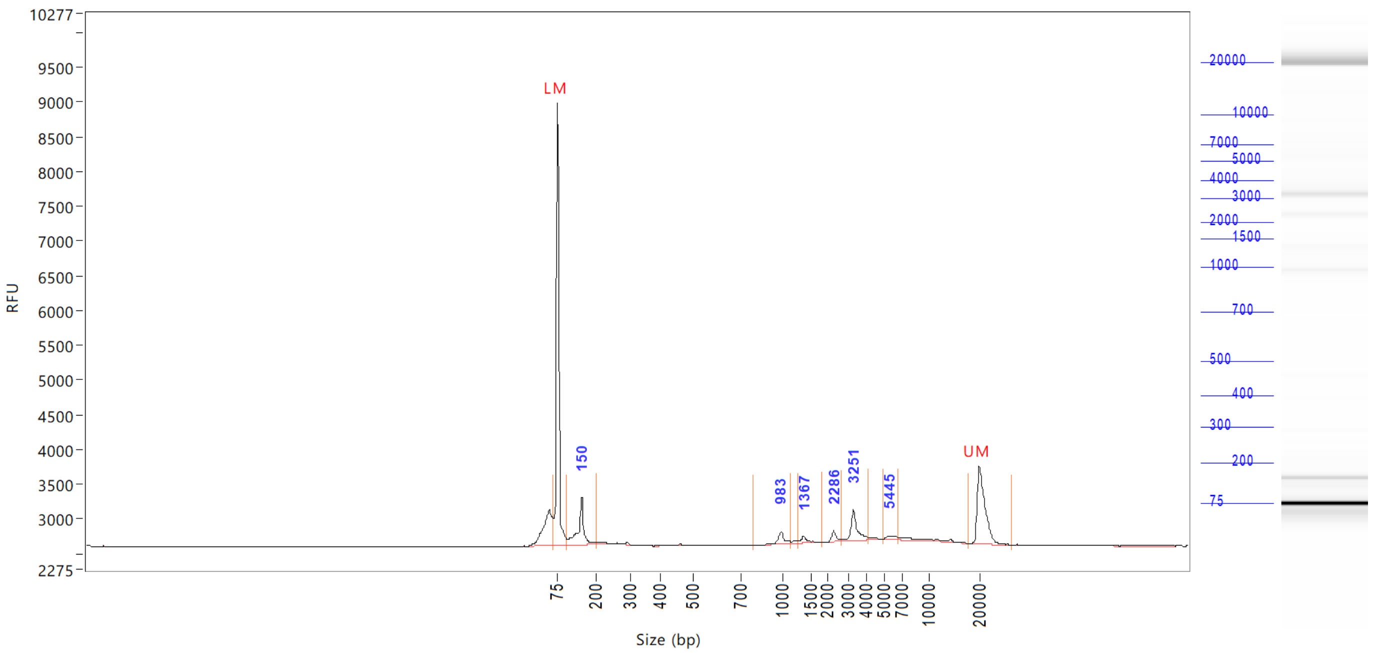

Peak	Size (bp)	Concentration (ng/uL)	From (bp)	To (bp)	Average size (bp)	CV%	RFU	Corrected peak area
1	75 (LM)	0.5000	64	105	74	7.66	6378	38.734
2	150	1.3598	105	200	144	12.72	672	8.779
3	983	0.2899	792	1145	991	5.50	182	1.872
4	1367	0.1499	1267	1827	1391	6.80	92	0.968
5	2286	0.1644	1827	2629	2283	5.53	162	1.062
6	3251	0.6485	2629	4104	3318	8.69	439	4.186
7	5445	0.1381	4931	6630	5734	8.27	63	0.892
8	20000 (UM)	0.1483	17927	26408	20571	4.77	1115	11.489

TIC:	2.7507	ng/uL
TIM:	16.6579	nmole/L
Total concentration:	3.3776	ng/uL

```
Sample peak width (sec): 5    Sample min peak height: 50    Sample baseline V to V?: Y    Sample baseline V to V points: 3
Sample filter: Binomial       Number of points for filter: 3  Sample start region (min): 0  Sample end region (min): 30
Marker peak width (sec): 5    Marker min peak height: 200   Marker baseline V to V?: Y    Marker baseline V to V points: 3
Lower marker selection: First peak > 500 RFU                Upper marker selection: Last peak > 200 RFU
Ladder size (bp)75, 200, 300, 400, 500, 700, 1000, 1500, 2000, 3000, 4000, 5000, 7000, 10000, 20000
Quantification using: Lower Marker          Final concentration (ng/uL): 0.5000          Dilution factor: 12.0
```

Sample: SampG4
Well location: G4
Created: Thursday, August 12, 2021 6:46:05 PM

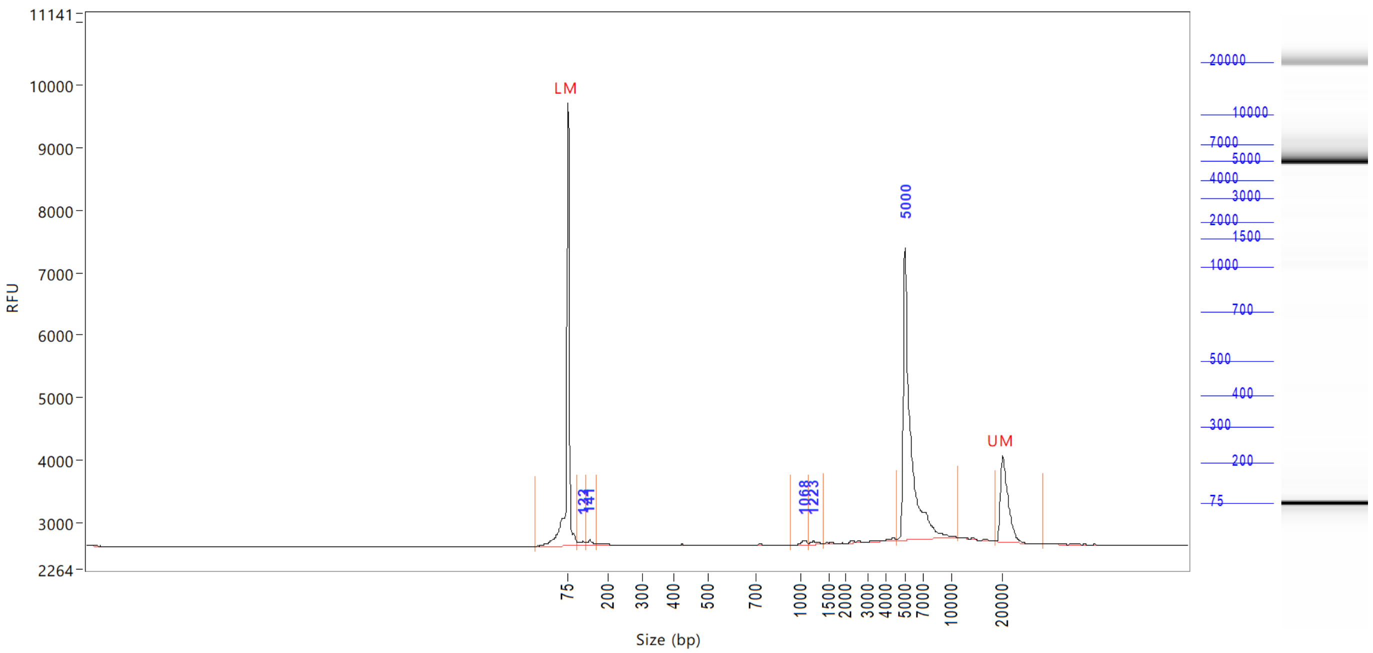

Peak	Size (bp)	Concentration (ng/uL)	From (bp)	To (bp)	Average size (bp)	CV%	RFU	Corrected peak area
1	75 (LM)	0.5000	0	105	69	20.25	7086	49.735
2	122	0.1469	105	133	119	7.28	69	1.218
3	141	0.1405	133	164	145	6.03	95	1.165
4	1068	0.0882	935	1134	1039	4.39	74	0.731
5	1223	0.0814	1134	1400	1245	5.32	56	0.675
6	5000	5.7749	4552	11342	5643	17.27	4668	47.869
7	20000 (UM)	0.1349	18659	27859	20526	4.36	1388	13.422

TIC:	6.2320	ng/uL
TIM:	5.5530	nmole/L
Total concentration:	6.6973	ng/uL

Sample peak width (sec): 5   Sample min peak height: 50   Sample baseline V to V?: Y   Sample baseline V to V points: 3
Sample filter: Binomial   Number of points for filter: 3 Sample start region (min): 0 Sample end region (min): 30
Marker peak width (sec): 5   Marker min peak height: 200   Marker baseline V to V?: Y   Marker baseline V to V points: 3
Lower marker selection: First peak > 500 RFU   Upper marker selection: Last peak > 200 RFU
Ladder size (bp)75, 200, 300, 400, 500, 700, 1000, 1500, 2000, 3000, 4000, 5000, 7000, 10000, 20000
Quantification using: Lower Marker   Final concentration (ng/uL): 0.5000   Dilution factor: 12.0

Sample: SampH4
Well location: H4
Created: Thursday, August 12, 2021 6:46:05 PM

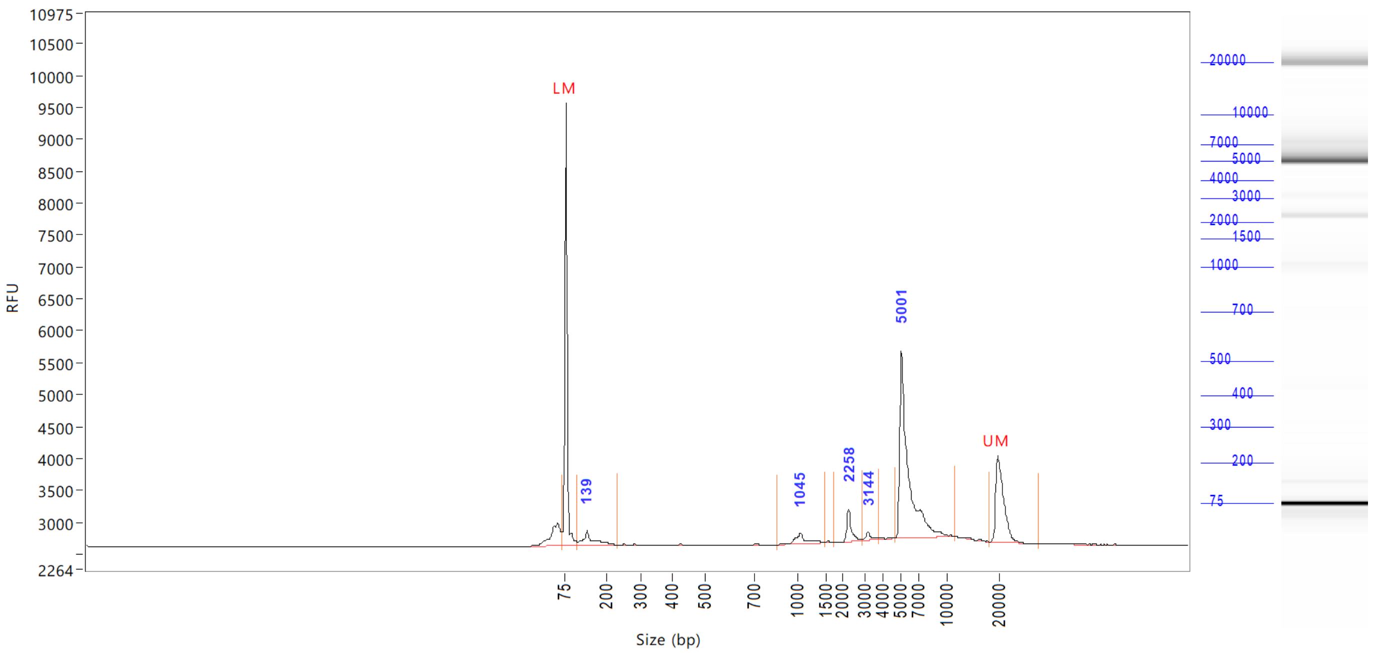

Peak	Size (bp)	Concentration (ng/uL)	From (bp)	To (bp)	Average size (bp)	CV%	RFU	Corrected peak area
1	75 (LM)	0.5000	64	108	73	7.46	6948	40.210
2	139	0.7530	108	230	149	16.69	239	5.047
3	1045	0.4482	861	1489	1104	12.71	166	3.004
4	2258	0.5460	1731	2886	2289	7.15	518	3.659
5	3144	0.1519	2886	3750	3201	6.17	127	1.018
6	5001	5.2891	4656	11586	5806	18.55	2936	35.446
7	20000 (UM)	0.1686	18293	27859	20487	4.61	1355	13.561

TIC:	7.1882	ng/uL
TIM:	10.9447	nmole/L
Total concentration:	7.5034	ng/uL

Sample peak width (sec): 5 Sample min peak height: 50 Sample baseline V to V?: Y Sample baseline V to V points: 3
Sample filter: Binomial Number of points for filter: 3 Sample start region (min): 0 Sample end region (min): 30
Marker peak width (sec): 5 Marker min peak height: 200 Marker baseline V to V?: Y Marker baseline V to V points: 3
Lower marker selection: First peak > 500 RFU Upper marker selection: Last peak > 200 RFU
Ladder size (bp)75, 200, 300, 400, 500, 700, 1000, 1500, 2000, 3000, 4000, 5000, 7000, 10000, 20000
Quantification using: Lower Marker Final concentration (ng/uL): 0.5000 Dilution factor: 12.0

Sample: SampA5
Well location: A5
Created: Thursday, August 12, 2021 6:46:05 PM

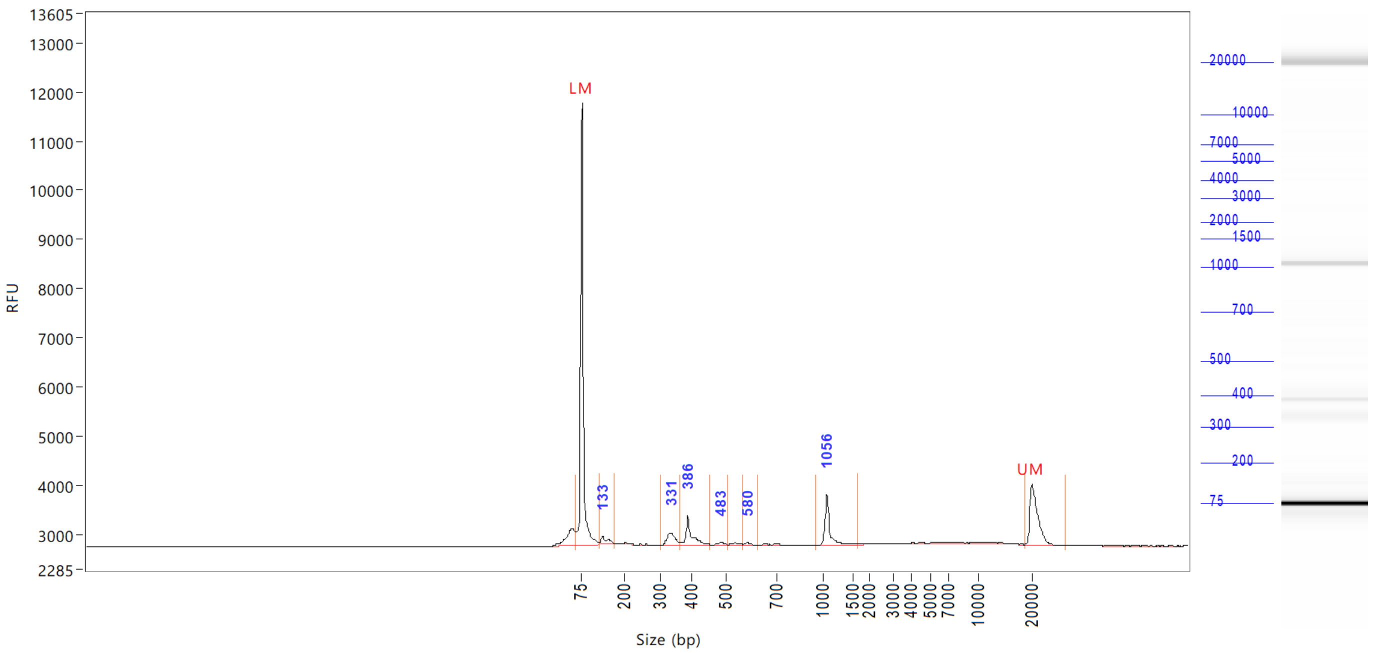

Peak	Size (bp)	Concentration (ng/uL)	From (bp)	To (bp)	Average size (bp)	CV%	RFU	Corrected peak area
1	75 (LM)	0.5000	56	126	74	11.02	9018	59.424
2	133	0.2240	126	169	142	7.61	156	2.219
3	331	0.4249	301	360	331	3.67	259	4.208
4	386	0.6333	360	452	392	4.39	603	6.272
5	483	0.0539	452	504	480	1.93	55	0.534
6	580	0.0373	562	621	582	2.14	51	0.370
7	1056	0.7542	952	1635	1091	8.70	1022	7.469
8	20000 (UM)	0.0996	18659	26287	20512	4.26	1233	11.841

TIC:	2.1276	ng/uL
TIM:	8.7904	nmole/L
Total concentration:	2.5392	ng/uL

```
Sample peak width (sec): 5    Sample min peak height: 50    Sample baseline V to V?: Y    Sample baseline V to V points: 3
Sample filter: Binomial       Number of points for filter: 3  Sample start region (min): 0  Sample end region (min): 30
Marker peak width (sec): 5    Marker min peak height: 200   Marker baseline V to V?: Y    Marker baseline V to V points: 3
Lower marker selection: First peak > 500 RFU                Upper marker selection: Last peak > 200 RFU
Ladder size (bp)75, 200, 300, 400, 500, 700, 1000, 1500, 2000, 3000, 4000, 5000, 7000, 10000, 20000
Quantification using: Lower Marker          Final concentration (ng/uL): 0.5000          Dilution factor: 12.0
```

Sample: SampB5
Well location: B5
Created: Thursday, August 12, 2021 6:46:05 PM

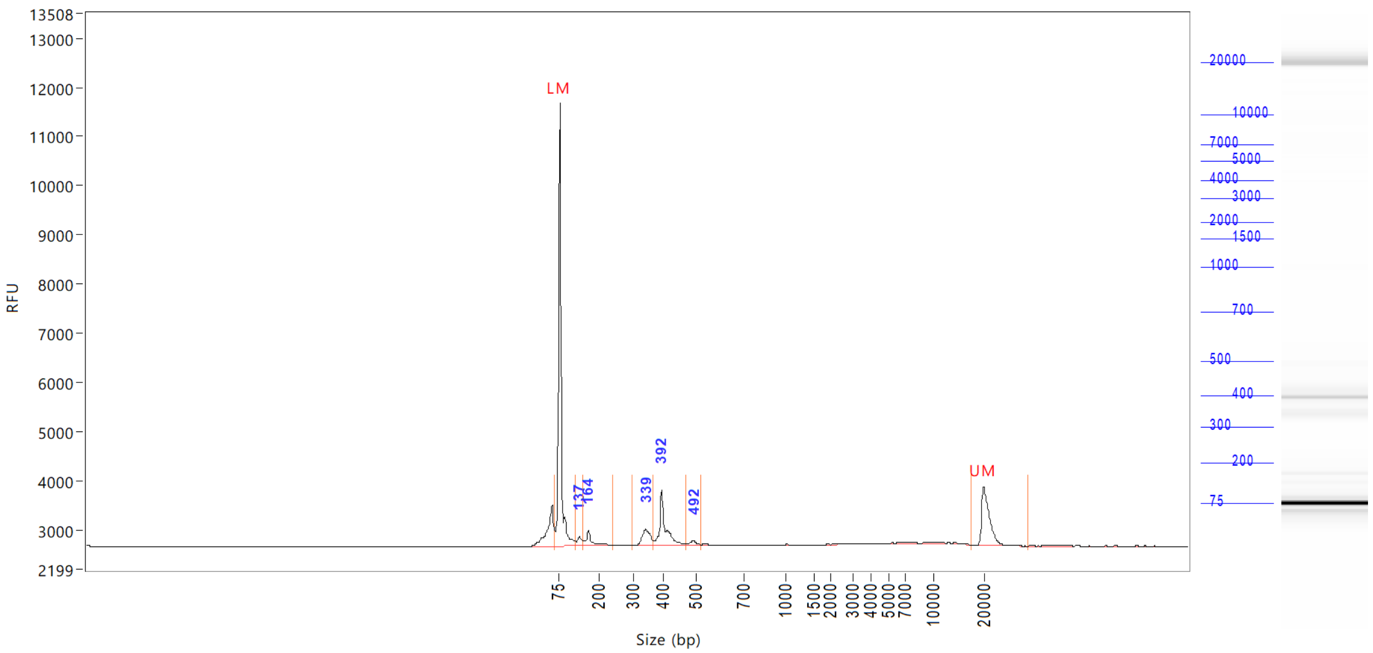

Peak	Size (bp)	Concentration (ng/uL)	From (bp)	To (bp)	Average size (bp)	CV%	RFU	Corrected peak area
1	75 (LM)	0.5000	62	126	76	11.76	9029	59.636
2	137	0.2298	126	150	137	5.04	202	2.285
3	164	0.3927	150	239	174	11.44	305	3.903
4	339	0.5509	296	366	340	3.63	327	5.475
5	392	1.2852	366	468	400	4.92	1115	12.774
6	492	0.1416	468	521	489	2.44	105	1.408
7	20000 (UM)	0.0993	17439	28705	20482	4.80	1193	11.849

TIC:	2.6002	ng/uL
TIM:	14.8952	nmole/L
Total concentration:	2.8643	ng/uL

Sample peak width (sec): 5   Sample min peak height: 50   Sample baseline V to V?: Y   Sample baseline V to V points: 3
Sample filter: Binomial   Number of points for filter: 3   Sample start region (min): 0   Sample end region (min): 30
Marker peak width (sec): 5   Marker min peak height: 200   Marker baseline V to V?: Y   Marker baseline V to V points: 3
Lower marker selection: First peak > 500 RFU   Upper marker selection: Last peak > 200 RFU
Ladder size (bp)75, 200, 300, 400, 500, 700, 1000, 1500, 2000, 3000, 4000, 5000, 7000, 10000, 20000
Quantification using: Lower Marker   Final concentration (ng/uL): 0.5000   Dilution factor: 12.0

Sample: SampC5
Well location: C5
Created: Thursday, August 12, 2021 6:46:05 PM

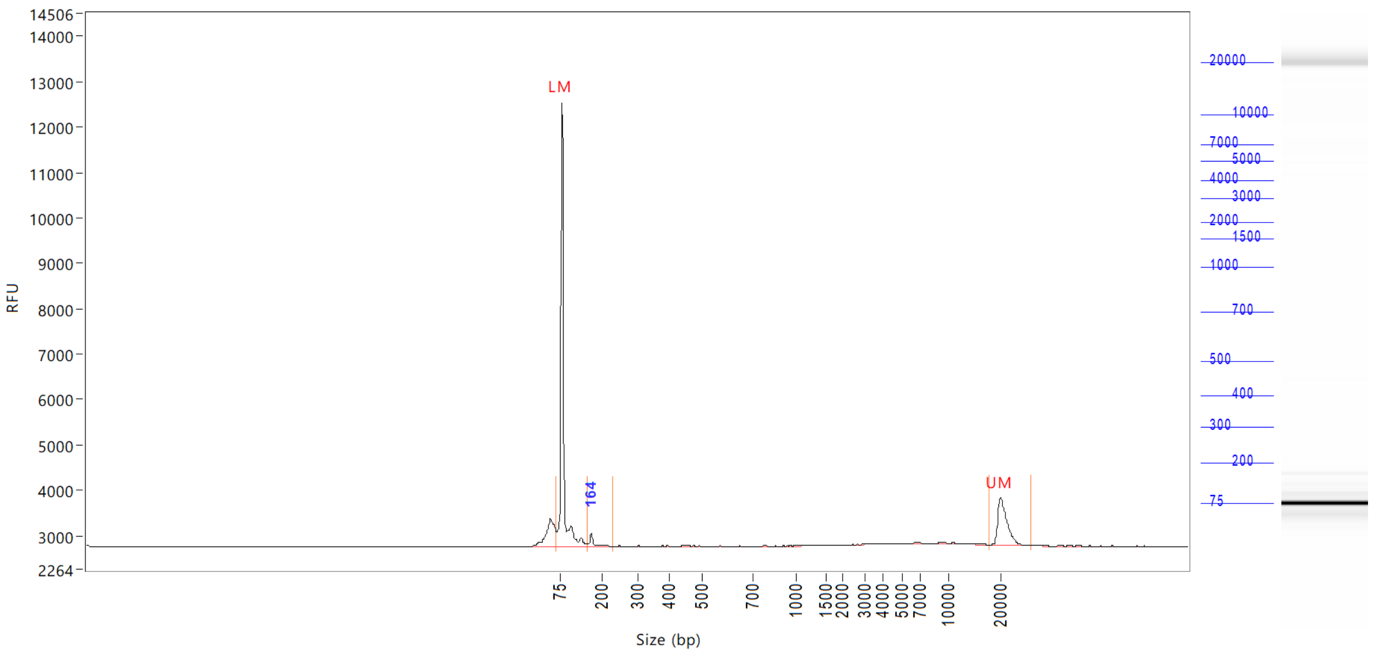

Peak	Size (bp)	Concentration (ng/uL)	From (bp)	To (bp)	Average size (bp)	CV%	RFU	Corrected peak area
1	75 (LM)	0.5000	58	154	78	18.18	9786	68.158
2	164	0.1954	154	226	168	8.28	279	2.219
3	20000 (UM)	0.0883	17805	26045	20528	4.98	1047	12.042

TIC:	0.1954	ng/uL
TIM:	1.9117	nmole/L
Total concentration:	0.5013	ng/uL

Sample peak width (sec): 5 Sample min peak height: 50 Sample baseline V to V?: Y Sample baseline V to V points: 3
Sample filter: Binomial Number of points for filter: 3 Sample start region (min): 0 Sample end region (min): 30
Marker peak width (sec): 5 Marker min peak height: 200 Marker baseline V to V?: Y Marker baseline V to V points: 3
Lower marker selection: First peak > 500 RFU Upper marker selection: Last peak > 200 RFU
Ladder size (bp)75, 200, 300, 400, 500, 700, 1000, 1500, 2000, 3000, 4000, 5000, 7000, 10000, 20000
Quantification using: Lower Marker Final concentration (ng/uL): 0.5000 Dilution factor: 12.0

Sample: SampD5
Well location: D5
Created: Thursday, August 12, 2021 6:46:05 PM

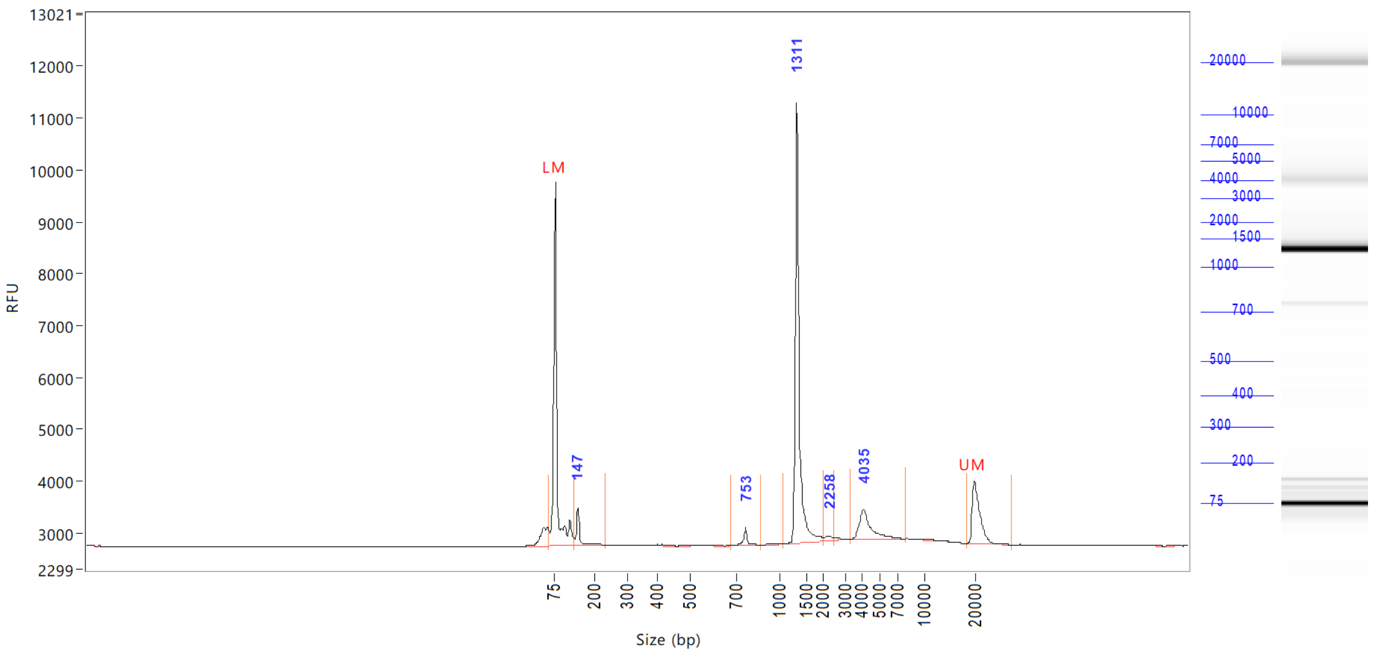

Peak	Size (bp)	Concentration (ng/uL)	From (bp)	To (bp)	Average size (bp)	CV%	RFU	Corrected peak area
1	75 (LM)	0.5000	58	137	79	19.84	7010	57.833
2	147	0.5867	137	232	152	11.32	733	5.655
3	753	0.2531	674	866	749	3.83	346	2.440
4	1311	5.5754	1068	2001	1340	7.98	8507	53.741
5	2258	0.1181	2001	2515	2222	6.22	98	1.139
6	4035	1.0079	3358	7867	4346	15.28	574	9.715
7	20000 (UM)	0.1031	18659	27617	20557	4.88	1196	11.922

TIC:	7.5413	ng/uL
TIM:	14.2182	nmole/L
Total concentration:	7.8164	ng/uL

Sample peak width (sec): 5 Sample min peak height: 50 Sample baseline V to V?: Y Sample baseline V to V points: 3
Sample filter: Binomial Number of points for filter: 3 Sample start region (min): 0 Sample end region (min): 30
Marker peak width (sec): 5 Marker min peak height: 200 Marker baseline V to V?: Y Marker baseline V to V points: 3
Lower marker selection: First peak > 500 RFU Upper marker selection: Last peak > 200 RFU
Ladder size (bp)75, 200, 300, 400, 500, 700, 1000, 1500, 2000, 3000, 4000, 5000, 7000, 10000, 20000
Quantification using: Lower Marker Final concentration (ng/uL): 0.5000 Dilution factor: 12.0

Sample: SampE5
Well location: E5
Created: Thursday, August 12, 2021 6:46:05 PM

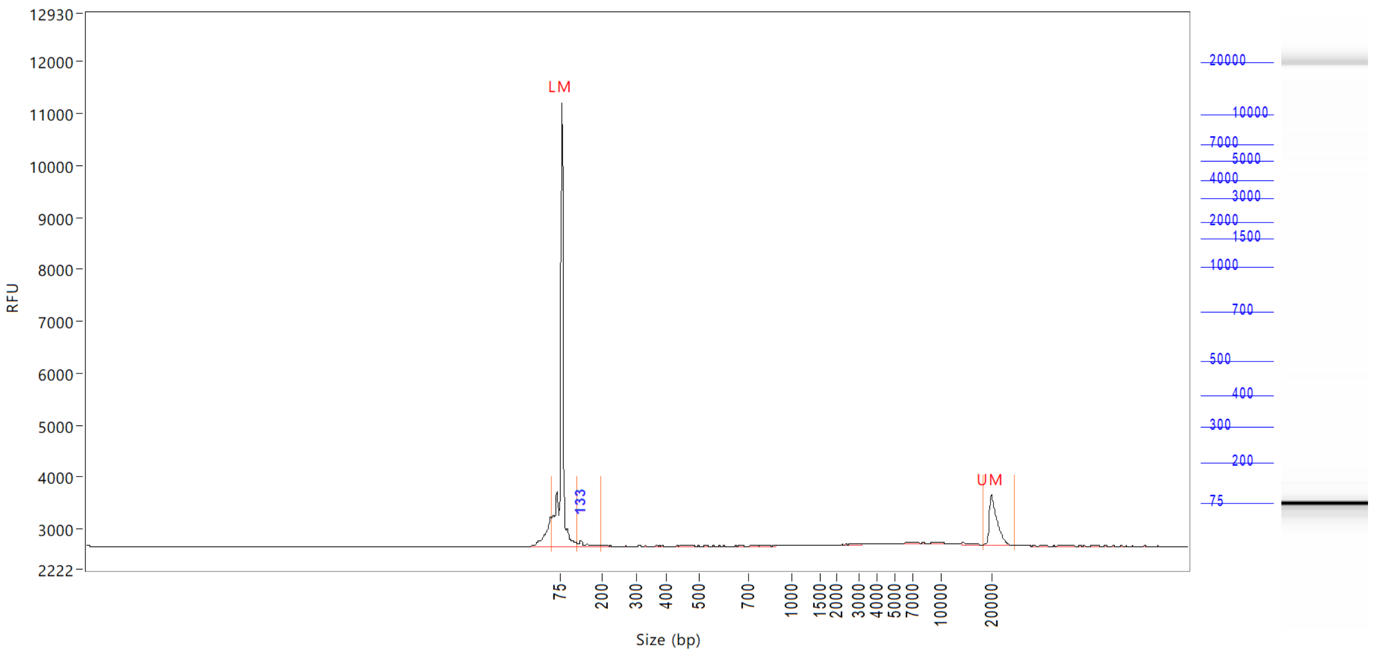

Peak	Size (bp)	Concentration (ng/uL)	From (bp)	To (bp)	Average size (bp)	CV%	RFU	Corrected peak area
1	75 (LM)	0.5000	43	124	71	15.13	8554	64.458
2	133	0.1392	124	196	144	12.50	115	1.495
3	20000 (UM)	0.0742	18293	24594	20496	4.30	970	9.564

TIC:	0.1392	ng/uL
TIM:	1.5887	nmole/L
Total concentration:	0.5077	ng/uL

```
Sample peak width (sec): 5    Sample min peak height: 50    Sample baseline V to V?: Y    Sample baseline V to V points: 3
Sample filter: Binomial       Number of points for filter: 3  Sample start region (min): 0  Sample end region (min): 30
Marker peak width (sec): 5    Marker min peak height: 200   Marker baseline V to V?: Y    Marker baseline V to V points: 3
Lower marker selection: First peak > 500 RFU                Upper marker selection: Last peak > 200 RFU
Ladder size (bp)75, 200, 300, 400, 500, 700, 1000, 1500, 2000, 3000, 4000, 5000, 7000, 10000, 20000
Quantification using: Lower Marker          Final concentration (ng/uL): 0.5000          Dilution factor: 12.0
```

Sample: SampF5
Well location: F5
Created: Thursday, August 12, 2021 6:46:05 PM

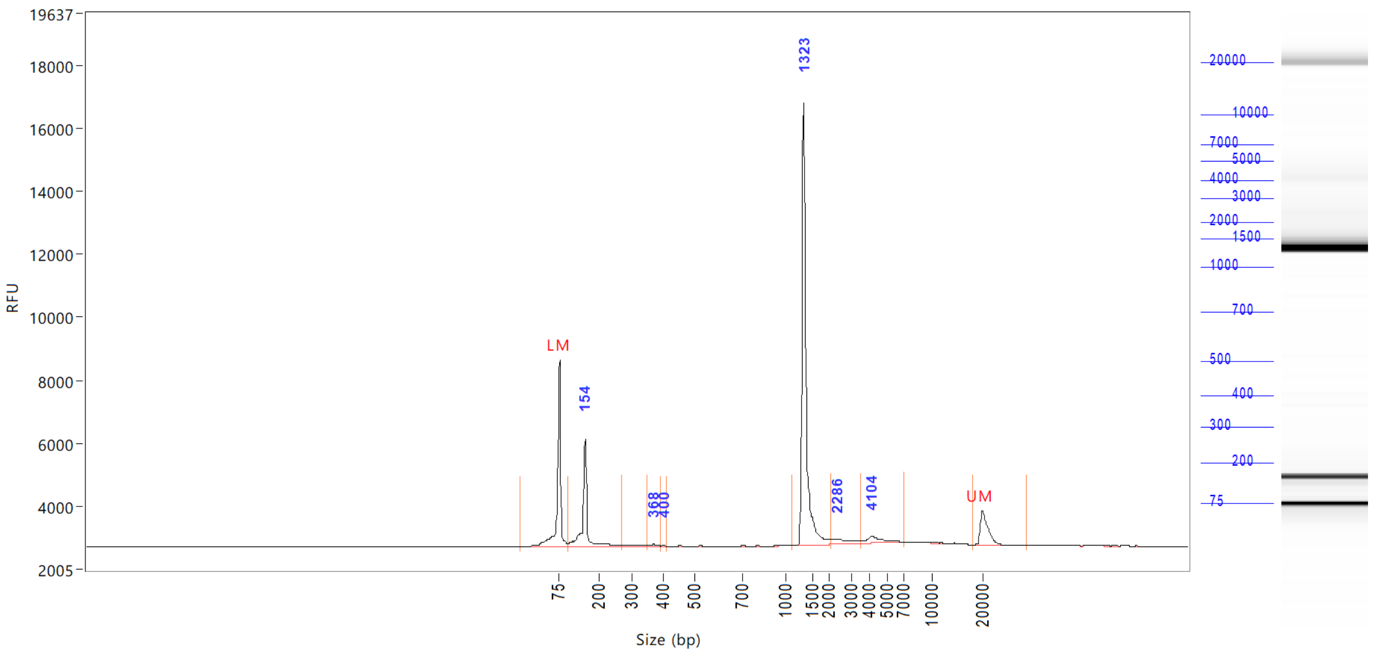

Peak	Size (bp)	Concentration (ng/uL)	From (bp)	To (bp)	Average size (bp)	CV%	RFU	Corrected peak area
1	75 (LM)	0.5000	0	101	66	26.39	5935	46.268
2	154	4.0321	101	268	154	15.00	3416	31.093
3	368	0.0468	349	392	369	2.55	59	0.361
4	400	0.0310	392	409	398	0.76	52	0.239
5	1323	11.7179	1101	2001	1355	8.06	14060	90.359
6	2286	0.6174	2001	3536	2598	16.01	173	4.761
7	4104	0.6040	3536	7000	4387	12.48	208	4.658
8	20000 (UM)	0.1160	18293	28826	20572	4.89	1065	10.733

TIC:	17.0491	ng/uL
TIM:	58.2226	nmole/L
Total concentration:	17.2876	ng/uL

Sample peak width (sec): 5   Sample min peak height: 50   Sample baseline V to V?: Y   Sample baseline V to V points: 3
Sample filter: Binomial   Number of points for filter: 3 Sample start region (min): 0 Sample end region (min): 30
Marker peak width (sec): 5   Marker min peak height: 200   Marker baseline V to V?: Y   Marker baseline V to V points: 3
Lower marker selection: First peak > 500 RFU   Upper marker selection: Last peak > 200 RFU
Ladder size (bp)75, 200, 300, 400, 500, 700, 1000, 1500, 2000, 3000, 4000, 5000, 7000, 10000, 20000
Quantification using: Lower Marker   Final concentration (ng/uL): 0.5000   Dilution factor: 12.0

Sample: SampG5
Well location: G5
Created: Thursday, August 12, 2021 6:46:05 PM

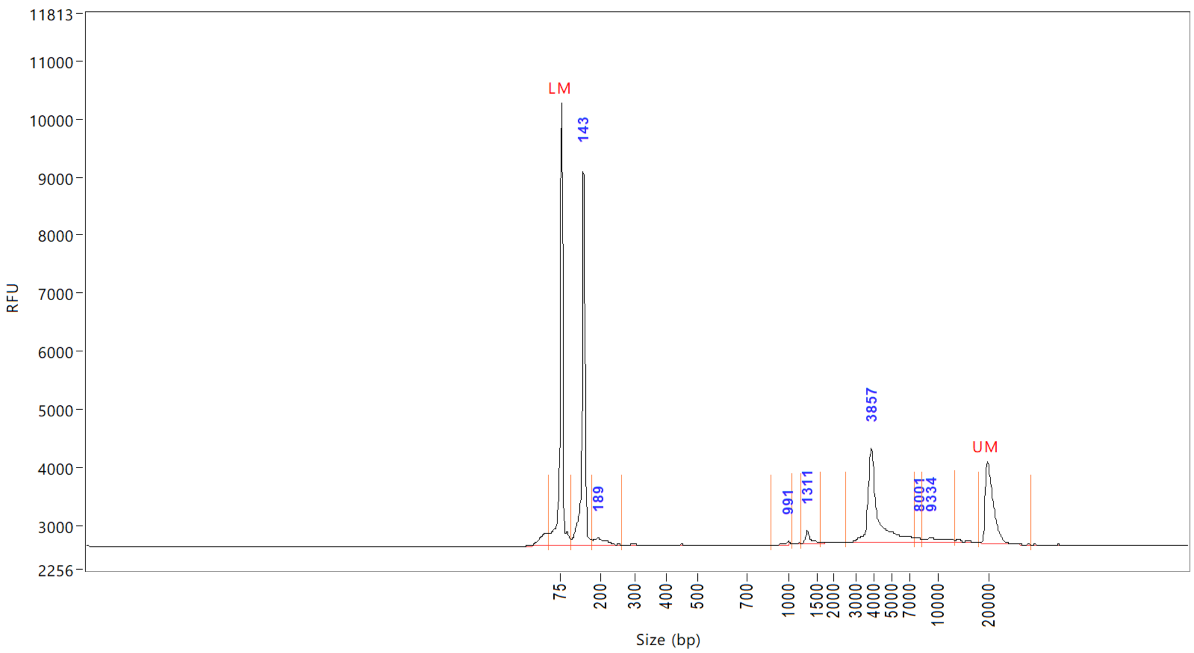

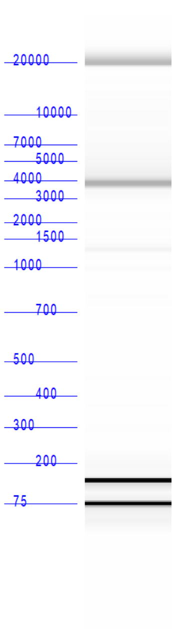

Peak	Size (bp)	Concentration (ng/uL)	From (bp)	To (bp)	Average size (bp)	CV%	RFU	Corrected peak area
1	75 (LM)	0.5000	37	105	72	12.62	7623	53.940
2	143	5.1768	105	171	140	6.39	6417	46.540
3	189	0.3654	171	260	197	8.70	117	3.285
4	991	0.0430	870	1045	978	2.86	52	0.386
5	1311	0.2068	1212	1597	1340	5.54	227	1.859
6	3857	2.5702	2543	7534	4217	20.12	1609	23.106
7	8001	0.0686	7534	8401	7941	3.26	66	0.617
8	9334	0.2071	8401	13415	10305	13.38	64	1.862
9	20000 (UM)	0.1330	18293	28584	20499	4.80	1415	14.353

TIC:	8.6379	ng/uL
TIM:	65.1916	nmole/L
Total concentration:	8.8427	ng/uL

Sample peak width (sec): 5   Sample min peak height: 50   Sample baseline V to V?: Y   Sample baseline V to V points: 3
Sample filter: Binomial   Number of points for filter: 3   Sample start region (min): 0   Sample end region (min): 30
Marker peak width (sec): 5   Marker min peak height: 200   Marker baseline V to V?: Y   Marker baseline V to V points: 3
Lower marker selection: First peak > 500 RFU   Upper marker selection: Last peak > 200 RFU
Ladder size (bp)75, 200, 300, 400, 500, 700, 1000, 1500, 2000, 3000, 4000, 5000, 7000, 10000, 20000
Quantification using: Lower Marker   Final concentration (ng/uL): 0.5000   Dilution factor: 12.0

Sample: SampH5
Well location: H5
Created: Thursday, August 12, 2021 6:46:05 PM

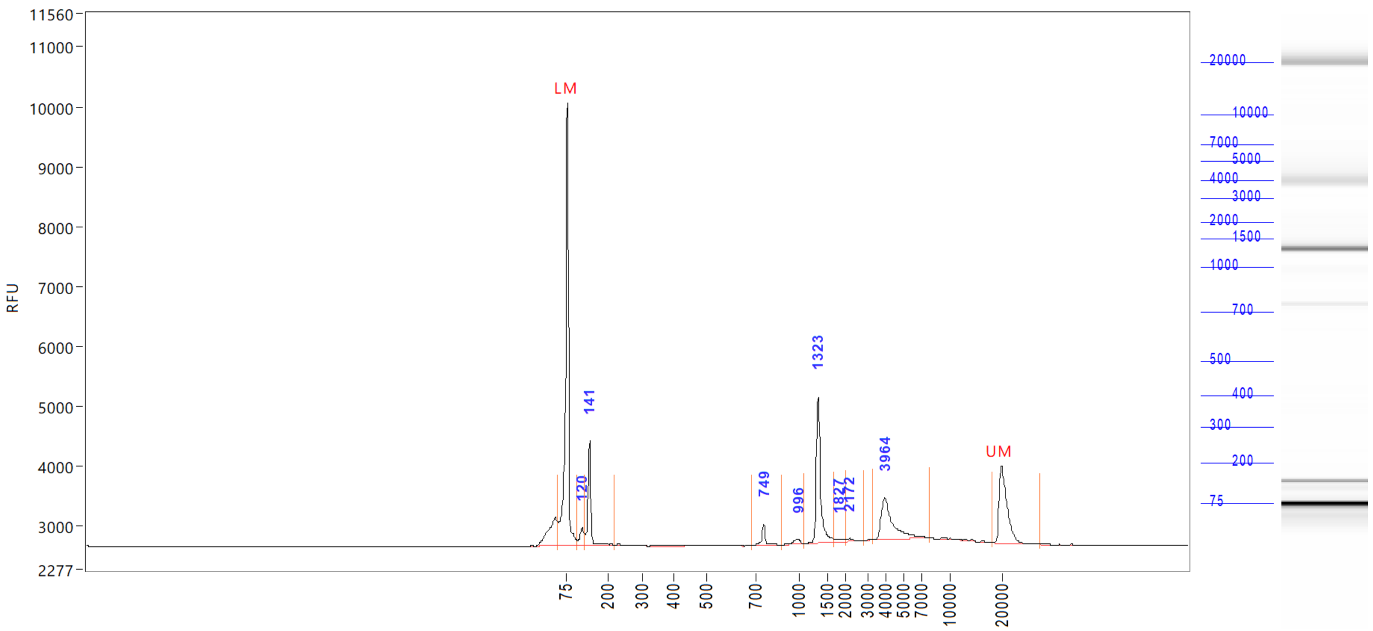

Peak	Size (bp)	Concentration (ng/uL)	From (bp)	To (bp)	Average size (bp)	CV%	RFU	Corrected peak area
1	75 (LM)	0.5000	47	107	71	11.42	7403	56.684
2	120	0.3338	107	129	119	5.22	306	3.153
3	141	1.2202	129	217	141	6.88	1735	11.527
4	749	0.2515	682	874	747	3.60	365	2.376
5	996	0.1056	874	1079	968	3.12	83	0.998
6	1323	1.7080	1079	1693	1334	5.34	2445	16.136
7	1827	0.0800	1693	2058	1863	5.69	51	0.755
8	2172	0.0540	2058	2829	2255	6.88	52	0.510
9	3964	1.2312	3322	7867	4317	15.83	693	11.631
10	20000 (UM)	0.1129	18293	27496	20566	4.66	1291	12.799

TIC:	4.9843	ng/uL
TIM:	22.2496	nmole/L
Total concentration:	5.1808	ng/uL

```
Sample peak width (sec): 5    Sample min peak height: 50    Sample baseline V to V?: Y    Sample baseline V to V points: 3
Sample filter: Binomial       Number of points for filter: 3  Sample start region (min): 0  Sample end region (min): 30
Marker peak width (sec): 5    Marker min peak height: 200   Marker baseline V to V?: Y    Marker baseline V to V points: 3
Lower marker selection: First peak > 500 RFU                Upper marker selection: Last peak > 200 RFU
Ladder size (bp)75, 200, 300, 400, 500, 700, 1000, 1500, 2000, 3000, 4000, 5000, 7000, 10000, 20000
Quantification using: Lower Marker          Final concentration (ng/uL): 0.5000          Dilution factor: 12.0
```

Sample: SampA6
Well location: A6
Created: Thursday, August 12, 2021 6:46:05 PM

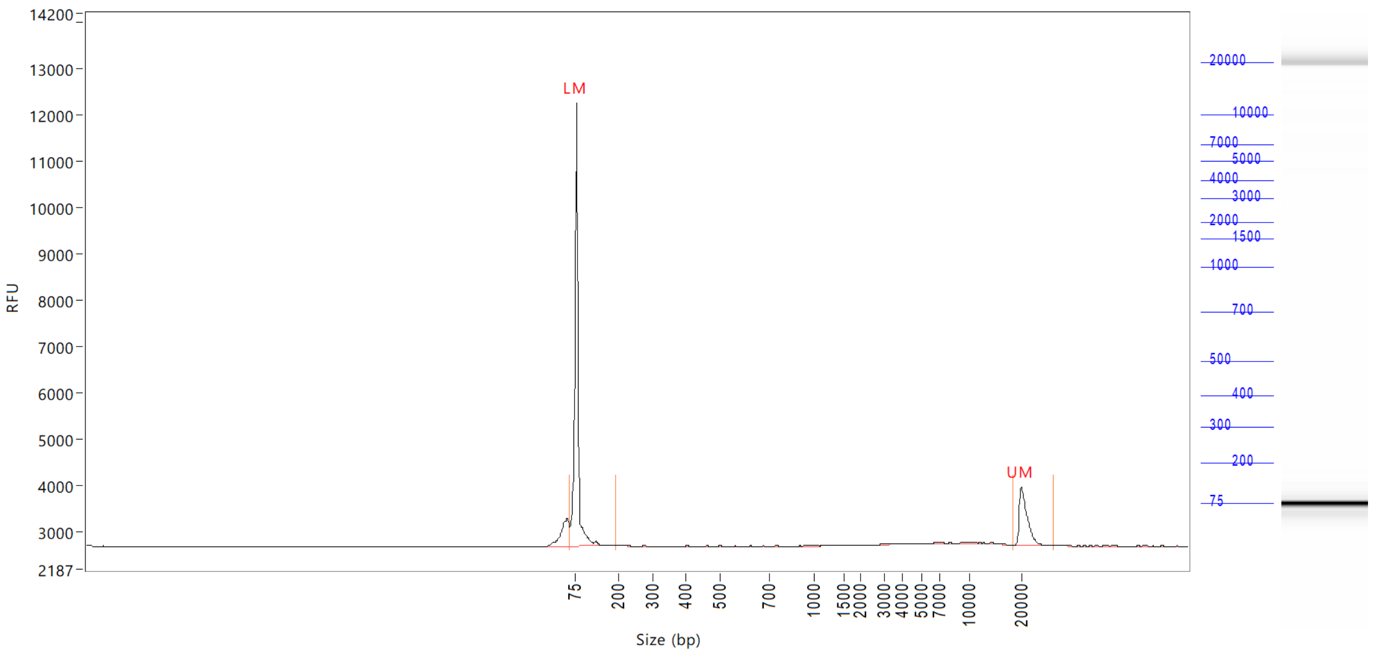

Peak	Size<br>(bp)	Concentration<br>(ng/uL)	From<br>(bp)	To<br>(bp)	Average size<br>(bp)	CV%	RFU	Corrected peak area
1	75 (LM)	0.5000	54	192	74	15.81	9592	75.080
2	20000 (UM)	0.0833	18415	26045	20524	4.54	1274	12.507

TIC:	0.0000	ng/uL
TIM:	0.0000	nmole/L
Total concentration:	0.2391	ng/uL

Sample peak width (sec): 5   Sample min peak height: 50   Sample baseline V to V?: Y   Sample baseline V to V points: 3
Sample filter: Binomial   Number of points for filter: 3 Sample start region (min): 0 Sample end region (min): 30
Marker peak width (sec): 5   Marker min peak height: 200   Marker baseline V to V?: Y   Marker baseline V to V points: 3
Lower marker selection: First peak > 500 RFU   Upper marker selection: Last peak > 200 RFU
Ladder size (bp)75, 200, 300, 400, 500, 700, 1000, 1500, 2000, 3000, 4000, 5000, 7000, 10000, 20000
Quantification using: Lower Marker   Final concentration (ng/uL): 0.5000   Dilution factor: 12.0

Sample: SampB6
Well location: B6
Created: Thursday, August 12, 2021 6:46:05 PM

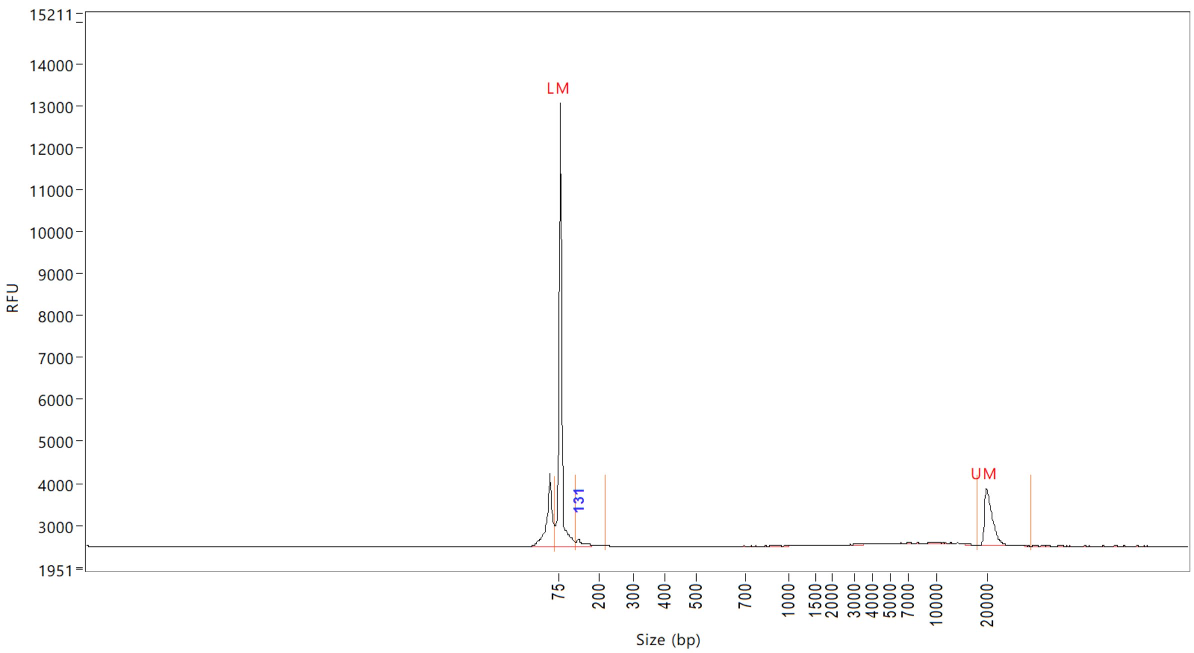

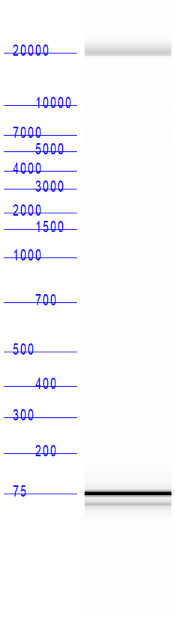

Peak	Size (bp)	Concentration (ng/uL)	From (bp)	To (bp)	Average size (bp)	CV%	RFU	Corrected peak area
1	75 (LM)	0.5000	60	124	75	11.51	10591	73.707
2	131	0.2106	124	217	144	13.29	173	2.587
3	20000 (UM)	0.0905	18049	28705	20578	4.76	1345	13.337

TIC:	0.2106	ng/uL
TIM:	2.4030	nmole/L
Total concentration:	0.4282	ng/uL

Sample peak width (sec): 5   Sample min peak height: 50   Sample baseline V to V?: Y   Sample baseline V to V points: 3
Sample filter: Binomial   Number of points for filter: 3 Sample start region (min): 0 Sample end region (min): 30
Marker peak width (sec): 5   Marker min peak height: 200   Marker baseline V to V?: Y   Marker baseline V to V points: 3
Lower marker selection: First peak > 500 RFU   Upper marker selection: Last peak > 200 RFU
Ladder size (bp)75, 200, 300, 400, 500, 700, 1000, 1500, 2000, 3000, 4000, 5000, 7000, 10000, 20000
Quantification using: Lower Marker   Final concentration (ng/uL): 0.5000   Dilution factor: 12.0

Sample: SampC6
Well location: C6
Created: Thursday, August 12, 2021 6:46:05 PM

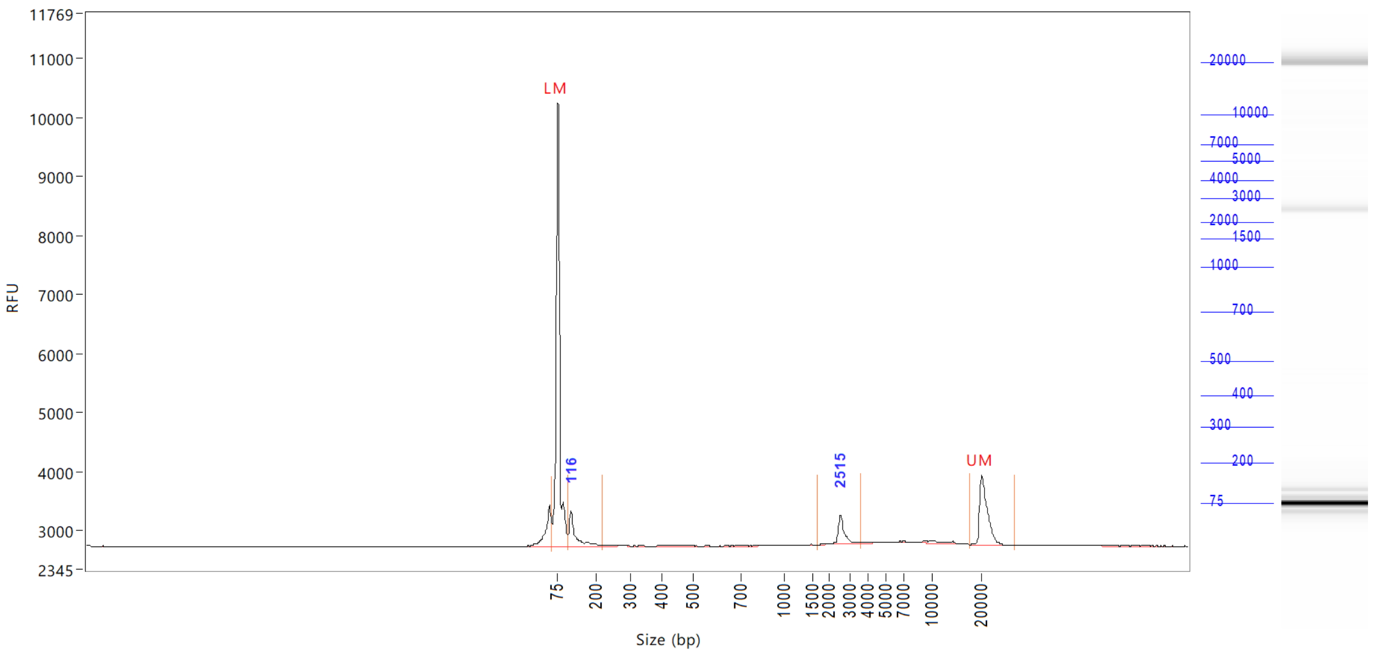

Peak	Size (bp)	Concentration (ng/uL)	From (bp)	To (bp)	Average size (bp)	CV%	RFU	Corrected peak area
1	75 (LM)	0.5000	58	107	75	10.58	7526	61.493
2	116	0.8116	107	214	131	18.71	606	8.318
3	2515	0.4580	1655	3536	2579	9.31	497	4.694
4	20000 (UM)	0.0973	17561	26408	20549	4.59	1195	11.973

TIC:	1.2696	ng/uL
TIM:	10.4721	nmole/L
Total concentration:	1.6090	ng/uL

Sample peak width (sec): 5 Sample min peak height: 50 Sample baseline V to V?: Y Sample baseline V to V points: 3
Sample filter: Binomial Number of points for filter: 3 Sample start region (min): 0 Sample end region (min): 30
Marker peak width (sec): 5 Marker min peak height: 200 Marker baseline V to V?: Y Marker baseline V to V points: 3
Lower marker selection: First peak > 500 RFU Upper marker selection: Last peak > 200 RFU
Ladder size (bp)75, 200, 300, 400, 500, 700, 1000, 1500, 2000, 3000, 4000, 5000, 7000, 10000, 20000
Quantification using: Lower Marker Final concentration (ng/uL): 0.5000 Dilution factor: 12.0

Sample: SampD6
Well location: D6
Created: Thursday, August 12, 2021 6:46:05 PM

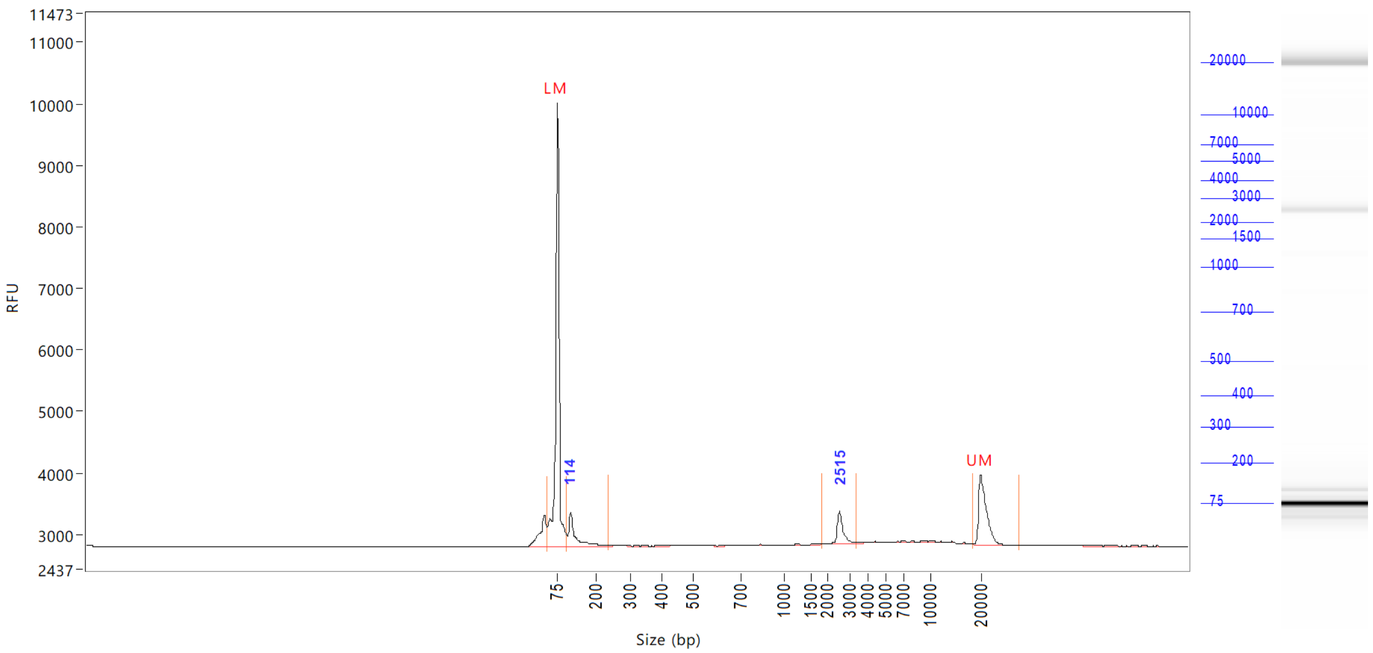

Peak	Size (bp)	Concentration (ng/uL)	From (bp)	To (bp)	Average size (bp)	CV%	RFU	Corrected peak area
1	75 (LM)	0.5000	43	105	72	13.14	7220	58.804
2	114	0.8420	105	233	133	21.49	559	8.253
3	2515	0.4922	1808	3322	2551	7.90	533	4.824
4	20000 (UM)	0.0984	18293	27617	20504	4.81	1138	11.568

TIC:	1.3343	ng/uL
TIM:	10.7207	nmole/L
Total concentration:	1.6500	ng/uL

Sample peak width (sec): 5 Sample min peak height: 50 Sample baseline V to V?: Y Sample baseline V to V points: 3
Sample filter: Binomial Number of points for filter: 3 Sample start region (min): 0 Sample end region (min): 30
Marker peak width (sec): 5 Marker min peak height: 200 Marker baseline V to V?: Y Marker baseline V to V points: 3
Lower marker selection: First peak > 500 RFU Upper marker selection: Last peak > 200 RFU
Ladder size (bp)75, 200, 300, 400, 500, 700, 1000, 1500, 2000, 3000, 4000, 5000, 7000, 10000, 20000
Quantification using: Lower Marker Final concentration (ng/uL): 0.5000 Dilution factor: 12.0

Sample: SampE6
Well location: E6
Created: Thursday, August 12, 2021 6:46:05 PM

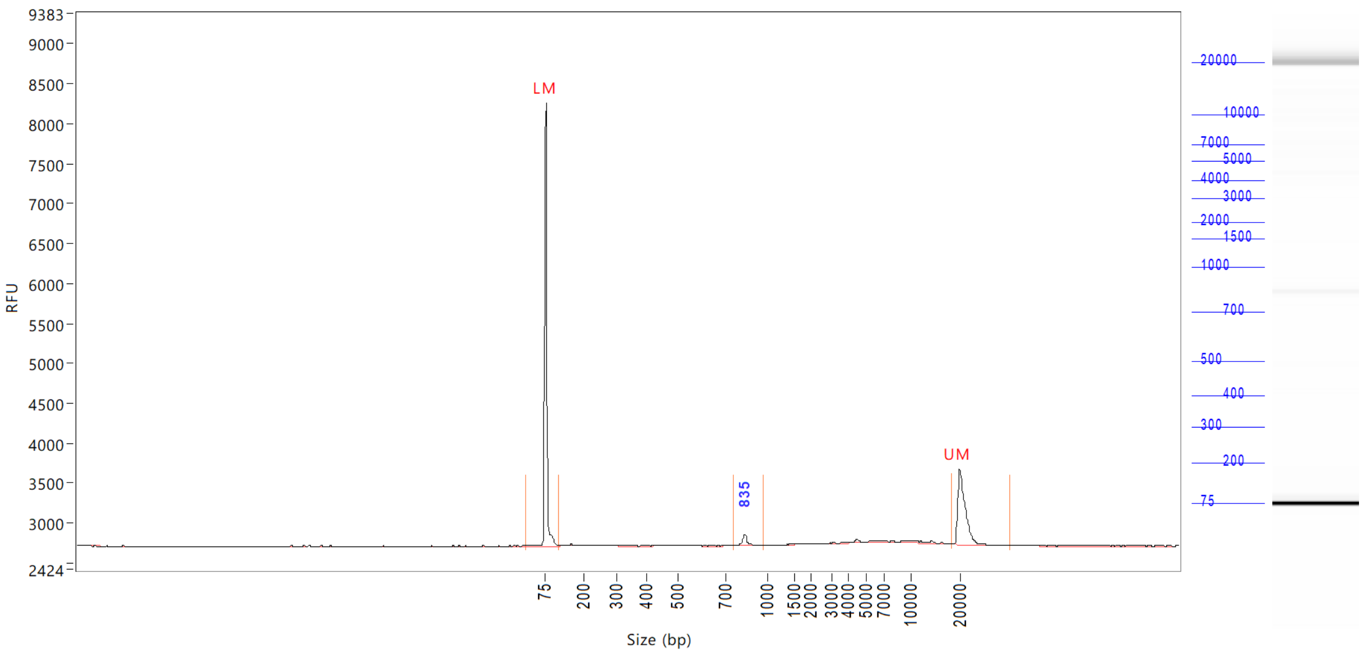

Peak	Size (bp)	Concentration (ng/uL)	From (bp)	To (bp)	Average size (bp)	CV%	RFU	Corrected peak area
1	75 (LM)	0.5000	12	118	73	8.42	5557	31.942
2	835	0.2202	757	970	837	3.60	143	1.172
3	20000 (UM)	0.1466	18537	30398	20666	5.66	944	9.367

TIC:	0.2202	ng/uL
TIM:	0.4331	nmole/L
Total concentration:	0.9843	ng/uL

Sample peak width (sec): 5   Sample min peak height: 50   Sample baseline V to V?: Y   Sample baseline V to V points: 3
Sample filter: Binomial   Number of points for filter: 3   Sample start region (min): 0   Sample end region (min): 30
Marker peak width (sec): 5   Marker min peak height: 200   Marker baseline V to V?: Y   Marker baseline V to V points: 3
Lower marker selection: First peak > 500 RFU   Upper marker selection: Last peak > 200 RFU
Ladder size (bp)75, 200, 300, 400, 500, 700, 1000, 1500, 2000, 3000, 4000, 5000, 7000, 10000, 20000
Quantification using: Lower Marker   Final concentration (ng/uL): 0.5000   Dilution factor: 12.0

Sample: SampF6
Well location: F6
Created: Thursday, August 12, 2021 6:46:05 PM

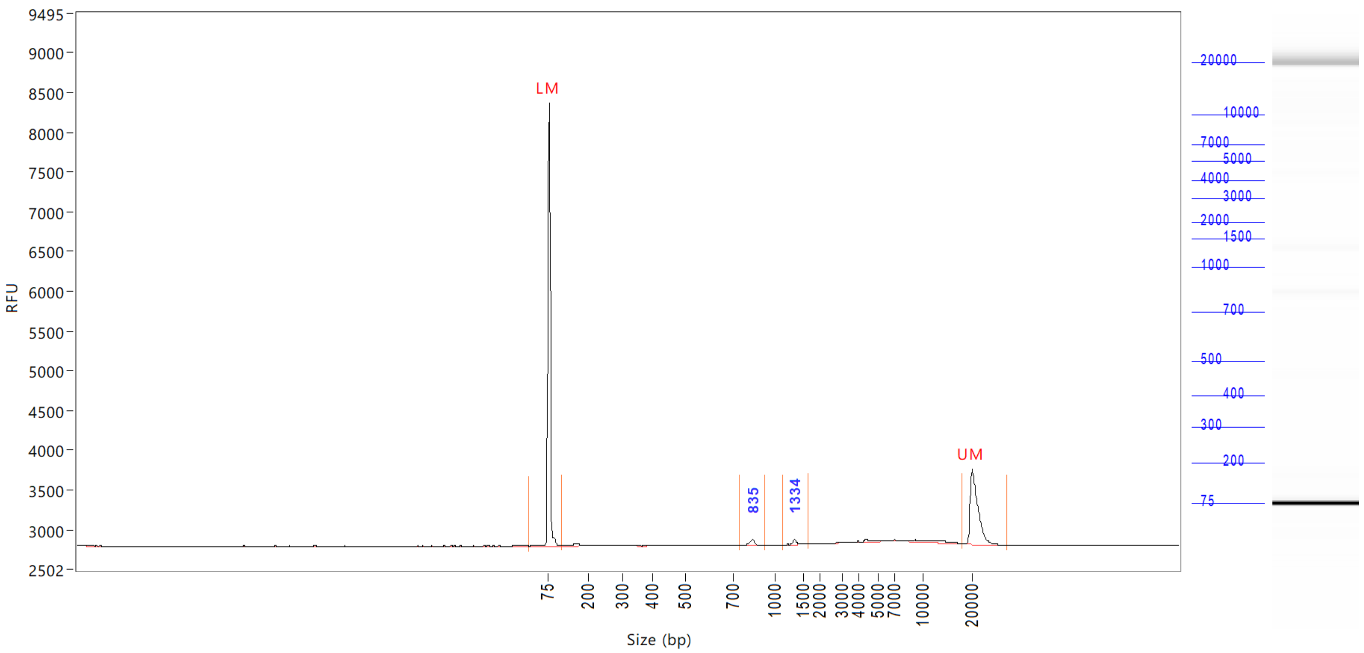

Peak	Size (bp)	Concentration (ng/uL)	From (bp)	To (bp)	Average size (bp)	CV%	RFU	Corrected peak area
1	75 (LM)	0.5000	14	114	73	8.12	5586	31.200
2	835	0.1514	740	926	827	3.26	85	0.787
3	1334	0.0969	1134	1635	1352	5.89	63	0.504
4	20000 (UM)	0.1532	17927	26891	20528	4.87	943	9.561

TIC:	0.2483	ng/uL
TIM:	0.4193	nmole/L
Total concentration:	0.9824	ng/uL

```
Sample peak width (sec): 5    Sample min peak height: 50    Sample baseline V to V?: Y    Sample baseline V to V points: 3
Sample filter: Binomial       Number of points for filter: 3  Sample start region (min): 0  Sample end region (min): 30
Marker peak width (sec): 5    Marker min peak height: 200   Marker baseline V to V?: Y    Marker baseline V to V points: 3
Lower marker selection: First peak > 500 RFU                Upper marker selection: Last peak > 200 RFU
Ladder size (bp)75, 200, 300, 400, 500, 700, 1000, 1500, 2000, 3000, 4000, 5000, 7000, 10000, 20000
Quantification using: Lower Marker          Final concentration (ng/uL): 0.5000          Dilution factor: 12.0
```

Sample: SampG6
Well location: G6
Created: Thursday, August 12, 2021 6:46:05 PM

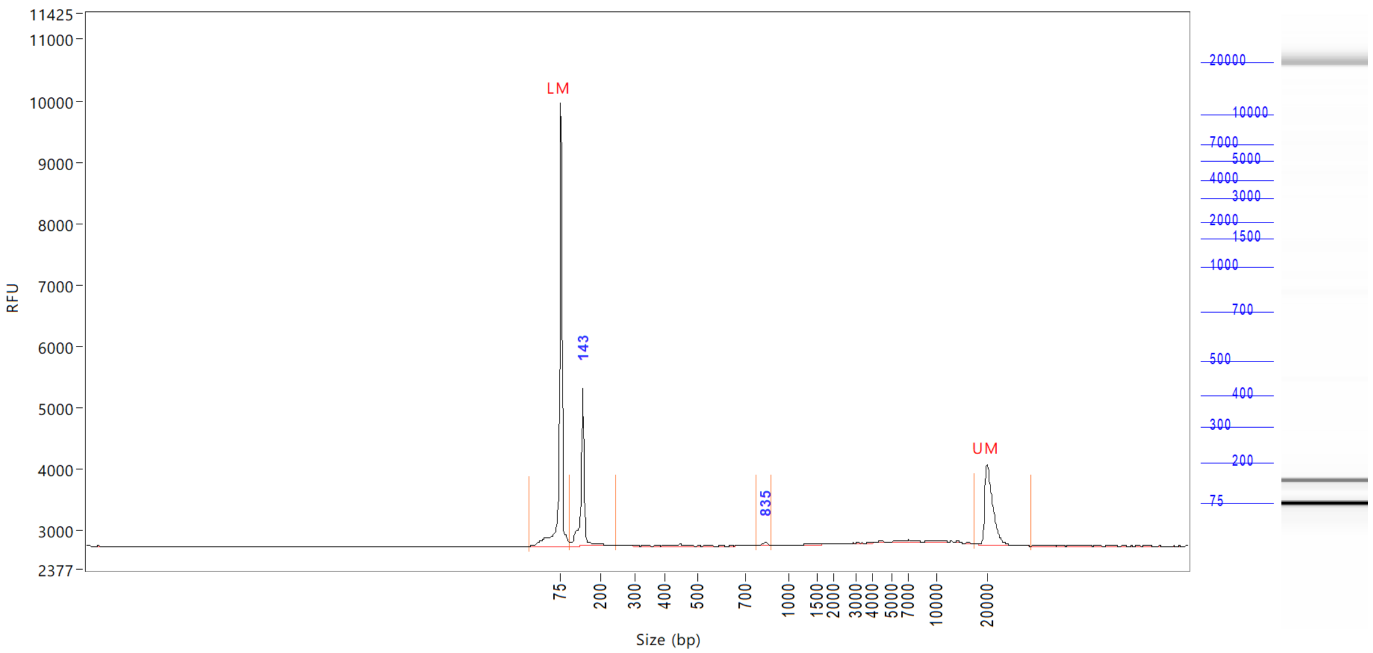

Peak	Size (bp)	Concentration (ng/uL)	From (bp)	To (bp)	Average size (bp)	CV%	RFU	Corrected peak area
1	75 (LM)	0.5000	0	105	68	23.17	7226	49.932
2	143	2.4593	105	246	141	10.81	2556	20.467
3	835	0.0700	770	879	825	2.59	61	0.583
4	20000 (UM)	0.1336	17561	28584	20556	5.20	1316	13.345

TIC:	2.5294	ng/uL
TIM:	28.8030	nmole/L
Total concentration:	2.9805	ng/uL

Sample peak width (sec): 5 Sample min peak height: 50 Sample baseline V to V?: Y Sample baseline V to V points: 3
Sample filter: Binomial Number of points for filter: 3 Sample start region (min): 0 Sample end region (min): 30
Marker peak width (sec): 5 Marker min peak height: 200 Marker baseline V to V?: Y Marker baseline V to V points: 3
Lower marker selection: First peak > 500 RFU Upper marker selection: Last peak > 200 RFU
Ladder size (bp)75, 200, 300, 400, 500, 700, 1000, 1500, 2000, 3000, 4000, 5000, 7000, 10000, 20000
Quantification using: Lower Marker Final concentration (ng/uL): 0.5000 Dilution factor: 12.0

Sample: SampH6
Well location: H6
Created: Thursday, August 12, 2021 6:46:05 PM

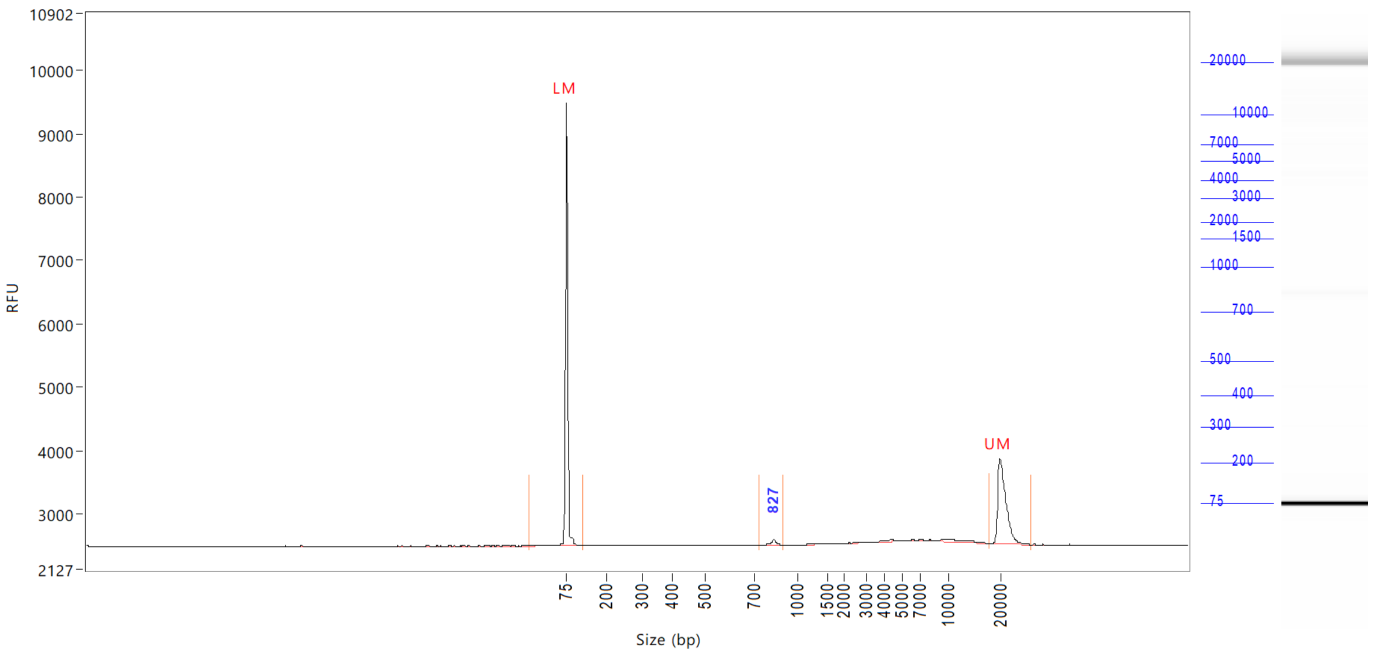

Peak	Size (bp)	Concentration (ng/uL)	From (bp)	To (bp)	Average size (bp)	CV%	RFU	Corrected peak area
1	75 (LM)	0.5000	0	128	72	11.52	7003	40.071
2	827	0.1183	723	896	820	2.83	85	0.790
3	20000 (UM)	0.1723	17805	25924	20564	4.66	1357	13.812

TIC:	0.1183	ng/uL
TIM:	0.2373	nmole/L
Total concentration:	0.7940	ng/uL

Sample peak width (sec): 5 Sample min peak height: 50 Sample baseline V to V?: Y Sample baseline V to V points: 3
Sample filter: Binomial Number of points for filter: 3 Sample start region (min): 0 Sample end region (min): 30
Marker peak width (sec): 5 Marker min peak height: 200 Marker baseline V to V?: Y Marker baseline V to V points: 3
Lower marker selection: First peak > 500 RFU Upper marker selection: Last peak > 200 RFU
Ladder size (bp)75, 200, 300, 400, 500, 700, 1000, 1500, 2000, 3000, 4000, 5000, 7000, 10000, 20000
Quantification using: Lower Marker Final concentration (ng/uL): 0.5000 Dilution factor: 12.0

Sample: SampA7
Well location: A7
Created: Thursday, August 12, 2021 6:46:05 PM

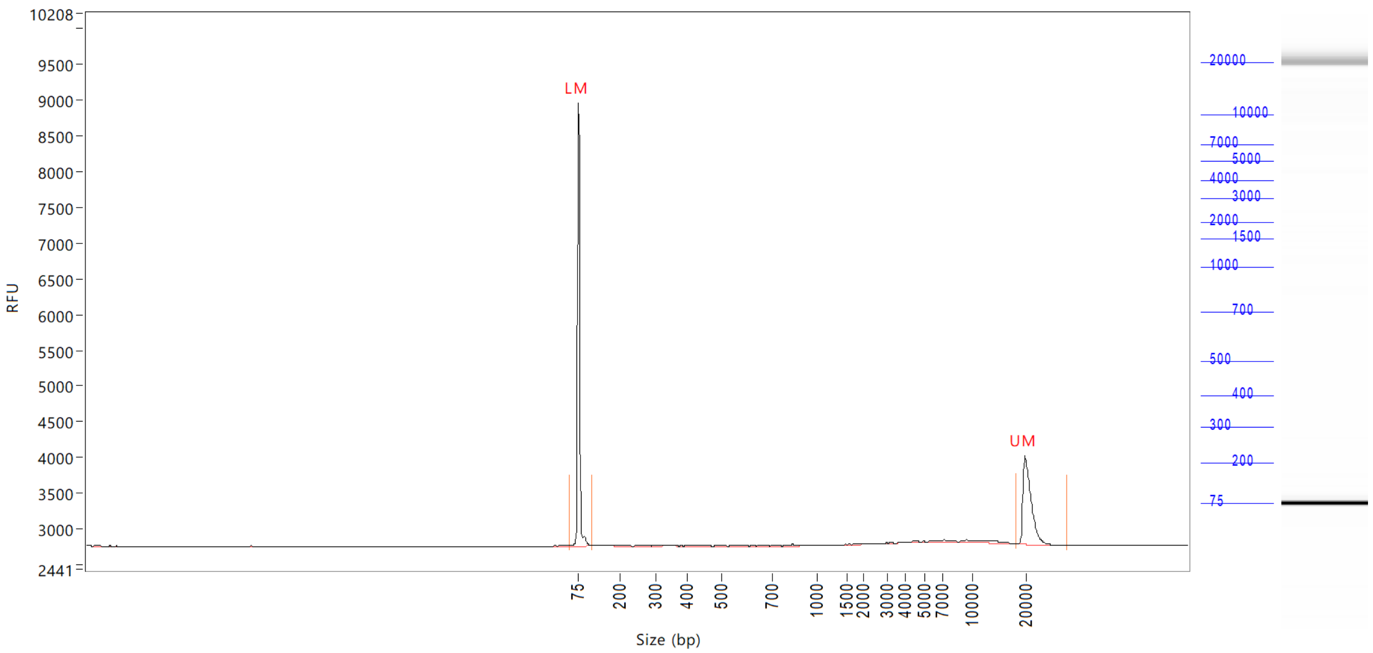

Peak	Size (bp)	Concentration (ng/uL)	From (bp)	To (bp)	Average size (bp)	CV%	RFU	Corrected peak area
1	75 (LM)	0.5000	52	118	74	6.24	6201	33.737
2	20000 (UM)	0.1839	18293	27859	20560	4.75	1240	12.409

TIC:	0.0000	ng/uL
TIM:	0.0000	nmole/L
Total concentration:	0.7793	ng/uL

```
Sample peak width (sec): 5    Sample min peak height: 50    Sample baseline V to V?: Y    Sample baseline V to V points: 3
Sample filter: Binomial       Number of points for filter: 3  Sample start region (min): 0  Sample end region (min): 30
Marker peak width (sec): 5    Marker min peak height: 200   Marker baseline V to V?: Y    Marker baseline V to V points: 3
Lower marker selection: First peak > 500 RFU                Upper marker selection: Last peak > 200 RFU
Ladder size (bp)75, 200, 300, 400, 500, 700, 1000, 1500, 2000, 3000, 4000, 5000, 7000, 10000, 20000
Quantification using: Lower Marker          Final concentration (ng/uL): 0.5000          Dilution factor: 12.0
```

Sample: SampB7
Well location: B7
Created: Thursday, August 12, 2021 6:46:05 PM

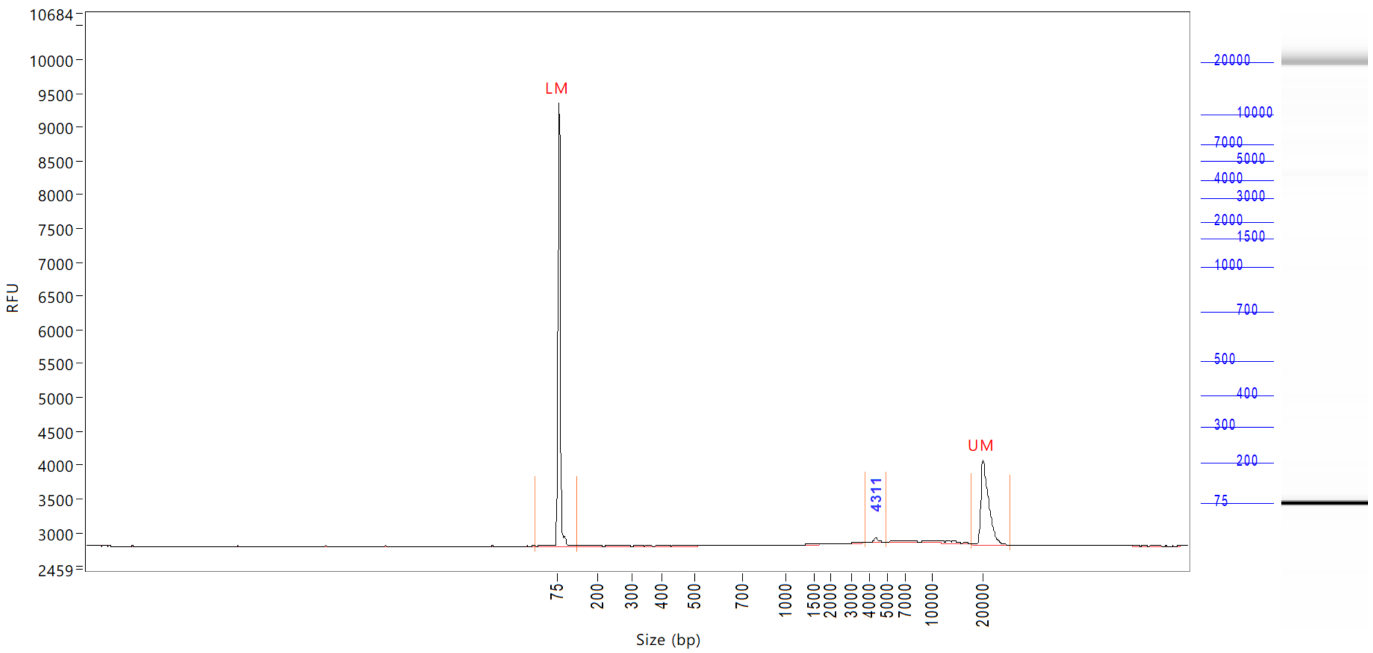

Peak	Size (bp)	Concentration (ng/uL)	From (bp)	To (bp)	Average size (bp)	CV%	RFU	Corrected peak area
1	75 (LM)	0.5000	5	131	74	9.35	6570	37.367
2	4311	0.1019	3715	4931	4258	5.66	70	0.635
3	20000 (UM)	0.1708	17683	25440	20575	4.55	1251	12.763

TIC:	0.1019	ng/uL
TIM:	0.0394	nmole/L
Total concentration:	0.8358	ng/uL

```
Sample peak width (sec): 5    Sample min peak height: 50    Sample baseline V to V?: Y    Sample baseline V to V points: 3
Sample filter: Binomial       Number of points for filter: 3  Sample start region (min): 0  Sample end region (min): 30
Marker peak width (sec): 5    Marker min peak height: 200   Marker baseline V to V?: Y    Marker baseline V to V points: 3
Lower marker selection: First peak > 500 RFU                Upper marker selection: Last peak > 200 RFU
Ladder size (bp)75, 200, 300, 400, 500, 700, 1000, 1500, 2000, 3000, 4000, 5000, 7000, 10000, 20000
Quantification using: Lower Marker          Final concentration (ng/uL): 0.5000          Dilution factor: 12.0
```

Sample: SampC7
Well location: C7
Created: Thursday, August 12, 2021 6:46:05 PM

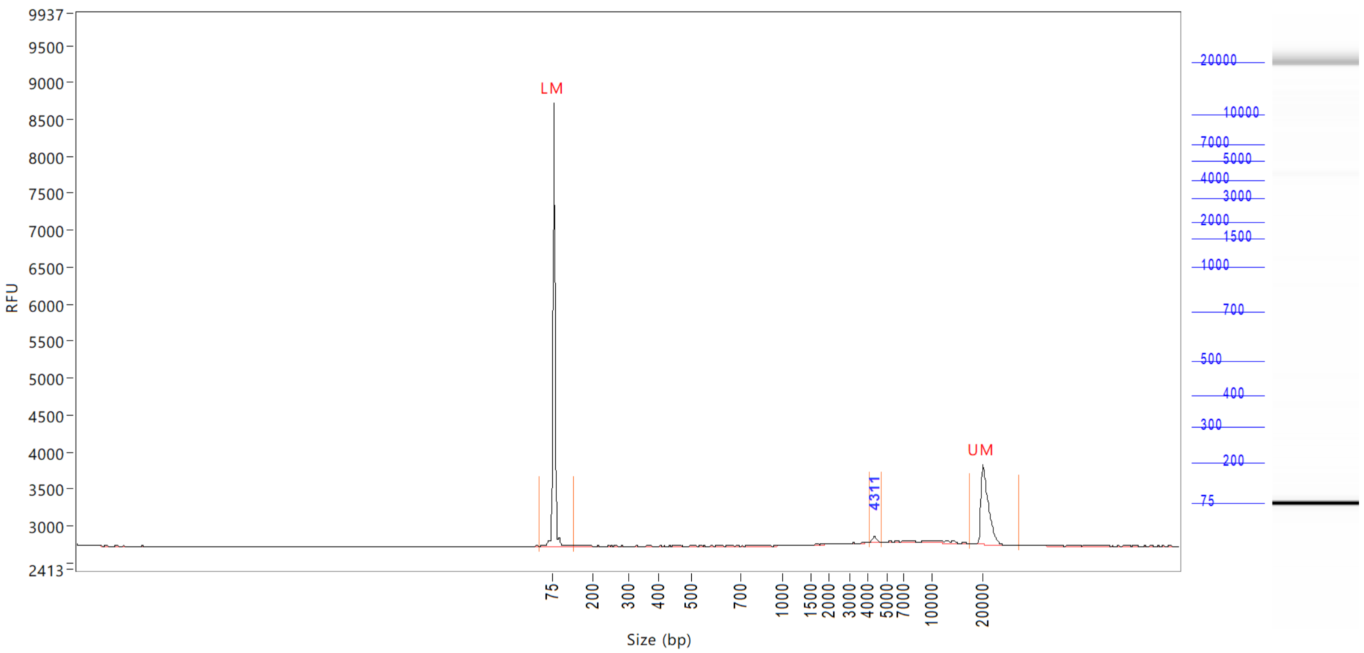

Peak	Size (bp)	Concentration (ng/uL)	From (bp)	To (bp)	Average size (bp)	CV%	RFU	Corrected peak area
1	75 (LM)	0.5000	30	135	73	8.53	6013	33.650
2	4311	0.0915	4035	4724	4303	2.74	79	0.513
3	20000 (UM)	0.1565	17317	27254	20514	4.51	1079	10.534

TIC:	0.0915	ng/uL
TIM:	0.0350	nmole/L
Total concentration:	0.7678	ng/uL

Sample peak width (sec): 5 Sample min peak height: 50 Sample baseline V to V?: Y Sample baseline V to V points: 3
Sample filter: Binomial Number of points for filter: 3 Sample start region (min): 0 Sample end region (min): 30
Marker peak width (sec): 5 Marker min peak height: 200 Marker baseline V to V?: Y Marker baseline V to V points: 3
Lower marker selection: First peak > 500 RFU Upper marker selection: Last peak > 200 RFU
Ladder size (bp)75, 200, 300, 400, 500, 700, 1000, 1500, 2000, 3000, 4000, 5000, 7000, 10000, 20000
Quantification using: Lower Marker Final concentration (ng/uL): 0.5000 Dilution factor: 12.0

Sample: SampD7
Well location: D7
Created: Thursday, August 12, 2021 6:46:05 PM

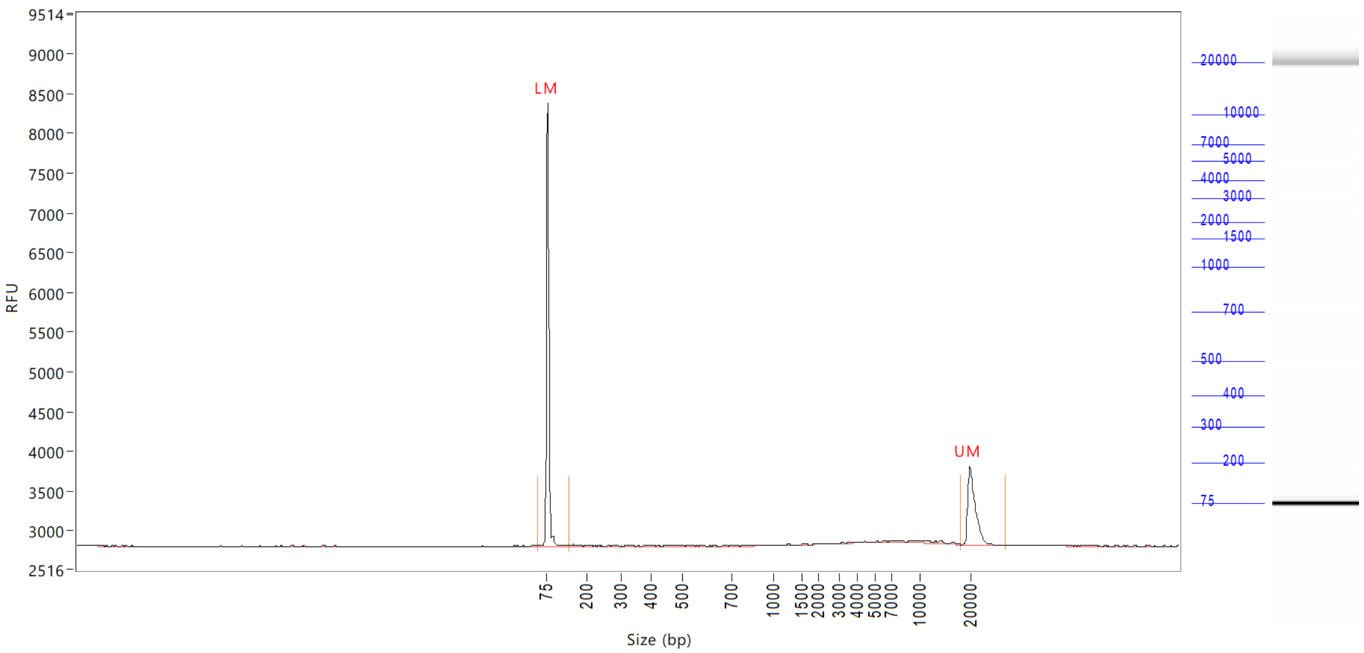

Peak	Size (bp)	Concentration (ng/uL)	From (bp)	To (bp)	Average size (bp)	CV%	RFU	Corrected peak area
1	75 (LM)	0.5000	47	145	74	9.26	5590	31.781
2	20000 (UM)	0.1551	18293	27012	20483	4.64	984	9.856

TIC:	0.0000	ng/uL
TIM:	0.0000	nmole/L
Total concentration:	0.7979	ng/uL

Sample peak width (sec): 5 Sample min peak height: 50 Sample baseline V to V?: Y Sample baseline V to V points: 3
Sample filter: Binomial Number of points for filter: 3 Sample start region (min): 0 Sample end region (min): 30
Marker peak width (sec): 5 Marker min peak height: 200 Marker baseline V to V?: Y Marker baseline V to V points: 3
Lower marker selection: First peak > 500 RFU Upper marker selection: Last peak > 200 RFU
Ladder size (bp)75, 200, 300, 400, 500, 700, 1000, 1500, 2000, 3000, 4000, 5000, 7000, 10000, 20000
Quantification using: Lower Marker Final concentration (ng/uL): 0.5000 Dilution factor: 12.0

Sample: SampE7
Well location: E7
Created: Thursday, August 12, 2021 6:46:05 PM

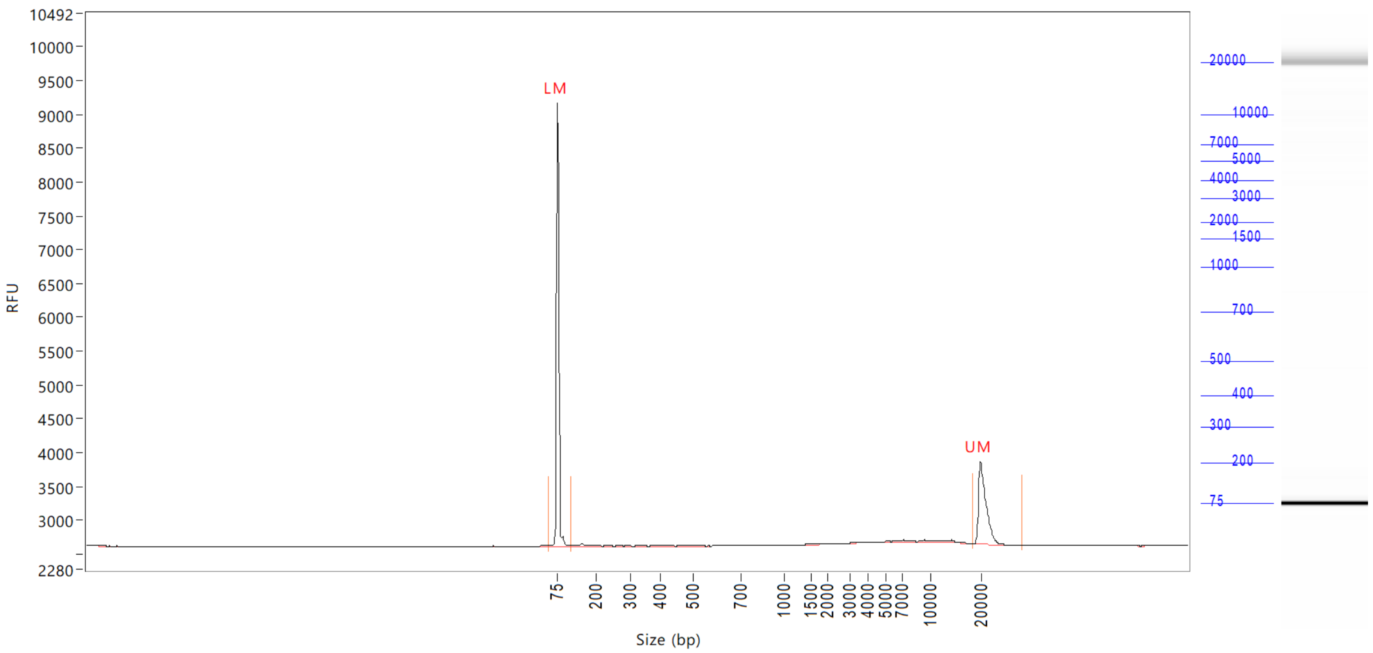

Peak	Size (bp)	Concentration (ng/uL)	From (bp)	To (bp)	Average size (bp)	CV%	RFU	Corrected peak area
1	75 (LM)	0.5000	47	116	73	6.44	6561	36.279
2	20000 (UM)	0.1667	18415	28342	20592	4.95	1214	12.095

TIC:	0.0000	ng/uL
TIM:	0.0000	nmole/L
Total concentration:	0.7019	ng/uL

```
Sample peak width (sec): 5    Sample min peak height: 50    Sample baseline V to V?: Y    Sample baseline V to V points: 3
Sample filter: Binomial       Number of points for filter: 3  Sample start region (min): 0  Sample end region (min): 30
Marker peak width (sec): 5    Marker min peak height: 200   Marker baseline V to V?: Y    Marker baseline V to V points: 3
Lower marker selection: First peak > 500 RFU                Upper marker selection: Last peak > 200 RFU
Ladder size (bp)75, 200, 300, 400, 500, 700, 1000, 1500, 2000, 3000, 4000, 5000, 7000, 10000, 20000
Quantification using: Lower Marker          Final concentration (ng/uL): 0.5000          Dilution factor: 12.0
```

Sample: SampF7
Well location: F7
Created: Thursday, August 12, 2021 6:46:05 PM

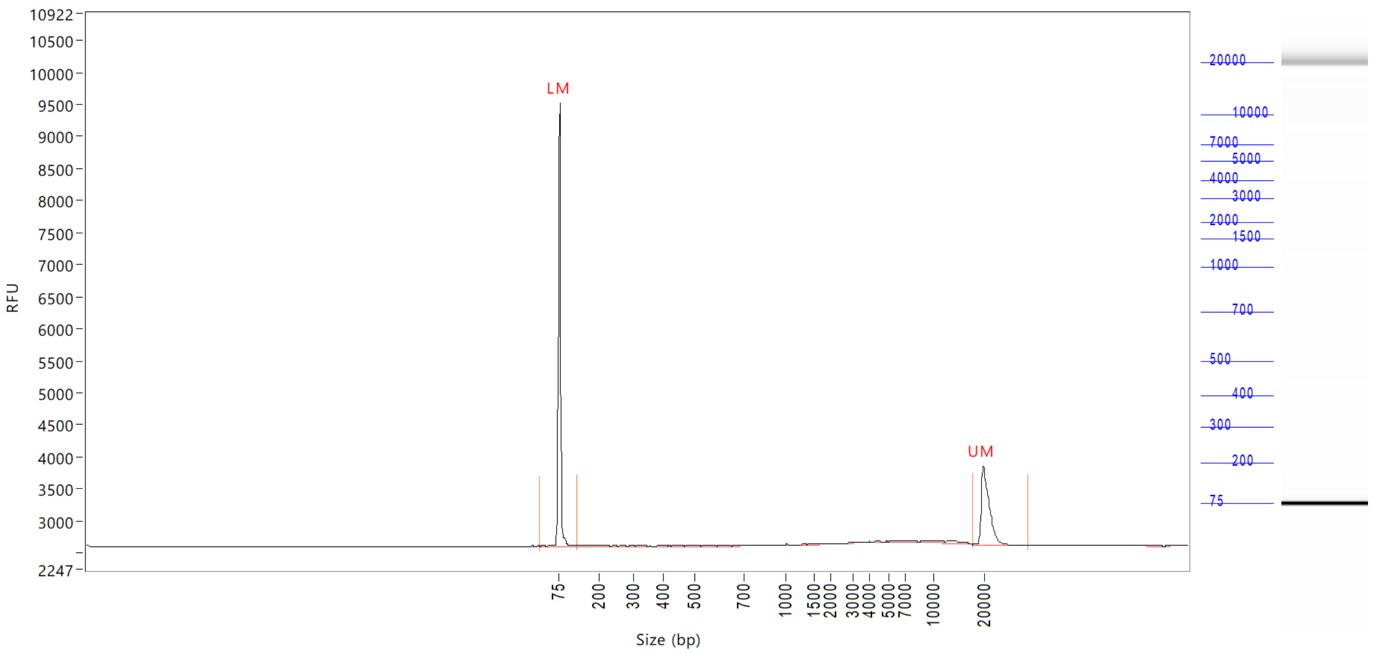

Peak	Size (bp)	Concentration (ng/uL)	From (bp)	To (bp)	Average size (bp)	CV%	RFU	Corrected peak area
1	75 (LM)	0.5000	14	129	73	8.06	6929	38.625
2	20000 (UM)	0.1621	17805	28826	20576	5.14	1226	12.524

TIC:	0.0000	ng/uL
TIM:	0.0000	nmole/L
Total concentration:	0.6973	ng/uL

Sample peak width (sec): 5 Sample min peak height: 50 Sample baseline V to V?: Y Sample baseline V to V points: 3
Sample filter: Binomial Number of points for filter: 3 Sample start region (min): 0 Sample end region (min): 30
Marker peak width (sec): 5 Marker min peak height: 200 Marker baseline V to V?: Y Marker baseline V to V points: 3
Lower marker selection: First peak > 500 RFU Upper marker selection: Last peak > 200 RFU
Ladder size (bp)75, 200, 300, 400, 500, 700, 1000, 1500, 2000, 3000, 4000, 5000, 7000, 10000, 20000
Quantification using: Lower Marker Final concentration (ng/uL): 0.5000 Dilution factor: 12.0

Sample: SampG7
Well location: G7
Created: Thursday, August 12, 2021 6:46:05 PM

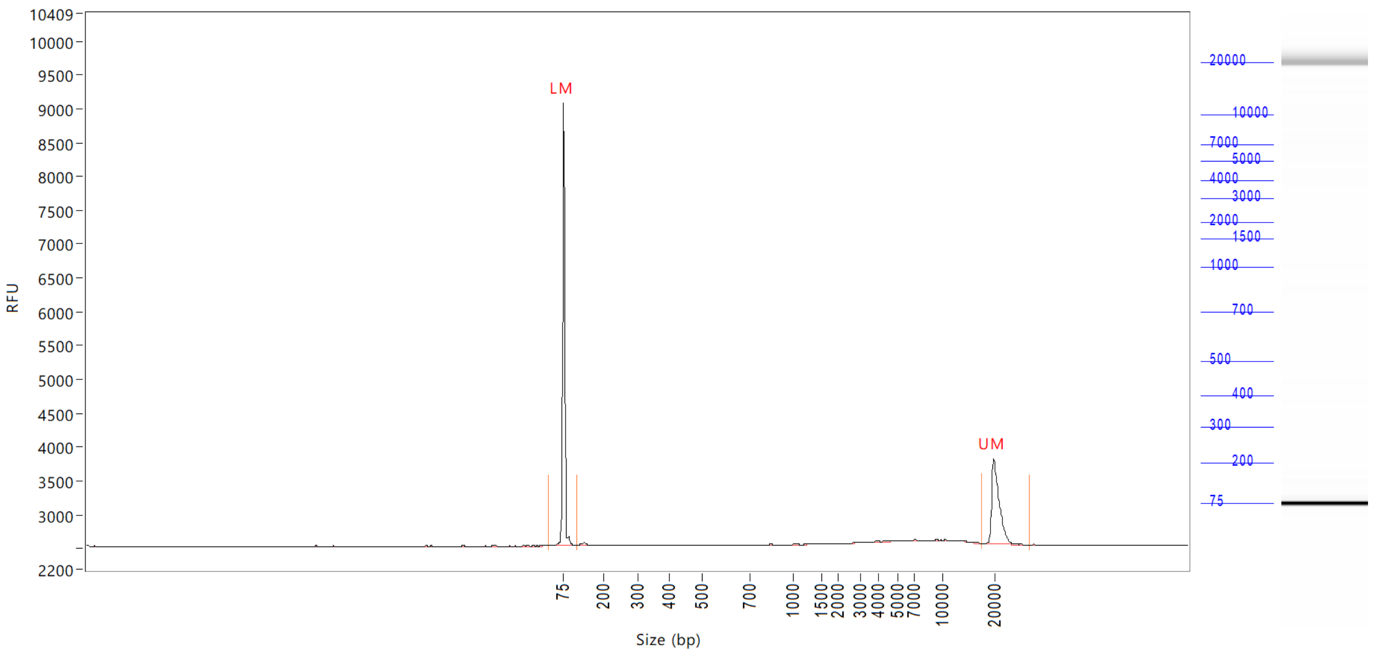

Peak	Size (bp)	Concentration (ng/uL)	From (bp)	To (bp)	Average size (bp)	CV%	RFU	Corrected peak area
1	75 (LM)	0.5000	28	118	73	7.06	6550	36.592
2	20000 (UM)	0.1672	17683	27133	20479	4.52	1263	12.239

TIC:	0.0000	ng/uL
TIM:	0.0000	nmole/L
Total concentration:	0.6663	ng/uL

Sample peak width (sec): 5   Sample min peak height: 50   Sample baseline V to V?: Y   Sample baseline V to V points: 3
Sample filter: Binomial   Number of points for filter: 3 Sample start region (min): 0 Sample end region (min): 30
Marker peak width (sec): 5   Marker min peak height: 200   Marker baseline V to V?: Y   Marker baseline V to V points: 3
Lower marker selection: First peak > 500 RFU   Upper marker selection: Last peak > 200 RFU
Ladder size (bp)75, 200, 300, 400, 500, 700, 1000, 1500, 2000, 3000, 4000, 5000, 7000, 10000, 20000
Quantification using: Lower Marker   Final concentration (ng/uL): 0.5000   Dilution factor: 12.0

Sample: SampH7
Well location: H7
Created: Thursday, August 12, 2021 6:46:05 PM

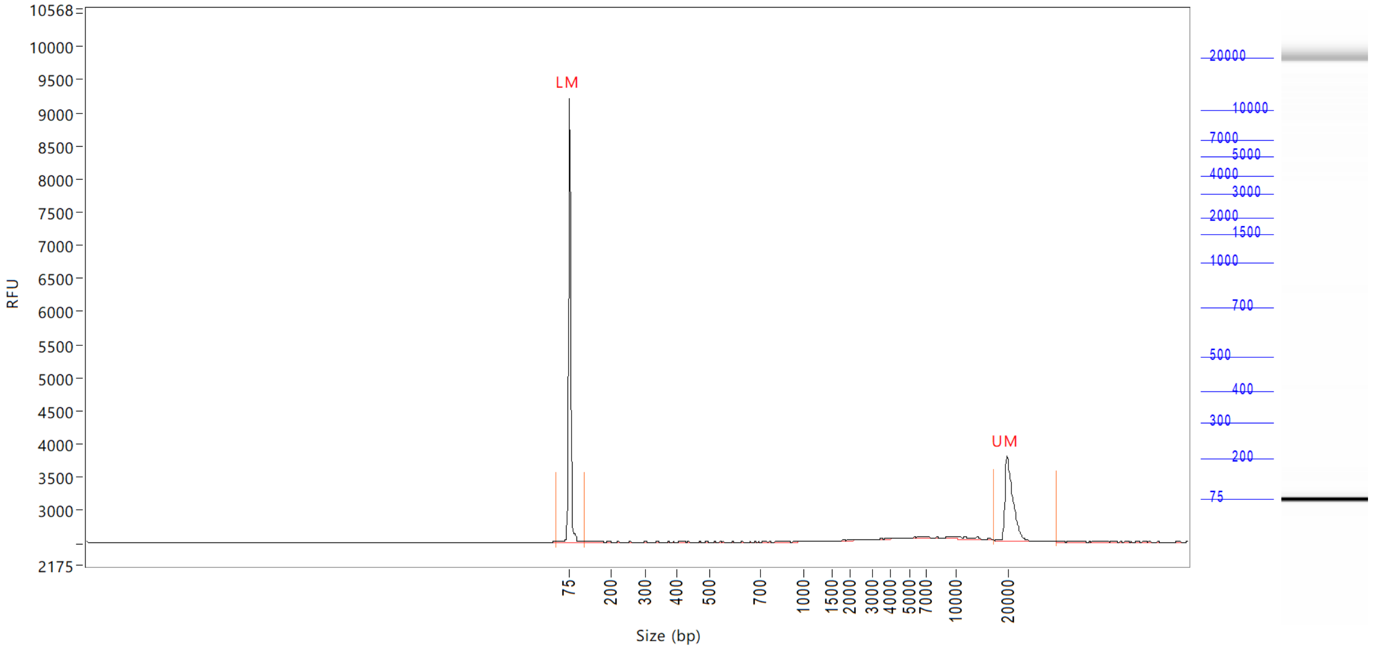

Peak	Size (bp)	Concentration (ng/uL)	From (bp)	To (bp)	Average size (bp)	CV%	RFU	Corrected peak area
1	75 (LM)	0.5000	35	118	73	6.64	6706	37.471
2	20000 (UM)	0.1708	17439	29430	20521	5.23	1269	12.800

TIC:	0.0000	ng/uL
TIM:	0.0000	nmole/L
Total concentration:	0.6700	ng/uL

Sample peak width (sec): 5   Sample min peak height: 50   Sample baseline V to V?: Y   Sample baseline V to V points: 3
Sample filter: Binomial   Number of points for filter: 3   Sample start region (min): 0   Sample end region (min): 30
Marker peak width (sec): 5   Marker min peak height: 200   Marker baseline V to V?: Y   Marker baseline V to V points: 3
Lower marker selection: First peak > 500 RFU   Upper marker selection: Last peak > 200 RFU
Ladder size (bp)75, 200, 300, 400, 500, 700, 1000, 1500, 2000, 3000, 4000, 5000, 7000, 10000, 20000
Quantification using: Lower Marker   Final concentration (ng/uL): 0.5000   Dilution factor: 12.0

Sample: SampA8
Well location: A8
Created: Thursday, August 12, 2021 6:46:05 PM

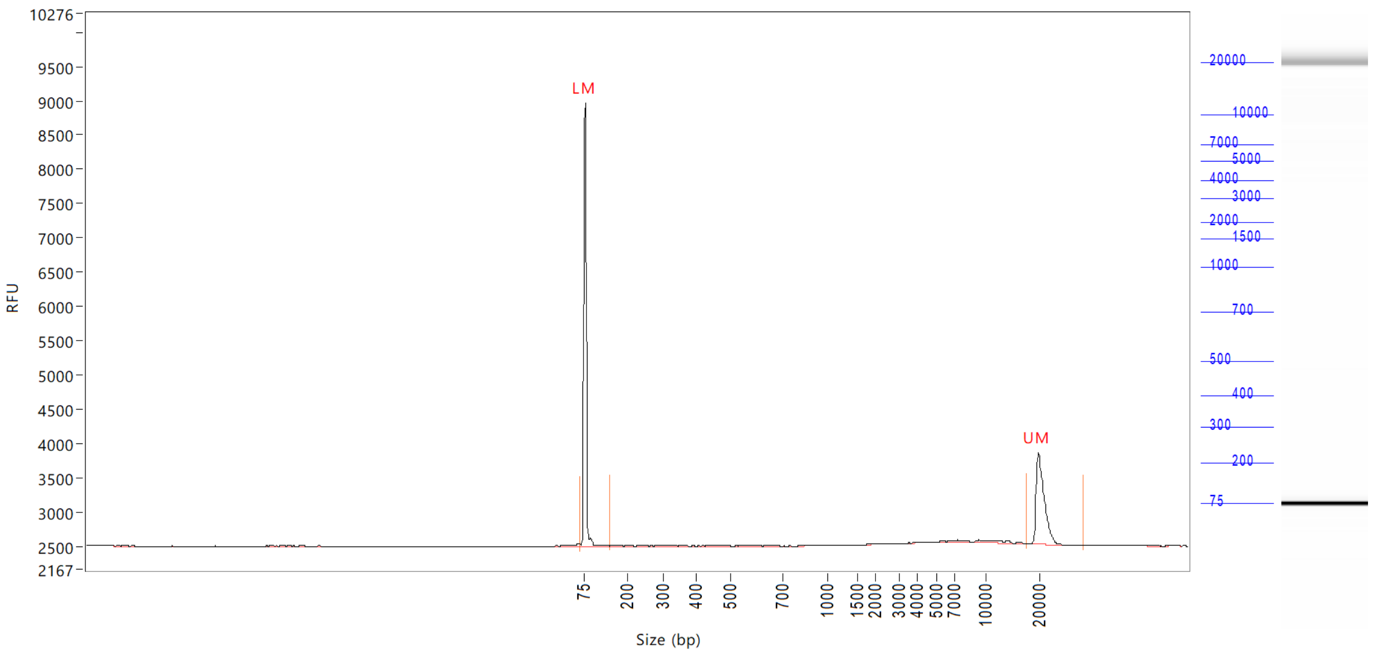

Peak	Size (bp)	Concentration (ng/uL)	From (bp)	To (bp)	Average size (bp)	CV%	RFU	Corrected peak area
1	75 (LM)	0.5000	62	147	73	6.92	6478	35.166
2	20000 (UM)	0.1860	17805	28342	20471	4.62	1333	13.078

TIC:	0.0000	ng/uL
TIM:	0.0000	nmole/L
Total concentration:	0.6665	ng/uL

Sample peak width (sec): 5 Sample min peak height: 50 Sample baseline V to V?: Y Sample baseline V to V points: 3
Sample filter: Binomial Number of points for filter: 3 Sample start region (min): 0 Sample end region (min): 30
Marker peak width (sec): 5 Marker min peak height: 200 Marker baseline V to V?: Y Marker baseline V to V points: 3
Lower marker selection: First peak > 500 RFU Upper marker selection: Last peak > 200 RFU
Ladder size (bp)75, 200, 300, 400, 500, 700, 1000, 1500, 2000, 3000, 4000, 5000, 7000, 10000, 20000
Quantification using: Lower Marker Final concentration (ng/uL): 0.5000 Dilution factor: 12.0

Sample: SampB8
Well location: B8
Created: Thursday, August 12, 2021 6:46:05 PM

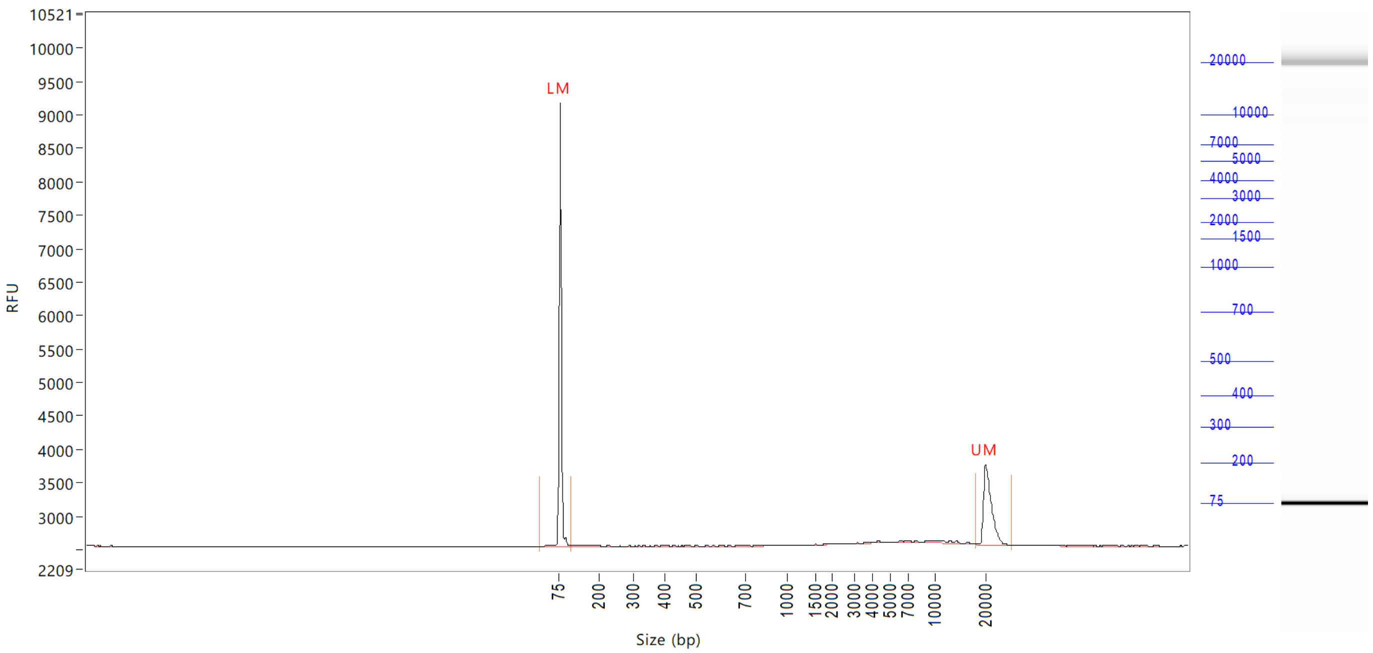

Peak	Size (bp)	Concentration (ng/uL)	From (bp)	To (bp)	Average size (bp)	CV%	RFU	Corrected peak area
1	75 (LM)	0.5000	14	108	73	6.98	6641	36.595
2	20000 (UM)	0.1646	18049	25199	20480	4.39	1201	12.050

TIC:	0.0000	ng/uL
TIM:	0.0000	nmole/L
Total concentration:	0.6927	ng/uL

Sample peak width (sec): 5 Sample min peak height: 50 Sample baseline V to V?: Y Sample baseline V to V points: 3
Sample filter: Binomial Number of points for filter: 3 Sample start region (min): 0 Sample end region (min): 30
Marker peak width (sec): 5 Marker min peak height: 200 Marker baseline V to V?: Y Marker baseline V to V points: 3
Lower marker selection: First peak > 500 RFU Upper marker selection: Last peak > 200 RFU
Ladder size (bp)75, 200, 300, 400, 500, 700, 1000, 1500, 2000, 3000, 4000, 5000, 7000, 10000, 20000
Quantification using: Lower Marker Final concentration (ng/uL): 0.5000 Dilution factor: 12.0

Sample: SampC8
Well location: C8
Created: Thursday, August 12, 2021 6:46:05 PM

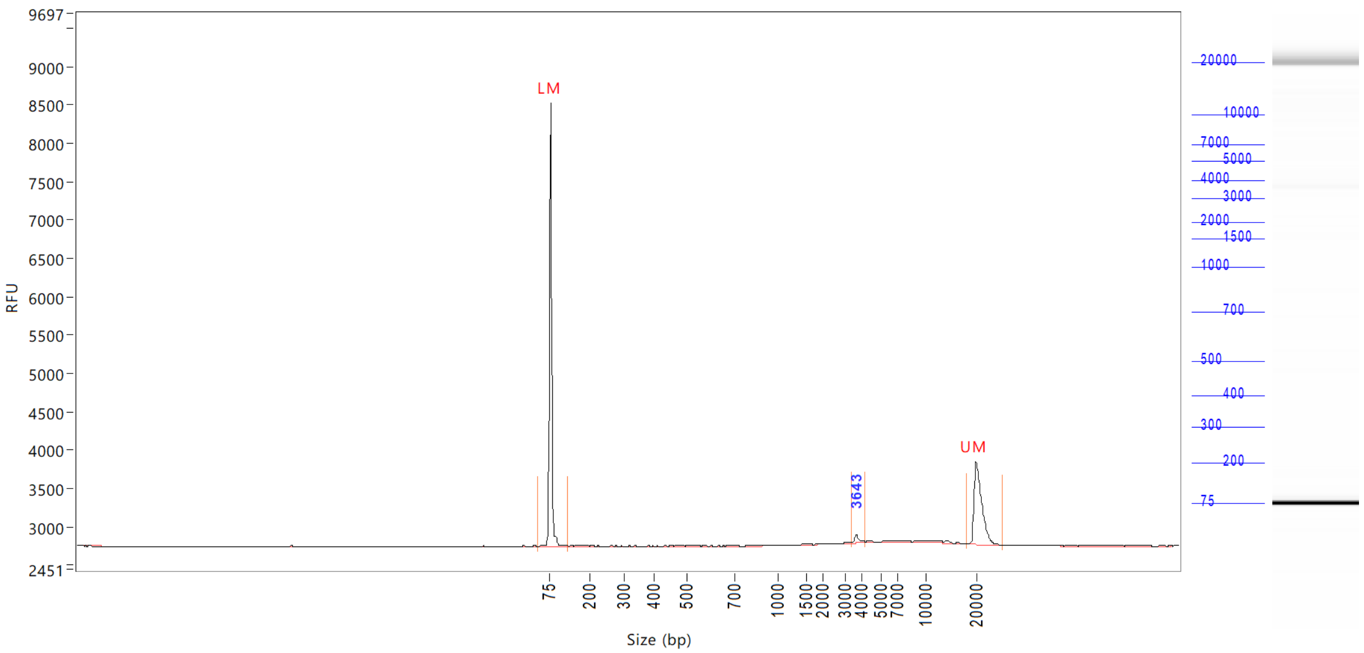

Peak	Size (bp)	Concentration (ng/uL)	From (bp)	To (bp)	Average size (bp)	CV%	RFU	Corrected peak area
1	75 (LM)	0.5000	37	131	74	7.13	5790	33.224
2	3643	0.1395	3358	4139	3707	4.75	100	0.773
3	20000 (UM)	0.1610	18049	25320	20526	4.45	1089	10.700

TIC:	0.1395	ng/uL
TIM:	0.0620	nmole/L
Total concentration:	0.8062	ng/uL

Sample peak width (sec): 5   Sample min peak height: 50   Sample baseline V to V?: Y   Sample baseline V to V points: 3
Sample filter: Binomial   Number of points for filter: 3   Sample start region (min): 0   Sample end region (min): 30
Marker peak width (sec): 5   Marker min peak height: 200   Marker baseline V to V?: Y   Marker baseline V to V points: 3
Lower marker selection: First peak > 500 RFU   Upper marker selection: Last peak > 200 RFU
Ladder size (bp)75, 200, 300, 400, 500, 700, 1000, 1500, 2000, 3000, 4000, 5000, 7000, 10000, 20000
Quantification using: Lower Marker   Final concentration (ng/uL): 0.5000   Dilution factor: 12.0

Sample: SampD8
Well location: D8
Created: Thursday, August 12, 2021 6:46:05 PM

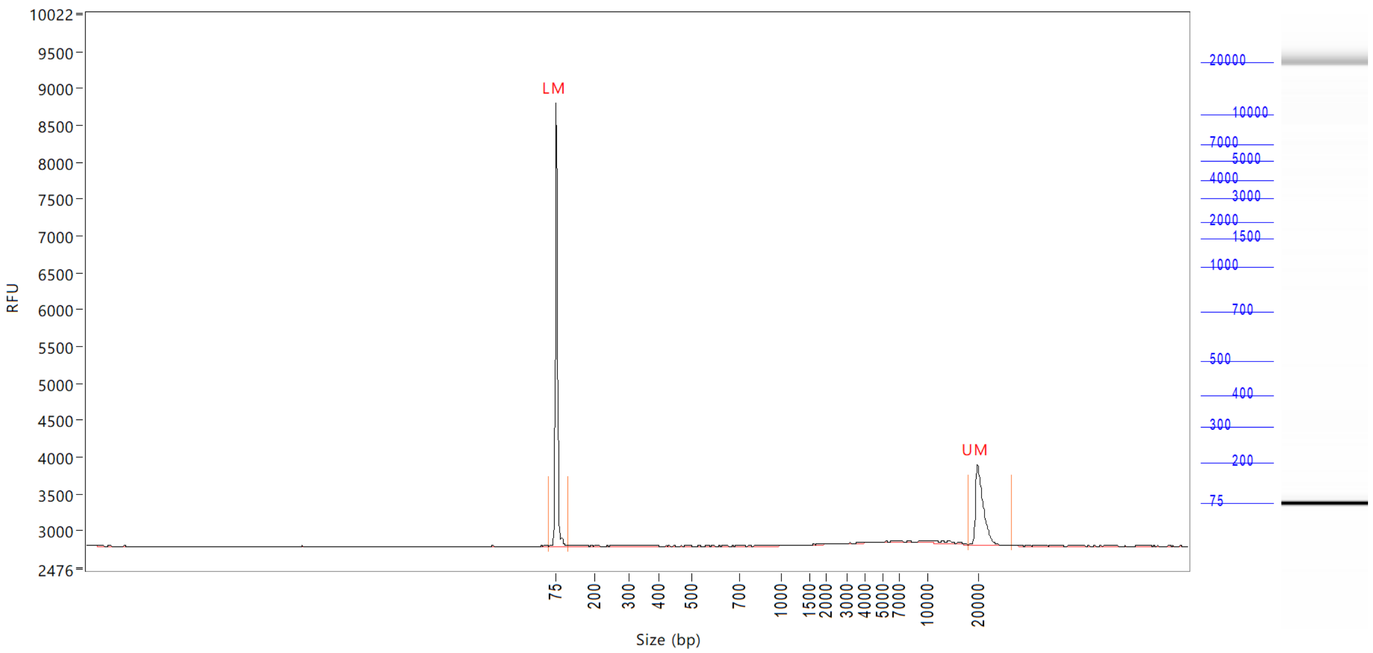

Peak	Size (bp)	Concentration (ng/uL)	From (bp)	To (bp)	Average size (bp)	CV%	RFU	Corrected peak area
1	75 (LM)	0.5000	50	110	73	5.78	6028	33.196
2	20000 (UM)	0.1666	18171	27012	20611	4.90	1085	11.061

TIC:	0.0000	ng/uL
TIM:	0.0000	nmole/L
Total concentration:	0.7795	ng/uL

Sample peak width (sec): 5   Sample min peak height: 50   Sample baseline V to V?: Y   Sample baseline V to V points: 3
Sample filter: Binomial   Number of points for filter: 3 Sample start region (min): 0 Sample end region (min): 30
Marker peak width (sec): 5   Marker min peak height: 200   Marker baseline V to V?: Y   Marker baseline V to V points: 3
Lower marker selection: First peak > 500 RFU   Upper marker selection: Last peak > 200 RFU
Ladder size (bp)75, 200, 300, 400, 500, 700, 1000, 1500, 2000, 3000, 4000, 5000, 7000, 10000, 20000
Quantification using: Lower Marker   Final concentration (ng/uL): 0.5000   Dilution factor: 12.0

Sample: SampE8
Well location: E8
Created: Thursday, August 12, 2021 6:46:05 PM

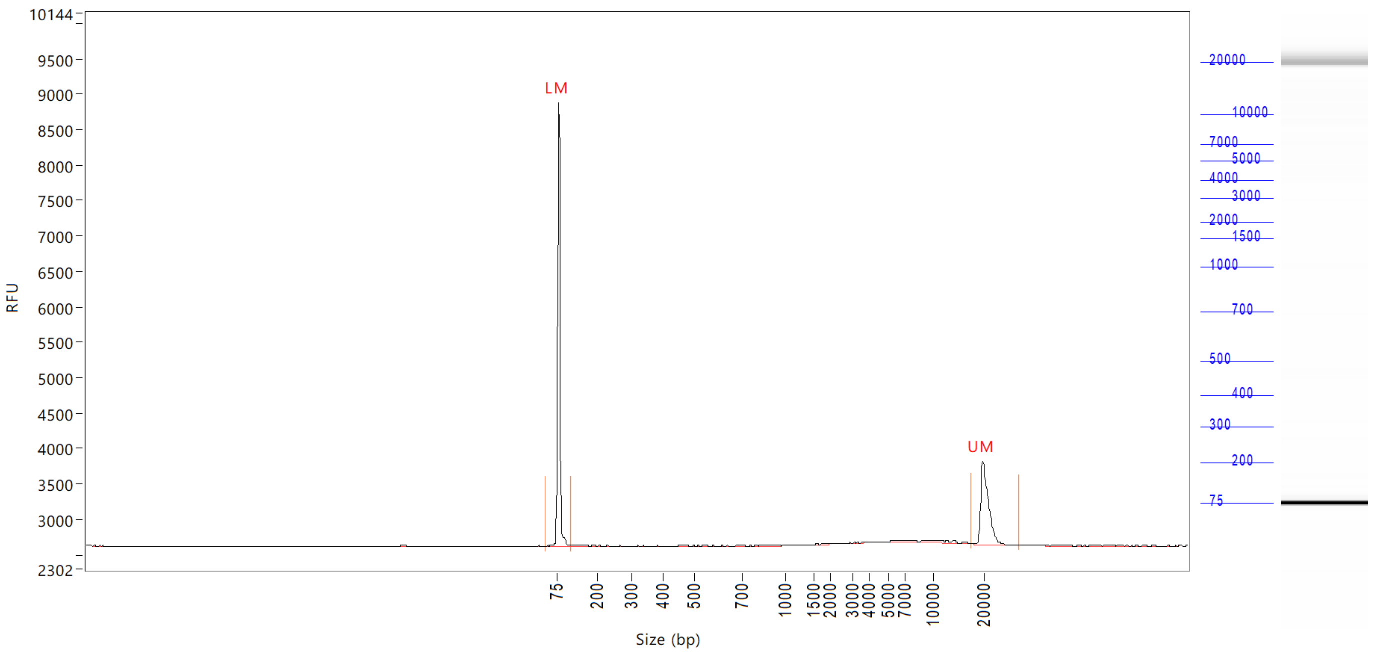

Peak	Size (bp)	Concentration (ng/uL)	From (bp)	To (bp)	Average size (bp)	CV%	RFU	Corrected peak area
1	75 (LM)	0.5000	35	114	74	6.20	6266	34.850
2	20000 (UM)	0.1697	17561	27133	20451	4.64	1182	11.825

TIC:	0.0000	ng/uL
TIM:	0.0000	nmole/L
Total concentration:	0.7626	ng/uL

Sample peak width (sec): 5 Sample min peak height: 50 Sample baseline V to V?: Y Sample baseline V to V points: 3
Sample filter: Binomial Number of points for filter: 3 Sample start region (min): 0 Sample end region (min): 30
Marker peak width (sec): 5 Marker min peak height: 200 Marker baseline V to V?: Y Marker baseline V to V points: 3
Lower marker selection: First peak > 500 RFU Upper marker selection: Last peak > 200 RFU
Ladder size (bp)75, 200, 300, 400, 500, 700, 1000, 1500, 2000, 3000, 4000, 5000, 7000, 10000, 20000
Quantification using: Lower Marker Final concentration (ng/uL): 0.5000 Dilution factor: 12.0

Sample: SampF8
Well location: F8
Created: Thursday, August 12, 2021 6:46:05 PM

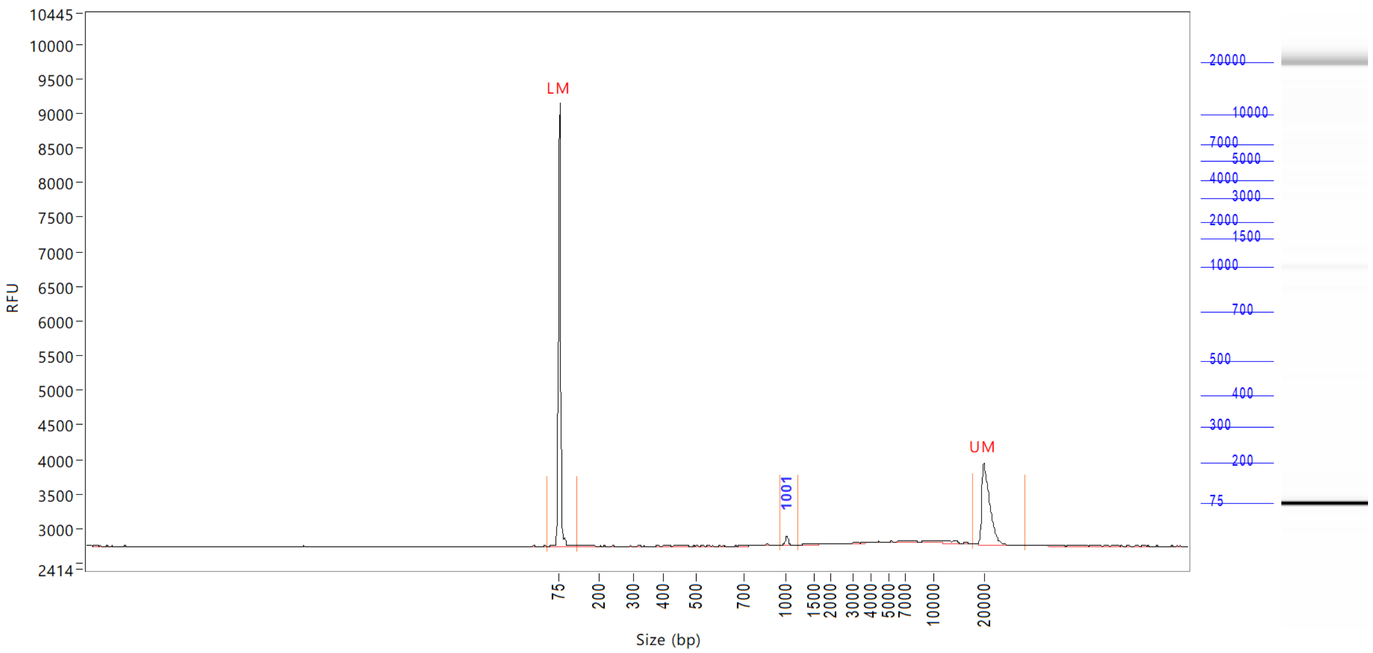

Peak	Size (bp)	Concentration (ng/uL)	From (bp)	To (bp)	Average size (bp)	CV%	RFU	Corrected peak area
1	75 (LM)	0.5000	39	131	73	6.96	6417	35.473
2	1001	0.1330	952	1201	1010	3.93	128	0.786
3	20000 (UM)	0.1697	17927	27979	20534	4.84	1188	12.038

TIC:	0.1330	ng/uL
TIM:	0.2167	nmole/L
Total concentration:	0.8576	ng/uL

```
Sample peak width (sec): 5    Sample min peak height: 50    Sample baseline V to V?: Y    Sample baseline V to V points: 3
Sample filter: Binomial       Number of points for filter: 3  Sample start region (min): 0  Sample end region (min): 30
Marker peak width (sec): 5    Marker min peak height: 200   Marker baseline V to V?: Y    Marker baseline V to V points: 3
Lower marker selection: First peak > 500 RFU                Upper marker selection: Last peak > 200 RFU
Ladder size (bp)75, 200, 300, 400, 500, 700, 1000, 1500, 2000, 3000, 4000, 5000, 7000, 10000, 20000
Quantification using: Lower Marker          Final concentration (ng/uL): 0.5000          Dilution factor: 12.0
```

Sample: SampG8
Well location: G8
Created: Thursday, August 12, 2021 6:46:05 PM

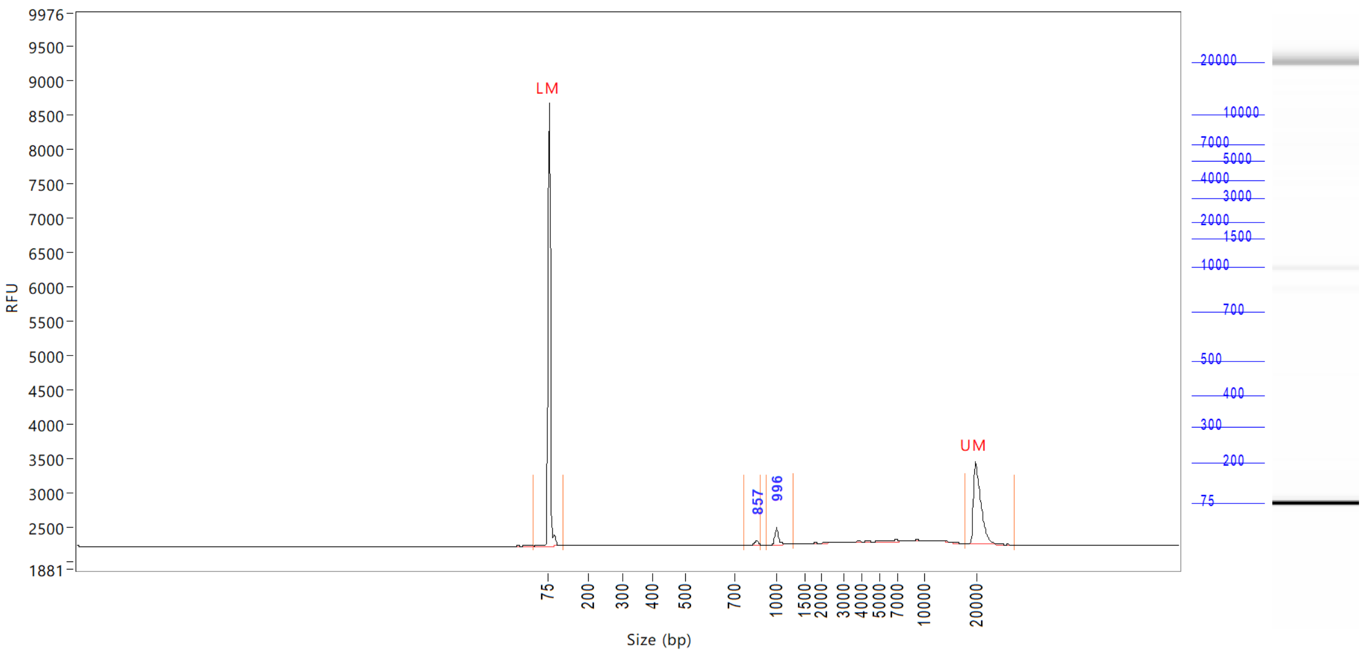

Peak	Size (bp)	Concentration (ng/uL)	From (bp)	To (bp)	Average size (bp)	CV%	RFU	Corrected peak area
1	75 (LM)	0.5000	30	118	74	6.81	6463	36.356
2	857	0.0836	770	887	845	1.93	73	0.507
3	996	0.2625	931	1289	1007	4.49	249	1.591
4	20000 (UM)	0.1571	18049	27738	20505	4.52	1211	11.423

TIC:	0.3461	ng/uL
TIM:	0.5919	nmole/L
Total concentration:	0.8914	ng/uL

Sample peak width (sec): 5 Sample min peak height: 50 Sample baseline V to V?: Y Sample baseline V to V points: 3
Sample filter: Binomial Number of points for filter: 3 Sample start region (min): 0 Sample end region (min): 30
Marker peak width (sec): 5 Marker min peak height: 200 Marker baseline V to V?: Y Marker baseline V to V points: 3
Lower marker selection: First peak > 500 RFU Upper marker selection: Last peak > 200 RFU
Ladder size (bp)75, 200, 300, 400, 500, 700, 1000, 1500, 2000, 3000, 4000, 5000, 7000, 10000, 20000
Quantification using: Lower Marker Final concentration (ng/uL): 0.5000 Dilution factor: 12.0

Sample: SampH8
Well location: H8
Created: Thursday, August 12, 2021 6:46:05 PM

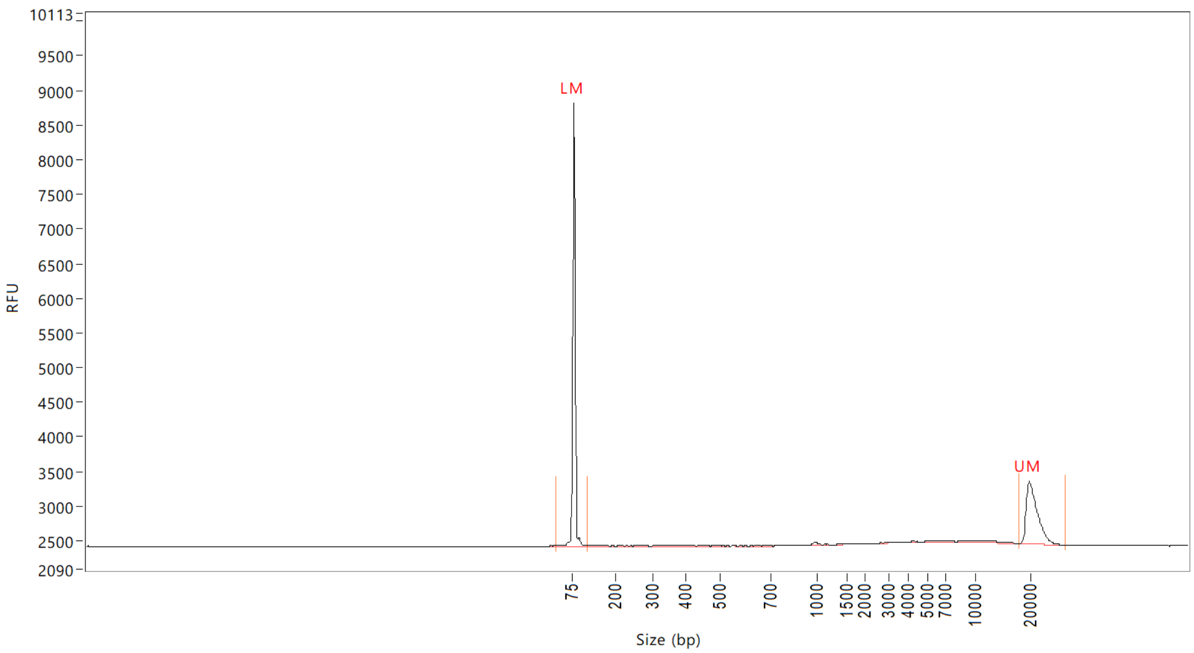

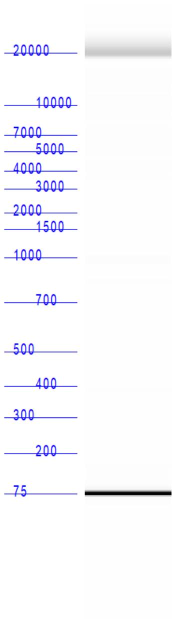

Peak	Size (bp)	Concentration (ng/uL)	From (bp)	To (bp)	Average size (bp)	CV%	RFU	Corrected peak area
1	75 (LM)	0.5000	28	116	74	6.77	6409	38.600
2	20000 (UM)	0.1611	18049	26770	20692	5.72	907	12.440

TIC:	0.0000	ng/uL
TIM:	0.0000	nmole/L
Total concentration:	0.7020	ng/uL

```
Sample peak width (sec): 5    Sample min peak height: 50    Sample baseline V to V?: Y    Sample baseline V to V points: 3
Sample filter: Binomial       Number of points for filter: 3  Sample start region (min): 0  Sample end region (min): 30
Marker peak width (sec): 5    Marker min peak height: 200   Marker baseline V to V?: Y    Marker baseline V to V points: 3
Lower marker selection: First peak > 500 RFU                Upper marker selection: Last peak > 200 RFU
Ladder size (bp)75, 200, 300, 400, 500, 700, 1000, 1500, 2000, 3000, 4000, 5000, 7000, 10000, 20000
Quantification using: Lower Marker          Final concentration (ng/uL): 0.5000          Dilution factor: 12.0
```

Sample: SampA9
Well location: A9
Created: Thursday, August 12, 2021 6:46:05 PM

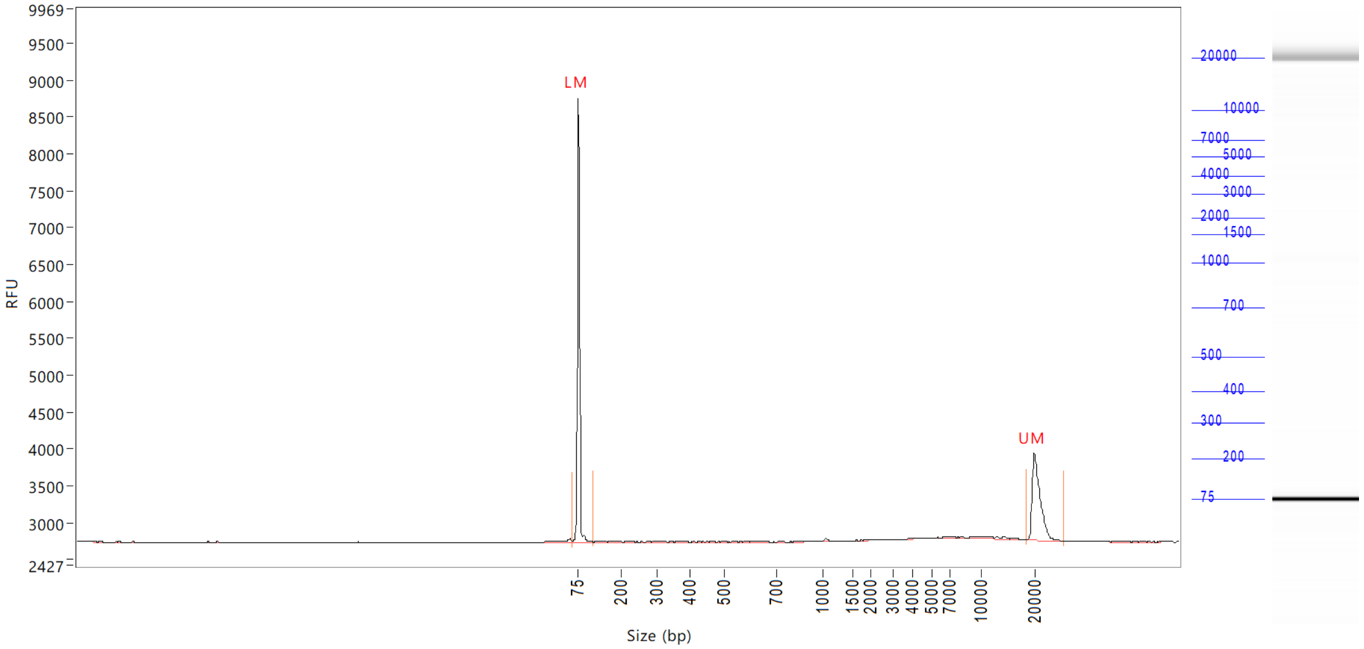

Peak	Size (bp)	Concentration (ng/uL)	From (bp)	To (bp)	Average size (bp)	CV%	RFU	Corrected peak area
1	75 (LM)	0.5000	60	118	73	5.49	6024	31.678
2	20000 (UM)	0.1806	18537	25440	20472	4.29	1185	11.443

TIC:	0.0000	ng/uL
TIM:	0.0000	nmole/L
Total concentration:	0.7447	ng/uL

Sample peak width (sec): 5   Sample min peak height: 50   Sample baseline V to V?: Y   Sample baseline V to V points: 3
Sample filter: Binomial   Number of points for filter: 3 Sample start region (min): 0 Sample end region (min): 30
Marker peak width (sec): 5   Marker min peak height: 200   Marker baseline V to V?: Y   Marker baseline V to V points: 3
Lower marker selection: First peak > 500 RFU   Upper marker selection: Last peak > 200 RFU
Ladder size (bp)75, 200, 300, 400, 500, 700, 1000, 1500, 2000, 3000, 4000, 5000, 7000, 10000, 20000
Quantification using: Lower Marker   Final concentration (ng/uL): 0.5000   Dilution factor: 12.0

Sample: SampB9
Well location: B9
Created: Thursday, August 12, 2021 6:46:05 PM

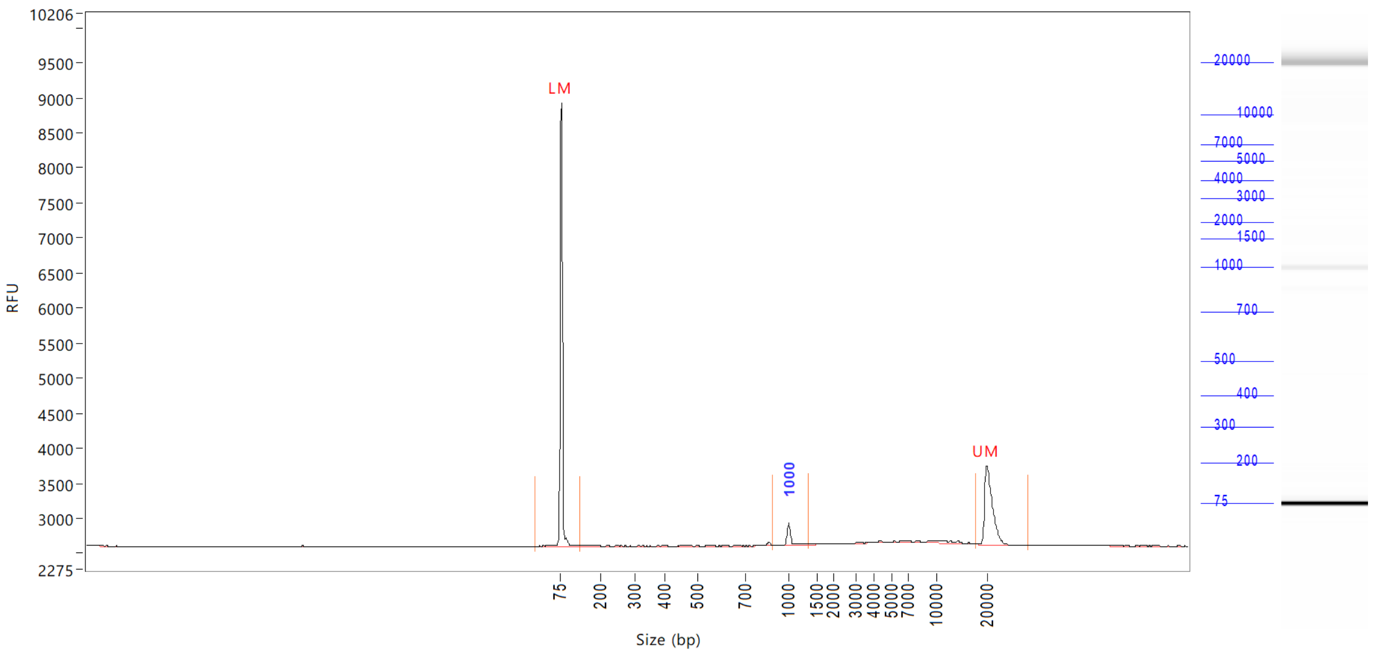

Peak	Size (bp)	Concentration (ng/uL)	From (bp)	To (bp)	Average size (bp)	CV%	RFU	Corrected peak area
1	75 (LM)	0.5000	0	133	73	9.62	6338	35.932
2	1000	0.3533	883	1345	1021	6.58	319	2.116
3	20000 (UM)	0.1564	17805	27979	20486	4.91	1121	11.243

TIC:	0.3533	ng/uL
TIM:	0.5696	nmole/L
Total concentration:	1.0530	ng/uL

Sample peak width (sec): 5   Sample min peak height: 50   Sample baseline V to V?: Y   Sample baseline V to V points: 3
Sample filter: Binomial   Number of points for filter: 3 Sample start region (min): 0 Sample end region (min): 30
Marker peak width (sec): 5   Marker min peak height: 200   Marker baseline V to V?: Y   Marker baseline V to V points: 3
Lower marker selection: First peak > 500 RFU   Upper marker selection: Last peak > 200 RFU
Ladder size (bp)75, 200, 300, 400, 500, 700, 1000, 1500, 2000, 3000, 4000, 5000, 7000, 10000, 20000
Quantification using: Lower Marker   Final concentration (ng/uL): 0.5000   Dilution factor: 12.0

Sample: SampC9
Well location: C9
Created: Thursday, August 12, 2021 6:46:05 PM

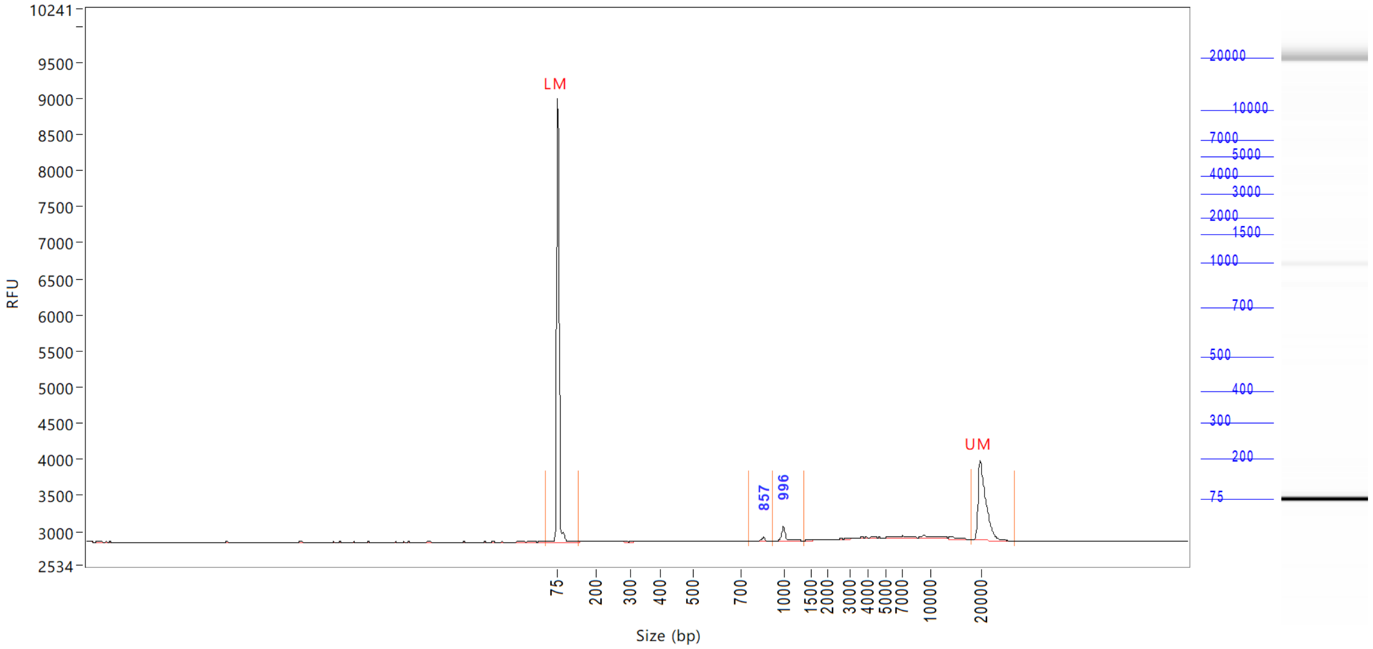

Peak	Size (bp)	Concentration (ng/uL)	From (bp)	To (bp)	Average size (bp)	CV%	RFU	Corrected peak area
1	75 (LM)	0.5000	39	139	74	7.71	6153	34.738
2	857	0.0733	753	922	846	2.81	57	0.425
3	996	0.2517	922	1356	1018	6.91	206	1.457
4	20000 (UM)	0.1600	18171	27012	20509	4.69	1109	11.120

TIC:	0.3250	ng/uL
TIM:	0.5495	nmole/L
Total concentration:	1.0320	ng/uL

Sample peak width (sec): 5   Sample min peak height: 50   Sample baseline V to V?: Y   Sample baseline V to V points: 3
Sample filter: Binomial   Number of points for filter: 3 Sample start region (min): 0 Sample end region (min): 30
Marker peak width (sec): 5   Marker min peak height: 200   Marker baseline V to V?: Y   Marker baseline V to V points: 3
Lower marker selection: First peak > 500 RFU   Upper marker selection: Last peak > 200 RFU
Ladder size (bp)75, 200, 300, 400, 500, 700, 1000, 1500, 2000, 3000, 4000, 5000, 7000, 10000, 20000
Quantification using: Lower Marker   Final concentration (ng/uL): 0.5000   Dilution factor: 12.0

Sample: SampD9
Well location: D9
Created: Thursday, August 12, 2021 6:46:05 PM

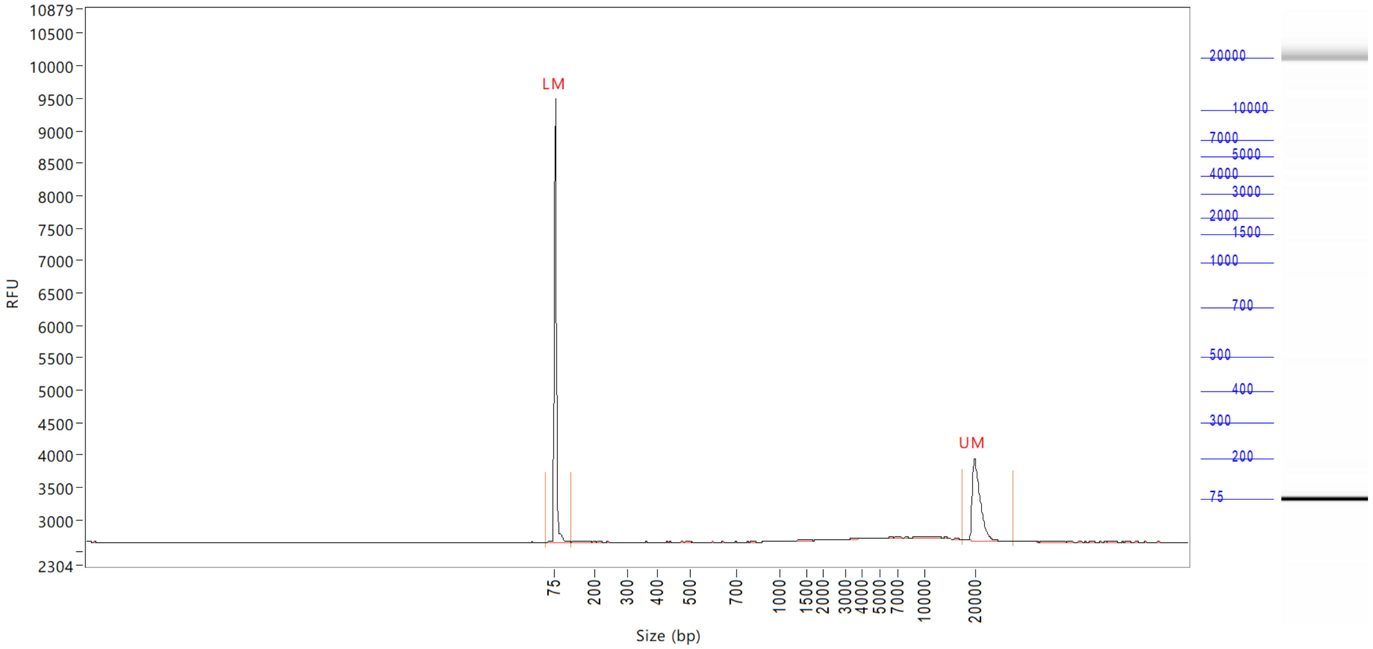

Peak	Size (bp)	Concentration (ng/uL)	From (bp)	To (bp)	Average size (bp)	CV%	RFU	Corrected peak area
1	75 (LM)	0.5000	47	126	74	6.52	6852	38.415
2	20000 (UM)	0.1683	17561	27738	20578	4.68	1269	12.931

TIC:	0.0000	ng/uL
TIM:	0.0000	nmole/L
Total concentration:	0.5924	ng/uL

```
Sample peak width (sec): 5    Sample min peak height: 50    Sample baseline V to V?: Y    Sample baseline V to V points: 3
Sample filter: Binomial       Number of points for filter: 3  Sample start region (min): 0  Sample end region (min): 30
Marker peak width (sec): 5    Marker min peak height: 200   Marker baseline V to V?: Y    Marker baseline V to V points: 3
Lower marker selection: First peak > 500 RFU                Upper marker selection: Last peak > 200 RFU
Ladder size (bp)75, 200, 300, 400, 500, 700, 1000, 1500, 2000, 3000, 4000, 5000, 7000, 10000, 20000
Quantification using: Lower Marker          Final concentration (ng/uL): 0.5000          Dilution factor: 12.0
```

Sample: SampE9
Well location: E9
Created: Thursday, August 12, 2021 6:46:05 PM

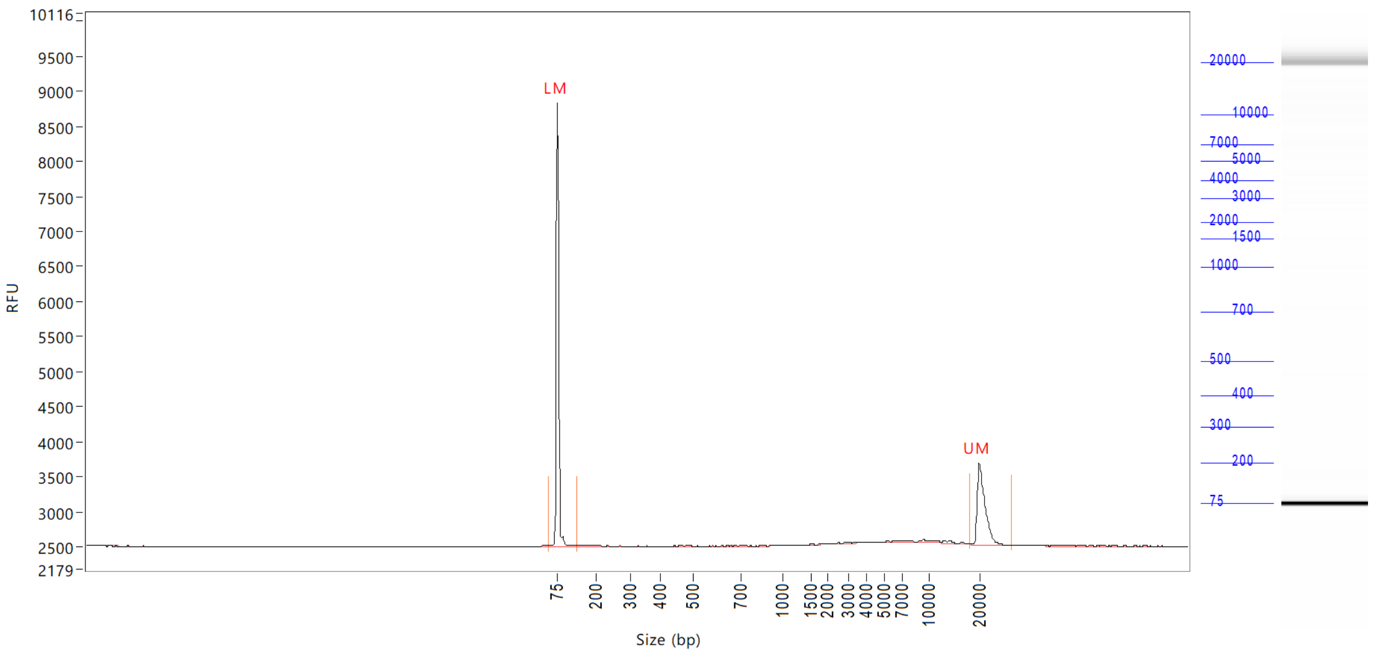

Peak	Size (bp)	Concentration (ng/uL)	From (bp)	To (bp)	Average size (bp)	CV%	RFU	Corrected peak area
1	75 (LM)	0.5000	47	137	73	7.22	6341	36.317
2	20000 (UM)	0.1628	18049	26529	20580	4.68	1164	11.828

TIC:	0.0000	ng/uL
TIM:	0.0000	nmole/L
Total concentration:	0.6654	ng/uL

Sample peak width (sec): 5 Sample min peak height: 50 Sample baseline V to V?: Y Sample baseline V to V points: 3
Sample filter: Binomial Number of points for filter: 3 Sample start region (min): 0 Sample end region (min): 30
Marker peak width (sec): 5 Marker min peak height: 200 Marker baseline V to V?: Y Marker baseline V to V points: 3
Lower marker selection: First peak > 500 RFU Upper marker selection: Last peak > 200 RFU
Ladder size (bp)75, 200, 300, 400, 500, 700, 1000, 1500, 2000, 3000, 4000, 5000, 7000, 10000, 20000
Quantification using: Lower Marker Final concentration (ng/uL): 0.5000 Dilution factor: 12.0

Sample: SampF9
Well location: F9
Created: Thursday, August 12, 2021 6:46:05 PM

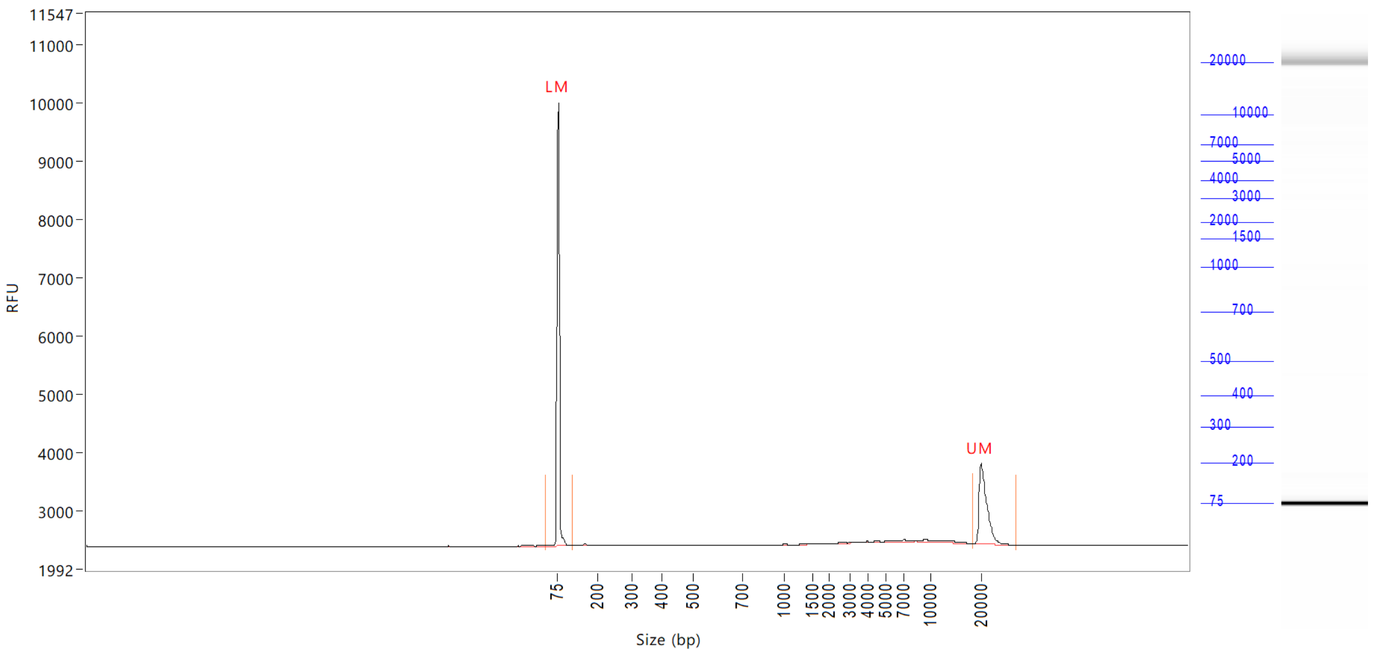

Peak	Size (bp)	Concentration (ng/uL)	From (bp)	To (bp)	Average size (bp)	CV%	RFU	Corrected peak area
1	75 (LM)	0.5000	39	118	73	6.19	7628	42.357
2	20000 (UM)	0.1659	18537	27012	20570	4.58	1403	14.053

TIC:	0.0000	ng/uL
TIM:	0.0000	nmole/L
Total concentration:	0.6864	ng/uL

Sample peak width (sec): 5   Sample min peak height: 50   Sample baseline V to V?: Y   Sample baseline V to V points: 3
Sample filter: Binomial   Number of points for filter: 3 Sample start region (min): 0 Sample end region (min): 30
Marker peak width (sec): 5   Marker min peak height: 200   Marker baseline V to V?: Y   Marker baseline V to V points: 3
Lower marker selection: First peak > 500 RFU   Upper marker selection: Last peak > 200 RFU
Ladder size (bp)75, 200, 300, 400, 500, 700, 1000, 1500, 2000, 3000, 4000, 5000, 7000, 10000, 20000
Quantification using: Lower Marker   Final concentration (ng/uL): 0.5000   Dilution factor: 12.0

Sample: SampG9
Well location: G9
Created: Thursday, August 12, 2021 6:46:05 PM

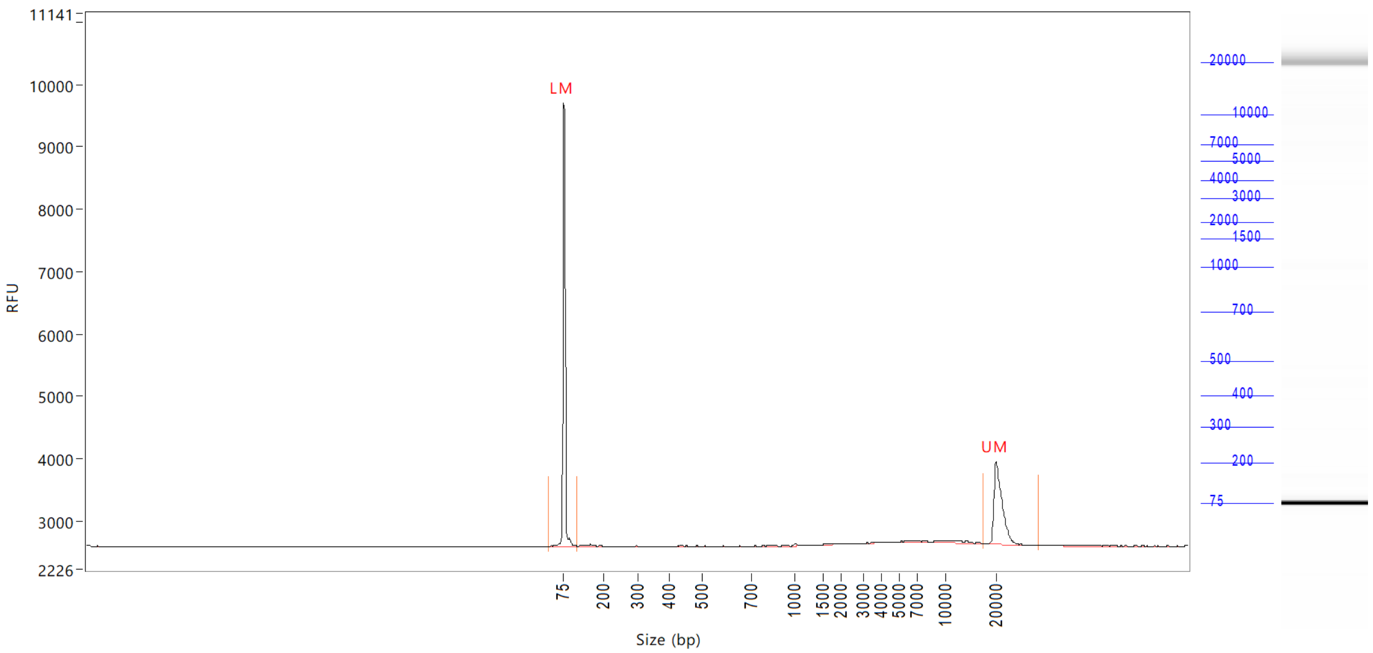

Peak	Size (bp)	Concentration (ng/uL)	From (bp)	To (bp)	Average size (bp)	CV%	RFU	Corrected peak area
1	75 (LM)	0.5000	31	116	74	6.59	7125	40.653
2	20000 (UM)	0.1703	17317	28221	20549	4.94	1322	13.847

TIC:	0.0000	ng/uL
TIM:	0.0000	nmole/L
Total concentration:	0.8010	ng/uL

Sample peak width (sec): 5 Sample min peak height: 50 Sample baseline V to V?: Y Sample baseline V to V points: 3
Sample filter: Binomial Number of points for filter: 3 Sample start region (min): 0 Sample end region (min): 30
Marker peak width (sec): 5 Marker min peak height: 200 Marker baseline V to V?: Y Marker baseline V to V points: 3
Lower marker selection: First peak > 500 RFU Upper marker selection: Last peak > 200 RFU
Ladder size (bp)75, 200, 300, 400, 500, 700, 1000, 1500, 2000, 3000, 4000, 5000, 7000, 10000, 20000
Quantification using: Lower Marker Final concentration (ng/uL): 0.5000 Dilution factor: 12.0

Sample: SampH9
Well location: H9
Created: Thursday, August 12, 2021 6:46:05 PM

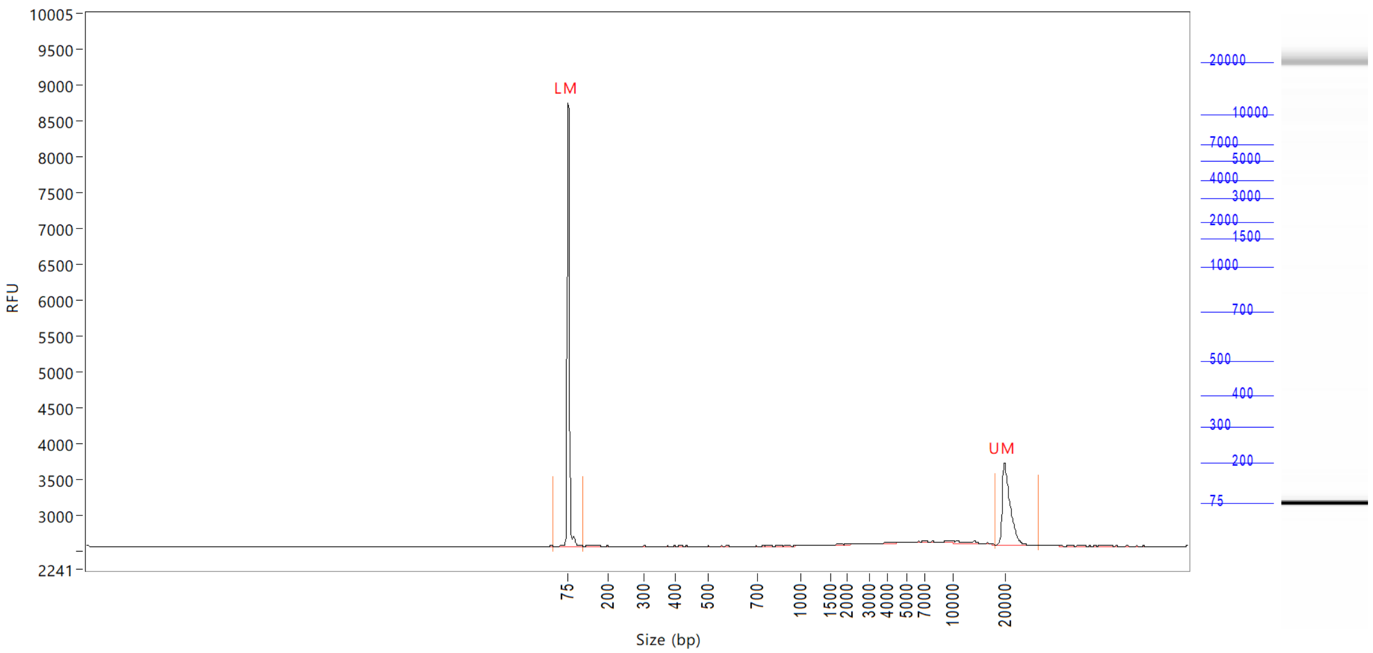

Peak	Size (bp)	Concentration (ng/uL)	From (bp)	To (bp)	Average size (bp)	CV%	RFU	Corrected peak area
1	75 (LM)	0.5000	31	122	74	6.77	6205	35.021
2	20000 (UM)	0.1641	18171	26529	20564	4.58	1147	11.495

TIC:	0.0000	ng/uL
TIM:	0.0000	nmole/L
Total concentration:	0.6212	ng/uL

Sample peak width (sec): 5   Sample min peak height: 50   Sample baseline V to V?: Y   Sample baseline V to V points: 3
Sample filter: Binomial   Number of points for filter: 3 Sample start region (min): 0 Sample end region (min): 30
Marker peak width (sec): 5   Marker min peak height: 200   Marker baseline V to V?: Y   Marker baseline V to V points: 3
Lower marker selection: First peak > 500 RFU   Upper marker selection: Last peak > 200 RFU
Ladder size (bp)75, 200, 300, 400, 500, 700, 1000, 1500, 2000, 3000, 4000, 5000, 7000, 10000, 20000
Quantification using: Lower Marker   Final concentration (ng/uL): 0.5000   Dilution factor: 12.0

Sample: SampA10
Well location: A10
Created: Thursday, August 12, 2021 6:46:05 PM

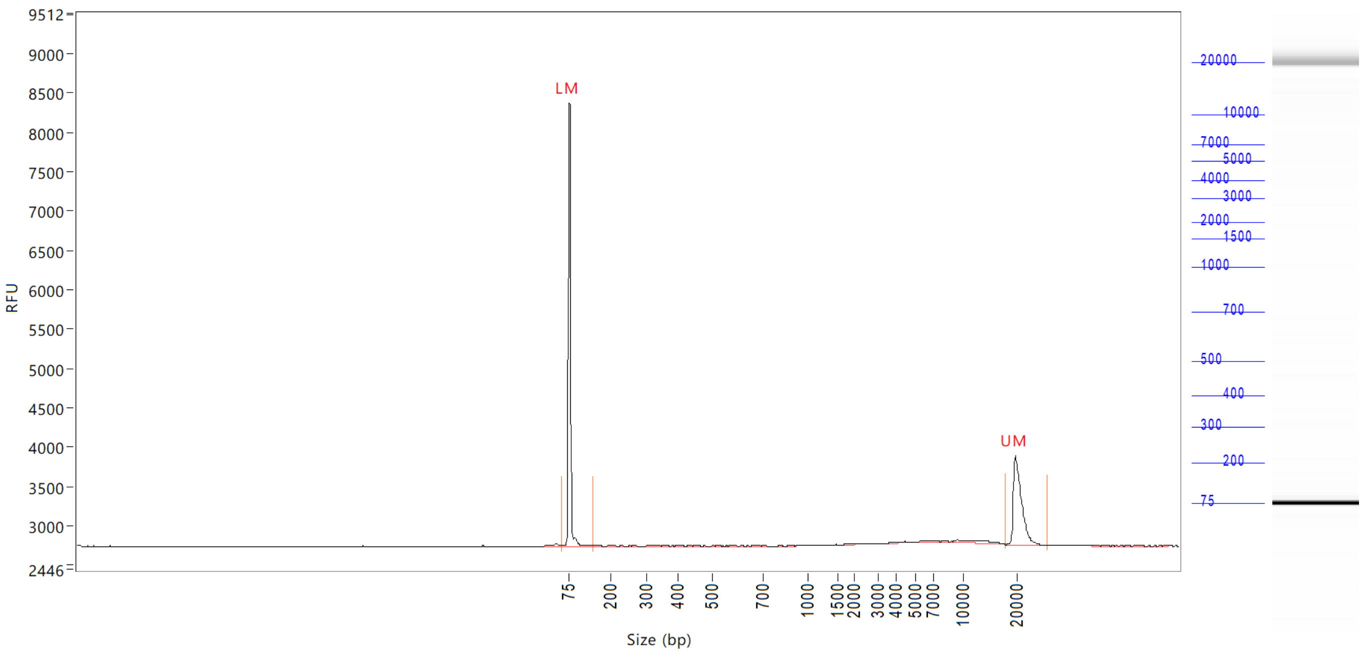

Peak	Size (bp)	Concentration (ng/uL)	From (bp)	To (bp)	Average size (bp)	CV%	RFU	Corrected peak area
1	75 (LM)	0.5000	54	145	75	8.26	5646	31.656
2	20000 (UM)	0.1787	18171	26045	20479	4.73	1135	11.314

TIC:	0.0000	ng/uL
TIM:	0.0000	nmole/L
Total concentration:	0.8078	ng/uL

Sample peak width (sec): 5   Sample min peak height: 50   Sample baseline V to V?: Y   Sample baseline V to V points: 3
Sample filter: Binomial   Number of points for filter: 3 Sample start region (min): 0 Sample end region (min): 30
Marker peak width (sec): 5   Marker min peak height: 200   Marker baseline V to V?: Y   Marker baseline V to V points: 3
Lower marker selection: First peak > 500 RFU   Upper marker selection: Last peak > 200 RFU
Ladder size (bp)75, 200, 300, 400, 500, 700, 1000, 1500, 2000, 3000, 4000, 5000, 7000, 10000, 20000
Quantification using: Lower Marker   Final concentration (ng/uL): 0.5000   Dilution factor: 12.0

Sample: SampB10
Well location: B10
Created: Thursday, August 12, 2021 6:46:05 PM

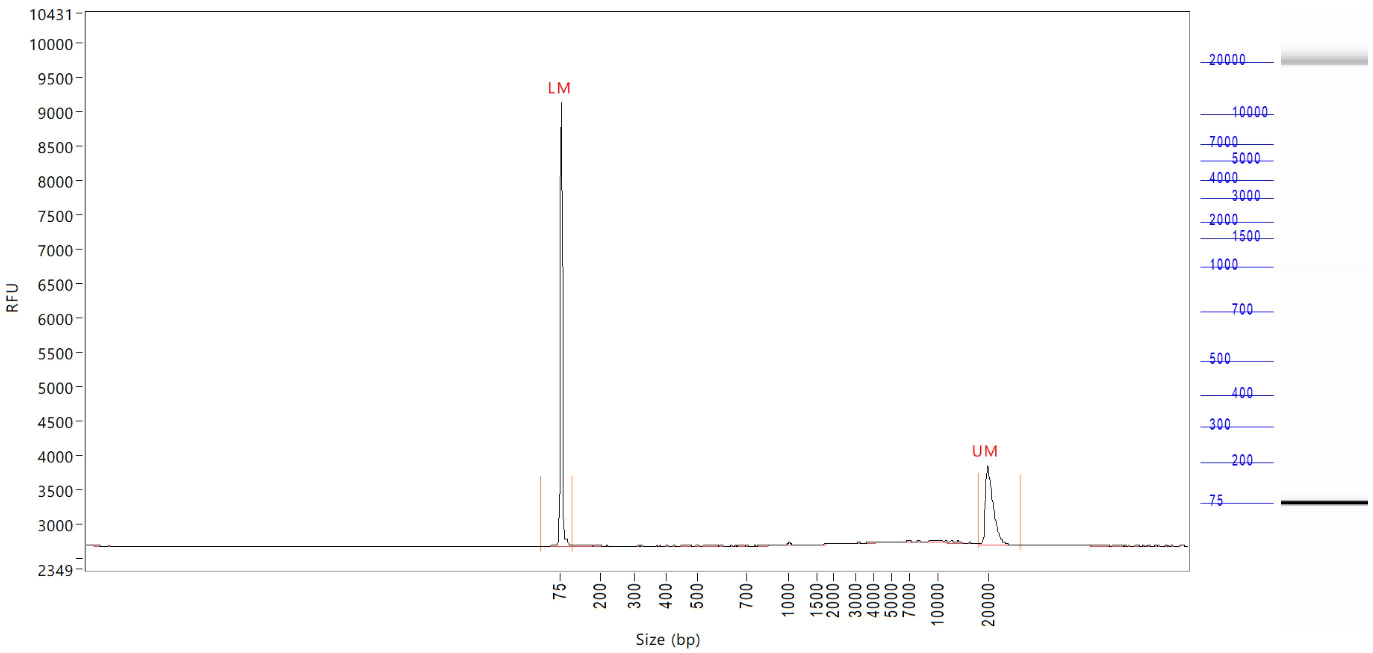

Peak	Size (bp)	Concentration (ng/uL)	From (bp)	To (bp)	Average size (bp)	CV%	RFU	Corrected peak area
1	75 (LM)	0.5000	14	108	73	6.62	6457	35.578
2	20000 (UM)	0.1594	18293	26408	20553	4.60	1142	11.342

TIC:	0.0000	ng/uL
TIM:	0.0000	nmole/L
Total concentration:	0.6822	ng/uL

```
Sample peak width (sec): 5    Sample min peak height: 50    Sample baseline V to V?: Y    Sample baseline V to V points: 3
Sample filter: Binomial       Number of points for filter: 3  Sample start region (min): 0  Sample end region (min): 30
Marker peak width (sec): 5    Marker min peak height: 200   Marker baseline V to V?: Y    Marker baseline V to V points: 3
Lower marker selection: First peak > 500 RFU                Upper marker selection: Last peak > 200 RFU
Ladder size (bp)75, 200, 300, 400, 500, 700, 1000, 1500, 2000, 3000, 4000, 5000, 7000, 10000, 20000
Quantification using: Lower Marker          Final concentration (ng/uL): 0.5000          Dilution factor: 12.0
```

Sample: SampC10
Well location: C10
Created: Thursday, August 12, 2021 6:46:05 PM

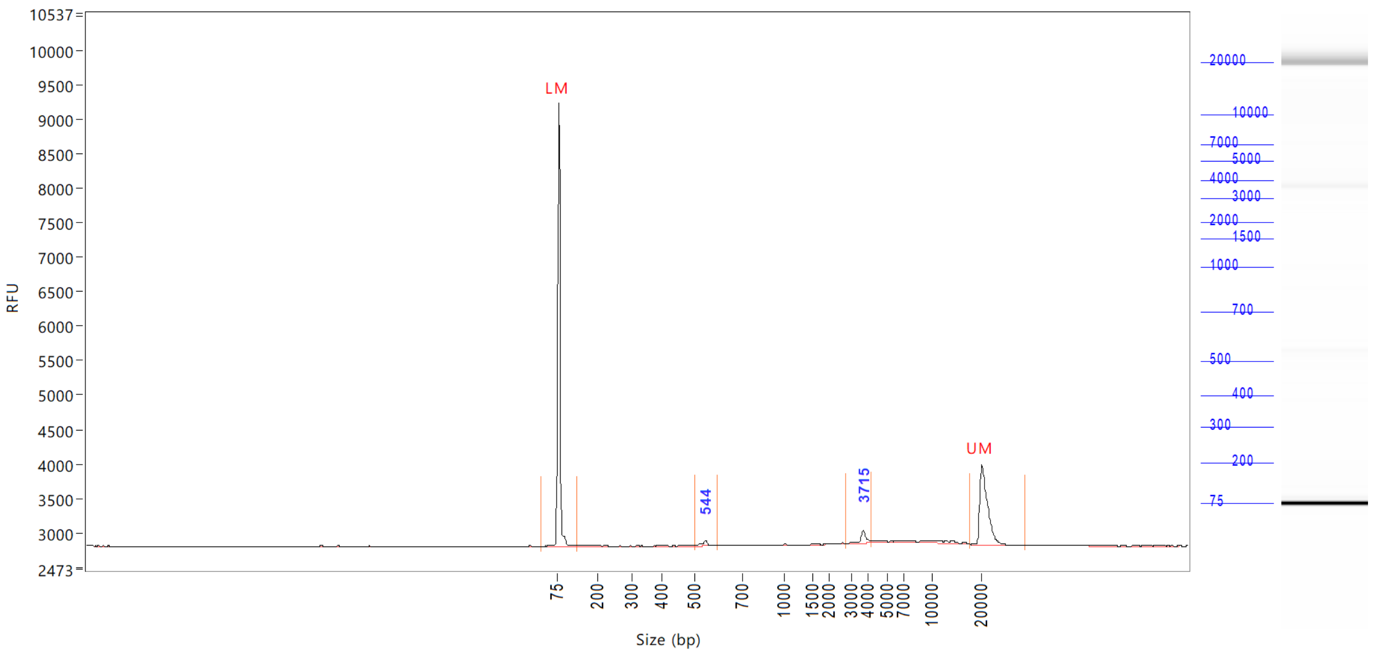

Peak	Size (bp)	Concentration (ng/uL)	From (bp)	To (bp)	Average size (bp)	CV%	RFU	Corrected peak area
1	75 (LM)	0.5000	24	131	74	9.37	6444	36.188
2	544	0.1378	500	593	538	3.46	81	0.831
3	3715	0.2557	2772	4139	3655	7.15	196	1.542
4	20000 (UM)	0.1636	17805	28584	20621	5.11	1159	11.844

TIC:	0.3935	ng/uL
TIM:	0.5366	nmole/L
Total concentration:	1.2859	ng/uL

```
Sample peak width (sec): 5    Sample min peak height: 50    Sample baseline V to V?: Y    Sample baseline V to V points: 3
Sample filter: Binomial       Number of points for filter: 3  Sample start region (min): 0  Sample end region (min): 30
Marker peak width (sec): 5    Marker min peak height: 200   Marker baseline V to V?: Y    Marker baseline V to V points: 3
Lower marker selection: First peak > 500 RFU                Upper marker selection: Last peak > 200 RFU
Ladder size (bp)75, 200, 300, 400, 500, 700, 1000, 1500, 2000, 3000, 4000, 5000, 7000, 10000, 20000
Quantification using: Lower Marker          Final concentration (ng/uL): 0.5000          Dilution factor: 12.0
```

Sample: SampD10
Well location: D10
Created: Thursday, August 12, 2021 6:46:05 PM

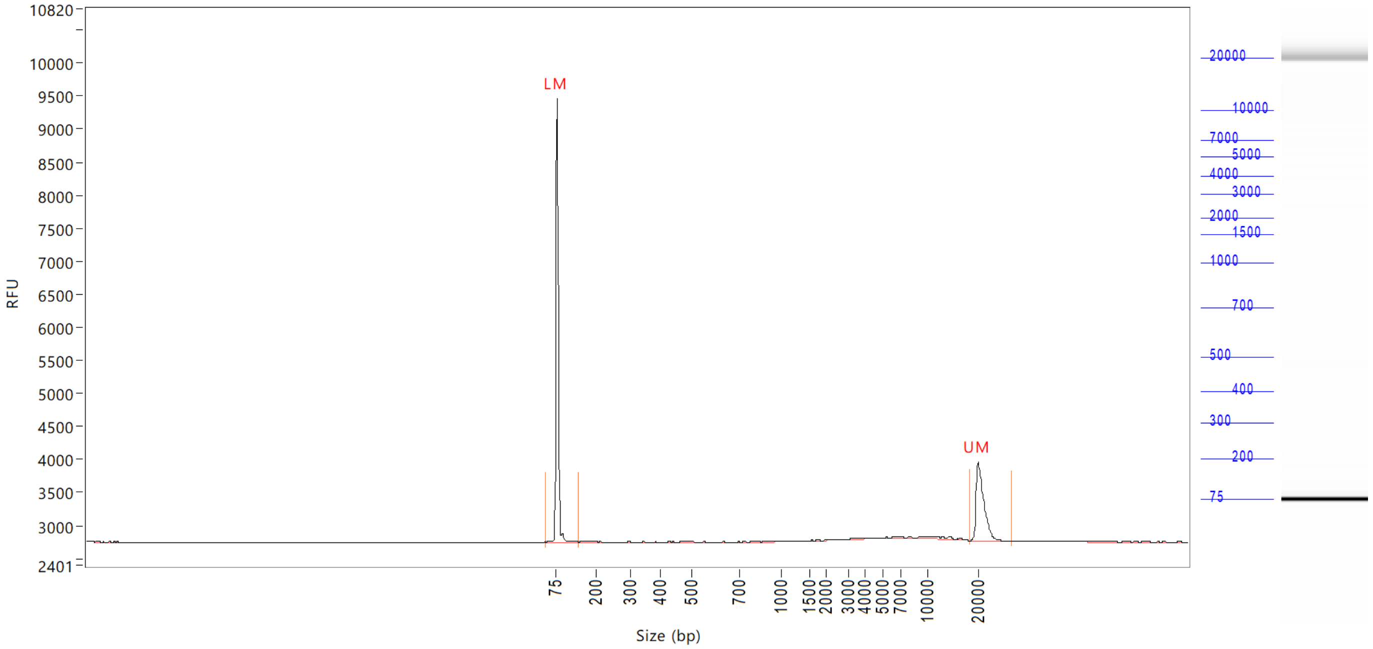

Peak	Size (bp)	Concentration (ng/uL)	From (bp)	To (bp)	Average size (bp)	CV%	RFU	Corrected peak area
1	75 (LM)	0.5000	43	145	74	7.72	6727	37.273
2	20000 (UM)	0.1633	18537	26770	20593	4.75	1202	12.174

TIC:	0.0000	ng/uL
TIM:	0.0000	nmole/L
Total concentration:	0.7037	ng/uL

Sample peak width (sec): 5   Sample min peak height: 50   Sample baseline V to V?: Y   Sample baseline V to V points: 3
Sample filter: Binomial   Number of points for filter: 3 Sample start region (min): 0 Sample end region (min): 30
Marker peak width (sec): 5   Marker min peak height: 200   Marker baseline V to V?: Y   Marker baseline V to V points: 3
Lower marker selection: First peak > 500 RFU   Upper marker selection: Last peak > 200 RFU
Ladder size (bp)75, 200, 300, 400, 500, 700, 1000, 1500, 2000, 3000, 4000, 5000, 7000, 10000, 20000
Quantification using: Lower Marker   Final concentration (ng/uL): 0.5000   Dilution factor: 12.0

Sample: SampE10
Well location: E10
Created: Thursday, August 12, 2021 6:46:05 PM

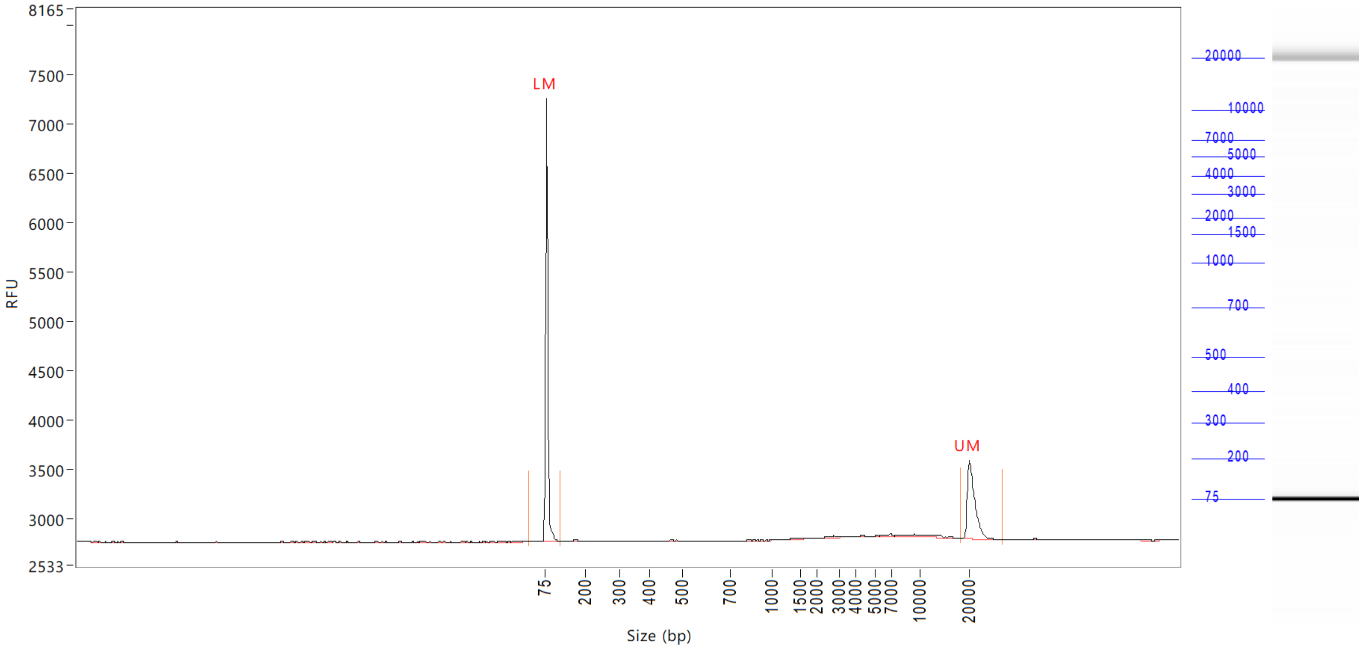

Peak	Size (bp)	Concentration (ng/uL)	From (bp)	To (bp)	Average size (bp)	CV%	RFU	Corrected peak area
1	75 (LM)	0.5000	22	120	74	7.56	4495	25.937
2	20000 (UM)	0.1510	18415	26770	20502	4.65	801	7.832

TIC:	0.0000	ng/uL
TIM:	0.0000	nmole/L
Total concentration:	0.9041	ng/uL

```
Sample peak width (sec): 5    Sample min peak height: 50    Sample baseline V to V?: Y    Sample baseline V to V points: 3
Sample filter: Binomial       Number of points for filter: 3  Sample start region (min): 0  Sample end region (min): 30
Marker peak width (sec): 5    Marker min peak height: 200   Marker baseline V to V?: Y    Marker baseline V to V points: 3
Lower marker selection: First peak > 500 RFU                Upper marker selection: Last peak > 200 RFU
Ladder size (bp)75, 200, 300, 400, 500, 700, 1000, 1500, 2000, 3000, 4000, 5000, 7000, 10000, 20000
Quantification using: Lower Marker          Final concentration (ng/uL): 0.5000          Dilution factor: 12.0
```

Sample: SampF10
Well location: F10
Created: Thursday, August 12, 2021 6:46:05 PM

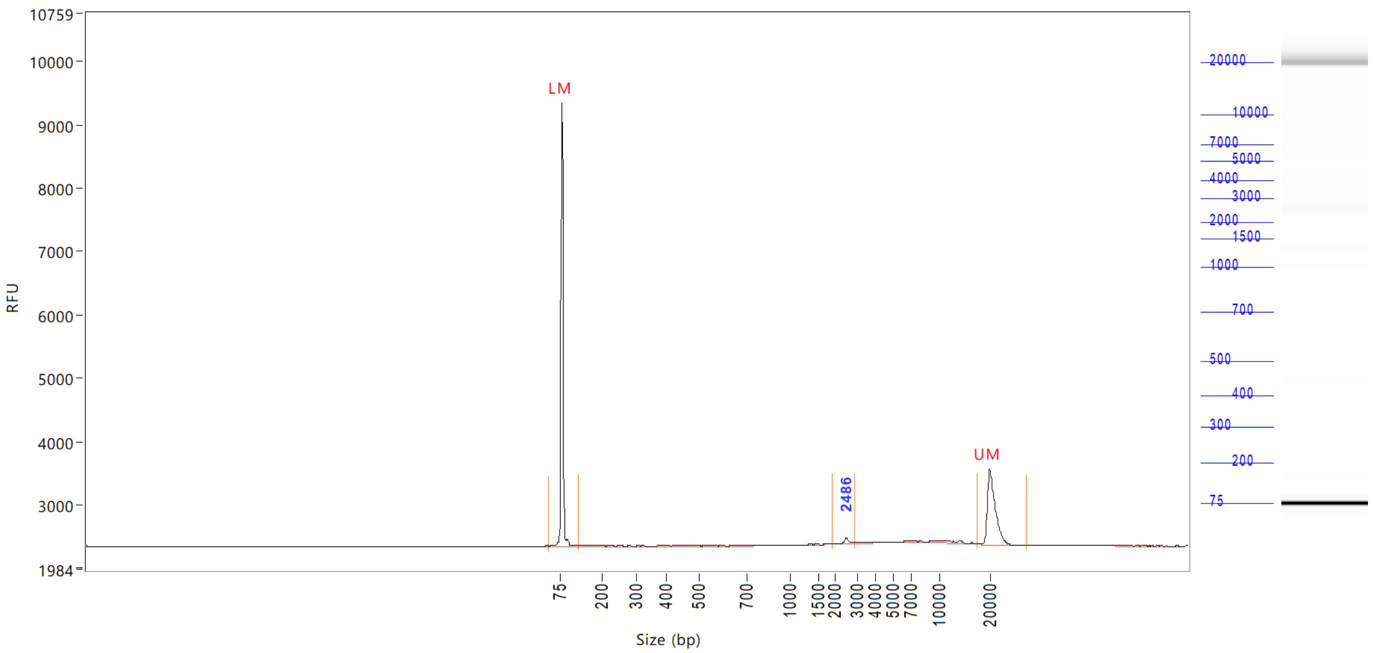

Peak	Size (bp)	Concentration (ng/uL)	From (bp)	To (bp)	Average size (bp)	CV%	RFU	Corrected peak area
1	75 (LM)	0.5000	35	128	73	6.60	7008	38.161
2	2486	0.1103	1923	2914	2492	6.55	100	0.702
3	20000 (UM)	0.1586	17683	27254	20512	4.60	1195	12.102

TIC:	0.1103	ng/uL
TIM:	0.0729	nmole/L
Total concentration:	0.7503	ng/uL

```
Sample peak width (sec): 5    Sample min peak height: 50    Sample baseline V to V?: Y    Sample baseline V to V points: 3
Sample filter: Binomial       Number of points for filter: 3  Sample start region (min): 0  Sample end region (min): 30
Marker peak width (sec): 5    Marker min peak height: 200   Marker baseline V to V?: Y    Marker baseline V to V points: 3
Lower marker selection: First peak > 500 RFU                Upper marker selection: Last peak > 200 RFU
Ladder size (bp)75, 200, 300, 400, 500, 700, 1000, 1500, 2000, 3000, 4000, 5000, 7000, 10000, 20000
Quantification using: Lower Marker          Final concentration (ng/uL): 0.5000          Dilution factor: 12.0
```

Sample: SampG10
Well location: G10
Created: Thursday, August 12, 2021 6:46:05 PM

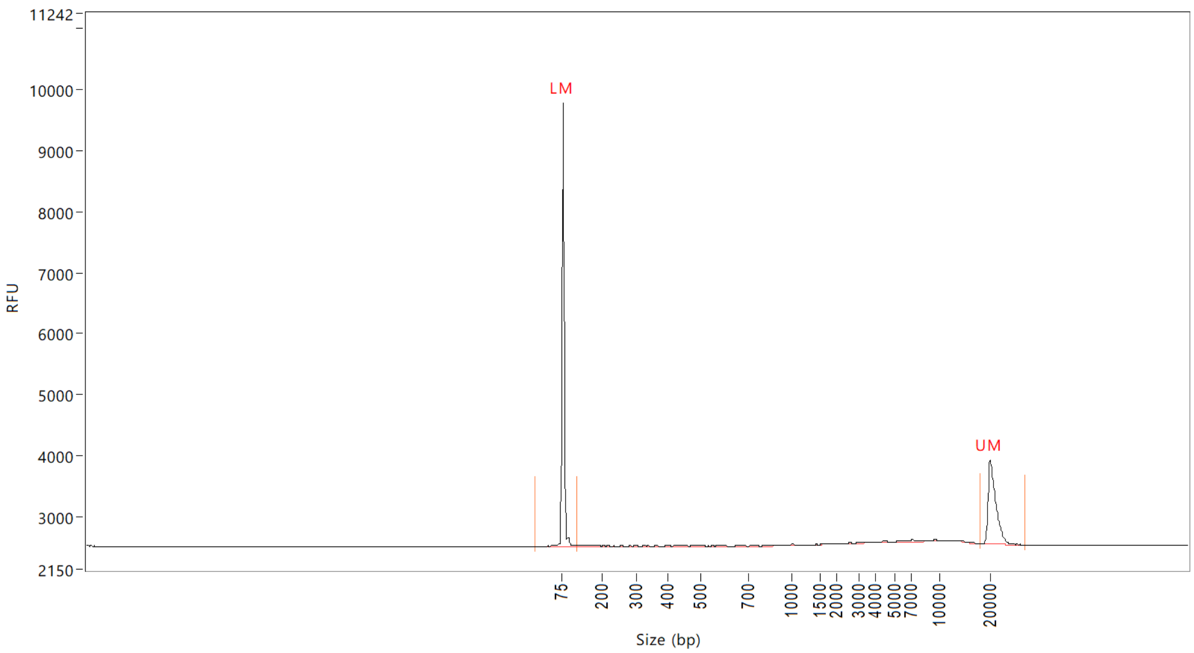

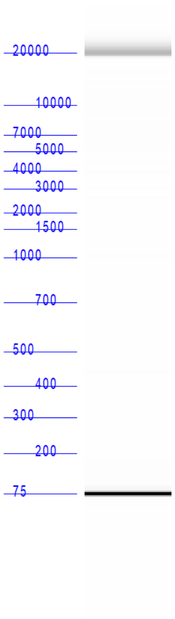

Peak	Size (bp)	Concentration (ng/uL)	From (bp)	To (bp)	Average size (bp)	CV%	RFU	Corrected peak area
1	75 (LM)	0.5000	0	118	73	9.15	7266	40.511
2	20000 (UM)	0.1691	18049	26891	20536	4.64	1365	13.699

TIC:	0.0000	ng/uL
TIM:	0.0000	nmole/L
Total concentration:	0.5672	ng/uL

Sample peak width (sec): 5 Sample min peak height: 50 Sample baseline V to V?: Y Sample baseline V to V points: 3
Sample filter: Binomial Number of points for filter: 3 Sample start region (min): 0 Sample end region (min): 30
Marker peak width (sec): 5 Marker min peak height: 200 Marker baseline V to V?: Y Marker baseline V to V points: 3
Lower marker selection: First peak > 500 RFU Upper marker selection: Last peak > 200 RFU
Ladder size (bp)75, 200, 300, 400, 500, 700, 1000, 1500, 2000, 3000, 4000, 5000, 7000, 10000, 20000
Quantification using: Lower Marker Final concentration (ng/uL): 0.5000 Dilution factor: 12.0

Sample: SampH10
Well location: H10
Created: Thursday, August 12, 2021 6:46:05 PM

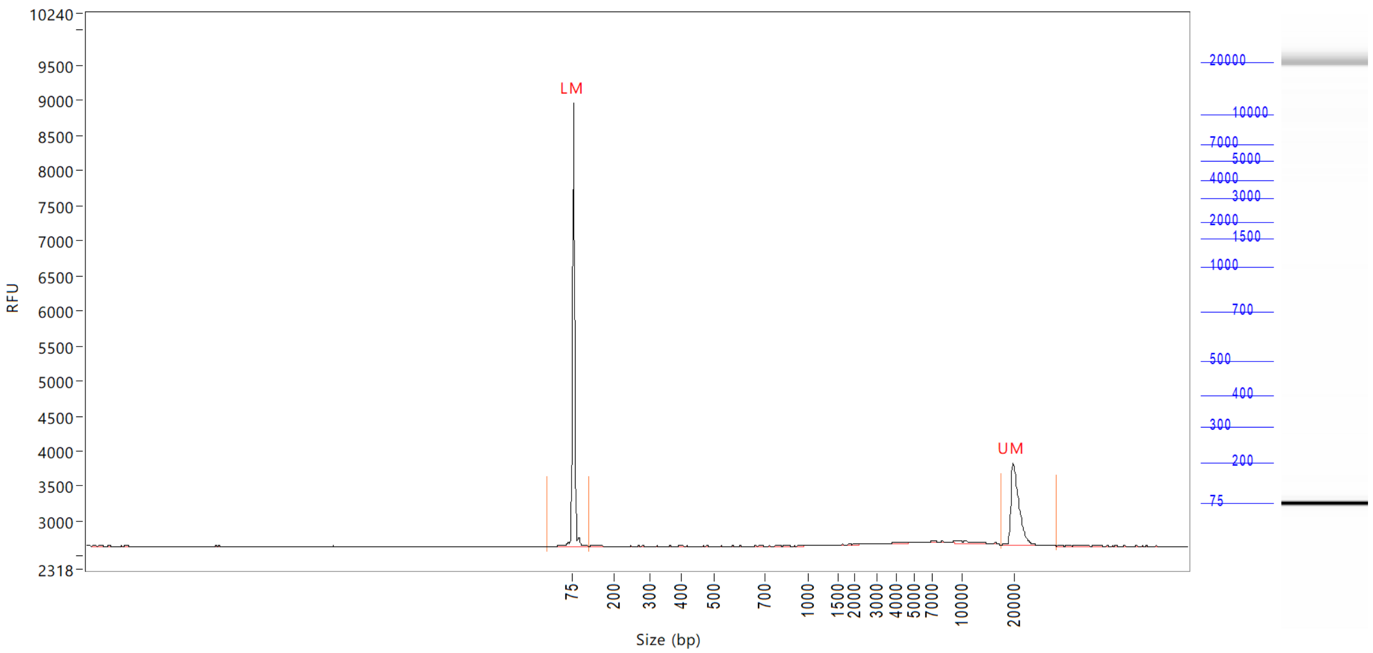

Peak	Size (bp)	Concentration (ng/uL)	From (bp)	To (bp)	Average size (bp)	CV%	RFU	Corrected peak area
1	75 (LM)	0.5000	0	120	73	8.68	6331	34.660
2	20000 (UM)	0.1676	17561	28342	20498	4.96	1161	11.621

TIC:	0.0000	ng/uL
TIM:	0.0000	nmole/L
Total concentration:	0.6368	ng/uL

```
Sample peak width (sec): 5    Sample min peak height: 50    Sample baseline V to V?: Y    Sample baseline V to V points: 3
Sample filter: Binomial       Number of points for filter: 3  Sample start region (min): 0  Sample end region (min): 30
Marker peak width (sec): 5    Marker min peak height: 200   Marker baseline V to V?: Y    Marker baseline V to V points: 3
Lower marker selection: First peak > 500 RFU                Upper marker selection: Last peak > 200 RFU
Ladder size (bp)75, 200, 300, 400, 500, 700, 1000, 1500, 2000, 3000, 4000, 5000, 7000, 10000, 20000
Quantification using: Lower Marker          Final concentration (ng/uL): 0.5000          Dilution factor: 12.0
```

Sample: SampA11
Well location: A11
Created: Thursday, August 12, 2021 6:46:05 PM

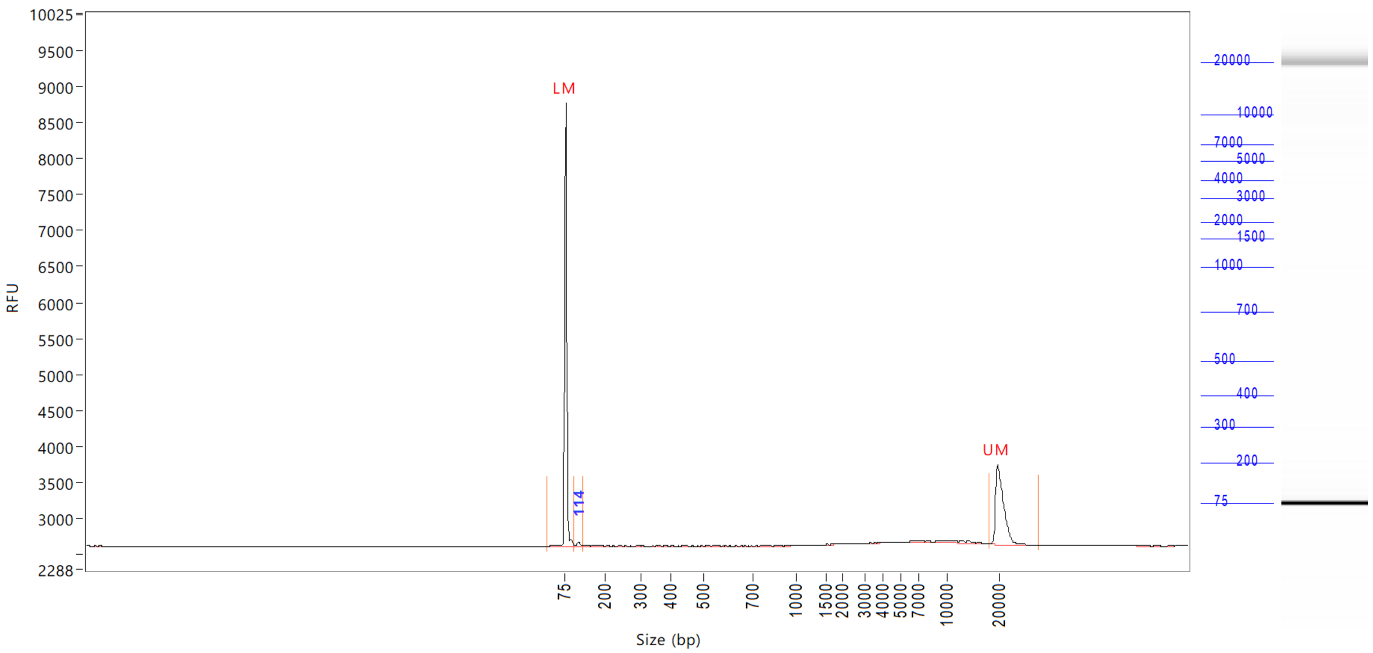

Peak	Size (bp)	Concentration (ng/uL)	From (bp)	To (bp)	Average size (bp)	CV%	RFU	Corrected peak area
1	75 (LM)	0.5000	20	103	73	6.09	6180	33.468
2	114	0.0866	103	128	114	5.35	57	0.483
3	20000 (UM)	0.1681	18171	27979	20491	4.98	1115	11.250

TIC:	0.0866	ng/uL
TIM:	1.2478	nmole/L
Total concentration:	0.7696	ng/uL

```
Sample peak width (sec): 5    Sample min peak height: 50    Sample baseline V to V?: Y    Sample baseline V to V points: 3
Sample filter: Binomial       Number of points for filter: 3  Sample start region (min): 0  Sample end region (min): 30
Marker peak width (sec): 5    Marker min peak height: 200   Marker baseline V to V?: Y    Marker baseline V to V points: 3
Lower marker selection: First peak > 500 RFU                Upper marker selection: Last peak > 200 RFU
Ladder size (bp)75, 200, 300, 400, 500, 700, 1000, 1500, 2000, 3000, 4000, 5000, 7000, 10000, 20000
Quantification using: Lower Marker          Final concentration (ng/uL): 0.5000          Dilution factor: 12.0
```

Sample: SampB11
Well location: B11
Created: Thursday, August 12, 2021 6:46:05 PM

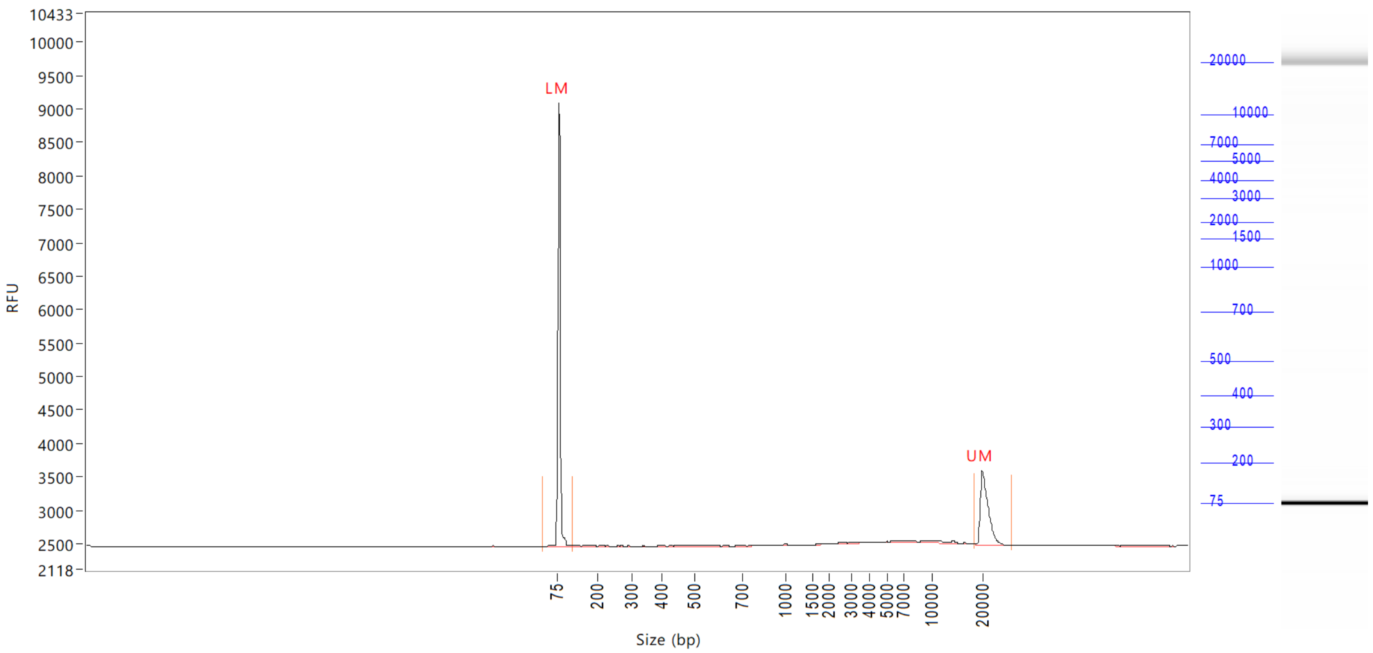

Peak	Size (bp)	Concentration (ng/uL)	From (bp)	To (bp)	Average size (bp)	CV%	RFU	Corrected peak area
1	75 (LM)	0.5000	26	118	74	6.69	6644	36.919
2	20000 (UM)	0.1508	18415	25924	20582	4.53	1099	11.133

TIC:	0.0000	ng/uL
TIM:	0.0000	nmole/L
Total concentration:	0.7798	ng/uL

Sample peak width (sec): 5   Sample min peak height: 50   Sample baseline V to V?: Y   Sample baseline V to V points: 3
Sample filter: Binomial   Number of points for filter: 3   Sample start region (min): 0   Sample end region (min): 30
Marker peak width (sec): 5   Marker min peak height: 200   Marker baseline V to V?: Y   Marker baseline V to V points: 3
Lower marker selection: First peak > 500 RFU   Upper marker selection: Last peak > 200 RFU
Ladder size (bp)75, 200, 300, 400, 500, 700, 1000, 1500, 2000, 3000, 4000, 5000, 7000, 10000, 20000
Quantification using: Lower Marker   Final concentration (ng/uL): 0.5000   Dilution factor: 12.0

Sample: SampC11
Well location: C11
Created: Thursday, August 12, 2021 6:46:05 PM

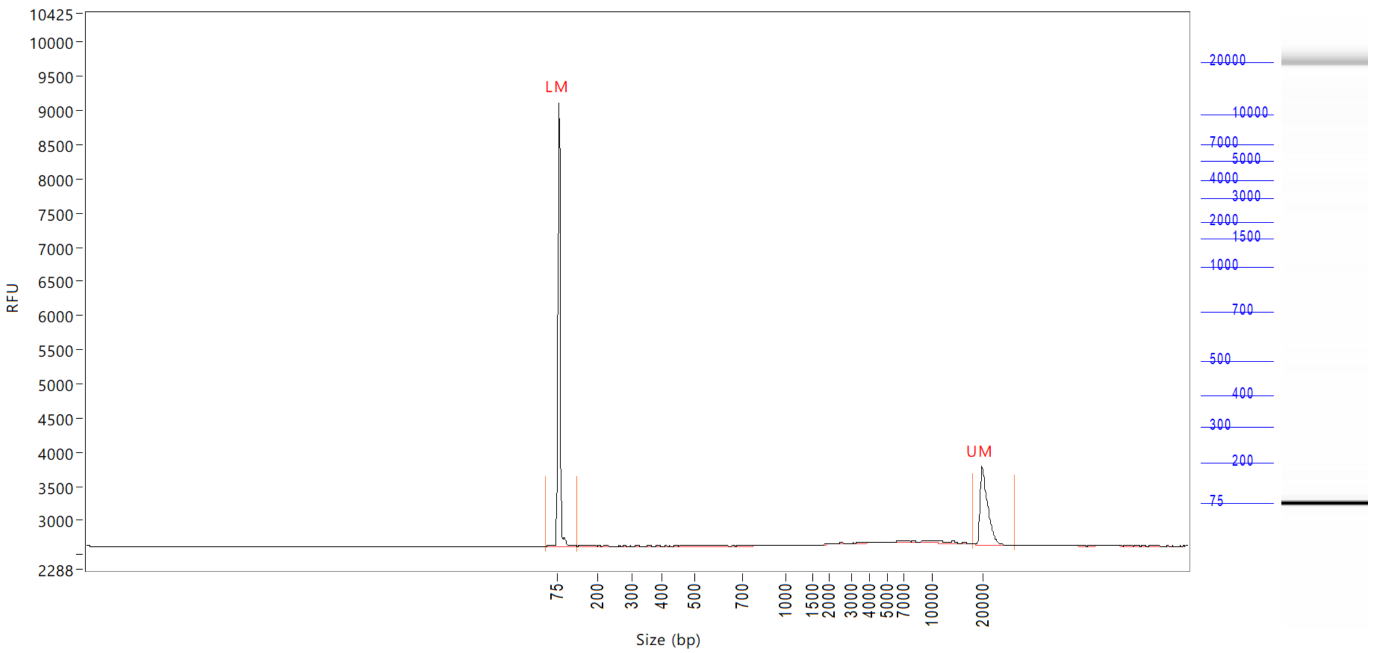

Peak	Size (bp)	Concentration (ng/uL)	From (bp)	To (bp)	Average size (bp)	CV%	RFU	Corrected peak area
1	75 (LM)	0.5000	33	133	74	7.35	6501	35.943
2	20000 (UM)	0.1577	18049	26408	20521	4.57	1141	11.334

TIC:	0.0000	ng/uL
TIM:	0.0000	nmole/L
Total concentration:	0.7184	ng/uL

Sample peak width (sec): 5 Sample min peak height: 50 Sample baseline V to V?: Y Sample baseline V to V points: 3
Sample filter: Binomial Number of points for filter: 3 Sample start region (min): 0 Sample end region (min): 30
Marker peak width (sec): 5 Marker min peak height: 200 Marker baseline V to V?: Y Marker baseline V to V points: 3
Lower marker selection: First peak > 500 RFU Upper marker selection: Last peak > 200 RFU
Ladder size (bp)75, 200, 300, 400, 500, 700, 1000, 1500, 2000, 3000, 4000, 5000, 7000, 10000, 20000
Quantification using: Lower Marker Final concentration (ng/uL): 0.5000 Dilution factor: 12.0

Sample: SampD11
Well location: D11
Created: Thursday, August 12, 2021 6:46:05 PM

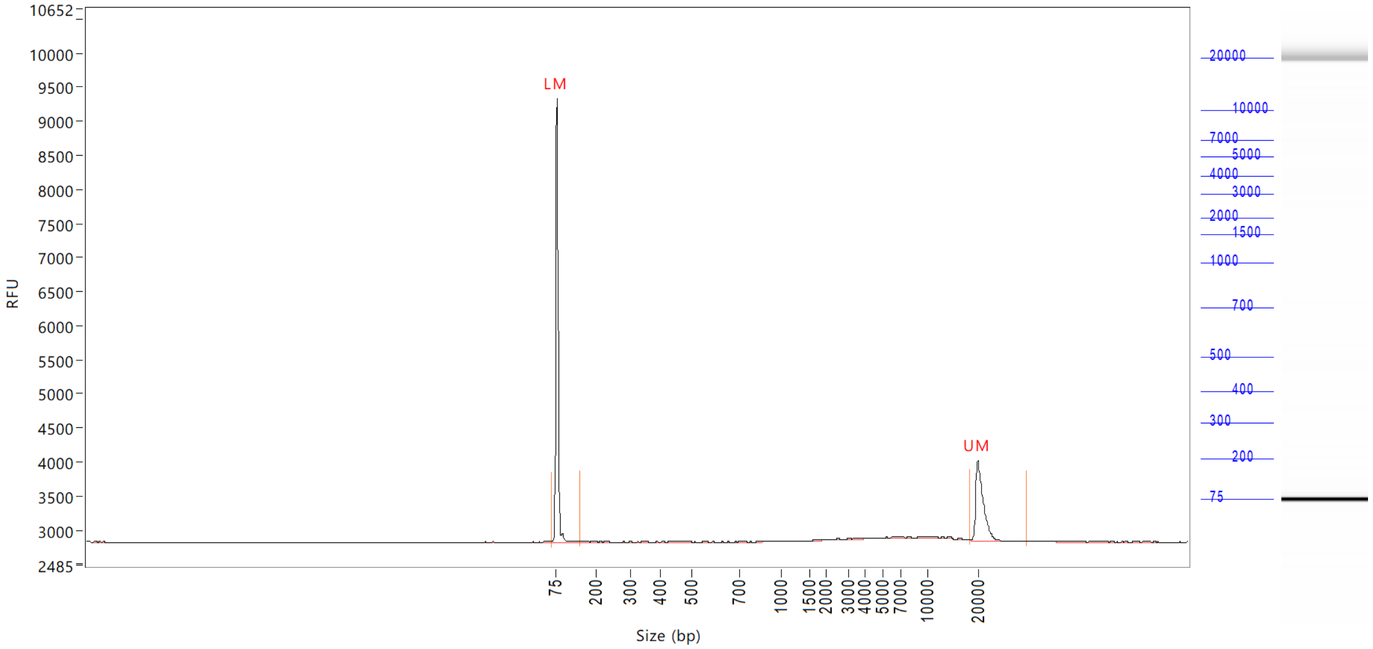

Peak	Size (bp)	Concentration (ng/uL)	From (bp)	To (bp)	Average size (bp)	CV%	RFU	Corrected peak area
1	75 (LM)	0.5000	62	150	73	8.29	6524	36.397
2	20000 (UM)	0.1619	18293	29793	20551	5.36	1171	11.784

TIC:	0.0000	ng/uL
TIM:	0.0000	nmole/L
Total concentration:	0.6225	ng/uL

Sample peak width (sec): 5   Sample min peak height: 50   Sample baseline V to V?: Y   Sample baseline V to V points: 3
Sample filter: Binomial   Number of points for filter: 3 Sample start region (min): 0 Sample end region (min): 30
Marker peak width (sec): 5   Marker min peak height: 200   Marker baseline V to V?: Y   Marker baseline V to V points: 3
Lower marker selection: First peak > 500 RFU   Upper marker selection: Last peak > 200 RFU
Ladder size (bp)75, 200, 300, 400, 500, 700, 1000, 1500, 2000, 3000, 4000, 5000, 7000, 10000, 20000
Quantification using: Lower Marker   Final concentration (ng/uL): 0.5000   Dilution factor: 12.0

Sample: SampE11
Well location: E11
Created: Thursday, August 12, 2021 6:46:05 PM

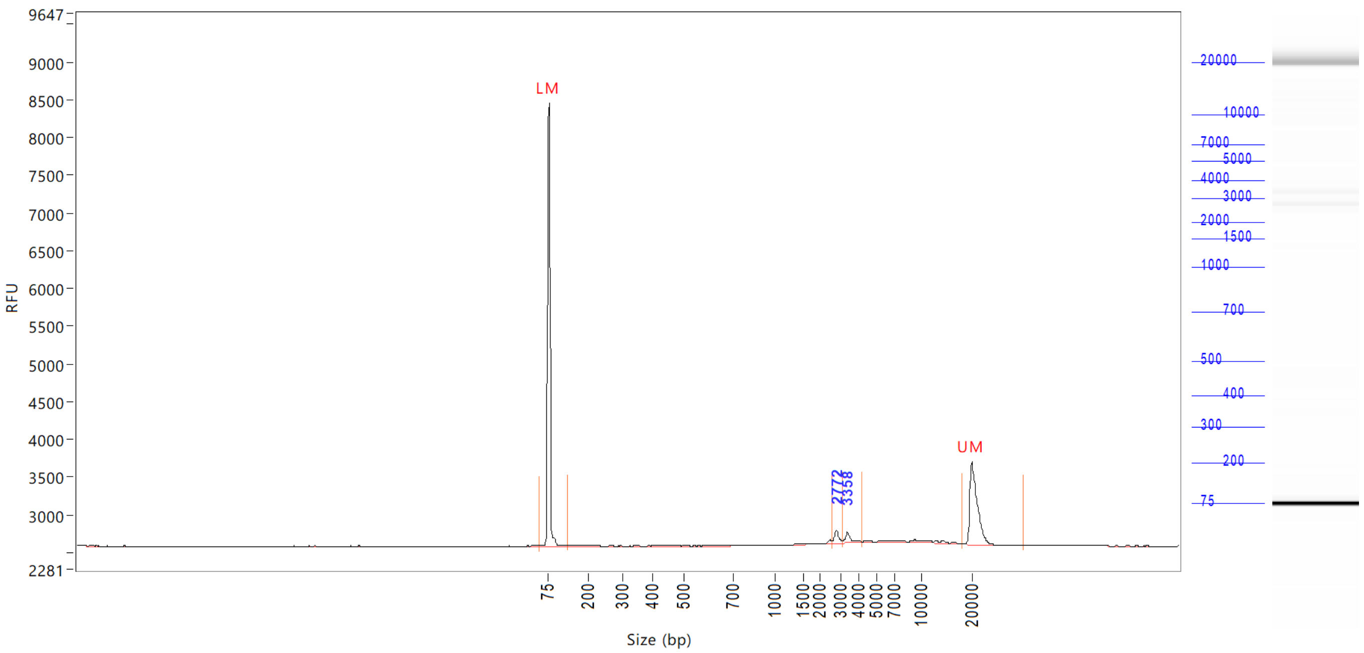

Peak	Size (bp)	Concentration (ng/uL)	From (bp)	To (bp)	Average size (bp)	CV%	RFU	Corrected peak area
1	75 (LM)	0.5000	45	135	73	7.59	5884	33.620
2	2772	0.2112	2572	3072	2784	4.34	174	1.184
3	3358	0.2228	3072	4173	3460	7.58	139	1.249
4	20000 (UM)	0.1610	18171	30277	20558	5.18	1093	10.824

TIC:	0.4340	ng/uL
TIM:	0.2309	nmole/L
Total concentration:	1.1658	ng/uL

Sample peak width (sec): 5   Sample min peak height: 50   Sample baseline V to V?: Y   Sample baseline V to V points: 3
Sample filter: Binomial   Number of points for filter: 3 Sample start region (min): 0 Sample end region (min): 30
Marker peak width (sec): 5   Marker min peak height: 200   Marker baseline V to V?: Y   Marker baseline V to V points: 3
Lower marker selection: First peak > 500 RFU   Upper marker selection: Last peak > 200 RFU
Ladder size (bp)75, 200, 300, 400, 500, 700, 1000, 1500, 2000, 3000, 4000, 5000, 7000, 10000, 20000
Quantification using: Lower Marker   Final concentration (ng/uL): 0.5000   Dilution factor: 12.0

Sample: SampF11
Well location: F11
Created: Thursday, August 12, 2021 6:46:05 PM

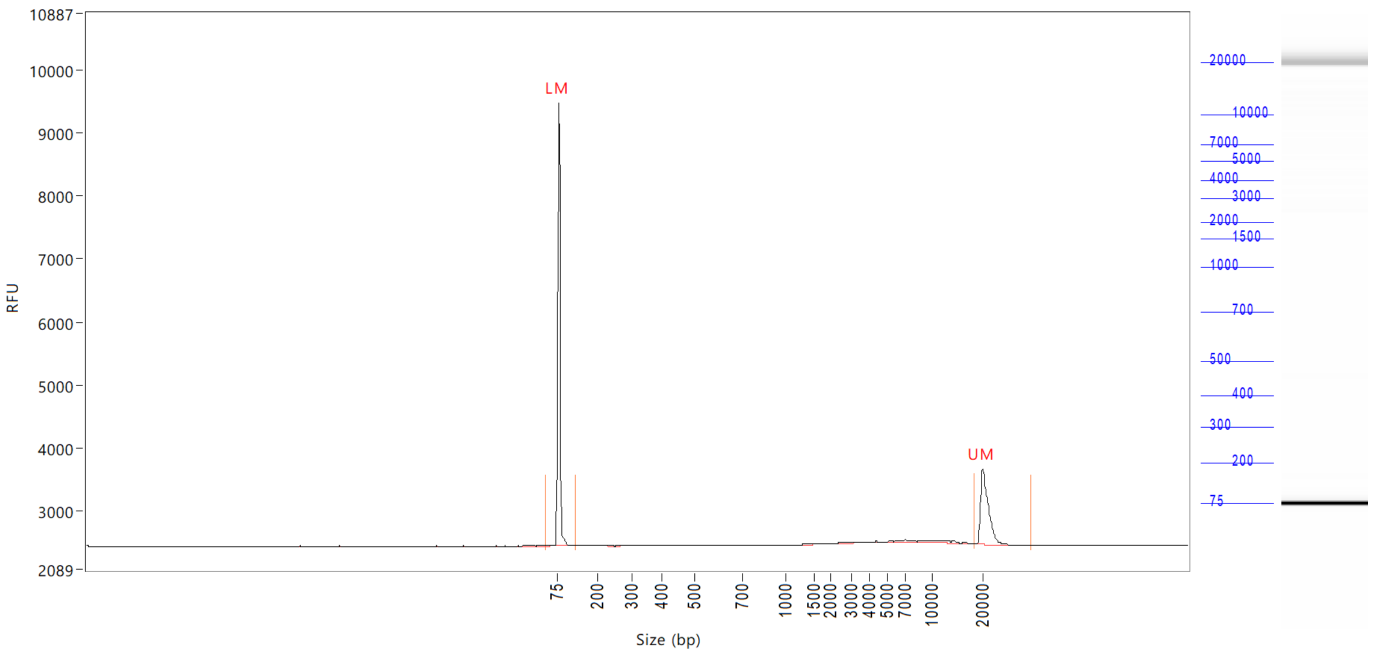

Peak	Size (bp)	Concentration (ng/uL)	From (bp)	To (bp)	Average size (bp)	CV%	RFU	Corrected peak area
1	75 (LM)	0.5000	35	128	73	6.57	7024	38.458
2	20000 (UM)	0.1576	18415	29672	20522	4.95	1202	12.124

TIC:	0.0000	ng/uL
TIM:	0.0000	nmole/L
Total concentration:	0.6693	ng/uL

Sample peak width (sec): 5   Sample min peak height: 50   Sample baseline V to V?: Y   Sample baseline V to V points: 3
Sample filter: Binomial   Number of points for filter: 3 Sample start region (min): 0 Sample end region (min): 30
Marker peak width (sec): 5   Marker min peak height: 200   Marker baseline V to V?: Y   Marker baseline V to V points: 3
Lower marker selection: First peak > 500 RFU   Upper marker selection: Last peak > 200 RFU
Ladder size (bp)75, 200, 300, 400, 500, 700, 1000, 1500, 2000, 3000, 4000, 5000, 7000, 10000, 20000
Quantification using: Lower Marker   Final concentration (ng/uL): 0.5000   Dilution factor: 12.0

Sample: SampG11
Well location: G11
Created: Thursday, August 12, 2021 6:46:05 PM

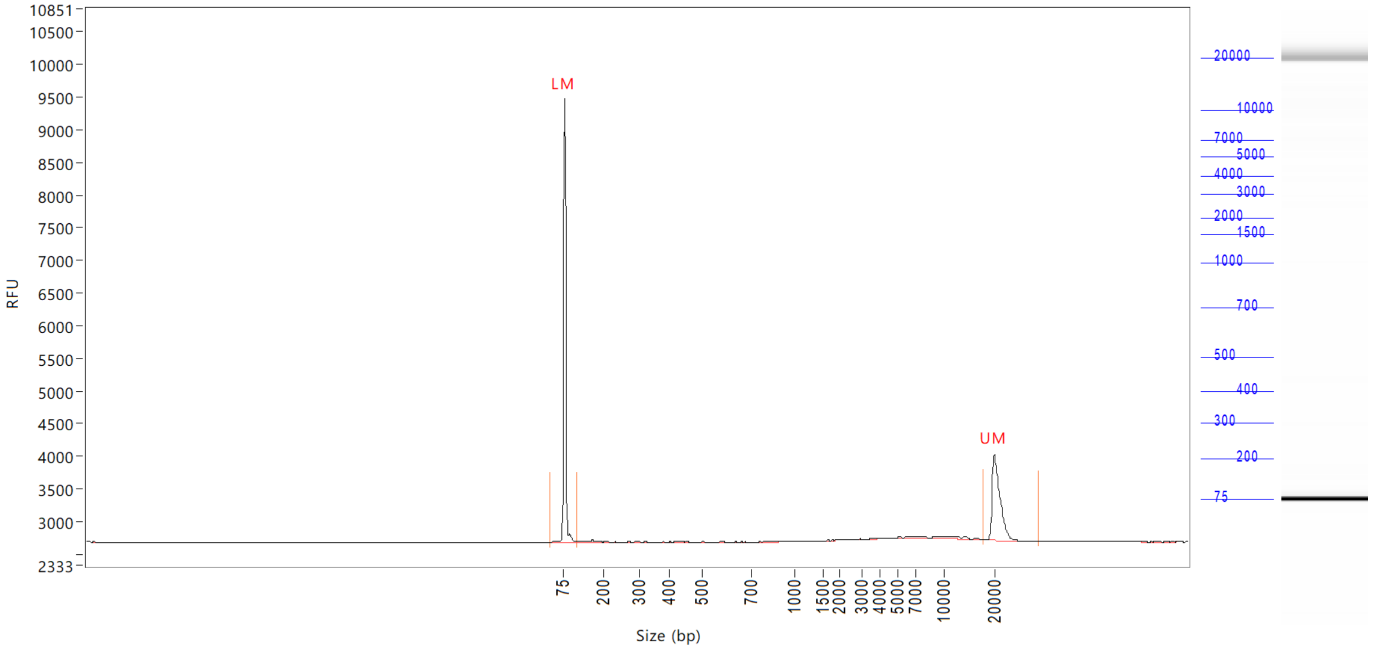

Peak	Size (bp)	Concentration (ng/uL)	From (bp)	To (bp)	Average size (bp)	CV%	RFU	Corrected peak area
1	75 (LM)	0.5000	31	116	74	6.72	6807	38.758
2	20000 (UM)	0.1723	17683	28584	20528	5.06	1326	13.359

TIC:	0.0000	ng/uL
TIM:	0.0000	nmole/L
Total concentration:	0.6716	ng/uL

Sample peak width (sec): 5 Sample min peak height: 50 Sample baseline V to V?: Y Sample baseline V to V points: 3
Sample filter: Binomial Number of points for filter: 3 Sample start region (min): 0 Sample end region (min): 30
Marker peak width (sec): 5 Marker min peak height: 200 Marker baseline V to V?: Y Marker baseline V to V points: 3
Lower marker selection: First peak > 500 RFU Upper marker selection: Last peak > 200 RFU
Ladder size (bp)75, 200, 300, 400, 500, 700, 1000, 1500, 2000, 3000, 4000, 5000, 7000, 10000, 20000
Quantification using: Lower Marker Final concentration (ng/uL): 0.5000 Dilution factor: 12.0

Sample: SampH11
Well location: H11
Created: Thursday, August 12, 2021 6:46:05 PM

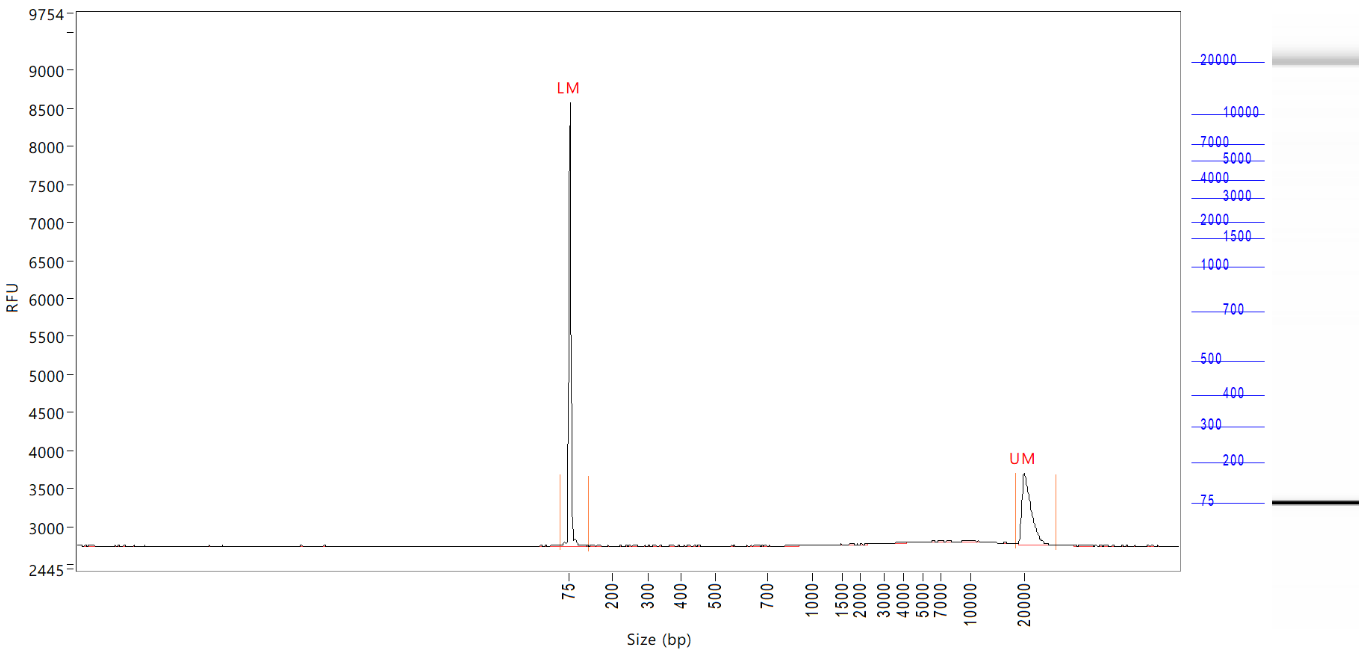

Peak	Size (bp)	Concentration (ng/uL)	From (bp)	To (bp)	Average size (bp)	CV%	RFU	Corrected peak area
1	75 (LM)	0.5000	50	131	73	5.99	5838	31.763
2	20000 (UM)	0.1675	18293	25803	20569	4.91	932	10.641

TIC:	0.0000	ng/uL
TIM:	0.0000	nmole/L
Total concentration:	0.4959	ng/uL

Sample peak width (sec): 5 Sample min peak height: 50 Sample baseline V to V?: Y Sample baseline V to V points: 3
Sample filter: Binomial Number of points for filter: 3 Sample start region (min): 0 Sample end region (min): 30
Marker peak width (sec): 5 Marker min peak height: 200 Marker baseline V to V?: Y Marker baseline V to V points: 3
Lower marker selection: First peak > 500 RFU Upper marker selection: Last peak > 200 RFU
Ladder size (bp)75, 200, 300, 400, 500, 700, 1000, 1500, 2000, 3000, 4000, 5000, 7000, 10000, 20000
Quantification using: Lower Marker Final concentration (ng/uL): 0.5000 Dilution factor: 12.0

Sample: SampA12
Well location: A12
Created: Thursday, August 12, 2021 6:46:05 PM

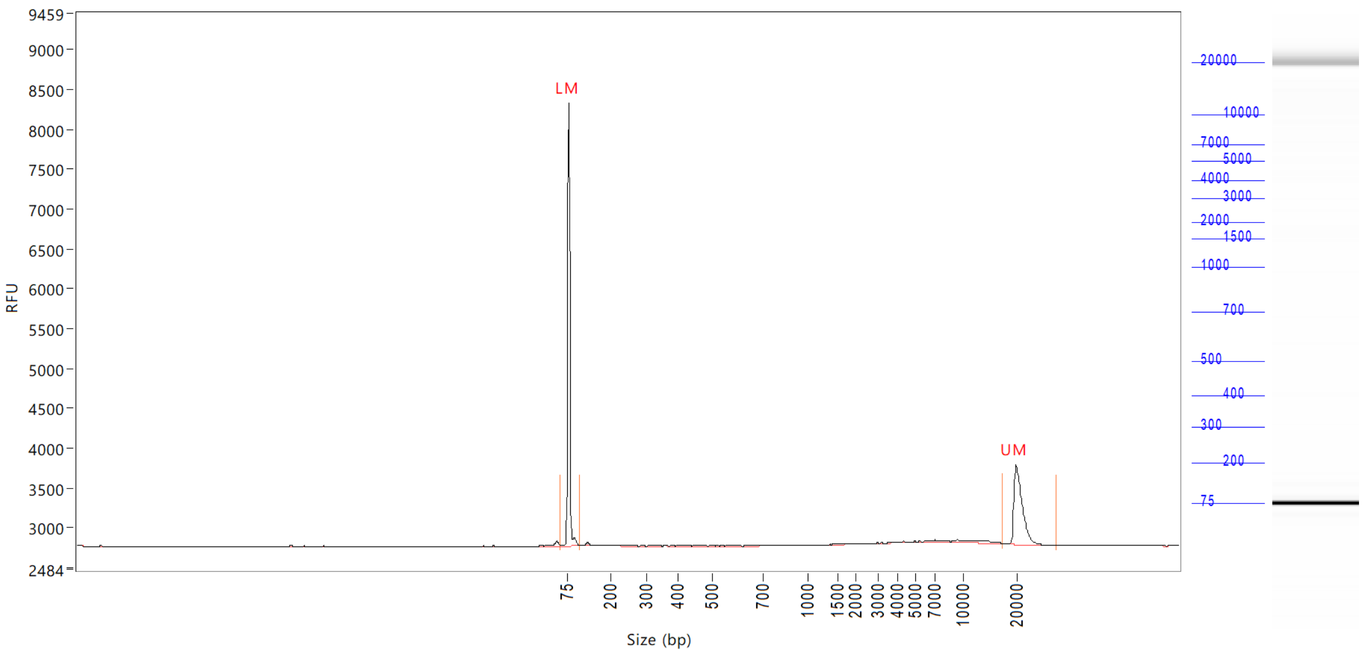

Peak	Size (bp)	Concentration (ng/uL)	From (bp)	To (bp)	Average size (bp)	CV%	RFU	Corrected peak area
1	75 (LM)	0.5000	54	110	74	5.82	5569	30.122
2	20000 (UM)	0.1689	17439	27617	20462	4.79	1003	10.174

TIC:	0.0000	ng/uL
TIM:	0.0000	nmole/L
Total concentration:	0.8182	ng/uL

Sample peak width (sec): 5   Sample min peak height: 50   Sample baseline V to V?: Y   Sample baseline V to V points: 3
Sample filter: Binomial   Number of points for filter: 3 Sample start region (min): 0 Sample end region (min): 30
Marker peak width (sec): 5   Marker min peak height: 200   Marker baseline V to V?: Y   Marker baseline V to V points: 3
Lower marker selection: First peak > 500 RFU   Upper marker selection: Last peak > 200 RFU
Ladder size (bp)75, 200, 300, 400, 500, 700, 1000, 1500, 2000, 3000, 4000, 5000, 7000, 10000, 20000
Quantification using: Lower Marker   Final concentration (ng/uL): 0.5000   Dilution factor: 12.0

Sample: SampB12
Well location: B12
Created: Thursday, August 12, 2021 6:46:05 PM

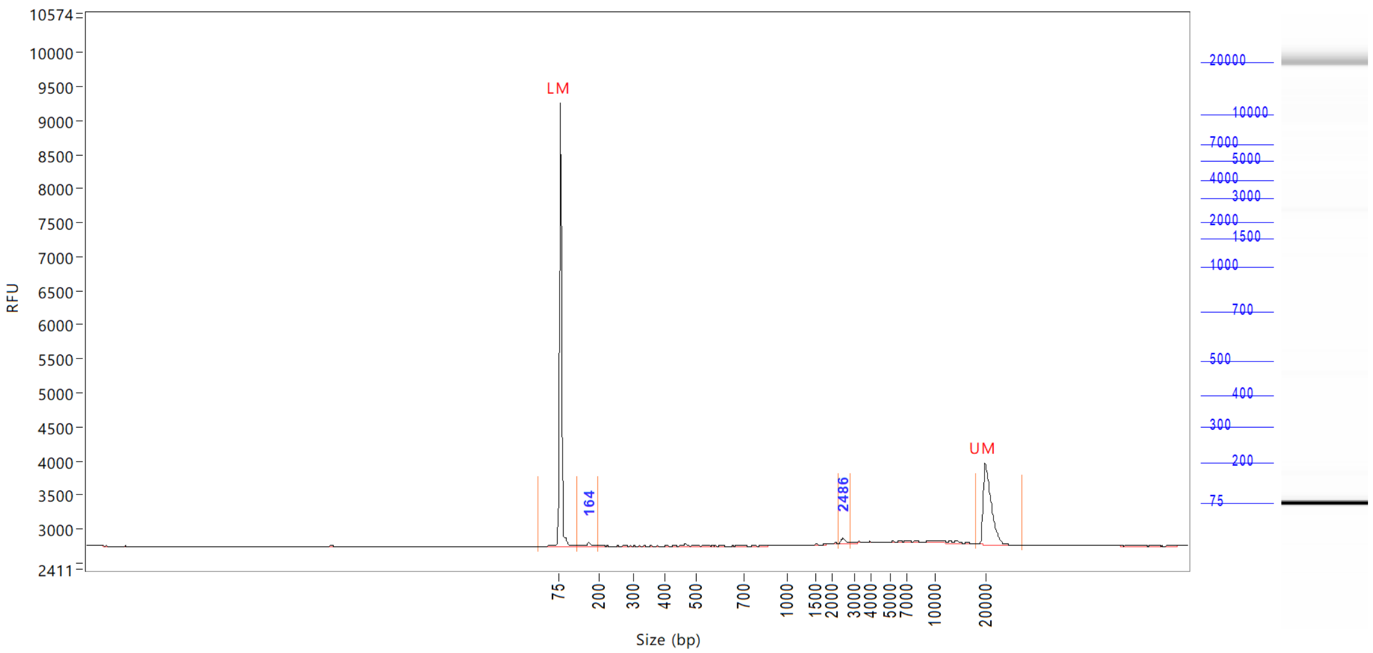

Peak	Size (bp)	Concentration (ng/uL)	From (bp)	To (bp)	Average size (bp)	CV%	RFU	Corrected peak area
1	75 (LM)	0.5000	7	128	74	8.62	6523	37.473
2	164	0.1392	128	192	159	9.60	53	0.870
3	2486	0.0924	2286	2829	2510	4.69	81	0.577
4	20000 (UM)	0.1626	18049	27375	20562	4.60	1193	12.185

TIC:	0.2316	ng/uL
TIM:	1.4998	nmole/L
Total concentration:	0.8744	ng/uL

Sample peak width (sec): 5   Sample min peak height: 50   Sample baseline V to V?: Y   Sample baseline V to V points: 3
Sample filter: Binomial   Number of points for filter: 3   Sample start region (min): 0   Sample end region (min): 30
Marker peak width (sec): 5   Marker min peak height: 200   Marker baseline V to V?: Y   Marker baseline V to V points: 3
Lower marker selection: First peak > 500 RFU   Upper marker selection: Last peak > 200 RFU
Ladder size (bp)75, 200, 300, 400, 500, 700, 1000, 1500, 2000, 3000, 4000, 5000, 7000, 10000, 20000
Quantification using: Lower Marker   Final concentration (ng/uL): 0.5000   Dilution factor: 12.0

Sample: SampC12
Well location: C12
Created: Thursday, August 12, 2021 6:46:05 PM

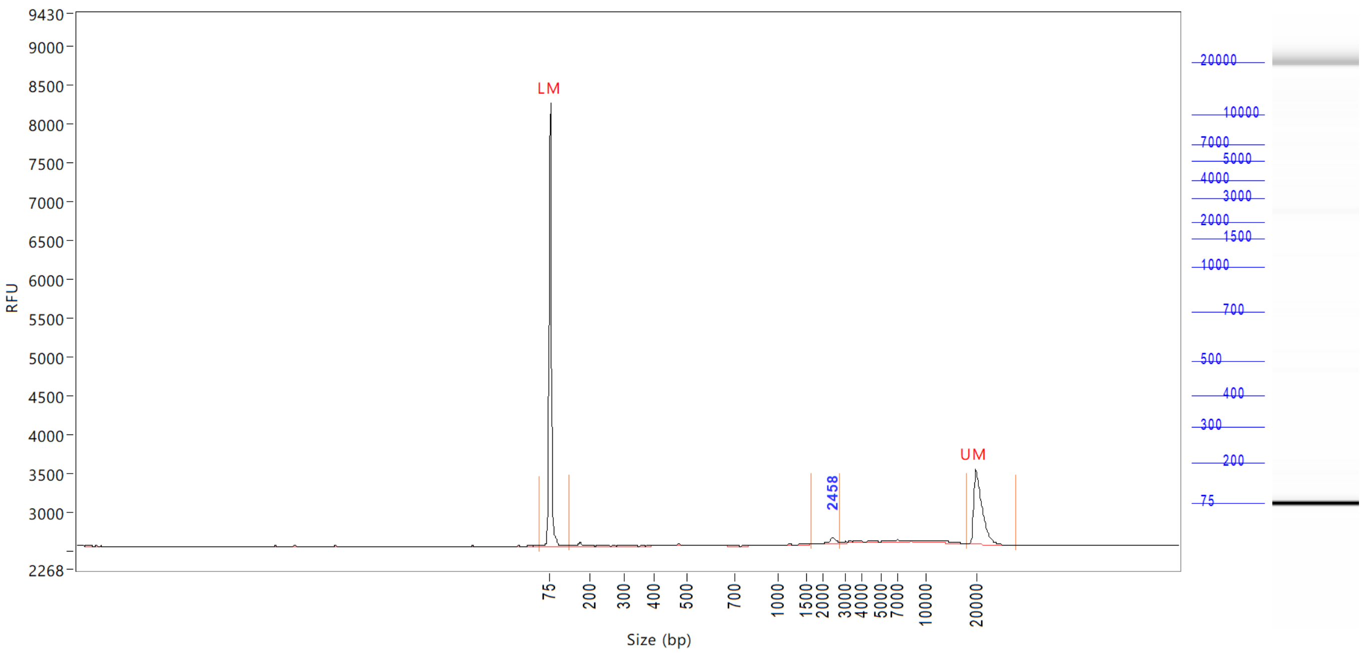

Peak	Size (bp)	Concentration (ng/uL)	From (bp)	To (bp)	Average size (bp)	CV%	RFU	Corrected peak area
1	75 (LM)	0.5000	41	135	73	7.85	5721	34.218
2	2458	0.1506	1635	2772	2372	9.66	81	0.859
3	20000 (UM)	0.1513	18049	27738	20618	5.39	964	10.354

TIC:	0.1506	ng/uL
TIM:	0.1045	nmole/L
Total concentration:	0.9595	ng/uL

```
Sample peak width (sec): 5    Sample min peak height: 50    Sample baseline V to V?: Y    Sample baseline V to V points: 3
Sample filter: Binomial       Number of points for filter: 3  Sample start region (min): 0  Sample end region (min): 30
Marker peak width (sec): 5    Marker min peak height: 200   Marker baseline V to V?: Y    Marker baseline V to V points: 3
Lower marker selection: First peak > 500 RFU                Upper marker selection: Last peak > 200 RFU
Ladder size (bp)75, 200, 300, 400, 500, 700, 1000, 1500, 2000, 3000, 4000, 5000, 7000, 10000, 20000
Quantification using: Lower Marker          Final concentration (ng/uL): 0.5000          Dilution factor: 12.0
```

Sample: SampD12
Well location: D12
Created: Thursday, August 12, 2021 6:46:05 PM

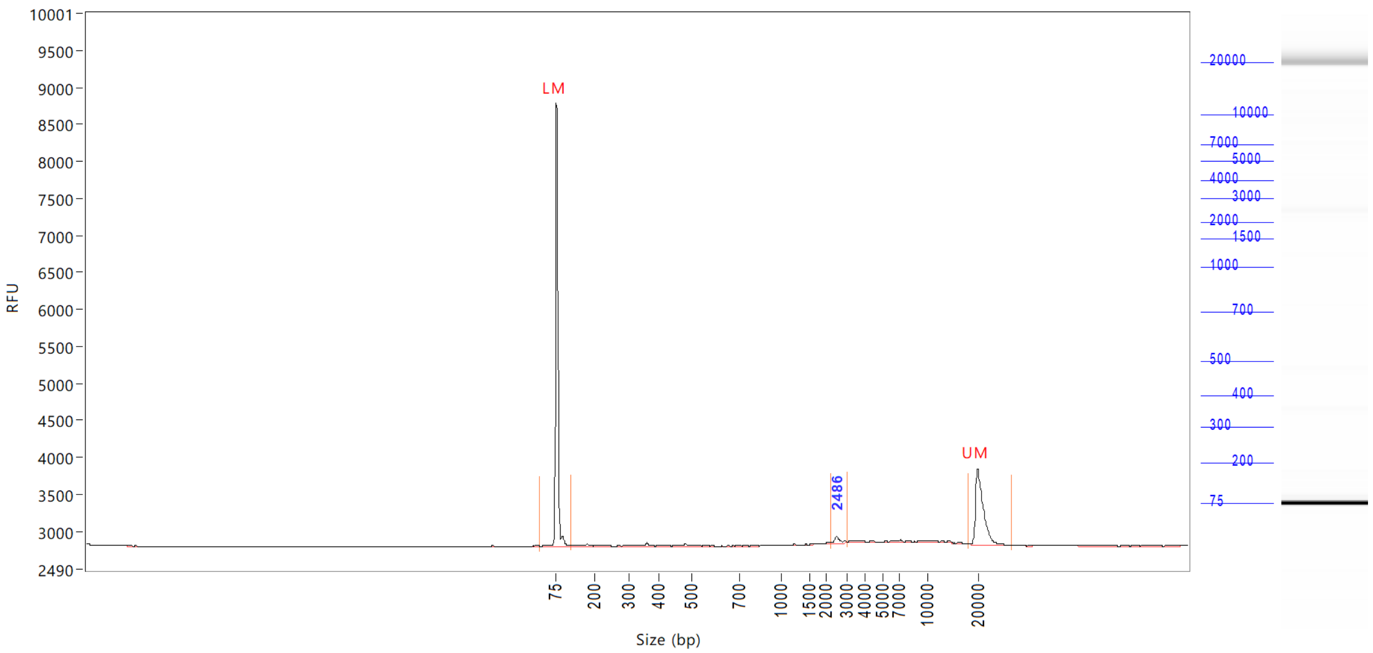

Peak	Size (bp)	Concentration (ng/uL)	From (bp)	To (bp)	Average size (bp)	CV%	RFU	Corrected peak area
1	75 (LM)	0.5000	24	120	74	7.15	5999	34.607
2	2486	0.1490	2258	3000	2551	7.12	98	0.859
3	20000 (UM)	0.1505	18293	26891	20638	5.05	1015	10.418

TIC:	0.1490	ng/uL
TIM:	0.0961	nmole/L
Total concentration:	0.8657	ng/uL

Sample peak width (sec): 5 Sample min peak height: 50 Sample baseline V to V?: Y Sample baseline V to V points: 3
Sample filter: Binomial Number of points for filter: 3 Sample start region (min): 0 Sample end region (min): 30
Marker peak width (sec): 5 Marker min peak height: 200 Marker baseline V to V?: Y Marker baseline V to V points: 3
Lower marker selection: First peak > 500 RFU Upper marker selection: Last peak > 200 RFU
Ladder size (bp)75, 200, 300, 400, 500, 700, 1000, 1500, 2000, 3000, 4000, 5000, 7000, 10000, 20000
Quantification using: Lower Marker Final concentration (ng/uL): 0.5000 Dilution factor: 12.0

Sample: SampE12
Well location: E12
Created: Thursday, August 12, 2021 6:46:05 PM

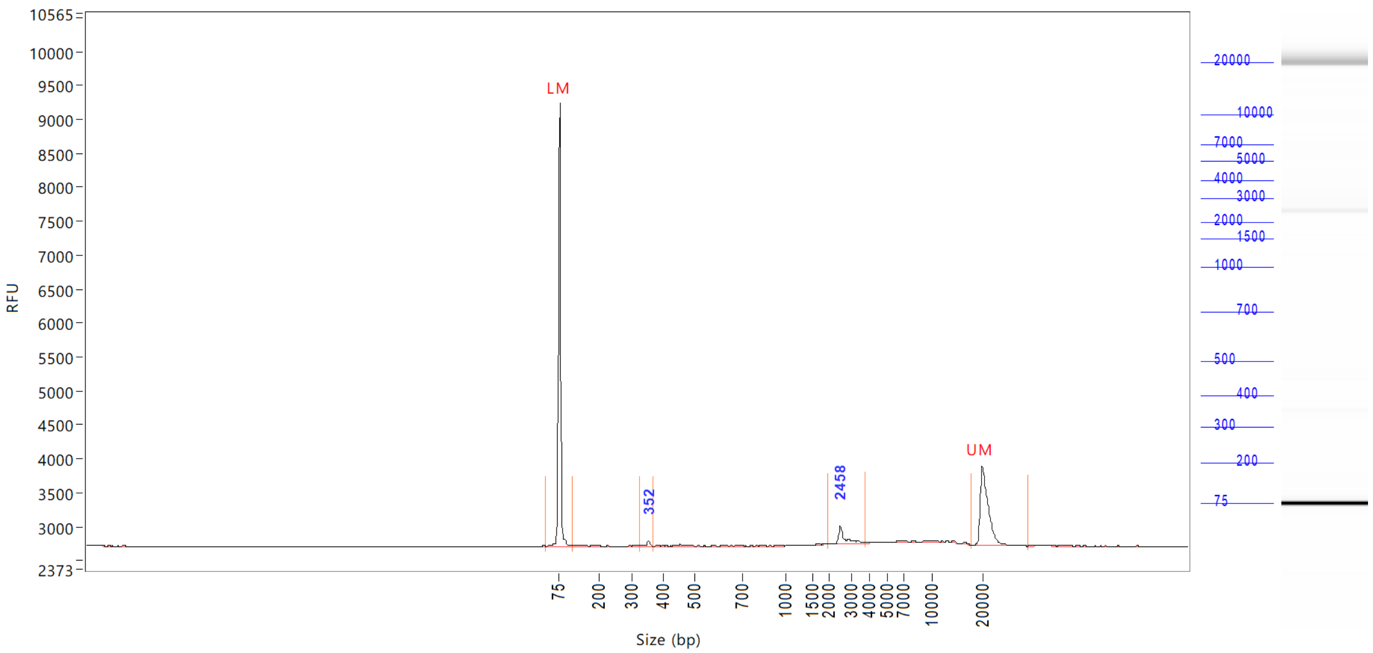

Peak	Size (bp)	Concentration (ng/uL)	From (bp)	To (bp)	Average size (bp)	CV%	RFU	Corrected peak area
1	75 (LM)	0.5000	35	116	73	6.65	6546	36.139
2	352	0.0862	321	368	345	2.49	74	0.519
3	2458	0.4246	1962	3786	2685	13.76	259	2.557
4	20000 (UM)	0.1642	17805	29068	20620	5.26	1164	11.867

TIC:	0.5108	ng/uL
TIM:	0.6715	nmole/L
Total concentration:	1.0653	ng/uL

Sample peak width (sec): 5   Sample min peak height: 50   Sample baseline V to V?: Y   Sample baseline V to V points: 3
Sample filter: Binomial   Number of points for filter: 3 Sample start region (min): 0 Sample end region (min): 30
Marker peak width (sec): 5   Marker min peak height: 200   Marker baseline V to V?: Y   Marker baseline V to V points: 3
Lower marker selection: First peak > 500 RFU   Upper marker selection: Last peak > 200 RFU
Ladder size (bp)75, 200, 300, 400, 500, 700, 1000, 1500, 2000, 3000, 4000, 5000, 7000, 10000, 20000
Quantification using: Lower Marker   Final concentration (ng/uL): 0.5000   Dilution factor: 12.0

Sample: SampF12
Well location: F12
Created: Thursday, August 12, 2021 6:46:05 PM

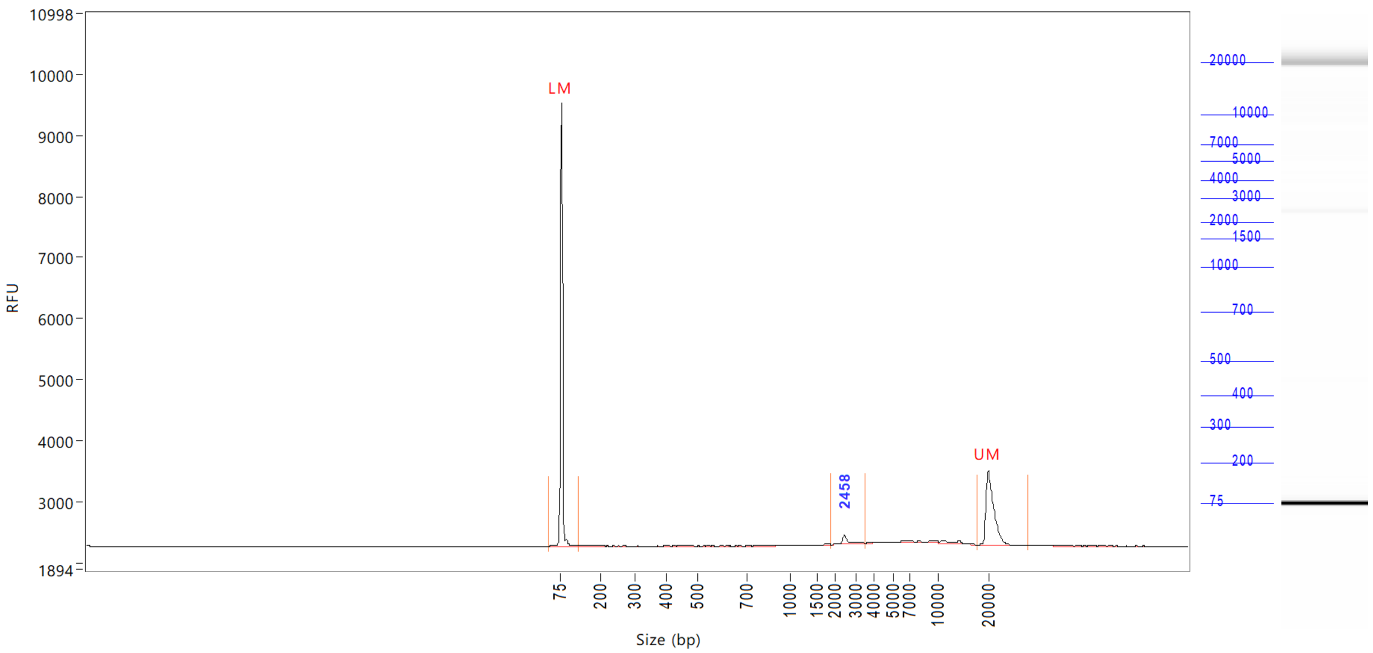

Peak	Size (bp)	Concentration (ng/uL)	From (bp)	To (bp)	Average size (bp)	CV%	RFU	Corrected peak area
1	75 (LM)	0.5000	35	129	73	7.13	7274	39.716
2	2458	0.1877	1923	3500	2546	10.80	147	1.243
3	20000 (UM)	0.1530	17683	27738	20515	4.79	1215	12.155

TIC:	0.1877	ng/uL
TIM:	0.1214	nmole/L
Total concentration:	0.7562	ng/uL

Sample peak width (sec): 5 Sample min peak height: 50 Sample baseline V to V?: Y Sample baseline V to V points: 3
Sample filter: Binomial Number of points for filter: 3 Sample start region (min): 0 Sample end region (min): 30
Marker peak width (sec): 5 Marker min peak height: 200 Marker baseline V to V?: Y Marker baseline V to V points: 3
Lower marker selection: First peak > 500 RFU Upper marker selection: Last peak > 200 RFU
Ladder size (bp)75, 200, 300, 400, 500, 700, 1000, 1500, 2000, 3000, 4000, 5000, 7000, 10000, 20000
Quantification using: Lower Marker Final concentration (ng/uL): 0.5000 Dilution factor: 12.0

Sample: SampG12
Well location: G12
Created: Thursday, August 12, 2021 6:46:05 PM

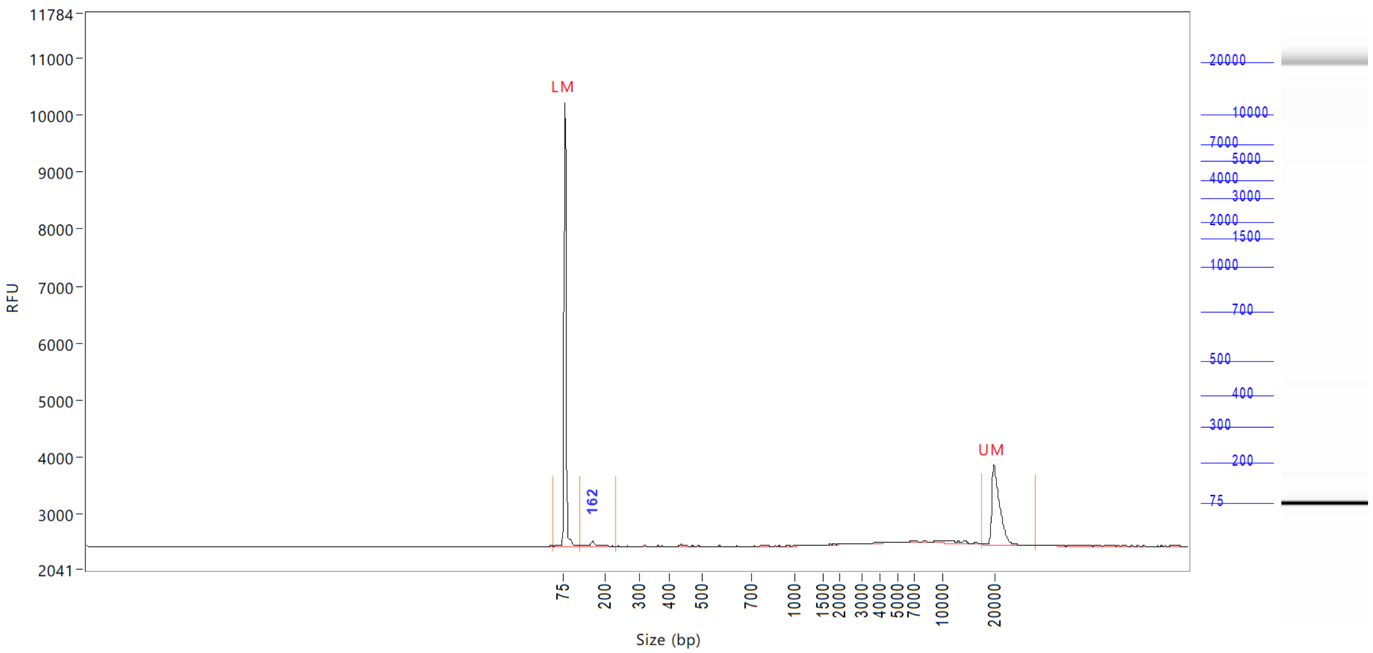

Peak	Size (bp)	Concentration (ng/uL)	From (bp)	To (bp)	Average size (bp)	CV%	RFU	Corrected peak area
1	75 (LM)	0.5000	39	122	74	6.60	7785	42.363
2	162	0.1913	122	230	162	12.03	97	1.351
3	20000 (UM)	0.1657	17683	28221	20566	4.78	1409	14.040

TIC:	0.1913	ng/uL
TIM:	1.9412	nmole/L
Total concentration:	0.7672	ng/uL

Sample peak width (sec): 5 Sample min peak height: 50 Sample baseline V to V?: Y Sample baseline V to V points: 3
Sample filter: Binomial Number of points for filter: 3 Sample start region (min): 0 Sample end region (min): 30
Marker peak width (sec): 5 Marker min peak height: 200 Marker baseline V to V?: Y Marker baseline V to V points: 3
Lower marker selection: First peak > 500 RFU Upper marker selection: Last peak > 200 RFU
Ladder size (bp)75, 200, 300, 400, 500, 700, 1000, 1500, 2000, 3000, 4000, 5000, 7000, 10000, 20000
Quantification using: Lower Marker Final concentration (ng/uL): 0.5000 Dilution factor: 12.0

Sample: SampH12
Well location: H12
Created: Thursday, August 12, 2021 6:46:05 PM

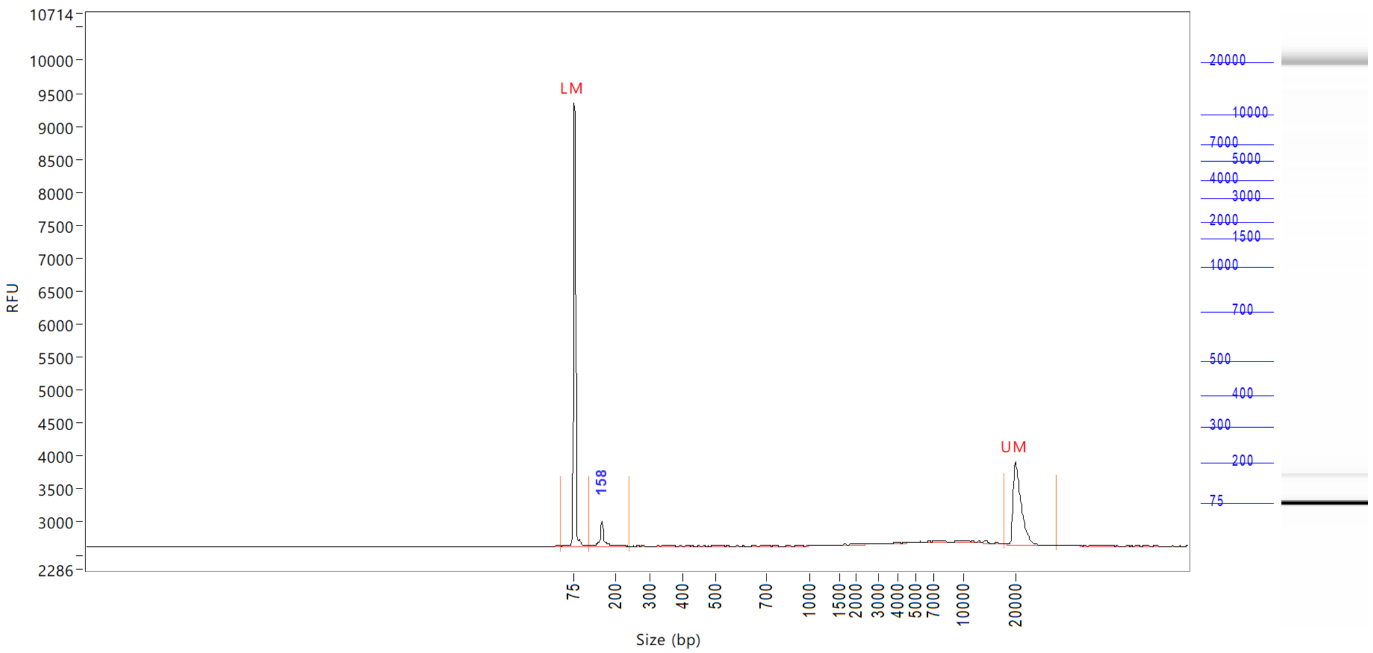

Peak	Size (bp)	Concentration (ng/uL)	From (bp)	To (bp)	Average size (bp)	CV%	RFU	Corrected peak area
1	75 (LM)	0.5000	37	118	74	6.45	6737	37.091
2	158	0.5938	118	237	161	11.14	382	3.671
3	20000 (UM)	0.1695	18049	27859	20573	5.00	1272	12.572

TIC:	0.5938	ng/uL
TIM:	6.0619	nmole/L
Total concentration:	1.1602	ng/uL

```
Sample peak width (sec): 5    Sample min peak height: 200   Sample baseline V to V?: Y    Sample baseline V to V points: 3
Sample filter: Binomial       Number of points for filter: 3  Sample start region (min): 0  Sample end region (min): 30
Marker peak width (sec): 5    Marker min peak height: 200   Marker baseline V to V?: Y    Marker baseline V to V points: 3
Lower marker selection: First peak > 500 RFU                Upper marker selection: Last peak > 200 RFU
Ladder size (bp)75, 200, 300, 400, 500, 700, 1000, 1500, 2000, 3000, 4000, 5000, 7000, 10000, 20000
Quantification using: Lower Marker          Final concentration (ng/uL): 0.5000          Dilution factor: 12.0
```

Sample: DNA size ladder
Well location: A7
Created: Thursday, August 12, 2021 6:46:05 PM
Import from: C:\AATI\Data\2021 08 12\17-56-24\210811_alberto_ladder.SCAL
Fit type: Point to point

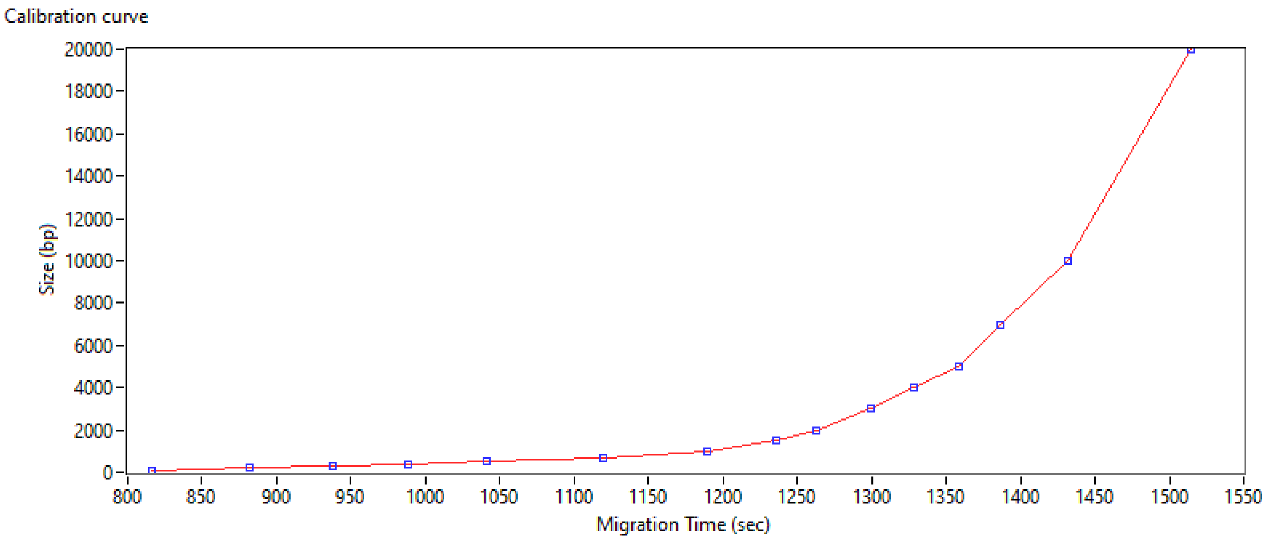

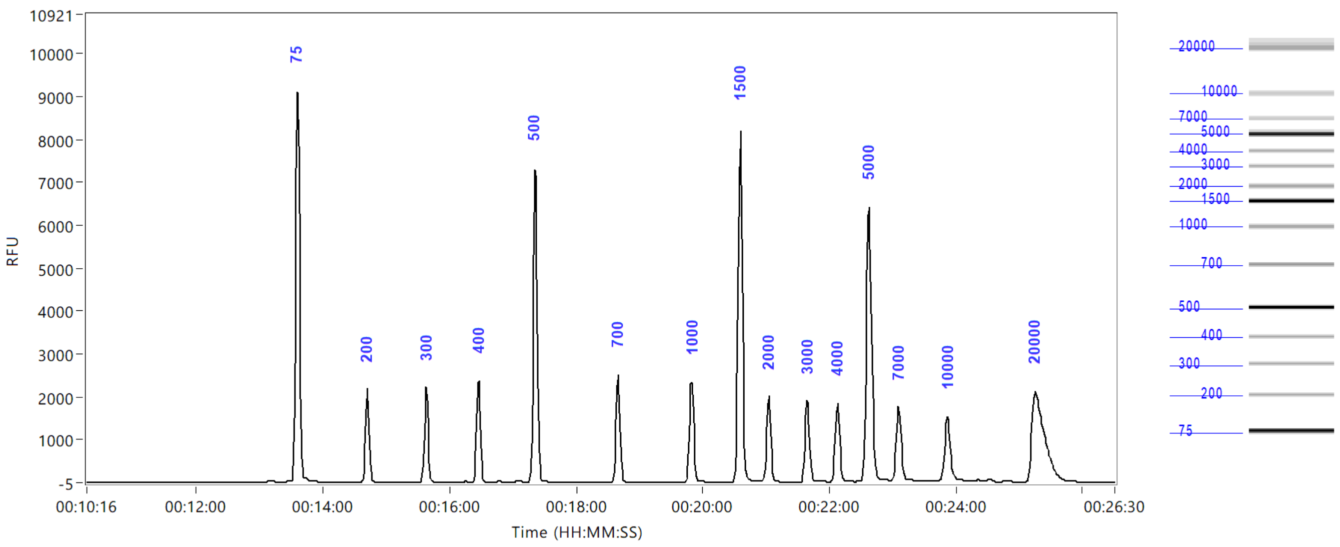

Supplement: Supplementary file 2 — sb3c00292_si_002.zip [file sb3c00292_si_002.zip › dnada_supplementary_material_pks_library_build/ZAG_trials/ZAG_Trial_3/18-36-28_redoplate2/210813_trial3_redoplate2.pdf]
