# Supplementary material for: Automated Platform for the Plasmid Construction Process
Source: ACS Synth Biol. 2023 Nov 10;12(12):3506–13. doi: 10.1021/acssynbio.3c00292 (PMC10729297; doi:10.1021/acssynbio.3c00292)

Instrument controller software run summary:

Filename and data path: C:\AATI\Data\2021 08 12\17-56-24\2021 08 12 17H 56M..raw
Created: Thursday, August 12, 2021 6:06:18 PM
Number of capillaries: 96
Array serial number: 111920-09SFS
Effect length: 33 cm
Array usage count: 187
Instrument type: ZAG DNA Analyzer
Instrument controller software version: 1.0.1.9
Device serial number: Z7597

Method Information

Method name: ZAG130GP33 - DNA 75-20000bp - Gel Prime Only.mthds
Gel prime: Yes
Full conditioning: No
Gel prime to buffer: No
Gel selection: Gel 1
Perform prerun: 8.0 kV, 30 sec.
Rinse: No
Marker 1: Row: A, 5.0 kV, 5 sec.
Rinse: No
Sample injection: 5.0 kV, 5 sec.
Separation: 8.0 kV, 30.0 min.
Tray name: Sample Tray-1

Analysis mode: DNA

Notes

Gel Image

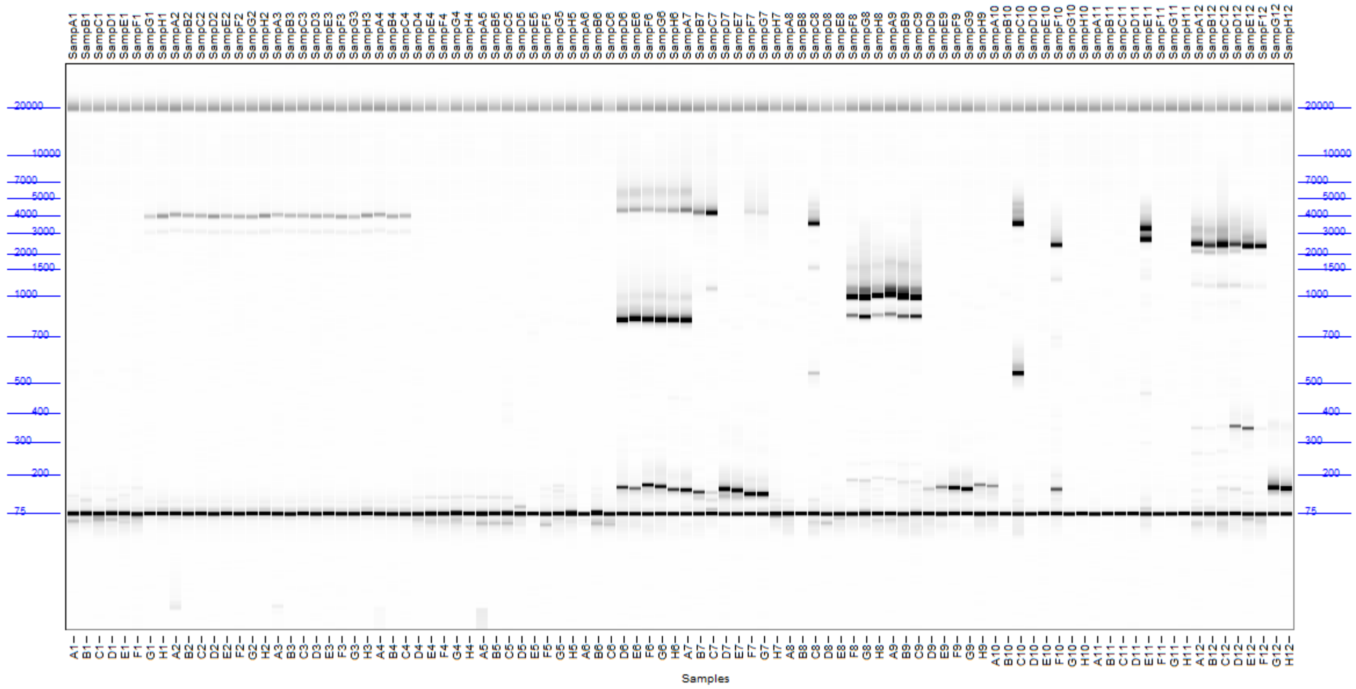
20000
10000
7000
5000
4000
3000
2000
1500
1000
700
500
400
300
200
75
Samples

Filename and data path: C:\AATI\Data\2021 08 12\17-56-24\2021 08 12 17H 56M..raw

A1: SampA1

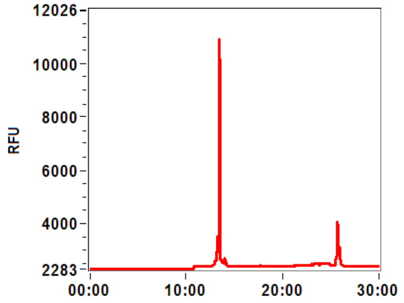

B1: SampB1

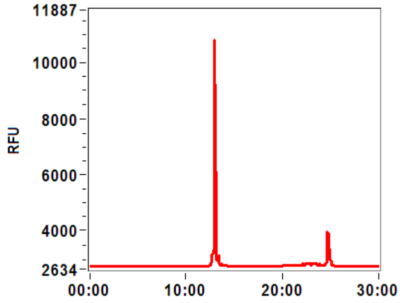

C1: SampC1

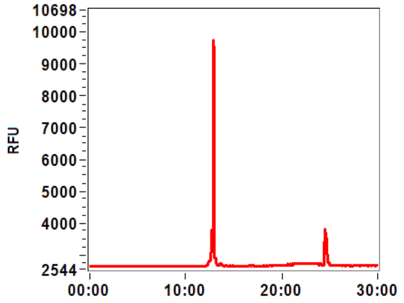

D1: SampD1

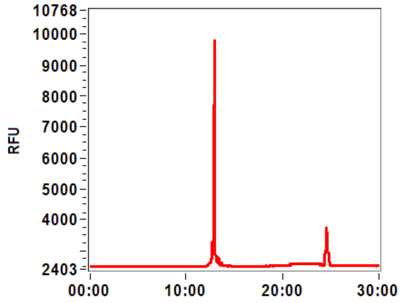

E1: SampE1

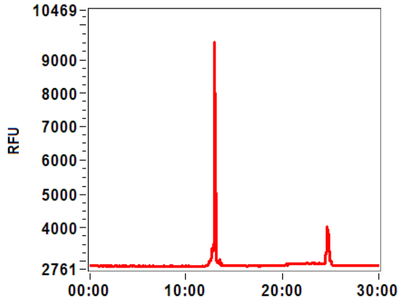

F1: SampF1

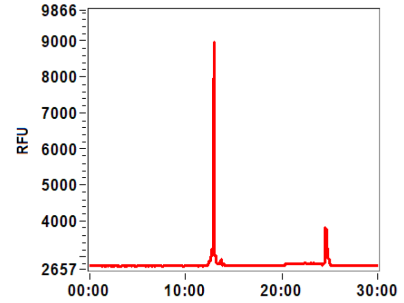

G1: SampG1

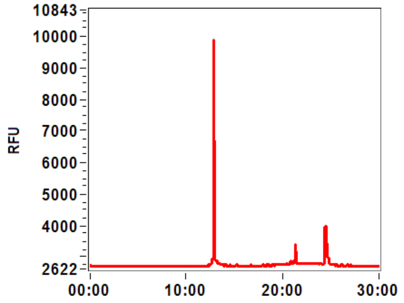

H1: SampH1

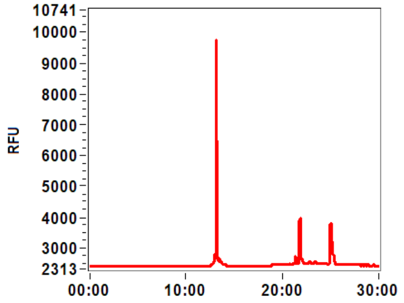

A2: SampA2

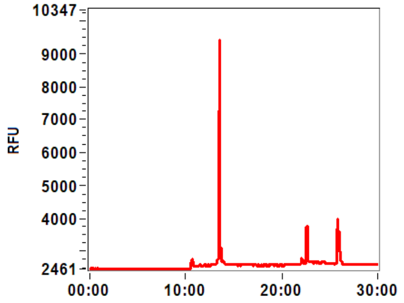

B2: SampB2

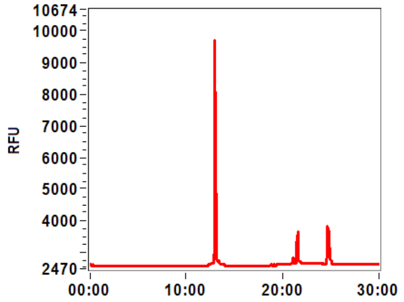

C2: SampC2

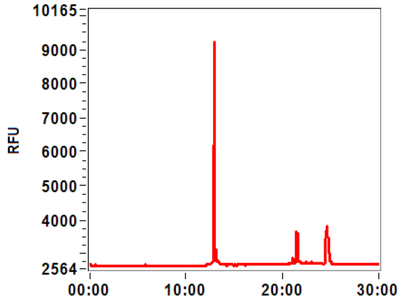

D2: SampD2

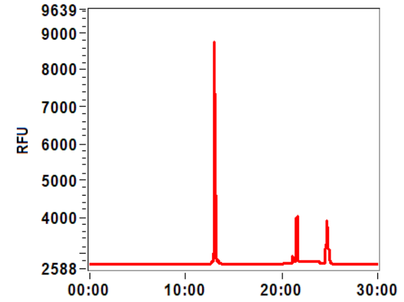

Filename and data path: C:\AATI\Data\2021 08 12\17-56-24\2021 08 12 17H 56M..raw

E2: SampE2

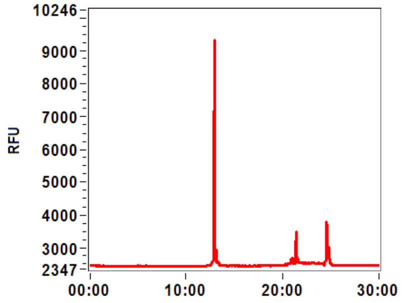

F2: SampF2

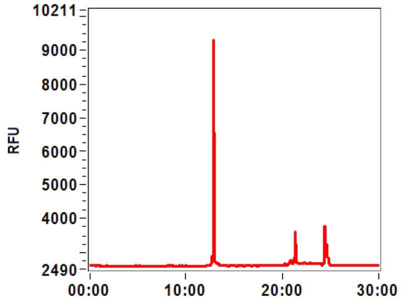

G2: SampG2

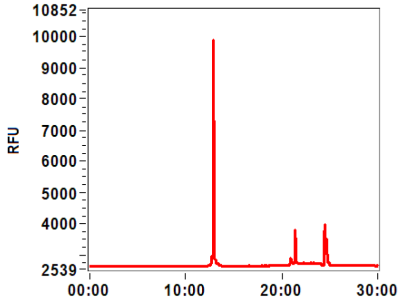

H2: SampH2

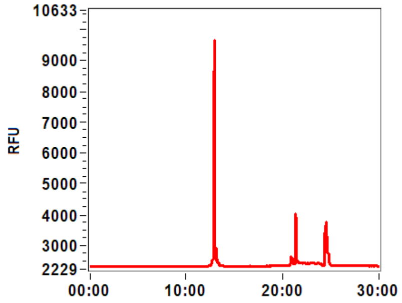

A3: SampA3

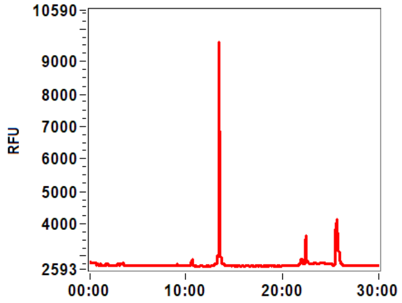

B3: SampB3

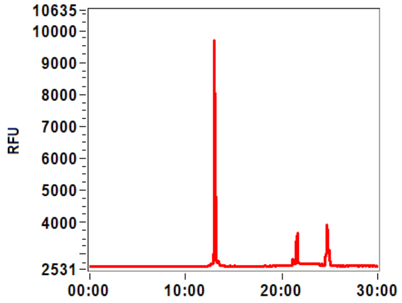

C3: SampC3

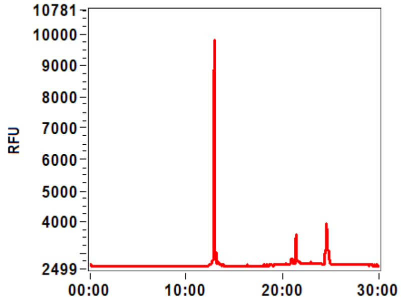

D3: SampD3

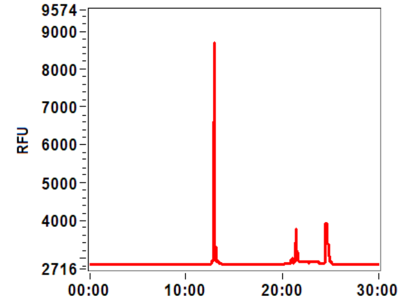

E3: SampE3

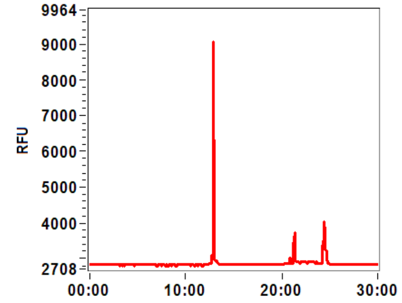

F3: SampF3

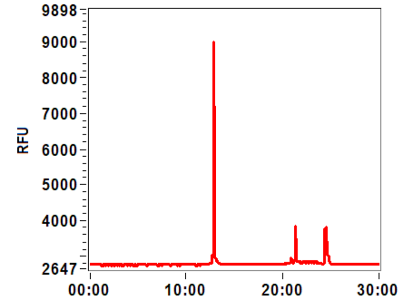

G3: SampG3

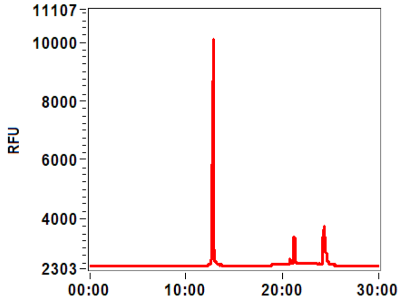

H3: SampH3

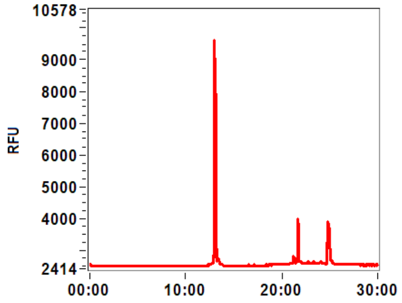

Filename and data path: C:\AATI\Data\2021 08 12\17-56-24\2021 08 12 17H 56M..raw

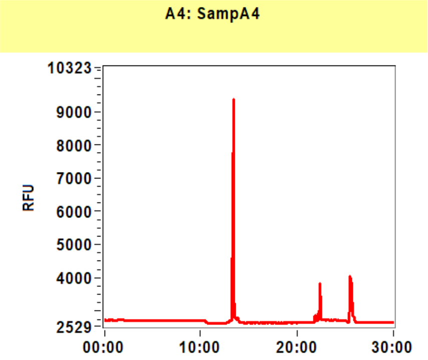

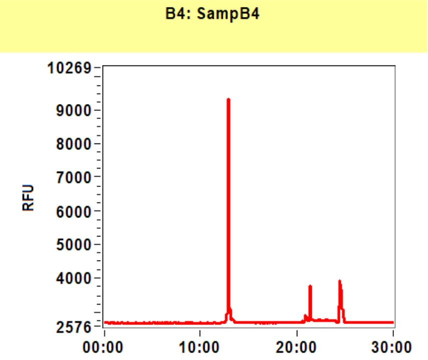

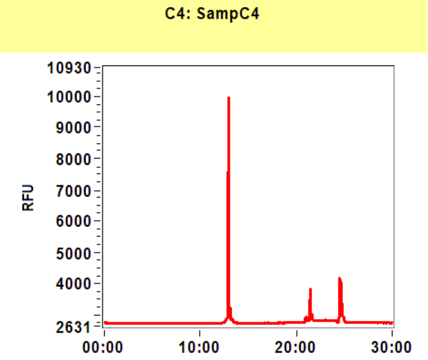

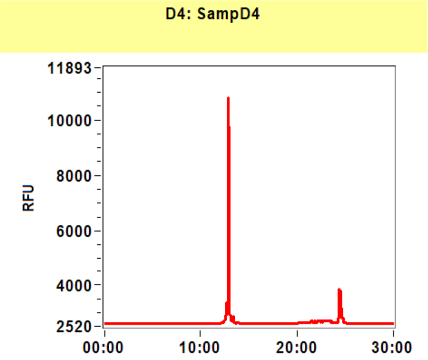

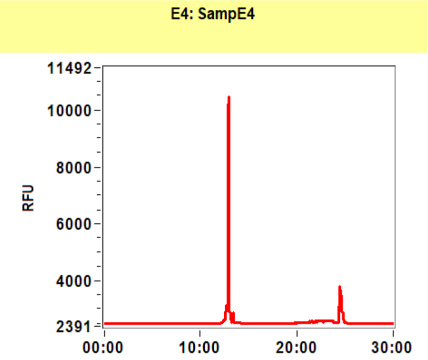

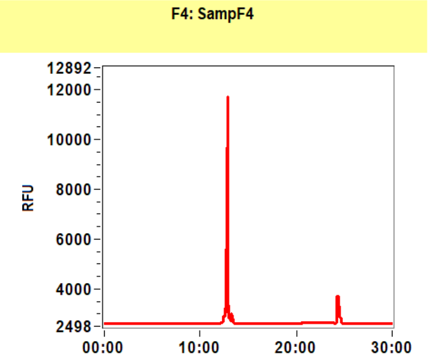

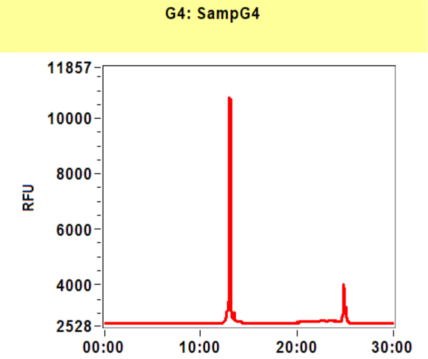

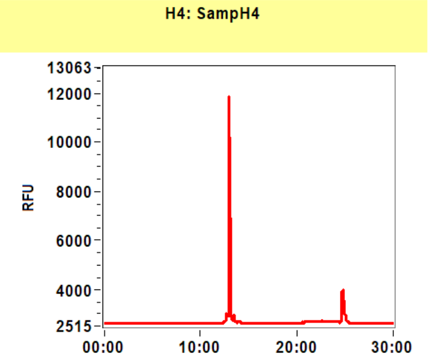

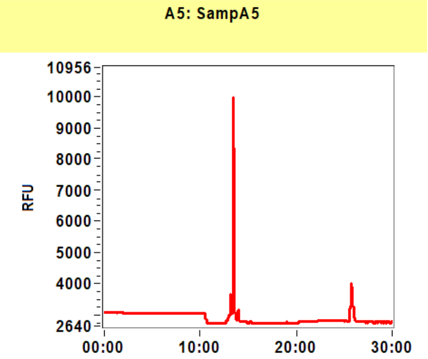

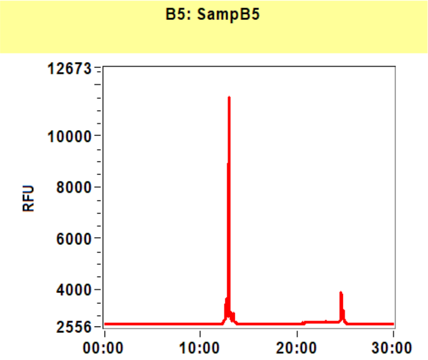

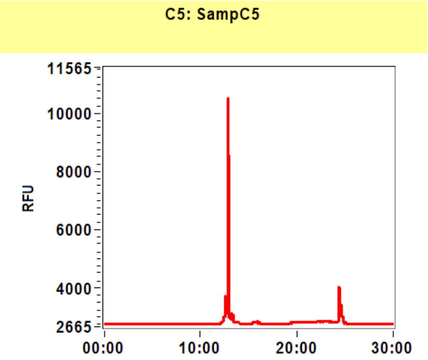

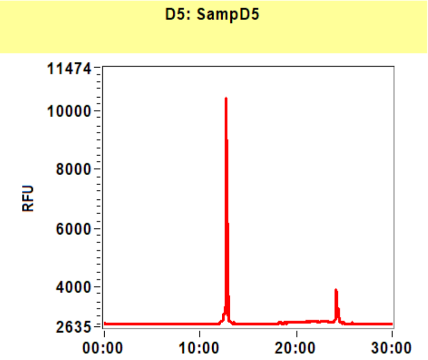

Filename and data path: C:\AATI\Data\2021 08 12\17-56-24\2021 08 12 17H 56M..raw

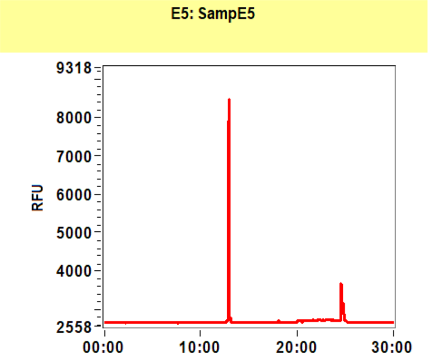

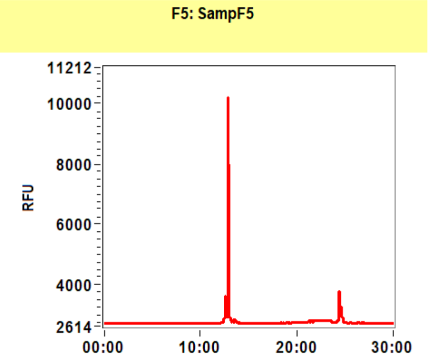

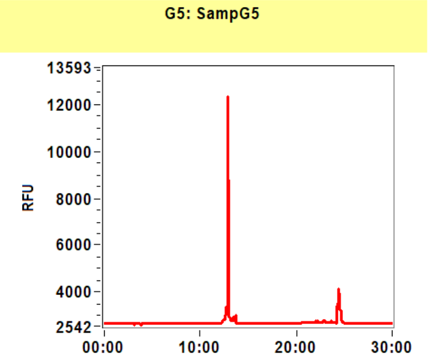

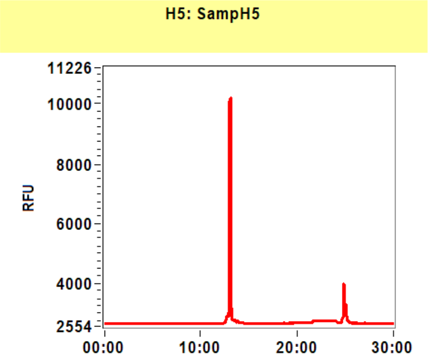

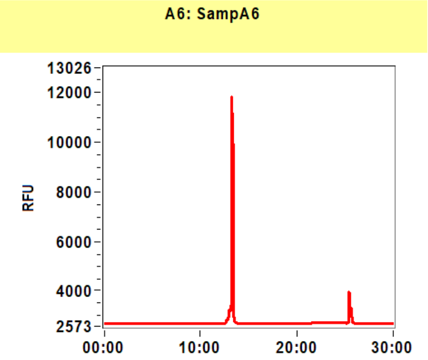

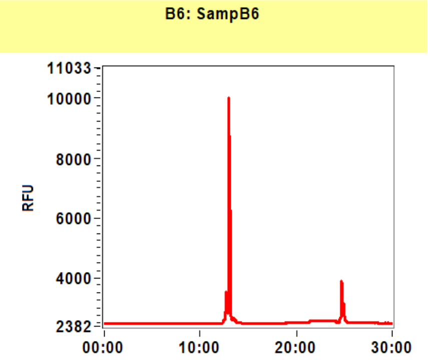

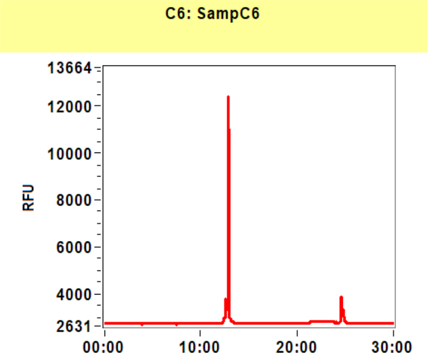

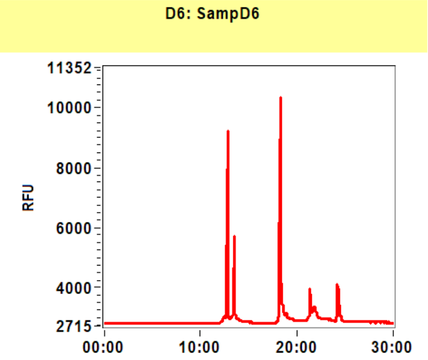

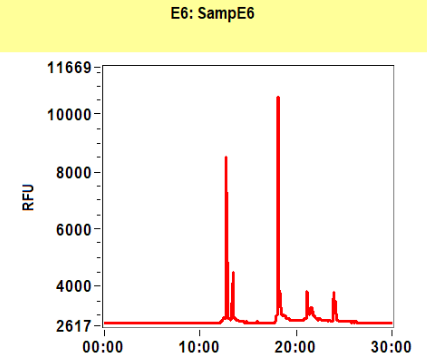

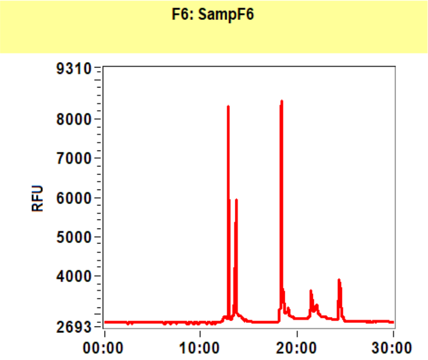

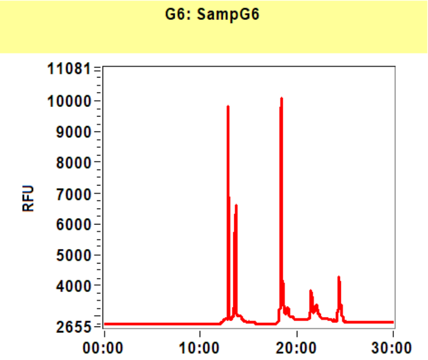

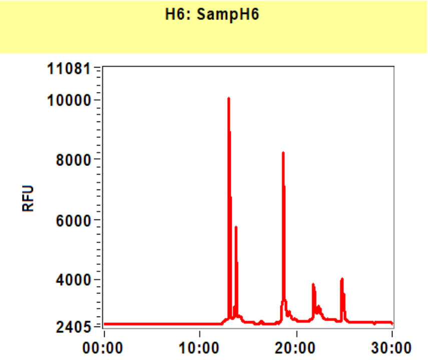

Filename and data path: C:\AATI\Data\2021 08 12\17-56-24\2021 08 12 17H 56M..raw

A7: SampA7

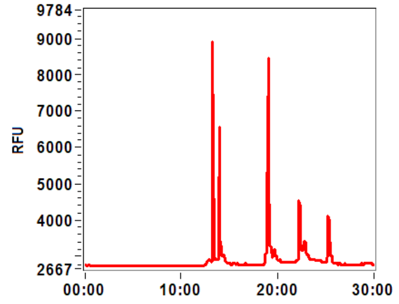

B7: SampB7

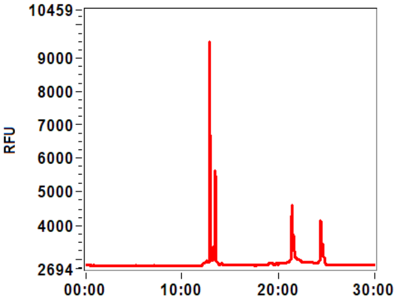

C7: SampC7

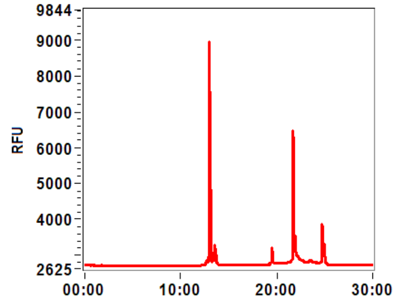

D7: SampD7

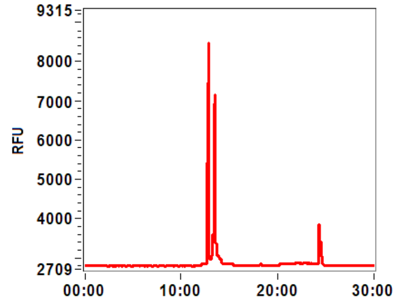

E7: SampE7

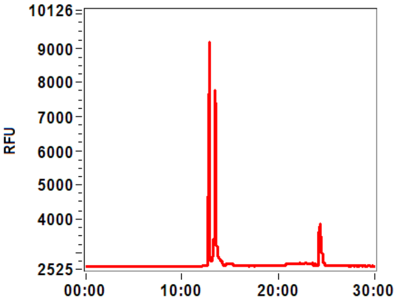

F7: SampF7

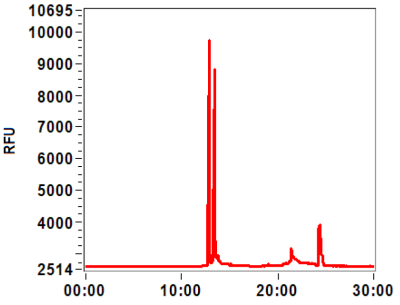

G7: SampG7

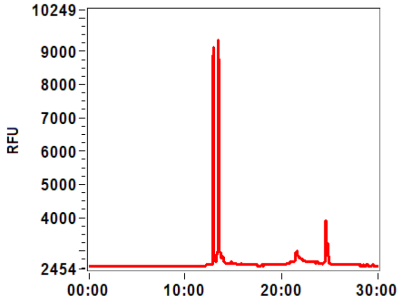

H7: SampH7

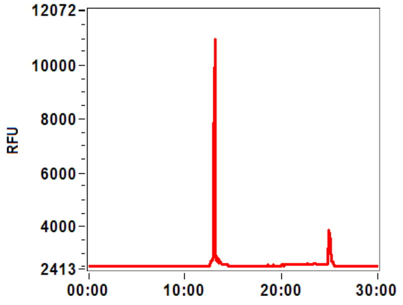

A8: SampA8

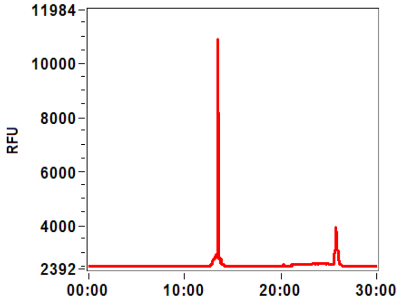

B8: SampB8

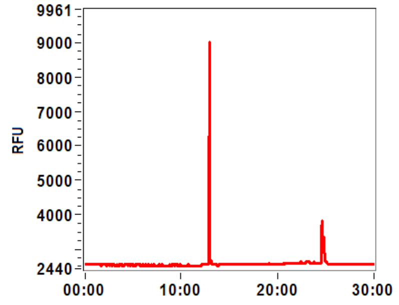

C8: SampC8

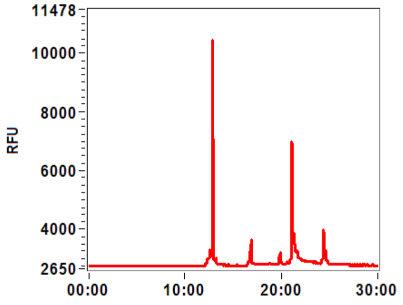

D8: SampD8

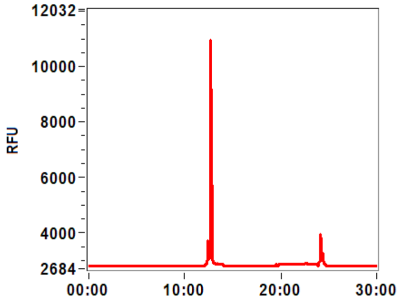

Filename and data path: C:\AATI\Data\2021 08 12\17-56-24\2021 08 12 17H 56M..raw

E8: SampE8

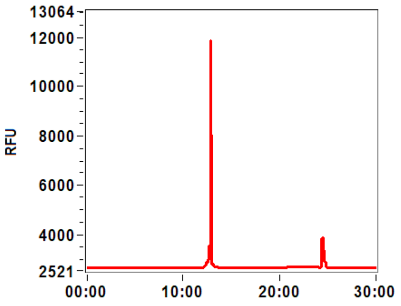

F8: SampF8

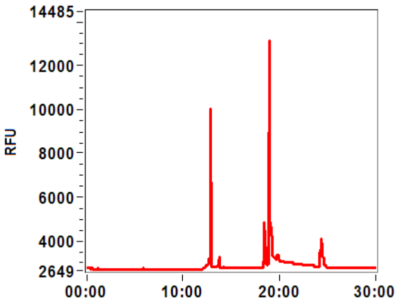

G8: SampG8

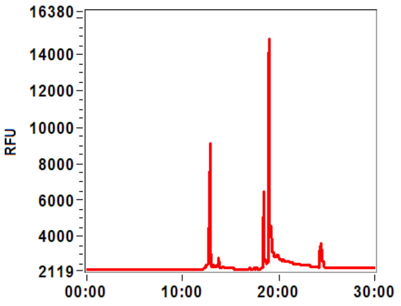

H8: SampH8

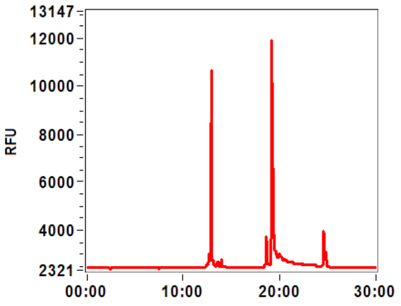

A9: SampA9

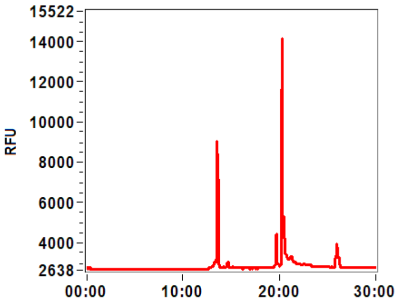

B9: SampB9

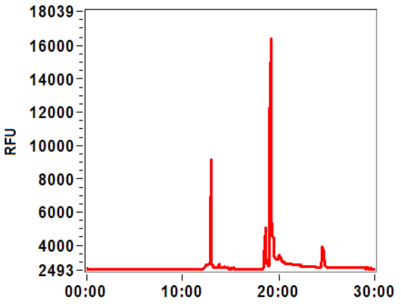

C9: SampC9

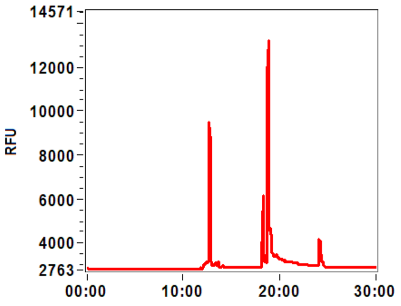

D9: SampD9

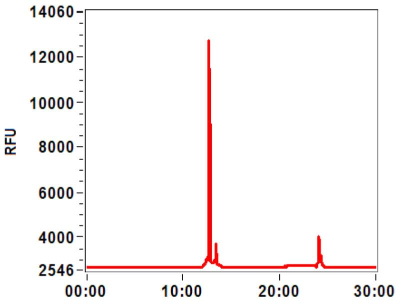

E9: SampE9

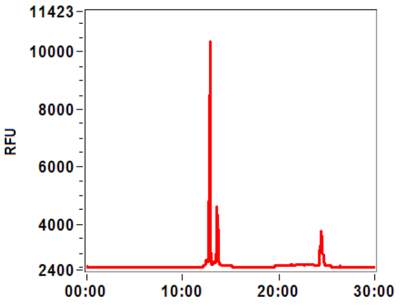

F9: SampF9

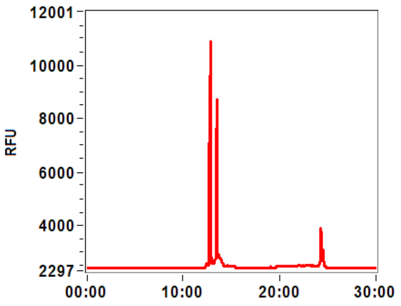

G9: SampG9

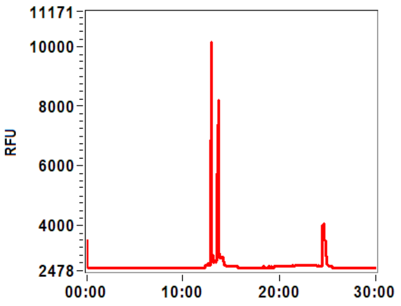

H9: SampH9

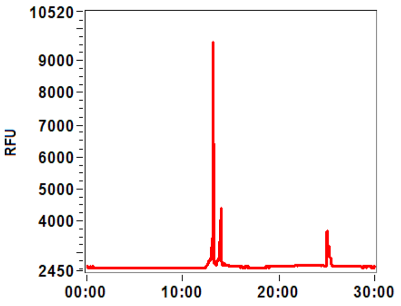

Filename and data path: C:\AATI\Data\2021 08 12\17-56-24\2021 08 12 17H 56M..raw

A10: SampA10

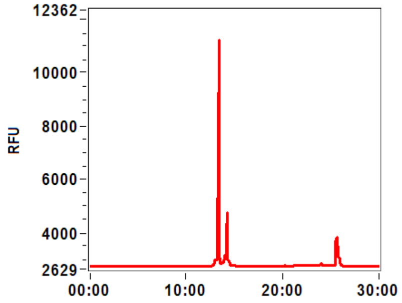

B10: SampB10

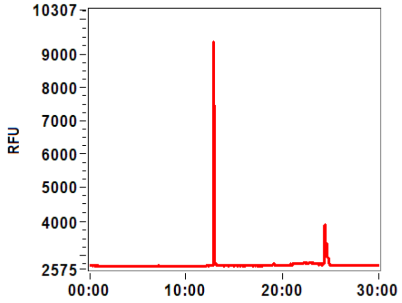

C10: SampC10

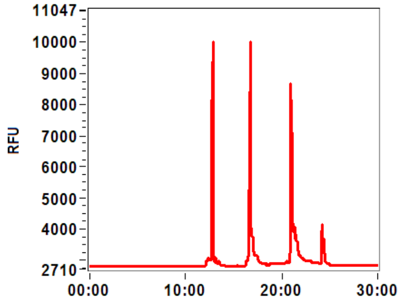

D10: SampD10

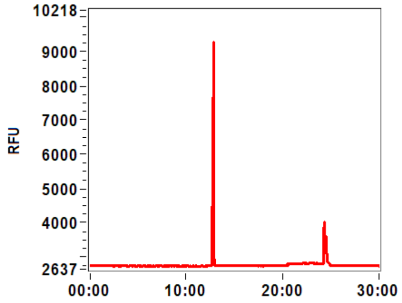

E10: SampE10

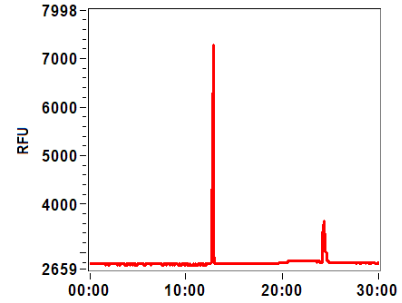

F10: SampF10

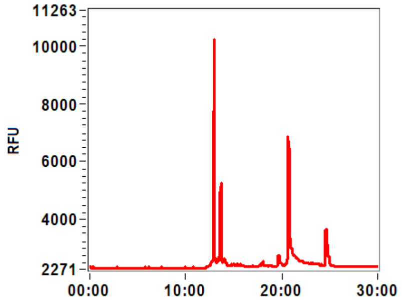

G10: SampG10

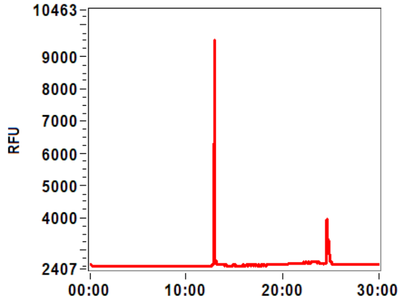

H10: SampH10

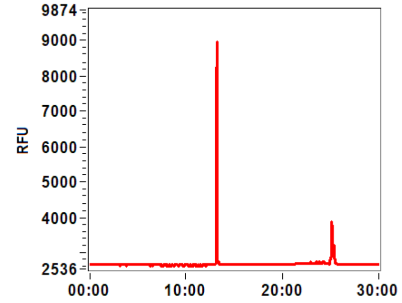

A11: SampA11

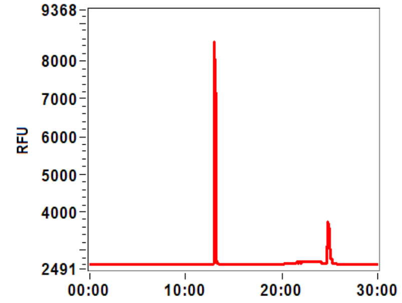

B11: SampB11

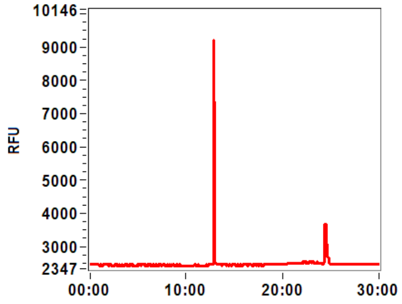

C11: SampC11

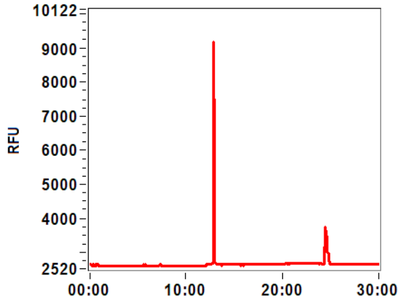

D11: SampD11

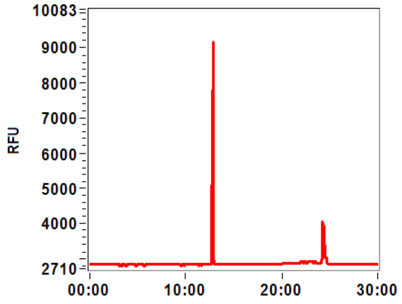

Filename and data path: C:\AATI\Data\2021 08 12\17-56-24\2021 08 12 17H 56M..raw

E11: SampE11

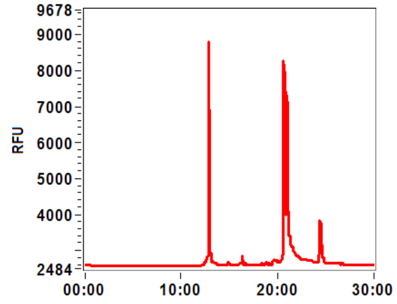

F11: SampF11

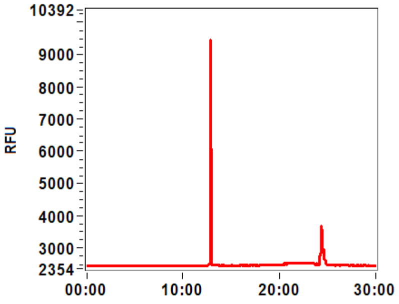

G11: SampG11

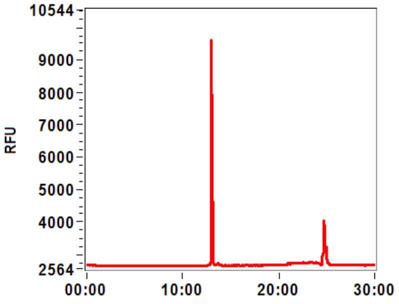

H11: SampH11

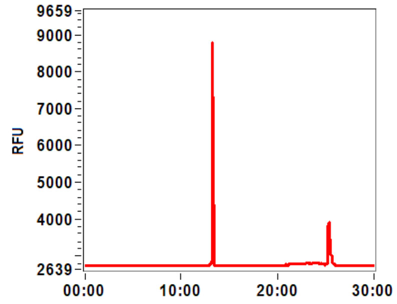

A12: SampA12

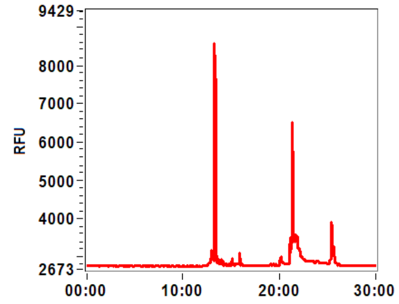

B12: SampB12

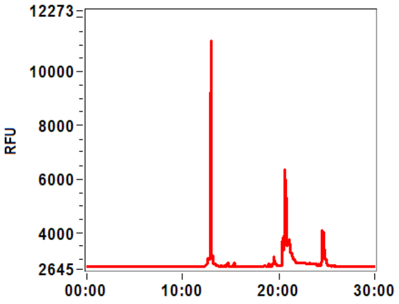

C12: SampC12

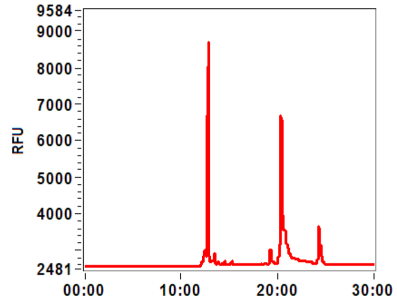

D12: SampD12

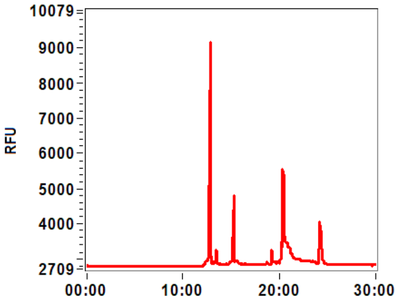

E12: SampE12

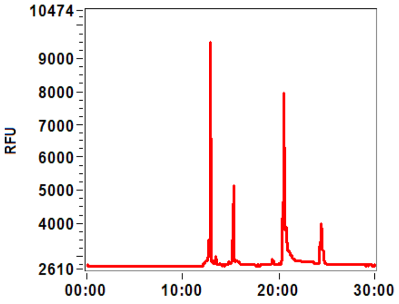

F12: SampF12

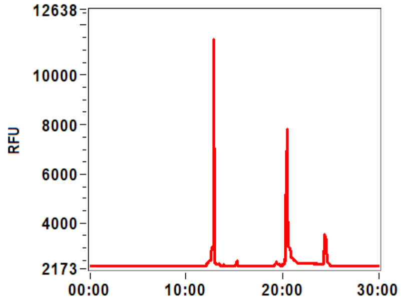

G12: SampG12

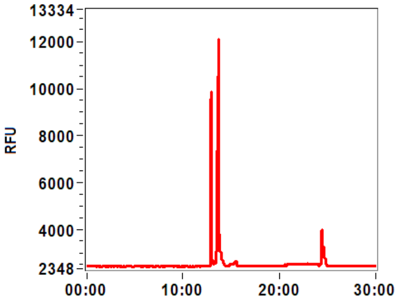

H12: SampH12

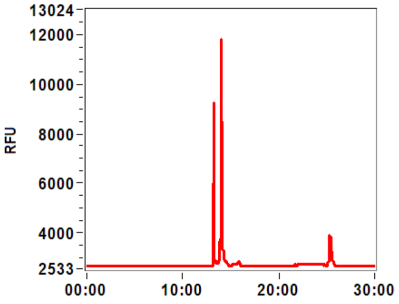

Sample: SampA1
Well location: A1
Created: Thursday, August 12, 2021 6:06:18 PM

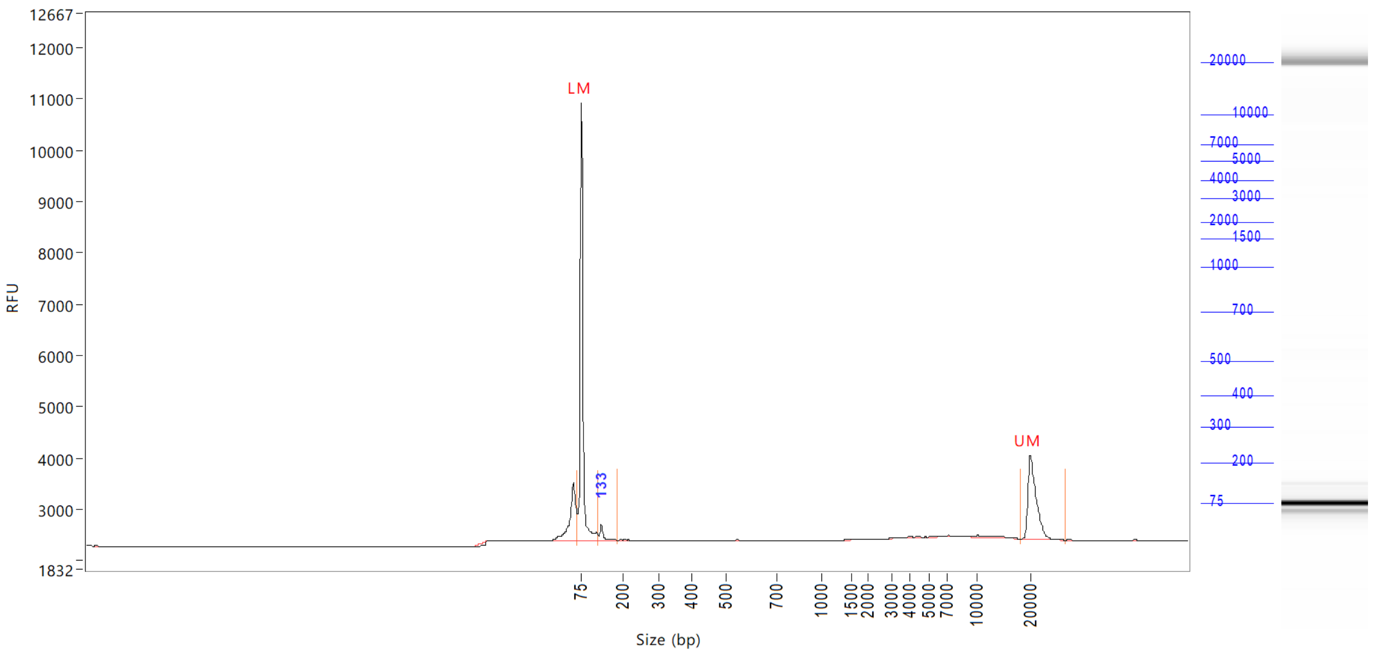

Peak	Size (bp)	Concentration (ng/uL)	From (bp)	To (bp)	Average size (bp)	CV%	RFU	Corrected peak area
1	75 (LM)	0.5000	62	126	75	12.35	8542	57.056
2	133	0.2457	126	181	136	7.66	304	2.337
3	20000 (UM)	0.1443	18171	26770	20550	4.50	1635	16.469

TIC:	0.2457	ng/uL
TIM:	2.9689	nmole/L
Total concentration:	0.7053	ng/uL

Sample peak width (sec): 5   Sample min peak height: 50   Sample baseline V to V?: Y   Sample baseline V to V points: 3
Sample filter: Binomial   Number of points for filter: 3 Sample start region (min): 0 Sample end region (min): 30
Marker peak width (sec): 5   Marker min peak height: 200   Marker baseline V to V?: Y   Marker baseline V to V points: 3
Lower marker selection: First peak > 500 RFU   Upper marker selection: Last peak > 200 RFU
Ladder size (bp)75, 200, 300, 400, 500, 700, 1000, 1500, 2000, 3000, 4000, 5000, 7000, 10000, 20000
Quantification using: Lower Marker   Final concentration (ng/uL): 0.5000   Dilution factor: 12.0

Sample: SampB1
Well location: B1
Created: Thursday, August 12, 2021 6:06:18 PM

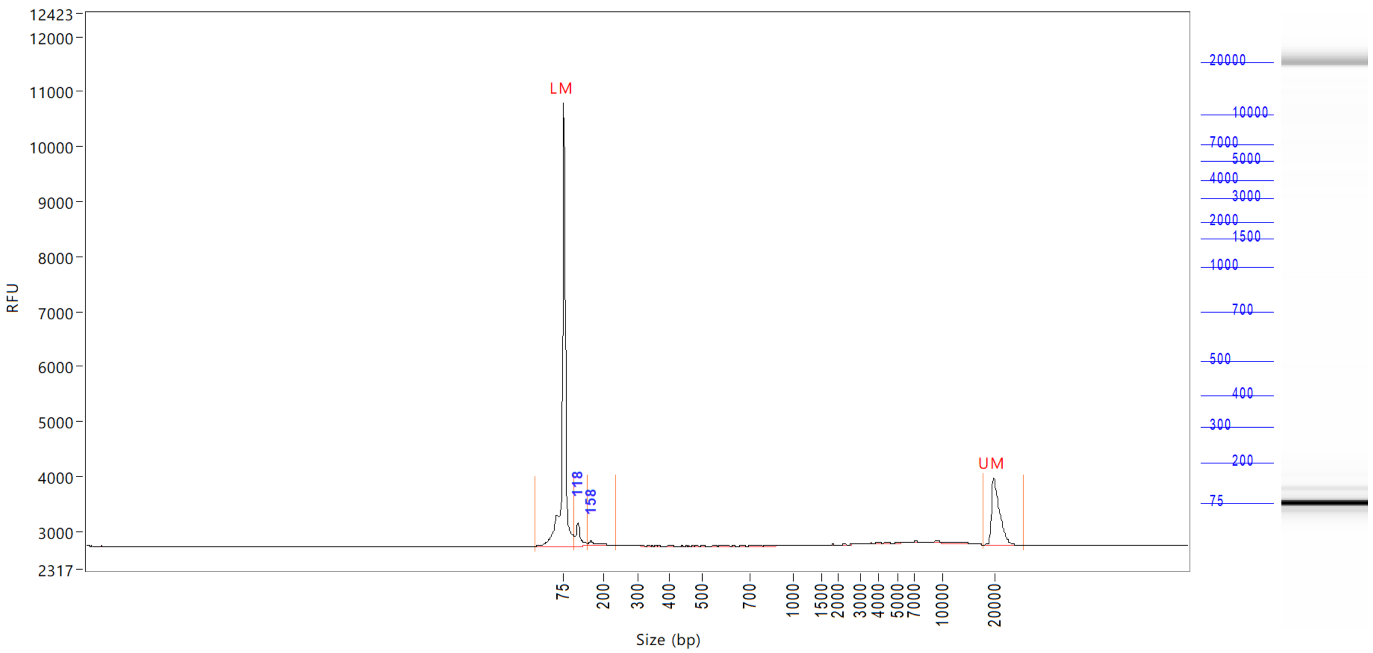

Peak	Size (bp)	Concentration (ng/uL)	From (bp)	To (bp)	Average size (bp)	CV%	RFU	Corrected peak area
1	75 (LM)	0.5000	0	108	70	19.19	8071	67.263
2	118	0.4013	108	148	121	7.95	414	4.499
3	158	0.1734	148	237	176	11.69	93	1.944
4	20000 (UM)	0.0933	17805	25803	20509	4.54	1218	12.547

TIC:	0.5748	ng/uL
TIM:	7.0691	nmole/L
Total concentration:	0.8868	ng/uL

Sample peak width (sec): 5   Sample min peak height: 50   Sample baseline V to V?: Y   Sample baseline V to V points: 3
Sample filter: Binomial   Number of points for filter: 3 Sample start region (min): 0 Sample end region (min): 30
Marker peak width (sec): 5   Marker min peak height: 200   Marker baseline V to V?: Y   Marker baseline V to V points: 3
Lower marker selection: First peak > 500 RFU   Upper marker selection: Last peak > 200 RFU
Ladder size (bp)75, 200, 300, 400, 500, 700, 1000, 1500, 2000, 3000, 4000, 5000, 7000, 10000, 20000
Quantification using: Lower Marker   Final concentration (ng/uL): 0.5000   Dilution factor: 12.0

Sample: SampC1
Well location: C1
Created: Thursday, August 12, 2021 6:06:18 PM

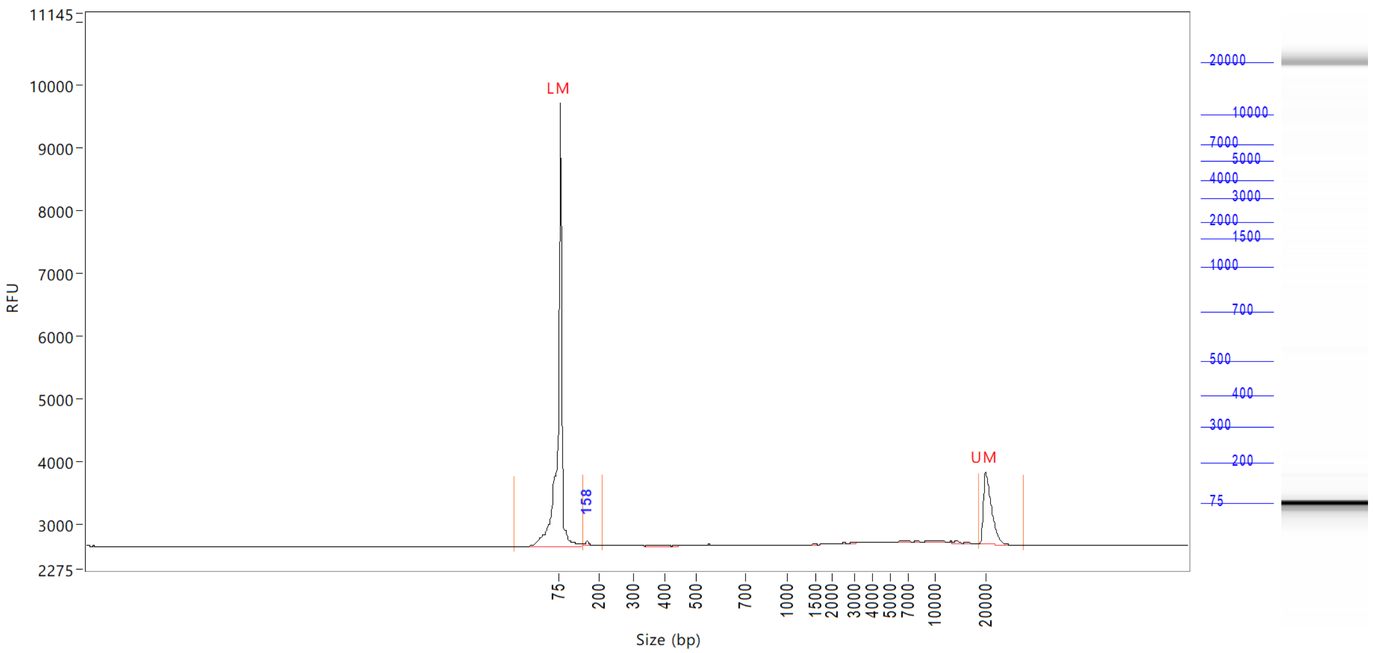

Peak	Size (bp)	Concentration (ng/uL)	From (bp)	To (bp)	Average size (bp)	CV%	RFU	Corrected peak area
1	75 (LM)	0.5000	0	148	66	28.75	7083	67.993
2	158	0.0841	148	206	163	7.88	88	0.953
3	20000 (UM)	0.0877	18537	27375	20504	4.68	1152	11.925

TIC:	0.0841	ng/uL
TIM:	0.8476	nmole/L
Total concentration:	0.3907	ng/uL

Sample peak width (sec): 5 Sample min peak height: 50 Sample baseline V to V?: Y Sample baseline V to V points: 3
Sample filter: Binomial Number of points for filter: 3 Sample start region (min): 0 Sample end region (min): 30
Marker peak width (sec): 5 Marker min peak height: 200 Marker baseline V to V?: Y Marker baseline V to V points: 3
Lower marker selection: First peak > 500 RFU Upper marker selection: Last peak > 200 RFU
Ladder size (bp)75, 200, 300, 400, 500, 700, 1000, 1500, 2000, 3000, 4000, 5000, 7000, 10000, 20000
Quantification using: Lower Marker Final concentration (ng/uL): 0.5000 Dilution factor: 12.0

Sample: SampD1
Well location: D1
Created: Thursday, August 12, 2021 6:06:18 PM

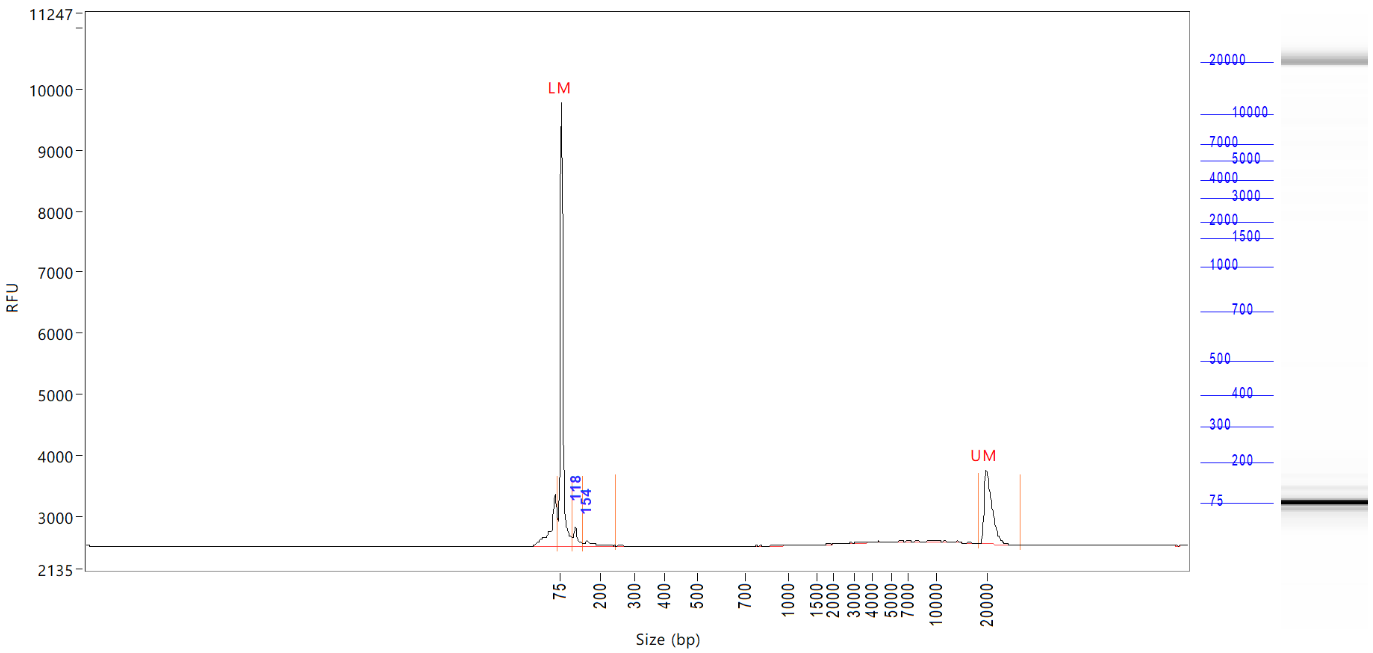

Peak	Size (bp)	Concentration (ng/uL)	From (bp)	To (bp)	Average size (bp)	CV%	RFU	Corrected peak area
1	75 (LM)	0.5000	66	110	76	9.10	7281	51.139
2	118	0.3555	110	145	121	7.30	299	3.030
3	154	0.2060	145	244	173	13.26	96	1.756
4	20000 (UM)	0.1200	18537	26770	20545	4.49	1208	12.276

TIC:	0.5615	ng/uL
TIM:	6.7841	nmole/L
Total concentration:	0.8988	ng/uL

Sample peak width (sec): 5 Sample min peak height: 50 Sample baseline V to V?: Y Sample baseline V to V points: 3
Sample filter: Binomial Number of points for filter: 3 Sample start region (min): 0 Sample end region (min): 30
Marker peak width (sec): 5 Marker min peak height: 200 Marker baseline V to V?: Y Marker baseline V to V points: 3
Lower marker selection: First peak > 500 RFU Upper marker selection: Last peak > 200 RFU
Ladder size (bp)75, 200, 300, 400, 500, 700, 1000, 1500, 2000, 3000, 4000, 5000, 7000, 10000, 20000
Quantification using: Lower Marker Final concentration (ng/uL): 0.5000 Dilution factor: 12.0

Sample: SampE1
Well location: E1
Created: Thursday, August 12, 2021 6:06:18 PM

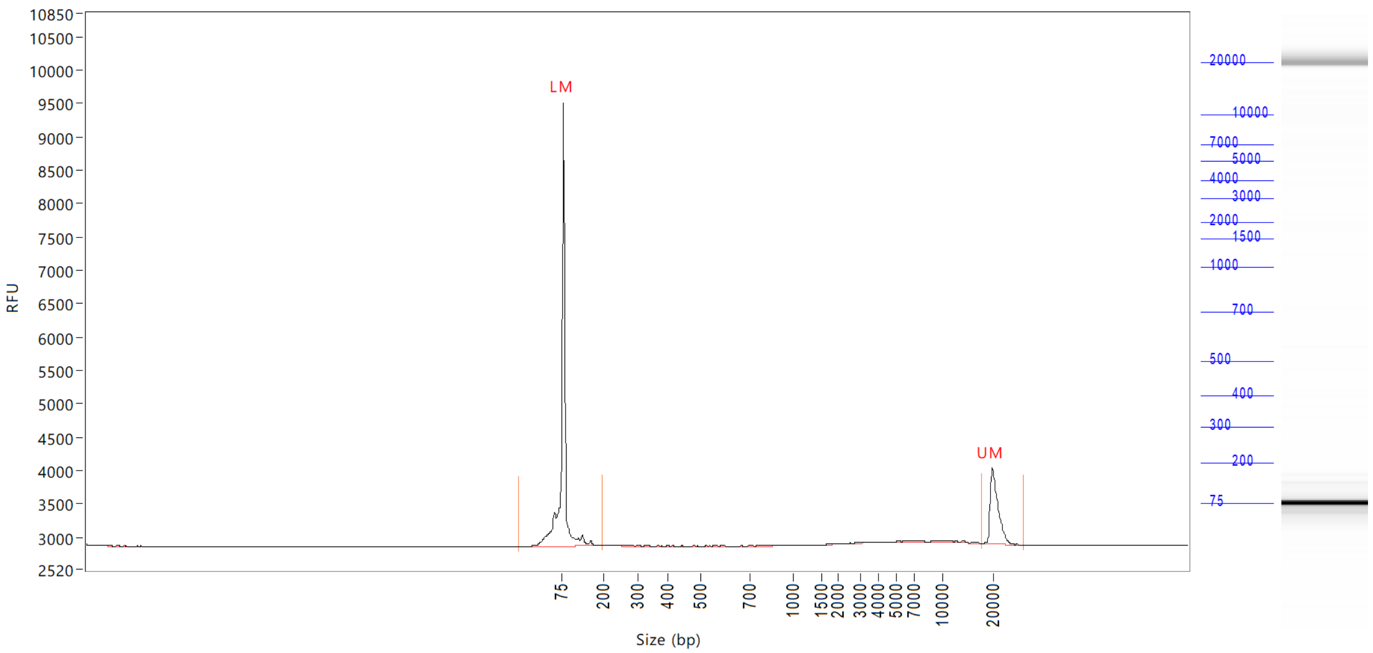

Peak	Size (bp)	Concentration (ng/uL)	From (bp)	To (bp)	Average size (bp)	CV%	RFU	Corrected peak area
1	75 (LM)	0.5000	0	200	70	33.45	6653	59.773
2	20000 (UM)	0.0969	17927	26045	20454	4.56	1147	11.587

TIC:	0.0000	ng/uL
TIM:	0.0000	nmole/L
Total concentration:	0.3544	ng/uL

Sample peak width (sec): 5 Sample min peak height: 50 Sample baseline V to V?: Y Sample baseline V to V points: 3
Sample filter: Binomial Number of points for filter: 3 Sample start region (min): 0 Sample end region (min): 30
Marker peak width (sec): 5 Marker min peak height: 200 Marker baseline V to V?: Y Marker baseline V to V points: 3
Lower marker selection: First peak > 500 RFU Upper marker selection: Last peak > 200 RFU
Ladder size (bp)75, 200, 300, 400, 500, 700, 1000, 1500, 2000, 3000, 4000, 5000, 7000, 10000, 20000
Quantification using: Lower Marker Final concentration (ng/uL): 0.5000 Dilution factor: 12.0

Sample: SampF1
Well location: F1
Created: Thursday, August 12, 2021 6:06:18 PM

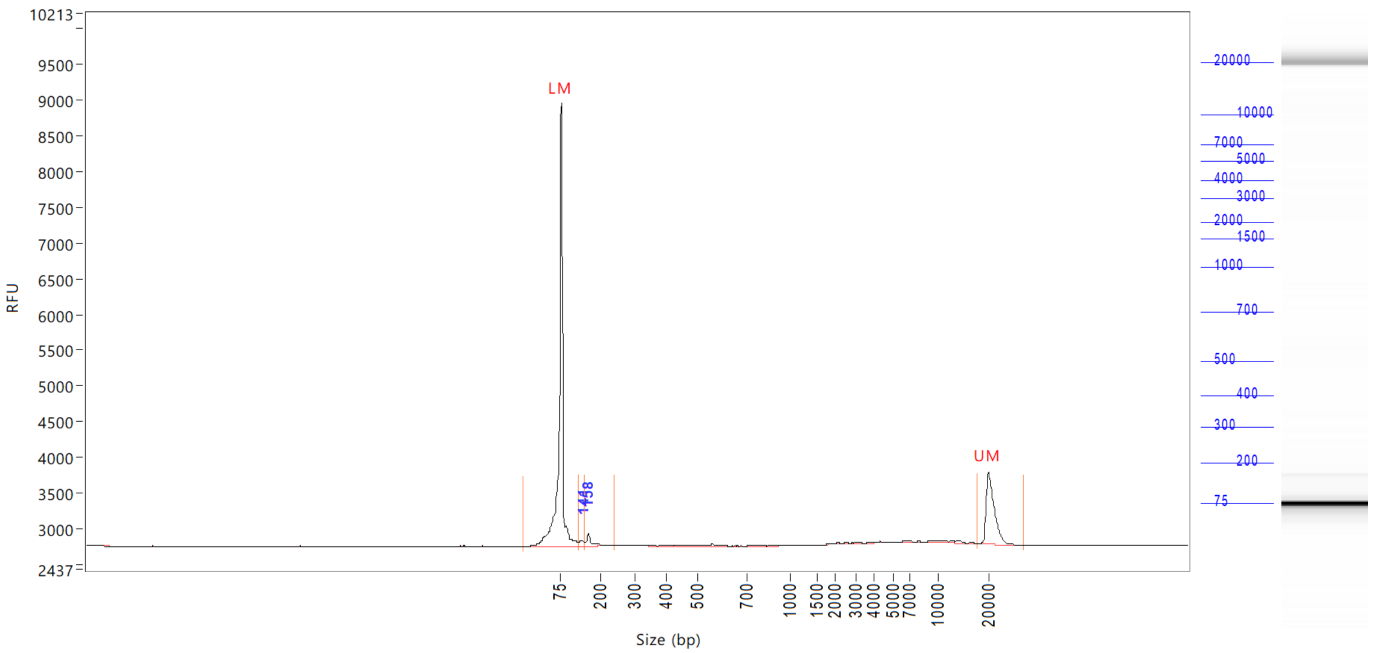

Peak	Size<br>(bp)	Concentration<br>(ng/uL)	From<br>(bp)	To<br>(bp)	Average size<br>(bp)	CV%	RFU	Corrected peak area
1	75 (LM)	0.5000	0	128	68	25.68	6211	54.022
2	141	0.1026	128	148	138	4.70	66	0.924
3	158	0.2323	148	237	172	12.95	170	2.092
4	20000 (UM)	0.0972	17683	26891	20558	4.82	1020	10.503

TIC:	0.3349	ng/uL
TIM:	3.4424	nmole/L
Total concentration:	0.7549	ng/uL

Sample peak width (sec): 5   Sample min peak height: 50   Sample baseline V to V?: Y   Sample baseline V to V points: 3
Sample filter: Binomial   Number of points for filter: 3 Sample start region (min): 0 Sample end region (min): 30
Marker peak width (sec): 5   Marker min peak height: 200   Marker baseline V to V?: Y   Marker baseline V to V points: 3
Lower marker selection: First peak > 500 RFU   Upper marker selection: Last peak > 200 RFU
Ladder size (bp)75, 200, 300, 400, 500, 700, 1000, 1500, 2000, 3000, 4000, 5000, 7000, 10000, 20000
Quantification using: Lower Marker   Final concentration (ng/uL): 0.5000   Dilution factor: 12.0

Sample: SampG1
Well location: G1
Created: Thursday, August 12, 2021 6:06:18 PM

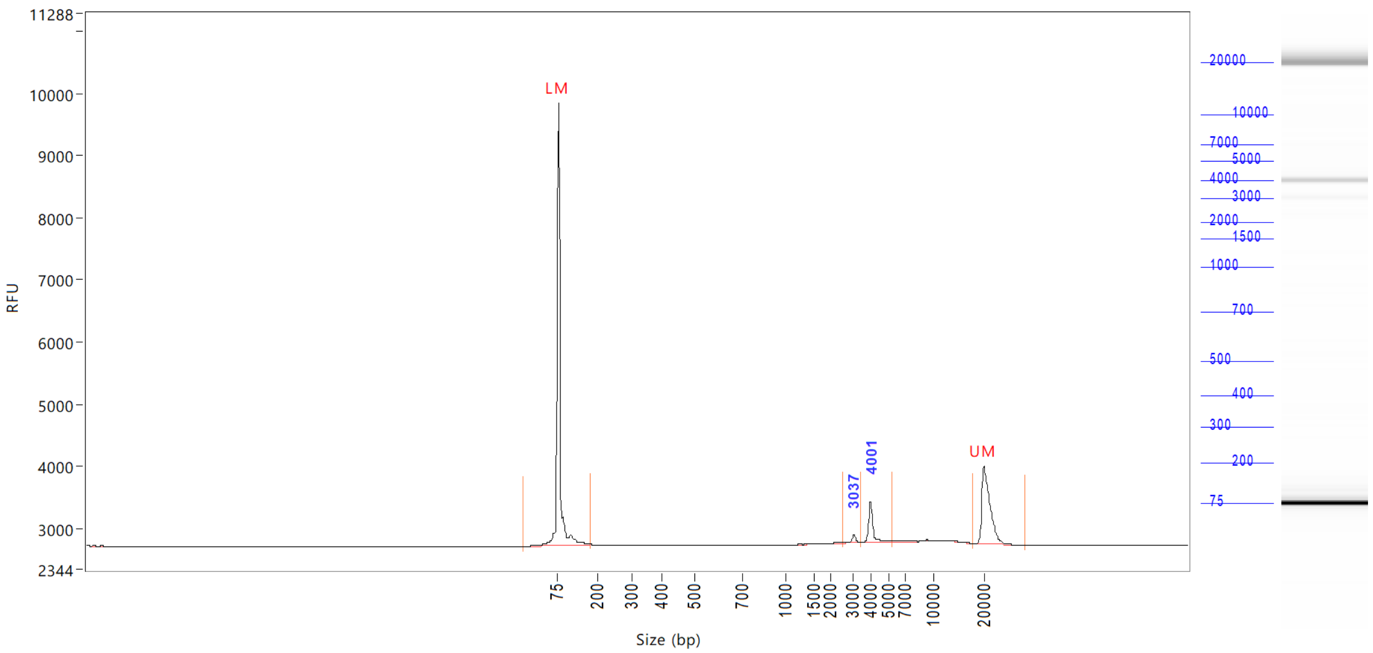

Peak	Size (bp)	Concentration (ng/uL)	From (bp)	To (bp)	Average size (bp)	CV%	RFU	Corrected peak area
1	75 (LM)	0.5000	0	177	76	20.66	7132	49.552
2	3037	0.0989	2572	3465	3031	4.67	127	0.817
3	4001	0.4992	3465	5445	4047	6.09	655	4.123
4	20000 (UM)	0.1329	17927	27979	20560	4.74	1257	13.174

TIC:	0.5982	ng/uL
TIM:	0.2568	nmole/L
Total concentration:	0.9380	ng/uL

Sample peak width (sec): 5   Sample min peak height: 50   Sample baseline V to V?: Y   Sample baseline V to V points: 3
Sample filter: Binomial   Number of points for filter: 3 Sample start region (min): 0 Sample end region (min): 30
Marker peak width (sec): 5   Marker min peak height: 200   Marker baseline V to V?: Y   Marker baseline V to V points: 3
Lower marker selection: First peak > 500 RFU   Upper marker selection: Last peak > 200 RFU
Ladder size (bp)75, 200, 300, 400, 500, 700, 1000, 1500, 2000, 3000, 4000, 5000, 7000, 10000, 20000
Quantification using: Lower Marker   Final concentration (ng/uL): 0.5000   Dilution factor: 12.0

Sample: SampH1
Well location: H1
Created: Thursday, August 12, 2021 6:06:18 PM

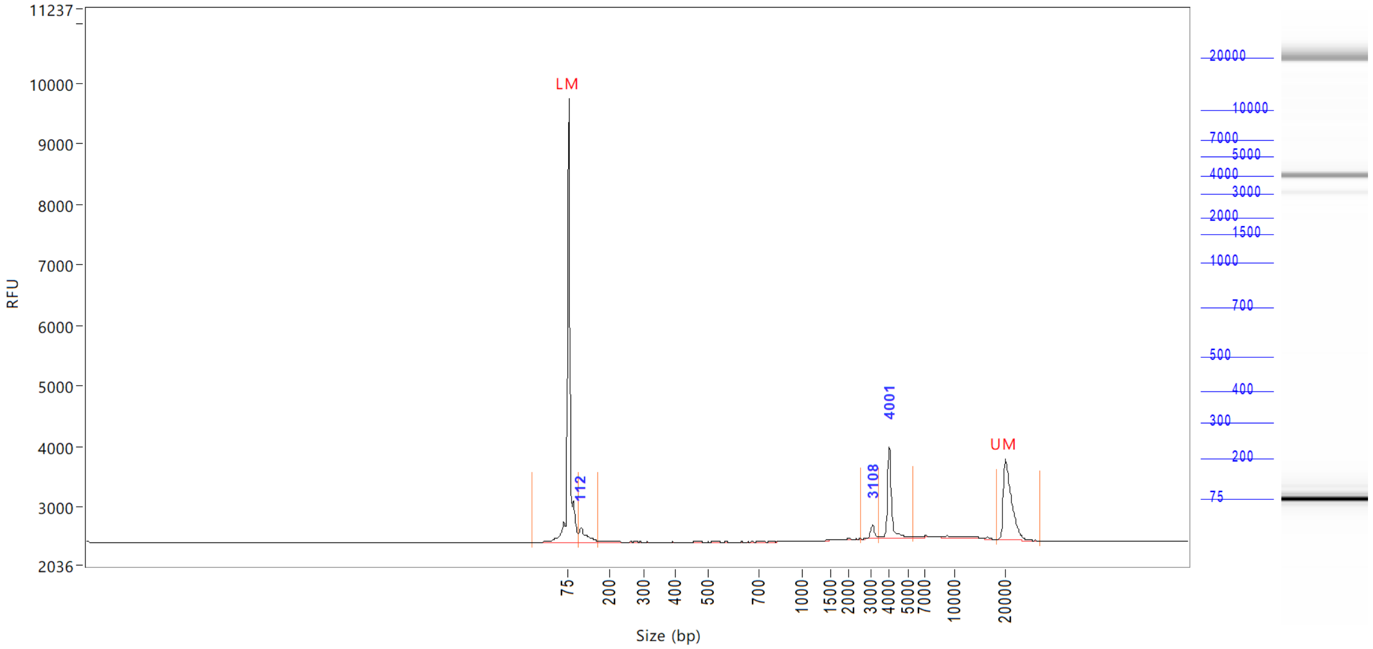

Peak	Size (bp)	Concentration (ng/uL)	From (bp)	To (bp)	Average size (bp)	CV%	RFU	Corrected peak area
1	75 (LM)	0.5000	0	105	73	17.11	7353	50.853
2	112	0.4935	105	166	123	11.99	246	4.183
3	3108	0.1645	2600	3429	3069	3.97	224	1.395
4	4001	1.0920	3429	5667	4061	6.13	1510	9.255
5	20000 (UM)	0.1382	18171	26649	20569	4.74	1348	14.058

TIC:	1.7500	ng/uL
TIM:	7.1229	nmole/L
Total concentration:	2.1298	ng/uL

Sample peak width (sec): 5   Sample min peak height: 50   Sample baseline V to V?: Y   Sample baseline V to V points: 3
Sample filter: Binomial   Number of points for filter: 3   Sample start region (min): 0   Sample end region (min): 30
Marker peak width (sec): 5   Marker min peak height: 200   Marker baseline V to V?: Y   Marker baseline V to V points: 3
Lower marker selection: First peak > 500 RFU   Upper marker selection: Last peak > 200 RFU
Ladder size (bp)75, 200, 300, 400, 500, 700, 1000, 1500, 2000, 3000, 4000, 5000, 7000, 10000, 20000
Quantification using: Lower Marker   Final concentration (ng/uL): 0.5000   Dilution factor: 12.0

Sample: SampA2
Well location: A2
Created: Thursday, August 12, 2021 6:06:18 PM

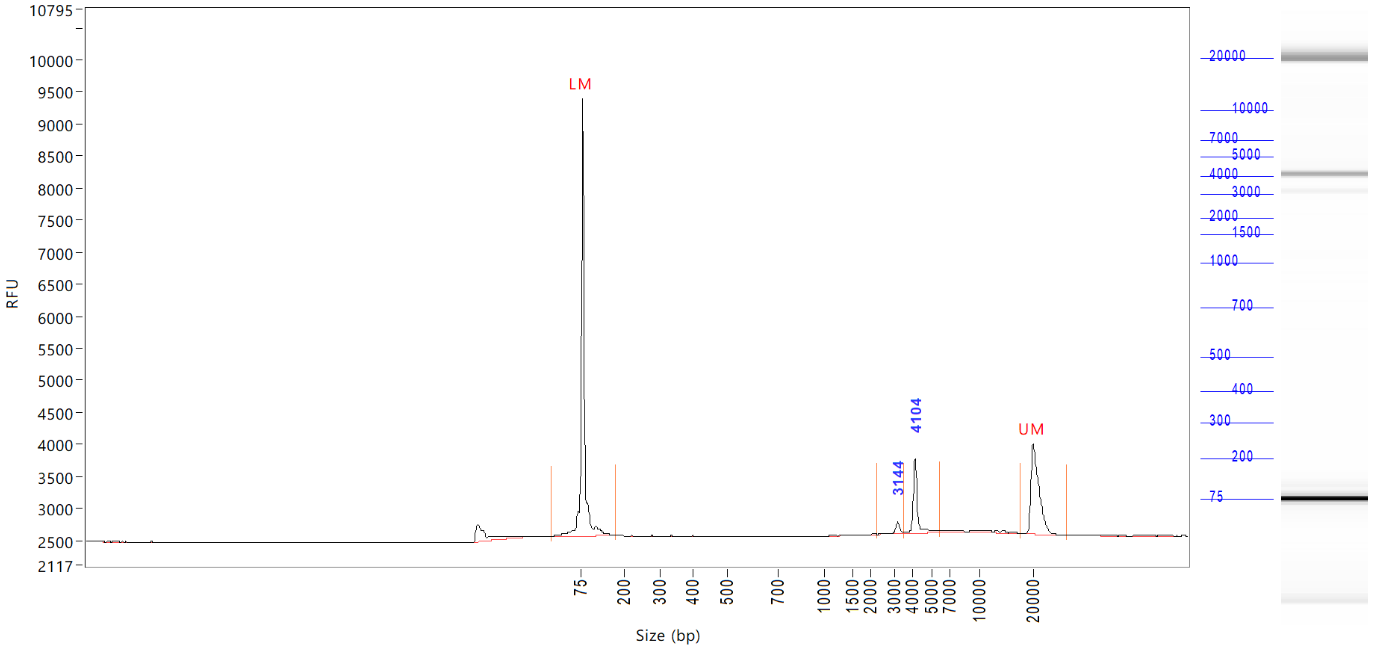

Peak	Size (bp)	Concentration (ng/uL)	From (bp)	To (bp)	Average size (bp)	CV%	RFU	Corrected peak area
1	75 (LM)	0.5000	0	173	75	20.28	6837	50.417
2	3144	0.1386	2258	3536	3132	5.54	185	1.165
3	4104	0.8526	3536	5889	4170	7.78	1150	7.164
4	20000 (UM)	0.1378	17561	26287	20437	4.44	1401	13.890

TIC:	0.9912	ng/uL
TIM:	0.4094	nmole/L
Total concentration:	1.3706	ng/uL

Sample peak width (sec): 5   Sample min peak height: 50   Sample baseline V to V?: Y   Sample baseline V to V points: 3
Sample filter: Binomial   Number of points for filter: 3 Sample start region (min): 0 Sample end region (min): 30
Marker peak width (sec): 5   Marker min peak height: 200   Marker baseline V to V?: Y   Marker baseline V to V points: 3
Lower marker selection: First peak > 500 RFU   Upper marker selection: Last peak > 200 RFU
Ladder size (bp)75, 200, 300, 400, 500, 700, 1000, 1500, 2000, 3000, 4000, 5000, 7000, 10000, 20000
Quantification using: Lower Marker   Final concentration (ng/uL): 0.5000   Dilution factor: 12.0

Sample: SampB2
Well location: B2
Created: Thursday, August 12, 2021 6:06:18 PM

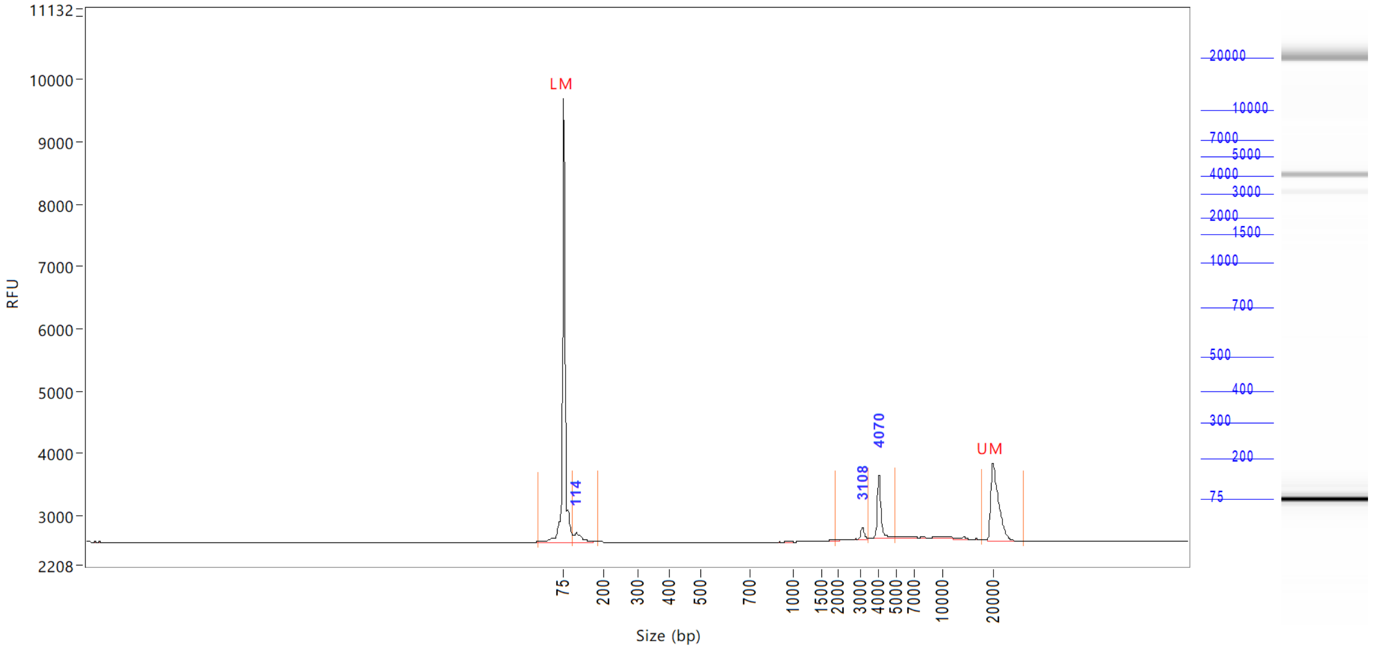

Peak	Size (bp)	Concentration (ng/uL)	From (bp)	To (bp)	Average size (bp)	CV%	RFU	Corrected peak area
1	75 (LM)	0.5000	0	105	72	14.07	7128	48.151
2	114	0.3317	105	183	121	10.92	161	2.662
3	3108	0.1513	1942	3500	3035	8.55	196	1.214
4	4070	0.7252	3500	4966	4069	4.30	1014	5.820
5	20000 (UM)	0.1318	17805	26045	20547	4.36	1245	12.689

TIC:	1.2082	ng/uL
TIM:	4.8789	nmole/L
Total concentration:	1.5741	ng/uL

```
Sample peak width (sec): 5    Sample min peak height: 50    Sample baseline V to V?: Y    Sample baseline V to V points: 3
Sample filter: Binomial       Number of points for filter: 3  Sample start region (min): 0  Sample end region (min): 30
Marker peak width (sec): 5    Marker min peak height: 200   Marker baseline V to V?: Y    Marker baseline V to V points: 3
Lower marker selection: First peak > 500 RFU                Upper marker selection: Last peak > 200 RFU
Ladder size (bp)75, 200, 300, 400, 500, 700, 1000, 1500, 2000, 3000, 4000, 5000, 7000, 10000, 20000
Quantification using: Lower Marker          Final concentration (ng/uL): 0.5000          Dilution factor: 12.0
```

Sample: SampC2
Well location: C2
Created: Thursday, August 12, 2021 6:06:18 PM

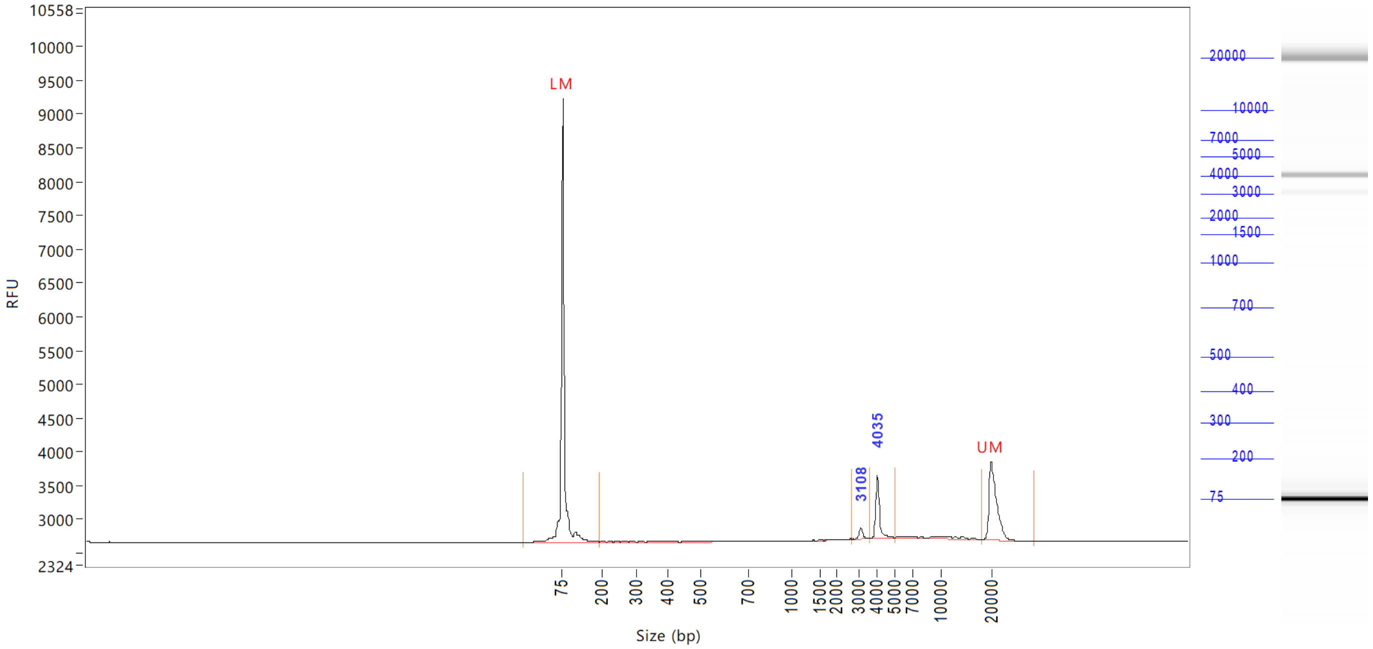

Peak	Size (bp)	Concentration (ng/uL)	From (bp)	To (bp)	Average size (bp)	CV%	RFU	Corrected peak area
1	75 (LM)	0.5000	0	189	75	23.61	6580	48.498
2	3108	0.1250	2715	3643	3108	4.78	169	1.010
3	4035	0.6744	3643	4966	4058	4.36	943	5.451
4	20000 (UM)	0.1188	18049	28463	20453	4.68	1150	11.524

TIC:	0.7993	ng/uL
TIM:	0.3398	nmole/L
Total concentration:	1.2387	ng/uL

Sample peak width (sec): 5 Sample min peak height: 50 Sample baseline V to V?: Y Sample baseline V to V points: 3
Sample filter: Binomial Number of points for filter: 3 Sample start region (min): 0 Sample end region (min): 30
Marker peak width (sec): 5 Marker min peak height: 200 Marker baseline V to V?: Y Marker baseline V to V points: 3
Lower marker selection: First peak > 500 RFU Upper marker selection: Last peak > 200 RFU
Ladder size (bp)75, 200, 300, 400, 500, 700, 1000, 1500, 2000, 3000, 4000, 5000, 7000, 10000, 20000
Quantification using: Lower Marker Final concentration (ng/uL): 0.5000 Dilution factor: 12.0

Sample: SampD2
Well location: D2
Created: Thursday, August 12, 2021 6:06:18 PM

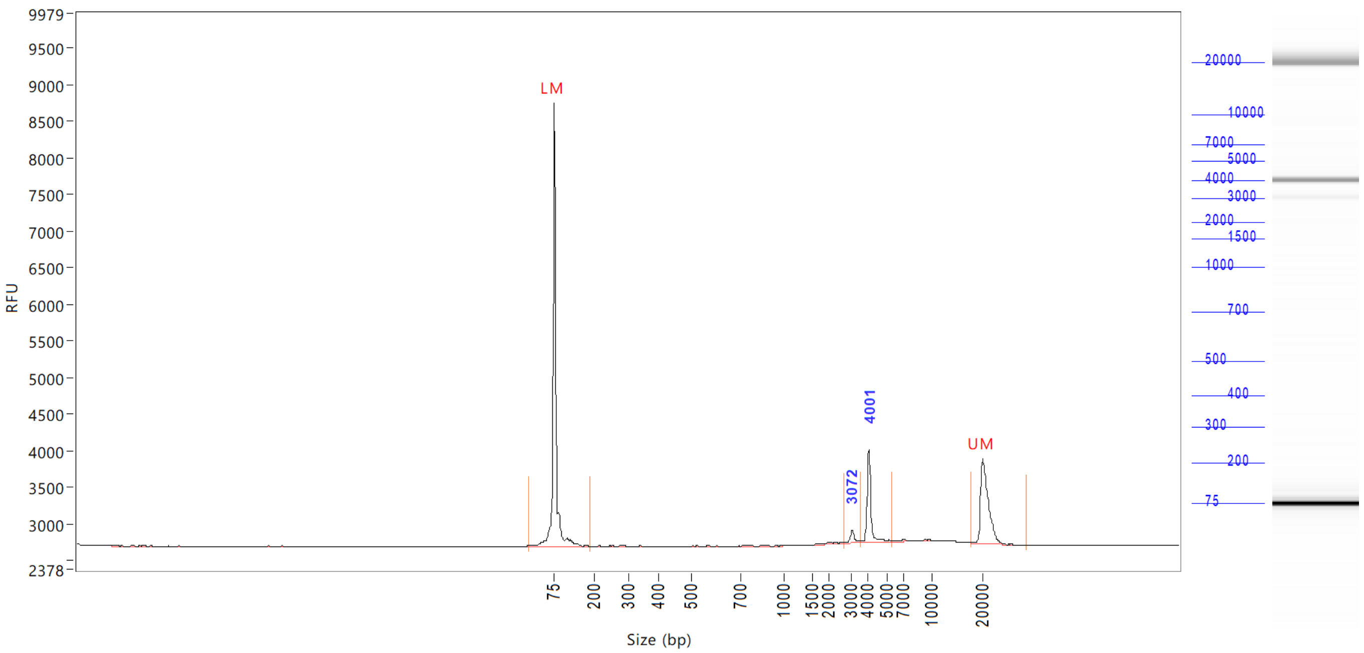

Peak	Size (bp)	Concentration (ng/uL)	From (bp)	To (bp)	Average size (bp)	CV%	RFU	Corrected peak area
1	75 (LM)	0.5000	0	187	74	21.67	6076	44.502
2	3072	0.1557	2686	3536	3061	4.93	176	1.155
3	4001	0.9916	3536	5593	4056	6.46	1257	7.355
4	20000 (UM)	0.1311	17561	28463	20490	4.80	1165	11.667

TIC:	1.1473	ng/uL
TIM:	0.4862	nmole/L
Total concentration:	1.5482	ng/uL

Sample peak width (sec): 5   Sample min peak height: 50   Sample baseline V to V?: Y   Sample baseline V to V points: 3
Sample filter: Binomial   Number of points for filter: 3 Sample start region (min): 0 Sample end region (min): 30
Marker peak width (sec): 5   Marker min peak height: 200   Marker baseline V to V?: Y   Marker baseline V to V points: 3
Lower marker selection: First peak > 500 RFU   Upper marker selection: Last peak > 200 RFU
Ladder size (bp)75, 200, 300, 400, 500, 700, 1000, 1500, 2000, 3000, 4000, 5000, 7000, 10000, 20000
Quantification using: Lower Marker   Final concentration (ng/uL): 0.5000   Dilution factor: 12.0

Sample: SampE2
Well location: E2
Created: Thursday, August 12, 2021 6:06:18 PM

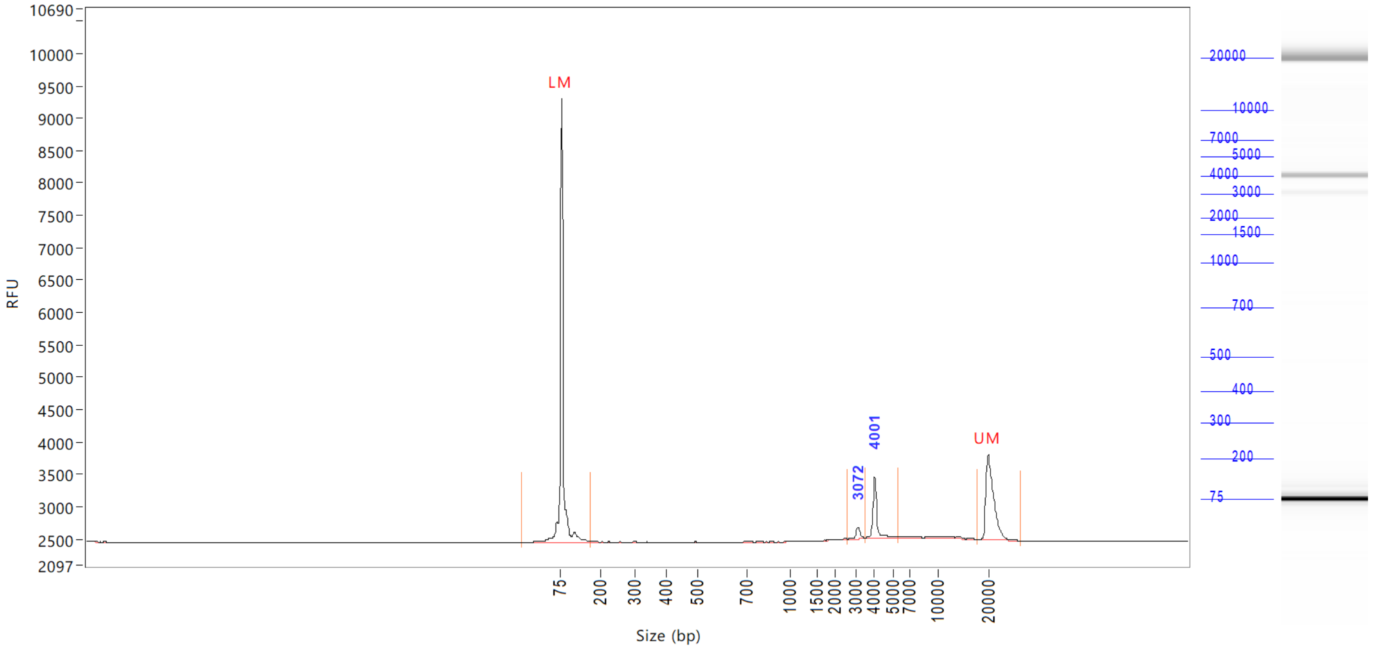

Peak	Size (bp)	Concentration (ng/uL)	From (bp)	To (bp)	Average size (bp)	CV%	RFU	Corrected peak area
1	75 (LM)	0.5000	0	166	75	21.98	6869	50.328
2	3072	0.1440	2600	3500	3054	4.93	185	1.208
3	4001	0.6913	3500	5667	4079	6.92	959	5.799
4	20000 (UM)	0.1302	17805	26287	20458	4.57	1308	13.109

TIC:	0.8354	ng/uL
TIM:	0.3567	nmole/L
Total concentration:	1.2500	ng/uL

Sample peak width (sec): 5 Sample min peak height: 50 Sample baseline V to V?: Y Sample baseline V to V points: 3
Sample filter: Binomial Number of points for filter: 3 Sample start region (min): 0 Sample end region (min): 30
Marker peak width (sec): 5 Marker min peak height: 200 Marker baseline V to V?: Y Marker baseline V to V points: 3
Lower marker selection: First peak > 500 RFU Upper marker selection: Last peak > 200 RFU
Ladder size (bp)75, 200, 300, 400, 500, 700, 1000, 1500, 2000, 3000, 4000, 5000, 7000, 10000, 20000
Quantification using: Lower Marker Final concentration (ng/uL): 0.5000 Dilution factor: 12.0

Sample: SampF2
Well location: F2
Created: Thursday, August 12, 2021 6:06:18 PM

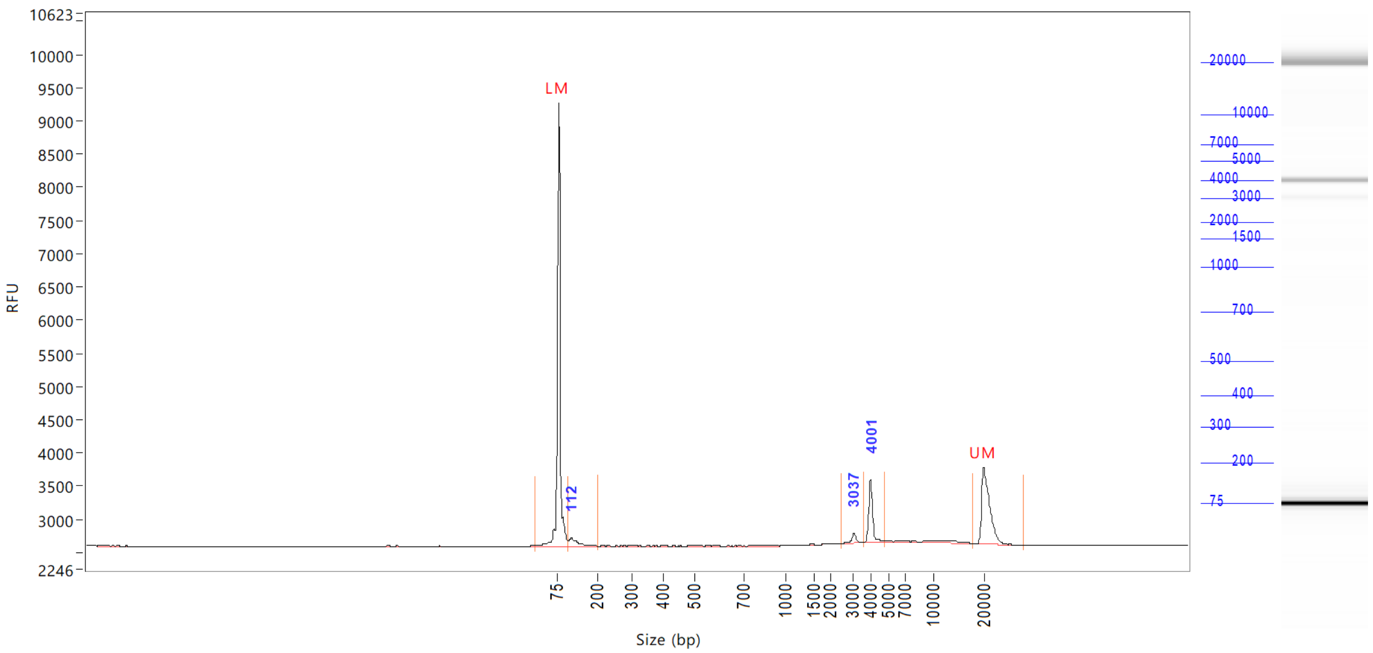

Peak	Size (bp)	Concentration (ng/uL)	From (bp)	To (bp)	Average size (bp)	CV%	RFU	Corrected peak area
1	75 (LM)	0.5000	1	105	72	13.77	6695	44.105
2	112	0.3316	105	196	126	14.50	127	2.438
3	3037	0.1387	2458	3608	3041	5.68	150	1.020
4	4001	0.7325	3608	4828	4010	3.96	941	5.384
5	20000 (UM)	0.1357	17683	27738	20500	4.68	1163	11.968

TIC:	1.2028	ng/uL
TIM:	4.7000	nmole/L
Total concentration:	1.6765	ng/uL

Sample peak width (sec): 5 Sample min peak height: 50 Sample baseline V to V?: Y Sample baseline V to V points: 3
Sample filter: Binomial Number of points for filter: 3 Sample start region (min): 0 Sample end region (min): 30
Marker peak width (sec): 5 Marker min peak height: 200 Marker baseline V to V?: Y Marker baseline V to V points: 3
Lower marker selection: First peak > 500 RFU Upper marker selection: Last peak > 200 RFU
Ladder size (bp)75, 200, 300, 400, 500, 700, 1000, 1500, 2000, 3000, 4000, 5000, 7000, 10000, 20000
Quantification using: Lower Marker Final concentration (ng/uL): 0.5000 Dilution factor: 12.0

Sample: SampG2
Well location: G2
Created: Thursday, August 12, 2021 6:06:18 PM

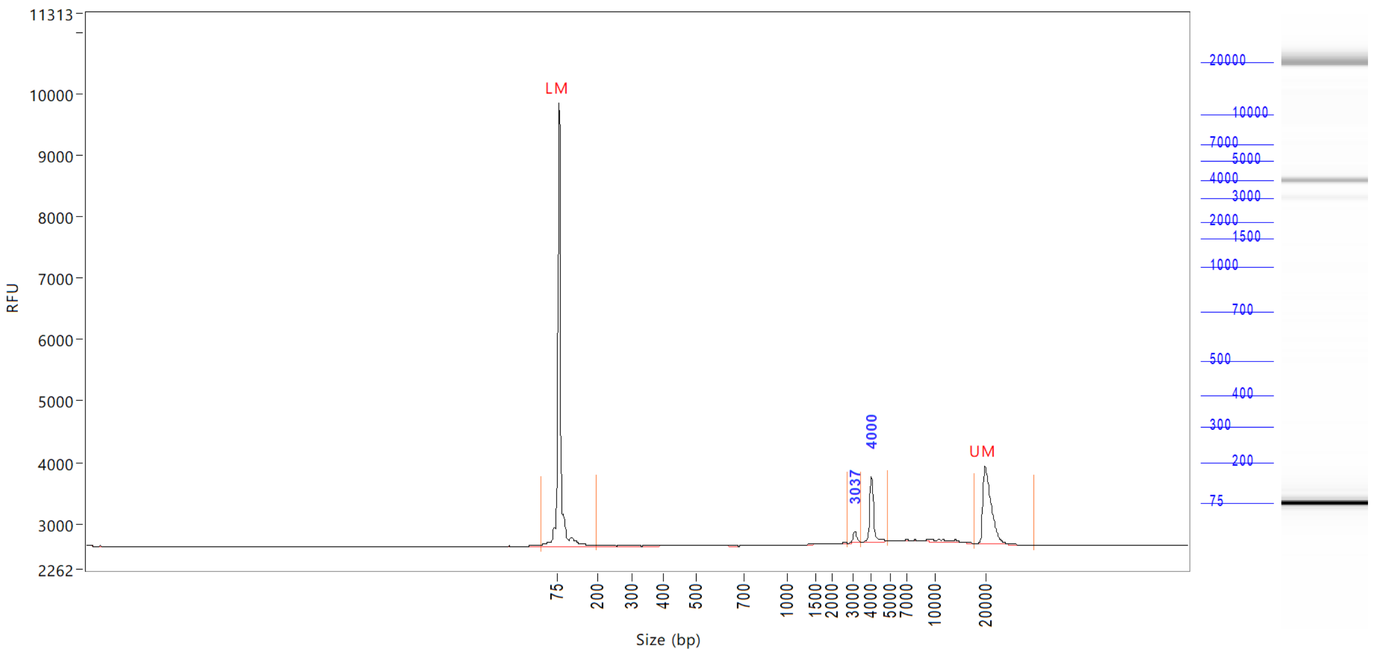

Peak	Size (bp)	Concentration (ng/uL)	From (bp)	To (bp)	Average size (bp)	CV%	RFU	Corrected peak area
1	75 (LM)	0.5000	20	194	76	20.63	7231	51.681
2	3037	0.1280	2743	3429	3041	3.47	189	1.102
3	4000	0.7144	3429	4931	4002	4.35	1070	6.153
4	20000 (UM)	0.1307	17805	29551	20563	5.20	1272	13.512

TIC:	0.8423	ng/uL
TIM:	0.3631	nmole/L
Total concentration:	1.2653	ng/uL

```
Sample peak width (sec): 5    Sample min peak height: 50    Sample baseline V to V?: Y    Sample baseline V to V points: 3
Sample filter: Binomial       Number of points for filter: 3  Sample start region (min): 0  Sample end region (min): 30
Marker peak width (sec): 5    Marker min peak height: 200   Marker baseline V to V?: Y    Marker baseline V to V points: 3
Lower marker selection: First peak > 500 RFU                Upper marker selection: Last peak > 200 RFU
Ladder size (bp)75, 200, 300, 400, 500, 700, 1000, 1500, 2000, 3000, 4000, 5000, 7000, 10000, 20000
Quantification using: Lower Marker          Final concentration (ng/uL): 0.5000          Dilution factor: 12.0
```

Sample: SampH2
Well location: H2
Created: Thursday, August 12, 2021 6:06:18 PM

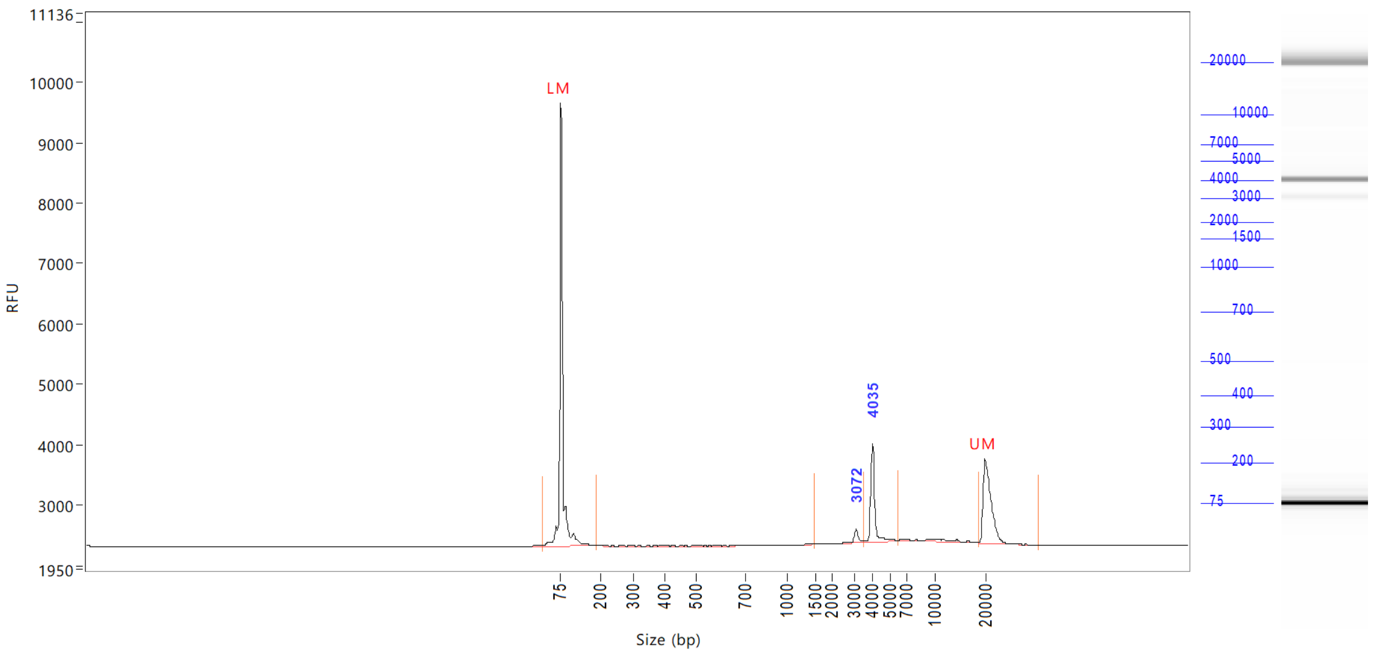

Peak	Size (bp)	Concentration (ng/uL)	From (bp)	To (bp)	Average size (bp)	CV%	RFU	Corrected peak area
1	75 (LM)	0.5000	22	187	77	19.78	7335	53.738
2	3072	0.1752	1489	3572	2994	11.09	233	1.569
3	4035	1.0548	3572	5889	4095	6.79	1618	9.447
4	20000 (UM)	0.1329	18659	30519	20567	4.99	1397	14.282

TIC:	1.2299	ng/uL
TIM:	0.5203	nmole/L
Total concentration:	1.5496	ng/uL

Sample peak width (sec): 5 Sample min peak height: 50 Sample baseline V to V?: Y Sample baseline V to V points: 3
Sample filter: Binomial Number of points for filter: 3 Sample start region (min): 0 Sample end region (min): 30
Marker peak width (sec): 5 Marker min peak height: 200 Marker baseline V to V?: Y Marker baseline V to V points: 3
Lower marker selection: First peak > 500 RFU Upper marker selection: Last peak > 200 RFU
Ladder size (bp)75, 200, 300, 400, 500, 700, 1000, 1500, 2000, 3000, 4000, 5000, 7000, 10000, 20000
Quantification using: Lower Marker Final concentration (ng/uL): 0.5000 Dilution factor: 12.0

Sample: SampA3
Well location: A3
Created: Thursday, August 12, 2021 6:06:18 PM

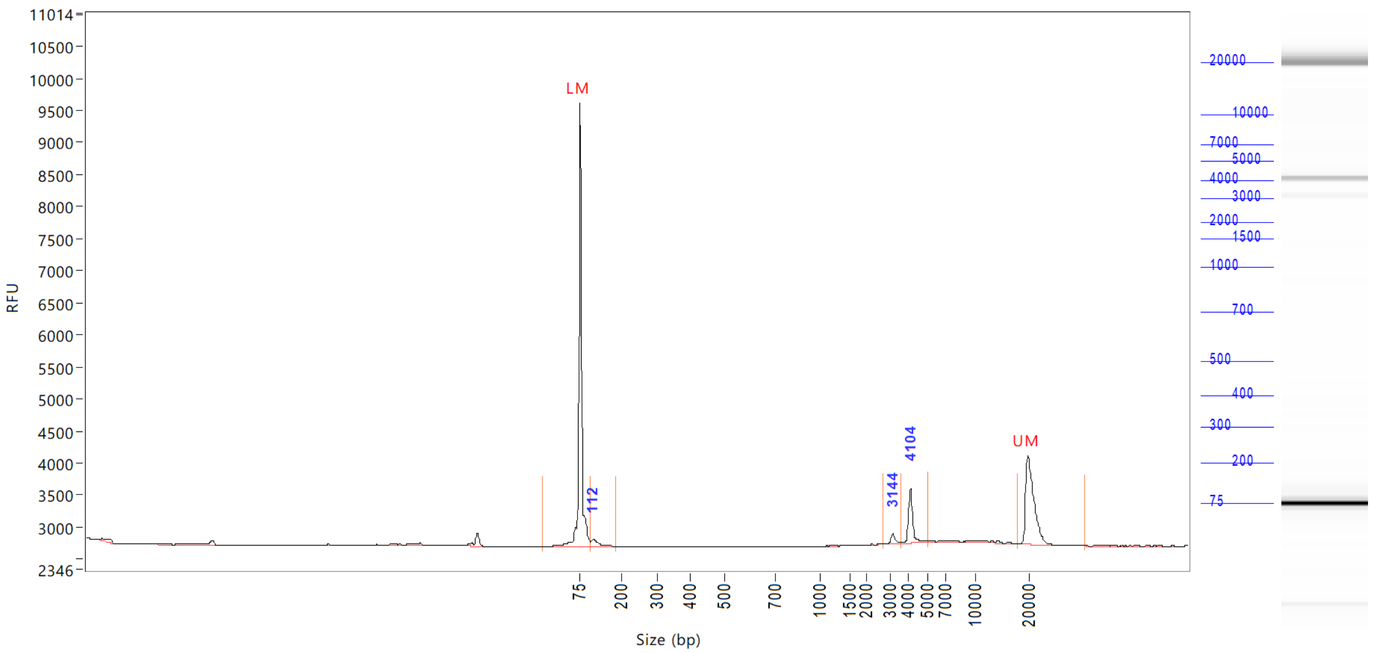

Peak	Size (bp)	Concentration (ng/uL)	From (bp)	To (bp)	Average size (bp)	CV%	RFU	Corrected peak area
1	75 (LM)	0.5000	0	105	72	15.95	6931	45.658
2	112	0.2828	105	181	125	13.87	119	2.152
3	3144	0.1266	2743	3572	3141	4.37	155	0.964
4	4104	0.6740	3572	5001	4116	4.89	853	5.129
5	20000 (UM)	0.1544	18171	30760	20519	5.06	1382	14.099

TIC:	1.0835	ng/uL
TIM:	4.0531	nmole/L
Total concentration:	1.5714	ng/uL

```
Sample peak width (sec): 5    Sample min peak height: 50    Sample baseline V to V?: Y    Sample baseline V to V points: 3
Sample filter: Binomial       Number of points for filter: 3  Sample start region (min): 0  Sample end region (min): 30
Marker peak width (sec): 5    Marker min peak height: 200   Marker baseline V to V?: Y    Marker baseline V to V points: 3
Lower marker selection: First peak > 500 RFU                Upper marker selection: Last peak > 200 RFU
Ladder size (bp)75, 200, 300, 400, 500, 700, 1000, 1500, 2000, 3000, 4000, 5000, 7000, 10000, 20000
Quantification using: Lower Marker          Final concentration (ng/uL): 0.5000          Dilution factor: 12.0
```

Sample: SampB3
Well location: B3
Created: Thursday, August 12, 2021 6:06:18 PM

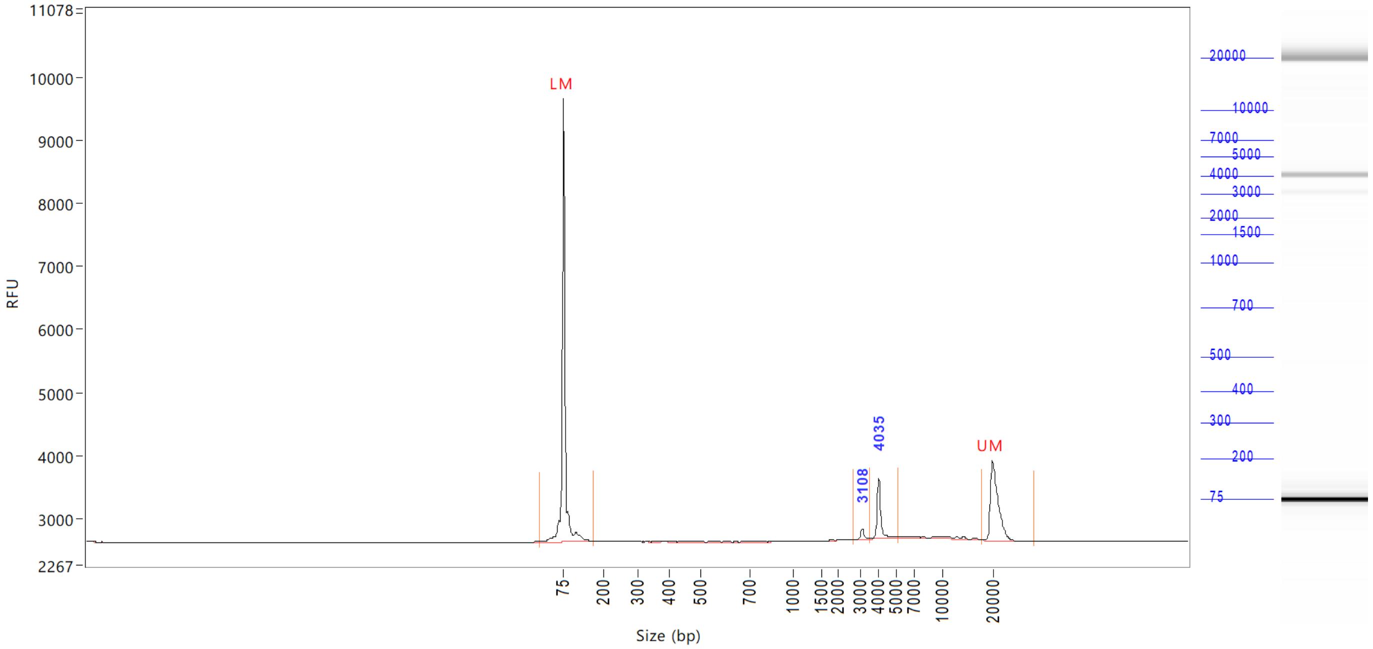

Peak	Size (bp)	Concentration (ng/uL)	From (bp)	To (bp)	Average size (bp)	CV%	RFU	Corrected peak area
1	75 (LM)	0.5000	3	169	74	19.27	7034	51.054
2	3108	0.1086	2686	3536	3105	4.14	162	0.924
3	4035	0.6356	3536	5075	4064	4.66	958	5.409
4	20000 (UM)	0.1250	17805	28100	20500	4.79	1260	12.762

TIC:	0.7442	ng/uL
TIM:	0.3150	nmole/L
Total concentration:	1.1079	ng/uL

```
Sample peak width (sec): 5    Sample min peak height: 50    Sample baseline V to V?: Y    Sample baseline V to V points: 3
Sample filter: Binomial       Number of points for filter: 3  Sample start region (min): 0  Sample end region (min): 30
Marker peak width (sec): 5    Marker min peak height: 200   Marker baseline V to V?: Y    Marker baseline V to V points: 3
Lower marker selection: First peak > 500 RFU                Upper marker selection: Last peak > 200 RFU
Ladder size (bp)75, 200, 300, 400, 500, 700, 1000, 1500, 2000, 3000, 4000, 5000, 7000, 10000, 20000
Quantification using: Lower Marker          Final concentration (ng/uL): 0.5000          Dilution factor: 12.0
```

Sample: SampC3
Well location: C3
Created: Thursday, August 12, 2021 6:06:18 PM

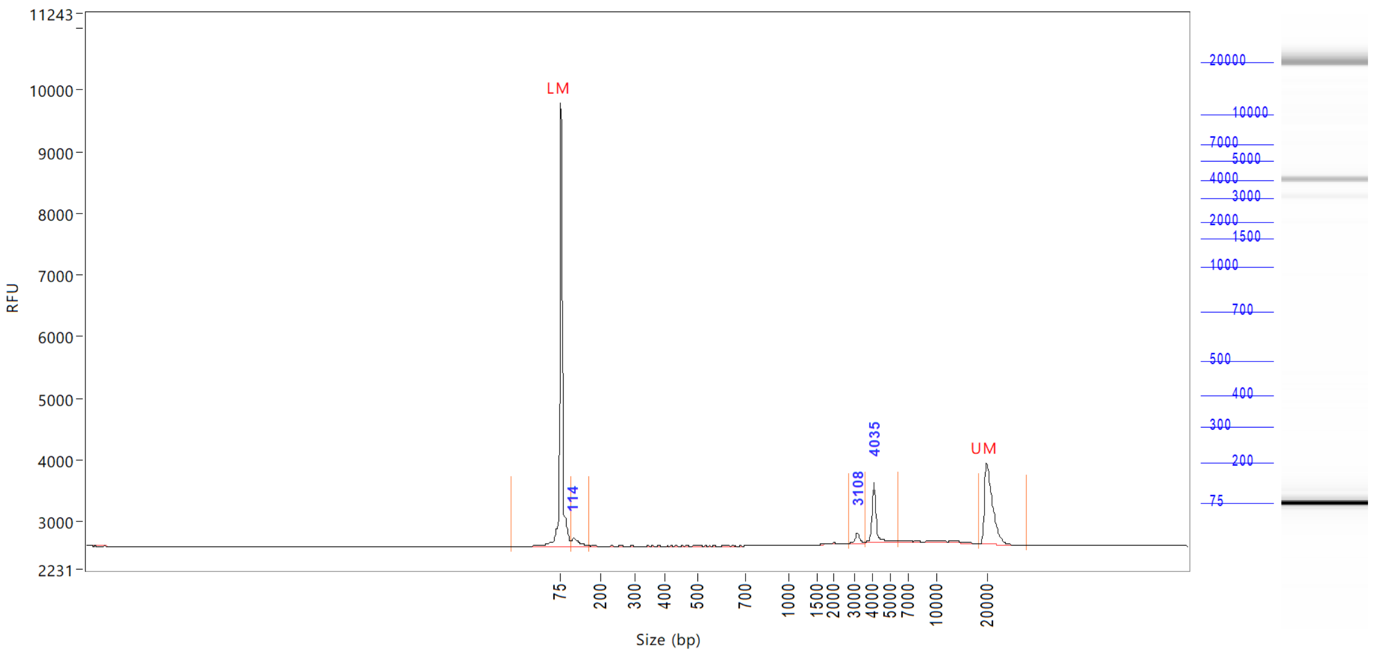

Peak	Size (bp)	Concentration (ng/uL)	From (bp)	To (bp)	Average size (bp)	CV%	RFU	Corrected peak area
1	75 (LM)	0.5000	0	107	73	17.95	7203	49.003
2	114	0.3010	107	166	124	10.92	145	2.458
3	3108	0.1371	2715	3536	3108	4.67	173	1.119
4	4035	0.7333	3536	5741	4097	6.63	967	5.989
5	20000 (UM)	0.1385	18537	27979	20595	4.65	1304	13.578

TIC:	1.1714	ng/uL
TIM:	4.3554	nmole/L
Total concentration:	1.5732	ng/uL

Sample peak width (sec): 5   Sample min peak height: 50   Sample baseline V to V?: Y   Sample baseline V to V points: 3
Sample filter: Binomial   Number of points for filter: 3 Sample start region (min): 0 Sample end region (min): 30
Marker peak width (sec): 5   Marker min peak height: 200   Marker baseline V to V?: Y   Marker baseline V to V points: 3
Lower marker selection: First peak > 500 RFU   Upper marker selection: Last peak > 200 RFU
Ladder size (bp)75, 200, 300, 400, 500, 700, 1000, 1500, 2000, 3000, 4000, 5000, 7000, 10000, 20000
Quantification using: Lower Marker   Final concentration (ng/uL): 0.5000   Dilution factor: 12.0

Sample: SampD3
Well location: D3
Created: Thursday, August 12, 2021 6:06:18 PM

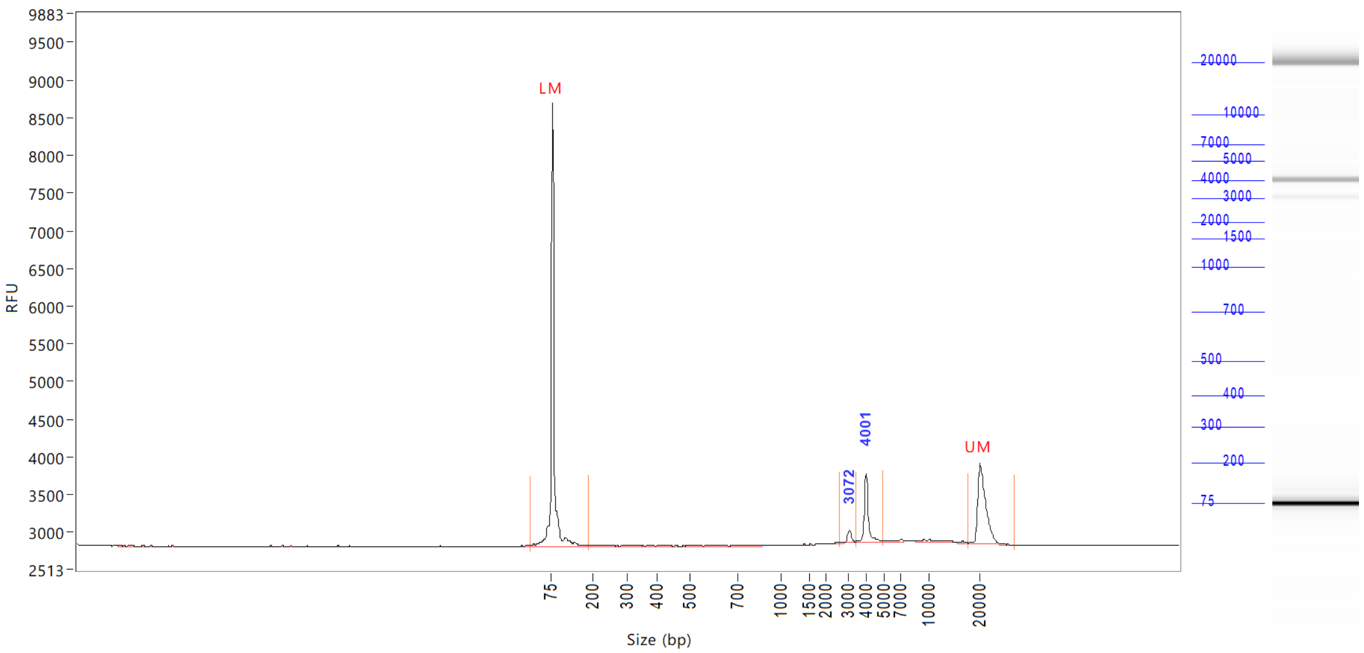

Peak	Size (bp)	Concentration (ng/uL)	From (bp)	To (bp)	Average size (bp)	CV%	RFU	Corrected peak area
1	75 (LM)	0.5000	7	187	75	21.33	5888	43.157
2	3072	0.1431	2657	3429	3057	4.09	165	1.029
3	4001	0.7754	3429	5000	4044	4.97	914	5.577
4	20000 (UM)	0.1275	17683	26770	20568	4.80	1064	11.003

TIC:	0.9185	ng/uL
TIM:	0.3927	nmole/L
Total concentration:	1.4147	ng/uL

Sample peak width (sec): 5 Sample min peak height: 50 Sample baseline V to V?: Y Sample baseline V to V points: 3
Sample filter: Binomial Number of points for filter: 3 Sample start region (min): 0 Sample end region (min): 30
Marker peak width (sec): 5 Marker min peak height: 200 Marker baseline V to V?: Y Marker baseline V to V points: 3
Lower marker selection: First peak > 500 RFU Upper marker selection: Last peak > 200 RFU
Ladder size (bp)75, 200, 300, 400, 500, 700, 1000, 1500, 2000, 3000, 4000, 5000, 7000, 10000, 20000
Quantification using: Lower Marker Final concentration (ng/uL): 0.5000 Dilution factor: 12.0

Sample: SampE3
Well location: E3
Created: Thursday, August 12, 2021 6:06:18 PM

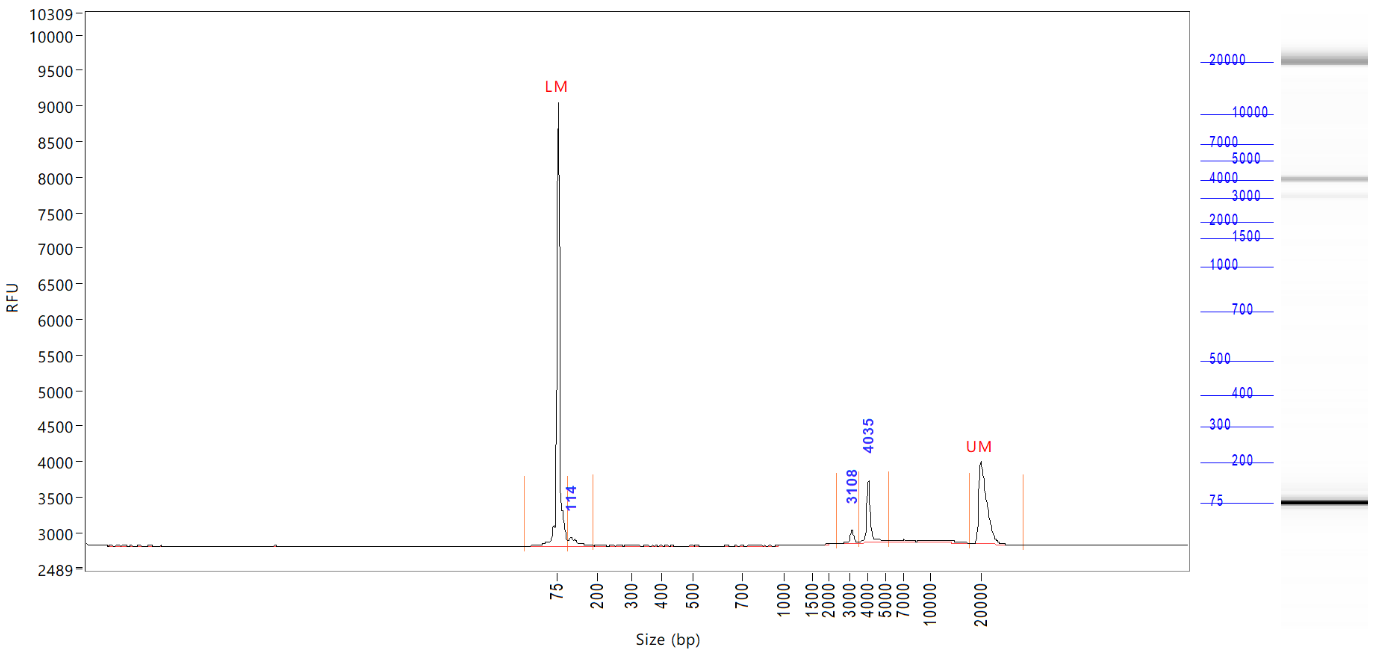

Peak	Size (bp)	Concentration (ng/uL)	From (bp)	To (bp)	Average size (bp)	CV%	RFU	Corrected peak area
1	75 (LM)	0.5000	0	107	73	16.52	6250	43.429
2	114	0.3312	107	189	128	13.80	127	2.397
3	3108	0.1742	2401	3536	3049	6.51	185	1.261
4	4035	0.7482	3536	5371	4087	6.36	871	5.415
5	20000 (UM)	0.1392	17805	28584	20568	4.68	1168	12.090

TIC:	1.2536	ng/uL
TIM:	4.6464	nmole/L
Total concentration:	1.7445	ng/uL

Sample peak width (sec): 5   Sample min peak height: 50   Sample baseline V to V?: Y   Sample baseline V to V points: 3
Sample filter: Binomial   Number of points for filter: 3   Sample start region (min): 0   Sample end region (min): 30
Marker peak width (sec): 5   Marker min peak height: 200   Marker baseline V to V?: Y   Marker baseline V to V points: 3
Lower marker selection: First peak > 500 RFU   Upper marker selection: Last peak > 200 RFU
Ladder size (bp)75, 200, 300, 400, 500, 700, 1000, 1500, 2000, 3000, 4000, 5000, 7000, 10000, 20000
Quantification using: Lower Marker   Final concentration (ng/uL): 0.5000   Dilution factor: 12.0

Sample: SampF3
Well location: F3
Created: Thursday, August 12, 2021 6:06:18 PM

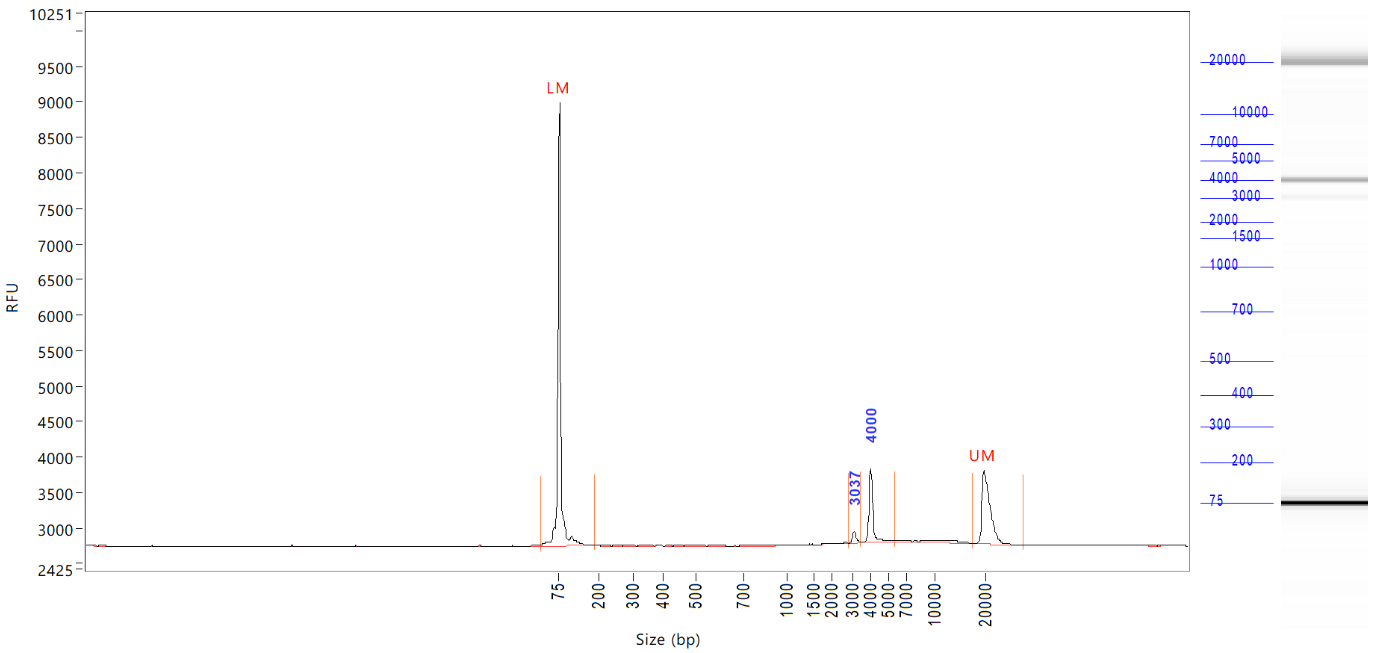

Peak	Size (bp)	Concentration (ng/uL)	From (bp)	To (bp)	Average size (bp)	CV%	RFU	Corrected peak area
1	75 (LM)	0.5000	20	185	75	18.93	6249	43.528
2	3037	0.1293	2800	3429	3055	3.65	159	0.938
3	4000	0.8490	3429	5593	4041	6.53	1035	6.159
4	20000 (UM)	0.1262	17683	27617	20539	5.18	1042	10.982

TIC:	0.9783	ng/uL
TIM:	0.4155	nmole/L
Total concentration:	1.3841	ng/uL

Sample peak width (sec): 5 Sample min peak height: 50 Sample baseline V to V?: Y Sample baseline V to V points: 3
Sample filter: Binomial Number of points for filter: 3 Sample start region (min): 0 Sample end region (min): 30
Marker peak width (sec): 5 Marker min peak height: 200 Marker baseline V to V?: Y Marker baseline V to V points: 3
Lower marker selection: First peak > 500 RFU Upper marker selection: Last peak > 200 RFU
Ladder size (bp)75, 200, 300, 400, 500, 700, 1000, 1500, 2000, 3000, 4000, 5000, 7000, 10000, 20000
Quantification using: Lower Marker Final concentration (ng/uL): 0.5000 Dilution factor: 12.0

Sample: SampG3
Well location: G3
Created: Thursday, August 12, 2021 6:06:18 PM

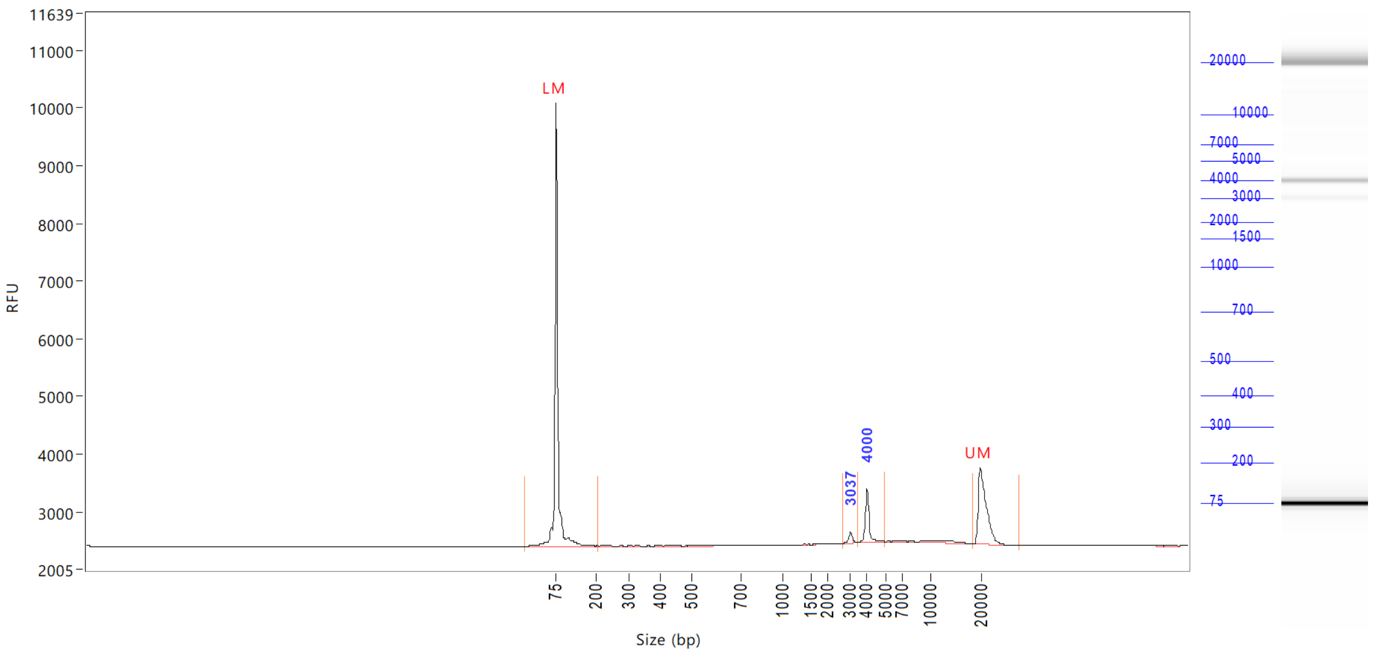

Peak	Size (bp)	Concentration (ng/uL)	From (bp)	To (bp)	Average size (bp)	CV%	RFU	Corrected peak area
1	75 (LM)	0.5000	0	206	75	23.77	7699	55.614
2	3037	0.1207	2715	3465	3043	3.92	190	1.118
3	4000	0.5925	3465	5001	4003	4.95	925	5.492
4	20000 (UM)	0.1251	18537	27859	20576	4.76	1317	13.911

TIC:	0.7132	ng/uL
TIM:	0.3090	nmole/L
Total concentration:	1.0655	ng/uL

Sample peak width (sec): 5 Sample min peak height: 50 Sample baseline V to V?: Y Sample baseline V to V points: 3
Sample filter: Binomial Number of points for filter: 3 Sample start region (min): 0 Sample end region (min): 30
Marker peak width (sec): 5 Marker min peak height: 200 Marker baseline V to V?: Y Marker baseline V to V points: 3
Lower marker selection: First peak > 500 RFU Upper marker selection: Last peak > 200 RFU
Ladder size (bp)75, 200, 300, 400, 500, 700, 1000, 1500, 2000, 3000, 4000, 5000, 7000, 10000, 20000
Quantification using: Lower Marker Final concentration (ng/uL): 0.5000 Dilution factor: 12.0

Sample: SampH3
Well location: H3
Created: Thursday, August 12, 2021 6:06:18 PM

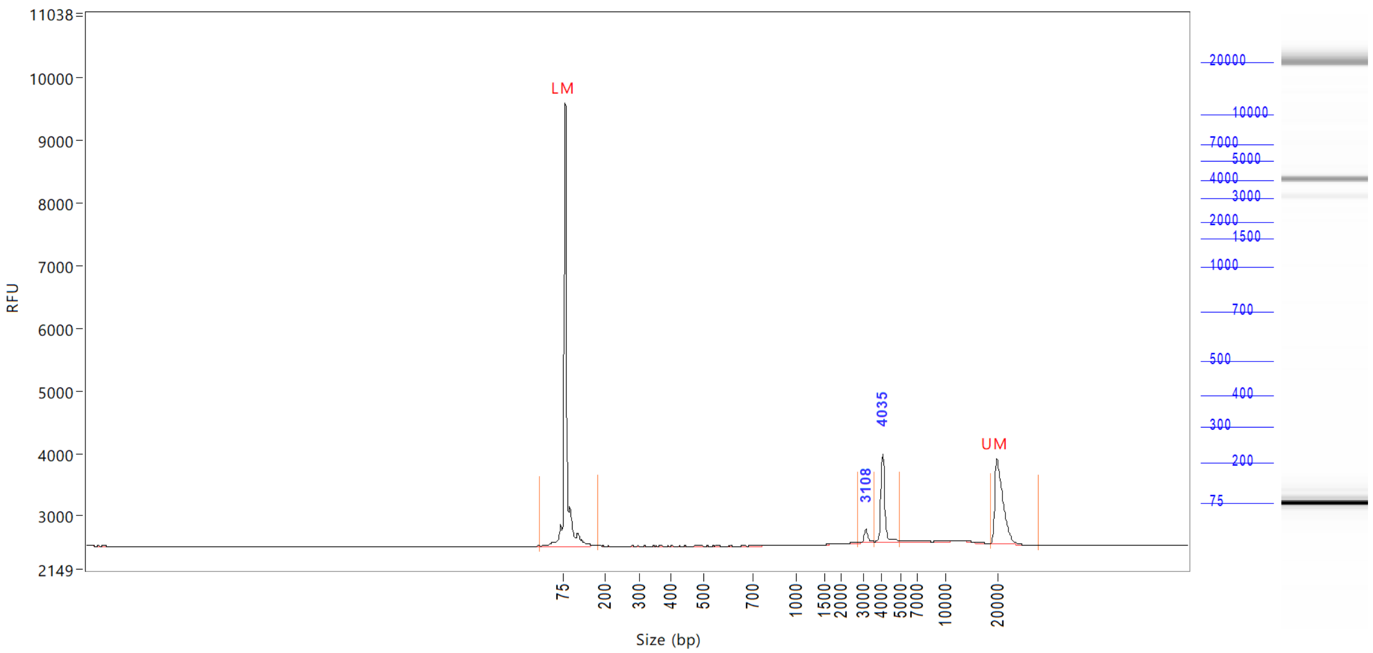

Peak	Size (bp)	Concentration (ng/uL)	From (bp)	To (bp)	Average size (bp)	CV%	RFU	Corrected peak area
1	75 (LM)	0.5000	0	175	76	19.92	7102	52.474
2	3108	0.1560	2772	3608	3119	4.67	225	1.364
3	4035	0.9130	3608	5001	4067	4.27	1415	7.985
4	20000 (UM)	0.1346	18659	28221	20586	4.62	1359	14.127

TIC:	1.0690	ng/uL
TIM:	0.4519	nmole/L
Total concentration:	1.3724	ng/uL

Sample peak width (sec): 5 Sample min peak height: 50 Sample baseline V to V?: Y Sample baseline V to V points: 3
Sample filter: Binomial Number of points for filter: 3 Sample start region (min): 0 Sample end region (min): 30
Marker peak width (sec): 5 Marker min peak height: 200 Marker baseline V to V?: Y Marker baseline V to V points: 3
Lower marker selection: First peak > 500 RFU Upper marker selection: Last peak > 200 RFU
Ladder size (bp)75, 200, 300, 400, 500, 700, 1000, 1500, 2000, 3000, 4000, 5000, 7000, 10000, 20000
Quantification using: Lower Marker Final concentration (ng/uL): 0.5000 Dilution factor: 12.0

Sample: SampA4
Well location: A4
Created: Thursday, August 12, 2021 6:06:18 PM

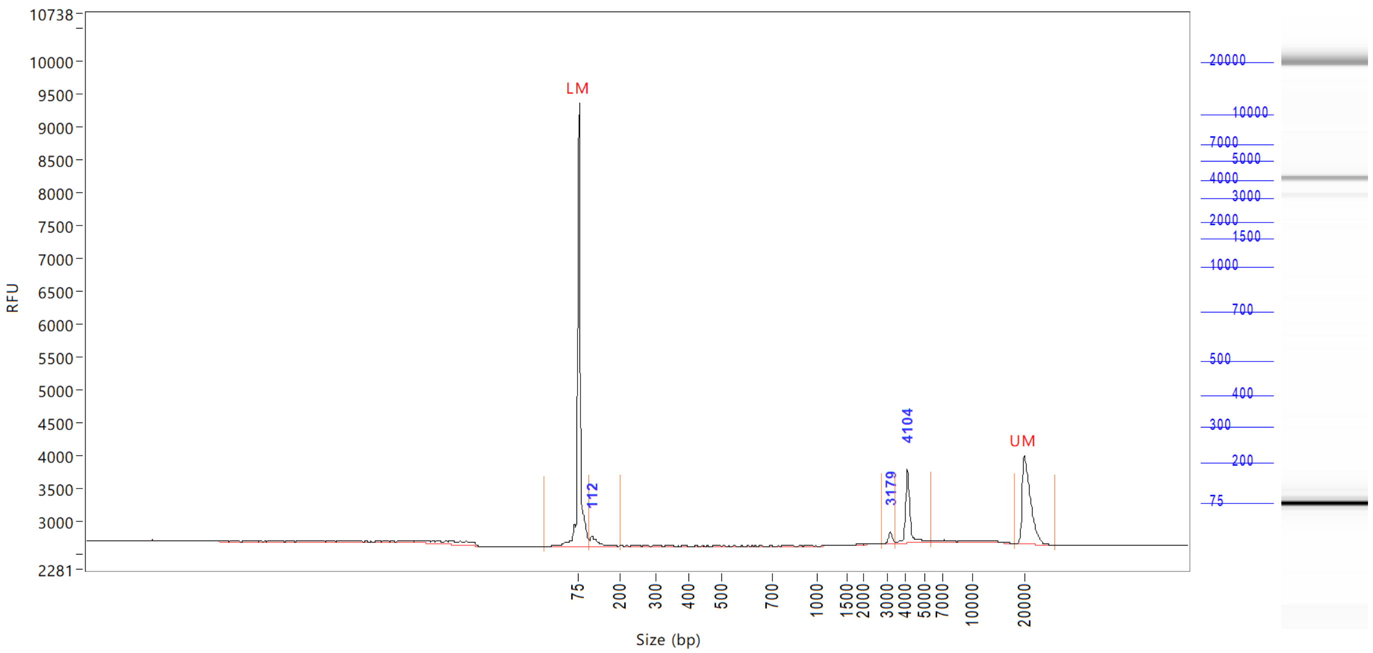

Peak	Size (bp)	Concentration (ng/uL)	From (bp)	To (bp)	Average size (bp)	CV%	RFU	Corrected peak area
1	75 (LM)	0.5000	0	105	72	15.33	6755	45.487
2	112	0.3362	105	196	123	13.21	160	2.549
3	3179	0.1386	2772	3429	3144	3.69	181	1.051
4	4104	0.9331	3429	5667	4153	7.31	1121	7.074
5	20000 (UM)	0.1510	18293	25682	20531	4.45	1352	13.733

TIC:	1.4079	ng/uL
TIM:	4.9329	nmole/L
Total concentration:	1.8466	ng/uL

Sample peak width (sec): 5   Sample min peak height: 50   Sample baseline V to V?: Y   Sample baseline V to V points: 3
Sample filter: Binomial   Number of points for filter: 3 Sample start region (min): 0 Sample end region (min): 30
Marker peak width (sec): 5   Marker min peak height: 200   Marker baseline V to V?: Y   Marker baseline V to V points: 3
Lower marker selection: First peak > 500 RFU   Upper marker selection: Last peak > 200 RFU
Ladder size (bp)75, 200, 300, 400, 500, 700, 1000, 1500, 2000, 3000, 4000, 5000, 7000, 10000, 20000
Quantification using: Lower Marker   Final concentration (ng/uL): 0.5000   Dilution factor: 12.0

Sample: SampB4
Well location: B4
Created: Thursday, August 12, 2021 6:06:18 PM

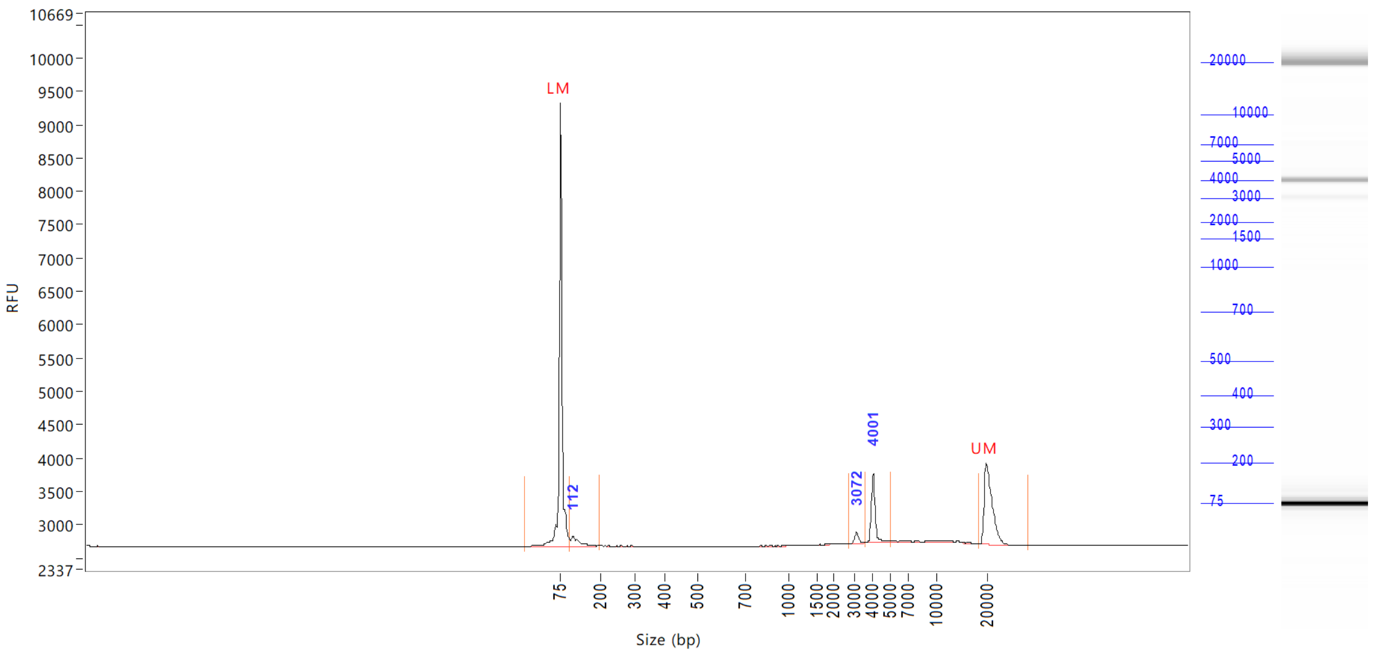

Peak	Size (bp)	Concentration (ng/uL)	From (bp)	To (bp)	Average size (bp)	CV%	RFU	Corrected peak area
1	75 (LM)	0.5000	0	105	71	16.30	6656	47.357
2	112	0.3281	105	196	123	12.42	149	2.590
3	3072	0.1298	2715	3572	3065	4.13	171	1.024
4	4001	0.7522	3572	4966	4025	4.33	1046	5.937
5	20000 (UM)	0.1308	18415	28100	20504	4.55	1214	12.386

TIC:	1.2101	ng/uL
TIM:	4.7601	nmole/L
Total concentration:	1.4875	ng/uL

Sample peak width (sec): 5 Sample min peak height: 50 Sample baseline V to V?: Y Sample baseline V to V points: 3
Sample filter: Binomial Number of points for filter: 3 Sample start region (min): 0 Sample end region (min): 30
Marker peak width (sec): 5 Marker min peak height: 200 Marker baseline V to V?: Y Marker baseline V to V points: 3
Lower marker selection: First peak > 500 RFU Upper marker selection: Last peak > 200 RFU
Ladder size (bp)75, 200, 300, 400, 500, 700, 1000, 1500, 2000, 3000, 4000, 5000, 7000, 10000, 20000
Quantification using: Lower Marker Final concentration (ng/uL): 0.5000 Dilution factor: 12.0

Sample: SampC4
Well location: C4
Created: Thursday, August 12, 2021 6:06:18 PM

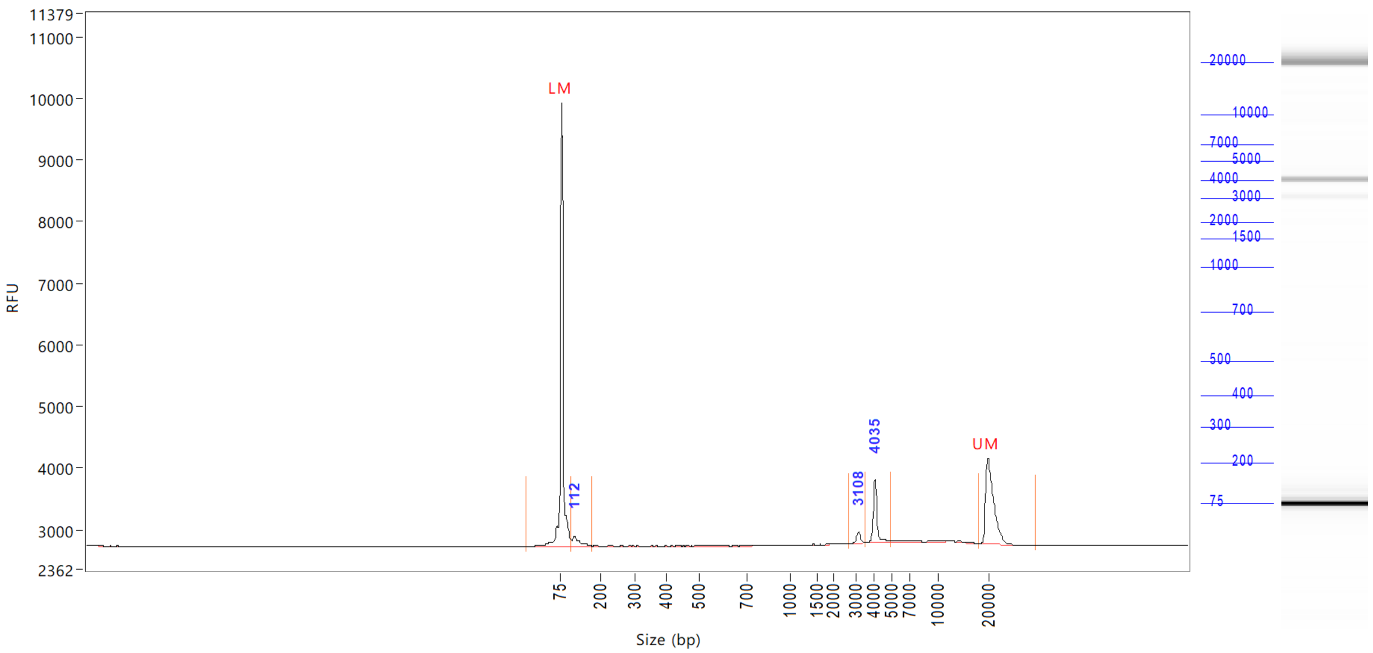

Peak	Size (bp)	Concentration (ng/uL)	From (bp)	To (bp)	Average size (bp)	CV%	RFU	Corrected peak area
1	75 (LM)	0.5000	0	105	71	15.85	7206	50.387
2	112	0.3235	105	168	122	11.41	175	2.717
3	3108	0.1288	2657	3500	3084	4.05	179	1.082
4	4035	0.6875	3500	4931	4049	4.35	1020	5.774
5	20000 (UM)	0.1433	18293	29309	20582	4.92	1400	14.439

TIC:	1.1399	ng/uL
TIM:	4.7046	nmole/L
Total concentration:	1.4739	ng/uL

Sample peak width (sec): 5   Sample min peak height: 50   Sample baseline V to V?: Y   Sample baseline V to V points: 3
Sample filter: Binomial   Number of points for filter: 3   Sample start region (min): 0   Sample end region (min): 30
Marker peak width (sec): 5   Marker min peak height: 200   Marker baseline V to V?: Y   Marker baseline V to V points: 3
Lower marker selection: First peak > 500 RFU   Upper marker selection: Last peak > 200 RFU
Ladder size (bp)75, 200, 300, 400, 500, 700, 1000, 1500, 2000, 3000, 4000, 5000, 7000, 10000, 20000
Quantification using: Lower Marker   Final concentration (ng/uL): 0.5000   Dilution factor: 12.0

Sample: SampD4
Well location: D4
Created: Thursday, August 12, 2021 6:06:18 PM

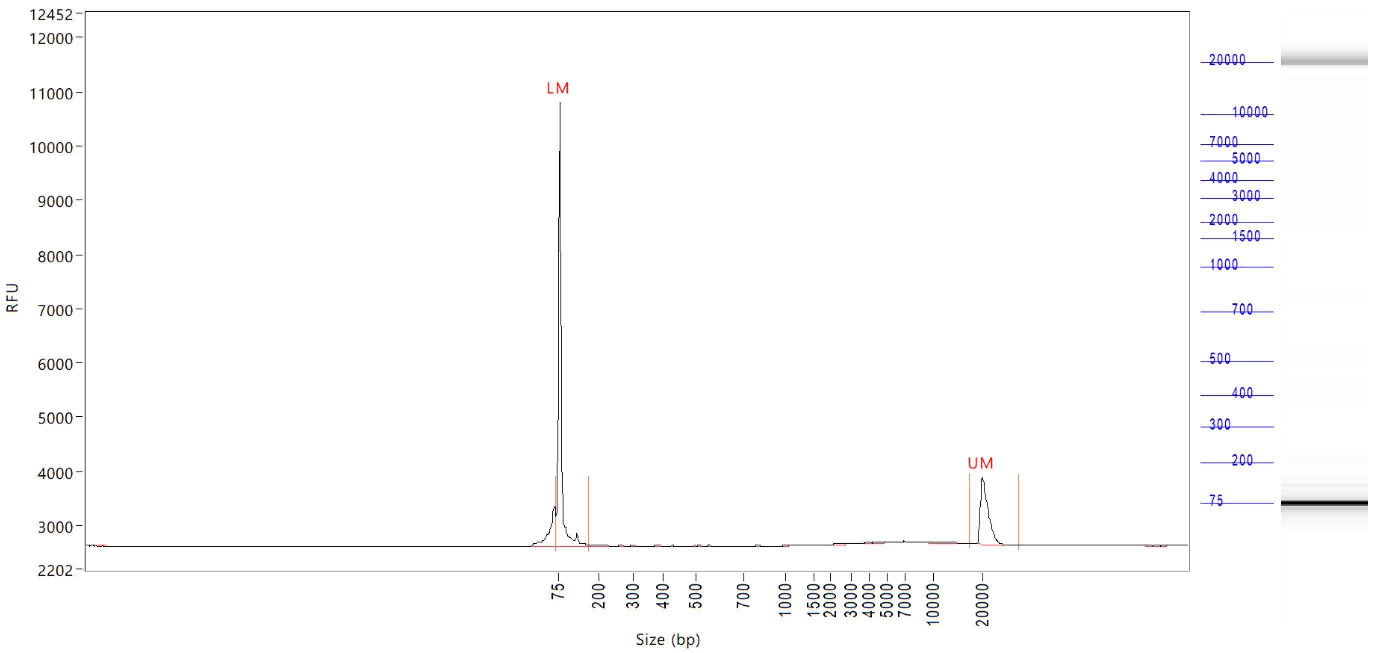

Peak	Size (bp)	Concentration (ng/uL)	From (bp)	To (bp)	Average size (bp)	CV%	RFU	Corrected peak area
1	75 (LM)	0.5000	64	168	77	18.70	8193	57.690
2	20000 (UM)	0.1094	17439	27254	20499	4.62	1239	12.627

TIC:	0.0000	ng/uL
TIM:	0.0000	nmole/L
Total concentration:	0.4785	ng/uL

Sample peak width (sec): 5 Sample min peak height: 50 Sample baseline V to V?: Y Sample baseline V to V points: 3
Sample filter: Binomial Number of points for filter: 3 Sample start region (min): 0 Sample end region (min): 30
Marker peak width (sec): 5 Marker min peak height: 200 Marker baseline V to V?: Y Marker baseline V to V points: 3
Lower marker selection: First peak > 500 RFU Upper marker selection: Last peak > 200 RFU
Ladder size (bp)75, 200, 300, 400, 500, 700, 1000, 1500, 2000, 3000, 4000, 5000, 7000, 10000, 20000
Quantification using: Lower Marker Final concentration (ng/uL): 0.5000 Dilution factor: 12.0

Sample: SampE4
Well location: E4
Created: Thursday, August 12, 2021 6:06:18 PM

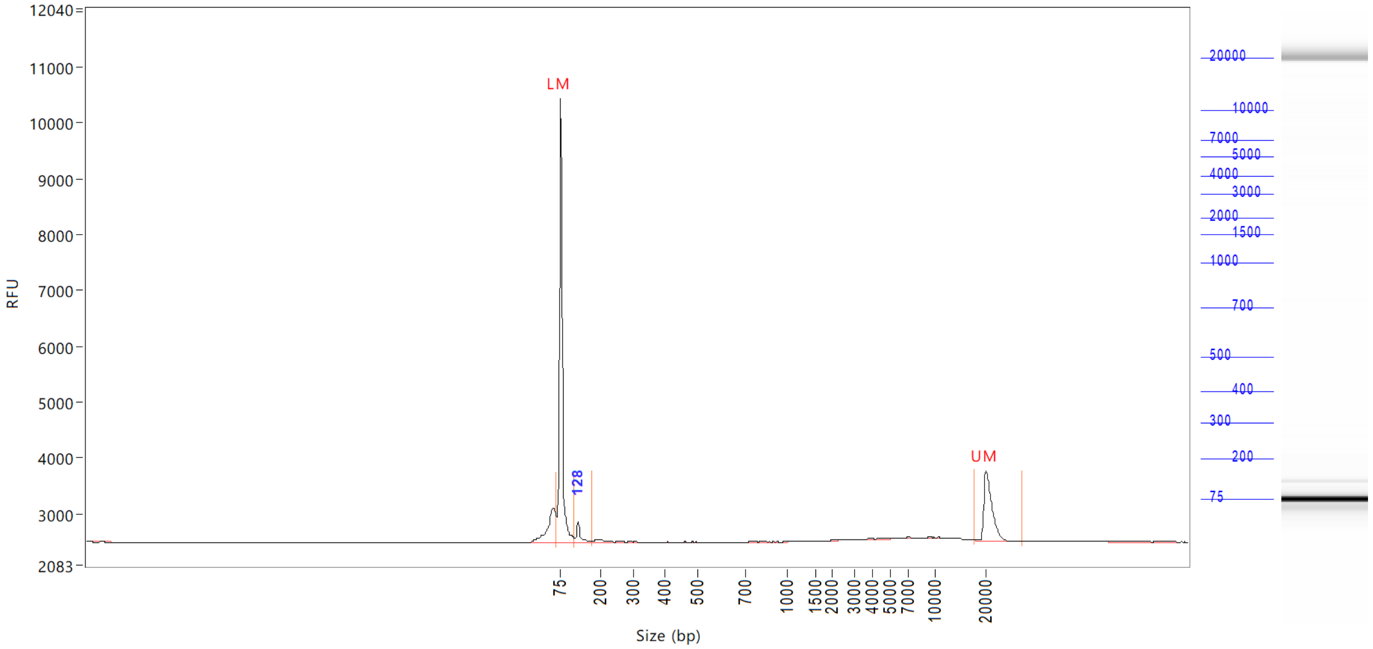

Peak	Size (bp)	Concentration (ng/uL)	From (bp)	To (bp)	Average size (bp)	CV%	RFU	Corrected peak area
1	75 (LM)	0.5000	64	118	75	9.92	7953	58.329
2	128	0.3318	118	171	132	9.45	367	3.225
3	20000 (UM)	0.1107	17805	27254	20590	4.68	1260	12.910

TIC:	0.3318	ng/uL
TIM:	4.1297	nmole/L
Total concentration:	0.7215	ng/uL

```
Sample peak width (sec): 5    Sample min peak height: 50    Sample baseline V to V?: Y    Sample baseline V to V points: 3
Sample filter: Binomial       Number of points for filter: 3  Sample start region (min): 0  Sample end region (min): 30
Marker peak width (sec): 5    Marker min peak height: 200   Marker baseline V to V?: Y    Marker baseline V to V points: 3
Lower marker selection: First peak > 500 RFU                Upper marker selection: Last peak > 200 RFU
Ladder size (bp)75, 200, 300, 400, 500, 700, 1000, 1500, 2000, 3000, 4000, 5000, 7000, 10000, 20000
Quantification using: Lower Marker          Final concentration (ng/uL): 0.5000          Dilution factor: 12.0
```

Sample: SampF4
Well location: F4
Created: Thursday, August 12, 2021 6:06:18 PM

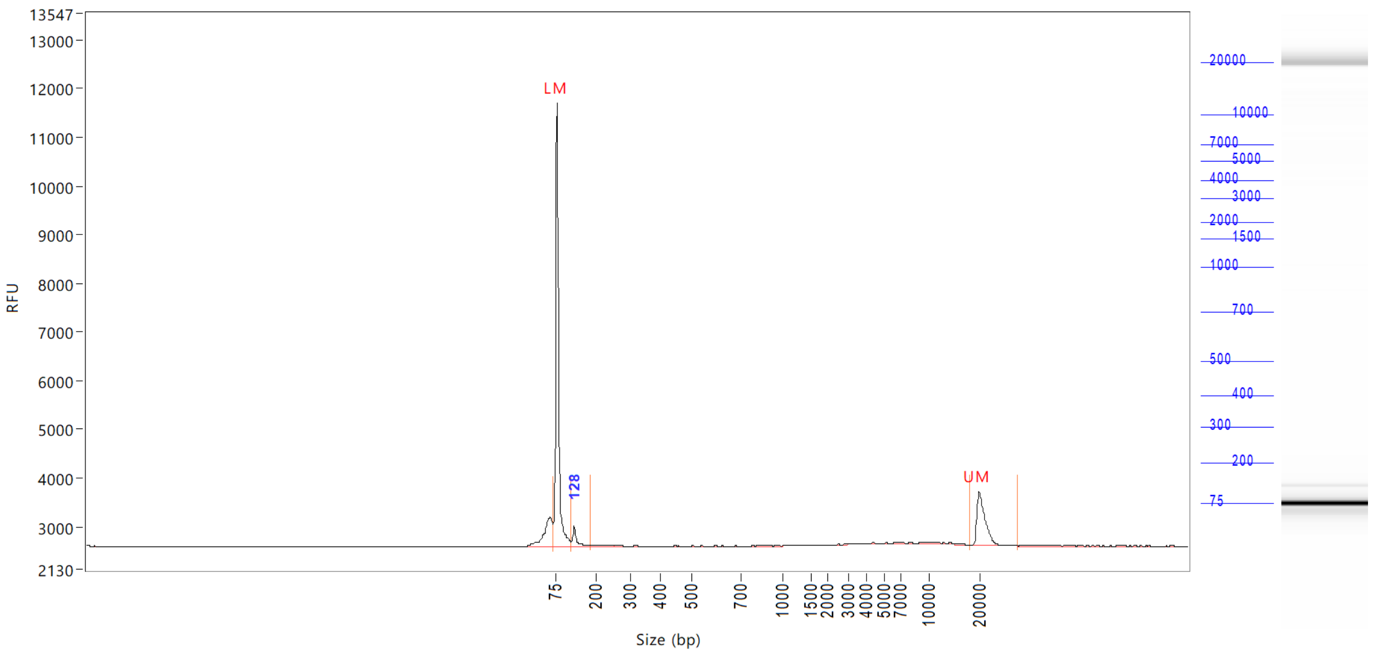

Peak	Size (bp)	Concentration (ng/uL)	From (bp)	To (bp)	Average size (bp)	CV%	RFU	Corrected peak area
1	75 (LM)	0.5000	64	118	75	10.60	9120	61.931
2	128	0.3686	118	181	134	11.21	406	3.804
3	20000 (UM)	0.0950	18171	27859	20575	5.22	1108	11.765

TIC:	0.3686	ng/uL
TIM:	4.5193	nmole/L
Total concentration:	0.7397	ng/uL

```
Sample peak width (sec): 5    Sample min peak height: 50    Sample baseline V to V?: Y    Sample baseline V to V points: 3
Sample filter: Binomial       Number of points for filter: 3  Sample start region (min): 0  Sample end region (min): 30
Marker peak width (sec): 5    Marker min peak height: 200   Marker baseline V to V?: Y    Marker baseline V to V points: 3
Lower marker selection: First peak > 500 RFU                Upper marker selection: Last peak > 200 RFU
Ladder size (bp)75, 200, 300, 400, 500, 700, 1000, 1500, 2000, 3000, 4000, 5000, 7000, 10000, 20000
Quantification using: Lower Marker          Final concentration (ng/uL): 0.5000          Dilution factor: 12.0
```

Sample: SampG4
Well location: G4
Created: Thursday, August 12, 2021 6:06:18 PM

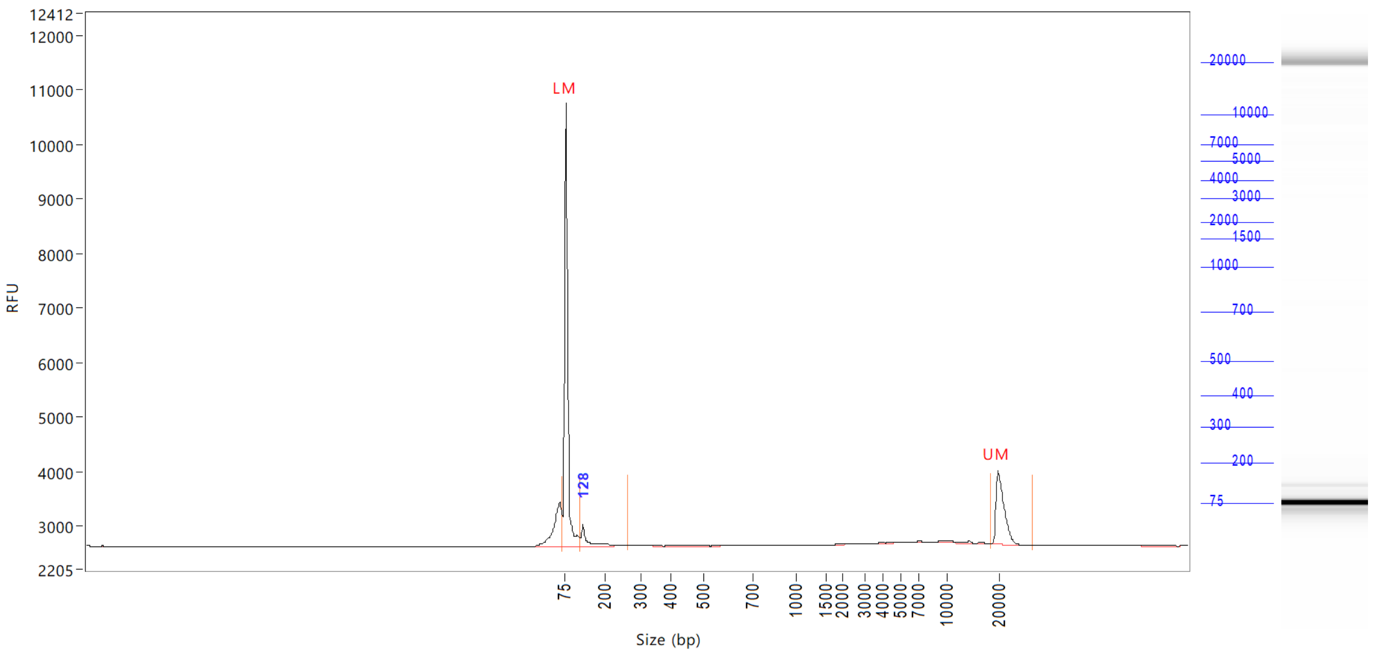

Peak	Size<br>(bp)	Concentration<br>(ng/uL)	From<br>(bp)	To<br>(bp)	Average size<br>(bp)	CV%	RFU	Corrected peak area
1	75 (LM)	0.5000	66	120	77	11.09	8155	63.755
2	128	0.4789	120	262	146	19.42	395	5.089
3	20000 (UM)	0.1076	18537	26770	20558	4.44	1356	13.726

TIC:	0.4789	ng/uL
TIM:	5.3910	nmole/L
Total concentration:	0.7968	ng/uL

Sample peak width (sec): 5 Sample min peak height: 50 Sample baseline V to V?: Y Sample baseline V to V points: 3
Sample filter: Binomial Number of points for filter: 3 Sample start region (min): 0 Sample end region (min): 30
Marker peak width (sec): 5 Marker min peak height: 200 Marker baseline V to V?: Y Marker baseline V to V points: 3
Lower marker selection: First peak > 500 RFU Upper marker selection: Last peak > 200 RFU
Ladder size (bp)75, 200, 300, 400, 500, 700, 1000, 1500, 2000, 3000, 4000, 5000, 7000, 10000, 20000
Quantification using: Lower Marker Final concentration (ng/uL): 0.5000 Dilution factor: 12.0

Sample: SampH4
Well location: H4
Created: Thursday, August 12, 2021 6:06:18 PM

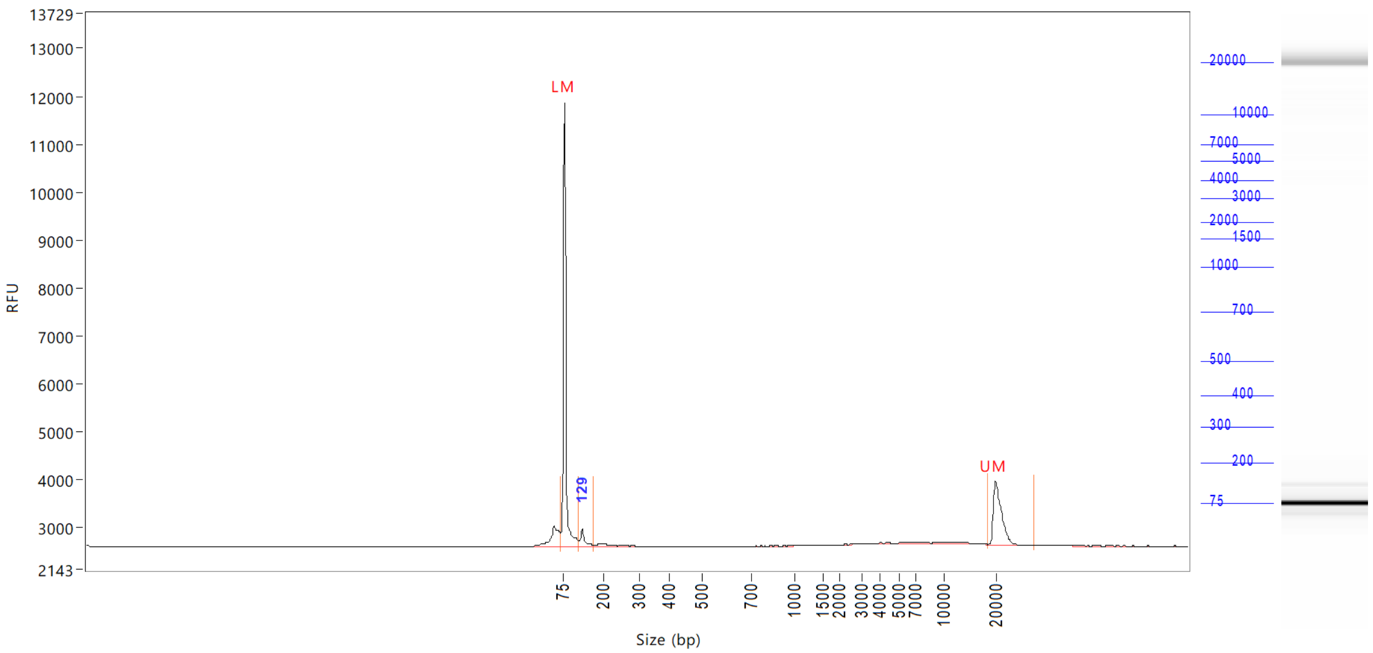

Peak	Size<br><br>(bp)	Concentration<br><br>(ng/uL)	From<br><br>(bp)	To<br><br>(bp)	Average size<br>(bp)	CV%	RFU	Corrected peak area
1	75 (LM)	0.5000	64	120	75	10.89	9258	59.159
2	129	0.3516	120	166	134	8.13	358	3.466
3	20000 (UM)	0.1174	18415	27496	20527	4.64	1331	13.893

TIC:	0.3516	ng/uL
TIM:	4.3112	nmole/L
Total concentration:	0.8400	ng/uL

Sample peak width (sec): 5 Sample min peak height: 50 Sample baseline V to V?: Y Sample baseline V to V points: 3
Sample filter: Binomial Number of points for filter: 3 Sample start region (min): 0 Sample end region (min): 30
Marker peak width (sec): 5 Marker min peak height: 200 Marker baseline V to V?: Y Marker baseline V to V points: 3
Lower marker selection: First peak > 500 RFU Upper marker selection: Last peak > 200 RFU
Ladder size (bp)75, 200, 300, 400, 500, 700, 1000, 1500, 2000, 3000, 4000, 5000, 7000, 10000, 20000
Quantification using: Lower Marker Final concentration (ng/uL): 0.5000 Dilution factor: 12.0

Sample: SampA5
Well location: A5
Created: Thursday, August 12, 2021 6:06:18 PM

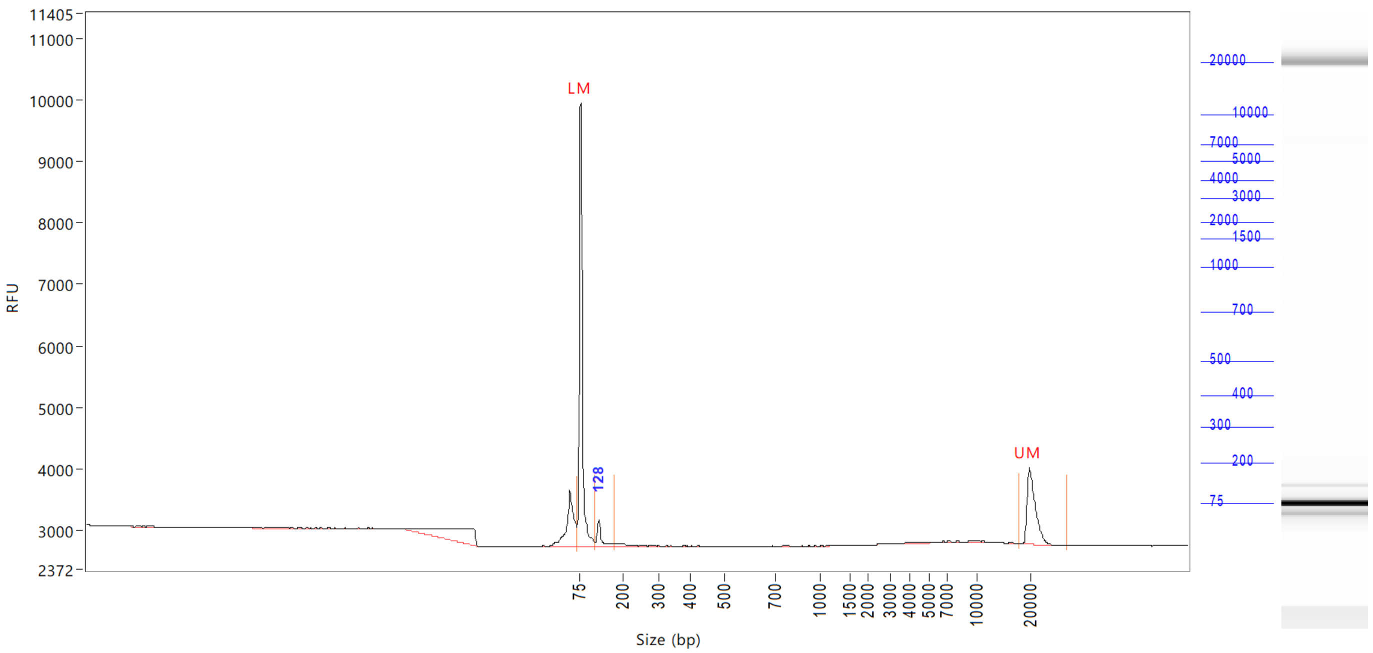

Peak	Size (bp)	Concentration (ng/uL)	From (bp)	To (bp)	Average size (bp)	CV%	RFU	Corrected peak area
1	75 (LM)	0.5000	64	116	74	9.69	7215	52.929
2	128	0.4382	116	175	133	10.81	434	3.866
3	20000 (UM)	0.1182	18049	27012	20512	4.52	1245	12.511

TIC:	0.4382	ng/uL
TIM:	5.4141	nmole/L
Total concentration:	0.8245	ng/uL

Sample peak width (sec): 5   Sample min peak height: 50   Sample baseline V to V?: Y   Sample baseline V to V points: 3
Sample filter: Binomial   Number of points for filter: 3   Sample start region (min): 0   Sample end region (min): 30
Marker peak width (sec): 5   Marker min peak height: 200   Marker baseline V to V?: Y   Marker baseline V to V points: 3
Lower marker selection: First peak > 500 RFU   Upper marker selection: Last peak > 200 RFU
Ladder size (bp)75, 200, 300, 400, 500, 700, 1000, 1500, 2000, 3000, 4000, 5000, 7000, 10000, 20000
Quantification using: Lower Marker   Final concentration (ng/uL): 0.5000   Dilution factor: 12.0

Sample: SampB5
Well location: B5
Created: Thursday, August 12, 2021 6:06:18 PM

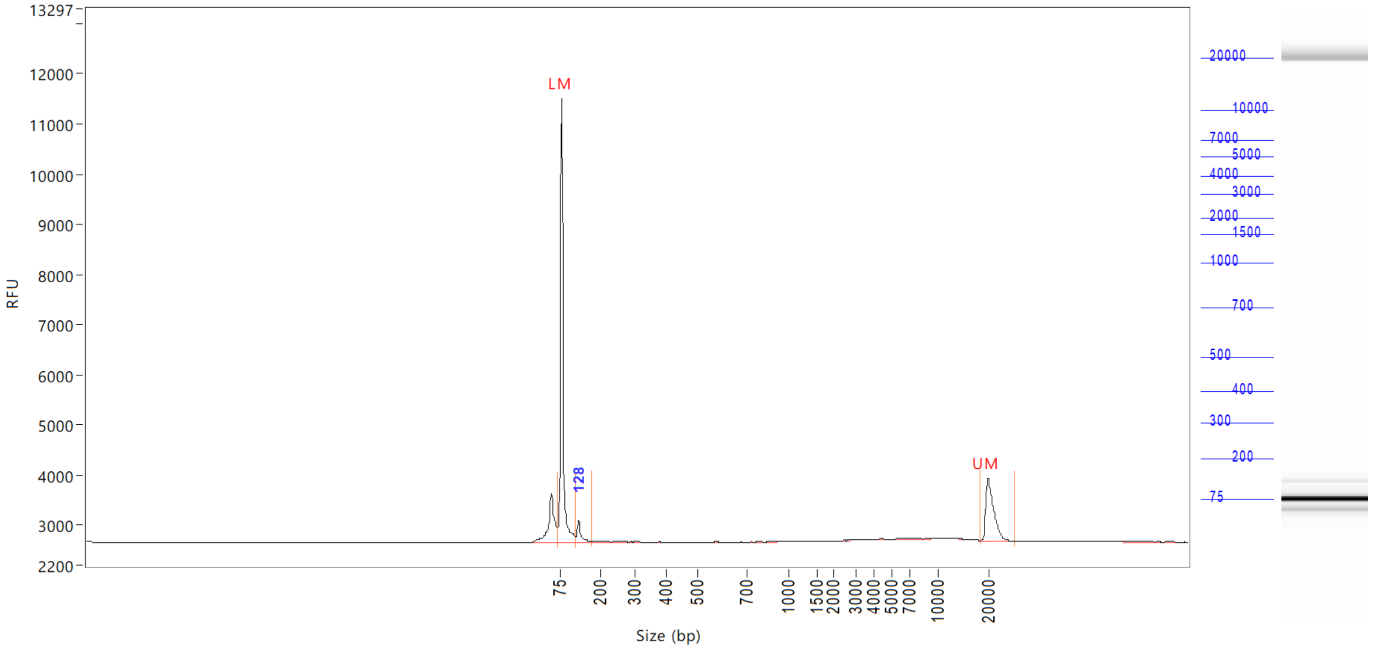

Peak	Size<br>(bp)	Concentration<br>(ng/uL)	From<br>(bp)	To<br>(bp)	Average size<br>(bp)	CV%	RFU	Corrected peak area
1	75 (LM)	0.5000	64	118	76	10.46	8864	59.553
2	128	0.4185	118	173	132	9.13	449	4.154
3	20000 (UM)	0.1048	18415	25320	20581	4.44	1231	12.484

TIC:	0.4185	ng/uL
TIM:	5.2096	nmole/L
Total concentration:	0.7706	ng/uL

Sample peak width (sec): 5   Sample min peak height: 50   Sample baseline V to V?: Y   Sample baseline V to V points: 3
Sample filter: Binomial   Number of points for filter: 3   Sample start region (min): 0   Sample end region (min): 30
Marker peak width (sec): 5   Marker min peak height: 200   Marker baseline V to V?: Y   Marker baseline V to V points: 3
Lower marker selection: First peak > 500 RFU   Upper marker selection: Last peak > 200 RFU
Ladder size (bp)75, 200, 300, 400, 500, 700, 1000, 1500, 2000, 3000, 4000, 5000, 7000, 10000, 20000
Quantification using: Lower Marker   Final concentration (ng/uL): 0.5000   Dilution factor: 12.0

Sample: SampC5
Well location: C5
Created: Thursday, August 12, 2021 6:06:18 PM

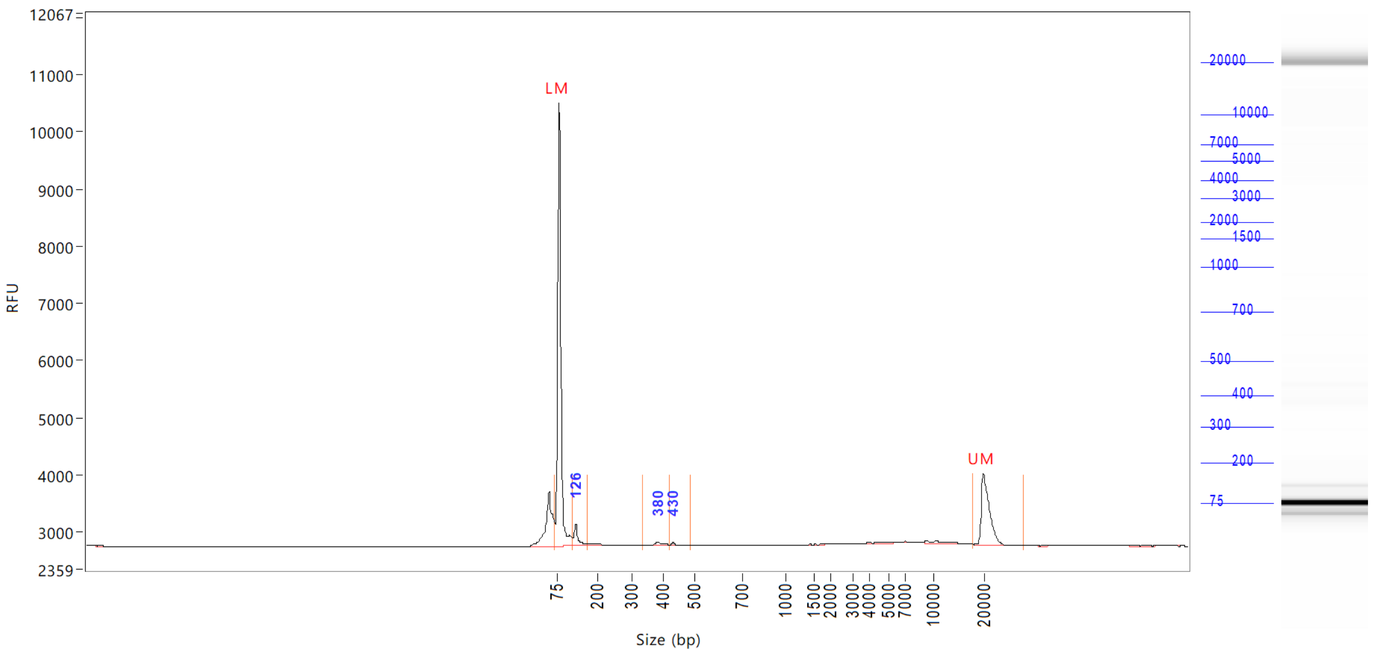

Peak	Size (bp)	Concentration (ng/uL)	From (bp)	To (bp)	Average size (bp)	CV%	RFU	Corrected peak area
1	75 (LM)	0.5000	64	118	76	10.77	7751	59.302
2	126	0.3806	118	166	130	8.64	383	3.761
3	380	0.1250	331	418	384	4.66	58	1.235
4	430	0.0810	418	485	441	4.01	68	0.800
5	20000 (UM)	0.1084	18049	27859	20572	4.67	1243	12.856

TIC:	0.5865	ng/uL
TIM:	5.6475	nmole/L
Total concentration:	1.0281	ng/uL

Sample peak width (sec): 5 Sample min peak height: 50 Sample baseline V to V?: Y Sample baseline V to V points: 3
Sample filter: Binomial Number of points for filter: 3 Sample start region (min): 0 Sample end region (min): 30
Marker peak width (sec): 5 Marker min peak height: 200 Marker baseline V to V?: Y Marker baseline V to V points: 3
Lower marker selection: First peak > 500 RFU Upper marker selection: Last peak > 200 RFU
Ladder size (bp)75, 200, 300, 400, 500, 700, 1000, 1500, 2000, 3000, 4000, 5000, 7000, 10000, 20000
Quantification using: Lower Marker Final concentration (ng/uL): 0.5000 Dilution factor: 12.0

Sample: SampD5
Well location: D5
Created: Thursday, August 12, 2021 6:06:18 PM

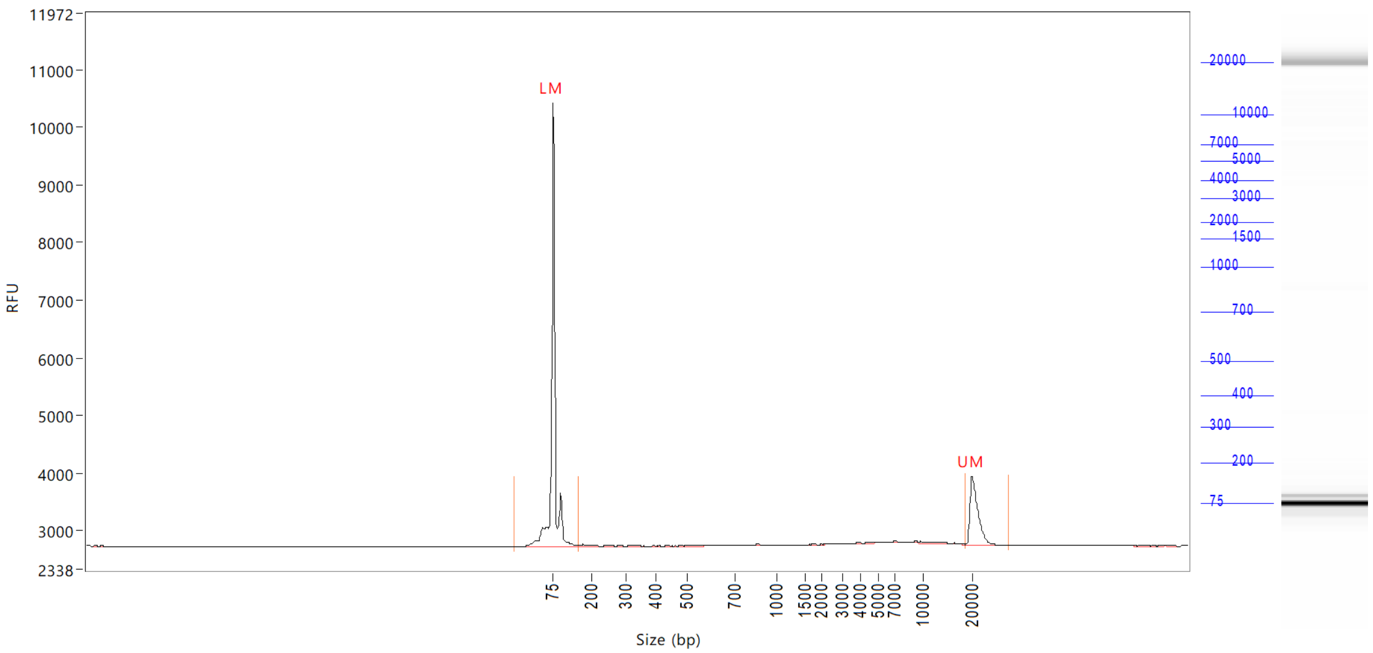

Peak	Size (bp)	Concentration (ng/uL)	From (bp)	To (bp)	Average size (bp)	CV%	RFU	Corrected peak area
1	75 (LM)	0.5000	0	156	72	24.60	7699	66.941
2	20000 (UM)	0.0912	18537	27254	20513	4.86	1171	12.216

TIC:	0.0000	ng/uL
TIM:	0.0000	nmole/L
Total concentration:	0.3736	ng/uL

```
Sample peak width (sec): 5    Sample min peak height: 50    Sample baseline V to V?: Y    Sample baseline V to V points: 3
Sample filter: Binomial       Number of points for filter: 3  Sample start region (min): 0  Sample end region (min): 30
Marker peak width (sec): 5    Marker min peak height: 200   Marker baseline V to V?: Y    Marker baseline V to V points: 3
Lower marker selection: First peak > 500 RFU                Upper marker selection: Last peak > 200 RFU
Ladder size (bp)75, 200, 300, 400, 500, 700, 1000, 1500, 2000, 3000, 4000, 5000, 7000, 10000, 20000
Quantification using: Lower Marker          Final concentration (ng/uL): 0.5000          Dilution factor: 12.0
```

Sample: SampE5
Well location: E5
Created: Thursday, August 12, 2021 6:06:18 PM

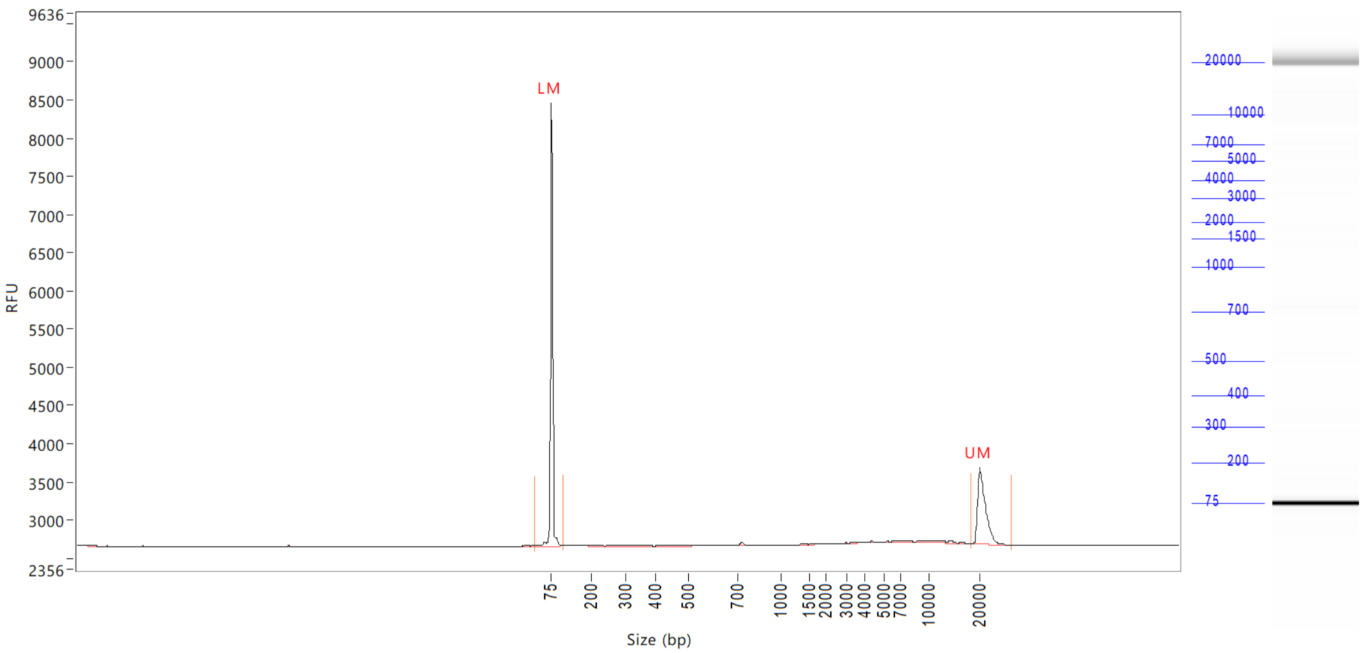

Peak	Size (bp)	Concentration (ng/uL)	From (bp)	To (bp)	Average size (bp)	CV%	RFU	Corrected peak area
1	75 (LM)	0.5000	26	114	73	7.43	5813	32.376
2	20000 (UM)	0.1538	18171	26166	20531	4.52	996	9.957

TIC:	0.0000	ng/uL
TIM:	0.0000	nmole/L
Total concentration:	0.7787	ng/uL

Sample peak width (sec): 5 Sample min peak height: 50 Sample baseline V to V?: Y Sample baseline V to V points: 3
Sample filter: Binomial Number of points for filter: 3 Sample start region (min): 0 Sample end region (min): 30
Marker peak width (sec): 5 Marker min peak height: 200 Marker baseline V to V?: Y Marker baseline V to V points: 3
Lower marker selection: First peak > 500 RFU Upper marker selection: Last peak > 200 RFU
Ladder size (bp)75, 200, 300, 400, 500, 700, 1000, 1500, 2000, 3000, 4000, 5000, 7000, 10000, 20000
Quantification using: Lower Marker Final concentration (ng/uL): 0.5000 Dilution factor: 12.0

Sample: SampF5
Well location: F5
Created: Thursday, August 12, 2021 6:06:18 PM

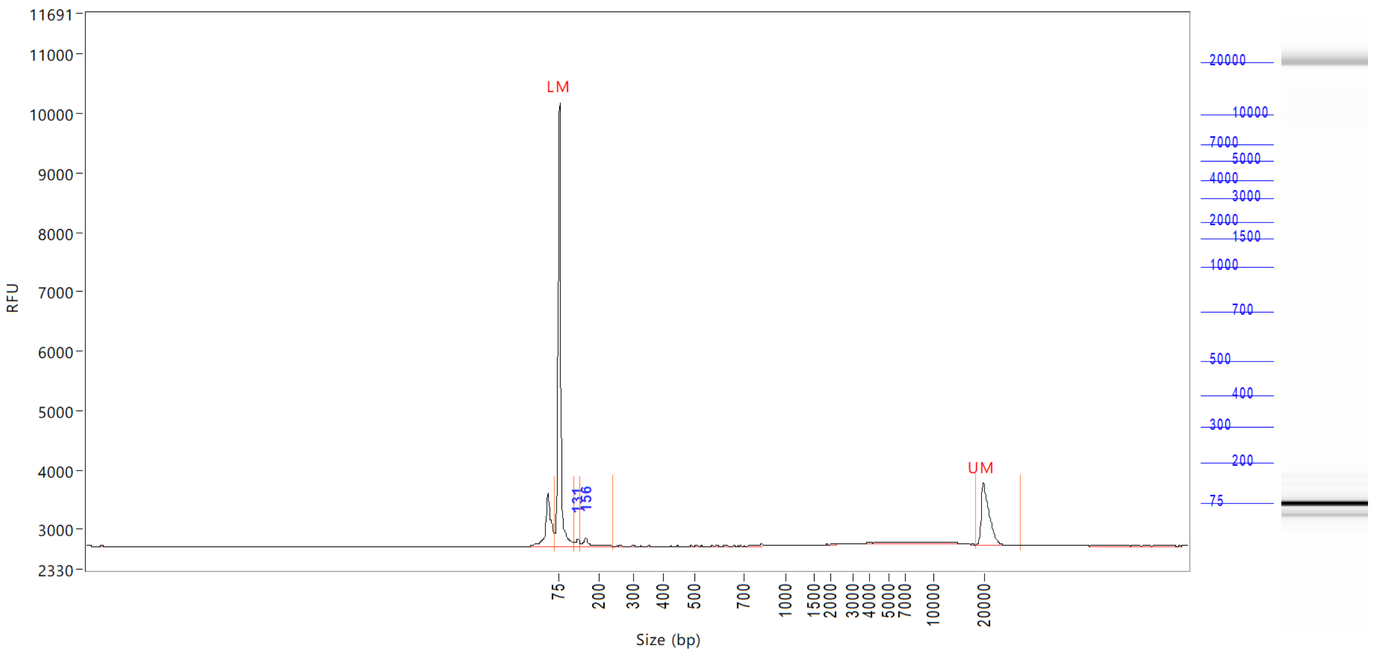

Peak	Size<br>(bp)	Concentration<br>(ng/uL)	From<br>(bp)	To<br>(bp)	Average size<br>(bp)	CV%	RFU	Corrected peak area
1	75 (LM)	0.5000	60	122	74	10.60	7481	49.836
2	131	0.1325	122	141	131	4.08	123	1.101
3	156	0.2293	141	242	164	12.46	145	1.905
4	20000 (UM)	0.1060	18415	27375	20529	4.54	1039	10.562

TIC:	0.3618	ng/uL
TIM:	3.9605	nmole/L
Total concentration:	0.7799	ng/uL

Sample peak width (sec): 5 Sample min peak height: 50 Sample baseline V to V?: Y Sample baseline V to V points: 3
Sample filter: Binomial Number of points for filter: 3 Sample start region (min): 0 Sample end region (min): 30
Marker peak width (sec): 5 Marker min peak height: 200 Marker baseline V to V?: Y Marker baseline V to V points: 3
Lower marker selection: First peak > 500 RFU Upper marker selection: Last peak > 200 RFU
Ladder size (bp)75, 200, 300, 400, 500, 700, 1000, 1500, 2000, 3000, 4000, 5000, 7000, 10000, 20000
Quantification using: Lower Marker Final concentration (ng/uL): 0.5000 Dilution factor: 12.0

Sample: SampG5
Well location: G5
Created: Thursday, August 12, 2021 6:06:18 PM

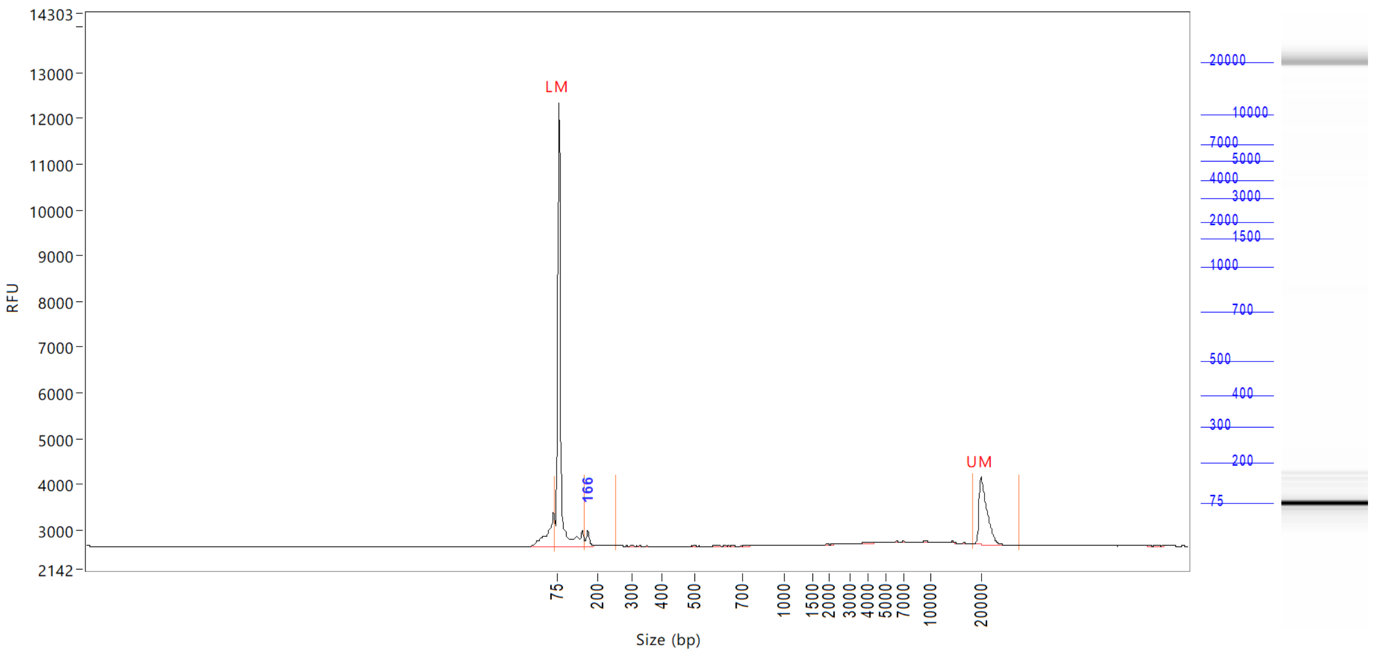

Peak	Size (bp)	Concentration (ng/uL)	From (bp)	To (bp)	Average size (bp)	CV%	RFU	Corrected peak area
1	75 (LM)	0.5000	66	158	80	23.65	9715	68.520
2	166	0.2782	158	253	175	11.08	360	3.178
3	20000 (UM)	0.1118	18537	27738	20581	4.65	1477	15.325

TIC:	0.2782	ng/uL
TIM:	2.6138	nmole/L
Total concentration:	0.5502	ng/uL

```
Sample peak width (sec): 5    Sample min peak height: 50    Sample baseline V to V?: Y    Sample baseline V to V points: 3
Sample filter: Binomial       Number of points for filter: 3  Sample start region (min): 0  Sample end region (min): 30
Marker peak width (sec): 5    Marker min peak height: 200   Marker baseline V to V?: Y    Marker baseline V to V points: 3
Lower marker selection: First peak > 500 RFU                Upper marker selection: Last peak > 200 RFU
Ladder size (bp)75, 200, 300, 400, 500, 700, 1000, 1500, 2000, 3000, 4000, 5000, 7000, 10000, 20000
Quantification using: Lower Marker          Final concentration (ng/uL): 0.5000          Dilution factor: 12.0
```

Sample: SampH5
Well location: H5
Created: Thursday, August 12, 2021 6:06:18 PM

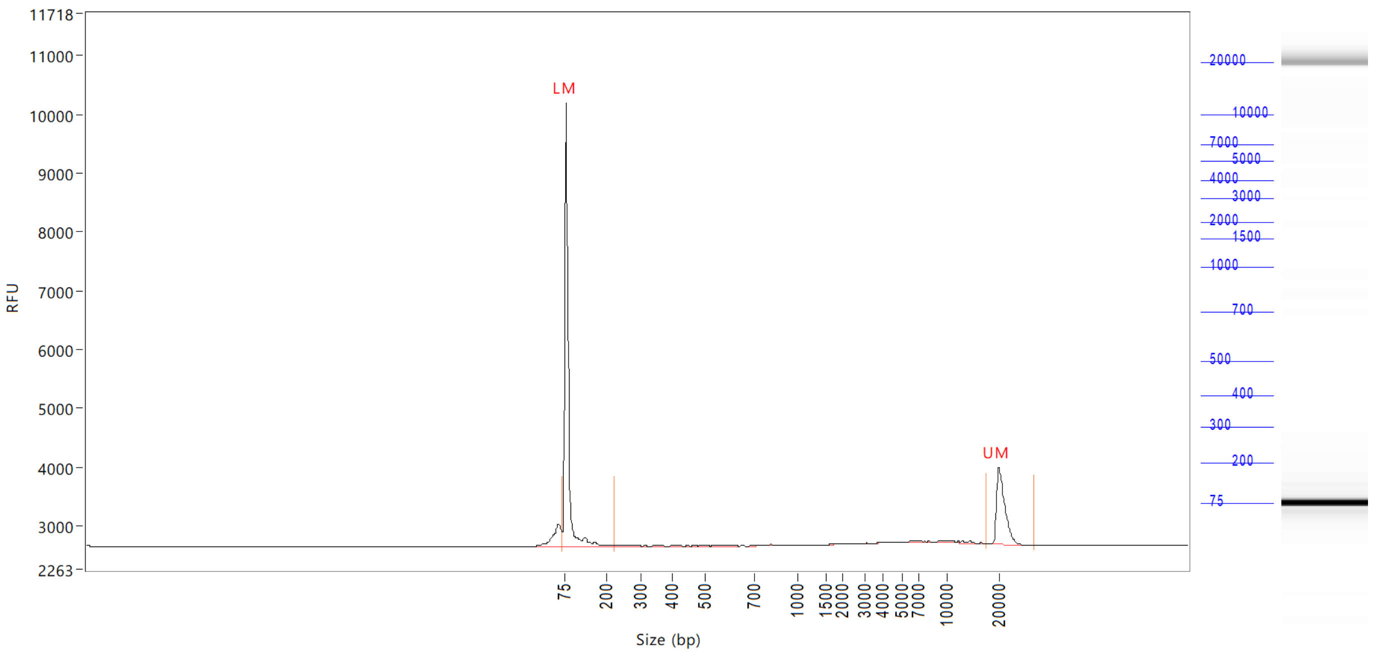

Peak	Size (bp)	Concentration (ng/uL)	From (bp)	To (bp)	Average size (bp)	CV%	RFU	Corrected peak area
1	75 (LM)	0.5000	62	221	80	25.66	7559	66.299
2	20000 (UM)	0.1031	17439	26891	20575	4.79	1309	13.667

TIC:	0.0000	ng/uL
TIM:	0.0000	nmole/L
Total concentration:	0.3233	ng/uL

Sample peak width (sec): 5   Sample min peak height: 50   Sample baseline V to V?: Y   Sample baseline V to V points: 3
Sample filter: Binomial   Number of points for filter: 3 Sample start region (min): 0 Sample end region (min): 30
Marker peak width (sec): 5   Marker min peak height: 200   Marker baseline V to V?: Y   Marker baseline V to V points: 3
Lower marker selection: First peak > 500 RFU   Upper marker selection: Last peak > 200 RFU
Ladder size (bp)75, 200, 300, 400, 500, 700, 1000, 1500, 2000, 3000, 4000, 5000, 7000, 10000, 20000
Quantification using: Lower Marker   Final concentration (ng/uL): 0.5000   Dilution factor: 12.0

Sample: SampA6
Well location: A6
Created: Thursday, August 12, 2021 6:06:18 PM

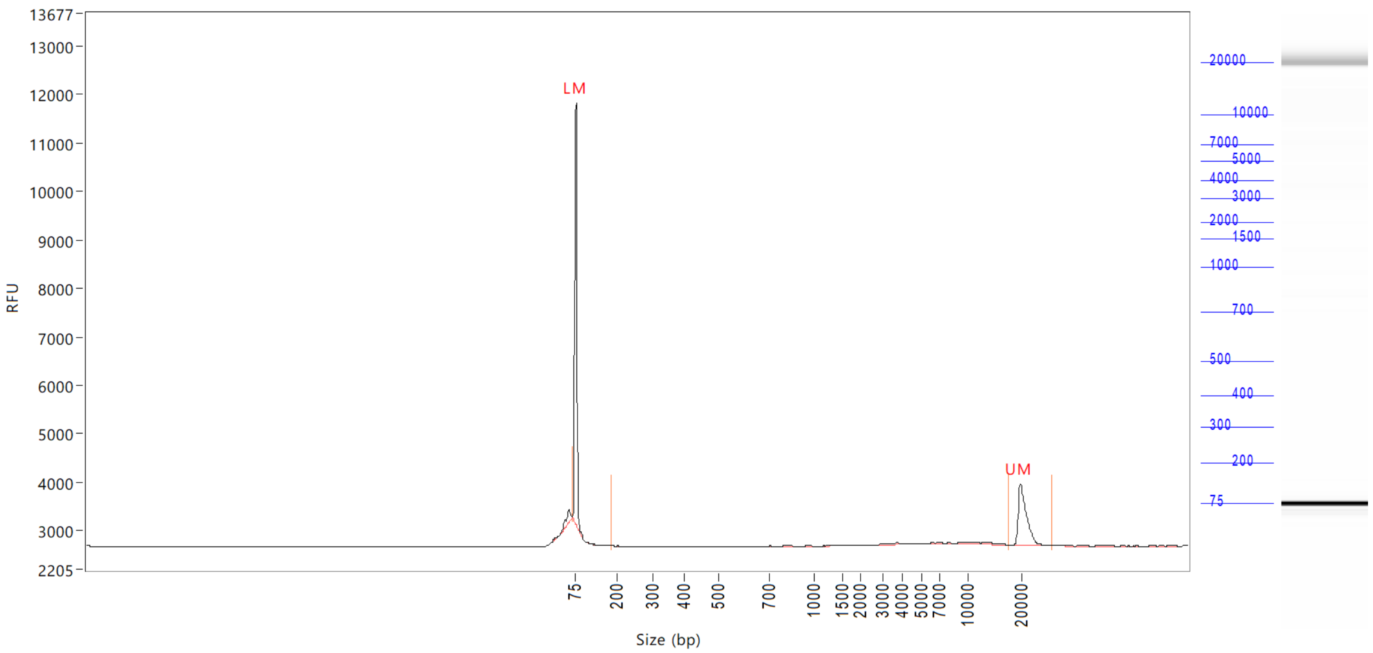

Peak	Size	Concentration	From	To	Average size	CV%	RFU	Corrected peak area
	(bp)	(ng/uL)	(bp)	(bp)	(bp)			
1	75 (LM)	0.5000	64	183	73	6.05	8723	47.127
2	20000 (UM)	0.1372	17683	26045	20471	4.56	1267	12.933

TIC:	0.0000	ng/uL
TIM:	0.0000	nmole/L
Total concentration:	0.5563	ng/uL

Sample peak width (sec): 5 Sample min peak height: 50 Sample baseline V to V?: Y Sample baseline V to V points: 3
Sample filter: Binomial Number of points for filter: 3 Sample start region (min): 0 Sample end region (min): 30
Marker peak width (sec): 5 Marker min peak height: 200 Marker baseline V to V?: Y Marker baseline V to V points: 3
Lower marker selection: First peak > 500 RFU Upper marker selection: Last peak > 200 RFU
Ladder size (bp)75, 200, 300, 400, 500, 700, 1000, 1500, 2000, 3000, 4000, 5000, 7000, 10000, 20000
Quantification using: Lower Marker Final concentration (ng/uL): 0.5000 Dilution factor: 12.0

Sample: SampB6
Well location: B6
Created: Thursday, August 12, 2021 6:06:18 PM

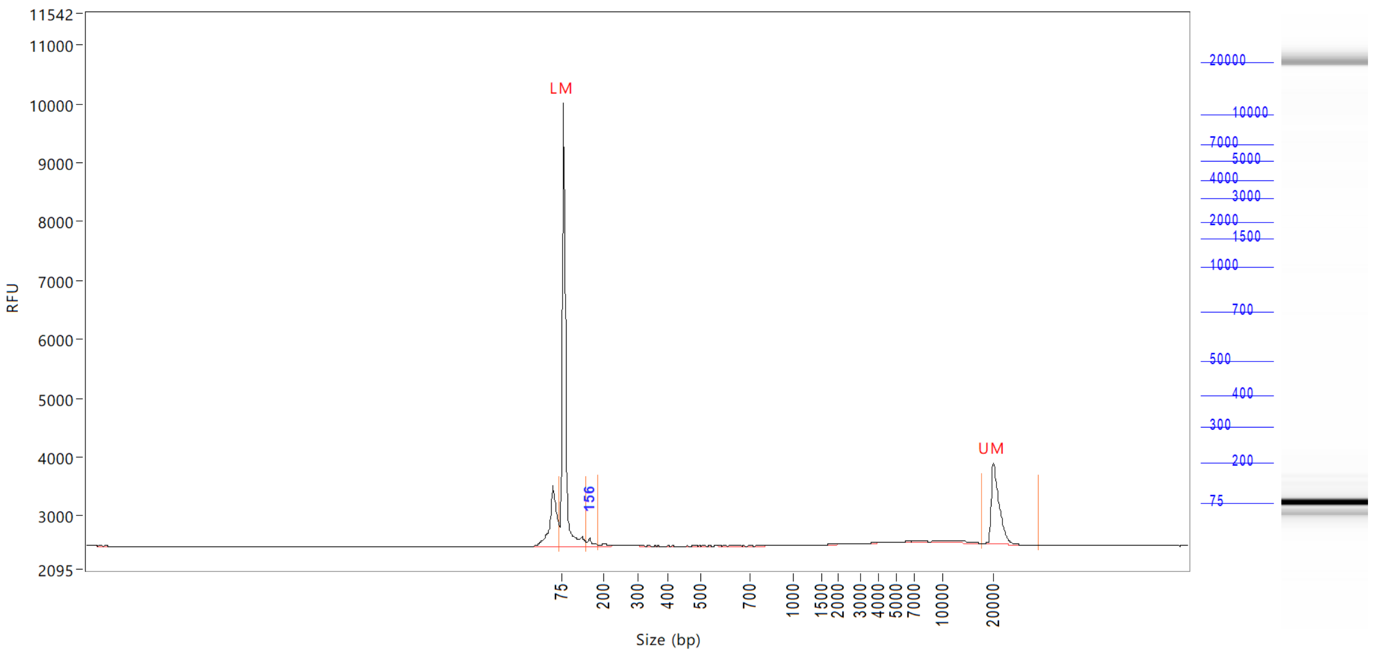

Peak	Size	Concentration	From	To	Average size	CV%	RFU	Corrected peak area
	(bp)	(ng/uL)	(bp)	(bp)	(bp)			
1	75 (LM)	0.5000	64	148	79	16.31	7549	64.568
2	156	0.1322	148	185	160	6.20	145	1.423
3	20000 (UM)	0.1093	17683	29068	20574	4.88	1387	14.114

TIC:	0.1322	ng/uL
TIM:	1.3586	nmole/L
Total concentration:	0.5018	ng/uL

```
Sample peak width (sec): 5    Sample min peak height: 50    Sample baseline V to V?: Y    Sample baseline V to V points: 3
Sample filter: Binomial       Number of points for filter: 3  Sample start region (min): 0  Sample end region (min): 30
Marker peak width (sec): 5    Marker min peak height: 200   Marker baseline V to V?: Y    Marker baseline V to V points: 3
Lower marker selection: First peak > 500 RFU                Upper marker selection: Last peak > 200 RFU
Ladder size (bp)75, 200, 300, 400, 500, 700, 1000, 1500, 2000, 3000, 4000, 5000, 7000, 10000, 20000
Quantification using: Lower Marker          Final concentration (ng/uL): 0.5000          Dilution factor: 12.0
```

Sample: SampC6
Well location: C6
Created: Thursday, August 12, 2021 6:06:18 PM

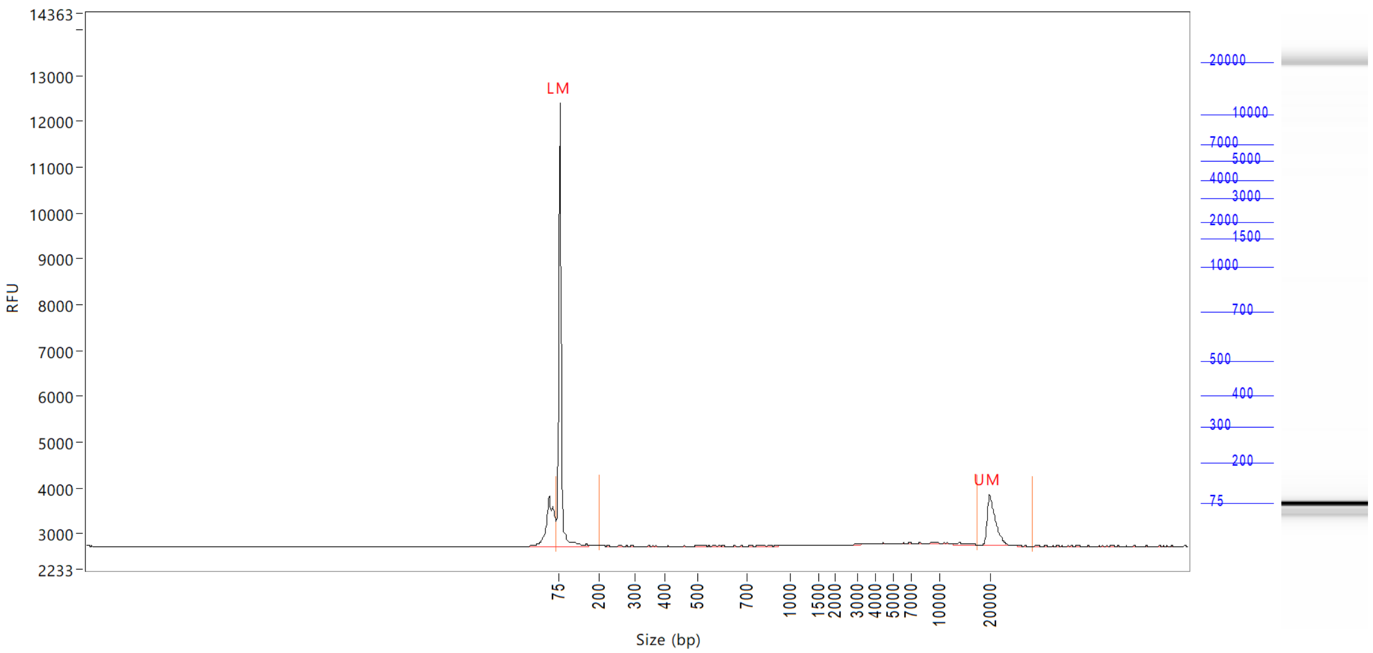

Peak	Size (bp)	Concentration (ng/uL)	From (bp)	To (bp)	Average size (bp)	CV%	RFU	Corrected peak area
1	75 (LM)	0.5000	64	200	75	15.33	9689	61.784
2	20000 (UM)	0.0958	17561	28463	20554	5.07	1122	11.840

TIC:	0.0000	ng/uL
TIM:	0.0000	nmole/L
Total concentration:	0.3478	ng/uL

```
Sample peak width (sec): 5    Sample min peak height: 50    Sample baseline V to V?: Y    Sample baseline V to V points: 3
Sample filter: Binomial       Number of points for filter: 3  Sample start region (min): 0  Sample end region (min): 30
Marker peak width (sec): 5    Marker min peak height: 200   Marker baseline V to V?: Y    Marker baseline V to V points: 3
Lower marker selection: First peak > 500 RFU                Upper marker selection: Last peak > 200 RFU
Ladder size (bp)75, 200, 300, 400, 500, 700, 1000, 1500, 2000, 3000, 4000, 5000, 7000, 10000, 20000
Quantification using: Lower Marker          Final concentration (ng/uL): 0.5000          Dilution factor: 12.0
```

Sample: SampD6
Well location: D6
Created: Thursday, August 12, 2021 6:06:18 PM

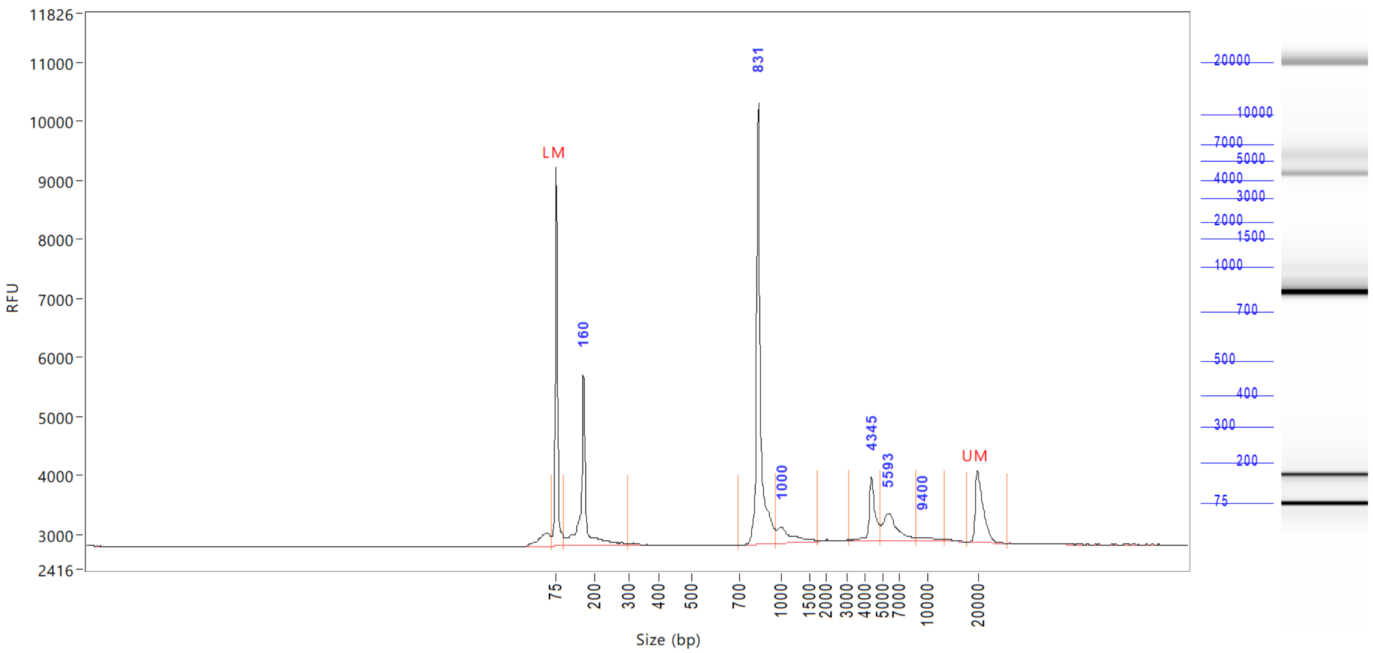

Peak	Size (bp)	Concentration (ng/uL)	From (bp)	To (bp)	Average size (bp)	CV%	RFU	Corrected peak area
1	75 (LM)	0.5000	60	99	74	7.23	6423	38.048
2	160	5.1362	99	296	163	19.86	2884	32.570
3	831	8.5610	697	961	839	4.03	7484	54.287
4	1000	1.0946	961	1770	1137	16.21	281	6.941
5	4345	1.4743	3108	4862	4384	5.69	1070	9.349
6	5593	1.4988	4862	8800	6072	15.79	453	9.504
7	9400	0.2362	8800	13415	10439	12.63	63	1.498
8	20000 (UM)	0.1647	17927	25924	20545	4.60	1214	12.529

TIC:	18.0011	ng/uL
TIM:	71.1709	nmole/L
Total concentration:	18.2369	ng/uL

```
Sample peak width (sec): 5    Sample min peak height: 50    Sample baseline V to V?: Y    Sample baseline V to V points: 3
Sample filter: Binomial       Number of points for filter: 3  Sample start region (min): 0  Sample end region (min): 30
Marker peak width (sec): 5    Marker min peak height: 200   Marker baseline V to V?: Y    Marker baseline V to V points: 3
Lower marker selection: First peak > 500 RFU                Upper marker selection: Last peak > 200 RFU
Ladder size (bp)75, 200, 300, 400, 500, 700, 1000, 1500, 2000, 3000, 4000, 5000, 7000, 10000, 20000
Quantification using: Lower Marker          Final concentration (ng/uL): 0.5000          Dilution factor: 12.0
```

Sample: SampE6
Well location: E6
Created: Thursday, August 12, 2021 6:06:18 PM

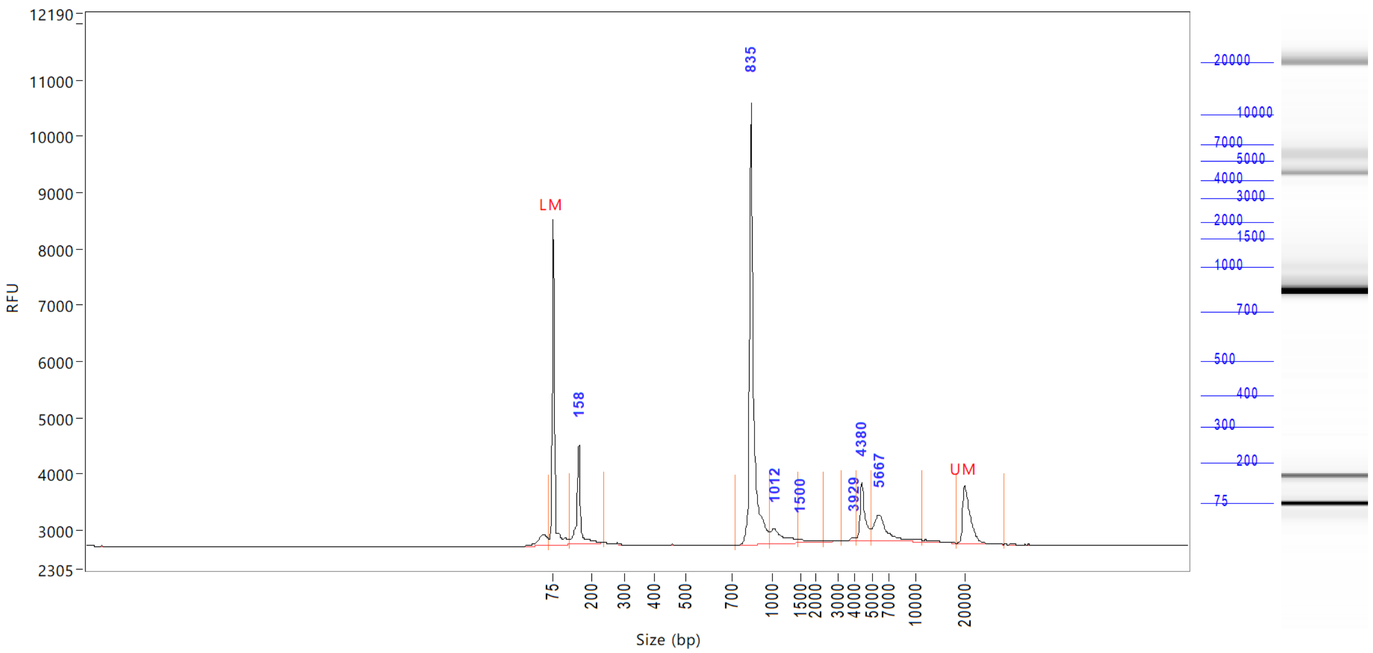

Peak	Size (bp)	Concentration (ng/uL)	From (bp)	To (bp)	Average size (bp)	CV%	RFU	Corrected peak area
1	75 (LM)	0.5000	64	128	76	13.87	5780	35.461
2	158	2.6542	128	237	157	9.74	1757	15.687
3	835	9.6264	723	970	847	3.87	7870	56.894
4	1012	1.0323	970	1467	1121	12.64	271	6.101
5	1500	0.1829	1467	2344	1724	13.18	60	1.081
6	3929	0.0985	3251	4104	3853	4.82	57	0.582
7	4380	1.3685	4104	4931	4468	4.33	1036	8.088
8	5667	1.6212	4931	11342	6433	21.17	466	9.582
9	20000 (UM)	0.1482	18293	28221	20663	5.43	1032	10.509

TIC:	16.5840	ng/uL
TIM:	49.1438	nmole/L
Total concentration:	16.8404	ng/uL

Sample peak width (sec): 5 Sample min peak height: 50 Sample baseline V to V?: Y Sample baseline V to V points: 3
Sample filter: Binomial Number of points for filter: 3 Sample start region (min): 0 Sample end region (min): 30
Marker peak width (sec): 5 Marker min peak height: 200 Marker baseline V to V?: Y Marker baseline V to V points: 3
Lower marker selection: First peak > 500 RFU Upper marker selection: Last peak > 200 RFU
Ladder size (bp)75, 200, 300, 400, 500, 700, 1000, 1500, 2000, 3000, 4000, 5000, 7000, 10000, 20000
Quantification using: Lower Marker Final concentration (ng/uL): 0.5000 Dilution factor: 12.0

Sample: SampF6
Well location: F6
Created: Thursday, August 12, 2021 6:06:18 PM

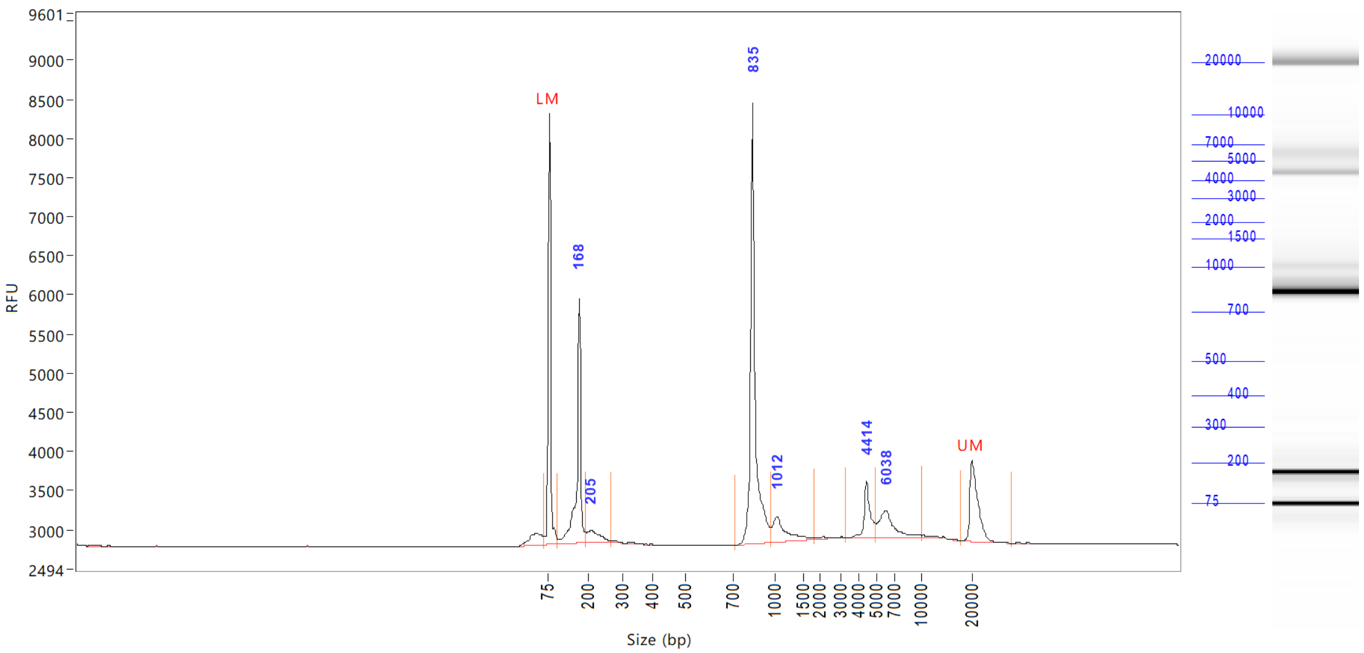

Peak	Size (bp)	Concentration (ng/uL)	From (bp)	To (bp)	Average size (bp)	CV%	RFU	Corrected peak area
1	75 (LM)	0.5000	58	99	73	6.72	5517	32.652
2	168	5.2313	99	190	160	9.84	3128	28.469
3	205	0.8179	190	262	216	8.05	152	4.451
4	835	8.4026	710	965	843	4.18	5647	45.726
5	1012	1.3783	965	1866	1149	17.19	332	7.501
6	4414	1.2241	3215	4966	4458	5.71	715	6.661
7	6038	1.3130	4966	10001	6373	17.78	342	7.145
8	20000 (UM)	0.1664	17683	27979	20571	4.90	1039	10.865

TIC:	18.3672	ng/uL
TIM:	79.1388	nmole/L
Total concentration:	18.6326	ng/uL

```
Sample peak width (sec): 5     Sample min peak height: 50     Sample baseline V to V?: Y     Sample baseline V to V points: 3
Sample filter: Binomial        Number of points for filter: 3  Sample start region (min): 0  Sample end region (min): 30
Marker peak width (sec): 5     Marker min peak height: 200    Marker baseline V to V?: Y     Marker baseline V to V points: 3
Lower marker selection: First peak > 500 RFU                  Upper marker selection: Last peak > 200 RFU
Ladder size (bp)75, 200, 300, 400, 500, 700, 1000, 1500, 2000, 3000, 4000, 5000, 7000, 10000, 20000
Quantification using: Lower Marker          Final concentration (ng/uL): 0.5000          Dilution factor: 12.0
```

Sample: SampG6
Well location: G6
Created: Thursday, August 12, 2021 6:06:18 PM

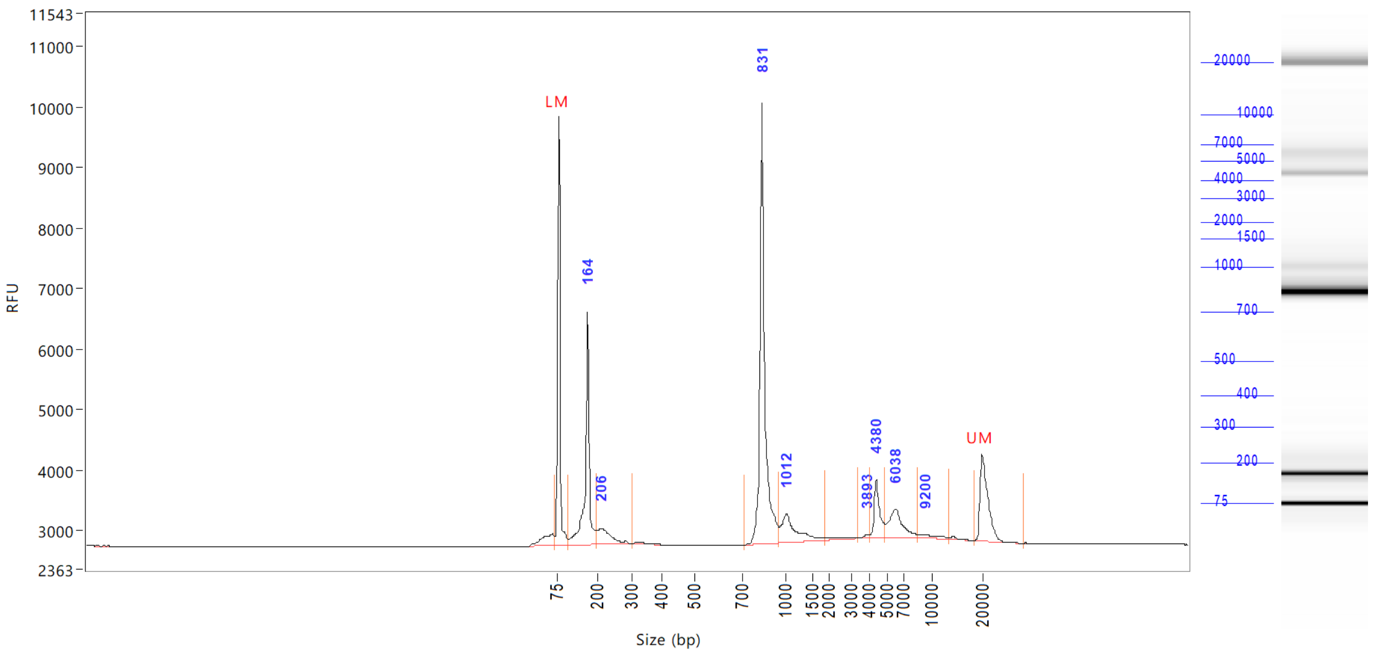

Peak	Size (bp)	Concentration (ng/uL)	From (bp)	To (bp)	Average size (bp)	CV%	RFU	Corrected peak area
1	75 (LM)	0.5000	64	103	74	7.29	7079	40.997
2	164	5.2816	103	192	158	9.84	3841	36.088
3	206	1.2083	192	298	220	9.75	263	8.256
4	831	8.9736	705	957	839	4.04	7295	61.316
5	1012	1.6777	957	1846	1141	16.89	477	11.464
6	3893	0.0777	3322	4001	3807	4.09	61	0.531
7	4380	1.2301	4001	4897	4440	4.49	967	8.405
8	6038	1.3764	4897	8467	6110	13.90	477	9.405
9	9200	0.2335	8467	13293	10204	12.96	60	1.596
10	20000 (UM)	0.1803	18415	28221	20532	4.81	1434	14.781

TIC:	20.0590	ng/uL
TIM:	84.8975	nmole/L
Total concentration:	20.2894	ng/uL

```
Sample peak width (sec): 5    Sample min peak height: 50    Sample baseline V to V?: Y    Sample baseline V to V points: 3
Sample filter: Binomial       Number of points for filter: 3  Sample start region (min): 0  Sample end region (min): 30
Marker peak width (sec): 5    Marker min peak height: 200   Marker baseline V to V?: Y    Marker baseline V to V points: 3
Lower marker selection: First peak > 500 RFU                Upper marker selection: Last peak > 200 RFU
Ladder size (bp)75, 200, 300, 400, 500, 700, 1000, 1500, 2000, 3000, 4000, 5000, 7000, 10000, 20000
Quantification using: Lower Marker          Final concentration (ng/uL): 0.5000          Dilution factor: 12.0
```

Sample: SampH6
Well location: H6
Created: Thursday, August 12, 2021 6:06:18 PM

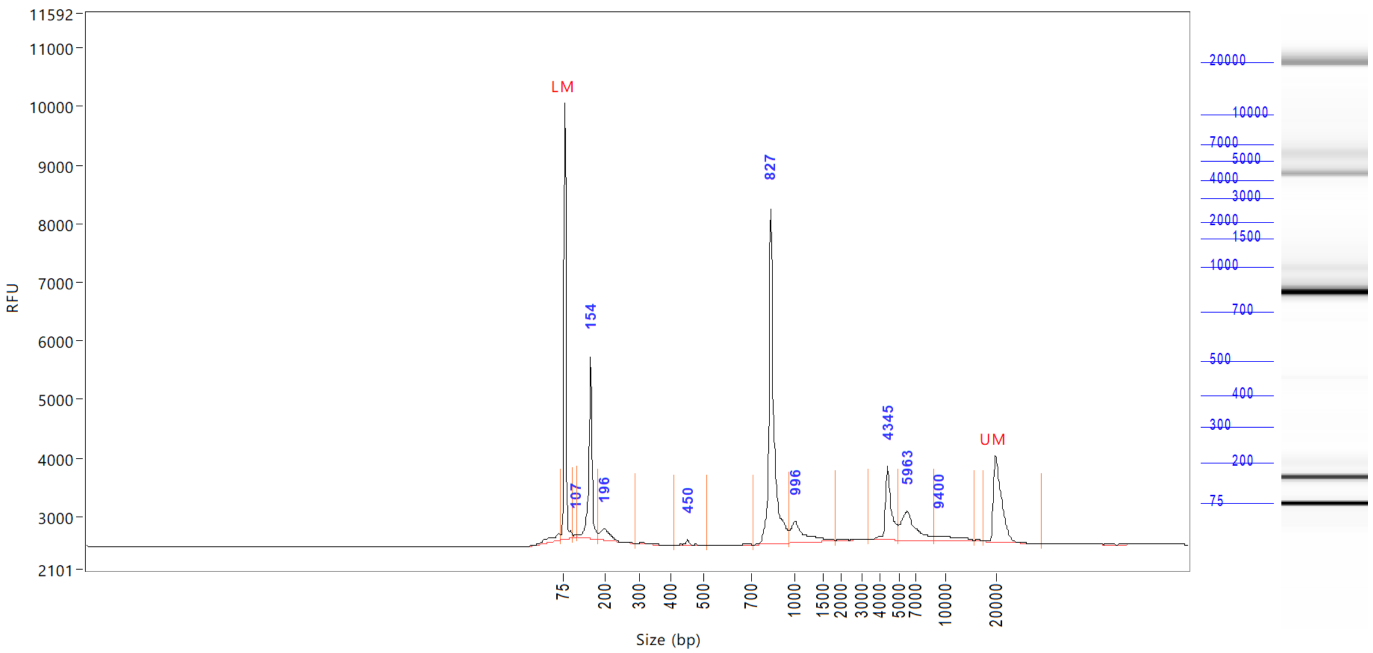

Peak	Size (bp)	Concentration (ng/uL)	From (bp)	To (bp)	Average size (bp)	CV%	RFU	Corrected peak area
1	75 (LM)	0.5000	62	99	73	5.47	7461	41.133
2	107	0.0584	99	112	105	3.90	50	0.400
3	154	3.5659	112	179	151	5.95	3104	24.446
4	196	0.6332	179	286	202	9.37	192	4.341
5	450	0.1284	409	511	450	4.28	89	0.880
6	827	7.1061	710	952	837	3.92	5722	48.717
7	996	1.1867	952	1846	1122	16.66	365	8.136
8	4345	1.6712	3286	4966	4411	5.82	1256	11.457
9	5963	1.4237	4966	8934	6257	15.32	489	9.761
10	9400	0.3021	8934	15732	11045	15.52	77	2.071
11	20000 (UM)	0.1851	17683	28826	20533	4.80	1483	15.230

TIC:	16.0757	ng/uL
TIM:	62.1068	nmole/L
Total concentration:	16.1950	ng/uL

Sample peak width (sec): 5   Sample min peak height: 50   Sample baseline V to V?: Y   Sample baseline V to V points: 3
Sample filter: Binomial   Number of points for filter: 3 Sample start region (min): 0 Sample end region (min): 30
Marker peak width (sec): 5   Marker min peak height: 200   Marker baseline V to V?: Y   Marker baseline V to V points: 3
Lower marker selection: First peak > 500 RFU   Upper marker selection: Last peak > 200 RFU
Ladder size (bp)75, 200, 300, 400, 500, 700, 1000, 1500, 2000, 3000, 4000, 5000, 7000, 10000, 20000
Quantification using: Lower Marker   Final concentration (ng/uL): 0.5000   Dilution factor: 12.0

Sample: SampA7
Well location: A7
Created: Thursday, August 12, 2021 6:06:18 PM

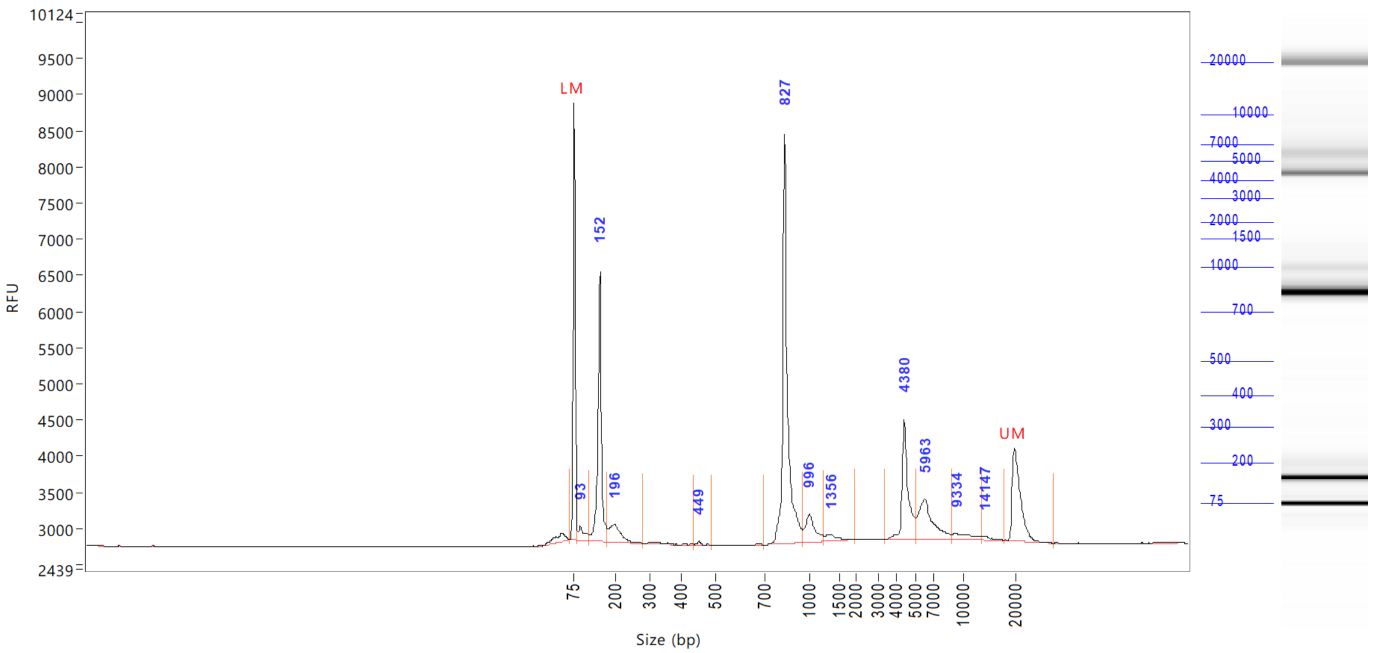

Peak	Size	Concentration	From	To	Average size	CV%	RFU	Corrected peak area
	(bp)	(ng/uL)	(bp)	(bp)	(bp)			
1	75 (LM)	0.5000	62	86	73	4.31	6051	31.847
2	93	0.5738	86	122	101	10.72	192	3.045
3	152	5.5288	122	175	150	5.47	3735	29.346
4	196	1.2067	175	278	201	9.63	243	6.405
5	449	0.0760	433	485	451	2.68	51	0.403
6	827	8.6830	692	952	834	3.94	5651	46.088
7	996	1.1440	952	1245	1033	7.19	391	6.072
8	1356	0.3210	1245	1981	1411	10.81	91	1.704
9	4380	2.7432	3358	5001	4445	5.69	1663	14.560
10	5963	2.0996	5001	8867	6359	14.85	561	11.144
11	9334	0.4235	8867	13659	10640	13.14	87	2.248
12	14147	0.1229	13659	17927	15039	7.19	53	0.652
13	20000 (UM)	0.2063	17927	27375	20534	5.03	1288	13.142

TIC:	22.9225	ng/uL
TIM:	101.0262	nmole/L
Total concentration:	23.0309	ng/uL

```
Sample peak width (sec): 5    Sample min peak height: 50    Sample baseline V to V?: Y    Sample baseline V to V points: 3
Sample filter: Binomial       Number of points for filter: 3  Sample start region (min): 0  Sample end region (min): 30
Marker peak width (sec): 5    Marker min peak height: 200    Marker baseline V to V?: Y    Marker baseline V to V points: 3
Lower marker selection: First peak > 500 RFU                 Upper marker selection: Last peak > 200 RFU
Ladder size (bp)75, 200, 300, 400, 500, 700, 1000, 1500, 2000, 3000, 4000, 5000, 7000, 10000, 20000
Quantification using: Lower Marker          Final concentration (ng/uL): 0.5000          Dilution factor: 12.0
```

Sample: SampB7
Well location: B7
Created: Thursday, August 12, 2021 6:06:18 PM

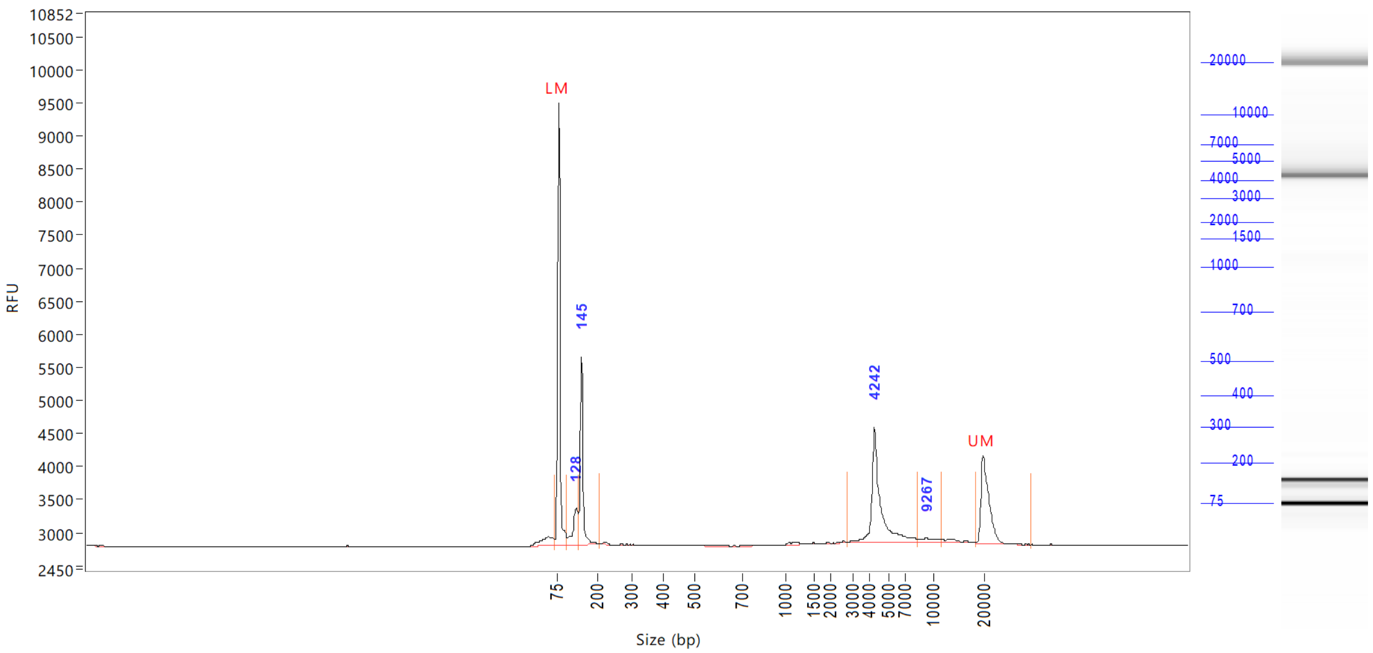

Peak	Size<br>(bp)	Concentration<br>(ng/uL)	From<br>(bp)	To<br>(bp)	Average size<br>(bp)	CV%	RFU	Corrected peak area
1	75 (LM)	0.5000	64	101	74	7.24	6699	39.033
2	128	1.1106	101	135	123	7.17	554	7.225
3	145	2.9381	135	201	145	5.21	2832	19.114
4	4242	3.3580	2800	8334	4598	18.99	1745	21.846
5	9267	0.1845	8334	11586	9728	9.16	52	1.200
6	20000 (UM)	0.1727	18415	29430	20479	4.82	1325	13.484

TIC:	7.5912	ng/uL
TIM:	49.3675	nmole/L
Total concentration:	8.0917	ng/uL

Sample peak width (sec): 5 Sample min peak height: 50 Sample baseline V to V?: Y Sample baseline V to V points: 3
Sample filter: Binomial Number of points for filter: 3 Sample start region (min): 0 Sample end region (min): 30
Marker peak width (sec): 5 Marker min peak height: 200 Marker baseline V to V?: Y Marker baseline V to V points: 3
Lower marker selection: First peak > 500 RFU Upper marker selection: Last peak > 200 RFU
Ladder size (bp)75, 200, 300, 400, 500, 700, 1000, 1500, 2000, 3000, 4000, 5000, 7000, 10000, 20000
Quantification using: Lower Marker Final concentration (ng/uL): 0.5000 Dilution factor: 12.0

Sample: SampC7
Well location: C7
Created: Thursday, August 12, 2021 6:06:18 PM

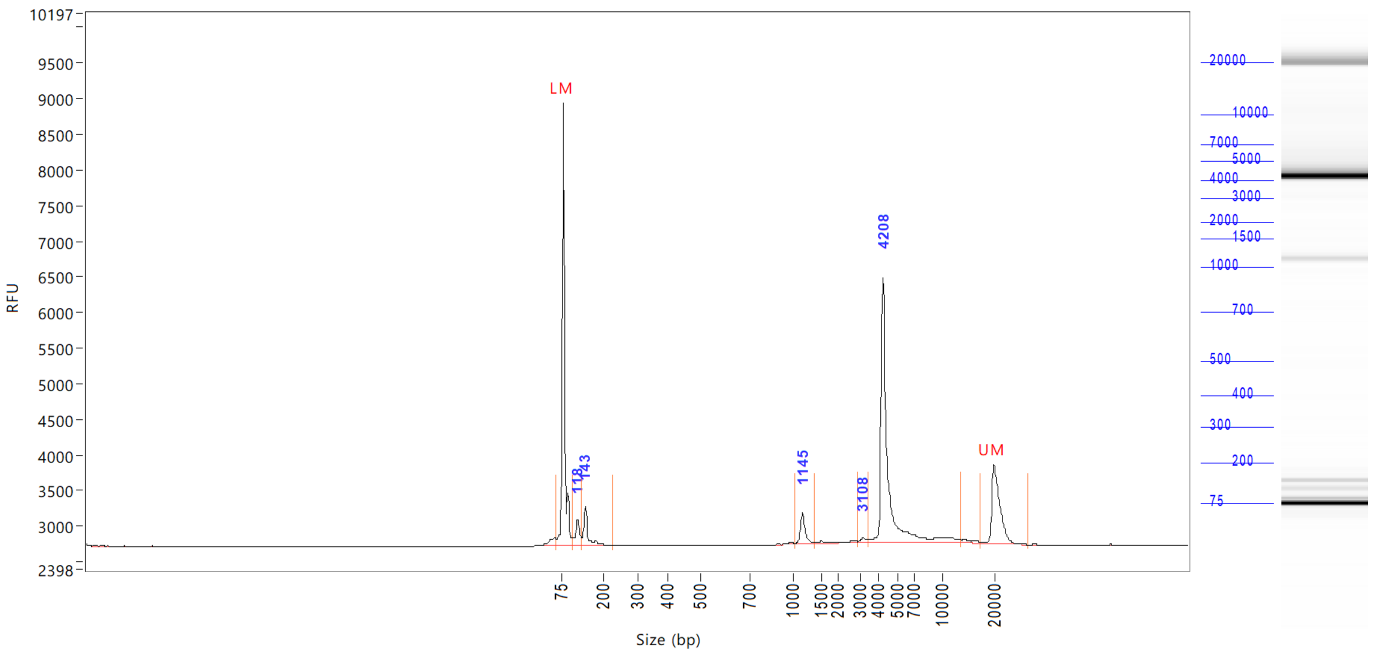

Peak	Size (bp)	Concentration (ng/uL)	From (bp)	To (bp)	Average size (bp)	CV%	RFU	Corrected peak area
1	75 (LM)	0.5000	54	107	75	9.26	6214	39.940
2	118	0.5374	107	133	119	5.70	360	3.577
3	143	0.7421	133	228	150	10.69	547	4.940
4	1145	0.5054	1012	1378	1156	5.17	445	3.364
5	3108	0.1028	2886	3429	3145	4.82	64	0.684
6	4208	5.3018	3429	13537	4927	34.77	3721	35.292
7	20000 (UM)	0.1420	17439	26891	20553	4.81	1100	11.341

TIC:	7.1894	ng/uL
TIM:	18.0948	nmole/L
Total concentration:	7.6266	ng/uL

```
Sample peak width (sec): 5    Sample min peak height: 50    Sample baseline V to V?: Y    Sample baseline V to V points: 3
Sample filter: Binomial       Number of points for filter: 3  Sample start region (min): 0  Sample end region (min): 30
Marker peak width (sec): 5    Marker min peak height: 200   Marker baseline V to V?: Y    Marker baseline V to V points: 3
Lower marker selection: First peak > 500 RFU                Upper marker selection: Last peak > 200 RFU
Ladder size (bp)75, 200, 300, 400, 500, 700, 1000, 1500, 2000, 3000, 4000, 5000, 7000, 10000, 20000
Quantification using: Lower Marker          Final concentration (ng/uL): 0.5000          Dilution factor: 12.0
```

Sample: SampD7
Well location: D7
Created: Thursday, August 12, 2021 6:06:18 PM

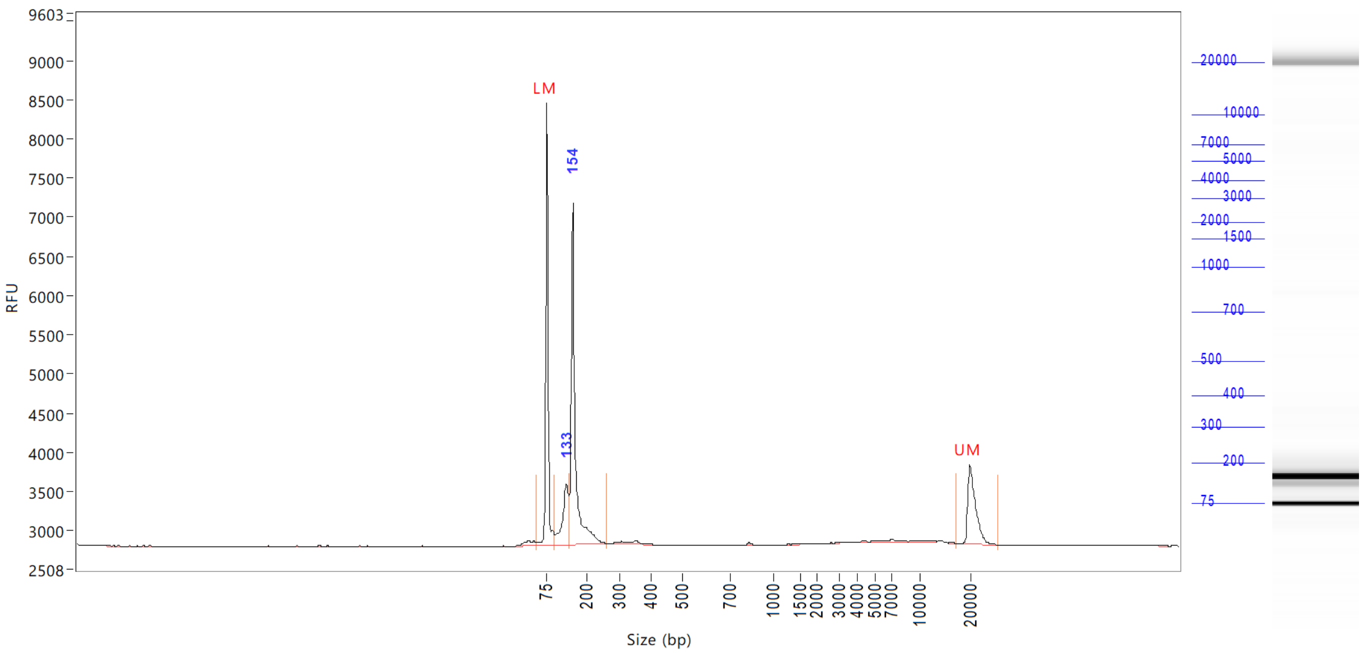

Peak	Size (bp)	Concentration (ng/uL)	From (bp)	To (bp)	Average size (bp)	CV%	RFU	Corrected peak area
1	75 (LM)	0.5000	45	97	73	7.69	5656	33.787
2	133	2.1277	97	143	127	9.09	768	11.982
3	154	7.2196	143	259	161	11.07	4356	40.655
4	20000 (UM)	0.1532	17317	25561	20486	4.58	1013	10.355

TIC:	9.3473	ng/uL
TIM:	101.2334	nmole/L
Total concentration:	9.8769	ng/uL

Sample peak width (sec): 5 Sample min peak height: 50 Sample baseline V to V?: Y Sample baseline V to V points: 3
Sample filter: Binomial Number of points for filter: 3 Sample start region (min): 0 Sample end region (min): 30
Marker peak width (sec): 5 Marker min peak height: 200 Marker baseline V to V?: Y Marker baseline V to V points: 3
Lower marker selection: First peak > 500 RFU Upper marker selection: Last peak > 200 RFU
Ladder size (bp)75, 200, 300, 400, 500, 700, 1000, 1500, 2000, 3000, 4000, 5000, 7000, 10000, 20000
Quantification using: Lower Marker Final concentration (ng/uL): 0.5000 Dilution factor: 12.0

Sample: SampE7
Well location: E7
Created: Thursday, August 12, 2021 6:06:18 PM

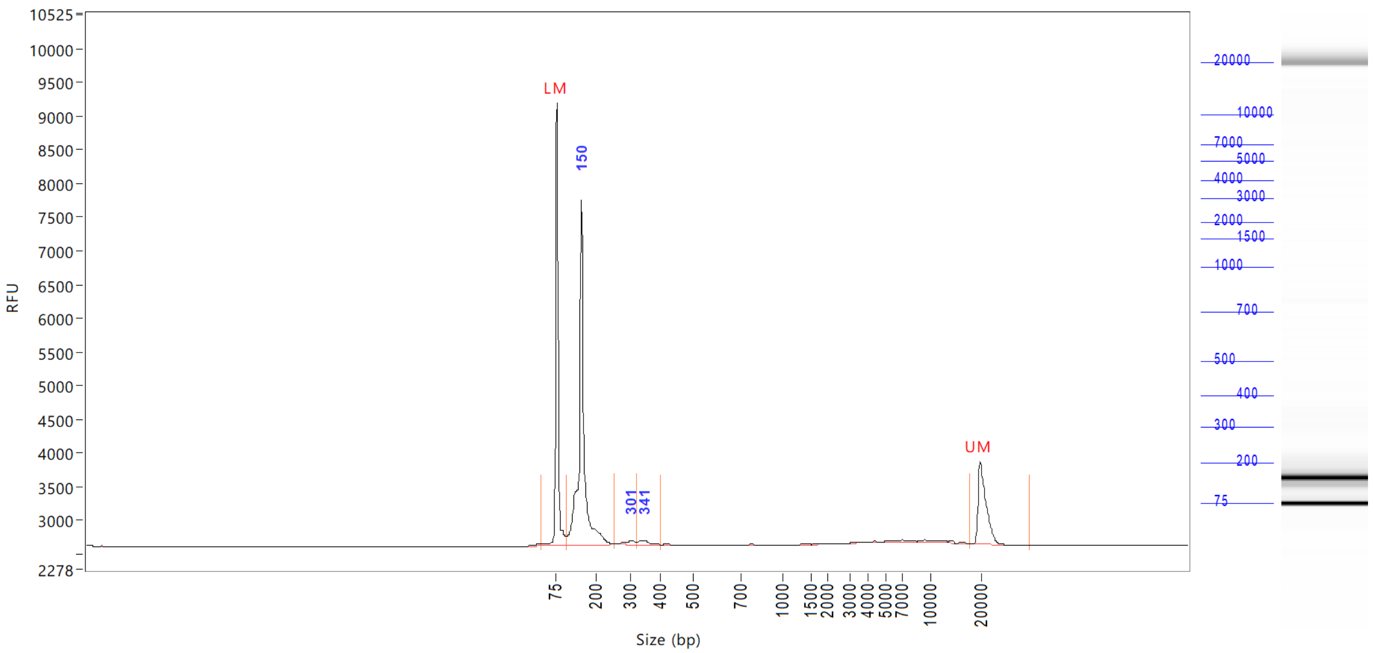

Peak	Size (bp)	Concentration (ng/uL)	From (bp)	To (bp)	Average size (bp)	CV%	RFU	Corrected peak area
1	75 (LM)	0.5000	28	105	73	10.11	6575	39.935
2	150	8.8617	105	251	151	13.56	5119	58.981
3	301	0.1895	251	317	292	5.46	53	1.261
4	341	0.1915	317	400	342	5.08	56	1.275
5	20000 (UM)	0.1601	18049	29914	20542	4.96	1219	12.788

TIC:	9.2427	ng/uL
TIM:	98.4420	nmole/L
Total concentration:	9.7713	ng/uL

Sample peak width (sec): 5 Sample min peak height: 50 Sample baseline V to V?: Y Sample baseline V to V points: 3
Sample filter: Binomial Number of points for filter: 3 Sample start region (min): 0 Sample end region (min): 30
Marker peak width (sec): 5 Marker min peak height: 200 Marker baseline V to V?: Y Marker baseline V to V points: 3
Lower marker selection: First peak > 500 RFU Upper marker selection: Last peak > 200 RFU
Ladder size (bp)75, 200, 300, 400, 500, 700, 1000, 1500, 2000, 3000, 4000, 5000, 7000, 10000, 20000
Quantification using: Lower Marker Final concentration (ng/uL): 0.5000 Dilution factor: 12.0

Sample: SampF7
Well location: F7
Created: Thursday, August 12, 2021 6:06:18 PM

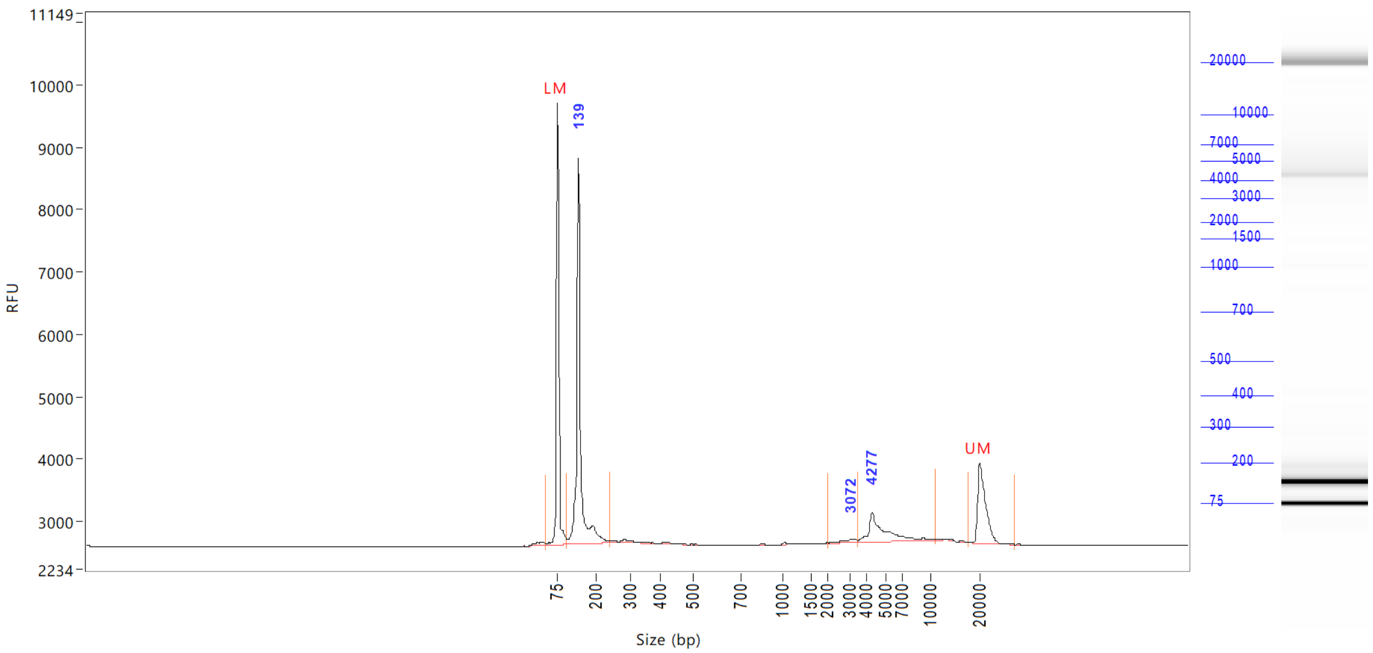

Peak	Size (bp)	Concentration (ng/uL)	From (bp)	To (bp)	Average size (bp)	CV%	RFU	Corrected peak area
1	75 (LM)	0.5000	39	105	73	8.41	7097	41.970
2	139	7.9447	105	239	143	13.39	6190	55.573
3	3072	0.1669	2030	3465	2911	11.62	54	1.168
4	4277	1.5340	3465	11098	5246	28.44	481	10.730
5	20000 (UM)	0.1581	17683	27133	20556	4.71	1277	13.271

TIC:	9.6456	ng/uL
TIM:	91.8771	nmole/L
Total concentration:	10.1456	ng/uL

Sample peak width (sec): 5 Sample min peak height: 50 Sample baseline V to V?: Y Sample baseline V to V points: 3
Sample filter: Binomial Number of points for filter: 3 Sample start region (min): 0 Sample end region (min): 30
Marker peak width (sec): 5 Marker min peak height: 200 Marker baseline V to V?: Y Marker baseline V to V points: 3
Lower marker selection: First peak > 500 RFU Upper marker selection: Last peak > 200 RFU
Ladder size (bp)75, 200, 300, 400, 500, 700, 1000, 1500, 2000, 3000, 4000, 5000, 7000, 10000, 20000
Quantification using: Lower Marker Final concentration (ng/uL): 0.5000 Dilution factor: 12.0

Sample: SampG7
Well location: G7
Created: Thursday, August 12, 2021 6:06:18 PM

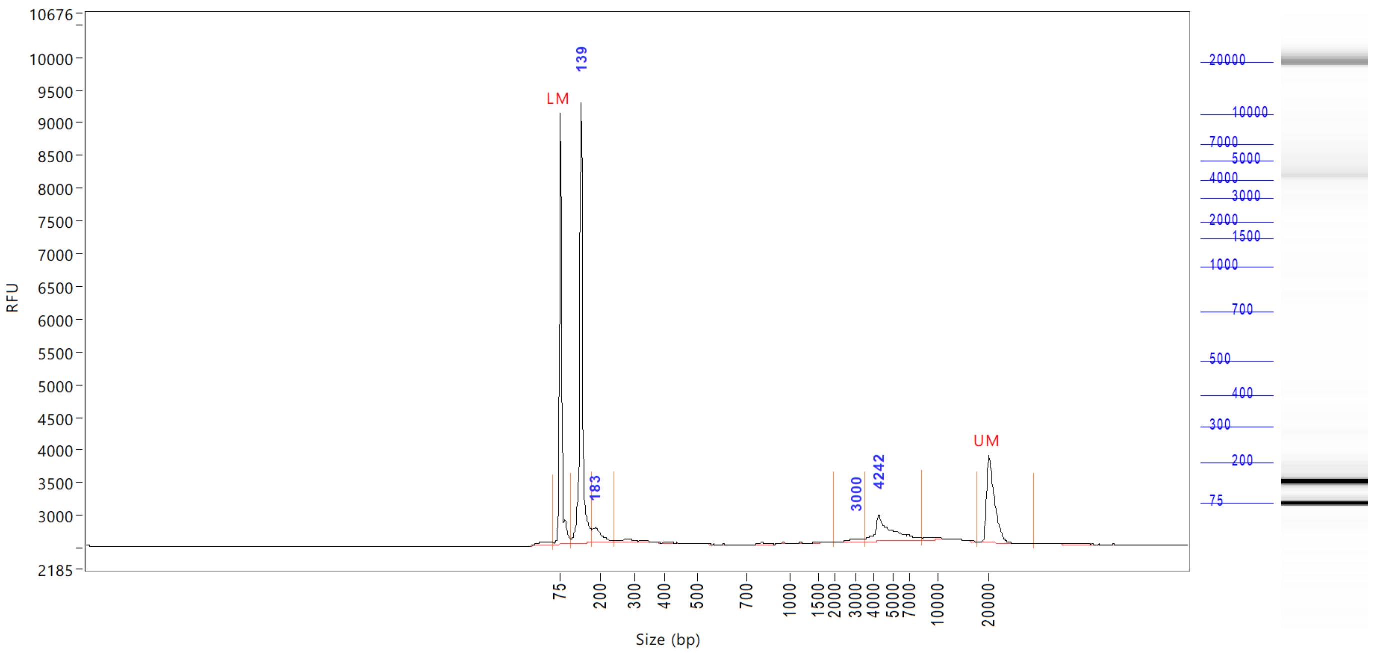

Peak	Size (bp)	Concentration (ng/uL)	From (bp)	To (bp)	Average size (bp)	CV%	RFU	Corrected peak area
1	75 (LM)	0.5000	54	108	74	8.74	6576	39.889
2	139	7.6506	108	171	138	6.43	6737	50.862
3	183	0.7219	171	241	191	7.70	225	4.799
4	3000	0.1663	2000	3429	2917	10.74	53	1.105
5	4242	1.3997	3429	8334	4944	21.91	401	9.305
6	20000 (UM)	0.1700	17561	28826	20525	4.74	1323	13.565

TIC:	9.9385	ng/uL
TIM:	97.8751	nmole/L
Total concentration:	10.5121	ng/uL

Sample peak width (sec): 5 Sample min peak height: 50 Sample baseline V to V?: Y Sample baseline V to V points: 3
Sample filter: Binomial Number of points for filter: 3 Sample start region (min): 0 Sample end region (min): 30
Marker peak width (sec): 5 Marker min peak height: 200 Marker baseline V to V?: Y Marker baseline V to V points: 3
Lower marker selection: First peak > 500 RFU Upper marker selection: Last peak > 200 RFU
Ladder size (bp)75, 200, 300, 400, 500, 700, 1000, 1500, 2000, 3000, 4000, 5000, 7000, 10000, 20000
Quantification using: Lower Marker Final concentration (ng/uL): 0.5000 Dilution factor: 12.0

Sample: SampH7
Well location: H7
Created: Thursday, August 12, 2021 6:06:18 PM

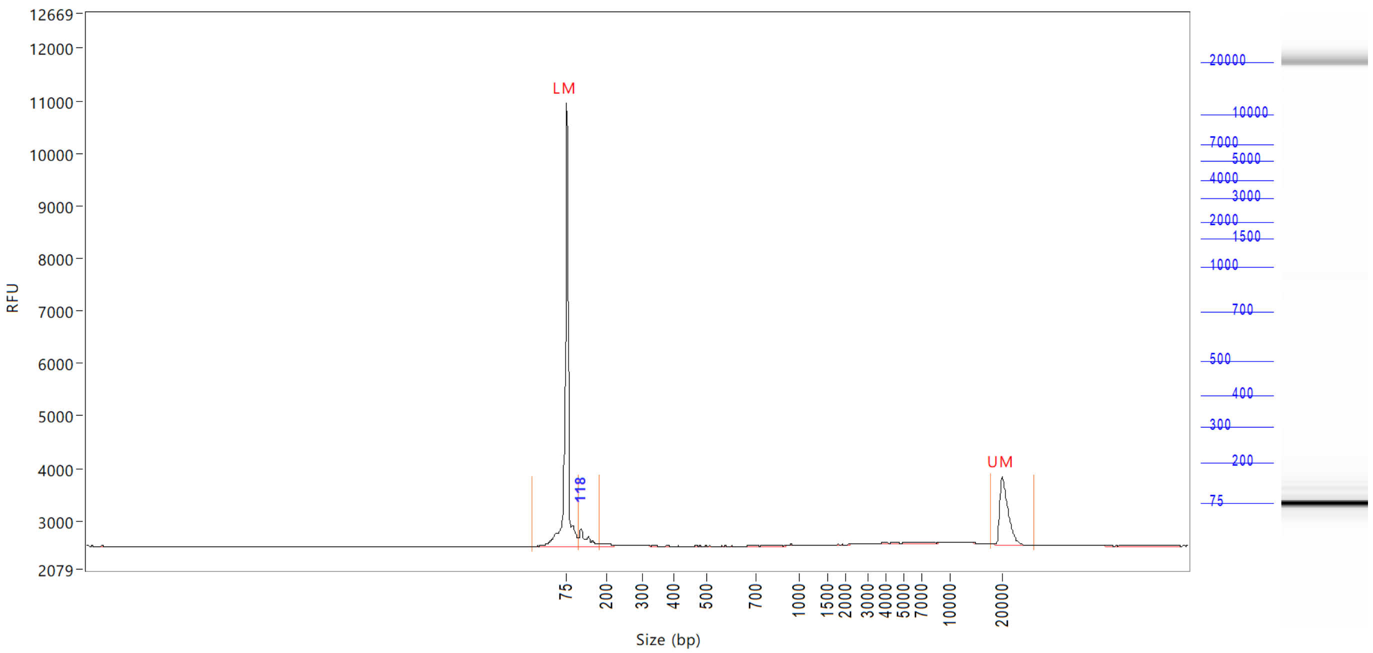

Peak	Size (bp)	Concentration (ng/uL)	From (bp)	To (bp)	Average size (bp)	CV%	RFU	Corrected peak area
1	75 (LM)	0.5000	0	110	71	19.65	8459	68.990
2	118	0.4930	110	173	132	12.73	332	5.669
3	20000 (UM)	0.0989	17805	26166	20588	4.57	1314	13.646

TIC:	0.4930	ng/uL
TIM:	6.1374	nmole/L
Total concentration:	0.8936	ng/uL

```
Sample peak width (sec): 5    Sample min peak height: 50    Sample baseline V to V?: Y    Sample baseline V to V points: 3
Sample filter: Binomial       Number of points for filter: 3  Sample start region (min): 0  Sample end region (min): 30
Marker peak width (sec): 5    Marker min peak height: 200   Marker baseline V to V?: Y    Marker baseline V to V points: 3
Lower marker selection: First peak > 500 RFU                Upper marker selection: Last peak > 200 RFU
Ladder size (bp)75, 200, 300, 400, 500, 700, 1000, 1500, 2000, 3000, 4000, 5000, 7000, 10000, 20000
Quantification using: Lower Marker          Final concentration (ng/uL): 0.5000          Dilution factor: 12.0
```

Sample: SampA8
Well location: A8
Created: Thursday, August 12, 2021 6:06:18 PM

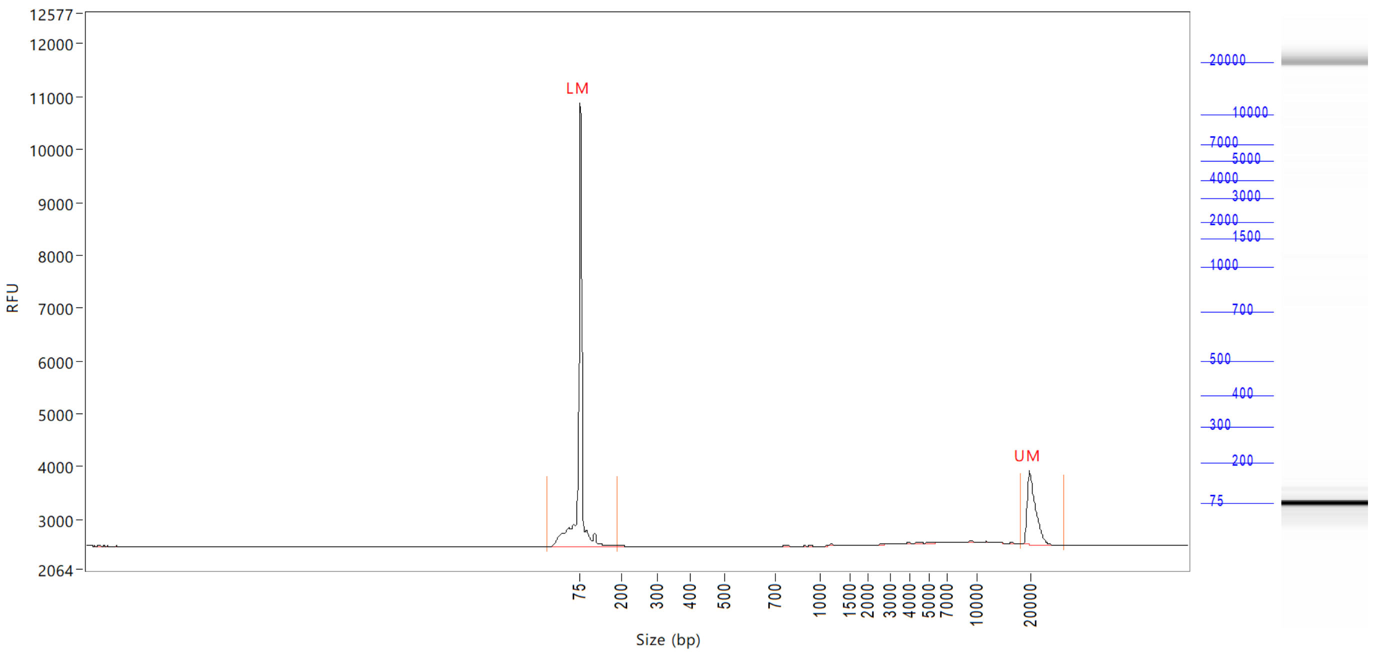

Peak	Size	Concentration	From	To	Average size	CV%	RFU	Corrected peak area
	(bp)	(ng/uL)	(bp)	(bp)	(bp)			
1	75 (LM)	0.5000	0	185	70	31.01	8404	71.761
2	20000 (UM)	0.0985	18293	26408	20539	4.44	1389	14.143

TIC:	0.0000	ng/uL
TIM:	0.0000	nmole/L
Total concentration:	0.2537	ng/uL

Sample peak width (sec): 5 Sample min peak height: 50 Sample baseline V to V?: Y Sample baseline V to V points: 3
Sample filter: Binomial Number of points for filter: 3 Sample start region (min): 0 Sample end region (min): 30
Marker peak width (sec): 5 Marker min peak height: 200 Marker baseline V to V?: Y Marker baseline V to V points: 3
Lower marker selection: First peak > 500 RFU Upper marker selection: Last peak > 200 RFU
Ladder size (bp)75, 200, 300, 400, 500, 700, 1000, 1500, 2000, 3000, 4000, 5000, 7000, 10000, 20000
Quantification using: Lower Marker Final concentration (ng/uL): 0.5000 Dilution factor: 12.0

Sample: SampB8
Well location: B8
Created: Thursday, August 12, 2021 6:06:18 PM

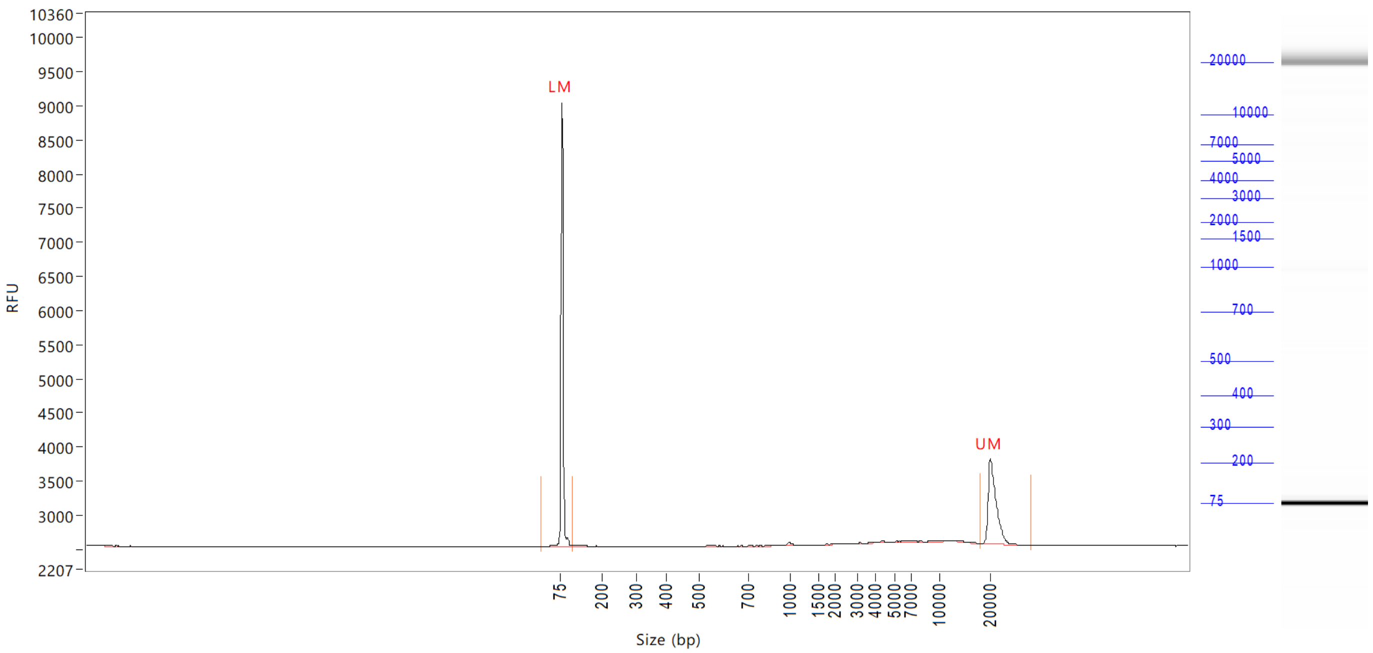

Peak	Size (bp)	Concentration (ng/uL)	From (bp)	To (bp)	Average size (bp)	CV%	RFU	Corrected peak area
1	75 (LM)	0.5000	12	108	73	7.56	6518	36.171
2	20000 (UM)	0.1788	18049	27979	20574	4.94	1254	12.931

TIC:	0.0000	ng/uL
TIM:	0.0000	nmole/L
Total concentration:	0.5713	ng/uL

Sample peak width (sec): 5   Sample min peak height: 50   Sample baseline V to V?: Y   Sample baseline V to V points: 3
Sample filter: Binomial   Number of points for filter: 3   Sample start region (min): 0   Sample end region (min): 30
Marker peak width (sec): 5   Marker min peak height: 200   Marker baseline V to V?: Y   Marker baseline V to V points: 3
Lower marker selection: First peak > 500 RFU   Upper marker selection: Last peak > 200 RFU
Ladder size (bp)75, 200, 300, 400, 500, 700, 1000, 1500, 2000, 3000, 4000, 5000, 7000, 10000, 20000
Quantification using: Lower Marker   Final concentration (ng/uL): 0.5000   Dilution factor: 12.0

Sample: SampC8
Well location: C8
Created: Thursday, August 12, 2021 6:06:18 PM

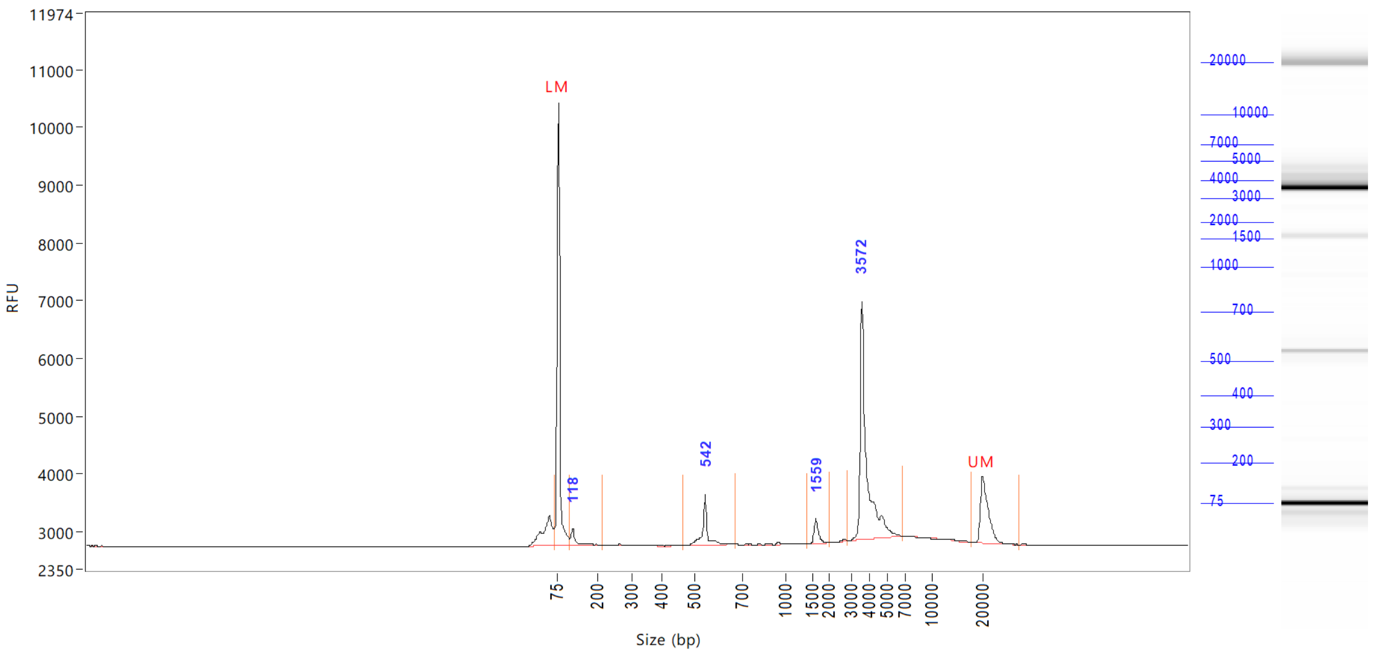

Peak	Size (bp)	Concentration (ng/uL)	From (bp)	To (bp)	Average size (bp)	CV%	RFU	Corrected peak area
1	75 (LM)	0.5000	64	110	75	9.31	7677	49.542
2	118	0.3994	110	214	129	15.84	287	3.298
3	542	0.9267	464	667	540	4.88	889	7.652
4	1559	0.3582	1378	1981	1574	5.09	425	2.958
5	3572	4.8069	2800	6852	3843	11.87	4111	39.691
6	20000 (UM)	0.1180	17683	27254	20468	4.75	1148	11.688

TIC:	6.4913	ng/uL
TIM:	10.3453	nmole/L
Total concentration:	6.7354	ng/uL

Sample peak width (sec): 5   Sample min peak height: 50   Sample baseline V to V?: Y   Sample baseline V to V points: 3
Sample filter: Binomial   Number of points for filter: 3 Sample start region (min): 0 Sample end region (min): 30
Marker peak width (sec): 5   Marker min peak height: 200   Marker baseline V to V?: Y   Marker baseline V to V points: 3
Lower marker selection: First peak > 500 RFU   Upper marker selection: Last peak > 200 RFU
Ladder size (bp)75, 200, 300, 400, 500, 700, 1000, 1500, 2000, 3000, 4000, 5000, 7000, 10000, 20000
Quantification using: Lower Marker   Final concentration (ng/uL): 0.5000   Dilution factor: 12.0

Sample: SampD8
Well location: D8
Created: Thursday, August 12, 2021 6:06:18 PM

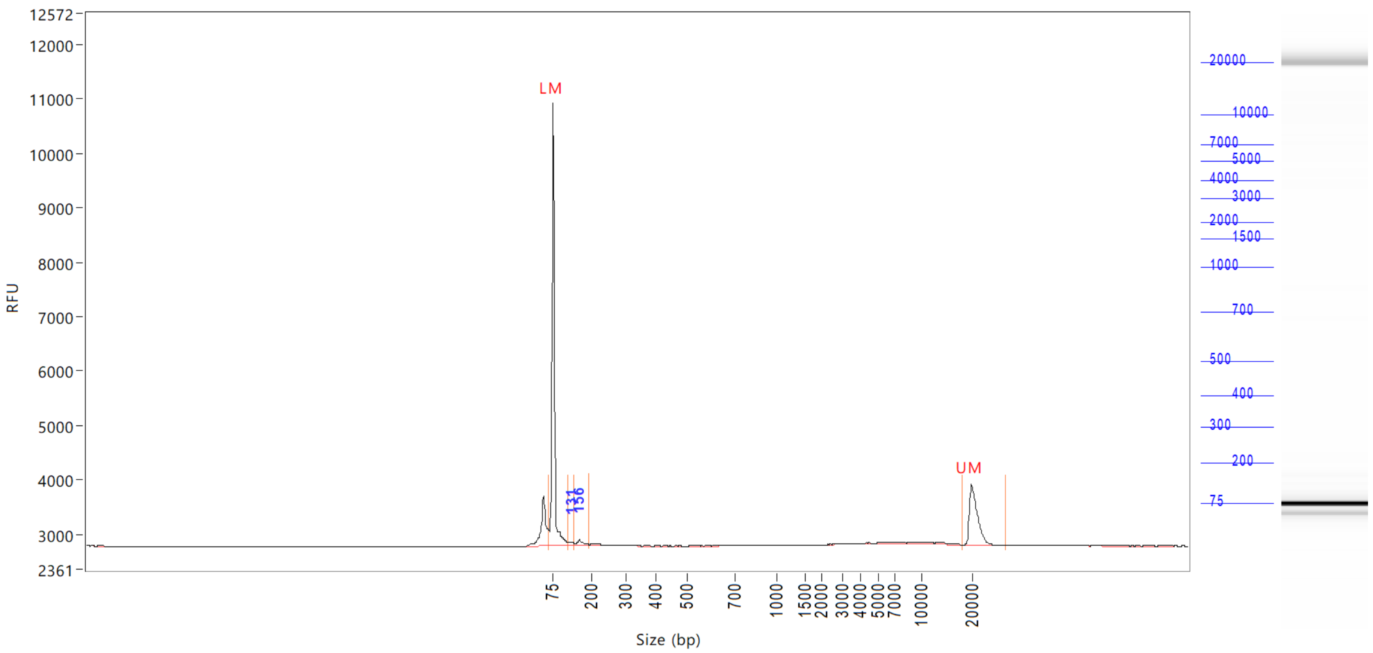

Peak	Size (bp)	Concentration (ng/uL)	From (bp)	To (bp)	Average size (bp)	CV%	RFU	Corrected peak area
1	75 (LM)	0.5000	64	124	74	10.31	8143	51.817
2	131	0.0714	124	143	132	4.19	64	0.617
3	156	0.1220	143	189	158	5.48	89	1.053
4	20000 (UM)	0.1121	18171	26891	20519	4.84	1110	11.614

TIC:	0.1934	ng/uL
TIM:	2.1579	nmole/L
Total concentration:	0.6820	ng/uL

Sample peak width (sec): 5 Sample min peak height: 50 Sample baseline V to V?: Y Sample baseline V to V points: 3
Sample filter: Binomial Number of points for filter: 3 Sample start region (min): 0 Sample end region (min): 30
Marker peak width (sec): 5 Marker min peak height: 200 Marker baseline V to V?: Y Marker baseline V to V points: 3
Lower marker selection: First peak > 500 RFU Upper marker selection: Last peak > 200 RFU
Ladder size (bp)75, 200, 300, 400, 500, 700, 1000, 1500, 2000, 3000, 4000, 5000, 7000, 10000, 20000
Quantification using: Lower Marker Final concentration (ng/uL): 0.5000 Dilution factor: 12.0

Sample: SampE8
Well location: E8
Created: Thursday, August 12, 2021 6:06:18 PM

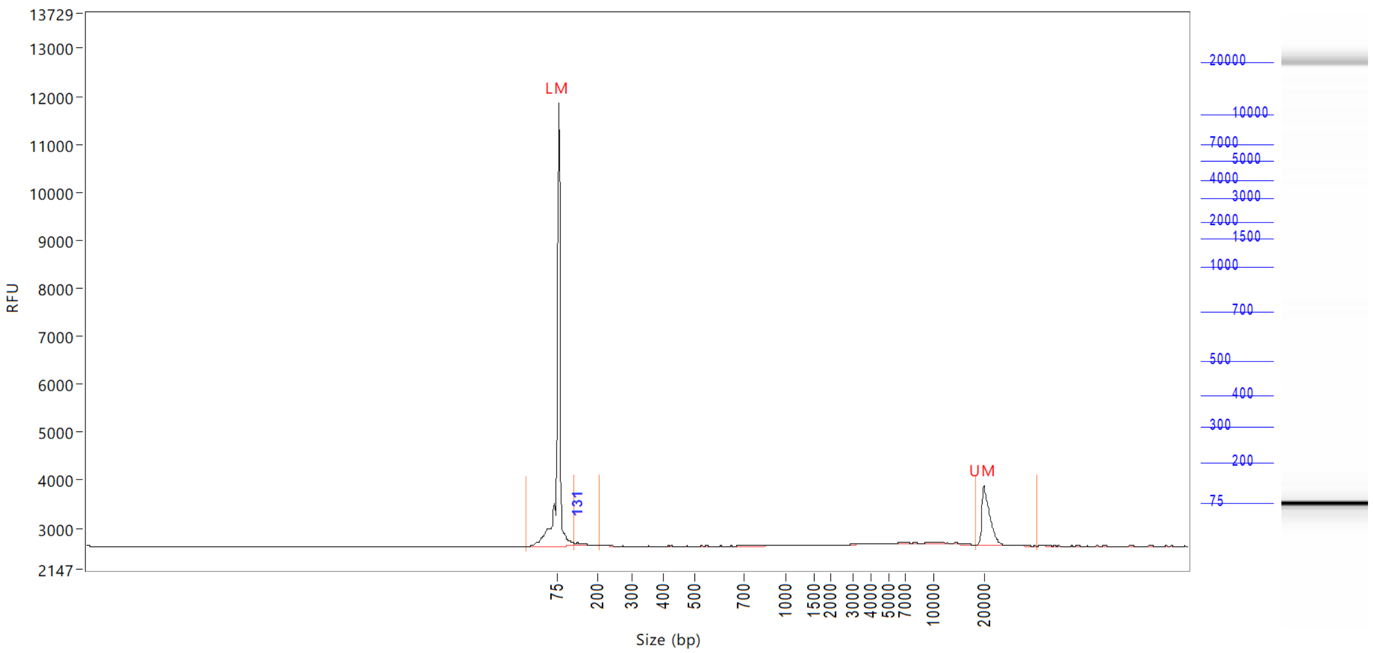

Peak	Size (bp)	Concentration (ng/uL)	From (bp)	To (bp)	Average size (bp)	CV%	RFU	Corrected peak area
1	75 (LM)	0.5000	0	124	68	24.49	9249	71.058
2	131	0.1024	124	201	142	10.14	74	1.212
3	20000 (UM)	0.0875	18415	30398	20579	5.23	1232	12.439

TIC:	0.1024	ng/uL
TIM:	1.1846	nmole/L
Total concentration:	0.4328	ng/uL

```
Sample peak width (sec): 5    Sample min peak height: 50    Sample baseline V to V?: Y    Sample baseline V to V points: 3
Sample filter: Binomial       Number of points for filter: 3  Sample start region (min): 0  Sample end region (min): 30
Marker peak width (sec): 5    Marker min peak height: 200   Marker baseline V to V?: Y    Marker baseline V to V points: 3
Lower marker selection: First peak > 500 RFU                Upper marker selection: Last peak > 200 RFU
Ladder size (bp)75, 200, 300, 400, 500, 700, 1000, 1500, 2000, 3000, 4000, 5000, 7000, 10000, 20000
Quantification using: Lower Marker          Final concentration (ng/uL): 0.5000          Dilution factor: 12.0
```

Sample: SampF8
Well location: F8
Created: Thursday, August 12, 2021 6:06:18 PM

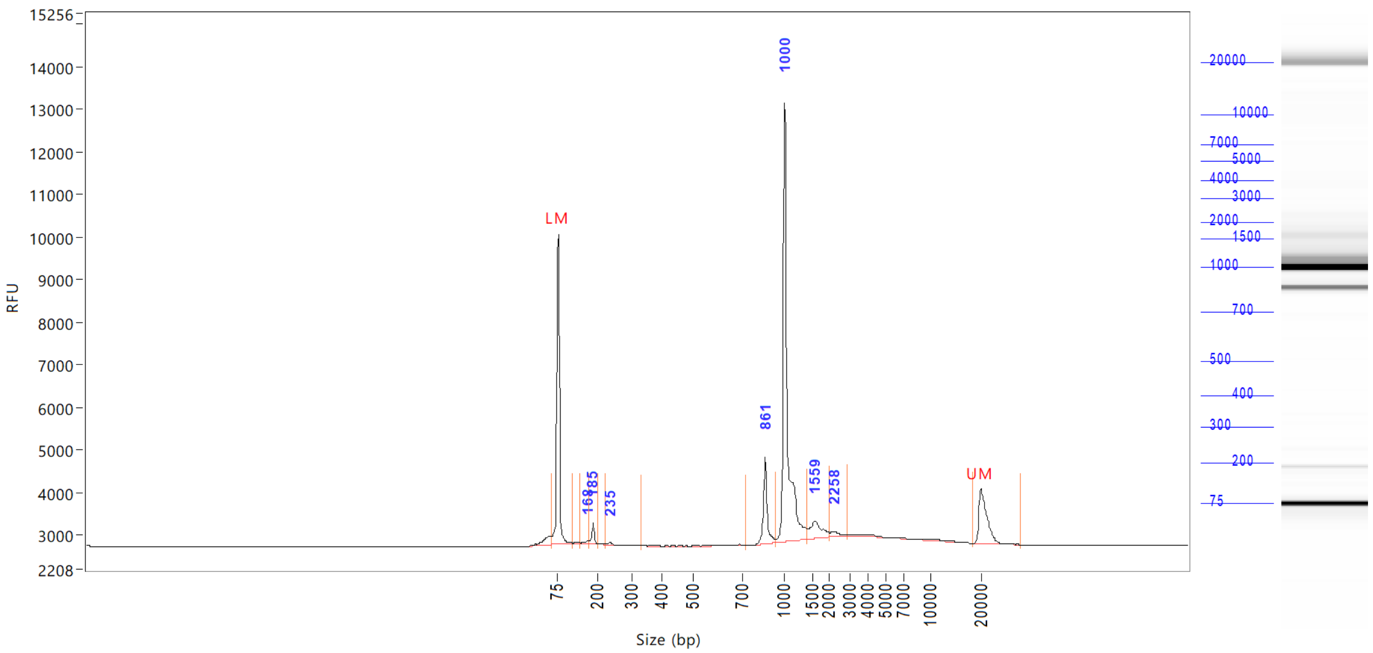

Peak	Size (bp)	Concentration (ng/uL)	From (bp)	To (bp)	Average size (bp)	CV%	RFU	Corrected peak area
1	75 (LM)	0.5000	56	120	73	7.48	7274	46.083
2	168	0.1112	145	175	164	5.21	75	0.854
3	185	0.3543	175	201	183	1.93	490	2.721
4	235	0.0774	224	331	253	11.84	55	0.594
5	861	1.7027	727	939	862	2.48	2042	13.078
6	1000	9.8071	939	1422	1046	8.86	10315	75.323
7	1559	0.9788	1422	2030	1639	10.03	417	7.518
8	2258	0.2121	2030	2829	2294	8.51	126	1.629
9	20000 (UM)	0.1445	18415	27859	20575	4.90	1274	13.316

TIC:	13.2435	ng/uL
TIM:	24.6181	nmole/L
Total concentration:	13.7431	ng/uL

Sample peak width (sec): 5 Sample min peak height: 50 Sample baseline V to V?: Y Sample baseline V to V points: 3
Sample filter: Binomial Number of points for filter: 3 Sample start region (min): 0 Sample end region (min): 30
Marker peak width (sec): 5 Marker min peak height: 200 Marker baseline V to V?: Y Marker baseline V to V points: 3
Lower marker selection: First peak > 500 RFU Upper marker selection: Last peak > 200 RFU
Ladder size (bp)75, 200, 300, 400, 500, 700, 1000, 1500, 2000, 3000, 4000, 5000, 7000, 10000, 20000
Quantification using: Lower Marker Final concentration (ng/uL): 0.5000 Dilution factor: 12.0

Sample: SampG8
Well location: G8
Created: Thursday, August 12, 2021 6:06:18 PM

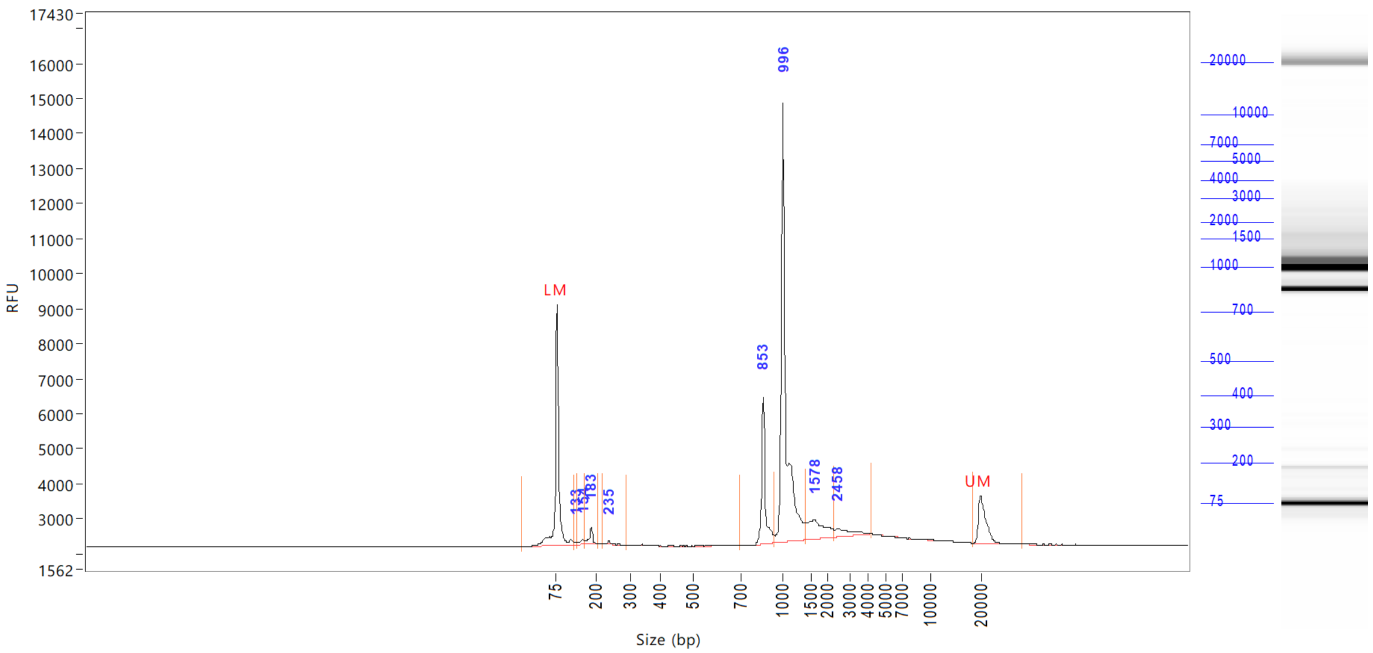

Peak	Size (bp)	Concentration (ng/uL)	From (bp)	To (bp)	Average size (bp)	CV%	RFU	Corrected peak area
1	75 (LM)	0.5000	0	128	72	21.75	6877	54.006
2	133	0.0809	128	139	133	2.84	89	0.728
3	154	0.1837	139	162	151	4.49	136	1.653
4	183	0.4465	162	203	178	4.18	493	4.019
5	235	0.0922	215	287	237	5.32	98	0.830
6	853	3.0039	695	939	857	2.88	4205	27.038
7	996	11.1794	939	1389	1048	9.11	12552	100.626
8	1578	1.7685	1389	2315	1703	14.83	547	15.918
9	2458	0.6431	2315	4173	2874	14.91	246	5.789
10	20000 (UM)	0.1237	18537	28342	20533	4.79	1346	13.359

TIC:	17.3981	ng/uL
TIM:	33.1669	nmole/L
Total concentration:	17.4557	ng/uL

Sample peak width (sec): 5   Sample min peak height: 50   Sample baseline V to V?: Y   Sample baseline V to V points: 3
Sample filter: Binomial   Number of points for filter: 3 Sample start region (min): 0 Sample end region (min): 30
Marker peak width (sec): 5   Marker min peak height: 200   Marker baseline V to V?: Y   Marker baseline V to V points: 3
Lower marker selection: First peak > 500 RFU   Upper marker selection: Last peak > 200 RFU
Ladder size (bp)75, 200, 300, 400, 500, 700, 1000, 1500, 2000, 3000, 4000, 5000, 7000, 10000, 20000
Quantification using: Lower Marker   Final concentration (ng/uL): 0.5000   Dilution factor: 12.0

Sample: SampH8
Well location: H8
Created: Thursday, August 12, 2021 6:06:18 PM

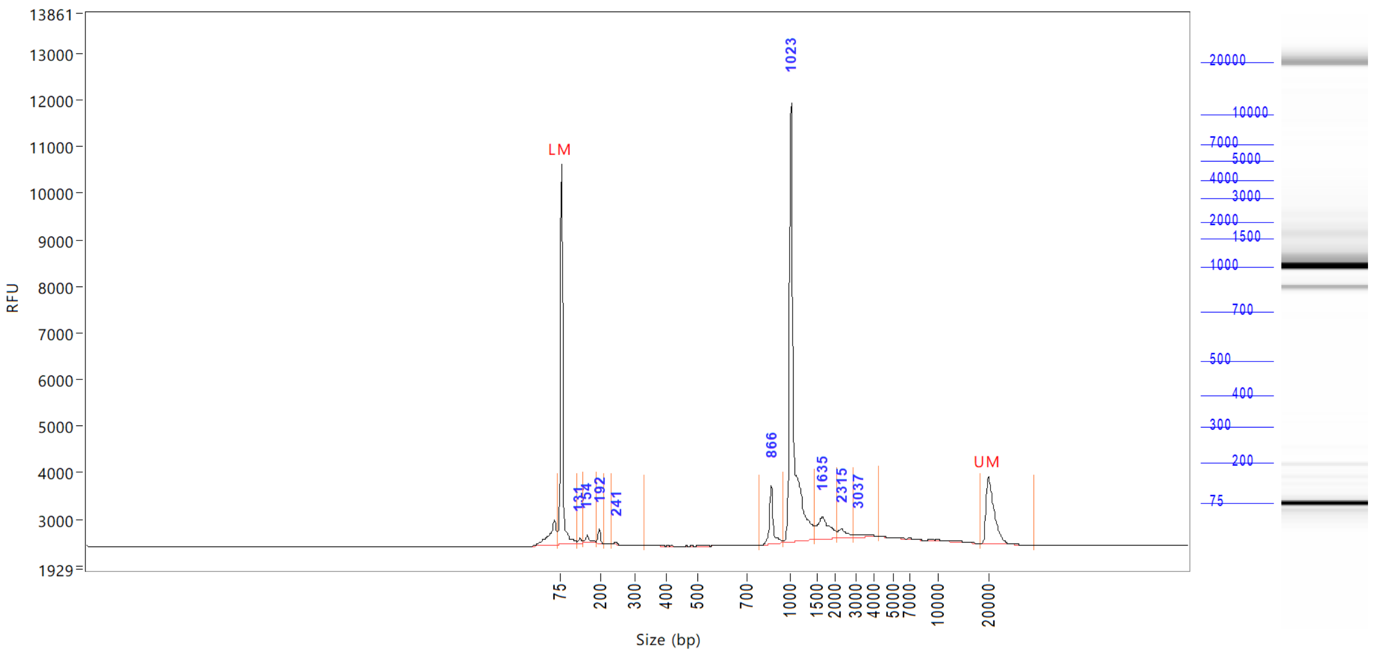

Peak	Size (bp)	Concentration (ng/uL)	From (bp)	To (bp)	Average size (bp)	CV%	RFU	Corrected peak area
1	75 (LM)	0.5000	64	124	75	9.60	8176	50.452
2	131	0.1154	124	145	133	4.33	113	0.970
3	154	0.1986	145	183	158	6.35	182	1.670
4	192	0.1849	183	208	190	1.72	298	1.555
5	241	0.0522	228	329	250	10.42	52	0.439
6	866	0.9837	783	957	868	2.79	1269	8.271
7	1023	8.5632	957	1456	1071	9.80	9437	72.005
8	1635	1.0743	1456	2087	1694	10.13	464	9.034
9	2315	0.3321	2087	2857	2390	8.81	187	2.793
10	3037	0.1638	2857	4208	3342	10.65	64	1.378
11	20000 (UM)	0.1477	18293	29068	20560	4.95	1440	14.901

TIC:	11.6683	ng/uL
TIM:	21.8140	nmole/L
Total concentration:	11.8571	ng/uL

```
Sample peak width (sec): 5     Sample min peak height: 50     Sample baseline V to V?: Y     Sample baseline V to V points: 3
Sample filter: Binomial        Number of points for filter: 3  Sample start region (min): 0  Sample end region (min): 30
Marker peak width (sec): 5     Marker min peak height: 200    Marker baseline V to V?: Y     Marker baseline V to V points: 3
Lower marker selection: First peak > 500 RFU                  Upper marker selection: Last peak > 200 RFU
Ladder size (bp)75, 200, 300, 400, 500, 700, 1000, 1500, 2000, 3000, 4000, 5000, 7000, 10000, 20000
Quantification using: Lower Marker          Final concentration (ng/uL): 0.5000          Dilution factor: 12.0
```

Sample: SampA9
Well location: A9
Created: Thursday, August 12, 2021 6:06:18 PM

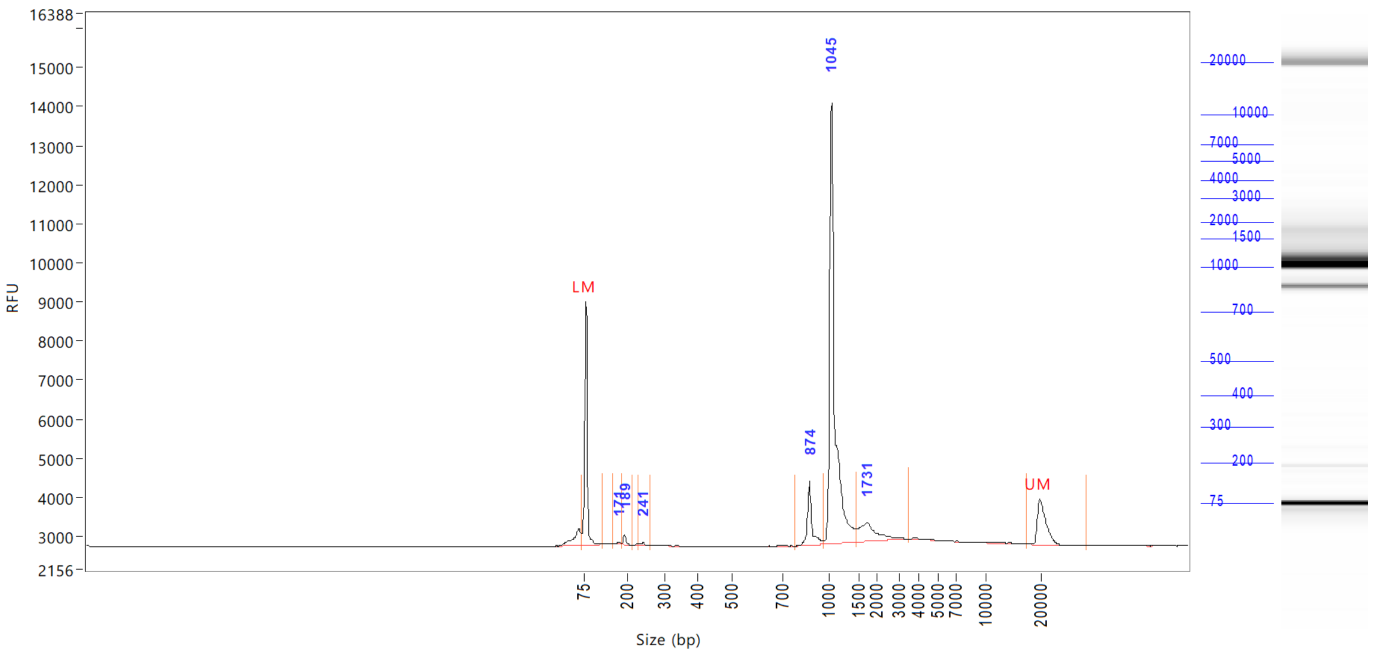

Peak	Size (bp)	Concentration (ng/uL)	From (bp)	To (bp)	Average size (bp)	CV%	RFU	Corrected peak area
1	75 (LM)	0.5000	64	122	74	6.74	6241	35.922
2	171	0.0956	156	181	171	4.07	60	0.572
3	189	0.2251	181	212	187	2.87	244	1.348
4	241	0.0927	226	262	237	2.68	79	0.555
5	874	1.9624	775	965	875	3.12	1626	11.749
6	1045	15.1402	965	1456	1084	9.00	11302	90.646
7	1731	2.0906	1456	3429	1845	18.92	479	12.517
8	20000 (UM)	0.1690	17561	28826	20497	4.53	1189	12.145

TIC:	19.6067	ng/uL
TIM:	32.0873	nmole/L
Total concentration:	20.0406	ng/uL

```
Sample peak width (sec): 5    Sample min peak height: 50    Sample baseline V to V?: Y    Sample baseline V to V points: 3
Sample filter: Binomial       Number of points for filter: 3  Sample start region (min): 0  Sample end region (min): 30
Marker peak width (sec): 5    Marker min peak height: 200   Marker baseline V to V?: Y    Marker baseline V to V points: 3
Lower marker selection: First peak > 500 RFU                Upper marker selection: Last peak > 200 RFU
Ladder size (bp)75, 200, 300, 400, 500, 700, 1000, 1500, 2000, 3000, 4000, 5000, 7000, 10000, 20000
Quantification using: Lower Marker          Final concentration (ng/uL): 0.5000          Dilution factor: 12.0
```

Sample: SampB9
Well location: B9
Created: Thursday, August 12, 2021 6:06:18 PM

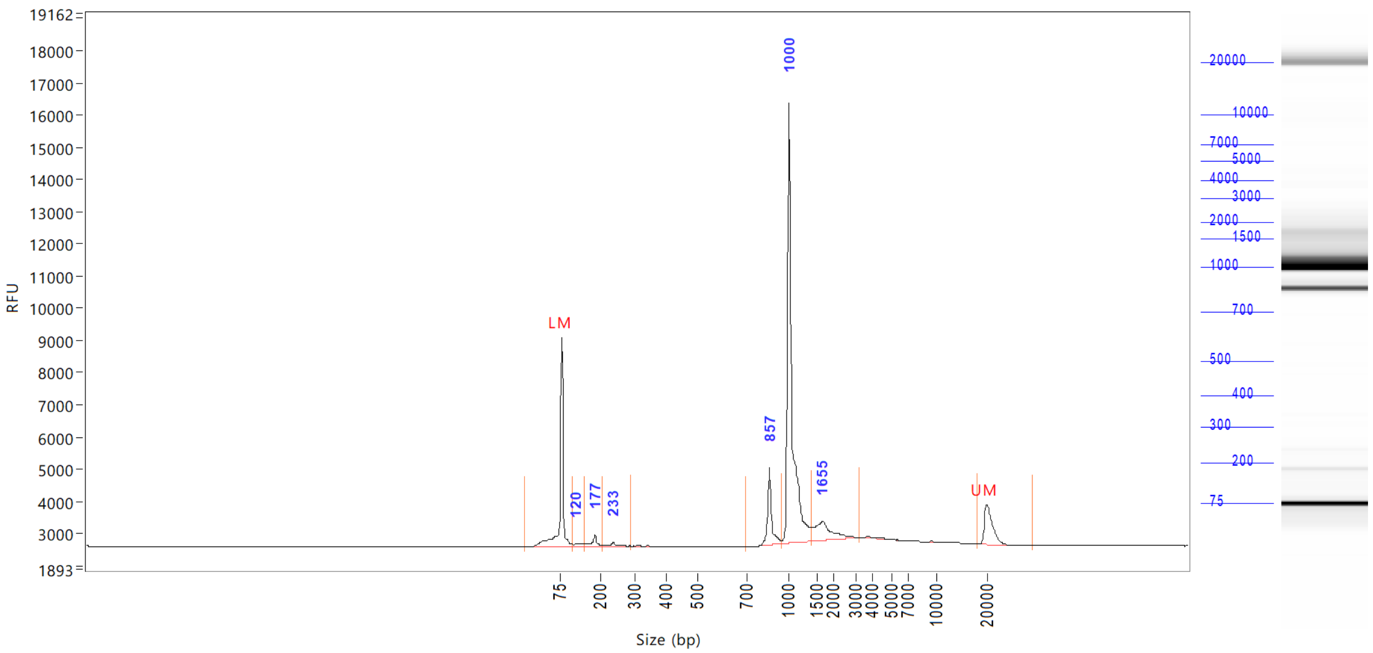

Peak	Size (bp)	Concentration (ng/uL)	From (bp)	To (bp)	Average size (bp)	CV%	RFU	Corrected peak area
1	75 (LM)	0.5000	0	110	65	29.54	6509	50.197
2	120	0.2050	110	145	128	7.96	79	1.715
3	177	0.4874	145	203	172	7.65	336	4.077
4	233	0.1979	203	287	229	6.83	113	1.655
5	857	1.9294	700	952	861	3.08	2398	16.141
6	1000	12.7143	952	1411	1049	8.78	13693	106.370
7	1655	1.8361	1411	3215	1761	18.21	593	15.361
8	20000 (UM)	0.1276	18293	29189	20600	5.09	1247	12.812

TIC:	17.3699	ng/uL
TIM:	34.0641	nmole/L
Total concentration:	17.5577	ng/uL

```
Sample peak width (sec): 5    Sample min peak height: 50    Sample baseline V to V?: Y    Sample baseline V to V points: 3
Sample filter: Binomial       Number of points for filter: 3  Sample start region (min): 0  Sample end region (min): 30
Marker peak width (sec): 5    Marker min peak height: 200   Marker baseline V to V?: Y    Marker baseline V to V points: 3
Lower marker selection: First peak > 500 RFU                Upper marker selection: Last peak > 200 RFU
Ladder size (bp)75, 200, 300, 400, 500, 700, 1000, 1500, 2000, 3000, 4000, 5000, 7000, 10000, 20000
Quantification using: Lower Marker          Final concentration (ng/uL): 0.5000          Dilution factor: 12.0
```

Sample: SampC9
Well location: C9
Created: Thursday, August 12, 2021 6:06:18 PM

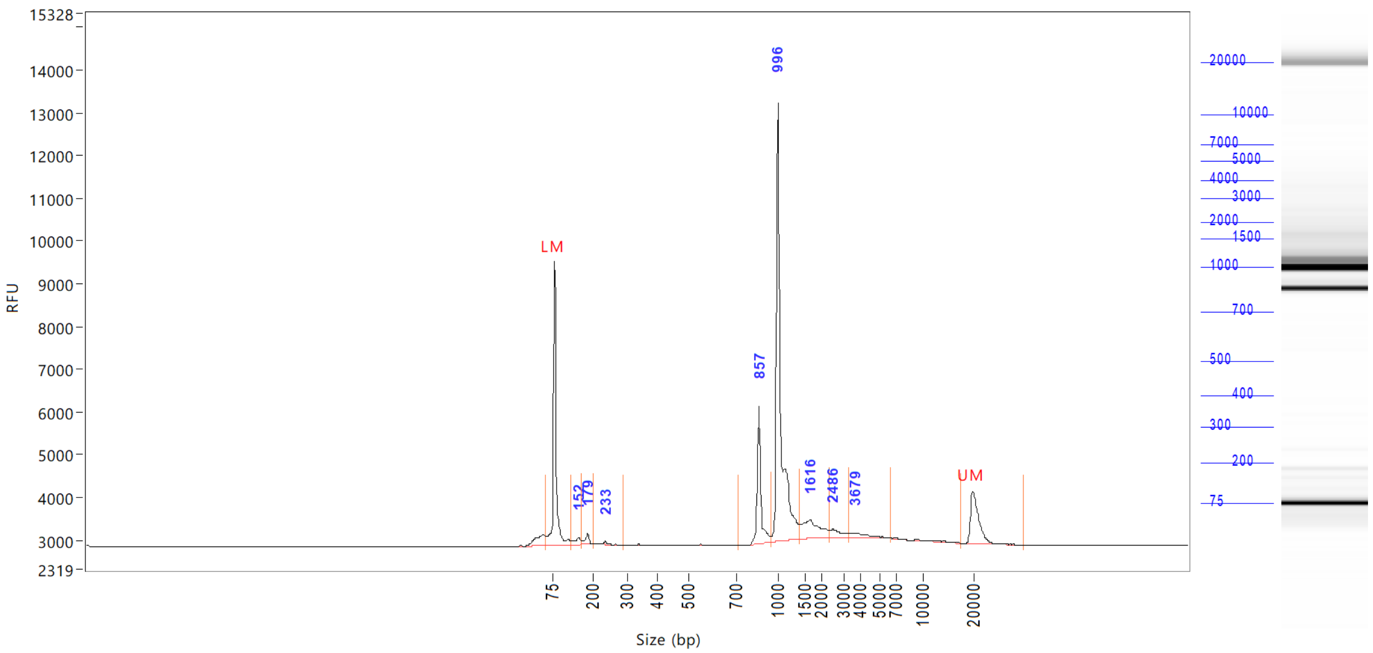

Peak	Size (bp)	Concentration (ng/uL)	From (bp)	To (bp)	Average size (bp)	CV%	RFU	Corrected peak area
1	75 (LM)	0.5000	48	129	75	14.72	6653	47.319
2	152	0.3017	129	162	146	6.13	171	2.379
3	179	0.3010	162	200	175	3.59	272	2.374
4	233	0.0866	200	289	233	6.27	79	0.683
5	857	2.6152	718	948	860	2.92	3233	20.625
6	996	10.0455	948	1378	1045	8.67	10275	79.224
7	1616	1.6137	1378	2344	1706	15.16	433	12.726
8	2486	0.4450	2344	3286	2746	9.97	189	3.510
9	3679	0.3890	3286	6408	4035	14.50	119	3.068
10	20000 (UM)	0.1374	17683	30277	20543	5.38	1223	13.002

TIC:	15.7975	ng/uL
TIM:	29.6438	nmole/L
Total concentration:	15.9855	ng/uL

Sample peak width (sec): 5 Sample min peak height: 50 Sample baseline V to V?: Y Sample baseline V to V points: 3
Sample filter: Binomial Number of points for filter: 3 Sample start region (min): 0 Sample end region (min): 30
Marker peak width (sec): 5 Marker min peak height: 200 Marker baseline V to V?: Y Marker baseline V to V points: 3
Lower marker selection: First peak > 500 RFU Upper marker selection: Last peak > 200 RFU
Ladder size (bp)75, 200, 300, 400, 500, 700, 1000, 1500, 2000, 3000, 4000, 5000, 7000, 10000, 20000
Quantification using: Lower Marker Final concentration (ng/uL): 0.5000 Dilution factor: 12.0

Sample: SampD9
Well location: D9
Created: Thursday, August 12, 2021 6:06:18 PM

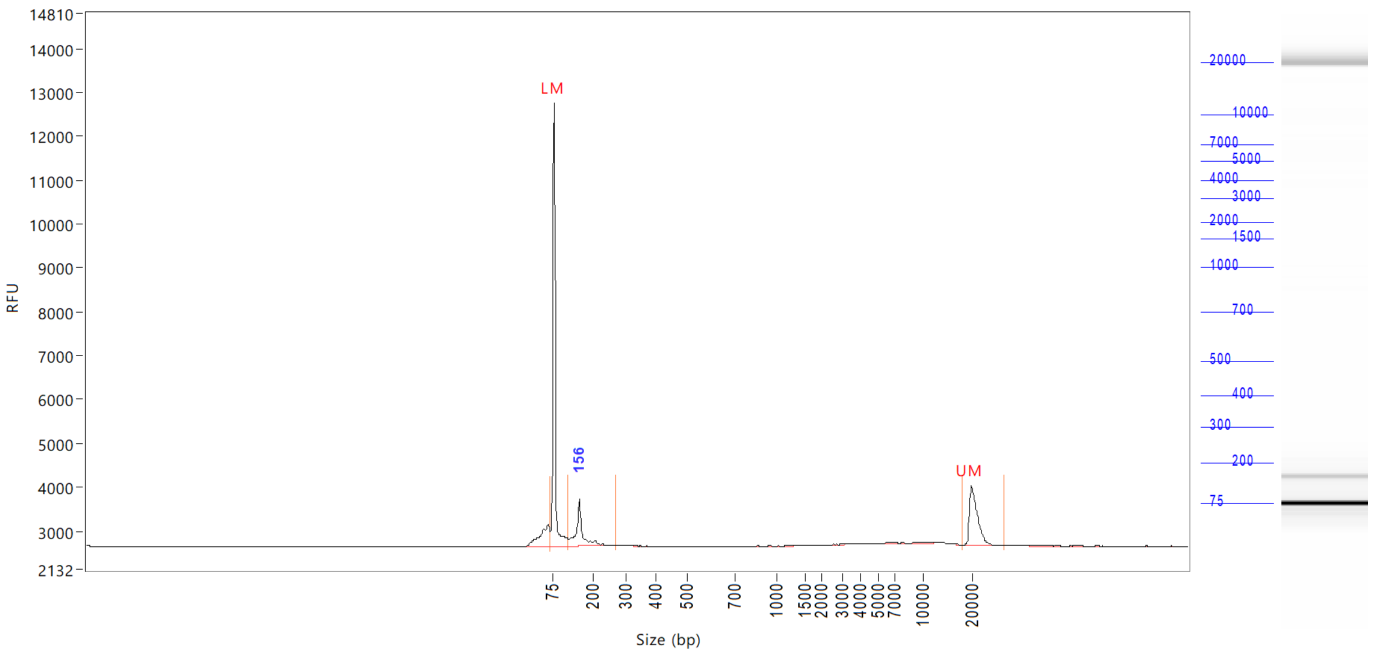

Peak	Size (bp)	Concentration (ng/uL)	From (bp)	To (bp)	Average size (bp)	CV%	RFU	Corrected peak area
1	75 (LM)	0.5000	64	124	76	12.67	10126	62.963
2	156	1.4371	124	268	161	15.39	1075	15.081
3	20000 (UM)	0.1132	18049	26529	20537	4.90	1356	14.250

TIC:	1.4371	ng/uL
TIM:	14.6723	nmole/L
Total concentration:	1.7184	ng/uL

Sample peak width (sec): 5 Sample min peak height: 50 Sample baseline V to V?: Y Sample baseline V to V points: 3
Sample filter: Binomial Number of points for filter: 3 Sample start region (min): 0 Sample end region (min): 30
Marker peak width (sec): 5 Marker min peak height: 200 Marker baseline V to V?: Y Marker baseline V to V points: 3
Lower marker selection: First peak > 500 RFU Upper marker selection: Last peak > 200 RFU
Ladder size (bp)75, 200, 300, 400, 500, 700, 1000, 1500, 2000, 3000, 4000, 5000, 7000, 10000, 20000
Quantification using: Lower Marker Final concentration (ng/uL): 0.5000 Dilution factor: 12.0

Sample: SampE9
Well location: E9
Created: Thursday, August 12, 2021 6:06:18 PM

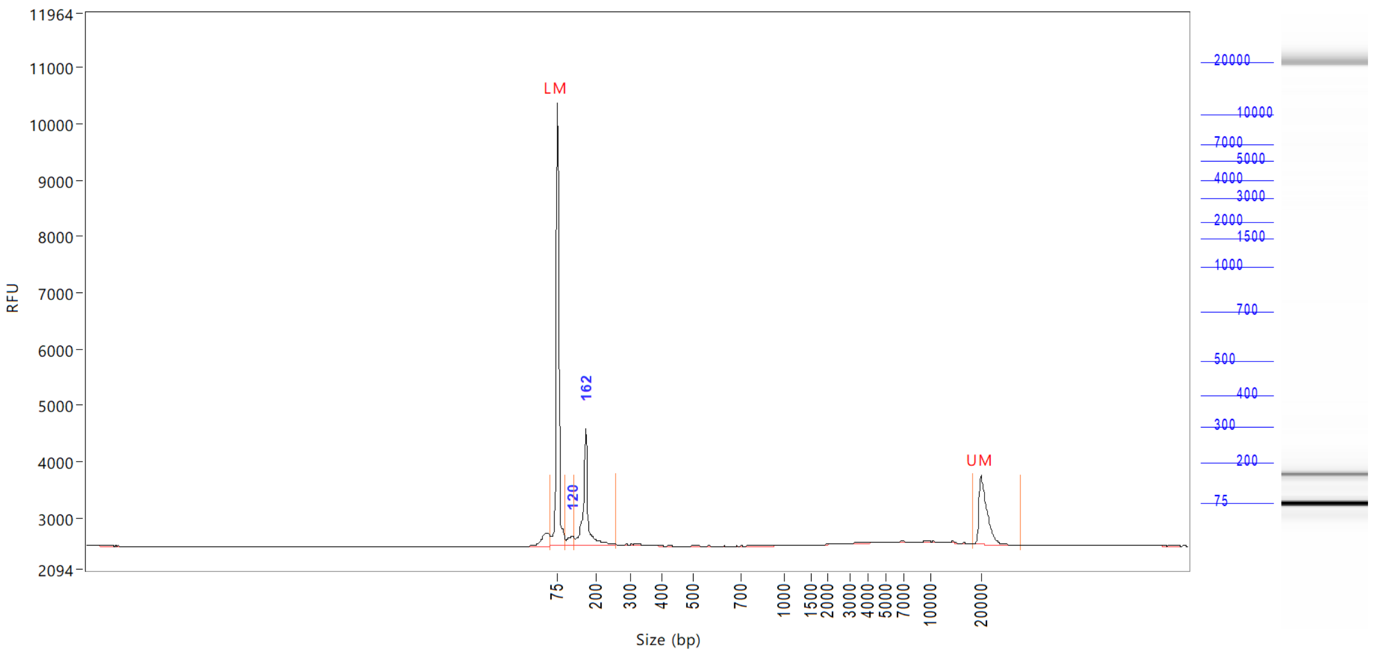

Peak	Size (bp)	Concentration (ng/uL)	From (bp)	To (bp)	Average size (bp)	CV%	RFU	Corrected peak area
1	75 (LM)	0.5000	54	101	73	7.73	7876	52.148
2	120	0.2948	101	128	114	6.81	163	2.562
3	162	2.3836	128	253	162	11.04	2079	20.717
4	20000 (UM)	0.1219	18293	27859	20619	5.14	1220	12.710

TIC:	2.6785	ng/uL
TIM:	28.4334	nmole/L
Total concentration:	3.0042	ng/uL

Sample peak width (sec): 5 Sample min peak height: 50 Sample baseline V to V?: Y Sample baseline V to V points: 3
Sample filter: Binomial Number of points for filter: 3 Sample start region (min): 0 Sample end region (min): 30
Marker peak width (sec): 5 Marker min peak height: 200 Marker baseline V to V?: Y Marker baseline V to V points: 3
Lower marker selection: First peak > 500 RFU Upper marker selection: Last peak > 200 RFU
Ladder size (bp)75, 200, 300, 400, 500, 700, 1000, 1500, 2000, 3000, 4000, 5000, 7000, 10000, 20000
Quantification using: Lower Marker Final concentration (ng/uL): 0.5000 Dilution factor: 12.0

Sample: SampF9
Well location: F9
Created: Thursday, August 12, 2021 6:06:18 PM

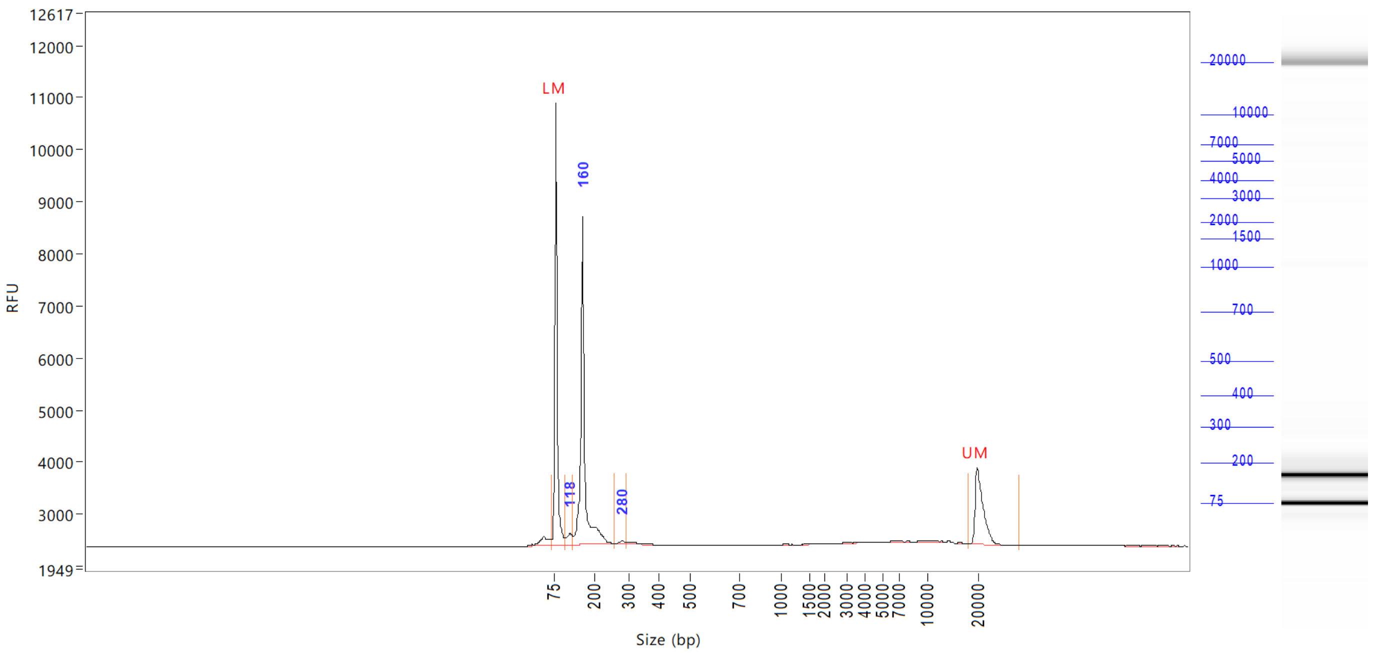

Peak	Size (bp)	Concentration (ng/uL)	From (bp)	To (bp)	Average size (bp)	CV%	RFU	Corrected peak area
1	75 (LM)	0.5000	64	103	75	7.26	8501	51.120
2	118	0.3521	103	128	116	6.27	211	3.000
3	160	6.6529	128	255	163	11.05	6307	56.683
4	280	0.0675	255	291	278	2.73	51	0.575
5	20000 (UM)	0.1501	18171	28463	20563	4.91	1465	15.346

TIC:	7.0725	ng/uL
TIM:	72.4756	nmole/L
Total concentration:	7.5061	ng/uL

```
Sample peak width (sec): 5    Sample min peak height: 50    Sample baseline V to V?: Y    Sample baseline V to V points: 3
Sample filter: Binomial       Number of points for filter: 3  Sample start region (min): 0  Sample end region (min): 30
Marker peak width (sec): 5    Marker min peak height: 200   Marker baseline V to V?: Y    Marker baseline V to V points: 3
Lower marker selection: First peak > 500 RFU                Upper marker selection: Last peak > 200 RFU
Ladder size (bp)75, 200, 300, 400, 500, 700, 1000, 1500, 2000, 3000, 4000, 5000, 7000, 10000, 20000
Quantification using: Lower Marker          Final concentration (ng/uL): 0.5000          Dilution factor: 12.0
```

Sample: SampG9
Well location: G9
Created: Thursday, August 12, 2021 6:06:18 PM

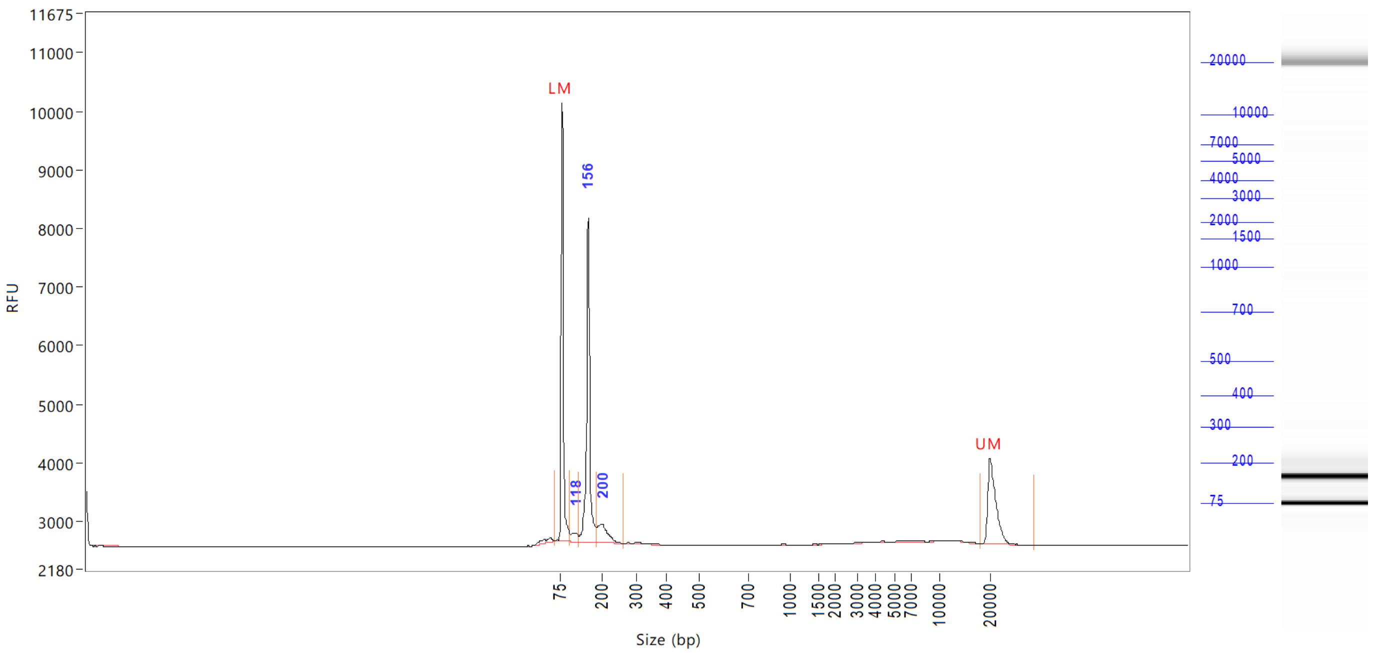

Peak	Size (bp)	Concentration (ng/uL)	From (bp)	To (bp)	Average size (bp)	CV%	RFU	Corrected peak area
1	75 (LM)	0.5000	54	101	75	6.95	7498	43.395
2	118	0.3447	101	128	114	6.93	154	2.493
3	156	5.9093	128	183	154	5.45	5540	42.739
4	200	1.0000	183	264	202	6.74	313	7.232
5	20000 (UM)	0.1751	18171	28705	20500	4.78	1467	15.195

TIC:	7.2539	ng/uL
TIM:	76.1736	nmole/L
Total concentration:	7.7372	ng/uL

Sample peak width (sec): 5 Sample min peak height: 50 Sample baseline V to V?: Y Sample baseline V to V points: 3
Sample filter: Binomial Number of points for filter: 3 Sample start region (min): 0 Sample end region (min): 30
Marker peak width (sec): 5 Marker min peak height: 200 Marker baseline V to V?: Y Marker baseline V to V points: 3
Lower marker selection: First peak > 500 RFU Upper marker selection: Last peak > 200 RFU
Ladder size (bp)75, 200, 300, 400, 500, 700, 1000, 1500, 2000, 3000, 4000, 5000, 7000, 10000, 20000
Quantification using: Lower Marker Final concentration (ng/uL): 0.5000 Dilution factor: 12.0

Sample: SampH9
Well location: H9
Created: Thursday, August 12, 2021 6:06:18 PM

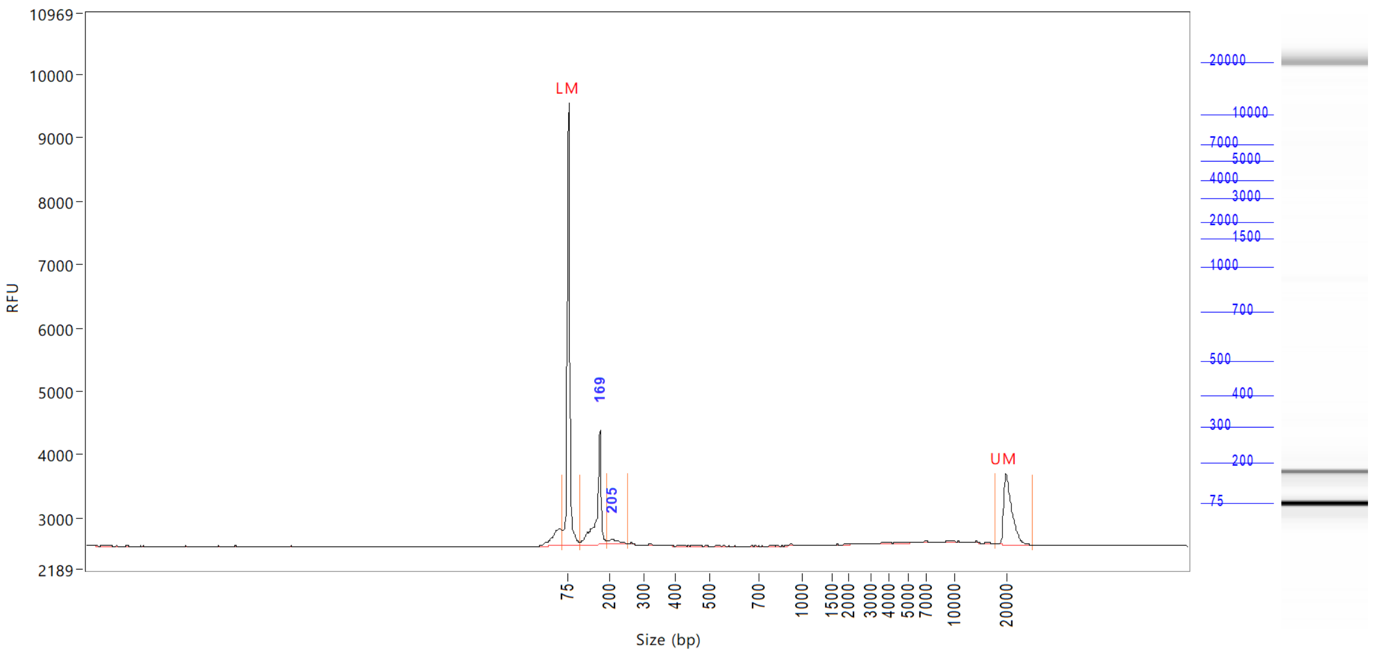

Peak	Size (bp)	Concentration (ng/uL)	From (bp)	To (bp)	Average size (bp)	CV%	RFU	Corrected peak area
1	75 (LM)	0.5000	56	112	73	8.69	6999	46.055
2	169	2.3283	112	194	158	10.06	1815	17.871
3	205	0.2208	194	251	214	6.47	67	1.695
4	20000 (UM)	0.1262	17927	25199	20480	4.53	1131	11.626

TIC:	2.5491	ng/uL
TIM:	25.9176	nmole/L
Total concentration:	2.9770	ng/uL

Sample peak width (sec): 5 Sample min peak height: 50 Sample baseline V to V?: Y Sample baseline V to V points: 3
Sample filter: Binomial Number of points for filter: 3 Sample start region (min): 0 Sample end region (min): 30
Marker peak width (sec): 5 Marker min peak height: 200 Marker baseline V to V?: Y Marker baseline V to V points: 3
Lower marker selection: First peak > 500 RFU Upper marker selection: Last peak > 200 RFU
Ladder size (bp)75, 200, 300, 400, 500, 700, 1000, 1500, 2000, 3000, 4000, 5000, 7000, 10000, 20000
Quantification using: Lower Marker Final concentration (ng/uL): 0.5000 Dilution factor: 12.0

Sample: SampA10
Well location: A10
Created: Thursday, August 12, 2021 6:06:18 PM

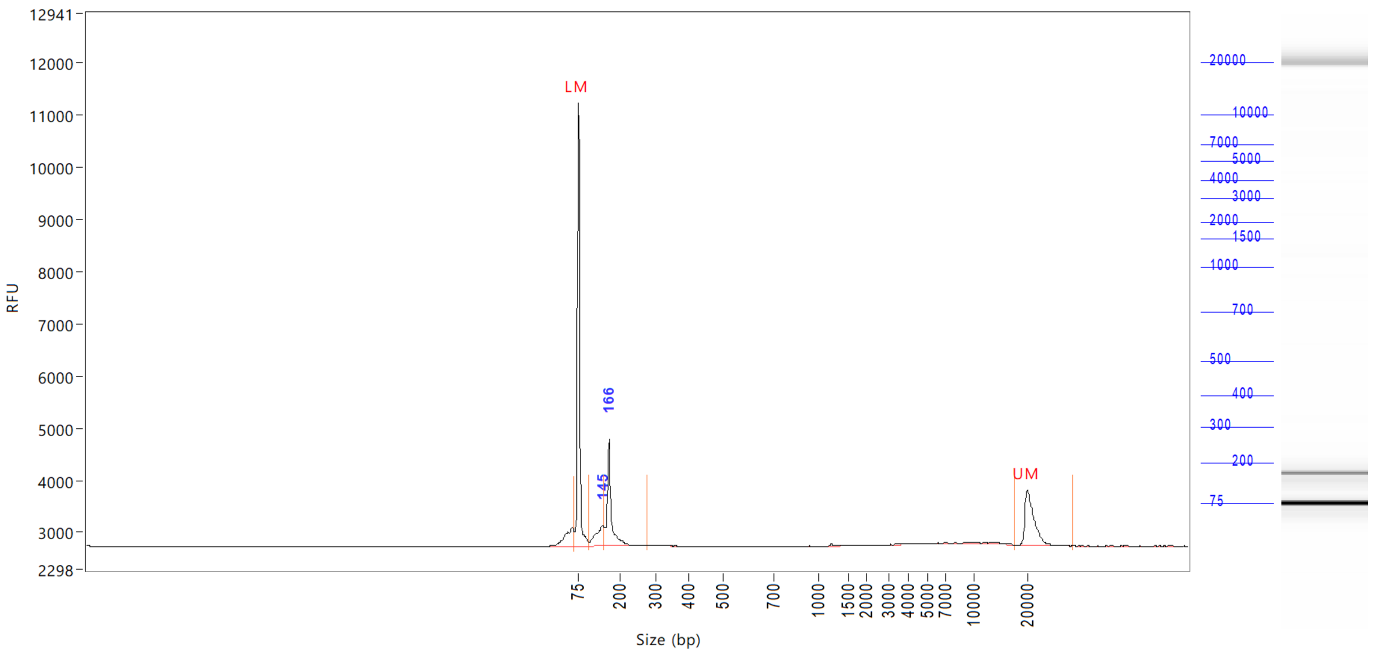

Peak	Size (bp)	Concentration (ng/uL)	From (bp)	To (bp)	Average size (bp)	CV%	RFU	Corrected peak area
1	75 (LM)	0.5000	64	105	74	6.82	8500	49.090
2	145	0.8966	105	150	133	9.42	384	7.336
3	166	2.2230	150	275	170	9.64	2032	18.188
4	20000 (UM)	0.1175	17683	28584	20584	5.17	1048	11.532

TIC:	3.1195	ng/uL
TIM:	32.5720	nmole/L
Total concentration:	3.4732	ng/uL

Sample peak width (sec): 5   Sample min peak height: 50   Sample baseline V to V?: Y   Sample baseline V to V points: 3
Sample filter: Binomial   Number of points for filter: 3   Sample start region (min): 0   Sample end region (min): 30
Marker peak width (sec): 5   Marker min peak height: 200   Marker baseline V to V?: Y   Marker baseline V to V points: 3
Lower marker selection: First peak > 500 RFU   Upper marker selection: Last peak > 200 RFU
Ladder size (bp)75, 200, 300, 400, 500, 700, 1000, 1500, 2000, 3000, 4000, 5000, 7000, 10000, 20000
Quantification using: Lower Marker   Final concentration (ng/uL): 0.5000   Dilution factor: 12.0

Sample: SampB10
Well location: B10
Created: Thursday, August 12, 2021 6:06:18 PM

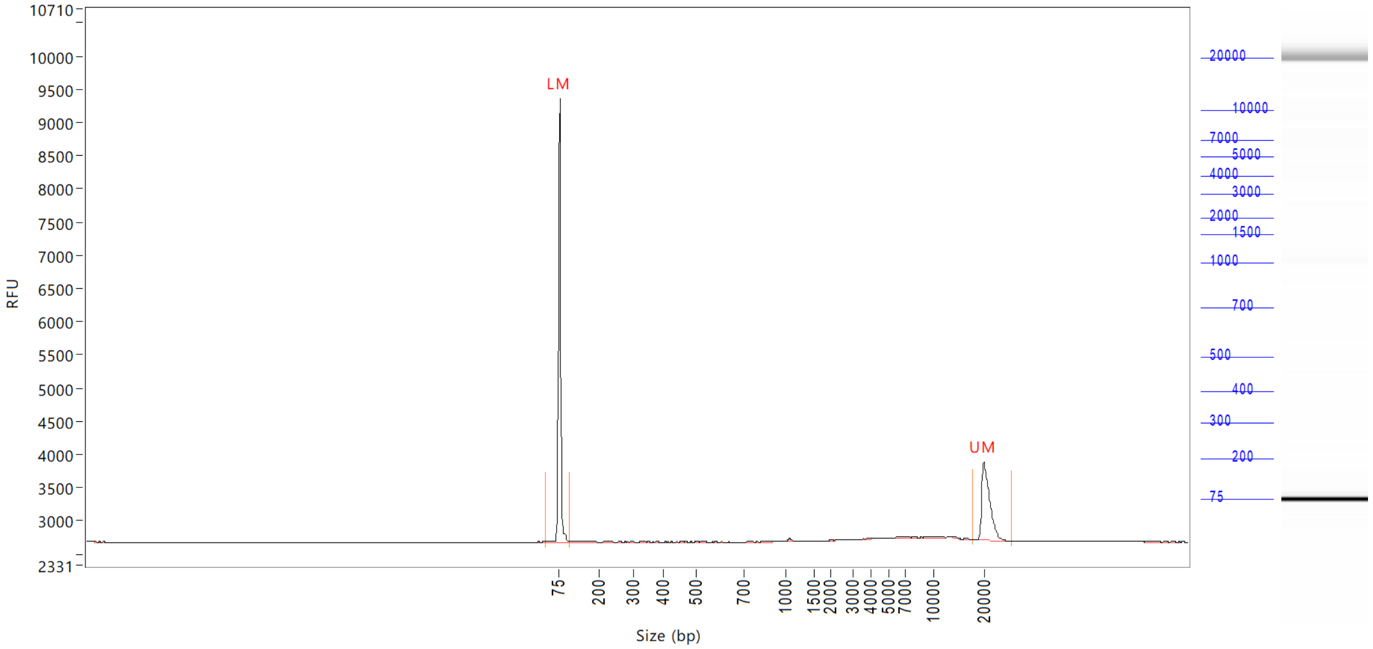

Peak	Size (bp)	Concentration (ng/uL)	From (bp)	To (bp)	Average size (bp)	CV%	RFU	Corrected peak area
1	75 (LM)	0.5000	31	107	73	6.35	6694	36.980
2	20000 (UM)	0.1634	17805	25561	20478	4.31	1184	12.084

TIC:	0.0000	ng/uL
TIM:	0.0000	nmole/L
Total concentration:	0.5835	ng/uL

Sample peak width (sec): 5 Sample min peak height: 50 Sample baseline V to V?: Y Sample baseline V to V points: 3
Sample filter: Binomial Number of points for filter: 3 Sample start region (min): 0 Sample end region (min): 30
Marker peak width (sec): 5 Marker min peak height: 200 Marker baseline V to V?: Y Marker baseline V to V points: 3
Lower marker selection: First peak > 500 RFU Upper marker selection: Last peak > 200 RFU
Ladder size (bp)75, 200, 300, 400, 500, 700, 1000, 1500, 2000, 3000, 4000, 5000, 7000, 10000, 20000
Quantification using: Lower Marker Final concentration (ng/uL): 0.5000 Dilution factor: 12.0

Sample: SampC10
Well location: C10
Created: Thursday, August 12, 2021 6:06:18 PM

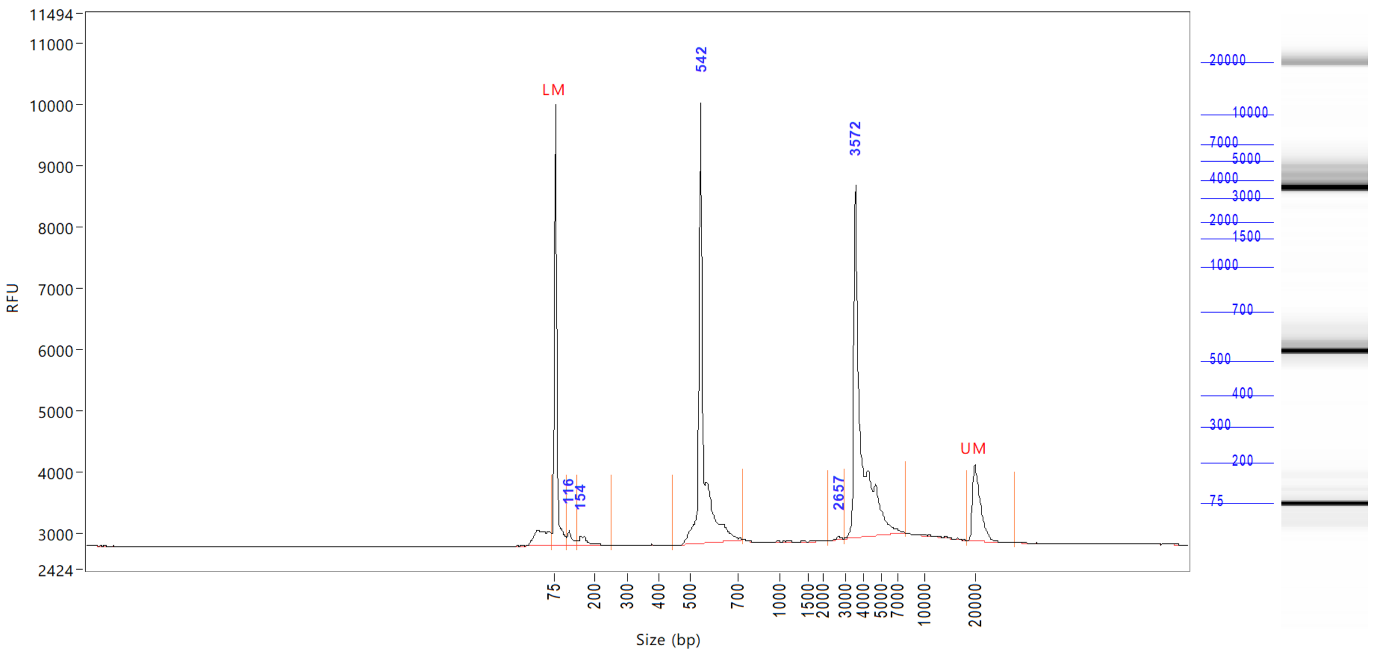

Peak	Size (bp)	Concentration (ng/uL)	From (bp)	To (bp)	Average size (bp)	CV%	RFU	Corrected peak area
1	75 (LM)	0.5000	62	108	74	9.45	7211	46.768
2	116	0.3976	108	145	122	8.66	244	3.099
3	154	0.4026	145	250	170	12.51	154	3.138
4	542	8.2067	445	731	554	6.51	7196	63.969
5	2657	0.0478	2201	2943	2630	4.79	53	0.372
6	3572	8.0360	2943	7801	3977	15.77	5757	62.638
7	20000 (UM)	0.1375	18293	27979	20529	4.93	1237	12.859

TIC:	17.0907	ng/uL
TIM:	36.9810	nmole/L
Total concentration:	17.2990	ng/uL

```
Sample peak width (sec): 5    Sample min peak height: 50    Sample baseline V to V?: Y    Sample baseline V to V points: 3
Sample filter: Binomial       Number of points for filter: 3  Sample start region (min): 0  Sample end region (min): 30
Marker peak width (sec): 5    Marker min peak height: 200   Marker baseline V to V?: Y    Marker baseline V to V points: 3
Lower marker selection: First peak > 500 RFU                Upper marker selection: Last peak > 200 RFU
Ladder size (bp)75, 200, 300, 400, 500, 700, 1000, 1500, 2000, 3000, 4000, 5000, 7000, 10000, 20000
Quantification using: Lower Marker          Final concentration (ng/uL): 0.5000          Dilution factor: 12.0
```

Sample: SampD10
Well location: D10
Created: Thursday, August 12, 2021 6:06:18 PM

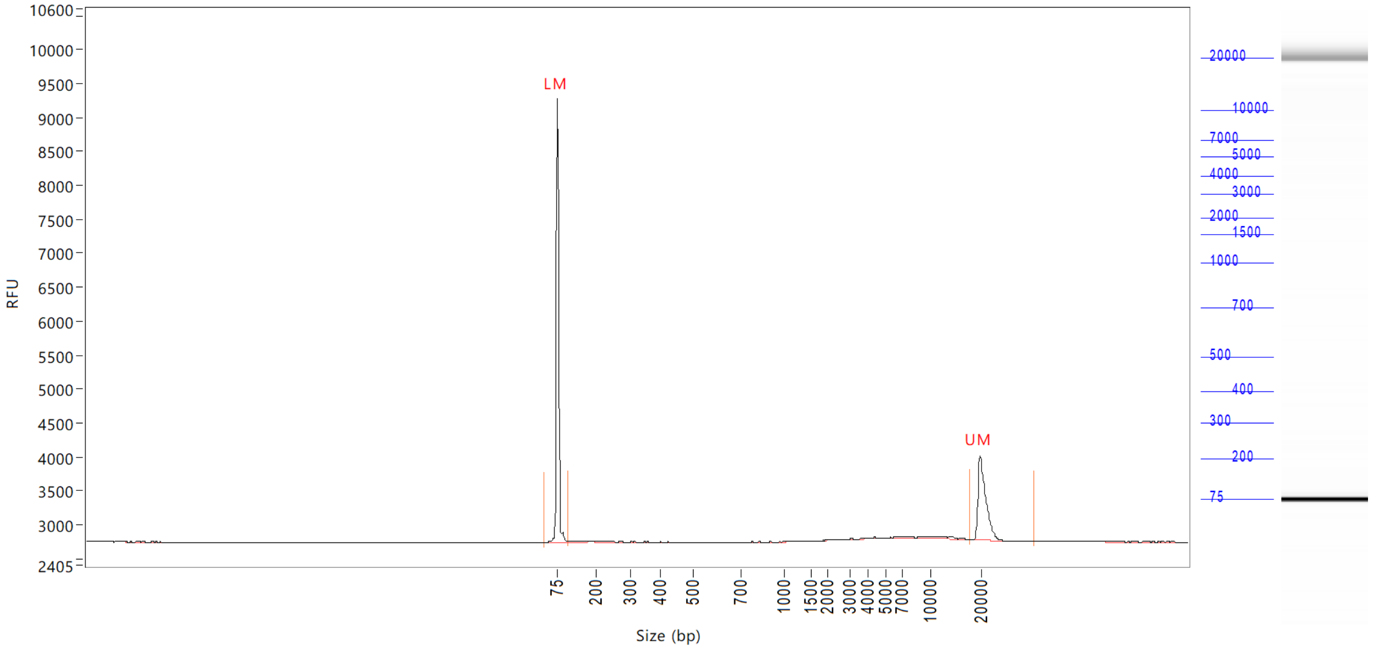

Peak	Size (bp)	Concentration (ng/uL)	From (bp)	To (bp)	Average size (bp)	CV%	RFU	Corrected peak area
1	75 (LM)	0.5000	35	110	73	6.46	6547	37.321
2	20000 (UM)	0.1701	18049	30639	20511	5.17	1247	12.699

TIC:	0.0000	ng/uL
TIM:	0.0000	nmole/L
Total concentration:	0.6542	ng/uL

Sample peak width (sec): 5   Sample min peak height: 50   Sample baseline V to V?: Y   Sample baseline V to V points: 3
Sample filter: Binomial   Number of points for filter: 3 Sample start region (min): 0 Sample end region (min): 30
Marker peak width (sec): 5   Marker min peak height: 200   Marker baseline V to V?: Y   Marker baseline V to V points: 3
Lower marker selection: First peak > 500 RFU   Upper marker selection: Last peak > 200 RFU
Ladder size (bp)75, 200, 300, 400, 500, 700, 1000, 1500, 2000, 3000, 4000, 5000, 7000, 10000, 20000
Quantification using: Lower Marker   Final concentration (ng/uL): 0.5000   Dilution factor: 12.0

Sample: SampE10
Well location: E10
Created: Thursday, August 12, 2021 6:06:18 PM

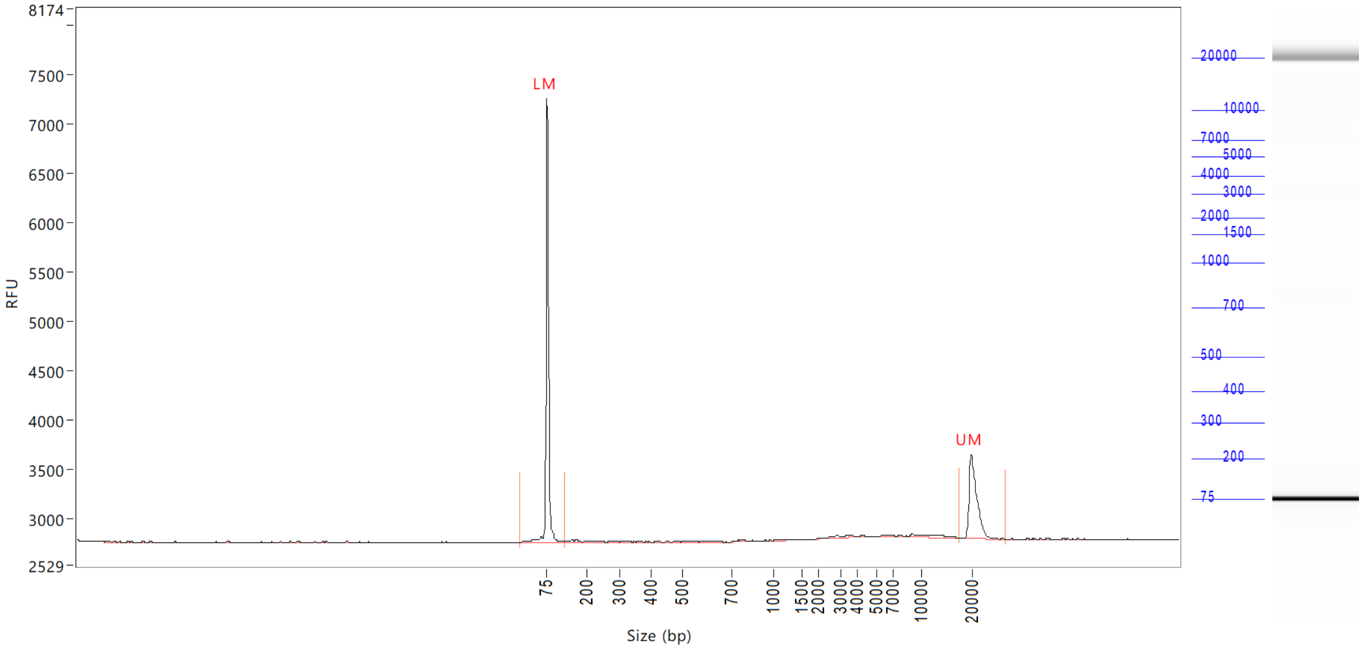

Peak	Size (bp)	Concentration (ng/uL)	From (bp)	To (bp)	Average size (bp)	CV%	RFU	Corrected peak area
1	75 (LM)	0.5000	0	129	73	14.86	4512	27.595
2	20000 (UM)	0.1555	17561	26770	20499	4.62	856	8.581

TIC:	0.0000	ng/uL
TIM:	0.0000	nmole/L
Total concentration:	1.0110	ng/uL

Sample peak width (sec): 5   Sample min peak height: 50   Sample baseline V to V?: Y   Sample baseline V to V points: 3
Sample filter: Binomial   Number of points for filter: 3   Sample start region (min): 0   Sample end region (min): 30
Marker peak width (sec): 5   Marker min peak height: 200   Marker baseline V to V?: Y   Marker baseline V to V points: 3
Lower marker selection: First peak > 500 RFU   Upper marker selection: Last peak > 200 RFU
Ladder size (bp)75, 200, 300, 400, 500, 700, 1000, 1500, 2000, 3000, 4000, 5000, 7000, 10000, 20000
Quantification using: Lower Marker   Final concentration (ng/uL): 0.5000   Dilution factor: 12.0

Sample: SampF10
Well location: F10
Created: Thursday, August 12, 2021 6:06:18 PM

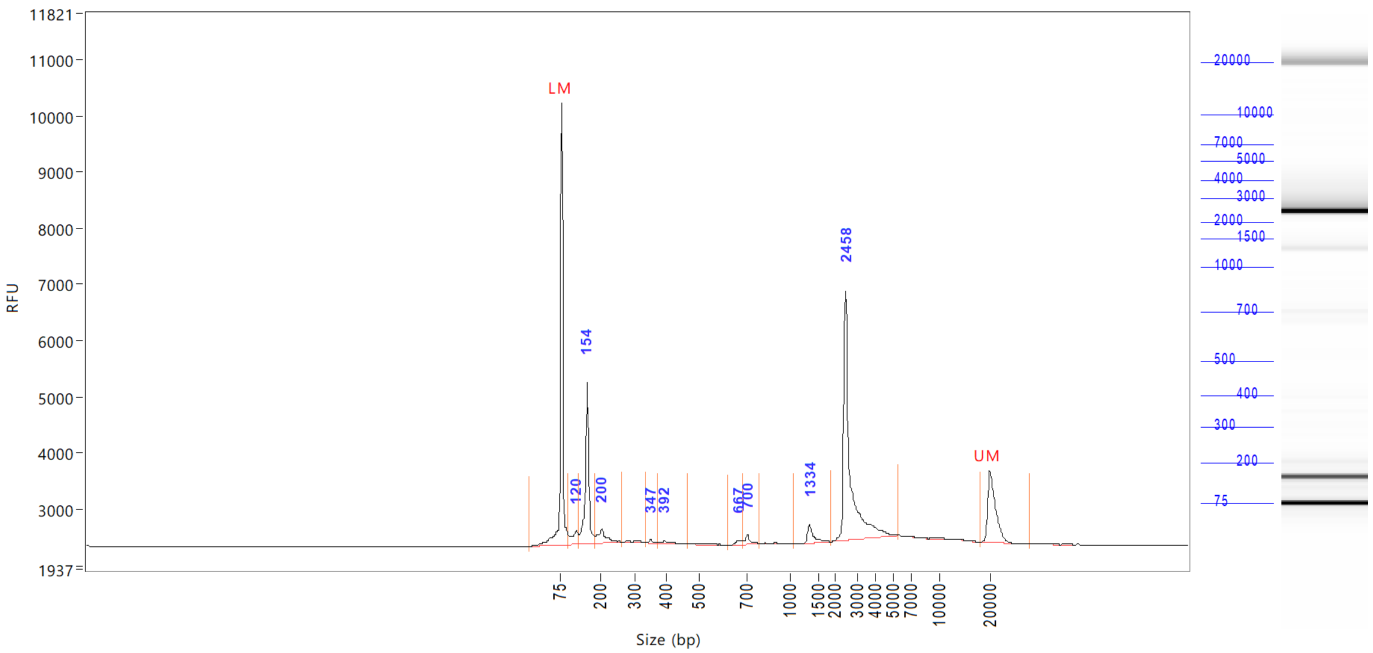

Peak	Size (bp)	Concentration (ng/uL)	From (bp)	To (bp)	Average size (bp)	CV%	RFU	Corrected peak area
1	75 (LM)	0.5000	0	99	71	17.47	7872	52.340
2	120	0.4538	99	131	116	8.18	242	3.958
3	154	2.6993	131	181	153	5.52	2874	23.547
4	200	0.5179	181	260	202	7.52	247	4.518
5	347	0.0503	333	370	347	2.34	59	0.439
6	392	0.1016	370	466	401	4.19	62	0.886
7	667	0.1007	623	682	664	1.66	82	0.878
8	700	0.1953	682	783	712	3.46	168	1.703
9	1334	0.3865	1056	1846	1369	7.20	352	3.371
10	2458	4.8672	1846	5593	2728	19.03	4435	42.458
11	20000 (UM)	0.1224	18293	27979	20583	4.64	1267	12.813

TIC:	9.3725	ng/uL
TIM:	44.3966	nmole/L
Total concentration:	9.5927	ng/uL

Sample peak width (sec): 5 Sample min peak height: 50 Sample baseline V to V?: Y Sample baseline V to V points: 3
Sample filter: Binomial Number of points for filter: 3 Sample start region (min): 0 Sample end region (min): 30
Marker peak width (sec): 5 Marker min peak height: 200 Marker baseline V to V?: Y Marker baseline V to V points: 3
Lower marker selection: First peak > 500 RFU Upper marker selection: Last peak > 200 RFU
Ladder size (bp)75, 200, 300, 400, 500, 700, 1000, 1500, 2000, 3000, 4000, 5000, 7000, 10000, 20000
Quantification using: Lower Marker Final concentration (ng/uL): 0.5000 Dilution factor: 12.0

Sample: SampG10
Well location: G10
Created: Thursday, August 12, 2021 6:06:18 PM

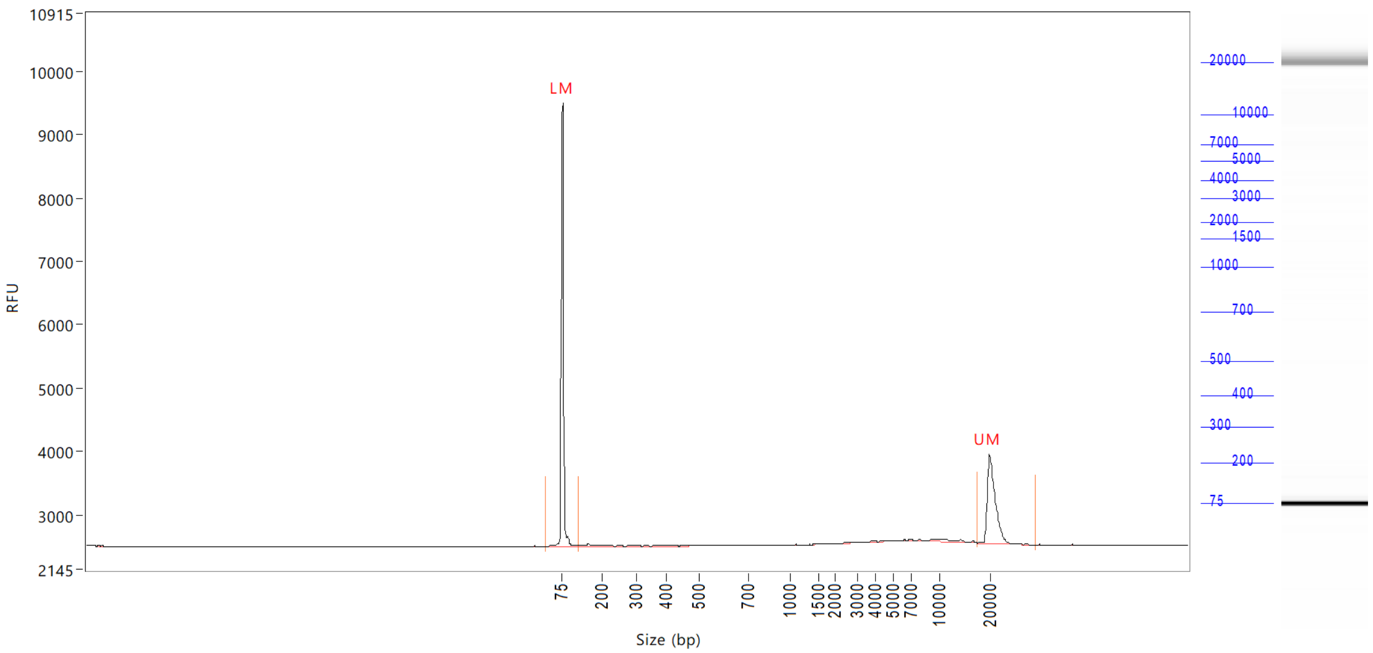

Peak	Size (bp)	Concentration (ng/uL)	From (bp)	To (bp)	Average size (bp)	CV%	RFU	Corrected peak area
1	75 (LM)	0.5000	26	124	73	7.89	7008	40.148
2	20000 (UM)	0.1787	17561	28947	20514	4.73	1408	14.352

TIC:	0.0000	ng/uL
TIM:	0.0000	nmole/L
Total concentration:	0.6777	ng/uL

Sample peak width (sec): 5 Sample min peak height: 50 Sample baseline V to V?: Y Sample baseline V to V points: 3
Sample filter: Binomial Number of points for filter: 3 Sample start region (min): 0 Sample end region (min): 30
Marker peak width (sec): 5 Marker min peak height: 200 Marker baseline V to V?: Y Marker baseline V to V points: 3
Lower marker selection: First peak > 500 RFU Upper marker selection: Last peak > 200 RFU
Ladder size (bp)75, 200, 300, 400, 500, 700, 1000, 1500, 2000, 3000, 4000, 5000, 7000, 10000, 20000
Quantification using: Lower Marker Final concentration (ng/uL): 0.5000 Dilution factor: 12.0

Sample: SampH10
Well location: H10
Created: Thursday, August 12, 2021 6:06:18 PM

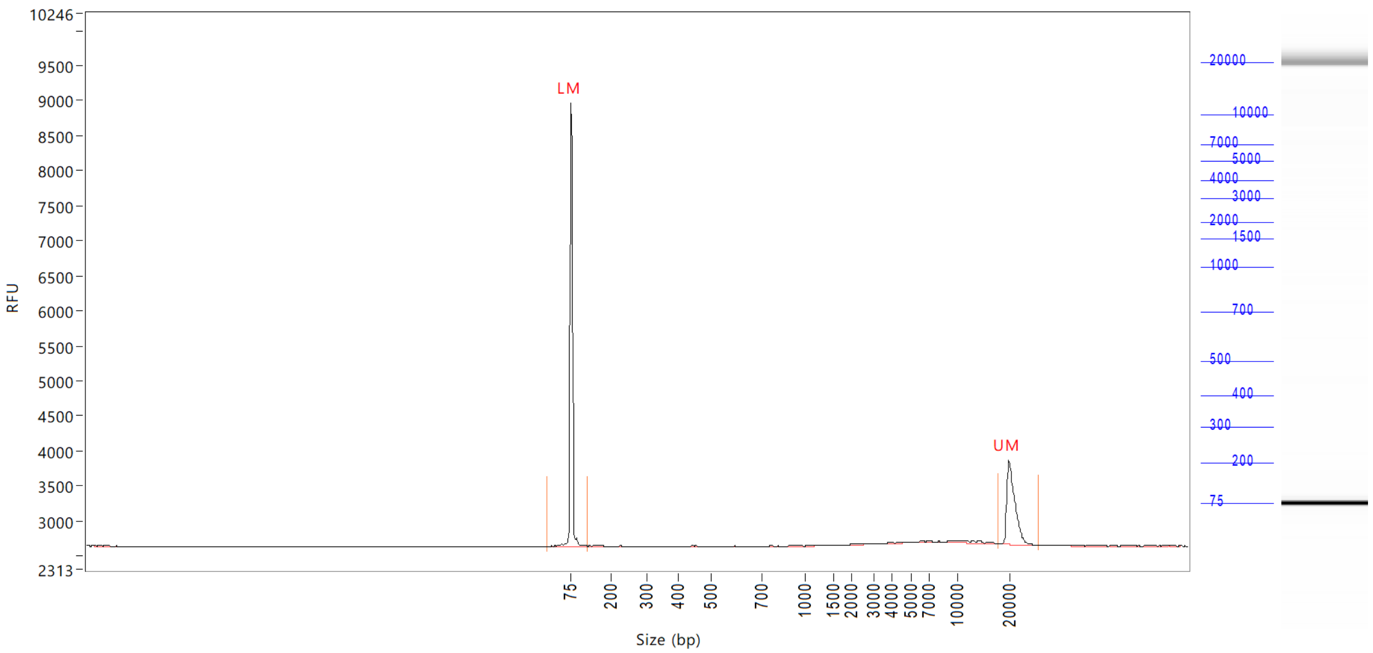

Peak	Size (bp)	Concentration (ng/uL)	From (bp)	To (bp)	Average size (bp)	CV%	RFU	Corrected peak area
1	75 (LM)	0.5000	3	124	73	8.84	6341	35.255
2	20000 (UM)	0.1749	17927	25803	20502	4.52	1207	12.335

TIC:	0.0000	ng/uL
TIM:	0.0000	nmole/L
Total concentration:	0.6953	ng/uL

Sample peak width (sec): 5   Sample min peak height: 50   Sample baseline V to V?: Y   Sample baseline V to V points: 3
Sample filter: Binomial   Number of points for filter: 3 Sample start region (min): 0 Sample end region (min): 30
Marker peak width (sec): 5   Marker min peak height: 200   Marker baseline V to V?: Y   Marker baseline V to V points: 3
Lower marker selection: First peak > 500 RFU   Upper marker selection: Last peak > 200 RFU
Ladder size (bp)75, 200, 300, 400, 500, 700, 1000, 1500, 2000, 3000, 4000, 5000, 7000, 10000, 20000
Quantification using: Lower Marker   Final concentration (ng/uL): 0.5000   Dilution factor: 12.0

Sample: SampA11
Well location: A11
Created: Thursday, August 12, 2021 6:06:18 PM

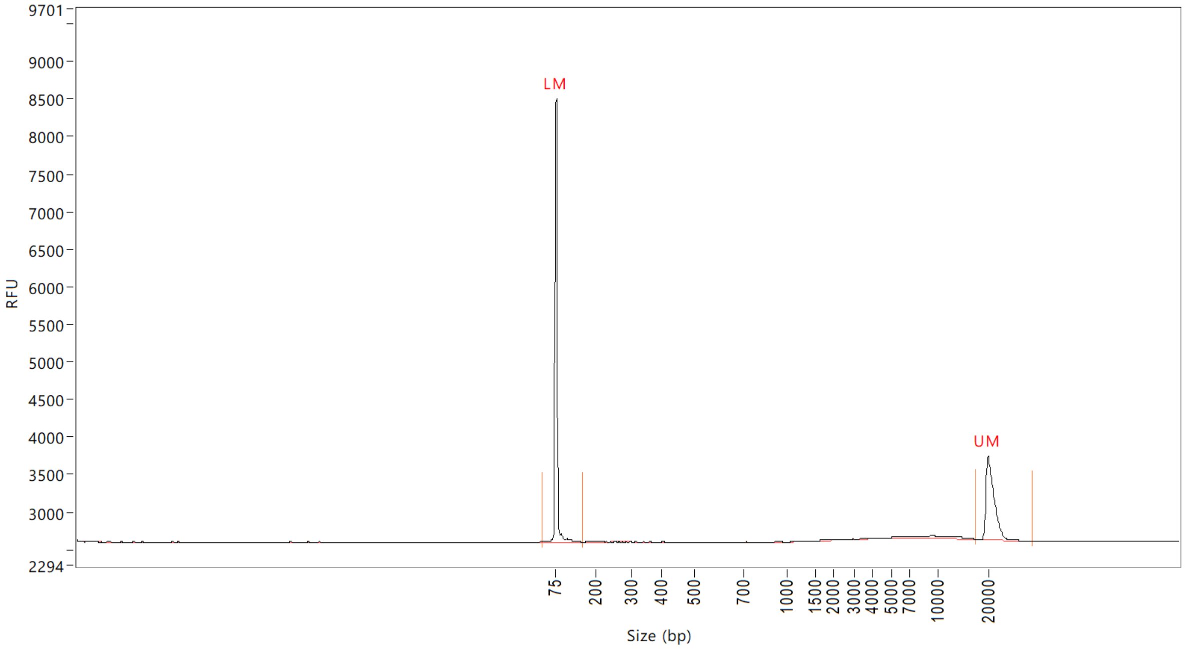

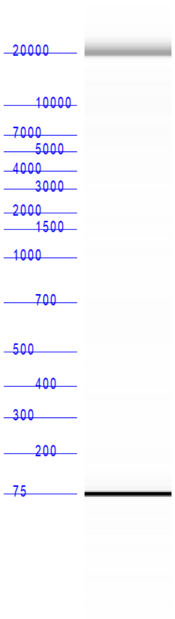

Peak	Size<br>(bp)	Concentration<br>(ng/uL)	From<br>(bp)	To<br>(bp)	Average size<br>(bp)	CV%	RFU	Corrected peak area
1	75 (LM)	0.5000	33	158	73	10.99	5920	34.382
2	20000 (UM)	0.1681	17439	28584	20525	5.05	1110	11.563

TIC:	0.0000	ng/uL
TIM:	0.0000	nmole/L
Total concentration:	0.7558	ng/uL

Sample peak width (sec): 5   Sample min peak height: 50   Sample baseline V to V?: Y   Sample baseline V to V points: 3
Sample filter: Binomial   Number of points for filter: 3 Sample start region (min): 0 Sample end region (min): 30
Marker peak width (sec): 5   Marker min peak height: 200   Marker baseline V to V?: Y   Marker baseline V to V points: 3
Lower marker selection: First peak > 500 RFU   Upper marker selection: Last peak > 200 RFU
Ladder size (bp)75, 200, 300, 400, 500, 700, 1000, 1500, 2000, 3000, 4000, 5000, 7000, 10000, 20000
Quantification using: Lower Marker   Final concentration (ng/uL): 0.5000   Dilution factor: 12.0

Sample: SampB11
Well location: B11
Created: Thursday, August 12, 2021 6:06:18 PM

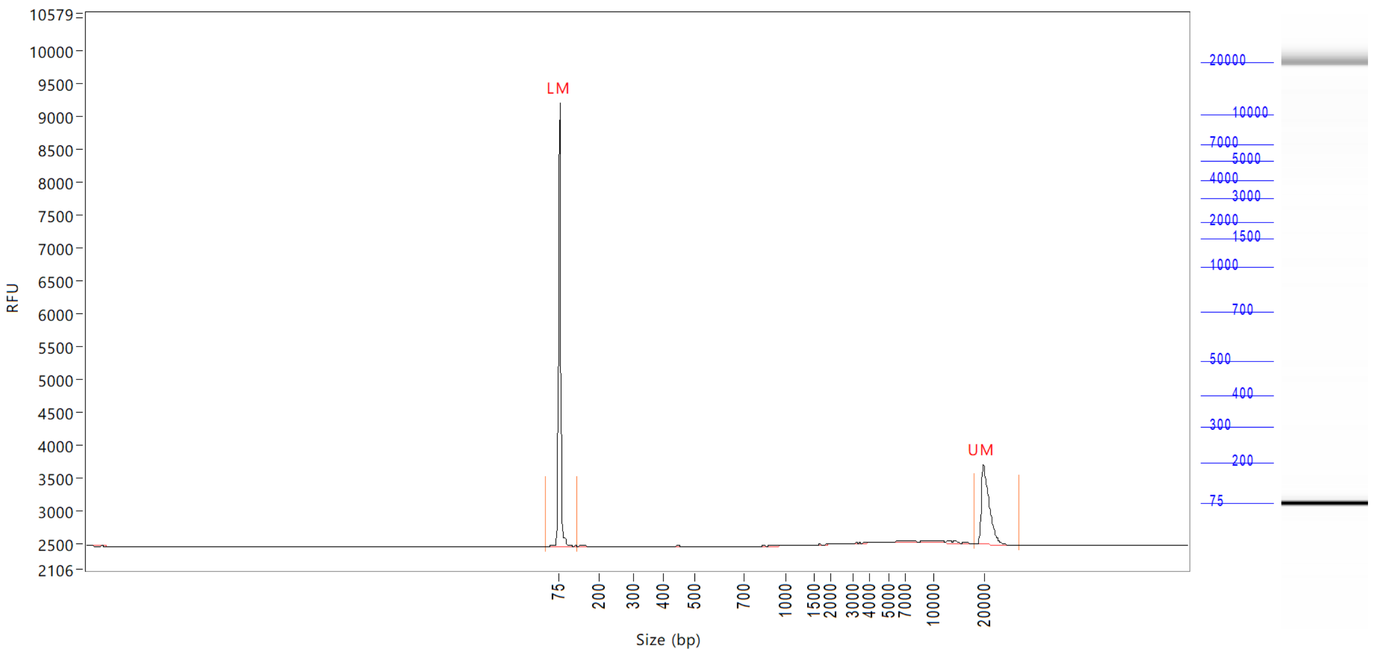

Peak	Size (bp)	Concentration (ng/uL)	From (bp)	To (bp)	Average size (bp)	CV%	RFU	Corrected peak area
1	75 (LM)	0.5000	31	131	73	7.19	6773	37.467
2	20000 (UM)	0.1647	18171	27254	20550	4.54	1216	12.338

TIC:	0.0000	ng/uL
TIM:	0.0000	nmole/L
Total concentration:	0.7154	ng/uL

Sample peak width (sec): 5   Sample min peak height: 50   Sample baseline V to V?: Y   Sample baseline V to V points: 3
Sample filter: Binomial   Number of points for filter: 3 Sample start region (min): 0 Sample end region (min): 30
Marker peak width (sec): 5   Marker min peak height: 200   Marker baseline V to V?: Y   Marker baseline V to V points: 3
Lower marker selection: First peak > 500 RFU   Upper marker selection: Last peak > 200 RFU
Ladder size (bp)75, 200, 300, 400, 500, 700, 1000, 1500, 2000, 3000, 4000, 5000, 7000, 10000, 20000
Quantification using: Lower Marker   Final concentration (ng/uL): 0.5000   Dilution factor: 12.0

Sample: SampC11
Well location: C11
Created: Thursday, August 12, 2021 6:06:18 PM

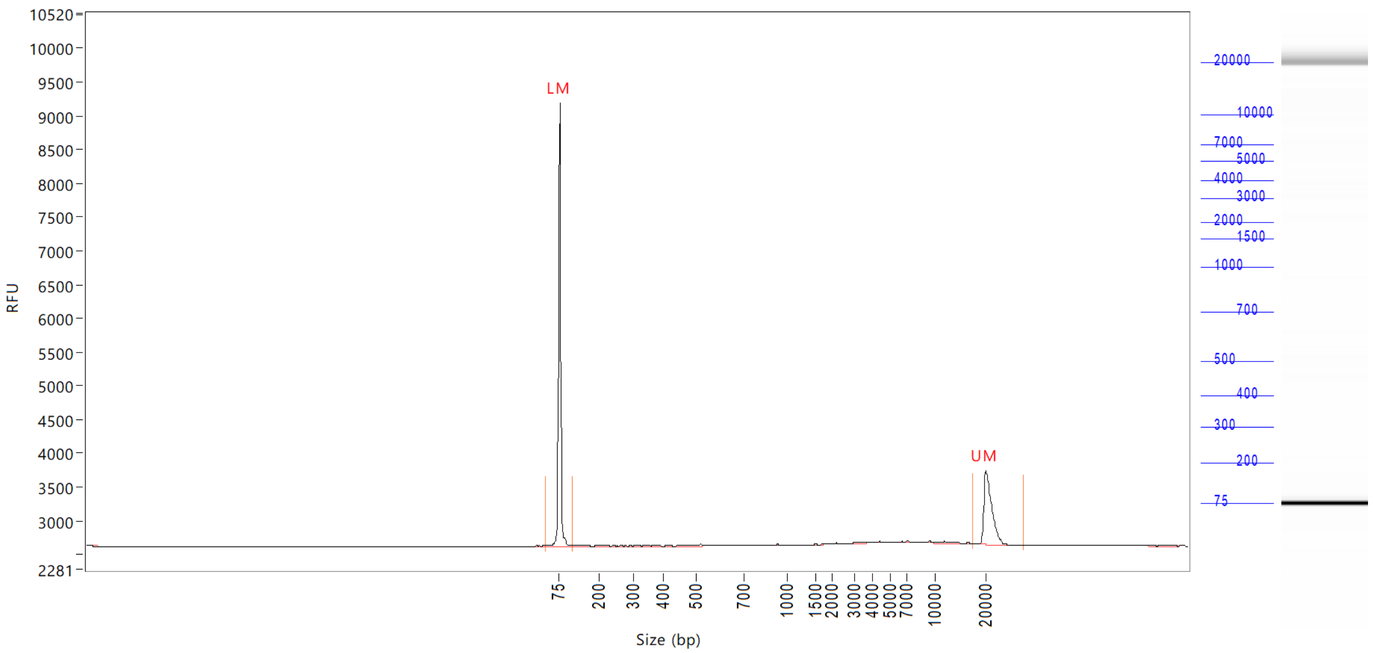

Peak	Size<br>(bp)	Concentration<br>(ng/uL)	From<br>(bp)	To<br>(bp)	Average size<br>(bp)	CV%	RFU	Corrected peak area
1	75 (LM)	0.5000	33	114	73	7.10	6584	36.628
2	20000 (UM)	0.1510	17439	27496	20570	4.54	1088	11.062

TIC:	0.0000	ng/uL
TIM:	0.0000	nmole/L
Total concentration:	0.6886	ng/uL

Sample peak width (sec): 5 Sample min peak height: 50 Sample baseline V to V?: Y Sample baseline V to V points: 3
Sample filter: Binomial Number of points for filter: 3 Sample start region (min): 0 Sample end region (min): 30
Marker peak width (sec): 5 Marker min peak height: 200 Marker baseline V to V?: Y Marker baseline V to V points: 3
Lower marker selection: First peak > 500 RFU Upper marker selection: Last peak > 200 RFU
Ladder size (bp)75, 200, 300, 400, 500, 700, 1000, 1500, 2000, 3000, 4000, 5000, 7000, 10000, 20000
Quantification using: Lower Marker Final concentration (ng/uL): 0.5000 Dilution factor: 12.0

Sample: SampD11
Well location: D11
Created: Thursday, August 12, 2021 6:06:18 PM

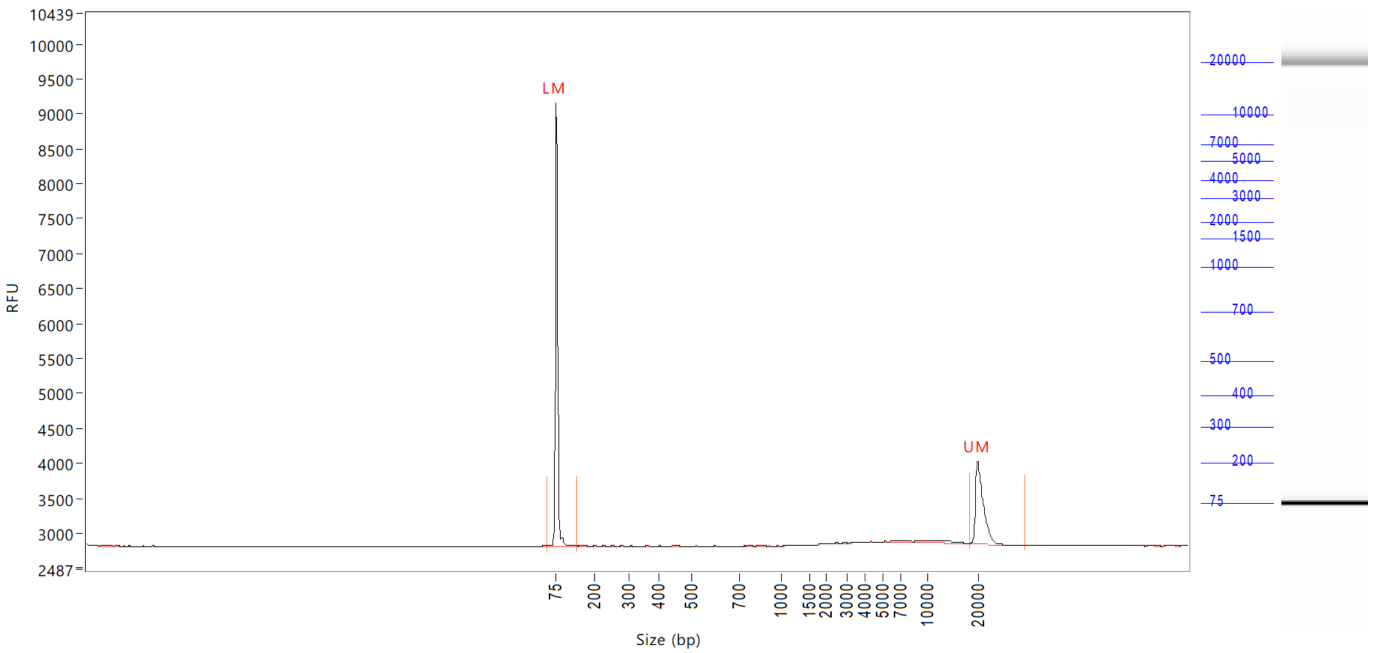

Peak	Size (bp)	Concentration (ng/uL)	From (bp)	To (bp)	Average size (bp)	CV%	RFU	Corrected peak area
1	75 (LM)	0.5000	48	143	74	7.71	6355	35.496
2	20000 (UM)	0.1729	18293	29430	20490	5.04	1193	12.271

TIC:	0.0000	ng/uL
TIM:	0.0000	nmole/L
Total concentration:	0.7152	ng/uL

```
Sample peak width (sec): 5    Sample min peak height: 50    Sample baseline V to V?: Y    Sample baseline V to V points: 3
Sample filter: Binomial       Number of points for filter: 3  Sample start region (min): 0  Sample end region (min): 30
Marker peak width (sec): 5    Marker min peak height: 200   Marker baseline V to V?: Y    Marker baseline V to V points: 3
Lower marker selection: First peak > 500 RFU                Upper marker selection: Last peak > 200 RFU
Ladder size (bp)75, 200, 300, 400, 500, 700, 1000, 1500, 2000, 3000, 4000, 5000, 7000, 10000, 20000
Quantification using: Lower Marker          Final concentration (ng/uL): 0.5000          Dilution factor: 12.0
```

Sample: SampE11
Well location: E11
Created: Thursday, August 12, 2021 6:06:18 PM

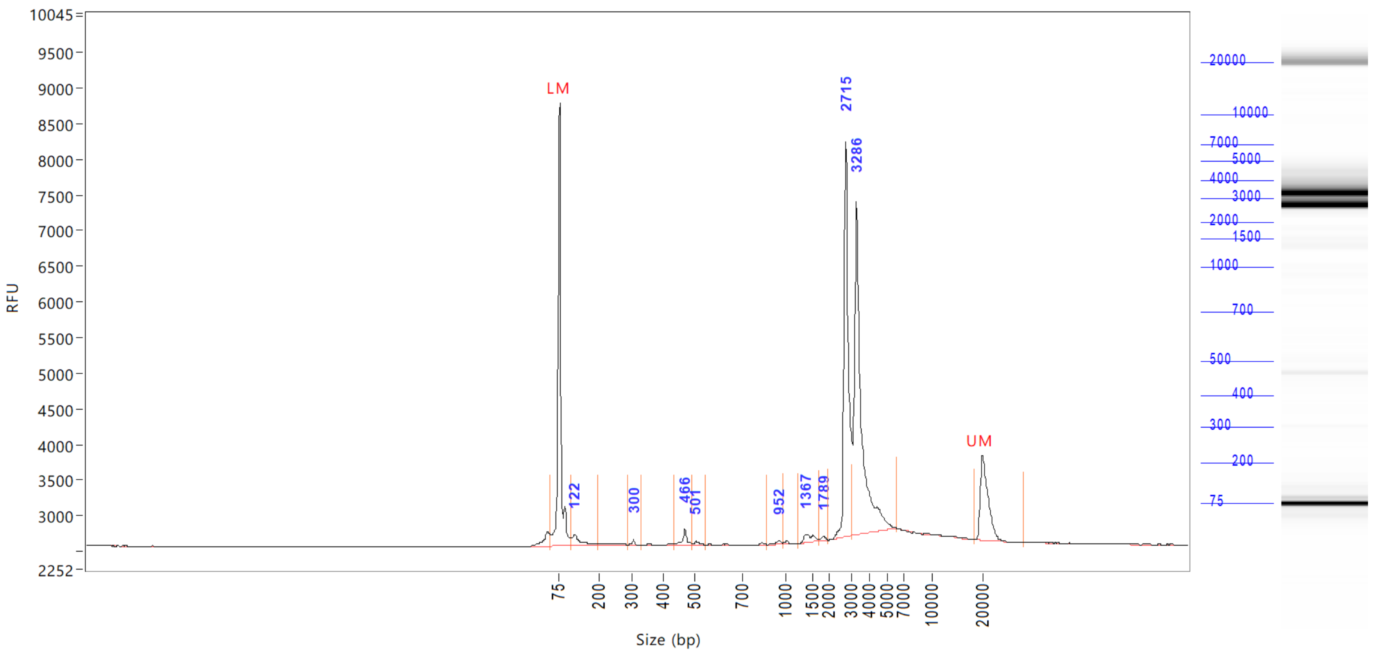

Peak	Size (bp)	Concentration (ng/uL)	From (bp)	To (bp)	Average size (bp)	CV%	RFU	Corrected peak area
1	75 (LM)	0.5000	48	112	74	11.80	6218	43.911
2	122	0.3775	112	196	134	14.65	147	2.763
3	300	0.0666	284	327	297	2.01	78	0.488
4	466	0.2652	435	492	464	2.52	231	1.941
5	501	0.0763	492	542	507	2.08	52	0.558
6	952	0.0661	870	978	940	2.44	57	0.484
7	1367	0.2298	1234	1655	1420	5.84	113	1.682
8	1789	0.0546	1655	1962	1769	3.37	57	0.399
9	2715	5.2229	1962	3037	2745	5.15	5542	38.224
10	3286	6.5739	3037	6038	3521	12.58	4659	48.112
11	20000 (UM)	0.1374	18415	28221	20553	4.52	1186	12.071

TIC:	12.9329	ng/uL
TIM:	12.8251	nmole/L
Total concentration:	13.1120	ng/uL

```
Sample peak width (sec): 5    Sample min peak height: 50    Sample baseline V to V?: Y    Sample baseline V to V points: 3
Sample filter: Binomial       Number of points for filter: 3  Sample start region (min): 0  Sample end region (min): 30
Marker peak width (sec): 5    Marker min peak height: 200   Marker baseline V to V?: Y    Marker baseline V to V points: 3
Lower marker selection: First peak > 500 RFU                Upper marker selection: Last peak > 200 RFU
Ladder size (bp)75, 200, 300, 400, 500, 700, 1000, 1500, 2000, 3000, 4000, 5000, 7000, 10000, 20000
Quantification using: Lower Marker          Final concentration (ng/uL): 0.5000          Dilution factor: 12.0
```

Sample: SampF11
Well location: F11
Created: Thursday, August 12, 2021 6:06:18 PM

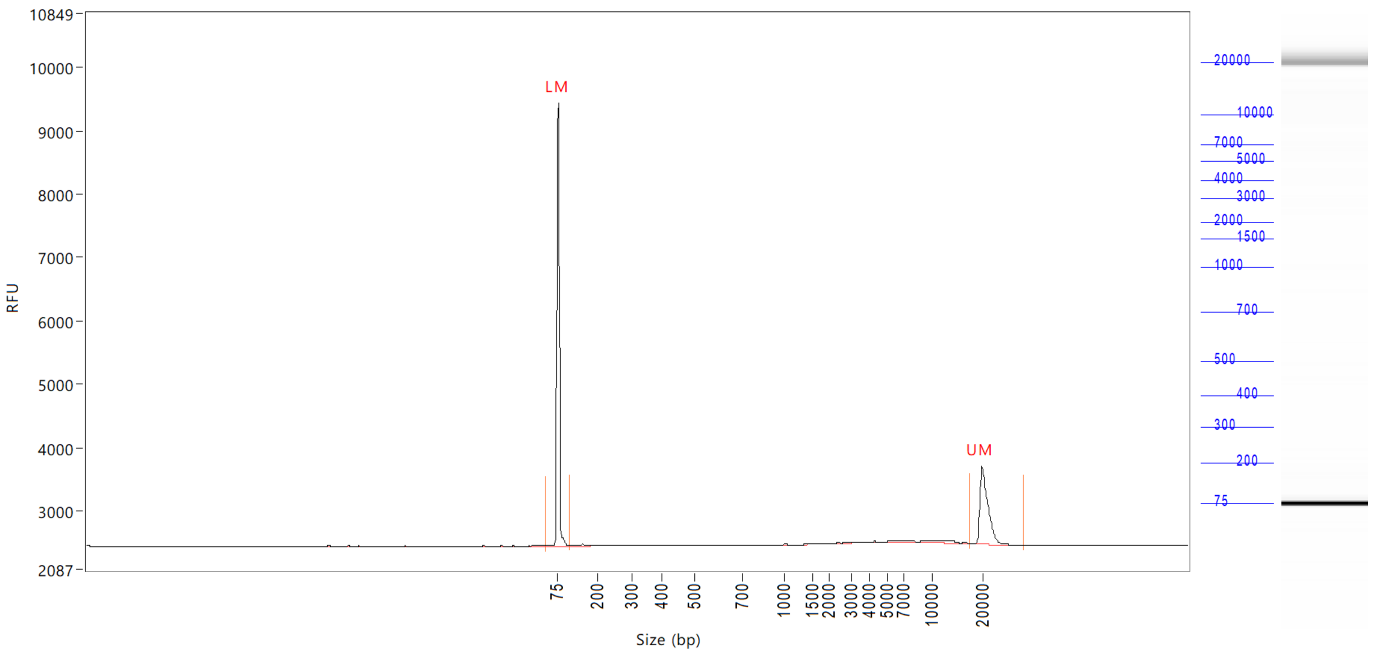

Peak	Size (bp)	Concentration (ng/uL)	From (bp)	To (bp)	Average size (bp)	CV%	RFU	Corrected peak area
1	75 (LM)	0.5000	37	110	73	6.48	6998	39.395
2	20000 (UM)	0.1614	17561	28221	20531	4.87	1228	12.717

TIC:	0.0000	ng/uL
TIM:	0.0000	nmole/L
Total concentration:	0.7163	ng/uL

Sample peak width (sec): 5   Sample min peak height: 50   Sample baseline V to V?: Y   Sample baseline V to V points: 3
Sample filter: Binomial   Number of points for filter: 3 Sample start region (min): 0 Sample end region (min): 30
Marker peak width (sec): 5   Marker min peak height: 200   Marker baseline V to V?: Y   Marker baseline V to V points: 3
Lower marker selection: First peak > 500 RFU   Upper marker selection: Last peak > 200 RFU
Ladder size (bp)75, 200, 300, 400, 500, 700, 1000, 1500, 2000, 3000, 4000, 5000, 7000, 10000, 20000
Quantification using: Lower Marker   Final concentration (ng/uL): 0.5000   Dilution factor: 12.0

Sample: SampG11
Well location: G11
Created: Thursday, August 12, 2021 6:06:18 PM

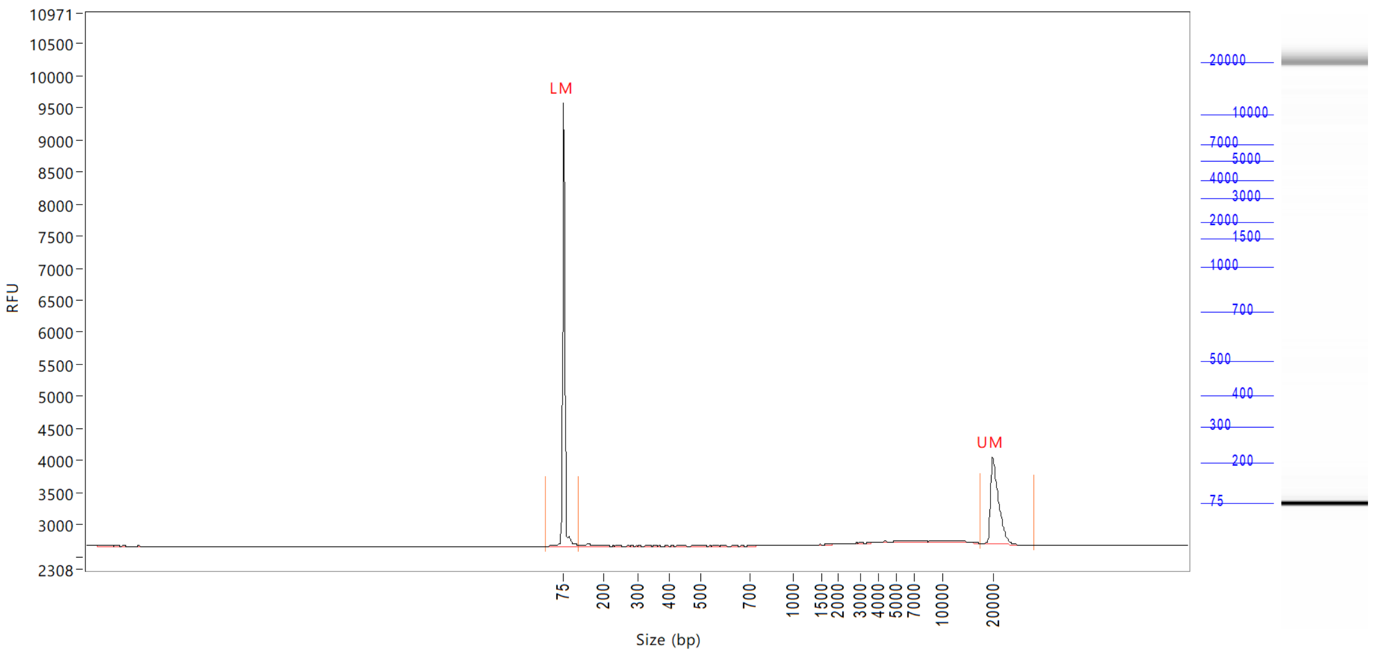

Peak	Size (bp)	Concentration (ng/uL)	From (bp)	To (bp)	Average size (bp)	CV%	RFU	Corrected peak area
1	75 (LM)	0.5000	20	120	73	8.68	6922	39.265
2	20000 (UM)	0.1782	17561	28100	20539	4.57	1364	13.993

TIC:	0.0000	ng/uL
TIM:	0.0000	nmole/L
Total concentration:	0.7043	ng/uL

```
Sample peak width (sec): 5    Sample min peak height: 50    Sample baseline V to V?: Y    Sample baseline V to V points: 3
Sample filter: Binomial       Number of points for filter: 3  Sample start region (min): 0  Sample end region (min): 30
Marker peak width (sec): 5    Marker min peak height: 200   Marker baseline V to V?: Y    Marker baseline V to V points: 3
Lower marker selection: First peak > 500 RFU                Upper marker selection: Last peak > 200 RFU
Ladder size (bp)75, 200, 300, 400, 500, 700, 1000, 1500, 2000, 3000, 4000, 5000, 7000, 10000, 20000
Quantification using: Lower Marker          Final concentration (ng/uL): 0.5000          Dilution factor: 12.0
```

Sample: SampH11
Well location: H11
Created: Thursday, August 12, 2021 6:06:18 PM

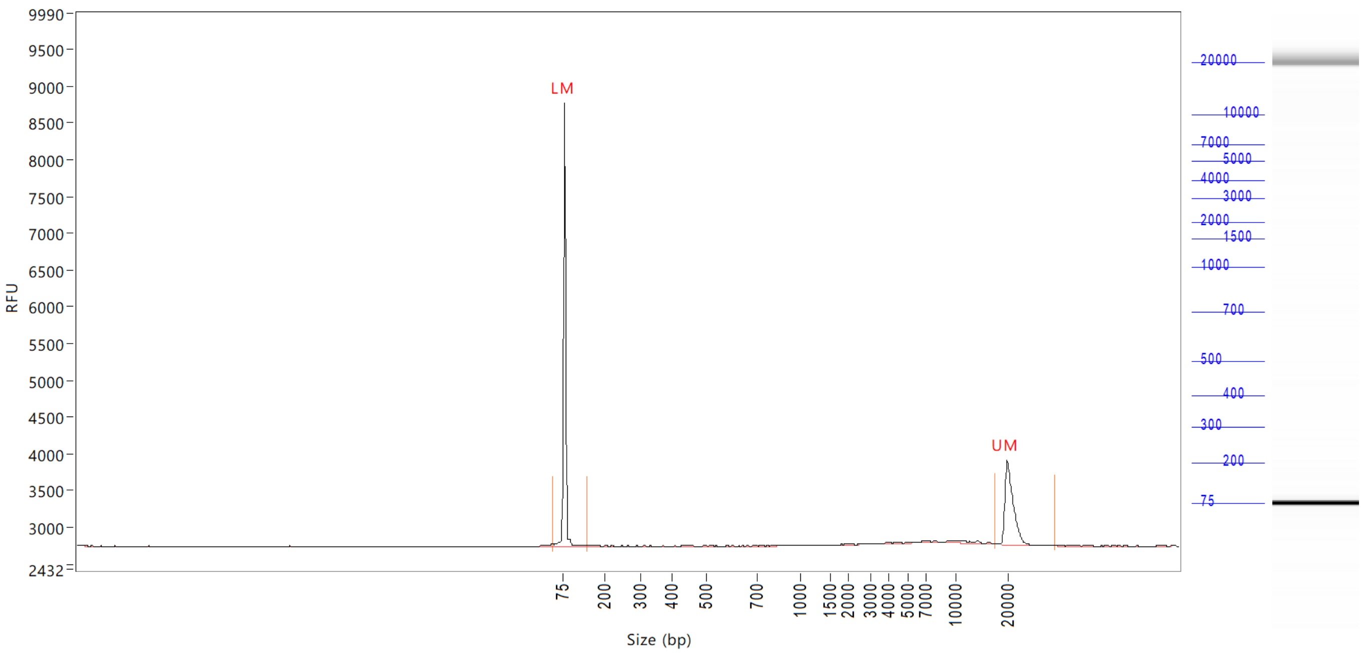

Peak	Size (bp)	Concentration (ng/uL)	From (bp)	To (bp)	Average size (bp)	CV%	RFU	Corrected peak area
1	75 (LM)	0.5000	45	147	74	8.45	6039	33.845
2	20000 (UM)	0.1744	17683	28947	20499	4.86	1140	11.806

TIC:	0.0000	ng/uL
TIM:	0.0000	nmole/L
Total concentration:	0.6684	ng/uL

Sample peak width (sec): 5 Sample min peak height: 50 Sample baseline V to V?: Y Sample baseline V to V points: 3
Sample filter: Binomial Number of points for filter: 3 Sample start region (min): 0 Sample end region (min): 30
Marker peak width (sec): 5 Marker min peak height: 200 Marker baseline V to V?: Y Marker baseline V to V points: 3
Lower marker selection: First peak > 500 RFU Upper marker selection: Last peak > 200 RFU
Ladder size (bp)75, 200, 300, 400, 500, 700, 1000, 1500, 2000, 3000, 4000, 5000, 7000, 10000, 20000
Quantification using: Lower Marker Final concentration (ng/uL): 0.5000 Dilution factor: 12.0

Sample: SampA12
Well location: A12
Created: Thursday, August 12, 2021 6:06:18 PM

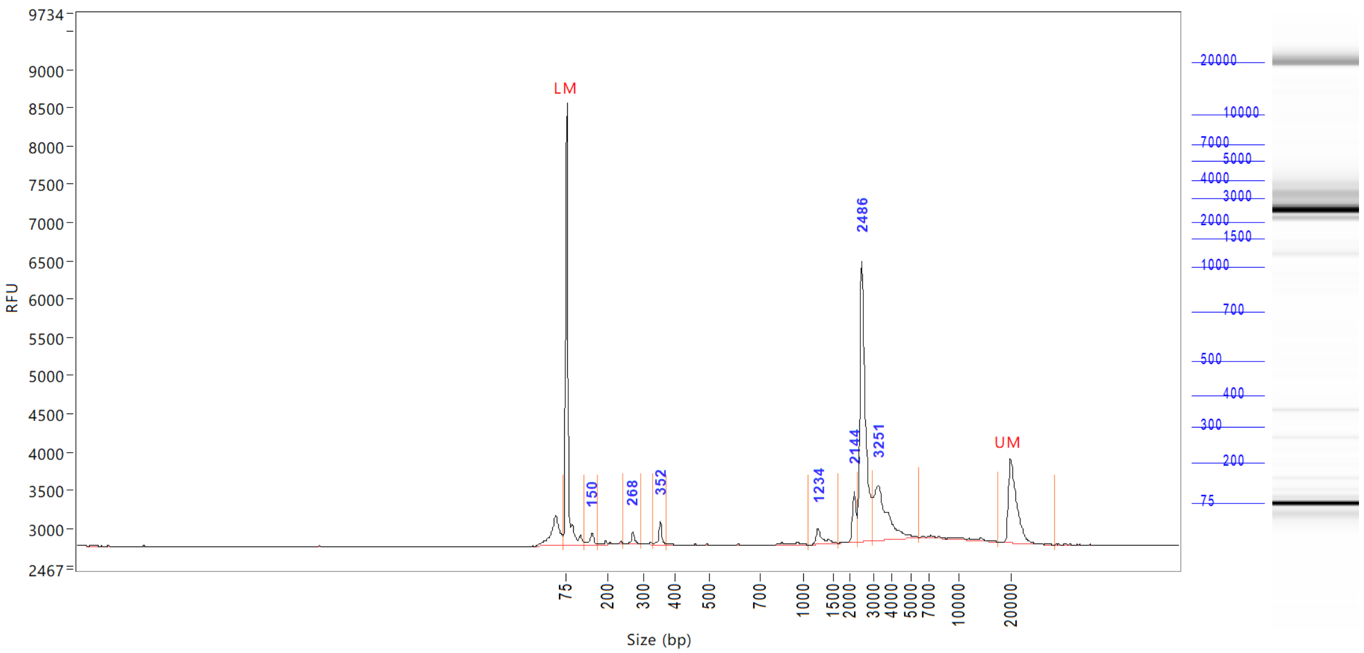

Peak	Size (bp)	Concentration (ng/uL)	From (bp)	To (bp)	Average size (bp)	CV%	RFU	Corrected peak area
1	75 (LM)	0.5000	64	128	75	12.19	5794	35.008
2	150	0.2591	128	168	145	5.52	153	1.512
3	268	0.2007	242	295	267	3.07	159	1.171
4	352	0.3206	329	372	348	2.01	307	1.871
5	1234	0.3578	1068	1616	1283	7.15	207	2.088
6	2144	0.6649	1616	2286	2125	4.58	666	3.879
7	2486	5.1365	2286	2971	2551	6.19	3670	29.970
8	3251	2.4497	2971	5889	3535	14.01	720	14.293
9	20000 (UM)	0.1627	17805	28342	20607	5.18	1092	11.394

TIC:	9.3893	ng/uL
TIM:	11.1180	nmole/L
Total concentration:	10.0098	ng/uL

```
Sample peak width (sec): 5    Sample min peak height: 50    Sample baseline V to V?: Y    Sample baseline V to V points: 3
Sample filter: Binomial       Number of points for filter: 3  Sample start region (min): 0  Sample end region (min): 30
Marker peak width (sec): 5    Marker min peak height: 200   Marker baseline V to V?: Y    Marker baseline V to V points: 3
Lower marker selection: First peak > 500 RFU                Upper marker selection: Last peak > 200 RFU
Ladder size (bp)75, 200, 300, 400, 500, 700, 1000, 1500, 2000, 3000, 4000, 5000, 7000, 10000, 20000
Quantification using: Lower Marker          Final concentration (ng/uL): 0.5000          Dilution factor: 12.0
```

Sample: SampB12
Well location: B12
Created: Thursday, August 12, 2021 6:06:18 PM

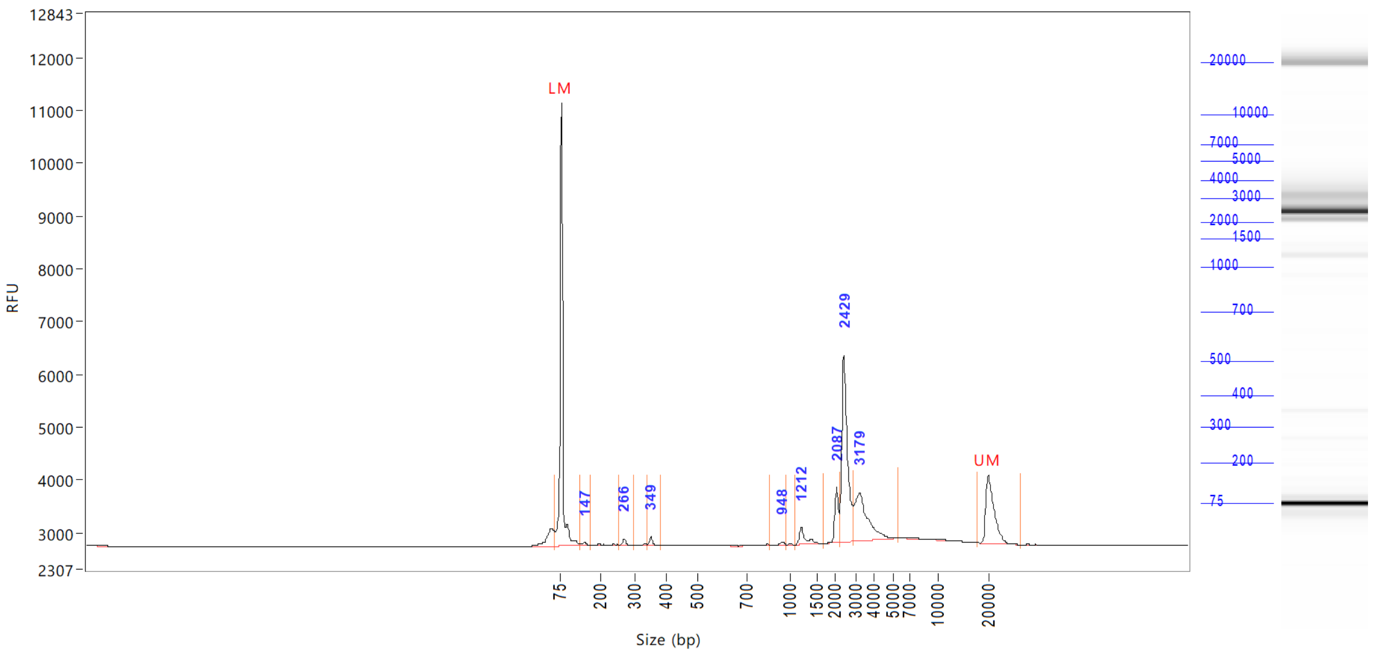

Peak	Size (bp)	Concentration (ng/uL)	From (bp)	To (bp)	Average size (bp)	CV%	RFU	Corrected peak area
1	75 (LM)	0.5000	58	135	75	12.17	8410	55.437
2	147	0.0590	135	166	145	3.90	53	0.545
3	266	0.0908	251	295	265	2.56	123	0.839
4	349	0.1138	335	384	347	1.81	169	1.051
5	948	0.0549	861	978	933	2.75	68	0.507
6	1212	0.3329	1090	1674	1249	7.19	317	3.075
7	2087	0.6858	1674	2229	2081	3.93	1046	6.337
8	2429	3.3373	2229	2857	2479	6.10	3533	30.835
9	3179	1.9752	2857	5593	3391	13.31	919	18.250
10	20000 (UM)	0.1225	17683	26408	20503	4.63	1291	13.577

TIC:	6.6496	ng/uL
TIM:	6.0245	nmole/L
Total concentration:	6.8190	ng/uL

Sample peak width (sec): 5   Sample min peak height: 50   Sample baseline V to V?: Y   Sample baseline V to V points: 3
Sample filter: Binomial   Number of points for filter: 3   Sample start region (min): 0   Sample end region (min): 30
Marker peak width (sec): 5   Marker min peak height: 200   Marker baseline V to V?: Y   Marker baseline V to V points: 3
Lower marker selection: First peak > 500 RFU   Upper marker selection: Last peak > 200 RFU
Ladder size (bp)75, 200, 300, 400, 500, 700, 1000, 1500, 2000, 3000, 4000, 5000, 7000, 10000, 20000
Quantification using: Lower Marker   Final concentration (ng/uL): 0.5000   Dilution factor: 12.0

Sample: SampC12
Well location: C12
Created: Thursday, August 12, 2021 6:06:18 PM

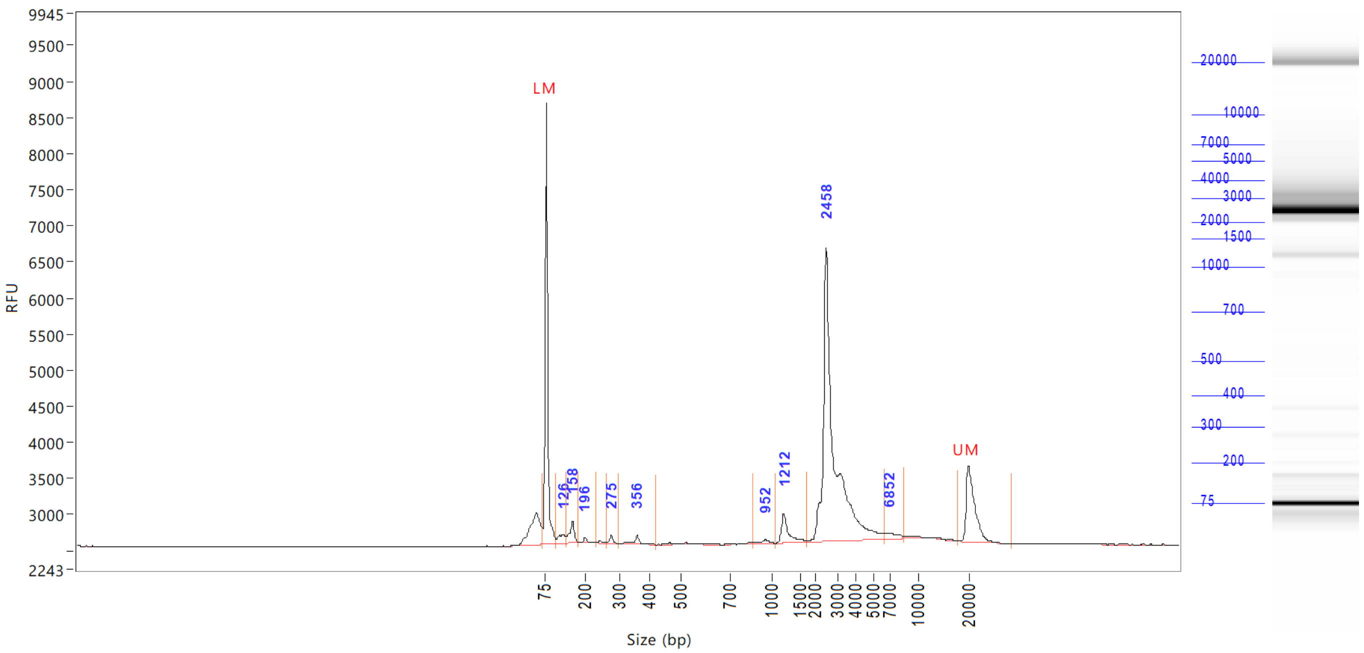

Peak	Size (bp)	Concentration (ng/uL)	From (bp)	To (bp)	Average size (bp)	CV%	RFU	Corrected peak area
1	75 (LM)	0.5000	64	105	74	7.62	6124	38.839
2	126	0.3327	105	135	120	7.29	123	2.154
3	158	0.5062	135	177	152	5.30	302	3.277
4	196	0.0959	177	228	198	4.61	71	0.621
5	275	0.1258	259	296	273	2.18	115	0.814
6	356	0.2103	296	418	350	5.93	127	1.361
7	952	0.1116	866	1045	936	3.36	53	0.722
8	1212	0.7030	1045	1712	1268	9.55	412	4.551
9	2458	10.2695	1712	6334	2863	24.96	4073	66.476
10	6852	0.2259	6334	8401	7222	7.89	82	1.462
11	20000 (UM)	0.1518	17805	28584	20687	5.35	1056	11.793

TIC:	12.5809	ng/uL
TIM:	19.6369	nmole/L
Total concentration:	12.8896	ng/uL

```
Sample peak width (sec): 5    Sample min peak height: 50    Sample baseline V to V?: Y    Sample baseline V to V points: 3
Sample filter: Binomial       Number of points for filter: 3  Sample start region (min): 0  Sample end region (min): 30
Marker peak width (sec): 5    Marker min peak height: 200   Marker baseline V to V?: Y    Marker baseline V to V points: 3
Lower marker selection: First peak > 500 RFU                Upper marker selection: Last peak > 200 RFU
Ladder size (bp)75, 200, 300, 400, 500, 700, 1000, 1500, 2000, 3000, 4000, 5000, 7000, 10000, 20000
Quantification using: Lower Marker          Final concentration (ng/uL): 0.5000          Dilution factor: 12.0
```

Sample: SampD12
Well location: D12
Created: Thursday, August 12, 2021 6:06:18 PM

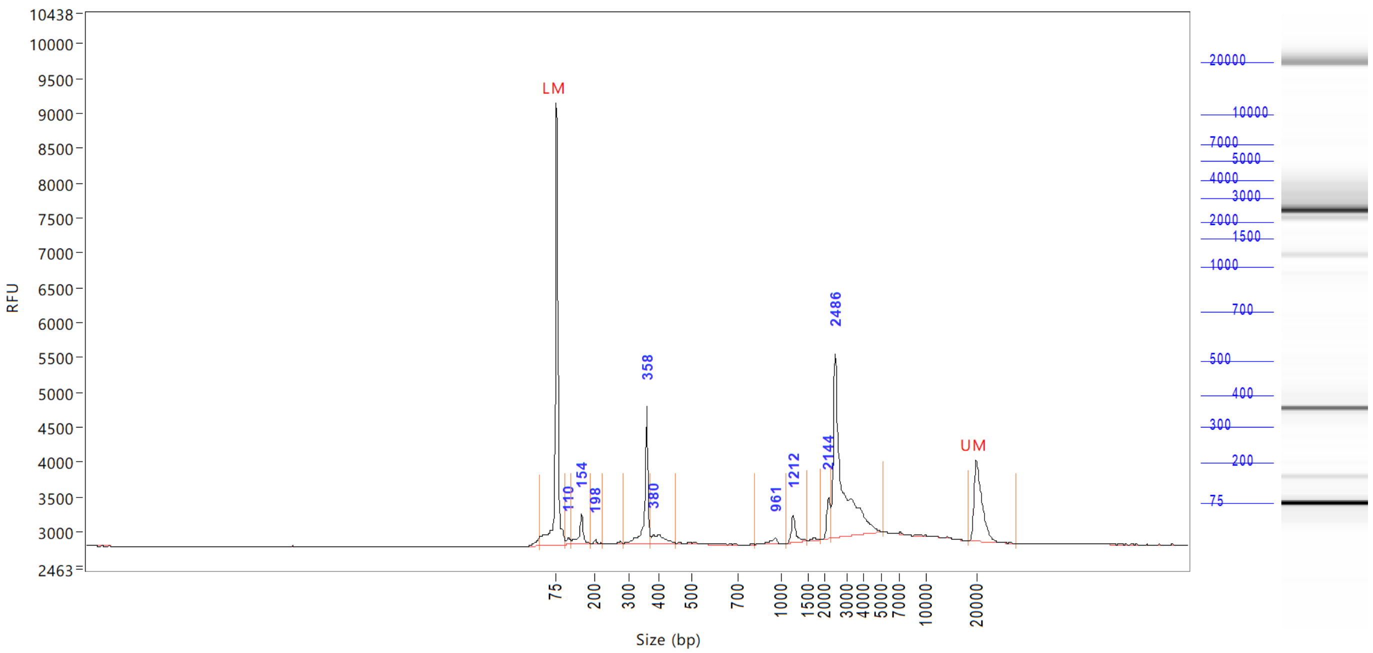

Peak	Size (bp)	Concentration (ng/uL)	From (bp)	To (bp)	Average size (bp)	CV%	RFU	Corrected peak area
1	75 (LM)	0.5000	22	105	71	16.47	6352	43.687
2	110	0.1183	105	122	113	4.68	87	0.862
3	154	0.5713	122	185	148	7.32	424	4.159
4	198	0.0508	185	221	198	3.30	54	0.370
5	358	1.8640	280	372	349	4.34	1965	13.572
6	380	0.4750	372	452	400	4.88	129	3.458
7	961	0.1389	809	1079	933	4.23	90	1.012
8	1212	0.4006	1079	1489	1232	5.51	398	2.917
9	2144	0.4858	1866	2286	2145	4.10	596	3.537
10	2486	4.9890	2286	5149	2899	19.34	2637	36.326
11	20000 (UM)	0.1389	18537	28100	20566	4.86	1172	12.132

TIC:	9.0937	ng/uL
TIM:	23.2119	nmole/L
Total concentration:	9.3840	ng/uL

```
Sample peak width (sec): 5     Sample min peak height: 50     Sample baseline V to V?: Y     Sample baseline V to V points: 3
Sample filter: Binomial        Number of points for filter: 3  Sample start region (min): 0  Sample end region (min): 30
Marker peak width (sec): 5     Marker min peak height: 200    Marker baseline V to V?: Y     Marker baseline V to V points: 3
Lower marker selection: First peak > 500 RFU                  Upper marker selection: Last peak > 200 RFU
Ladder size (bp)75, 200, 300, 400, 500, 700, 1000, 1500, 2000, 3000, 4000, 5000, 7000, 10000, 20000
Quantification using: Lower Marker          Final concentration (ng/uL): 0.5000          Dilution factor: 12.0
```

Sample: SampE12
Well location: E12
Created: Thursday, August 12, 2021 6:06:18 PM

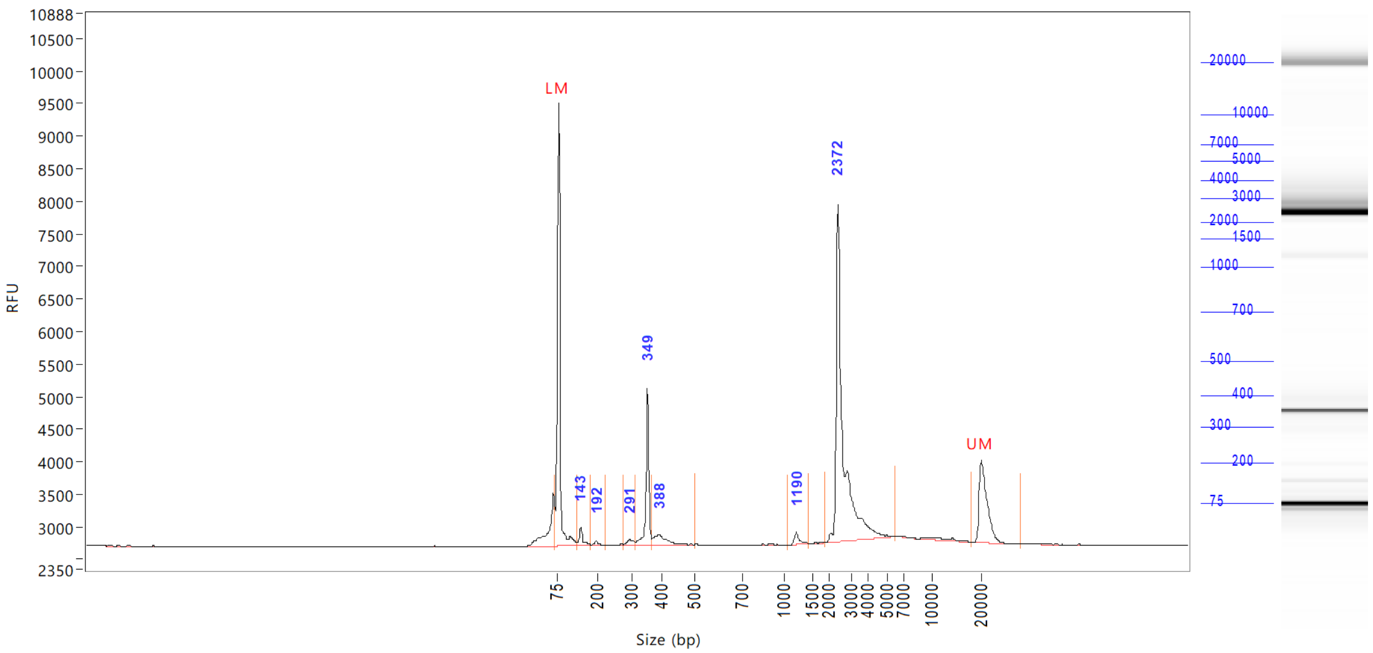

Peak	Size (bp)	Concentration (ng/uL)	From (bp)	To (bp)	Average size (bp)	CV%	RFU	Corrected peak area
1	75 (LM)	0.5000	64	131	75	13.31	6815	44.072
2	143	0.3453	131	177	145	7.05	274	2.536
3	192	0.1070	177	221	192	3.98	85	0.786
4	291	0.1802	271	307	291	3.03	87	1.323
5	349	1.9437	307	364	345	2.62	2398	14.277
6	388	0.5618	364	501	397	6.35	154	4.126
7	1190	0.1989	1045	1433	1213	5.20	189	1.461
8	2372	7.3742	1866	5963	2677	19.08	5176	54.167
9	20000 (UM)	0.1495	17927	27979	20484	4.84	1259	13.178

TIC:	10.7110	ng/uL
TIM:	22.2492	nmole/L
Total concentration:	11.0265	ng/uL

Sample peak width (sec): 5   Sample min peak height: 50   Sample baseline V to V?: Y   Sample baseline V to V points: 3
Sample filter: Binomial   Number of points for filter: 3   Sample start region (min): 0   Sample end region (min): 30
Marker peak width (sec): 5   Marker min peak height: 200   Marker baseline V to V?: Y   Marker baseline V to V points: 3
Lower marker selection: First peak > 500 RFU   Upper marker selection: Last peak > 200 RFU
Ladder size (bp)75, 200, 300, 400, 500, 700, 1000, 1500, 2000, 3000, 4000, 5000, 7000, 10000, 20000
Quantification using: Lower Marker   Final concentration (ng/uL): 0.5000   Dilution factor: 12.0

Sample: SampF12
Well location: F12
Created: Thursday, August 12, 2021 6:06:18 PM

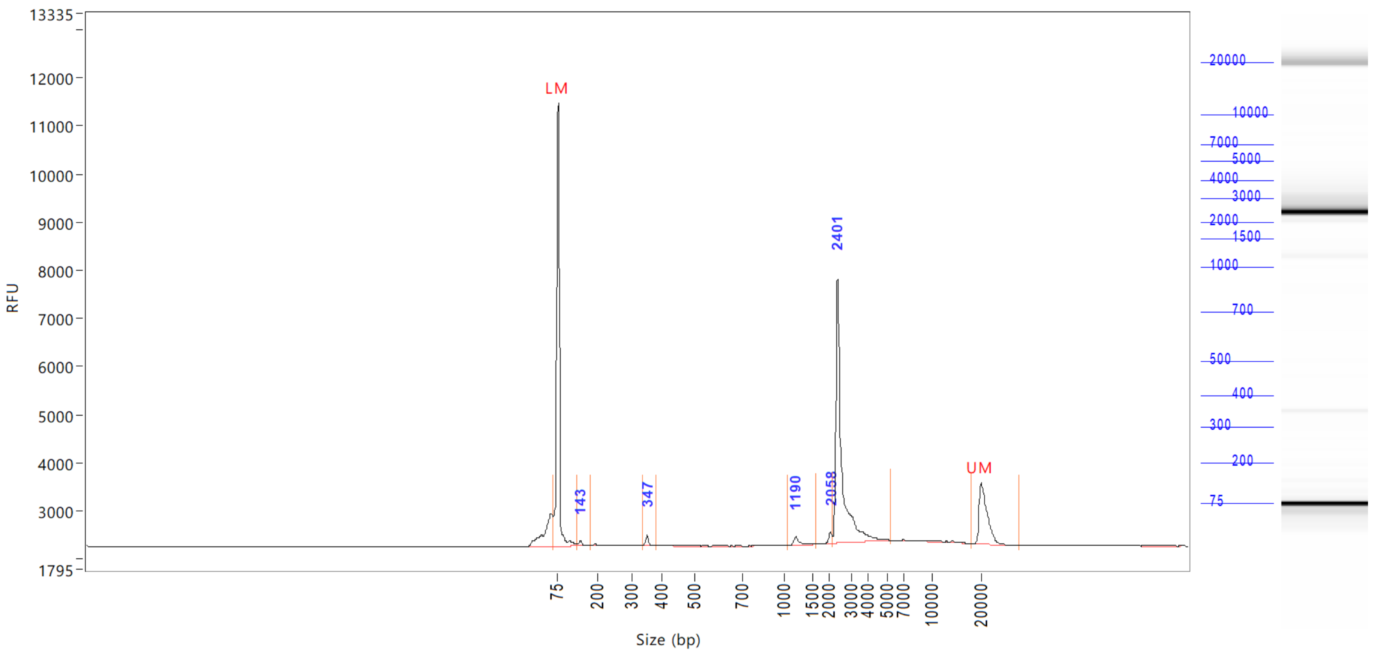

Peak	Size (bp)	Concentration (ng/uL)	From (bp)	To (bp)	Average size (bp)	CV%	RFU	Corrected peak area
1	75 (LM)	0.5000	60	133	73	11.39	9215	59.666
2	143	0.1104	133	179	146	7.17	103	1.098
3	347	0.1421	333	382	347	2.11	242	1.413
4	1190	0.1587	1056	1578	1229	7.14	177	1.578
5	2058	0.1296	1578	2172	2043	3.83	229	1.288
6	2401	4.9502	2172	5519	2611	16.67	5473	49.226
7	20000 (UM)	0.1125	17927	27617	20486	4.76	1286	13.428

TIC:	5.4909	ng/uL
TIM:	5.3545	nmole/L
Total concentration:	5.7508	ng/uL

Sample peak width (sec): 5 Sample min peak height: 50 Sample baseline V to V?: Y Sample baseline V to V points: 3
Sample filter: Binomial Number of points for filter: 3 Sample start region (min): 0 Sample end region (min): 30
Marker peak width (sec): 5 Marker min peak height: 200 Marker baseline V to V?: Y Marker baseline V to V points: 3
Lower marker selection: First peak > 500 RFU Upper marker selection: Last peak > 200 RFU
Ladder size (bp)75, 200, 300, 400, 500, 700, 1000, 1500, 2000, 3000, 4000, 5000, 7000, 10000, 20000
Quantification using: Lower Marker Final concentration (ng/uL): 0.5000 Dilution factor: 12.0

Sample: SampG12
Well location: G12
Created: Thursday, August 12, 2021 6:06:18 PM

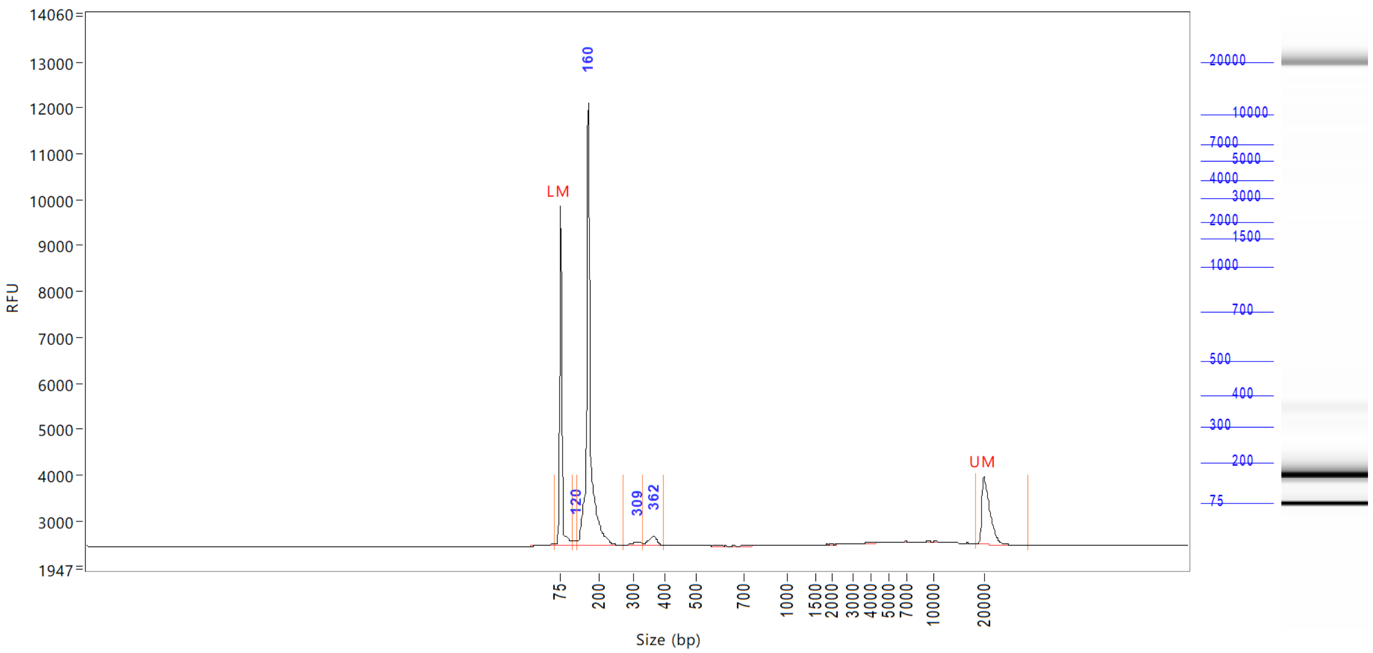

Peak	Size (bp)	Concentration (ng/uL)	From (bp)	To (bp)	Average size (bp)	CV%	RFU	Corrected peak area
1	75 (LM)	0.5000	58	110	74	8.70	7389	42.766
2	120	0.1509	110	126	118	4.09	103	1.076
3	160	12.4107	126	268	163	9.74	9655	88.458
4	309	0.2035	268	327	304	4.46	66	1.451
5	362	0.4945	327	396	357	3.70	200	3.525
6	20000 (UM)	0.1786	18415	28705	20568	4.92	1489	15.277

TIC:	13.2596	ng/uL
TIM:	130.6339	nmole/L
Total concentration:	13.5944	ng/uL

Sample peak width (sec): 5   Sample min peak height: 50   Sample baseline V to V?: Y   Sample baseline V to V points: 3
Sample filter: Binomial   Number of points for filter: 3 Sample start region (min): 0 Sample end region (min): 30
Marker peak width (sec): 5   Marker min peak height: 200   Marker baseline V to V?: Y   Marker baseline V to V points: 3
Lower marker selection: First peak > 500 RFU   Upper marker selection: Last peak > 200 RFU
Ladder size (bp)75, 200, 300, 400, 500, 700, 1000, 1500, 2000, 3000, 4000, 5000, 7000, 10000, 20000
Quantification using: Lower Marker   Final concentration (ng/uL): 0.5000   Dilution factor: 12.0

Sample: SampH12
Well location: H12
Created: Thursday, August 12, 2021 6:06:18 PM

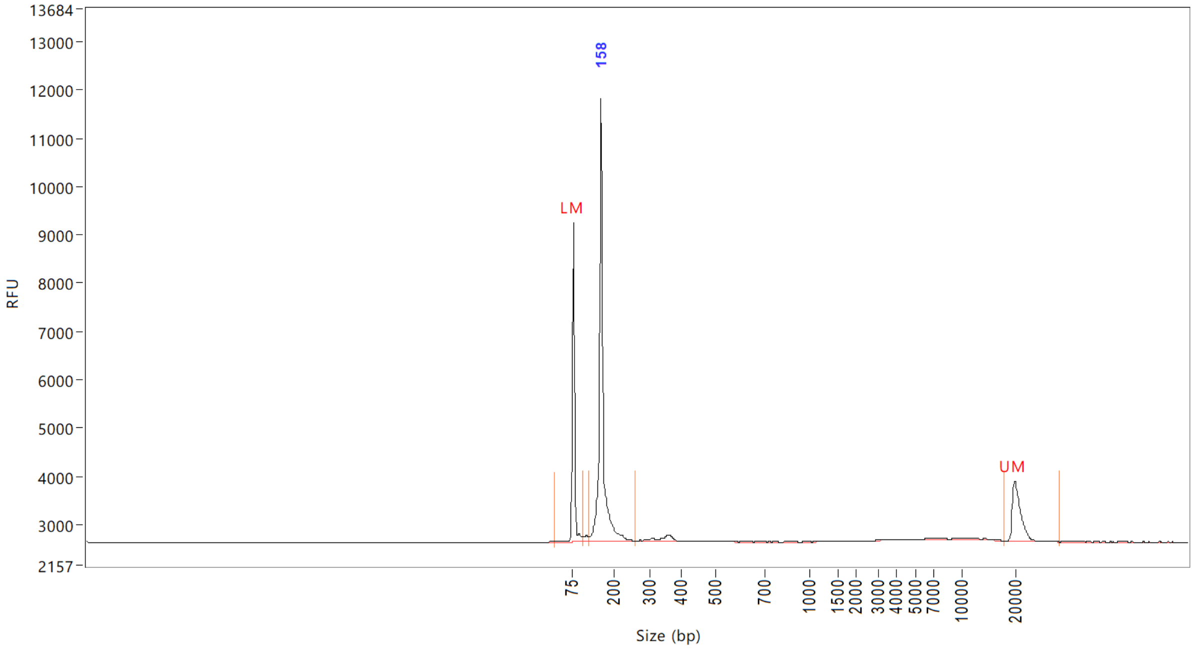

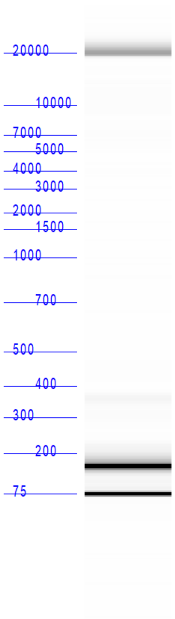

Peak	Size (bp)	Concentration (ng/uL)	From (bp)	To (bp)	Average size (bp)	CV%	RFU	Corrected peak area
1	75 (LM)	0.5000	18	105	73	9.39	6620	37.359
2	158	12.4841	120	257	159	9.41	9193	77.732
3	20000 (UM)	0.1702	18049	28705	20542	4.98	1250	12.720

TIC:	12.4841	ng/uL
TIM:	129.0552	nmole/L
Total concentration:	13.7294	ng/uL

Sample peak width (sec): 5 Sample min peak height: 200 Sample baseline V to V?: Y Sample baseline V to V points: 3
Sample filter: Binomial Number of points for filter: 3 Sample start region (min): 0 Sample end region (min): 30
Marker peak width (sec): 5 Marker min peak height: 200 Marker baseline V to V?: Y Marker baseline V to V points: 3
Lower marker selection: First peak > 500 RFU Upper marker selection: Last peak > 200 RFU
Ladder size (bp)75, 200, 300, 400, 500, 700, 1000, 1500, 2000, 3000, 4000, 5000, 7000, 10000, 20000
Quantification using: Lower Marker Final concentration (ng/uL): 0.5000 Dilution factor: 12.0

Sample: DNA size ladder
Well location: A7
Created: Thursday, August 12, 2021 6:06:18 PM
Import from: C:\AATI\Data\2021 08 11\17-13-47\210811_alberto_ladder.SCAL
Fit type: Point to point

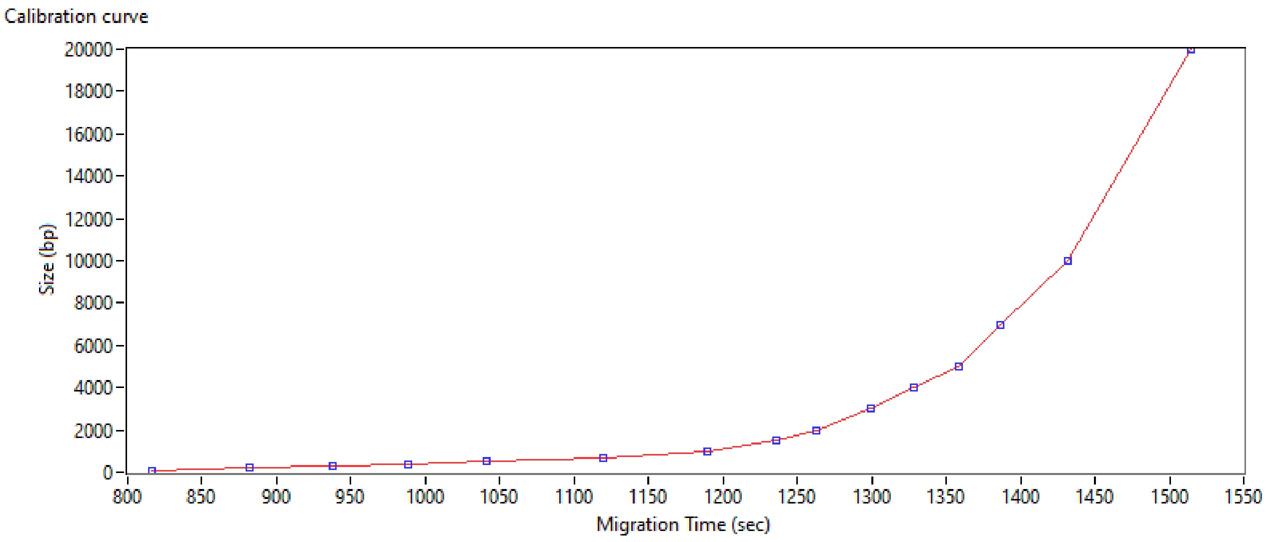

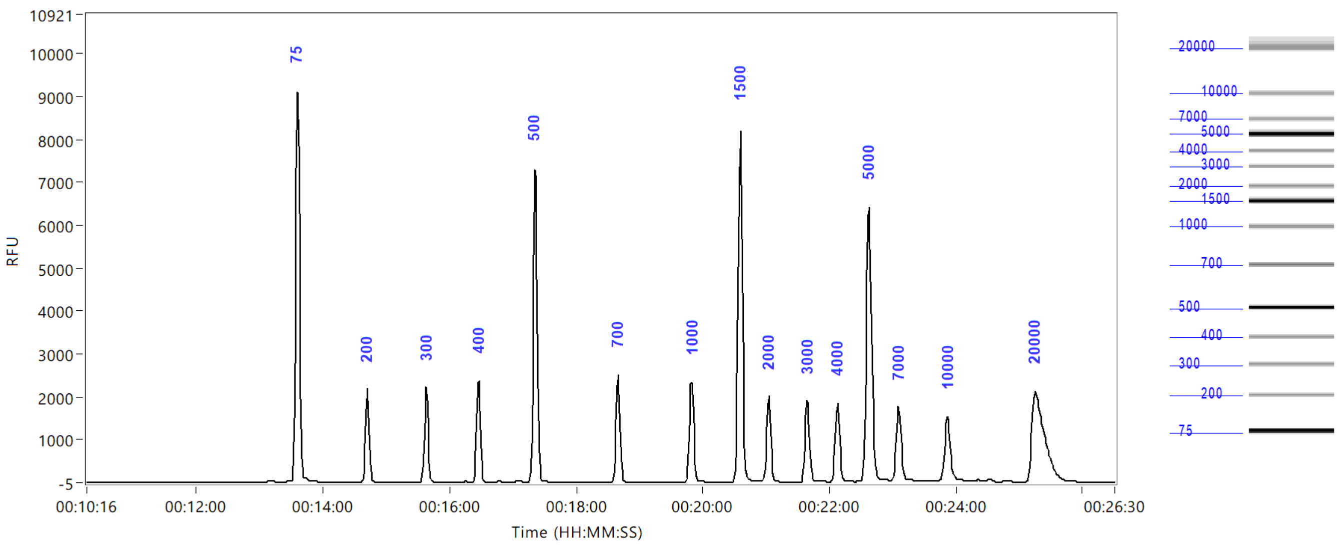

Supplement: Supplementary file 2 — sb3c00292_si_002.zip [file sb3c00292_si_002.zip › dnada_supplementary_material_pks_library_build/ZAG_trials/ZAG_Trial_3/17-56-24_redoplate1/210813_trial3_redoplate1.pdf]
