# Supplementary material for: Automated Platform for the Plasmid Construction Process
Source: ACS Synth Biol. 2023 Nov 10;12(12):3506–13. doi: 10.1021/acssynbio.3c00292 (PMC10729297; doi:10.1021/acssynbio.3c00292)

Instrument controller software run summary:

Filename and data path: C:\AATI\Data\2021 08 10\21-51-18\2021 08 10 21H 51M..raw
Created: Tuesday, August 10, 2021 10:01:13 PM
Number of capillaries: 96
Array serial number: 111920-09SFS
Effect length: 33 cm
Array usage count: 169
Instrument type: ZAG DNA Analyzer
Instrument controller software version: 1.0.1.9
Device serial number: Z7597

Method Information

Method name: ZAG130GP33 - DNA 75-20000bp - Gel Prime Only.mthds
Gel prime: Yes
Full conditioning: No
Gel prime to buffer: No
Gel selection: Gel 1
Perform prerun: 8.0 kV, 30 sec.
Rinse: No
Marker 1: Row: A, 5.0 kV, 5 sec.
Rinse: No
Sample injection: 5.0 kV, 5 sec.
Separation: 8.0 kV, 30.0 min.
Tray name: Sample Tray-6

Analysis mode: DNA

Notes

Gel Image

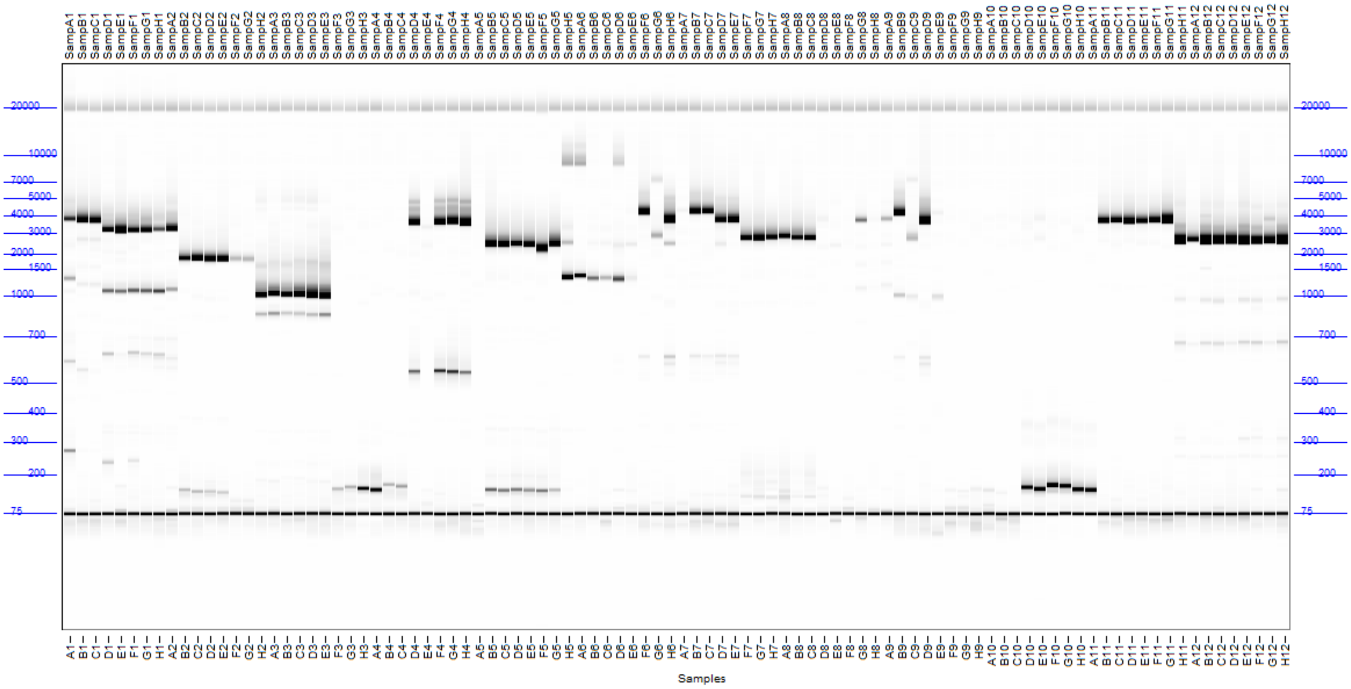

Filename and data path: C:\AATI\Data\2021 08 10\21-51-18\2021 08 10 21H 51M..raw

A1: SampA1

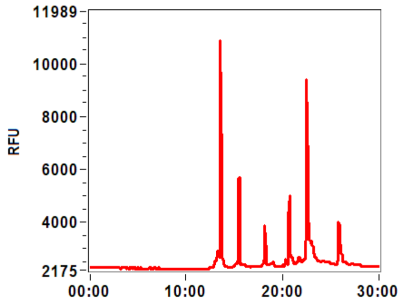

B1: SampB1

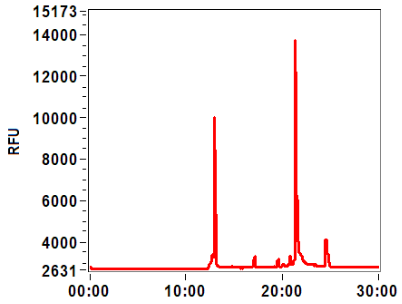

C1: SampC1

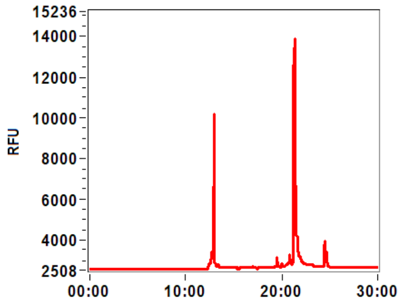

D1: SampD1

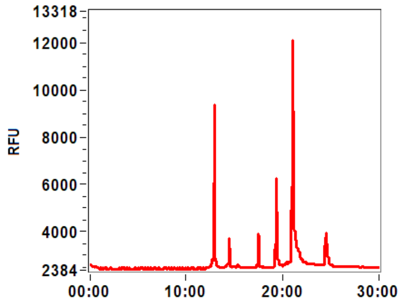

E1: SampE1

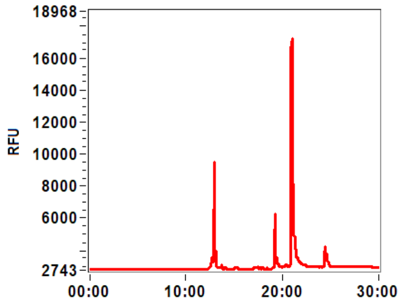

F1: SampF1

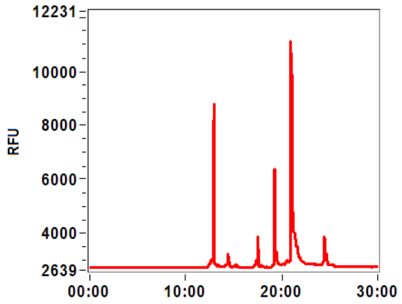

G1: SampG1

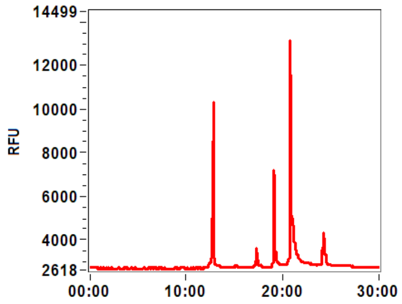

H1: SampH1

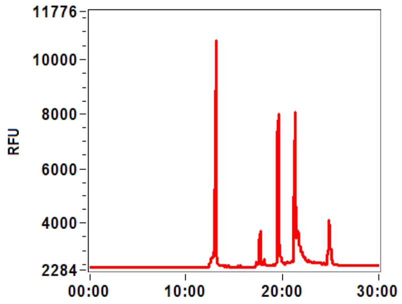

A2: SampA2

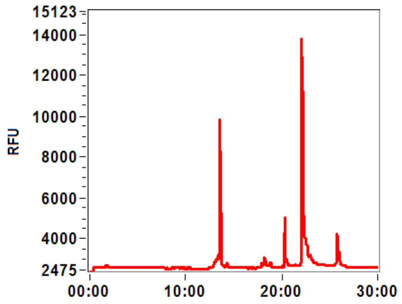

B2: SampB2

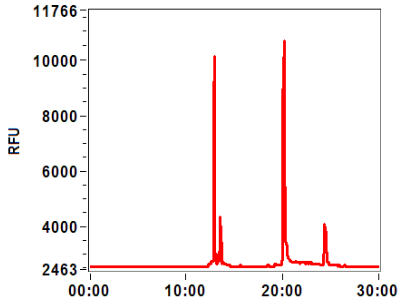

C2: SampC2

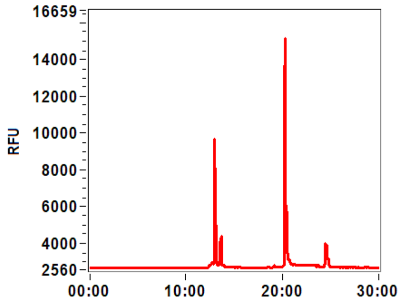

D2: SampD2

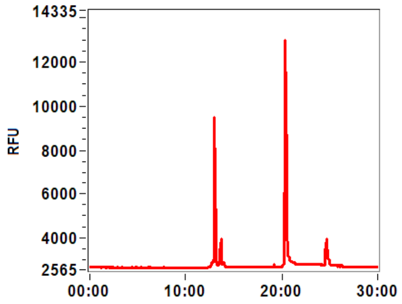

Filename and data path: C:\AATI\Data\2021 08 10\21-51-18\2021 08 10 21H 51M..raw

E2: SampE2

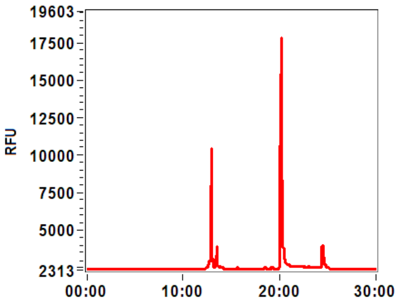

F2: SampF2

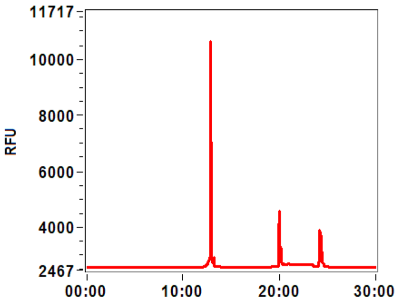

G2: SampG2

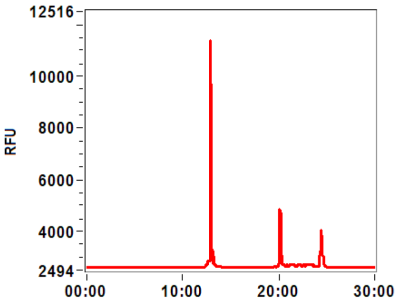

H2: SampH2

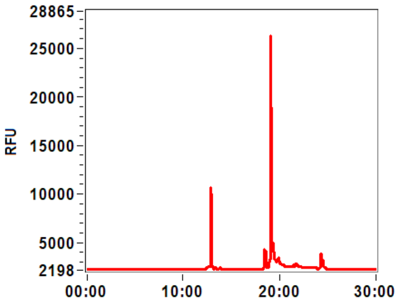

A3: SampA3

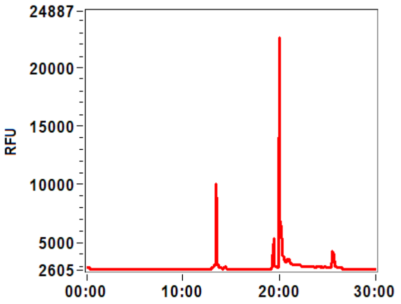

B3: SampB3

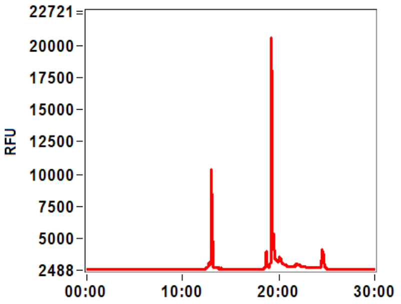

C3: SampC3

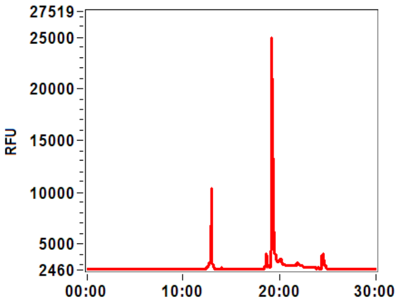

D3: SampD3

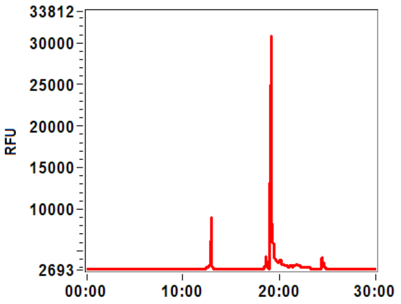

E3: SampE3

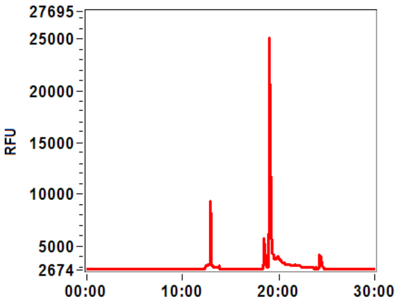

F3: SampF3

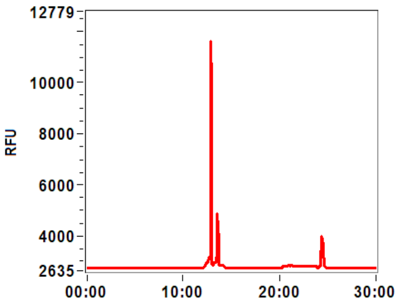

G3: SampG3

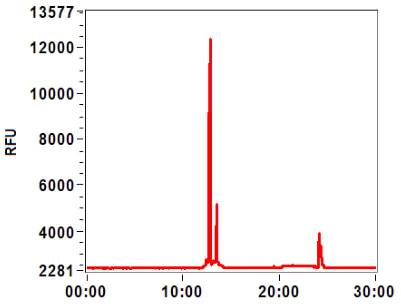

H3: SampH3

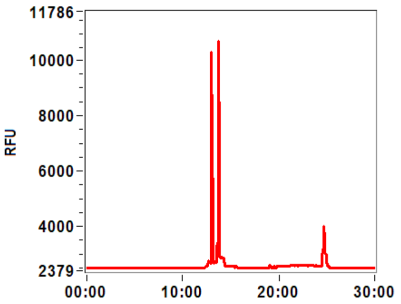

Filename and data path: C:\AATI\Data\2021 08 10\21-51-18\2021 08 10 21H 51M..raw

A4: SampA4

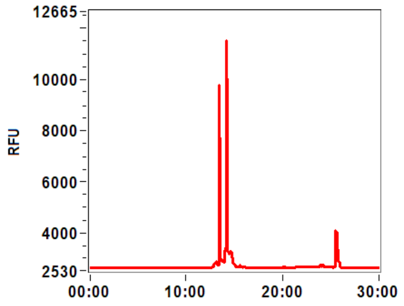

B4: SampB4

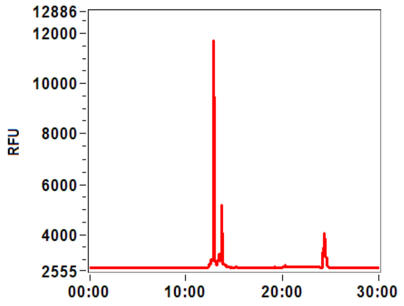

C4: SampC4

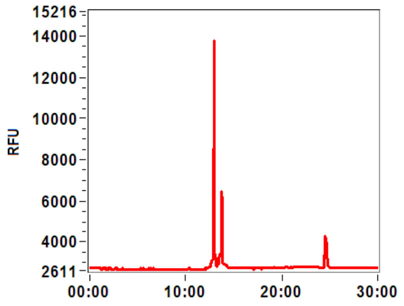

D4: SampD4

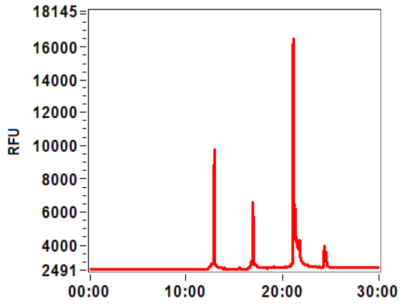

E4: SampE4

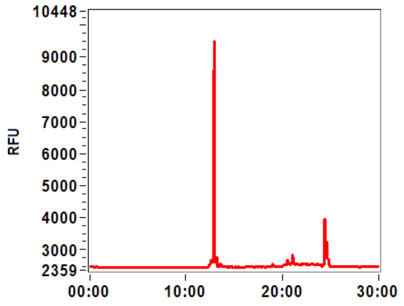

F4: SampF4

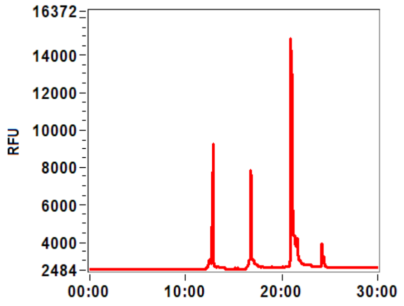

G4: SampG4

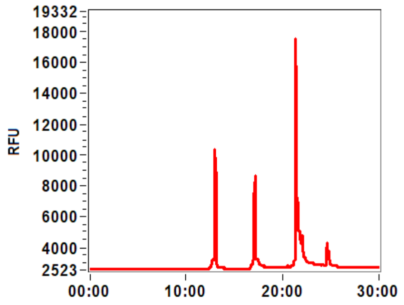

H4: SampH4

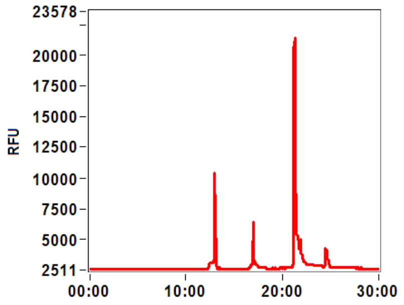

A5: SampA5

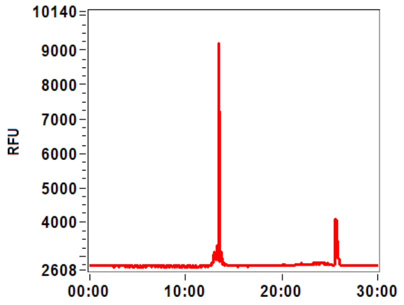

B5: SampB5

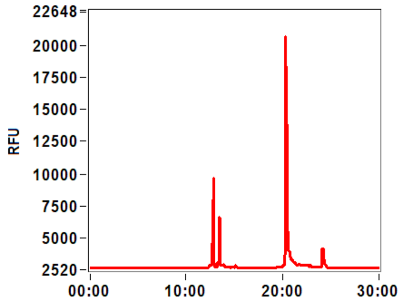

C5: SampC5

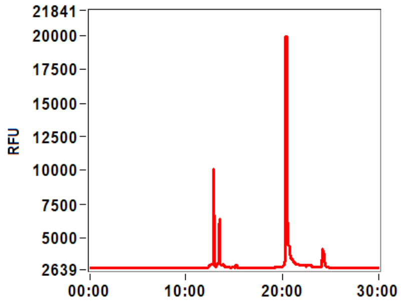

D5: SampD5

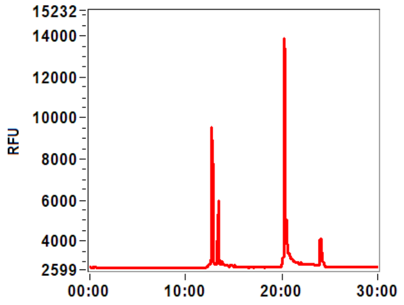

Filename and data path: C:\AATI\Data\2021 08 10\21-51-18\2021 08 10 21H 51M..raw

E5: SampE5

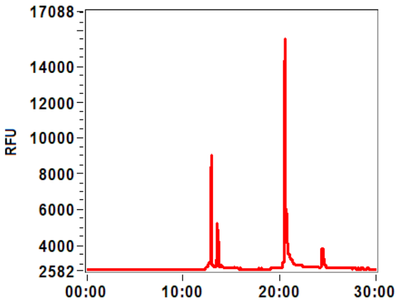

F5: SampF5

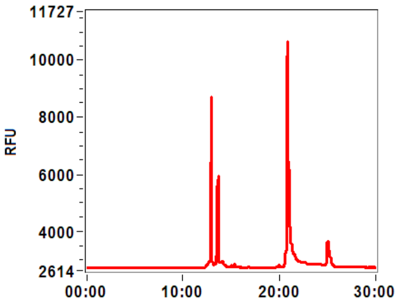

G5: SampG5

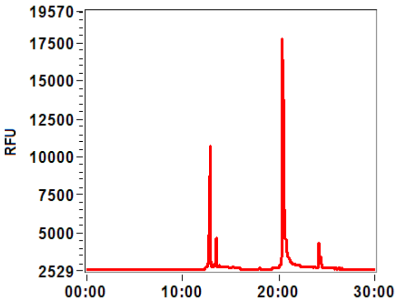

H5: SampH5

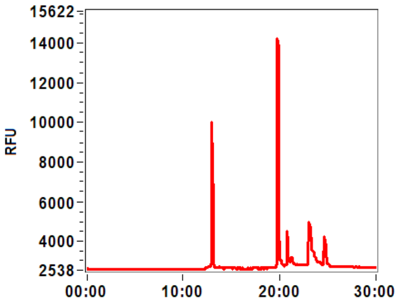

A6: SampA6

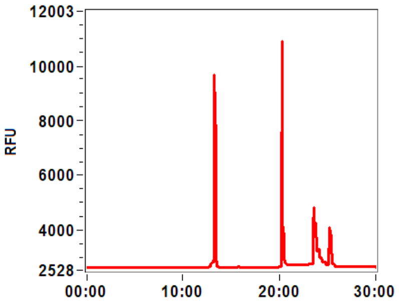

B6: SampB6

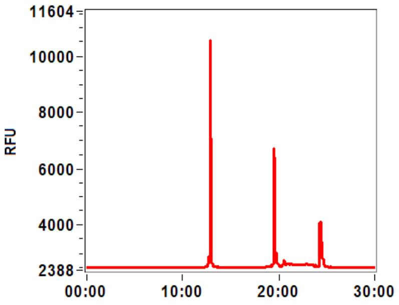

C6: SampC6

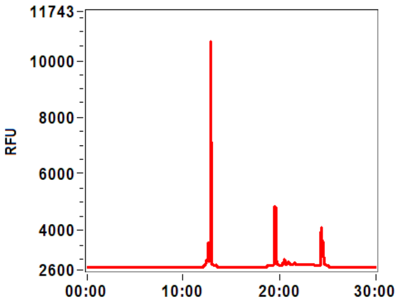

D6: SampD6

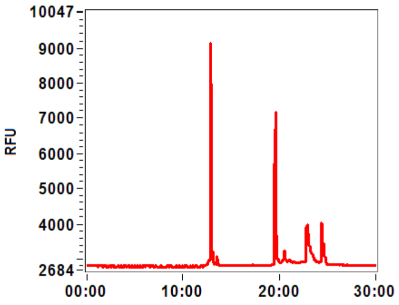

E6: SampE6

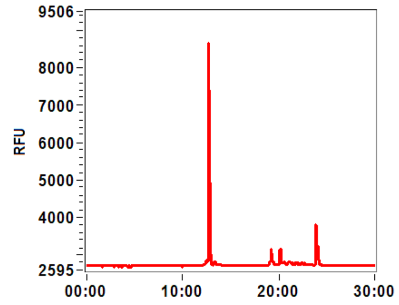

F6: SampF6

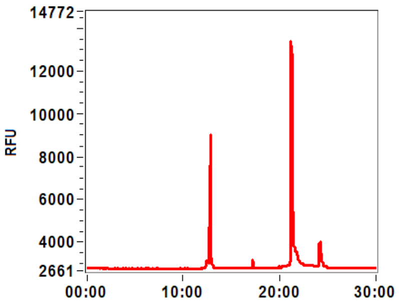

G6: SampG6

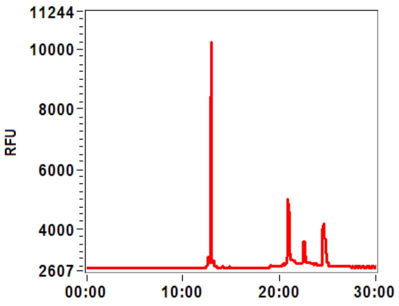

H6: SampH6

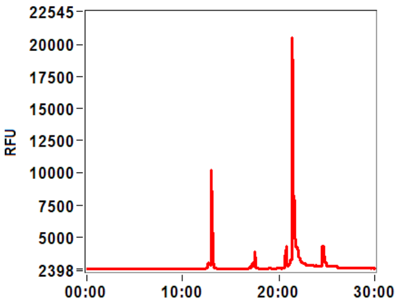

Filename and data path: C:\AATI\Data\2021 08 10\21-51-18\2021 08 10 21H 51M..raw

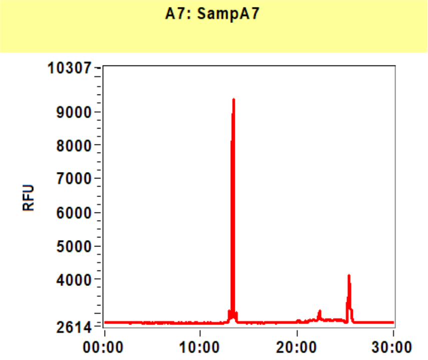

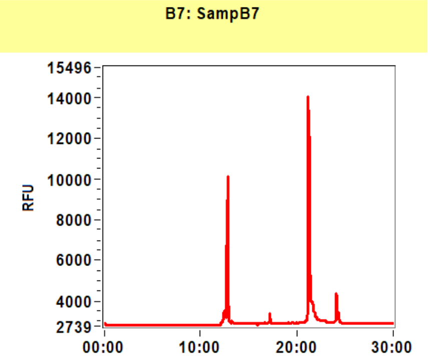

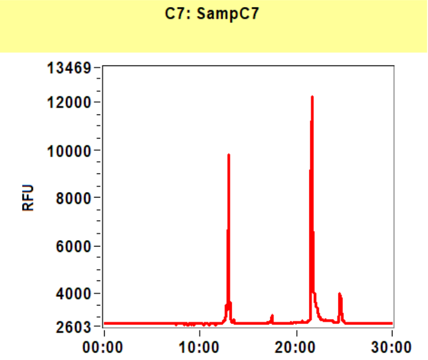

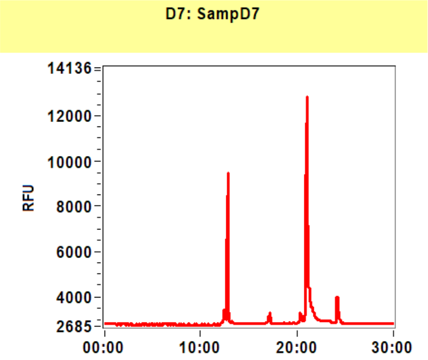

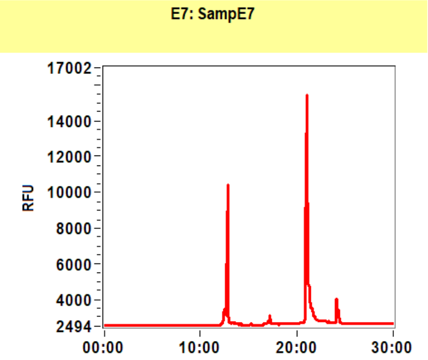

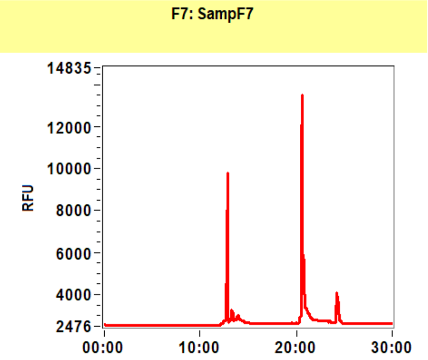

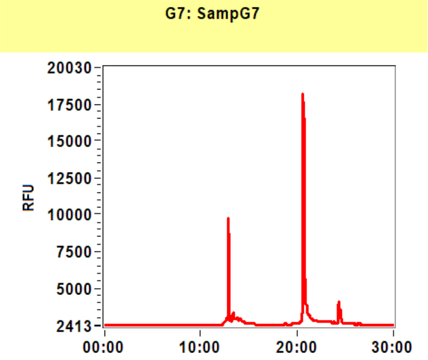

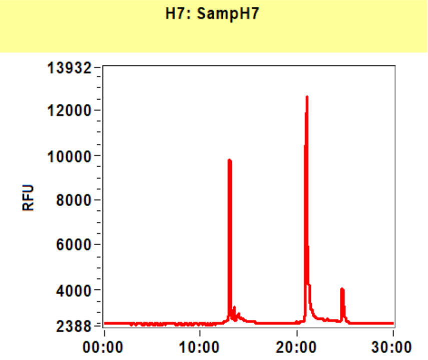

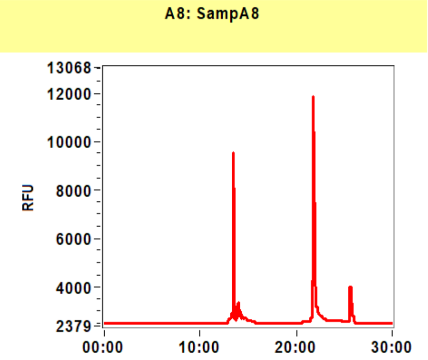

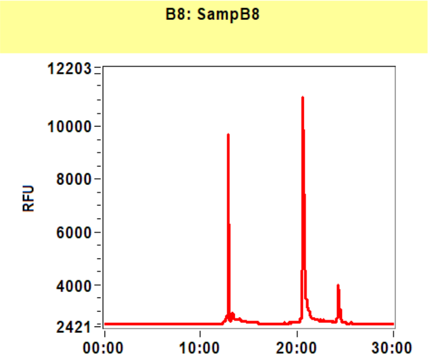

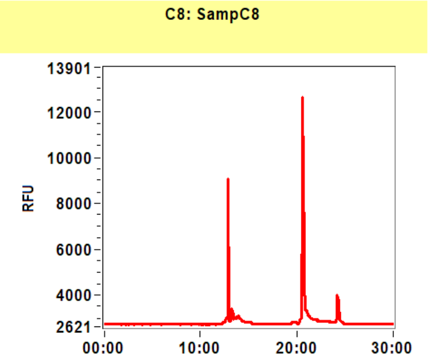

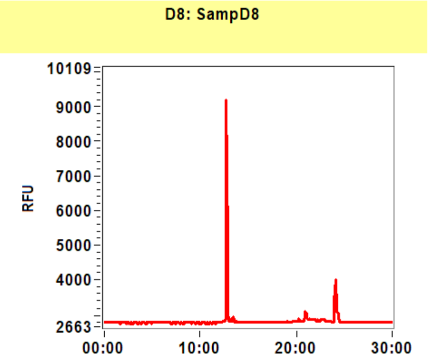

Filename and data path: C:\AATI\Data\2021 08 10\21-51-18\2021 08 10 21H 51M..raw

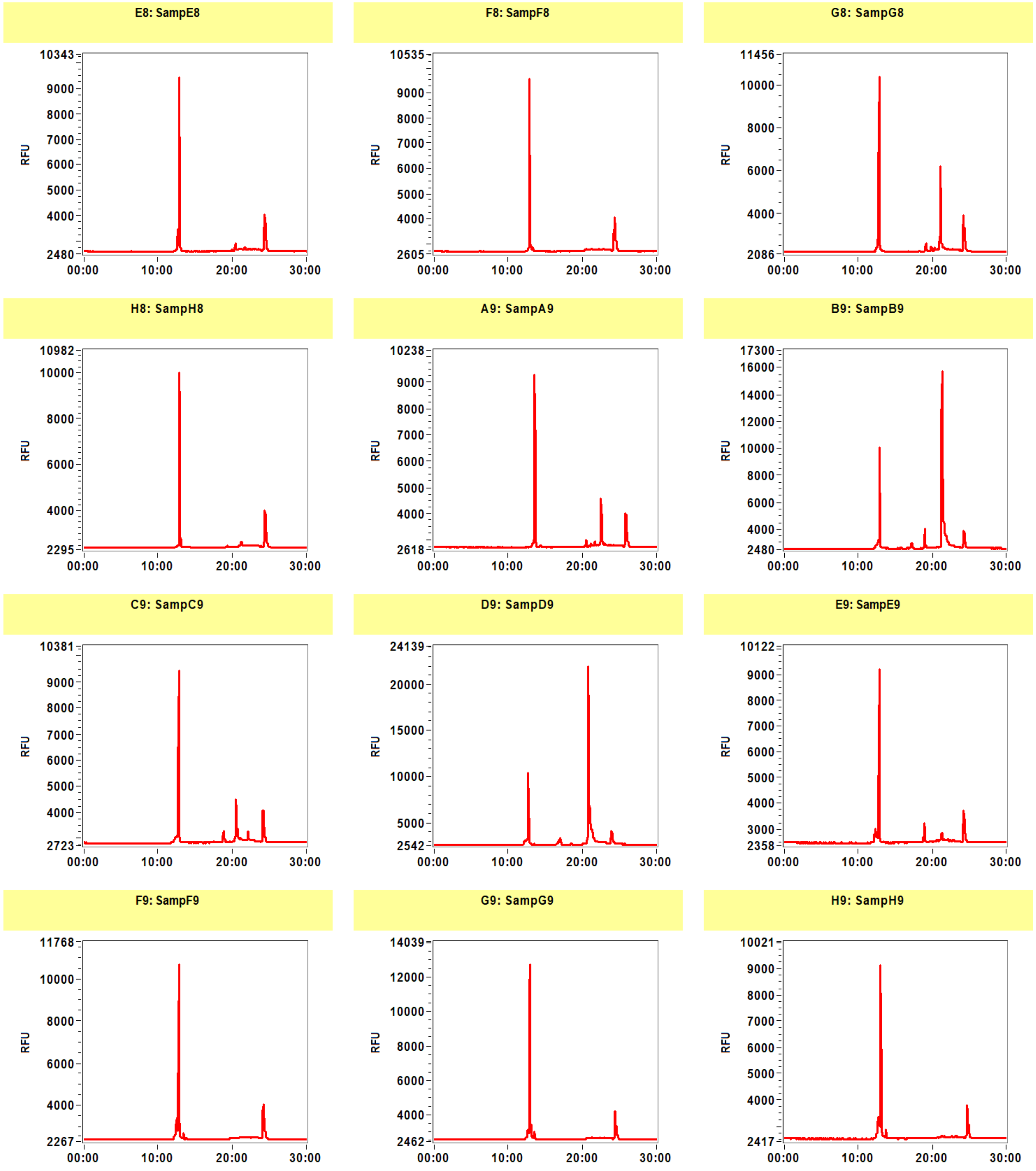

Filename and data path: C:\AATI\Data\2021 08 10\21-51-18\2021 08 10 21H 51M..raw

A10: SampA10

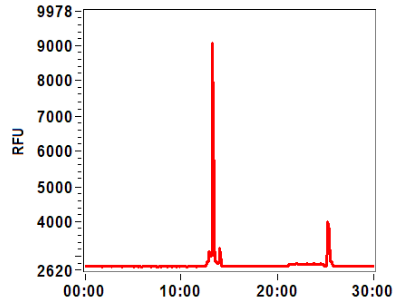

B10: SampB10

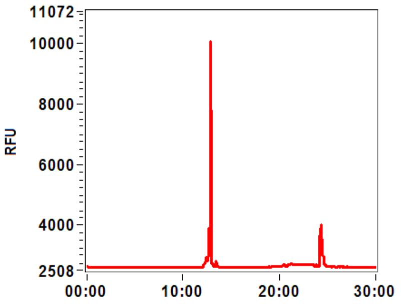

C10: SampC10

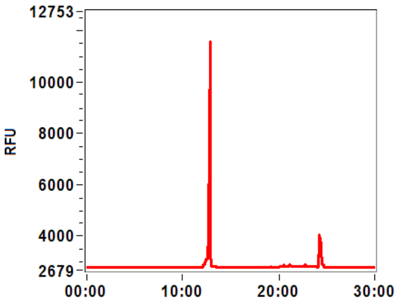

D10: SampD10

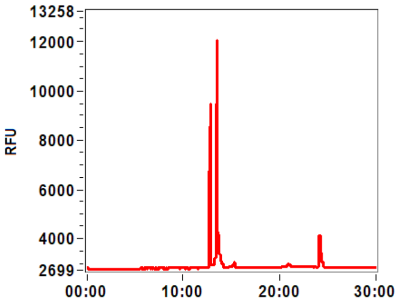

E10: SampE10

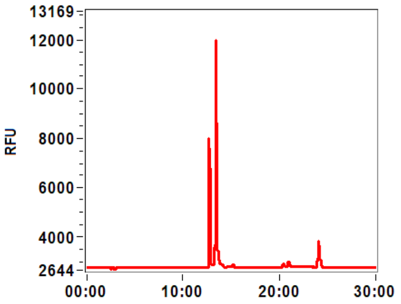

F10: SampF10

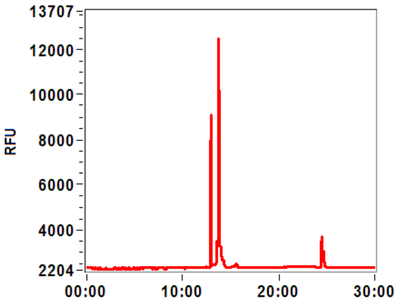

G10: SampG10

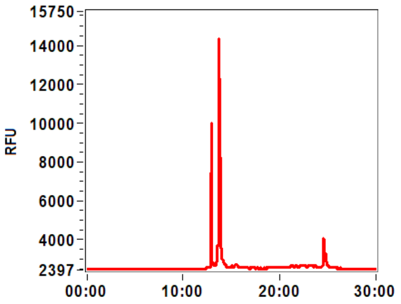

H10: SampH10

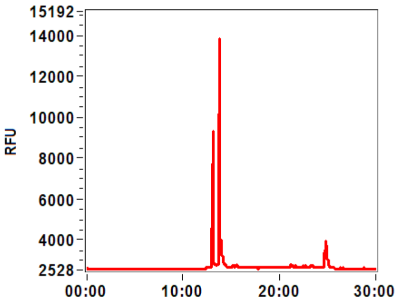

A11: SampA11

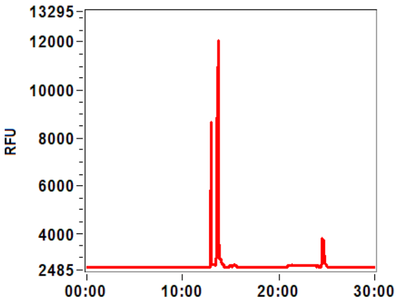

B11: SampB11

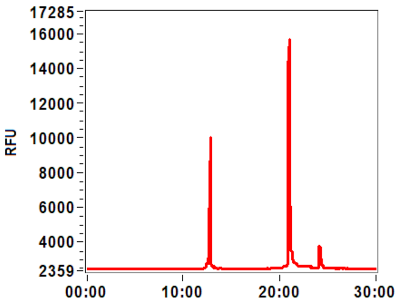

C11: SampC11

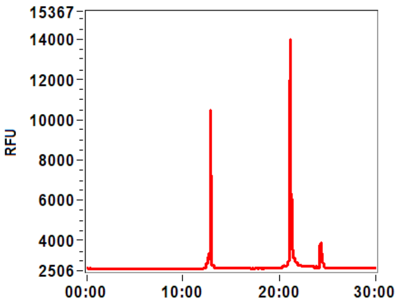

D11: SampD11

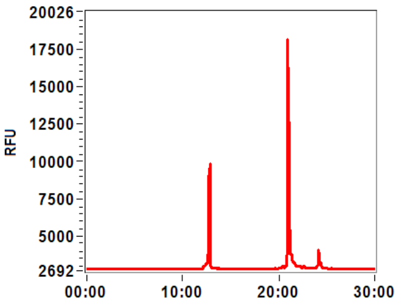

Filename and data path: C:\AATI\Data\2021 08 10\21-51-18\2021 08 10 21H 51M..raw

E11: SampE11

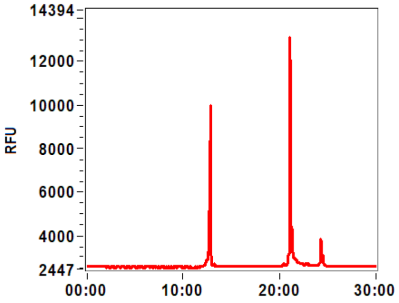

F11: SampF11

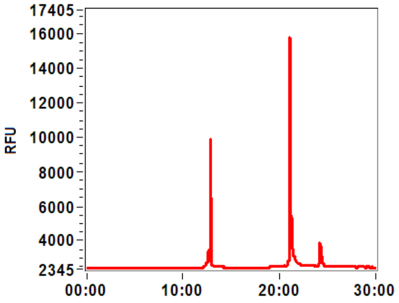

G11: SampG11

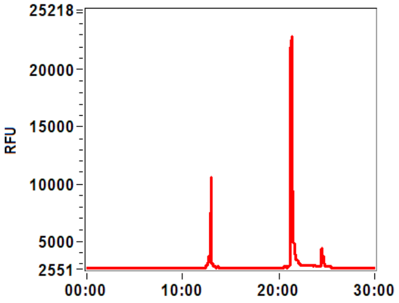

H11: SampH11

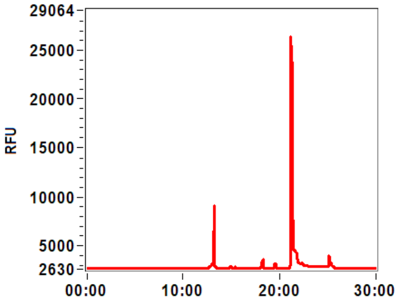

A12: SampA12

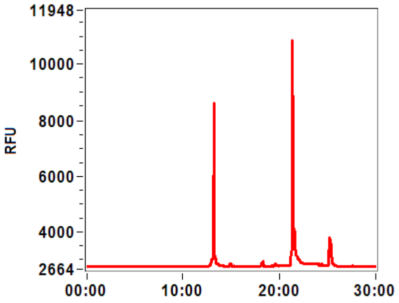

B12: SampB12

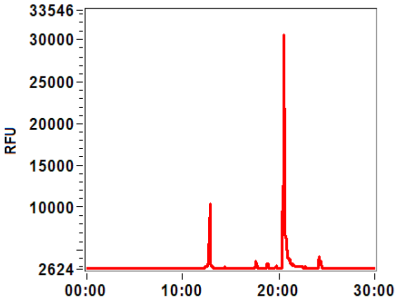

C12: SampC12

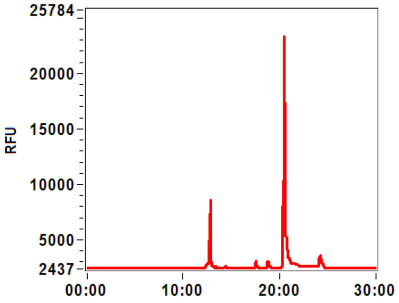

D12: SampD12

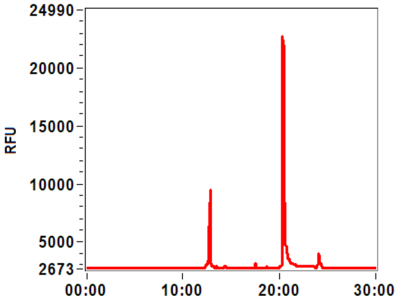

E12: SampE12

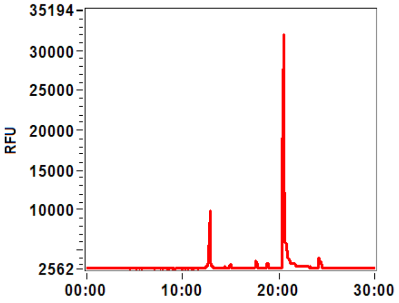

F12: SampF12

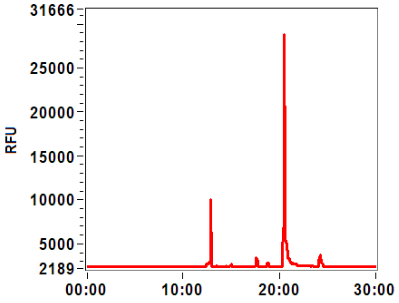

G12: SampG12

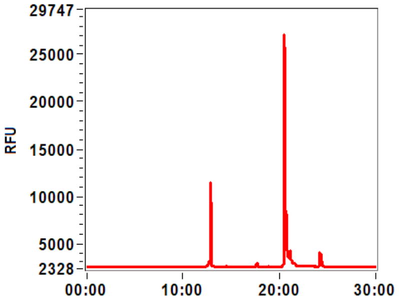

H12: SampH12

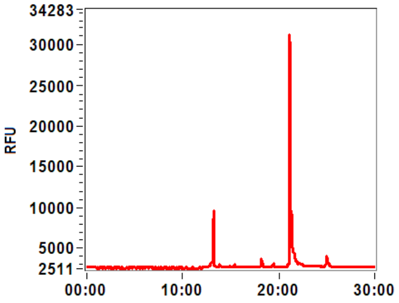

Sample: SampA1
Well location: A1
Created: Tuesday, August 10, 2021 10:01:13 PM

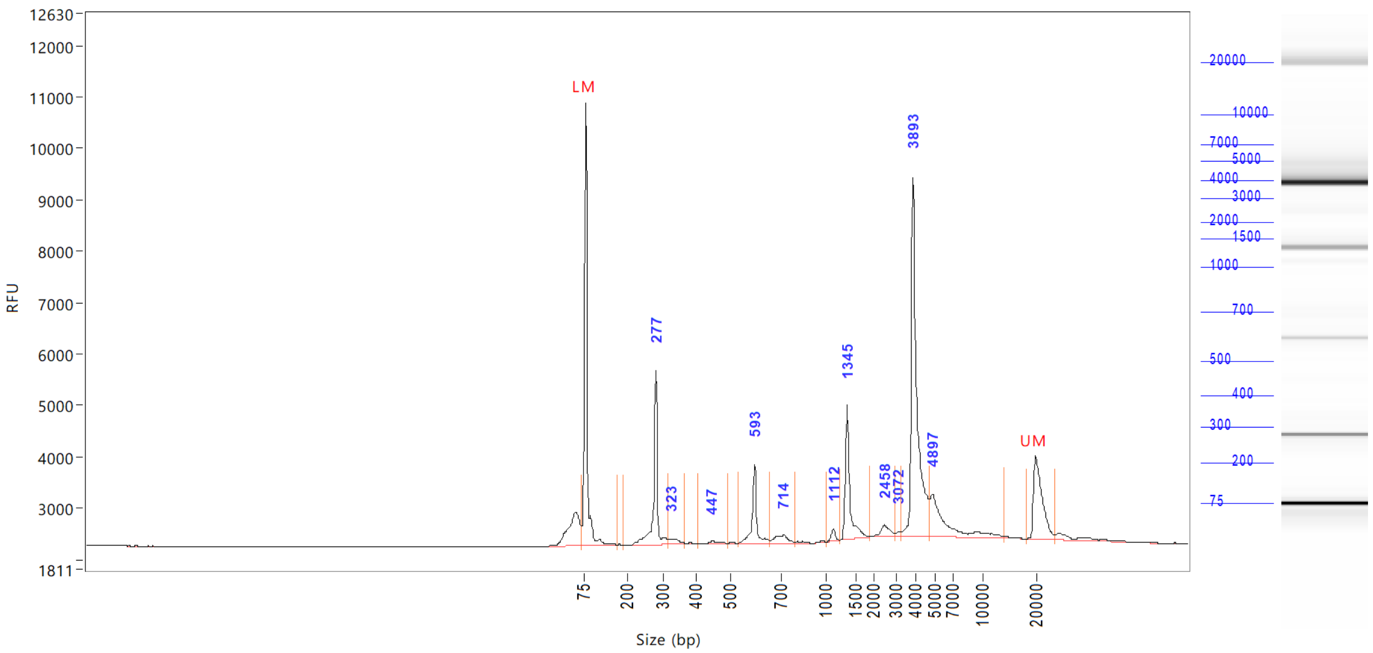

Peak	Size (bp)	Concentration (ng/uL)	From (bp)	To (bp)	Average size (bp)	CV%	RFU	Corrected peak area
1	75 (LM)	0.5000	62	169	75	13.88	8627	53.749
2	277	2.9132	185	311	270	5.73	3387	26.097
3	323	0.2445	311	364	330	3.93	112	2.190
4	447	0.0923	405	492	448	3.91	52	0.827
5	593	1.2683	529	654	592	3.40	1538	11.362
6	714	0.3857	654	788	702	4.29	155	3.455
7	1112	0.1621	1000	1223	1095	2.91	248	1.452
8	1345	1.9585	1223	1904	1372	6.22	2607	17.545
9	2458	0.3405	1904	2914	2491	8.34	232	3.051
10	3072	0.0829	2914	3215	3061	2.95	107	0.742
11	3893	6.5260	3215	4724	3995	6.57	6977	58.461
12	4897	1.9329	4724	14025	6422	32.07	826	17.315
13	20000 (UM)	0.1590	18293	23627	20636	4.55	1623	17.093

TIC:	15.9069	ng/uL
TIM:	29.7818	nmole/L
Total concentration:	15.9782	ng/uL

Sample peak width (sec): 5   Sample min peak height: 50   Sample baseline V to V?: Y   Sample baseline V to V points: 3
Sample filter: Binomial   Number of points for filter: 3   Sample start region (min): 0   Sample end region (min): 30
Marker peak width (sec): 5   Marker min peak height: 200   Marker baseline V to V?: Y   Marker baseline V to V points: 3
Lower marker selection: First peak > 500 RFU   Upper marker selection: Last peak > 200 RFU
Ladder size (bp)75, 200, 300, 400, 500, 700, 1000, 1500, 2000, 3000, 4000, 5000, 7000, 10000, 20000
Quantification using: Lower Marker   Final concentration (ng/uL): 0.5000   Dilution factor: 12.0

Sample: SampB1
Well location: B1
Created: Tuesday, August 10, 2021 10:01:13 PM

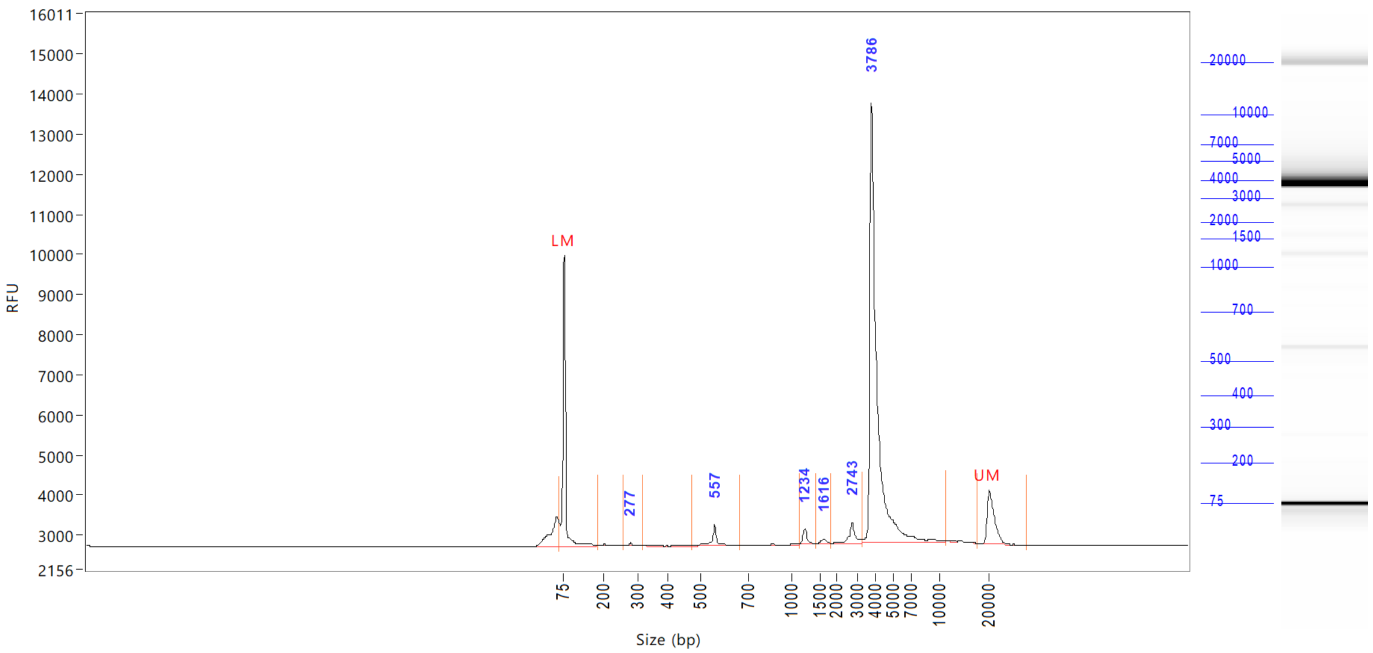

Peak	Size (bp)	Concentration (ng/uL)	From (bp)	To (bp)	Average size (bp)	CV%	RFU	Corrected peak area
1	75 (LM)	0.5000	62	179	76	19.25	7260	48.114
2	277	0.0751	255	313	277	3.49	86	0.602
3	557	0.6106	473	664	551	5.28	547	4.896
4	1234	0.3263	1134	1445	1239	3.92	390	2.616
5	1616	0.1468	1445	1866	1604	5.56	129	1.177
6	2743	0.6183	1866	3251	2695	9.65	520	4.958
7	3786	13.6975	3251	11342	4146	19.76	10982	109.841
8	20000 (UM)	0.1292	17683	27375	20514	4.24	1328	12.431

TIC:	15.4745	ng/uL
TIM:	8.6701	nmole/L
Total concentration:	15.7626	ng/uL

```
Sample peak width (sec): 5    Sample min peak height: 50    Sample baseline V to V?: Y    Sample baseline V to V points: 3
Sample filter: Binomial       Number of points for filter: 3  Sample start region (min): 0  Sample end region (min): 30
Marker peak width (sec): 5    Marker min peak height: 200   Marker baseline V to V?: Y    Marker baseline V to V points: 3
Lower marker selection: First peak > 500 RFU                Upper marker selection: Last peak > 200 RFU
Ladder size (bp)75, 200, 300, 400, 500, 700, 1000, 1500, 2000, 3000, 4000, 5000, 7000, 10000, 20000
Quantification using: Lower Marker          Final concentration (ng/uL): 0.5000          Dilution factor: 12.0
```

Sample: SampC1
Well location: C1
Created: Tuesday, August 10, 2021 10:01:13 PM

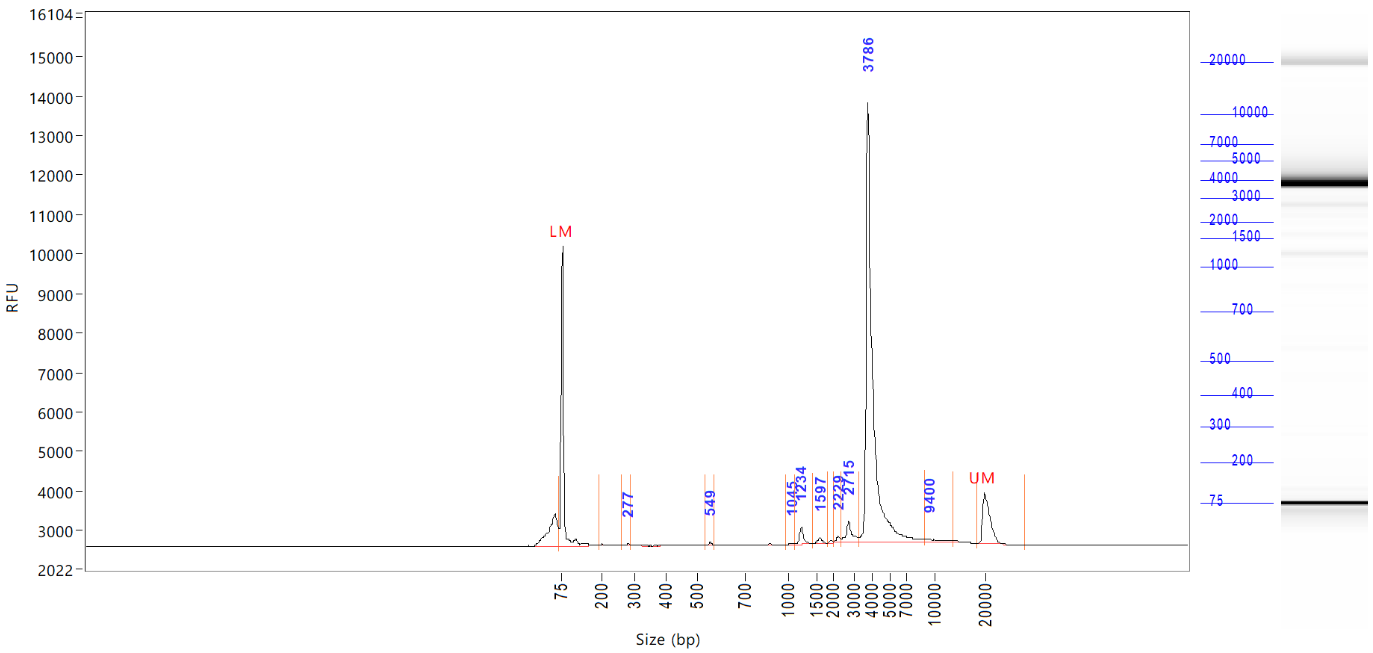

Peak	Size (bp)	Concentration (ng/uL)	From (bp)	To (bp)	Average size (bp)	CV%	RFU	Corrected peak area
1	75 (LM)	0.5000	64	192	78	21.97	7602	49.161
2	277	0.0364	257	286	273	1.97	54	0.299
3	549	0.0739	527	567	548	1.49	96	0.606
4	1045	0.0467	978	1112	1041	3.13	51	0.382
5	1234	0.3610	1112	1445	1228	4.09	443	2.958
6	1597	0.1226	1445	1827	1577	3.96	154	1.004
7	2229	0.1280	2001	2372	2208	4.36	148	1.048
8	2715	0.6021	2372	3251	2767	7.35	525	4.933
9	3786	12.9863	3251	8867	4052	16.23	11142	106.404
10	9400	0.1448	8867	13537	10465	12.74	52	1.187
11	20000 (UM)	0.1225	18537	28100	20394	4.11	1287	12.043

TIC:	14.5018	ng/uL
TIM:	6.8797	nmole/L
Total concentration:	14.8162	ng/uL

Sample peak width (sec): 5   Sample min peak height: 50   Sample baseline V to V?: Y   Sample baseline V to V points: 3
Sample filter: Binomial   Number of points for filter: 3   Sample start region (min): 0   Sample end region (min): 30
Marker peak width (sec): 5   Marker min peak height: 200   Marker baseline V to V?: Y   Marker baseline V to V points: 3
Lower marker selection: First peak > 500 RFU   Upper marker selection: Last peak > 200 RFU
Ladder size (bp)75, 200, 300, 400, 500, 700, 1000, 1500, 2000, 3000, 4000, 5000, 7000, 10000, 20000
Quantification using: Lower Marker   Final concentration (ng/uL): 0.5000   Dilution factor: 12.0

Sample: SampD1
Well location: D1
Created: Tuesday, August 10, 2021 10:01:13 PM

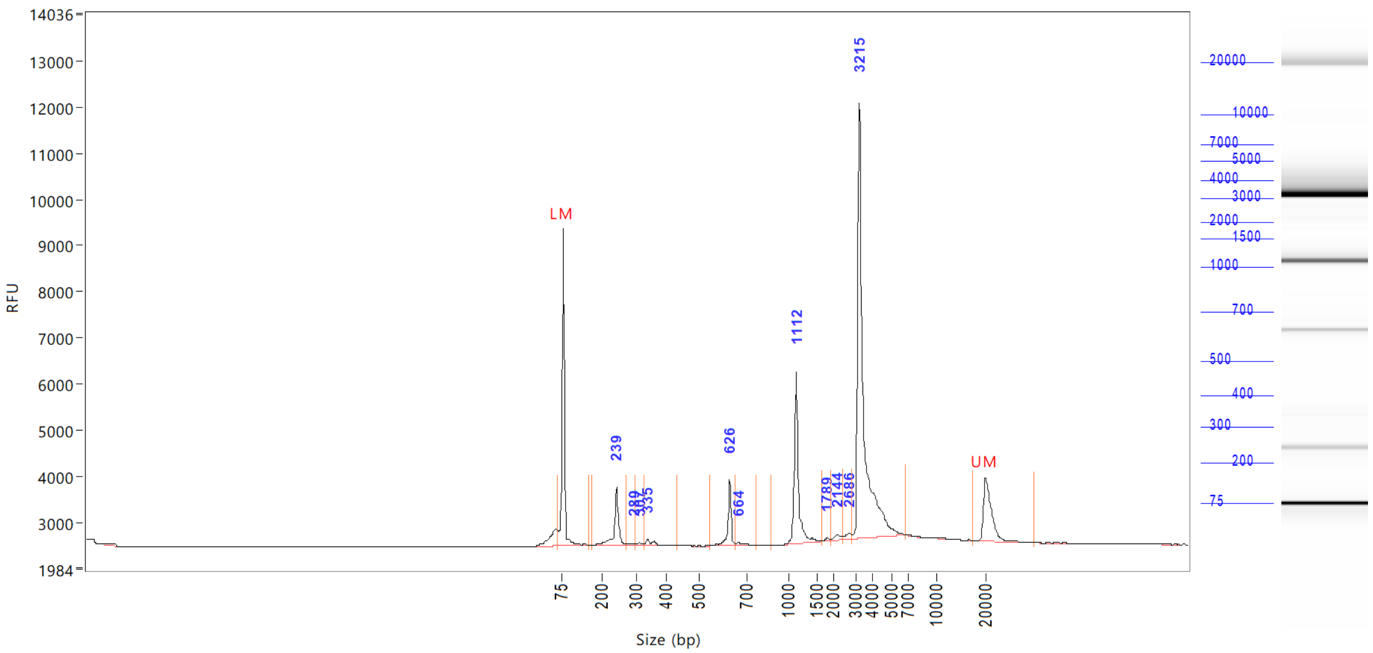

Peak	Size (bp)	Concentration (ng/uL)	From (bp)	To (bp)	Average size (bp)	CV%	RFU	Corrected peak area
1	75 (LM)	0.5000	60	160	74	12.02	6865	41.472
2	239	1.7474	166	271	234	6.48	1252	12.078
3	289	0.1042	271	296	285	2.62	52	0.720
4	307	0.1084	296	323	308	2.62	59	0.750
5	335	0.2907	323	431	346	4.82	148	2.010
6	626	1.2242	547	649	620	2.26	1424	8.461
7	664	0.1580	649	770	681	3.93	63	1.092
8	1112	3.5317	870	1635	1126	6.91	3726	24.412
9	1789	0.0606	1635	1904	1796	3.46	61	0.419
10	2144	0.1986	1904	2429	2156	6.68	126	1.372
11	2686	0.1804	2429	2857	2657	4.66	116	1.247
12	3215	11.8873	2857	6630	3497	13.80	9457	82.165
13	20000 (UM)	0.1693	17561	29672	20568	5.88	1381	14.045

TIC:	19.4914	ng/uL
TIM:	29.5519	nmole/L
Total concentration:	19.5929	ng/uL

```
Sample peak width (sec): 5     Sample min peak height: 50    Sample baseline V to V?: Y    Sample baseline V to V points: 3
Sample filter: Binomial        Number of points for filter: 3  Sample start region (min): 0  Sample end region (min): 30
Marker peak width (sec): 5     Marker min peak height: 200   Marker baseline V to V?: Y    Marker baseline V to V points: 3
Lower marker selection: First peak > 500 RFU                 Upper marker selection: Last peak > 200 RFU
Ladder size (bp)75, 200, 300, 400, 500, 700, 1000, 1500, 2000, 3000, 4000, 5000, 7000, 10000, 20000
Quantification using: Lower Marker          Final concentration (ng/uL): 0.5000          Dilution factor: 12.0
```

Sample: SampE1
Well location: E1
Created: Tuesday, August 10, 2021 10:01:13 PM

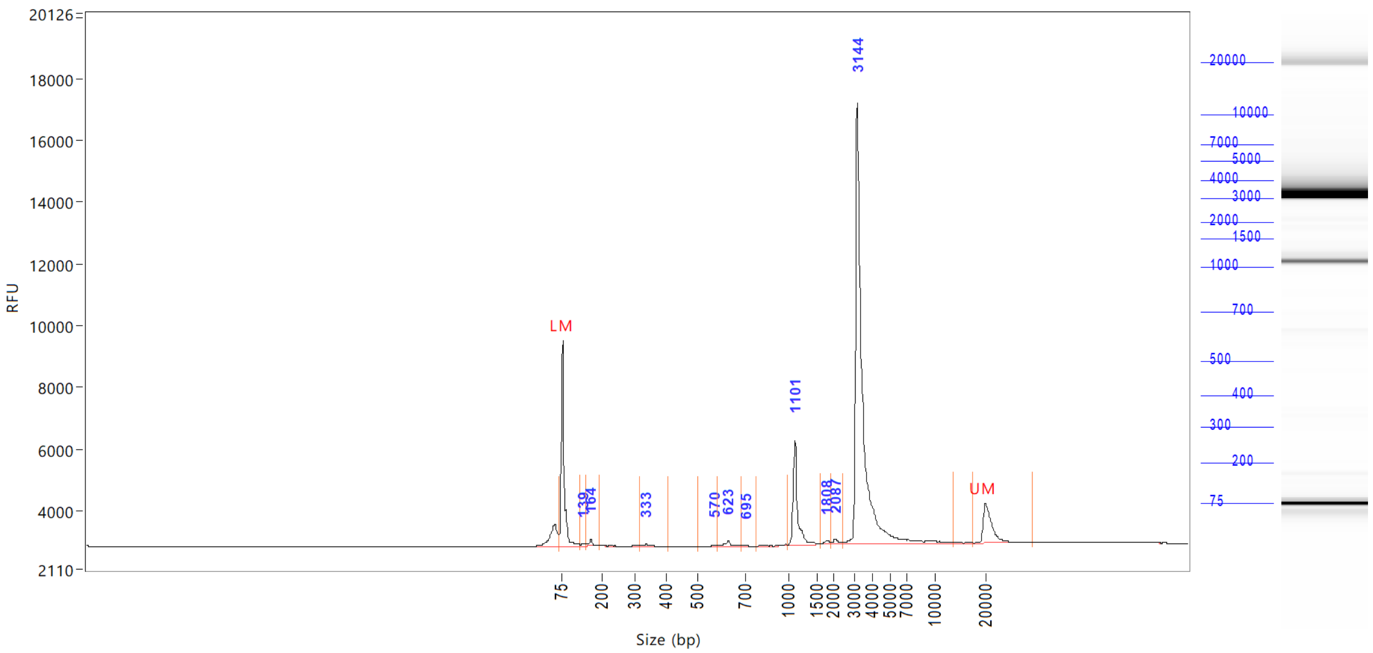

Peak	Size (bp)	Concentration (ng/uL)	From (bp)	To (bp)	Average size (bp)	CV%	RFU	Corrected peak area
1	75 (LM)	0.5000	64	129	76	11.94	6679	47.245
2	139	0.1114	129	150	140	4.44	76	0.877
3	164	0.2021	150	194	162	4.06	228	1.591
4	333	0.1348	309	407	338	5.36	70	1.061
5	570	0.0736	498	580	556	3.64	57	0.579
6	623	0.2766	580	677	620	3.39	182	2.178
7	695	0.0530	677	770	698	2.70	51	0.417
8	1101	3.0505	996	1635	1130	8.14	3386	24.020
9	1808	0.1297	1635	1962	1804	4.41	124	1.021
10	2087	0.1788	1962	2458	2144	6.56	166	1.408
11	3144	17.9982	2458	13659	3560	30.18	14313	141.722
12	20000 (UM)	0.1509	17683	29551	20847	6.97	1300	14.257

TIC:	22.2086	ng/uL
TIM:	18.1128	nmole/L
Total concentration:	22.3784	ng/uL

```
Sample peak width (sec): 5     Sample min peak height: 50     Sample baseline V to V?: Y     Sample baseline V to V points: 3
Sample filter: Binomial        Number of points for filter: 3  Sample start region (min): 0  Sample end region (min): 30
Marker peak width (sec): 5     Marker min peak height: 200    Marker baseline V to V?: Y     Marker baseline V to V points: 3
Lower marker selection: First peak > 500 RFU                  Upper marker selection: Last peak > 200 RFU
Ladder size (bp)75, 200, 300, 400, 500, 700, 1000, 1500, 2000, 3000, 4000, 5000, 7000, 10000, 20000
Quantification using: Lower Marker          Final concentration (ng/uL): 0.5000          Dilution factor: 12.0
```

Sample: SampF1
Well location: F1
Created: Tuesday, August 10, 2021 10:01:13 PM

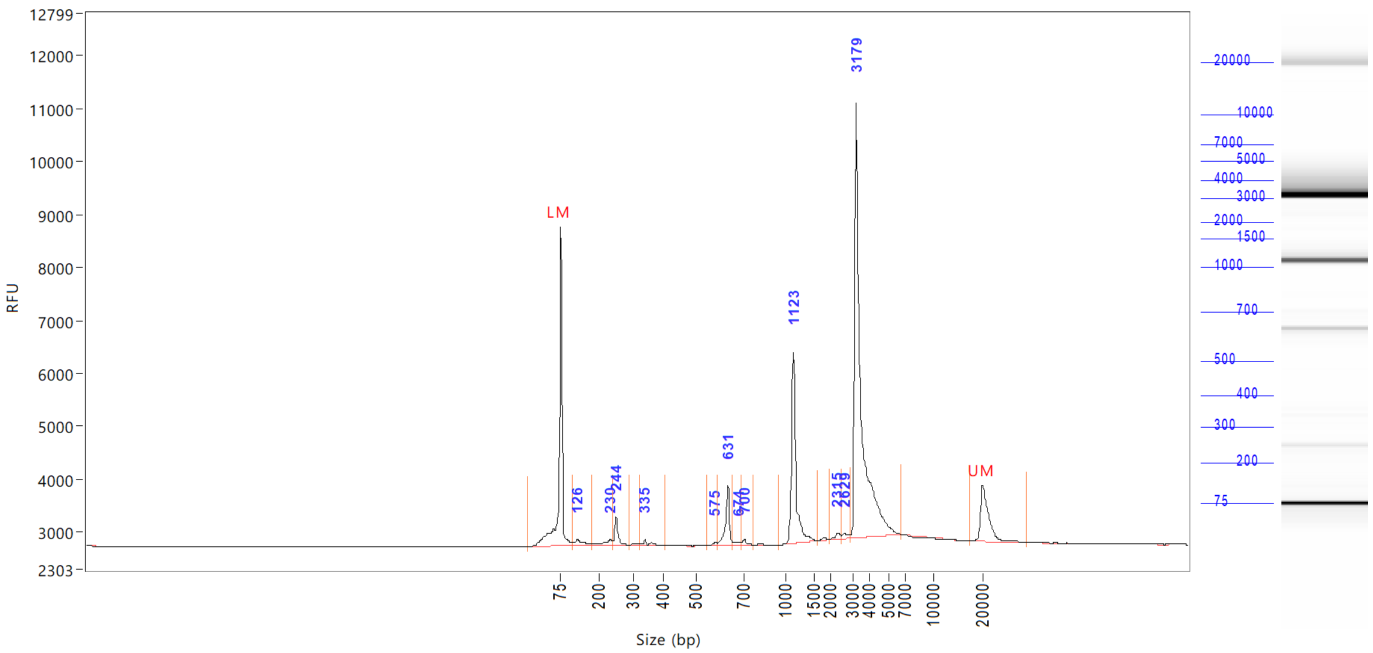

Peak	Size (bp)	Concentration (ng/uL)	From (bp)	To (bp)	Average size (bp)	CV%	RFU	Corrected peak area
1	75 (LM)	0.5000	0	110	66	27.21	6028	45.463
2	126	0.3168	110	175	135	12.63	118	2.400
3	230	0.2578	175	235	213	7.79	101	1.953
4	244	0.5222	235	286	246	3.03	520	3.957
5	335	0.1675	317	401	345	4.63	104	1.269
6	575	0.0629	544	585	572	1.54	66	0.477
7	631	0.9964	585	654	624	1.99	1125	7.550
8	674	0.0920	654	687	670	1.51	56	0.697
9	700	0.0941	687	762	701	1.72	105	0.713
10	1123	3.3413	944	1578	1134	6.64	3611	25.317
11	2315	0.1256	1962	2486	2278	5.61	116	0.952
12	2629	0.1227	2486	2886	2675	4.40	94	0.930
13	3179	10.3624	2886	6482	3503	14.26	8224	78.518
14	20000 (UM)	0.1231	17561	28826	20637	6.29	1066	11.189

TIC:	16.4619	ng/uL
TIM:	23.2779	nmole/L
Total concentration:	16.5814	ng/uL

```
Sample peak width (sec): 5    Sample min peak height: 50    Sample baseline V to V?: Y    Sample baseline V to V points: 3
Sample filter: Binomial       Number of points for filter: 3  Sample start region (min): 0  Sample end region (min): 30
Marker peak width (sec): 5    Marker min peak height: 200   Marker baseline V to V?: Y    Marker baseline V to V points: 3
Lower marker selection: First peak > 500 RFU               Upper marker selection: Last peak > 200 RFU
Ladder size (bp)75, 200, 300, 400, 500, 700, 1000, 1500, 2000, 3000, 4000, 5000, 7000, 10000, 20000
Quantification using: Lower Marker          Final concentration (ng/uL): 0.5000          Dilution factor: 12.0
```

Sample: SampG1
Well location: G1
Created: Tuesday, August 10, 2021 10:01:13 PM

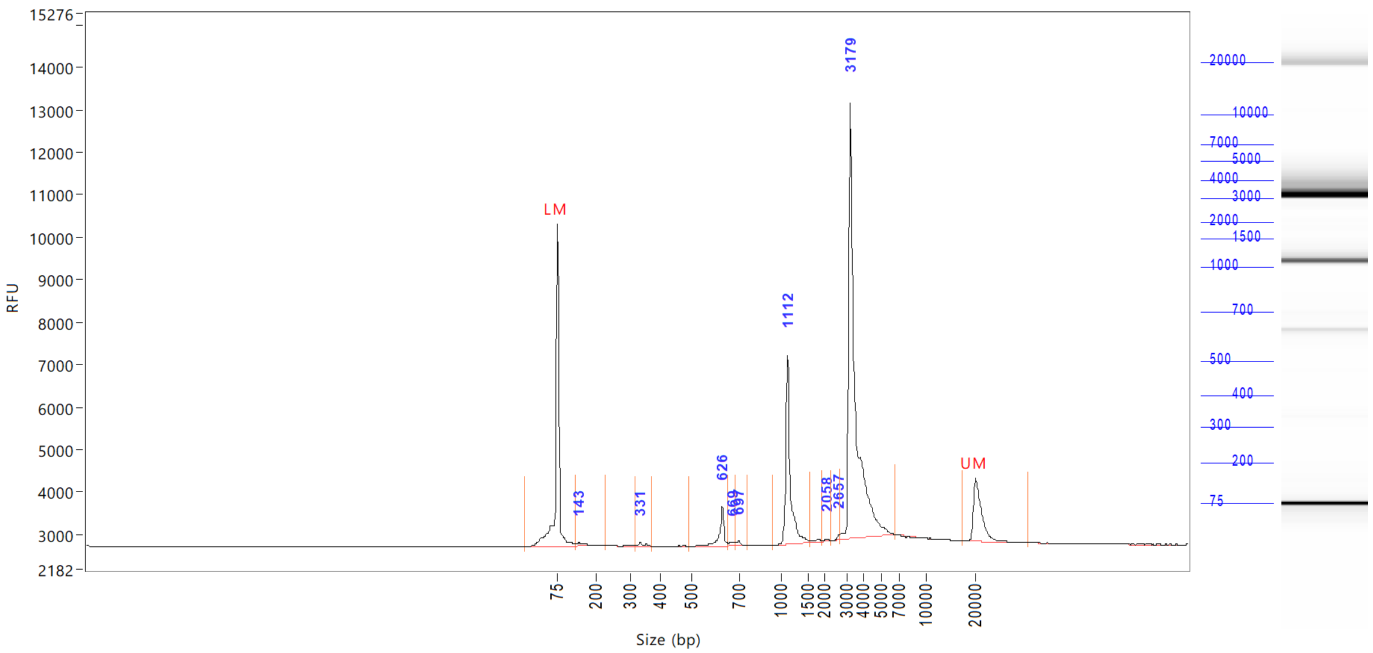

Peak	Size (bp)	Concentration (ng/uL)	From (bp)	To (bp)	Average size (bp)	CV%	RFU	Corrected peak area
1	75 (LM)	0.5000	0	135	68	27.39	7603	59.344
2	143	0.1781	135	228	161	12.69	78	1.762
3	331	0.1114	315	372	337	3.67	103	1.102
4	626	0.7823	492	654	615	3.71	935	7.738
5	669	0.0869	654	682	668	1.29	82	0.859
6	697	0.1147	682	757	701	2.56	130	1.135
7	1112	3.3592	944	1559	1137	7.47	4439	33.225
8	2058	0.0352	1923	2229	2026	2.79	60	0.348
9	2657	0.0478	2229	2686	2598	3.03	110	0.472
10	3179	10.5678	2686	6556	3494	13.88	10275	104.522
11	20000 (UM)	0.1343	17195	30639	20764	6.67	1492	15.937

TIC:	15.2836	ng/uL
TIM:	14.8406	nmole/L
Total concentration:	15.4406	ng/uL

```
Sample peak width (sec): 5     Sample min peak height: 50    Sample baseline V to V?: Y    Sample baseline V to V points: 3
Sample filter: Binomial        Number of points for filter: 3  Sample start region (min): 0  Sample end region (min): 30
Marker peak width (sec): 5     Marker min peak height: 200   Marker baseline V to V?: Y    Marker baseline V to V points: 3
Lower marker selection: First peak > 500 RFU                 Upper marker selection: Last peak > 200 RFU
Ladder size (bp)75, 200, 300, 400, 500, 700, 1000, 1500, 2000, 3000, 4000, 5000, 7000, 10000, 20000
Quantification using: Lower Marker          Final concentration (ng/uL): 0.5000          Dilution factor: 12.0
```

Sample: SampH1
Well location: H1
Created: Tuesday, August 10, 2021 10:01:13 PM

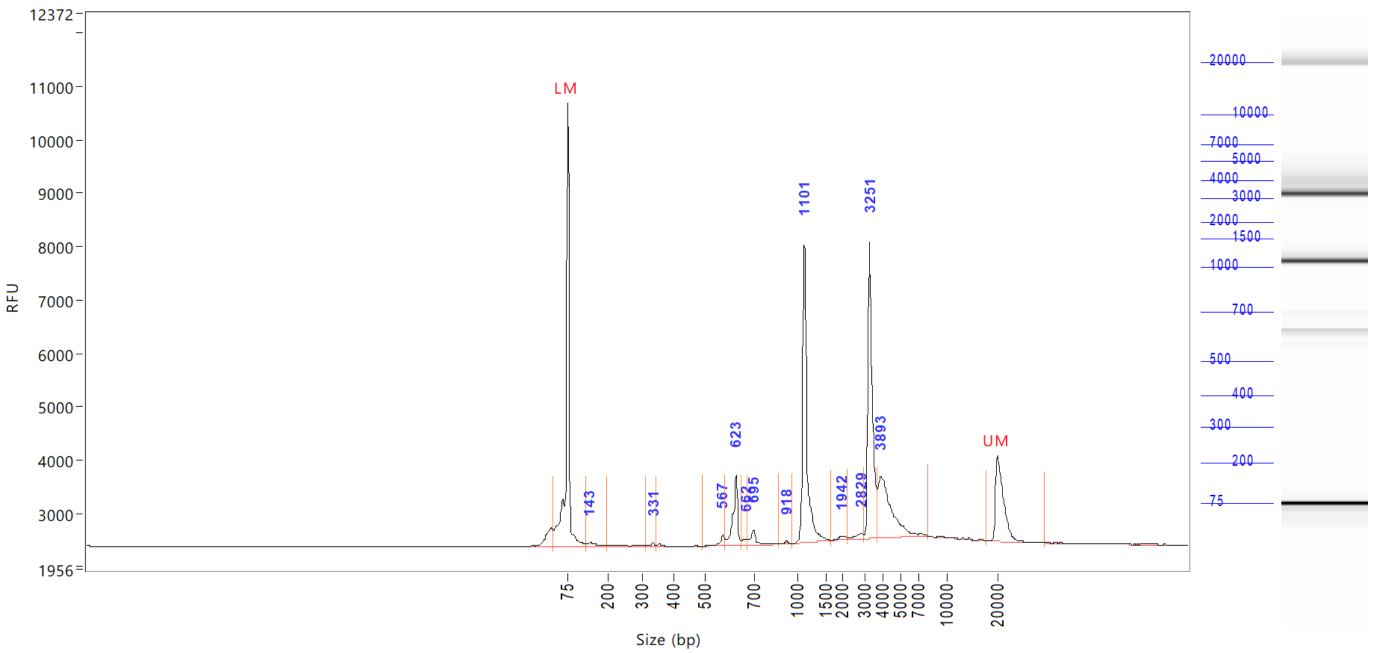

Peak	Size (bp)	Concentration (ng/uL)	From (bp)	To (bp)	Average size (bp)	CV%	RFU	Corrected peak area
1	75 (LM)	0.5000	30	131	69	19.32	8325	63.975
2	143	0.1465	131	196	156	11.46	71	1.562
3	331	0.0562	309	343	327	2.66	65	0.599
4	567	0.1660	490	580	559	3.10	200	1.770
5	623	1.0052	580	644	615	2.02	1326	10.718
6	662	0.1136	644	672	658	1.29	124	1.211
7	695	0.2594	672	866	705	4.94	285	2.766
8	918	0.0278	866	957	912	1.34	55	0.296
9	1101	3.5361	957	1635	1123	6.63	5587	37.704
10	1942	0.0753	1635	2229	1971	5.82	74	0.803
11	2829	0.1220	2229	2971	2687	7.25	101	1.300
12	3251	3.4256	2971	3679	3290	4.63	5565	36.526
13	3893	2.1871	3679	8001	4320	14.91	1145	23.320
14	20000 (UM)	0.1277	17805	29430	20649	5.50	1619	16.337

TIC:	11.1208	ng/uL
TIM:	13.8129	nmole/L
Total concentration:	11.2299	ng/uL

```
Sample peak width (sec): 5    Sample min peak height: 50    Sample baseline V to V?: Y    Sample baseline V to V points: 3
Sample filter: Binomial       Number of points for filter: 3  Sample start region (min): 0  Sample end region (min): 30
Marker peak width (sec): 5    Marker min peak height: 200   Marker baseline V to V?: Y    Marker baseline V to V points: 3
Lower marker selection: First peak > 500 RFU                Upper marker selection: Last peak > 200 RFU
Ladder size (bp)75, 200, 300, 400, 500, 700, 1000, 1500, 2000, 3000, 4000, 5000, 7000, 10000, 20000
Quantification using: Lower Marker          Final concentration (ng/uL): 0.5000          Dilution factor: 12.0
```

Sample: SampA2
Well location: A2
Created: Tuesday, August 10, 2021 10:01:13 PM

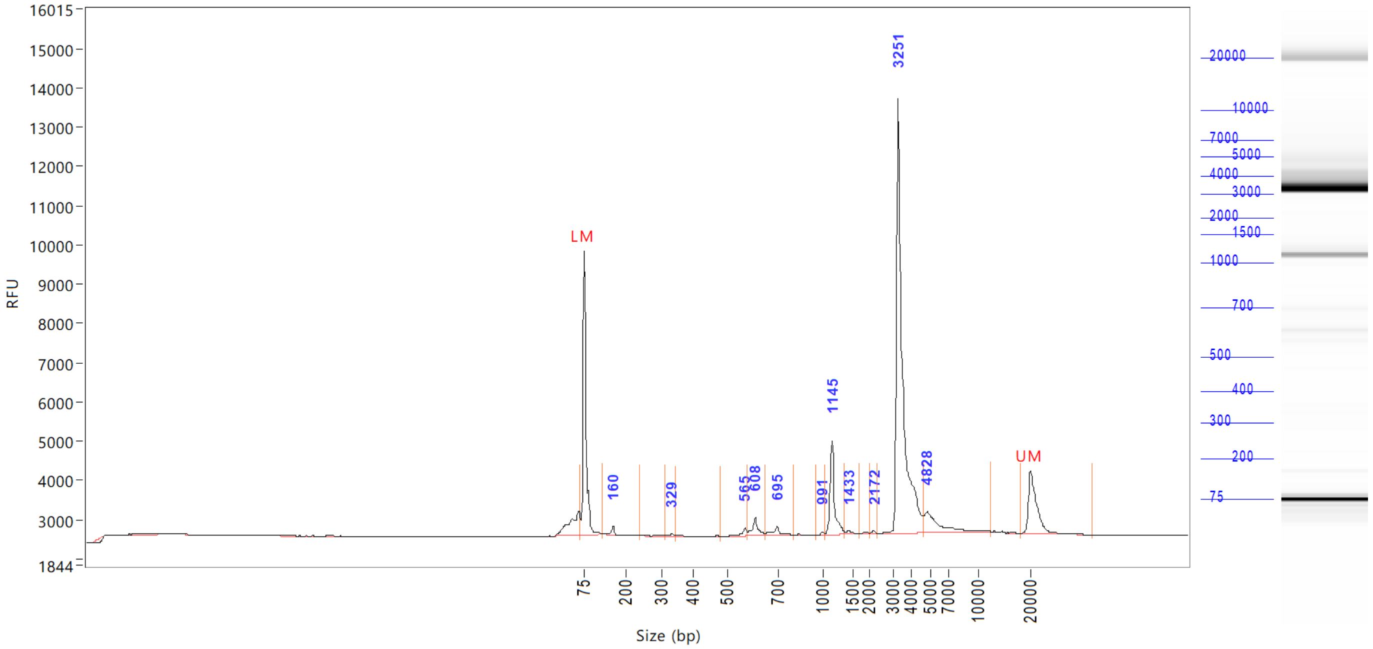

Peak	Size (bp)	Concentration (ng/uL)	From (bp)	To (bp)	Average size (bp)	CV%	RFU	Corrected peak area
1	75 (LM)	0.5000	64	131	75	9.47	7213	48.136
2	160	0.2102	131	237	161	11.68	213	1.687
3	329	0.0684	309	341	326	2.63	57	0.549
4	565	0.2502	479	578	554	3.43	209	2.007
5	608	0.5581	578	646	607	2.64	468	4.478
6	695	0.3607	646	801	690	4.29	226	2.894
7	991	0.0589	948	1023	992	1.28	90	0.473
8	1145	1.9973	1023	1367	1153	5.19	2404	16.023
9	1433	0.1012	1367	1693	1449	4.59	84	0.812
10	2172	0.0436	2000	2315	2110	2.84	72	0.349
11	3251	12.2438	2315	4621	3468	10.11	11092	98.227
12	4828	1.4272	4621	12562	6046	26.93	534	11.450
13	20000 (UM)	0.1691	18171	31607	20693	6.46	1594	16.283

TIC:	17.3195	ng/uL
TIM:	14.9066	nmole/L
Total concentration:	17.4602	ng/uL

```
Sample peak width (sec): 5    Sample min peak height: 50    Sample baseline V to V?: Y    Sample baseline V to V points: 3
Sample filter: Binomial       Number of points for filter: 3  Sample start region (min): 0  Sample end region (min): 30
Marker peak width (sec): 5    Marker min peak height: 200   Marker baseline V to V?: Y    Marker baseline V to V points: 3
Lower marker selection: First peak > 500 RFU                Upper marker selection: Last peak > 200 RFU
Ladder size (bp)75, 200, 300, 400, 500, 700, 1000, 1500, 2000, 3000, 4000, 5000, 7000, 10000, 20000
Quantification using: Lower Marker          Final concentration (ng/uL): 0.5000          Dilution factor: 12.0
```

Sample: SampB2
Well location: B2
Created: Tuesday, August 10, 2021 10:01:13 PM

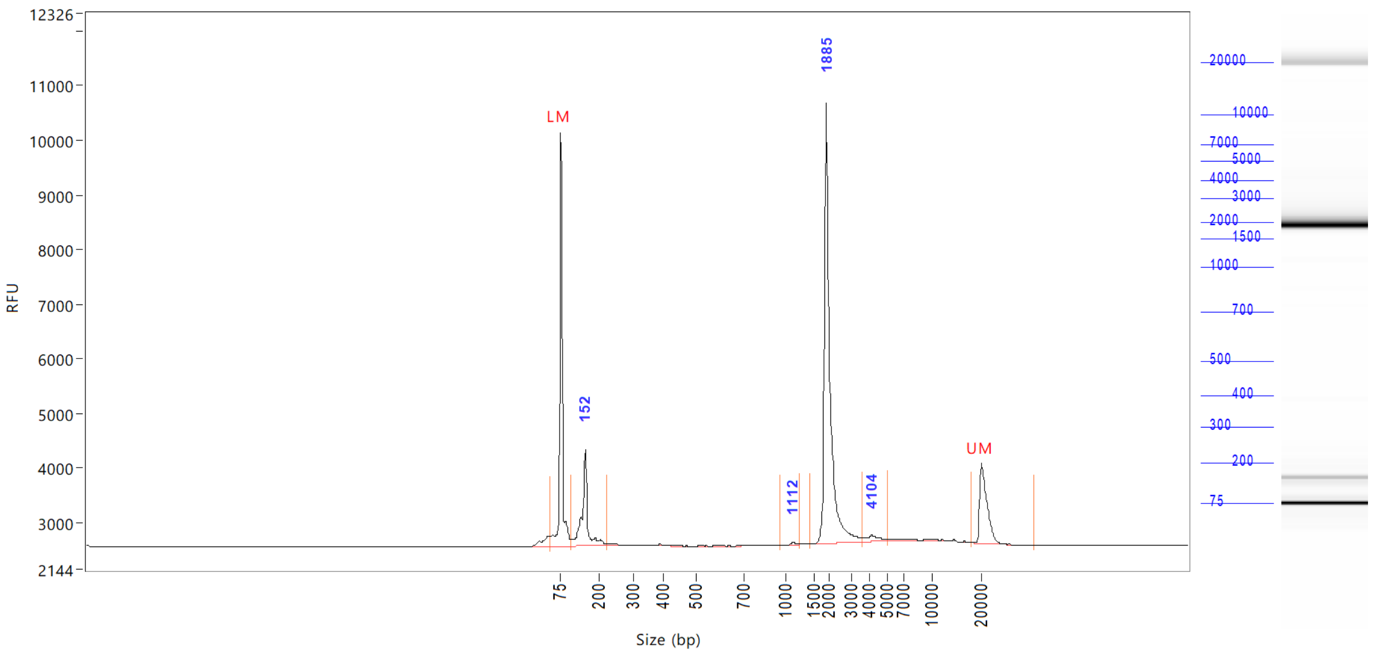

Peak	Size	Concentration	From	To	Average size	CV%	RFU	Corrected peak area
	(bp)	(ng/uL)	(bp)	(bp)	(bp)			
1	75 (LM)	0.5000	45	107	74	10.93	7563	49.639
2	152	2.5064	107	219	150	13.68	1769	20.735
3	1112	0.0458	965	1234	1112	3.96	60	0.379
4	1885	8.4942	1456	3608	1993	13.97	8082	70.273
5	4104	0.2731	3608	5075	4201	8.82	111	2.259
6	20000 (UM)	0.1417	18049	30398	20549	5.03	1466	14.067

TIC:	11.3194	ng/uL
TIM:	34.6523	nmole/L
Total concentration:	11.7139	ng/uL

```
Sample peak width (sec): 5    Sample min peak height: 50    Sample baseline V to V?: Y    Sample baseline V to V points: 3
Sample filter: Binomial       Number of points for filter: 3  Sample start region (min): 0  Sample end region (min): 30
Marker peak width (sec): 5    Marker min peak height: 200   Marker baseline V to V?: Y    Marker baseline V to V points: 3
Lower marker selection: First peak > 500 RFU                Upper marker selection: Last peak > 200 RFU
Ladder size (bp)75, 200, 300, 400, 500, 700, 1000, 1500, 2000, 3000, 4000, 5000, 7000, 10000, 20000
Quantification using: Lower Marker          Final concentration (ng/uL): 0.5000          Dilution factor: 12.0
```

Sample: SampC2
Well location: C2
Created: Tuesday, August 10, 2021 10:01:13 PM

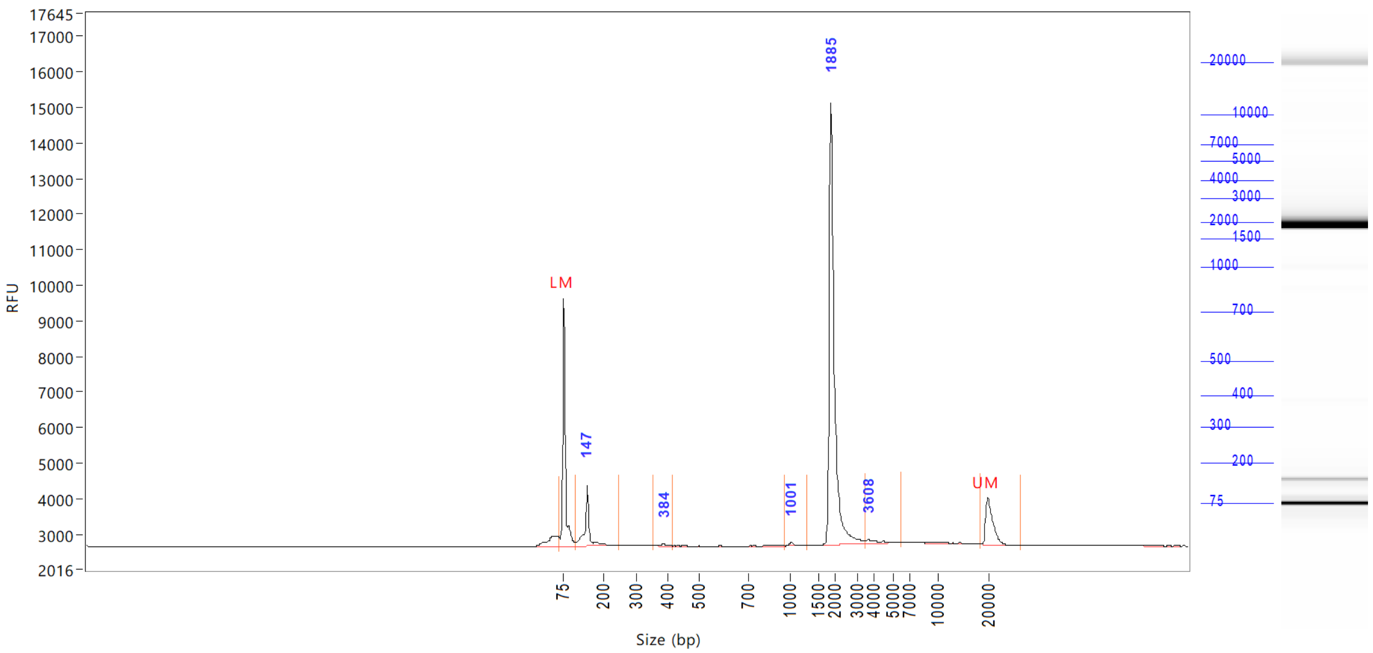

Peak	Size (bp)	Concentration (ng/uL)	From (bp)	To (bp)	Average size (bp)	CV%	RFU	Corrected peak area
1	75 (LM)	0.5000	64	110	76	9.77	6964	45.318
2	147	2.2560	110	244	148	14.43	1708	17.039
3	384	0.0716	352	414	379	3.19	69	0.541
4	1001	0.1141	961	1300	1042	6.48	106	0.862
5	1885	12.6966	1300	3429	1964	11.56	12443	95.897
6	3608	0.2756	3429	6038	4060	12.25	106	2.082
7	20000 (UM)	0.1325	18415	26408	20455	4.10	1315	12.010

TIC:	15.4138	ng/uL
TIM:	36.2958	nmole/L
Total concentration:	15.6804	ng/uL

Sample peak width (sec): 5 Sample min peak height: 50 Sample baseline V to V?: Y Sample baseline V to V points: 3
Sample filter: Binomial Number of points for filter: 3 Sample start region (min): 0 Sample end region (min): 30
Marker peak width (sec): 5 Marker min peak height: 200 Marker baseline V to V?: Y Marker baseline V to V points: 3
Lower marker selection: First peak > 500 RFU Upper marker selection: Last peak > 200 RFU
Ladder size (bp)75, 200, 300, 400, 500, 700, 1000, 1500, 2000, 3000, 4000, 5000, 7000, 10000, 20000
Quantification using: Lower Marker Final concentration (ng/uL): 0.5000 Dilution factor: 12.0

Sample: SampD2
Well location: D2
Created: Tuesday, August 10, 2021 10:01:13 PM

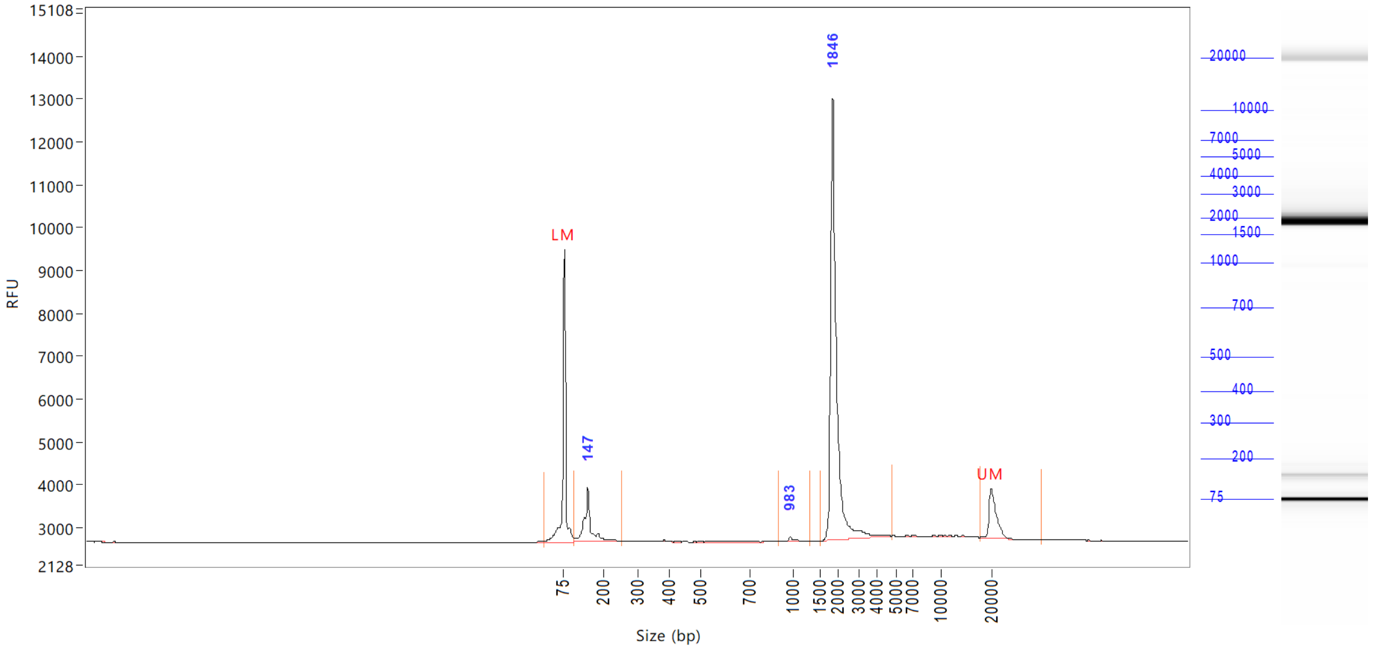

Peak	Size (bp)	Concentration (ng/uL)	From (bp)	To (bp)	Average size (bp)	CV%	RFU	Corrected peak area
1	75 (LM)	0.5000	12	107	71	16.33	6839	47.810
2	147	2.1728	107	251	149	14.65	1259	17.314
3	983	0.1088	905	1300	1015	6.40	108	0.867
4	1846	11.4213	1500	4828	1975	17.41	10319	91.008
5	20000 (UM)	0.1140	17683	29914	20426	5.03	1162	10.904

TIC:	13.7029	ng/uL
TIM:	33.6627	nmole/L
Total concentration:	13.9467	ng/uL

Sample peak width (sec): 5 Sample min peak height: 50 Sample baseline V to V?: Y Sample baseline V to V points: 3
Sample filter: Binomial Number of points for filter: 3 Sample start region (min): 0 Sample end region (min): 30
Marker peak width (sec): 5 Marker min peak height: 200 Marker baseline V to V?: Y Marker baseline V to V points: 3
Lower marker selection: First peak > 500 RFU Upper marker selection: Last peak > 200 RFU
Ladder size (bp)75, 200, 300, 400, 500, 700, 1000, 1500, 2000, 3000, 4000, 5000, 7000, 10000, 20000
Quantification using: Lower Marker Final concentration (ng/uL): 0.5000 Dilution factor: 12.0

Sample: SampE2
Well location: E2
Created: Tuesday, August 10, 2021 10:01:13 PM

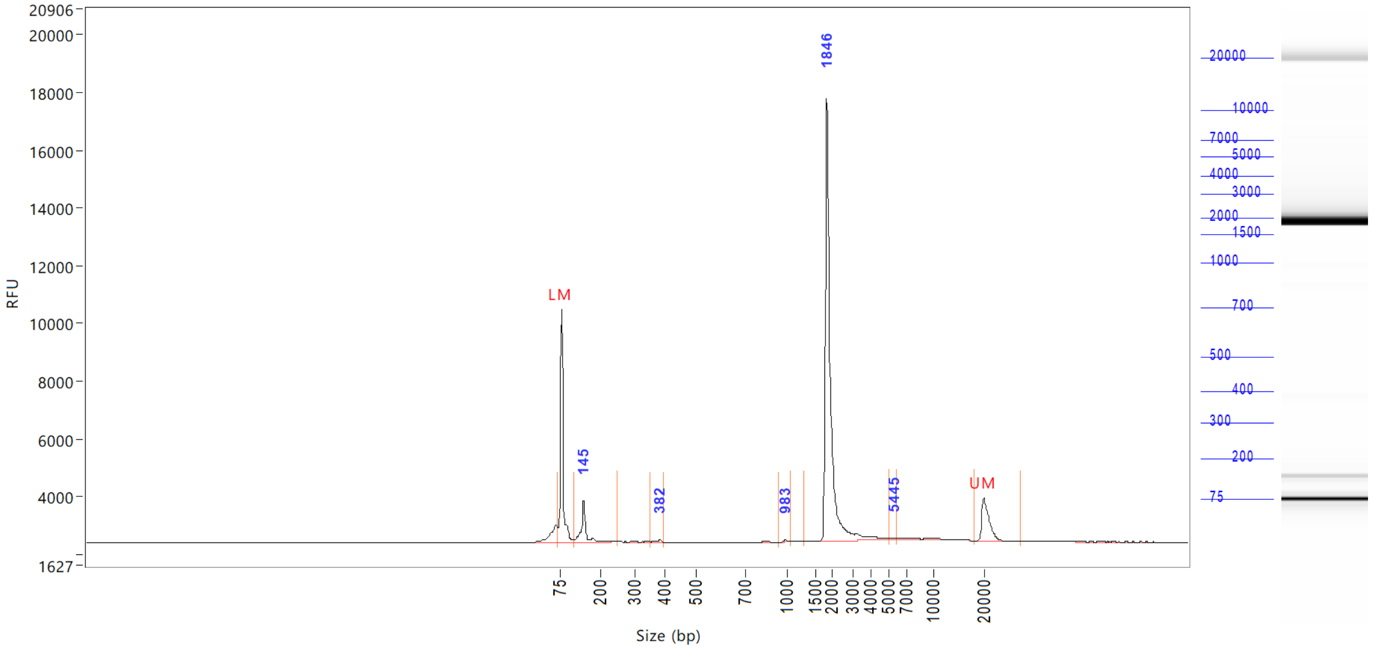

Peak	Size (bp)	Concentration (ng/uL)	From (bp)	To (bp)	Average size (bp)	CV%	RFU	Corrected peak area
1	75 (LM)	0.5000	64	114	76	9.66	8074	55.125
2	145	1.5860	114	248	145	13.10	1419	14.571
3	382	0.0759	354	396	376	2.30	98	0.697
4	983	0.0459	935	1045	984	1.86	70	0.422
5	1846	13.2250	1289	5001	2010	21.91	15363	121.506
6	5445	0.0525	5001	5889	5433	4.96	53	0.482
7	20000 (UM)	0.1255	18049	27254	20435	4.26	1465	13.838

TIC:	14.9853	ng/uL
TIM:	29.2312	nmole/L
Total concentration:	15.3603	ng/uL

Sample peak width (sec): 5   Sample min peak height: 50   Sample baseline V to V?: Y   Sample baseline V to V points: 3
Sample filter: Binomial   Number of points for filter: 3   Sample start region (min): 0   Sample end region (min): 30
Marker peak width (sec): 5   Marker min peak height: 200   Marker baseline V to V?: Y   Marker baseline V to V points: 3
Lower marker selection: First peak > 500 RFU   Upper marker selection: Last peak > 200 RFU
Ladder size (bp)75, 200, 300, 400, 500, 700, 1000, 1500, 2000, 3000, 4000, 5000, 7000, 10000, 20000
Quantification using: Lower Marker   Final concentration (ng/uL): 0.5000   Dilution factor: 12.0

Sample: SampF2
Well location: F2
Created: Tuesday, August 10, 2021 10:01:13 PM

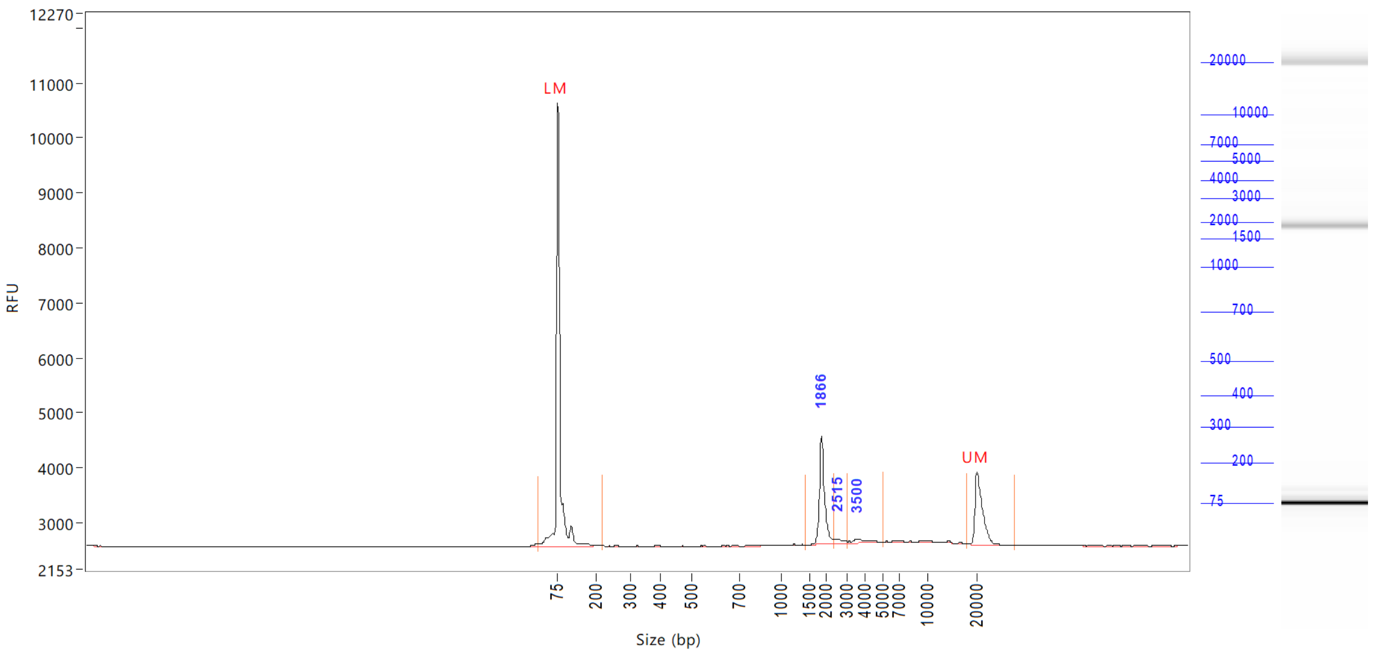

Peak	Size (bp)	Concentration (ng/uL)	From (bp)	To (bp)	Average size (bp)	CV%	RFU	Corrected peak area
1	75 (LM)	0.5000	12	214	78	24.37	8081	66.236
2	1866	1.4614	1433	2372	1892	5.93	1970	16.133
3	2515	0.1063	2372	3001	2635	6.95	92	1.173
4	3500	0.1523	3001	5075	3808	12.43	74	1.682
5	20000 (UM)	0.0978	18049	27738	20567	4.63	1319	12.959

TIC:	1.7200	ng/uL
TIM:	1.4037	nmole/L
Total concentration:	1.9295	ng/uL

Sample peak width (sec): 5 Sample min peak height: 50 Sample baseline V to V?: Y Sample baseline V to V points: 3
Sample filter: Binomial Number of points for filter: 3 Sample start region (min): 0 Sample end region (min): 30
Marker peak width (sec): 5 Marker min peak height: 200 Marker baseline V to V?: Y Marker baseline V to V points: 3
Lower marker selection: First peak > 500 RFU Upper marker selection: Last peak > 200 RFU
Ladder size (bp)75, 200, 300, 400, 500, 700, 1000, 1500, 2000, 3000, 4000, 5000, 7000, 10000, 20000
Quantification using: Lower Marker Final concentration (ng/uL): 0.5000 Dilution factor: 12.0

Sample: SampG2
Well location: G2
Created: Tuesday, August 10, 2021 10:01:13 PM

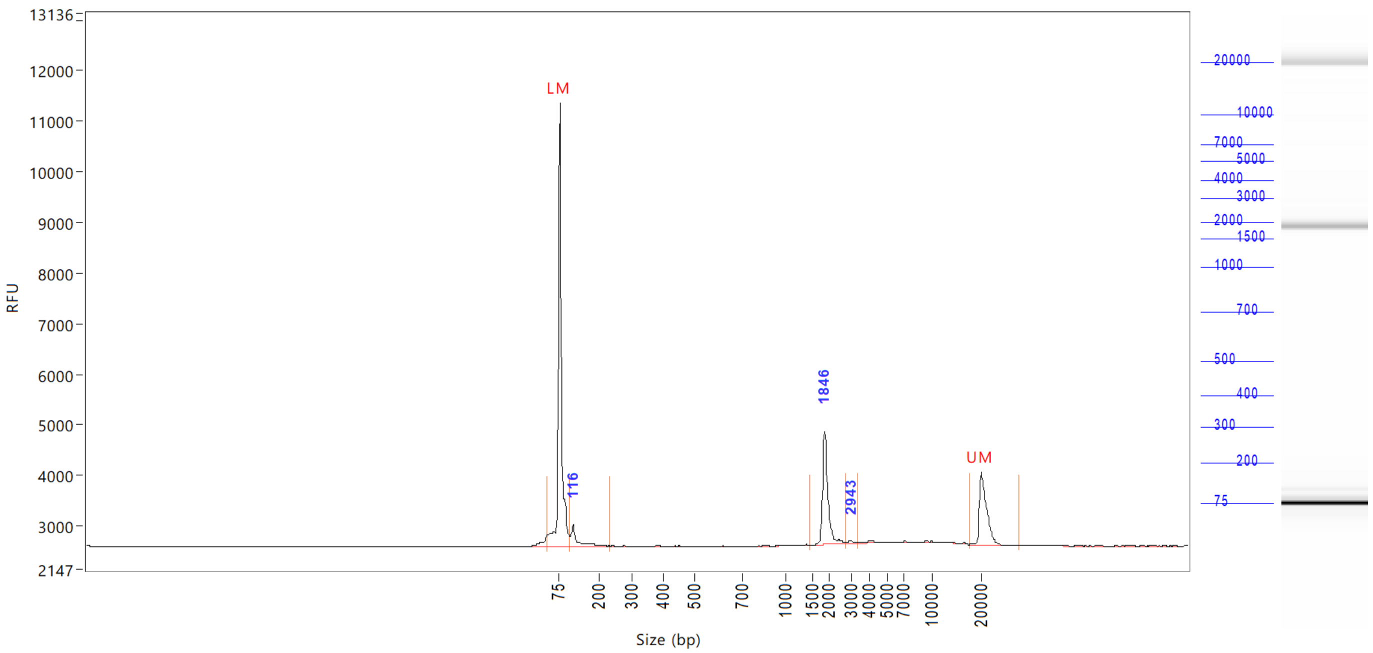

Peak	Size (bp)	Concentration (ng/uL)	From (bp)	To (bp)	Average size (bp)	CV%	RFU	Corrected peak area
1	75 (LM)	0.5000	39	108	74	13.25	8783	68.204
2	116	0.5378	108	232	136	21.19	436	6.113
3	1846	1.6855	1456	2743	1893	8.17	2241	19.159
4	2943	0.0518	2743	3358	2989	5.34	55	0.588
5	20000 (UM)	0.0989	17561	27617	20443	4.41	1425	13.492

TIC:	2.2750	ng/uL
TIM:	7.9916	nmole/L
Total concentration:	2.5385	ng/uL

Sample peak width (sec): 5 Sample min peak height: 50 Sample baseline V to V?: Y Sample baseline V to V points: 3
Sample filter: Binomial Number of points for filter: 3 Sample start region (min): 0 Sample end region (min): 30
Marker peak width (sec): 5 Marker min peak height: 200 Marker baseline V to V?: Y Marker baseline V to V points: 3
Lower marker selection: First peak > 500 RFU Upper marker selection: Last peak > 200 RFU
Ladder size (bp)75, 200, 300, 400, 500, 700, 1000, 1500, 2000, 3000, 4000, 5000, 7000, 10000, 20000
Quantification using: Lower Marker Final concentration (ng/uL): 0.5000 Dilution factor: 12.0

Sample: SampH2
Well location: H2
Created: Tuesday, August 10, 2021 10:01:13 PM

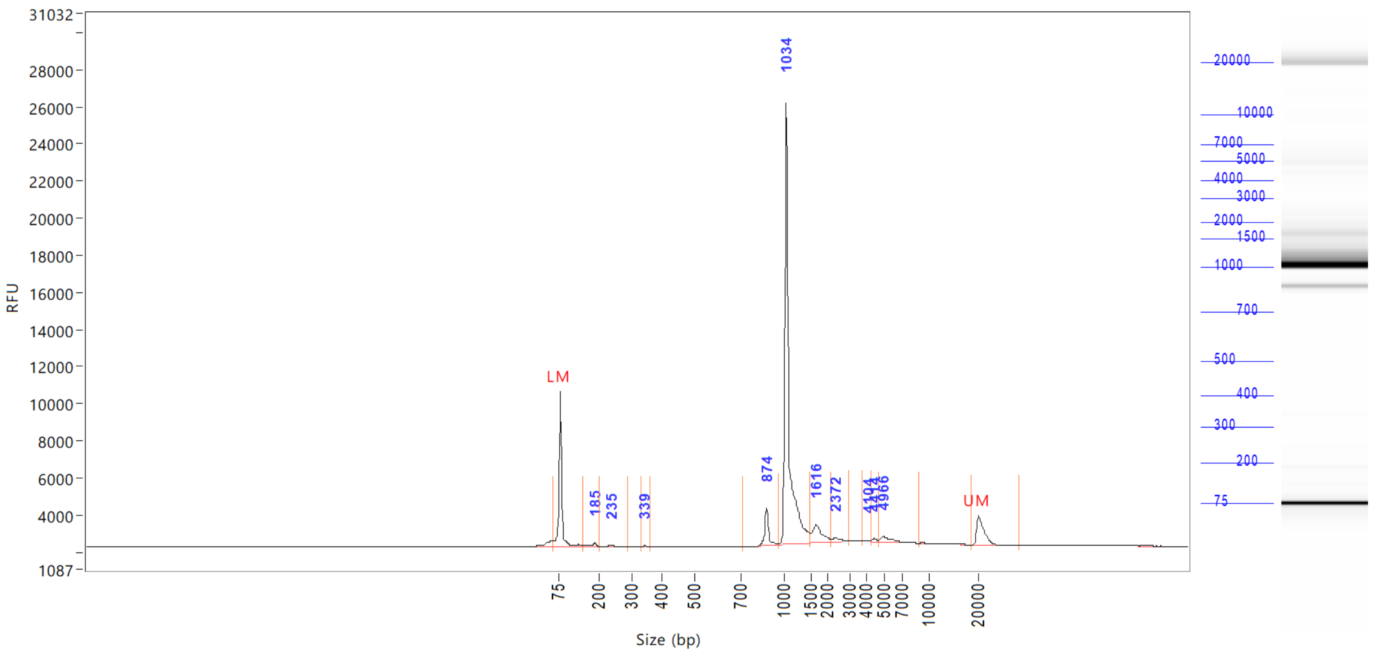

Peak	Size (bp)	Concentration (ng/uL)	From (bp)	To (bp)	Average size (bp)	CV%	RFU	Corrected peak area
1	75 (LM)	0.5000	56	147	76	15.94	8395	57.522
2	185	0.2963	147	201	173	7.58	201	2.841
3	235	0.1142	201	286	231	7.60	64	1.095
4	339	0.0250	329	356	338	0.98	52	0.240
5	874	1.3323	710	961	872	2.65	2029	12.772
6	1034	17.4311	961	1478	1080	9.66	23812	167.110
7	1616	1.5689	1478	2144	1697	9.73	990	15.041
8	2372	0.2842	2144	2971	2417	8.00	190	2.725
9	4104	0.0696	3750	4208	4012	3.18	75	0.668
10	4414	0.1200	4208	4621	4409	2.60	169	1.151
11	4966	0.5005	4621	8867	5506	14.82	301	4.798
12	20000 (UM)	0.1332	18537	28221	20587	4.84	1561	15.325

TIC:	21.7421	ng/uL
TIM:	34.7693	nmole/L
Total concentration:	21.8388	ng/uL

Sample peak width (sec): 5 Sample min peak height: 50 Sample baseline V to V?: Y Sample baseline V to V points: 3
Sample filter: Binomial Number of points for filter: 3 Sample start region (min): 0 Sample end region (min): 30
Marker peak width (sec): 5 Marker min peak height: 200 Marker baseline V to V?: Y Marker baseline V to V points: 3
Lower marker selection: First peak > 500 RFU Upper marker selection: Last peak > 200 RFU
Ladder size (bp)75, 200, 300, 400, 500, 700, 1000, 1500, 2000, 3000, 4000, 5000, 7000, 10000, 20000
Quantification using: Lower Marker Final concentration (ng/uL): 0.5000 Dilution factor: 12.0

Sample: SampA3
Well location: A3
Created: Tuesday, August 10, 2021 10:01:13 PM

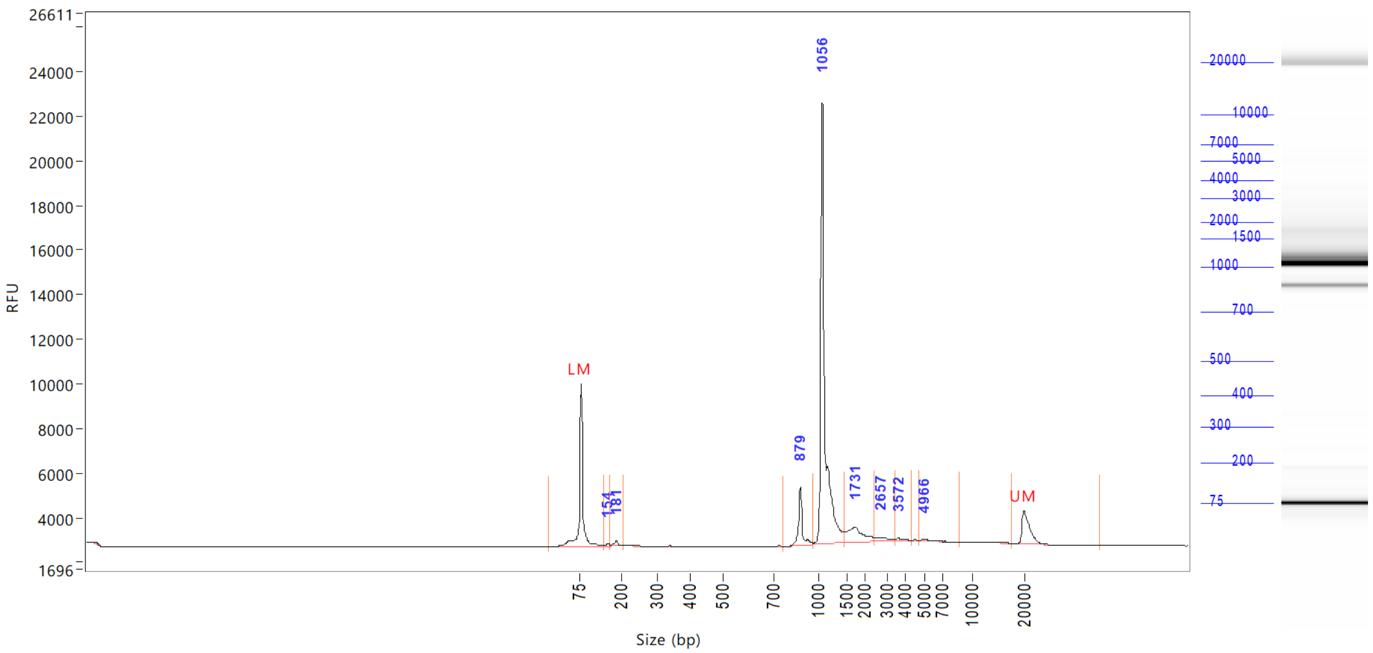

Peak	Size (bp)	Concentration (ng/uL)	From (bp)	To (bp)	Average size (bp)	CV%	RFU	Corrected peak area
1	75 (LM)	0.5000	0	147	72	23.30	7317	57.779
2	154	0.0826	147	162	154	3.01	87	0.796
3	181	0.2097	162	201	176	3.86	252	2.019
4	879	1.6763	762	965	878	2.67	2603	16.143
5	1056	14.1010	965	1456	1097	8.23	19793	135.792
6	1731	1.7044	1456	2458	1777	14.13	684	16.414
7	2657	0.3289	2458	3393	2830	9.15	181	3.168
8	3572	0.1212	3393	4277	3678	5.54	106	1.167
9	4966	0.0971	4690	8667	5645	15.85	55	0.935
10	20000 (UM)	0.1211	17683	34388	20497	6.08	1470	14.000

TIC:	18.3213	ng/uL
TIM:	28.9931	nmole/L
Total concentration:	18.5214	ng/uL

Sample peak width (sec): 5   Sample min peak height: 50   Sample baseline V to V?: Y   Sample baseline V to V points: 3
Sample filter: Binomial   Number of points for filter: 3 Sample start region (min): 0 Sample end region (min): 30
Marker peak width (sec): 5   Marker min peak height: 200   Marker baseline V to V?: Y   Marker baseline V to V points: 3
Lower marker selection: First peak > 500 RFU   Upper marker selection: Last peak > 200 RFU
Ladder size (bp)75, 200, 300, 400, 500, 700, 1000, 1500, 2000, 3000, 4000, 5000, 7000, 10000, 20000
Quantification using: Lower Marker   Final concentration (ng/uL): 0.5000   Dilution factor: 12.0

Sample: SampB3
Well location: B3
Created: Tuesday, August 10, 2021 10:01:13 PM

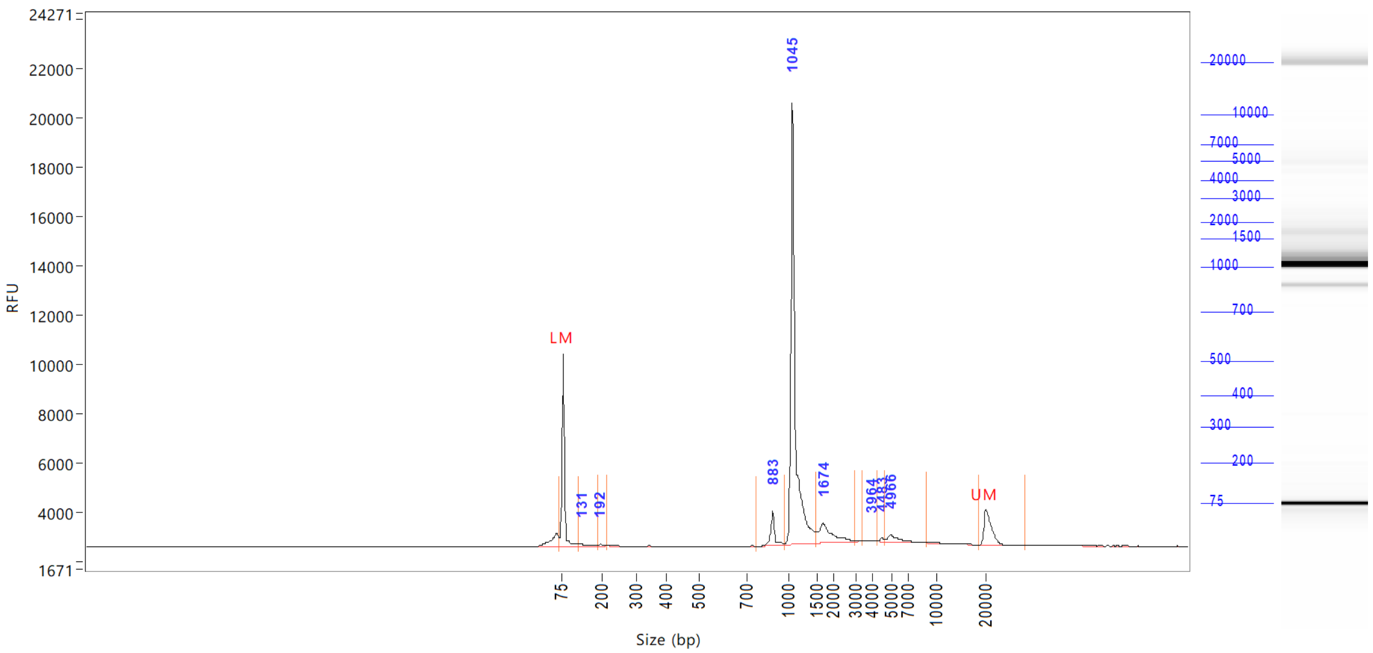

Peak	Size (bp)	Concentration (ng/uL)	From (bp)	To (bp)	Average size (bp)	CV%	RFU	Corrected peak area
1	75 (LM)	0.5000	64	122	75	10.40	7831	48.126
2	131	0.2815	122	185	148	12.59	102	2.258
3	192	0.0721	185	214	192	2.60	94	0.578
4	883	1.1703	762	965	879	2.83	1380	9.387
5	1045	15.9373	965	1478	1091	9.37	17978	127.833
6	1674	2.0929	1478	2971	1843	17.45	793	16.787
7	3964	0.0931	3393	4242	3814	5.96	50	0.746
8	4483	0.1108	4242	4690	4479	2.61	137	0.889
9	4966	0.5486	4690	9000	5600	15.49	282	4.400
10	20000 (UM)	0.1422	18537	27979	20528	4.25	1446	13.685

TIC:	20.3065	ng/uL
TIM:	32.0905	nmole/L
Total concentration:	20.5318	ng/uL

Sample peak width (sec): 5 Sample min peak height: 50 Sample baseline V to V?: Y Sample baseline V to V points: 3
Sample filter: Binomial Number of points for filter: 3 Sample start region (min): 0 Sample end region (min): 30
Marker peak width (sec): 5 Marker min peak height: 200 Marker baseline V to V?: Y Marker baseline V to V points: 3
Lower marker selection: First peak > 500 RFU Upper marker selection: Last peak > 200 RFU
Ladder size (bp)75, 200, 300, 400, 500, 700, 1000, 1500, 2000, 3000, 4000, 5000, 7000, 10000, 20000
Quantification using: Lower Marker Final concentration (ng/uL): 0.5000 Dilution factor: 12.0

Sample: SampC3
Well location: C3
Created: Tuesday, August 10, 2021 10:01:13 PM

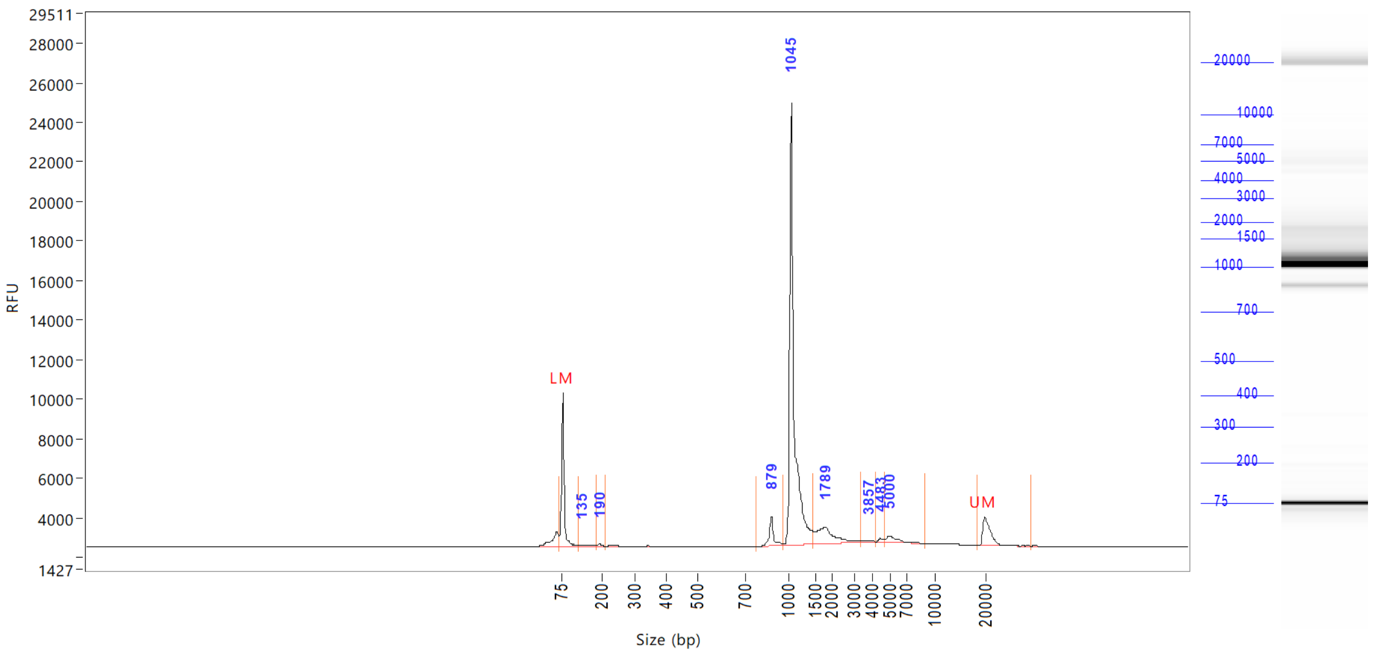

Peak	Size (bp)	Concentration (ng/uL)	From (bp)	To (bp)	Average size (bp)	CV%	RFU	Corrected peak area
1	75 (LM)	0.5000	64	124	75	10.25	7785	50.586
2	135	0.2504	124	183	151	11.54	74	2.111
3	190	0.0872	183	208	189	2.01	129	0.735
4	879	1.3063	770	970	879	3.18	1502	11.013
5	1045	19.7900	970	1456	1090	8.89	22369	166.848
6	1789	2.7355	1456	3322	1872	20.48	857	23.063
7	3857	0.1126	3322	4208	3712	6.23	60	0.949
8	4483	0.1426	4208	4690	4482	2.60	186	1.202
9	5000	0.6131	4690	8934	5537	14.64	325	5.169
10	20000 (UM)	0.1354	18537	29430	20541	4.62	1445	13.697

TIC:	25.0375	ng/uL
TIM:	38.5033	nmole/L
Total concentration:	25.1820	ng/uL

```
Sample peak width (sec): 5     Sample min peak height: 50     Sample baseline V to V?: Y     Sample baseline V to V points: 3
Sample filter: Binomial        Number of points for filter: 3  Sample start region (min): 0  Sample end region (min): 30
Marker peak width (sec): 5     Marker min peak height: 200    Marker baseline V to V?: Y     Marker baseline V to V points: 3
Lower marker selection: First peak > 500 RFU                  Upper marker selection: Last peak > 200 RFU
Ladder size (bp)75, 200, 300, 400, 500, 700, 1000, 1500, 2000, 3000, 4000, 5000, 7000, 10000, 20000
Quantification using: Lower Marker          Final concentration (ng/uL): 0.5000          Dilution factor: 12.0
```

Sample: SampD3
Well location: D3
Created: Tuesday, August 10, 2021 10:01:13 PM

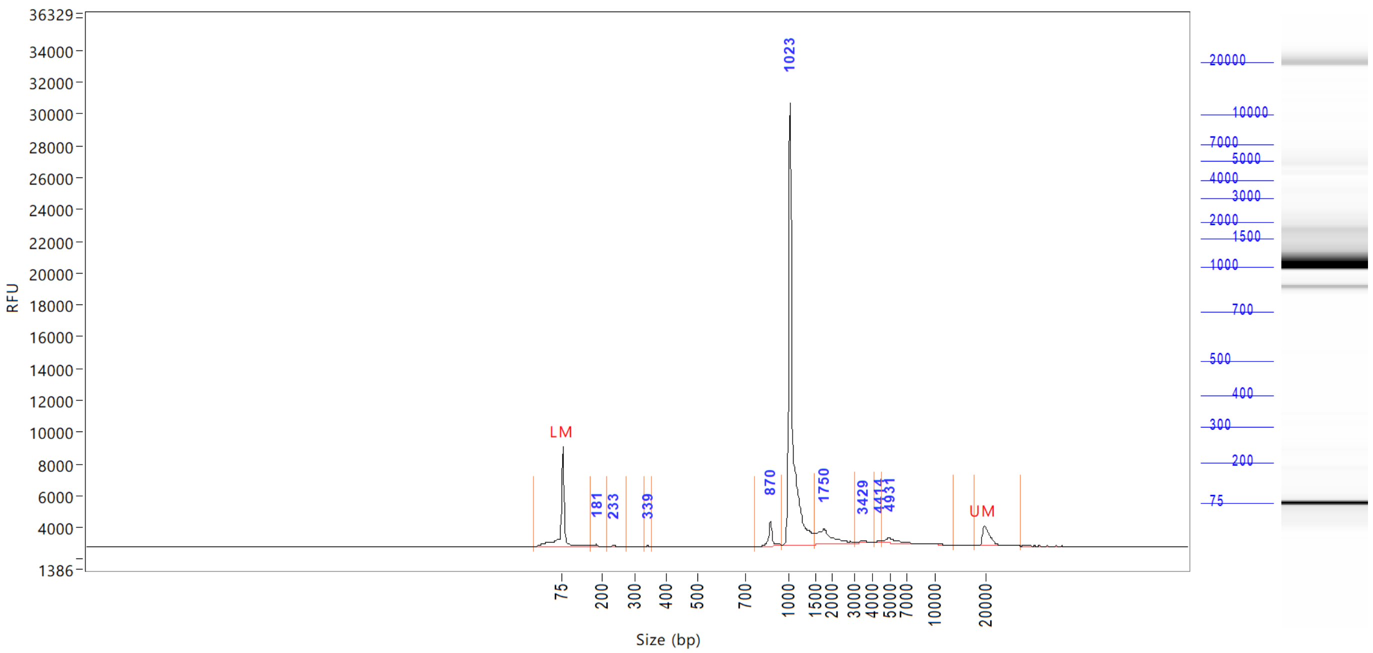

Peak	Size (bp)	Concentration (ng/uL)	From (bp)	To (bp)	Average size (bp)	CV%	RFU	Corrected peak area
1	75 (LM)	0.5000	0	164	69	35.74	6277	55.445
2	181	0.1955	164	214	182	6.94	141	1.806
3	233	0.1152	214	273	234	5.84	80	1.065
4	339	0.0268	327	352	337	1.07	55	0.247
5	870	1.1128	757	957	870	2.99	1514	10.283
6	1023	21.1251	957	1467	1071	9.39	27856	195.214
7	1750	2.4655	1467	3000	1846	18.52	935	22.783
8	3429	0.2238	3000	4139	3441	8.07	142	2.068
9	4414	0.1005	4139	4587	4404	2.36	157	0.929
10	4931	0.5958	4587	13781	5753	26.07	315	5.505
11	20000 (UM)	0.1050	17927	27254	20465	4.33	1247	11.641

TIC:	25.9609	ng/uL
TIM:	39.7909	nmole/L
Total concentration:	25.9995	ng/uL

```
Sample peak width (sec): 5     Sample min peak height: 50     Sample baseline V to V?: Y     Sample baseline V to V points: 3
Sample filter: Binomial        Number of points for filter: 3  Sample start region (min): 0  Sample end region (min): 30
Marker peak width (sec): 5     Marker min peak height: 200    Marker baseline V to V?: Y     Marker baseline V to V points: 3
Lower marker selection: First peak > 500 RFU                  Upper marker selection: Last peak > 200 RFU
Ladder size (bp)75, 200, 300, 400, 500, 700, 1000, 1500, 2000, 3000, 4000, 5000, 7000, 10000, 20000
Quantification using: Lower Marker          Final concentration (ng/uL): 0.5000          Dilution factor: 12.0
```

Sample: SampE3
Well location: E3
Created: Tuesday, August 10, 2021 10:01:13 PM

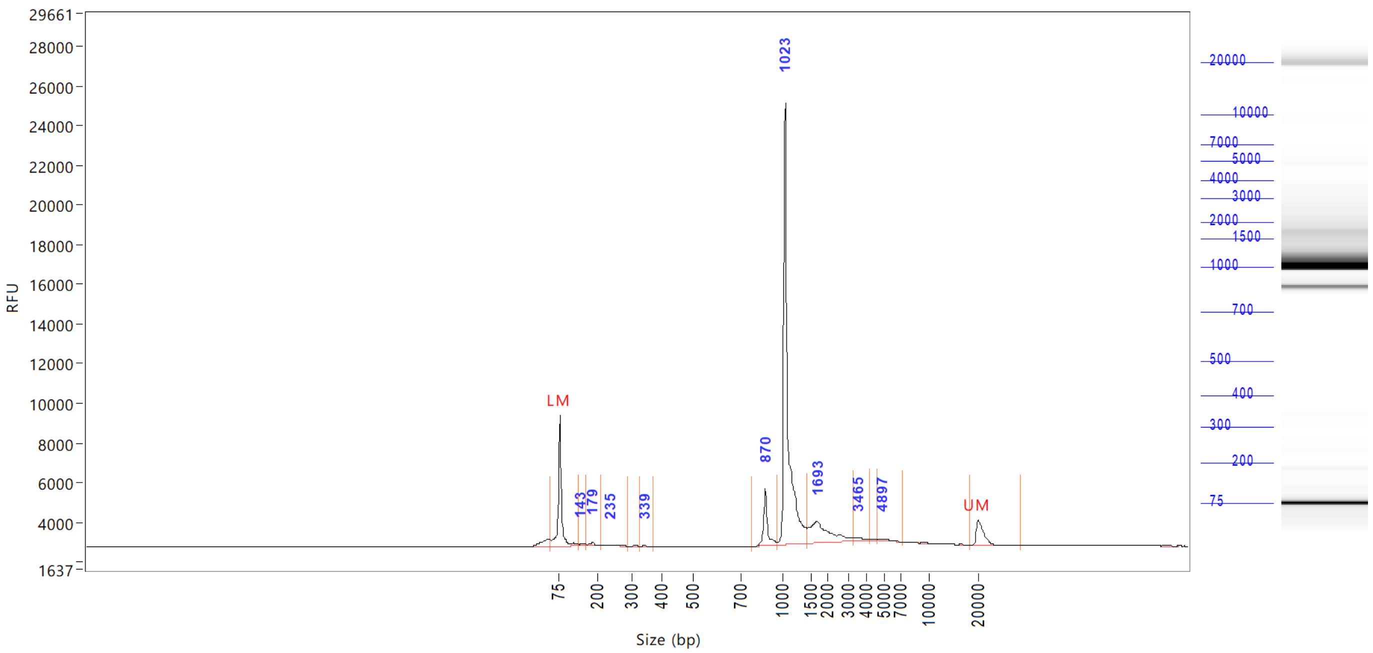

Peak	Size (bp)	Concentration (ng/uL)	From (bp)	To (bp)	Average size (bp)	CV%	RFU	Corrected peak area
1	75 (LM)	0.5000	48	139	76	16.97	6625	51.355
2	143	0.1281	139	162	150	4.71	73	1.097
3	179	0.2264	162	205	176	4.18	209	1.938
4	235	0.0872	205	286	237	7.05	52	0.746
5	339	0.0308	327	368	337	1.42	53	0.264
6	870	2.2091	775	957	872	2.88	2866	18.908
7	1023	19.1495	957	1411	1065	8.96	22299	163.902
8	1693	3.9713	1411	3251	1892	23.25	1076	33.991
9	3465	0.2445	3251	4173	3585	6.39	144	2.092
10	4897	0.1280	4621	7001	5228	9.27	87	1.096
11	20000 (UM)	0.1163	18415	28342	20448	4.57	1258	11.949

TIC:	26.1750	ng/uL
TIM:	41.6473	nmole/L
Total concentration:	26.2389	ng/uL

```
Sample peak width (sec): 5    Sample min peak height: 50    Sample baseline V to V?: Y    Sample baseline V to V points: 3
Sample filter: Binomial       Number of points for filter: 3  Sample start region (min): 0  Sample end region (min): 30
Marker peak width (sec): 5    Marker min peak height: 200   Marker baseline V to V?: Y    Marker baseline V to V points: 3
Lower marker selection: First peak > 500 RFU                Upper marker selection: Last peak > 200 RFU
Ladder size (bp)75, 200, 300, 400, 500, 700, 1000, 1500, 2000, 3000, 4000, 5000, 7000, 10000, 20000
Quantification using: Lower Marker          Final concentration (ng/uL): 0.5000          Dilution factor: 12.0
```

Sample: SampF3
Well location: F3
Created: Tuesday, August 10, 2021 10:01:13 PM

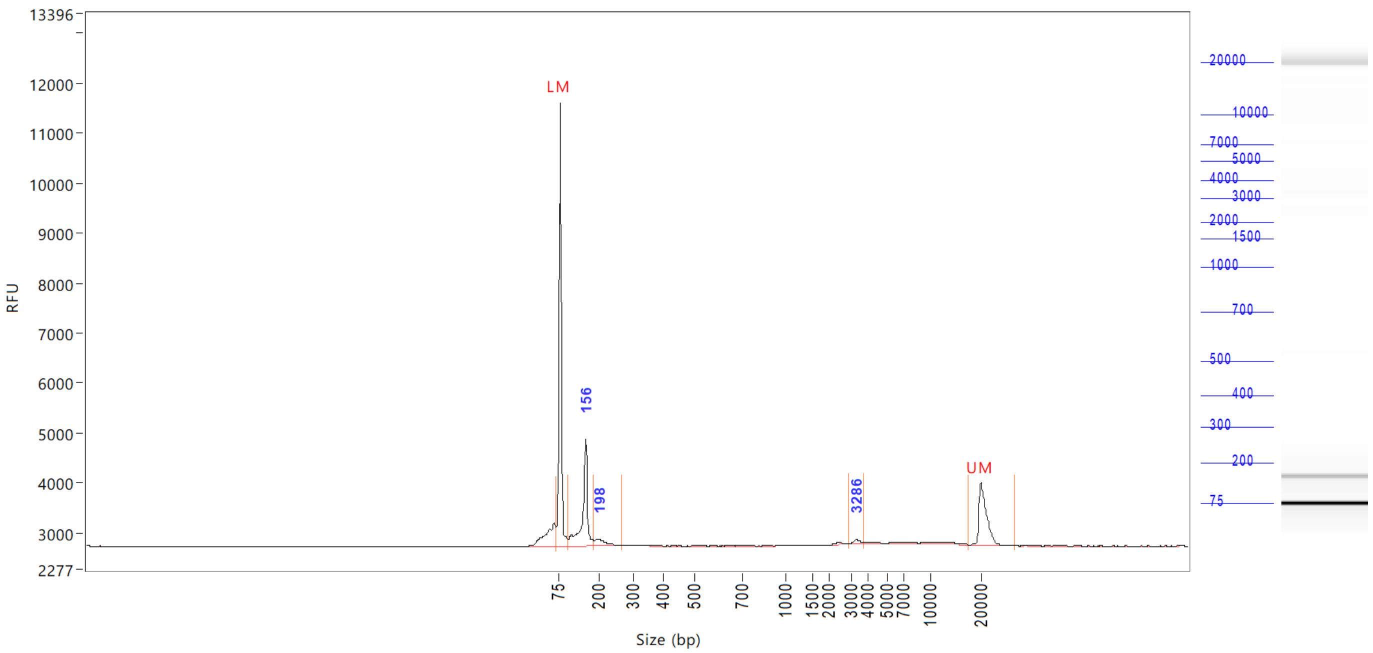

Peak	Size (bp)	Concentration (ng/uL)	From (bp)	To (bp)	Average size (bp)	CV%	RFU	Corrected peak area
1	75 (LM)	0.5000	64	99	73	6.44	8882	51.673
2	156	2.6706	99	181	145	12.00	2127	23.000
3	198	0.4024	181	268	205	8.72	128	3.466
4	3286	0.1041	2943	3715	3326	5.77	90	0.896
5	20000 (UM)	0.1188	17561	26649	20501	4.50	1259	12.280

TIC:	3.1771	ng/uL
TIM:	33.5479	nmole/L
Total concentration:	3.6561	ng/uL

Sample peak width (sec): 5 Sample min peak height: 50 Sample baseline V to V?: Y Sample baseline V to V points: 3
Sample filter: Binomial Number of points for filter: 3 Sample start region (min): 0 Sample end region (min): 30
Marker peak width (sec): 5 Marker min peak height: 200 Marker baseline V to V?: Y Marker baseline V to V points: 3
Lower marker selection: First peak > 500 RFU Upper marker selection: Last peak > 200 RFU
Ladder size (bp)75, 200, 300, 400, 500, 700, 1000, 1500, 2000, 3000, 4000, 5000, 7000, 10000, 20000
Quantification using: Lower Marker Final concentration (ng/uL): 0.5000 Dilution factor: 12.0

Sample: SampG3
Well location: G3
Created: Tuesday, August 10, 2021 10:01:13 PM

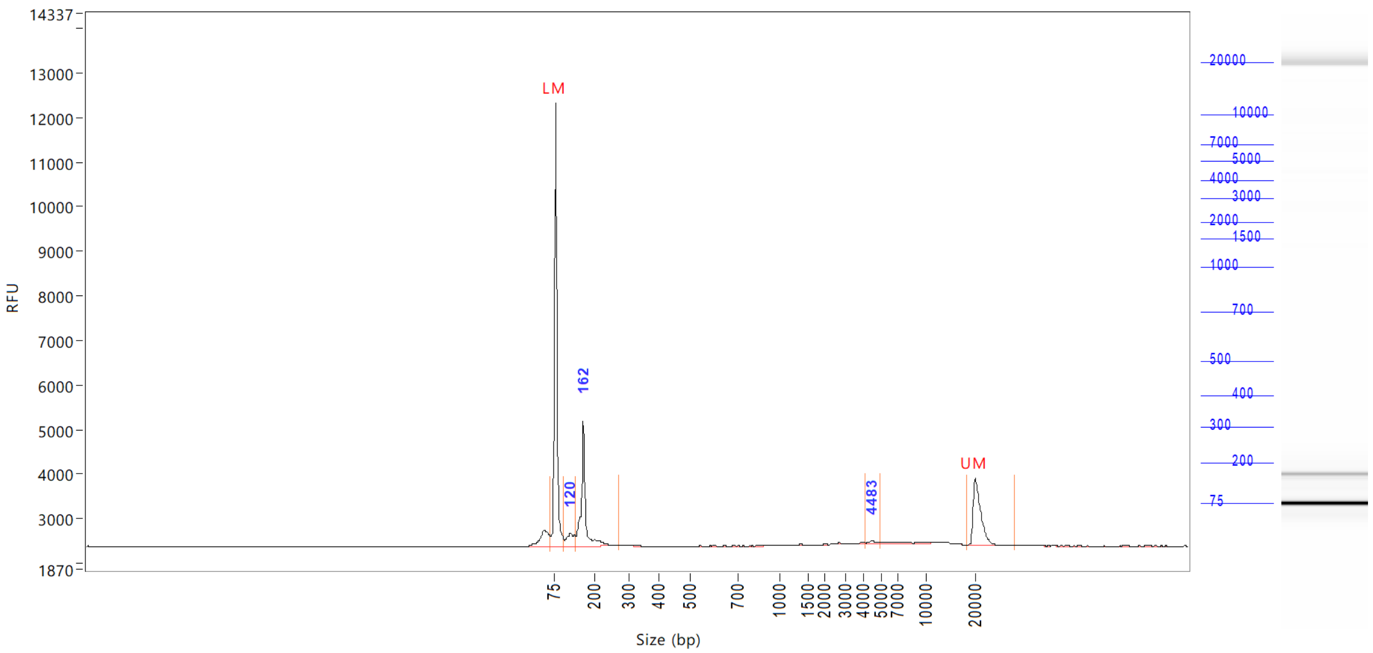

Peak	Size (bp)	Concentration (ng/uL)	From (bp)	To (bp)	Average size (bp)	CV%	RFU	Corrected peak area
1	75 (LM)	0.5000	58	101	74	7.39	9961	64.464
2	120	0.5037	101	137	120	8.63	293	5.412
3	162	2.5420	137	271	165	11.91	2804	27.311
4	4483	0.0441	4139	4931	4508	3.96	58	0.473
5	20000 (UM)	0.1144	18415	27859	20486	4.70	1503	14.746

TIC:	3.0898	ng/uL
TIM:	32.2363	nmole/L
Total concentration:	3.3795	ng/uL

Sample peak width (sec): 5   Sample min peak height: 50   Sample baseline V to V?: Y   Sample baseline V to V points: 3
Sample filter: Binomial   Number of points for filter: 3 Sample start region (min): 0 Sample end region (min): 30
Marker peak width (sec): 5   Marker min peak height: 200   Marker baseline V to V?: Y   Marker baseline V to V points: 3
Lower marker selection: First peak > 500 RFU   Upper marker selection: Last peak > 200 RFU
Ladder size (bp)75, 200, 300, 400, 500, 700, 1000, 1500, 2000, 3000, 4000, 5000, 7000, 10000, 20000
Quantification using: Lower Marker   Final concentration (ng/uL): 0.5000   Dilution factor: 12.0

Sample: SampH3
Well location: H3
Created: Tuesday, August 10, 2021 10:01:13 PM

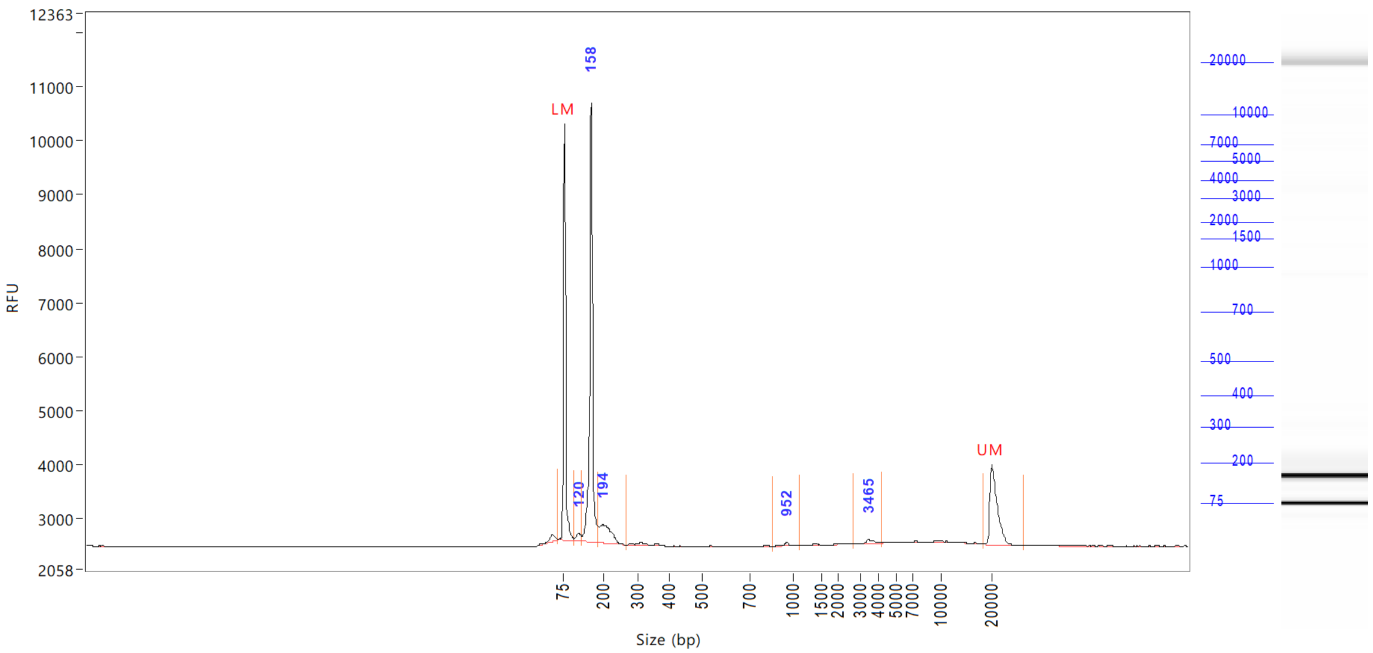

Peak	Size (bp)	Concentration (ng/uL)	From (bp)	To (bp)	Average size (bp)	CV%	RFU	Corrected peak area
1	75 (LM)	0.5000	56	105	74	6.38	7726	43.313
2	120	0.2455	105	129	118	5.74	144	1.772
3	158	7.9644	129	181	155	4.69	8152	57.495
4	194	1.2668	181	262	203	7.09	331	9.145
5	952	0.0762	861	1112	955	3.34	74	0.550
6	3465	0.1249	2715	4208	3566	8.32	68	0.902
7	20000 (UM)	0.1633	18171	26287	20456	4.25	1480	14.147

TIC:	9.6779	ng/uL
TIM:	98.3220	nmole/L
Total concentration:	10.1237	ng/uL

Sample peak width (sec): 5 Sample min peak height: 50 Sample baseline V to V?: Y Sample baseline V to V points: 3
Sample filter: Binomial Number of points for filter: 3 Sample start region (min): 0 Sample end region (min): 30
Marker peak width (sec): 5 Marker min peak height: 200 Marker baseline V to V?: Y Marker baseline V to V points: 3
Lower marker selection: First peak > 500 RFU Upper marker selection: Last peak > 200 RFU
Ladder size (bp)75, 200, 300, 400, 500, 700, 1000, 1500, 2000, 3000, 4000, 5000, 7000, 10000, 20000
Quantification using: Lower Marker Final concentration (ng/uL): 0.5000 Dilution factor: 12.0

Sample: SampA4
Well location: A4
Created: Tuesday, August 10, 2021 10:01:13 PM

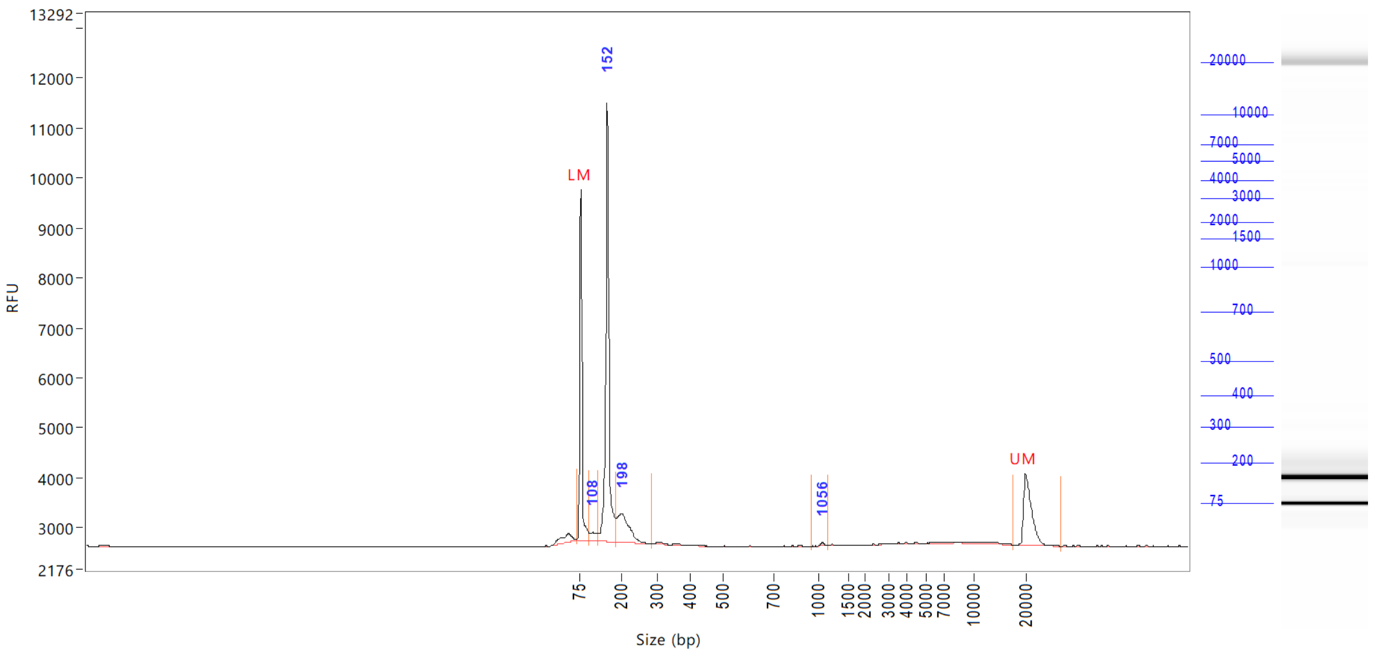

Peak	Size (bp)	Concentration (ng/uL)	From (bp)	To (bp)	Average size (bp)	CV%	RFU	Corrected peak area
1	75 (LM)	0.5000	64	101	74	7.49	7026	39.307
2	108	0.3991	101	126	113	6.72	166	2.615
3	152	9.9741	126	179	152	5.33	8786	65.343
4	198	2.3516	179	282	203	8.41	564	15.406
5	1056	0.0672	948	1145	1051	3.64	71	0.440
6	20000 (UM)	0.1767	17683	26770	20519	4.49	1430	13.889

TIC:	12.7921	ng/uL
TIM:	132.8030	nmole/L
Total concentration:	13.2934	ng/uL

Sample peak width (sec): 5 Sample min peak height: 50 Sample baseline V to V?: Y Sample baseline V to V points: 3
Sample filter: Binomial Number of points for filter: 3 Sample start region (min): 0 Sample end region (min): 30
Marker peak width (sec): 5 Marker min peak height: 200 Marker baseline V to V?: Y Marker baseline V to V points: 3
Lower marker selection: First peak > 500 RFU Upper marker selection: Last peak > 200 RFU
Ladder size (bp)75, 200, 300, 400, 500, 700, 1000, 1500, 2000, 3000, 4000, 5000, 7000, 10000, 20000
Quantification using: Lower Marker Final concentration (ng/uL): 0.5000 Dilution factor: 12.0

Sample: SampB4
Well location: B4
Created: Tuesday, August 10, 2021 10:01:13 PM

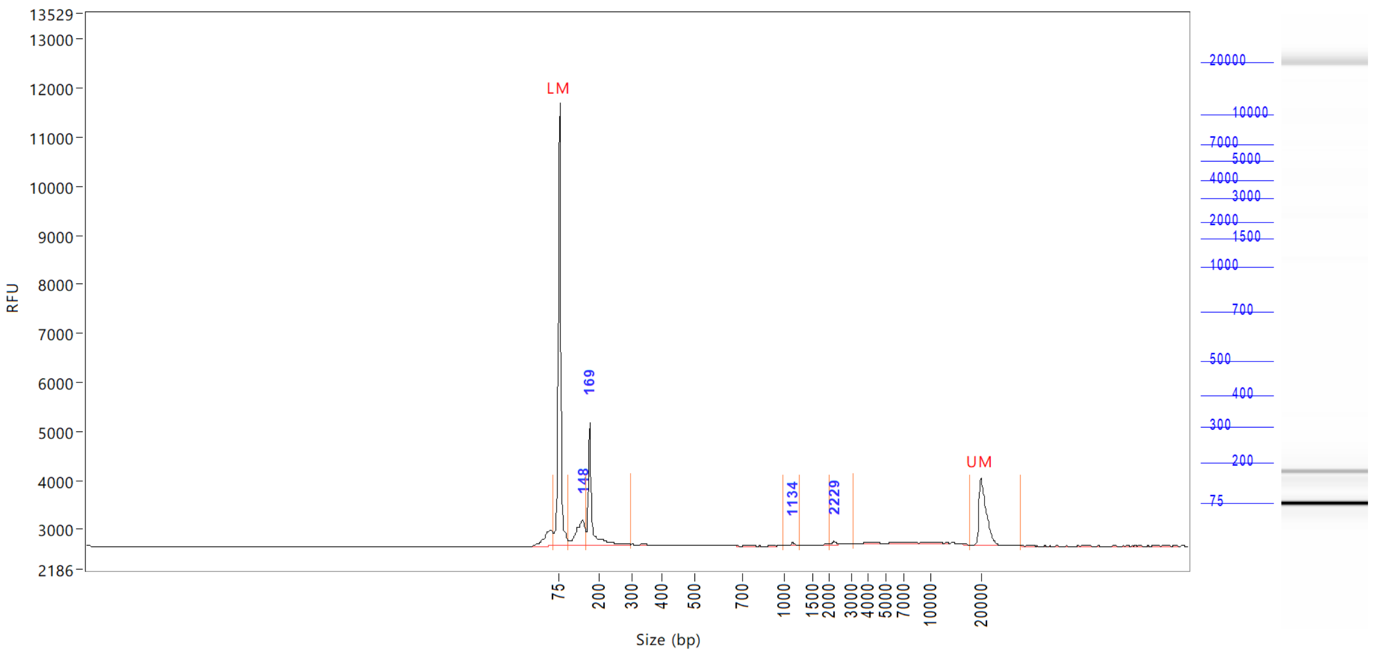

Peak	Size (bp)	Concentration (ng/uL)	From (bp)	To (bp)	Average size (bp)	CV%	RFU	Corrected peak area
1	75 (LM)	0.5000	56	105	73	7.98	9051	59.352
2	148	1.0977	105	160	138	10.00	521	10.858
3	169	2.0287	160	296	177	10.95	2497	20.068
4	1134	0.0382	987	1278	1140	4.39	56	0.377
5	2229	0.0693	2001	3144	2352	10.67	63	0.686
6	20000 (UM)	0.1096	17927	27979	20483	4.40	1366	13.009

TIC:	3.2339	ng/uL
TIM:	32.0170	nmole/L
Total concentration:	3.4717	ng/uL

Sample peak width (sec): 5   Sample min peak height: 50   Sample baseline V to V?: Y   Sample baseline V to V points: 3
Sample filter: Binomial   Number of points for filter: 3 Sample start region (min): 0 Sample end region (min): 30
Marker peak width (sec): 5   Marker min peak height: 200   Marker baseline V to V?: Y   Marker baseline V to V points: 3
Lower marker selection: First peak > 500 RFU   Upper marker selection: Last peak > 200 RFU
Ladder size (bp)75, 200, 300, 400, 500, 700, 1000, 1500, 2000, 3000, 4000, 5000, 7000, 10000, 20000
Quantification using: Lower Marker   Final concentration (ng/uL): 0.5000   Dilution factor: 12.0

Sample: SampC4
Well location: C4
Created: Tuesday, August 10, 2021 10:01:13 PM

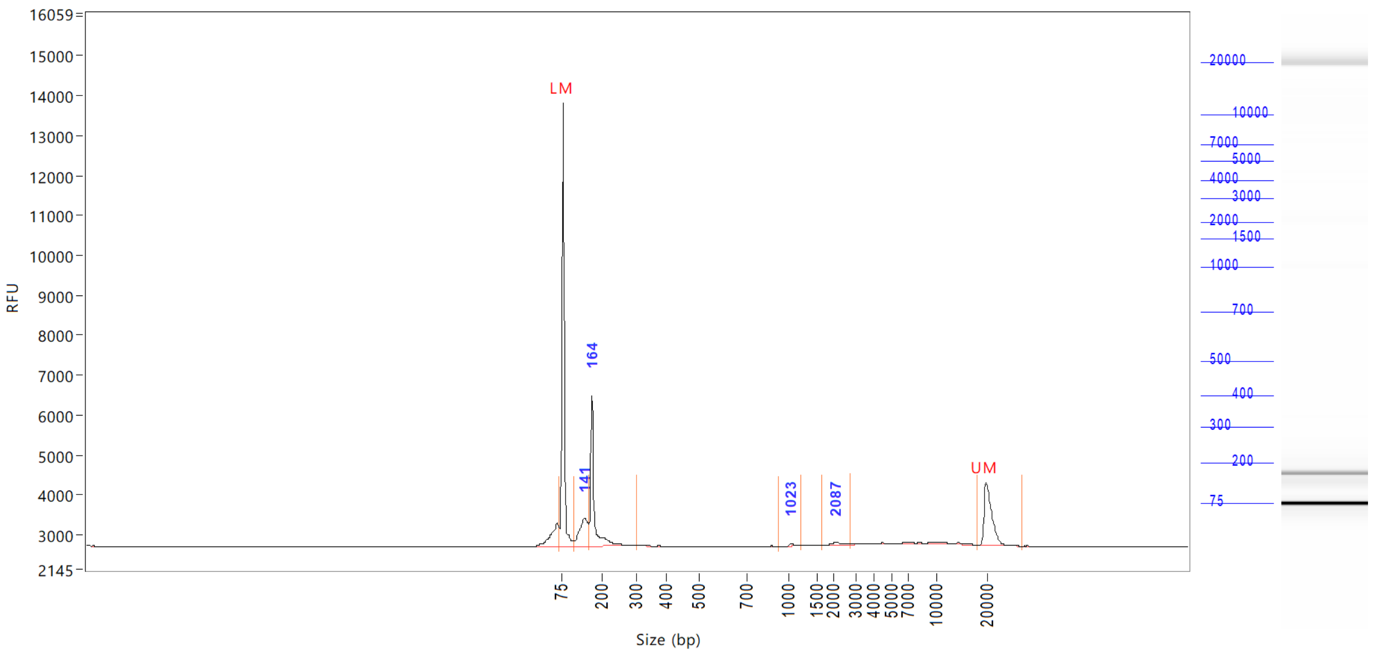

Peak	Size (bp)	Concentration (ng/uL)	From (bp)	To (bp)	Average size (bp)	CV%	RFU	Corrected peak area
1	75 (LM)	0.5000	64	108	74	7.92	11116	65.782
2	141	1.2944	108	154	136	8.74	698	14.191
3	164	2.8017	154	300	172	12.19	3748	30.717
4	1023	0.0507	926	1201	1044	5.26	72	0.556
5	2087	0.0890	1635	2772	2143	10.38	76	0.976
6	20000 (UM)	0.1156	18171	27254	20445	4.25	1577	15.206

TIC:	4.2358	ng/uL
TIM:	42.5649	nmole/L
Total concentration:	4.5032	ng/uL

```
Sample peak width (sec): 5    Sample min peak height: 50    Sample baseline V to V?: Y    Sample baseline V to V points: 3
Sample filter: Binomial       Number of points for filter: 3  Sample start region (min): 0  Sample end region (min): 30
Marker peak width (sec): 5    Marker min peak height: 200   Marker baseline V to V?: Y    Marker baseline V to V points: 3
Lower marker selection: First peak > 500 RFU                Upper marker selection: Last peak > 200 RFU
Ladder size (bp)75, 200, 300, 400, 500, 700, 1000, 1500, 2000, 3000, 4000, 5000, 7000, 10000, 20000
Quantification using: Lower Marker          Final concentration (ng/uL): 0.5000          Dilution factor: 12.0
```

Sample: SampD4
Well location: D4
Created: Tuesday, August 10, 2021 10:01:13 PM

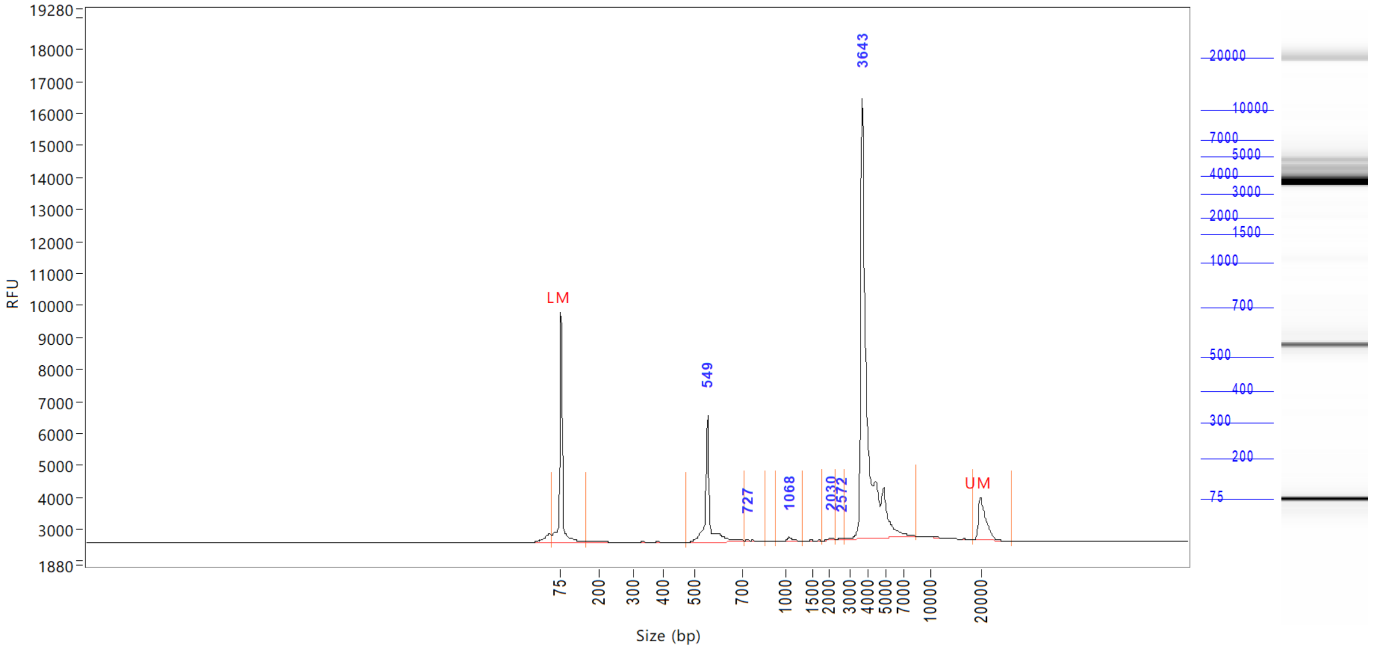

Peak	Size (bp)	Concentration (ng/uL)	From (bp)	To (bp)	Average size (bp)	CV%	RFU	Corrected peak area
1	75 (LM)	0.5000	47	158	75	17.32	7217	50.054
2	549	3.7865	471	705	556	6.39	3964	31.589
3	727	0.0963	705	857	745	3.97	62	0.803
4	1068	0.1549	939	1300	1075	6.42	121	1.292
5	2030	0.0769	1770	2315	2064	5.37	81	0.641
6	2572	0.0522	2315	2686	2510	4.08	60	0.436
7	3643	16.0948	2686	8401	3971	15.24	13790	134.268
8	20000 (UM)	0.1276	18415	26408	20494	4.23	1339	12.777

TIC:	20.2615	ng/uL
TIM:	18.4247	nmole/L
Total concentration:	20.5614	ng/uL

Sample peak width (sec): 5 Sample min peak height: 50 Sample baseline V to V?: Y Sample baseline V to V points: 3
Sample filter: Binomial Number of points for filter: 3 Sample start region (min): 0 Sample end region (min): 30
Marker peak width (sec): 5 Marker min peak height: 200 Marker baseline V to V?: Y Marker baseline V to V points: 3
Lower marker selection: First peak > 500 RFU Upper marker selection: Last peak > 200 RFU
Ladder size (bp)75, 200, 300, 400, 500, 700, 1000, 1500, 2000, 3000, 4000, 5000, 7000, 10000, 20000
Quantification using: Lower Marker Final concentration (ng/uL): 0.5000 Dilution factor: 12.0

Sample: SampE4
Well location: E4
Created: Tuesday, August 10, 2021 10:01:13 PM

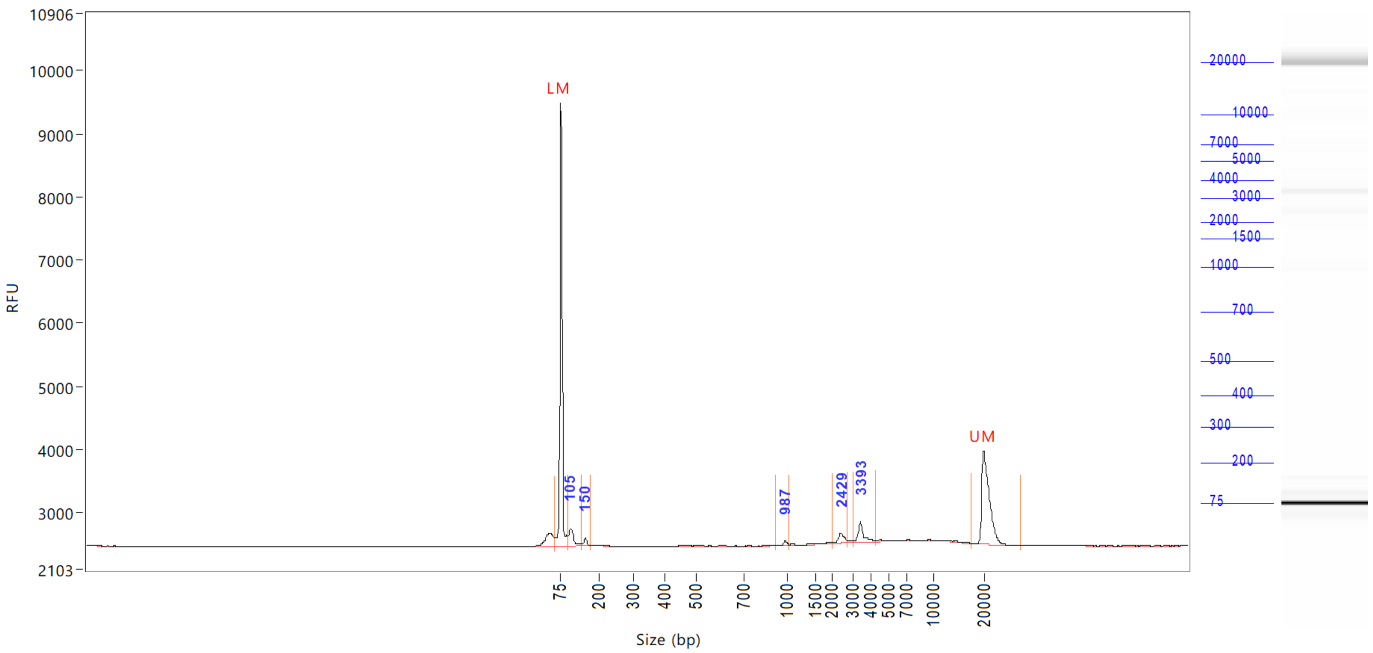

Peak	Size (bp)	Concentration (ng/uL)	From (bp)	To (bp)	Average size (bp)	CV%	RFU	Corrected peak area
1	75 (LM)	0.5000	58	97	74	6.50	7031	40.582
2	105	0.5051	97	141	109	9.73	285	3.416
3	150	0.1231	141	171	149	3.09	121	0.833
4	987	0.0727	926	1034	985	1.61	83	0.491
5	2429	0.2149	2001	2772	2428	6.09	160	1.454
6	3393	0.3747	3000	4277	3457	7.28	323	2.534
7	20000 (UM)	0.1772	17561	27496	20464	4.44	1485	14.385

TIC:	1.2905	ng/uL
TIM:	9.4148	nmole/L
Total concentration:	1.7405	ng/uL

Sample peak width (sec): 5 Sample min peak height: 50 Sample baseline V to V?: Y Sample baseline V to V points: 3
Sample filter: Binomial Number of points for filter: 3 Sample start region (min): 0 Sample end region (min): 30
Marker peak width (sec): 5 Marker min peak height: 200 Marker baseline V to V?: Y Marker baseline V to V points: 3
Lower marker selection: First peak > 500 RFU Upper marker selection: Last peak > 200 RFU
Ladder size (bp)75, 200, 300, 400, 500, 700, 1000, 1500, 2000, 3000, 4000, 5000, 7000, 10000, 20000
Quantification using: Lower Marker Final concentration (ng/uL): 0.5000 Dilution factor: 12.0

Sample: SampF4
Well location: F4
Created: Tuesday, August 10, 2021 10:01:13 PM

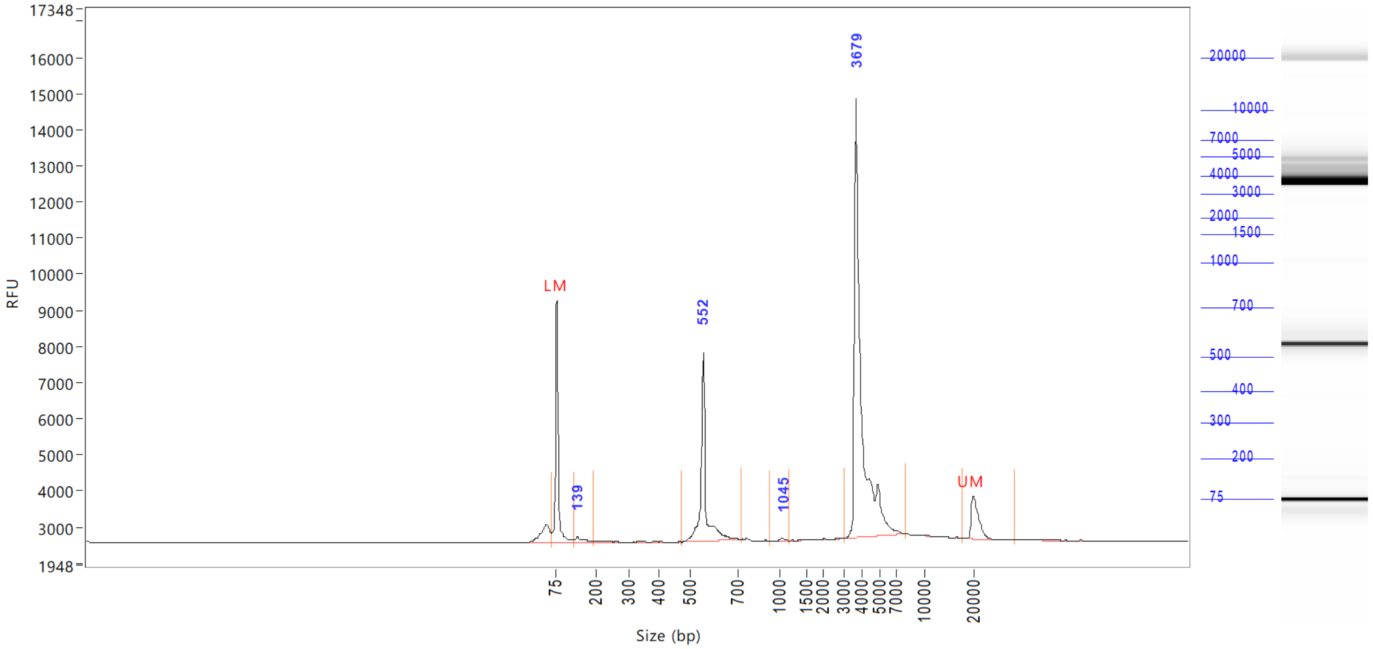

Peak	Size (bp)	Concentration (ng/uL)	From (bp)	To (bp)	Average size (bp)	CV%	RFU	Corrected peak area
1	75 (LM)	0.5000	58	129	75	13.13	6686	44.461
2	139	0.3358	129	192	152	11.42	160	2.488
3	552	5.8933	471	723	557	5.84	5198	43.671
4	1045	0.0732	935	1178	1029	3.91	72	0.542
5	3679	16.7362	3001	8001	4013	14.56	12173	124.019
6	20000 (UM)	0.1292	17683	28584	20561	4.66	1200	11.490

TIC:	23.0384	ng/uL
TIM:	28.0247	nmole/L
Total concentration:	23.3405	ng/uL

```
Sample peak width (sec): 5    Sample min peak height: 50    Sample baseline V to V?: Y    Sample baseline V to V points: 3
Sample filter: Binomial       Number of points for filter: 3  Sample start region (min): 0  Sample end region (min): 30
Marker peak width (sec): 5    Marker min peak height: 200   Marker baseline V to V?: Y    Marker baseline V to V points: 3
Lower marker selection: First peak > 500 RFU                Upper marker selection: Last peak > 200 RFU
Ladder size (bp)75, 200, 300, 400, 500, 700, 1000, 1500, 2000, 3000, 4000, 5000, 7000, 10000, 20000
Quantification using: Lower Marker          Final concentration (ng/uL): 0.5000          Dilution factor: 12.0
```

Sample: SampG4
Well location: G4
Created: Tuesday, August 10, 2021 10:01:13 PM

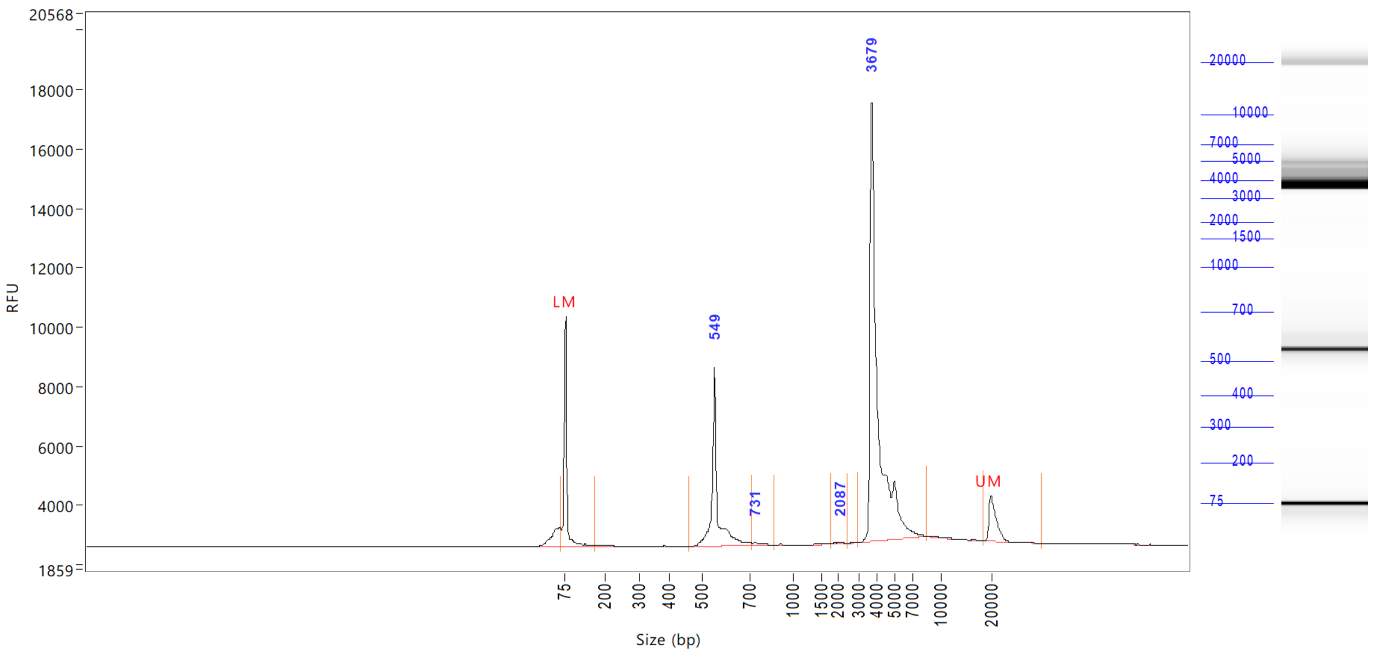

Peak	Size (bp)	Concentration (ng/uL)	From (bp)	To (bp)	Average size (bp)	CV%	RFU	Corrected peak area
1	75 (LM)	0.5000	62	166	76	18.35	7724	51.539
2	549	6.7252	458	714	559	7.15	5988	57.768
3	731	0.1935	714	870	766	5.21	99	1.662
4	2087	0.0774	1770	2486	2036	6.51	64	0.665
5	3679	19.1787	2971	8401	4095	16.00	14782	164.741
6	20000 (UM)	0.1431	18537	30035	20569	5.21	1547	14.750

TIC:	26.1748	ng/uL
TIM:	27.9862	nmole/L
Total concentration:	26.5426	ng/uL

```
Sample peak width (sec): 5    Sample min peak height: 50    Sample baseline V to V?: Y    Sample baseline V to V points: 3
Sample filter: Binomial       Number of points for filter: 3  Sample start region (min): 0  Sample end region (min): 30
Marker peak width (sec): 5    Marker min peak height: 200   Marker baseline V to V?: Y    Marker baseline V to V points: 3
Lower marker selection: First peak > 500 RFU                Upper marker selection: Last peak > 200 RFU
Ladder size (bp)75, 200, 300, 400, 500, 700, 1000, 1500, 2000, 3000, 4000, 5000, 7000, 10000, 20000
Quantification using: Lower Marker          Final concentration (ng/uL): 0.5000          Dilution factor: 12.0
```

Sample: SampH4
Well location: H4
Created: Tuesday, August 10, 2021 10:01:13 PM

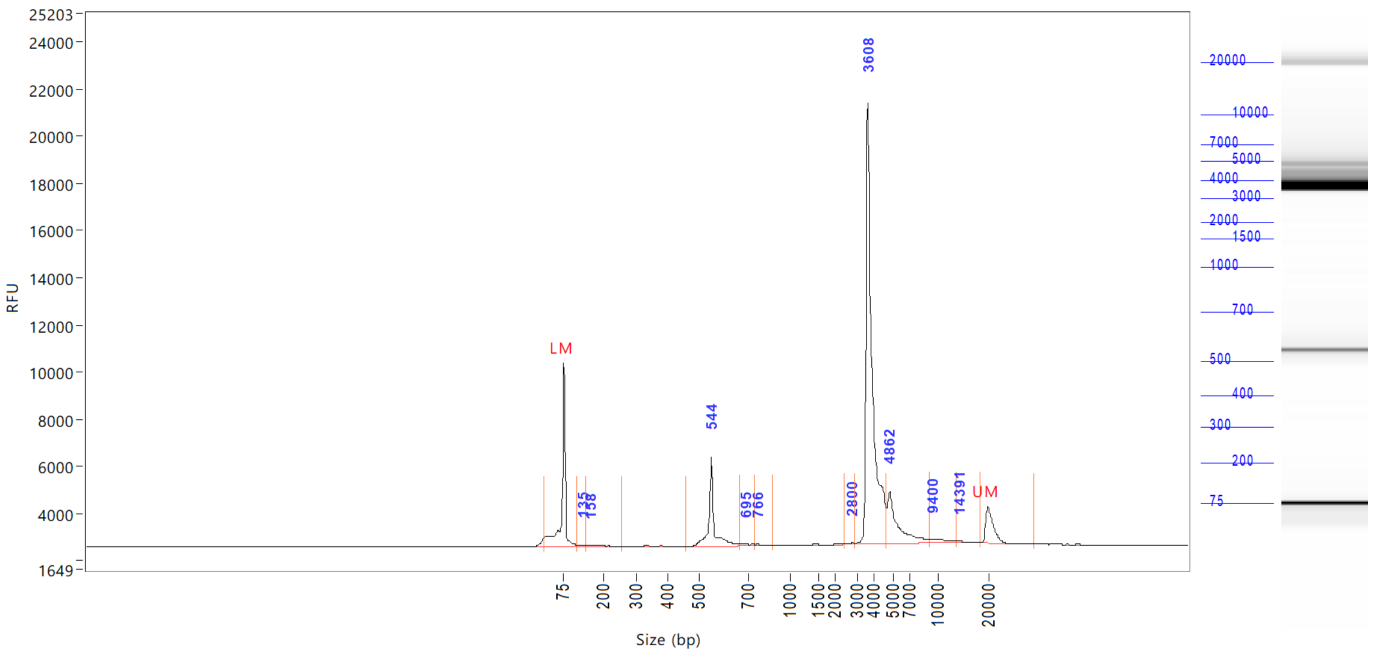

Peak	Size (bp)	Concentration (ng/uL)	From (bp)	To (bp)	Average size (bp)	CV%	RFU	Corrected peak area
1	75 (LM)	0.5000	16	116	66	26.18	7756	65.726
2	135	0.1192	116	145	129	6.40	79	1.306
3	158	0.1693	145	253	177	13.61	66	1.855
4	544	3.2396	458	664	550	5.91	3795	35.487
5	695	0.1587	664	749	700	3.41	95	1.739
6	766	0.0716	749	874	784	3.95	77	0.784
7	2800	0.0415	2429	2914	2730	4.41	62	0.454
8	3608	15.7250	2914	4690	3791	8.28	18710	172.257
9	4862	2.9804	4690	9067	5717	20.11	2223	32.648
10	9400	0.3712	9067	13781	10738	12.56	171	4.066
11	14391	0.0972	13781	18415	15237	7.26	87	1.065
12	20000 (UM)	0.1119	18415	29309	20551	4.73	1531	14.711

TIC:	22.9737	ng/uL
TIM:	21.0864	nmole/L
Total concentration:	23.0809	ng/uL

```
Sample peak width (sec): 5    Sample min peak height: 50    Sample baseline V to V?: Y    Sample baseline V to V points: 3
Sample filter: Binomial       Number of points for filter: 3  Sample start region (min): 0  Sample end region (min): 30
Marker peak width (sec): 5    Marker min peak height: 200   Marker baseline V to V?: Y    Marker baseline V to V points: 3
Lower marker selection: First peak > 500 RFU                Upper marker selection: Last peak > 200 RFU
Ladder size (bp)75, 200, 300, 400, 500, 700, 1000, 1500, 2000, 3000, 4000, 5000, 7000, 10000, 20000
Quantification using: Lower Marker          Final concentration (ng/uL): 0.5000          Dilution factor: 12.0
```

Sample: SampA5
Well location: A5
Created: Tuesday, August 10, 2021 10:01:13 PM

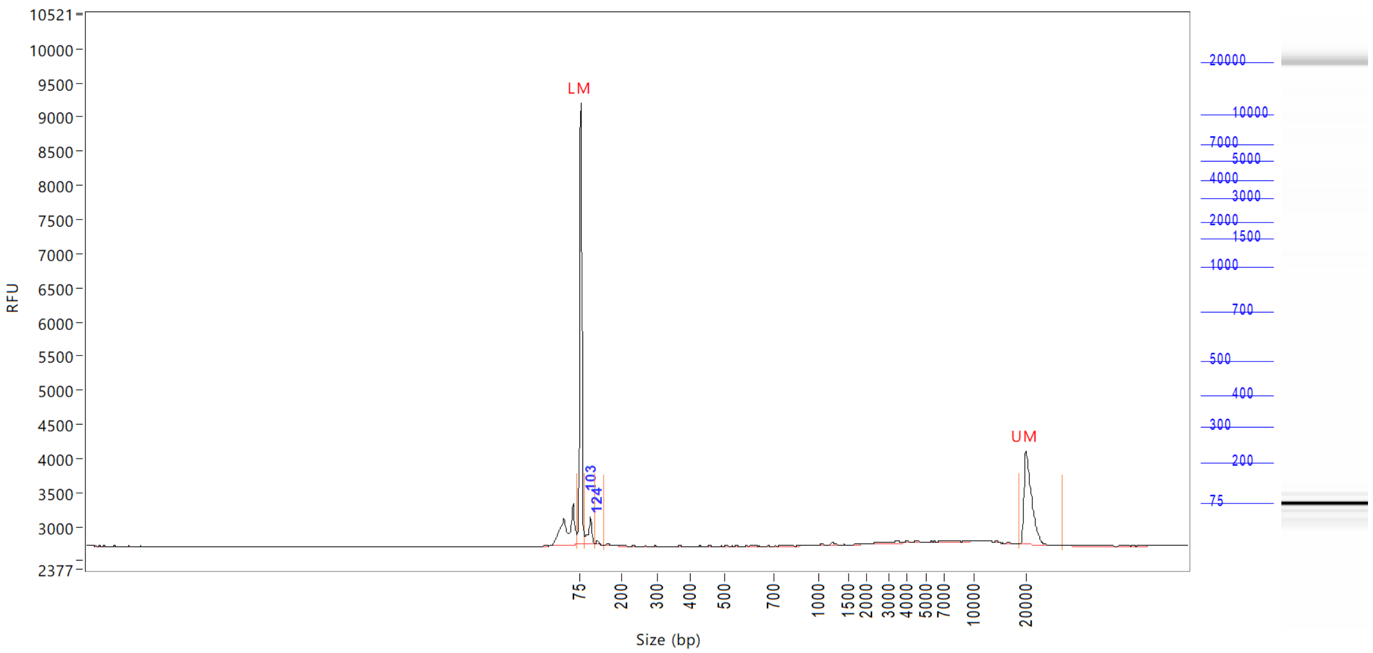

Peak	Size (bp)	Concentration (ng/uL)	From (bp)	To (bp)	Average size (bp)	CV%	RFU	Corrected peak area
1	75 (LM)	0.5000	64	86	73	4.45	6475	34.384
2	103	0.5767	86	116	97	5.93	391	3.305
3	124	0.0512	116	145	123	4.09	58	0.293
4	20000 (UM)	0.1824	18537	26891	20447	4.24	1363	12.543

TIC:	0.6279	ng/uL
TIM:	10.4460	nmole/L
Total concentration:	1.3792	ng/uL

Sample peak width (sec): 5   Sample min peak height: 50   Sample baseline V to V?: Y   Sample baseline V to V points: 3
Sample filter: Binomial   Number of points for filter: 3 Sample start region (min): 0 Sample end region (min): 30
Marker peak width (sec): 5   Marker min peak height: 200   Marker baseline V to V?: Y   Marker baseline V to V points: 3
Lower marker selection: First peak > 500 RFU   Upper marker selection: Last peak > 200 RFU
Ladder size (bp)75, 200, 300, 400, 500, 700, 1000, 1500, 2000, 3000, 4000, 5000, 7000, 10000, 20000
Quantification using: Lower Marker   Final concentration (ng/uL): 0.5000   Dilution factor: 12.0

Sample: SampB5
Well location: B5
Created: Tuesday, August 10, 2021 10:01:13 PM

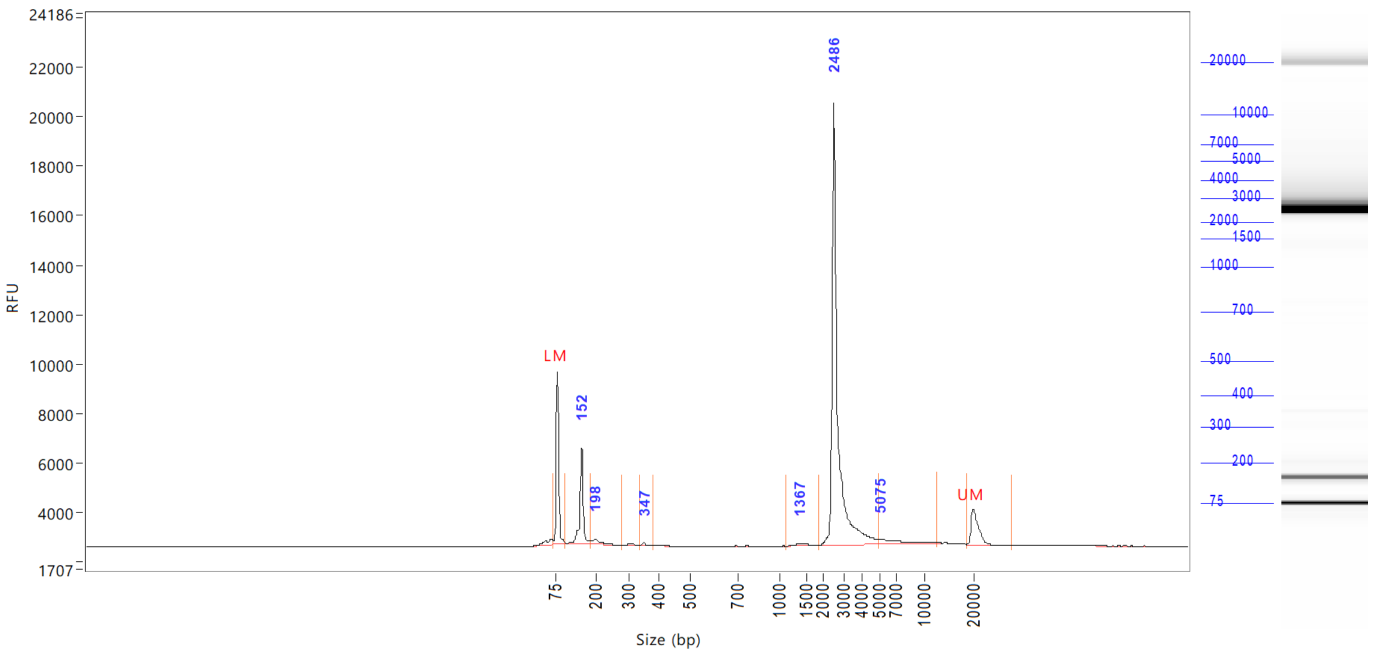

Peak	Size (bp)	Concentration (ng/uL)	From (bp)	To (bp)	Average size (bp)	CV%	RFU	Corrected peak area
1	75 (LM)	0.5000	66	103	74	5.46	6957	40.966
2	152	4.5563	103	183	150	6.28	3864	31.109
3	198	0.6032	183	277	203	7.60	228	4.119
4	347	0.1264	333	378	346	1.84	146	0.863
5	1367	0.2976	1101	1866	1433	10.13	106	2.032
6	2486	22.5175	1866	4931	2712	17.59	17910	153.742
7	5075	0.9116	4931	12562	7025	24.33	186	6.224
8	20000 (UM)	0.1659	18659	27979	20536	4.55	1450	13.596

TIC:	29.0127	ng/uL
TIM:	69.6335	nmole/L
Total concentration:	29.2781	ng/uL

Sample peak width (sec): 5 Sample min peak height: 50 Sample baseline V to V?: Y Sample baseline V to V points: 3
Sample filter: Binomial Number of points for filter: 3 Sample start region (min): 0 Sample end region (min): 30
Marker peak width (sec): 5 Marker min peak height: 200 Marker baseline V to V?: Y Marker baseline V to V points: 3
Lower marker selection: First peak > 500 RFU Upper marker selection: Last peak > 200 RFU
Ladder size (bp)75, 200, 300, 400, 500, 700, 1000, 1500, 2000, 3000, 4000, 5000, 7000, 10000, 20000
Quantification using: Lower Marker Final concentration (ng/uL): 0.5000 Dilution factor: 12.0

Sample: SampC5
Well location: C5
Created: Tuesday, August 10, 2021 10:01:13 PM

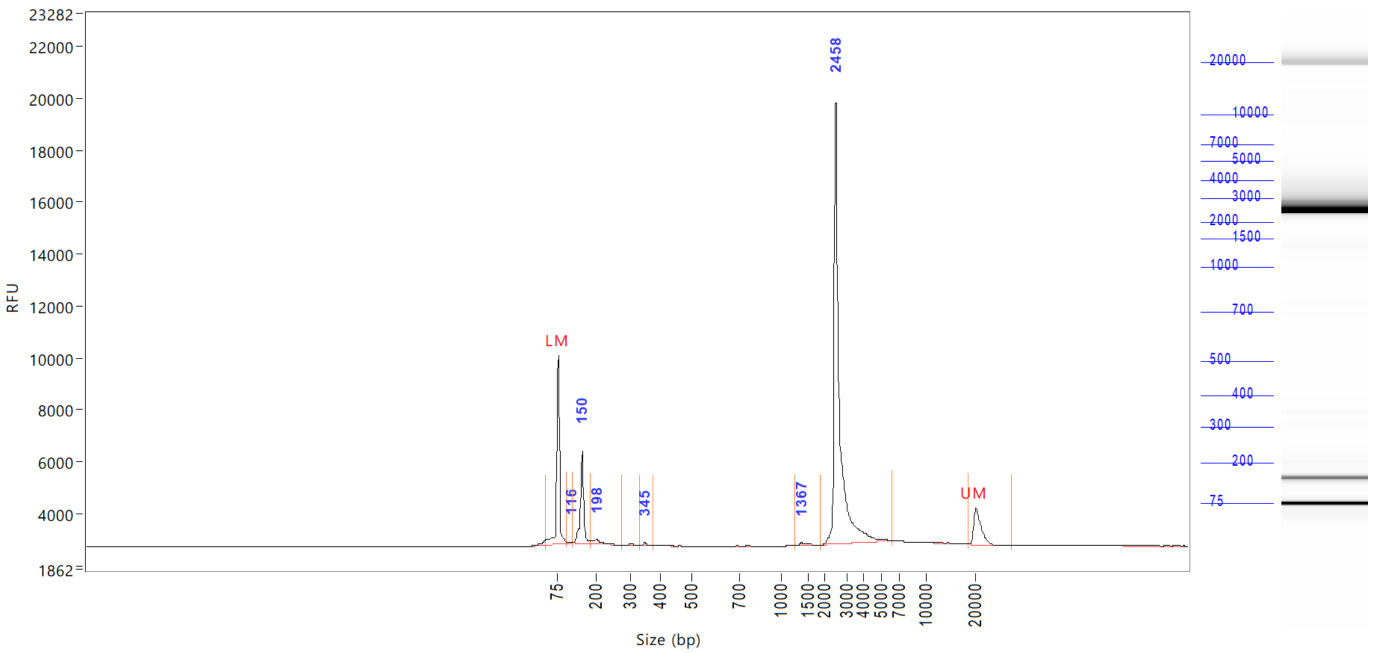

Peak	Size (bp)	Concentration (ng/uL)	From (bp)	To (bp)	Average size (bp)	CV%	RFU	Corrected peak area
1	75 (LM)	0.5000	37	101	71	11.58	7279	46.613
2	116	0.0635	101	122	112	5.18	51	0.493
3	150	3.5740	122	179	148	5.40	3548	27.766
4	198	0.4015	179	275	200	7.82	177	3.119
5	345	0.0893	329	378	343	1.50	135	0.694
6	1367	0.1713	1245	1866	1418	7.26	110	1.330
7	2458	18.3544	1866	6260	2673	16.63	17023	142.592
8	20000 (UM)	0.1441	18537	27254	20544	4.33	1418	13.437

TIC:	22.6539	ng/uL
TIM:	55.8500	nmole/L
Total concentration:	22.9838	ng/uL

```
Sample peak width (sec): 5    Sample min peak height: 50    Sample baseline V to V?: Y    Sample baseline V to V points: 3
Sample filter: Binomial       Number of points for filter: 3  Sample start region (min): 0  Sample end region (min): 30
Marker peak width (sec): 5    Marker min peak height: 200   Marker baseline V to V?: Y    Marker baseline V to V points: 3
Lower marker selection: First peak > 500 RFU                Upper marker selection: Last peak > 200 RFU
Ladder size (bp)75, 200, 300, 400, 500, 700, 1000, 1500, 2000, 3000, 4000, 5000, 7000, 10000, 20000
Quantification using: Lower Marker          Final concentration (ng/uL): 0.5000          Dilution factor: 12.0
```

Sample: SampD5
Well location: D5
Created: Tuesday, August 10, 2021 10:01:13 PM

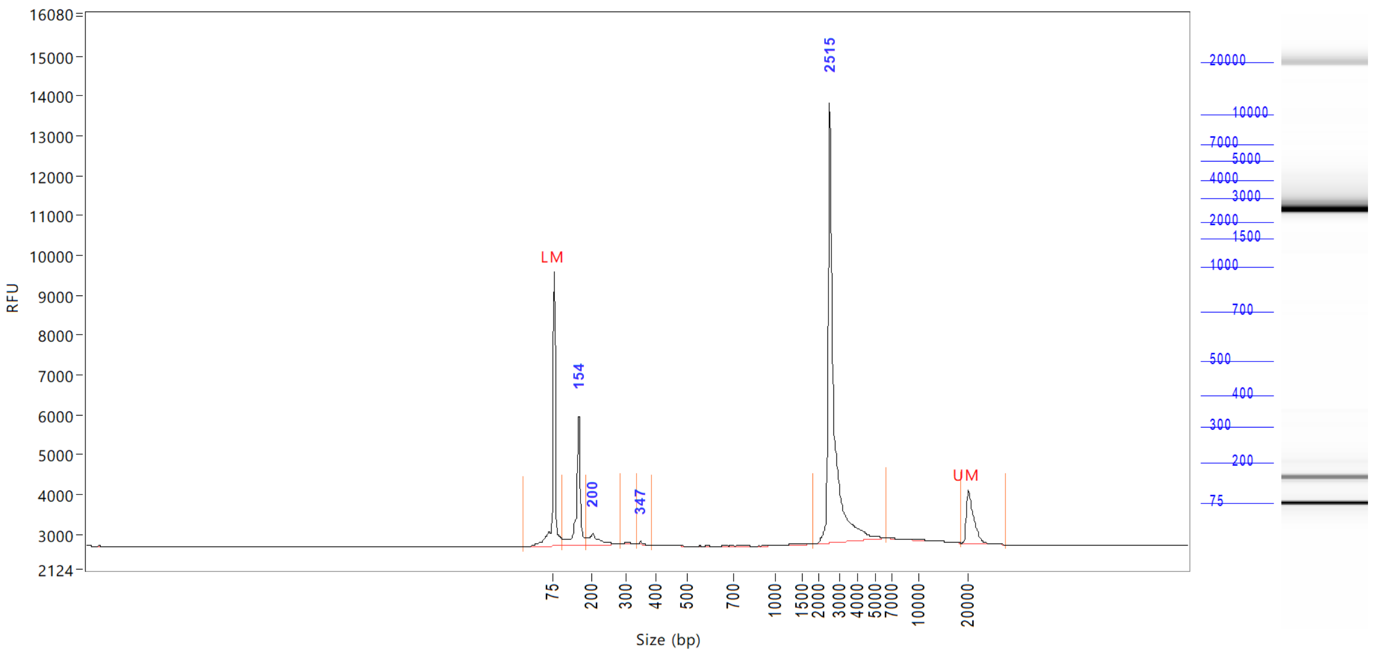

Peak	Size (bp)	Concentration (ng/uL)	From (bp)	To (bp)	Average size (bp)	CV%	RFU	Corrected peak area
1	75 (LM)	0.5000	0	103	71	18.99	6869	48.758
2	154	3.7501	103	179	148	9.00	3230	30.475
3	200	0.8713	179	284	209	11.34	296	7.081
4	347	0.0575	337	386	348	2.13	80	0.467
5	2515	11.9497	1827	6408	2740	18.13	11065	97.108
6	20000 (UM)	0.1316	18659	27617	20576	4.55	1333	12.831

TIC:	16.6287	ng/uL
TIM:	55.9498	nmole/L
Total concentration:	16.9871	ng/uL

Sample peak width (sec): 5   Sample min peak height: 50   Sample baseline V to V?: Y   Sample baseline V to V points: 3
Sample filter: Binomial   Number of points for filter: 3   Sample start region (min): 0   Sample end region (min): 30
Marker peak width (sec): 5   Marker min peak height: 200   Marker baseline V to V?: Y   Marker baseline V to V points: 3
Lower marker selection: First peak > 500 RFU   Upper marker selection: Last peak > 200 RFU
Ladder size (bp)75, 200, 300, 400, 500, 700, 1000, 1500, 2000, 3000, 4000, 5000, 7000, 10000, 20000
Quantification using: Lower Marker   Final concentration (ng/uL): 0.5000   Dilution factor: 12.0

Sample: SampE5
Well location: E5
Created: Tuesday, August 10, 2021 10:01:13 PM

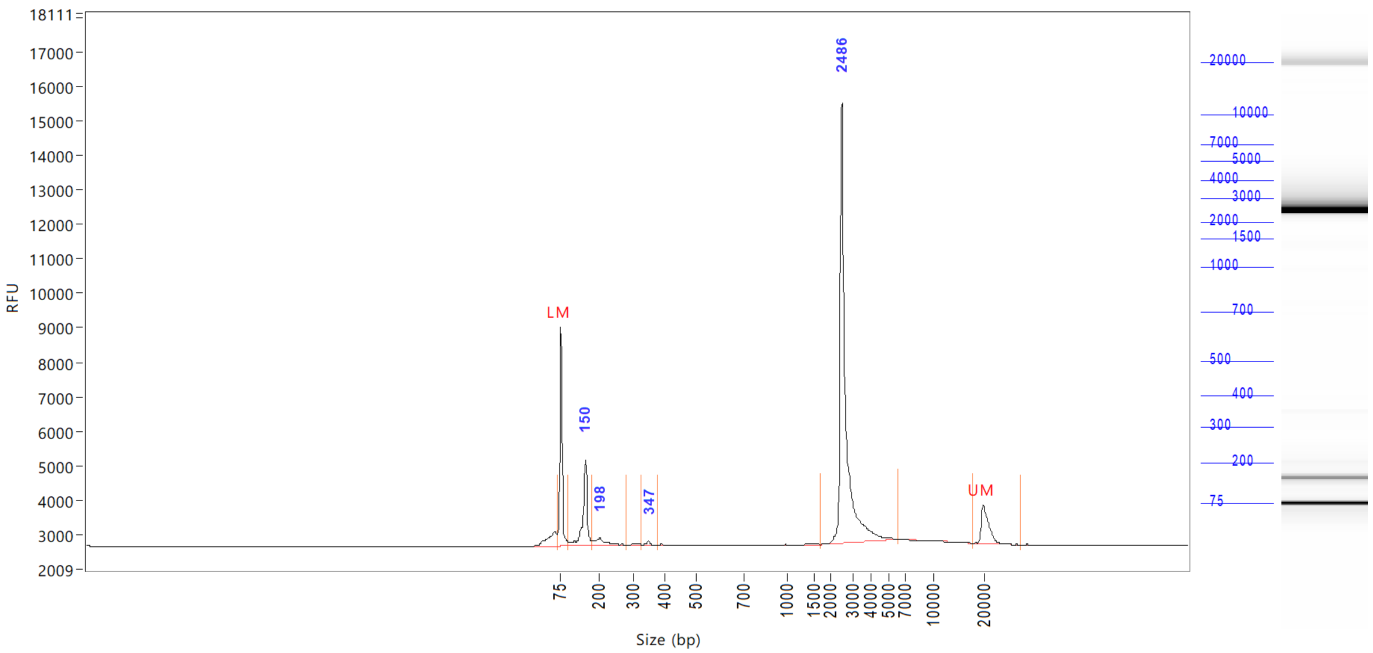

Peak	Size (bp)	Concentration (ng/uL)	From (bp)	To (bp)	Average size (bp)	CV%	RFU	Corrected peak area
1	75 (LM)	0.5000	66	101	74	6.73	6359	37.787
2	150	3.7836	101	175	146	8.90	2490	23.828
3	198	0.8175	175	278	203	9.94	223	5.148
4	347	0.1238	325	376	345	2.81	116	0.779
5	2486	17.1214	1655	6038	2678	16.71	12771	107.827
6	20000 (UM)	0.1414	17805	27617	20464	4.43	1106	10.687

TIC:	21.8462	ng/uL
TIM:	60.3253	nmole/L
Total concentration:	22.2061	ng/uL

Sample peak width (sec): 5   Sample min peak height: 50   Sample baseline V to V?: Y   Sample baseline V to V points: 3
Sample filter: Binomial   Number of points for filter: 3 Sample start region (min): 0 Sample end region (min): 30
Marker peak width (sec): 5   Marker min peak height: 200   Marker baseline V to V?: Y   Marker baseline V to V points: 3
Lower marker selection: First peak > 500 RFU   Upper marker selection: Last peak > 200 RFU
Ladder size (bp)75, 200, 300, 400, 500, 700, 1000, 1500, 2000, 3000, 4000, 5000, 7000, 10000, 20000
Quantification using: Lower Marker   Final concentration (ng/uL): 0.5000   Dilution factor: 12.0

Sample: SampF5
Well location: F5
Created: Tuesday, August 10, 2021 10:01:13 PM

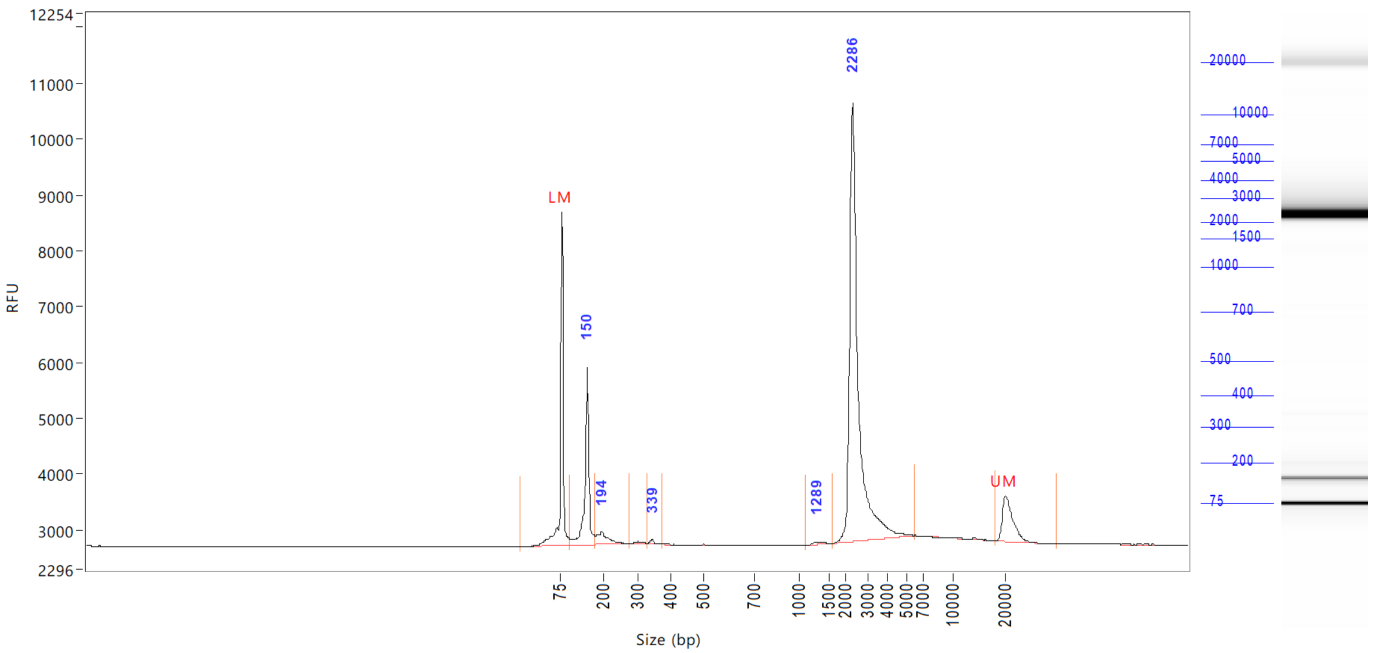

Peak	Size (bp)	Concentration (ng/uL)	From (bp)	To (bp)	Average size (bp)	CV%	RFU	Corrected peak area
1	75 (LM)	0.5000	0	99	69	20.91	5961	41.906
2	150	4.1884	99	175	145	8.97	3173	29.253
3	194	0.7888	175	273	200	9.43	231	5.509
4	339	0.1041	323	374	338	2.44	94	0.727
5	1289	0.1208	1101	1616	1313	6.60	52	0.844
6	2286	14.7570	1616	5963	2478	18.59	7870	103.067
7	20000 (UM)	0.1303	18171	29793	20617	5.95	824	10.923

TIC:	19.9591	ng/uL
TIM:	64.4176	nmole/L
Total concentration:	20.2415	ng/uL

Sample peak width (sec): 5 Sample min peak height: 50 Sample baseline V to V?: Y Sample baseline V to V points: 3
Sample filter: Binomial Number of points for filter: 3 Sample start region (min): 0 Sample end region (min): 30
Marker peak width (sec): 5 Marker min peak height: 200 Marker baseline V to V?: Y Marker baseline V to V points: 3
Lower marker selection: First peak > 500 RFU Upper marker selection: Last peak > 200 RFU
Ladder size (bp)75, 200, 300, 400, 500, 700, 1000, 1500, 2000, 3000, 4000, 5000, 7000, 10000, 20000
Quantification using: Lower Marker Final concentration (ng/uL): 0.5000 Dilution factor: 12.0

Sample: SampG5
Well location: G5
Created: Tuesday, August 10, 2021 10:01:13 PM

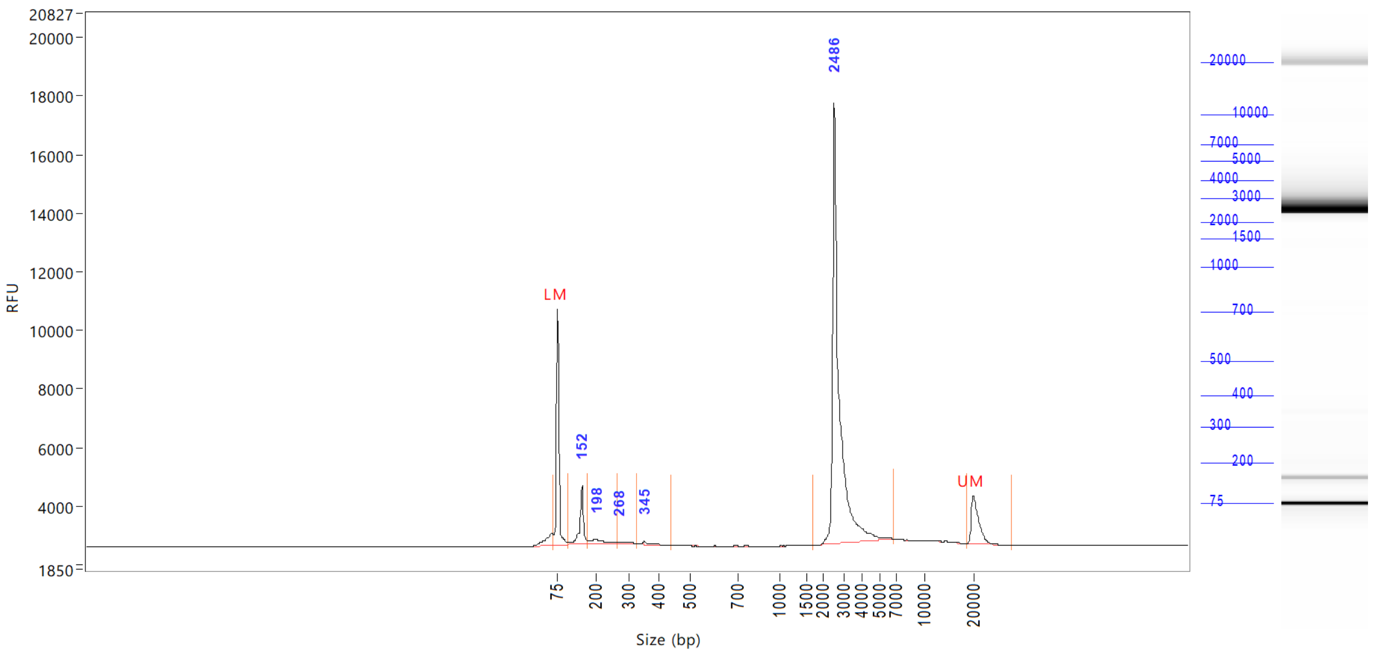

Peak	Size (bp)	Concentration (ng/uL)	From (bp)	To (bp)	Average size (bp)	CV%	RFU	Corrected peak area
1	75 (LM)	0.5000	64	108	74	7.58	8054	47.691
2	152	2.1726	108	171	147	7.37	1975	17.269
3	198	0.6460	171	264	210	12.57	160	5.135
4	268	0.2235	264	325	291	6.00	62	1.777
5	345	0.2195	325	435	357	6.00	126	1.744
6	2486	18.8367	1693	6630	2748	17.77	15065	149.725
7	20000 (UM)	0.1635	18781	27979	20598	4.59	1615	15.591

TIC:	22.0983	ng/uL
TIM:	42.9073	nmole/L
Total concentration:	22.2950	ng/uL

Sample peak width (sec): 5 Sample min peak height: 50 Sample baseline V to V?: Y Sample baseline V to V points: 3
Sample filter: Binomial Number of points for filter: 3 Sample start region (min): 0 Sample end region (min): 30
Marker peak width (sec): 5 Marker min peak height: 200 Marker baseline V to V?: Y Marker baseline V to V points: 3
Lower marker selection: First peak > 500 RFU Upper marker selection: Last peak > 200 RFU
Ladder size (bp)75, 200, 300, 400, 500, 700, 1000, 1500, 2000, 3000, 4000, 5000, 7000, 10000, 20000
Quantification using: Lower Marker Final concentration (ng/uL): 0.5000 Dilution factor: 12.0

Sample: SampH5
Well location: H5
Created: Tuesday, August 10, 2021 10:01:13 PM

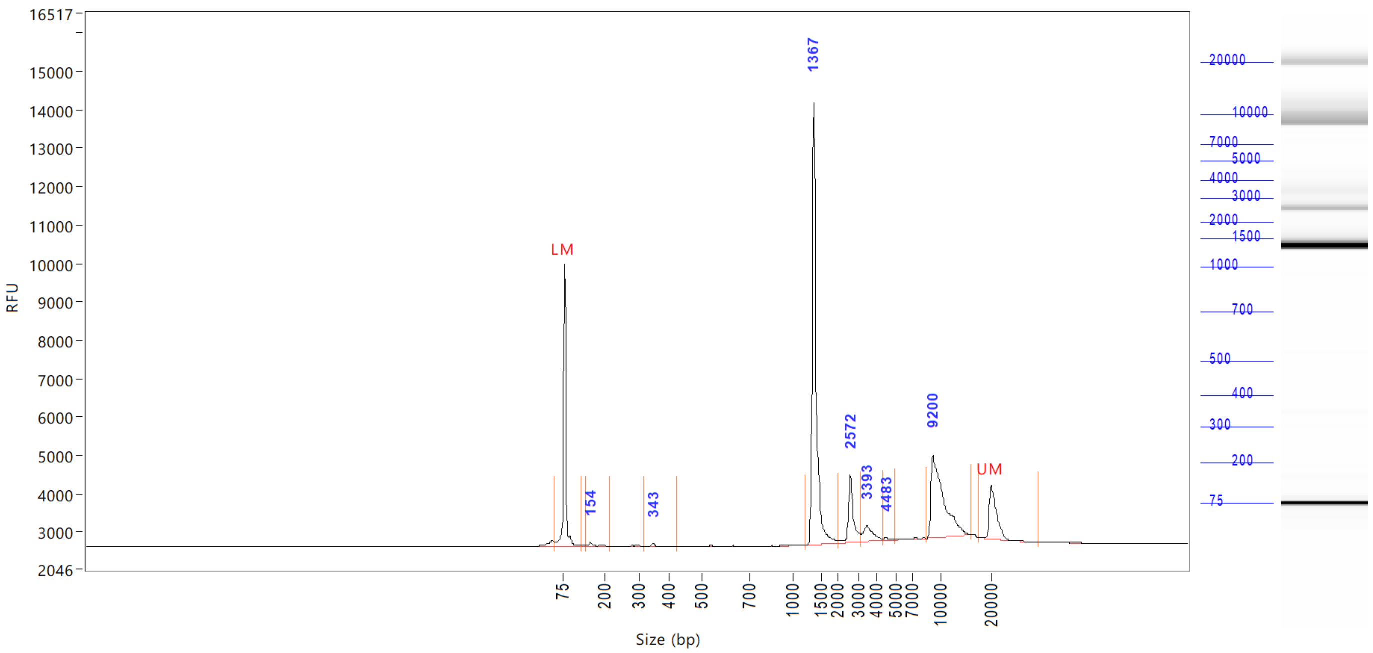

Peak	Size (bp)	Concentration (ng/uL)	From (bp)	To (bp)	Average size (bp)	CV%	RFU	Corrected peak area
1	75 (LM)	0.5000	45	128	73	10.53	7363	45.487
2	154	0.1401	143	215	162	10.04	92	1.062
3	343	0.0739	313	422	349	6.45	71	0.560
4	1367	9.7586	1212	2001	1389	6.30	11527	73.983
5	2572	1.7835	2001	3037	2594	6.98	1759	13.521
6	3393	0.7850	3037	4277	3498	8.39	403	5.951
7	4483	0.0724	4277	5001	4514	3.89	71	0.549
8	9200	4.8142	8467	16098	10232	13.03	2152	36.498
9	20000 (UM)	0.1439	17439	29189	20505	4.66	1387	13.093

TIC:	17.4277	ng/uL
TIM:	15.6363	nmole/L
Total concentration:	17.6606	ng/uL

Sample peak width (sec): 5 Sample min peak height: 50 Sample baseline V to V?: Y Sample baseline V to V points: 3
Sample filter: Binomial Number of points for filter: 3 Sample start region (min): 0 Sample end region (min): 30
Marker peak width (sec): 5 Marker min peak height: 200 Marker baseline V to V?: Y Marker baseline V to V points: 3
Lower marker selection: First peak > 500 RFU Upper marker selection: Last peak > 200 RFU
Ladder size (bp)75, 200, 300, 400, 500, 700, 1000, 1500, 2000, 3000, 4000, 5000, 7000, 10000, 20000
Quantification using: Lower Marker Final concentration (ng/uL): 0.5000 Dilution factor: 12.0

Sample: SampA6
Well location: A6
Created: Tuesday, August 10, 2021 10:01:13 PM

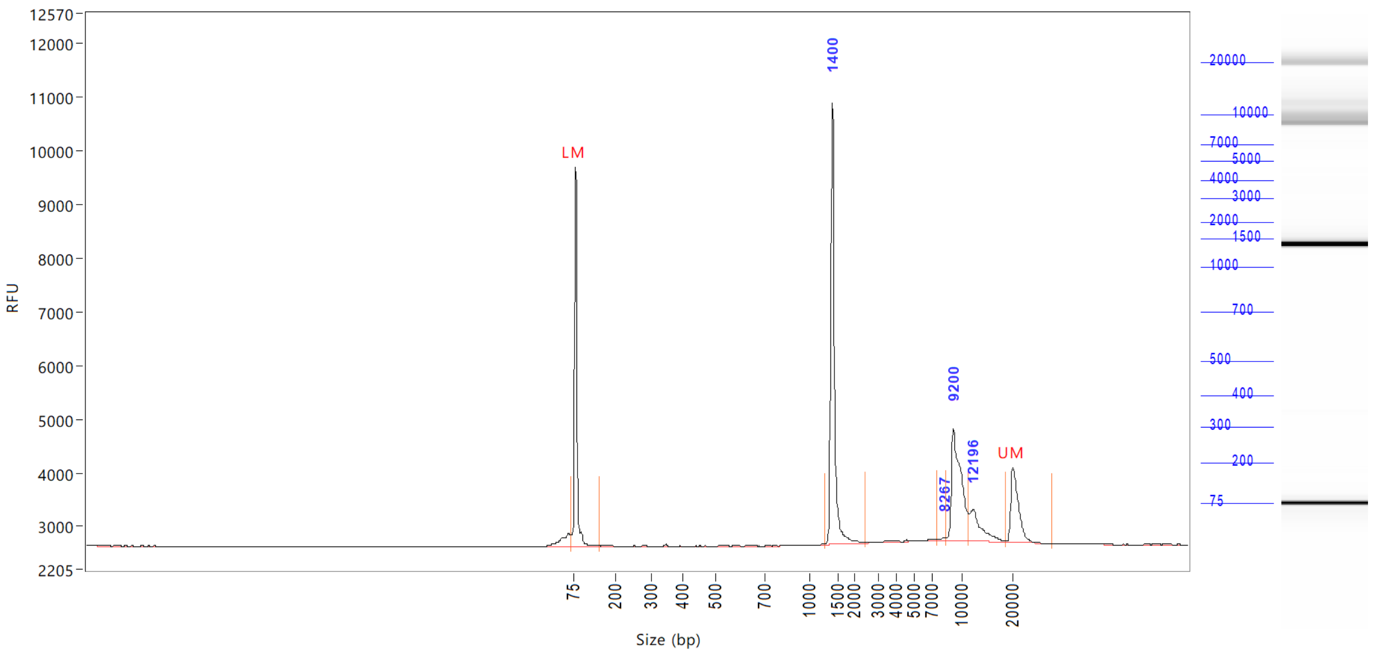

Peak	Size (bp)	Concentration (ng/uL)	From (bp)	To (bp)	Average size (bp)	CV%	RFU	Corrected peak area
1	75 (LM)	0.5000	62	150	75	10.16	7068	43.186
2	1400	6.4856	1267	2458	1419	7.71	8245	46.681
3	8267	0.0655	7534	8467	8055	3.34	57	0.471
4	9200	3.7277	8467	11464	9675	6.63	2094	26.831
5	12196	1.2793	11464	18659	13342	12.00	583	9.208
6	20000 (UM)	0.1532	18659	27738	20539	4.57	1404	13.232

TIC:	11.5580	ng/uL
TIM:	8.3289	nmole/L
Total concentration:	11.9662	ng/uL

Sample peak width (sec): 5 Sample min peak height: 50 Sample baseline V to V?: Y Sample baseline V to V points: 3
Sample filter: Binomial Number of points for filter: 3 Sample start region (min): 0 Sample end region (min): 30
Marker peak width (sec): 5 Marker min peak height: 200 Marker baseline V to V?: Y Marker baseline V to V points: 3
Lower marker selection: First peak > 500 RFU Upper marker selection: Last peak > 200 RFU
Ladder size (bp)75, 200, 300, 400, 500, 700, 1000, 1500, 2000, 3000, 4000, 5000, 7000, 10000, 20000
Quantification using: Lower Marker Final concentration (ng/uL): 0.5000 Dilution factor: 12.0

Sample: SampB6
Well location: B6
Created: Tuesday, August 10, 2021 10:01:13 PM

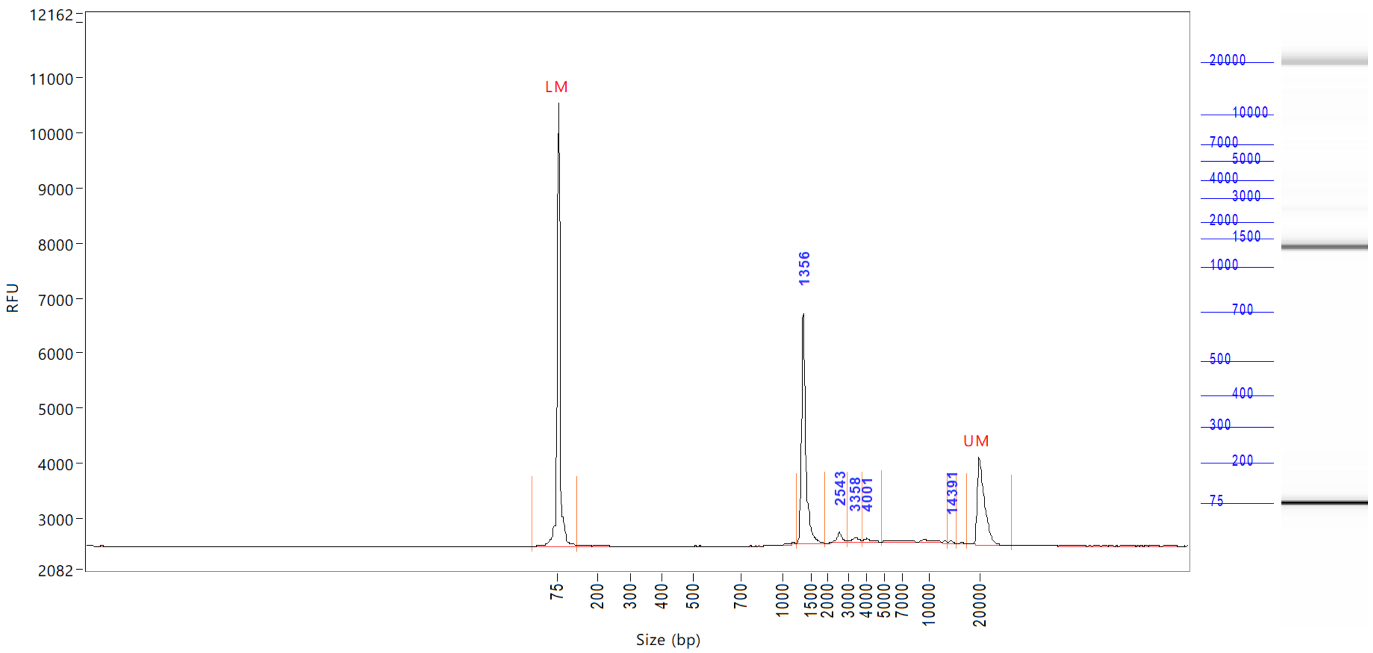

Peak	Size (bp)	Concentration (ng/uL)	From (bp)	To (bp)	Average size (bp)	CV%	RFU	Corrected peak area
1	75 (LM)	0.5000	0	137	74	15.37	8060	56.346
2	1356	3.0085	1223	1923	1365	4.96	4171	28.253
3	2543	0.1790	1923	2943	2521	7.02	192	1.681
4	3358	0.1072	2943	3750	3328	6.35	83	1.007
5	4001	0.0753	3750	4793	4069	5.18	65	0.707
6	14391	0.0322	13659	15488	14288	2.95	51	0.303
7	20000 (UM)	0.1331	17683	26649	20490	4.34	1585	15.004

TIC:	3.4023	ng/uL
TIM:	3.8320	nmole/L
Total concentration:	3.8748	ng/uL

Sample peak width (sec): 5 Sample min peak height: 50 Sample baseline V to V?: Y Sample baseline V to V points: 3
Sample filter: Binomial Number of points for filter: 3 Sample start region (min): 0 Sample end region (min): 30
Marker peak width (sec): 5 Marker min peak height: 200 Marker baseline V to V?: Y Marker baseline V to V points: 3
Lower marker selection: First peak > 500 RFU Upper marker selection: Last peak > 200 RFU
Ladder size (bp)75, 200, 300, 400, 500, 700, 1000, 1500, 2000, 3000, 4000, 5000, 7000, 10000, 20000
Quantification using: Lower Marker Final concentration (ng/uL): 0.5000 Dilution factor: 12.0

Sample: SampC6
Well location: C6
Created: Tuesday, August 10, 2021 10:01:13 PM

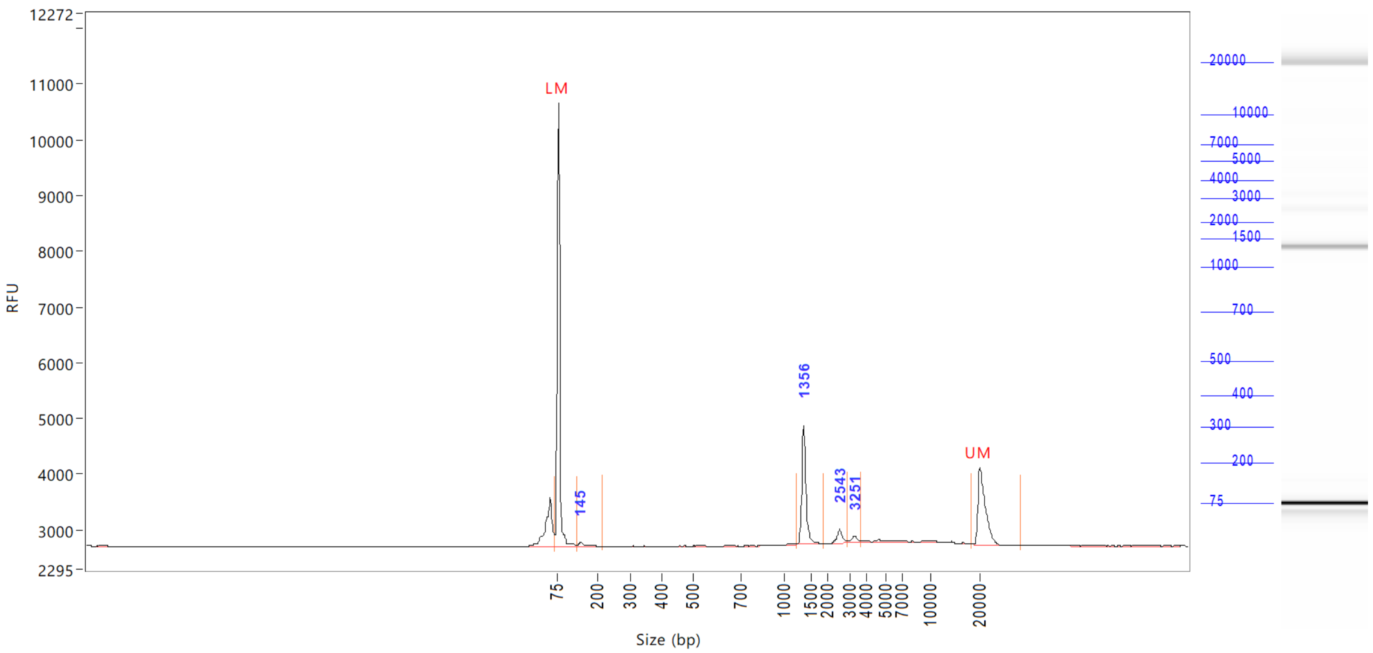

Peak	Size (bp)	Concentration (ng/uL)	From (bp)	To (bp)	Average size (bp)	CV%	RFU	Corrected peak area
1	75 (LM)	0.5000	64	133	75	9.40	7971	48.279
2	145	0.1135	133	212	150	9.74	72	0.913
3	1356	1.7087	1223	1885	1361	4.19	2129	13.749
4	2543	0.2690	1885	2914	2527	6.31	251	2.165
5	3251	0.1353	2914	3643	3234	5.38	114	1.089
6	20000 (UM)	0.1400	18415	28342	20558	4.57	1381	13.520

TIC:	2.2265	ng/uL
TIM:	3.5540	nmole/L
Total concentration:	2.6558	ng/uL

Sample peak width (sec): 5 Sample min peak height: 50 Sample baseline V to V?: Y Sample baseline V to V points: 3
Sample filter: Binomial Number of points for filter: 3 Sample start region (min): 0 Sample end region (min): 30
Marker peak width (sec): 5 Marker min peak height: 200 Marker baseline V to V?: Y Marker baseline V to V points: 3
Lower marker selection: First peak > 500 RFU Upper marker selection: Last peak > 200 RFU
Ladder size (bp)75, 200, 300, 400, 500, 700, 1000, 1500, 2000, 3000, 4000, 5000, 7000, 10000, 20000
Quantification using: Lower Marker Final concentration (ng/uL): 0.5000 Dilution factor: 12.0

Sample: SampD6
Well location: D6
Created: Tuesday, August 10, 2021 10:01:13 PM

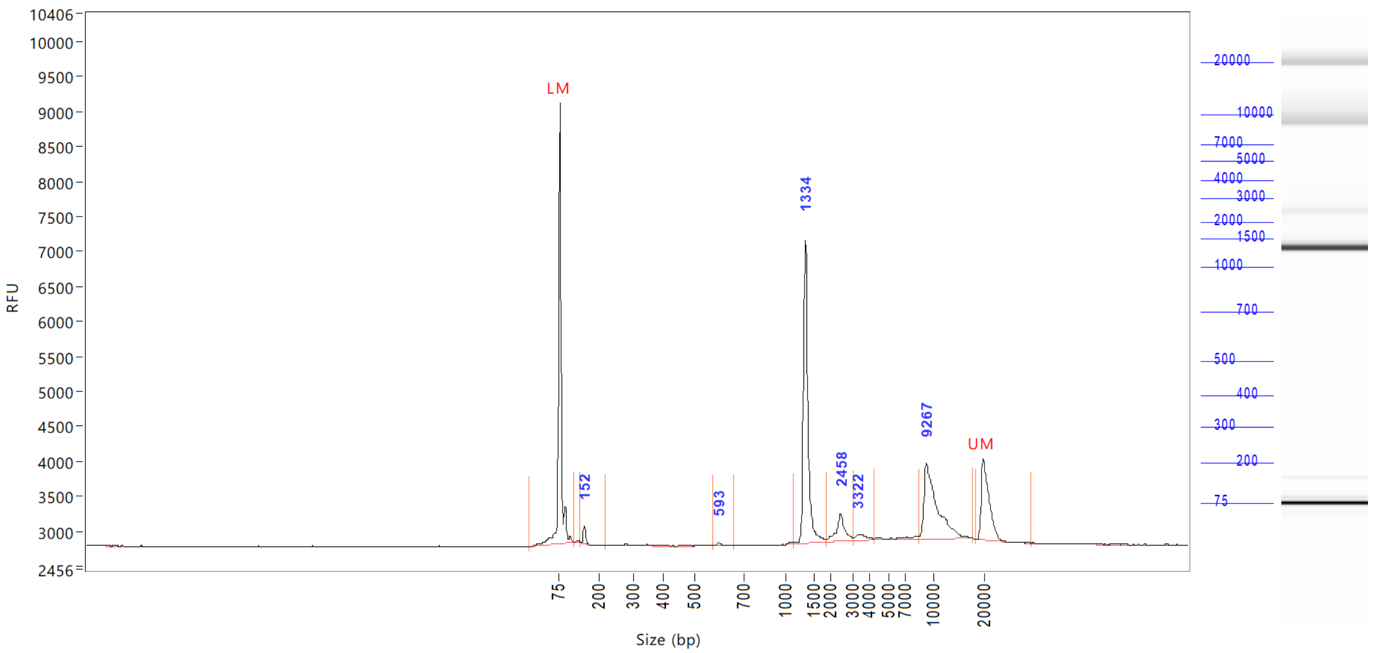

Peak	Size (bp)	Concentration (ng/uL)	From (bp)	To (bp)	Average size (bp)	CV%	RFU	Corrected peak area
1	75 (LM)	0.5000	0	120	73	15.96	6313	42.669
2	152	0.2135	141	221	154	9.11	259	1.518
3	593	0.0729	572	657	603	3.32	51	0.518
4	1334	4.3897	1123	1885	1349	5.71	4328	31.217
5	2458	0.7062	1885	3001	2415	10.24	396	5.022
6	3322	0.1993	3001	4173	3488	8.45	80	1.417
7	9267	2.7280	8401	17805	10407	15.47	1075	19.400
8	20000 (UM)	0.1369	18415	29672	20542	4.90	1160	11.686

TIC:	8.3096	ng/uL
TIM:	8.8409	nmole/L
Total concentration:	8.6826	ng/uL

Sample peak width (sec): 5 Sample min peak height: 50 Sample baseline V to V?: Y Sample baseline V to V points: 3
Sample filter: Binomial Number of points for filter: 3 Sample start region (min): 0 Sample end region (min): 30
Marker peak width (sec): 5 Marker min peak height: 200 Marker baseline V to V?: Y Marker baseline V to V points: 3
Lower marker selection: First peak > 500 RFU Upper marker selection: Last peak > 200 RFU
Ladder size (bp)75, 200, 300, 400, 500, 700, 1000, 1500, 2000, 3000, 4000, 5000, 7000, 10000, 20000
Quantification using: Lower Marker Final concentration (ng/uL): 0.5000 Dilution factor: 12.0

Sample: SampE6
Well location: E6
Created: Tuesday, August 10, 2021 10:01:13 PM

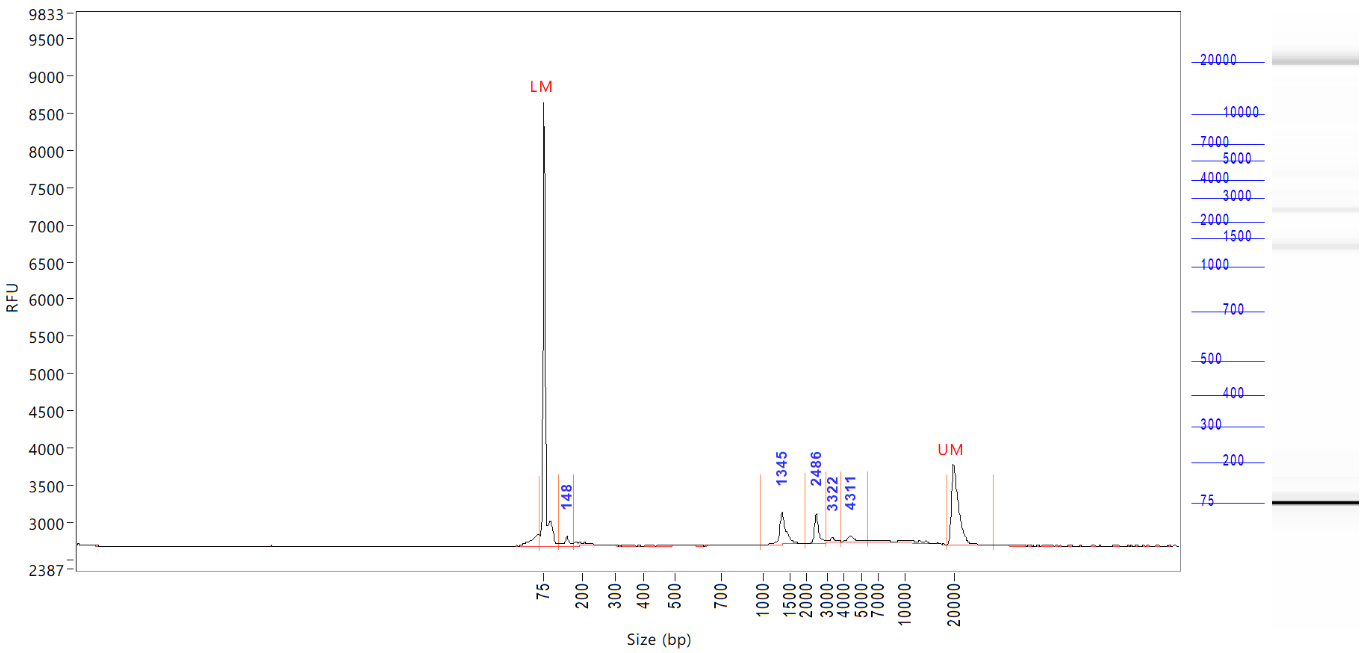

Peak	Size (bp)	Concentration (ng/uL)	From (bp)	To (bp)	Average size (bp)	CV%	RFU	Corrected peak area
1	75 (LM)	0.5000	64	124	76	11.23	5948	36.919
2	148	0.2592	124	169	147	7.81	134	1.595
3	1345	0.7361	983	1981	1371	7.77	434	4.529
4	2486	0.4421	1981	3000	2516	6.28	400	2.720
5	3322	0.1182	3000	3893	3323	6.72	71	0.727
6	4311	0.1710	3893	5815	4476	8.40	83	1.052
7	20000 (UM)	0.1392	18659	28221	20585	5.22	1085	10.278

TIC:	1.7266	ng/uL
TIM:	4.1919	nmole/L
Total concentration:	2.3464	ng/uL

Sample peak width (sec): 5 Sample min peak height: 50 Sample baseline V to V?: Y Sample baseline V to V points: 3
Sample filter: Binomial Number of points for filter: 3 Sample start region (min): 0 Sample end region (min): 30
Marker peak width (sec): 5 Marker min peak height: 200 Marker baseline V to V?: Y Marker baseline V to V points: 3
Lower marker selection: First peak > 500 RFU Upper marker selection: Last peak > 200 RFU
Ladder size (bp)75, 200, 300, 400, 500, 700, 1000, 1500, 2000, 3000, 4000, 5000, 7000, 10000, 20000
Quantification using: Lower Marker Final concentration (ng/uL): 0.5000 Dilution factor: 12.0

Sample: SampF6
Well location: F6
Created: Tuesday, August 10, 2021 10:01:13 PM

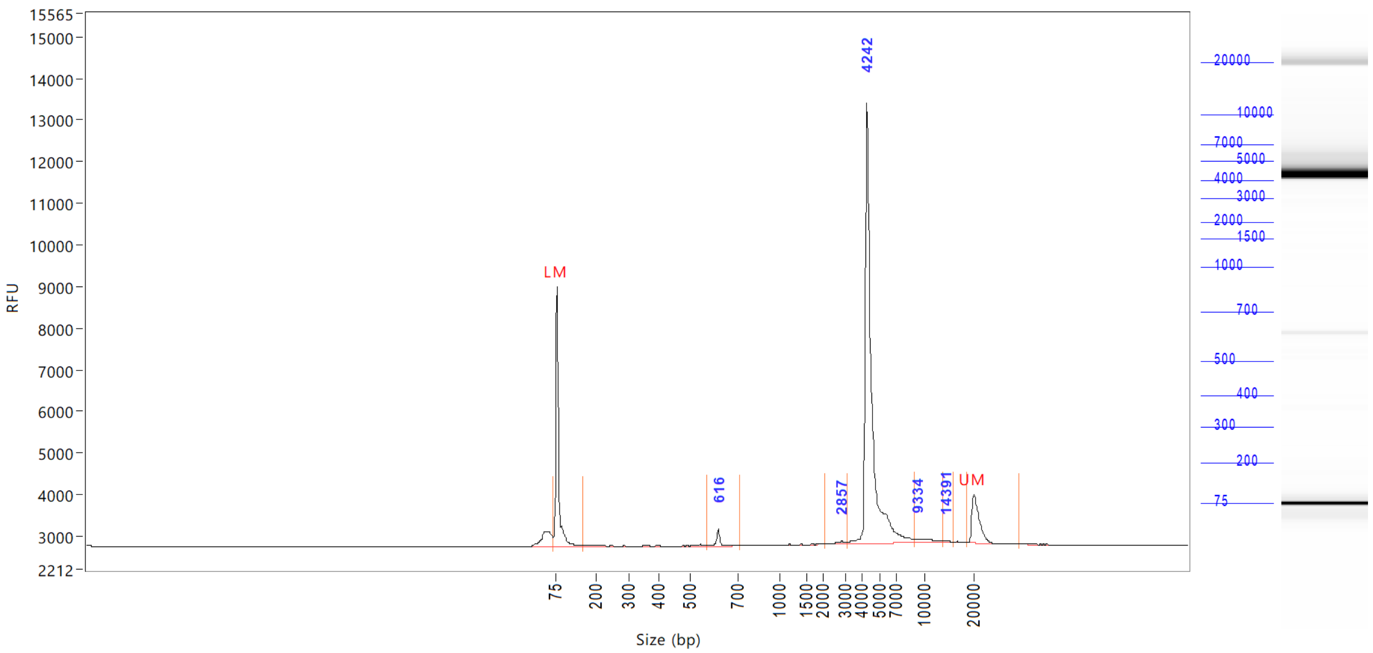

Peak	Size (bp)	Concentration (ng/uL)	From (bp)	To (bp)	Average size (bp)	CV%	RFU	Corrected peak area
1	75 (LM)	0.5000	64	156	77	15.16	6226	41.454
2	616	0.3934	565	710	613	3.09	415	2.718
3	2857	0.1149	2058	3108	2725	8.96	59	0.794
4	4242	14.4619	3108	8867	4566	16.52	10598	99.917
5	9334	0.3655	8867	13903	10713	13.68	93	2.525
6	14391	0.0654	13903	15976	14714	3.97	54	0.452
7	20000 (UM)	0.1344	18781	29309	20581	4.68	1159	11.143

TIC:	15.4011	ng/uL
TIM:	6.4032	nmole/L
Total concentration:	15.8657	ng/uL

Sample peak width (sec): 5   Sample min peak height: 50   Sample baseline V to V?: Y   Sample baseline V to V points: 3
Sample filter: Binomial   Number of points for filter: 3   Sample start region (min): 0   Sample end region (min): 30
Marker peak width (sec): 5   Marker min peak height: 200   Marker baseline V to V?: Y   Marker baseline V to V points: 3
Lower marker selection: First peak > 500 RFU   Upper marker selection: Last peak > 200 RFU
Ladder size (bp)75, 200, 300, 400, 500, 700, 1000, 1500, 2000, 3000, 4000, 5000, 7000, 10000, 20000
Quantification using: Lower Marker   Final concentration (ng/uL): 0.5000   Dilution factor: 12.0

Sample: SampG6
Well location: G6
Created: Tuesday, August 10, 2021 10:01:13 PM

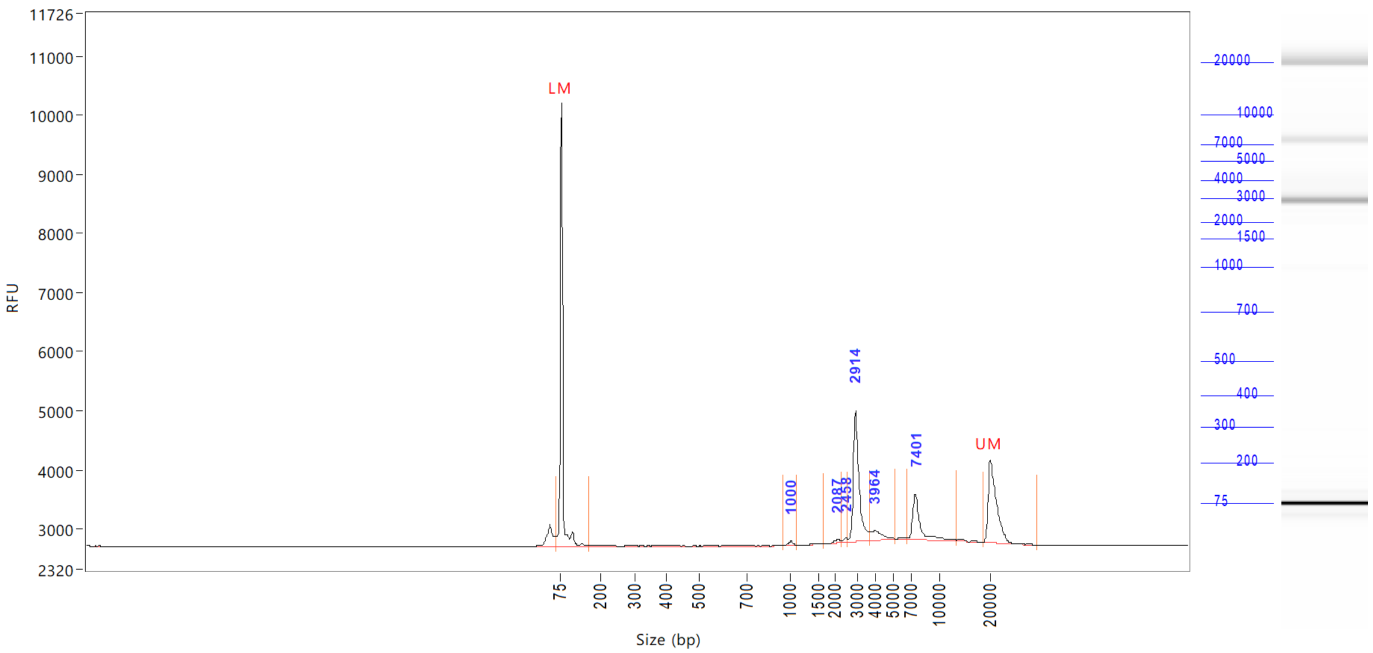

Peak	Size (bp)	Concentration (ng/uL)	From (bp)	To (bp)	Average size (bp)	CV%	RFU	Corrected peak area
1	75 (LM)	0.5000	62	162	76	15.02	7519	46.347
2	1000	0.0670	948	1101	996	2.09	69	0.518
3	2087	0.0780	1655	2286	2058	5.46	54	0.603
4	2458	0.0627	2286	2572	2441	3.39	71	0.485
5	2914	2.4972	2572	3679	2971	6.65	2208	19.290
6	3964	0.3818	3679	5075	4106	7.70	159	2.949
7	7401	1.0743	6556	13293	7883	14.72	778	8.298
8	20000 (UM)	0.1626	18659	29189	20643	5.15	1399	15.071

TIC:	4.1611	ng/uL
TIM:	1.9766	nmole/L
Total concentration:	4.4718	ng/uL

```
Sample peak width (sec): 5    Sample min peak height: 50    Sample baseline V to V?: Y    Sample baseline V to V points: 3
Sample filter: Binomial       Number of points for filter: 3  Sample start region (min): 0  Sample end region (min): 30
Marker peak width (sec): 5    Marker min peak height: 200   Marker baseline V to V?: Y    Marker baseline V to V points: 3
Lower marker selection: First peak > 500 RFU                Upper marker selection: Last peak > 200 RFU
Ladder size (bp)75, 200, 300, 400, 500, 700, 1000, 1500, 2000, 3000, 4000, 5000, 7000, 10000, 20000
Quantification using: Lower Marker         Final concentration (ng/uL): 0.5000         Dilution factor: 12.0
```

Sample: SampH6
Well location: H6
Created: Tuesday, August 10, 2021 10:01:13 PM

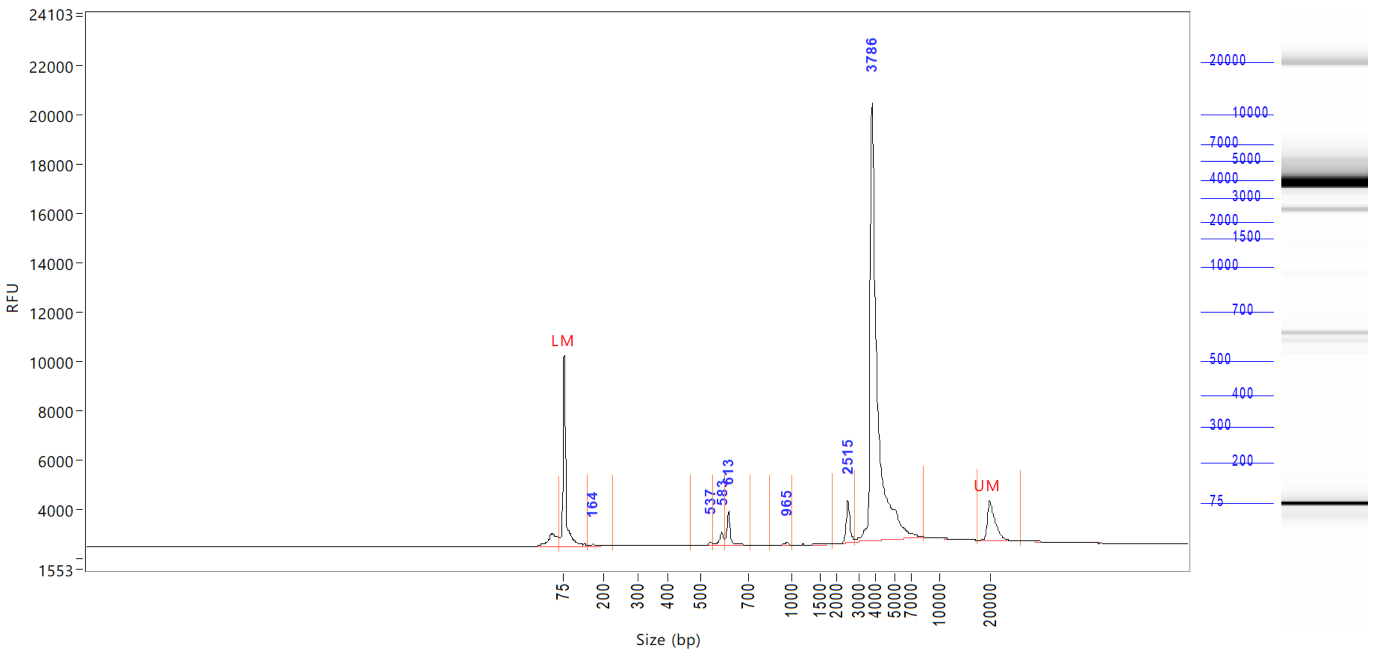

Peak	Size (bp)	Concentration (ng/uL)	From (bp)	To (bp)	Average size (bp)	CV%	RFU	Corrected peak area
1	75 (LM)	0.5000	62	147	77	16.19	7747	55.674
2	164	0.1726	147	223	173	11.30	68	1.601
3	537	0.1286	470	547	529	2.73	166	1.193
4	583	0.4781	547	598	579	2.08	572	4.436
5	613	1.0213	598	710	619	3.21	1399	9.477
6	965	0.1153	848	1001	951	2.16	119	1.070
7	2515	1.0772	1885	2886	2524	4.32	1687	9.995
8	3786	19.9731	2886	8334	4070	14.56	17786	185.329
9	20000 (UM)	0.1438	17561	26287	20537	4.86	1601	16.016

TIC:	22.9661	ng/uL
TIM:	15.0947	nmole/L
Total concentration:	23.2436	ng/uL

Sample peak width (sec): 5 Sample min peak height: 50 Sample baseline V to V?: Y Sample baseline V to V points: 3
Sample filter: Binomial Number of points for filter: 3 Sample start region (min): 0 Sample end region (min): 30
Marker peak width (sec): 5 Marker min peak height: 200 Marker baseline V to V?: Y Marker baseline V to V points: 3
Lower marker selection: First peak > 500 RFU Upper marker selection: Last peak > 200 RFU
Ladder size (bp)75, 200, 300, 400, 500, 700, 1000, 1500, 2000, 3000, 4000, 5000, 7000, 10000, 20000
Quantification using: Lower Marker Final concentration (ng/uL): 0.5000 Dilution factor: 12.0

Sample: SampA7
Well location: A7
Created: Tuesday, August 10, 2021 10:01:13 PM

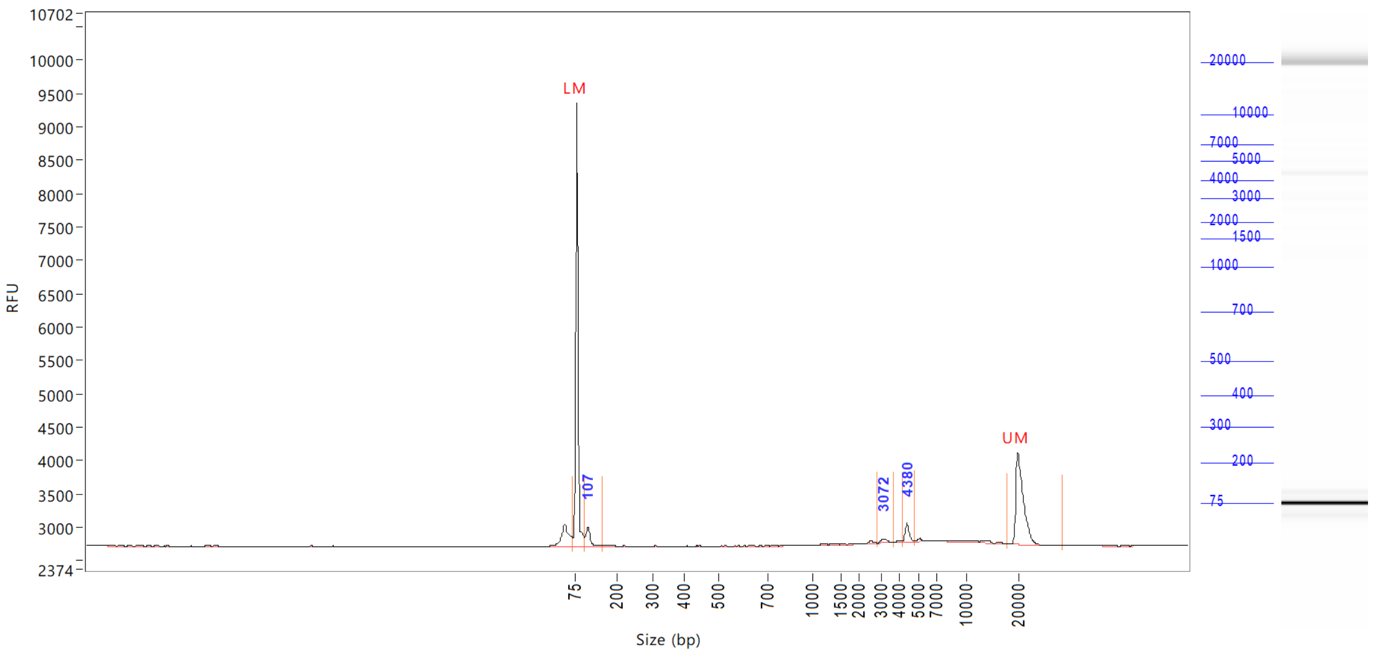

Peak	Size (bp)	Concentration (ng/uL)	From (bp)	To (bp)	Average size (bp)	CV%	RFU	Corrected peak area
1	75 (LM)	0.5000	64	99	74	6.66	6654	37.654
2	107	0.5197	99	156	110	9.58	295	3.262
3	3072	0.0872	2829	3679	3117	4.81	64	0.547
4	4380	0.2586	4104	4759	4365	2.79	279	1.623
5	20000 (UM)	0.1735	18049	28705	20530	4.52	1372	13.063

TIC:	0.8656	ng/uL
TIM:	7.9043	nmole/L
Total concentration:	1.5858	ng/uL

Sample peak width (sec): 5   Sample min peak height: 50   Sample baseline V to V?: Y   Sample baseline V to V points: 3
Sample filter: Binomial   Number of points for filter: 3   Sample start region (min): 0   Sample end region (min): 30
Marker peak width (sec): 5   Marker min peak height: 200   Marker baseline V to V?: Y   Marker baseline V to V points: 3
Lower marker selection: First peak > 500 RFU   Upper marker selection: Last peak > 200 RFU
Ladder size (bp)75, 200, 300, 400, 500, 700, 1000, 1500, 2000, 3000, 4000, 5000, 7000, 10000, 20000
Quantification using: Lower Marker   Final concentration (ng/uL): 0.5000   Dilution factor: 12.0

Sample: SampB7
Well location: B7
Created: Tuesday, August 10, 2021 10:01:13 PM

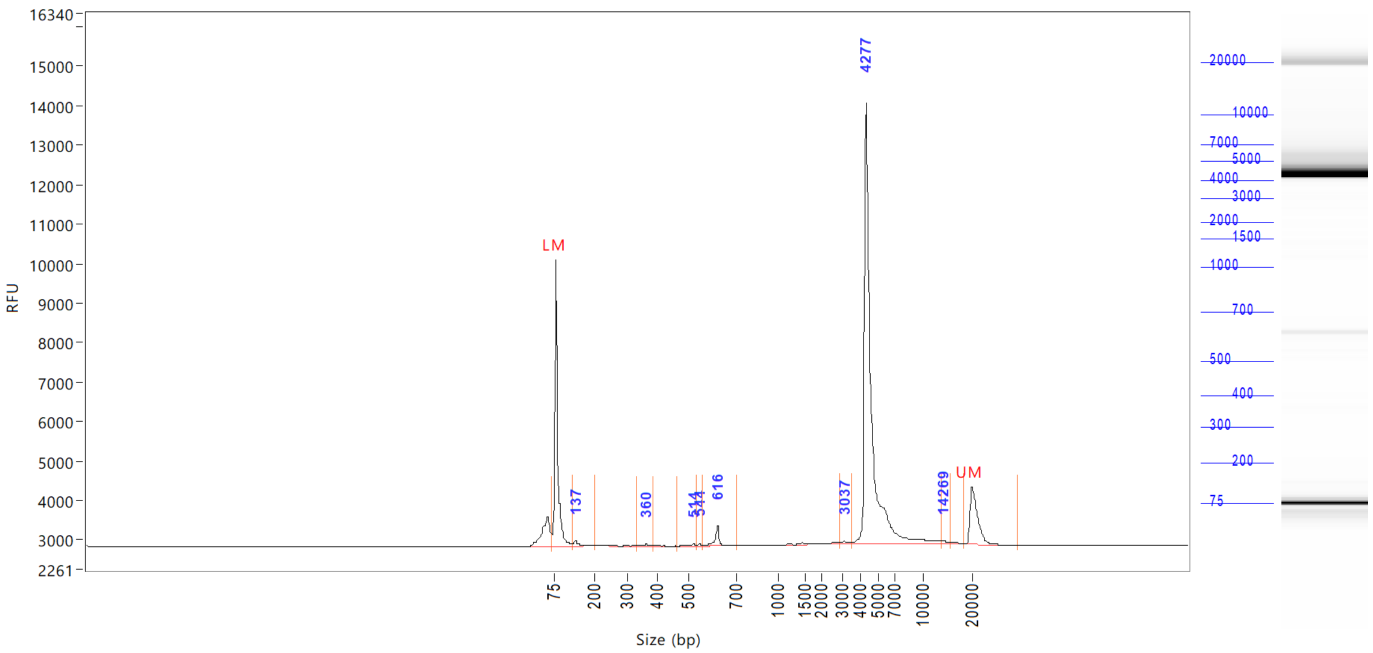

Peak	Size (bp)	Concentration (ng/uL)	From (bp)	To (bp)	Average size (bp)	CV%	RFU	Corrected peak area
1	75 (LM)	0.5000	64	128	77	11.74	7236	51.388
2	137	0.2350	128	200	150	13.11	143	2.013
3	360	0.0700	329	382	355	3.37	57	0.599
4	514	0.0604	462	527	500	3.29	53	0.517
5	544	0.0494	527	557	541	1.28	76	0.423
6	616	0.4807	557	700	612	3.44	519	4.117
7	3037	0.0779	2829	3536	3126	6.21	55	0.667
8	4277	13.1619	3536	13781	4831	26.91	11177	112.726
9	14269	0.0752	13781	15488	14518	3.47	77	0.644
10	20000 (UM)	0.1402	18293	29430	20594	4.89	1455	14.413

TIC:	14.2105	ng/uL
TIM:	9.0759	nmole/L
Total concentration:	14.6134	ng/uL

```
Sample peak width (sec): 5    Sample min peak height: 50    Sample baseline V to V?: Y    Sample baseline V to V points: 3
Sample filter: Binomial       Number of points for filter: 3  Sample start region (min): 0  Sample end region (min): 30
Marker peak width (sec): 5    Marker min peak height: 200   Marker baseline V to V?: Y    Marker baseline V to V points: 3
Lower marker selection: First peak > 500 RFU                Upper marker selection: Last peak > 200 RFU
Ladder size (bp)75, 200, 300, 400, 500, 700, 1000, 1500, 2000, 3000, 4000, 5000, 7000, 10000, 20000
Quantification using: Lower Marker          Final concentration (ng/uL): 0.5000          Dilution factor: 12.0
```

Sample: SampC7
Well location: C7
Created: Tuesday, August 10, 2021 10:01:13 PM

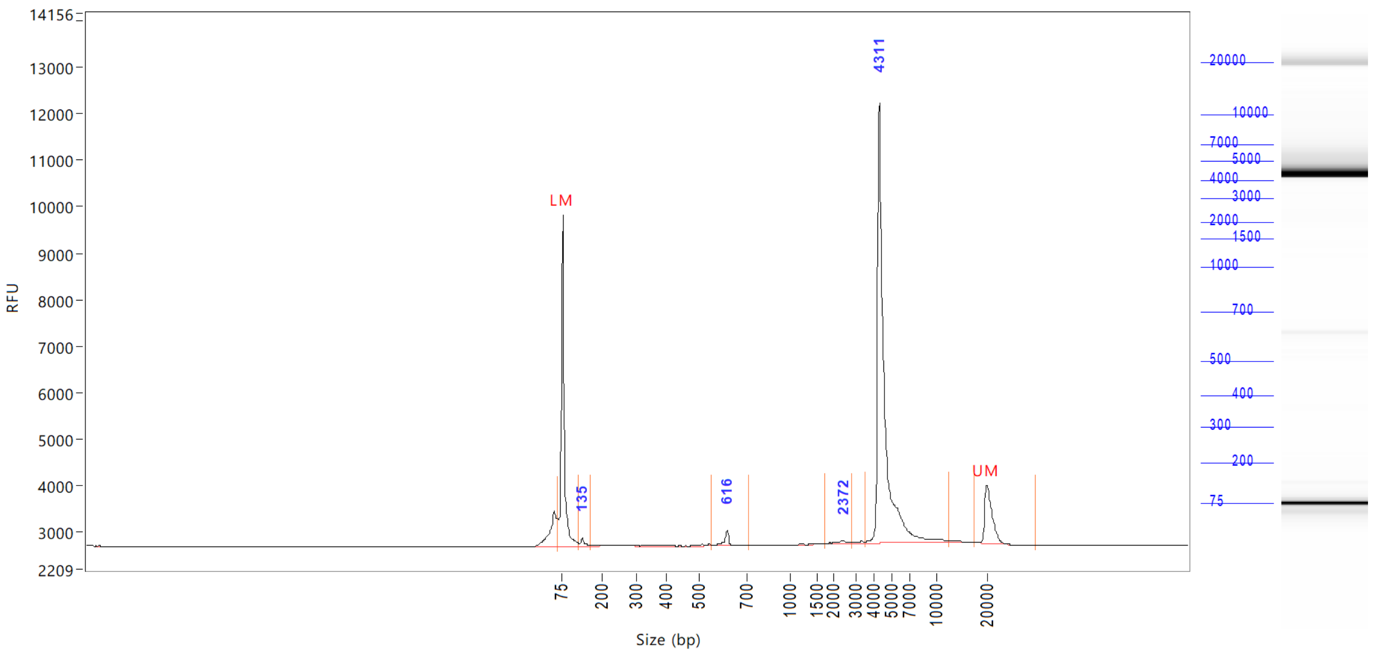

Peak	Size (bp)	Concentration (ng/uL)	From (bp)	To (bp)	Average size (bp)	CV%	RFU	Corrected peak area
1	75 (LM)	0.5000	62	128	76	12.38	7131	51.396
2	135	0.1899	128	164	139	6.53	171	1.626
3	616	0.3423	555	710	612	3.98	339	2.932
4	2372	0.1296	1712	2800	2234	11.46	61	1.110
5	4311	10.9475	3536	12318	4764	22.41	9474	93.776
6	20000 (UM)	0.1163	17561	29793	20436	4.85	1259	11.954

TIC:	11.6093	ng/uL
TIM:	7.0436	nmole/L
Total concentration:	12.0968	ng/uL

Sample peak width (sec): 5 Sample min peak height: 50 Sample baseline V to V?: Y Sample baseline V to V points: 3
Sample filter: Binomial Number of points for filter: 3 Sample start region (min): 0 Sample end region (min): 30
Marker peak width (sec): 5 Marker min peak height: 200 Marker baseline V to V?: Y Marker baseline V to V points: 3
Lower marker selection: First peak > 500 RFU Upper marker selection: Last peak > 200 RFU
Ladder size (bp)75, 200, 300, 400, 500, 700, 1000, 1500, 2000, 3000, 4000, 5000, 7000, 10000, 20000
Quantification using: Lower Marker Final concentration (ng/uL): 0.5000 Dilution factor: 12.0

Sample: SampD7
Well location: D7
Created: Tuesday, August 10, 2021 10:01:13 PM

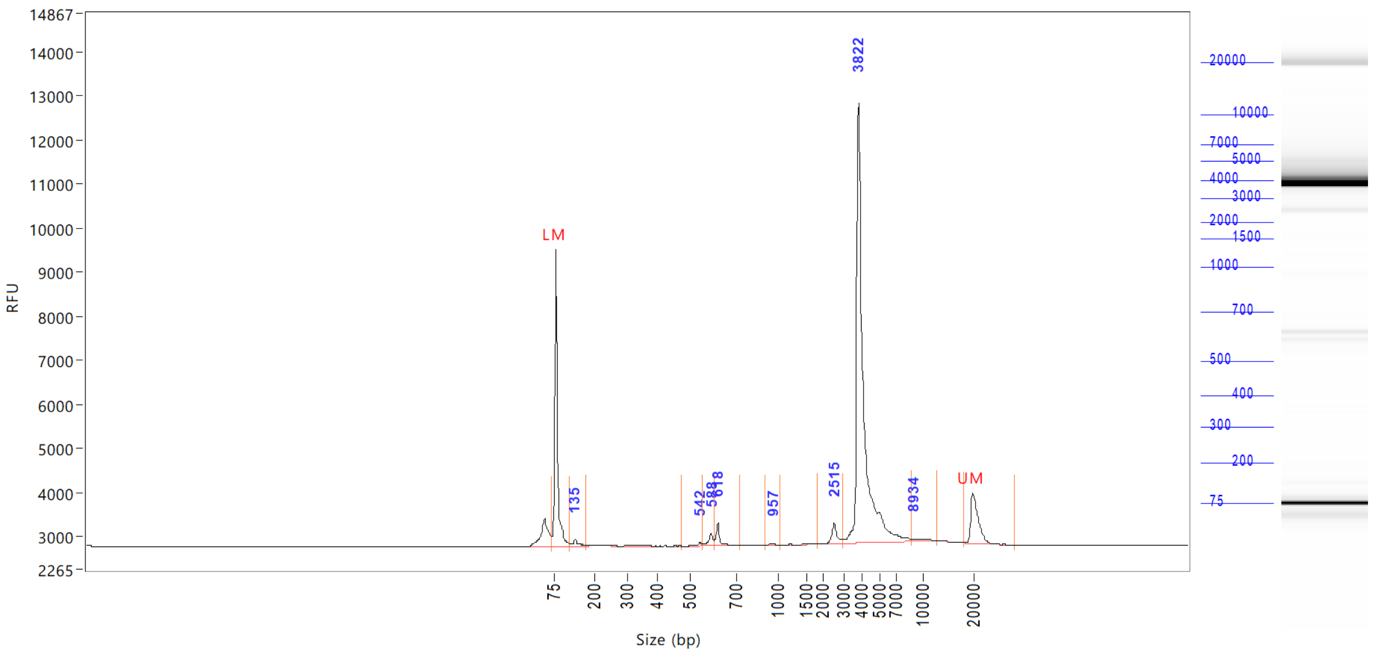

Peak	Size (bp)	Concentration (ng/uL)	From (bp)	To (bp)	Average size (bp)	CV%	RFU	Corrected peak area
1	75 (LM)	0.5000	64	122	76	10.63	6719	45.576
2	135	0.2571	122	173	141	9.28	147	1.953
3	542	0.0808	475	552	535	2.70	82	0.614
4	588	0.3253	552	600	582	2.03	294	2.471
5	618	0.4883	600	723	623	3.34	529	3.709
6	957	0.0616	900	1001	944	1.86	52	0.468
7	2515	0.4979	1846	2943	2534	6.80	471	3.782
8	3822	13.2818	2943	8734	4142	17.44	9992	100.889
9	8934	0.1372	8734	12806	9736	8.09	56	1.042
10	20000 (UM)	0.1204	18049	28342	20481	4.68	1135	10.971

TIC:	15.1301	ng/uL
TIM:	11.1876	nmole/L
Total concentration:	15.3319	ng/uL

Sample peak width (sec): 5   Sample min peak height: 50   Sample baseline V to V?: Y   Sample baseline V to V points: 3
Sample filter: Binomial   Number of points for filter: 3   Sample start region (min): 0   Sample end region (min): 30
Marker peak width (sec): 5   Marker min peak height: 200   Marker baseline V to V?: Y   Marker baseline V to V points: 3
Lower marker selection: First peak > 500 RFU   Upper marker selection: Last peak > 200 RFU
Ladder size (bp)75, 200, 300, 400, 500, 700, 1000, 1500, 2000, 3000, 4000, 5000, 7000, 10000, 20000
Quantification using: Lower Marker   Final concentration (ng/uL): 0.5000   Dilution factor: 12.0

Sample: SampE7
Well location: E7
Created: Tuesday, August 10, 2021 10:01:13 PM

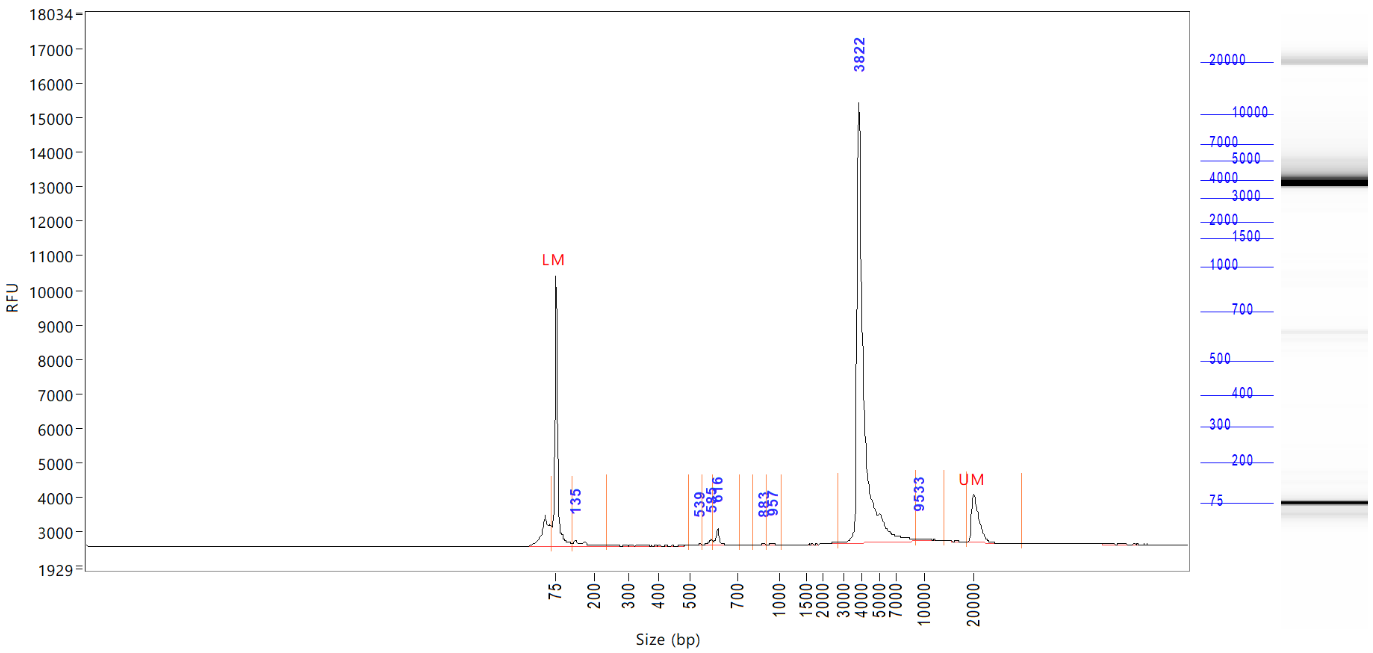

Peak	Size (bp)	Concentration (ng/uL)	From (bp)	To (bp)	Average size (bp)	CV%	RFU	Corrected peak area
1	75 (LM)	0.5000	62	128	75	12.74	7820	54.354
2	135	0.4217	128	233	159	16.10	163	3.820
3	539	0.0602	496	552	535	2.28	73	0.545
4	585	0.1811	552	595	579	1.90	186	1.640
5	616	0.3970	595	718	619	3.27	490	3.596
6	883	0.0488	809	913	878	2.36	62	0.442
7	957	0.0657	913	1023	946	1.78	63	0.595
8	3822	14.3118	2743	9134	4145	18.01	12775	129.651
9	9533	0.1424	9134	14147	10320	10.39	76	1.290
10	20000 (UM)	0.1215	18659	29793	20571	4.65	1383	13.210

TIC:	15.6286	ng/uL
TIM:	12.0273	nmole/L
Total concentration:	15.8059	ng/uL

Sample peak width (sec): 5   Sample min peak height: 50   Sample baseline V to V?: Y   Sample baseline V to V points: 3
Sample filter: Binomial   Number of points for filter: 3   Sample start region (min): 0   Sample end region (min): 30
Marker peak width (sec): 5   Marker min peak height: 200   Marker baseline V to V?: Y   Marker baseline V to V points: 3
Lower marker selection: First peak > 500 RFU   Upper marker selection: Last peak > 200 RFU
Ladder size (bp)75, 200, 300, 400, 500, 700, 1000, 1500, 2000, 3000, 4000, 5000, 7000, 10000, 20000
Quantification using: Lower Marker   Final concentration (ng/uL): 0.5000   Dilution factor: 12.0

Sample: SampF7
Well location: F7
Created: Tuesday, August 10, 2021 10:01:13 PM

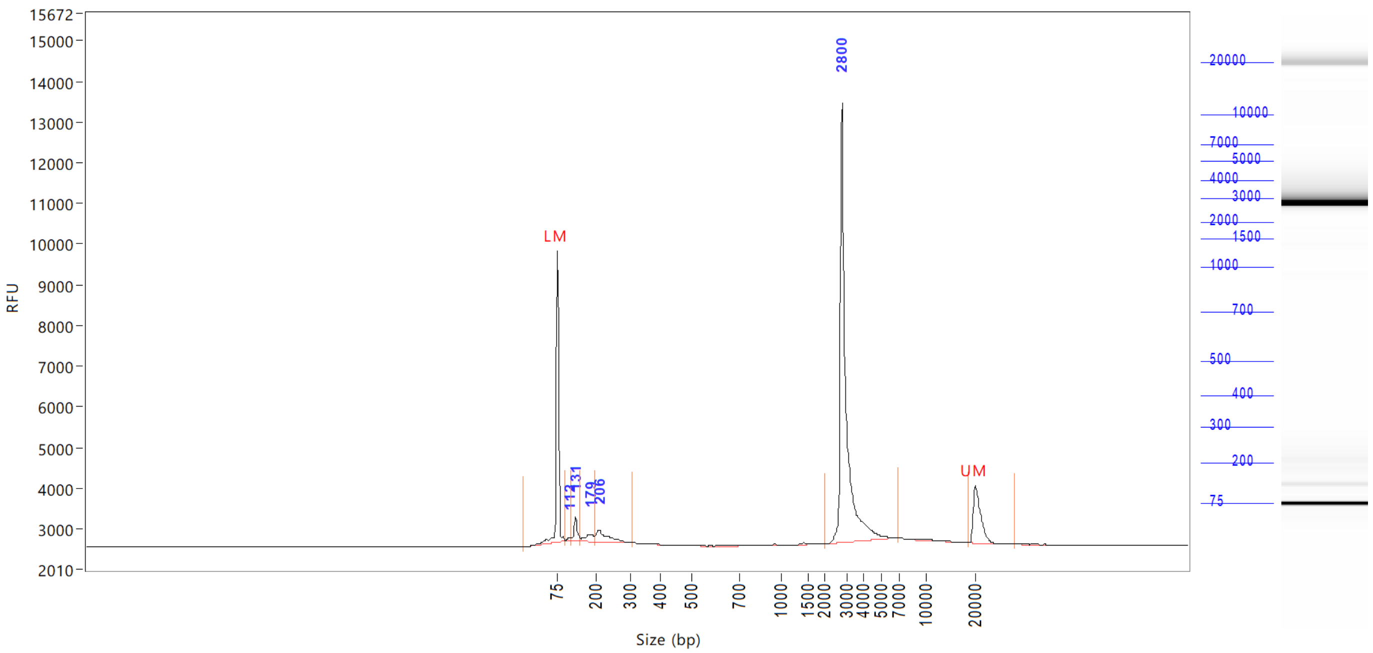

Peak	Size (bp)	Concentration (ng/uL)	From (bp)	To (bp)	Average size (bp)	CV%	RFU	Corrected peak area
1	75 (LM)	0.5000	0	99	70	15.24	7164	42.452
2	112	0.1248	99	120	111	4.97	96	0.883
3	131	0.6592	120	148	131	4.73	588	4.664
4	179	0.5417	148	192	173	6.94	184	3.833
5	206	1.0588	192	307	223	10.34	292	7.492
6	2800	13.3012	2000	6926	3015	15.65	10823	94.111
7	20000 (UM)	0.1606	18537	27738	20466	4.41	1425	13.632

TIC:	15.6858	ng/uL
TIM:	30.3329	nmole/L
Total concentration:	16.0308	ng/uL

Sample peak width (sec): 5 Sample min peak height: 50 Sample baseline V to V?: Y Sample baseline V to V points: 3
Sample filter: Binomial Number of points for filter: 3 Sample start region (min): 0 Sample end region (min): 30
Marker peak width (sec): 5 Marker min peak height: 200 Marker baseline V to V?: Y Marker baseline V to V points: 3
Lower marker selection: First peak > 500 RFU Upper marker selection: Last peak > 200 RFU
Ladder size (bp)75, 200, 300, 400, 500, 700, 1000, 1500, 2000, 3000, 4000, 5000, 7000, 10000, 20000
Quantification using: Lower Marker Final concentration (ng/uL): 0.5000 Dilution factor: 12.0

Sample: SampG7
Well location: G7
Created: Tuesday, August 10, 2021 10:01:13 PM

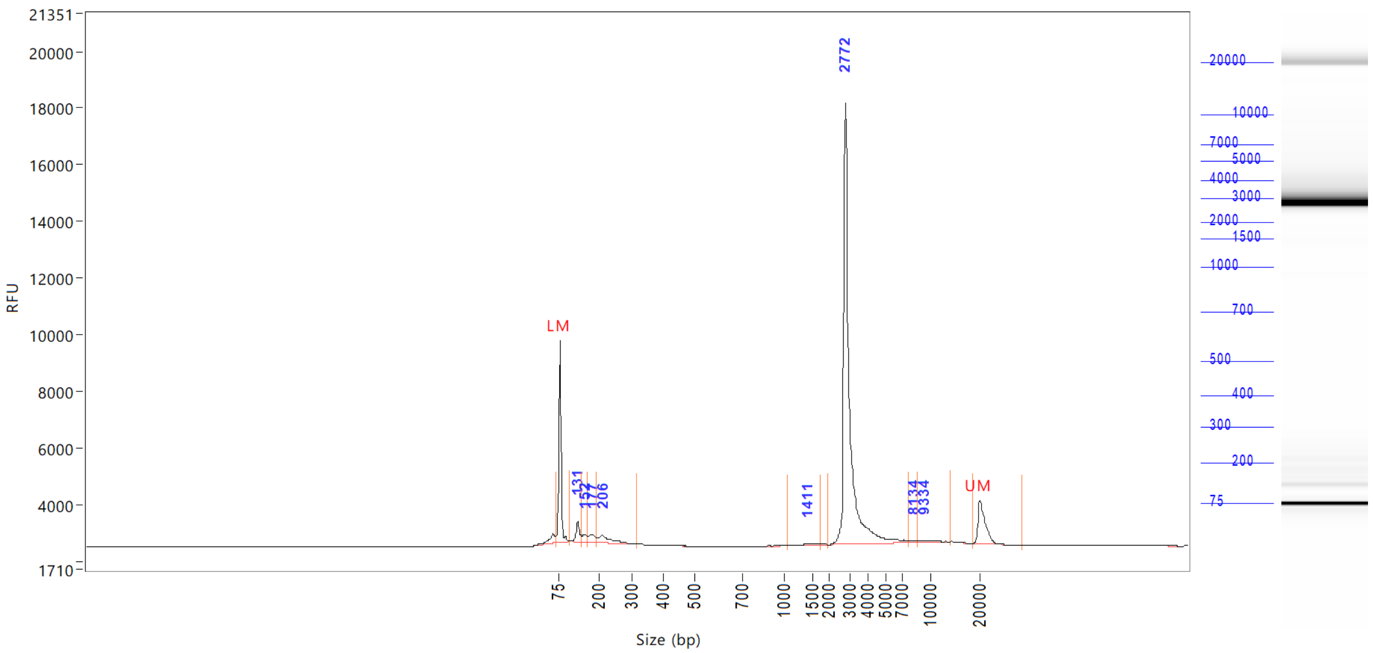

Peak	Size (bp)	Concentration (ng/uL)	From (bp)	To (bp)	Average size (bp)	CV%	RFU	Corrected peak area
1	75 (LM)	0.5000	64	105	73	5.69	7130	40.868
2	131	0.9327	105	145	129	5.83	716	6.353
3	152	0.4116	145	162	153	3.46	267	2.804
4	177	0.6876	162	192	176	4.94	277	4.683
5	206	1.0393	192	313	223	10.33	259	7.079
6	1411	0.1359	1056	1731	1449	7.53	62	0.926
7	2772	20.0271	1981	7601	3048	21.14	15616	136.410
8	8134	0.1170	7601	8667	8094	3.87	74	0.797
9	9334	0.2009	8667	14025	10063	11.15	65	1.369
10	20000 (UM)	0.1740	18537	28463	20520	4.27	1517	14.222

TIC:	23.5522	ng/uL
TIM:	41.4162	nmole/L
Total concentration:	23.6181	ng/uL

Sample peak width (sec): 5 Sample min peak height: 50 Sample baseline V to V?: Y Sample baseline V to V points: 3
Sample filter: Binomial Number of points for filter: 3 Sample start region (min): 0 Sample end region (min): 30
Marker peak width (sec): 5 Marker min peak height: 200 Marker baseline V to V?: Y Marker baseline V to V points: 3
Lower marker selection: First peak > 500 RFU Upper marker selection: Last peak > 200 RFU
Ladder size (bp)75, 200, 300, 400, 500, 700, 1000, 1500, 2000, 3000, 4000, 5000, 7000, 10000, 20000
Quantification using: Lower Marker Final concentration (ng/uL): 0.5000 Dilution factor: 12.0

Sample: SampH7
Well location: H7
Created: Tuesday, August 10, 2021 10:01:13 PM

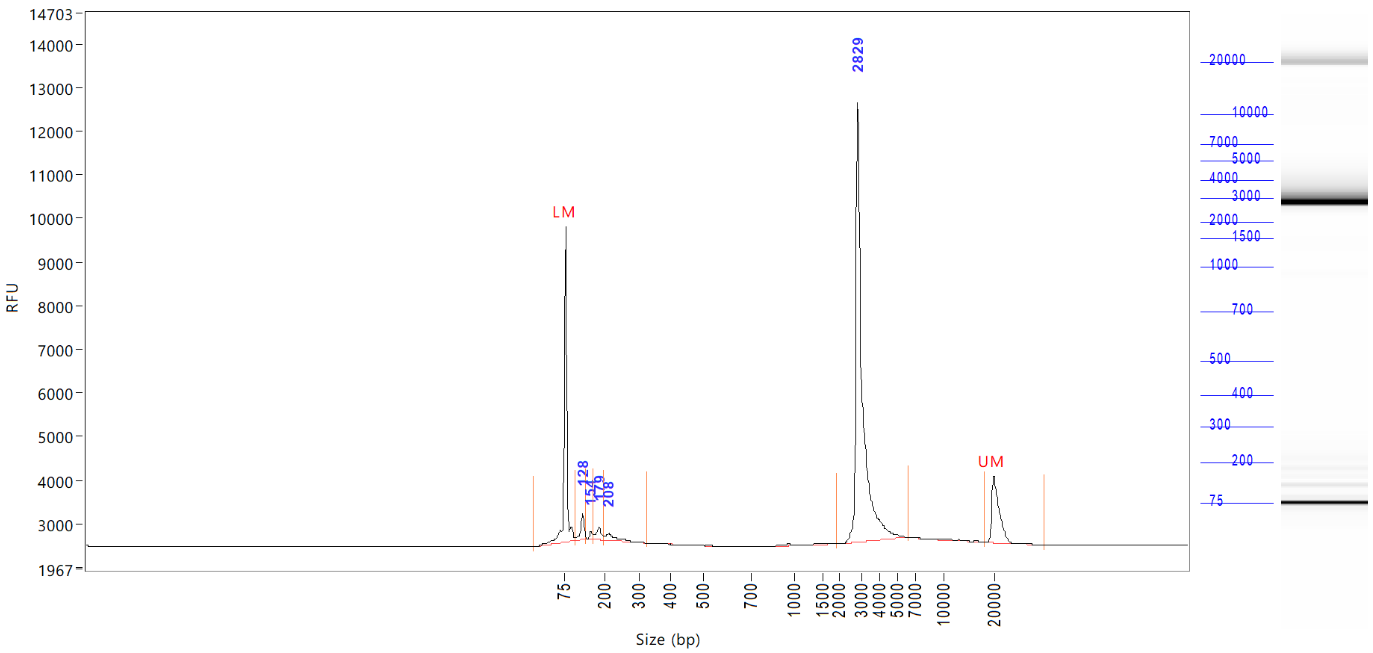

Peak	Size (bp)	Concentration (ng/uL)	From (bp)	To (bp)	Average size (bp)	CV%	RFU	Corrected peak area
1	75 (LM)	0.5000	0	105	72	17.12	7220	48.647
2	128	0.6147	105	141	124	5.31	607	4.984
3	154	0.1550	141	162	154	3.06	177	1.257
4	179	0.4261	162	196	178	4.92	281	3.455
5	208	0.4470	196	325	224	10.22	158	3.624
6	2829	11.6836	1923	6186	3045	14.04	10085	94.729
7	20000 (UM)	0.1492	18293	29914	20568	4.71	1513	14.512

TIC:	13.3265	ng/uL
TIM:	23.3326	nmole/L
Total concentration:	13.6212	ng/uL

```
Sample peak width (sec): 5    Sample min peak height: 50    Sample baseline V to V?: Y    Sample baseline V to V points: 3
Sample filter: Binomial       Number of points for filter: 3  Sample start region (min): 0  Sample end region (min): 30
Marker peak width (sec): 5    Marker min peak height: 200   Marker baseline V to V?: Y    Marker baseline V to V points: 3
Lower marker selection: First peak > 500 RFU                Upper marker selection: Last peak > 200 RFU
Ladder size (bp)75, 200, 300, 400, 500, 700, 1000, 1500, 2000, 3000, 4000, 5000, 7000, 10000, 20000
Quantification using: Lower Marker          Final concentration (ng/uL): 0.5000          Dilution factor: 12.0
```

Sample: SampA8
Well location: A8
Created: Tuesday, August 10, 2021 10:01:13 PM

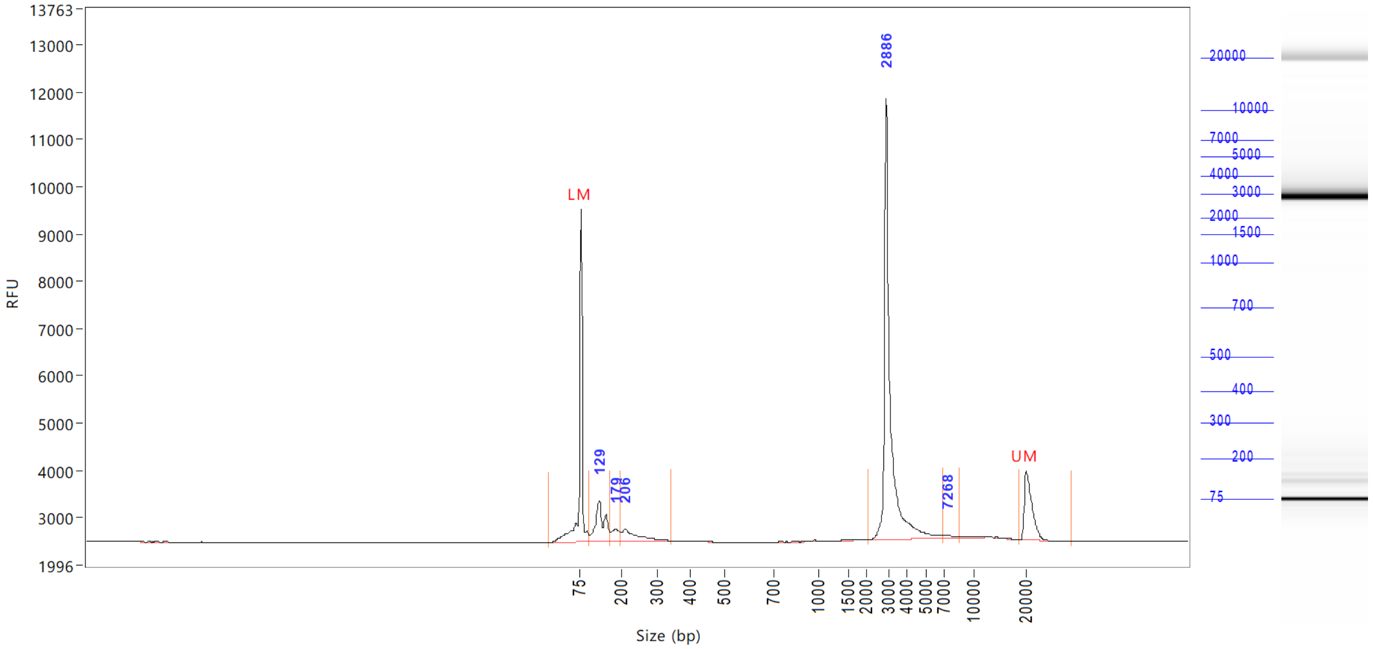

Peak	Size (bp)	Concentration (ng/uL)	From (bp)	To (bp)	Average size (bp)	CV%	RFU	Corrected peak area
1	75 (LM)	0.5000	0	101	68	23.58	7034	49.014
2	129	2.0626	101	162	132	11.13	856	16.849
3	179	0.5870	162	194	177	5.33	255	4.795
4	206	0.9711	194	339	230	12.72	249	7.933
5	2886	9.6554	2058	6926	3124	18.23	9343	78.875
6	7268	0.1072	6926	8600	7611	6.39	60	0.875
7	20000 (UM)	0.1376	18537	28463	20513	4.36	1446	13.484

TIC:	13.3832	ng/uL
TIM:	43.1813	nmole/L
Total concentration:	13.5820	ng/uL

Sample peak width (sec): 5 Sample min peak height: 50 Sample baseline V to V?: Y Sample baseline V to V points: 3
Sample filter: Binomial Number of points for filter: 3 Sample start region (min): 0 Sample end region (min): 30
Marker peak width (sec): 5 Marker min peak height: 200 Marker baseline V to V?: Y Marker baseline V to V points: 3
Lower marker selection: First peak > 500 RFU Upper marker selection: Last peak > 200 RFU
Ladder size (bp)75, 200, 300, 400, 500, 700, 1000, 1500, 2000, 3000, 4000, 5000, 7000, 10000, 20000
Quantification using: Lower Marker Final concentration (ng/uL): 0.5000 Dilution factor: 12.0

Sample: SampB8
Well location: B8
Created: Tuesday, August 10, 2021 10:01:13 PM

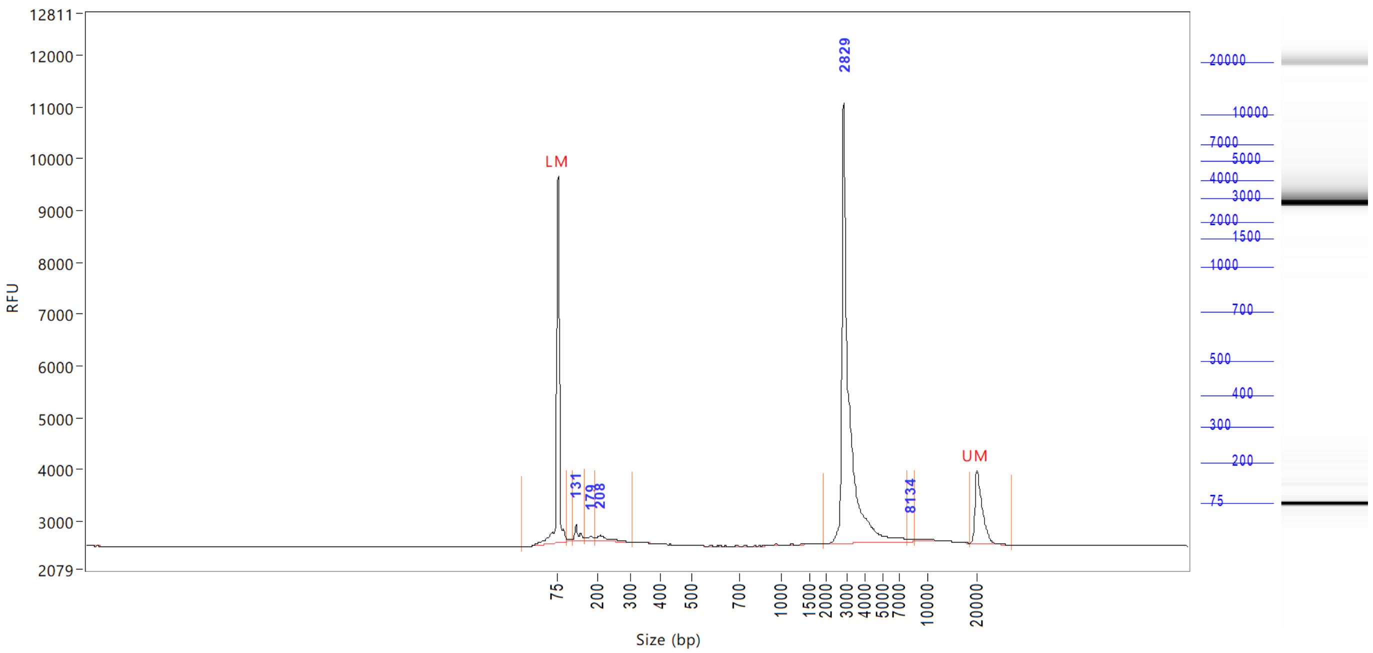

Peak	Size (bp)	Concentration (ng/uL)	From (bp)	To (bp)	Average size (bp)	CV%	RFU	Corrected peak area
1	75 (LM)	0.5000	0	101	70	17.72	7084	46.858
2	131	0.3663	122	158	136	6.75	301	2.861
3	179	0.1672	158	192	176	5.51	72	1.306
4	208	0.3325	192	305	222	10.39	105	2.597
5	2829	11.2426	1962	7734	3168	22.23	8514	87.801
6	8134	0.0582	7734	8667	8153	3.29	54	0.454
7	20000 (UM)	0.1429	18537	27133	20521	4.28	1419	13.397

TIC:	12.1667	ng/uL
TIM:	14.3046	nmole/L
Total concentration:	12.5064	ng/uL

Sample peak width (sec): 5 Sample min peak height: 50 Sample baseline V to V?: Y Sample baseline V to V points: 3
Sample filter: Binomial Number of points for filter: 3 Sample start region (min): 0 Sample end region (min): 30
Marker peak width (sec): 5 Marker min peak height: 200 Marker baseline V to V?: Y Marker baseline V to V points: 3
Lower marker selection: First peak > 500 RFU Upper marker selection: Last peak > 200 RFU
Ladder size (bp)75, 200, 300, 400, 500, 700, 1000, 1500, 2000, 3000, 4000, 5000, 7000, 10000, 20000
Quantification using: Lower Marker Final concentration (ng/uL): 0.5000 Dilution factor: 12.0

Sample: SampC8
Well location: C8
Created: Tuesday, August 10, 2021 10:01:13 PM

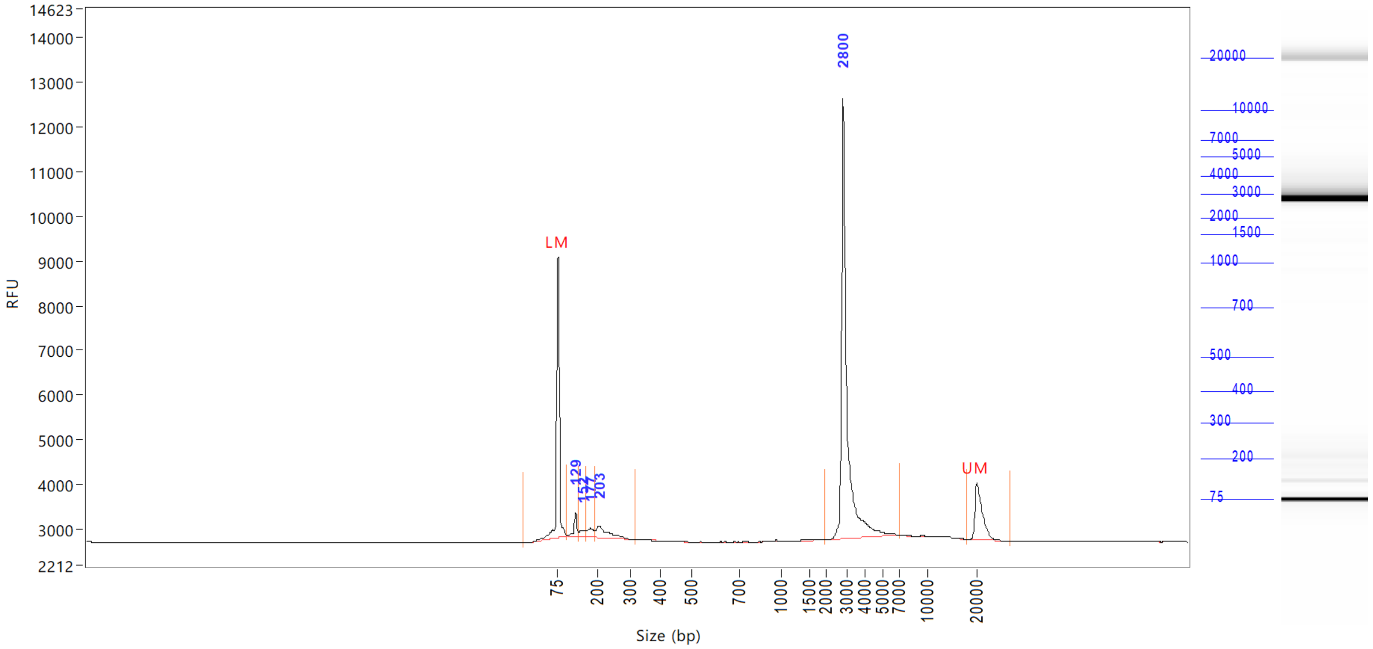

Peak	Size (bp)	Concentration (ng/uL)	From (bp)	To (bp)	Average size (bp)	CV%	RFU	Corrected peak area
1	75 (LM)	0.5000	0	101	70	16.21	6267	40.994
2	129	0.5282	101	139	127	5.17	535	3.609
3	152	0.2940	139	162	151	4.48	150	2.008
4	177	0.4616	162	190	176	4.75	196	3.154
5	203	1.0376	190	315	221	10.58	267	7.089
6	2800	11.3403	1981	7000	3003	15.61	9837	77.480
7	20000 (UM)	0.1482	18171	26649	20550	4.43	1253	12.149

TIC:	13.6617	ng/uL
TIM:	28.2817	nmole/L
Total concentration:	13.8956	ng/uL

```
Sample peak width (sec): 5    Sample min peak height: 50    Sample baseline V to V?: Y    Sample baseline V to V points: 3
Sample filter: Binomial       Number of points for filter: 3  Sample start region (min): 0  Sample end region (min): 30
Marker peak width (sec): 5    Marker min peak height: 200   Marker baseline V to V?: Y    Marker baseline V to V points: 3
Lower marker selection: First peak > 500 RFU                Upper marker selection: Last peak > 200 RFU
Ladder size (bp)75, 200, 300, 400, 500, 700, 1000, 1500, 2000, 3000, 4000, 5000, 7000, 10000, 20000
Quantification using: Lower Marker          Final concentration (ng/uL): 0.5000          Dilution factor: 12.0
```

Sample: SampD8
Well location: D8
Created: Tuesday, August 10, 2021 10:01:13 PM

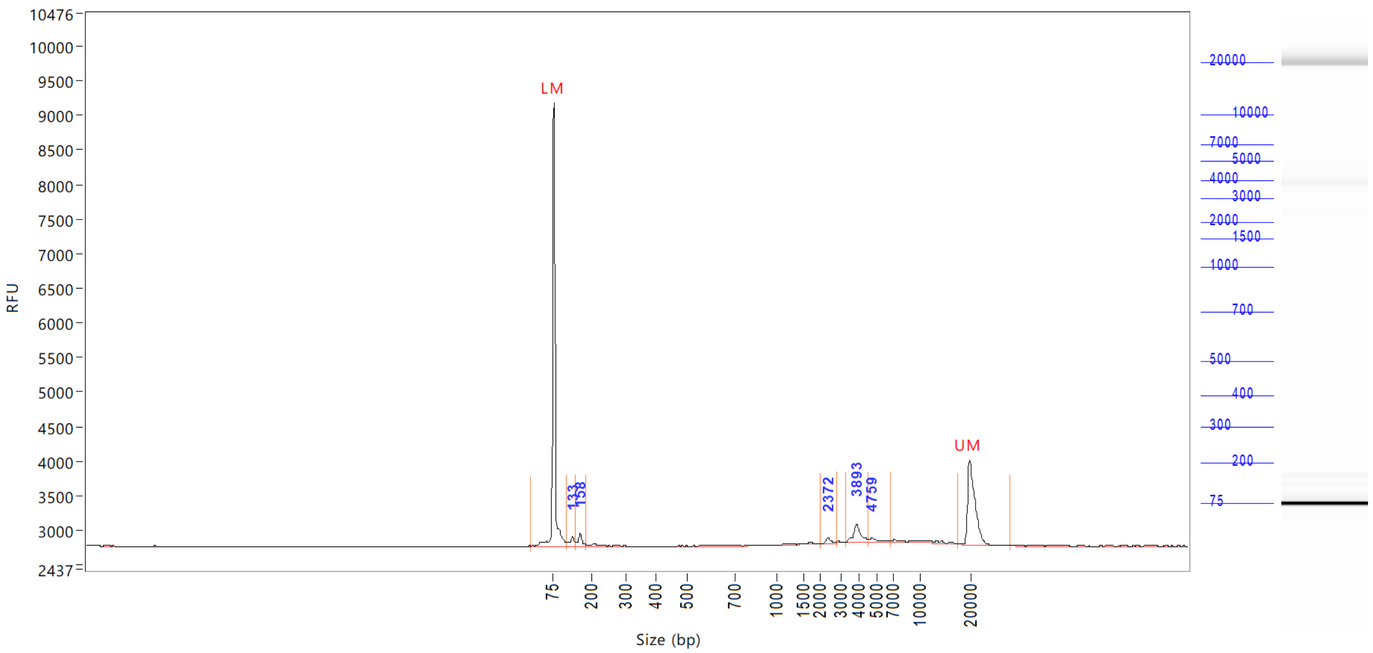

Peak	Size (bp)	Concentration (ng/uL)	From (bp)	To (bp)	Average size (bp)	CV%	RFU	Corrected peak area
1	75 (LM)	0.5000	1	116	73	13.62	6419	40.491
2	133	0.2062	116	143	130	5.50	133	1.391
3	158	0.2704	143	181	157	5.16	190	1.824
4	2372	0.1010	2001	2772	2400	6.48	86	0.682
5	3893	0.4435	3215	4518	3883	6.91	268	2.993
6	4759	0.1231	4518	6630	5154	11.66	56	0.831
7	20000 (UM)	0.1464	17439	28221	20447	4.65	1219	11.853

TIC:	1.1442	ng/uL
TIM:	5.7328	nmole/L
Total concentration:	1.7073	ng/uL

Sample peak width (sec): 5 Sample min peak height: 50 Sample baseline V to V?: Y Sample baseline V to V points: 3
Sample filter: Binomial Number of points for filter: 3 Sample start region (min): 0 Sample end region (min): 30
Marker peak width (sec): 5 Marker min peak height: 200 Marker baseline V to V?: Y Marker baseline V to V points: 3
Lower marker selection: First peak > 500 RFU Upper marker selection: Last peak > 200 RFU
Ladder size (bp)75, 200, 300, 400, 500, 700, 1000, 1500, 2000, 3000, 4000, 5000, 7000, 10000, 20000
Quantification using: Lower Marker Final concentration (ng/uL): 0.5000 Dilution factor: 12.0

Sample: SampE8
Well location: E8
Created: Tuesday, August 10, 2021 10:01:13 PM

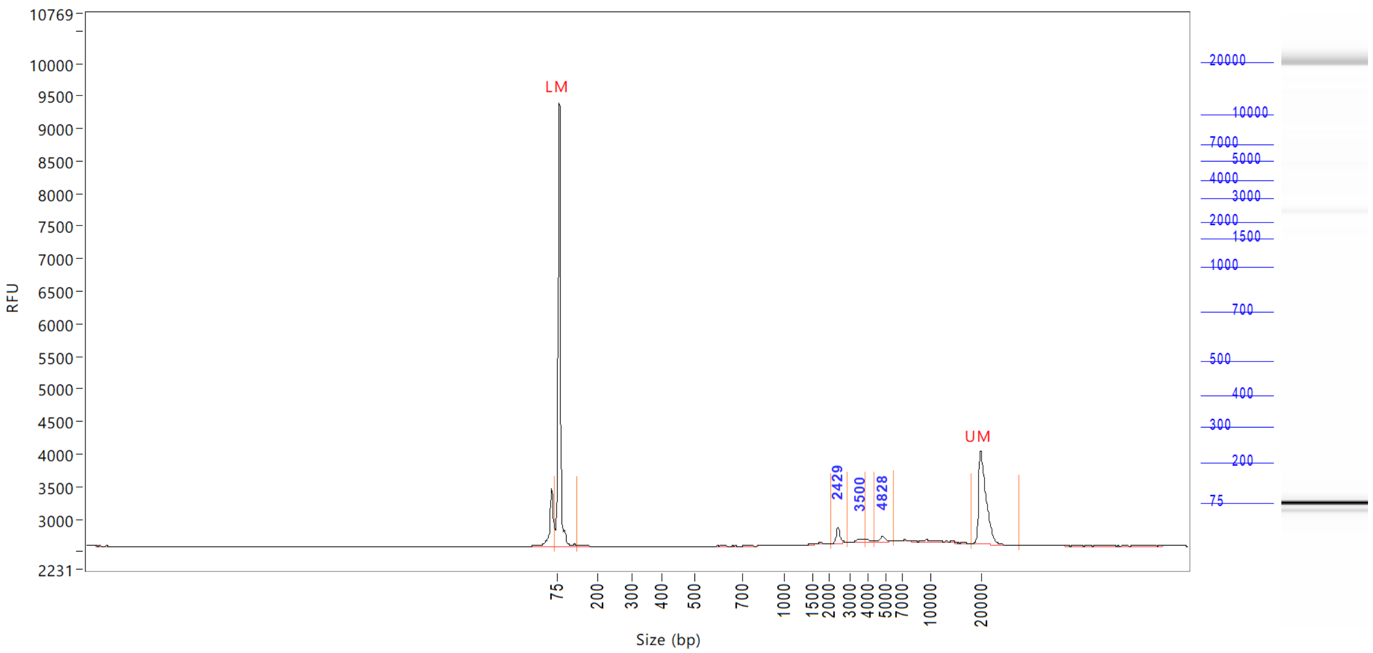

Peak	Size (bp)	Concentration (ng/uL)	From (bp)	To (bp)	Average size (bp)	CV%	RFU	Corrected peak area
1	75 (LM)	0.5000	66	131	75	8.67	6824	41.528
2	2429	0.2243	2087	2886	2436	4.52	254	1.553
3	3500	0.0845	2886	3822	3492	5.91	57	0.585
4	4828	0.1039	4311	5963	4879	6.74	76	0.719
5	20000 (UM)	0.1678	18293	27859	20550	4.36	1438	13.935

TIC:	0.4128	ng/uL
TIM:	0.2265	nmole/L
Total concentration:	0.9541	ng/uL

Sample peak width (sec): 5 Sample min peak height: 50 Sample baseline V to V?: Y Sample baseline V to V points: 3
Sample filter: Binomial Number of points for filter: 3 Sample start region (min): 0 Sample end region (min): 30
Marker peak width (sec): 5 Marker min peak height: 200 Marker baseline V to V?: Y Marker baseline V to V points: 3
Lower marker selection: First peak > 500 RFU Upper marker selection: Last peak > 200 RFU
Ladder size (bp)75, 200, 300, 400, 500, 700, 1000, 1500, 2000, 3000, 4000, 5000, 7000, 10000, 20000
Quantification using: Lower Marker Final concentration (ng/uL): 0.5000 Dilution factor: 12.0

Sample: SampF8
Well location: F8
Created: Tuesday, August 10, 2021 10:01:13 PM

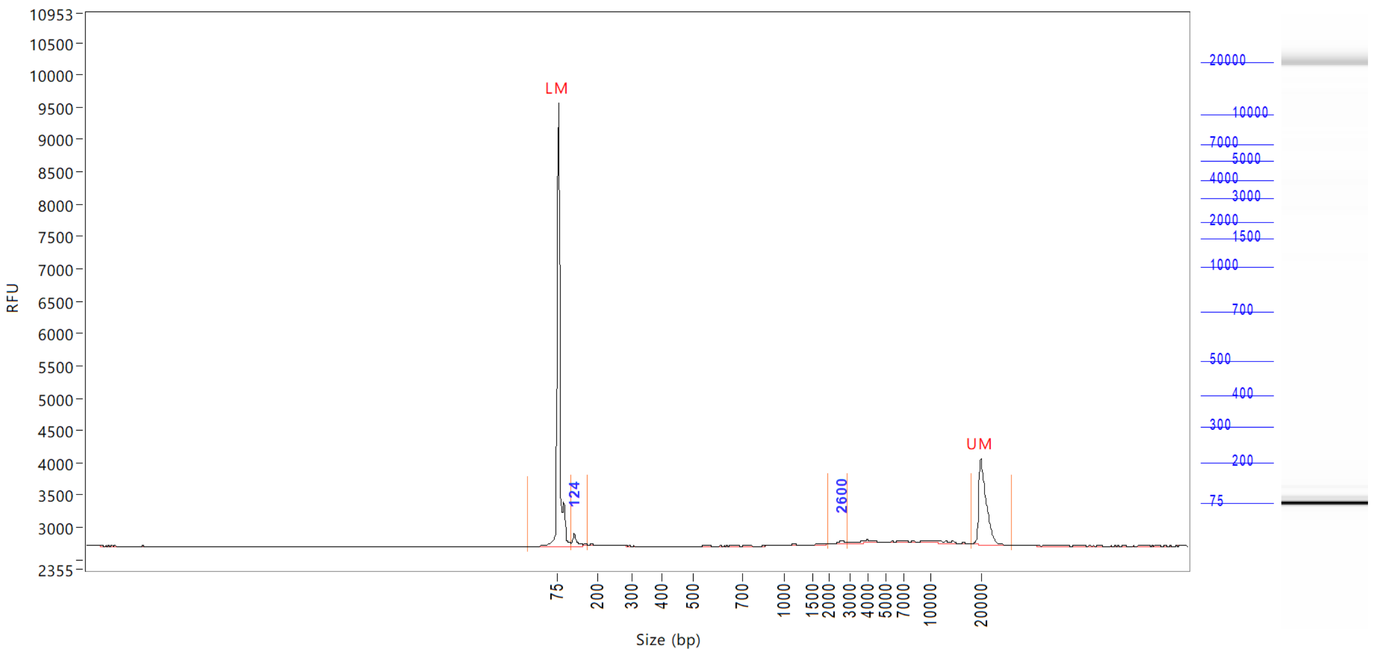

Peak	Size (bp)	Concentration (ng/uL)	From (bp)	To (bp)	Average size (bp)	CV%	RFU	Corrected peak area
1	75 (LM)	0.5000	0	114	75	11.88	6868	45.505
2	124	0.2874	114	168	131	10.59	203	2.180
3	2600	0.0825	2000	2886	2539	7.26	59	0.625
4	20000 (UM)	0.1430	18049	26287	20465	4.54	1329	13.016

TIC:	0.3698	ng/uL
TIM:	3.6580	nmole/L
Total concentration:	1.0276	ng/uL

Sample peak width (sec): 5 Sample min peak height: 50 Sample baseline V to V?: Y Sample baseline V to V points: 3
Sample filter: Binomial Number of points for filter: 3 Sample start region (min): 0 Sample end region (min): 30
Marker peak width (sec): 5 Marker min peak height: 200 Marker baseline V to V?: Y Marker baseline V to V points: 3
Lower marker selection: First peak > 500 RFU Upper marker selection: Last peak > 200 RFU
Ladder size (bp)75, 200, 300, 400, 500, 700, 1000, 1500, 2000, 3000, 4000, 5000, 7000, 10000, 20000
Quantification using: Lower Marker Final concentration (ng/uL): 0.5000 Dilution factor: 12.0

Sample: SampG8
Well location: G8
Created: Tuesday, August 10, 2021 10:01:13 PM

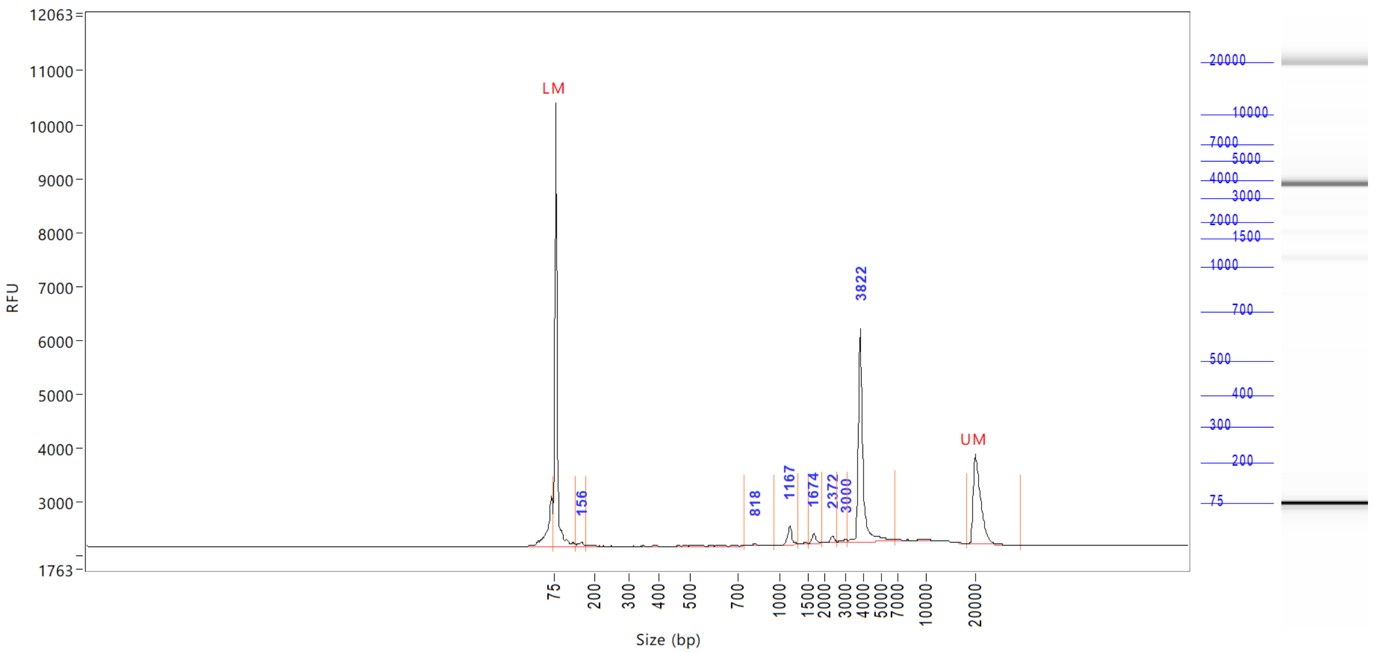

Peak	Size<br>(bp)	Concentration<br>(ng/uL)	From<br>(bp)	To<br>(bp)	Average size<br>(bp)	CV%	RFU	Corrected peak area
1	75 (LM)	0.5000	67	139	76	12.53	8227	49.816
2	156	0.1039	139	173	152	5.10	70	0.863
3	818	0.0569	740	957	821	4.88	51	0.472
4	1167	0.3015	957	1334	1150	4.33	349	2.503
5	1674	0.1332	1501	1942	1664	3.89	173	1.106
6	2372	0.0914	1942	2600	2353	4.39	128	0.759
7	3000	0.0570	2600	3108	2896	4.83	51	0.474
8	3822	3.2563	3108	6704	3914	9.93	3941	27.037
9	20000 (UM)	0.1602	18415	28947	20464	4.55	1664	15.962

TIC:	4.0002	ng/uL
TIM:	3.2672	nmole/L
Total concentration:	4.2943	ng/uL

```
Sample peak width (sec): 5    Sample min peak height: 50    Sample baseline V to V?: Y    Sample baseline V to V points: 3
Sample filter: Binomial       Number of points for filter: 3  Sample start region (min): 0  Sample end region (min): 30
Marker peak width (sec): 5    Marker min peak height: 200   Marker baseline V to V?: Y    Marker baseline V to V points: 3
Lower marker selection: First peak > 500 RFU                Upper marker selection: Last peak > 200 RFU
Ladder size (bp)75, 200, 300, 400, 500, 700, 1000, 1500, 2000, 3000, 4000, 5000, 7000, 10000, 20000
Quantification using: Lower Marker          Final concentration (ng/uL): 0.5000          Dilution factor: 12.0
```

Sample: SampH8
Well location: H8
Created: Tuesday, August 10, 2021 10:01:13 PM

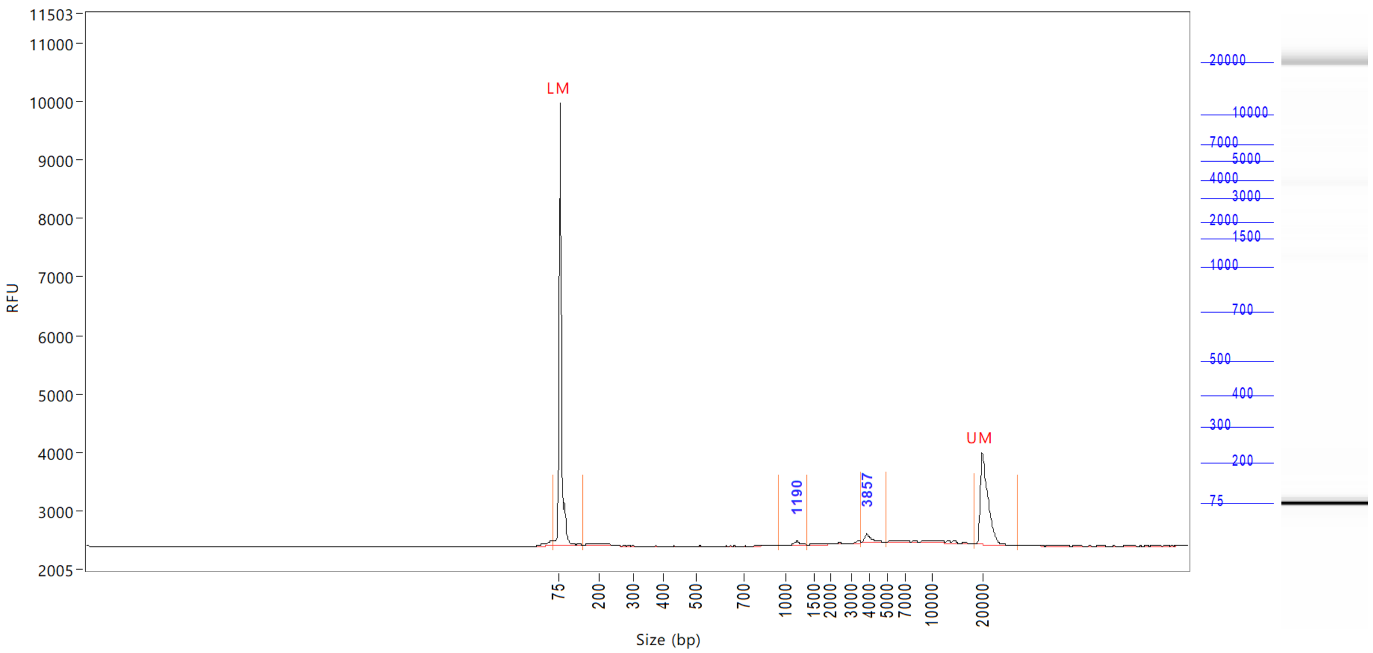

Peak	Size	Concentration	From	To	Average size	CV%	RFU	Corrected peak area
	(bp)	(ng/uL)	(bp)	(bp)	(bp)			
1	75 (LM)	0.5000	54	150	75	10.12	7575	46.941
2	1190	0.0936	948	1367	1179	6.74	72	0.732
3	3857	0.2006	3536	4966	3973	6.82	155	1.569
4	20000 (UM)	0.1651	18415	27133	20563	4.56	1568	15.498

TIC:	0.2942	ng/uL
TIM:	0.2138	nmole/L
Total concentration:	0.9199	ng/uL

Sample peak width (sec): 5 Sample min peak height: 50 Sample baseline V to V?: Y Sample baseline V to V points: 3
Sample filter: Binomial Number of points for filter: 3 Sample start region (min): 0 Sample end region (min): 30
Marker peak width (sec): 5 Marker min peak height: 200 Marker baseline V to V?: Y Marker baseline V to V points: 3
Lower marker selection: First peak > 500 RFU Upper marker selection: Last peak > 200 RFU
Ladder size (bp)75, 200, 300, 400, 500, 700, 1000, 1500, 2000, 3000, 4000, 5000, 7000, 10000, 20000
Quantification using: Lower Marker Final concentration (ng/uL): 0.5000 Dilution factor: 12.0

Sample: SampA9
Well location: A9
Created: Tuesday, August 10, 2021 10:01:13 PM

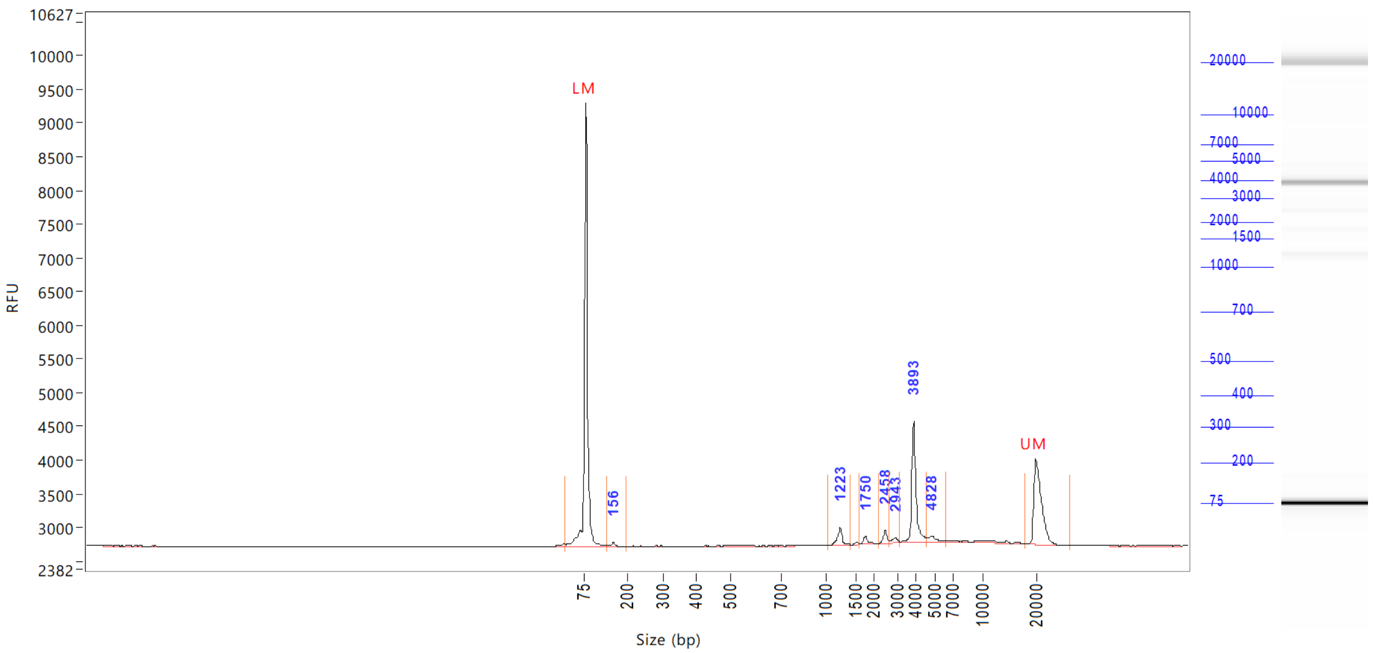

Peak	Size (bp)	Concentration (ng/uL)	From (bp)	To (bp)	Average size (bp)	CV%	RFU	Corrected peak area
1	75 (LM)	0.5000	18	137	73	14.45	6589	45.084
2	156	0.0785	137	196	155	7.51	60	0.590
3	1223	0.2568	1023	1411	1202	4.43	259	1.930
4	1750	0.1053	1578	2172	1760	5.22	113	0.791
5	2458	0.1406	2172	2686	2463	3.61	191	1.057
6	2943	0.0776	2686	3144	2880	3.91	70	0.583
7	3893	1.4811	3144	4587	3896	4.69	1789	11.129
8	4828	0.1284	4587	6112	4977	7.21	87	0.965
9	20000 (UM)	0.1316	18049	26287	20438	4.34	1268	11.866

TIC:	2.2683	ng/uL
TIM:	2.0887	nmole/L
Total concentration:	2.6195	ng/uL

```
Sample peak width (sec): 5    Sample min peak height: 50    Sample baseline V to V?: Y    Sample baseline V to V points: 3
Sample filter: Binomial       Number of points for filter: 3  Sample start region (min): 0  Sample end region (min): 30
Marker peak width (sec): 5    Marker min peak height: 200   Marker baseline V to V?: Y    Marker baseline V to V points: 3
Lower marker selection: First peak > 500 RFU                Upper marker selection: Last peak > 200 RFU
Ladder size (bp)75, 200, 300, 400, 500, 700, 1000, 1500, 2000, 3000, 4000, 5000, 7000, 10000, 20000
Quantification using: Lower Marker          Final concentration (ng/uL): 0.5000          Dilution factor: 12.0
```

Sample: SampB9
Well location: B9
Created: Tuesday, August 10, 2021 10:01:13 PM

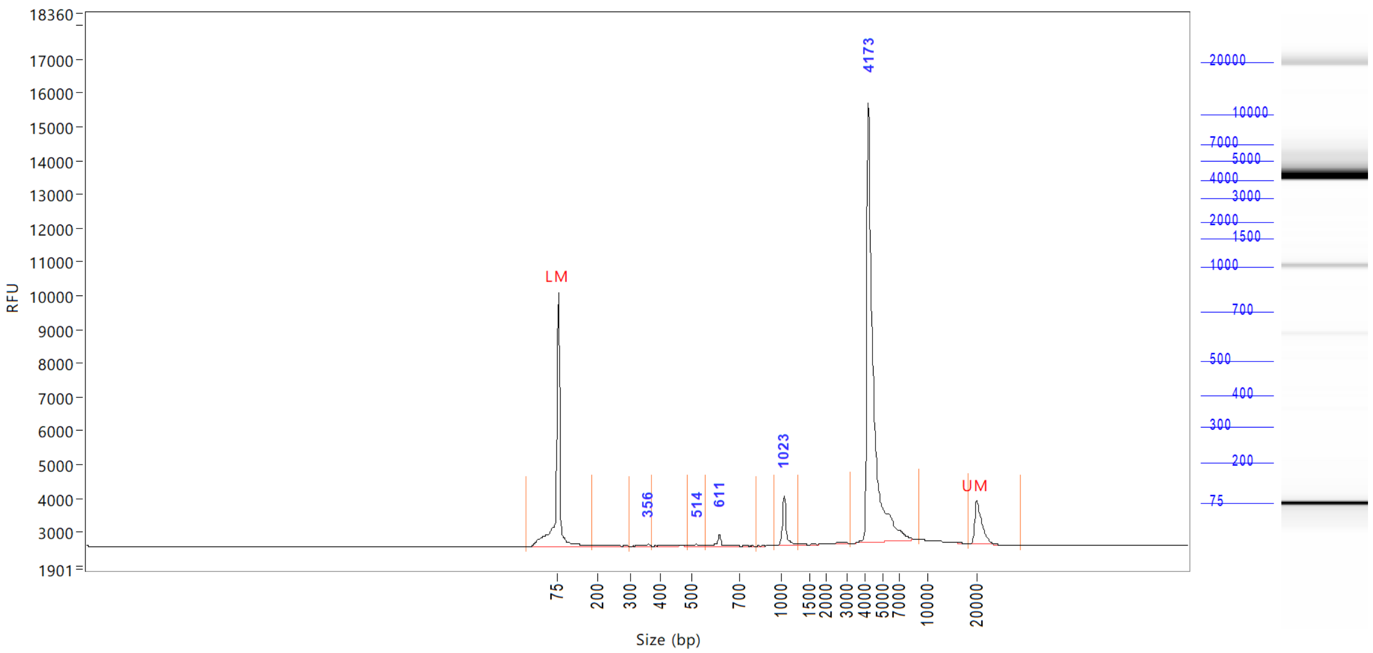

Peak	Size (bp)	Concentration (ng/uL)	From (bp)	To (bp)	Average size (bp)	CV%	RFU	Corrected peak area
1	75 (LM)	0.5000	0	183	67	35.46	7508	65.876
2	356	0.0460	296	370	343	4.92	63	0.505
3	514	0.0485	487	549	516	2.77	54	0.532
4	611	0.2642	549	809	615	6.02	351	2.901
5	1023	0.8672	944	1300	1035	5.20	1487	9.521
6	4173	11.4023	3108	9134	4502	14.75	13032	125.190
7	20000 (UM)	0.0916	18415	29068	20496	4.67	1284	12.073

TIC:	12.6282	ng/uL
TIM:	6.6310	nmole/L
Total concentration:	12.8792	ng/uL

Sample peak width (sec): 5 Sample min peak height: 50 Sample baseline V to V?: Y Sample baseline V to V points: 3
Sample filter: Binomial Number of points for filter: 3 Sample start region (min): 0 Sample end region (min): 30
Marker peak width (sec): 5 Marker min peak height: 200 Marker baseline V to V?: Y Marker baseline V to V points: 3
Lower marker selection: First peak > 500 RFU Upper marker selection: Last peak > 200 RFU
Ladder size (bp)75, 200, 300, 400, 500, 700, 1000, 1500, 2000, 3000, 4000, 5000, 7000, 10000, 20000
Quantification using: Lower Marker Final concentration (ng/uL): 0.5000 Dilution factor: 12.0

Sample: SampC9
Well location: C9
Created: Tuesday, August 10, 2021 10:01:13 PM

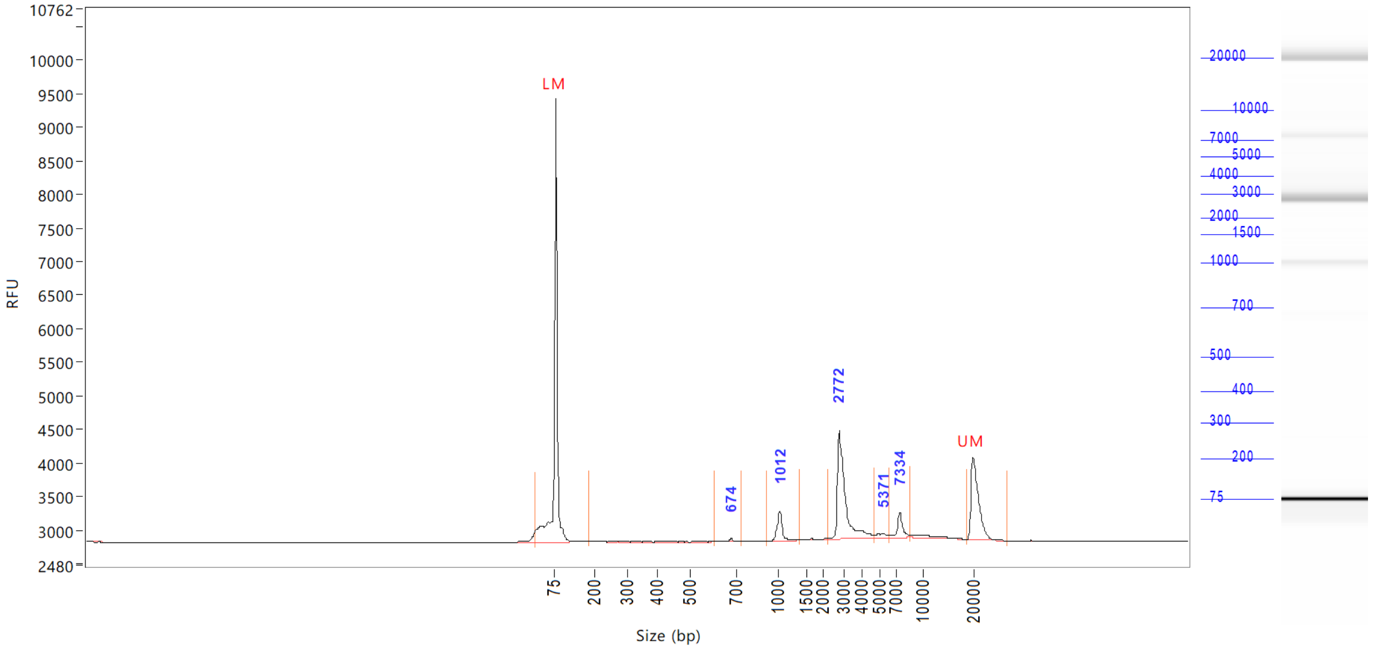

Peak	Size (bp)	Concentration (ng/uL)	From (bp)	To (bp)	Average size (bp)	CV%	RFU	Corrected peak area
1	75 (LM)	0.5000	11	179	68	25.81	6613	49.743
2	674	0.0671	608	731	664	3.54	53	0.556
3	1012	0.4541	909	1378	1024	7.03	449	3.765
4	2772	2.3199	2258	4759	3011	14.76	1625	19.233
5	5371	0.1048	4759	6112	5270	7.43	60	0.868
6	7334	0.3663	6112	8534	7335	5.91	378	3.037
7	20000 (UM)	0.1196	18659	26891	20544	4.62	1231	11.899

TIC:	3.3122	ng/uL
TIM:	2.2796	nmole/L
Total concentration:	3.6190	ng/uL

Sample peak width (sec): 5 Sample min peak height: 50 Sample baseline V to V?: Y Sample baseline V to V points: 3
Sample filter: Binomial Number of points for filter: 3 Sample start region (min): 0 Sample end region (min): 30
Marker peak width (sec): 5 Marker min peak height: 200 Marker baseline V to V?: Y Marker baseline V to V points: 3
Lower marker selection: First peak > 500 RFU Upper marker selection: Last peak > 200 RFU
Ladder size (bp)75, 200, 300, 400, 500, 700, 1000, 1500, 2000, 3000, 4000, 5000, 7000, 10000, 20000
Quantification using: Lower Marker Final concentration (ng/uL): 0.5000 Dilution factor: 12.0

Sample: SampD9
Well location: D9
Created: Tuesday, August 10, 2021 10:01:13 PM

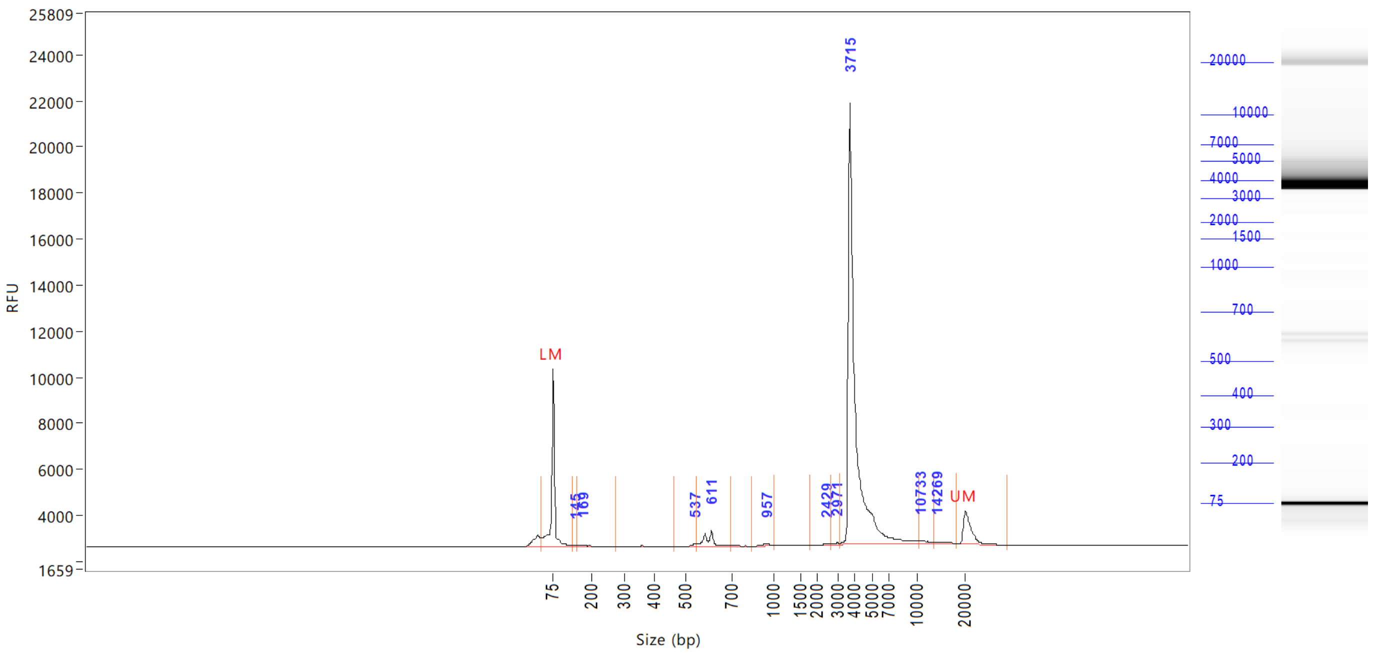

Peak	Size (bp)	Concentration (ng/uL)	From (bp)	To (bp)	Average size (bp)	CV%	RFU	Corrected peak area
1	75 (LM)	0.5000	37	135	71	17.90	7744	59.682
2	145	0.0616	135	152	144	3.71	61	0.613
3	169	0.1987	152	273	187	15.73	82	1.976
4	537	0.1250	464	547	528	3.21	128	1.244
5	611	0.9858	547	695	596	4.51	676	9.806
6	957	0.1069	844	1001	935	2.72	90	1.063
7	2429	0.0611	1808	2715	2421	7.05	71	0.608
8	2971	0.0657	2715	3108	2932	3.58	75	0.654
9	3715	20.3032	3108	10367	4159	23.51	19212	201.954
10	10733	0.2226	10367	13659	11904	8.28	134	2.215
11	14269	0.1951	13659	18293	15607	8.54	108	1.941
12	20000 (UM)	0.1254	18293	28584	20675	5.52	1452	14.973

TIC:	22.3257	ng/uL
TIM:	13.9160	nmole/L
Total concentration:	22.4589	ng/uL

Sample peak width (sec): 5 Sample min peak height: 50 Sample baseline V to V?: Y Sample baseline V to V points: 3
Sample filter: Binomial Number of points for filter: 3 Sample start region (min): 0 Sample end region (min): 30
Marker peak width (sec): 5 Marker min peak height: 200 Marker baseline V to V?: Y Marker baseline V to V points: 3
Lower marker selection: First peak > 500 RFU Upper marker selection: Last peak > 200 RFU
Ladder size (bp)75, 200, 300, 400, 500, 700, 1000, 1500, 2000, 3000, 4000, 5000, 7000, 10000, 20000
Quantification using: Lower Marker Final concentration (ng/uL): 0.5000 Dilution factor: 12.0

Sample: SampE9
Well location: E9
Created: Tuesday, August 10, 2021 10:01:13 PM

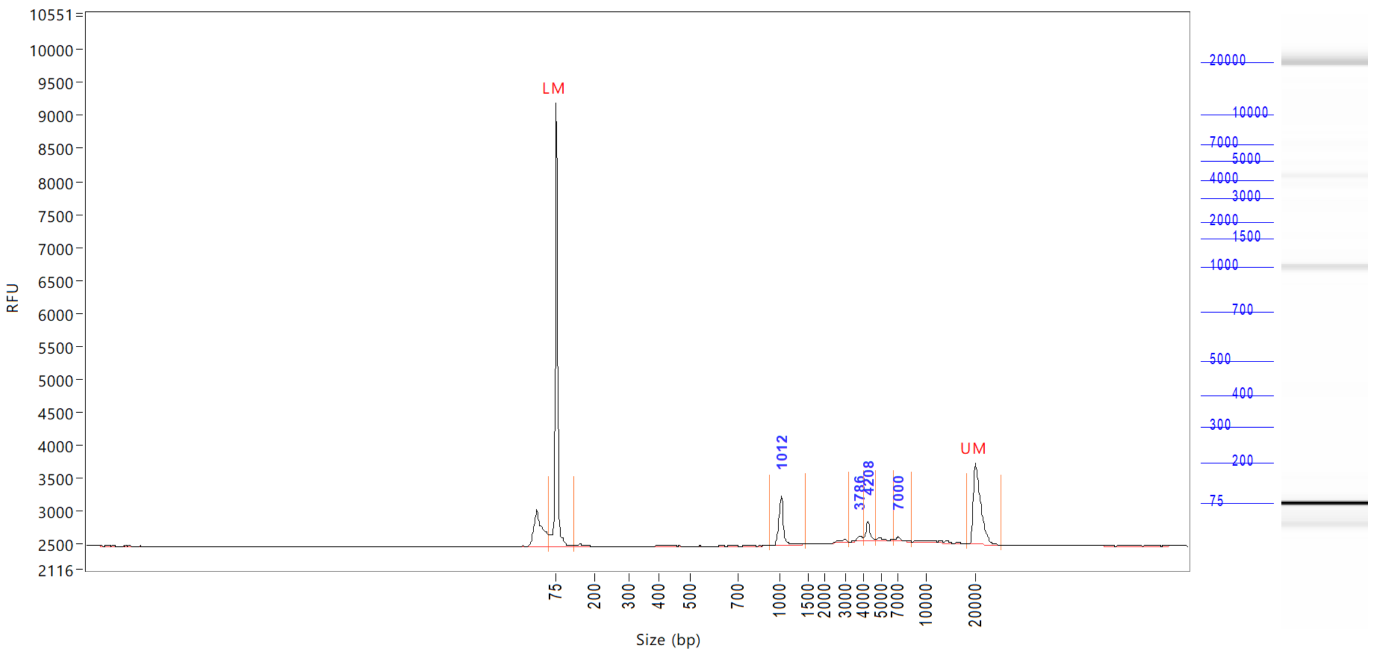

Peak	Size (bp)	Concentration (ng/uL)	From (bp)	To (bp)	Average size (bp)	CV%	RFU	Corrected peak area
1	75 (LM)	0.5000	54	133	74	9.24	6743	40.010
2	1012	0.8443	922	1456	1016	5.90	748	5.630
3	3786	0.0950	3144	4000	3752	4.22	70	0.633
4	4208	0.2777	4000	4690	4224	3.28	300	1.852
5	7000	0.0721	6482	8467	7154	6.70	60	0.481
6	20000 (UM)	0.1478	18293	25320	20445	4.15	1239	11.825

TIC:	1.2891	ng/uL
TIM:	1.5343	nmole/L
Total concentration:	1.9373	ng/uL

Sample peak width (sec): 5 Sample min peak height: 50 Sample baseline V to V?: Y Sample baseline V to V points: 3
Sample filter: Binomial Number of points for filter: 3 Sample start region (min): 0 Sample end region (min): 30
Marker peak width (sec): 5 Marker min peak height: 200 Marker baseline V to V?: Y Marker baseline V to V points: 3
Lower marker selection: First peak > 500 RFU Upper marker selection: Last peak > 200 RFU
Ladder size (bp)75, 200, 300, 400, 500, 700, 1000, 1500, 2000, 3000, 4000, 5000, 7000, 10000, 20000
Quantification using: Lower Marker Final concentration (ng/uL): 0.5000 Dilution factor: 12.0

Sample: SampF9
Well location: F9
Created: Tuesday, August 10, 2021 10:01:13 PM

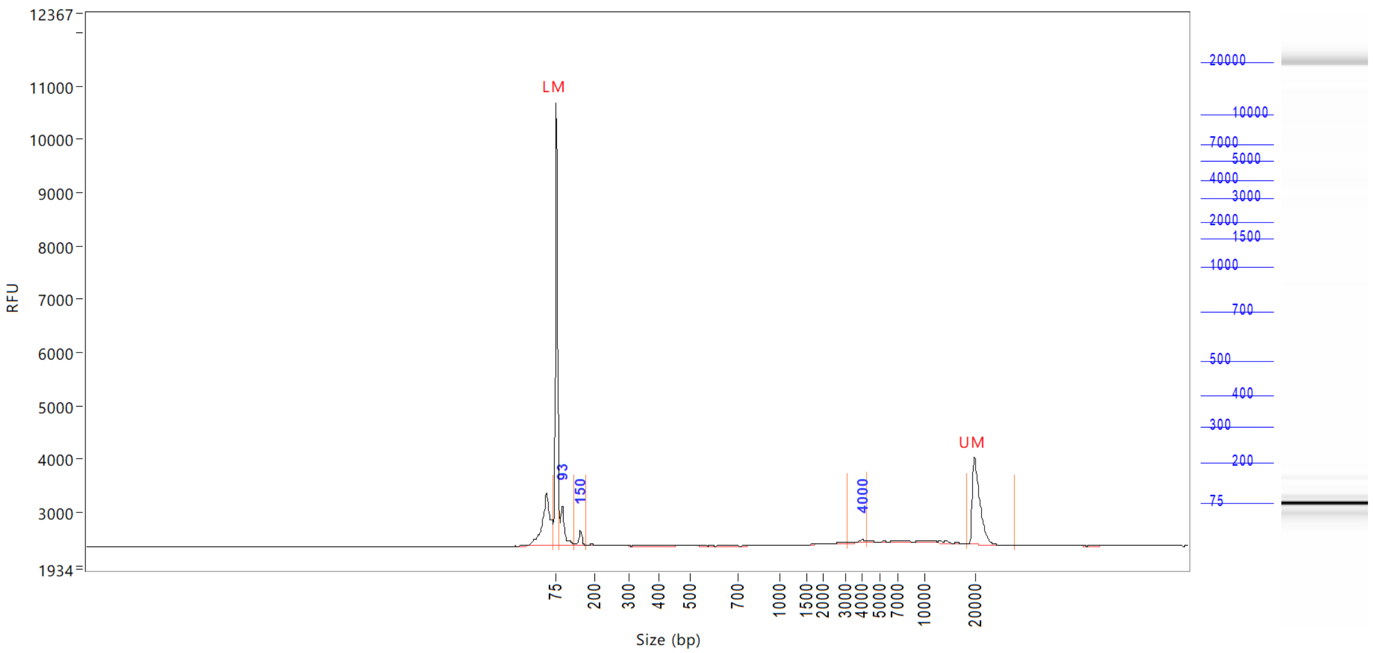

Peak	Size (bp)	Concentration (ng/uL)	From (bp)	To (bp)	Average size (bp)	CV%	RFU	Corrected peak area
1	75 (LM)	0.5000	66	86	74	4.78	8322	46.876
2	93	0.9684	86	133	96	11.38	730	7.566
3	150	0.2826	133	169	148	3.86	270	2.208
4	4000	0.0779	3108	4208	3818	7.11	54	0.609
5	20000 (UM)	0.1690	18537	28342	20551	4.46	1643	15.845

TIC:	1.3289	ng/uL
TIM:	19.7345	nmole/L
Total concentration:	1.9139	ng/uL

Sample peak width (sec): 5 Sample min peak height: 50 Sample baseline V to V?: Y Sample baseline V to V points: 3
Sample filter: Binomial Number of points for filter: 3 Sample start region (min): 0 Sample end region (min): 30
Marker peak width (sec): 5 Marker min peak height: 200 Marker baseline V to V?: Y Marker baseline V to V points: 3
Lower marker selection: First peak > 500 RFU Upper marker selection: Last peak > 200 RFU
Ladder size (bp)75, 200, 300, 400, 500, 700, 1000, 1500, 2000, 3000, 4000, 5000, 7000, 10000, 20000
Quantification using: Lower Marker Final concentration (ng/uL): 0.5000 Dilution factor: 12.0

Sample: SampG9
Well location: G9
Created: Tuesday, August 10, 2021 10:01:13 PM

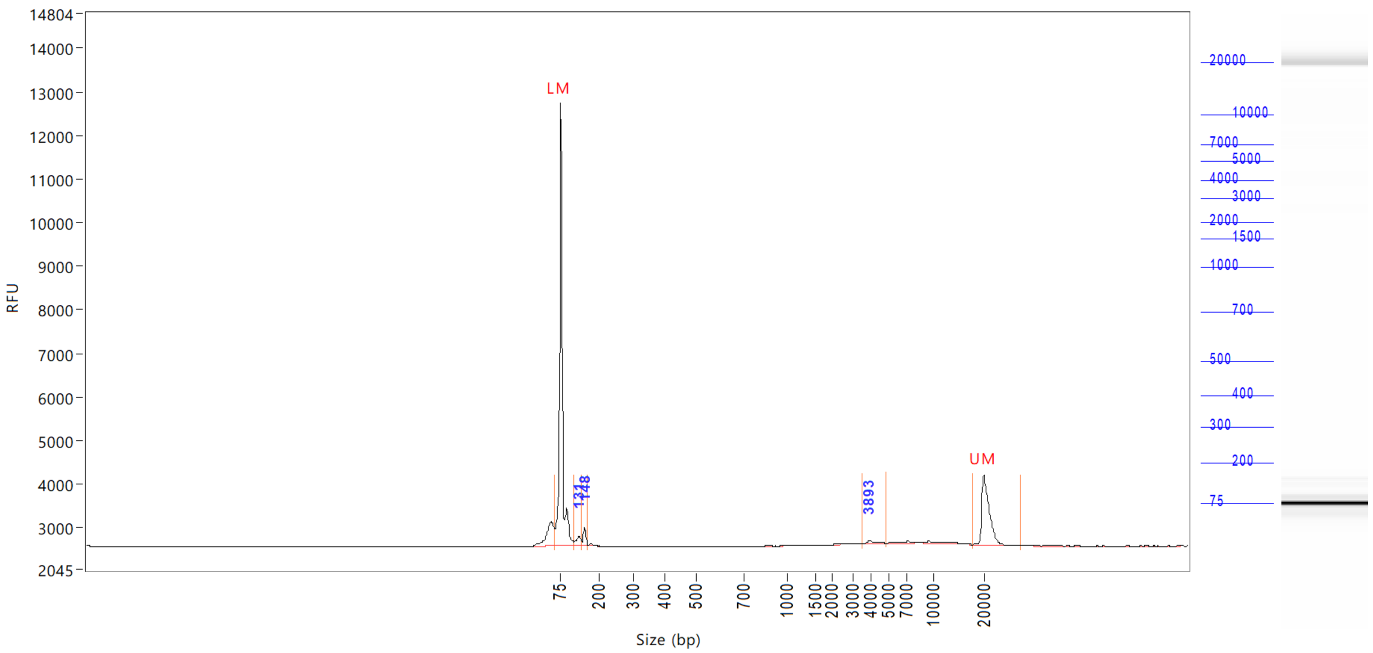

Peak	Size (bp)	Concentration (ng/uL)	From (bp)	To (bp)	Average size (bp)	CV%	RFU	Corrected peak area
1	75 (LM)	0.5000	58	116	75	10.95	10177	70.532
2	131	0.1960	116	141	129	5.21	217	2.303
3	148	0.1907	141	160	147	2.20	403	2.241
4	3893	0.0619	3500	4862	4021	6.83	55	0.728
5	20000 (UM)	0.1086	17683	27254	20462	4.46	1590	15.325

TIC:	0.4485	ng/uL
TIM:	4.6527	nmole/L
Total concentration:	0.7884	ng/uL

Sample peak width (sec): 5 Sample min peak height: 50 Sample baseline V to V?: Y Sample baseline V to V points: 3
Sample filter: Binomial Number of points for filter: 3 Sample start region (min): 0 Sample end region (min): 30
Marker peak width (sec): 5 Marker min peak height: 200 Marker baseline V to V?: Y Marker baseline V to V points: 3
Lower marker selection: First peak > 500 RFU Upper marker selection: Last peak > 200 RFU
Ladder size (bp)75, 200, 300, 400, 500, 700, 1000, 1500, 2000, 3000, 4000, 5000, 7000, 10000, 20000
Quantification using: Lower Marker Final concentration (ng/uL): 0.5000 Dilution factor: 12.0

Sample: SampH9
Well location: H9
Created: Tuesday, August 10, 2021 10:01:13 PM

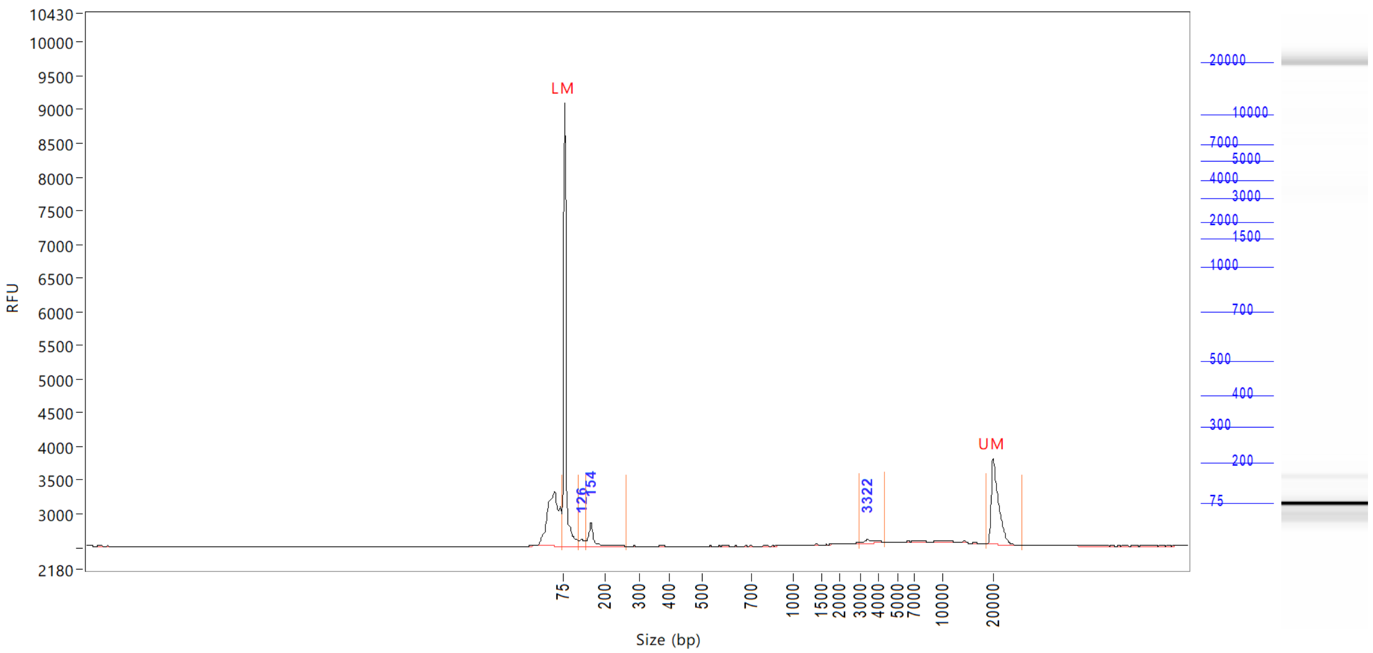

Peak	Size (bp)	Concentration (ng/uL)	From (bp)	To (bp)	Average size (bp)	CV%	RFU	Corrected peak area
1	75 (LM)	0.5000	66	120	75	11.09	6586	41.789
2	126	0.1851	120	141	130	4.97	104	1.289
3	154	0.5547	141	262	161	12.51	349	3.864
4	3322	0.1303	2943	4277	3549	9.53	53	0.907
5	20000 (UM)	0.1428	18659	25682	20497	4.27	1270	11.932

TIC:	0.8701	ng/uL
TIM:	8.0635	nmole/L
Total concentration:	1.1523	ng/uL

```
Sample peak width (sec): 5    Sample min peak height: 50    Sample baseline V to V?: Y    Sample baseline V to V points: 3
Sample filter: Binomial       Number of points for filter: 3  Sample start region (min): 0  Sample end region (min): 30
Marker peak width (sec): 5    Marker min peak height: 200   Marker baseline V to V?: Y    Marker baseline V to V points: 3
Lower marker selection: First peak > 500 RFU                Upper marker selection: Last peak > 200 RFU
Ladder size (bp)75, 200, 300, 400, 500, 700, 1000, 1500, 2000, 3000, 4000, 5000, 7000, 10000, 20000
Quantification using: Lower Marker          Final concentration (ng/uL): 0.5000          Dilution factor: 12.0
```

Sample: SampA10
Well location: A10
Created: Tuesday, August 10, 2021 10:01:13 PM

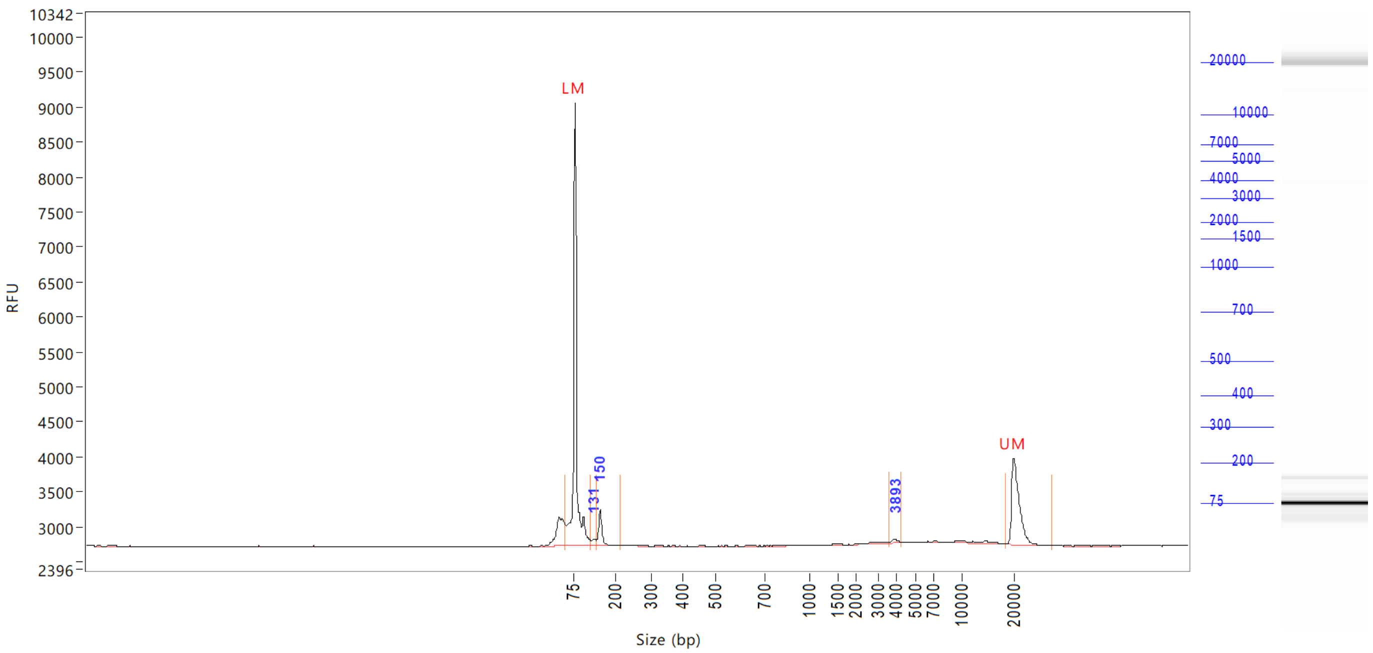

Peak	Size (bp)	Concentration (ng/uL)	From (bp)	To (bp)	Average size (bp)	CV%	RFU	Corrected peak area
1	75 (LM)	0.5000	48	122	75	14.36	6341	46.656
2	131	0.1256	122	139	130	4.13	86	0.976
3	150	0.5296	139	212	152	6.18	528	4.118
4	3893	0.0591	3572	4242	3879	3.63	58	0.460
5	20000 (UM)	0.1246	18293	27375	20464	4.61	1237	11.627

TIC:	0.7143	ng/uL
TIM:	7.3386	nmole/L
Total concentration:	1.1846	ng/uL

Sample peak width (sec): 5 Sample min peak height: 50 Sample baseline V to V?: Y Sample baseline V to V points: 3
Sample filter: Binomial Number of points for filter: 3 Sample start region (min): 0 Sample end region (min): 30
Marker peak width (sec): 5 Marker min peak height: 200 Marker baseline V to V?: Y Marker baseline V to V points: 3
Lower marker selection: First peak > 500 RFU Upper marker selection: Last peak > 200 RFU
Ladder size (bp)75, 200, 300, 400, 500, 700, 1000, 1500, 2000, 3000, 4000, 5000, 7000, 10000, 20000
Quantification using: Lower Marker Final concentration (ng/uL): 0.5000 Dilution factor: 12.0

Sample: SampB10
Well location: B10
Created: Tuesday, August 10, 2021 10:01:13 PM

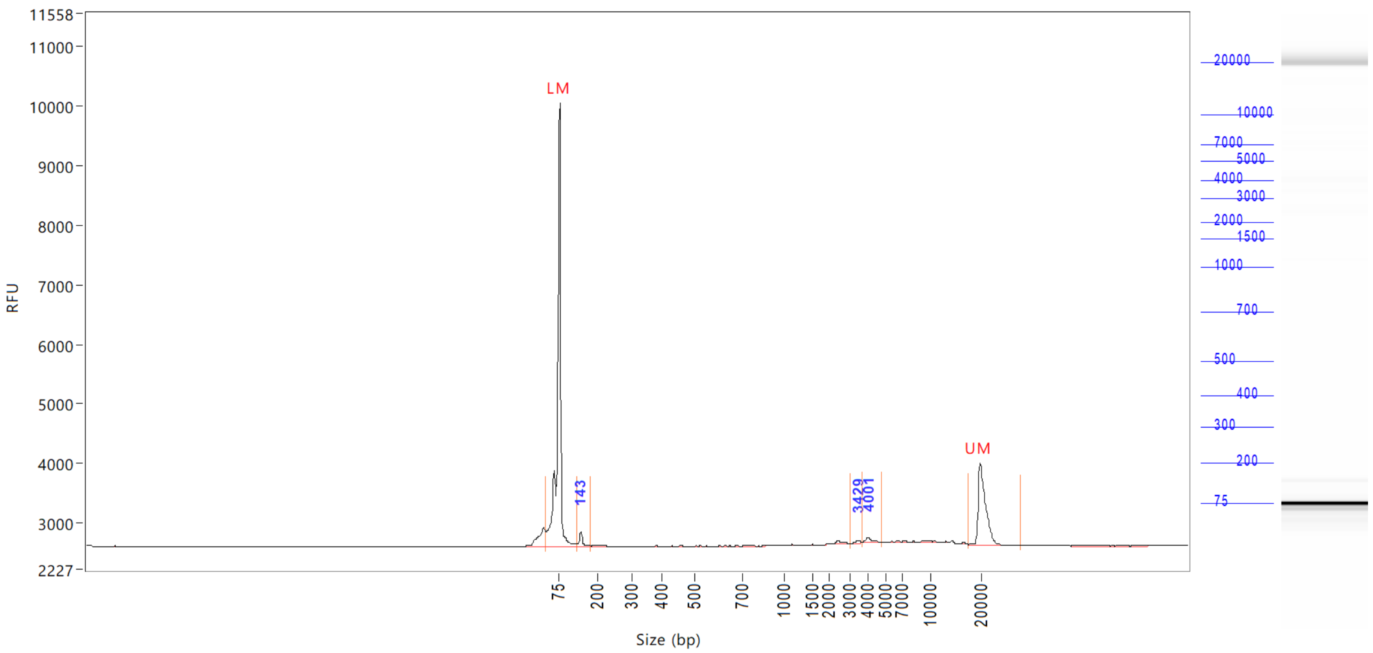

Peak	Size (bp)	Concentration (ng/uL)	From (bp)	To (bp)	Average size (bp)	CV%	RFU	Corrected peak area
1	75 (LM)	0.5000	33	129	69	17.02	7455	59.943
2	143	0.1807	129	175	142	5.66	238	1.805
3	3429	0.0342	3001	3679	3410	4.23	50	0.342
4	4001	0.0877	3679	4759	4068	5.39	81	0.877
5	20000 (UM)	0.1093	17683	28100	20461	4.47	1365	13.107

TIC:	0.3027	ng/uL
TIM:	2.1429	nmole/L
Total concentration:	0.6067	ng/uL

Sample peak width (sec): 5   Sample min peak height: 50   Sample baseline V to V?: Y   Sample baseline V to V points: 3
Sample filter: Binomial   Number of points for filter: 3 Sample start region (min): 0 Sample end region (min): 30
Marker peak width (sec): 5   Marker min peak height: 200   Marker baseline V to V?: Y   Marker baseline V to V points: 3
Lower marker selection: First peak > 500 RFU   Upper marker selection: Last peak > 200 RFU
Ladder size (bp)75, 200, 300, 400, 500, 700, 1000, 1500, 2000, 3000, 4000, 5000, 7000, 10000, 20000
Quantification using: Lower Marker   Final concentration (ng/uL): 0.5000   Dilution factor: 12.0

Sample: SampC10
Well location: C10
Created: Tuesday, August 10, 2021 10:01:13 PM

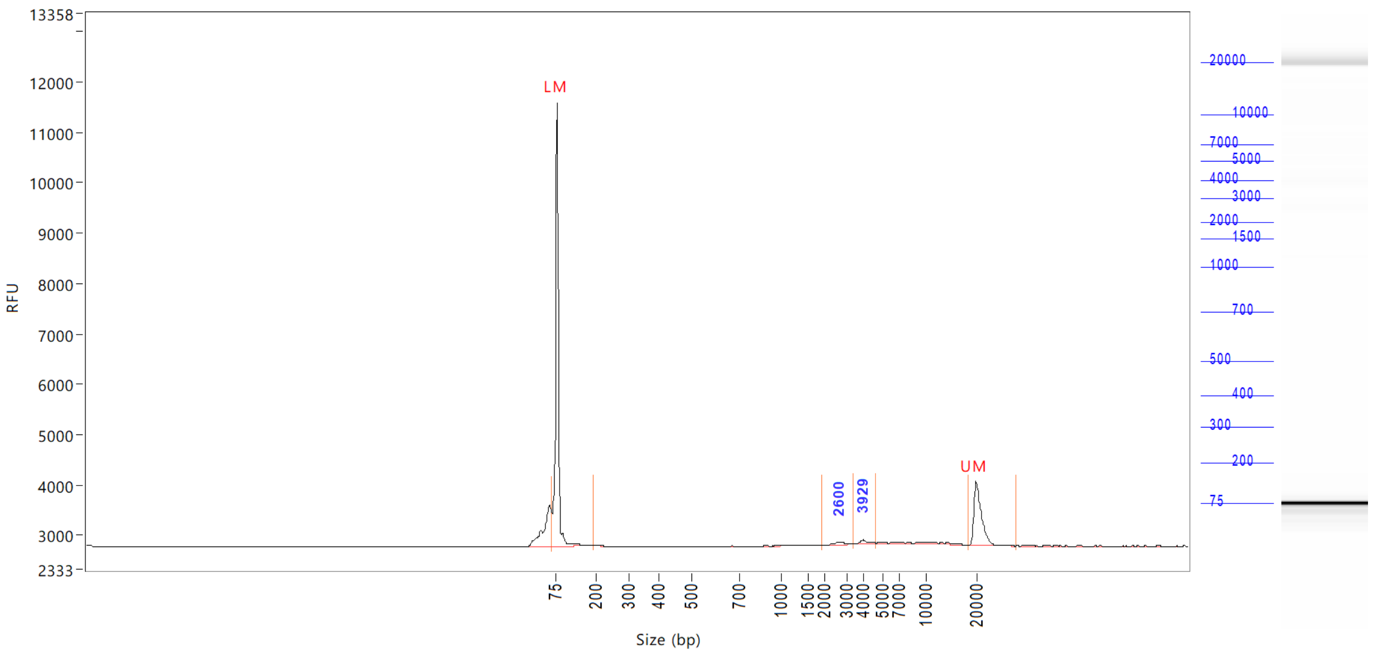

Peak	Size (bp)	Concentration (ng/uL)	From (bp)	To (bp)	Average size (bp)	CV%	RFU	Corrected peak area
1	75 (LM)	0.5000	60	190	74	14.29	8805	58.742
2	2600	0.0892	1923	3358	2669	10.22	59	0.873
3	3929	0.1059	3358	4690	4006	7.28	82	1.037
4	20000 (UM)	0.1048	18415	27979	20472	4.56	1266	12.308

TIC:	0.1950	ng/uL
TIM:	0.0985	nmole/L
Total concentration:	0.5869	ng/uL

Sample peak width (sec): 5   Sample min peak height: 50   Sample baseline V to V?: Y   Sample baseline V to V points: 3
Sample filter: Binomial   Number of points for filter: 3 Sample start region (min): 0 Sample end region (min): 30
Marker peak width (sec): 5   Marker min peak height: 200   Marker baseline V to V?: Y   Marker baseline V to V points: 3
Lower marker selection: First peak > 500 RFU   Upper marker selection: Last peak > 200 RFU
Ladder size (bp)75, 200, 300, 400, 500, 700, 1000, 1500, 2000, 3000, 4000, 5000, 7000, 10000, 20000
Quantification using: Lower Marker   Final concentration (ng/uL): 0.5000   Dilution factor: 12.0

Sample: SampD10
Well location: D10
Created: Tuesday, August 10, 2021 10:01:13 PM

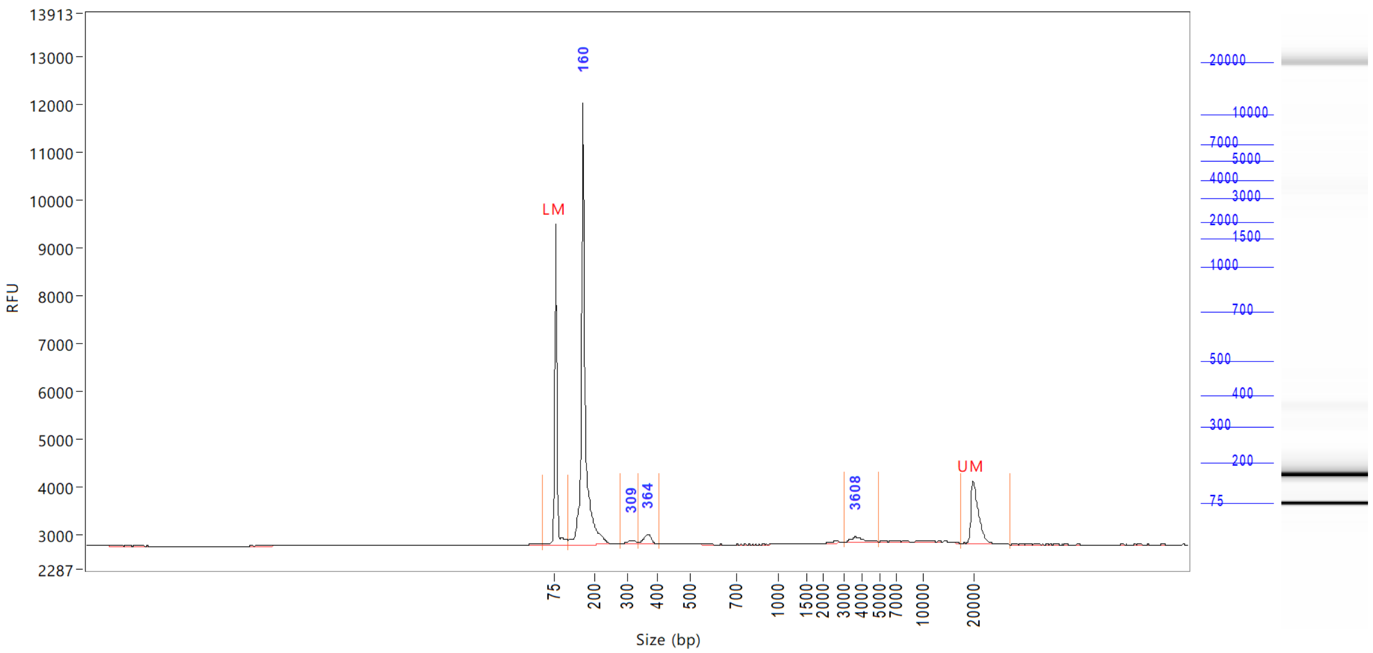

Peak	Size (bp)	Concentration (ng/uL)	From (bp)	To (bp)	Average size (bp)	CV%	RFU	Corrected peak area
1	75 (LM)	0.5000	37	112	74	10.41	6705	39.192
2	160	13.1057	112	275	164	10.32	9253	85.607
3	309	0.2540	275	333	308	4.19	85	1.659
4	364	0.5224	333	401	361	3.58	208	3.413
5	3608	0.3742	3037	4966	3789	11.12	117	2.445
6	20000 (UM)	0.1616	17683	27617	20542	4.85	1308	12.669

TIC:	14.2564	ng/uL
TIM:	135.2572	nmole/L
Total concentration:	14.7005	ng/uL

Sample peak width (sec): 5   Sample min peak height: 50   Sample baseline V to V?: Y   Sample baseline V to V points: 3
Sample filter: Binomial   Number of points for filter: 3   Sample start region (min): 0   Sample end region (min): 30
Marker peak width (sec): 5   Marker min peak height: 200   Marker baseline V to V?: Y   Marker baseline V to V points: 3
Lower marker selection: First peak > 500 RFU   Upper marker selection: Last peak > 200 RFU
Ladder size (bp)75, 200, 300, 400, 500, 700, 1000, 1500, 2000, 3000, 4000, 5000, 7000, 10000, 20000
Quantification using: Lower Marker   Final concentration (ng/uL): 0.5000   Dilution factor: 12.0

Sample: SampE10
Well location: E10
Created: Tuesday, August 10, 2021 10:01:13 PM

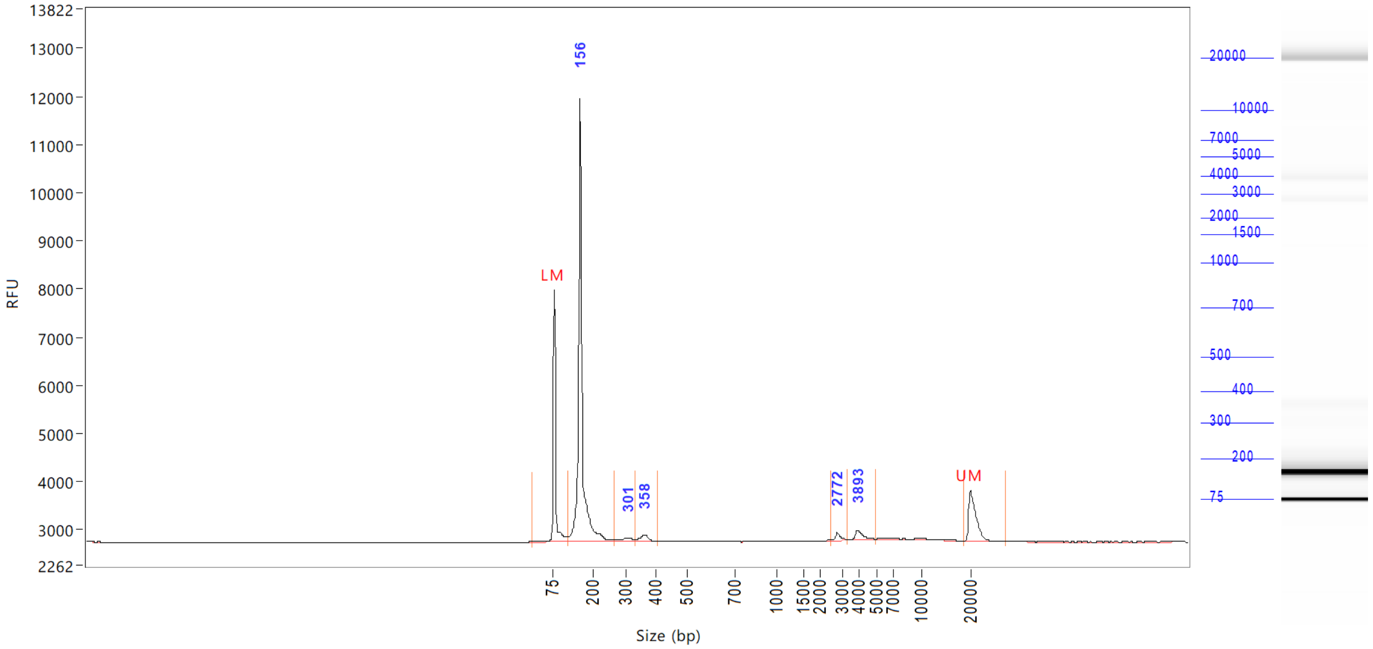

Peak	Size (bp)	Concentration (ng/uL)	From (bp)	To (bp)	Average size (bp)	CV%	RFU	Corrected peak area
1	75 (LM)	0.5000	5	118	74	14.54	5218	33.348
2	156	14.4575	118	262	159	10.94	9219	80.355
3	301	0.2760	262	327	298	5.64	60	1.534
4	358	0.3932	327	401	353	3.79	124	2.185
5	2772	0.2405	2486	3251	2824	5.46	159	1.337
6	3893	0.4660	3251	4931	4011	7.98	210	2.590
7	20000 (UM)	0.1526	18659	27254	20585	4.86	1045	10.179

TIC:	15.8332	ng/uL
TIM:	153.1429	nmole/L
Total concentration:	16.2698	ng/uL

Sample peak width (sec): 5   Sample min peak height: 50   Sample baseline V to V?: Y   Sample baseline V to V points: 3
Sample filter: Binomial   Number of points for filter: 3   Sample start region (min): 0   Sample end region (min): 30
Marker peak width (sec): 5   Marker min peak height: 200   Marker baseline V to V?: Y   Marker baseline V to V points: 3
Lower marker selection: First peak > 500 RFU   Upper marker selection: Last peak > 200 RFU
Ladder size (bp)75, 200, 300, 400, 500, 700, 1000, 1500, 2000, 3000, 4000, 5000, 7000, 10000, 20000
Quantification using: Lower Marker   Final concentration (ng/uL): 0.5000   Dilution factor: 12.0

Sample: SampF10
Well location: F10
Created: Tuesday, August 10, 2021 10:01:13 PM

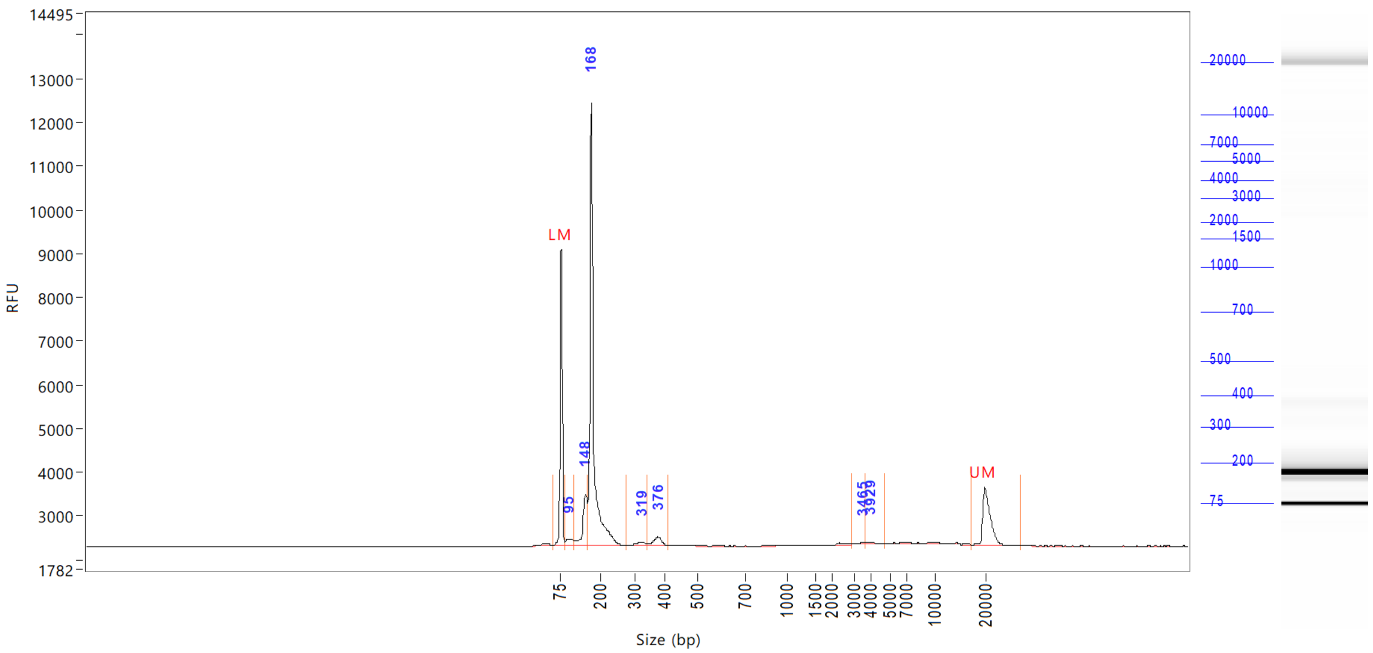

Peak	Size (bp)	Concentration (ng/uL)	From (bp)	To (bp)	Average size (bp)	CV%	RFU	Corrected peak area
1	75 (LM)	0.5000	50	86	72	4.57	6759	37.384
2	95	0.4065	86	116	100	8.74	139	2.533
3	148	1.9697	116	156	146	6.01	1164	12.273
4	168	13.7440	156	277	174	9.23	10131	85.634
5	319	0.2480	277	339	315	4.24	79	1.545
6	376	0.5578	339	411	371	3.79	198	3.475
7	3465	0.0802	2914	3643	3347	6.05	54	0.500
8	3929	0.1032	3643	4724	3998	6.40	53	0.643
9	20000 (UM)	0.1675	17439	27133	20521	4.38	1325	12.521

TIC:	17.1094	ng/uL
TIM:	162.5473	nmole/L
Total concentration:	17.3617	ng/uL

Sample peak width (sec): 5 Sample min peak height: 50 Sample baseline V to V?: Y Sample baseline V to V points: 3
Sample filter: Binomial Number of points for filter: 3 Sample start region (min): 0 Sample end region (min): 30
Marker peak width (sec): 5 Marker min peak height: 200 Marker baseline V to V?: Y Marker baseline V to V points: 3
Lower marker selection: First peak > 500 RFU Upper marker selection: Last peak > 200 RFU
Ladder size (bp)75, 200, 300, 400, 500, 700, 1000, 1500, 2000, 3000, 4000, 5000, 7000, 10000, 20000
Quantification using: Lower Marker Final concentration (ng/uL): 0.5000 Dilution factor: 12.0

Sample: SampG10
Well location: G10
Created: Tuesday, August 10, 2021 10:01:13 PM

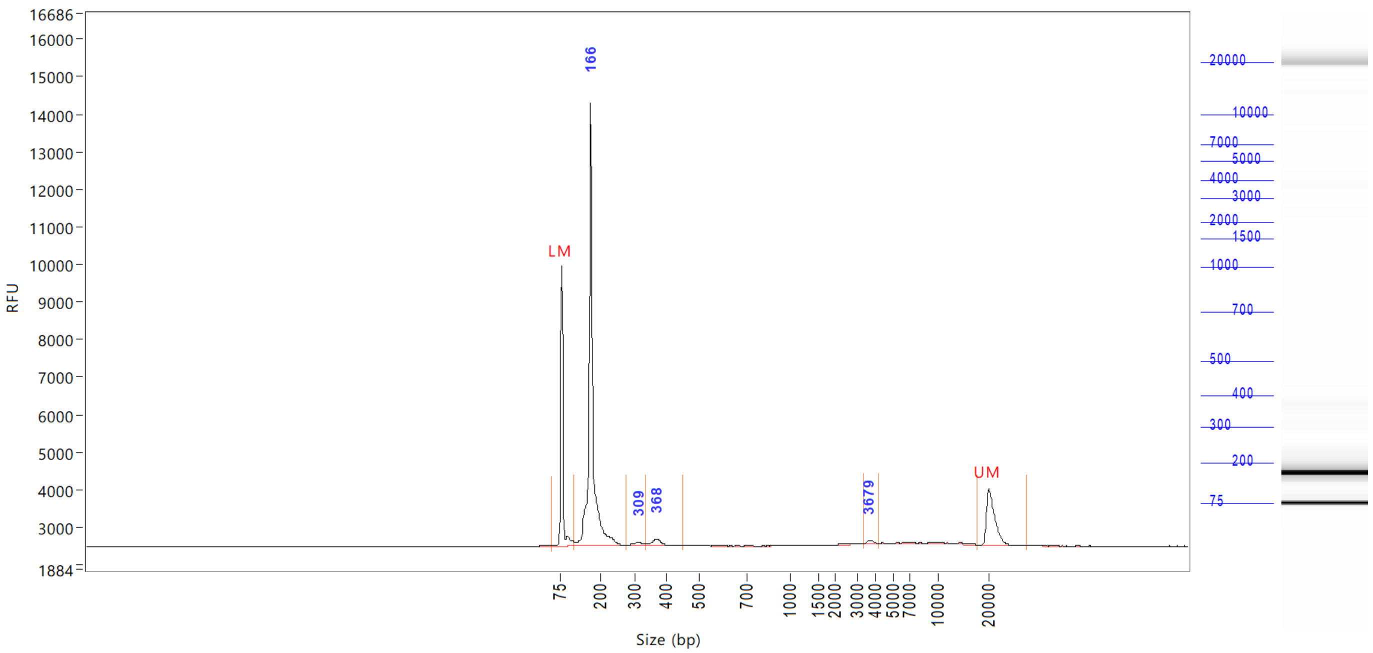

Peak	Size (bp)	Concentration (ng/uL)	From (bp)	To (bp)	Average size (bp)	CV%	RFU	Corrected peak area
1	75 (LM)	0.5000	47	116	76	10.30	7444	45.360
2	166	14.2447	116	275	168	10.65	11810	107.691
3	309	0.2212	275	333	306	4.71	71	1.672
4	368	0.4614	333	452	370	6.30	168	3.488
5	3679	0.1133	3358	4173	3746	4.94	73	0.856
6	20000 (UM)	0.1739	17683	27375	20518	4.66	1527	15.778

TIC:	15.0405	ng/uL
TIM:	142.6696	nmole/L
Total concentration:	15.5266	ng/uL

Sample peak width (sec): 5 Sample min peak height: 50 Sample baseline V to V?: Y Sample baseline V to V points: 3
Sample filter: Binomial Number of points for filter: 3 Sample start region (min): 0 Sample end region (min): 30
Marker peak width (sec): 5 Marker min peak height: 200 Marker baseline V to V?: Y Marker baseline V to V points: 3
Lower marker selection: First peak > 500 RFU Upper marker selection: Last peak > 200 RFU
Ladder size (bp)75, 200, 300, 400, 500, 700, 1000, 1500, 2000, 3000, 4000, 5000, 7000, 10000, 20000
Quantification using: Lower Marker Final concentration (ng/uL): 0.5000 Dilution factor: 12.0

Sample: SampH10
Well location: H10
Created: Tuesday, August 10, 2021 10:01:13 PM

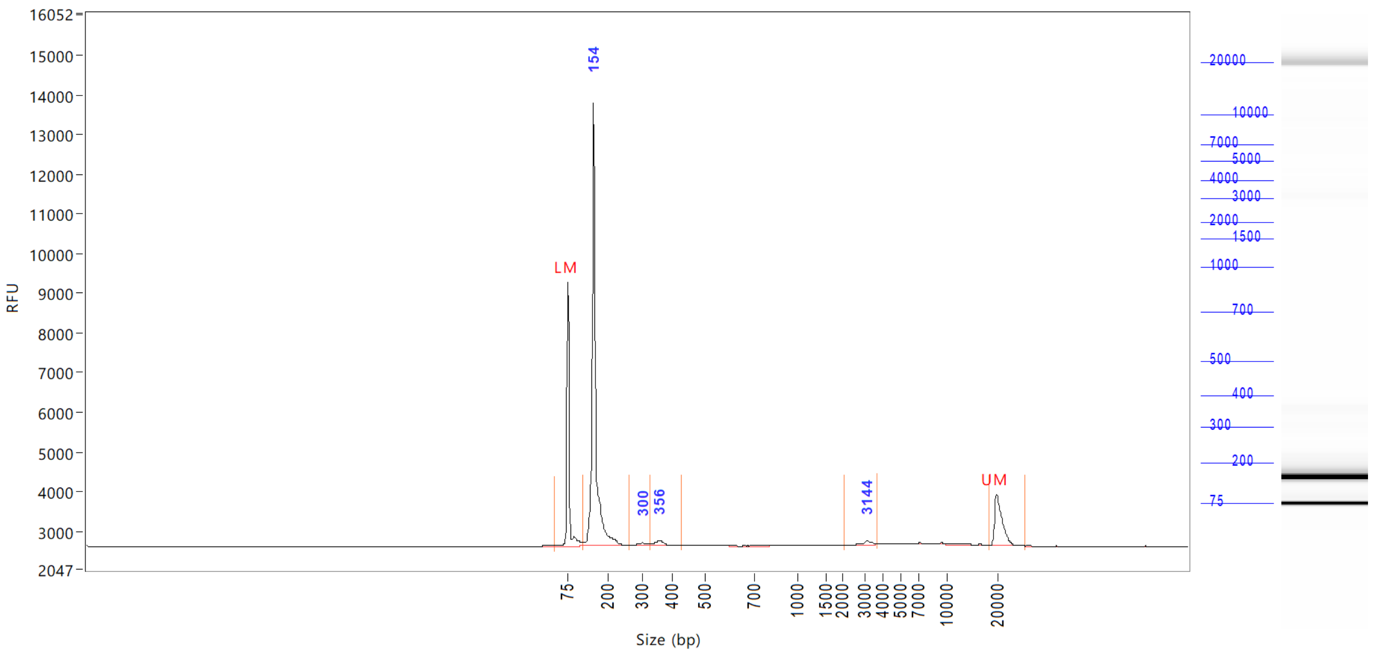

Peak	Size (bp)	Concentration (ng/uL)	From (bp)	To (bp)	Average size (bp)	CV%	RFU	Corrected peak area
1	75 (LM)	0.5000	37	124	75	12.87	6641	39.455
2	154	14.2685	124	264	157	10.23	11178	93.829
3	300	0.2234	264	323	297	4.75	68	1.469
4	356	0.3796	323	426	355	5.37	128	2.496
5	3144	0.1783	2115	3715	3146	8.65	103	1.172
6	20000 (UM)	0.1556	18415	25682	20442	4.14	1303	12.276

TIC:	15.0497	ng/uL
TIM:	152.4671	nmole/L
Total concentration:	15.5200	ng/uL

Sample peak width (sec): 5 Sample min peak height: 50 Sample baseline V to V?: Y Sample baseline V to V points: 3
Sample filter: Binomial Number of points for filter: 3 Sample start region (min): 0 Sample end region (min): 30
Marker peak width (sec): 5 Marker min peak height: 200 Marker baseline V to V?: Y Marker baseline V to V points: 3
Lower marker selection: First peak > 500 RFU Upper marker selection: Last peak > 200 RFU
Ladder size (bp)75, 200, 300, 400, 500, 700, 1000, 1500, 2000, 3000, 4000, 5000, 7000, 10000, 20000
Quantification using: Lower Marker Final concentration (ng/uL): 0.5000 Dilution factor: 12.0

Sample: SampA11
Well location: A11
Created: Tuesday, August 10, 2021 10:01:13 PM

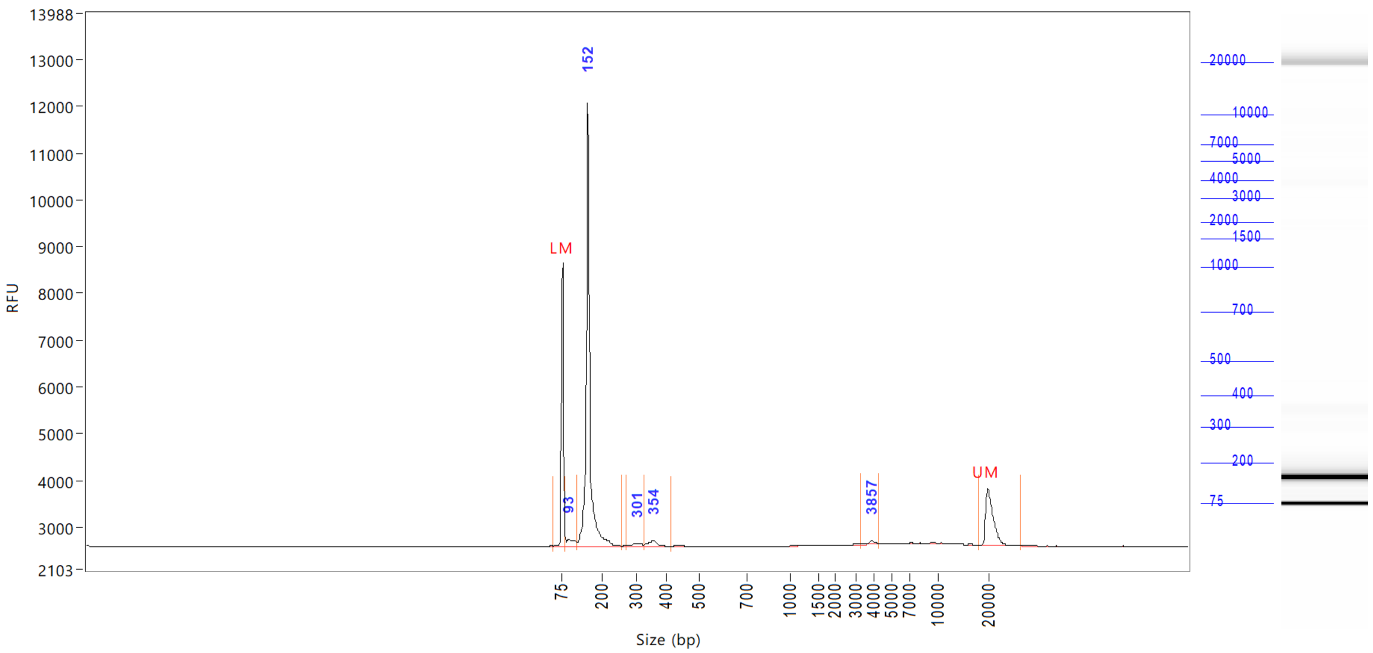

Peak	Size (bp)	Concentration (ng/uL)	From (bp)	To (bp)	Average size (bp)	CV%	RFU	Corrected peak area
1	75 (LM)	0.5000	47	86	73	4.56	6065	32.707
2	93	0.5162	86	120	102	9.93	147	2.814
3	152	14.6578	120	257	155	9.40	9491	79.901
4	301	0.1861	269	323	301	4.01	55	1.015
5	354	0.3766	323	411	350	4.03	118	2.053
6	3857	0.1103	3286	4242	3861	4.53	72	0.601
7	20000 (UM)	0.1782	18171	26649	20525	4.56	1218	11.659

TIC:	15.8471	ng/uL
TIM:	166.5754	nmole/L
Total concentration:	16.2723	ng/uL

Sample peak width (sec): 5 Sample min peak height: 50 Sample baseline V to V?: Y Sample baseline V to V points: 3
Sample filter: Binomial Number of points for filter: 3 Sample start region (min): 0 Sample end region (min): 30
Marker peak width (sec): 5 Marker min peak height: 200 Marker baseline V to V?: Y Marker baseline V to V points: 3
Lower marker selection: First peak > 500 RFU Upper marker selection: Last peak > 200 RFU
Ladder size (bp)75, 200, 300, 400, 500, 700, 1000, 1500, 2000, 3000, 4000, 5000, 7000, 10000, 20000
Quantification using: Lower Marker Final concentration (ng/uL): 0.5000 Dilution factor: 12.0

Sample: SampB11
Well location: B11
Created: Tuesday, August 10, 2021 10:01:13 PM

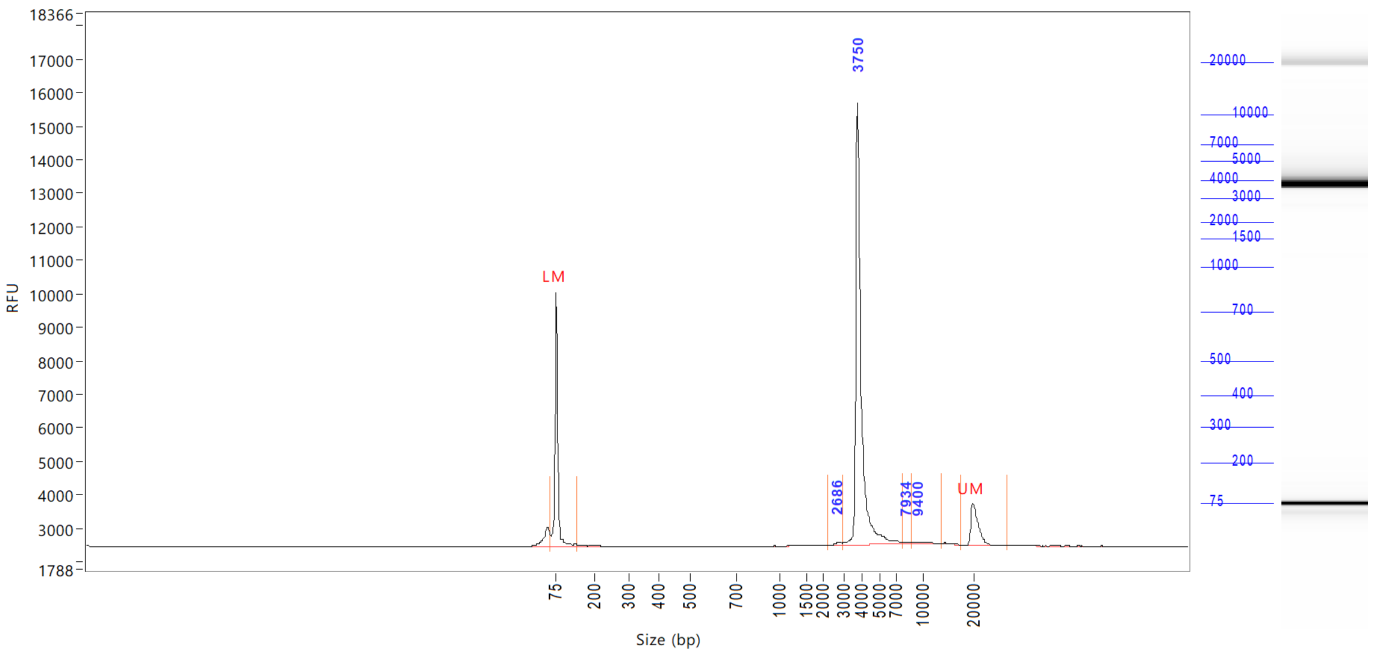

Peak	Size (bp)	Concentration (ng/uL)	From (bp)	To (bp)	Average size (bp)	CV%	RFU	Corrected peak area
1	75 (LM)	0.5000	58	143	75	14.20	7581	51.908
2	2686	0.1022	2201	2914	2678	5.57	98	0.884
3	3750	11.9811	2914	7601	3936	12.59	13196	103.652
4	7934	0.0710	7601	8667	8081	3.90	55	0.614
5	9400	0.1489	8667	13537	10417	12.85	52	1.288
6	20000 (UM)	0.1148	17439	26770	20479	4.51	1263	11.919

TIC:	12.3032	ng/uL
TIM:	5.1120	nmole/L
Total concentration:	12.6147	ng/uL

Sample peak width (sec): 5   Sample min peak height: 50   Sample baseline V to V?: Y   Sample baseline V to V points: 3
Sample filter: Binomial   Number of points for filter: 3   Sample start region (min): 0   Sample end region (min): 30
Marker peak width (sec): 5   Marker min peak height: 200   Marker baseline V to V?: Y   Marker baseline V to V points: 3
Lower marker selection: First peak > 500 RFU   Upper marker selection: Last peak > 200 RFU
Ladder size (bp)75, 200, 300, 400, 500, 700, 1000, 1500, 2000, 3000, 4000, 5000, 7000, 10000, 20000
Quantification using: Lower Marker   Final concentration (ng/uL): 0.5000   Dilution factor: 12.0

Sample: SampC11
Well location: C11
Created: Tuesday, August 10, 2021 10:01:13 PM

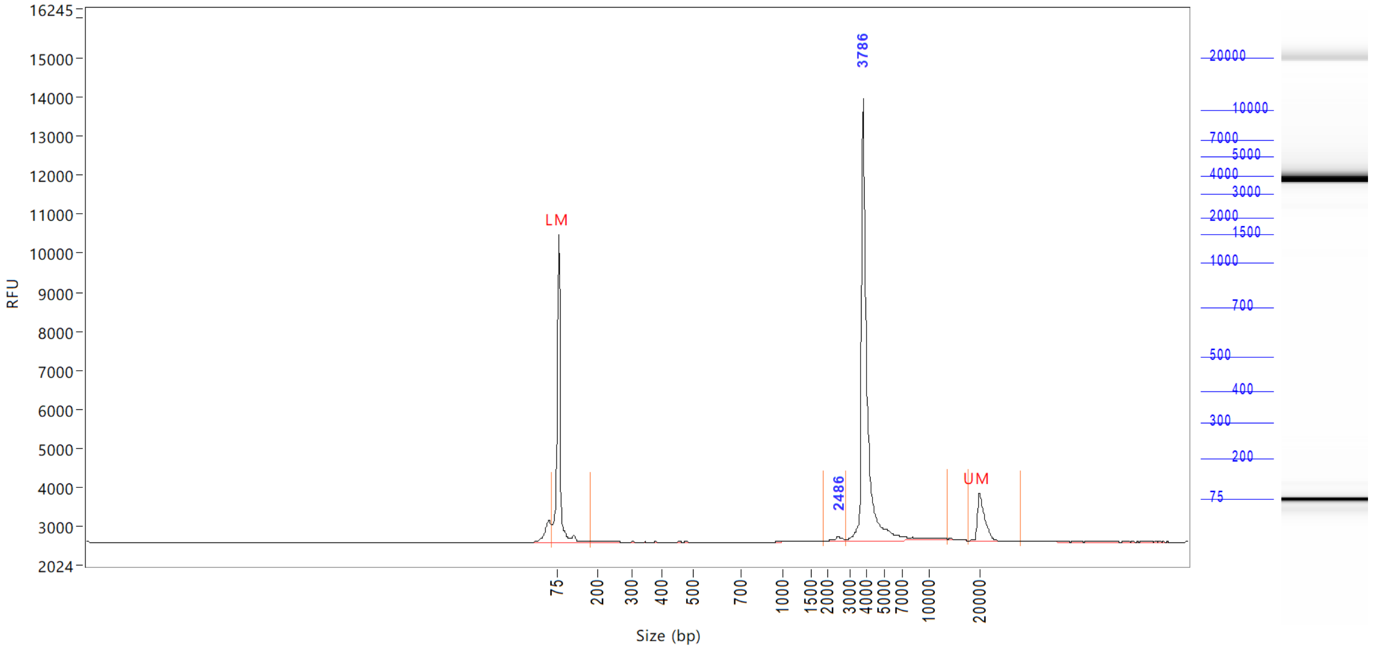

Peak	Size (bp)	Concentration (ng/uL)	From (bp)	To (bp)	Average size (bp)	CV%	RFU	Corrected peak area
1	75 (LM)	0.5000	56	175	75	18.53	7887	59.189
2	2486	0.1332	1866	2857	2469	8.89	108	1.314
3	3786	9.8938	2857	13659	4137	26.48	11325	97.601
4	20000 (UM)	0.0987	17805	28342	20554	4.58	1205	11.684

TIC:	10.0270	ng/uL
TIM:	4.0259	nmole/L
Total concentration:	10.3250	ng/uL

Sample peak width (sec): 5   Sample min peak height: 50   Sample baseline V to V?: Y   Sample baseline V to V points: 3
Sample filter: Binomial   Number of points for filter: 3 Sample start region (min): 0 Sample end region (min): 30
Marker peak width (sec): 5   Marker min peak height: 200   Marker baseline V to V?: Y   Marker baseline V to V points: 3
Lower marker selection: First peak > 500 RFU   Upper marker selection: Last peak > 200 RFU
Ladder size (bp)75, 200, 300, 400, 500, 700, 1000, 1500, 2000, 3000, 4000, 5000, 7000, 10000, 20000
Quantification using: Lower Marker   Final concentration (ng/uL): 0.5000   Dilution factor: 12.0

Sample: SampD11
Well location: D11
Created: Tuesday, August 10, 2021 10:01:13 PM

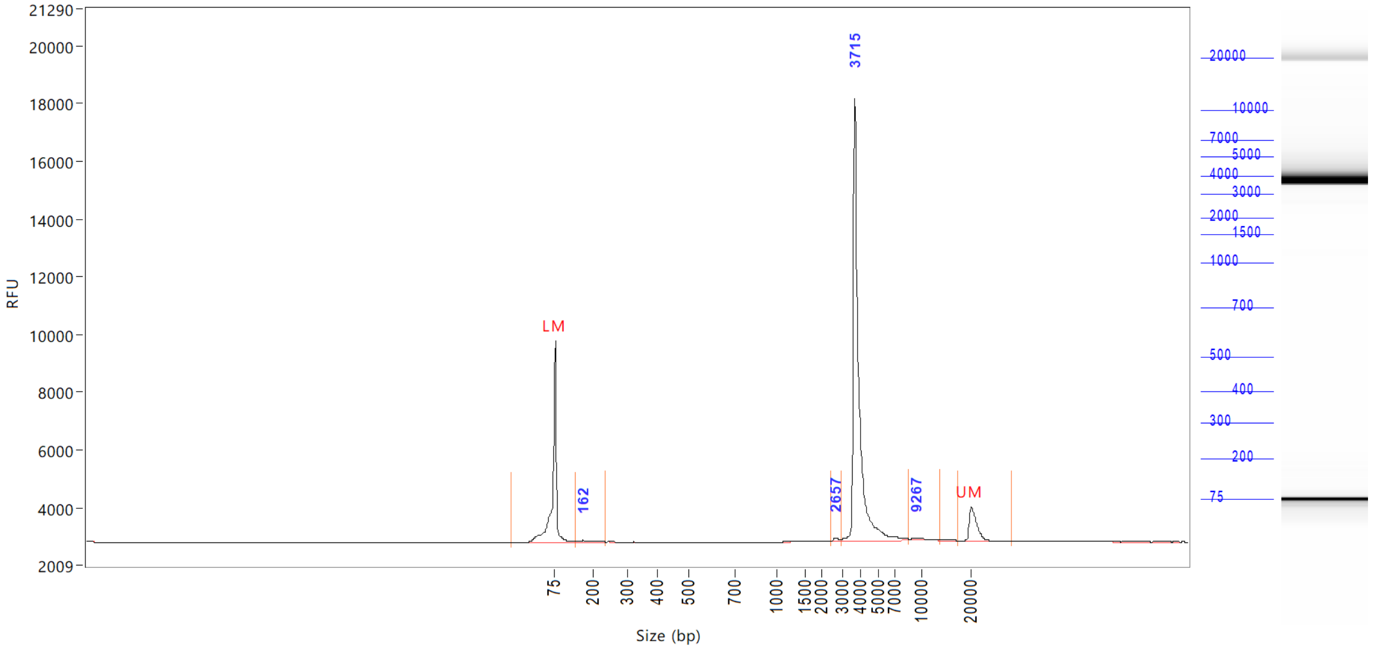

Peak	Size (bp)	Concentration (ng/uL)	From (bp)	To (bp)	Average size (bp)	CV%	RFU	Corrected peak area
1	75 (LM)	0.5000	0	139	64	31.89	6990	64.339
2	162	0.1675	139	235	172	12.89	66	1.796
3	2657	0.0866	2458	2914	2702	4.40	107	0.929
4	3715	11.9949	2914	8600	3946	15.45	15359	128.623
5	9267	0.1604	8600	13537	10457	13.35	60	1.720
6	20000 (UM)	0.0895	17195	28221	20569	4.99	1207	11.514

TIC:	12.4094	ng/uL
TIM:	6.6830	nmole/L
Total concentration:	12.6052	ng/uL

```
Sample peak width (sec): 5    Sample min peak height: 50    Sample baseline V to V?: Y    Sample baseline V to V points: 3
Sample filter: Binomial       Number of points for filter: 3  Sample start region (min): 0  Sample end region (min): 30
Marker peak width (sec): 5    Marker min peak height: 200   Marker baseline V to V?: Y    Marker baseline V to V points: 3
Lower marker selection: First peak > 500 RFU                Upper marker selection: Last peak > 200 RFU
Ladder size (bp)75, 200, 300, 400, 500, 700, 1000, 1500, 2000, 3000, 4000, 5000, 7000, 10000, 20000
Quantification using: Lower Marker          Final concentration (ng/uL): 0.5000          Dilution factor: 12.0
```

Sample: SampE11
Well location: E11
Created: Tuesday, August 10, 2021 10:01:13 PM

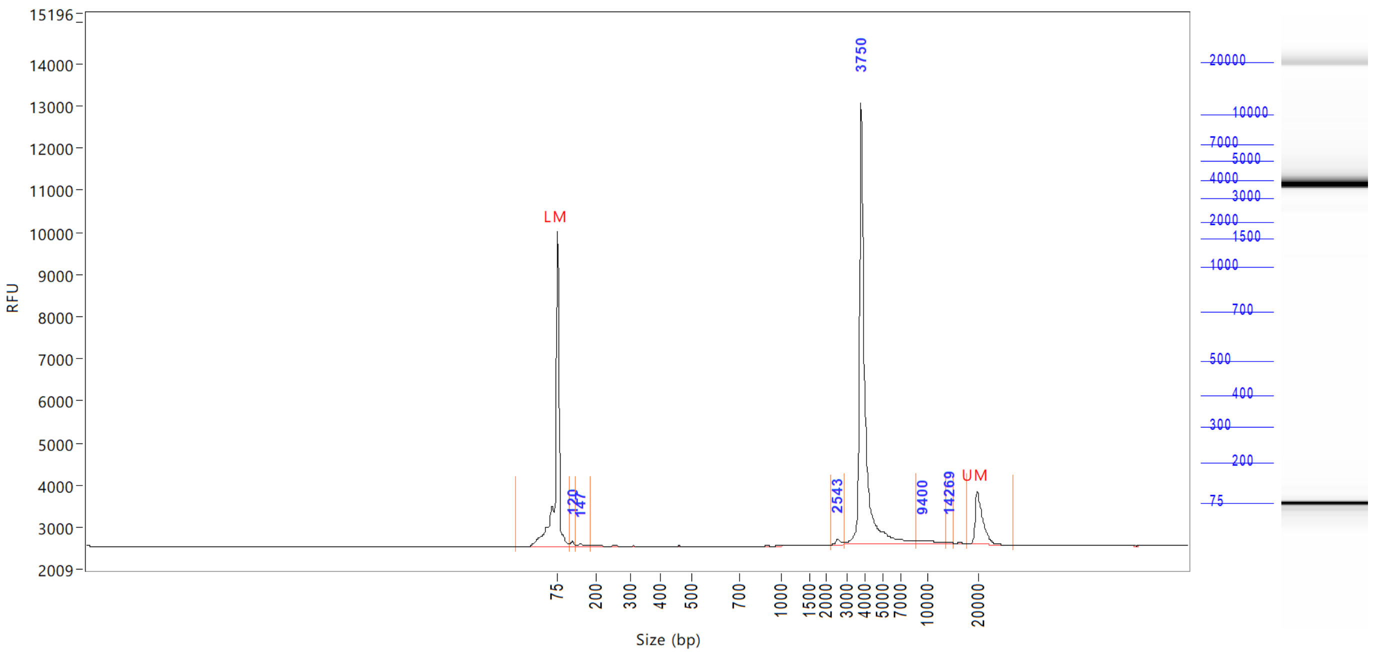

Peak	Size (bp)	Concentration (ng/uL)	From (bp)	To (bp)	Average size (bp)	CV%	RFU	Corrected peak area
1	75 (LM)	0.5000	0	112	64	30.50	7472	66.676
2	120	0.0975	112	135	121	5.06	123	1.084
3	147	0.0888	135	181	153	8.20	64	0.987
4	2543	0.1019	2172	2886	2576	5.64	127	1.132
5	3750	7.7976	2886	8667	3992	16.87	10486	86.652
6	9400	0.1781	8667	13537	10534	13.26	74	1.979
7	14269	0.0355	13537	15244	14243	3.21	51	0.394
8	20000 (UM)	0.0914	17927	27133	20494	4.61	1244	12.190

TIC:	8.2994	ng/uL
TIM:	5.5911	nmole/L
Total concentration:	8.4901	ng/uL

Sample peak width (sec): 5 Sample min peak height: 50 Sample baseline V to V?: Y Sample baseline V to V points: 3
Sample filter: Binomial Number of points for filter: 3 Sample start region (min): 0 Sample end region (min): 30
Marker peak width (sec): 5 Marker min peak height: 200 Marker baseline V to V?: Y Marker baseline V to V points: 3
Lower marker selection: First peak > 500 RFU Upper marker selection: Last peak > 200 RFU
Ladder size (bp)75, 200, 300, 400, 500, 700, 1000, 1500, 2000, 3000, 4000, 5000, 7000, 10000, 20000
Quantification using: Lower Marker Final concentration (ng/uL): 0.5000 Dilution factor: 12.0

Sample: SampF11
Well location: F11
Created: Tuesday, August 10, 2021 10:01:13 PM

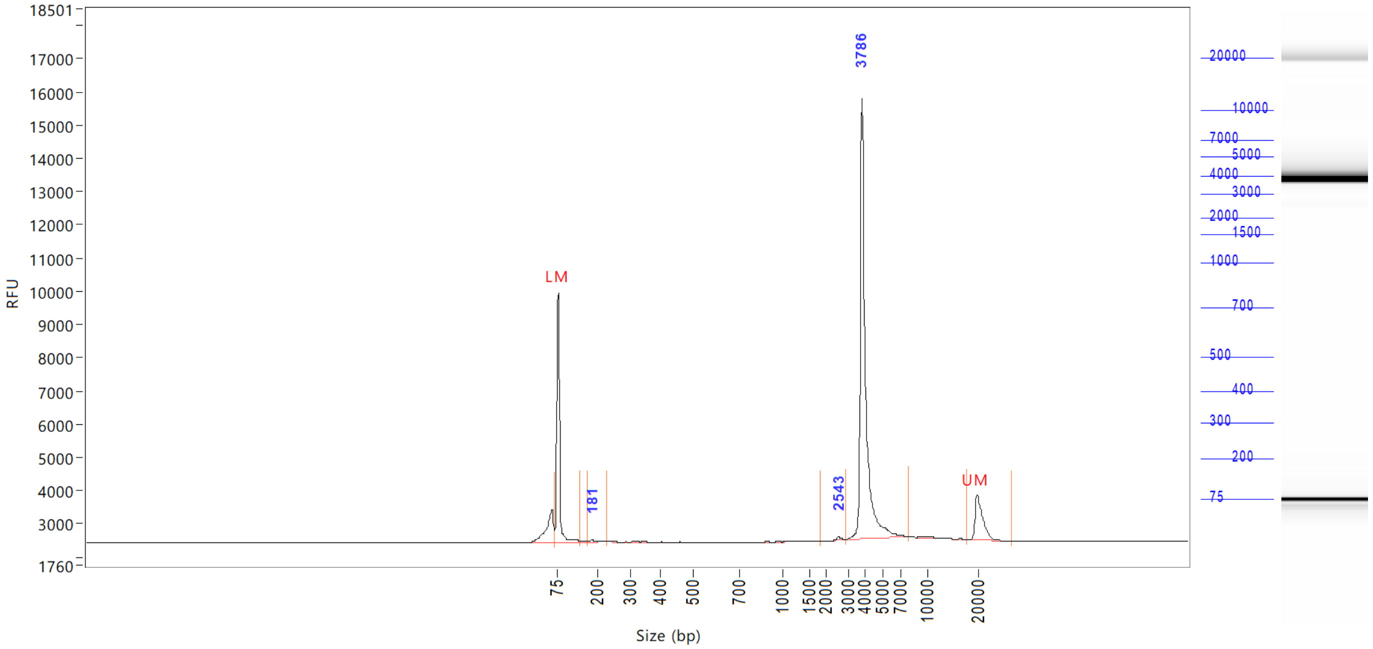

Peak	Size (bp)	Concentration (ng/uL)	From (bp)	To (bp)	Average size (bp)	CV%	RFU	Corrected peak area
1	75 (LM)	0.5000	64	145	75	14.24	7481	48.886
2	181	0.1007	169	228	188	6.85	56	0.821
3	2543	0.0853	1846	2914	2567	5.23	97	0.695
4	3786	13.0368	2914	7867	3957	10.70	13272	106.219
5	20000 (UM)	0.1386	18049	27012	20484	4.57	1364	13.551

TIC:	13.2229	ng/uL
TIM:	6.3594	nmole/L
Total concentration:	13.5701	ng/uL

```
Sample peak width (sec): 5    Sample min peak height: 50    Sample baseline V to V?: Y    Sample baseline V to V points: 3
Sample filter: Binomial       Number of points for filter: 3  Sample start region (min): 0  Sample end region (min): 30
Marker peak width (sec): 5    Marker min peak height: 200   Marker baseline V to V?: Y    Marker baseline V to V points: 3
Lower marker selection: First peak > 500 RFU                Upper marker selection: Last peak > 200 RFU
Ladder size (bp)75, 200, 300, 400, 500, 700, 1000, 1500, 2000, 3000, 4000, 5000, 7000, 10000, 20000
Quantification using: Lower Marker          Final concentration (ng/uL): 0.5000          Dilution factor: 12.0
```

Sample: SampG11
Well location: G11
Created: Tuesday, August 10, 2021 10:01:13 PM

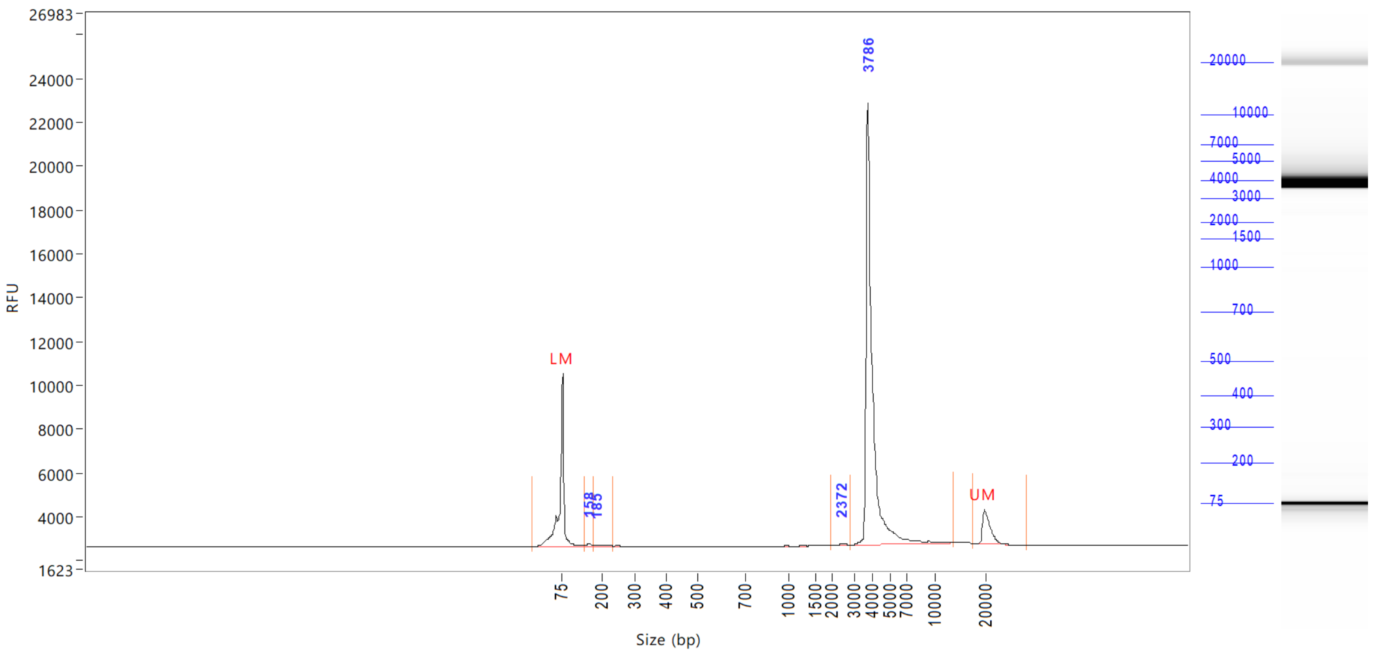

Peak	Size (bp)	Concentration (ng/uL)	From (bp)	To (bp)	Average size (bp)	CV%	RFU	Corrected peak area
1	75 (LM)	0.5000	0	143	65	29.18	7911	77.731
2	158	0.1206	143	173	157	5.15	118	1.562
3	185	0.1164	173	232	196	8.10	71	1.508
4	2372	0.0593	1962	2857	2448	7.07	79	0.768
5	3786	14.5445	2857	13659	4087	22.92	20190	188.426
6	20000 (UM)	0.0968	17561	28342	20495	4.36	1572	15.051

TIC:	14.8408	ng/uL
TIM:	8.1377	nmole/L
Total concentration:	15.0067	ng/uL

Sample peak width (sec): 5   Sample min peak height: 50   Sample baseline V to V?: Y   Sample baseline V to V points: 3
Sample filter: Binomial   Number of points for filter: 3 Sample start region (min): 0 Sample end region (min): 30
Marker peak width (sec): 5   Marker min peak height: 200   Marker baseline V to V?: Y   Marker baseline V to V points: 3
Lower marker selection: First peak > 500 RFU   Upper marker selection: Last peak > 200 RFU
Ladder size (bp)75, 200, 300, 400, 500, 700, 1000, 1500, 2000, 3000, 4000, 5000, 7000, 10000, 20000
Quantification using: Lower Marker   Final concentration (ng/uL): 0.5000   Dilution factor: 12.0

Sample: SampH11
Well location: H11
Created: Tuesday, August 10, 2021 10:01:13 PM

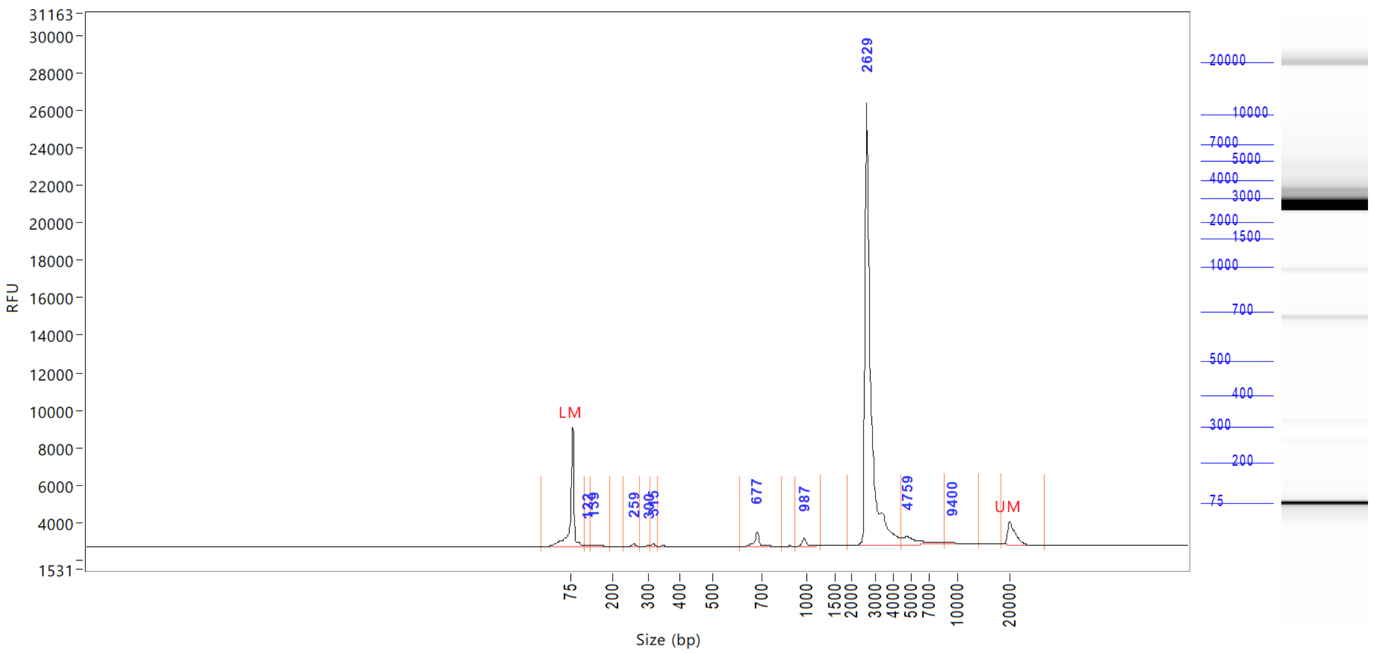

Peak	Size (bp)	Concentration (ng/uL)	From (bp)	To (bp)	Average size (bp)	CV%	RFU	Corrected peak area
1	75 (LM)	0.5000	0	114	67	24.79	6386	52.876
2	122	0.0806	114	131	122	4.27	69	0.710
3	139	0.1597	131	189	148	8.63	90	1.407
4	259	0.1086	230	277	255	2.93	154	0.957
5	300	0.0569	277	307	297	2.51	65	0.501
6	315	0.1079	307	331	314	1.54	172	0.951
7	677	0.7732	608	831	677	3.84	819	6.814
8	987	0.3636	922	1256	996	4.58	415	3.204
9	2629	23.3344	1846	4449	2807	12.81	23624	205.639
10	4759	1.2171	4449	8600	5624	20.27	439	10.726
11	9400	0.1565	8600	13903	9793	9.96	72	1.379
12	20000 (UM)	0.1104	18293	26891	20498	4.40	1208	11.677

TIC:	26.3585	ng/uL
TIM:	20.9871	nmole/L
Total concentration:	26.4995	ng/uL

```
Sample peak width (sec): 5    Sample min peak height: 50    Sample baseline V to V?: Y    Sample baseline V to V points: 3
Sample filter: Binomial       Number of points for filter: 3  Sample start region (min): 0  Sample end region (min): 30
Marker peak width (sec): 5    Marker min peak height: 200   Marker baseline V to V?: Y    Marker baseline V to V points: 3
Lower marker selection: First peak > 500 RFU                Upper marker selection: Last peak > 200 RFU
Ladder size (bp)75, 200, 300, 400, 500, 700, 1000, 1500, 2000, 3000, 4000, 5000, 7000, 10000, 20000
Quantification using: Lower Marker          Final concentration (ng/uL): 0.5000          Dilution factor: 12.0
```

Sample: SampA12
Well location: A12
Created: Tuesday, August 10, 2021 10:01:13 PM

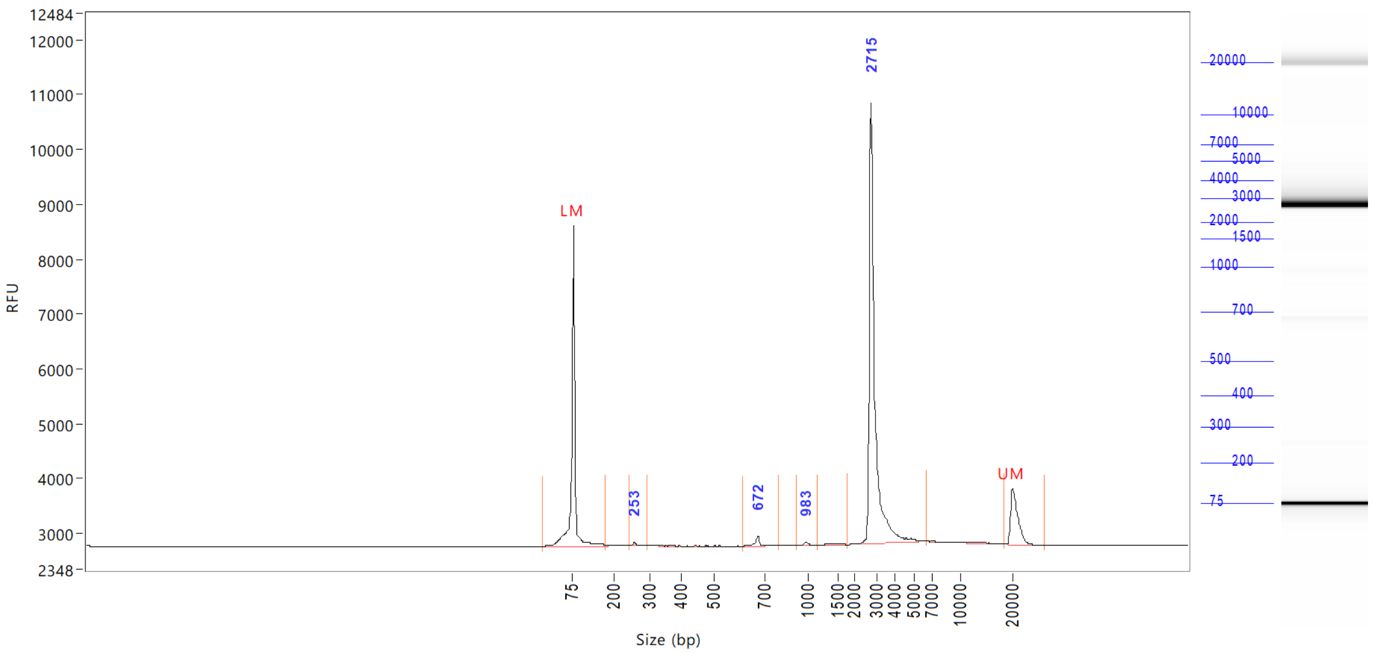

Peak	Size (bp)	Concentration (ng/uL)	From (bp)	To (bp)	Average size (bp)	CV%	RFU	Corrected peak area
1	75 (LM)	0.5000	0	175	71	26.48	5853	44.625
2	253	0.0685	242	291	255	3.38	69	0.510
3	672	0.2081	611	792	669	3.52	187	1.548
4	983	0.0619	913	1156	990	3.88	59	0.460
5	2715	8.5104	1770	6482	2891	13.95	8048	63.296
6	20000 (UM)	0.1045	18293	26166	20451	4.23	1025	9.327

TIC:	8.8489	ng/uL
TIM:	5.9029	nmole/L
Total concentration:	9.2073	ng/uL

Sample peak width (sec): 5   Sample min peak height: 50   Sample baseline V to V?: Y   Sample baseline V to V points: 3
Sample filter: Binomial   Number of points for filter: 3 Sample start region (min): 0 Sample end region (min): 30
Marker peak width (sec): 5   Marker min peak height: 200   Marker baseline V to V?: Y   Marker baseline V to V points: 3
Lower marker selection: First peak > 500 RFU   Upper marker selection: Last peak > 200 RFU
Ladder size (bp)75, 200, 300, 400, 500, 700, 1000, 1500, 2000, 3000, 4000, 5000, 7000, 10000, 20000
Quantification using: Lower Marker   Final concentration (ng/uL): 0.5000   Dilution factor: 12.0

Sample: SampB12
Well location: B12
Created: Tuesday, August 10, 2021 10:01:13 PM

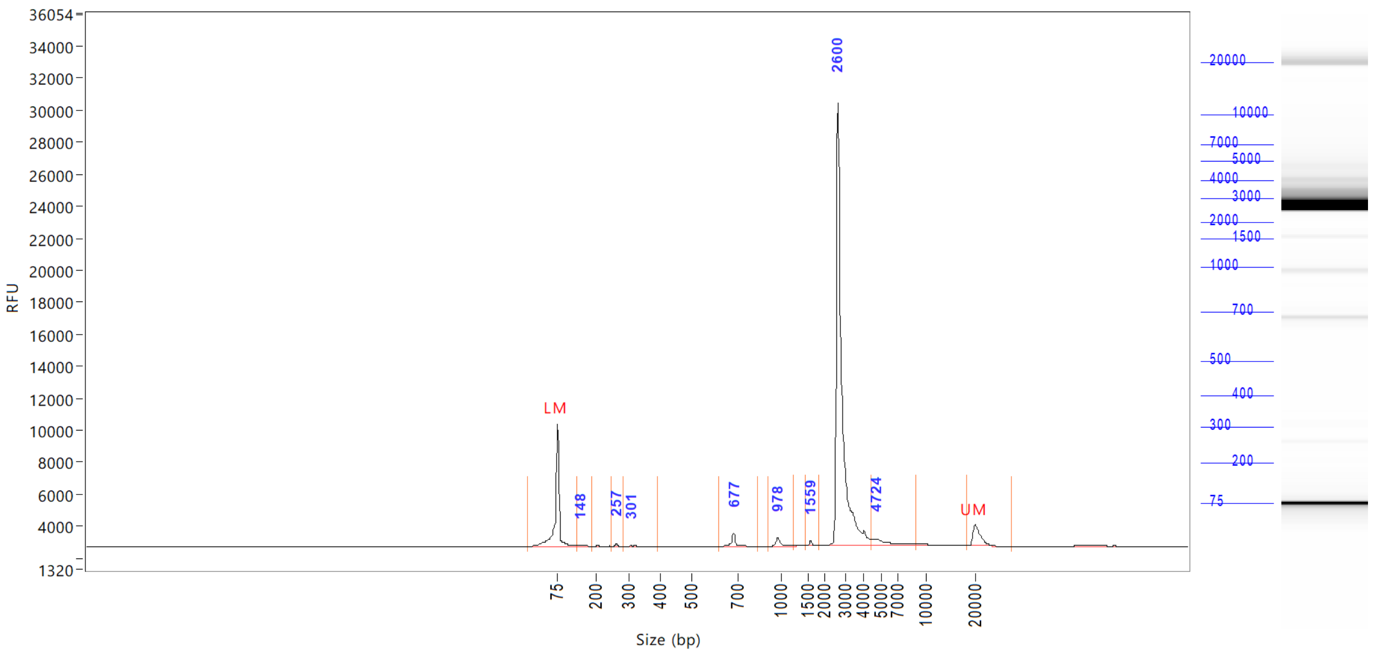

Peak	Size (bp)	Concentration (ng/uL)	From (bp)	To (bp)	Average size (bp)	CV%	RFU	Corrected peak area
1	75 (LM)	0.5000	0	139	67	28.83	7613	68.522
2	148	0.1116	139	187	155	7.47	74	1.274
3	257	0.1061	244	282	256	2.46	207	1.211
4	301	0.0671	282	390	316	7.35	57	0.767
5	677	0.5883	616	831	676	3.51	843	6.719
6	978	0.3843	909	1245	997	5.57	557	4.389
7	1559	0.1251	1456	1827	1570	5.04	343	1.428
8	2600	21.1700	1827	4483	2796	13.21	27709	241.769
9	4724	0.7915	4483	9000	5593	20.86	421	9.040
10	20000 (UM)	0.0929	18537	27617	20530	4.41	1331	12.725

TIC:	23.3440	ng/uL
TIM:	17.1092	nmole/L
Total concentration:	23.5162	ng/uL

Sample peak width (sec): 5 Sample min peak height: 50 Sample baseline V to V?: Y Sample baseline V to V points: 3
Sample filter: Binomial Number of points for filter: 3 Sample start region (min): 0 Sample end region (min): 30
Marker peak width (sec): 5 Marker min peak height: 200 Marker baseline V to V?: Y Marker baseline V to V points: 3
Lower marker selection: First peak > 500 RFU Upper marker selection: Last peak > 200 RFU
Ladder size (bp)75, 200, 300, 400, 500, 700, 1000, 1500, 2000, 3000, 4000, 5000, 7000, 10000, 20000
Quantification using: Lower Marker Final concentration (ng/uL): 0.5000 Dilution factor: 12.0

Sample: SampC12
Well location: C12
Created: Tuesday, August 10, 2021 10:01:13 PM

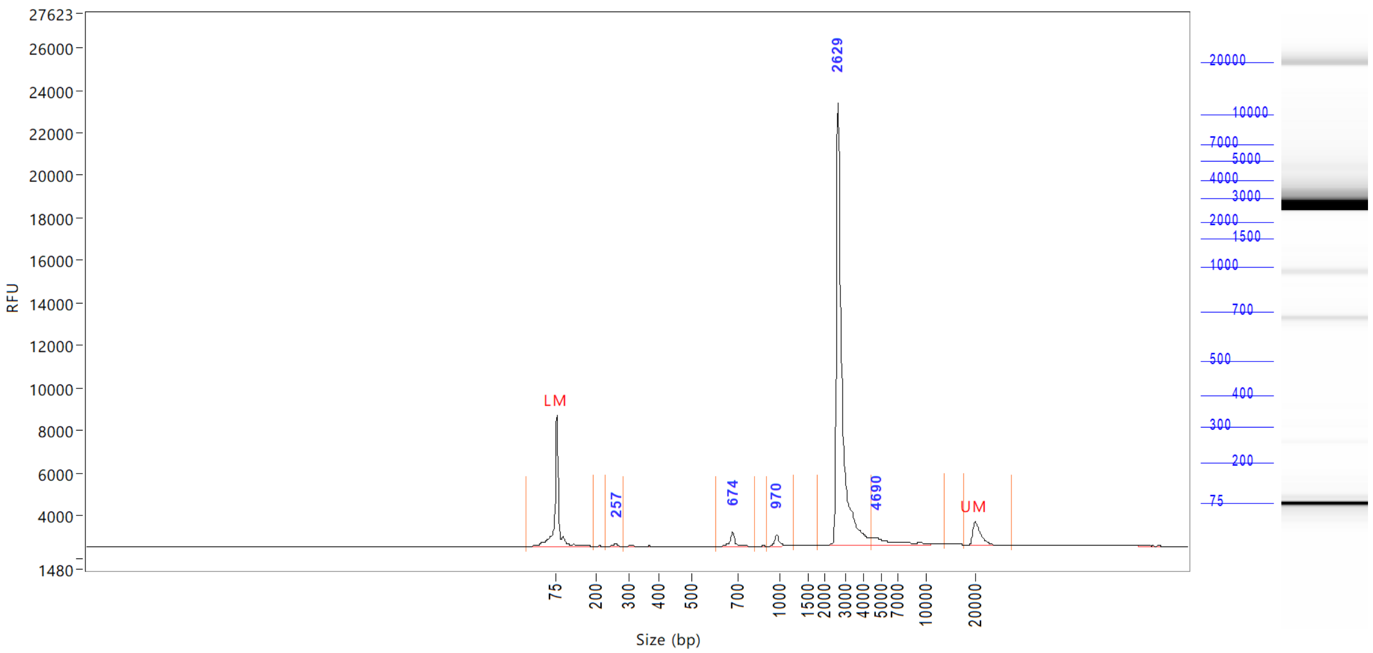

Peak	Size (bp)	Concentration (ng/uL)	From (bp)	To (bp)	Average size (bp)	CV%	RFU	Corrected peak area
1	75 (LM)	0.5000	0	192	70	30.56	6146	57.394
2	257	0.1012	230	282	254	2.95	133	0.968
3	674	0.6795	603	818	674	3.72	694	6.500
4	970	0.4839	900	1234	990	5.72	518	4.629
5	2629	19.5296	1789	4414	2805	12.56	20840	186.813
6	4690	1.1994	4414	13781	6552	34.24	368	11.473
7	20000 (UM)	0.0971	17683	27496	20630	5.17	1091	11.145

TIC:	21.9936	ng/uL
TIM:	14.8817	nmole/L
Total concentration:	22.1818	ng/uL

Sample peak width (sec): 5   Sample min peak height: 50   Sample baseline V to V?: Y   Sample baseline V to V points: 3
Sample filter: Binomial   Number of points for filter: 3 Sample start region (min): 0 Sample end region (min): 30
Marker peak width (sec): 5   Marker min peak height: 200   Marker baseline V to V?: Y   Marker baseline V to V points: 3
Lower marker selection: First peak > 500 RFU   Upper marker selection: Last peak > 200 RFU
Ladder size (bp)75, 200, 300, 400, 500, 700, 1000, 1500, 2000, 3000, 4000, 5000, 7000, 10000, 20000
Quantification using: Lower Marker   Final concentration (ng/uL): 0.5000   Dilution factor: 12.0

Sample: SampD12
Well location: D12
Created: Tuesday, August 10, 2021 10:01:13 PM

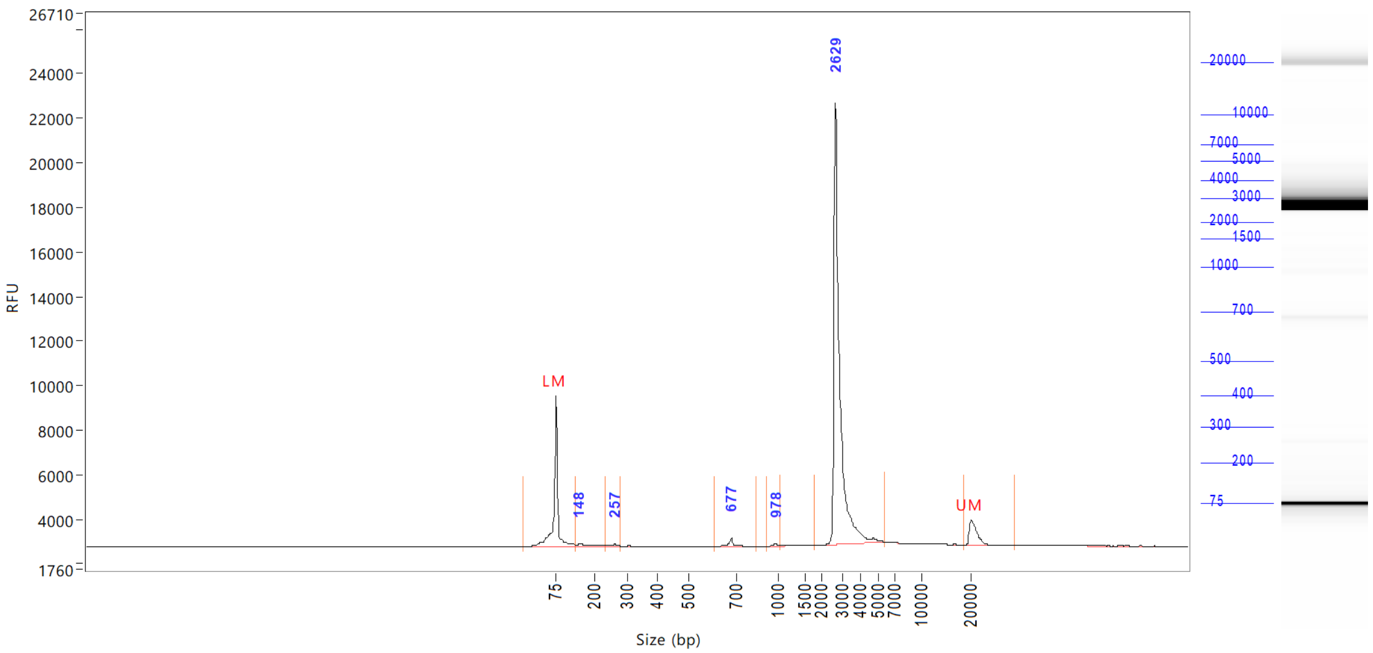

Peak	Size (bp)	Concentration (ng/uL)	From (bp)	To (bp)	Average size (bp)	CV%	RFU	Corrected peak area
1	75 (LM)	0.5000	0	139	69	28.30	6751	60.756
2	148	0.3343	139	233	176	15.05	95	3.385
3	257	0.1122	233	277	253	3.73	119	1.136
4	677	0.3340	608	844	675	4.26	368	3.382
5	978	0.0832	922	1034	977	2.08	110	0.842
6	2629	17.6470	1770	5889	2823	13.85	19853	178.694
7	20000 (UM)	0.0921	18415	28826	20575	4.85	1152	11.189

TIC:	18.5107	ng/uL
TIM:	15.0972	nmole/L
Total concentration:	18.7480	ng/uL

```
Sample peak width (sec): 5     Sample min peak height: 50     Sample baseline V to V?: Y     Sample baseline V to V points: 3
Sample filter: Binomial        Number of points for filter: 3  Sample start region (min): 0  Sample end region (min): 30
Marker peak width (sec): 5     Marker min peak height: 200    Marker baseline V to V?: Y     Marker baseline V to V points: 3
Lower marker selection: First peak > 500 RFU                  Upper marker selection: Last peak > 200 RFU
Ladder size (bp)75, 200, 300, 400, 500, 700, 1000, 1500, 2000, 3000, 4000, 5000, 7000, 10000, 20000
Quantification using: Lower Marker          Final concentration (ng/uL): 0.5000          Dilution factor: 12.0
```

Sample: SampE12
Well location: E12
Created: Tuesday, August 10, 2021 10:01:13 PM

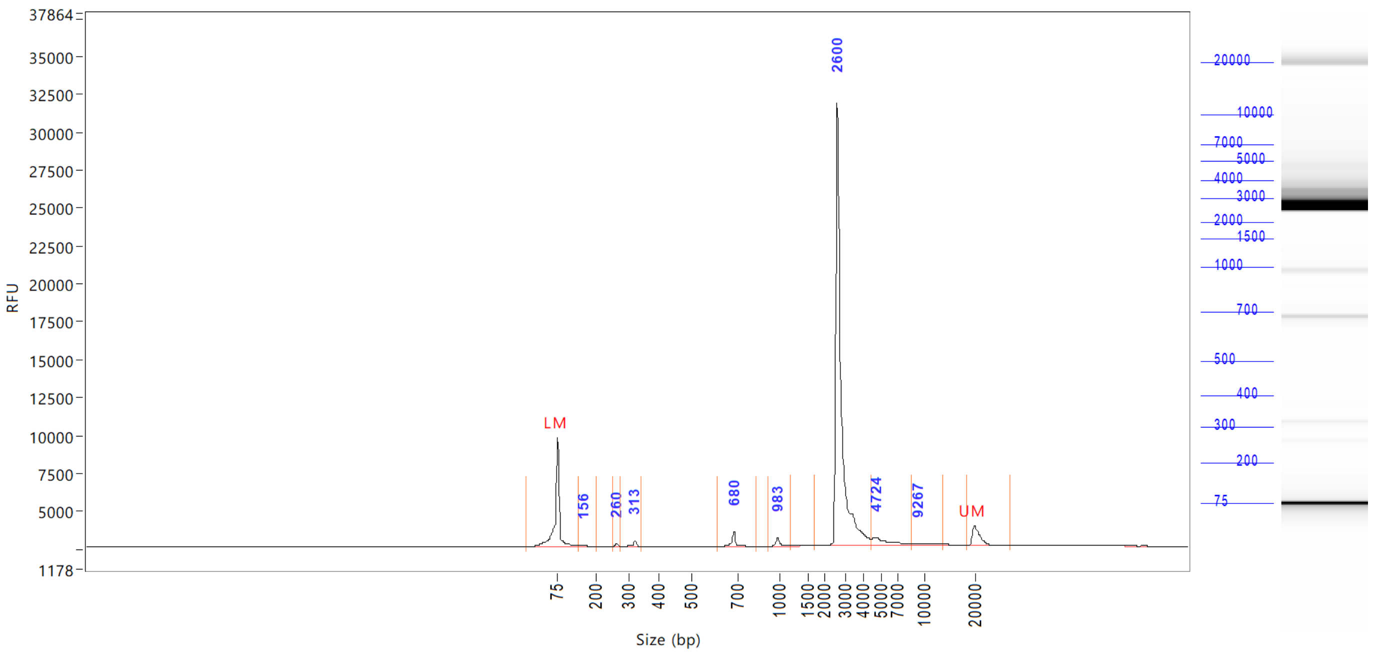

Peak	Size (bp)	Concentration (ng/uL)	From (bp)	To (bp)	Average size (bp)	CV%	RFU	Corrected peak area
1	75 (LM)	0.5000	0	141	68	28.26	7182	68.763
2	156	0.1277	141	201	157	8.57	85	1.463
3	260	0.0974	246	273	258	1.84	193	1.116
4	313	0.2600	273	339	308	3.37	389	2.979
5	680	0.6454	611	827	678	3.36	972	7.396
6	983	0.3636	909	1190	990	4.08	583	4.167
7	2600	21.7298	1712	4449	2792	13.49	29268	249.035
8	4724	1.1361	4449	8467	5631	20.22	529	13.020
9	9267	0.2642	8467	13781	10293	14.14	106	3.028
10	20000 (UM)	0.0886	18659	27254	20548	4.35	1270	12.190

TIC:	24.6240	ng/uL
TIM:	18.7035	nmole/L
Total concentration:	24.7472	ng/uL

```
Sample peak width (sec): 5    Sample min peak height: 50    Sample baseline V to V?: Y    Sample baseline V to V points: 3
Sample filter: Binomial       Number of points for filter: 3  Sample start region (min): 0  Sample end region (min): 30
Marker peak width (sec): 5    Marker min peak height: 200   Marker baseline V to V?: Y    Marker baseline V to V points: 3
Lower marker selection: First peak > 500 RFU                Upper marker selection: Last peak > 200 RFU
Ladder size (bp)75, 200, 300, 400, 500, 700, 1000, 1500, 2000, 3000, 4000, 5000, 7000, 10000, 20000
Quantification using: Lower Marker          Final concentration (ng/uL): 0.5000          Dilution factor: 12.0
```

Sample: SampF12
Well location: F12
Created: Tuesday, August 10, 2021 10:01:13 PM

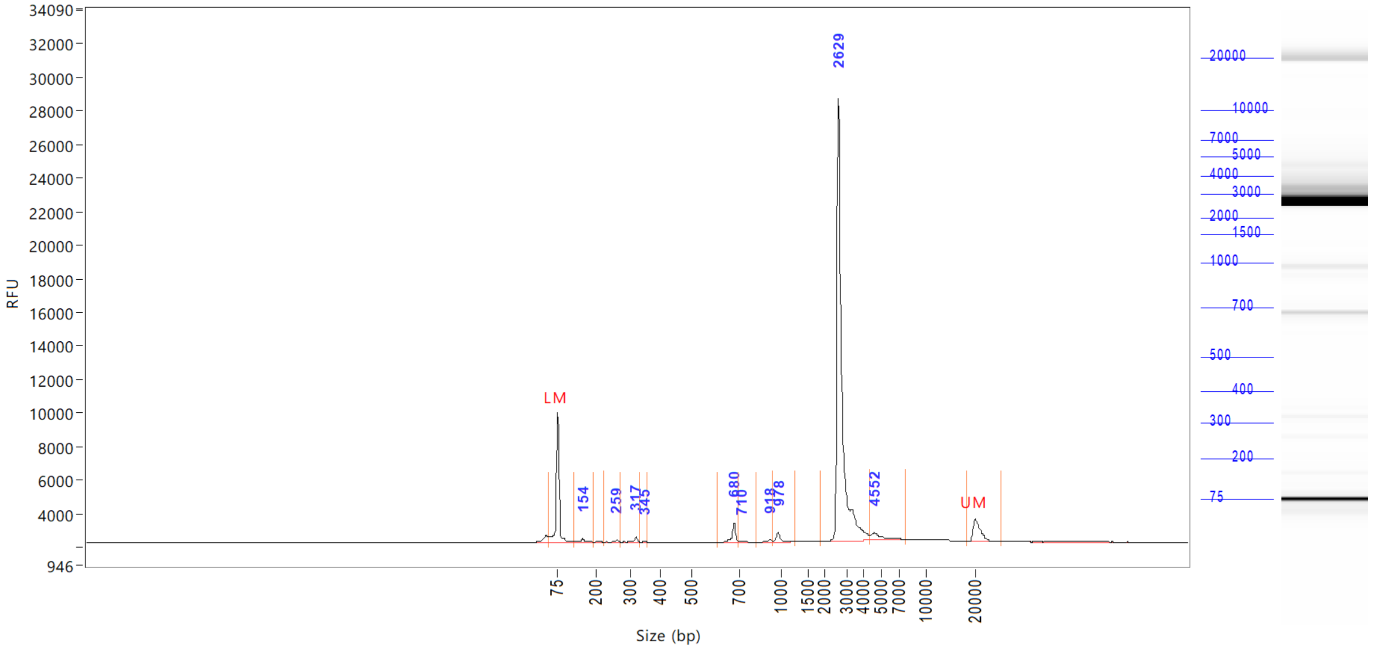

Peak	Size (bp)	Concentration (ng/uL)	From (bp)	To (bp)	Average size (bp)	CV%	RFU	Corrected peak area
1	75 (LM)	0.5000	47	129	73	13.64	7727	53.209
2	154	0.2532	129	189	151	7.67	214	2.245
3	259	0.1298	221	271	254	3.69	175	1.151
4	317	0.2686	271	331	309	3.77	294	2.382
5	345	0.0650	331	356	343	1.54	92	0.577
6	680	0.8669	606	697	671	1.98	1158	7.688
7	710	0.1144	697	827	724	3.45	89	1.015
8	918	0.1312	827	939	906	2.27	152	1.164
9	978	0.4799	939	1245	990	4.57	572	4.256
10	2629	23.4023	1866	4345	2780	12.04	26414	207.534
11	4552	0.7653	4345	7801	4998	14.03	442	6.787
12	20000 (UM)	0.1185	18415	25320	20429	4.16	1330	12.611

TIC:	26.4768	ng/uL
TIM:	22.8707	nmole/L
Total concentration:	26.6349	ng/uL

Sample peak width (sec): 5 Sample min peak height: 50 Sample baseline V to V?: Y Sample baseline V to V points: 3
Sample filter: Binomial Number of points for filter: 3 Sample start region (min): 0 Sample end region (min): 30
Marker peak width (sec): 5 Marker min peak height: 200 Marker baseline V to V?: Y Marker baseline V to V points: 3
Lower marker selection: First peak > 500 RFU Upper marker selection: Last peak > 200 RFU
Ladder size (bp)75, 200, 300, 400, 500, 700, 1000, 1500, 2000, 3000, 4000, 5000, 7000, 10000, 20000
Quantification using: Lower Marker Final concentration (ng/uL): 0.5000 Dilution factor: 12.0

Sample: SampG12
Well location: G12
Created: Tuesday, August 10, 2021 10:01:13 PM

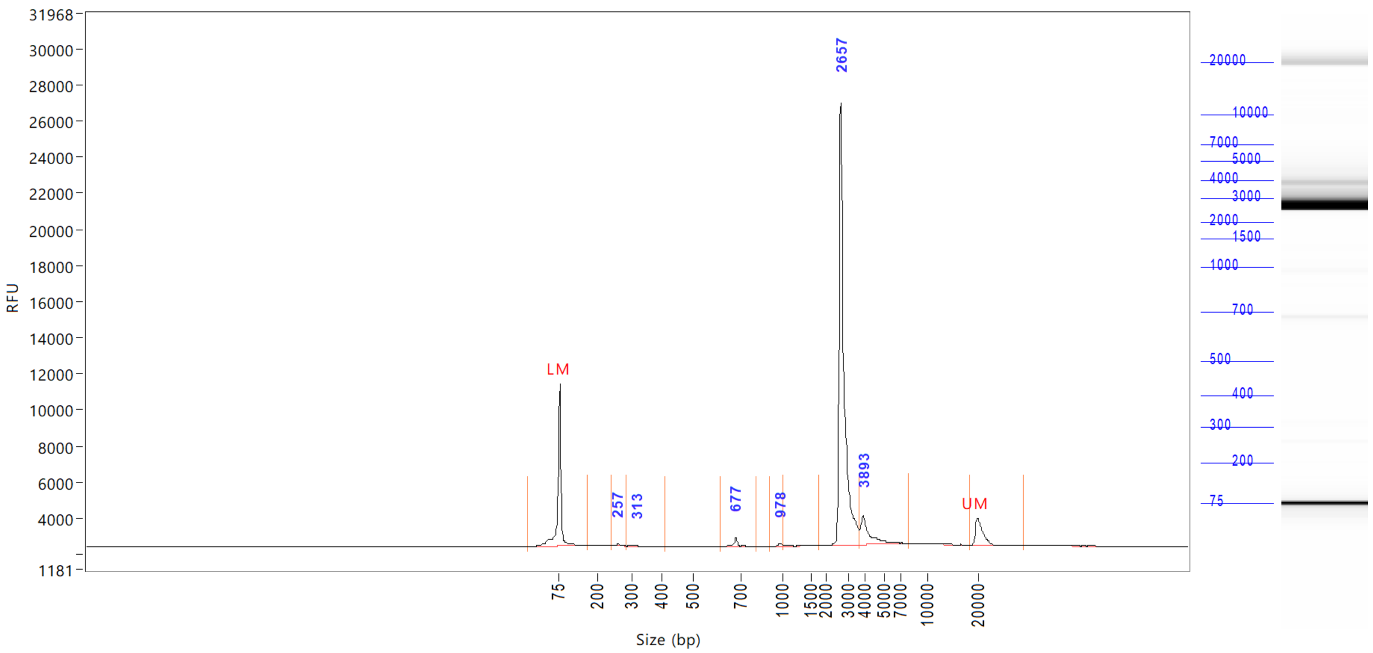

Peak	Size (bp)	Concentration (ng/uL)	From (bp)	To (bp)	Average size (bp)	CV%	RFU	Corrected peak area
1	75 (LM)	0.5000	0	164	70	23.22	8986	68.959
2	257	0.0583	239	284	256	2.27	123	0.669
3	313	0.0670	284	409	319	8.47	62	0.770
4	677	0.3229	611	809	676	3.49	457	3.711
5	978	0.0800	905	1012	975	1.60	148	0.919
6	2657	16.3316	1750	3608	2746	7.98	24533	187.702
7	3893	1.9009	3608	7867	4213	15.22	1655	21.848
8	20000 (UM)	0.1058	18415	29430	20547	4.66	1523	14.586

TIC:	18.7606	ng/uL
TIM:	12.1743	nmole/L
Total concentration:	18.8982	ng/uL

```
Sample peak width (sec): 5    Sample min peak height: 50    Sample baseline V to V?: Y    Sample baseline V to V points: 3
Sample filter: Binomial       Number of points for filter: 3  Sample start region (min): 0  Sample end region (min): 30
Marker peak width (sec): 5    Marker min peak height: 200   Marker baseline V to V?: Y    Marker baseline V to V points: 3
Lower marker selection: First peak > 500 RFU                Upper marker selection: Last peak > 200 RFU
Ladder size (bp)75, 200, 300, 400, 500, 700, 1000, 1500, 2000, 3000, 4000, 5000, 7000, 10000, 20000
Quantification using: Lower Marker          Final concentration (ng/uL): 0.5000          Dilution factor: 12.0
```

Sample: SampH12
Well location: H12
Created: Tuesday, August 10, 2021 10:01:13 PM

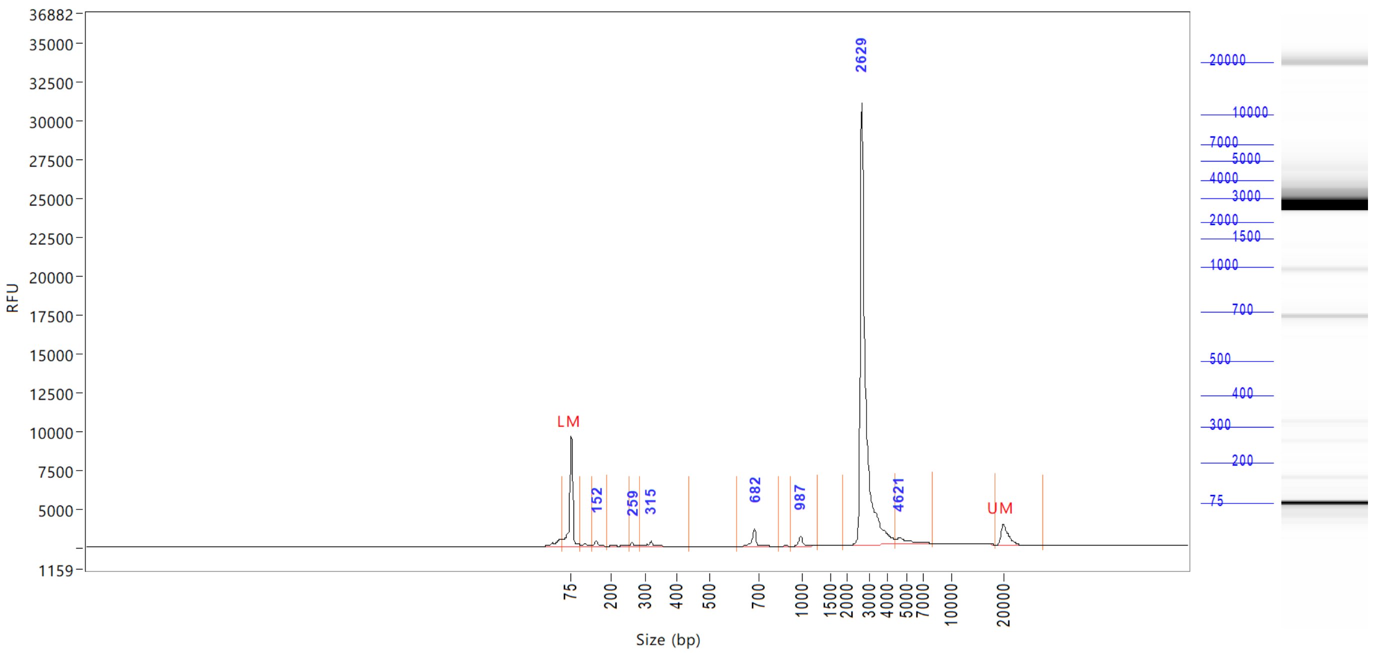

Peak	Size (bp)	Concentration (ng/uL)	From (bp)	To (bp)	Average size (bp)	CV%	RFU	Corrected peak area
1	75 (LM)	0.5000	50	105	72	10.70	7082	51.421
2	152	0.3482	141	185	152	4.46	396	2.984
3	259	0.1990	250	282	261	3.18	242	1.705
4	315	0.4396	282	435	320	8.43	317	3.768
5	682	1.0182	611	835	681	3.64	1140	8.726
6	987	0.5402	922	1278	1001	4.99	632	4.630
7	2629	27.7072	1866	4414	2797	12.25	28464	237.453
8	4621	0.8173	4414	7867	5083	13.60	419	7.004
9	20000 (UM)	0.1183	18537	27738	20486	4.36	1304	12.165

TIC:	31.0697	ng/uL
TIM:	27.1997	nmole/L
Total concentration:	31.5882	ng/uL

```
Sample peak width (sec): 5     Sample min peak height: 200   Sample baseline V to V?: Y    Sample baseline V to V points: 3
Sample filter: Binomial        Number of points for filter: 3  Sample start region (min): 0  Sample end region (min): 30
Marker peak width (sec): 5     Marker min peak height: 200   Marker baseline V to V?: Y    Marker baseline V to V points: 3
Lower marker selection: First peak > 500 RFU                 Upper marker selection: Last peak > 200 RFU
Ladder size (bp)75, 200, 300, 400, 500, 700, 1000, 1500, 2000, 3000, 4000, 5000, 7000, 10000, 20000
Quantification using: Lower Marker          Final concentration (ng/uL): 0.5000         Dilution factor: 12.0
```

Sample: DNA size ladder
Well location: A7
Created: Tuesday, August 10, 2021 10:01:13 PM
Import from: C:\Users\admin\Desktop\210811_alberto_ladder.SCAL
Fit type: Point to point

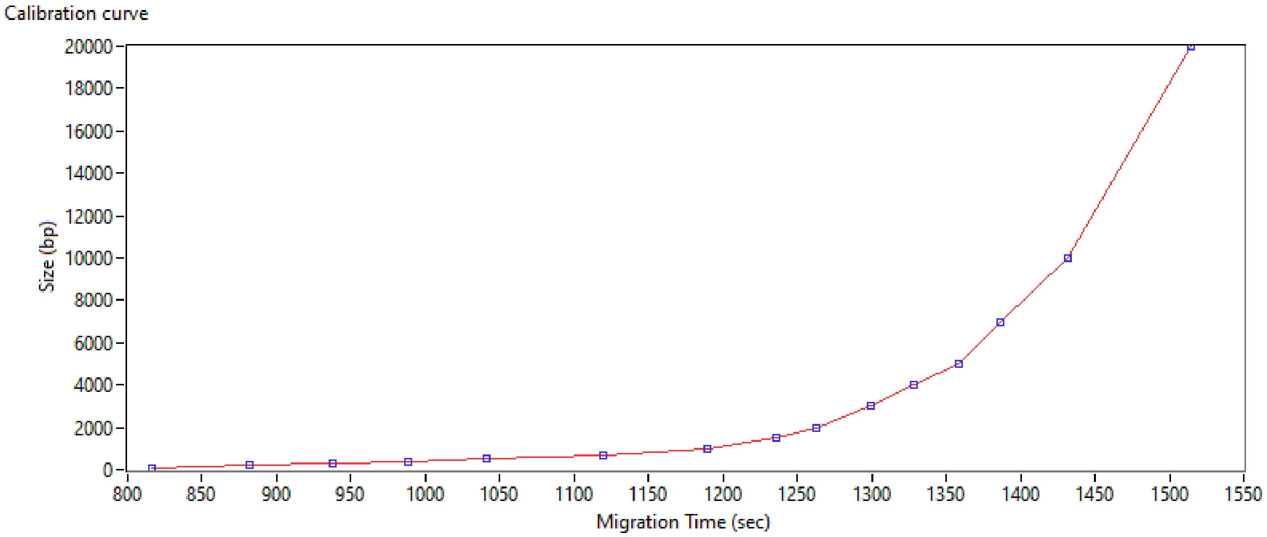

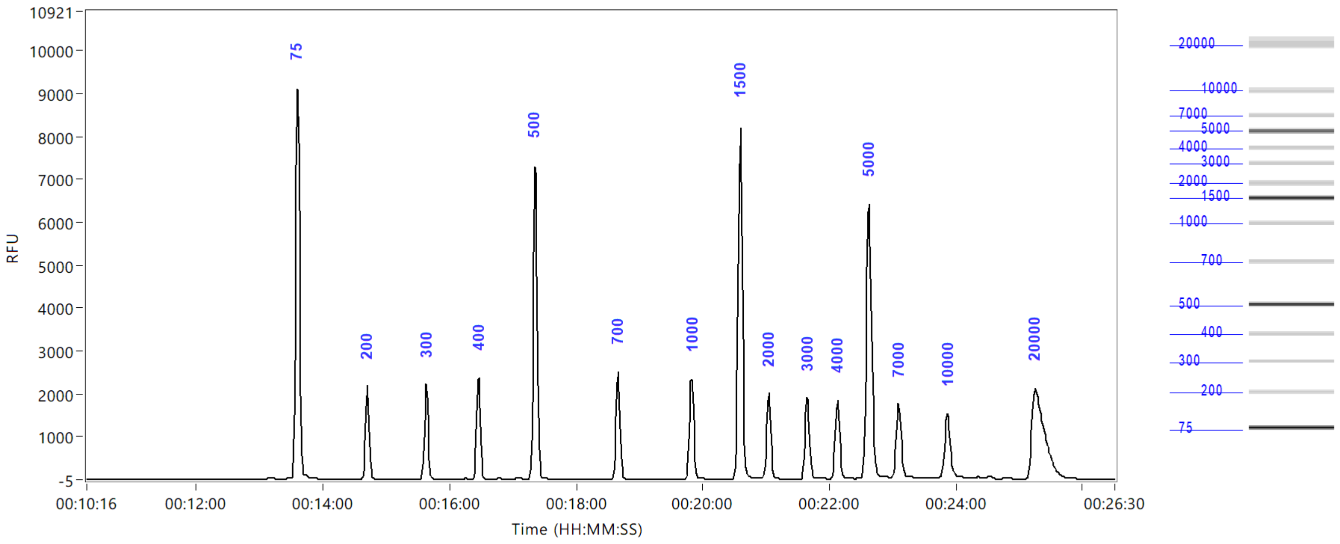

Supplement: Supplementary file 2 — sb3c00292_si_002.zip [file sb3c00292_si_002.zip › dnada_supplementary_material_pks_library_build/ZAG_trials/ZAG_Trial_1/21-51-18_plate6/plate6_report.pdf]
