# Supplementary material for: Automated Platform for the Plasmid Construction Process
Source: ACS Synth Biol. 2023 Nov 10;12(12):3506–13. doi: 10.1021/acssynbio.3c00292 (PMC10729297; doi:10.1021/acssynbio.3c00292)

Instrument controller software run summary:

Filename and data path: C:\AATI\Data\2021 08 10\18-20-33\2021 08 10 18H 20M..raw
Created: Tuesday, August 10, 2021 6:41:11 PM
Number of capillaries: 96
Array serial number: 111920-09SFS
Effect length: 33 cm
Array usage count: 164
Instrument type: ZAG DNA Analyzer
Instrument controller software version: 1.0.1.9
Device serial number: Z7597

Method Information

Method name: ZAG130FC33 - DNA 75-20000bp - Full Conditioning.mthds
Gel prime: No
Full conditioning: Yes
Gel prime to buffer: No
Gel selection: Gel 1
Perform prerun: 8.0 kV, 30 sec.
Rinse: No
Marker 1: Row: A, 5.0 kV, 5 sec.
Rinse: No
Sample injection: 5.0 kV, 5 sec.
Separation: 8.0 kV, 30.0 min.
Tray name: Sample Tray-1

Analysis mode: DNA

Notes

Gel Image

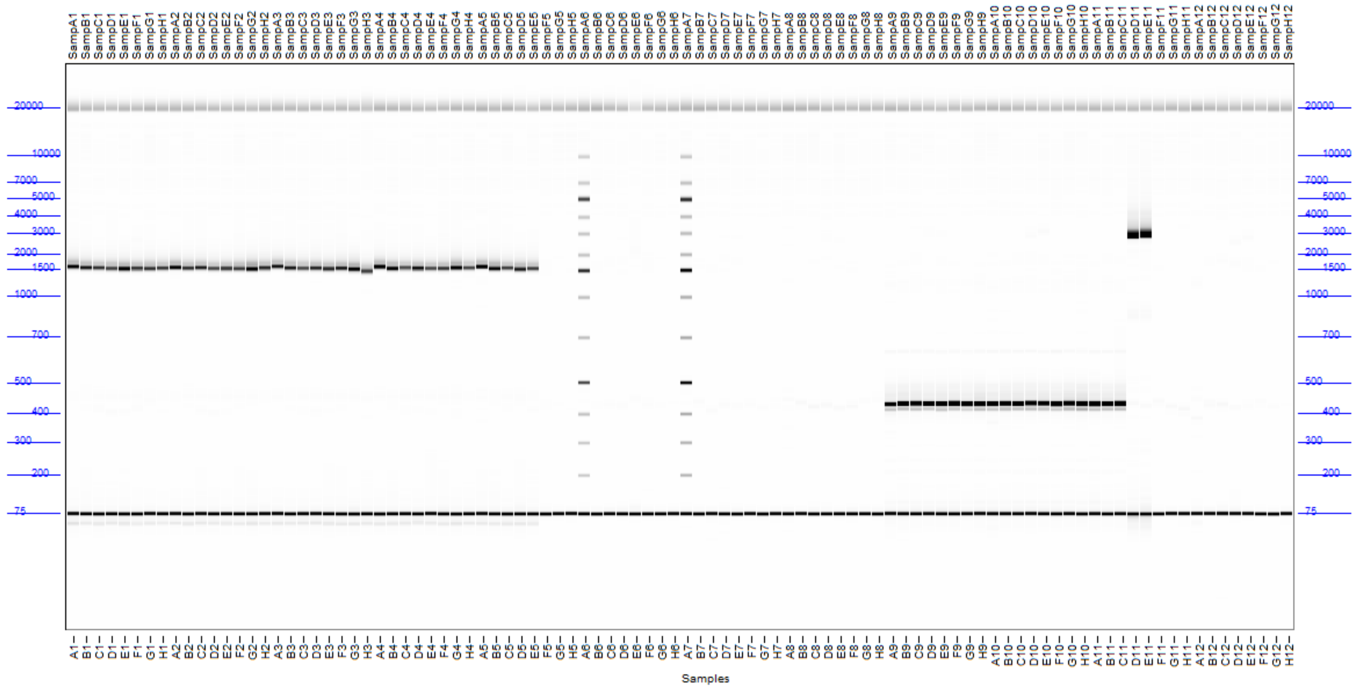

Filename and data path: C:\AATI\Data\2021 08 10\18-20-33\2021 08 10 18H 20M..raw

A1: SampA1

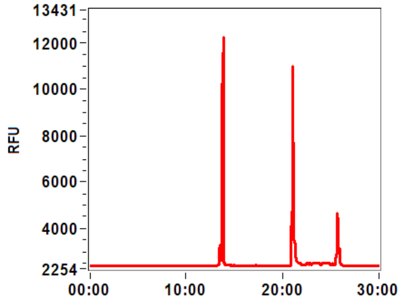

B1: SampB1

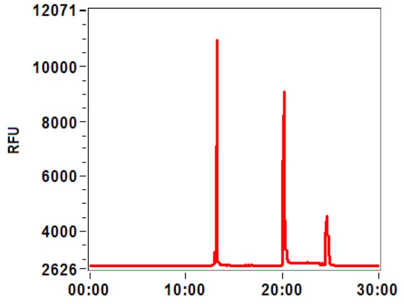

C1: SampC1

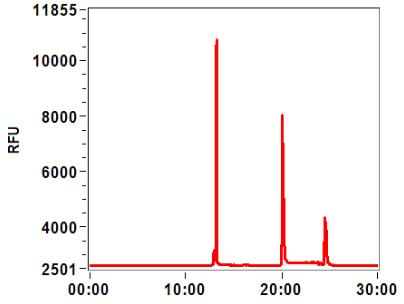

D1: SampD1

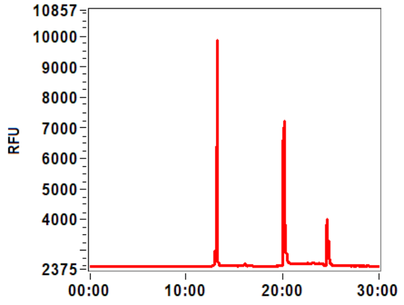

E1: SampE1

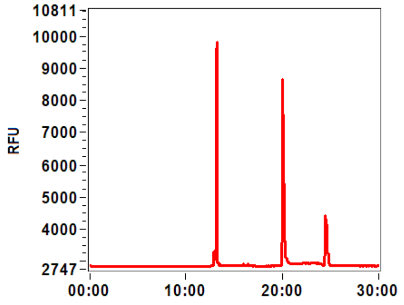

F1: SampF1

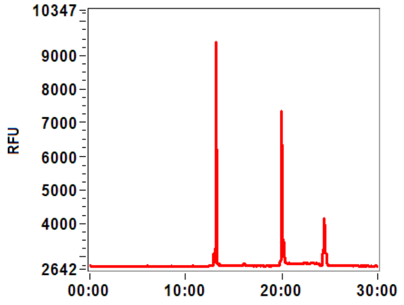

G1: SampG1

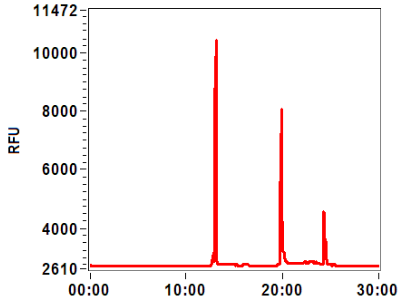

H1: SampH1

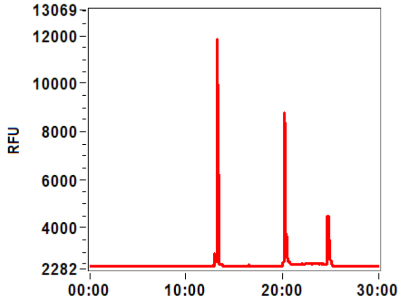

A2: SampA2

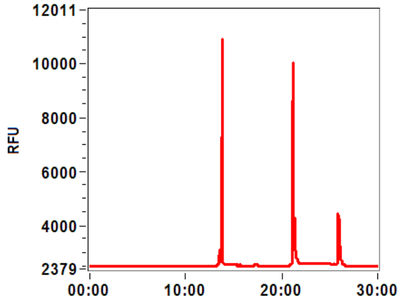

B2: SampB2

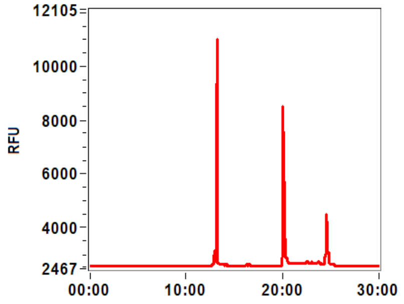

C2: SampC2

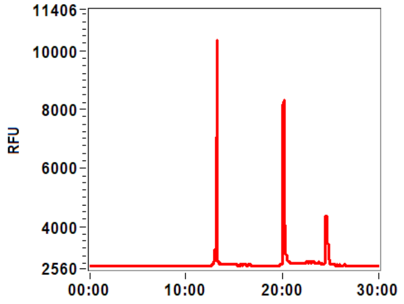

D2: SampD2

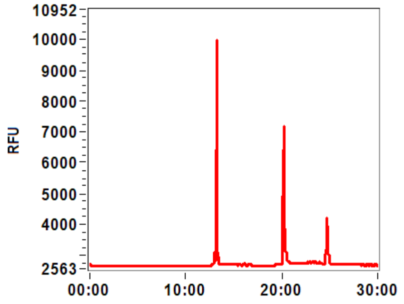

Filename and data path: C:\AATI\Data\2021 08 10\18-20-33\2021 08 10 18H 20M..raw

E2: SampE2

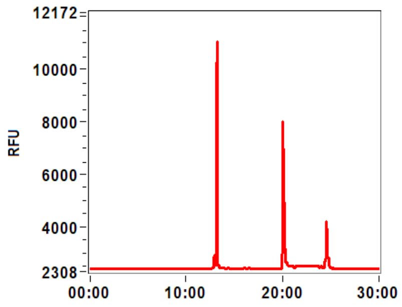

F2: SampF2

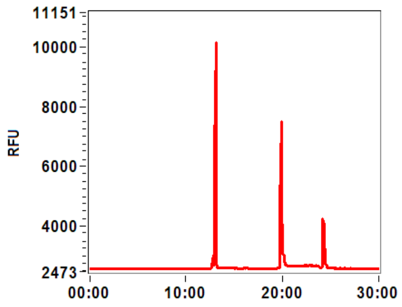

G2: SampG2

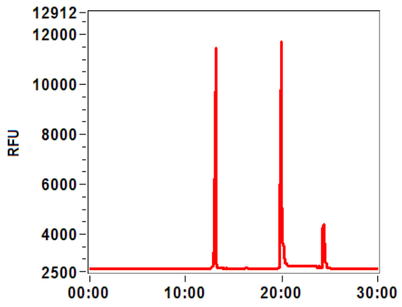

H2: SampH2

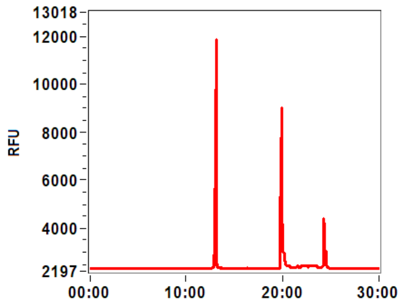

A3: SampA3

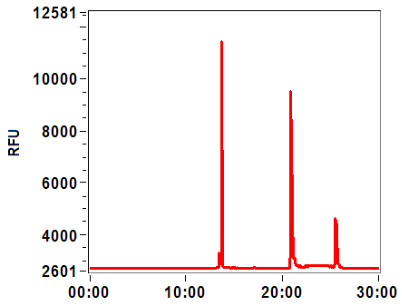

B3: SampB3

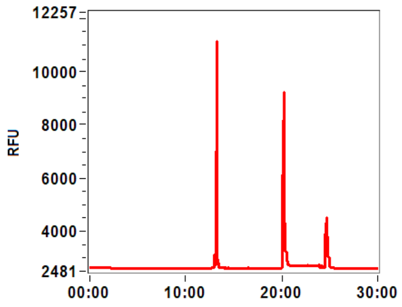

C3: SampC3

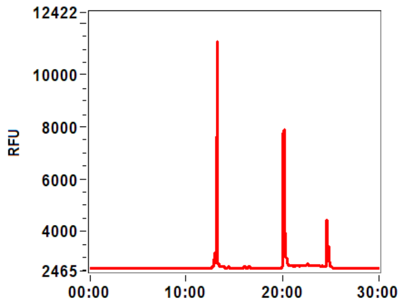

D3: SampD3

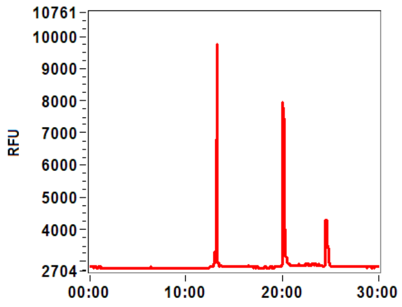

E3: SampE3

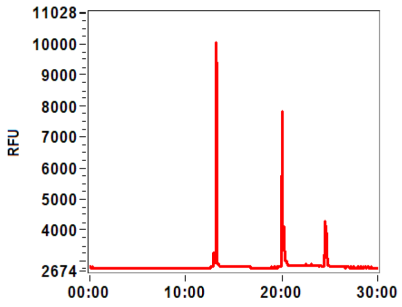

F3: SampF3

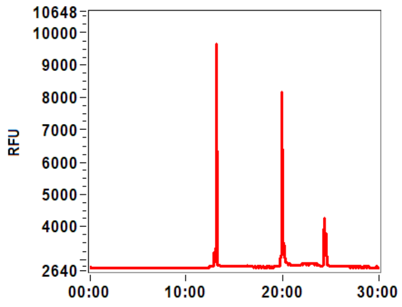

G3: SampG3

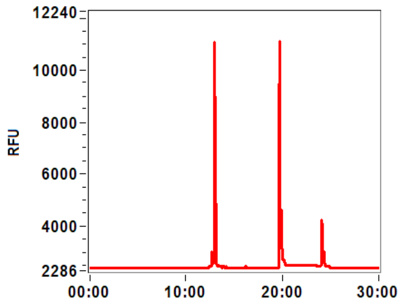

H3: SampH3

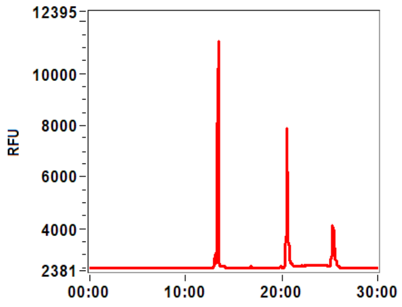

Filename and data path: C:\AATI\Data\2021 08 10\18-20-33\2021 08 10 18H 20M..raw

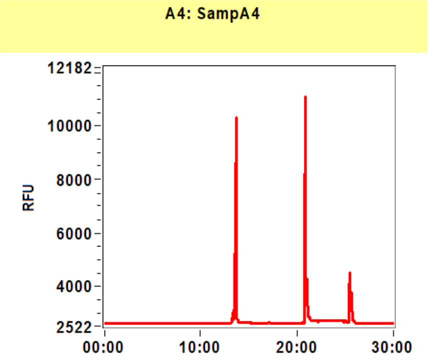

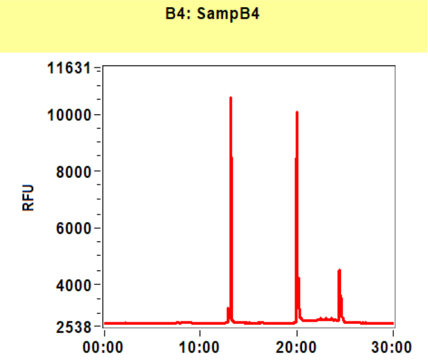

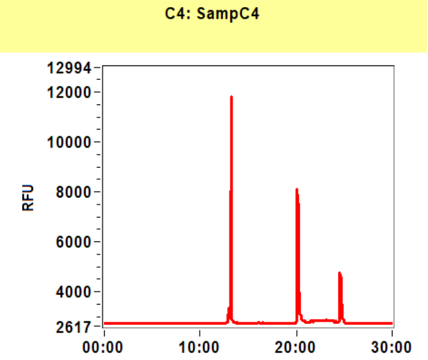

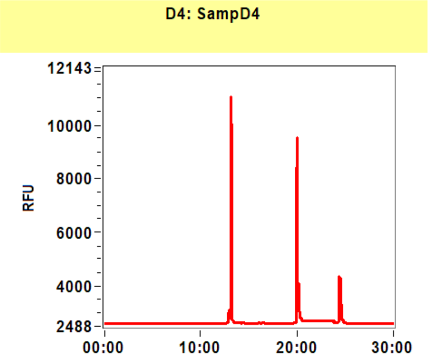

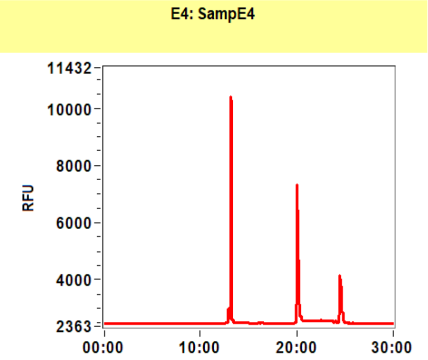

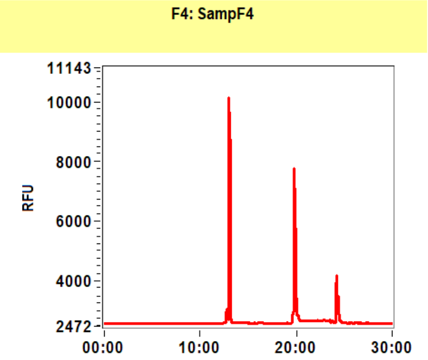

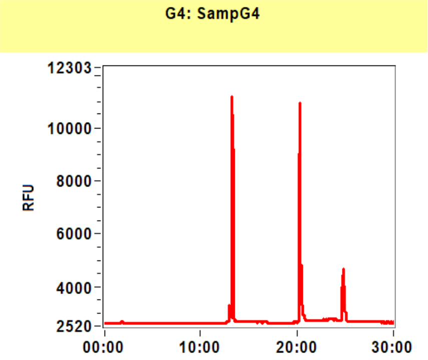

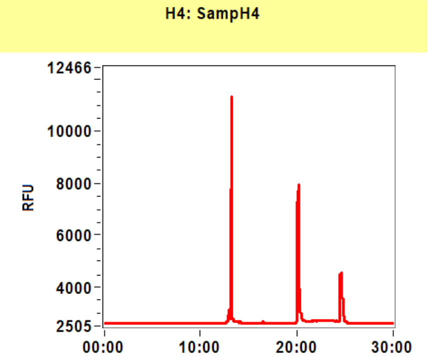

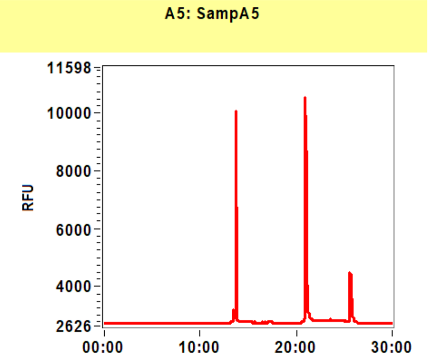

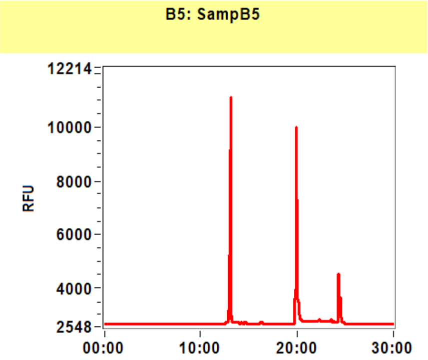

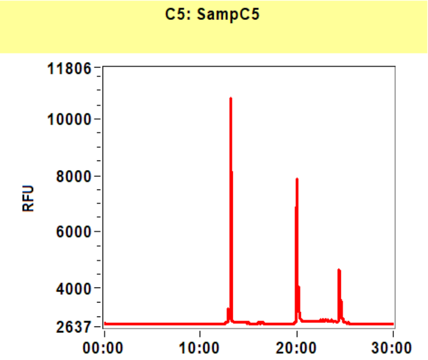

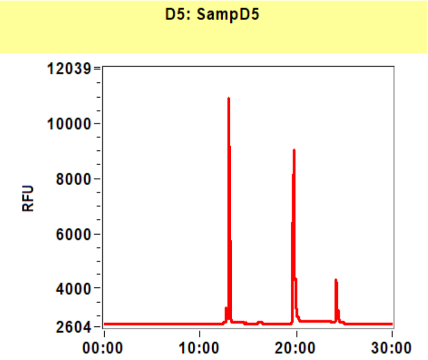

Filename and data path: C:\AATI\Data\2021 08 10\18-20-33\2021 08 10 18H 20M..raw

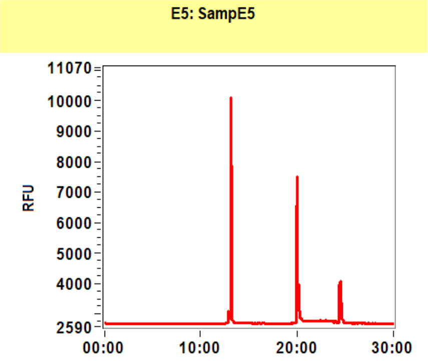

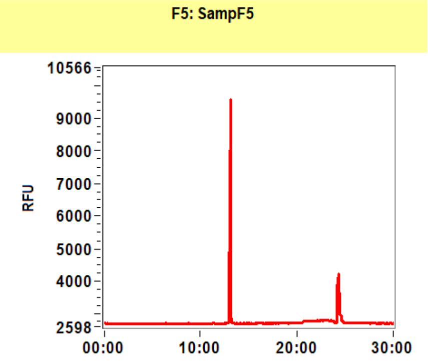

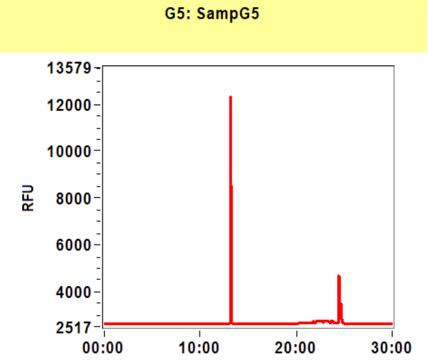

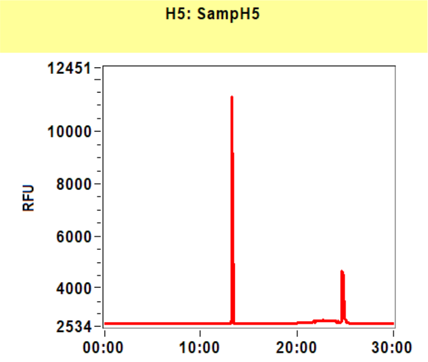

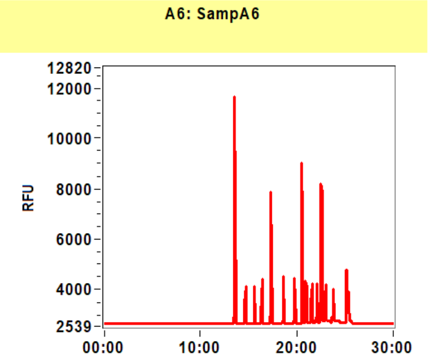

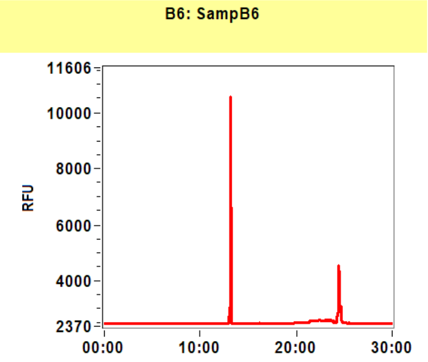

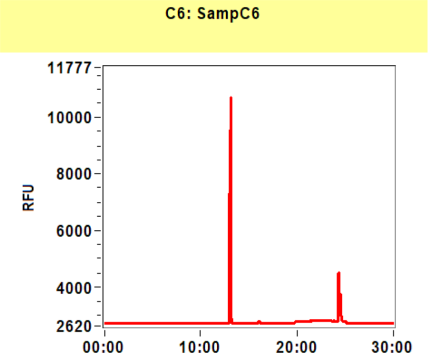

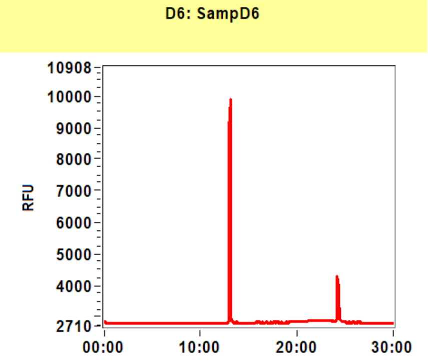

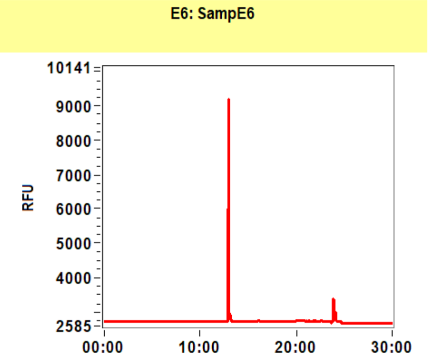

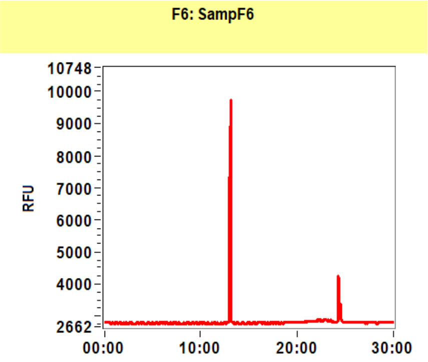

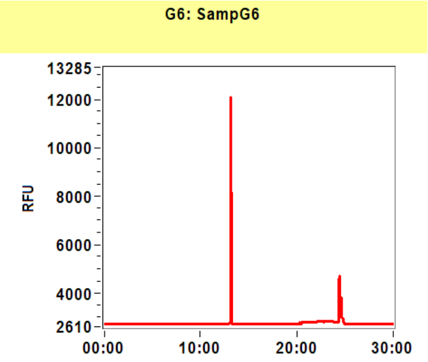

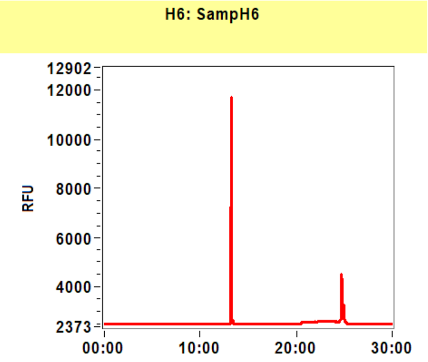

Filename and data path: C:\AATI\Data\2021 08 10\18-20-33\2021 08 10 18H 20M..raw

A7: SampA7

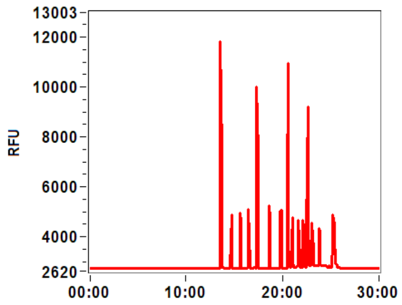

B7: SampB7

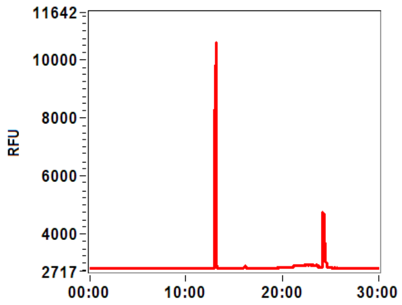

C7: SampC7

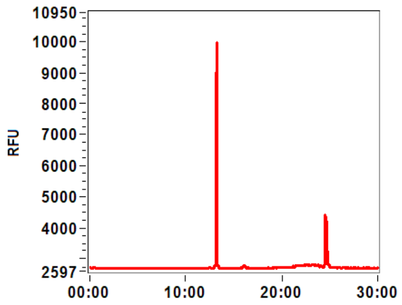

D7: SampD7

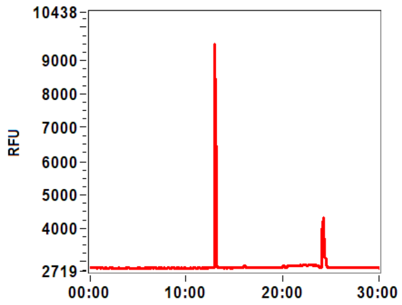

E7: SampE7

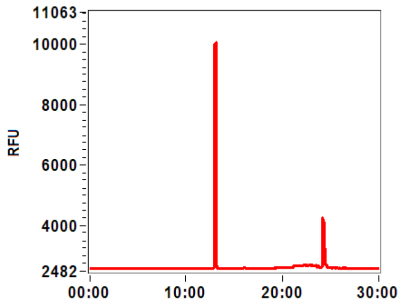

F7: SampF7

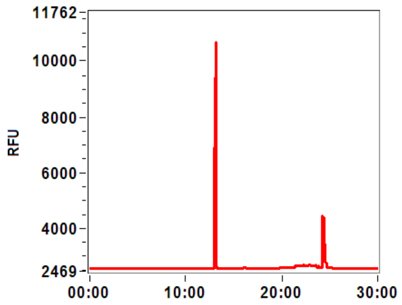

G7: SampG7

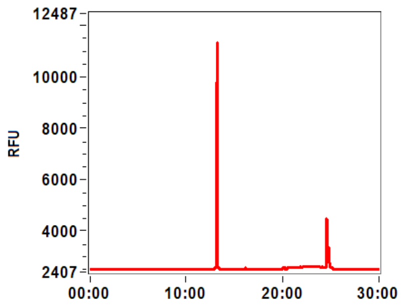

H7: SampH7

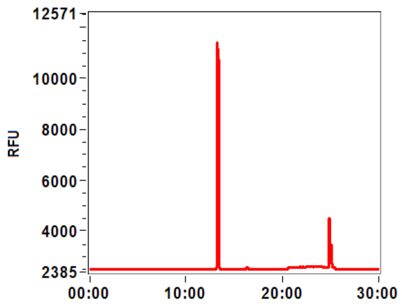

A8: SampA8

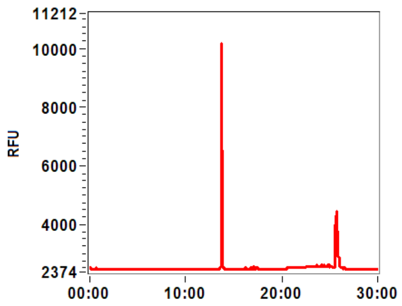

B8: SampB8

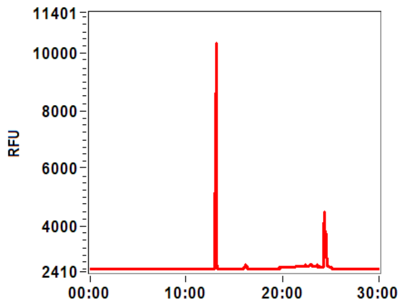

C8: SampC8

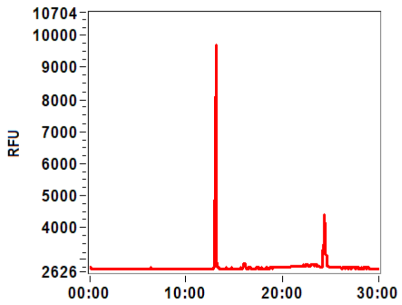

D8: SampD8

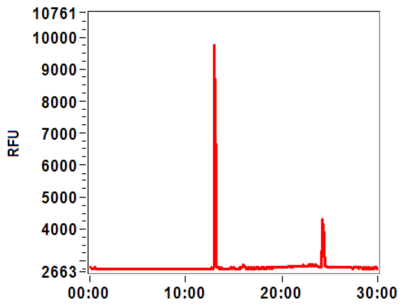

Filename and data path: C:\AATI\Data\2021 08 10\18-20-33\2021 08 10 18H 20M..raw

E8: SampE8

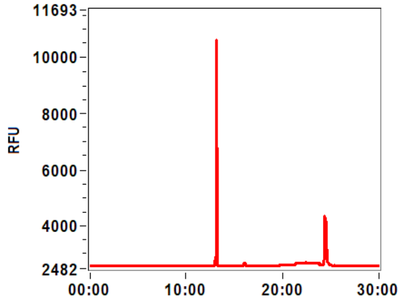

F8: SampF8

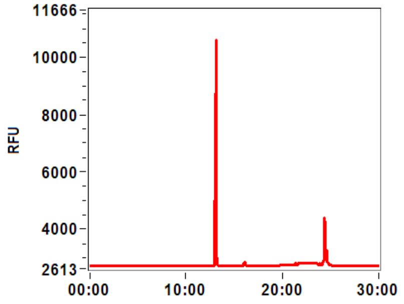

G8: SampG8

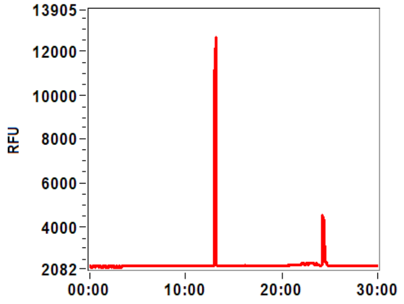

H8: SampH8

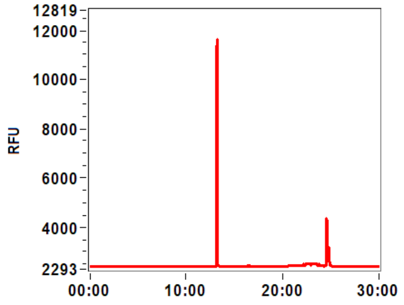

A9: SampA9

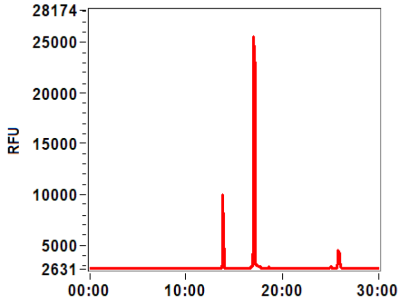

B9: SampB9

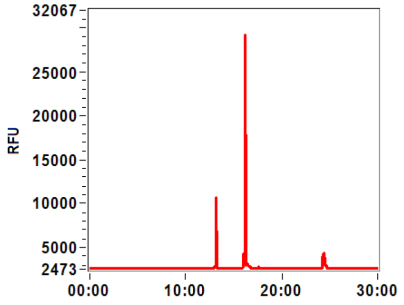

C9: SampC9

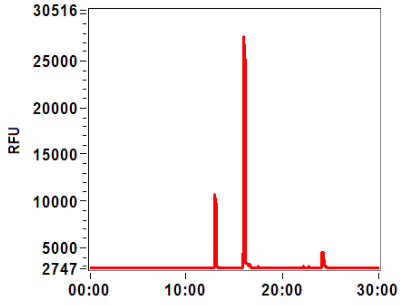

D9: SampD9

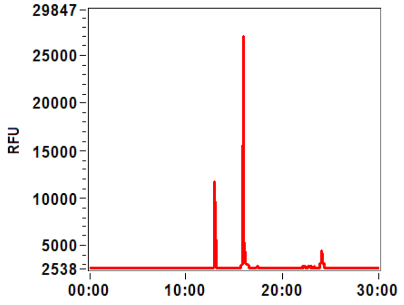

E9: SampE9

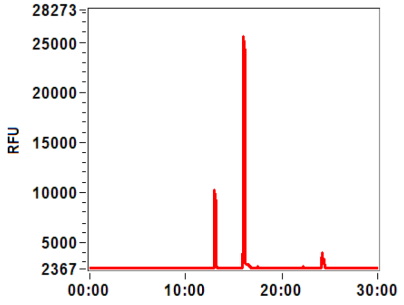

F9: SampF9

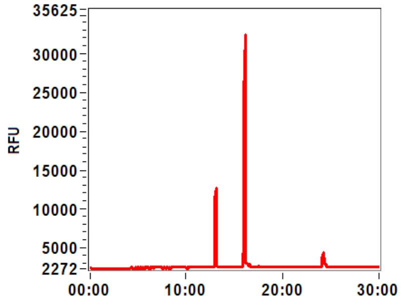

G9: SampG9

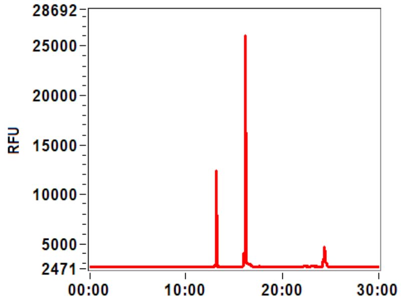

H9: SampH9

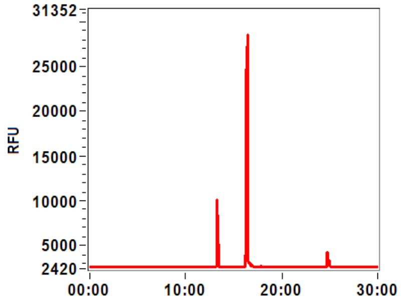

Filename and data path: C:\AATI\Data\2021 08 10\18-20-33\2021 08 10 18H 20M..raw

A10: SampA10

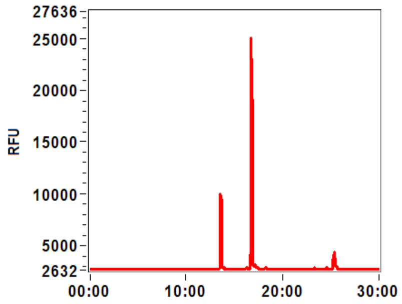

B10: SampB10

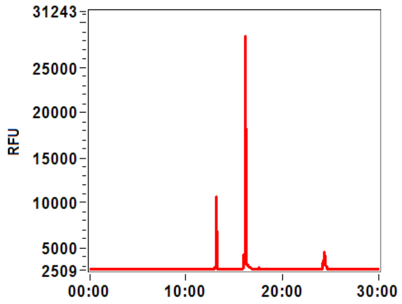

C10: SampC10

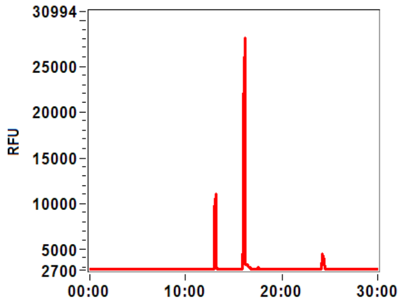

D10: SampD10

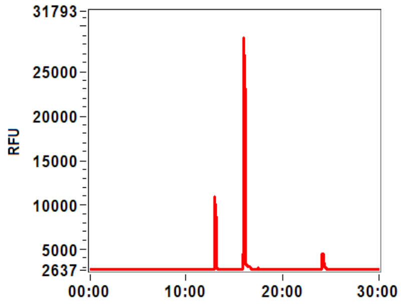

E10: SampE10

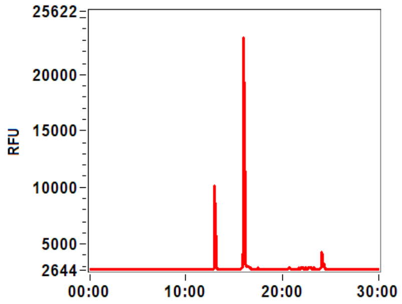

F10: SampF10

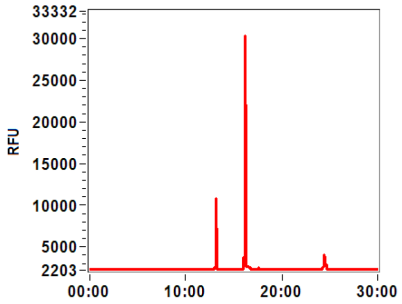

G10: SampG10

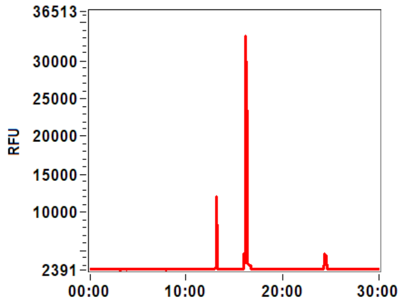

H10: SampH10

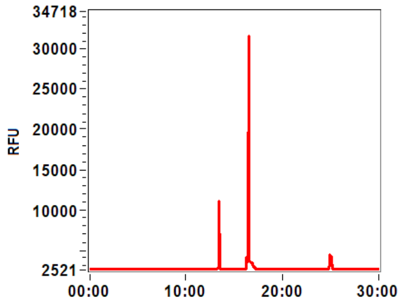

A11: SampA11

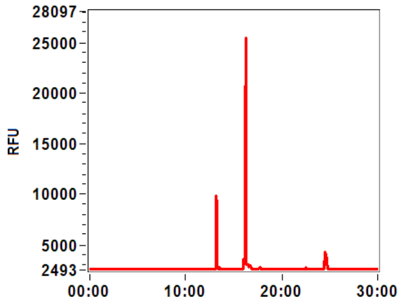

B11: SampB11

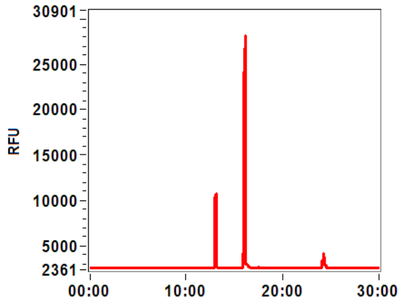

C11: SampC11

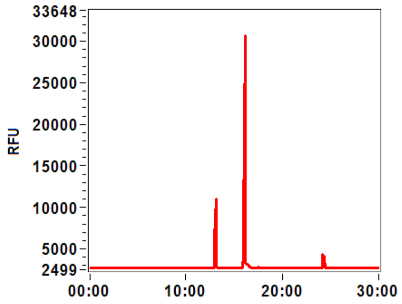

D11: SampD11

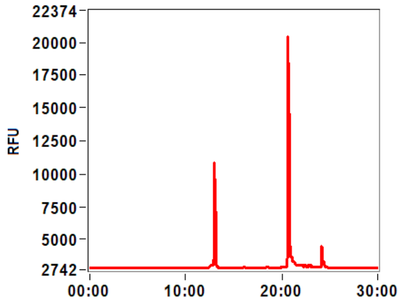

Filename and data path: C:\AATI\Data\2021 08 10\18-20-33\2021 08 10 18H 20M..raw

E11: SampE11

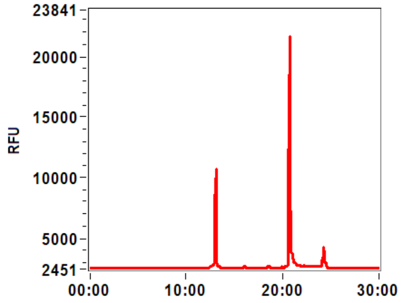

F11: SampF11

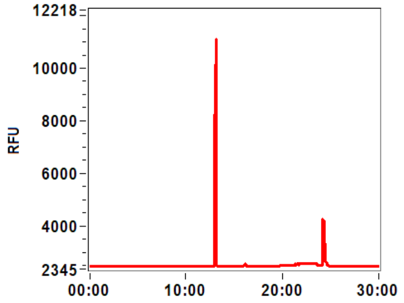

G11: SampG11

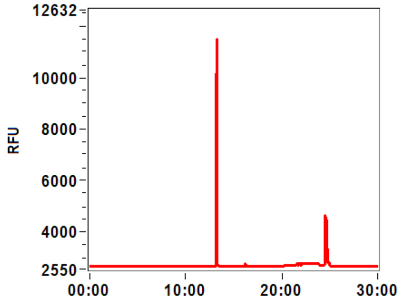

H11: SampH11

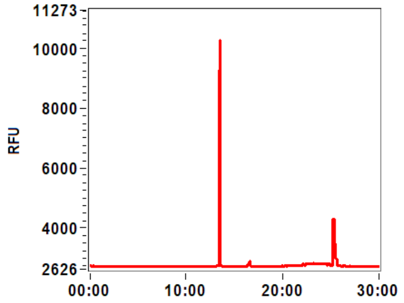

A12: SampA12

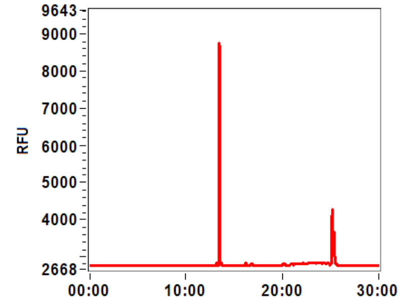

B12: SampB12

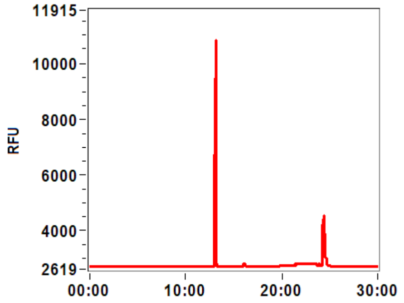

C12: SampC12

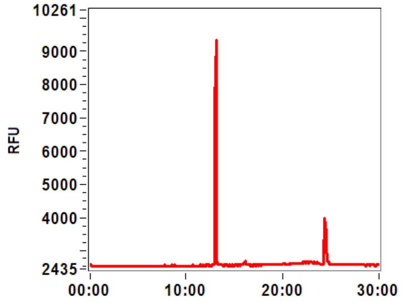

D12: SampD12

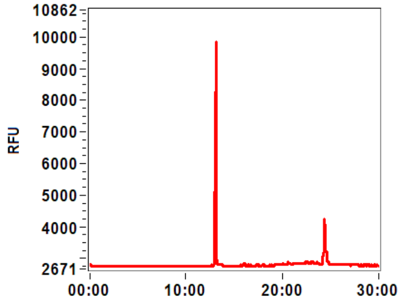

E12: SampE12

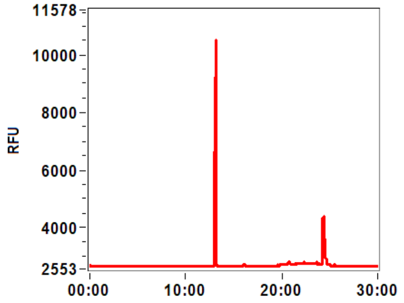

F12: SampF12

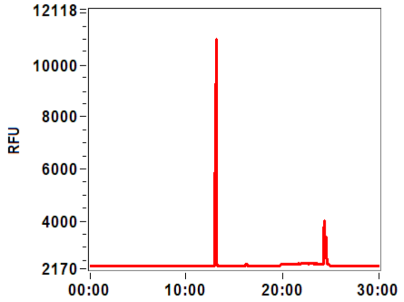

G12: SampG12

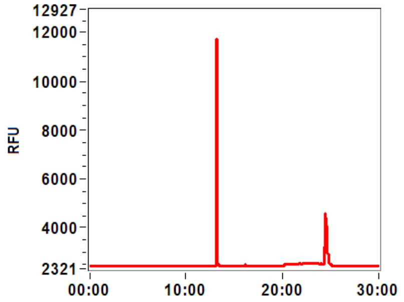

H12: SampH12

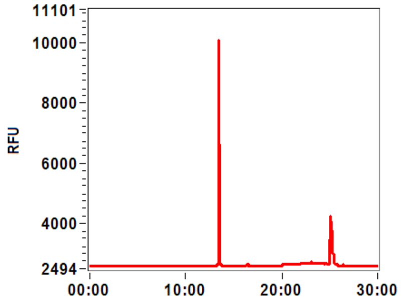

Sample: SampA1
Well location: A1
Created: Tuesday, August 10, 2021 6:41:11 PM

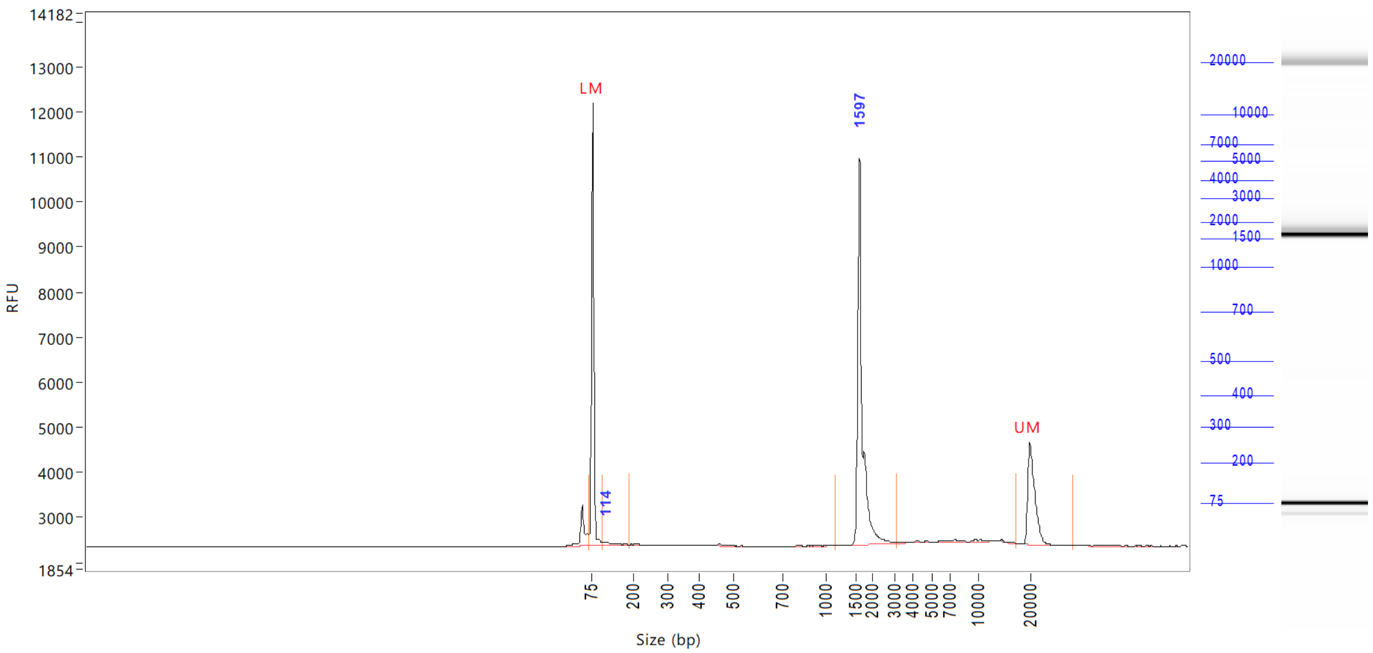

Peak	Size (bp)	Concentration (ng/uL)	From (bp)	To (bp)	Average size (bp)	CV%	RFU	Corrected peak area
1	75 (LM)	0.5000	64	107	74	6.19	9835	54.020
2	114	0.1693	107	187	130	13.86	63	1.524
3	1597	6.4453	1145	3108	1679	11.85	8586	58.029
4	20000 (UM)	0.1821	17317	28221	20387	4.19	2259	19.675

TIC:	6.6146	ng/uL
TIM:	8.4587	nmole/L
Total concentration:	7.0728	ng/uL

Sample peak width (sec): 5 Sample min peak height: 50 Sample baseline V to V?: Y Sample baseline V to V points: 3
Sample filter: Binomial Number of points for filter: 3 Sample start region (min): 0 Sample end region (min): 30
Marker peak width (sec): 5 Marker min peak height: 200 Marker baseline V to V?: Y Marker baseline V to V points: 3
Lower marker selection: First peak > 500 RFU Upper marker selection: Last peak > 200 RFU
Ladder size (bp)75, 200, 300, 400, 500, 700, 1000, 1500, 2000, 3000, 4000, 5000, 7000, 10000, 20000
Quantification using: Lower Marker Final concentration (ng/uL): 0.5000 Dilution factor: 12.0

Sample: SampB1
Well location: B1
Created: Tuesday, August 10, 2021 6:41:11 PM

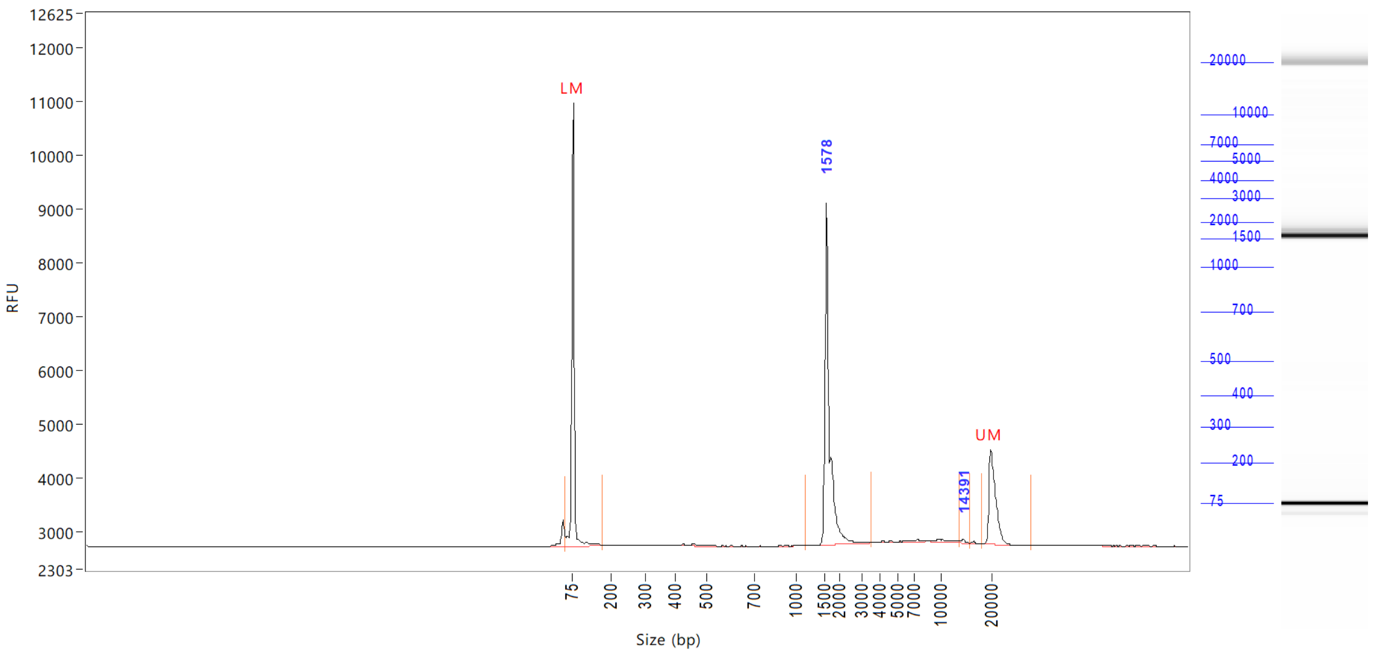

Peak	Size (bp)	Concentration (ng/uL)	From (bp)	To (bp)	Average size (bp)	CV%	RFU	Corrected peak area
1	75 (LM)	0.5000	52	168	75	16.20	8244	47.964
2	1578	5.5607	1156	3536	1653	13.71	6360	44.452
3	14391	0.0460	13781	15732	14430	3.14	63	0.368
4	20000 (UM)	0.1604	18293	28342	20432	4.21	1772	15.388

TIC:	5.6067	ng/uL
TIM:	5.5427	nmole/L
Total concentration:	6.2231	ng/uL

```
Sample peak width (sec): 5    Sample min peak height: 50    Sample baseline V to V?: Y    Sample baseline V to V points: 3
Sample filter: Binomial       Number of points for filter: 3  Sample start region (min): 0  Sample end region (min): 30
Marker peak width (sec): 5    Marker min peak height: 200   Marker baseline V to V?: Y    Marker baseline V to V points: 3
Lower marker selection: First peak > 500 RFU                Upper marker selection: Last peak > 200 RFU
Ladder size (bp)75, 200, 300, 400, 500, 700, 1000, 1500, 2000, 3000, 4000, 5000, 7000, 10000, 20000
Quantification using: Lower Marker          Final concentration (ng/uL): 0.5000          Dilution factor: 12.0
```

Sample: SampC1
Well location: C1
Created: Tuesday, August 10, 2021 6:41:11 PM

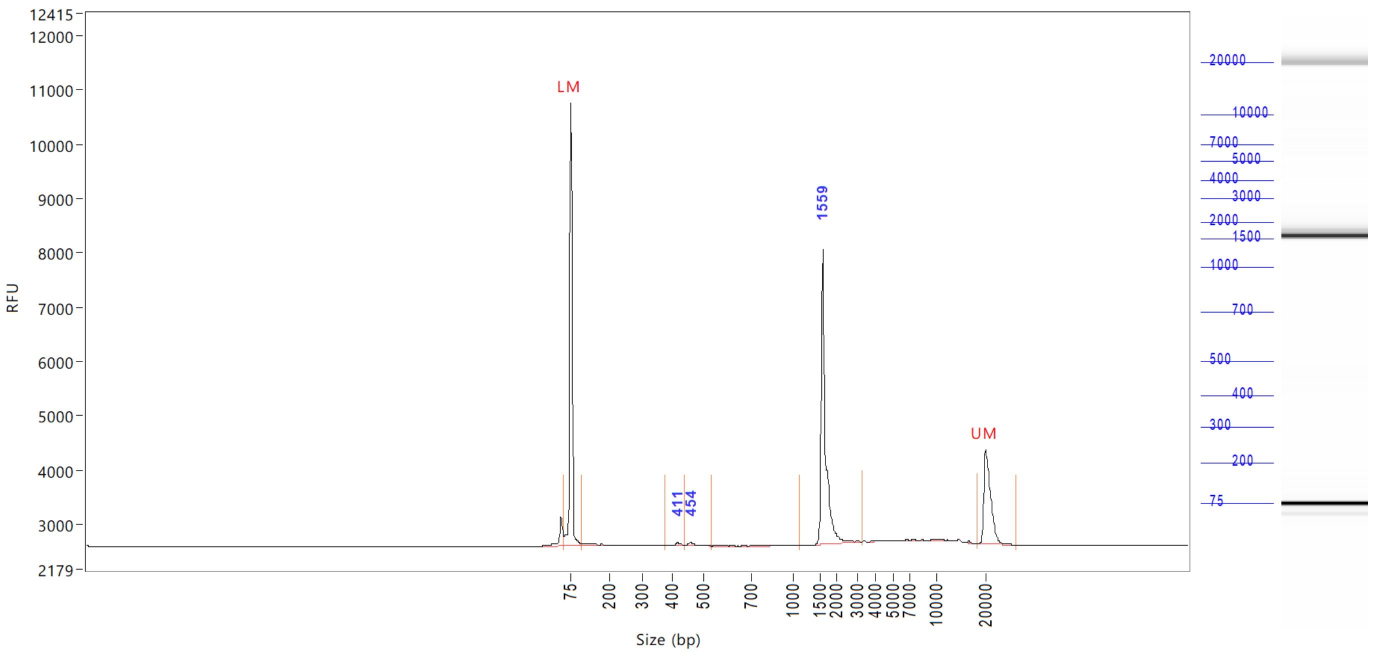

Peak	Size (bp)	Concentration (ng/uL)	From (bp)	To (bp)	Average size (bp)	CV%	RFU	Corrected peak area
1	75 (LM)	0.5000	52	108	72	7.51	8168	47.522
2	411	0.0833	376	437	413	2.96	52	0.660
3	454	0.1048	437	532	464	4.00	54	0.830
4	1559	4.7001	1123	3286	1626	10.33	5432	37.227
5	20000 (UM)	0.1558	18171	26287	20405	3.94	1723	14.810

TIC:	4.8882	ng/uL
TIM:	5.4617	nmole/L
Total concentration:	5.4027	ng/uL

Sample peak width (sec): 5 Sample min peak height: 50 Sample baseline V to V?: Y Sample baseline V to V points: 3
Sample filter: Binomial Number of points for filter: 3 Sample start region (min): 0 Sample end region (min): 30
Marker peak width (sec): 5 Marker min peak height: 200 Marker baseline V to V?: Y Marker baseline V to V points: 3
Lower marker selection: First peak > 500 RFU Upper marker selection: Last peak > 200 RFU
Ladder size (bp)75, 200, 300, 400, 500, 700, 1000, 1500, 2000, 3000, 4000, 5000, 7000, 10000, 20000
Quantification using: Lower Marker Final concentration (ng/uL): 0.5000 Dilution factor: 12.0

Sample: SampD1
Well location: D1
Created: Tuesday, August 10, 2021 6:41:11 PM

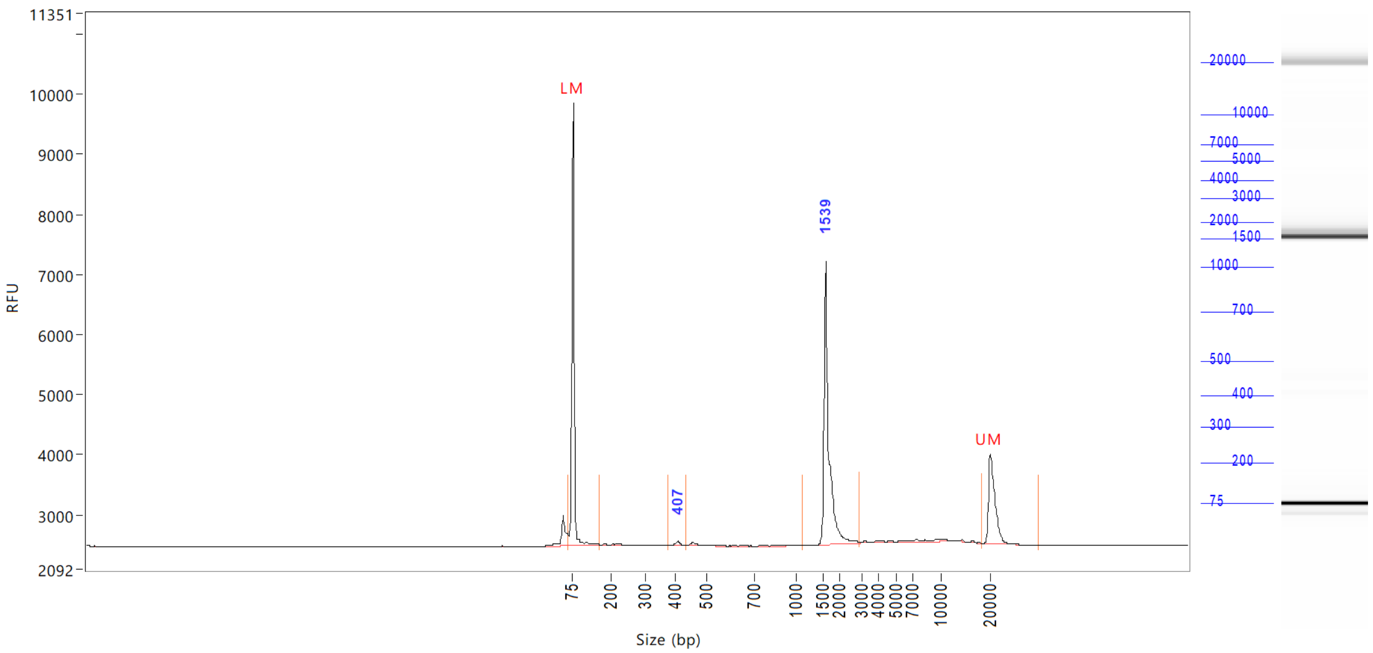

Peak	Size<br>(bp)	Concentration<br>(ng/uL)	From<br>(bp)	To<br>(bp)	Average size<br>(bp)	CV%	RFU	Corrected peak area
1	75 (LM)	0.5000	64	160	75	12.92	7388	41.238
2	407	0.0941	376	433	403	2.25	75	0.647
3	1539	5.0673	1112	2914	1631	11.93	4716	34.828
4	20000 (UM)	0.1514	18415	29793	20449	4.32	1486	12.483

TIC:	5.1615	ng/uL
TIM:	5.4986	nmole/L
Total concentration:	5.7847	ng/uL

Sample peak width (sec): 5 Sample min peak height: 50 Sample baseline V to V?: Y Sample baseline V to V points: 3
Sample filter: Binomial Number of points for filter: 3 Sample start region (min): 0 Sample end region (min): 30
Marker peak width (sec): 5 Marker min peak height: 200 Marker baseline V to V?: Y Marker baseline V to V points: 3
Lower marker selection: First peak > 500 RFU Upper marker selection: Last peak > 200 RFU
Ladder size (bp)75, 200, 300, 400, 500, 700, 1000, 1500, 2000, 3000, 4000, 5000, 7000, 10000, 20000
Quantification using: Lower Marker Final concentration (ng/uL): 0.5000 Dilution factor: 12.0

Sample: SampE1
Well location: E1
Created: Tuesday, August 10, 2021 6:41:11 PM

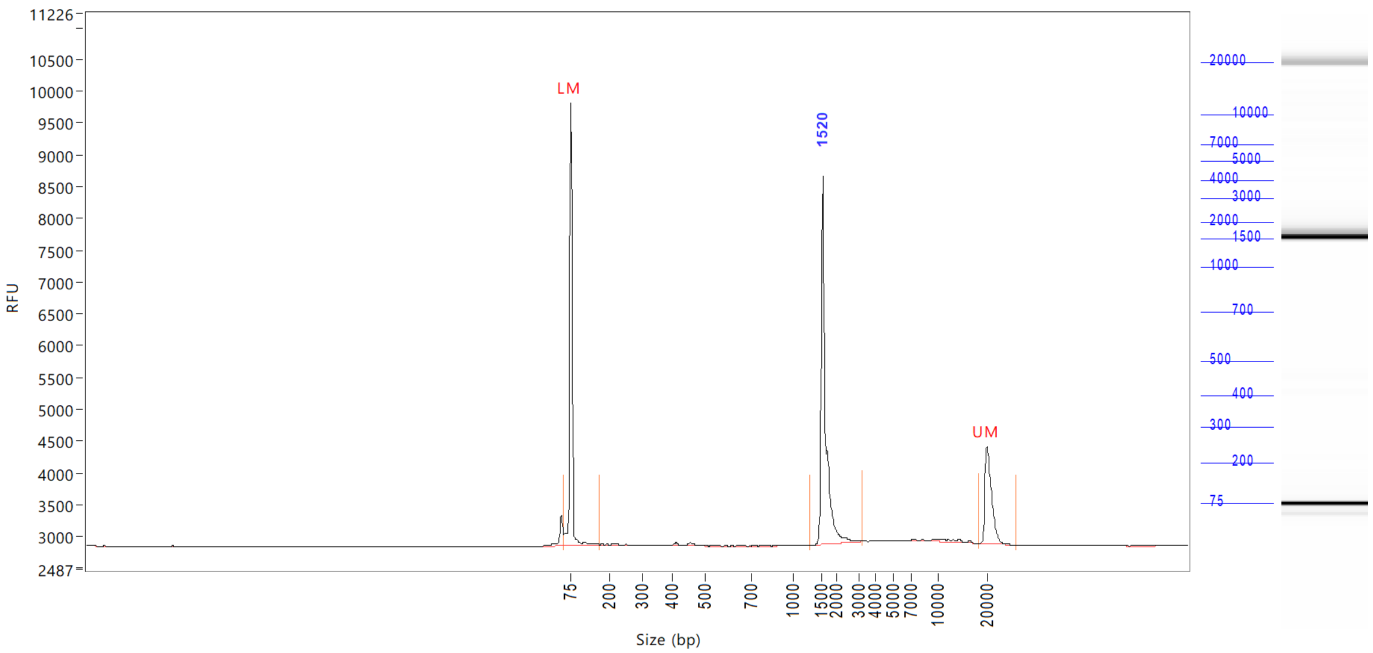

Peak	Size (bp)	Concentration (ng/uL)	From (bp)	To (bp)	Average size (bp)	CV%	RFU	Corrected peak area
1	75 (LM)	0.5000	52	166	74	14.63	6972	41.689
2	1520	5.7916	1289	3215	1604	10.83	5783	40.241
3	20000 (UM)	0.1554	18415	25803	20353	3.86	1525	12.960

TIC:	5.7916	ng/uL
TIM:	5.9436	nmole/L
Total concentration:	6.4419	ng/uL

```
Sample peak width (sec): 5    Sample min peak height: 50    Sample baseline V to V?: Y    Sample baseline V to V points: 3
Sample filter: Binomial       Number of points for filter: 3  Sample start region (min): 0  Sample end region (min): 30
Marker peak width (sec): 5    Marker min peak height: 200   Marker baseline V to V?: Y    Marker baseline V to V points: 3
Lower marker selection: First peak > 500 RFU                Upper marker selection: Last peak > 200 RFU
Ladder size (bp)75, 200, 300, 400, 500, 700, 1000, 1500, 2000, 3000, 4000, 5000, 7000, 10000, 20000
Quantification using: Lower Marker          Final concentration (ng/uL): 0.5000          Dilution factor: 12.0
```

Sample: SampF1
Well location: F1
Created: Tuesday, August 10, 2021 6:41:11 PM

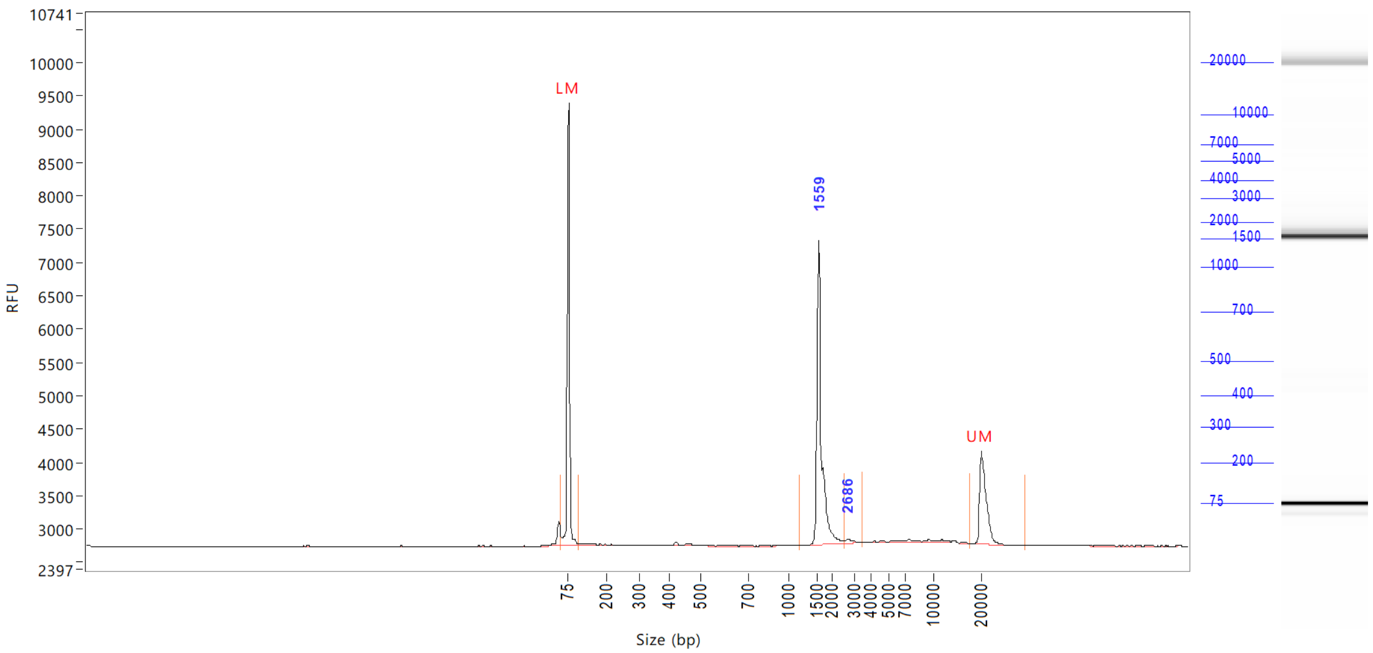

Peak	Size (bp)	Concentration (ng/uL)	From (bp)	To (bp)	Average size (bp)	CV%	RFU	Corrected peak area
1	75 (LM)	0.5000	52	108	72	7.12	6655	38.188
2	1559	4.9102	1167	2543	1604	9.52	4563	31.252
3	2686	0.1108	2543	3500	2828	7.92	65	0.705
4	20000 (UM)	0.1568	17439	29068	20419	4.47	1388	11.978

TIC:	5.0210	ng/uL
TIM:	5.1036	nmole/L
Total concentration:	5.6728	ng/uL

Sample peak width (sec): 5   Sample min peak height: 50   Sample baseline V to V?: Y   Sample baseline V to V points: 3
Sample filter: Binomial   Number of points for filter: 3   Sample start region (min): 0   Sample end region (min): 30
Marker peak width (sec): 5   Marker min peak height: 200   Marker baseline V to V?: Y   Marker baseline V to V points: 3
Lower marker selection: First peak > 500 RFU   Upper marker selection: Last peak > 200 RFU
Ladder size (bp)75, 200, 300, 400, 500, 700, 1000, 1500, 2000, 3000, 4000, 5000, 7000, 10000, 20000
Quantification using: Lower Marker   Final concentration (ng/uL): 0.5000   Dilution factor: 12.0

Sample: SampG1
Well location: G1
Created: Tuesday, August 10, 2021 6:41:11 PM

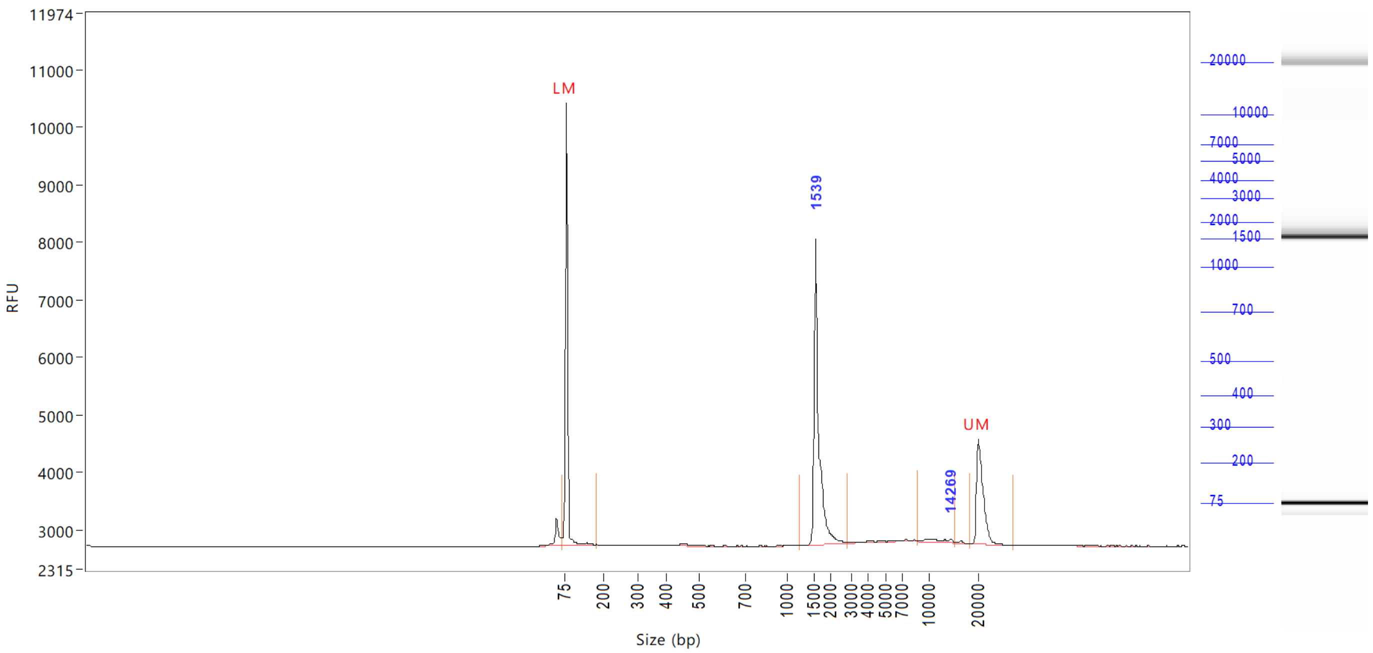

Peak	Size (bp)	Concentration (ng/uL)	From (bp)	To (bp)	Average size (bp)	CV%	RFU	Corrected peak area
1	75 (LM)	0.5000	64	171	75	11.54	7701	44.072
2	1539	5.3253	1223	2857	1623	11.40	5316	39.116
3	14269	0.2171	8667	15122	11697	15.41	70	1.595
4	20000 (UM)	0.1793	18415	27133	20415	4.23	1819	15.801

TIC:	5.5424	ng/uL
TIM:	5.4316	nmole/L
Total concentration:	6.0273	ng/uL

Sample peak width (sec): 5 Sample min peak height: 50 Sample baseline V to V?: Y Sample baseline V to V points: 3
Sample filter: Binomial Number of points for filter: 3 Sample start region (min): 0 Sample end region (min): 30
Marker peak width (sec): 5 Marker min peak height: 200 Marker baseline V to V?: Y Marker baseline V to V points: 3
Lower marker selection: First peak > 500 RFU Upper marker selection: Last peak > 200 RFU
Ladder size (bp)75, 200, 300, 400, 500, 700, 1000, 1500, 2000, 3000, 4000, 5000, 7000, 10000, 20000
Quantification using: Lower Marker Final concentration (ng/uL): 0.5000 Dilution factor: 12.0

Sample: SampH1
Well location: H1
Created: Tuesday, August 10, 2021 6:41:11 PM

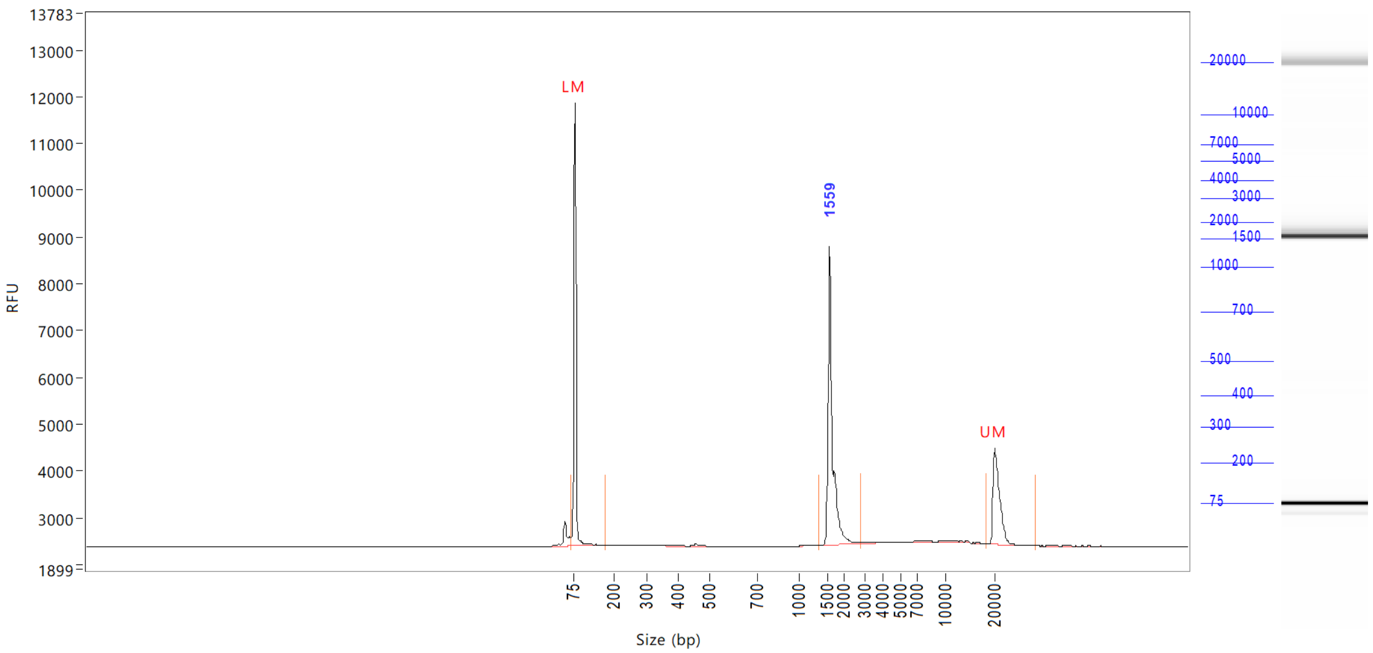

Peak	Size (bp)	Concentration (ng/uL)	From (bp)	To (bp)	Average size (bp)	CV%	RFU	Corrected peak area
1	75 (LM)	0.5000	64	171	74	10.26	9484	51.573
2	1559	4.9237	1345	2800	1619	9.76	6378	42.322
3	20000 (UM)	0.1703	18293	27979	20373	4.00	2055	17.569

TIC:	4.9237	ng/uL
TIM:	5.0061	nmole/L
Total concentration:	5.3834	ng/uL

```
Sample peak width (sec): 5    Sample min peak height: 50    Sample baseline V to V?: Y    Sample baseline V to V points: 3
Sample filter: Binomial       Number of points for filter: 3  Sample start region (min): 0  Sample end region (min): 30
Marker peak width (sec): 5    Marker min peak height: 200   Marker baseline V to V?: Y    Marker baseline V to V points: 3
Lower marker selection: First peak > 500 RFU                Upper marker selection: Last peak > 200 RFU
Ladder size (bp)75, 200, 300, 400, 500, 700, 1000, 1500, 2000, 3000, 4000, 5000, 7000, 10000, 20000
Quantification using: Lower Marker          Final concentration (ng/uL): 0.5000          Dilution factor: 12.0
```

Sample: SampA2
Well location: A2
Created: Tuesday, August 10, 2021 6:41:11 PM

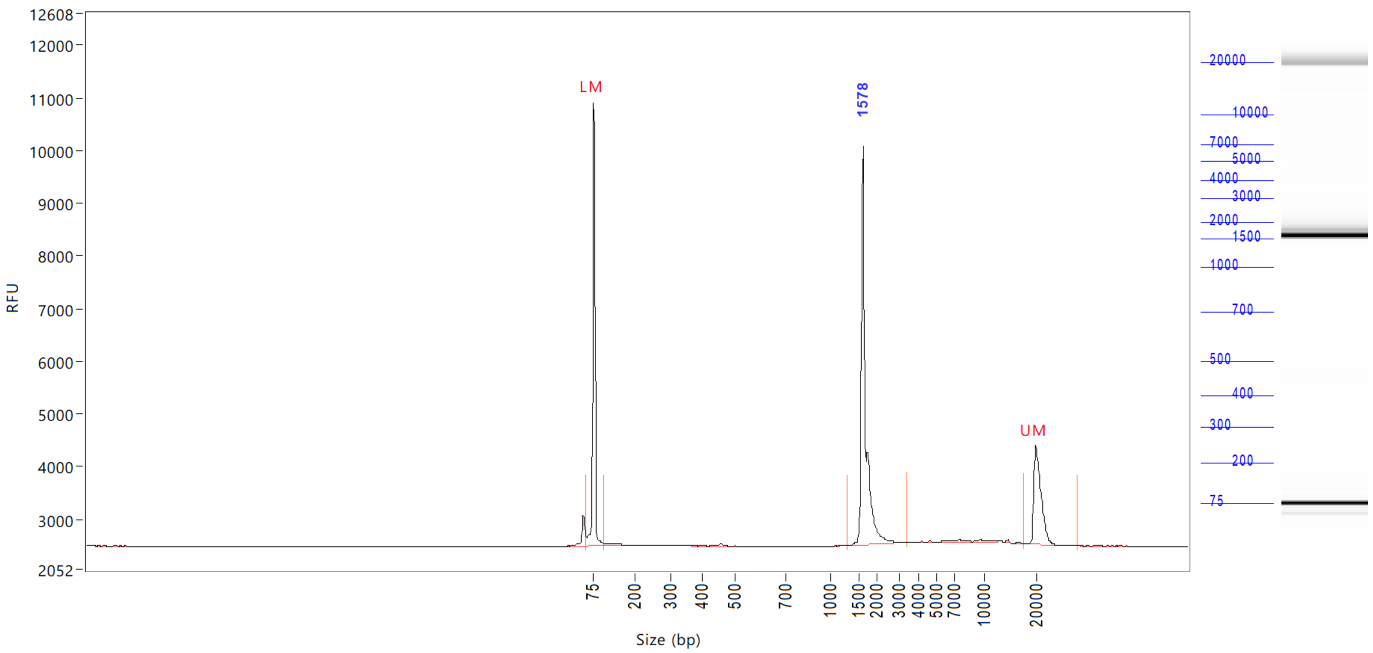

Peak	Size	Concentration	From	To	Average size	CV%	RFU	Corrected peak area
	(bp)	(ng/uL)	(bp)	(bp)	(bp)			
1	75 (LM)	0.5000	52	105	73	6.98	8424	46.701
2	1578	6.2946	1300	3393	1650	11.11	7561	48.994
3	20000 (UM)	0.1771	17561	27859	20372	3.94	1891	16.543

TIC:	6.2946	ng/uL
TIM:	6.2798	nmole/L
Total concentration:	6.9062	ng/uL

```
Sample peak width (sec): 5    Sample min peak height: 50    Sample baseline V to V?: Y    Sample baseline V to V points: 3
Sample filter: Binomial       Number of points for filter: 3  Sample start region (min): 0  Sample end region (min): 30
Marker peak width (sec): 5    Marker min peak height: 200   Marker baseline V to V?: Y    Marker baseline V to V points: 3
Lower marker selection: First peak > 500 RFU                Upper marker selection: Last peak > 200 RFU
Ladder size (bp)75, 200, 300, 400, 500, 700, 1000, 1500, 2000, 3000, 4000, 5000, 7000, 10000, 20000
Quantification using: Lower Marker          Final concentration (ng/uL): 0.5000          Dilution factor: 12.0
```

Sample: SampB2
Well location: B2
Created: Tuesday, August 10, 2021 6:41:11 PM

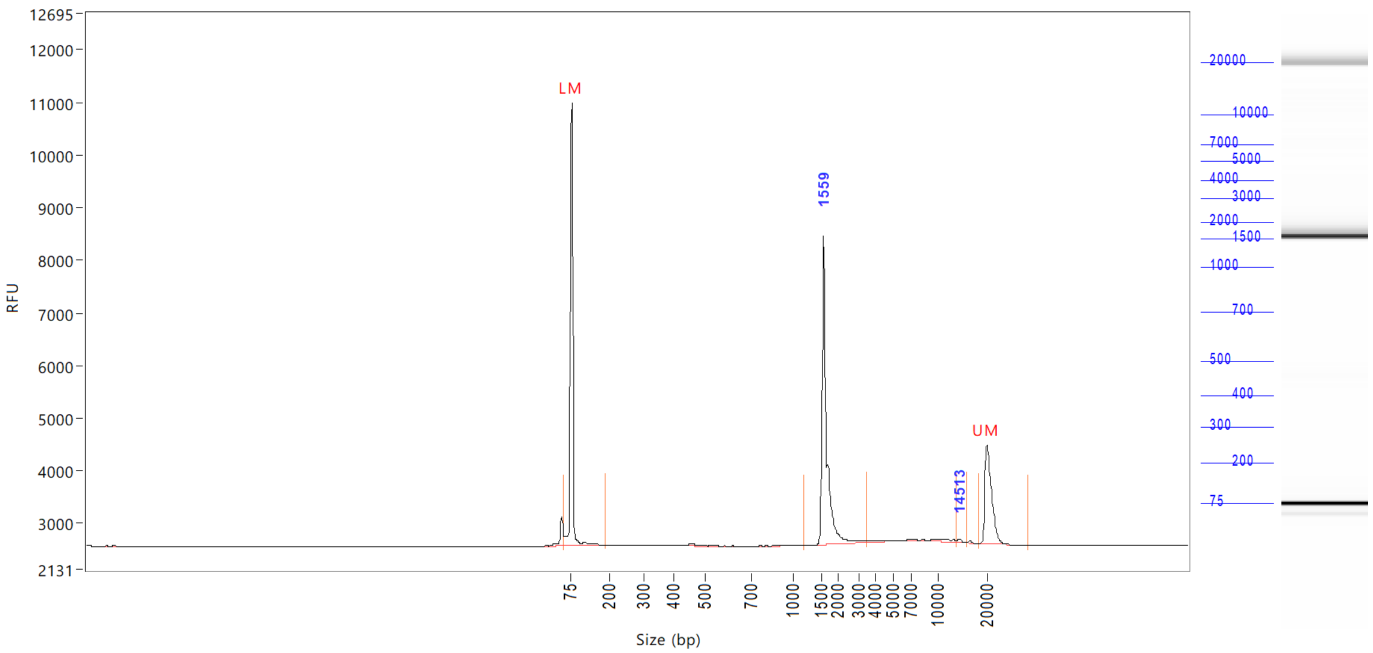

Peak	Size (bp)	Concentration (ng/uL)	From (bp)	To (bp)	Average size (bp)	CV%	RFU	Corrected peak area
1	75 (LM)	0.5000	52	185	74	12.75	8426	48.985
2	1559	5.0489	1167	3465	1647	14.49	5875	41.221
3	14513	0.0480	13781	15854	14499	3.59	64	0.392
4	20000 (UM)	0.1647	18293	28342	20370	4.06	1868	16.138

TIC:	5.0969	ng/uL
TIM:	5.0516	nmole/L
Total concentration:	5.6015	ng/uL

Sample peak width (sec): 5   Sample min peak height: 50   Sample baseline V to V?: Y   Sample baseline V to V points: 3
Sample filter: Binomial   Number of points for filter: 3 Sample start region (min): 0 Sample end region (min): 30
Marker peak width (sec): 5   Marker min peak height: 200   Marker baseline V to V?: Y   Marker baseline V to V points: 3
Lower marker selection: First peak > 500 RFU   Upper marker selection: Last peak > 200 RFU
Ladder size (bp)75, 200, 300, 400, 500, 700, 1000, 1500, 2000, 3000, 4000, 5000, 7000, 10000, 20000
Quantification using: Lower Marker   Final concentration (ng/uL): 0.5000   Dilution factor: 12.0

Sample: SampC2
Well location: C2
Created: Tuesday, August 10, 2021 6:41:11 PM

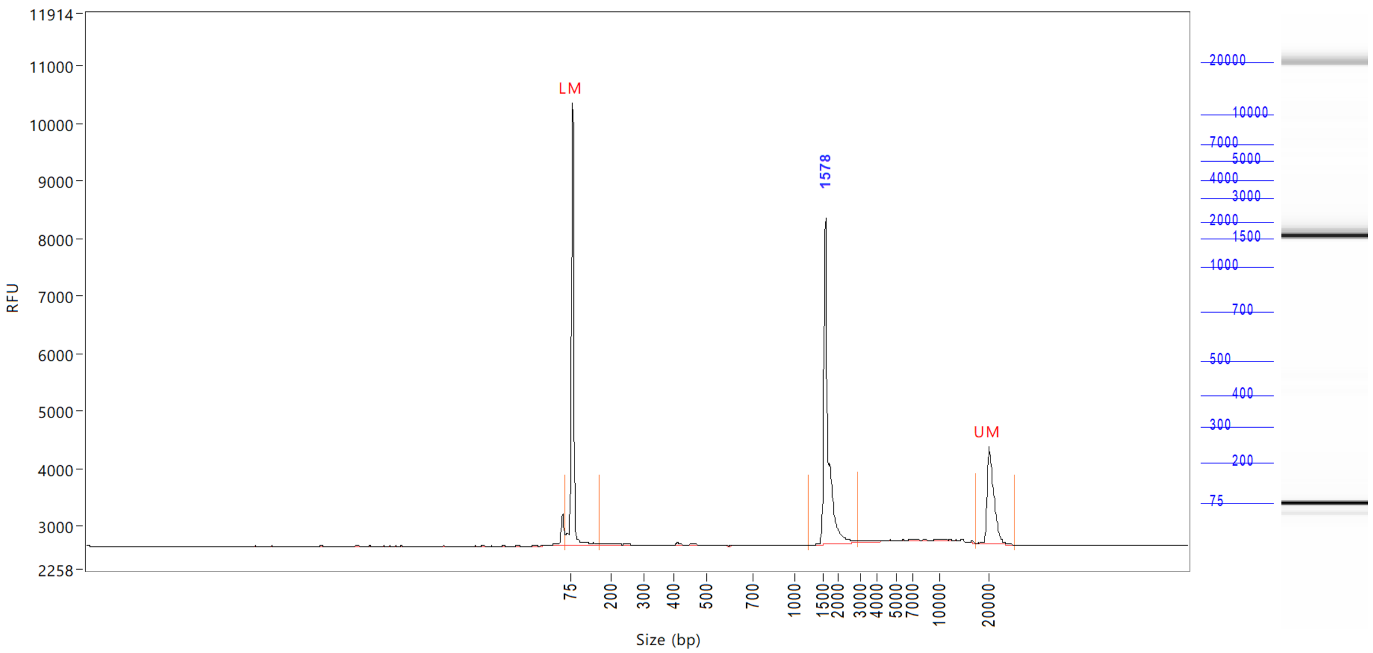

Peak	Size (bp)	Concentration (ng/uL)	From (bp)	To (bp)	Average size (bp)	CV%	RFU	Corrected peak area
1	75 (LM)	0.5000	52	164	75	15.40	7702	46.195
2	1578	5.0830	1245	2857	1644	11.45	5659	39.135
3	20000 (UM)	0.1568	17317	25078	20422	3.92	1680	14.487

TIC:	5.0830	ng/uL
TIM:	5.0895	nmole/L
Total concentration:	5.7277	ng/uL

Sample peak width (sec): 5 Sample min peak height: 50 Sample baseline V to V?: Y Sample baseline V to V points: 3
Sample filter: Binomial Number of points for filter: 3 Sample start region (min): 0 Sample end region (min): 30
Marker peak width (sec): 5 Marker min peak height: 200 Marker baseline V to V?: Y Marker baseline V to V points: 3
Lower marker selection: First peak > 500 RFU Upper marker selection: Last peak > 200 RFU
Ladder size (bp)75, 200, 300, 400, 500, 700, 1000, 1500, 2000, 3000, 4000, 5000, 7000, 10000, 20000
Quantification using: Lower Marker Final concentration (ng/uL): 0.5000 Dilution factor: 12.0

Sample: SampD2
Well location: D2
Created: Tuesday, August 10, 2021 6:41:11 PM

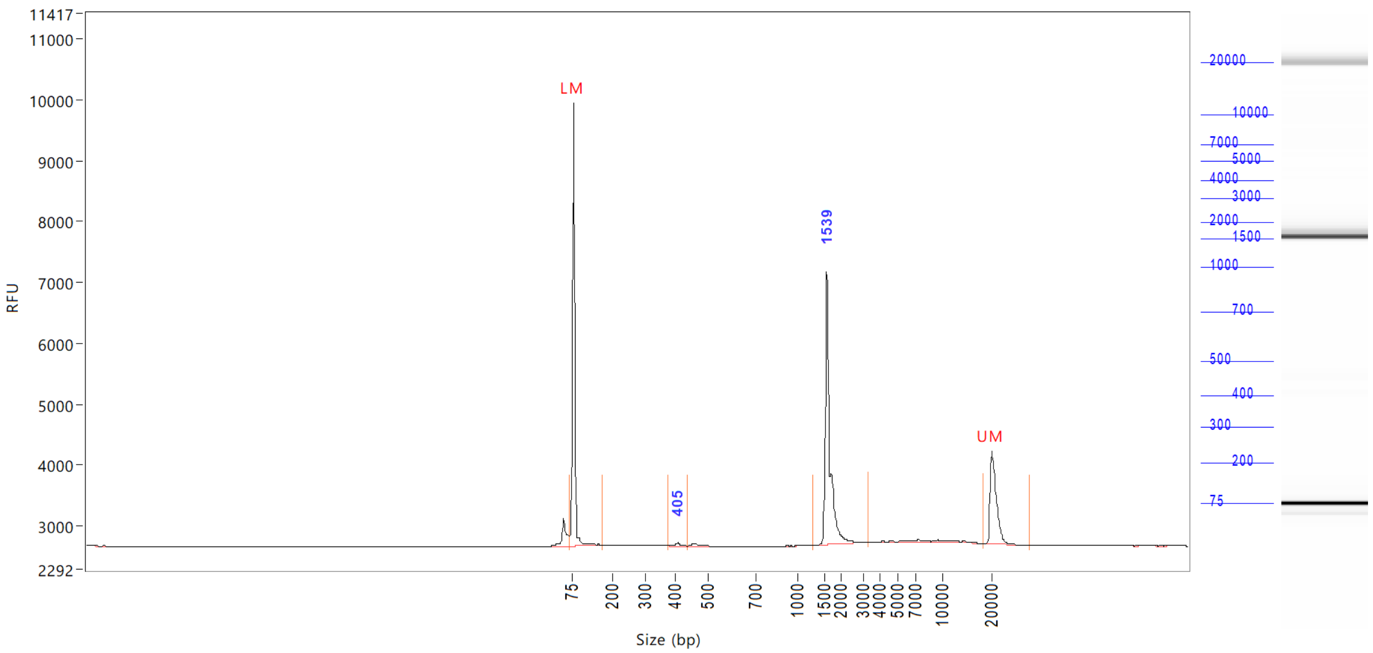

Peak	Size (bp)	Concentration (ng/uL)	From (bp)	To (bp)	Average size (bp)	CV%	RFU	Corrected peak area
1	75 (LM)	0.5000	64	166	75	12.99	7284	41.053
2	405	0.0730	370	433	401	2.72	51	0.500
3	1539	4.5010	1278	3286	1612	10.69	4470	30.797
4	20000 (UM)	0.1505	18415	27496	20344	3.86	1523	12.357

TIC:	4.5740	ng/uL
TIM:	4.8959	nmole/L
Total concentration:	5.0739	ng/uL

```
Sample peak width (sec): 5    Sample min peak height: 50    Sample baseline V to V?: Y    Sample baseline V to V points: 3
Sample filter: Binomial       Number of points for filter: 3  Sample start region (min): 0  Sample end region (min): 30
Marker peak width (sec): 5    Marker min peak height: 200   Marker baseline V to V?: Y    Marker baseline V to V points: 3
Lower marker selection: First peak > 500 RFU                Upper marker selection: Last peak > 200 RFU
Ladder size (bp)75, 200, 300, 400, 500, 700, 1000, 1500, 2000, 3000, 4000, 5000, 7000, 10000, 20000
Quantification using: Lower Marker          Final concentration (ng/uL): 0.5000          Dilution factor: 12.0
```

Sample: SampE2
Well location: E2
Created: Tuesday, August 10, 2021 6:41:11 PM

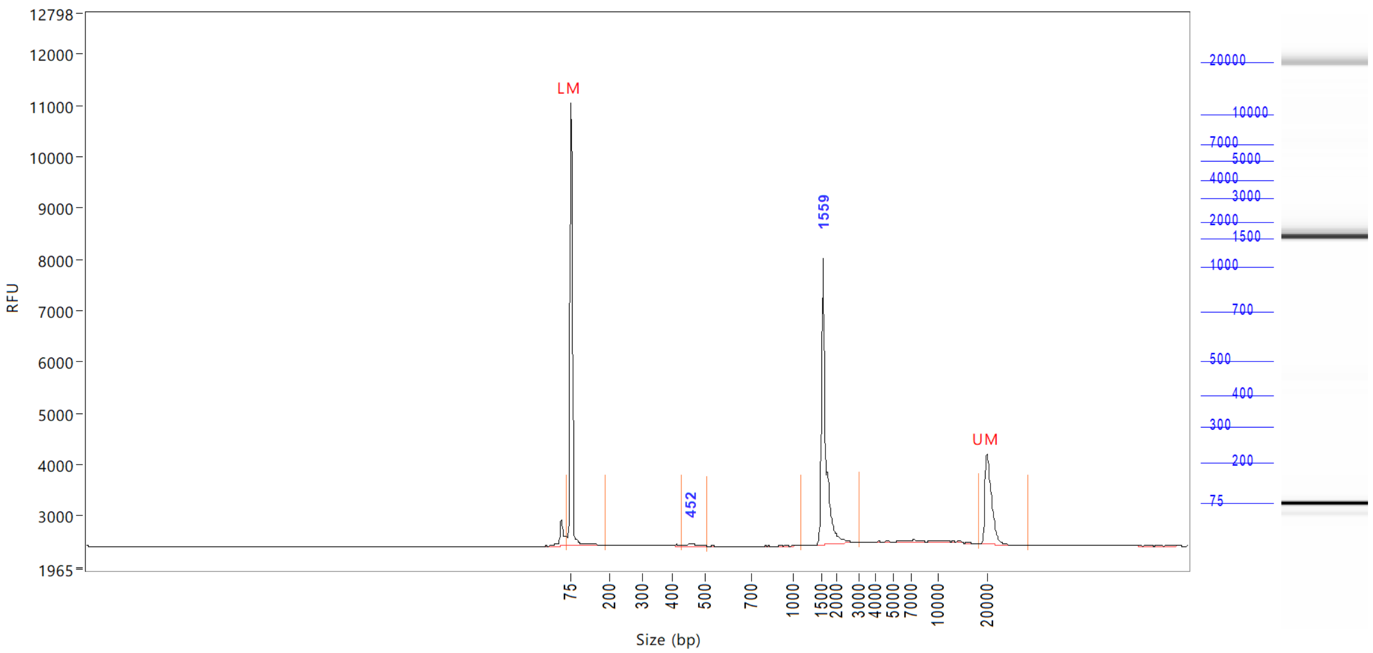

Peak	Size (bp)	Concentration (ng/uL)	From (bp)	To (bp)	Average size (bp)	CV%	RFU	Corrected peak area
1	75 (LM)	0.5000	64	185	75	11.93	8644	48.076
2	452	0.1105	424	509	460	3.67	54	0.885
3	1559	4.7332	1145	3037	1603	9.86	5584	37.925
4	20000 (UM)	0.1542	18415	28463	20371	4.20	1769	14.825

TIC:	4.8437	ng/uL
TIM:	5.2557	nmole/L
Total concentration:	5.2265	ng/uL

```
Sample peak width (sec): 5    Sample min peak height: 50    Sample baseline V to V?: Y    Sample baseline V to V points: 3
Sample filter: Binomial       Number of points for filter: 3  Sample start region (min): 0  Sample end region (min): 30
Marker peak width (sec): 5    Marker min peak height: 200   Marker baseline V to V?: Y    Marker baseline V to V points: 3
Lower marker selection: First peak > 500 RFU                Upper marker selection: Last peak > 200 RFU
Ladder size (bp)75, 200, 300, 400, 500, 700, 1000, 1500, 2000, 3000, 4000, 5000, 7000, 10000, 20000
Quantification using: Lower Marker          Final concentration (ng/uL): 0.5000          Dilution factor: 12.0
```

Sample: SampF2
Well location: F2
Created: Tuesday, August 10, 2021 6:41:11 PM

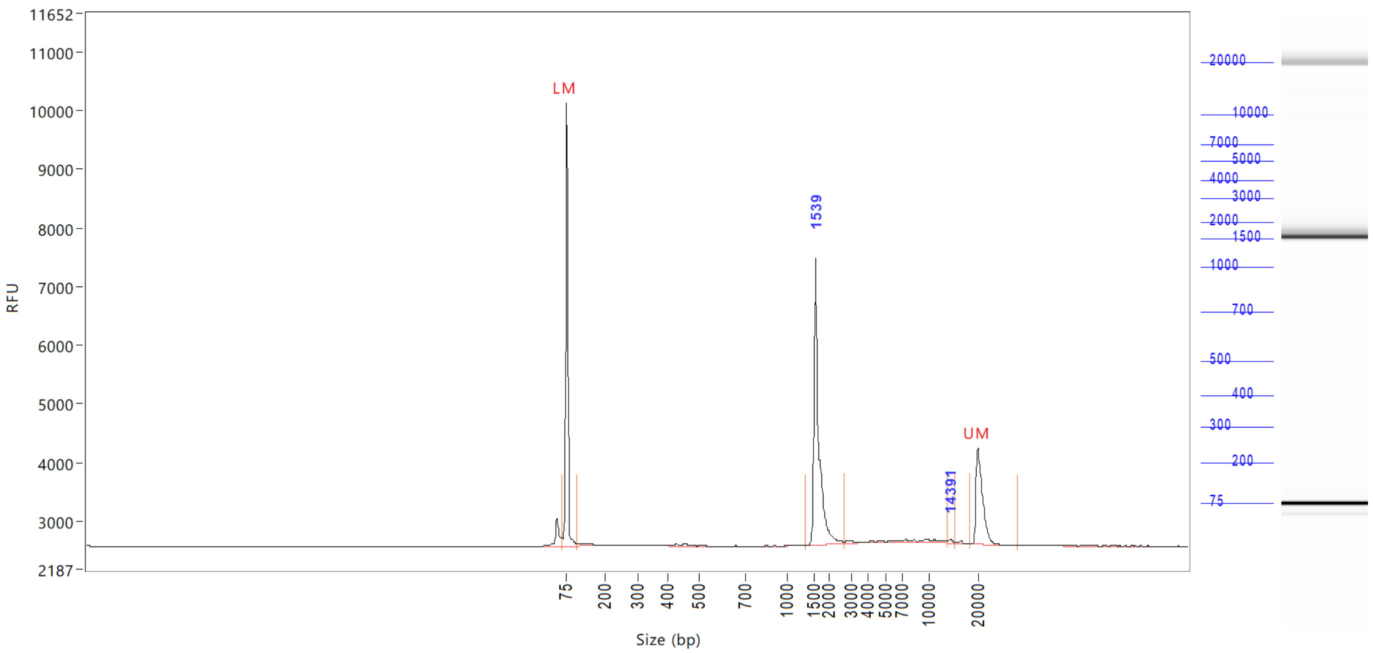

Peak	Size (bp)	Concentration (ng/uL)	From (bp)	To (bp)	Average size (bp)	CV%	RFU	Corrected peak area
1	75 (LM)	0.5000	64	110	73	6.66	7558	41.701
2	1539	5.2561	1345	2657	1620	10.67	4886	36.531
3	14391	0.0515	13659	15366	14299	2.85	65	0.358
4	20000 (UM)	0.1707	18293	28221	20426	4.20	1628	14.234

TIC:	5.3076	ng/uL
TIM:	5.3467	nmole/L
Total concentration:	6.2382	ng/uL

```
Sample peak width (sec): 5    Sample min peak height: 50    Sample baseline V to V?: Y    Sample baseline V to V points: 3
Sample filter: Binomial       Number of points for filter: 3  Sample start region (min): 0  Sample end region (min): 30
Marker peak width (sec): 5    Marker min peak height: 200   Marker baseline V to V?: Y    Marker baseline V to V points: 3
Lower marker selection: First peak > 500 RFU                Upper marker selection: Last peak > 200 RFU
Ladder size (bp)75, 200, 300, 400, 500, 700, 1000, 1500, 2000, 3000, 4000, 5000, 7000, 10000, 20000
Quantification using: Lower Marker          Final concentration (ng/uL): 0.5000          Dilution factor: 12.0
```

Sample: SampG2
Well location: G2
Created: Tuesday, August 10, 2021 6:41:11 PM

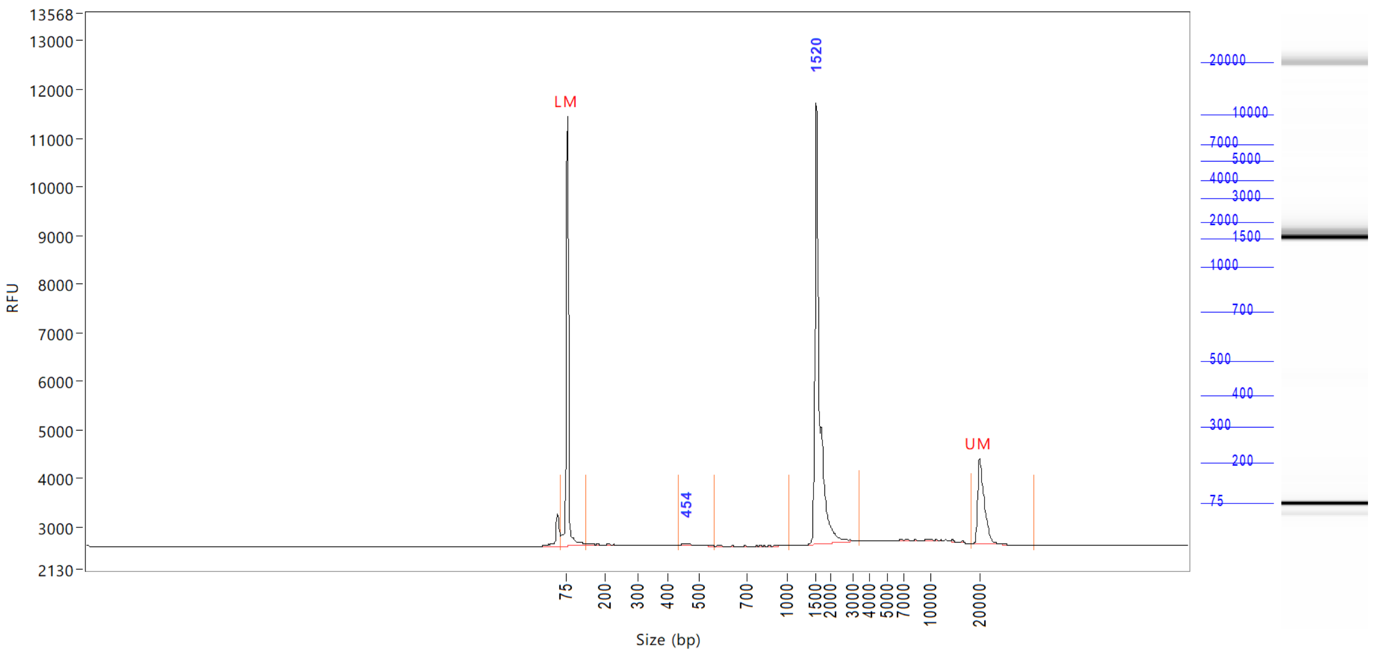

Peak	Size (bp)	Concentration (ng/uL)	From (bp)	To (bp)	Average size (bp)	CV%	RFU	Corrected peak area
1	75 (LM)	0.5000	54	135	74	11.73	8835	51.922
2	454	0.1102	433	565	464	5.42	51	0.953
3	1520	7.5129	1012	3393	1614	11.97	9096	65.013
4	20000 (UM)	0.1465	18171	31002	20409	4.63	1751	15.214

TIC:	7.6231	ng/uL
TIM:	8.0529	nmole/L
Total concentration:	8.0898	ng/uL

Sample peak width (sec): 5 Sample min peak height: 50 Sample baseline V to V?: Y Sample baseline V to V points: 3
Sample filter: Binomial Number of points for filter: 3 Sample start region (min): 0 Sample end region (min): 30
Marker peak width (sec): 5 Marker min peak height: 200 Marker baseline V to V?: Y Marker baseline V to V points: 3
Lower marker selection: First peak > 500 RFU Upper marker selection: Last peak > 200 RFU
Ladder size (bp)75, 200, 300, 400, 500, 700, 1000, 1500, 2000, 3000, 4000, 5000, 7000, 10000, 20000
Quantification using: Lower Marker Final concentration (ng/uL): 0.5000 Dilution factor: 12.0

Sample: SampH2
Well location: H2
Created: Tuesday, August 10, 2021 6:41:11 PM

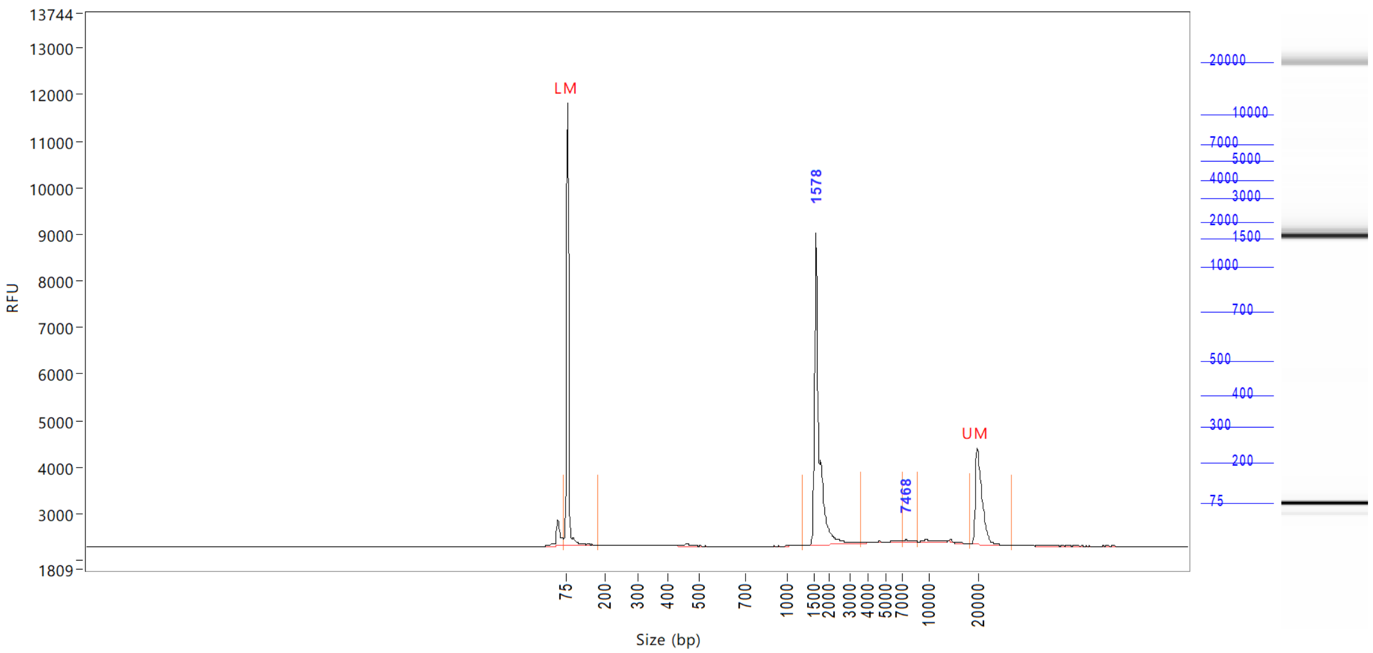

Peak	Size (bp)	Concentration (ng/uL)	From (bp)	To (bp)	Average size (bp)	CV%	RFU	Corrected peak area
1	75 (LM)	0.5000	64	173	75	11.40	9522	53.367
2	1578	5.4939	1289	3643	1655	14.82	6702	48.866
3	7468	0.0545	7068	8800	7798	5.94	50	0.484
4	20000 (UM)	0.1700	18415	27012	20431	4.15	2081	18.146

TIC:	5.5484	ng/uL
TIM:	5.4759	nmole/L
Total concentration:	5.9236	ng/uL

Sample peak width (sec): 5   Sample min peak height: 50   Sample baseline V to V?: Y   Sample baseline V to V points: 3
Sample filter: Binomial   Number of points for filter: 3   Sample start region (min): 0   Sample end region (min): 30
Marker peak width (sec): 5   Marker min peak height: 200   Marker baseline V to V?: Y   Marker baseline V to V points: 3
Lower marker selection: First peak > 500 RFU   Upper marker selection: Last peak > 200 RFU
Ladder size (bp)75, 200, 300, 400, 500, 700, 1000, 1500, 2000, 3000, 4000, 5000, 7000, 10000, 20000
Quantification using: Lower Marker   Final concentration (ng/uL): 0.5000   Dilution factor: 12.0

Sample: SampA3
Well location: A3
Created: Tuesday, August 10, 2021 6:41:11 PM

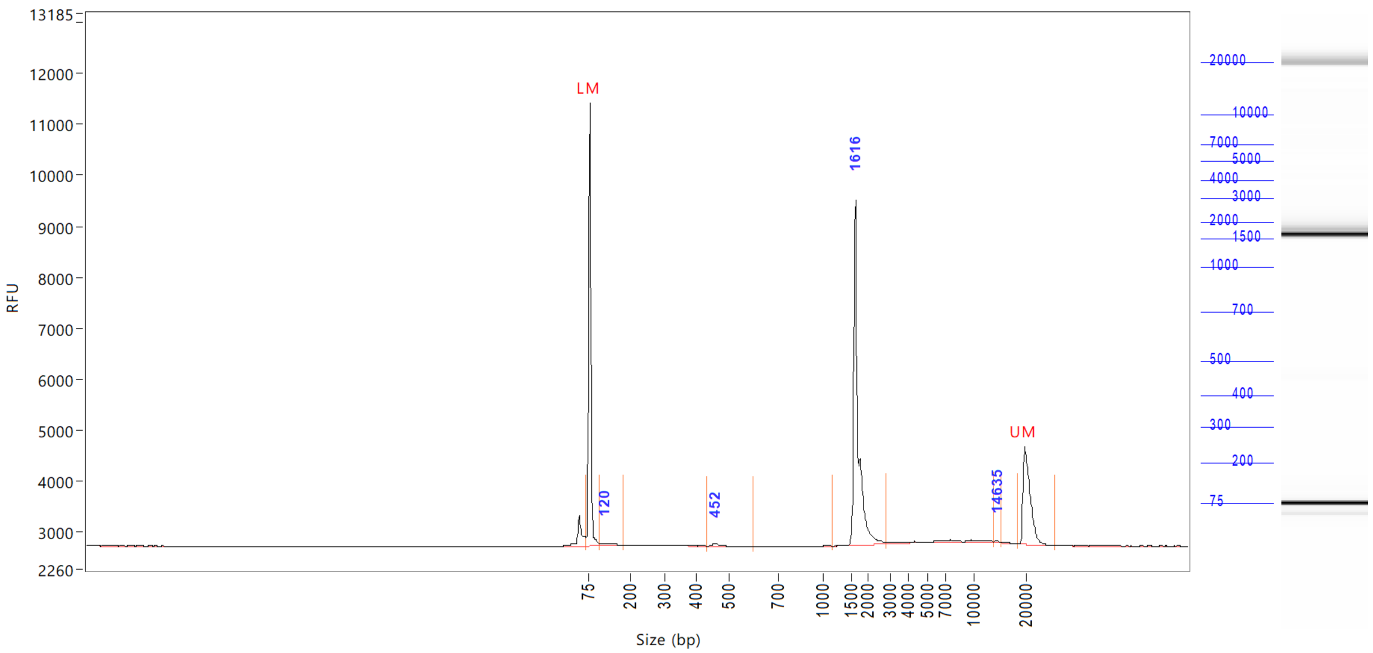

Peak	Size (bp)	Concentration (ng/uL)	From (bp)	To (bp)	Average size (bp)	CV%	RFU	Corrected peak area
1	75 (LM)	0.5000	64	107	74	5.96	8722	48.193
2	120	0.1438	107	179	130	13.78	51	1.155
3	452	0.1107	431	595	474	7.53	51	0.889
4	1616	5.6871	1178	2829	1681	10.59	6784	45.680
5	14635	0.0333	13903	15366	14427	2.14	53	0.267
6	20000 (UM)	0.1723	18415	25682	20454	3.97	1910	16.609

TIC:	5.9749	ng/uL
TIM:	7.7749	nmole/L
Total concentration:	6.4724	ng/uL

Sample peak width (sec): 5 Sample min peak height: 50 Sample baseline V to V?: Y Sample baseline V to V points: 3
Sample filter: Binomial Number of points for filter: 3 Sample start region (min): 0 Sample end region (min): 30
Marker peak width (sec): 5 Marker min peak height: 200 Marker baseline V to V?: Y Marker baseline V to V points: 3
Lower marker selection: First peak > 500 RFU Upper marker selection: Last peak > 200 RFU
Ladder size (bp)75, 200, 300, 400, 500, 700, 1000, 1500, 2000, 3000, 4000, 5000, 7000, 10000, 20000
Quantification using: Lower Marker Final concentration (ng/uL): 0.5000 Dilution factor: 12.0

Sample: SampB3
Well location: B3
Created: Tuesday, August 10, 2021 6:41:11 PM

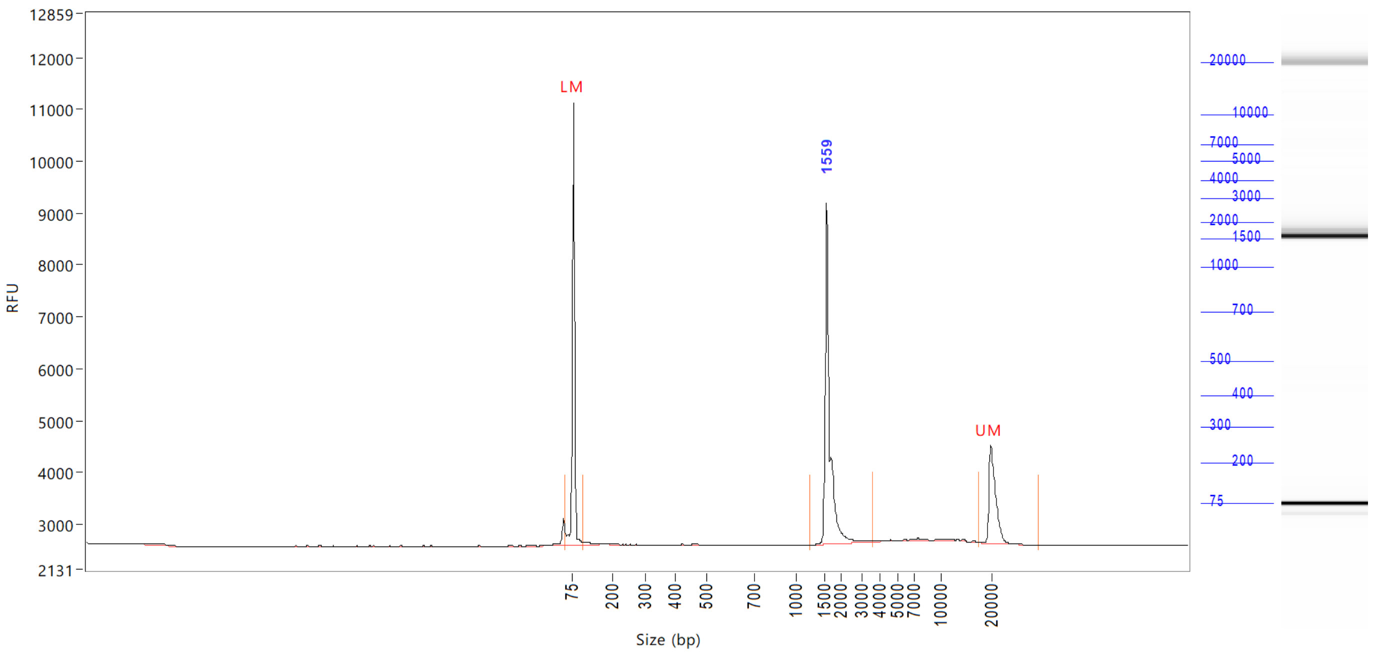

Peak	Size	Concentration	From	To	Average size	CV%	RFU	Corrected peak area
	(bp)	(ng/uL)	(bp)	(bp)	(bp)			
1	75 (LM)	0.5000	52	107	73	7.17	8549	48.090
2	1559	5.7752	1245	3643	1655	14.99	6599	46.289
3	20000 (UM)	0.1706	17683	29672	20450	4.33	1887	16.410

TIC:	5.7752	ng/uL
TIM:	5.7442	nmole/L
Total concentration:	6.3438	ng/uL

```
Sample peak width (sec): 5    Sample min peak height: 50    Sample baseline V to V?: Y    Sample baseline V to V points: 3
Sample filter: Binomial       Number of points for filter: 3  Sample start region (min): 0  Sample end region (min): 30
Marker peak width (sec): 5    Marker min peak height: 200   Marker baseline V to V?: Y    Marker baseline V to V points: 3
Lower marker selection: First peak > 500 RFU                Upper marker selection: Last peak > 200 RFU
Ladder size (bp)75, 200, 300, 400, 500, 700, 1000, 1500, 2000, 3000, 4000, 5000, 7000, 10000, 20000
Quantification using: Lower Marker          Final concentration (ng/uL): 0.5000          Dilution factor: 12.0
```

Sample: SampC3
Well location: C3
Created: Tuesday, August 10, 2021 6:41:11 PM

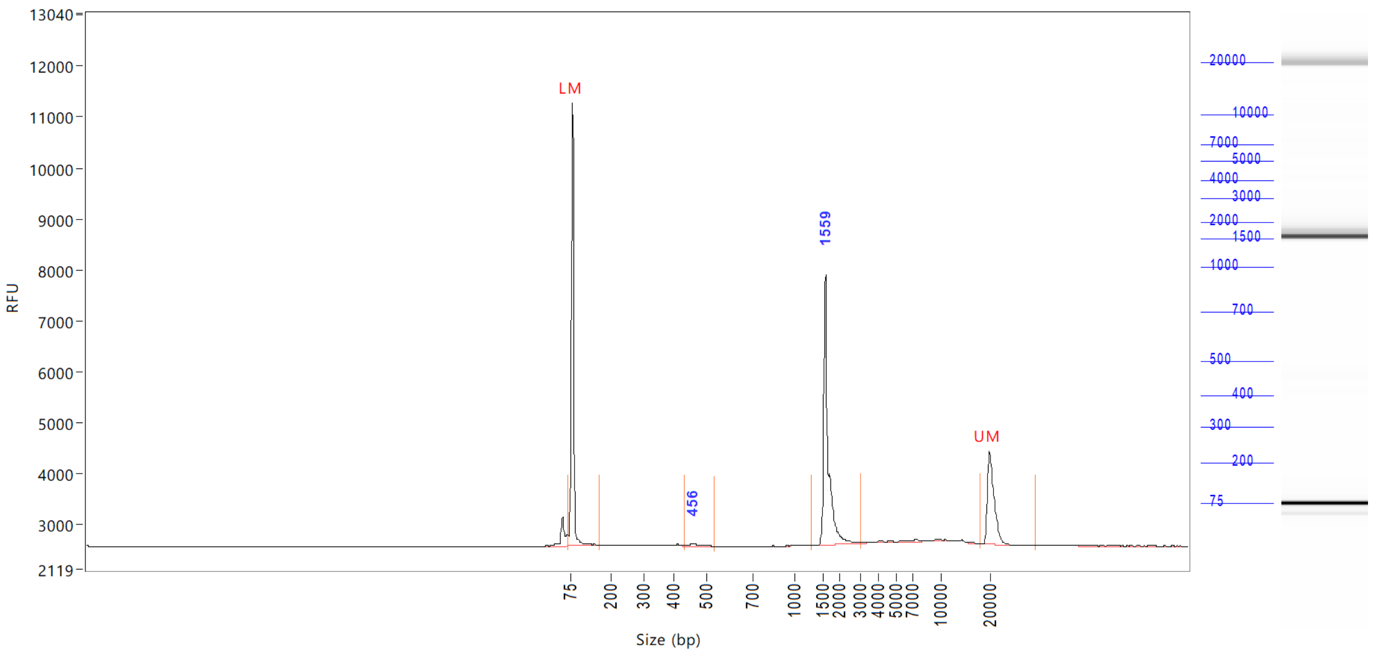

Peak	Size (bp)	Concentration (ng/uL)	From (bp)	To (bp)	Average size (bp)	CV%	RFU	Corrected peak area
1	75 (LM)	0.5000	64	162	75	11.90	8717	49.176
2	456	0.1099	433	534	464	4.11	58	0.901
3	1559	4.5857	1300	3037	1634	12.40	5317	37.584
4	20000 (UM)	0.1627	18049	29189	20380	4.42	1837	16.006

TIC:	4.6956	ng/uL
TIM:	5.0093	nmole/L
Total concentration:	5.1602	ng/uL

Sample peak width (sec): 5 Sample min peak height: 50 Sample baseline V to V?: Y Sample baseline V to V points: 3
Sample filter: Binomial Number of points for filter: 3 Sample start region (min): 0 Sample end region (min): 30
Marker peak width (sec): 5 Marker min peak height: 200 Marker baseline V to V?: Y Marker baseline V to V points: 3
Lower marker selection: First peak > 500 RFU Upper marker selection: Last peak > 200 RFU
Ladder size (bp)75, 200, 300, 400, 500, 700, 1000, 1500, 2000, 3000, 4000, 5000, 7000, 10000, 20000
Quantification using: Lower Marker Final concentration (ng/uL): 0.5000 Dilution factor: 12.0

Sample: SampD3
Well location: D3
Created: Tuesday, August 10, 2021 6:41:11 PM

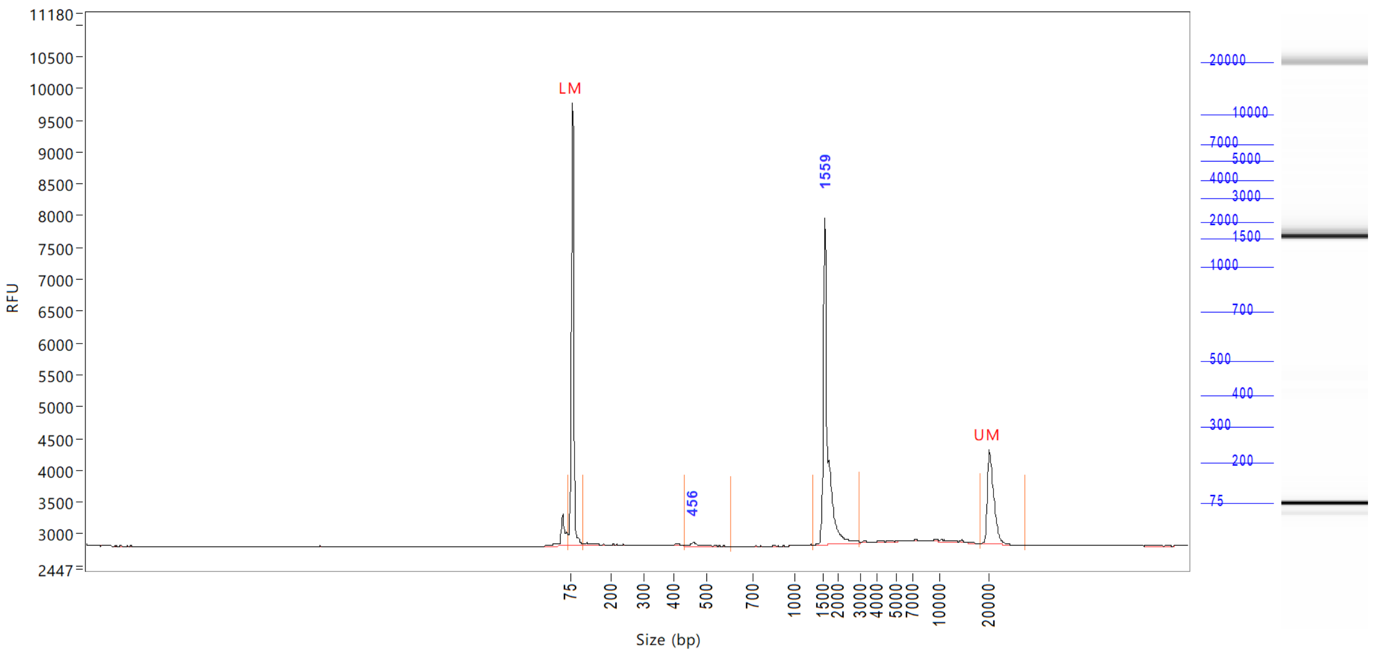

Peak	Size (bp)	Concentration (ng/uL)	From (bp)	To (bp)	Average size (bp)	CV%	RFU	Corrected peak area
1	75 (LM)	0.5000	64	107	74	6.07	6965	39.307
2	456	0.1460	431	603	477	7.63	51	0.956
3	1559	5.4784	1334	2971	1634	12.14	5137	35.890
4	20000 (UM)	0.1567	18171	27375	20441	4.01	1475	12.315

TIC:	5.6244	ng/uL
TIM:	6.0226	nmole/L
Total concentration:	6.2640	ng/uL

Sample peak width (sec): 5   Sample min peak height: 50   Sample baseline V to V?: Y   Sample baseline V to V points: 3
Sample filter: Binomial   Number of points for filter: 3   Sample start region (min): 0   Sample end region (min): 30
Marker peak width (sec): 5   Marker min peak height: 200   Marker baseline V to V?: Y   Marker baseline V to V points: 3
Lower marker selection: First peak > 500 RFU   Upper marker selection: Last peak > 200 RFU
Ladder size (bp)75, 200, 300, 400, 500, 700, 1000, 1500, 2000, 3000, 4000, 5000, 7000, 10000, 20000
Quantification using: Lower Marker   Final concentration (ng/uL): 0.5000   Dilution factor: 12.0

Sample: SampE3
Well location: E3
Created: Tuesday, August 10, 2021 6:41:11 PM

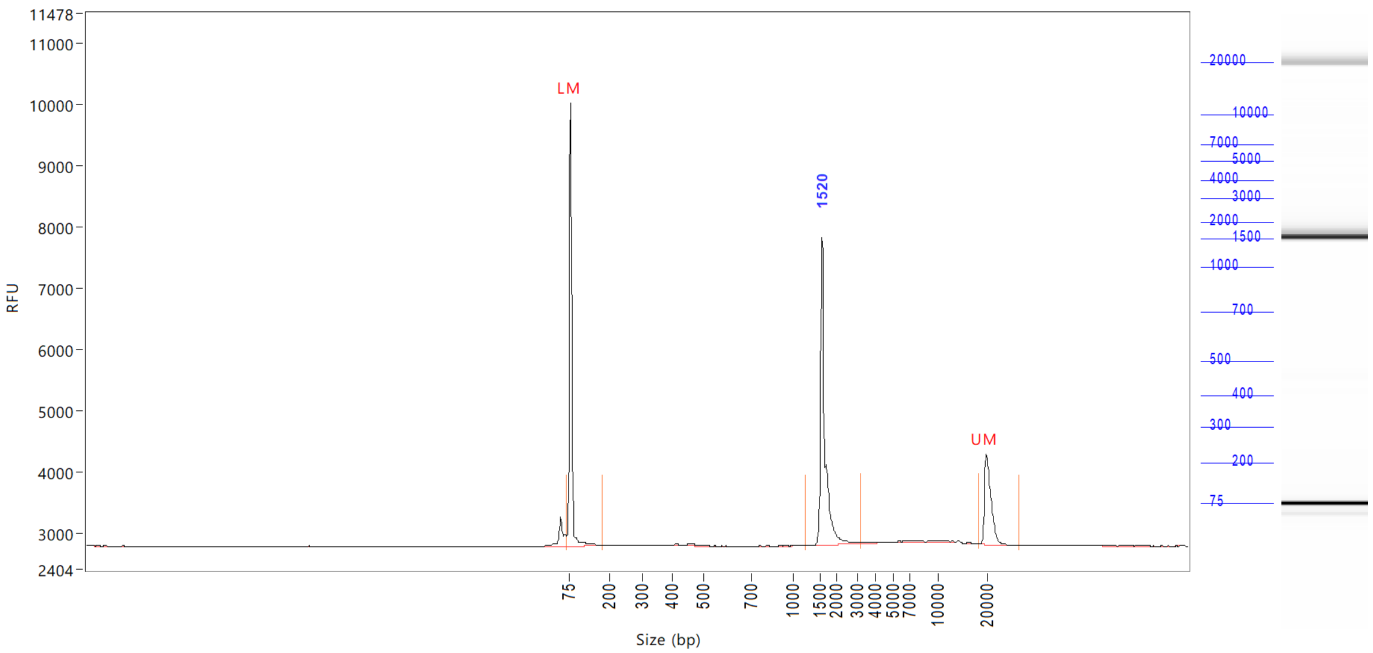

Peak	Size<br>(bp)	Concentration<br>(ng/uL)	From<br>(bp)	To<br>(bp)	Average size<br>(bp)	CV%	RFU	Corrected peak area
1	75 (LM)	0.5000	64	181	76	16.54	7246	41.820
2	1520	5.0293	1223	3144	1610	12.44	5024	35.054
3	20000 (UM)	0.1458	18415	26529	20378	3.84	1469	12.198

TIC:	5.0293	ng/uL
TIM:	5.1421	nmole/L
Total concentration:	5.6102	ng/uL

```
Sample peak width (sec): 5    Sample min peak height: 50    Sample baseline V to V?: Y    Sample baseline V to V points: 3
Sample filter: Binomial       Number of points for filter: 3  Sample start region (min): 0  Sample end region (min): 30
Marker peak width (sec): 5    Marker min peak height: 200   Marker baseline V to V?: Y    Marker baseline V to V points: 3
Lower marker selection: First peak > 500 RFU                Upper marker selection: Last peak > 200 RFU
Ladder size (bp)75, 200, 300, 400, 500, 700, 1000, 1500, 2000, 3000, 4000, 5000, 7000, 10000, 20000
Quantification using: Lower Marker          Final concentration (ng/uL): 0.5000          Dilution factor: 12.0
```

Sample: SampF3
Well location: F3
Created: Tuesday, August 10, 2021 6:41:11 PM

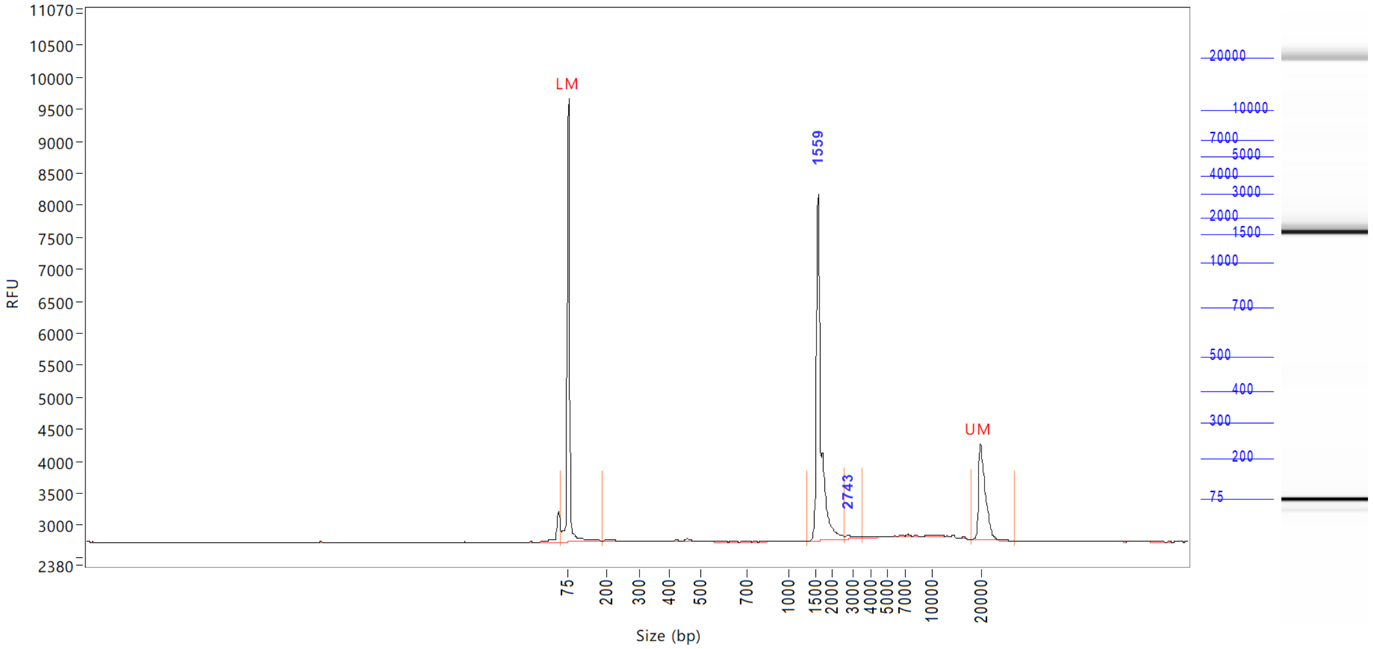

Peak	Size (bp)	Concentration (ng/uL)	From (bp)	To (bp)	Average size (bp)	CV%	RFU	Corrected peak area
1	75 (LM)	0.5000	52	187	75	18.48	6937	42.320
2	1559	5.3224	1334	2600	1621	10.48	5414	37.541
3	2743	0.1139	2600	3536	2948	8.62	53	0.803
4	20000 (UM)	0.1559	18049	27133	20479	4.25	1502	13.199

TIC:	5.4362	ng/uL
TIM:	5.4684	nmole/L
Total concentration:	6.0125	ng/uL

```
Sample peak width (sec): 5    Sample min peak height: 50    Sample baseline V to V?: Y    Sample baseline V to V points: 3
Sample filter: Binomial       Number of points for filter: 3  Sample start region (min): 0  Sample end region (min): 30
Marker peak width (sec): 5    Marker min peak height: 200   Marker baseline V to V?: Y    Marker baseline V to V points: 3
Lower marker selection: First peak > 500 RFU                Upper marker selection: Last peak > 200 RFU
Ladder size (bp)75, 200, 300, 400, 500, 700, 1000, 1500, 2000, 3000, 4000, 5000, 7000, 10000, 20000
Quantification using: Lower Marker          Final concentration (ng/uL): 0.5000          Dilution factor: 12.0
```

Sample: SampG3
Well location: G3
Created: Tuesday, August 10, 2021 6:41:11 PM

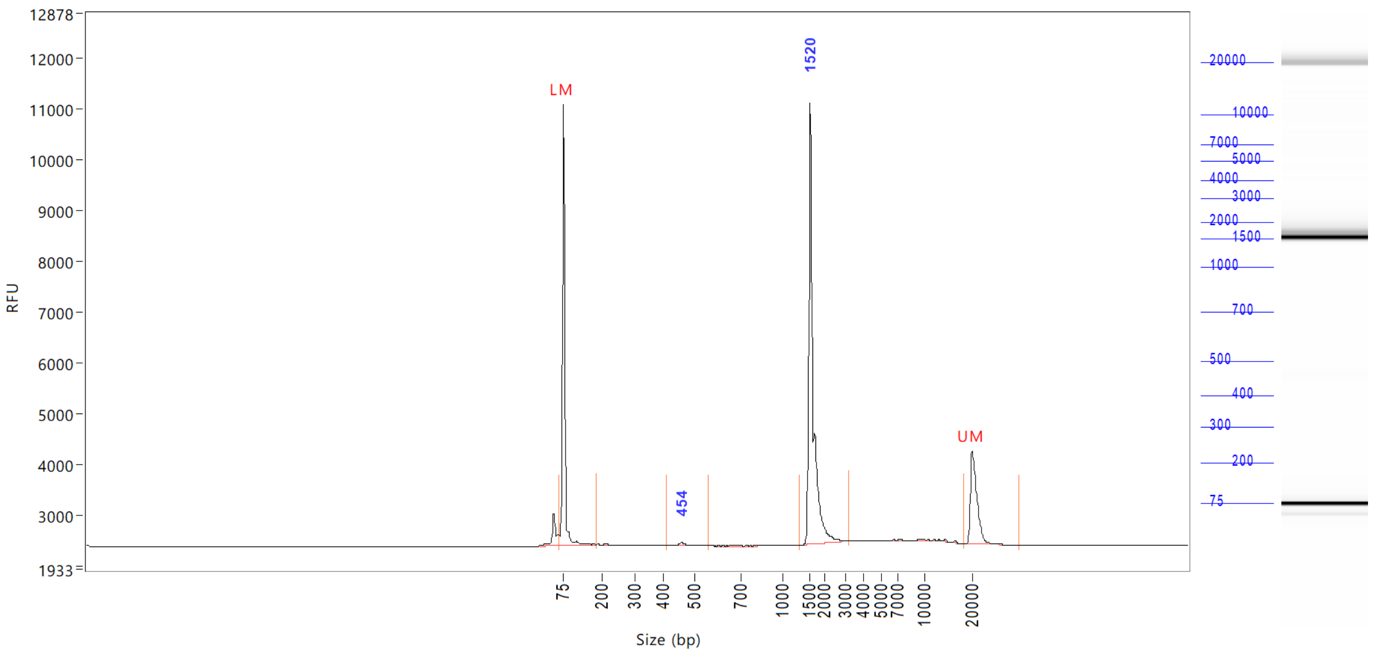

Peak	Size (bp)	Concentration (ng/uL)	From (bp)	To (bp)	Average size (bp)	CV%	RFU	Corrected peak area
1	75 (LM)	0.5000	64	183	74	13.53	8684	52.358
2	454	0.1016	409	557	459	4.34	62	0.886
3	1520	6.8757	1311	3179	1598	10.95	8703	60.000
4	20000 (UM)	0.1461	18171	29793	20436	4.40	1818	15.296

TIC:	6.9773	ng/uL
TIM:	7.4468	nmole/L
Total concentration:	7.2637	ng/uL

```
Sample peak width (sec): 5    Sample min peak height: 50    Sample baseline V to V?: Y    Sample baseline V to V points: 3
Sample filter: Binomial       Number of points for filter: 3  Sample start region (min): 0  Sample end region (min): 30
Marker peak width (sec): 5    Marker min peak height: 200   Marker baseline V to V?: Y    Marker baseline V to V points: 3
Lower marker selection: First peak > 500 RFU                Upper marker selection: Last peak > 200 RFU
Ladder size (bp)75, 200, 300, 400, 500, 700, 1000, 1500, 2000, 3000, 4000, 5000, 7000, 10000, 20000
Quantification using: Lower Marker          Final concentration (ng/uL): 0.5000          Dilution factor: 12.0
```

Sample: SampH3
Well location: H3
Created: Tuesday, August 10, 2021 6:41:11 PM

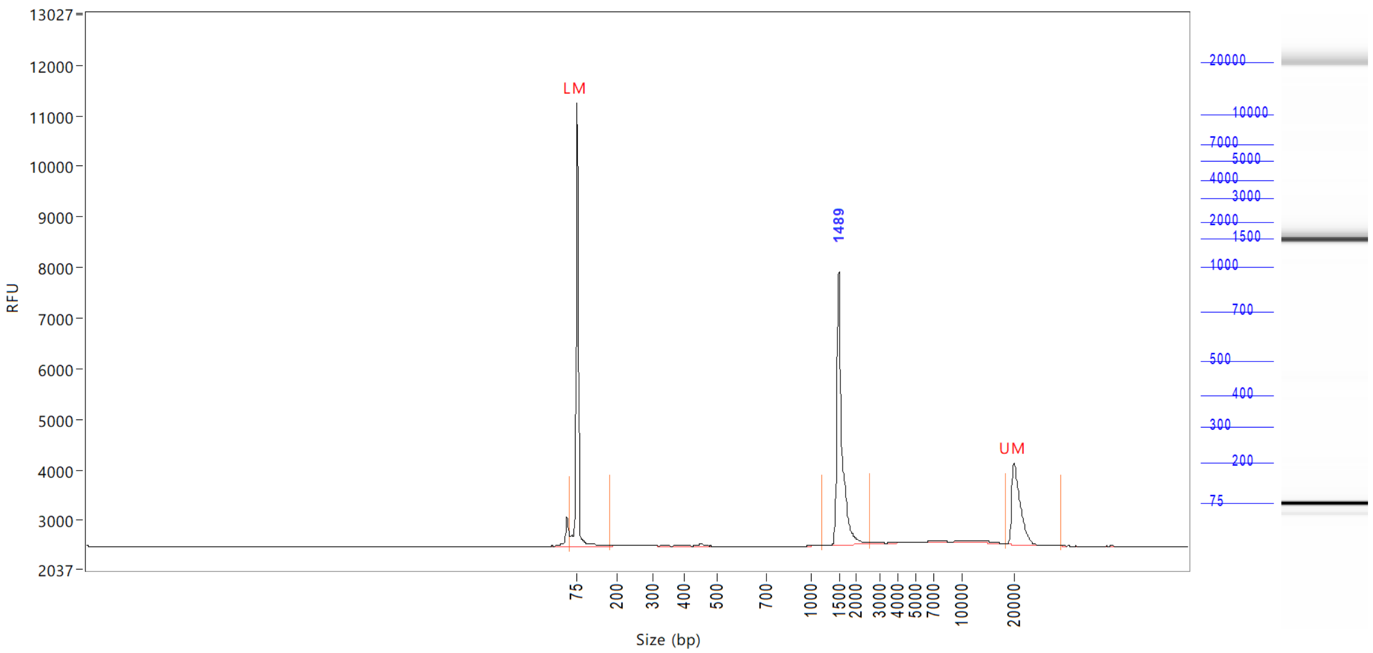

Peak	Size (bp)	Concentration (ng/uL)	From (bp)	To (bp)	Average size (bp)	CV%	RFU	Corrected peak area
1	75 (LM)	0.5000	52	177	74	15.10	8780	51.183
2	1489	5.0949	1201	2600	1536	9.15	5400	43.462
3	20000 (UM)	0.1672	18415	28947	20609	4.96	1620	17.120

TIC:	5.0949	ng/uL
TIM:	5.4601	nmole/L
Total concentration:	5.6880	ng/uL

Sample peak width (sec): 5   Sample min peak height: 50   Sample baseline V to V?: Y   Sample baseline V to V points: 3
Sample filter: Binomial   Number of points for filter: 3   Sample start region (min): 0   Sample end region (min): 30
Marker peak width (sec): 5   Marker min peak height: 200   Marker baseline V to V?: Y   Marker baseline V to V points: 3
Lower marker selection: First peak > 500 RFU   Upper marker selection: Last peak > 200 RFU
Ladder size (bp)75, 200, 300, 400, 500, 700, 1000, 1500, 2000, 3000, 4000, 5000, 7000, 10000, 20000
Quantification using: Lower Marker   Final concentration (ng/uL): 0.5000   Dilution factor: 12.0

Sample: SampA4
Well location: A4
Created: Tuesday, August 10, 2021 6:41:11 PM

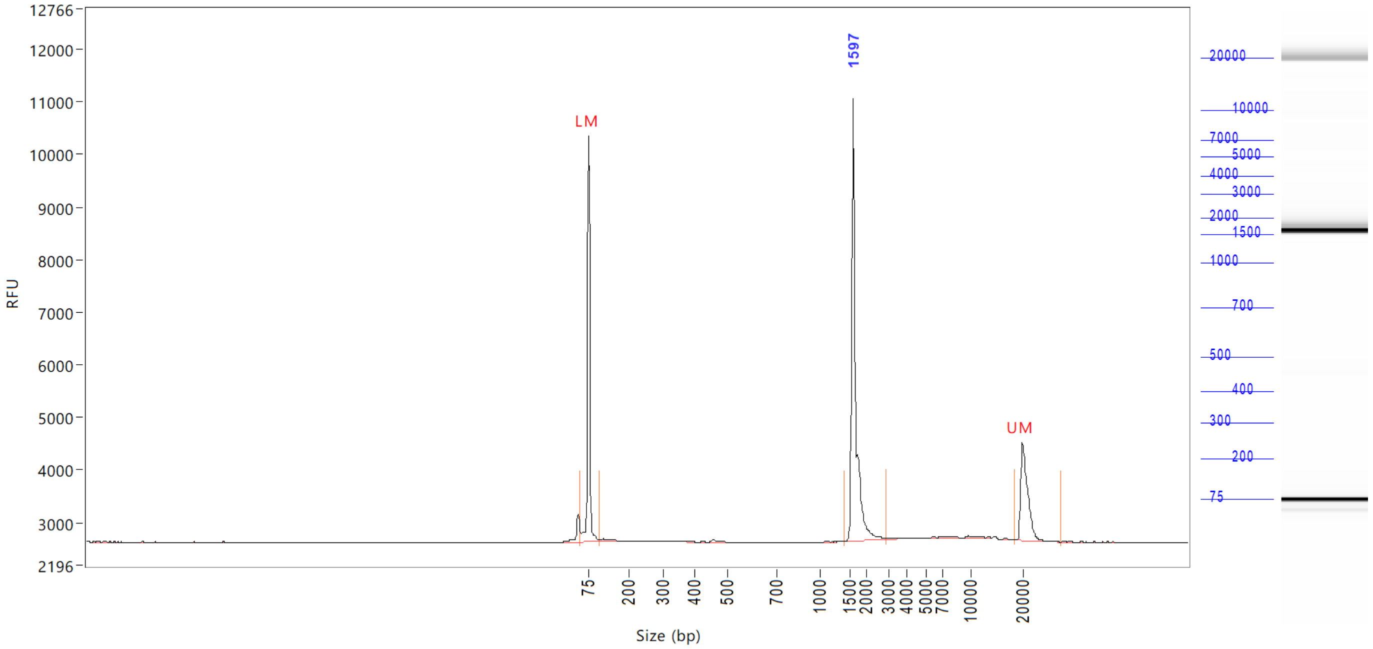

Peak	Size (bp)	Concentration (ng/uL)	From (bp)	To (bp)	Average size (bp)	CV%	RFU	Corrected peak area
1	75 (LM)	0.5000	50	107	72	7.80	7700	44.783
2	1597	6.8469	1422	2914	1656	10.17	8420	51.105
3	20000 (UM)	0.1785	18537	27375	20464	4.16	1856	15.988

TIC:	6.8469	ng/uL
TIM:	6.8060	nmole/L
Total concentration:	7.5312	ng/uL

```
Sample peak width (sec): 5    Sample min peak height: 50    Sample baseline V to V?: Y    Sample baseline V to V points: 3
Sample filter: Binomial       Number of points for filter: 3  Sample start region (min): 0  Sample end region (min): 30
Marker peak width (sec): 5    Marker min peak height: 200   Marker baseline V to V?: Y    Marker baseline V to V points: 3
Lower marker selection: First peak > 500 RFU                Upper marker selection: Last peak > 200 RFU
Ladder size (bp)75, 200, 300, 400, 500, 700, 1000, 1500, 2000, 3000, 4000, 5000, 7000, 10000, 20000
Quantification using: Lower Marker          Final concentration (ng/uL): 0.5000          Dilution factor: 12.0
```

Sample: SampB4
Well location: B4
Created: Tuesday, August 10, 2021 6:41:11 PM

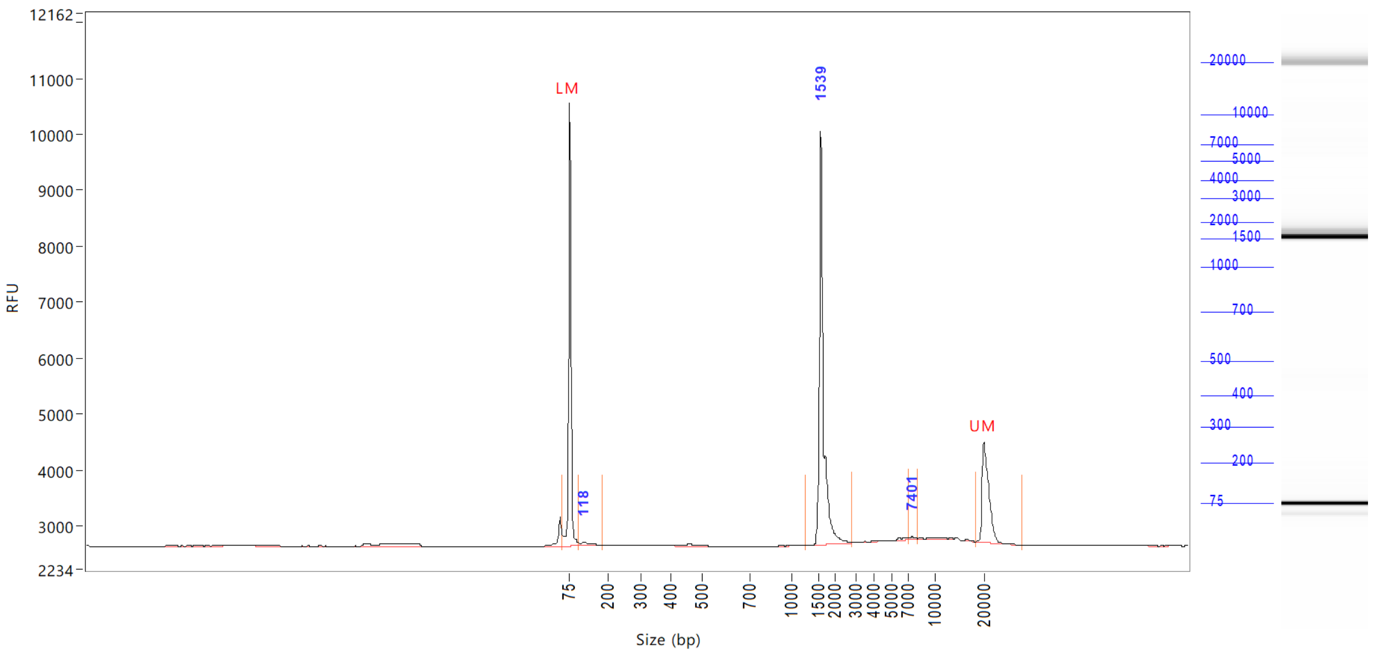

Peak	Size (bp)	Concentration (ng/uL)	From (bp)	To (bp)	Average size (bp)	CV%	RFU	Corrected peak area
1	75 (LM)	0.5000	52	107	73	7.39	7927	45.163
2	118	0.2212	107	183	134	14.65	55	1.665
3	1539	6.2751	1267	2829	1606	10.10	7390	47.234
4	7401	0.0446	7068	8001	7434	3.33	52	0.336
5	20000 (UM)	0.1732	18293	27859	20420	4.22	1800	15.641

TIC:	6.5410	ng/uL
TIM:	9.1543	nmole/L
Total concentration:	7.0611	ng/uL

```
Sample peak width (sec): 5    Sample min peak height: 50    Sample baseline V to V?: Y    Sample baseline V to V points: 3
Sample filter: Binomial       Number of points for filter: 3  Sample start region (min): 0  Sample end region (min): 30
Marker peak width (sec): 5    Marker min peak height: 200   Marker baseline V to V?: Y    Marker baseline V to V points: 3
Lower marker selection: First peak > 500 RFU                Upper marker selection: Last peak > 200 RFU
Ladder size (bp)75, 200, 300, 400, 500, 700, 1000, 1500, 2000, 3000, 4000, 5000, 7000, 10000, 20000
Quantification using: Lower Marker          Final concentration (ng/uL): 0.5000          Dilution factor: 12.0
```

Sample: SampC4
Well location: C4
Created: Tuesday, August 10, 2021 6:41:11 PM

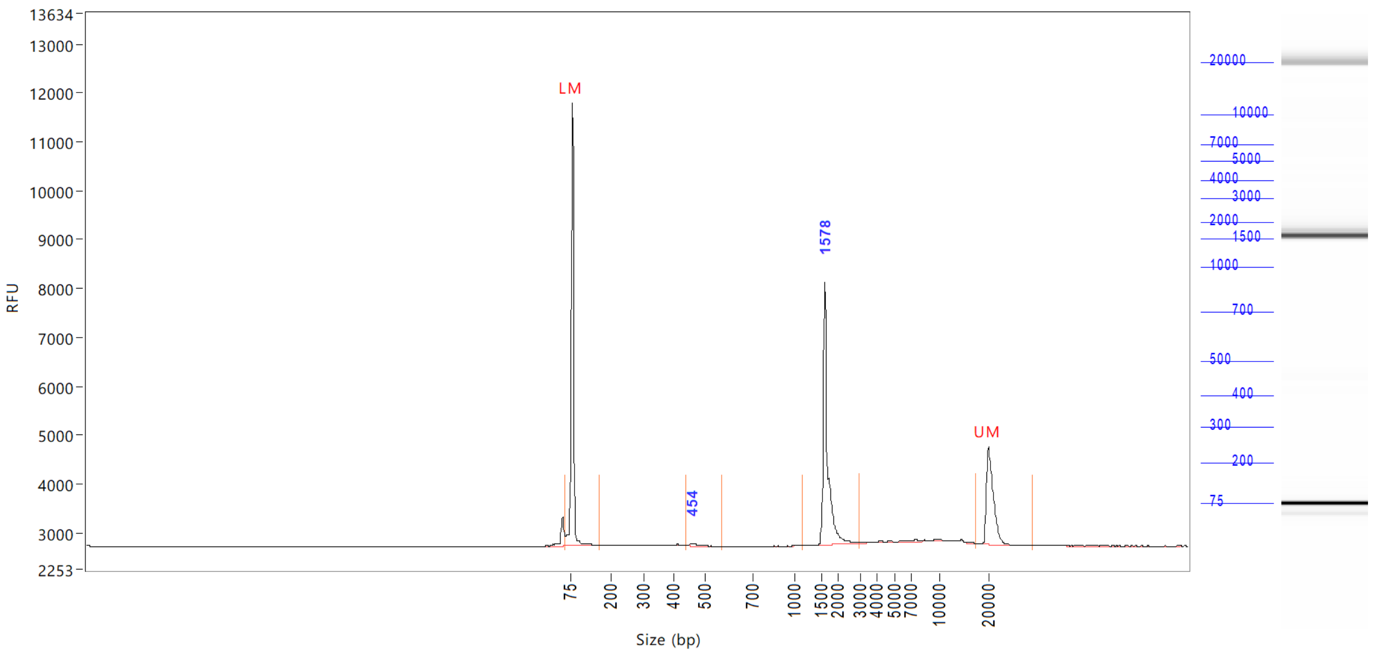

Peak	Size (bp)	Concentration (ng/uL)	From (bp)	To (bp)	Average size (bp)	CV%	RFU	Corrected peak area
1	75 (LM)	0.5000	52	162	74	11.91	9082	52.289
2	454	0.0952	437	567	463	4.21	60	0.830
3	1578	4.2498	1145	3000	1636	11.67	5363	37.036
4	20000 (UM)	0.1690	17439	29068	20464	4.43	1999	17.670

TIC:	4.3450	ng/uL
TIM:	4.6144	nmole/L
Total concentration:	4.7886	ng/uL

Sample peak width (sec): 5   Sample min peak height: 50   Sample baseline V to V?: Y   Sample baseline V to V points: 3
Sample filter: Binomial   Number of points for filter: 3 Sample start region (min): 0 Sample end region (min): 30
Marker peak width (sec): 5   Marker min peak height: 200   Marker baseline V to V?: Y   Marker baseline V to V points: 3
Lower marker selection: First peak > 500 RFU   Upper marker selection: Last peak > 200 RFU
Ladder size (bp)75, 200, 300, 400, 500, 700, 1000, 1500, 2000, 3000, 4000, 5000, 7000, 10000, 20000
Quantification using: Lower Marker   Final concentration (ng/uL): 0.5000   Dilution factor: 12.0

Sample: SampD4
Well location: D4
Created: Tuesday, August 10, 2021 6:41:11 PM

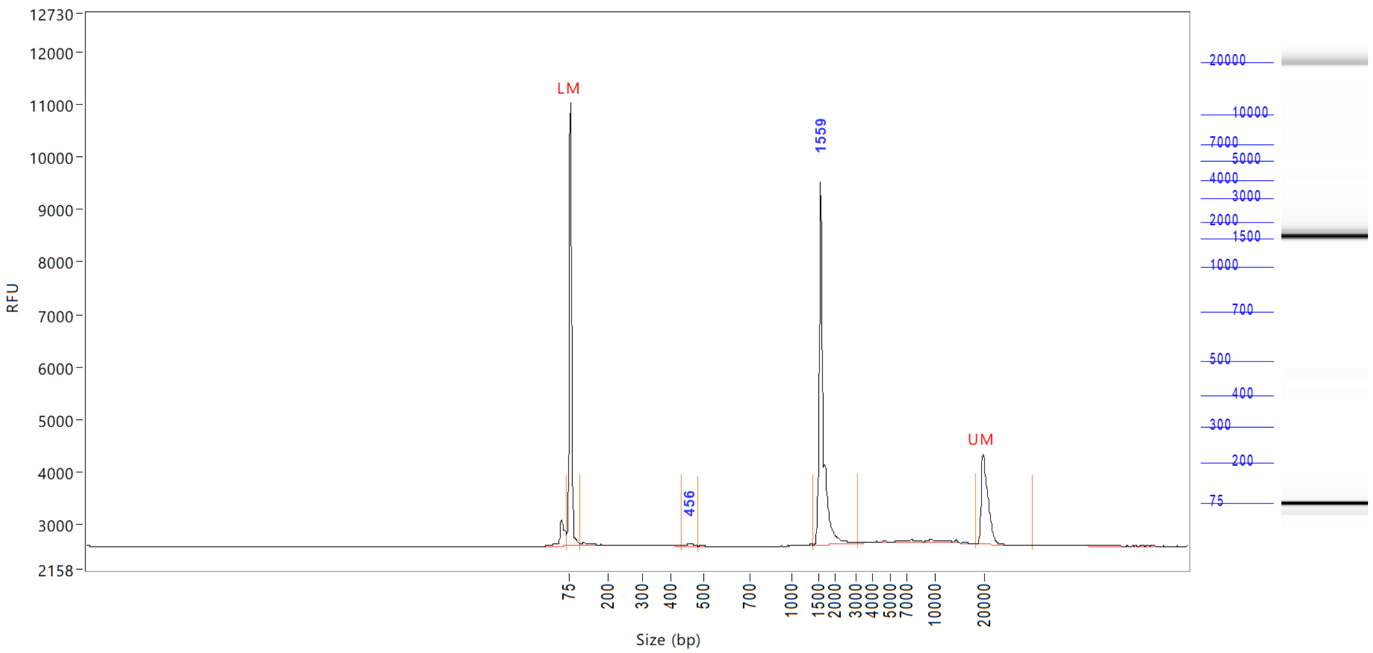

Peak	Size (bp)	Concentration (ng/uL)	From (bp)	To (bp)	Average size (bp)	CV%	RFU	Corrected peak area
1	75 (LM)	0.5000	64	107	73	6.18	8436	46.952
2	456	0.0888	433	485	457	2.20	56	0.695
3	1559	5.7002	1411	3108	1615	11.87	6904	44.605
4	20000 (UM)	0.1586	18415	30035	20401	4.49	1707	14.896

TIC:	5.7890	ng/uL
TIM:	6.1298	nmole/L
Total concentration:	6.3671	ng/uL

```
Sample peak width (sec): 5    Sample min peak height: 50    Sample baseline V to V?: Y    Sample baseline V to V points: 3
Sample filter: Binomial       Number of points for filter: 3  Sample start region (min): 0  Sample end region (min): 30
Marker peak width (sec): 5    Marker min peak height: 200   Marker baseline V to V?: Y    Marker baseline V to V points: 3
Lower marker selection: First peak > 500 RFU                Upper marker selection: Last peak > 200 RFU
Ladder size (bp)75, 200, 300, 400, 500, 700, 1000, 1500, 2000, 3000, 4000, 5000, 7000, 10000, 20000
Quantification using: Lower Marker          Final concentration (ng/uL): 0.5000          Dilution factor: 12.0
```

Sample: SampE4
Well location: E4
Created: Tuesday, August 10, 2021 6:41:11 PM

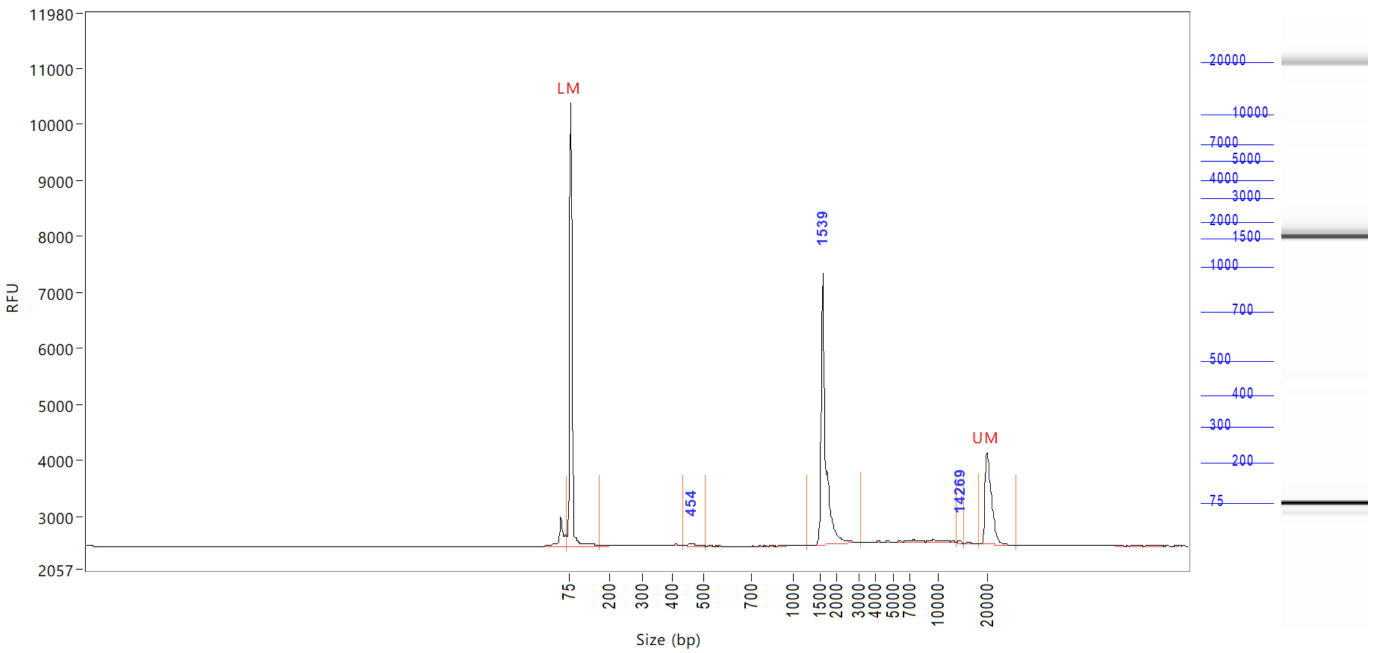

Peak	Size (bp)	Concentration (ng/uL)	From (bp)	To (bp)	Average size (bp)	CV%	RFU	Corrected peak area
1	75 (LM)	0.5000	64	168	76	14.74	7926	46.519
2	454	0.0936	431	504	460	3.14	51	0.726
3	1539	4.4584	1256	3179	1620	11.10	4833	34.567
4	14269	0.0322	13659	15122	14187	2.11	51	0.250
5	20000 (UM)	0.1439	18415	25803	20345	3.76	1631	13.390

TIC:	4.5843	ng/uL
TIM:	4.8688	nmole/L
Total concentration:	5.1186	ng/uL

Sample peak width (sec): 5   Sample min peak height: 50   Sample baseline V to V?: Y   Sample baseline V to V points: 3
Sample filter: Binomial   Number of points for filter: 3 Sample start region (min): 0 Sample end region (min): 30
Marker peak width (sec): 5   Marker min peak height: 200   Marker baseline V to V?: Y   Marker baseline V to V points: 3
Lower marker selection: First peak > 500 RFU   Upper marker selection: Last peak > 200 RFU
Ladder size (bp)75, 200, 300, 400, 500, 700, 1000, 1500, 2000, 3000, 4000, 5000, 7000, 10000, 20000
Quantification using: Lower Marker   Final concentration (ng/uL): 0.5000   Dilution factor: 12.0

Sample: SampF4
Well location: F4
Created: Tuesday, August 10, 2021 6:41:11 PM

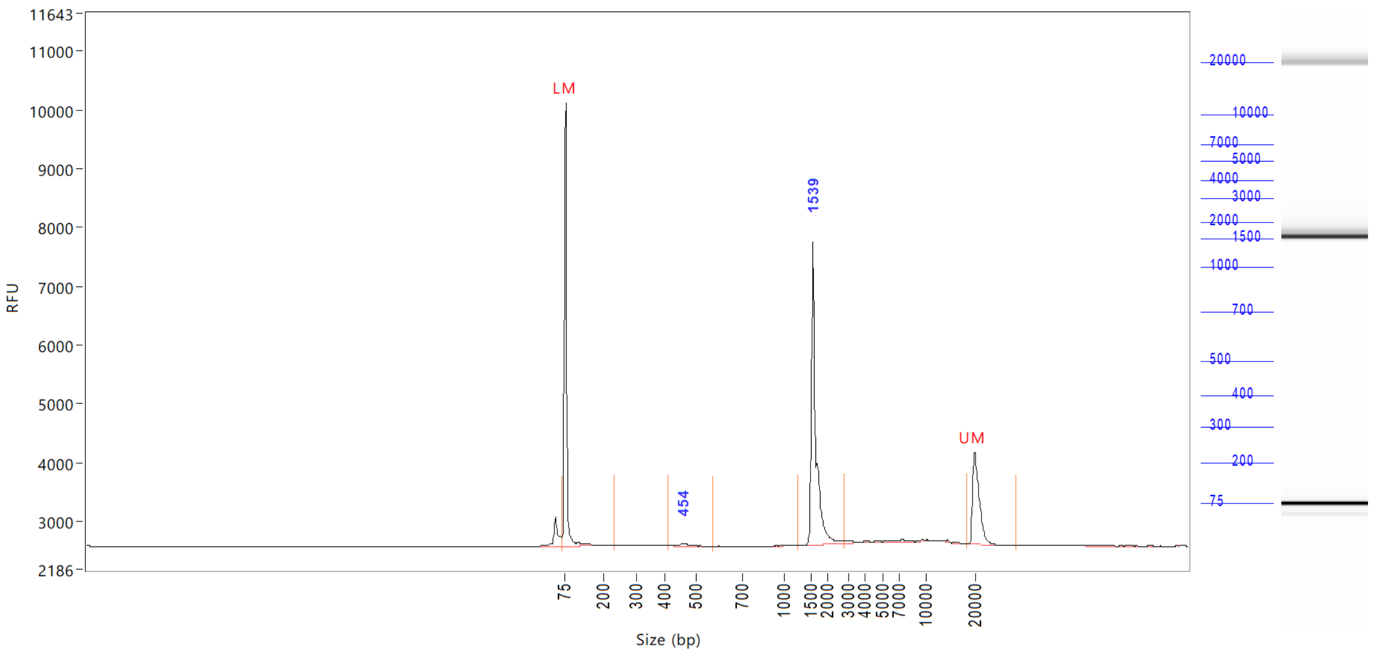

Peak	Size (bp)	Concentration (ng/uL)	From (bp)	To (bp)	Average size (bp)	CV%	RFU	Corrected peak area
1	75 (LM)	0.5000	64	232	77	23.22	7553	45.116
2	454	0.1379	407	570	462	6.39	51	1.037
3	1539	4.8897	1267	2800	1624	11.52	5152	36.768
4	20000 (UM)	0.1496	18293	28584	20527	4.36	1557	13.495

TIC:	5.0276	ng/uL
TIM:	5.4474	nmole/L
Total concentration:	5.4371	ng/uL

Sample peak width (sec): 5   Sample min peak height: 50   Sample baseline V to V?: Y   Sample baseline V to V points: 3
Sample filter: Binomial   Number of points for filter: 3   Sample start region (min): 0   Sample end region (min): 30
Marker peak width (sec): 5   Marker min peak height: 200   Marker baseline V to V?: Y   Marker baseline V to V points: 3
Lower marker selection: First peak > 500 RFU   Upper marker selection: Last peak > 200 RFU
Ladder size (bp)75, 200, 300, 400, 500, 700, 1000, 1500, 2000, 3000, 4000, 5000, 7000, 10000, 20000
Quantification using: Lower Marker   Final concentration (ng/uL): 0.5000   Dilution factor: 12.0

Sample: SampG4
Well location: G4
Created: Tuesday, August 10, 2021 6:41:11 PM

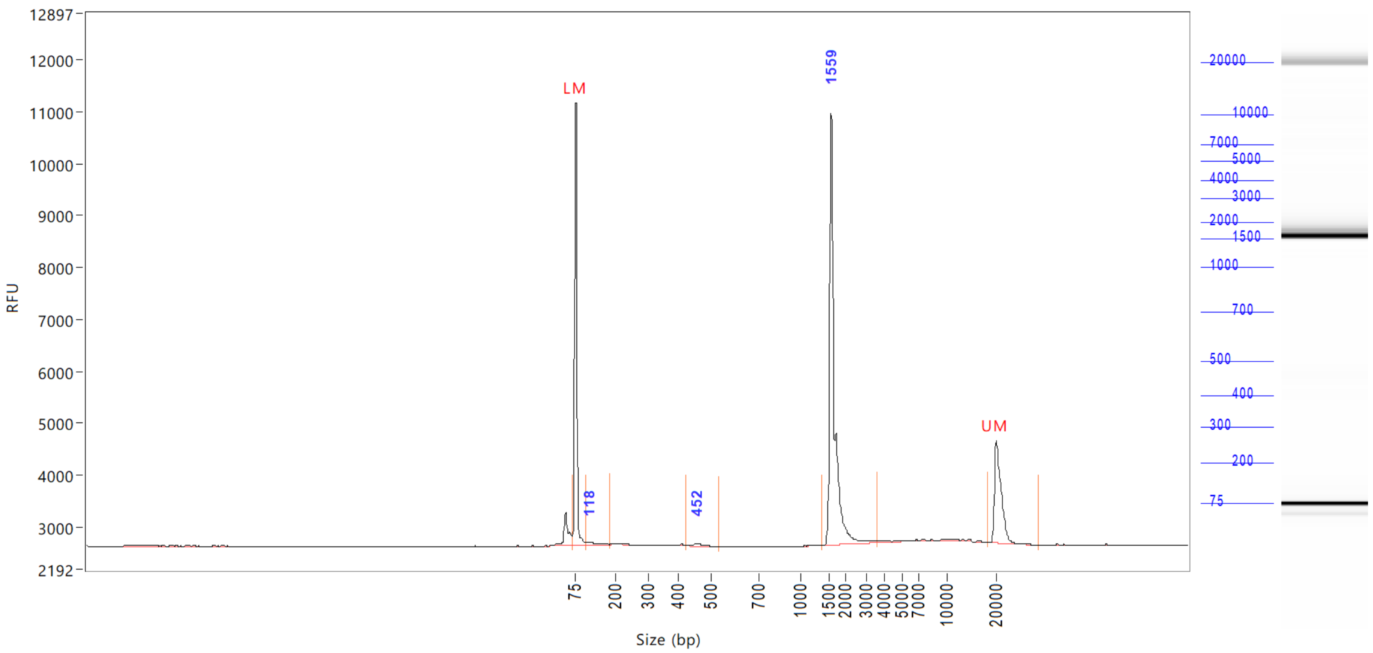

Peak	Size (bp)	Concentration (ng/uL)	From (bp)	To (bp)	Average size (bp)	CV%	RFU	Corrected peak area
1	75 (LM)	0.5000	64	105	73	6.19	8534	48.421
2	118	0.1619	105	181	129	14.00	54	1.306
3	452	0.1435	420	532	459	4.06	61	1.158
4	1559	7.2457	1389	3643	1660	14.61	8300	58.474
5	20000 (UM)	0.1719	18415	28584	20436	4.02	1963	16.651

TIC:	7.5511	ng/uL
TIM:	9.7612	nmole/L
Total concentration:	8.0032	ng/uL

```
Sample peak width (sec): 5    Sample min peak height: 50    Sample baseline V to V?: Y    Sample baseline V to V points: 3
Sample filter: Binomial       Number of points for filter: 3  Sample start region (min): 0  Sample end region (min): 30
Marker peak width (sec): 5    Marker min peak height: 200   Marker baseline V to V?: Y    Marker baseline V to V points: 3
Lower marker selection: First peak > 500 RFU                Upper marker selection: Last peak > 200 RFU
Ladder size (bp)75, 200, 300, 400, 500, 700, 1000, 1500, 2000, 3000, 4000, 5000, 7000, 10000, 20000
Quantification using: Lower Marker          Final concentration (ng/uL): 0.5000          Dilution factor: 12.0
```

Sample: SampH4
Well location: H4
Created: Tuesday, August 10, 2021 6:41:11 PM

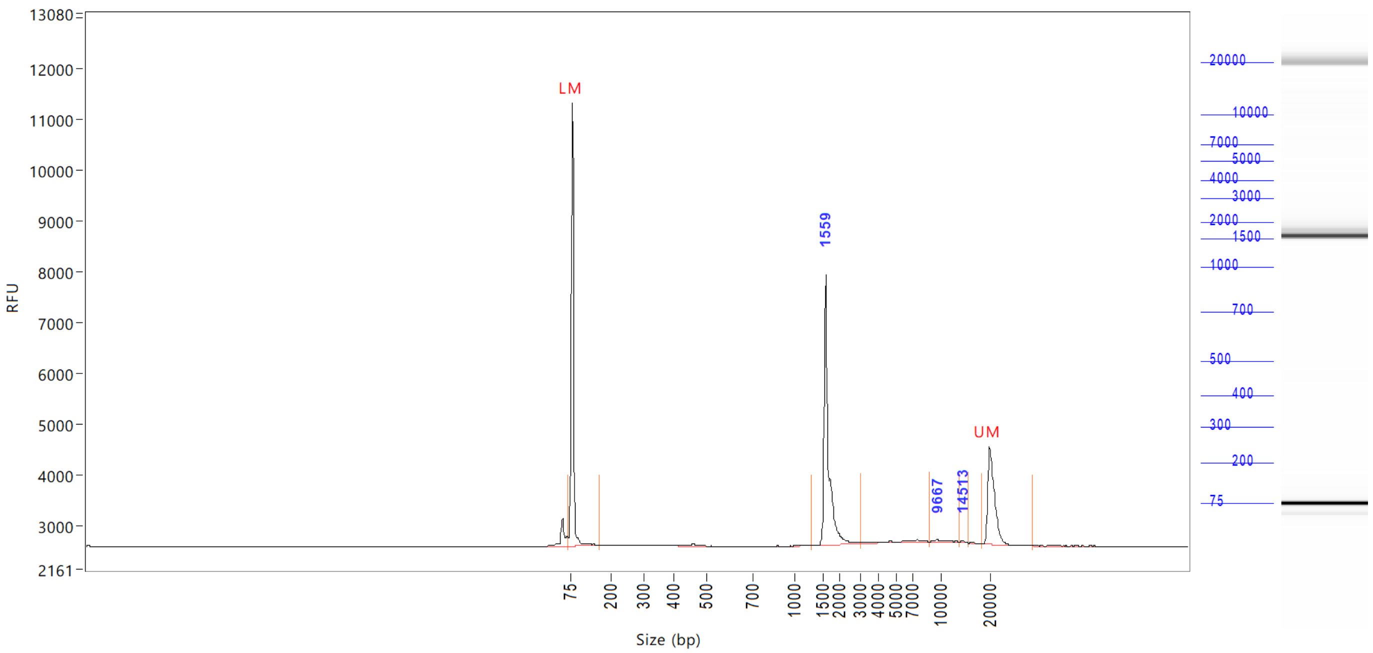

Peak	Size (bp)	Concentration (ng/uL)	From (bp)	To (bp)	Average size (bp)	CV%	RFU	Corrected peak area
1	75 (LM)	0.5000	64	164	75	12.76	8718	48.764
2	1559	4.4906	1289	3037	1642	12.02	5298	36.496
3	9667	0.1494	8800	13903	11017	12.78	50	1.214
4	14513	0.0288	13903	15732	14371	2.03	52	0.234
5	20000 (UM)	0.1710	18537	28705	20443	4.25	1925	16.675

TIC:	4.6688	ng/uL
TIM:	4.5274	nmole/L
Total concentration:	5.1057	ng/uL

Sample peak width (sec): 5 Sample min peak height: 50 Sample baseline V to V?: Y Sample baseline V to V points: 3
Sample filter: Binomial Number of points for filter: 3 Sample start region (min): 0 Sample end region (min): 30
Marker peak width (sec): 5 Marker min peak height: 200 Marker baseline V to V?: Y Marker baseline V to V points: 3
Lower marker selection: First peak > 500 RFU Upper marker selection: Last peak > 200 RFU
Ladder size (bp)75, 200, 300, 400, 500, 700, 1000, 1500, 2000, 3000, 4000, 5000, 7000, 10000, 20000
Quantification using: Lower Marker Final concentration (ng/uL): 0.5000 Dilution factor: 12.0

Sample: SampA5
Well location: A5
Created: Tuesday, August 10, 2021 6:41:11 PM

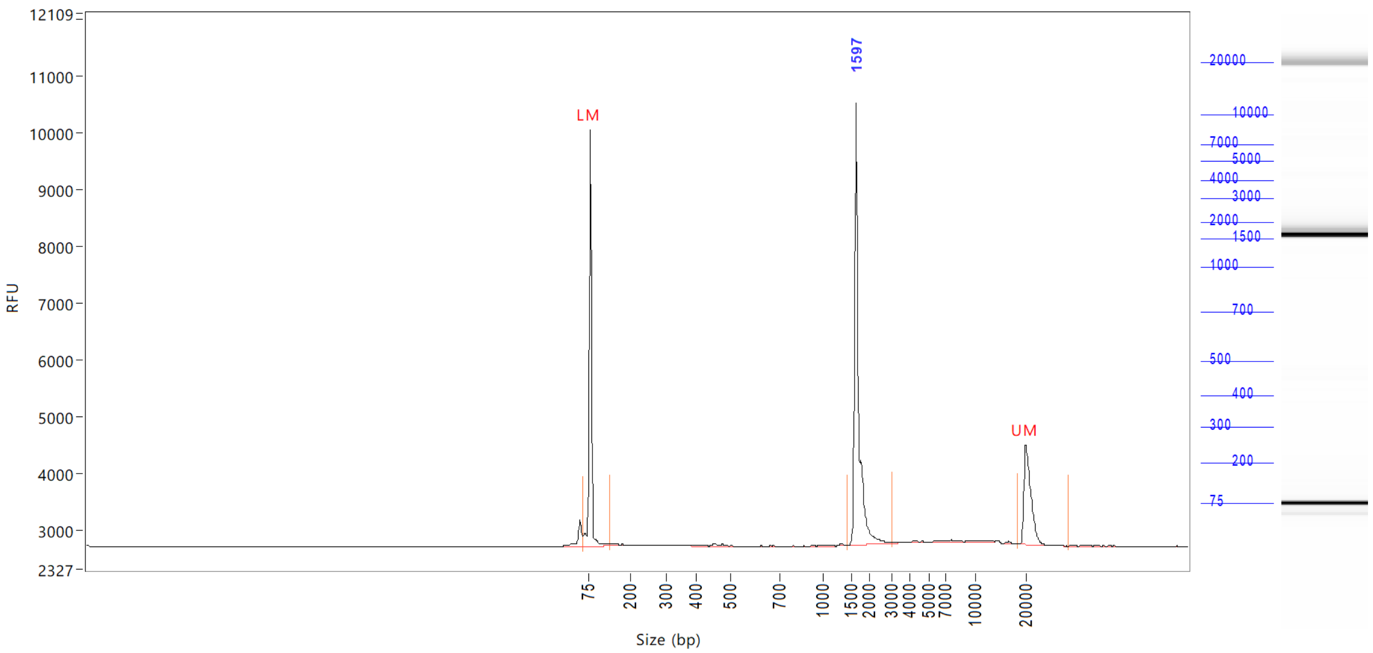

Peak	Size<br>(bp)	Concentration<br>(ng/uL)	From<br>(bp)	To<br>(bp)	Average size<br>(bp)	CV%	RFU	Corrected peak area
1	75 (LM)	0.5000	52	133	74	10.87	7320	42.445
2	1597	6.6638	1433	3037	1665	10.93	7792	47.141
3	20000 (UM)	0.1753	18293	28100	20366	4.06	1746	14.877

TIC:	6.6638	ng/uL
TIM:	6.5882	nmole/L
Total concentration:	7.4457	ng/uL

Sample peak width (sec): 5 Sample min peak height: 50 Sample baseline V to V?: Y Sample baseline V to V points: 3
Sample filter: Binomial Number of points for filter: 3 Sample start region (min): 0 Sample end region (min): 30
Marker peak width (sec): 5 Marker min peak height: 200 Marker baseline V to V?: Y Marker baseline V to V points: 3
Lower marker selection: First peak > 500 RFU Upper marker selection: Last peak > 200 RFU
Ladder size (bp)75, 200, 300, 400, 500, 700, 1000, 1500, 2000, 3000, 4000, 5000, 7000, 10000, 20000
Quantification using: Lower Marker Final concentration (ng/uL): 0.5000 Dilution factor: 12.0

Sample: SampB5
Well location: B5
Created: Tuesday, August 10, 2021 6:41:11 PM

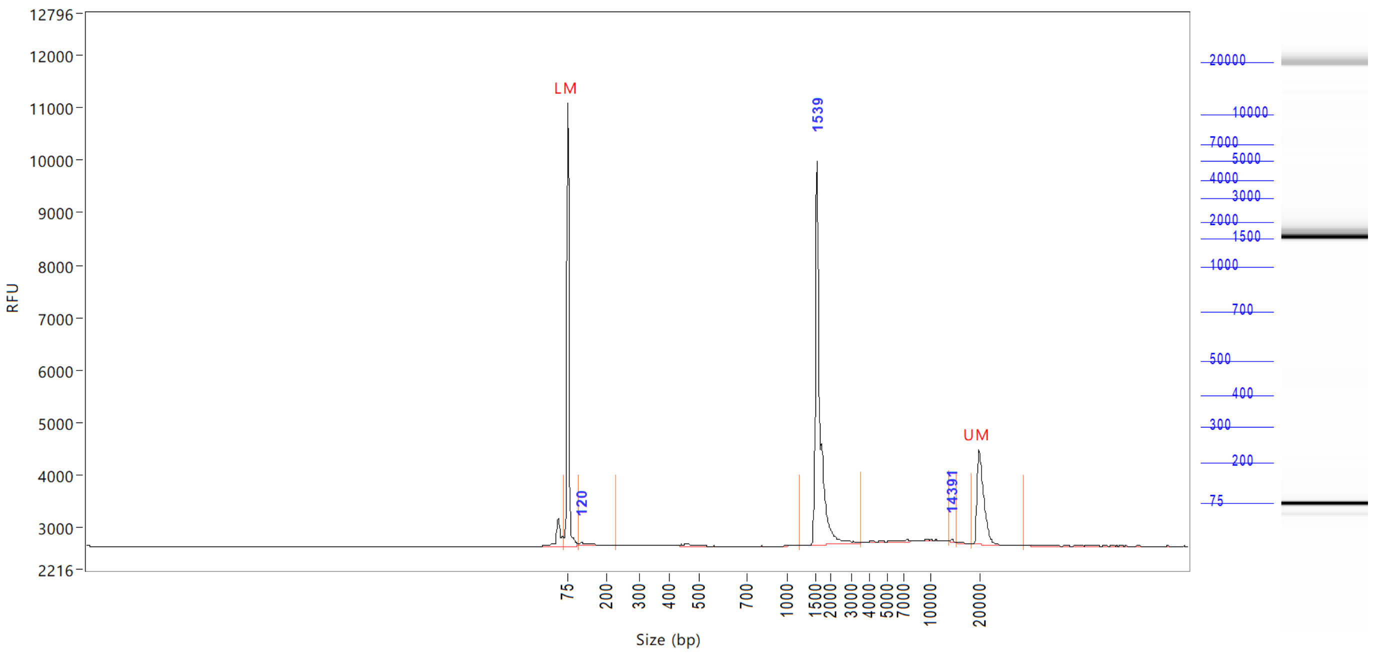

Peak	Size (bp)	Concentration (ng/uL)	From (bp)	To (bp)	Average size (bp)	CV%	RFU	Corrected peak area
1	75 (LM)	0.5000	64	108	73	6.68	8448	46.977
2	120	0.2695	108	228	149	21.48	58	2.110
3	1539	6.6168	1201	3536	1637	14.32	7300	51.806
4	14391	0.0242	13781	15366	14264	1.76	51	0.189
5	20000 (UM)	0.1699	18293	29068	20410	4.46	1812	15.960

TIC:	6.9105	ng/uL
TIM:	9.6294	nmole/L
Total concentration:	7.3920	ng/uL

```
Sample peak width (sec): 5    Sample min peak height: 50    Sample baseline V to V?: Y    Sample baseline V to V points: 3
Sample filter: Binomial       Number of points for filter: 3  Sample start region (min): 0  Sample end region (min): 30
Marker peak width (sec): 5    Marker min peak height: 200   Marker baseline V to V?: Y    Marker baseline V to V points: 3
Lower marker selection: First peak > 500 RFU                Upper marker selection: Last peak > 200 RFU
Ladder size (bp)75, 200, 300, 400, 500, 700, 1000, 1500, 2000, 3000, 4000, 5000, 7000, 10000, 20000
Quantification using: Lower Marker          Final concentration (ng/uL): 0.5000          Dilution factor: 12.0
```

Sample: SampC5
Well location: C5
Created: Tuesday, August 10, 2021 6:41:11 PM

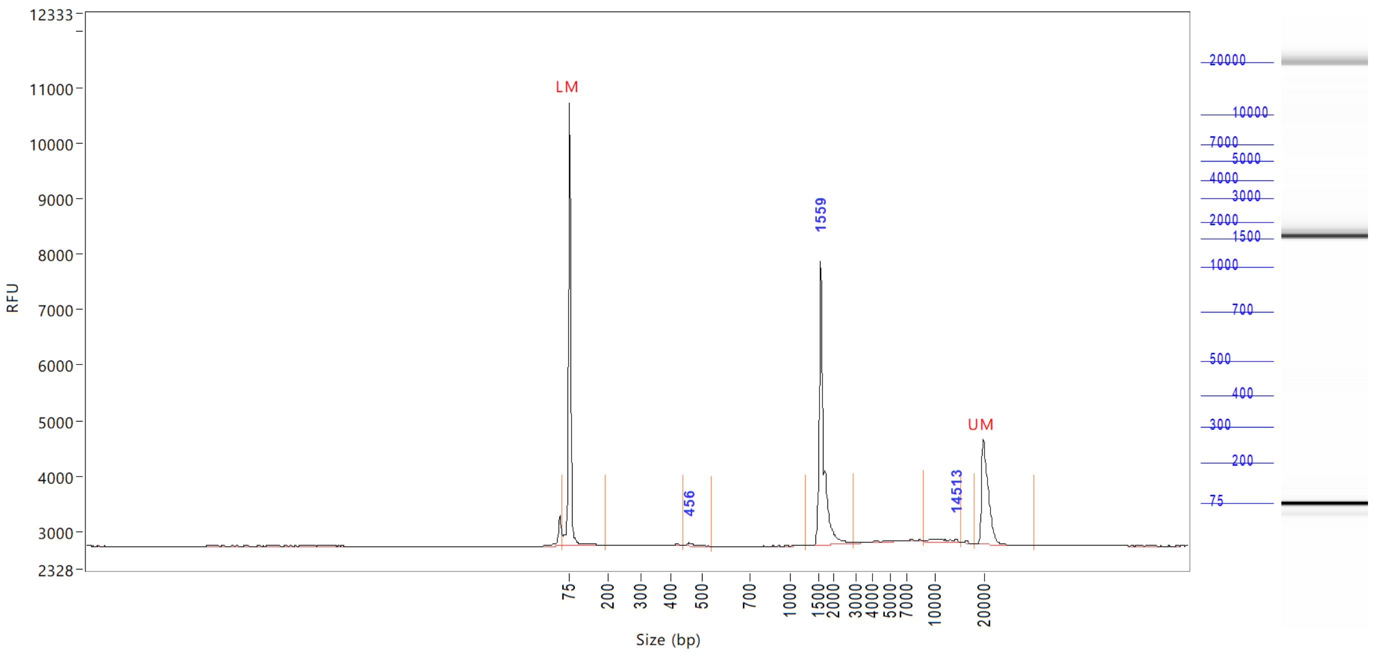

Peak	Size (bp)	Concentration (ng/uL)	From (bp)	To (bp)	Average size (bp)	CV%	RFU	Corrected peak area
1	75 (LM)	0.5000	52	189	73	12.56	7980	47.995
2	456	0.0979	437	534	463	3.97	54	0.783
3	1559	4.4087	1267	2886	1631	11.26	5112	35.266
4	14513	0.1859	8800	15488	11606	15.26	59	1.487
5	20000 (UM)	0.1709	18293	30277	20481	4.74	1889	16.406

TIC:	4.6925	ng/uL
TIM:	4.8238	nmole/L
Total concentration:	5.0031	ng/uL

Sample peak width (sec): 5 Sample min peak height: 50 Sample baseline V to V?: Y Sample baseline V to V points: 3
Sample filter: Binomial Number of points for filter: 3 Sample start region (min): 0 Sample end region (min): 30
Marker peak width (sec): 5 Marker min peak height: 200 Marker baseline V to V?: Y Marker baseline V to V points: 3
Lower marker selection: First peak > 500 RFU Upper marker selection: Last peak > 200 RFU
Ladder size (bp)75, 200, 300, 400, 500, 700, 1000, 1500, 2000, 3000, 4000, 5000, 7000, 10000, 20000
Quantification using: Lower Marker Final concentration (ng/uL): 0.5000 Dilution factor: 12.0

Sample: SampD5
Well location: D5
Created: Tuesday, August 10, 2021 6:41:11 PM

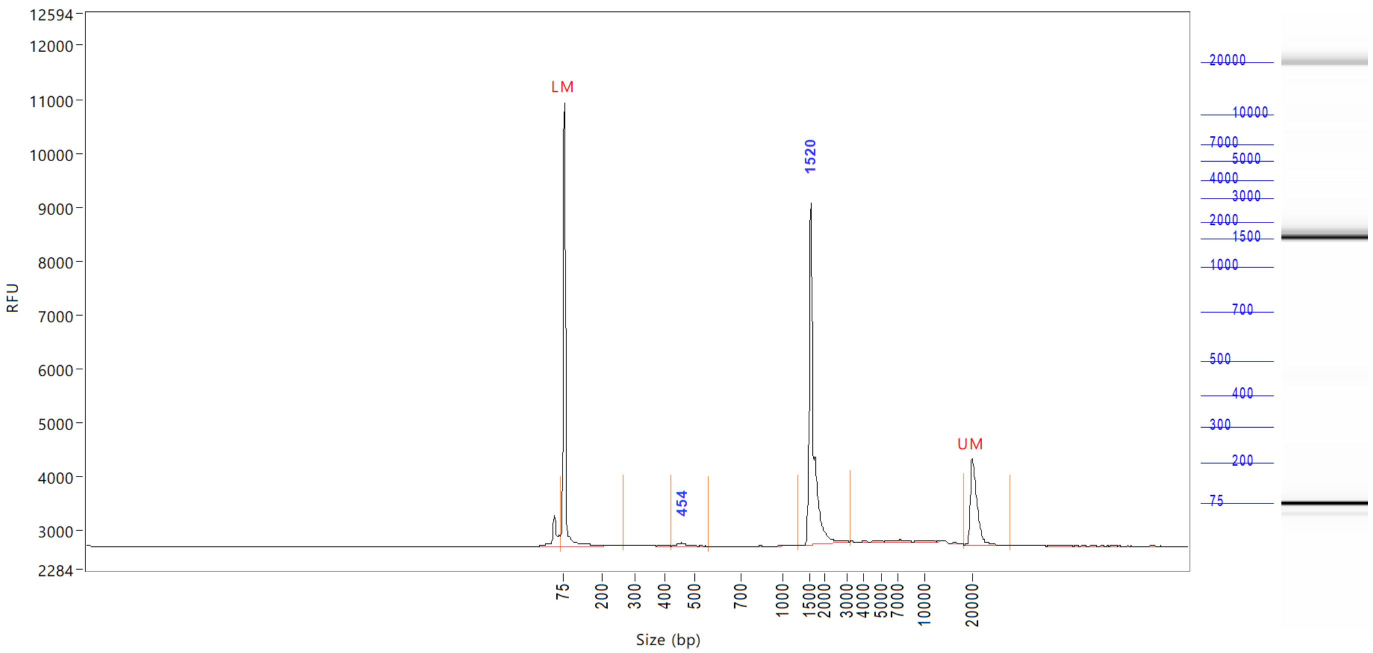

Peak	Size (bp)	Concentration (ng/uL)	From (bp)	To (bp)	Average size (bp)	CV%	RFU	Corrected peak area
1	75 (LM)	0.5000	64	262	78	30.34	8239	49.867
2	454	0.1481	418	552	461	5.45	60	1.231
3	1520	5.3047	1278	3286	1597	10.96	6335	44.089
4	20000 (UM)	0.1371	18293	27738	20431	4.41	1607	13.676

TIC:	5.4528	ng/uL
TIM:	5.9962	nmole/L
Total concentration:	5.7298	ng/uL

Sample peak width (sec): 5   Sample min peak height: 50   Sample baseline V to V?: Y   Sample baseline V to V points: 3
Sample filter: Binomial   Number of points for filter: 3 Sample start region (min): 0 Sample end region (min): 30
Marker peak width (sec): 5   Marker min peak height: 200   Marker baseline V to V?: Y   Marker baseline V to V points: 3
Lower marker selection: First peak > 500 RFU   Upper marker selection: Last peak > 200 RFU
Ladder size (bp)75, 200, 300, 400, 500, 700, 1000, 1500, 2000, 3000, 4000, 5000, 7000, 10000, 20000
Quantification using: Lower Marker   Final concentration (ng/uL): 0.5000   Dilution factor: 12.0

Sample: SampE5
Well location: E5
Created: Tuesday, August 10, 2021 6:41:11 PM

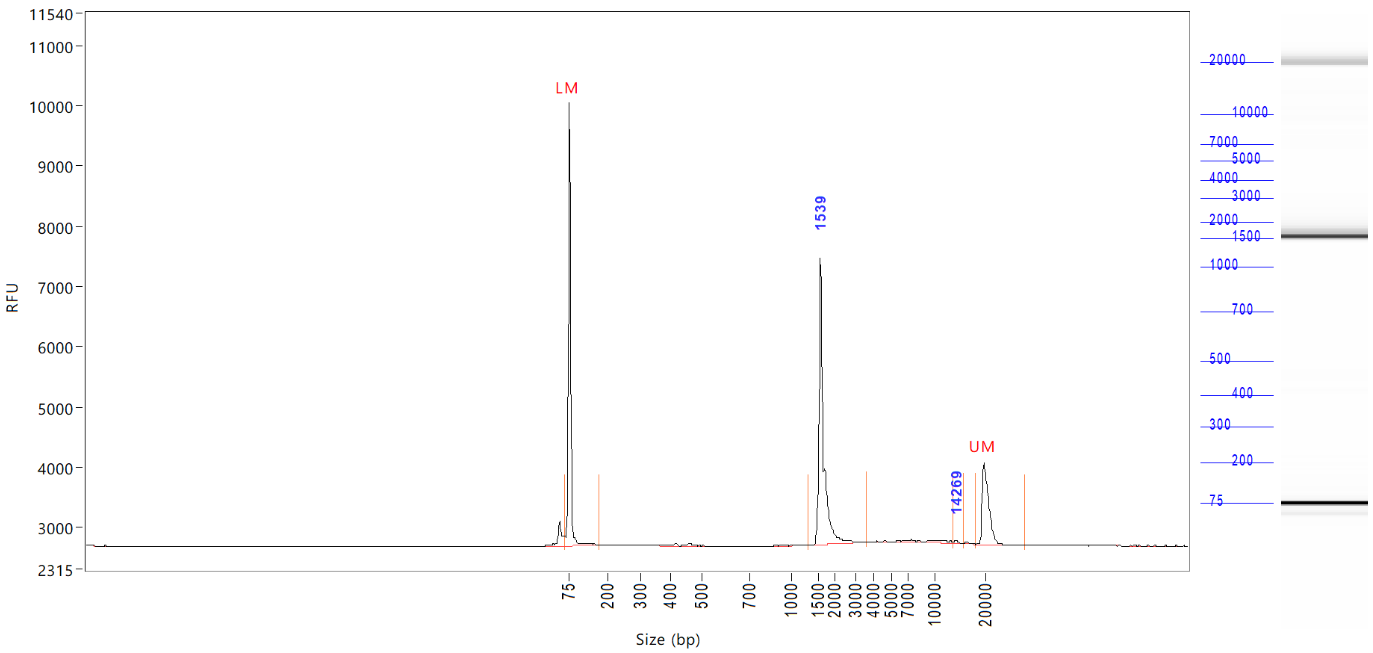

Peak	Size (bp)	Concentration (ng/uL)	From (bp)	To (bp)	Average size (bp)	CV%	RFU	Corrected peak area
1	75 (LM)	0.5000	64	171	75	13.99	7366	42.022
2	1539	4.7846	1311	3572	1625	12.87	4752	33.510
3	14269	0.0471	13537	15732	14364	4.00	54	0.330
4	20000 (UM)	0.1355	18171	28342	20357	4.19	1351	11.391

TIC:	4.8317	ng/uL
TIM:	4.8521	nmole/L
Total concentration:	5.3977	ng/uL

Sample peak width (sec): 5 Sample min peak height: 50 Sample baseline V to V?: Y Sample baseline V to V points: 3
Sample filter: Binomial Number of points for filter: 3 Sample start region (min): 0 Sample end region (min): 30
Marker peak width (sec): 5 Marker min peak height: 200 Marker baseline V to V?: Y Marker baseline V to V points: 3
Lower marker selection: First peak > 500 RFU Upper marker selection: Last peak > 200 RFU
Ladder size (bp)75, 200, 300, 400, 500, 700, 1000, 1500, 2000, 3000, 4000, 5000, 7000, 10000, 20000
Quantification using: Lower Marker Final concentration (ng/uL): 0.5000 Dilution factor: 12.0

Sample: SampF5
Well location: F5
Created: Tuesday, August 10, 2021 6:41:11 PM

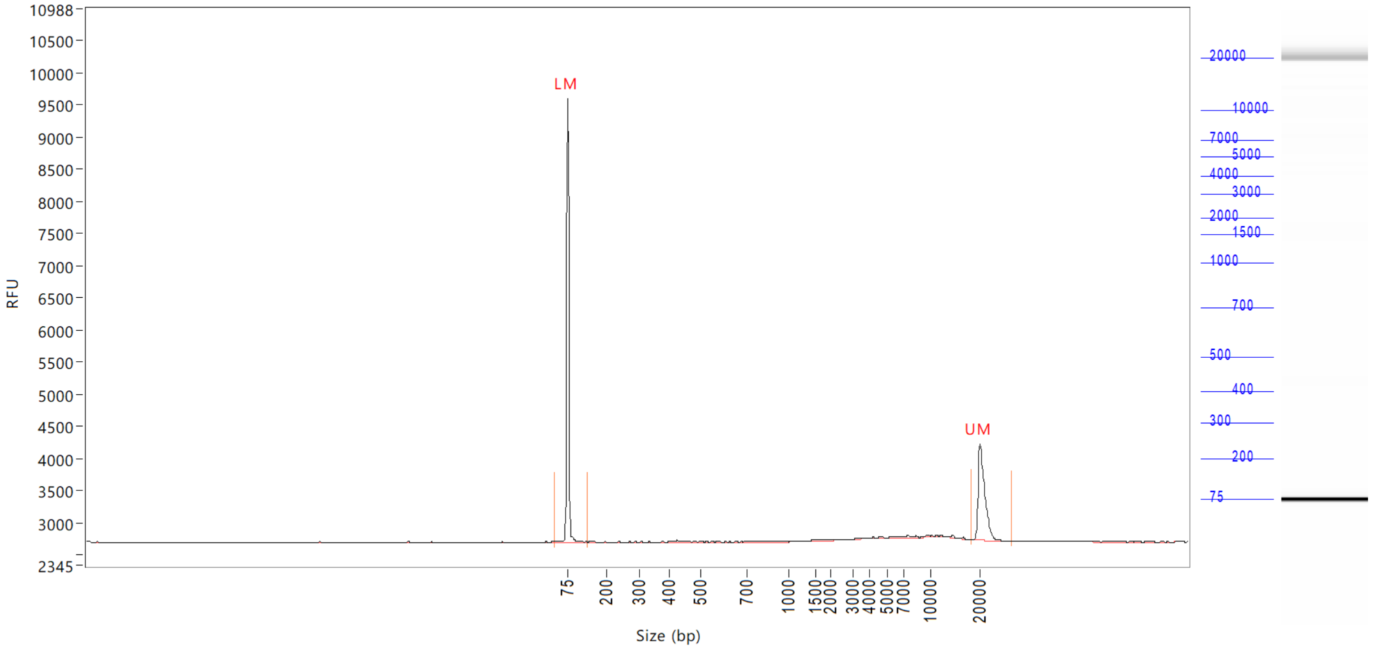

Peak	Size (bp)	Concentration (ng/uL)	From (bp)	To (bp)	Average size (bp)	CV%	RFU	Corrected peak area
1	75 (LM)	0.5000	31	137	72	7.02	6906	38.506
2	20000 (UM)	0.1702	18171	26408	20508	4.25	1500	13.107

TIC:	0.0000	ng/uL
TIM:	0.0000	nmole/L
Total concentration:	0.5941	ng/uL

Sample peak width (sec): 5   Sample min peak height: 50   Sample baseline V to V?: Y   Sample baseline V to V points: 3
Sample filter: Binomial   Number of points for filter: 3 Sample start region (min): 0 Sample end region (min): 30
Marker peak width (sec): 5   Marker min peak height: 200   Marker baseline V to V?: Y   Marker baseline V to V points: 3
Lower marker selection: First peak > 500 RFU   Upper marker selection: Last peak > 200 RFU
Ladder size (bp)75, 200, 300, 400, 500, 700, 1000, 1500, 2000, 3000, 4000, 5000, 7000, 10000, 20000
Quantification using: Lower Marker   Final concentration (ng/uL): 0.5000   Dilution factor: 12.0

Sample: SampG5
Well location: G5
Created: Tuesday, August 10, 2021 6:41:11 PM

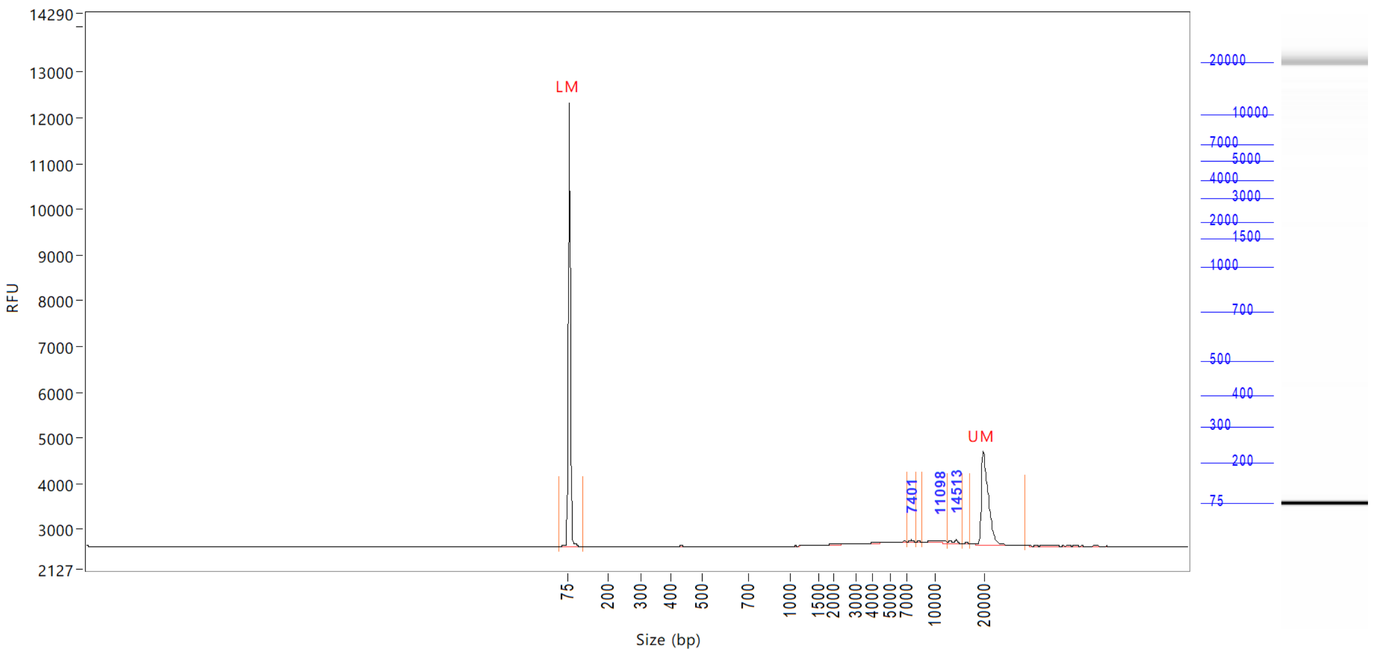

Peak	Size<br>(bp)	Concentration<br>(ng/uL)	From<br>(bp)	To<br>(bp)	Average size<br>(bp)	CV%	RFU	Corrected peak area
1	75 (LM)	0.5000	47	120	73	5.47	9725	51.570
2	7401	0.0392	7000	8001	7410	3.60	51	0.337
3	11098	0.1279	8734	12684	10613	9.91	51	1.099
4	14513	0.0801	12684	15732	13966	5.34	80	0.688
5	20000 (UM)	0.1770	17317	28584	20454	4.38	2055	18.258

TIC:	0.2471	ng/uL
TIM:	0.0380	nmole/L
Total concentration:	0.5783	ng/uL

Sample peak width (sec): 5 Sample min peak height: 50 Sample baseline V to V?: Y Sample baseline V to V points: 3
Sample filter: Binomial Number of points for filter: 3 Sample start region (min): 0 Sample end region (min): 30
Marker peak width (sec): 5 Marker min peak height: 200 Marker baseline V to V?: Y Marker baseline V to V points: 3
Lower marker selection: First peak > 500 RFU Upper marker selection: Last peak > 200 RFU
Ladder size (bp)75, 200, 300, 400, 500, 700, 1000, 1500, 2000, 3000, 4000, 5000, 7000, 10000, 20000
Quantification using: Lower Marker Final concentration (ng/uL): 0.5000 Dilution factor: 12.0

Sample: SampH5
Well location: H5
Created: Tuesday, August 10, 2021 6:41:11 PM

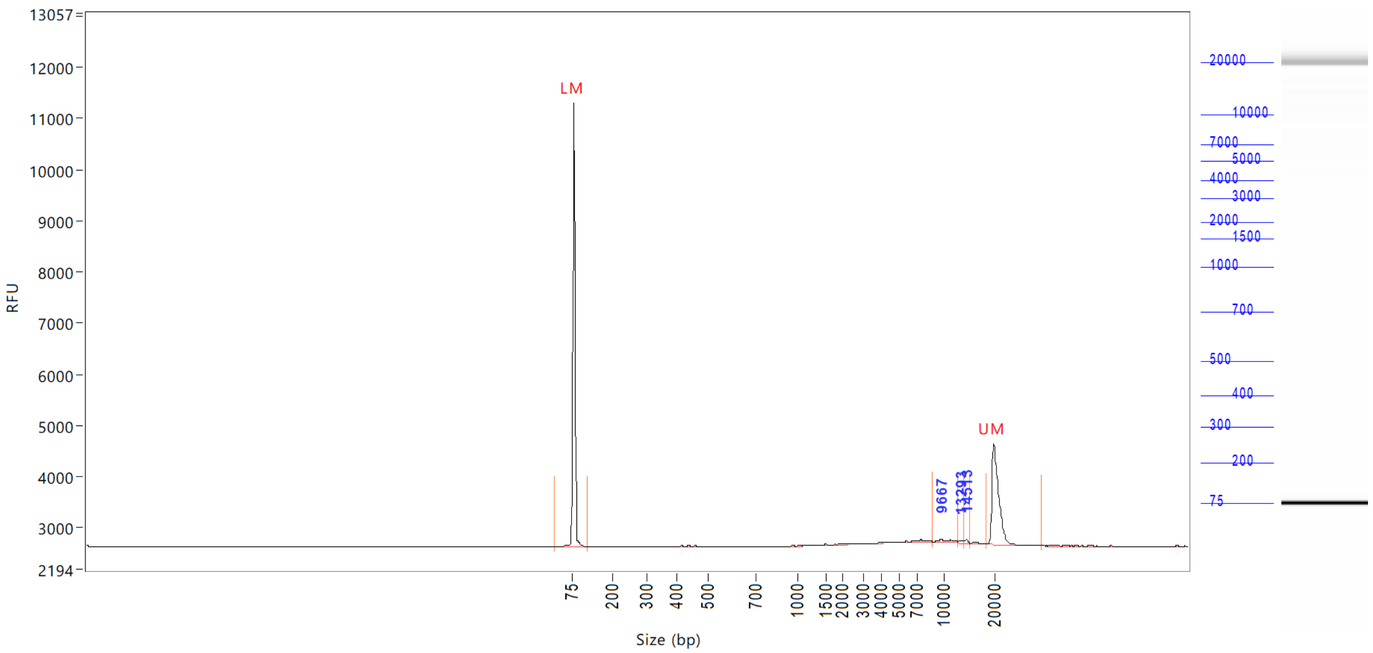

Peak	Size (bp)	Concentration (ng/uL)	From (bp)	To (bp)	Average size (bp)	CV%	RFU	Corrected peak area
1	75 (LM)	0.5000	14	118	74	6.22	8684	47.568
2	9667	0.1240	8800	12684	10635	9.80	51	0.983
3	13293	0.0337	12684	13781	13235	2.47	51	0.267
4	14513	0.0487	13781	15244	14339	2.37	74	0.386
5	20000 (UM)	0.1793	18415	29430	20452	4.49	1971	17.057

TIC:	0.2064	ng/uL
TIM:	0.0290	nmole/L
Total concentration:	0.6075	ng/uL

Sample peak width (sec): 5   Sample min peak height: 50   Sample baseline V to V?: Y   Sample baseline V to V points: 3
Sample filter: Binomial   Number of points for filter: 3   Sample start region (min): 0   Sample end region (min): 30
Marker peak width (sec): 5   Marker min peak height: 200   Marker baseline V to V?: Y   Marker baseline V to V points: 3
Lower marker selection: First peak > 500 RFU   Upper marker selection: Last peak > 200 RFU
Ladder size (bp)75, 200, 300, 400, 500, 700, 1000, 1500, 2000, 3000, 4000, 5000, 7000, 10000, 20000
Quantification using: Lower Marker   Final concentration (ng/uL): 0.5000   Dilution factor: 12.0

Sample: SampA6
Well location: A6
Created: Tuesday, August 10, 2021 6:41:11 PM

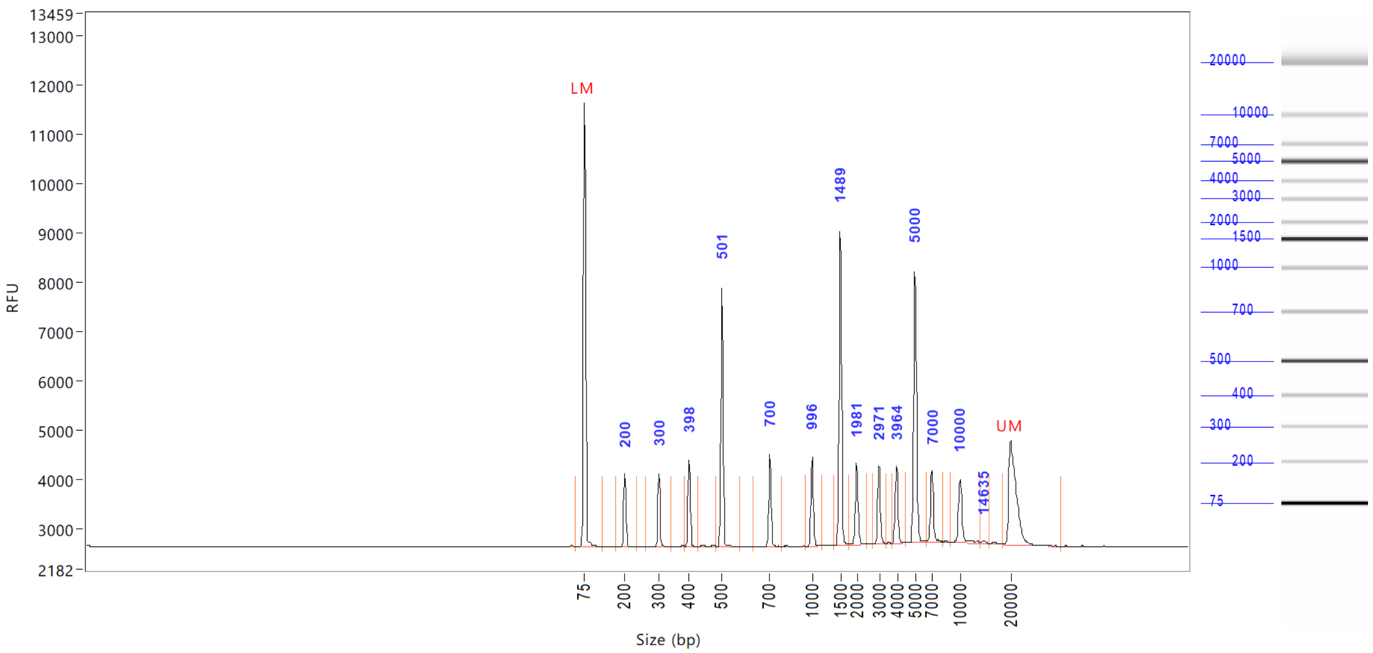

Peak	Size (bp)	Concentration (ng/uL)	From (bp)	To (bp)	Average size (bp)	CV%	RFU	Corrected peak area
1	75 (LM)	0.5000	50	131	73	5.81	9014	46.866
2	200	0.8831	171	235	198	1.98	1476	6.898
3	300	0.8699	262	337	298	1.44	1492	6.795
4	398	0.9474	386	428	396	0.97	1752	7.400
5	501	2.8352	485	575	499	1.03	5243	22.146
6	700	0.9719	634	783	697	1.09	1871	7.591
7	996	0.9350	952	1156	993	1.36	1808	7.303
8	1489	3.2664	1378	1731	1481	1.76	6364	25.514
9	1981	0.8679	1731	2401	1962	2.78	1650	6.779
10	2971	0.8102	2715	3358	2955	2.00	1573	6.329
11	3964	0.7890	3679	4518	3938	2.16	1550	6.163
12	5000	3.0558	4518	6334	4998	2.89	5506	23.869
13	7000	0.8103	6334	8201	6949	3.33	1457	6.329
14	10000	0.8136	8934	13903	10198	7.97	1297	6.355
15	14635	0.0451	13903	15610	14544	2.63	62	0.352
16	20000 (UM)	0.2110	18415	29793	20497	4.74	2112	19.773

TIC:	17.9008	ng/uL
TIM:	35.7399	nmole/L
Total concentration:	18.0634	ng/uL

```
Sample peak width (sec): 5    Sample min peak height: 50    Sample baseline V to V?: Y    Sample baseline V to V points: 3
Sample filter: Binomial       Number of points for filter: 3  Sample start region (min): 0  Sample end region (min): 30
Marker peak width (sec): 5    Marker min peak height: 200   Marker baseline V to V?: Y    Marker baseline V to V points: 3
Lower marker selection: First peak > 500 RFU                Upper marker selection: Last peak > 200 RFU
Ladder size (bp)75, 200, 300, 400, 500, 700, 1000, 1500, 2000, 3000, 4000, 5000, 7000, 10000, 20000
Quantification using: Lower Marker          Final concentration (ng/uL): 0.5000          Dilution factor: 12.0
```

Sample: SampB6
Well location: B6
Created: Tuesday, August 10, 2021 6:41:11 PM

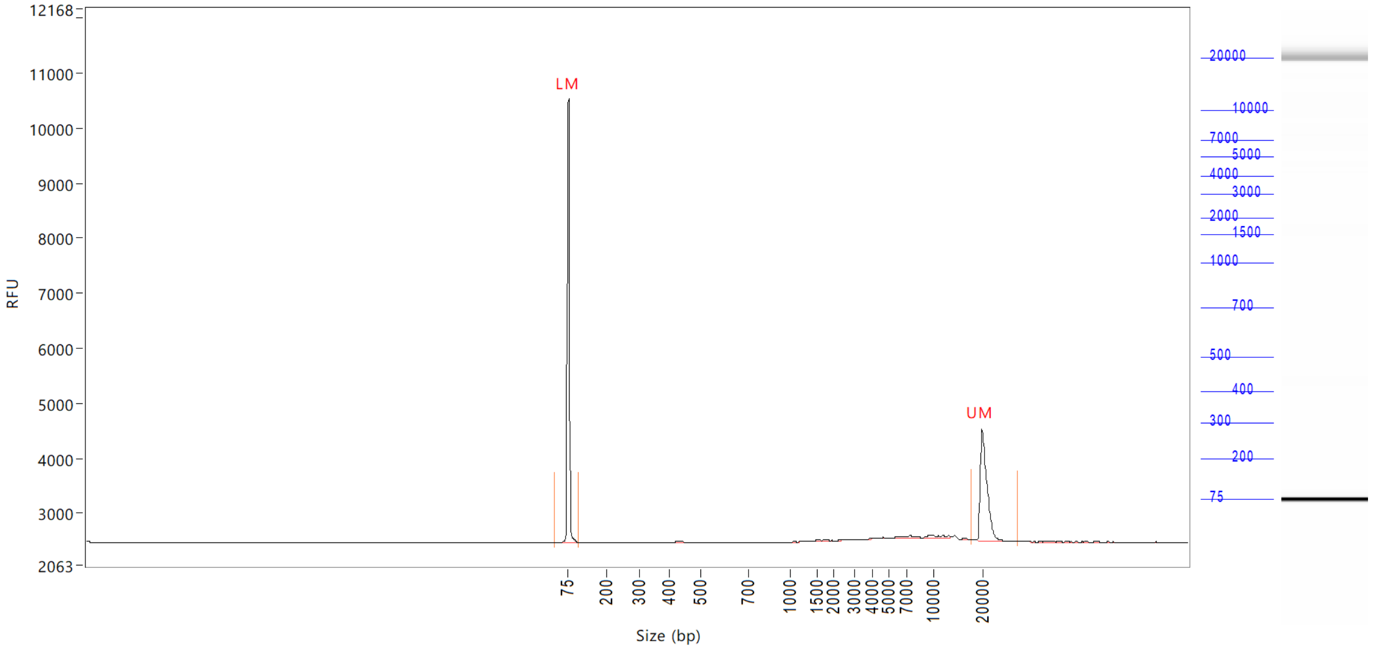

Peak	Size<br>(bp)	Concentration<br>(ng/uL)	From<br>(bp)	To<br>(bp)	Average size<br>(bp)	CV%	RFU	Corrected peak area
1	75 (LM)	0.5000	31	108	72	5.38	8079	44.339
2	20000 (UM)	0.1981	17683	27375	20416	4.03	2022	17.564

TIC:	0.0000	ng/uL
TIM:	0.0000	nmole/L
Total concentration:	0.5453	ng/uL

Sample peak width (sec): 5 Sample min peak height: 50 Sample baseline V to V?: Y Sample baseline V to V points: 3
Sample filter: Binomial Number of points for filter: 3 Sample start region (min): 0 Sample end region (min): 30
Marker peak width (sec): 5 Marker min peak height: 200 Marker baseline V to V?: Y Marker baseline V to V points: 3
Lower marker selection: First peak > 500 RFU Upper marker selection: Last peak > 200 RFU
Ladder size (bp)75, 200, 300, 400, 500, 700, 1000, 1500, 2000, 3000, 4000, 5000, 7000, 10000, 20000
Quantification using: Lower Marker Final concentration (ng/uL): 0.5000 Dilution factor: 12.0

Sample: SampC6
Well location: C6
Created: Tuesday, August 10, 2021 6:41:11 PM

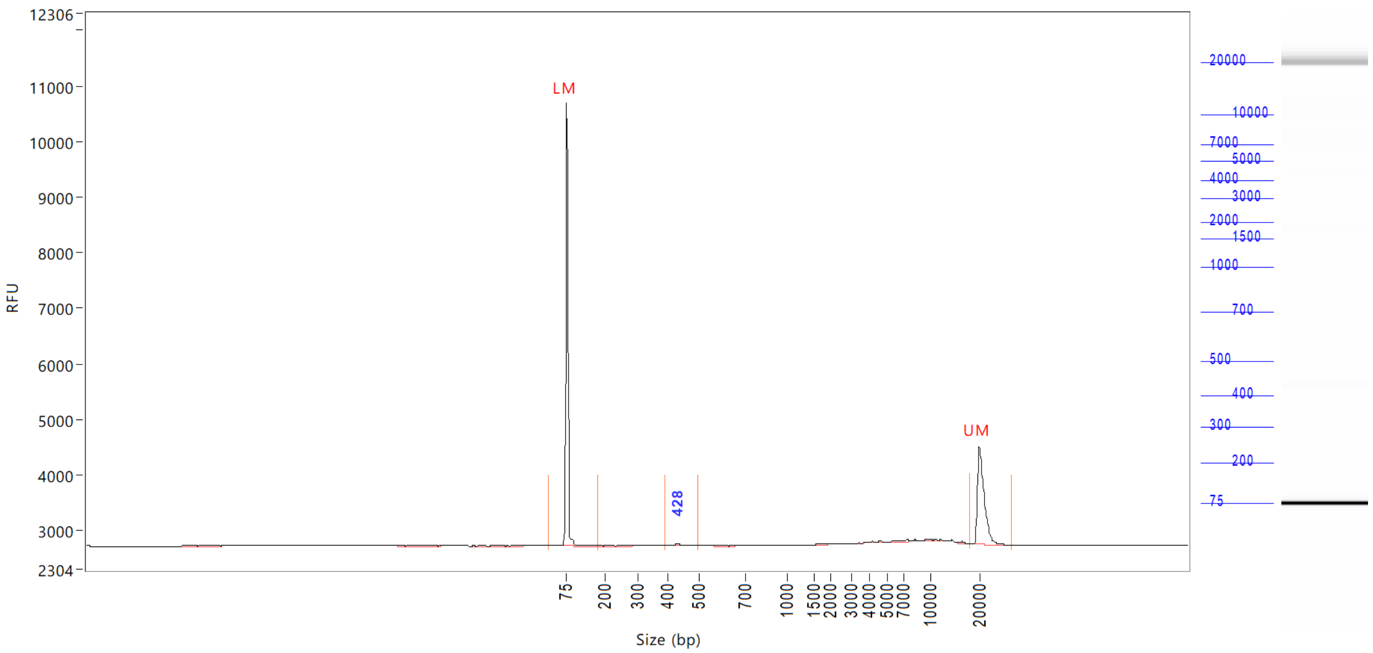

Peak	Size (bp)	Concentration (ng/uL)	From (bp)	To (bp)	Average size (bp)	CV%	RFU	Corrected peak area
1	75 (LM)	0.5000	20	177	74	9.48	7985	43.215
2	428	0.0957	392	494	432	5.05	51	0.690
3	20000 (UM)	0.1807	18049	26529	20501	4.36	1767	15.614

TIC:	0.0957	ng/uL
TIM:	0.3646	nmole/L
Total concentration:	0.5959	ng/uL

```
Sample peak width (sec): 5    Sample min peak height: 50    Sample baseline V to V?: Y    Sample baseline V to V points: 3
Sample filter: Binomial       Number of points for filter: 3  Sample start region (min): 0  Sample end region (min): 30
Marker peak width (sec): 5    Marker min peak height: 200   Marker baseline V to V?: Y    Marker baseline V to V points: 3
Lower marker selection: First peak > 500 RFU                Upper marker selection: Last peak > 200 RFU
Ladder size (bp)75, 200, 300, 400, 500, 700, 1000, 1500, 2000, 3000, 4000, 5000, 7000, 10000, 20000
Quantification using: Lower Marker          Final concentration (ng/uL): 0.5000          Dilution factor: 12.0
```

Sample: SampD6
Well location: D6
Created: Tuesday, August 10, 2021 6:41:11 PM

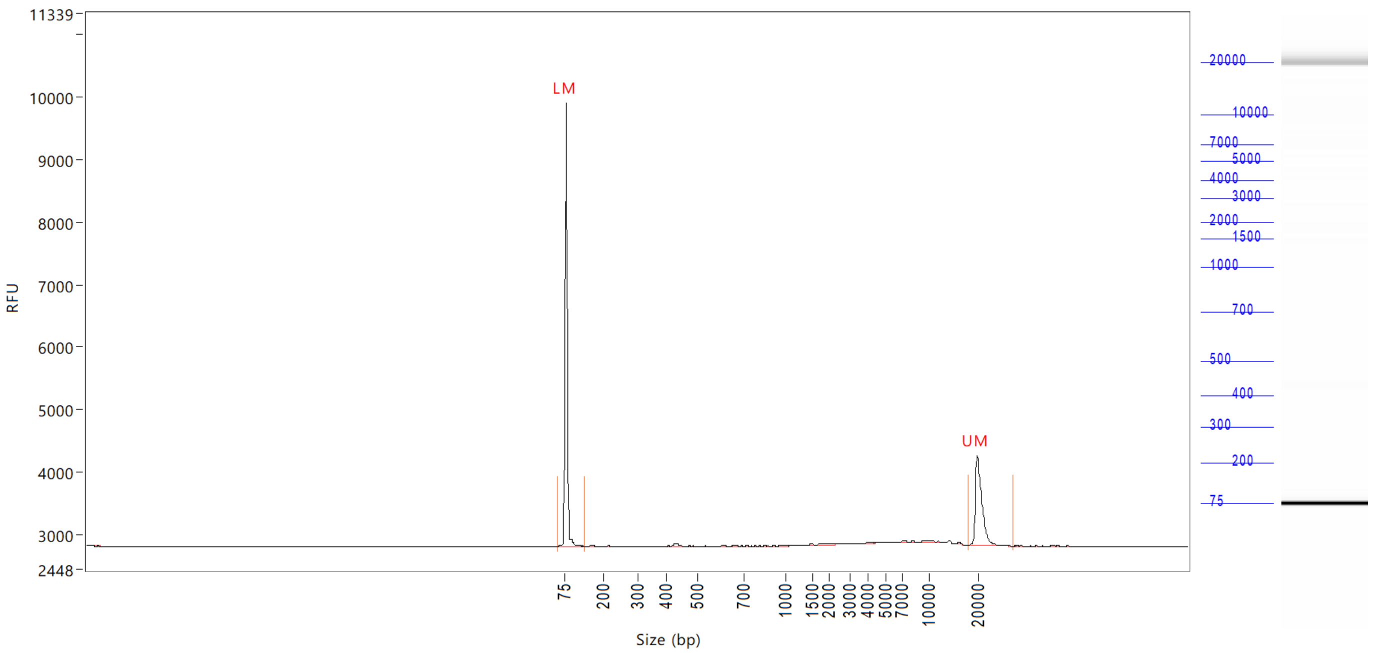

Peak	Size (bp)	Concentration (ng/uL)	From (bp)	To (bp)	Average size (bp)	CV%	RFU	Corrected peak area
1	75 (LM)	0.5000	47	135	73	6.93	7105	38.862
2	20000 (UM)	0.1581	18049	27375	20412	4.32	1437	12.285

TIC:	0.0000	ng/uL
TIM:	0.0000	nmole/L
Total concentration:	0.6171	ng/uL

Sample peak width (sec): 5   Sample min peak height: 50   Sample baseline V to V?: Y   Sample baseline V to V points: 3
Sample filter: Binomial   Number of points for filter: 3   Sample start region (min): 0   Sample end region (min): 30
Marker peak width (sec): 5   Marker min peak height: 200   Marker baseline V to V?: Y   Marker baseline V to V points: 3
Lower marker selection: First peak > 500 RFU   Upper marker selection: Last peak > 200 RFU
Ladder size (bp)75, 200, 300, 400, 500, 700, 1000, 1500, 2000, 3000, 4000, 5000, 7000, 10000, 20000
Quantification using: Lower Marker   Final concentration (ng/uL): 0.5000   Dilution factor: 12.0

Sample: SampE6
Well location: E6
Created: Tuesday, August 10, 2021 6:41:11 PM

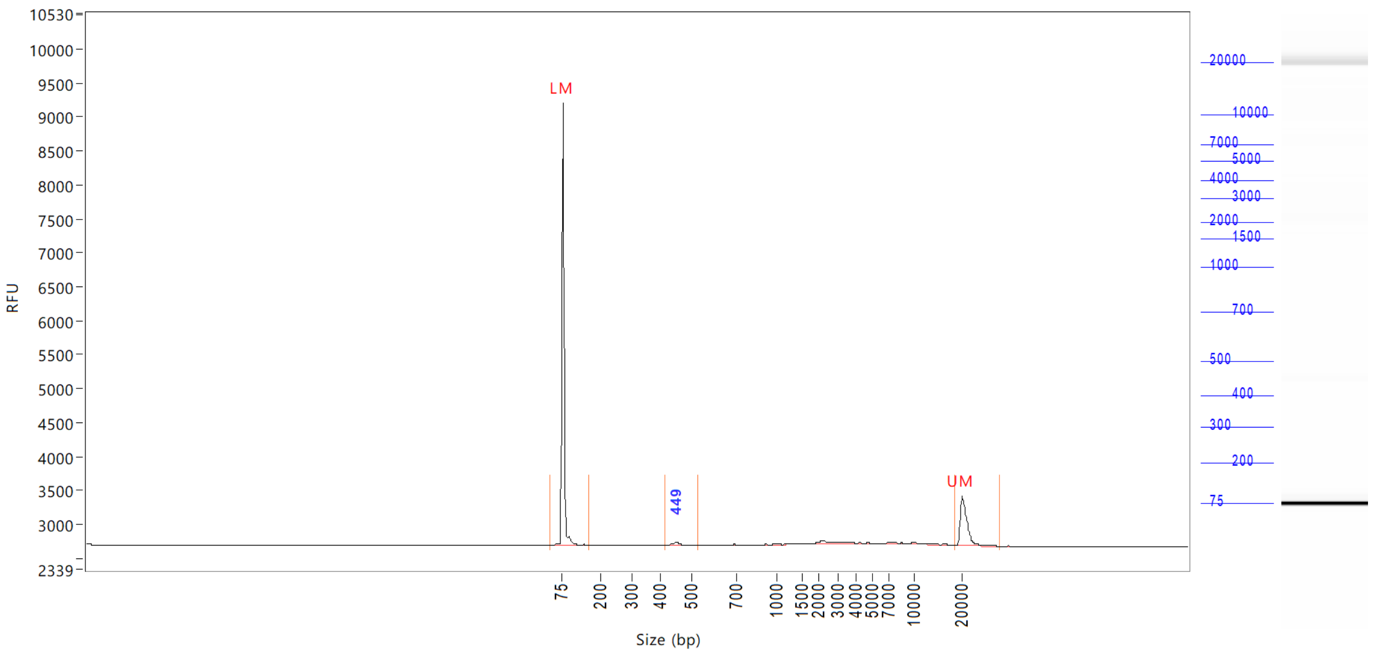

Peak	Size (bp)	Concentration (ng/uL)	From (bp)	To (bp)	Average size (bp)	CV%	RFU	Corrected peak area
1	75 (LM)	0.5000	31	160	74	10.36	6526	37.024
2	449	0.1011	414	529	456	4.94	51	0.624
3	20000 (UM)	0.0893	18415	27859	20624	5.68	728	6.615

TIC:	0.1011	ng/uL
TIM:	0.3649	nmole/L
Total concentration:	0.7048	ng/uL

```
Sample peak width (sec): 5    Sample min peak height: 50    Sample baseline V to V?: Y    Sample baseline V to V points: 3
Sample filter: Binomial       Number of points for filter: 3  Sample start region (min): 0  Sample end region (min): 30
Marker peak width (sec): 5    Marker min peak height: 200   Marker baseline V to V?: Y    Marker baseline V to V points: 3
Lower marker selection: First peak > 500 RFU                Upper marker selection: Last peak > 200 RFU
Ladder size (bp)75, 200, 300, 400, 500, 700, 1000, 1500, 2000, 3000, 4000, 5000, 7000, 10000, 20000
Quantification using: Lower Marker          Final concentration (ng/uL): 0.5000          Dilution factor: 12.0
```

Sample: SampF6
Well location: F6
Created: Tuesday, August 10, 2021 6:41:11 PM

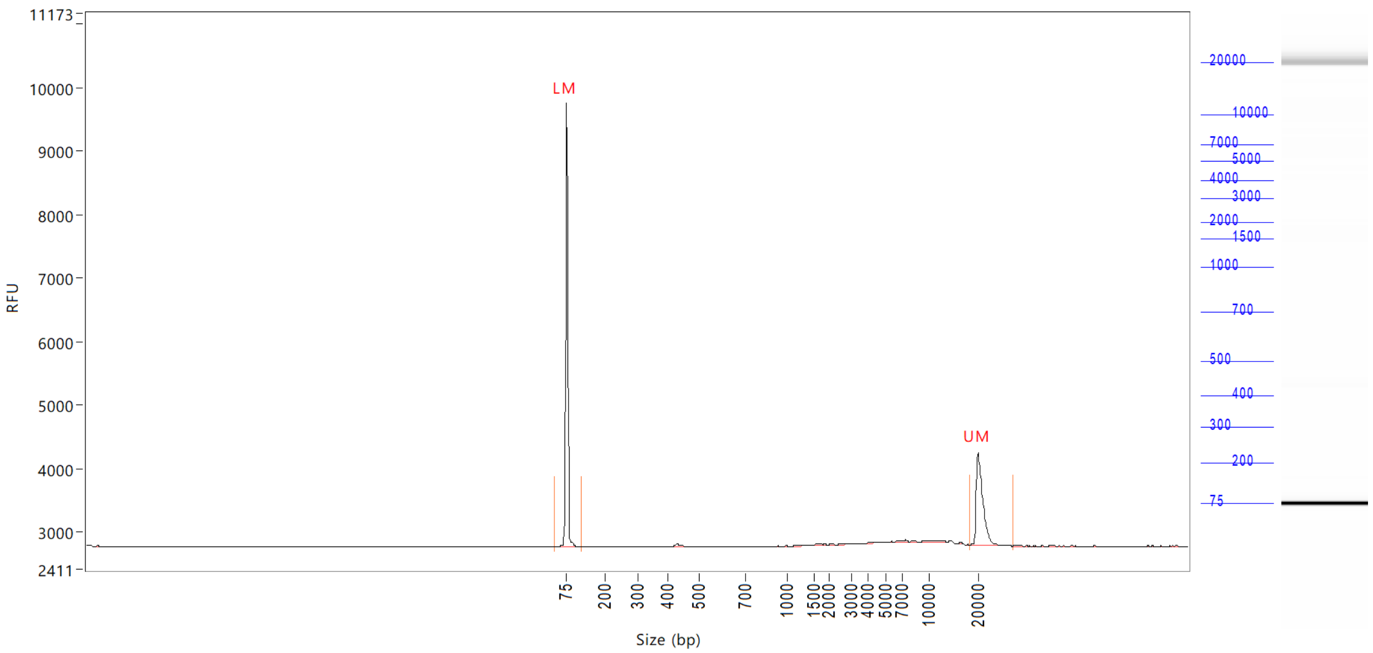

Peak	Size (bp)	Concentration (ng/uL)	From (bp)	To (bp)	Average size (bp)	CV%	RFU	Corrected peak area
1	75 (LM)	0.5000	35	124	73	6.31	7008	38.012
2	20000 (UM)	0.1664	18293	27133	20506	4.54	1450	12.653

TIC:	0.0000	ng/uL
TIM:	0.0000	nmole/L
Total concentration:	0.6292	ng/uL

Sample peak width (sec): 5 Sample min peak height: 50 Sample baseline V to V?: Y Sample baseline V to V points: 3
Sample filter: Binomial Number of points for filter: 3 Sample start region (min): 0 Sample end region (min): 30
Marker peak width (sec): 5 Marker min peak height: 200 Marker baseline V to V?: Y Marker baseline V to V points: 3
Lower marker selection: First peak > 500 RFU Upper marker selection: Last peak > 200 RFU
Ladder size (bp)75, 200, 300, 400, 500, 700, 1000, 1500, 2000, 3000, 4000, 5000, 7000, 10000, 20000
Quantification using: Lower Marker Final concentration (ng/uL): 0.5000 Dilution factor: 12.0

Sample: SampG6
Well location: G6
Created: Tuesday, August 10, 2021 6:41:11 PM

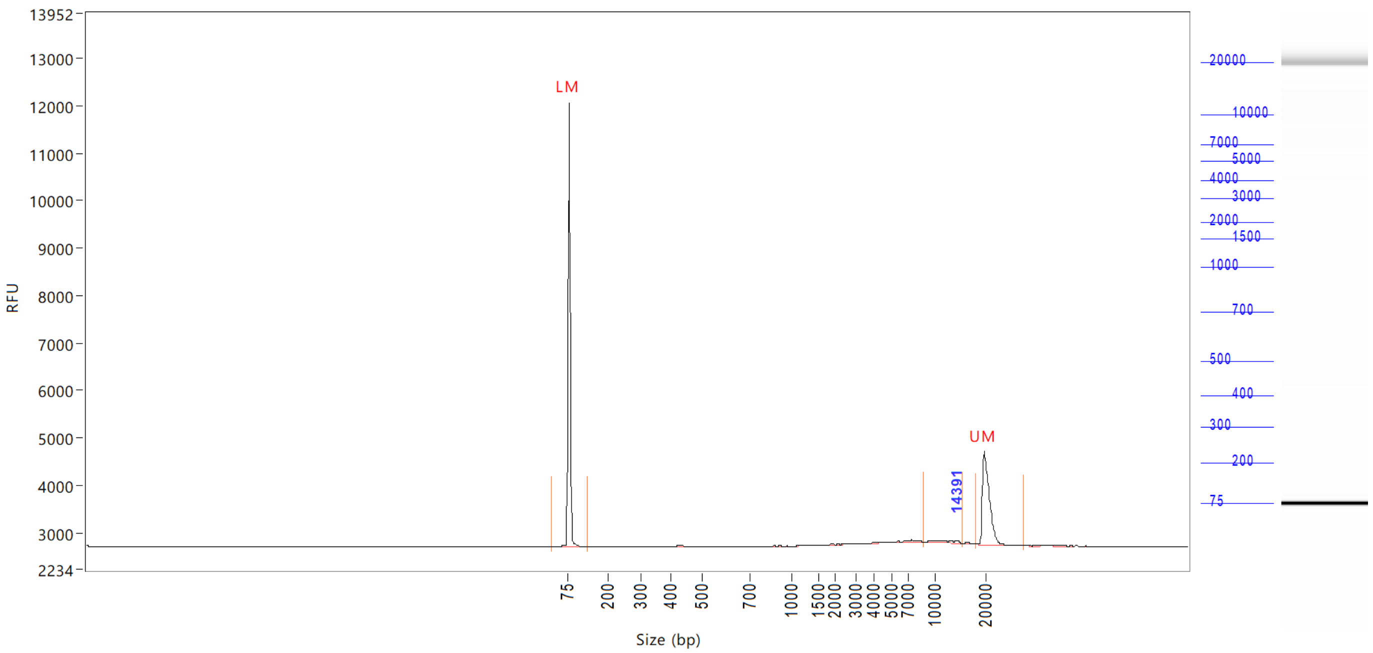

Peak	Size (bp)	Concentration (ng/uL)	From (bp)	To (bp)	Average size (bp)	CV%	RFU	Corrected peak area
1	75 (LM)	0.5000	18	135	73	6.39	9367	49.701
2	14391	0.1935	8667	15488	11555	15.45	63	1.603
3	20000 (UM)	0.1763	18293	27979	20410	4.28	1966	17.524

TIC:	0.1935	ng/uL
TIM:	0.0276	nmole/L
Total concentration:	0.5863	ng/uL

```
Sample peak width (sec): 5    Sample min peak height: 50    Sample baseline V to V?: Y    Sample baseline V to V points: 3
Sample filter: Binomial       Number of points for filter: 3  Sample start region (min): 0  Sample end region (min): 30
Marker peak width (sec): 5    Marker min peak height: 200   Marker baseline V to V?: Y    Marker baseline V to V points: 3
Lower marker selection: First peak > 500 RFU                Upper marker selection: Last peak > 200 RFU
Ladder size (bp)75, 200, 300, 400, 500, 700, 1000, 1500, 2000, 3000, 4000, 5000, 7000, 10000, 20000
Quantification using: Lower Marker          Final concentration (ng/uL): 0.5000          Dilution factor: 12.0
```

Sample: SampH6
Well location: H6
Created: Tuesday, August 10, 2021 6:41:11 PM

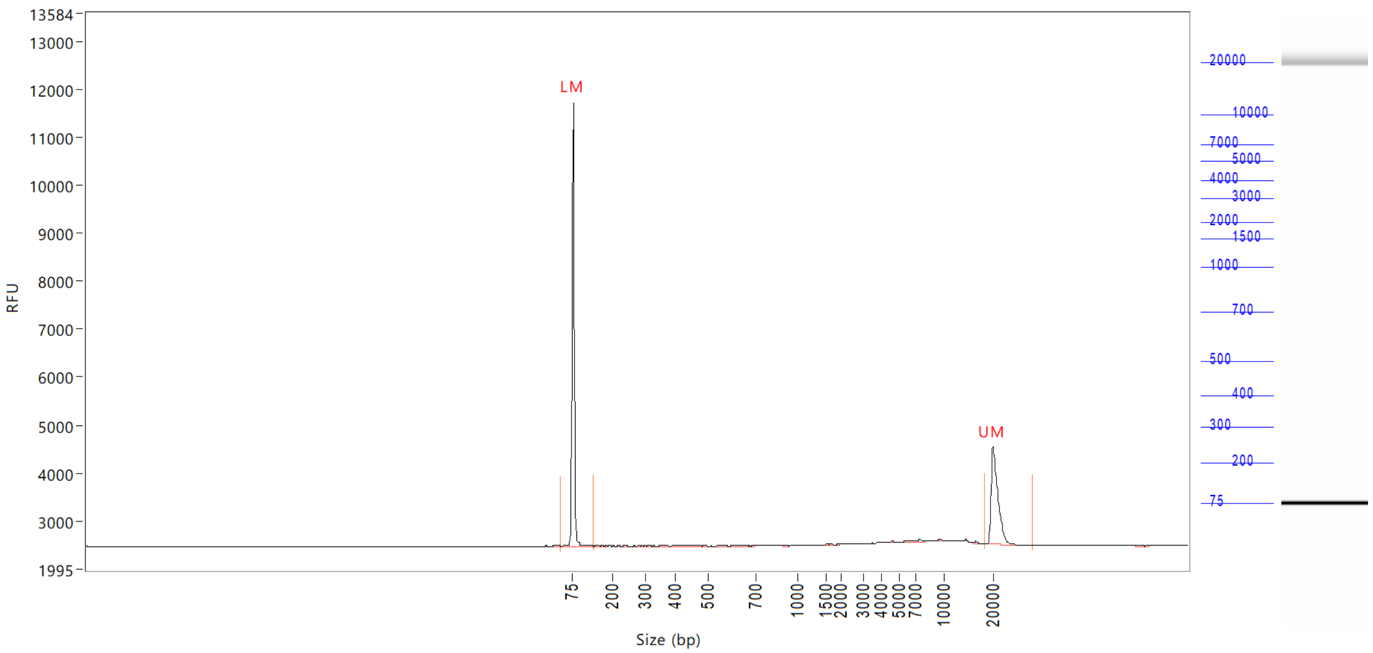

Peak	Size	Concentration	From	To	Average size	CV%	RFU	Corrected peak area
	(bp)	(ng/uL)	(bp)	(bp)	(bp)			
1	75 (LM)	0.5000	39	141	74	6.26	9255	49.389
2	20000 (UM)	0.1808	18415	27859	20401	4.18	2022	17.860

TIC:	0.0000	ng/uL
TIM:	0.0000	nmole/L
Total concentration:	0.4132	ng/uL

Sample peak width (sec): 5 Sample min peak height: 50 Sample baseline V to V?: Y Sample baseline V to V points: 3
Sample filter: Binomial Number of points for filter: 3 Sample start region (min): 0 Sample end region (min): 30
Marker peak width (sec): 5 Marker min peak height: 200 Marker baseline V to V?: Y Marker baseline V to V points: 3
Lower marker selection: First peak > 500 RFU Upper marker selection: Last peak > 200 RFU
Ladder size (bp)75, 200, 300, 400, 500, 700, 1000, 1500, 2000, 3000, 4000, 5000, 7000, 10000, 20000
Quantification using: Lower Marker Final concentration (ng/uL): 0.5000 Dilution factor: 12.0

Sample: SampA7
Well location: A7
Created: Tuesday, August 10, 2021 6:41:11 PM

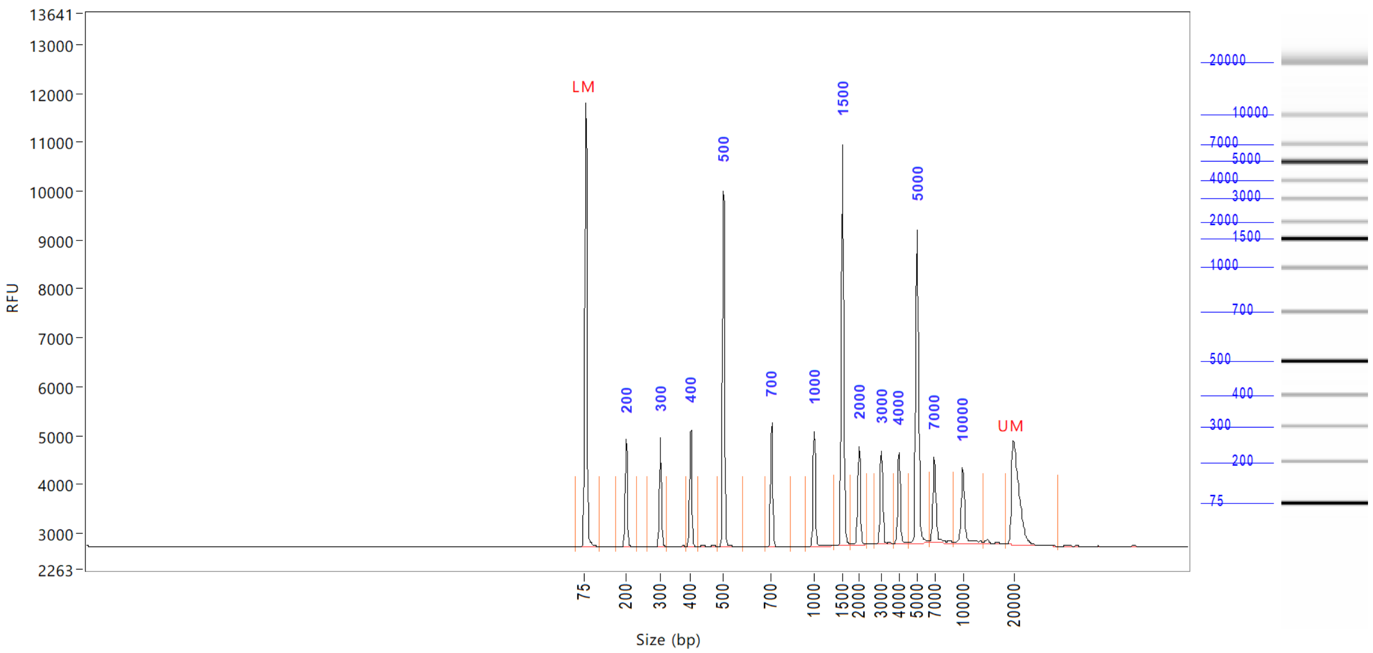

Peak	Size (bp)	Concentration (ng/uL)	From (bp)	To (bp)	Average size (bp)	CV%	RFU	Corrected peak area
1	75 (LM)	0.5000	43	118	73	5.62	9101	47.197
2	200	1.2867	168	232	198	1.92	2191	10.121
3	300	1.2338	264	321	298	1.07	2218	9.705
4	400	1.3064	386	420	397	0.82	2382	10.277
5	500	3.8588	481	580	499	1.05	7286	30.354
6	700	1.2716	674	835	698	1.44	2525	10.003
7	1000	1.2175	935	1334	997	2.50	2341	9.577
8	1500	4.0463	1334	1731	1489	1.76	8207	31.829
9	2000	1.0164	1731	2315	1973	2.53	2016	7.996
10	3000	0.9653	2715	3679	2986	2.91	1912	7.593
11	4000	0.9286	3679	4518	3965	2.17	1860	7.305
12	5000	3.6072	4518	6408	4986	3.39	6405	28.375
13	7000	1.0146	6408	9067	7022	5.63	1762	7.981
14	10000	0.9914	9067	14147	10207	8.16	1543	7.799
15	20000 (UM)	0.2098	18537	28947	20553	4.39	2136	19.806

TIC:	22.7448	ng/uL
TIM:	48.4700	nmole/L
Total concentration:	22.9450	ng/uL

```
Sample peak width (sec): 5    Sample min peak height: 50    Sample baseline V to V?: Y    Sample baseline V to V points: 3
Sample filter: Binomial       Number of points for filter: 3  Sample start region (min): 0  Sample end region (min): 30
Marker peak width (sec): 5    Marker min peak height: 200   Marker baseline V to V?: Y    Marker baseline V to V points: 3
Lower marker selection: First peak > 500 RFU                Upper marker selection: Last peak > 200 RFU
Ladder size (bp)75, 200, 300, 400, 500, 700, 1000, 1500, 2000, 3000, 4000, 5000, 7000, 10000, 20000
Quantification using: Lower Marker          Final concentration (ng/uL): 0.5000          Dilution factor: 12.0
```

Sample: SampB7
Well location: B7
Created: Tuesday, August 10, 2021 6:41:11 PM

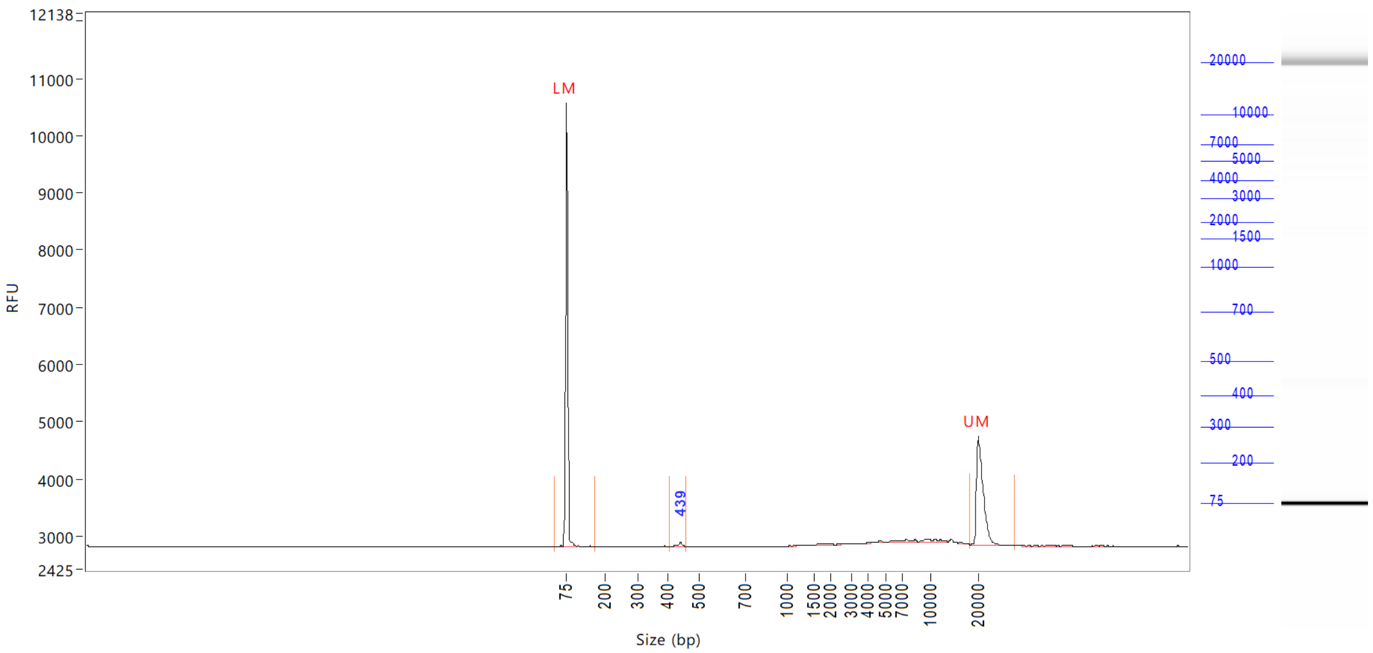

Peak	Size	Concentration	From	To	Average size	CV%	RFU	Corrected peak area
	(bp)	(ng/uL)	(bp)	(bp)	(bp)			
1	75 (LM)	0.5000	35	166	73	7.34	7763	42.367
2	439	0.0918	403	460	433	2.36	72	0.648
3	20000 (UM)	0.1963	18293	27496	20423	4.34	1895	16.637

TIC:	0.0918	ng/uL
TIM:	0.3488	nmole/L
Total concentration:	0.6507	ng/uL

Sample peak width (sec): 5 Sample min peak height: 50 Sample baseline V to V?: Y Sample baseline V to V points: 3
Sample filter: Binomial Number of points for filter: 3 Sample start region (min): 0 Sample end region (min): 30
Marker peak width (sec): 5 Marker min peak height: 200 Marker baseline V to V?: Y Marker baseline V to V points: 3
Lower marker selection: First peak > 500 RFU Upper marker selection: Last peak > 200 RFU
Ladder size (bp)75, 200, 300, 400, 500, 700, 1000, 1500, 2000, 3000, 4000, 5000, 7000, 10000, 20000
Quantification using: Lower Marker Final concentration (ng/uL): 0.5000 Dilution factor: 12.0

Sample: SampC7
Well location: C7
Created: Tuesday, August 10, 2021 6:41:11 PM

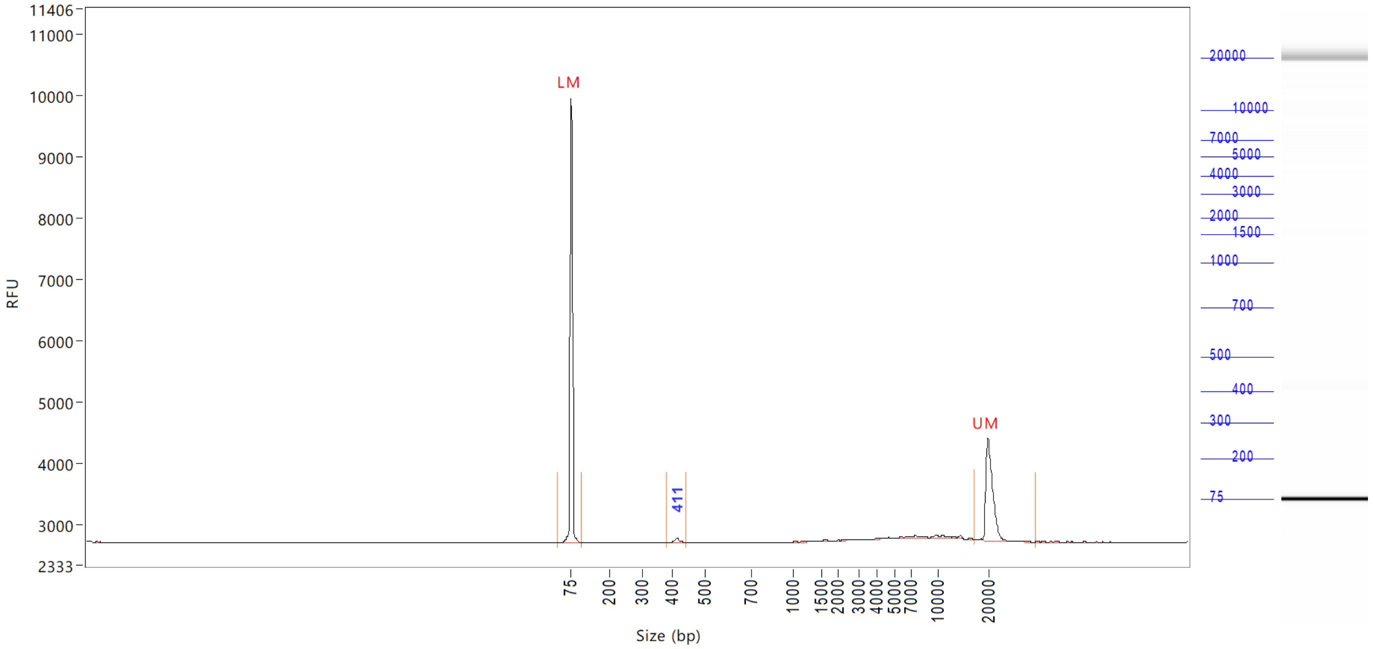

Peak	Size<br>(bp)	Concentration<br>(ng/uL)	From<br>(bp)	To<br>(bp)	Average size<br>(bp)	CV%	RFU	Corrected peak area
1	75 (LM)	0.5000	33	107	73	5.69	7257	39.446
2	411	0.1063	378	439	408	2.63	76	0.699
3	20000 (UM)	0.1838	17317	29551	20457	4.67	1677	14.503

TIC:	0.1063	ng/uL
TIM:	0.4286	nmole/L
Total concentration:	0.6435	ng/uL

Sample peak width (sec): 5   Sample min peak height: 50   Sample baseline V to V?: Y   Sample baseline V to V points: 3
Sample filter: Binomial   Number of points for filter: 3 Sample start region (min): 0 Sample end region (min): 30
Marker peak width (sec): 5   Marker min peak height: 200   Marker baseline V to V?: Y   Marker baseline V to V points: 3
Lower marker selection: First peak > 500 RFU   Upper marker selection: Last peak > 200 RFU
Ladder size (bp)75, 200, 300, 400, 500, 700, 1000, 1500, 2000, 3000, 4000, 5000, 7000, 10000, 20000
Quantification using: Lower Marker   Final concentration (ng/uL): 0.5000   Dilution factor: 12.0

Sample: SampD7
Well location: D7
Created: Tuesday, August 10, 2021 6:41:11 PM

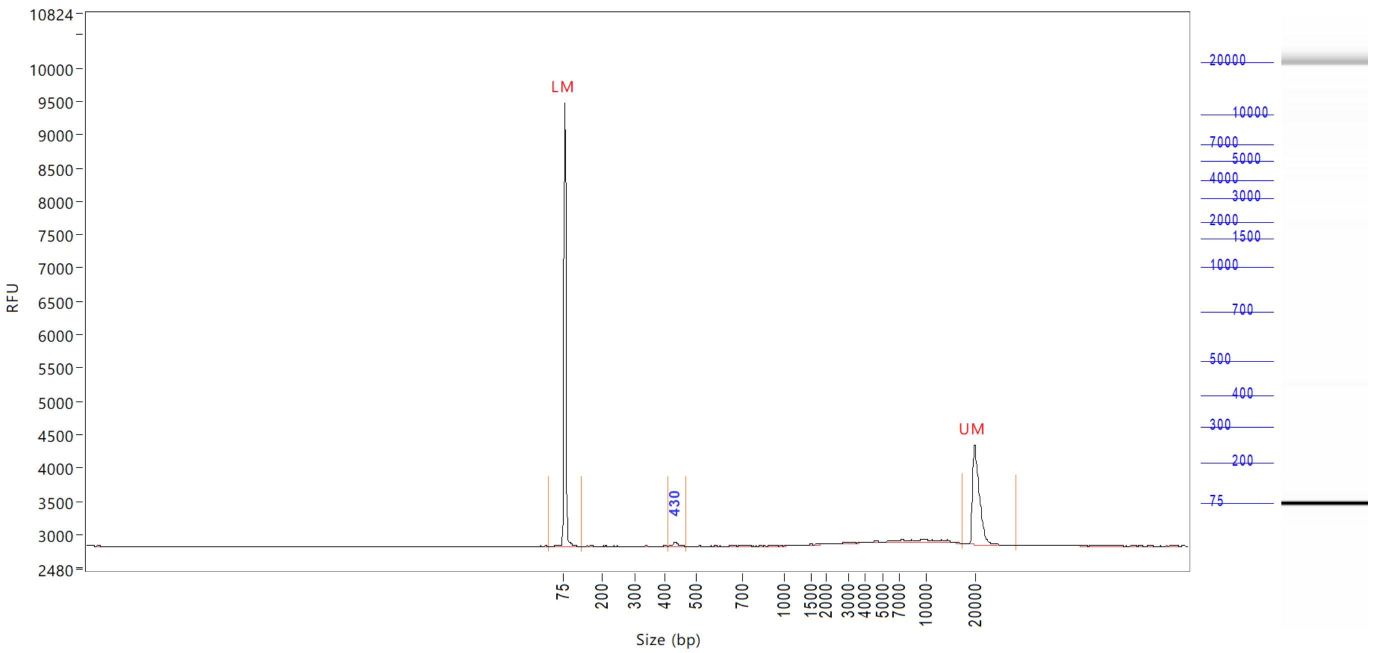

Peak	Size<br>(bp)	Concentration<br>(ng/uL)	From<br>(bp)	To<br>(bp)	Average size<br>(bp)	CV%	RFU	Corrected peak area
1	75 (LM)	0.5000	24	128	73	7.13	6671	36.242
2	430	0.1048	407	466	431	2.57	60	0.633
3	20000 (UM)	0.1827	17561	28584	20562	4.81	1493	13.244

TIC:	0.1048	ng/uL
TIM:	0.3999	nmole/L
Total concentration:	0.7841	ng/uL

Sample peak width (sec): 5 Sample min peak height: 50 Sample baseline V to V?: Y Sample baseline V to V points: 3
Sample filter: Binomial Number of points for filter: 3 Sample start region (min): 0 Sample end region (min): 30
Marker peak width (sec): 5 Marker min peak height: 200 Marker baseline V to V?: Y Marker baseline V to V points: 3
Lower marker selection: First peak > 500 RFU Upper marker selection: Last peak > 200 RFU
Ladder size (bp)75, 200, 300, 400, 500, 700, 1000, 1500, 2000, 3000, 4000, 5000, 7000, 10000, 20000
Quantification using: Lower Marker Final concentration (ng/uL): 0.5000 Dilution factor: 12.0

Sample: SampE7
Well location: E7
Created: Tuesday, August 10, 2021 6:41:11 PM

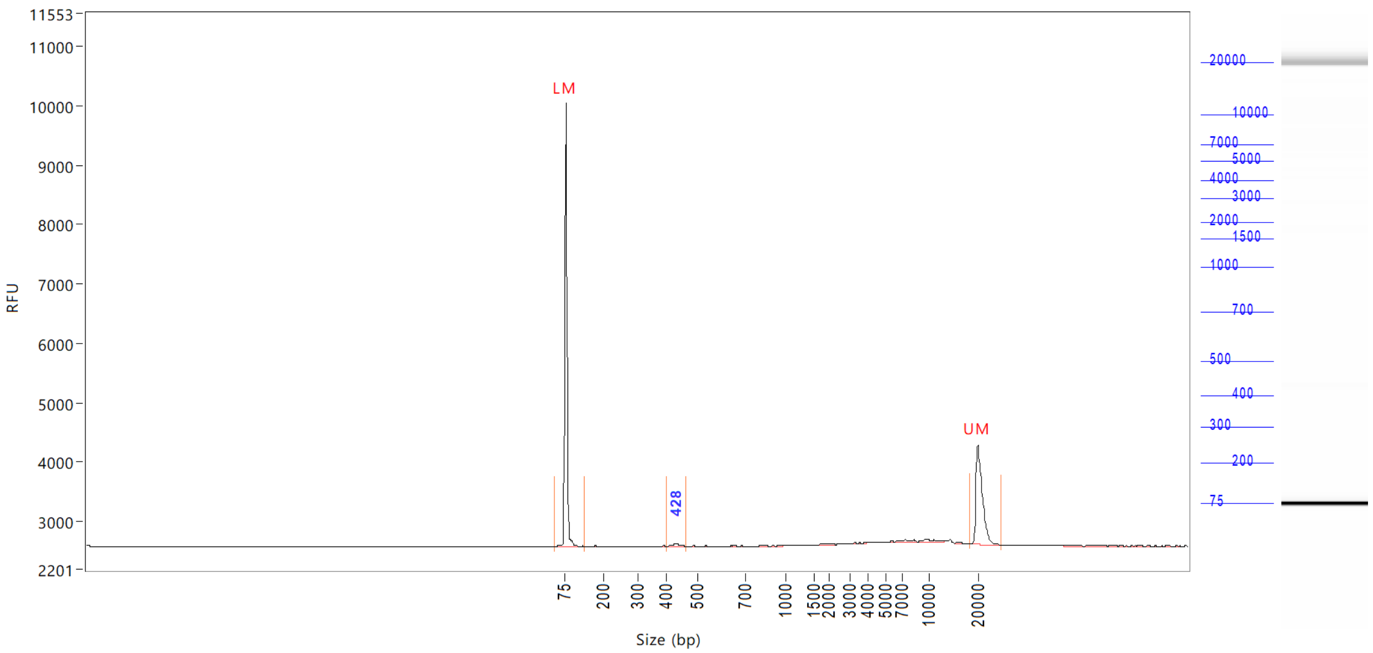

Peak	Size (bp)	Concentration (ng/uL)	From (bp)	To (bp)	Average size (bp)	CV%	RFU	Corrected peak area
1	75 (LM)	0.5000	37	133	73	6.66	7474	41.839
2	428	0.0800	398	462	426	2.85	57	0.558
3	20000 (UM)	0.1694	18293	24594	20363	3.82	1668	14.175

TIC:	0.0800	ng/uL
TIM:	0.3092	nmole/L
Total concentration:	0.5986	ng/uL

Sample peak width (sec): 5 Sample min peak height: 50 Sample baseline V to V?: Y Sample baseline V to V points: 3
Sample filter: Binomial Number of points for filter: 3 Sample start region (min): 0 Sample end region (min): 30
Marker peak width (sec): 5 Marker min peak height: 200 Marker baseline V to V?: Y Marker baseline V to V points: 3
Lower marker selection: First peak > 500 RFU Upper marker selection: Last peak > 200 RFU
Ladder size (bp)75, 200, 300, 400, 500, 700, 1000, 1500, 2000, 3000, 4000, 5000, 7000, 10000, 20000
Quantification using: Lower Marker Final concentration (ng/uL): 0.5000 Dilution factor: 12.0

Sample: SampF7
Well location: F7
Created: Tuesday, August 10, 2021 6:41:11 PM

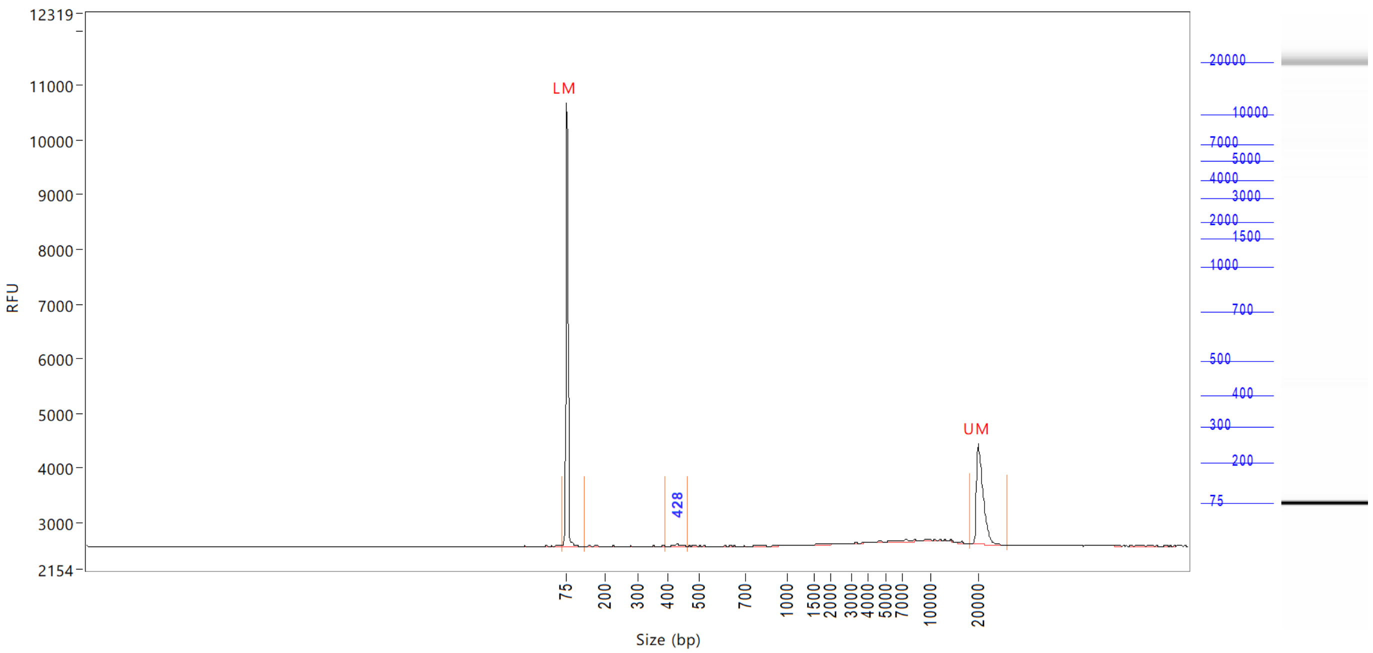

Peak	Size<br>(bp)	Concentration<br>(ng/uL)	From<br>(bp)	To<br>(bp)	Average size<br>(bp)	CV%	RFU	Corrected peak area
1	75 (LM)	0.5000	62	133	74	5.34	8120	43.516
2	428	0.0783	392	462	430	2.99	51	0.568
3	20000 (UM)	0.1859	18293	25924	20398	4.04	1834	16.180

TIC:	0.0783	ng/uL
TIM:	0.2997	nmole/L
Total concentration:	0.6131	ng/uL

Sample peak width (sec): 5 Sample min peak height: 50 Sample baseline V to V?: Y Sample baseline V to V points: 3
Sample filter: Binomial Number of points for filter: 3 Sample start region (min): 0 Sample end region (min): 30
Marker peak width (sec): 5 Marker min peak height: 200 Marker baseline V to V?: Y Marker baseline V to V points: 3
Lower marker selection: First peak > 500 RFU Upper marker selection: Last peak > 200 RFU
Ladder size (bp)75, 200, 300, 400, 500, 700, 1000, 1500, 2000, 3000, 4000, 5000, 7000, 10000, 20000
Quantification using: Lower Marker Final concentration (ng/uL): 0.5000 Dilution factor: 12.0

Sample: SampG7
Well location: G7
Created: Tuesday, August 10, 2021 6:41:11 PM

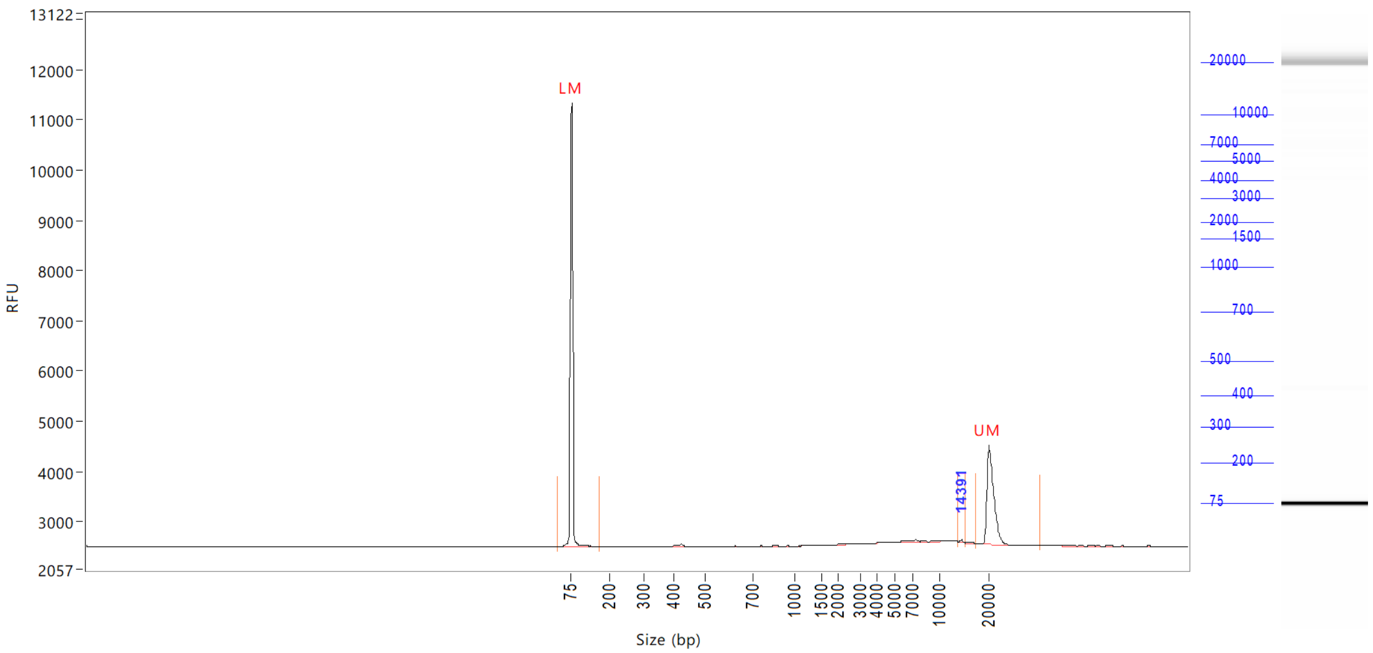

Peak	Size (bp)	Concentration (ng/uL)	From (bp)	To (bp)	Average size (bp)	CV%	RFU	Corrected peak area
1	75 (LM)	0.5000	31	166	73	8.90	8843	48.453
2	14391	0.0222	13781	15122	14305	1.71	51	0.179
3	20000 (UM)	0.1785	17317	30277	20433	4.69	1986	17.300

TIC:	0.0222	ng/uL
TIM:	0.0026	nmole/L
Total concentration:	0.4433	ng/uL

Sample peak width (sec): 5   Sample min peak height: 50   Sample baseline V to V?: Y   Sample baseline V to V points: 3
Sample filter: Binomial   Number of points for filter: 3 Sample start region (min): 0 Sample end region (min): 30
Marker peak width (sec): 5   Marker min peak height: 200   Marker baseline V to V?: Y   Marker baseline V to V points: 3
Lower marker selection: First peak > 500 RFU   Upper marker selection: Last peak > 200 RFU
Ladder size (bp)75, 200, 300, 400, 500, 700, 1000, 1500, 2000, 3000, 4000, 5000, 7000, 10000, 20000
Quantification using: Lower Marker   Final concentration (ng/uL): 0.5000   Dilution factor: 12.0

Sample: SampH7
Well location: H7
Created: Tuesday, August 10, 2021 6:41:11 PM

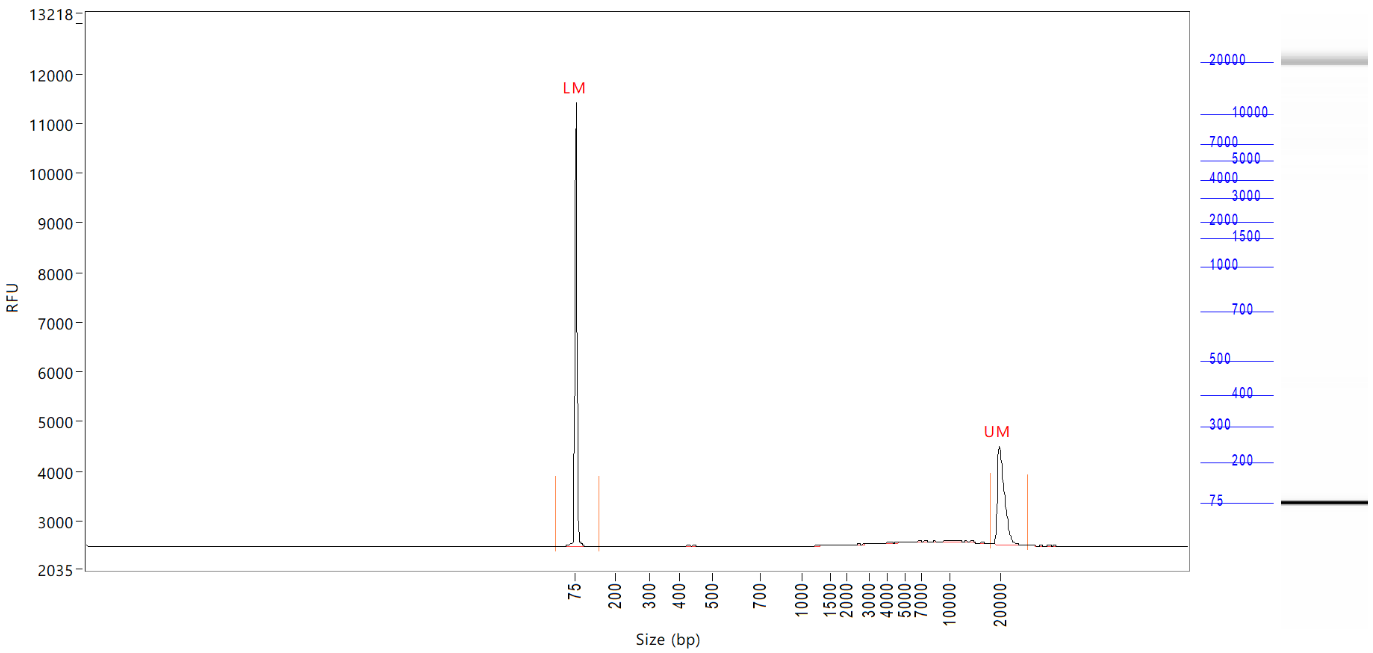

Peak	Size (bp)	Concentration (ng/uL)	From (bp)	To (bp)	Average size (bp)	CV%	RFU	Corrected peak area
1	75 (LM)	0.5000	16	148	73	6.88	8941	47.808
2	20000 (UM)	0.1814	18171	25561	20370	3.91	1970	17.343

TIC:	0.0000	ng/uL
TIM:	0.0000	nmole/L
Total concentration:	0.4908	ng/uL

Sample peak width (sec): 5 Sample min peak height: 50 Sample baseline V to V?: Y Sample baseline V to V points: 3
Sample filter: Binomial Number of points for filter: 3 Sample start region (min): 0 Sample end region (min): 30
Marker peak width (sec): 5 Marker min peak height: 200 Marker baseline V to V?: Y Marker baseline V to V points: 3
Lower marker selection: First peak > 500 RFU Upper marker selection: Last peak > 200 RFU
Ladder size (bp)75, 200, 300, 400, 500, 700, 1000, 1500, 2000, 3000, 4000, 5000, 7000, 10000, 20000
Quantification using: Lower Marker Final concentration (ng/uL): 0.5000 Dilution factor: 12.0

Sample: SampA8
Well location: A8
Created: Tuesday, August 10, 2021 6:41:11 PM

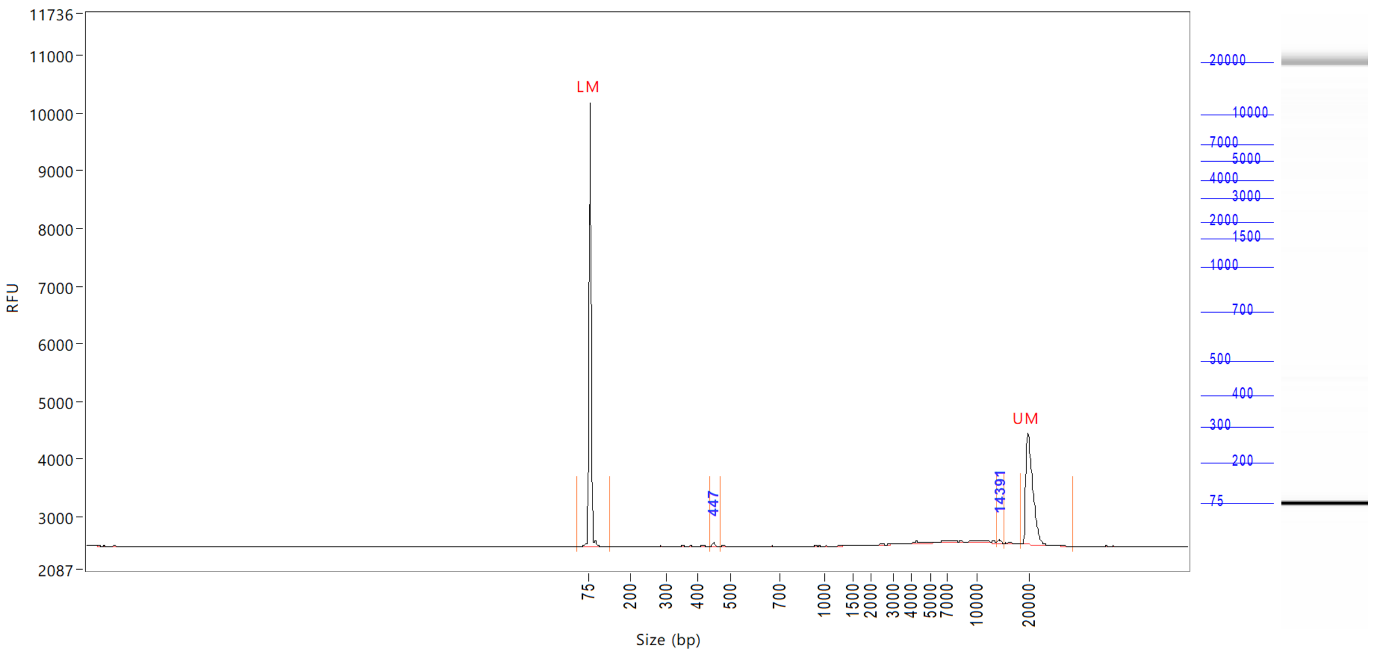

Peak	Size (bp)	Concentration (ng/uL)	From (bp)	To (bp)	Average size (bp)	CV%	RFU	Corrected peak area
1	75 (LM)	0.5000	35	137	73	6.48	7719	40.289
2	447	0.0653	435	468	447	1.45	76	0.438
3	14391	0.0408	13781	15244	14319	2.25	58	0.274
4	20000 (UM)	0.2028	18537	28705	20366	3.90	1938	16.338

TIC:	0.1061	ng/uL
TIM:	0.2450	nmole/L
Total concentration:	0.8319	ng/uL

Sample peak width (sec): 5   Sample min peak height: 50   Sample baseline V to V?: Y   Sample baseline V to V points: 3
Sample filter: Binomial   Number of points for filter: 3   Sample start region (min): 0   Sample end region (min): 30
Marker peak width (sec): 5   Marker min peak height: 200   Marker baseline V to V?: Y   Marker baseline V to V points: 3
Lower marker selection: First peak > 500 RFU   Upper marker selection: Last peak > 200 RFU
Ladder size (bp)75, 200, 300, 400, 500, 700, 1000, 1500, 2000, 3000, 4000, 5000, 7000, 10000, 20000
Quantification using: Lower Marker   Final concentration (ng/uL): 0.5000   Dilution factor: 12.0

Sample: SampB8
Well location: B8
Created: Tuesday, August 10, 2021 6:41:11 PM

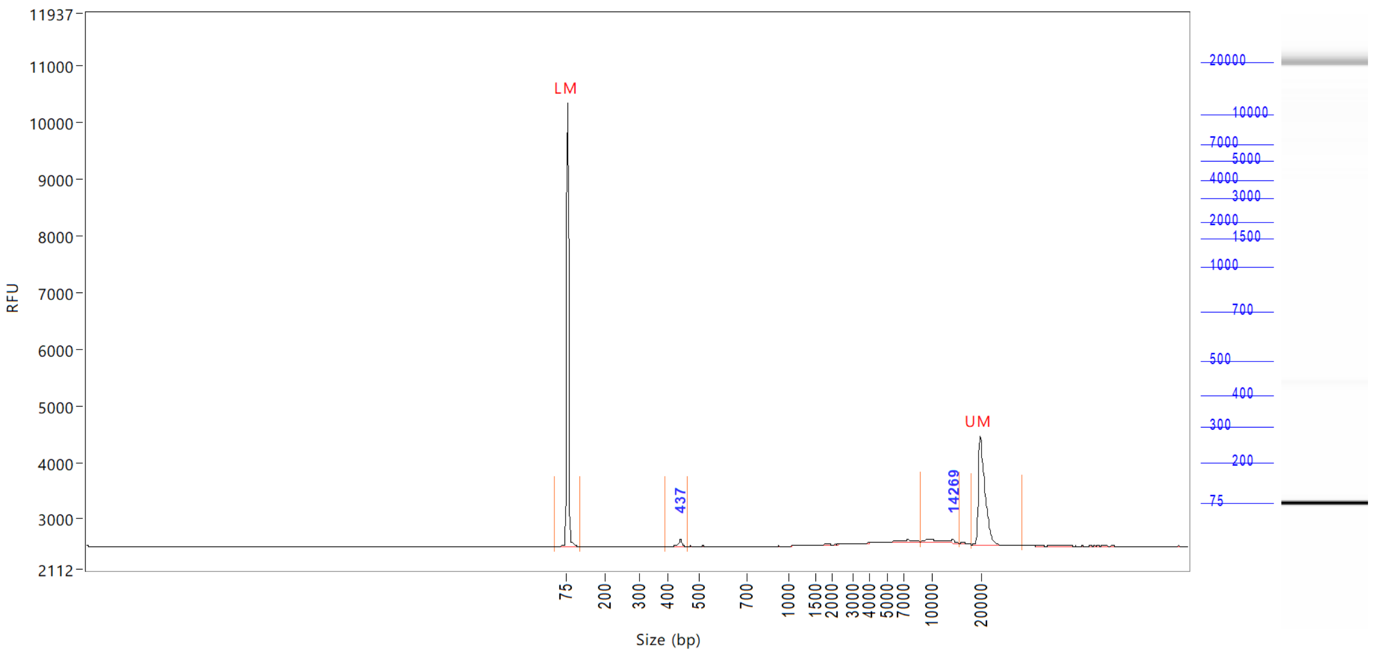

Peak	Size (bp)	Concentration (ng/uL)	From (bp)	To (bp)	Average size (bp)	CV%	RFU	Corrected peak area
1	75 (LM)	0.5000	37	118	74	4.98	7853	42.191
2	437	0.1382	390	458	431	2.20	132	0.972
3	14269	0.2444	8800	15610	11430	15.73	61	1.718
4	20000 (UM)	0.1963	18293	28463	20423	4.21	1912	16.561

TIC:	0.3826	ng/uL
TIM:	0.5629	nmole/L
Total concentration:	0.7825	ng/uL

Sample peak width (sec): 5   Sample min peak height: 50   Sample baseline V to V?: Y   Sample baseline V to V points: 3
Sample filter: Binomial   Number of points for filter: 3 Sample start region (min): 0 Sample end region (min): 30
Marker peak width (sec): 5   Marker min peak height: 200   Marker baseline V to V?: Y   Marker baseline V to V points: 3
Lower marker selection: First peak > 500 RFU   Upper marker selection: Last peak > 200 RFU
Ladder size (bp)75, 200, 300, 400, 500, 700, 1000, 1500, 2000, 3000, 4000, 5000, 7000, 10000, 20000
Quantification using: Lower Marker   Final concentration (ng/uL): 0.5000   Dilution factor: 12.0

Sample: SampC8
Well location: C8
Created: Tuesday, August 10, 2021 6:41:11 PM

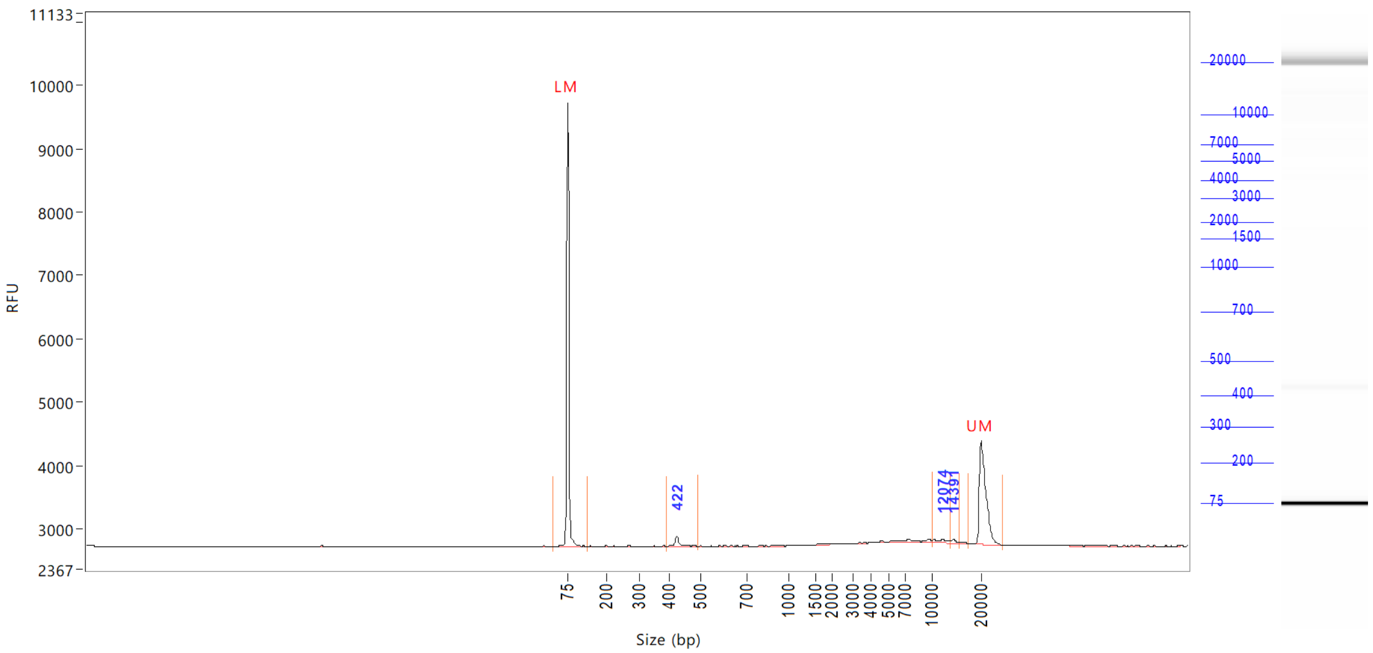

Peak	Size (bp)	Concentration (ng/uL)	From (bp)	To (bp)	Average size (bp)	CV%	RFU	Corrected peak area
1	75 (LM)	0.5000	30	137	73	6.94	7004	38.435
2	422	0.1974	392	490	423	3.19	159	1.265
3	12074	0.1378	10000	13781	11813	9.58	51	0.882
4	14391	0.0592	13781	15732	14480	3.41	62	0.379
5	20000 (UM)	0.1835	17439	24473	20453	3.85	1631	14.105

TIC:	0.3944	ng/uL
TIM:	0.7939	nmole/L
Total concentration:	0.8105	ng/uL

Sample peak width (sec): 5 Sample min peak height: 50 Sample baseline V to V?: Y Sample baseline V to V points: 3
Sample filter: Binomial Number of points for filter: 3 Sample start region (min): 0 Sample end region (min): 30
Marker peak width (sec): 5 Marker min peak height: 200 Marker baseline V to V?: Y Marker baseline V to V points: 3
Lower marker selection: First peak > 500 RFU Upper marker selection: Last peak > 200 RFU
Ladder size (bp)75, 200, 300, 400, 500, 700, 1000, 1500, 2000, 3000, 4000, 5000, 7000, 10000, 20000
Quantification using: Lower Marker Final concentration (ng/uL): 0.5000 Dilution factor: 12.0

Sample: SampD8
Well location: D8
Created: Tuesday, August 10, 2021 6:41:11 PM

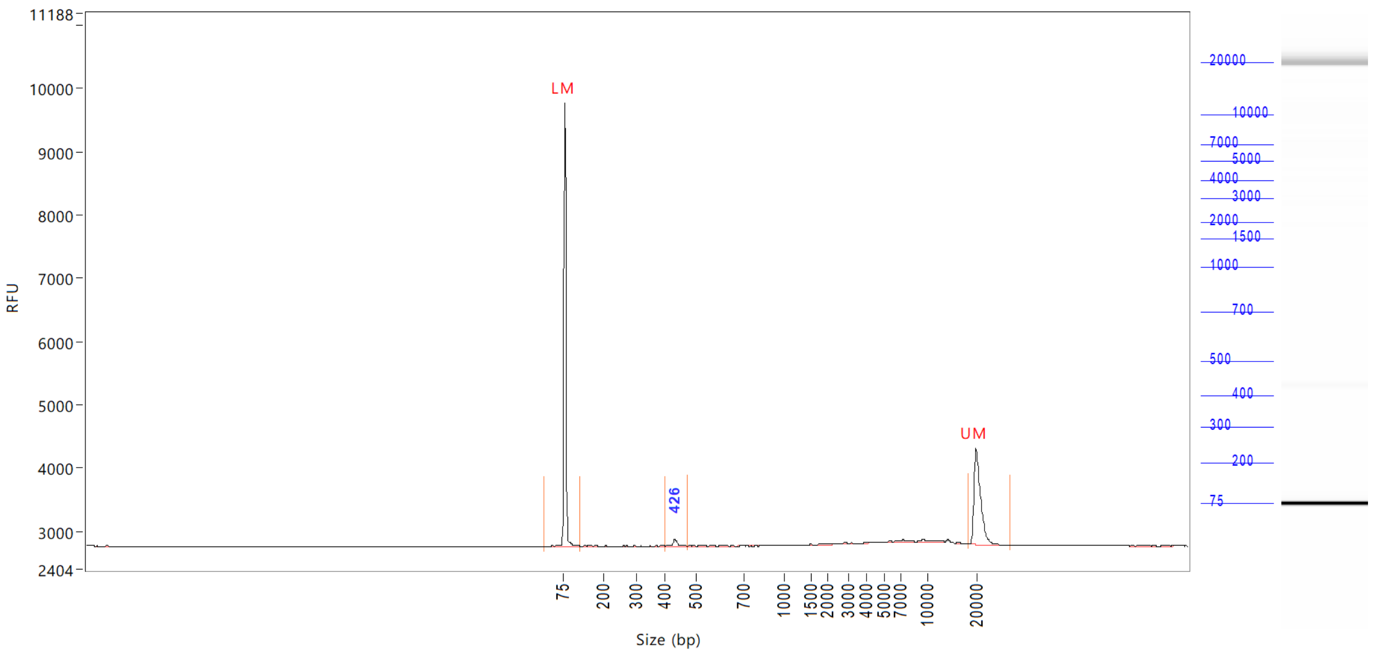

Peak	Size (bp)	Concentration (ng/uL)	From (bp)	To (bp)	Average size (bp)	CV%	RFU	Corrected peak area
1	75 (LM)	0.5000	9	126	73	7.27	7019	37.737
2	426	0.1581	398	468	427	2.53	115	0.994
3	20000 (UM)	0.1759	18293	27012	20396	4.40	1533	13.274

TIC:	0.1581	ng/uL
TIM:	0.6092	nmole/L
Total concentration:	0.6601	ng/uL

Sample peak width (sec): 5 Sample min peak height: 50 Sample baseline V to V?: Y Sample baseline V to V points: 3
Sample filter: Binomial Number of points for filter: 3 Sample start region (min): 0 Sample end region (min): 30
Marker peak width (sec): 5 Marker min peak height: 200 Marker baseline V to V?: Y Marker baseline V to V points: 3
Lower marker selection: First peak > 500 RFU Upper marker selection: Last peak > 200 RFU
Ladder size (bp)75, 200, 300, 400, 500, 700, 1000, 1500, 2000, 3000, 4000, 5000, 7000, 10000, 20000
Quantification using: Lower Marker Final concentration (ng/uL): 0.5000 Dilution factor: 12.0

Sample: SampE8
Well location: E8
Created: Tuesday, August 10, 2021 6:41:11 PM

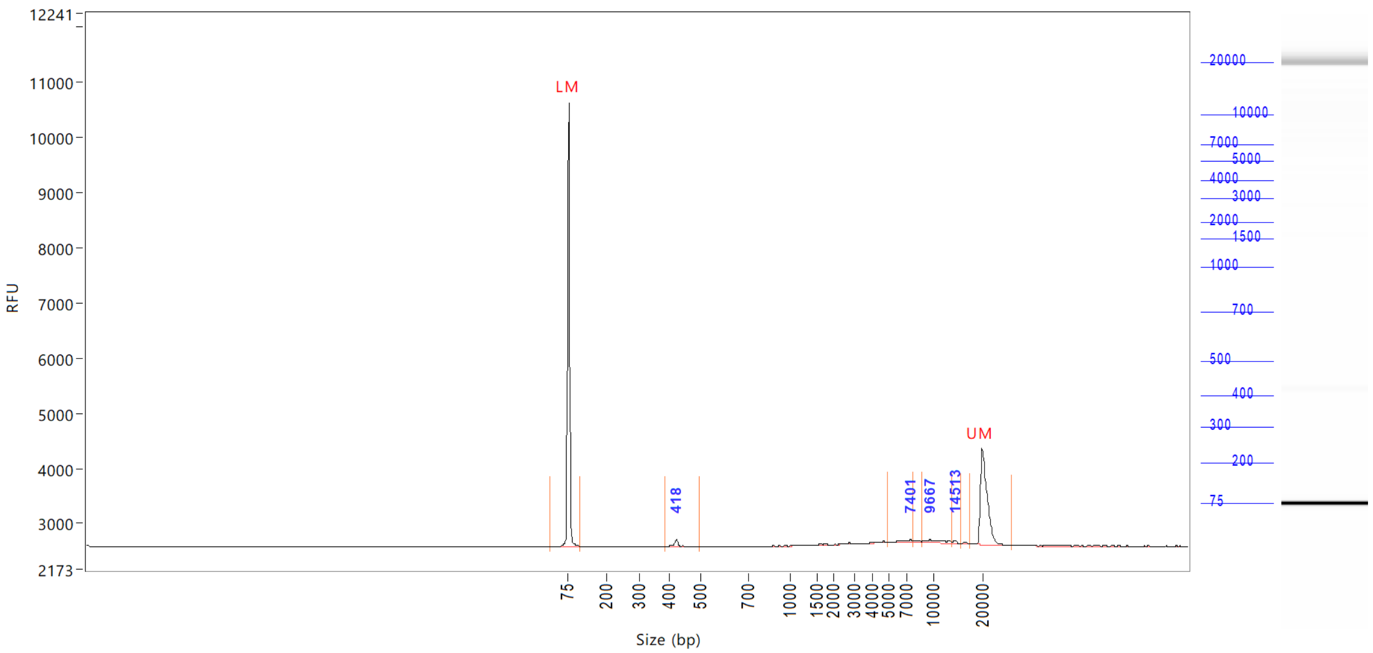

Peak	Size<br>(bp)	Concentration<br>(ng/uL)	From<br>(bp)	To<br>(bp)	Average size<br>(bp)	CV%	RFU	Corrected peak area
1	75 (LM)	0.5000	18	114	73	6.20	8049	42.805
2	418	0.1364	382	492	421	4.55	119	0.973
3	7401	0.1125	4897	7801	6654	11.52	51	0.802
4	9667	0.1943	8734	13659	10789	12.77	53	1.386
5	14513	0.0399	13659	15610	14274	2.33	57	0.285
6	20000 (UM)	0.1781	17561	26045	20456	4.09	1744	15.245

TIC:	0.4831	ng/uL
TIM:	0.5952	nmole/L
Total concentration:	0.7895	ng/uL

```
Sample peak width (sec): 5    Sample min peak height: 50    Sample baseline V to V?: Y    Sample baseline V to V points: 3
Sample filter: Binomial       Number of points for filter: 3  Sample start region (min): 0  Sample end region (min): 30
Marker peak width (sec): 5    Marker min peak height: 200   Marker baseline V to V?: Y    Marker baseline V to V points: 3
Lower marker selection: First peak > 500 RFU                Upper marker selection: Last peak > 200 RFU
Ladder size (bp)75, 200, 300, 400, 500, 700, 1000, 1500, 2000, 3000, 4000, 5000, 7000, 10000, 20000
Quantification using: Lower Marker          Final concentration (ng/uL): 0.5000          Dilution factor: 12.0
```

Sample: SampF8
Well location: F8
Created: Tuesday, August 10, 2021 6:41:11 PM

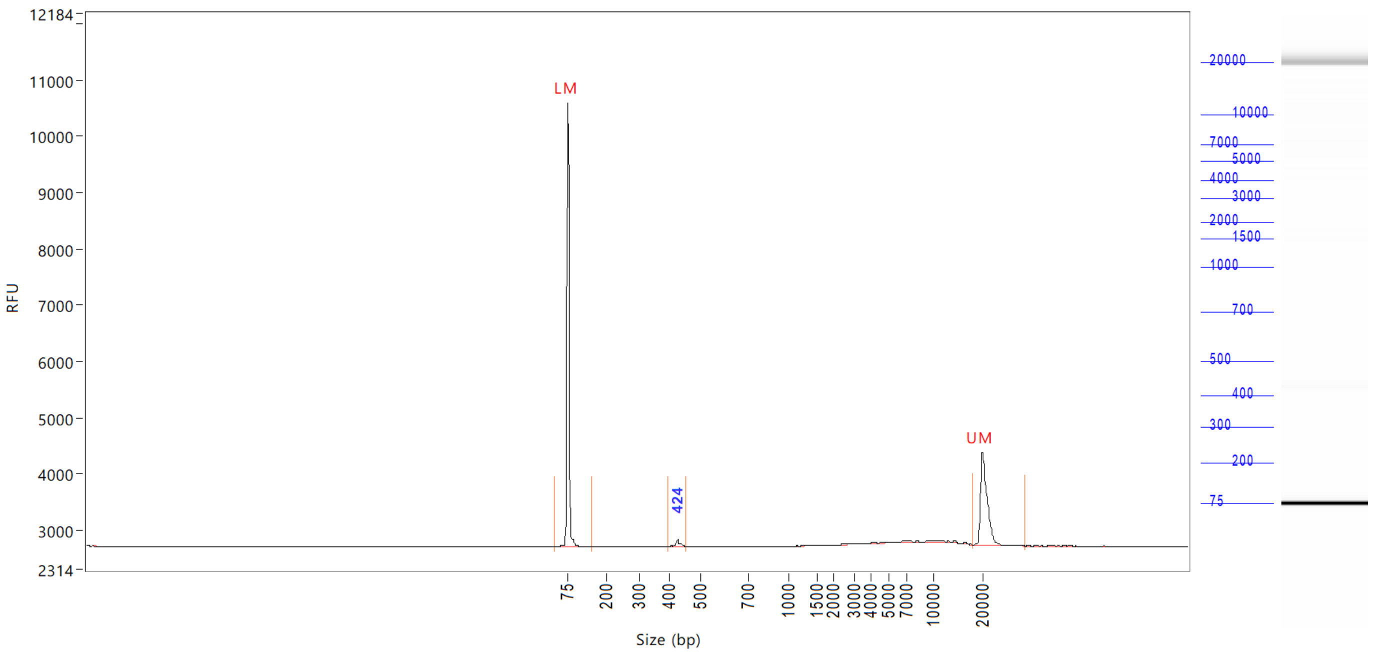

Peak	Size (bp)	Concentration (ng/uL)	From (bp)	To (bp)	Average size (bp)	CV%	RFU	Corrected peak area
1	75 (LM)	0.5000	31	154	73	7.86	7893	42.933
2	424	0.1358	392	452	423	2.36	117	0.972
3	20000 (UM)	0.1648	18293	28826	20485	4.44	1646	14.146

TIC:	0.1358	ng/uL
TIM:	0.5284	nmole/L
Total concentration:	0.5770	ng/uL

Sample peak width (sec): 5 Sample min peak height: 50 Sample baseline V to V?: Y Sample baseline V to V points: 3
Sample filter: Binomial Number of points for filter: 3 Sample start region (min): 0 Sample end region (min): 30
Marker peak width (sec): 5 Marker min peak height: 200 Marker baseline V to V?: Y Marker baseline V to V points: 3
Lower marker selection: First peak > 500 RFU Upper marker selection: Last peak > 200 RFU
Ladder size (bp)75, 200, 300, 400, 500, 700, 1000, 1500, 2000, 3000, 4000, 5000, 7000, 10000, 20000
Quantification using: Lower Marker Final concentration (ng/uL): 0.5000 Dilution factor: 12.0

Sample: SampG8
Well location: G8
Created: Tuesday, August 10, 2021 6:41:11 PM

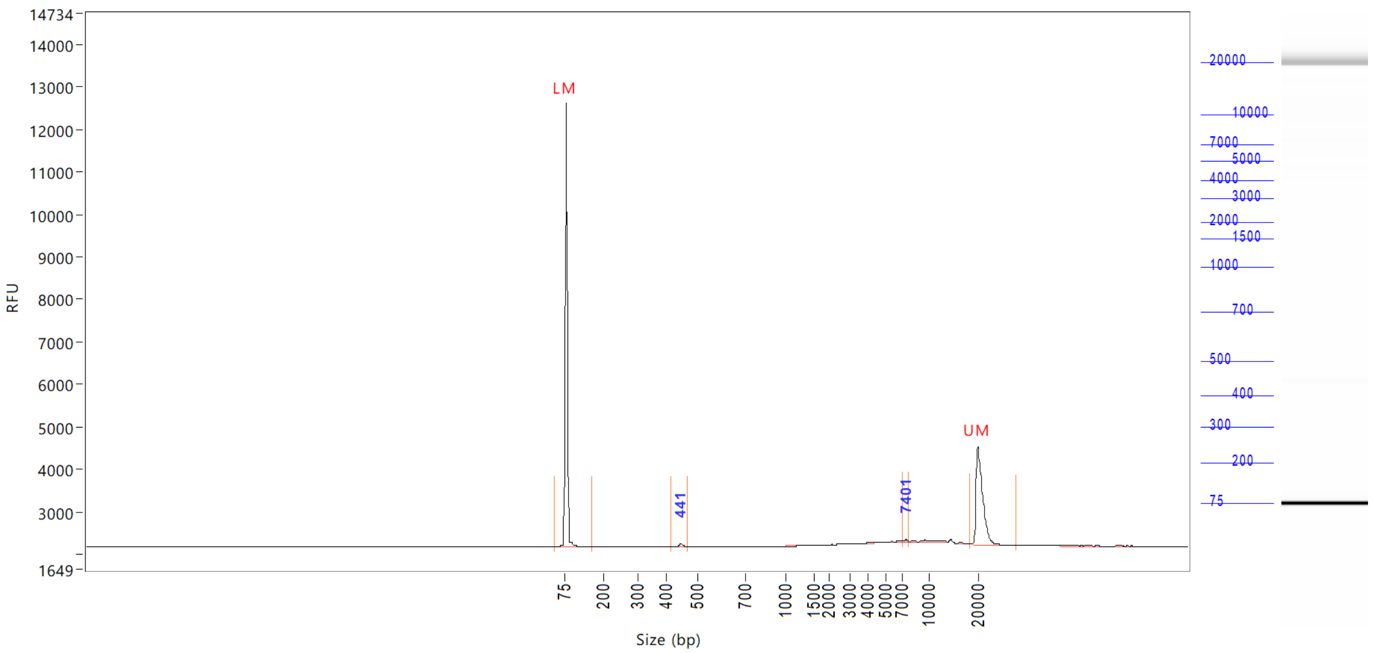

Peak	Size (bp)	Concentration (ng/uL)	From (bp)	To (bp)	Average size (bp)	CV%	RFU	Corrected peak area
1	75 (LM)	0.5000	41	162	73	7.07	10458	55.282
2	441	0.0533	412	464	439	1.90	67	0.491
3	7401	0.0308	7001	7801	7340	2.71	52	0.284
4	20000 (UM)	0.1827	18537	27859	20459	4.36	2303	20.203

TIC:	0.0841	ng/uL
TIM:	0.2068	nmole/L
Total concentration:	0.4200	ng/uL

```
Sample peak width (sec): 5    Sample min peak height: 50    Sample baseline V to V?: Y    Sample baseline V to V points: 3
Sample filter: Binomial       Number of points for filter: 3  Sample start region (min): 0  Sample end region (min): 30
Marker peak width (sec): 5    Marker min peak height: 200   Marker baseline V to V?: Y    Marker baseline V to V points: 3
Lower marker selection: First peak > 500 RFU                Upper marker selection: Last peak > 200 RFU
Ladder size (bp)75, 200, 300, 400, 500, 700, 1000, 1500, 2000, 3000, 4000, 5000, 7000, 10000, 20000
Quantification using: Lower Marker          Final concentration (ng/uL): 0.5000          Dilution factor: 12.0
```

Sample: SampH8
Well location: H8
Created: Tuesday, August 10, 2021 6:41:11 PM

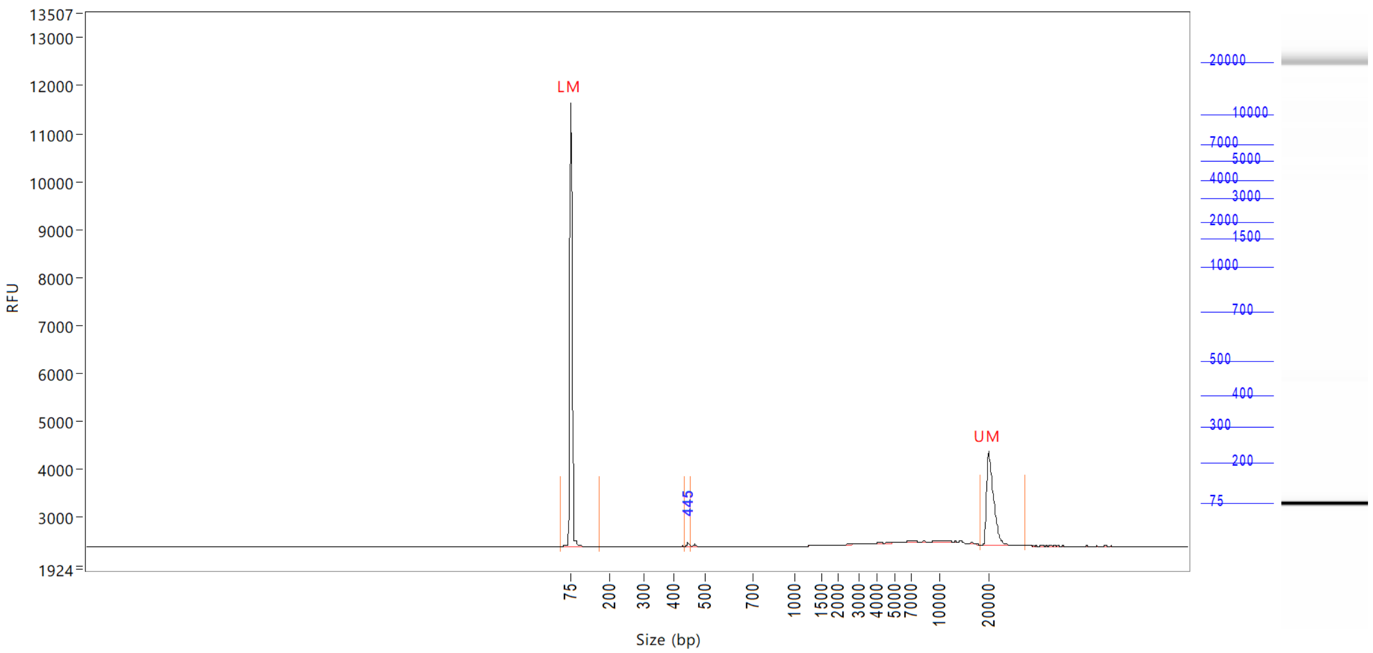

Peak	Size (bp)	Concentration (ng/uL)	From (bp)	To (bp)	Average size (bp)	CV%	RFU	Corrected peak area
1	75 (LM)	0.5000	45	166	73	7.86	9261	50.144
2	445	0.0475	433	456	444	1.01	74	0.397
3	20000 (UM)	0.1736	18293	27617	20498	4.22	1965	17.412

TIC:	0.0475	ng/uL
TIM:	0.1759	nmole/L
Total concentration:	0.4808	ng/uL

```
Sample peak width (sec): 5    Sample min peak height: 50    Sample baseline V to V?: Y    Sample baseline V to V points: 3
Sample filter: Binomial       Number of points for filter: 3  Sample start region (min): 0  Sample end region (min): 30
Marker peak width (sec): 5    Marker min peak height: 200   Marker baseline V to V?: Y    Marker baseline V to V points: 3
Lower marker selection: First peak > 500 RFU                Upper marker selection: Last peak > 200 RFU
Ladder size (bp)75, 200, 300, 400, 500, 700, 1000, 1500, 2000, 3000, 4000, 5000, 7000, 10000, 20000
Quantification using: Lower Marker          Final concentration (ng/uL): 0.5000          Dilution factor: 12.0
```

Sample: SampA9
Well location: A9
Created: Tuesday, August 10, 2021 6:41:11 PM

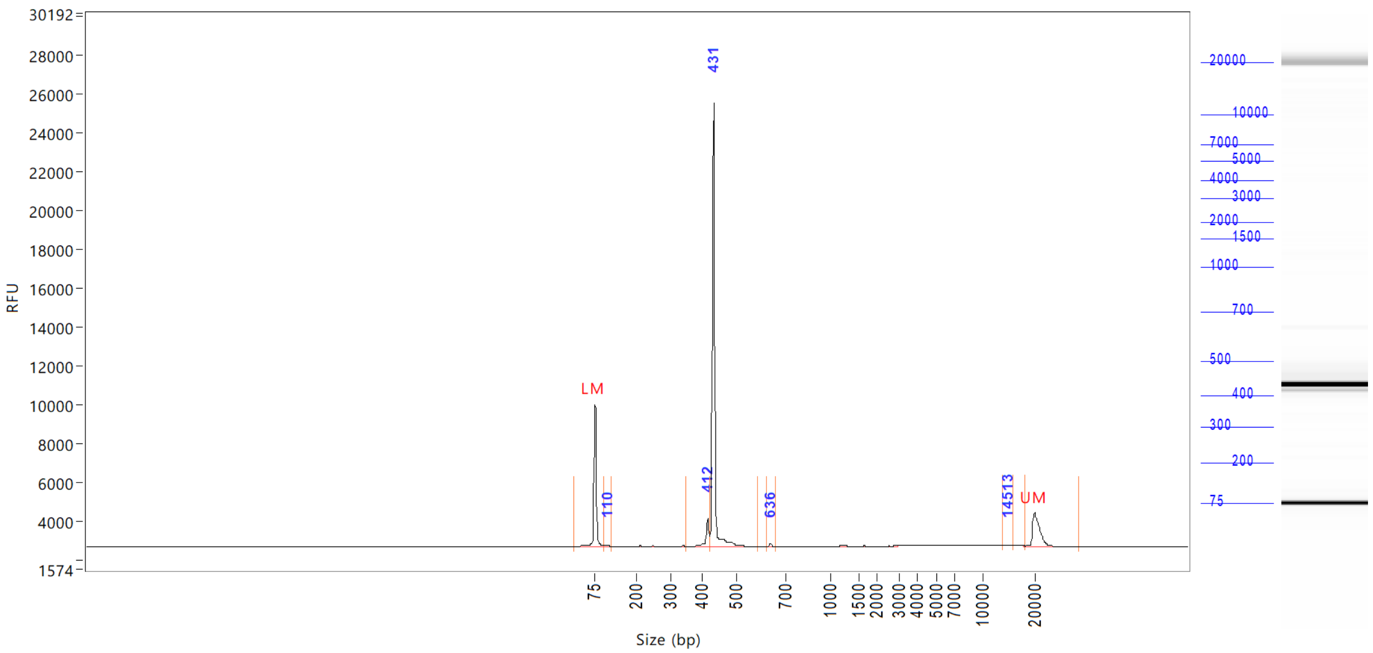

Peak	Size (bp)	Concentration (ng/uL)	From (bp)	To (bp)	Average size (bp)	CV%	RFU	Corrected peak area
1	75 (LM)	0.5000	14	103	73	10.27	7329	42.082
2	110	0.1173	103	124	110	4.48	100	0.823
3	412	1.2227	345	420	408	2.70	1419	8.576
4	431	15.5126	420	588	433	3.12	22877	108.800
5	636	0.0974	621	662	633	0.90	156	0.683
6	14513	0.0489	13781	15732	14467	3.17	60	0.343
7	20000 (UM)	0.1733	18171	28584	20371	4.04	1719	14.584

TIC:	16.9988	ng/uL
TIM:	65.8879	nmole/L
Total concentration:	17.5927	ng/uL

```
Sample peak width (sec): 5    Sample min peak height: 50    Sample baseline V to V?: Y    Sample baseline V to V points: 3
Sample filter: Binomial       Number of points for filter: 3  Sample start region (min): 0  Sample end region (min): 30
Marker peak width (sec): 5    Marker min peak height: 200   Marker baseline V to V?: Y    Marker baseline V to V points: 3
Lower marker selection: First peak > 500 RFU                Upper marker selection: Last peak > 200 RFU
Ladder size (bp)75, 200, 300, 400, 500, 700, 1000, 1500, 2000, 3000, 4000, 5000, 7000, 10000, 20000
Quantification using: Lower Marker          Final concentration (ng/uL): 0.5000          Dilution factor: 12.0
```

Sample: SampB9
Well location: B9
Created: Tuesday, August 10, 2021 6:41:11 PM

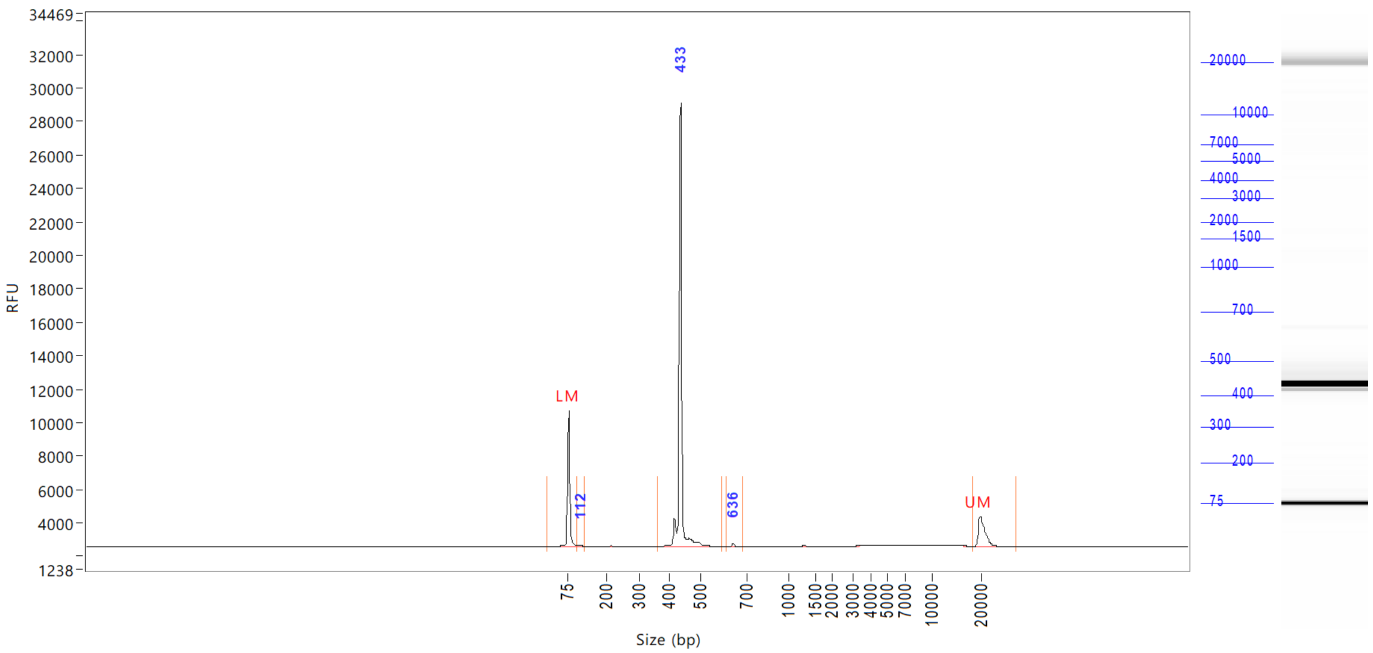

Peak	Size (bp)	Concentration (ng/uL)	From (bp)	To (bp)	Average size (bp)	CV%	RFU	Corrected peak area
1	75 (LM)	0.5000	9	103	73	10.98	8098	47.087
2	112	0.1436	103	126	111	5.18	126	1.127
3	433	18.3745	360	588	433	3.41	26567	144.199
4	636	0.0920	608	680	636	1.29	162	0.722
5	20000 (UM)	0.1660	18293	27254	20424	4.19	1800	15.629

TIC:	18.6101	ng/uL
TIM:	72.1850	nmole/L
Total concentration:	19.1763	ng/uL

Sample peak width (sec): 5 Sample min peak height: 50 Sample baseline V to V?: Y Sample baseline V to V points: 3
Sample filter: Binomial Number of points for filter: 3 Sample start region (min): 0 Sample end region (min): 30
Marker peak width (sec): 5 Marker min peak height: 200 Marker baseline V to V?: Y Marker baseline V to V points: 3
Lower marker selection: First peak > 500 RFU Upper marker selection: Last peak > 200 RFU
Ladder size (bp)75, 200, 300, 400, 500, 700, 1000, 1500, 2000, 3000, 4000, 5000, 7000, 10000, 20000
Quantification using: Lower Marker Final concentration (ng/uL): 0.5000 Dilution factor: 12.0

Sample: SampC9
Well location: C9
Created: Tuesday, August 10, 2021 6:41:11 PM

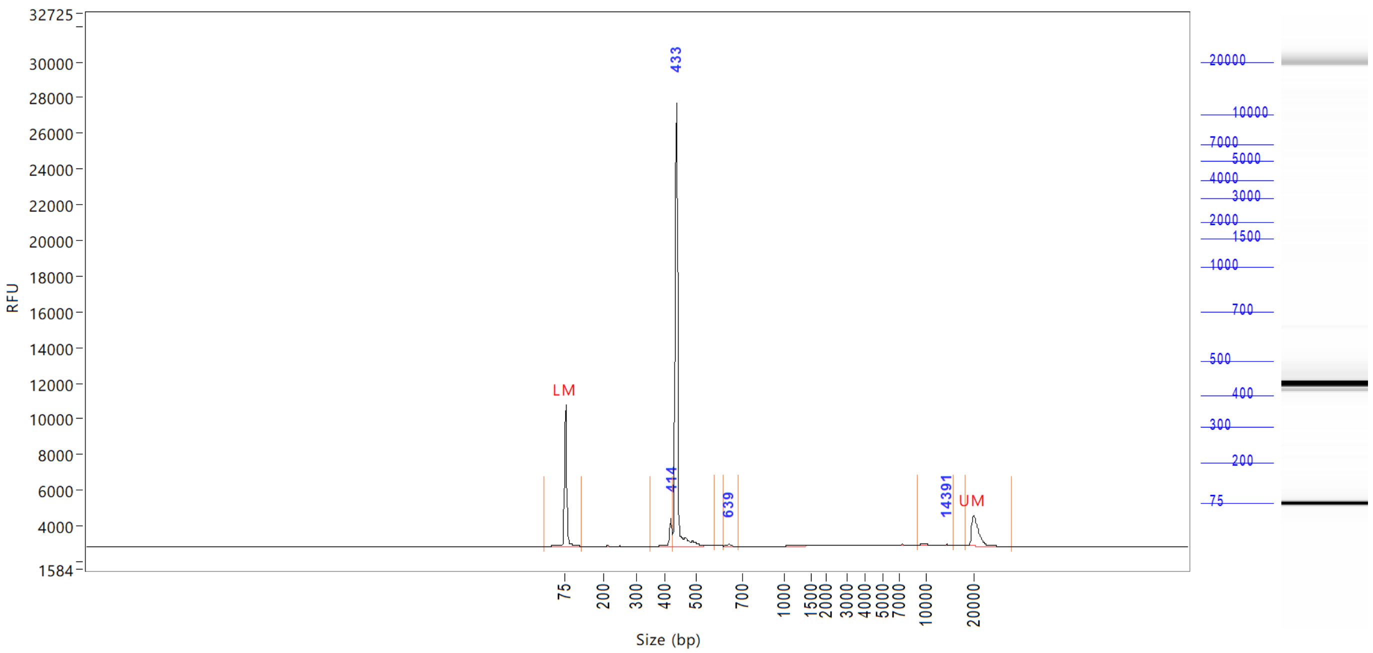

Peak	Size (bp)	Concentration (ng/uL)	From (bp)	To (bp)	Average size (bp)	CV%	RFU	Corrected peak area
1	75 (LM)	0.5000	7	126	73	12.63	7963	47.228
2	414	1.1128	347	420	410	2.43	1563	8.760
3	433	15.8361	420	578	435	3.08	24874	124.650
4	639	0.0805	613	682	636	1.11	142	0.633
5	14391	0.1684	9134	15610	11660	14.71	52	1.326
6	20000 (UM)	0.1581	18171	27738	20506	4.73	1687	14.931

TIC:	17.1978	ng/uL
TIM:	64.5974	nmole/L
Total concentration:	17.6119	ng/uL

Sample peak width (sec): 5   Sample min peak height: 50   Sample baseline V to V?: Y   Sample baseline V to V points: 3
Sample filter: Binomial   Number of points for filter: 3 Sample start region (min): 0 Sample end region (min): 30
Marker peak width (sec): 5   Marker min peak height: 200   Marker baseline V to V?: Y   Marker baseline V to V points: 3
Lower marker selection: First peak > 500 RFU   Upper marker selection: Last peak > 200 RFU
Ladder size (bp)75, 200, 300, 400, 500, 700, 1000, 1500, 2000, 3000, 4000, 5000, 7000, 10000, 20000
Quantification using: Lower Marker   Final concentration (ng/uL): 0.5000   Dilution factor: 12.0

Sample: SampD9
Well location: D9
Created: Tuesday, August 10, 2021 6:41:11 PM

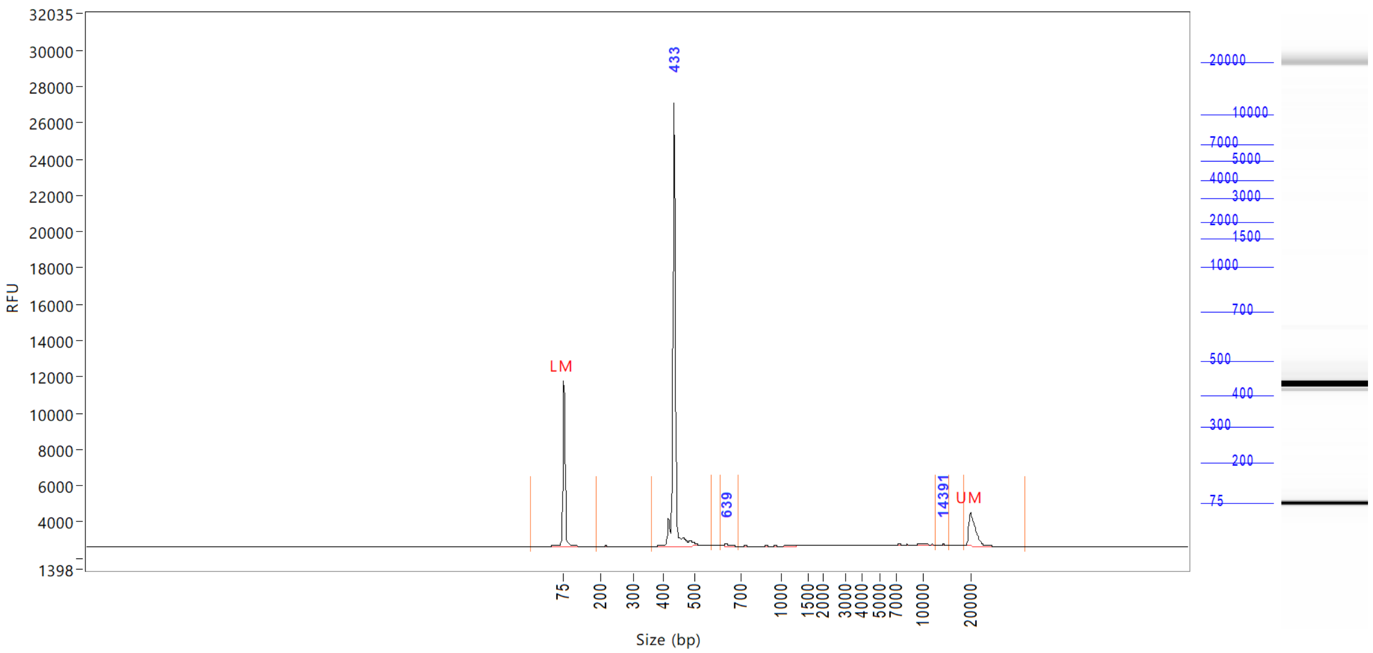

Peak	Size (bp)	Concentration (ng/uL)	From (bp)	To (bp)	Average size (bp)	CV%	RFU	Corrected peak area
1	75 (LM)	0.5000	0	183	74	15.66	9133	54.362
2	433	14.8928	358	570	432	3.31	24472	134.933
3	639	0.0736	611	692	636	1.53	143	0.667
4	14391	0.0689	12562	15366	13818	5.09	68	0.625
5	20000 (UM)	0.1476	18537	31486	20547	5.37	1813	16.050

TIC:	15.0353	ng/uL
TIM:	56.9212	nmole/L
Total concentration:	15.4648	ng/uL

Sample peak width (sec): 5   Sample min peak height: 50   Sample baseline V to V?: Y   Sample baseline V to V points: 3
Sample filter: Binomial   Number of points for filter: 3 Sample start region (min): 0 Sample end region (min): 30
Marker peak width (sec): 5   Marker min peak height: 200   Marker baseline V to V?: Y   Marker baseline V to V points: 3
Lower marker selection: First peak > 500 RFU   Upper marker selection: Last peak > 200 RFU
Ladder size (bp)75, 200, 300, 400, 500, 700, 1000, 1500, 2000, 3000, 4000, 5000, 7000, 10000, 20000
Quantification using: Lower Marker   Final concentration (ng/uL): 0.5000   Dilution factor: 12.0

Sample: SampE9
Well location: E9
Created: Tuesday, August 10, 2021 6:41:11 PM

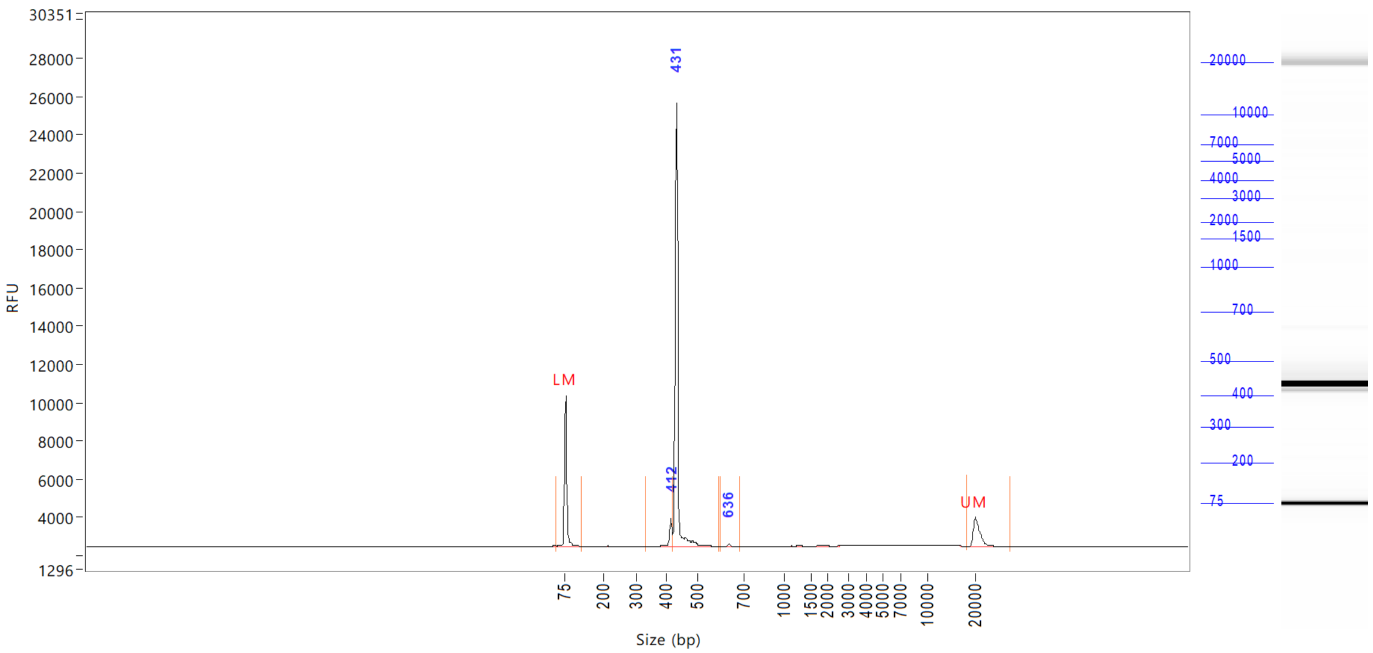

Peak	Size (bp)	Concentration (ng/uL)	From (bp)	To (bp)	Average size (bp)	CV%	RFU	Corrected peak area
1	75 (LM)	0.5000	47	126	74	9.86	7861	46.357
2	412	1.0603	331	420	409	2.48	1469	8.192
3	431	15.2656	420	590	434	3.26	23222	117.944
4	636	0.0777	598	682	634	1.49	125	0.600
5	20000 (UM)	0.1364	18171	27133	20385	4.26	1501	12.648

TIC:	16.4037	ng/uL
TIM:	62.3420	nmole/L
Total concentration:	16.8375	ng/uL

Sample peak width (sec): 5 Sample min peak height: 50 Sample baseline V to V?: Y Sample baseline V to V points: 3
Sample filter: Binomial Number of points for filter: 3 Sample start region (min): 0 Sample end region (min): 30
Marker peak width (sec): 5 Marker min peak height: 200 Marker baseline V to V?: Y Marker baseline V to V points: 3
Lower marker selection: First peak > 500 RFU Upper marker selection: Last peak > 200 RFU
Ladder size (bp)75, 200, 300, 400, 500, 700, 1000, 1500, 2000, 3000, 4000, 5000, 7000, 10000, 20000
Quantification using: Lower Marker Final concentration (ng/uL): 0.5000 Dilution factor: 12.0

Sample: SampF9
Well location: F9
Created: Tuesday, August 10, 2021 6:41:11 PM

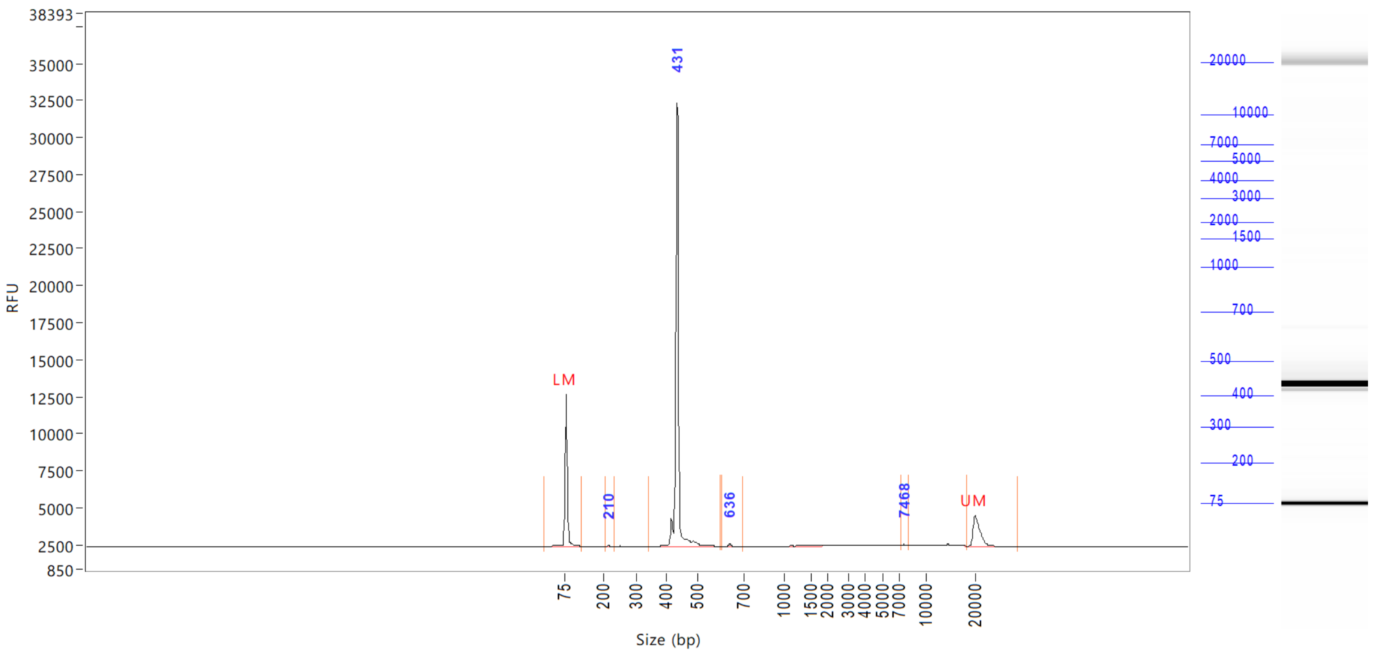

Peak	Size (bp)	Concentration (ng/uL)	From (bp)	To (bp)	Average size (bp)	CV%	RFU	Corrected peak area
1	75 (LM)	0.5000	3	126	73	12.51	10241	60.676
2	210	0.0324	201	232	209	2.39	60	0.328
3	431	16.1916	339	600	433	3.32	30001	163.741
4	636	0.0743	608	692	636	1.22	171	0.751
5	7468	0.0350	7068	8067	7489	3.65	52	0.354
6	20000 (UM)	0.1509	18415	28947	20514	4.30	2069	18.307

TIC:	16.3333	ng/uL
TIM:	61.9822	nmole/L
Total concentration:	16.7467	ng/uL

```
Sample peak width (sec): 5    Sample min peak height: 50    Sample baseline V to V?: Y    Sample baseline V to V points: 3
Sample filter: Binomial       Number of points for filter: 3  Sample start region (min): 0  Sample end region (min): 30
Marker peak width (sec): 5    Marker min peak height: 200   Marker baseline V to V?: Y    Marker baseline V to V points: 3
Lower marker selection: First peak > 500 RFU                Upper marker selection: Last peak > 200 RFU
Ladder size (bp)75, 200, 300, 400, 500, 700, 1000, 1500, 2000, 3000, 4000, 5000, 7000, 10000, 20000
Quantification using: Lower Marker          Final concentration (ng/uL): 0.5000          Dilution factor: 12.0
```

Sample: SampG9
Well location: G9
Created: Tuesday, August 10, 2021 6:41:11 PM

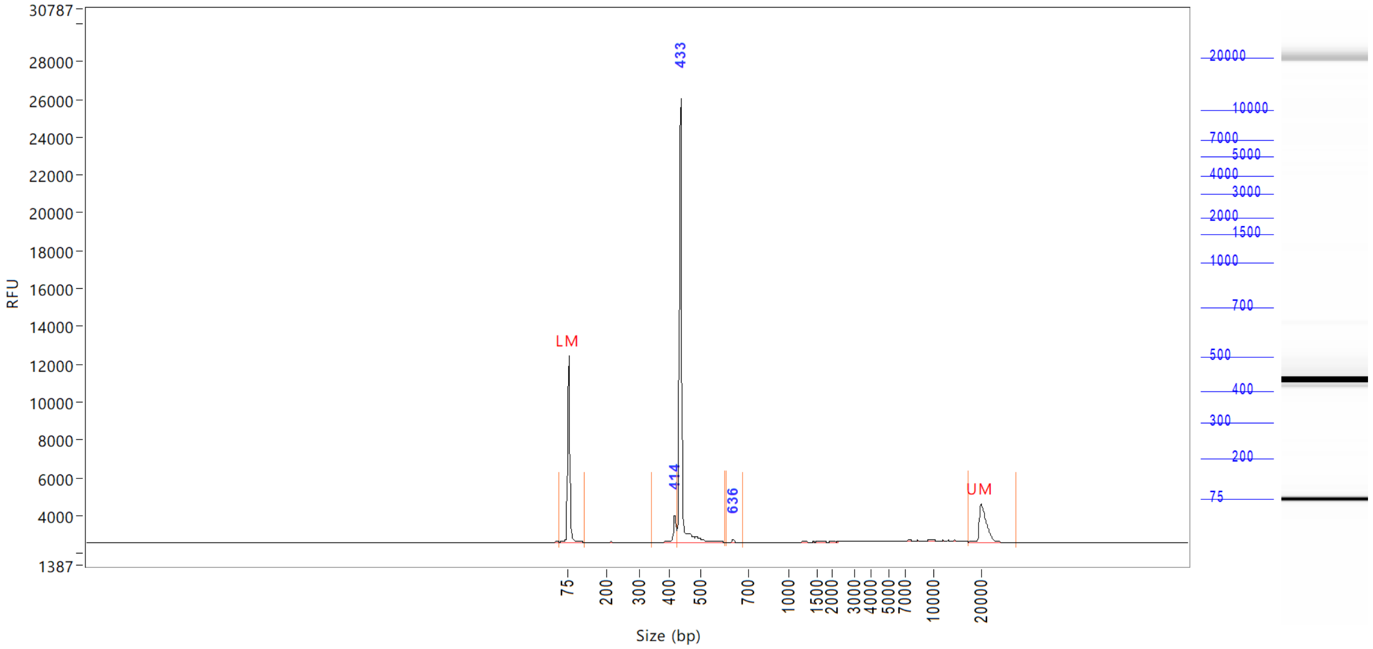

Peak	Size (bp)	Concentration (ng/uL)	From (bp)	To (bp)	Average size (bp)	CV%	RFU	Corrected peak area
1	75 (LM)	0.5000	45	126	74	9.04	9916	56.245
2	414	0.9059	339	420	410	2.58	1404	8.492
3	433	12.6505	420	600	434	3.35	23495	118.588
4	636	0.0638	608	680	636	1.37	130	0.598
5	20000 (UM)	0.1624	17317	27012	20399	4.20	2056	18.273

TIC:	13.6201	ng/uL
TIM:	51.7607	nmole/L
Total concentration:	13.9783	ng/uL

Sample peak width (sec): 5 Sample min peak height: 50 Sample baseline V to V?: Y Sample baseline V to V points: 3
Sample filter: Binomial Number of points for filter: 3 Sample start region (min): 0 Sample end region (min): 30
Marker peak width (sec): 5 Marker min peak height: 200 Marker baseline V to V?: Y Marker baseline V to V points: 3
Lower marker selection: First peak > 500 RFU Upper marker selection: Last peak > 200 RFU
Ladder size (bp)75, 200, 300, 400, 500, 700, 1000, 1500, 2000, 3000, 4000, 5000, 7000, 10000, 20000
Quantification using: Lower Marker Final concentration (ng/uL): 0.5000 Dilution factor: 12.0

Sample: SampH9
Well location: H9
Created: Tuesday, August 10, 2021 6:41:11 PM

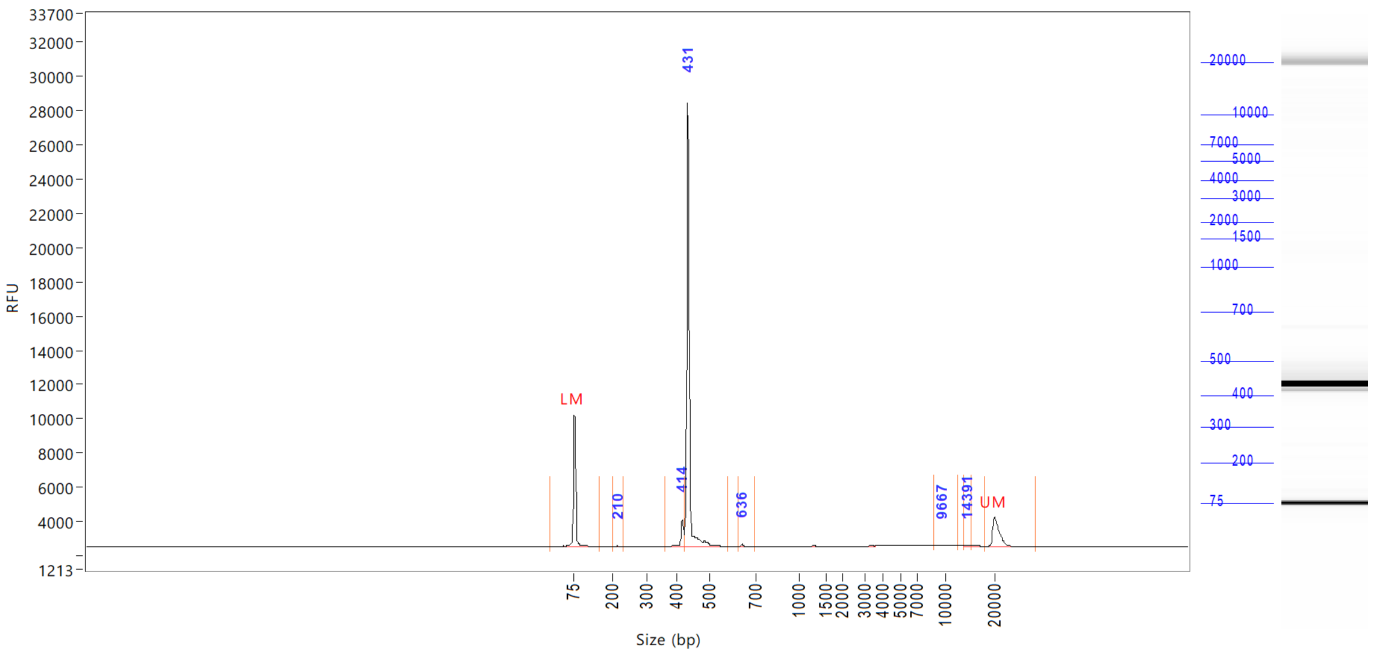

Peak	Size (bp)	Concentration (ng/uL)	From (bp)	To (bp)	Average size (bp)	CV%	RFU	Corrected peak area
1	75 (LM)	0.5000	0	156	74	15.15	7670	46.975
2	210	0.0463	200	230	210	3.03	51	0.363
3	414	1.1744	362	420	410	2.27	1606	9.195
4	431	16.6568	420	580	434	3.24	25972	130.408
5	636	0.0736	623	690	636	1.27	130	0.577
6	9667	0.1142	8800	12684	10592	9.87	50	0.894
7	14391	0.0423	13781	15244	14369	2.52	62	0.332
8	20000 (UM)	0.1565	18171	28100	20477	4.42	1702	14.703

TIC:	18.1078	ng/uL
TIM:	68.4380	nmole/L
Total concentration:	18.4539	ng/uL

Sample peak width (sec): 5 Sample min peak height: 50 Sample baseline V to V?: Y Sample baseline V to V points: 3
Sample filter: Binomial Number of points for filter: 3 Sample start region (min): 0 Sample end region (min): 30
Marker peak width (sec): 5 Marker min peak height: 200 Marker baseline V to V?: Y Marker baseline V to V points: 3
Lower marker selection: First peak > 500 RFU Upper marker selection: Last peak > 200 RFU
Ladder size (bp)75, 200, 300, 400, 500, 700, 1000, 1500, 2000, 3000, 4000, 5000, 7000, 10000, 20000
Quantification using: Lower Marker Final concentration (ng/uL): 0.5000 Dilution factor: 12.0

Sample: SampA10
Well location: A10
Created: Tuesday, August 10, 2021 6:41:11 PM

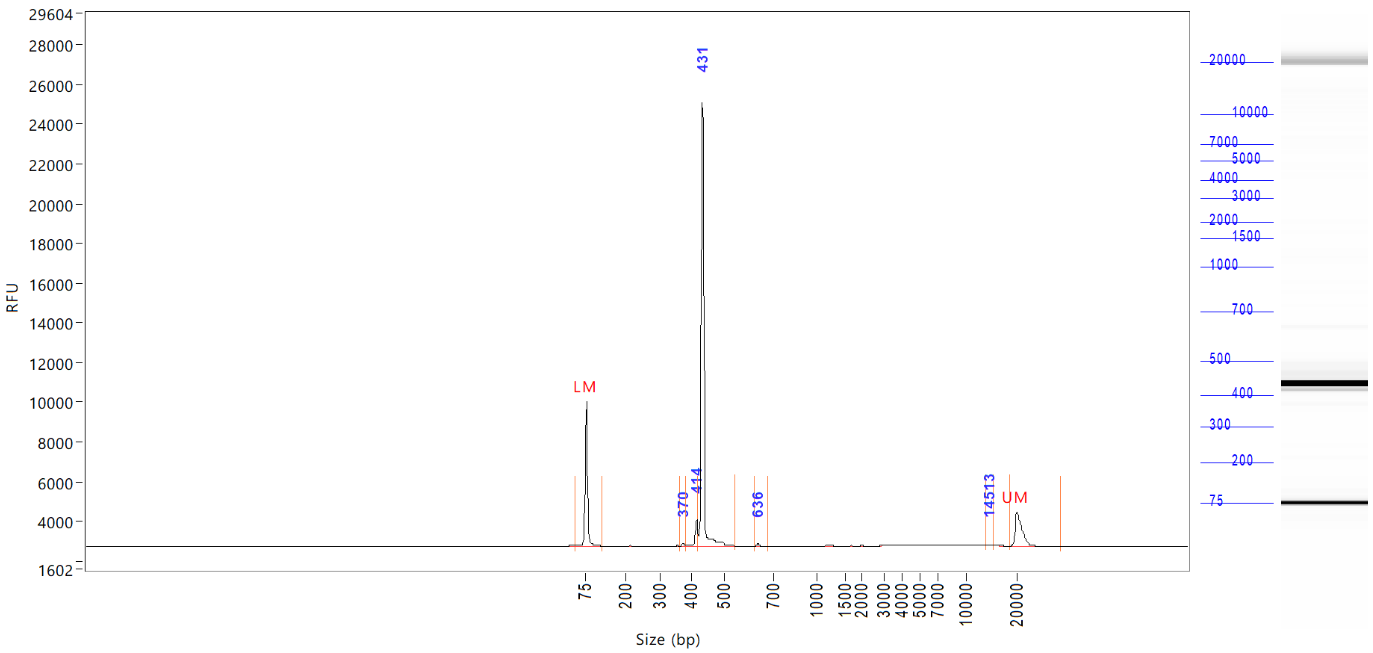

Peak	Size (bp)	Concentration (ng/uL)	From (bp)	To (bp)	Average size (bp)	CV%	RFU	Corrected peak area
1	75 (LM)	0.5000	45	126	74	10.10	7321	41.943
2	370	0.0949	360	380	369	1.37	116	0.664
3	414	1.0154	380	420	411	1.77	1314	7.098
4	431	15.6470	420	539	433	2.62	22370	109.380
5	636	0.0934	621	680	634	0.91	155	0.653
6	14513	0.0502	13903	15488	14497	2.68	64	0.351
7	20000 (UM)	0.1695	18537	28463	20482	4.06	1668	14.217

TIC:	16.9010	ng/uL
TIM:	64.1940	nmole/L
Total concentration:	17.5043	ng/uL

Sample peak width (sec): 5 Sample min peak height: 50 Sample baseline V to V?: Y Sample baseline V to V points: 3
Sample filter: Binomial Number of points for filter: 3 Sample start region (min): 0 Sample end region (min): 30
Marker peak width (sec): 5 Marker min peak height: 200 Marker baseline V to V?: Y Marker baseline V to V points: 3
Lower marker selection: First peak > 500 RFU Upper marker selection: Last peak > 200 RFU
Ladder size (bp)75, 200, 300, 400, 500, 700, 1000, 1500, 2000, 3000, 4000, 5000, 7000, 10000, 20000
Quantification using: Lower Marker Final concentration (ng/uL): 0.5000 Dilution factor: 12.0

Sample: SampB10
Well location: B10
Created: Tuesday, August 10, 2021 6:41:11 PM

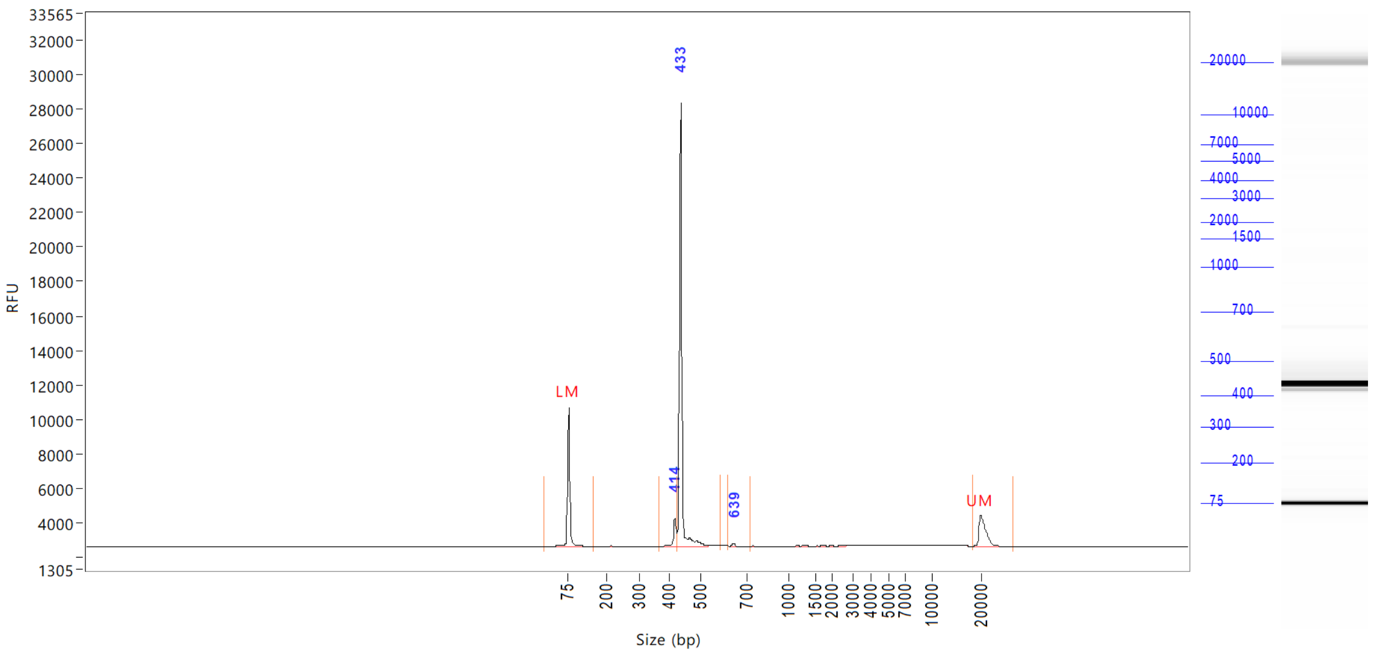

Peak	Size (bp)	Concentration (ng/uL)	From (bp)	To (bp)	Average size (bp)	CV%	RFU	Corrected peak area
1	75 (LM)	0.5000	0	158	74	15.52	8062	48.000
2	414	1.1695	362	422	412	2.15	1624	9.356
3	433	15.9681	422	580	435	3.14	25772	127.745
4	639	0.0839	616	714	639	2.20	141	0.671
5	20000 (UM)	0.1645	18171	26529	20440	4.09	1827	15.789

TIC:	17.2215	ng/uL
TIM:	65.2854	nmole/L
Total concentration:	17.6717	ng/uL

Sample peak width (sec): 5 Sample min peak height: 50 Sample baseline V to V?: Y Sample baseline V to V points: 3
Sample filter: Binomial Number of points for filter: 3 Sample start region (min): 0 Sample end region (min): 30
Marker peak width (sec): 5 Marker min peak height: 200 Marker baseline V to V?: Y Marker baseline V to V points: 3
Lower marker selection: First peak > 500 RFU Upper marker selection: Last peak > 200 RFU
Ladder size (bp)75, 200, 300, 400, 500, 700, 1000, 1500, 2000, 3000, 4000, 5000, 7000, 10000, 20000
Quantification using: Lower Marker Final concentration (ng/uL): 0.5000 Dilution factor: 12.0

Sample: SampC10
Well location: C10
Created: Tuesday, August 10, 2021 6:41:11 PM

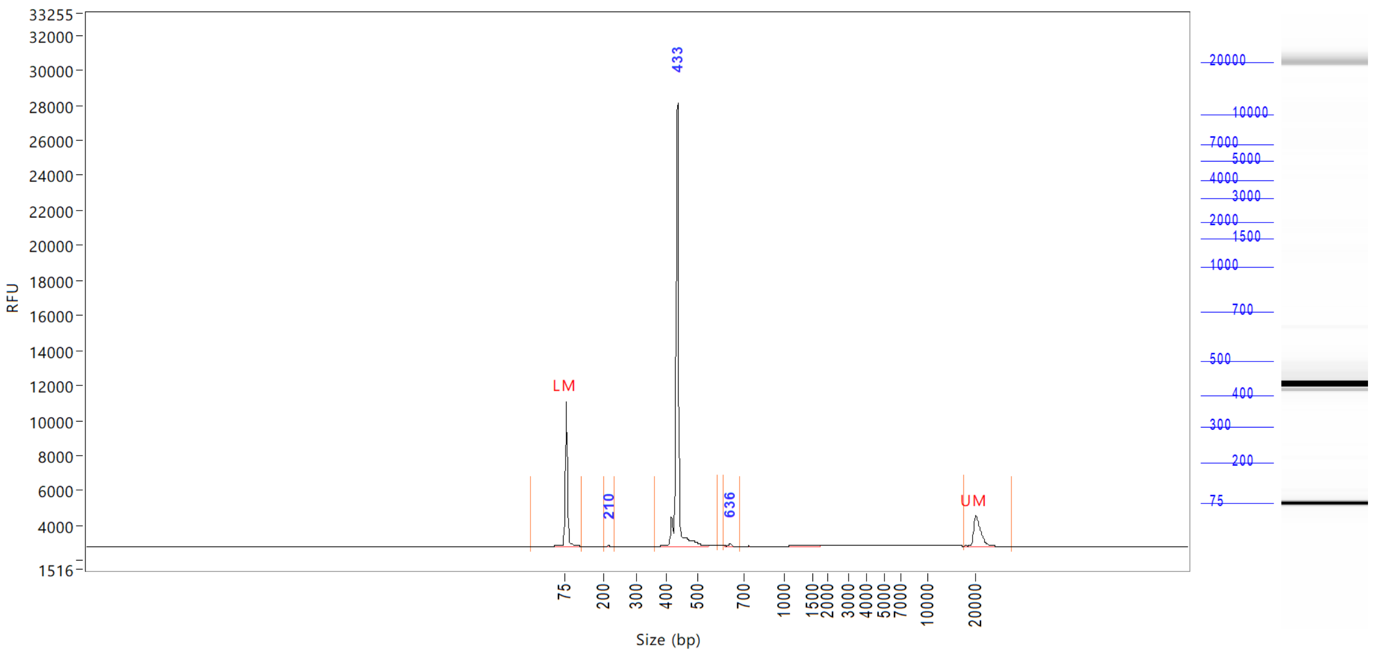

Peak	Size (bp)	Concentration (ng/uL)	From (bp)	To (bp)	Average size (bp)	CV%	RFU	Corrected peak area
1	75 (LM)	0.5000	0	126	73	14.40	8263	48.967
2	210	0.0430	198	232	208	2.48	55	0.351
3	433	17.1441	362	585	433	3.55	25356	139.915
4	636	0.0861	611	682	636	1.11	158	0.703
5	20000 (UM)	0.1602	17439	27375	20518	4.55	1760	15.689

TIC:	17.2732	ng/uL
TIM:	65.7094	nmole/L
Total concentration:	17.8205	ng/uL

Sample peak width (sec): 5   Sample min peak height: 50   Sample baseline V to V?: Y   Sample baseline V to V points: 3
Sample filter: Binomial   Number of points for filter: 3   Sample start region (min): 0   Sample end region (min): 30
Marker peak width (sec): 5   Marker min peak height: 200   Marker baseline V to V?: Y   Marker baseline V to V points: 3
Lower marker selection: First peak > 500 RFU   Upper marker selection: Last peak > 200 RFU
Ladder size (bp)75, 200, 300, 400, 500, 700, 1000, 1500, 2000, 3000, 4000, 5000, 7000, 10000, 20000
Quantification using: Lower Marker   Final concentration (ng/uL): 0.5000   Dilution factor: 12.0

Sample: SampD10
Well location: D10
Created: Tuesday, August 10, 2021 6:41:11 PM

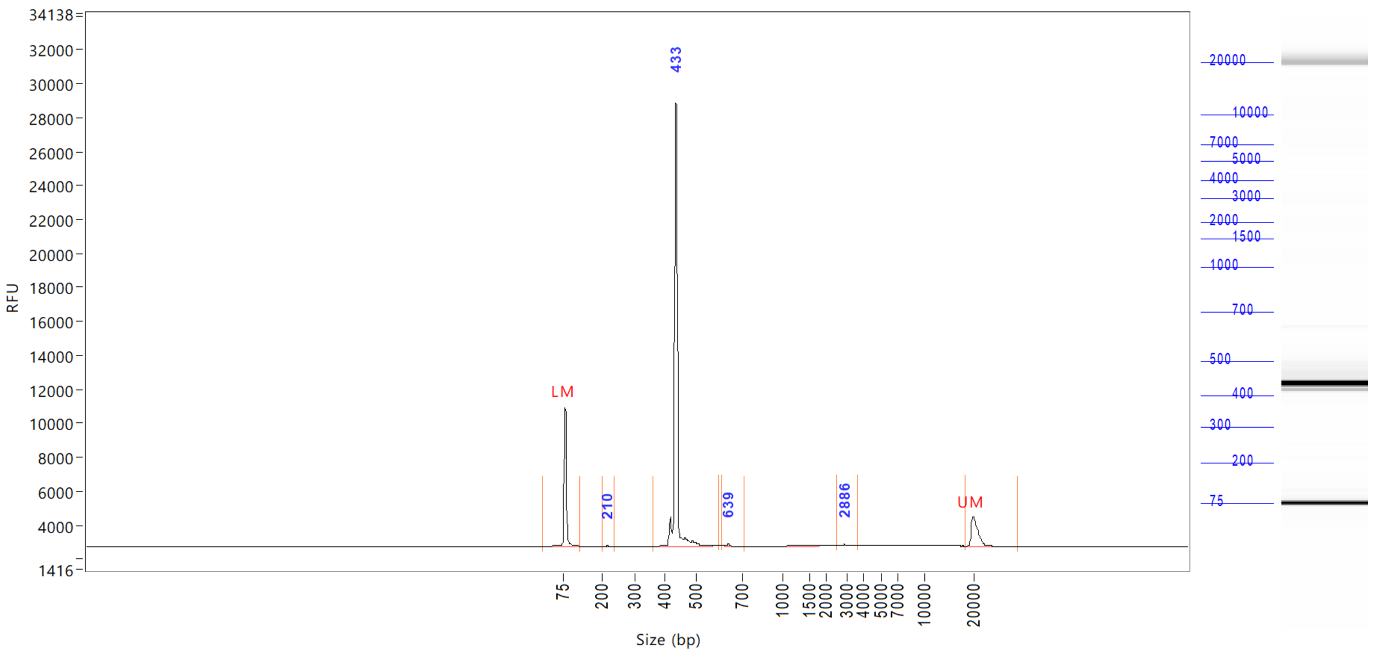

Peak	Size (bp)	Concentration (ng/uL)	From (bp)	To (bp)	Average size (bp)	CV%	RFU	Corrected peak area
1	75 (LM)	0.5000	3	126	74	12.95	8204	49.972
2	210	0.0469	198	233	211	3.39	58	0.391
3	433	17.4020	362	598	434	3.54	26144	144.935
4	639	0.0843	613	714	638	1.68	157	0.702
5	2886	0.0776	2486	3643	2966	7.05	76	0.646
6	20000 (UM)	0.1530	18293	29068	20511	4.55	1753	15.288

TIC:	17.6108	ng/uL
TIM:	66.6003	nmole/L
Total concentration:	18.1198	ng/uL

```
Sample peak width (sec): 5    Sample min peak height: 50    Sample baseline V to V?: Y    Sample baseline V to V points: 3
Sample filter: Binomial       Number of points for filter: 3  Sample start region (min): 0  Sample end region (min): 30
Marker peak width (sec): 5    Marker min peak height: 200   Marker baseline V to V?: Y    Marker baseline V to V points: 3
Lower marker selection: First peak > 500 RFU                Upper marker selection: Last peak > 200 RFU
Ladder size (bp)75, 200, 300, 400, 500, 700, 1000, 1500, 2000, 3000, 4000, 5000, 7000, 10000, 20000
Quantification using: Lower Marker          Final concentration (ng/uL): 0.5000          Dilution factor: 12.0
```

Sample: SampE10
Well location: E10
Created: Tuesday, August 10, 2021 6:41:11 PM

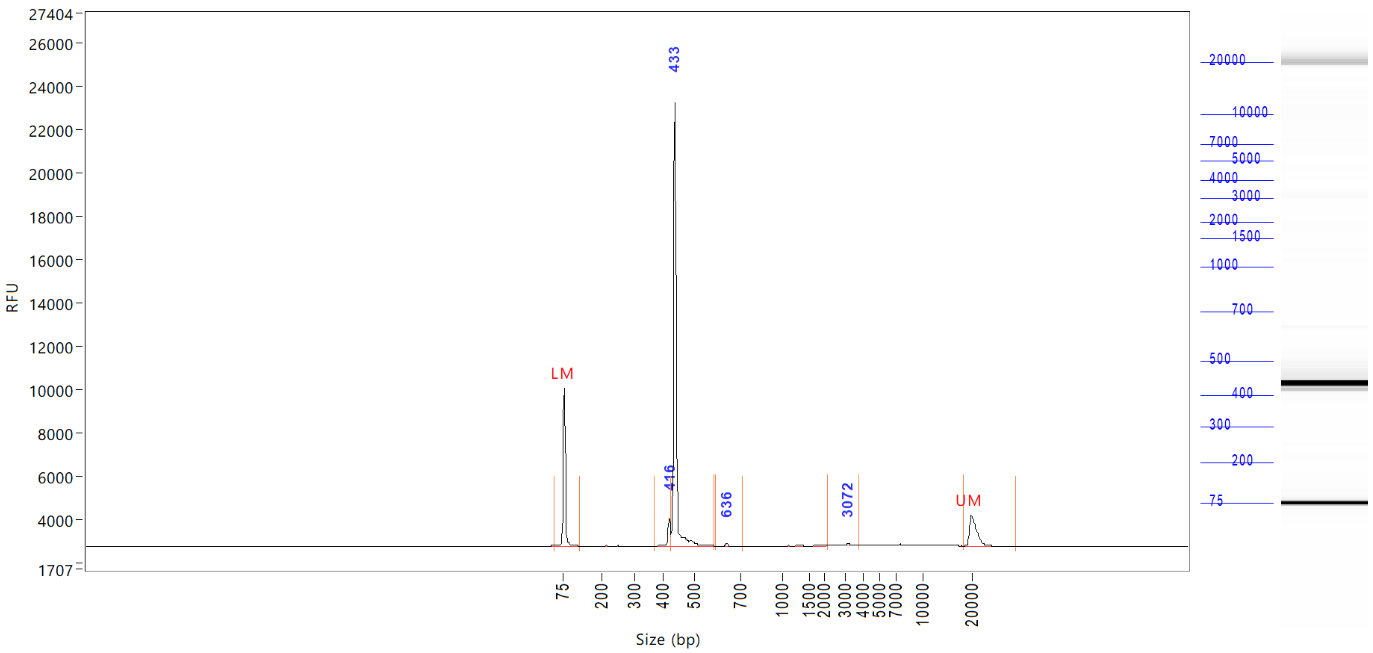

Peak	Size (bp)	Concentration (ng/uL)	From (bp)	To (bp)	Average size (bp)	CV%	RFU	Corrected peak area
1	75 (LM)	0.5000	45	126	74	9.57	7337	42.555
2	416	1.0427	366	422	412	2.08	1316	7.395
3	433	14.7668	422	583	436	3.33	20534	104.733
4	636	0.0775	590	714	638	2.27	111	0.549
5	3072	0.1012	2144	3822	3107	8.18	90	0.718
6	20000 (UM)	0.1507	18293	29189	20512	4.71	1425	12.828

TIC:	15.9881	ng/uL
TIM:	60.1444	nmole/L
Total concentration:	16.5810	ng/uL

Sample peak width (sec): 5 Sample min peak height: 50 Sample baseline V to V?: Y Sample baseline V to V points: 3
Sample filter: Binomial Number of points for filter: 3 Sample start region (min): 0 Sample end region (min): 30
Marker peak width (sec): 5 Marker min peak height: 200 Marker baseline V to V?: Y Marker baseline V to V points: 3
Lower marker selection: First peak > 500 RFU Upper marker selection: Last peak > 200 RFU
Ladder size (bp)75, 200, 300, 400, 500, 700, 1000, 1500, 2000, 3000, 4000, 5000, 7000, 10000, 20000
Quantification using: Lower Marker Final concentration (ng/uL): 0.5000 Dilution factor: 12.0

Sample: SampF10
Well location: F10
Created: Tuesday, August 10, 2021 6:41:11 PM

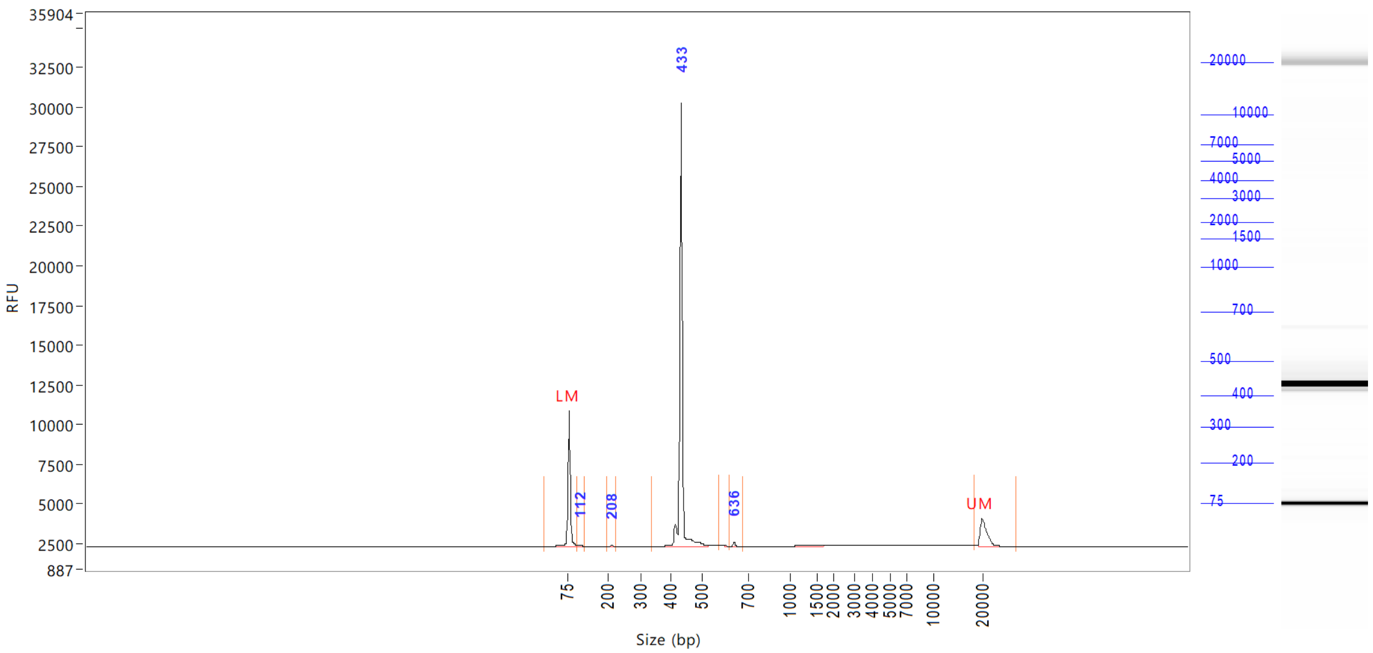

Peak	Size (bp)	Concentration (ng/uL)	From (bp)	To (bp)	Average size (bp)	CV%	RFU	Corrected peak area
1	75 (LM)	0.5000	0	101	72	11.29	8602	50.102
2	112	0.1337	101	126	110	5.51	129	1.117
3	208	0.0373	196	223	208	1.76	59	0.311
4	433	17.3347	335	575	432	3.07	27974	144.750
5	636	0.1127	618	674	634	0.82	225	0.941
6	20000 (UM)	0.1497	18415	27012	20407	4.05	1758	15.002

TIC:	17.6184	ng/uL
TIM:	68.6074	nmole/L
Total concentration:	18.1548	ng/uL

Sample peak width (sec): 5 Sample min peak height: 50 Sample baseline V to V?: Y Sample baseline V to V points: 3
Sample filter: Binomial Number of points for filter: 3 Sample start region (min): 0 Sample end region (min): 30
Marker peak width (sec): 5 Marker min peak height: 200 Marker baseline V to V?: Y Marker baseline V to V points: 3
Lower marker selection: First peak > 500 RFU Upper marker selection: Last peak > 200 RFU
Ladder size (bp)75, 200, 300, 400, 500, 700, 1000, 1500, 2000, 3000, 4000, 5000, 7000, 10000, 20000
Quantification using: Lower Marker Final concentration (ng/uL): 0.5000 Dilution factor: 12.0

Sample: SampG10
Well location: G10
Created: Tuesday, August 10, 2021 6:41:11 PM

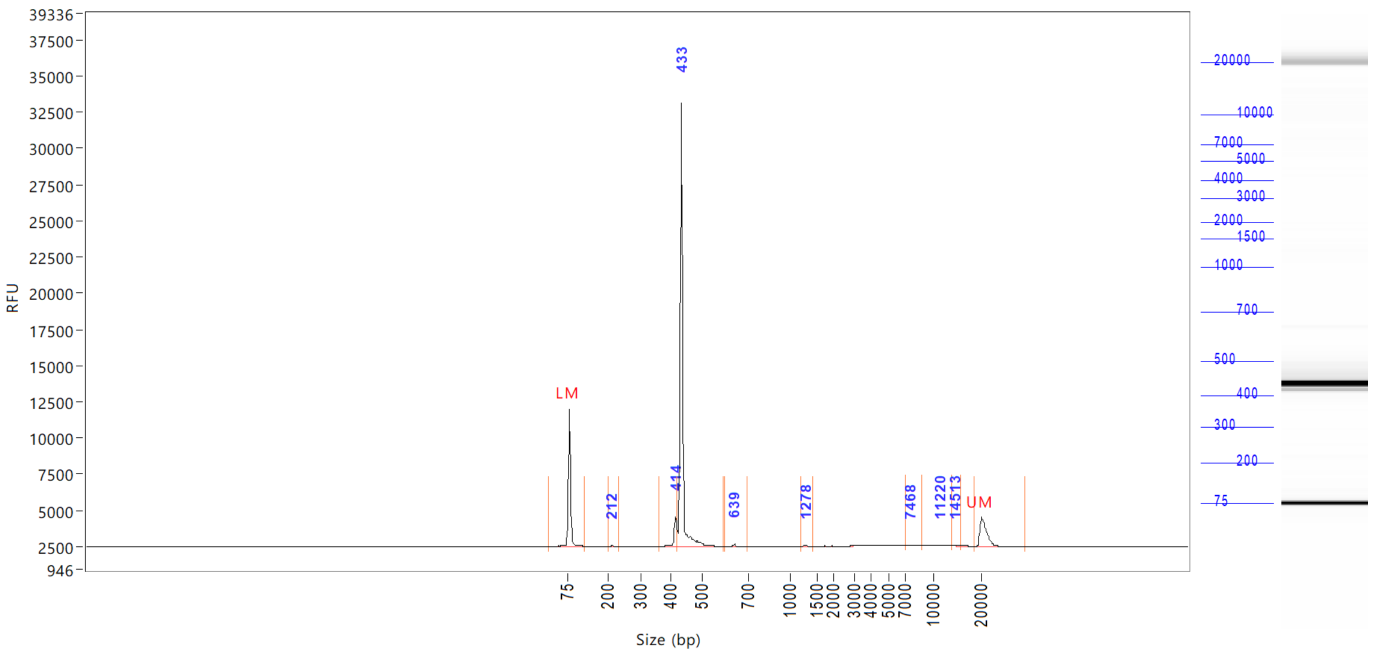

Peak	Size (bp)	Concentration (ng/uL)	From (bp)	To (bp)	Average size (bp)	CV%	RFU	Corrected peak area
1	75 (LM)	0.5000	9	126	74	12.12	9545	55.942
2	212	0.0428	201	233	211	2.57	70	0.399
3	414	1.2394	360	422	412	2.22	2054	11.555
4	433	17.0479	422	590	436	3.33	30689	158.948
5	639	0.0782	598	697	638	2.00	143	0.729
6	1278	0.0499	1212	1422	1296	4.24	52	0.466
7	7468	0.0597	7001	8800	7777	6.32	51	0.556
8	11220	0.1553	8800	13781	11046	12.59	55	1.448
9	14513	0.0464	13781	15854	14502	3.44	75	0.432
10	20000 (UM)	0.1556	18537	28826	20505	4.30	1989	17.406

TIC:	18.7195	ng/uL
TIM:	69.9244	nmole/L
Total concentration:	18.9737	ng/uL

Sample peak width (sec): 5   Sample min peak height: 50   Sample baseline V to V?: Y   Sample baseline V to V points: 3
Sample filter: Binomial   Number of points for filter: 3 Sample start region (min): 0 Sample end region (min): 30
Marker peak width (sec): 5   Marker min peak height: 200   Marker baseline V to V?: Y   Marker baseline V to V points: 3
Lower marker selection: First peak > 500 RFU   Upper marker selection: Last peak > 200 RFU
Ladder size (bp)75, 200, 300, 400, 500, 700, 1000, 1500, 2000, 3000, 4000, 5000, 7000, 10000, 20000
Quantification using: Lower Marker   Final concentration (ng/uL): 0.5000   Dilution factor: 12.0

Sample: SampH10
Well location: H10
Created: Tuesday, August 10, 2021 6:41:11 PM

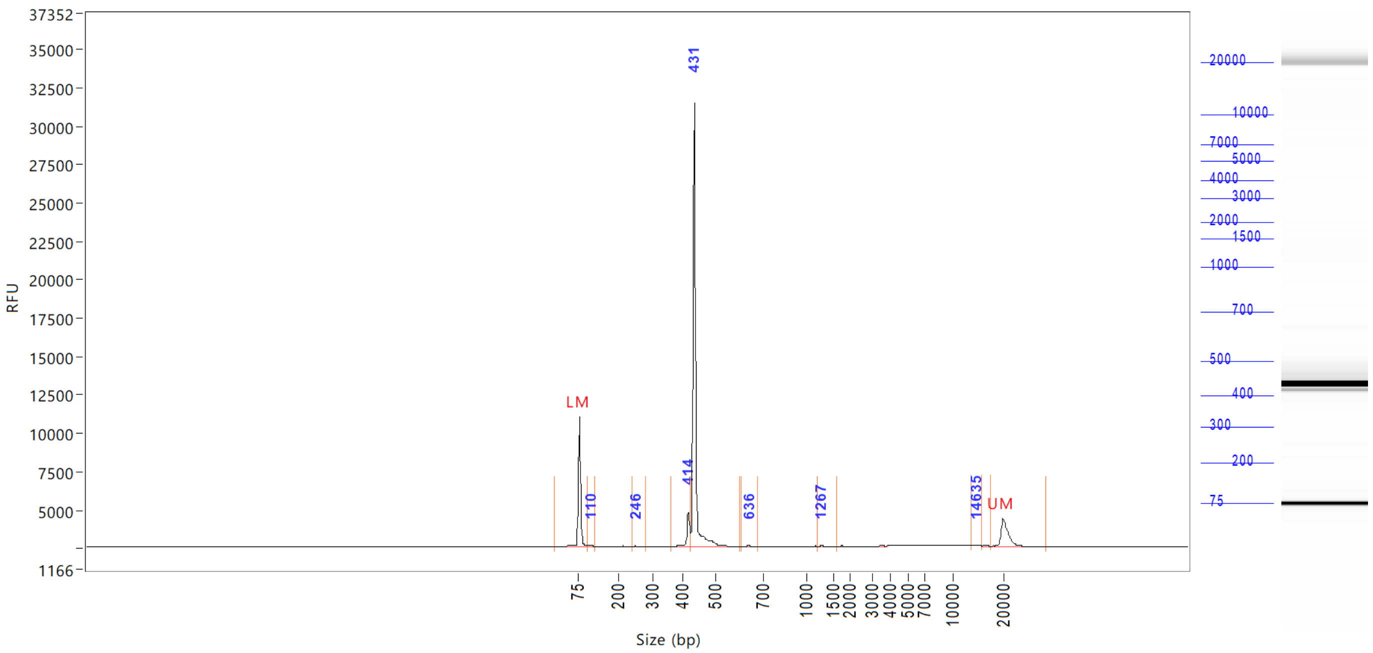

Peak	Size (bp)	Concentration (ng/uL)	From (bp)	To (bp)	Average size (bp)	CV%	RFU	Corrected peak area
1	75 (LM)	0.5000	0	101	72	10.64	8490	48.887
2	110	0.1231	101	124	109	5.02	123	1.003
3	246	0.0450	237	278	249	3.25	53	0.366
4	414	1.5489	362	420	410	2.32	2275	12.620
5	431	18.6488	420	600	434	3.35	28931	151.946
6	636	0.0703	608	677	636	1.53	108	0.573
7	1267	0.0820	1201	1559	1328	7.02	52	0.668
8	14635	0.0339	13659	15854	14359	2.75	50	0.276
9	20000 (UM)	0.1605	17561	28705	20479	4.65	1765	15.691

TIC:	20.5519	ng/uL
TIM:	79.3556	nmole/L
Total concentration:	20.9616	ng/uL

```
Sample peak width (sec): 5    Sample min peak height: 50    Sample baseline V to V?: Y    Sample baseline V to V points: 3
Sample filter: Binomial       Number of points for filter: 3  Sample start region (min): 0  Sample end region (min): 30
Marker peak width (sec): 5    Marker min peak height: 200   Marker baseline V to V?: Y    Marker baseline V to V points: 3
Lower marker selection: First peak > 500 RFU                Upper marker selection: Last peak > 200 RFU
Ladder size (bp)75, 200, 300, 400, 500, 700, 1000, 1500, 2000, 3000, 4000, 5000, 7000, 10000, 20000
Quantification using: Lower Marker          Final concentration (ng/uL): 0.5000          Dilution factor: 12.0
```

Sample: SampA11
Well location: A11
Created: Tuesday, August 10, 2021 6:41:11 PM

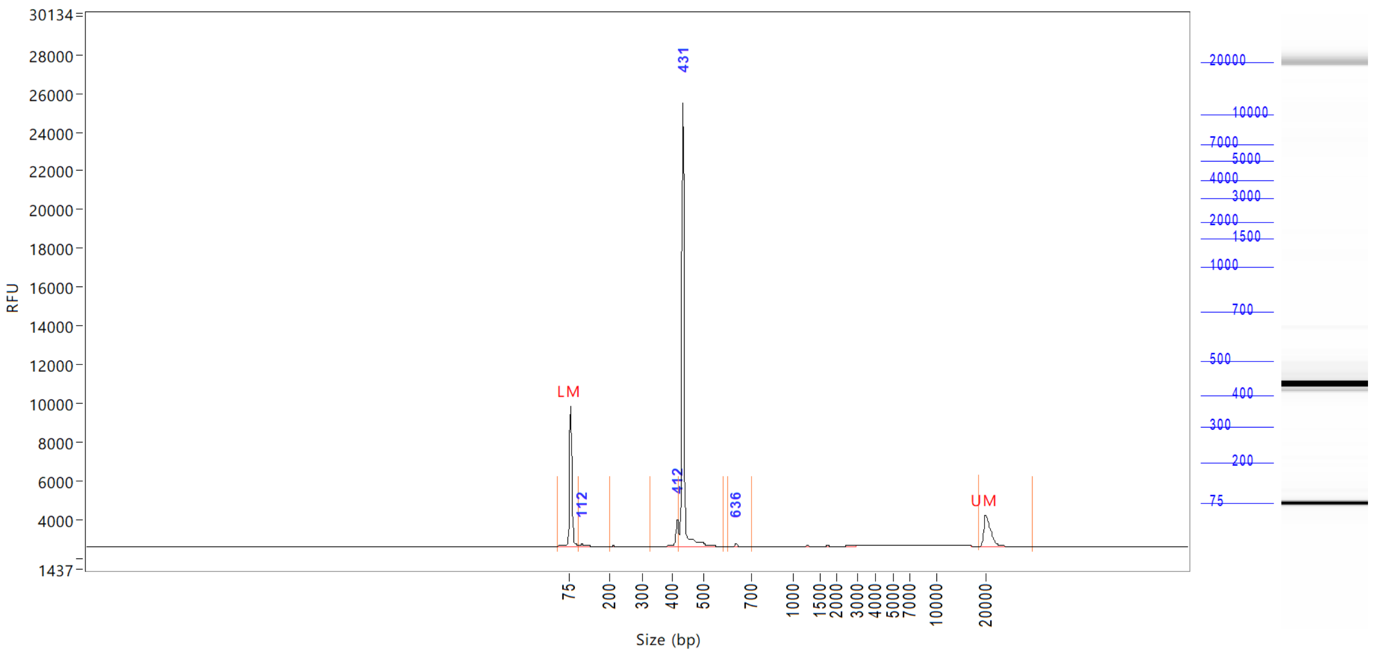

Peak	Size (bp)	Concentration (ng/uL)	From (bp)	To (bp)	Average size (bp)	CV%	RFU	Corrected peak area
1	75 (LM)	0.5000	35	101	73	7.99	7247	41.426
2	112	0.3186	101	200	127	18.12	137	2.199
3	412	1.1979	327	420	410	2.68	1374	8.271
4	431	16.5466	420	583	433	3.14	22941	114.243
5	636	0.0980	606	701	635	1.60	151	0.677
6	20000 (UM)	0.1688	18659	29430	20470	5.04	1619	13.987

TIC:	18.1611	ng/uL
TIM:	72.0586	nmole/L
Total concentration:	18.5509	ng/uL

```
Sample peak width (sec): 5    Sample min peak height: 50    Sample baseline V to V?: Y    Sample baseline V to V points: 3
Sample filter: Binomial       Number of points for filter: 3  Sample start region (min): 0  Sample end region (min): 30
Marker peak width (sec): 5    Marker min peak height: 200   Marker baseline V to V?: Y    Marker baseline V to V points: 3
Lower marker selection: First peak > 500 RFU                Upper marker selection: Last peak > 200 RFU
Ladder size (bp)75, 200, 300, 400, 500, 700, 1000, 1500, 2000, 3000, 4000, 5000, 7000, 10000, 20000
Quantification using: Lower Marker          Final concentration (ng/uL): 0.5000          Dilution factor: 12.0
```

Sample: SampB11
Well location: B11
Created: Tuesday, August 10, 2021 6:41:11 PM

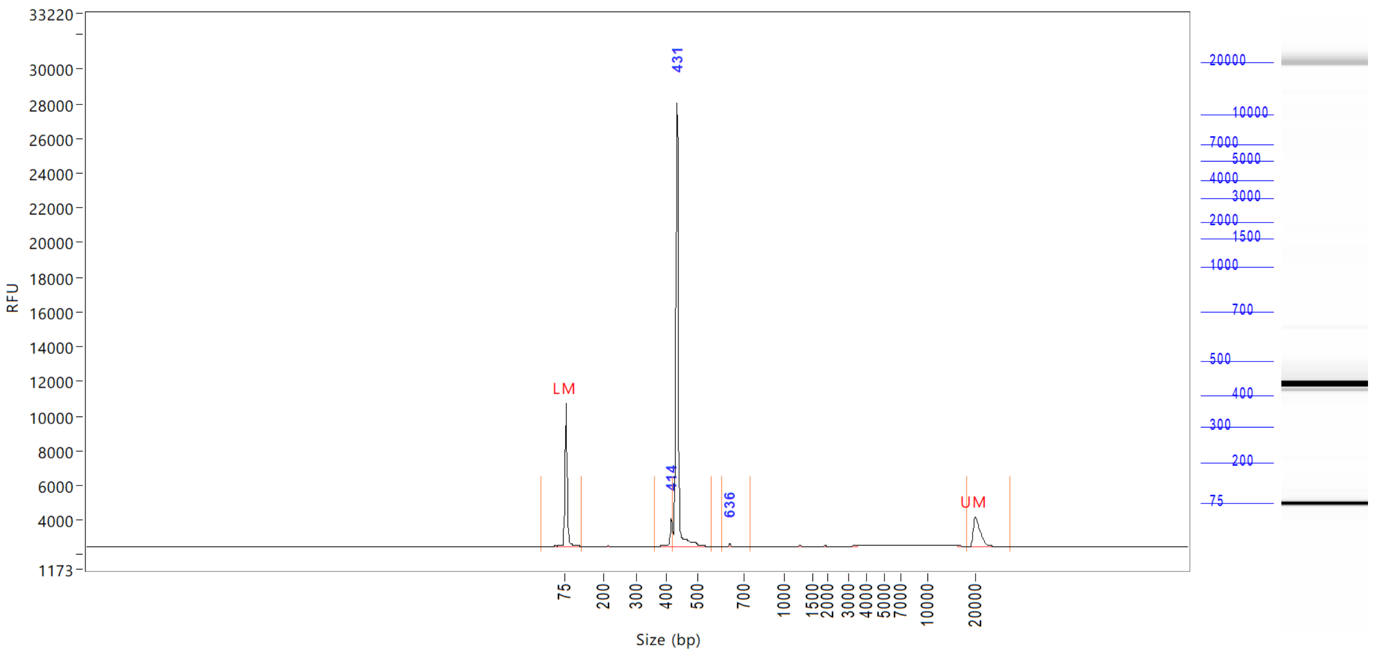

Peak	Size (bp)	Concentration (ng/uL)	From (bp)	To (bp)	Average size (bp)	CV%	RFU	Corrected peak area
1	75 (LM)	0.5000	0	124	73	12.70	8283	49.372
2	414	1.1126	360	420	410	2.31	1653	9.156
3	431	15.5168	420	557	433	2.82	25617	127.684
4	636	0.0849	606	740	637	2.61	153	0.699
5	20000 (UM)	0.1548	18293	27254	20445	4.54	1738	15.287

TIC:	16.7143	ng/uL
TIM:	63.6472	nmole/L
Total concentration:	17.1649	ng/uL

```
Sample peak width (sec): 5    Sample min peak height: 50    Sample baseline V to V?: Y    Sample baseline V to V points: 3
Sample filter: Binomial       Number of points for filter: 3  Sample start region (min): 0  Sample end region (min): 30
Marker peak width (sec): 5    Marker min peak height: 200   Marker baseline V to V?: Y    Marker baseline V to V points: 3
Lower marker selection: First peak > 500 RFU                Upper marker selection: Last peak > 200 RFU
Ladder size (bp)75, 200, 300, 400, 500, 700, 1000, 1500, 2000, 3000, 4000, 5000, 7000, 10000, 20000
Quantification using: Lower Marker          Final concentration (ng/uL): 0.5000          Dilution factor: 12.0
```

Sample: SampC11
Well location: C11
Created: Tuesday, August 10, 2021 6:41:11 PM

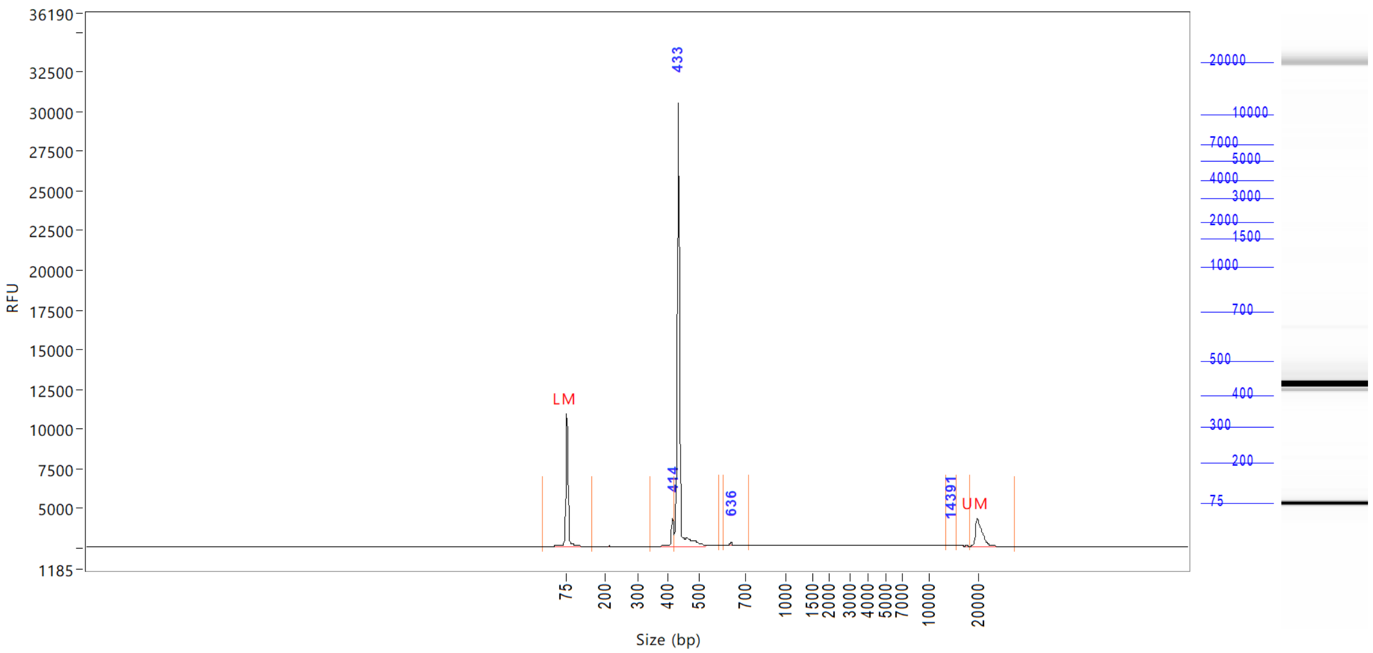

Peak	Size (bp)	Concentration (ng/uL)	From (bp)	To (bp)	Average size (bp)	CV%	RFU	Corrected peak area
1	75 (LM)	0.5000	0	156	74	15.55	8357	50.061
2	414	1.1899	343	420	410	2.59	1781	9.928
3	433	17.0184	420	588	435	3.62	27968	141.992
4	636	0.1764	608	718	646	3.86	179	1.472
5	14391	0.0327	13659	15610	14211	2.14	53	0.273
6	20000 (UM)	0.1494	18293	27859	20463	4.27	1715	14.954

TIC:	18.4174	ng/uL
TIM:	69.6000	nmole/L
Total concentration:	18.8562	ng/uL

```
Sample peak width (sec): 5    Sample min peak height: 50    Sample baseline V to V?: Y    Sample baseline V to V points: 3
Sample filter: Binomial       Number of points for filter: 3  Sample start region (min): 0  Sample end region (min): 30
Marker peak width (sec): 5    Marker min peak height: 200   Marker baseline V to V?: Y    Marker baseline V to V points: 3
Lower marker selection: First peak > 500 RFU                Upper marker selection: Last peak > 200 RFU
Ladder size (bp)75, 200, 300, 400, 500, 700, 1000, 1500, 2000, 3000, 4000, 5000, 7000, 10000, 20000
Quantification using: Lower Marker          Final concentration (ng/uL): 0.5000          Dilution factor: 12.0
```

Sample: SampD11
Well location: D11
Created: Tuesday, August 10, 2021 6:41:11 PM

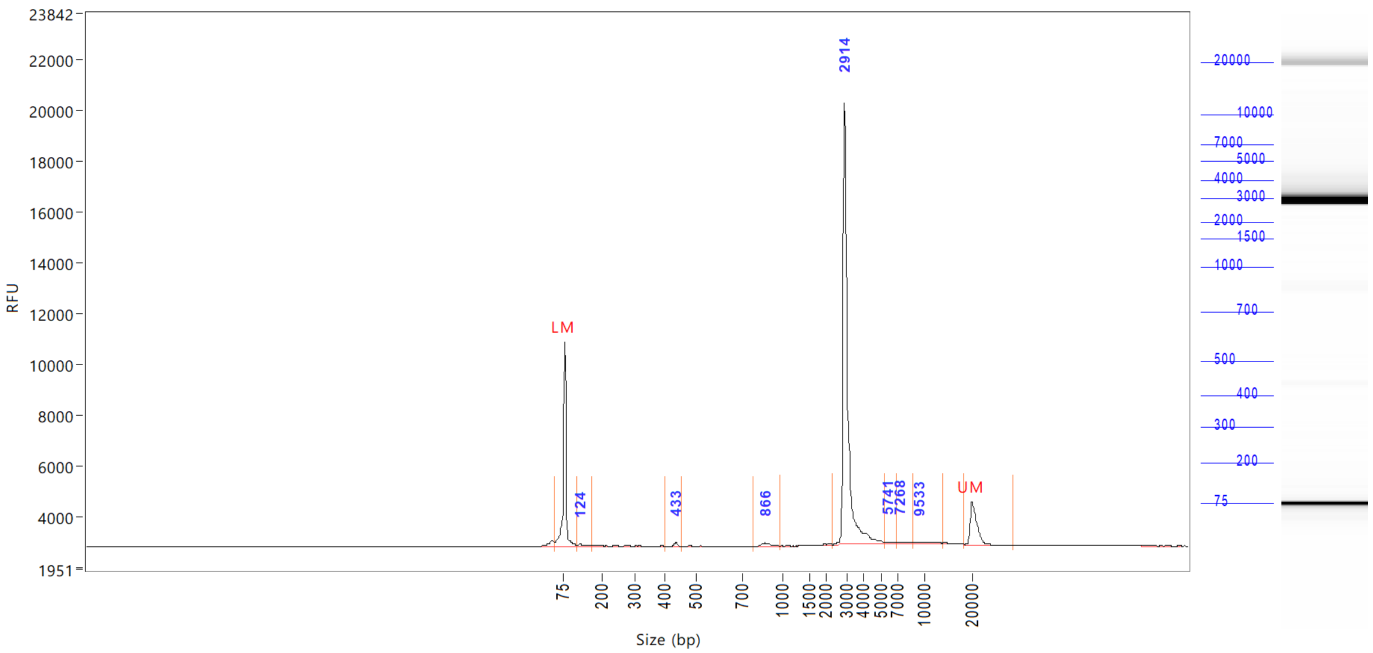

Peak	Size (bp)	Concentration (ng/uL)	From (bp)	To (bp)	Average size (bp)	CV%	RFU	Corrected peak area
1	75 (LM)	0.5000	41	116	71	12.44	8024	55.285
2	124	0.1606	116	164	134	9.99	105	1.480
3	433	0.1094	401	450	430	1.84	138	1.008
4	866	0.2116	779	974	874	3.99	133	1.950
5	2914	12.9404	2344	5445	3050	12.56	17414	119.234
6	5741	0.1343	5445	7000	6185	7.46	84	1.237
7	7268	0.1162	7000	8734	7714	6.82	77	1.070
8	9533	0.1514	8734	13781	10643	12.92	60	1.395
9	20000 (UM)	0.1321	18293	28463	20421	4.59	1664	14.609

TIC:	13.8238	ng/uL
TIM:	9.8551	nmole/L
Total concentration:	14.1838	ng/uL

```
Sample peak width (sec): 5     Sample min peak height: 50    Sample baseline V to V?: Y    Sample baseline V to V points: 3
Sample filter: Binomial        Number of points for filter: 3  Sample start region (min): 0  Sample end region (min): 30
Marker peak width (sec): 5     Marker min peak height: 200   Marker baseline V to V?: Y    Marker baseline V to V points: 3
Lower marker selection: First peak > 500 RFU                 Upper marker selection: Last peak > 200 RFU
Ladder size (bp)75, 200, 300, 400, 500, 700, 1000, 1500, 2000, 3000, 4000, 5000, 7000, 10000, 20000
Quantification using: Lower Marker          Final concentration (ng/uL): 0.5000          Dilution factor: 12.0
```

Sample: SampE11
Well location: E11
Created: Tuesday, August 10, 2021 6:41:11 PM

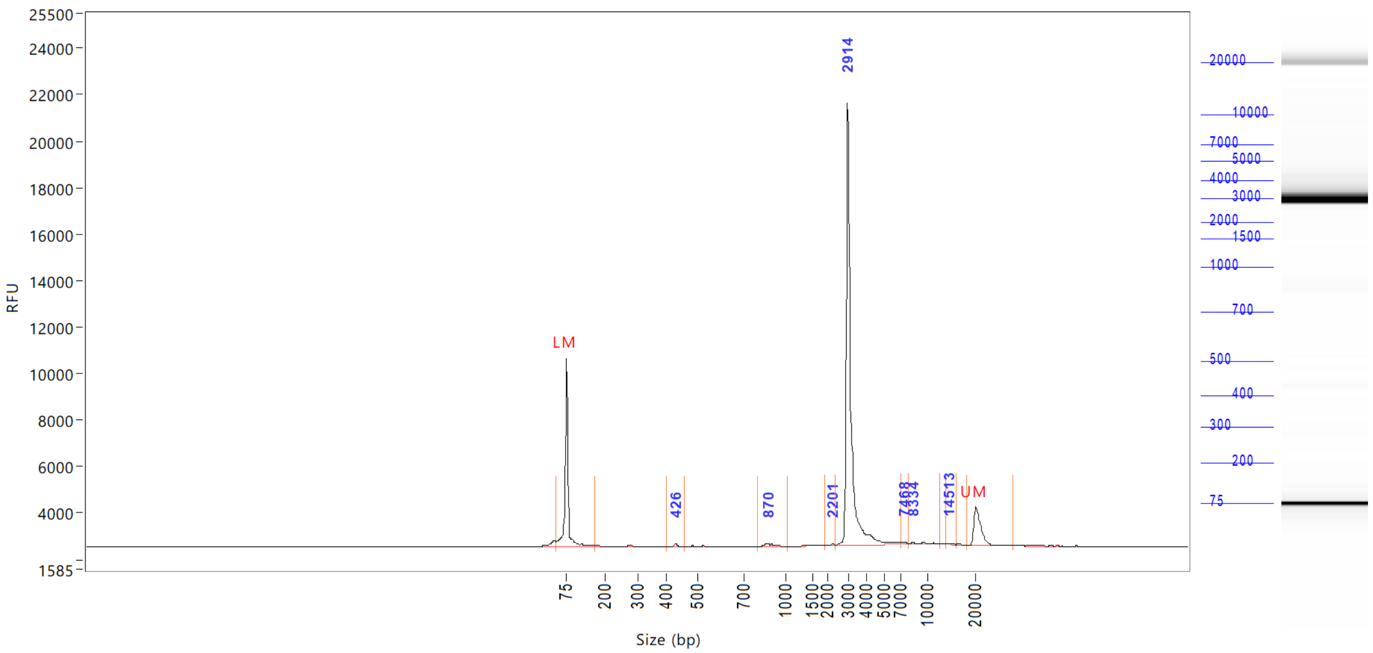

Peak	Size (bp)	Concentration (ng/uL)	From (bp)	To (bp)	Average size (bp)	CV%	RFU	Corrected peak area
1	75 (LM)	0.5000	41	166	74	19.66	8076	58.837
2	426	0.0807	400	456	425	2.15	107	0.792
3	870	0.1833	792	1034	881	4.51	106	1.798
4	2201	0.0437	1904	2344	2127	5.31	65	0.428
5	2914	13.4847	2344	7068	3099	15.83	19051	132.233
6	7468	0.0625	7068	7934	7453	3.36	81	0.613
7	8334	0.1785	7934	12684	9787	13.05	67	1.750
8	14513	0.0310	13903	15976	14562	3.36	56	0.304
9	20000 (UM)	0.1267	18293	27738	20515	4.15	1690	14.912

TIC:	14.0644	ng/uL
TIM:	7.8994	nmole/L
Total concentration:	14.3920	ng/uL

```
Sample peak width (sec): 5     Sample min peak height: 50     Sample baseline V to V?: Y     Sample baseline V to V points: 3
Sample filter: Binomial        Number of points for filter: 3  Sample start region (min): 0  Sample end region (min): 30
Marker peak width (sec): 5     Marker min peak height: 200    Marker baseline V to V?: Y     Marker baseline V to V points: 3
Lower marker selection: First peak > 500 RFU                  Upper marker selection: Last peak > 200 RFU
Ladder size (bp)75, 200, 300, 400, 500, 700, 1000, 1500, 2000, 3000, 4000, 5000, 7000, 10000, 20000
Quantification using: Lower Marker          Final concentration (ng/uL): 0.5000          Dilution factor: 12.0
```

Sample: SampF11
Well location: F11
Created: Tuesday, August 10, 2021 6:41:11 PM

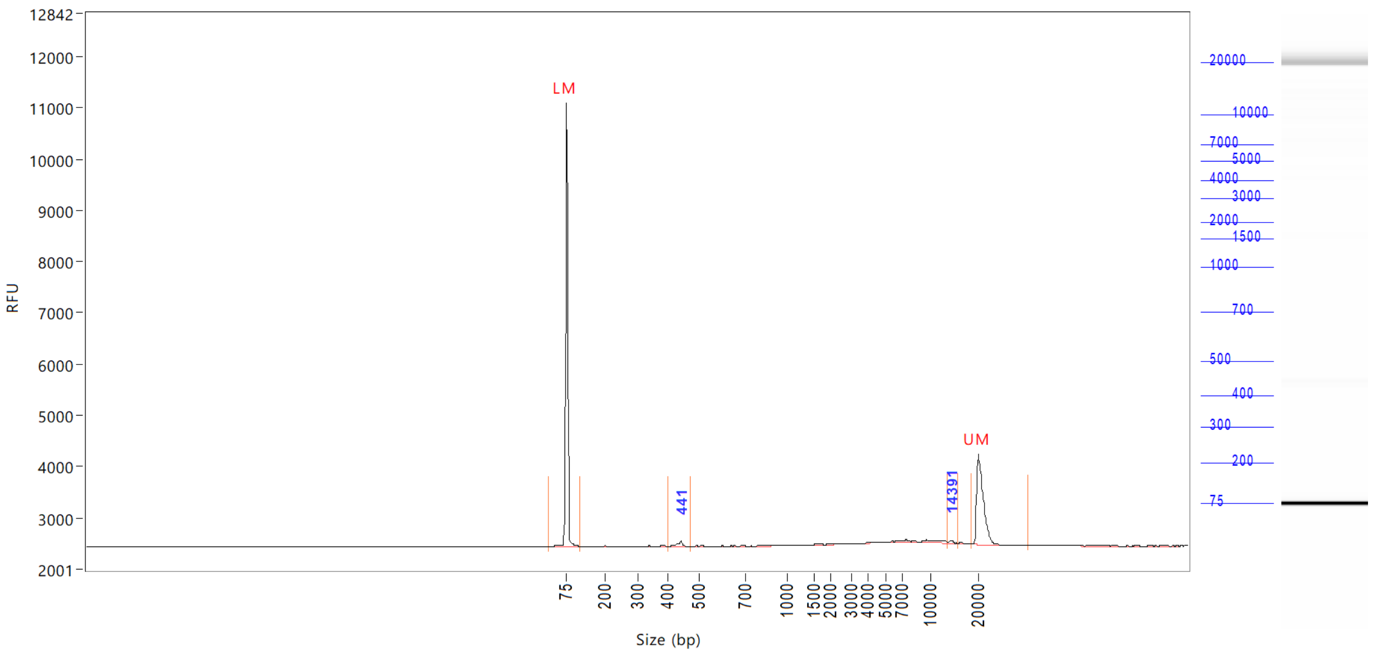

Peak	Size (bp)	Concentration (ng/uL)	From (bp)	To (bp)	Average size (bp)	CV%	RFU	Corrected peak area
1	75 (LM)	0.5000	18	118	73	6.00	8662	46.336
2	441	0.1221	398	473	434	2.79	107	0.943
3	14391	0.0483	13659	15854	14419	3.87	63	0.373
4	20000 (UM)	0.1669	18537	30156	20451	4.25	1756	15.465

TIC:	0.1704	ng/uL
TIM:	0.4683	nmole/L
Total concentration:	0.6858	ng/uL

```
Sample peak width (sec): 5    Sample min peak height: 50    Sample baseline V to V?: Y    Sample baseline V to V points: 3
Sample filter: Binomial       Number of points for filter: 3  Sample start region (min): 0  Sample end region (min): 30
Marker peak width (sec): 5    Marker min peak height: 200   Marker baseline V to V?: Y    Marker baseline V to V points: 3
Lower marker selection: First peak > 500 RFU                Upper marker selection: Last peak > 200 RFU
Ladder size (bp)75, 200, 300, 400, 500, 700, 1000, 1500, 2000, 3000, 4000, 5000, 7000, 10000, 20000
Quantification using: Lower Marker          Final concentration (ng/uL): 0.5000          Dilution factor: 12.0
```

Sample: SampG11
Well location: G11
Created: Tuesday, August 10, 2021 6:41:11 PM

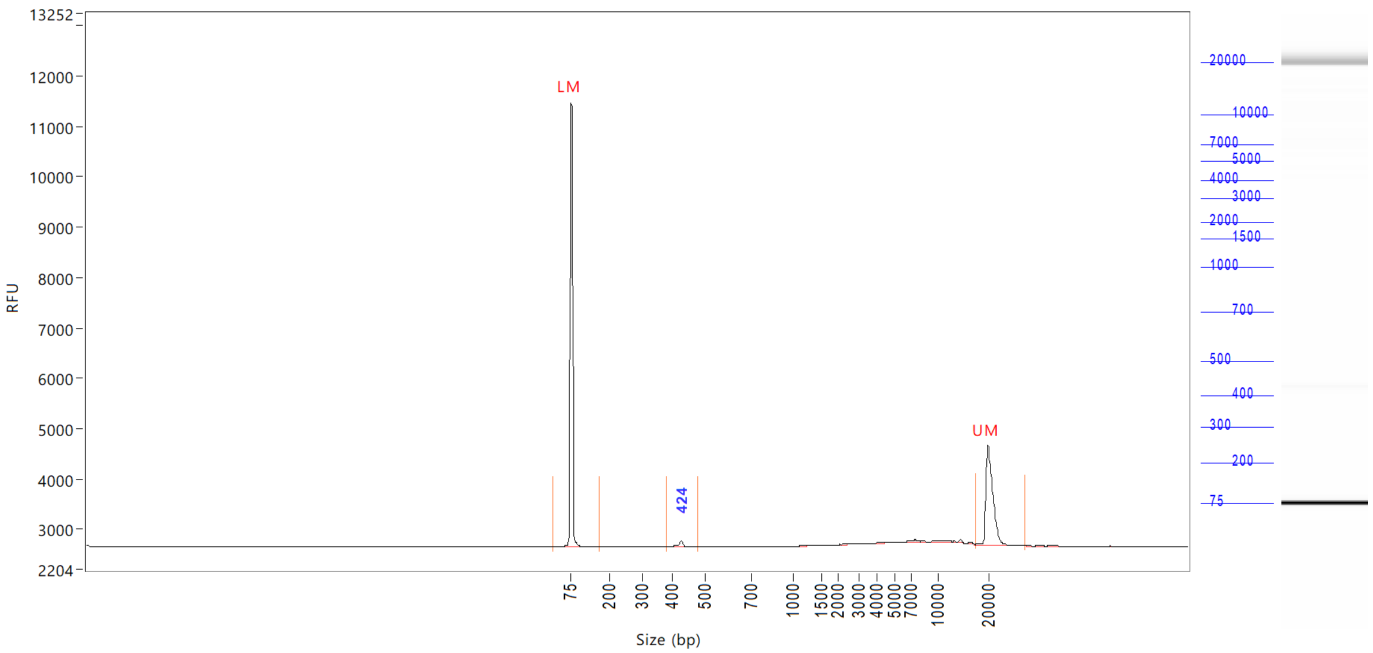

Peak	Size (bp)	Concentration (ng/uL)	From (bp)	To (bp)	Average size (bp)	CV%	RFU	Corrected peak area
1	75 (LM)	0.5000	20	168	74	8.02	8835	48.945
2	424	0.1274	378	475	422	3.68	131	1.039
3	20000 (UM)	0.1781	17439	27617	20483	4.31	1970	17.433

TIC:	0.1274	ng/uL
TIM:	0.4968	nmole/L
Total concentration:	0.5281	ng/uL

Sample peak width (sec): 5 Sample min peak height: 50 Sample baseline V to V?: Y Sample baseline V to V points: 3
Sample filter: Binomial Number of points for filter: 3 Sample start region (min): 0 Sample end region (min): 30
Marker peak width (sec): 5 Marker min peak height: 200 Marker baseline V to V?: Y Marker baseline V to V points: 3
Lower marker selection: First peak > 500 RFU Upper marker selection: Last peak > 200 RFU
Ladder size (bp)75, 200, 300, 400, 500, 700, 1000, 1500, 2000, 3000, 4000, 5000, 7000, 10000, 20000
Quantification using: Lower Marker Final concentration (ng/uL): 0.5000 Dilution factor: 12.0

Sample: SampH11
Well location: H11
Created: Tuesday, August 10, 2021 6:41:11 PM

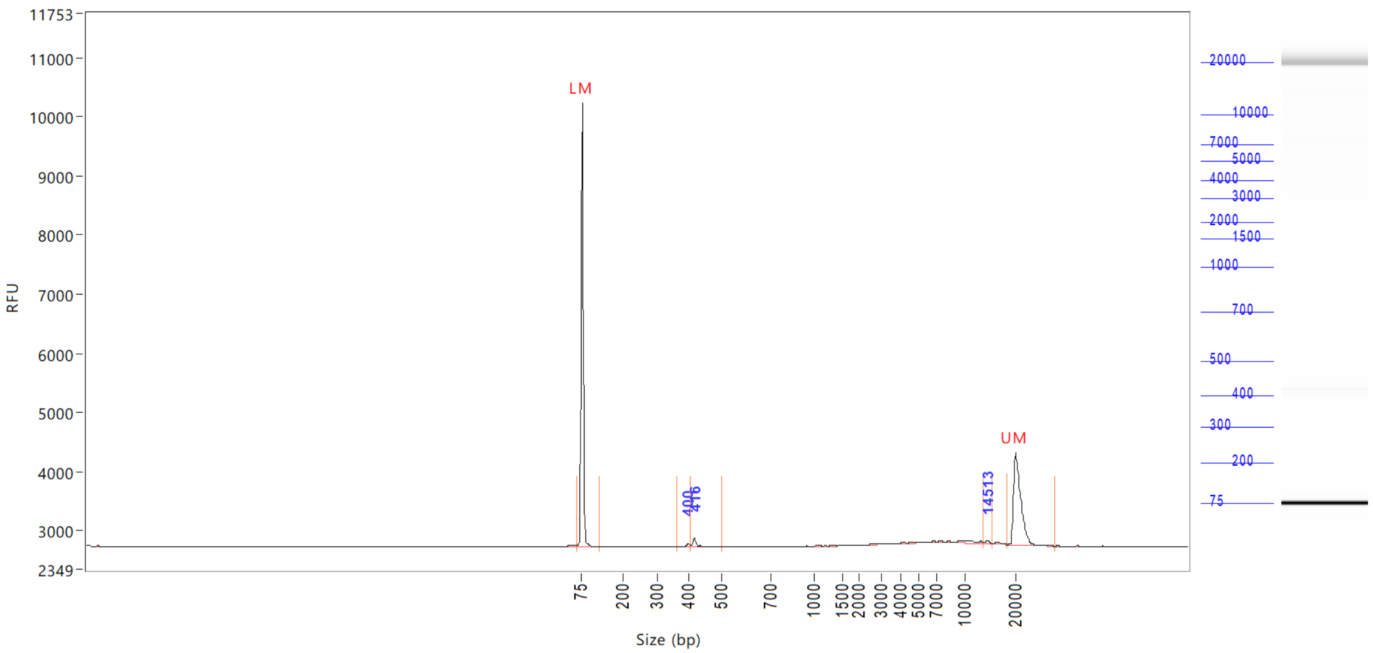

Peak	Size	Concentration	From	To	Average size	CV%	RFU	Corrected peak area
	(bp)	(ng/uL)	(bp)	(bp)	(bp)			
1	75 (LM)	0.5000	60	129	74	5.03	7518	39.806
2	400	0.0623	366	405	394	2.25	60	0.414
3	416	0.1661	405	500	424	4.84	156	1.102
4	14513	0.0465	13659	15366	14367	2.70	56	0.309
5	20000 (UM)	0.1751	18415	27738	20418	4.27	1548	13.937

TIC:	0.2750	ng/uL
TIM:	0.9102	nmole/L
Total concentration:	0.8430	ng/uL

```
Sample peak width (sec): 5    Sample min peak height: 50    Sample baseline V to V?: Y    Sample baseline V to V points: 3
Sample filter: Binomial       Number of points for filter: 3  Sample start region (min): 0  Sample end region (min): 30
Marker peak width (sec): 5    Marker min peak height: 200   Marker baseline V to V?: Y    Marker baseline V to V points: 3
Lower marker selection: First peak > 500 RFU                Upper marker selection: Last peak > 200 RFU
Ladder size (bp)75, 200, 300, 400, 500, 700, 1000, 1500, 2000, 3000, 4000, 5000, 7000, 10000, 20000
Quantification using: Lower Marker          Final concentration (ng/uL): 0.5000          Dilution factor: 12.0
```

Sample: SampA12
Well location: A12
Created: Tuesday, August 10, 2021 6:41:11 PM

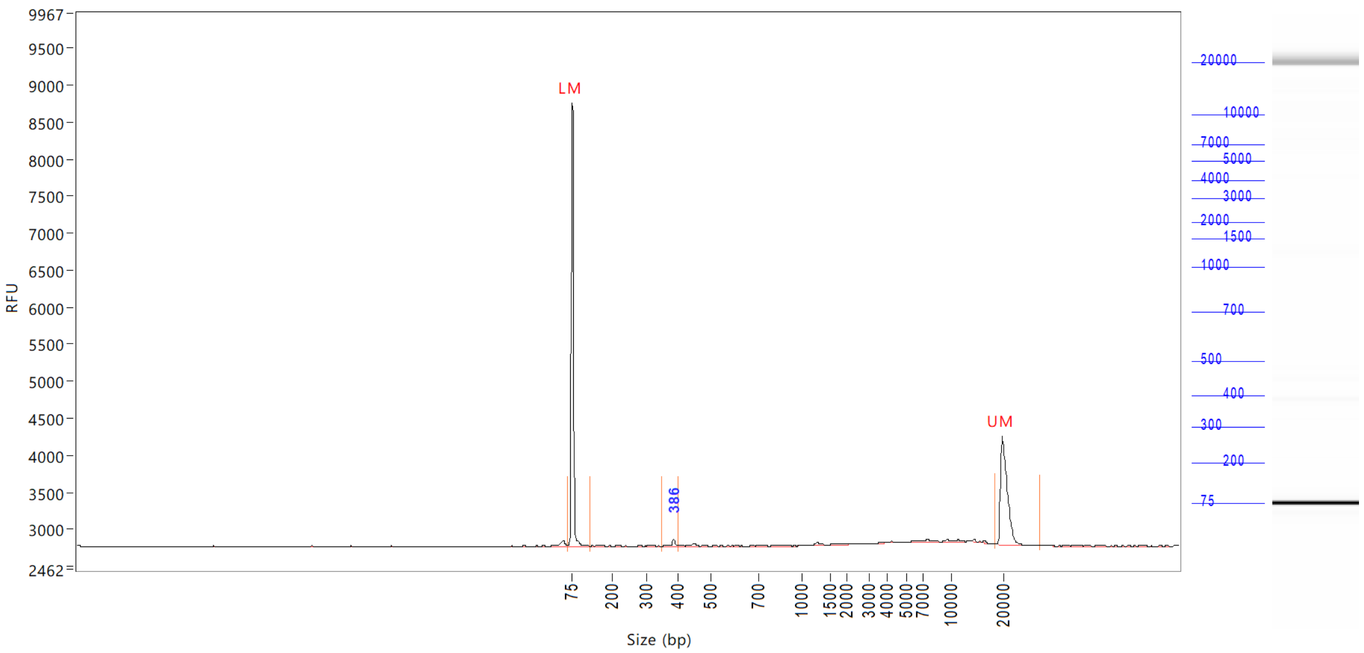

Peak	Size (bp)	Concentration (ng/uL)	From (bp)	To (bp)	Average size (bp)	CV%	RFU	Corrected peak area
1	75 (LM)	0.5000	62	129	74	6.54	5996	32.566
2	386	0.1209	345	400	379	2.74	95	0.656
3	20000 (UM)	0.1882	18537	27496	20409	3.88	1474	12.256

TIC:	0.1209	ng/uL
TIM:	0.5247	nmole/L
Total concentration:	0.8344	ng/uL

Sample peak width (sec): 5   Sample min peak height: 50   Sample baseline V to V?: Y   Sample baseline V to V points: 3
Sample filter: Binomial   Number of points for filter: 3 Sample start region (min): 0 Sample end region (min): 30
Marker peak width (sec): 5   Marker min peak height: 200   Marker baseline V to V?: Y   Marker baseline V to V points: 3
Lower marker selection: First peak > 500 RFU   Upper marker selection: Last peak > 200 RFU
Ladder size (bp)75, 200, 300, 400, 500, 700, 1000, 1500, 2000, 3000, 4000, 5000, 7000, 10000, 20000
Quantification using: Lower Marker   Final concentration (ng/uL): 0.5000   Dilution factor: 12.0

Sample: SampB12
Well location: B12
Created: Tuesday, August 10, 2021 6:41:11 PM

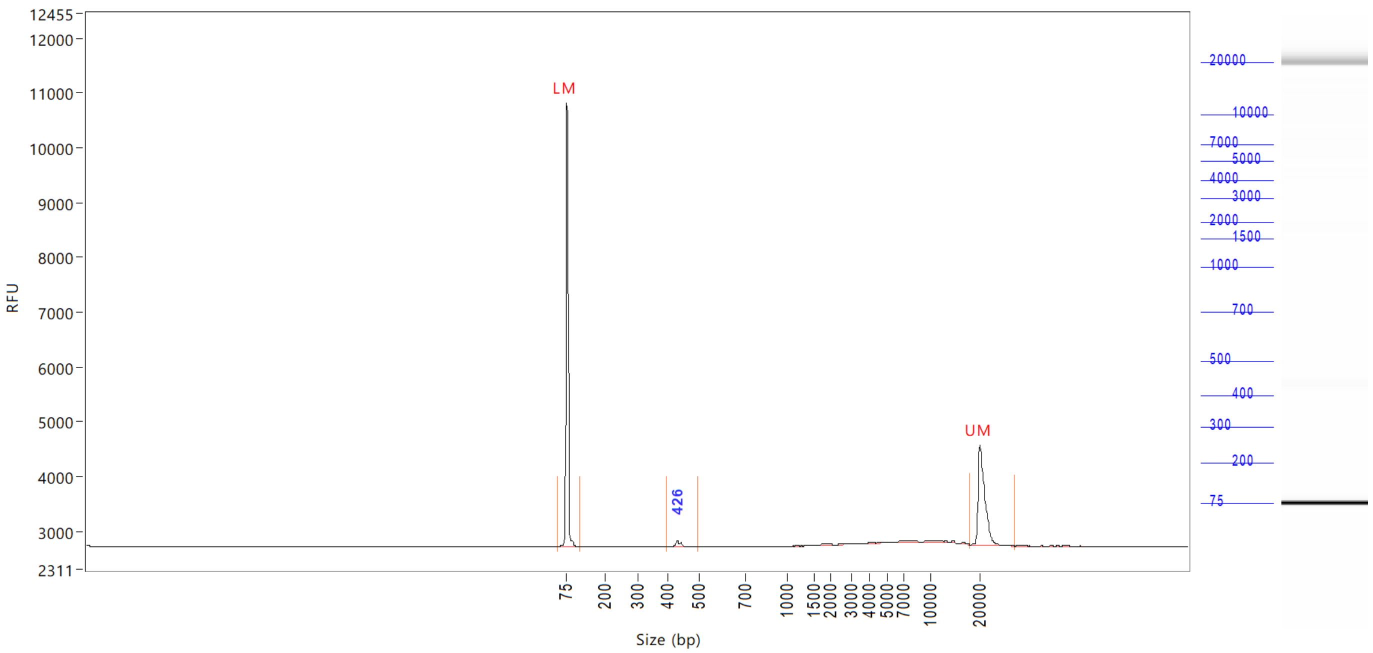

Peak	Size (bp)	Concentration (ng/uL)	From (bp)	To (bp)	Average size (bp)	CV%	RFU	Corrected peak area
1	75 (LM)	0.5000	48	120	74	5.15	8110	44.614
2	426	0.1648	396	494	433	4.03	104	1.225
3	20000 (UM)	0.1780	18049	27254	20507	4.34	1812	15.886

TIC:	0.1648	ng/uL
TIM:	0.6262	nmole/L
Total concentration:	0.6785	ng/uL

Sample peak width (sec): 5   Sample min peak height: 50   Sample baseline V to V?: Y   Sample baseline V to V points: 3
Sample filter: Binomial   Number of points for filter: 3   Sample start region (min): 0   Sample end region (min): 30
Marker peak width (sec): 5   Marker min peak height: 200   Marker baseline V to V?: Y   Marker baseline V to V points: 3
Lower marker selection: First peak > 500 RFU   Upper marker selection: Last peak > 200 RFU
Ladder size (bp)75, 200, 300, 400, 500, 700, 1000, 1500, 2000, 3000, 4000, 5000, 7000, 10000, 20000
Quantification using: Lower Marker   Final concentration (ng/uL): 0.5000   Dilution factor: 12.0

Sample: SampC12
Well location: C12
Created: Tuesday, August 10, 2021 6:41:11 PM

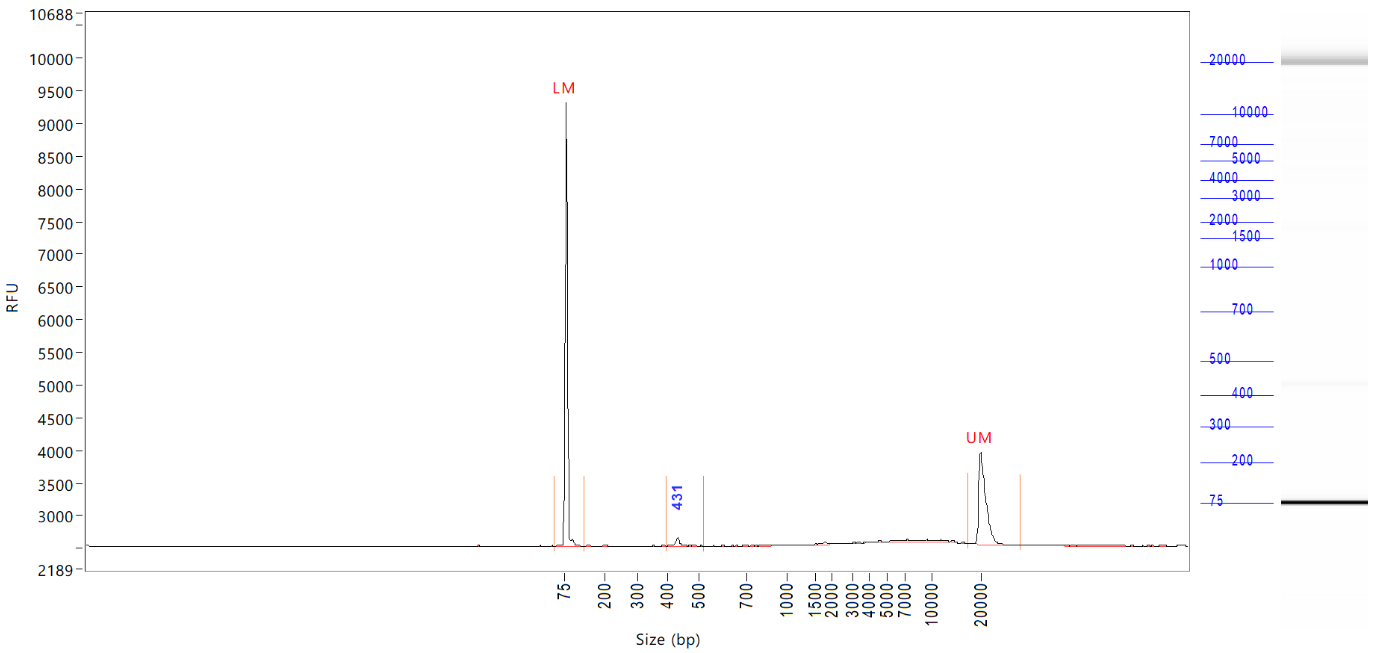

Peak	Size (bp)	Concentration (ng/uL)	From (bp)	To (bp)	Average size (bp)	CV%	RFU	Corrected peak area
1	75 (LM)	0.5000	37	133	74	6.67	6792	40.244
2	431	0.1908	396	519	433	4.48	142	1.280
3	20000 (UM)	0.1634	17439	28221	20514	4.83	1417	13.154

TIC:	0.1908	ng/uL
TIM:	0.7252	nmole/L
Total concentration:	0.7011	ng/uL

Sample peak width (sec): 5   Sample min peak height: 50   Sample baseline V to V?: Y   Sample baseline V to V points: 3
Sample filter: Binomial   Number of points for filter: 3   Sample start region (min): 0   Sample end region (min): 30
Marker peak width (sec): 5   Marker min peak height: 200   Marker baseline V to V?: Y   Marker baseline V to V points: 3
Lower marker selection: First peak > 500 RFU   Upper marker selection: Last peak > 200 RFU
Ladder size (bp)75, 200, 300, 400, 500, 700, 1000, 1500, 2000, 3000, 4000, 5000, 7000, 10000, 20000
Quantification using: Lower Marker   Final concentration (ng/uL): 0.5000   Dilution factor: 12.0

Sample: SampD12
Well location: D12
Created: Tuesday, August 10, 2021 6:41:11 PM

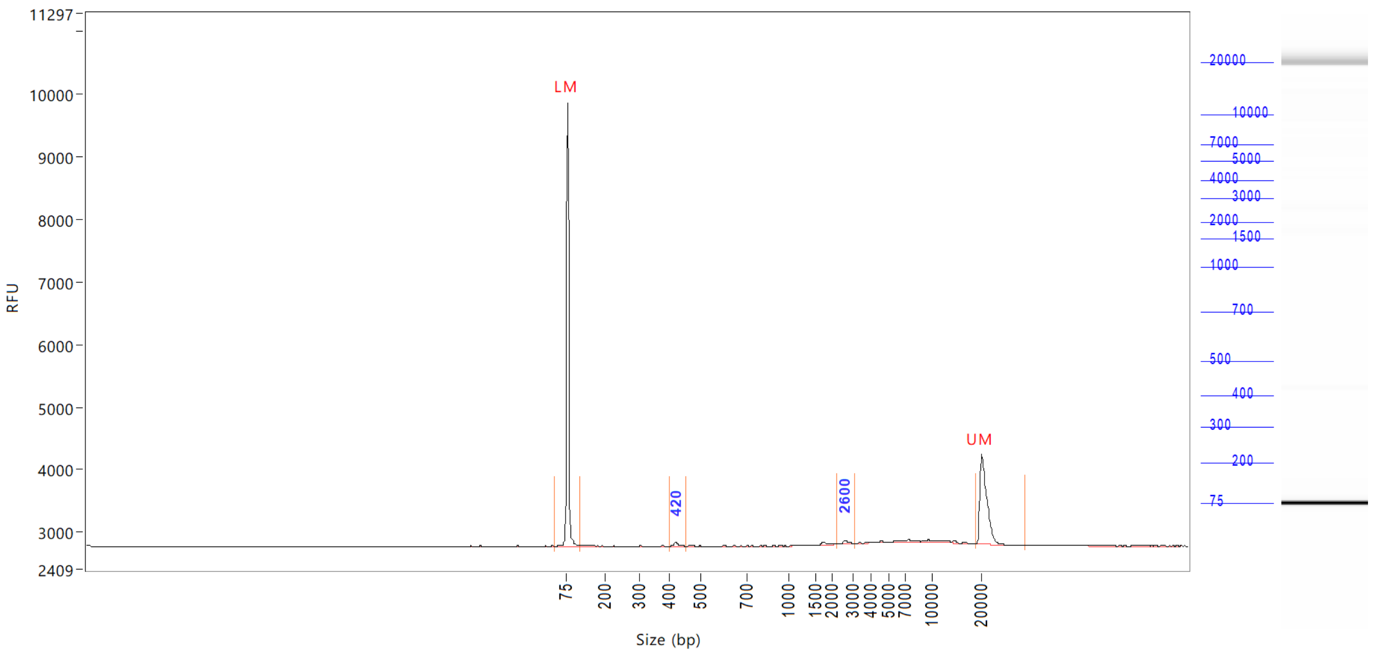

Peak	Size (bp)	Concentration (ng/uL)	From (bp)	To (bp)	Average size (bp)	CV%	RFU	Corrected peak area
1	75 (LM)	0.5000	33	118	74	6.78	7102	39.882
2	420	0.0809	400	454	420	2.24	65	0.538
3	2600	0.0712	2172	3001	2613	5.80	61	0.474
4	20000 (UM)	0.1640	18781	28947	20534	4.53	1451	13.083

TIC:	0.1521	ng/uL
TIM:	0.3617	nmole/L
Total concentration:	0.7516	ng/uL

Sample peak width (sec): 5   Sample min peak height: 50   Sample baseline V to V?: Y   Sample baseline V to V points: 3
Sample filter: Binomial   Number of points for filter: 3 Sample start region (min): 0 Sample end region (min): 30
Marker peak width (sec): 5   Marker min peak height: 200   Marker baseline V to V?: Y   Marker baseline V to V points: 3
Lower marker selection: First peak > 500 RFU   Upper marker selection: Last peak > 200 RFU
Ladder size (bp)75, 200, 300, 400, 500, 700, 1000, 1500, 2000, 3000, 4000, 5000, 7000, 10000, 20000
Quantification using: Lower Marker   Final concentration (ng/uL): 0.5000   Dilution factor: 12.0

Sample: SampE12
Well location: E12
Created: Tuesday, August 10, 2021 6:41:11 PM

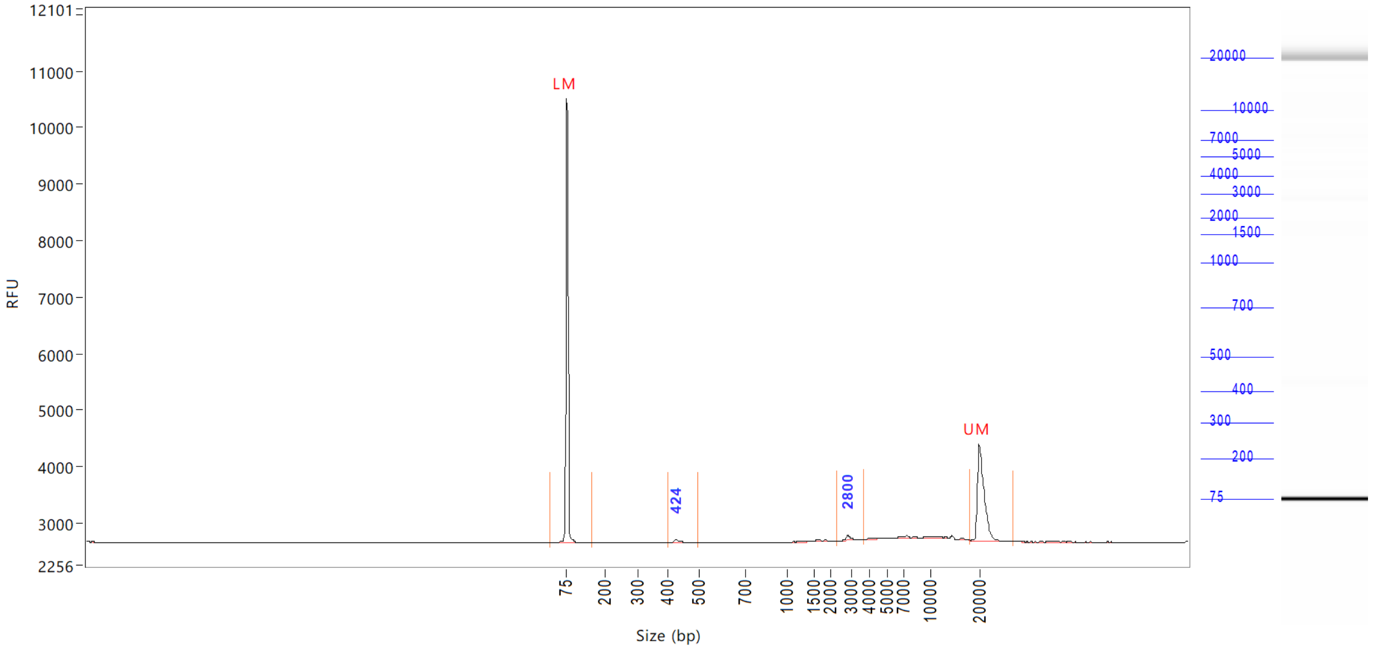

Peak	Size (bp)	Concentration (ng/uL)	From (bp)	To (bp)	Average size (bp)	CV%	RFU	Corrected peak area
1	75 (LM)	0.5000	24	156	74	6.07	7870	43.057
2	424	0.0923	398	496	434	4.76	53	0.662
3	2800	0.0988	2258	3715	2887	7.46	86	0.709
4	20000 (UM)	0.1737	18171	26891	20479	4.21	1699	14.956

TIC:	0.1912	ng/uL
TIM:	0.4064	nmole/L
Total concentration:	0.6453	ng/uL

Sample peak width (sec): 5 Sample min peak height: 50 Sample baseline V to V?: Y Sample baseline V to V points: 3
Sample filter: Binomial Number of points for filter: 3 Sample start region (min): 0 Sample end region (min): 30
Marker peak width (sec): 5 Marker min peak height: 200 Marker baseline V to V?: Y Marker baseline V to V points: 3
Lower marker selection: First peak > 500 RFU Upper marker selection: Last peak > 200 RFU
Ladder size (bp)75, 200, 300, 400, 500, 700, 1000, 1500, 2000, 3000, 4000, 5000, 7000, 10000, 20000
Quantification using: Lower Marker Final concentration (ng/uL): 0.5000 Dilution factor: 12.0

Sample: SampF12
Well location: F12
Created: Tuesday, August 10, 2021 6:41:11 PM

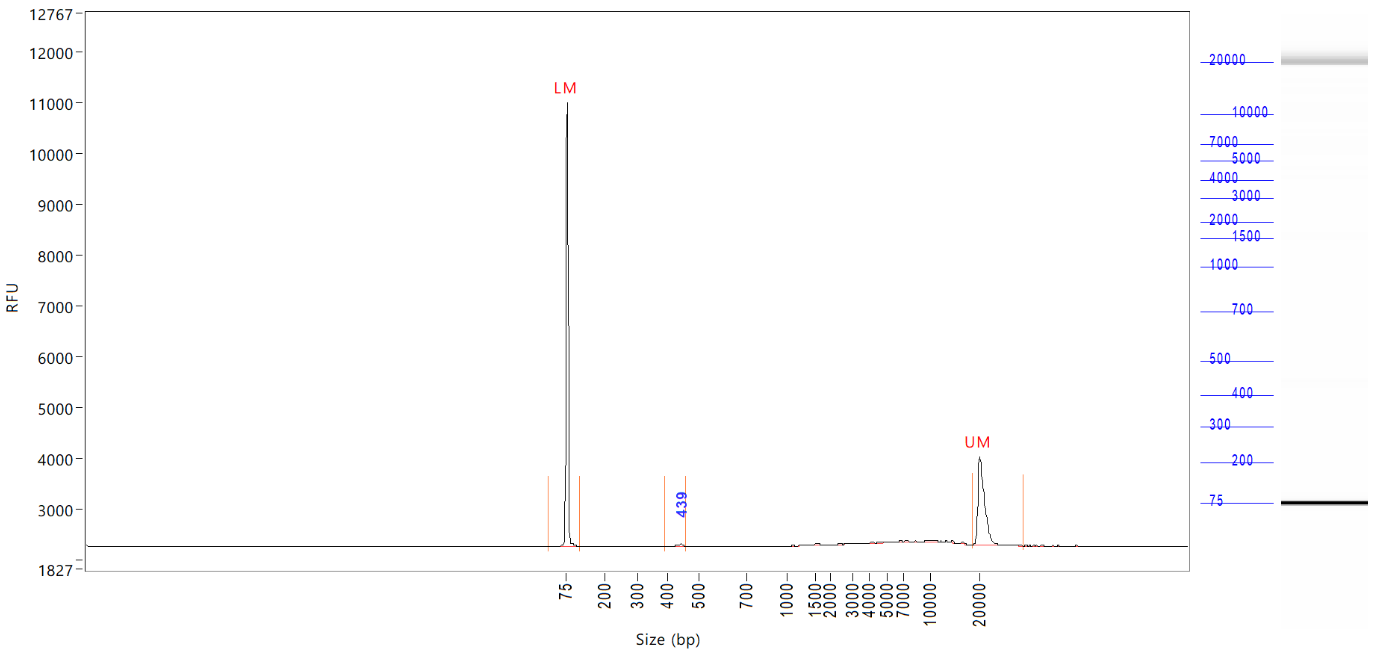

Peak	Size (bp)	Concentration (ng/uL)	From (bp)	To (bp)	Average size (bp)	CV%	RFU	Corrected peak area
1	75 (LM)	0.5000	18	118	73	5.91	8746	46.409
2	439	0.0679	388	456	429	2.96	53	0.526
3	20000 (UM)	0.1601	18537	29068	20492	4.19	1715	14.858

TIC:	0.0679	ng/uL
TIM:	0.2606	nmole/L
Total concentration:	0.4646	ng/uL

```
Sample peak width (sec): 5    Sample min peak height: 50    Sample baseline V to V?: Y    Sample baseline V to V points: 3
Sample filter: Binomial       Number of points for filter: 3  Sample start region (min): 0  Sample end region (min): 30
Marker peak width (sec): 5    Marker min peak height: 200   Marker baseline V to V?: Y    Marker baseline V to V points: 3
Lower marker selection: First peak > 500 RFU                Upper marker selection: Last peak > 200 RFU
Ladder size (bp)75, 200, 300, 400, 500, 700, 1000, 1500, 2000, 3000, 4000, 5000, 7000, 10000, 20000
Quantification using: Lower Marker          Final concentration (ng/uL): 0.5000          Dilution factor: 12.0
```

Sample: SampG12
Well location: G12
Created: Tuesday, August 10, 2021 6:41:11 PM

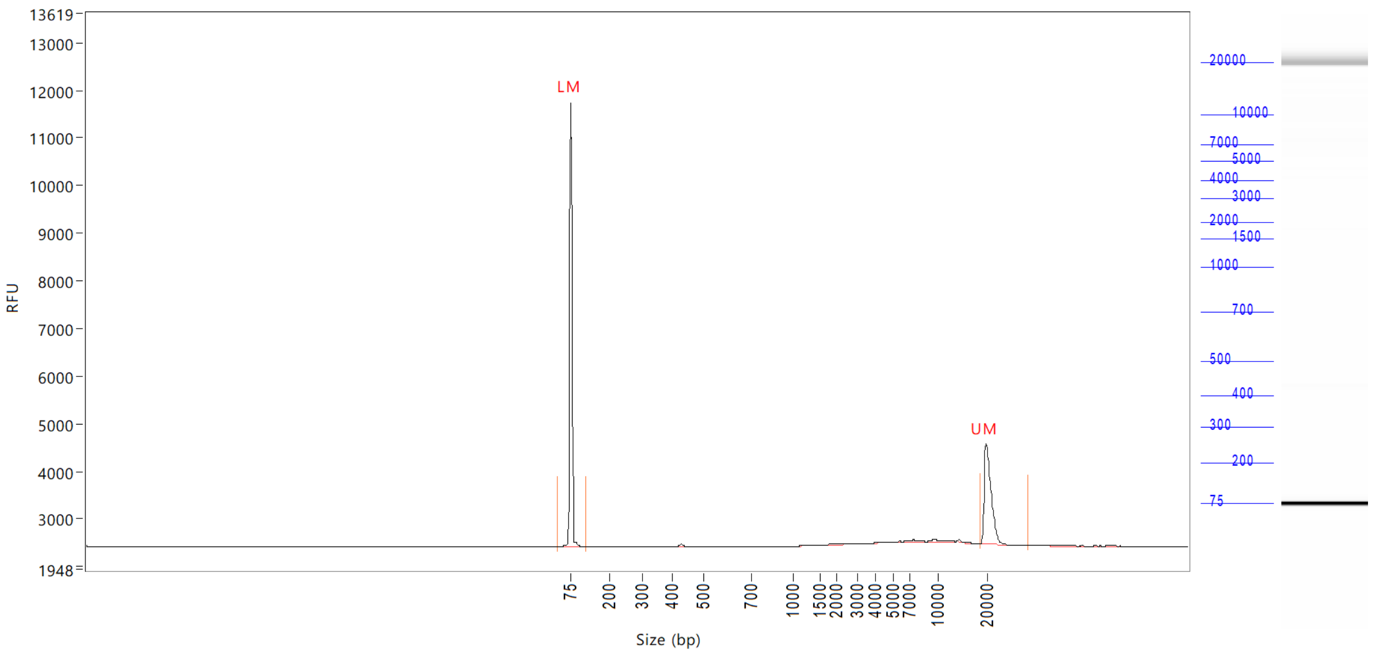

Peak	Size (bp)	Concentration (ng/uL)	From (bp)	To (bp)	Average size (bp)	CV%	RFU	Corrected peak area
1	75 (LM)	0.5000	33	126	72	5.75	9329	51.255
2	20000 (UM)	0.1803	18659	28584	20382	4.02	2110	18.478

TIC:	0.0000	ng/uL
TIM:	0.0000	nmole/L
Total concentration:	0.4873	ng/uL

Sample peak width (sec): 5 Sample min peak height: 50 Sample baseline V to V?: Y Sample baseline V to V points: 3
Sample filter: Binomial Number of points for filter: 3 Sample start region (min): 0 Sample end region (min): 30
Marker peak width (sec): 5 Marker min peak height: 200 Marker baseline V to V?: Y Marker baseline V to V points: 3
Lower marker selection: First peak > 500 RFU Upper marker selection: Last peak > 200 RFU
Ladder size (bp)75, 200, 300, 400, 500, 700, 1000, 1500, 2000, 3000, 4000, 5000, 7000, 10000, 20000
Quantification using: Lower Marker Final concentration (ng/uL): 0.5000 Dilution factor: 12.0

Sample: SampH12
Well location: H12
Created: Tuesday, August 10, 2021 6:41:11 PM

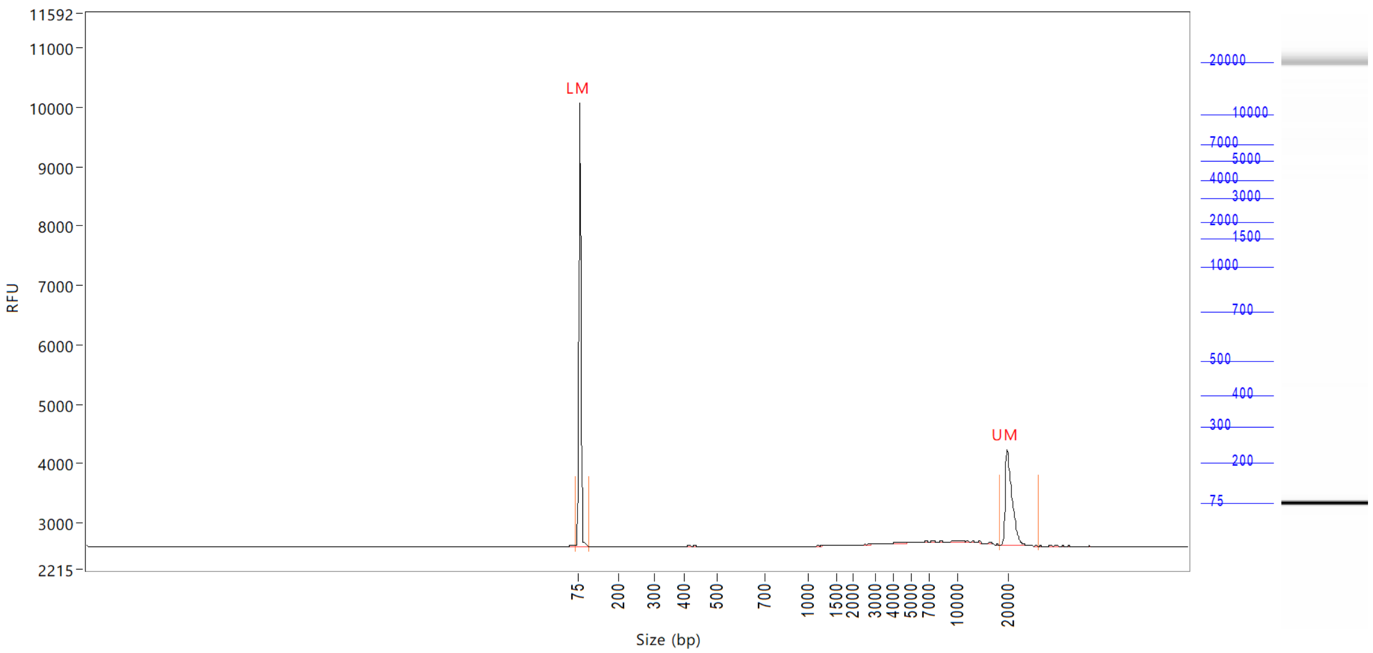

Peak	Size (bp)	Concentration (ng/uL)	From (bp)	To (bp)	Average size (bp)	CV%	RFU	Corrected peak area
1	75 (LM)	0.5000	64	103	74	4.50	7499	39.565
2	20000 (UM)	0.1755	18537	26166	20413	3.98	1616	13.887

TIC:	0.0000	ng/uL
TIM:	0.0000	nmole/L
Total concentration:	0.5661	ng/uL

```
Sample peak width (sec): 5    Sample min peak height: 200   Sample baseline V to V?: Y    Sample baseline V to V points: 3
Sample filter: Binomial       Number of points for filter: 3  Sample start region (min): 0  Sample end region (min): 30
Marker peak width (sec): 5    Marker min peak height: 200   Marker baseline V to V?: Y    Marker baseline V to V points: 3
Lower marker selection: First peak > 500 RFU                Upper marker selection: Last peak > 200 RFU
Ladder size (bp)75, 200, 300, 400, 500, 700, 1000, 1500, 2000, 3000, 4000, 5000, 7000, 10000, 20000
Quantification using: Lower Marker          Final concentration (ng/uL): 0.5000          Dilution factor: 12.0
```

Sample: SampA7
Well location: A7
Created: Tuesday, August 10, 2021 6:41:11 PM
Fit type: Point to point

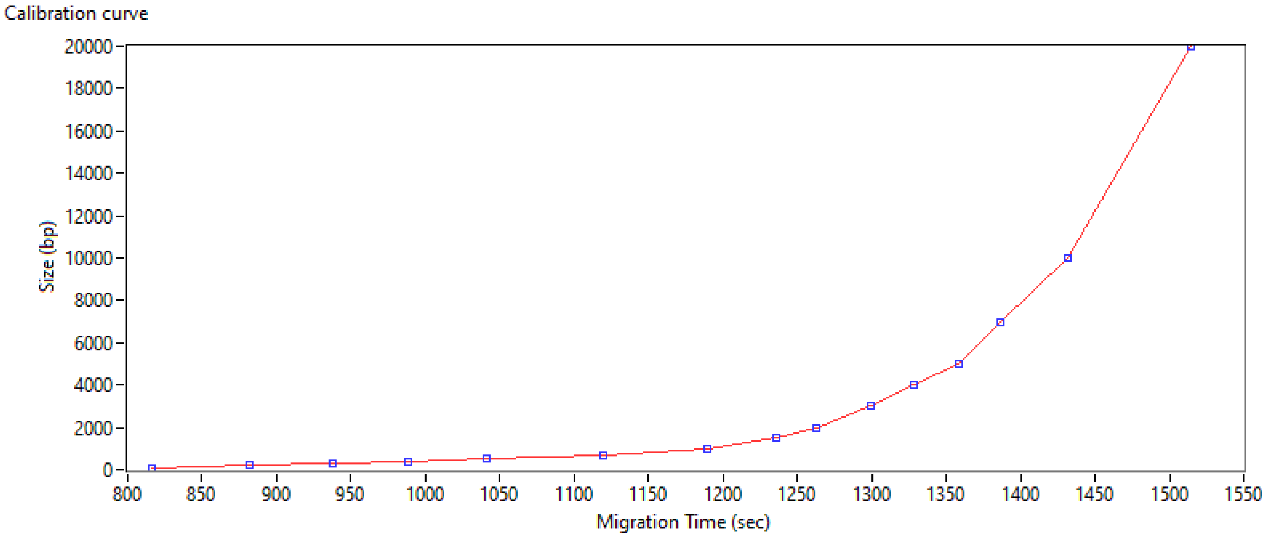

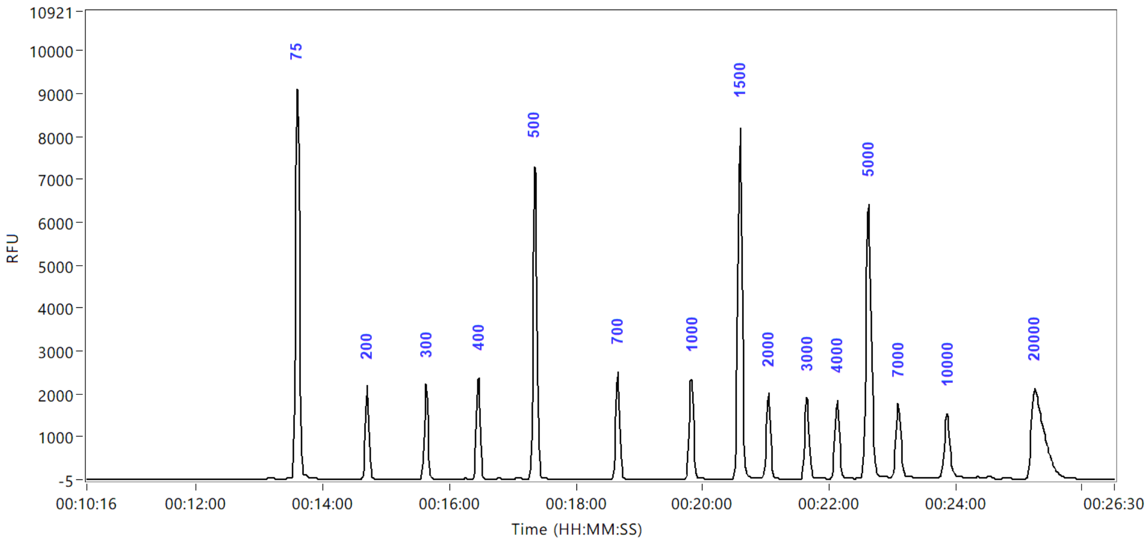

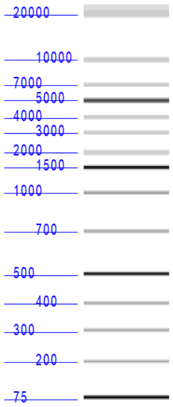

Supplement: Supplementary file 2 — sb3c00292_si_002.zip [file sb3c00292_si_002.zip › dnada_supplementary_material_pks_library_build/ZAG_trials/ZAG_Trial_1/18-20-33_plate1/plate1_report.pdf]
