# Supplementary material for: Automated Platform for the Plasmid Construction Process
Source: ACS Synth Biol. 2023 Nov 10;12(12):3506–13. doi: 10.1021/acssynbio.3c00292 (PMC10729297; doi:10.1021/acssynbio.3c00292)

Instrument controller software run summary:

Filename and data path: C:\AATI\Data\2021 08 10\19-11-21\2021 08 10 19H 11M..raw
Created: Tuesday, August 10, 2021 7:21:14 PM
Number of capillaries: 96
Array serial number: 111920-09SFS
Effect length: 33 cm
Array usage count: 165
Instrument type: ZAG DNA Analyzer
Instrument controller software version: 1.0.1.9
Device serial number: Z7597

Method Information

Method name: ZAG130GP33 - DNA 75-20000bp - Gel Prime Only.mthds
Gel prime: Yes
Full conditioning: No
Gel prime to buffer: No
Gel selection: Gel 1
Perform prerun: 8.0 kV, 30 sec.
Rinse: No
Marker 1: Row: A, 5.0 kV, 5 sec.
Rinse: No
Sample injection: 5.0 kV, 5 sec.
Separation: 8.0 kV, 30.0 min.
Tray name: Sample Tray-2

Analysis mode: DNA

Notes

Gel Image

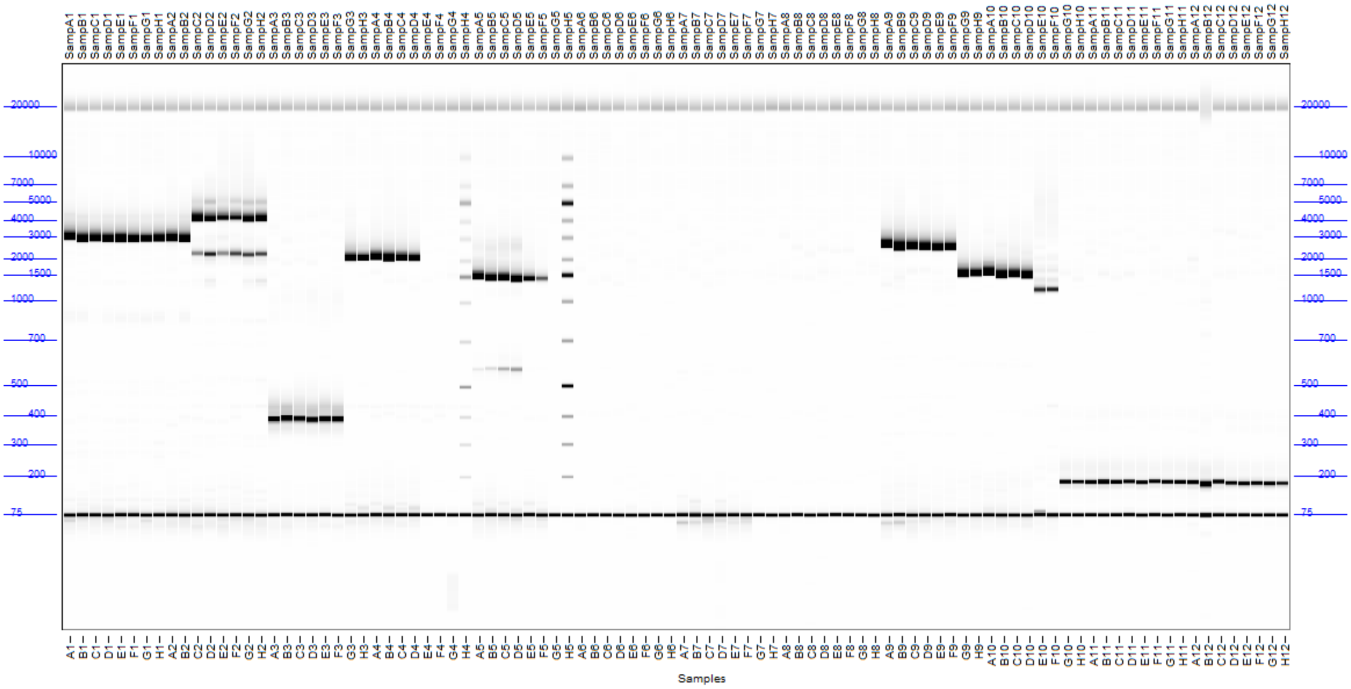

Filename and data path: C:\AATI\Data\2021 08 10\19-11-21\2021 08 10 19H 11M..raw

A1: SampA1

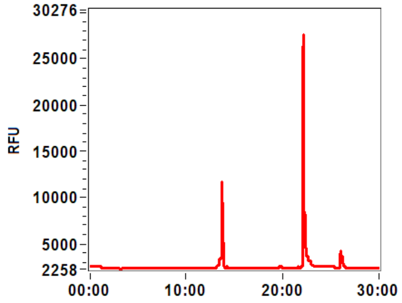

B1: SampB1

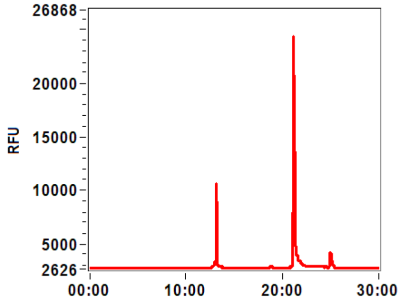

C1: SampC1

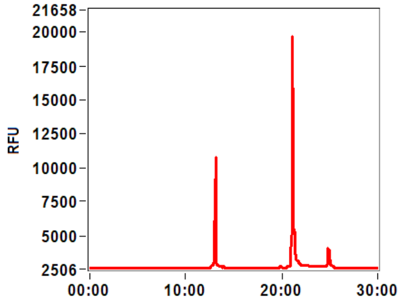

D1: SampD1

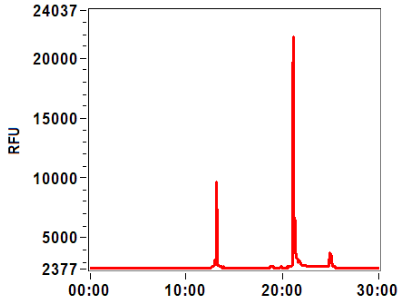

E1: SampE1

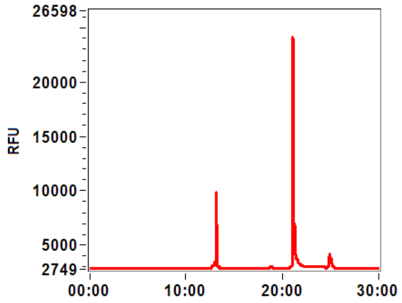

F1: SampF1

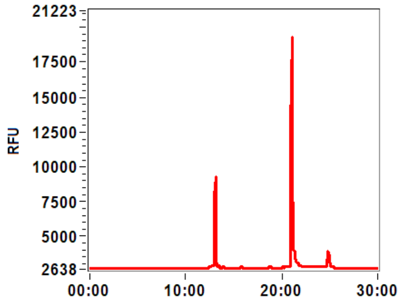

G1: SampG1

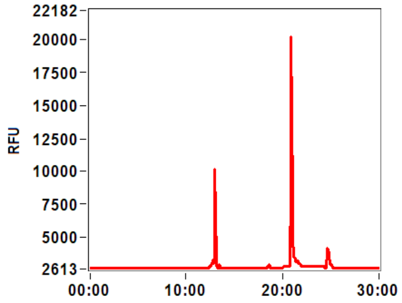

H1: SampH1

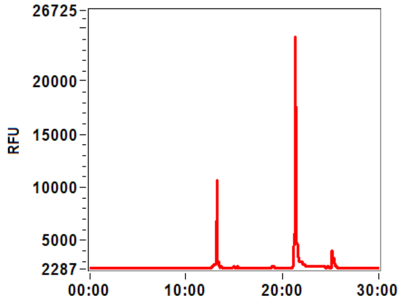

A2: SampA2

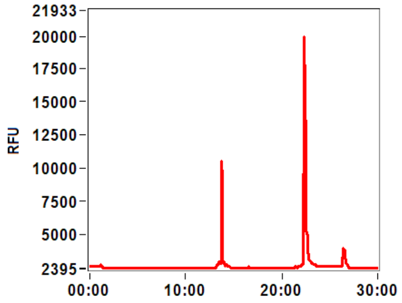

B2: SampB2

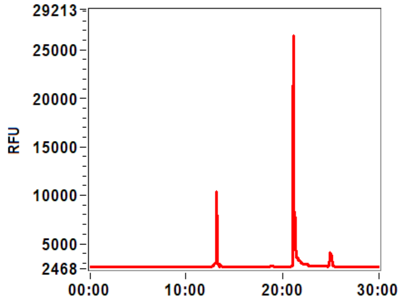

C2: SampC2

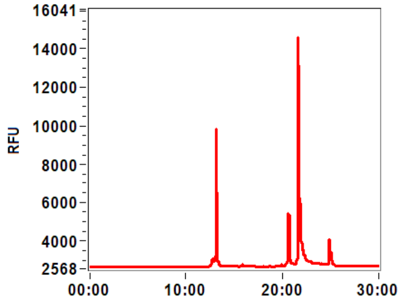

D2: SampD2

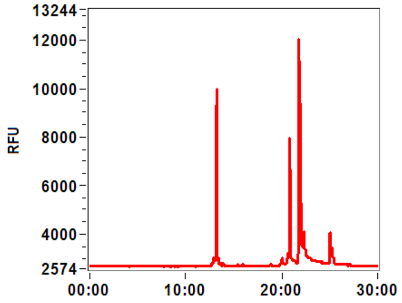

Filename and data path: C:\AATI\Data\2021 08 10\19-11-21\2021 08 10 19H 11M..raw

E2: SampE2

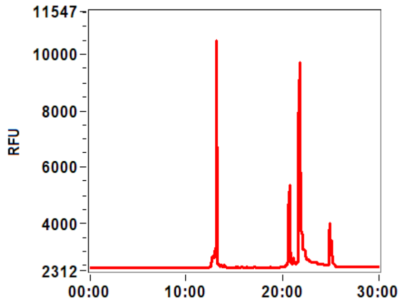

F2: SampF2

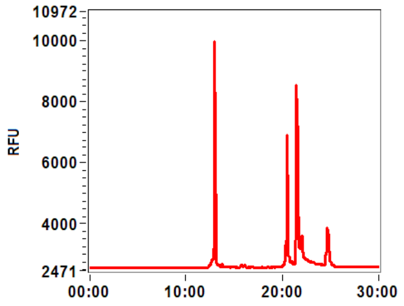

G2: SampG2

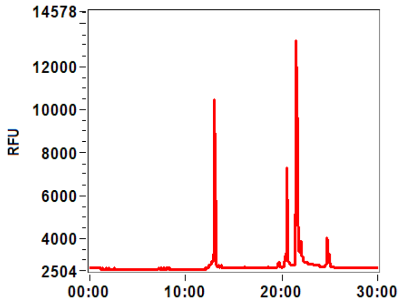

H2: SampH2

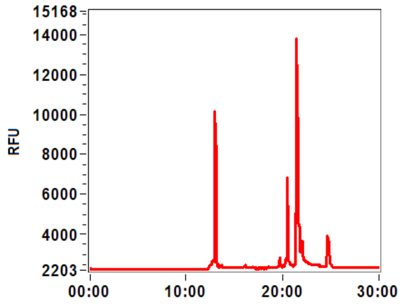

A3: SampA3

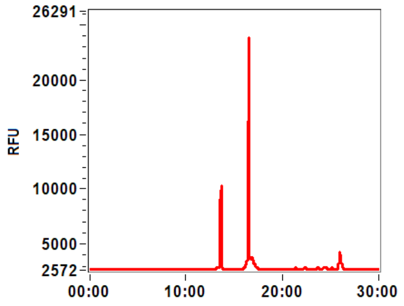

B3: SampB3

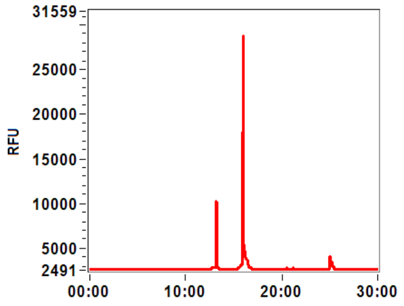

C3: SampC3

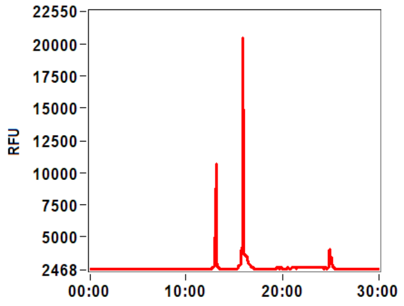

D3: SampD3

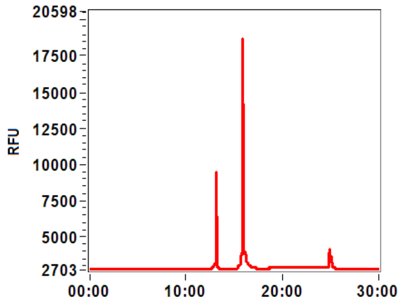

E3: SampE3

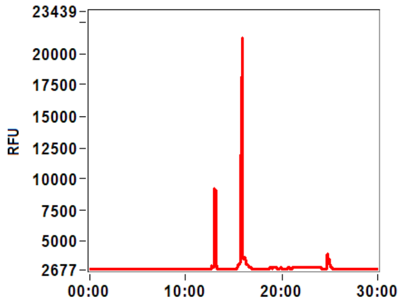

F3: SampF3

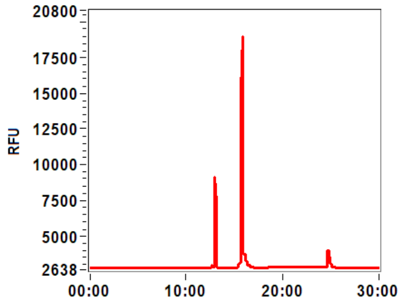

G3: SampG3

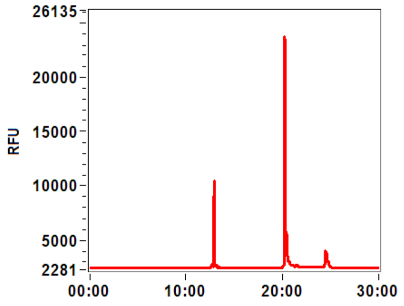

H3: SampH3

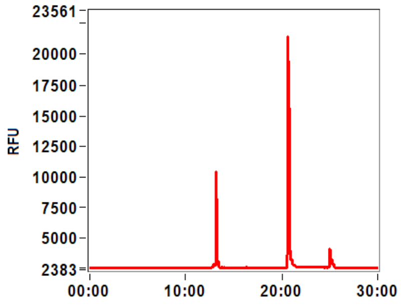

Filename and data path: C:\AATI\Data\2021 08 10\19-11-21\2021 08 10 19H 11M..raw

A4: SampA4

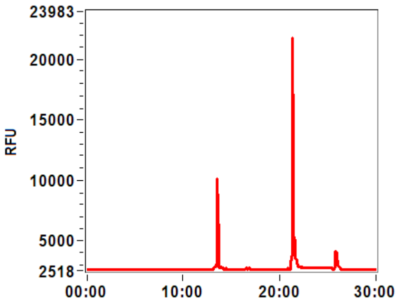

B4: SampB4

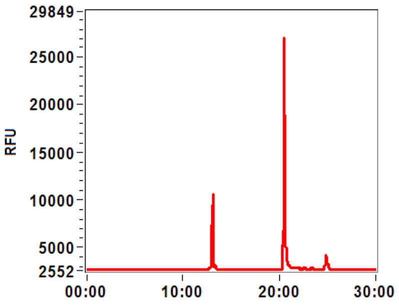

C4: SampC4

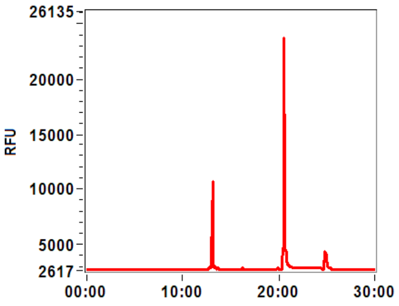

D4: SampD4

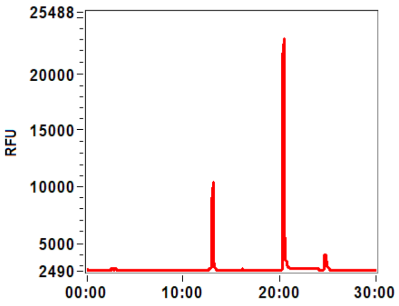

E4: SampE4

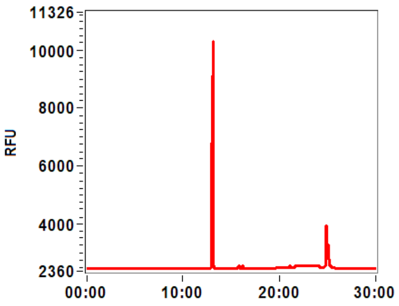

F4: SampF4

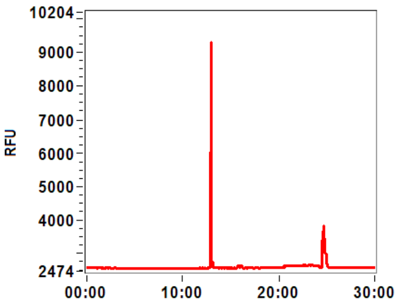

G4: SampG4

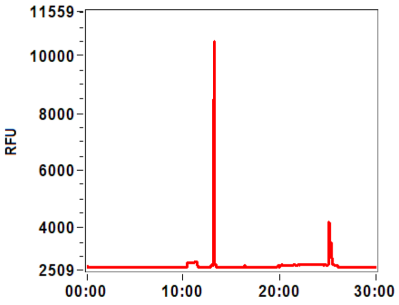

H4: SampH4

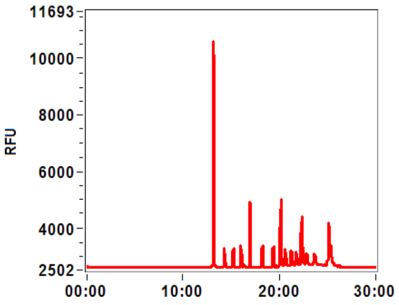

A5: SampA5

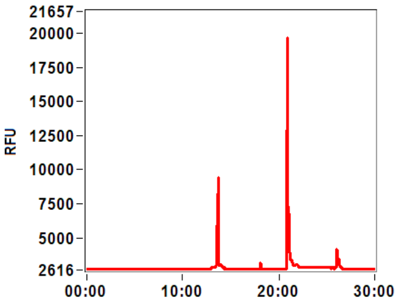

B5: SampB5

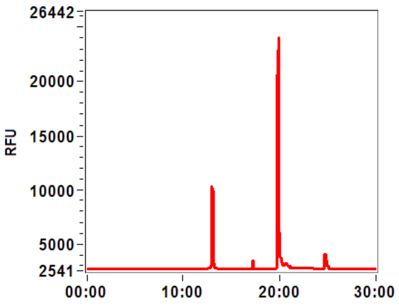

C5: SampC5

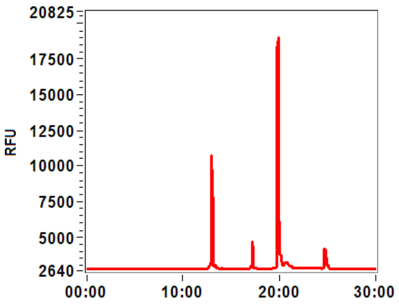

D5: SampD5

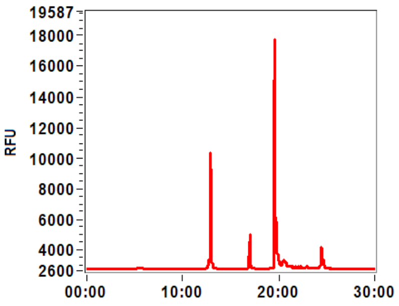

Filename and data path: C:\AATI\Data\2021 08 10\19-11-21\2021 08 10 19H 11M..raw

E5: SampE5

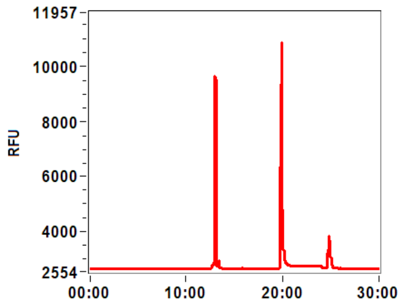

F5: SampF5

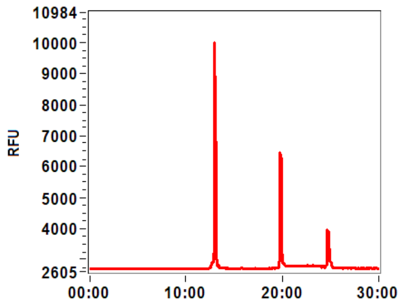

G5: SampG5

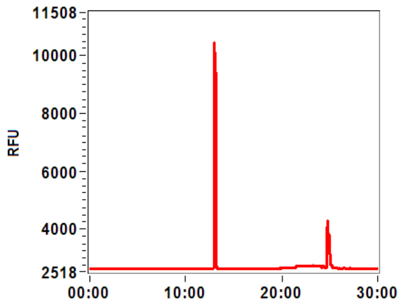

H5: SampH5

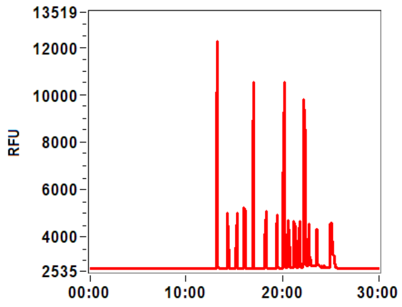

A6: SampA6

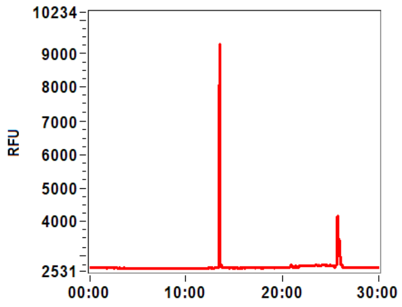

B6: SampB6

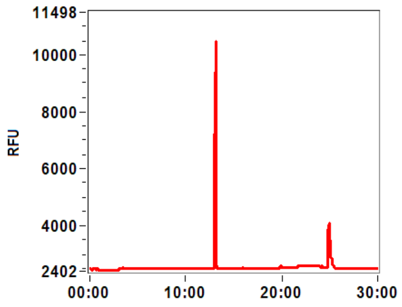

C6: SampC6

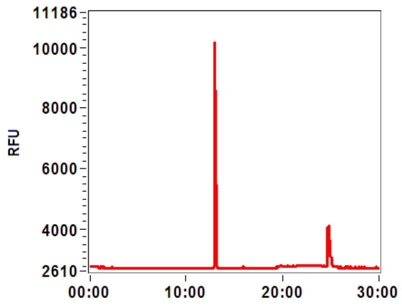

D6: SampD6

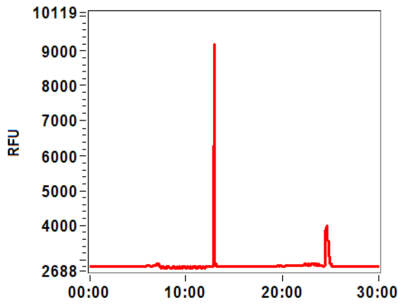

E6: SampE6

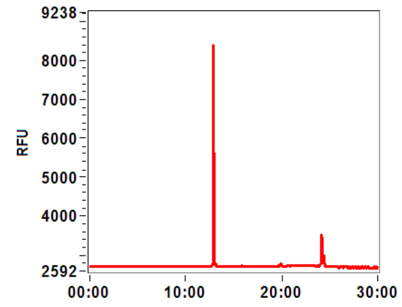

F6: SampF6

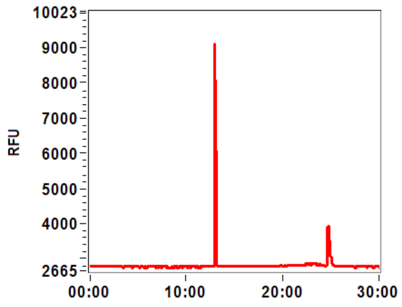

G6: SampG6

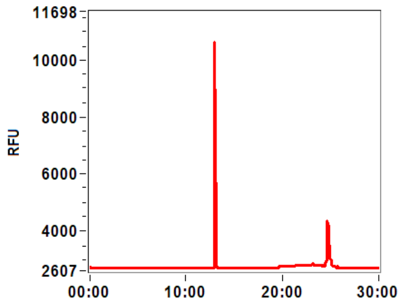

H6: SampH6

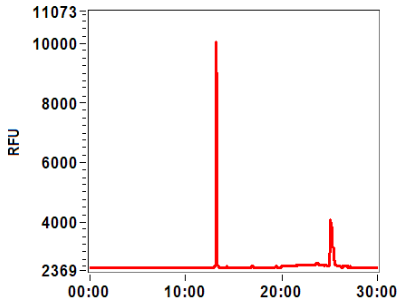

Filename and data path: C:\AATI\Data\2021 08 10\19-11-21\2021 08 10 19H 11M..raw

A7: SampA7

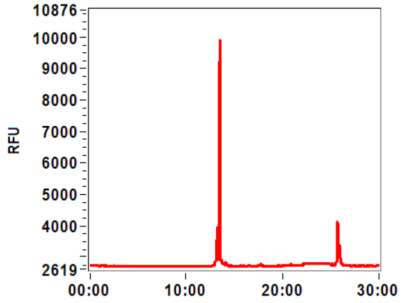

B7: SampB7

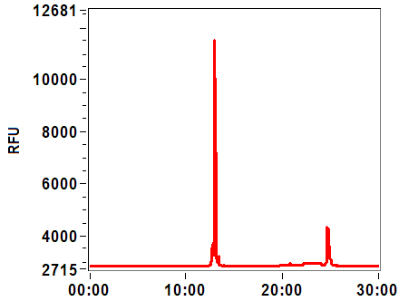

C7: SampC7

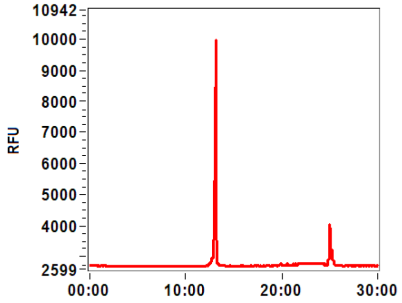

D7: SampD7

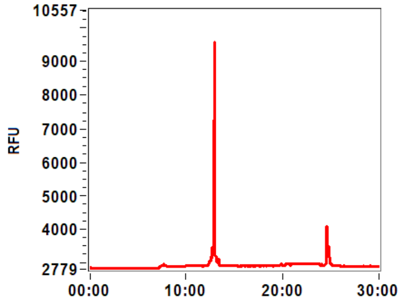

E7: SampE7

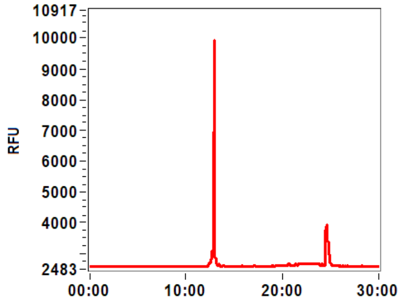

F7: SampF7

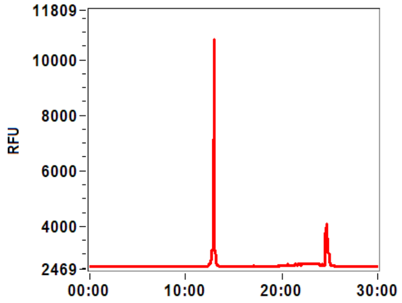

G7: SampG7

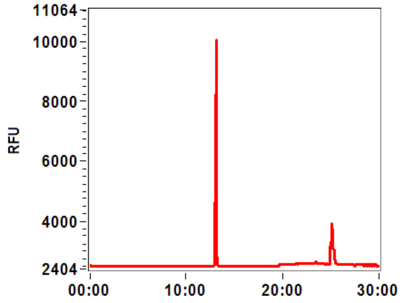

H7: SampH7

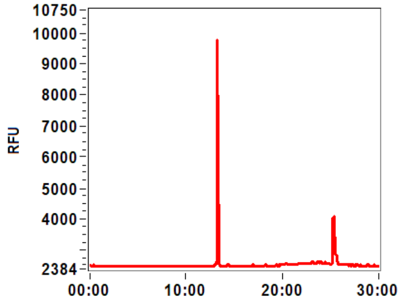

A8: SampA8

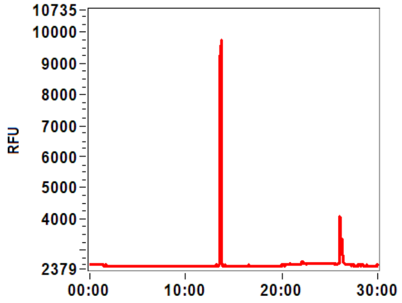

B8: SampB8

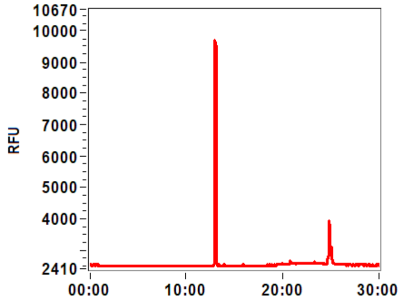

C8: SampC8

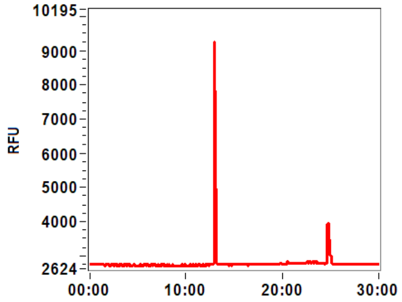

D8: SampD8

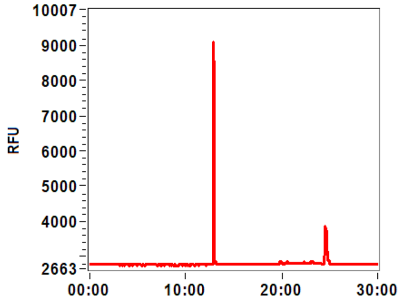

Filename and data path: C:\AATI\Data\2021 08 10\19-11-21\2021 08 10 19H 11M..raw

E8: SampE8

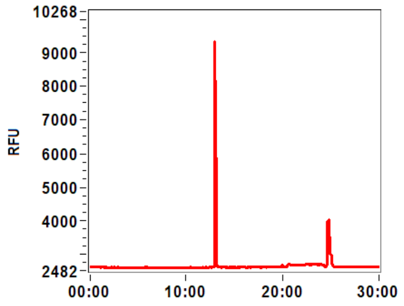

F8: SampF8

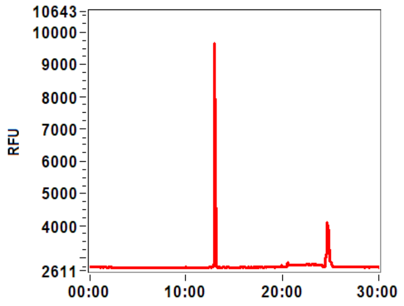

G8: SampG8

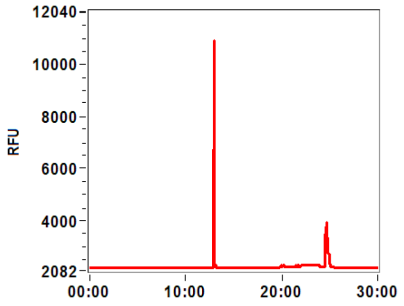

H8: SampH8

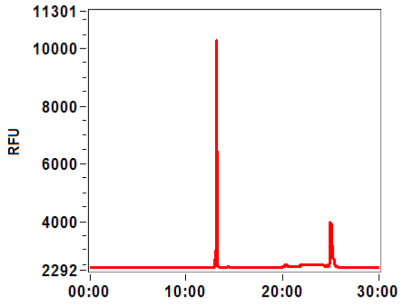

A9: SampA9

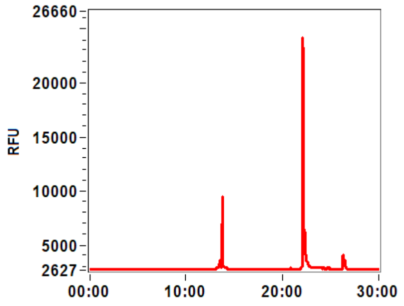

B9: SampB9

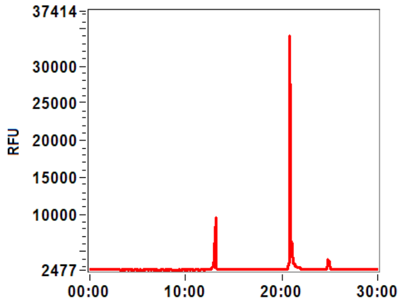

C9: SampC9

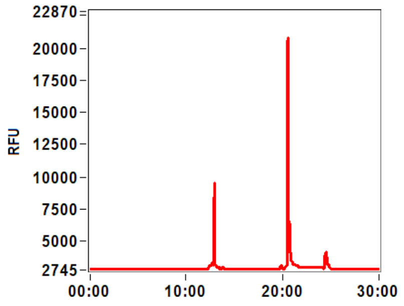

D9: SampD9

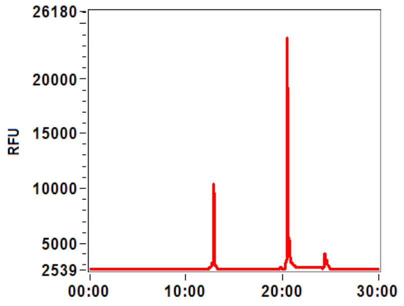

E9: SampE9

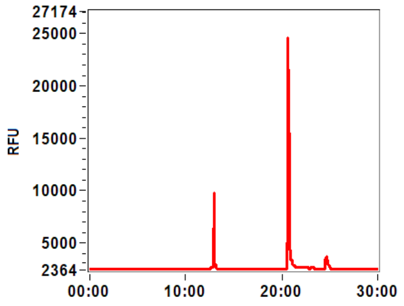

F9: SampF9

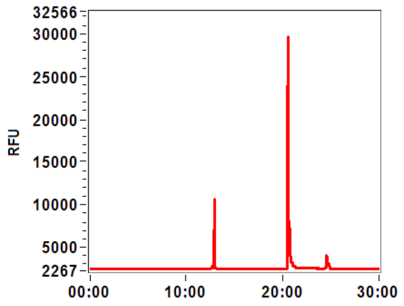

G9: SampG9

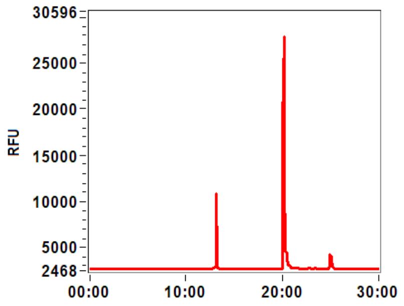

H9: SampH9

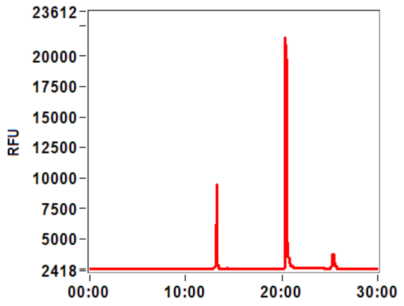

Filename and data path: C:\AATI\Data\2021 08 10\19-11-21\2021 08 10 19H 11M..raw

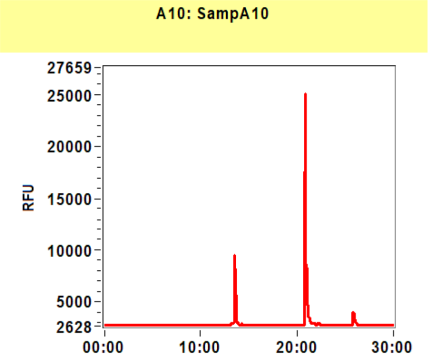

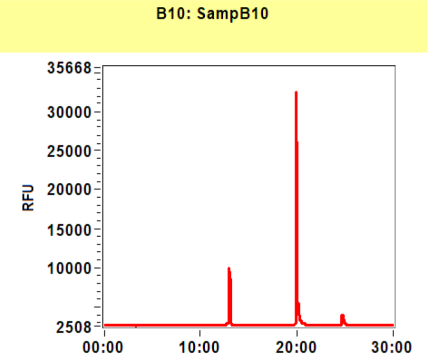

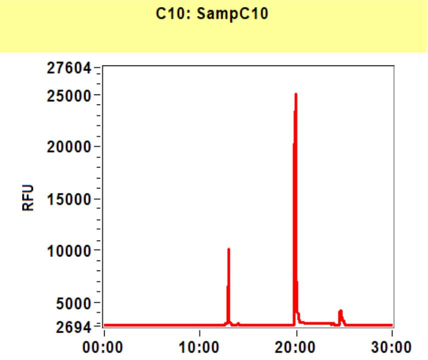

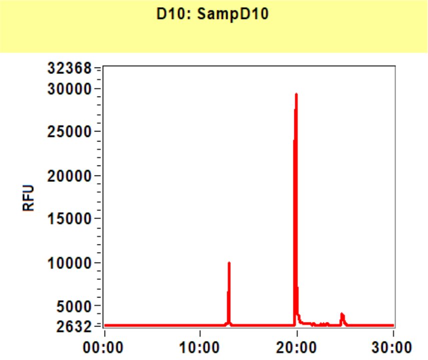

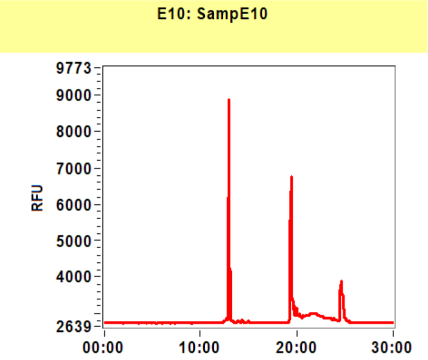

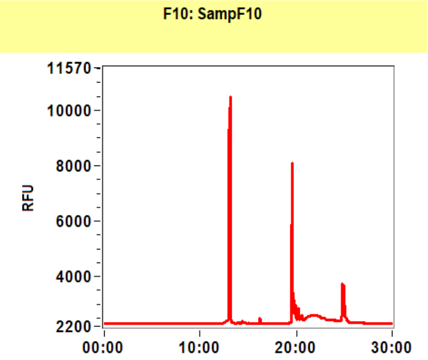

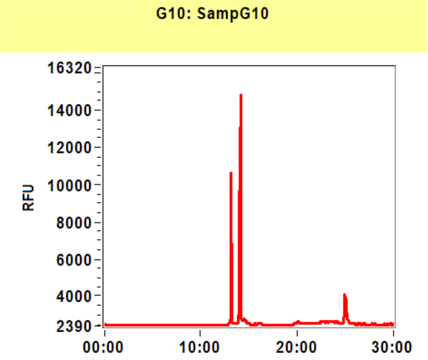

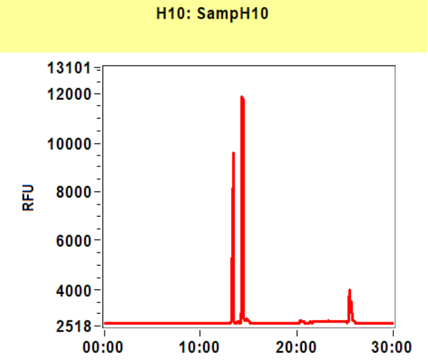

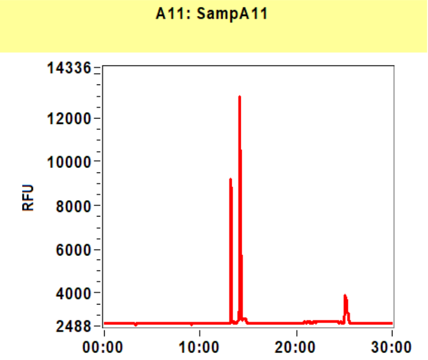

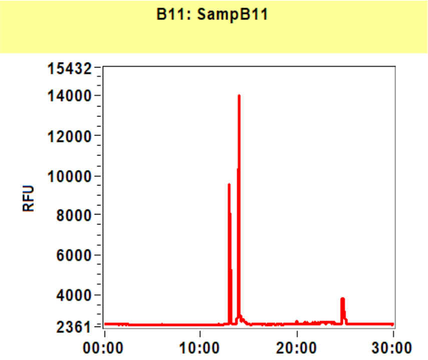

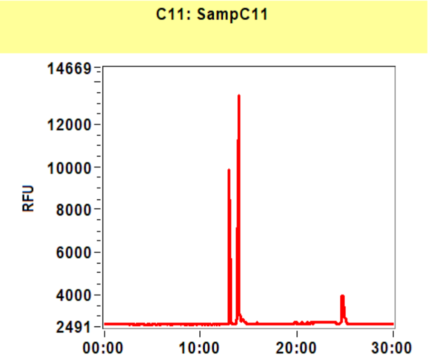

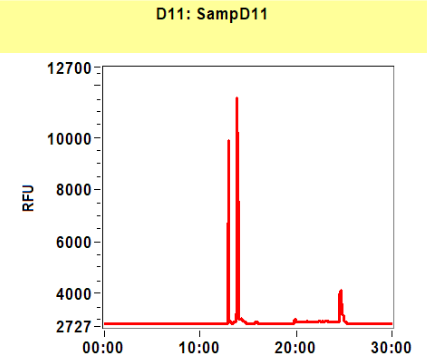

Filename and data path: C:\AATI\Data\2021 08 10\19-11-21\2021 08 10 19H 11M..raw

E11: SampE11

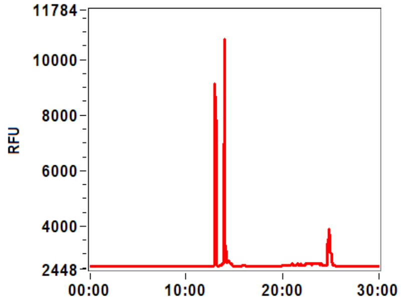

F11: SampF11

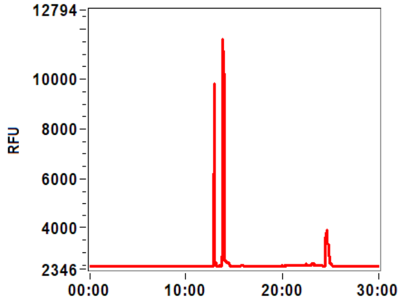

G11: SampG11

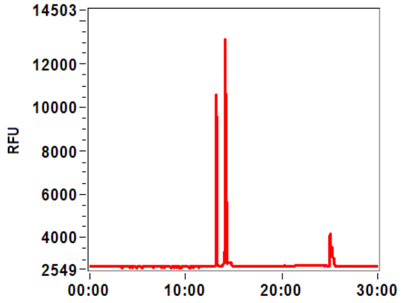

H11: SampH11

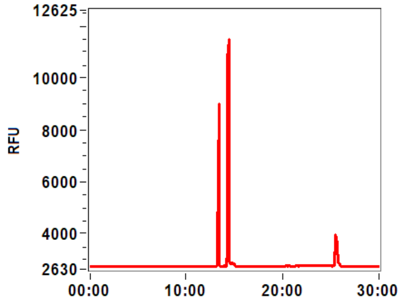

A12: SampA12

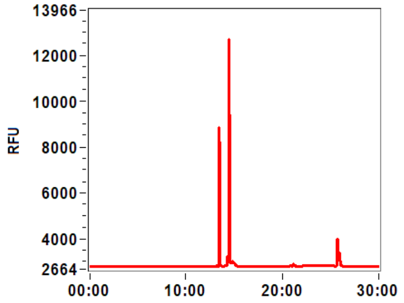

B12: SampB12

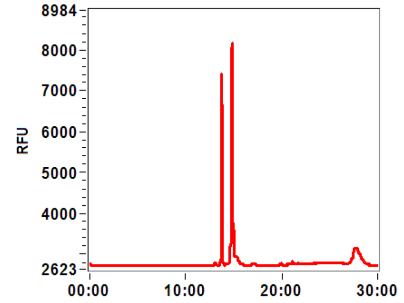

C12: SampC12

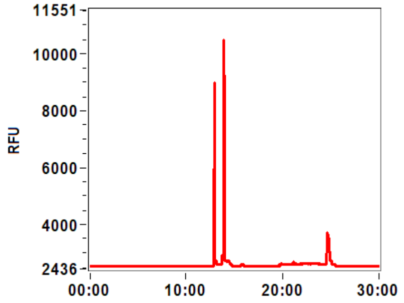

D12: SampD12

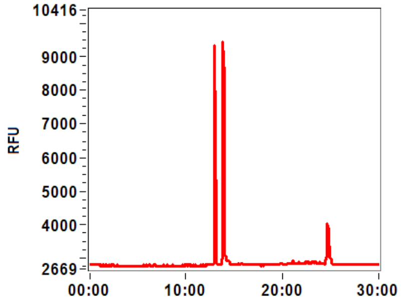

E12: SampE12

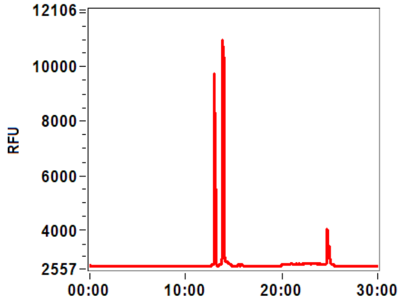

F12: SampF12

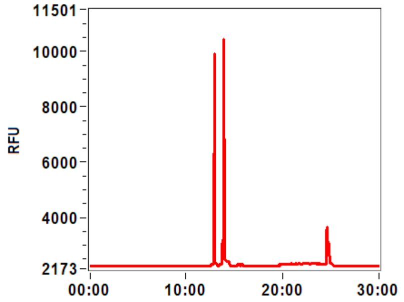

G12: SampG12

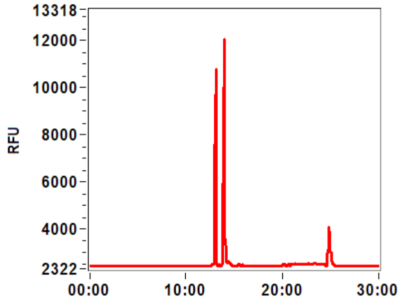

H12: SampH12

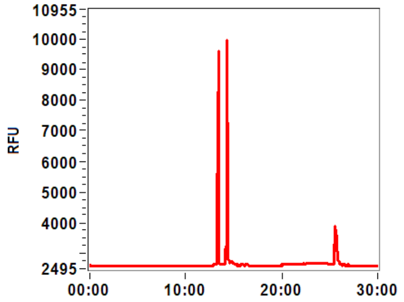

Sample: SampA1
Well location: A1
Created: Tuesday, August 10, 2021 7:21:14 PM

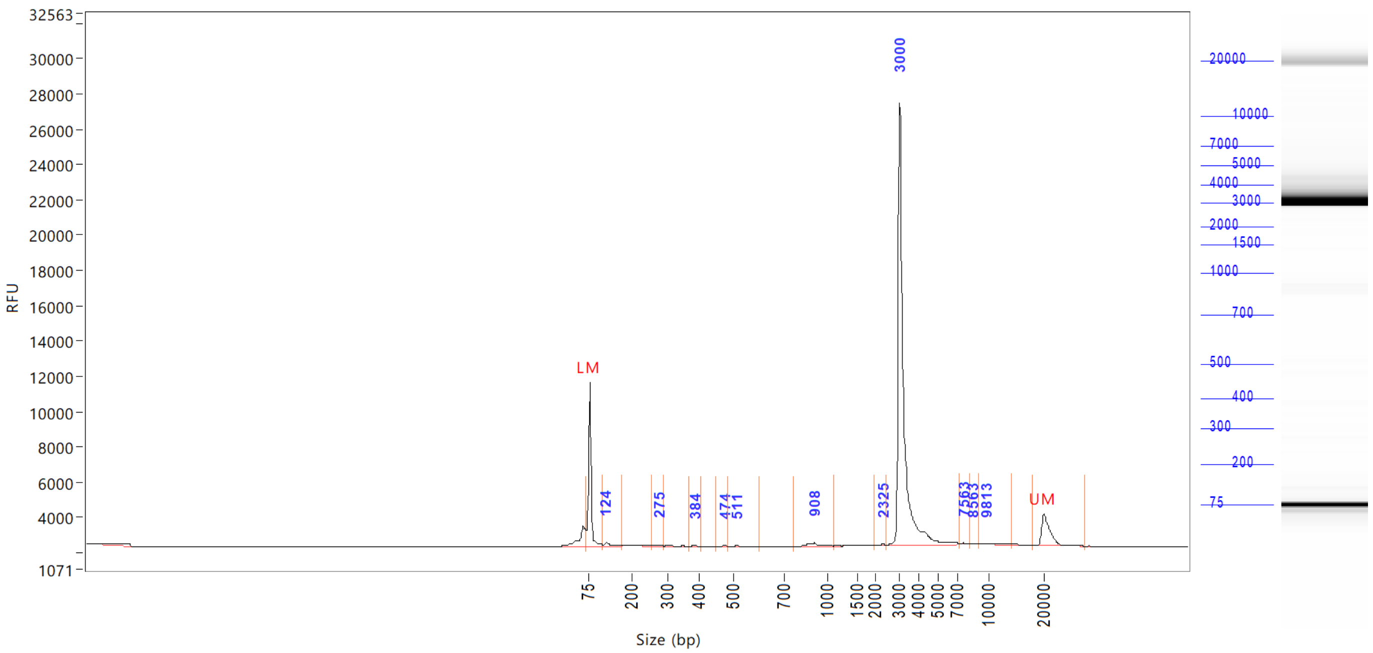

Peak	Size (bp)	Concentration (ng/uL)	From (bp)	To (bp)	Average size (bp)	CV%	RFU	Corrected peak area
1	75 (LM)	0.5000	64	114	73	8.93	9308	57.449
2	124	0.2891	114	169	132	10.58	219	2.768
3	275	0.0660	255	289	271	2.68	83	0.632
4	384	0.0472	369	405	382	1.51	68	0.452
5	474	0.0377	450	483	470	1.50	51	0.361
6	511	0.0565	483	599	516	4.49	55	0.541
7	908	0.3280	767	1080	890	4.68	183	3.141
8	2325	0.0389	1982	2433	2242	5.29	58	0.373
9	3000	20.5312	2433	7251	3238	15.99	25085	196.582
10	7563	0.0813	7251	8188	7660	3.60	82	0.778
11	8563	0.0504	8188	9063	8577	2.95	59	0.482
12	9813	0.1682	9063	14070	11113	13.06	56	1.611
13	20000 (UM)	0.1504	18023	27381	20440	4.22	1760	17.285

TIC:	21.6946	ng/uL
TIM:	15.6399	nmole/L
Total concentration:	21.9179	ng/uL

```
Sample peak width (sec): 5    Sample min peak height: 50    Sample baseline V to V?: Y    Sample baseline V to V points: 3
Sample filter: Binomial       Number of points for filter: 3  Sample start region (min): 0  Sample end region (min): 30
Marker peak width (sec): 5    Marker min peak height: 200   Marker baseline V to V?: Y    Marker baseline V to V points: 3
Lower marker selection: First peak > 500 RFU                Upper marker selection: Last peak > 200 RFU
Ladder size (bp)75, 200, 300, 400, 500, 700, 1000, 1500, 2000, 3000, 4000, 5000, 7000, 10000, 20000
Quantification using: Lower Marker          Final concentration (ng/uL): 0.5000          Dilution factor: 12.0
```

Sample: SampB1
Well location: B1
Created: Tuesday, August 10, 2021 7:21:14 PM

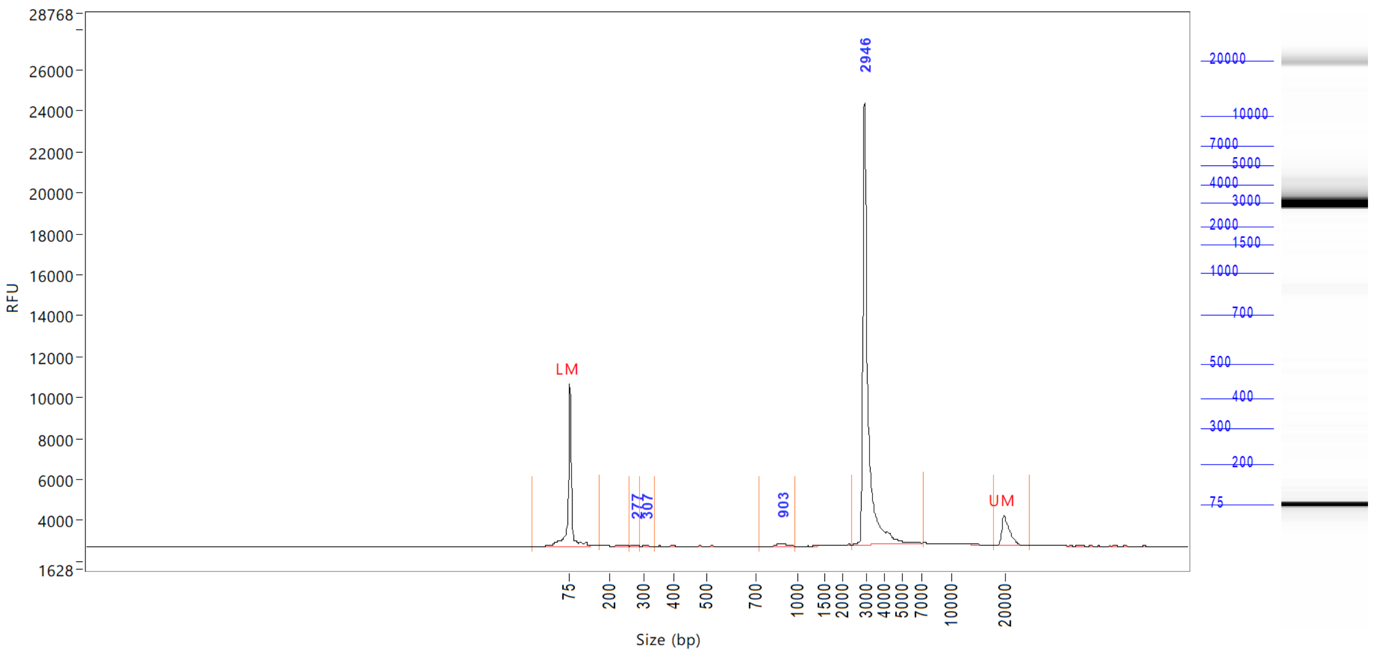

Peak	Size (bp)	Concentration (ng/uL)	From (bp)	To (bp)	Average size (bp)	CV%	RFU	Corrected peak area
1	75 (LM)	0.5000	0	166	71	26.39	7965	63.403
2	277	0.0657	255	287	270	2.86	67	0.694
3	307	0.0534	287	334	305	2.71	64	0.565
4	903	0.2388	727	982	882	4.49	146	2.523
5	2946	15.7094	2379	7188	3096	13.07	21603	166.004
6	20000 (UM)	0.1103	18023	24728	20420	4.06	1423	13.986

TIC:	16.0673	ng/uL
TIM:	9.4871	nmole/L
Total concentration:	16.4802	ng/uL

Sample peak width (sec): 5 Sample min peak height: 50 Sample baseline V to V?: Y Sample baseline V to V points: 3
Sample filter: Binomial Number of points for filter: 3 Sample start region (min): 0 Sample end region (min): 30
Marker peak width (sec): 5 Marker min peak height: 200 Marker baseline V to V?: Y Marker baseline V to V points: 3
Lower marker selection: First peak > 500 RFU Upper marker selection: Last peak > 200 RFU
Ladder size (bp)75, 200, 300, 400, 500, 700, 1000, 1500, 2000, 3000, 4000, 5000, 7000, 10000, 20000
Quantification using: Lower Marker Final concentration (ng/uL): 0.5000 Dilution factor: 12.0

Sample: SampC1
Well location: C1
Created: Tuesday, August 10, 2021 7:21:14 PM

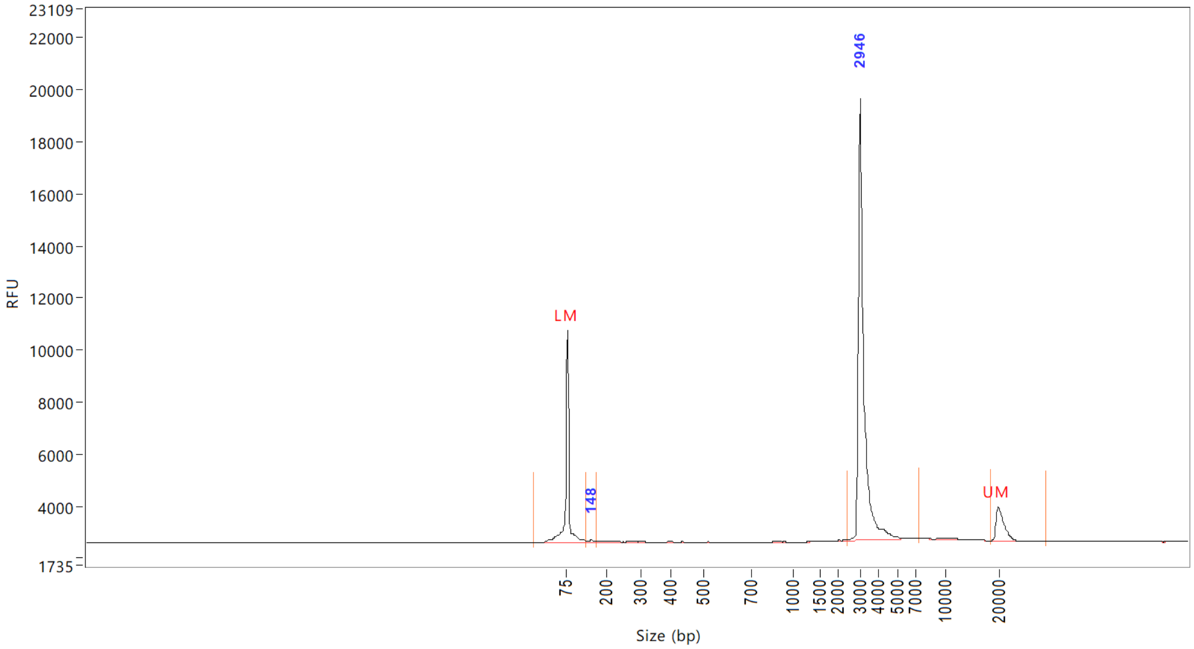

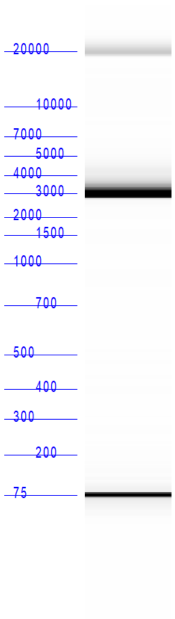

Peak	Size (bp)	Concentration (ng/uL)	From (bp)	To (bp)	Average size (bp)	CV%	RFU	Corrected peak area
1	75 (LM)	0.5000	0	133	71	23.32	8161	62.254
2	148	0.1158	133	164	147	5.74	77	1.201
3	2946	13.1412	2379	7313	3123	12.72	17001	136.348
4	20000 (UM)	0.1061	18489	29111	20561	4.75	1330	13.204

TIC:	13.2570	ng/uL
TIM:	8.2215	nmole/L
Total concentration:	13.7826	ng/uL

Sample peak width (sec): 5 Sample min peak height: 50 Sample baseline V to V?: Y Sample baseline V to V points: 3
Sample filter: Binomial Number of points for filter: 3 Sample start region (min): 0 Sample end region (min): 30
Marker peak width (sec): 5 Marker min peak height: 200 Marker baseline V to V?: Y Marker baseline V to V points: 3
Lower marker selection: First peak > 500 RFU Upper marker selection: Last peak > 200 RFU
Ladder size (bp)75, 200, 300, 400, 500, 700, 1000, 1500, 2000, 3000, 4000, 5000, 7000, 10000, 20000
Quantification using: Lower Marker Final concentration (ng/uL): 0.5000 Dilution factor: 12.0

Sample: SampD1
Well location: D1
Created: Tuesday, August 10, 2021 7:21:14 PM

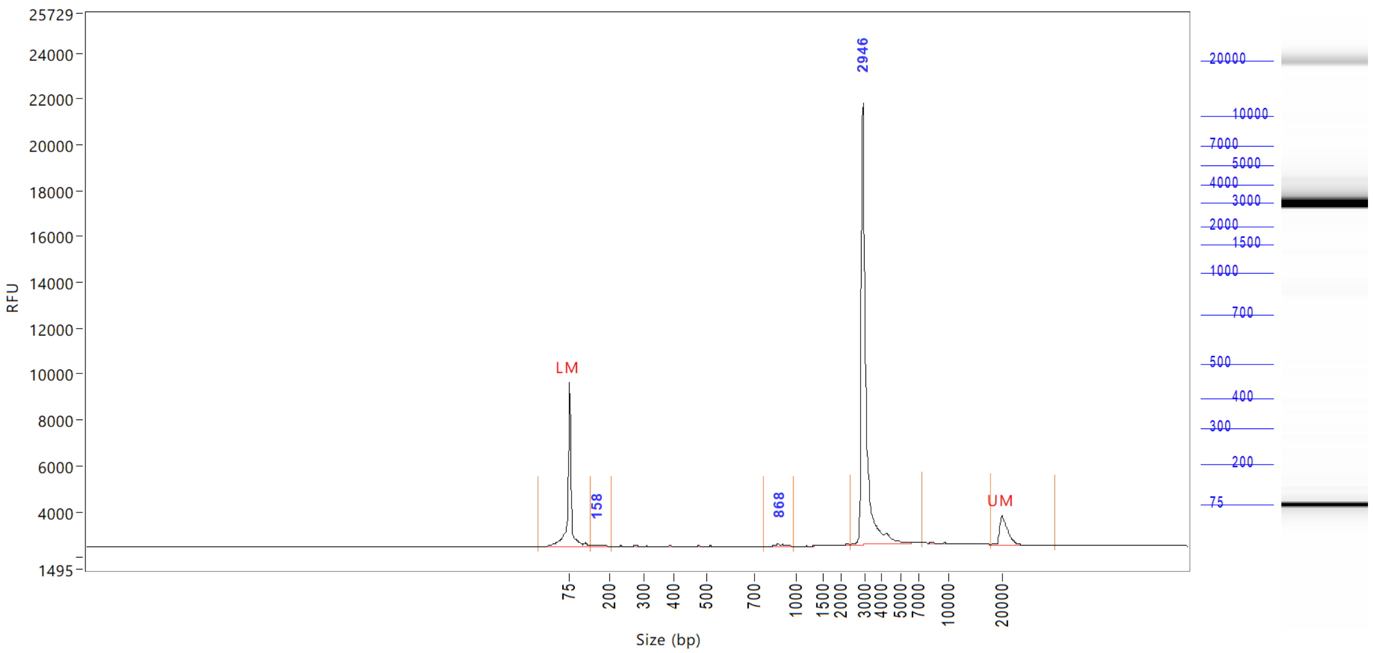

Peak	Size (bp)	Concentration (ng/uL)	From (bp)	To (bp)	Average size (bp)	CV%	RFU	Corrected peak area
1	75 (LM)	0.5000	0	139	70	24.79	7173	59.044
2	158	0.1606	139	205	163	10.27	75	1.581
3	868	0.1577	767	982	878	4.25	97	1.552
4	2946	14.1901	2379	7188	3084	12.75	19283	139.640
5	20000 (UM)	0.1055	17791	30149	20532	4.63	1253	12.460

TIC:	14.5084	ng/uL
TIM:	9.4899	nmole/L
Total concentration:	14.8414	ng/uL

Sample peak width (sec): 5 Sample min peak height: 50 Sample baseline V to V?: Y Sample baseline V to V points: 3
Sample filter: Binomial Number of points for filter: 3 Sample start region (min): 0 Sample end region (min): 30
Marker peak width (sec): 5 Marker min peak height: 200 Marker baseline V to V?: Y Marker baseline V to V points: 3
Lower marker selection: First peak > 500 RFU Upper marker selection: Last peak > 200 RFU
Ladder size (bp)75, 200, 300, 400, 500, 700, 1000, 1500, 2000, 3000, 4000, 5000, 7000, 10000, 20000
Quantification using: Lower Marker Final concentration (ng/uL): 0.5000 Dilution factor: 12.0

Sample: SampE1
Well location: E1
Created: Tuesday, August 10, 2021 7:21:14 PM

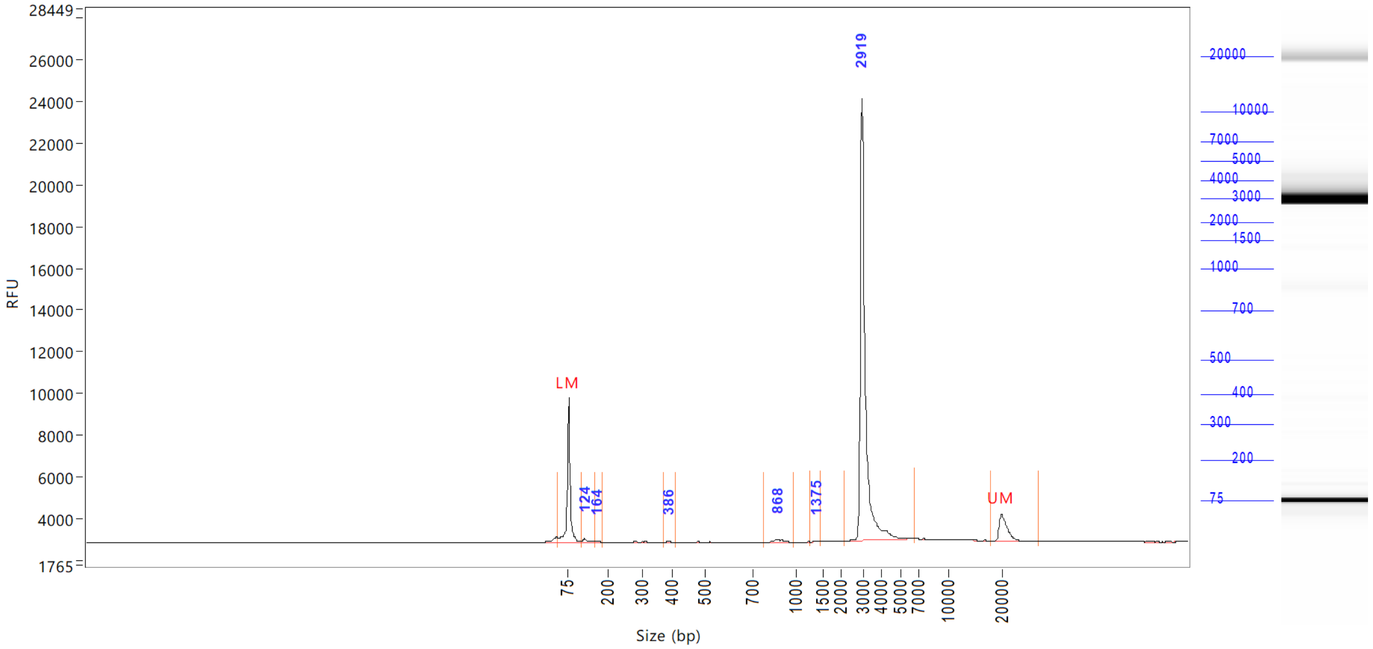

Peak	Size (bp)	Concentration (ng/uL)	From (bp)	To (bp)	Average size (bp)	CV%	RFU	Corrected peak area
1	75 (LM)	0.5000	43	114	73	11.77	6971	49.051
2	124	0.1903	114	156	129	9.09	161	1.556
3	164	0.0521	156	183	163	2.78	58	0.426
4	386	0.0634	367	408	384	1.97	55	0.518
5	868	0.2477	767	982	871	3.93	144	2.025
6	1375	0.0410	1273	1466	1359	2.34	55	0.335
7	2919	19.5297	2136	6517	3057	11.13	21232	159.659
8	20000 (UM)	0.1305	17791	27035	20450	4.58	1269	12.807

TIC:	20.1242	ng/uL
TIM:	14.2558	nmole/L
Total concentration:	20.5217	ng/uL

```
Sample peak width (sec): 5    Sample min peak height: 50    Sample baseline V to V?: Y    Sample baseline V to V points: 3
Sample filter: Binomial       Number of points for filter: 3  Sample start region (min): 0  Sample end region (min): 30
Marker peak width (sec): 5    Marker min peak height: 200   Marker baseline V to V?: Y    Marker baseline V to V points: 3
Lower marker selection: First peak > 500 RFU                Upper marker selection: Last peak > 200 RFU
Ladder size (bp)75, 200, 300, 400, 500, 700, 1000, 1500, 2000, 3000, 4000, 5000, 7000, 10000, 20000
Quantification using: Lower Marker          Final concentration (ng/uL): 0.5000          Dilution factor: 12.0
```

Sample: SampF1
Well location: F1
Created: Tuesday, August 10, 2021 7:21:14 PM

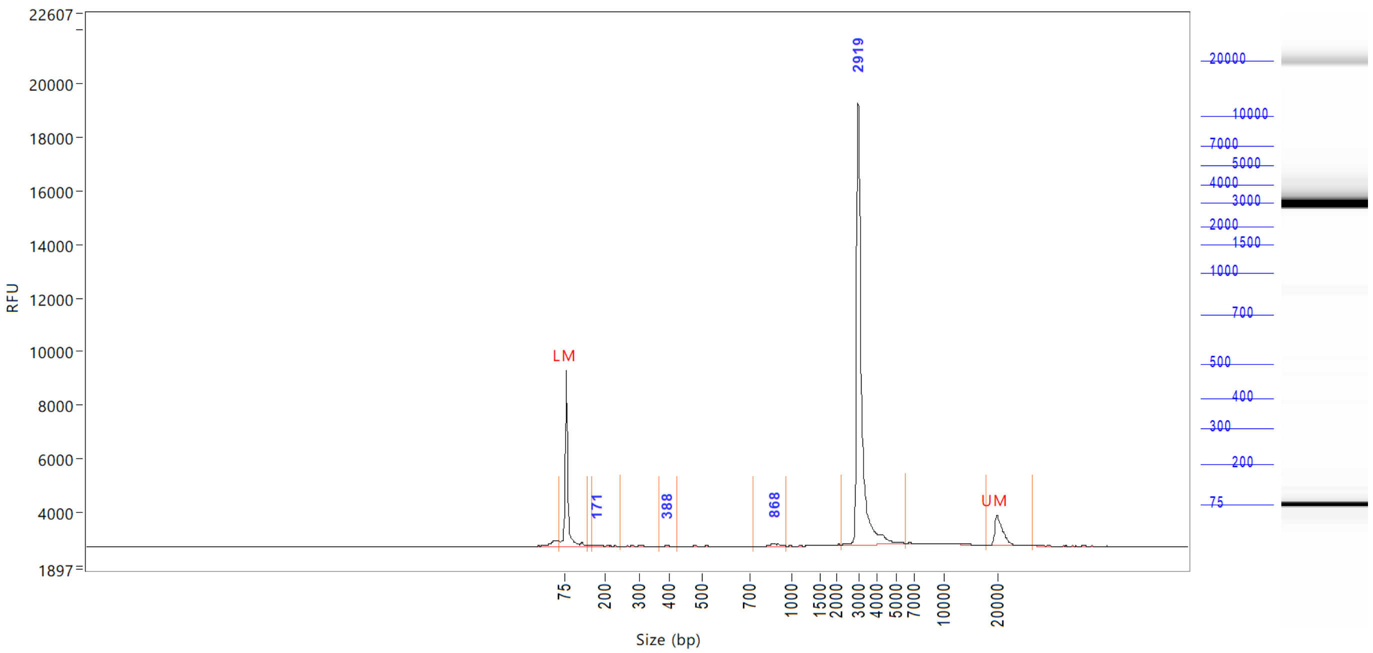

Peak	Size (bp)	Concentration (ng/uL)	From (bp)	To (bp)	Average size (bp)	CV%	RFU	Corrected peak area
1	75 (LM)	0.5000	56	141	76	14.32	6545	44.099
2	171	0.1364	154	242	180	11.25	54	1.003
3	388	0.0695	363	422	388	2.82	54	0.511
4	868	0.2215	723	960	868	4.43	102	1.628
5	2919	16.5575	2190	6035	3073	12.41	16490	121.694
6	20000 (UM)	0.1286	17907	26689	20551	4.75	1109	11.345

TIC:	16.9850	ng/uL
TIM:	10.8308	nmole/L
Total concentration:	17.5393	ng/uL

Sample peak width (sec): 5   Sample min peak height: 50   Sample baseline V to V?: Y   Sample baseline V to V points: 3
Sample filter: Binomial   Number of points for filter: 3 Sample start region (min): 0 Sample end region (min): 30
Marker peak width (sec): 5   Marker min peak height: 200   Marker baseline V to V?: Y   Marker baseline V to V points: 3
Lower marker selection: First peak > 500 RFU   Upper marker selection: Last peak > 200 RFU
Ladder size (bp)75, 200, 300, 400, 500, 700, 1000, 1500, 2000, 3000, 4000, 5000, 7000, 10000, 20000
Quantification using: Lower Marker   Final concentration (ng/uL): 0.5000   Dilution factor: 12.0

Sample: SampG1
Well location: G1
Created: Tuesday, August 10, 2021 7:21:14 PM

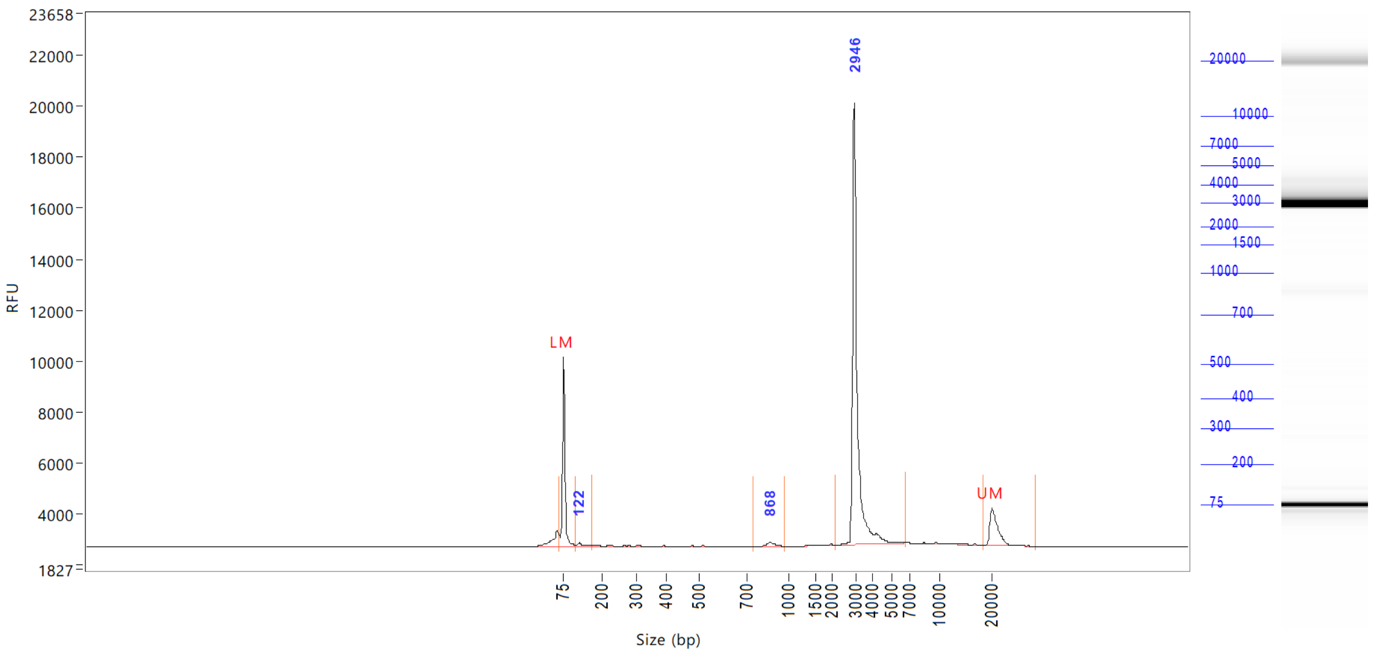

Peak	Size (bp)	Concentration (ng/uL)	From (bp)	To (bp)	Average size (bp)	CV%	RFU	Corrected peak area
1	75 (LM)	0.5000	64	114	74	8.11	7429	46.284
2	122	0.2324	114	168	132	11.05	152	1.793
3	868	0.2988	745	978	869	3.91	183	2.305
4	2946	15.8552	2109	6517	3068	11.40	17371	122.306
5	20000 (UM)	0.1608	18256	28303	20514	4.57	1465	14.888

TIC:	16.3864	ng/uL
TIM:	11.9667	nmole/L
Total concentration:	16.9770	ng/uL

Sample peak width (sec): 5 Sample min peak height: 50 Sample baseline V to V?: Y Sample baseline V to V points: 3
Sample filter: Binomial Number of points for filter: 3 Sample start region (min): 0 Sample end region (min): 30
Marker peak width (sec): 5 Marker min peak height: 200 Marker baseline V to V?: Y Marker baseline V to V points: 3
Lower marker selection: First peak > 500 RFU Upper marker selection: Last peak > 200 RFU
Ladder size (bp)75, 200, 300, 400, 500, 700, 1000, 1500, 2000, 3000, 4000, 5000, 7000, 10000, 20000
Quantification using: Lower Marker Final concentration (ng/uL): 0.5000 Dilution factor: 12.0

Sample: SampH1
Well location: H1
Created: Tuesday, August 10, 2021 7:21:14 PM

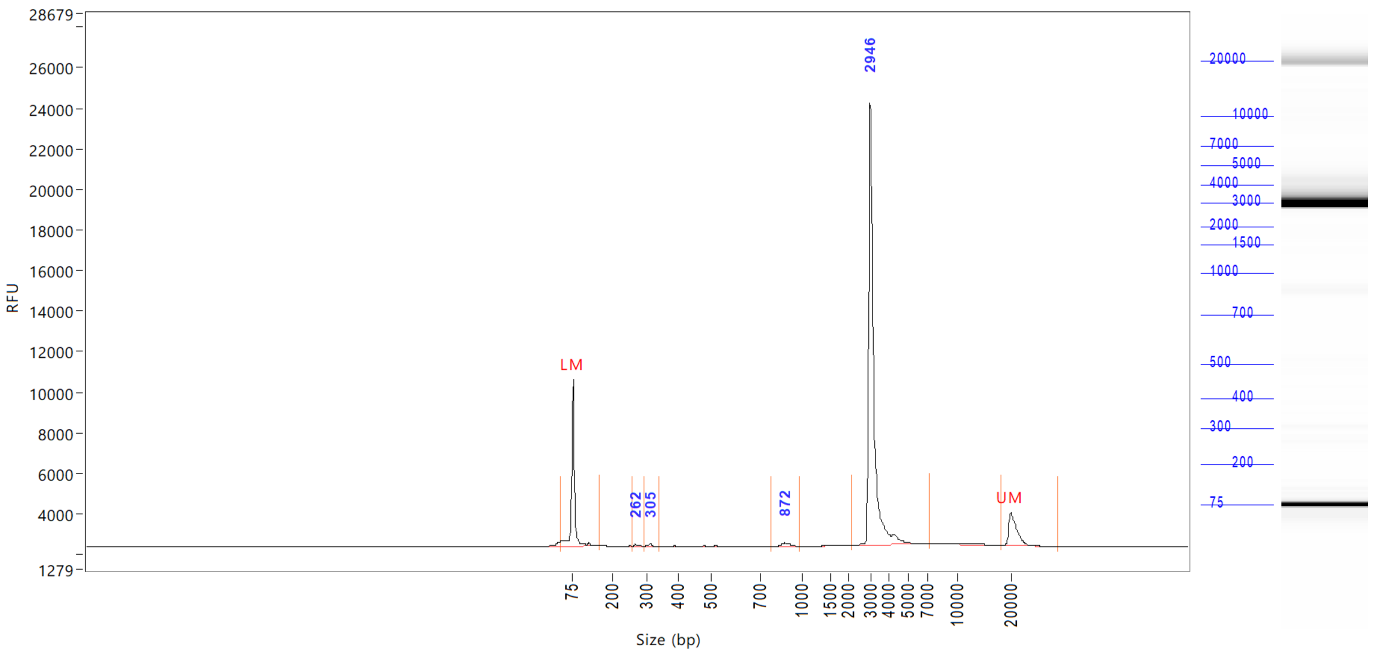

Peak	Size (bp)	Concentration (ng/uL)	From (bp)	To (bp)	Average size (bp)	CV%	RFU	Corrected peak area
1	75 (LM)	0.5000	37	160	74	17.41	8206	56.706
2	262	0.0877	253	287	266	3.05	93	0.829
3	305	0.0900	287	336	303	2.29	119	0.851
4	872	0.2456	780	982	877	3.42	170	2.321
5	2946	16.6860	2163	7126	3090	12.07	21826	157.699
6	20000 (UM)	0.1418	18023	28649	20527	4.51	1605	16.079

TIC:	17.1093	ng/uL
TIM:	10.3817	nmole/L
Total concentration:	17.5704	ng/uL

```
Sample peak width (sec): 5    Sample min peak height: 50    Sample baseline V to V?: Y    Sample baseline V to V points: 3
Sample filter: Binomial       Number of points for filter: 3  Sample start region (min): 0  Sample end region (min): 30
Marker peak width (sec): 5    Marker min peak height: 200   Marker baseline V to V?: Y    Marker baseline V to V points: 3
Lower marker selection: First peak > 500 RFU                Upper marker selection: Last peak > 200 RFU
Ladder size (bp)75, 200, 300, 400, 500, 700, 1000, 1500, 2000, 3000, 4000, 5000, 7000, 10000, 20000
Quantification using: Lower Marker         Final concentration (ng/uL): 0.5000         Dilution factor: 12.0
```

Sample: SampA2
Well location: A2
Created: Tuesday, August 10, 2021 7:21:14 PM

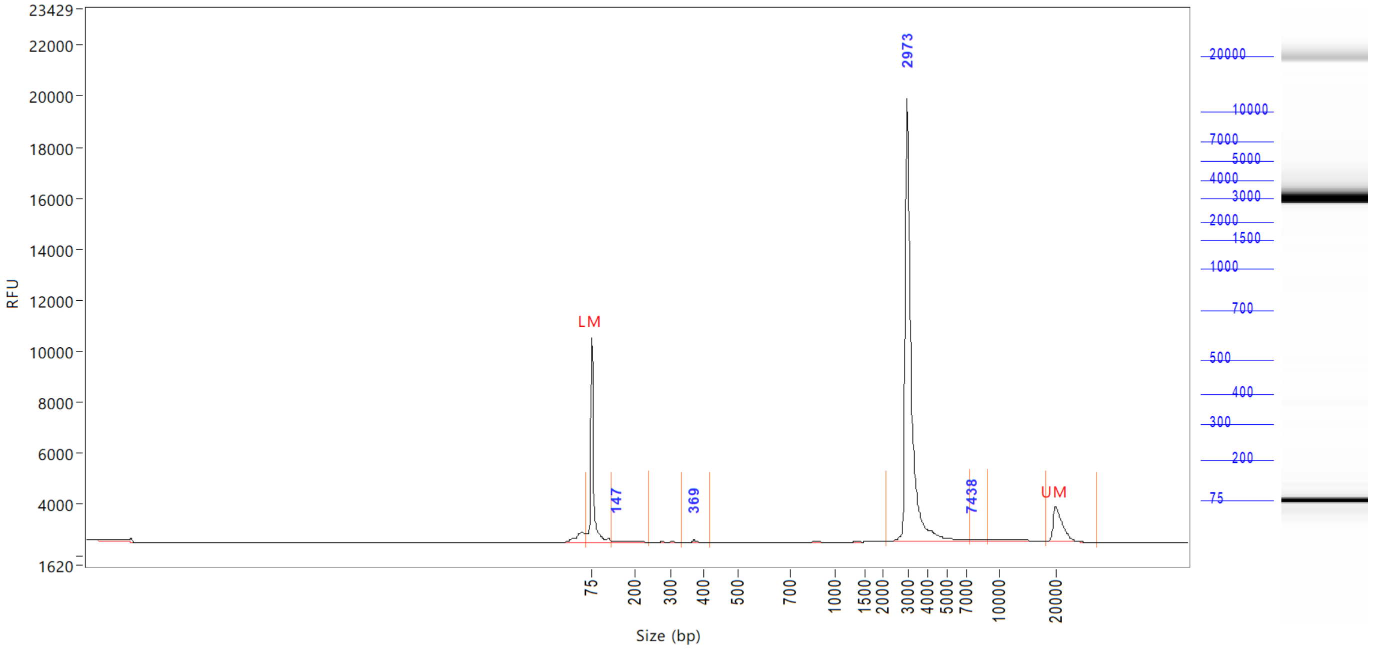

Peak	Size (bp)	Concentration (ng/uL)	From (bp)	To (bp)	Average size (bp)	CV%	RFU	Corrected peak area
1	75 (LM)	0.5000	60	133	76	13.78	8016	54.451
2	147	0.2266	133	237	167	15.48	68	2.057
3	369	0.0957	330	418	371	4.19	100	0.869
4	2973	16.4724	2109	7251	3143	15.12	17388	149.490
5	7438	0.0836	7251	8938	7946	6.20	55	0.758
6	20000 (UM)	0.1391	18256	27265	20531	4.54	1380	15.144

TIC:	16.8784	ng/uL
TIM:	11.3005	nmole/L
Total concentration:	17.3303	ng/uL

```
Sample peak width (sec): 5    Sample min peak height: 50    Sample baseline V to V?: Y    Sample baseline V to V points: 3
Sample filter: Binomial       Number of points for filter: 3  Sample start region (min): 0  Sample end region (min): 30
Marker peak width (sec): 5    Marker min peak height: 200   Marker baseline V to V?: Y    Marker baseline V to V points: 3
Lower marker selection: First peak > 500 RFU                Upper marker selection: Last peak > 200 RFU
Ladder size (bp)75, 200, 300, 400, 500, 700, 1000, 1500, 2000, 3000, 4000, 5000, 7000, 10000, 20000
Quantification using: Lower Marker          Final concentration (ng/uL): 0.5000          Dilution factor: 12.0
```

Sample: SampB2
Well location: B2
Created: Tuesday, August 10, 2021 7:21:14 PM

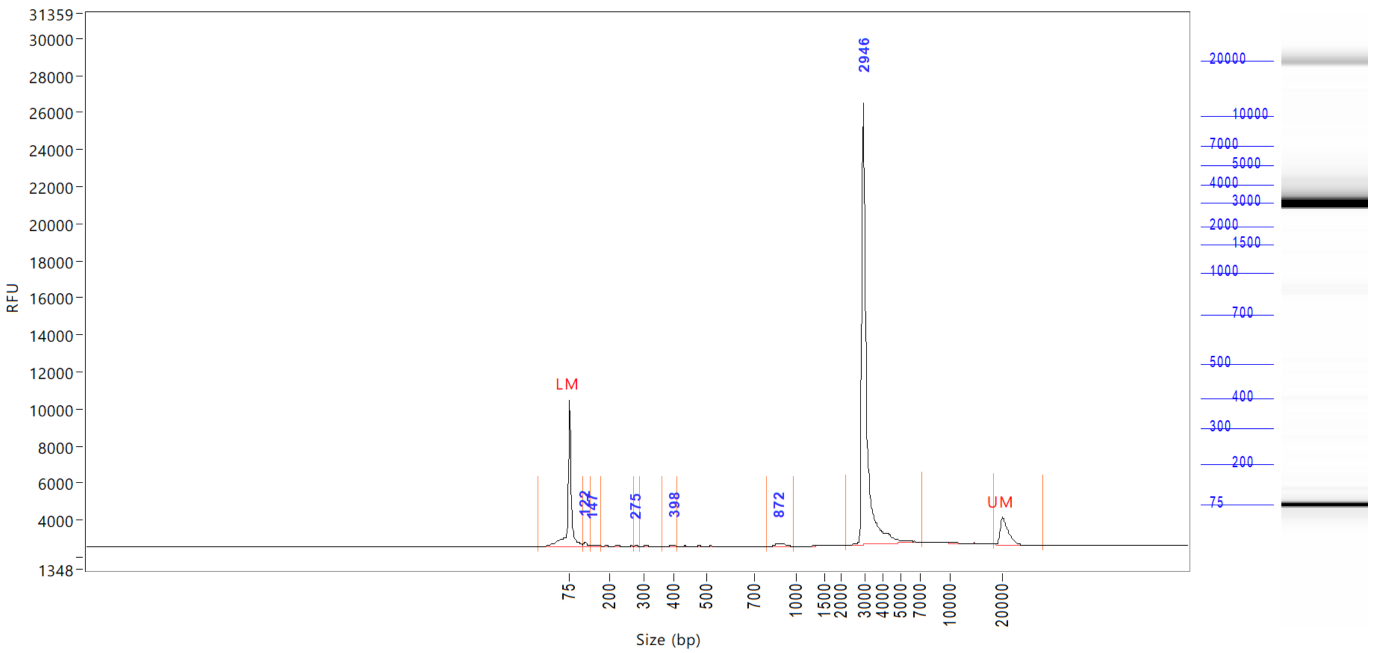

Peak	Size (bp)	Concentration (ng/uL)	From (bp)	To (bp)	Average size (bp)	CV%	RFU	Corrected peak area
1	75 (LM)	0.5000	0	114	69	23.58	7917	63.960
2	122	0.1838	114	139	122	4.89	245	1.960
3	147	0.0869	139	173	152	5.68	69	0.927
4	275	0.0308	268	287	274	1.51	61	0.328
5	398	0.0683	359	412	392	2.27	92	0.728
6	872	0.2298	780	974	877	3.58	157	2.450
7	2946	16.5245	2163	7313	3100	13.98	23879	176.150
8	20000 (UM)	0.1151	18372	27611	20505	4.34	1484	14.725

TIC:	17.1242	ng/uL
TIM:	13.0935	nmole/L
Total concentration:	17.5013	ng/uL

```
Sample peak width (sec): 5     Sample min peak height: 50     Sample baseline V to V?: Y     Sample baseline V to V points: 3
Sample filter: Binomial        Number of points for filter: 3  Sample start region (min): 0  Sample end region (min): 30
Marker peak width (sec): 5     Marker min peak height: 200    Marker baseline V to V?: Y     Marker baseline V to V points: 3
Lower marker selection: First peak > 500 RFU                  Upper marker selection: Last peak > 200 RFU
Ladder size (bp)75, 200, 300, 400, 500, 700, 1000, 1500, 2000, 3000, 4000, 5000, 7000, 10000, 20000
Quantification using: Lower Marker          Final concentration (ng/uL): 0.5000          Dilution factor: 12.0
```

Sample: SampC2
Well location: C2
Created: Tuesday, August 10, 2021 7:21:14 PM

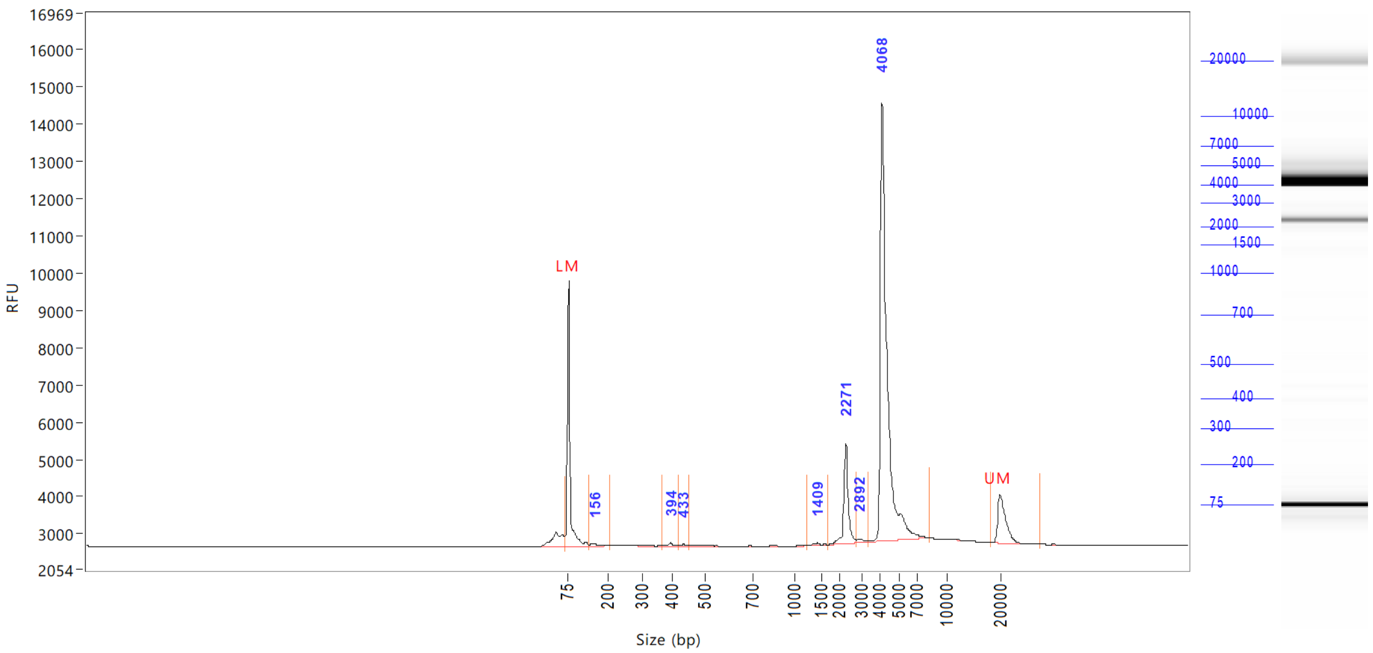

Peak	Size (bp)	Concentration (ng/uL)	From (bp)	To (bp)	Average size (bp)	CV%	RFU	Corrected peak area
1	75 (LM)	0.5000	64	137	77	14.45	7144	46.264
2	156	0.1743	137	201	159	9.62	69	1.344
3	394	0.0890	365	420	391	2.29	91	0.686
4	433	0.0427	420	450	432	1.33	58	0.329
5	1409	0.1007	1205	1667	1391	6.10	65	0.777
6	2271	2.3315	1667	2757	2258	6.54	2700	17.977
7	2892	0.1177	2757	3286	2926	4.29	105	0.907
8	4068	14.4826	3286	8188	4317	11.24	11775	111.671
9	20000 (UM)	0.1400	18140	27611	20520	4.55	1294	12.957

TIC:	17.3384	ng/uL
TIM:	9.7470	nmole/L
Total concentration:	17.6100	ng/uL

```
Sample peak width (sec): 5    Sample min peak height: 50    Sample baseline V to V?: Y    Sample baseline V to V points: 3
Sample filter: Binomial       Number of points for filter: 3  Sample start region (min): 0  Sample end region (min): 30
Marker peak width (sec): 5    Marker min peak height: 200   Marker baseline V to V?: Y    Marker baseline V to V points: 3
Lower marker selection: First peak > 500 RFU                Upper marker selection: Last peak > 200 RFU
Ladder size (bp)75, 200, 300, 400, 500, 700, 1000, 1500, 2000, 3000, 4000, 5000, 7000, 10000, 20000
Quantification using: Lower Marker          Final concentration (ng/uL): 0.5000          Dilution factor: 12.0
```

Sample: SampD2
Well location: D2
Created: Tuesday, August 10, 2021 7:21:14 PM

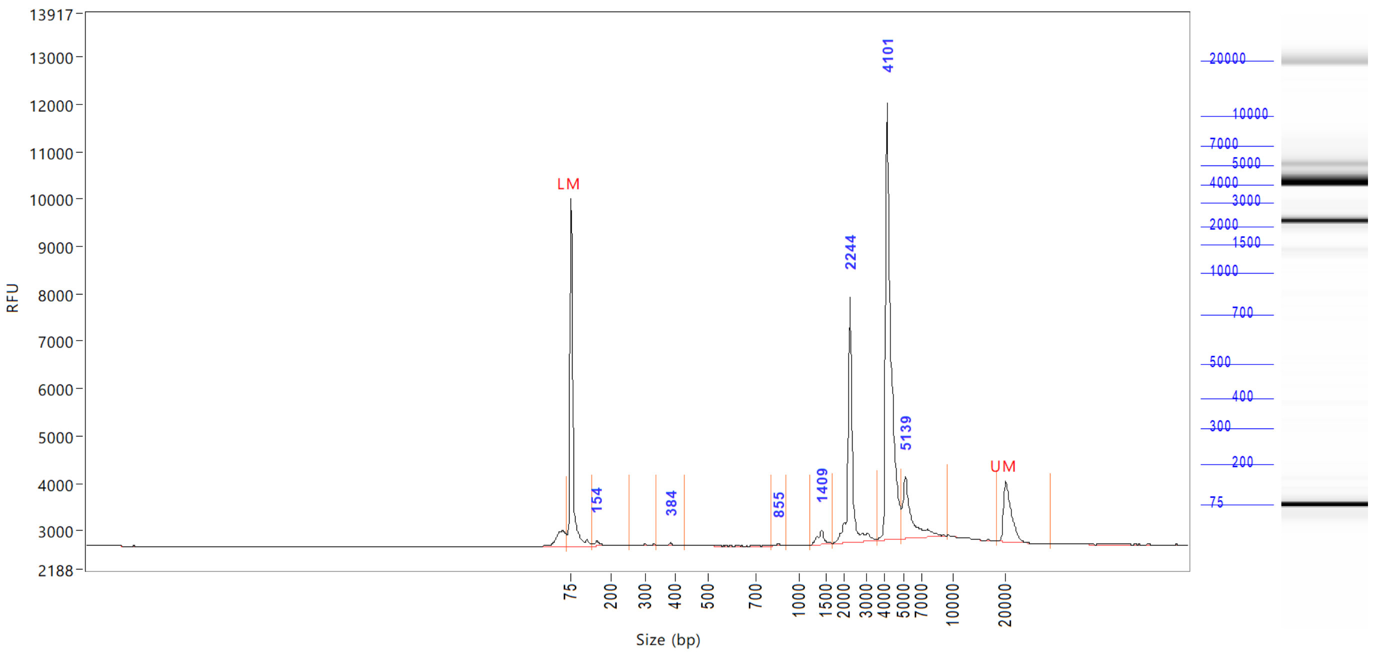

Peak	Size (bp)	Concentration (ng/uL)	From (bp)	To (bp)	Average size (bp)	CV%	RFU	Corrected peak area
1	75 (LM)	0.5000	62	139	77	14.25	7331	48.987
2	154	0.2275	139	255	167	13.79	124	1.858
3	384	0.0644	336	425	379	3.46	69	0.525
4	855	0.0469	807	912	851	2.44	55	0.383
5	1409	0.3864	1194	1649	1373	5.34	313	3.155
6	2244	4.2691	1649	3536	2264	10.49	5170	34.855
7	4101	9.2525	3536	4867	4198	5.09	9233	75.543
8	5139	1.9771	4867	9500	5725	16.16	1305	16.142
9	20000 (UM)	0.1289	18489	28534	20536	4.45	1273	12.627

TIC:	16.2239	ng/uL
TIM:	10.3739	nmole/L
Total concentration:	16.3922	ng/uL

```
Sample peak width (sec): 5     Sample min peak height: 50     Sample baseline V to V?: Y     Sample baseline V to V points: 3
Sample filter: Binomial        Number of points for filter: 3  Sample start region (min): 0  Sample end region (min): 30
Marker peak width (sec): 5     Marker min peak height: 200    Marker baseline V to V?: Y     Marker baseline V to V points: 3
Lower marker selection: First peak > 500 RFU                  Upper marker selection: Last peak > 200 RFU
Ladder size (bp)75, 200, 300, 400, 500, 700, 1000, 1500, 2000, 3000, 4000, 5000, 7000, 10000, 20000
Quantification using: Lower Marker          Final concentration (ng/uL): 0.5000          Dilution factor: 12.0
```

Sample: SampE2
Well location: E2
Created: Tuesday, August 10, 2021 7:21:14 PM

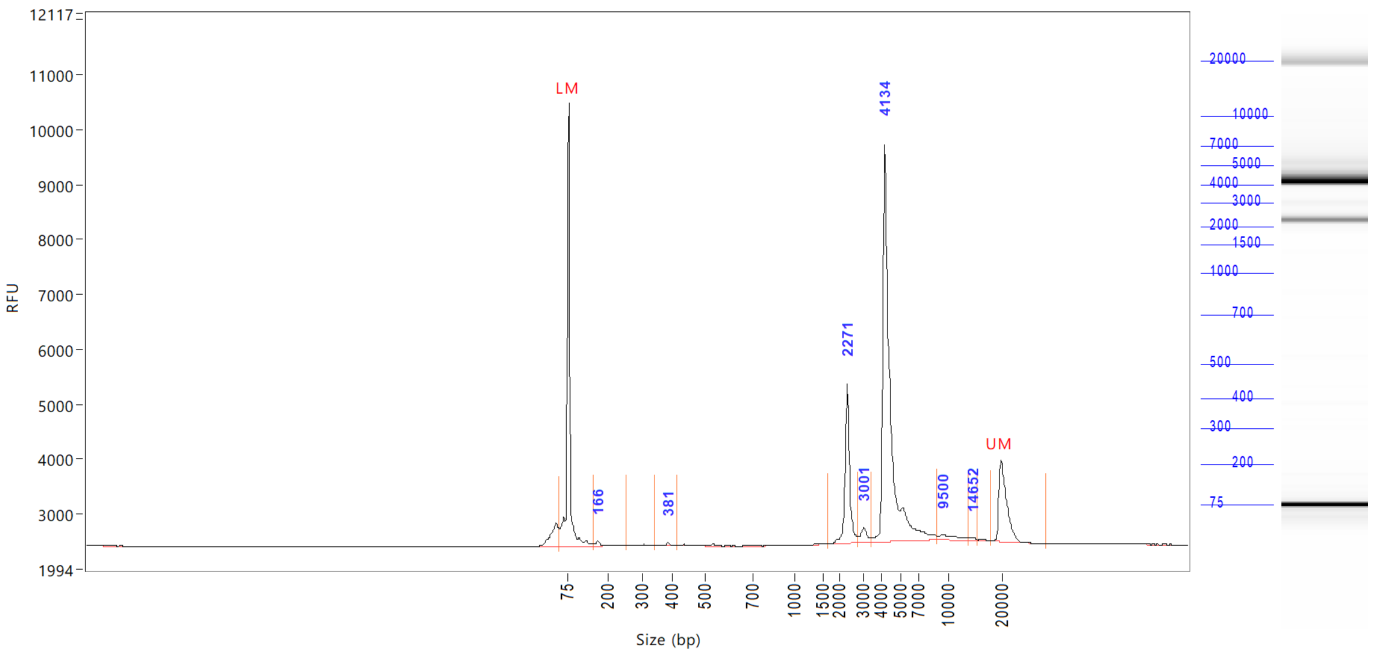

Peak	Size (bp)	Concentration (ng/uL)	From (bp)	To (bp)	Average size (bp)	CV%	RFU	Corrected peak area
1	75 (LM)	0.5000	47	154	74	17.86	8087	56.052
2	166	0.1399	154	253	179	12.75	91	1.307
3	381	0.0607	340	416	378	3.63	66	0.567
4	2271	2.1578	1649	2757	2269	6.38	2912	20.158
5	3001	0.2999	2757	3465	3032	5.79	265	2.801
6	4134	8.2009	3465	8875	4538	17.98	7230	76.613
7	9500	0.2558	8875	13838	10662	13.30	89	2.390
8	14652	0.0452	13838	15582	14542	3.06	56	0.422
9	20000 (UM)	0.1296	17907	28534	20485	4.59	1472	14.531

TIC:	11.1602	ng/uL
TIM:	6.2969	nmole/L
Total concentration:	11.3481	ng/uL

Sample peak width (sec): 5 Sample min peak height: 50 Sample baseline V to V?: Y Sample baseline V to V points: 3
Sample filter: Binomial Number of points for filter: 3 Sample start region (min): 0 Sample end region (min): 30
Marker peak width (sec): 5 Marker min peak height: 200 Marker baseline V to V?: Y Marker baseline V to V points: 3
Lower marker selection: First peak > 500 RFU Upper marker selection: Last peak > 200 RFU
Ladder size (bp)75, 200, 300, 400, 500, 700, 1000, 1500, 2000, 3000, 4000, 5000, 7000, 10000, 20000
Quantification using: Lower Marker Final concentration (ng/uL): 0.5000 Dilution factor: 12.0

Sample: SampF2
Well location: F2
Created: Tuesday, August 10, 2021 7:21:14 PM

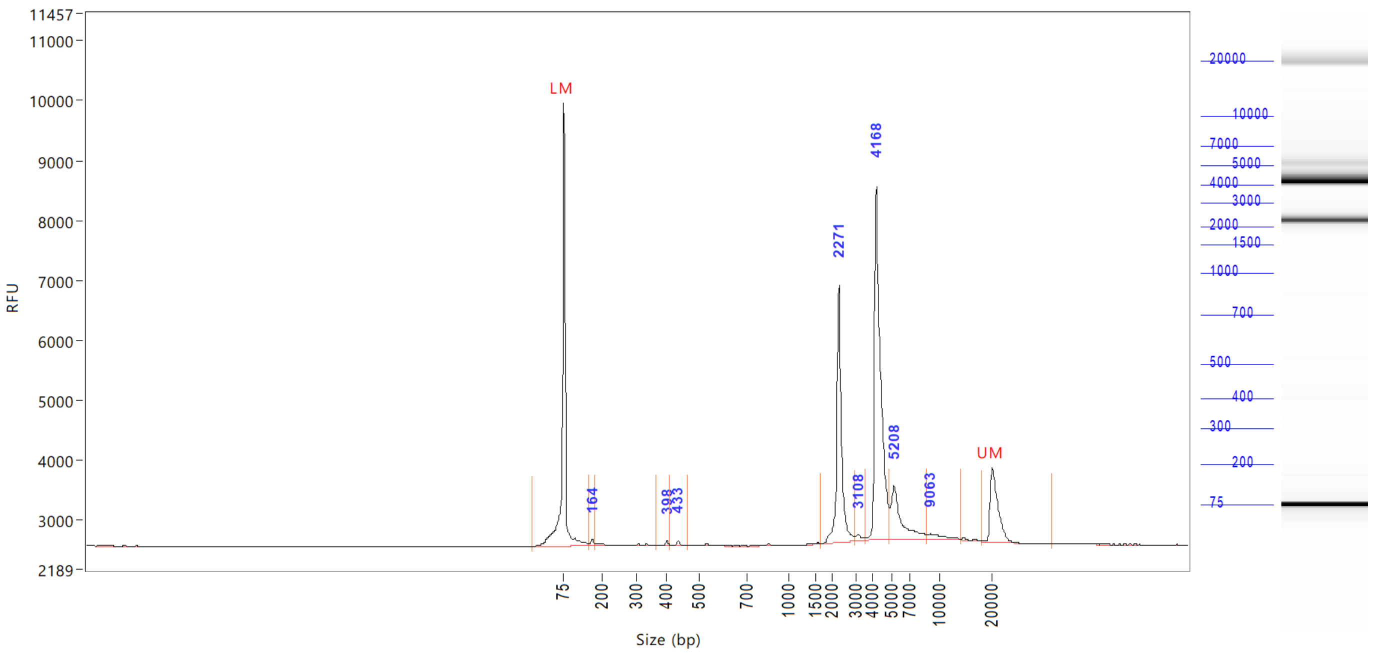

Peak	Size (bp)	Concentration (ng/uL)	From (bp)	To (bp)	Average size (bp)	CV%	RFU	Corrected peak area
1	75 (LM)	0.5000	0	154	71	25.60	7400	56.820
2	164	0.0867	154	173	163	3.05	107	0.821
3	398	0.0604	365	408	394	2.17	80	0.572
4	433	0.0729	408	463	429	2.38	93	0.690
5	2271	3.3212	1649	2973	2266	7.39	4281	31.452
6	3108	0.0971	2973	3536	3168	5.03	105	0.919
7	4168	5.6451	3536	4900	4265	5.10	5874	53.459
8	5208	1.3752	4900	8750	5929	17.42	891	13.023
9	9063	0.2327	8750	13954	10351	13.44	91	2.203
10	20000 (UM)	0.1112	18023	31417	20594	5.24	1248	12.633

TIC:	10.8911	ng/uL
TIM:	6.4669	nmole/L
Total concentration:	11.1592	ng/uL

Sample peak width (sec): 5   Sample min peak height: 50   Sample baseline V to V?: Y   Sample baseline V to V points: 3
Sample filter: Binomial   Number of points for filter: 3 Sample start region (min): 0 Sample end region (min): 30
Marker peak width (sec): 5   Marker min peak height: 200   Marker baseline V to V?: Y   Marker baseline V to V points: 3
Lower marker selection: First peak > 500 RFU   Upper marker selection: Last peak > 200 RFU
Ladder size (bp)75, 200, 300, 400, 500, 700, 1000, 1500, 2000, 3000, 4000, 5000, 7000, 10000, 20000
Quantification using: Lower Marker   Final concentration (ng/uL): 0.5000   Dilution factor: 12.0

Sample: SampG2
Well location: G2
Created: Tuesday, August 10, 2021 7:21:14 PM

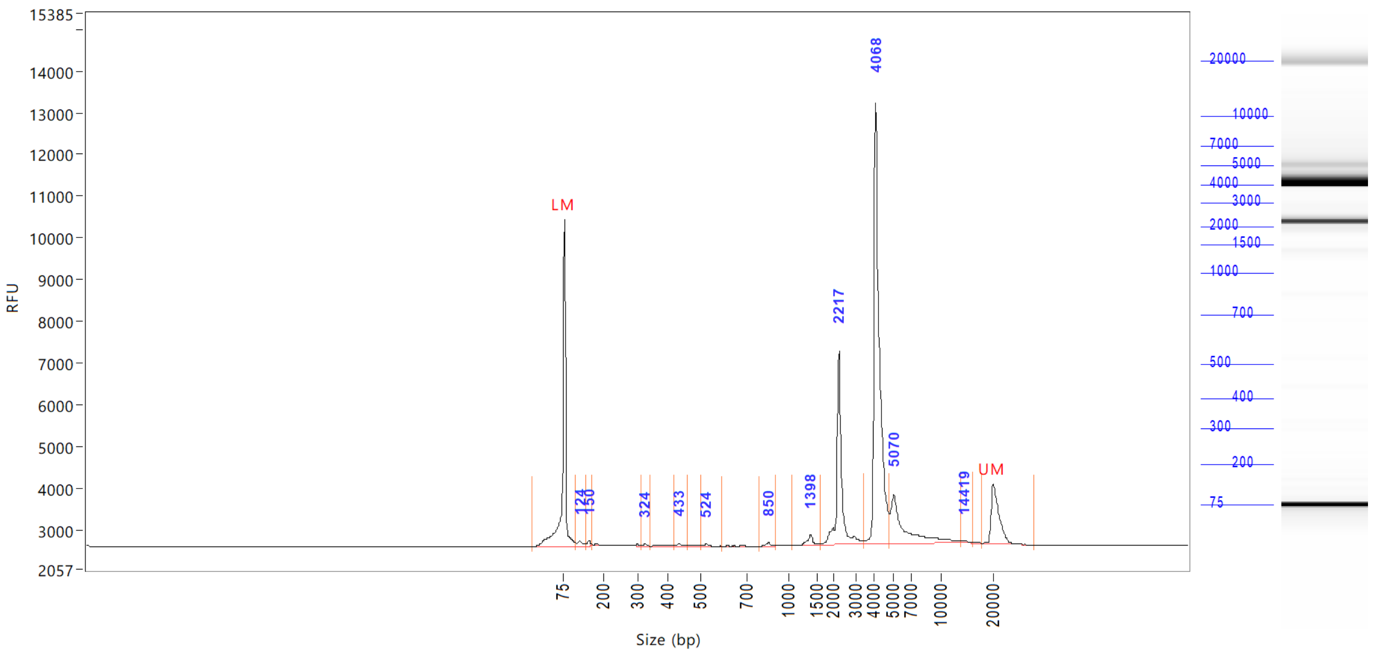

Peak	Size (bp)	Concentration (ng/uL)	From (bp)	To (bp)	Average size (bp)	CV%	RFU	Corrected peak area
1	75 (LM)	0.5000	0	112	66	26.78	7835	61.060
2	124	0.1520	112	141	125	6.47	129	1.547
3	150	0.1080	141	166	151	4.32	125	1.099
4	324	0.0316	311	340	321	1.61	50	0.322
5	433	0.0390	422	459	433	1.62	70	0.397
6	524	0.0387	501	591	525	3.06	54	0.394
7	850	0.0549	785	899	841	1.81	88	0.558
8	1398	0.2393	1035	1630	1369	6.12	243	2.435
9	2217	3.3575	1630	3393	2241	10.85	4642	34.168
10	4068	8.5640	3393	4834	4161	5.06	10581	87.153
11	5070	2.1462	4834	13838	6639	30.71	1184	21.841
12	14419	0.0514	13838	16047	14683	4.21	61	0.523
13	20000 (UM)	0.1178	17907	27842	20524	4.44	1413	14.392

TIC:	14.7825	ng/uL
TIM:	10.3924	nmole/L
Total concentration:	14.9415	ng/uL

```
Sample peak width (sec): 5    Sample min peak height: 50    Sample baseline V to V?: Y    Sample baseline V to V points: 3
Sample filter: Binomial       Number of points for filter: 3  Sample start region (min): 0  Sample end region (min): 30
Marker peak width (sec): 5    Marker min peak height: 200   Marker baseline V to V?: Y    Marker baseline V to V points: 3
Lower marker selection: First peak > 500 RFU                Upper marker selection: Last peak > 200 RFU
Ladder size (bp)75, 200, 300, 400, 500, 700, 1000, 1500, 2000, 3000, 4000, 5000, 7000, 10000, 20000
Quantification using: Lower Marker          Final concentration (ng/uL): 0.5000          Dilution factor: 12.0
```

Sample: SampH2
Well location: H2
Created: Tuesday, August 10, 2021 7:21:14 PM

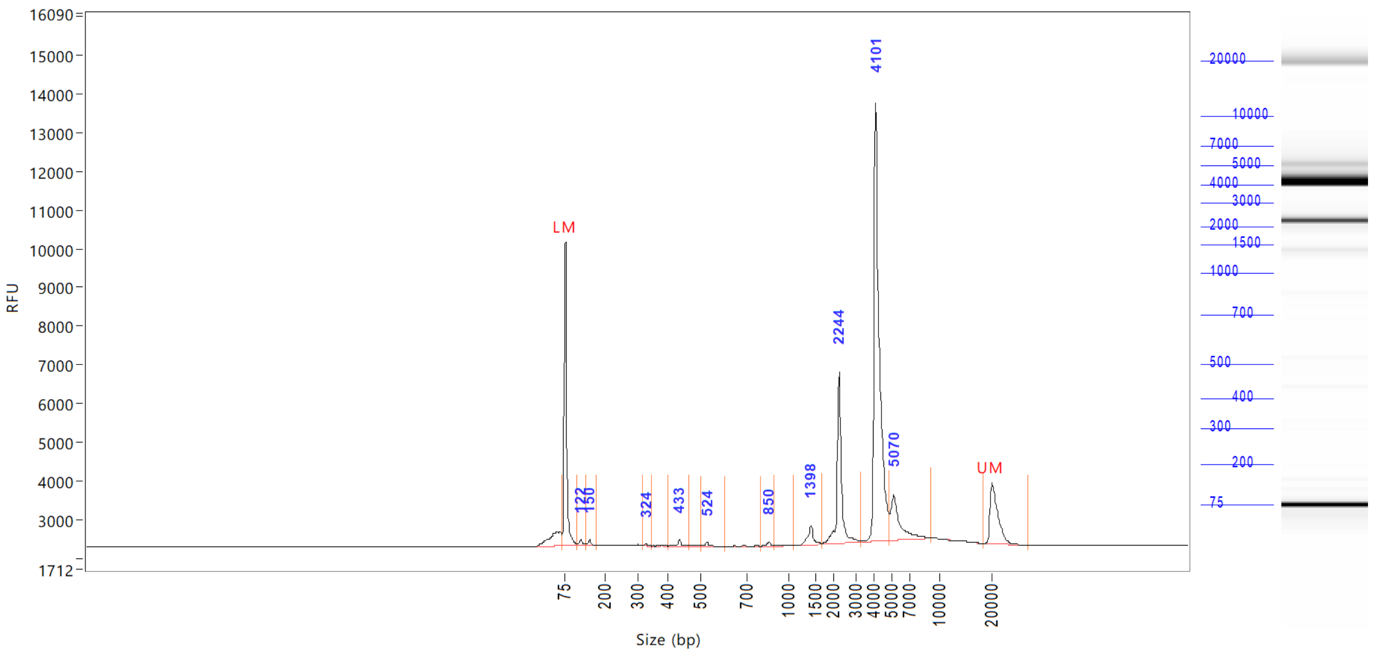

Peak	Size (bp)	Concentration (ng/uL)	From (bp)	To (bp)	Average size (bp)	CV%	RFU	Corrected peak area
1	75 (LM)	0.5000	64	110	74	7.92	7841	49.523
2	122	0.1504	110	141	122	6.13	141	1.241
3	150	0.1131	141	173	150	3.45	138	0.933
4	324	0.0431	311	342	320	1.46	58	0.356
5	433	0.1165	400	463	431	2.22	158	0.961
6	524	0.0948	501	602	525	2.93	109	0.782
7	850	0.0893	789	890	844	1.83	119	0.737
8	1398	0.5696	1058	1649	1378	5.44	512	4.701
9	2244	3.6575	1649	3286	2244	8.66	4448	30.188
10	4101	11.1876	3286	4867	4191	4.97	11356	92.341
11	5070	1.8235	4867	9063	5643	14.79	1196	15.051
12	20000 (UM)	0.1626	18372	26919	20554	4.56	1586	16.104

TIC:	17.8452	ng/uL
TIM:	12.6919	nmole/L
Total concentration:	17.9662	ng/uL

```
Sample peak width (sec): 5     Sample min peak height: 50    Sample baseline V to V?: Y    Sample baseline V to V points: 3
Sample filter: Binomial        Number of points for filter: 3  Sample start region (min): 0  Sample end region (min): 30
Marker peak width (sec): 5     Marker min peak height: 200   Marker baseline V to V?: Y    Marker baseline V to V points: 3
Lower marker selection: First peak > 500 RFU                 Upper marker selection: Last peak > 200 RFU
Ladder size (bp)75, 200, 300, 400, 500, 700, 1000, 1500, 2000, 3000, 4000, 5000, 7000, 10000, 20000
Quantification using: Lower Marker          Final concentration (ng/uL): 0.5000          Dilution factor: 12.0
```

Sample: SampA3
Well location: A3
Created: Tuesday, August 10, 2021 7:21:14 PM

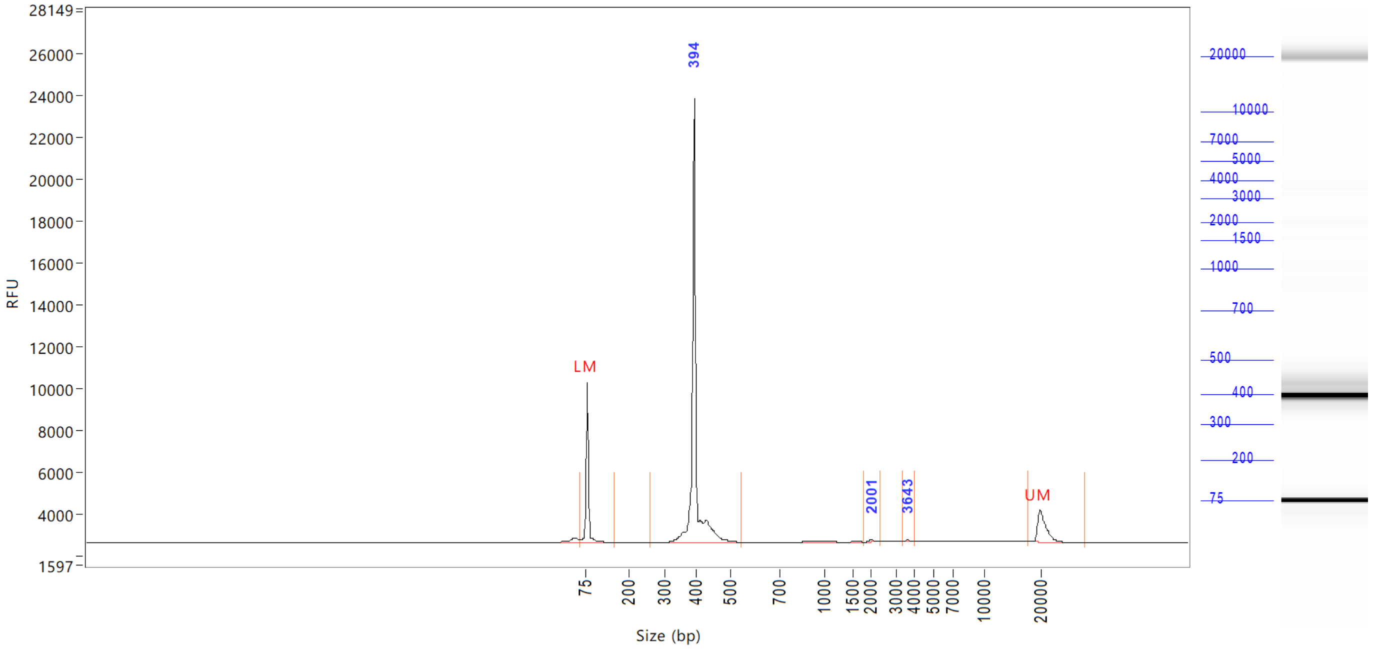

Peak	Size (bp)	Concentration (ng/uL)	From (bp)	To (bp)	Average size (bp)	CV%	RFU	Corrected peak area
1	75 (LM)	0.5000	54	156	75	12.20	7641	44.667
2	394	20.4264	262	540	396	5.67	21219	152.065
3	2001	0.0861	1797	2352	2015	4.23	94	0.641
4	3643	0.0748	3393	4001	3661	4.36	65	0.557
5	20000 (UM)	0.1704	17907	28073	20543	4.76	1527	15.225

TIC:	20.5874	ng/uL
TIM:	84.9708	nmole/L
Total concentration:	21.1954	ng/uL

Sample peak width (sec): 5   Sample min peak height: 50   Sample baseline V to V?: Y   Sample baseline V to V points: 3
Sample filter: Binomial   Number of points for filter: 3 Sample start region (min): 0 Sample end region (min): 30
Marker peak width (sec): 5   Marker min peak height: 200   Marker baseline V to V?: Y   Marker baseline V to V points: 3
Lower marker selection: First peak > 500 RFU   Upper marker selection: Last peak > 200 RFU
Ladder size (bp)75, 200, 300, 400, 500, 700, 1000, 1500, 2000, 3000, 4000, 5000, 7000, 10000, 20000
Quantification using: Lower Marker   Final concentration (ng/uL): 0.5000   Dilution factor: 12.0

Sample: SampB3
Well location: B3
Created: Tuesday, August 10, 2021 7:21:14 PM

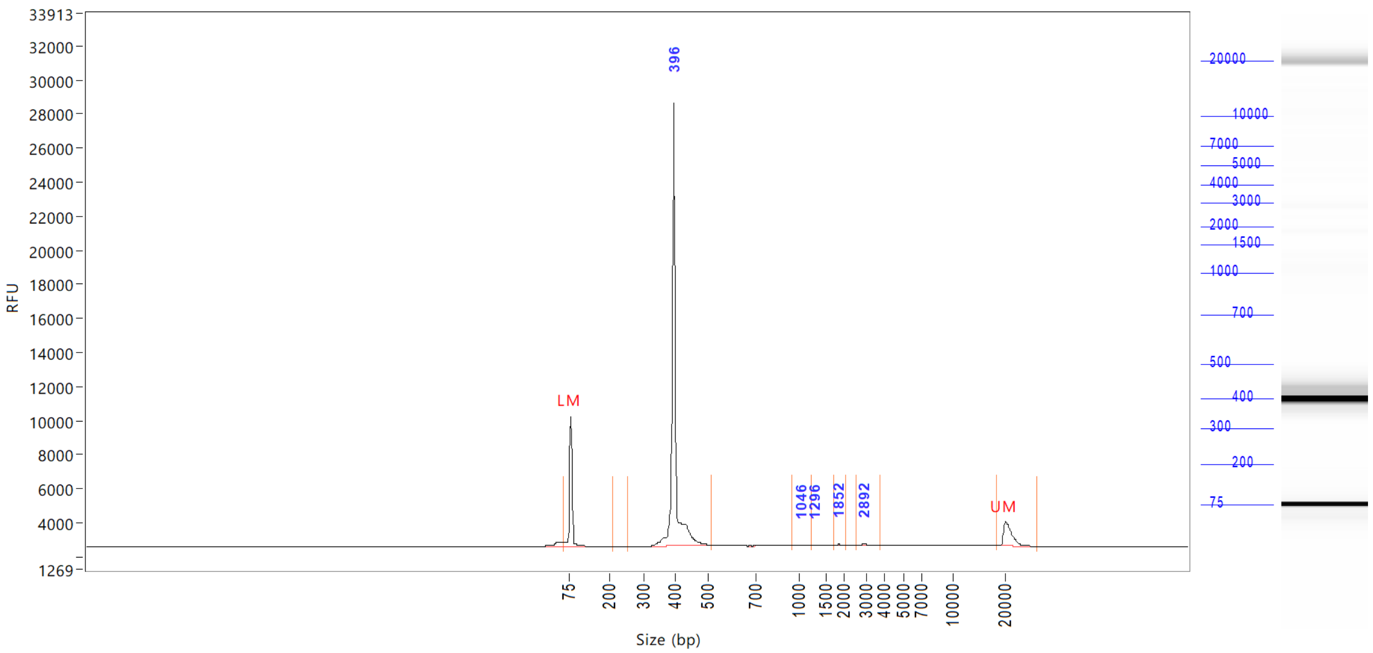

Peak	Size (bp)	Concentration (ng/uL)	From (bp)	To (bp)	Average size (bp)	CV%	RFU	Corrected peak area
1	75 (LM)	0.5000	54	208	76	17.18	7609	47.650
2	396	22.6385	251	511	397	4.77	26052	179.786
3	1046	0.1651	956	1228	1065	7.12	68	1.311
4	1296	0.0646	1228	1723	1325	6.73	50	0.513
5	1852	0.0700	1723	2109	1873	3.90	84	0.556
6	2892	0.1001	2568	3786	2950	6.69	89	0.795
7	20000 (UM)	0.1545	18489	25881	20563	4.37	1468	14.726

TIC:	23.0383	ng/uL
TIM:	94.2733	nmole/L
Total concentration:	23.4343	ng/uL

```
Sample peak width (sec): 5    Sample min peak height: 50    Sample baseline V to V?: Y    Sample baseline V to V points: 3
Sample filter: Binomial       Number of points for filter: 3  Sample start region (min): 0  Sample end region (min): 30
Marker peak width (sec): 5    Marker min peak height: 200   Marker baseline V to V?: Y    Marker baseline V to V points: 3
Lower marker selection: First peak > 500 RFU                Upper marker selection: Last peak > 200 RFU
Ladder size (bp)75, 200, 300, 400, 500, 700, 1000, 1500, 2000, 3000, 4000, 5000, 7000, 10000, 20000
Quantification using: Lower Marker          Final concentration (ng/uL): 0.5000          Dilution factor: 12.0
```

Sample: SampC3
Well location: C3
Created: Tuesday, August 10, 2021 7:21:14 PM

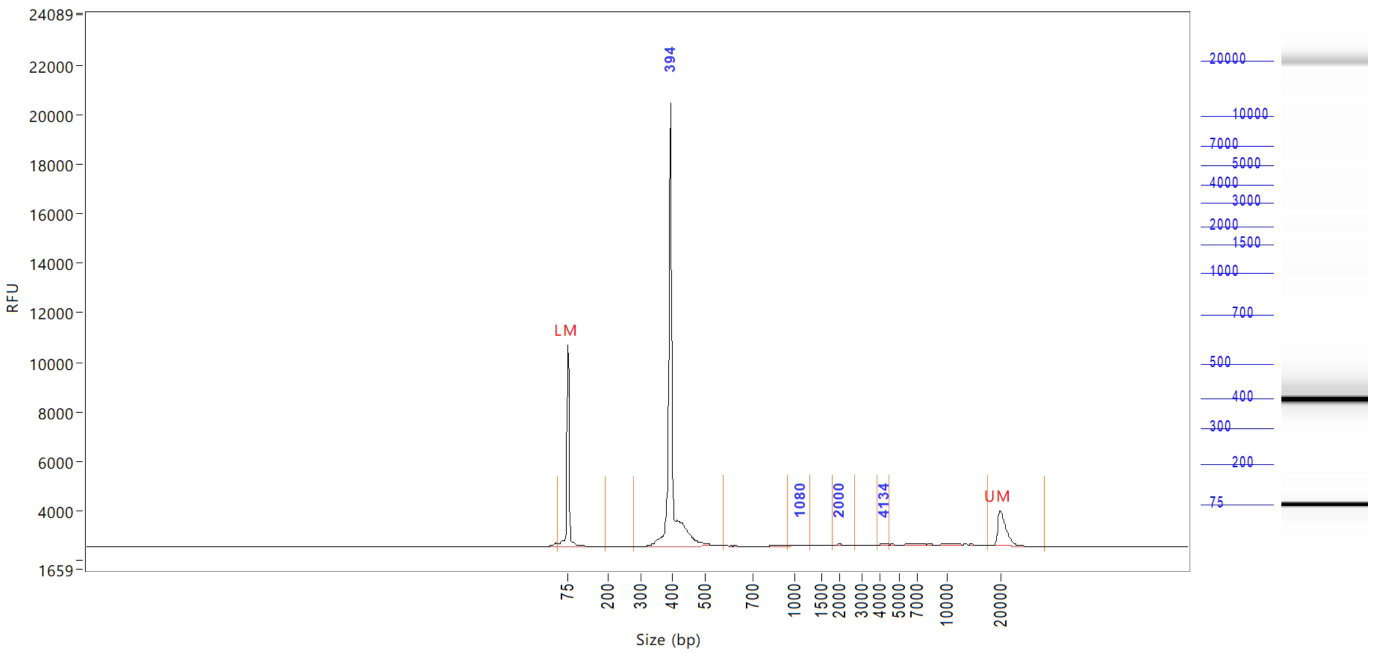

Peak	Size (bp)	Concentration (ng/uL)	From (bp)	To (bp)	Average size (bp)	CV%	RFU	Corrected peak area
1	75 (LM)	0.5000	45	190	74	14.35	8154	49.250
2	394	16.8264	273	579	399	6.30	17918	138.116
3	1080	0.1568	952	1296	1089	8.88	55	1.287
4	2000	0.0860	1815	2649	2096	9.22	83	0.706
5	4134	0.0580	3822	4500	4162	3.72	53	0.476
6	20000 (UM)	0.1479	17791	28534	20546	4.59	1439	14.567

TIC:	17.1272	ng/uL
TIM:	69.7115	nmole/L
Total concentration:	17.5332	ng/uL

Sample peak width (sec): 5   Sample min peak height: 50   Sample baseline V to V?: Y   Sample baseline V to V points: 3
Sample filter: Binomial   Number of points for filter: 3   Sample start region (min): 0   Sample end region (min): 30
Marker peak width (sec): 5   Marker min peak height: 200   Marker baseline V to V?: Y   Marker baseline V to V points: 3
Lower marker selection: First peak > 500 RFU   Upper marker selection: Last peak > 200 RFU
Ladder size (bp)75, 200, 300, 400, 500, 700, 1000, 1500, 2000, 3000, 4000, 5000, 7000, 10000, 20000
Quantification using: Lower Marker   Final concentration (ng/uL): 0.5000   Dilution factor: 12.0

Sample: SampD3
Well location: D3
Created: Tuesday, August 10, 2021 7:21:14 PM

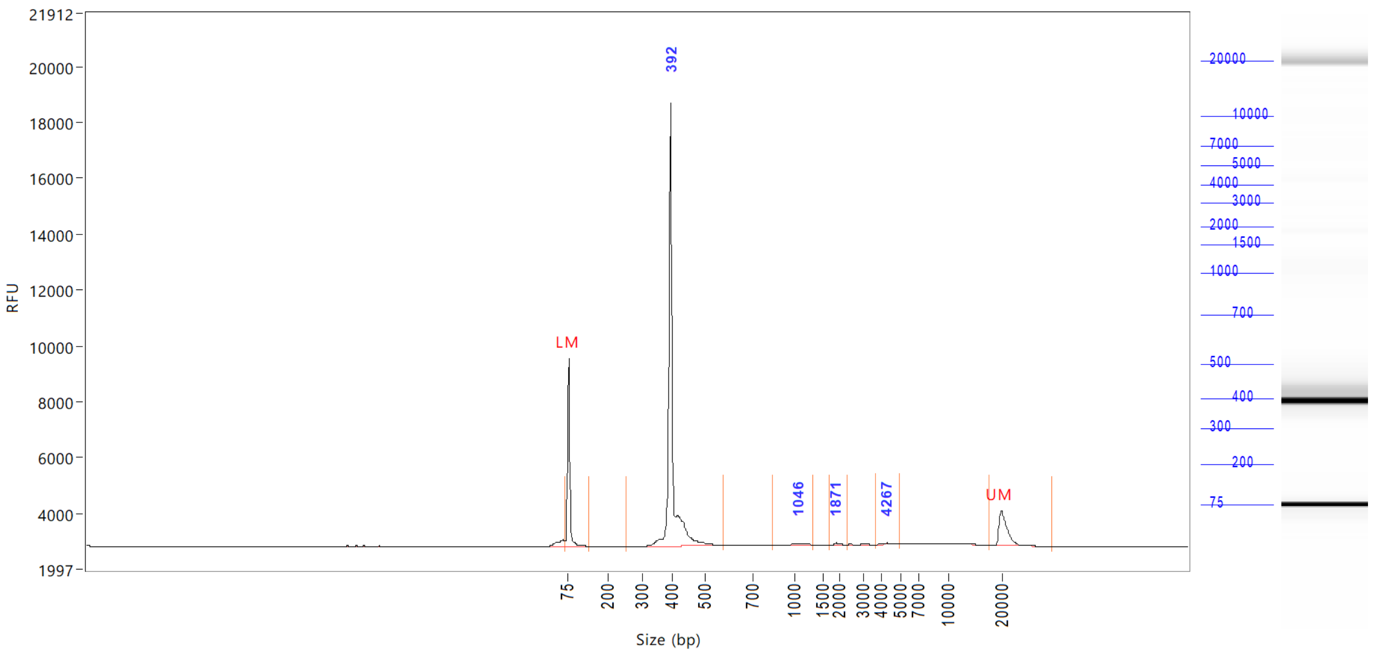

Peak	Size (bp)	Concentration (ng/uL)	From (bp)	To (bp)	Average size (bp)	CV%	RFU	Corrected peak area
1	75 (LM)	0.5000	64	137	74	9.33	6719	38.935
2	392	18.9507	251	573	396	6.29	15904	122.973
3	1046	0.3902	833	1307	1043	10.82	84	2.532
4	1871	0.0967	1667	2271	1917	5.70	79	0.628
5	4267	0.0877	3715	5000	4386	5.55	54	0.569
6	20000 (UM)	0.1617	17791	29572	20550	4.75	1251	12.590

TIC:	19.5253	ng/uL
TIM:	79.4672	nmole/L
Total concentration:	19.9143	ng/uL

```
Sample peak width (sec): 5    Sample min peak height: 50    Sample baseline V to V?: Y    Sample baseline V to V points: 3
Sample filter: Binomial       Number of points for filter: 3  Sample start region (min): 0  Sample end region (min): 30
Marker peak width (sec): 5    Marker min peak height: 200   Marker baseline V to V?: Y    Marker baseline V to V points: 3
Lower marker selection: First peak > 500 RFU                Upper marker selection: Last peak > 200 RFU
Ladder size (bp)75, 200, 300, 400, 500, 700, 1000, 1500, 2000, 3000, 4000, 5000, 7000, 10000, 20000
Quantification using: Lower Marker          Final concentration (ng/uL): 0.5000          Dilution factor: 12.0
```

Sample: SampE3
Well location: E3
Created: Tuesday, August 10, 2021 7:21:14 PM

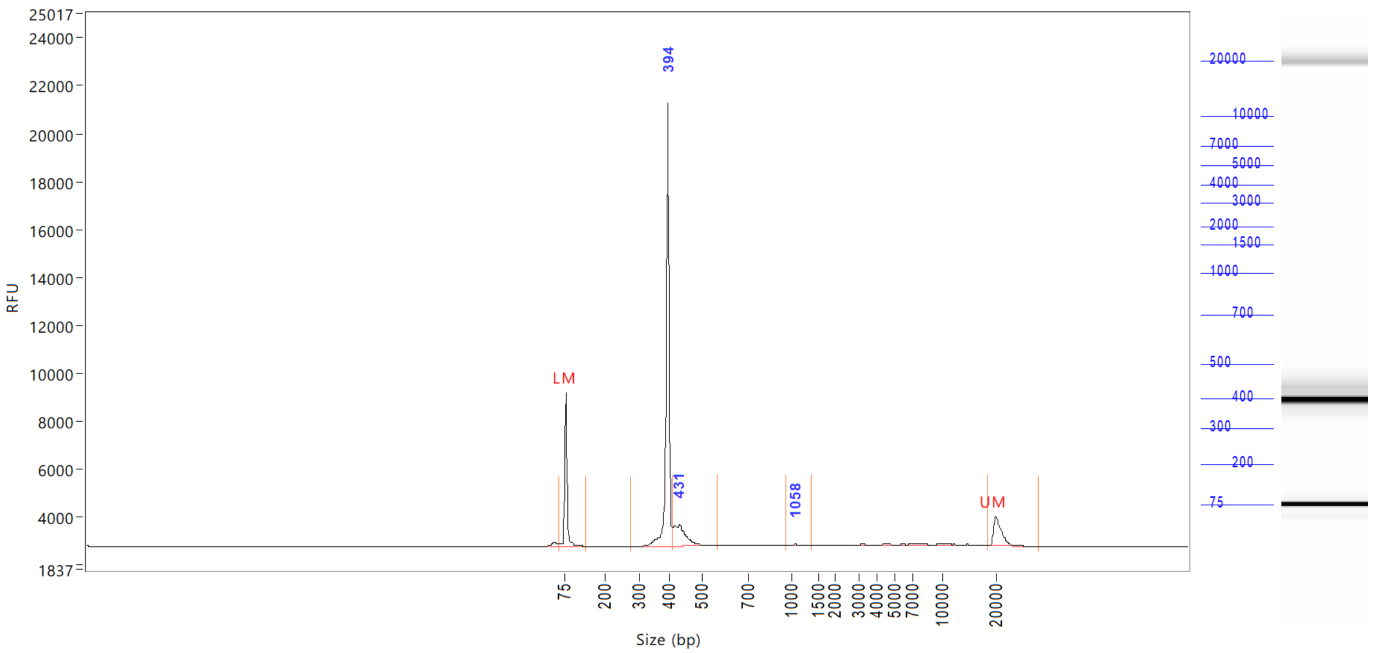

Peak	Size (bp)	Concentration (ng/uL)	From (bp)	To (bp)	Average size (bp)	CV%	RFU	Corrected peak area
1	75 (LM)	0.5000	54	141	75	10.54	6408	39.535
2	394	16.8014	277	408	389	2.97	18512	110.706
3	431	3.2837	408	563	436	5.03	879	21.637
4	1058	0.1764	960	1375	1090	9.49	52	1.162
5	20000 (UM)	0.1526	18605	28303	20533	4.72	1211	12.063

TIC:	20.2616	ng/uL
TIM:	83.7196	nmole/L
Total concentration:	20.8160	ng/uL

Sample peak width (sec): 5 Sample min peak height: 50 Sample baseline V to V?: Y Sample baseline V to V points: 3
Sample filter: Binomial Number of points for filter: 3 Sample start region (min): 0 Sample end region (min): 30
Marker peak width (sec): 5 Marker min peak height: 200 Marker baseline V to V?: Y Marker baseline V to V points: 3
Lower marker selection: First peak > 500 RFU Upper marker selection: Last peak > 200 RFU
Ladder size (bp)75, 200, 300, 400, 500, 700, 1000, 1500, 2000, 3000, 4000, 5000, 7000, 10000, 20000
Quantification using: Lower Marker Final concentration (ng/uL): 0.5000 Dilution factor: 12.0

Sample: SampF3
Well location: F3
Created: Tuesday, August 10, 2021 7:21:14 PM

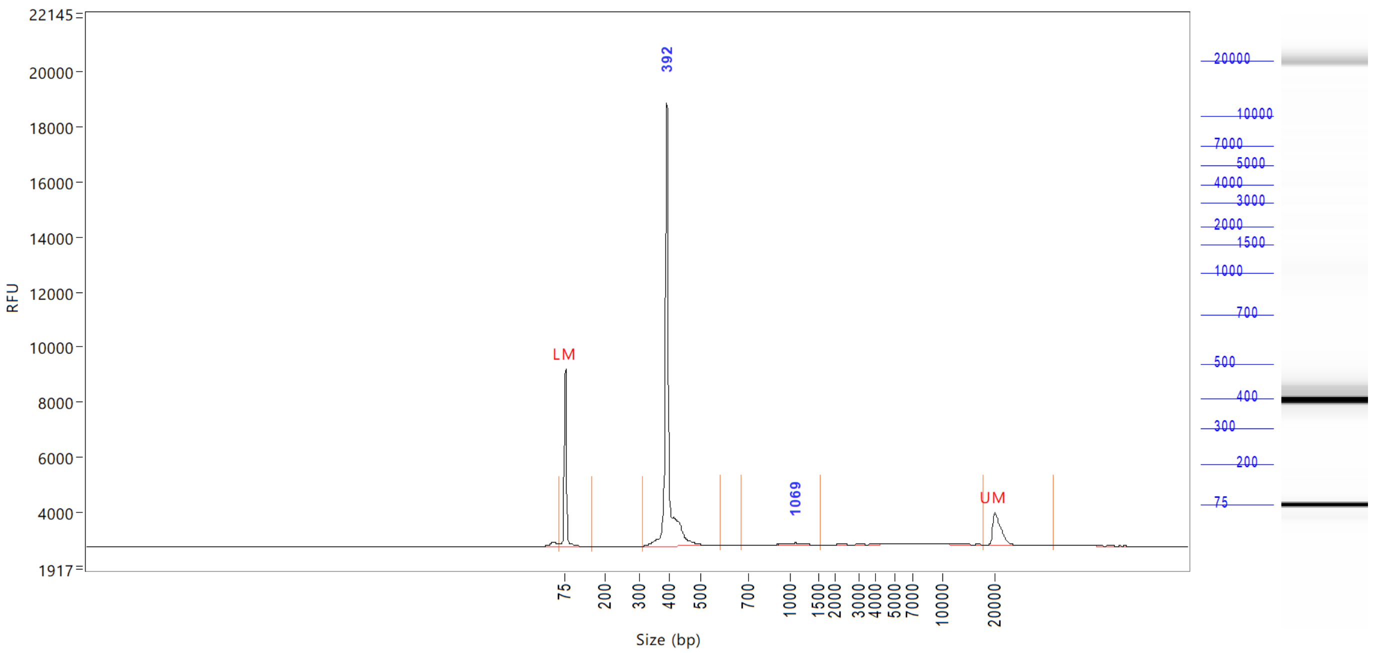

Peak	Size (bp)	Concentration (ng/uL)	From (bp)	To (bp)	Average size (bp)	CV%	RFU	Corrected peak area
1	75 (LM)	0.5000	58	158	73	11.11	6443	37.955
2	392	19.4117	309	581	396	5.42	16153	122.795
3	1069	0.4834	669	1575	1052	15.83	84	3.058
4	20000 (UM)	0.1643	17791	31071	20565	5.67	1199	12.471

TIC:	19.8951	ng/uL
TIM:	81.4072	nmole/L
Total concentration:	20.4088	ng/uL

Sample peak width (sec): 5 Sample min peak height: 50 Sample baseline V to V?: Y Sample baseline V to V points: 3
Sample filter: Binomial Number of points for filter: 3 Sample start region (min): 0 Sample end region (min): 30
Marker peak width (sec): 5 Marker min peak height: 200 Marker baseline V to V?: Y Marker baseline V to V points: 3
Lower marker selection: First peak > 500 RFU Upper marker selection: Last peak > 200 RFU
Ladder size (bp)75, 200, 300, 400, 500, 700, 1000, 1500, 2000, 3000, 4000, 5000, 7000, 10000, 20000
Quantification using: Lower Marker Final concentration (ng/uL): 0.5000 Dilution factor: 12.0

Sample: SampG3
Well location: G3
Created: Tuesday, August 10, 2021 7:21:14 PM

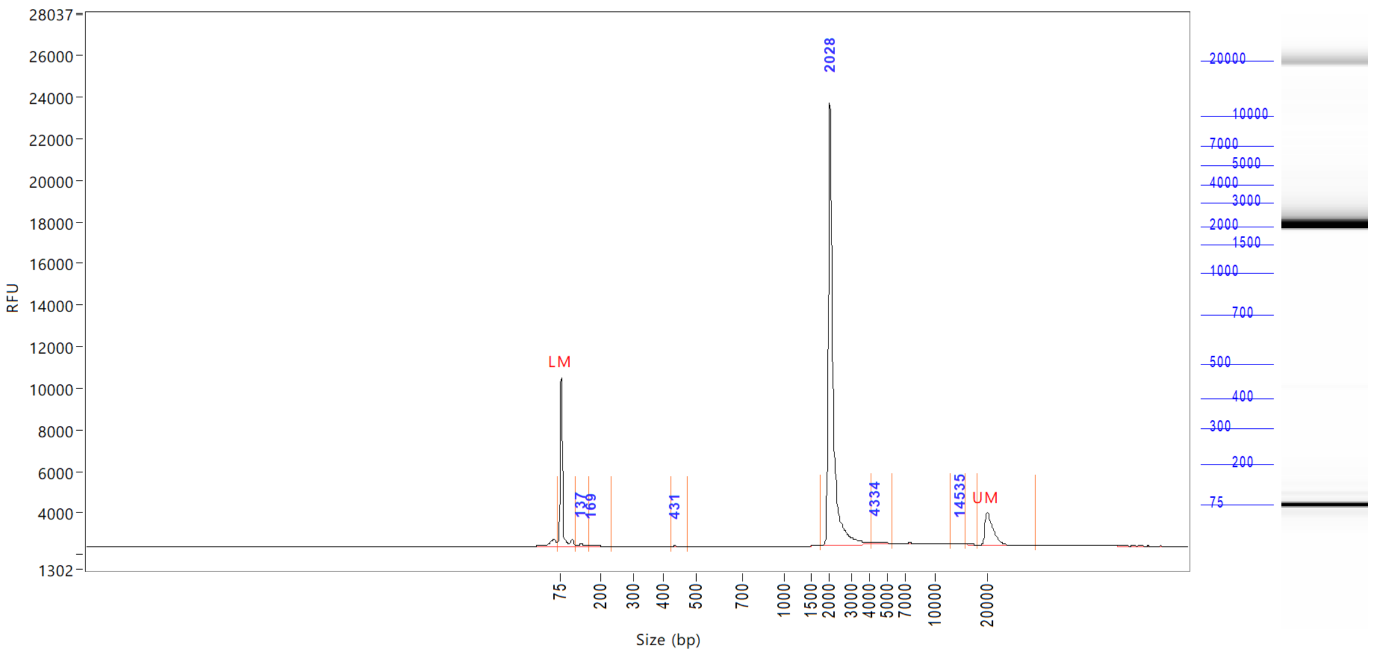

Peak	Size (bp)	Concentration (ng/uL)	From (bp)	To (bp)	Average size (bp)	CV%	RFU	Corrected peak area
1	75 (LM)	0.5000	64	122	75	12.32	8095	51.056
2	137	0.2213	122	162	138	7.48	124	1.883
3	169	0.1306	162	232	183	8.31	50	1.112
4	431	0.0473	422	472	434	2.22	64	0.403
5	2028	18.0143	1741	4068	2152	13.60	21319	153.291
6	4334	0.2332	4068	5415	4508	6.89	114	1.985
7	14535	0.0766	12791	15698	14077	5.35	51	0.651
8	20000 (UM)	0.1575	18023	29226	20597	4.89	1560	16.088

TIC:	18.7234	ng/uL
TIM:	17.8624	nmole/L
Total concentration:	19.0217	ng/uL

```
Sample peak width (sec): 5    Sample min peak height: 50    Sample baseline V to V?: Y    Sample baseline V to V points: 3
Sample filter: Binomial       Number of points for filter: 3  Sample start region (min): 0  Sample end region (min): 30
Marker peak width (sec): 5    Marker min peak height: 200   Marker baseline V to V?: Y    Marker baseline V to V points: 3
Lower marker selection: First peak > 500 RFU                Upper marker selection: Last peak > 200 RFU
Ladder size (bp)75, 200, 300, 400, 500, 700, 1000, 1500, 2000, 3000, 4000, 5000, 7000, 10000, 20000
Quantification using: Lower Marker          Final concentration (ng/uL): 0.5000          Dilution factor: 12.0
```

Sample: SampH3
Well location: H3
Created: Tuesday, August 10, 2021 7:21:14 PM

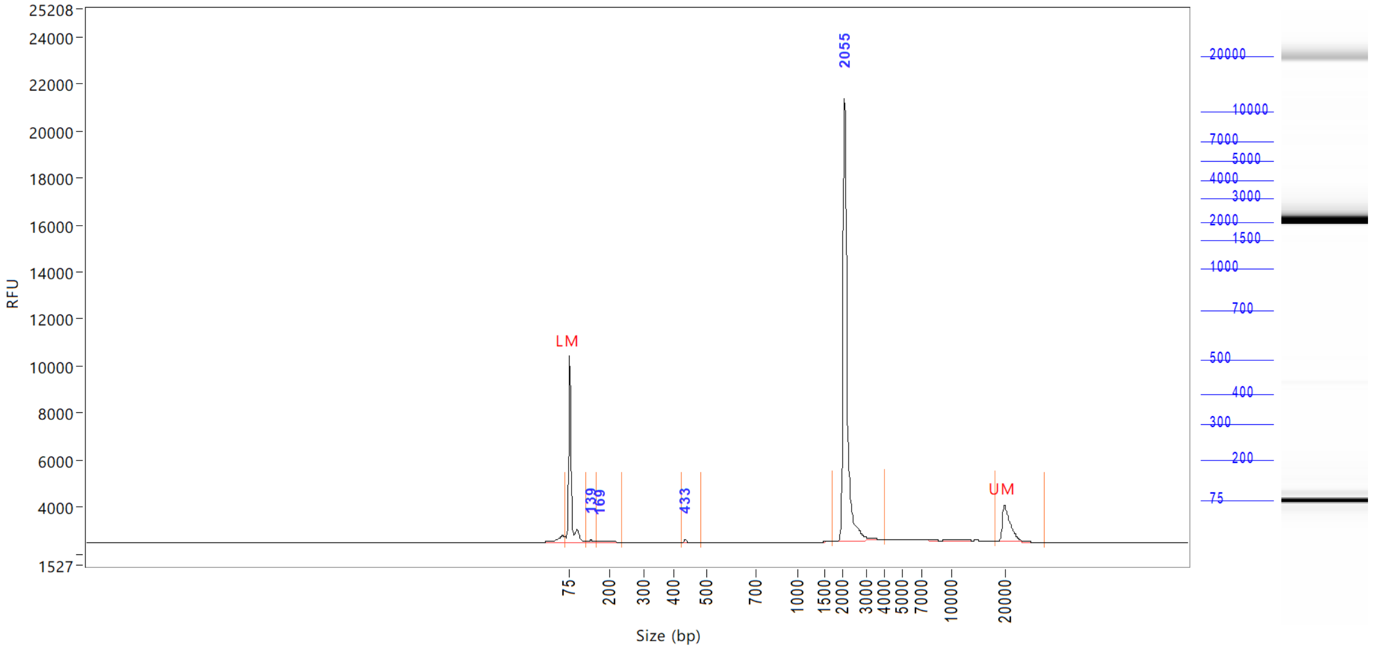

Peak	Size (bp)	Concentration (ng/uL)	From (bp)	To (bp)	Average size (bp)	CV%	RFU	Corrected peak area
1	75 (LM)	0.5000	64	128	76	12.52	7943	50.244
2	139	0.1673	128	160	142	6.50	95	1.401
3	169	0.1623	160	235	184	9.62	62	1.359
4	433	0.0784	420	479	432	2.02	116	0.656
5	2055	14.1723	1704	4001	2121	9.65	18875	118.678
6	20000 (UM)	0.1567	18372	27727	20592	4.60	1537	15.748

TIC:	14.5803	ng/uL
TIM:	14.6844	nmole/L
Total concentration:	14.8730	ng/uL

Sample peak width (sec): 5   Sample min peak height: 50   Sample baseline V to V?: Y   Sample baseline V to V points: 3
Sample filter: Binomial   Number of points for filter: 3 Sample start region (min): 0 Sample end region (min): 30
Marker peak width (sec): 5   Marker min peak height: 200   Marker baseline V to V?: Y   Marker baseline V to V points: 3
Lower marker selection: First peak > 500 RFU   Upper marker selection: Last peak > 200 RFU
Ladder size (bp)75, 200, 300, 400, 500, 700, 1000, 1500, 2000, 3000, 4000, 5000, 7000, 10000, 20000
Quantification using: Lower Marker   Final concentration (ng/uL): 0.5000   Dilution factor: 12.0

Sample: SampA4
Well location: A4
Created: Tuesday, August 10, 2021 7:21:14 PM

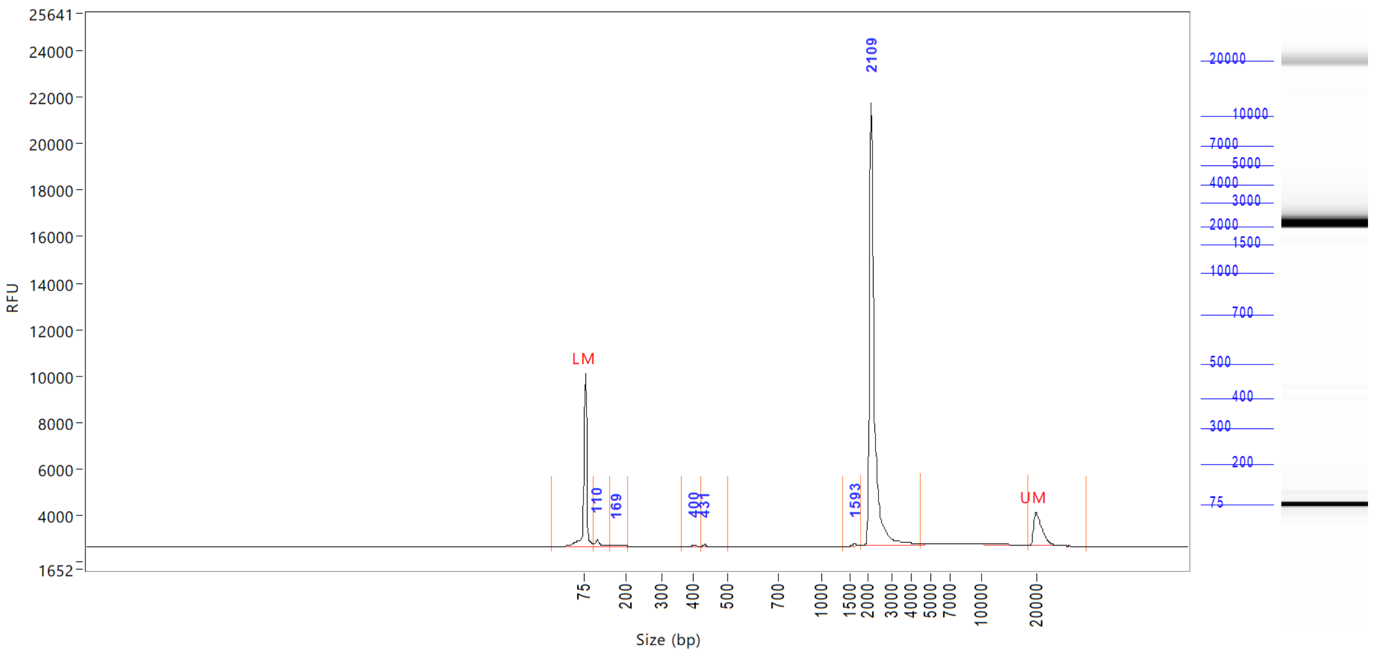

Peak	Size (bp)	Concentration (ng/uL)	From (bp)	To (bp)	Average size (bp)	CV%	RFU	Corrected peak area
1	75 (LM)	0.5000	0	99	71	16.18	7483	50.298
2	110	0.4705	99	150	117	11.73	296	3.945
3	169	0.1865	150	203	173	8.01	73	1.564
4	400	0.0872	363	420	397	2.70	90	0.731
5	431	0.0840	420	498	437	3.98	103	0.704
6	1593	0.0761	1375	1815	1599	5.13	87	0.638
7	2109	15.9242	1815	4500	2199	13.37	19136	133.493
8	20000 (UM)	0.1432	18489	28995	20487	4.51	1442	14.405

TIC:	16.8286	ng/uL
TIM:	21.0560	nmole/L
Total concentration:	17.2282	ng/uL

Sample peak width (sec): 5   Sample min peak height: 50   Sample baseline V to V?: Y   Sample baseline V to V points: 3
Sample filter: Binomial   Number of points for filter: 3 Sample start region (min): 0 Sample end region (min): 30
Marker peak width (sec): 5   Marker min peak height: 200   Marker baseline V to V?: Y   Marker baseline V to V points: 3
Lower marker selection: First peak > 500 RFU   Upper marker selection: Last peak > 200 RFU
Ladder size (bp)75, 200, 300, 400, 500, 700, 1000, 1500, 2000, 3000, 4000, 5000, 7000, 10000, 20000
Quantification using: Lower Marker   Final concentration (ng/uL): 0.5000   Dilution factor: 12.0

Sample: SampB4
Well location: B4
Created: Tuesday, August 10, 2021 7:21:14 PM

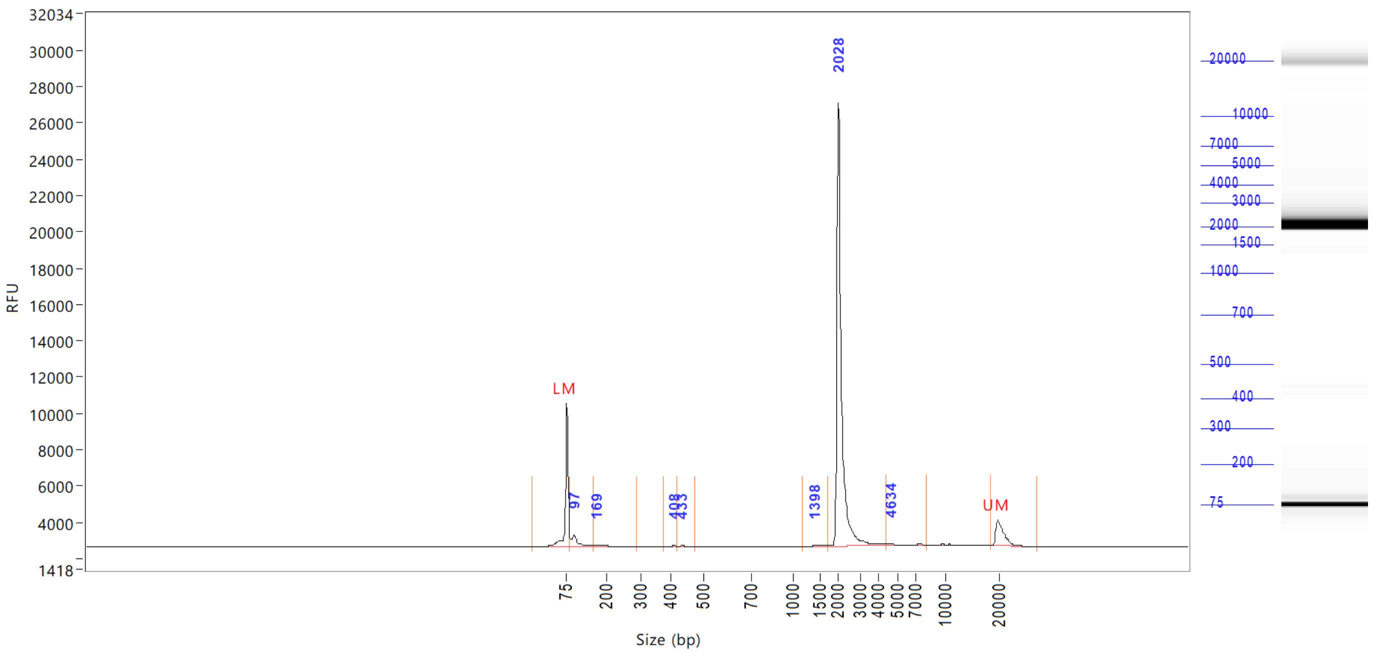

Peak	Size (bp)	Concentration (ng/uL)	From (bp)	To (bp)	Average size (bp)	CV%	RFU	Corrected peak area
1	75 (LM)	0.5000	0	86	69	17.08	7890	52.922
2	97	1.1690	86	158	106	17.64	636	10.311
3	169	0.3231	158	287	195	15.80	97	2.850
4	408	0.0713	375	422	406	2.05	88	0.629
5	433	0.0743	422	474	435	2.46	102	0.656
6	1398	0.0920	1171	1704	1439	7.07	79	0.811
7	2028	19.5903	1704	4401	2133	13.30	24448	172.793
8	4634	0.2705	4401	8063	5831	19.56	74	2.386
9	20000 (UM)	0.1344	18489	27381	20504	4.50	1418	14.228

TIC:	21.5905	ng/uL
TIM:	36.7063	nmole/L
Total concentration:	21.7874	ng/uL

Sample peak width (sec): 5   Sample min peak height: 50   Sample baseline V to V?: Y   Sample baseline V to V points: 3
Sample filter: Binomial   Number of points for filter: 3 Sample start region (min): 0 Sample end region (min): 30
Marker peak width (sec): 5   Marker min peak height: 200   Marker baseline V to V?: Y   Marker baseline V to V points: 3
Lower marker selection: First peak > 500 RFU   Upper marker selection: Last peak > 200 RFU
Ladder size (bp)75, 200, 300, 400, 500, 700, 1000, 1500, 2000, 3000, 4000, 5000, 7000, 10000, 20000
Quantification using: Lower Marker   Final concentration (ng/uL): 0.5000   Dilution factor: 12.0

Sample: SampC4
Well location: C4
Created: Tuesday, August 10, 2021 7:21:14 PM

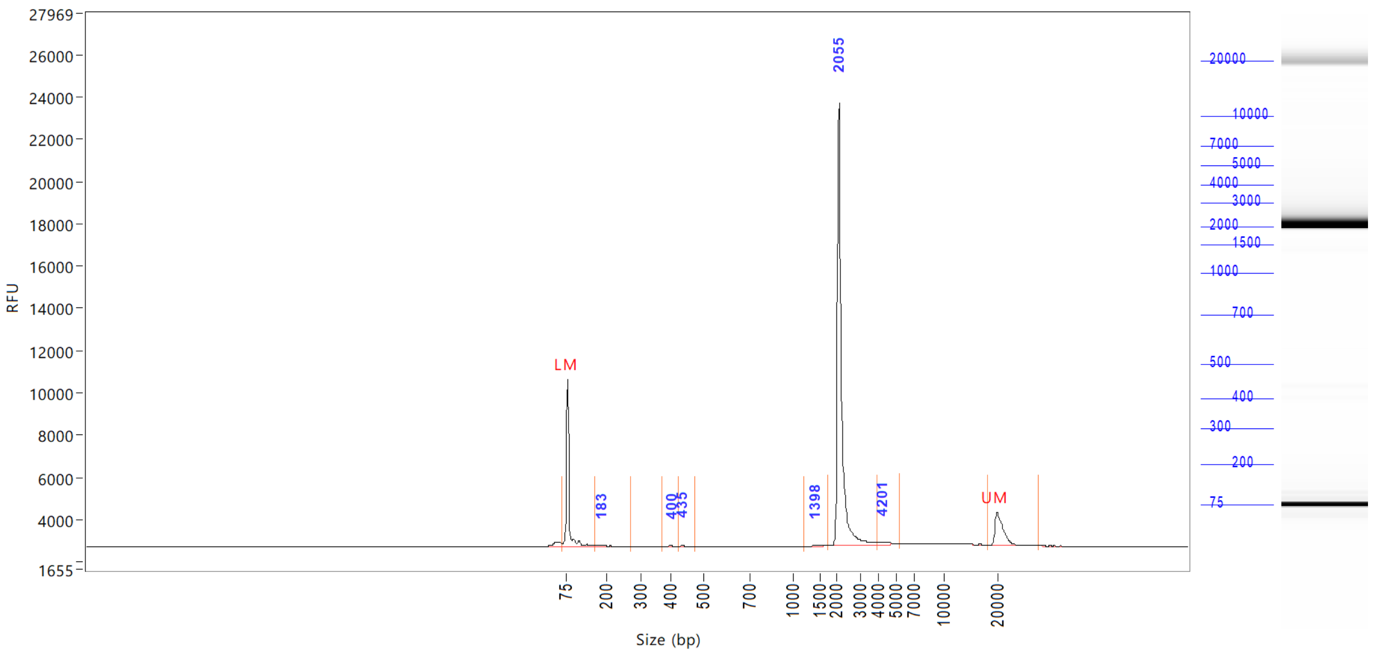

Peak	Size (bp)	Concentration (ng/uL)	From (bp)	To (bp)	Average size (bp)	CV%	RFU	Corrected peak area
1	75 (LM)	0.5000	58	162	78	19.12	7906	52.472
2	183	0.1752	162	271	192	12.43	58	1.532
3	400	0.0783	371	423	398	2.79	68	0.685
4	435	0.0738	423	476	437	2.54	102	0.645
5	1398	0.0762	1194	1741	1424	6.02	66	0.666
6	2055	15.7225	1741	3964	2143	12.22	20990	137.499
7	4201	0.2450	3964	5277	4452	7.69	102	2.143
8	20000 (UM)	0.1529	18256	27957	20600	4.54	1557	16.041

TIC:	16.3709	ng/uL
TIM:	14.3575	nmole/L
Total concentration:	16.6599	ng/uL

```
Sample peak width (sec): 5    Sample min peak height: 50    Sample baseline V to V?: Y    Sample baseline V to V points: 3
Sample filter: Binomial       Number of points for filter: 3  Sample start region (min): 0  Sample end region (min): 30
Marker peak width (sec): 5    Marker min peak height: 200   Marker baseline V to V?: Y    Marker baseline V to V points: 3
Lower marker selection: First peak > 500 RFU                Upper marker selection: Last peak > 200 RFU
Ladder size (bp)75, 200, 300, 400, 500, 700, 1000, 1500, 2000, 3000, 4000, 5000, 7000, 10000, 20000
Quantification using: Lower Marker          Final concentration (ng/uL): 0.5000          Dilution factor: 12.0
```

Sample: SampD4
Well location: D4
Created: Tuesday, August 10, 2021 7:21:14 PM

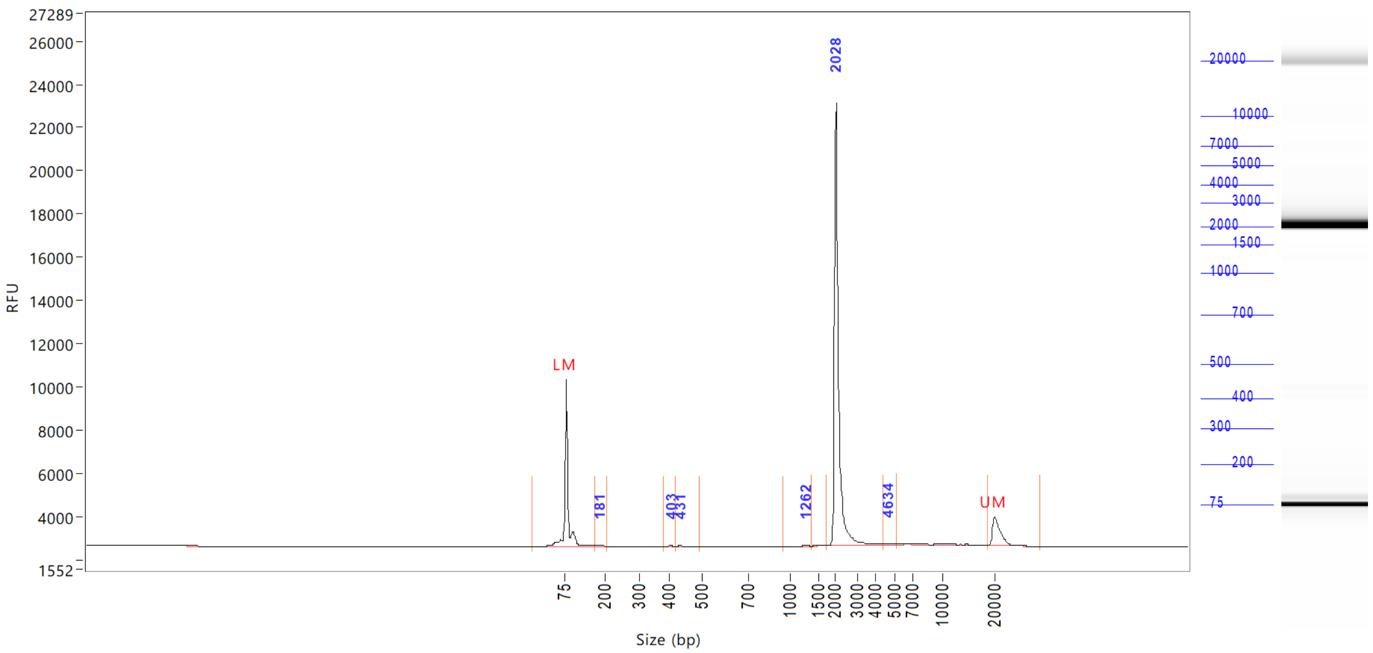

Peak	Size (bp)	Concentration (ng/uL)	From (bp)	To (bp)	Average size (bp)	CV%	RFU	Corrected peak area
1	75 (LM)	0.5000	0	166	76	25.18	7746	59.258
2	181	0.1200	166	203	182	5.73	61	1.185
3	403	0.0605	379	420	401	2.15	65	0.598
4	431	0.0607	420	493	435	2.99	85	0.599
5	1262	0.0384	943	1375	1234	6.13	56	0.380
6	2028	13.3547	1741	4401	2109	11.85	20532	131.896
7	4634	0.0552	4401	5277	4685	4.16	52	0.545
8	20000 (UM)	0.1123	18605	28765	20560	4.58	1315	13.310

TIC:	13.6895	ng/uL
TIM:	12.0561	nmole/L
Total concentration:	13.9664	ng/uL

Sample peak width (sec): 5   Sample min peak height: 50   Sample baseline V to V?: Y   Sample baseline V to V points: 3
Sample filter: Binomial   Number of points for filter: 3 Sample start region (min): 0 Sample end region (min): 30
Marker peak width (sec): 5   Marker min peak height: 200   Marker baseline V to V?: Y   Marker baseline V to V points: 3
Lower marker selection: First peak > 500 RFU   Upper marker selection: Last peak > 200 RFU
Ladder size (bp)75, 200, 300, 400, 500, 700, 1000, 1500, 2000, 3000, 4000, 5000, 7000, 10000, 20000
Quantification using: Lower Marker   Final concentration (ng/uL): 0.5000   Dilution factor: 12.0

Sample: SampE4
Well location: E4
Created: Tuesday, August 10, 2021 7:21:14 PM

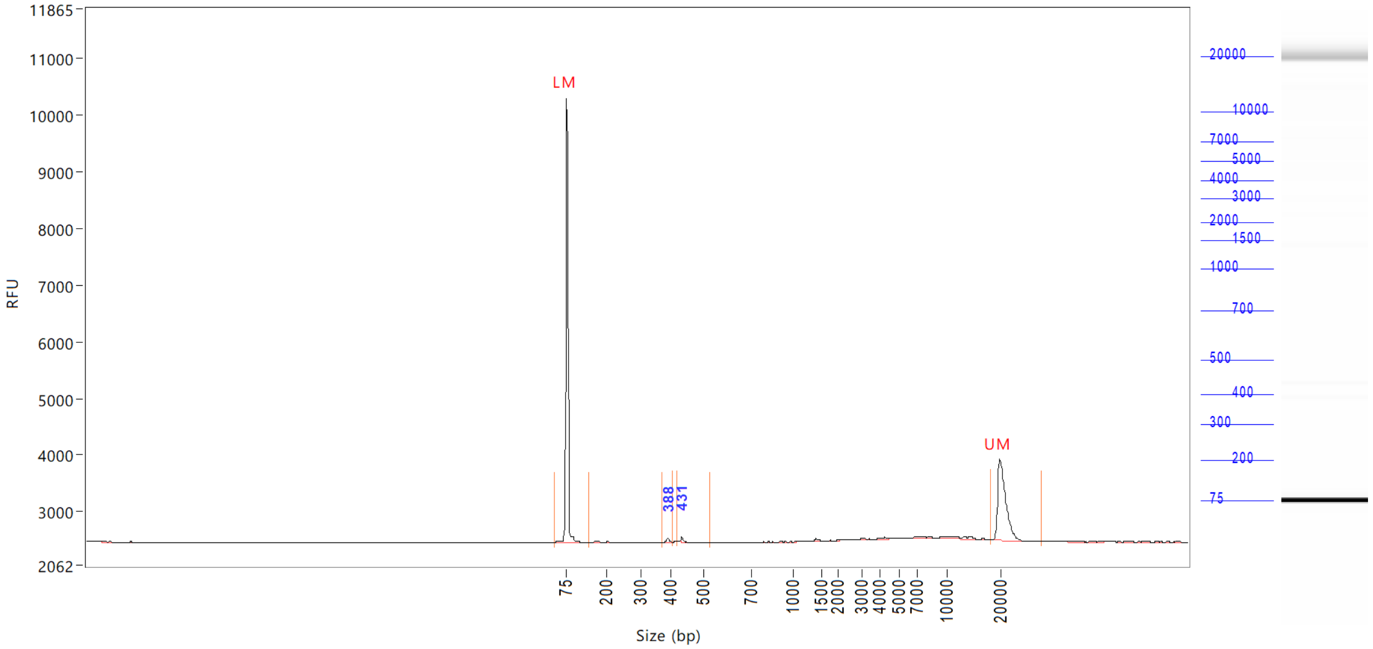

Peak	Size (bp)	Concentration (ng/uL)	From (bp)	To (bp)	Average size (bp)	CV%	RFU	Corrected peak area
1	75 (LM)	0.5000	37	143	74	7.04	7835	42.890
2	388	0.0567	369	403	386	1.25	72	0.405
3	431	0.0686	420	522	438	4.69	85	0.490
4	20000 (UM)	0.1690	18140	27957	20529	4.57	1423	14.493

TIC:	0.1252	ng/uL
TIM:	0.4991	nmole/L
Total concentration:	0.7563	ng/uL

Sample peak width (sec): 5   Sample min peak height: 50   Sample baseline V to V?: Y   Sample baseline V to V points: 3
Sample filter: Binomial   Number of points for filter: 3 Sample start region (min): 0 Sample end region (min): 30
Marker peak width (sec): 5   Marker min peak height: 200   Marker baseline V to V?: Y   Marker baseline V to V points: 3
Lower marker selection: First peak > 500 RFU   Upper marker selection: Last peak > 200 RFU
Ladder size (bp)75, 200, 300, 400, 500, 700, 1000, 1500, 2000, 3000, 4000, 5000, 7000, 10000, 20000
Quantification using: Lower Marker   Final concentration (ng/uL): 0.5000   Dilution factor: 12.0

Sample: SampF4
Well location: F4
Created: Tuesday, August 10, 2021 7:21:14 PM

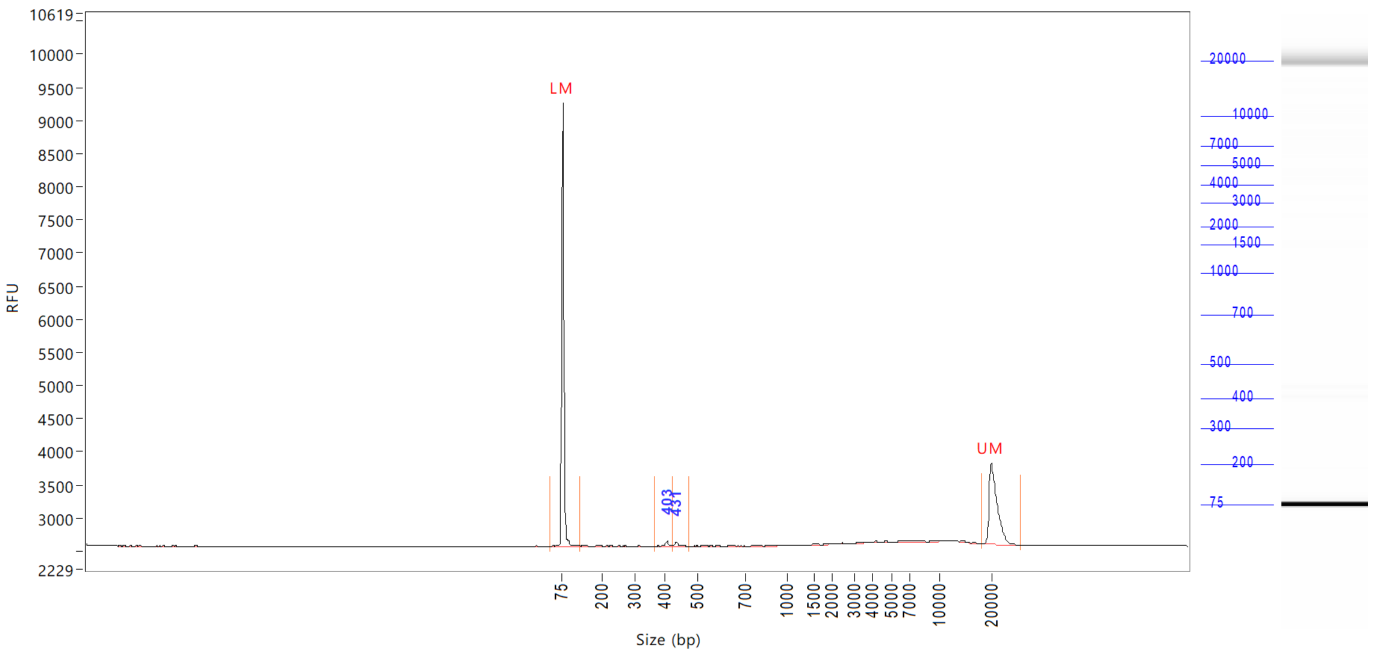

Peak	Size (bp)	Concentration (ng/uL)	From (bp)	To (bp)	Average size (bp)	CV%	RFU	Corrected peak area
1	75 (LM)	0.5000	39	131	74	6.42	6702	37.147
2	403	0.0898	363	420	400	2.33	72	0.556
3	431	0.0710	420	472	433	2.06	65	0.440
4	20000 (UM)	0.1712	18256	25651	20488	4.34	1230	12.722

TIC:	0.1608	ng/uL
TIM:	0.6394	nmole/L
Total concentration:	0.6583	ng/uL

Sample peak width (sec): 5 Sample min peak height: 50 Sample baseline V to V?: Y Sample baseline V to V points: 3
Sample filter: Binomial Number of points for filter: 3 Sample start region (min): 0 Sample end region (min): 30
Marker peak width (sec): 5 Marker min peak height: 200 Marker baseline V to V?: Y Marker baseline V to V points: 3
Lower marker selection: First peak > 500 RFU Upper marker selection: Last peak > 200 RFU
Ladder size (bp)75, 200, 300, 400, 500, 700, 1000, 1500, 2000, 3000, 4000, 5000, 7000, 10000, 20000
Quantification using: Lower Marker Final concentration (ng/uL): 0.5000 Dilution factor: 12.0

Sample: SampG4
Well location: G4
Created: Tuesday, August 10, 2021 7:21:14 PM

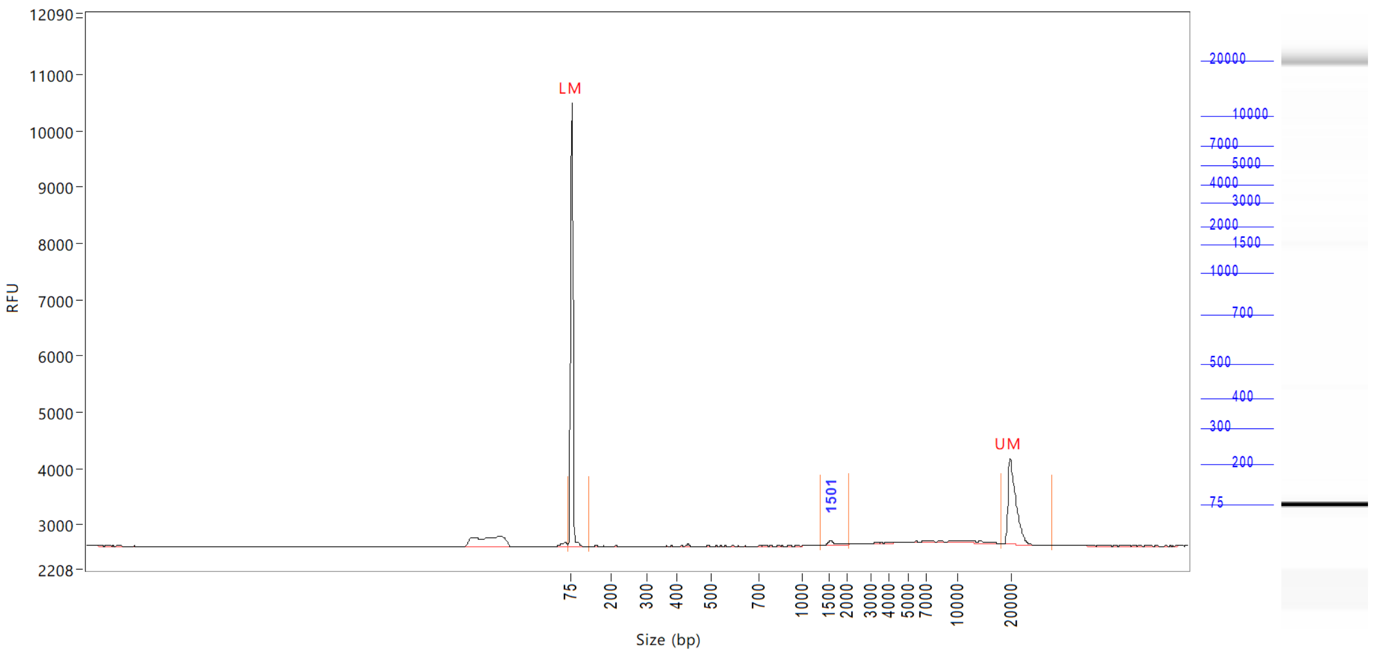

Peak	Size (bp)	Concentration (ng/uL)	From (bp)	To (bp)	Average size (bp)	CV%	RFU	Corrected peak area
1	75 (LM)	0.5000	64	129	73	5.48	7898	41.793
2	1501	0.1364	1341	2055	1584	9.94	90	0.950
3	20000 (UM)	0.1813	18372	27727	20495	4.44	1535	15.152

TIC:	0.1364	ng/uL
TIM:	0.1418	nmole/L
Total concentration:	0.8397	ng/uL

Sample peak width (sec): 5   Sample min peak height: 50   Sample baseline V to V?: Y   Sample baseline V to V points: 3
Sample filter: Binomial   Number of points for filter: 3   Sample start region (min): 0   Sample end region (min): 30
Marker peak width (sec): 5   Marker min peak height: 200   Marker baseline V to V?: Y   Marker baseline V to V points: 3
Lower marker selection: First peak > 500 RFU   Upper marker selection: Last peak > 200 RFU
Ladder size (bp)75, 200, 300, 400, 500, 700, 1000, 1500, 2000, 3000, 4000, 5000, 7000, 10000, 20000
Quantification using: Lower Marker   Final concentration (ng/uL): 0.5000   Dilution factor: 12.0

Sample: SampH4
Well location: H4
Created: Tuesday, August 10, 2021 7:21:14 PM

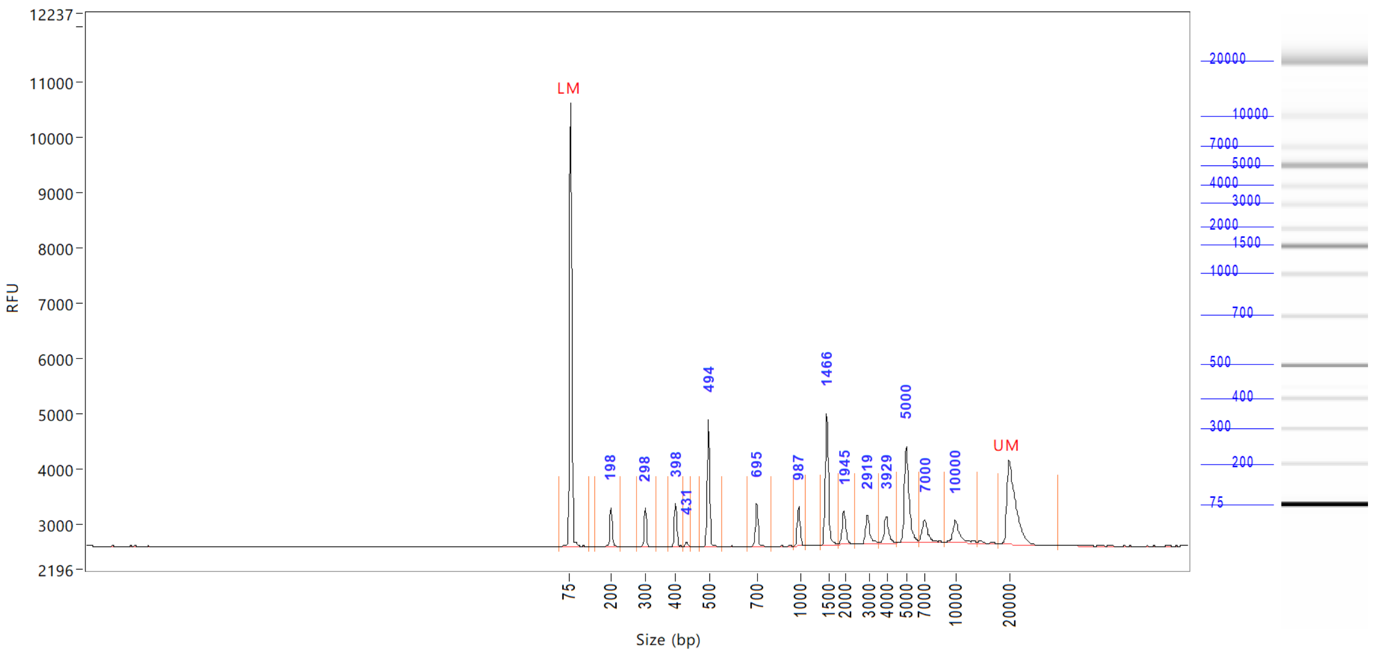

Peak	Size (bp)	Concentration (ng/uL)	From (bp)	To (bp)	Average size (bp)	CV%	RFU	Corrected peak area
1	75 (LM)	0.5000	39	135	73	6.03	8027	43.264
2	198	0.5159	152	226	196	3.47	709	3.720
3	298	0.4802	277	332	297	1.40	699	3.463
4	398	0.5038	375	420	396	1.09	766	3.633
5	431	0.0612	420	444	429	0.88	87	0.441
6	494	1.5122	470	548	495	1.00	2284	10.904
7	695	0.5271	656	793	694	1.44	770	3.801
8	987	0.4856	947	1092	983	1.15	713	3.501
9	1466	1.7409	1353	1778	1462	2.95	2367	12.553
10	1945	0.4781	1778	2406	1936	3.50	596	3.447
11	2919	0.4644	2406	3536	2942	4.54	509	3.349
12	3929	0.4492	3536	4467	3929	3.68	490	3.239
13	5000	1.6434	4467	6311	5064	5.10	1751	11.850
14	7000	0.4916	6311	8875	7057	6.42	424	3.545
15	10000	0.5353	8875	13954	10421	9.57	403	3.860
16	20000 (UM)	0.1961	17907	29111	20672	5.43	1530	16.966

TIC:	9.8889	ng/uL
TIM:	19.9534	nmole/L
Total concentration:	10.0286	ng/uL

```
Sample peak width (sec): 5     Sample min peak height: 50     Sample baseline V to V?: Y     Sample baseline V to V points: 3
Sample filter: Binomial        Number of points for filter: 3  Sample start region (min): 0  Sample end region (min): 30
Marker peak width (sec): 5     Marker min peak height: 200    Marker baseline V to V?: Y     Marker baseline V to V points: 3
Lower marker selection: First peak > 500 RFU                  Upper marker selection: Last peak > 200 RFU
Ladder size (bp)75, 200, 300, 400, 500, 700, 1000, 1500, 2000, 3000, 4000, 5000, 7000, 10000, 20000
Quantification using: Lower Marker          Final concentration (ng/uL): 0.5000          Dilution factor: 12.0
```

Sample: SampA5
Well location: A5
Created: Tuesday, August 10, 2021 7:21:14 PM

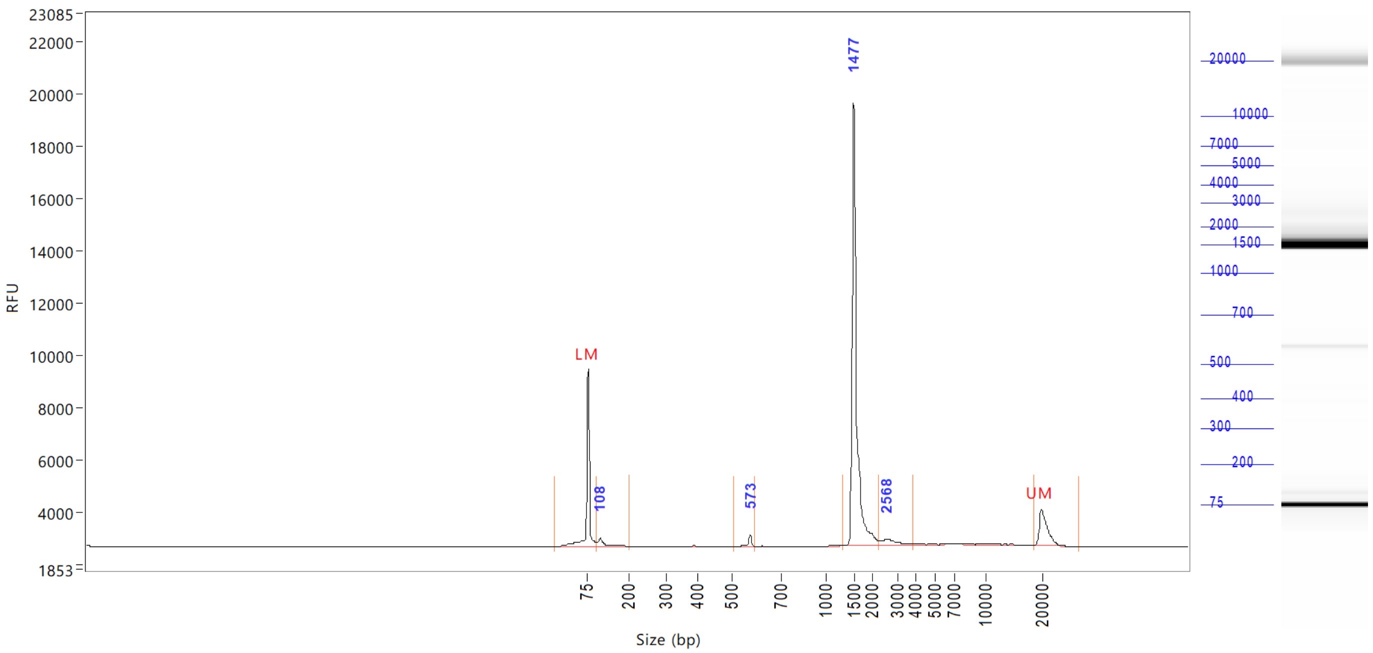

Peak	Size (bp)	Concentration (ng/uL)	From (bp)	To (bp)	Average size (bp)	CV%	RFU	Corrected peak area
1	75 (LM)	0.5000	0	99	69	22.11	6749	44.799
2	108	0.6276	99	196	120	16.00	316	4.686
3	573	0.3478	506	591	564	2.44	429	2.597
4	1477	15.2066	1296	2217	1523	8.17	16942	113.540
5	2568	0.7416	2217	3893	2745	14.13	245	5.537
6	20000 (UM)	0.1520	18605	26804	20448	4.20	1399	13.620

TIC:	16.9236	ng/uL
TIM:	26.4865	nmole/L
Total concentration:	17.4090	ng/uL

Sample peak width (sec): 5   Sample min peak height: 50   Sample baseline V to V?: Y   Sample baseline V to V points: 3
Sample filter: Binomial   Number of points for filter: 3 Sample start region (min): 0 Sample end region (min): 30
Marker peak width (sec): 5   Marker min peak height: 200   Marker baseline V to V?: Y   Marker baseline V to V points: 3
Lower marker selection: First peak > 500 RFU   Upper marker selection: Last peak > 200 RFU
Ladder size (bp)75, 200, 300, 400, 500, 700, 1000, 1500, 2000, 3000, 4000, 5000, 7000, 10000, 20000
Quantification using: Lower Marker   Final concentration (ng/uL): 0.5000   Dilution factor: 12.0

Sample: SampB5
Well location: B5
Created: Tuesday, August 10, 2021 7:21:14 PM

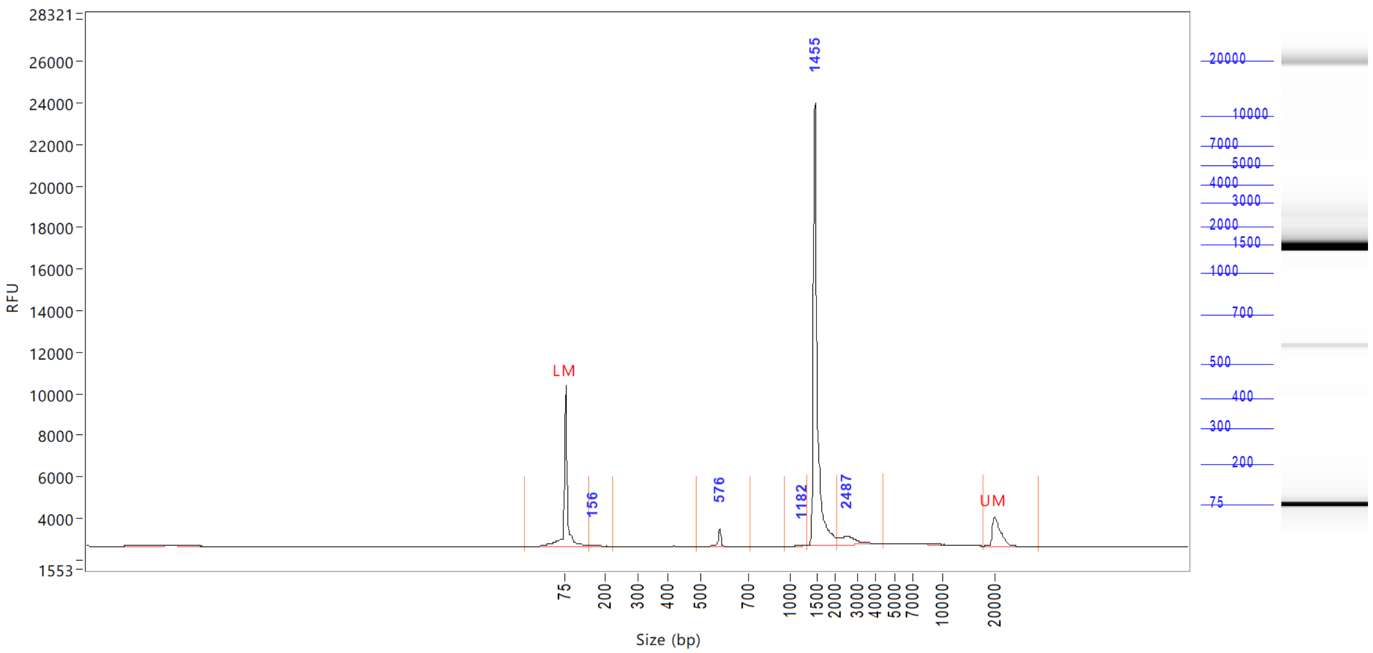

Peak	Size (bp)	Concentration (ng/uL)	From (bp)	To (bp)	Average size (bp)	CV%	RFU	Corrected peak area
1	75 (LM)	0.5000	0	147	73	27.18	7748	61.756
2	156	0.1711	147	224	170	11.79	89	1.761
3	576	0.5077	483	705	573	3.77	822	5.225
4	1182	0.0650	960	1285	1165	5.70	56	0.670
5	1455	13.5267	1285	2055	1486	7.67	21351	139.226
6	2487	1.1079	2055	4401	2650	17.11	441	11.403
7	20000 (UM)	0.1219	17791	28303	20567	4.67	1449	15.061

TIC:	15.3785	ng/uL
TIM:	18.8765	nmole/L
Total concentration:	15.6498	ng/uL

Sample peak width (sec): 5 Sample min peak height: 50 Sample baseline V to V?: Y Sample baseline V to V points: 3
Sample filter: Binomial Number of points for filter: 3 Sample start region (min): 0 Sample end region (min): 30
Marker peak width (sec): 5 Marker min peak height: 200 Marker baseline V to V?: Y Marker baseline V to V points: 3
Lower marker selection: First peak > 500 RFU Upper marker selection: Last peak > 200 RFU
Ladder size (bp)75, 200, 300, 400, 500, 700, 1000, 1500, 2000, 3000, 4000, 5000, 7000, 10000, 20000
Quantification using: Lower Marker Final concentration (ng/uL): 0.5000 Dilution factor: 12.0

Sample: SampC5
Well location: C5
Created: Tuesday, August 10, 2021 7:21:14 PM

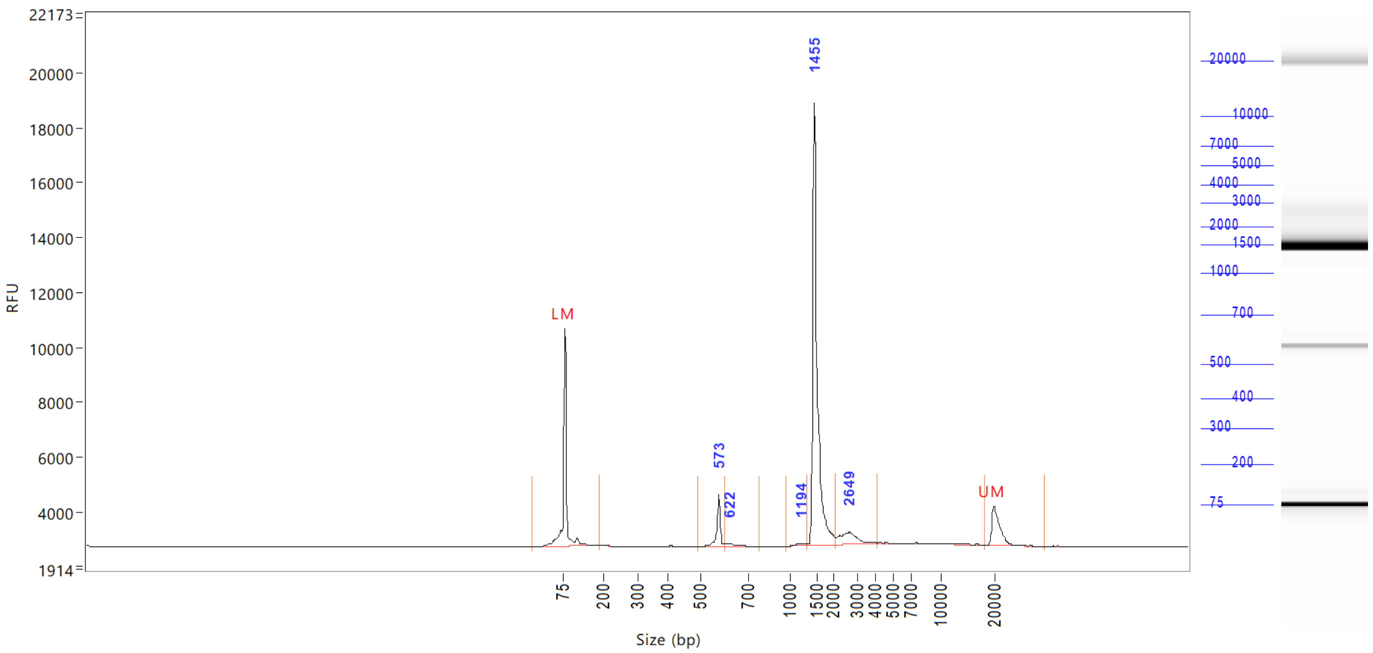

Peak	Size (bp)	Concentration (ng/uL)	From (bp)	To (bp)	Average size (bp)	CV%	RFU	Corrected peak area
1	75 (LM)	0.5000	0	183	73	23.36	7931	57.882
2	573	1.2109	493	599	568	2.22	1892	11.681
3	622	0.1769	599	785	631	4.31	101	1.707
4	1194	0.1169	969	1307	1175	6.00	84	1.127
5	1455	12.1482	1307	2055	1493	7.52	16148	117.195
6	2649	1.2409	2055	4134	2672	16.02	435	11.971
7	20000 (UM)	0.1255	18256	29687	20573	4.86	1420	14.526

TIC:	14.8938	ng/uL
TIM:	18.2915	nmole/L
Total concentration:	15.2485	ng/uL

Sample peak width (sec): 5   Sample min peak height: 50   Sample baseline V to V?: Y   Sample baseline V to V points: 3
Sample filter: Binomial   Number of points for filter: 3 Sample start region (min): 0 Sample end region (min): 30
Marker peak width (sec): 5   Marker min peak height: 200   Marker baseline V to V?: Y   Marker baseline V to V points: 3
Lower marker selection: First peak > 500 RFU   Upper marker selection: Last peak > 200 RFU
Ladder size (bp)75, 200, 300, 400, 500, 700, 1000, 1500, 2000, 3000, 4000, 5000, 7000, 10000, 20000
Quantification using: Lower Marker   Final concentration (ng/uL): 0.5000   Dilution factor: 12.0

Sample: SampD5
Well location: D5
Created: Tuesday, August 10, 2021 7:21:14 PM

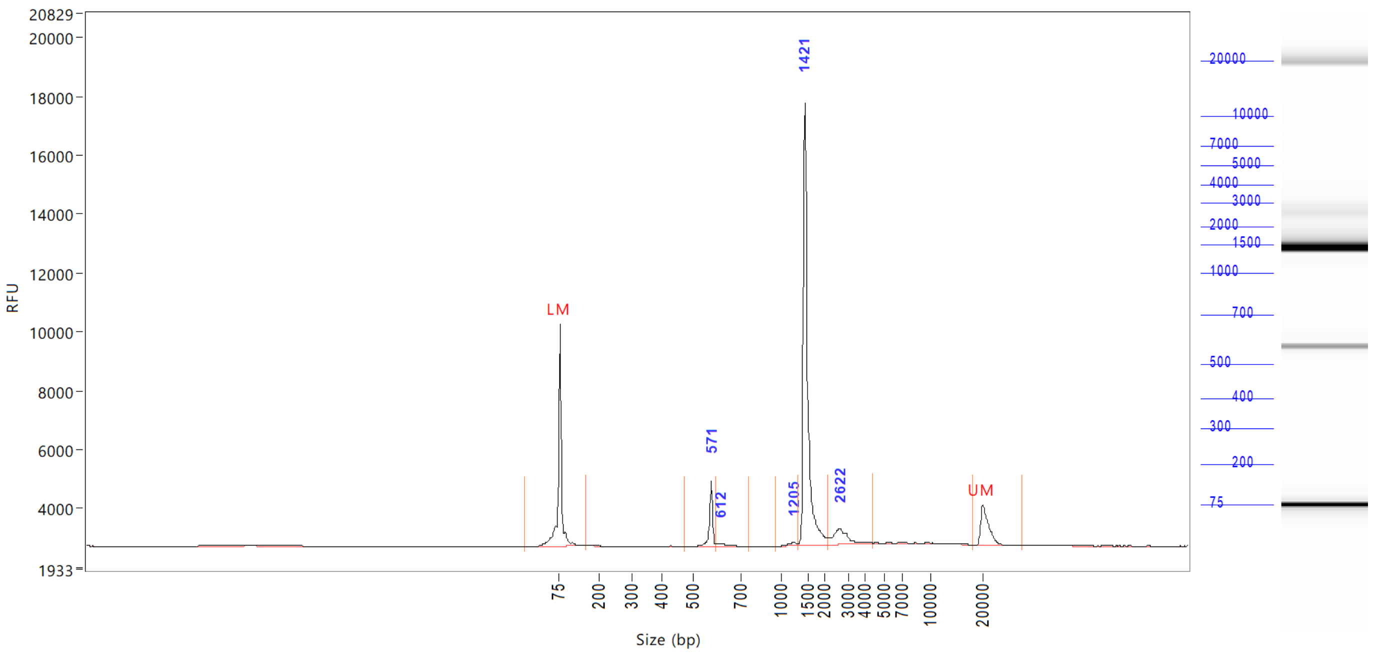

Peak	Size (bp)	Concentration (ng/uL)	From (bp)	To (bp)	Average size (bp)	CV%	RFU	Corrected peak area
1	75 (LM)	0.5000	0	158	70	21.72	7563	59.455
2	571	1.3468	472	594	567	2.22	2250	13.345
3	612	0.2052	594	754	629	5.02	104	2.033
4	1205	0.1432	952	1296	1177	6.15	96	1.419
5	1421	10.6403	1296	2055	1464	7.68	15066	105.436
6	2622	1.2578	2055	4367	2720	16.60	539	12.464
7	20000 (UM)	0.1192	18023	27727	20600	4.99	1354	14.177

TIC:	13.5932	ng/uL
TIM:	17.3705	nmole/L
Total concentration:	13.7797	ng/uL

Sample peak width (sec): 5   Sample min peak height: 50   Sample baseline V to V?: Y   Sample baseline V to V points: 3
Sample filter: Binomial   Number of points for filter: 3 Sample start region (min): 0 Sample end region (min): 30
Marker peak width (sec): 5   Marker min peak height: 200   Marker baseline V to V?: Y   Marker baseline V to V points: 3
Lower marker selection: First peak > 500 RFU   Upper marker selection: Last peak > 200 RFU
Ladder size (bp)75, 200, 300, 400, 500, 700, 1000, 1500, 2000, 3000, 4000, 5000, 7000, 10000, 20000
Quantification using: Lower Marker   Final concentration (ng/uL): 0.5000   Dilution factor: 12.0

Sample: SampE5
Well location: E5
Created: Tuesday, August 10, 2021 7:21:14 PM

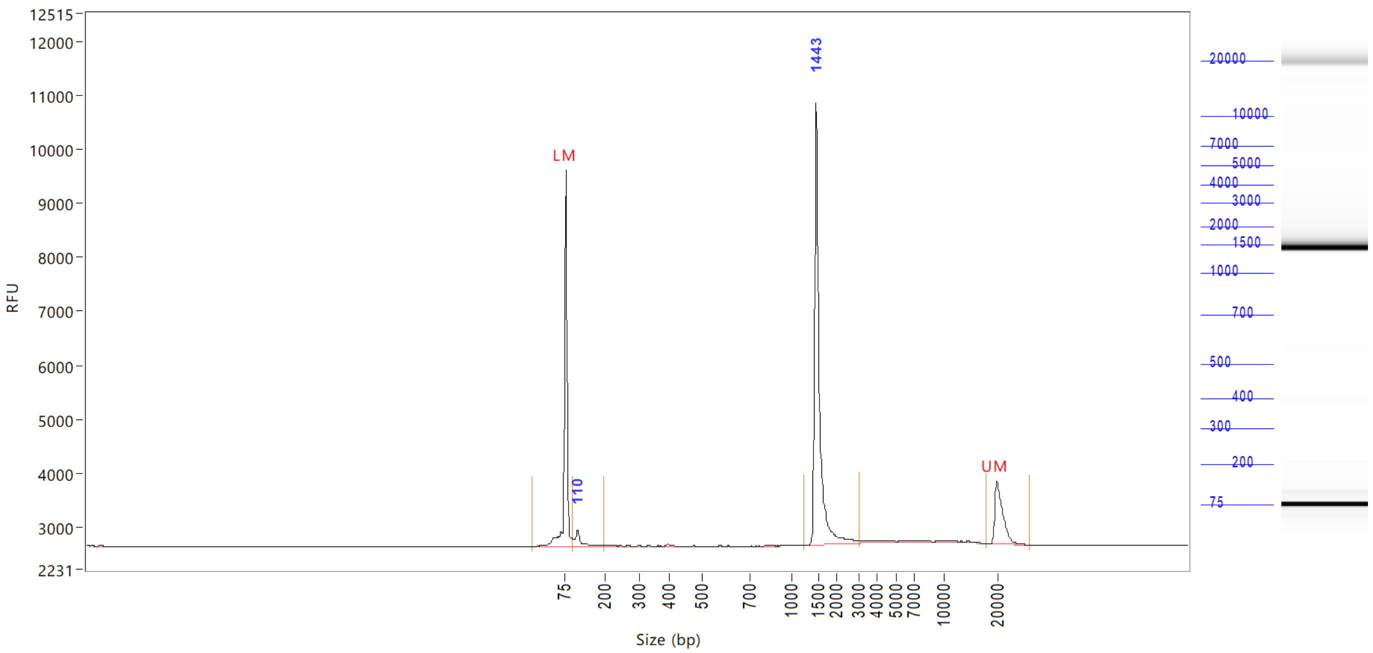

Peak	Size (bp)	Concentration (ng/uL)	From (bp)	To (bp)	Average size (bp)	CV%	RFU	Corrected peak area
1	75 (LM)	0.5000	0	99	71	17.34	6966	46.867
2	110	0.5166	99	196	121	17.48	287	4.035
3	1443	7.6552	1205	3000	1514	14.32	8189	59.796
4	20000 (UM)	0.1249	17907	26112	20532	4.36	1155	11.705

TIC:	8.1718	ng/uL
TIM:	15.3367	nmole/L
Total concentration:	8.6399	ng/uL

Sample peak width (sec): 5 Sample min peak height: 50 Sample baseline V to V?: Y Sample baseline V to V points: 3
Sample filter: Binomial Number of points for filter: 3 Sample start region (min): 0 Sample end region (min): 30
Marker peak width (sec): 5 Marker min peak height: 200 Marker baseline V to V?: Y Marker baseline V to V points: 3
Lower marker selection: First peak > 500 RFU Upper marker selection: Last peak > 200 RFU
Ladder size (bp)75, 200, 300, 400, 500, 700, 1000, 1500, 2000, 3000, 4000, 5000, 7000, 10000, 20000
Quantification using: Lower Marker Final concentration (ng/uL): 0.5000 Dilution factor: 12.0

Sample: SampF5
Well location: F5
Created: Tuesday, August 10, 2021 7:21:14 PM

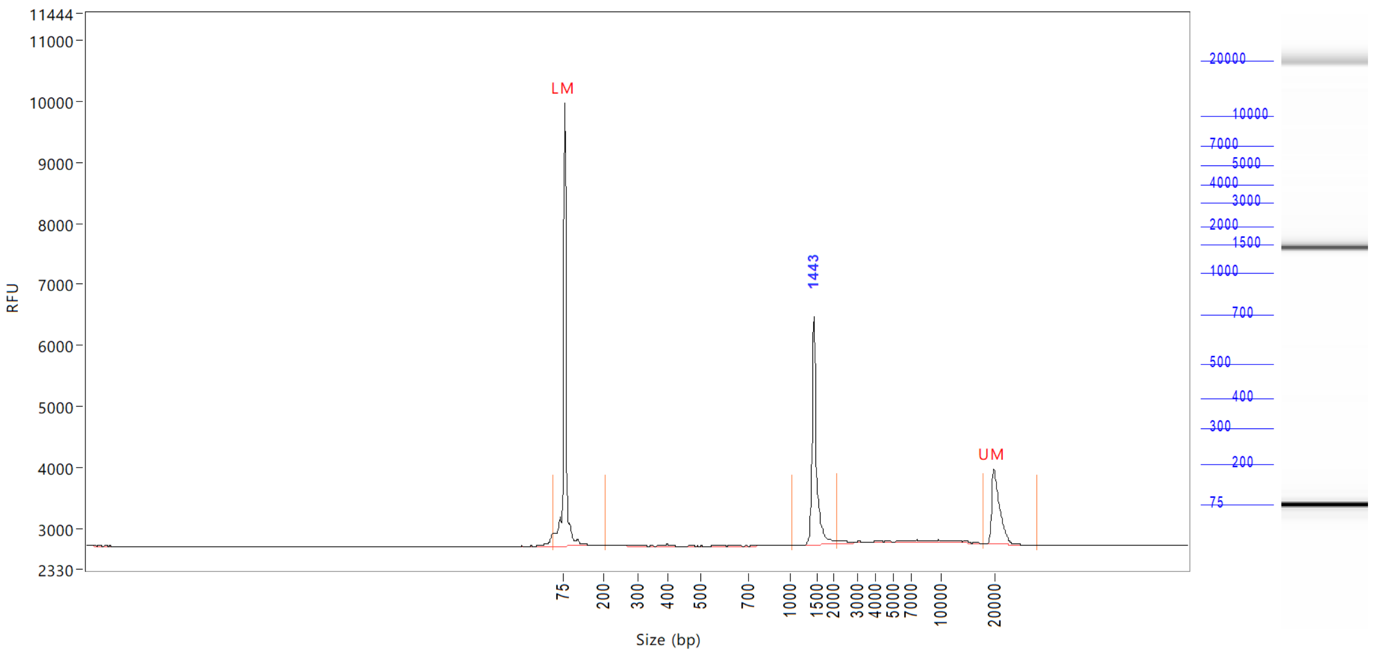

Peak	Size (bp)	Concentration (ng/uL)	From (bp)	To (bp)	Average size (bp)	CV%	RFU	Corrected peak area
1	75 (LM)	0.5000	41	201	73	17.98	7276	53.077
2	1443	2.9973	1024	2082	1467	7.14	3737	26.515
3	20000 (UM)	0.1179	17907	28188	20533	4.53	1228	12.515

TIC:	2.9973	ng/uL
TIM:	3.3632	nmole/L
Total concentration:	3.4970	ng/uL

```
Sample peak width (sec): 5    Sample min peak height: 50    Sample baseline V to V?: Y    Sample baseline V to V points: 3
Sample filter: Binomial       Number of points for filter: 3  Sample start region (min): 0  Sample end region (min): 30
Marker peak width (sec): 5    Marker min peak height: 200   Marker baseline V to V?: Y    Marker baseline V to V points: 3
Lower marker selection: First peak > 500 RFU                Upper marker selection: Last peak > 200 RFU
Ladder size (bp)75, 200, 300, 400, 500, 700, 1000, 1500, 2000, 3000, 4000, 5000, 7000, 10000, 20000
Quantification using: Lower Marker          Final concentration (ng/uL): 0.5000          Dilution factor: 12.0
```

Sample: SampG5
Well location: G5
Created: Tuesday, August 10, 2021 7:21:14 PM

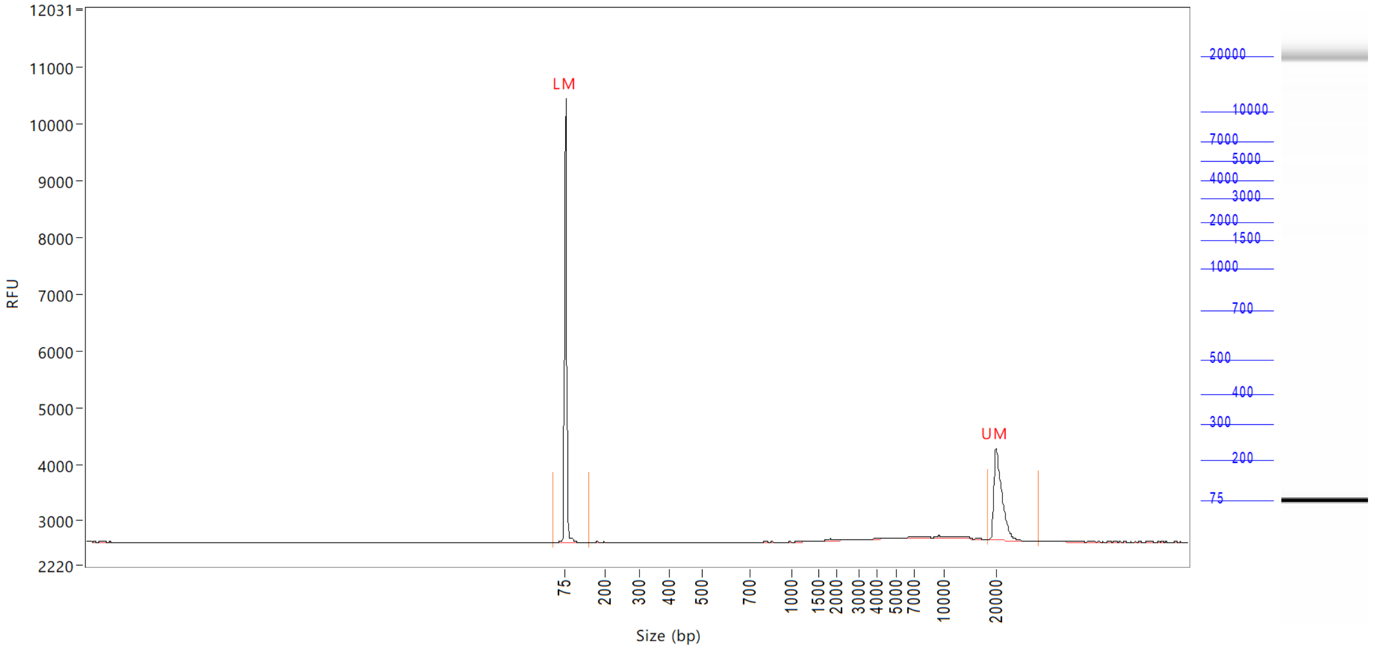

Peak	Size (bp)	Concentration (ng/uL)	From (bp)	To (bp)	Average size (bp)	CV%	RFU	Corrected peak area
1	75 (LM)	0.5000	37	148	73	6.64	7842	42.283
2	20000 (UM)	0.1973	18256	27957	20506	4.70	1617	16.686

TIC:	0.0000	ng/uL
TIM:	0.0000	nmole/L
Total concentration:	0.5992	ng/uL

Sample peak width (sec): 5   Sample min peak height: 50   Sample baseline V to V?: Y   Sample baseline V to V points: 3
Sample filter: Binomial   Number of points for filter: 3 Sample start region (min): 0 Sample end region (min): 30
Marker peak width (sec): 5   Marker min peak height: 200   Marker baseline V to V?: Y   Marker baseline V to V points: 3
Lower marker selection: First peak > 500 RFU   Upper marker selection: Last peak > 200 RFU
Ladder size (bp)75, 200, 300, 400, 500, 700, 1000, 1500, 2000, 3000, 4000, 5000, 7000, 10000, 20000
Quantification using: Lower Marker   Final concentration (ng/uL): 0.5000   Dilution factor: 12.0

Sample: SampH5
Well location: H5
Created: Tuesday, August 10, 2021 7:21:14 PM

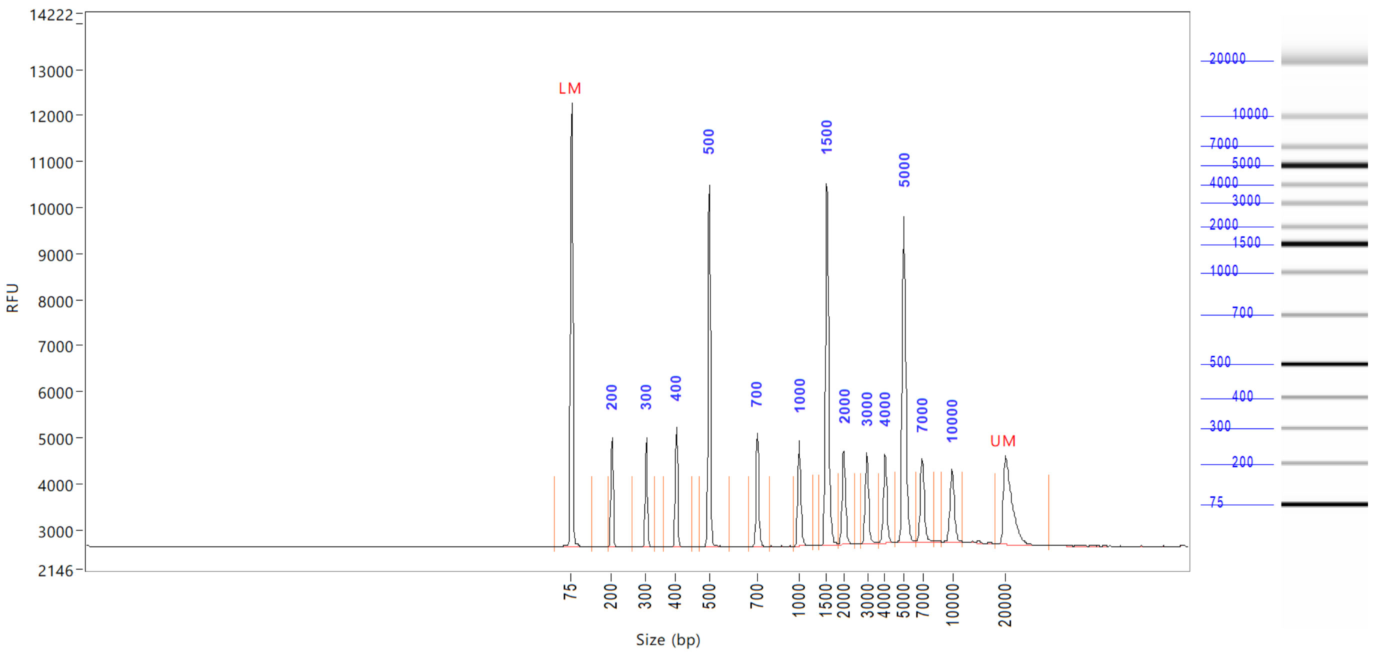

Peak	Size (bp)	Concentration (ng/uL)	From (bp)	To (bp)	Average size (bp)	CV%	RFU	Corrected peak area
1	75 (LM)	0.5000	24	137	73	5.98	9655	51.658
2	200	1.3143	189	260	198	2.27	2360	11.316
3	300	1.2902	260	328	298	1.24	2380	11.109
4	400	1.3908	357	448	398	1.09	2580	11.975
5	500	4.2054	470	584	498	1.05	7866	36.207
6	700	1.4096	664	785	700	1.12	2447	12.136
7	1000	1.3831	956	1262	1000	2.10	2287	11.908
8	1500	4.8451	1364	1834	1500	2.75	7857	41.715
9	2000	1.2236	1834	2433	1982	2.84	2027	10.535
10	3000	1.2120	2730	3643	2975	3.23	1961	10.435
11	4000	1.1667	3643	4500	3971	2.29	1933	10.045
12	5000	4.4871	4500	6242	5007	3.13	7073	38.633
13	7000	1.1721	6242	8188	6944	3.25	1814	10.092
14	10000	1.0995	8938	11861	9983	3.18	1575	9.466
15	20000 (UM)	0.2047	18140	28188	20668	5.06	1929	21.153

TIC:	26.1996	ng/uL
TIM:	52.6938	nmole/L
Total concentration:	26.3593	ng/uL

```
Sample peak width (sec): 5    Sample min peak height: 50    Sample baseline V to V?: Y    Sample baseline V to V points: 3
Sample filter: Binomial       Number of points for filter: 3  Sample start region (min): 0  Sample end region (min): 30
Marker peak width (sec): 5    Marker min peak height: 200   Marker baseline V to V?: Y    Marker baseline V to V points: 3
Lower marker selection: First peak > 500 RFU                Upper marker selection: Last peak > 200 RFU
Ladder size (bp)75, 200, 300, 400, 500, 700, 1000, 1500, 2000, 3000, 4000, 5000, 7000, 10000, 20000
Quantification using: Lower Marker          Final concentration (ng/uL): 0.5000          Dilution factor: 12.0
```

Sample: SampA6
Well location: A6
Created: Tuesday, August 10, 2021 7:21:14 PM

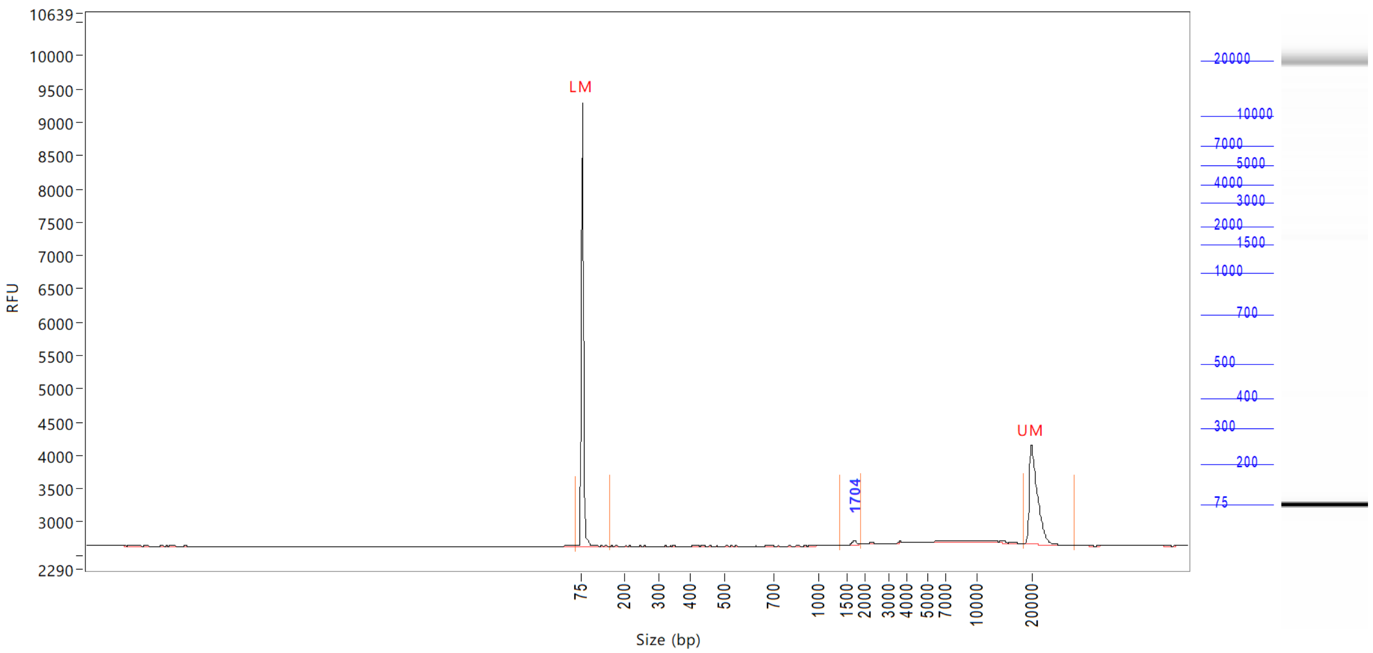

Peak	Size (bp)	Concentration (ng/uL)	From (bp)	To (bp)	Average size (bp)	CV%	RFU	Corrected peak area
1	75 (LM)	0.5000	52	156	73	6.54	6669	35.995
2	1704	0.0828	1387	1908	1693	5.21	70	0.497
3	20000 (UM)	0.2082	18372	27727	20470	4.57	1498	14.989

TIC:	0.0828	ng/uL
TIM:	0.0806	nmole/L
Total concentration:	0.7075	ng/uL

Sample peak width (sec): 5 Sample min peak height: 50 Sample baseline V to V?: Y Sample baseline V to V points: 3
Sample filter: Binomial Number of points for filter: 3 Sample start region (min): 0 Sample end region (min): 30
Marker peak width (sec): 5 Marker min peak height: 200 Marker baseline V to V?: Y Marker baseline V to V points: 3
Lower marker selection: First peak > 500 RFU Upper marker selection: Last peak > 200 RFU
Ladder size (bp)75, 200, 300, 400, 500, 700, 1000, 1500, 2000, 3000, 4000, 5000, 7000, 10000, 20000
Quantification using: Lower Marker Final concentration (ng/uL): 0.5000 Dilution factor: 12.0

Sample: SampB6
Well location: B6
Created: Tuesday, August 10, 2021 7:21:14 PM

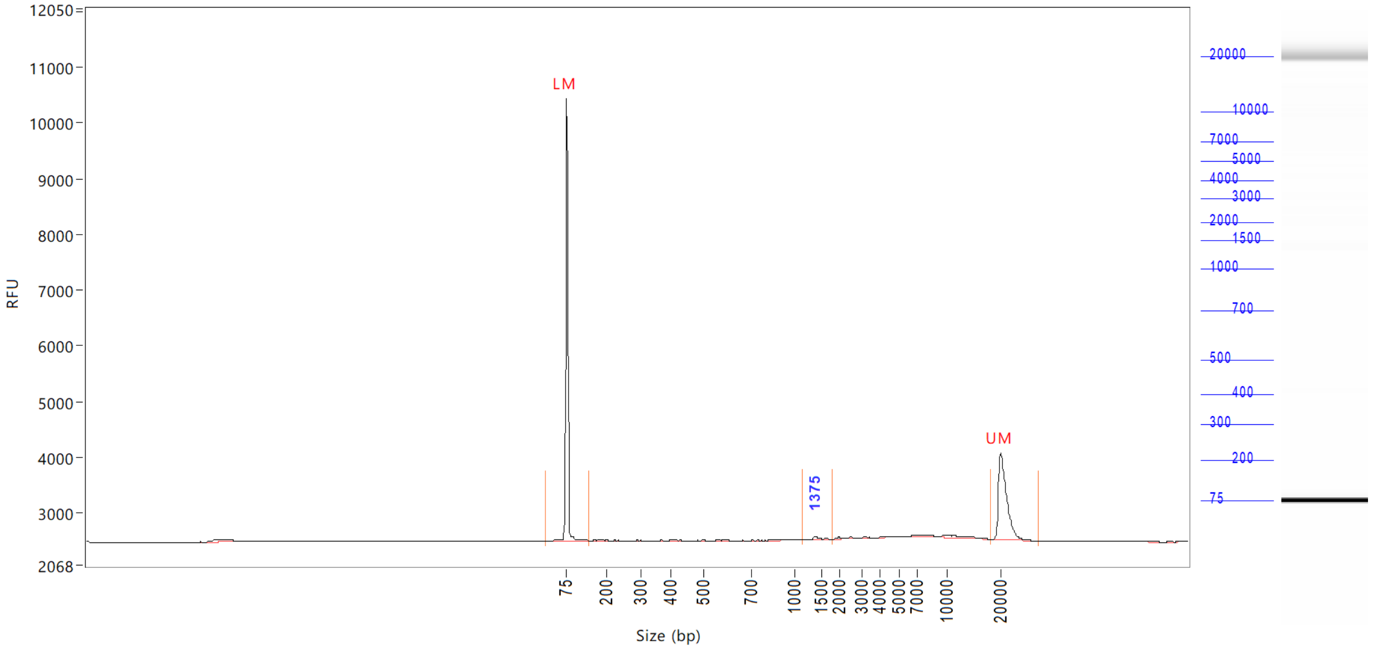

Peak	Size (bp)	Concentration (ng/uL)	From (bp)	To (bp)	Average size (bp)	CV%	RFU	Corrected peak area
1	75 (LM)	0.5000	11	147	73	7.81	7948	42.928
2	1375	0.0849	1148	1797	1426	7.52	60	0.607
3	20000 (UM)	0.1864	18140	27150	20577	4.64	1557	16.000

TIC:	0.0849	ng/uL
TIM:	0.0980	nmole/L
Total concentration:	0.7528	ng/uL

```
Sample peak width (sec): 5    Sample min peak height: 50    Sample baseline V to V?: Y    Sample baseline V to V points: 3
Sample filter: Binomial       Number of points for filter: 3  Sample start region (min): 0  Sample end region (min): 30
Marker peak width (sec): 5    Marker min peak height: 200   Marker baseline V to V?: Y    Marker baseline V to V points: 3
Lower marker selection: First peak > 500 RFU                Upper marker selection: Last peak > 200 RFU
Ladder size (bp)75, 200, 300, 400, 500, 700, 1000, 1500, 2000, 3000, 4000, 5000, 7000, 10000, 20000
Quantification using: Lower Marker          Final concentration (ng/uL): 0.5000          Dilution factor: 12.0
```

Sample: SampC6
Well location: C6
Created: Tuesday, August 10, 2021 7:21:14 PM

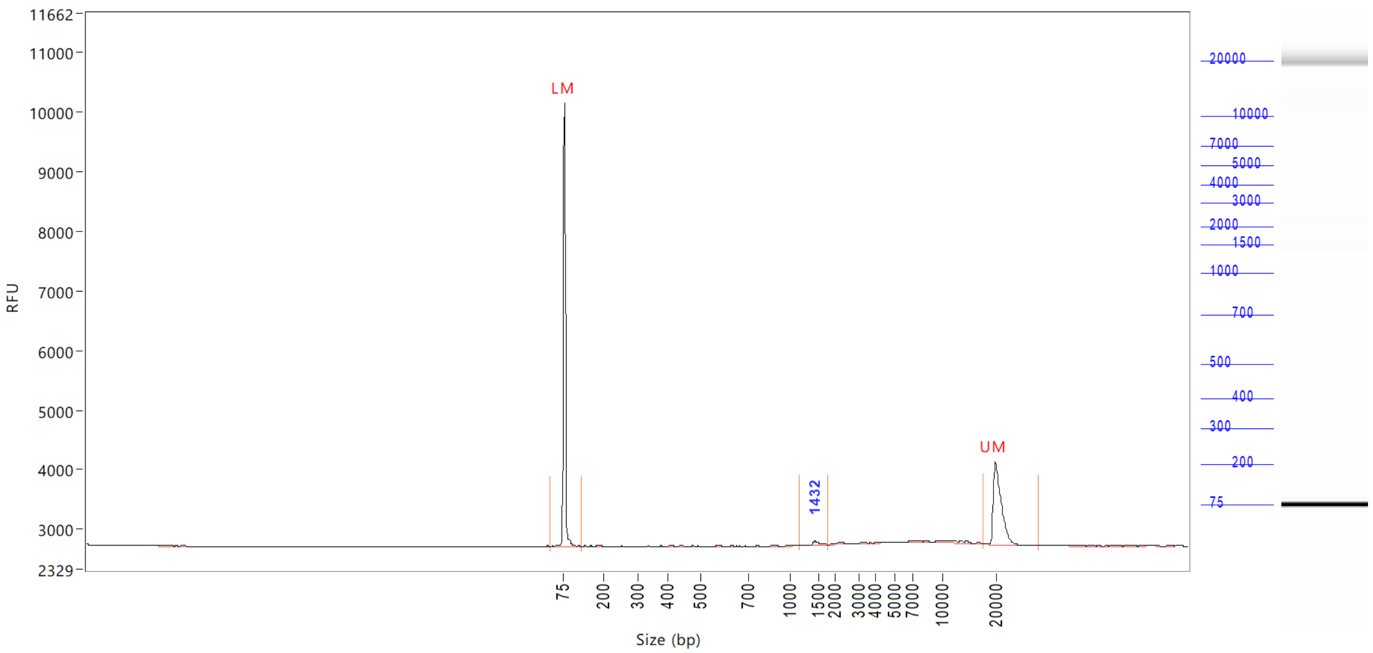

Peak	Size (bp)	Concentration (ng/uL)	From (bp)	To (bp)	Average size (bp)	CV%	RFU	Corrected peak area
1	75 (LM)	0.5000	33	128	73	6.28	7457	40.626
2	1432	0.0963	1171	1797	1464	6.00	68	0.652
3	20000 (UM)	0.1760	17791	27957	20489	4.61	1399	14.298

TIC:	0.0963	ng/uL
TIM:	0.1083	nmole/L
Total concentration:	0.8183	ng/uL

Sample peak width (sec): 5   Sample min peak height: 50   Sample baseline V to V?: Y   Sample baseline V to V points: 3
Sample filter: Binomial   Number of points for filter: 3   Sample start region (min): 0   Sample end region (min): 30
Marker peak width (sec): 5   Marker min peak height: 200   Marker baseline V to V?: Y   Marker baseline V to V points: 3
Lower marker selection: First peak > 500 RFU   Upper marker selection: Last peak > 200 RFU
Ladder size (bp)75, 200, 300, 400, 500, 700, 1000, 1500, 2000, 3000, 4000, 5000, 7000, 10000, 20000
Quantification using: Lower Marker   Final concentration (ng/uL): 0.5000   Dilution factor: 12.0

Sample: SampD6
Well location: D6
Created: Tuesday, August 10, 2021 7:21:14 PM

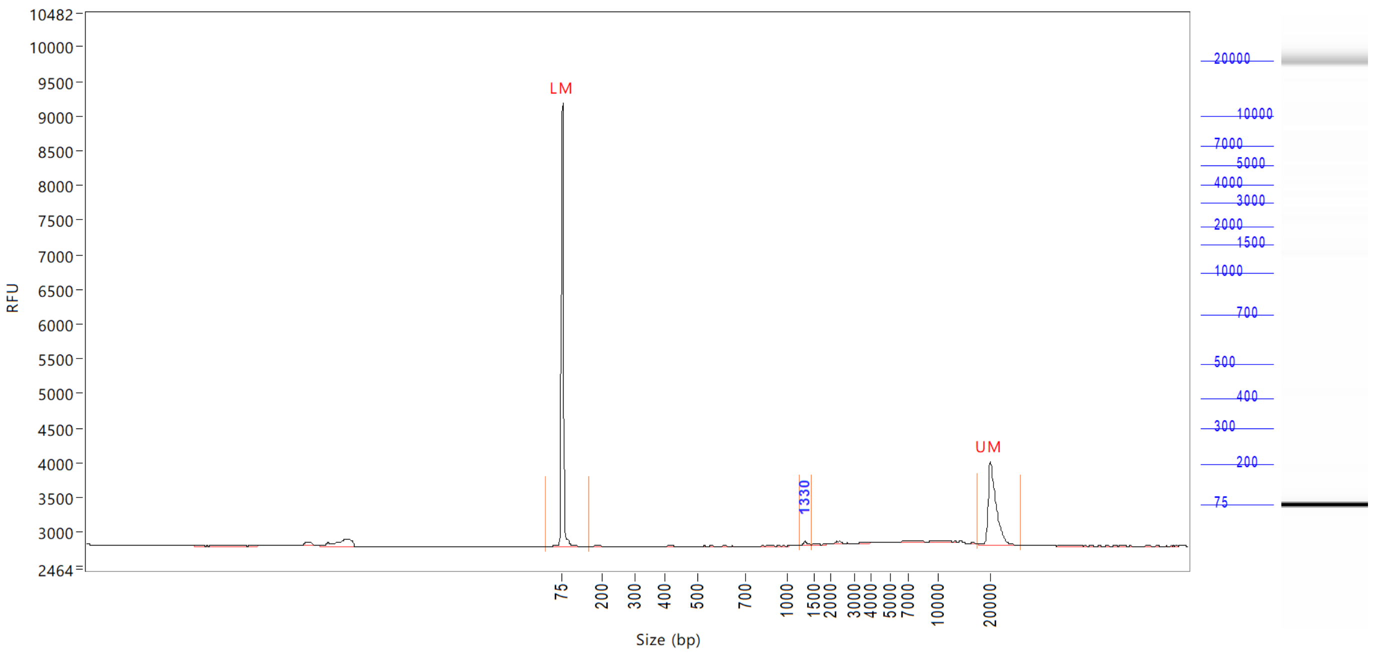

Peak	Size (bp)	Concentration (ng/uL)	From (bp)	To (bp)	Average size (bp)	CV%	RFU	Corrected peak area
1	75 (LM)	0.5000	26	158	73	9.42	6411	36.796
2	1330	0.0597	1250	1455	1342	3.09	56	0.366
3	20000 (UM)	0.1659	17675	25997	20547	4.52	1200	12.209

TIC:	0.0597	ng/uL
TIM:	0.0732	nmole/L
Total concentration:	0.7025	ng/uL

Sample peak width (sec): 5 Sample min peak height: 50 Sample baseline V to V?: Y Sample baseline V to V points: 3
Sample filter: Binomial Number of points for filter: 3 Sample start region (min): 0 Sample end region (min): 30
Marker peak width (sec): 5 Marker min peak height: 200 Marker baseline V to V?: Y Marker baseline V to V points: 3
Lower marker selection: First peak > 500 RFU Upper marker selection: Last peak > 200 RFU
Ladder size (bp)75, 200, 300, 400, 500, 700, 1000, 1500, 2000, 3000, 4000, 5000, 7000, 10000, 20000
Quantification using: Lower Marker Final concentration (ng/uL): 0.5000 Dilution factor: 12.0

Sample: SampE6
Well location: E6
Created: Tuesday, August 10, 2021 7:21:14 PM

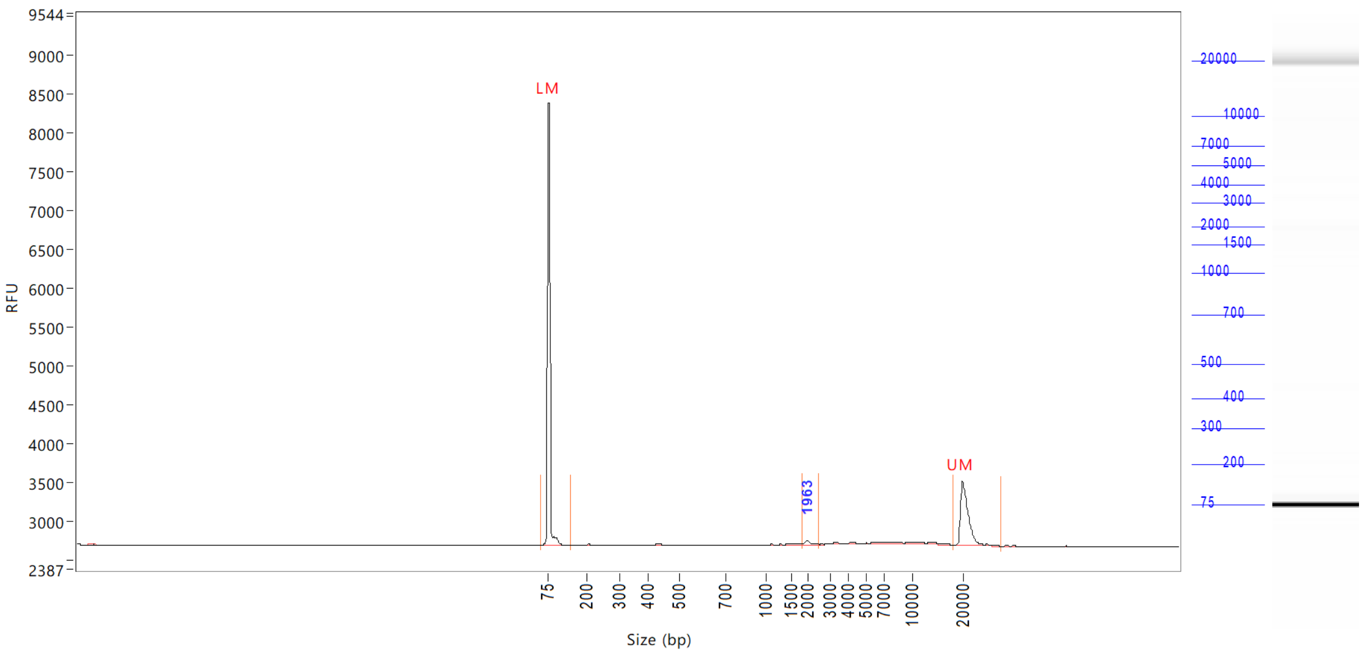

Peak	Size (bp)	Concentration (ng/uL)	From (bp)	To (bp)	Average size (bp)	CV%	RFU	Corrected peak area
1	75 (LM)	0.5000	48	147	73	9.26	5707	33.089
2	1963	0.0815	1797	2487	1986	6.60	51	0.450
3	20000 (UM)	0.1249	18140	27496	20637	5.82	834	8.265

TIC:	0.0815	ng/uL
TIM:	0.0676	nmole/L
Total concentration:	0.7853	ng/uL

Sample peak width (sec): 5 Sample min peak height: 50 Sample baseline V to V?: Y Sample baseline V to V points: 3
Sample filter: Binomial Number of points for filter: 3 Sample start region (min): 0 Sample end region (min): 30
Marker peak width (sec): 5 Marker min peak height: 200 Marker baseline V to V?: Y Marker baseline V to V points: 3
Lower marker selection: First peak > 500 RFU Upper marker selection: Last peak > 200 RFU
Ladder size (bp)75, 200, 300, 400, 500, 700, 1000, 1500, 2000, 3000, 4000, 5000, 7000, 10000, 20000
Quantification using: Lower Marker Final concentration (ng/uL): 0.5000 Dilution factor: 12.0

Sample: SampF6
Well location: F6
Created: Tuesday, August 10, 2021 7:21:14 PM

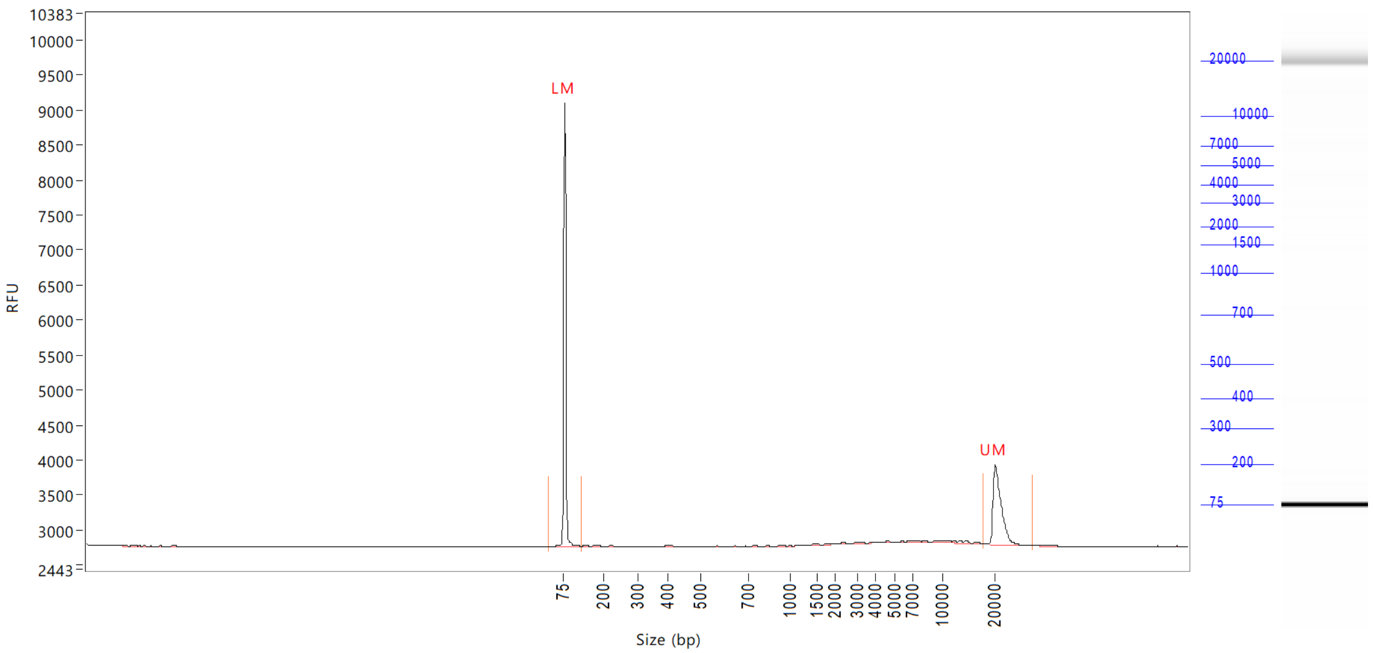

Peak	Size (bp)	Concentration (ng/uL)	From (bp)	To (bp)	Average size (bp)	CV%	RFU	Corrected peak area
1	75 (LM)	0.5000	28	126	73	6.99	6347	35.610
2	20000 (UM)	0.1681	17907	27150	20602	4.92	1147	11.969

TIC:	0.0000	ng/uL
TIM:	0.0000	nmole/L
Total concentration:	0.7388	ng/uL

Sample peak width (sec): 5 Sample min peak height: 50 Sample baseline V to V?: Y Sample baseline V to V points: 3
Sample filter: Binomial Number of points for filter: 3 Sample start region (min): 0 Sample end region (min): 30
Marker peak width (sec): 5 Marker min peak height: 200 Marker baseline V to V?: Y Marker baseline V to V points: 3
Lower marker selection: First peak > 500 RFU Upper marker selection: Last peak > 200 RFU
Ladder size (bp)75, 200, 300, 400, 500, 700, 1000, 1500, 2000, 3000, 4000, 5000, 7000, 10000, 20000
Quantification using: Lower Marker Final concentration (ng/uL): 0.5000 Dilution factor: 12.0

Sample: SampG6
Well location: G6
Created: Tuesday, August 10, 2021 7:21:14 PM

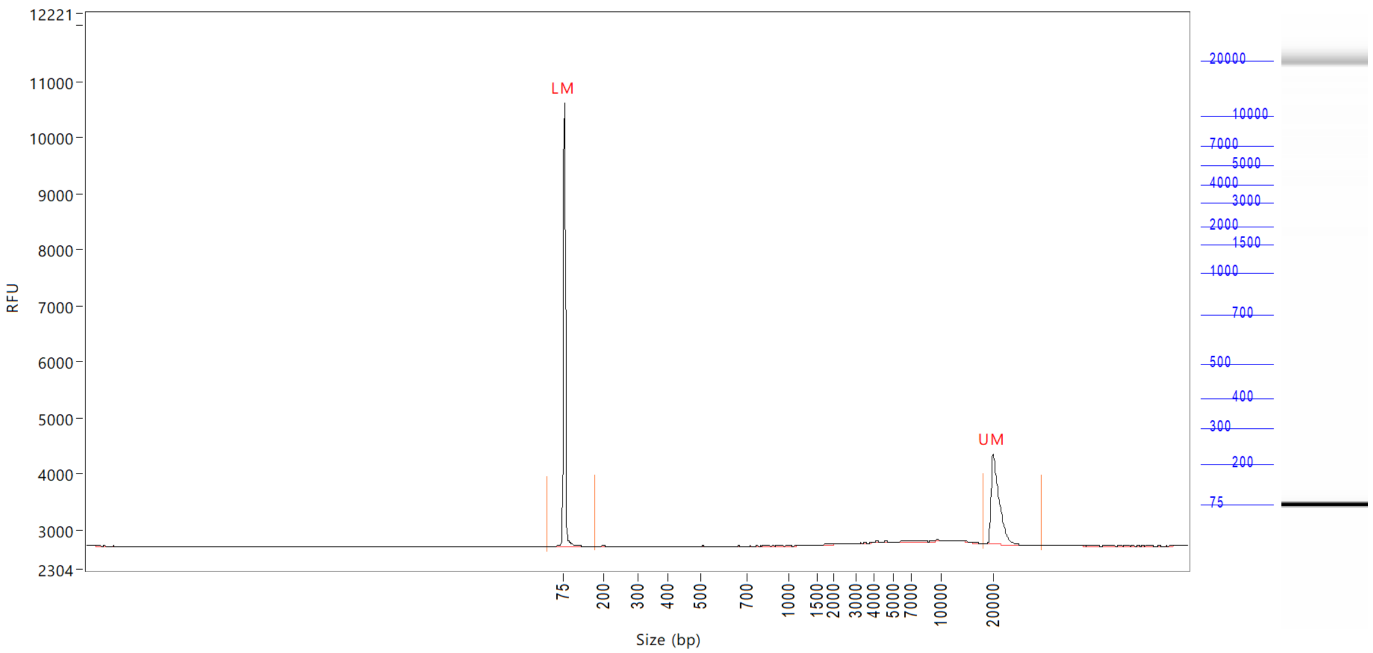

Peak	Size (bp)	Concentration (ng/uL)	From (bp)	To (bp)	Average size (bp)	CV%	RFU	Corrected peak area
1	75 (LM)	0.5000	24	173	73	8.21	7925	42.906
2	20000 (UM)	0.1961	18256	29111	20584	4.95	1601	16.830

TIC:	0.0000	ng/uL
TIM:	0.0000	nmole/L
Total concentration:	0.5004	ng/uL

Sample peak width (sec): 5 Sample min peak height: 50 Sample baseline V to V?: Y Sample baseline V to V points: 3
Sample filter: Binomial Number of points for filter: 3 Sample start region (min): 0 Sample end region (min): 30
Marker peak width (sec): 5 Marker min peak height: 200 Marker baseline V to V?: Y Marker baseline V to V points: 3
Lower marker selection: First peak > 500 RFU Upper marker selection: Last peak > 200 RFU
Ladder size (bp)75, 200, 300, 400, 500, 700, 1000, 1500, 2000, 3000, 4000, 5000, 7000, 10000, 20000
Quantification using: Lower Marker Final concentration (ng/uL): 0.5000 Dilution factor: 12.0

Sample: SampH6
Well location: H6
Created: Tuesday, August 10, 2021 7:21:14 PM

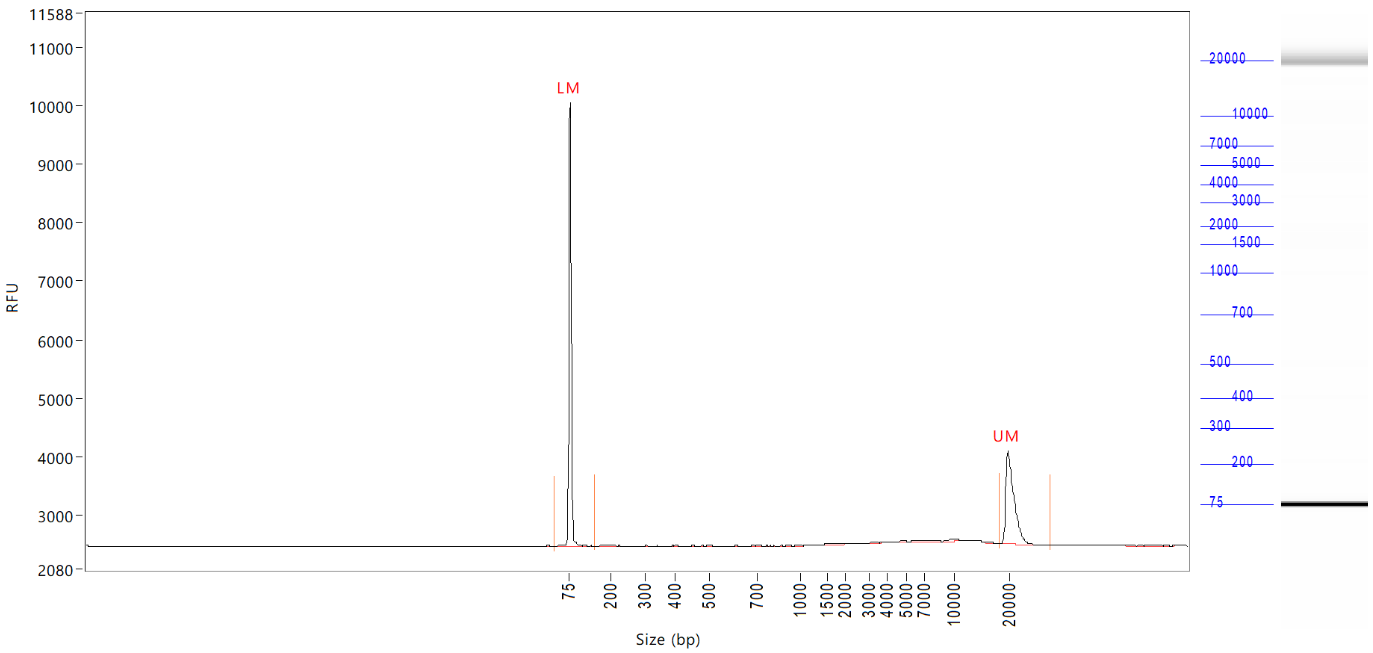

Peak	Size (bp)	Concentration (ng/uL)	From (bp)	To (bp)	Average size (bp)	CV%	RFU	Corrected peak area
1	75 (LM)	0.5000	30	152	73	6.09	7596	41.453
2	20000 (UM)	0.1987	18372	27842	20499	4.64	1604	16.474

TIC:	0.0000	ng/uL
TIM:	0.0000	nmole/L
Total concentration:	0.5947	ng/uL

Sample peak width (sec): 5   Sample min peak height: 50   Sample baseline V to V?: Y   Sample baseline V to V points: 3
Sample filter: Binomial   Number of points for filter: 3 Sample start region (min): 0 Sample end region (min): 30
Marker peak width (sec): 5   Marker min peak height: 200   Marker baseline V to V?: Y   Marker baseline V to V points: 3
Lower marker selection: First peak > 500 RFU   Upper marker selection: Last peak > 200 RFU
Ladder size (bp)75, 200, 300, 400, 500, 700, 1000, 1500, 2000, 3000, 4000, 5000, 7000, 10000, 20000
Quantification using: Lower Marker   Final concentration (ng/uL): 0.5000   Dilution factor: 12.0

Sample: SampA7
Well location: A7
Created: Tuesday, August 10, 2021 7:21:14 PM

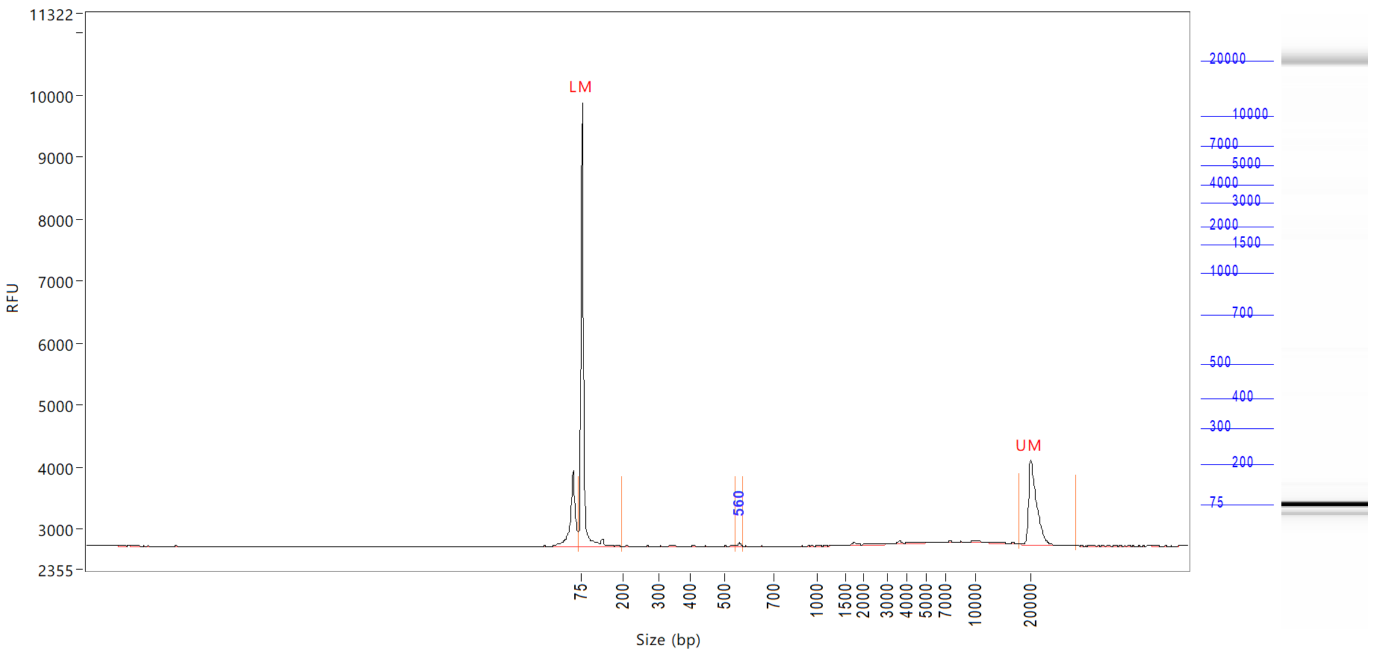

Peak	Size	Concentration	From	To	Average size	CV%	RFU	Corrected peak area
	(bp)	(ng/uL)	(bp)	(bp)	(bp)			
1	75 (LM)	0.5000	64	196	77	16.46	7165	44.785
2	560	0.0374	548	573	557	0.83	59	0.279
3	20000 (UM)	0.1513	18023	28188	20544	4.44	1373	13.549

TIC:	0.0374	ng/uL
TIM:	0.1106	nmole/L
Total concentration:	0.6113	ng/uL

```
Sample peak width (sec): 5    Sample min peak height: 50    Sample baseline V to V?: Y    Sample baseline V to V points: 3
Sample filter: Binomial       Number of points for filter: 3  Sample start region (min): 0  Sample end region (min): 30
Marker peak width (sec): 5    Marker min peak height: 200   Marker baseline V to V?: Y    Marker baseline V to V points: 3
Lower marker selection: First peak > 500 RFU                Upper marker selection: Last peak > 200 RFU
Ladder size (bp)75, 200, 300, 400, 500, 700, 1000, 1500, 2000, 3000, 4000, 5000, 7000, 10000, 20000
Quantification using: Lower Marker          Final concentration (ng/uL): 0.5000          Dilution factor: 12.0
```

Sample: SampB7
Well location: B7
Created: Tuesday, August 10, 2021 7:21:14 PM

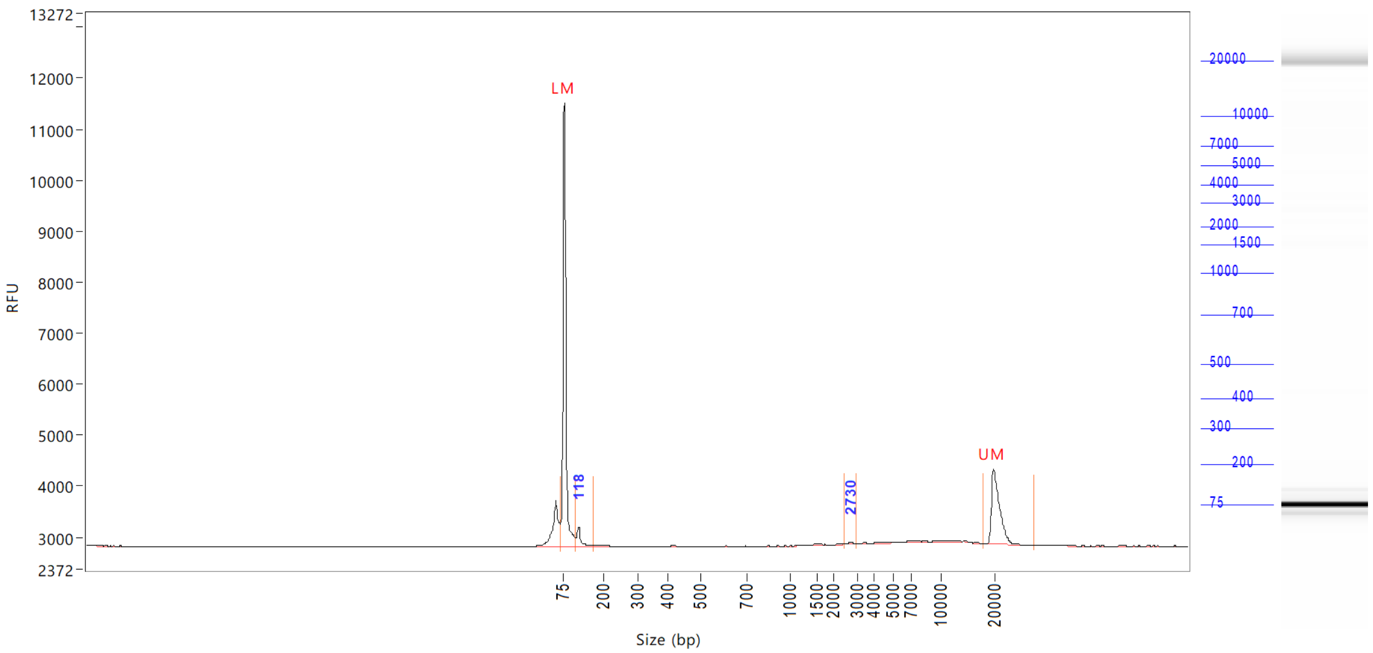

Peak	Size (bp)	Concentration (ng/uL)	From (bp)	To (bp)	Average size (bp)	CV%	RFU	Corrected peak area
1	75 (LM)	0.5000	64	110	75	9.62	8708	63.341
2	118	0.3548	110	168	123	10.86	383	3.746
3	2730	0.0392	2433	3000	2724	4.50	53	0.414
4	20000 (UM)	0.1206	18023	27611	20493	4.49	1476	15.276

TIC:	0.3941	ng/uL
TIM:	4.7632	nmole/L
Total concentration:	0.8569	ng/uL

```
Sample peak width (sec): 5    Sample min peak height: 50    Sample baseline V to V?: Y    Sample baseline V to V points: 3
Sample filter: Binomial       Number of points for filter: 3  Sample start region (min): 0  Sample end region (min): 30
Marker peak width (sec): 5    Marker min peak height: 200   Marker baseline V to V?: Y    Marker baseline V to V points: 3
Lower marker selection: First peak > 500 RFU                Upper marker selection: Last peak > 200 RFU
Ladder size (bp)75, 200, 300, 400, 500, 700, 1000, 1500, 2000, 3000, 4000, 5000, 7000, 10000, 20000
Quantification using: Lower Marker          Final concentration (ng/uL): 0.5000          Dilution factor: 12.0
```

Sample: SampC7
Well location: C7
Created: Tuesday, August 10, 2021 7:21:14 PM

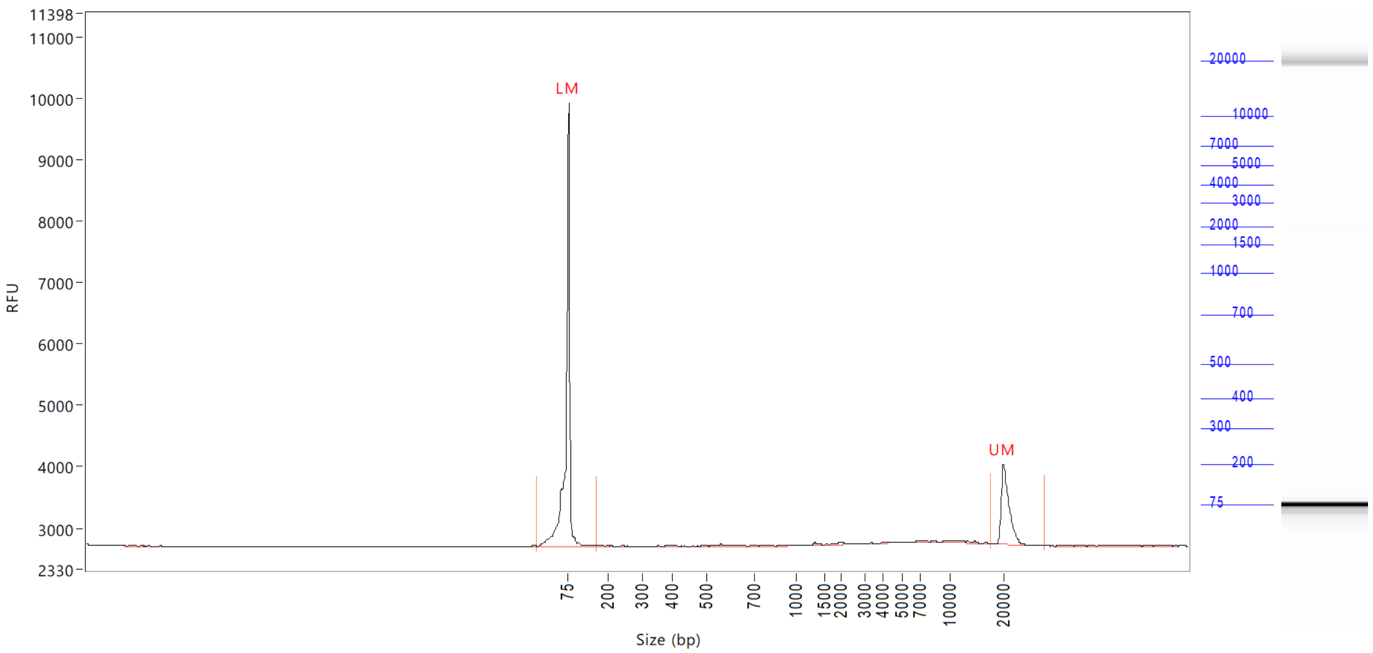

Peak	Size (bp)	Concentration (ng/uL)	From (bp)	To (bp)	Average size (bp)	CV%	RFU	Corrected peak area
1	75 (LM)	0.5000	0	160	65	28.02	7245	64.248
2	20000 (UM)	0.1010	17675	27611	20444	4.36	1310	12.972

TIC:	0.0000	ng/uL
TIM:	0.0000	nmole/L
Total concentration:	0.5320	ng/uL

```
Sample peak width (sec): 5    Sample min peak height: 50    Sample baseline V to V?: Y    Sample baseline V to V points: 3
Sample filter: Binomial       Number of points for filter: 3  Sample start region (min): 0  Sample end region (min): 30
Marker peak width (sec): 5    Marker min peak height: 200   Marker baseline V to V?: Y    Marker baseline V to V points: 3
Lower marker selection: First peak > 500 RFU                Upper marker selection: Last peak > 200 RFU
Ladder size (bp)75, 200, 300, 400, 500, 700, 1000, 1500, 2000, 3000, 4000, 5000, 7000, 10000, 20000
Quantification using: Lower Marker          Final concentration (ng/uL): 0.5000          Dilution factor: 12.0
```

Sample: SampD7
Well location: D7
Created: Tuesday, August 10, 2021 7:21:14 PM

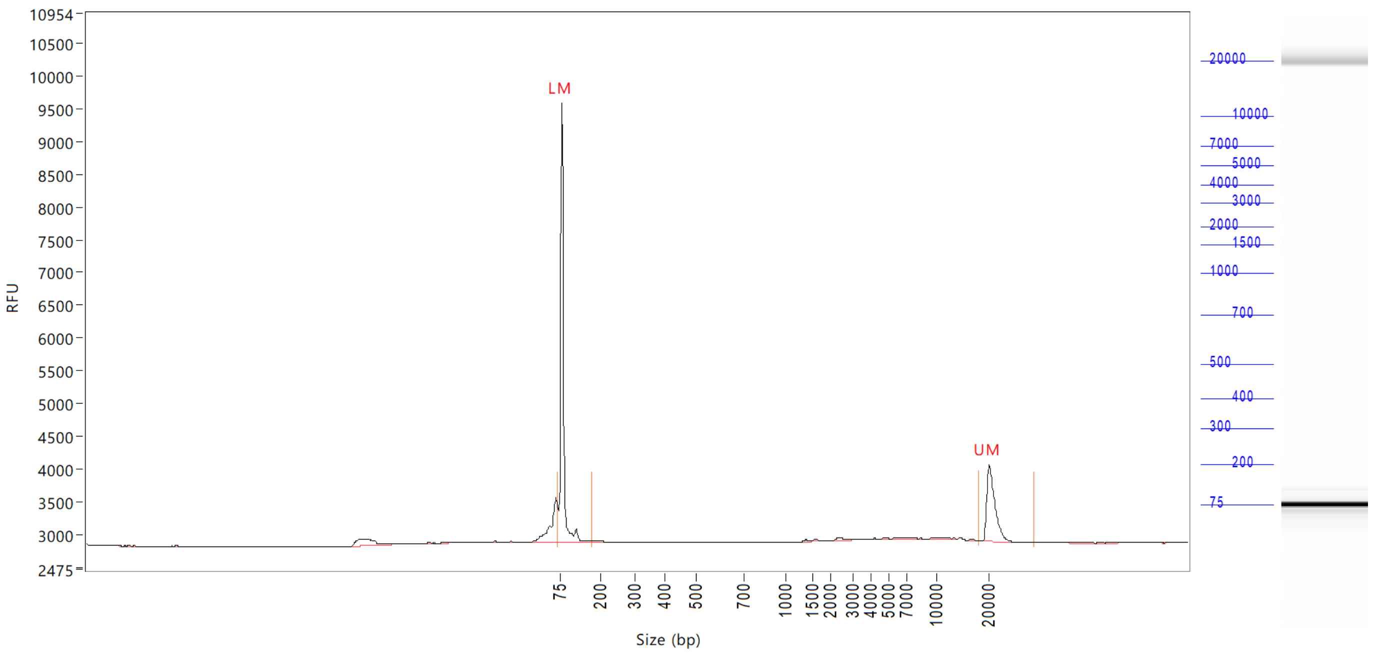

Peak	Size (bp)	Concentration (ng/uL)	From (bp)	To (bp)	Average size (bp)	CV%	RFU	Corrected peak area
1	75 (LM)	0.5000	64	171	76	16.45	6713	48.252
2	20000 (UM)	0.1258	18023	28534	20534	4.63	1181	12.139

TIC:	0.0000	ng/uL
TIM:	0.0000	nmole/L
Total concentration:	0.5447	ng/uL

```
Sample peak width (sec): 5     Sample min peak height: 50     Sample baseline V to V?: Y     Sample baseline V to V points: 3
Sample filter: Binomial        Number of points for filter: 3  Sample start region (min): 0  Sample end region (min): 30
Marker peak width (sec): 5     Marker min peak height: 200    Marker baseline V to V?: Y     Marker baseline V to V points: 3
Lower marker selection: First peak > 500 RFU                  Upper marker selection: Last peak > 200 RFU
Ladder size (bp)75, 200, 300, 400, 500, 700, 1000, 1500, 2000, 3000, 4000, 5000, 7000, 10000, 20000
Quantification using: Lower Marker          Final concentration (ng/uL): 0.5000          Dilution factor: 12.0
```

Sample: SampE7
Well location: E7
Created: Tuesday, August 10, 2021 7:21:14 PM

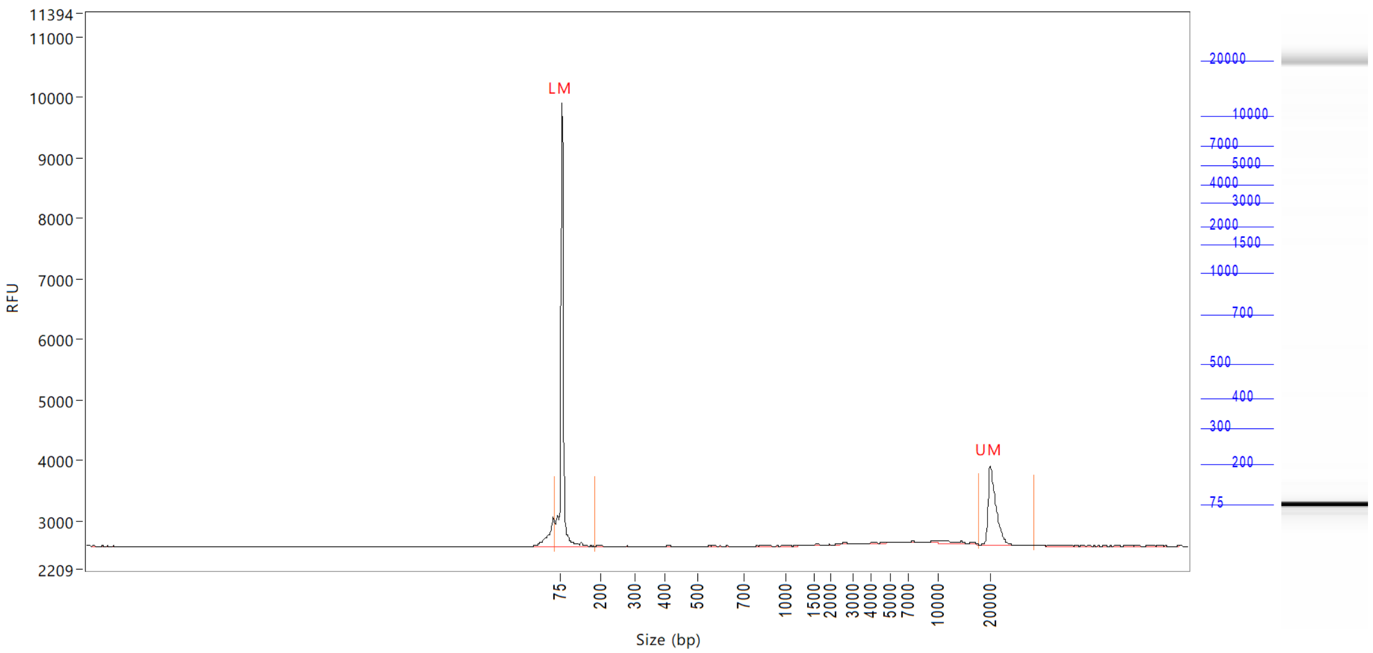

Peak	Size (bp)	Concentration (ng/uL)	From (bp)	To (bp)	Average size (bp)	CV%	RFU	Corrected peak area
1	75 (LM)	0.5000	54	179	75	15.11	7341	49.190
2	20000 (UM)	0.1350	17907	28419	20547	4.68	1310	13.284

TIC:	0.0000	ng/uL
TIM:	0.0000	nmole/L
Total concentration:	0.5917	ng/uL

Sample peak width (sec): 5 Sample min peak height: 50 Sample baseline V to V?: Y Sample baseline V to V points: 3
Sample filter: Binomial Number of points for filter: 3 Sample start region (min): 0 Sample end region (min): 30
Marker peak width (sec): 5 Marker min peak height: 200 Marker baseline V to V?: Y Marker baseline V to V points: 3
Lower marker selection: First peak > 500 RFU Upper marker selection: Last peak > 200 RFU
Ladder size (bp)75, 200, 300, 400, 500, 700, 1000, 1500, 2000, 3000, 4000, 5000, 7000, 10000, 20000
Quantification using: Lower Marker Final concentration (ng/uL): 0.5000 Dilution factor: 12.0

Sample: SampF7
Well location: F7
Created: Tuesday, August 10, 2021 7:21:14 PM

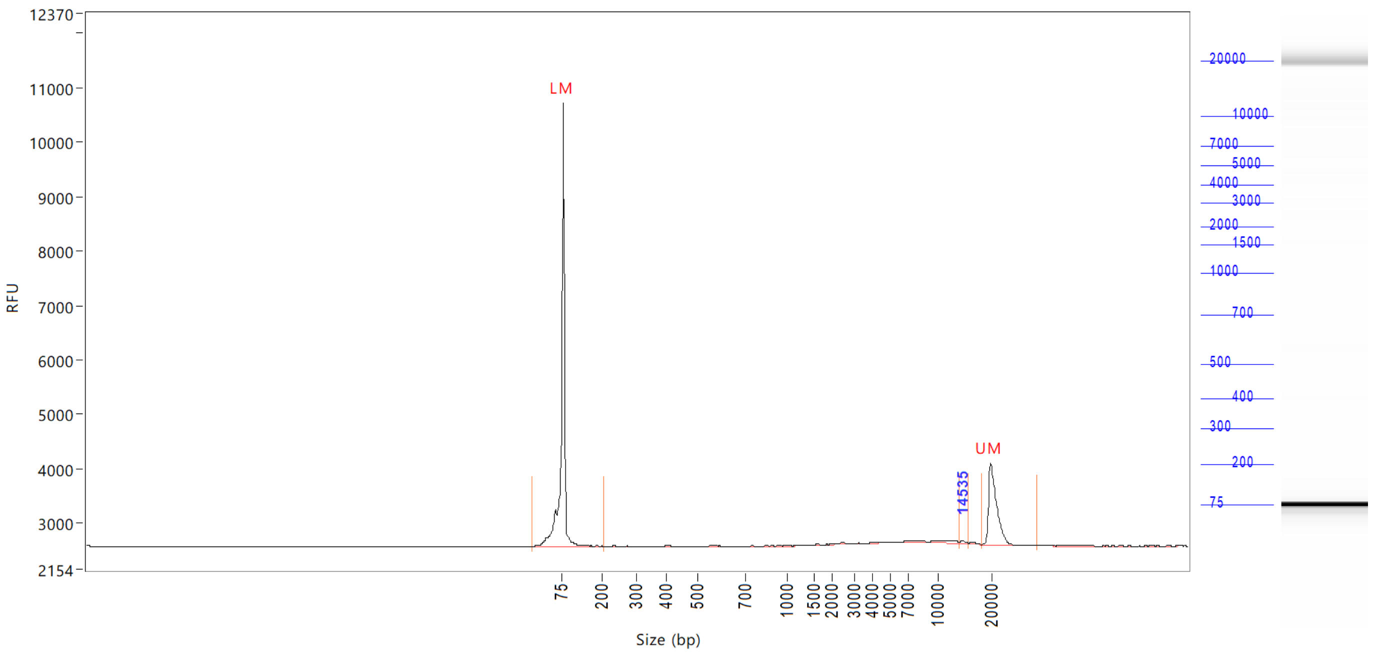

Peak	Size	Concentration	From	To	Average size	CV%	RFU	Corrected peak area
	(bp)	(ng/uL)	(bp)	(bp)	(bp)			
1	75 (LM)	0.5000	0	203	68	25.70	8166	65.308
2	14535	0.0380	13838	15698	14593	3.43	52	0.414
3	20000 (UM)	0.1204	18256	28880	20553	4.97	1500	15.727

TIC:	0.0380	ng/uL
TIM:	0.0043	nmole/L
Total concentration:	0.4759	ng/uL

Sample peak width (sec): 5 Sample min peak height: 50 Sample baseline V to V?: Y Sample baseline V to V points: 3
Sample filter: Binomial Number of points for filter: 3 Sample start region (min): 0 Sample end region (min): 30
Marker peak width (sec): 5 Marker min peak height: 200 Marker baseline V to V?: Y Marker baseline V to V points: 3
Lower marker selection: First peak > 500 RFU Upper marker selection: Last peak > 200 RFU
Ladder size (bp)75, 200, 300, 400, 500, 700, 1000, 1500, 2000, 3000, 4000, 5000, 7000, 10000, 20000
Quantification using: Lower Marker Final concentration (ng/uL): 0.5000 Dilution factor: 12.0

Sample: SampG7
Well location: G7
Created: Tuesday, August 10, 2021 7:21:14 PM

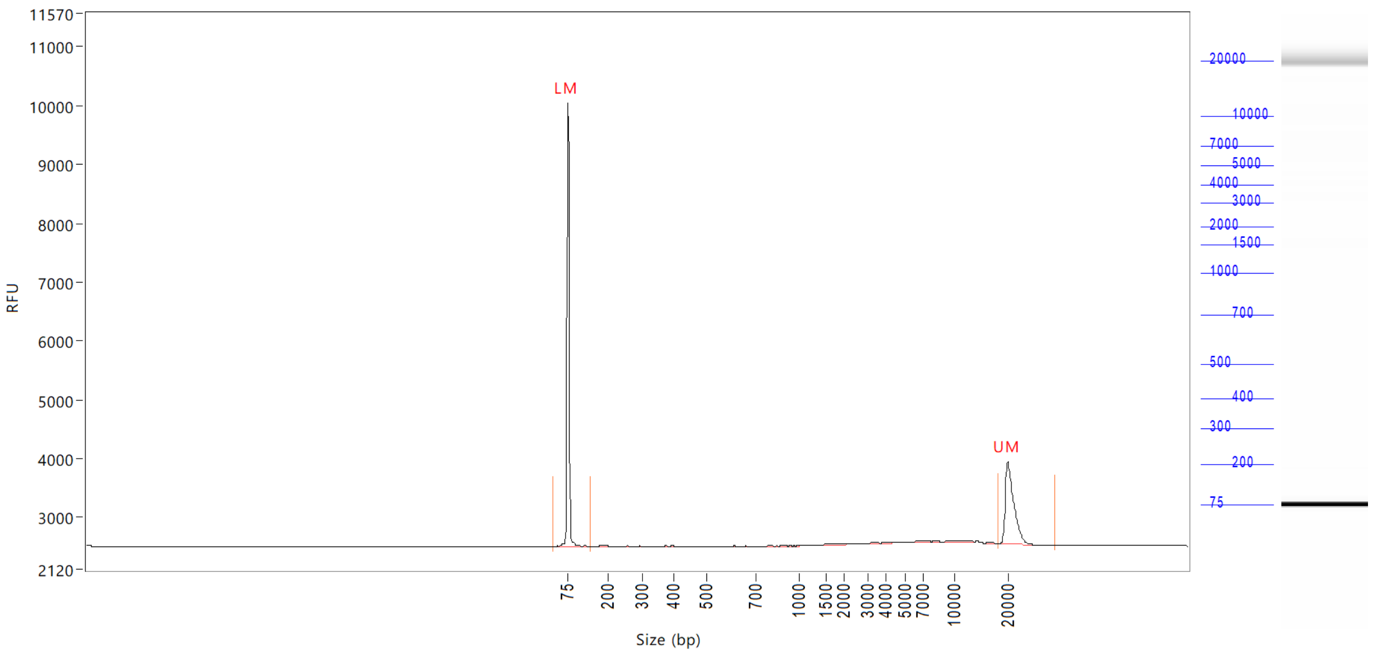

Peak	Size (bp)	Concentration (ng/uL)	From (bp)	To (bp)	Average size (bp)	CV%	RFU	Corrected peak area
1	75 (LM)	0.5000	31	143	73	6.26	7550	40.918
2	20000 (UM)	0.1839	18140	28880	20503	4.81	1395	15.048

TIC:	0.0000	ng/uL
TIM:	0.0000	nmole/L
Total concentration:	0.5841	ng/uL

Sample peak width (sec): 5 Sample min peak height: 50 Sample baseline V to V?: Y Sample baseline V to V points: 3
Sample filter: Binomial Number of points for filter: 3 Sample start region (min): 0 Sample end region (min): 30
Marker peak width (sec): 5 Marker min peak height: 200 Marker baseline V to V?: Y Marker baseline V to V points: 3
Lower marker selection: First peak > 500 RFU Upper marker selection: Last peak > 200 RFU
Ladder size (bp)75, 200, 300, 400, 500, 700, 1000, 1500, 2000, 3000, 4000, 5000, 7000, 10000, 20000
Quantification using: Lower Marker Final concentration (ng/uL): 0.5000 Dilution factor: 12.0

Sample: SampH7
Well location: H7
Created: Tuesday, August 10, 2021 7:21:14 PM

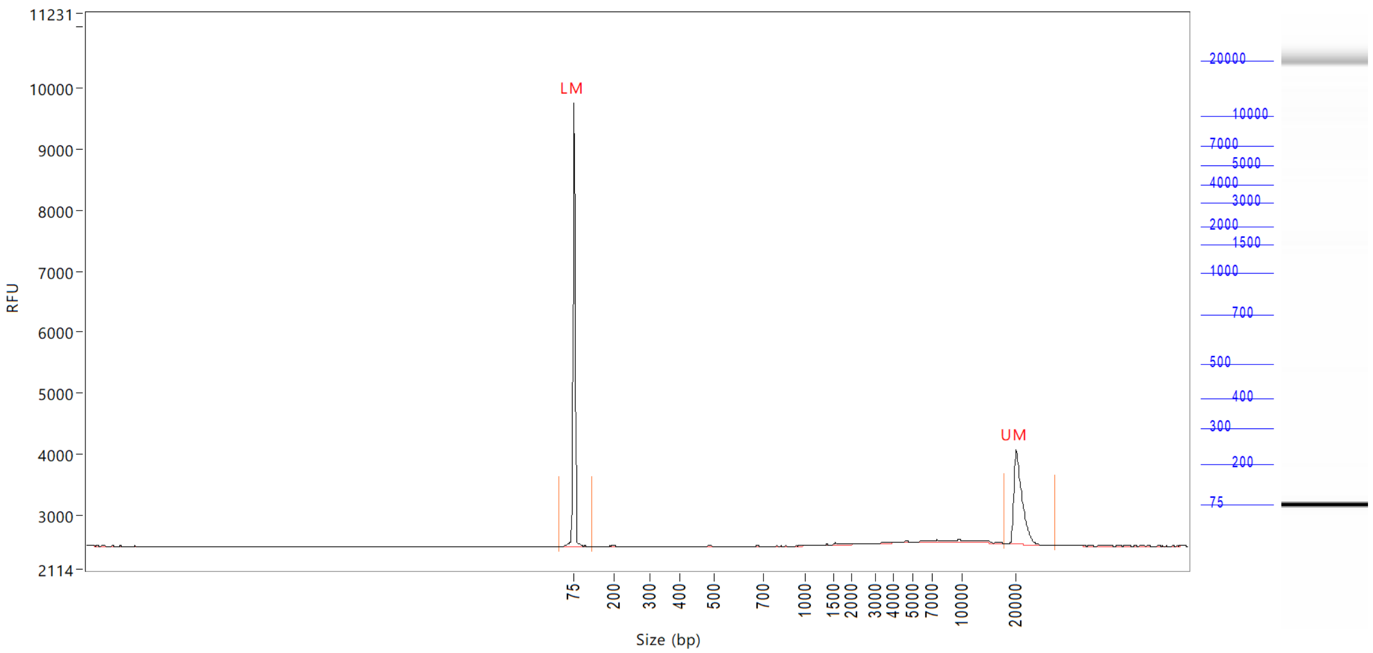

Peak	Size (bp)	Concentration (ng/uL)	From (bp)	To (bp)	Average size (bp)	CV%	RFU	Corrected peak area
1	75 (LM)	0.5000	28	131	72	6.55	7288	39.779
2	20000 (UM)	0.1987	17907	27381	20586	4.56	1555	15.808

TIC:	0.0000	ng/uL
TIM:	0.0000	nmole/L
Total concentration:	0.7105	ng/uL

Sample peak width (sec): 5   Sample min peak height: 50   Sample baseline V to V?: Y   Sample baseline V to V points: 3
Sample filter: Binomial   Number of points for filter: 3 Sample start region (min): 0 Sample end region (min): 30
Marker peak width (sec): 5   Marker min peak height: 200   Marker baseline V to V?: Y   Marker baseline V to V points: 3
Lower marker selection: First peak > 500 RFU   Upper marker selection: Last peak > 200 RFU
Ladder size (bp)75, 200, 300, 400, 500, 700, 1000, 1500, 2000, 3000, 4000, 5000, 7000, 10000, 20000
Quantification using: Lower Marker   Final concentration (ng/uL): 0.5000   Dilution factor: 12.0

Sample: SampA8
Well location: A8
Created: Tuesday, August 10, 2021 7:21:14 PM

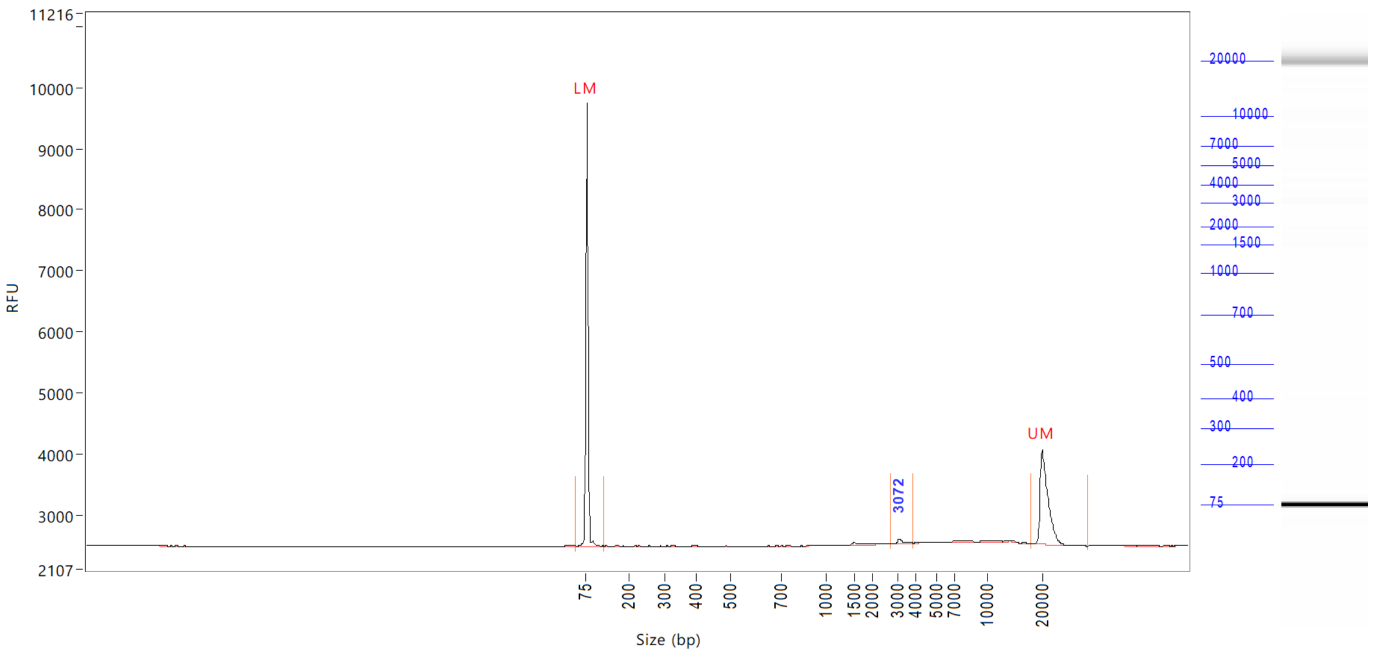

Peak	Size (bp)	Concentration (ng/uL)	From (bp)	To (bp)	Average size (bp)	CV%	RFU	Corrected peak area
1	75 (LM)	0.5000	45	126	73	6.24	7281	38.668
2	3072	0.0965	2730	3857	3175	6.74	75	0.622
3	20000 (UM)	0.1948	17907	28188	20495	4.52	1545	15.066

TIC:	0.0965	ng/uL
TIM:	0.0500	nmole/L
Total concentration:	0.6114	ng/uL

Sample peak width (sec): 5 Sample min peak height: 50 Sample baseline V to V?: Y Sample baseline V to V points: 3
Sample filter: Binomial Number of points for filter: 3 Sample start region (min): 0 Sample end region (min): 30
Marker peak width (sec): 5 Marker min peak height: 200 Marker baseline V to V?: Y Marker baseline V to V points: 3
Lower marker selection: First peak > 500 RFU Upper marker selection: Last peak > 200 RFU
Ladder size (bp)75, 200, 300, 400, 500, 700, 1000, 1500, 2000, 3000, 4000, 5000, 7000, 10000, 20000
Quantification using: Lower Marker Final concentration (ng/uL): 0.5000 Dilution factor: 12.0

Sample: SampB8
Well location: B8
Created: Tuesday, August 10, 2021 7:21:14 PM

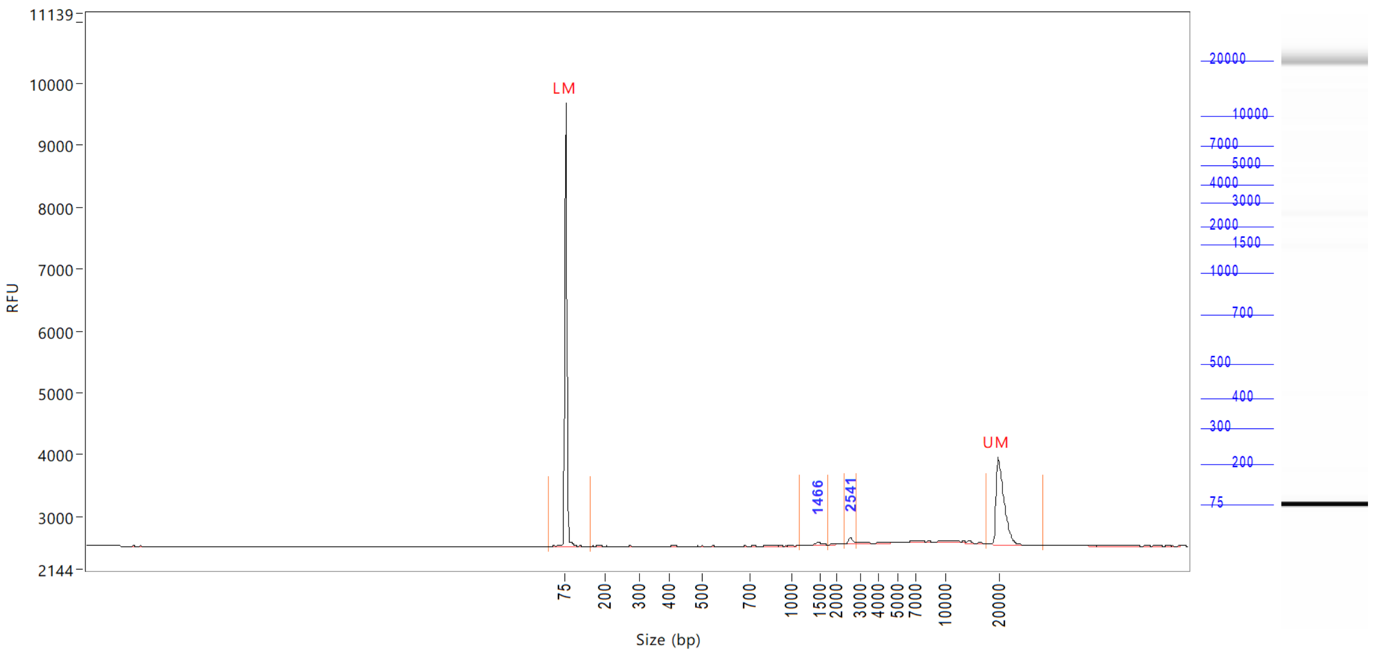

Peak	Size (bp)	Concentration (ng/uL)	From (bp)	To (bp)	Average size (bp)	CV%	RFU	Corrected peak area
1	75 (LM)	0.5000	24	152	74	7.16	7188	39.805
2	1466	0.0840	1137	1741	1462	5.84	57	0.557
3	2541	0.1125	2325	2838	2565	4.17	107	0.746
4	20000 (UM)	0.1793	17791	28534	20552	4.54	1418	14.271

TIC:	0.1965	ng/uL
TIM:	0.1667	nmole/L
Total concentration:	0.7906	ng/uL

Sample peak width (sec): 5 Sample min peak height: 50 Sample baseline V to V?: Y Sample baseline V to V points: 3
Sample filter: Binomial Number of points for filter: 3 Sample start region (min): 0 Sample end region (min): 30
Marker peak width (sec): 5 Marker min peak height: 200 Marker baseline V to V?: Y Marker baseline V to V points: 3
Lower marker selection: First peak > 500 RFU Upper marker selection: Last peak > 200 RFU
Ladder size (bp)75, 200, 300, 400, 500, 700, 1000, 1500, 2000, 3000, 4000, 5000, 7000, 10000, 20000
Quantification using: Lower Marker Final concentration (ng/uL): 0.5000 Dilution factor: 12.0

Sample: SampC8
Well location: C8
Created: Tuesday, August 10, 2021 7:21:14 PM

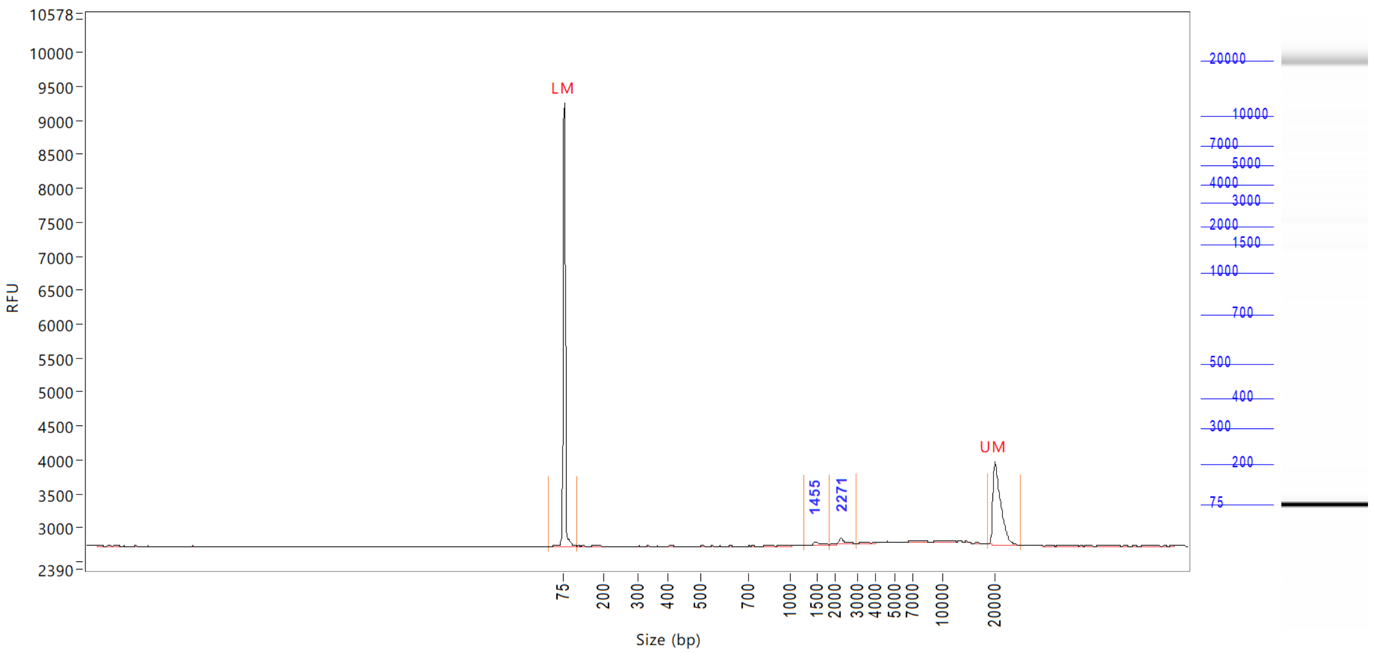

Peak	Size (bp)	Concentration (ng/uL)	From (bp)	To (bp)	Average size (bp)	CV%	RFU	Corrected peak area
1	75 (LM)	0.5000	26	114	73	6.40	6543	36.404
2	1455	0.0918	1239	1834	1488	7.27	53	0.557
3	2271	0.1482	1834	2946	2323	9.28	88	0.899
4	20000 (UM)	0.1670	18489	24959	20492	4.28	1222	12.158

TIC:	0.2399	ng/uL
TIM:	0.2065	nmole/L
Total concentration:	0.7655	ng/uL

```
Sample peak width (sec): 5    Sample min peak height: 50    Sample baseline V to V?: Y    Sample baseline V to V points: 3
Sample filter: Binomial       Number of points for filter: 3  Sample start region (min): 0  Sample end region (min): 30
Marker peak width (sec): 5    Marker min peak height: 200   Marker baseline V to V?: Y    Marker baseline V to V points: 3
Lower marker selection: First peak > 500 RFU                Upper marker selection: Last peak > 200 RFU
Ladder size (bp)75, 200, 300, 400, 500, 700, 1000, 1500, 2000, 3000, 4000, 5000, 7000, 10000, 20000
Quantification using: Lower Marker          Final concentration (ng/uL): 0.5000          Dilution factor: 12.0
```

Sample: SampD8
Well location: D8
Created: Tuesday, August 10, 2021 7:21:14 PM

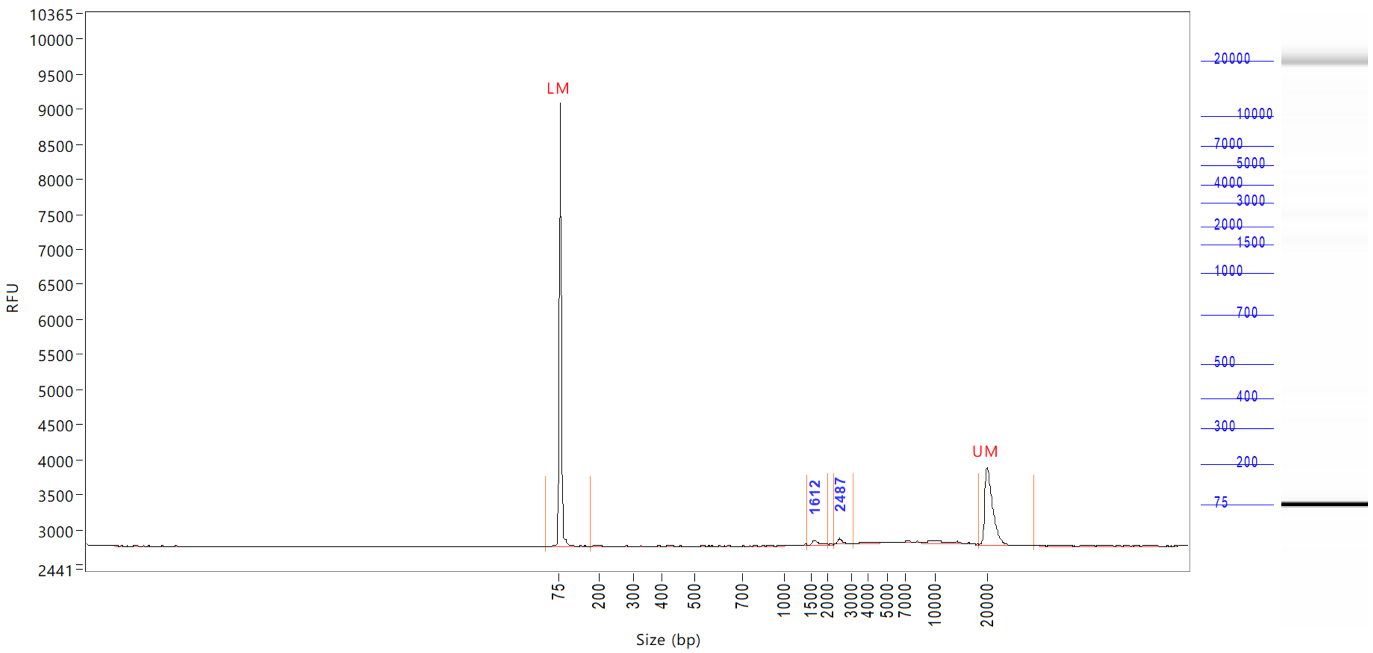

Peak	Size (bp)	Concentration (ng/uL)	From (bp)	To (bp)	Average size (bp)	CV%	RFU	Corrected peak area
1	75 (LM)	0.5000	33	171	74	8.79	6334	35.015
2	1612	0.1201	1432	2001	1628	7.63	68	0.701
3	2487	0.1253	2217	3144	2547	7.22	84	0.731
4	20000 (UM)	0.1585	18489	28995	20517	5.03	1109	11.099

TIC:	0.2454	ng/uL
TIM:	0.2024	nmole/L
Total concentration:	0.9241	ng/uL

```
Sample peak width (sec): 5    Sample min peak height: 50    Sample baseline V to V?: Y    Sample baseline V to V points: 3
Sample filter: Binomial       Number of points for filter: 3  Sample start region (min): 0  Sample end region (min): 30
Marker peak width (sec): 5    Marker min peak height: 200   Marker baseline V to V?: Y    Marker baseline V to V points: 3
Lower marker selection: First peak > 500 RFU               Upper marker selection: Last peak > 200 RFU
Ladder size (bp)75, 200, 300, 400, 500, 700, 1000, 1500, 2000, 3000, 4000, 5000, 7000, 10000, 20000
Quantification using: Lower Marker          Final concentration (ng/uL): 0.5000          Dilution factor: 12.0
```

Sample: SampE8
Well location: E8
Created: Tuesday, August 10, 2021 7:21:14 PM

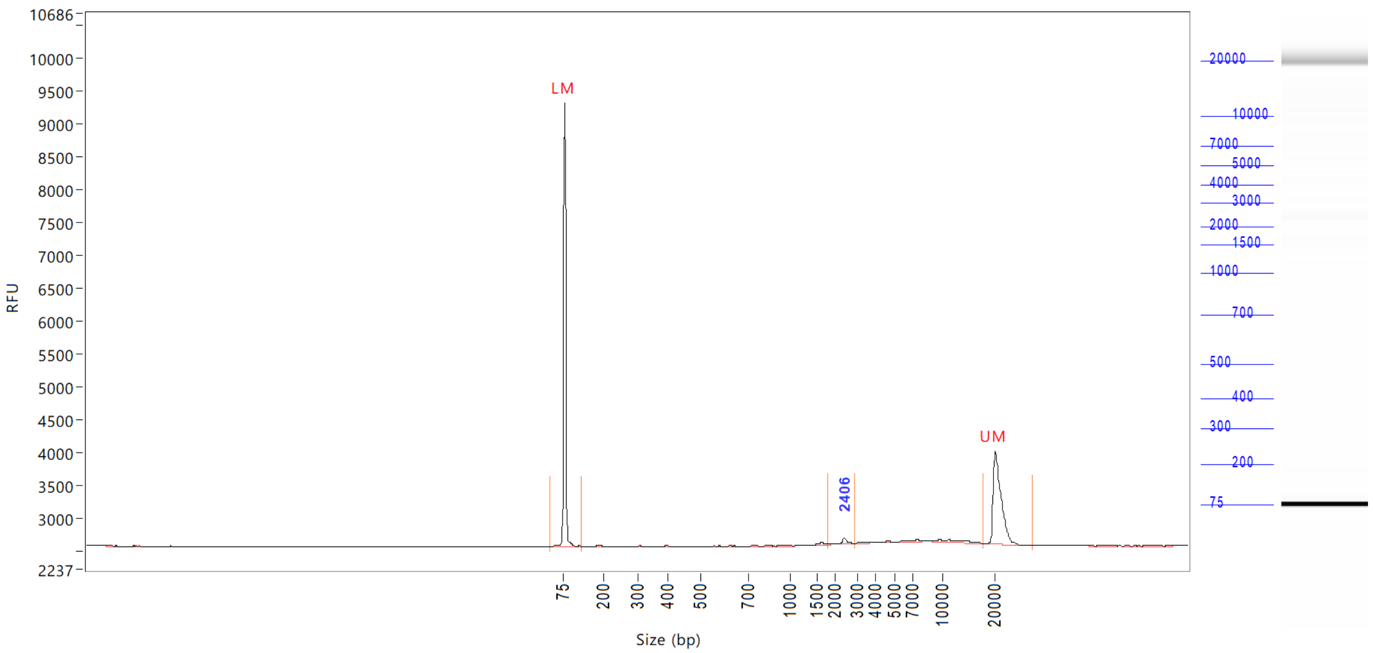

Peak	Size (bp)	Concentration (ng/uL)	From (bp)	To (bp)	Average size (bp)	CV%	RFU	Corrected peak area
1	75 (LM)	0.5000	31	129	74	6.09	6752	37.974
2	2406	0.1057	1815	2865	2385	7.27	87	0.669
3	20000 (UM)	0.1909	17791	27150	20563	4.45	1417	14.502

TIC:	0.1057	ng/uL
TIM:	0.0729	nmole/L
Total concentration:	0.7790	ng/uL

```
Sample peak width (sec): 5    Sample min peak height: 50    Sample baseline V to V?: Y    Sample baseline V to V points: 3
Sample filter: Binomial       Number of points for filter: 3  Sample start region (min): 0  Sample end region (min): 30
Marker peak width (sec): 5    Marker min peak height: 200   Marker baseline V to V?: Y    Marker baseline V to V points: 3
Lower marker selection: First peak > 500 RFU                Upper marker selection: Last peak > 200 RFU
Ladder size (bp)75, 200, 300, 400, 500, 700, 1000, 1500, 2000, 3000, 4000, 5000, 7000, 10000, 20000
Quantification using: Lower Marker          Final concentration (ng/uL): 0.5000          Dilution factor: 12.0
```

Sample: SampF8
Well location: F8
Created: Tuesday, August 10, 2021 7:21:14 PM

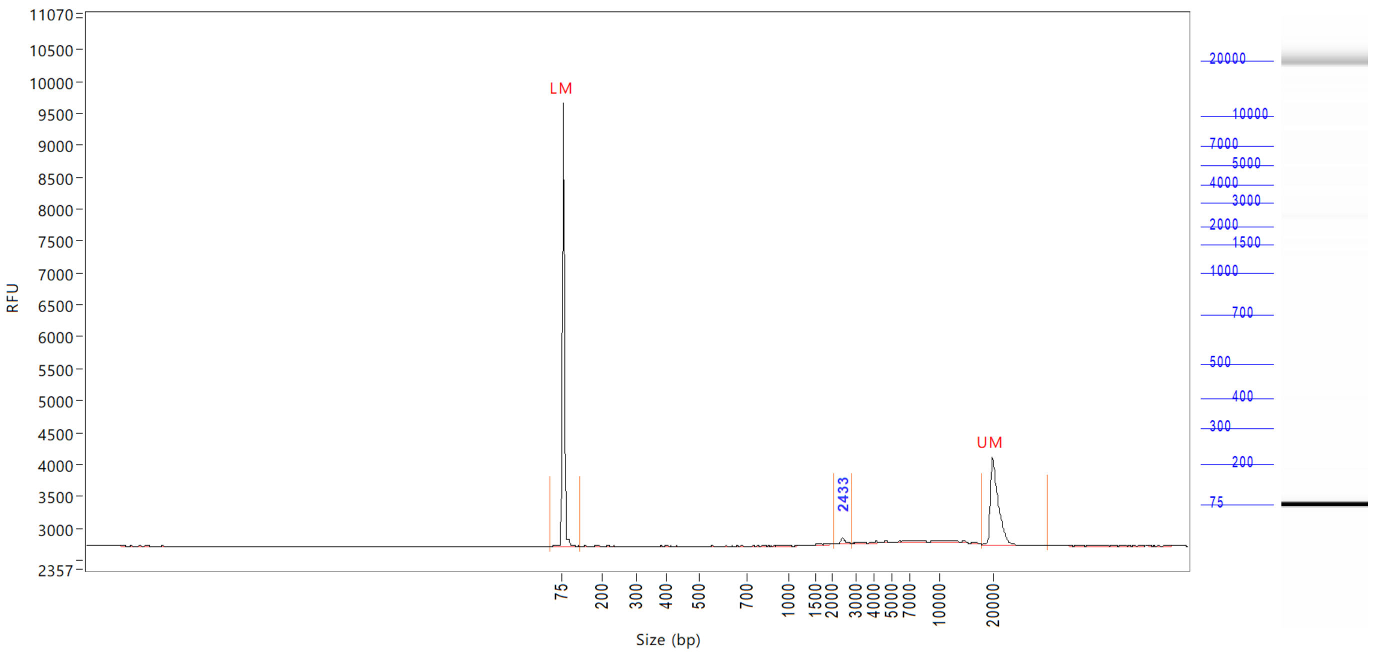

Peak	Size<br>(bp)	Concentration<br>(ng/uL)	From<br>(bp)	To<br>(bp)	Average size<br>(bp)	CV%	RFU	Corrected peak area
1	75 (LM)	0.5000	37	129	74	6.32	6963	38.404
2	2433	0.1116	2028	2838	2443	5.82	99	0.714
3	20000 (UM)	0.1826	18023	30495	20554	5.34	1364	14.022

TIC:	0.1116	ng/uL
TIM:	0.0752	nmole/L
Total concentration:	0.7894	ng/uL

```
Sample peak width (sec): 5    Sample min peak height: 50    Sample baseline V to V?: Y    Sample baseline V to V points: 3
Sample filter: Binomial       Number of points for filter: 3  Sample start region (min): 0  Sample end region (min): 30
Marker peak width (sec): 5    Marker min peak height: 200   Marker baseline V to V?: Y    Marker baseline V to V points: 3
Lower marker selection: First peak > 500 RFU                Upper marker selection: Last peak > 200 RFU
Ladder size (bp)75, 200, 300, 400, 500, 700, 1000, 1500, 2000, 3000, 4000, 5000, 7000, 10000, 20000
Quantification using: Lower Marker          Final concentration (ng/uL): 0.5000          Dilution factor: 12.0
```

Sample: SampG8
Well location: G8
Created: Tuesday, August 10, 2021 7:21:14 PM

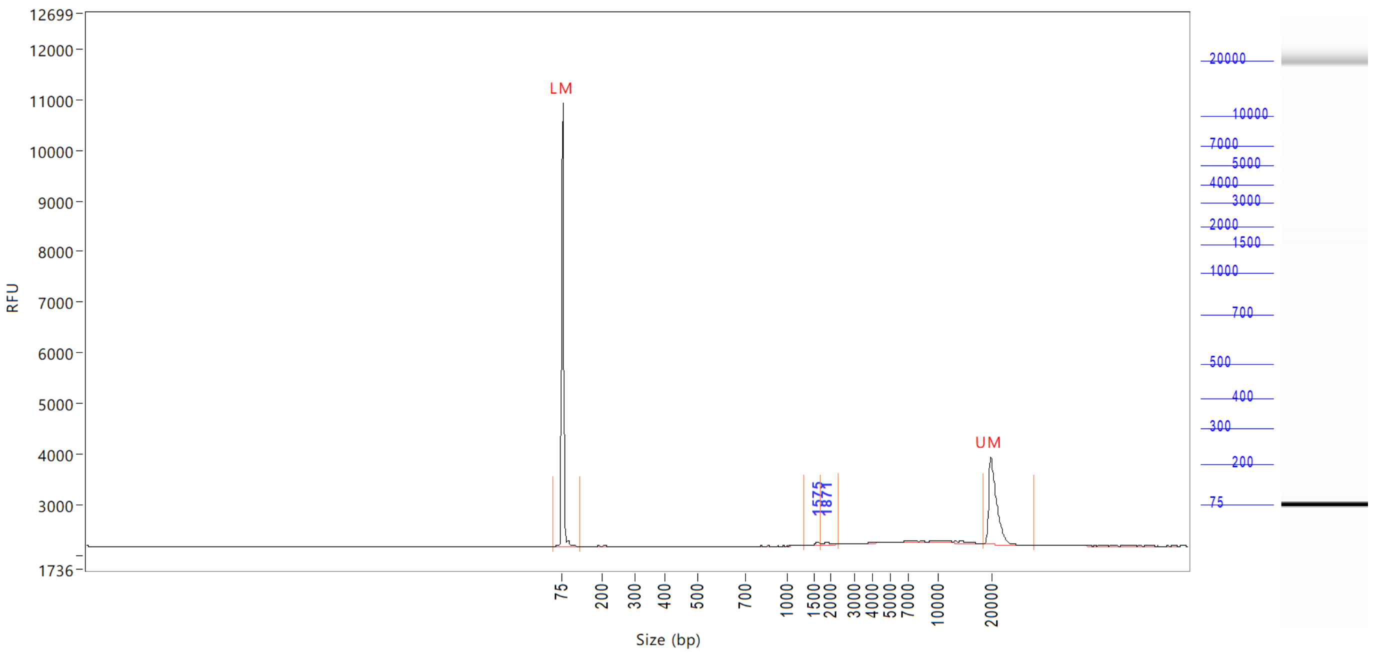

Peak	Size (bp)	Concentration (ng/uL)	From (bp)	To (bp)	Average size (bp)	CV%	RFU	Corrected peak area
1	75 (LM)	0.5000	47	131	73	5.98	8762	46.935
2	1575	0.0508	1307	1686	1550	4.10	60	0.397
3	1871	0.0776	1686	2325	1902	7.08	56	0.607
4	20000 (UM)	0.1860	18605	28188	20542	4.58	1723	17.462

TIC:	0.1284	ng/uL
TIM:	0.1211	nmole/L
Total concentration:	0.6413	ng/uL

```
Sample peak width (sec): 5    Sample min peak height: 50    Sample baseline V to V?: Y    Sample baseline V to V points: 3
Sample filter: Binomial       Number of points for filter: 3  Sample start region (min): 0  Sample end region (min): 30
Marker peak width (sec): 5    Marker min peak height: 200   Marker baseline V to V?: Y    Marker baseline V to V points: 3
Lower marker selection: First peak > 500 RFU                Upper marker selection: Last peak > 200 RFU
Ladder size (bp)75, 200, 300, 400, 500, 700, 1000, 1500, 2000, 3000, 4000, 5000, 7000, 10000, 20000
Quantification using: Lower Marker          Final concentration (ng/uL): 0.5000          Dilution factor: 12.0
```

Sample: SampH8
Well location: H8
Created: Tuesday, August 10, 2021 7:21:14 PM

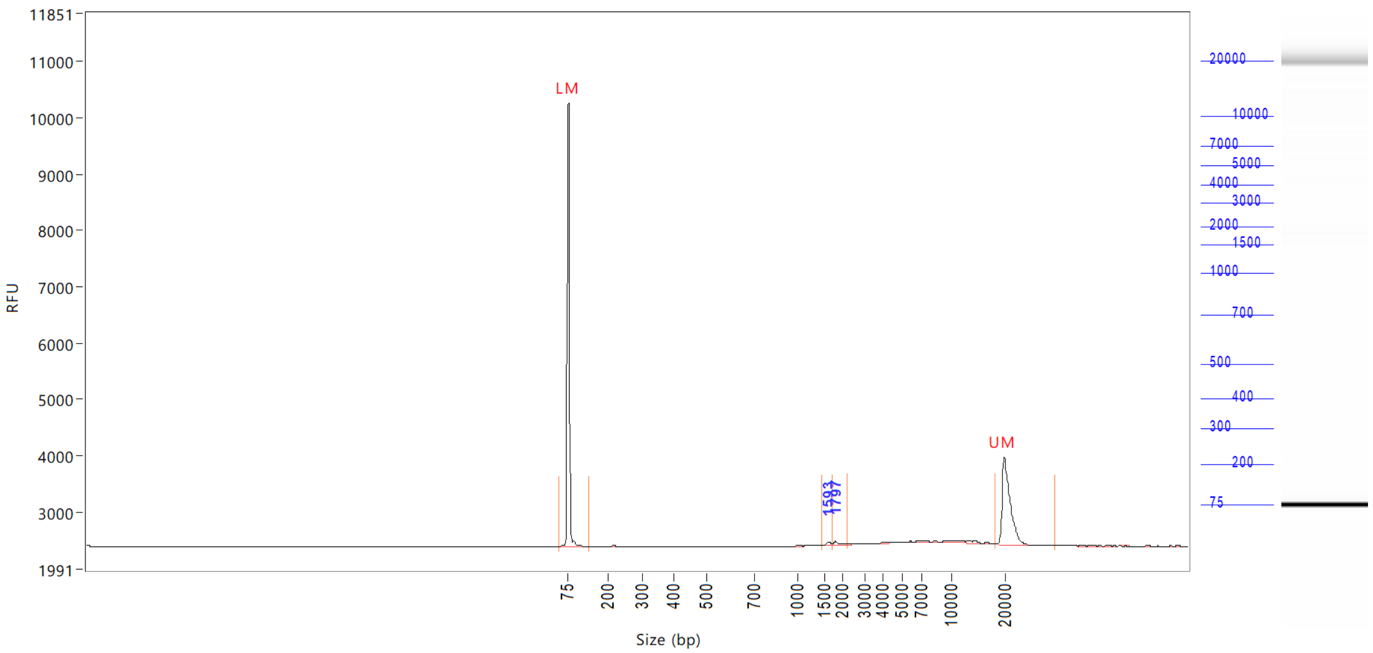

Peak	Size (bp)	Concentration (ng/uL)	From (bp)	To (bp)	Average size (bp)	CV%	RFU	Corrected peak area
1	75 (LM)	0.5000	47	139	73	6.34	7882	44.028
2	1593	0.0525	1443	1704	1586	3.94	53	0.385
3	1797	0.0779	1704	2244	1863	6.98	63	0.571
4	20000 (UM)	0.1796	18256	29687	20562	4.68	1556	15.811

TIC:	0.1304	ng/uL
TIM:	0.1233	nmole/L
Total concentration:	0.6662	ng/uL

Sample peak width (sec): 5   Sample min peak height: 50   Sample baseline V to V?: Y   Sample baseline V to V points: 3
Sample filter: Binomial   Number of points for filter: 3   Sample start region (min): 0   Sample end region (min): 30
Marker peak width (sec): 5   Marker min peak height: 200   Marker baseline V to V?: Y   Marker baseline V to V points: 3
Lower marker selection: First peak > 500 RFU   Upper marker selection: Last peak > 200 RFU
Ladder size (bp)75, 200, 300, 400, 500, 700, 1000, 1500, 2000, 3000, 4000, 5000, 7000, 10000, 20000
Quantification using: Lower Marker   Final concentration (ng/uL): 0.5000   Dilution factor: 12.0

Sample: SampA9
Well location: A9
Created: Tuesday, August 10, 2021 7:21:14 PM

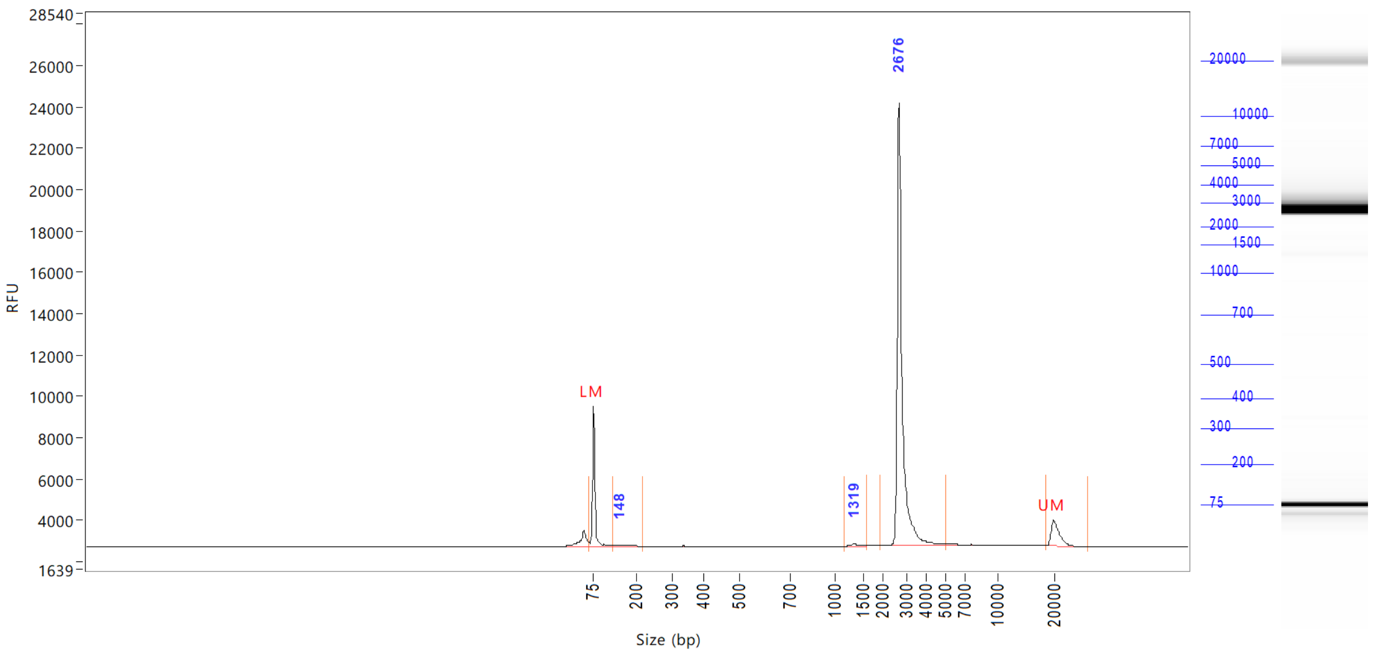

Peak	Size (bp)	Concentration (ng/uL)	From (bp)	To (bp)	Average size (bp)	CV%	RFU	Corrected peak area
1	75 (LM)	0.5000	64	129	75	12.06	6763	39.839
2	148	0.3318	129	217	161	13.97	78	2.203
3	1319	0.1788	1160	1575	1355	6.44	114	1.187
4	2676	22.9978	1945	5001	2775	10.84	21461	152.701
5	20000 (UM)	0.1491	18721	26112	20475	4.05	1241	11.881

TIC:	23.5084	ng/uL
TIM:	17.2474	nmole/L
Total concentration:	24.1859	ng/uL

Sample peak width (sec): 5   Sample min peak height: 50   Sample baseline V to V?: Y   Sample baseline V to V points: 3
Sample filter: Binomial   Number of points for filter: 3   Sample start region (min): 0   Sample end region (min): 30
Marker peak width (sec): 5   Marker min peak height: 200   Marker baseline V to V?: Y   Marker baseline V to V points: 3
Lower marker selection: First peak > 500 RFU   Upper marker selection: Last peak > 200 RFU
Ladder size (bp)75, 200, 300, 400, 500, 700, 1000, 1500, 2000, 3000, 4000, 5000, 7000, 10000, 20000
Quantification using: Lower Marker   Final concentration (ng/uL): 0.5000   Dilution factor: 12.0

Sample: SampB9
Well location: B9
Created: Tuesday, August 10, 2021 7:21:14 PM

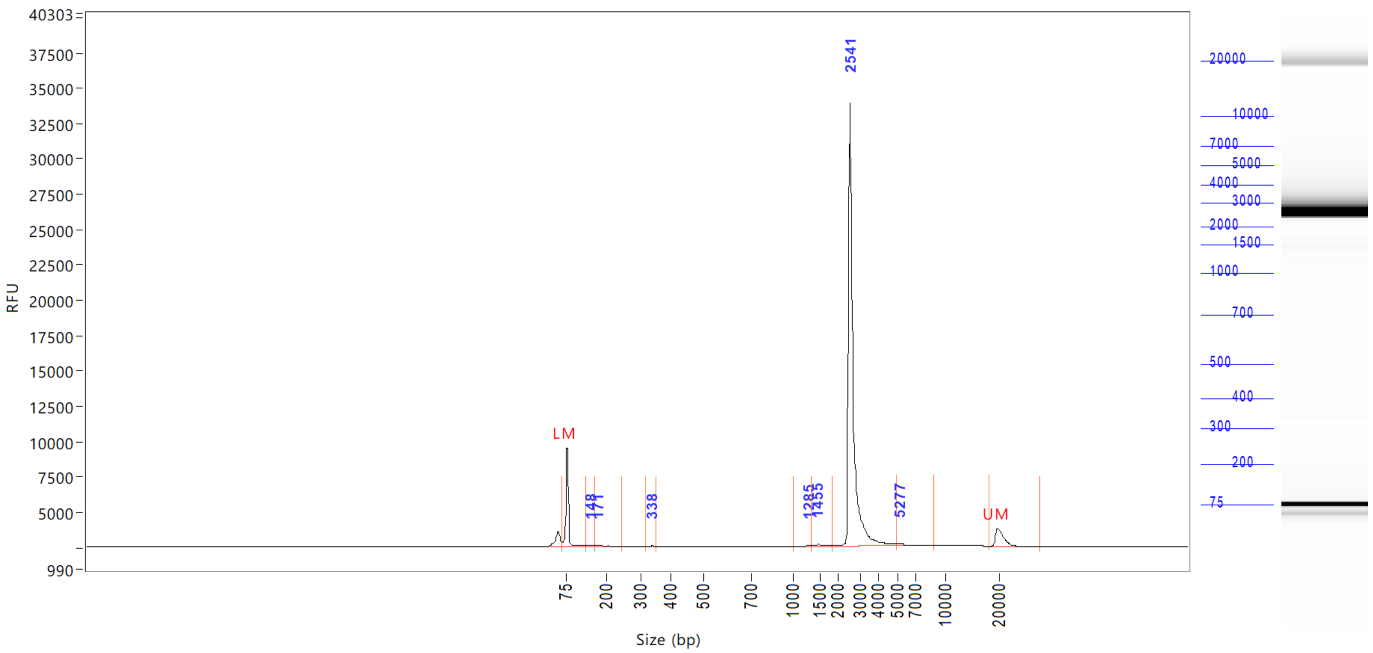

Peak	Size (bp)	Concentration (ng/uL)	From (bp)	To (bp)	Average size (bp)	CV%	RFU	Corrected peak area
1	75 (LM)	0.5000	62	133	76	12.92	7004	44.502
2	148	0.1885	133	164	148	6.17	77	1.398
3	171	0.2205	164	244	188	10.11	82	1.636
4	338	0.0464	318	350	334	1.57	56	0.344
5	1285	0.0847	1000	1330	1234	5.26	69	0.628
6	1455	0.2752	1330	1834	1511	7.75	139	2.041
7	2541	31.0469	1834	5000	2658	12.53	31390	230.275
8	5277	0.3175	5000	8875	6321	15.40	97	2.355
9	20000 (UM)	0.1442	18256	27957	20437	4.51	1281	12.837

TIC:	32.1798	ng/uL
TIM:	23.9744	nmole/L
Total concentration:	32.4020	ng/uL

```
Sample peak width (sec): 5    Sample min peak height: 50    Sample baseline V to V?: Y    Sample baseline V to V points: 3
Sample filter: Binomial       Number of points for filter: 3  Sample start region (min): 0  Sample end region (min): 30
Marker peak width (sec): 5    Marker min peak height: 200   Marker baseline V to V?: Y    Marker baseline V to V points: 3
Lower marker selection: First peak > 500 RFU                Upper marker selection: Last peak > 200 RFU
Ladder size (bp)75, 200, 300, 400, 500, 700, 1000, 1500, 2000, 3000, 4000, 5000, 7000, 10000, 20000
Quantification using: Lower Marker          Final concentration (ng/uL): 0.5000          Dilution factor: 12.0
```

Sample: SampC9
Well location: C9
Created: Tuesday, August 10, 2021 7:21:14 PM

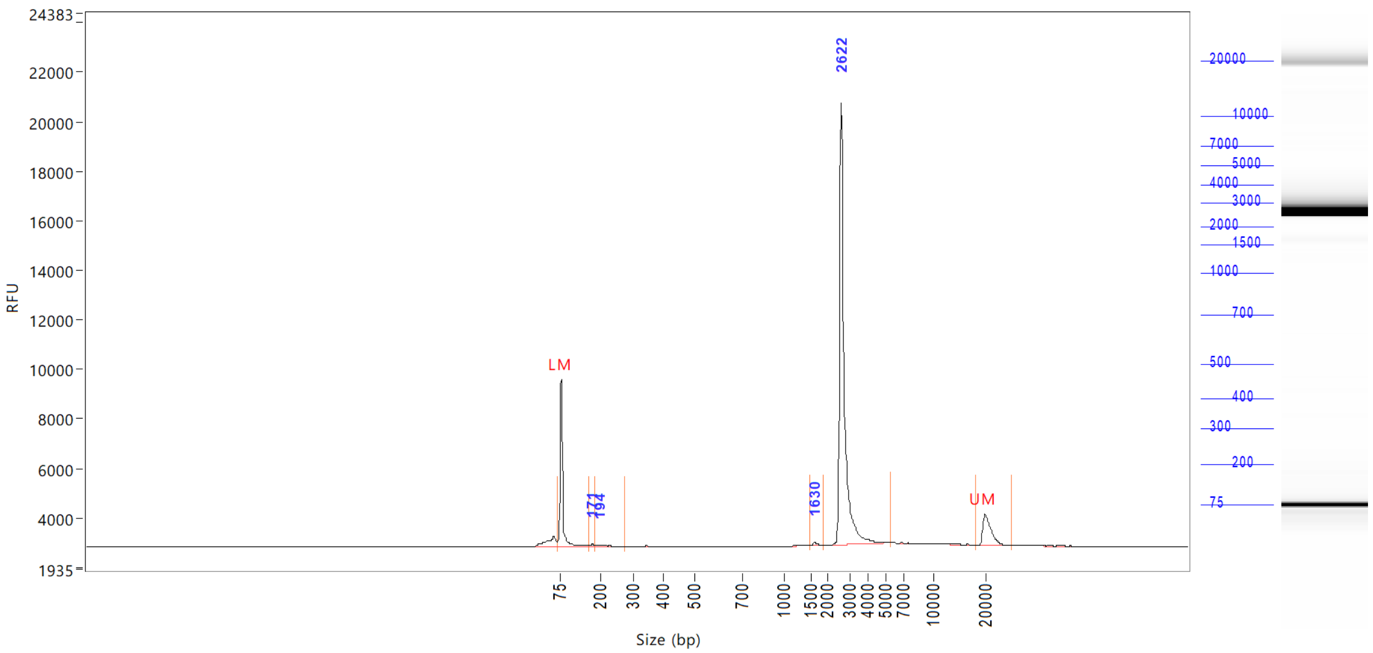

Peak	Size (bp)	Concentration (ng/uL)	From (bp)	To (bp)	Average size (bp)	CV%	RFU	Corrected peak area
1	75 (LM)	0.5000	64	164	77	19.47	6723	44.502
2	171	0.1045	164	183	172	3.05	92	0.775
3	194	0.1426	183	271	208	9.71	50	1.058
4	1630	0.1197	1477	1871	1620	4.32	128	0.888
5	2622	17.6245	1871	5415	2699	10.59	17869	130.719
6	20000 (UM)	0.1474	18140	25190	20508	4.53	1281	13.117

TIC:	17.9913	ng/uL
TIM:	12.9979	nmole/L
Total concentration:	18.3389	ng/uL

Sample peak width (sec): 5   Sample min peak height: 50   Sample baseline V to V?: Y   Sample baseline V to V points: 3
Sample filter: Binomial   Number of points for filter: 3 Sample start region (min): 0 Sample end region (min): 30
Marker peak width (sec): 5   Marker min peak height: 200   Marker baseline V to V?: Y   Marker baseline V to V points: 3
Lower marker selection: First peak > 500 RFU   Upper marker selection: Last peak > 200 RFU
Ladder size (bp)75, 200, 300, 400, 500, 700, 1000, 1500, 2000, 3000, 4000, 5000, 7000, 10000, 20000
Quantification using: Lower Marker   Final concentration (ng/uL): 0.5000   Dilution factor: 12.0

Sample: SampD9
Well location: D9
Created: Tuesday, August 10, 2021 7:21:14 PM

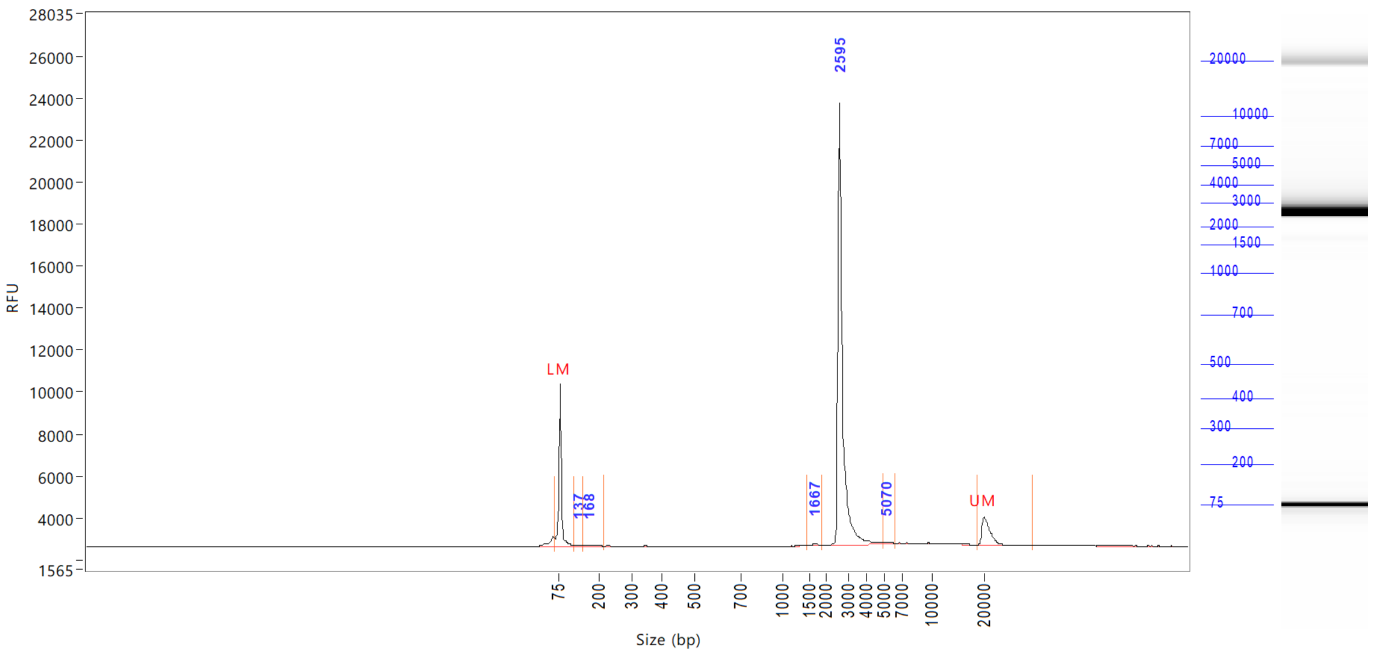

Peak	Size (bp)	Concentration (ng/uL)	From (bp)	To (bp)	Average size (bp)	CV%	RFU	Corrected peak area
1	75 (LM)	0.5000	60	120	74	10.13	7715	49.344
2	137	0.1419	120	150	133	6.29	71	1.167
3	168	0.1294	150	214	175	8.80	56	1.064
4	1667	0.1075	1466	1871	1639	4.44	112	0.884
5	2595	18.3966	1871	4900	2687	11.53	21087	151.293
6	5070	0.1127	4900	6173	5417	7.12	66	0.927
7	20000 (UM)	0.1410	18605	29457	20565	4.75	1359	13.915

TIC:	18.8881	ng/uL
TIM:	14.3816	nmole/L
Total concentration:	19.3918	ng/uL

Sample peak width (sec): 5 Sample min peak height: 50 Sample baseline V to V?: Y Sample baseline V to V points: 3
Sample filter: Binomial Number of points for filter: 3 Sample start region (min): 0 Sample end region (min): 30
Marker peak width (sec): 5 Marker min peak height: 200 Marker baseline V to V?: Y Marker baseline V to V points: 3
Lower marker selection: First peak > 500 RFU Upper marker selection: Last peak > 200 RFU
Ladder size (bp)75, 200, 300, 400, 500, 700, 1000, 1500, 2000, 3000, 4000, 5000, 7000, 10000, 20000
Quantification using: Lower Marker Final concentration (ng/uL): 0.5000 Dilution factor: 12.0

Sample: SampE9
Well location: E9
Created: Tuesday, August 10, 2021 7:21:14 PM

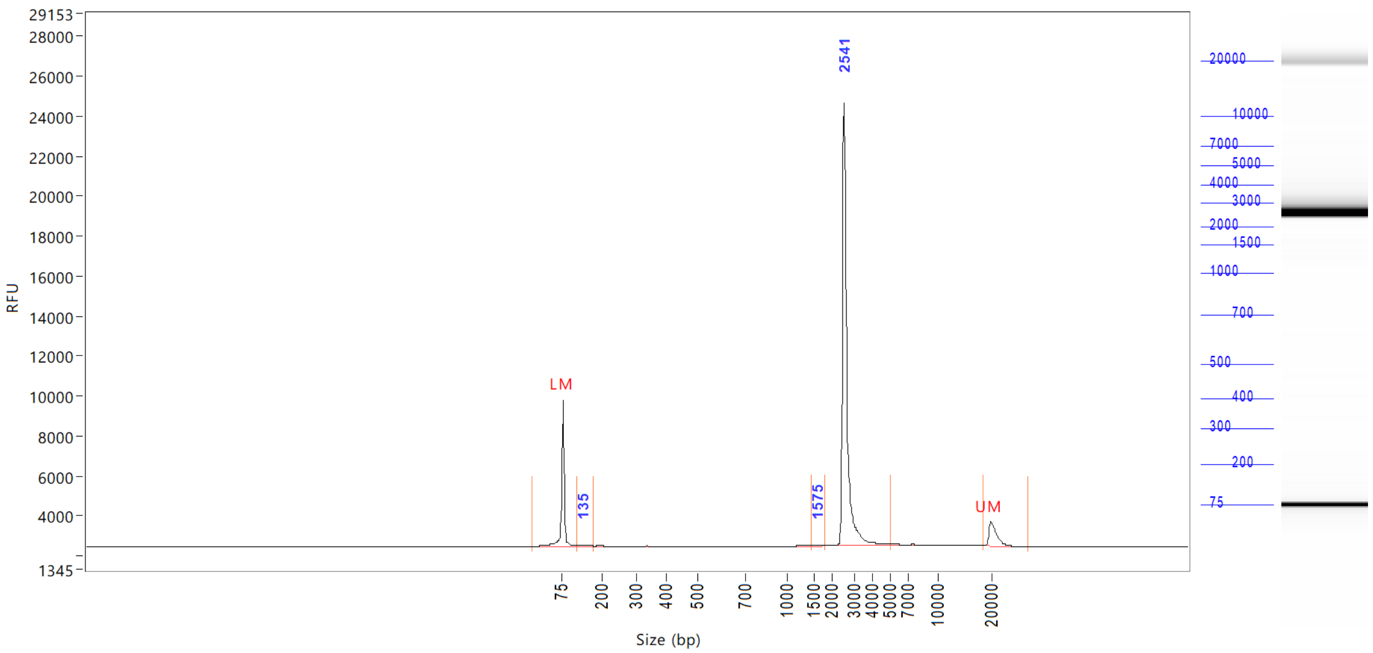

Peak	Size (bp)	Concentration (ng/uL)	From (bp)	To (bp)	Average size (bp)	CV%	RFU	Corrected peak area
1	75 (LM)	0.5000	0	118	69	21.26	7303	50.323
2	135	0.1811	118	173	141	10.80	61	1.519
3	1575	0.0600	1443	1797	1574	5.12	61	0.503
4	2541	17.7061	1797	5001	2636	11.43	22188	148.504
5	20000 (UM)	0.1192	18489	27035	20538	4.49	1197	12.000

TIC:	17.9472	ng/uL
TIM:	13.2310	nmole/L
Total concentration:	18.5333	ng/uL

```
Sample peak width (sec): 5    Sample min peak height: 50    Sample baseline V to V?: Y    Sample baseline V to V points: 3
Sample filter: Binomial       Number of points for filter: 3  Sample start region (min): 0  Sample end region (min): 30
Marker peak width (sec): 5    Marker min peak height: 200   Marker baseline V to V?: Y    Marker baseline V to V points: 3
Lower marker selection: First peak > 500 RFU                Upper marker selection: Last peak > 200 RFU
Ladder size (bp)75, 200, 300, 400, 500, 700, 1000, 1500, 2000, 3000, 4000, 5000, 7000, 10000, 20000
Quantification using: Lower Marker          Final concentration (ng/uL): 0.5000          Dilution factor: 12.0
```

Sample: SampF9
Well location: F9
Created: Tuesday, August 10, 2021 7:21:14 PM

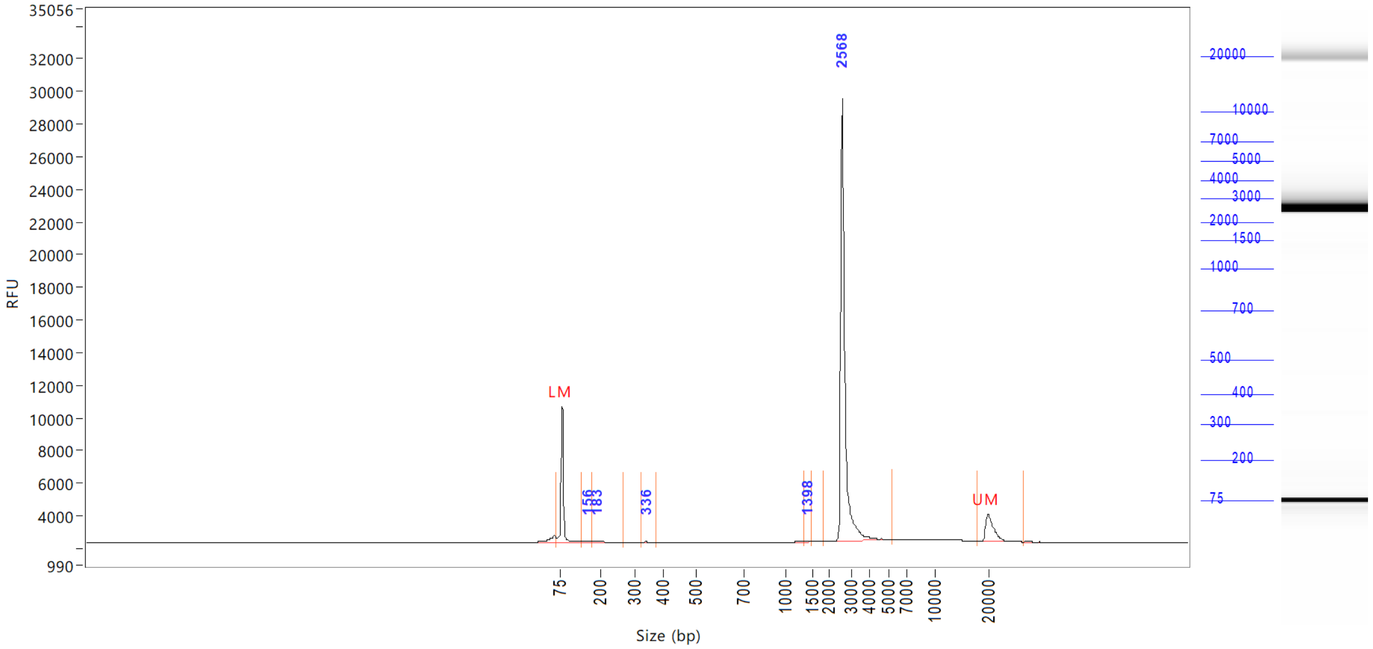

Peak	Size (bp)	Concentration (ng/uL)	From (bp)	To (bp)	Average size (bp)	CV%	RFU	Corrected peak area
1	75 (LM)	0.5000	60	139	75	12.13	8292	51.345
2	156	0.1553	139	173	155	6.09	76	1.329
3	183	0.1299	173	266	198	9.94	51	1.112
4	336	0.0420	322	373	335	2.05	60	0.359
5	1398	0.0424	1330	1500	1397	2.56	57	0.363
6	2568	20.4777	1834	5346	2653	10.19	27148	175.238
7	20000 (UM)	0.1647	18140	26804	20581	4.60	1638	16.913

TIC:	20.8473	ng/uL
TIM:	15.6883	nmole/L
Total concentration:	21.1289	ng/uL

Sample peak width (sec): 5   Sample min peak height: 50   Sample baseline V to V?: Y   Sample baseline V to V points: 3
Sample filter: Binomial   Number of points for filter: 3   Sample start region (min): 0   Sample end region (min): 30
Marker peak width (sec): 5   Marker min peak height: 200   Marker baseline V to V?: Y   Marker baseline V to V points: 3
Lower marker selection: First peak > 500 RFU   Upper marker selection: Last peak > 200 RFU
Ladder size (bp)75, 200, 300, 400, 500, 700, 1000, 1500, 2000, 3000, 4000, 5000, 7000, 10000, 20000
Quantification using: Lower Marker   Final concentration (ng/uL): 0.5000   Dilution factor: 12.0

Sample: SampG9
Well location: G9
Created: Tuesday, August 10, 2021 7:21:14 PM

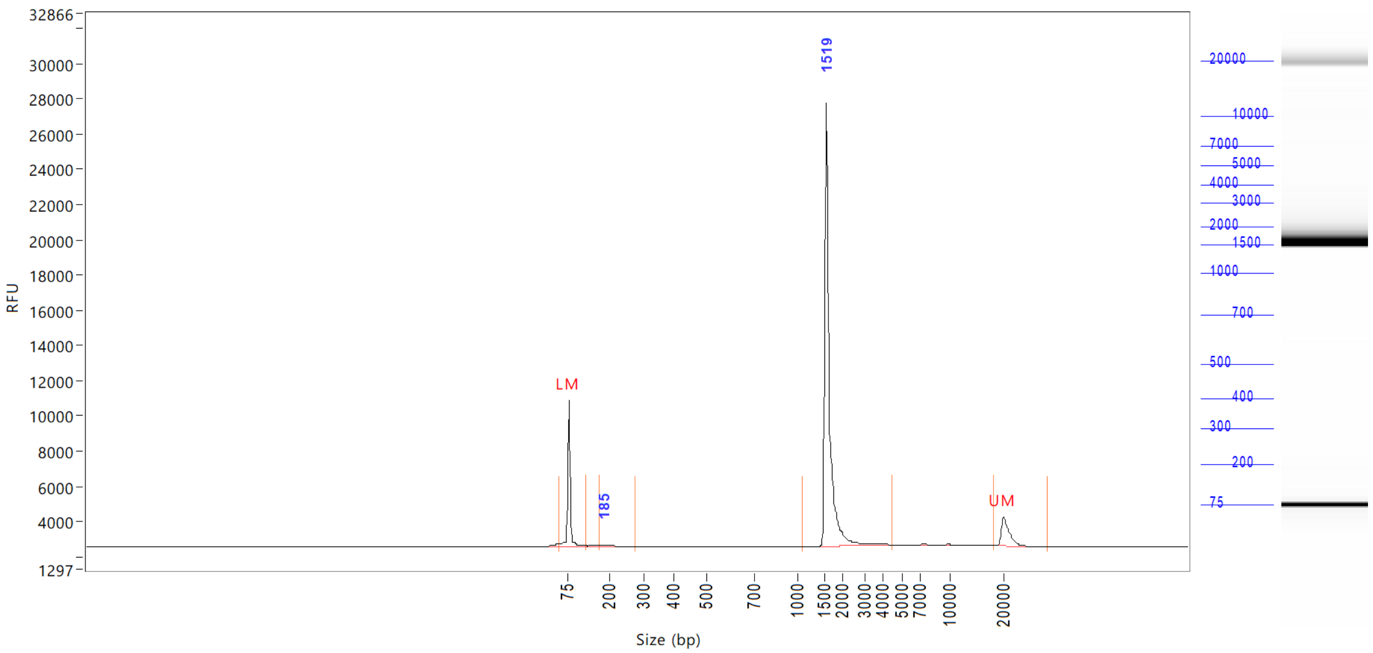

Peak	Size (bp)	Concentration (ng/uL)	From (bp)	To (bp)	Average size (bp)	CV%	RFU	Corrected peak area
1	75 (LM)	0.5000	45	129	73	10.26	8321	50.718
2	185	0.1379	171	277	193	8.14	66	1.166
3	1519	21.9281	1092	4434	1625	18.23	25213	185.357
4	20000 (UM)	0.1630	18256	28303	20506	4.74	1617	16.535

TIC:	22.0660	ng/uL
TIM:	23.3874	nmole/L
Total concentration:	22.5938	ng/uL

Sample peak width (sec): 5   Sample min peak height: 50   Sample baseline V to V?: Y   Sample baseline V to V points: 3
Sample filter: Binomial   Number of points for filter: 3 Sample start region (min): 0 Sample end region (min): 30
Marker peak width (sec): 5   Marker min peak height: 200   Marker baseline V to V?: Y   Marker baseline V to V points: 3
Lower marker selection: First peak > 500 RFU   Upper marker selection: Last peak > 200 RFU
Ladder size (bp)75, 200, 300, 400, 500, 700, 1000, 1500, 2000, 3000, 4000, 5000, 7000, 10000, 20000
Quantification using: Lower Marker   Final concentration (ng/uL): 0.5000   Dilution factor: 12.0

Sample: SampH9
Well location: H9
Created: Tuesday, August 10, 2021 7:21:14 PM

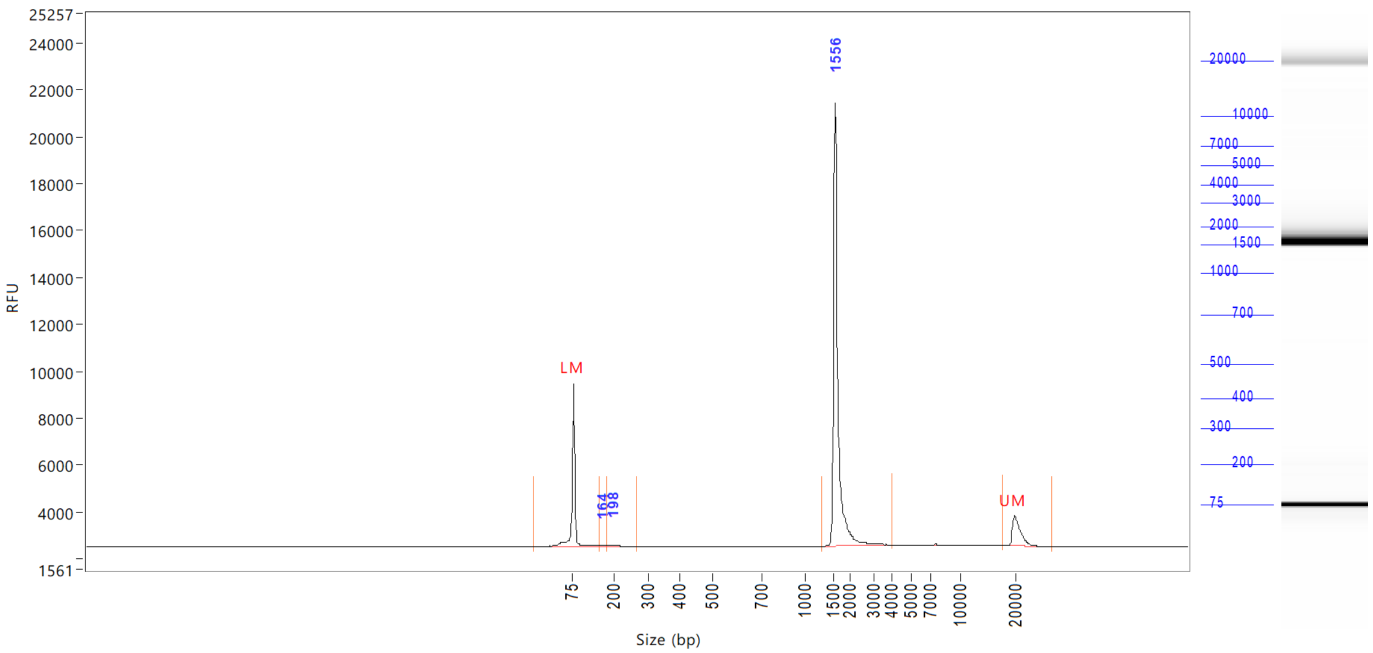

Peak	Size (bp)	Concentration (ng/uL)	From (bp)	To (bp)	Average size (bp)	CV%	RFU	Corrected peak area
1	75 (LM)	0.5000	0	152	71	24.69	6968	47.493
2	164	0.1032	152	179	165	4.78	58	0.817
3	198	0.1992	179	262	204	9.00	88	1.577
4	1556	16.3517	1296	4001	1612	12.23	18917	129.432
5	20000 (UM)	0.1330	17675	26919	20510	4.35	1280	12.632

TIC:	16.6540	ng/uL
TIM:	19.3308	nmole/L
Total concentration:	16.9914	ng/uL

Sample peak width (sec): 5   Sample min peak height: 50   Sample baseline V to V?: Y   Sample baseline V to V points: 3
Sample filter: Binomial   Number of points for filter: 3   Sample start region (min): 0   Sample end region (min): 30
Marker peak width (sec): 5   Marker min peak height: 200   Marker baseline V to V?: Y   Marker baseline V to V points: 3
Lower marker selection: First peak > 500 RFU   Upper marker selection: Last peak > 200 RFU
Ladder size (bp)75, 200, 300, 400, 500, 700, 1000, 1500, 2000, 3000, 4000, 5000, 7000, 10000, 20000
Quantification using: Lower Marker   Final concentration (ng/uL): 0.5000   Dilution factor: 12.0

Sample: SampA10
Well location: A10
Created: Tuesday, August 10, 2021 7:21:14 PM

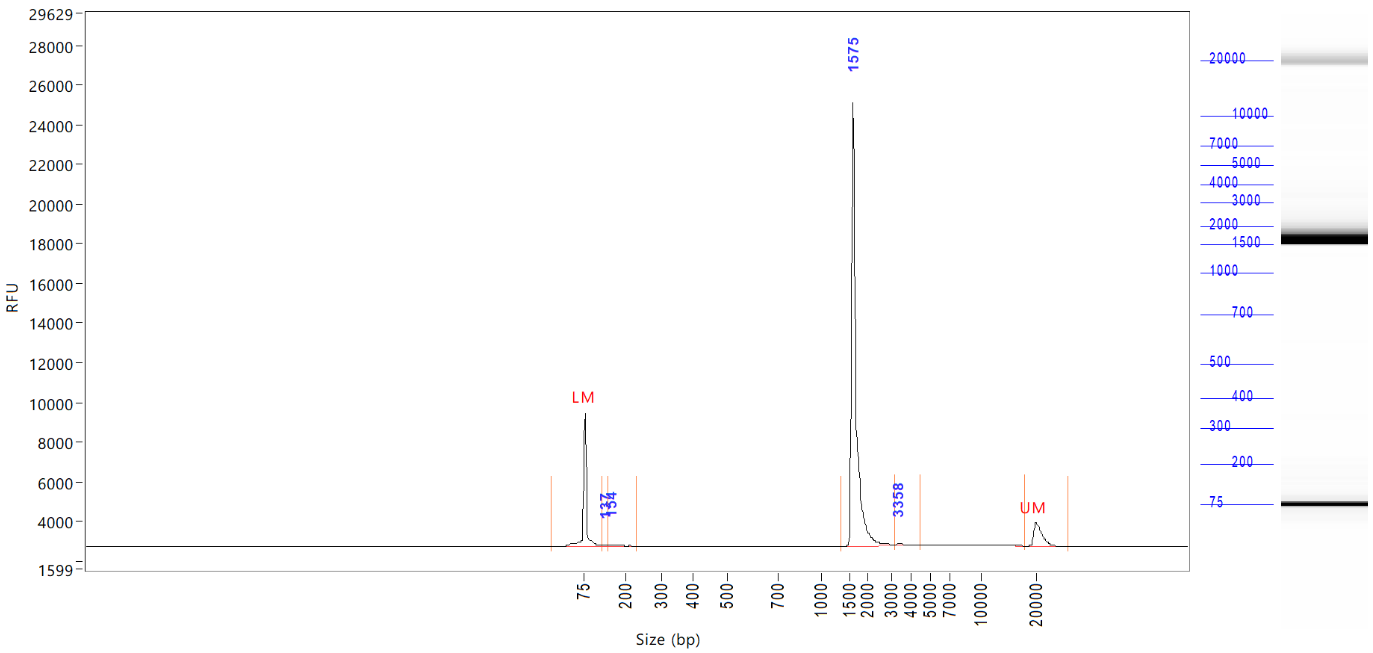

Peak	Size (bp)	Concentration (ng/uL)	From (bp)	To (bp)	Average size (bp)	CV%	RFU	Corrected peak area
1	75 (LM)	0.5000	0	128	72	19.71	6688	46.122
2	137	0.0942	128	145	136	3.63	90	0.724
3	154	0.2940	145	230	173	11.62	97	2.260
4	1575	20.3928	1330	3108	1633	11.04	22392	156.761
5	3358	0.2130	3108	4467	3624	9.74	108	1.637
6	20000 (UM)	0.1274	18140	25997	20493	4.18	1205	11.751

TIC:	20.9940	ng/uL
TIM:	24.5854	nmole/L
Total concentration:	21.3897	ng/uL

Sample peak width (sec): 5   Sample min peak height: 50   Sample baseline V to V?: Y   Sample baseline V to V points: 3
Sample filter: Binomial   Number of points for filter: 3 Sample start region (min): 0 Sample end region (min): 30
Marker peak width (sec): 5   Marker min peak height: 200   Marker baseline V to V?: Y   Marker baseline V to V points: 3
Lower marker selection: First peak > 500 RFU   Upper marker selection: Last peak > 200 RFU
Ladder size (bp)75, 200, 300, 400, 500, 700, 1000, 1500, 2000, 3000, 4000, 5000, 7000, 10000, 20000
Quantification using: Lower Marker   Final concentration (ng/uL): 0.5000   Dilution factor: 12.0

Sample: SampB10
Well location: B10
Created: Tuesday, August 10, 2021 7:21:14 PM

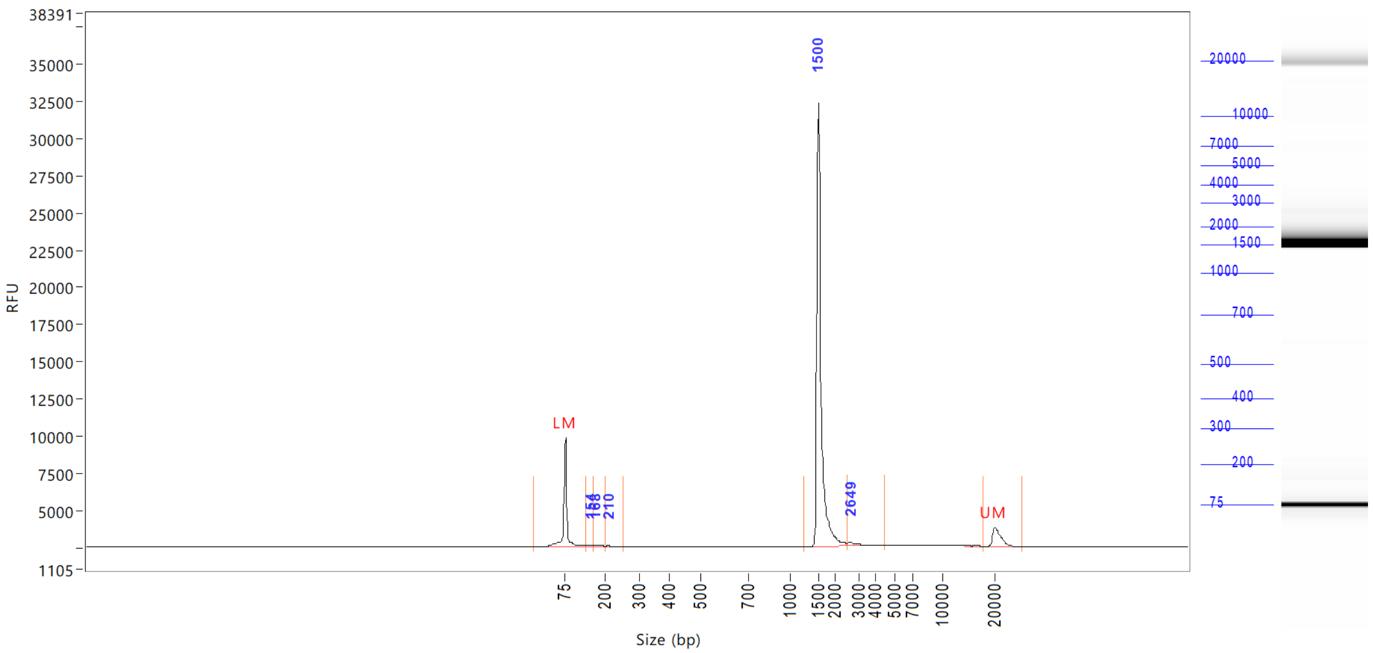

Peak	Size (bp)	Concentration (ng/uL)	From (bp)	To (bp)	Average size (bp)	CV%	RFU	Corrected peak area
1	75 (LM)	0.5000	0	139	71	21.03	7359	54.659
2	154	0.1400	139	162	151	4.14	128	1.275
3	168	0.1817	162	201	179	6.05	83	1.655
4	210	0.0774	201	251	213	5.00	71	0.705
5	1500	21.5820	1239	2514	1553	9.15	29788	196.610
6	2649	0.4077	2514	4467	2953	14.04	245	3.714
7	20000 (UM)	0.1205	17675	25190	20437	4.23	1322	13.173

TIC:	22.3887	ng/uL
TIM:	26.8924	nmole/L
Total concentration:	22.6602	ng/uL

Sample peak width (sec): 5   Sample min peak height: 50   Sample baseline V to V?: Y   Sample baseline V to V points: 3
Sample filter: Binomial   Number of points for filter: 3 Sample start region (min): 0 Sample end region (min): 30
Marker peak width (sec): 5   Marker min peak height: 200   Marker baseline V to V?: Y   Marker baseline V to V points: 3
Lower marker selection: First peak > 500 RFU   Upper marker selection: Last peak > 200 RFU
Ladder size (bp)75, 200, 300, 400, 500, 700, 1000, 1500, 2000, 3000, 4000, 5000, 7000, 10000, 20000
Quantification using: Lower Marker   Final concentration (ng/uL): 0.5000   Dilution factor: 12.0

Sample: SampC10
Well location: C10
Created: Tuesday, August 10, 2021 7:21:14 PM

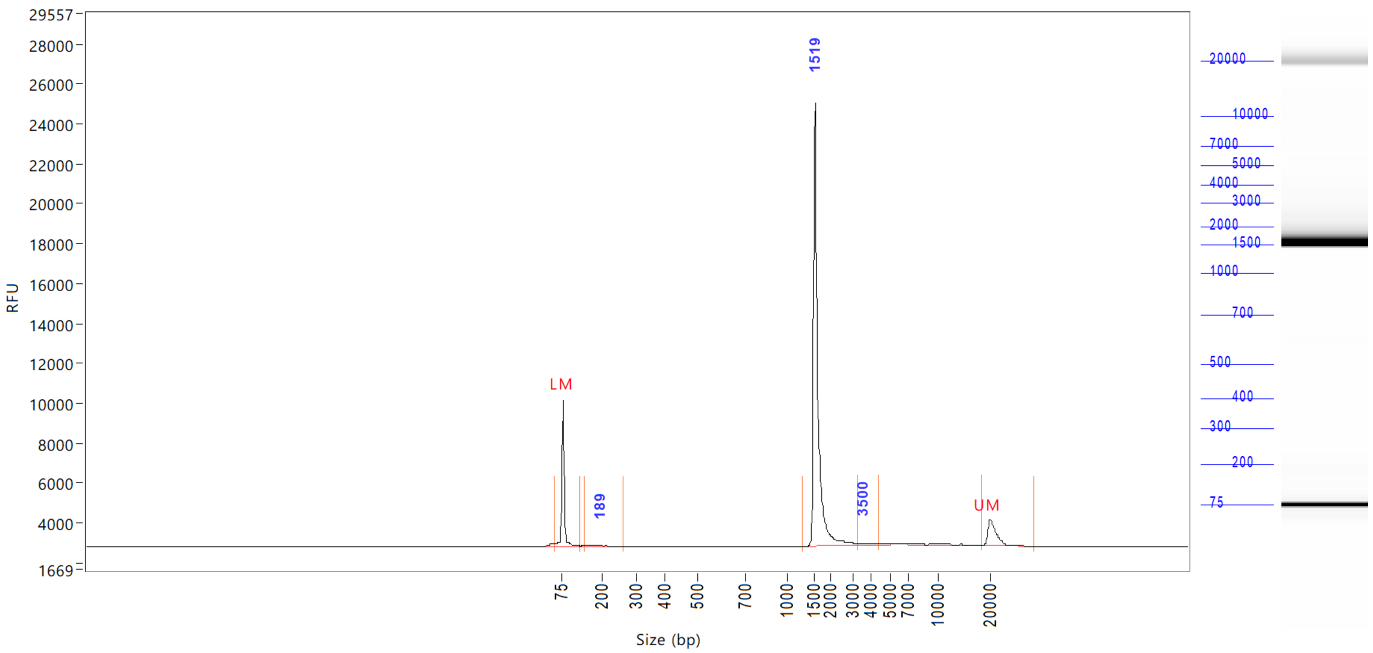

Peak	Size (bp)	Concentration (ng/uL)	From (bp)	To (bp)	Average size (bp)	CV%	RFU	Corrected peak area
1	75 (LM)	0.5000	52	128	74	11.09	7377	45.958
2	189	0.3526	143	264	183	13.48	94	2.701
3	1519	20.0964	1285	3251	1601	14.14	22265	153.931
4	3500	0.2346	3251	4434	3735	8.69	93	1.797
5	20000 (UM)	0.1523	18605	28534	20620	4.70	1332	13.997

TIC:	20.6836	ng/uL
TIM:	23.9333	nmole/L
Total concentration:	21.1521	ng/uL

Sample peak width (sec): 5 Sample min peak height: 50 Sample baseline V to V?: Y Sample baseline V to V points: 3
Sample filter: Binomial Number of points for filter: 3 Sample start region (min): 0 Sample end region (min): 30
Marker peak width (sec): 5 Marker min peak height: 200 Marker baseline V to V?: Y Marker baseline V to V points: 3
Lower marker selection: First peak > 500 RFU Upper marker selection: Last peak > 200 RFU
Ladder size (bp)75, 200, 300, 400, 500, 700, 1000, 1500, 2000, 3000, 4000, 5000, 7000, 10000, 20000
Quantification using: Lower Marker Final concentration (ng/uL): 0.5000 Dilution factor: 12.0

Sample: SampD10
Well location: D10
Created: Tuesday, August 10, 2021 7:21:14 PM

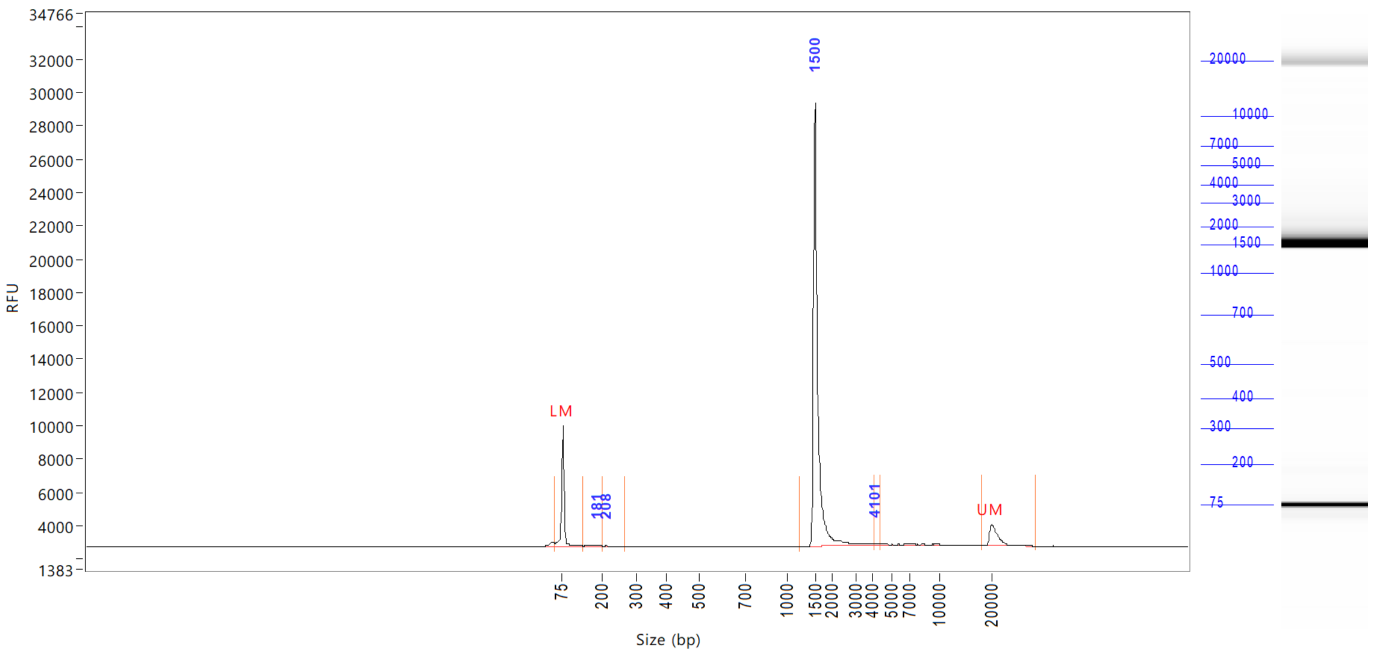

Peak	Size (bp)	Concentration (ng/uL)	From (bp)	To (bp)	Average size (bp)	CV%	RFU	Corrected peak area
1	75 (LM)	0.5000	50	137	73	13.45	7250	48.128
2	181	0.2954	137	201	169	9.99	94	2.369
3	208	0.0809	201	266	216	6.08	50	0.649
4	1500	22.8225	1194	4034	1576	16.99	26658	183.068
5	4101	0.0409	4034	4367	4167	2.39	51	0.328
6	20000 (UM)	0.1374	18023	28419	20496	4.80	1270	13.222

TIC:	23.2397	ng/uL
TIM:	27.3426	nmole/L
Total concentration:	23.5857	ng/uL

Sample peak width (sec): 5 Sample min peak height: 50 Sample baseline V to V?: Y Sample baseline V to V points: 3
Sample filter: Binomial Number of points for filter: 3 Sample start region (min): 0 Sample end region (min): 30
Marker peak width (sec): 5 Marker min peak height: 200 Marker baseline V to V?: Y Marker baseline V to V points: 3
Lower marker selection: First peak > 500 RFU Upper marker selection: Last peak > 200 RFU
Ladder size (bp)75, 200, 300, 400, 500, 700, 1000, 1500, 2000, 3000, 4000, 5000, 7000, 10000, 20000
Quantification using: Lower Marker Final concentration (ng/uL): 0.5000 Dilution factor: 12.0

Sample: SampE10
Well location: E10
Created: Tuesday, August 10, 2021 7:21:14 PM

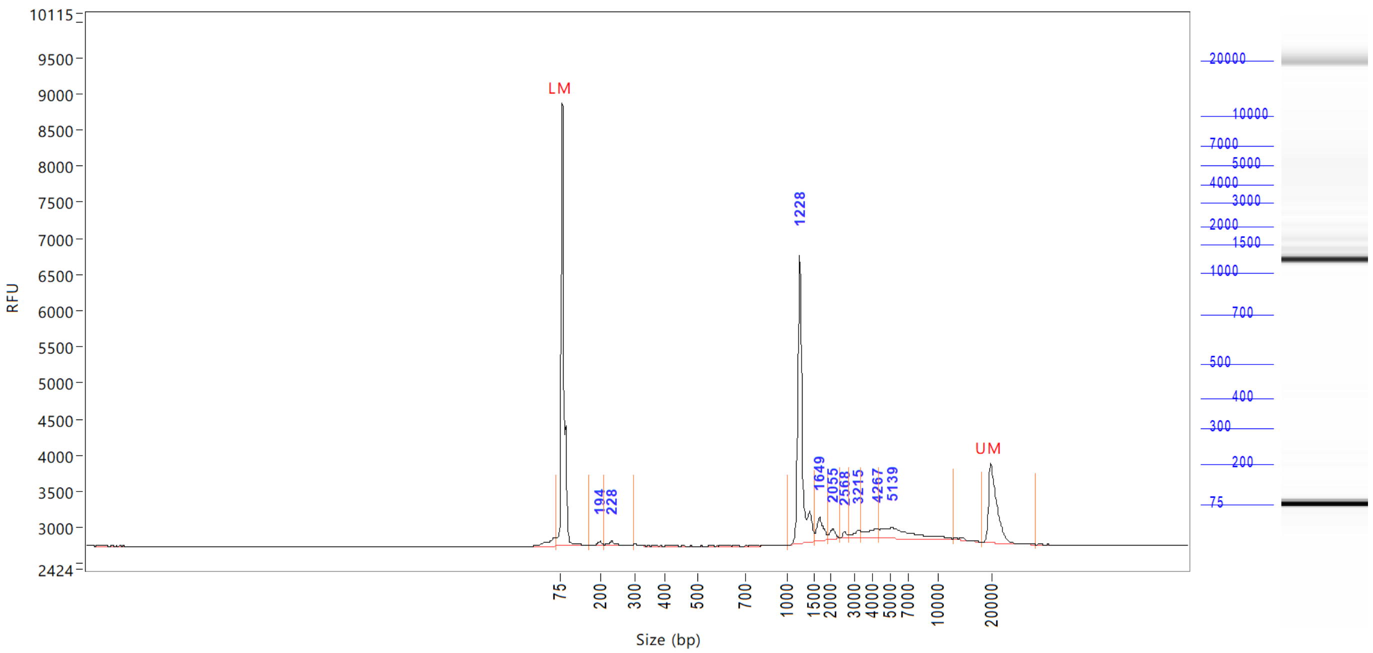

Peak	Size (bp)	Concentration (ng/uL)	From (bp)	To (bp)	Average size (bp)	CV%	RFU	Corrected peak area
1	75 (LM)	0.5000	60	160	77	10.67	6139	45.480
2	194	0.0812	160	206	190	5.31	55	0.615
3	228	0.1202	206	293	234	7.60	62	0.911
4	1228	3.9679	1012	1519	1245	6.19	3991	30.077
5	1649	0.4353	1519	1908	1675	5.71	323	3.300
6	2055	0.1629	1908	2379	2042	4.33	140	1.235
7	2568	0.0748	2379	2757	2579	3.54	89	0.567
8	3215	0.1701	2757	3429	3123	5.97	109	1.289
9	4267	0.3309	3429	4401	3958	7.18	129	2.508
10	5139	0.8639	4401	12675	6475	29.58	151	6.548
11	20000 (UM)	0.1281	18372	28649	20578	5.23	1097	11.654

TIC:	6.2071	ng/uL
TIM:	7.8467	nmole/L
Total concentration:	6.3513	ng/uL

```
Sample peak width (sec): 5    Sample min peak height: 50    Sample baseline V to V?: Y    Sample baseline V to V points: 3
Sample filter: Binomial       Number of points for filter: 3  Sample start region (min): 0  Sample end region (min): 30
Marker peak width (sec): 5    Marker min peak height: 200   Marker baseline V to V?: Y    Marker baseline V to V points: 3
Lower marker selection: First peak > 500 RFU                Upper marker selection: Last peak > 200 RFU
Ladder size (bp)75, 200, 300, 400, 500, 700, 1000, 1500, 2000, 3000, 4000, 5000, 7000, 10000, 20000
Quantification using: Lower Marker          Final concentration (ng/uL): 0.5000          Dilution factor: 12.0
```

Sample: SampF10
Well location: F10
Created: Tuesday, August 10, 2021 7:21:14 PM

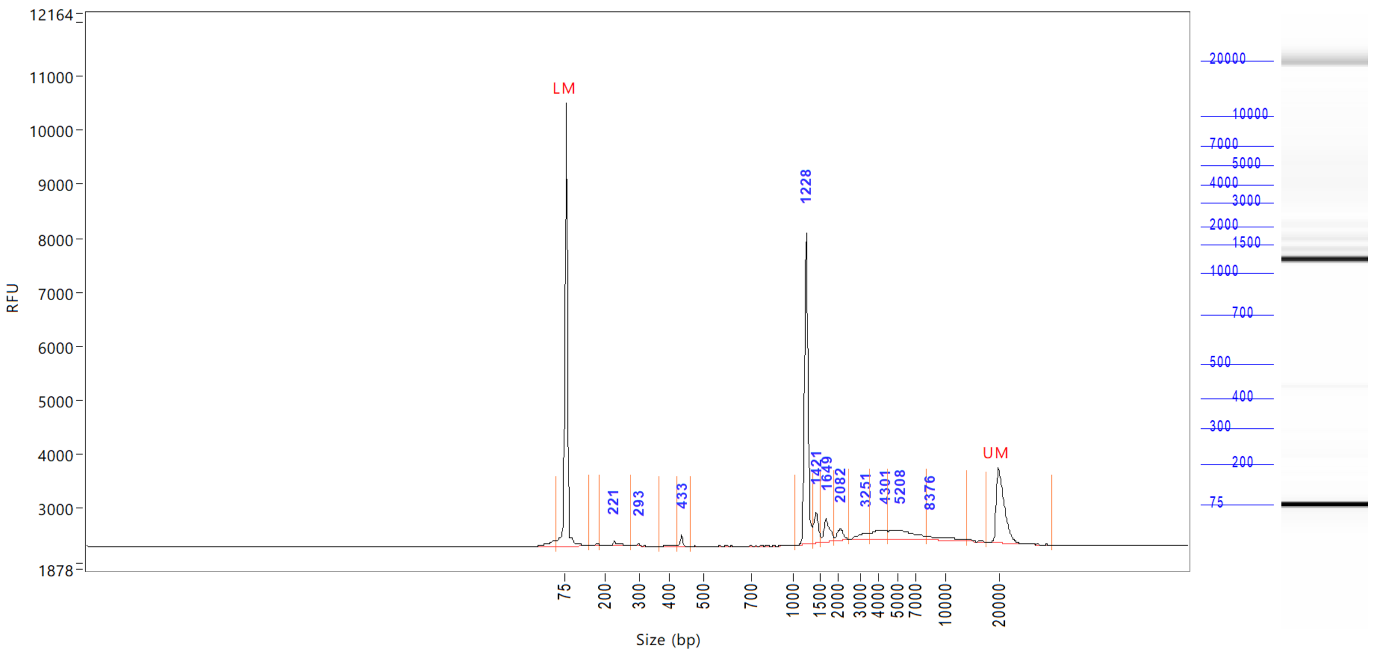

Peak	Size (bp)	Concentration (ng/uL)	From (bp)	To (bp)	Average size (bp)	CV%	RFU	Corrected peak area
1	75 (LM)	0.5000	45	148	73	10.84	8214	54.385
2	221	0.1496	181	271	227	8.46	82	1.356
3	293	0.0427	271	361	294	6.01	52	0.387
4	433	0.1193	422	461	433	1.30	204	1.081
5	1228	3.6269	1024	1353	1225	2.97	5759	32.874
6	1421	0.4676	1353	1501	1412	2.68	572	4.239
7	1649	0.4469	1501	1908	1674	5.58	446	4.051
8	2082	0.2211	1908	2460	2071	4.89	203	2.005
9	3251	0.2312	2460	3500	3095	8.11	121	2.096
10	4301	0.3966	3500	4467	4009	6.85	170	3.594
11	5208	0.6616	4467	8126	5769	17.99	166	5.997
12	8376	0.2171	8126	13954	10222	15.50	62	1.968
13	20000 (UM)	0.1303	17675	30033	20483	4.87	1394	14.168

TIC:	6.5807	ng/uL
TIM:	8.3195	nmole/L
Total concentration:	6.6805	ng/uL

```
Sample peak width (sec): 5    Sample min peak height: 50    Sample baseline V to V?: Y    Sample baseline V to V points: 3
Sample filter: Binomial       Number of points for filter: 3  Sample start region (min): 0  Sample end region (min): 30
Marker peak width (sec): 5    Marker min peak height: 200   Marker baseline V to V?: Y    Marker baseline V to V points: 3
Lower marker selection: First peak > 500 RFU                Upper marker selection: Last peak > 200 RFU
Ladder size (bp)75, 200, 300, 400, 500, 700, 1000, 1500, 2000, 3000, 4000, 5000, 7000, 10000, 20000
Quantification using: Lower Marker          Final concentration (ng/uL): 0.5000          Dilution factor: 12.0
```

Sample: SampG10
Well location: G10
Created: Tuesday, August 10, 2021 7:21:14 PM

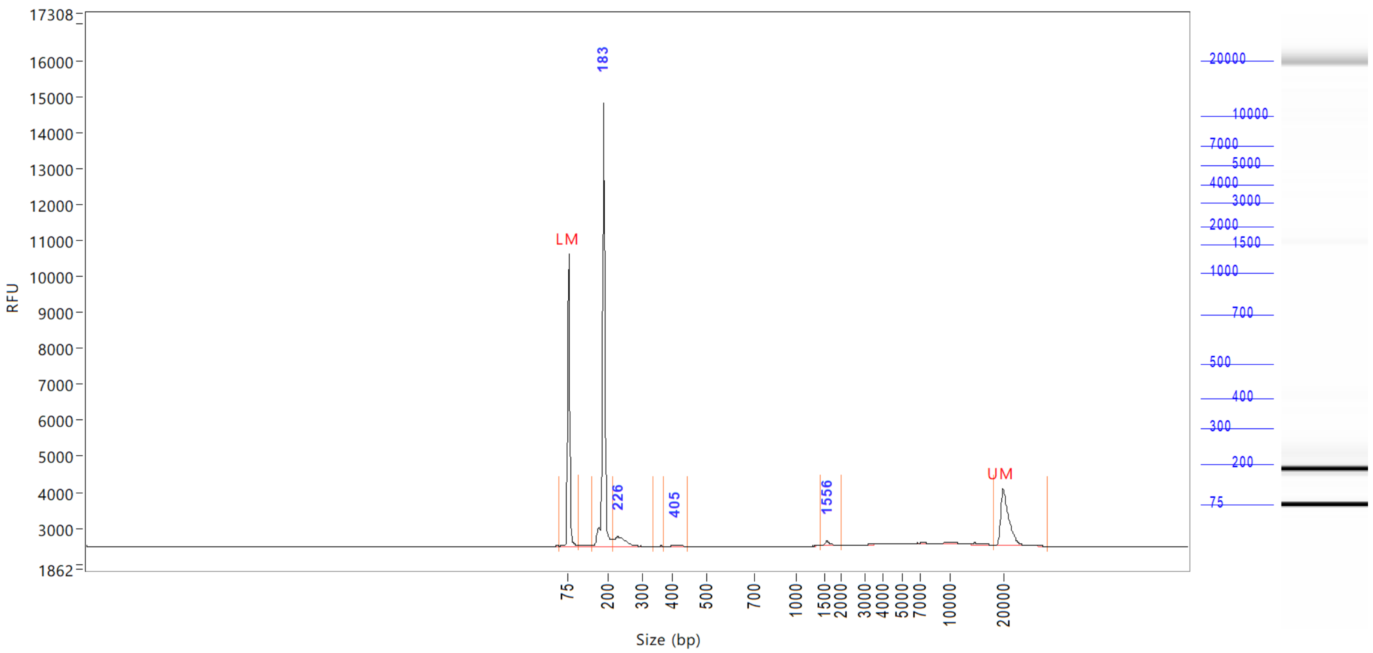

Peak	Size (bp)	Concentration (ng/uL)	From (bp)	To (bp)	Average size (bp)	CV%	RFU	Corrected peak area
1	75 (LM)	0.5000	47	107	73	5.20	8133	43.886
2	183	9.8799	148	212	181	3.79	12338	72.266
3	226	0.9173	212	332	236	7.69	262	6.709
4	405	0.1290	371	444	407	3.23	52	0.943
5	1556	0.1458	1421	1982	1613	7.36	119	1.066
6	20000 (UM)	0.1812	18140	28303	20508	4.59	1581	15.906

TIC:	11.0719	ng/uL
TIM:	96.8002	nmole/L
Total concentration:	11.6461	ng/uL

Sample peak width (sec): 5 Sample min peak height: 50 Sample baseline V to V?: Y Sample baseline V to V points: 3
Sample filter: Binomial Number of points for filter: 3 Sample start region (min): 0 Sample end region (min): 30
Marker peak width (sec): 5 Marker min peak height: 200 Marker baseline V to V?: Y Marker baseline V to V points: 3
Lower marker selection: First peak > 500 RFU Upper marker selection: Last peak > 200 RFU
Ladder size (bp)75, 200, 300, 400, 500, 700, 1000, 1500, 2000, 3000, 4000, 5000, 7000, 10000, 20000
Quantification using: Lower Marker Final concentration (ng/uL): 0.5000 Dilution factor: 12.0

Sample: SampH10
Well location: H10
Created: Tuesday, August 10, 2021 7:21:14 PM

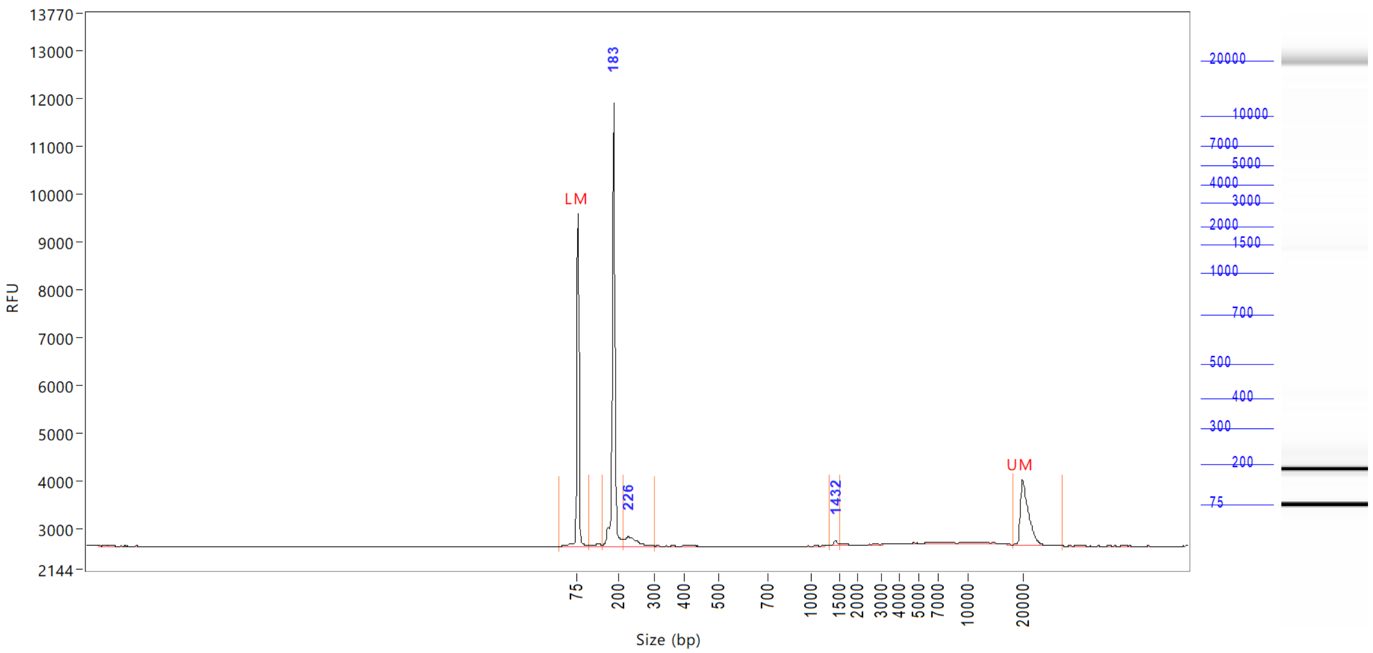

Peak	Size (bp)	Concentration (ng/uL)	From (bp)	To (bp)	Average size (bp)	CV%	RFU	Corrected peak area
1	75 (LM)	0.5000	22	110	73	7.13	6944	37.369
2	183	8.8766	150	212	181	3.85	9284	55.284
3	226	0.7840	212	303	235	7.38	192	4.883
4	1432	0.1106	1330	1501	1426	2.69	101	0.689
5	20000 (UM)	0.1831	18140	27381	20526	4.49	1362	13.682

TIC:	9.7713	ng/uL
TIM:	86.2394	nmole/L
Total concentration:	10.5911	ng/uL

```
Sample peak width (sec): 5    Sample min peak height: 50    Sample baseline V to V?: Y    Sample baseline V to V points: 3
Sample filter: Binomial       Number of points for filter: 3  Sample start region (min): 0  Sample end region (min): 30
Marker peak width (sec): 5    Marker min peak height: 200   Marker baseline V to V?: Y    Marker baseline V to V points: 3
Lower marker selection: First peak > 500 RFU                Upper marker selection: Last peak > 200 RFU
Ladder size (bp)75, 200, 300, 400, 500, 700, 1000, 1500, 2000, 3000, 4000, 5000, 7000, 10000, 20000
Quantification using: Lower Marker          Final concentration (ng/uL): 0.5000          Dilution factor: 12.0
```

Sample: SampA11
Well location: A11
Created: Tuesday, August 10, 2021 7:21:14 PM

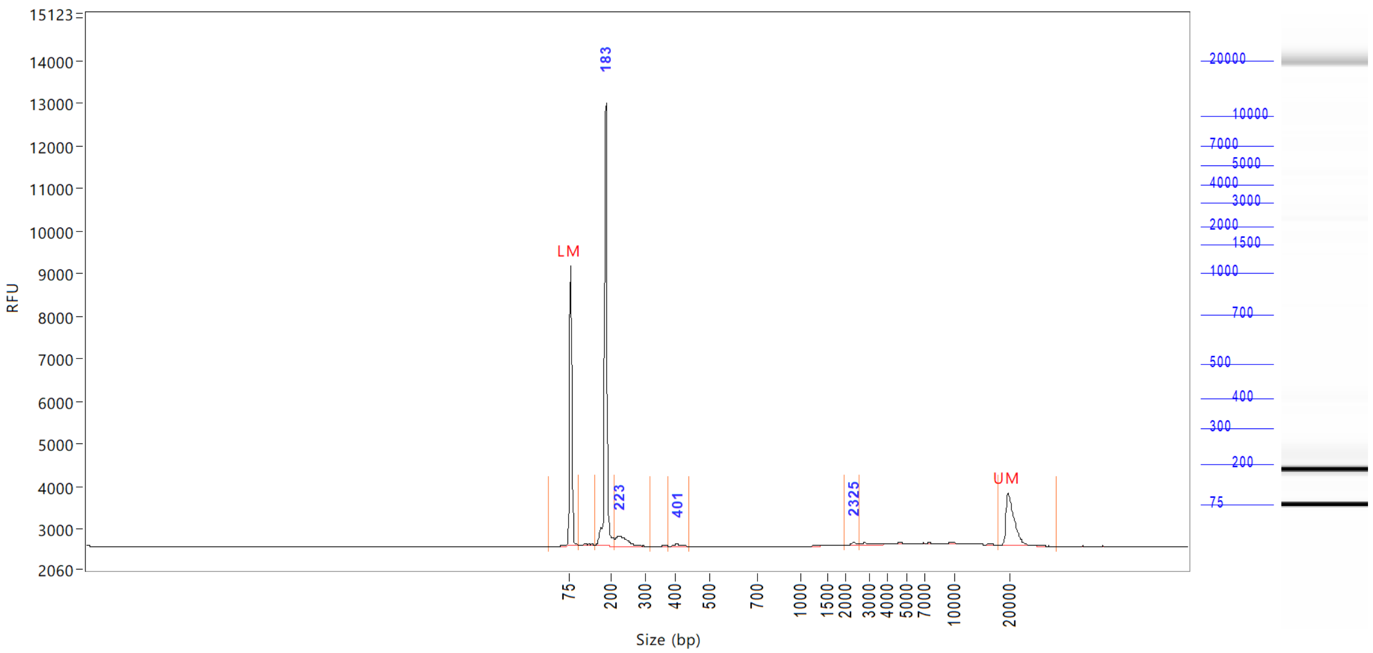

Peak	Size (bp)	Concentration (ng/uL)	From (bp)	To (bp)	Average size (bp)	CV%	RFU	Corrected peak area
1	75 (LM)	0.5000	11	101	73	5.28	6572	35.219
2	183	10.5948	150	208	180	3.49	10427	62.190
3	223	1.0353	208	313	232	7.64	230	6.077
4	401	0.1547	375	438	405	3.05	50	0.908
5	2325	0.0975	1963	2568	2321	5.26	81	0.572
6	20000 (UM)	0.1826	18023	28995	20530	5.20	1248	12.861

TIC:	11.8822	ng/uL
TIM:	104.8009	nmole/L
Total concentration:	12.5570	ng/uL

Sample peak width (sec): 5   Sample min peak height: 50   Sample baseline V to V?: Y   Sample baseline V to V points: 3
Sample filter: Binomial   Number of points for filter: 3   Sample start region (min): 0   Sample end region (min): 30
Marker peak width (sec): 5   Marker min peak height: 200   Marker baseline V to V?: Y   Marker baseline V to V points: 3
Lower marker selection: First peak > 500 RFU   Upper marker selection: Last peak > 200 RFU
Ladder size (bp)75, 200, 300, 400, 500, 700, 1000, 1500, 2000, 3000, 4000, 5000, 7000, 10000, 20000
Quantification using: Lower Marker   Final concentration (ng/uL): 0.5000   Dilution factor: 12.0

Sample: SampB11
Well location: B11
Created: Tuesday, August 10, 2021 7:21:14 PM

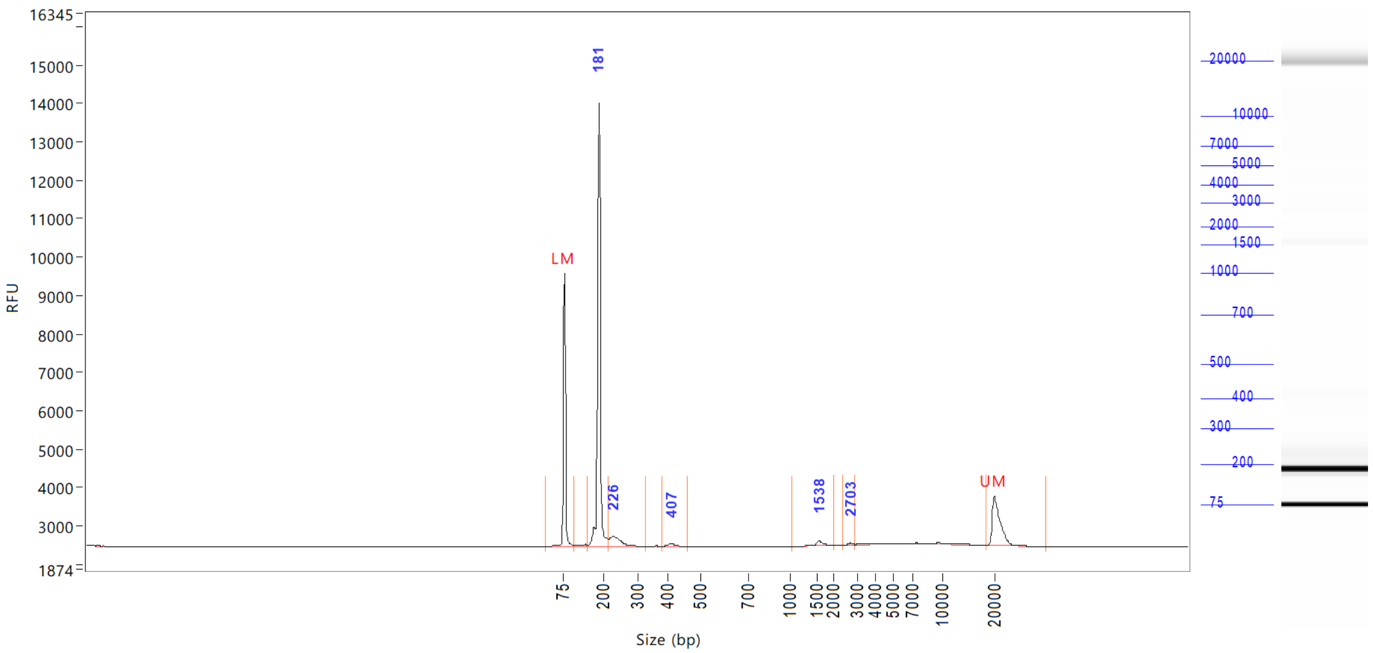

Peak	Size (bp)	Concentration (ng/uL)	From (bp)	To (bp)	Average size (bp)	CV%	RFU	Corrected peak area
1	75 (LM)	0.5000	20	105	73	6.20	7117	38.741
2	181	11.0196	148	212	181	3.96	11562	71.152
3	226	1.0532	212	324	236	7.89	264	6.800
4	407	0.1822	381	461	410	3.42	67	1.177
5	1538	0.1942	1024	2000	1567	7.84	141	1.254
6	2703	0.0517	2379	2919	2671	3.75	50	0.334
7	20000 (UM)	0.1664	18489	29918	20524	5.04	1279	12.895

TIC:	12.5010	ng/uL
TIM:	108.3959	nmole/L
Total concentration:	13.1022	ng/uL

Sample peak width (sec): 5   Sample min peak height: 50   Sample baseline V to V?: Y   Sample baseline V to V points: 3
Sample filter: Binomial   Number of points for filter: 3 Sample start region (min): 0 Sample end region (min): 30
Marker peak width (sec): 5   Marker min peak height: 200   Marker baseline V to V?: Y   Marker baseline V to V points: 3
Lower marker selection: First peak > 500 RFU   Upper marker selection: Last peak > 200 RFU
Ladder size (bp)75, 200, 300, 400, 500, 700, 1000, 1500, 2000, 3000, 4000, 5000, 7000, 10000, 20000
Quantification using: Lower Marker   Final concentration (ng/uL): 0.5000   Dilution factor: 12.0

Sample: SampC11
Well location: C11
Created: Tuesday, August 10, 2021 7:21:14 PM

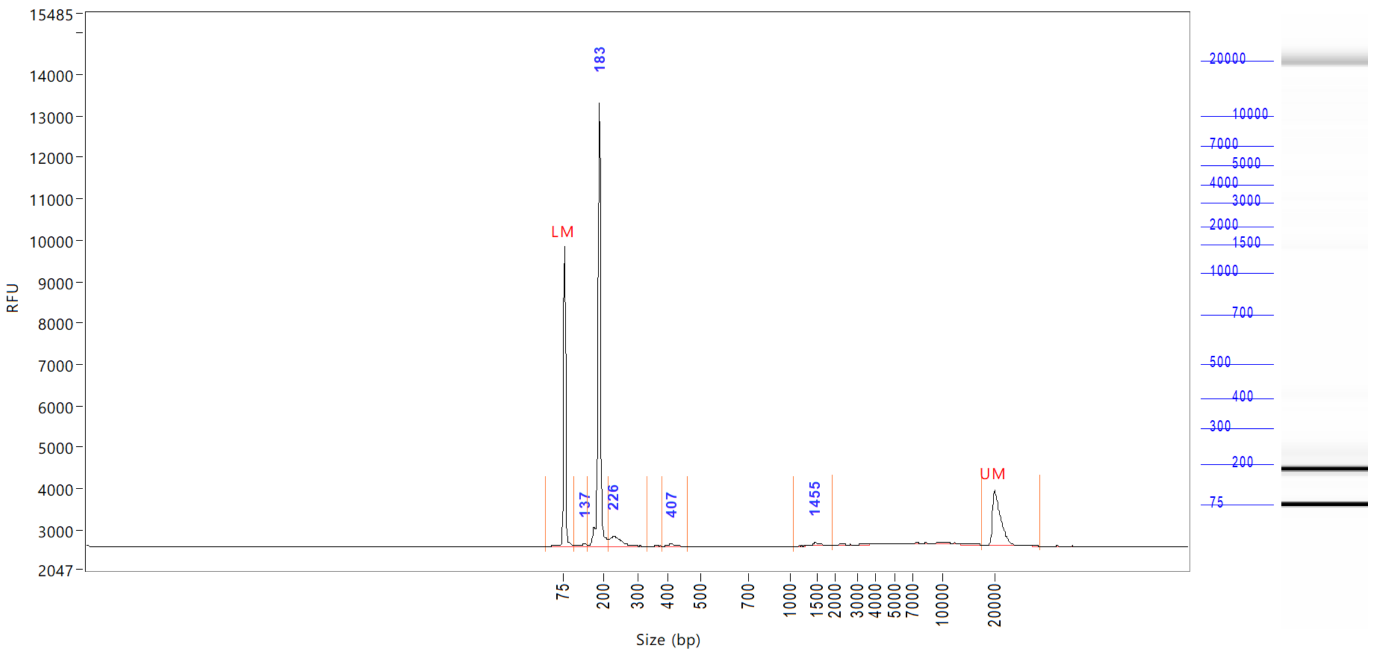

Peak	Size (bp)	Concentration (ng/uL)	From (bp)	To (bp)	Average size (bp)	CV%	RFU	Corrected peak area
1	75 (LM)	0.5000	18	105	73	6.40	7254	39.798
2	137	0.1481	105	148	129	9.17	50	0.982
3	183	9.5467	148	214	181	4.03	10736	63.323
4	226	0.8956	214	328	237	7.53	239	5.941
5	407	0.1478	381	459	411	3.43	60	0.980
6	1455	0.1395	1058	1926	1509	8.83	88	0.925
7	20000 (UM)	0.1740	17558	28534	20484	4.73	1328	13.847

TIC:	10.8776	ng/uL
TIM:	95.5555	nmole/L
Total concentration:	11.4242	ng/uL

Sample peak width (sec): 5   Sample min peak height: 50   Sample baseline V to V?: Y   Sample baseline V to V points: 3
Sample filter: Binomial   Number of points for filter: 3 Sample start region (min): 0 Sample end region (min): 30
Marker peak width (sec): 5   Marker min peak height: 200   Marker baseline V to V?: Y   Marker baseline V to V points: 3
Lower marker selection: First peak > 500 RFU   Upper marker selection: Last peak > 200 RFU
Ladder size (bp)75, 200, 300, 400, 500, 700, 1000, 1500, 2000, 3000, 4000, 5000, 7000, 10000, 20000
Quantification using: Lower Marker   Final concentration (ng/uL): 0.5000   Dilution factor: 12.0

Sample: SampD11
Well location: D11
Created: Tuesday, August 10, 2021 7:21:14 PM

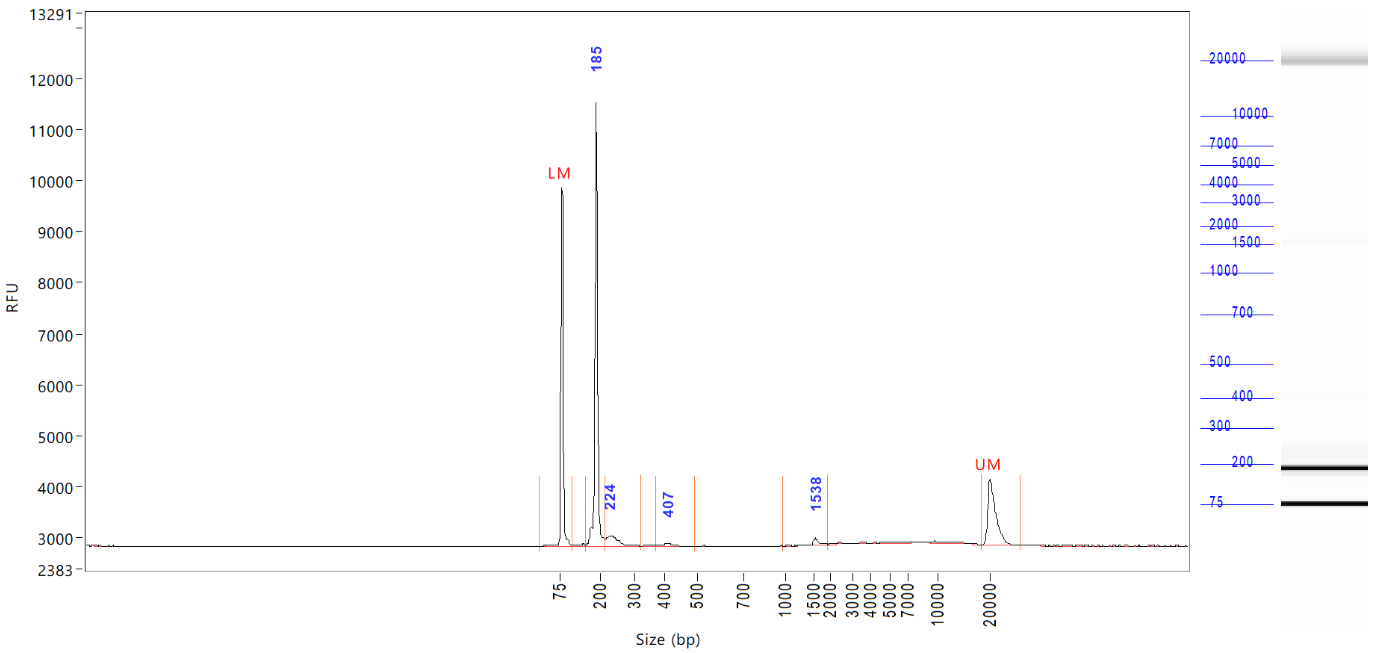

Peak	Size (bp)	Concentration (ng/uL)	From (bp)	To (bp)	Average size (bp)	CV%	RFU	Corrected peak area
1	75 (LM)	0.5000	7	108	74	7.70	7022	40.495
2	185	7.8562	152	210	182	3.64	8713	53.023
3	224	0.7817	210	320	235	8.03	200	5.276
4	407	0.1456	369	494	410	4.52	55	0.983
5	1538	0.1688	982	1926	1528	10.20	134	1.139
6	20000 (UM)	0.1638	18489	25881	20541	4.57	1279	13.270

TIC:	8.9523	ng/uL
TIM:	77.2018	nmole/L
Total concentration:	9.5647	ng/uL

Sample peak width (sec): 5   Sample min peak height: 50   Sample baseline V to V?: Y   Sample baseline V to V points: 3
Sample filter: Binomial   Number of points for filter: 3 Sample start region (min): 0 Sample end region (min): 30
Marker peak width (sec): 5   Marker min peak height: 200   Marker baseline V to V?: Y   Marker baseline V to V points: 3
Lower marker selection: First peak > 500 RFU   Upper marker selection: Last peak > 200 RFU
Ladder size (bp)75, 200, 300, 400, 500, 700, 1000, 1500, 2000, 3000, 4000, 5000, 7000, 10000, 20000
Quantification using: Lower Marker   Final concentration (ng/uL): 0.5000   Dilution factor: 12.0

Sample: SampE11
Well location: E11
Created: Tuesday, August 10, 2021 7:21:14 PM

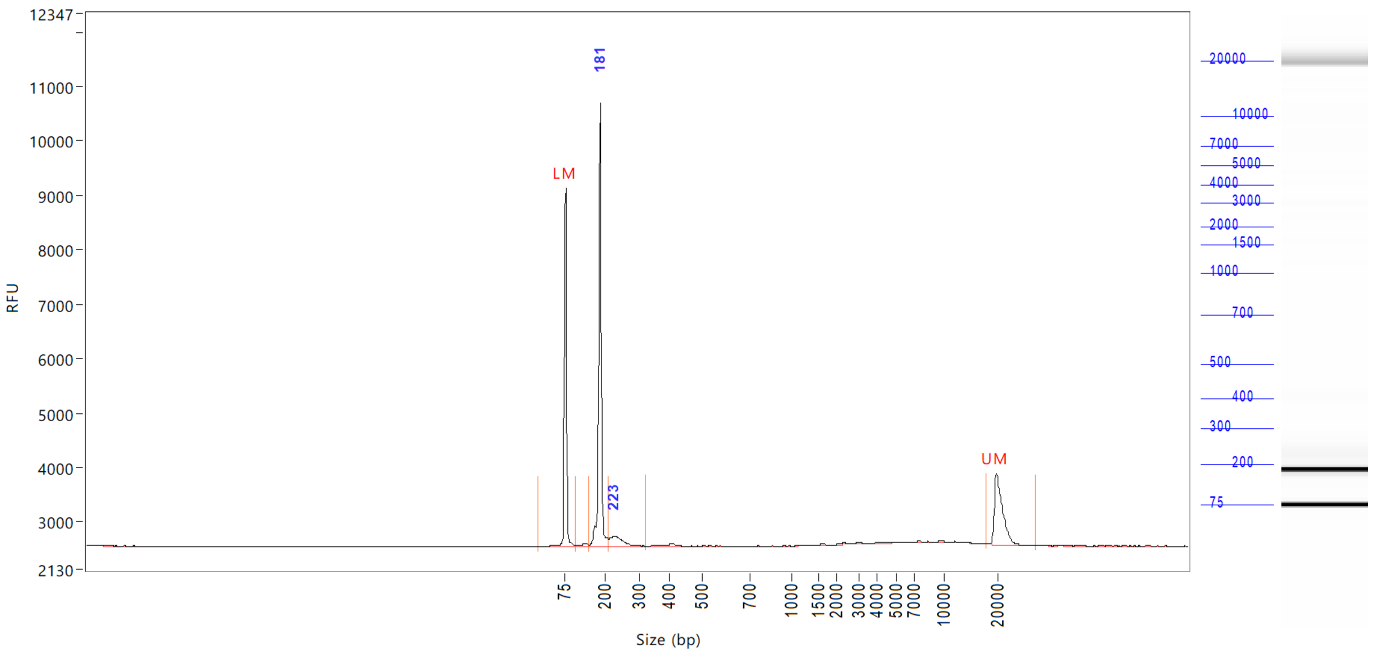

Peak	Size (bp)	Concentration (ng/uL)	From (bp)	To (bp)	Average size (bp)	CV%	RFU	Corrected peak area
1	75 (LM)	0.5000	0	105	72	8.44	6580	37.013
2	181	7.8385	147	208	179	3.86	8162	48.355
3	223	0.8268	208	320	234	8.14	189	5.100
4	20000 (UM)	0.1826	17907	27381	20487	4.65	1306	13.521

TIC:	8.6653	ng/uL
TIM:	77.8014	nmole/L
Total concentration:	9.3870	ng/uL

Sample peak width (sec): 5   Sample min peak height: 50   Sample baseline V to V?: Y   Sample baseline V to V points: 3
Sample filter: Binomial   Number of points for filter: 3 Sample start region (min): 0 Sample end region (min): 30
Marker peak width (sec): 5   Marker min peak height: 200   Marker baseline V to V?: Y   Marker baseline V to V points: 3
Lower marker selection: First peak > 500 RFU   Upper marker selection: Last peak > 200 RFU
Ladder size (bp)75, 200, 300, 400, 500, 700, 1000, 1500, 2000, 3000, 4000, 5000, 7000, 10000, 20000
Quantification using: Lower Marker   Final concentration (ng/uL): 0.5000   Dilution factor: 12.0

Sample: SampF11
Well location: F11
Created: Tuesday, August 10, 2021 7:21:14 PM

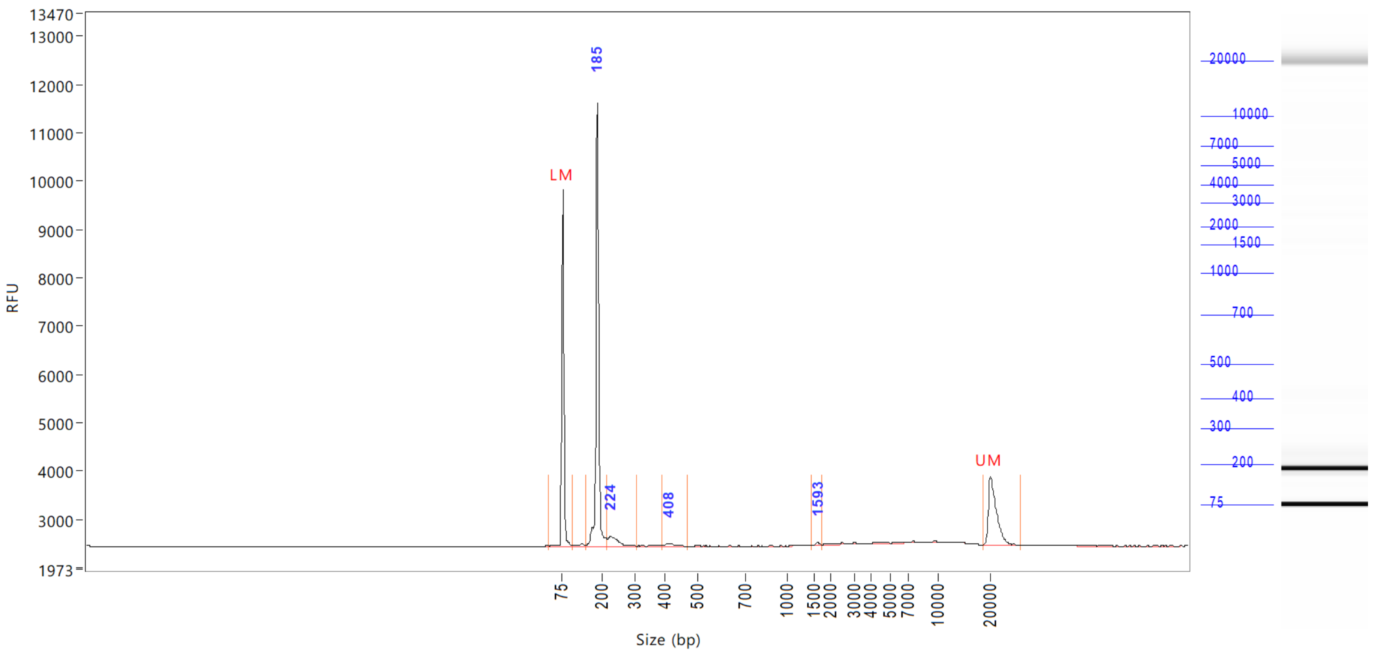

Peak	Size (bp)	Concentration (ng/uL)	From (bp)	To (bp)	Average size (bp)	CV%	RFU	Corrected peak area
1	75 (LM)	0.5000	33	107	74	5.49	7365	40.668
2	185	8.1421	148	214	183	3.95	9180	55.186
3	224	0.6395	214	303	234	6.65	194	4.335
4	408	0.1382	388	466	412	3.39	54	0.936
5	1593	0.0477	1455	1723	1582	3.84	51	0.324
6	20000 (UM)	0.1821	18605	25766	20604	4.53	1424	14.809

TIC:	8.9675	ng/uL
TIM:	78.2423	nmole/L
Total concentration:	9.5752	ng/uL

Sample peak width (sec): 5   Sample min peak height: 50   Sample baseline V to V?: Y   Sample baseline V to V points: 3
Sample filter: Binomial   Number of points for filter: 3 Sample start region (min): 0 Sample end region (min): 30
Marker peak width (sec): 5   Marker min peak height: 200   Marker baseline V to V?: Y   Marker baseline V to V points: 3
Lower marker selection: First peak > 500 RFU   Upper marker selection: Last peak > 200 RFU
Ladder size (bp)75, 200, 300, 400, 500, 700, 1000, 1500, 2000, 3000, 4000, 5000, 7000, 10000, 20000
Quantification using: Lower Marker   Final concentration (ng/uL): 0.5000   Dilution factor: 12.0

Sample: SampG11
Well location: G11
Created: Tuesday, August 10, 2021 7:21:14 PM

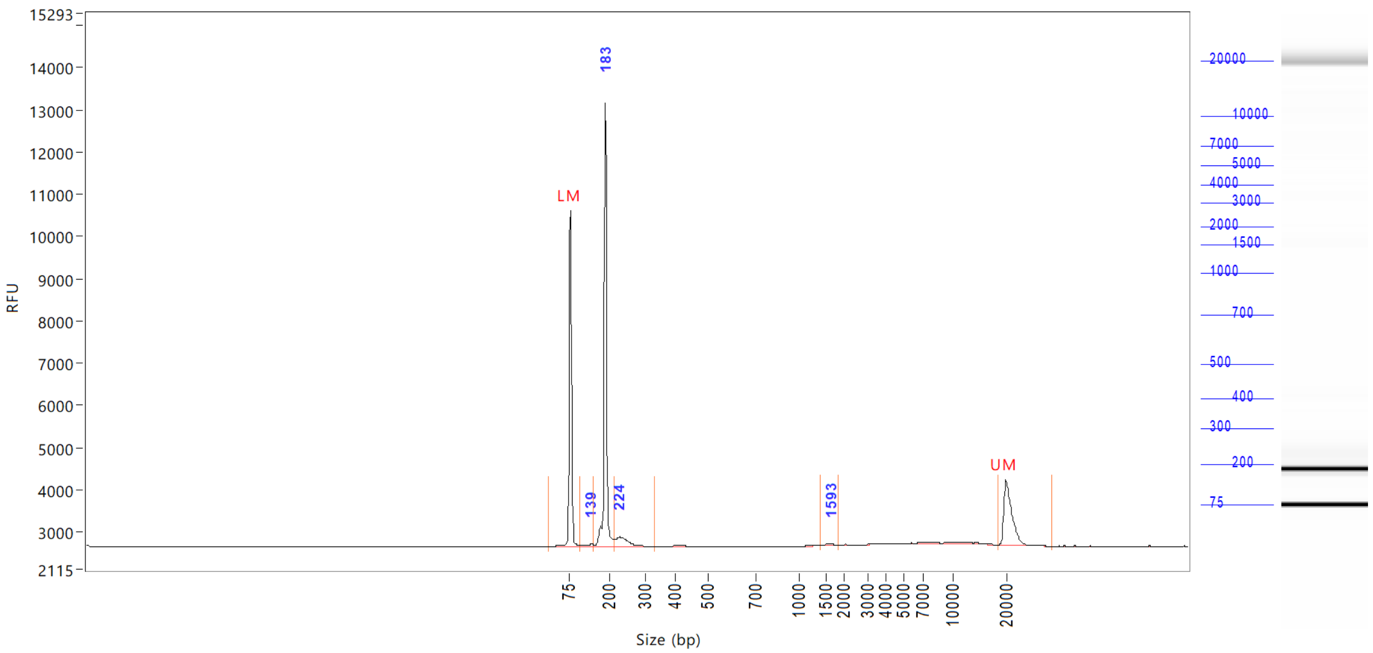

Peak	Size (bp)	Concentration (ng/uL)	From (bp)	To (bp)	Average size (bp)	CV%	RFU	Corrected peak area
1	75 (LM)	0.5000	9	107	72	7.50	7964	43.939
2	139	0.1434	107	148	130	8.92	57	1.050
3	183	8.4182	148	210	181	3.83	10533	61.647
4	224	0.8346	210	330	236	8.20	228	6.112
5	1593	0.0893	1387	1852	1573	6.65	53	0.654
6	20000 (UM)	0.1787	18489	28765	20518	4.57	1539	15.701

TIC:	9.4855	ng/uL
TIM:	84.1837	nmole/L
Total concentration:	10.0549	ng/uL

Sample peak width (sec): 5 Sample min peak height: 50 Sample baseline V to V?: Y Sample baseline V to V points: 3
Sample filter: Binomial Number of points for filter: 3 Sample start region (min): 0 Sample end region (min): 30
Marker peak width (sec): 5 Marker min peak height: 200 Marker baseline V to V?: Y Marker baseline V to V points: 3
Lower marker selection: First peak > 500 RFU Upper marker selection: Last peak > 200 RFU
Ladder size (bp)75, 200, 300, 400, 500, 700, 1000, 1500, 2000, 3000, 4000, 5000, 7000, 10000, 20000
Quantification using: Lower Marker Final concentration (ng/uL): 0.5000 Dilution factor: 12.0

Sample: SampH11
Well location: H11
Created: Tuesday, August 10, 2021 7:21:14 PM

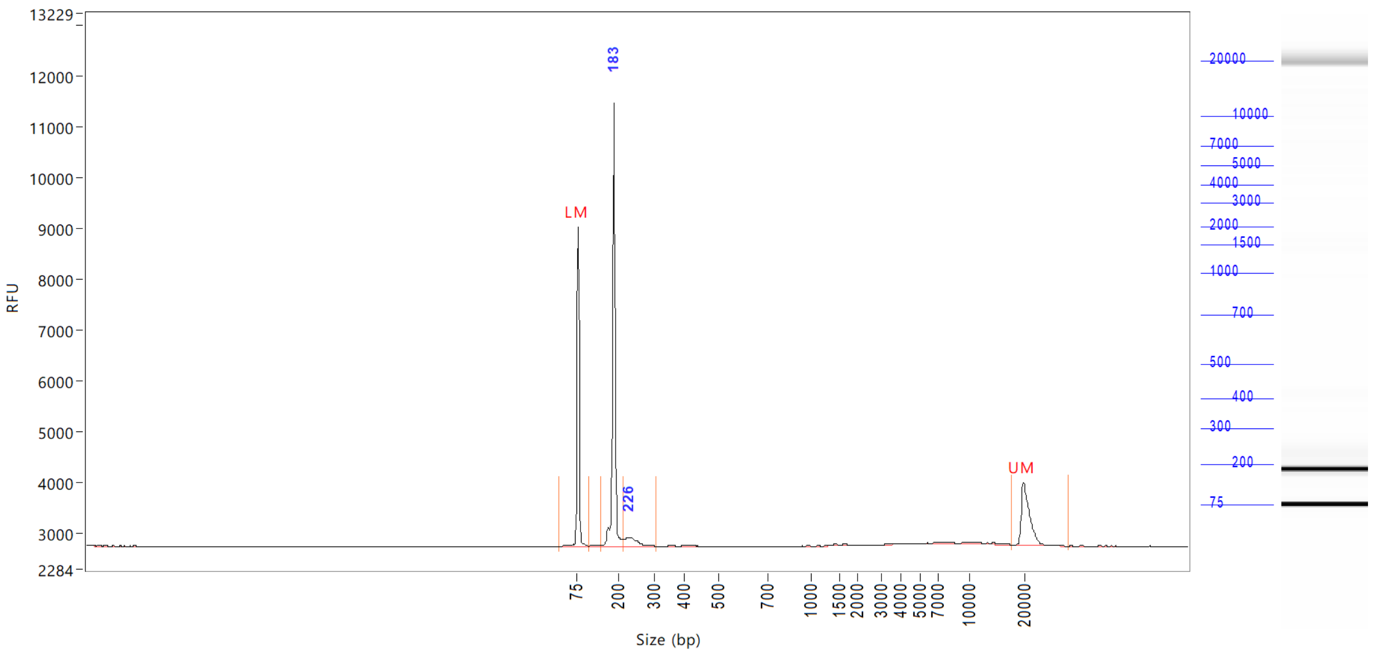

Peak	Size (bp)	Concentration (ng/uL)	From (bp)	To (bp)	Average size (bp)	CV%	RFU	Corrected peak area
1	75 (LM)	0.5000	22	108	73	6.97	6279	34.440
2	183	8.8918	147	212	181	3.80	8743	51.039
3	226	0.8002	212	303	235	7.40	176	4.593
4	20000 (UM)	0.1802	17907	28073	20475	4.62	1236	12.413

TIC:	9.6920	ng/uL
TIM:	86.3629	nmole/L
Total concentration:	10.5873	ng/uL

```
Sample peak width (sec): 5    Sample min peak height: 50    Sample baseline V to V?: Y    Sample baseline V to V points: 3
Sample filter: Binomial       Number of points for filter: 3  Sample start region (min): 0  Sample end region (min): 30
Marker peak width (sec): 5    Marker min peak height: 200   Marker baseline V to V?: Y    Marker baseline V to V points: 3
Lower marker selection: First peak > 500 RFU                Upper marker selection: Last peak > 200 RFU
Ladder size (bp)75, 200, 300, 400, 500, 700, 1000, 1500, 2000, 3000, 4000, 5000, 7000, 10000, 20000
Quantification using: Lower Marker          Final concentration (ng/uL): 0.5000          Dilution factor: 12.0
```

Sample: SampA12
Well location: A12
Created: Tuesday, August 10, 2021 7:21:14 PM

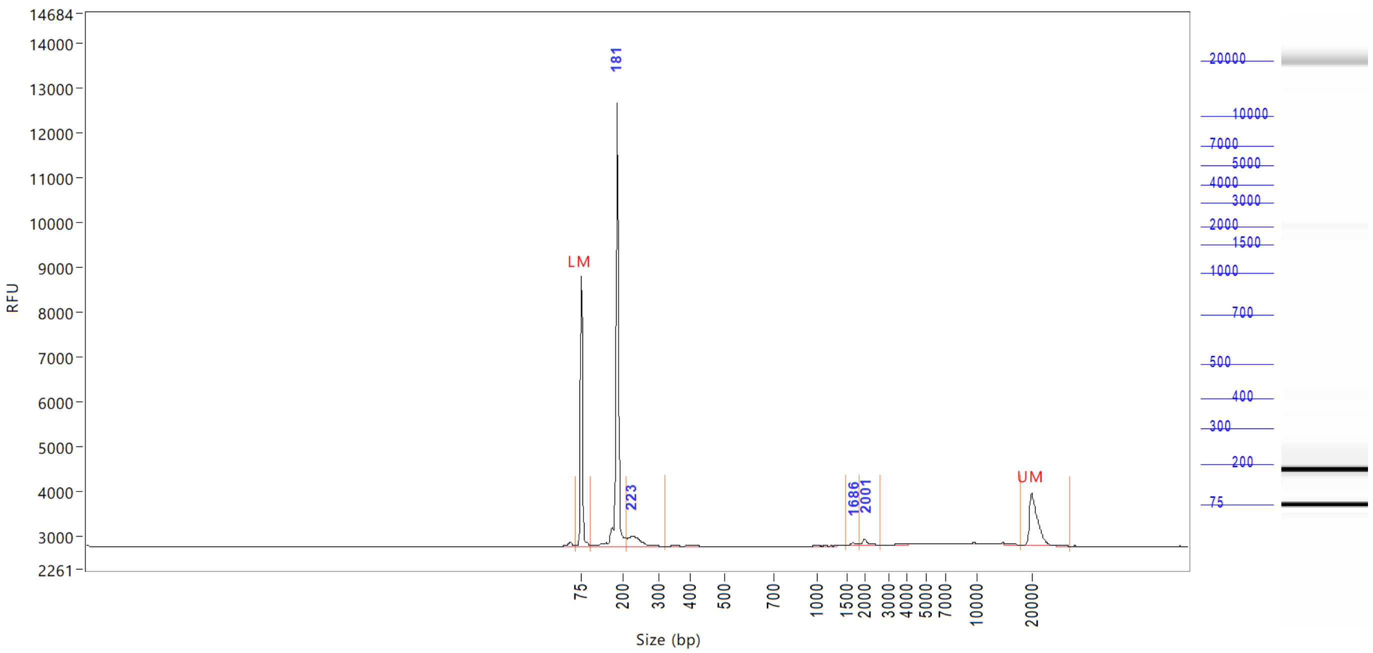

Peak	Size (bp)	Concentration (ng/uL)	From (bp)	To (bp)	Average size (bp)	CV%	RFU	Corrected peak area
1	75 (LM)	0.5000	60	103	73	5.50	6041	32.327
2	181	11.1408	103	208	179	5.64	9925	60.024
3	223	1.1245	208	320	233	7.90	229	6.059
4	1686	0.1075	1489	1852	1677	5.16	62	0.579
5	2001	0.2244	1852	2649	2060	7.50	140	1.209
6	20000 (UM)	0.1818	18023	26919	20491	4.69	1183	11.751

TIC:	12.5972	ng/uL
TIM:	110.5415	nmole/L
Total concentration:	13.2972	ng/uL

Sample peak width (sec): 5 Sample min peak height: 50 Sample baseline V to V?: Y Sample baseline V to V points: 3
Sample filter: Binomial Number of points for filter: 3 Sample start region (min): 0 Sample end region (min): 30
Marker peak width (sec): 5 Marker min peak height: 200 Marker baseline V to V?: Y Marker baseline V to V points: 3
Lower marker selection: First peak > 500 RFU Upper marker selection: Last peak > 200 RFU
Ladder size (bp)75, 200, 300, 400, 500, 700, 1000, 1500, 2000, 3000, 4000, 5000, 7000, 10000, 20000
Quantification using: Lower Marker Final concentration (ng/uL): 0.5000 Dilution factor: 12.0

Sample: SampB12
Well location: B12
Created: Tuesday, August 10, 2021 7:21:14 PM

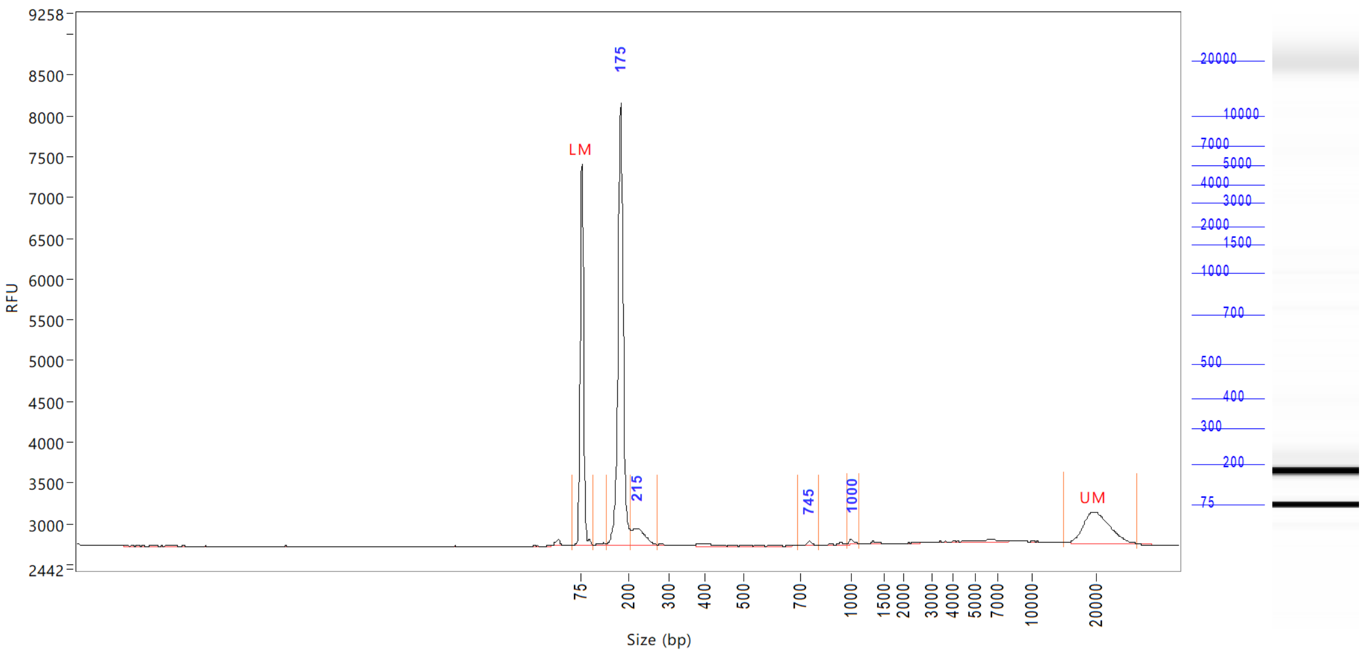

Peak	Size (bp)	Concentration (ng/uL)	From (bp)	To (bp)	Average size (bp)	CV%	RFU	Corrected peak area
1	75 (LM)	0.5000	48	103	73	6.23	4669	38.105
2	175	9.8917	139	205	174	4.87	5432	62.820
3	215	0.8961	205	268	224	6.31	210	5.691
4	745	0.0745	690	807	741	2.86	53	0.473
5	1000	0.0759	974	1114	1012	3.07	66	0.482
6	20000 (UM)	0.1511	15117	26689	20390	9.68	379	11.515

TIC:	10.9382	ng/uL
TIM:	100.3215	nmole/L
Total concentration:	11.5962	ng/uL

Sample peak width (sec): 5   Sample min peak height: 50   Sample baseline V to V?: Y   Sample baseline V to V points: 3
Sample filter: Binomial   Number of points for filter: 3 Sample start region (min): 0 Sample end region (min): 30
Marker peak width (sec): 5   Marker min peak height: 200   Marker baseline V to V?: Y   Marker baseline V to V points: 3
Lower marker selection: First peak > 500 RFU   Upper marker selection: Last peak > 200 RFU
Ladder size (bp)75, 200, 300, 400, 500, 700, 1000, 1500, 2000, 3000, 4000, 5000, 7000, 10000, 20000
Quantification using: Lower Marker   Final concentration (ng/uL): 0.5000   Dilution factor: 12.0

Sample: SampC12
Well location: C12
Created: Tuesday, August 10, 2021 7:21:14 PM

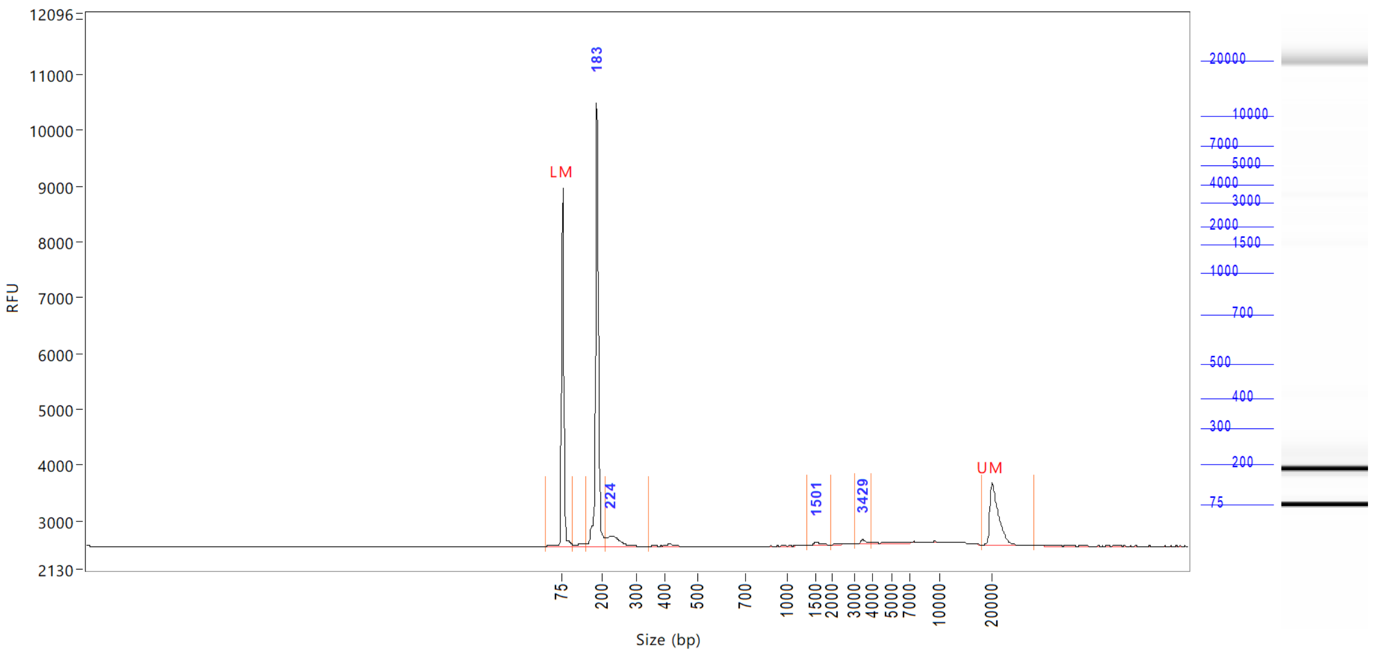

Peak	Size<br>(bp)	Concentration<br>(ng/uL)	From<br>(bp)	To<br>(bp)	Average size<br>(bp)	CV%	RFU	Corrected peak area
1	75 (LM)	0.5000	22	108	74	7.06	6414	37.917
2	183	8.4014	148	208	181	3.90	7960	53.093
3	224	0.8596	208	342	234	8.35	189	5.432
4	1501	0.0970	1364	1982	1573	7.63	55	0.613
5	3429	0.0992	3000	3893	3457	4.99	84	0.627
6	20000 (UM)	0.1653	18140	28073	20665	5.41	1126	12.533

TIC:	9.4571	ng/uL
TIM:	82.4984	nmole/L
Total concentration:	10.1135	ng/uL

Sample peak width (sec): 5   Sample min peak height: 50   Sample baseline V to V?: Y   Sample baseline V to V points: 3
Sample filter: Binomial   Number of points for filter: 3 Sample start region (min): 0 Sample end region (min): 30
Marker peak width (sec): 5   Marker min peak height: 200   Marker baseline V to V?: Y   Marker baseline V to V points: 3
Lower marker selection: First peak > 500 RFU   Upper marker selection: Last peak > 200 RFU
Ladder size (bp)75, 200, 300, 400, 500, 700, 1000, 1500, 2000, 3000, 4000, 5000, 7000, 10000, 20000
Quantification using: Lower Marker   Final concentration (ng/uL): 0.5000   Dilution factor: 12.0

Sample: SampD12
Well location: D12
Created: Tuesday, August 10, 2021 7:21:14 PM

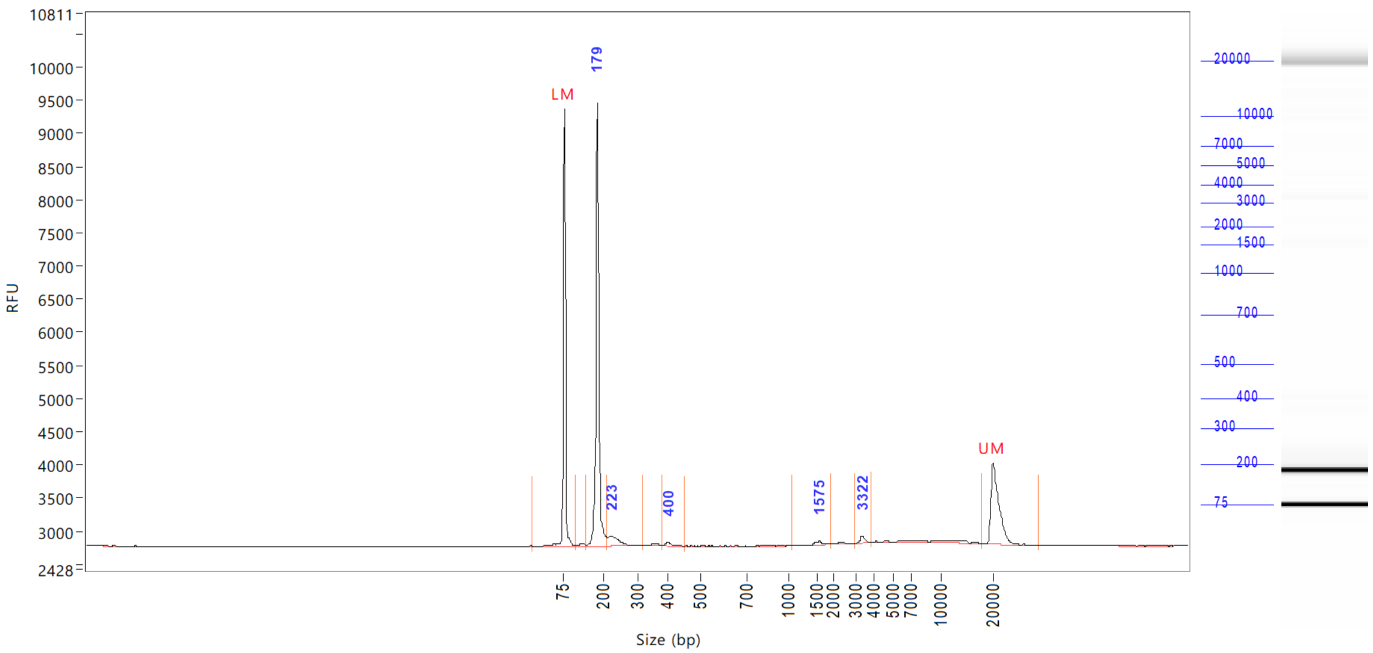

Peak	Size (bp)	Concentration (ng/uL)	From (bp)	To (bp)	Average size (bp)	CV%	RFU	Corrected peak area
1	75 (LM)	0.5000	0	112	73	10.32	6585	36.885
2	179	7.0306	143	208	178	4.18	6693	43.220
3	223	0.6090	208	315	232	8.10	139	3.744
4	400	0.1179	384	450	403	2.90	62	0.725
5	1575	0.1116	1046	1926	1532	8.83	53	0.686
6	3322	0.1276	2919	3857	3335	5.12	105	0.785
7	20000 (UM)	0.1741	17907	28649	20560	5.21	1217	12.844

TIC:	7.9966	ng/uL
TIM:	69.9131	nmole/L
Total concentration:	8.6268	ng/uL

Sample peak width (sec): 5 Sample min peak height: 50 Sample baseline V to V?: Y Sample baseline V to V points: 3
Sample filter: Binomial Number of points for filter: 3 Sample start region (min): 0 Sample end region (min): 30
Marker peak width (sec): 5 Marker min peak height: 200 Marker baseline V to V?: Y Marker baseline V to V points: 3
Lower marker selection: First peak > 500 RFU Upper marker selection: Last peak > 200 RFU
Ladder size (bp)75, 200, 300, 400, 500, 700, 1000, 1500, 2000, 3000, 4000, 5000, 7000, 10000, 20000
Quantification using: Lower Marker Final concentration (ng/uL): 0.5000 Dilution factor: 12.0

Sample: SampE12
Well location: E12
Created: Tuesday, August 10, 2021 7:21:14 PM

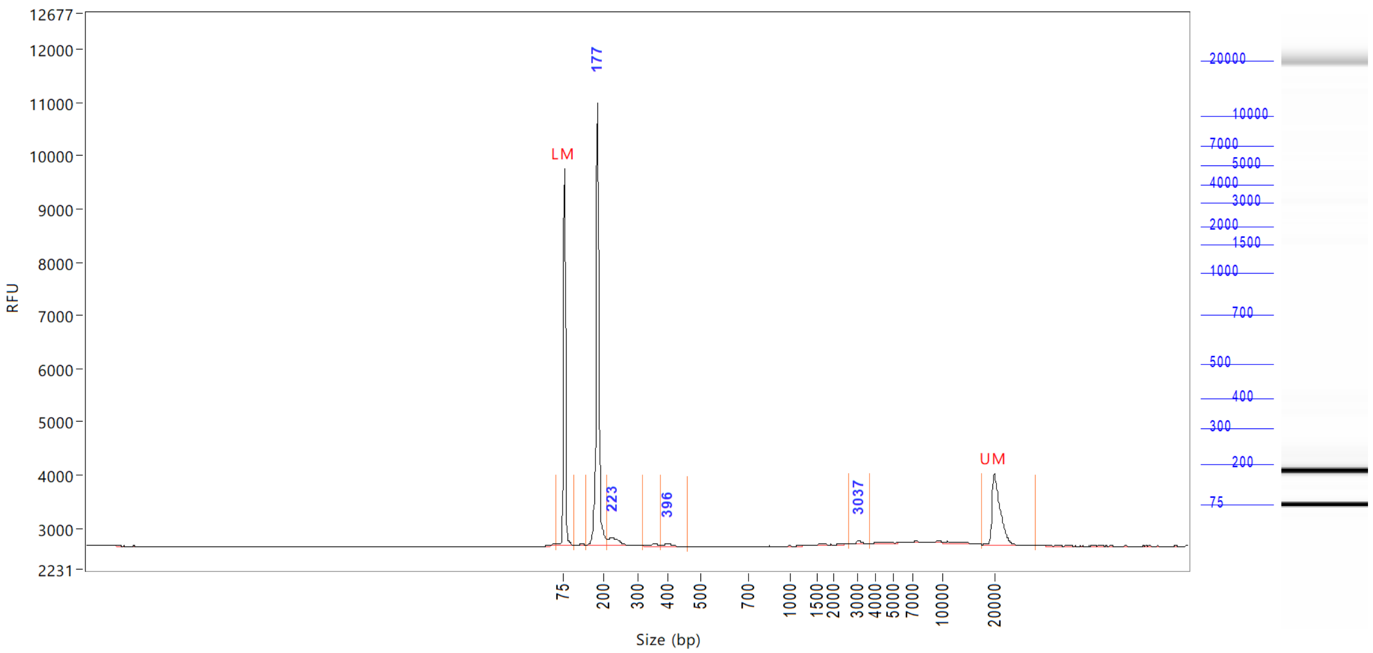

Peak	Size (bp)	Concentration (ng/uL)	From (bp)	To (bp)	Average size (bp)	CV%	RFU	Corrected peak area
1	75 (LM)	0.5000	50	105	73	5.03	7083	38.384
2	177	8.3232	145	210	176	4.20	8330	53.245
3	223	0.5570	210	313	232	7.12	142	3.563
4	396	0.1082	377	461	401	3.55	52	0.692
5	3037	0.0809	2649	3679	3099	5.70	65	0.518
6	20000 (UM)	0.1820	17675	27842	20509	4.74	1343	13.970

TIC:	9.0693	ng/uL
TIM:	82.1781	nmole/L
Total concentration:	9.7683	ng/uL

Sample peak width (sec): 5   Sample min peak height: 50   Sample baseline V to V?: Y   Sample baseline V to V points: 3
Sample filter: Binomial   Number of points for filter: 3 Sample start region (min): 0 Sample end region (min): 30
Marker peak width (sec): 5   Marker min peak height: 200   Marker baseline V to V?: Y   Marker baseline V to V points: 3
Lower marker selection: First peak > 500 RFU   Upper marker selection: Last peak > 200 RFU
Ladder size (bp)75, 200, 300, 400, 500, 700, 1000, 1500, 2000, 3000, 4000, 5000, 7000, 10000, 20000
Quantification using: Lower Marker   Final concentration (ng/uL): 0.5000   Dilution factor: 12.0

Sample: SampF12
Well location: F12
Created: Tuesday, August 10, 2021 7:21:14 PM

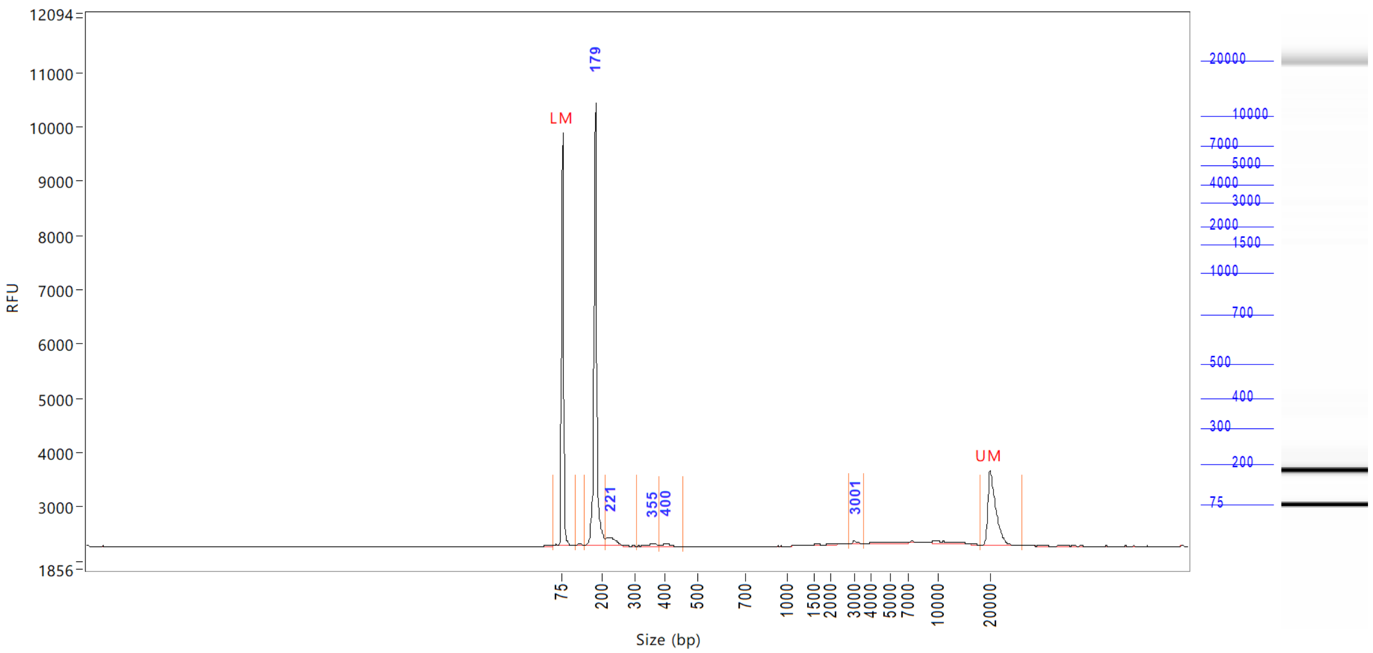

Peak	Size (bp)	Concentration (ng/uL)	From (bp)	To (bp)	Average size (bp)	CV%	RFU	Corrected peak area
1	75 (LM)	0.5000	48	116	73	5.14	7605	41.055
2	179	7.8447	145	208	177	4.06	8166	53.678
3	221	0.5171	208	301	228	6.42	150	3.538
4	355	0.1191	301	377	351	4.13	55	0.815
5	400	0.1176	377	455	401	3.25	57	0.804
6	3001	0.0574	2784	3572	3065	4.74	51	0.393
7	20000 (UM)	0.1713	18256	26112	20489	4.42	1368	14.067

TIC:	8.6560	ng/uL
TIM:	77.6618	nmole/L
Total concentration:	9.2233	ng/uL

Sample peak width (sec): 5   Sample min peak height: 50   Sample baseline V to V?: Y   Sample baseline V to V points: 3
Sample filter: Binomial   Number of points for filter: 3 Sample start region (min): 0 Sample end region (min): 30
Marker peak width (sec): 5   Marker min peak height: 200   Marker baseline V to V?: Y   Marker baseline V to V points: 3
Lower marker selection: First peak > 500 RFU   Upper marker selection: Last peak > 200 RFU
Ladder size (bp)75, 200, 300, 400, 500, 700, 1000, 1500, 2000, 3000, 4000, 5000, 7000, 10000, 20000
Quantification using: Lower Marker   Final concentration (ng/uL): 0.5000   Dilution factor: 12.0

Sample: SampG12
Well location: G12
Created: Tuesday, August 10, 2021 7:21:14 PM

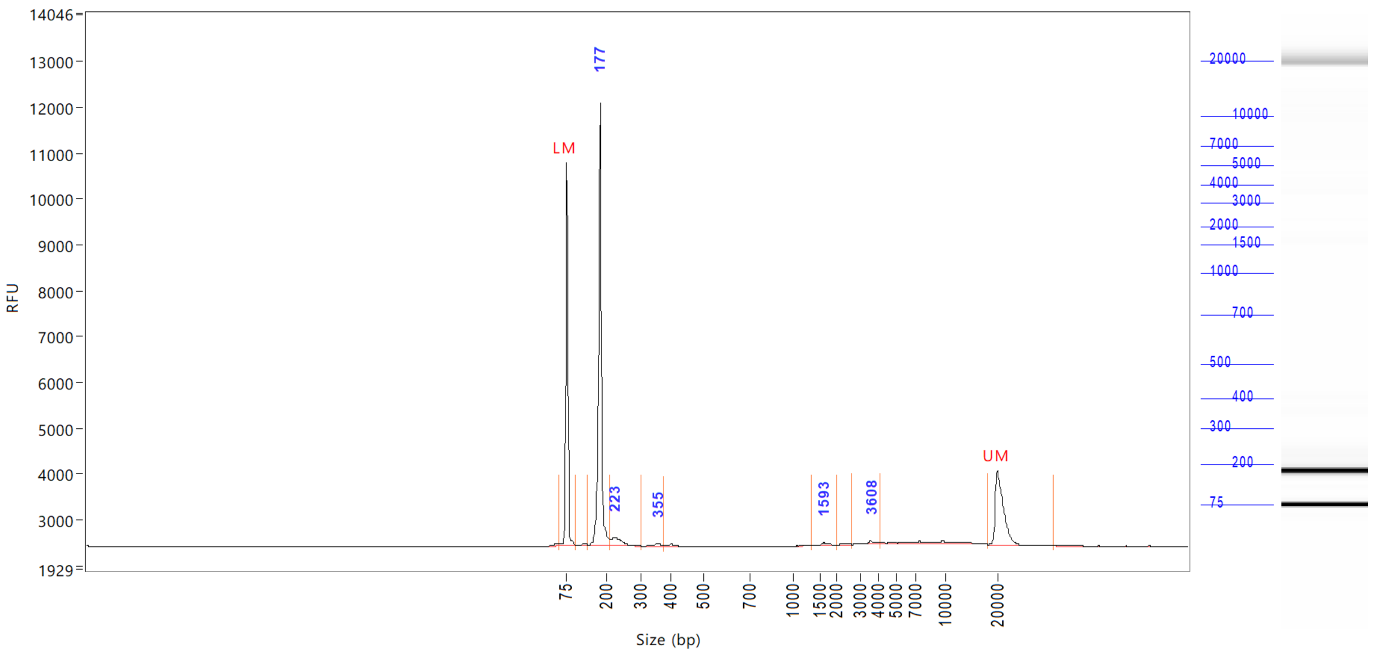

Peak	Size (bp)	Concentration (ng/uL)	From (bp)	To (bp)	Average size (bp)	CV%	RFU	Corrected peak area
1	75 (LM)	0.5000	52	105	73	5.25	8354	45.152
2	177	8.3098	141	206	176	4.01	9661	62.534
3	223	0.5820	206	303	230	7.31	168	4.380
4	355	0.1077	303	377	351	3.60	62	0.811
5	1593	0.0824	1341	2000	1645	7.98	55	0.620
6	3608	0.0743	2622	4101	3630	8.14	54	0.559
7	20000 (UM)	0.1849	18140	30725	20574	5.11	1618	16.694

TIC:	9.1562	ng/uL
TIM:	82.4008	nmole/L
Total concentration:	9.7412	ng/uL

```
Sample peak width (sec): 5    Sample min peak height: 50    Sample baseline V to V?: Y    Sample baseline V to V points: 3
Sample filter: Binomial       Number of points for filter: 3  Sample start region (min): 0  Sample end region (min): 30
Marker peak width (sec): 5    Marker min peak height: 200   Marker baseline V to V?: Y    Marker baseline V to V points: 3
Lower marker selection: First peak > 500 RFU                Upper marker selection: Last peak > 200 RFU
Ladder size (bp)75, 200, 300, 400, 500, 700, 1000, 1500, 2000, 3000, 4000, 5000, 7000, 10000, 20000
Quantification using: Lower Marker          Final concentration (ng/uL): 0.5000          Dilution factor: 12.0
```

Sample: SampH12
Well location: H12
Created: Tuesday, August 10, 2021 7:21:14 PM

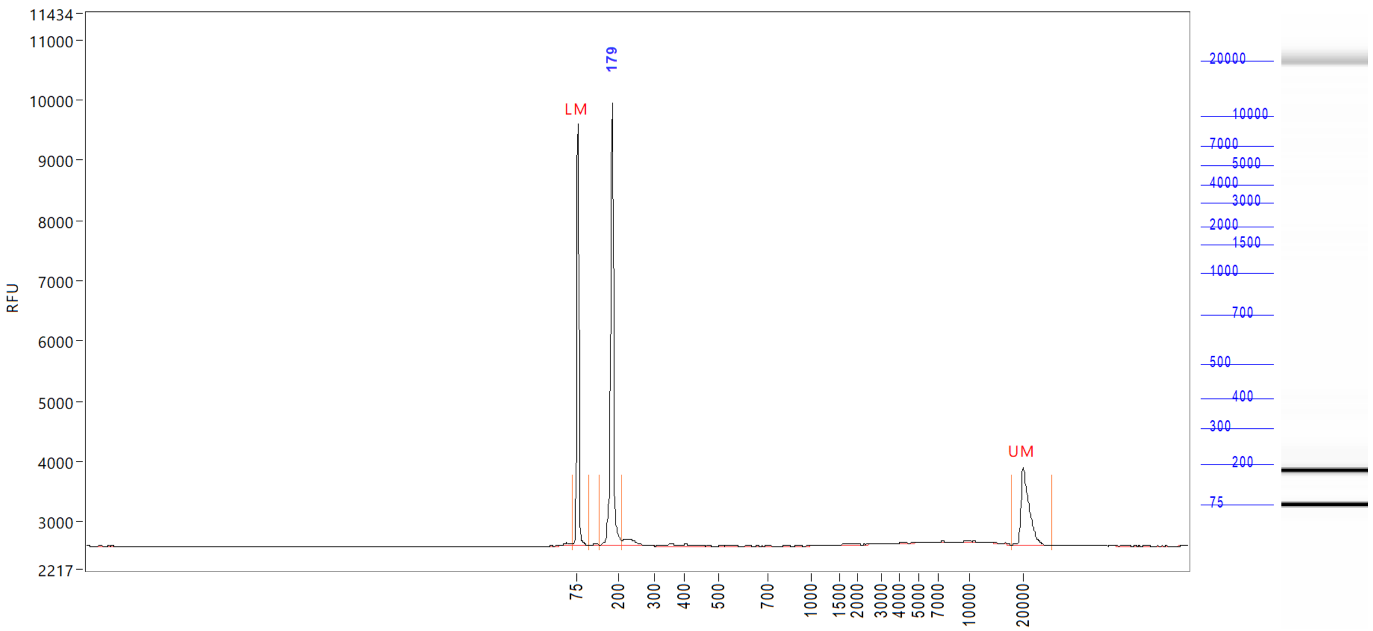

Peak	Size (bp)	Concentration (ng/uL)	From (bp)	To (bp)	Average size (bp)	CV%	RFU	Corrected peak area
1	75 (LM)	0.5000	62	108	73	4.75	7000	37.210
2	179	7.4229	141	206	176	3.97	7351	46.035
3	20000 (UM)	0.1723	17791	25420	20523	4.37	1279	12.825

TIC:	7.4229	ng/uL
TIM:	69.3338	nmole/L
Total concentration:	8.6756	ng/uL

Sample peak width (sec): 5 Sample min peak height: 200 Sample baseline V to V?: Y Sample baseline V to V points: 3
Sample filter: Binomial Number of points for filter: 3 Sample start region (min): 0 Sample end region (min): 30
Marker peak width (sec): 5 Marker min peak height: 200 Marker baseline V to V?: Y Marker baseline V to V points: 3
Lower marker selection: First peak > 500 RFU Upper marker selection: Last peak > 200 RFU
Ladder size (bp)75, 200, 300, 400, 500, 700, 1000, 1500, 2000, 3000, 4000, 5000, 7000, 10000, 20000
Quantification using: Lower Marker Final concentration (ng/uL): 0.5000 Dilution factor: 12.0

Sample: SampH5
Well location: H5
Created: Tuesday, August 10, 2021 7:21:14 PM
Fit type: Point to point

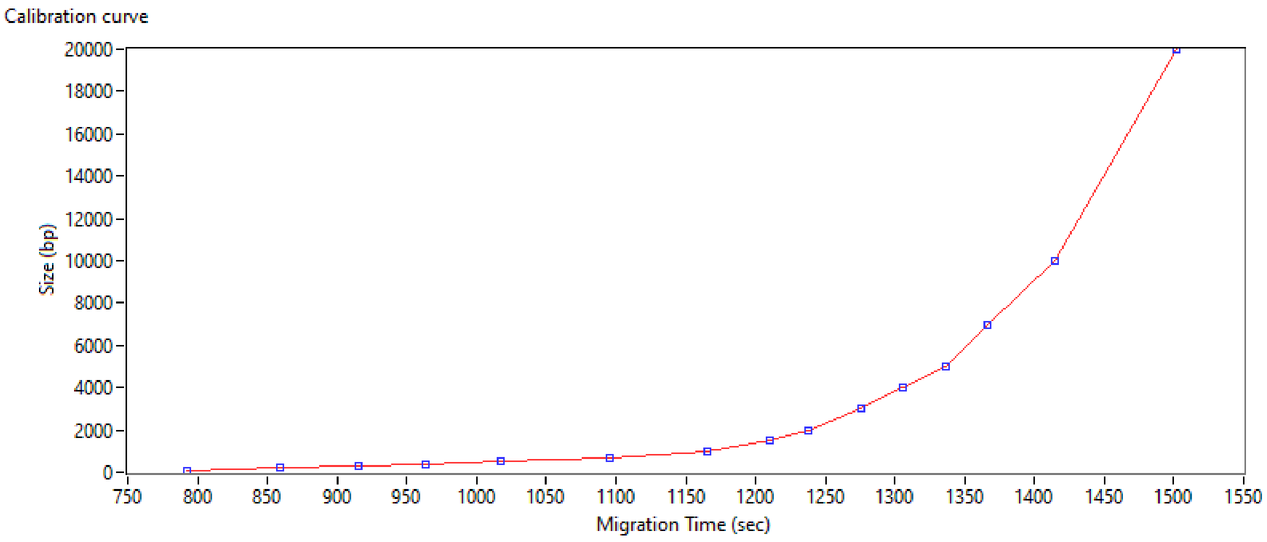

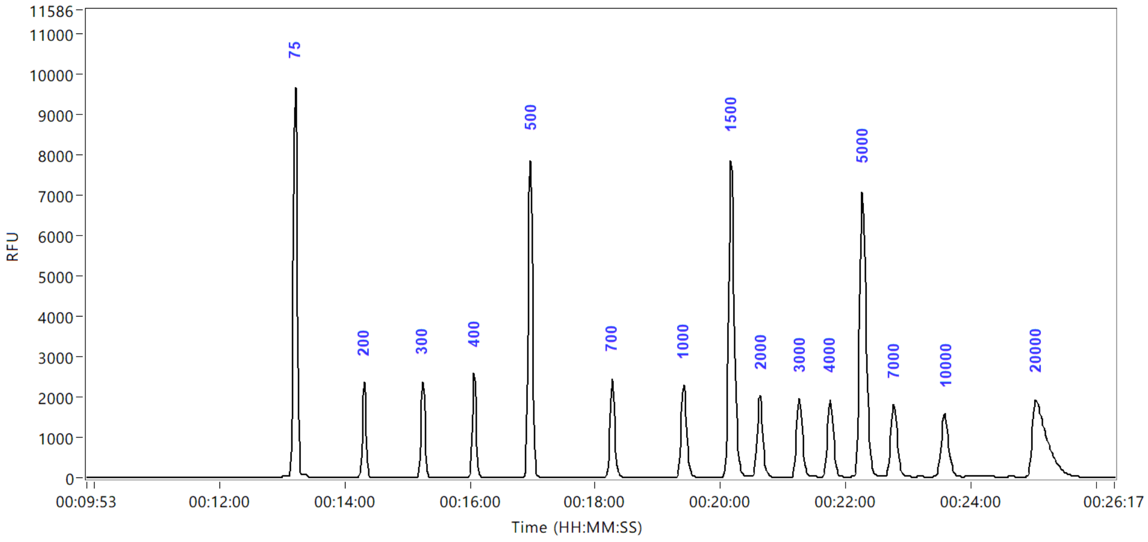

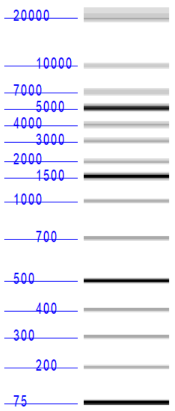

Supplement: Supplementary file 2 — sb3c00292_si_002.zip [file sb3c00292_si_002.zip › dnada_supplementary_material_pks_library_build/ZAG_trials/ZAG_Trial_1/19-11-21_plate2/plate2_report.pdf]
