# Supplementary material for: Automated Platform for the Plasmid Construction Process
Source: ACS Synth Biol. 2023 Nov 10;12(12):3506–13. doi: 10.1021/acssynbio.3c00292 (PMC10729297; doi:10.1021/acssynbio.3c00292)

Instrument controller software run summary:

Filename and data path: C:\AATI\Data\2021 08 10\21-11-26\2021 08 10 21H 11M..raw
Created: Tuesday, August 10, 2021 9:21:07 PM
Number of capillaries: 96
Array serial number: 111920-09SFS
Effect length: 33 cm
Array usage count: 168
Instrument type: ZAG DNA Analyzer
Instrument controller software version: 1.0.1.9
Device serial number: Z7597

Method Information

Method name: ZAG130GP33 - DNA 75-20000bp - Gel Prime Only.mthds
Gel prime: Yes
Full conditioning: No
Gel prime to buffer: No
Gel selection: Gel 1
Perform prerun: 8.0 kV, 30 sec.
Rinse: No
Marker 1: Row: A, 5.0 kV, 5 sec.
Rinse: No
Sample injection: 5.0 kV, 5 sec.
Separation: 8.0 kV, 30.0 min.
Tray name: Sample Tray-5

Analysis mode: DNA

Notes

Gel Image

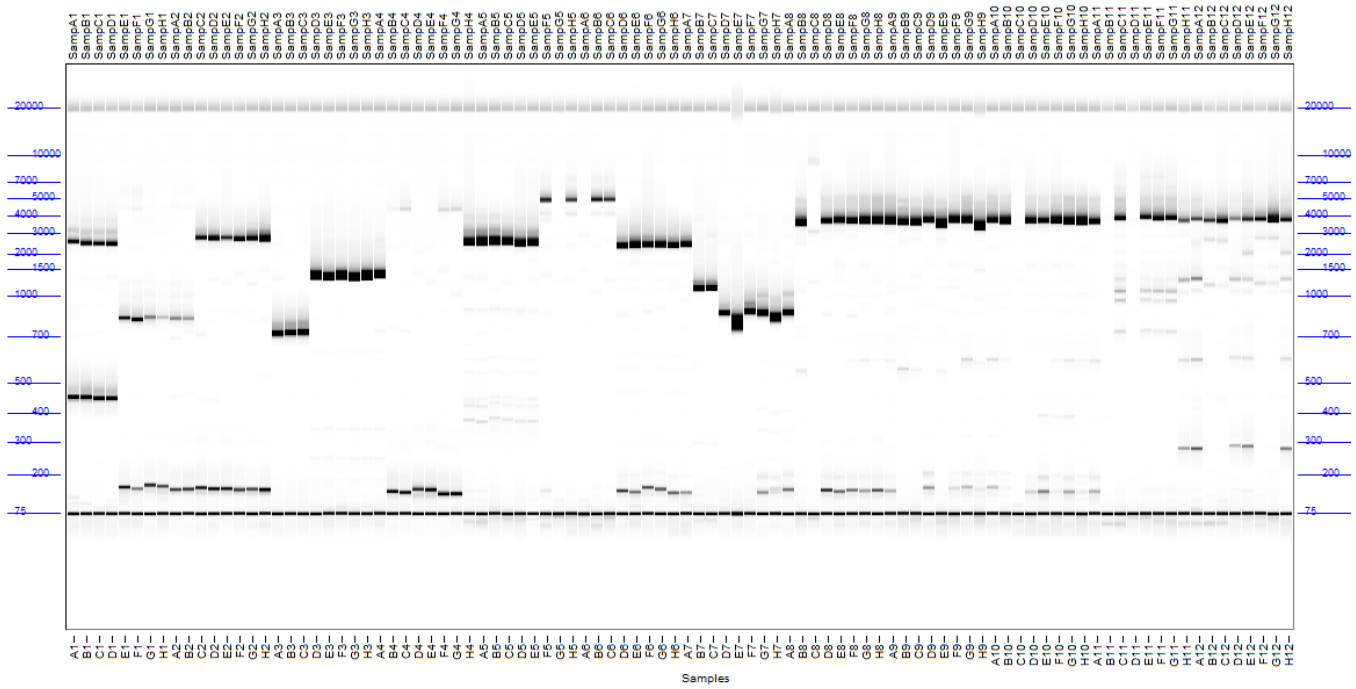
20000
10000
7000
5000
4000
3000
2000
1500
1000
700
500
400
300
200
75
Samples

Filename and data path: C:\AATI\Data\2021 08 10\21-11-26\2021 08 10 21H 11M..raw

A1: SampA1

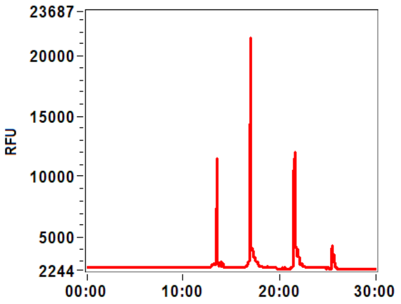

B1: SampB1

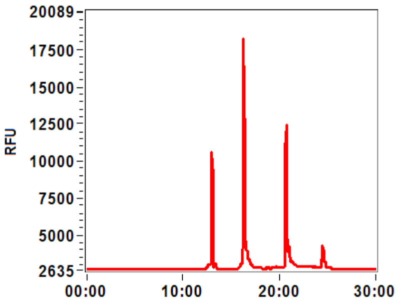

C1: SampC1

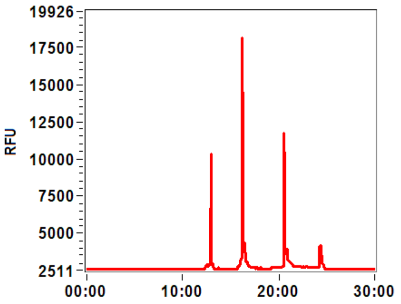

D1: SampD1

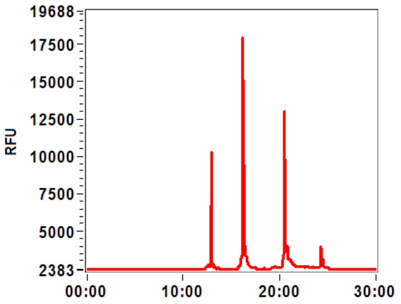

E1: SampE1

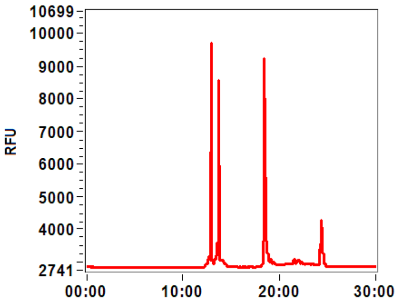

F1: SampF1

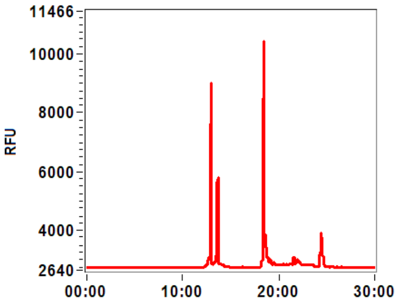

G1: SampG1

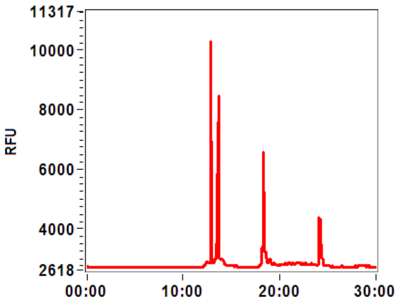

H1: SampH1

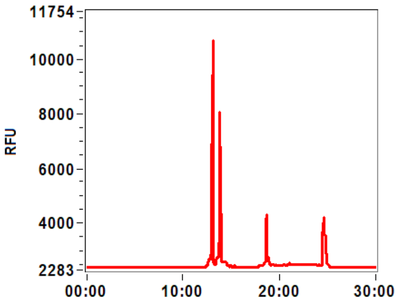

A2: SampA2

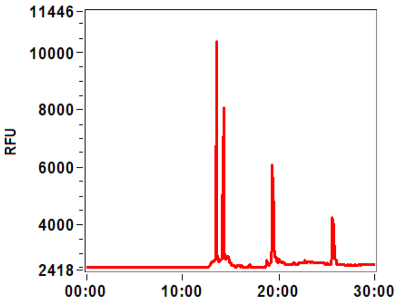

B2: SampB2

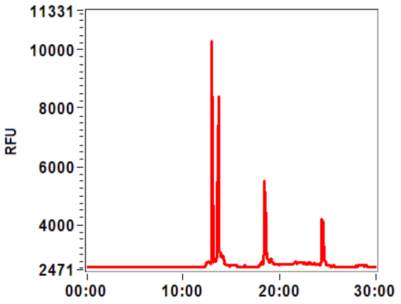

C2: SampC2

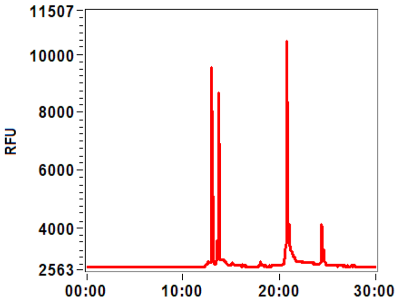

D2: SampD2

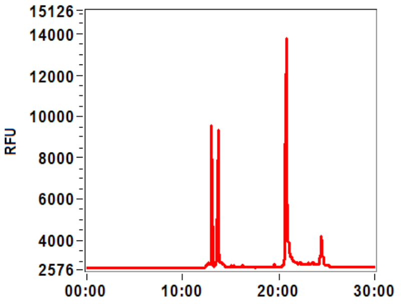

Filename and data path: C:\AATI\Data\2021 08 10\21-11-26\2021 08 10 21H 11M..raw

E2: SampE2

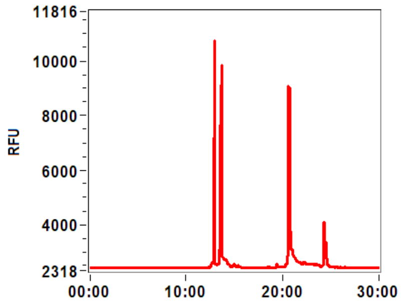

F2: SampF2

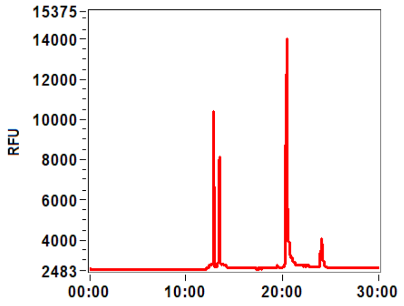

G2: SampG2

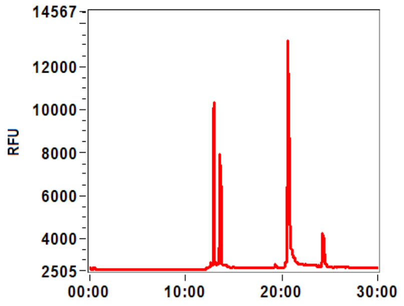

H2: SampH2

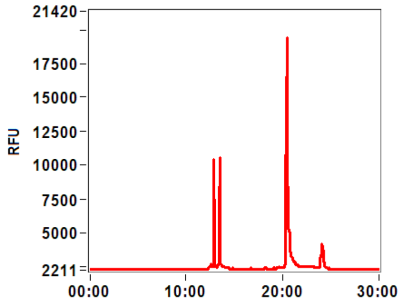

A3: SampA3

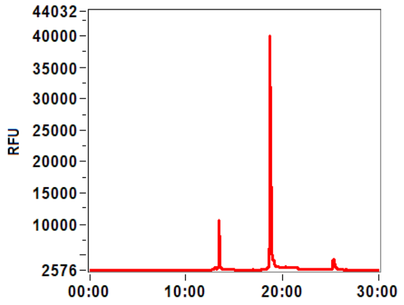

B3: SampB3

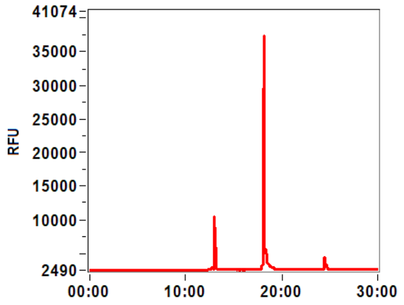

C3: SampC3

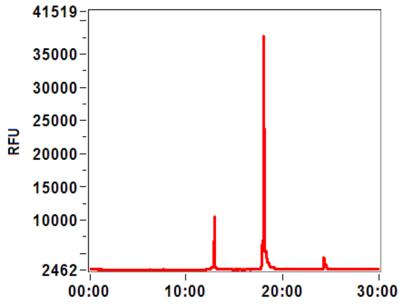

D3: SampD3

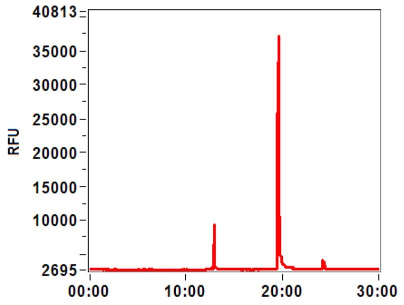

E3: SampE3

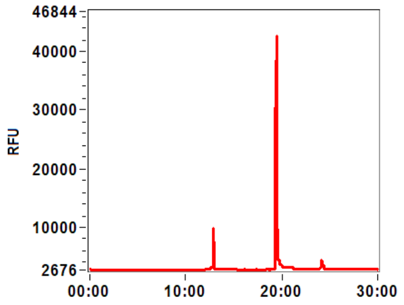

F3: SampF3

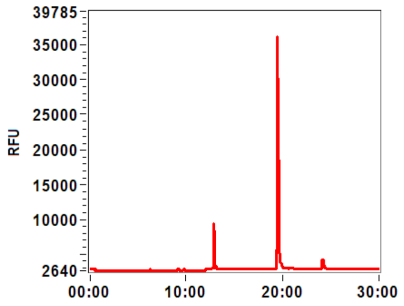

G3: SampG3

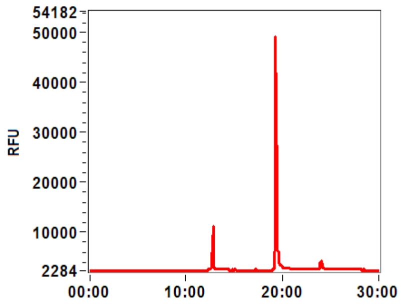

H3: SampH3

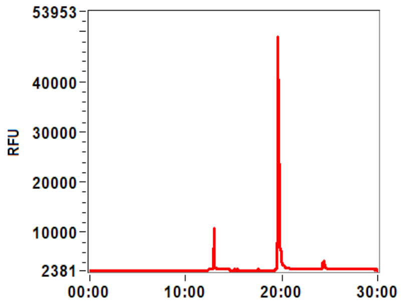

Filename and data path: C:\AATI\Data\2021 08 10\21-11-26\2021 08 10 21H 11M..raw

A4: SampA4

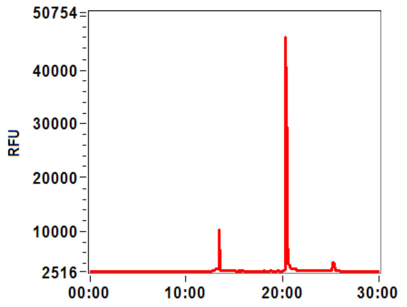

B4: SampB4

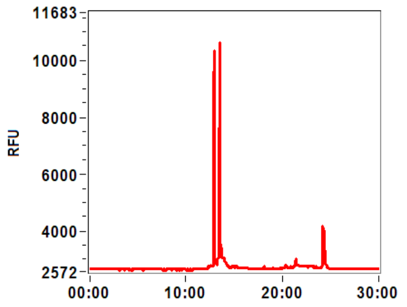

C4: SampC4

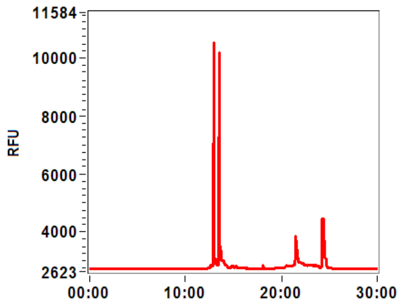

D4: SampD4

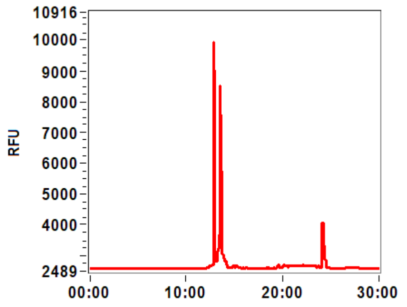

E4: SampE4

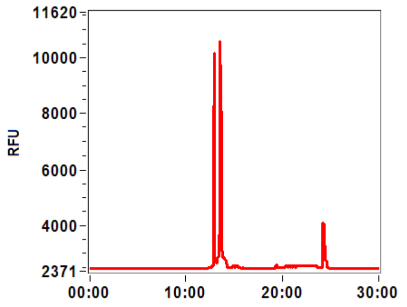

F4: SampF4

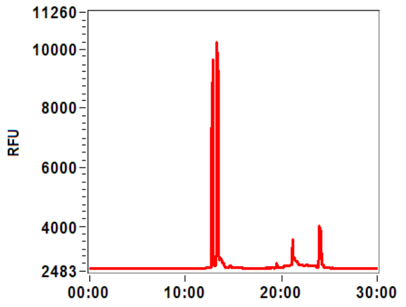

G4: SampG4

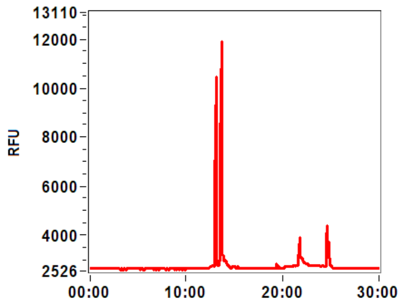

H4: SampH4

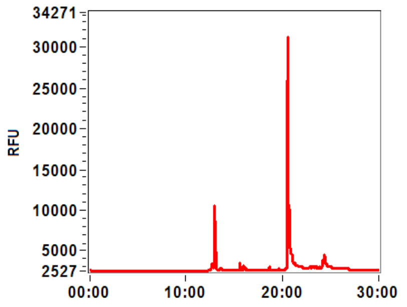

A5: SampA5

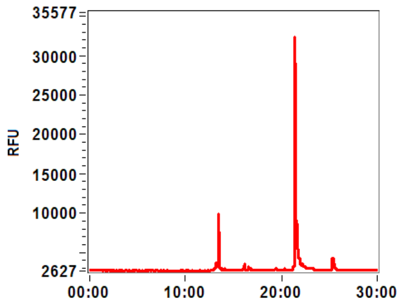

B5: SampB5

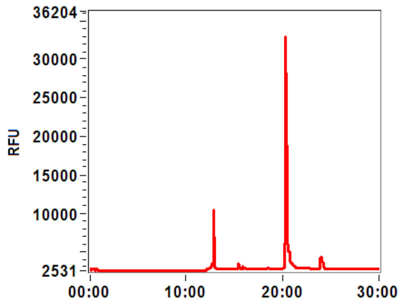

C5: SampC5

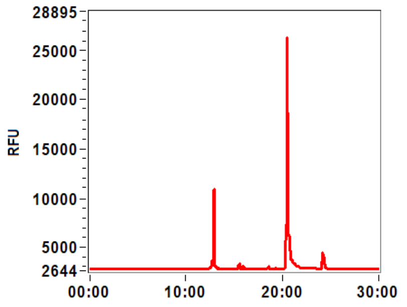

D5: SampD5

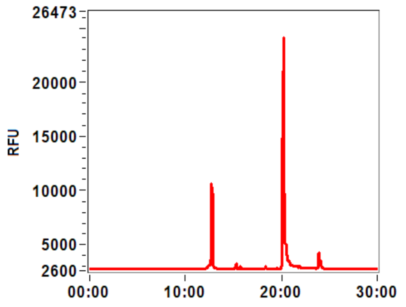

Filename and data path: C:\AATI\Data\2021 08 10\21-11-26\2021 08 10 21H 11M..raw

E5: SampE5

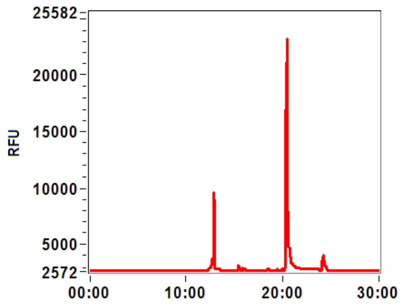

F5: SampF5

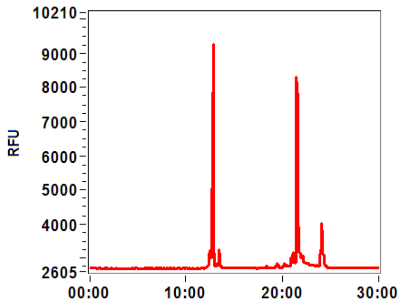

G5: SampG5

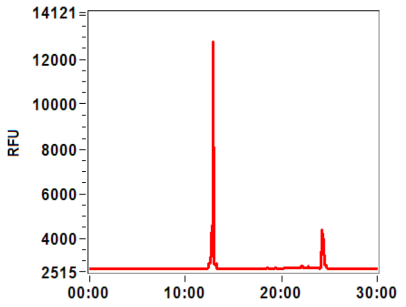

H5: SampH5

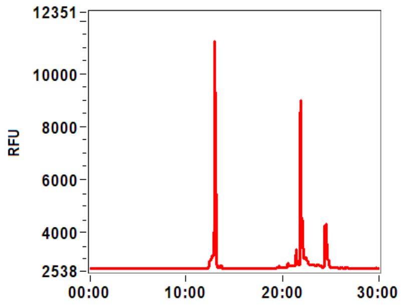

A6: SampA6

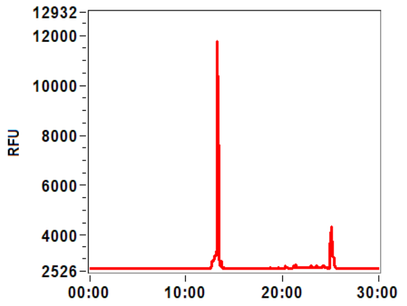

B6: SampB6

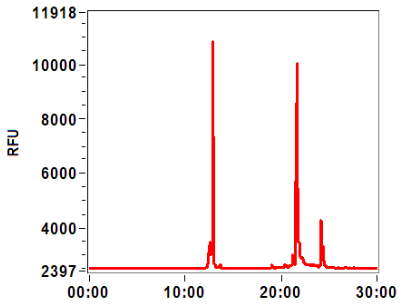

C6: SampC6

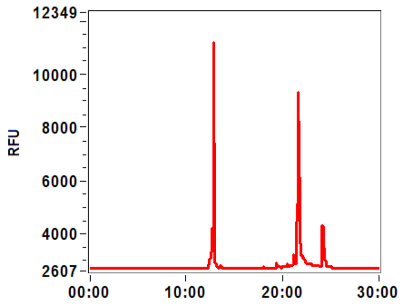

D6: SampD6

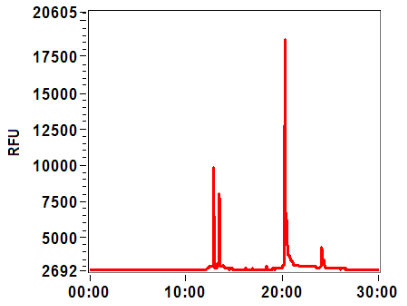

E6: SampE6

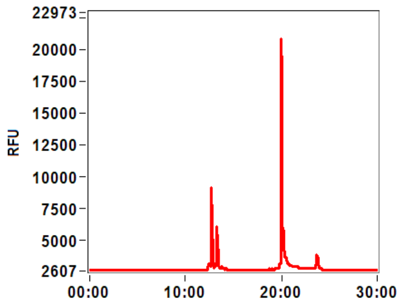

F6: SampF6

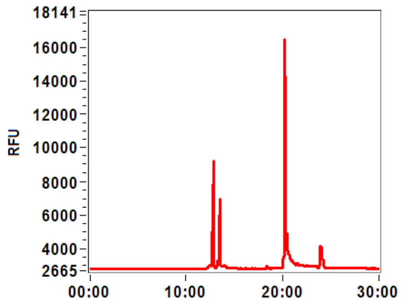

G6: SampG6

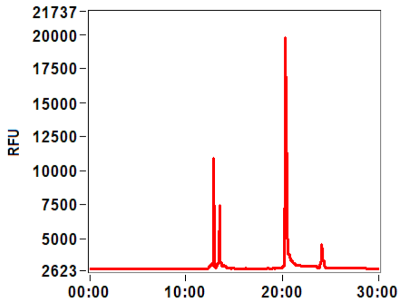

H6: SampH6

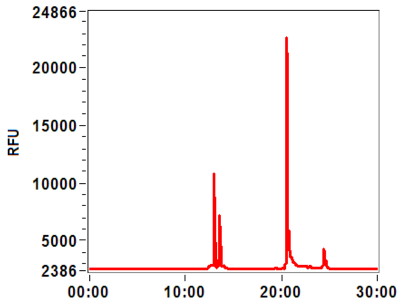

Filename and data path: C:\AATI\Data\2021 08 10\21-11-26\2021 08 10 21H 11M..raw

A7: SampA7

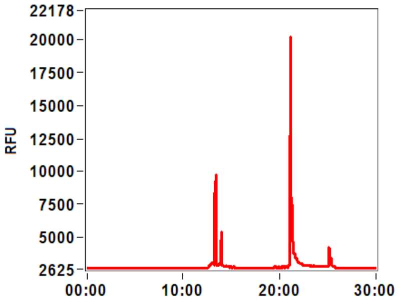

B7: SampB7

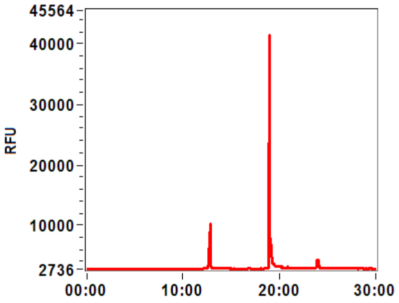

C7: SampC7

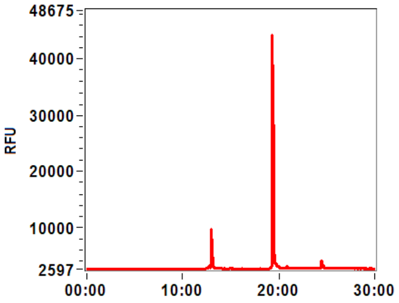

D7: SampD7

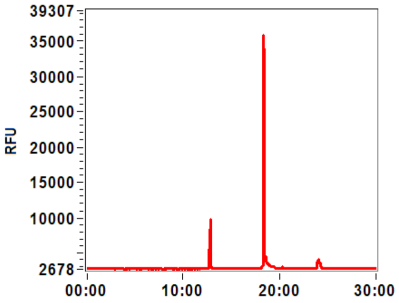

E7: SampE7

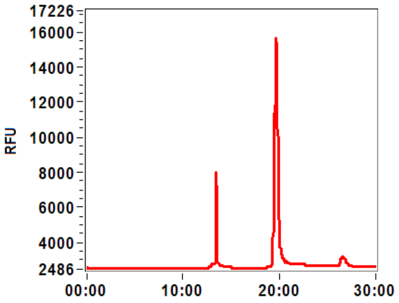

F7: SampF7

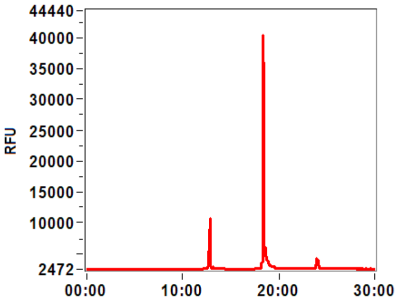

G7: SampG7

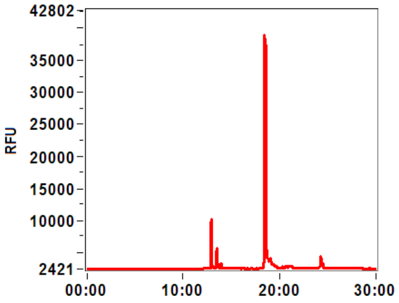

H7: SampH7

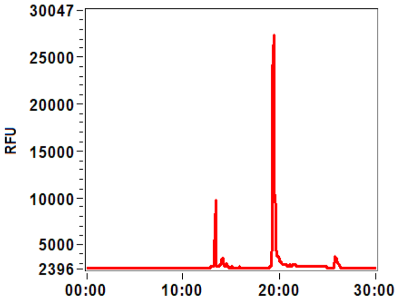

A8: SampA8

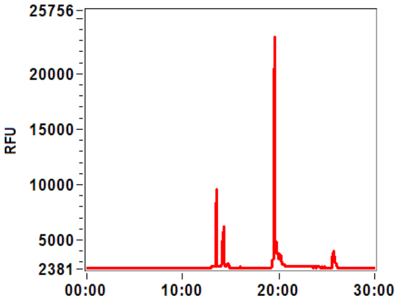

B8: SampB8

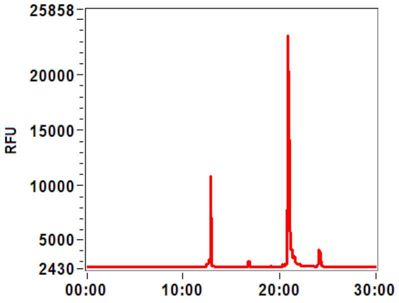

C8: SampC8

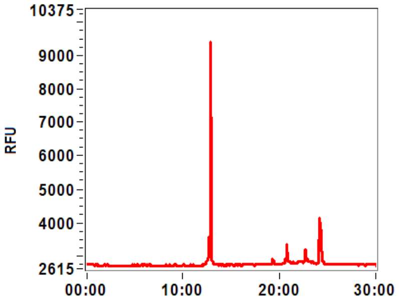

D8: SampD8

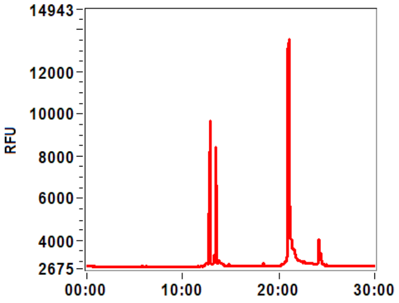

Filename and data path: C:\AATI\Data\2021 08 10\21-11-26\2021 08 10 21H 11M..raw

E8: SampE8

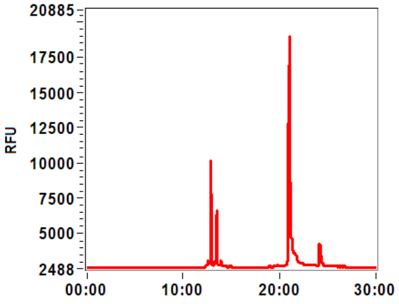

F8: SampF8

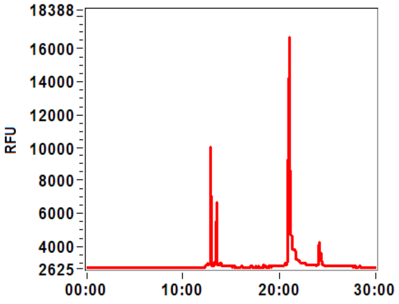

G8: SampG8

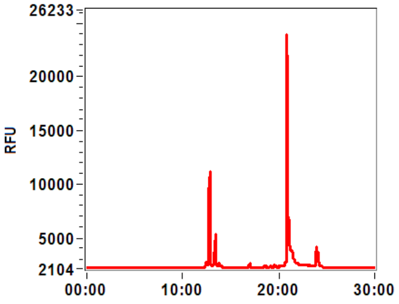

H8: SampH8

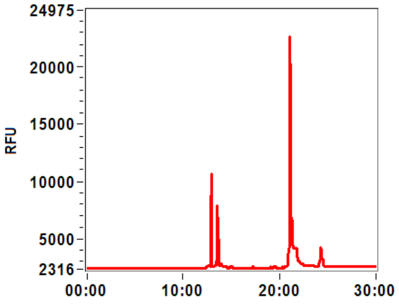

A9: SampA9

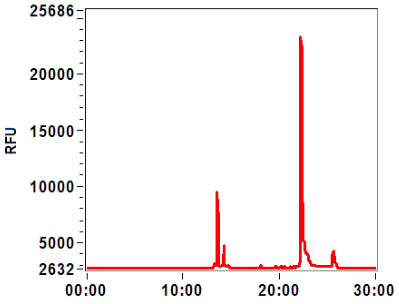

B9: SampB9

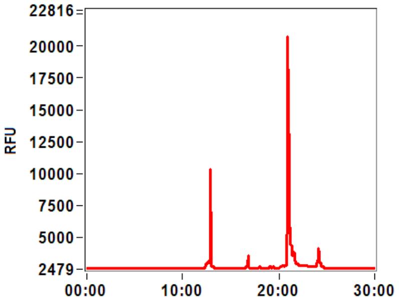

C9: SampC9

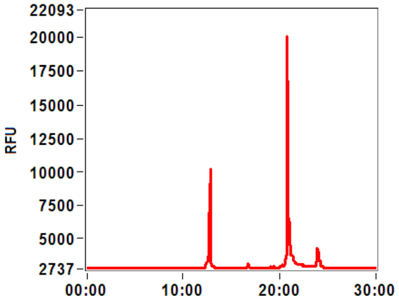

D9: SampD9

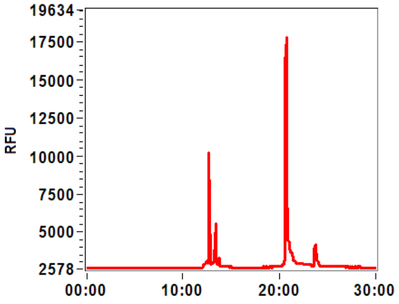

E9: SampE9

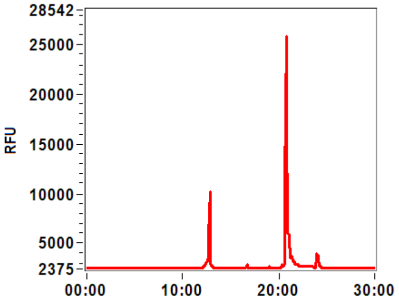

F9: SampF9

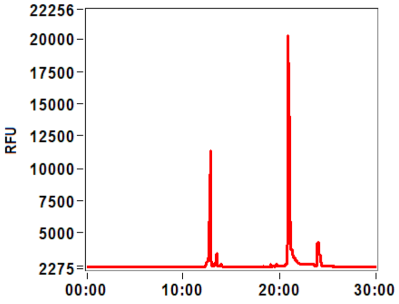

G9: SampG9

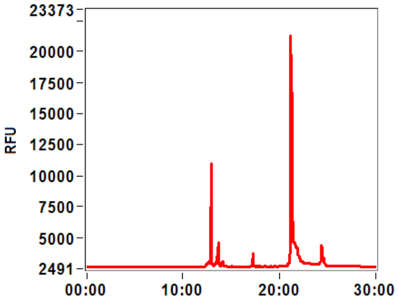

H9: SampH9

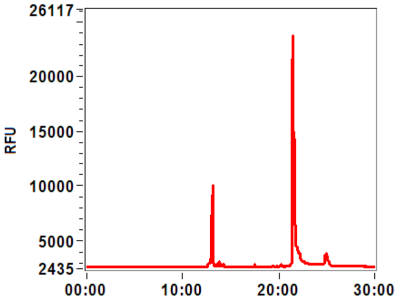

Filename and data path: C:\AATI\Data\2021 08 10\21-11-26\2021 08 10 21H 11M..raw

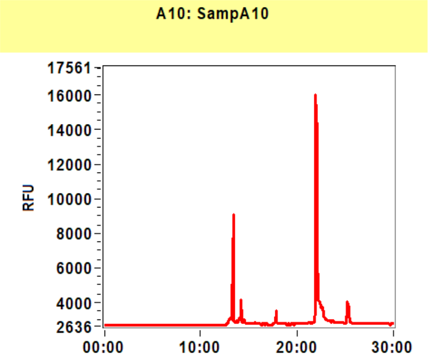

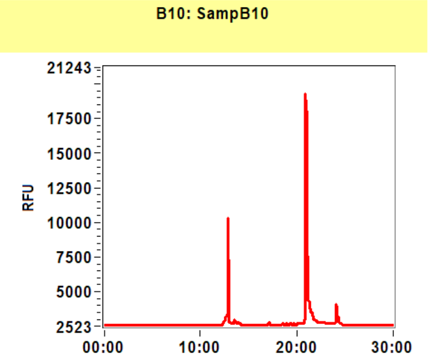

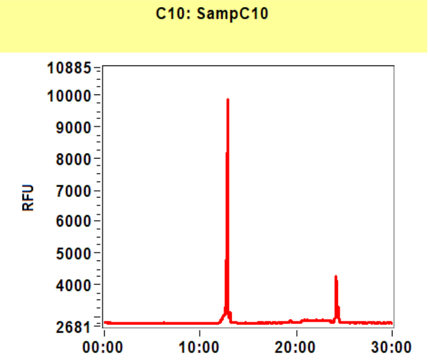

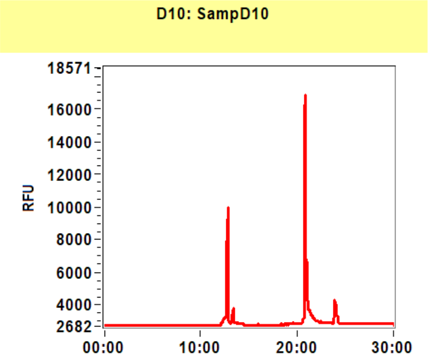

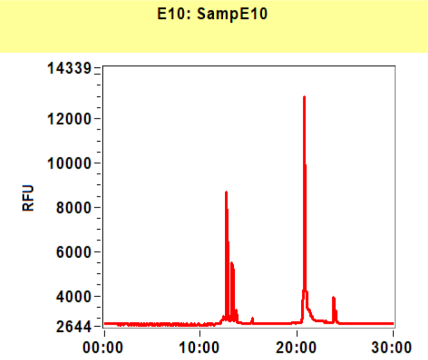

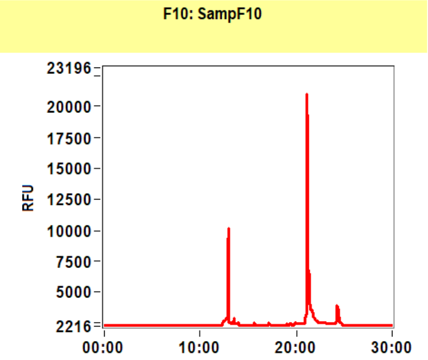

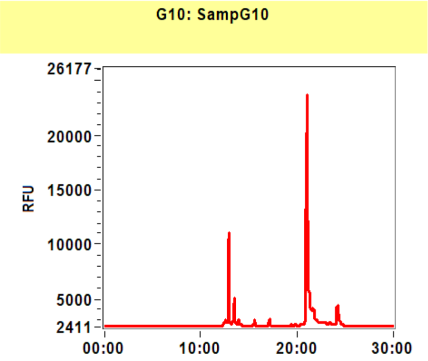

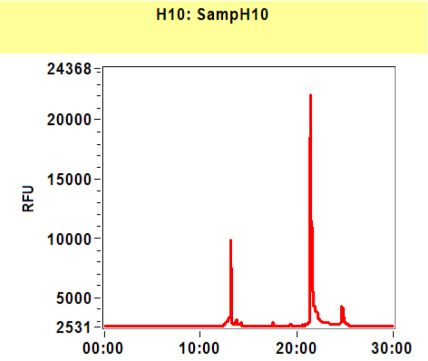

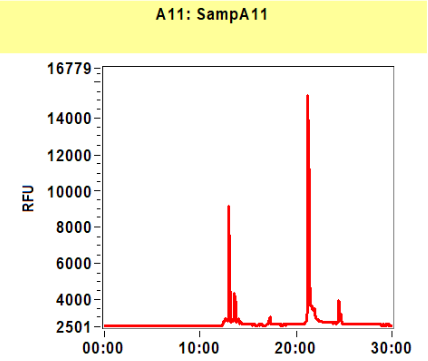

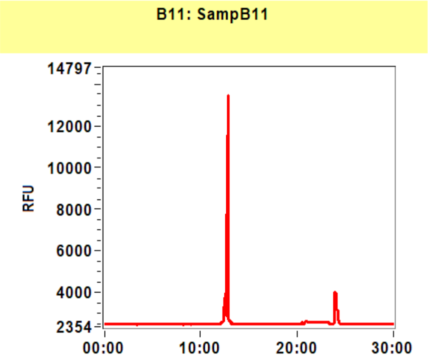

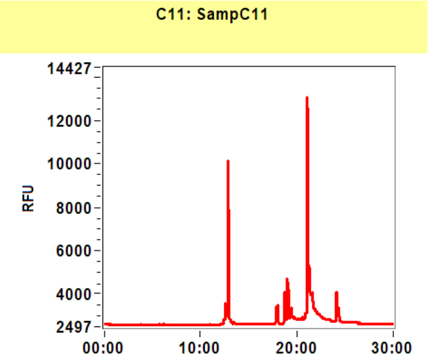

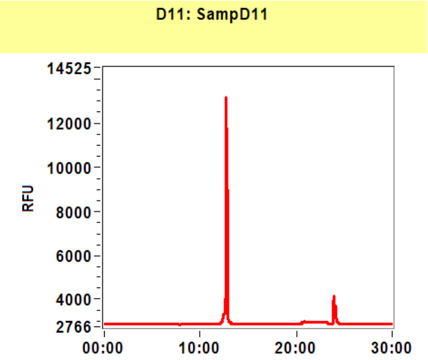

Filename and data path: C:\AATI\Data\2021 08 10\21-11-26\2021 08 10 21H 11M..raw

E11: SampE11

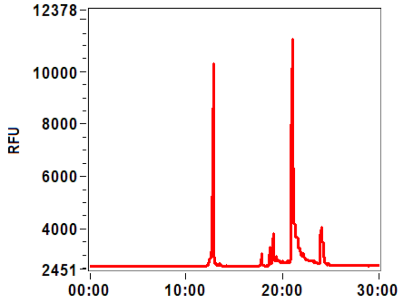

F11: SampF11

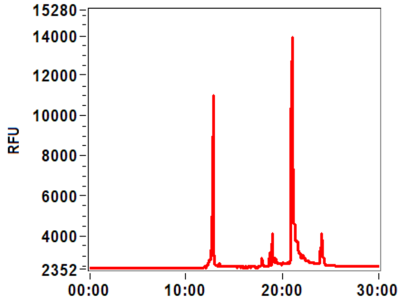

G11: SampG11

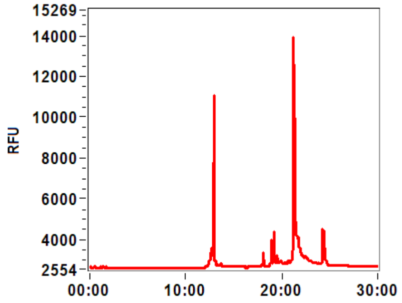

H11: SampH11

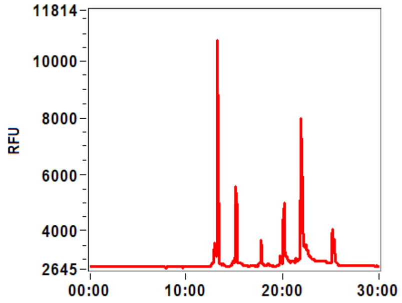

A12: SampA12

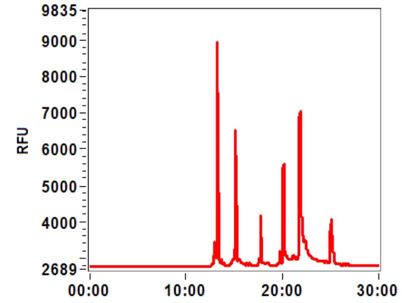

B12: SampB12

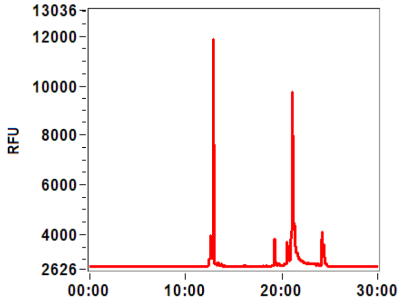

C12: SampC12

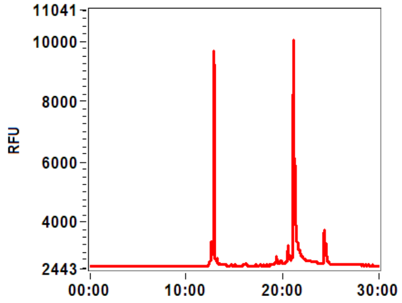

D12: SampD12

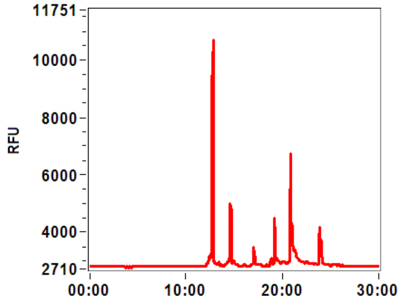

E12: SampE12

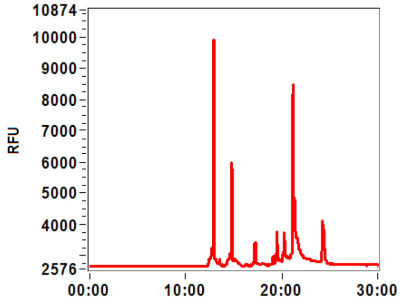

F12: SampF12

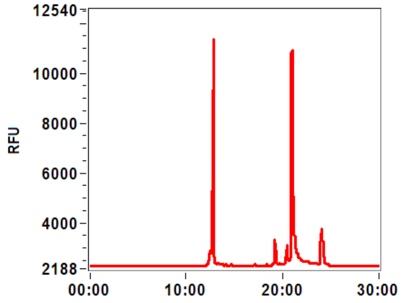

G12: SampG12

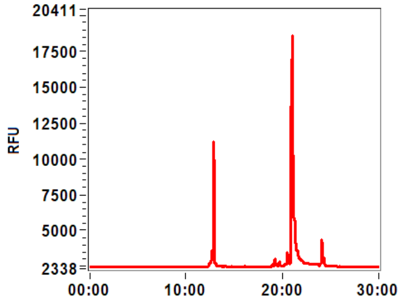

H12: SampH12

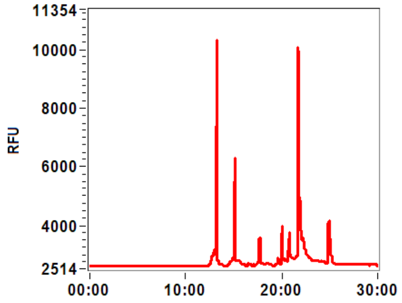

Sample: SampA1
Well location: A1
Created: Tuesday, August 10, 2021 9:21:07 PM

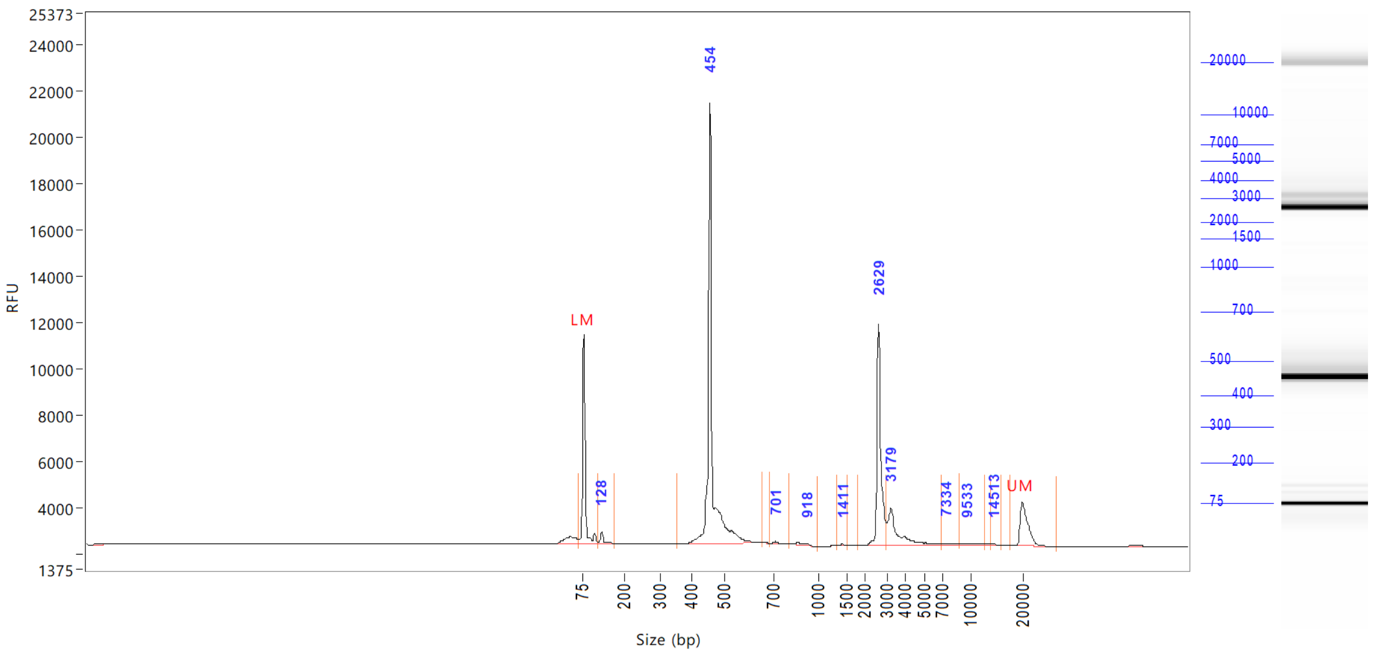

Peak	Size (bp)	Concentration (ng/uL)	From (bp)	To (bp)	Average size (bp)	CV%	RFU	Corrected peak area
1	75 (LM)	0.5000	62	116	75	11.60	9049	53.903
2	128	0.4696	116	169	131	9.05	528	4.219
3	454	15.4248	354	652	462	6.07	19049	138.574
4	701	0.0689	680	805	720	4.71	85	0.619
5	918	0.2002	805	996	880	3.94	100	1.799
6	1411	0.0341	1323	1500	1389	1.92	58	0.306
7	2629	6.3561	1808	3000	2635	5.13	9578	57.102
8	3179	2.5431	3000	7000	3667	21.38	1594	22.847
9	7334	0.1083	7000	8934	7720	7.13	76	0.973
10	9533	0.1150	8934	12684	10433	9.93	50	1.033
11	14513	0.0344	13781	15732	14442	3.16	56	0.309
12	20000 (UM)	0.1665	17683	26649	20411	4.18	1910	17.945

TIC:	25.3547	ng/uL
TIM:	66.5569	nmole/L
Total concentration:	25.4378	ng/uL

```
Sample peak width (sec): 5     Sample min peak height: 50     Sample baseline V to V?: Y     Sample baseline V to V points: 3
Sample filter: Binomial        Number of points for filter: 3  Sample start region (min): 0  Sample end region (min): 30
Marker peak width (sec): 5     Marker min peak height: 200    Marker baseline V to V?: Y     Marker baseline V to V points: 3
Lower marker selection: First peak > 500 RFU                  Upper marker selection: Last peak > 200 RFU
Ladder size (bp)75, 200, 300, 400, 500, 700, 1000, 1500, 2000, 3000, 4000, 5000, 7000, 10000, 20000
Quantification using: Lower Marker          Final concentration (ng/uL): 0.5000          Dilution factor: 12.0
```

Sample: SampB1
Well location: B1
Created: Tuesday, August 10, 2021 9:21:07 PM

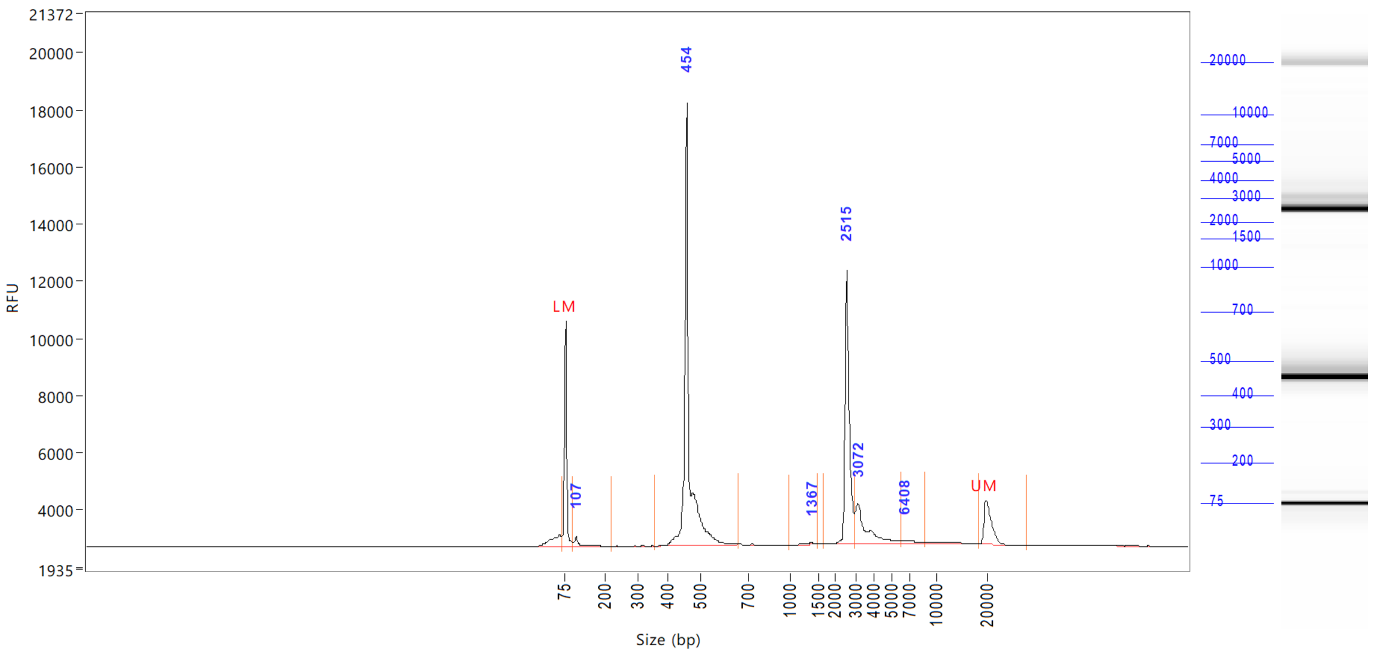

Peak	Size (bp)	Concentration (ng/uL)	From (bp)	To (bp)	Average size (bp)	CV%	RFU	Corrected peak area
1	75 (LM)	0.5000	64	99	74	6.59	7878	45.045
2	107	0.5772	99	219	123	21.71	381	4.333
3	454	17.3426	352	659	463	6.04	15500	130.199
4	1367	0.1257	991	1500	1303	8.07	81	0.944
5	2515	8.2124	1635	2914	2554	5.36	9582	61.654
6	3072	3.1329	2914	6112	3574	19.11	1395	23.520
7	6408	0.2332	6112	8667	7175	9.63	85	1.750
8	20000 (UM)	0.1602	18415	28100	20399	4.24	1546	14.433

TIC:	29.6239	ng/uL
TIM:	76.2909	nmole/L
Total concentration:	29.8530	ng/uL

Sample peak width (sec): 5 Sample min peak height: 50 Sample baseline V to V?: Y Sample baseline V to V points: 3
Sample filter: Binomial Number of points for filter: 3 Sample start region (min): 0 Sample end region (min): 30
Marker peak width (sec): 5 Marker min peak height: 200 Marker baseline V to V?: Y Marker baseline V to V points: 3
Lower marker selection: First peak > 500 RFU Upper marker selection: Last peak > 200 RFU
Ladder size (bp)75, 200, 300, 400, 500, 700, 1000, 1500, 2000, 3000, 4000, 5000, 7000, 10000, 20000
Quantification using: Lower Marker Final concentration (ng/uL): 0.5000 Dilution factor: 12.0

Sample: SampC1
Well location: C1
Created: Tuesday, August 10, 2021 9:21:07 PM

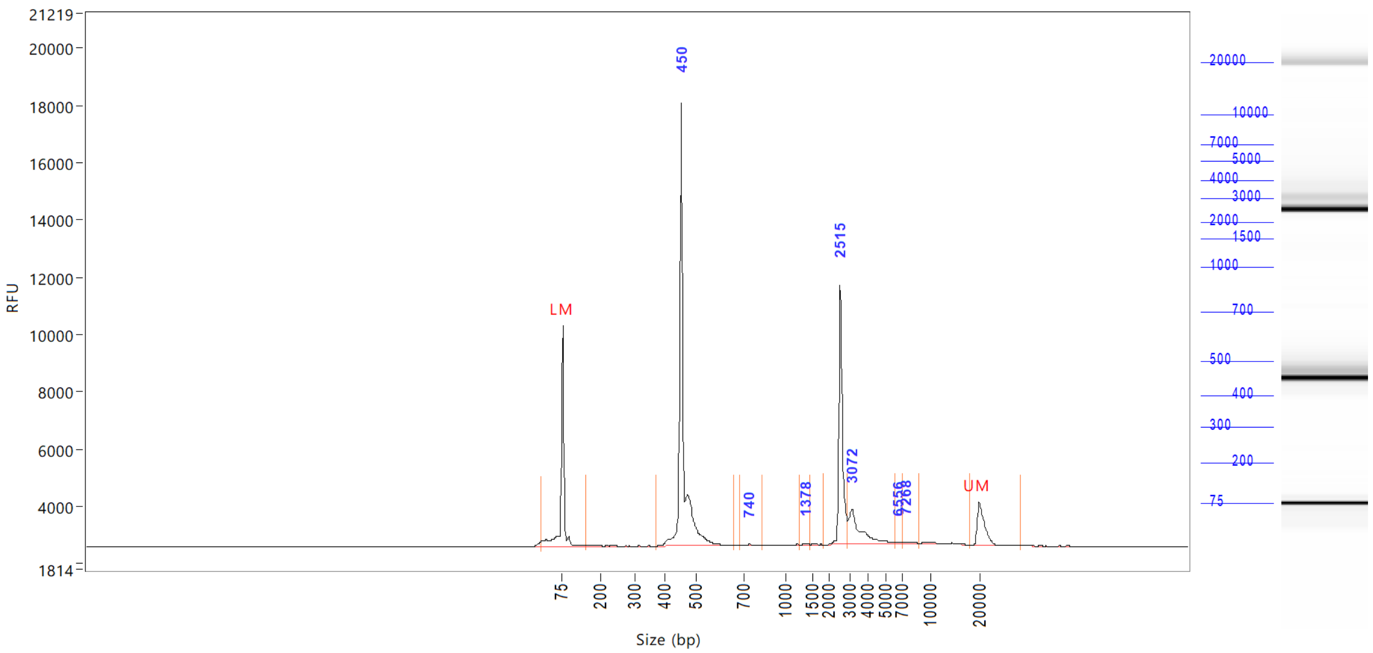

Peak	Size<br>(bp)	Concentration<br>(ng/uL)	From<br>(bp)	To<br>(bp)	Average size<br>(bp)	CV%	RFU	Corrected peak area
1	75 (LM)	0.5000	9	152	68	25.52	7705	55.429
2	450	12.9438	370	659	458	5.58	15485	119.577
3	740	0.0406	685	840	738	2.50	62	0.375
4	1378	0.0557	1267	1456	1356	3.11	69	0.515
5	2515	5.5526	1827	2857	2532	4.50	9037	51.296
6	3072	2.4006	2857	6112	3505	18.31	1221	22.177
7	6556	0.0514	6112	7000	6534	4.09	58	0.475
8	7268	0.0713	7000	8734	7544	5.83	62	0.658
9	20000 (UM)	0.1252	18293	28584	20492	4.23	1491	13.880

TIC:	21.1160	ng/uL
TIM:	51.4267	nmole/L
Total concentration:	21.2984	ng/uL

```
Sample peak width (sec): 5    Sample min peak height: 50    Sample baseline V to V?: Y    Sample baseline V to V points: 3
Sample filter: Binomial       Number of points for filter: 3  Sample start region (min): 0  Sample end region (min): 30
Marker peak width (sec): 5    Marker min peak height: 200   Marker baseline V to V?: Y    Marker baseline V to V points: 3
Lower marker selection: First peak > 500 RFU                Upper marker selection: Last peak > 200 RFU
Ladder size (bp)75, 200, 300, 400, 500, 700, 1000, 1500, 2000, 3000, 4000, 5000, 7000, 10000, 20000
Quantification using: Lower Marker          Final concentration (ng/uL): 0.5000          Dilution factor: 12.0
```

Sample: SampD1
Well location: D1
Created: Tuesday, August 10, 2021 9:21:07 PM

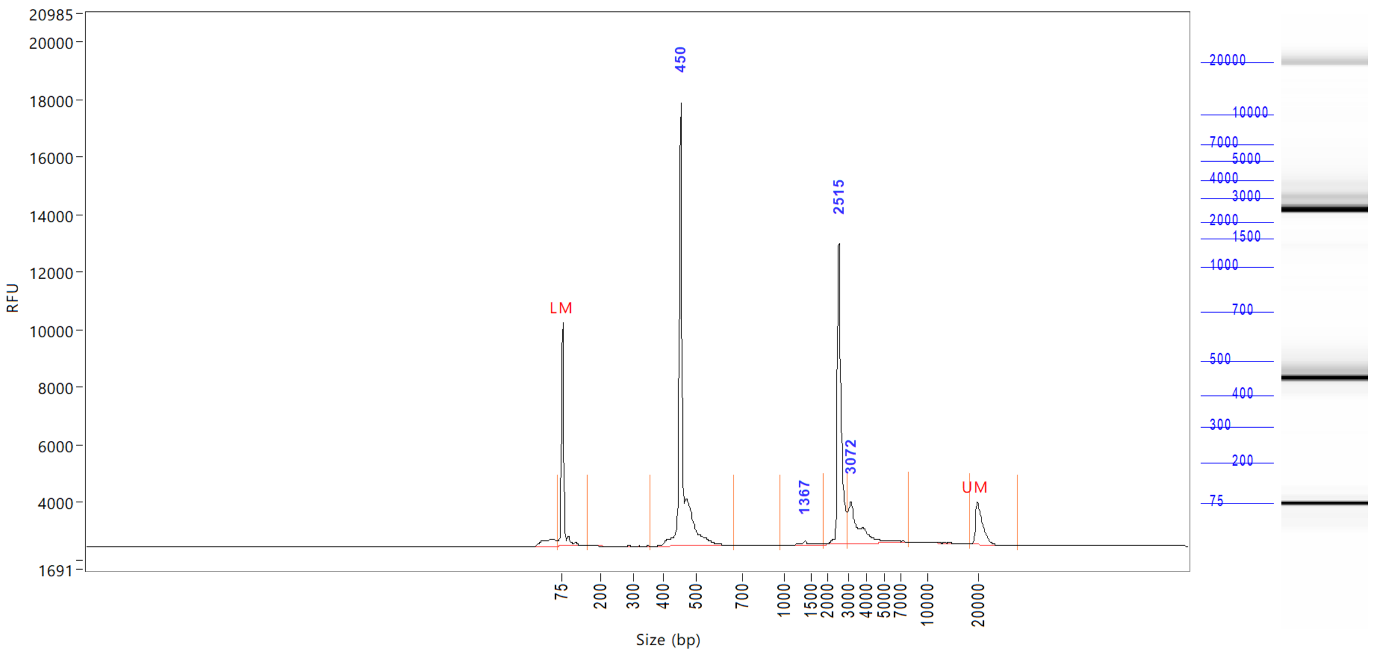

Peak	Size (bp)	Concentration (ng/uL)	From (bp)	To (bp)	Average size (bp)	CV%	RFU	Corrected peak area
1	75 (LM)	0.5000	60	154	75	11.90	7746	47.207
2	450	14.9674	354	662	459	6.01	15400	117.761
3	1367	0.2434	965	1866	1359	12.14	133	1.915
4	2515	8.3578	1866	2886	2528	5.15	10448	65.758
5	3072	3.2728	2886	7934	3566	21.18	1464	25.750
6	20000 (UM)	0.1424	18537	28221	20501	4.25	1452	13.440

TIC:	26.8415	ng/uL
TIM:	60.9037	nmole/L
Total concentration:	26.9227	ng/uL

```
Sample peak width (sec): 5    Sample min peak height: 50    Sample baseline V to V?: Y    Sample baseline V to V points: 3
Sample filter: Binomial       Number of points for filter: 3  Sample start region (min): 0  Sample end region (min): 30
Marker peak width (sec): 5    Marker min peak height: 200   Marker baseline V to V?: Y    Marker baseline V to V points: 3
Lower marker selection: First peak > 500 RFU                Upper marker selection: Last peak > 200 RFU
Ladder size (bp)75, 200, 300, 400, 500, 700, 1000, 1500, 2000, 3000, 4000, 5000, 7000, 10000, 20000
Quantification using: Lower Marker          Final concentration (ng/uL): 0.5000          Dilution factor: 12.0
```

Sample: SampE1
Well location: E1
Created: Tuesday, August 10, 2021 9:21:07 PM

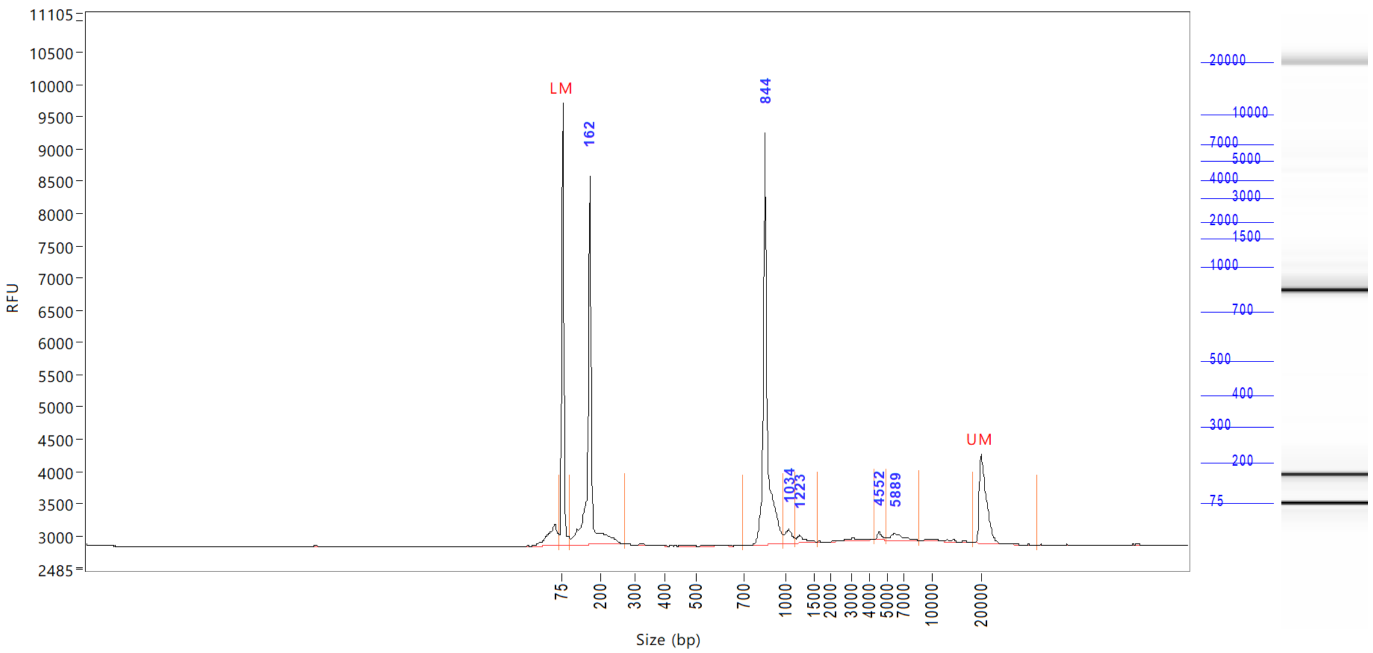

Peak	Size (bp)	Concentration (ng/uL)	From (bp)	To (bp)	Average size (bp)	CV%	RFU	Corrected peak area
1	75 (LM)	0.5000	64	99	74	5.98	6872	38.648
2	162	7.7817	99	268	159	13.84	5713	50.124
3	844	7.0807	695	978	854	4.12	6387	45.609
4	1034	0.4176	978	1156	1037	4.73	216	2.690
5	1223	0.2379	1156	1578	1278	7.28	122	1.532
6	4552	0.1401	4242	5000	4603	3.83	128	0.902
7	5889	0.2913	5000	8667	6325	13.36	103	1.876
8	20000 (UM)	0.1659	18537	31244	20552	5.21	1375	12.827

TIC:	15.9493	ng/uL
TIM:	95.1852	nmole/L
Total concentration:	16.3872	ng/uL

```
Sample peak width (sec): 5     Sample min peak height: 50     Sample baseline V to V?: Y     Sample baseline V to V points: 3
Sample filter: Binomial        Number of points for filter: 3  Sample start region (min): 0  Sample end region (min): 30
Marker peak width (sec): 5     Marker min peak height: 200    Marker baseline V to V?: Y     Marker baseline V to V points: 3
Lower marker selection: First peak > 500 RFU                  Upper marker selection: Last peak > 200 RFU
Ladder size (bp)75, 200, 300, 400, 500, 700, 1000, 1500, 2000, 3000, 4000, 5000, 7000, 10000, 20000
Quantification using: Lower Marker          Final concentration (ng/uL): 0.5000          Dilution factor: 12.0
```

Sample: SampF1
Well location: F1
Created: Tuesday, August 10, 2021 9:21:07 PM

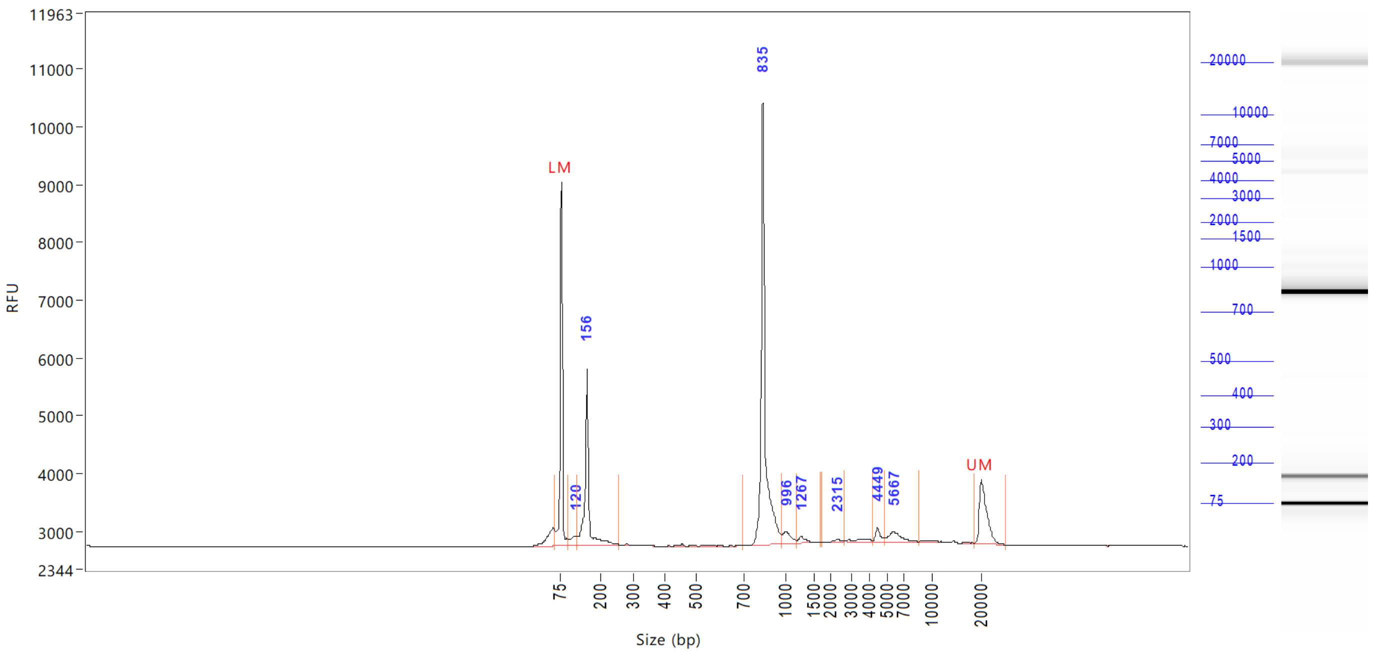

Peak	Size (bp)	Concentration (ng/uL)	From (bp)	To (bp)	Average size (bp)	CV%	RFU	Corrected peak area
1	75 (LM)	0.5000	58	99	73	7.01	6288	36.684
2	120	0.4343	99	126	113	6.96	167	2.655
3	156	4.2420	126	253	157	11.88	3060	25.936
4	835	8.9167	697	965	842	3.91	7660	54.517
5	996	0.5001	965	1190	1028	5.74	214	3.058
6	1267	0.2156	1190	1674	1302	6.25	112	1.318
7	2315	0.0710	1750	2657	2238	7.36	56	0.434
8	4449	0.2844	4173	4828	4478	3.43	247	1.739
9	5667	0.4942	4828	8534	5946	13.13	173	3.022
10	20000 (UM)	0.1451	18537	24957	20424	4.15	1114	10.648

TIC:	15.1584	ng/uL
TIM:	69.5191	nmole/L
Total concentration:	15.5280	ng/uL

Sample peak width (sec): 5   Sample min peak height: 50   Sample baseline V to V?: Y   Sample baseline V to V points: 3
Sample filter: Binomial   Number of points for filter: 3 Sample start region (min): 0 Sample end region (min): 30
Marker peak width (sec): 5   Marker min peak height: 200   Marker baseline V to V?: Y   Marker baseline V to V points: 3
Lower marker selection: First peak > 500 RFU   Upper marker selection: Last peak > 200 RFU
Ladder size (bp)75, 200, 300, 400, 500, 700, 1000, 1500, 2000, 3000, 4000, 5000, 7000, 10000, 20000
Quantification using: Lower Marker   Final concentration (ng/uL): 0.5000   Dilution factor: 12.0

Sample: SampG1
Well location: G1
Created: Tuesday, August 10, 2021 9:21:07 PM

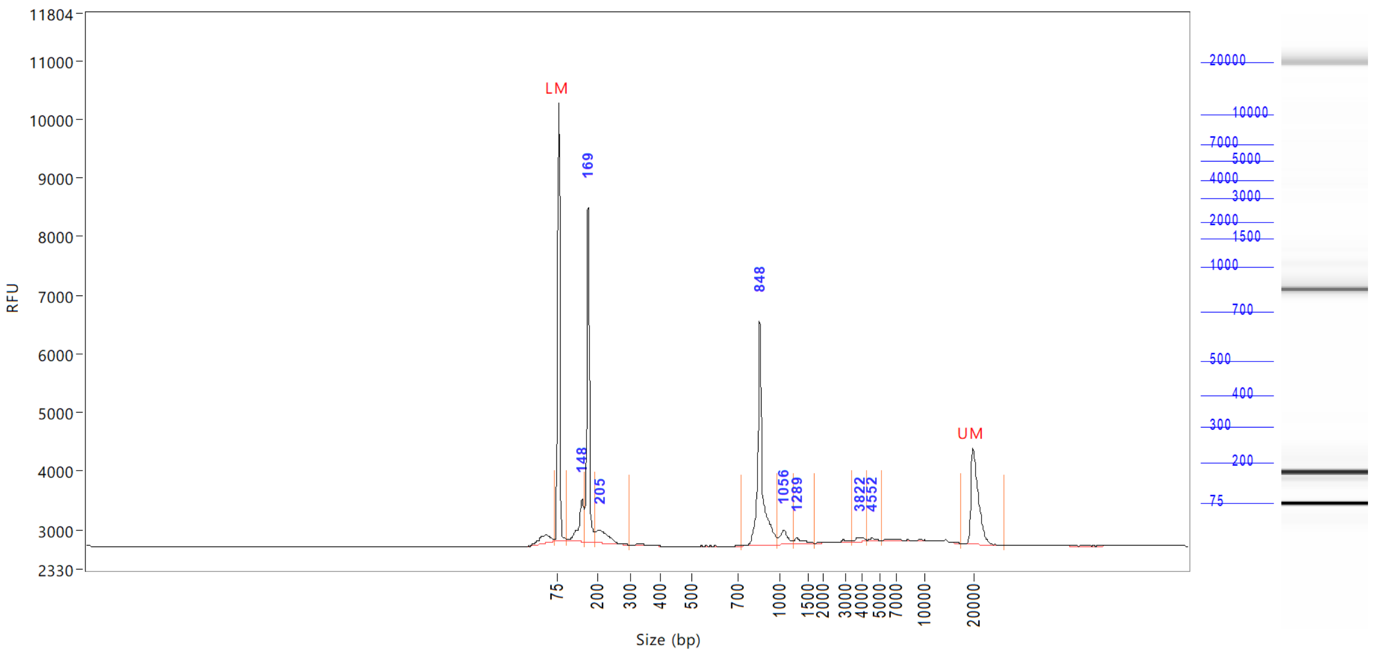

Peak	Size (bp)	Concentration (ng/uL)	From (bp)	To (bp)	Average size (bp)	CV%	RFU	Corrected peak area
1	75 (LM)	0.5000	62	99	72	4.59	7476	40.644
2	148	1.3783	99	156	142	8.25	730	9.337
3	169	5.3109	156	190	167	3.33	5689	35.976
4	205	0.9144	190	298	216	8.58	214	6.194
5	848	4.6599	714	983	856	4.35	3831	31.566
6	1056	0.4622	983	1223	1062	5.97	237	3.131
7	1289	0.2025	1223	1731	1351	7.03	101	1.372
8	3822	0.1331	3429	4242	3854	5.03	69	0.902
9	4552	0.0849	4242	5149	4606	4.93	54	0.575
10	20000 (UM)	0.1924	17439	26529	20502	4.41	1638	15.641

TIC:	13.1464	ng/uL
TIM:	85.1990	nmole/L
Total concentration:	13.4594	ng/uL

```
Sample peak width (sec): 5    Sample min peak height: 50    Sample baseline V to V?: Y    Sample baseline V to V points: 3
Sample filter: Binomial       Number of points for filter: 3  Sample start region (min): 0  Sample end region (min): 30
Marker peak width (sec): 5    Marker min peak height: 200   Marker baseline V to V?: Y    Marker baseline V to V points: 3
Lower marker selection: First peak > 500 RFU                Upper marker selection: Last peak > 200 RFU
Ladder size (bp)75, 200, 300, 400, 500, 700, 1000, 1500, 2000, 3000, 4000, 5000, 7000, 10000, 20000
Quantification using: Lower Marker          Final concentration (ng/uL): 0.5000          Dilution factor: 12.0
```

Sample: SampH1
Well location: H1
Created: Tuesday, August 10, 2021 9:21:07 PM

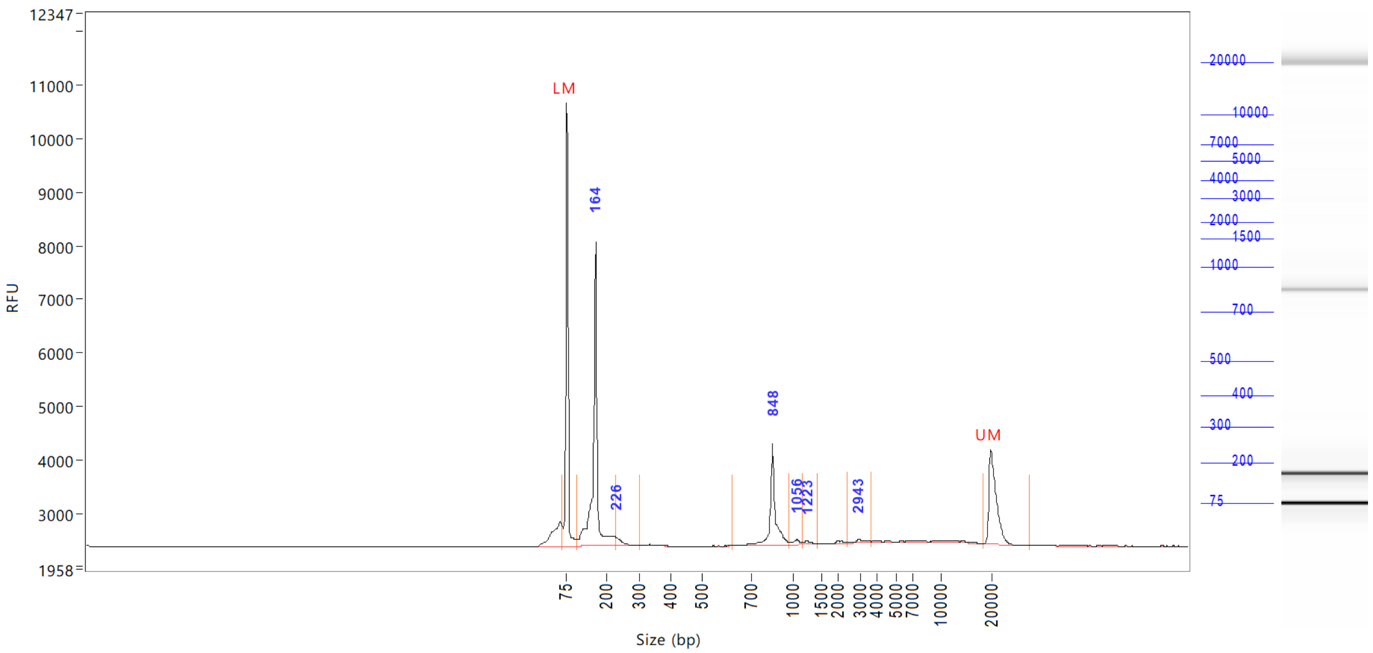

Peak	Size (bp)	Concentration (ng/uL)	From (bp)	To (bp)	Average size (bp)	CV%	RFU	Corrected peak area
1	75 (LM)	0.5000	64	108	74	7.97	8297	47.710
2	164	6.4631	108	226	161	12.40	5679	51.392
3	226	0.2825	226	298	242	5.89	162	2.247
4	848	2.0340	618	974	847	5.75	1900	16.173
5	1056	0.1373	974	1156	1044	4.88	89	1.092
6	1223	0.0834	1156	1433	1238	4.58	73	0.663
7	2943	0.1148	2458	3643	3052	8.90	65	0.912
8	20000 (UM)	0.1753	18659	27859	20503	4.23	1767	16.723

TIC:	9.1150	ng/uL
TIM:	72.2457	nmole/L
Total concentration:	9.4433	ng/uL

```
Sample peak width (sec): 5    Sample min peak height: 50    Sample baseline V to V?: Y    Sample baseline V to V points: 3
Sample filter: Binomial       Number of points for filter: 3  Sample start region (min): 0  Sample end region (min): 30
Marker peak width (sec): 5    Marker min peak height: 200   Marker baseline V to V?: Y    Marker baseline V to V points: 3
Lower marker selection: First peak > 500 RFU                Upper marker selection: Last peak > 200 RFU
Ladder size (bp)75, 200, 300, 400, 500, 700, 1000, 1500, 2000, 3000, 4000, 5000, 7000, 10000, 20000
Quantification using: Lower Marker          Final concentration (ng/uL): 0.5000          Dilution factor: 12.0
```

Sample: SampA2
Well location: A2
Created: Tuesday, August 10, 2021 9:21:07 PM

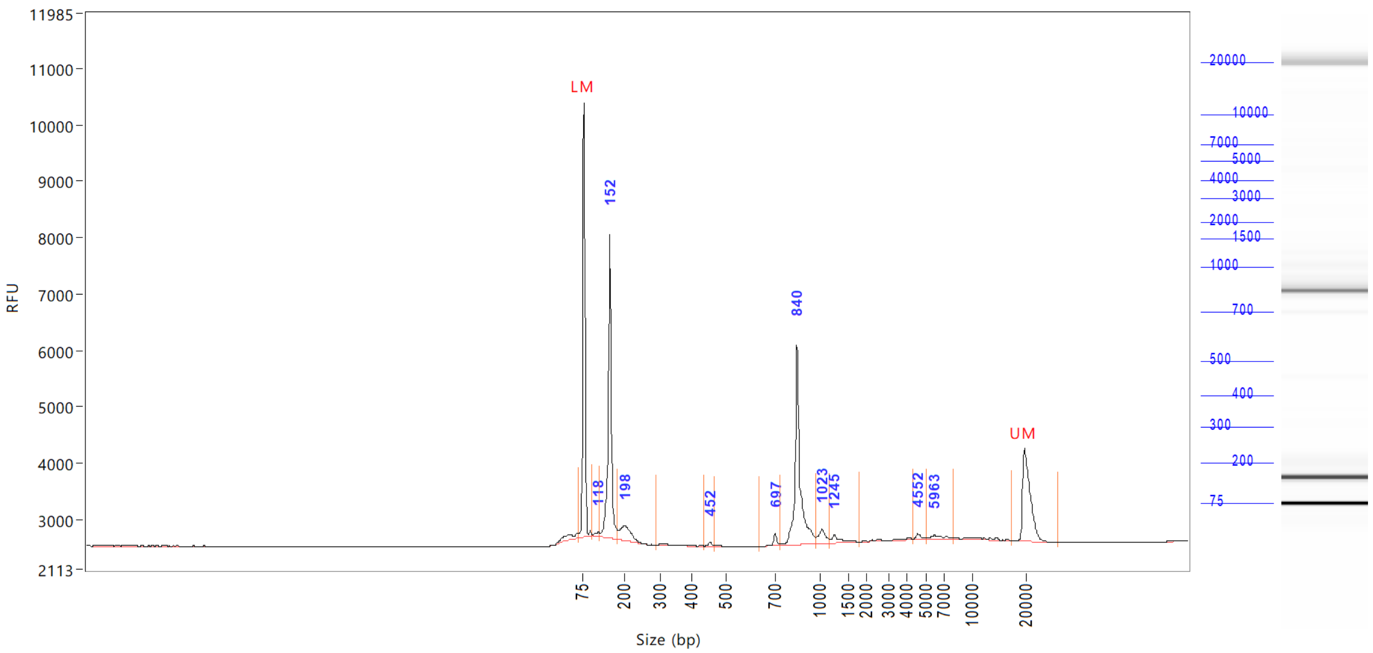

Peak	Size (bp)	Concentration (ng/uL)	From (bp)	To (bp)	Average size (bp)	CV%	RFU	Corrected peak area
1	75 (LM)	0.5000	62	101	73	4.99	7721	39.893
2	118	0.1211	101	124	114	5.68	71	0.805
3	152	5.7478	124	177	151	4.95	5400	38.216
4	198	0.9481	177	287	200	7.65	262	6.304
5	452	0.0697	433	466	451	1.31	76	0.463
6	697	0.1762	636	723	695	1.30	226	1.172
7	840	4.4166	723	970	850	4.29	3557	29.366
8	1023	0.4615	970	1167	1035	5.07	250	3.068
9	1245	0.2879	1167	1808	1316	9.52	146	1.915
10	4552	0.1278	4311	5075	4639	4.19	99	0.850
11	5963	0.2008	5075	8001	6384	11.81	70	1.335
12	20000 (UM)	0.1919	17561	26166	20401	4.12	1657	15.312

TIC:	12.5576	ng/uL
TIM:	82.5147	nmole/L
Total concentration:	12.9998	ng/uL

Sample peak width (sec): 5 Sample min peak height: 50 Sample baseline V to V?: Y Sample baseline V to V points: 3
Sample filter: Binomial Number of points for filter: 3 Sample start region (min): 0 Sample end region (min): 30
Marker peak width (sec): 5 Marker min peak height: 200 Marker baseline V to V?: Y Marker baseline V to V points: 3
Lower marker selection: First peak > 500 RFU Upper marker selection: Last peak > 200 RFU
Ladder size (bp)75, 200, 300, 400, 500, 700, 1000, 1500, 2000, 3000, 4000, 5000, 7000, 10000, 20000
Quantification using: Lower Marker Final concentration (ng/uL): 0.5000 Dilution factor: 12.0

Sample: SampB2
Well location: B2
Created: Tuesday, August 10, 2021 9:21:07 PM

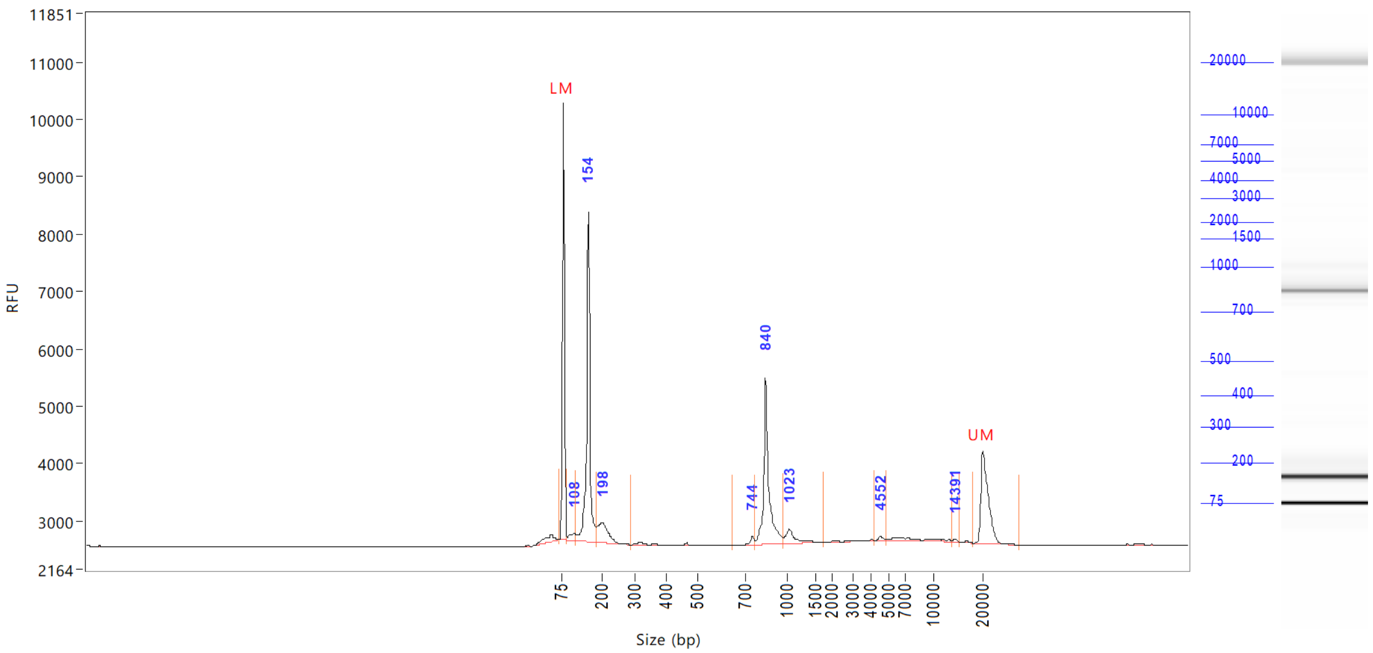

Peak	Size (bp)	Concentration (ng/uL)	From (bp)	To (bp)	Average size (bp)	CV%	RFU	Corrected peak area
1	75 (LM)	0.5000	64	87	74	3.92	7626	40.251
2	108	0.2759	87	114	102	7.86	121	1.851
3	154	6.6669	114	179	152	6.12	5743	44.725
4	198	1.2657	179	284	201	7.71	344	8.491
5	744	0.1657	646	766	733	3.50	149	1.111
6	840	3.6574	766	965	850	4.19	2896	24.536
7	1023	0.6157	965	1731	1098	12.82	247	4.131
8	4552	0.0790	4208	4897	4556	3.18	78	0.530
9	14391	0.0503	13659	15366	14325	2.80	54	0.337
10	20000 (UM)	0.1912	18171	27254	20507	4.39	1611	15.396

TIC:	12.7766	ng/uL
TIM:	95.2956	nmole/L
Total concentration:	13.3709	ng/uL

```
Sample peak width (sec): 5    Sample min peak height: 50    Sample baseline V to V?: Y    Sample baseline V to V points: 3
Sample filter: Binomial       Number of points for filter: 3  Sample start region (min): 0  Sample end region (min): 30
Marker peak width (sec): 5    Marker min peak height: 200   Marker baseline V to V?: Y    Marker baseline V to V points: 3
Lower marker selection: First peak > 500 RFU                Upper marker selection: Last peak > 200 RFU
Ladder size (bp)75, 200, 300, 400, 500, 700, 1000, 1500, 2000, 3000, 4000, 5000, 7000, 10000, 20000
Quantification using: Lower Marker          Final concentration (ng/uL): 0.5000          Dilution factor: 12.0
```

Sample: SampC2
Well location: C2
Created: Tuesday, August 10, 2021 9:21:07 PM

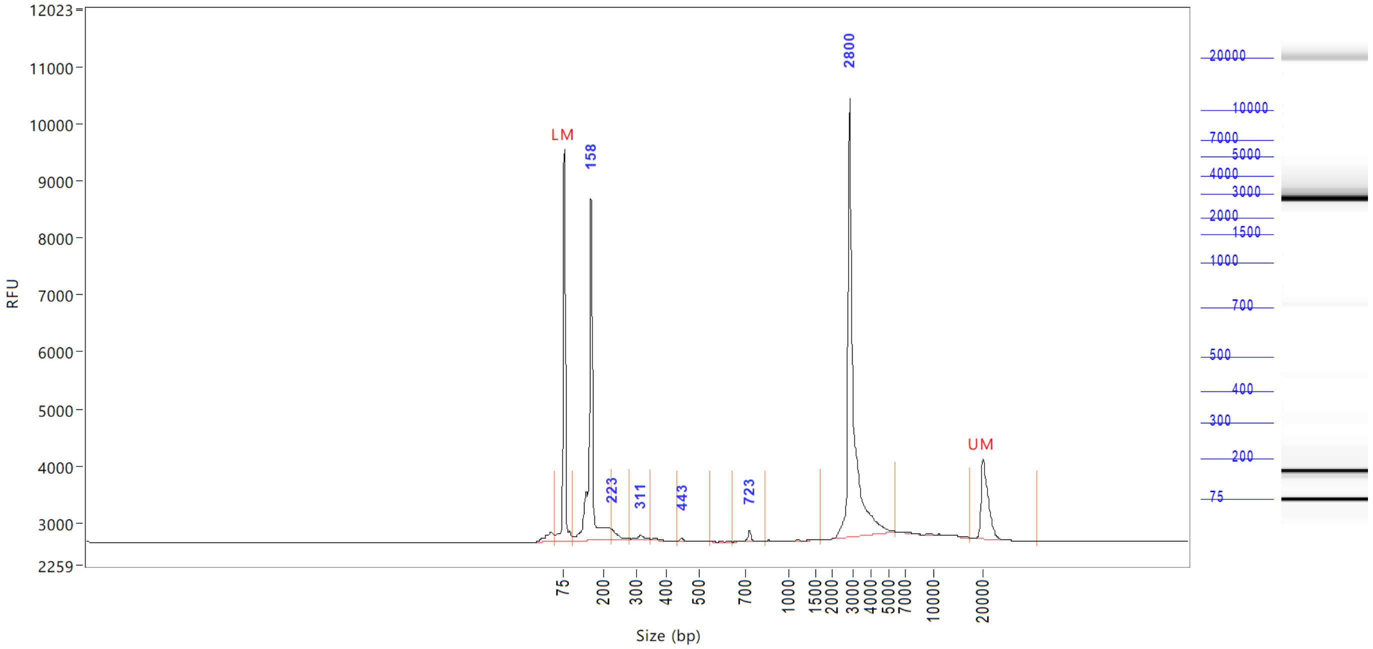

Peak	Size (bp)	Concentration (ng/uL)	From (bp)	To (bp)	Average size (bp)	CV%	RFU	Corrected peak area
1	75 (LM)	0.5000	45	103	72	8.60	6868	41.006
2	158	7.7883	103	223	159	11.80	6006	53.227
3	223	0.3463	223	278	237	5.49	185	2.367
4	311	0.1956	278	345	312	4.82	79	1.337
5	443	0.0898	431	544	465	6.83	62	0.614
6	723	0.2055	644	831	724	4.24	200	1.404
7	2800	9.7490	1635	5815	3014	14.83	7710	66.627
8	20000 (UM)	0.1570	17561	31002	20553	5.48	1399	12.879

TIC:	18.3745	ng/uL
TIM:	90.0564	nmole/L
Total concentration:	18.5901	ng/uL

Sample peak width (sec): 5 Sample min peak height: 50 Sample baseline V to V?: Y Sample baseline V to V points: 3
Sample filter: Binomial Number of points for filter: 3 Sample start region (min): 0 Sample end region (min): 30
Marker peak width (sec): 5 Marker min peak height: 200 Marker baseline V to V?: Y Marker baseline V to V points: 3
Lower marker selection: First peak > 500 RFU Upper marker selection: Last peak > 200 RFU
Ladder size (bp)75, 200, 300, 400, 500, 700, 1000, 1500, 2000, 3000, 4000, 5000, 7000, 10000, 20000
Quantification using: Lower Marker Final concentration (ng/uL): 0.5000 Dilution factor: 12.0

Sample: SampD2
Well location: D2
Created: Tuesday, August 10, 2021 9:21:07 PM

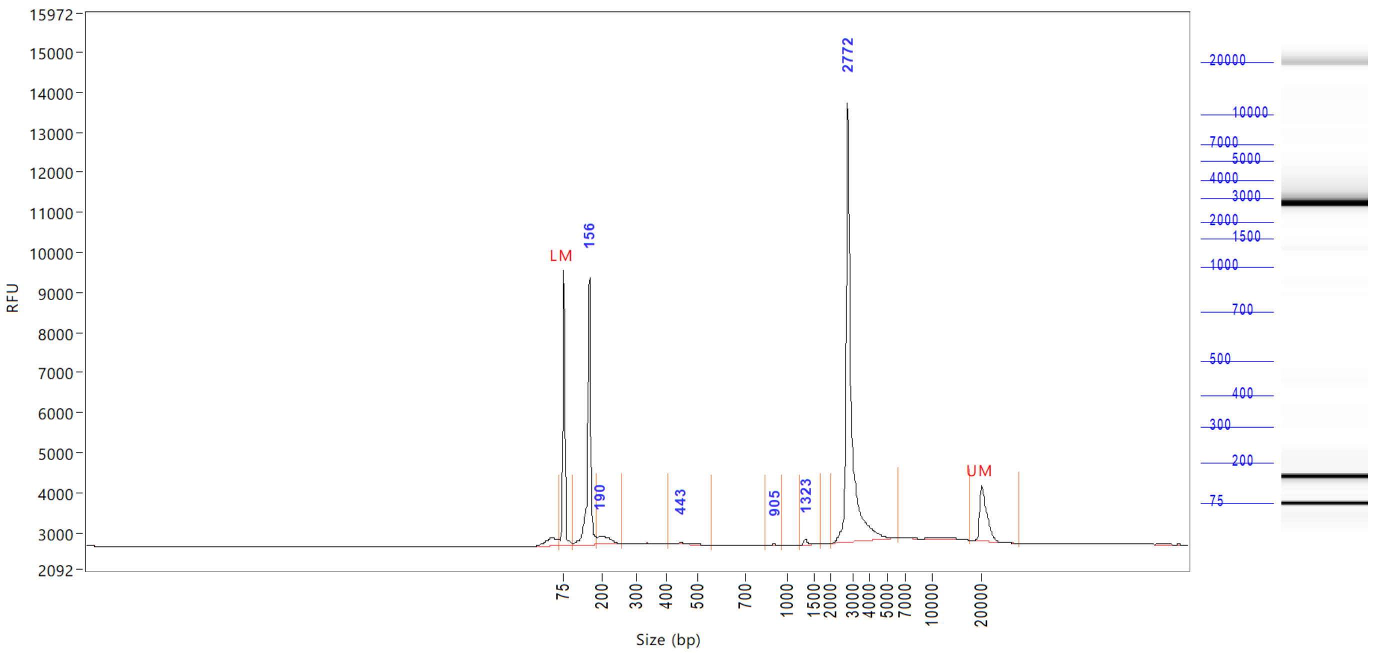

Peak	Size (bp)	Concentration (ng/uL)	From (bp)	To (bp)	Average size (bp)	CV%	RFU	Corrected peak area
1	75 (LM)	0.5000	64	103	73	5.97	6875	37.873
2	156	7.8887	103	181	152	6.61	6667	49.795
3	190	0.8650	181	255	203	7.30	203	5.460
4	443	0.1450	407	557	455	6.14	68	0.915
5	905	0.0511	844	957	897	1.95	56	0.322
6	1323	0.1417	1234	1674	1340	4.74	133	0.895
7	2772	13.8988	1981	6260	2938	13.76	10982	87.732
8	20000 (UM)	0.1689	17561	27617	20461	4.12	1400	12.794

TIC:	22.9903	ng/uL
TIM:	100.8857	nmole/L
Total concentration:	23.3597	ng/uL

```
Sample peak width (sec): 5    Sample min peak height: 50    Sample baseline V to V?: Y    Sample baseline V to V points: 3
Sample filter: Binomial       Number of points for filter: 3  Sample start region (min): 0  Sample end region (min): 30
Marker peak width (sec): 5    Marker min peak height: 200   Marker baseline V to V?: Y    Marker baseline V to V points: 3
Lower marker selection: First peak > 500 RFU                Upper marker selection: Last peak > 200 RFU
Ladder size (bp)75, 200, 300, 400, 500, 700, 1000, 1500, 2000, 3000, 4000, 5000, 7000, 10000, 20000
Quantification using: Lower Marker          Final concentration (ng/uL): 0.5000          Dilution factor: 12.0
```

Sample: SampE2
Well location: E2
Created: Tuesday, August 10, 2021 9:21:07 PM

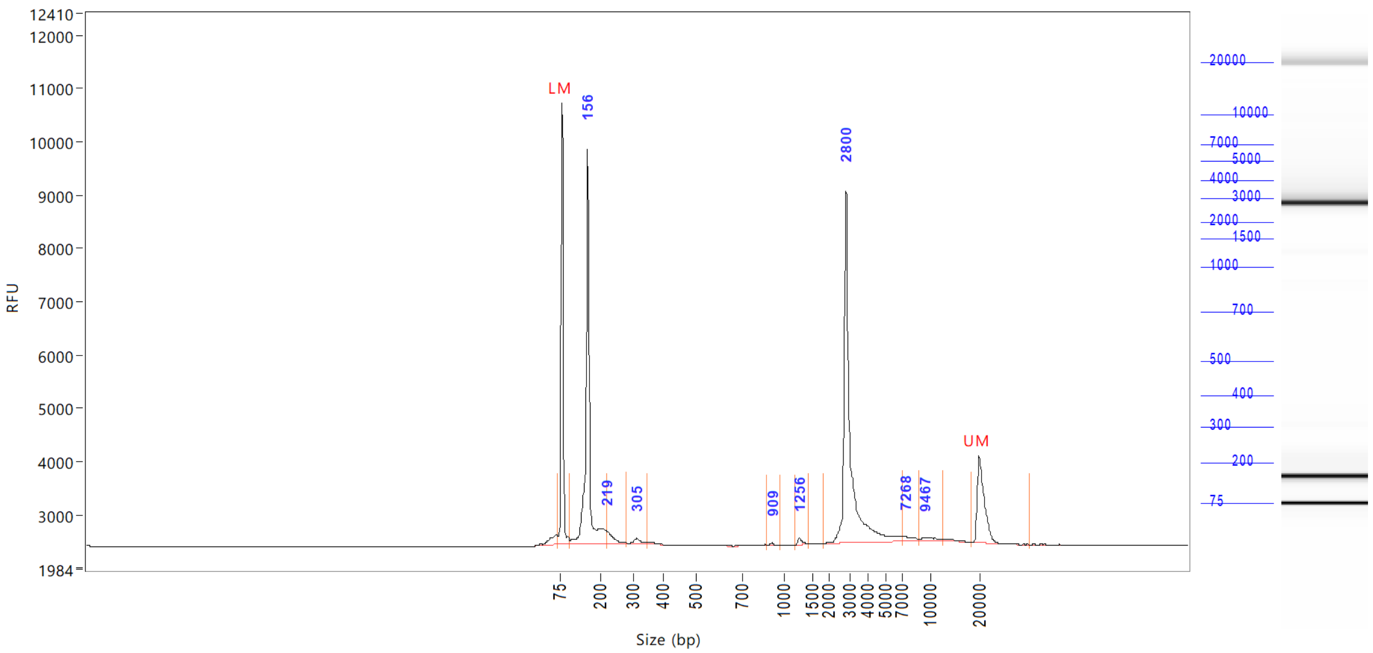

Peak	Size (bp)	Concentration (ng/uL)	From (bp)	To (bp)	Average size (bp)	CV%	RFU	Corrected peak area
1	75 (LM)	0.5000	64	101	74	5.87	8293	45.908
2	156	8.2711	101	219	157	11.53	7400	63.285
3	219	0.3489	219	278	232	4.89	227	2.670
4	305	0.1611	278	341	309	4.06	93	1.233
5	909	0.0393	870	961	901	1.72	52	0.301
6	1256	0.1229	1167	1433	1276	4.04	136	0.941
7	2800	7.8320	1846	7000	3112	22.85	6588	59.926
8	7268	0.1725	7000	8800	7708	6.83	90	1.320
9	9467	0.1573	8800	12684	10182	9.84	60	1.204
10	20000 (UM)	0.1637	18537	30277	20494	4.72	1623	15.031

TIC:	17.1052	ng/uL
TIM:	94.3571	nmole/L
Total concentration:	17.2780	ng/uL

```
Sample peak width (sec): 5    Sample min peak height: 50    Sample baseline V to V?: Y    Sample baseline V to V points: 3
Sample filter: Binomial       Number of points for filter: 3  Sample start region (min): 0  Sample end region (min): 30
Marker peak width (sec): 5    Marker min peak height: 200   Marker baseline V to V?: Y    Marker baseline V to V points: 3
Lower marker selection: First peak > 500 RFU                Upper marker selection: Last peak > 200 RFU
Ladder size (bp)75, 200, 300, 400, 500, 700, 1000, 1500, 2000, 3000, 4000, 5000, 7000, 10000, 20000
Quantification using: Lower Marker          Final concentration (ng/uL): 0.5000          Dilution factor: 12.0
```

Sample: SampF2
Well location: F2
Created: Tuesday, August 10, 2021 9:21:07 PM

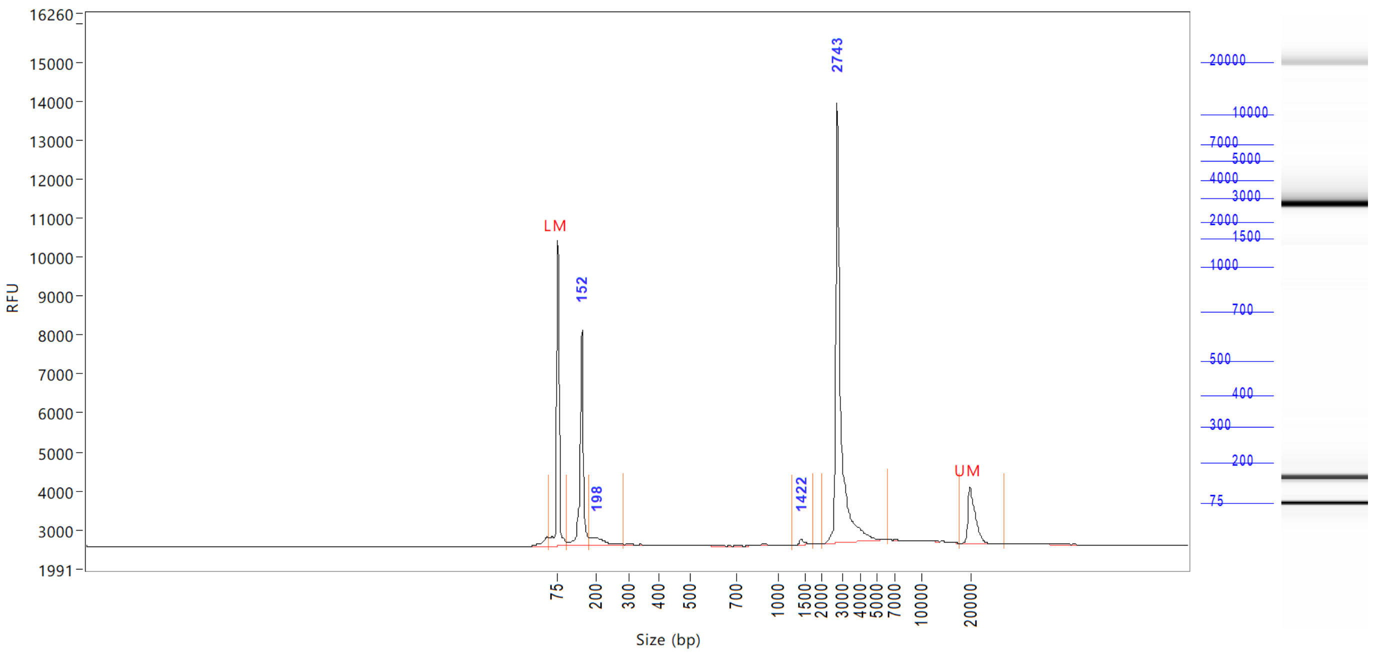

Peak	Size<br>(bp)	Concentration<br>(ng/uL)	From<br>(bp)	To<br>(bp)	Average size<br>(bp)	CV%	RFU	Corrected peak area
1	75 (LM)	0.5000	50	105	74	8.49	7833	47.912
2	152	5.4461	105	179	149	6.48	5515	43.489
3	198	0.7372	179	284	205	9.63	200	5.887
4	1422	0.1454	1245	1712	1440	3.86	157	1.161
5	2743	11.0086	2030	6186	2920	13.90	11311	87.908
6	20000 (UM)	0.1437	17927	27133	20514	4.44	1446	13.771

TIC:	17.3373	ng/uL
TIM:	72.3569	nmole/L
Total concentration:	17.6543	ng/uL

```
Sample peak width (sec): 5    Sample min peak height: 50    Sample baseline V to V?: Y    Sample baseline V to V points: 3
Sample filter: Binomial       Number of points for filter: 3  Sample start region (min): 0  Sample end region (min): 30
Marker peak width (sec): 5    Marker min peak height: 200   Marker baseline V to V?: Y    Marker baseline V to V points: 3
Lower marker selection: First peak > 500 RFU                Upper marker selection: Last peak > 200 RFU
Ladder size (bp)75, 200, 300, 400, 500, 700, 1000, 1500, 2000, 3000, 4000, 5000, 7000, 10000, 20000
Quantification using: Lower Marker          Final concentration (ng/uL): 0.5000          Dilution factor: 12.0
```

Sample: SampG2
Well location: G2
Created: Tuesday, August 10, 2021 9:21:07 PM

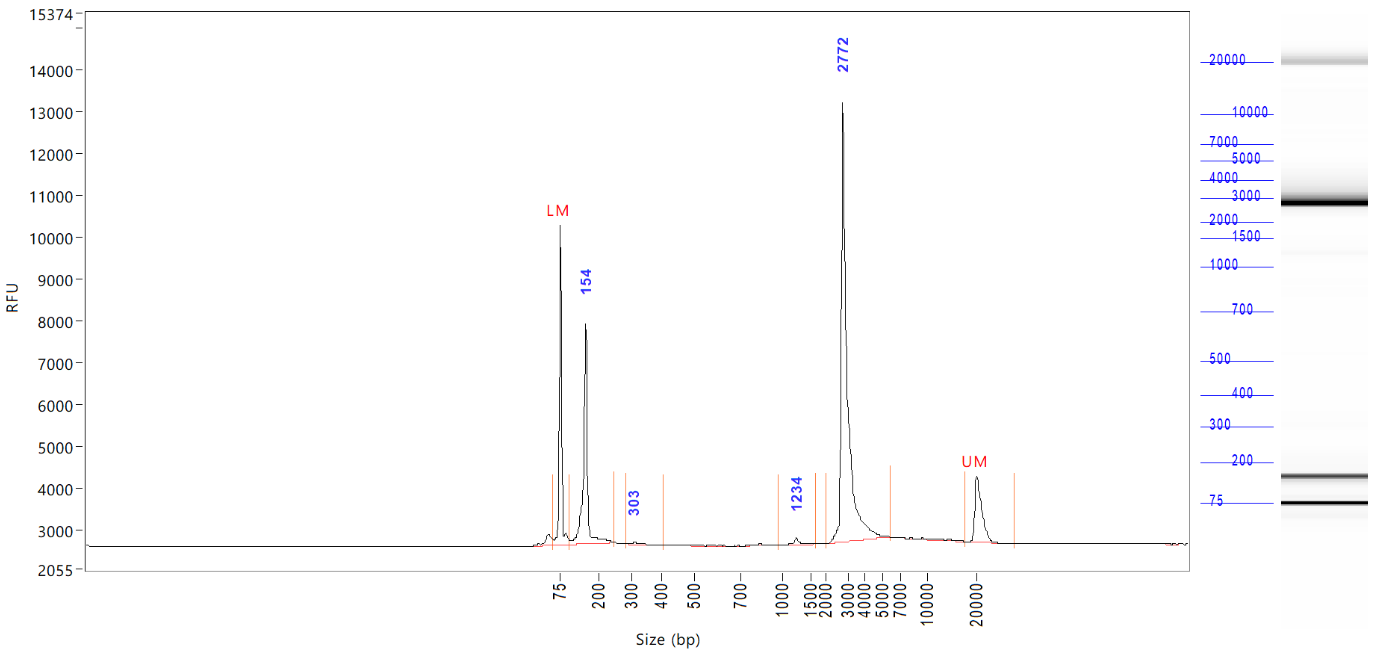

Peak	Size (bp)	Concentration (ng/uL)	From (bp)	To (bp)	Average size (bp)	CV%	RFU	Corrected peak area
1	75 (LM)	0.5000	54	105	73	8.30	7656	45.651
2	154	6.1952	105	244	153	11.53	5283	47.136
3	303	0.1280	278	401	315	7.05	56	0.974
4	1234	0.2025	970	1674	1265	8.84	161	1.541
5	2772	12.5679	2000	5815	2957	13.51	10540	95.622
6	20000 (UM)	0.1617	17683	27859	20509	4.30	1583	14.761

TIC:	19.0936	ng/uL
TIM:	74.4794	nmole/L
Total concentration:	19.4335	ng/uL

```
Sample peak width (sec): 5    Sample min peak height: 50    Sample baseline V to V?: Y    Sample baseline V to V points: 3
Sample filter: Binomial       Number of points for filter: 3  Sample start region (min): 0  Sample end region (min): 30
Marker peak width (sec): 5    Marker min peak height: 200   Marker baseline V to V?: Y    Marker baseline V to V points: 3
Lower marker selection: First peak > 500 RFU                Upper marker selection: Last peak > 200 RFU
Ladder size (bp)75, 200, 300, 400, 500, 700, 1000, 1500, 2000, 3000, 4000, 5000, 7000, 10000, 20000
Quantification using: Lower Marker          Final concentration (ng/uL): 0.5000          Dilution factor: 12.0
```

Sample: SampH2
Well location: H2
Created: Tuesday, August 10, 2021 9:21:07 PM

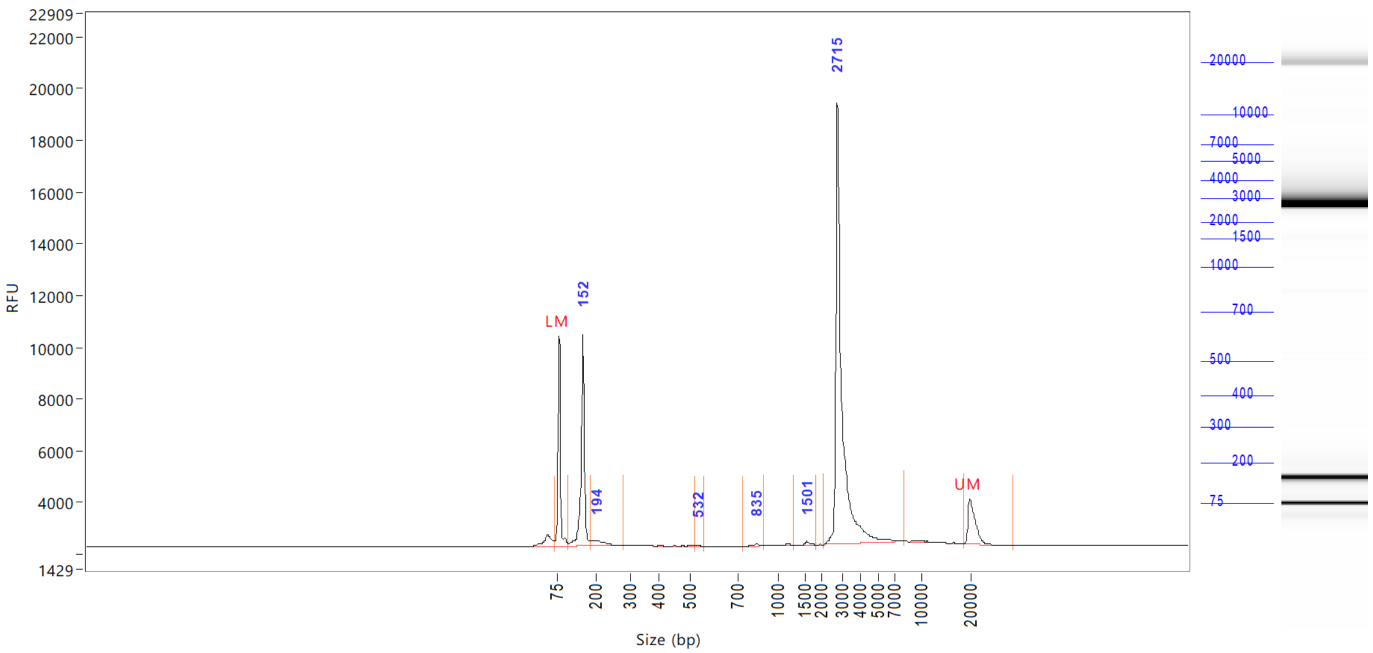

Peak	Size (bp)	Concentration (ng/uL)	From (bp)	To (bp)	Average size (bp)	CV%	RFU	Corrected peak area
1	75 (LM)	0.5000	66	107	75	8.00	8123	47.657
2	152	7.9693	107	177	148	6.20	8172	63.299
3	194	0.7383	177	280	205	9.76	198	5.864
4	532	0.0417	519	555	530	1.25	63	0.331
5	835	0.1078	740	896	822	2.73	113	0.856
6	1501	0.1383	1267	1808	1510	6.43	135	1.099
7	2715	19.6424	2030	8067	2980	18.08	17071	156.017
8	20000 (UM)	0.1740	18659	28826	20522	4.39	1758	16.586

TIC:	28.6378	ng/uL
TIM:	105.7646	nmole/L
Total concentration:	28.9184	ng/uL

```
Sample peak width (sec): 5    Sample min peak height: 50    Sample baseline V to V?: Y    Sample baseline V to V points: 3
Sample filter: Binomial       Number of points for filter: 3  Sample start region (min): 0  Sample end region (min): 30
Marker peak width (sec): 5    Marker min peak height: 200   Marker baseline V to V?: Y    Marker baseline V to V points: 3
Lower marker selection: First peak > 500 RFU                Upper marker selection: Last peak > 200 RFU
Ladder size (bp)75, 200, 300, 400, 500, 700, 1000, 1500, 2000, 3000, 4000, 5000, 7000, 10000, 20000
Quantification using: Lower Marker          Final concentration (ng/uL): 0.5000          Dilution factor: 12.0
```

Sample: SampA3
Well location: A3
Created: Tuesday, August 10, 2021 9:21:07 PM

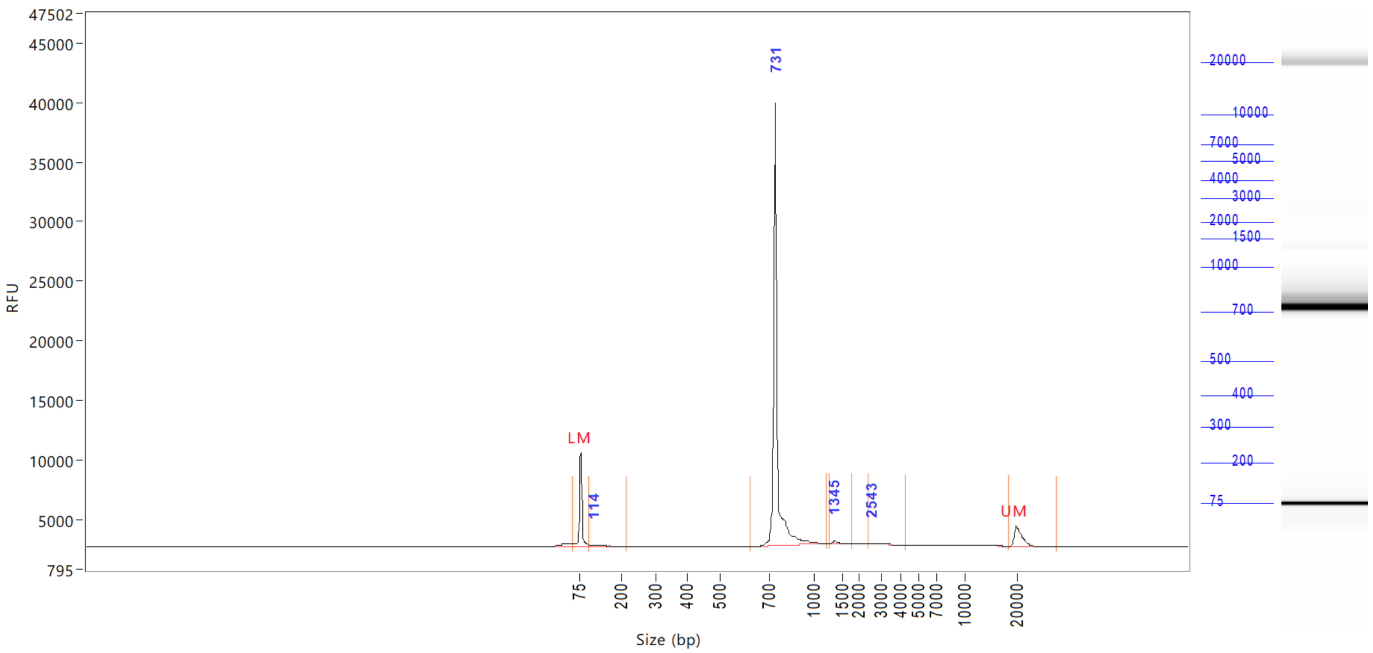

Peak	Size (bp)	Concentration (ng/uL)	From (bp)	To (bp)	Average size (bp)	CV%	RFU	Corrected peak area
1	75 (LM)	0.5000	48	103	73	9.04	7960	49.677
2	114	0.4322	103	212	135	19.11	133	3.578
3	731	26.8994	621	1212	743	6.42	37258	222.715
4	1345	0.1896	1267	1770	1373	5.48	234	1.570
5	2543	0.1424	2344	4311	2892	17.65	77	1.179
6	20000 (UM)	0.1574	18537	27738	20479	4.28	1671	15.634

TIC:	27.6636	ng/uL
TIM:	65.1522	nmole/L
Total concentration:	28.0366	ng/uL

Sample peak width (sec): 5 Sample min peak height: 50 Sample baseline V to V?: Y Sample baseline V to V points: 3
Sample filter: Binomial Number of points for filter: 3 Sample start region (min): 0 Sample end region (min): 30
Marker peak width (sec): 5 Marker min peak height: 200 Marker baseline V to V?: Y Marker baseline V to V points: 3
Lower marker selection: First peak > 500 RFU Upper marker selection: Last peak > 200 RFU
Ladder size (bp)75, 200, 300, 400, 500, 700, 1000, 1500, 2000, 3000, 4000, 5000, 7000, 10000, 20000
Quantification using: Lower Marker Final concentration (ng/uL): 0.5000 Dilution factor: 12.0

Sample: SampB3
Well location: B3
Created: Tuesday, August 10, 2021 9:21:07 PM

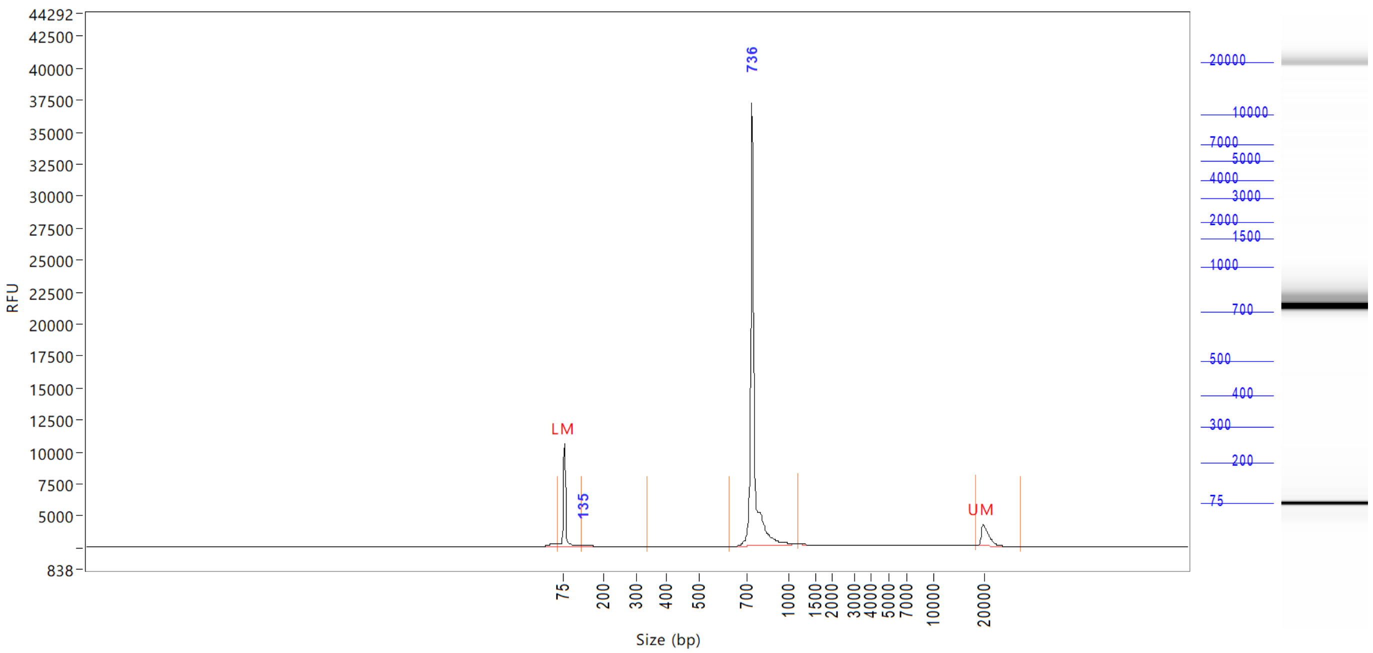

Peak	Size (bp)	Concentration (ng/uL)	From (bp)	To (bp)	Average size (bp)	CV%	RFU	Corrected peak area
1	75 (LM)	0.5000	54	129	76	14.01	8035	50.535
2	135	0.4850	129	335	181	26.08	100	4.085
3	736	25.9097	631	1167	750	6.23	34677	218.225
4	20000 (UM)	0.1551	18537	27375	20470	4.35	1656	15.674

TIC:	26.3948	ng/uL
TIM:	61.2615	nmole/L
Total concentration:	26.7518	ng/uL

```
Sample peak width (sec): 5    Sample min peak height: 50    Sample baseline V to V?: Y    Sample baseline V to V points: 3
Sample filter: Binomial       Number of points for filter: 3  Sample start region (min): 0  Sample end region (min): 30
Marker peak width (sec): 5    Marker min peak height: 200   Marker baseline V to V?: Y    Marker baseline V to V points: 3
Lower marker selection: First peak > 500 RFU                Upper marker selection: Last peak > 200 RFU
Ladder size (bp)75, 200, 300, 400, 500, 700, 1000, 1500, 2000, 3000, 4000, 5000, 7000, 10000, 20000
Quantification using: Lower Marker          Final concentration (ng/uL): 0.5000          Dilution factor: 12.0
```

Sample: SampC3
Well location: C3
Created: Tuesday, August 10, 2021 9:21:07 PM

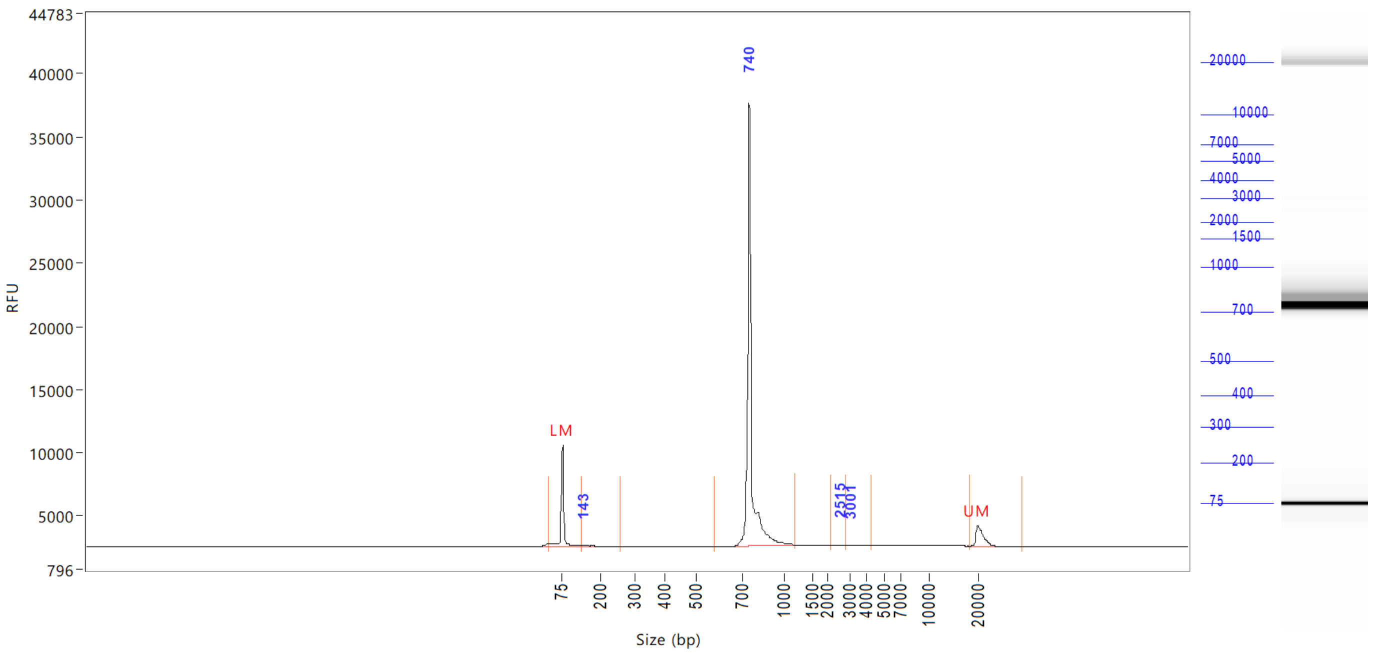

Peak	Size (bp)	Concentration (ng/uL)	From (bp)	To (bp)	Average size (bp)	CV%	RFU	Corrected peak area
1	75 (LM)	0.5000	30	135	73	20.14	7994	56.155
2	143	0.3617	135	255	170	16.06	115	3.385
3	740	25.4450	578	1190	755	6.47	35114	238.144
4	2515	0.0764	2115	2857	2523	6.93	64	0.715
5	3001	0.1168	2857	4277	3413	11.71	56	1.093
6	20000 (UM)	0.1395	18415	29068	20520	4.64	1647	15.669

TIC:	25.9999	ng/uL
TIM:	59.0702	nmole/L
Total concentration:	26.2480	ng/uL

Sample peak width (sec): 5   Sample min peak height: 50   Sample baseline V to V?: Y   Sample baseline V to V points: 3
Sample filter: Binomial   Number of points for filter: 3 Sample start region (min): 0 Sample end region (min): 30
Marker peak width (sec): 5   Marker min peak height: 200   Marker baseline V to V?: Y   Marker baseline V to V points: 3
Lower marker selection: First peak > 500 RFU   Upper marker selection: Last peak > 200 RFU
Ladder size (bp)75, 200, 300, 400, 500, 700, 1000, 1500, 2000, 3000, 4000, 5000, 7000, 10000, 20000
Quantification using: Lower Marker   Final concentration (ng/uL): 0.5000   Dilution factor: 12.0

Sample: SampD3
Well location: D3
Created: Tuesday, August 10, 2021 9:21:07 PM

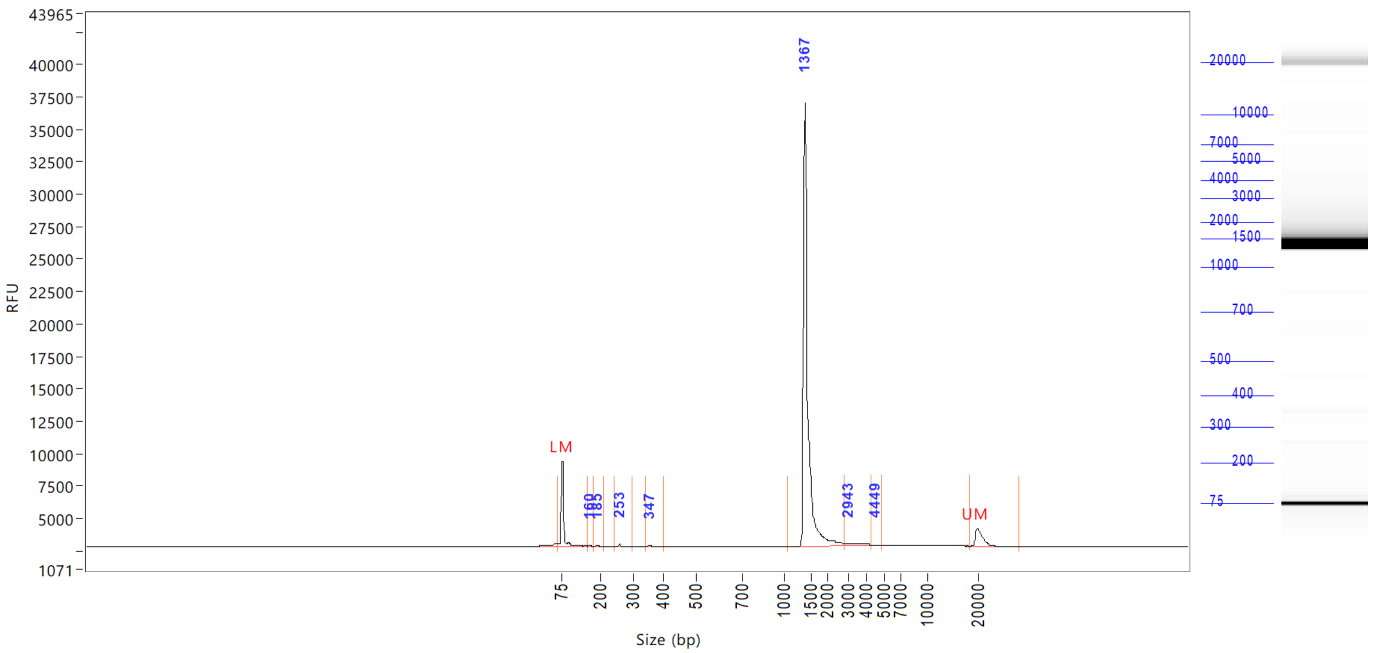

Peak	Size (bp)	Concentration (ng/uL)	From (bp)	To (bp)	Average size (bp)	CV%	RFU	Corrected peak area
1	75 (LM)	0.5000	62	154	76	16.24	6600	42.359
2	160	0.0675	154	175	163	3.75	52	0.476
3	185	0.0762	175	208	184	3.19	70	0.538
4	253	0.1313	239	293	252	2.90	164	0.927
5	347	0.1114	335	396	347	2.53	145	0.786
6	1367	33.0281	1056	2800	1438	13.36	34273	233.173
7	2943	0.6100	2800	4277	3387	12.71	187	4.306
8	4449	0.1058	4277	4897	4540	3.95	64	0.747
9	20000 (UM)	0.1511	18537	28584	20490	4.37	1364	12.801

TIC:	34.1303	ng/uL
TIM:	40.8878	nmole/L
Total concentration:	34.5236	ng/uL

Sample peak width (sec): 5   Sample min peak height: 50   Sample baseline V to V?: Y   Sample baseline V to V points: 3
Sample filter: Binomial   Number of points for filter: 3 Sample start region (min): 0 Sample end region (min): 30
Marker peak width (sec): 5   Marker min peak height: 200   Marker baseline V to V?: Y   Marker baseline V to V points: 3
Lower marker selection: First peak > 500 RFU   Upper marker selection: Last peak > 200 RFU
Ladder size (bp)75, 200, 300, 400, 500, 700, 1000, 1500, 2000, 3000, 4000, 5000, 7000, 10000, 20000
Quantification using: Lower Marker   Final concentration (ng/uL): 0.5000   Dilution factor: 12.0

Sample: SampE3
Well location: E3
Created: Tuesday, August 10, 2021 9:21:07 PM

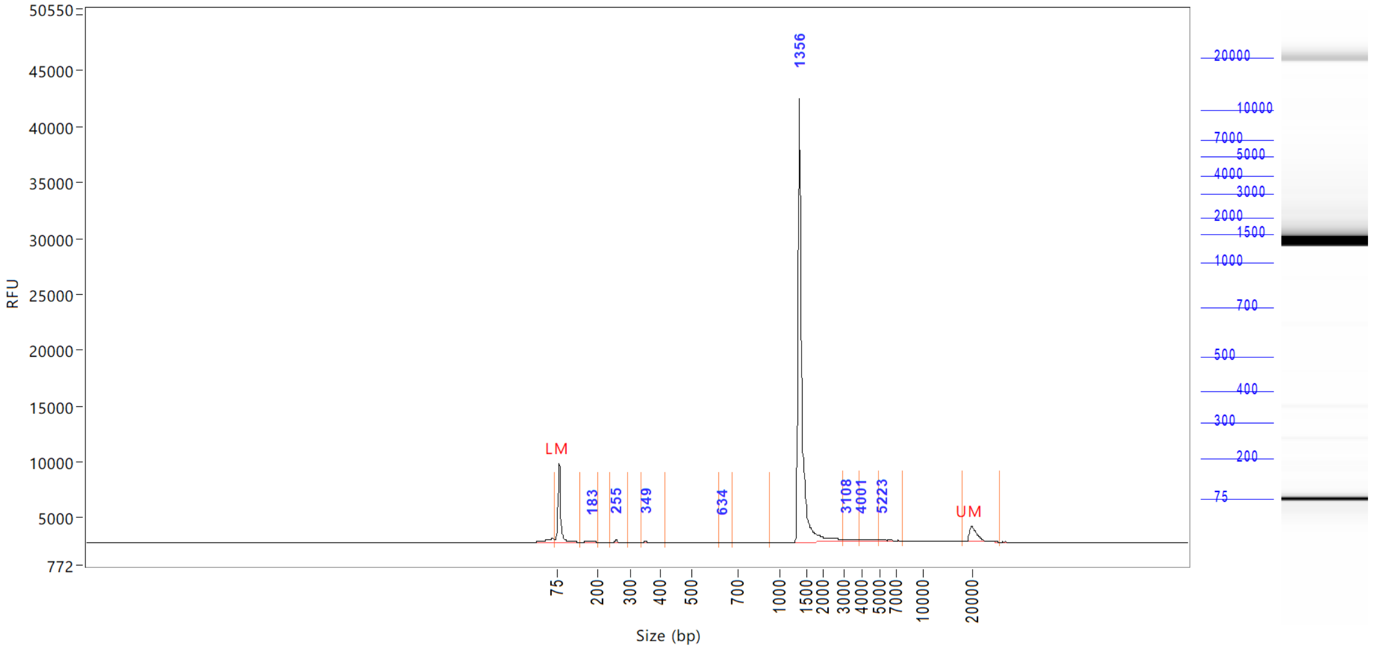

Peak	Size (bp)	Concentration (ng/uL)	From (bp)	To (bp)	Average size (bp)	CV%	RFU	Corrected peak area
1	75 (LM)	0.5000	66	145	77	13.12	7061	46.708
2	183	0.2211	145	201	170	8.16	75	1.721
3	255	0.1389	239	291	252	1.99	196	1.081
4	349	0.1187	335	414	348	2.20	184	0.924
5	634	0.0362	616	677	632	1.43	52	0.282
6	1356	32.5176	926	2914	1420	14.46	39767	253.136
7	3108	0.4986	2914	3893	3346	8.81	200	3.881
8	4001	0.3207	3893	5000	4336	7.32	142	2.497
9	5223	0.1336	5000	7667	5967	12.14	57	1.040
10	20000 (UM)	0.1430	18049	25803	20445	4.36	1398	13.363

TIC:	33.9854	ng/uL
TIM:	41.7976	nmole/L
Total concentration:	34.2062	ng/uL

```
Sample peak width (sec): 5    Sample min peak height: 50    Sample baseline V to V?: Y    Sample baseline V to V points: 3
Sample filter: Binomial       Number of points for filter: 3  Sample start region (min): 0  Sample end region (min): 30
Marker peak width (sec): 5    Marker min peak height: 200   Marker baseline V to V?: Y    Marker baseline V to V points: 3
Lower marker selection: First peak > 500 RFU                Upper marker selection: Last peak > 200 RFU
Ladder size (bp)75, 200, 300, 400, 500, 700, 1000, 1500, 2000, 3000, 4000, 5000, 7000, 10000, 20000
Quantification using: Lower Marker          Final concentration (ng/uL): 0.5000          Dilution factor: 12.0
```

Sample: SampF3
Well location: F3
Created: Tuesday, August 10, 2021 9:21:07 PM

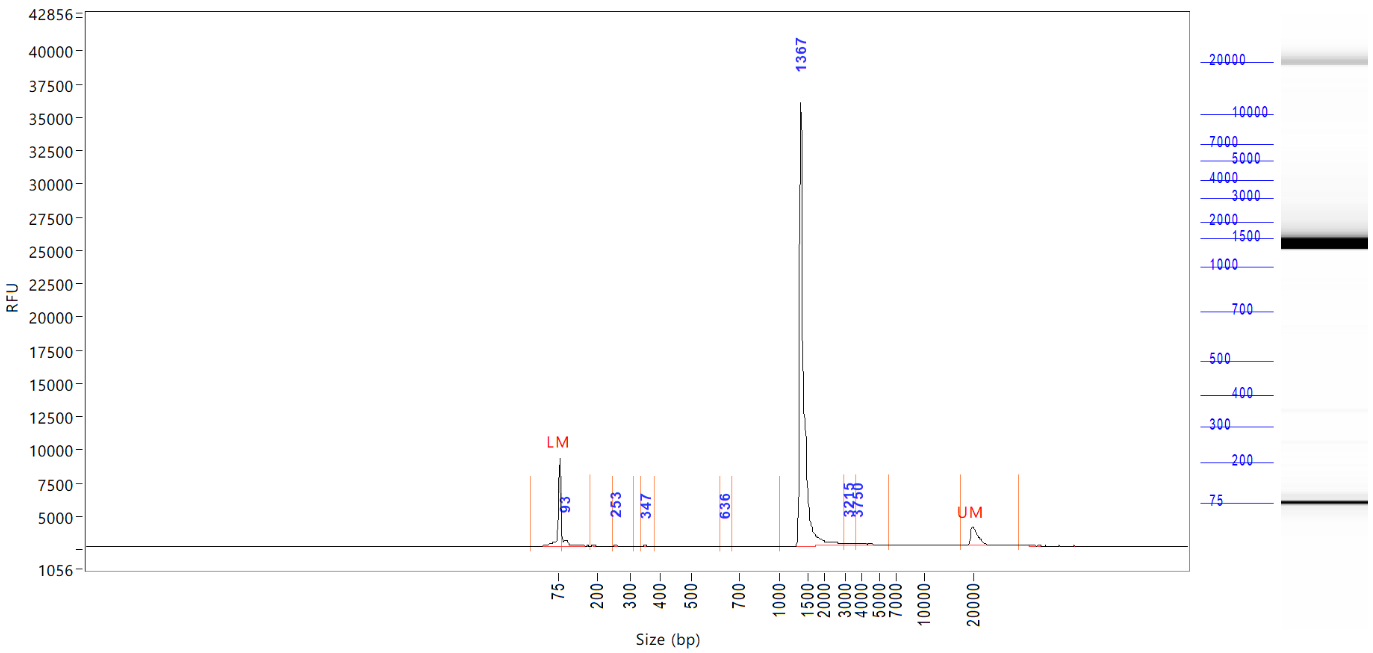

Peak	Size (bp)	Concentration (ng/uL)	From (bp)	To (bp)	Average size (bp)	CV%	RFU	Corrected peak area
1	75 (LM)	0.5000	0	86	70	18.61	6638	44.837
2	93	0.9557	86	175	103	17.13	468	7.142
3	253	0.0905	244	305	254	4.19	130	0.676
4	347	0.0802	335	378	347	1.64	116	0.599
5	636	0.0383	618	674	634	1.34	51	0.286
6	1367	30.9802	991	3000	1431	13.15	33387	231.510
7	3215	0.2197	3000	3643	3292	6.03	124	1.642
8	3750	0.3020	3643	6112	4292	12.15	97	2.257
9	20000 (UM)	0.1491	17439	29430	20547	5.05	1378	13.374

TIC:	32.6666	ng/uL
TIM:	52.1646	nmole/L
Total concentration:	33.0153	ng/uL

Sample peak width (sec): 5   Sample min peak height: 50   Sample baseline V to V?: Y   Sample baseline V to V points: 3
Sample filter: Binomial   Number of points for filter: 3 Sample start region (min): 0 Sample end region (min): 30
Marker peak width (sec): 5   Marker min peak height: 200   Marker baseline V to V?: Y   Marker baseline V to V points: 3
Lower marker selection: First peak > 500 RFU   Upper marker selection: Last peak > 200 RFU
Ladder size (bp)75, 200, 300, 400, 500, 700, 1000, 1500, 2000, 3000, 4000, 5000, 7000, 10000, 20000
Quantification using: Lower Marker   Final concentration (ng/uL): 0.5000   Dilution factor: 12.0

Sample: SampG3
Well location: G3
Created: Tuesday, August 10, 2021 9:21:07 PM

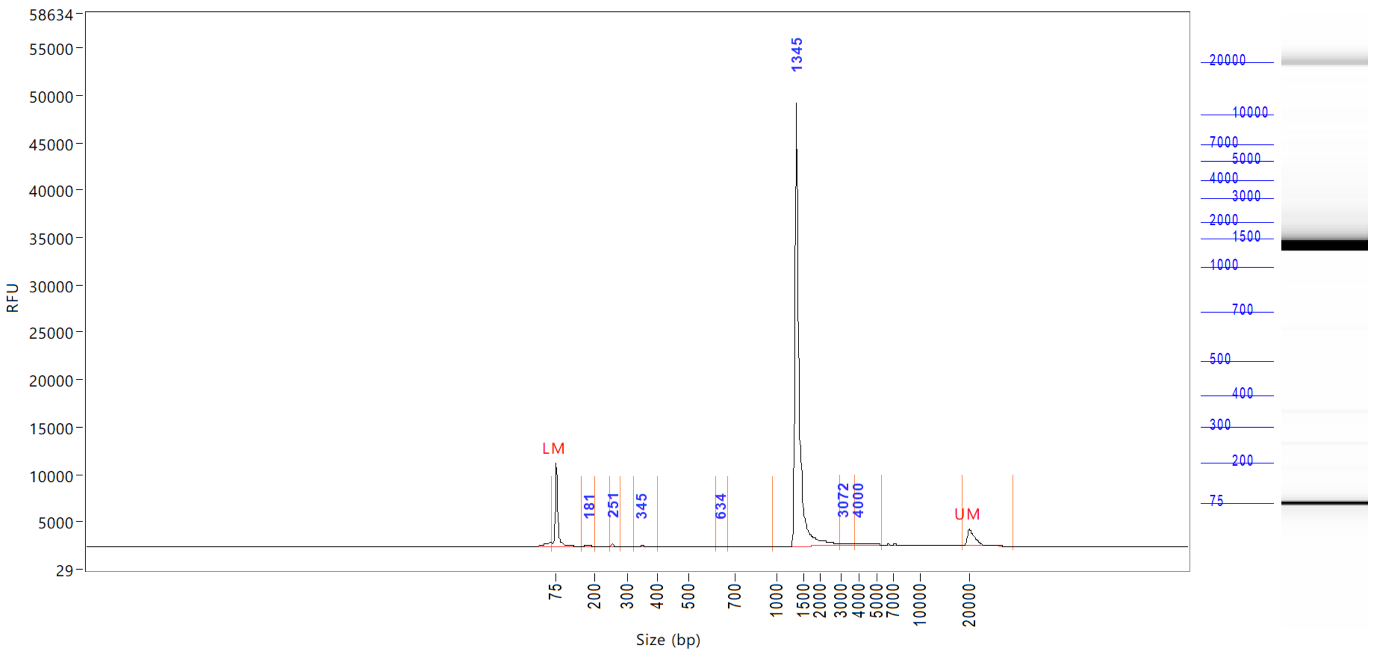

Peak	Size (bp)	Concentration (ng/uL)	From (bp)	To (bp)	Average size (bp)	CV%	RFU	Corrected peak area
1	75 (LM)	0.5000	64	154	76	13.95	8746	56.876
2	181	0.1402	154	200	175	5.35	103	1.329
3	251	0.1289	242	275	250	1.52	243	1.222
4	345	0.1475	321	398	344	3.15	211	1.399
5	634	0.0398	616	669	630	1.32	70	0.378
6	1345	34.0672	970	2914	1410	13.90	46817	322.933
7	3072	0.3316	2914	3750	3269	7.63	193	3.143
8	4000	0.2762	3750	5667	4300	9.77	128	2.618
9	20000 (UM)	0.1497	18537	28947	20542	4.68	1737	17.030

TIC:	35.1314	ng/uL
TIM:	43.0182	nmole/L
Total concentration:	35.4248	ng/uL

Sample peak width (sec): 5   Sample min peak height: 50   Sample baseline V to V?: Y   Sample baseline V to V points: 3
Sample filter: Binomial   Number of points for filter: 3 Sample start region (min): 0 Sample end region (min): 30
Marker peak width (sec): 5   Marker min peak height: 200   Marker baseline V to V?: Y   Marker baseline V to V points: 3
Lower marker selection: First peak > 500 RFU   Upper marker selection: Last peak > 200 RFU
Ladder size (bp)75, 200, 300, 400, 500, 700, 1000, 1500, 2000, 3000, 4000, 5000, 7000, 10000, 20000
Quantification using: Lower Marker   Final concentration (ng/uL): 0.5000   Dilution factor: 12.0

Sample: SampH3
Well location: H3
Created: Tuesday, August 10, 2021 9:21:07 PM

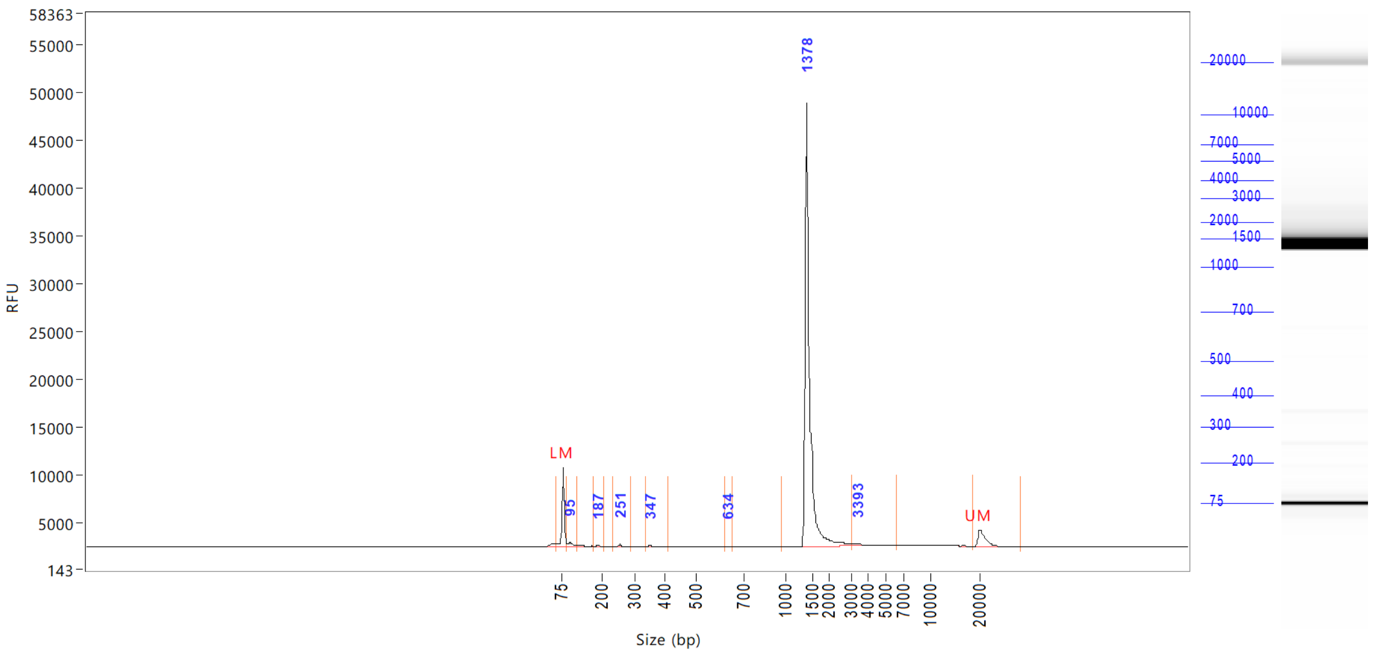

Peak	Size (bp)	Concentration (ng/uL)	From (bp)	To (bp)	Average size (bp)	CV%	RFU	Corrected peak area
1	75 (LM)	0.5000	54	86	73	6.05	8342	48.248
2	95	0.6208	86	120	97	8.54	385	4.992
3	187	0.0711	171	206	184	3.26	77	0.572
4	251	0.1586	232	286	249	2.50	220	1.275
5	347	0.1377	335	407	348	3.04	206	1.107
6	634	0.0436	618	649	631	0.86	73	0.350
7	1378	41.2579	965	3001	1434	13.97	46523	331.770
8	3393	0.5267	3001	6260	3740	15.24	165	4.235
9	20000 (UM)	0.1669	18659	28463	20538	4.39	1714	16.105

TIC:	42.8163	ng/uL
TIM:	60.5471	nmole/L
Total concentration:	43.1894	ng/uL

```
Sample peak width (sec): 5    Sample min peak height: 50    Sample baseline V to V?: Y    Sample baseline V to V points: 3
Sample filter: Binomial       Number of points for filter: 3  Sample start region (min): 0  Sample end region (min): 30
Marker peak width (sec): 5    Marker min peak height: 200   Marker baseline V to V?: Y    Marker baseline V to V points: 3
Lower marker selection: First peak > 500 RFU                Upper marker selection: Last peak > 200 RFU
Ladder size (bp)75, 200, 300, 400, 500, 700, 1000, 1500, 2000, 3000, 4000, 5000, 7000, 10000, 20000
Quantification using: Lower Marker          Final concentration (ng/uL): 0.5000          Dilution factor: 12.0
```

Sample: SampA4
Well location: A4
Created: Tuesday, August 10, 2021 9:21:07 PM

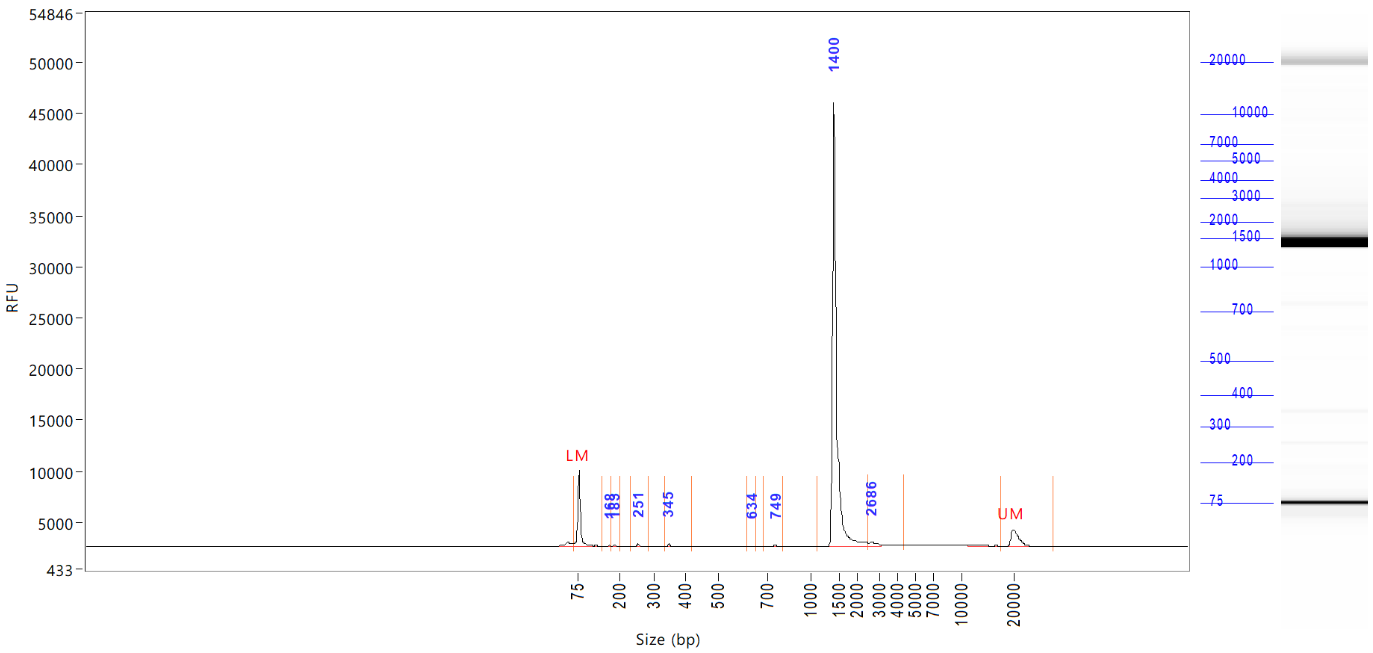

Peak	Size (bp)	Concentration (ng/uL)	From (bp)	To (bp)	Average size (bp)	CV%	RFU	Corrected peak area
1	75 (LM)	0.5000	60	147	76	13.85	7500	50.460
2	168	0.0746	147	173	161	4.99	53	0.627
3	183	0.0835	173	201	182	3.08	80	0.702
4	251	0.1664	233	282	249	2.29	244	1.399
5	345	0.1346	335	420	347	3.22	218	1.132
6	634	0.0424	616	654	631	1.15	67	0.357
7	749	0.1223	685	805	746	2.78	164	1.028
8	1400	32.8247	1101	2543	1444	10.77	43488	276.053
9	2686	0.7626	2543	4380	3124	16.17	350	6.413
10	20000 (UM)	0.1525	17683	27859	20510	4.32	1627	15.391

TIC:	34.2110	ng/uL
TIM:	41.4527	nmole/L
Total concentration:	34.6964	ng/uL

```
Sample peak width (sec): 5    Sample min peak height: 50    Sample baseline V to V?: Y    Sample baseline V to V points: 3
Sample filter: Binomial       Number of points for filter: 3  Sample start region (min): 0  Sample end region (min): 30
Marker peak width (sec): 5    Marker min peak height: 200   Marker baseline V to V?: Y    Marker baseline V to V points: 3
Lower marker selection: First peak > 500 RFU                Upper marker selection: Last peak > 200 RFU
Ladder size (bp)75, 200, 300, 400, 500, 700, 1000, 1500, 2000, 3000, 4000, 5000, 7000, 10000, 20000
Quantification using: Lower Marker          Final concentration (ng/uL): 0.5000          Dilution factor: 12.0
```

Sample: SampB4
Well location: B4
Created: Tuesday, August 10, 2021 9:21:07 PM

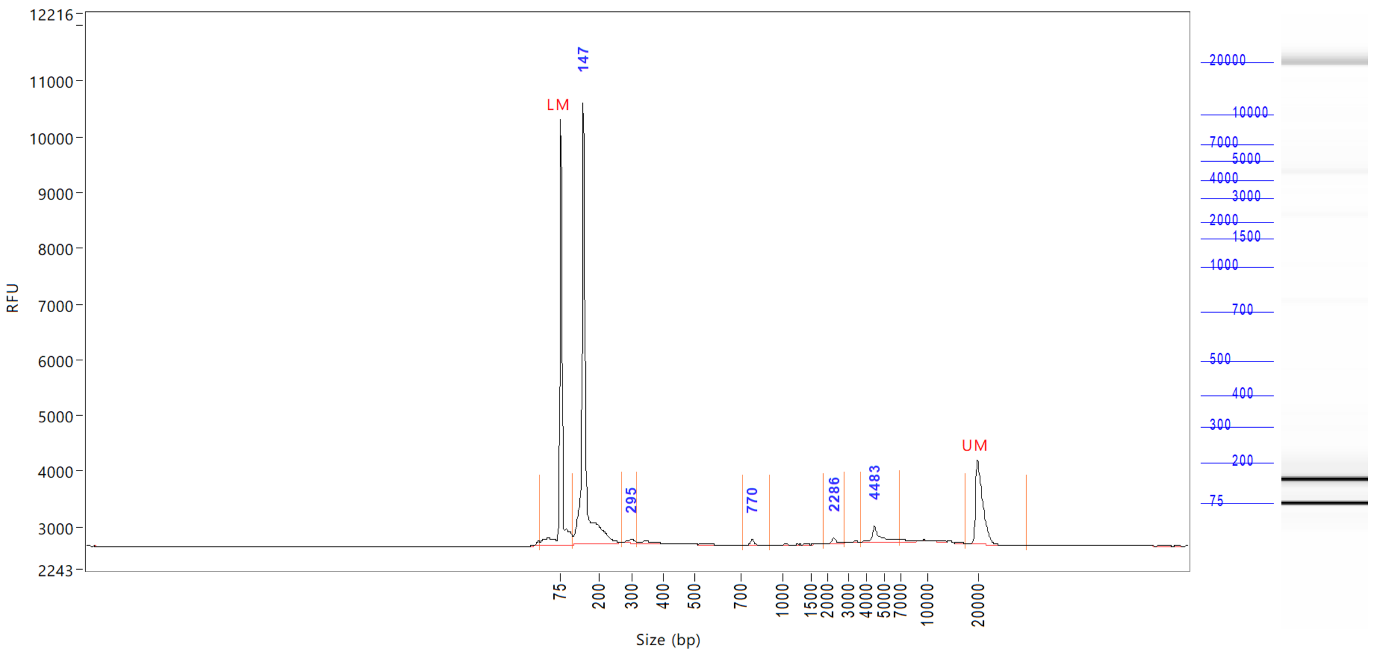

Peak	Size (bp)	Concentration (ng/uL)	From (bp)	To (bp)	Average size (bp)	CV%	RFU	Corrected peak area
1	75 (LM)	0.5000	11	116	73	19.71	7631	49.286
2	147	8.3570	116	268	153	14.52	7930	68.647
3	295	0.1363	268	313	291	3.66	69	1.119
4	770	0.1010	710	900	779	4.47	112	0.830
5	2286	0.1009	1885	2772	2307	6.85	105	0.829
6	4483	0.4505	3715	7000	4859	15.07	292	3.701
7	20000 (UM)	0.1438	17561	30035	20530	4.65	1509	14.172

TIC:	9.1458	ng/uL
TIM:	90.9816	nmole/L
Total concentration:	9.5642	ng/uL

Sample peak width (sec): 5   Sample min peak height: 50   Sample baseline V to V?: Y   Sample baseline V to V points: 3
Sample filter: Binomial   Number of points for filter: 3 Sample start region (min): 0 Sample end region (min): 30
Marker peak width (sec): 5   Marker min peak height: 200   Marker baseline V to V?: Y   Marker baseline V to V points: 3
Lower marker selection: First peak > 500 RFU   Upper marker selection: Last peak > 200 RFU
Ladder size (bp)75, 200, 300, 400, 500, 700, 1000, 1500, 2000, 3000, 4000, 5000, 7000, 10000, 20000
Quantification using: Lower Marker   Final concentration (ng/uL): 0.5000   Dilution factor: 12.0

Sample: SampC4
Well location: C4
Created: Tuesday, August 10, 2021 9:21:07 PM

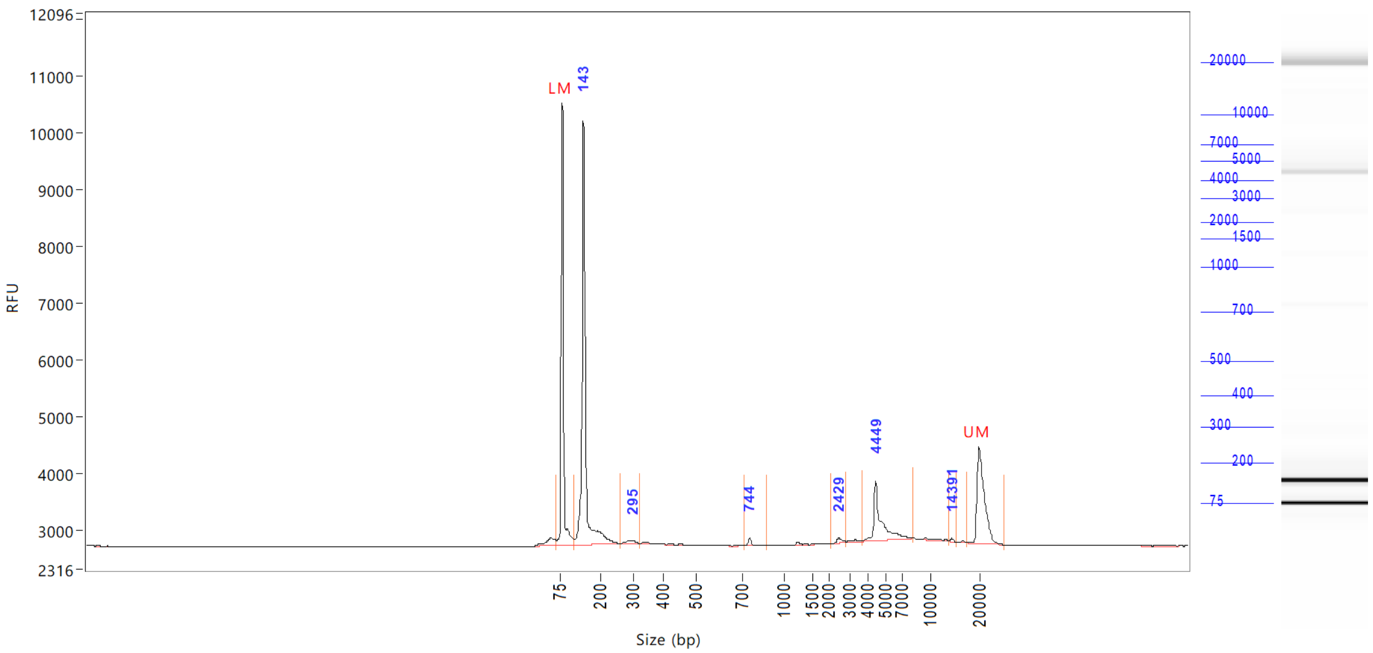

Peak	Size (bp)	Concentration (ng/uL)	From (bp)	To (bp)	Average size (bp)	CV%	RFU	Corrected peak area
1	75 (LM)	0.5000	60	112	75	9.09	7795	46.770
2	143	7.7068	112	255	148	13.03	7449	60.075
3	295	0.1533	255	319	287	4.82	56	1.195
4	744	0.1129	701	866	748	3.70	136	0.880
5	2429	0.0937	2087	2800	2487	5.45	90	0.730
6	4449	1.4183	3679	8067	4832	14.90	1059	11.055
7	14391	0.0339	13781	15366	14323	2.09	56	0.264
8	20000 (UM)	0.1731	17561	25199	20441	4.18	1700	16.197

TIC:	9.5189	ng/uL
TIM:	87.2565	nmole/L
Total concentration:	9.9705	ng/uL

Sample peak width (sec): 5   Sample min peak height: 50   Sample baseline V to V?: Y   Sample baseline V to V points: 3
Sample filter: Binomial   Number of points for filter: 3 Sample start region (min): 0 Sample end region (min): 30
Marker peak width (sec): 5   Marker min peak height: 200   Marker baseline V to V?: Y   Marker baseline V to V points: 3
Lower marker selection: First peak > 500 RFU   Upper marker selection: Last peak > 200 RFU
Ladder size (bp)75, 200, 300, 400, 500, 700, 1000, 1500, 2000, 3000, 4000, 5000, 7000, 10000, 20000
Quantification using: Lower Marker   Final concentration (ng/uL): 0.5000   Dilution factor: 12.0

Sample: SampD4
Well location: D4
Created: Tuesday, August 10, 2021 9:21:07 PM

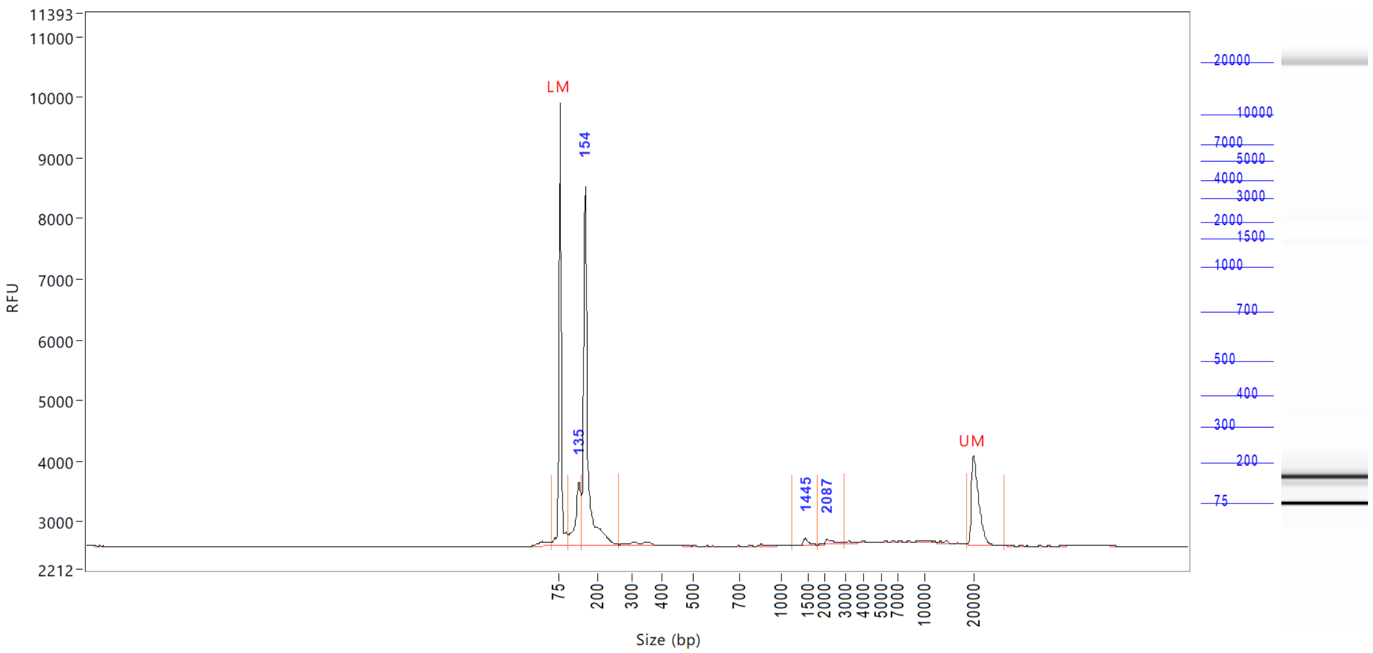

Peak	Size (bp)	Concentration (ng/uL)	From (bp)	To (bp)	Average size (bp)	CV%	RFU	Corrected peak area
1	75 (LM)	0.5000	50	99	73	7.89	7324	42.417
2	135	2.0468	99	143	128	8.76	1046	14.469
3	154	7.6234	143	260	162	11.44	5921	53.894
4	1445	0.1392	1201	1750	1435	5.60	113	0.984
5	2087	0.1537	1750	2914	2267	11.31	88	1.087
6	20000 (UM)	0.1632	18537	26166	20464	4.23	1463	13.847

TIC:	9.9631	ng/uL
TIM:	103.8943	nmole/L
Total concentration:	10.5358	ng/uL

```
Sample peak width (sec): 5    Sample min peak height: 50    Sample baseline V to V?: Y    Sample baseline V to V points: 3
Sample filter: Binomial       Number of points for filter: 3  Sample start region (min): 0  Sample end region (min): 30
Marker peak width (sec): 5    Marker min peak height: 200   Marker baseline V to V?: Y    Marker baseline V to V points: 3
Lower marker selection: First peak > 500 RFU                Upper marker selection: Last peak > 200 RFU
Ladder size (bp)75, 200, 300, 400, 500, 700, 1000, 1500, 2000, 3000, 4000, 5000, 7000, 10000, 20000
Quantification using: Lower Marker          Final concentration (ng/uL): 0.5000          Dilution factor: 12.0
```

Sample: SampE4
Well location: E4
Created: Tuesday, August 10, 2021 9:21:07 PM

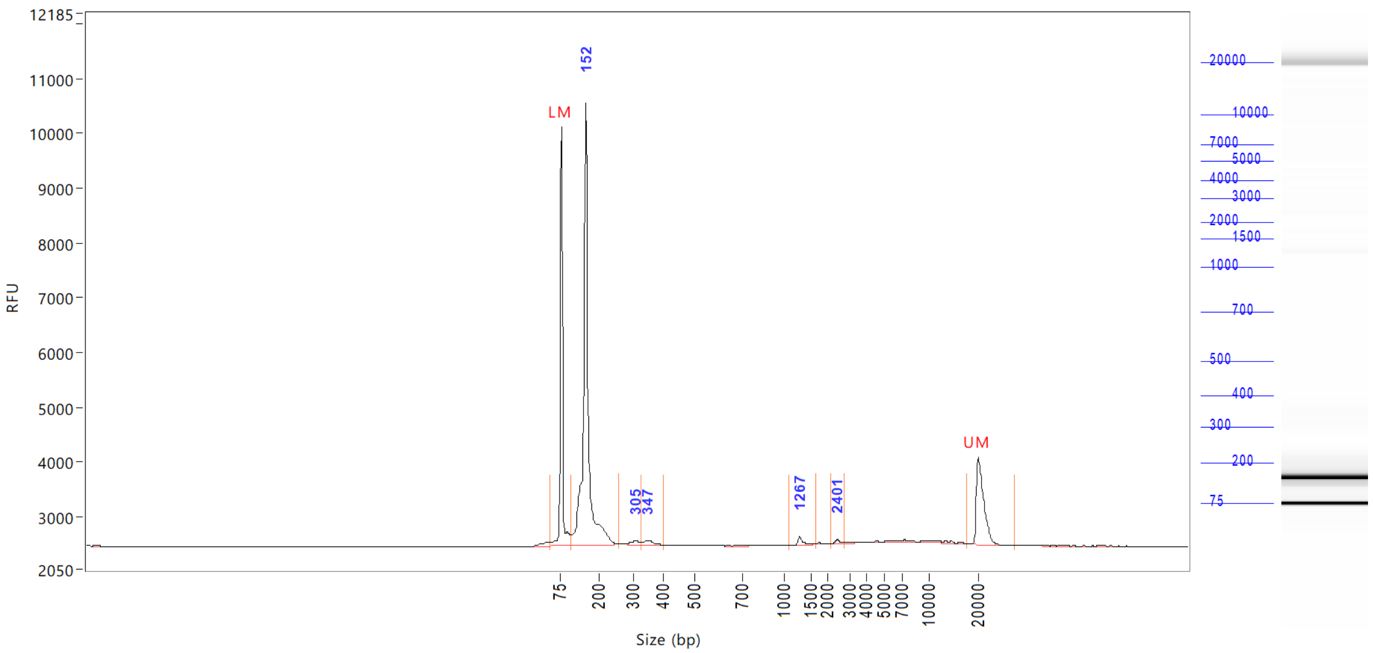

Peak	Size (bp)	Concentration (ng/uL)	From (bp)	To (bp)	Average size (bp)	CV%	RFU	Corrected peak area
1	75 (LM)	0.5000	43	105	74	9.94	7650	45.563
2	152	11.3833	105	255	154	13.23	8076	86.443
3	305	0.1866	255	323	300	4.50	79	1.417
4	347	0.2100	323	398	348	4.44	83	1.595
5	1267	0.1591	1090	1597	1309	6.14	151	1.208
6	2401	0.0768	2087	2772	2440	5.80	77	0.583
7	20000 (UM)	0.1704	17561	27375	20466	4.57	1608	15.527

TIC:	12.0158	ng/uL
TIM:	123.7581	nmole/L
Total concentration:	12.5394	ng/uL

```
Sample peak width (sec): 5    Sample min peak height: 50    Sample baseline V to V?: Y    Sample baseline V to V points: 3
Sample filter: Binomial       Number of points for filter: 3  Sample start region (min): 0  Sample end region (min): 30
Marker peak width (sec): 5    Marker min peak height: 200   Marker baseline V to V?: Y    Marker baseline V to V points: 3
Lower marker selection: First peak > 500 RFU                Upper marker selection: Last peak > 200 RFU
Ladder size (bp)75, 200, 300, 400, 500, 700, 1000, 1500, 2000, 3000, 4000, 5000, 7000, 10000, 20000
Quantification using: Lower Marker          Final concentration (ng/uL): 0.5000          Dilution factor: 12.0
```

Sample: SampF4
Well location: F4
Created: Tuesday, August 10, 2021 9:21:07 PM

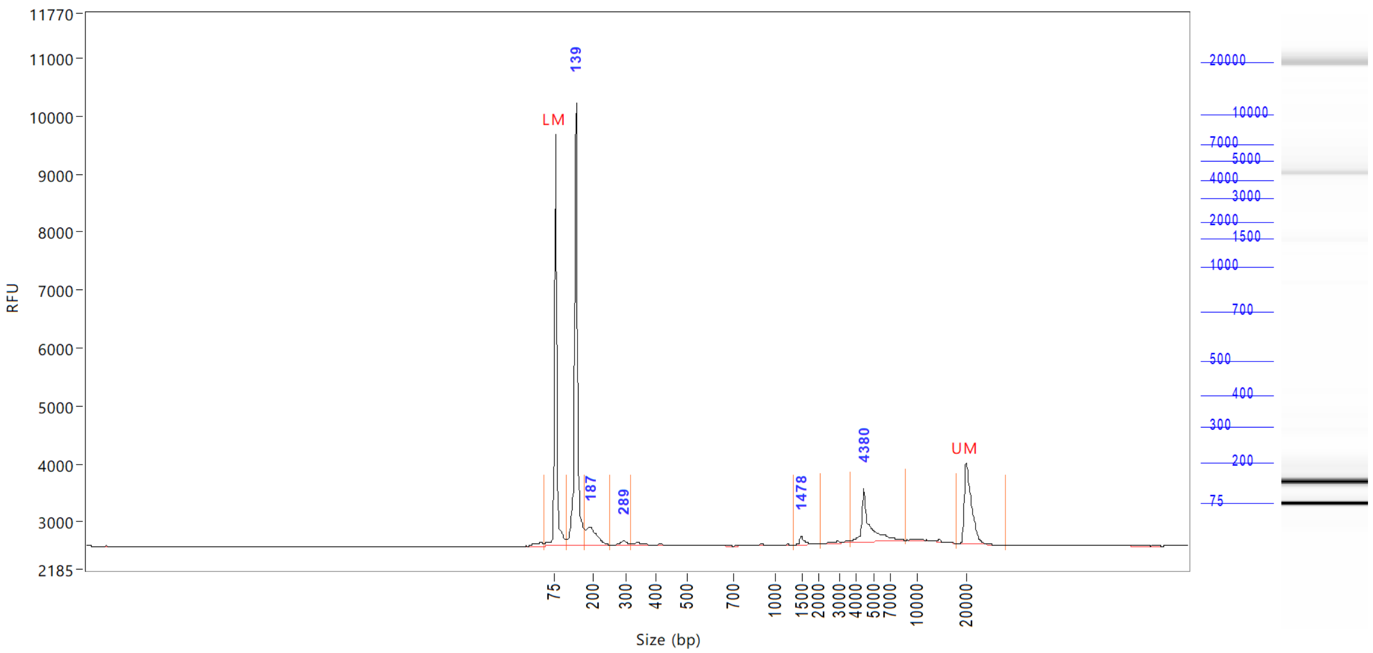

Peak	Size (bp)	Concentration (ng/uL)	From (bp)	To (bp)	Average size (bp)	CV%	RFU	Corrected peak area
1	75 (LM)	0.5000	37	110	74	10.42	7084	42.850
2	139	8.0642	110	169	138	6.16	7641	57.591
3	187	1.0593	169	246	191	7.80	306	7.565
4	289	0.1475	246	313	287	4.00	69	1.054
5	1478	0.1654	1345	2087	1531	7.78	137	1.181
6	4380	1.4985	3679	8734	4891	18.11	913	10.701
7	20000 (UM)	0.1551	18049	28100	20494	4.44	1387	13.292

TIC:	10.9349	ng/uL
TIM:	106.6719	nmole/L
Total concentration:	11.3886	ng/uL

Sample peak width (sec): 5   Sample min peak height: 50   Sample baseline V to V?: Y   Sample baseline V to V points: 3
Sample filter: Binomial   Number of points for filter: 3 Sample start region (min): 0 Sample end region (min): 30
Marker peak width (sec): 5   Marker min peak height: 200   Marker baseline V to V?: Y   Marker baseline V to V points: 3
Lower marker selection: First peak > 500 RFU   Upper marker selection: Last peak > 200 RFU
Ladder size (bp)75, 200, 300, 400, 500, 700, 1000, 1500, 2000, 3000, 4000, 5000, 7000, 10000, 20000
Quantification using: Lower Marker   Final concentration (ng/uL): 0.5000   Dilution factor: 12.0

Sample: SampG4
Well location: G4
Created: Tuesday, August 10, 2021 9:21:07 PM

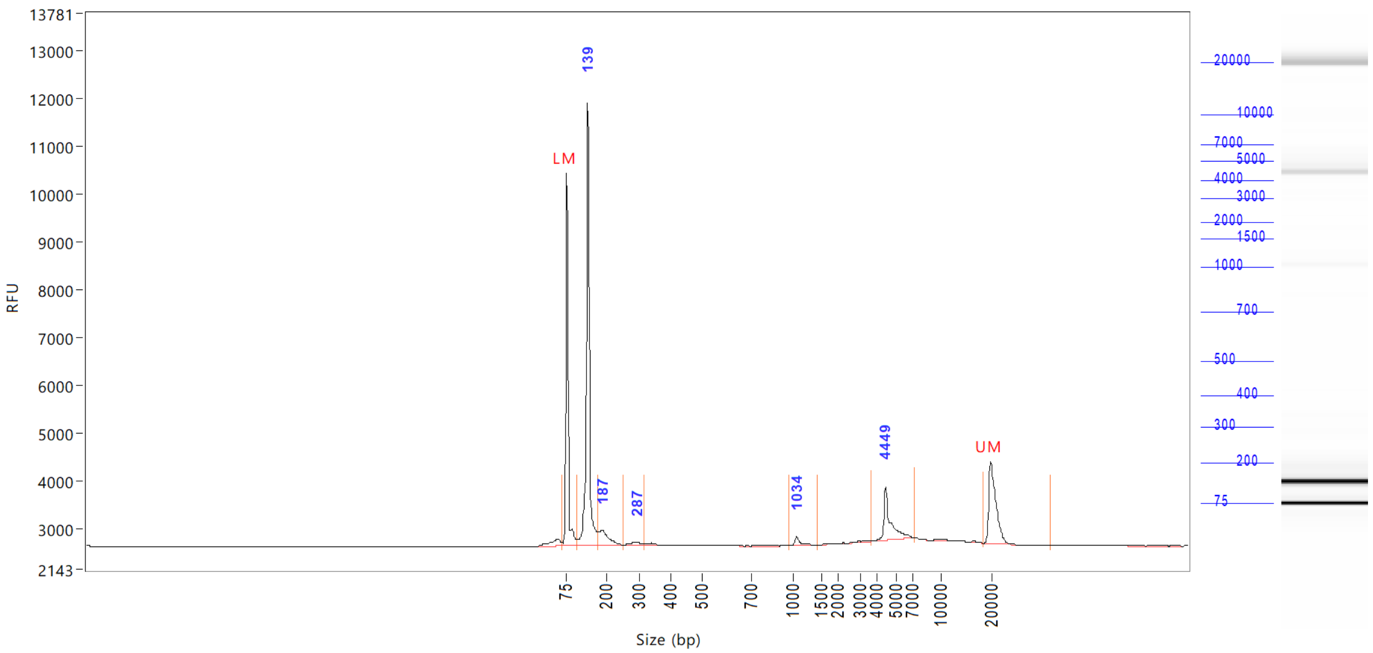

Peak	Size (bp)	Concentration (ng/uL)	From (bp)	To (bp)	Average size (bp)	CV%	RFU	Corrected peak area
1	75 (LM)	0.5000	64	108	75	8.67	7792	44.784
2	139	9.1003	108	171	138	6.16	9280	67.925
3	187	0.9220	171	248	192	7.84	311	6.882
4	287	0.1505	248	313	283	4.74	59	1.123
5	1034	0.2045	970	1422	1085	8.15	187	1.526
6	4449	1.3910	3679	7134	4699	10.85	1124	10.382
7	20000 (UM)	0.1793	18659	31969	20488	5.02	1716	16.063

TIC:	11.7682	ng/uL
TIM:	117.9309	nmole/L
Total concentration:	12.1252	ng/uL

Sample peak width (sec): 5   Sample min peak height: 50   Sample baseline V to V?: Y   Sample baseline V to V points: 3
Sample filter: Binomial   Number of points for filter: 3 Sample start region (min): 0 Sample end region (min): 30
Marker peak width (sec): 5   Marker min peak height: 200   Marker baseline V to V?: Y   Marker baseline V to V points: 3
Lower marker selection: First peak > 500 RFU   Upper marker selection: Last peak > 200 RFU
Ladder size (bp)75, 200, 300, 400, 500, 700, 1000, 1500, 2000, 3000, 4000, 5000, 7000, 10000, 20000
Quantification using: Lower Marker   Final concentration (ng/uL): 0.5000   Dilution factor: 12.0

Sample: SampH4
Well location: H4
Created: Tuesday, August 10, 2021 9:21:07 PM

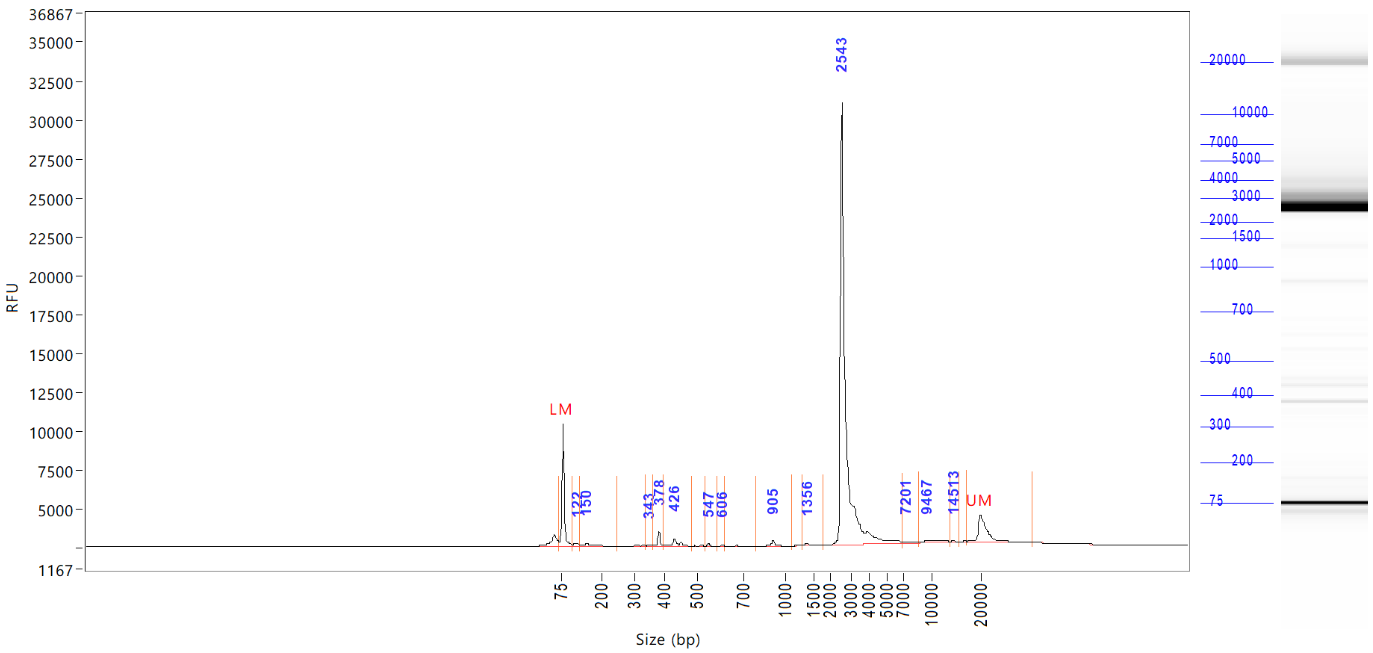

Peak	Size (bp)	Concentration (ng/uL)	From (bp)	To (bp)	Average size (bp)	CV%	RFU	Corrected peak area
1	75 (LM)	0.5000	66	103	74	7.39	7883	45.762
2	122	0.3210	103	129	115	6.89	160	2.448
3	150	0.5400	129	246	160	15.17	218	4.119
4	343	0.1077	333	360	346	2.01	78	0.821
5	378	0.5752	360	392	376	1.29	891	4.387
6	426	0.6475	392	485	431	3.58	485	4.938
7	547	0.0896	532	583	546	1.51	143	0.683
8	606	0.0626	583	618	602	0.82	113	0.477
9	905	0.3169	783	1090	899	3.40	367	2.417
10	1356	0.1806	1289	1770	1399	6.12	169	1.378
11	2543	29.8357	1770	6778	2799	20.32	28441	227.555
12	7201	0.2635	6778	8600	7582	7.11	118	2.010
13	9467	0.3693	8600	13781	10580	13.45	94	2.817
14	14513	0.0424	13781	15732	14364	2.59	53	0.323
15	20000 (UM)	0.2016	17195	30519	20830	8.05	1694	18.447

TIC:	33.3520	ng/uL
TIM:	34.5343	nmole/L
Total concentration:	33.5142	ng/uL

```
Sample peak width (sec): 5    Sample min peak height: 50    Sample baseline V to V?: Y    Sample baseline V to V points: 3
Sample filter: Binomial       Number of points for filter: 3  Sample start region (min): 0  Sample end region (min): 30
Marker peak width (sec): 5    Marker min peak height: 200   Marker baseline V to V?: Y    Marker baseline V to V points: 3
Lower marker selection: First peak > 500 RFU                Upper marker selection: Last peak > 200 RFU
Ladder size (bp)75, 200, 300, 400, 500, 700, 1000, 1500, 2000, 3000, 4000, 5000, 7000, 10000, 20000
Quantification using: Lower Marker          Final concentration (ng/uL): 0.5000          Dilution factor: 12.0
```

Sample: SampA5
Well location: A5
Created: Tuesday, August 10, 2021 9:21:07 PM

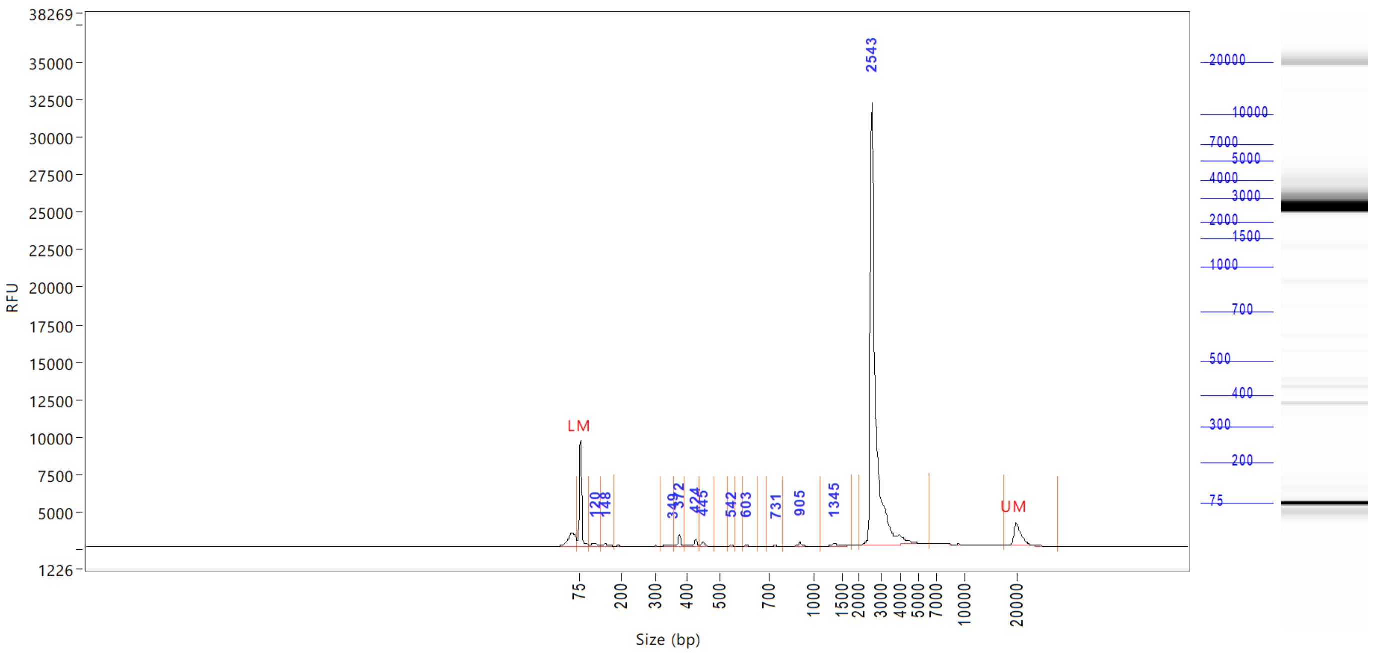

Peak	Size (bp)	Concentration (ng/uL)	From (bp)	To (bp)	Average size (bp)	CV%	RFU	Corrected peak area
1	75 (LM)	0.5000	64	101	73	6.81	7058	40.749
2	120	0.4011	101	135	116	7.87	180	2.724
3	148	0.2358	135	177	148	5.30	159	1.601
4	349	0.1493	315	358	341	3.27	71	1.014
5	372	0.5919	358	390	370	1.34	793	4.020
6	424	0.4536	390	435	418	2.29	435	3.080
7	445	0.2439	435	483	446	1.85	261	1.656
8	542	0.0699	529	555	538	0.74	114	0.475
9	603	0.0912	588	652	605	2.03	121	0.619
10	731	0.0604	682	788	728	1.89	73	0.410
11	905	0.2570	788	1101	902	3.58	244	1.746
12	1345	0.2266	1101	1770	1370	6.43	179	1.539
13	2543	33.5865	2000	6260	2720	14.26	29530	228.100
14	20000 (UM)	0.1698	17683	28221	20418	4.40	1495	13.841

TIC:	36.3671	ng/uL
TIM:	36.0028	nmole/L
Total concentration:	36.6343	ng/uL

```
Sample peak width (sec): 5    Sample min peak height: 50    Sample baseline V to V?: Y    Sample baseline V to V points: 3
Sample filter: Binomial       Number of points for filter: 3  Sample start region (min): 0  Sample end region (min): 30
Marker peak width (sec): 5    Marker min peak height: 200   Marker baseline V to V?: Y    Marker baseline V to V points: 3
Lower marker selection: First peak > 500 RFU                Upper marker selection: Last peak > 200 RFU
Ladder size (bp)75, 200, 300, 400, 500, 700, 1000, 1500, 2000, 3000, 4000, 5000, 7000, 10000, 20000
Quantification using: Lower Marker          Final concentration (ng/uL): 0.5000          Dilution factor: 12.0
```

Sample: SampB5
Well location: B5
Created: Tuesday, August 10, 2021 9:21:07 PM

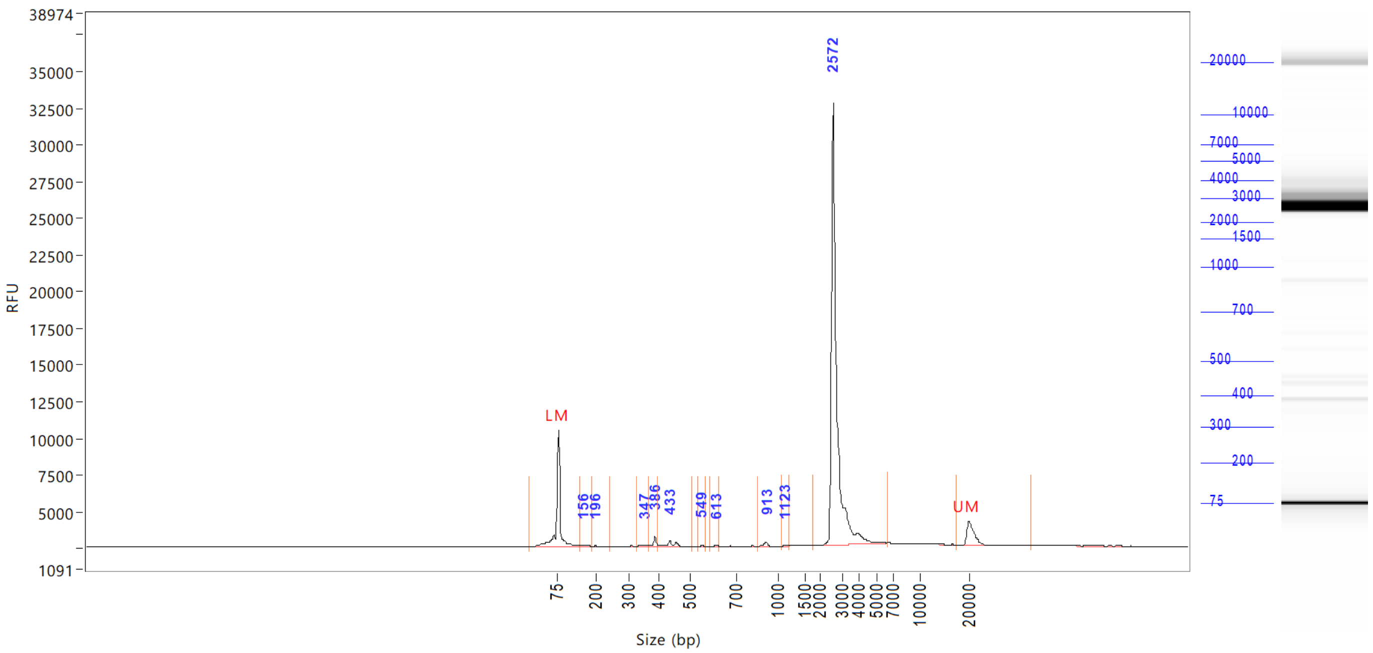

Peak	Size (bp)	Concentration (ng/uL)	From (bp)	To (bp)	Average size (bp)	CV%	RFU	Corrected peak area
1	75 (LM)	0.5000	0	148	71	28.83	7898	66.884
2	156	0.1334	148	185	163	5.96	87	1.487
3	196	0.0660	185	242	204	7.02	50	0.736
4	347	0.1443	321	364	345	3.24	111	1.608
5	386	0.3569	364	398	382	1.68	715	3.978
6	433	0.4331	398	506	436	4.01	403	4.828
7	549	0.0430	537	565	547	0.73	120	0.479
8	613	0.0438	583	626	608	1.26	102	0.488
9	913	0.1852	853	1056	904	2.31	325	2.064
10	1123	0.0246	1056	1223	1126	2.83	56	0.275
11	2572	21.7247	1750	6334	2751	14.24	30183	242.175
12	20000 (UM)	0.1182	17561	32695	20560	5.57	1673	15.813

TIC:	23.1550	ng/uL
TIM:	19.3583	nmole/L
Total concentration:	23.3821	ng/uL

Sample peak width (sec): 5 Sample min peak height: 50 Sample baseline V to V?: Y Sample baseline V to V points: 3
Sample filter: Binomial Number of points for filter: 3 Sample start region (min): 0 Sample end region (min): 30
Marker peak width (sec): 5 Marker min peak height: 200 Marker baseline V to V?: Y Marker baseline V to V points: 3
Lower marker selection: First peak > 500 RFU Upper marker selection: Last peak > 200 RFU
Ladder size (bp)75, 200, 300, 400, 500, 700, 1000, 1500, 2000, 3000, 4000, 5000, 7000, 10000, 20000
Quantification using: Lower Marker Final concentration (ng/uL): 0.5000 Dilution factor: 12.0

Sample: SampC5
Well location: C5
Created: Tuesday, August 10, 2021 9:21:07 PM

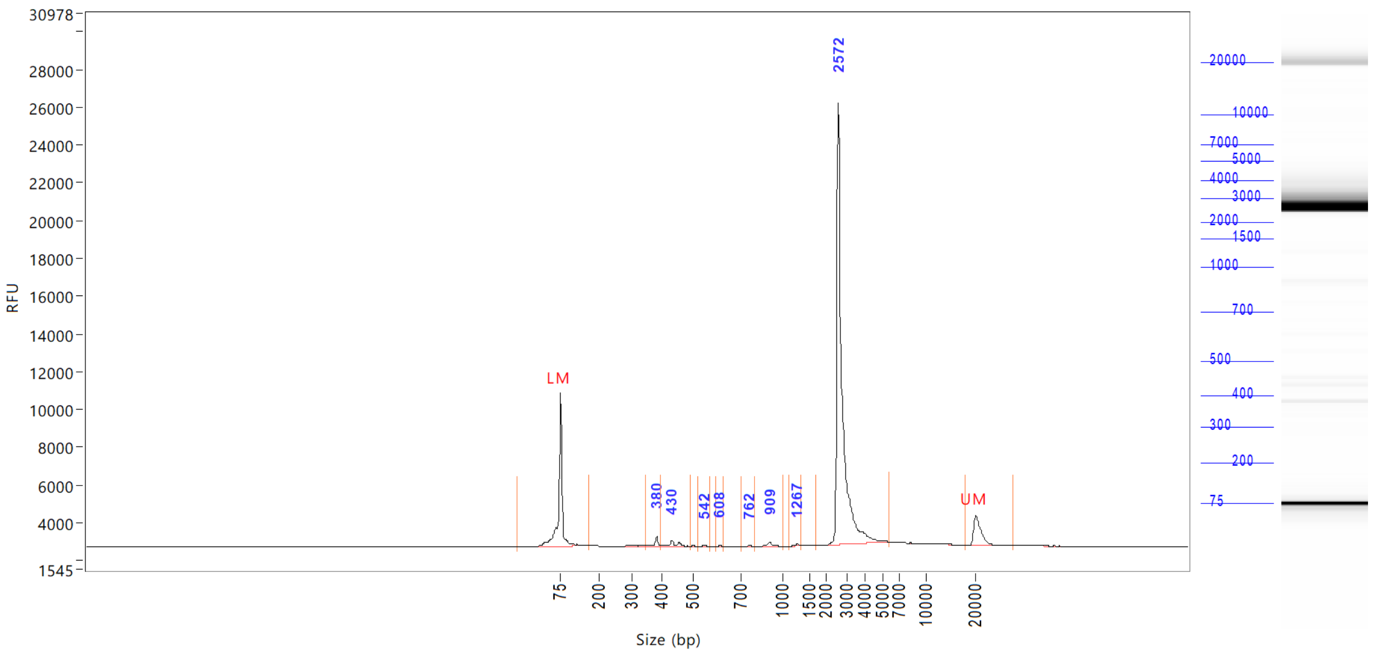

Peak	Size (bp)	Concentration (ng/uL)	From (bp)	To (bp)	Average size (bp)	CV%	RFU	Corrected peak area
1	75 (LM)	0.5000	0	166	68	25.61	8124	69.920
2	380	0.2933	343	394	373	2.66	547	3.418
3	430	0.3138	394	489	433	3.84	294	3.657
4	542	0.0320	519	567	540	1.76	67	0.373
5	608	0.0340	595	623	606	0.78	91	0.396
6	762	0.0269	700	801	754	1.91	53	0.314
7	909	0.1551	801	1000	897	2.79	250	1.808
8	1267	0.0406	1123	1334	1259	2.65	86	0.474
9	2572	16.7405	1693	5741	2757	14.25	23423	195.081
10	20000 (UM)	0.1083	17805	27738	20568	4.69	1588	15.146

TIC:	17.6362	ng/uL
TIM:	13.0683	nmole/L
Total concentration:	17.8721	ng/uL

```
Sample peak width (sec): 5    Sample min peak height: 50    Sample baseline V to V?: Y    Sample baseline V to V points: 3
Sample filter: Binomial       Number of points for filter: 3  Sample start region (min): 0  Sample end region (min): 30
Marker peak width (sec): 5    Marker min peak height: 200   Marker baseline V to V?: Y    Marker baseline V to V points: 3
Lower marker selection: First peak > 500 RFU                Upper marker selection: Last peak > 200 RFU
Ladder size (bp)75, 200, 300, 400, 500, 700, 1000, 1500, 2000, 3000, 4000, 5000, 7000, 10000, 20000
Quantification using: Lower Marker          Final concentration (ng/uL): 0.5000          Dilution factor: 12.0
```

Sample: SampD5
Well location: D5
Created: Tuesday, August 10, 2021 9:21:07 PM

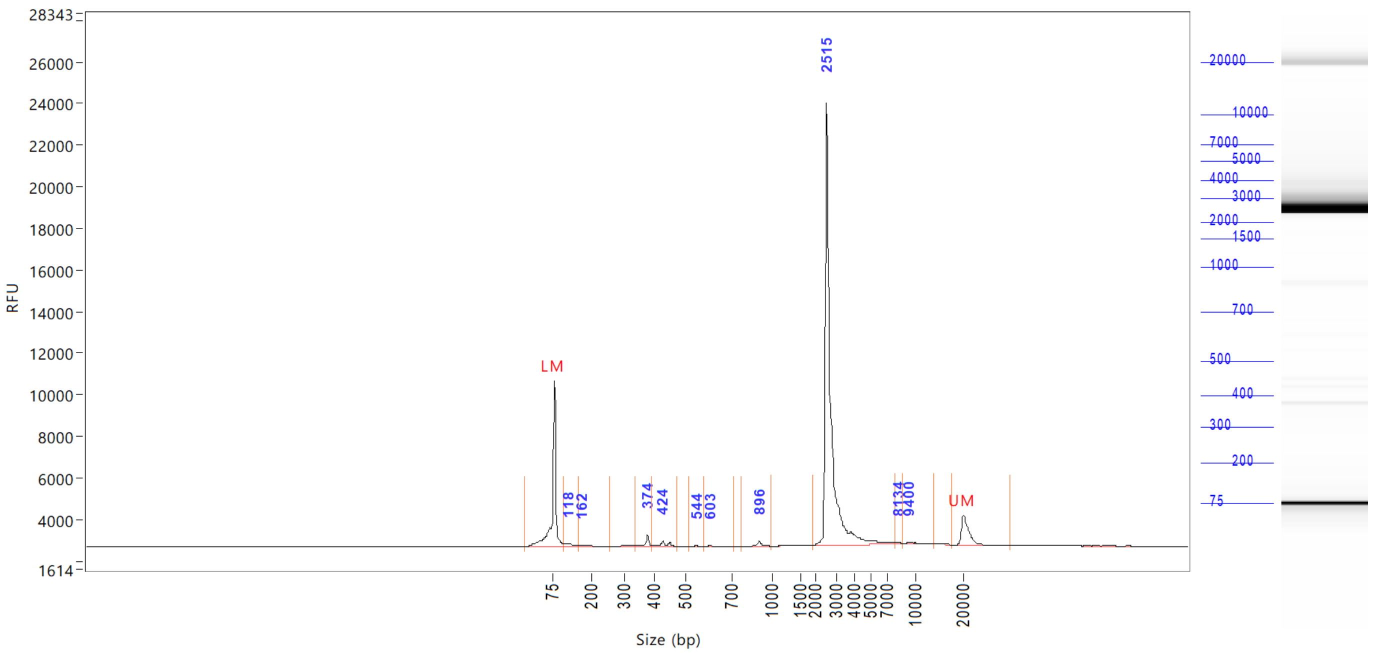

Peak	Size (bp)	Concentration (ng/uL)	From (bp)	To (bp)	Average size (bp)	CV%	RFU	Corrected peak area
1	75 (LM)	0.5000	0	107	65	28.61	7946	67.967
2	118	0.2630	107	152	125	10.05	138	2.980
3	162	0.1551	152	253	182	12.21	64	1.757
4	374	0.3263	333	388	366	3.20	539	3.696
5	424	0.2732	388	471	427	3.94	244	3.095
6	544	0.0296	514	578	541	2.27	55	0.335
7	603	0.0495	578	710	617	4.23	75	0.560
8	896	0.1715	766	983	889	2.88	245	1.942
9	2515	15.7709	1923	7867	2786	24.48	21295	178.650
10	8134	0.0532	7867	8600	8185	2.70	80	0.603
11	9400	0.1163	8600	14025	10020	11.19	65	1.317
12	20000 (UM)	0.1022	17561	29793	20601	5.32	1448	13.898

TIC:	17.2085	ng/uL
TIM:	17.2660	nmole/L
Total concentration:	17.3368	ng/uL

```
Sample peak width (sec): 5    Sample min peak height: 50    Sample baseline V to V?: Y    Sample baseline V to V points: 3
Sample filter: Binomial       Number of points for filter: 3  Sample start region (min): 0  Sample end region (min): 30
Marker peak width (sec): 5    Marker min peak height: 200   Marker baseline V to V?: Y    Marker baseline V to V points: 3
Lower marker selection: First peak > 500 RFU                Upper marker selection: Last peak > 200 RFU
Ladder size (bp)75, 200, 300, 400, 500, 700, 1000, 1500, 2000, 3000, 4000, 5000, 7000, 10000, 20000
Quantification using: Lower Marker          Final concentration (ng/uL): 0.5000          Dilution factor: 12.0
```

Sample: SampE5
Well location: E5
Created: Tuesday, August 10, 2021 9:21:07 PM

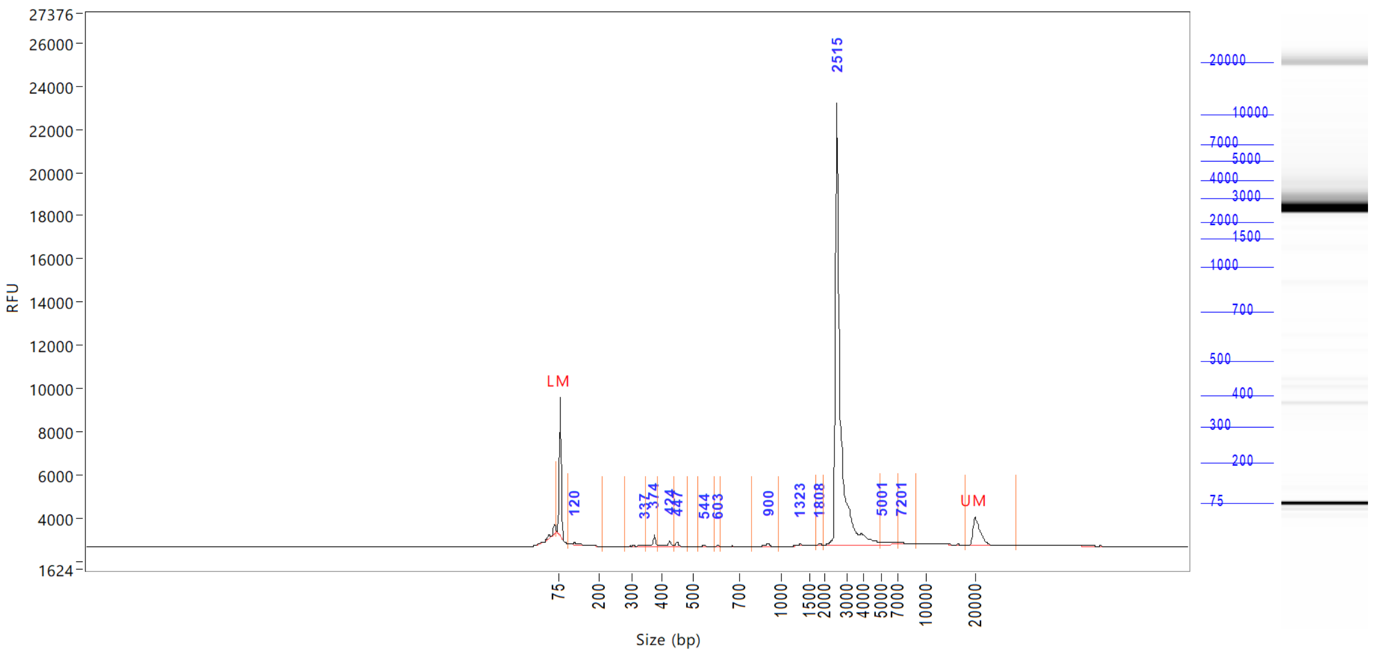

Peak	Size (bp)	Concentration (ng/uL)	From (bp)	To (bp)	Average size (bp)	CV%	RFU	Corrected peak area
1	75 (LM)	0.5000	66	101	73	3.80	6399	32.574
2	120	0.2728	101	212	132	15.37	83	1.481
3	337	0.1717	277	343	319	5.93	61	0.932
4	374	0.5620	343	386	369	2.28	512	3.051
5	424	0.3451	386	437	418	2.77	228	1.874
6	447	0.2053	437	481	447	1.53	183	1.114
7	544	0.0828	521	590	547	2.88	66	0.449
8	603	0.0550	590	616	601	0.80	67	0.299
9	900	0.1887	788	978	886	2.95	148	1.025
10	1323	0.1630	978	1693	1356	7.68	78	0.885
11	1808	0.0732	1693	1942	1806	2.99	72	0.398
12	2515	29.4968	1942	4897	2715	15.24	20514	160.136
13	5001	0.4351	4897	6926	5726	10.87	127	2.362
14	7201	0.2538	6926	8934	7713	7.45	92	1.378
15	20000 (UM)	0.1857	18171	28342	20538	4.40	1277	12.099

TIC:	32.3053	ng/uL
TIM:	27.9795	nmole/L
Total concentration:	32.5824	ng/uL

```
Sample peak width (sec): 5    Sample min peak height: 50    Sample baseline V to V?: Y    Sample baseline V to V points: 3
Sample filter: Binomial       Number of points for filter: 3  Sample start region (min): 0  Sample end region (min): 30
Marker peak width (sec): 5    Marker min peak height: 200   Marker baseline V to V?: Y    Marker baseline V to V points: 3
Lower marker selection: First peak > 500 RFU                Upper marker selection: Last peak > 200 RFU
Ladder size (bp)75, 200, 300, 400, 500, 700, 1000, 1500, 2000, 3000, 4000, 5000, 7000, 10000, 20000
Quantification using: Lower Marker          Final concentration (ng/uL): 0.5000          Dilution factor: 12.0
```

Sample: SampF5
Well location: F5
Created: Tuesday, August 10, 2021 9:21:07 PM

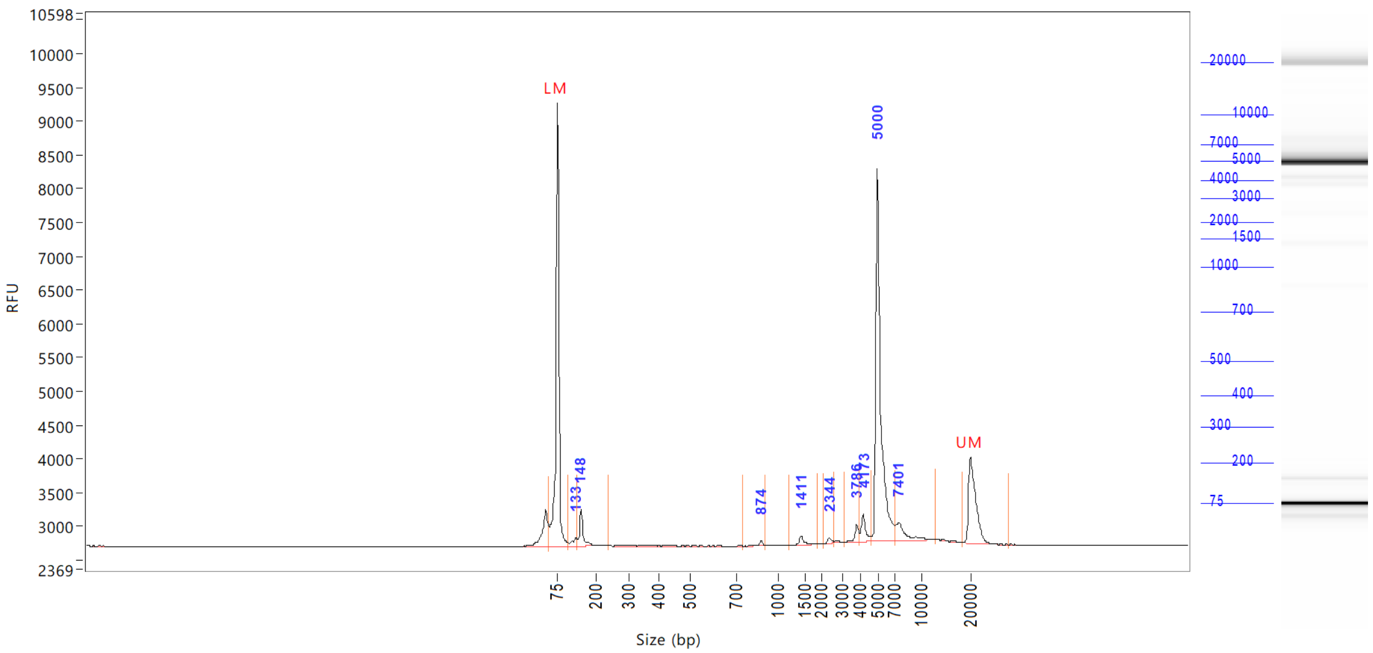

Peak	Size (bp)	Concentration (ng/uL)	From (bp)	To (bp)	Average size (bp)	CV%	RFU	Corrected peak area
1	75 (LM)	0.5000	48	112	72	10.72	6572	46.105
2	133	0.1988	112	139	128	6.00	128	1.527
3	148	0.5916	139	237	154	9.65	536	4.546
4	874	0.0650	744	905	860	3.19	73	0.499
5	1411	0.1361	1212	1846	1434	5.92	137	1.046
6	2344	0.0649	2030	2572	2345	4.32	79	0.499
7	3786	0.1882	3072	4000	3773	3.99	253	1.446
8	4173	0.3622	4000	4587	4195	3.67	414	2.783
9	5000	5.3137	4587	7068	5200	9.35	5509	40.831
10	7401	0.4903	7068	12928	8097	12.77	262	3.768
11	20000 (UM)	0.1320	18293	27859	20495	4.51	1282	12.168

TIC:	7.4108	ng/uL
TIM:	11.1976	nmole/L
Total concentration:	7.5615	ng/uL

```
Sample peak width (sec): 5     Sample min peak height: 50    Sample baseline V to V?: Y    Sample baseline V to V points: 3
Sample filter: Binomial        Number of points for filter: 3  Sample start region (min): 0  Sample end region (min): 30
Marker peak width (sec): 5     Marker min peak height: 200   Marker baseline V to V?: Y    Marker baseline V to V points: 3
Lower marker selection: First peak > 500 RFU                 Upper marker selection: Last peak > 200 RFU
Ladder size (bp)75, 200, 300, 400, 500, 700, 1000, 1500, 2000, 3000, 4000, 5000, 7000, 10000, 20000
Quantification using: Lower Marker          Final concentration (ng/uL): 0.5000          Dilution factor: 12.0
```

Sample: SampG5
Well location: G5
Created: Tuesday, August 10, 2021 9:21:07 PM

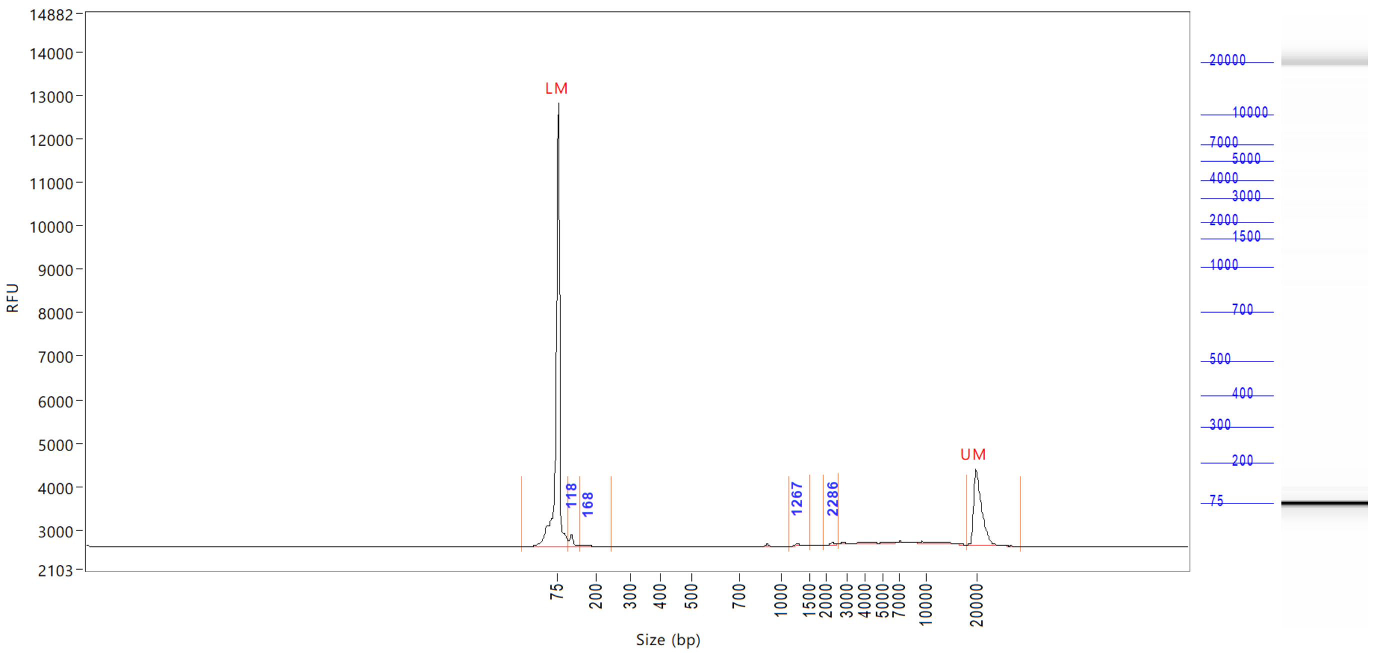

Peak	Size (bp)	Concentration (ng/uL)	From (bp)	To (bp)	Average size (bp)	CV%	RFU	Corrected peak area
1	75 (LM)	0.5000	0	108	68	21.71	10219	86.409
2	118	0.1670	108	145	119	7.49	272	2.405
3	168	0.0853	145	242	173	13.01	51	1.228
4	1267	0.0366	1145	1520	1295	5.38	66	0.527
5	2286	0.0264	1923	2600	2319	5.26	52	0.381
6	20000 (UM)	0.1049	18049	29068	20502	4.93	1741	18.128

TIC:	0.3153	ng/uL
TIM:	3.1813	nmole/L
Total concentration:	0.6357	ng/uL

```
Sample peak width (sec): 5    Sample min peak height: 50    Sample baseline V to V?: Y    Sample baseline V to V points: 3
Sample filter: Binomial       Number of points for filter: 3  Sample start region (min): 0  Sample end region (min): 30
Marker peak width (sec): 5    Marker min peak height: 200   Marker baseline V to V?: Y    Marker baseline V to V points: 3
Lower marker selection: First peak > 500 RFU                Upper marker selection: Last peak > 200 RFU
Ladder size (bp)75, 200, 300, 400, 500, 700, 1000, 1500, 2000, 3000, 4000, 5000, 7000, 10000, 20000
Quantification using: Lower Marker          Final concentration (ng/uL): 0.5000          Dilution factor: 12.0
```

Sample: SampH5
Well location: H5
Created: Tuesday, August 10, 2021 9:21:07 PM

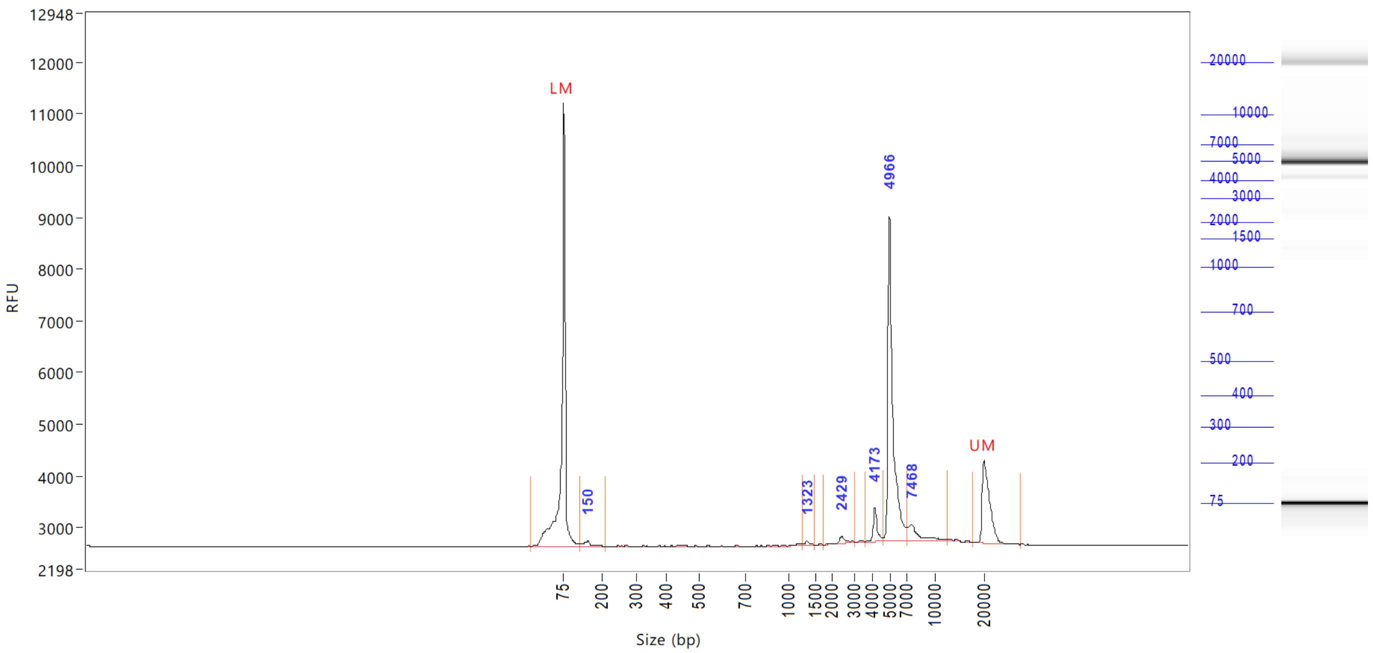

Peak	Size (bp)	Concentration (ng/uL)	From (bp)	To (bp)	Average size (bp)	CV%	RFU	Corrected peak area
1	75 (LM)	0.5000	0	126	64	31.32	8591	77.040
2	150	0.1335	126	205	149	10.45	104	1.714
3	1323	0.0507	1256	1478	1334	3.70	83	0.651
4	2429	0.0983	1712	3037	2448	8.71	140	1.262
5	4173	0.3027	3608	4621	4180	3.70	646	3.886
6	4966	3.6191	4621	7001	5229	9.21	6268	46.469
7	7468	0.4156	7001	12684	8342	15.65	314	5.337
8	20000 (UM)	0.0997	17805	27496	20519	4.27	1618	15.356

TIC:	4.6199	ng/uL
TIM:	2.9418	nmole/L
Total concentration:	4.7795	ng/uL

```
Sample peak width (sec): 5    Sample min peak height: 50    Sample baseline V to V?: Y    Sample baseline V to V points: 3
Sample filter: Binomial       Number of points for filter: 3  Sample start region (min): 0  Sample end region (min): 30
Marker peak width (sec): 5    Marker min peak height: 200   Marker baseline V to V?: Y    Marker baseline V to V points: 3
Lower marker selection: First peak > 500 RFU                Upper marker selection: Last peak > 200 RFU
Ladder size (bp)75, 200, 300, 400, 500, 700, 1000, 1500, 2000, 3000, 4000, 5000, 7000, 10000, 20000
Quantification using: Lower Marker          Final concentration (ng/uL): 0.5000          Dilution factor: 12.0
```

Sample: SampA6
Well location: A6
Created: Tuesday, August 10, 2021 9:21:07 PM

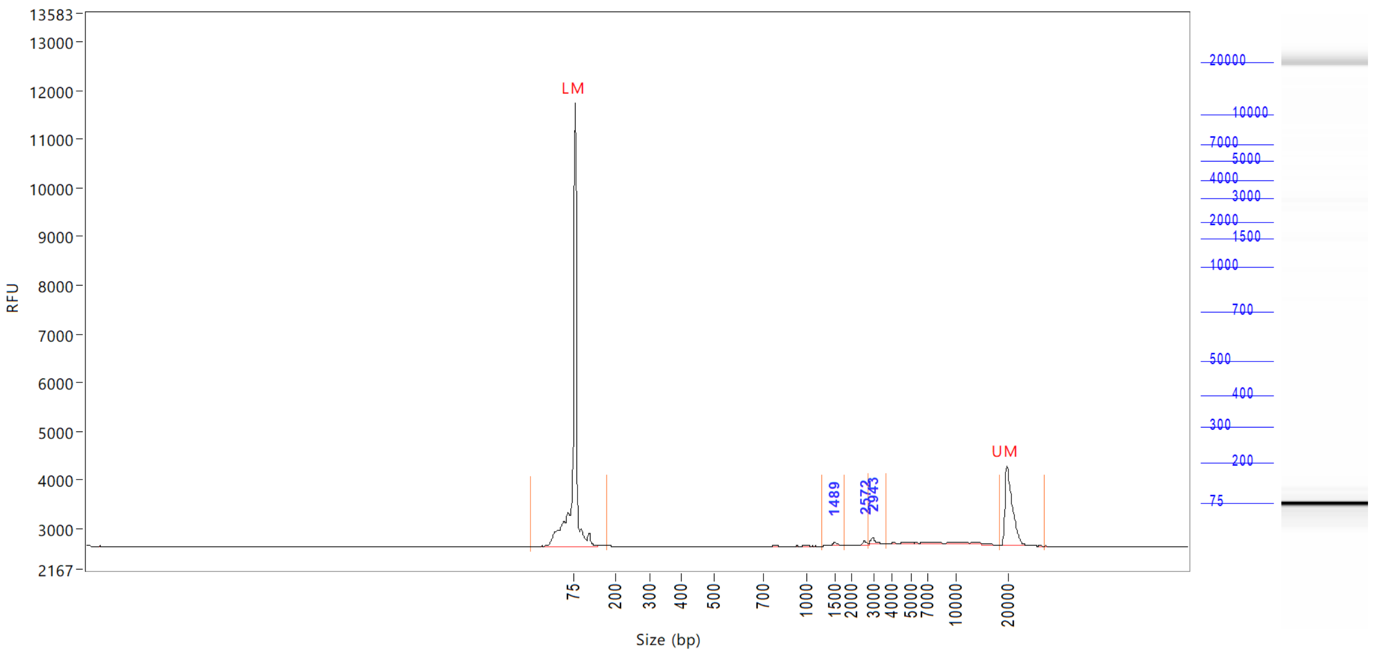

Peak	Size (bp)	Concentration (ng/uL)	From (bp)	To (bp)	Average size (bp)	CV%	RFU	Corrected peak area
1	75 (LM)	0.5000	0	171	65	33.51	9119	83.660
2	1489	0.0405	1278	1808	1513	5.30	74	0.565
3	2572	0.0345	1808	2743	2506	7.69	71	0.481
4	2943	0.0801	2743	3715	3003	6.44	137	1.117
5	20000 (UM)	0.0908	18537	27133	20443	4.33	1631	15.196

TIC:	0.1551	ng/uL
TIM:	0.1107	nmole/L
Total concentration:	0.4547	ng/uL

```
Sample peak width (sec): 5    Sample min peak height: 50    Sample baseline V to V?: Y    Sample baseline V to V points: 3
Sample filter: Binomial       Number of points for filter: 3  Sample start region (min): 0  Sample end region (min): 30
Marker peak width (sec): 5    Marker min peak height: 200   Marker baseline V to V?: Y    Marker baseline V to V points: 3
Lower marker selection: First peak > 500 RFU                Upper marker selection: Last peak > 200 RFU
Ladder size (bp)75, 200, 300, 400, 500, 700, 1000, 1500, 2000, 3000, 4000, 5000, 7000, 10000, 20000
Quantification using: Lower Marker          Final concentration (ng/uL): 0.5000          Dilution factor: 12.0
```

Sample: SampB6
Well location: B6
Created: Tuesday, August 10, 2021 9:21:07 PM

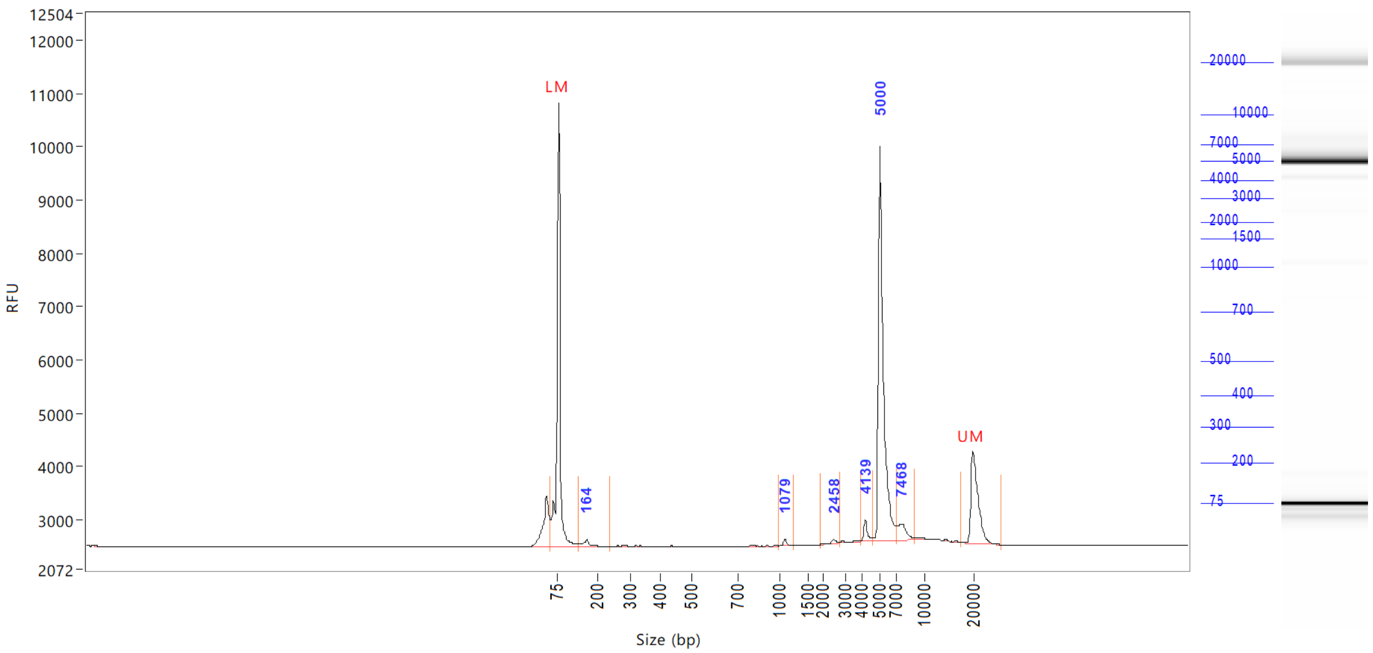

Peak	Size (bp)	Concentration (ng/uL)	From (bp)	To (bp)	Average size (bp)	CV%	RFU	Corrected peak area
1	75 (LM)	0.5000	48	141	72	15.01	8336	61.581
2	164	0.1560	141	235	162	8.92	127	1.601
3	1079	0.0707	991	1256	1083	4.44	129	0.725
4	2458	0.0578	1885	2772	2462	5.80	82	0.593
5	4139	0.2181	3893	4587	4168	3.52	399	2.239
6	5000	5.2476	4587	7000	5217	8.49	7408	53.858
7	7468	0.4000	7000	9000	7613	6.40	304	4.106
8	20000 (UM)	0.1294	17561	25682	20462	4.29	1718	15.938

TIC:	6.1503	ng/uL
TIM:	3.5579	nmole/L
Total concentration:	6.3918	ng/uL

Sample peak width (sec): 5   Sample min peak height: 50   Sample baseline V to V?: Y   Sample baseline V to V points: 3
Sample filter: Binomial   Number of points for filter: 3 Sample start region (min): 0 Sample end region (min): 30
Marker peak width (sec): 5   Marker min peak height: 200   Marker baseline V to V?: Y   Marker baseline V to V points: 3
Lower marker selection: First peak > 500 RFU   Upper marker selection: Last peak > 200 RFU
Ladder size (bp)75, 200, 300, 400, 500, 700, 1000, 1500, 2000, 3000, 4000, 5000, 7000, 10000, 20000
Quantification using: Lower Marker   Final concentration (ng/uL): 0.5000   Dilution factor: 12.0

Sample: SampC6
Well location: C6
Created: Tuesday, August 10, 2021 9:21:07 PM

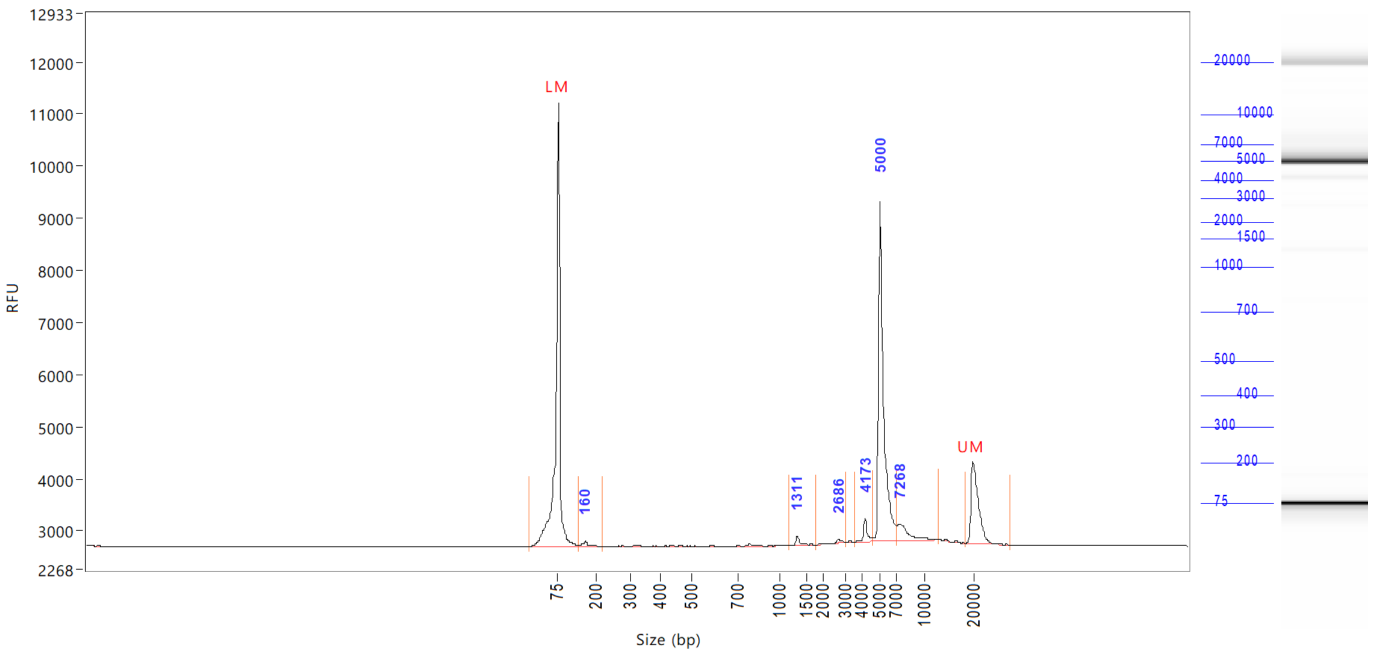

Peak	Size (bp)	Concentration (ng/uL)	From (bp)	To (bp)	Average size (bp)	CV%	RFU	Corrected peak area
1	75 (LM)	0.5000	0	143	67	26.72	8524	79.780
2	160	0.0885	143	217	162	8.63	96	1.177
3	1311	0.0896	1156	1770	1344	7.11	179	1.191
4	2686	0.0438	1770	3037	2606	10.32	79	0.582
5	4173	0.1989	3536	4621	4169	4.13	435	2.645
6	5000	3.6357	4621	7000	5258	8.97	6505	48.342
7	7268	0.4073	7000	12928	8046	12.80	316	5.415
8	20000 (UM)	0.0938	18415	27496	20523	4.51	1568	14.959

TIC:	4.4637	ng/uL
TIM:	2.3353	nmole/L
Total concentration:	4.5755	ng/uL

```
Sample peak width (sec): 5    Sample min peak height: 50    Sample baseline V to V?: Y    Sample baseline V to V points: 3
Sample filter: Binomial       Number of points for filter: 3  Sample start region (min): 0  Sample end region (min): 30
Marker peak width (sec): 5    Marker min peak height: 200   Marker baseline V to V?: Y    Marker baseline V to V points: 3
Lower marker selection: First peak > 500 RFU                Upper marker selection: Last peak > 200 RFU
Ladder size (bp)75, 200, 300, 400, 500, 700, 1000, 1500, 2000, 3000, 4000, 5000, 7000, 10000, 20000
Quantification using: Lower Marker          Final concentration (ng/uL): 0.5000          Dilution factor: 12.0
```

Sample: SampD6
Well location: D6
Created: Tuesday, August 10, 2021 9:21:07 PM

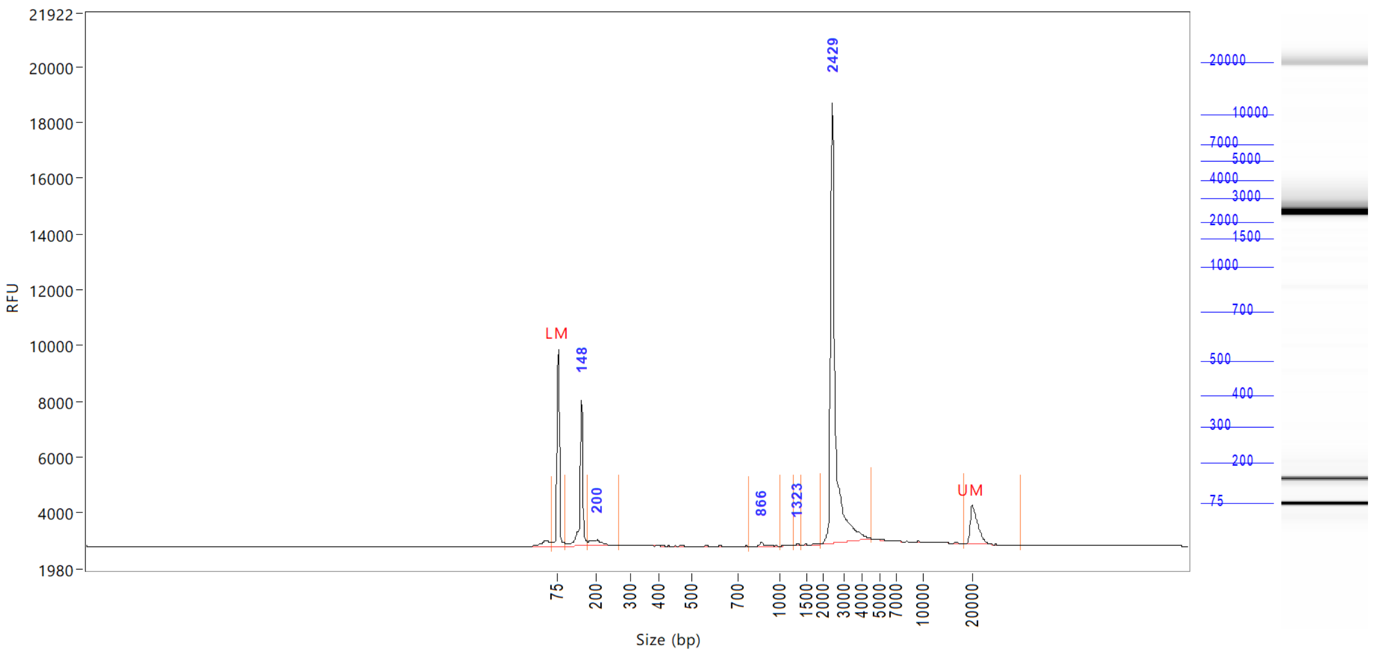

Peak	Size (bp)	Concentration (ng/uL)	From (bp)	To (bp)	Average size (bp)	CV%	RFU	Corrected peak area
1	75 (LM)	0.5000	58	99	73	6.47	7072	40.518
2	148	5.4040	99	169	144	7.09	5225	36.493
3	200	0.8430	169	264	197	9.62	215	5.693
4	866	0.1555	775	1001	871	3.37	157	1.050
5	1323	0.0344	1234	1389	1312	1.82	52	0.232
6	2429	17.1527	1923	4552	2541	13.44	15818	115.833
7	20000 (UM)	0.1663	18171	30035	20528	4.97	1392	13.477

TIC:	23.5897	ng/uL
TIM:	80.1584	nmole/L
Total concentration:	24.0078	ng/uL

Sample peak width (sec): 5   Sample min peak height: 50   Sample baseline V to V?: Y   Sample baseline V to V points: 3
Sample filter: Binomial   Number of points for filter: 3 Sample start region (min): 0 Sample end region (min): 30
Marker peak width (sec): 5   Marker min peak height: 200   Marker baseline V to V?: Y   Marker baseline V to V points: 3
Lower marker selection: First peak > 500 RFU   Upper marker selection: Last peak > 200 RFU
Ladder size (bp)75, 200, 300, 400, 500, 700, 1000, 1500, 2000, 3000, 4000, 5000, 7000, 10000, 20000
Quantification using: Lower Marker   Final concentration (ng/uL): 0.5000   Dilution factor: 12.0

Sample: SampE6
Well location: E6
Created: Tuesday, August 10, 2021 9:21:07 PM

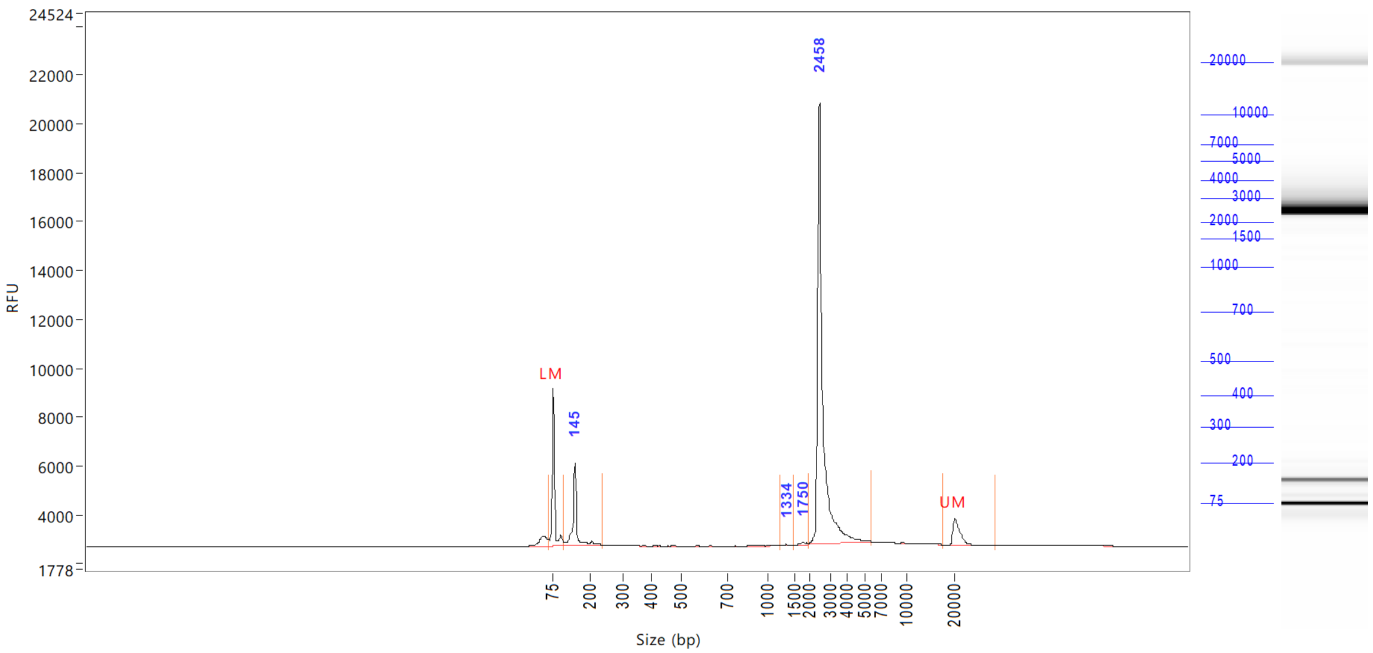

Peak	Size (bp)	Concentration (ng/uL)	From (bp)	To (bp)	Average size (bp)	CV%	RFU	Corrected peak area
1	75 (LM)	0.5000	64	110	75	11.14	6448	40.113
2	145	4.3146	110	233	146	12.02	3364	28.845
3	1334	0.0472	1223	1489	1330	2.97	53	0.316
4	1750	0.1775	1489	1962	1759	6.35	101	1.187
5	2458	21.5589	1962	5815	2604	15.71	18076	144.134
6	20000 (UM)	0.1268	17561	28463	20585	5.22	1104	10.170

TIC:	26.0982	ng/uL
TIM:	62.4201	nmole/L
Total concentration:	26.5265	ng/uL

```
Sample peak width (sec): 5    Sample min peak height: 50    Sample baseline V to V?: Y    Sample baseline V to V points: 3
Sample filter: Binomial       Number of points for filter: 3  Sample start region (min): 0  Sample end region (min): 30
Marker peak width (sec): 5    Marker min peak height: 200   Marker baseline V to V?: Y    Marker baseline V to V points: 3
Lower marker selection: First peak > 500 RFU                Upper marker selection: Last peak > 200 RFU
Ladder size (bp)75, 200, 300, 400, 500, 700, 1000, 1500, 2000, 3000, 4000, 5000, 7000, 10000, 20000
Quantification using: Lower Marker          Final concentration (ng/uL): 0.5000          Dilution factor: 12.0
```

Sample: SampF6
Well location: F6
Created: Tuesday, August 10, 2021 9:21:07 PM

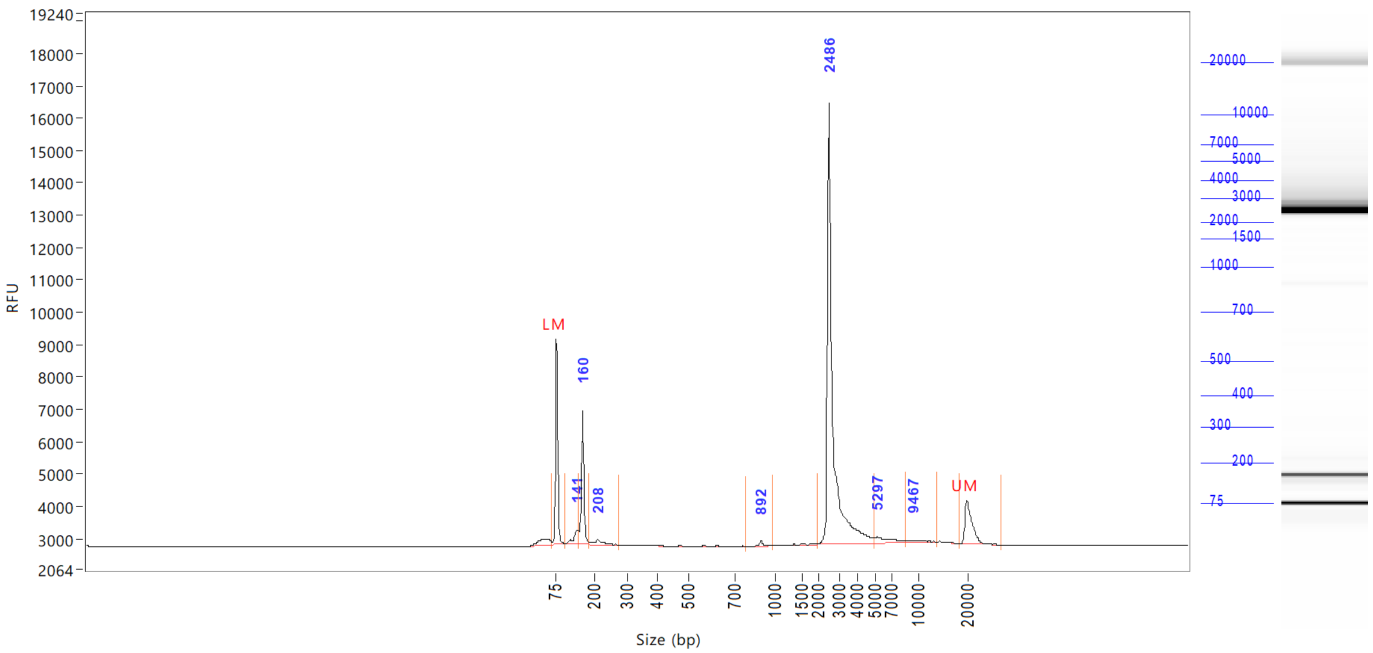

Peak	Size (bp)	Concentration (ng/uL)	From (bp)	To (bp)	Average size (bp)	CV%	RFU	Corrected peak area
1	75 (LM)	0.5000	64	103	74	4.78	6335	35.118
2	141	0.9906	103	148	135	7.66	454	5.798
3	160	4.2827	148	181	158	3.05	4135	25.067
4	208	0.6056	181	273	210	9.37	170	3.544
5	892	0.1760	779	983	890	2.55	160	1.030
6	2486	19.5379	1942	4966	2712	18.86	13658	114.356
7	5297	0.8136	4966	8667	6438	16.57	192	4.762
8	9467	0.2260	8667	13781	10032	11.29	67	1.323
9	20000 (UM)	0.1784	18659	27012	20497	4.36	1317	12.528

TIC:	26.6324	ng/uL
TIM:	73.7813	nmole/L
Total concentration:	26.9147	ng/uL

```
Sample peak width (sec): 5    Sample min peak height: 50    Sample baseline V to V?: Y    Sample baseline V to V points: 3
Sample filter: Binomial       Number of points for filter: 3  Sample start region (min): 0  Sample end region (min): 30
Marker peak width (sec): 5    Marker min peak height: 200   Marker baseline V to V?: Y    Marker baseline V to V points: 3
Lower marker selection: First peak > 500 RFU                Upper marker selection: Last peak > 200 RFU
Ladder size (bp)75, 200, 300, 400, 500, 700, 1000, 1500, 2000, 3000, 4000, 5000, 7000, 10000, 20000
Quantification using: Lower Marker          Final concentration (ng/uL): 0.5000          Dilution factor: 12.0
```

Sample: SampG6
Well location: G6
Created: Tuesday, August 10, 2021 9:21:07 PM

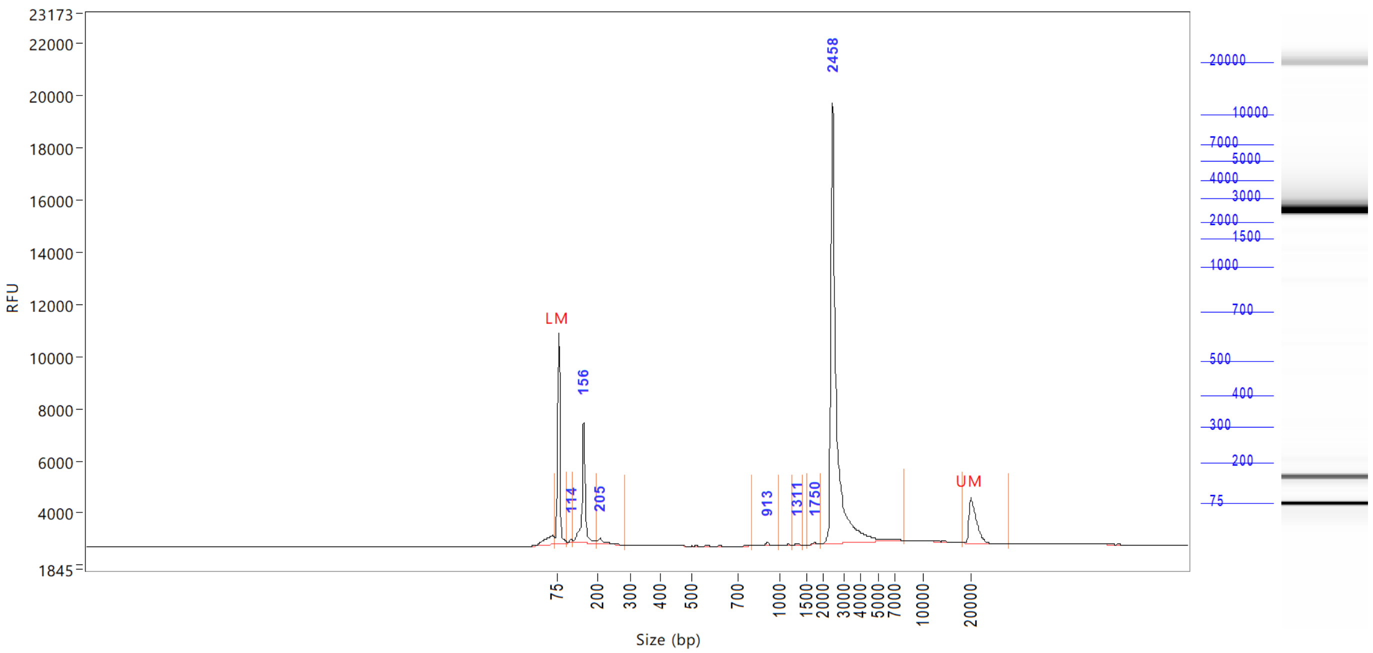

Peak	Size (bp)	Concentration (ng/uL)	From (bp)	To (bp)	Average size (bp)	CV%	RFU	Corrected peak area
1	75 (LM)	0.5000	64	99	73	5.61	8098	46.201
2	114	0.1413	99	122	112	5.44	113	1.088
3	156	4.5568	122	196	152	6.79	4602	35.088
4	205	0.3739	196	282	215	7.45	203	2.879
5	913	0.1039	796	996	904	2.70	123	0.800
6	1311	0.0534	1234	1422	1313	2.40	76	0.411
7	1750	0.0485	1501	1942	1733	3.71	64	0.374
8	2458	18.6821	1942	8001	2696	20.97	16929	143.855
9	20000 (UM)	0.1810	18049	27738	20482	4.36	1732	16.722

TIC:	23.9599	ng/uL
TIM:	65.9126	nmole/L
Total concentration:	24.3081	ng/uL

Sample peak width (sec): 5   Sample min peak height: 50   Sample baseline V to V?: Y   Sample baseline V to V points: 3
Sample filter: Binomial   Number of points for filter: 3 Sample start region (min): 0 Sample end region (min): 30
Marker peak width (sec): 5   Marker min peak height: 200   Marker baseline V to V?: Y   Marker baseline V to V points: 3
Lower marker selection: First peak > 500 RFU   Upper marker selection: Last peak > 200 RFU
Ladder size (bp)75, 200, 300, 400, 500, 700, 1000, 1500, 2000, 3000, 4000, 5000, 7000, 10000, 20000
Quantification using: Lower Marker   Final concentration (ng/uL): 0.5000   Dilution factor: 12.0

Sample: SampH6
Well location: H6
Created: Tuesday, August 10, 2021 9:21:07 PM

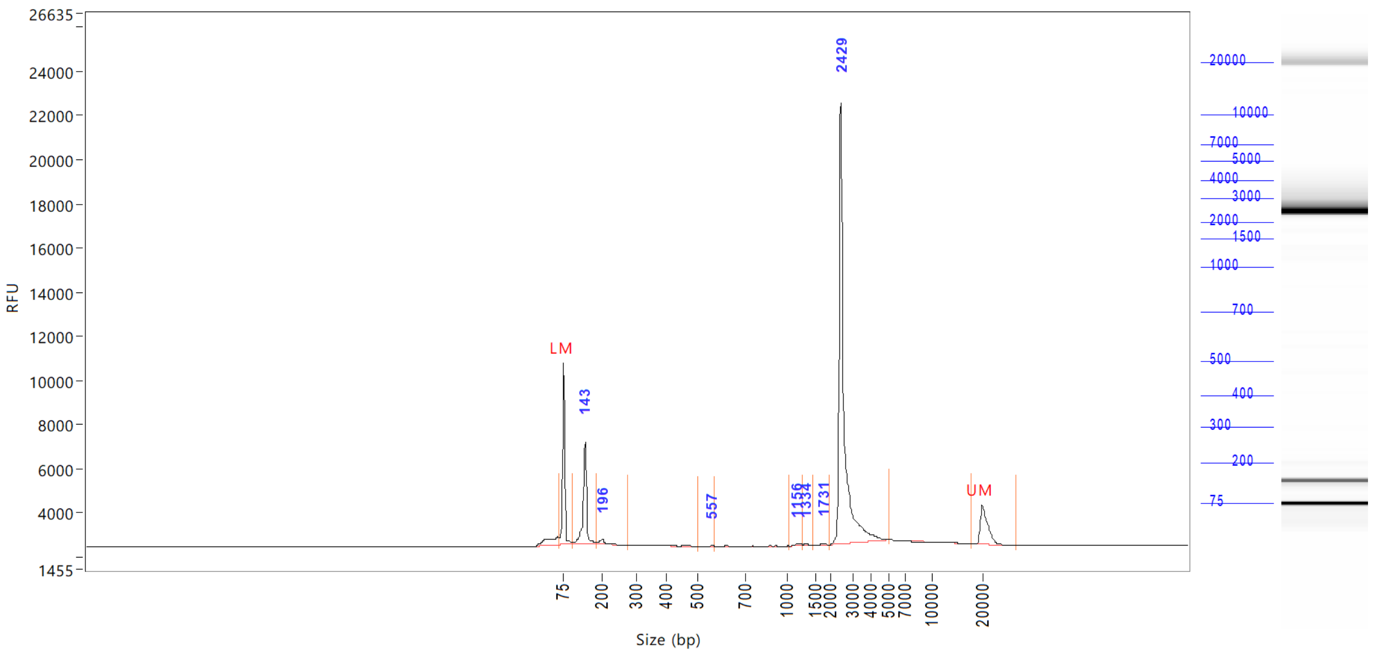

Peak	Size (bp)	Concentration (ng/uL)	From (bp)	To (bp)	Average size (bp)	CV%	RFU	Corrected peak area
1	75 (LM)	0.5000	64	105	73	6.31	8187	46.246
2	143	4.4587	105	179	139	6.91	4613	34.366
3	196	0.3227	179	273	199	8.05	209	2.487
4	557	0.0513	498	570	545	3.16	66	0.396
5	1156	0.1088	1034	1278	1180	4.42	84	0.839
6	1334	0.0687	1278	1478	1331	2.62	83	0.529
7	1731	0.0647	1478	1942	1715	3.65	89	0.499
8	2429	19.5026	1942	5001	2597	15.14	20003	150.319
9	20000 (UM)	0.1822	17805	26770	20509	4.25	1760	16.851

TIC:	24.5775	ng/uL
TIM:	68.1942	nmole/L
Total concentration:	24.7930	ng/uL

```
Sample peak width (sec): 5    Sample min peak height: 50    Sample baseline V to V?: Y    Sample baseline V to V points: 3
Sample filter: Binomial       Number of points for filter: 3  Sample start region (min): 0  Sample end region (min): 30
Marker peak width (sec): 5    Marker min peak height: 200   Marker baseline V to V?: Y    Marker baseline V to V points: 3
Lower marker selection: First peak > 500 RFU                Upper marker selection: Last peak > 200 RFU
Ladder size (bp)75, 200, 300, 400, 500, 700, 1000, 1500, 2000, 3000, 4000, 5000, 7000, 10000, 20000
Quantification using: Lower Marker          Final concentration (ng/uL): 0.5000          Dilution factor: 12.0
```

Sample: SampA7
Well location: A7
Created: Tuesday, August 10, 2021 9:21:07 PM

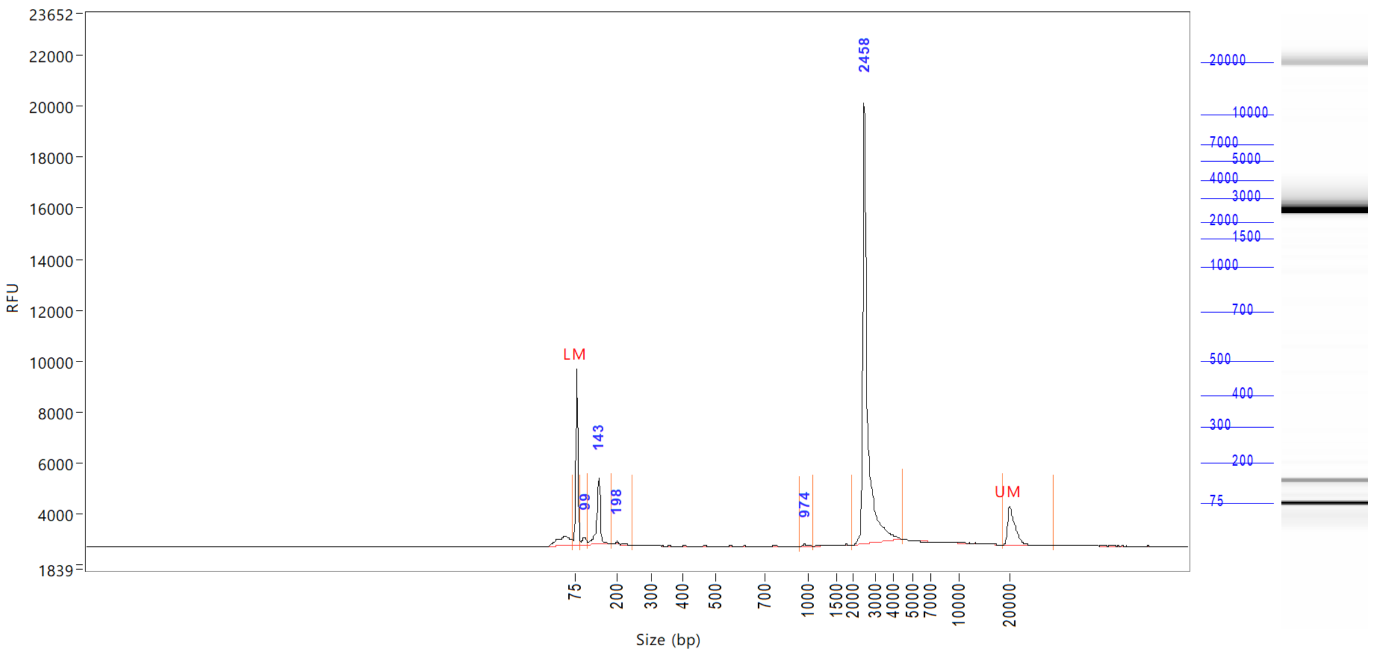

Peak	Size (bp)	Concentration (ng/uL)	From (bp)	To (bp)	Average size (bp)	CV%	RFU	Corrected peak area
1	75 (LM)	0.5000	64	87	74	4.52	6907	37.338
2	99	0.4708	87	108	97	5.78	299	2.930
3	143	3.2953	108	181	138	6.63	2609	20.506
4	198	0.1415	181	244	203	6.95	113	0.881
5	974	0.0745	939	1079	974	1.86	73	0.464
6	2458	19.5867	1962	4483	2592	11.76	17326	121.887
7	20000 (UM)	0.1849	18537	28463	20475	4.35	1502	13.804

TIC:	23.5688	ng/uL
TIM:	60.9209	nmole/L
Total concentration:	24.0821	ng/uL

Sample peak width (sec): 5 Sample min peak height: 50 Sample baseline V to V?: Y Sample baseline V to V points: 3
Sample filter: Binomial Number of points for filter: 3 Sample start region (min): 0 Sample end region (min): 30
Marker peak width (sec): 5 Marker min peak height: 200 Marker baseline V to V?: Y Marker baseline V to V points: 3
Lower marker selection: First peak > 500 RFU Upper marker selection: Last peak > 200 RFU
Ladder size (bp)75, 200, 300, 400, 500, 700, 1000, 1500, 2000, 3000, 4000, 5000, 7000, 10000, 20000
Quantification using: Lower Marker Final concentration (ng/uL): 0.5000 Dilution factor: 12.0

Sample: SampB7
Well location: B7
Created: Tuesday, August 10, 2021 9:21:07 PM

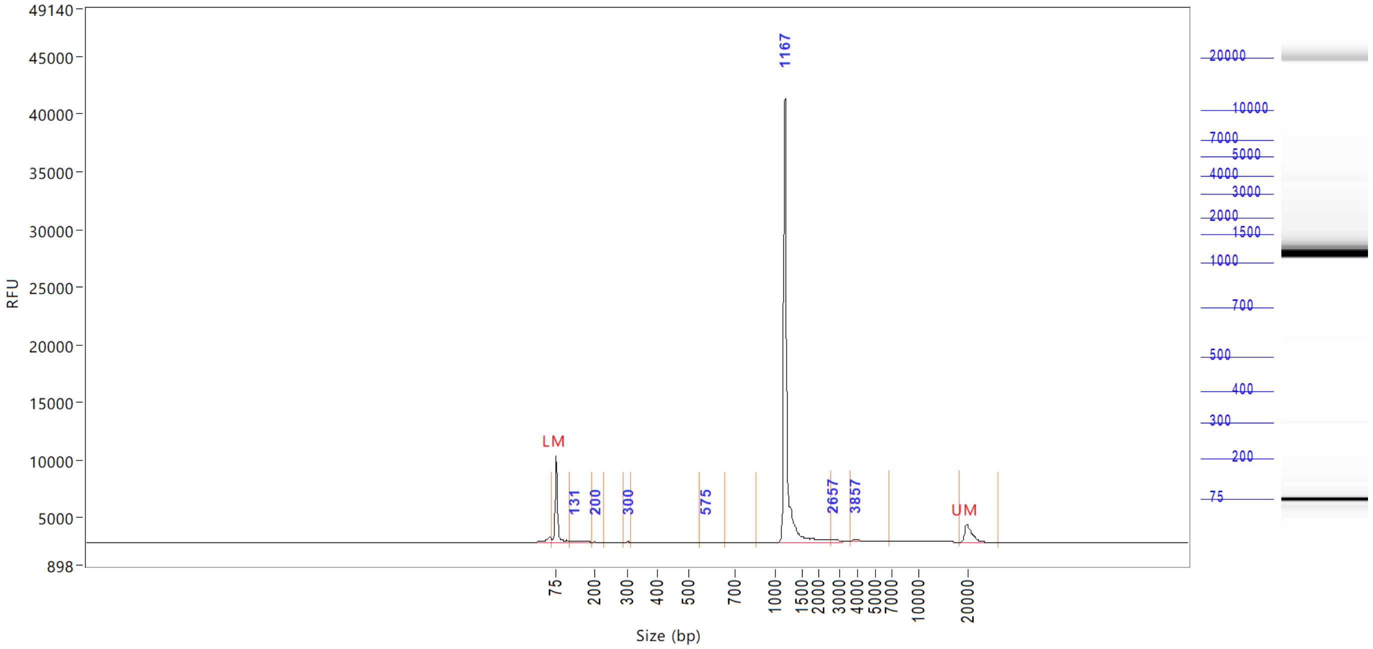

Peak	Size (bp)	Concentration (ng/uL)	From (bp)	To (bp)	Average size (bp)	CV%	RFU	Corrected peak area
1	75 (LM)	0.5000	64	120	75	11.09	7475	46.156
2	131	0.4249	120	190	149	13.41	146	3.268
3	200	0.0605	190	228	199	3.63	53	0.466
4	300	0.0828	289	309	298	1.05	132	0.637
5	575	0.0781	549	662	587	4.41	59	0.601
6	1167	28.9594	861	2543	1201	14.35	38557	222.773
7	2657	0.4659	2543	3608	2965	10.38	187	3.584
8	3857	0.5516	3608	6778	4543	18.20	192	4.243
9	20000 (UM)	0.1573	18659	26529	20483	4.62	1513	14.520

TIC:	30.6232	ng/uL
TIM:	46.0106	nmole/L
Total concentration:	30.8824	ng/uL

Sample peak width (sec): 5   Sample min peak height: 50   Sample baseline V to V?: Y   Sample baseline V to V points: 3
Sample filter: Binomial   Number of points for filter: 3   Sample start region (min): 0   Sample end region (min): 30
Marker peak width (sec): 5   Marker min peak height: 200   Marker baseline V to V?: Y   Marker baseline V to V points: 3
Lower marker selection: First peak > 500 RFU   Upper marker selection: Last peak > 200 RFU
Ladder size (bp)75, 200, 300, 400, 500, 700, 1000, 1500, 2000, 3000, 4000, 5000, 7000, 10000, 20000
Quantification using: Lower Marker   Final concentration (ng/uL): 0.5000   Dilution factor: 12.0

Sample: SampC7
Well location: C7
Created: Tuesday, August 10, 2021 9:21:07 PM

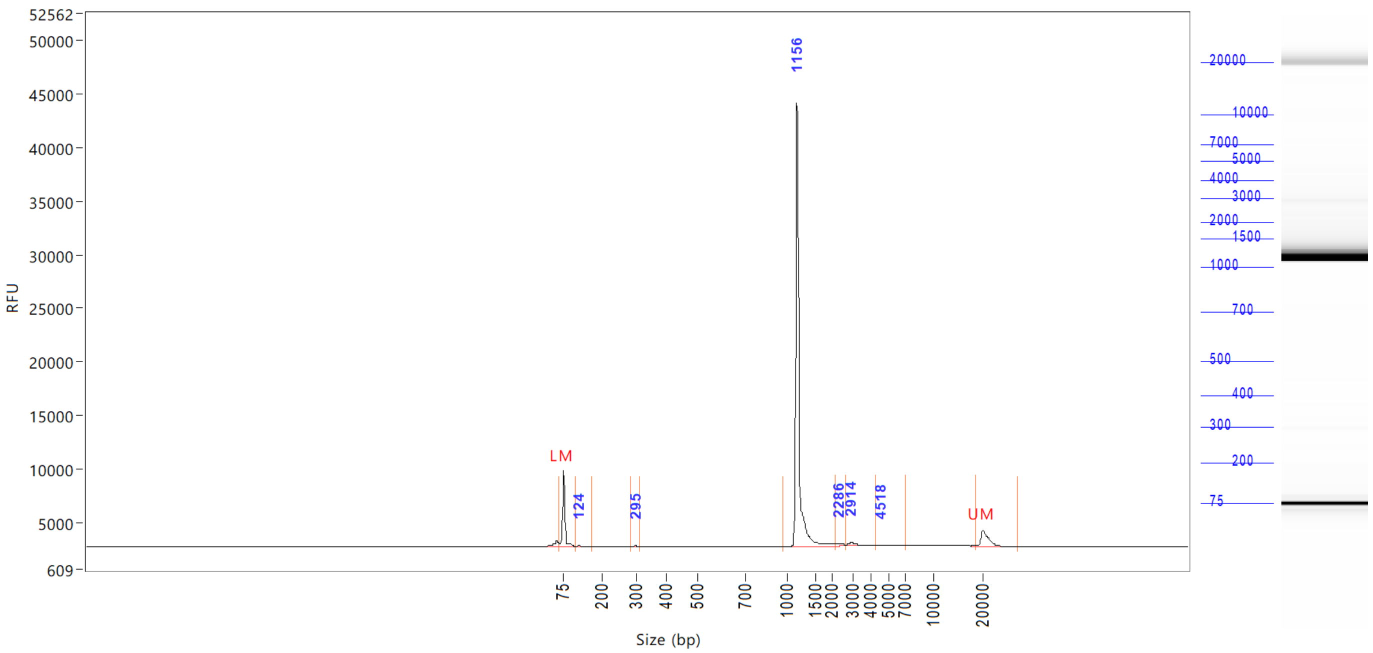

Peak	Size (bp)	Concentration (ng/uL)	From (bp)	To (bp)	Average size (bp)	CV%	RFU	Corrected peak area
1	75 (LM)	0.5000	64	112	74	9.33	7136	43.497
2	124	0.2559	112	164	135	11.39	84	1.855
3	295	0.0762	284	309	293	1.44	111	0.552
4	1156	31.2871	965	2144	1188	9.81	41523	226.818
5	2286	0.3084	2144	2629	2370	6.12	178	2.235
6	2914	0.7003	2629	4277	3218	13.95	312	5.077
7	4518	0.1722	4277	7000	4956	13.25	75	1.249
8	20000 (UM)	0.1536	18659	26891	20526	4.23	1423	13.362

TIC:	32.8001	ng/uL
TIM:	47.5212	nmole/L
Total concentration:	33.1968	ng/uL

Sample peak width (sec): 5 Sample min peak height: 50 Sample baseline V to V?: Y Sample baseline V to V points: 3
Sample filter: Binomial Number of points for filter: 3 Sample start region (min): 0 Sample end region (min): 30
Marker peak width (sec): 5 Marker min peak height: 200 Marker baseline V to V?: Y Marker baseline V to V points: 3
Lower marker selection: First peak > 500 RFU Upper marker selection: Last peak > 200 RFU
Ladder size (bp)75, 200, 300, 400, 500, 700, 1000, 1500, 2000, 3000, 4000, 5000, 7000, 10000, 20000
Quantification using: Lower Marker Final concentration (ng/uL): 0.5000 Dilution factor: 12.0

Sample: SampD7
Well location: D7
Created: Tuesday, August 10, 2021 9:21:07 PM

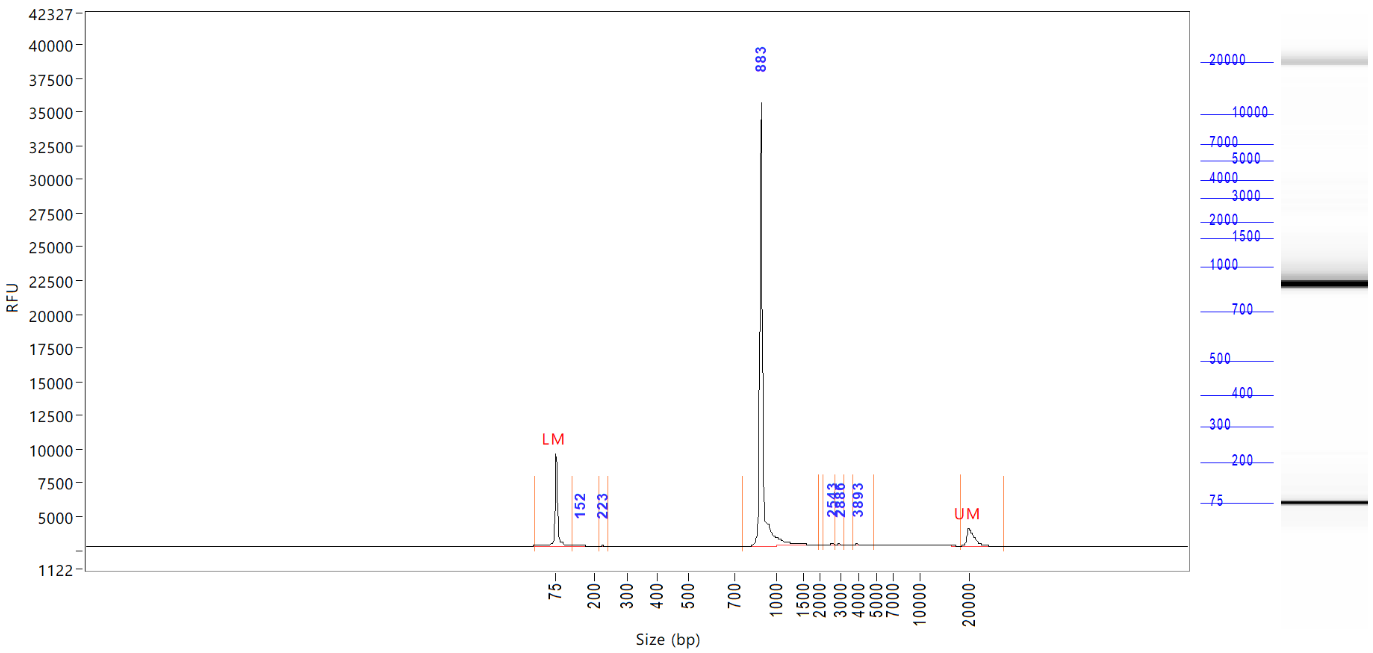

Peak	Size (bp)	Concentration (ng/uL)	From (bp)	To (bp)	Average size (bp)	CV%	RFU	Corrected peak area
1	75 (LM)	0.5000	9	129	72	23.98	6919	50.775
2	152	0.3819	129	214	158	13.16	113	3.232
3	223	0.0471	214	241	221	1.75	72	0.398
4	883	21.6560	744	1942	903	9.17	32917	183.264
5	2543	0.0831	2144	2715	2478	5.52	83	0.703
6	2886	0.0612	2715	3108	2890	3.56	73	0.518
7	3893	0.0757	3643	4862	3994	5.94	63	0.640
8	20000 (UM)	0.1205	18293	27133	20490	4.65	1266	12.236

TIC:	22.3051	ng/uL
TIM:	43.9169	nmole/L
Total concentration:	22.6078	ng/uL

```
Sample peak width (sec): 5    Sample min peak height: 50    Sample baseline V to V?: Y    Sample baseline V to V points: 3
Sample filter: Binomial       Number of points for filter: 3  Sample start region (min): 0  Sample end region (min): 30
Marker peak width (sec): 5    Marker min peak height: 200   Marker baseline V to V?: Y    Marker baseline V to V points: 3
Lower marker selection: First peak > 500 RFU                Upper marker selection: Last peak > 200 RFU
Ladder size (bp)75, 200, 300, 400, 500, 700, 1000, 1500, 2000, 3000, 4000, 5000, 7000, 10000, 20000
Quantification using: Lower Marker          Final concentration (ng/uL): 0.5000          Dilution factor: 12.0
```

Sample: SampE7
Well location: E7
Created: Tuesday, August 10, 2021 9:21:07 PM

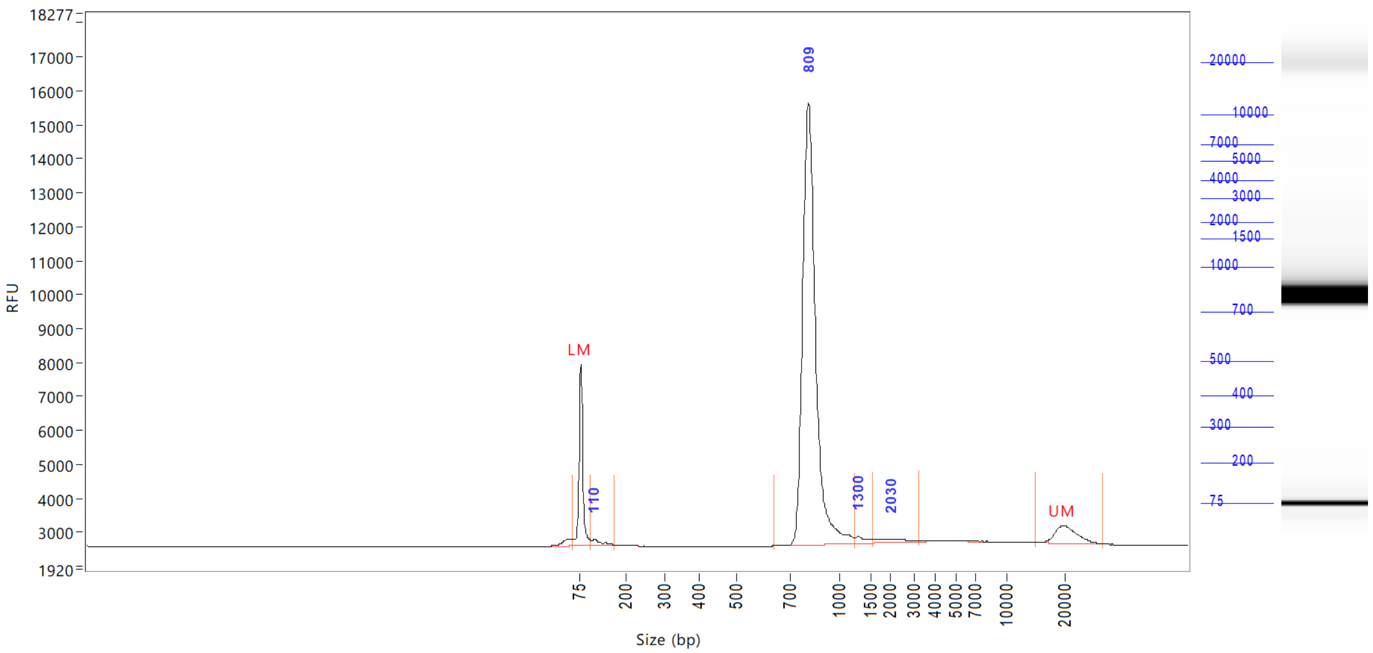

Peak	Size (bp)	Concentration (ng/uL)	From (bp)	To (bp)	Average size (bp)	CV%	RFU	Corrected peak area
1	75 (LM)	0.5000	52	101	73	9.10	5356	48.482
2	110	0.5466	101	169	123	14.22	185	4.417
3	809	33.6153	639	1256	820	7.54	13029	271.625
4	1300	0.3988	1256	1539	1373	6.00	201	3.222
5	2030	0.5422	1539	3215	2146	20.23	100	4.381
6	20000 (UM)	0.1388	15122	26891	20550	9.03	513	13.460

TIC:	35.1029	ng/uL
TIM:	75.6653	nmole/L
Total concentration:	35.3511	ng/uL

```
Sample peak width (sec): 5    Sample min peak height: 50    Sample baseline V to V?: Y    Sample baseline V to V points: 3
Sample filter: Binomial       Number of points for filter: 3  Sample start region (min): 0  Sample end region (min): 30
Marker peak width (sec): 5    Marker min peak height: 200   Marker baseline V to V?: Y    Marker baseline V to V points: 3
Lower marker selection: First peak > 500 RFU                Upper marker selection: Last peak > 200 RFU
Ladder size (bp)75, 200, 300, 400, 500, 700, 1000, 1500, 2000, 3000, 4000, 5000, 7000, 10000, 20000
Quantification using: Lower Marker          Final concentration (ng/uL): 0.5000          Dilution factor: 12.0
```

Sample: SampF7
Well location: F7
Created: Tuesday, August 10, 2021 9:21:07 PM

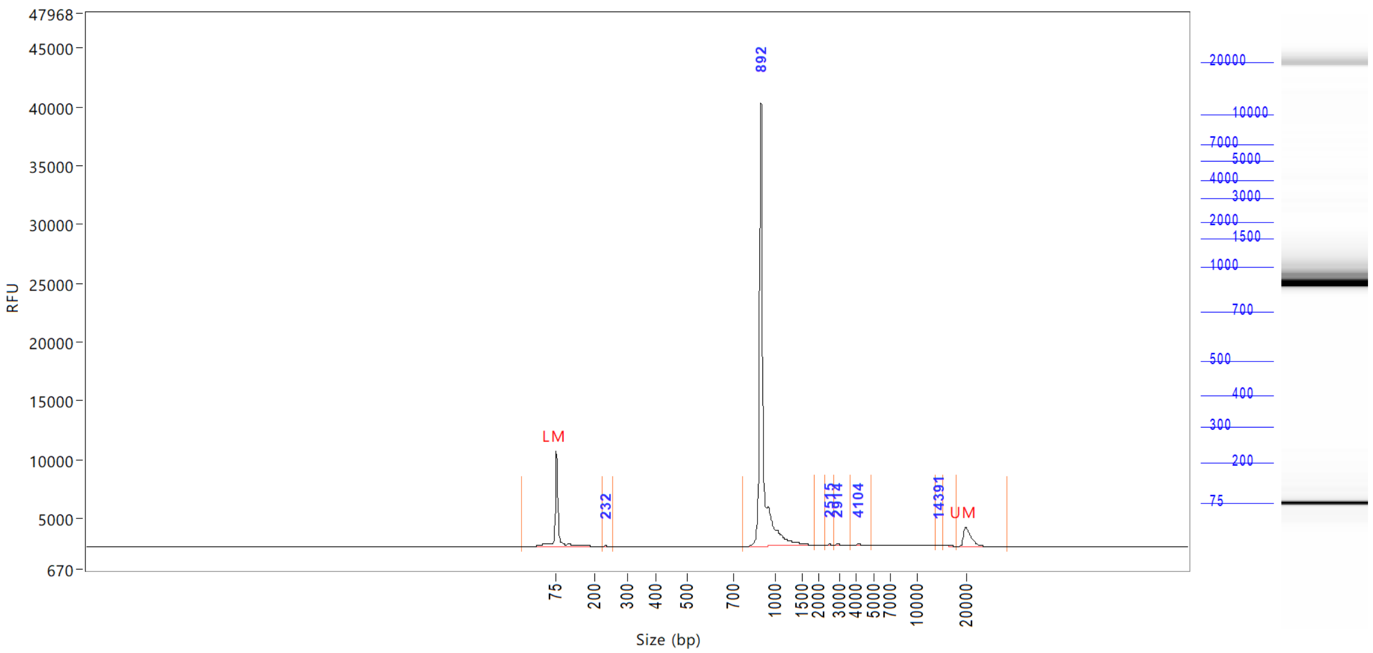

Peak	Size (bp)	Concentration (ng/uL)	From (bp)	To (bp)	Average size (bp)	CV%	RFU	Corrected peak area
1	75 (LM)	0.5000	0	221	78	37.62	8140	66.008
2	232	0.0384	221	257	230	2.37	71	0.422
3	892	23.6652	757	1885	922	9.99	37773	260.348
4	2515	0.0435	2315	2743	2543	4.33	60	0.478
5	2914	0.0695	2743	3715	3019	7.77	91	0.764
6	4104	0.0821	3715	4862	4162	5.31	82	0.904
7	14391	0.0268	13781	15244	14340	2.46	55	0.295
8	20000 (UM)	0.1211	18171	28584	20477	4.57	1647	15.986

TIC:	23.9255	ng/uL
TIM:	42.6217	nmole/L
Total concentration:	24.1416	ng/uL

Sample peak width (sec): 5 Sample min peak height: 50 Sample baseline V to V?: Y Sample baseline V to V points: 3
Sample filter: Binomial Number of points for filter: 3 Sample start region (min): 0 Sample end region (min): 30
Marker peak width (sec): 5 Marker min peak height: 200 Marker baseline V to V?: Y Marker baseline V to V points: 3
Lower marker selection: First peak > 500 RFU Upper marker selection: Last peak > 200 RFU
Ladder size (bp)75, 200, 300, 400, 500, 700, 1000, 1500, 2000, 3000, 4000, 5000, 7000, 10000, 20000
Quantification using: Lower Marker Final concentration (ng/uL): 0.5000 Dilution factor: 12.0

Sample: SampG7
Well location: G7
Created: Tuesday, August 10, 2021 9:21:07 PM

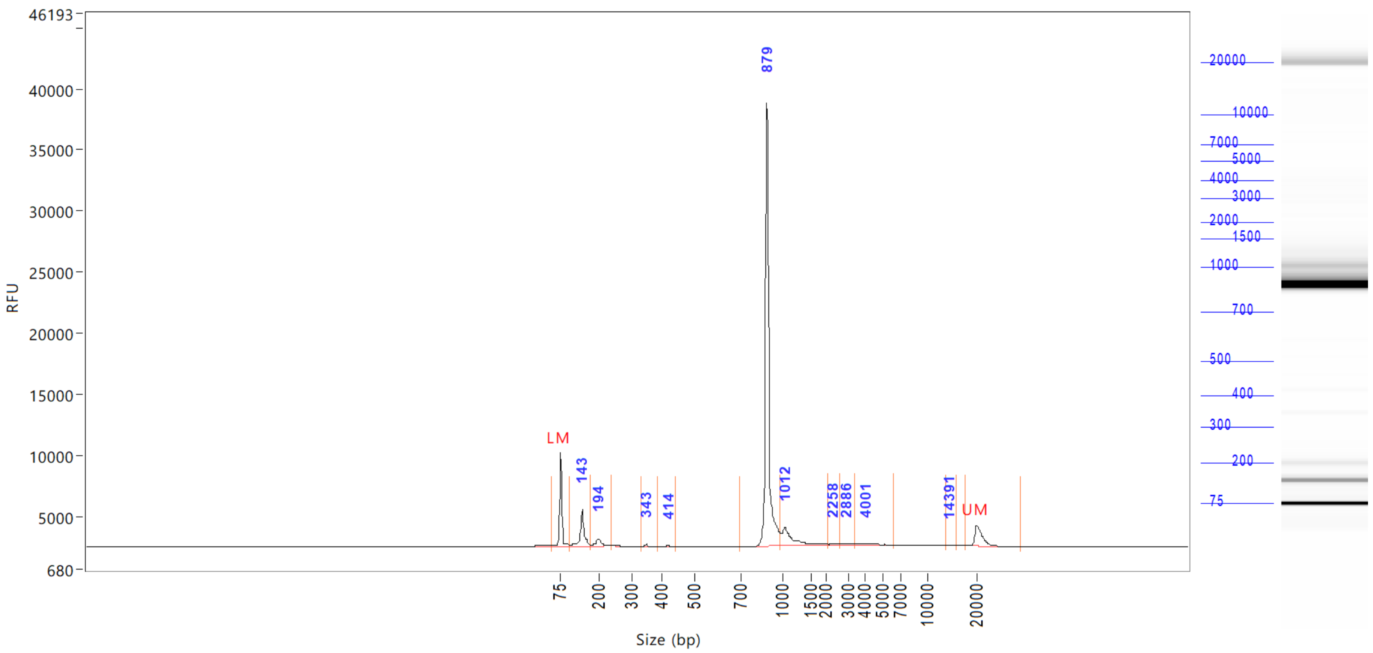

Peak	Size (bp)	Concentration (ng/uL)	From (bp)	To (bp)	Average size (bp)	CV%	RFU	Corrected peak area
1	75 (LM)	0.5000	50	103	73	8.17	7624	44.612
2	143	3.6184	103	171	141	7.91	3004	26.904
3	194	1.0326	171	235	195	5.87	613	7.678
4	343	0.1138	327	380	342	1.86	164	0.846
5	414	0.0714	380	439	410	1.84	100	0.531
6	879	28.2787	695	983	884	2.78	36337	210.262
7	1012	3.3882	983	2058	1130	14.87	1503	25.192
8	2258	0.0595	2058	2600	2308	5.93	58	0.442
9	2886	0.1592	2600	3393	3011	6.73	85	1.183
10	4001	0.2249	3393	6186	4038	11.57	76	1.672
11	14391	0.0471	13659	15976	14474	4.03	50	0.350
12	20000 (UM)	0.1849	17561	29068	20539	4.90	1706	16.495

TIC:	36.9938	ng/uL
TIM:	109.5252	nmole/L
Total concentration:	37.1590	ng/uL

```
Sample peak width (sec): 5     Sample min peak height: 50     Sample baseline V to V?: Y     Sample baseline V to V points: 3
Sample filter: Binomial        Number of points for filter: 3  Sample start region (min): 0  Sample end region (min): 30
Marker peak width (sec): 5     Marker min peak height: 200    Marker baseline V to V?: Y     Marker baseline V to V points: 3
Lower marker selection: First peak > 500 RFU                  Upper marker selection: Last peak > 200 RFU
Ladder size (bp)75, 200, 300, 400, 500, 700, 1000, 1500, 2000, 3000, 4000, 5000, 7000, 10000, 20000
Quantification using: Lower Marker          Final concentration (ng/uL): 0.5000          Dilution factor: 12.0
```

Sample: SampH7
Well location: H7
Created: Tuesday, August 10, 2021 9:21:07 PM

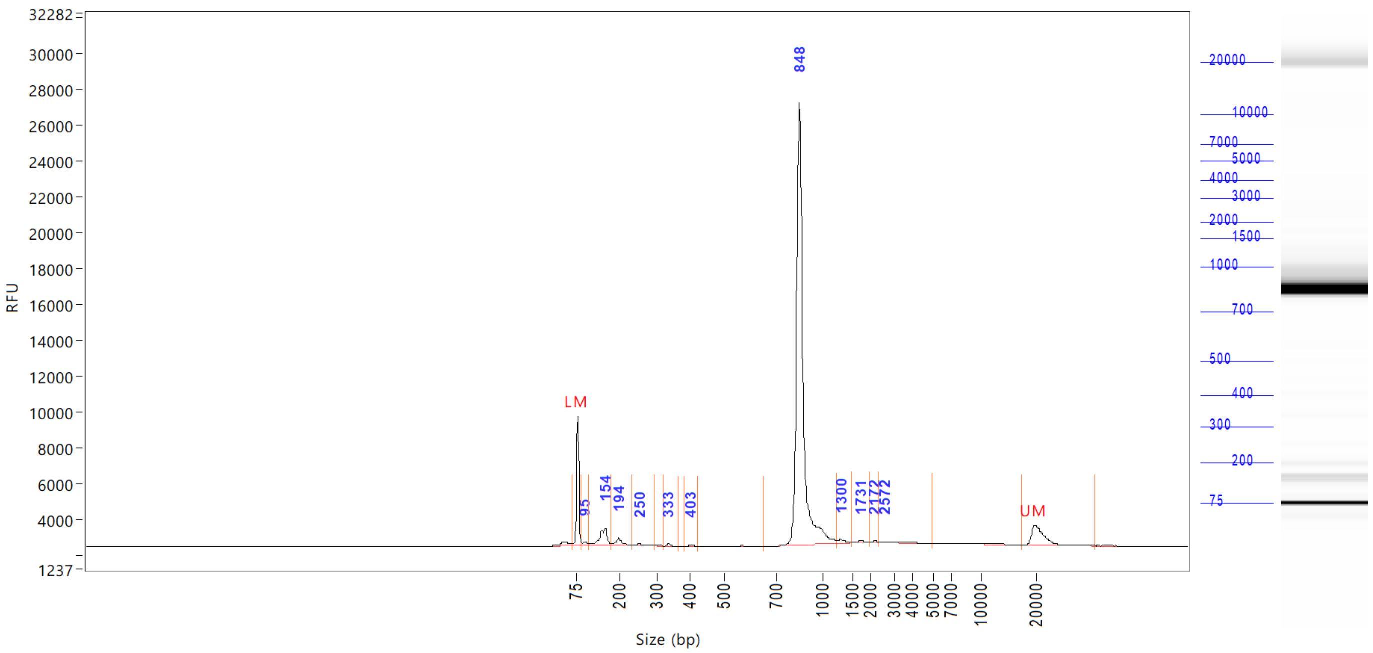

Peak	Size (bp)	Concentration (ng/uL)	From (bp)	To (bp)	Average size (bp)	CV%	RFU	Corrected peak area
1	75 (LM)	0.5000	62	87	73	4.68	7184	41.089
2	95	0.2273	87	108	95	5.91	149	1.556
3	154	2.1038	108	173	146	7.88	922	14.407
4	194	0.6024	173	232	193	4.76	362	4.126
5	250	0.0968	232	293	247	2.67	82	0.663
6	333	0.1269	319	364	334	2.08	112	0.869
7	403	0.1176	382	420	400	1.50	102	0.806
8	848	39.0192	649	1245	860	5.96	24737	267.208
9	1300	0.3374	1245	1500	1334	5.10	198	2.311
10	1731	0.1650	1500	2000	1713	7.44	89	1.130
11	2172	0.0922	2000	2372	2181	5.09	69	0.631
12	2572	0.2583	2372	5000	3090	18.59	66	1.769
13	20000 (UM)	0.1969	17439	31244	20741	6.72	1115	16.180

TIC:	43.1470	ng/uL
TIM:	109.9516	nmole/L
Total concentration:	43.4196	ng/uL

```
Sample peak width (sec): 5     Sample min peak height: 50    Sample baseline V to V?: Y    Sample baseline V to V points: 3
Sample filter: Binomial        Number of points for filter: 3  Sample start region (min): 0  Sample end region (min): 30
Marker peak width (sec): 5     Marker min peak height: 200   Marker baseline V to V?: Y    Marker baseline V to V points: 3
Lower marker selection: First peak > 500 RFU                 Upper marker selection: Last peak > 200 RFU
Ladder size (bp)75, 200, 300, 400, 500, 700, 1000, 1500, 2000, 3000, 4000, 5000, 7000, 10000, 20000
Quantification using: Lower Marker          Final concentration (ng/uL): 0.5000          Dilution factor: 12.0
```

Sample: SampA8
Well location: A8
Created: Tuesday, August 10, 2021 9:21:07 PM

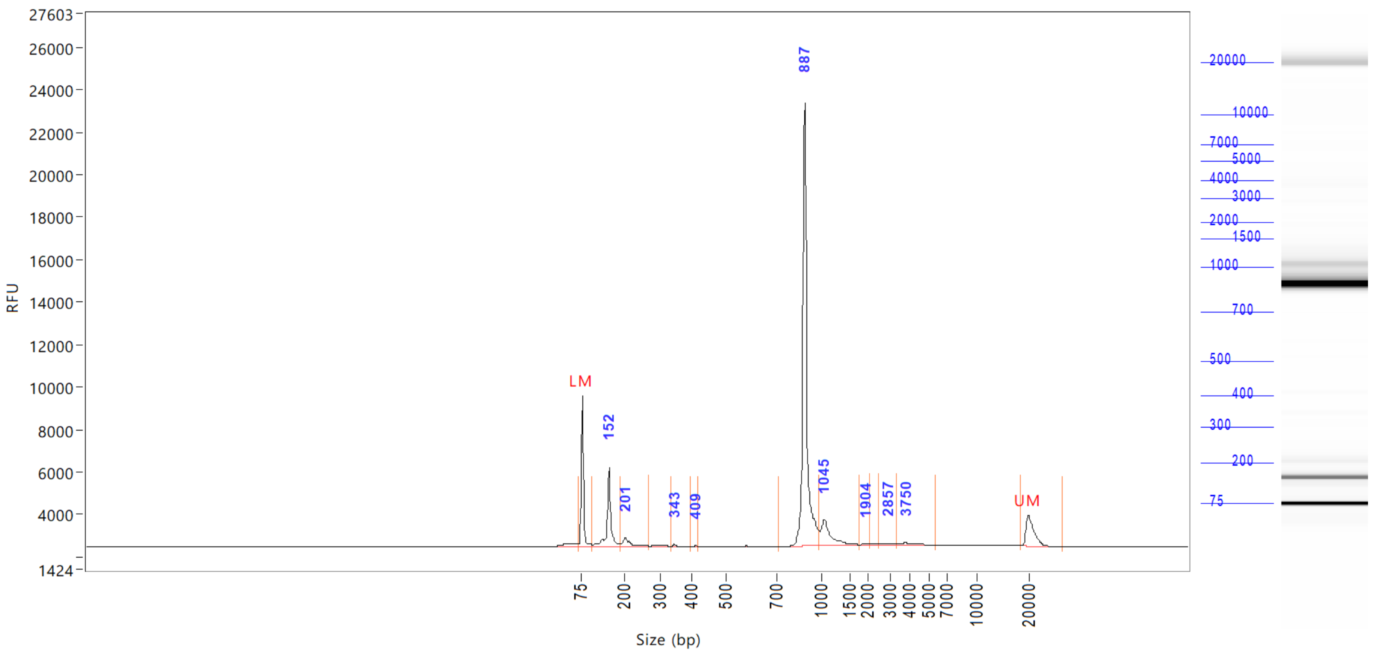

Peak	Size (bp)	Concentration (ng/uL)	From (bp)	To (bp)	Average size (bp)	CV%	RFU	Corrected peak area
1	75 (LM)	0.5000	62	105	73	7.34	7095	40.018
2	152	4.5486	105	185	149	8.15	3714	30.338
3	201	0.8360	185	269	206	7.31	388	5.576
4	343	0.0723	333	394	344	2.55	91	0.482
5	409	0.0474	394	418	406	0.84	65	0.316
6	887	20.4009	714	987	889	2.97	20897	136.066
7	1045	2.8462	987	1750	1116	12.12	1220	18.983
8	1904	0.0581	1750	2058	1894	3.69	56	0.387
9	2857	0.0991	2486	3322	2917	6.51	72	0.661
10	3750	0.2594	3322	5815	4046	11.62	99	1.730
11	20000 (UM)	0.1732	18415	26408	20391	4.27	1445	13.861

TIC:	29.1679	ng/uL
TIM:	99.5627	nmole/L
Total concentration:	29.4797	ng/uL

```
Sample peak width (sec): 5    Sample min peak height: 50    Sample baseline V to V?: Y    Sample baseline V to V points: 3
Sample filter: Binomial       Number of points for filter: 3  Sample start region (min): 0  Sample end region (min): 30
Marker peak width (sec): 5    Marker min peak height: 200   Marker baseline V to V?: Y    Marker baseline V to V points: 3
Lower marker selection: First peak > 500 RFU                Upper marker selection: Last peak > 200 RFU
Ladder size (bp)75, 200, 300, 400, 500, 700, 1000, 1500, 2000, 3000, 4000, 5000, 7000, 10000, 20000
Quantification using: Lower Marker          Final concentration (ng/uL): 0.5000          Dilution factor: 12.0
```

Sample: SampB8
Well location: B8
Created: Tuesday, August 10, 2021 9:21:07 PM

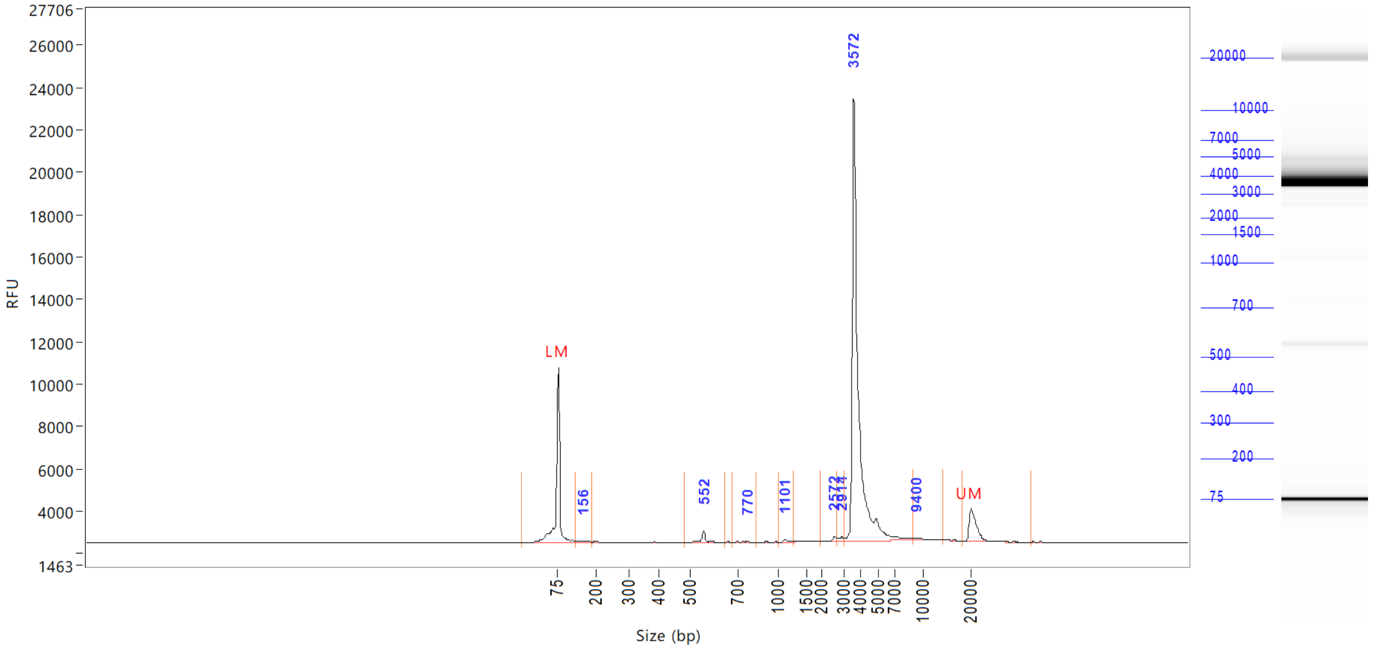

Peak	Size (bp)	Concentration (ng/uL)	From (bp)	To (bp)	Average size (bp)	CV%	RFU	Corrected peak area
1	75 (LM)	0.5000	0	131	68	26.33	8286	66.442
2	156	0.1611	131	185	155	8.94	84	1.784
3	552	0.3666	481	644	549	4.16	572	4.059
4	770	0.0794	674	835	732	4.69	64	0.880
5	1101	0.0952	1000	1267	1126	5.00	138	1.054
6	2572	0.1256	1942	2686	2515	5.21	225	1.391
7	2914	0.1536	2686	3037	2864	3.72	184	1.701
8	3572	17.0131	3037	8934	3877	16.85	20907	188.398
9	9400	0.1213	8934	14025	10251	11.19	69	1.343
10	20000 (UM)	0.1079	18293	32090	20518	5.11	1545	14.342

TIC:	18.1159	ng/uL
TIM:	10.5388	nmole/L
Total concentration:	18.3266	ng/uL

Sample peak width (sec): 5 Sample min peak height: 50 Sample baseline V to V?: Y Sample baseline V to V points: 3
Sample filter: Binomial Number of points for filter: 3 Sample start region (min): 0 Sample end region (min): 30
Marker peak width (sec): 5 Marker min peak height: 200 Marker baseline V to V?: Y Marker baseline V to V points: 3
Lower marker selection: First peak > 500 RFU Upper marker selection: Last peak > 200 RFU
Ladder size (bp)75, 200, 300, 400, 500, 700, 1000, 1500, 2000, 3000, 4000, 5000, 7000, 10000, 20000
Quantification using: Lower Marker Final concentration (ng/uL): 0.5000 Dilution factor: 12.0

Sample: SampC8
Well location: C8
Created: Tuesday, August 10, 2021 9:21:07 PM

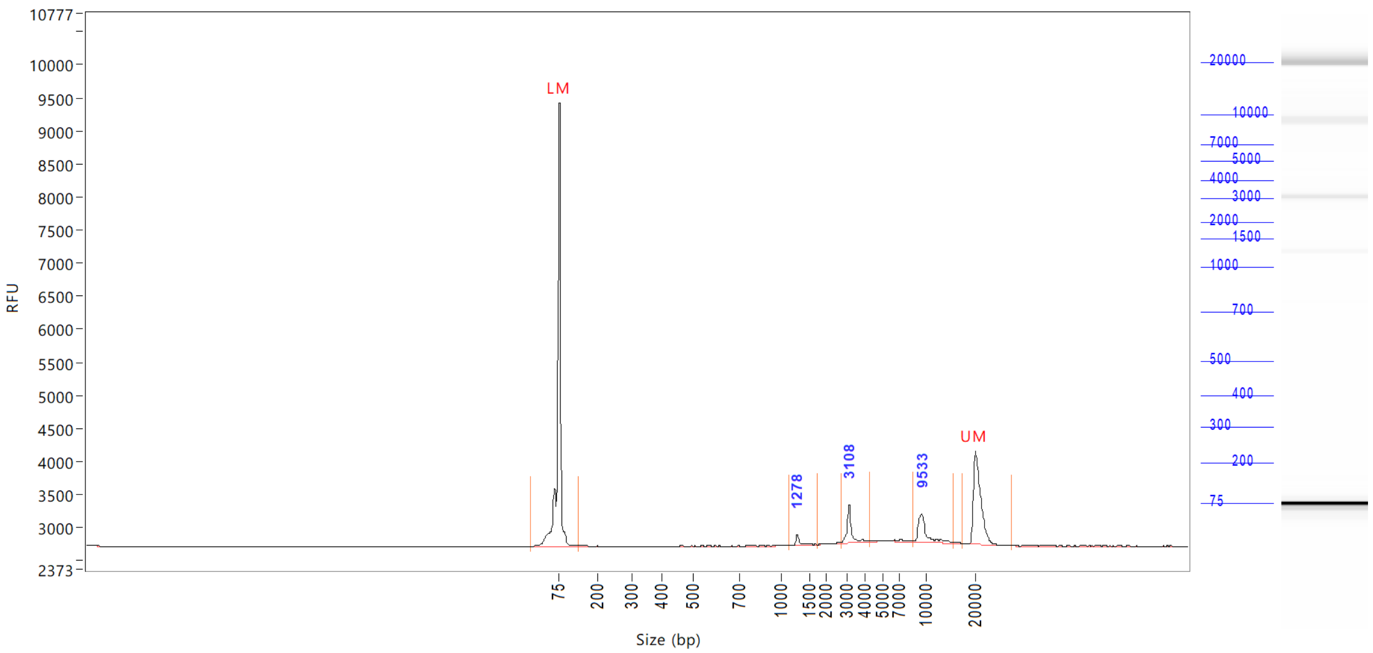

Peak	Size (bp)	Concentration (ng/uL)	From (bp)	To (bp)	Average size (bp)	CV%	RFU	Corrected peak area
1	75 (LM)	0.5000	0	137	68	19.39	6718	50.781
2	1278	0.1278	1134	1750	1310	6.94	164	1.081
3	3108	0.4625	2743	4277	3161	8.86	574	3.914
4	9533	0.5633	8600	15610	10109	14.22	425	4.767
5	20000 (UM)	0.1323	17317	27496	20480	4.39	1418	13.435

TIC:	1.1535	ng/uL
TIM:	0.4931	nmole/L
Total concentration:	1.4786	ng/uL

Sample peak width (sec): 5 Sample min peak height: 50 Sample baseline V to V?: Y Sample baseline V to V points: 3
Sample filter: Binomial Number of points for filter: 3 Sample start region (min): 0 Sample end region (min): 30
Marker peak width (sec): 5 Marker min peak height: 200 Marker baseline V to V?: Y Marker baseline V to V points: 3
Lower marker selection: First peak > 500 RFU Upper marker selection: Last peak > 200 RFU
Ladder size (bp)75, 200, 300, 400, 500, 700, 1000, 1500, 2000, 3000, 4000, 5000, 7000, 10000, 20000
Quantification using: Lower Marker Final concentration (ng/uL): 0.5000 Dilution factor: 12.0

Sample: SampD8
Well location: D8
Created: Tuesday, August 10, 2021 9:21:07 PM

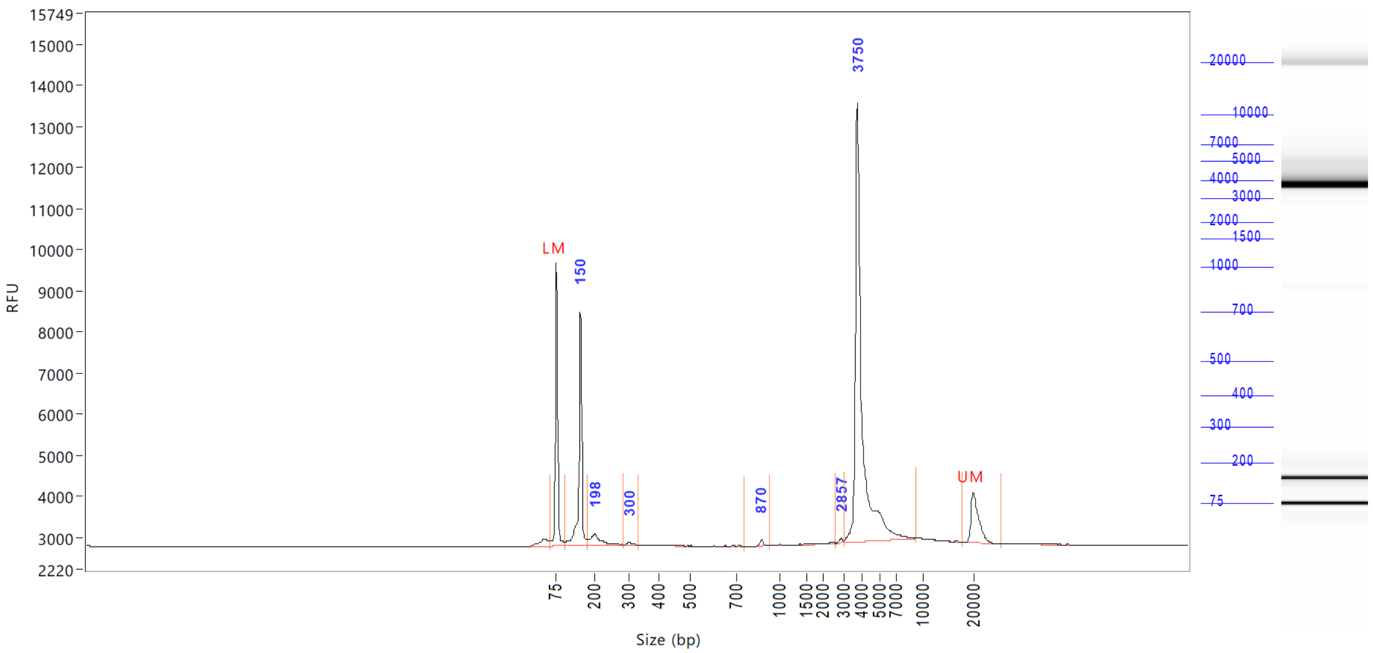

Peak	Size (bp)	Concentration (ng/uL)	From (bp)	To (bp)	Average size (bp)	CV%	RFU	Corrected peak area
1	75 (LM)	0.5000	58	101	74	6.64	6897	39.332
2	150	6.2966	101	175	147	7.28	5674	41.277
3	198	0.9708	175	286	203	9.88	276	6.364
4	300	0.0964	286	331	302	2.80	51	0.632
5	870	0.1445	744	935	867	2.52	169	0.947
6	2857	0.0947	2600	2971	2828	2.95	108	0.621
7	3750	15.5253	2971	9200	4052	16.93	10703	101.775
8	20000 (UM)	0.1542	17805	25803	20524	4.41	1214	12.130

TIC:	23.1283	ng/uL
TIM:	85.4212	nmole/L
Total concentration:	23.3985	ng/uL

Sample peak width (sec): 5   Sample min peak height: 50   Sample baseline V to V?: Y   Sample baseline V to V points: 3
Sample filter: Binomial   Number of points for filter: 3 Sample start region (min): 0 Sample end region (min): 30
Marker peak width (sec): 5   Marker min peak height: 200   Marker baseline V to V?: Y   Marker baseline V to V points: 3
Lower marker selection: First peak > 500 RFU   Upper marker selection: Last peak > 200 RFU
Ladder size (bp)75, 200, 300, 400, 500, 700, 1000, 1500, 2000, 3000, 4000, 5000, 7000, 10000, 20000
Quantification using: Lower Marker   Final concentration (ng/uL): 0.5000   Dilution factor: 12.0

Sample: SampE8
Well location: E8
Created: Tuesday, August 10, 2021 9:21:07 PM

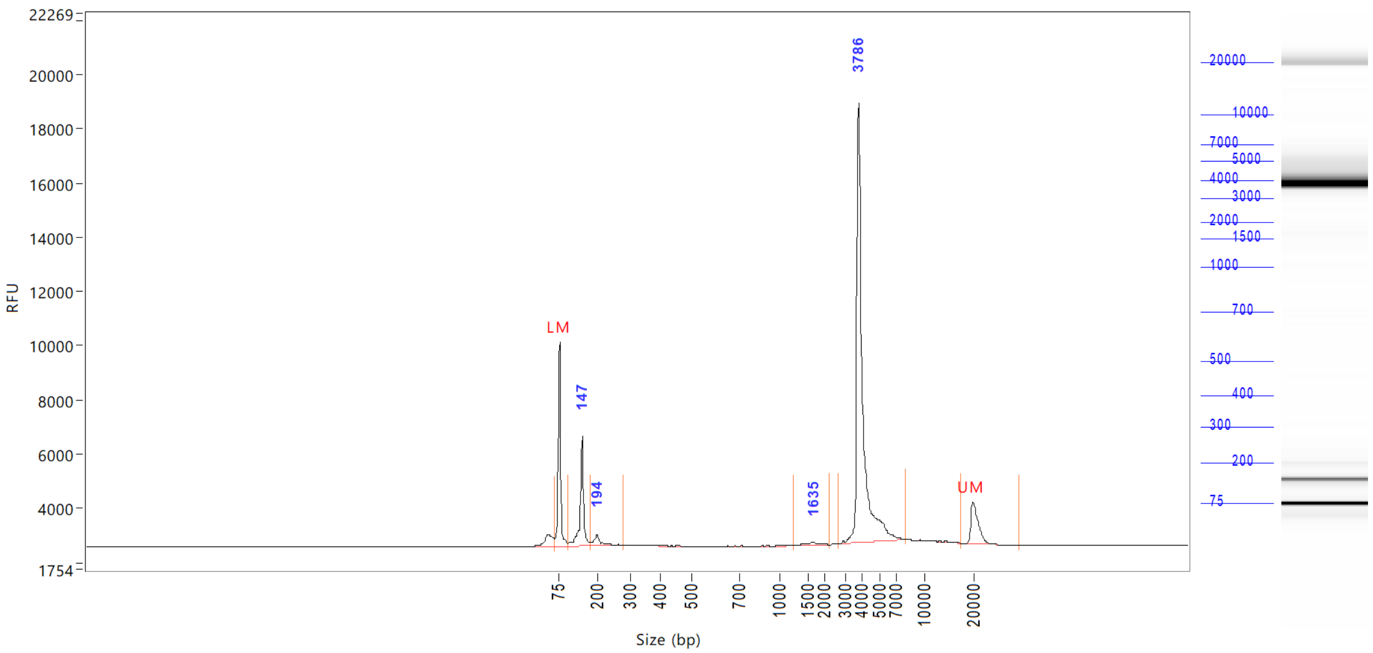

Peak	Size (bp)	Concentration (ng/uL)	From (bp)	To (bp)	Average size (bp)	CV%	RFU	Corrected peak area
1	75 (LM)	0.5000	60	101	73	7.39	7549	45.894
2	147	4.3891	101	177	142	8.42	4035	33.572
3	194	0.8773	177	278	203	10.16	424	6.711
4	1635	0.2946	1234	2258	1581	12.35	127	2.253
5	3786	18.2222	2715	8001	3998	13.31	16249	139.381
6	20000 (UM)	0.1602	17561	29430	20470	4.46	1560	14.700

TIC:	23.7833	ng/uL
TIM:	65.7110	nmole/L
Total concentration:	24.1106	ng/uL

Sample peak width (sec): 5   Sample min peak height: 50   Sample baseline V to V?: Y   Sample baseline V to V points: 3
Sample filter: Binomial   Number of points for filter: 3   Sample start region (min): 0   Sample end region (min): 30
Marker peak width (sec): 5   Marker min peak height: 200   Marker baseline V to V?: Y   Marker baseline V to V points: 3
Lower marker selection: First peak > 500 RFU   Upper marker selection: Last peak > 200 RFU
Ladder size (bp)75, 200, 300, 400, 500, 700, 1000, 1500, 2000, 3000, 4000, 5000, 7000, 10000, 20000
Quantification using: Lower Marker   Final concentration (ng/uL): 0.5000   Dilution factor: 12.0

Sample: SampF8
Well location: F8
Created: Tuesday, August 10, 2021 9:21:07 PM

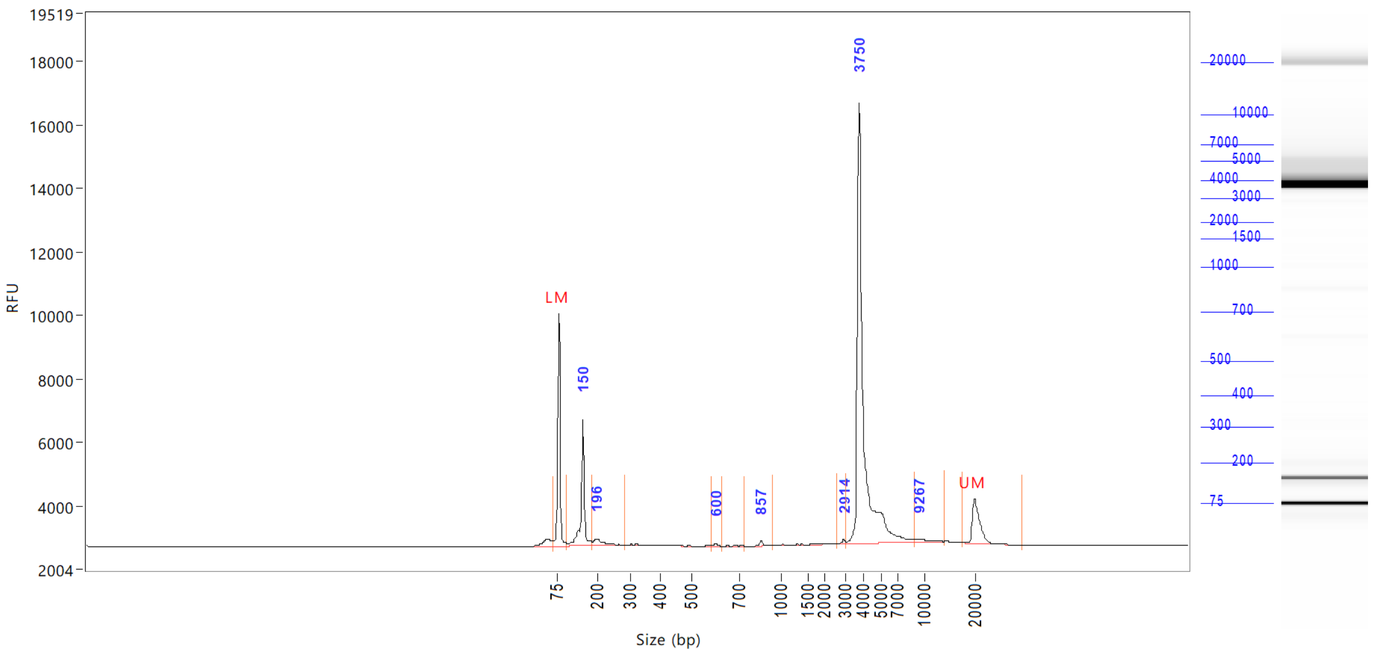

Peak	Size (bp)	Concentration (ng/uL)	From (bp)	To (bp)	Average size (bp)	CV%	RFU	Corrected peak area
1	75 (LM)	0.5000	58	99	73	6.30	7321	41.864
2	150	4.3662	99	181	146	8.41	3961	30.465
3	196	0.6798	181	280	206	9.99	220	4.743
4	600	0.0750	583	626	596	1.15	96	0.524
5	857	0.1504	736	935	853	3.01	167	1.049
6	2914	0.1023	2600	3001	2865	3.22	126	0.714
7	3750	18.0698	3001	8800	4096	18.58	13905	126.080
8	9267	0.2267	8800	14025	10092	10.88	79	1.582
9	20000 (UM)	0.1560	17561	29672	20517	4.55	1387	13.064

TIC:	23.6702	ng/uL
TIM:	62.4294	nmole/L
Total concentration:	24.0568	ng/uL

Sample peak width (sec): 5   Sample min peak height: 50   Sample baseline V to V?: Y   Sample baseline V to V points: 3
Sample filter: Binomial   Number of points for filter: 3 Sample start region (min): 0 Sample end region (min): 30
Marker peak width (sec): 5   Marker min peak height: 200   Marker baseline V to V?: Y   Marker baseline V to V points: 3
Lower marker selection: First peak > 500 RFU   Upper marker selection: Last peak > 200 RFU
Ladder size (bp)75, 200, 300, 400, 500, 700, 1000, 1500, 2000, 3000, 4000, 5000, 7000, 10000, 20000
Quantification using: Lower Marker   Final concentration (ng/uL): 0.5000   Dilution factor: 12.0

Sample: SampG8
Well location: G8
Created: Tuesday, August 10, 2021 9:21:07 PM

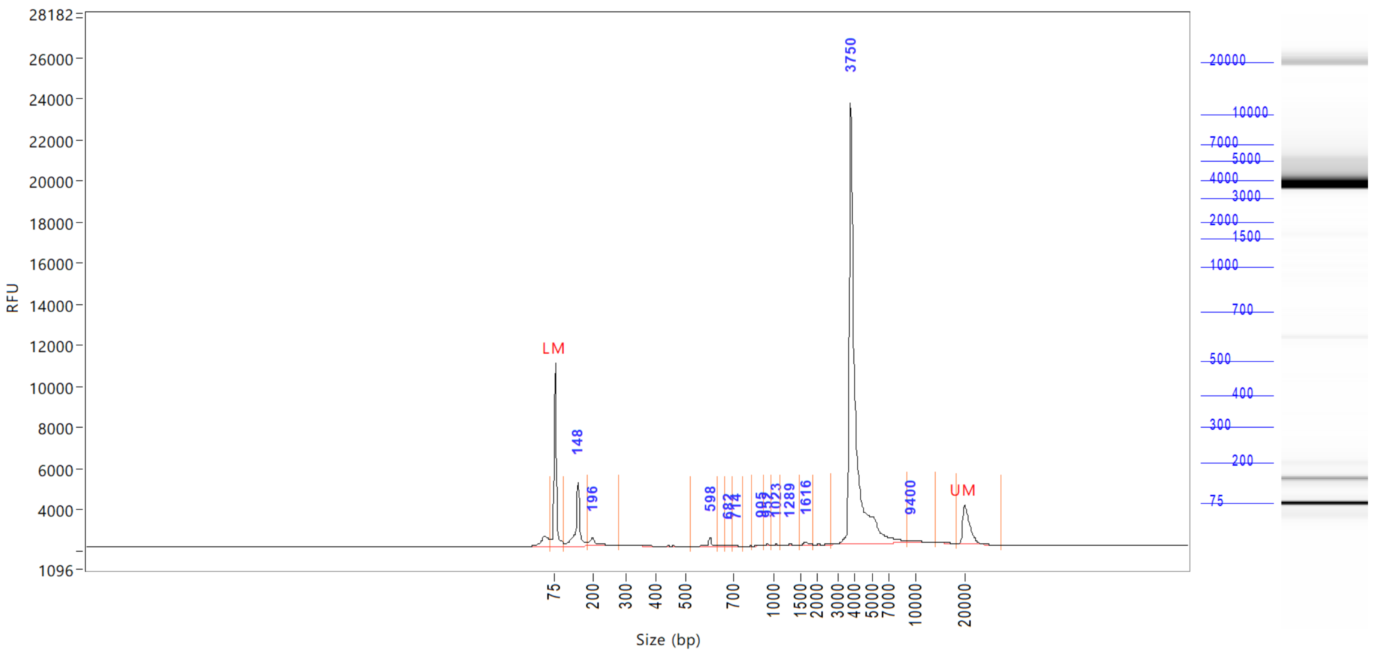

Peak	Size (bp)	Concentration (ng/uL)	From (bp)	To (bp)	Average size (bp)	CV%	RFU	Corrected peak area
1	75 (LM)	0.5000	62	105	74	7.77	8962	54.314
2	148	3.2885	105	179	144	9.17	3107	29.769
3	196	0.7966	179	278	205	10.40	410	7.211
4	598	0.3298	514	631	591	2.70	440	2.986
5	682	0.0387	669	697	682	1.02	57	0.350
6	714	0.0307	697	775	713	1.83	52	0.278
7	905	0.0413	840	922	892	1.75	62	0.374
8	952	0.0425	922	978	946	1.15	79	0.385
9	1023	0.0382	978	1090	1015	1.87	70	0.346
10	1289	0.0767	1090	1467	1282	6.95	63	0.694
11	1616	0.1413	1467	1904	1638	5.52	171	1.279
12	3750	22.1037	2629	9067	4094	18.65	21529	200.091
13	9400	0.1844	9067	14025	10154	10.04	107	1.669
14	20000 (UM)	0.1636	18415	27738	20537	4.33	1889	17.773

TIC:	27.1124	ng/uL
TIM:	54.3729	nmole/L
Total concentration:	27.2734	ng/uL

Sample peak width (sec): 5 Sample min peak height: 50 Sample baseline V to V?: Y Sample baseline V to V points: 3
Sample filter: Binomial Number of points for filter: 3 Sample start region (min): 0 Sample end region (min): 30
Marker peak width (sec): 5 Marker min peak height: 200 Marker baseline V to V?: Y Marker baseline V to V points: 3
Lower marker selection: First peak > 500 RFU Upper marker selection: Last peak > 200 RFU
Ladder size (bp)75, 200, 300, 400, 500, 700, 1000, 1500, 2000, 3000, 4000, 5000, 7000, 10000, 20000
Quantification using: Lower Marker Final concentration (ng/uL): 0.5000 Dilution factor: 12.0

Sample: SampH8
Well location: H8
Created: Tuesday, August 10, 2021 9:21:07 PM

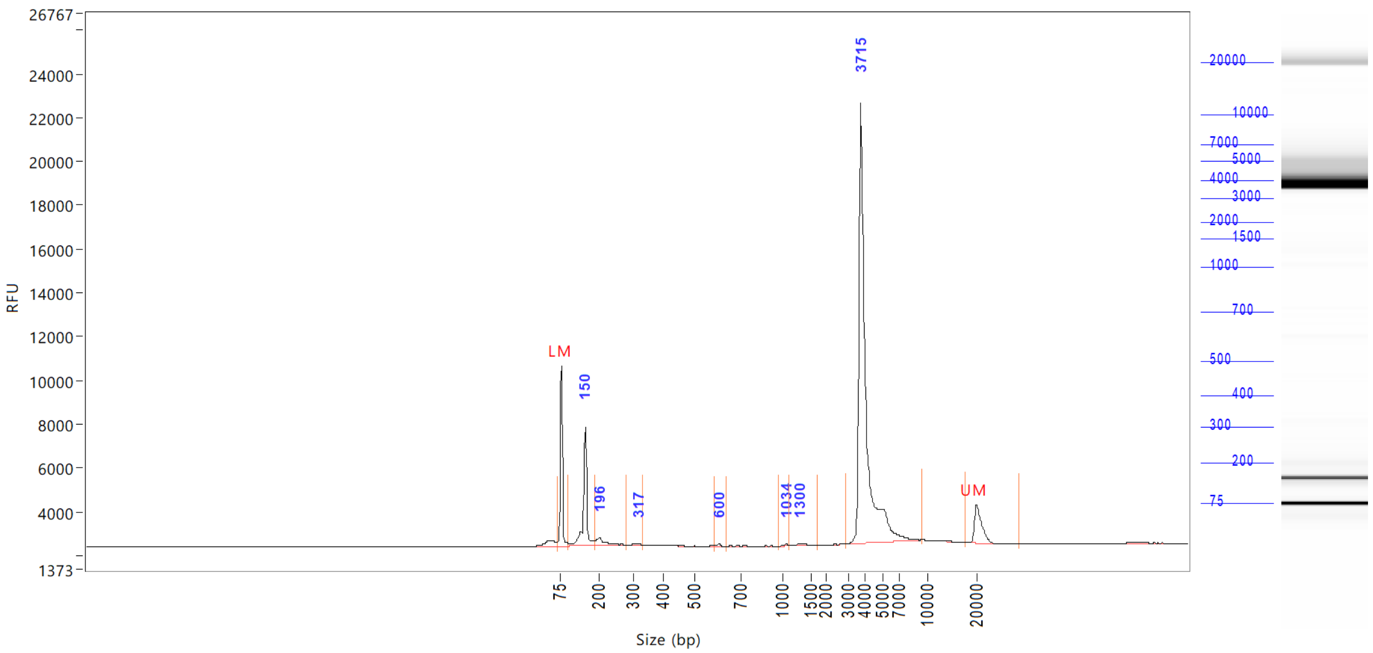

Peak	Size (bp)	Concentration (ng/uL)	From (bp)	To (bp)	Average size (bp)	CV%	RFU	Corrected peak area
1	75 (LM)	0.5000	64	99	73	6.01	8206	45.870
2	150	5.4854	99	183	147	8.57	5446	41.936
3	196	0.9420	183	280	209	10.84	331	7.202
4	317	0.1520	280	329	305	3.80	97	1.162
5	600	0.0884	588	634	600	1.28	134	0.676
6	1034	0.0615	970	1123	1033	3.04	85	0.470
7	1300	0.1994	1123	1693	1333	8.11	101	1.525
8	3715	27.0993	2886	9400	4067	16.64	20150	207.172
9	20000 (UM)	0.1814	17805	28705	20542	4.32	1741	16.642

TIC:	34.0280	ng/uL
TIM:	81.1142	nmole/L
Total concentration:	34.2783	ng/uL

```
Sample peak width (sec): 5    Sample min peak height: 50    Sample baseline V to V?: Y    Sample baseline V to V points: 3
Sample filter: Binomial       Number of points for filter: 3  Sample start region (min): 0  Sample end region (min): 30
Marker peak width (sec): 5    Marker min peak height: 200   Marker baseline V to V?: Y    Marker baseline V to V points: 3
Lower marker selection: First peak > 500 RFU                Upper marker selection: Last peak > 200 RFU
Ladder size (bp)75, 200, 300, 400, 500, 700, 1000, 1500, 2000, 3000, 4000, 5000, 7000, 10000, 20000
Quantification using: Lower Marker          Final concentration (ng/uL): 0.5000          Dilution factor: 12.0
```

Sample: SampA9
Well location: A9
Created: Tuesday, August 10, 2021 9:21:07 PM

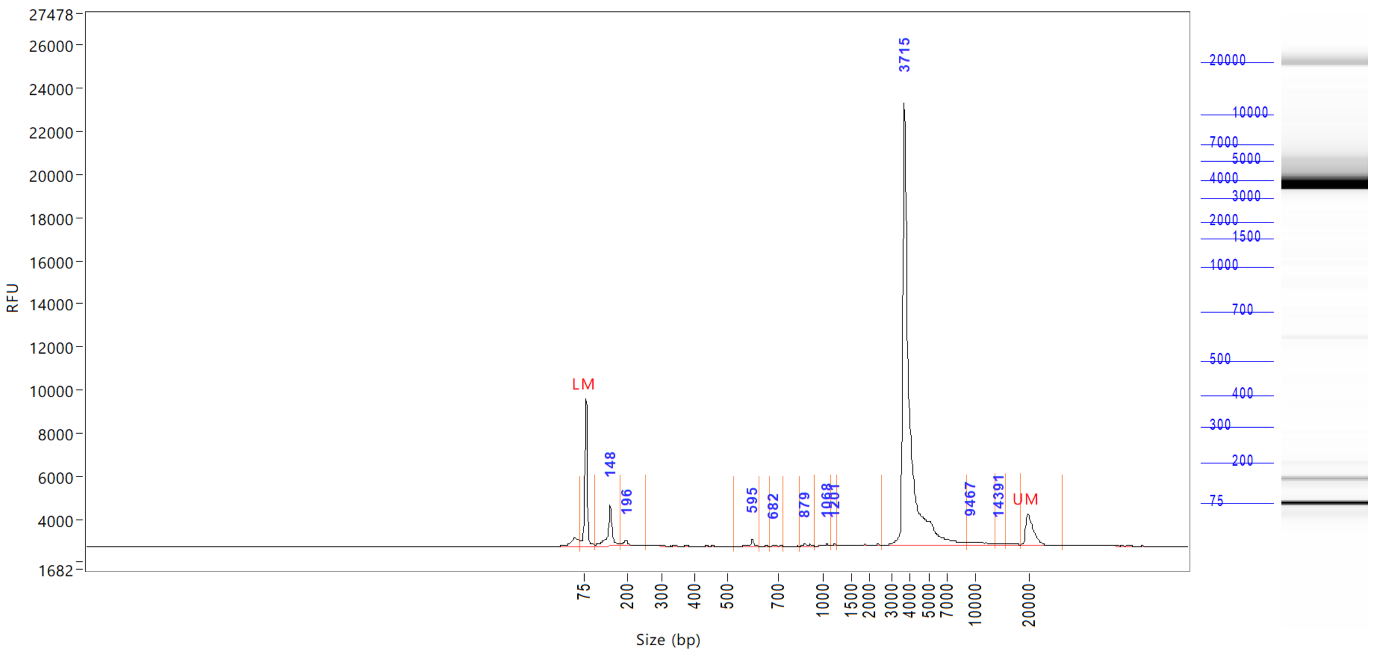

Peak	Size (bp)	Concentration (ng/uL)	From (bp)	To (bp)	Average size (bp)	CV%	RFU	Corrected peak area
1	75 (LM)	0.5000	58	103	74	7.18	6827	40.291
2	148	2.8198	103	181	144	9.70	1939	18.935
3	196	0.5111	181	253	200	7.72	246	3.432
4	595	0.3327	521	629	587	3.03	309	2.234
5	682	0.0914	667	736	693	2.41	56	0.614
6	879	0.1352	844	948	891	2.35	86	0.908
7	1068	0.0517	948	1134	1055	2.85	65	0.347
8	1201	0.0463	1134	1256	1195	2.27	61	0.311
9	3715	28.7318	2572	9000	4051	18.81	20548	192.939
10	9467	0.4304	9000	13781	10732	12.91	120	2.890
11	14391	0.0764	13781	15610	14502	3.55	65	0.513
12	20000 (UM)	0.1643	18415	26408	20443	4.12	1439	13.237

TIC:	33.2266	ng/uL
TIM:	49.6771	nmole/L
Total concentration:	33.6449	ng/uL

```
Sample peak width (sec): 5    Sample min peak height: 50    Sample baseline V to V?: Y    Sample baseline V to V points: 3
Sample filter: Binomial       Number of points for filter: 3  Sample start region (min): 0  Sample end region (min): 30
Marker peak width (sec): 5    Marker min peak height: 200   Marker baseline V to V?: Y    Marker baseline V to V points: 3
Lower marker selection: First peak > 500 RFU                Upper marker selection: Last peak > 200 RFU
Ladder size (bp)75, 200, 300, 400, 500, 700, 1000, 1500, 2000, 3000, 4000, 5000, 7000, 10000, 20000
Quantification using: Lower Marker          Final concentration (ng/uL): 0.5000          Dilution factor: 12.0
```

Sample: SampB9
Well location: B9
Created: Tuesday, August 10, 2021 9:21:07 PM

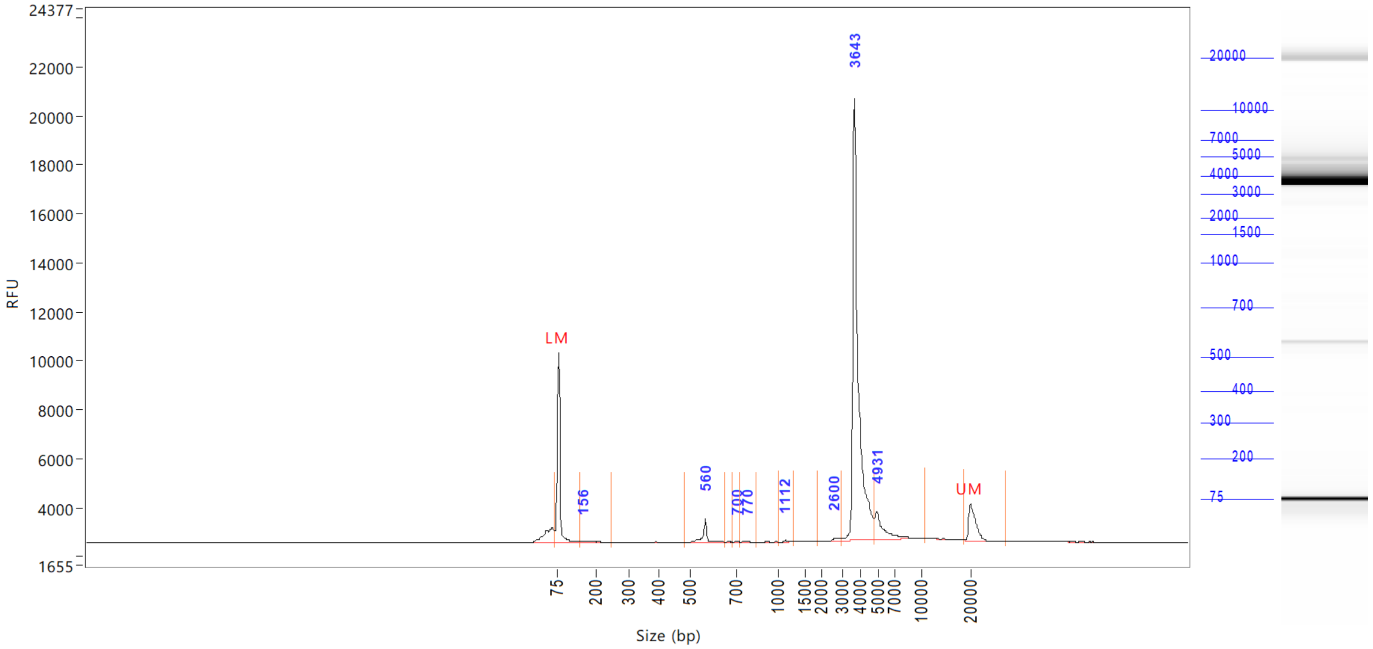

Peak	Size (bp)	Concentration (ng/uL)	From (bp)	To (bp)	Average size (bp)	CV%	RFU	Corrected peak area
1	75 (LM)	0.5000	64	145	76	13.33	7759	51.301
2	156	0.1769	145	242	176	13.24	59	1.513
3	560	0.8978	481	646	556	4.75	994	7.676
4	700	0.0404	682	718	697	1.18	54	0.345
5	770	0.0673	718	840	760	3.08	57	0.576
6	1112	0.0693	1000	1267	1143	4.40	66	0.592
7	2600	0.1681	1846	2914	2638	6.98	136	1.437
8	3643	17.8079	2914	4759	3799	7.80	18073	152.260
9	4931	1.8579	4759	10611	5713	19.80	1176	15.885
10	20000 (UM)	0.1341	18537	27133	20481	4.18	1503	13.757

TIC:	21.0856	ng/uL
TIM:	13.0078	nmole/L
Total concentration:	21.2348	ng/uL

```
Sample peak width (sec): 5    Sample min peak height: 50    Sample baseline V to V?: Y    Sample baseline V to V points: 3
Sample filter: Binomial       Number of points for filter: 3  Sample start region (min): 0  Sample end region (min): 30
Marker peak width (sec): 5    Marker min peak height: 200   Marker baseline V to V?: Y    Marker baseline V to V points: 3
Lower marker selection: First peak > 500 RFU                Upper marker selection: Last peak > 200 RFU
Ladder size (bp)75, 200, 300, 400, 500, 700, 1000, 1500, 2000, 3000, 4000, 5000, 7000, 10000, 20000
Quantification using: Lower Marker          Final concentration (ng/uL): 0.5000          Dilution factor: 12.0
```

Sample: SampC9
Well location: C9
Created: Tuesday, August 10, 2021 9:21:07 PM

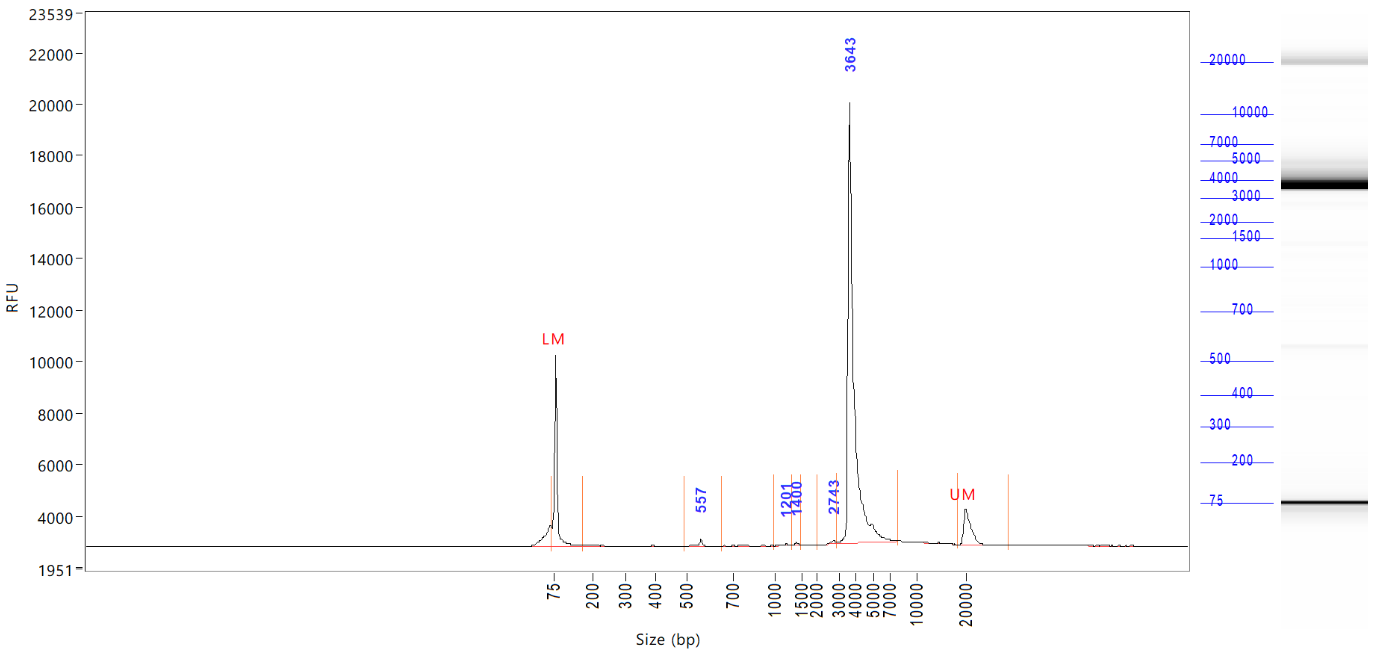

Peak	Size (bp)	Concentration (ng/uL)	From (bp)	To (bp)	Average size (bp)	CV%	RFU	Corrected peak area
1	75 (LM)	0.5000	64	164	77	17.55	7396	51.635
2	557	0.2554	489	649	549	4.42	282	2.198
3	1201	0.0557	996	1311	1170	5.70	59	0.479
4	1400	0.0731	1311	1478	1394	2.43	108	0.629
5	2743	0.1086	1962	2886	2674	5.62	133	0.935
6	3643	18.1362	2886	7934	3861	12.47	17130	156.078
7	20000 (UM)	0.1280	18293	28947	20588	4.74	1375	13.218

TIC:	18.6290	ng/uL
TIM:	8.7300	nmole/L
Total concentration:	19.0261	ng/uL

Sample peak width (sec): 5 Sample min peak height: 50 Sample baseline V to V?: Y Sample baseline V to V points: 3
Sample filter: Binomial Number of points for filter: 3 Sample start region (min): 0 Sample end region (min): 30
Marker peak width (sec): 5 Marker min peak height: 200 Marker baseline V to V?: Y Marker baseline V to V points: 3
Lower marker selection: First peak > 500 RFU Upper marker selection: Last peak > 200 RFU
Ladder size (bp)75, 200, 300, 400, 500, 700, 1000, 1500, 2000, 3000, 4000, 5000, 7000, 10000, 20000
Quantification using: Lower Marker Final concentration (ng/uL): 0.5000 Dilution factor: 12.0

Sample: SampD9
Well location: D9
Created: Tuesday, August 10, 2021 9:21:07 PM

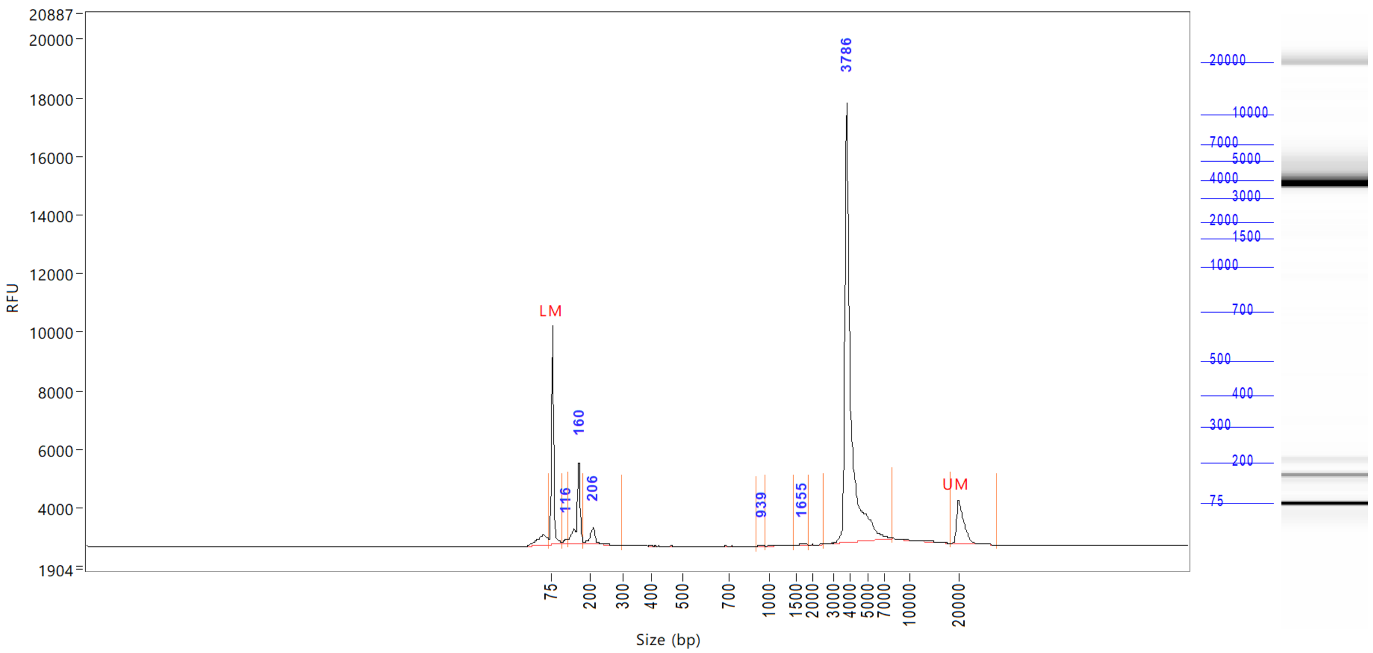

Peak	Size (bp)	Concentration (ng/uL)	From (bp)	To (bp)	Average size (bp)	CV%	RFU	Corrected peak area
1	75 (LM)	0.5000	64	105	73	6.39	7495	43.264
2	116	0.1970	105	124	115	4.59	144	1.421
3	160	3.2567	124	177	154	6.28	2758	23.483
4	206	1.0128	177	295	205	6.98	567	7.303
5	939	0.0425	896	970	932	1.41	53	0.306
6	1655	0.0849	1445	1904	1672	6.36	58	0.612
7	3786	18.8194	2543	8001	4059	13.68	15035	135.700
8	20000 (UM)	0.1585	18415	27979	20549	4.18	1459	13.711

TIC:	23.4134	ng/uL
TIM:	53.4872	nmole/L
Total concentration:	23.7615	ng/uL

Sample peak width (sec): 5   Sample min peak height: 50   Sample baseline V to V?: Y   Sample baseline V to V points: 3
Sample filter: Binomial   Number of points for filter: 3 Sample start region (min): 0 Sample end region (min): 30
Marker peak width (sec): 5   Marker min peak height: 200   Marker baseline V to V?: Y   Marker baseline V to V points: 3
Lower marker selection: First peak > 500 RFU   Upper marker selection: Last peak > 200 RFU
Ladder size (bp)75, 200, 300, 400, 500, 700, 1000, 1500, 2000, 3000, 4000, 5000, 7000, 10000, 20000
Quantification using: Lower Marker   Final concentration (ng/uL): 0.5000   Dilution factor: 12.0

Sample: SampE9
Well location: E9
Created: Tuesday, August 10, 2021 9:21:07 PM

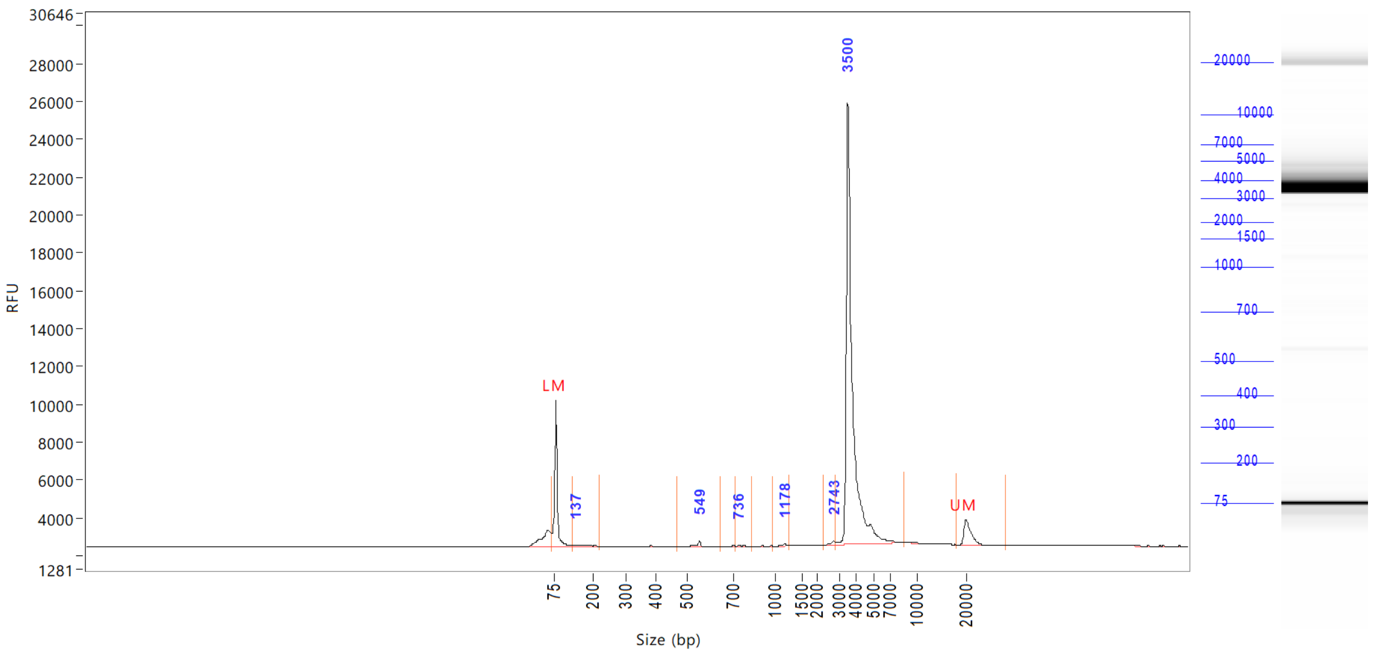

Peak	Size (bp)	Concentration (ng/uL)	From (bp)	To (bp)	Average size (bp)	CV%	RFU	Corrected peak area
1	75 (LM)	0.5000	62	129	75	11.70	7756	52.339
2	137	0.1987	129	215	154	12.33	78	1.734
3	549	0.2548	468	644	545	5.20	266	2.223
4	736	0.0834	714	835	754	3.33	64	0.728
5	1178	0.0795	987	1245	1149	3.63	119	0.693
6	2743	0.1823	2258	2857	2648	4.99	217	1.590
7	3500	25.1347	2857	8600	3759	14.16	23358	219.255
8	20000 (UM)	0.1201	18171	28221	20514	4.41	1351	12.570

TIC:	25.9335	ng/uL
TIM:	14.3074	nmole/L
Total concentration:	26.2697	ng/uL

```
Sample peak width (sec): 5    Sample min peak height: 50    Sample baseline V to V?: Y    Sample baseline V to V points: 3
Sample filter: Binomial       Number of points for filter: 3  Sample start region (min): 0  Sample end region (min): 30
Marker peak width (sec): 5    Marker min peak height: 200   Marker baseline V to V?: Y    Marker baseline V to V points: 3
Lower marker selection: First peak > 500 RFU                Upper marker selection: Last peak > 200 RFU
Ladder size (bp)75, 200, 300, 400, 500, 700, 1000, 1500, 2000, 3000, 4000, 5000, 7000, 10000, 20000
Quantification using: Lower Marker          Final concentration (ng/uL): 0.5000          Dilution factor: 12.0
```

Sample: SampF9
Well location: F9
Created: Tuesday, August 10, 2021 9:21:07 PM

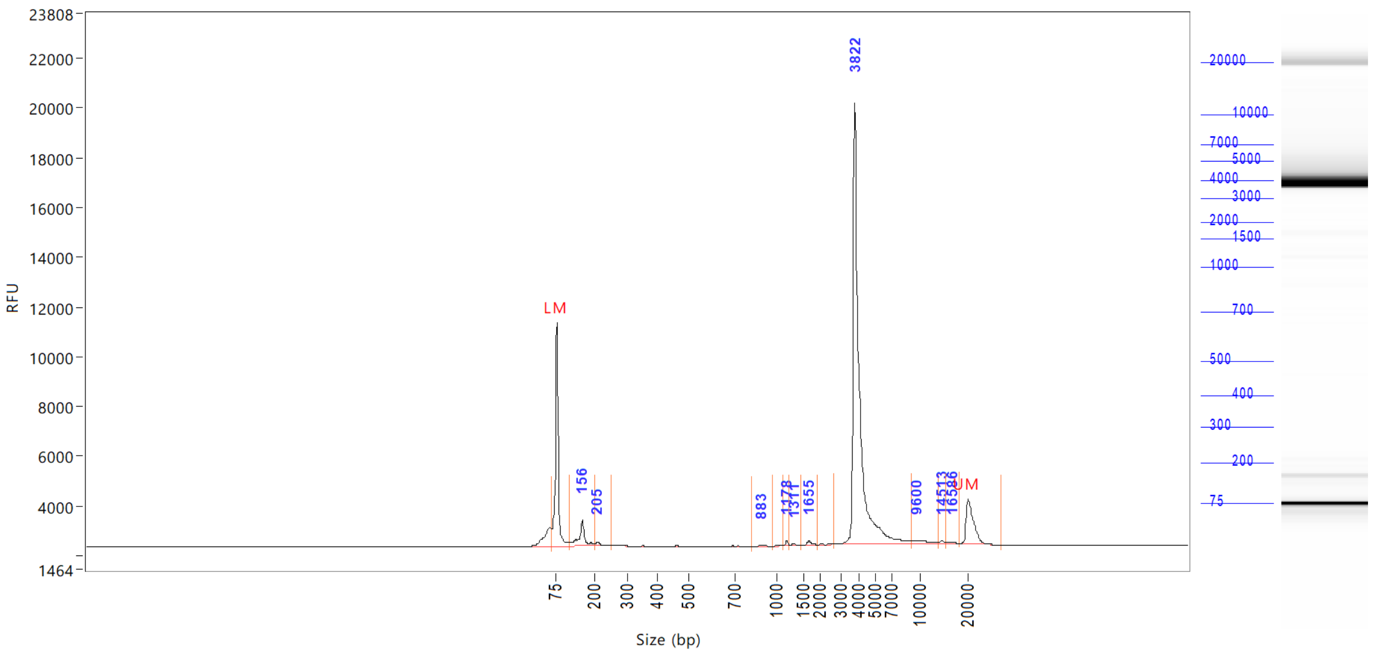

Peak	Size (bp)	Concentration (ng/uL)	From (bp)	To (bp)	Average size (bp)	CV%	RFU	Corrected peak area
1	75 (LM)	0.5000	58	116	73	11.09	9008	63.119
2	156	1.2798	116	196	152	10.83	1054	13.463
3	205	0.1410	196	250	207	4.36	164	1.483
4	883	0.0491	818	970	889	2.43	56	0.517
5	1178	0.0795	1101	1234	1166	2.18	180	0.836
6	1311	0.0503	1234	1445	1322	4.08	56	0.529
7	1655	0.1160	1445	1923	1636	5.43	173	1.221
8	3822	15.7313	2686	9134	4097	18.20	17766	165.490
9	9600	0.2548	9134	13781	10889	12.72	115	2.680
10	14513	0.0570	13781	15366	14510	3.10	86	0.599
11	16586	0.0568	15366	18293	16254	3.40	67	0.598
12	20000 (UM)	0.1375	18293	26891	20503	4.29	1811	17.359

TIC:	17.8155	ng/uL
TIM:	21.7119	nmole/L
Total concentration:	17.9740	ng/uL

```
Sample peak width (sec): 5    Sample min peak height: 50    Sample baseline V to V?: Y    Sample baseline V to V points: 3
Sample filter: Binomial       Number of points for filter: 3  Sample start region (min): 0  Sample end region (min): 30
Marker peak width (sec): 5    Marker min peak height: 200   Marker baseline V to V?: Y    Marker baseline V to V points: 3
Lower marker selection: First peak > 500 RFU                Upper marker selection: Last peak > 200 RFU
Ladder size (bp)75, 200, 300, 400, 500, 700, 1000, 1500, 2000, 3000, 4000, 5000, 7000, 10000, 20000
Quantification using: Lower Marker          Final concentration (ng/uL): 0.5000          Dilution factor: 12.0
```

Sample: SampG9
Well location: G9
Created: Tuesday, August 10, 2021 9:21:07 PM

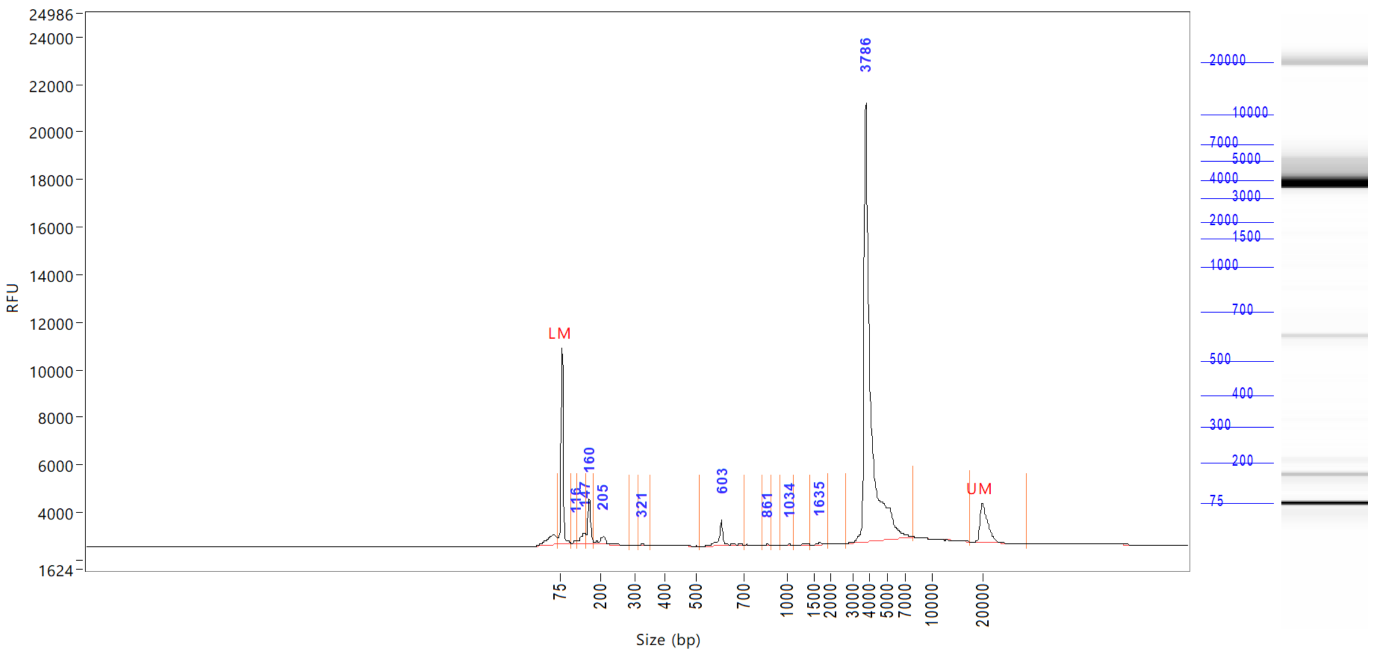

Peak	Size (bp)	Concentration (ng/uL)	From (bp)	To (bp)	Average size (bp)	CV%	RFU	Corrected peak area
1	75 (LM)	0.5000	62	105	73	5.44	8261	46.530
2	116	0.1430	105	124	116	4.10	132	1.109
3	147	0.7163	124	152	141	5.63	439	5.555
4	160	1.6880	152	177	160	2.82	1881	13.090
5	205	0.6411	177	286	203	7.72	346	4.972
6	321	0.0712	309	354	323	3.05	89	0.552
7	603	1.1695	511	697	602	4.85	1092	9.069
8	861	0.0369	831	887	858	1.28	54	0.286
9	1034	0.0399	948	1112	1022	2.50	54	0.310
10	1635	0.0786	1433	1904	1643	5.42	79	0.610
11	3786	23.6603	2686	7934	4063	14.04	18474	183.484
12	20000 (UM)	0.1738	17561	28947	20466	4.47	1661	16.173

TIC:	28.2449	ng/uL
TIM:	46.2677	nmole/L
Total concentration:	28.4498	ng/uL

Sample peak width (sec): 5   Sample min peak height: 50   Sample baseline V to V?: Y   Sample baseline V to V points: 3
Sample filter: Binomial   Number of points for filter: 3   Sample start region (min): 0   Sample end region (min): 30
Marker peak width (sec): 5   Marker min peak height: 200   Marker baseline V to V?: Y   Marker baseline V to V points: 3
Lower marker selection: First peak > 500 RFU   Upper marker selection: Last peak > 200 RFU
Ladder size (bp)75, 200, 300, 400, 500, 700, 1000, 1500, 2000, 3000, 4000, 5000, 7000, 10000, 20000
Quantification using: Lower Marker   Final concentration (ng/uL): 0.5000   Dilution factor: 12.0

Sample: SampH9
Well location: H9
Created: Tuesday, August 10, 2021 9:21:07 PM

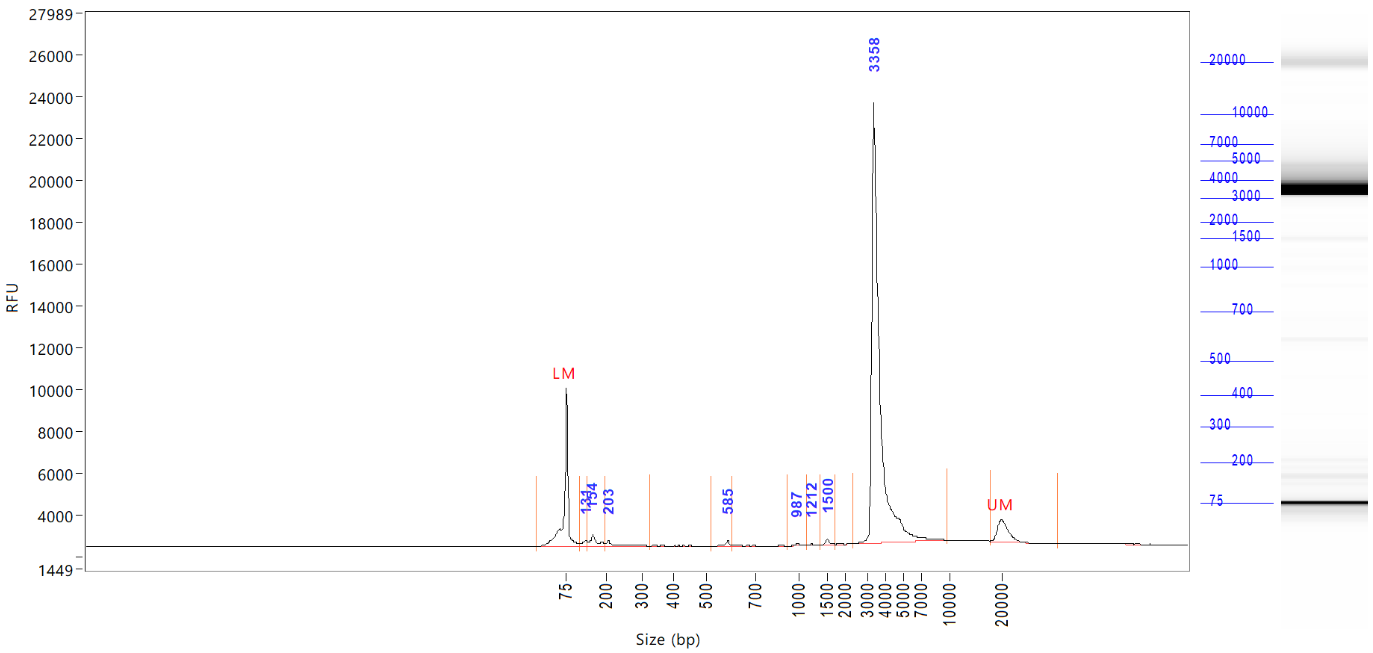

Peak	Size (bp)	Concentration (ng/uL)	From (bp)	To (bp)	Average size (bp)	CV%	RFU	Corrected peak area
1	75 (LM)	0.5000	0	116	66	26.55	7560	69.462
2	131	0.2796	116	139	128	5.10	283	3.237
3	154	0.7033	139	194	161	9.05	536	8.142
4	203	0.3405	194	323	222	11.91	276	3.942
5	585	0.1755	516	606	575	2.77	271	2.032
6	987	0.0672	918	1134	1002	4.03	87	0.778
7	1212	0.0352	1134	1356	1212	3.60	52	0.408
8	1500	0.1532	1356	1731	1499	3.89	269	1.774
9	3358	20.7683	2344	9733	3679	18.04	21061	240.436
10	20000 (UM)	0.1051	17683	30760	20433	6.14	1051	14.596

TIC:	22.5229	ng/uL
TIM:	23.4137	nmole/L
Total concentration:	22.7446	ng/uL

```
Sample peak width (sec): 5    Sample min peak height: 50    Sample baseline V to V?: Y    Sample baseline V to V points: 3
Sample filter: Binomial       Number of points for filter: 3  Sample start region (min): 0  Sample end region (min): 30
Marker peak width (sec): 5    Marker min peak height: 200   Marker baseline V to V?: Y    Marker baseline V to V points: 3
Lower marker selection: First peak > 500 RFU                Upper marker selection: Last peak > 200 RFU
Ladder size (bp)75, 200, 300, 400, 500, 700, 1000, 1500, 2000, 3000, 4000, 5000, 7000, 10000, 20000
Quantification using: Lower Marker          Final concentration (ng/uL): 0.5000          Dilution factor: 12.0
```

Sample: SampA10
Well location: A10
Created: Tuesday, August 10, 2021 9:21:07 PM

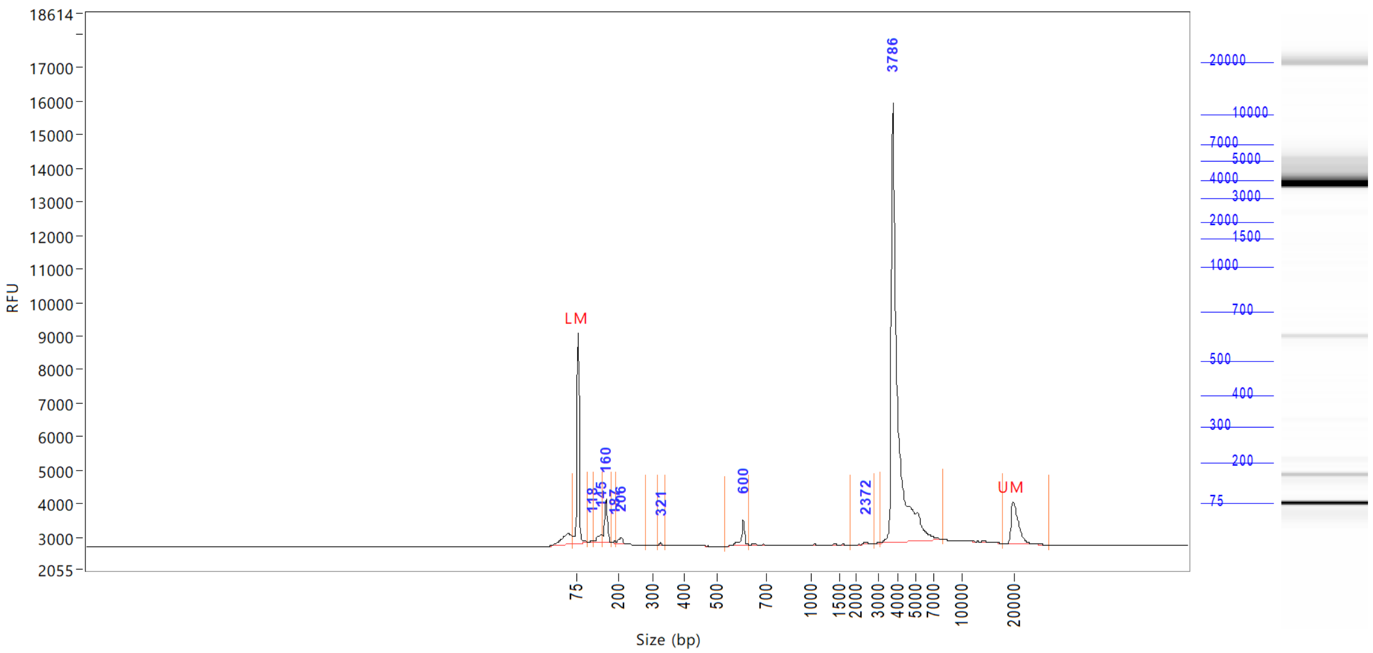

Peak	Size<br>(bp)	Concentration<br>(ng/uL)	From<br>(bp)	To<br>(bp)	Average size<br>(bp)	CV%	RFU	Corrected peak area
1	75 (LM)	0.5000	62	107	74	5.62	6284	35.142
2	118	0.0980	107	126	118	4.00	77	0.574
3	145	0.5093	126	150	140	4.91	252	2.983
4	160	1.4287	150	177	159	2.57	1296	8.368
5	187	0.0550	177	192	186	2.08	63	0.322
6	206	0.3214	192	278	207	7.52	187	1.882
7	321	0.0718	311	337	320	1.47	83	0.421
8	600	0.7500	527	629	592	2.32	741	4.393
9	2372	0.0840	1846	2772	2359	6.07	76	0.492
10	3786	20.1499	3072	8067	4076	13.93	13112	118.019
11	20000 (UM)	0.1603	17927	27012	20440	4.37	1264	11.270

TIC:	23.4680	ng/uL
TIM:	35.8002	nmole/L
Total concentration:	23.8873	ng/uL

```
Sample peak width (sec): 5    Sample min peak height: 50    Sample baseline V to V?: Y    Sample baseline V to V points: 3
Sample filter: Binomial       Number of points for filter: 3  Sample start region (min): 0  Sample end region (min): 30
Marker peak width (sec): 5    Marker min peak height: 200   Marker baseline V to V?: Y    Marker baseline V to V points: 3
Lower marker selection: First peak > 500 RFU                Upper marker selection: Last peak > 200 RFU
Ladder size (bp)75, 200, 300, 400, 500, 700, 1000, 1500, 2000, 3000, 4000, 5000, 7000, 10000, 20000
Quantification using: Lower Marker          Final concentration (ng/uL): 0.5000         Dilution factor: 12.0
```

Sample: SampB10
Well location: B10
Created: Tuesday, August 10, 2021 9:21:07 PM

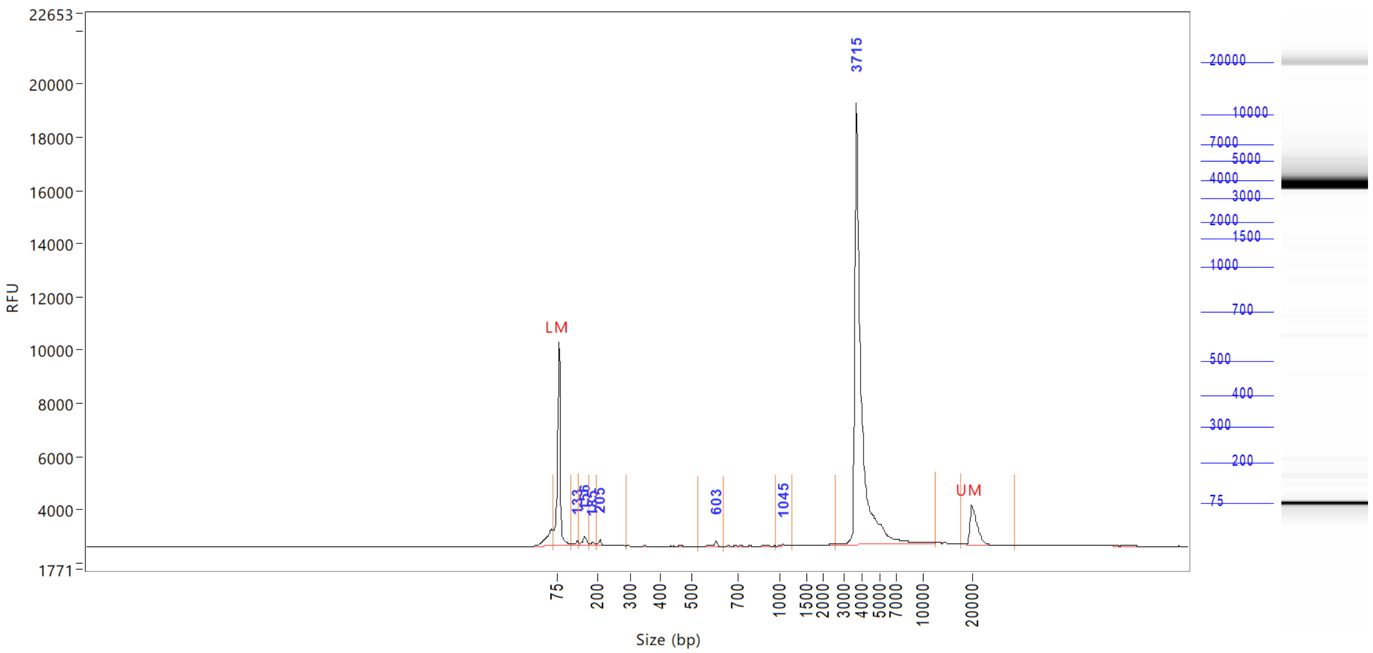

Peak	Size (bp)	Concentration (ng/uL)	From (bp)	To (bp)	Average size (bp)	CV%	RFU	Corrected peak area
1	75 (LM)	0.5000	60	116	73	8.54	7662	50.324
2	133	0.1223	116	141	130	4.72	122	1.026
3	156	0.3120	141	175	156	4.49	291	2.617
4	185	0.1009	175	198	184	3.16	112	0.847
5	205	0.1568	198	287	214	9.86	196	1.315
6	603	0.1637	529	636	592	2.88	193	1.373
7	1045	0.0713	978	1223	1069	5.39	61	0.598
8	3715	18.7880	2572	12562	4048	20.16	16616	157.581
9	20000 (UM)	0.1355	17683	28947	20512	4.32	1478	13.635

TIC:	19.7151	ng/uL
TIM:	15.1458	nmole/L
Total concentration:	20.0216	ng/uL

Sample peak width (sec): 5   Sample min peak height: 50   Sample baseline V to V?: Y   Sample baseline V to V points: 3
Sample filter: Binomial   Number of points for filter: 3 Sample start region (min): 0 Sample end region (min): 30
Marker peak width (sec): 5   Marker min peak height: 200   Marker baseline V to V?: Y   Marker baseline V to V points: 3
Lower marker selection: First peak > 500 RFU   Upper marker selection: Last peak > 200 RFU
Ladder size (bp)75, 200, 300, 400, 500, 700, 1000, 1500, 2000, 3000, 4000, 5000, 7000, 10000, 20000
Quantification using: Lower Marker   Final concentration (ng/uL): 0.5000   Dilution factor: 12.0

Sample: SampC10
Well location: C10
Created: Tuesday, August 10, 2021 9:21:07 PM

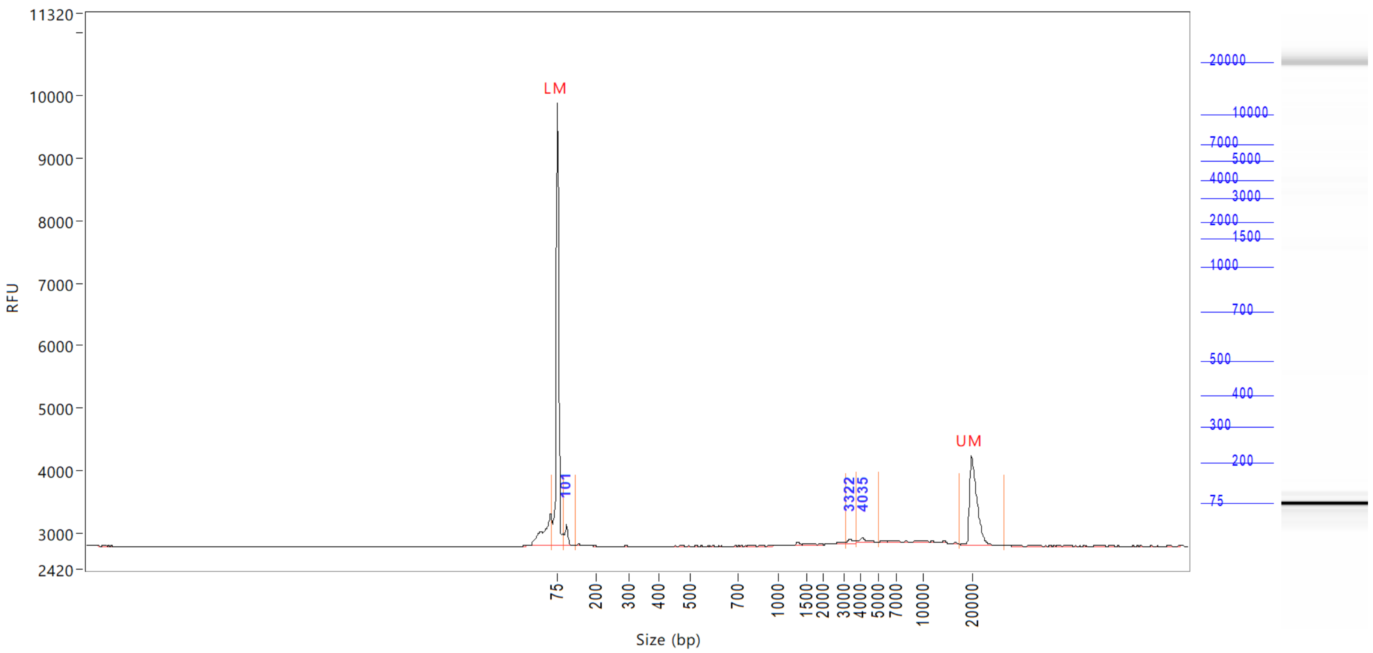

Peak	Size (bp)	Concentration (ng/uL)	From (bp)	To (bp)	Average size (bp)	CV%	RFU	Corrected peak area
1	75 (LM)	0.5000	60	95	73	6.81	7098	42.630
2	101	0.3328	95	133	102	5.19	337	2.364
3	3322	0.0923	3072	3750	3391	5.26	75	0.656
4	4035	0.1358	3750	5000	4173	7.02	78	0.965
5	20000 (UM)	0.1602	17561	26529	20459	4.45	1432	13.659

TIC:	0.5609	ng/uL
TIM:	5.4557	nmole/L
Total concentration:	1.0867	ng/uL

Sample peak width (sec): 5 Sample min peak height: 50 Sample baseline V to V?: Y Sample baseline V to V points: 3
Sample filter: Binomial Number of points for filter: 3 Sample start region (min): 0 Sample end region (min): 30
Marker peak width (sec): 5 Marker min peak height: 200 Marker baseline V to V?: Y Marker baseline V to V points: 3
Lower marker selection: First peak > 500 RFU Upper marker selection: Last peak > 200 RFU
Ladder size (bp)75, 200, 300, 400, 500, 700, 1000, 1500, 2000, 3000, 4000, 5000, 7000, 10000, 20000
Quantification using: Lower Marker Final concentration (ng/uL): 0.5000 Dilution factor: 12.0

Sample: SampD10
Well location: D10
Created: Tuesday, August 10, 2021 9:21:07 PM

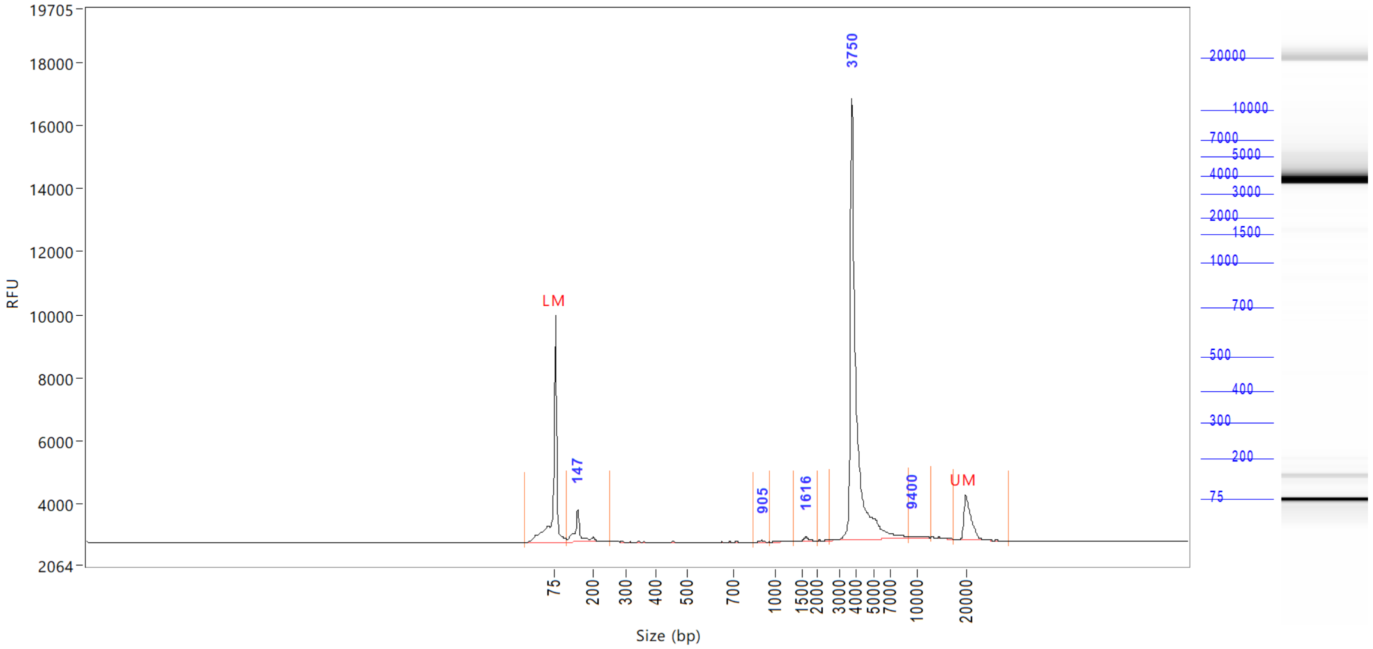

Peak	Size<br>(bp)	Concentration<br>(ng/uL)	From<br>(bp)	To<br>(bp)	Average size<br>(bp)	CV%	RFU	Corrected peak area
1	75 (LM)	0.5000	0	112	63	32.48	7176	61.006
2	147	1.3525	112	246	149	15.35	1017	13.752
3	905	0.0427	840	965	897	2.24	64	0.435
4	1616	0.1009	1356	1962	1612	6.86	131	1.026
5	3750	12.4494	2543	9067	4072	18.20	14017	126.581
6	9400	0.0877	9067	12928	9959	7.65	64	0.892
7	20000 (UM)	0.1107	17561	28947	20555	4.91	1404	13.501

TIC:	14.0333	ng/uL
TIM:	20.1479	nmole/L
Total concentration:	14.2246	ng/uL

Sample peak width (sec): 5   Sample min peak height: 50   Sample baseline V to V?: Y   Sample baseline V to V points: 3
Sample filter: Binomial   Number of points for filter: 3 Sample start region (min): 0 Sample end region (min): 30
Marker peak width (sec): 5   Marker min peak height: 200   Marker baseline V to V?: Y   Marker baseline V to V points: 3
Lower marker selection: First peak > 500 RFU   Upper marker selection: Last peak > 200 RFU
Ladder size (bp)75, 200, 300, 400, 500, 700, 1000, 1500, 2000, 3000, 4000, 5000, 7000, 10000, 20000
Quantification using: Lower Marker   Final concentration (ng/uL): 0.5000   Dilution factor: 12.0

Sample: SampE10
Well location: E10
Created: Tuesday, August 10, 2021 9:21:07 PM

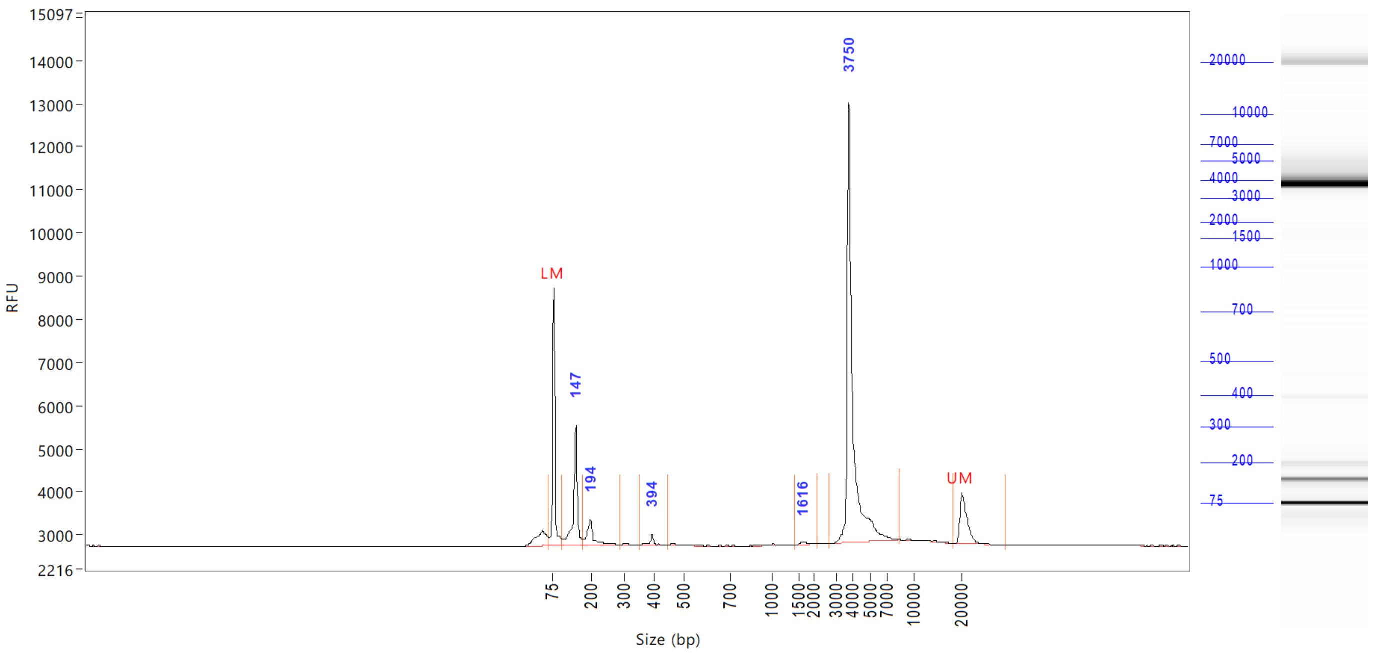

Peak	Size (bp)	Concentration (ng/uL)	From (bp)	To (bp)	Average size (bp)	CV%	RFU	Corrected peak area
1	75 (LM)	0.5000	62	103	74	8.26	5979	36.090
2	147	4.2844	103	169	140	8.54	2786	25.770
3	194	1.4675	169	287	197	10.06	599	8.827
4	394	0.3729	349	445	392	4.61	258	2.243
5	1616	0.1284	1422	2115	1666	8.89	55	0.772
6	3750	14.3607	2715	8467	4018	14.68	10205	86.379
7	20000 (UM)	0.1543	18293	29068	20557	4.68	1171	11.138

TIC:	20.6139	ng/uL
TIM:	70.1136	nmole/L
Total concentration:	21.0029	ng/uL

```
Sample peak width (sec): 5     Sample min peak height: 50    Sample baseline V to V?: Y    Sample baseline V to V points: 3
Sample filter: Binomial        Number of points for filter: 3  Sample start region (min): 0  Sample end region (min): 30
Marker peak width (sec): 5     Marker min peak height: 200   Marker baseline V to V?: Y    Marker baseline V to V points: 3
Lower marker selection: First peak > 500 RFU                 Upper marker selection: Last peak > 200 RFU
Ladder size (bp)75, 200, 300, 400, 500, 700, 1000, 1500, 2000, 3000, 4000, 5000, 7000, 10000, 20000
Quantification using: Lower Marker          Final concentration (ng/uL): 0.5000          Dilution factor: 12.0
```

Sample: SampF10
Well location: F10
Created: Tuesday, August 10, 2021 9:21:07 PM

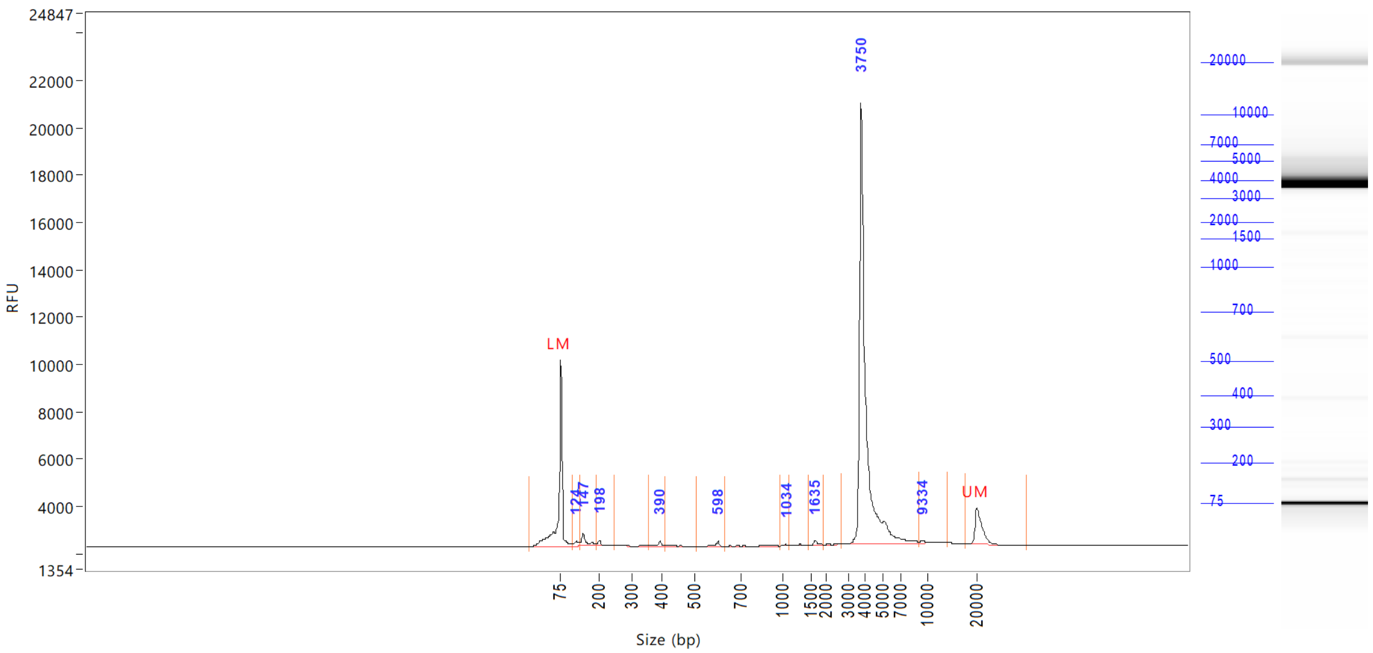

Peak	Size (bp)	Concentration (ng/uL)	From (bp)	To (bp)	Average size (bp)	CV%	RFU	Corrected peak area
1	75 (LM)	0.5000	0	114	64	31.61	7871	66.036
2	124	0.2249	114	137	126	5.26	202	2.476
3	147	0.5569	137	189	154	9.22	548	6.130
4	198	0.1764	189	244	201	5.27	243	1.941
5	390	0.1481	352	409	383	3.04	217	1.630
6	598	0.1381	509	631	587	3.25	207	1.520
7	1034	0.0285	974	1101	1022	2.32	63	0.313
8	1635	0.1102	1467	1904	1645	4.68	197	1.213
9	3750	15.6288	2657	9067	4065	17.52	18675	172.011
10	9334	0.1085	9067	14147	10189	10.00	72	1.195
11	20000 (UM)	0.1074	17683	30035	20538	4.76	1517	14.188

TIC:	17.1205	ng/uL
TIM:	17.8464	nmole/L
Total concentration:	17.3858	ng/uL

```
Sample peak width (sec): 5    Sample min peak height: 50    Sample baseline V to V?: Y    Sample baseline V to V points: 3
Sample filter: Binomial       Number of points for filter: 3  Sample start region (min): 0  Sample end region (min): 30
Marker peak width (sec): 5    Marker min peak height: 200   Marker baseline V to V?: Y    Marker baseline V to V points: 3
Lower marker selection: First peak > 500 RFU                Upper marker selection: Last peak > 200 RFU
Ladder size (bp)75, 200, 300, 400, 500, 700, 1000, 1500, 2000, 3000, 4000, 5000, 7000, 10000, 20000
Quantification using: Lower Marker          Final concentration (ng/uL): 0.5000          Dilution factor: 12.0
```

Sample: SampG10
Well location: G10
Created: Tuesday, August 10, 2021 9:21:07 PM

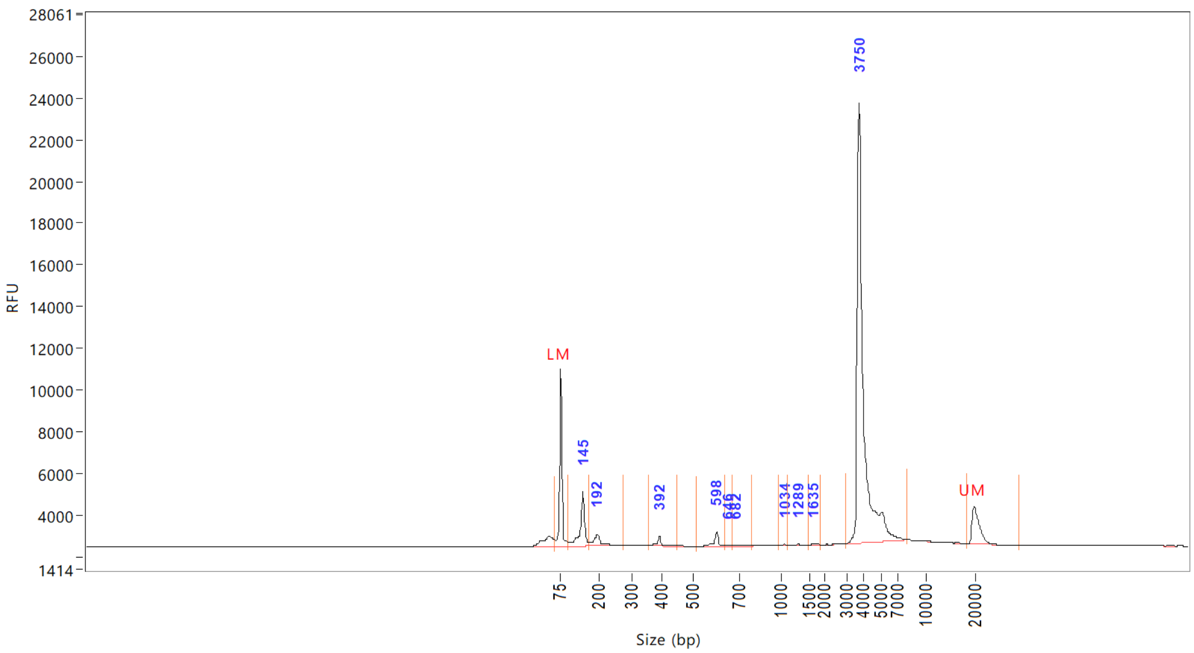

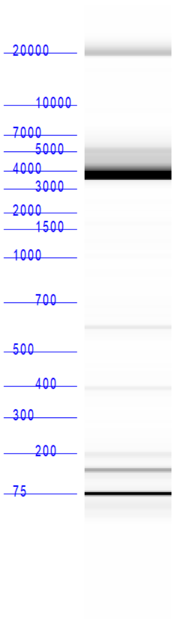

Peak	Size (bp)	Concentration (ng/uL)	From (bp)	To (bp)	Average size (bp)	CV%	RFU	Corrected peak area
1	75 (LM)	0.5000	60	101	74	7.58	8483	50.856
2	145	3.1858	101	169	139	9.35	2619	27.002
3	192	1.0970	169	275	197	10.24	557	9.298
4	392	0.4624	354	445	388	4.05	462	3.919
5	598	0.5868	511	631	588	3.14	659	4.973
6	646	0.0589	631	667	647	1.55	53	0.499
7	682	0.0896	667	788	697	3.59	64	0.760
8	1034	0.0364	974	1101	1024	2.51	60	0.308
9	1289	0.0859	1101	1500	1293	7.94	54	0.728
10	1635	0.0812	1500	1866	1640	4.99	88	0.688
11	3750	22.7454	2971	8001	3983	13.68	21138	192.788
12	20000 (UM)	0.1656	18415	29309	20474	4.55	1785	16.843

TIC:	28.4292	ng/uL
TIM:	60.4333	nmole/L
Total concentration:	28.6569	ng/uL

```
Sample peak width (sec): 5     Sample min peak height: 50    Sample baseline V to V?: Y    Sample baseline V to V points: 3
Sample filter: Binomial        Number of points for filter: 3  Sample start region (min): 0  Sample end region (min): 30
Marker peak width (sec): 5     Marker min peak height: 200   Marker baseline V to V?: Y    Marker baseline V to V points: 3
Lower marker selection: First peak > 500 RFU                 Upper marker selection: Last peak > 200 RFU
Ladder size (bp)75, 200, 300, 400, 500, 700, 1000, 1500, 2000, 3000, 4000, 5000, 7000, 10000, 20000
Quantification using: Lower Marker          Final concentration (ng/uL): 0.5000          Dilution factor: 12.0
```

Sample: SampH10
Well location: H10
Created: Tuesday, August 10, 2021 9:21:07 PM

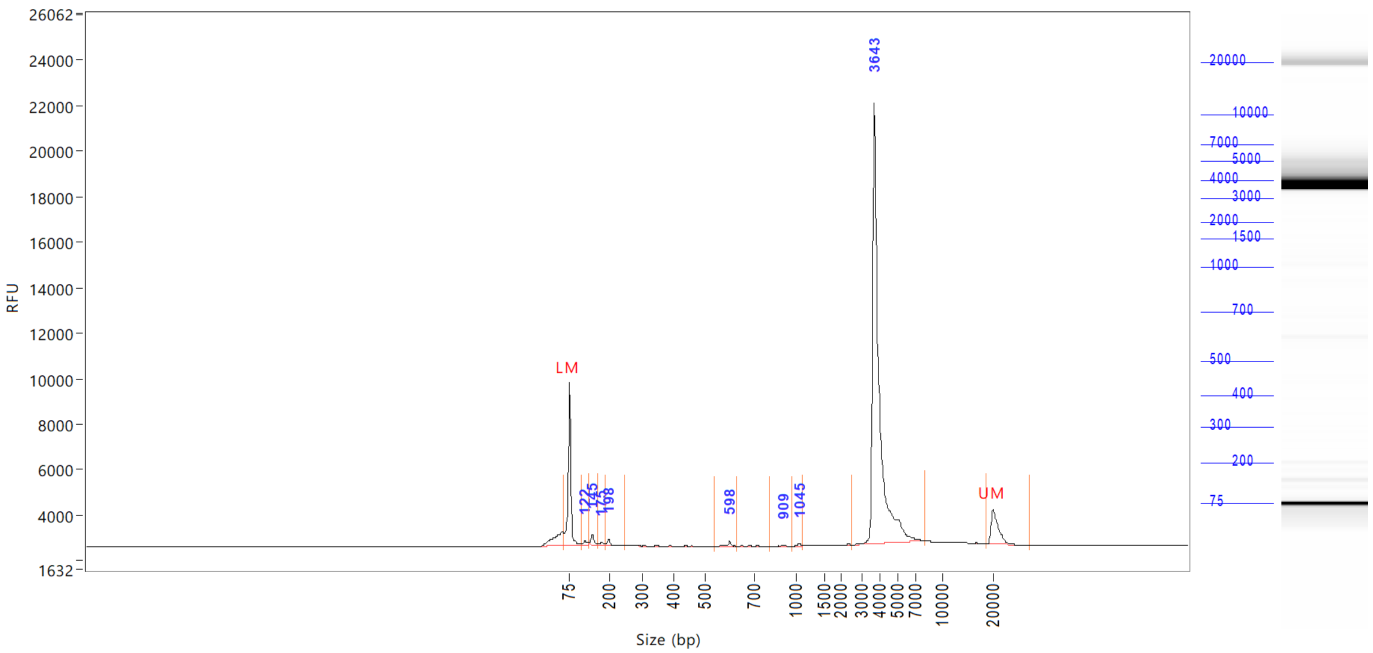

Peak	Size (bp)	Concentration (ng/uL)	From (bp)	To (bp)	Average size (bp)	CV%	RFU	Corrected peak area
1	75 (LM)	0.5000	58	112	72	8.34	7160	47.027
2	122	0.1753	112	135	124	5.19	147	1.374
3	145	0.3942	135	162	144	3.71	421	3.090
4	175	0.1089	162	187	173	3.44	112	0.853
5	198	0.1943	187	246	199	5.44	244	1.523
6	598	0.2015	537	631	590	2.92	218	1.579
7	909	0.0617	814	970	895	3.11	63	0.484
8	1045	0.0768	970	1112	1039	3.11	95	0.602
9	3643	23.5405	2515	8067	3922	13.34	19398	184.505
10	20000 (UM)	0.1499	18537	27375	20446	4.28	1506	14.100

TIC:	24.7532	ng/uL
TIM:	20.1395	nmole/L
Total concentration:	25.1255	ng/uL

```
Sample peak width (sec): 5    Sample min peak height: 50    Sample baseline V to V?: Y    Sample baseline V to V points: 3
Sample filter: Binomial       Number of points for filter: 3  Sample start region (min): 0  Sample end region (min): 30
Marker peak width (sec): 5    Marker min peak height: 200   Marker baseline V to V?: Y    Marker baseline V to V points: 3
Lower marker selection: First peak > 500 RFU                Upper marker selection: Last peak > 200 RFU
Ladder size (bp)75, 200, 300, 400, 500, 700, 1000, 1500, 2000, 3000, 4000, 5000, 7000, 10000, 20000
Quantification using: Lower Marker          Final concentration (ng/uL): 0.5000          Dilution factor: 12.0
```

Sample: SampA11
Well location: A11
Created: Tuesday, August 10, 2021 9:21:07 PM

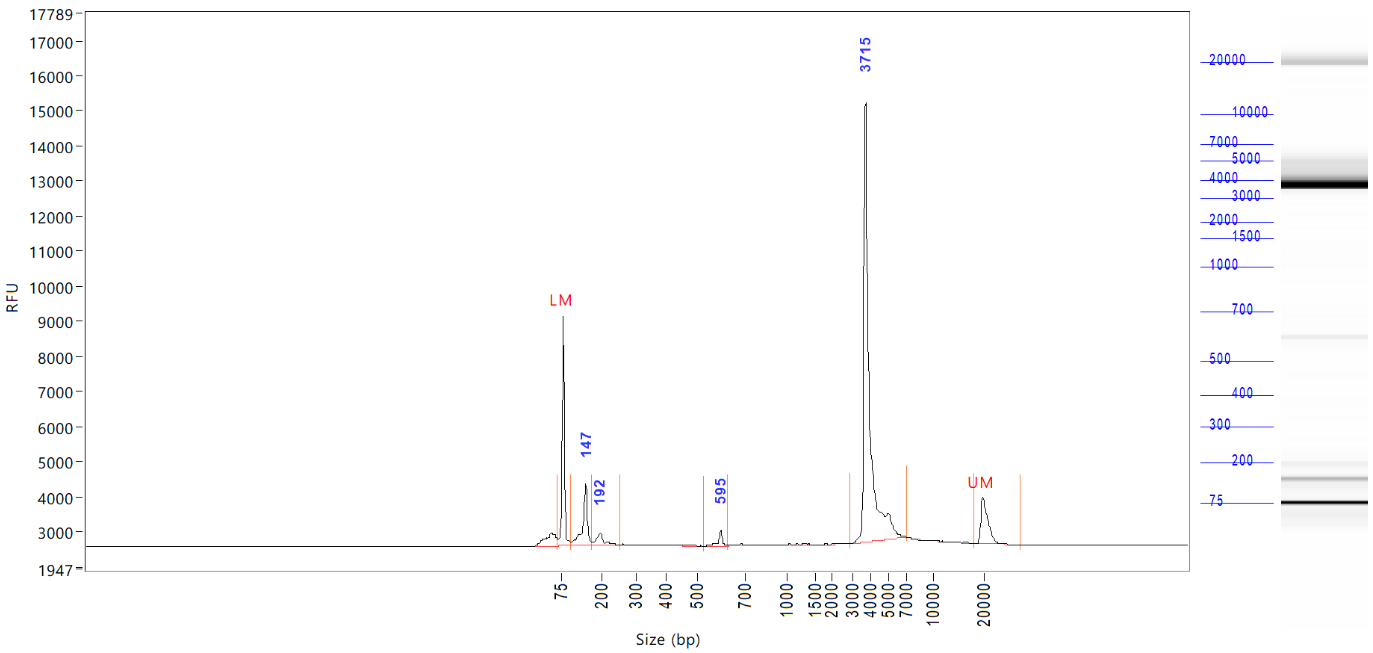

Peak	Size (bp)	Concentration (ng/uL)	From (bp)	To (bp)	Average size (bp)	CV%	RFU	Corrected peak area
1	75 (LM)	0.5000	60	101	74	6.89	6536	38.833
2	147	2.8714	101	168	139	9.78	1749	18.584
3	192	0.8589	168	253	192	7.74	362	5.559
4	595	0.5138	521	629	587	2.89	447	3.326
5	3715	16.3018	2886	7068	3922	11.78	12541	105.507
6	20000 (UM)	0.1537	18171	27617	20376	4.01	1307	11.938

TIC:	20.5460	ng/uL
TIM:	49.5847	nmole/L
Total concentration:	21.0987	ng/uL

```
Sample peak width (sec): 5    Sample min peak height: 50    Sample baseline V to V?: Y    Sample baseline V to V points: 3
Sample filter: Binomial       Number of points for filter: 3  Sample start region (min): 0  Sample end region (min): 30
Marker peak width (sec): 5    Marker min peak height: 200   Marker baseline V to V?: Y    Marker baseline V to V points: 3
Lower marker selection: First peak > 500 RFU                Upper marker selection: Last peak > 200 RFU
Ladder size (bp)75, 200, 300, 400, 500, 700, 1000, 1500, 2000, 3000, 4000, 5000, 7000, 10000, 20000
Quantification using: Lower Marker          Final concentration (ng/uL): 0.5000          Dilution factor: 12.0
```

Sample: SampB11
Well location: B11
Created: Tuesday, August 10, 2021 9:21:07 PM

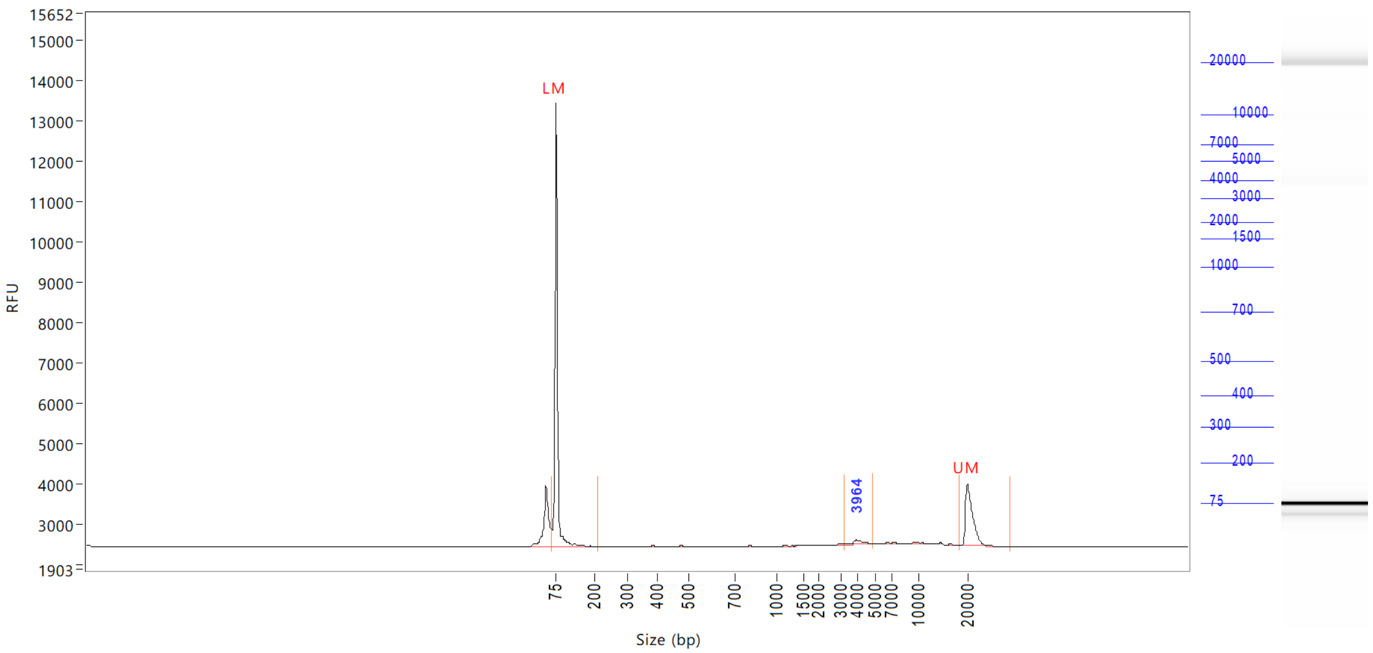

Peak	Size (bp)	Concentration (ng/uL)	From (bp)	To (bp)	Average size (bp)	CV%	RFU	Corrected peak area
1	75 (LM)	0.5000	64	210	75	14.97	10990	68.708
2	3964	0.1385	3251	4897	4045	7.71	110	1.586
3	20000 (UM)	0.1062	18537	28826	20513	4.65	1522	14.587

TIC:	0.1385	ng/uL
TIM:	0.0564	nmole/L
Total concentration:	0.4185	ng/uL

```
Sample peak width (sec): 5    Sample min peak height: 50    Sample baseline V to V?: Y    Sample baseline V to V points: 3
Sample filter: Binomial       Number of points for filter: 3  Sample start region (min): 0  Sample end region (min): 30
Marker peak width (sec): 5    Marker min peak height: 200   Marker baseline V to V?: Y    Marker baseline V to V points: 3
Lower marker selection: First peak > 500 RFU                Upper marker selection: Last peak > 200 RFU
Ladder size (bp)75, 200, 300, 400, 500, 700, 1000, 1500, 2000, 3000, 4000, 5000, 7000, 10000, 20000
Quantification using: Lower Marker          Final concentration (ng/uL): 0.5000          Dilution factor: 12.0
```

Sample: SampC11
Well location: C11
Created: Tuesday, August 10, 2021 9:21:07 PM

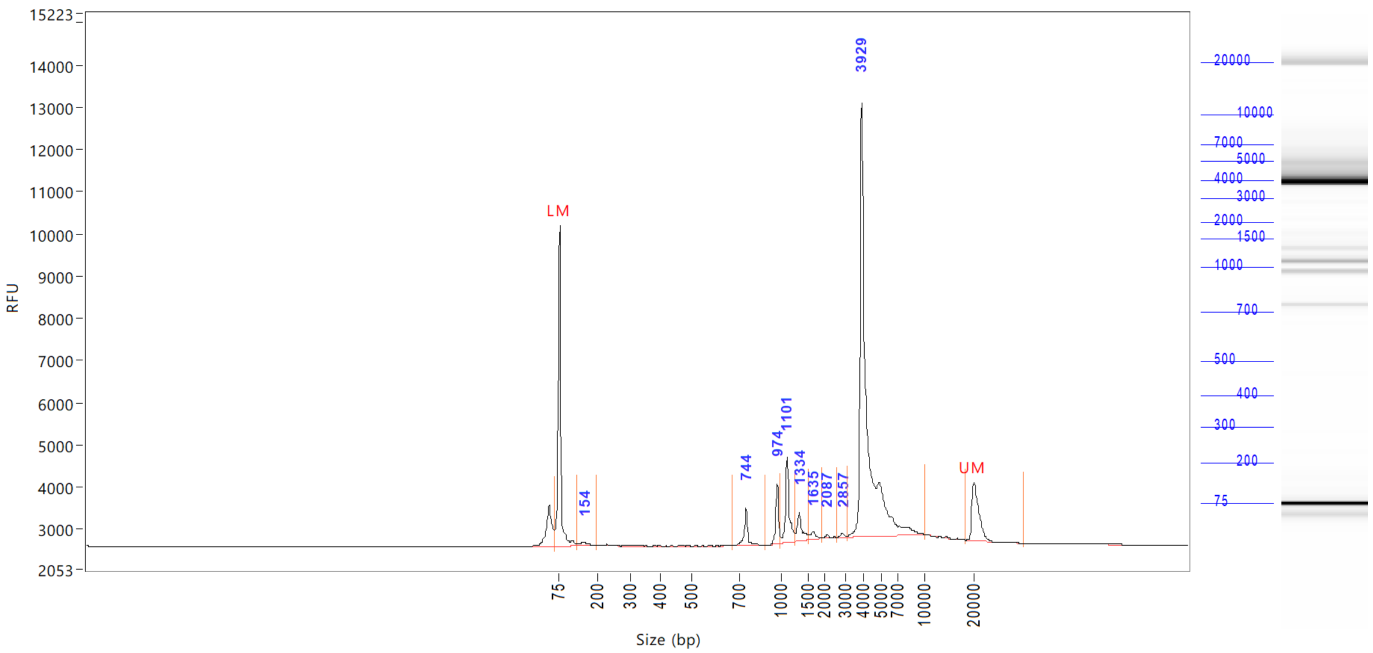

Peak	Size (bp)	Concentration (ng/uL)	From (bp)	To (bp)	Average size (bp)	CV%	RFU	Corrected peak area
1	75 (LM)	0.5000	60	131	75	12.99	7582	49.640
2	154	0.2200	131	192	153	8.95	85	1.820
3	744	0.7085	667	883	742	3.95	904	5.862
4	974	0.8669	883	1000	968	1.35	1421	7.172
5	1101	1.5611	1000	1256	1109	4.73	2017	12.916
6	1334	0.6923	1256	1501	1353	4.76	689	5.728
7	1635	0.2024	1501	1942	1632	5.27	182	1.675
8	2087	0.0620	1942	2572	2144	6.92	91	0.513
9	2857	0.0812	2572	3037	2831	3.60	107	0.672
10	3929	12.6787	3037	10000	4422	21.43	10295	104.896
11	20000 (UM)	0.1273	18415	30277	20521	4.62	1381	12.643

TIC:	17.0731	ng/uL
TIM:	13.5873	nmole/L
Total concentration:	17.2364	ng/uL

Sample peak width (sec): 5   Sample min peak height: 50   Sample baseline V to V?: Y   Sample baseline V to V points: 3
Sample filter: Binomial   Number of points for filter: 3 Sample start region (min): 0 Sample end region (min): 30
Marker peak width (sec): 5   Marker min peak height: 200   Marker baseline V to V?: Y   Marker baseline V to V points: 3
Lower marker selection: First peak > 500 RFU   Upper marker selection: Last peak > 200 RFU
Ladder size (bp)75, 200, 300, 400, 500, 700, 1000, 1500, 2000, 3000, 4000, 5000, 7000, 10000, 20000
Quantification using: Lower Marker   Final concentration (ng/uL): 0.5000   Dilution factor: 12.0

Sample: SampD11
Well location: D11
Created: Tuesday, August 10, 2021 9:21:07 PM

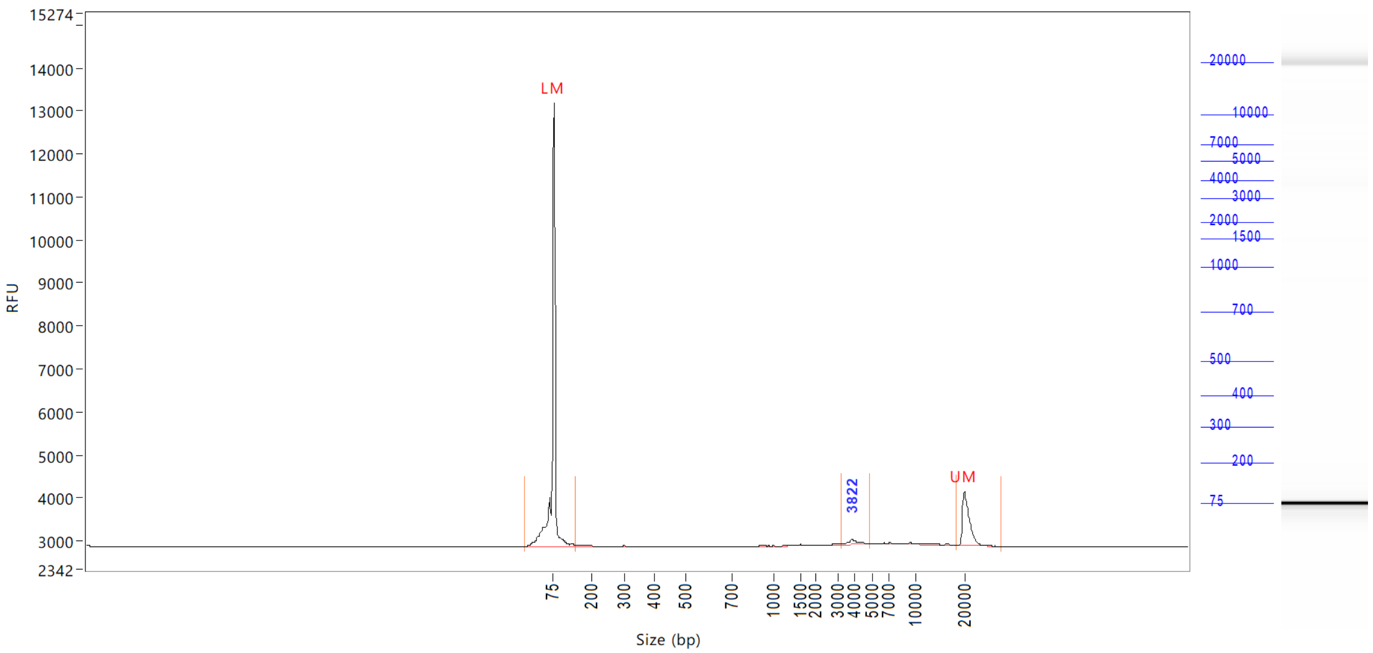

Peak	Size (bp)	Concentration (ng/uL)	From (bp)	To (bp)	Average size (bp)	CV%	RFU	Corrected peak area
1	75 (LM)	0.5000	0	143	68	26.27	10336	79.749
2	3822	0.1275	3251	4862	3958	8.62	113	1.695
3	20000 (UM)	0.0735	18415	27617	20460	4.50	1252	11.725

TIC:	0.1275	ng/uL
TIM:	0.0530	nmole/L
Total concentration:	0.5194	ng/uL

```
Sample peak width (sec): 5    Sample min peak height: 50    Sample baseline V to V?: Y    Sample baseline V to V points: 3
Sample filter: Binomial       Number of points for filter: 3  Sample start region (min): 0  Sample end region (min): 30
Marker peak width (sec): 5    Marker min peak height: 200   Marker baseline V to V?: Y    Marker baseline V to V points: 3
Lower marker selection: First peak > 500 RFU                Upper marker selection: Last peak > 200 RFU
Ladder size (bp)75, 200, 300, 400, 500, 700, 1000, 1500, 2000, 3000, 4000, 5000, 7000, 10000, 20000
Quantification using: Lower Marker          Final concentration (ng/uL): 0.5000          Dilution factor: 12.0
```

Sample: SampE11
Well location: E11
Created: Tuesday, August 10, 2021 9:21:07 PM

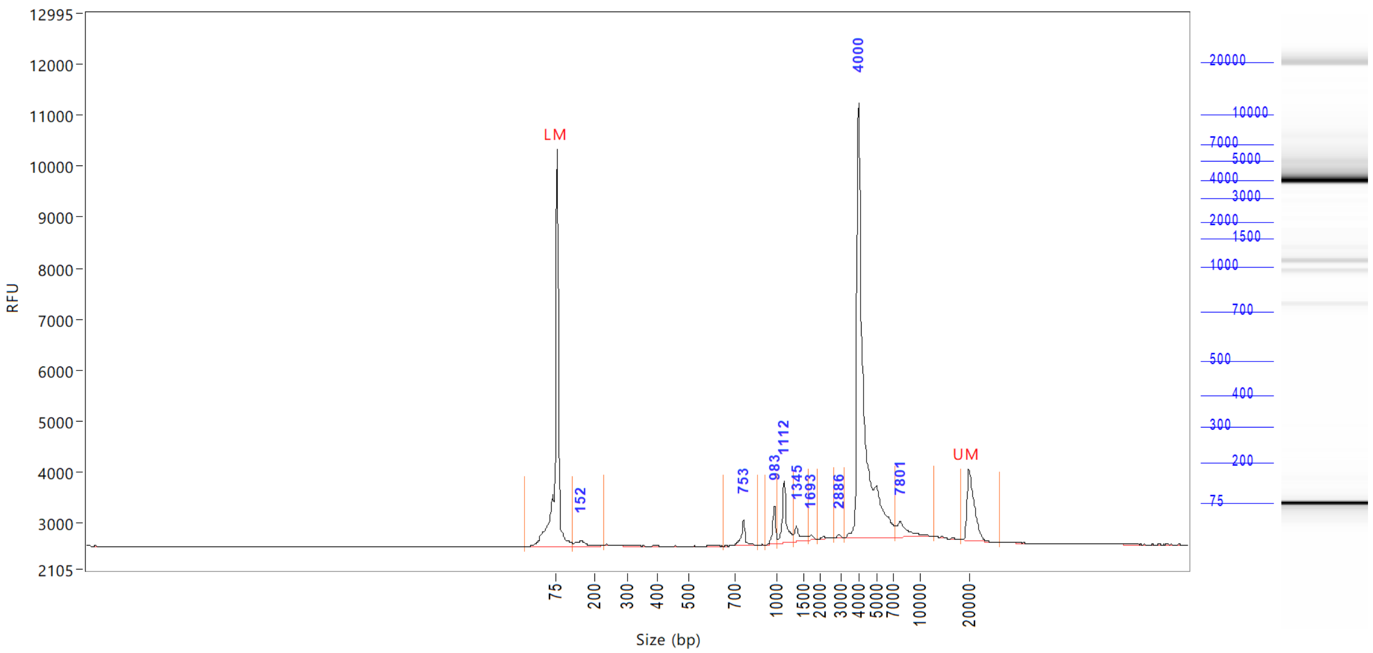

Peak	Size (bp)	Concentration (ng/uL)	From (bp)	To (bp)	Average size (bp)	CV%	RFU	Corrected peak area
1	75 (LM)	0.5000	0	128	67	26.14	7782	66.502
2	152	0.2367	128	224	157	14.01	118	2.624
3	753	0.3302	649	857	742	3.75	492	3.660
4	983	0.3730	909	1001	973	1.45	726	4.134
5	1112	0.7796	1001	1289	1126	5.40	1203	8.641
6	1345	0.2708	1289	1616	1399	6.24	300	3.001
7	1693	0.0612	1616	1885	1695	3.15	92	0.678
8	2886	0.0404	2686	3144	2869	2.95	73	0.448
9	4000	7.9512	3144	7268	4373	15.61	8533	88.129
10	7801	0.4420	7268	12928	8425	12.03	309	4.899
11	20000 (UM)	0.0992	18293	26408	20552	4.31	1386	13.192

TIC:	10.4852	ng/uL
TIM:	8.4621	nmole/L
Total concentration:	10.6046	ng/uL

```
Sample peak width (sec): 5     Sample min peak height: 50     Sample baseline V to V?: Y     Sample baseline V to V points: 3
Sample filter: Binomial        Number of points for filter: 3  Sample start region (min): 0  Sample end region (min): 30
Marker peak width (sec): 5     Marker min peak height: 200    Marker baseline V to V?: Y     Marker baseline V to V points: 3
Lower marker selection: First peak > 500 RFU                  Upper marker selection: Last peak > 200 RFU
Ladder size (bp)75, 200, 300, 400, 500, 700, 1000, 1500, 2000, 3000, 4000, 5000, 7000, 10000, 20000
Quantification using: Lower Marker          Final concentration (ng/uL): 0.5000          Dilution factor: 12.0
```

Sample: SampF11
Well location: F11
Created: Tuesday, August 10, 2021 9:21:07 PM

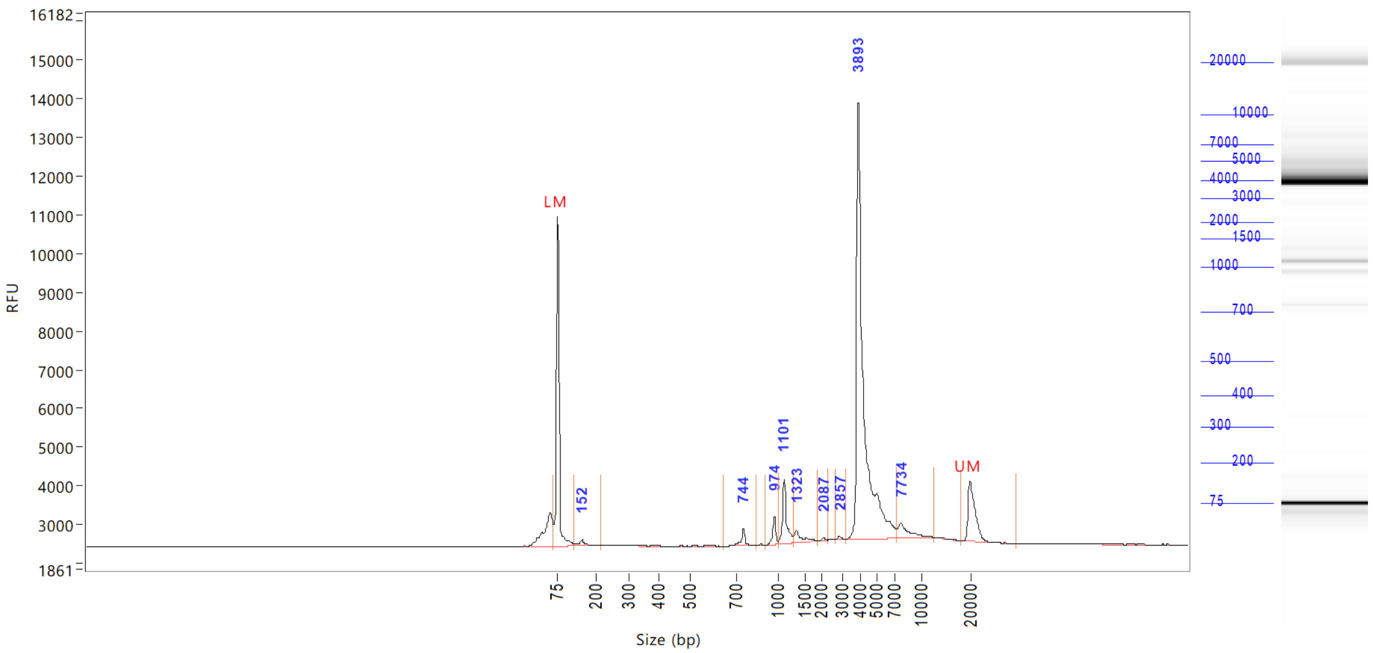

Peak	Size (bp)	Concentration (ng/uL)	From (bp)	To (bp)	Average size (bp)	CV%	RFU	Corrected peak area
1	75 (LM)	0.5000	64	128	75	11.21	8506	54.559
2	152	0.2631	128	214	153	10.91	159	2.393
3	744	0.3278	644	840	734	3.12	438	2.981
4	974	0.4437	905	1000	966	1.46	711	4.035
5	1101	1.2426	1000	1267	1113	5.05	1661	11.299
6	1323	0.4313	1267	1866	1409	9.00	334	3.921
7	2087	0.0343	1866	2258	2049	2.77	65	0.312
8	2857	0.0586	2657	3144	2843	3.04	96	0.533
9	3893	12.7674	3144	7268	4282	15.41	11259	116.095
10	7734	0.6748	7268	12562	8371	11.35	399	6.136
11	20000 (UM)	0.1328	18171	29430	20508	4.53	1548	14.493

TIC:	16.2436	ng/uL
TIM:	11.7618	nmole/L
Total concentration:	16.3685	ng/uL

```
Sample peak width (sec): 5    Sample min peak height: 50    Sample baseline V to V?: Y    Sample baseline V to V points: 3
Sample filter: Binomial       Number of points for filter: 3  Sample start region (min): 0  Sample end region (min): 30
Marker peak width (sec): 5    Marker min peak height: 200   Marker baseline V to V?: Y    Marker baseline V to V points: 3
Lower marker selection: First peak > 500 RFU                Upper marker selection: Last peak > 200 RFU
Ladder size (bp)75, 200, 300, 400, 500, 700, 1000, 1500, 2000, 3000, 4000, 5000, 7000, 10000, 20000
Quantification using: Lower Marker          Final concentration (ng/uL): 0.5000          Dilution factor: 12.0
```

Sample: SampG11
Well location: G11
Created: Tuesday, August 10, 2021 9:21:07 PM

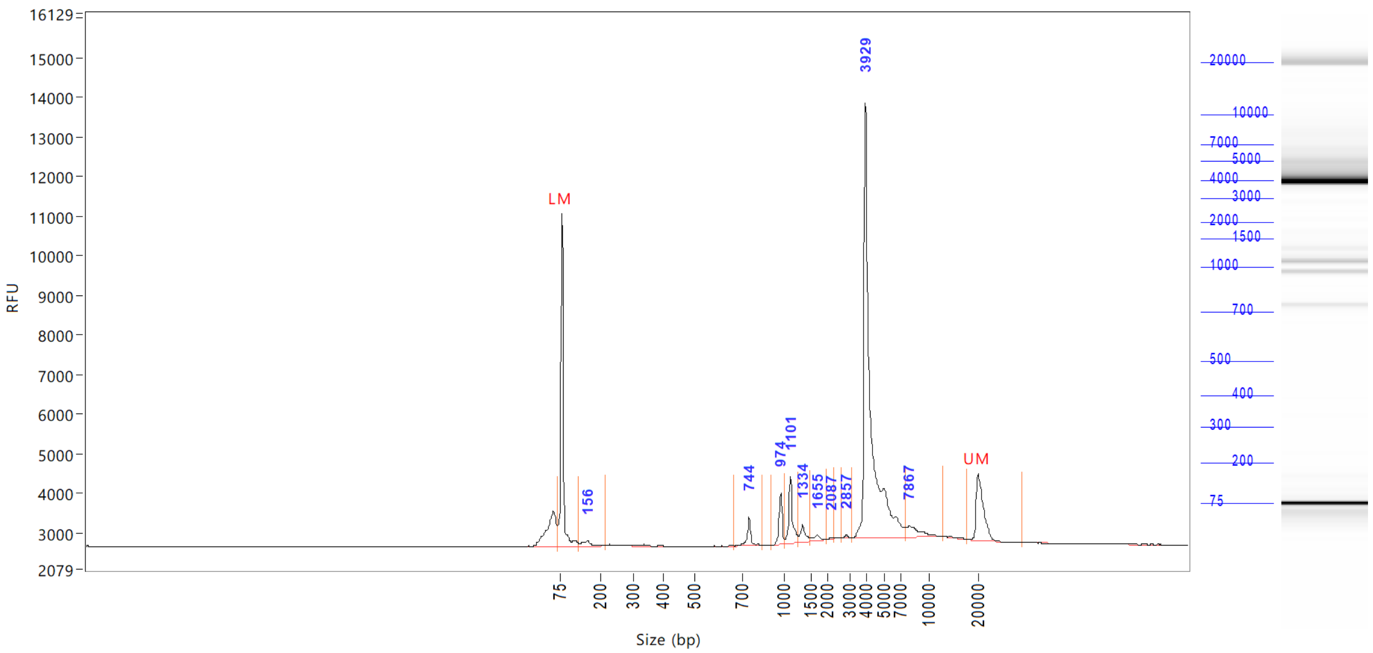

Peak	Size (bp)	Concentration (ng/uL)	From (bp)	To (bp)	Average size (bp)	CV%	RFU	Corrected peak area
1	75 (LM)	0.5000	62	131	75	13.27	8397	54.090
2	156	0.2935	131	214	155	10.87	146	2.646
3	744	0.5077	664	844	737	3.22	720	4.576
4	974	0.7353	905	1000	967	1.31	1288	6.629
5	1101	1.2081	1000	1256	1107	4.76	1685	10.891
6	1334	0.4010	1256	1467	1339	3.93	445	3.615
7	1655	0.1740	1467	1942	1608	6.01	134	1.569
8	2087	0.0247	1942	2286	2069	2.80	53	0.223
9	2857	0.0542	2629	3108	2837	2.98	94	0.488
10	3929	12.1083	3108	7468	4337	16.62	11013	109.155
11	7867	0.6149	7468	12928	8677	11.54	270	5.543
12	20000 (UM)	0.1498	17561	29068	20504	4.37	1701	16.203

TIC:	16.1216	ng/uL
TIM:	12.7292	nmole/L
Total concentration:	16.2767	ng/uL

Sample peak width (sec): 5 Sample min peak height: 50 Sample baseline V to V?: Y Sample baseline V to V points: 3
Sample filter: Binomial Number of points for filter: 3 Sample start region (min): 0 Sample end region (min): 30
Marker peak width (sec): 5 Marker min peak height: 200 Marker baseline V to V?: Y Marker baseline V to V points: 3
Lower marker selection: First peak > 500 RFU Upper marker selection: Last peak > 200 RFU
Ladder size (bp)75, 200, 300, 400, 500, 700, 1000, 1500, 2000, 3000, 4000, 5000, 7000, 10000, 20000
Quantification using: Lower Marker Final concentration (ng/uL): 0.5000 Dilution factor: 12.0

Sample: SampH11
Well location: H11
Created: Tuesday, August 10, 2021 9:21:07 PM

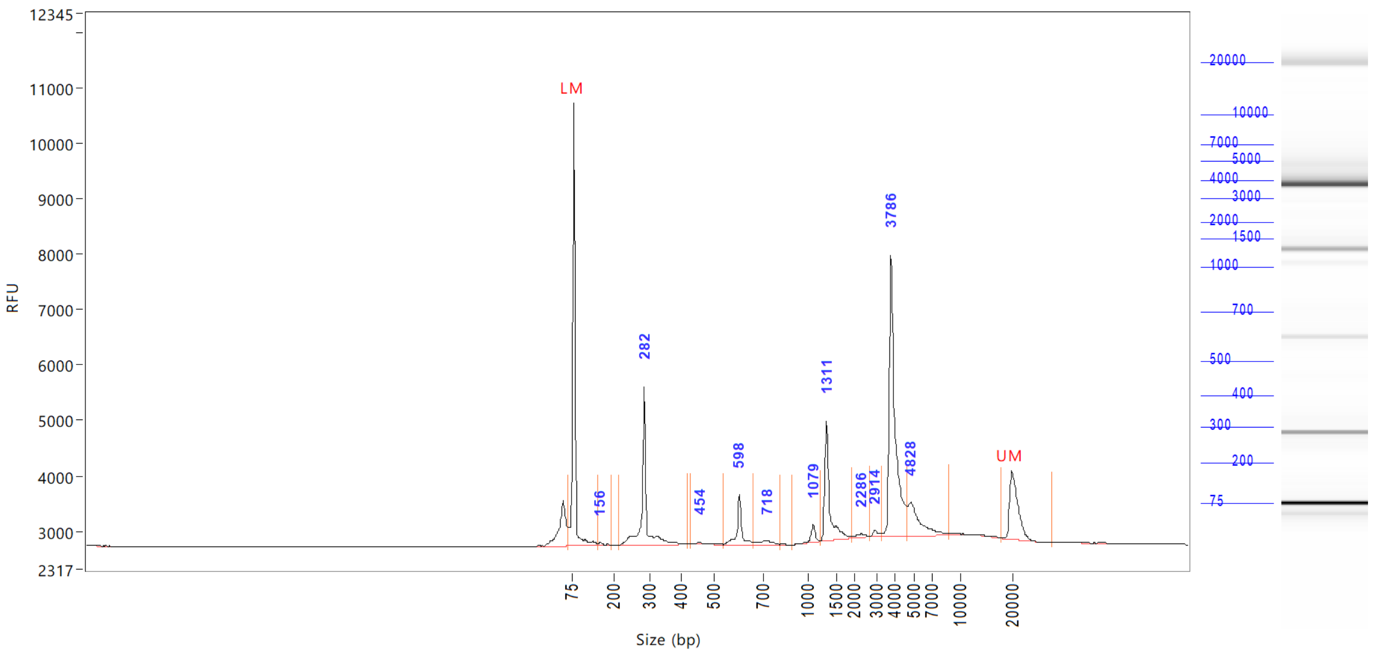

Peak	Size (bp)	Concentration (ng/uL)	From (bp)	To (bp)	Average size (bp)	CV%	RFU	Corrected peak area
1	75 (LM)	0.5000	60	148	76	14.76	8004	49.608
2	156	0.0795	148	190	160	6.27	62	0.658
3	282	3.0441	210	416	284	9.47	2842	25.169
4	454	0.0969	430	534	465	4.84	53	0.801
5	598	0.8615	534	659	596	3.55	896	7.123
6	718	0.2076	659	805	708	4.61	77	1.716
7	1079	0.2706	887	1201	1057	4.49	321	2.238
8	1311	2.0197	1201	1942	1360	8.55	2159	16.699
9	2286	0.1072	1942	2715	2232	7.88	62	0.886
10	2914	0.1172	2715	3251	2965	4.42	120	0.969
11	3786	5.3475	3251	4621	3905	6.46	5057	44.213
12	4828	1.3170	4621	8800	5516	17.65	603	10.889
13	20000 (UM)	0.1289	17805	27617	20467	4.65	1231	12.791

TIC:	13.4689	ng/uL
TIM:	27.3098	nmole/L
Total concentration:	13.5150	ng/uL

```
Sample peak width (sec): 5    Sample min peak height: 50    Sample baseline V to V?: Y    Sample baseline V to V points: 3
Sample filter: Binomial       Number of points for filter: 3  Sample start region (min): 0  Sample end region (min): 30
Marker peak width (sec): 5    Marker min peak height: 200   Marker baseline V to V?: Y    Marker baseline V to V points: 3
Lower marker selection: First peak > 500 RFU                Upper marker selection: Last peak > 200 RFU
Ladder size (bp)75, 200, 300, 400, 500, 700, 1000, 1500, 2000, 3000, 4000, 5000, 7000, 10000, 20000
Quantification using: Lower Marker          Final concentration (ng/uL): 0.5000          Dilution factor: 12.0
```

Sample: SampA12
Well location: A12
Created: Tuesday, August 10, 2021 9:21:07 PM

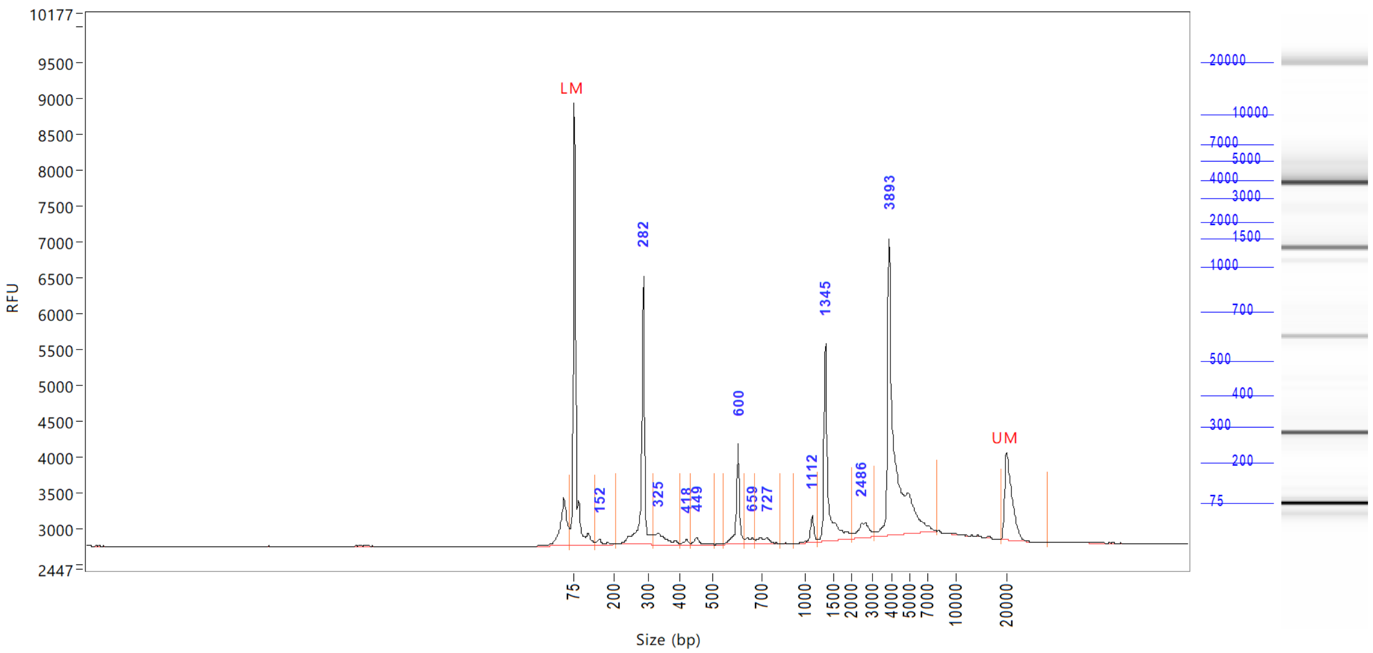

Peak	Size (bp)	Concentration (ng/uL)	From (bp)	To (bp)	Average size (bp)	CV%	RFU	Corrected peak area
1	75 (LM)	0.5000	64	139	77	14.93	6163	40.669
2	152	0.1558	139	201	156	8.31	77	1.056
3	282	3.8207	201	315	277	5.39	3732	25.897
4	325	0.5360	315	398	342	6.24	159	3.633
5	418	0.0977	398	431	415	1.97	80	0.663
6	449	0.1667	431	504	453	3.17	95	1.130
7	600	1.2252	539	629	595	2.24	1403	8.305
8	659	0.1879	629	672	649	1.97	86	1.273
9	727	0.2786	672	827	714	4.54	86	1.889
10	1112	0.3475	913	1212	1095	4.59	372	2.355
11	1345	2.7470	1212	2058	1406	11.08	2752	18.619
12	2486	0.5271	2058	3001	2546	9.26	202	3.572
13	3893	6.3264	3001	8067	4288	16.38	4126	42.882
14	20000 (UM)	0.1388	18781	27859	20487	4.30	1210	11.286

TIC:	16.4166	ng/uL
TIM:	38.9154	nmole/L
Total concentration:	16.4342	ng/uL

```
Sample peak width (sec): 5    Sample min peak height: 50    Sample baseline V to V?: Y    Sample baseline V to V points: 3
Sample filter: Binomial       Number of points for filter: 3  Sample start region (min): 0  Sample end region (min): 30
Marker peak width (sec): 5    Marker min peak height: 200   Marker baseline V to V?: Y    Marker baseline V to V points: 3
Lower marker selection: First peak > 500 RFU                Upper marker selection: Last peak > 200 RFU
Ladder size (bp)75, 200, 300, 400, 500, 700, 1000, 1500, 2000, 3000, 4000, 5000, 7000, 10000, 20000
Quantification using: Lower Marker          Final concentration (ng/uL): 0.5000          Dilution factor: 12.0
```

Sample: SampB12
Well location: B12
Created: Tuesday, August 10, 2021 9:21:07 PM

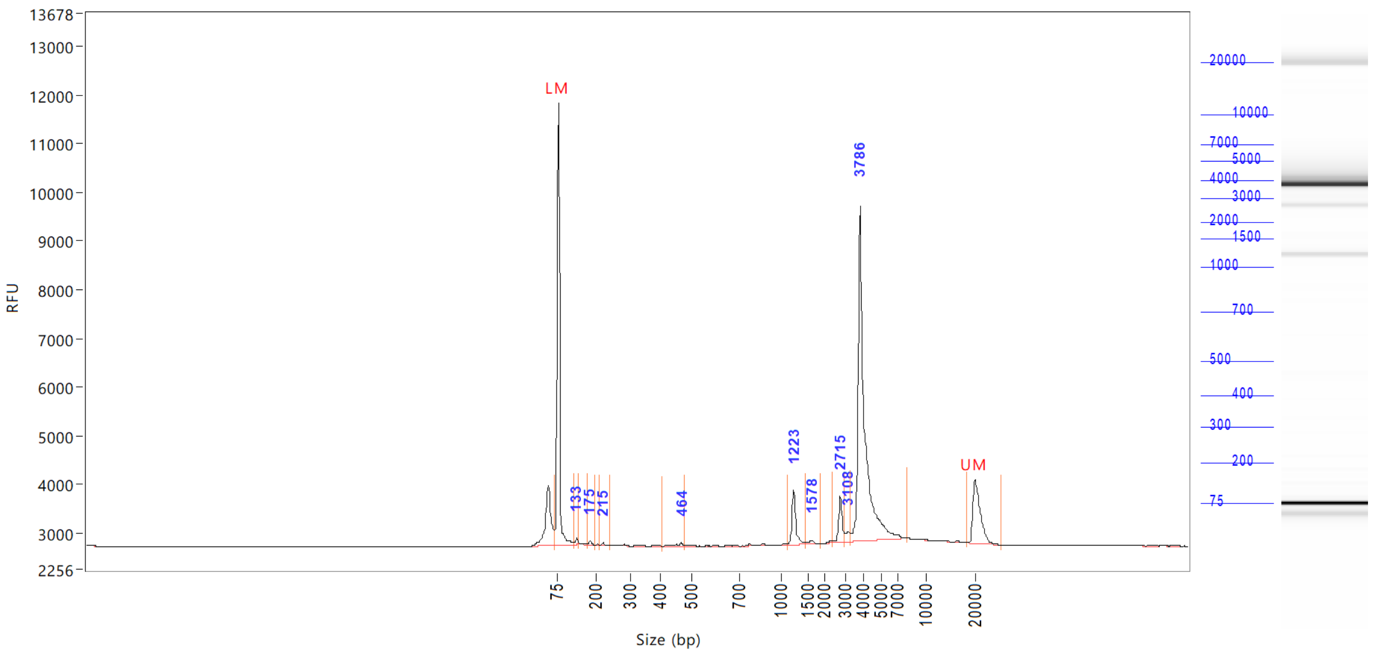

Peak	Size (bp)	Concentration (ng/uL)	From (bp)	To (bp)	Average size (bp)	CV%	RFU	Corrected peak area
1	75 (LM)	0.5000	64	126	75	9.45	9102	53.516
2	133	0.0941	126	143	132	2.53	145	0.839
3	175	0.0601	168	194	175	3.22	88	0.536
4	215	0.0316	208	237	215	2.20	57	0.282
5	464	0.0883	403	473	449	3.59	71	0.787
6	1223	0.7655	1112	1445	1227	4.36	1113	6.828
7	1578	0.0819	1445	1885	1557	5.11	79	0.731
8	2715	0.6087	2372	2943	2705	3.81	951	5.429
9	3108	0.1819	2943	3286	3095	3.45	206	1.623
10	3786	7.3755	3286	7934	4055	13.28	6892	65.784
11	20000 (UM)	0.1175	18415	25440	20493	4.21	1318	12.573

TIC:	9.2875	ng/uL
TIM:	6.8752	nmole/L
Total concentration:	9.6296	ng/uL

```
Sample peak width (sec): 5    Sample min peak height: 50    Sample baseline V to V?: Y    Sample baseline V to V points: 3
Sample filter: Binomial       Number of points for filter: 3  Sample start region (min): 0  Sample end region (min): 30
Marker peak width (sec): 5    Marker min peak height: 200   Marker baseline V to V?: Y    Marker baseline V to V points: 3
Lower marker selection: First peak > 500 RFU                Upper marker selection: Last peak > 200 RFU
Ladder size (bp)75, 200, 300, 400, 500, 700, 1000, 1500, 2000, 3000, 4000, 5000, 7000, 10000, 20000
Quantification using: Lower Marker          Final concentration (ng/uL): 0.5000          Dilution factor: 12.0
```

Sample: SampC12
Well location: C12
Created: Tuesday, August 10, 2021 9:21:07 PM

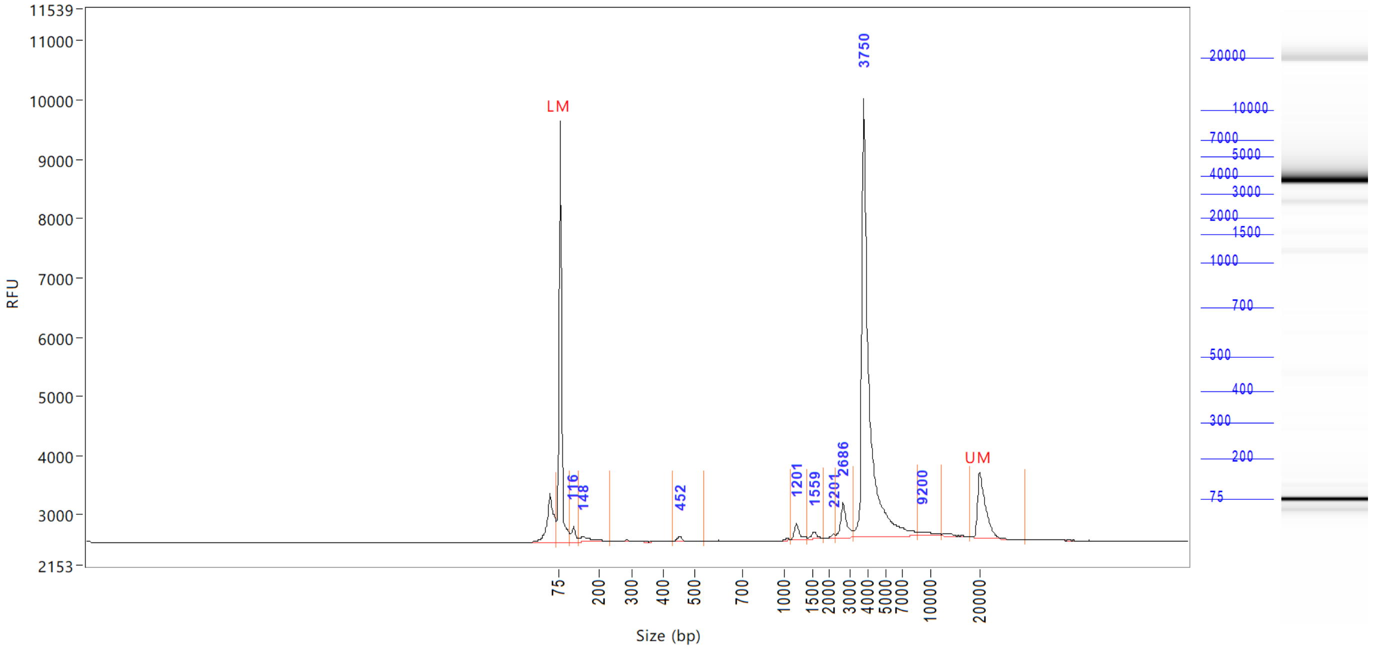

Peak	Size (bp)	Concentration (ng/uL)	From (bp)	To (bp)	Average size (bp)	CV%	RFU	Corrected peak area
1	75 (LM)	0.5000	64	107	75	8.68	7101	47.061
2	116	0.3253	107	133	117	5.93	254	2.551
3	148	0.3146	133	232	163	13.48	85	2.468
4	452	0.1490	431	534	455	3.50	93	1.168
5	1201	0.3053	1101	1422	1227	5.69	275	2.395
6	1559	0.1410	1422	1846	1542	4.75	120	1.106
7	2201	0.0593	1846	2344	2174	5.02	68	0.465
8	2686	0.6975	2344	3144	2716	6.33	597	5.471
9	3750	10.3132	3144	8667	4107	18.36	7412	80.892
10	9200	0.1886	8667	12318	9940	9.79	69	1.480
11	20000 (UM)	0.1221	18049	29068	20575	5.10	1095	11.489

TIC:	12.4939	ng/uL
TIM:	13.4712	nmole/L
Total concentration:	12.7807	ng/uL

```
Sample peak width (sec): 5    Sample min peak height: 50    Sample baseline V to V?: Y    Sample baseline V to V points: 3
Sample filter: Binomial       Number of points for filter: 3  Sample start region (min): 0  Sample end region (min): 30
Marker peak width (sec): 5    Marker min peak height: 200   Marker baseline V to V?: Y    Marker baseline V to V points: 3
Lower marker selection: First peak > 500 RFU                Upper marker selection: Last peak > 200 RFU
Ladder size (bp)75, 200, 300, 400, 500, 700, 1000, 1500, 2000, 3000, 4000, 5000, 7000, 10000, 20000
Quantification using: Lower Marker          Final concentration (ng/uL): 0.5000          Dilution factor: 12.0
```

Sample: SampD12
Well location: D12
Created: Tuesday, August 10, 2021 9:21:07 PM

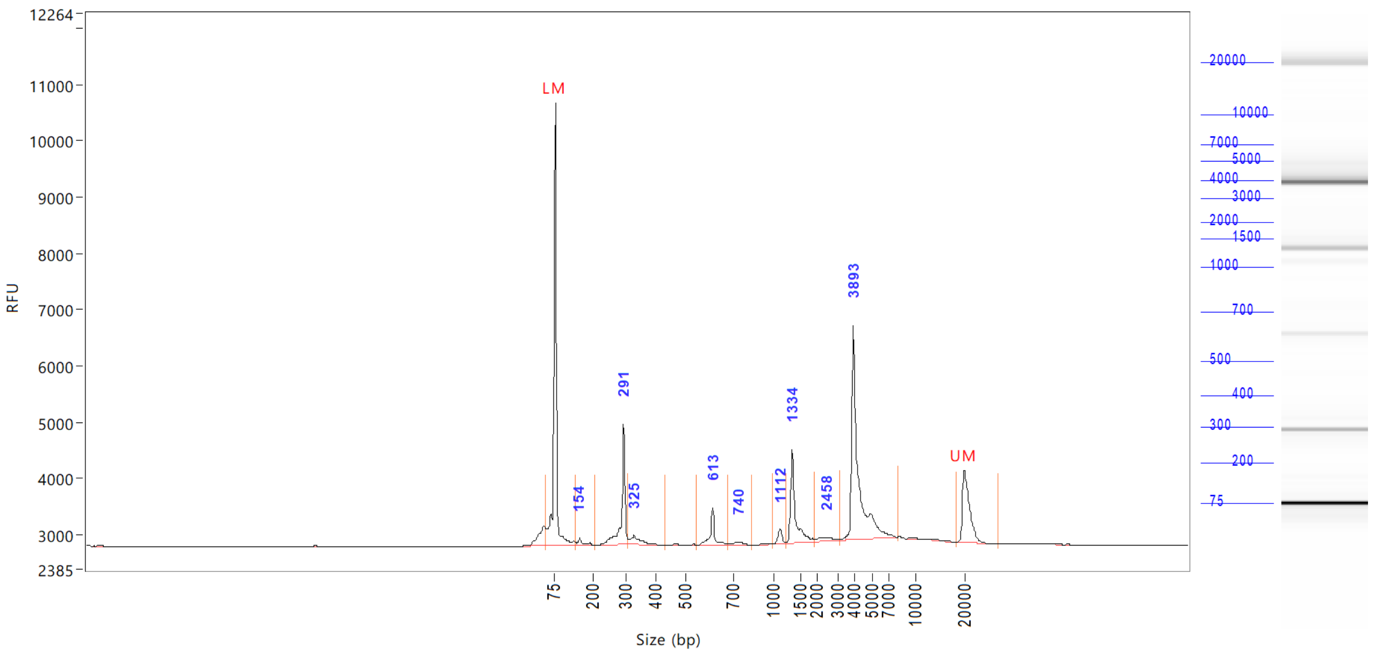

Peak	Size (bp)	Concentration (ng/uL)	From (bp)	To (bp)	Average size (bp)	CV%	RFU	Corrected peak area
1	75 (LM)	0.5000	45	145	74	16.59	7881	53.321
2	154	0.1587	145	203	162	8.84	125	1.411
3	291	1.7964	203	303	281	5.76	2148	15.965
4	325	0.3847	303	430	336	7.21	167	3.419
5	613	0.6049	539	680	608	3.65	673	5.375
6	740	0.1302	680	835	731	4.41	63	1.157
7	1112	0.2001	987	1223	1091	3.84	269	1.778
8	1334	1.4493	1223	1923	1388	9.08	1672	12.879
9	2458	0.1798	1923	3072	2357	12.31	69	1.598
10	3893	4.1607	3072	8001	4235	14.63	3791	36.975
11	20000 (UM)	0.1146	18415	26891	20484	4.46	1286	12.216

TIC:	9.0647	ng/uL
TIM:	19.7035	nmole/L
Total concentration:	9.1841	ng/uL

Sample peak width (sec): 5 Sample min peak height: 50 Sample baseline V to V?: Y Sample baseline V to V points: 3
Sample filter: Binomial Number of points for filter: 3 Sample start region (min): 0 Sample end region (min): 30
Marker peak width (sec): 5 Marker min peak height: 200 Marker baseline V to V?: Y Marker baseline V to V points: 3
Lower marker selection: First peak > 500 RFU Upper marker selection: Last peak > 200 RFU
Ladder size (bp)75, 200, 300, 400, 500, 700, 1000, 1500, 2000, 3000, 4000, 5000, 7000, 10000, 20000
Quantification using: Lower Marker Final concentration (ng/uL): 0.5000 Dilution factor: 12.0

Sample: SampE12
Well location: E12
Created: Tuesday, August 10, 2021 9:21:07 PM

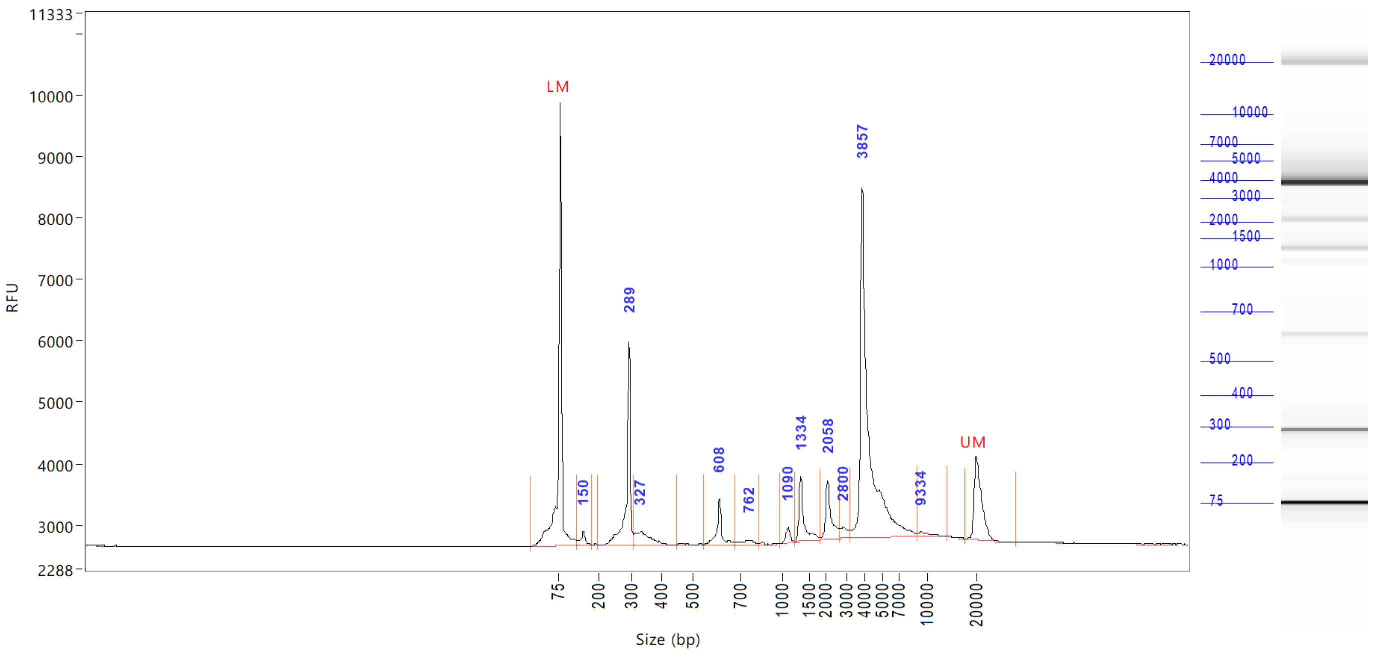

Peak	Size (bp)	Concentration (ng/uL)	From (bp)	To (bp)	Average size (bp)	CV%	RFU	Corrected peak area
1	75 (LM)	0.5000	0	131	69	26.95	7220	59.982
2	150	0.2457	131	181	147	6.53	222	2.456
3	289	2.6511	198	303	280	5.67	3316	26.503
4	327	0.6825	303	445	339	7.74	218	6.823
5	608	0.6565	542	677	607	3.81	744	6.563
6	762	0.1730	677	827	740	5.35	78	1.729
7	1090	0.1822	987	1223	1082	3.73	261	1.822
8	1334	0.8561	1223	1827	1381	8.27	1052	8.559
9	2058	0.8568	1827	2657	2153	9.14	957	8.565
10	2800	0.2234	2657	3179	2888	5.21	177	2.233
11	3857	6.8810	3179	8934	4360	20.78	5682	68.789
12	9334	0.1188	8934	14147	10112	9.99	67	1.188
13	20000 (UM)	0.1114	17683	28221	20575	4.68	1353	13.366

TIC:	13.5271	ng/uL
TIM:	28.4942	nmole/L
Total concentration:	13.5739	ng/uL

```
Sample peak width (sec): 5    Sample min peak height: 50    Sample baseline V to V?: Y    Sample baseline V to V points: 3
Sample filter: Binomial       Number of points for filter: 3  Sample start region (min): 0  Sample end region (min): 30
Marker peak width (sec): 5    Marker min peak height: 200   Marker baseline V to V?: Y    Marker baseline V to V points: 3
Lower marker selection: First peak > 500 RFU                Upper marker selection: Last peak > 200 RFU
Ladder size (bp)75, 200, 300, 400, 500, 700, 1000, 1500, 2000, 3000, 4000, 5000, 7000, 10000, 20000
Quantification using: Lower Marker          Final concentration (ng/uL): 0.5000          Dilution factor: 12.0
```

Sample: SampF12
Well location: F12
Created: Tuesday, August 10, 2021 9:21:07 PM

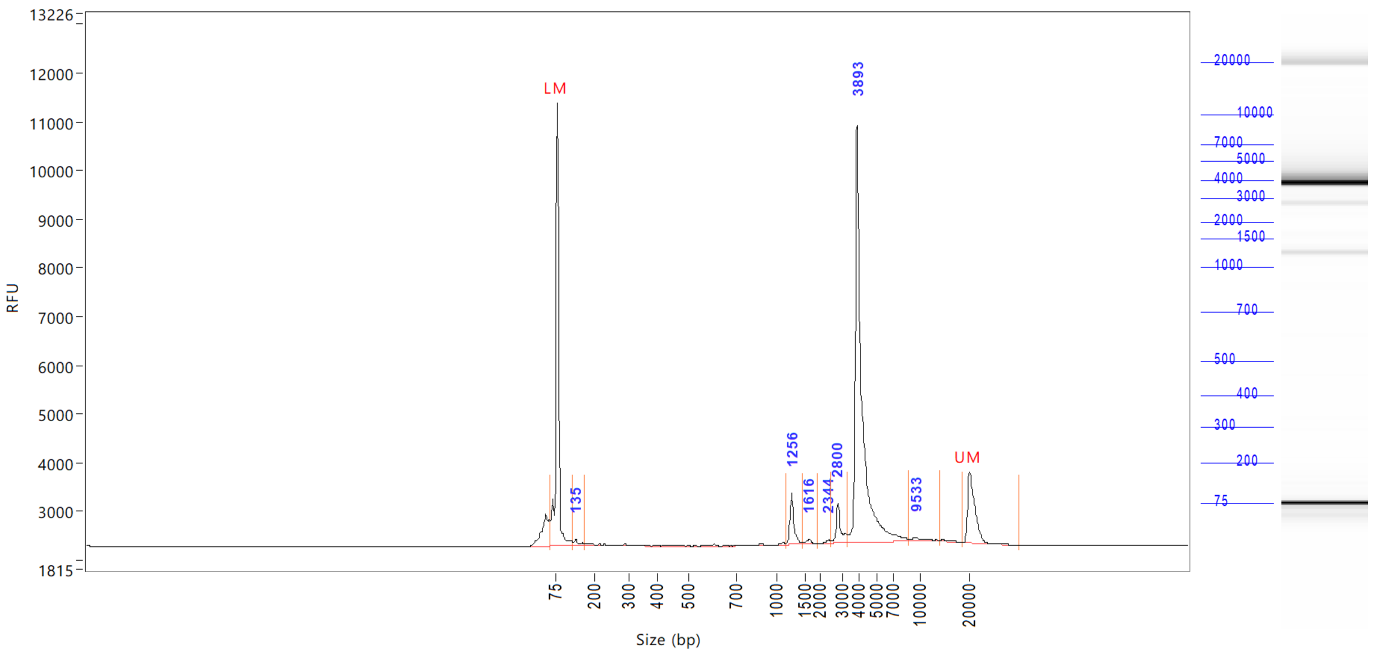

Peak	Size (bp)	Concentration (ng/uL)	From (bp)	To (bp)	Average size (bp)	CV%	RFU	Corrected peak area
1	75 (LM)	0.5000	54	128	74	12.39	9107	63.265
2	135	0.1434	128	166	142	7.95	133	1.512
3	1256	0.6489	1145	1467	1262	4.34	1052	6.842
4	1616	0.0762	1467	1885	1585	4.90	95	0.804
5	2344	0.0401	1885	2486	2310	4.57	64	0.423
6	2800	0.5668	2486	3358	2884	6.65	799	5.976
7	3893	7.7882	3358	8600	4214	17.26	8546	82.120
8	9533	0.1419	8600	13781	10226	13.18	65	1.497
9	20000 (UM)	0.1095	18659	30277	20578	4.86	1462	13.857

TIC:	9.4056	ng/uL
TIM:	6.0030	nmole/L
Total concentration:	9.7073	ng/uL

Sample peak width (sec): 5   Sample min peak height: 50   Sample baseline V to V?: Y   Sample baseline V to V points: 3
Sample filter: Binomial   Number of points for filter: 3 Sample start region (min): 0 Sample end region (min): 30
Marker peak width (sec): 5   Marker min peak height: 200   Marker baseline V to V?: Y   Marker baseline V to V points: 3
Lower marker selection: First peak > 500 RFU   Upper marker selection: Last peak > 200 RFU
Ladder size (bp)75, 200, 300, 400, 500, 700, 1000, 1500, 2000, 3000, 4000, 5000, 7000, 10000, 20000
Quantification using: Lower Marker   Final concentration (ng/uL): 0.5000   Dilution factor: 12.0

Sample: SampG12
Well location: G12
Created: Tuesday, August 10, 2021 9:21:07 PM

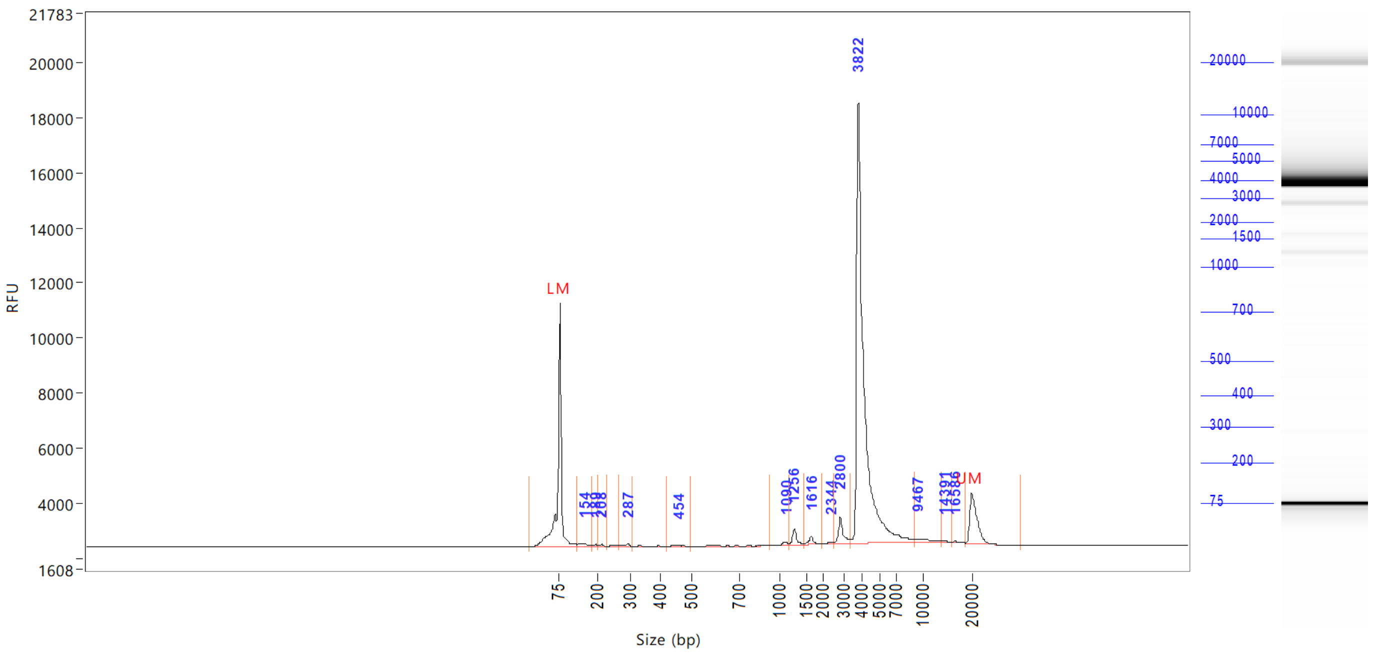

Peak	Size (bp)	Concentration (ng/uL)	From (bp)	To (bp)	Average size (bp)	CV%	RFU	Corrected peak area
1	75 (LM)	0.5000	0	129	66	27.84	8834	77.340
2	154	0.1501	129	181	151	9.30	83	1.934
3	189	0.0395	181	198	189	2.66	62	0.509
4	208	0.0367	198	224	206	2.11	63	0.473
5	287	0.0507	262	301	282	2.42	103	0.653
6	454	0.0474	418	492	454	3.27	53	0.611
7	1090	0.0802	922	1145	1061	3.84	132	1.034
8	1256	0.3020	1145	1433	1260	4.30	570	3.893
9	1616	0.1820	1433	1981	1587	5.52	288	2.346
10	2344	0.0493	1981	2458	2278	5.17	82	0.636
11	2800	0.5536	2458	3286	2838	5.82	992	7.136
12	3822	13.3196	3286	9000	4186	18.28	16005	171.690
13	9467	0.2094	9000	13781	10679	12.91	120	2.699
14	14391	0.0434	13781	15854	14631	4.07	73	0.560
15	16586	0.0260	15854	18659	16516	2.77	51	0.336
16	20000 (UM)	0.1099	18659	29914	20515	4.59	1817	17.004

TIC:	15.0900	ng/uL
TIM:	9.0794	nmole/L
Total concentration:	15.1916	ng/uL

```
Sample peak width (sec): 5    Sample min peak height: 50    Sample baseline V to V?: Y    Sample baseline V to V points: 3
Sample filter: Binomial       Number of points for filter: 3  Sample start region (min): 0  Sample end region (min): 30
Marker peak width (sec): 5    Marker min peak height: 200   Marker baseline V to V?: Y    Marker baseline V to V points: 3
Lower marker selection: First peak > 500 RFU                Upper marker selection: Last peak > 200 RFU
Ladder size (bp)75, 200, 300, 400, 500, 700, 1000, 1500, 2000, 3000, 4000, 5000, 7000, 10000, 20000
Quantification using: Lower Marker          Final concentration (ng/uL): 0.5000          Dilution factor: 12.0
```

Sample: SampH12
Well location: H12
Created: Tuesday, August 10, 2021 9:21:07 PM

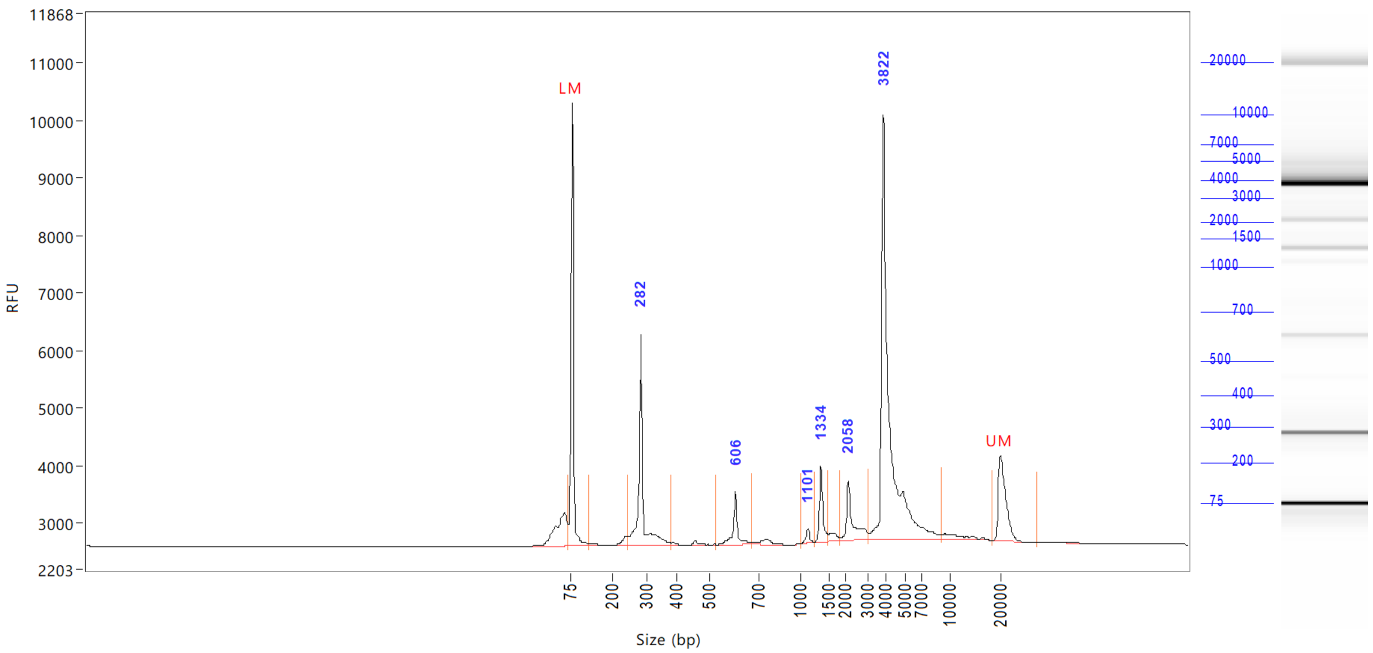

Peak	Size<br>(bp)	Concentration<br>(ng/uL)	From<br>(bp)	To<br>(bp)	Average size<br>(bp)	CV%	RFU	Corrected peak area
1	75 (LM)	0.5000	62	128	74	9.65	7714	45.636
2	282	4.0487	244	380	286	8.09	3660	30.795
3	606	0.9413	527	674	603	3.78	913	7.159
4	1101	0.1987	996	1234	1091	3.38	250	1.512
5	1334	0.9713	1234	1467	1340	3.18	1316	7.387
6	2058	1.3291	1808	3001	2265	13.55	1039	10.109
7	3822	10.4370	3001	9134	4312	21.36	7364	79.384
8	20000 (UM)	0.1531	18415	27254	20441	4.18	1489	13.974

TIC:	17.9261	ng/uL
TIM:	32.2977	nmole/L
Total concentration:	19.2057	ng/uL

Sample peak width (sec): 5 Sample min peak height: 200 Sample baseline V to V?: Y Sample baseline V to V points: 3
Sample filter: Binomial Number of points for filter: 3 Sample start region (min): 0 Sample end region (min): 30
Marker peak width (sec): 5 Marker min peak height: 200 Marker baseline V to V?: Y Marker baseline V to V points: 3
Lower marker selection: First peak > 500 RFU Upper marker selection: Last peak > 200 RFU
Ladder size (bp)75, 200, 300, 400, 500, 700, 1000, 1500, 2000, 3000, 4000, 5000, 7000, 10000, 20000
Quantification using: Lower Marker Final concentration (ng/uL): 0.5000 Dilution factor: 12.0

Sample: DNA size ladder
Well location: A7
Created: Tuesday, August 10, 2021 9:21:07 PM
Import from: C:\Users\admin\Desktop\210811_alberto_ladder.SCAL
Fit type: Point to point

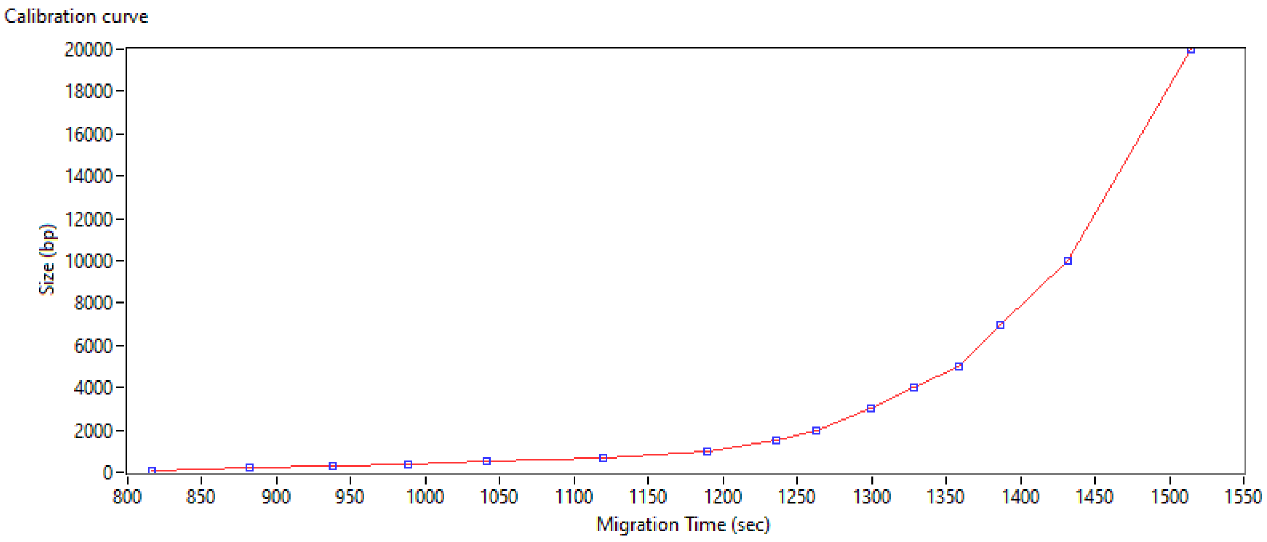

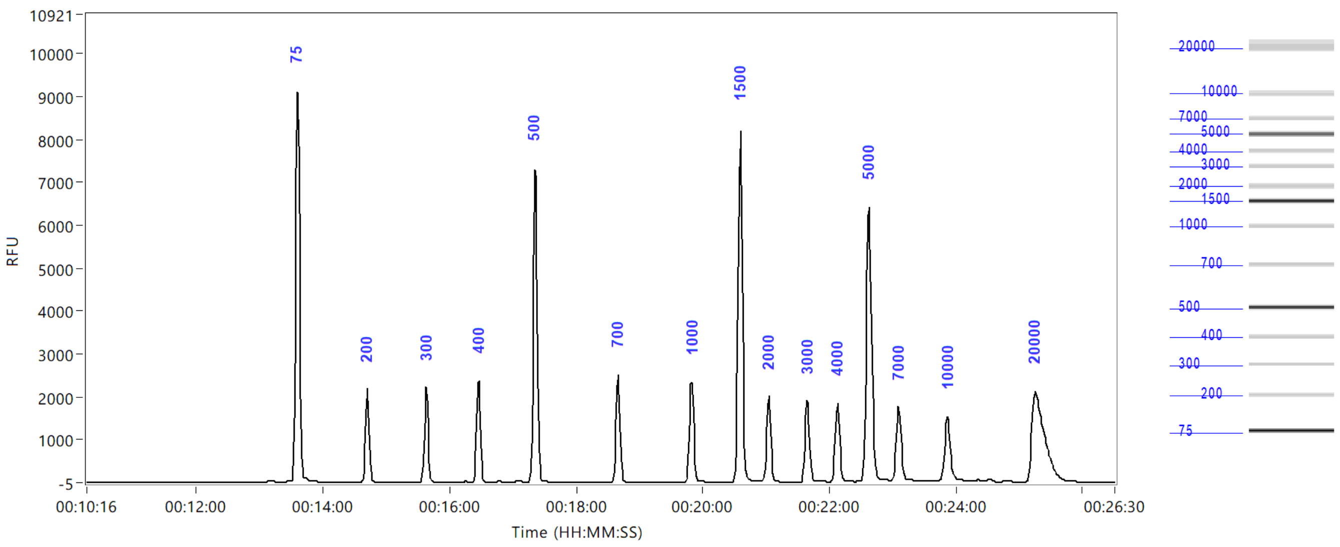

Supplement: Supplementary file 2 — sb3c00292_si_002.zip [file sb3c00292_si_002.zip › dnada_supplementary_material_pks_library_build/ZAG_trials/ZAG_Trial_1/21-11-26_plate5/plate5_report.pdf]
