# Supplementary material for: Automated Platform for the Plasmid Construction Process
Source: ACS Synth Biol. 2023 Nov 10;12(12):3506–13. doi: 10.1021/acssynbio.3c00292 (PMC10729297; doi:10.1021/acssynbio.3c00292)

Instrument controller software run summary:

Filename and data path: C:\AATI\Data\2021 08 10\22-31-23\2021 08 10 22H 31M..raw
Created: Tuesday, August 10, 2021 10:41:10 PM
Number of capillaries: 96
Array serial number: 111920-09SFS
Effect length: 33 cm
Array usage count: 170
Instrument type: ZAG DNA Analyzer
Instrument controller software version: 1.0.1.9
Device serial number: Z7597

Method Information

Method name: ZAG130GP33 - DNA 75-20000bp - Gel Prime Only.mthds
Gel prime: Yes
Full conditioning: No
Gel prime to buffer: No
Gel selection: Gel 1
Perform prerun: 8.0 kV, 30 sec.
Rinse: No
Marker 1: Row: A, 5.0 kV, 5 sec.
Rinse: No
Sample injection: 5.0 kV, 5 sec.
Separation: 8.0 kV, 30.0 min.
Tray name: Sample Tray-7

Analysis mode: DNA

Notes

Gel Image

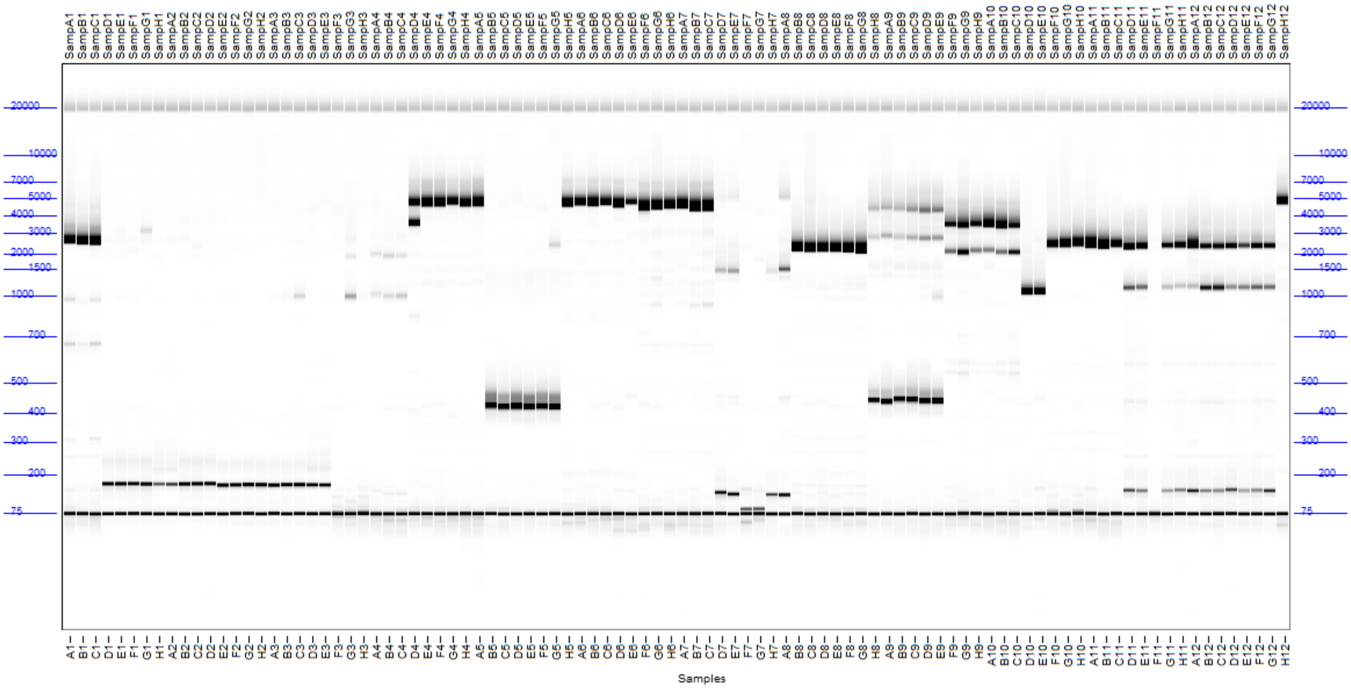

Filename and data path: C:\AATI\Data\2021 08 10\22-31-23\2021 08 10 22H 31M..raw

A1: SampA1

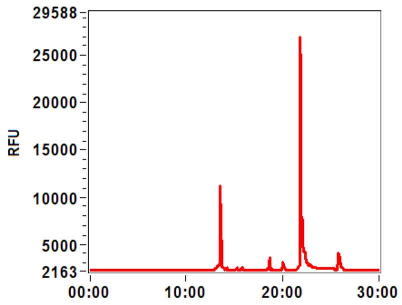

B1: SampB1

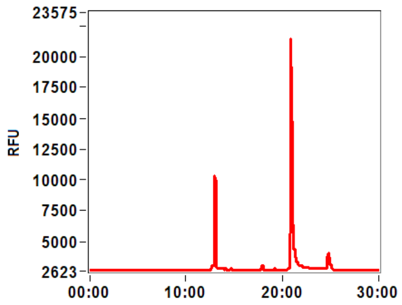

C1: SampC1

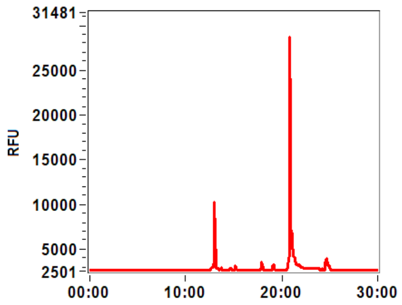

D1: SampD1

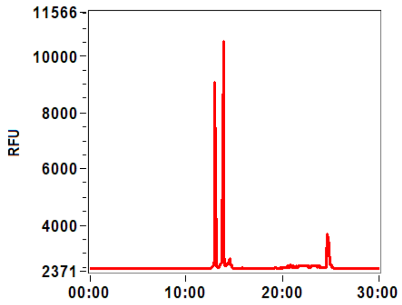

E1: SampE1

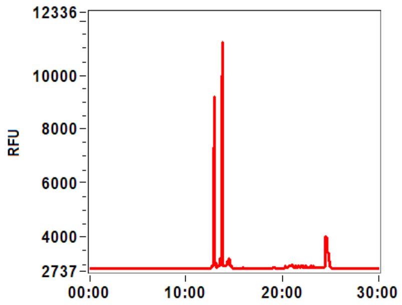

F1: SampF1

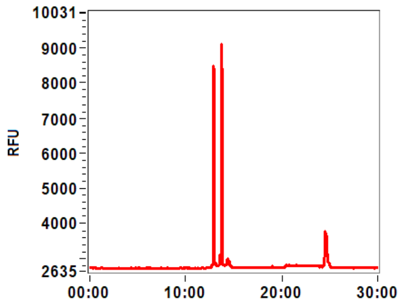

G1: SampG1

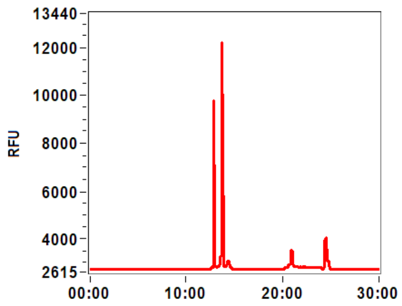

H1: SampH1

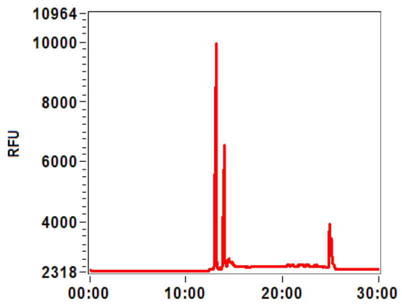

A2: SampA2

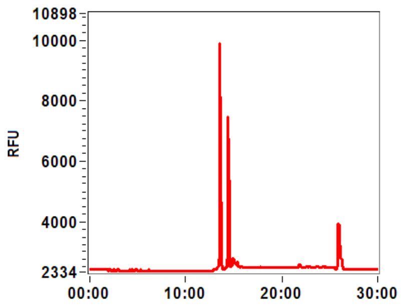

B2: SampB2

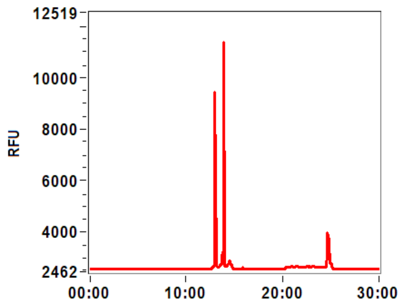

C2: SampC2

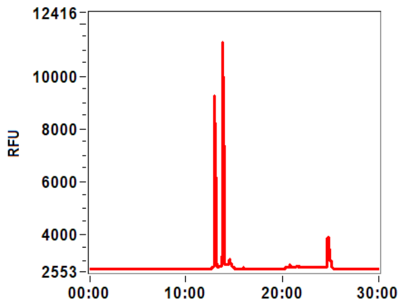

D2: SampD2

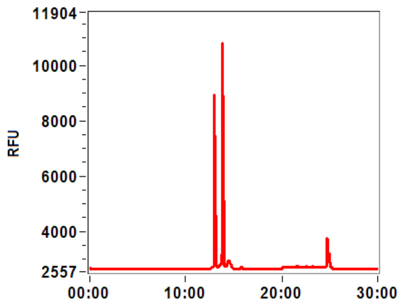

Filename and data path: C:\AATI\Data\2021 08 10\22-31-23\2021 08 10 22H 31M..raw

E2: SampE2

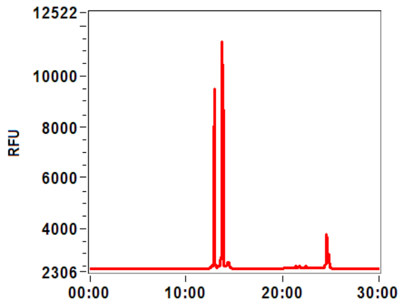

F2: SampF2

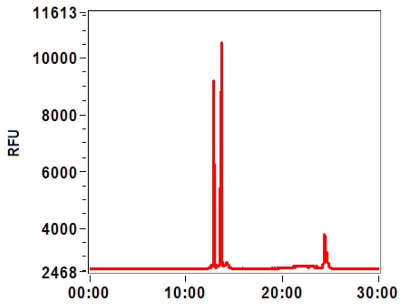

G2: SampG2

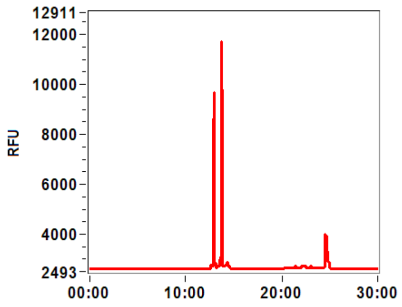

H2: SampH2

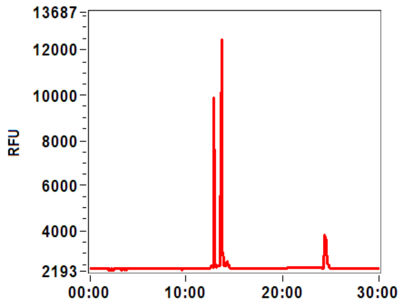

A3: SampA3

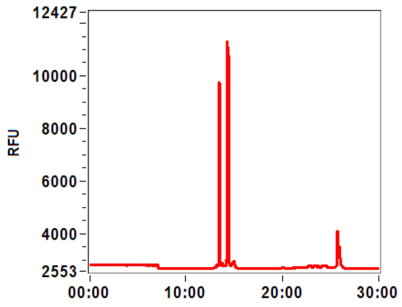

B3: SampB3

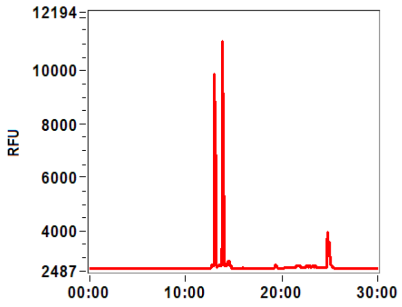

C3: SampC3

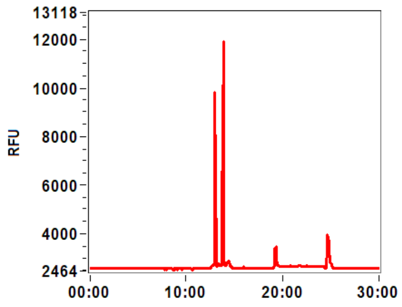

D3: SampD3

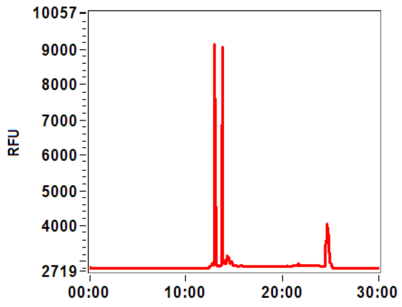

E3: SampE3

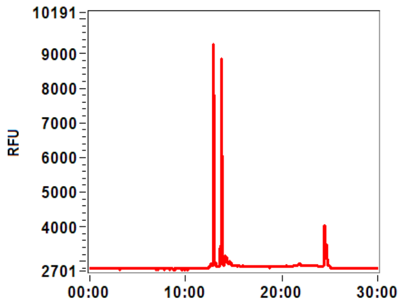

F3: SampF3

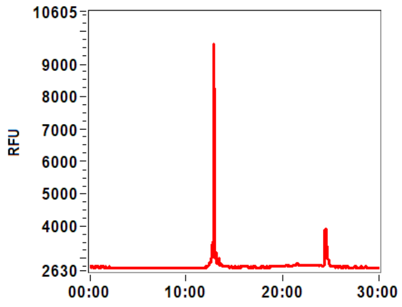

G3: SampG3

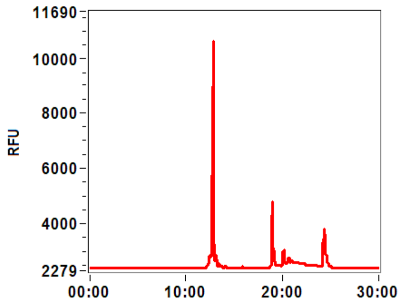

H3: SampH3

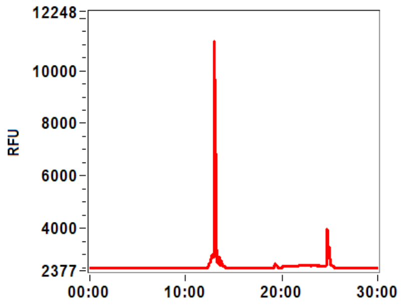

Filename and data path: C:\AATI\Data\2021 08 10\22-31-23\2021 08 10 22H 31M..raw

A4: SampA4

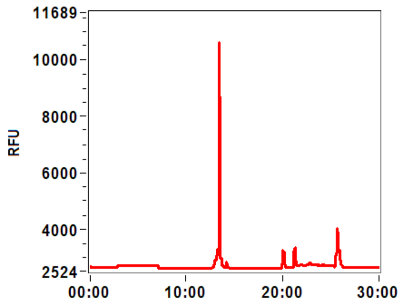

B4: SampB4

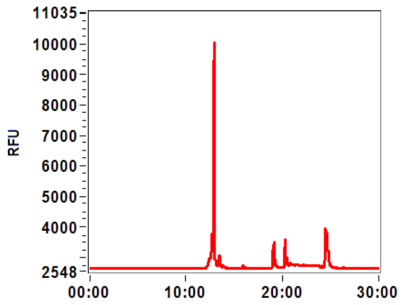

C4: SampC4

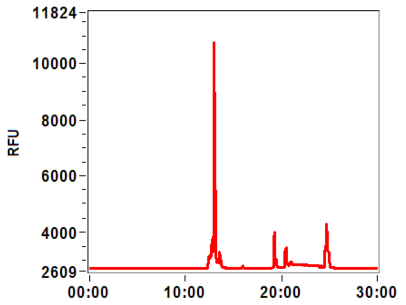

D4: SampD4

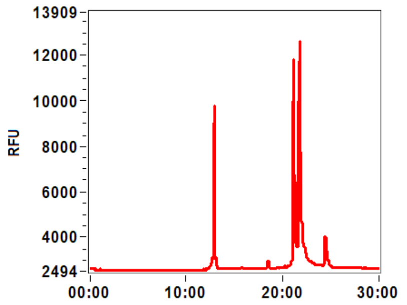

E4: SampE4

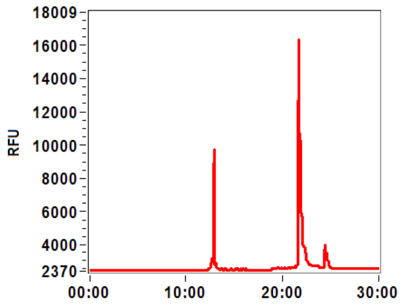

F4: SampF4

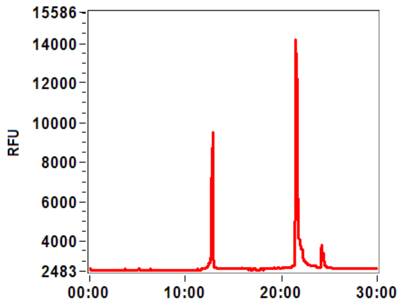

G4: SampG4

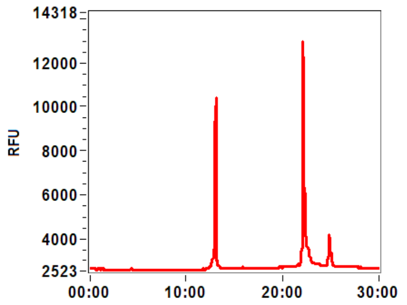

H4: SampH4

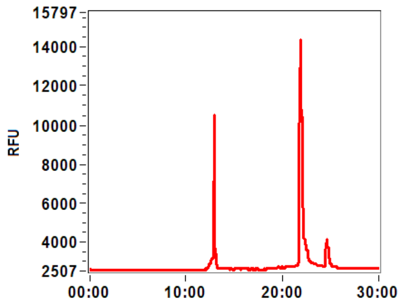

A5: SampA5

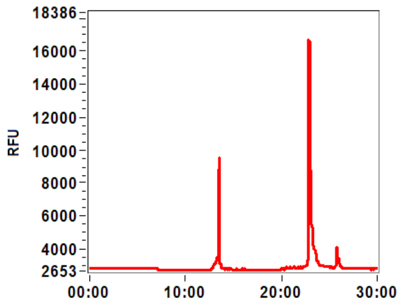

B5: SampB5

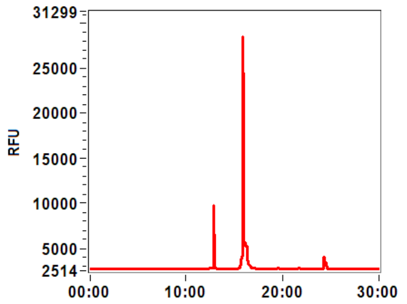

C5: SampC5

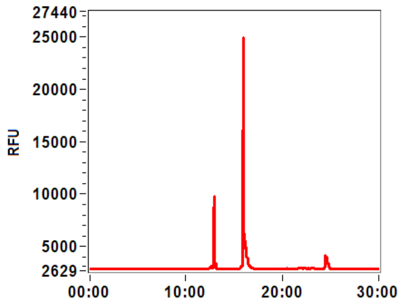

D5: SampD5

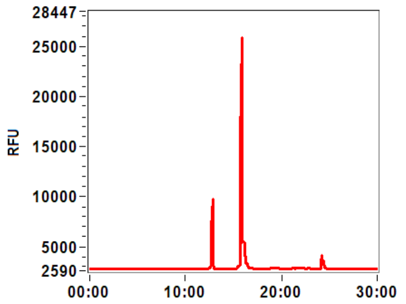

Filename and data path: C:\AATI\Data\2021 08 10\22-31-23\2021 08 10 22H 31M..raw

E5: SampE5

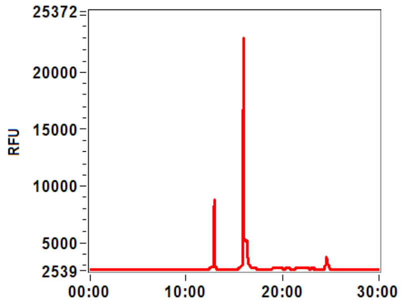

F5: SampF5

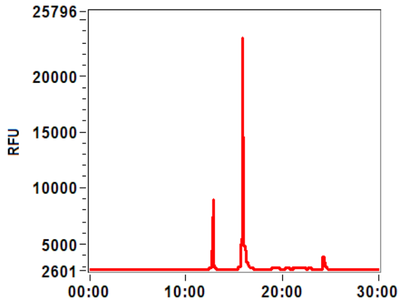

G5: SampG5

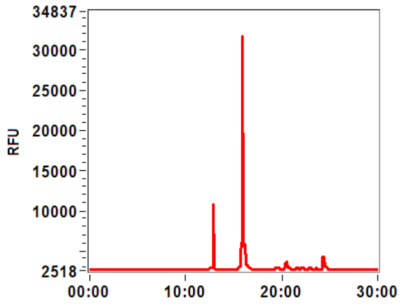

H5: SampH5

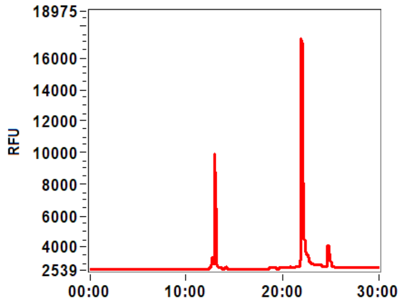

A6: SampA6

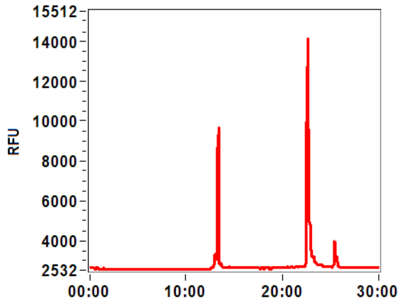

B6: SampB6

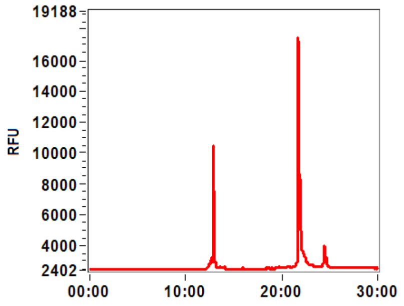

C6: SampC6

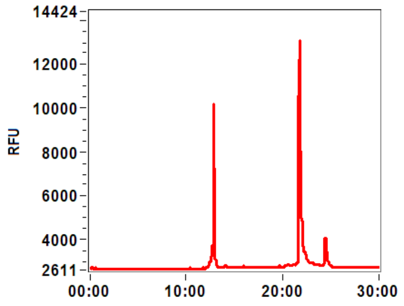

D6: SampD6

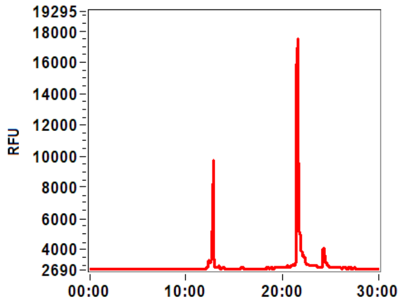

E6: SampE6

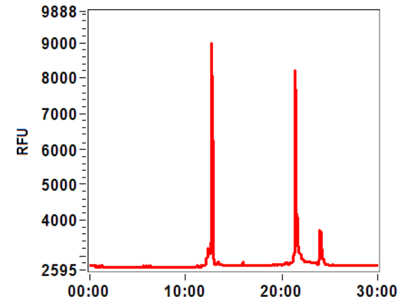

F6: SampF6

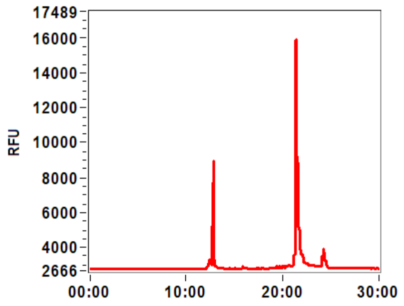

G6: SampG6

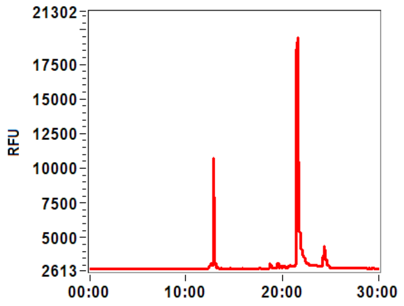

H6: SampH6

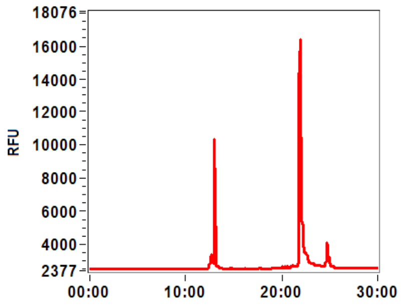

Filename and data path: C:\AATI\Data\2021 08 10\22-31-23\2021 08 10 22H 31M..raw

A7: SampA7

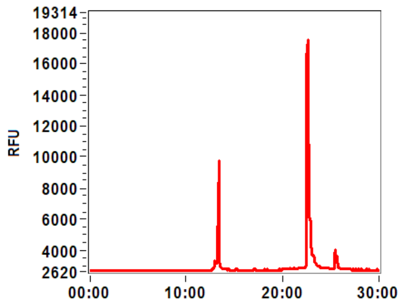

B7: SampB7

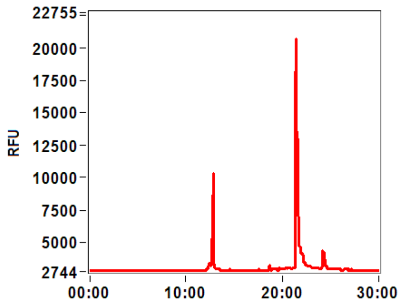

C7: SampC7

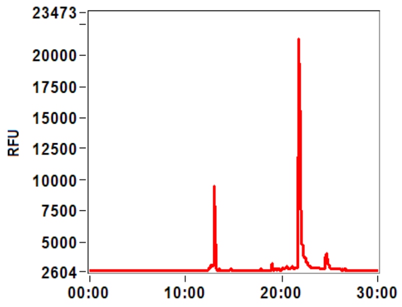

D7: SampD7

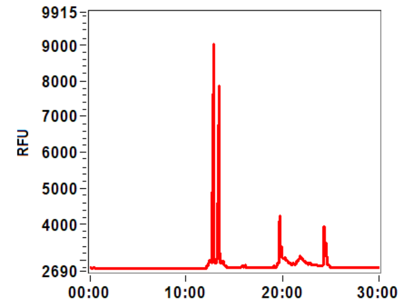

E7: SampE7

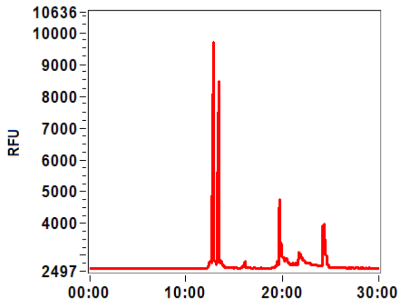

F7: SampF7

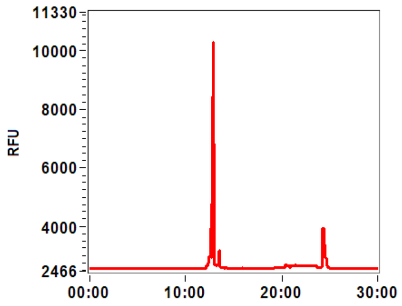

G7: SampG7

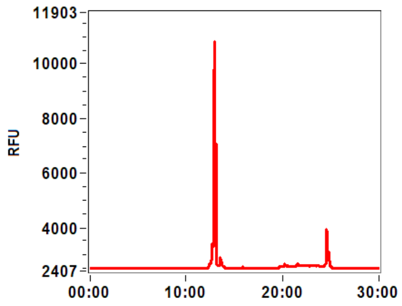

H7: SampH7

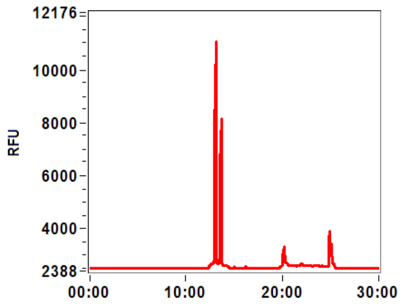

A8: SampA8

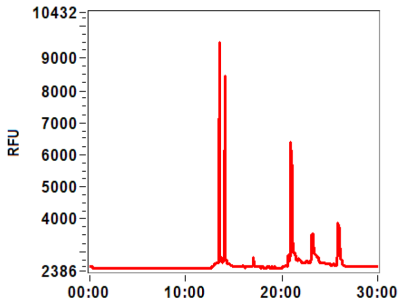

B8: SampB8

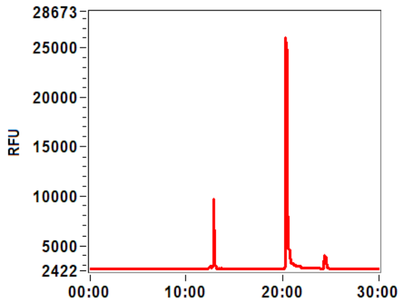

C8: SampC8

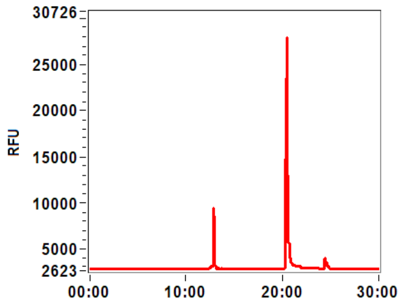

D8: SampD8

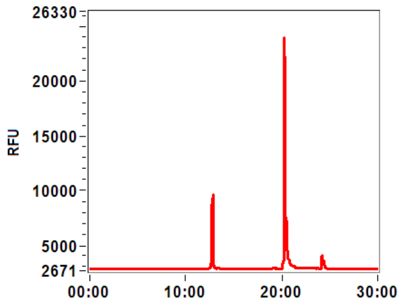

Filename and data path: C:\AATI\Data\2021 08 10\22-31-23\2021 08 10 22H 31M..raw

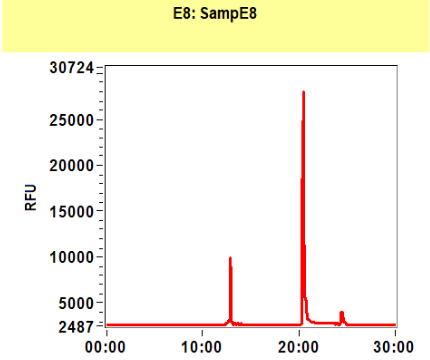

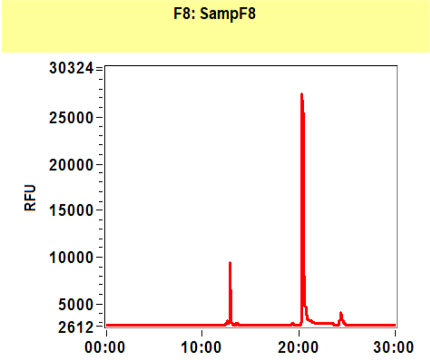

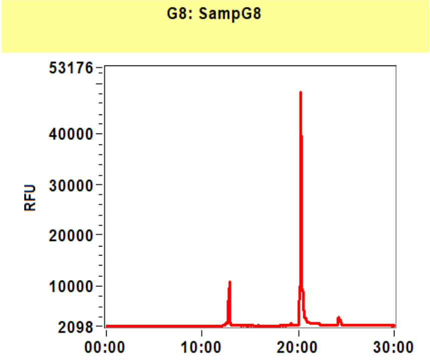

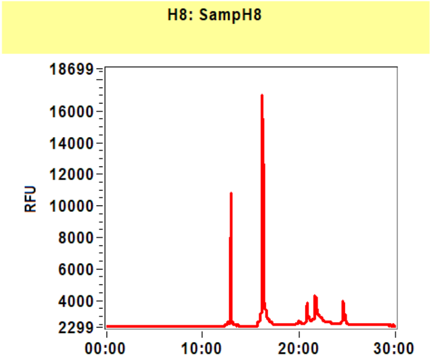

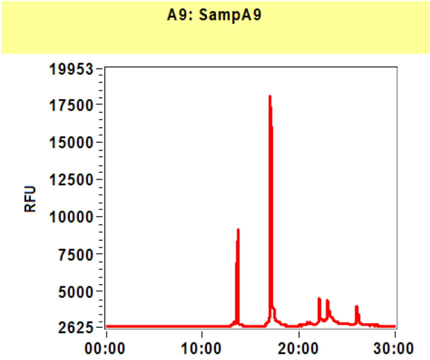

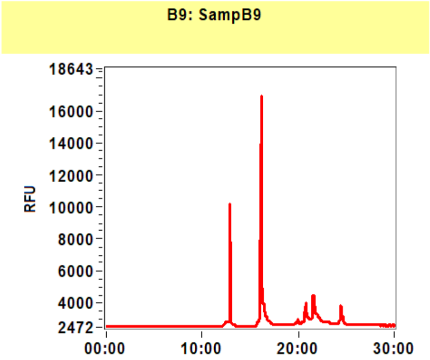

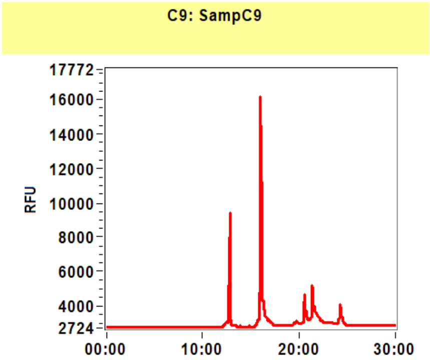

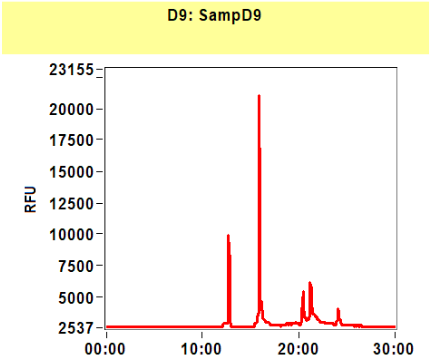

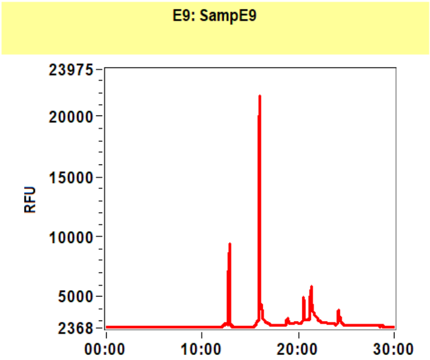

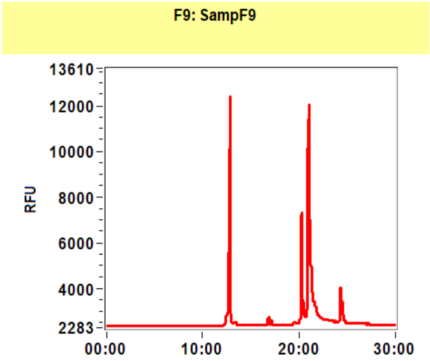

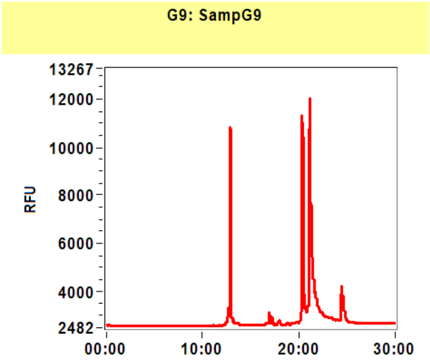

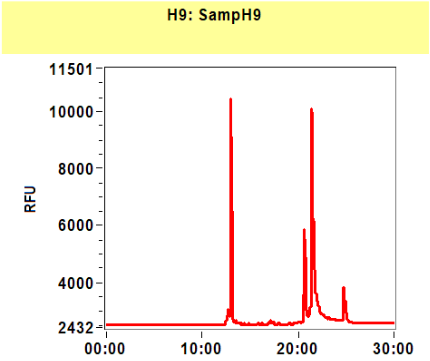

Filename and data path: C:\AATI\Data\2021 08 10\22-31-23\2021 08 10 22H 31M..raw

A10: SampA10

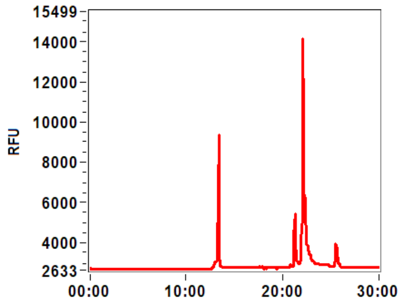

B10: SampB10

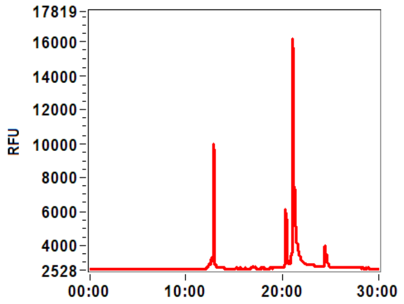

C10: SampC10

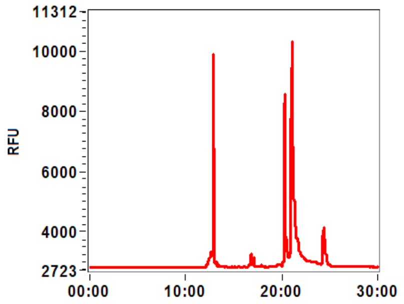

D10: SampD10

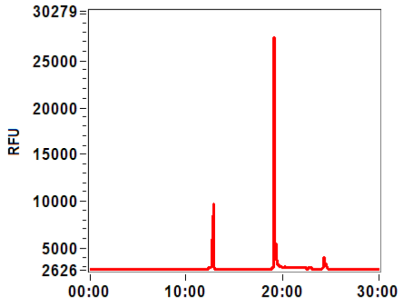

E10: SampE10

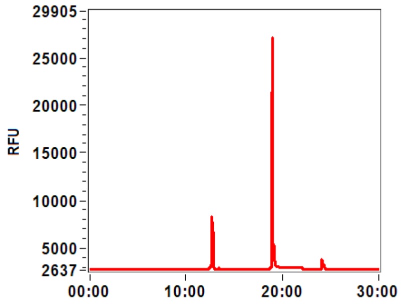

F10: SampF10

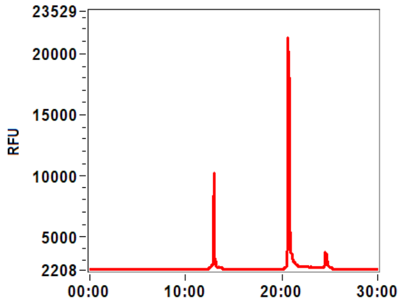

G10: SampG10

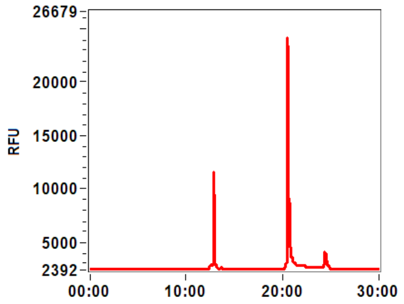

H10: SampH10

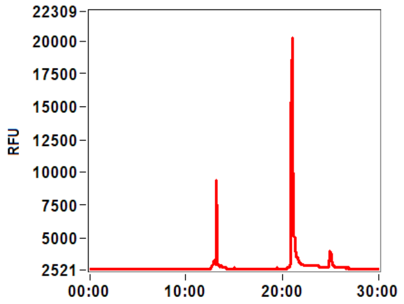

A11: SampA11

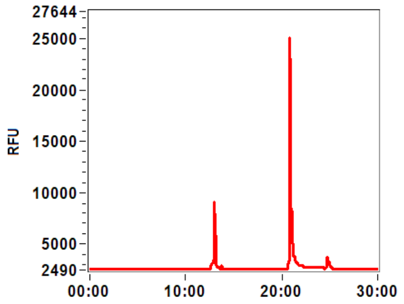

B11: SampB11

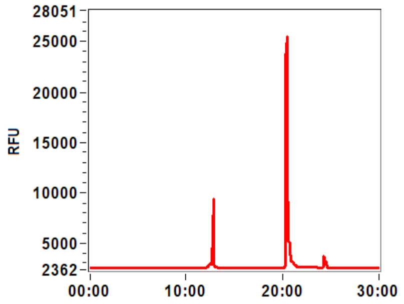

C11: SampC11

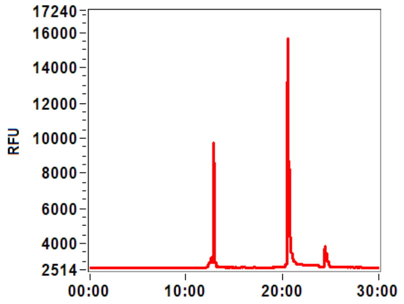

D11: SampD11

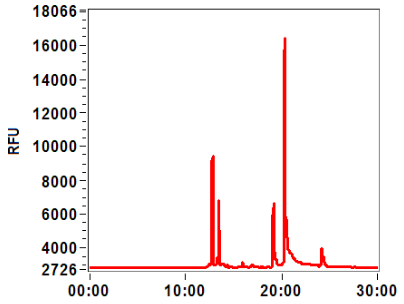

Filename and data path: C:\AATI\Data\2021 08 10\22-31-23\2021 08 10 22H 31M..raw

E11: SampE11

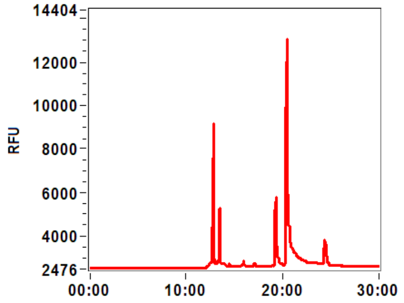

F11: SampF11

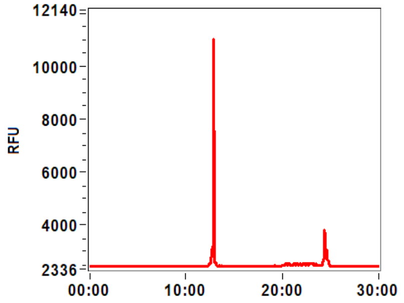

G11: SampG11

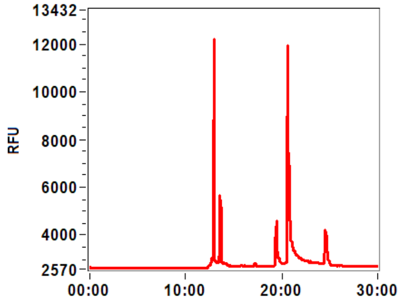

H11: SampH11

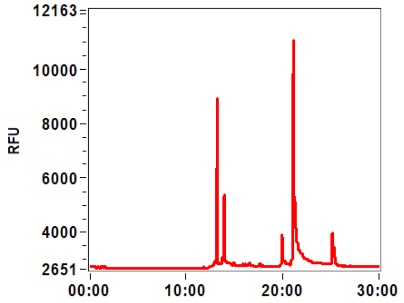

A12: SampA12

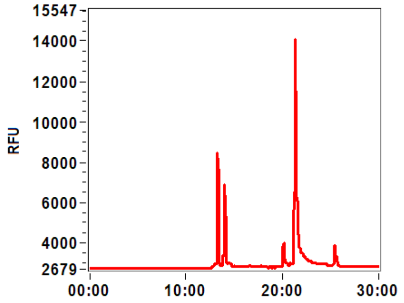

B12: SampB12

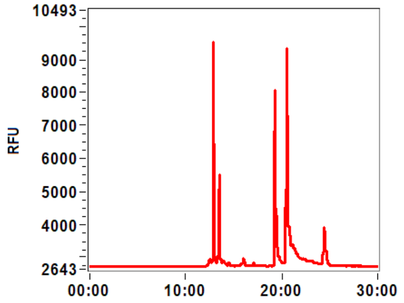

C12: SampC12

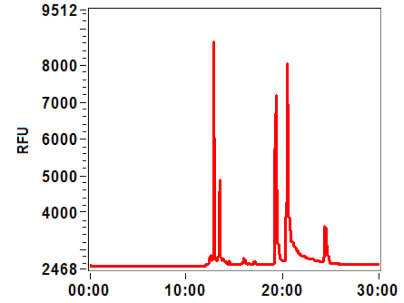

D12: SampD12

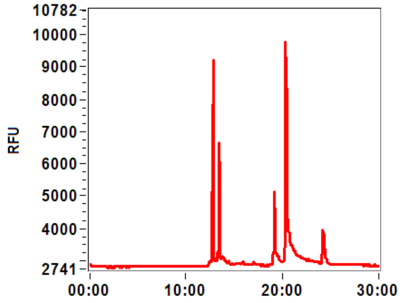

E12: SampE12

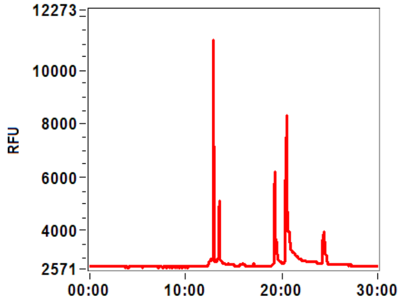

F12: SampF12

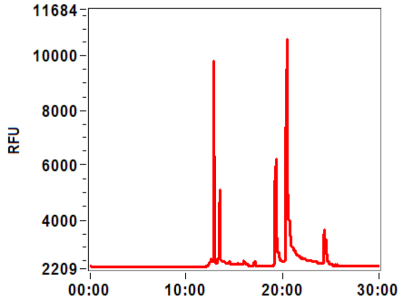

G12: SampG12

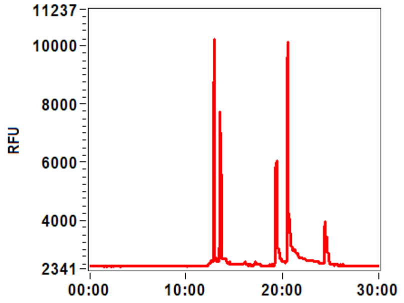

H12: SampH12

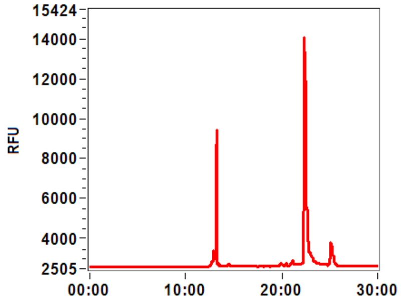

Sample: SampA1
Well location: A1
Created: Tuesday, August 10, 2021 10:41:10 PM

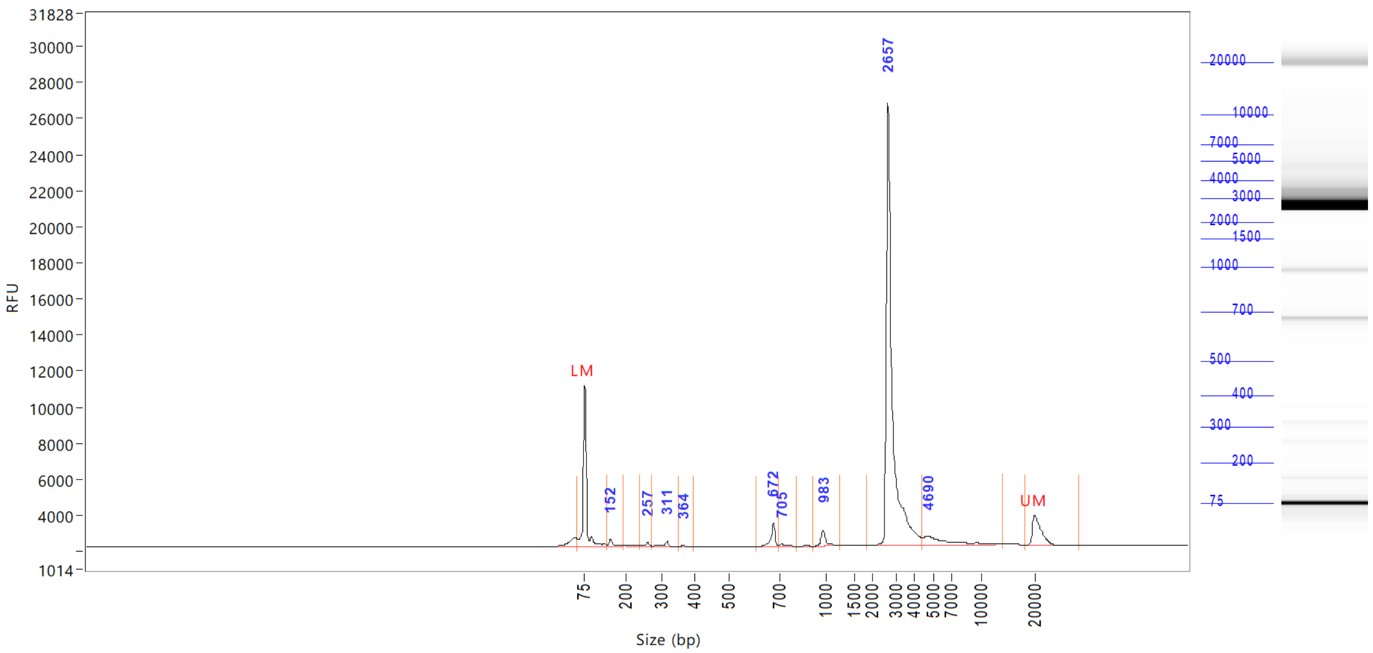

Peak	Size (bp)	Concentration (ng/uL)	From (bp)	To (bp)	Average size (bp)	CV%	RFU	Corrected peak area
1	75 (LM)	0.5000	56	141	76	15.49	8920	62.499
2	152	0.2908	141	192	152	5.09	378	3.029
3	257	0.1277	237	269	254	1.97	242	1.330
4	311	0.2074	269	347	305	3.64	265	2.161
5	364	0.0364	347	396	362	1.83	51	0.379
6	672	0.8860	608	692	665	1.92	1299	9.229
7	705	0.1265	692	809	721	3.81	112	1.318
8	983	0.6602	922	1234	996	4.66	902	6.877
9	2657	20.6489	1846	4414	2839	12.89	24561	215.089
10	4690	1.5086	4414	13903	6492	34.09	503	15.715
11	20000 (UM)	0.1396	18049	28342	20527	4.69	1657	17.444

TIC:	24.4926	ng/uL
TIM:	21.1840	nmole/L
Total concentration:	24.6298	ng/uL

Sample peak width (sec): 5 Sample min peak height: 50 Sample baseline V to V?: Y Sample baseline V to V points: 3
Sample filter: Binomial Number of points for filter: 3 Sample start region (min): 0 Sample end region (min): 30
Marker peak width (sec): 5 Marker min peak height: 200 Marker baseline V to V?: Y Marker baseline V to V points: 3
Lower marker selection: First peak > 500 RFU Upper marker selection: Last peak > 200 RFU
Ladder size (bp)75, 200, 300, 400, 500, 700, 1000, 1500, 2000, 3000, 4000, 5000, 7000, 10000, 20000
Quantification using: Lower Marker Final concentration (ng/uL): 0.5000 Dilution factor: 12.0

Sample: SampB1
Well location: B1
Created: Tuesday, August 10, 2021 10:41:10 PM

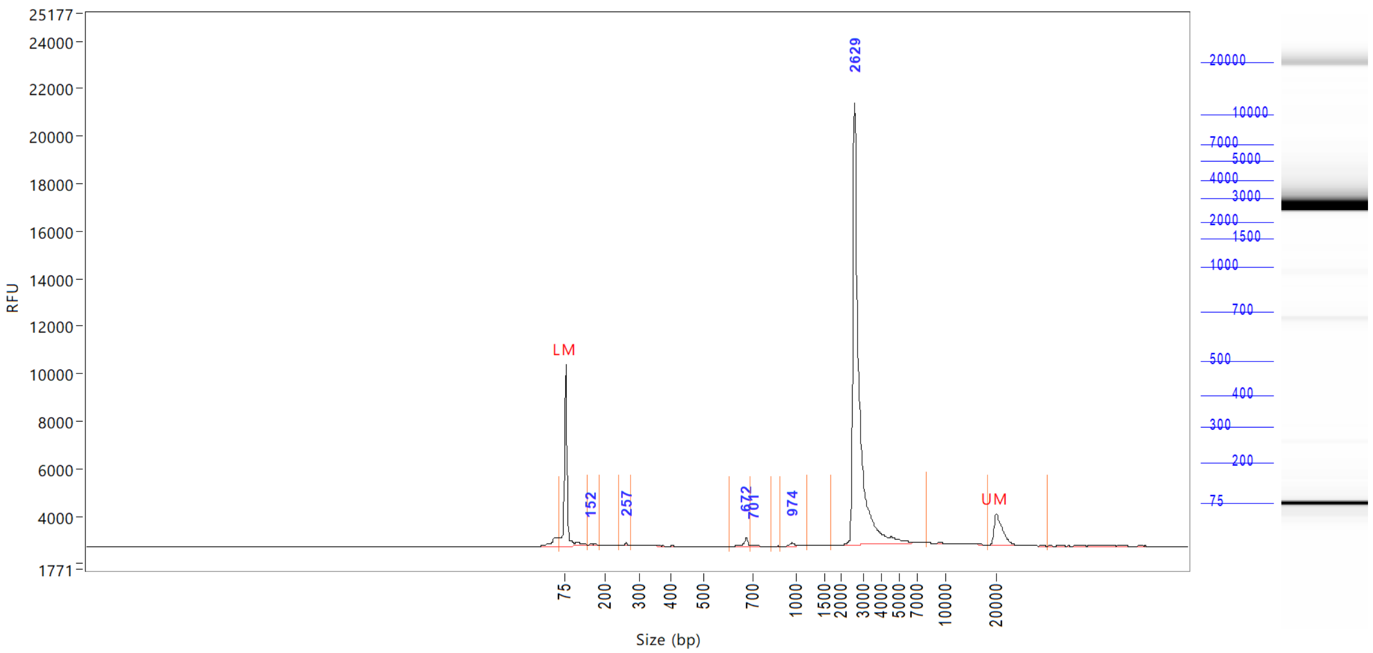

Peak	Size (bp)	Concentration (ng/uL)	From (bp)	To (bp)	Average size (bp)	CV%	RFU	Corrected peak area
1	75 (LM)	0.5000	58	143	76	14.77	7649	51.594
2	152	0.1641	143	179	158	6.00	103	1.411
3	257	0.0742	235	271	254	1.79	118	0.638
4	672	0.3605	603	692	663	2.36	368	3.100
5	701	0.0821	692	831	727	4.78	57	0.706
6	974	0.1541	896	1190	986	4.43	135	1.325
7	2629	20.7595	1712	7934	2841	18.35	18630	178.511
8	20000 (UM)	0.1346	18171	29914	20620	5.29	1316	13.886

TIC:	21.5945	ng/uL
TIM:	15.5547	nmole/L
Total concentration:	21.8262	ng/uL

```
Sample peak width (sec): 5    Sample min peak height: 50    Sample baseline V to V?: Y    Sample baseline V to V points: 3
Sample filter: Binomial       Number of points for filter: 3  Sample start region (min): 0  Sample end region (min): 30
Marker peak width (sec): 5    Marker min peak height: 200   Marker baseline V to V?: Y    Marker baseline V to V points: 3
Lower marker selection: First peak > 500 RFU                Upper marker selection: Last peak > 200 RFU
Ladder size (bp)75, 200, 300, 400, 500, 700, 1000, 1500, 2000, 3000, 4000, 5000, 7000, 10000, 20000
Quantification using: Lower Marker          Final concentration (ng/uL): 0.5000          Dilution factor: 12.0
```

Sample: SampC1
Well location: C1
Created: Tuesday, August 10, 2021 10:41:10 PM

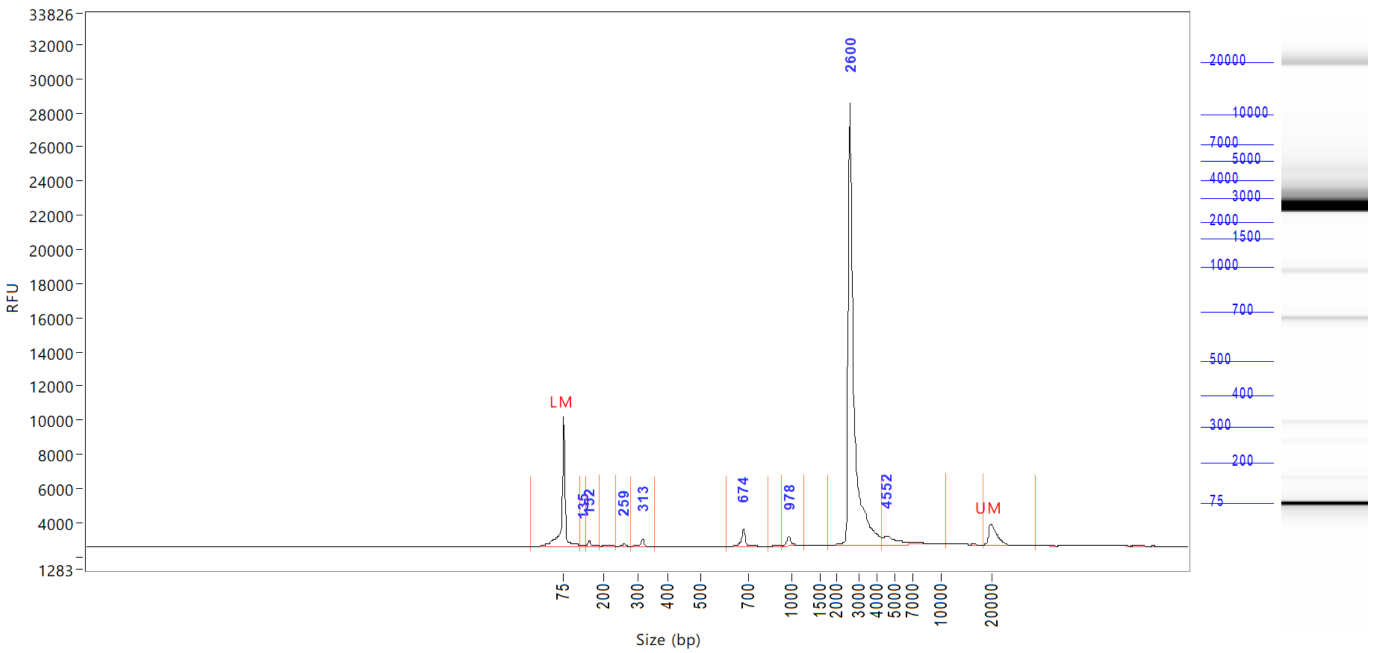

Peak	Size (bp)	Concentration (ng/uL)	From (bp)	To (bp)	Average size (bp)	CV%	RFU	Corrected peak area
1	75 (LM)	0.5000	0	126	68	24.85	7569	62.361
2	135	0.0956	126	143	134	4.05	88	0.994
3	152	0.2657	143	187	156	6.40	301	2.761
4	259	0.1031	235	278	257	2.53	179	1.071
5	313	0.3148	278	354	309	3.23	450	3.272
6	674	0.7564	603	831	672	3.04	1002	7.862
7	978	0.4299	931	1223	988	3.92	571	4.468
8	2600	22.2926	1712	4311	2762	12.86	25939	231.698
9	4552	1.2616	4311	10977	5710	26.95	532	13.113
10	20000 (UM)	0.1047	18415	28705	20596	4.78	1234	13.054

TIC:	25.5197	ng/uL
TIM:	22.5263	nmole/L
Total concentration:	25.6465	ng/uL

Sample peak width (sec): 5   Sample min peak height: 50   Sample baseline V to V?: Y   Sample baseline V to V points: 3
Sample filter: Binomial   Number of points for filter: 3   Sample start region (min): 0   Sample end region (min): 30
Marker peak width (sec): 5   Marker min peak height: 200   Marker baseline V to V?: Y   Marker baseline V to V points: 3
Lower marker selection: First peak > 500 RFU   Upper marker selection: Last peak > 200 RFU
Ladder size (bp)75, 200, 300, 400, 500, 700, 1000, 1500, 2000, 3000, 4000, 5000, 7000, 10000, 20000
Quantification using: Lower Marker   Final concentration (ng/uL): 0.5000   Dilution factor: 12.0

Sample: SampD1
Well location: D1
Created: Tuesday, August 10, 2021 10:41:10 PM

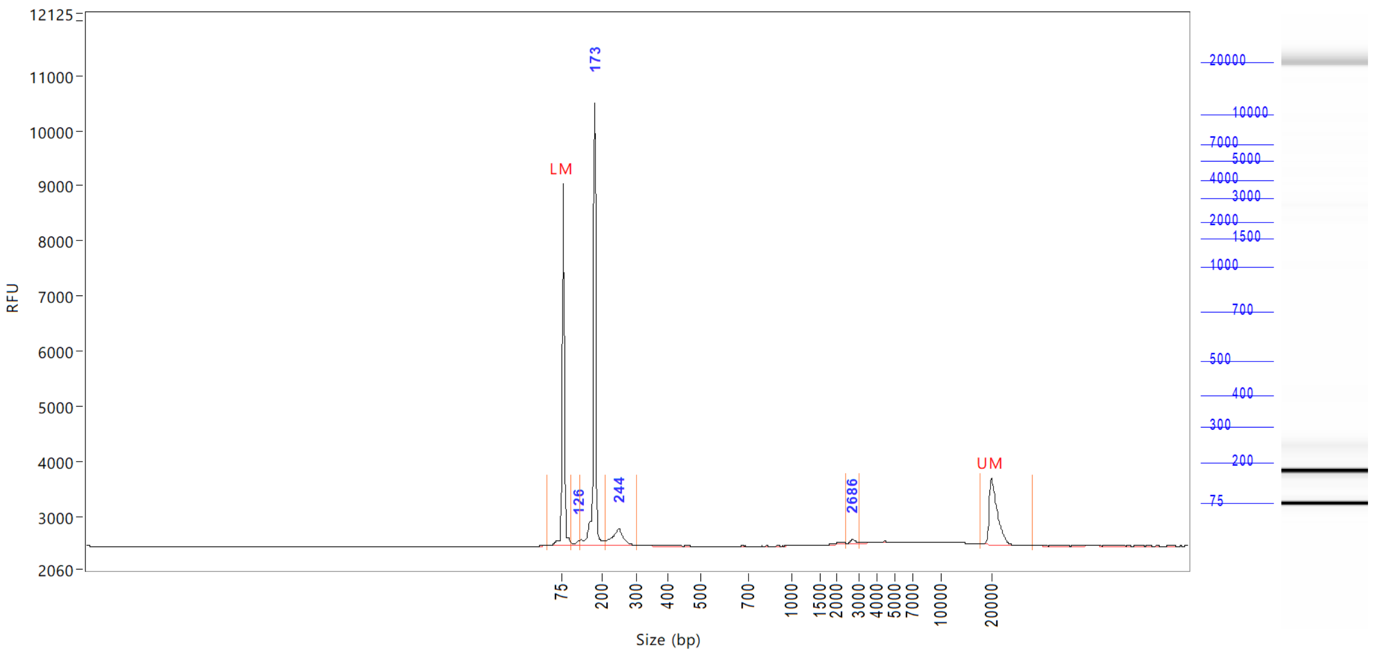

Peak	Size (bp)	Concentration (ng/uL)	From (bp)	To (bp)	Average size (bp)	CV%	RFU	Corrected peak area
1	75 (LM)	0.5000	28	101	73	8.02	6566	37.471
2	126	0.1845	101	128	117	6.91	90	1.152
3	173	8.1821	128	205	169	5.15	8030	51.099
4	244	1.2252	205	298	239	7.33	308	7.652
5	2686	0.1031	2401	3000	2691	4.94	78	0.644
6	20000 (UM)	0.1689	17927	28221	20558	4.96	1199	12.656

TIC:	9.6949	ng/uL
TIM:	90.6701	nmole/L
Total concentration:	10.1479	ng/uL

Sample peak width (sec): 5   Sample min peak height: 50   Sample baseline V to V?: Y   Sample baseline V to V points: 3
Sample filter: Binomial   Number of points for filter: 3 Sample start region (min): 0 Sample end region (min): 30
Marker peak width (sec): 5   Marker min peak height: 200   Marker baseline V to V?: Y   Marker baseline V to V points: 3
Lower marker selection: First peak > 500 RFU   Upper marker selection: Last peak > 200 RFU
Ladder size (bp)75, 200, 300, 400, 500, 700, 1000, 1500, 2000, 3000, 4000, 5000, 7000, 10000, 20000
Quantification using: Lower Marker   Final concentration (ng/uL): 0.5000   Dilution factor: 12.0

Sample: SampE1
Well location: E1
Created: Tuesday, August 10, 2021 10:41:10 PM

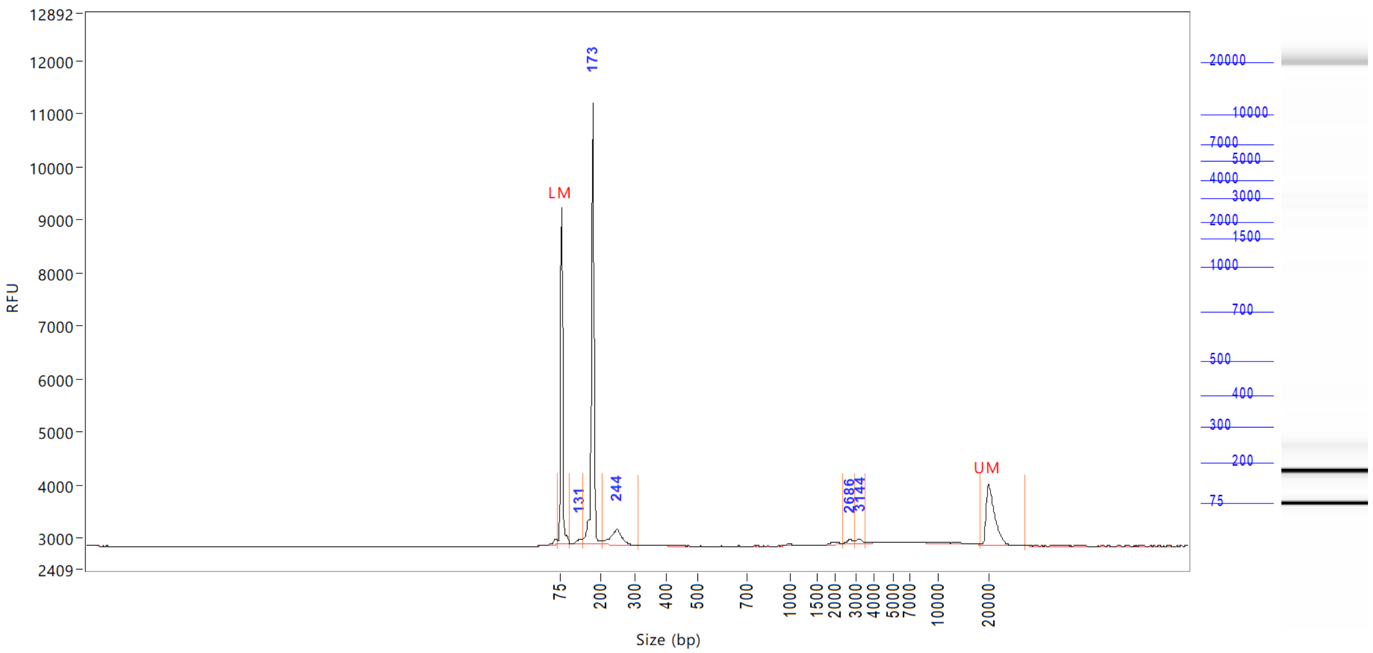

Peak	Size (bp)	Concentration (ng/uL)	From (bp)	To (bp)	Average size (bp)	CV%	RFU	Corrected peak area
1	75 (LM)	0.5000	64	103	74	5.58	6334	35.602
2	131	0.1928	103	141	129	6.45	83	1.144
3	173	8.6124	141	201	170	3.82	8330	51.103
4	244	1.2498	201	309	239	7.34	314	7.416
5	2686	0.1306	2401	2914	2685	4.83	81	0.775
6	3144	0.1397	2914	3536	3128	4.56	92	0.829
7	20000 (UM)	0.1744	18537	27375	20605	4.79	1152	12.417

TIC:	10.3253	ng/uL
TIM:	94.4880	nmole/L
Total concentration:	10.9811	ng/uL

Sample peak width (sec): 5   Sample min peak height: 50   Sample baseline V to V?: Y   Sample baseline V to V points: 3
Sample filter: Binomial   Number of points for filter: 3 Sample start region (min): 0 Sample end region (min): 30
Marker peak width (sec): 5   Marker min peak height: 200   Marker baseline V to V?: Y   Marker baseline V to V points: 3
Lower marker selection: First peak > 500 RFU   Upper marker selection: Last peak > 200 RFU
Ladder size (bp)75, 200, 300, 400, 500, 700, 1000, 1500, 2000, 3000, 4000, 5000, 7000, 10000, 20000
Quantification using: Lower Marker   Final concentration (ng/uL): 0.5000   Dilution factor: 12.0

Sample: SampF1
Well location: F1
Created: Tuesday, August 10, 2021 10:41:10 PM

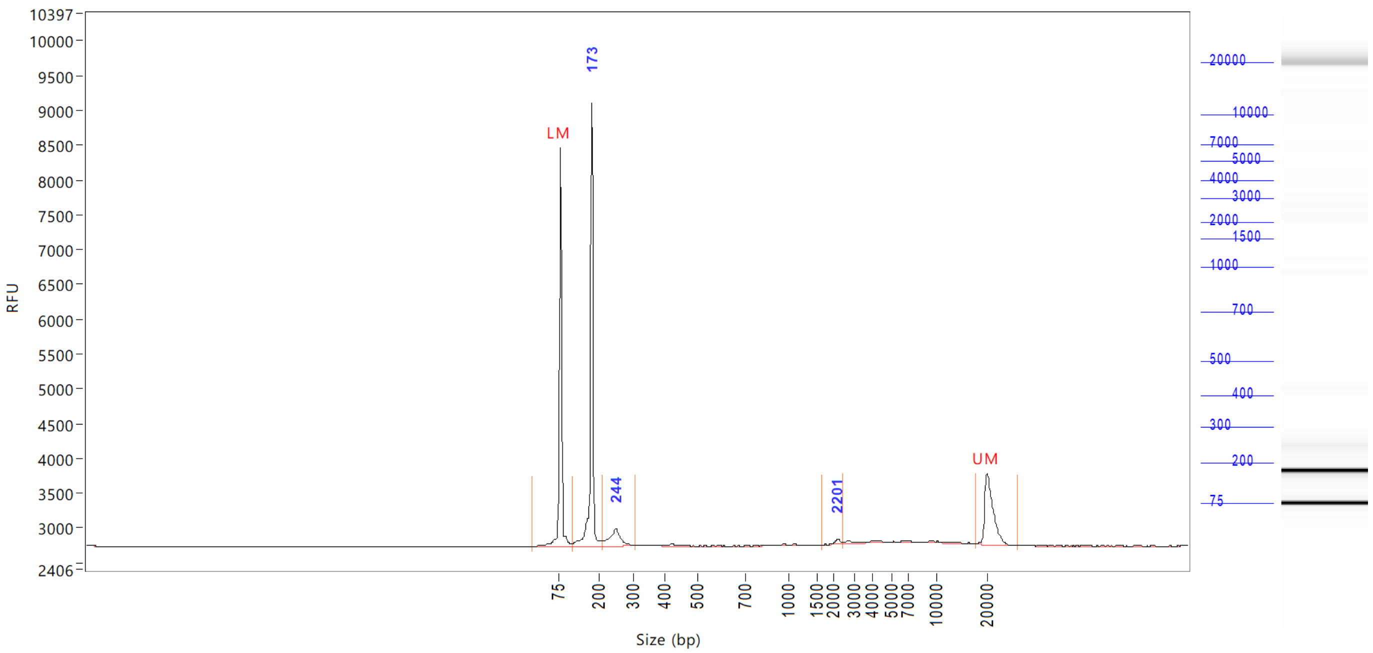

Peak	Size	Concentration	From	To	Average size	CV%	RFU	Corrected peak area
	(bp)	(ng/uL)	(bp)	(bp)	(bp)			
1	75 (LM)	0.5000	0	112	73	13.27	5740	35.853
2	173	6.8777	112	206	169	6.45	6383	41.098
3	244	0.9889	206	301	240	7.31	249	5.909
4	2201	0.1069	1674	2458	2173	6.64	74	0.639
5	20000 (UM)	0.1542	17805	25924	20537	4.85	1028	11.054

TIC:	7.9735	ng/uL
TIM:	73.7556	nmole/L
Total concentration:	8.5981	ng/uL

```
Sample peak width (sec): 5    Sample min peak height: 50    Sample baseline V to V?: Y    Sample baseline V to V points: 3
Sample filter: Binomial       Number of points for filter: 3  Sample start region (min): 0  Sample end region (min): 30
Marker peak width (sec): 5    Marker min peak height: 200   Marker baseline V to V?: Y    Marker baseline V to V points: 3
Lower marker selection: First peak > 500 RFU                Upper marker selection: Last peak > 200 RFU
Ladder size (bp)75, 200, 300, 400, 500, 700, 1000, 1500, 2000, 3000, 4000, 5000, 7000, 10000, 20000
Quantification using: Lower Marker          Final concentration (ng/uL): 0.5000          Dilution factor: 12.0
```

Sample: SampG1
Well location: G1
Created: Tuesday, August 10, 2021 10:41:10 PM

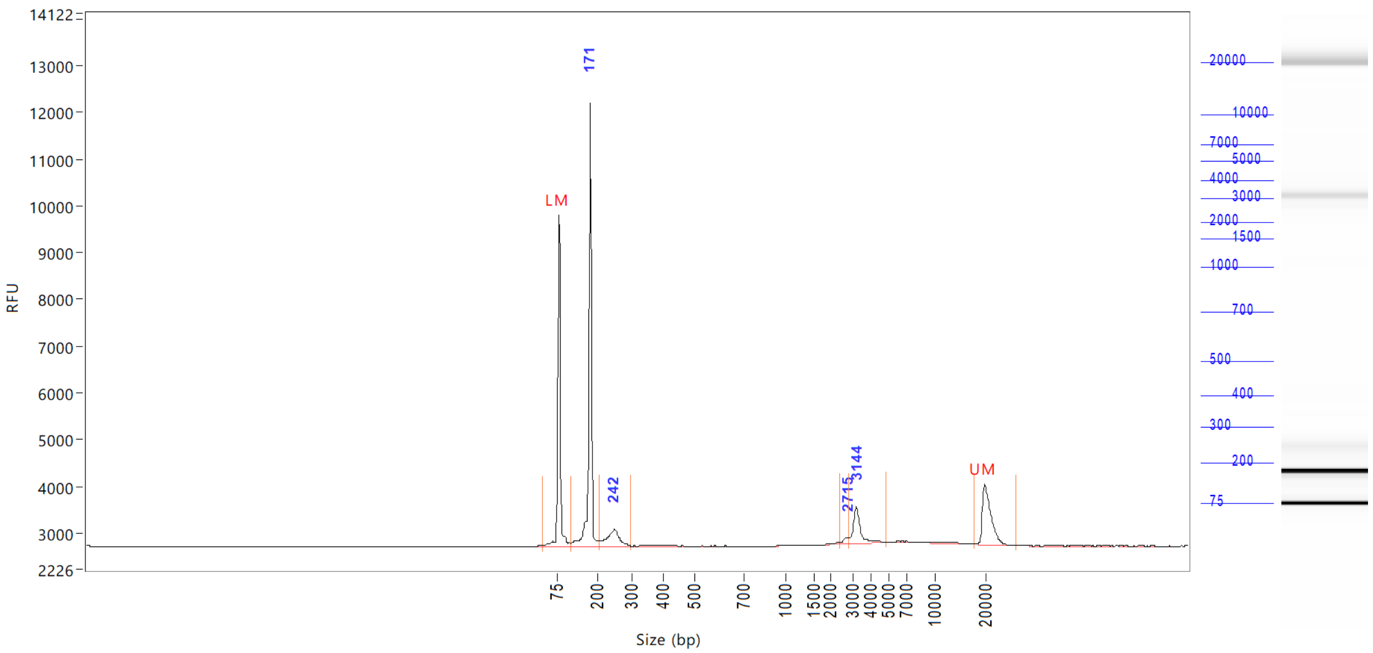

Peak	Size (bp)	Concentration (ng/uL)	From (bp)	To (bp)	Average size (bp)	CV%	RFU	Corrected peak area
1	75 (LM)	0.5000	28	112	74	10.68	7081	42.795
2	171	8.4944	112	201	168	6.40	9501	60.587
3	242	1.3245	201	296	238	7.70	362	9.447
4	2715	0.1708	2401	2829	2657	4.35	136	1.218
5	3144	1.1711	2829	4828	3279	11.36	790	8.353
6	20000 (UM)	0.1664	17927	26287	20586	4.88	1306	14.245

TIC:	11.1608	ng/uL
TIM:	92.9610	nmole/L
Total concentration:	11.7021	ng/uL

```
Sample peak width (sec): 5    Sample min peak height: 50    Sample baseline V to V?: Y    Sample baseline V to V points: 3
Sample filter: Binomial       Number of points for filter: 3  Sample start region (min): 0  Sample end region (min): 30
Marker peak width (sec): 5    Marker min peak height: 200   Marker baseline V to V?: Y    Marker baseline V to V points: 3
Lower marker selection: First peak > 500 RFU                Upper marker selection: Last peak > 200 RFU
Ladder size (bp)75, 200, 300, 400, 500, 700, 1000, 1500, 2000, 3000, 4000, 5000, 7000, 10000, 20000
Quantification using: Lower Marker          Final concentration (ng/uL): 0.5000          Dilution factor: 12.0
```

Sample: SampH1
Well location: H1
Created: Tuesday, August 10, 2021 10:41:10 PM

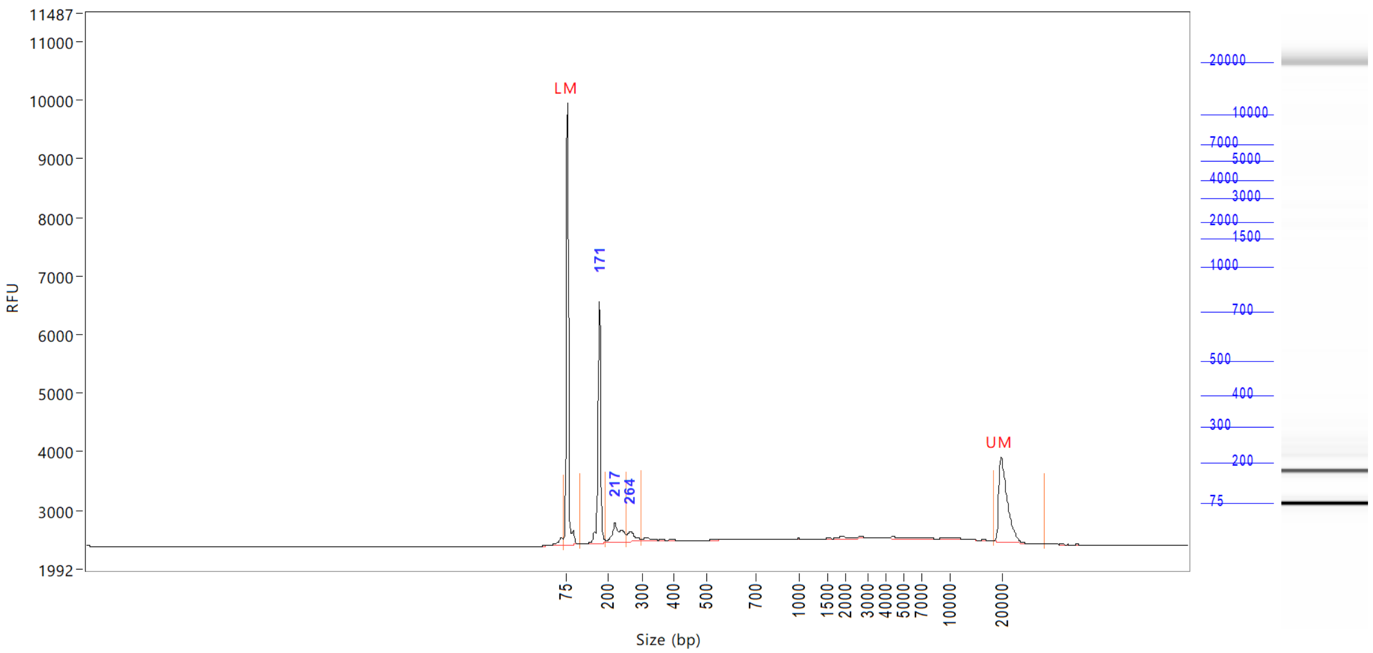

Peak	Size<br>(bp)	Concentration<br>(ng/uL)	From<br>(bp)	To<br>(bp)	Average size<br>(bp)	CV%	RFU	Corrected peak area
1	75 (LM)	0.5000	64	114	73	6.32	7560	42.665
2	171	3.4368	114	194	169	3.72	4130	24.439
3	217	0.9170	194	253	225	6.45	335	6.521
4	264	0.3924	253	295	268	4.00	178	2.790
5	20000 (UM)	0.1813	18659	28342	20569	4.73	1460	15.472

TIC:	4.7462	ng/uL
TIM:	42.5399	nmole/L
Total concentration:	5.4192	ng/uL

```
Sample peak width (sec): 5    Sample min peak height: 50    Sample baseline V to V?: Y    Sample baseline V to V points: 3
Sample filter: Binomial       Number of points for filter: 3  Sample start region (min): 0  Sample end region (min): 30
Marker peak width (sec): 5    Marker min peak height: 200   Marker baseline V to V?: Y    Marker baseline V to V points: 3
Lower marker selection: First peak > 500 RFU                Upper marker selection: Last peak > 200 RFU
Ladder size (bp)75, 200, 300, 400, 500, 700, 1000, 1500, 2000, 3000, 4000, 5000, 7000, 10000, 20000
Quantification using: Lower Marker          Final concentration (ng/uL): 0.5000          Dilution factor: 12.0
```

Sample: SampA2
Well location: A2
Created: Tuesday, August 10, 2021 10:41:10 PM

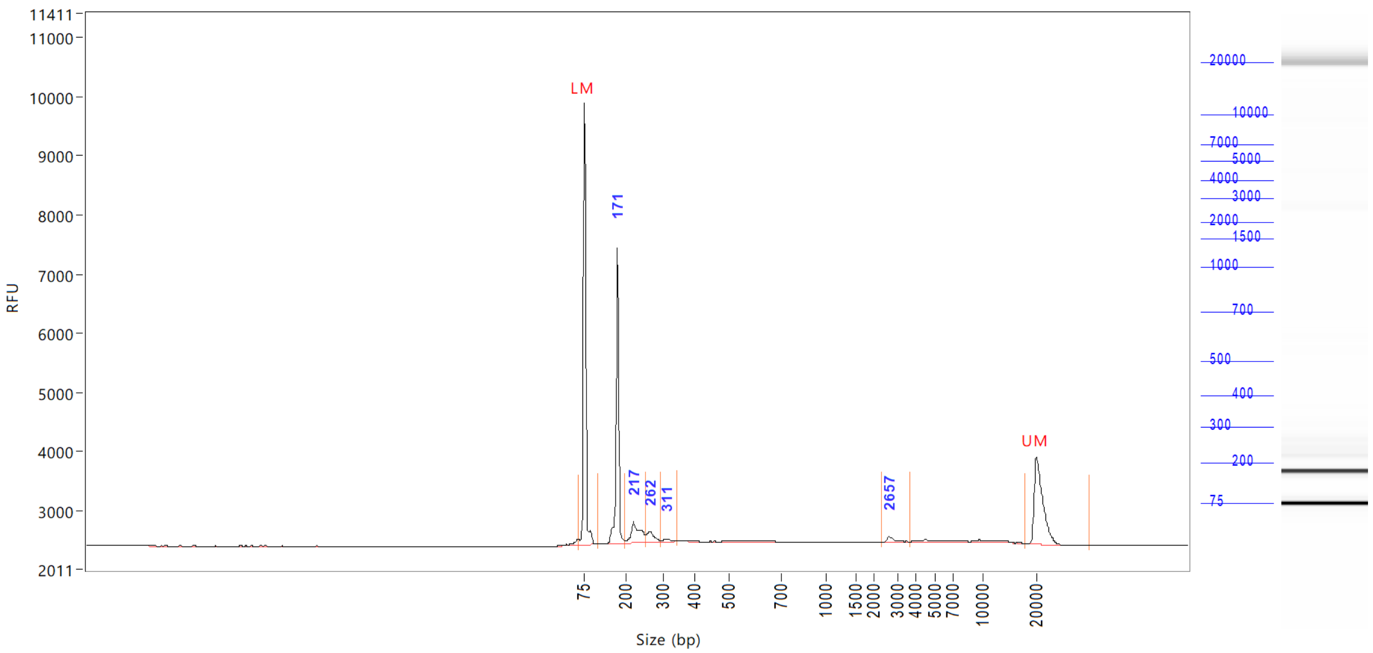

Peak	Size<br>(bp)	Concentration<br>(ng/uL)	From<br>(bp)	To<br>(bp)	Average size<br>(bp)	CV%	RFU	Corrected peak area
1	75 (LM)	0.5000	60	116	73	6.25	7484	41.391
2	171	4.2818	116	192	168	3.51	5006	29.538
3	217	0.9894	192	251	224	6.33	357	6.825
4	262	0.4123	251	293	265	3.83	183	2.844
5	311	0.1127	293	345	309	3.30	55	0.777
6	2657	0.1807	2315	3715	2819	10.87	96	1.247
7	20000 (UM)	0.1879	17683	29672	20540	5.14	1487	15.555

TIC:	5.9769	ng/uL
TIM:	52.4236	nmole/L
Total concentration:	6.5819	ng/uL

Sample peak width (sec): 5   Sample min peak height: 50   Sample baseline V to V?: Y   Sample baseline V to V points: 3
Sample filter: Binomial   Number of points for filter: 3 Sample start region (min): 0 Sample end region (min): 30
Marker peak width (sec): 5   Marker min peak height: 200   Marker baseline V to V?: Y   Marker baseline V to V points: 3
Lower marker selection: First peak > 500 RFU   Upper marker selection: Last peak > 200 RFU
Ladder size (bp)75, 200, 300, 400, 500, 700, 1000, 1500, 2000, 3000, 4000, 5000, 7000, 10000, 20000
Quantification using: Lower Marker   Final concentration (ng/uL): 0.5000   Dilution factor: 12.0

Sample: SampB2
Well location: B2
Created: Tuesday, August 10, 2021 10:41:10 PM

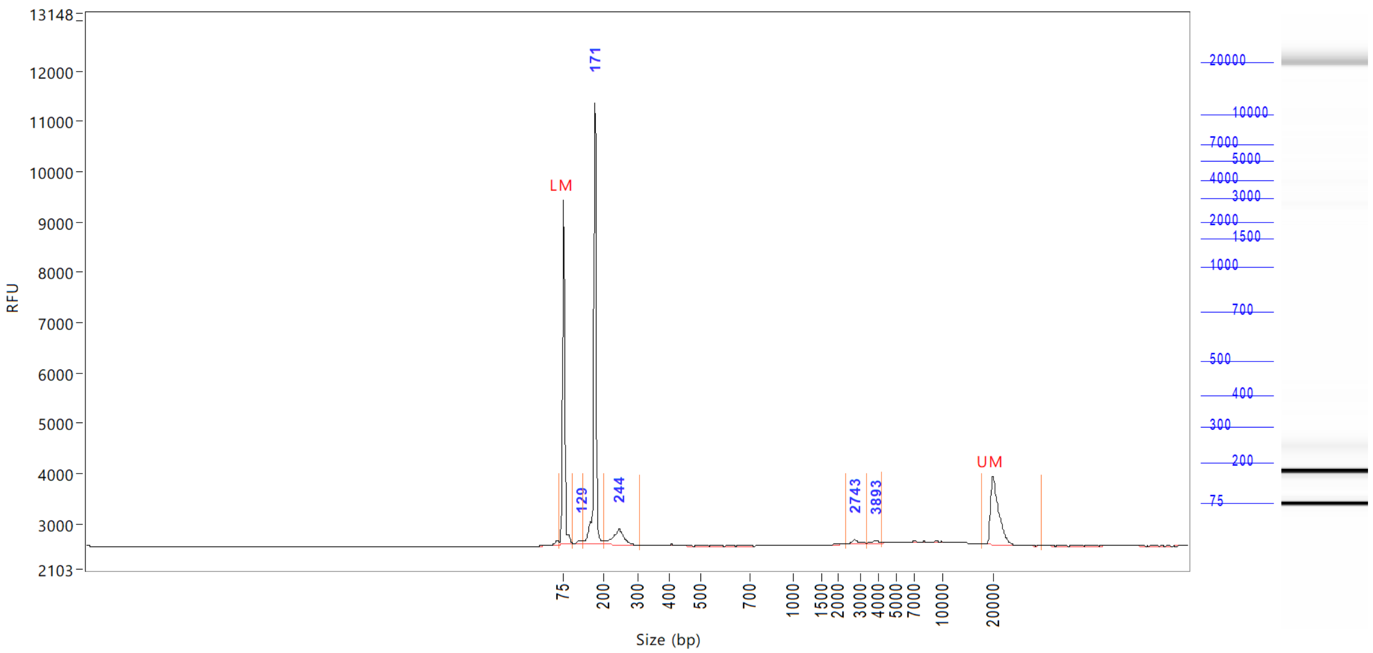

Peak	Size (bp)	Concentration (ng/uL)	From (bp)	To (bp)	Average size (bp)	CV%	RFU	Corrected peak area
1	75 (LM)	0.5000	62	101	73	5.79	6846	39.903
2	129	0.1329	101	135	124	6.51	70	0.884
3	171	7.8477	135	201	169	4.06	8777	52.191
4	244	1.0992	201	303	239	7.31	308	7.310
5	2743	0.1336	2344	3358	2793	7.11	85	0.889
6	3893	0.0891	3358	4173	3806	5.10	58	0.593
7	20000 (UM)	0.1801	17805	29551	20599	5.13	1355	14.370

TIC:	9.3025	ng/uL
TIM:	85.7744	nmole/L
Total concentration:	9.6996	ng/uL

Sample peak width (sec): 5 Sample min peak height: 50 Sample baseline V to V?: Y Sample baseline V to V points: 3
Sample filter: Binomial Number of points for filter: 3 Sample start region (min): 0 Sample end region (min): 30
Marker peak width (sec): 5 Marker min peak height: 200 Marker baseline V to V?: Y Marker baseline V to V points: 3
Lower marker selection: First peak > 500 RFU Upper marker selection: Last peak > 200 RFU
Ladder size (bp)75, 200, 300, 400, 500, 700, 1000, 1500, 2000, 3000, 4000, 5000, 7000, 10000, 20000
Quantification using: Lower Marker Final concentration (ng/uL): 0.5000 Dilution factor: 12.0

Sample: SampC2
Well location: C2
Created: Tuesday, August 10, 2021 10:41:10 PM

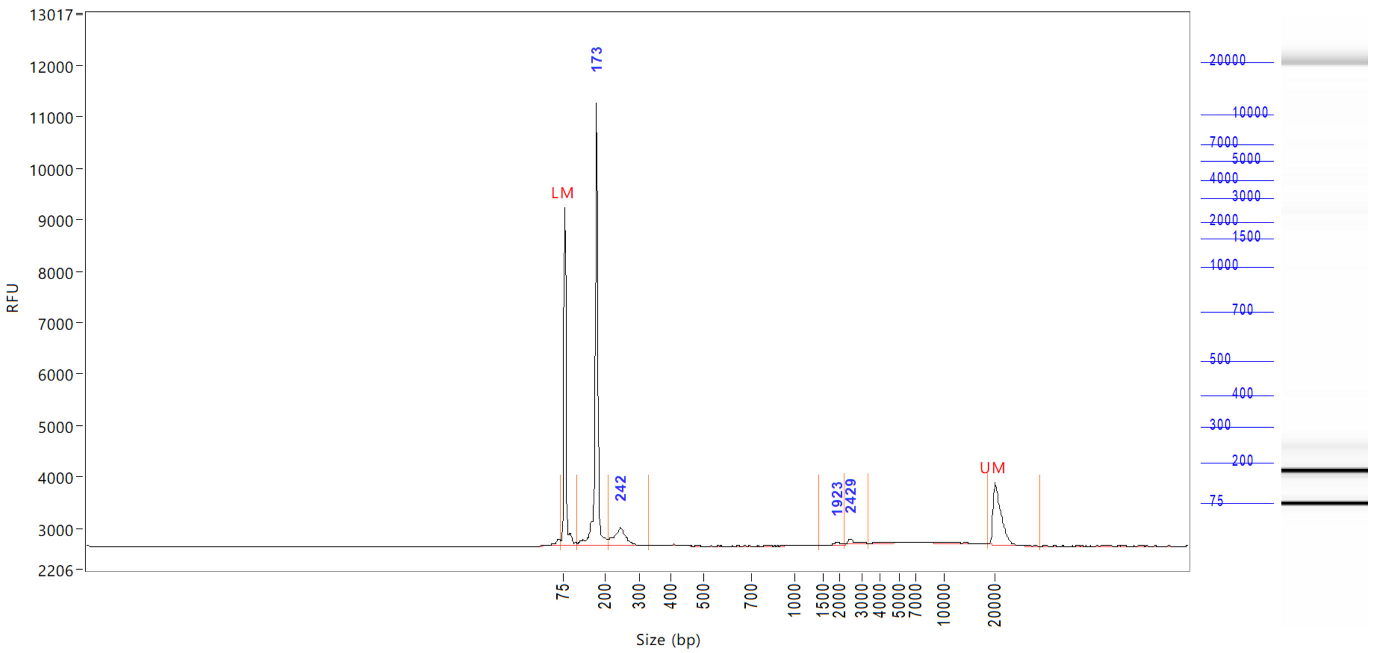

Peak	Size (bp)	Concentration (ng/uL)	From (bp)	To (bp)	Average size (bp)	CV%	RFU	Corrected peak area
1	75 (LM)	0.5000	64	112	74	7.75	6566	38.259
2	173	8.8017	112	208	169	6.45	8617	56.124
3	242	1.3346	208	327	240	7.44	340	8.510
4	1923	0.0867	1433	2144	1893	6.72	52	0.553
5	2429	0.2080	2144	3358	2595	10.66	107	1.326
6	20000 (UM)	0.1620	18537	28826	20601	4.96	1196	12.397

TIC:	10.4311	ng/uL
TIM:	94.9657	nmole/L
Total concentration:	10.9049	ng/uL

Sample peak width (sec): 5 Sample min peak height: 50 Sample baseline V to V?: Y Sample baseline V to V points: 3
Sample filter: Binomial Number of points for filter: 3 Sample start region (min): 0 Sample end region (min): 30
Marker peak width (sec): 5 Marker min peak height: 200 Marker baseline V to V?: Y Marker baseline V to V points: 3
Lower marker selection: First peak > 500 RFU Upper marker selection: Last peak > 200 RFU
Ladder size (bp)75, 200, 300, 400, 500, 700, 1000, 1500, 2000, 3000, 4000, 5000, 7000, 10000, 20000
Quantification using: Lower Marker Final concentration (ng/uL): 0.5000 Dilution factor: 12.0

Sample: SampD2
Well location: D2
Created: Tuesday, August 10, 2021 10:41:10 PM

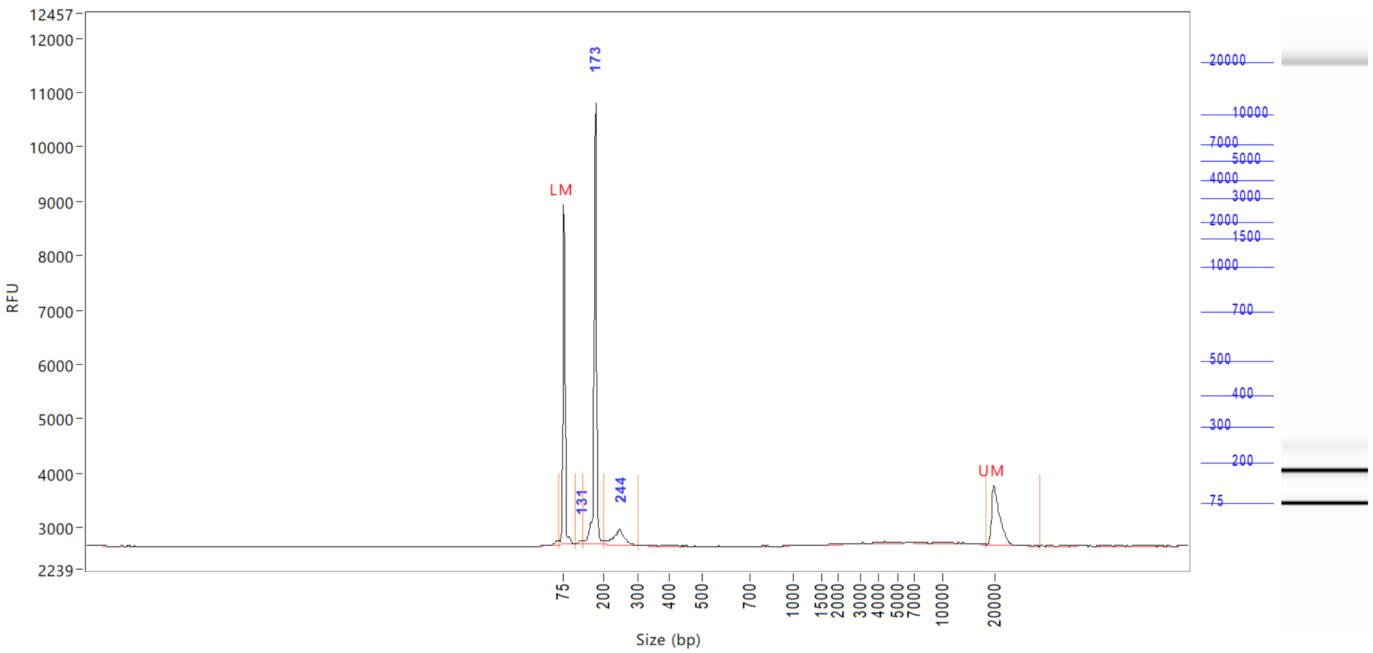

Peak	Size (bp)	Concentration (ng/uL)	From (bp)	To (bp)	Average size (bp)	CV%	RFU	Corrected peak area
1	75 (LM)	0.5000	64	112	74	5.59	6249	34.550
2	131	0.1088	112	137	127	4.94	58	0.626
3	173	8.3155	137	201	170	3.87	8123	47.883
4	244	1.2223	201	300	239	7.32	297	7.038
5	20000 (UM)	0.1634	18537	29189	20569	5.01	1093	11.292

TIC:	9.6465	ng/uL
TIM:	90.2259	nmole/L
Total concentration:	10.3475	ng/uL

```
Sample peak width (sec): 5    Sample min peak height: 50    Sample baseline V to V?: Y    Sample baseline V to V points: 3
Sample filter: Binomial       Number of points for filter: 3  Sample start region (min): 0  Sample end region (min): 30
Marker peak width (sec): 5    Marker min peak height: 200   Marker baseline V to V?: Y    Marker baseline V to V points: 3
Lower marker selection: First peak > 500 RFU                Upper marker selection: Last peak > 200 RFU
Ladder size (bp)75, 200, 300, 400, 500, 700, 1000, 1500, 2000, 3000, 4000, 5000, 7000, 10000, 20000
Quantification using: Lower Marker          Final concentration (ng/uL): 0.5000          Dilution factor: 12.0
```

Sample: SampE2
Well location: E2
Created: Tuesday, August 10, 2021 10:41:10 PM

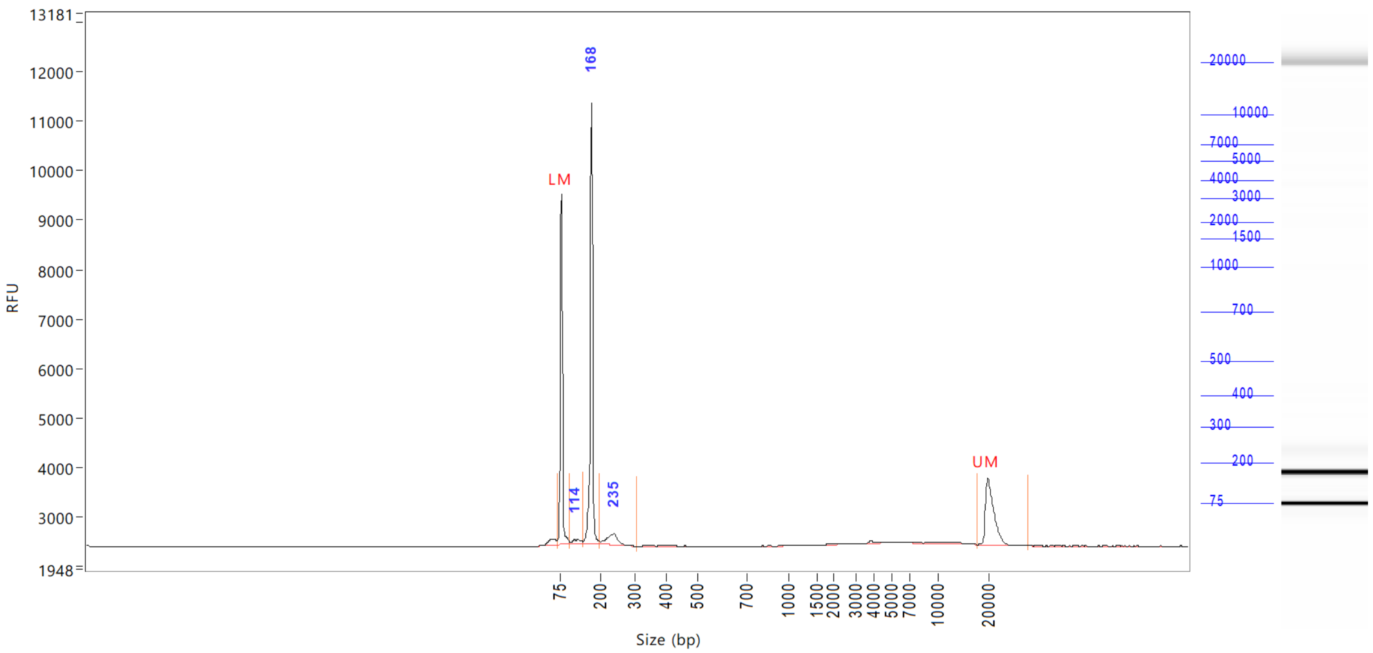

Peak	Size (bp)	Concentration (ng/uL)	From (bp)	To (bp)	Average size (bp)	CV%	RFU	Corrected peak area
1	75 (LM)	0.5000	64	101	73	5.74	7090	40.066
2	114	0.2128	101	141	120	8.81	81	1.421
3	168	7.8731	141	196	166	3.26	8915	52.574
4	235	0.7852	196	303	229	6.99	226	5.243
5	20000 (UM)	0.1781	18049	28100	20560	4.59	1350	14.275

TIC:	8.8711	ng/uL
TIM:	86.5141	nmole/L
Total concentration:	9.5277	ng/uL

Sample peak width (sec): 5 Sample min peak height: 50 Sample baseline V to V?: Y Sample baseline V to V points: 3
Sample filter: Binomial Number of points for filter: 3 Sample start region (min): 0 Sample end region (min): 30
Marker peak width (sec): 5 Marker min peak height: 200 Marker baseline V to V?: Y Marker baseline V to V points: 3
Lower marker selection: First peak > 500 RFU Upper marker selection: Last peak > 200 RFU
Ladder size (bp)75, 200, 300, 400, 500, 700, 1000, 1500, 2000, 3000, 4000, 5000, 7000, 10000, 20000
Quantification using: Lower Marker Final concentration (ng/uL): 0.5000 Dilution factor: 12.0

Sample: SampF2
Well location: F2
Created: Tuesday, August 10, 2021 10:41:10 PM

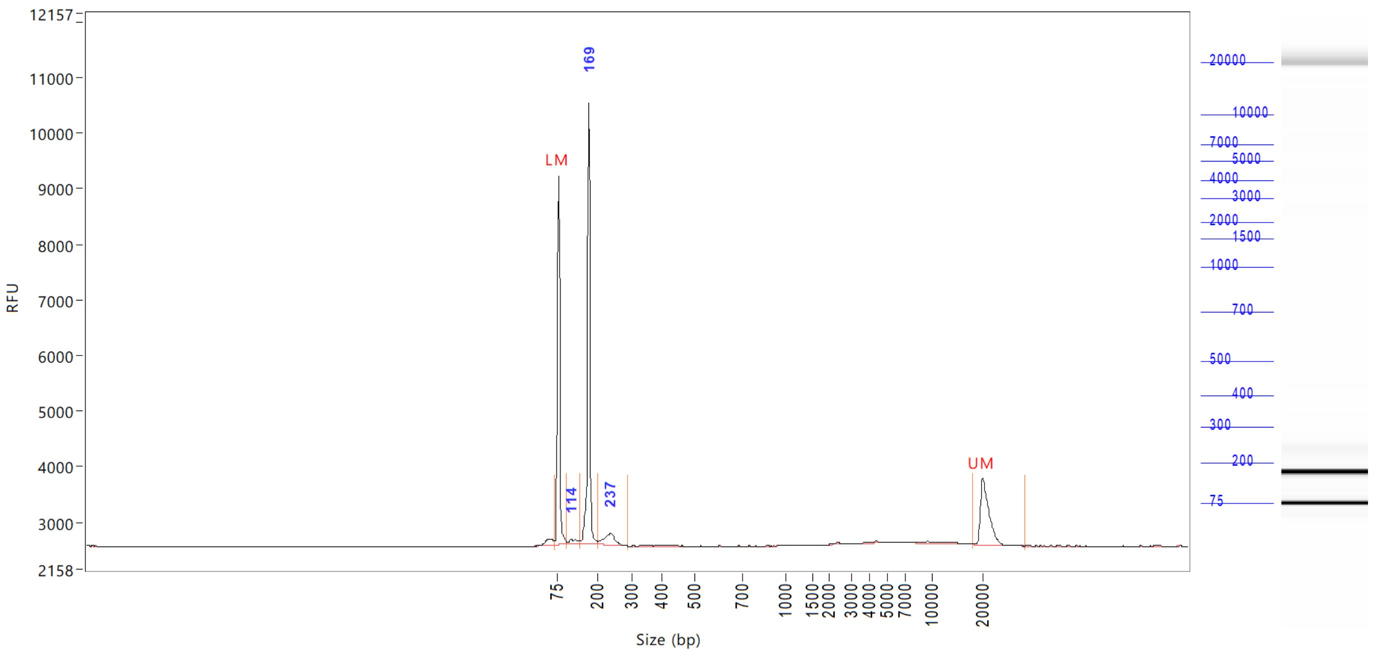

Peak	Size (bp)	Concentration (ng/uL)	From (bp)	To (bp)	Average size (bp)	CV%	RFU	Corrected peak area
1	75 (LM)	0.5000	64	103	74	5.63	6635	38.426
2	114	0.2184	103	143	122	8.99	77	1.399
3	169	7.4209	143	198	167	3.37	7940	47.526
4	237	0.7787	198	289	231	7.12	207	4.987
5	20000 (UM)	0.1697	17927	28221	20551	4.84	1201	13.040

TIC:	8.4181	ng/uL
TIM:	81.5303	nmole/L
Total concentration:	9.0542	ng/uL

Sample peak width (sec): 5   Sample min peak height: 50   Sample baseline V to V?: Y   Sample baseline V to V points: 3
Sample filter: Binomial   Number of points for filter: 3 Sample start region (min): 0 Sample end region (min): 30
Marker peak width (sec): 5   Marker min peak height: 200   Marker baseline V to V?: Y   Marker baseline V to V points: 3
Lower marker selection: First peak > 500 RFU   Upper marker selection: Last peak > 200 RFU
Ladder size (bp)75, 200, 300, 400, 500, 700, 1000, 1500, 2000, 3000, 4000, 5000, 7000, 10000, 20000
Quantification using: Lower Marker   Final concentration (ng/uL): 0.5000   Dilution factor: 12.0

Sample: SampG2
Well location: G2
Created: Tuesday, August 10, 2021 10:41:10 PM

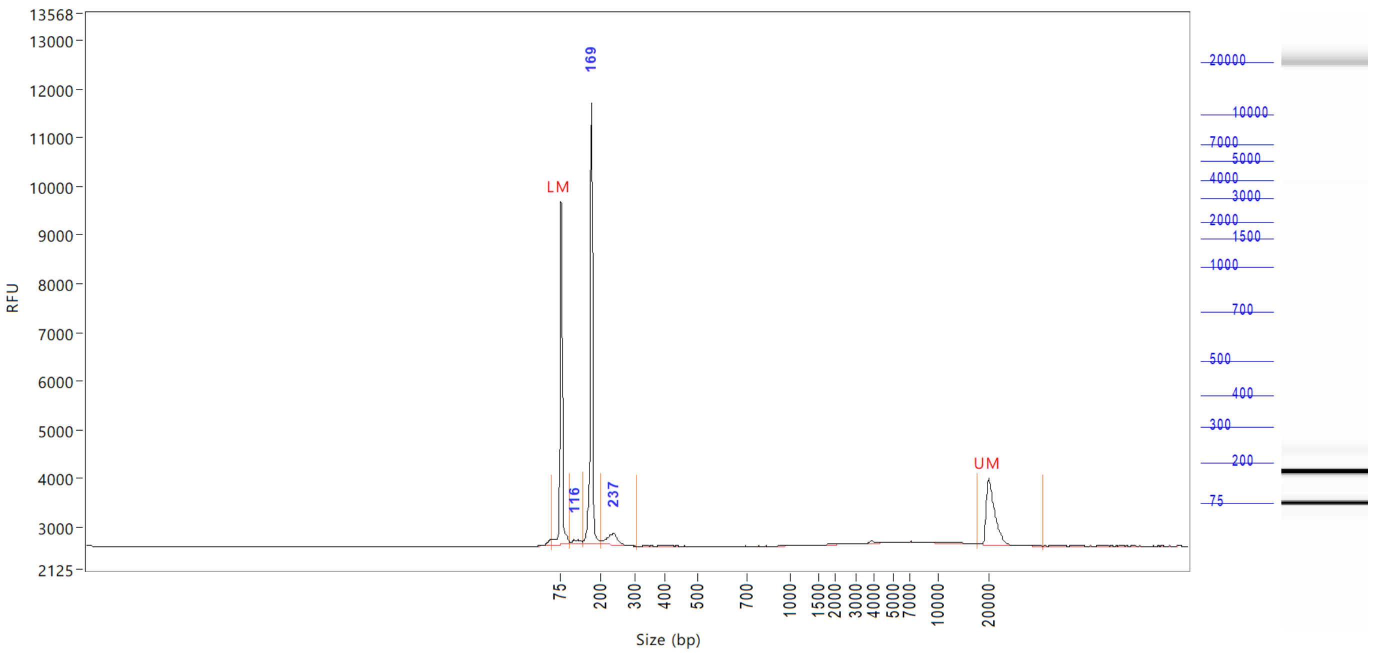

Peak	Size (bp)	Concentration (ng/uL)	From (bp)	To (bp)	Average size (bp)	CV%	RFU	Corrected peak area
1	75 (LM)	0.5000	50	103	74	7.13	7045	43.040
2	116	0.1949	103	143	122	8.78	80	1.398
3	169	7.4781	143	200	168	3.38	9083	53.643
4	237	0.7535	200	303	230	6.63	236	5.405
5	20000 (UM)	0.1721	17683	30639	20524	5.44	1361	14.816

TIC:	8.4265	ng/uL
TIM:	81.1828	nmole/L
Total concentration:	8.9925	ng/uL

Sample peak width (sec): 5 Sample min peak height: 50 Sample baseline V to V?: Y Sample baseline V to V points: 3
Sample filter: Binomial Number of points for filter: 3 Sample start region (min): 0 Sample end region (min): 30
Marker peak width (sec): 5 Marker min peak height: 200 Marker baseline V to V?: Y Marker baseline V to V points: 3
Lower marker selection: First peak > 500 RFU Upper marker selection: Last peak > 200 RFU
Ladder size (bp)75, 200, 300, 400, 500, 700, 1000, 1500, 2000, 3000, 4000, 5000, 7000, 10000, 20000
Quantification using: Lower Marker Final concentration (ng/uL): 0.5000 Dilution factor: 12.0

Sample: SampH2
Well location: H2
Created: Tuesday, August 10, 2021 10:41:10 PM

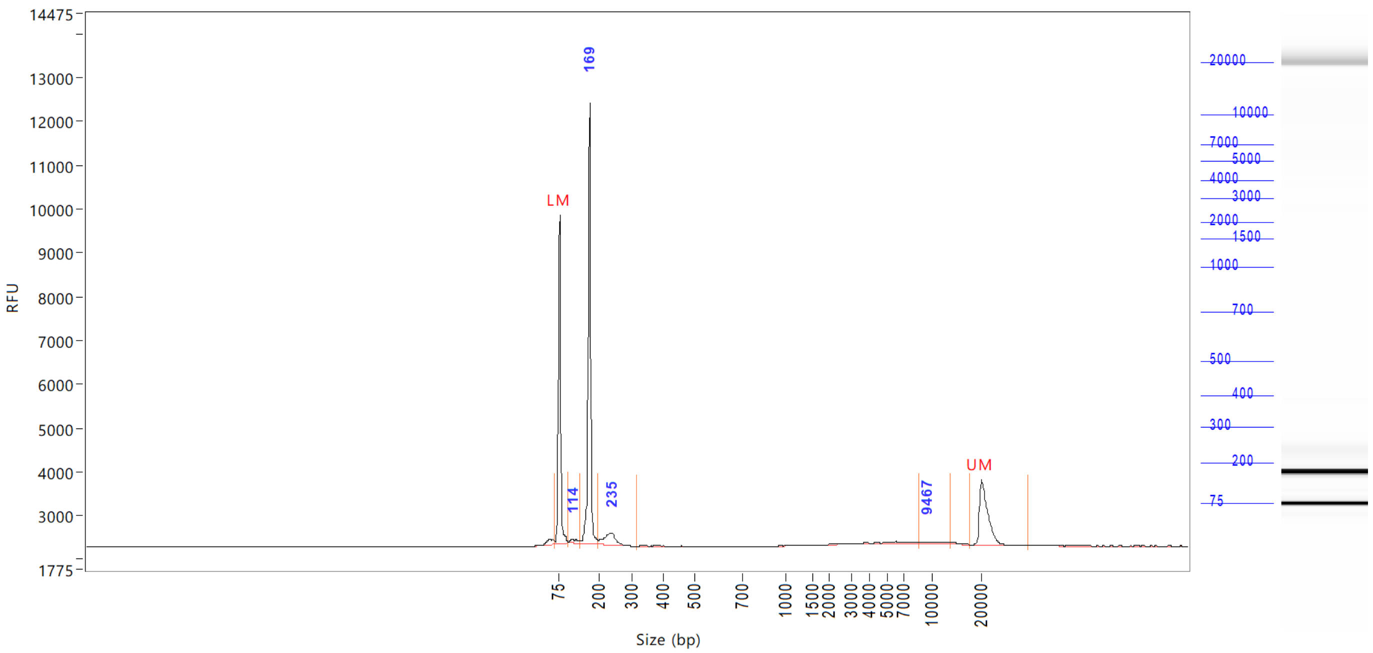

Peak	Size (bp)	Concentration (ng/uL)	From (bp)	To (bp)	Average size (bp)	CV%	RFU	Corrected peak area
1	75 (LM)	0.5000	64	105	73	6.16	7498	42.677
2	114	0.2094	105	141	122	8.43	90	1.490
3	169	8.4016	141	196	167	3.49	10087	59.760
4	235	1.0049	196	311	228	7.42	271	7.148
5	9467	0.2104	8667	13659	10766	13.67	52	1.496
6	20000 (UM)	0.1882	17683	29430	20631	5.08	1480	16.066

TIC:	9.8264	ng/uL
TIM:	92.7993	nmole/L
Total concentration:	10.3852	ng/uL

Sample peak width (sec): 5 Sample min peak height: 50 Sample baseline V to V?: Y Sample baseline V to V points: 3
Sample filter: Binomial Number of points for filter: 3 Sample start region (min): 0 Sample end region (min): 30
Marker peak width (sec): 5 Marker min peak height: 200 Marker baseline V to V?: Y Marker baseline V to V points: 3
Lower marker selection: First peak > 500 RFU Upper marker selection: Last peak > 200 RFU
Ladder size (bp)75, 200, 300, 400, 500, 700, 1000, 1500, 2000, 3000, 4000, 5000, 7000, 10000, 20000
Quantification using: Lower Marker Final concentration (ng/uL): 0.5000 Dilution factor: 12.0

Sample: SampA3
Well location: A3
Created: Tuesday, August 10, 2021 10:41:10 PM

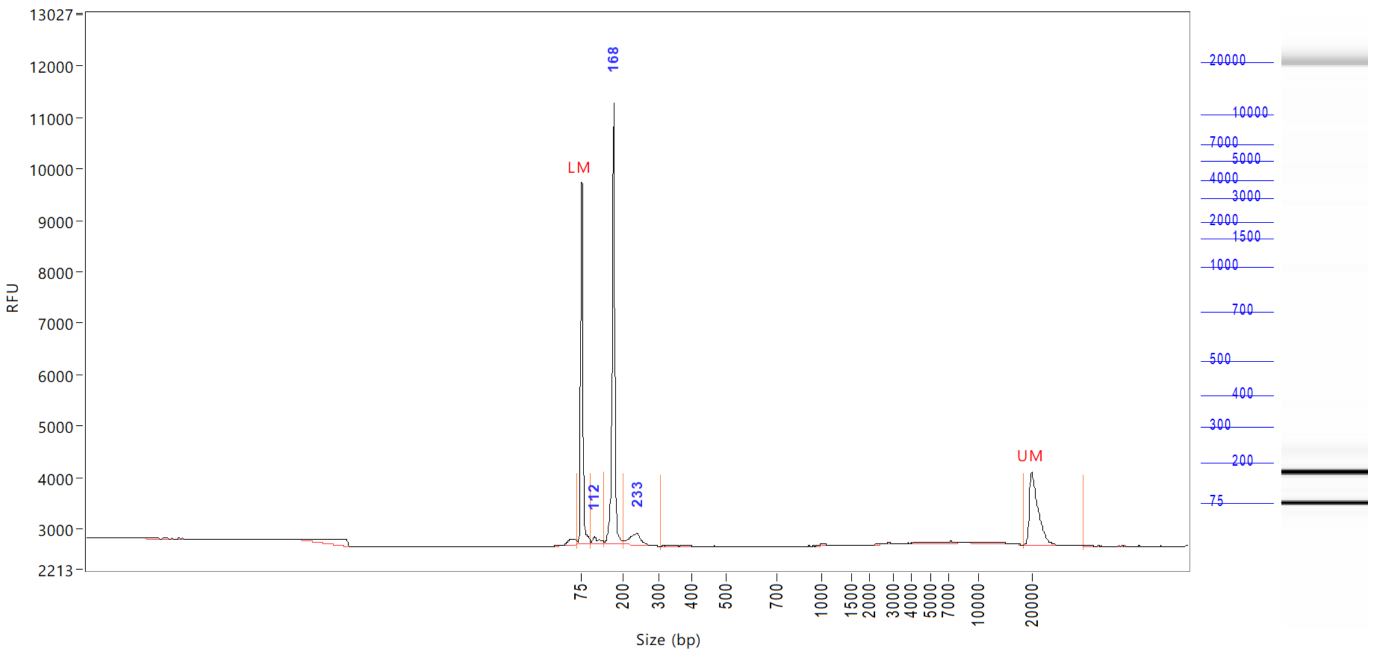

Peak	Size (bp)	Concentration (ng/uL)	From (bp)	To (bp)	Average size (bp)	CV%	RFU	Corrected peak area
1	75 (LM)	0.5000	64	101	74	5.51	7035	40.115
2	112	0.2543	101	141	118	9.09	127	1.700
3	168	7.4867	141	198	166	3.43	8587	50.055
4	233	0.8289	198	301	229	7.25	215	5.542
5	20000 (UM)	0.1907	18415	29793	20588	5.31	1423	15.300

TIC:	8.5699	ng/uL
TIM:	83.6280	nmole/L
Total concentration:	9.2903	ng/uL

```
Sample peak width (sec): 5    Sample min peak height: 50    Sample baseline V to V?: Y    Sample baseline V to V points: 3
Sample filter: Binomial       Number of points for filter: 3  Sample start region (min): 0  Sample end region (min): 30
Marker peak width (sec): 5    Marker min peak height: 200   Marker baseline V to V?: Y    Marker baseline V to V points: 3
Lower marker selection: First peak > 500 RFU                Upper marker selection: Last peak > 200 RFU
Ladder size (bp)75, 200, 300, 400, 500, 700, 1000, 1500, 2000, 3000, 4000, 5000, 7000, 10000, 20000
Quantification using: Lower Marker         Final concentration (ng/uL): 0.5000         Dilution factor: 12.0
```

Sample: SampB3
Well location: B3
Created: Tuesday, August 10, 2021 10:41:10 PM

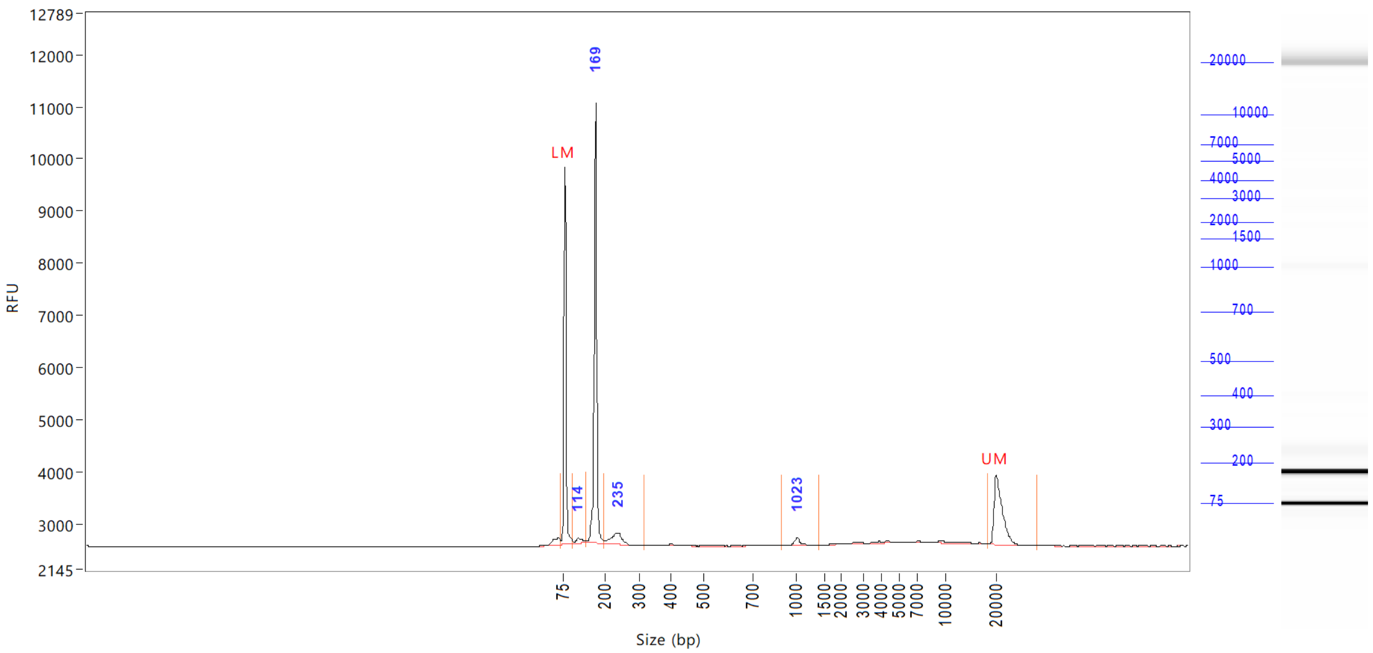

Peak	Size (bp)	Concentration (ng/uL)	From (bp)	To (bp)	Average size (bp)	CV%	RFU	Corrected peak area
1	75 (LM)	0.5000	64	101	73	5.60	7210	39.893
2	114	0.2466	101	143	120	9.07	91	1.639
3	169	7.3915	143	194	167	3.17	8439	49.144
4	235	0.8010	194	315	228	7.19	219	5.325
5	1023	0.2272	900	1400	1035	7.16	150	1.510
6	20000 (UM)	0.1757	18293	27979	20575	4.79	1323	14.022

TIC:	8.6662	ng/uL
TIM:	82.2691	nmole/L
Total concentration:	9.3447	ng/uL

```
Sample peak width (sec): 5    Sample min peak height: 50    Sample baseline V to V?: Y    Sample baseline V to V points: 3
Sample filter: Binomial       Number of points for filter: 3  Sample start region (min): 0  Sample end region (min): 30
Marker peak width (sec): 5    Marker min peak height: 200   Marker baseline V to V?: Y    Marker baseline V to V points: 3
Lower marker selection: First peak > 500 RFU                Upper marker selection: Last peak > 200 RFU
Ladder size (bp)75, 200, 300, 400, 500, 700, 1000, 1500, 2000, 3000, 4000, 5000, 7000, 10000, 20000
Quantification using: Lower Marker          Final concentration (ng/uL): 0.5000          Dilution factor: 12.0
```

Sample: SampC3
Well location: C3
Created: Tuesday, August 10, 2021 10:41:10 PM

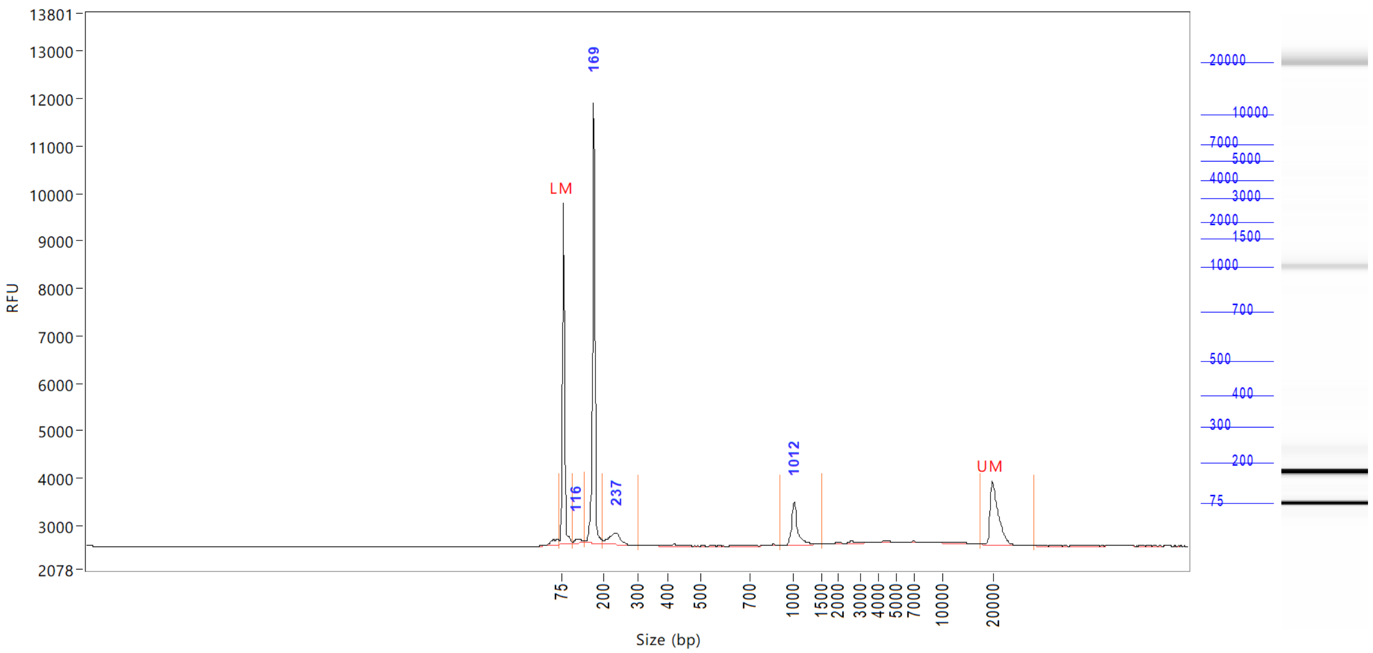

Peak	Size (bp)	Concentration (ng/uL)	From (bp)	To (bp)	Average size (bp)	CV%	RFU	Corrected peak area
1	75 (LM)	0.5000	64	103	74	5.75	7185	40.750
2	116	0.2405	103	143	121	8.72	95	1.633
3	169	8.0782	143	200	167	3.48	9298	54.864
4	237	0.9129	200	301	231	7.16	250	6.200
5	1012	1.2790	905	1520	1042	8.10	911	8.686
6	20000 (UM)	0.1749	17683	28221	20504	4.91	1352	14.256

TIC:	10.5106	ng/uL
TIM:	91.2990	nmole/L
Total concentration:	11.2137	ng/uL

Sample peak width (sec): 5   Sample min peak height: 50   Sample baseline V to V?: Y   Sample baseline V to V points: 3
Sample filter: Binomial   Number of points for filter: 3 Sample start region (min): 0 Sample end region (min): 30
Marker peak width (sec): 5   Marker min peak height: 200   Marker baseline V to V?: Y   Marker baseline V to V points: 3
Lower marker selection: First peak > 500 RFU   Upper marker selection: Last peak > 200 RFU
Ladder size (bp)75, 200, 300, 400, 500, 700, 1000, 1500, 2000, 3000, 4000, 5000, 7000, 10000, 20000
Quantification using: Lower Marker   Final concentration (ng/uL): 0.5000   Dilution factor: 12.0

Sample: SampD3
Well location: D3
Created: Tuesday, August 10, 2021 10:41:10 PM

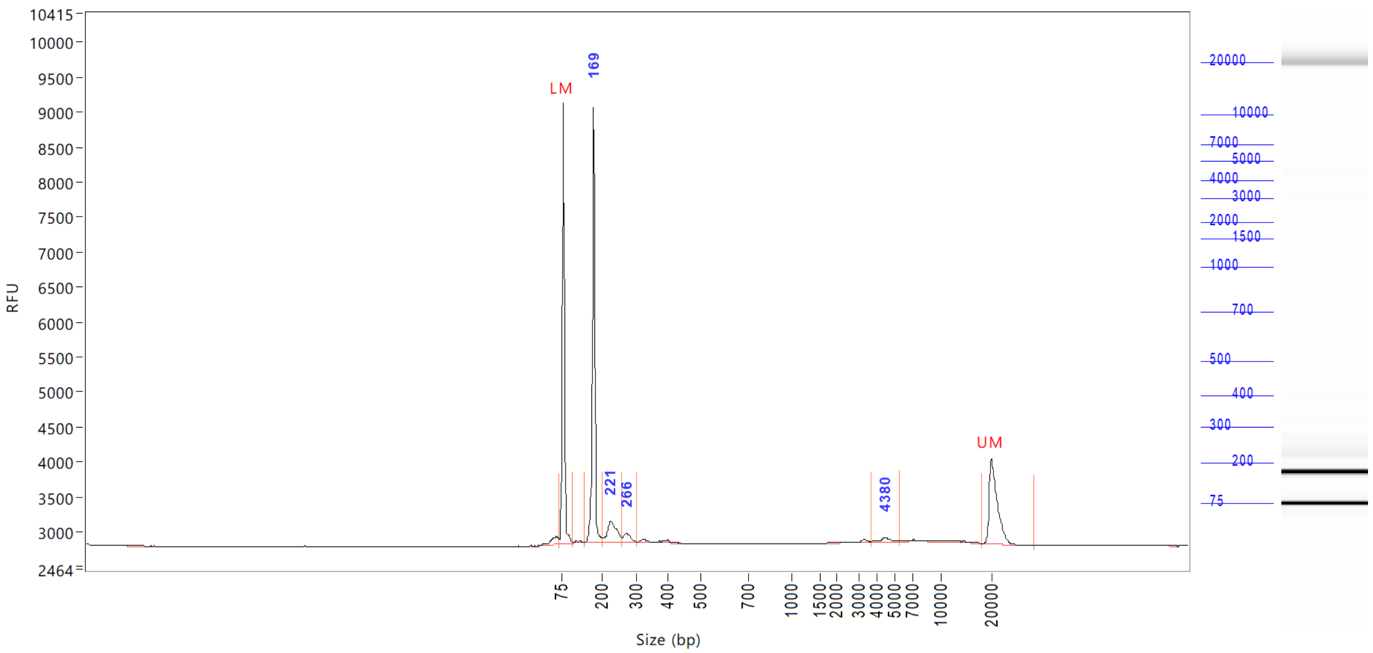

Peak	Size<br>(bp)	Concentration<br>(ng/uL)	From<br>(bp)	To<br>(bp)	Average size<br>(bp)	CV%	RFU	Corrected peak area
1	75 (LM)	0.5000	64	105	74	5.62	6314	35.231
2	169	6.3288	141	196	167	3.35	6213	37.162
3	221	1.0774	196	253	224	5.91	298	6.326
4	266	0.3471	253	298	268	3.67	131	2.038
5	4380	0.1696	3643	5519	4424	8.82	62	0.996
6	20000 (UM)	0.1815	18049	28342	20558	4.86	1221	12.792

TIC:	7.9230	ng/uL
TIM:	72.3987	nmole/L
Total concentration:	8.7050	ng/uL

```
Sample peak width (sec): 5    Sample min peak height: 50    Sample baseline V to V?: Y    Sample baseline V to V points: 3
Sample filter: Binomial       Number of points for filter: 3  Sample start region (min): 0  Sample end region (min): 30
Marker peak width (sec): 5    Marker min peak height: 200   Marker baseline V to V?: Y    Marker baseline V to V points: 3
Lower marker selection: First peak > 500 RFU                Upper marker selection: Last peak > 200 RFU
Ladder size (bp)75, 200, 300, 400, 500, 700, 1000, 1500, 2000, 3000, 4000, 5000, 7000, 10000, 20000
Quantification using: Lower Marker          Final concentration (ng/uL): 0.5000          Dilution factor: 12.0
```

Sample: SampE3
Well location: E3
Created: Tuesday, August 10, 2021 10:41:10 PM

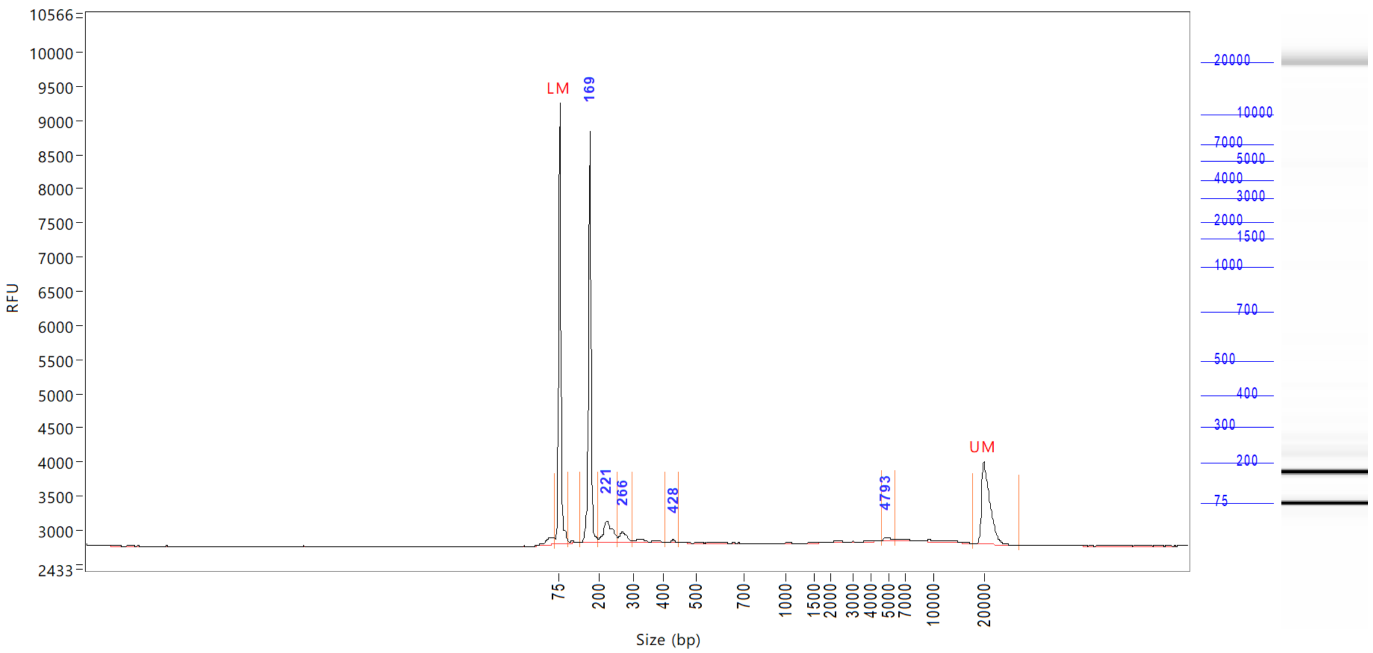

Peak	Size (bp)	Concentration (ng/uL)	From (bp)	To (bp)	Average size (bp)	CV%	RFU	Corrected peak area
1	75 (LM)	0.5000	64	105	74	6.21	6461	37.473
2	169	5.9199	141	194	167	3.23	6004	36.972
3	221	1.0262	194	253	224	6.05	315	6.409
4	266	0.3702	253	296	269	3.54	146	2.312
5	428	0.0562	403	445	425	1.53	50	0.351
6	4793	0.1066	4587	5815	4998	6.03	59	0.666
7	20000 (UM)	0.1731	17927	27012	20533	4.79	1206	12.972

TIC:	7.4791	ng/uL
TIM:	68.3201	nmole/L
Total concentration:	8.2715	ng/uL

```
Sample peak width (sec): 5    Sample min peak height: 50    Sample baseline V to V?: Y    Sample baseline V to V points: 3
Sample filter: Binomial       Number of points for filter: 3  Sample start region (min): 0  Sample end region (min): 30
Marker peak width (sec): 5    Marker min peak height: 200   Marker baseline V to V?: Y    Marker baseline V to V points: 3
Lower marker selection: First peak > 500 RFU                Upper marker selection: Last peak > 200 RFU
Ladder size (bp)75, 200, 300, 400, 500, 700, 1000, 1500, 2000, 3000, 4000, 5000, 7000, 10000, 20000
Quantification using: Lower Marker          Final concentration (ng/uL): 0.5000          Dilution factor: 12.0
```

Sample: SampF3
Well location: F3
Created: Tuesday, August 10, 2021 10:41:10 PM

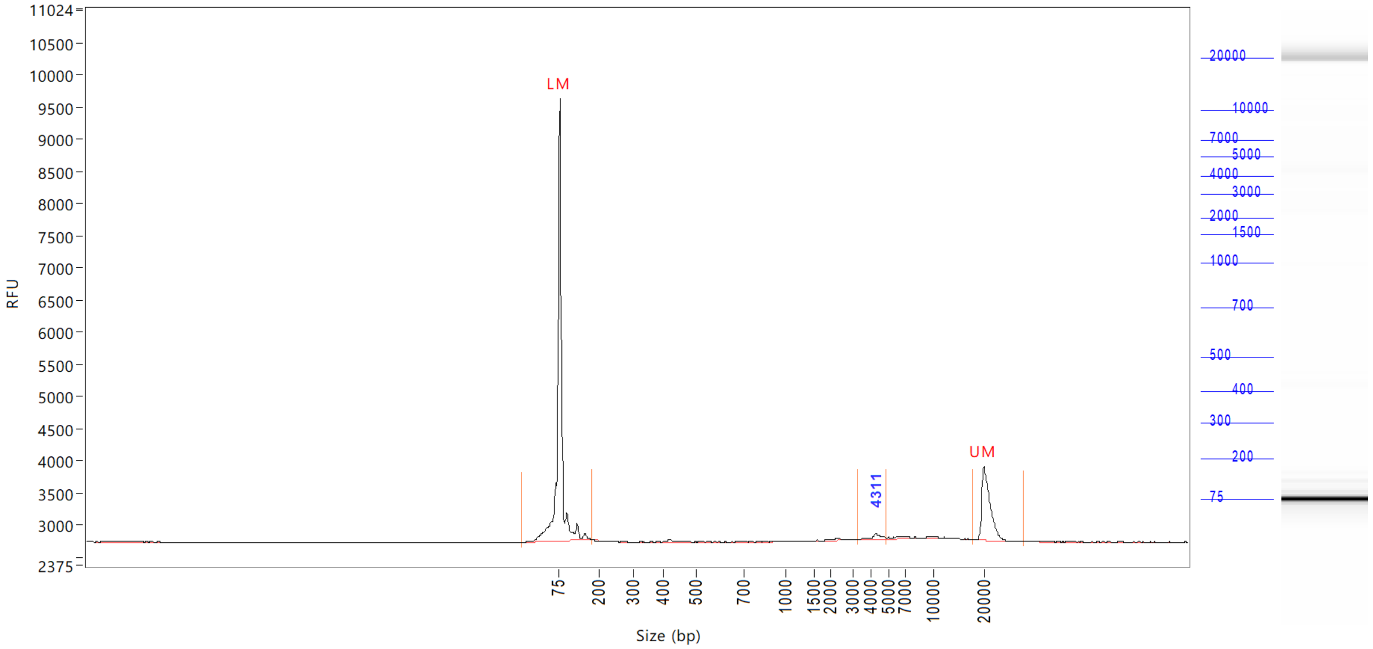

Peak	Size (bp)	Concentration (ng/uL)	From (bp)	To (bp)	Average size (bp)	CV%	RFU	Corrected peak area
1	75 (LM)	0.5000	0	175	73	29.10	6889	66.600
2	4311	0.1178	3286	4897	4239	8.57	86	1.307
3	20000 (UM)	0.0917	17683	27859	20570	4.81	1140	12.208

TIC:	0.1178	ng/uL
TIM:	0.0457	nmole/L
Total concentration:	0.4421	ng/uL

```
Sample peak width (sec): 5    Sample min peak height: 50    Sample baseline V to V?: Y    Sample baseline V to V points: 3
Sample filter: Binomial       Number of points for filter: 3  Sample start region (min): 0  Sample end region (min): 30
Marker peak width (sec): 5    Marker min peak height: 200   Marker baseline V to V?: Y    Marker baseline V to V points: 3
Lower marker selection: First peak > 500 RFU                Upper marker selection: Last peak > 200 RFU
Ladder size (bp)75, 200, 300, 400, 500, 700, 1000, 1500, 2000, 3000, 4000, 5000, 7000, 10000, 20000
Quantification using: Lower Marker          Final concentration (ng/uL): 0.5000          Dilution factor: 12.0
```

Sample: SampG3
Well location: G3
Created: Tuesday, August 10, 2021 10:41:10 PM

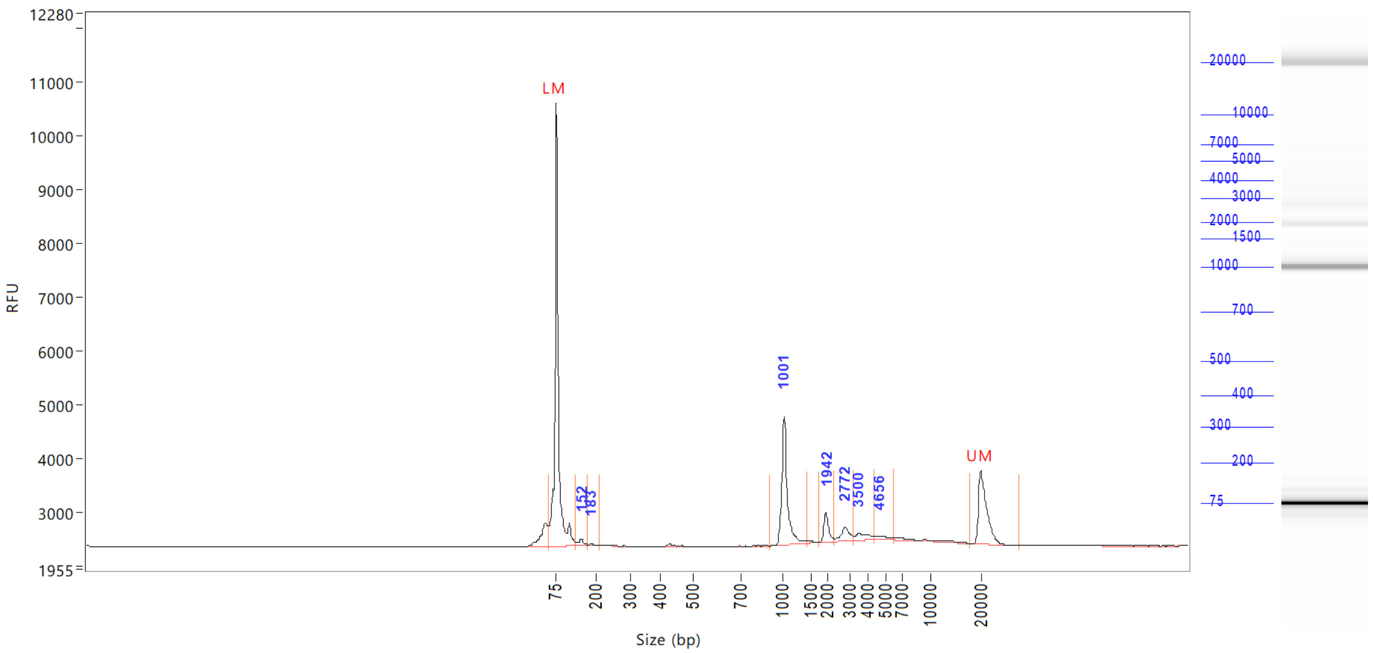

Peak	Size (bp)	Concentration (ng/uL)	From (bp)	To (bp)	Average size (bp)	CV%	RFU	Corrected peak area
1	75 (LM)	0.5000	52	137	76	17.06	8244	69.058
2	152	0.1365	137	173	150	5.81	135	1.571
3	183	0.0417	173	208	183	3.77	51	0.479
4	1001	1.9133	900	1411	1033	6.99	2373	22.022
5	1942	0.3688	1712	2258	1943	4.78	553	4.245
6	2772	0.3037	2258	3215	2758	8.06	257	3.496
7	3500	0.2149	3215	4345	3738	8.60	125	2.473
8	4656	0.1169	4345	5889	4845	7.98	69	1.346
9	20000 (UM)	0.1064	17683	27617	20514	4.92	1355	14.689

TIC:	3.0958	ng/uL
TIM:	5.5469	nmole/L
Total concentration:	3.3680	ng/uL

Sample peak width (sec): 5 Sample min peak height: 50 Sample baseline V to V?: Y Sample baseline V to V points: 3
Sample filter: Binomial Number of points for filter: 3 Sample start region (min): 0 Sample end region (min): 30
Marker peak width (sec): 5 Marker min peak height: 200 Marker baseline V to V?: Y Marker baseline V to V points: 3
Lower marker selection: First peak > 500 RFU Upper marker selection: Last peak > 200 RFU
Ladder size (bp)75, 200, 300, 400, 500, 700, 1000, 1500, 2000, 3000, 4000, 5000, 7000, 10000, 20000
Quantification using: Lower Marker Final concentration (ng/uL): 0.5000 Dilution factor: 12.0

Sample: SampH3
Well location: H3
Created: Tuesday, August 10, 2021 10:41:10 PM

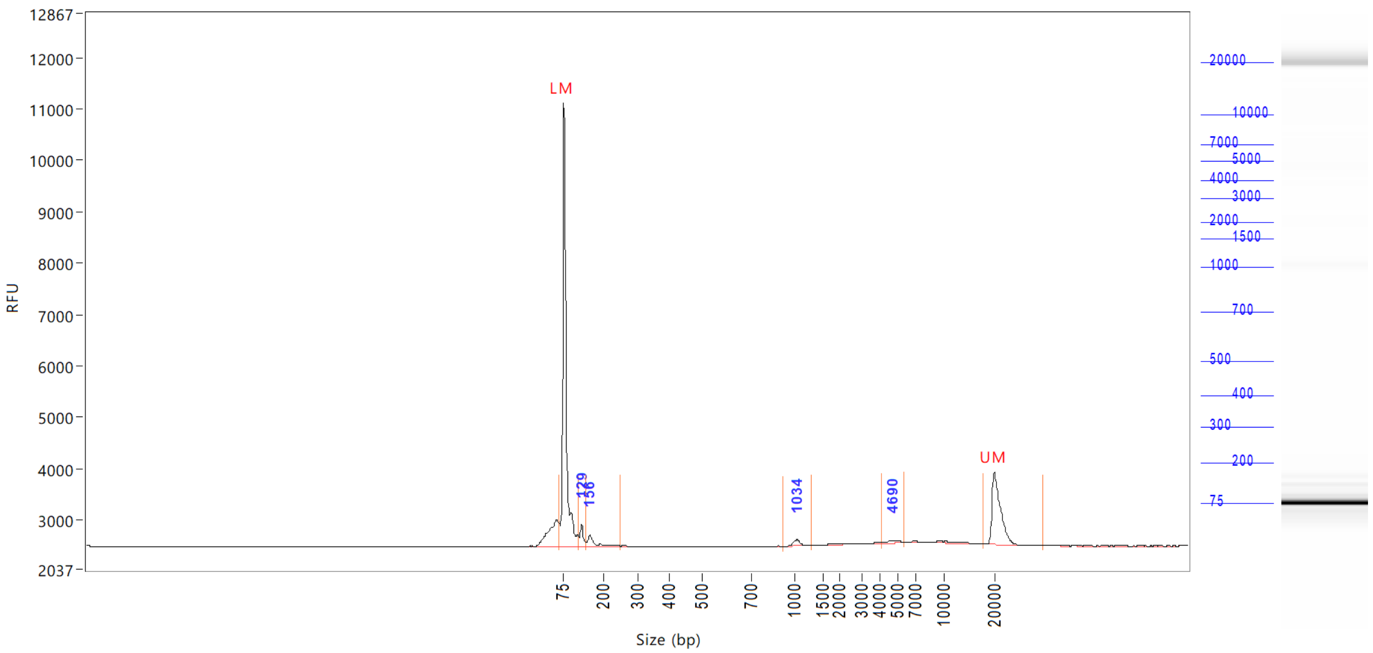

Peak	Size (bp)	Concentration (ng/uL)	From (bp)	To (bp)	Average size (bp)	CV%	RFU	Corrected peak area
1	75 (LM)	0.5000	64	122	78	12.63	8648	70.586
2	129	0.2858	122	145	130	4.42	420	3.362
3	156	0.2600	145	248	166	12.53	234	3.059
4	1034	0.1151	926	1300	1038	5.85	125	1.354
5	4690	0.0775	4104	5667	4768	7.13	59	0.911
6	20000 (UM)	0.1096	17683	29430	20580	5.17	1424	15.475

TIC:	0.7383	ng/uL
TIM:	6.3954	nmole/L
Total concentration:	1.0332	ng/uL

Sample peak width (sec): 5 Sample min peak height: 50 Sample baseline V to V?: Y Sample baseline V to V points: 3
Sample filter: Binomial Number of points for filter: 3 Sample start region (min): 0 Sample end region (min): 30
Marker peak width (sec): 5 Marker min peak height: 200 Marker baseline V to V?: Y Marker baseline V to V points: 3
Lower marker selection: First peak > 500 RFU Upper marker selection: Last peak > 200 RFU
Ladder size (bp)75, 200, 300, 400, 500, 700, 1000, 1500, 2000, 3000, 4000, 5000, 7000, 10000, 20000
Quantification using: Lower Marker Final concentration (ng/uL): 0.5000 Dilution factor: 12.0

Sample: SampA4
Well location: A4
Created: Tuesday, August 10, 2021 10:41:10 PM

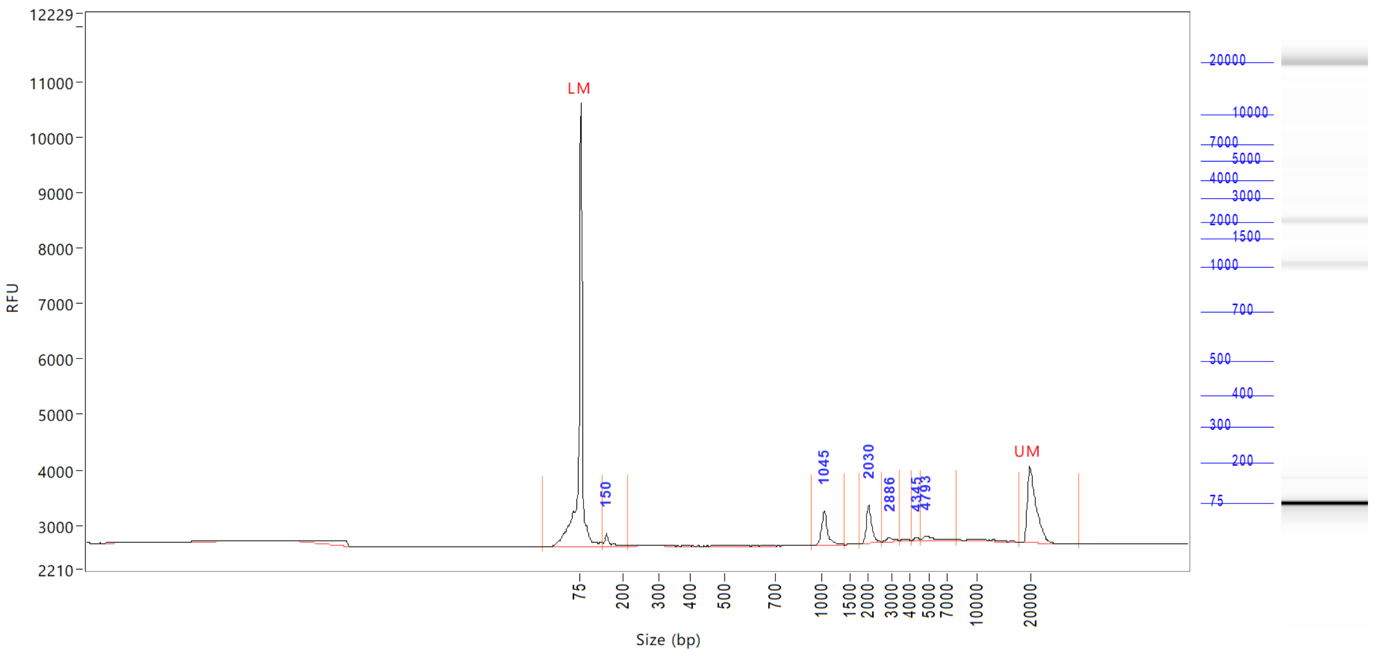

Peak	Size (bp)	Concentration (ng/uL)	From (bp)	To (bp)	Average size (bp)	CV%	RFU	Corrected peak area
1	75 (LM)	0.5000	0	141	68	27.40	8005	69.364
2	150	0.2016	141	214	158	10.33	237	2.331
3	1045	0.5195	935	1400	1056	6.52	609	6.006
4	2030	0.4646	1750	2600	2042	5.81	683	5.371
5	2886	0.0758	2600	3429	2953	6.46	73	0.876
6	4345	0.0367	4035	4552	4316	3.07	51	0.424
7	4793	0.1331	4552	7867	5624	16.96	77	1.539
8	20000 (UM)	0.1064	18049	29189	20538	4.95	1382	14.755

TIC:	1.4314	ng/uL
TIM:	3.3770	nmole/L
Total concentration:	1.6837	ng/uL

```
Sample peak width (sec): 5    Sample min peak height: 50    Sample baseline V to V?: Y    Sample baseline V to V points: 3
Sample filter: Binomial       Number of points for filter: 3  Sample start region (min): 0  Sample end region (min): 30
Marker peak width (sec): 5    Marker min peak height: 200   Marker baseline V to V?: Y    Marker baseline V to V points: 3
Lower marker selection: First peak > 500 RFU                Upper marker selection: Last peak > 200 RFU
Ladder size (bp)75, 200, 300, 400, 500, 700, 1000, 1500, 2000, 3000, 4000, 5000, 7000, 10000, 20000
Quantification using: Lower Marker          Final concentration (ng/uL): 0.5000          Dilution factor: 12.0
```

Sample: SampB4
Well location: B4
Created: Tuesday, August 10, 2021 10:41:10 PM

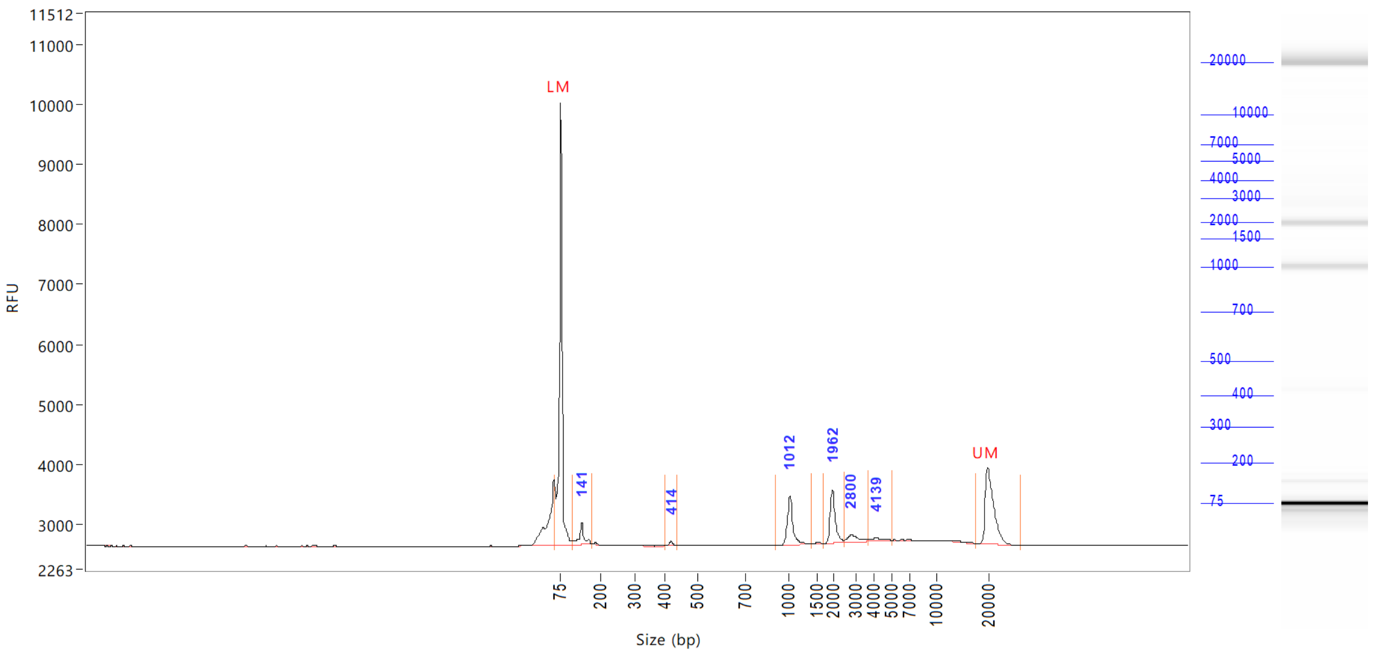

Peak	Size (bp)	Concentration (ng/uL)	From (bp)	To (bp)	Average size (bp)	CV%	RFU	Corrected peak area
1	75 (LM)	0.5000	60	110	73	8.89	7367	50.161
2	141	0.4907	110	173	138	9.10	366	4.102
3	414	0.0800	398	435	415	1.80	89	0.669
4	1012	0.9050	913	1400	1029	6.00	815	7.566
5	1962	0.8317	1674	2458	1982	5.81	890	6.953
6	2800	0.2572	2458	3715	2950	10.50	120	2.150
7	4139	0.1124	3715	5001	4204	7.29	55	0.939
8	20000 (UM)	0.1373	17683	26408	20530	4.80	1286	13.777

TIC:	2.6770	ng/uL
TIM:	8.4861	nmole/L
Total concentration:	2.9301	ng/uL

Sample peak width (sec): 5   Sample min peak height: 50   Sample baseline V to V?: Y   Sample baseline V to V points: 3
Sample filter: Binomial   Number of points for filter: 3 Sample start region (min): 0 Sample end region (min): 30
Marker peak width (sec): 5   Marker min peak height: 200   Marker baseline V to V?: Y   Marker baseline V to V points: 3
Lower marker selection: First peak > 500 RFU   Upper marker selection: Last peak > 200 RFU
Ladder size (bp)75, 200, 300, 400, 500, 700, 1000, 1500, 2000, 3000, 4000, 5000, 7000, 10000, 20000
Quantification using: Lower Marker   Final concentration (ng/uL): 0.5000   Dilution factor: 12.0

Sample: SampC4
Well location: C4
Created: Tuesday, August 10, 2021 10:41:10 PM

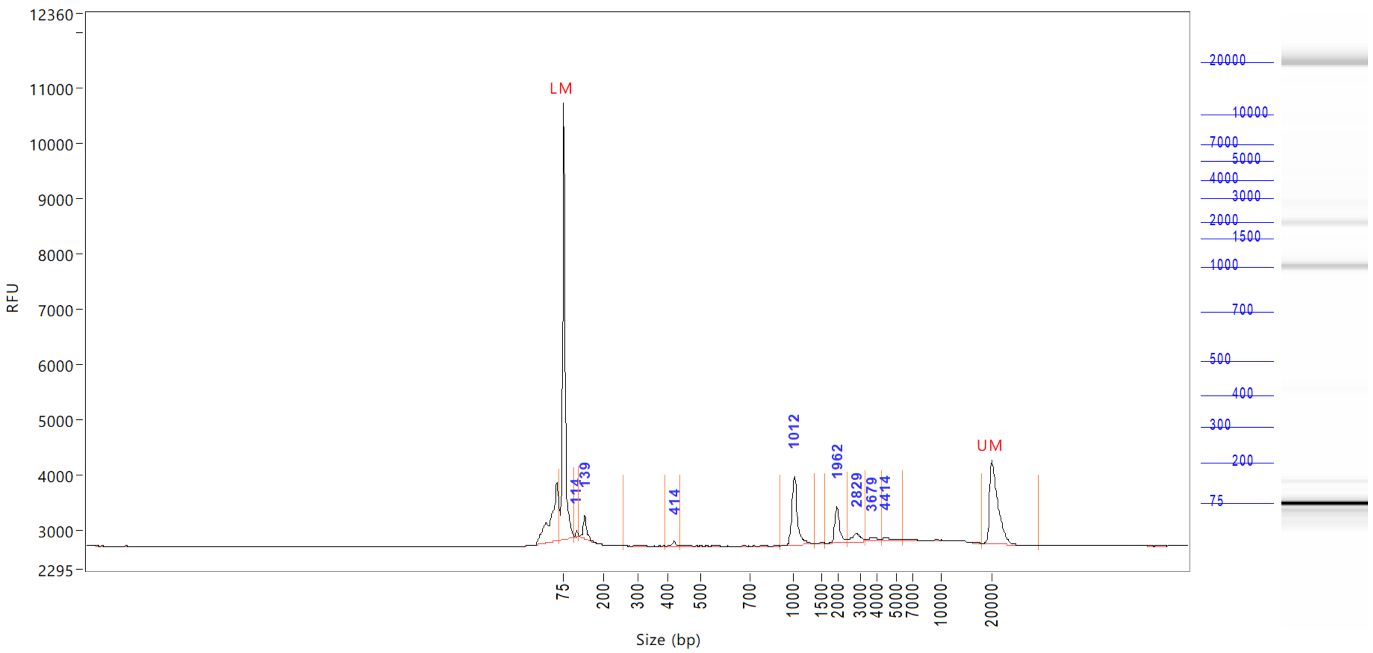

Peak	Size (bp)	Concentration (ng/uL)	From (bp)	To (bp)	Average size (bp)	CV%	RFU	Corrected peak area
1	75 (LM)	0.5000	64	107	74	7.74	7916	50.832
2	114	0.0744	107	122	112	2.79	116	0.631
3	139	0.3869	122	255	142	11.20	434	3.278
4	414	0.0805	388	433	414	1.96	89	0.682
5	1012	1.2916	909	1389	1030	5.82	1242	10.942
6	1962	0.6036	1616	2401	1986	5.63	667	5.113
7	2829	0.2449	2401	3251	2799	7.21	148	2.075
8	3679	0.1115	3251	4173	3690	6.86	56	0.945
9	4414	0.1003	4173	5815	4658	8.42	62	0.850
10	20000 (UM)	0.1589	17927	29068	20531	4.98	1515	16.158

TIC:	2.8937	ng/uL
TIM:	8.6830	nmole/L
Total concentration:	3.1162	ng/uL

Sample peak width (sec): 5   Sample min peak height: 50   Sample baseline V to V?: Y   Sample baseline V to V points: 3
Sample filter: Binomial   Number of points for filter: 3 Sample start region (min): 0 Sample end region (min): 30
Marker peak width (sec): 5   Marker min peak height: 200   Marker baseline V to V?: Y   Marker baseline V to V points: 3
Lower marker selection: First peak > 500 RFU   Upper marker selection: Last peak > 200 RFU
Ladder size (bp)75, 200, 300, 400, 500, 700, 1000, 1500, 2000, 3000, 4000, 5000, 7000, 10000, 20000
Quantification using: Lower Marker   Final concentration (ng/uL): 0.5000   Dilution factor: 12.0

Sample: SampD4
Well location: D4
Created: Tuesday, August 10, 2021 10:41:10 PM

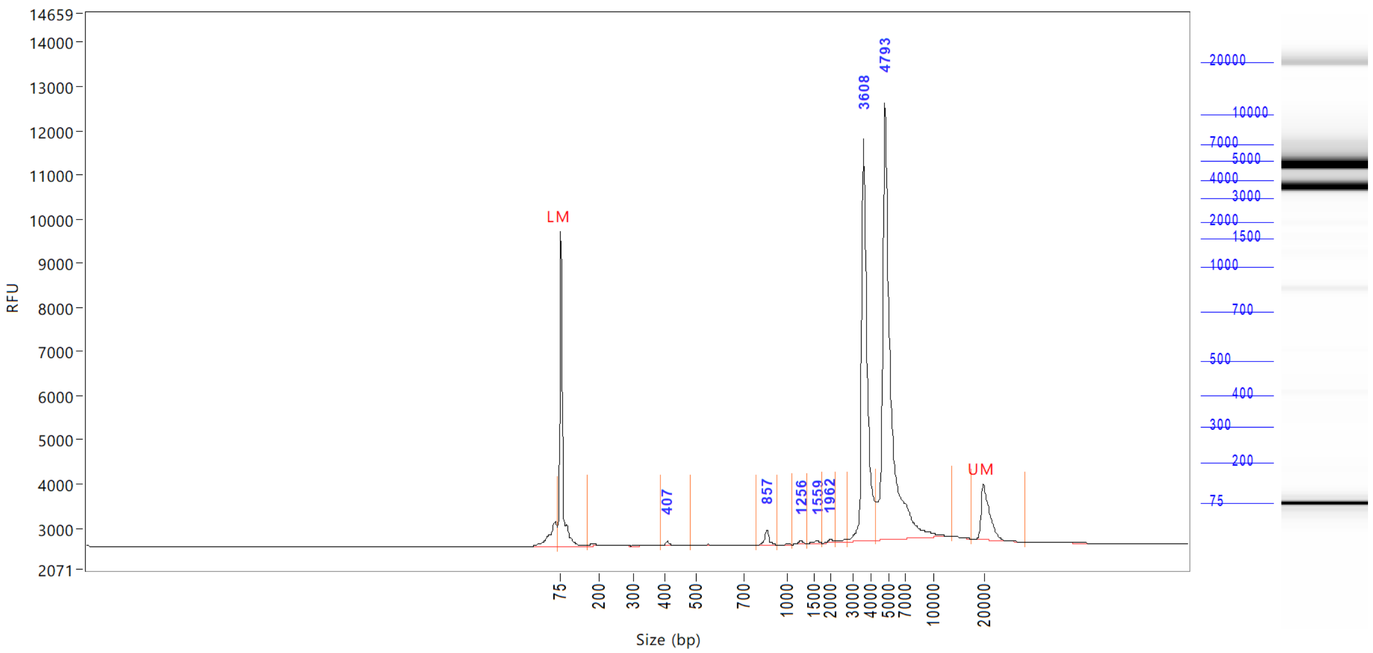

Peak	Size (bp)	Concentration (ng/uL)	From (bp)	To (bp)	Average size (bp)	CV%	RFU	Corrected peak area
1	75 (LM)	0.5000	66	160	77	14.90	7140	48.940
2	407	0.1729	386	483	418	4.80	107	1.410
3	857	0.3283	779	935	854	2.59	340	2.678
4	1256	0.0732	1090	1367	1232	3.83	69	0.597
5	1559	0.0655	1367	1731	1510	4.26	53	0.534
6	1962	0.0787	1731	2201	1982	4.36	81	0.642
7	3608	9.8343	2743	4311	3671	6.22	9101	80.215
8	4793	14.4674	4311	13659	5308	19.97	9908	118.005
9	20000 (UM)	0.1387	17561	28221	20594	5.14	1254	13.575

TIC:	25.0203	ng/uL
TIM:	10.4449	nmole/L
Total concentration:	25.3486	ng/uL

```
Sample peak width (sec): 5    Sample min peak height: 50    Sample baseline V to V?: Y    Sample baseline V to V points: 3
Sample filter: Binomial       Number of points for filter: 3  Sample start region (min): 0  Sample end region (min): 30
Marker peak width (sec): 5    Marker min peak height: 200   Marker baseline V to V?: Y    Marker baseline V to V points: 3
Lower marker selection: First peak > 500 RFU                Upper marker selection: Last peak > 200 RFU
Ladder size (bp)75, 200, 300, 400, 500, 700, 1000, 1500, 2000, 3000, 4000, 5000, 7000, 10000, 20000
Quantification using: Lower Marker          Final concentration (ng/uL): 0.5000          Dilution factor: 12.0
```

Sample: SampE4
Well location: E4
Created: Tuesday, August 10, 2021 10:41:10 PM

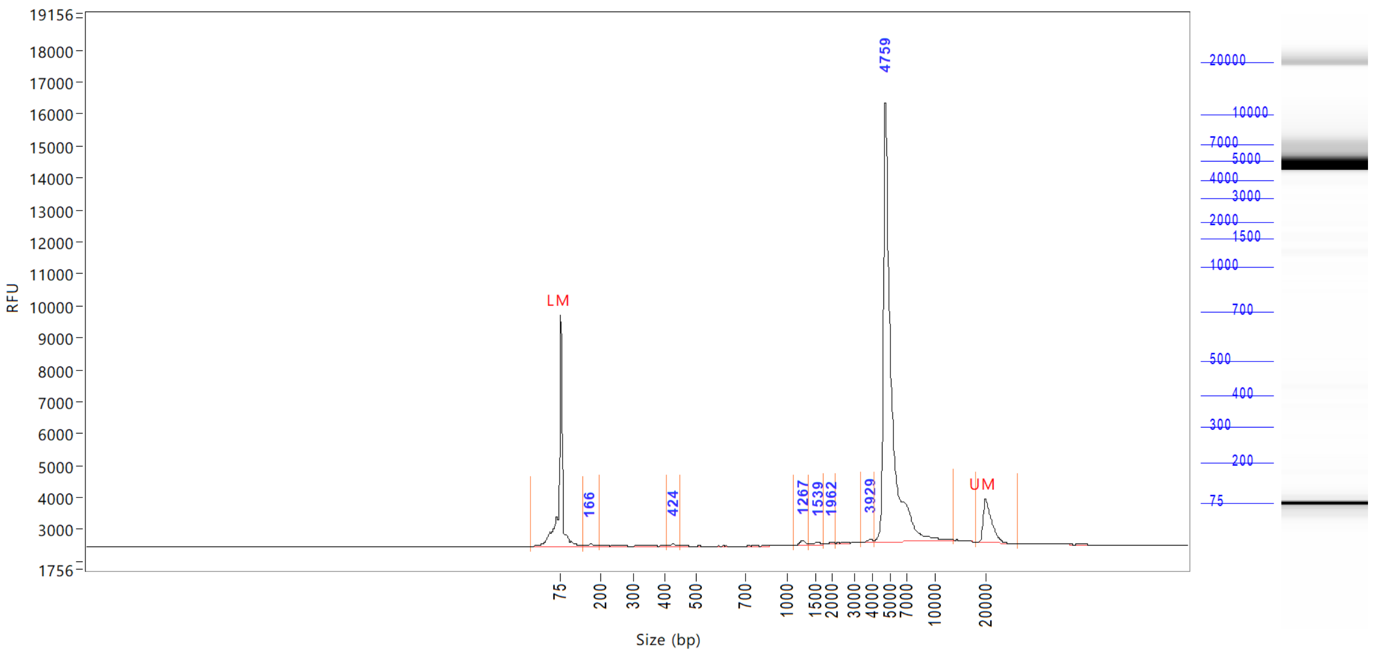

Peak	Size (bp)	Concentration (ng/uL)	From (bp)	To (bp)	Average size (bp)	CV%	RFU	Corrected peak area
1	75 (LM)	0.5000	0	147	69	25.39	7245	62.388
2	166	0.0937	147	194	168	6.61	71	0.974
3	424	0.0833	403	447	424	2.32	94	0.867
4	1267	0.1177	1112	1378	1247	3.85	140	1.224
5	1539	0.0753	1378	1731	1506	4.20	78	0.783
6	1962	0.0373	1731	2144	1964	3.98	50	0.388
7	3929	0.0860	3358	4104	3856	4.30	95	0.894
8	4759	15.2612	4104	13781	5237	18.74	13766	158.685
9	20000 (UM)	0.1154	18171	26287	20603	4.57	1355	14.400

TIC:	15.7545	ng/uL
TIM:	6.3434	nmole/L
Total concentration:	16.0821	ng/uL

Sample peak width (sec): 5 Sample min peak height: 50 Sample baseline V to V?: Y Sample baseline V to V points: 3
Sample filter: Binomial Number of points for filter: 3 Sample start region (min): 0 Sample end region (min): 30
Marker peak width (sec): 5 Marker min peak height: 200 Marker baseline V to V?: Y Marker baseline V to V points: 3
Lower marker selection: First peak > 500 RFU Upper marker selection: Last peak > 200 RFU
Ladder size (bp)75, 200, 300, 400, 500, 700, 1000, 1500, 2000, 3000, 4000, 5000, 7000, 10000, 20000
Quantification using: Lower Marker Final concentration (ng/uL): 0.5000 Dilution factor: 12.0

Sample: SampF4
Well location: F4
Created: Tuesday, August 10, 2021 10:41:10 PM

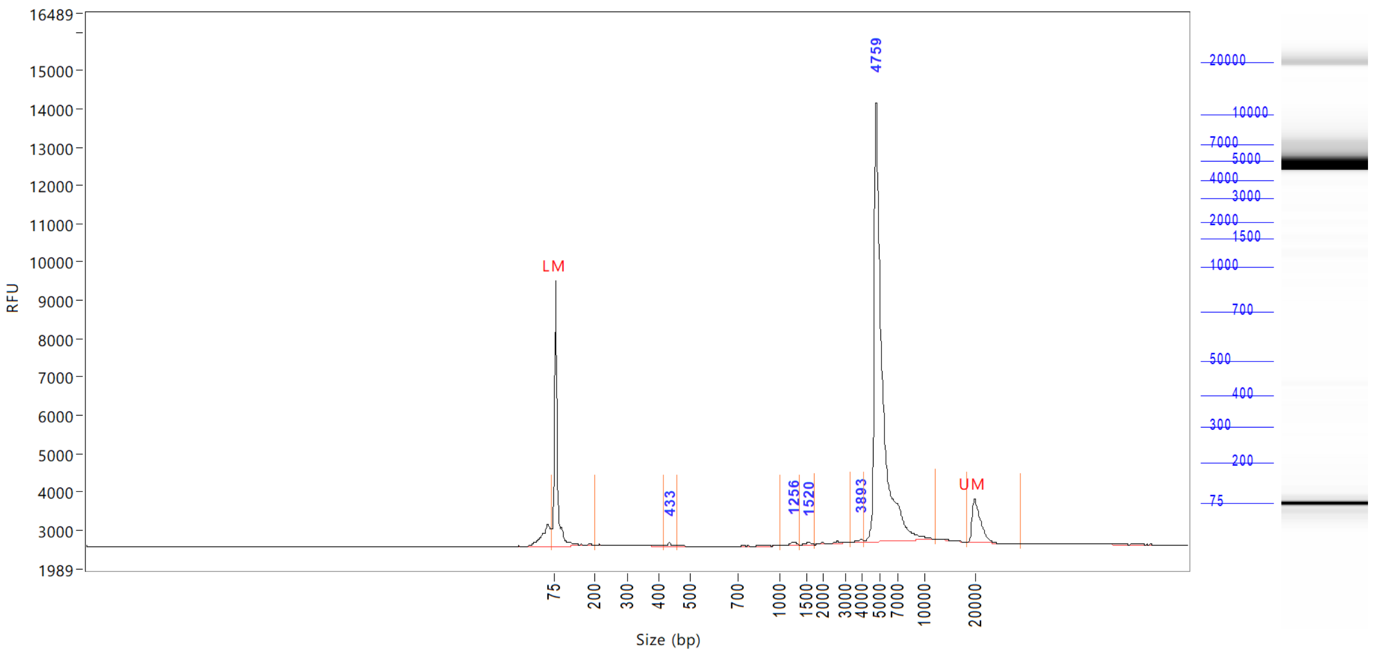

Peak	Size (bp)	Concentration (ng/uL)	From (bp)	To (bp)	Average size (bp)	CV%	RFU	Corrected peak area
1	75 (LM)	0.5000	64	200	79	22.96	6933	50.251
2	433	0.0827	418	456	433	1.73	92	0.692
3	1256	0.1159	1012	1367	1217	5.76	96	0.970
4	1520	0.0767	1367	1731	1505	4.14	68	0.643
5	3893	0.0796	3358	4070	3812	4.56	68	0.667
6	4759	16.3506	4070	12318	5250	18.10	11462	136.939
7	20000 (UM)	0.1218	18415	29551	20629	4.97	1141	12.242

TIC:	16.7055	ng/uL
TIM:	5.7164	nmole/L
Total concentration:	17.1470	ng/uL

Sample peak width (sec): 5   Sample min peak height: 50   Sample baseline V to V?: Y   Sample baseline V to V points: 3
Sample filter: Binomial   Number of points for filter: 3 Sample start region (min): 0 Sample end region (min): 30
Marker peak width (sec): 5   Marker min peak height: 200   Marker baseline V to V?: Y   Marker baseline V to V points: 3
Lower marker selection: First peak > 500 RFU   Upper marker selection: Last peak > 200 RFU
Ladder size (bp)75, 200, 300, 400, 500, 700, 1000, 1500, 2000, 3000, 4000, 5000, 7000, 10000, 20000
Quantification using: Lower Marker   Final concentration (ng/uL): 0.5000   Dilution factor: 12.0

Sample: SampG4
Well location: G4
Created: Tuesday, August 10, 2021 10:41:10 PM

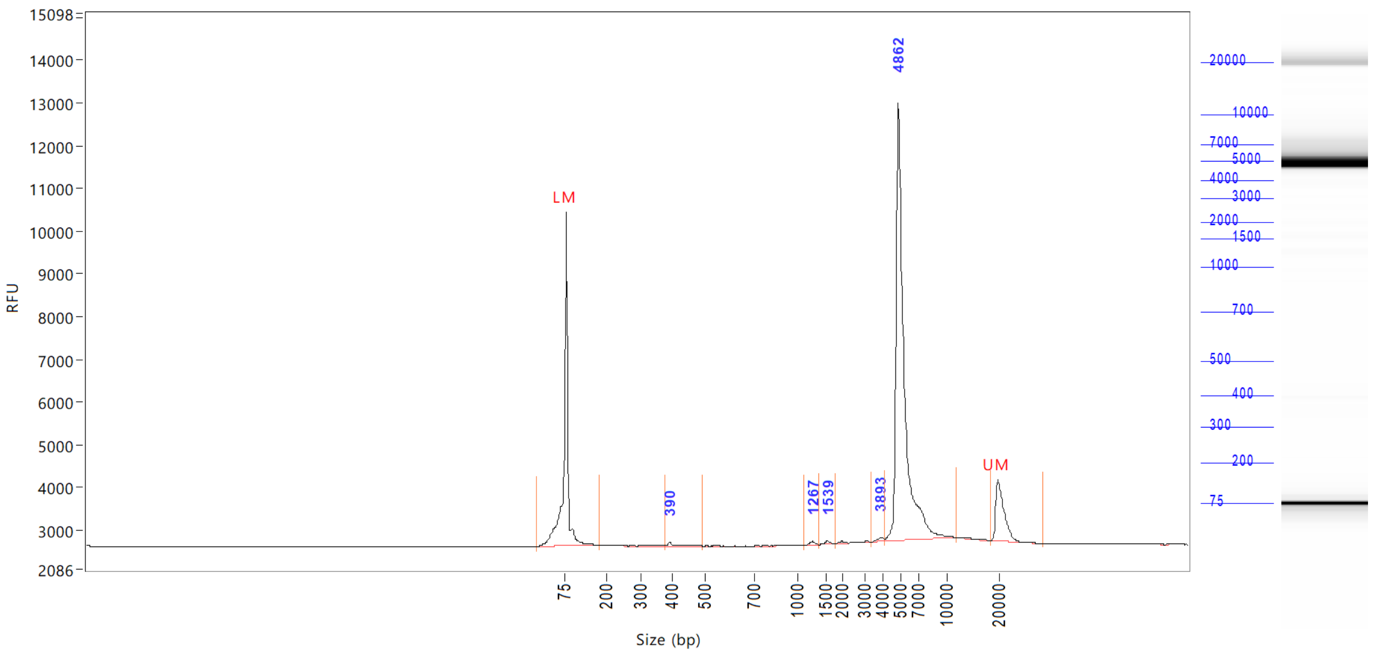

Peak	Size<br>(bp)	Concentration<br>(ng/uL)	From<br>(bp)	To<br>(bp)	Average size<br>(bp)	CV%	RFU	Corrected peak area
1	75 (LM)	0.5000	0	175	68	27.91	7796	71.475
2	390	0.0869	376	494	407	6.72	91	1.035
3	1267	0.0592	1112	1378	1244	3.60	84	0.706
4	1539	0.0472	1378	1789	1535	4.56	68	0.562
5	3893	0.0652	3322	4104	3847	4.31	77	0.777
6	4862	9.8942	4104	11708	5292	16.63	10257	117.865
7	20000 (UM)	0.1041	18415	28826	20543	4.76	1436	14.879

TIC:	10.1528	ng/uL
TIM:	3.5863	nmole/L
Total concentration:	10.3582	ng/uL

```
Sample peak width (sec): 5    Sample min peak height: 50    Sample baseline V to V?: Y    Sample baseline V to V points: 3
Sample filter: Binomial       Number of points for filter: 3  Sample start region (min): 0  Sample end region (min): 30
Marker peak width (sec): 5    Marker min peak height: 200   Marker baseline V to V?: Y    Marker baseline V to V points: 3
Lower marker selection: First peak > 500 RFU                Upper marker selection: Last peak > 200 RFU
Ladder size (bp)75, 200, 300, 400, 500, 700, 1000, 1500, 2000, 3000, 4000, 5000, 7000, 10000, 20000
Quantification using: Lower Marker          Final concentration (ng/uL): 0.5000          Dilution factor: 12.0
```

Sample: SampH4
Well location: H4
Created: Tuesday, August 10, 2021 10:41:10 PM

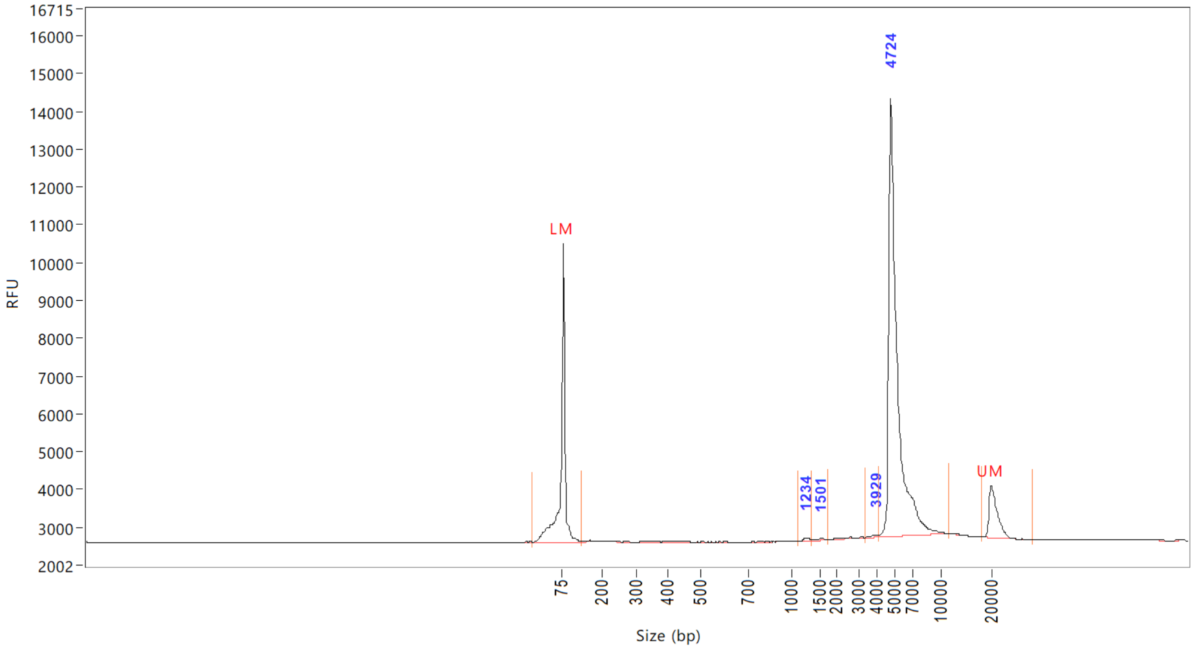

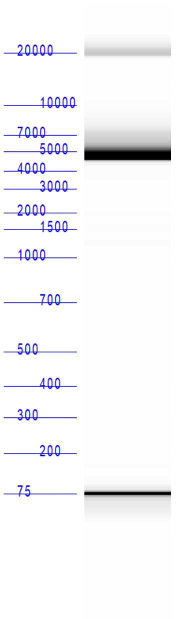

Peak	Size<br>(bp)	Concentration<br>(ng/uL)	From<br>(bp)	To<br>(bp)	Average size<br>(bp)	CV%	RFU	Corrected peak area
1	75 (LM)	0.5000	0	131	65	31.25	7895	76.312
2	1234	0.0616	1090	1334	1226	3.60	92	0.783
3	1501	0.0495	1334	1712	1489	4.50	61	0.630
4	3929	0.0552	3286	4035	3778	4.99	70	0.702
5	4724	11.1423	4035	11586	5223	17.16	11604	141.715
6	20000 (UM)	0.0975	18049	28221	20541	5.02	1387	14.876

TIC:	11.3086	ng/uL
TIM:	3.6735	nmole/L
Total concentration:	11.6474	ng/uL

```
Sample peak width (sec): 5    Sample min peak height: 50    Sample baseline V to V?: Y    Sample baseline V to V points: 3
Sample filter: Binomial       Number of points for filter: 3  Sample start region (min): 0  Sample end region (min): 30
Marker peak width (sec): 5    Marker min peak height: 200   Marker baseline V to V?: Y    Marker baseline V to V points: 3
Lower marker selection: First peak > 500 RFU                Upper marker selection: Last peak > 200 RFU
Ladder size (bp)75, 200, 300, 400, 500, 700, 1000, 1500, 2000, 3000, 4000, 5000, 7000, 10000, 20000
Quantification using: Lower Marker          Final concentration (ng/uL): 0.5000          Dilution factor: 12.0
```

Sample: SampA5
Well location: A5
Created: Tuesday, August 10, 2021 10:41:10 PM

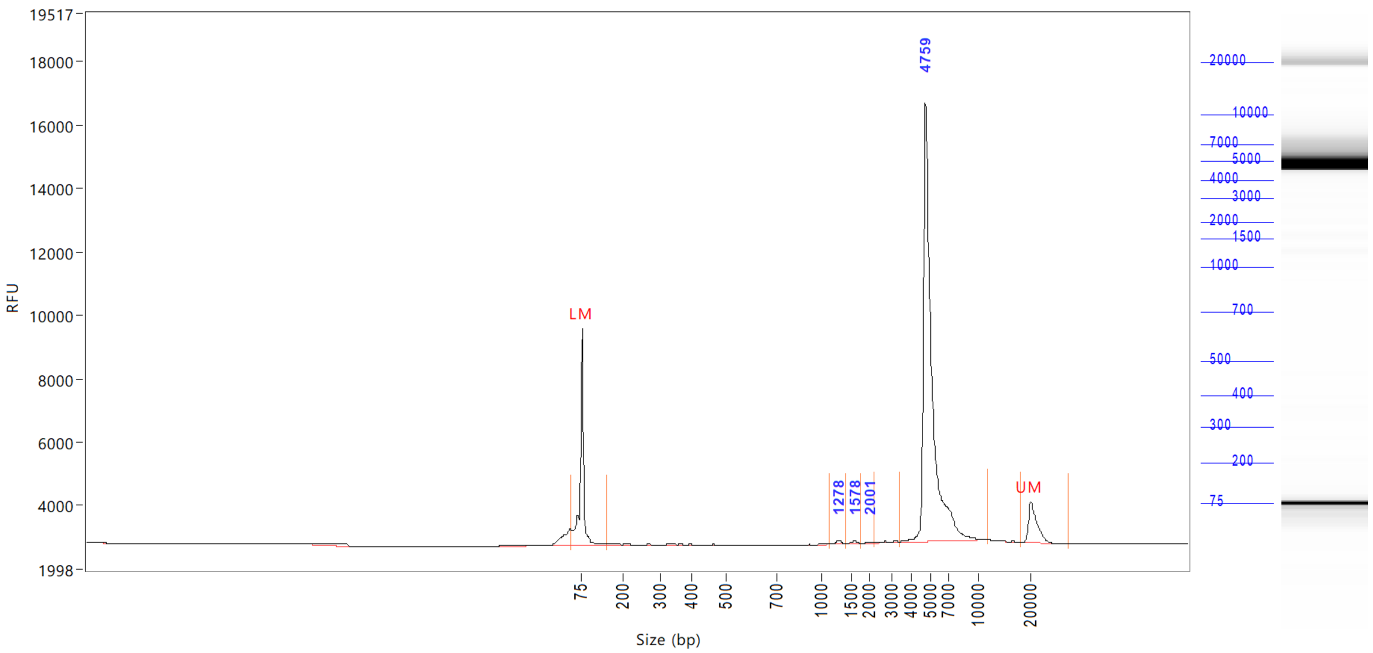

Peak	Size (bp)	Concentration (ng/uL)	From (bp)	To (bp)	Average size (bp)	CV%	RFU	Corrected peak area
1	75 (LM)	0.5000	45	148	72	15.80	6830	53.357
2	1278	0.1142	1123	1400	1266	3.65	118	1.015
3	1578	0.0867	1400	1770	1547	4.53	77	0.771
4	2001	0.0487	1770	2258	2019	4.73	53	0.433
5	4759	18.1108	3393	11708	5213	16.80	13848	161.056
6	20000 (UM)	0.1240	18049	27254	20579	4.65	1274	13.233

TIC:	18.3604	ng/uL
TIM:	5.9999	nmole/L
Total concentration:	18.8446	ng/uL

Sample peak width (sec): 5 Sample min peak height: 50 Sample baseline V to V?: Y Sample baseline V to V points: 3
Sample filter: Binomial Number of points for filter: 3 Sample start region (min): 0 Sample end region (min): 30
Marker peak width (sec): 5 Marker min peak height: 200 Marker baseline V to V?: Y Marker baseline V to V points: 3
Lower marker selection: First peak > 500 RFU Upper marker selection: Last peak > 200 RFU
Ladder size (bp)75, 200, 300, 400, 500, 700, 1000, 1500, 2000, 3000, 4000, 5000, 7000, 10000, 20000
Quantification using: Lower Marker Final concentration (ng/uL): 0.5000 Dilution factor: 12.0

Sample: SampB5
Well location: B5
Created: Tuesday, August 10, 2021 10:41:10 PM

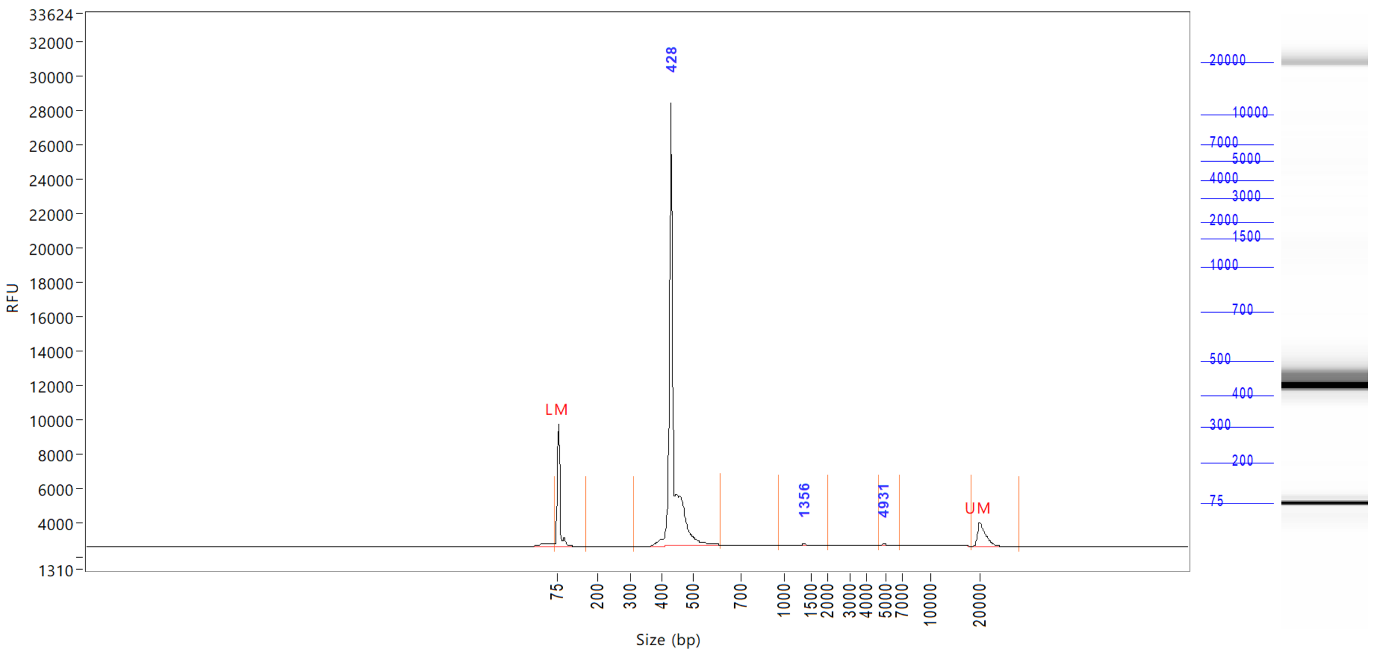

Peak	Size (bp)	Concentration (ng/uL)	From (bp)	To (bp)	Average size (bp)	CV%	RFU	Corrected peak area
1	75 (LM)	0.5000	64	166	76	11.23	7088	44.685
2	428	30.0313	309	611	436	5.41	25788	223.660
3	1356	0.3114	965	2001	1284	15.35	70	2.320
4	4931	0.1045	4690	6704	5372	10.90	55	0.779
5	20000 (UM)	0.1600	18293	27979	20547	4.64	1374	14.300

TIC:	30.4473	ng/uL
TIM:	113.7638	nmole/L
Total concentration:	30.7611	ng/uL

Sample peak width (sec): 5 Sample min peak height: 50 Sample baseline V to V?: Y Sample baseline V to V points: 3
Sample filter: Binomial Number of points for filter: 3 Sample start region (min): 0 Sample end region (min): 30
Marker peak width (sec): 5 Marker min peak height: 200 Marker baseline V to V?: Y Marker baseline V to V points: 3
Lower marker selection: First peak > 500 RFU Upper marker selection: Last peak > 200 RFU
Ladder size (bp)75, 200, 300, 400, 500, 700, 1000, 1500, 2000, 3000, 4000, 5000, 7000, 10000, 20000
Quantification using: Lower Marker Final concentration (ng/uL): 0.5000 Dilution factor: 12.0

Sample: SampC5
Well location: C5
Created: Tuesday, August 10, 2021 10:41:10 PM

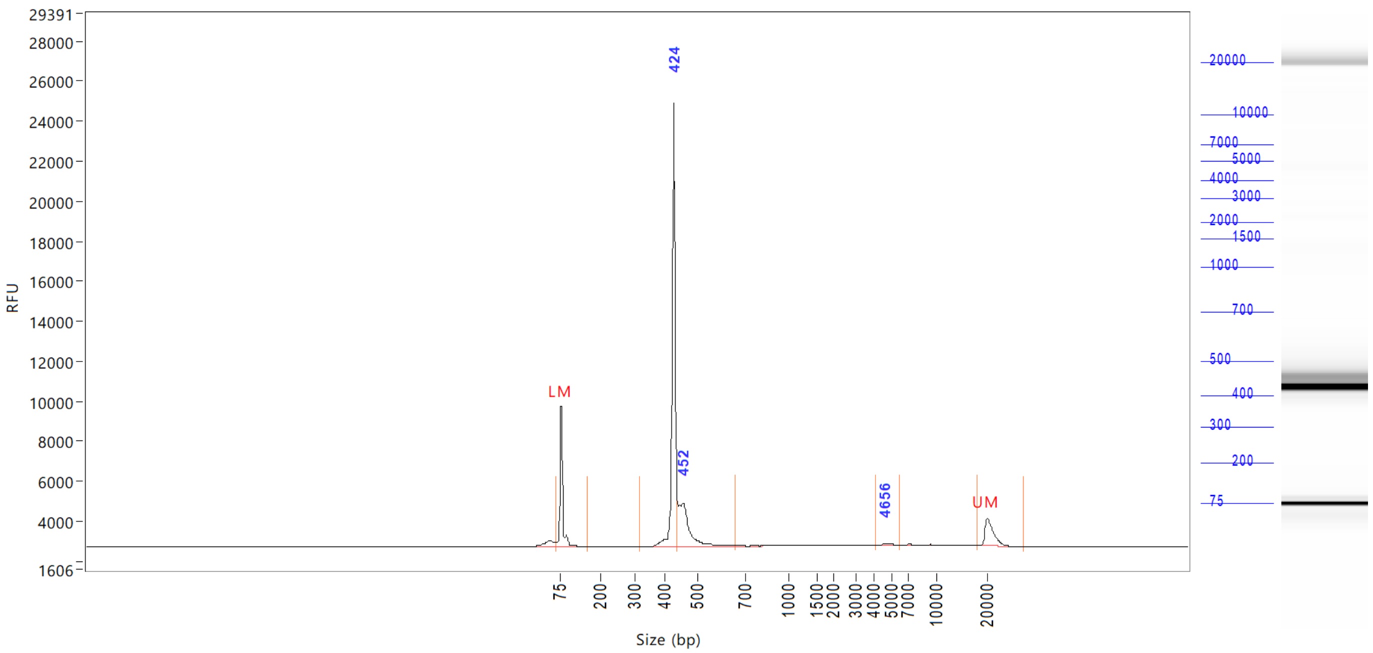

Peak	Size<br>(bp)	Concentration<br>(ng/uL)	From<br>(bp)	To<br>(bp)	Average size<br>(bp)	CV%	RFU	Corrected peak area
1	75 (LM)	0.5000	62	158	75	13.66	7032	47.184
2	424	17.5233	315	437	420	2.25	22205	137.803
3	452	5.7922	437	654	465	6.55	2166	45.550
4	4656	0.1415	4104	5963	4818	8.17	77	1.113
5	20000 (UM)	0.1528	18049	27254	20613	4.66	1361	14.418

TIC:	23.4569	ng/uL
TIM:	89.1915	nmole/L
Total concentration:	23.8119	ng/uL

Sample peak width (sec): 5 Sample min peak height: 50 Sample baseline V to V?: Y Sample baseline V to V points: 3
Sample filter: Binomial Number of points for filter: 3 Sample start region (min): 0 Sample end region (min): 30
Marker peak width (sec): 5 Marker min peak height: 200 Marker baseline V to V?: Y Marker baseline V to V points: 3
Lower marker selection: First peak > 500 RFU Upper marker selection: Last peak > 200 RFU
Ladder size (bp)75, 200, 300, 400, 500, 700, 1000, 1500, 2000, 3000, 4000, 5000, 7000, 10000, 20000
Quantification using: Lower Marker Final concentration (ng/uL): 0.5000 Dilution factor: 12.0

Sample: SampD5
Well location: D5
Created: Tuesday, August 10, 2021 10:41:10 PM

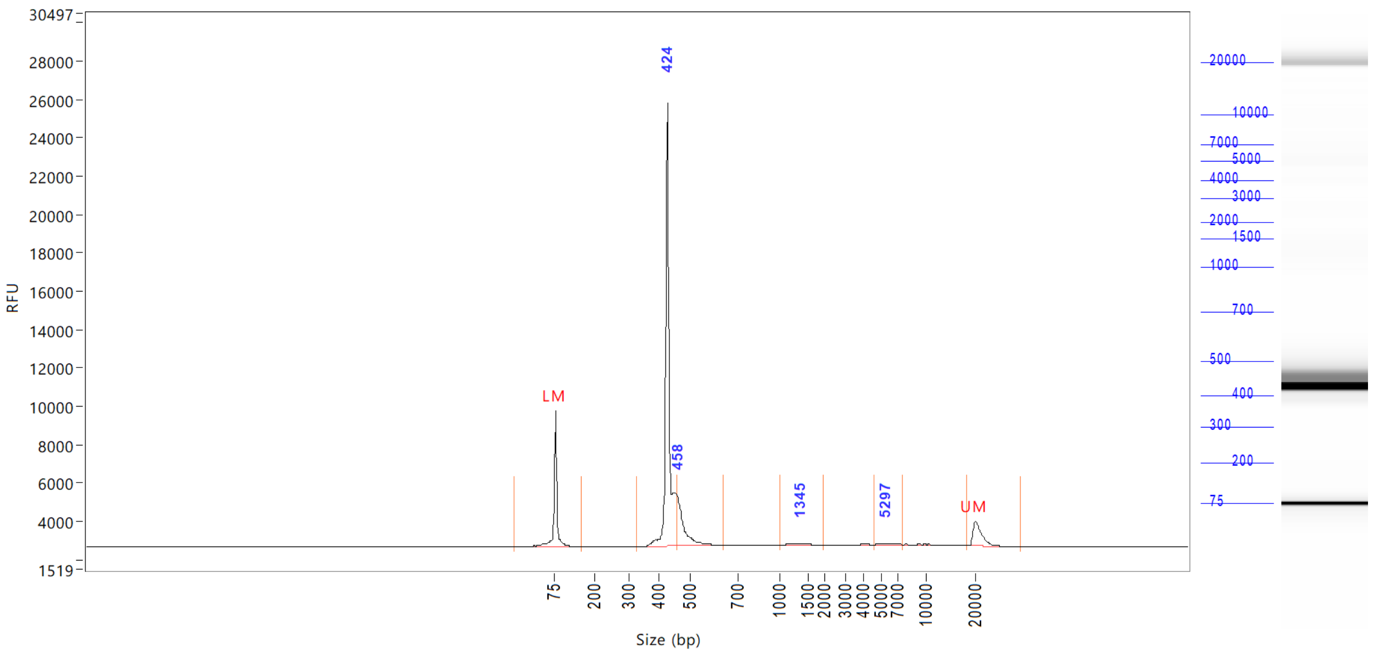

Peak	Size (bp)	Concentration (ng/uL)	From (bp)	To (bp)	Average size (bp)	CV%	RFU	Corrected peak area
1	75 (LM)	0.5000	0	158	70	24.80	7053	51.943
2	424	20.5731	325	458	426	3.11	23134	178.105
3	458	3.6717	458	636	481	5.55	2557	31.786
4	1345	0.2946	1001	1962	1321	13.53	77	2.550
5	5297	0.2182	4587	7534	5642	14.36	88	1.889
6	20000 (UM)	0.1379	18171	28947	20572	5.30	1312	14.328

TIC:	24.7576	ng/uL
TIM:	92.4518	nmole/L
Total concentration:	24.8355	ng/uL

Sample peak width (sec): 5 Sample min peak height: 50 Sample baseline V to V?: Y Sample baseline V to V points: 3
Sample filter: Binomial Number of points for filter: 3 Sample start region (min): 0 Sample end region (min): 30
Marker peak width (sec): 5 Marker min peak height: 200 Marker baseline V to V?: Y Marker baseline V to V points: 3
Lower marker selection: First peak > 500 RFU Upper marker selection: Last peak > 200 RFU
Ladder size (bp)75, 200, 300, 400, 500, 700, 1000, 1500, 2000, 3000, 4000, 5000, 7000, 10000, 20000
Quantification using: Lower Marker Final concentration (ng/uL): 0.5000 Dilution factor: 12.0

Sample: SampE5
Well location: E5
Created: Tuesday, August 10, 2021 10:41:10 PM

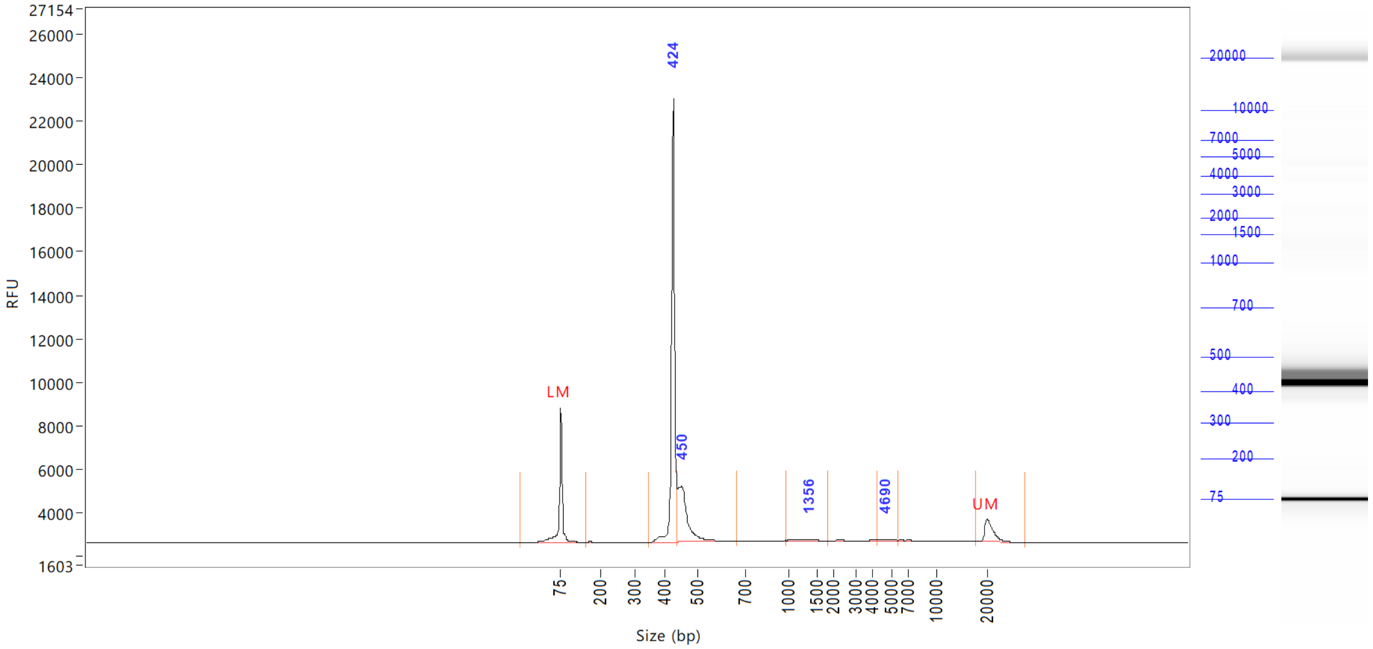

Peak	Size (bp)	Concentration (ng/uL)	From (bp)	To (bp)	Average size (bp)	CV%	RFU	Corrected peak area
1	75 (LM)	0.5000	0	154	70	25.29	6165	45.764
2	424	17.1977	343	437	421	2.35	20402	131.172
3	450	6.4160	437	664	461	5.94	2542	48.937
4	1356	0.3368	983	1808	1280	13.48	77	2.569
5	4690	0.1292	4208	5741	4804	7.18	69	0.985
6	20000 (UM)	0.1196	17683	27496	20557	4.85	1038	10.945

TIC:	24.0796	ng/uL
TIM:	90.5896	nmole/L
Total concentration:	24.1677	ng/uL

Sample peak width (sec): 5 Sample min peak height: 50 Sample baseline V to V?: Y Sample baseline V to V points: 3
Sample filter: Binomial Number of points for filter: 3 Sample start region (min): 0 Sample end region (min): 30
Marker peak width (sec): 5 Marker min peak height: 200 Marker baseline V to V?: Y Marker baseline V to V points: 3
Lower marker selection: First peak > 500 RFU Upper marker selection: Last peak > 200 RFU
Ladder size (bp)75, 200, 300, 400, 500, 700, 1000, 1500, 2000, 3000, 4000, 5000, 7000, 10000, 20000
Quantification using: Lower Marker Final concentration (ng/uL): 0.5000 Dilution factor: 12.0

Sample: SampF5
Well location: F5
Created: Tuesday, August 10, 2021 10:41:10 PM

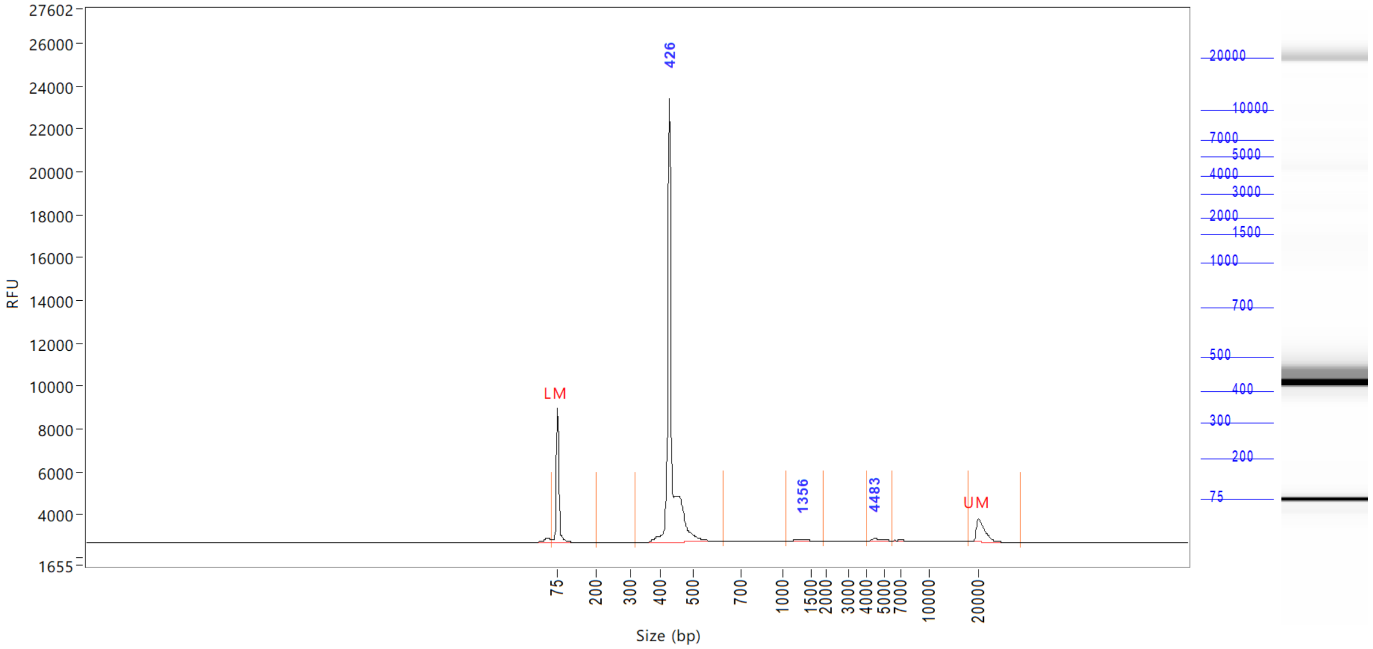

Peak	Size (bp)	Concentration (ng/uL)	From (bp)	To (bp)	Average size (bp)	CV%	RFU	Corrected peak area
1	75 (LM)	0.5000	60	198	76	17.17	6255	39.461
2	426	27.1822	315	629	433	5.62	20724	178.771
3	1356	0.2995	1056	1923	1336	12.37	64	1.970
4	4483	0.2564	4035	5963	4679	8.53	114	1.686
5	20000 (UM)	0.1489	17927	28463	20571	4.94	1094	11.752

TIC:	27.7382	ng/uL
TIM:	103.7500	nmole/L
Total concentration:	28.1321	ng/uL

```
Sample peak width (sec): 5    Sample min peak height: 50    Sample baseline V to V?: Y    Sample baseline V to V points: 3
Sample filter: Binomial       Number of points for filter: 3  Sample start region (min): 0  Sample end region (min): 30
Marker peak width (sec): 5    Marker min peak height: 200   Marker baseline V to V?: Y    Marker baseline V to V points: 3
Lower marker selection: First peak > 500 RFU                Upper marker selection: Last peak > 200 RFU
Ladder size (bp)75, 200, 300, 400, 500, 700, 1000, 1500, 2000, 3000, 4000, 5000, 7000, 10000, 20000
Quantification using: Lower Marker          Final concentration (ng/uL): 0.5000          Dilution factor: 12.0
```

Sample: SampG5
Well location: G5
Created: Tuesday, August 10, 2021 10:41:10 PM

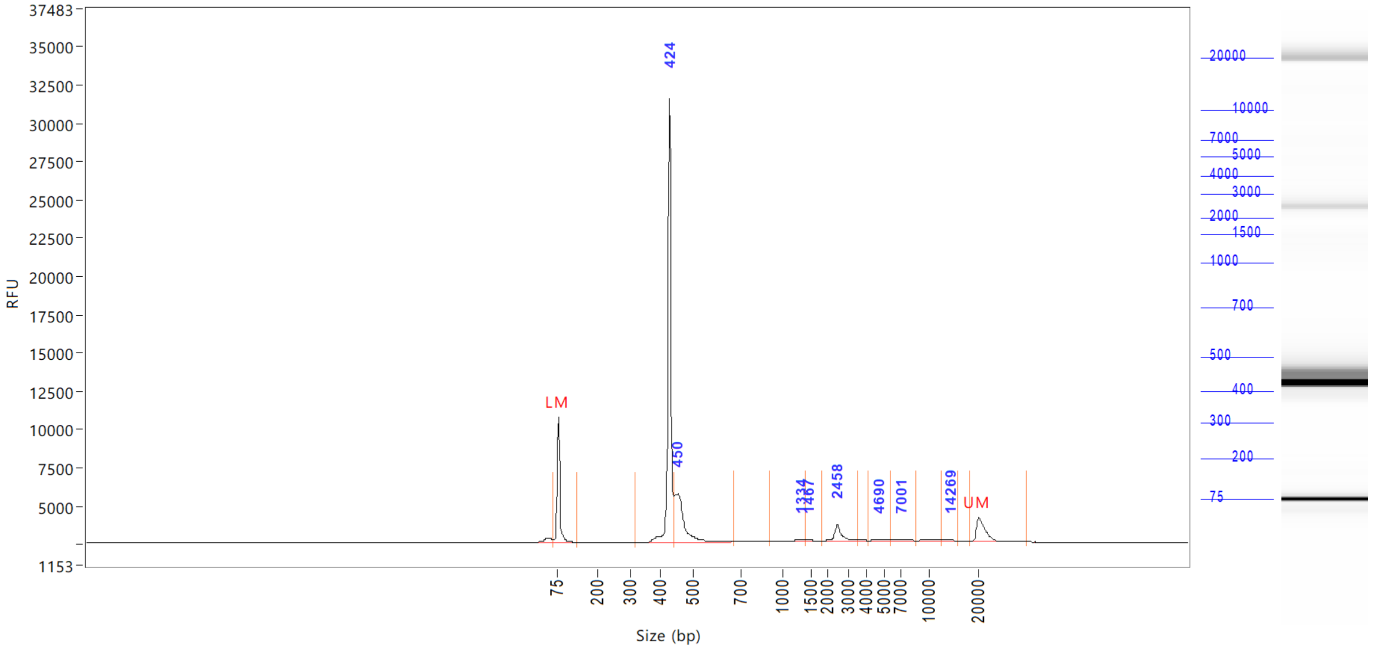

Peak	Size (bp)	Concentration (ng/uL)	From (bp)	To (bp)	Average size (bp)	CV%	RFU	Corrected peak area
1	75 (LM)	0.5000	62	135	75	10.75	8238	52.382
2	424	21.1447	313	437	420	2.29	29032	184.599
3	450	7.1294	437	672	463	6.38	3156	62.242
4	1334	0.2637	900	1400	1201	11.37	93	2.302
5	1467	0.1385	1400	1866	1494	5.50	101	1.209
6	2458	1.3714	1866	3572	2534	10.83	1048	11.973
7	4690	0.1289	4104	5815	4818	8.52	61	1.125
8	7001	0.1167	5815	8600	6937	9.52	54	1.019
9	14269	0.0830	12562	15732	14015	6.11	52	0.724
10	20000 (UM)	0.1661	18293	29551	20660	5.30	1566	17.397

TIC:	30.3763	ng/uL
TIM:	109.6574	nmole/L
Total concentration:	30.3924	ng/uL

```
Sample peak width (sec): 5     Sample min peak height: 50     Sample baseline V to V?: Y     Sample baseline V to V points: 3
Sample filter: Binomial        Number of points for filter: 3  Sample start region (min): 0  Sample end region (min): 30
Marker peak width (sec): 5     Marker min peak height: 200    Marker baseline V to V?: Y     Marker baseline V to V points: 3
Lower marker selection: First peak > 500 RFU                  Upper marker selection: Last peak > 200 RFU
Ladder size (bp)75, 200, 300, 400, 500, 700, 1000, 1500, 2000, 3000, 4000, 5000, 7000, 10000, 20000
Quantification using: Lower Marker          Final concentration (ng/uL): 0.5000          Dilution factor: 12.0
```

Sample: SampH5
Well location: H5
Created: Tuesday, August 10, 2021 10:41:10 PM

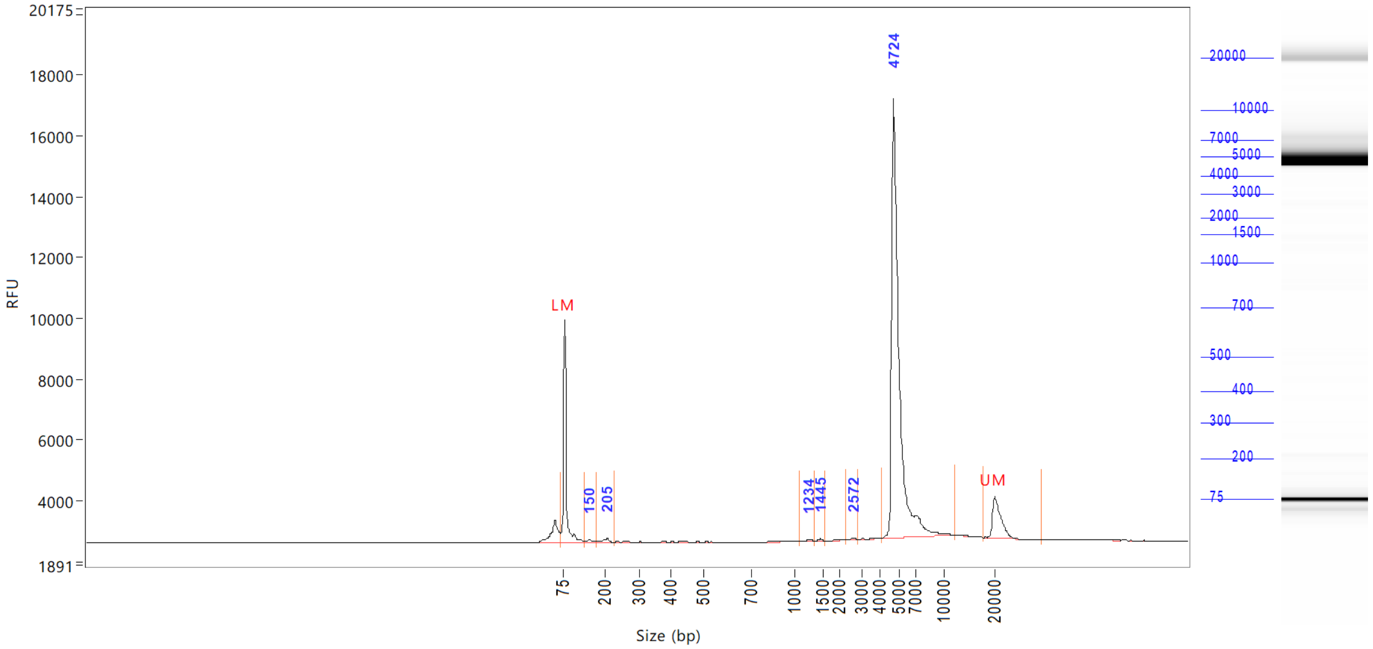

Peak	Size (bp)	Concentration (ng/uL)	From (bp)	To (bp)	Average size (bp)	CV%	RFU	Corrected peak area
1	75 (LM)	0.5000	62	135	75	13.34	7313	49.615
2	150	0.1270	135	173	149	5.97	95	1.050
3	205	0.1790	173	226	197	4.67	140	1.480
4	1234	0.0579	1090	1356	1245	3.78	51	0.479
5	1445	0.0525	1356	1539	1433	2.15	74	0.434
6	2572	0.0543	2229	2829	2551	4.23	59	0.449
7	4724	18.2726	4070	11952	5115	16.40	14465	151.099
8	20000 (UM)	0.1461	17683	29309	20500	4.72	1350	14.496

TIC:	18.7434	ng/uL
TIM:	8.9483	nmole/L
Total concentration:	19.1906	ng/uL

Sample peak width (sec): 5   Sample min peak height: 50   Sample baseline V to V?: Y   Sample baseline V to V points: 3
Sample filter: Binomial   Number of points for filter: 3   Sample start region (min): 0   Sample end region (min): 30
Marker peak width (sec): 5   Marker min peak height: 200   Marker baseline V to V?: Y   Marker baseline V to V points: 3
Lower marker selection: First peak > 500 RFU   Upper marker selection: Last peak > 200 RFU
Ladder size (bp)75, 200, 300, 400, 500, 700, 1000, 1500, 2000, 3000, 4000, 5000, 7000, 10000, 20000
Quantification using: Lower Marker   Final concentration (ng/uL): 0.5000   Dilution factor: 12.0

Sample: SampA6
Well location: A6
Created: Tuesday, August 10, 2021 10:41:10 PM

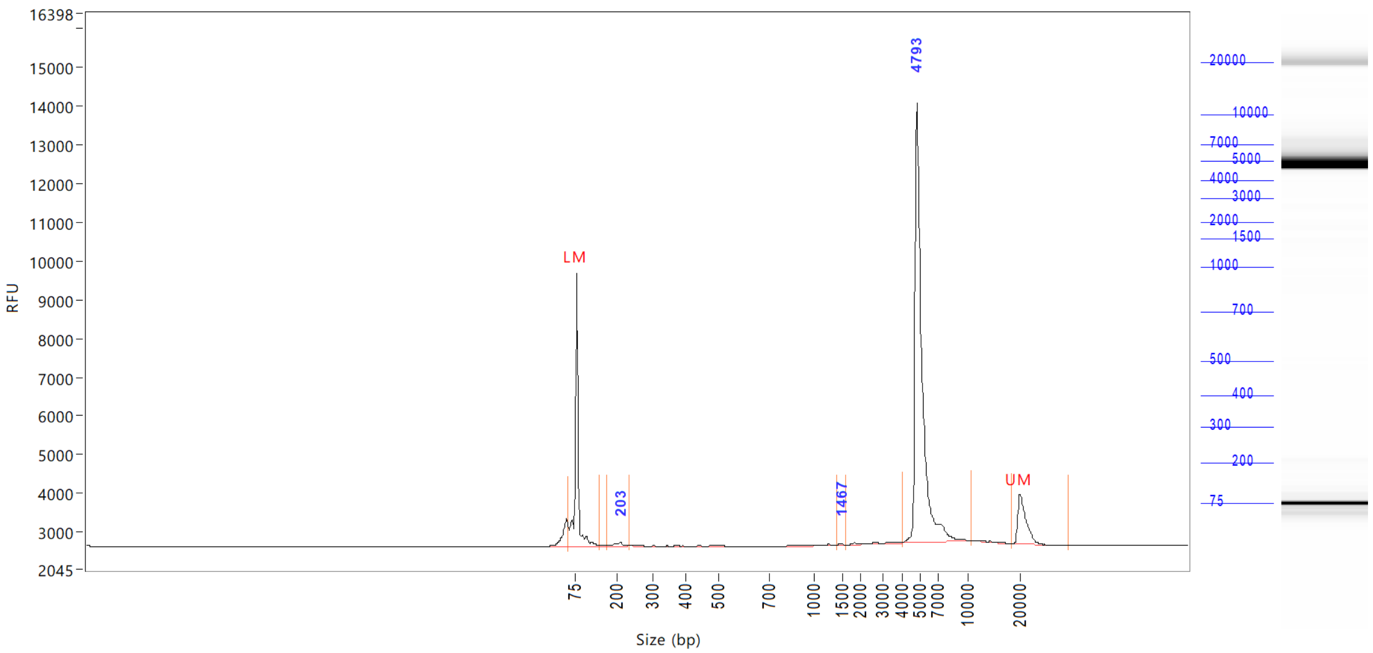

Peak	Size (bp)	Concentration (ng/uL)	From (bp)	To (bp)	Average size (bp)	CV%	RFU	Corrected peak area
1	75 (LM)	0.5000	52	143	75	15.95	7043	49.408
2	203	0.2032	166	233	193	6.97	105	1.673
3	1467	0.0398	1389	1578	1448	2.00	53	0.328
4	4793	13.5681	4070	10611	5127	14.09	11369	111.728
5	20000 (UM)	0.1356	18415	29189	20529	4.98	1290	13.401

TIC:	13.8110	ng/uL
TIM:	6.1326	nmole/L
Total concentration:	14.3651	ng/uL

```
Sample peak width (sec): 5    Sample min peak height: 50    Sample baseline V to V?: Y    Sample baseline V to V points: 3
Sample filter: Binomial       Number of points for filter: 3  Sample start region (min): 0  Sample end region (min): 30
Marker peak width (sec): 5    Marker min peak height: 200   Marker baseline V to V?: Y    Marker baseline V to V points: 3
Lower marker selection: First peak > 500 RFU                Upper marker selection: Last peak > 200 RFU
Ladder size (bp)75, 200, 300, 400, 500, 700, 1000, 1500, 2000, 3000, 4000, 5000, 7000, 10000, 20000
Quantification using: Lower Marker          Final concentration (ng/uL): 0.5000          Dilution factor: 12.0
```

Sample: SampB6
Well location: B6
Created: Tuesday, August 10, 2021 10:41:10 PM

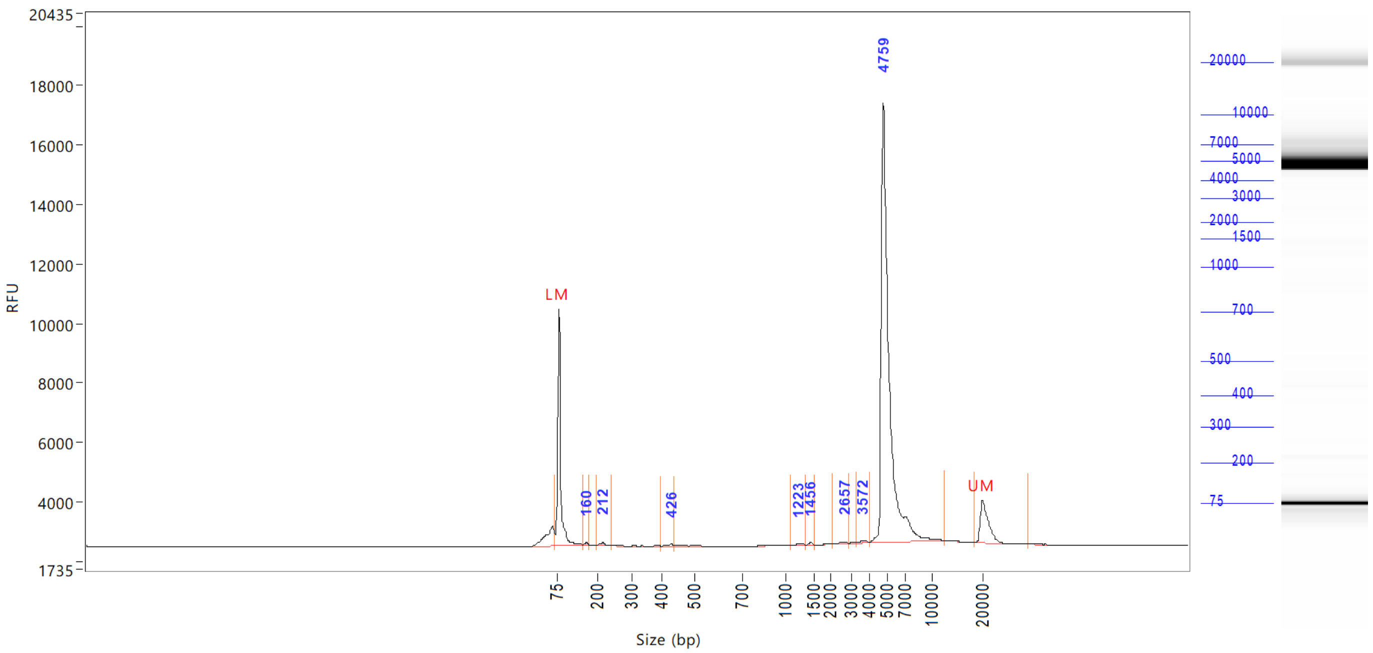

Peak	Size (bp)	Concentration (ng/uL)	From (bp)	To (bp)	Average size (bp)	CV%	RFU	Corrected peak area
1	75 (LM)	0.5000	64	148	77	14.04	7939	55.207
2	160	0.0750	148	169	158	2.93	100	0.690
3	212	0.1057	196	239	208	2.58	129	0.972
4	426	0.0696	392	437	421	2.32	73	0.641
5	1223	0.0598	1090	1356	1239	4.15	56	0.550
6	1456	0.0562	1356	1539	1437	2.28	80	0.517
7	2657	0.0857	2087	2857	2515	6.94	67	0.789
8	3572	0.0855	3251	4001	3643	5.28	76	0.787
9	4759	17.7909	4001	12196	5183	16.57	14807	163.696
10	20000 (UM)	0.1370	18293	29068	20506	5.00	1434	15.131

TIC:	18.3285	ng/uL
TIM:	7.7773	nmole/L
Total concentration:	18.6840	ng/uL

Sample peak width (sec): 5 Sample min peak height: 50 Sample baseline V to V?: Y Sample baseline V to V points: 3
Sample filter: Binomial Number of points for filter: 3 Sample start region (min): 0 Sample end region (min): 30
Marker peak width (sec): 5 Marker min peak height: 200 Marker baseline V to V?: Y Marker baseline V to V points: 3
Lower marker selection: First peak > 500 RFU Upper marker selection: Last peak > 200 RFU
Ladder size (bp)75, 200, 300, 400, 500, 700, 1000, 1500, 2000, 3000, 4000, 5000, 7000, 10000, 20000
Quantification using: Lower Marker Final concentration (ng/uL): 0.5000 Dilution factor: 12.0

Sample: SampC6
Well location: C6
Created: Tuesday, August 10, 2021 10:41:10 PM

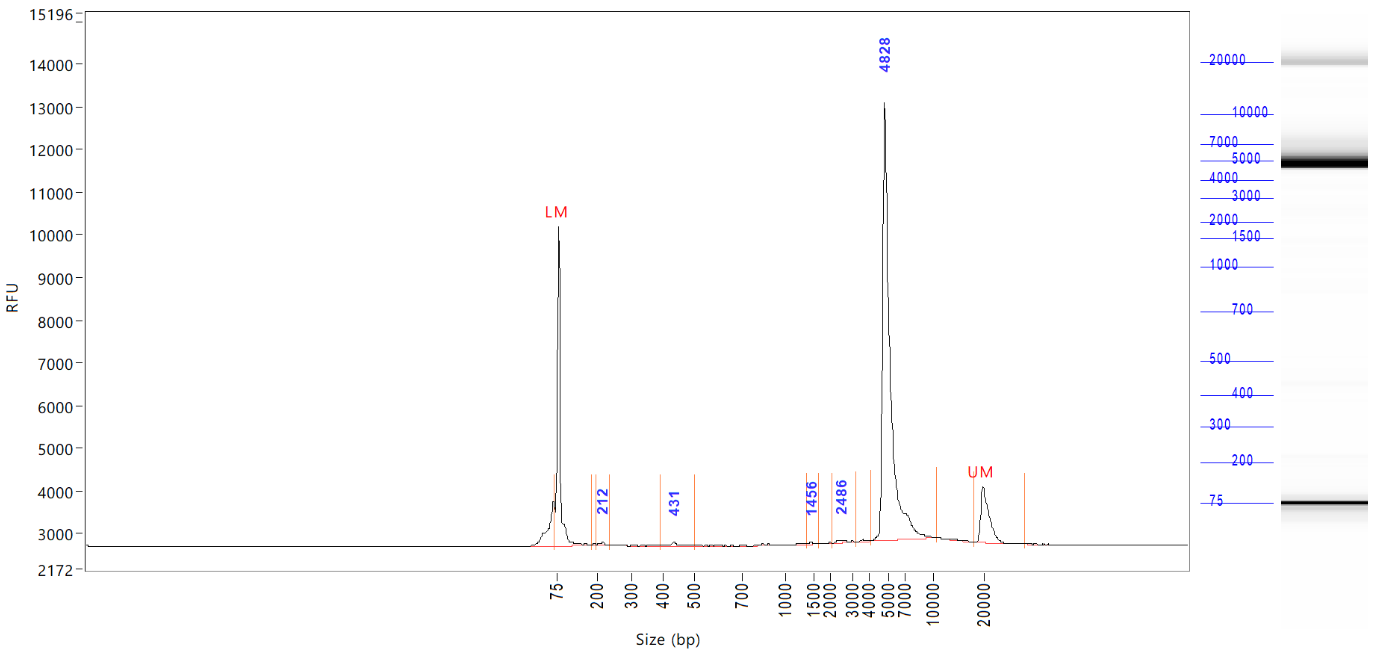

Peak	Size (bp)	Concentration (ng/uL)	From (bp)	To (bp)	Average size (bp)	CV%	RFU	Corrected peak area
1	75 (LM)	0.5000	66	177	77	18.12	7465	52.101
2	212	0.1241	192	233	207	3.68	102	1.078
3	431	0.1459	392	500	438	5.03	98	1.267
4	1456	0.0402	1367	1635	1442	2.46	54	0.349
5	2486	0.1201	2087	3251	2551	9.78	62	1.043
6	4828	12.8833	4035	10611	5223	15.28	10274	111.871
7	20000 (UM)	0.1353	18293	28100	20553	4.96	1304	14.098

TIC:	13.3137	ng/uL
TIM:	5.7183	nmole/L
Total concentration:	13.6699	ng/uL

```
Sample peak width (sec): 5    Sample min peak height: 50    Sample baseline V to V?: Y    Sample baseline V to V points: 3
Sample filter: Binomial       Number of points for filter: 3  Sample start region (min): 0  Sample end region (min): 30
Marker peak width (sec): 5    Marker min peak height: 200   Marker baseline V to V?: Y    Marker baseline V to V points: 3
Lower marker selection: First peak > 500 RFU                Upper marker selection: Last peak > 200 RFU
Ladder size (bp)75, 200, 300, 400, 500, 700, 1000, 1500, 2000, 3000, 4000, 5000, 7000, 10000, 20000
Quantification using: Lower Marker          Final concentration (ng/uL): 0.5000          Dilution factor: 12.0
```

Sample: SampD6
Well location: D6
Created: Tuesday, August 10, 2021 10:41:10 PM

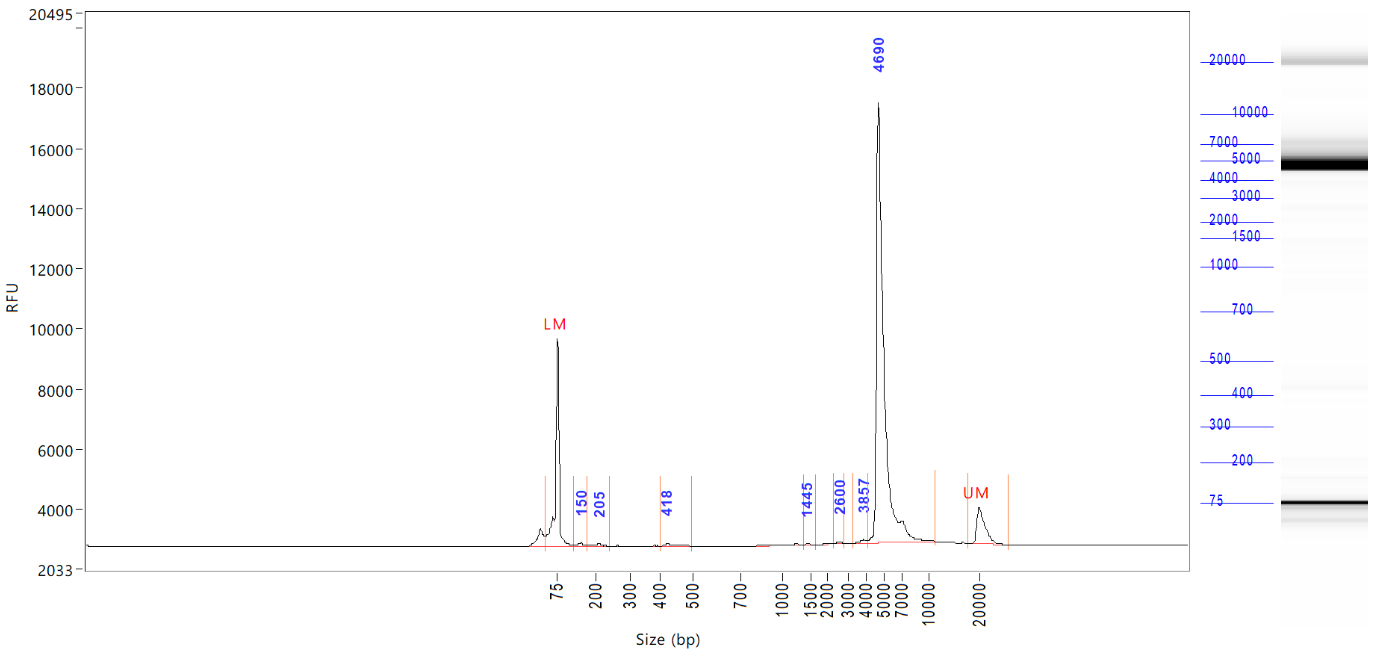

Peak	Size (bp)	Concentration (ng/uL)	From (bp)	To (bp)	Average size (bp)	CV%	RFU	Corrected peak area
1	75 (LM)	0.5000	37	128	70	16.92	6907	57.660
2	150	0.1567	128	169	146	6.97	122	1.506
3	205	0.1343	169	235	195	6.59	79	1.290
4	418	0.1548	398	498	432	5.01	104	1.487
5	1445	0.0448	1367	1635	1440	2.69	67	0.430
6	2600	0.0729	2286	2829	2541	4.19	82	0.700
7	3857	0.1198	3215	4035	3747	5.22	114	1.151
8	4690	16.1696	4035	11098	5027	14.91	14639	155.390
9	20000 (UM)	0.1131	17805	26045	20565	4.73	1215	13.041

TIC:	16.8528	ng/uL
TIM:	8.9321	nmole/L
Total concentration:	17.1287	ng/uL

```
Sample peak width (sec): 5    Sample min peak height: 50    Sample baseline V to V?: Y    Sample baseline V to V points: 3
Sample filter: Binomial       Number of points for filter: 3  Sample start region (min): 0  Sample end region (min): 30
Marker peak width (sec): 5    Marker min peak height: 200   Marker baseline V to V?: Y    Marker baseline V to V points: 3
Lower marker selection: First peak > 500 RFU                Upper marker selection: Last peak > 200 RFU
Ladder size (bp)75, 200, 300, 400, 500, 700, 1000, 1500, 2000, 3000, 4000, 5000, 7000, 10000, 20000
Quantification using: Lower Marker          Final concentration (ng/uL): 0.5000         Dilution factor: 12.0
```

Sample: SampE6
Well location: E6
Created: Tuesday, August 10, 2021 10:41:10 PM

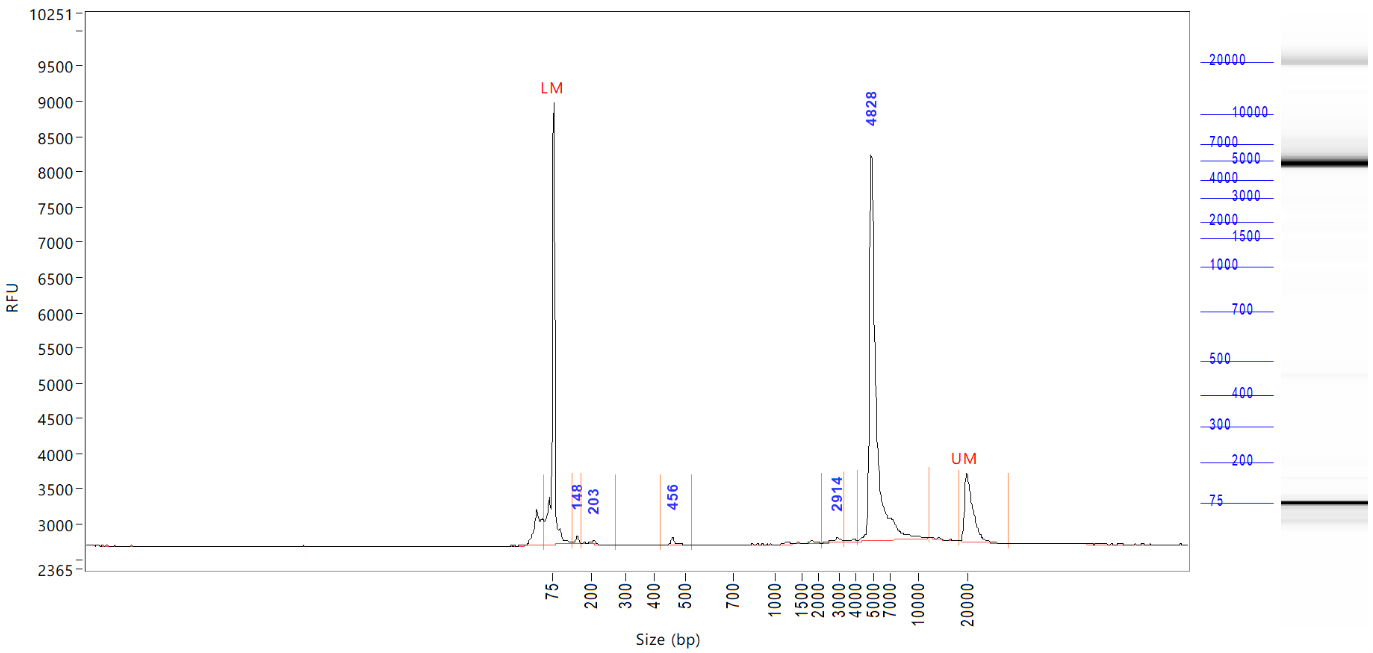

Peak	Size (bp)	Concentration (ng/uL)	From (bp)	To (bp)	Average size (bp)	CV%	RFU	Corrected peak area
1	75 (LM)	0.5000	45	137	72	14.49	6276	45.404
2	148	0.0927	137	166	147	3.14	108	0.702
3	203	0.1020	166	273	200	9.07	50	0.772
4	456	0.1650	418	521	460	3.92	120	1.248
5	2914	0.1010	2172	3251	2839	7.60	63	0.764
6	4828	7.0958	4070	12440	5264	17.64	5463	53.696
7	20000 (UM)	0.1183	18537	28463	20685	5.54	973	10.746

TIC:	7.5564	ng/uL
TIM:	4.7430	nmole/L
Total concentration:	7.9035	ng/uL

Sample peak width (sec): 5 Sample min peak height: 50 Sample baseline V to V?: Y Sample baseline V to V points: 3
Sample filter: Binomial Number of points for filter: 3 Sample start region (min): 0 Sample end region (min): 30
Marker peak width (sec): 5 Marker min peak height: 200 Marker baseline V to V?: Y Marker baseline V to V points: 3
Lower marker selection: First peak > 500 RFU Upper marker selection: Last peak > 200 RFU
Ladder size (bp)75, 200, 300, 400, 500, 700, 1000, 1500, 2000, 3000, 4000, 5000, 7000, 10000, 20000
Quantification using: Lower Marker Final concentration (ng/uL): 0.5000 Dilution factor: 12.0

Sample: SampF6
Well location: F6
Created: Tuesday, August 10, 2021 10:41:10 PM

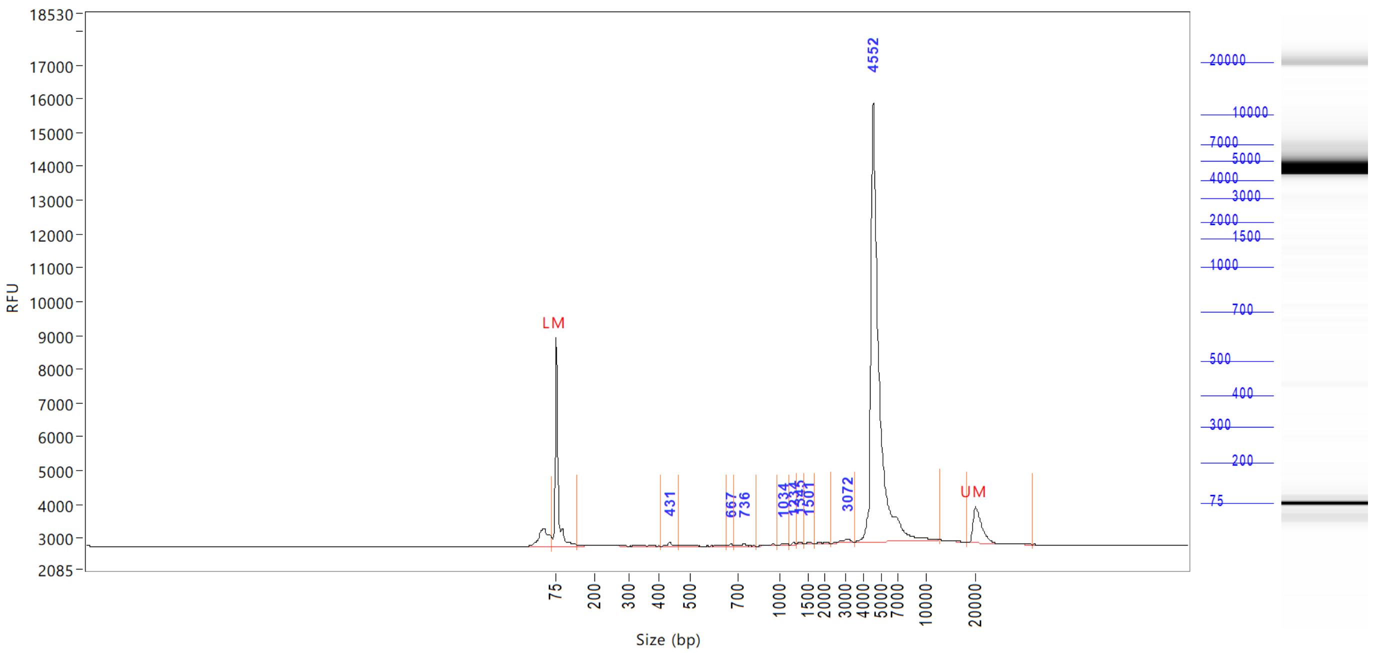

Peak	Size (bp)	Concentration (ng/uL)	From (bp)	To (bp)	Average size (bp)	CV%	RFU	Corrected peak area
1	75 (LM)	0.5000	64	141	77	14.69	6167	42.166
2	431	0.1509	403	460	433	2.48	127	1.061
3	667	0.0477	652	682	665	0.90	64	0.335
4	736	0.0781	682	822	726	3.04	72	0.549
5	1034	0.0663	978	1156	1045	3.81	51	0.466
6	1234	0.0441	1156	1278	1229	2.35	54	0.310
7	1345	0.0746	1278	1433	1331	2.27	81	0.524
8	1501	0.0535	1433	1712	1521	3.32	60	0.376
9	3072	0.2309	2286	3465	2910	8.39	119	1.623
10	4552	21.5946	3465	12684	4906	17.70	13008	151.759
11	20000 (UM)	0.1410	18171	31486	20645	6.13	1076	11.894

TIC:	22.3406	ng/uL
TIM:	8.5591	nmole/L
Total concentration:	22.7811	ng/uL

Sample peak width (sec): 5   Sample min peak height: 50   Sample baseline V to V?: Y   Sample baseline V to V points: 3
Sample filter: Binomial   Number of points for filter: 3 Sample start region (min): 0 Sample end region (min): 30
Marker peak width (sec): 5   Marker min peak height: 200   Marker baseline V to V?: Y   Marker baseline V to V points: 3
Lower marker selection: First peak > 500 RFU   Upper marker selection: Last peak > 200 RFU
Ladder size (bp)75, 200, 300, 400, 500, 700, 1000, 1500, 2000, 3000, 4000, 5000, 7000, 10000, 20000
Quantification using: Lower Marker   Final concentration (ng/uL): 0.5000   Dilution factor: 12.0

Sample: SampG6
Well location: G6
Created: Tuesday, August 10, 2021 10:41:10 PM

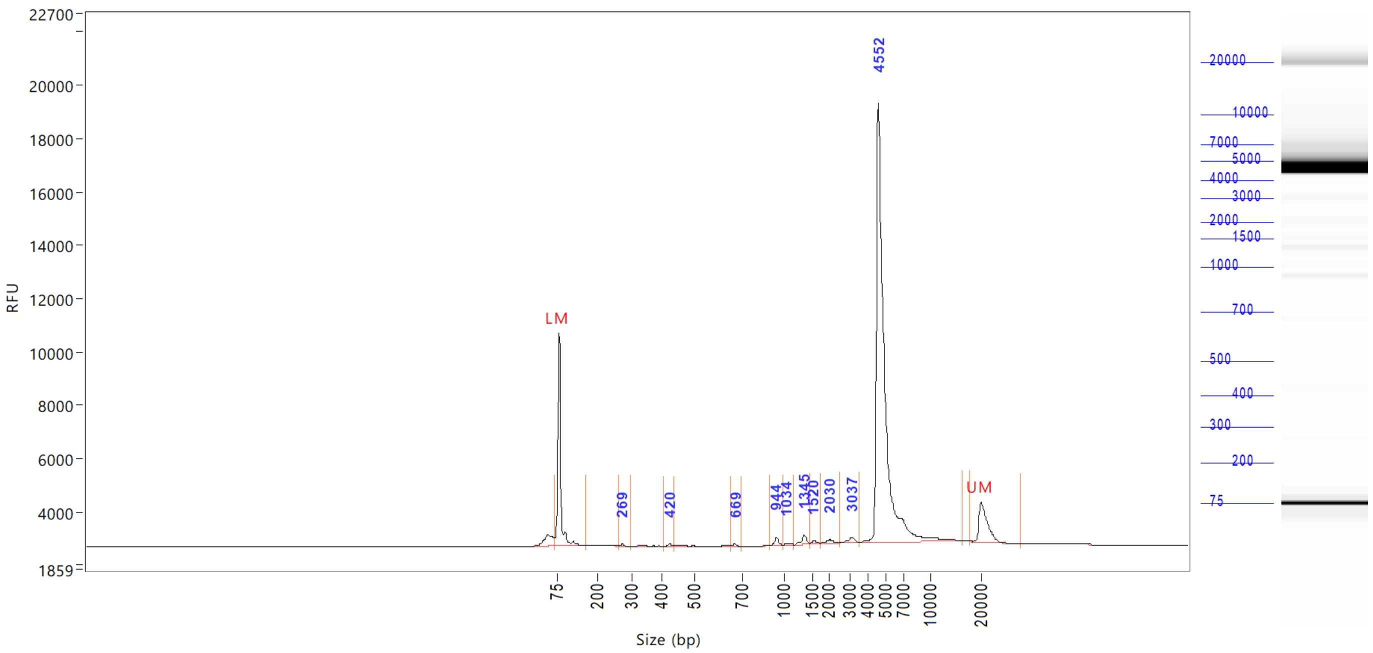

Peak	Size (bp)	Concentration (ng/uL)	From (bp)	To (bp)	Average size (bp)	CV%	RFU	Corrected peak area
1	75 (LM)	0.5000	64	164	77	13.60	7999	53.829
2	269	0.0438	260	296	268	1.99	66	0.393
3	420	0.0758	405	437	420	1.59	98	0.680
4	669	0.0611	654	695	667	1.04	91	0.548
5	944	0.2414	887	987	939	1.66	314	2.165
6	1034	0.0811	987	1167	1051	4.07	70	0.727
7	1345	0.2996	1167	1445	1324	4.22	333	2.688
8	1520	0.1157	1445	1731	1544	4.74	119	1.038
9	2030	0.1828	1731	2486	2007	7.09	134	1.640
10	3037	0.2170	2486	3500	2989	5.83	196	1.947
11	4552	21.0927	3500	16342	5064	23.76	16493	189.233
12	20000 (UM)	0.1521	17683	27859	20634	4.85	1490	16.376

TIC:	22.4109	ng/uL
TIM:	8.8889	nmole/L
Total concentration:	22.6512	ng/uL

Sample peak width (sec): 5 Sample min peak height: 50 Sample baseline V to V?: Y Sample baseline V to V points: 3
Sample filter: Binomial Number of points for filter: 3 Sample start region (min): 0 Sample end region (min): 30
Marker peak width (sec): 5 Marker min peak height: 200 Marker baseline V to V?: Y Marker baseline V to V points: 3
Lower marker selection: First peak > 500 RFU Upper marker selection: Last peak > 200 RFU
Ladder size (bp)75, 200, 300, 400, 500, 700, 1000, 1500, 2000, 3000, 4000, 5000, 7000, 10000, 20000
Quantification using: Lower Marker Final concentration (ng/uL): 0.5000 Dilution factor: 12.0

Sample: SampH6
Well location: H6
Created: Tuesday, August 10, 2021 10:41:10 PM

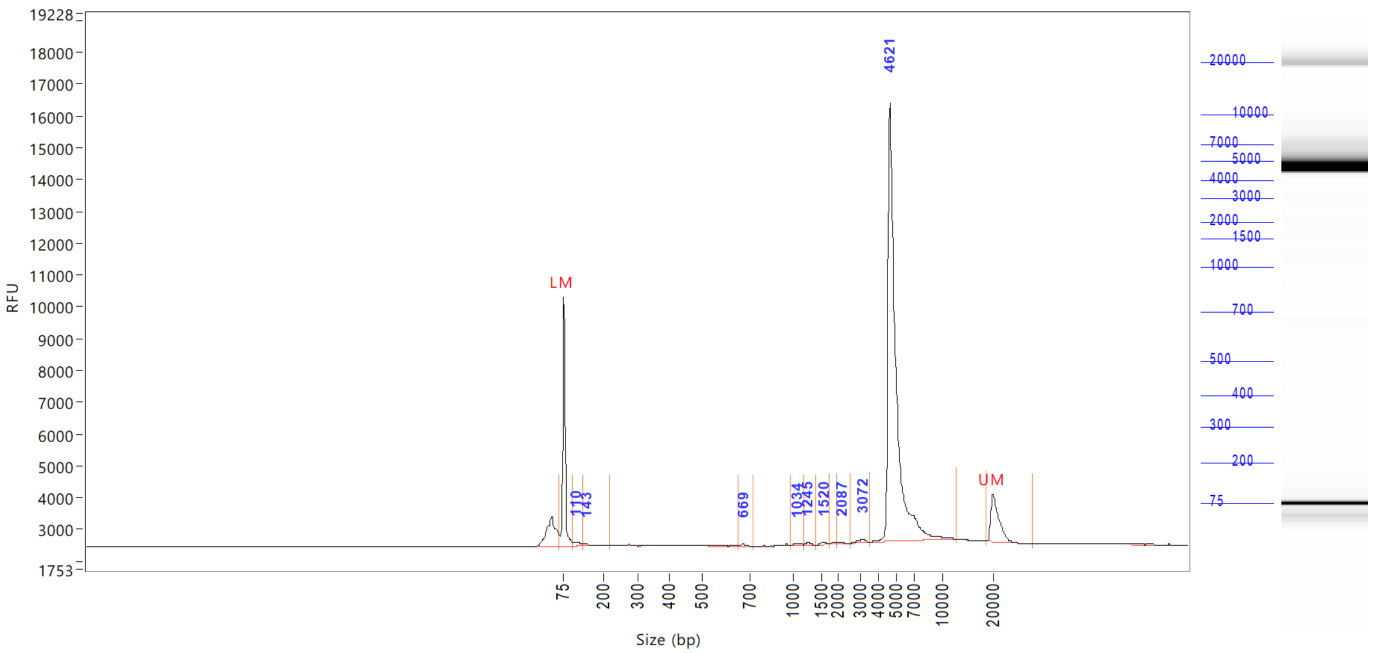

Peak	Size (bp)	Concentration (ng/uL)	From (bp)	To (bp)	Average size (bp)	CV%	RFU	Corrected peak area
1	75 (LM)	0.5000	64	105	74	7.54	7843	47.301
2	110	0.2521	105	135	117	7.30	121	1.987
3	143	0.1691	135	217	159	12.53	66	1.333
4	669	0.0602	654	718	668	1.40	70	0.475
5	1034	0.0855	983	1167	1060	4.81	61	0.674
6	1245	0.0804	1167	1389	1244	3.35	75	0.634
7	1520	0.0888	1389	1712	1545	4.73	76	0.700
8	2087	0.0610	1942	2515	2079	5.36	54	0.481
9	3072	0.1582	2515	3500	2992	6.00	107	1.247
10	4621	20.0234	3500	12684	5043	17.60	13804	157.854
11	20000 (UM)	0.1659	18537	27859	20515	4.86	1471	15.696

TIC:	20.9785	ng/uL
TIM:	12.4406	nmole/L
Total concentration:	21.3205	ng/uL

```
Sample peak width (sec): 5     Sample min peak height: 50    Sample baseline V to V?: Y    Sample baseline V to V points: 3
Sample filter: Binomial        Number of points for filter: 3  Sample start region (min): 0  Sample end region (min): 30
Marker peak width (sec): 5     Marker min peak height: 200   Marker baseline V to V?: Y    Marker baseline V to V points: 3
Lower marker selection: First peak > 500 RFU                 Upper marker selection: Last peak > 200 RFU
Ladder size (bp)75, 200, 300, 400, 500, 700, 1000, 1500, 2000, 3000, 4000, 5000, 7000, 10000, 20000
Quantification using: Lower Marker          Final concentration (ng/uL): 0.5000          Dilution factor: 12.0
```

Sample: SampA7
Well location: A7
Created: Tuesday, August 10, 2021 10:41:10 PM

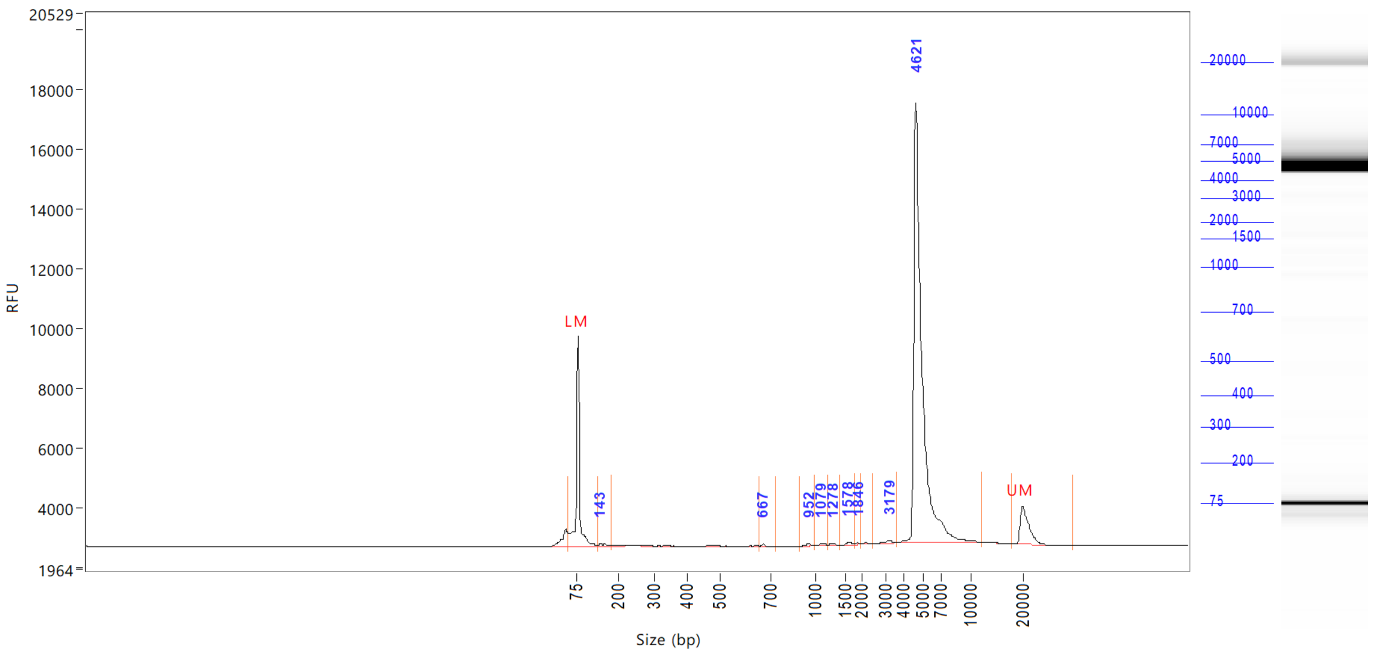

Peak	Size (bp)	Concentration (ng/uL)	From (bp)	To (bp)	Average size (bp)	CV%	RFU	Corrected peak area
1	75 (LM)	0.5000	50	135	75	14.67	7020	51.778
2	143	0.1528	135	177	151	7.10	77	1.319
3	667	0.0517	657	731	670	1.87	72	0.446
4	952	0.0747	896	991	945	1.82	83	0.645
5	1079	0.0698	991	1201	1091	4.70	56	0.602
6	1278	0.0671	1201	1411	1281	3.43	69	0.579
7	1578	0.0983	1411	1789	1614	5.55	90	0.849
8	1846	0.0440	1789	1962	1867	2.84	51	0.380
9	3179	0.1216	2458	3608	3087	6.50	90	1.050
10	4621	19.3423	3608	12318	5019	16.08	14710	166.917
11	20000 (UM)	0.1303	17927	29551	20542	4.82	1281	13.494

TIC:	20.0223	ng/uL
TIM:	8.6605	nmole/L
Total concentration:	20.3592	ng/uL

```
Sample peak width (sec): 5    Sample min peak height: 50    Sample baseline V to V?: Y    Sample baseline V to V points: 3
Sample filter: Binomial       Number of points for filter: 3  Sample start region (min): 0  Sample end region (min): 30
Marker peak width (sec): 5    Marker min peak height: 200   Marker baseline V to V?: Y    Marker baseline V to V points: 3
Lower marker selection: First peak > 500 RFU                Upper marker selection: Last peak > 200 RFU
Ladder size (bp)75, 200, 300, 400, 500, 700, 1000, 1500, 2000, 3000, 4000, 5000, 7000, 10000, 20000
Quantification using: Lower Marker          Final concentration (ng/uL): 0.5000          Dilution factor: 12.0
```

Sample: SampB7
Well location: B7
Created: Tuesday, August 10, 2021 10:41:10 PM

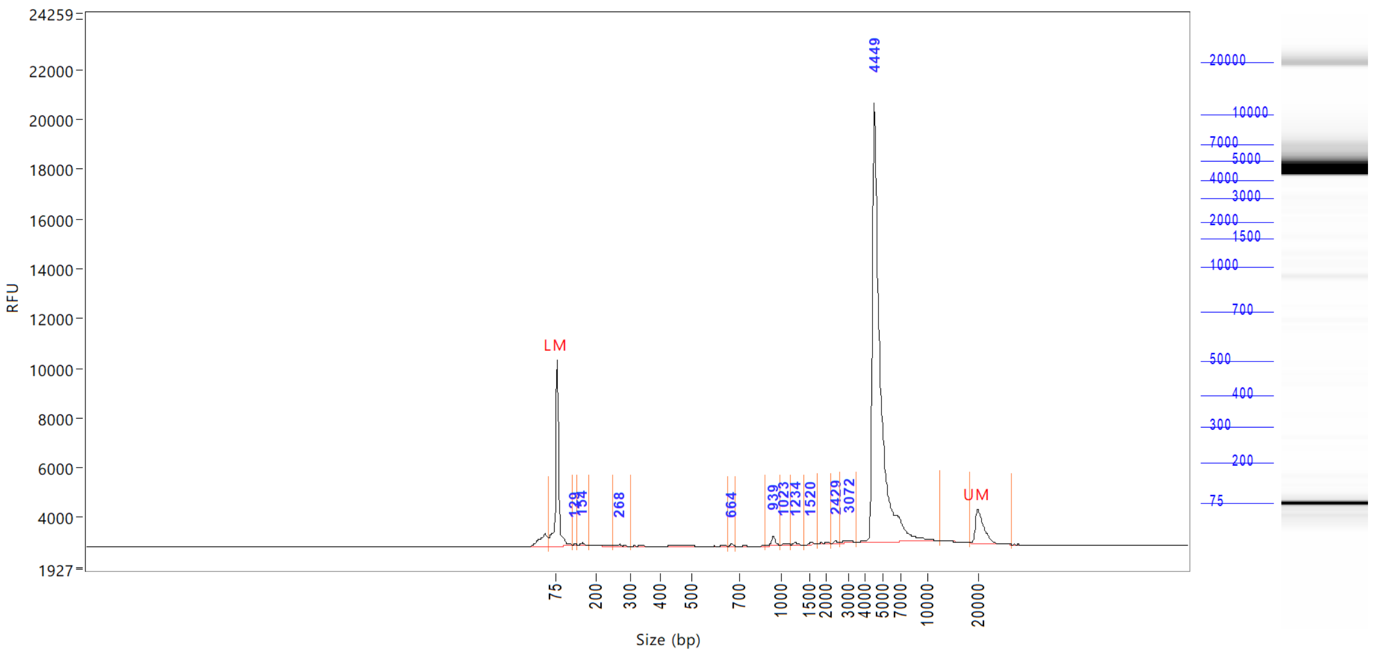

Peak	Size (bp)	Concentration (ng/uL)	From (bp)	To (bp)	Average size (bp)	CV%	RFU	Corrected peak area
1	75 (LM)	0.5000	48	122	74	12.71	7502	55.075
2	129	0.0796	122	139	129	3.87	95	0.731
3	154	0.1408	139	175	153	4.69	125	1.293
4	268	0.0545	248	301	266	3.33	67	0.500
5	664	0.0663	646	685	663	1.05	107	0.609
6	939	0.3018	879	987	935	2.01	378	2.770
7	1023	0.1227	987	1156	1051	4.42	100	1.126
8	1234	0.1001	1156	1389	1231	3.28	114	0.919
9	1520	0.0826	1389	1712	1532	3.65	106	0.758
10	2429	0.0618	2229	2600	2423	3.86	82	0.568
11	3072	0.1489	2600	3500	2953	6.22	111	1.367
12	4449	22.3643	3500	12318	4887	17.55	17675	205.285
13	20000 (UM)	0.1353	18415	26891	20512	4.77	1361	14.902

TIC:	23.5234	ng/uL
TIM:	11.6333	nmole/L
Total concentration:	23.8470	ng/uL

```
Sample peak width (sec): 5     Sample min peak height: 50     Sample baseline V to V?: Y     Sample baseline V to V points: 3
Sample filter: Binomial        Number of points for filter: 3  Sample start region (min): 0  Sample end region (min): 30
Marker peak width (sec): 5     Marker min peak height: 200    Marker baseline V to V?: Y     Marker baseline V to V points: 3
Lower marker selection: First peak > 500 RFU                  Upper marker selection: Last peak > 200 RFU
Ladder size (bp)75, 200, 300, 400, 500, 700, 1000, 1500, 2000, 3000, 4000, 5000, 7000, 10000, 20000
Quantification using: Lower Marker          Final concentration (ng/uL): 0.5000          Dilution factor: 12.0
```

Sample: SampC7
Well location: C7
Created: Tuesday, August 10, 2021 10:41:10 PM

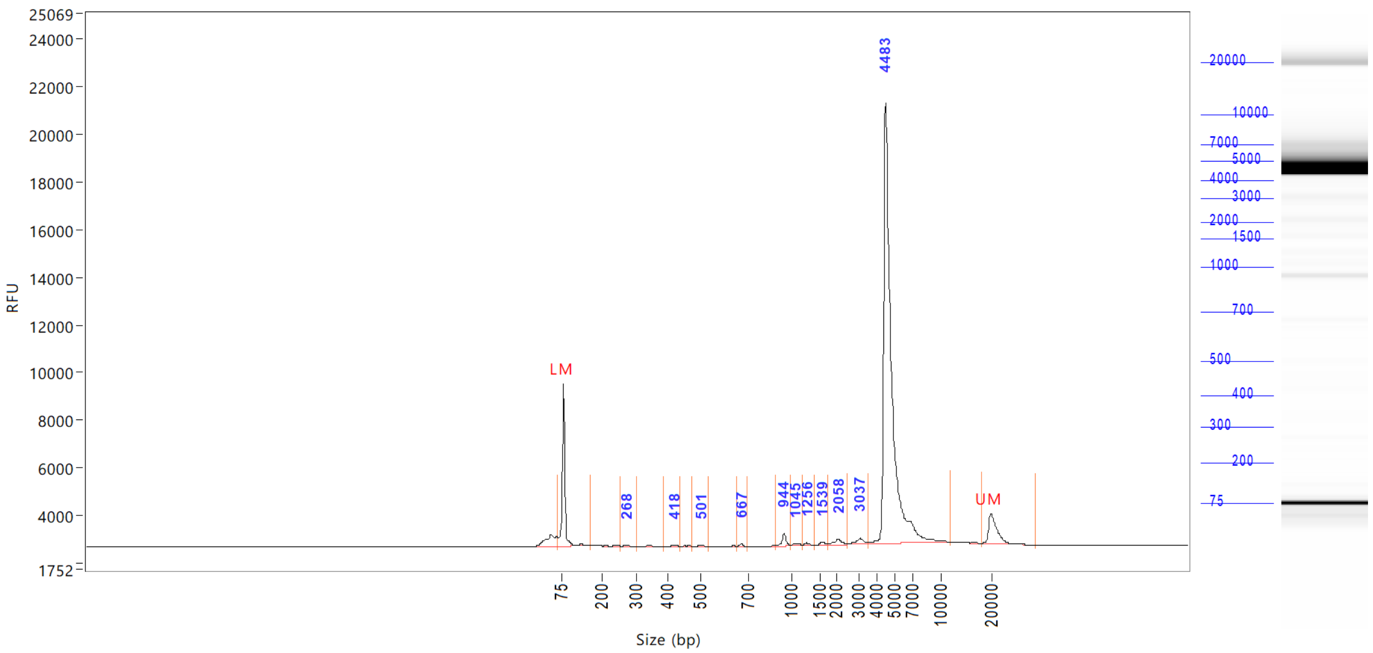

Peak	Size (bp)	Concentration (ng/uL)	From (bp)	To (bp)	Average size (bp)	CV%	RFU	Corrected peak area
1	75 (LM)	0.5000	60	160	76	13.23	6792	45.569
2	268	0.0594	250	296	266	2.70	62	0.451
3	418	0.0728	388	435	415	2.36	54	0.553
4	501	0.0616	475	532	498	2.01	52	0.468
5	667	0.0853	649	695	665	1.06	110	0.648
6	944	0.4706	892	991	940	1.83	516	3.575
7	1045	0.1508	991	1178	1067	4.73	104	1.145
8	1256	0.1218	1178	1411	1256	3.34	110	0.925
9	1539	0.1492	1411	1731	1564	4.75	130	1.133
10	2058	0.3658	1731	2515	2049	8.51	225	2.778
11	3037	0.4004	2515	3536	3012	7.33	245	3.041
12	4483	27.5604	3536	11830	4847	16.13	18507	209.317
13	20000 (UM)	0.1425	18171	28826	20557	4.76	1234	12.986

TIC:	29.4982	ng/uL
TIM:	12.3177	nmole/L
Total concentration:	29.7511	ng/uL

Sample peak width (sec): 5 Sample min peak height: 50 Sample baseline V to V?: Y Sample baseline V to V points: 3
Sample filter: Binomial Number of points for filter: 3 Sample start region (min): 0 Sample end region (min): 30
Marker peak width (sec): 5 Marker min peak height: 200 Marker baseline V to V?: Y Marker baseline V to V points: 3
Lower marker selection: First peak > 500 RFU Upper marker selection: Last peak > 200 RFU
Ladder size (bp)75, 200, 300, 400, 500, 700, 1000, 1500, 2000, 3000, 4000, 5000, 7000, 10000, 20000
Quantification using: Lower Marker Final concentration (ng/uL): 0.5000 Dilution factor: 12.0

Sample: SampD7
Well location: D7
Created: Tuesday, August 10, 2021 10:41:10 PM

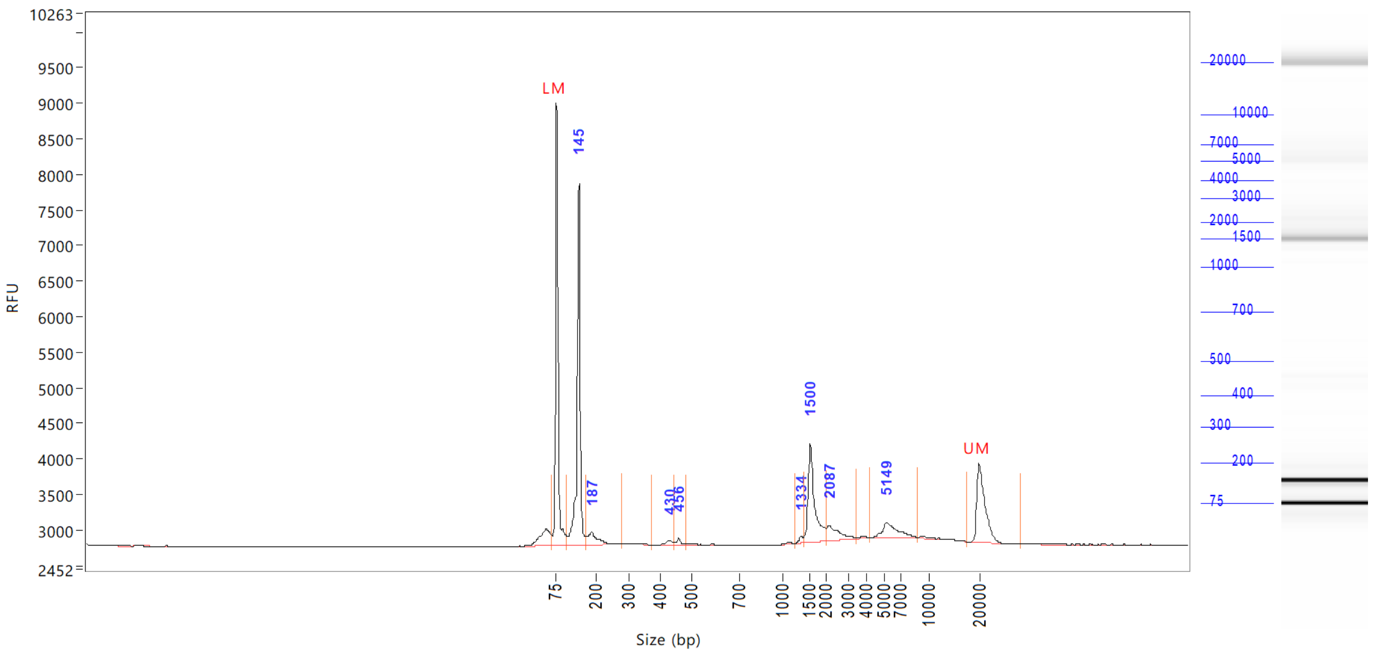

Peak	Size (bp)	Concentration (ng/uL)	From (bp)	To (bp)	Average size (bp)	CV%	RFU	Corrected peak area
1	75 (LM)	0.5000	64	108	75	8.83	6229	39.097
2	145	5.8613	108	169	140	6.32	5073	38.193
3	187	0.6370	169	275	196	10.65	176	4.151
4	430	0.1313	374	445	424	3.11	54	0.855
5	456	0.1103	445	479	456	1.58	101	0.719
6	1334	0.0985	1223	1378	1327	2.54	94	0.642
7	1500	2.3019	1378	1981	1571	9.01	1392	15.000
8	2087	0.6363	1981	3429	2395	14.58	220	4.146
9	5149	0.7728	4173	8734	5931	17.20	223	5.036
10	20000 (UM)	0.1524	17683	28342	20528	4.97	1108	11.920

TIC:	10.5494	ng/uL
TIM:	78.2368	nmole/L
Total concentration:	10.8203	ng/uL

```
Sample peak width (sec): 5    Sample min peak height: 50    Sample baseline V to V?: Y    Sample baseline V to V points: 3
Sample filter: Binomial       Number of points for filter: 3  Sample start region (min): 0  Sample end region (min): 30
Marker peak width (sec): 5    Marker min peak height: 200   Marker baseline V to V?: Y    Marker baseline V to V points: 3
Lower marker selection: First peak > 500 RFU                Upper marker selection: Last peak > 200 RFU
Ladder size (bp)75, 200, 300, 400, 500, 700, 1000, 1500, 2000, 3000, 4000, 5000, 7000, 10000, 20000
Quantification using: Lower Marker          Final concentration (ng/uL): 0.5000          Dilution factor: 12.0
```

Sample: SampE7
Well location: E7
Created: Tuesday, August 10, 2021 10:41:10 PM

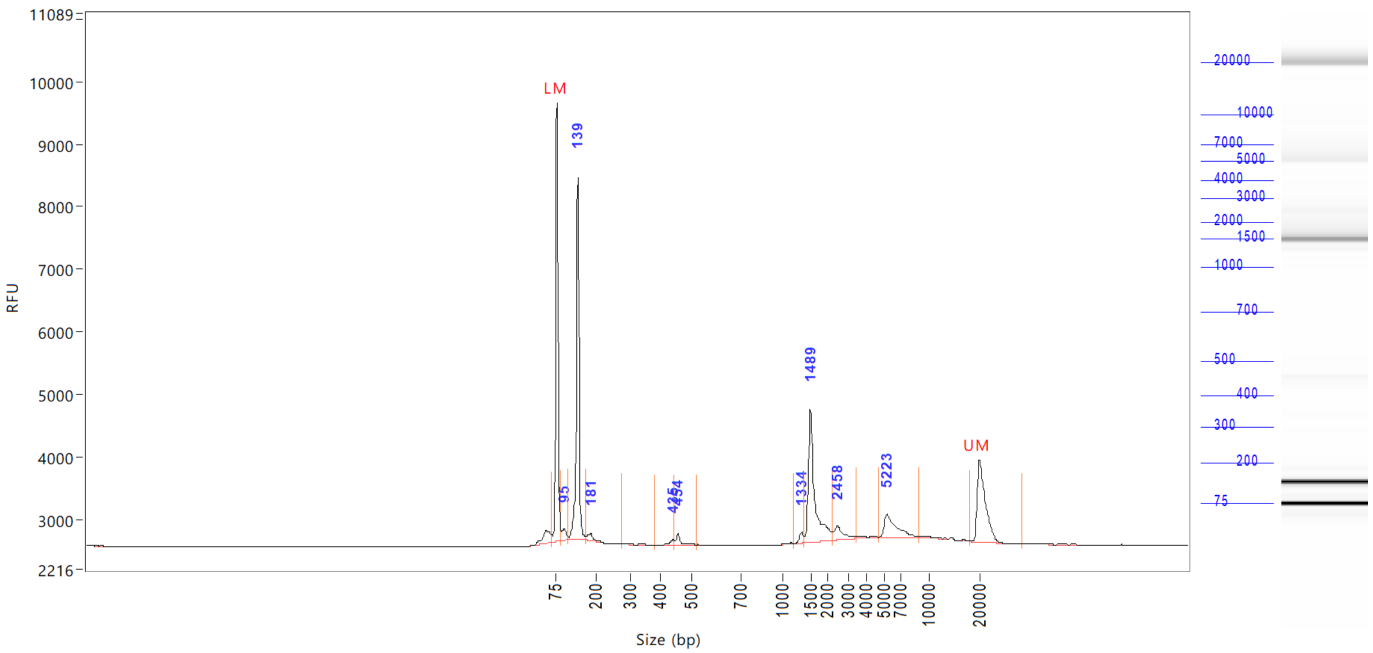

Peak	Size (bp)	Concentration (ng/uL)	From (bp)	To (bp)	Average size (bp)	CV%	RFU	Corrected peak area
1	75 (LM)	0.5000	62	87	73	4.79	7009	39.521
2	95	0.3170	87	110	96	6.29	178	2.088
3	139	5.8500	110	166	136	4.74	5771	38.533
4	181	0.2561	166	273	184	9.85	125	1.687
5	435	0.1478	382	445	429	2.88	86	0.974
6	454	0.2444	445	514	461	3.56	202	1.610
7	1334	0.1718	1201	1378	1320	2.64	170	1.132
8	1489	3.3582	1378	2258	1594	11.94	2125	22.120
9	2458	0.4753	2258	3393	2599	10.14	228	3.130
10	5223	1.0339	4656	8867	6035	16.24	376	6.810
11	20000 (UM)	0.1787	18171	28705	20623	4.92	1310	14.128

TIC:	11.8545	ng/uL
TIM:	84.0978	nmole/L
Total concentration:	12.1649	ng/uL

```
Sample peak width (sec): 5     Sample min peak height: 50    Sample baseline V to V?: Y    Sample baseline V to V points: 3
Sample filter: Binomial        Number of points for filter: 3  Sample start region (min): 0  Sample end region (min): 30
Marker peak width (sec): 5     Marker min peak height: 200   Marker baseline V to V?: Y    Marker baseline V to V points: 3
Lower marker selection: First peak > 500 RFU                 Upper marker selection: Last peak > 200 RFU
Ladder size (bp)75, 200, 300, 400, 500, 700, 1000, 1500, 2000, 3000, 4000, 5000, 7000, 10000, 20000
Quantification using: Lower Marker          Final concentration (ng/uL): 0.5000          Dilution factor: 12.0
```

Sample: SampF7
Well location: F7
Created: Tuesday, August 10, 2021 10:41:10 PM

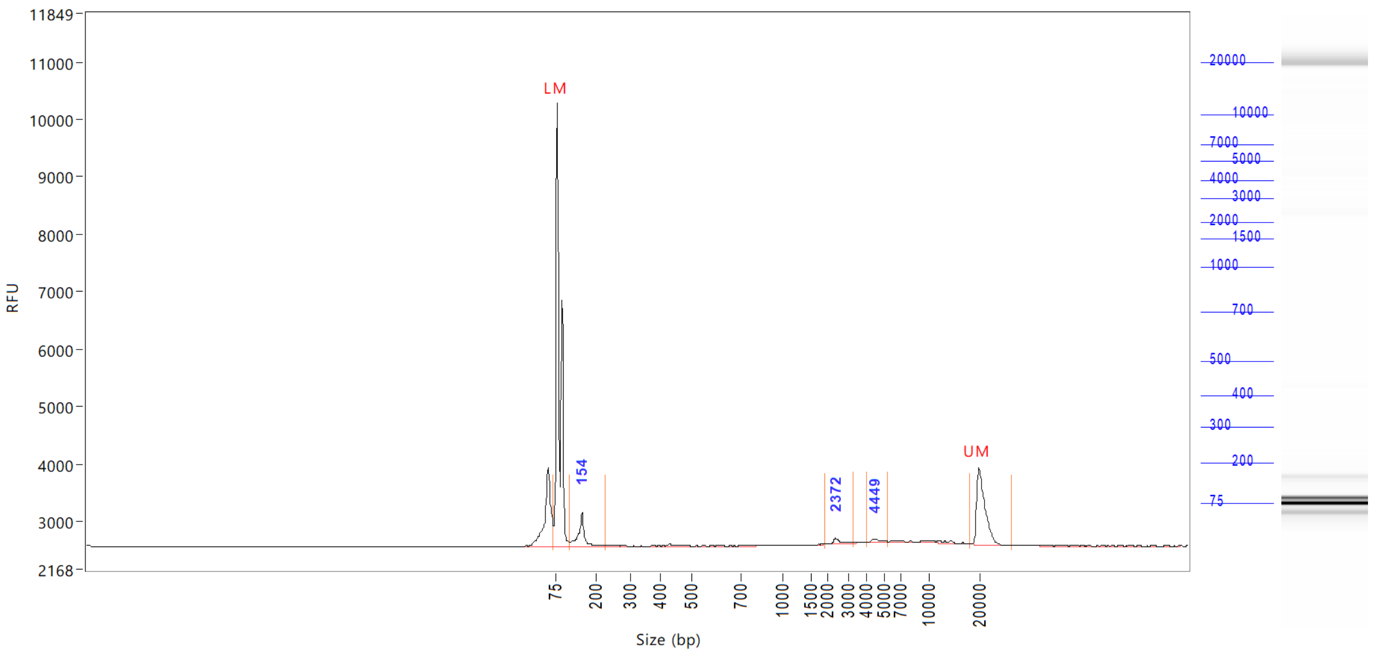

Peak	Size<br>(bp)	Concentration<br>(ng/uL)	From<br>(bp)	To<br>(bp)	Average size<br>(bp)	CV%	RFU	Corrected peak area
1	75 (LM)	0.5000	66	114	79	10.48	7731	69.641
2	154	0.6592	114	226	150	12.19	596	7.652
3	2372	0.1021	1962	3251	2509	10.38	97	1.185
4	4449	0.0646	4001	5371	4539	6.08	59	0.750
5	20000 (UM)	0.1058	18049	26408	20512	4.83	1349	14.730

TIC:	0.8259	ng/uL
TIM:	7.3135	nmole/L
Total concentration:	1.1411	ng/uL

Sample peak width (sec): 5   Sample min peak height: 50   Sample baseline V to V?: Y   Sample baseline V to V points: 3
Sample filter: Binomial   Number of points for filter: 3   Sample start region (min): 0   Sample end region (min): 30
Marker peak width (sec): 5   Marker min peak height: 200   Marker baseline V to V?: Y   Marker baseline V to V points: 3
Lower marker selection: First peak > 500 RFU   Upper marker selection: Last peak > 200 RFU
Ladder size (bp)75, 200, 300, 400, 500, 700, 1000, 1500, 2000, 3000, 4000, 5000, 7000, 10000, 20000
Quantification using: Lower Marker   Final concentration (ng/uL): 0.5000   Dilution factor: 12.0

Sample: SampG7
Well location: G7
Created: Tuesday, August 10, 2021 10:41:10 PM

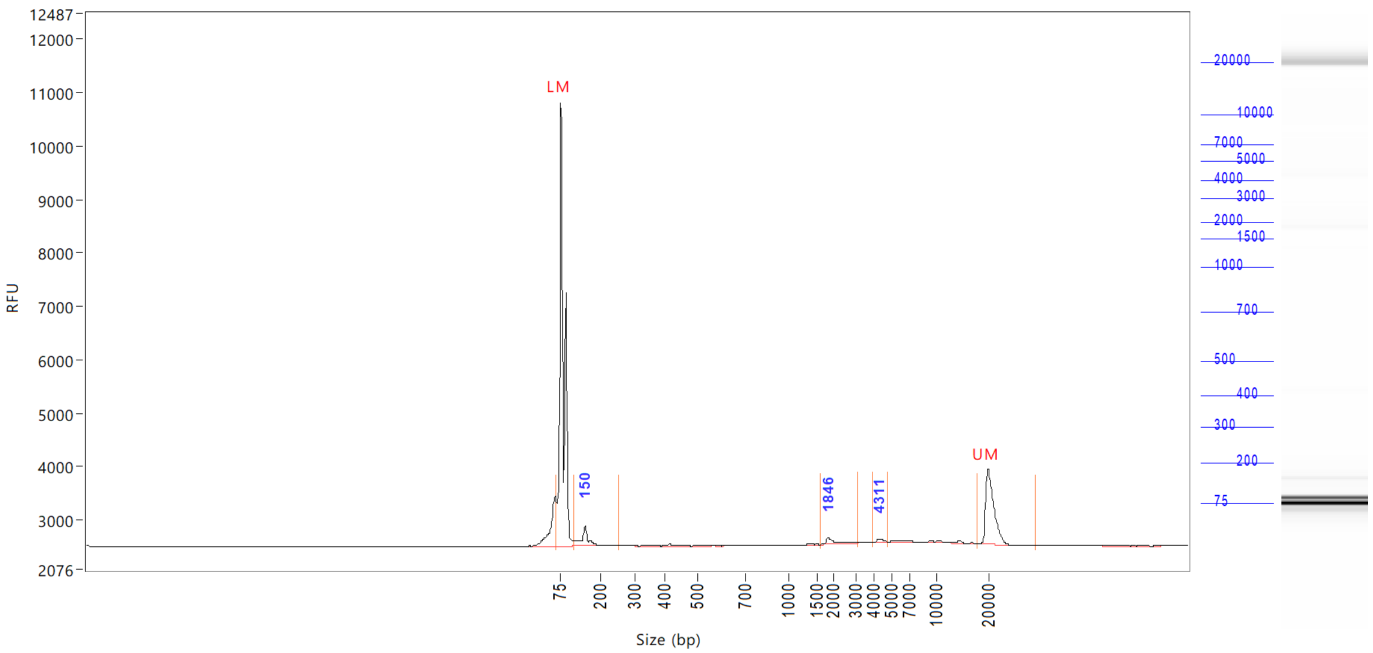

Peak	Size (bp)	Concentration (ng/uL)	From (bp)	To (bp)	Average size (bp)	CV%	RFU	Corrected peak area
1	75 (LM)	0.5000	64	116	79	11.13	8313	81.961
2	150	0.4244	116	251	151	15.08	387	5.798
3	1846	0.1210	1578	3144	2051	15.05	124	1.652
4	4311	0.0566	3964	4793	4356	4.40	73	0.773
5	20000 (UM)	0.0908	17805	29430	20600	4.91	1408	14.891

TIC:	0.6020	ng/uL
TIM:	4.7382	nmole/L
Total concentration:	0.8781	ng/uL

Sample peak width (sec): 5   Sample min peak height: 50   Sample baseline V to V?: Y   Sample baseline V to V points: 3
Sample filter: Binomial   Number of points for filter: 3 Sample start region (min): 0 Sample end region (min): 30
Marker peak width (sec): 5   Marker min peak height: 200   Marker baseline V to V?: Y   Marker baseline V to V points: 3
Lower marker selection: First peak > 500 RFU   Upper marker selection: Last peak > 200 RFU
Ladder size (bp)75, 200, 300, 400, 500, 700, 1000, 1500, 2000, 3000, 4000, 5000, 7000, 10000, 20000
Quantification using: Lower Marker   Final concentration (ng/uL): 0.5000   Dilution factor: 12.0

Sample: SampH7
Well location: H7
Created: Tuesday, August 10, 2021 10:41:10 PM

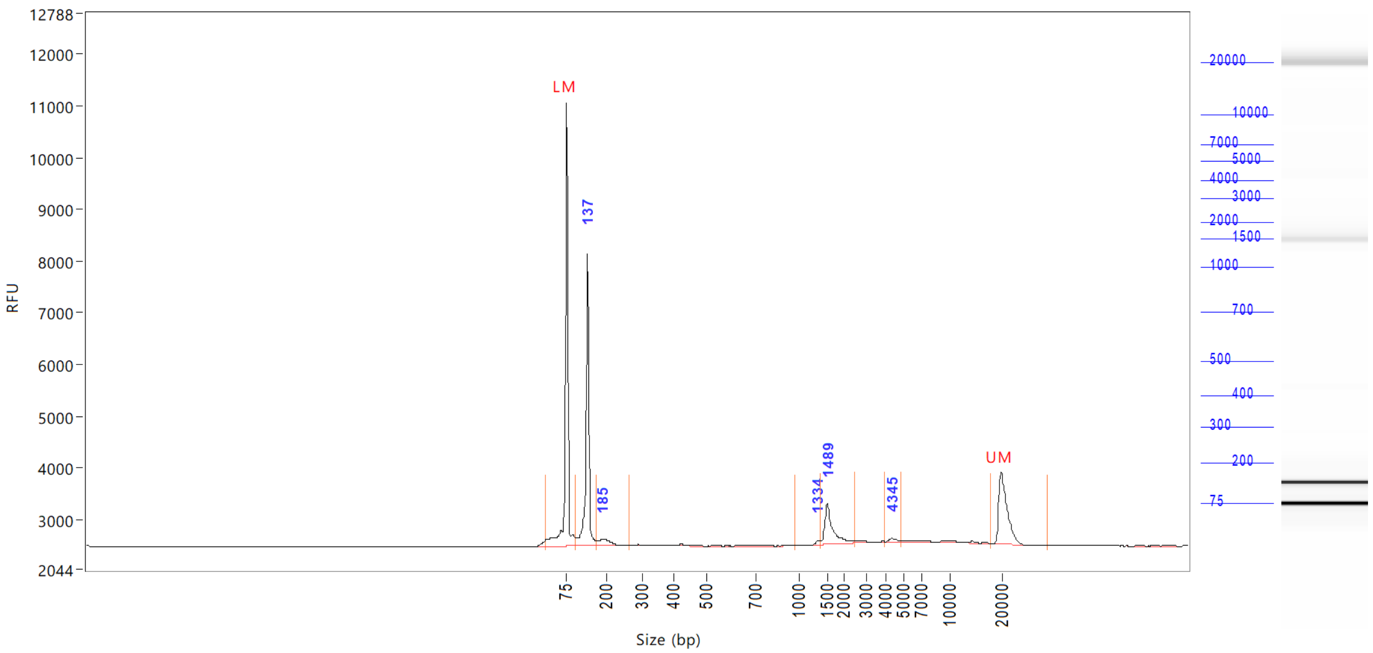

Peak	Size (bp)	Concentration (ng/uL)	From (bp)	To (bp)	Average size (bp)	CV%	RFU	Corrected peak area
1	75 (LM)	0.5000	14	103	70	17.73	8577	57.667
2	137	4.1679	103	164	134	6.29	5657	40.059
3	185	0.3765	164	262	190	9.51	119	3.618
4	1334	0.0698	970	1378	1305	5.54	91	0.671
5	1489	1.0054	1378	2486	1599	13.19	780	9.663
6	4345	0.0807	4001	4862	4411	4.62	68	0.776
7	20000 (UM)	0.1275	18049	29068	20556	4.92	1384	14.706

TIC:	5.7003	ng/uL
TIM:	55.5195	nmole/L
Total concentration:	6.1005	ng/uL

Sample peak width (sec): 5 Sample min peak height: 50 Sample baseline V to V?: Y Sample baseline V to V points: 3
Sample filter: Binomial Number of points for filter: 3 Sample start region (min): 0 Sample end region (min): 30
Marker peak width (sec): 5 Marker min peak height: 200 Marker baseline V to V?: Y Marker baseline V to V points: 3
Lower marker selection: First peak > 500 RFU Upper marker selection: Last peak > 200 RFU
Ladder size (bp)75, 200, 300, 400, 500, 700, 1000, 1500, 2000, 3000, 4000, 5000, 7000, 10000, 20000
Quantification using: Lower Marker Final concentration (ng/uL): 0.5000 Dilution factor: 12.0

Sample: SampA8
Well location: A8
Created: Tuesday, August 10, 2021 10:41:10 PM

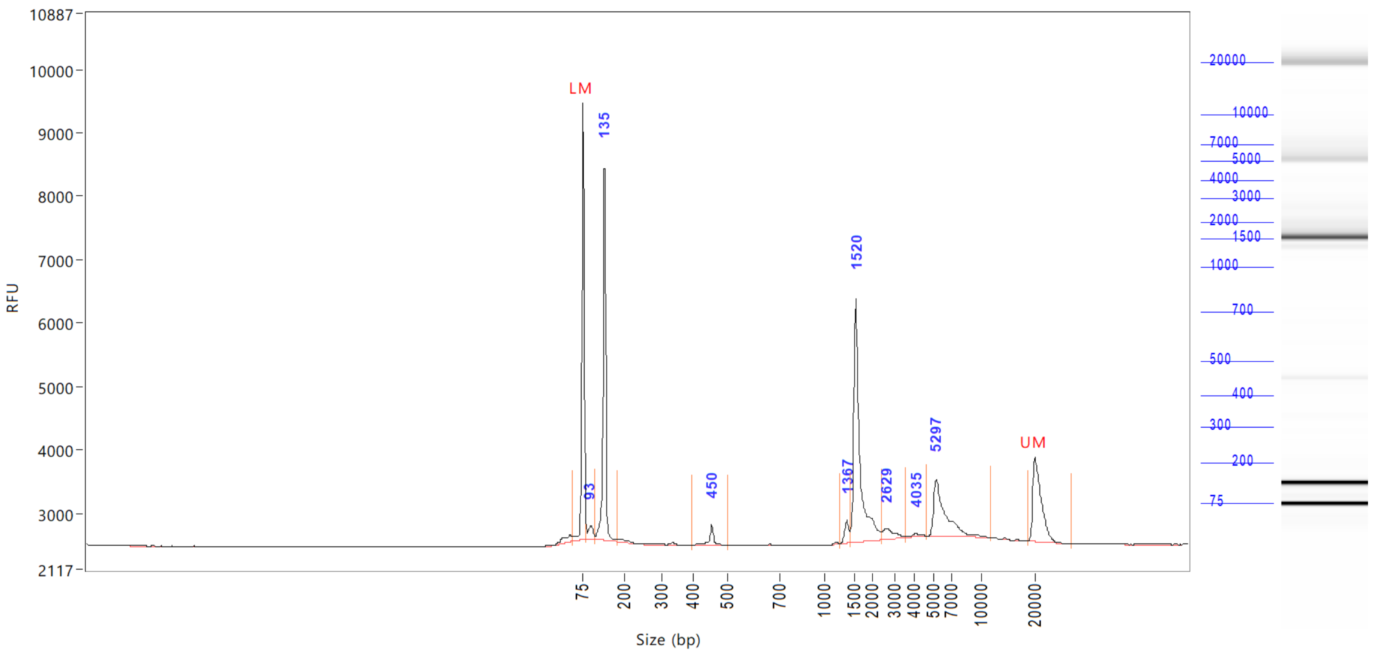

Peak	Size (bp)	Concentration (ng/uL)	From (bp)	To (bp)	Average size (bp)	CV%	RFU	Corrected peak area
1	75 (LM)	0.5000	45	86	72	6.14	6895	38.685
2	93	0.3656	86	108	95	6.17	203	2.357
3	135	5.8820	108	175	134	4.75	5868	37.925
4	450	0.4397	396	498	445	3.69	323	2.835
5	1367	0.3611	1245	1422	1358	2.60	358	2.328
6	1520	5.4872	1422	2372	1623	11.70	3850	35.379
7	2629	0.4493	2372	3500	2737	9.48	172	2.897
8	4035	0.0908	3500	4587	3990	5.53	55	0.586
9	5297	2.1716	4587	11708	6111	18.07	899	14.001
10	20000 (UM)	0.1771	18781	26891	20574	4.53	1325	13.706

TIC:	15.2474	ng/uL
TIM:	86.9694	nmole/L
Total concentration:	15.6752	ng/uL

```
Sample peak width (sec): 5    Sample min peak height: 50    Sample baseline V to V?: Y    Sample baseline V to V points: 3
Sample filter: Binomial       Number of points for filter: 3  Sample start region (min): 0  Sample end region (min): 30
Marker peak width (sec): 5    Marker min peak height: 200   Marker baseline V to V?: Y    Marker baseline V to V points: 3
Lower marker selection: First peak > 500 RFU                Upper marker selection: Last peak > 200 RFU
Ladder size (bp)75, 200, 300, 400, 500, 700, 1000, 1500, 2000, 3000, 4000, 5000, 7000, 10000, 20000
Quantification using: Lower Marker          Final concentration (ng/uL): 0.5000          Dilution factor: 12.0
```

Sample: SampB8
Well location: B8
Created: Tuesday, August 10, 2021 10:41:10 PM

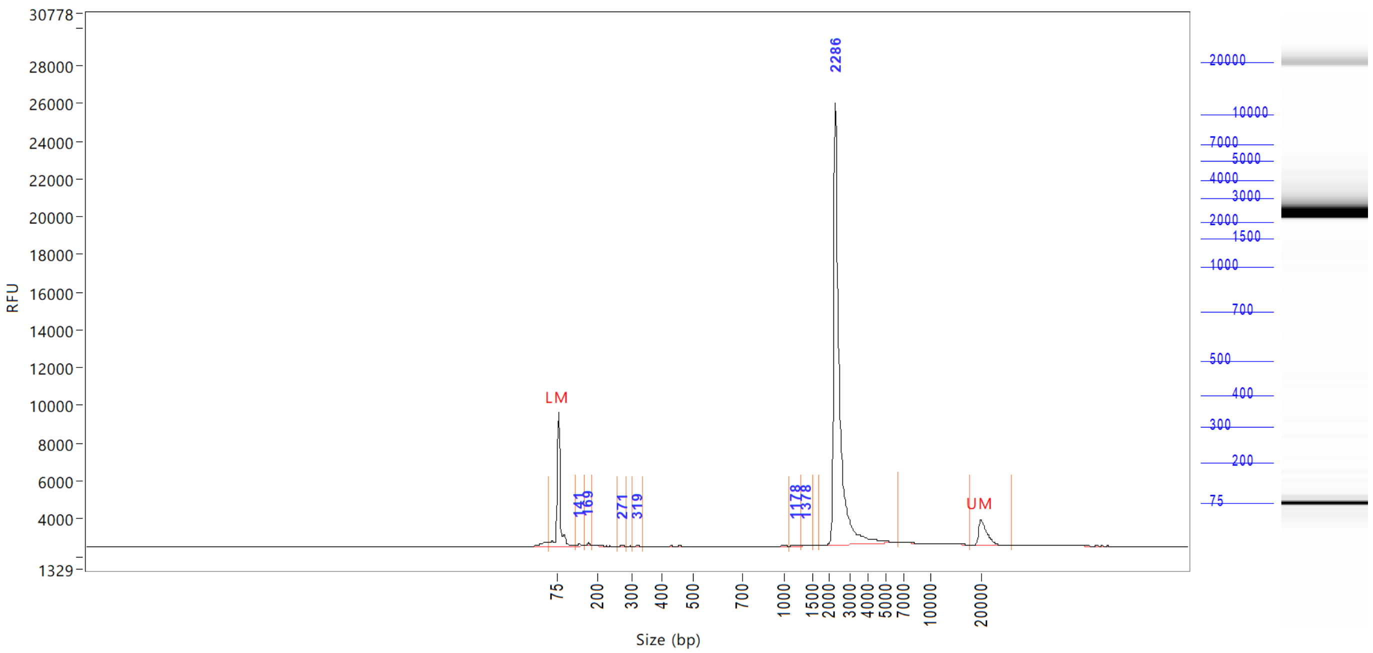

Peak	Size (bp)	Concentration (ng/uL)	From (bp)	To (bp)	Average size (bp)	CV%	RFU	Corrected peak area
1	75 (LM)	0.5000	48	129	75	13.00	7134	50.534
2	141	0.1376	129	160	141	5.10	139	1.159
3	169	0.1068	160	183	168	2.17	154	0.900
4	271	0.0441	259	282	269	1.54	65	0.371
5	319	0.0379	301	335	315	1.72	53	0.319
6	1178	0.0982	1068	1311	1201	4.89	72	0.827
7	1378	0.0563	1311	1539	1380	3.52	55	0.474
8	2286	25.0544	1693	6482	2463	17.54	23462	211.018
9	20000 (UM)	0.1419	17805	26045	20528	4.65	1362	14.339

TIC:	25.5353	ng/uL
TIM:	20.0632	nmole/L
Total concentration:	25.9561	ng/uL

Sample peak width (sec): 5 Sample min peak height: 50 Sample baseline V to V?: Y Sample baseline V to V points: 3
Sample filter: Binomial Number of points for filter: 3 Sample start region (min): 0 Sample end region (min): 30
Marker peak width (sec): 5 Marker min peak height: 200 Marker baseline V to V?: Y Marker baseline V to V points: 3
Lower marker selection: First peak > 500 RFU Upper marker selection: Last peak > 200 RFU
Ladder size (bp)75, 200, 300, 400, 500, 700, 1000, 1500, 2000, 3000, 4000, 5000, 7000, 10000, 20000
Quantification using: Lower Marker Final concentration (ng/uL): 0.5000 Dilution factor: 12.0

Sample: SampC8
Well location: C8
Created: Tuesday, August 10, 2021 10:41:10 PM

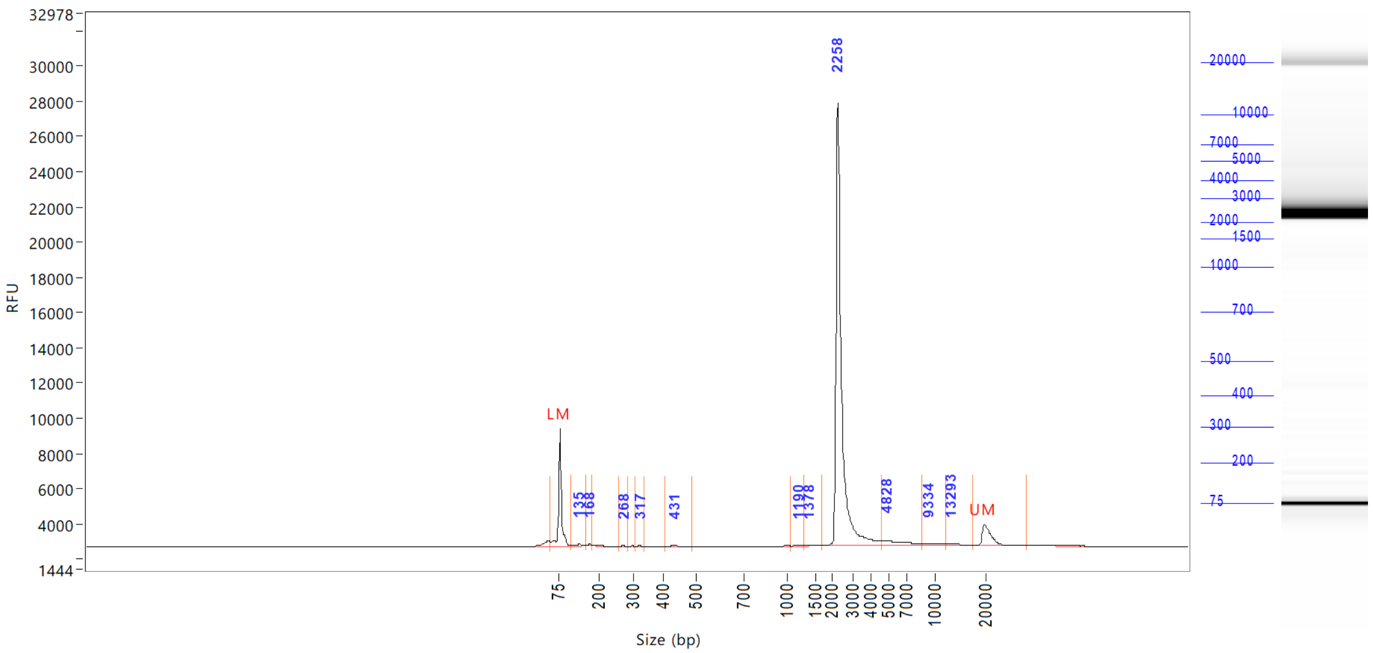

Peak	Size (bp)	Concentration (ng/uL)	From (bp)	To (bp)	Average size (bp)	CV%	RFU	Corrected peak area
1	75 (LM)	0.5000	47	110	73	11.17	6660	47.390
2	135	0.2115	110	156	128	7.97	130	1.670
3	168	0.1053	156	177	165	2.08	153	0.831
4	268	0.0407	255	282	267	1.99	52	0.321
5	317	0.0389	303	333	315	1.60	52	0.307
6	431	0.1238	403	487	433	3.52	86	0.978
7	1190	0.1156	1068	1311	1203	4.86	75	0.913
8	1378	0.0757	1311	1712	1398	5.16	62	0.598
9	2258	27.3861	1712	4587	2420	16.42	25160	216.305
10	4828	1.1298	4587	8600	6037	19.88	299	8.923
11	9334	0.3269	8600	12318	9988	10.38	109	2.582
12	13293	0.1551	12318	17683	14132	9.04	66	1.225
13	20000 (UM)	0.1365	17683	28221	20570	5.04	1217	12.941

TIC:	29.7092	ng/uL
TIM:	23.9436	nmole/L
Total concentration:	29.9626	ng/uL

```
Sample peak width (sec): 5    Sample min peak height: 50    Sample baseline V to V?: Y    Sample baseline V to V points: 3
Sample filter: Binomial       Number of points for filter: 3  Sample start region (min): 0  Sample end region (min): 30
Marker peak width (sec): 5    Marker min peak height: 200   Marker baseline V to V?: Y    Marker baseline V to V points: 3
Lower marker selection: First peak > 500 RFU                Upper marker selection: Last peak > 200 RFU
Ladder size (bp)75, 200, 300, 400, 500, 700, 1000, 1500, 2000, 3000, 4000, 5000, 7000, 10000, 20000
Quantification using: Lower Marker          Final concentration (ng/uL): 0.5000          Dilution factor: 12.0
```

Sample: SampD8
Well location: D8
Created: Tuesday, August 10, 2021 10:41:10 PM

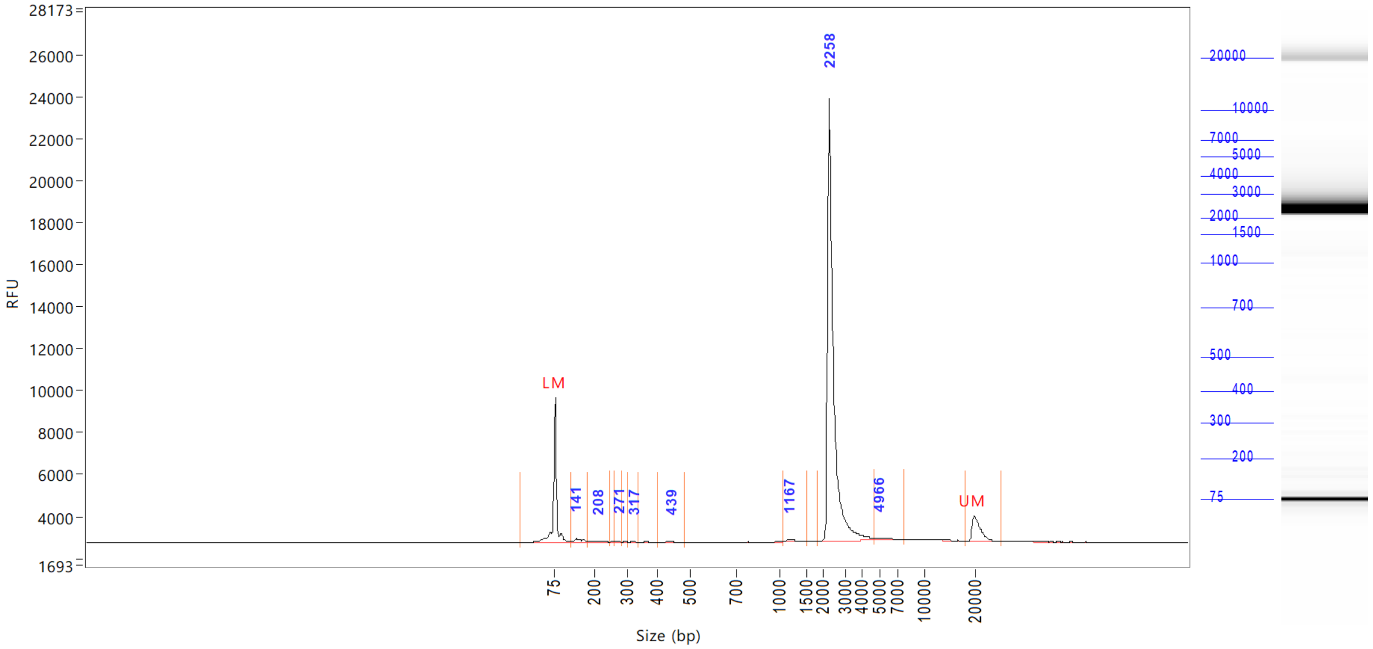

Peak	Size (bp)	Concentration (ng/uL)	From (bp)	To (bp)	Average size (bp)	CV%	RFU	Corrected peak area
1	75 (LM)	0.5000	0	126	71	21.89	6903	52.282
2	141	0.4069	126	179	149	9.22	176	3.546
3	208	0.1574	179	244	201	6.92	68	1.372
4	271	0.0442	260	280	269	1.40	69	0.385
5	317	0.0427	298	335	313	1.98	56	0.372
6	439	0.1084	400	485	439	3.20	75	0.945
7	1167	0.1894	1068	1489	1232	7.72	112	1.650
8	2258	21.9902	1846	4724	2428	15.15	21104	191.616
9	4966	0.2186	4724	7801	5530	12.90	98	1.905
10	20000 (UM)	0.1224	18293	25440	20609	4.74	1181	12.803

TIC:	23.1578	ng/uL
TIM:	21.9043	nmole/L
Total concentration:	23.4622	ng/uL

```
Sample peak width (sec): 5    Sample min peak height: 50    Sample baseline V to V?: Y    Sample baseline V to V points: 3
Sample filter: Binomial       Number of points for filter: 3  Sample start region (min): 0  Sample end region (min): 30
Marker peak width (sec): 5    Marker min peak height: 200   Marker baseline V to V?: Y    Marker baseline V to V points: 3
Lower marker selection: First peak > 500 RFU                Upper marker selection: Last peak > 200 RFU
Ladder size (bp)75, 200, 300, 400, 500, 700, 1000, 1500, 2000, 3000, 4000, 5000, 7000, 10000, 20000
Quantification using: Lower Marker          Final concentration (ng/uL): 0.5000          Dilution factor: 12.0
```

Sample: SampE8
Well location: E8
Created: Tuesday, August 10, 2021 10:41:10 PM

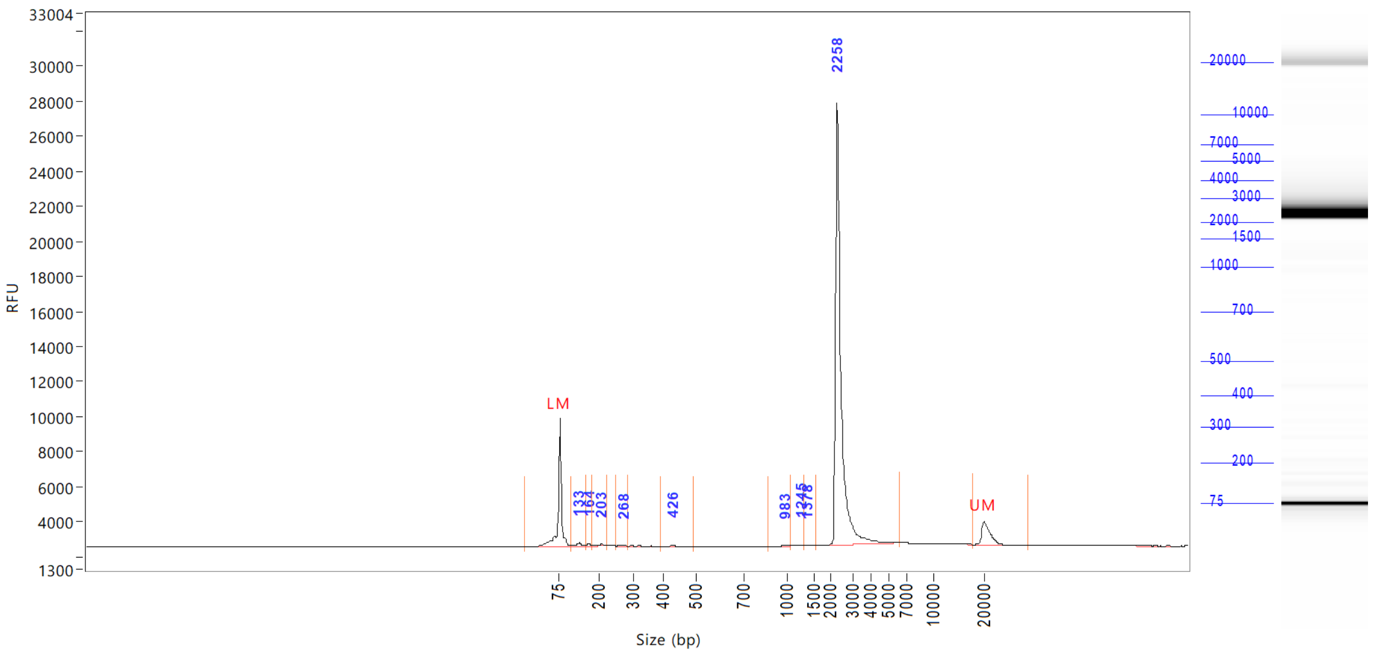

Peak	Size (bp)	Concentration (ng/uL)	From (bp)	To (bp)	Average size (bp)	CV%	RFU	Corrected peak area
1	75 (LM)	0.5000	0	110	68	21.79	7268	57.461
2	133	0.2661	110	154	130	7.91	184	2.549
3	164	0.1131	154	173	163	2.78	165	1.083
4	203	0.1509	173	221	194	5.08	121	1.445
5	268	0.0434	248	282	266	2.28	63	0.416
6	426	0.1029	390	492	429	3.58	105	0.985
7	983	0.0664	870	1068	979	2.95	71	0.636
8	1245	0.0973	1068	1311	1202	4.78	74	0.931
9	1378	0.0485	1311	1520	1374	3.00	58	0.464
10	2258	22.3499	1520	6260	2405	15.37	25259	214.043
11	20000 (UM)	0.1234	17927	28947	20556	4.86	1317	14.180

TIC:	23.2385	ng/uL
TIM:	22.0471	nmole/L
Total concentration:	23.4888	ng/uL

Sample peak width (sec): 5   Sample min peak height: 50   Sample baseline V to V?: Y   Sample baseline V to V points: 3
Sample filter: Binomial   Number of points for filter: 3 Sample start region (min): 0 Sample end region (min): 30
Marker peak width (sec): 5   Marker min peak height: 200   Marker baseline V to V?: Y   Marker baseline V to V points: 3
Lower marker selection: First peak > 500 RFU   Upper marker selection: Last peak > 200 RFU
Ladder size (bp)75, 200, 300, 400, 500, 700, 1000, 1500, 2000, 3000, 4000, 5000, 7000, 10000, 20000
Quantification using: Lower Marker   Final concentration (ng/uL): 0.5000   Dilution factor: 12.0

Sample: SampF8
Well location: F8
Created: Tuesday, August 10, 2021 10:41:10 PM

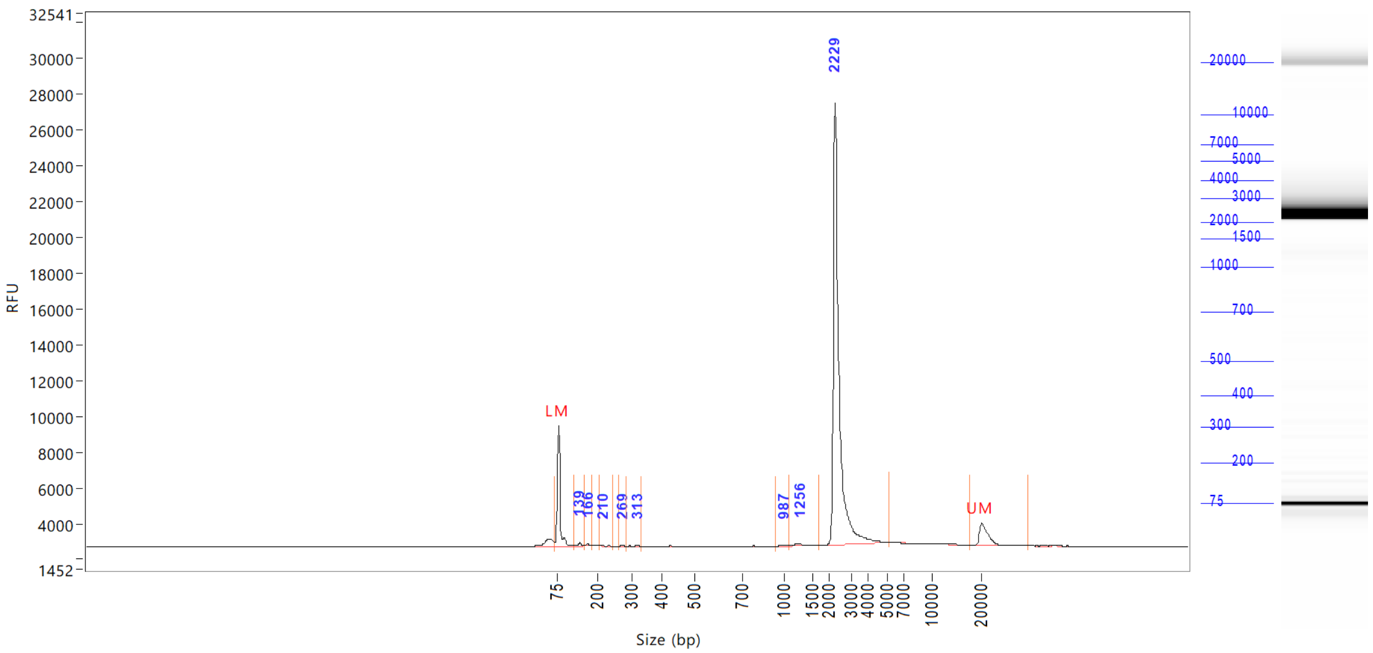

Peak	Size (bp)	Concentration (ng/uL)	From (bp)	To (bp)	Average size (bp)	CV%	RFU	Corrected peak area
1	75 (LM)	0.5000	62	124	74	10.76	6752	45.056
2	139	0.1883	124	154	137	4.51	179	1.414
3	166	0.1010	154	179	163	2.25	139	0.758
4	210	0.0477	201	241	212	4.29	55	0.358
5	269	0.0495	259	282	268	1.30	70	0.372
6	313	0.0712	282	329	307	3.27	69	0.534
7	987	0.0644	931	1068	987	2.21	52	0.483
8	1256	0.2585	1068	1674	1263	8.39	131	1.941
9	2229	29.0674	1674	5149	2394	14.92	24764	218.277
10	20000 (UM)	0.1515	17805	29309	20601	4.92	1248	13.650

TIC:	29.8479	ng/uL
TIM:	24.7639	nmole/L
Total concentration:	30.1405	ng/uL

```
Sample peak width (sec): 5    Sample min peak height: 50    Sample baseline V to V?: Y    Sample baseline V to V points: 3
Sample filter: Binomial       Number of points for filter: 3  Sample start region (min): 0  Sample end region (min): 30
Marker peak width (sec): 5    Marker min peak height: 200   Marker baseline V to V?: Y    Marker baseline V to V points: 3
Lower marker selection: First peak > 500 RFU                Upper marker selection: Last peak > 200 RFU
Ladder size (bp)75, 200, 300, 400, 500, 700, 1000, 1500, 2000, 3000, 4000, 5000, 7000, 10000, 20000
Quantification using: Lower Marker          Final concentration (ng/uL): 0.5000          Dilution factor: 12.0
```

Sample: SampG8
Well location: G8
Created: Tuesday, August 10, 2021 10:41:10 PM

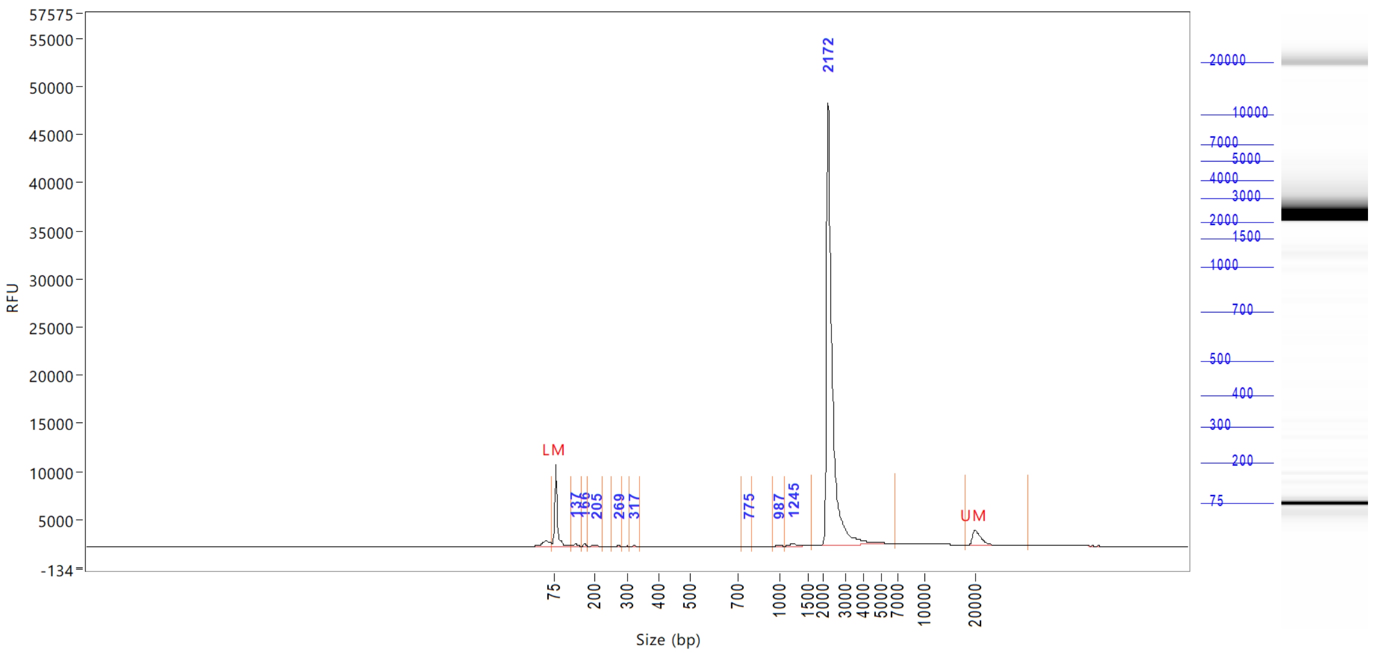

Peak	Size (bp)	Concentration (ng/uL)	From (bp)	To (bp)	Average size (bp)	CV%	RFU	Corrected peak area
1	75 (LM)	0.5000	64	126	76	10.80	8577	56.262
2	137	0.2166	126	156	137	4.95	231	2.031
3	166	0.1516	156	177	163	2.00	265	1.421
4	205	0.1070	177	223	197	4.30	96	1.004
5	269	0.0613	250	278	267	1.92	97	0.575
6	317	0.0710	303	335	315	1.69	108	0.666
7	775	0.0348	723	801	766	1.83	55	0.326
8	987	0.0787	944	1079	988	2.05	98	0.738
9	1245	0.3801	1079	1635	1255	7.34	266	3.564
10	2172	43.3229	1635	6704	2345	16.91	46022	406.239
11	20000 (UM)	0.1483	18171	30639	20581	5.18	1557	16.683

TIC:	44.4239	ng/uL
TIM:	36.8844	nmole/L
Total concentration:	44.7888	ng/uL

Sample peak width (sec): 5 Sample min peak height: 50 Sample baseline V to V?: Y Sample baseline V to V points: 3
Sample filter: Binomial Number of points for filter: 3 Sample start region (min): 0 Sample end region (min): 30
Marker peak width (sec): 5 Marker min peak height: 200 Marker baseline V to V?: Y Marker baseline V to V points: 3
Lower marker selection: First peak > 500 RFU Upper marker selection: Last peak > 200 RFU
Ladder size (bp)75, 200, 300, 400, 500, 700, 1000, 1500, 2000, 3000, 4000, 5000, 7000, 10000, 20000
Quantification using: Lower Marker Final concentration (ng/uL): 0.5000 Dilution factor: 12.0

Sample: SampH8
Well location: H8
Created: Tuesday, August 10, 2021 10:41:10 PM

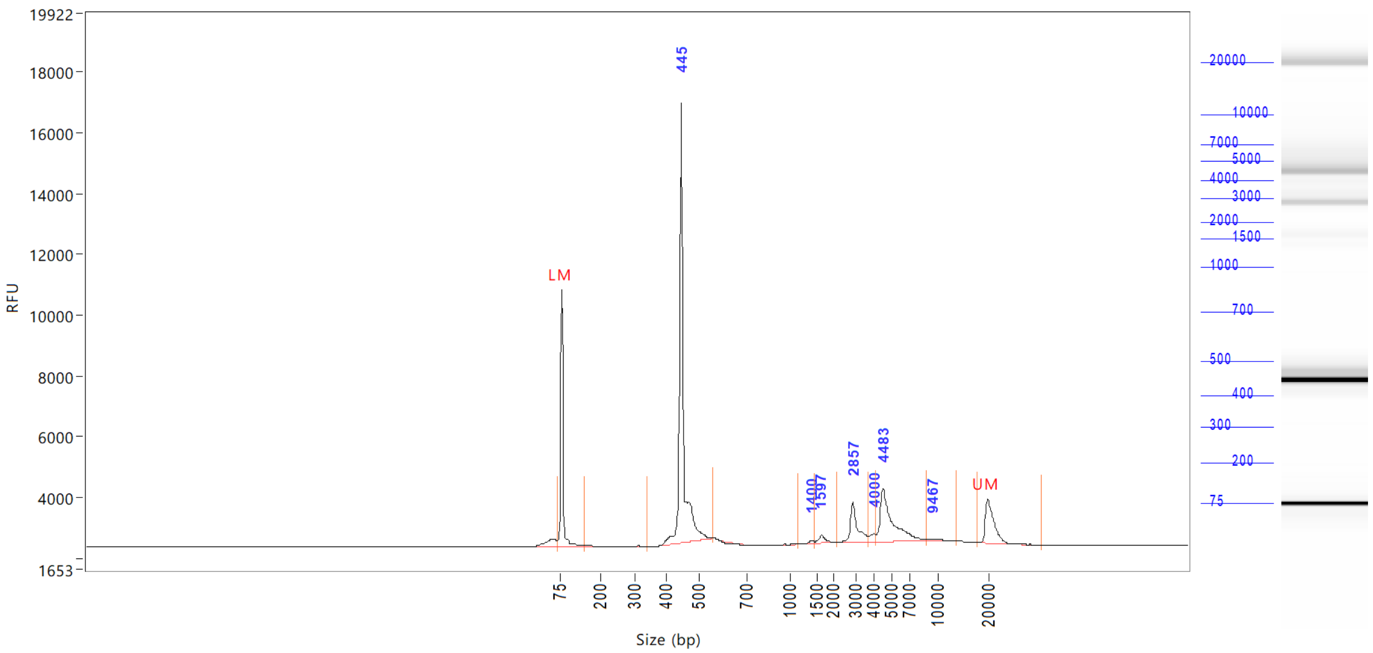

Peak	Size	Concentration	From	To	Average size	CV%	RFU	Corrected peak area
	(bp)	(ng/uL)	(bp)	(bp)	(bp)			
1	75 (LM)	0.5000	62	147	74	13.06	8431	52.779
2	445	12.6422	337	560	448	4.08	14481	111.206
3	1400	0.0954	1145	1456	1377	3.61	109	0.840
4	1597	0.3144	1456	2087	1626	6.92	253	2.766
5	2857	1.5693	2087	3679	2956	9.03	1306	13.804
6	4000	0.2800	3679	4104	3913	3.21	262	2.463
7	4483	3.4737	4104	8800	5126	19.05	1746	30.556
8	9467	0.1197	8800	13781	10034	9.90	52	1.053
9	20000 (UM)	0.1499	17927	30760	20613	5.29	1437	15.827

TIC:	18.4947	ng/uL
TIM:	48.9914	nmole/L
Total concentration:	18.6173	ng/uL

```
Sample peak width (sec): 5    Sample min peak height: 50    Sample baseline V to V?: Y    Sample baseline V to V points: 3
Sample filter: Binomial       Number of points for filter: 3  Sample start region (min): 0  Sample end region (min): 30
Marker peak width (sec): 5    Marker min peak height: 200   Marker baseline V to V?: Y    Marker baseline V to V points: 3
Lower marker selection: First peak > 500 RFU                Upper marker selection: Last peak > 200 RFU
Ladder size (bp)75, 200, 300, 400, 500, 700, 1000, 1500, 2000, 3000, 4000, 5000, 7000, 10000, 20000
Quantification using: Lower Marker          Final concentration (ng/uL): 0.5000          Dilution factor: 12.0
```

Sample: SampA9
Well location: A9
Created: Tuesday, August 10, 2021 10:41:10 PM

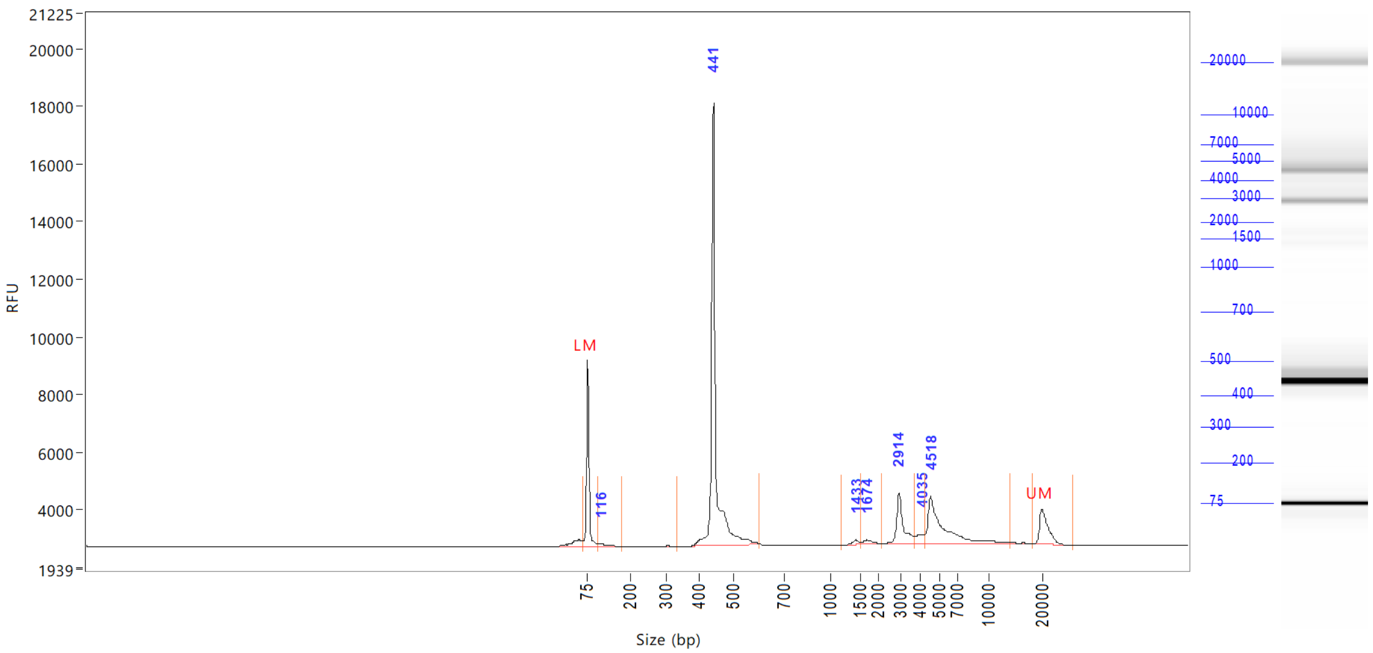

Peak	Size (bp)	Concentration (ng/uL)	From (bp)	To (bp)	Average size (bp)	CV%	RFU	Corrected peak area
1	75 (LM)	0.5000	64	107	74	7.58	6472	37.943
2	116	0.2613	107	175	125	13.05	98	1.653
3	441	19.2159	331	600	447	5.67	15378	121.518
4	1433	0.2346	1190	1500	1403	4.04	162	1.483
5	1674	0.3160	1500	2172	1698	7.59	144	1.998
6	2914	2.6308	2172	3715	3014	8.72	1742	16.637
7	4035	0.5690	3715	4208	3975	3.63	321	3.598
8	4518	5.0883	4208	14147	5700	31.31	1642	32.177
9	20000 (UM)	0.1649	18415	25682	20550	4.46	1217	12.516

TIC:	28.3159	ng/uL
TIM:	77.8923	nmole/L
Total concentration:	28.4676	ng/uL

```
Sample peak width (sec): 5    Sample min peak height: 50    Sample baseline V to V?: Y    Sample baseline V to V points: 3
Sample filter: Binomial       Number of points for filter: 3  Sample start region (min): 0  Sample end region (min): 30
Marker peak width (sec): 5    Marker min peak height: 200   Marker baseline V to V?: Y    Marker baseline V to V points: 3
Lower marker selection: First peak > 500 RFU                Upper marker selection: Last peak > 200 RFU
Ladder size (bp)75, 200, 300, 400, 500, 700, 1000, 1500, 2000, 3000, 4000, 5000, 7000, 10000, 20000
Quantification using: Lower Marker          Final concentration (ng/uL): 0.5000          Dilution factor: 12.0
```

Sample: SampB9
Well location: B9
Created: Tuesday, August 10, 2021 10:41:10 PM

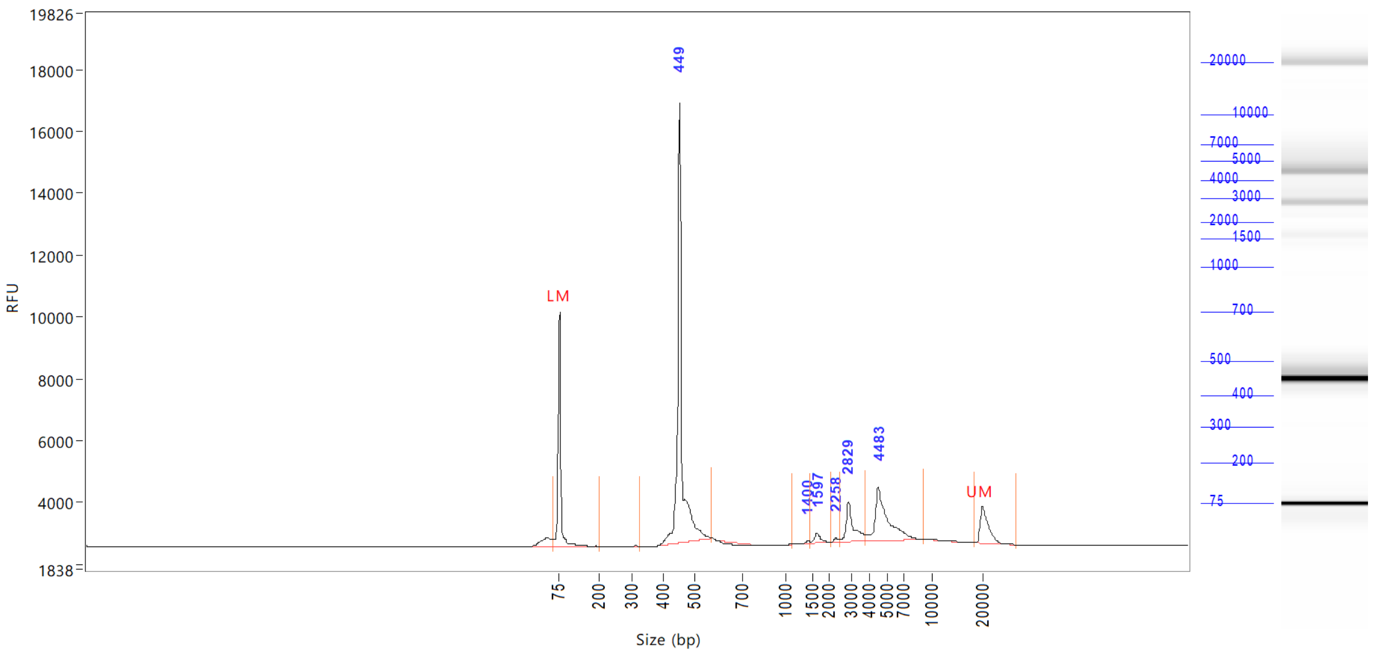

Peak	Size (bp)	Concentration (ng/uL)	From (bp)	To (bp)	Average size (bp)	CV%	RFU	Corrected peak area
1	75 (LM)	0.5000	58	200	76	19.42	7587	49.650
2	449	14.0957	323	570	453	4.55	14259	116.642
3	1400	0.0842	1112	1456	1380	4.01	90	0.697
4	1597	0.3644	1456	2001	1612	6.33	301	3.015
5	2258	0.1058	2001	2429	2270	4.23	118	0.875
6	2829	1.7931	2429	3786	3000	10.16	1301	14.838
7	4483	3.8611	3786	9134	5005	17.79	1729	31.951
8	20000 (UM)	0.1254	18415	26770	20592	4.65	1172	12.457

TIC:	20.3042	ng/uL
TIM:	54.0025	nmole/L
Total concentration:	20.4072	ng/uL

```
Sample peak width (sec): 5    Sample min peak height: 50    Sample baseline V to V?: Y    Sample baseline V to V points: 3
Sample filter: Binomial       Number of points for filter: 3  Sample start region (min): 0  Sample end region (min): 30
Marker peak width (sec): 5    Marker min peak height: 200   Marker baseline V to V?: Y    Marker baseline V to V points: 3
Lower marker selection: First peak > 500 RFU                Upper marker selection: Last peak > 200 RFU
Ladder size (bp)75, 200, 300, 400, 500, 700, 1000, 1500, 2000, 3000, 4000, 5000, 7000, 10000, 20000
Quantification using: Lower Marker          Final concentration (ng/uL): 0.5000          Dilution factor: 12.0
```

Sample: SampC9
Well location: C9
Created: Tuesday, August 10, 2021 10:41:10 PM

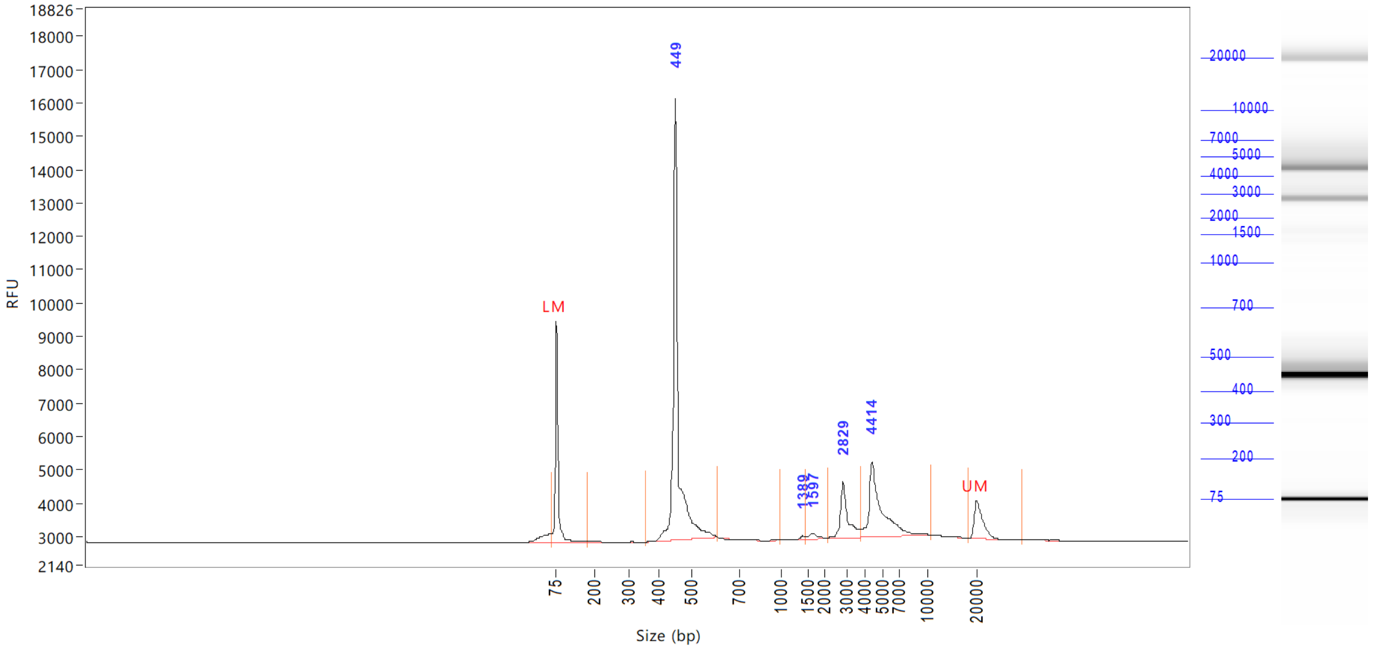

Peak	Size (bp)	Concentration (ng/uL)	From (bp)	To (bp)	Average size (bp)	CV%	RFU	Corrected peak area
1	75 (LM)	0.5000	64	175	77	16.59	6618	43.617
2	449	17.0505	354	608	456	6.15	13265	123.949
3	1389	0.1486	987	1445	1317	8.98	108	1.080
4	1597	0.3518	1445	2087	1649	9.53	175	2.558
5	2829	2.6116	2087	3786	2948	10.81	1687	18.985
6	4414	5.5093	3786	10733	5048	20.98	2263	40.050
7	20000 (UM)	0.1432	18293	29068	20558	5.24	1132	12.488

TIC:	25.6718	ng/uL
TIM:	65.3167	nmole/L
Total concentration:	25.8535	ng/uL

Sample peak width (sec): 5   Sample min peak height: 50   Sample baseline V to V?: Y   Sample baseline V to V points: 3
Sample filter: Binomial   Number of points for filter: 3   Sample start region (min): 0   Sample end region (min): 30
Marker peak width (sec): 5   Marker min peak height: 200   Marker baseline V to V?: Y   Marker baseline V to V points: 3
Lower marker selection: First peak > 500 RFU   Upper marker selection: Last peak > 200 RFU
Ladder size (bp)75, 200, 300, 400, 500, 700, 1000, 1500, 2000, 3000, 4000, 5000, 7000, 10000, 20000
Quantification using: Lower Marker   Final concentration (ng/uL): 0.5000   Dilution factor: 12.0

Sample: SampD9
Well location: D9
Created: Tuesday, August 10, 2021 10:41:10 PM

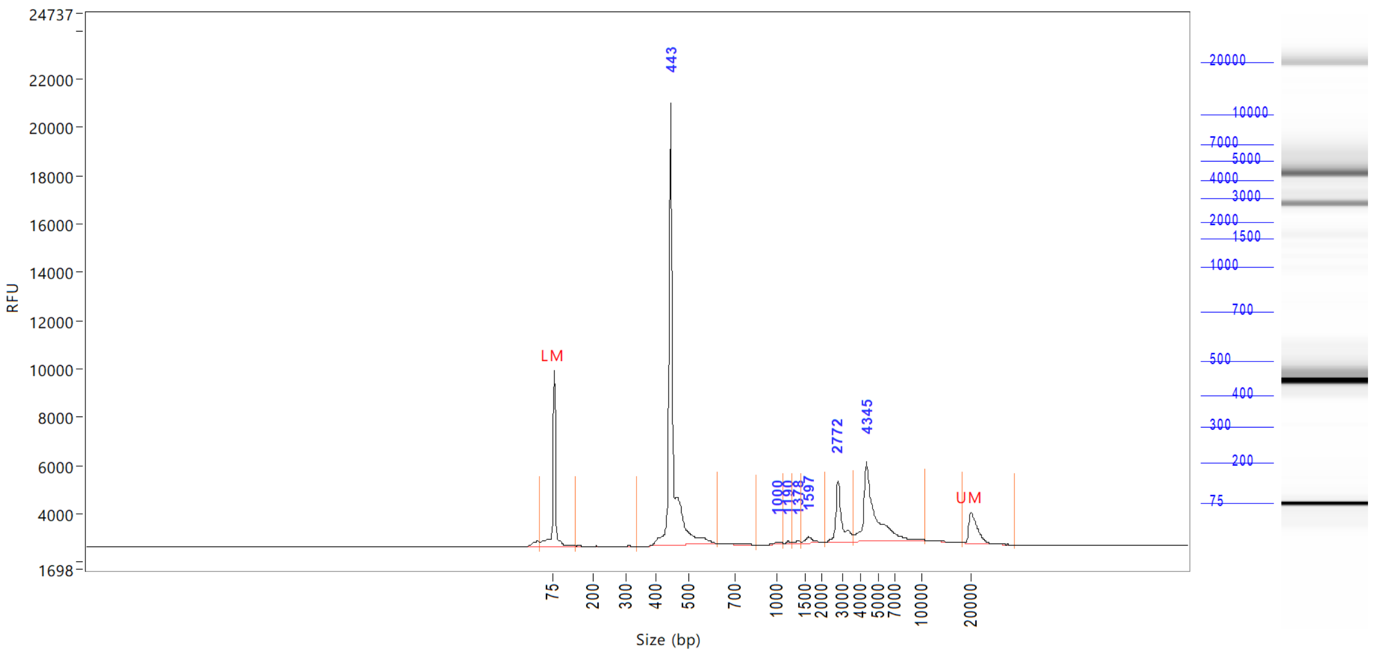

Peak	Size (bp)	Concentration (ng/uL)	From (bp)	To (bp)	Average size (bp)	CV%	RFU	Corrected peak area
1	75 (LM)	0.5000	28	145	71	18.89	7276	52.875
2	443	18.0128	331	621	451	6.24	18357	158.736
3	1000	0.1459	844	1112	1000	4.19	114	1.286
4	1190	0.0926	1112	1267	1186	3.18	113	0.816
5	1378	0.1137	1267	1445	1363	3.28	127	1.002
6	1597	0.3823	1445	2201	1638	8.44	253	3.369
7	2772	2.7599	2201	3608	2877	9.03	2550	24.321
8	4345	6.2420	3608	10611	4916	20.75	3329	55.007
9	20000 (UM)	0.1326	18171	28826	20593	5.03	1304	14.019

TIC:	27.7492	ng/uL
TIM:	70.2770	nmole/L
Total concentration:	27.9789	ng/uL

```
Sample peak width (sec): 5    Sample min peak height: 50    Sample baseline V to V?: Y    Sample baseline V to V points: 3
Sample filter: Binomial       Number of points for filter: 3  Sample start region (min): 0  Sample end region (min): 30
Marker peak width (sec): 5    Marker min peak height: 200   Marker baseline V to V?: Y    Marker baseline V to V points: 3
Lower marker selection: First peak > 500 RFU                Upper marker selection: Last peak > 200 RFU
Ladder size (bp)75, 200, 300, 400, 500, 700, 1000, 1500, 2000, 3000, 4000, 5000, 7000, 10000, 20000
Quantification using: Lower Marker          Final concentration (ng/uL): 0.5000          Dilution factor: 12.0
```

Sample: SampE9
Well location: E9
Created: Tuesday, August 10, 2021 10:41:10 PM

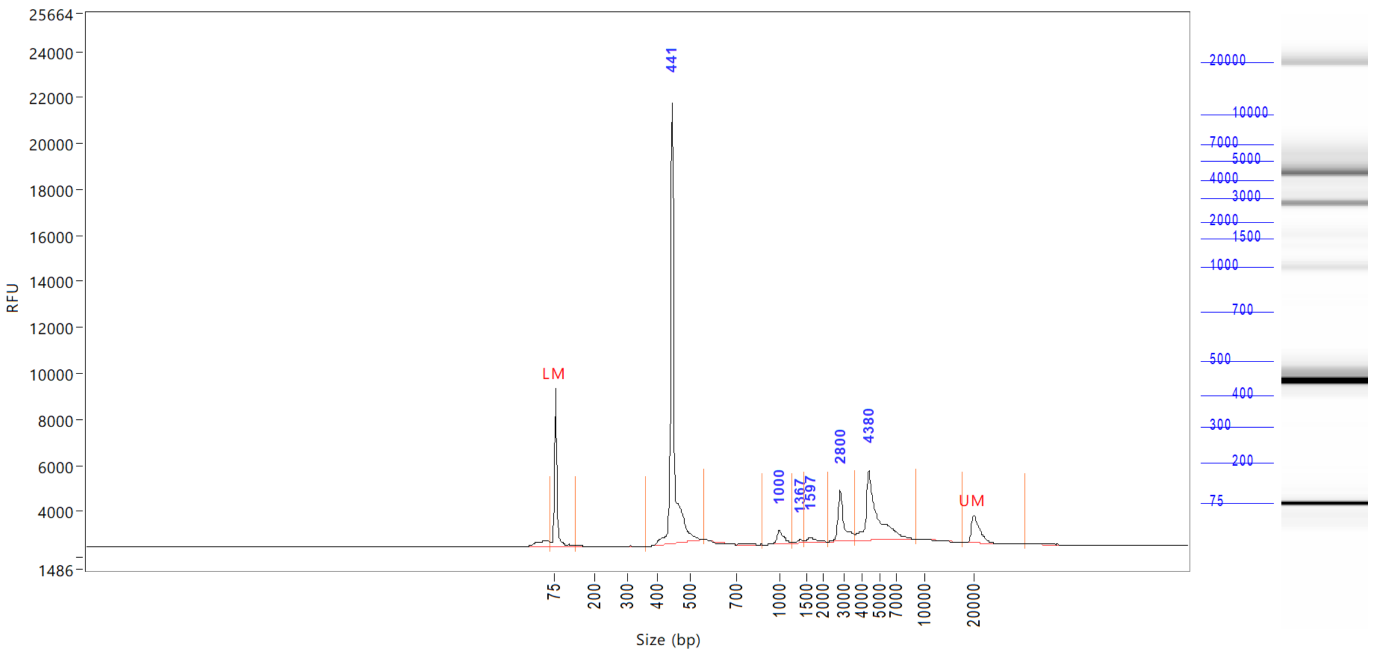

Peak	Size (bp)	Concentration (ng/uL)	From (bp)	To (bp)	Average size (bp)	CV%	RFU	Corrected peak area
1	75 (LM)	0.5000	60	139	75	12.31	6889	43.716
2	441	20.0605	358	560	445	3.85	19192	146.160
3	1000	0.9162	879	1234	1028	5.74	609	6.675
4	1367	0.1824	1234	1433	1348	3.95	137	1.329
5	1597	0.4665	1433	2201	1643	8.88	229	3.399
6	2800	2.8143	2201	3572	2887	8.34	2208	20.505
7	4380	6.8837	3572	9067	4865	18.24	3050	50.154
8	20000 (UM)	0.1467	17683	30398	20614	5.29	1183	12.827

TIC:	31.3236	ng/uL
TIM:	80.2656	nmole/L
Total concentration:	31.4661	ng/uL

Sample peak width (sec): 5 Sample min peak height: 50 Sample baseline V to V?: Y Sample baseline V to V points: 3
Sample filter: Binomial Number of points for filter: 3 Sample start region (min): 0 Sample end region (min): 30
Marker peak width (sec): 5 Marker min peak height: 200 Marker baseline V to V?: Y Marker baseline V to V points: 3
Lower marker selection: First peak > 500 RFU Upper marker selection: Last peak > 200 RFU
Ladder size (bp)75, 200, 300, 400, 500, 700, 1000, 1500, 2000, 3000, 4000, 5000, 7000, 10000, 20000
Quantification using: Lower Marker Final concentration (ng/uL): 0.5000 Dilution factor: 12.0

Sample: SampF9
Well location: F9
Created: Tuesday, August 10, 2021 10:41:10 PM

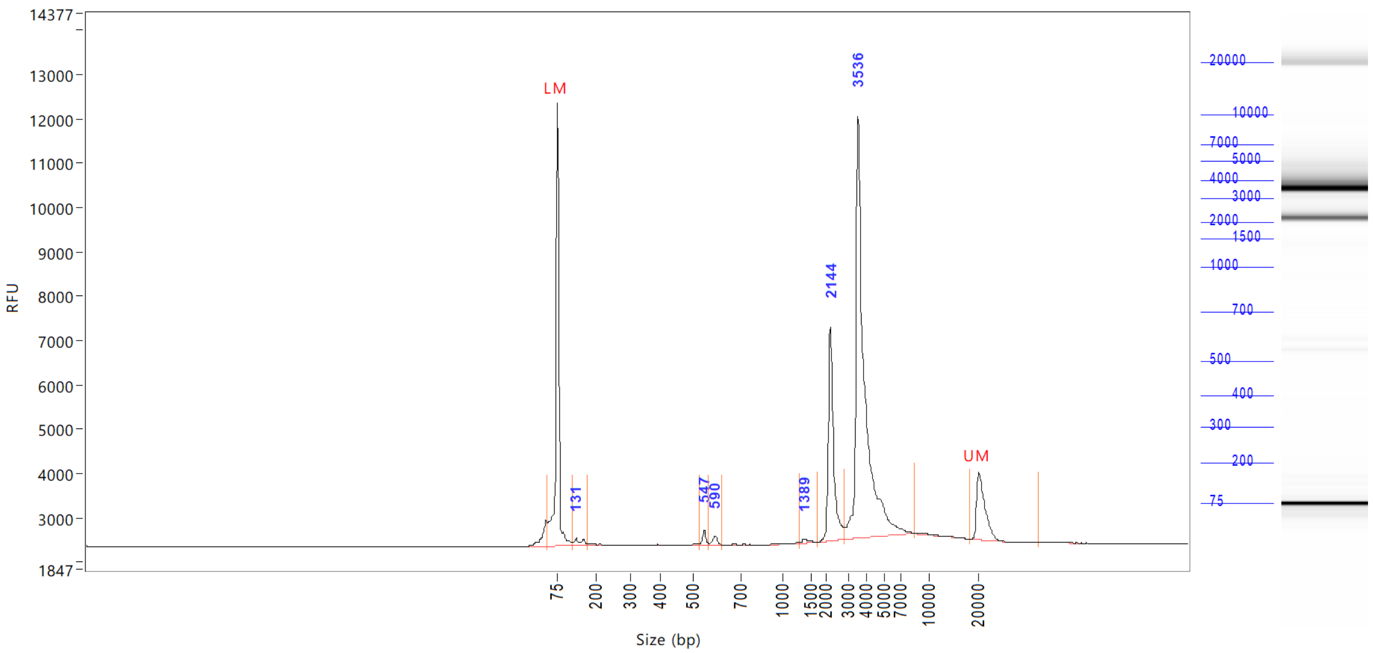

Peak	Size (bp)	Concentration (ng/uL)	From (bp)	To (bp)	Average size (bp)	CV%	RFU	Corrected peak area
1	75 (LM)	0.5000	45	122	72	13.54	10002	72.810
2	131	0.2460	122	169	142	8.98	175	2.985
3	547	0.1830	527	567	545	1.51	347	2.220
4	590	0.1433	567	618	587	1.52	206	1.739
5	1389	0.0865	1300	1693	1416	4.56	101	1.049
6	2144	3.2106	1693	2857	2194	8.46	4828	38.961
7	3536	9.9680	2857	8401	3889	16.77	9498	120.962
8	20000 (UM)	0.1158	18415	32090	20671	5.66	1540	16.864

TIC:	13.8374	ng/uL
TIM:	10.5298	nmole/L
Total concentration:	14.1019	ng/uL

Sample peak width (sec): 5 Sample min peak height: 50 Sample baseline V to V?: Y Sample baseline V to V points: 3
Sample filter: Binomial Number of points for filter: 3 Sample start region (min): 0 Sample end region (min): 30
Marker peak width (sec): 5 Marker min peak height: 200 Marker baseline V to V?: Y Marker baseline V to V points: 3
Lower marker selection: First peak > 500 RFU Upper marker selection: Last peak > 200 RFU
Ladder size (bp)75, 200, 300, 400, 500, 700, 1000, 1500, 2000, 3000, 4000, 5000, 7000, 10000, 20000
Quantification using: Lower Marker Final concentration (ng/uL): 0.5000 Dilution factor: 12.0

Sample: SampG9
Well location: G9
Created: Tuesday, August 10, 2021 10:41:10 PM

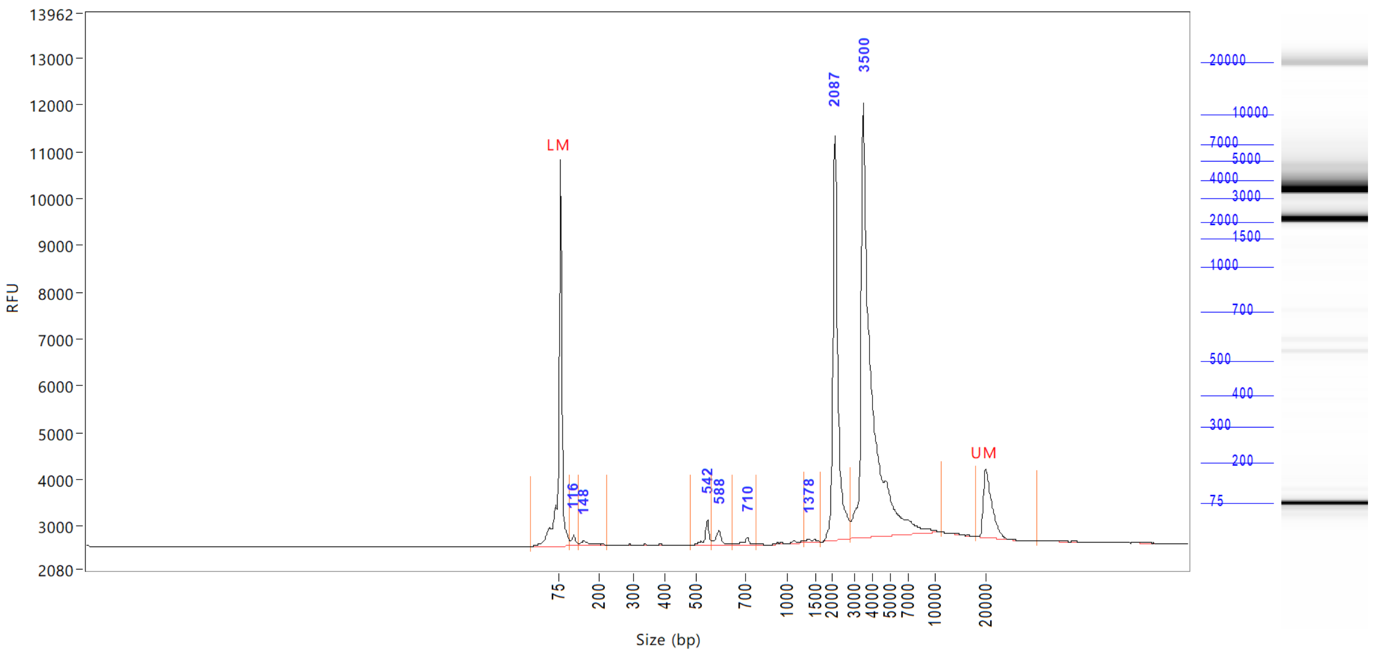

Peak	Size (bp)	Concentration (ng/uL)	From (bp)	To (bp)	Average size (bp)	CV%	RFU	Corrected peak area
1	75 (LM)	0.5000	0	107	69	20.79	8259	69.897
2	116	0.2099	107	135	117	6.41	234	2.446
3	148	0.2230	135	219	165	13.07	99	2.598
4	542	0.3819	479	560	533	2.93	537	4.449
5	588	0.3021	560	649	589	3.01	325	3.519
6	710	0.1570	649	775	701	3.70	159	1.829
7	1378	0.0838	1289	1655	1406	4.94	78	0.976
8	2087	6.0220	1655	2829	2144	8.42	8637	70.153
9	3500	12.3992	2829	11098	4017	24.56	9306	144.446
10	20000 (UM)	0.1146	17927	30156	20554	5.22	1464	16.027

TIC:	19.7790	ng/uL
TIM:	17.3643	nmole/L
Total concentration:	19.9802	ng/uL

Sample peak width (sec): 5   Sample min peak height: 50   Sample baseline V to V?: Y   Sample baseline V to V points: 3
Sample filter: Binomial   Number of points for filter: 3   Sample start region (min): 0   Sample end region (min): 30
Marker peak width (sec): 5   Marker min peak height: 200   Marker baseline V to V?: Y   Marker baseline V to V points: 3
Lower marker selection: First peak > 500 RFU   Upper marker selection: Last peak > 200 RFU
Ladder size (bp)75, 200, 300, 400, 500, 700, 1000, 1500, 2000, 3000, 4000, 5000, 7000, 10000, 20000
Quantification using: Lower Marker   Final concentration (ng/uL): 0.5000   Dilution factor: 12.0

Sample: SampH9
Well location: H9
Created: Tuesday, August 10, 2021 10:41:10 PM

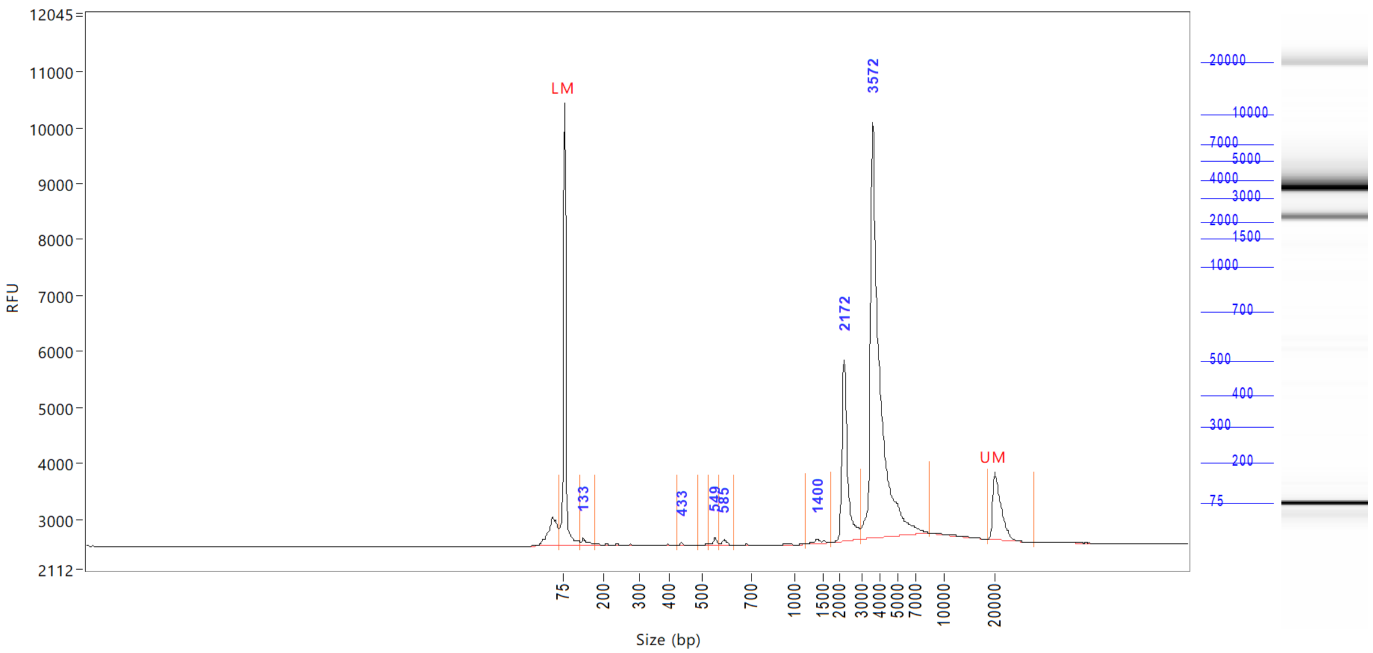

Peak	Size (bp)	Concentration (ng/uL)	From (bp)	To (bp)	Average size (bp)	CV%	RFU	Corrected peak area
1	75 (LM)	0.5000	58	126	75	10.71	7919	51.658
2	133	0.1752	126	169	140	7.76	124	1.509
3	433	0.0402	422	483	436	2.38	52	0.346
4	549	0.1077	524	567	546	1.70	128	0.927
5	585	0.1004	567	626	586	1.66	95	0.864
6	1400	0.1084	1178	1731	1401	6.44	76	0.933
7	2172	3.2109	1731	2943	2244	8.98	3251	27.645
8	3572	11.7173	2943	8401	3924	16.57	7427	100.882
9	20000 (UM)	0.1229	18537	27738	20569	4.84	1194	12.702

TIC:	15.4601	ng/uL
TIM:	10.2135	nmole/L
Total concentration:	15.7044	ng/uL

```
Sample peak width (sec): 5    Sample min peak height: 50    Sample baseline V to V?: Y    Sample baseline V to V points: 3
Sample filter: Binomial       Number of points for filter: 3  Sample start region (min): 0  Sample end region (min): 30
Marker peak width (sec): 5    Marker min peak height: 200   Marker baseline V to V?: Y    Marker baseline V to V points: 3
Lower marker selection: First peak > 500 RFU                Upper marker selection: Last peak > 200 RFU
Ladder size (bp)75, 200, 300, 400, 500, 700, 1000, 1500, 2000, 3000, 4000, 5000, 7000, 10000, 20000
Quantification using: Lower Marker          Final concentration (ng/uL): 0.5000          Dilution factor: 12.0
```

Sample: SampA10
Well location: A10
Created: Tuesday, August 10, 2021 10:41:10 PM

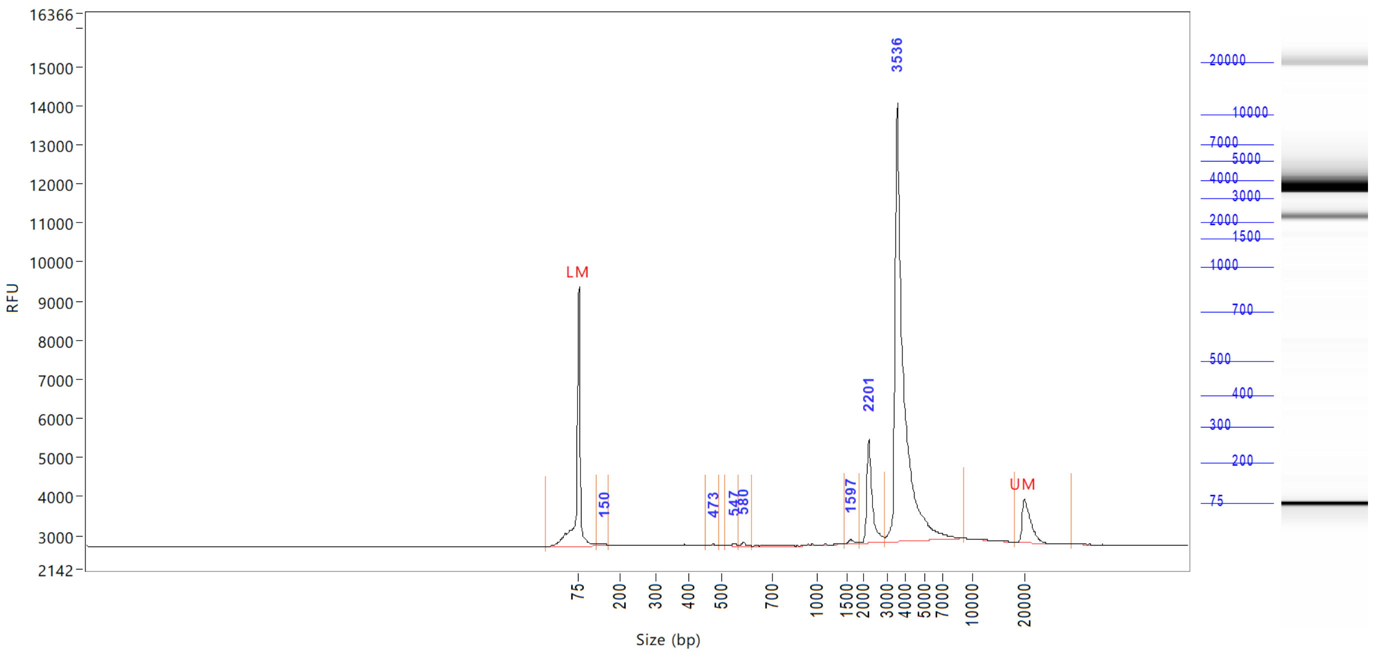

Peak	Size (bp)	Concentration (ng/uL)	From (bp)	To (bp)	Average size (bp)	CV%	RFU	Corrected peak area
1	75 (LM)	0.5000	0	126	66	26.21	6632	55.305
2	150	0.1299	126	164	142	6.93	62	1.197
3	473	0.0384	452	489	472	1.34	53	0.354
4	547	0.0813	509	567	543	2.64	73	0.750
5	580	0.0849	567	621	581	1.46	110	0.783
6	1597	0.1030	1478	1885	1631	5.84	105	0.949
7	2201	2.3341	1885	2914	2253	8.10	2668	21.515
8	3536	14.7270	2914	9134	3879	16.76	11243	135.745
9	20000 (UM)	0.1053	18171	28947	20564	4.72	1118	11.647

TIC:	17.4987	ng/uL
TIM:	10.1838	nmole/L
Total concentration:	17.8649	ng/uL

Sample peak width (sec): 5 Sample min peak height: 50 Sample baseline V to V?: Y Sample baseline V to V points: 3
Sample filter: Binomial Number of points for filter: 3 Sample start region (min): 0 Sample end region (min): 30
Marker peak width (sec): 5 Marker min peak height: 200 Marker baseline V to V?: Y Marker baseline V to V points: 3
Lower marker selection: First peak > 500 RFU Upper marker selection: Last peak > 200 RFU
Ladder size (bp)75, 200, 300, 400, 500, 700, 1000, 1500, 2000, 3000, 4000, 5000, 7000, 10000, 20000
Quantification using: Lower Marker Final concentration (ng/uL): 0.5000 Dilution factor: 12.0

Sample: SampB10
Well location: B10
Created: Tuesday, August 10, 2021 10:41:10 PM

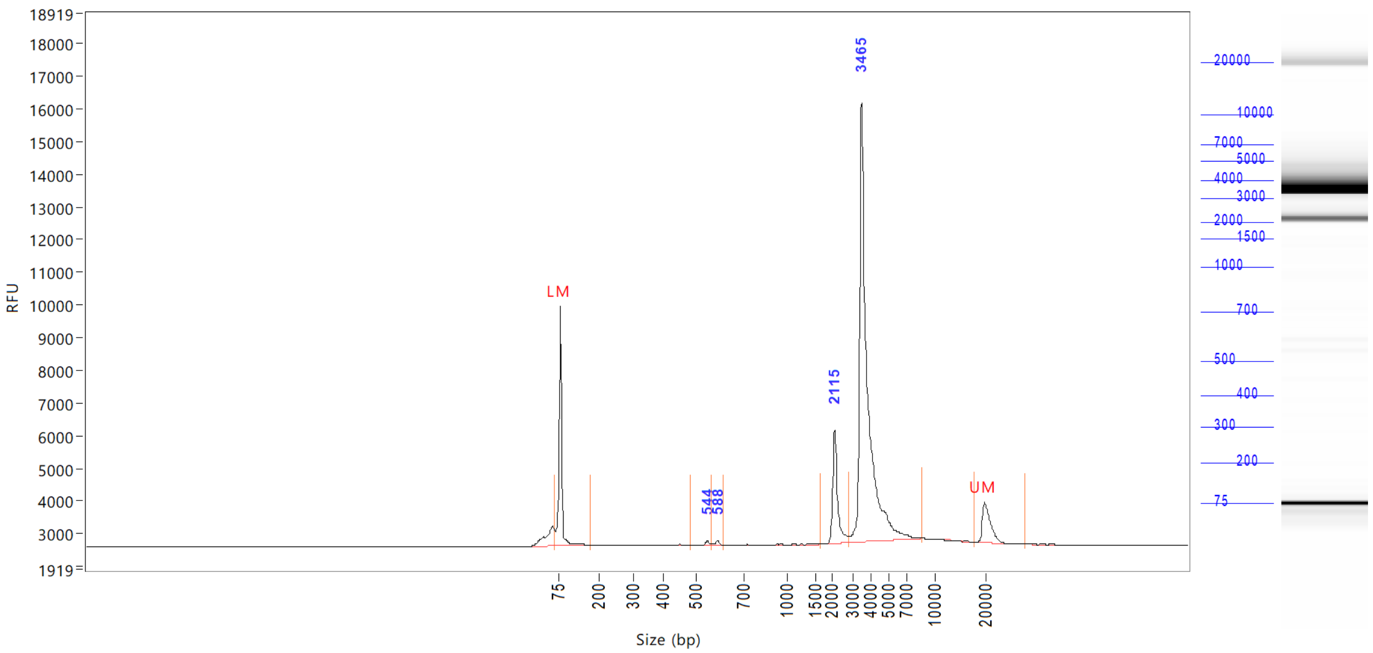

Peak	Size (bp)	Concentration (ng/uL)	From (bp)	To (bp)	Average size (bp)	CV%	RFU	Corrected peak area
1	75 (LM)	0.5000	60	169	75	14.05	7316	48.576
2	544	0.1889	481	562	536	3.12	166	1.529
3	588	0.1727	562	613	582	1.67	165	1.398
4	2115	3.3094	1635	2800	2158	8.15	3446	26.793
5	3465	19.4724	2800	8667	3775	16.26	13441	157.648
6	20000 (UM)	0.1314	18049	27979	20502	4.52	1242	12.763

TIC:	23.1434	ng/uL
TIM:	12.0844	nmole/L
Total concentration:	23.5651	ng/uL

Sample peak width (sec): 5   Sample min peak height: 50   Sample baseline V to V?: Y   Sample baseline V to V points: 3
Sample filter: Binomial   Number of points for filter: 3   Sample start region (min): 0   Sample end region (min): 30
Marker peak width (sec): 5   Marker min peak height: 200   Marker baseline V to V?: Y   Marker baseline V to V points: 3
Lower marker selection: First peak > 500 RFU   Upper marker selection: Last peak > 200 RFU
Ladder size (bp)75, 200, 300, 400, 500, 700, 1000, 1500, 2000, 3000, 4000, 5000, 7000, 10000, 20000
Quantification using: Lower Marker   Final concentration (ng/uL): 0.5000   Dilution factor: 12.0

Sample: SampC10
Well location: C10
Created: Tuesday, August 10, 2021 10:41:10 PM

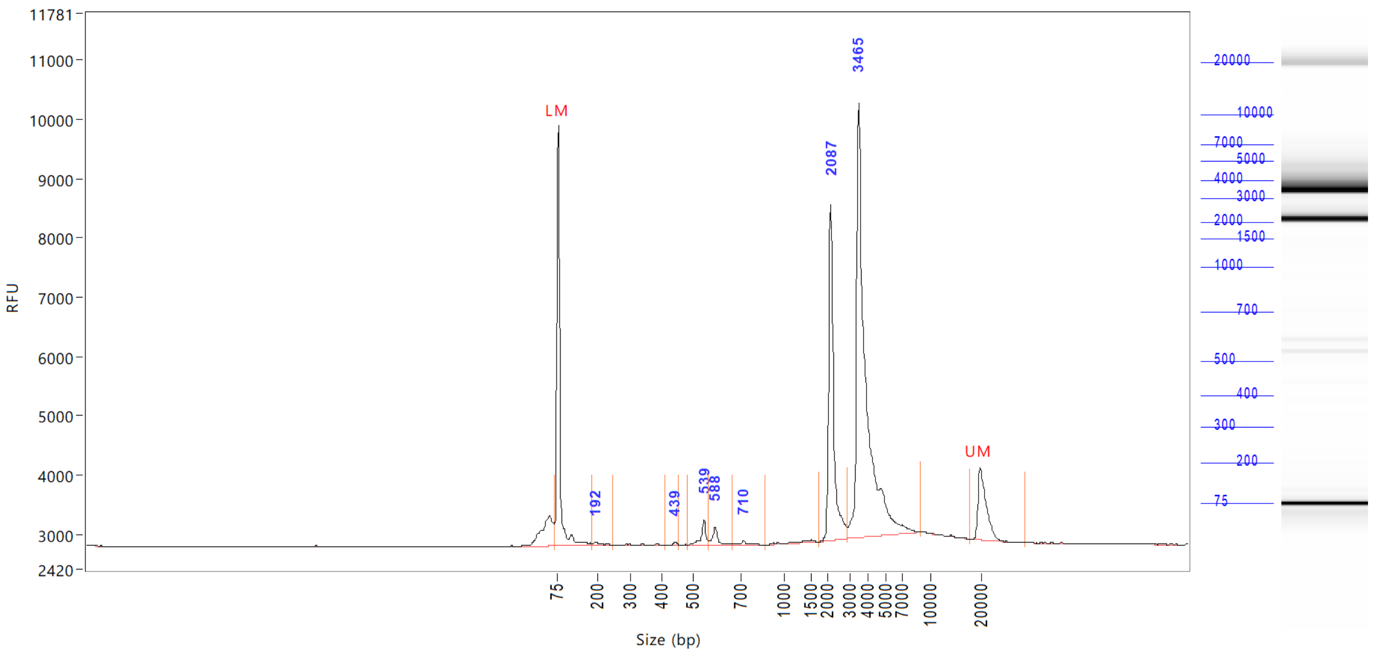

Peak	Size (bp)	Concentration (ng/uL)	From (bp)	To (bp)	Average size (bp)	CV%	RFU	Corrected peak area
1	75 (LM)	0.5000	64	183	78	22.15	7070	49.562
2	192	0.1007	183	242	202	7.43	50	0.832
3	439	0.0462	409	454	436	1.75	52	0.381
4	539	0.4356	481	560	532	3.07	428	3.598
5	588	0.3738	560	659	589	3.41	297	3.087
6	710	0.1346	659	866	719	6.09	75	1.112
7	2087	5.1666	1731	2857	2150	8.47	5657	42.677
8	3465	11.9420	2857	8934	3893	18.60	7331	98.644
9	20000 (UM)	0.1329	17927	29068	20536	4.99	1219	13.173

TIC:	18.1994	ng/uL
TIM:	12.6999	nmole/L
Total concentration:	18.4370	ng/uL

Sample peak width (sec): 5 Sample min peak height: 50 Sample baseline V to V?: Y Sample baseline V to V points: 3
Sample filter: Binomial Number of points for filter: 3 Sample start region (min): 0 Sample end region (min): 30
Marker peak width (sec): 5 Marker min peak height: 200 Marker baseline V to V?: Y Marker baseline V to V points: 3
Lower marker selection: First peak > 500 RFU Upper marker selection: Last peak > 200 RFU
Ladder size (bp)75, 200, 300, 400, 500, 700, 1000, 1500, 2000, 3000, 4000, 5000, 7000, 10000, 20000
Quantification using: Lower Marker Final concentration (ng/uL): 0.5000 Dilution factor: 12.0

Sample: SampD10
Well location: D10
Created: Tuesday, August 10, 2021 10:41:10 PM

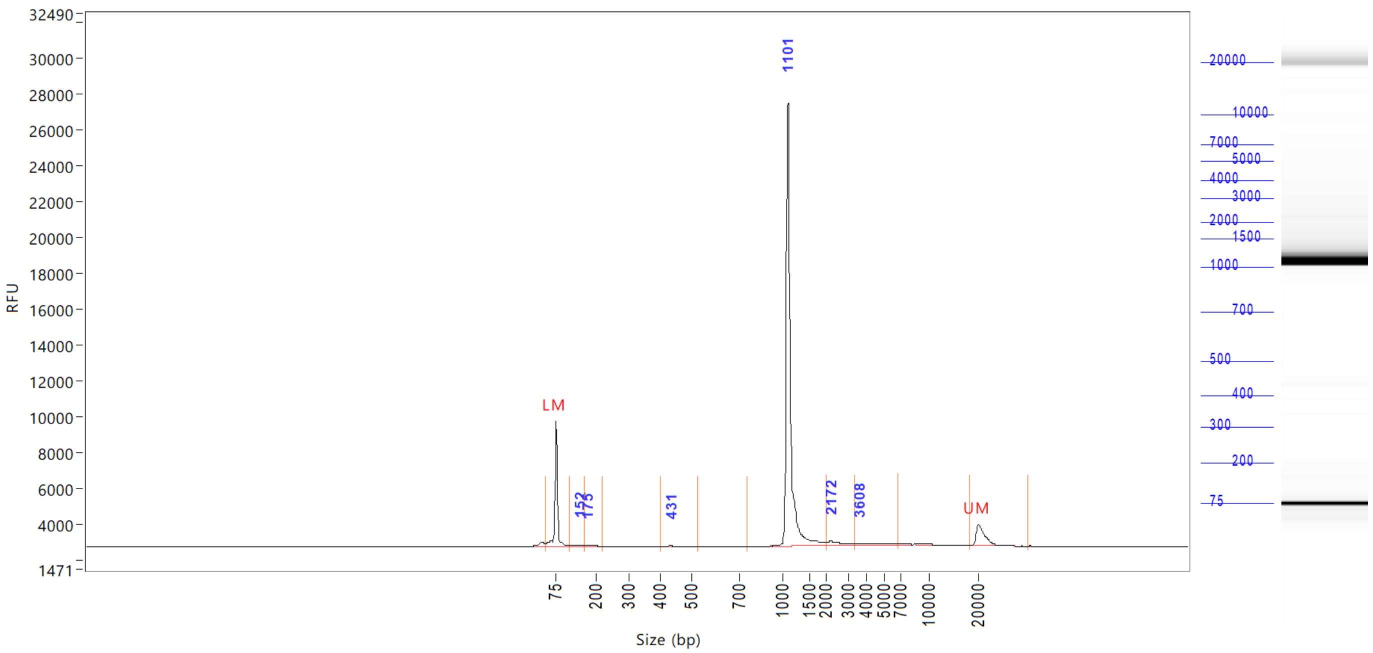

Peak	Size (bp)	Concentration (ng/uL)	From (bp)	To (bp)	Average size (bp)	CV%	RFU	Corrected peak area
1	75 (LM)	0.5000	41	118	72	12.06	6968	46.718
2	152	0.2270	118	166	143	8.15	98	1.768
3	175	0.1224	166	217	184	6.56	58	0.953
4	431	0.1030	403	521	439	4.70	78	0.802
5	1101	22.9859	753	2000	1130	11.44	24766	178.976
6	2172	0.7313	2000	3358	2522	15.30	236	5.694
7	3608	0.4630	3358	6704	4463	18.73	90	3.605
8	20000 (UM)	0.1394	18171	29914	20559	4.96	1180	13.029

TIC:	24.6326	ng/uL
TIM:	38.2183	nmole/L
Total concentration:	24.7996	ng/uL

```
Sample peak width (sec): 5     Sample min peak height: 50     Sample baseline V to V?: Y     Sample baseline V to V points: 3
Sample filter: Binomial        Number of points for filter: 3  Sample start region (min): 0  Sample end region (min): 30
Marker peak width (sec): 5     Marker min peak height: 200    Marker baseline V to V?: Y     Marker baseline V to V points: 3
Lower marker selection: First peak > 500 RFU                  Upper marker selection: Last peak > 200 RFU
Ladder size (bp)75, 200, 300, 400, 500, 700, 1000, 1500, 2000, 3000, 4000, 5000, 7000, 10000, 20000
Quantification using: Lower Marker          Final concentration (ng/uL): 0.5000          Dilution factor: 12.0
```

Sample: SampE10
Well location: E10
Created: Tuesday, August 10, 2021 10:41:10 PM

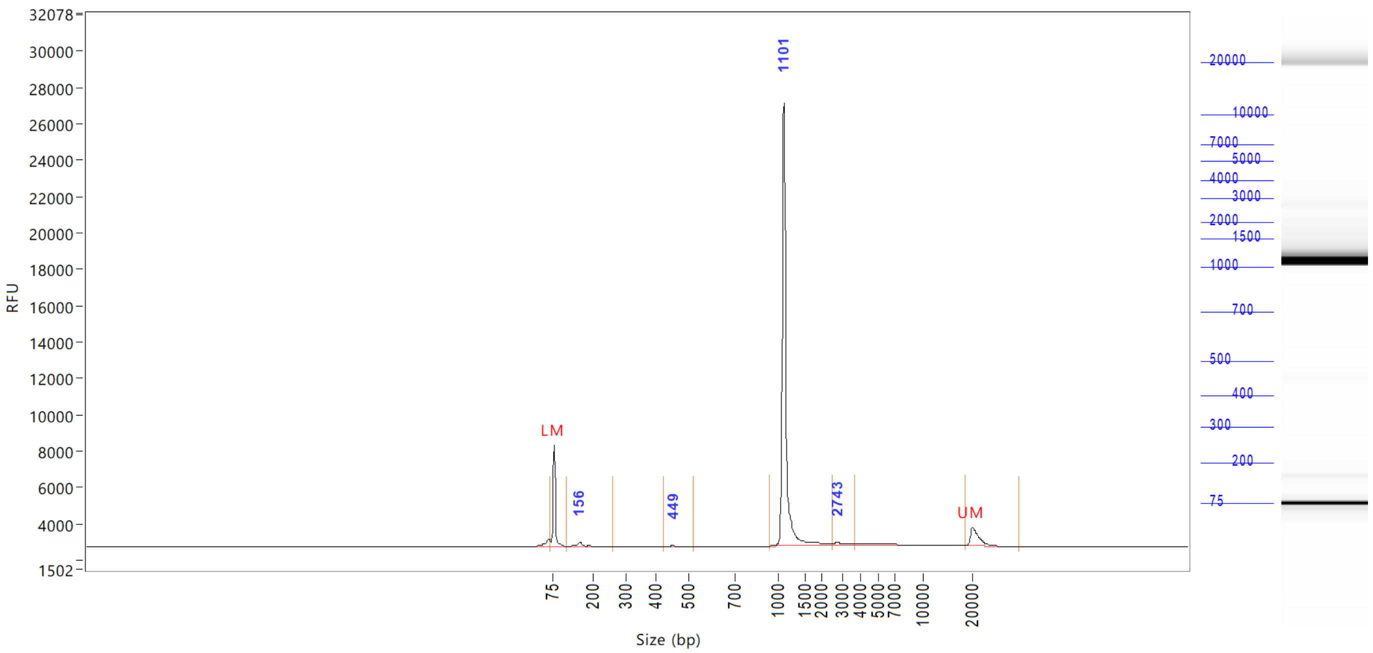

Peak	Size (bp)	Concentration (ng/uL)	From (bp)	To (bp)	Average size (bp)	CV%	RFU	Corrected peak area
1	75 (LM)	0.5000	66	114	75	7.72	5548	34.071
2	156	0.7671	114	257	165	18.25	257	4.356
3	449	0.1632	424	521	454	3.81	92	0.927
4	1101	27.9383	939	2486	1133	13.47	24404	158.645
5	2743	0.4253	2486	3679	2896	10.33	165	2.415
6	20000 (UM)	0.1606	18659	29430	20734	5.83	982	10.945

TIC:	29.2939	ng/uL
TIM:	49.0632	nmole/L
Total concentration:	29.7404	ng/uL

Sample peak width (sec): 5 Sample min peak height: 50 Sample baseline V to V?: Y Sample baseline V to V points: 3
Sample filter: Binomial Number of points for filter: 3 Sample start region (min): 0 Sample end region (min): 30
Marker peak width (sec): 5 Marker min peak height: 200 Marker baseline V to V?: Y Marker baseline V to V points: 3
Lower marker selection: First peak > 500 RFU Upper marker selection: Last peak > 200 RFU
Ladder size (bp)75, 200, 300, 400, 500, 700, 1000, 1500, 2000, 3000, 4000, 5000, 7000, 10000, 20000
Quantification using: Lower Marker Final concentration (ng/uL): 0.5000 Dilution factor: 12.0

Sample: SampF10
Well location: F10
Created: Tuesday, August 10, 2021 10:41:10 PM

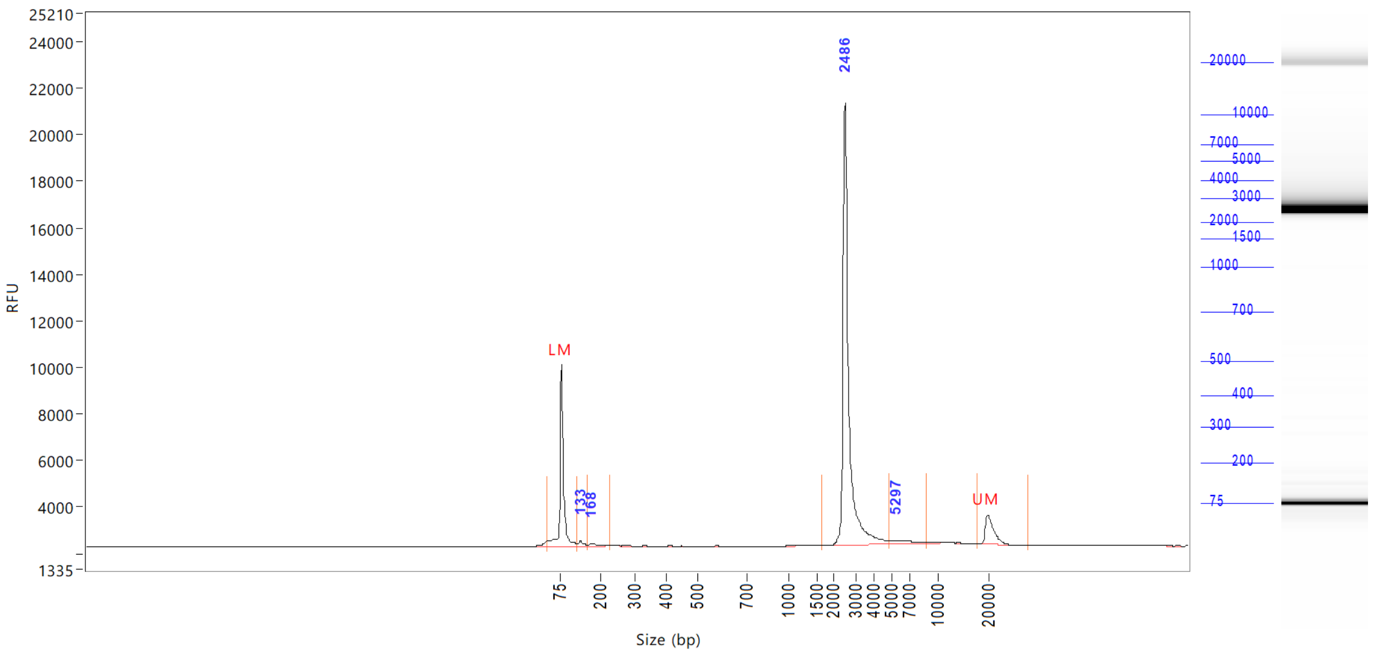

Peak	Size (bp)	Concentration (ng/uL)	From (bp)	To (bp)	Average size (bp)	CV%	RFU	Corrected peak area
1	75 (LM)	0.5000	31	124	74	17.28	7841	65.840
2	133	0.2384	124	156	136	6.42	249	2.616
3	168	0.1646	156	226	173	7.85	117	1.806
4	2486	14.7975	1635	4862	2634	15.41	19017	162.376
5	5297	0.3968	4862	8800	6366	17.71	143	4.354
6	20000 (UM)	0.1010	17927	27979	20550	4.69	1265	13.303

TIC:	15.5972	ng/uL
TIM:	13.7945	nmole/L
Total concentration:	15.8575	ng/uL

Sample peak width (sec): 5   Sample min peak height: 50   Sample baseline V to V?: Y   Sample baseline V to V points: 3
Sample filter: Binomial   Number of points for filter: 3 Sample start region (min): 0 Sample end region (min): 30
Marker peak width (sec): 5   Marker min peak height: 200   Marker baseline V to V?: Y   Marker baseline V to V points: 3
Lower marker selection: First peak > 500 RFU   Upper marker selection: Last peak > 200 RFU
Ladder size (bp)75, 200, 300, 400, 500, 700, 1000, 1500, 2000, 3000, 4000, 5000, 7000, 10000, 20000
Quantification using: Lower Marker   Final concentration (ng/uL): 0.5000   Dilution factor: 12.0

Sample: SampG10
Well location: G10
Created: Tuesday, August 10, 2021 10:41:10 PM

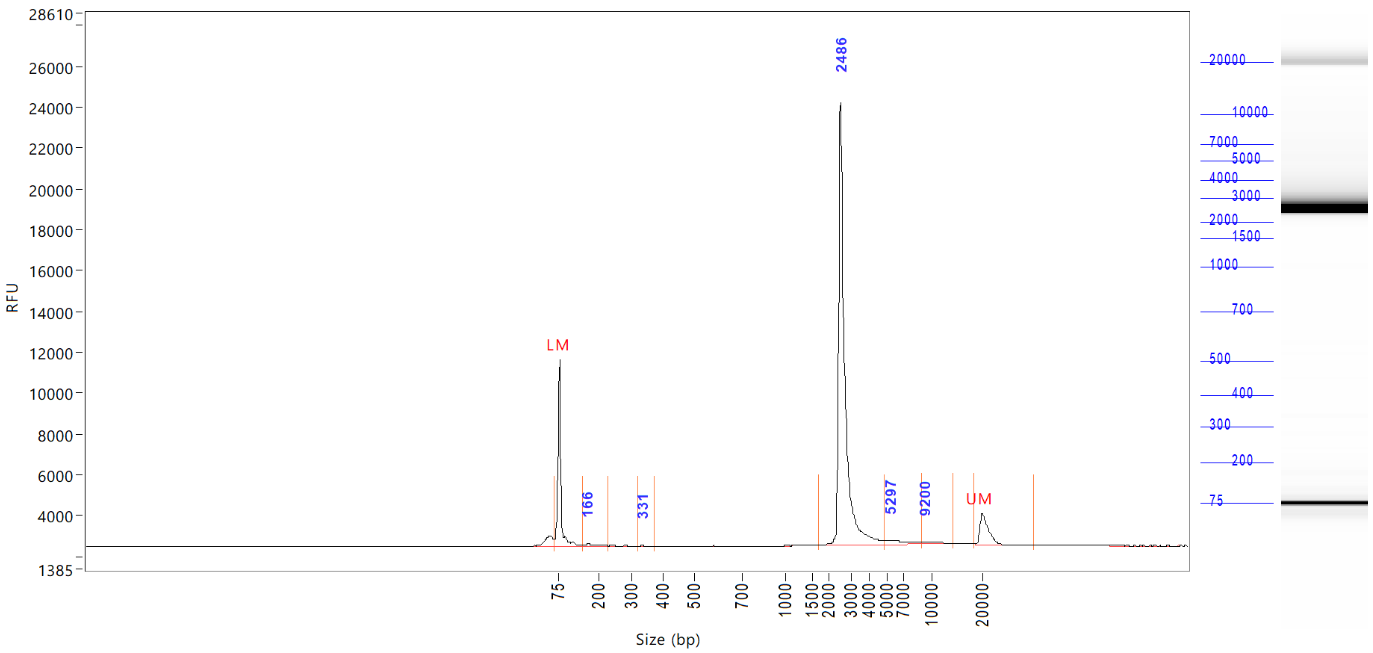

Peak	Size (bp)	Concentration (ng/uL)	From (bp)	To (bp)	Average size (bp)	CV%	RFU	Corrected peak area
1	75 (LM)	0.5000	60	147	77	16.25	9113	65.965
2	166	0.2099	147	228	172	11.27	121	2.308
3	331	0.0283	319	374	332	2.80	52	0.311
4	2486	18.9152	1655	4897	2659	15.33	21698	207.958
5	5297	0.5672	4897	8867	6501	17.86	208	6.236
6	9200	0.2258	8867	14025	10720	13.74	91	2.483
7	20000 (UM)	0.1257	18415	30398	20634	5.03	1528	16.587

TIC:	19.9464	ng/uL
TIM:	14.0353	nmole/L
Total concentration:	20.1356	ng/uL

Sample peak width (sec): 5 Sample min peak height: 50 Sample baseline V to V?: Y Sample baseline V to V points: 3
Sample filter: Binomial Number of points for filter: 3 Sample start region (min): 0 Sample end region (min): 30
Marker peak width (sec): 5 Marker min peak height: 200 Marker baseline V to V?: Y Marker baseline V to V points: 3
Lower marker selection: First peak > 500 RFU Upper marker selection: Last peak > 200 RFU
Ladder size (bp)75, 200, 300, 400, 500, 700, 1000, 1500, 2000, 3000, 4000, 5000, 7000, 10000, 20000
Quantification using: Lower Marker Final concentration (ng/uL): 0.5000 Dilution factor: 12.0

Sample: SampH10
Well location: H10
Created: Tuesday, August 10, 2021 10:41:10 PM

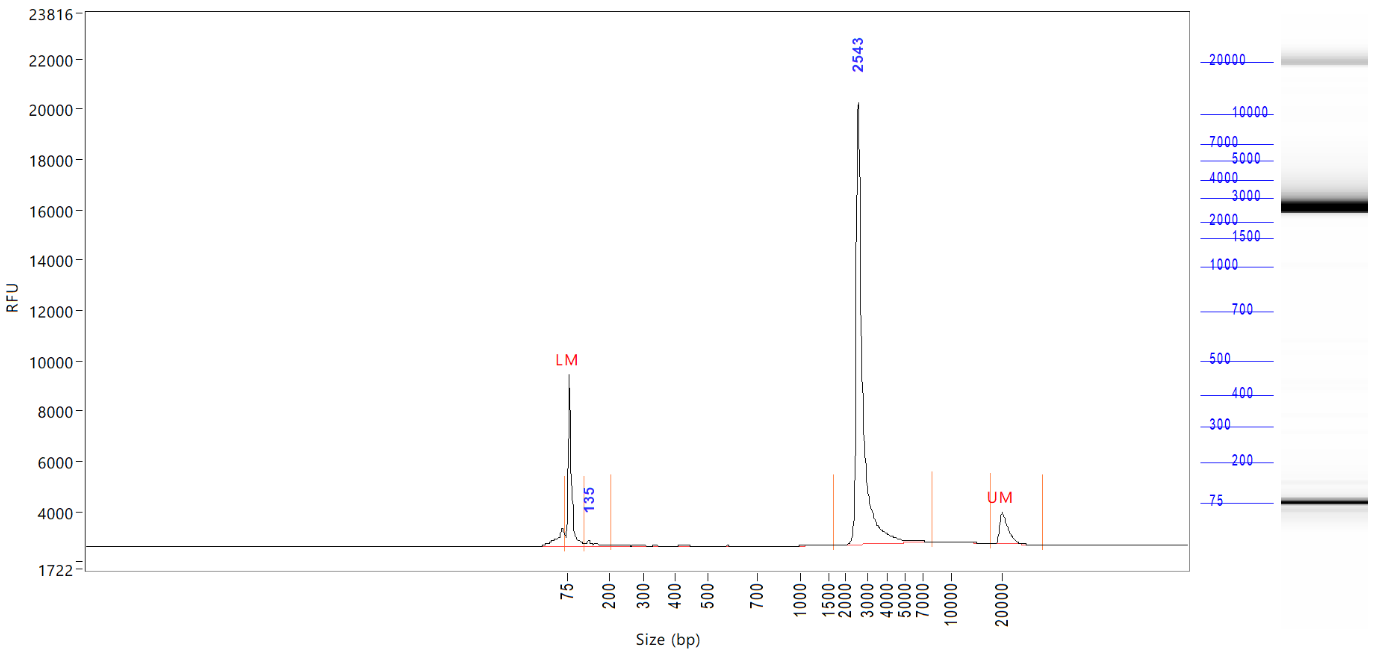

Peak	Size (bp)	Concentration (ng/uL)	From (bp)	To (bp)	Average size (bp)	CV%	RFU	Corrected peak area
1	75 (LM)	0.5000	66	124	78	12.10	6793	53.190
2	135	0.4605	124	205	149	13.82	244	4.082
3	2543	18.9894	1635	7934	2750	19.03	17585	168.341
4	20000 (UM)	0.1235	17683	27979	20529	4.75	1247	13.139

TIC:	19.4499	ng/uL
TIM:	16.4468	nmole/L
Total concentration:	19.8368	ng/uL

Sample peak width (sec): 5 Sample min peak height: 50 Sample baseline V to V?: Y Sample baseline V to V points: 3
Sample filter: Binomial Number of points for filter: 3 Sample start region (min): 0 Sample end region (min): 30
Marker peak width (sec): 5 Marker min peak height: 200 Marker baseline V to V?: Y Marker baseline V to V points: 3
Lower marker selection: First peak > 500 RFU Upper marker selection: Last peak > 200 RFU
Ladder size (bp)75, 200, 300, 400, 500, 700, 1000, 1500, 2000, 3000, 4000, 5000, 7000, 10000, 20000
Quantification using: Lower Marker Final concentration (ng/uL): 0.5000 Dilution factor: 12.0

Sample: SampA11
Well location: A11
Created: Tuesday, August 10, 2021 10:41:10 PM

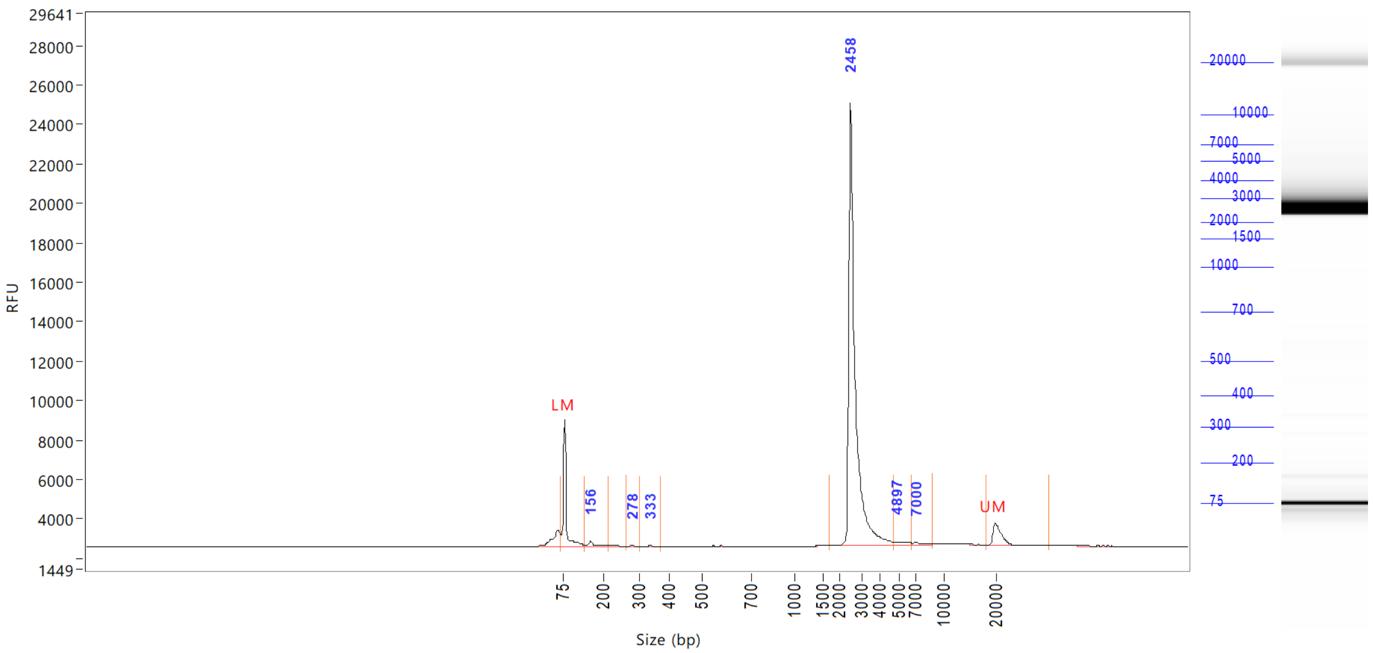

Peak	Size (bp)	Concentration (ng/uL)	From (bp)	To (bp)	Average size (bp)	CV%	RFU	Corrected peak area
1	75 (LM)	0.5000	66	139	78	16.50	6437	45.780
2	156	0.4896	139	208	162	10.16	290	3.736
3	278	0.0412	264	300	277	1.82	52	0.315
4	333	0.0539	300	372	332	3.53	61	0.411
5	2458	28.8565	1655	4724	2635	14.29	22482	220.177
6	4897	0.3959	4724	6482	5359	9.72	171	3.021
7	7000	0.2342	6482	8800	7459	8.74	94	1.787
8	20000 (UM)	0.1265	18049	30398	20536	5.59	1089	11.585

TIC:	30.0713	ng/uL
TIM:	23.6809	nmole/L
Total concentration:	30.4908	ng/uL

Sample peak width (sec): 5 Sample min peak height: 50 Sample baseline V to V?: Y Sample baseline V to V points: 3
Sample filter: Binomial Number of points for filter: 3 Sample start region (min): 0 Sample end region (min): 30
Marker peak width (sec): 5 Marker min peak height: 200 Marker baseline V to V?: Y Marker baseline V to V points: 3
Lower marker selection: First peak > 500 RFU Upper marker selection: Last peak > 200 RFU
Ladder size (bp)75, 200, 300, 400, 500, 700, 1000, 1500, 2000, 3000, 4000, 5000, 7000, 10000, 20000
Quantification using: Lower Marker Final concentration (ng/uL): 0.5000 Dilution factor: 12.0

Sample: SampB11
Well location: B11
Created: Tuesday, August 10, 2021 10:41:10 PM

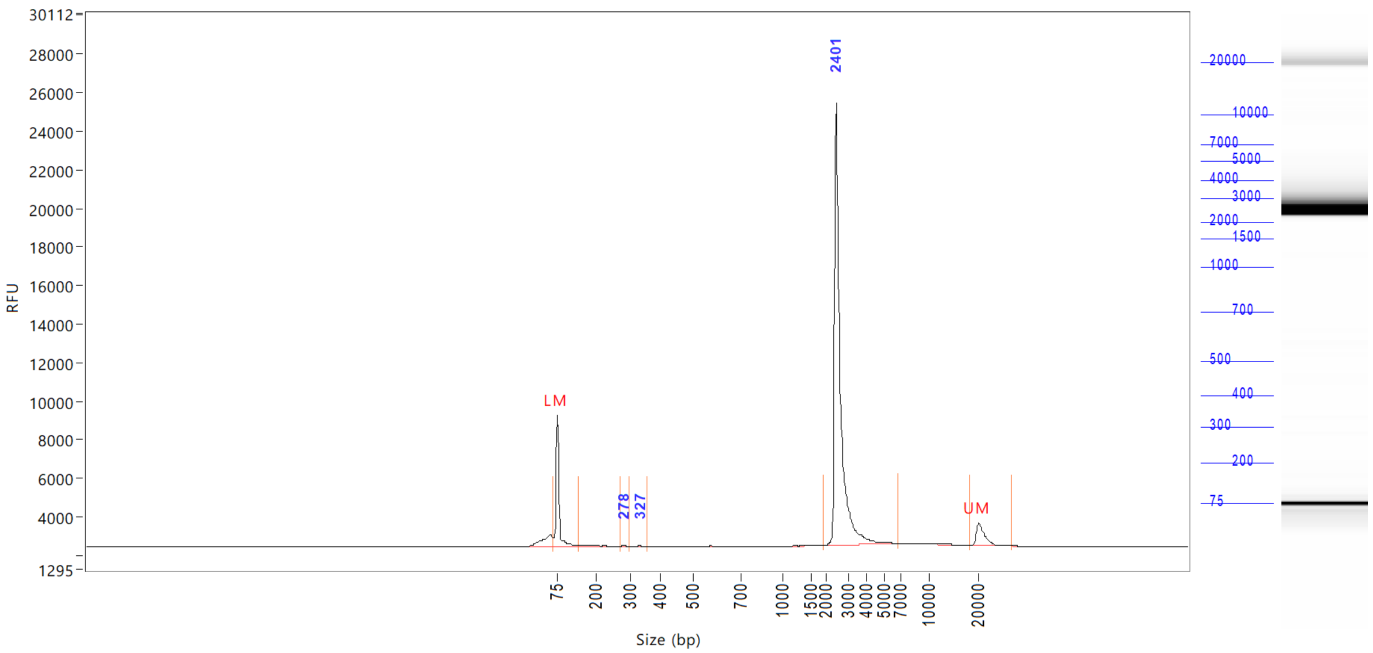

Peak	Size<br>(bp)	Concentration<br>(ng/uL)	From<br>(bp)	To<br>(bp)	Average size<br>(bp)	CV%	RFU	Corrected peak area
1	75 (LM)	0.5000	62	143	75	13.52	6834	46.571
2	278	0.0384	269	298	278	1.63	51	0.298
3	327	0.0414	298	356	324	2.43	52	0.321
4	2401	26.8834	1885	6778	2585	15.33	22964	208.665
5	20000 (UM)	0.1330	18171	26770	20531	4.63	1167	12.384

TIC:	26.9632	ng/uL
TIM:	17.5574	nmole/L
Total concentration:	27.4274	ng/uL

Sample peak width (sec): 5   Sample min peak height: 50   Sample baseline V to V?: Y   Sample baseline V to V points: 3
Sample filter: Binomial   Number of points for filter: 3 Sample start region (min): 0 Sample end region (min): 30
Marker peak width (sec): 5   Marker min peak height: 200   Marker baseline V to V?: Y   Marker baseline V to V points: 3
Lower marker selection: First peak > 500 RFU   Upper marker selection: Last peak > 200 RFU
Ladder size (bp)75, 200, 300, 400, 500, 700, 1000, 1500, 2000, 3000, 4000, 5000, 7000, 10000, 20000
Quantification using: Lower Marker   Final concentration (ng/uL): 0.5000   Dilution factor: 12.0

Sample: SampC11
Well location: C11
Created: Tuesday, August 10, 2021 10:41:10 PM

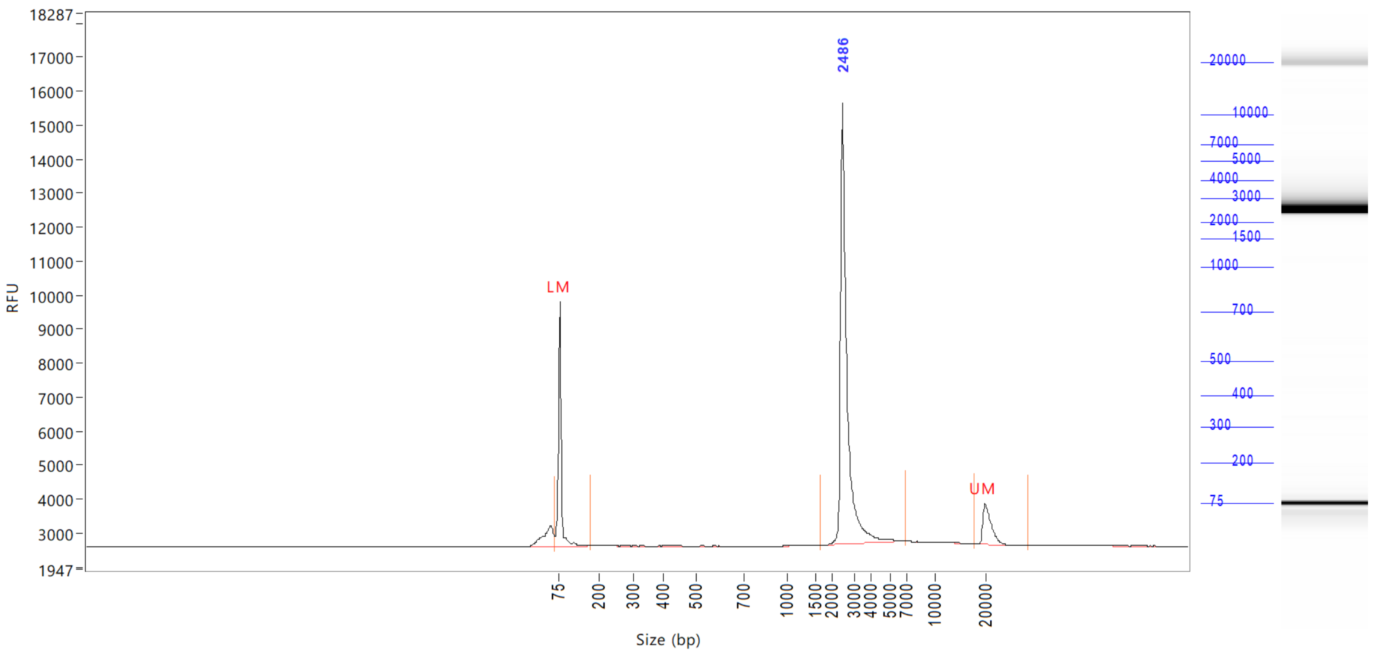

Peak	Size (bp)	Concentration (ng/uL)	From (bp)	To (bp)	Average size (bp)	CV%	RFU	Corrected peak area
1	75 (LM)	0.5000	60	171	77	17.22	7170	51.004
2	2486	14.7517	1635	6852	2649	15.62	12989	125.399
3	20000 (UM)	0.1257	17927	28463	20528	4.84	1201	12.823

TIC:	14.7517	ng/uL
TIM:	9.1673	nmole/L
Total concentration:	15.1890	ng/uL

Sample peak width (sec): 5 Sample min peak height: 50 Sample baseline V to V?: Y Sample baseline V to V points: 3
Sample filter: Binomial Number of points for filter: 3 Sample start region (min): 0 Sample end region (min): 30
Marker peak width (sec): 5 Marker min peak height: 200 Marker baseline V to V?: Y Marker baseline V to V points: 3
Lower marker selection: First peak > 500 RFU Upper marker selection: Last peak > 200 RFU
Ladder size (bp)75, 200, 300, 400, 500, 700, 1000, 1500, 2000, 3000, 4000, 5000, 7000, 10000, 20000
Quantification using: Lower Marker Final concentration (ng/uL): 0.5000 Dilution factor: 12.0

Sample: SampD11
Well location: D11
Created: Tuesday, August 10, 2021 10:41:10 PM

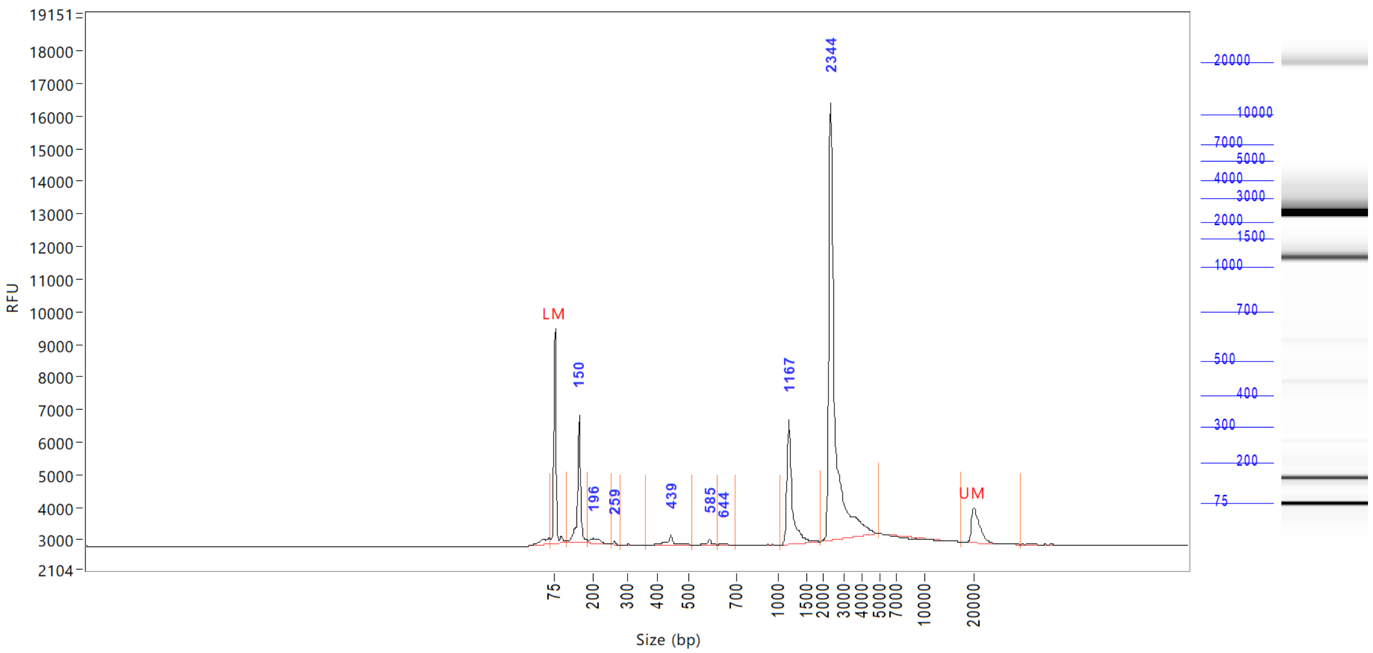

Peak	Size (bp)	Concentration (ng/uL)	From (bp)	To (bp)	Average size (bp)	CV%	RFU	Corrected peak area
1	75 (LM)	0.5000	62	112	73	7.66	6595	38.801
2	150	4.5182	112	179	148	5.95	3920	29.218
3	196	0.5506	179	250	201	6.63	150	3.560
4	259	0.1054	250	278	257	1.36	132	0.681
5	439	0.7407	360	511	435	6.57	316	4.790
6	585	0.3044	511	616	575	3.57	180	1.968
7	644	0.1450	616	695	648	3.13	54	0.937
8	1167	5.1967	1001	1904	1206	8.42	3810	33.606
9	2344	19.3712	1904	4966	2584	17.78	13432	125.269
10	20000 (UM)	0.1472	17561	29430	20619	5.72	1083	11.424

TIC:	30.9321	ng/uL
TIM:	78.8250	nmole/L
Total concentration:	31.1239	ng/uL

```
Sample peak width (sec): 5    Sample min peak height: 50    Sample baseline V to V?: Y    Sample baseline V to V points: 3
Sample filter: Binomial       Number of points for filter: 3  Sample start region (min): 0  Sample end region (min): 30
Marker peak width (sec): 5    Marker min peak height: 200   Marker baseline V to V?: Y    Marker baseline V to V points: 3
Lower marker selection: First peak > 500 RFU                Upper marker selection: Last peak > 200 RFU
Ladder size (bp)75, 200, 300, 400, 500, 700, 1000, 1500, 2000, 3000, 4000, 5000, 7000, 10000, 20000
Quantification using: Lower Marker          Final concentration (ng/uL): 0.5000          Dilution factor: 12.0
```

Sample: SampE11
Well location: E11
Created: Tuesday, August 10, 2021 10:41:10 PM

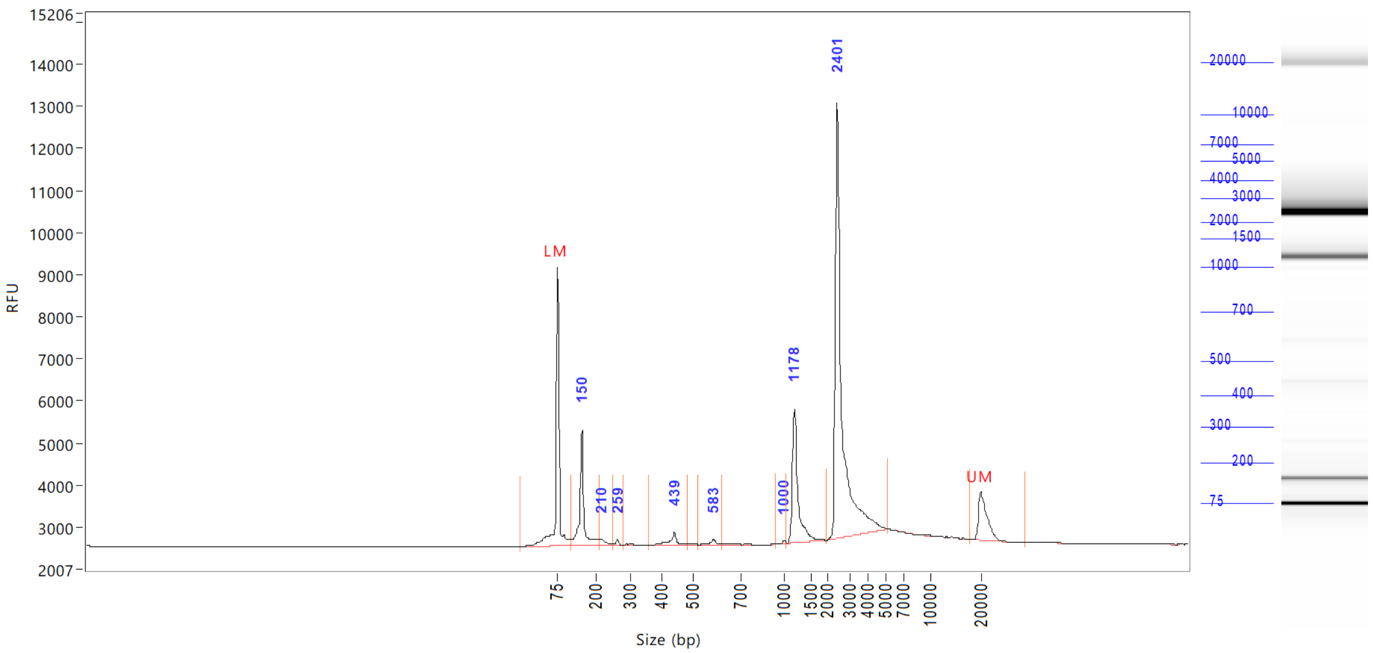

Peak	Size (bp)	Concentration (ng/uL)	From (bp)	To (bp)	Average size (bp)	CV%	RFU	Corrected peak area
1	75 (LM)	0.5000	0	118	69	26.90	6624	49.907
2	150	3.1775	118	206	151	11.32	2742	26.430
3	210	0.2354	206	248	220	5.03	133	1.958
4	259	0.1003	248	275	256	1.56	135	0.835
5	439	0.4861	358	481	429	5.05	300	4.043
6	583	0.1842	519	616	576	3.31	143	1.532
7	1000	0.0490	939	1045	992	1.45	79	0.407
8	1178	3.4238	1045	1942	1217	8.27	3161	28.479
9	2401	12.7305	1942	5149	2660	18.55	10354	105.891
10	20000 (UM)	0.1276	17927	28705	20555	5.11	1139	12.740

TIC:	20.3868	ng/uL
TIM:	51.9701	nmole/L
Total concentration:	20.6330	ng/uL

Sample peak width (sec): 5 Sample min peak height: 50 Sample baseline V to V?: Y Sample baseline V to V points: 3
Sample filter: Binomial Number of points for filter: 3 Sample start region (min): 0 Sample end region (min): 30
Marker peak width (sec): 5 Marker min peak height: 200 Marker baseline V to V?: Y Marker baseline V to V points: 3
Lower marker selection: First peak > 500 RFU Upper marker selection: Last peak > 200 RFU
Ladder size (bp)75, 200, 300, 400, 500, 700, 1000, 1500, 2000, 3000, 4000, 5000, 7000, 10000, 20000
Quantification using: Lower Marker Final concentration (ng/uL): 0.5000 Dilution factor: 12.0

Sample: SampF11
Well location: F11
Created: Tuesday, August 10, 2021 10:41:10 PM

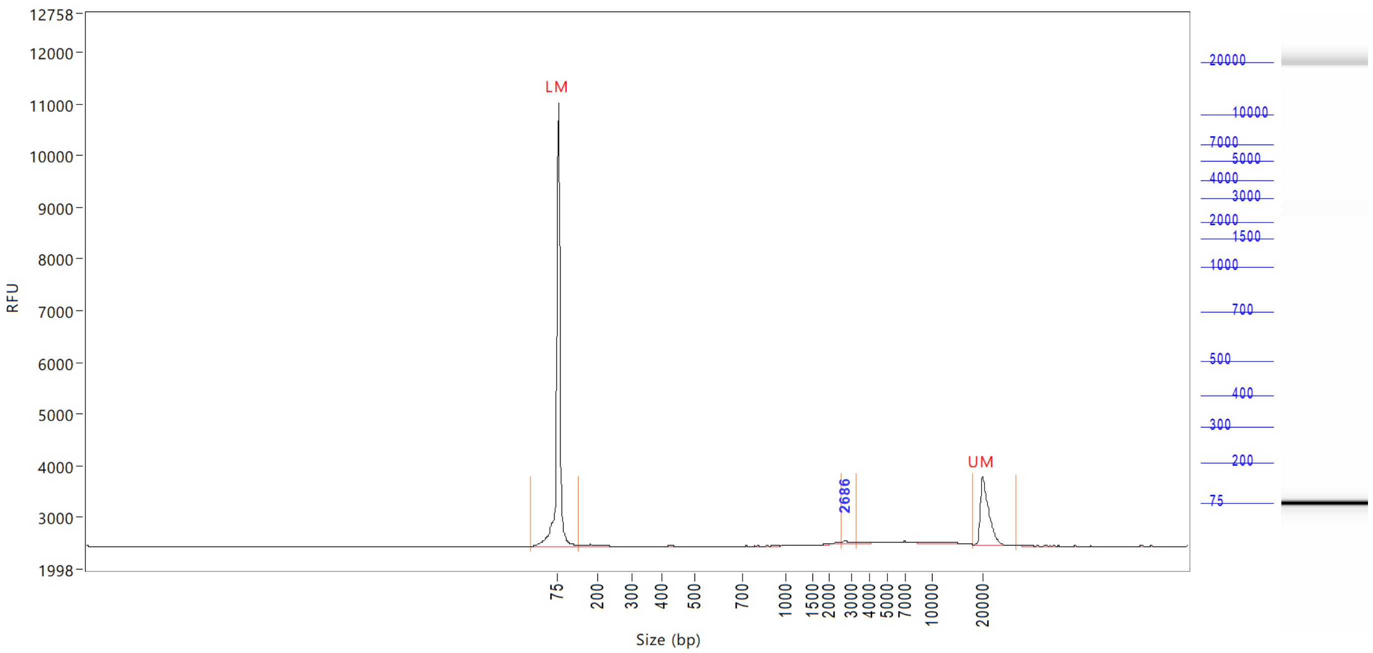

Peak	Size (bp)	Concentration (ng/uL)	From (bp)	To (bp)	Average size (bp)	CV%	RFU	Corrected peak area
1	75 (LM)	0.5000	0	141	71	18.80	8600	69.816
2	2686	0.0561	2515	3251	2768	6.62	73	0.653
3	20000 (UM)	0.1021	18171	26649	20534	4.74	1321	14.260

TIC:	0.0561	ng/uL
TIM:	0.0334	nmole/L
Total concentration:	0.5171	ng/uL

Sample peak width (sec): 5 Sample min peak height: 50 Sample baseline V to V?: Y Sample baseline V to V points: 3
Sample filter: Binomial Number of points for filter: 3 Sample start region (min): 0 Sample end region (min): 30
Marker peak width (sec): 5 Marker min peak height: 200 Marker baseline V to V?: Y Marker baseline V to V points: 3
Lower marker selection: First peak > 500 RFU Upper marker selection: Last peak > 200 RFU
Ladder size (bp)75, 200, 300, 400, 500, 700, 1000, 1500, 2000, 3000, 4000, 5000, 7000, 10000, 20000
Quantification using: Lower Marker Final concentration (ng/uL): 0.5000 Dilution factor: 12.0

Sample: SampG11
Well location: G11
Created: Tuesday, August 10, 2021 10:41:10 PM

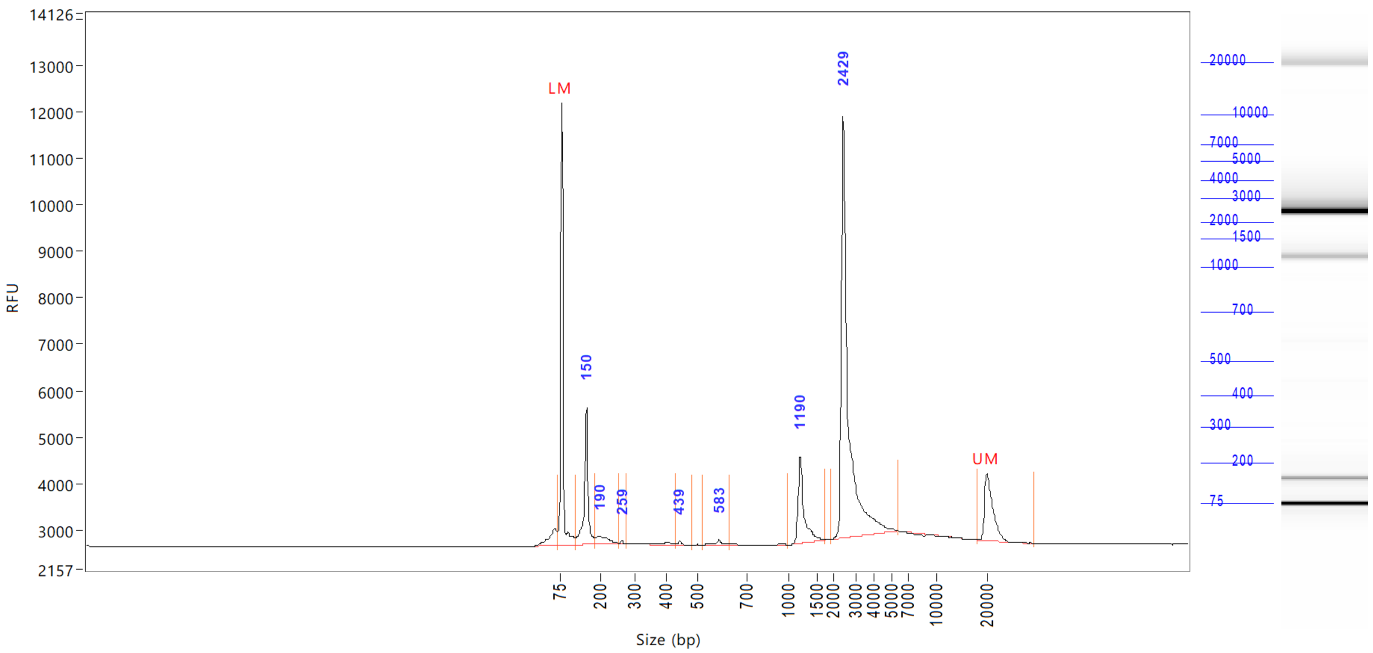

Peak	Size (bp)	Concentration (ng/uL)	From (bp)	To (bp)	Average size (bp)	CV%	RFU	Corrected peak area
1	75 (LM)	0.5000	62	116	74	10.87	9538	59.117
2	150	2.5656	116	177	147	7.14	2933	25.279
3	190	0.4455	177	251	201	7.97	161	4.389
4	259	0.0269	251	275	257	1.13	57	0.265
5	439	0.0529	430	479	440	1.87	77	0.521
6	583	0.1370	514	629	575	4.02	121	1.350
7	1190	1.8288	991	1712	1221	7.66	1866	18.019
8	2429	9.5009	1942	5667	2698	18.75	9086	93.611
9	20000 (UM)	0.1301	17927	29189	20530	5.11	1435	15.380

TIC:	14.5576	ng/uL
TIM:	41.3524	nmole/L
Total concentration:	14.7738	ng/uL

Sample peak width (sec): 5 Sample min peak height: 50 Sample baseline V to V?: Y Sample baseline V to V points: 3
Sample filter: Binomial Number of points for filter: 3 Sample start region (min): 0 Sample end region (min): 30
Marker peak width (sec): 5 Marker min peak height: 200 Marker baseline V to V?: Y Marker baseline V to V points: 3
Lower marker selection: First peak > 500 RFU Upper marker selection: Last peak > 200 RFU
Ladder size (bp)75, 200, 300, 400, 500, 700, 1000, 1500, 2000, 3000, 4000, 5000, 7000, 10000, 20000
Quantification using: Lower Marker Final concentration (ng/uL): 0.5000 Dilution factor: 12.0

Sample: SampH11
Well location: H11
Created: Tuesday, August 10, 2021 10:41:10 PM

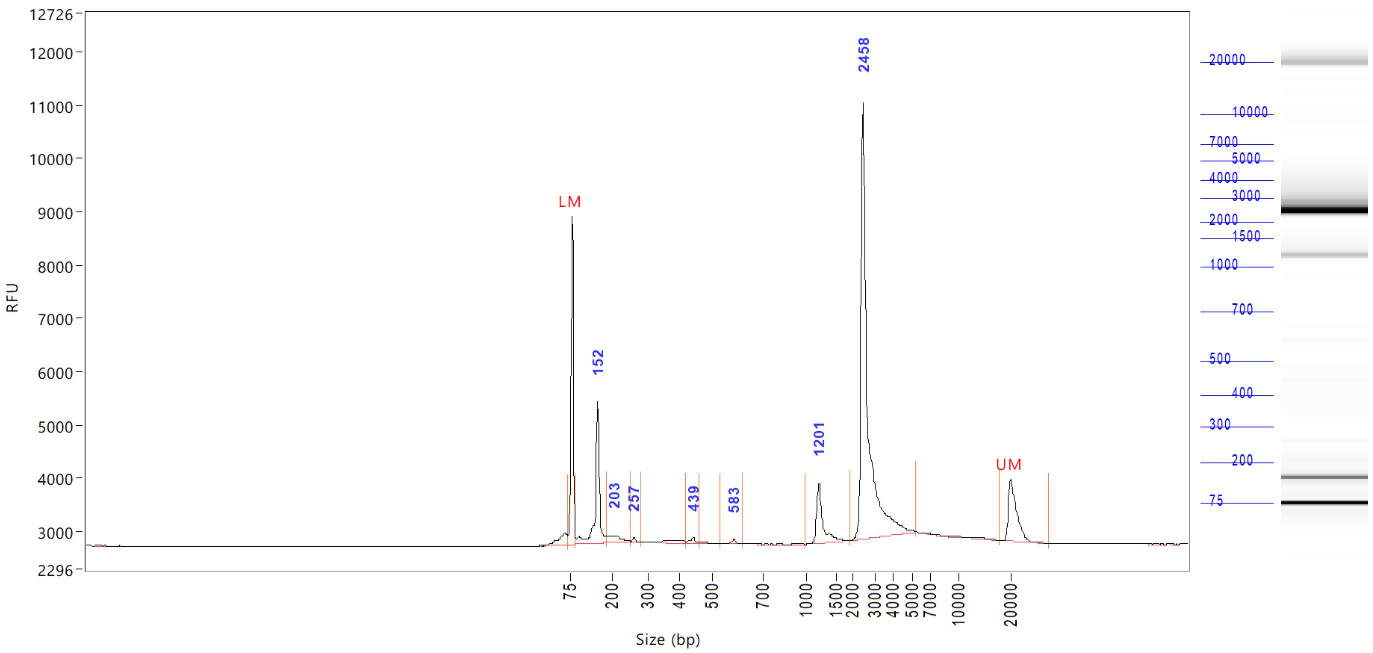

Peak	Size (bp)	Concentration (ng/uL)	From (bp)	To (bp)	Average size (bp)	CV%	RFU	Corrected peak area
1	75 (LM)	0.5000	62	87	73	4.69	6165	34.212
2	152	4.1153	87	181	145	11.92	2642	23.465
3	203	0.6639	181	250	204	7.77	130	3.786
4	257	0.0890	250	278	256	1.30	91	0.508
5	439	0.1881	418	458	437	2.26	109	1.073
6	583	0.1403	529	616	576	2.56	99	0.800
7	1201	1.9422	987	1942	1255	9.49	1131	11.074
8	2458	14.5141	1942	5445	2685	17.78	8200	82.758
9	20000 (UM)	0.1741	17805	27254	20527	4.75	1149	11.911

TIC:	21.6528	ng/uL
TIM:	65.1200	nmole/L
Total concentration:	22.3152	ng/uL

Sample peak width (sec): 5   Sample min peak height: 50   Sample baseline V to V?: Y   Sample baseline V to V points: 3
Sample filter: Binomial   Number of points for filter: 3 Sample start region (min): 0 Sample end region (min): 30
Marker peak width (sec): 5   Marker min peak height: 200   Marker baseline V to V?: Y   Marker baseline V to V points: 3
Lower marker selection: First peak > 500 RFU   Upper marker selection: Last peak > 200 RFU
Ladder size (bp)75, 200, 300, 400, 500, 700, 1000, 1500, 2000, 3000, 4000, 5000, 7000, 10000, 20000
Quantification using: Lower Marker   Final concentration (ng/uL): 0.5000   Dilution factor: 12.0

Sample: SampA12
Well location: A12
Created: Tuesday, August 10, 2021 10:41:10 PM

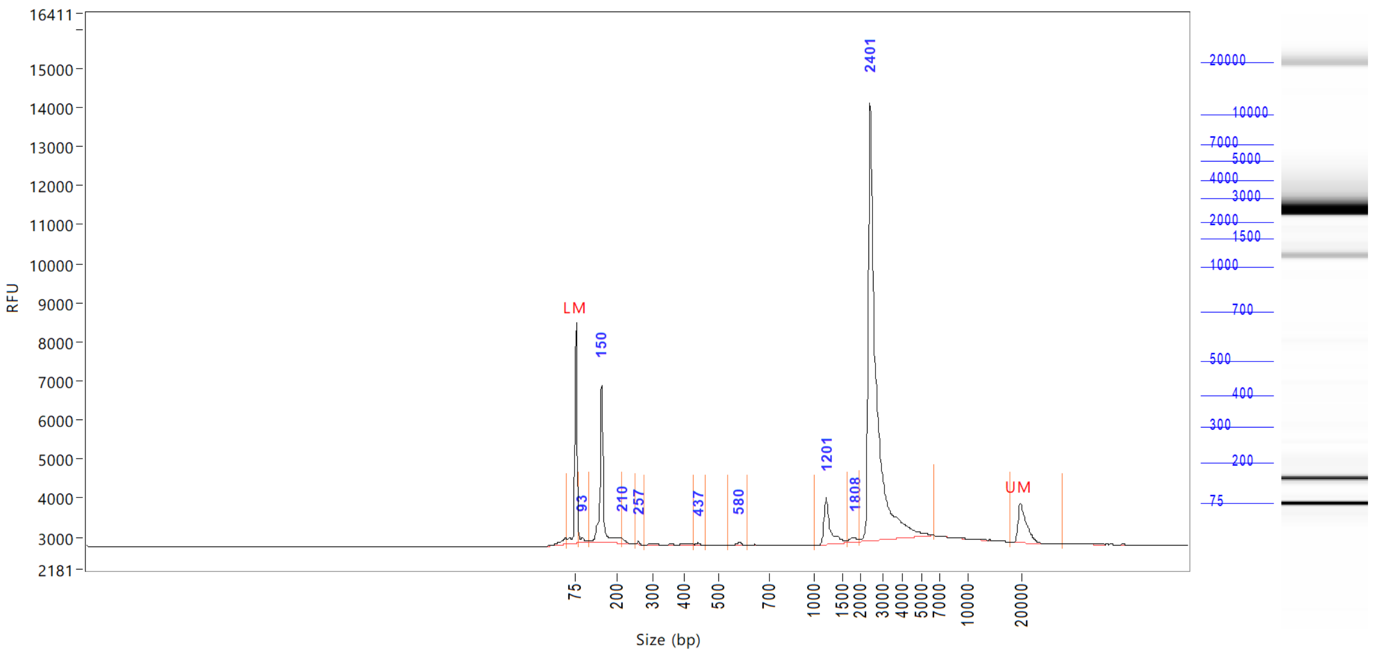

Peak	Size (bp)	Concentration (ng/uL)	From (bp)	To (bp)	Average size (bp)	CV%	RFU	Corrected peak area
1	75 (LM)	0.5000	50	86	72	6.36	5632	31.728
2	93	0.2364	86	112	94	7.15	125	1.250
3	150	6.1088	112	210	152	10.26	4030	32.303
4	210	0.1839	210	248	217	3.15	122	0.973
5	257	0.0719	248	277	255	1.35	79	0.380
6	437	0.0867	426	460	436	1.66	63	0.459
7	580	0.1559	534	613	574	2.67	96	0.824
8	1201	2.2244	1001	1597	1242	7.83	1195	11.763
9	1808	0.2636	1597	1962	1770	5.33	124	1.394
10	2401	24.5269	1962	6408	2714	19.57	11226	129.698
11	20000 (UM)	0.1655	18049	27979	20463	4.79	995	10.505

TIC:	33.8584	ng/uL
TIM:	90.8832	nmole/L
Total concentration:	34.3264	ng/uL

Sample peak width (sec): 5 Sample min peak height: 50 Sample baseline V to V?: Y Sample baseline V to V points: 3
Sample filter: Binomial Number of points for filter: 3 Sample start region (min): 0 Sample end region (min): 30
Marker peak width (sec): 5 Marker min peak height: 200 Marker baseline V to V?: Y Marker baseline V to V points: 3
Lower marker selection: First peak > 500 RFU Upper marker selection: Last peak > 200 RFU
Ladder size (bp)75, 200, 300, 400, 500, 700, 1000, 1500, 2000, 3000, 4000, 5000, 7000, 10000, 20000
Quantification using: Lower Marker Final concentration (ng/uL): 0.5000 Dilution factor: 12.0

Sample: SampB12
Well location: B12
Created: Tuesday, August 10, 2021 10:41:10 PM

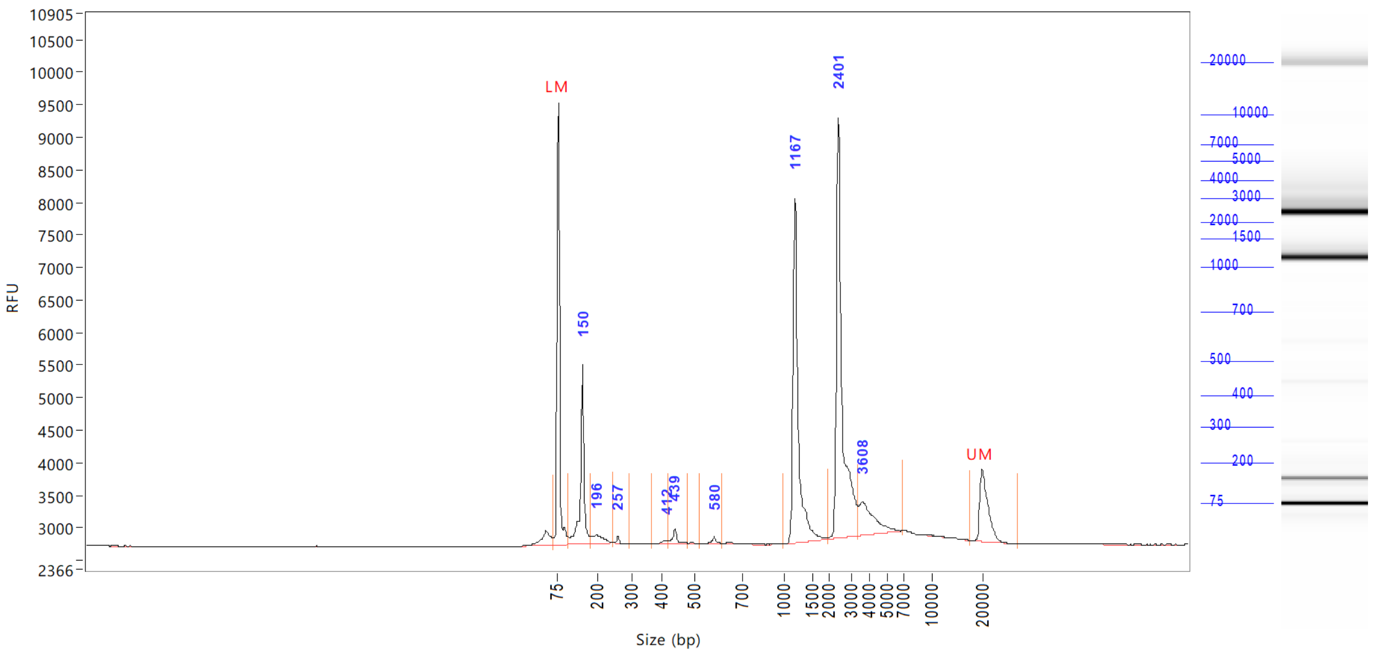

Peak	Size (bp)	Concentration (ng/uL)	From (bp)	To (bp)	Average size (bp)	CV%	RFU	Corrected peak area
1	75 (LM)	0.5000	62	108	74	8.89	6800	41.513
2	150	3.3781	108	177	145	7.89	2749	23.372
3	196	0.4646	177	244	200	7.39	131	3.215
4	257	0.0846	244	291	258	2.89	102	0.585
5	412	0.1125	366	418	402	2.89	54	0.778
6	439	0.3423	418	481	440	3.11	243	2.368
7	580	0.1567	519	611	577	2.63	116	1.084
8	1167	6.4376	987	1942	1206	8.98	5286	44.541
9	2401	8.6139	1942	3393	2536	10.90	6454	59.599
10	3608	1.6604	3393	6852	4043	13.25	522	11.488
11	20000 (UM)	0.1428	17683	27254	20482	4.68	1120	11.859

TIC:	21.2506	ng/uL
TIM:	59.8865	nmole/L
Total concentration:	21.4245	ng/uL

Sample peak width (sec): 5 Sample min peak height: 50 Sample baseline V to V?: Y Sample baseline V to V points: 3
Sample filter: Binomial Number of points for filter: 3 Sample start region (min): 0 Sample end region (min): 30
Marker peak width (sec): 5 Marker min peak height: 200 Marker baseline V to V?: Y Marker baseline V to V points: 3
Lower marker selection: First peak > 500 RFU Upper marker selection: Last peak > 200 RFU
Ladder size (bp)75, 200, 300, 400, 500, 700, 1000, 1500, 2000, 3000, 4000, 5000, 7000, 10000, 20000
Quantification using: Lower Marker Final concentration (ng/uL): 0.5000 Dilution factor: 12.0

Sample: SampC12
Well location: C12
Created: Tuesday, August 10, 2021 10:41:10 PM

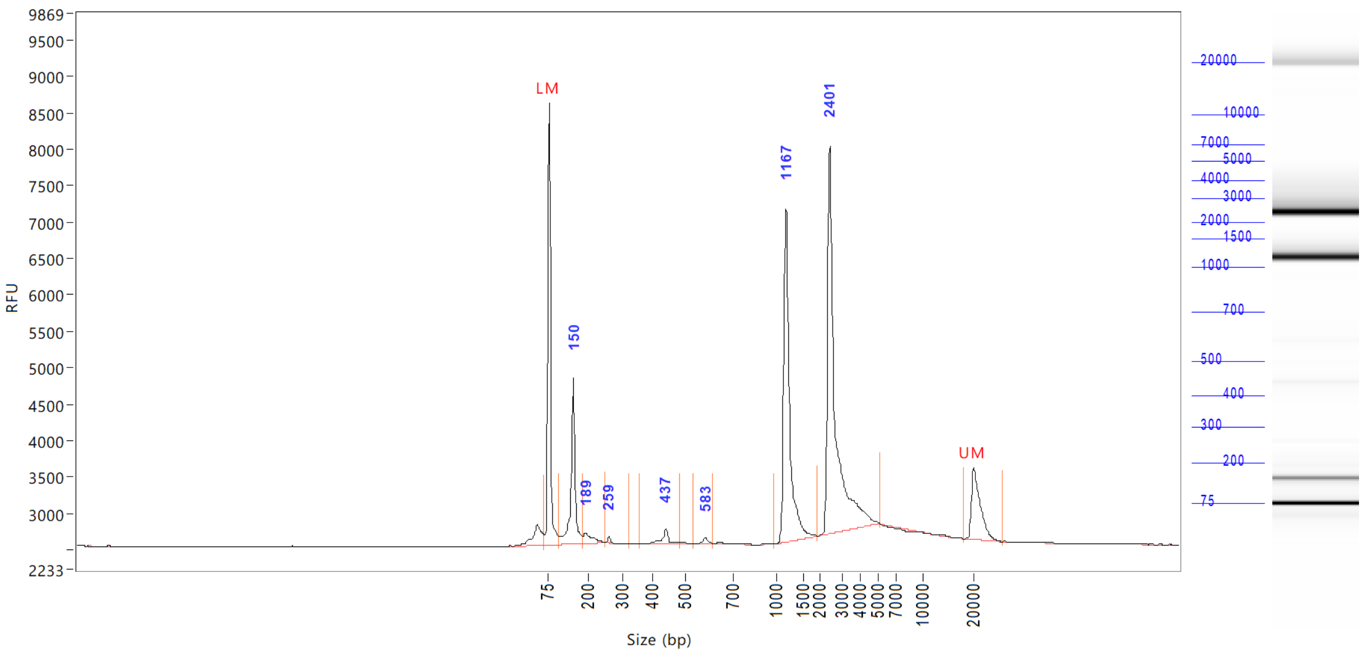

Peak	Size (bp)	Concentration (ng/uL)	From (bp)	To (bp)	Average size (bp)	CV%	RFU	Corrected peak area
1	75 (LM)	0.5000	62	108	75	8.85	6083	39.815
2	150	3.3142	108	179	146	8.16	2287	21.993
3	189	0.4522	179	248	200	7.63	148	3.001
4	259	0.0853	248	321	261	4.58	86	0.566
5	437	0.4826	354	485	430	5.42	212	3.203
6	583	0.1521	534	613	579	2.63	96	1.010
7	1167	6.5962	991	1904	1203	7.97	4575	43.772
8	2401	9.5056	1904	5075	2693	19.45	5324	63.078
9	20000 (UM)	0.1407	18171	25561	20662	5.07	979	11.205

TIC:	20.5884	ng/uL
TIM:	58.6769	nmole/L
Total concentration:	20.7578	ng/uL

```
Sample peak width (sec): 5     Sample min peak height: 50     Sample baseline V to V?: Y     Sample baseline V to V points: 3
Sample filter: Binomial        Number of points for filter: 3  Sample start region (min): 0  Sample end region (min): 30
Marker peak width (sec): 5     Marker min peak height: 200    Marker baseline V to V?: Y     Marker baseline V to V points: 3
Lower marker selection: First peak > 500 RFU                  Upper marker selection: Last peak > 200 RFU
Ladder size (bp)75, 200, 300, 400, 500, 700, 1000, 1500, 2000, 3000, 4000, 5000, 7000, 10000, 20000
Quantification using: Lower Marker          Final concentration (ng/uL): 0.5000          Dilution factor: 12.0
```

Sample: SampD12
Well location: D12
Created: Tuesday, August 10, 2021 10:41:10 PM

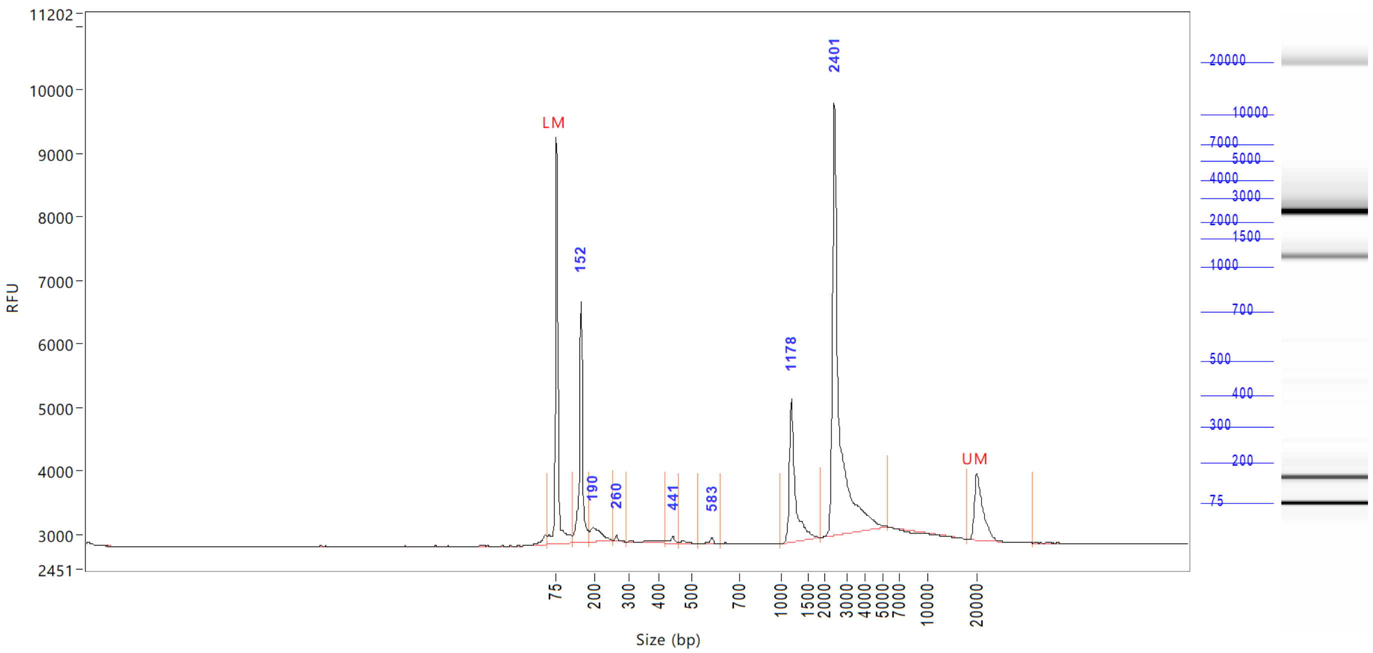

Peak	Size (bp)	Concentration (ng/uL)	From (bp)	To (bp)	Average size (bp)	CV%	RFU	Corrected peak area
1	75 (LM)	0.5000	47	124	76	13.87	6403	42.475
2	152	4.2242	124	177	151	5.58	3797	29.904
3	190	0.8114	177	250	201	8.08	223	5.744
4	260	0.0756	250	293	259	2.36	90	0.535
5	441	0.1664	416	462	437	2.48	111	1.178
6	583	0.1130	527	623	578	2.71	99	0.800
7	1178	3.0283	991	1846	1224	8.43	2257	21.438
8	2401	10.1179	1846	5519	2701	19.66	6819	71.627
9	20000 (UM)	0.1380	17927	31365	20639	6.10	1055	11.726

TIC:	18.5367	ng/uL
TIM:	64.2821	nmole/L
Total concentration:	18.8283	ng/uL

Sample peak width (sec): 5   Sample min peak height: 50   Sample baseline V to V?: Y   Sample baseline V to V points: 3
Sample filter: Binomial   Number of points for filter: 3 Sample start region (min): 0 Sample end region (min): 30
Marker peak width (sec): 5   Marker min peak height: 200   Marker baseline V to V?: Y   Marker baseline V to V points: 3
Lower marker selection: First peak > 500 RFU   Upper marker selection: Last peak > 200 RFU
Ladder size (bp)75, 200, 300, 400, 500, 700, 1000, 1500, 2000, 3000, 4000, 5000, 7000, 10000, 20000
Quantification using: Lower Marker   Final concentration (ng/uL): 0.5000   Dilution factor: 12.0

Sample: SampE12
Well location: E12
Created: Tuesday, August 10, 2021 10:41:10 PM

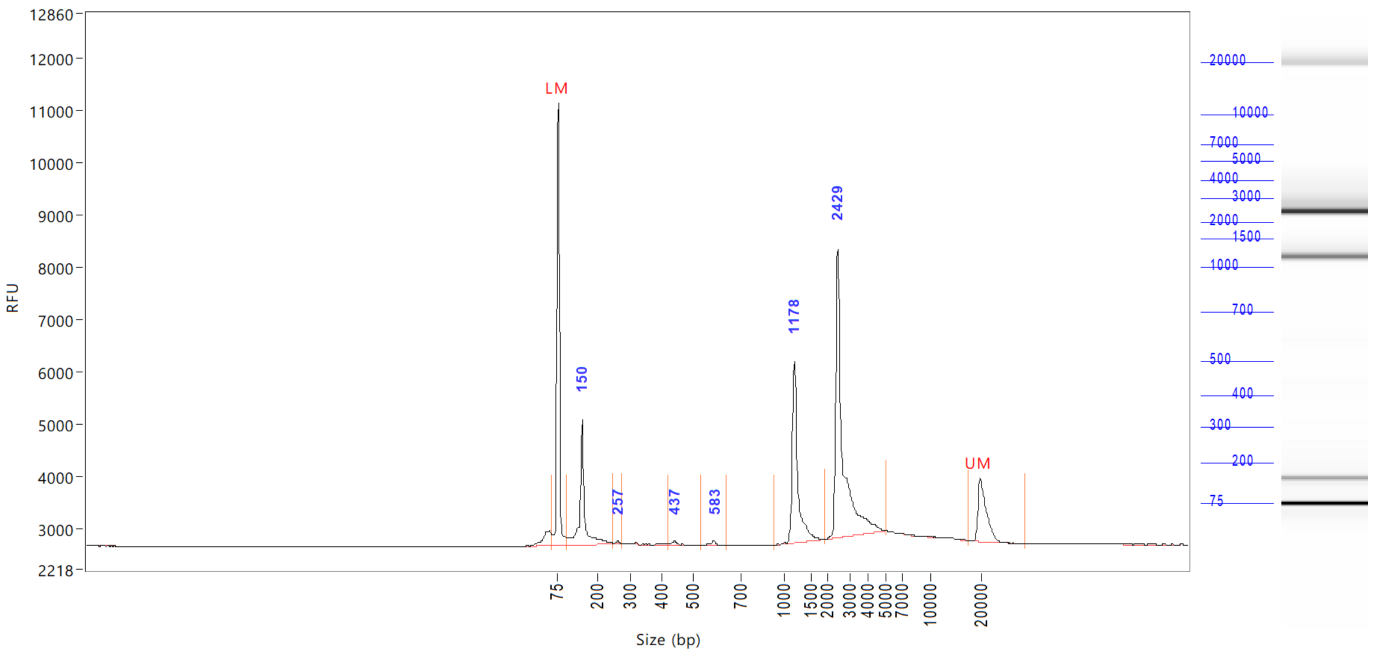

Peak	Size (bp)	Concentration (ng/uL)	From (bp)	To (bp)	Average size (bp)	CV%	RFU	Corrected peak area
1	75 (LM)	0.5000	58	101	73	7.14	8485	51.110
2	150	3.0900	101	244	152	15.49	2409	26.322
3	257	0.0542	244	271	254	1.91	64	0.462
4	437	0.1159	416	527	445	5.14	78	0.988
5	583	0.1080	527	639	581	3.75	95	0.920
6	1178	3.6017	935	1904	1211	8.08	3480	30.681
7	2429	7.0443	1904	5001	2715	18.69	5518	60.006
8	20000 (UM)	0.1279	17683	28947	20547	5.05	1205	13.076

TIC:	14.0142	ng/uL
TIM:	43.6641	nmole/L
Total concentration:	14.1916	ng/uL

Sample peak width (sec): 5   Sample min peak height: 50   Sample baseline V to V?: Y   Sample baseline V to V points: 3
Sample filter: Binomial   Number of points for filter: 3   Sample start region (min): 0   Sample end region (min): 30
Marker peak width (sec): 5   Marker min peak height: 200   Marker baseline V to V?: Y   Marker baseline V to V points: 3
Lower marker selection: First peak > 500 RFU   Upper marker selection: Last peak > 200 RFU
Ladder size (bp)75, 200, 300, 400, 500, 700, 1000, 1500, 2000, 3000, 4000, 5000, 7000, 10000, 20000
Quantification using: Lower Marker   Final concentration (ng/uL): 0.5000   Dilution factor: 12.0

Sample: SampF12
Well location: F12
Created: Tuesday, August 10, 2021 10:41:10 PM

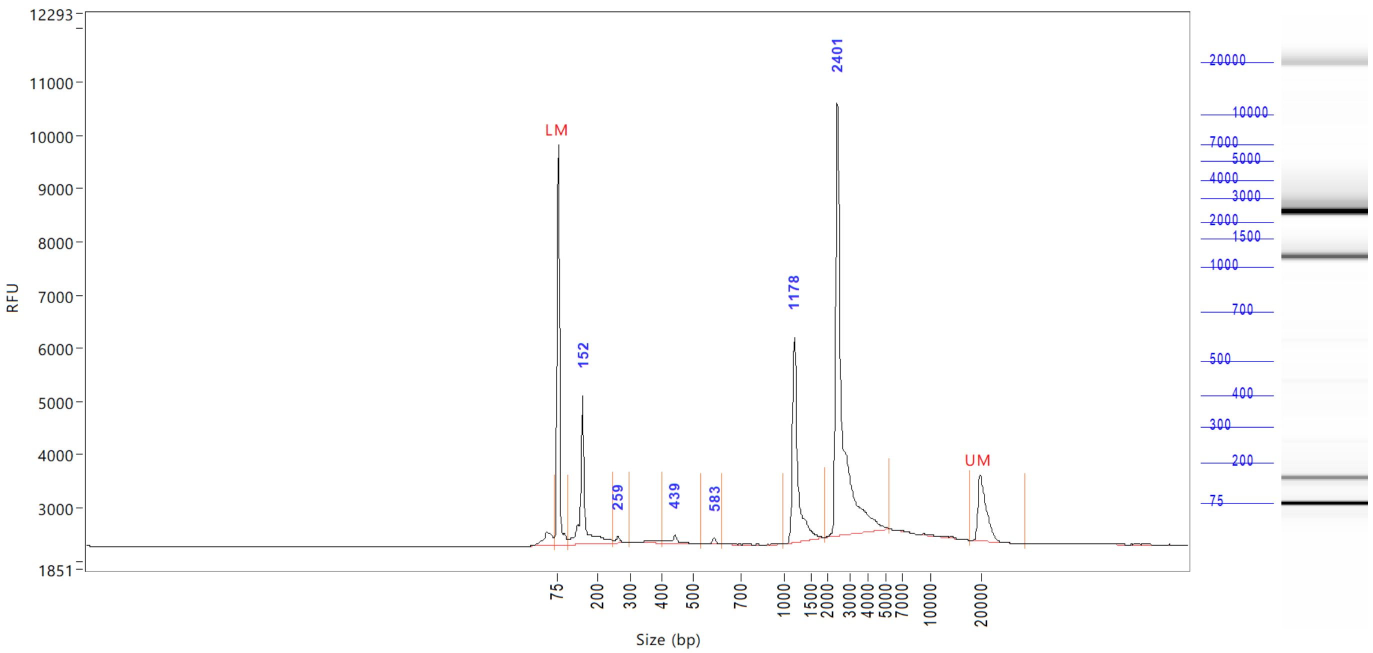

Peak	Size (bp)	Concentration (ng/uL)	From (bp)	To (bp)	Average size (bp)	CV%	RFU	Corrected peak area
1	75 (LM)	0.5000	64	108	74	8.36	7528	44.664
2	152	3.8510	108	244	155	15.53	2795	28.666
3	259	0.1724	244	296	257	3.52	130	1.284
4	439	0.3205	400	532	441	5.32	162	2.386
5	583	0.1380	532	616	579	2.43	130	1.027
6	1178	4.5013	996	1904	1219	8.45	3850	33.508
7	2401	11.3052	1904	5445	2702	19.32	8145	84.155
8	20000 (UM)	0.1508	18049	29068	20530	4.85	1242	13.470

TIC:	20.2884	ng/uL
TIM:	56.4927	nmole/L
Total concentration:	20.6879	ng/uL

```
Sample peak width (sec): 5    Sample min peak height: 50    Sample baseline V to V?: Y    Sample baseline V to V points: 3
Sample filter: Binomial       Number of points for filter: 3  Sample start region (min): 0  Sample end region (min): 30
Marker peak width (sec): 5    Marker min peak height: 200   Marker baseline V to V?: Y    Marker baseline V to V points: 3
Lower marker selection: First peak > 500 RFU                Upper marker selection: Last peak > 200 RFU
Ladder size (bp)75, 200, 300, 400, 500, 700, 1000, 1500, 2000, 3000, 4000, 5000, 7000, 10000, 20000
Quantification using: Lower Marker          Final concentration (ng/uL): 0.5000          Dilution factor: 12.0
```

Sample: SampG12
Well location: G12
Created: Tuesday, August 10, 2021 10:41:10 PM

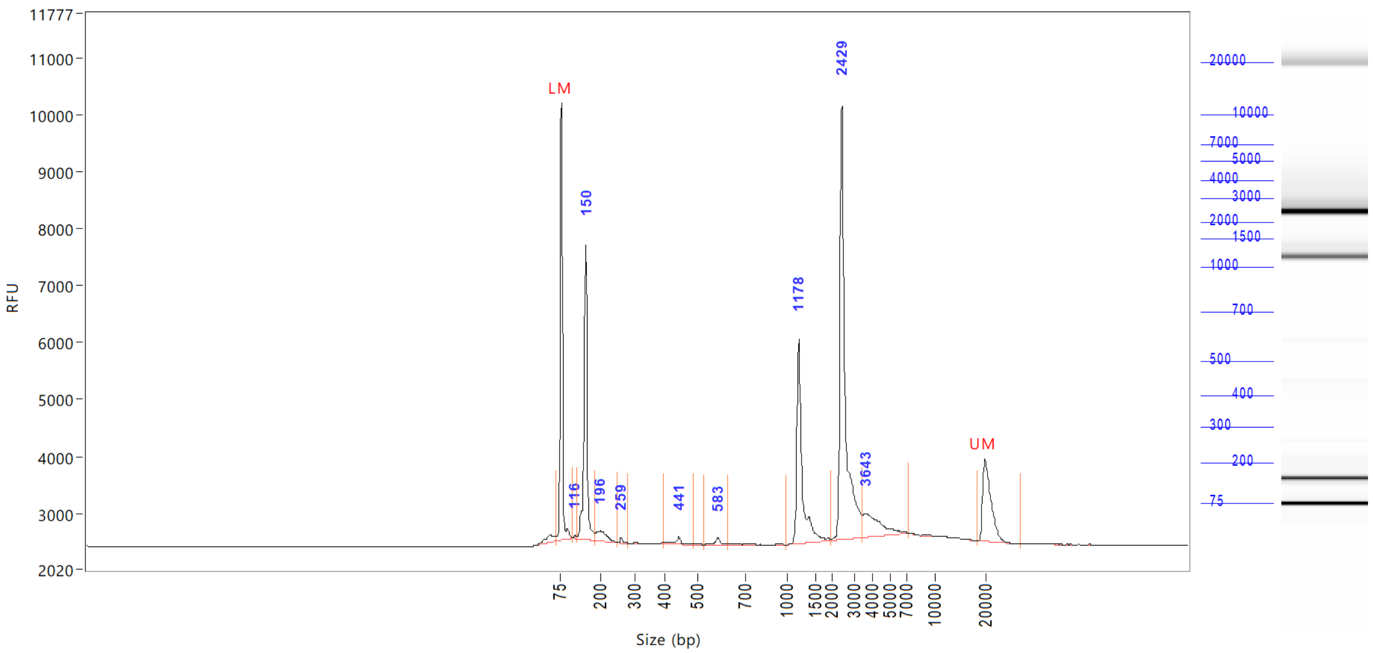

Peak	Size (bp)	Concentration (ng/uL)	From (bp)	To (bp)	Average size (bp)	CV%	RFU	Corrected peak area
1	75 (LM)	0.5000	62	108	73	6.71	7684	44.706
2	116	0.0534	108	124	116	3.90	53	0.398
3	150	4.9361	124	179	148	5.24	5183	36.779
4	196	0.5910	179	250	201	7.23	176	4.403
5	259	0.0725	250	280	258	1.80	104	0.540
6	441	0.2519	396	487	432	4.59	139	1.877
7	583	0.1665	524	623	579	3.25	128	1.240
8	1178	4.1778	991	1981	1232	10.37	3597	31.129
9	2429	8.5772	1981	3429	2534	10.36	7613	63.909
10	3643	1.4811	3429	7201	4198	15.66	417	11.036
11	20000 (UM)	0.1723	18537	26891	20487	4.68	1449	15.408

TIC:	20.3075	ng/uL
TIM:	74.0337	nmole/L
Total concentration:	20.5275	ng/uL

Sample peak width (sec): 5   Sample min peak height: 50   Sample baseline V to V?: Y   Sample baseline V to V points: 3
Sample filter: Binomial   Number of points for filter: 3 Sample start region (min): 0 Sample end region (min): 30
Marker peak width (sec): 5   Marker min peak height: 200   Marker baseline V to V?: Y   Marker baseline V to V points: 3
Lower marker selection: First peak > 500 RFU   Upper marker selection: Last peak > 200 RFU
Ladder size (bp)75, 200, 300, 400, 500, 700, 1000, 1500, 2000, 3000, 4000, 5000, 7000, 10000, 20000
Quantification using: Lower Marker   Final concentration (ng/uL): 0.5000   Dilution factor: 12.0

Sample: SampH12
Well location: H12
Created: Tuesday, August 10, 2021 10:41:10 PM

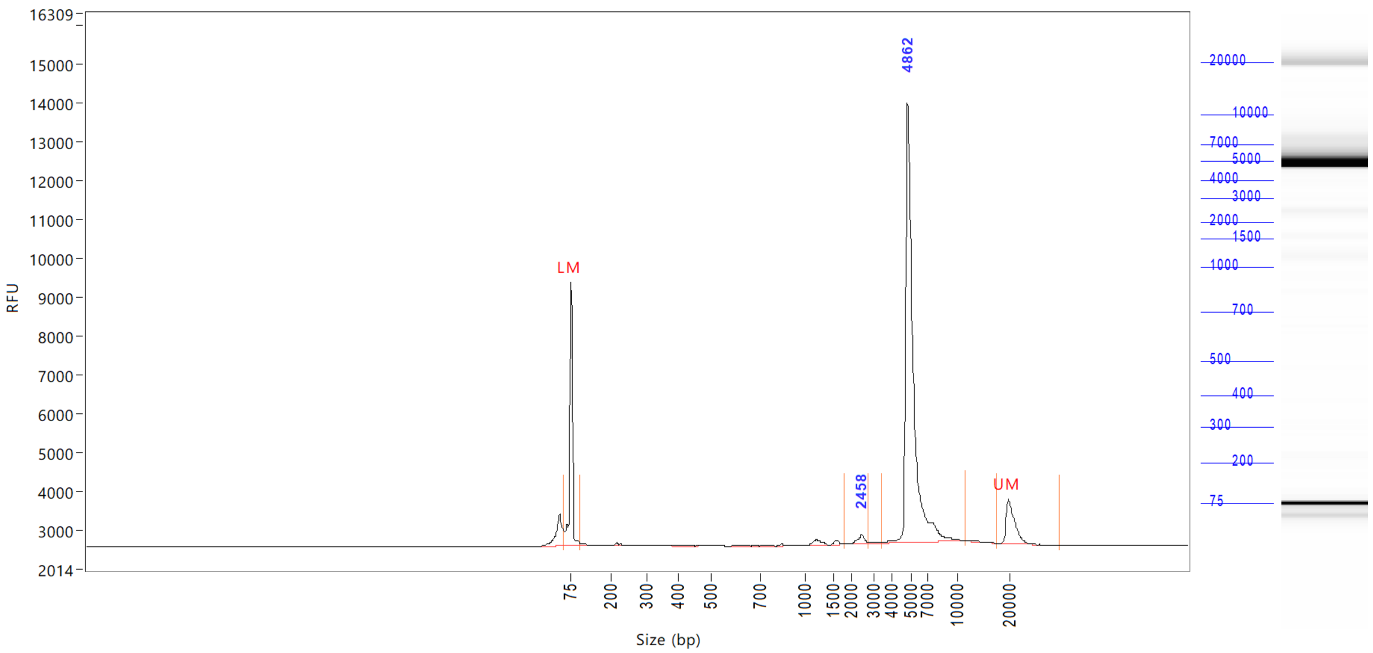

Peak	Size (bp)	Concentration (ng/uL)	From (bp)	To (bp)	Average size (bp)	CV%	RFU	Corrected peak area
1	75 (LM)	0.5000	54	105	73	8.45	6771	42.000
2	2458	0.3448	1808	2800	2391	7.26	233	2.414
3	4862	16.8781	3429	11586	5258	15.88	11321	118.147
4	20000 (UM)	0.1476	17805	29793	20563	5.13	1154	12.400

TIC:	17.2229	ng/uL
TIM:	5.5219	nmole/L
Total concentration:	18.3169	ng/uL

Sample peak width (sec): 5 Sample min peak height: 200 Sample baseline V to V?: Y Sample baseline V to V points: 3
Sample filter: Binomial Number of points for filter: 3 Sample start region (min): 0 Sample end region (min): 30
Marker peak width (sec): 5 Marker min peak height: 200 Marker baseline V to V?: Y Marker baseline V to V points: 3
Lower marker selection: First peak > 500 RFU Upper marker selection: Last peak > 200 RFU
Ladder size (bp)75, 200, 300, 400, 500, 700, 1000, 1500, 2000, 3000, 4000, 5000, 7000, 10000, 20000
Quantification using: Lower Marker Final concentration (ng/uL): 0.5000 Dilution factor: 12.0

Sample: DNA size ladder
Well location: A7
Created: Tuesday, August 10, 2021 10:41:10 PM
Import from: C:\AATI\Data\2021 08 10\21-51-18\210811_alberto_ladder.SCAL
Fit type: Point to point

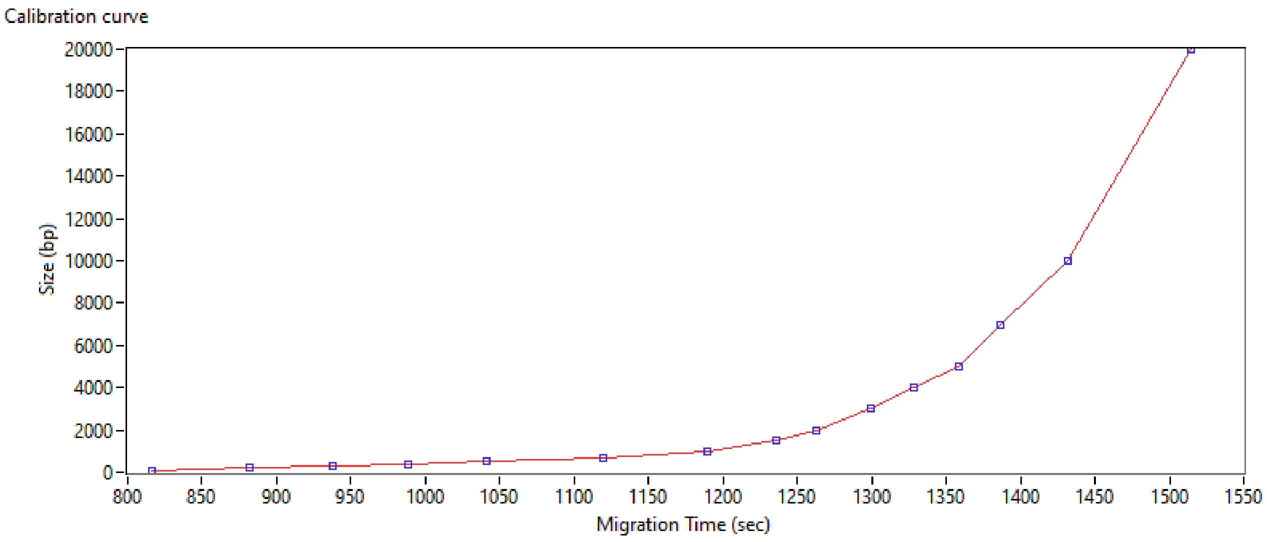

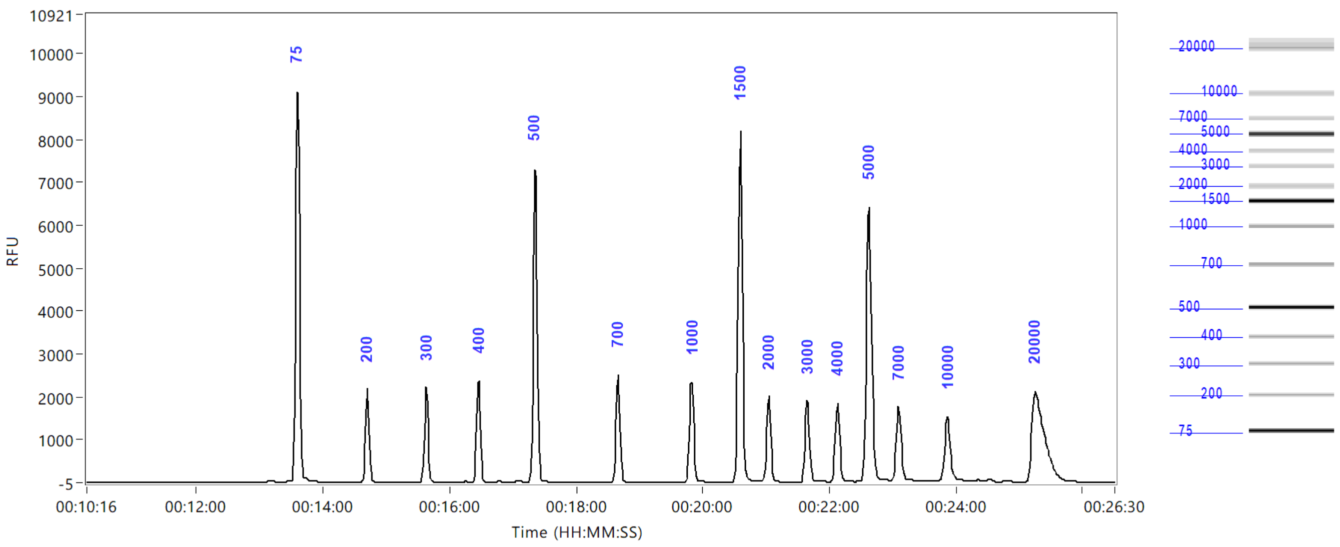

Supplement: Supplementary file 2 — sb3c00292_si_002.zip [file sb3c00292_si_002.zip › dnada_supplementary_material_pks_library_build/ZAG_trials/ZAG_Trial_1/22-31-23_plate7/plate7_report.pdf]
