# Supplementary material for: Automated Platform for the Plasmid Construction Process
Source: ACS Synth Biol. 2023 Nov 10;12(12):3506–13. doi: 10.1021/acssynbio.3c00292 (PMC10729297; doi:10.1021/acssynbio.3c00292)

Instrument controller software run summary:

Filename and data path: C:\AATI\Data\2021 08 10\23-11-21\2021 08 10 23H 11M..raw
Created: Tuesday, August 10, 2021 11:20:27 PM
Number of capillaries: 96
Array serial number: 111920-09SFS
Effect length: 33 cm
Array usage count: 171
Instrument type: ZAG DNA Analyzer
Instrument controller software version: 1.0.1.9
Device serial number: Z7597

Method Information

Method name: ZAG130GP33 - DNA 75-20000bp - Gel Prime Only.mthds
Gel prime: Yes
Full conditioning: No
Gel prime to buffer: No
Gel selection: Gel 1
Perform prerun: 8.0 kV, 30 sec.
Rinse: No
Marker 1: Row: A, 5.0 kV, 5 sec.
Rinse: No
Sample injection: 5.0 kV, 5 sec.
Separation: 8.0 kV, 30.0 min.
Tray name: Sample Tray-8

Analysis mode: DNA

Notes

Gel Image

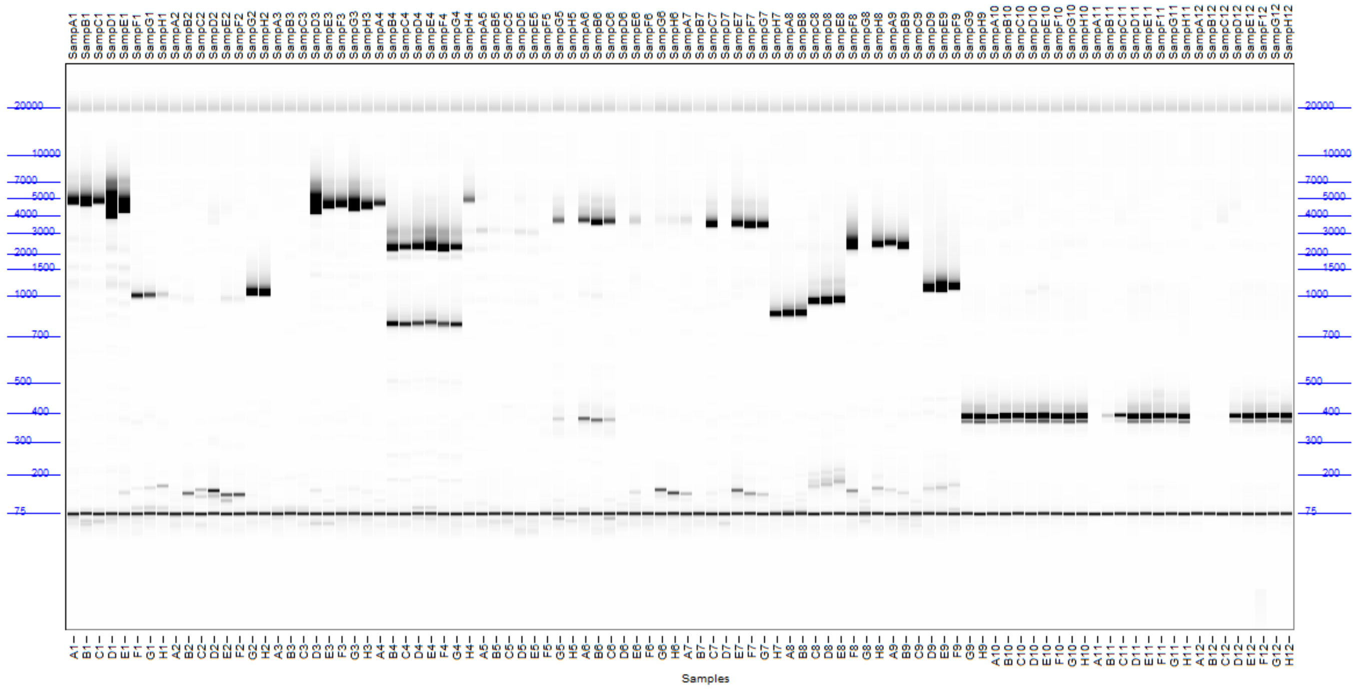

Filename and data path: C:\AATI\Data\2021 08 10\23-11-21\2021 08 10 23H 11M..raw

A1: SampA1

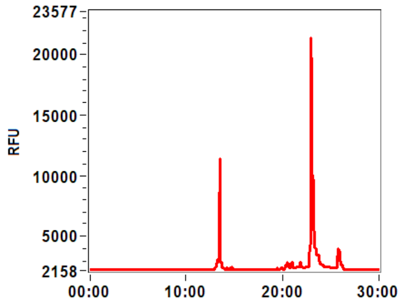

B1: SampB1

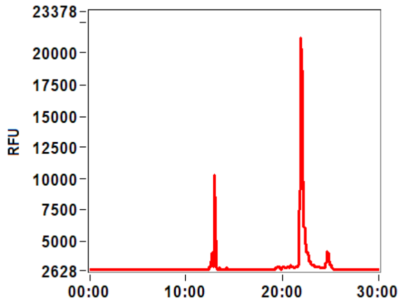

C1: SampC1

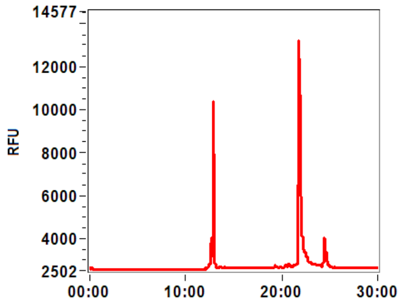

D1: SampD1

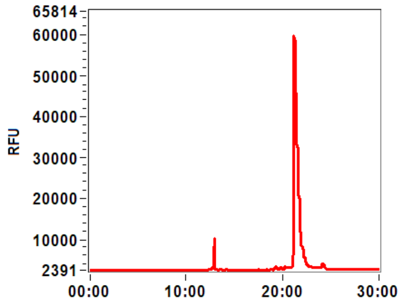

E1: SampE1

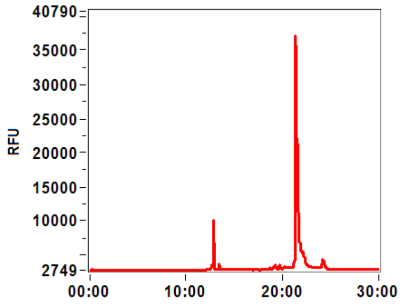

F1: SampF1

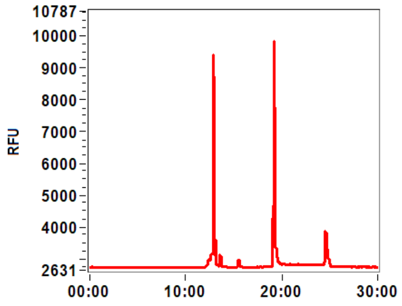

G1: SampG1

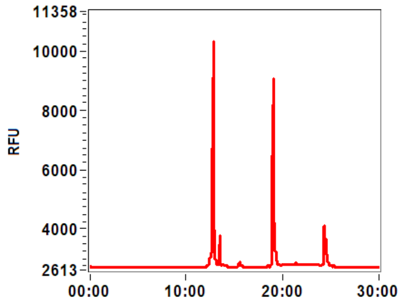

H1: SampH1

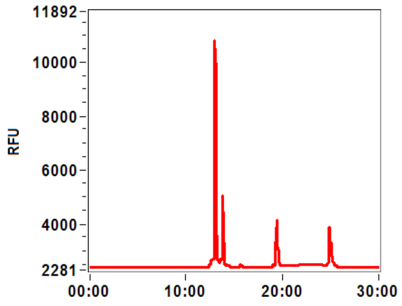

A2: SampA2

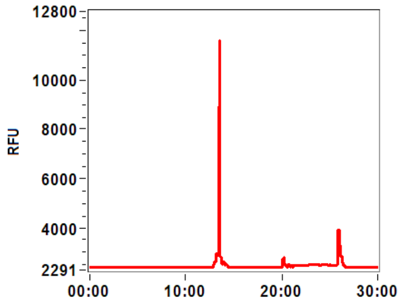

B2: SampB2

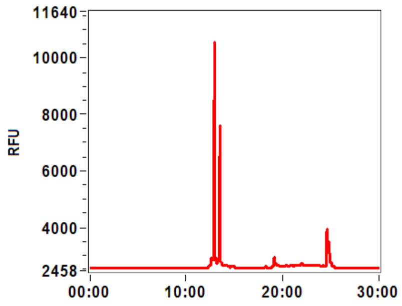

C2: SampC2

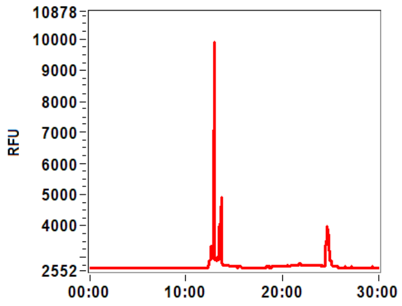

D2: SampD2

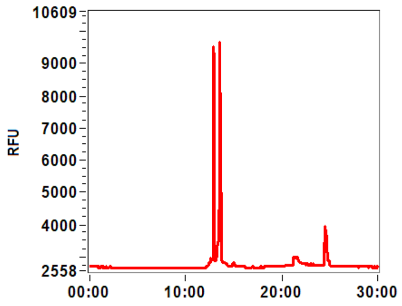

Filename and data path: C:\AATI\Data\2021 08 10\23-11-21\2021 08 10 23H 11M..raw

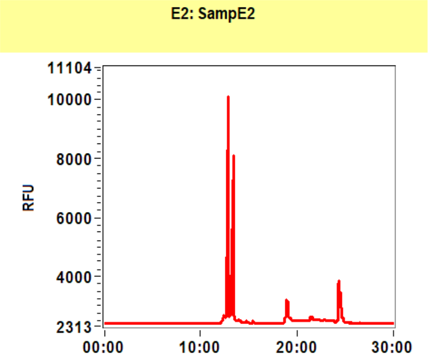

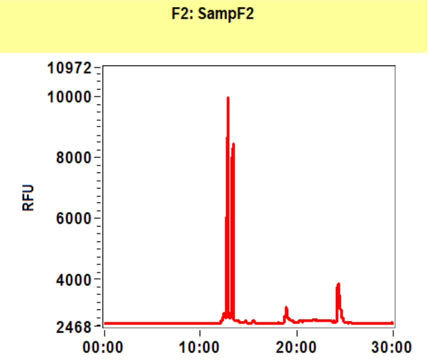

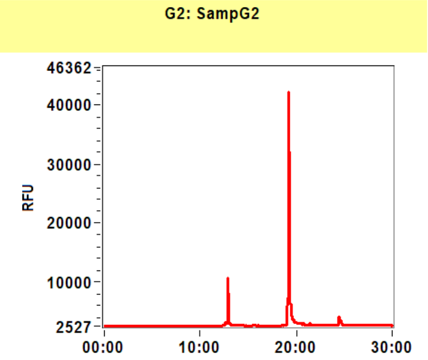

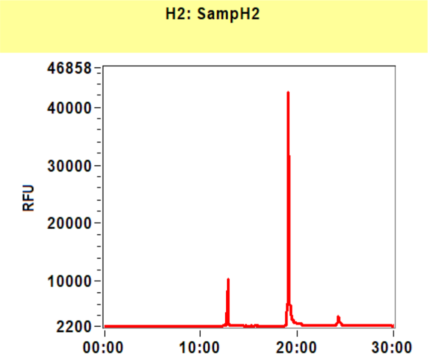

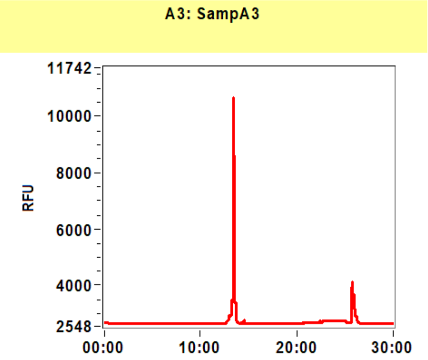

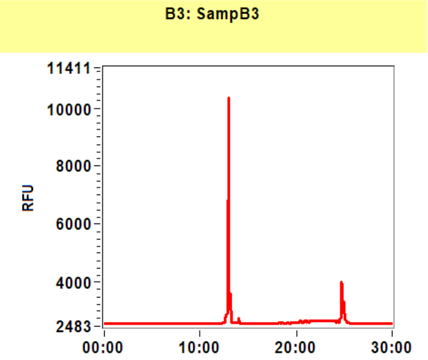

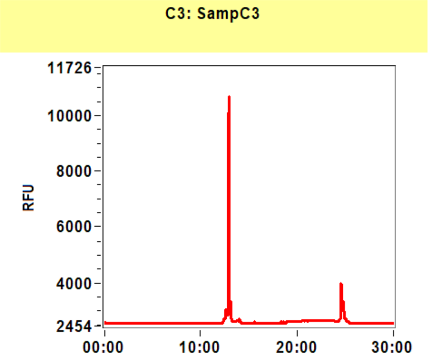

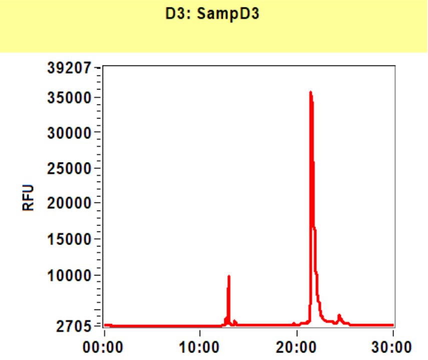

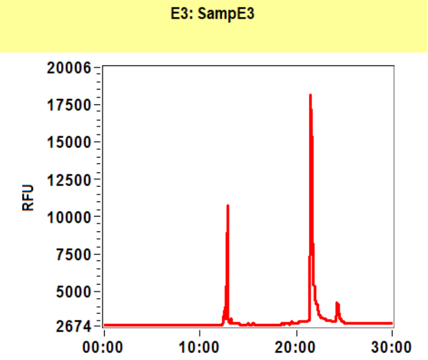

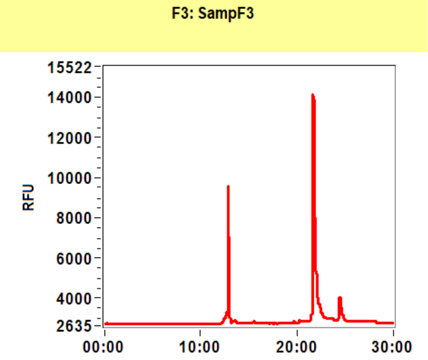

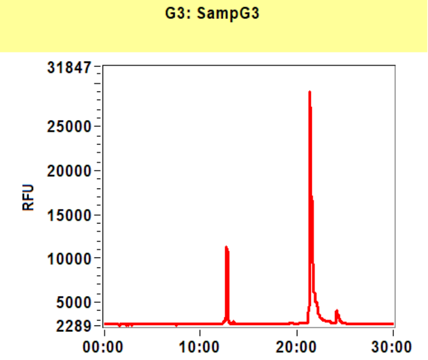

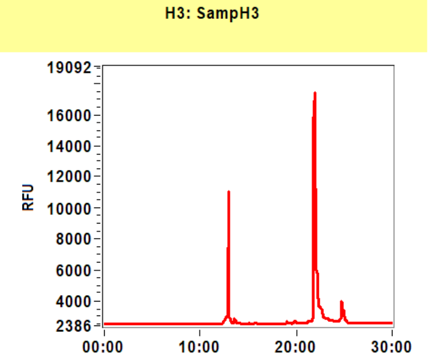

Filename and data path: C:\AATI\Data\2021 08 10\23-11-21\2021 08 10 23H 11M..raw

A4: SampA4

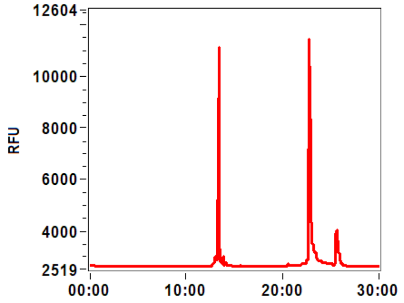

B4: SampB4

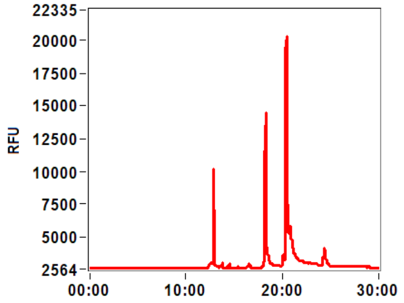

C4: SampC4

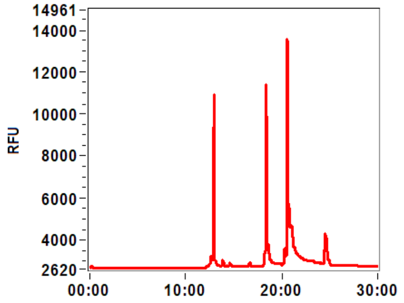

D4: SampD4

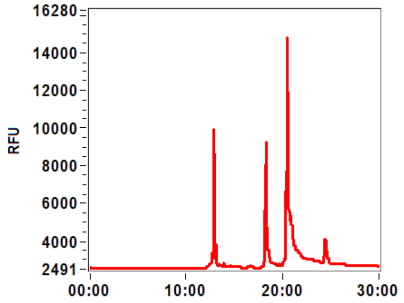

E4: SampE4

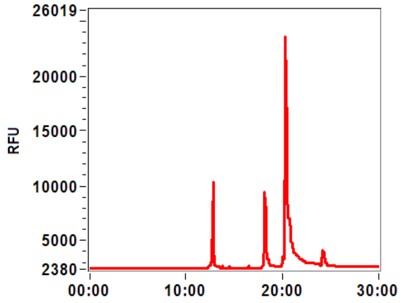

F4: SampF4

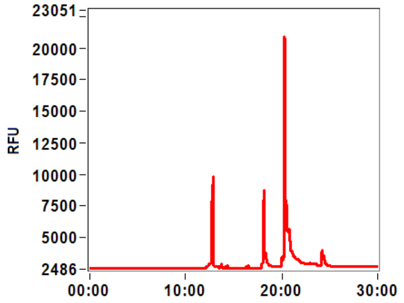

G4: SampG4

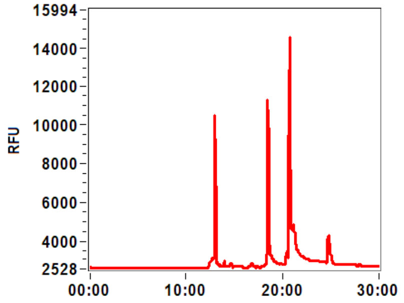

H4: SampH4

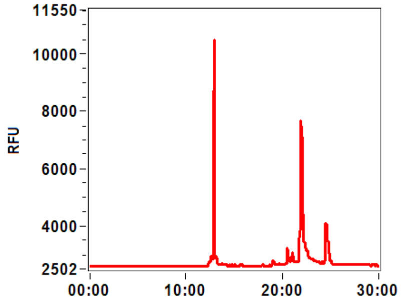

A5: SampA5

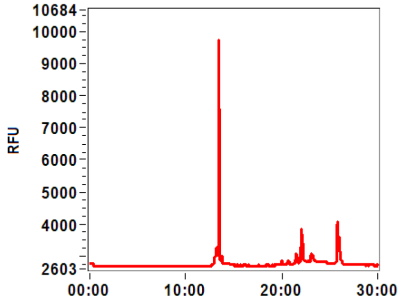

B5: SampB5

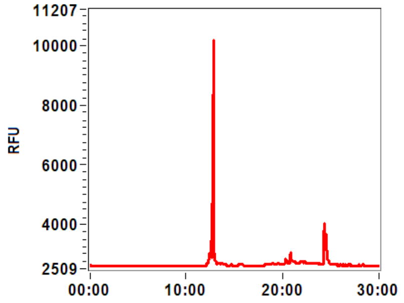

C5: SampC5

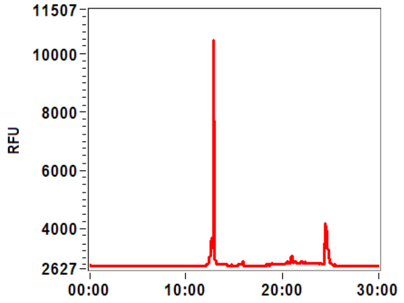

D5: SampD5

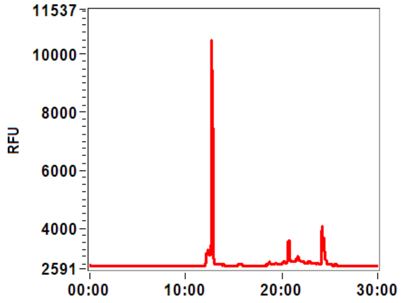

Filename and data path: C:\AATI\Data\2021 08 10\23-11-21\2021 08 10 23H 11M..raw

E5: SampE5

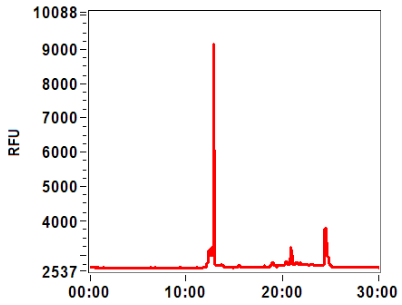

F5: SampF5

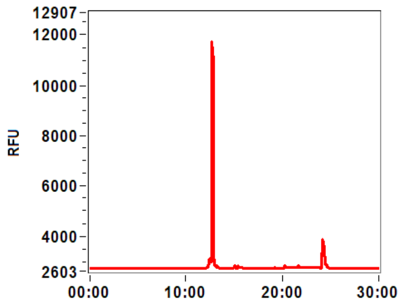

G5: SampG5

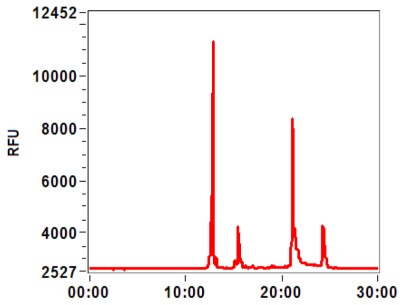

H5: SampH5

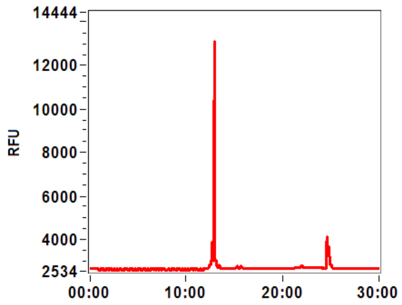

A6: SampA6

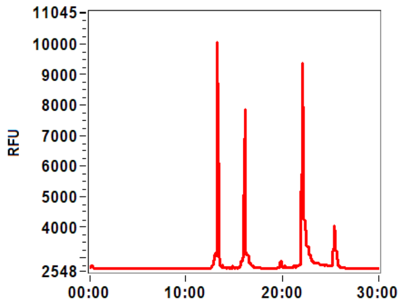

B6: SampB6

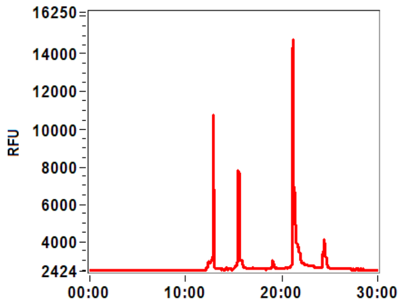

C6: SampC6

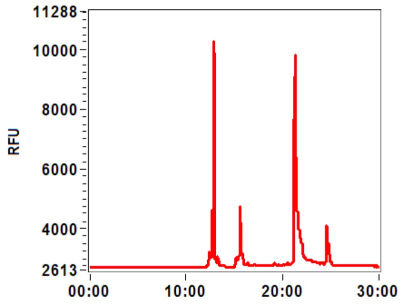

D6: SampD6

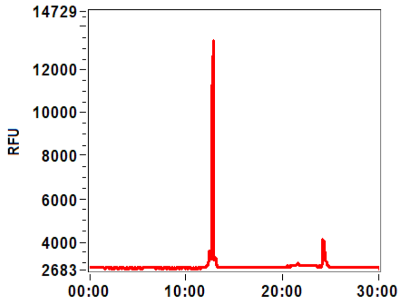

E6: SampE6

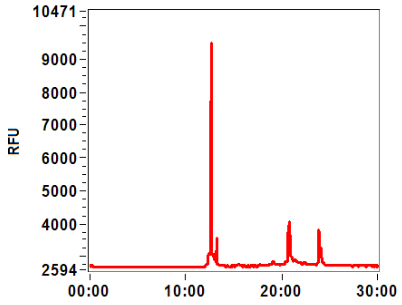

F6: SampF6

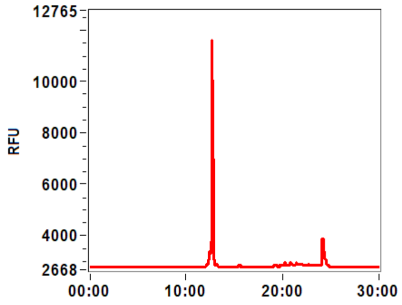

G6: SampG6

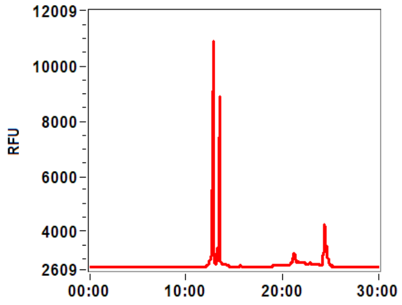

H6: SampH6

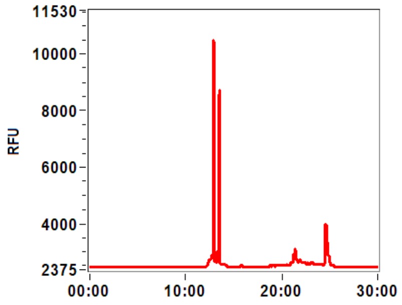

Filename and data path: C:\AATI\Data\2021 08 10\23-11-21\2021 08 10 23H 11M..raw

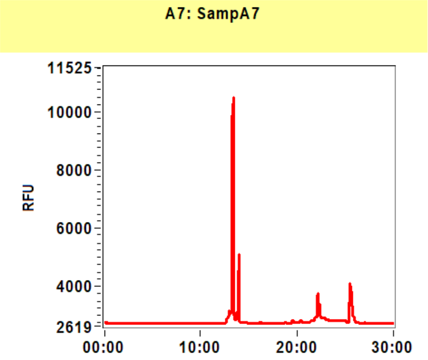

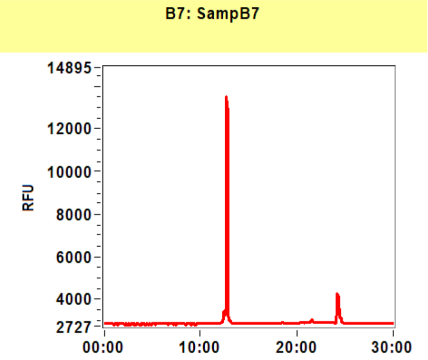

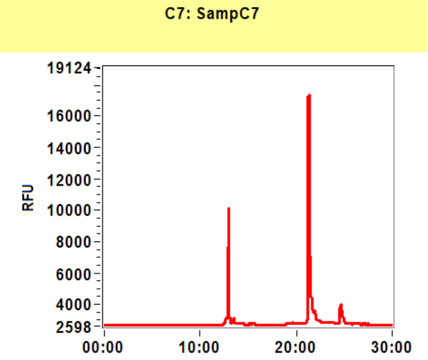

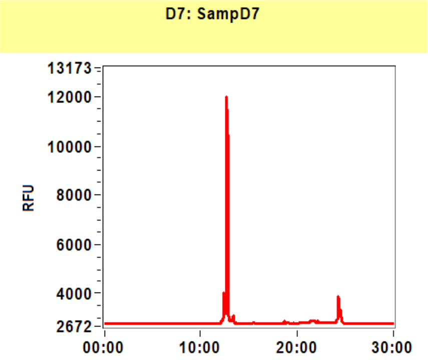

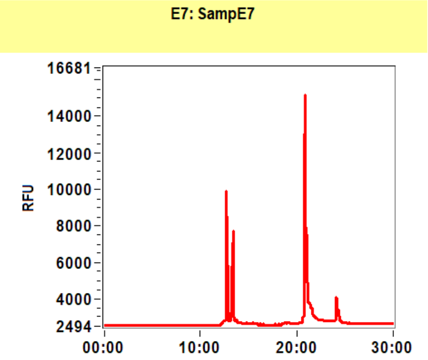

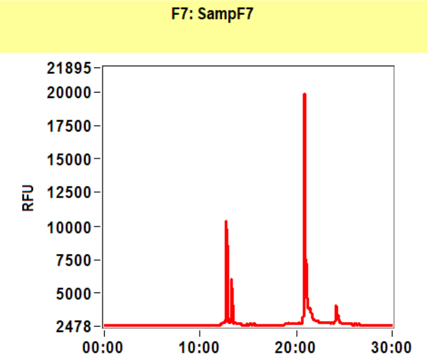

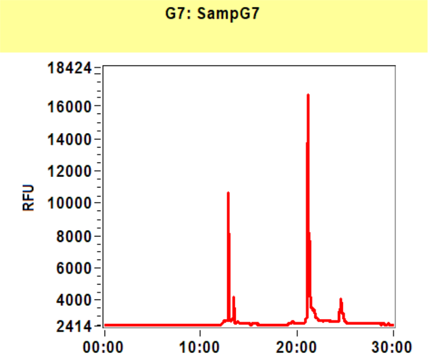

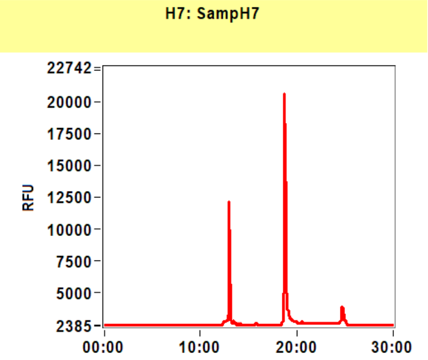

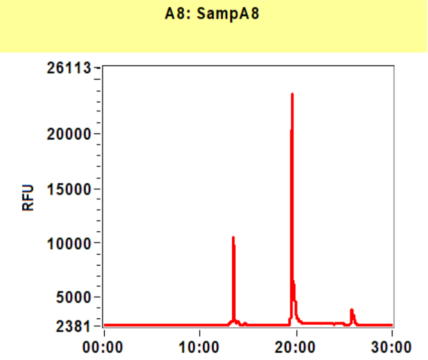

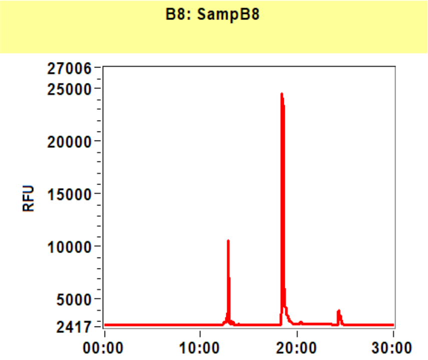

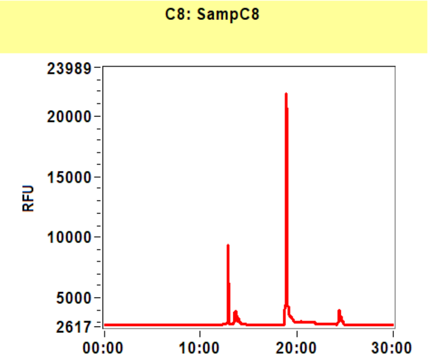

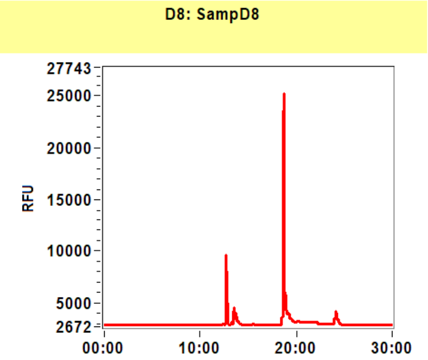

Filename and data path: C:\AATI\Data\2021 08 10\23-11-21\2021 08 10 23H 11M..raw

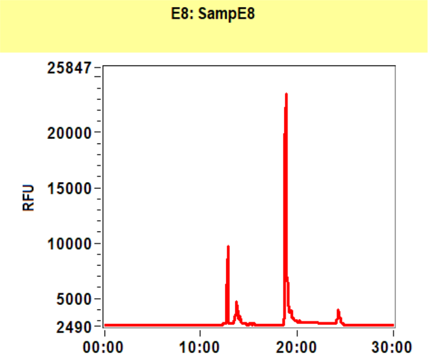

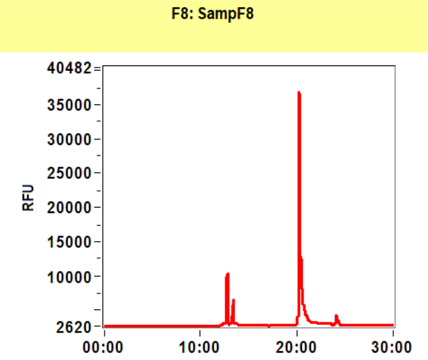

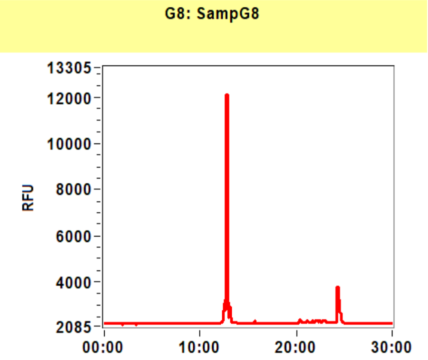

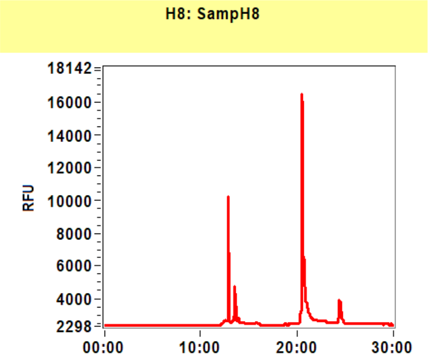

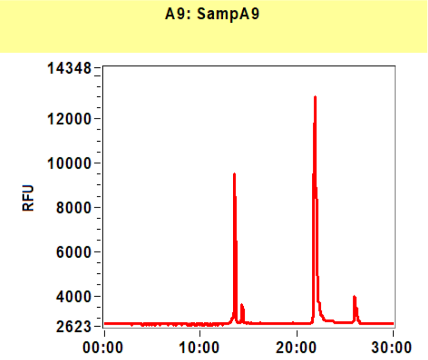

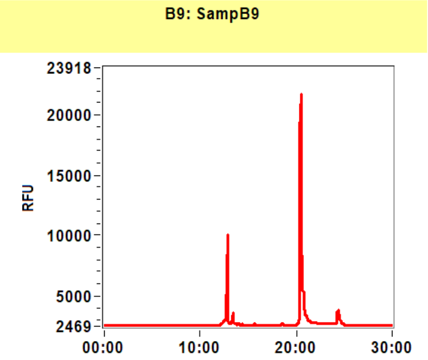

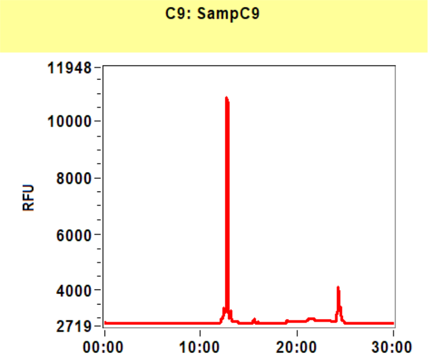

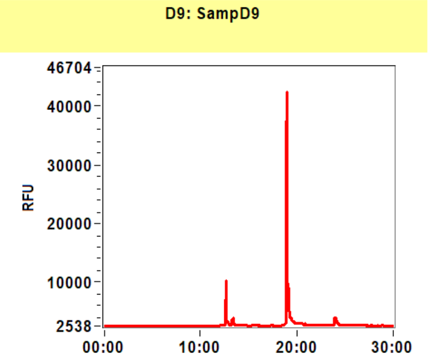

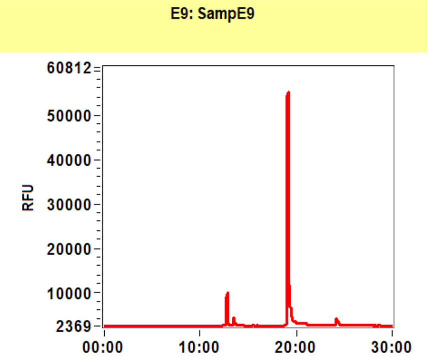

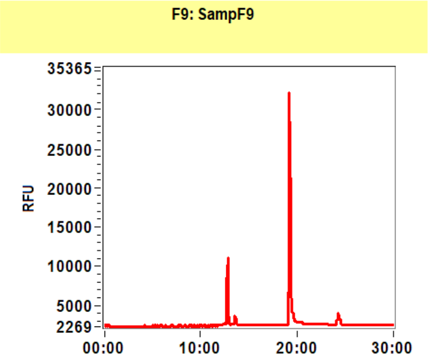

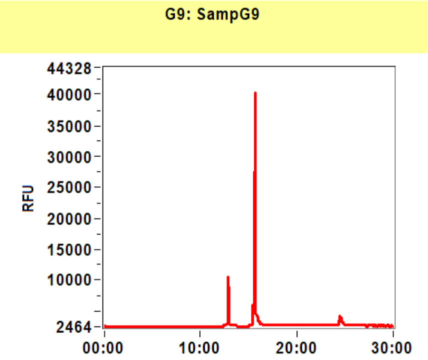

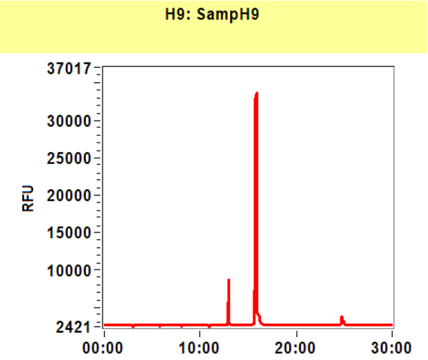

Filename and data path: C:\AATI\Data\2021 08 10\23-11-21\2021 08 10 23H 11M..raw

A10: SampA10

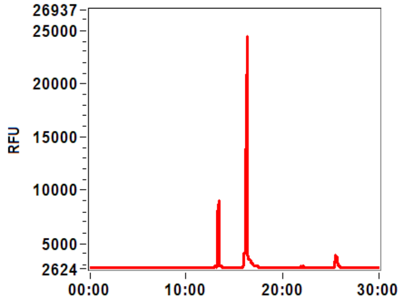

B10: SampB10

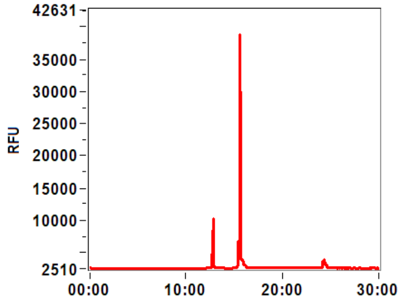

C10: SampC10

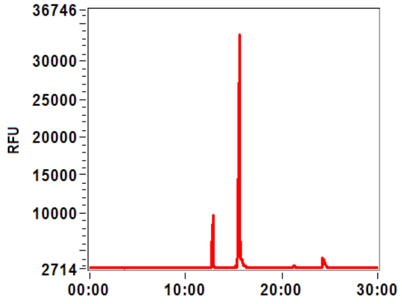

D10: SampD10

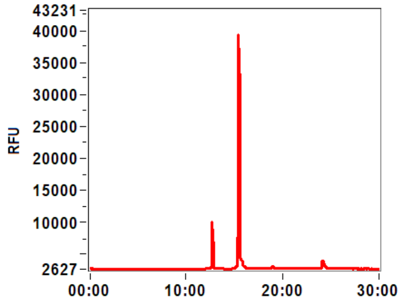

E10: SampE10

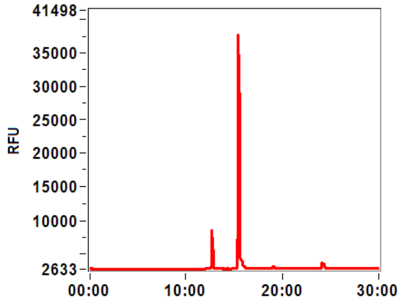

F10: SampF10

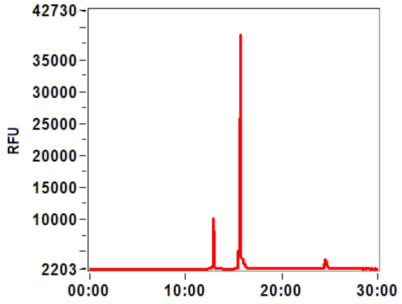

G10: SampG10

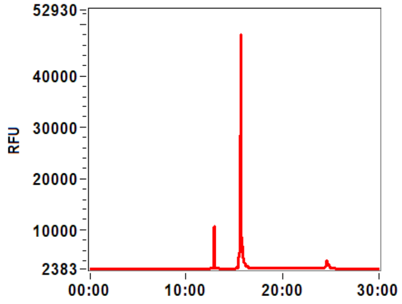

H10: SampH10

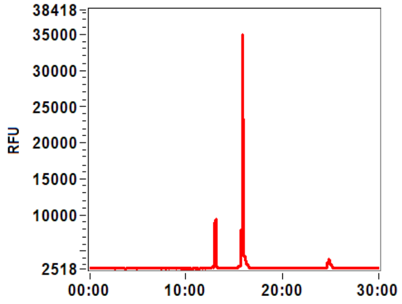

A11: SampA11

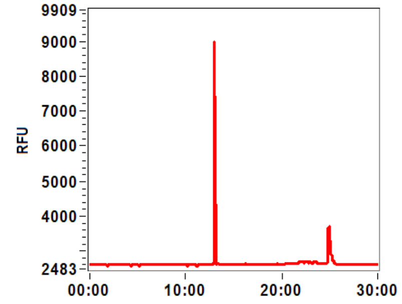

B11: SampB11

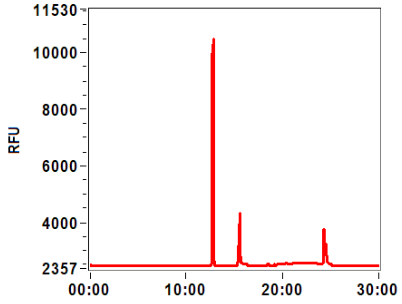

C11: SampC11

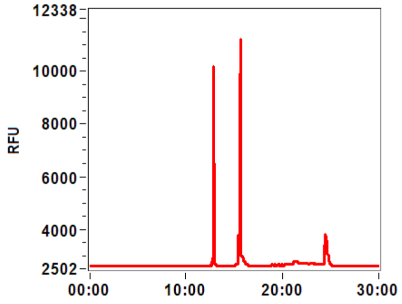

D11: SampD11

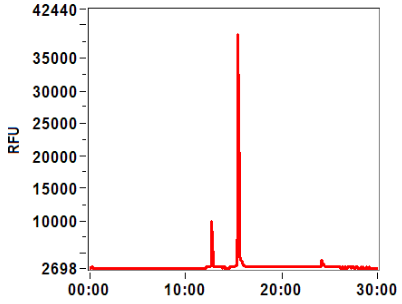

Filename and data path: C:\AATI\Data\2021 08 10\23-11-21\2021 08 10 23H 11M..raw

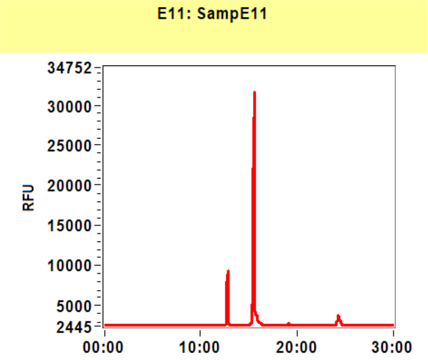

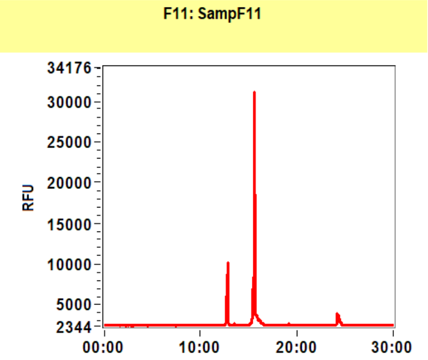

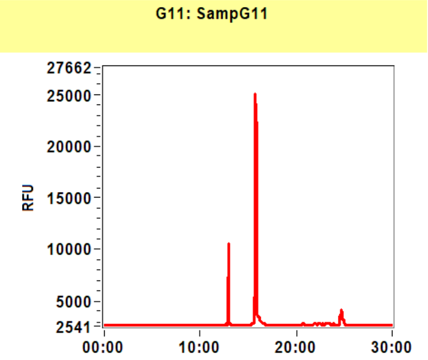

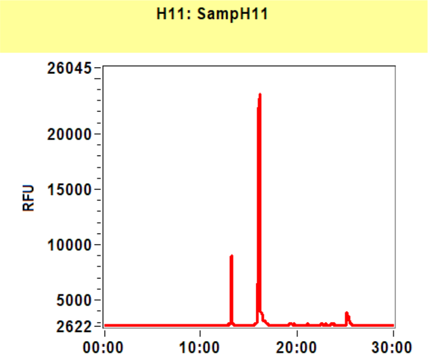

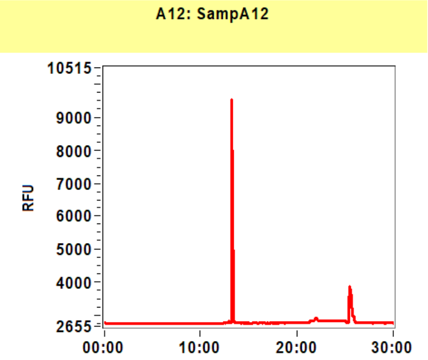

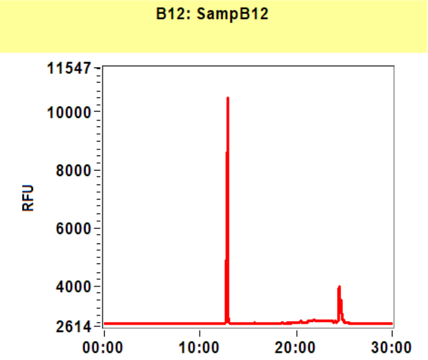

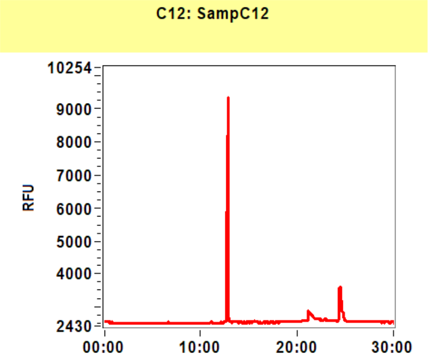

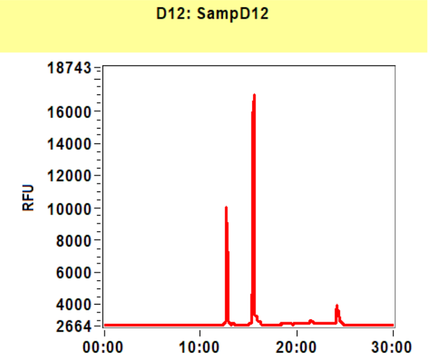

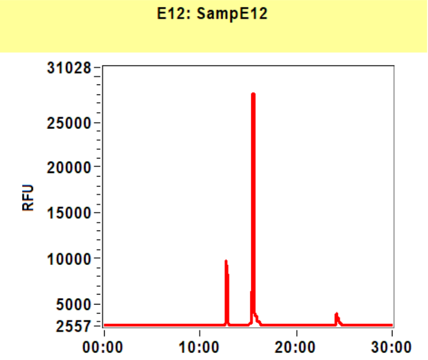

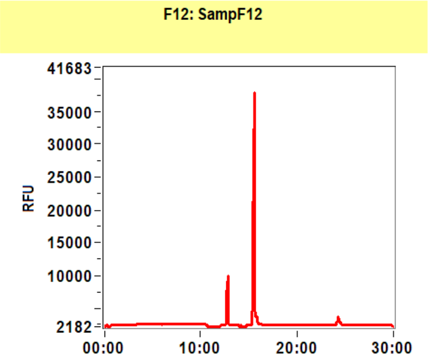

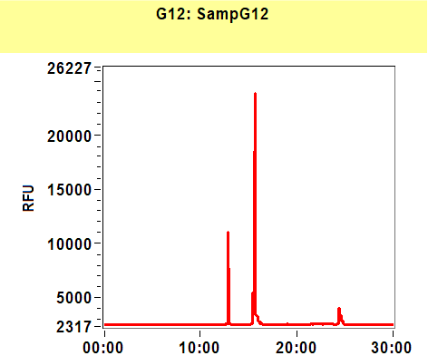

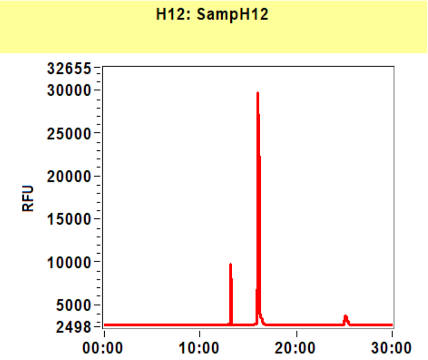

Sample: SampA1
Well location: A1
Created: Tuesday, August 10, 2021 11:20:27 PM

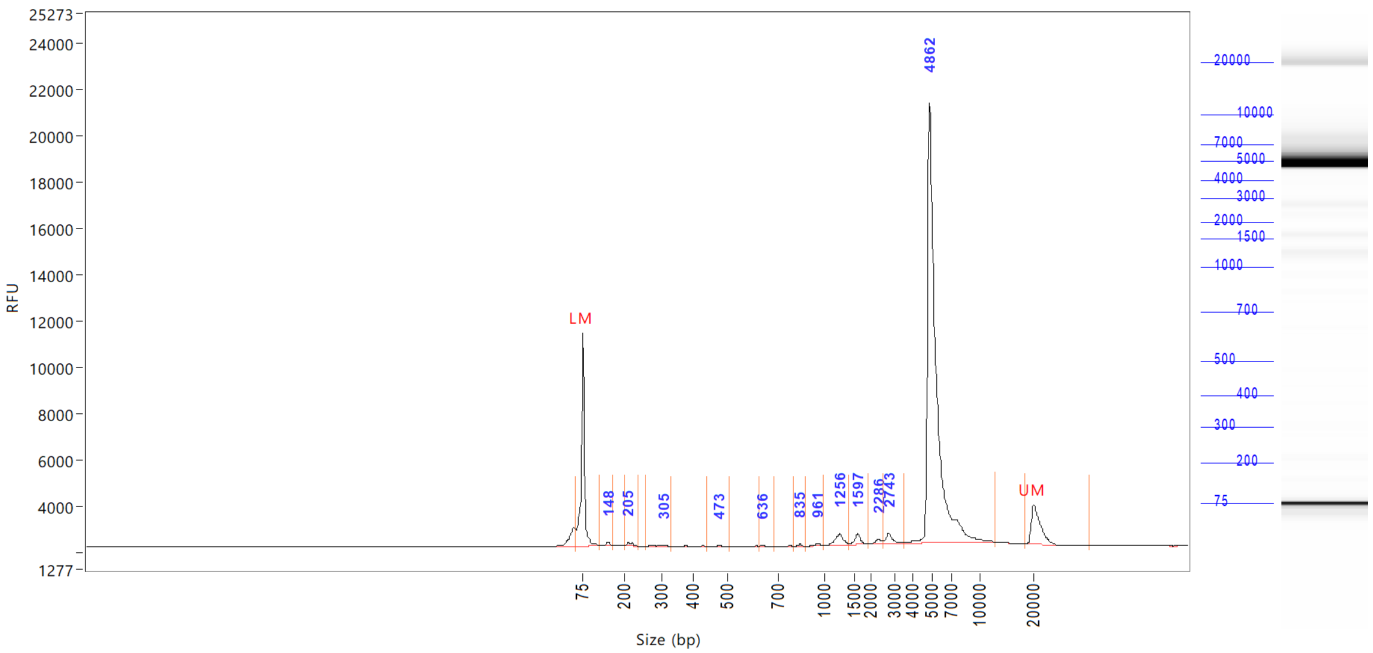

Peak	Size (bp)	Concentration (ng/uL)	From (bp)	To (bp)	Average size (bp)	CV%	RFU	Corrected peak area
1	75 (LM)	0.5000	56	124	72	10.77	9186	67.992
2	148	0.1288	124	164	144	5.96	144	1.460
3	205	0.1426	200	233	210	3.26	166	1.616
4	305	0.0742	257	327	292	5.28	52	0.841
5	473	0.0339	437	504	472	2.17	51	0.384
6	636	0.0388	621	682	635	1.66	66	0.439
7	835	0.0464	801	879	830	1.39	91	0.526
8	961	0.0770	879	991	945	1.91	101	0.873
9	1256	0.5510	991	1411	1231	6.02	484	6.244
10	1597	0.3485	1411	1923	1580	5.48	451	3.949
11	2286	0.1540	1923	2486	2255	5.91	186	1.745
12	2743	0.3798	2486	3500	2788	6.88	437	4.304
13	4862	19.6290	3500	12684	5353	17.65	19023	222.436
14	20000 (UM)	0.1391	18293	30277	20627	5.36	1692	18.922

TIC:	21.6040	ng/uL
TIM:	10.9226	nmole/L
Total concentration:	21.7579	ng/uL

Sample peak width (sec): 5   Sample min peak height: 50   Sample baseline V to V?: Y   Sample baseline V to V points: 3
Sample filter: Binomial   Number of points for filter: 3 Sample start region (min): 0 Sample end region (min): 30
Marker peak width (sec): 5   Marker min peak height: 200   Marker baseline V to V?: Y   Marker baseline V to V points: 3
Lower marker selection: First peak > 500 RFU   Upper marker selection: Last peak > 200 RFU
Ladder size (bp)75, 200, 300, 400, 500, 700, 1000, 1500, 2000, 3000, 4000, 5000, 7000, 10000, 20000
Quantification using: Lower Marker   Final concentration (ng/uL): 0.5000   Dilution factor: 12.0

Sample: SampB1
Well location: B1
Created: Tuesday, August 10, 2021 11:20:27 PM

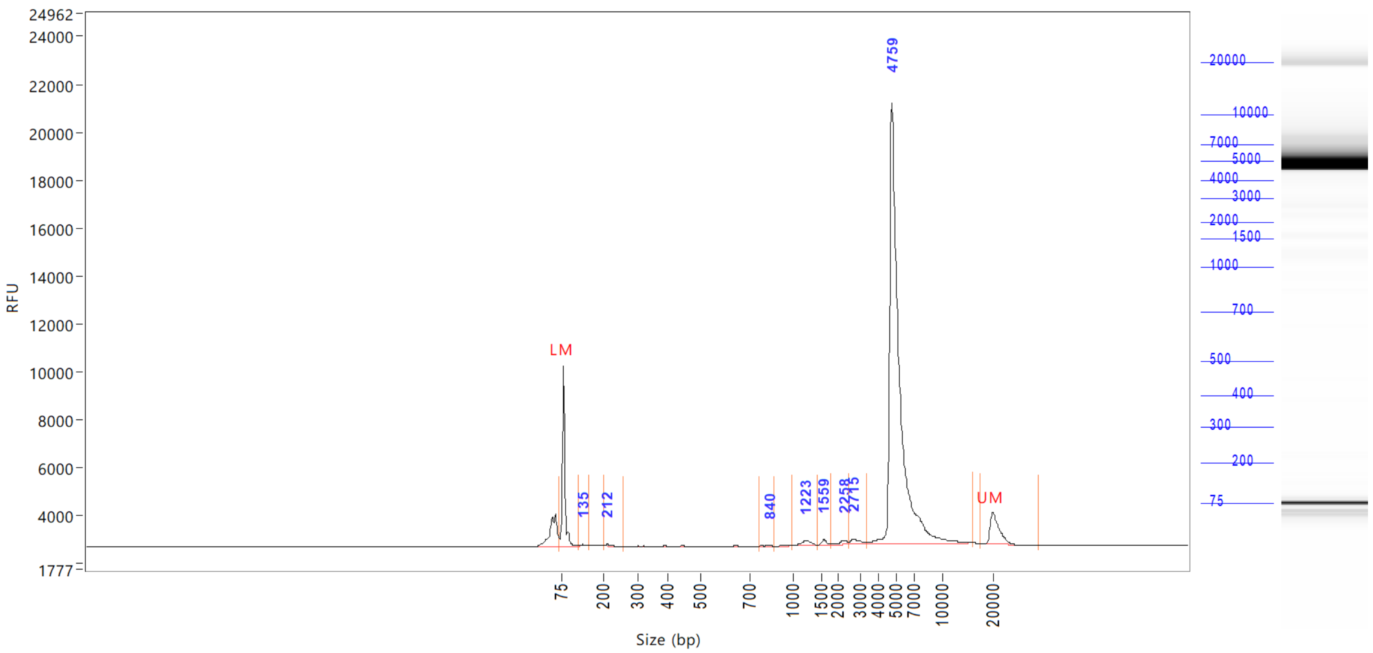

Peak	Size (bp)	Concentration (ng/uL)	From (bp)	To (bp)	Average size (bp)	CV%	RFU	Corrected peak area
1	75 (LM)	0.5000	66	122	75	8.69	7536	47.511
2	135	0.0969	122	154	134	5.89	61	0.768
3	212	0.0893	201	259	215	3.99	79	0.707
4	840	0.0593	762	874	822	3.01	54	0.469
5	1223	0.3682	991	1422	1206	6.44	187	2.916
6	1559	0.2060	1422	1808	1562	4.29	216	1.631
7	2258	0.2520	1808	2486	2210	7.19	185	1.995
8	2715	0.4323	2486	3393	2843	8.25	241	3.423
9	4759	31.9109	3393	16220	5360	21.73	18422	252.684
10	20000 (UM)	0.1581	17683	29068	20582	5.06	1339	15.019

TIC:	33.4149	ng/uL
TIM:	12.9496	nmole/L
Total concentration:	33.6903	ng/uL

```
Sample peak width (sec): 5    Sample min peak height: 50    Sample baseline V to V?: Y    Sample baseline V to V points: 3
Sample filter: Binomial       Number of points for filter: 3  Sample start region (min): 0  Sample end region (min): 30
Marker peak width (sec): 5    Marker min peak height: 200   Marker baseline V to V?: Y    Marker baseline V to V points: 3
Lower marker selection: First peak > 500 RFU                Upper marker selection: Last peak > 200 RFU
Ladder size (bp)75, 200, 300, 400, 500, 700, 1000, 1500, 2000, 3000, 4000, 5000, 7000, 10000, 20000
Quantification using: Lower Marker          Final concentration (ng/uL): 0.5000          Dilution factor: 12.0
```

Sample: SampC1
Well location: C1
Created: Tuesday, August 10, 2021 11:20:27 PM

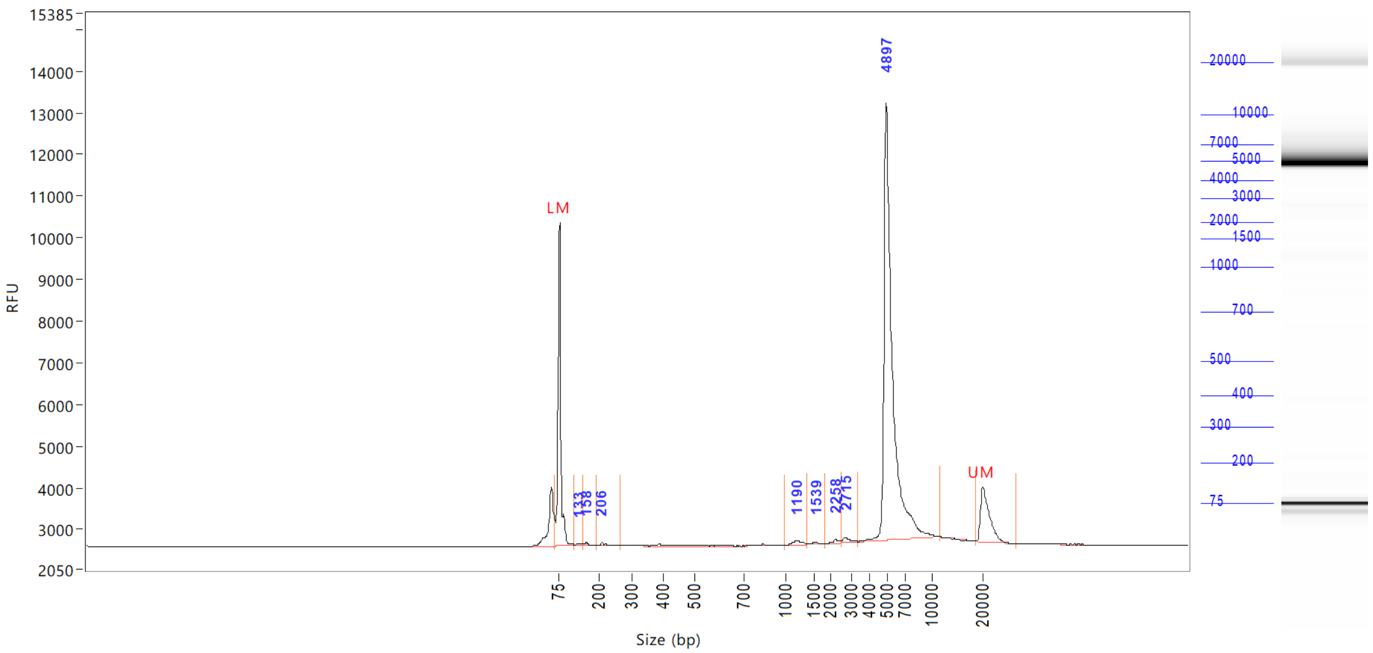

Peak	Size (bp)	Concentration (ng/uL)	From (bp)	To (bp)	Average size (bp)	CV%	RFU	Corrected peak area
1	75 (LM)	0.5000	64	122	74	8.97	7757	51.995
2	133	0.0768	122	147	134	5.24	52	0.665
3	158	0.0828	147	194	159	6.35	69	0.718
4	206	0.0729	194	260	211	4.49	68	0.632
5	1190	0.1844	991	1378	1186	6.79	102	1.598
6	1539	0.0607	1378	1827	1520	4.56	61	0.526
7	2258	0.1022	1827	2486	2223	6.82	89	0.886
8	2715	0.1587	2486	3322	2759	6.48	131	1.375
9	4897	14.6438	3322	11220	5389	16.60	10504	126.902
10	20000 (UM)	0.1465	18537	26649	20635	4.99	1331	15.237

TIC:	15.3823	ng/uL
TIM:	7.3309	nmole/L
Total concentration:	15.6257	ng/uL

Sample peak width (sec): 5 Sample min peak height: 50 Sample baseline V to V?: Y Sample baseline V to V points: 3
Sample filter: Binomial Number of points for filter: 3 Sample start region (min): 0 Sample end region (min): 30
Marker peak width (sec): 5 Marker min peak height: 200 Marker baseline V to V?: Y Marker baseline V to V points: 3
Lower marker selection: First peak > 500 RFU Upper marker selection: Last peak > 200 RFU
Ladder size (bp)75, 200, 300, 400, 500, 700, 1000, 1500, 2000, 3000, 4000, 5000, 7000, 10000, 20000
Quantification using: Lower Marker Final concentration (ng/uL): 0.5000 Dilution factor: 12.0

Sample: SampD1
Well location: D1
Created: Tuesday, August 10, 2021 11:20:27 PM

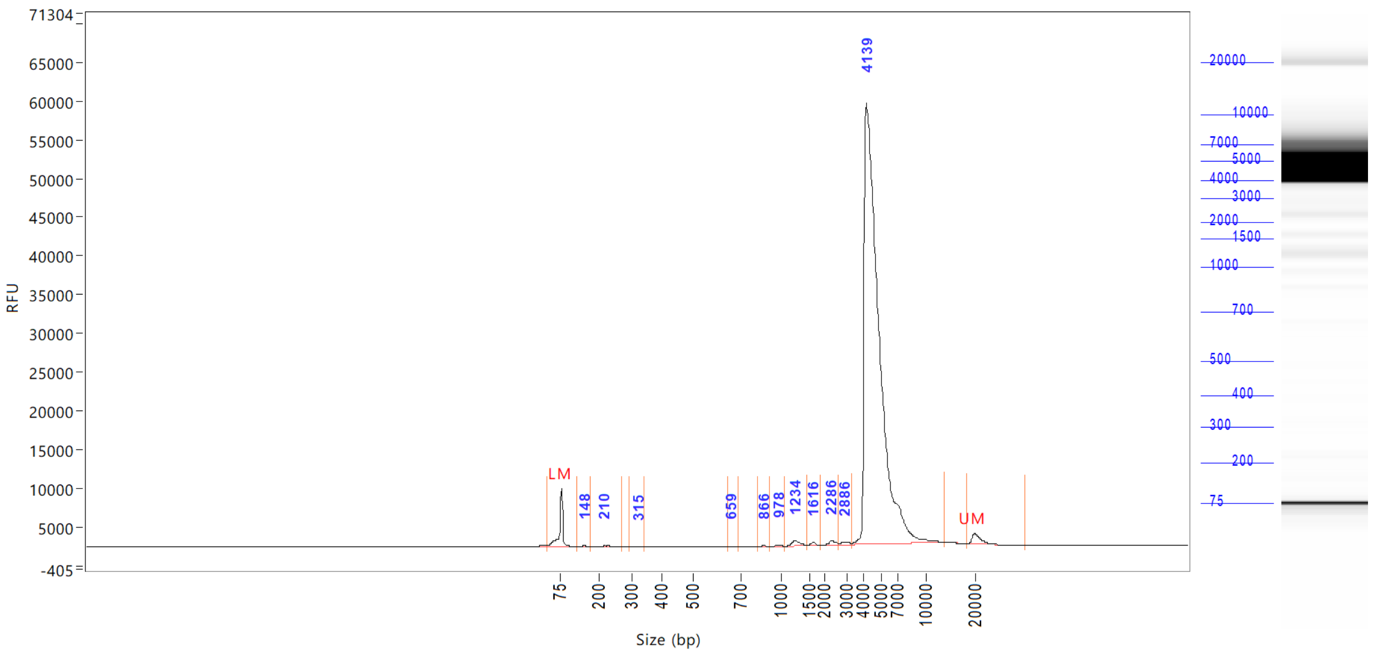

Peak	Size<br>(bp)	Concentration<br>(ng/uL)	From<br>(bp)	To<br>(bp)	Average size<br>(bp)	CV%	RFU	Corrected peak area
1	75 (LM)	0.5000	30	126	69	18.78	7593	63.035
2	148	0.1772	126	169	148	7.76	107	1.861
3	210	0.2595	169	268	206	8.92	160	2.727
4	315	0.0567	289	337	309	3.44	51	0.595
5	659	0.0508	644	687	656	0.96	88	0.533
6	866	0.1129	822	905	859	1.53	189	1.186
7	978	0.1994	905	1045	966	1.96	238	2.095
8	1234	0.9103	1045	1456	1243	6.07	703	9.564
9	1616	0.3081	1456	1866	1593	4.38	423	3.237
10	2286	0.6229	1866	2629	2304	7.12	620	6.545
11	2886	0.4075	2629	3286	2928	6.28	286	4.281
12	4139	117.8236	3286	13903	4745	20.04	57015	1237.829
13	20000 (UM)	0.1120	18537	30398	20649	5.28	1277	14.118

TIC:	120.9289	ng/uL
TIM:	48.1010	nmole/L
Total concentration:	121.1012	ng/uL

```
Sample peak width (sec): 5    Sample min peak height: 50    Sample baseline V to V?: Y    Sample baseline V to V points: 3
Sample filter: Binomial       Number of points for filter: 3  Sample start region (min): 0  Sample end region (min): 30
Marker peak width (sec): 5    Marker min peak height: 200   Marker baseline V to V?: Y    Marker baseline V to V points: 3
Lower marker selection: First peak > 500 RFU                Upper marker selection: Last peak > 200 RFU
Ladder size (bp)75, 200, 300, 400, 500, 700, 1000, 1500, 2000, 3000, 4000, 5000, 7000, 10000, 20000
Quantification using: Lower Marker          Final concentration (ng/uL): 0.5000          Dilution factor: 12.0
```

Sample: SampE1
Well location: E1
Created: Tuesday, August 10, 2021 11:20:27 PM

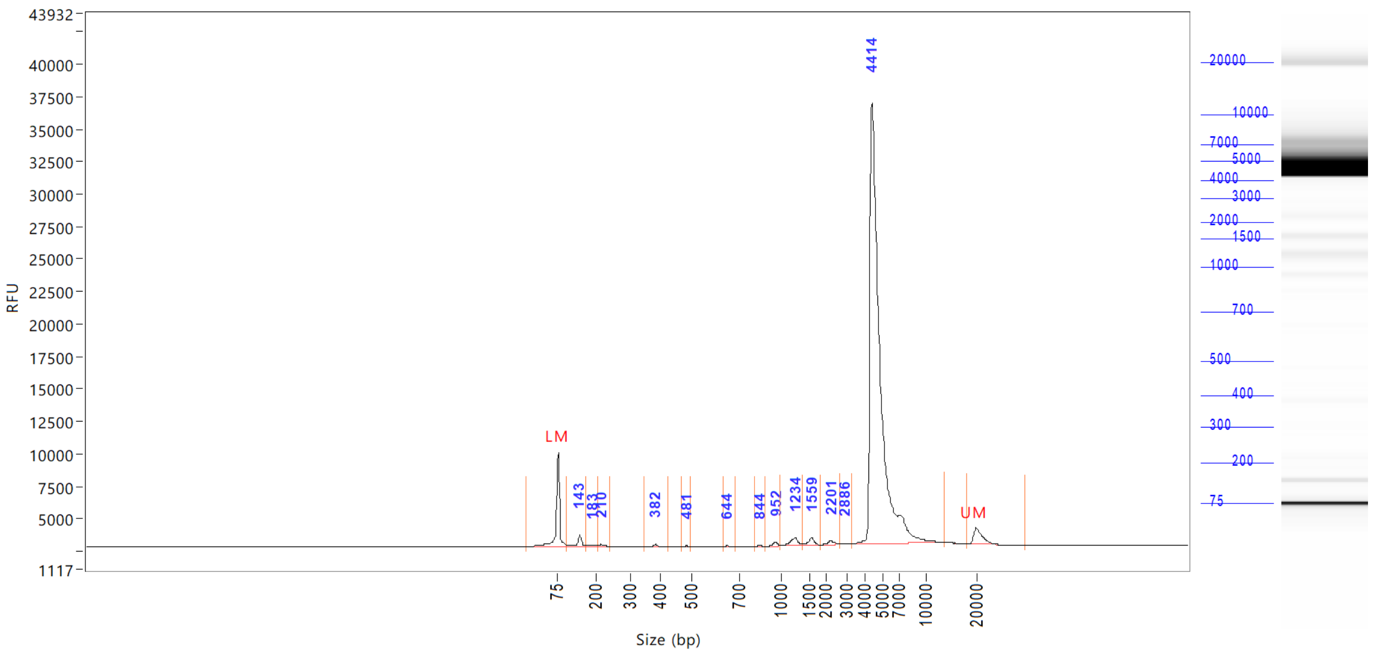

Peak	Size (bp)	Concentration (ng/uL)	From (bp)	To (bp)	Average size (bp)	CV%	RFU	Corrected peak area
1	75 (LM)	0.5000	0	103	67	25.13	7228	57.262
2	143	1.0140	103	164	137	9.05	876	9.678
3	183	0.1811	164	201	181	5.95	91	1.728
4	210	0.1834	201	239	213	3.56	159	1.751
5	382	0.1693	341	420	381	3.38	167	1.616
6	481	0.0335	468	494	479	1.04	51	0.320
7	644	0.0403	629	680	643	1.28	63	0.385
8	844	0.0679	809	883	837	1.42	101	0.648
9	952	0.2613	883	991	946	1.79	314	2.494
10	1234	0.7975	991	1378	1198	6.52	573	7.611
11	1559	0.5274	1378	1866	1533	4.95	544	5.033
12	2201	0.3301	1866	2686	2260	8.11	286	3.151
13	2886	0.1053	2686	3251	2906	5.04	85	1.005
14	4414	51.7102	3251	13537	4921	19.49	34007	493.505
15	20000 (UM)	0.1203	18171	29793	20579	5.19	1220	13.772

TIC:	55.4214	ng/uL
TIM:	36.0225	nmole/L
Total concentration:	55.5796	ng/uL

```
Sample peak width (sec): 5    Sample min peak height: 50    Sample baseline V to V?: Y    Sample baseline V to V points: 3
Sample filter: Binomial       Number of points for filter: 3  Sample start region (min): 0  Sample end region (min): 30
Marker peak width (sec): 5    Marker min peak height: 200   Marker baseline V to V?: Y    Marker baseline V to V points: 3
Lower marker selection: First peak > 500 RFU                Upper marker selection: Last peak > 200 RFU
Ladder size (bp)75, 200, 300, 400, 500, 700, 1000, 1500, 2000, 3000, 4000, 5000, 7000, 10000, 20000
Quantification using: Lower Marker          Final concentration (ng/uL): 0.5000          Dilution factor: 12.0
```

Sample: SampF1
Well location: F1
Created: Tuesday, August 10, 2021 11:20:27 PM

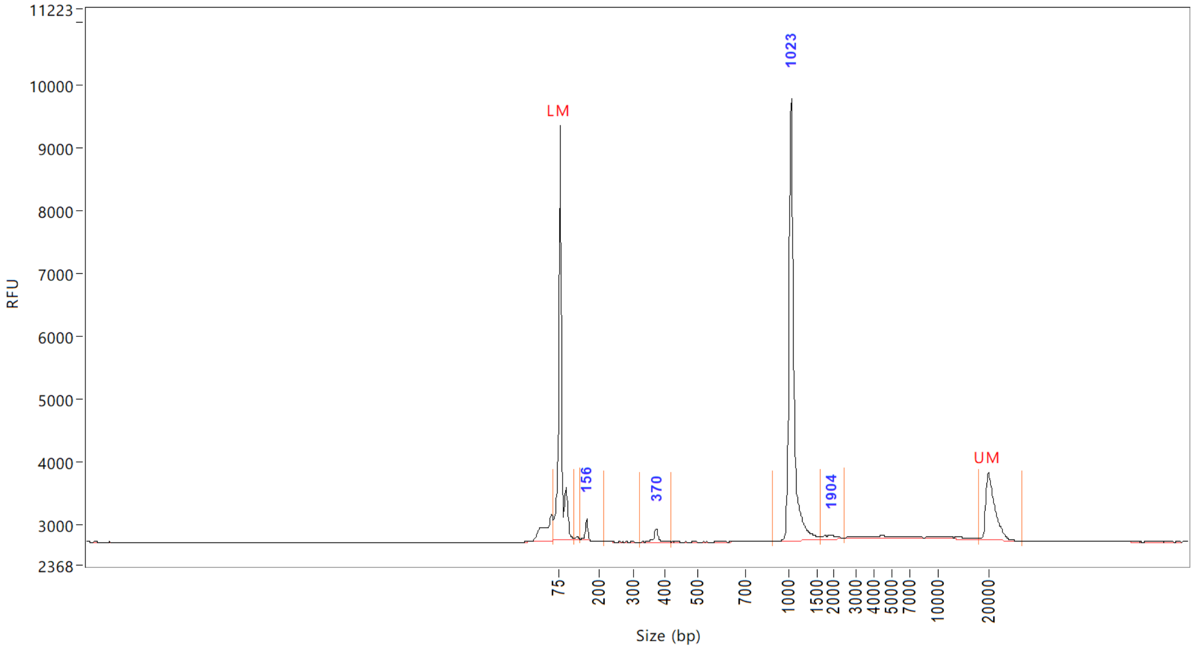

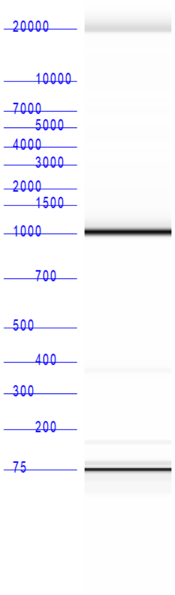

Peak	Size (bp)	Concentration (ng/uL)	From (bp)	To (bp)	Average size (bp)	CV%	RFU	Corrected peak area
1	75 (LM)	0.5000	54	118	75	12.03	6605	49.962
2	156	0.2419	139	212	155	4.42	335	2.015
3	370	0.2613	315	416	369	4.00	216	2.176
4	1023	6.8719	892	1597	1045	8.11	7052	57.223
5	1904	0.1583	1597	2429	1892	10.47	60	1.318
6	20000 (UM)	0.1245	18171	26770	20620	5.27	1072	12.441

TIC:	7.5335	ng/uL
TIM:	14.6919	nmole/L
Total concentration:	8.0338	ng/uL

```
Sample peak width (sec): 5    Sample min peak height: 50    Sample baseline V to V?: Y    Sample baseline V to V points: 3
Sample filter: Binomial       Number of points for filter: 3  Sample start region (min): 0  Sample end region (min): 30
Marker peak width (sec): 5    Marker min peak height: 200   Marker baseline V to V?: Y    Marker baseline V to V points: 3
Lower marker selection: First peak > 500 RFU                Upper marker selection: Last peak > 200 RFU
Ladder size (bp)75, 200, 300, 400, 500, 700, 1000, 1500, 2000, 3000, 4000, 5000, 7000, 10000, 20000
Quantification using: Lower Marker          Final concentration (ng/uL): 0.5000          Dilution factor: 12.0
```

Sample: SampG1
Well location: G1
Created: Tuesday, August 10, 2021 11:20:27 PM

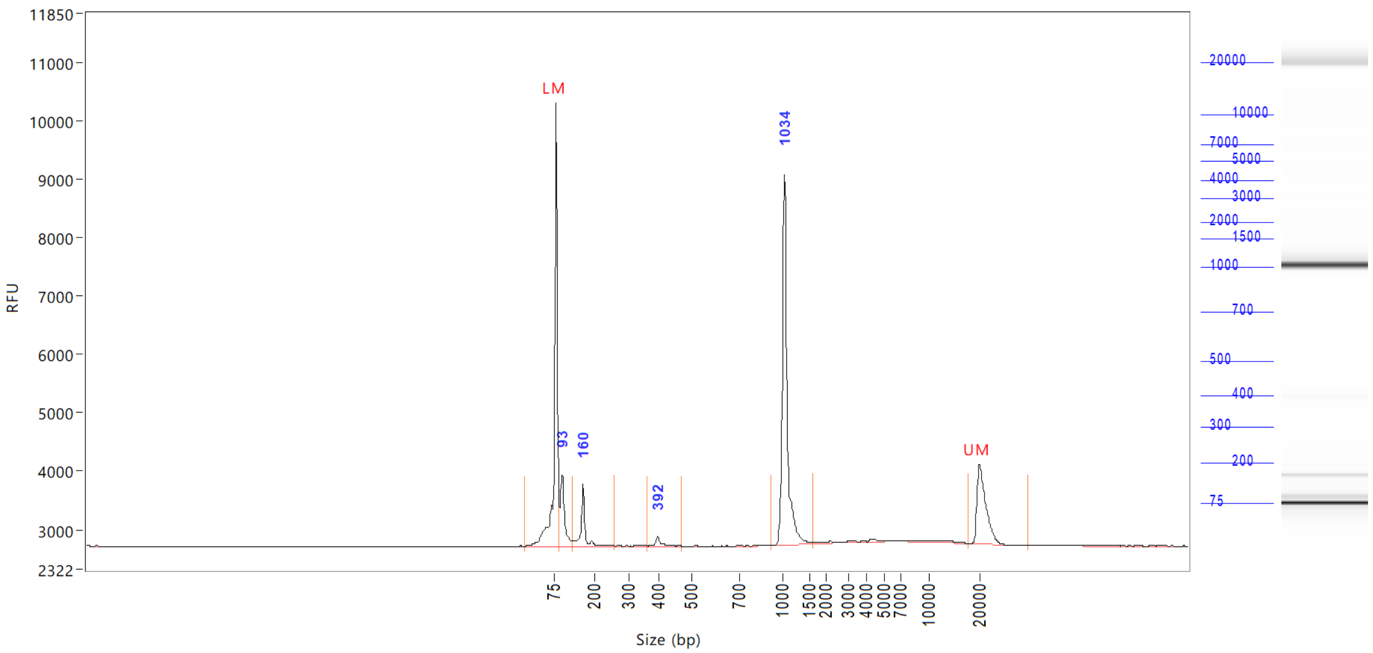

Peak	Size (bp)	Concentration (ng/uL)	From (bp)	To (bp)	Average size (bp)	CV%	RFU	Corrected peak area
1	75 (LM)	0.5000	0	86	68	21.63	7612	58.192
2	93	1.3837	86	126	96	9.29	1234	13.420
3	160	1.0389	126	257	162	13.48	1056	10.076
4	392	0.2037	362	470	398	4.62	170	1.975
5	1034	5.3420	926	1578	1052	7.31	6344	51.810
6	20000 (UM)	0.1370	17805	29914	20650	5.16	1364	15.943

TIC:	7.9683	ng/uL
TIM:	43.4075	nmole/L
Total concentration:	8.3978	ng/uL

```
Sample peak width (sec): 5    Sample min peak height: 50    Sample baseline V to V?: Y    Sample baseline V to V points: 3
Sample filter: Binomial       Number of points for filter: 3  Sample start region (min): 0  Sample end region (min): 30
Marker peak width (sec): 5    Marker min peak height: 200   Marker baseline V to V?: Y    Marker baseline V to V points: 3
Lower marker selection: First peak > 500 RFU                Upper marker selection: Last peak > 200 RFU
Ladder size (bp)75, 200, 300, 400, 500, 700, 1000, 1500, 2000, 3000, 4000, 5000, 7000, 10000, 20000
Quantification using: Lower Marker          Final concentration (ng/uL): 0.5000          Dilution factor: 12.0
```

Sample: SampH1
Well location: H1
Created: Tuesday, August 10, 2021 11:20:27 PM

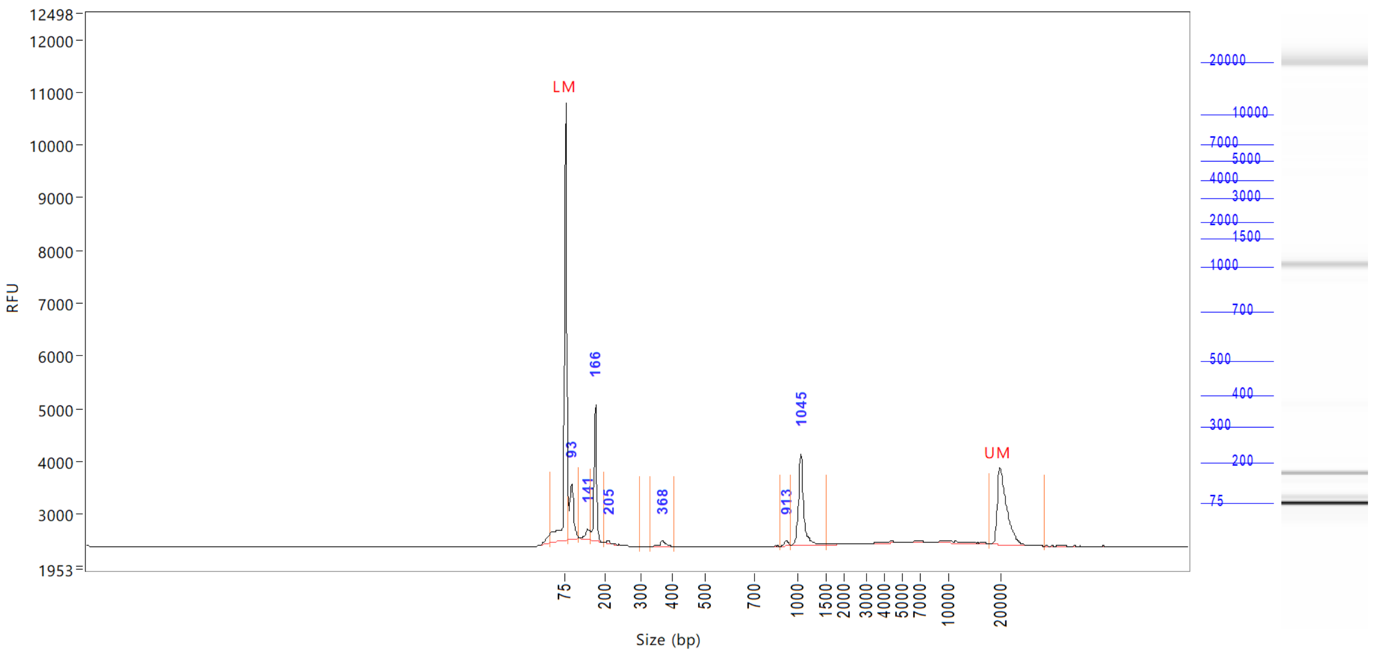

Peak	Size (bp)	Concentration (ng/uL)	From (bp)	To (bp)	Average size (bp)	CV%	RFU	Corrected peak area
1	75 (LM)	0.5000	30	86	71	12.89	8309	52.586
2	93	1.0424	86	116	93	5.85	1042	9.136
3	141	0.2769	116	152	141	5.66	196	2.427
4	166	1.7322	152	194	163	2.54	2592	15.182
5	205	0.0605	194	295	213	9.91	50	0.530
6	368	0.1463	331	403	367	3.47	108	1.282
7	913	0.0813	879	952	916	1.61	103	0.712
8	1045	1.7093	952	1500	1053	6.72	1742	14.981
9	20000 (UM)	0.1631	17927	28584	20634	5.38	1452	17.158

TIC:	5.0489	ng/uL
TIM:	43.0388	nmole/L
Total concentration:	5.4928	ng/uL

Sample peak width (sec): 5 Sample min peak height: 50 Sample baseline V to V?: Y Sample baseline V to V points: 3
Sample filter: Binomial Number of points for filter: 3 Sample start region (min): 0 Sample end region (min): 30
Marker peak width (sec): 5 Marker min peak height: 200 Marker baseline V to V?: Y Marker baseline V to V points: 3
Lower marker selection: First peak > 500 RFU Upper marker selection: Last peak > 200 RFU
Ladder size (bp)75, 200, 300, 400, 500, 700, 1000, 1500, 2000, 3000, 4000, 5000, 7000, 10000, 20000
Quantification using: Lower Marker Final concentration (ng/uL): 0.5000 Dilution factor: 12.0

Sample: SampA2
Well location: A2
Created: Tuesday, August 10, 2021 11:20:27 PM

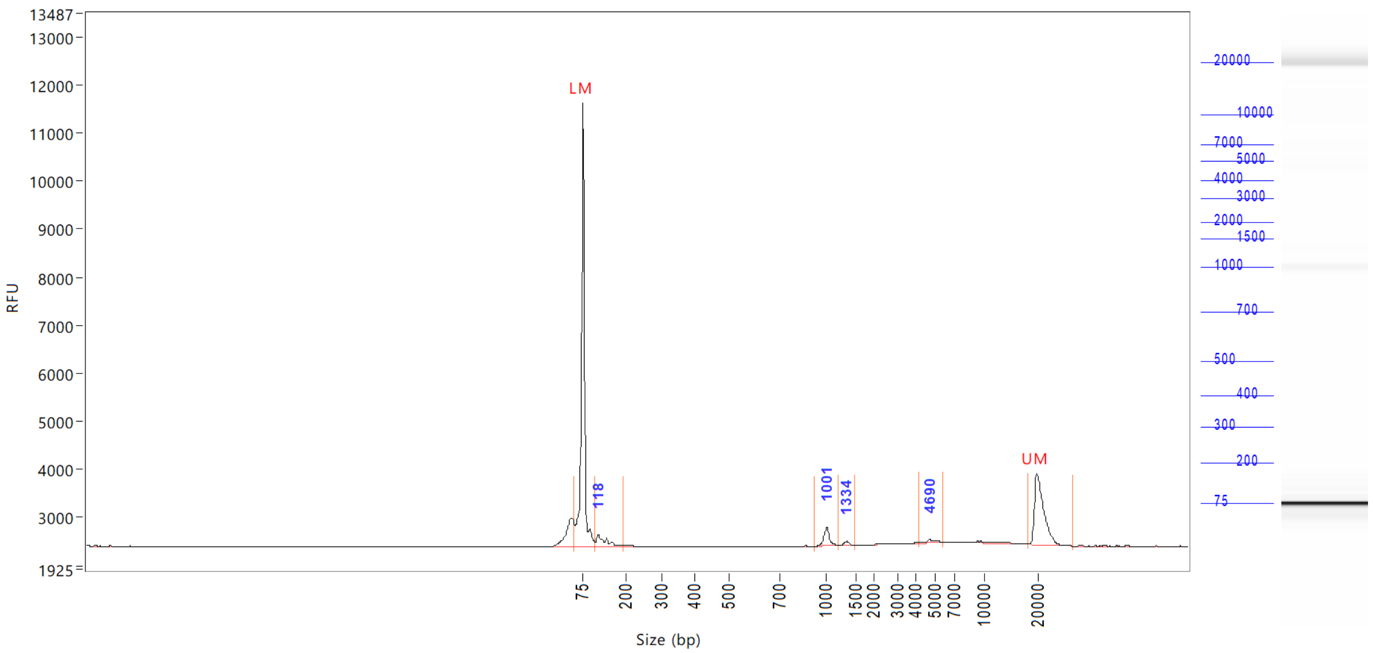

Peak	Size (bp)	Concentration (ng/uL)	From (bp)	To (bp)	Average size (bp)	CV%	RFU	Corrected peak area
1	75 (LM)	0.5000	50	108	72	10.72	9239	67.666
2	118	0.4292	108	192	134	13.68	237	4.840
3	1001	0.3027	922	1212	1009	3.86	394	3.414
4	1334	0.0589	1212	1467	1326	3.53	81	0.664
5	4690	0.0809	4139	5815	4816	7.31	67	0.913
6	20000 (UM)	0.1224	18171	26770	20520	4.85	1468	16.566

TIC:	0.8717	ng/uL
TIM:	5.8577	nmole/L
Total concentration:	1.1730	ng/uL

```
Sample peak width (sec): 5    Sample min peak height: 50    Sample baseline V to V?: Y    Sample baseline V to V points: 3
Sample filter: Binomial       Number of points for filter: 3  Sample start region (min): 0  Sample end region (min): 30
Marker peak width (sec): 5    Marker min peak height: 200   Marker baseline V to V?: Y    Marker baseline V to V points: 3
Lower marker selection: First peak > 500 RFU                Upper marker selection: Last peak > 200 RFU
Ladder size (bp)75, 200, 300, 400, 500, 700, 1000, 1500, 2000, 3000, 4000, 5000, 7000, 10000, 20000
Quantification using: Lower Marker          Final concentration (ng/uL): 0.5000          Dilution factor: 12.0
```

Sample: SampB2
Well location: B2
Created: Tuesday, August 10, 2021 11:20:27 PM

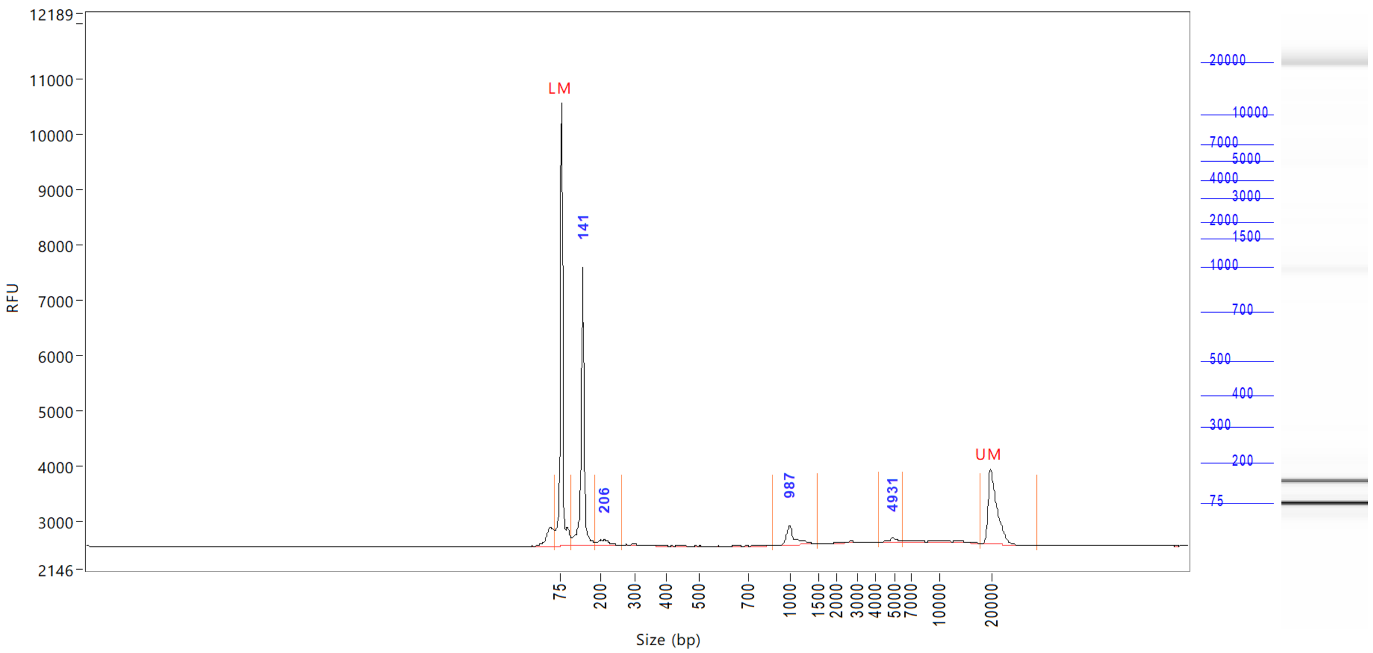

Peak	Size (bp)	Concentration (ng/uL)	From (bp)	To (bp)	Average size (bp)	CV%	RFU	Corrected peak area
1	75 (LM)	0.5000	56	107	74	9.58	8017	52.910
2	141	4.2156	107	181	138	6.99	5024	37.174
3	206	0.2966	181	259	205	7.61	103	2.615
4	987	0.5443	874	1478	1032	9.37	361	4.799
5	4931	0.1063	4104	5963	4985	8.01	70	0.937
6	20000 (UM)	0.1471	17805	29189	20564	5.21	1354	15.570

TIC:	5.1627	ng/uL
TIM:	53.4801	nmole/L
Total concentration:	5.5926	ng/uL

Sample peak width (sec): 5 Sample min peak height: 50 Sample baseline V to V?: Y Sample baseline V to V points: 3
Sample filter: Binomial Number of points for filter: 3 Sample start region (min): 0 Sample end region (min): 30
Marker peak width (sec): 5 Marker min peak height: 200 Marker baseline V to V?: Y Marker baseline V to V points: 3
Lower marker selection: First peak > 500 RFU Upper marker selection: Last peak > 200 RFU
Ladder size (bp)75, 200, 300, 400, 500, 700, 1000, 1500, 2000, 3000, 4000, 5000, 7000, 10000, 20000
Quantification using: Lower Marker Final concentration (ng/uL): 0.5000 Dilution factor: 12.0

Sample: SampC2
Well location: C2
Created: Tuesday, August 10, 2021 11:20:27 PM

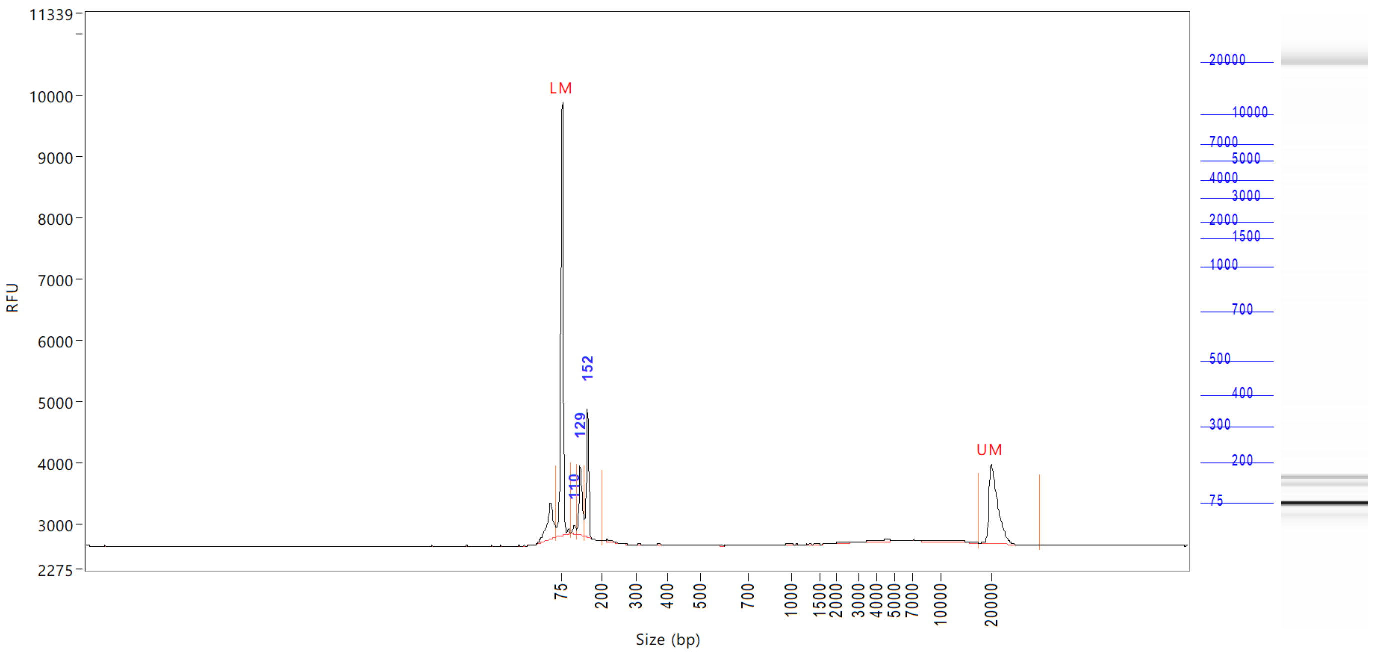

Peak	Size (bp)	Concentration (ng/uL)	From (bp)	To (bp)	Average size (bp)	CV%	RFU	Corrected peak area
1	75 (LM)	0.5000	58	101	72	5.93	7067	43.892
2	110	0.1574	101	120	111	4.76	140	1.151
3	129	1.2414	120	143	131	4.22	1136	9.081
4	152	1.6595	143	201	151	3.06	2096	12.140
5	20000 (UM)	0.1674	17561	29551	20636	5.11	1300	14.694

TIC:	3.0583	ng/uL
TIM:	35.9625	nmole/L
Total concentration:	3.6457	ng/uL

Sample peak width (sec): 5   Sample min peak height: 50   Sample baseline V to V?: Y   Sample baseline V to V points: 3
Sample filter: Binomial   Number of points for filter: 3 Sample start region (min): 0 Sample end region (min): 30
Marker peak width (sec): 5   Marker min peak height: 200   Marker baseline V to V?: Y   Marker baseline V to V points: 3
Lower marker selection: First peak > 500 RFU   Upper marker selection: Last peak > 200 RFU
Ladder size (bp)75, 200, 300, 400, 500, 700, 1000, 1500, 2000, 3000, 4000, 5000, 7000, 10000, 20000
Quantification using: Lower Marker   Final concentration (ng/uL): 0.5000   Dilution factor: 12.0

Sample: SampD2
Well location: D2
Created: Tuesday, August 10, 2021 11:20:27 PM

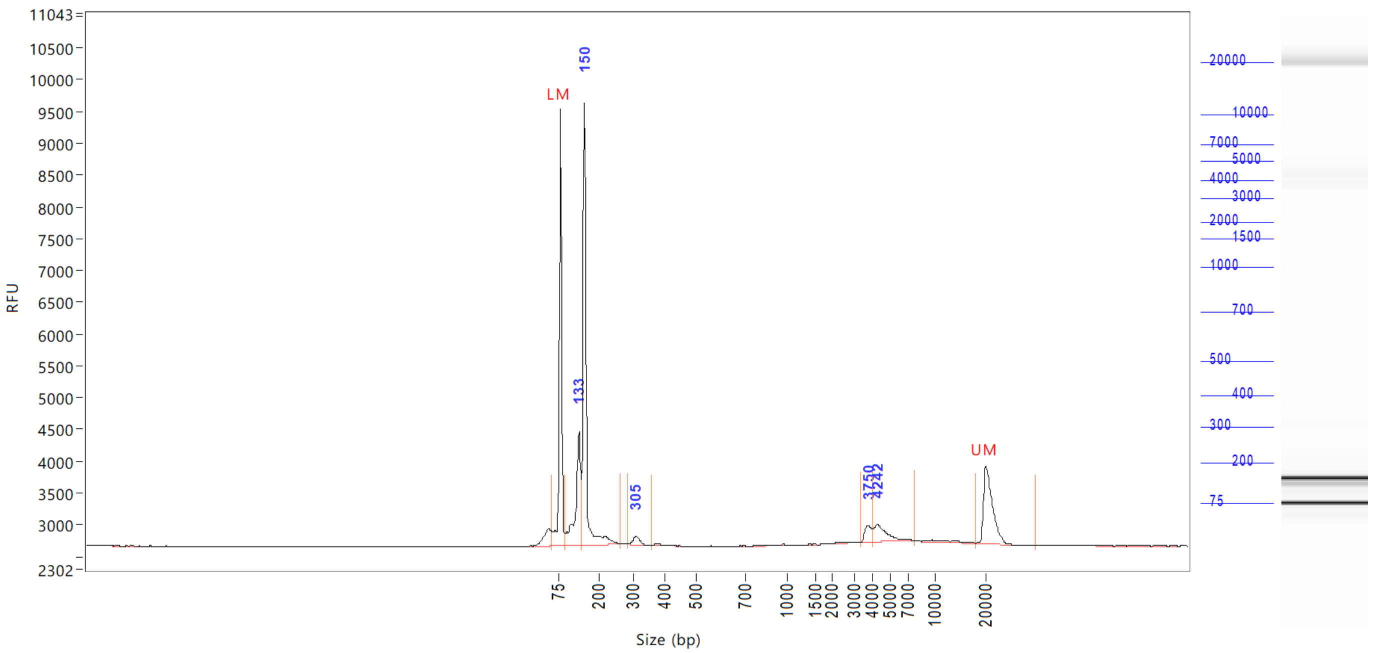

Peak	Size (bp)	Concentration (ng/uL)	From (bp)	To (bp)	Average size (bp)	CV%	RFU	Corrected peak area
1	75 (LM)	0.5000	52	89	73	6.86	6860	41.748
2	133	3.1952	89	141	125	10.34	1791	22.233
3	150	7.2040	141	260	154	11.56	6965	50.126
4	305	0.2755	282	358	306	3.89	127	1.917
5	3750	0.3851	3322	4001	3755	4.30	253	2.680
6	4242	0.7509	4001	7734	4661	14.18	273	5.225
7	20000 (UM)	0.1680	17927	29672	20644	5.28	1220	14.029

TIC:	11.8108	ng/uL
TIM:	120.7981	nmole/L
Total concentration:	12.0467	ng/uL

Sample peak width (sec): 5   Sample min peak height: 50   Sample baseline V to V?: Y   Sample baseline V to V points: 3
Sample filter: Binomial   Number of points for filter: 3 Sample start region (min): 0 Sample end region (min): 30
Marker peak width (sec): 5   Marker min peak height: 200   Marker baseline V to V?: Y   Marker baseline V to V points: 3
Lower marker selection: First peak > 500 RFU   Upper marker selection: Last peak > 200 RFU
Ladder size (bp)75, 200, 300, 400, 500, 700, 1000, 1500, 2000, 3000, 4000, 5000, 7000, 10000, 20000
Quantification using: Lower Marker   Final concentration (ng/uL): 0.5000   Dilution factor: 12.0

Sample: SampE2
Well location: E2
Created: Tuesday, August 10, 2021 11:20:27 PM

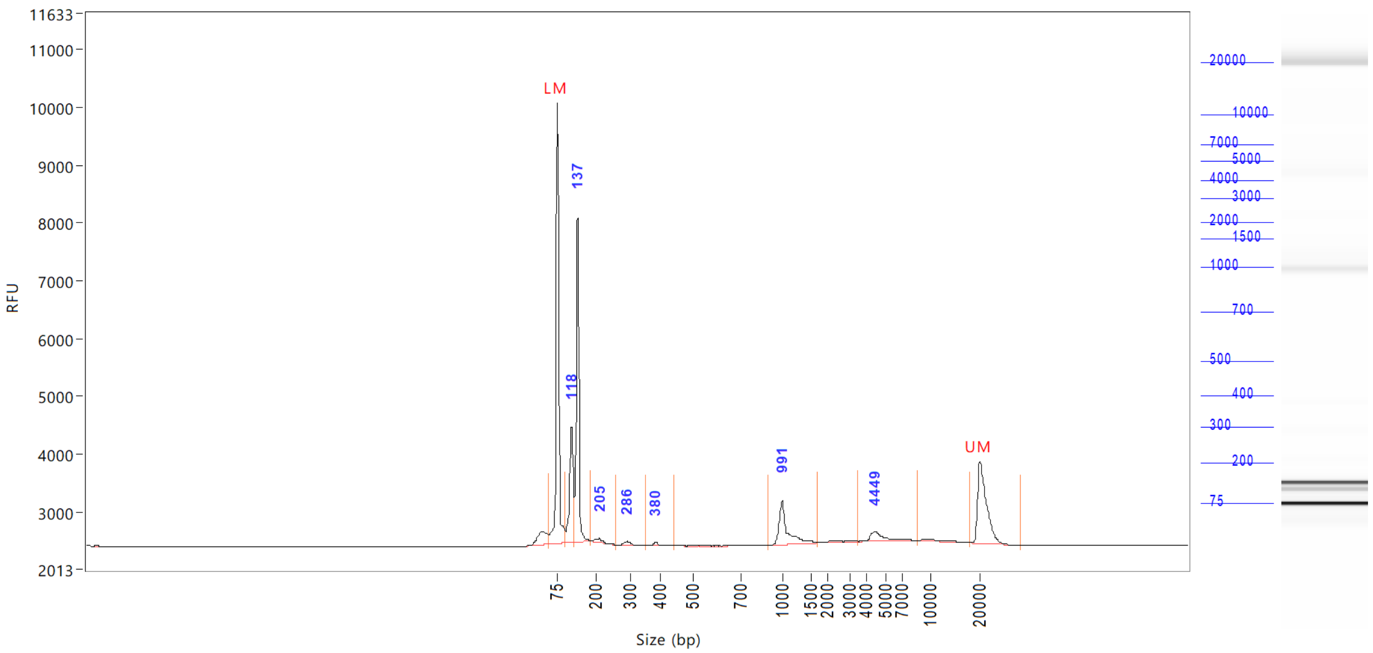

Peak	Size (bp)	Concentration (ng/uL)	From (bp)	To (bp)	Average size (bp)	CV%	RFU	Corrected peak area
1	75 (LM)	0.5000	48	99	73	9.11	7634	49.823
2	118	2.0763	99	126	115	5.33	1996	17.241
3	137	4.4563	126	179	135	4.20	5598	37.004
4	205	0.1295	179	253	204	6.51	70	1.076
5	286	0.1200	253	347	289	4.55	65	0.996
6	380	0.0809	347	437	381	3.55	65	0.672
7	991	1.2220	887	1712	1069	13.39	771	10.148
8	4449	0.4375	3500	8667	4990	20.66	167	3.633
9	20000 (UM)	0.1651	18171	28100	20660	5.10	1419	16.455

TIC:	8.5225	ng/uL
TIM:	88.0008	nmole/L
Total concentration:	8.6800	ng/uL

```
Sample peak width (sec): 5    Sample min peak height: 50    Sample baseline V to V?: Y    Sample baseline V to V points: 3
Sample filter: Binomial       Number of points for filter: 3  Sample start region (min): 0  Sample end region (min): 30
Marker peak width (sec): 5    Marker min peak height: 200   Marker baseline V to V?: Y    Marker baseline V to V points: 3
Lower marker selection: First peak > 500 RFU                Upper marker selection: Last peak > 200 RFU
Ladder size (bp)75, 200, 300, 400, 500, 700, 1000, 1500, 2000, 3000, 4000, 5000, 7000, 10000, 20000
Quantification using: Lower Marker          Final concentration (ng/uL): 0.5000          Dilution factor: 12.0
```

Sample: SampF2
Well location: F2
Created: Tuesday, August 10, 2021 11:20:27 PM

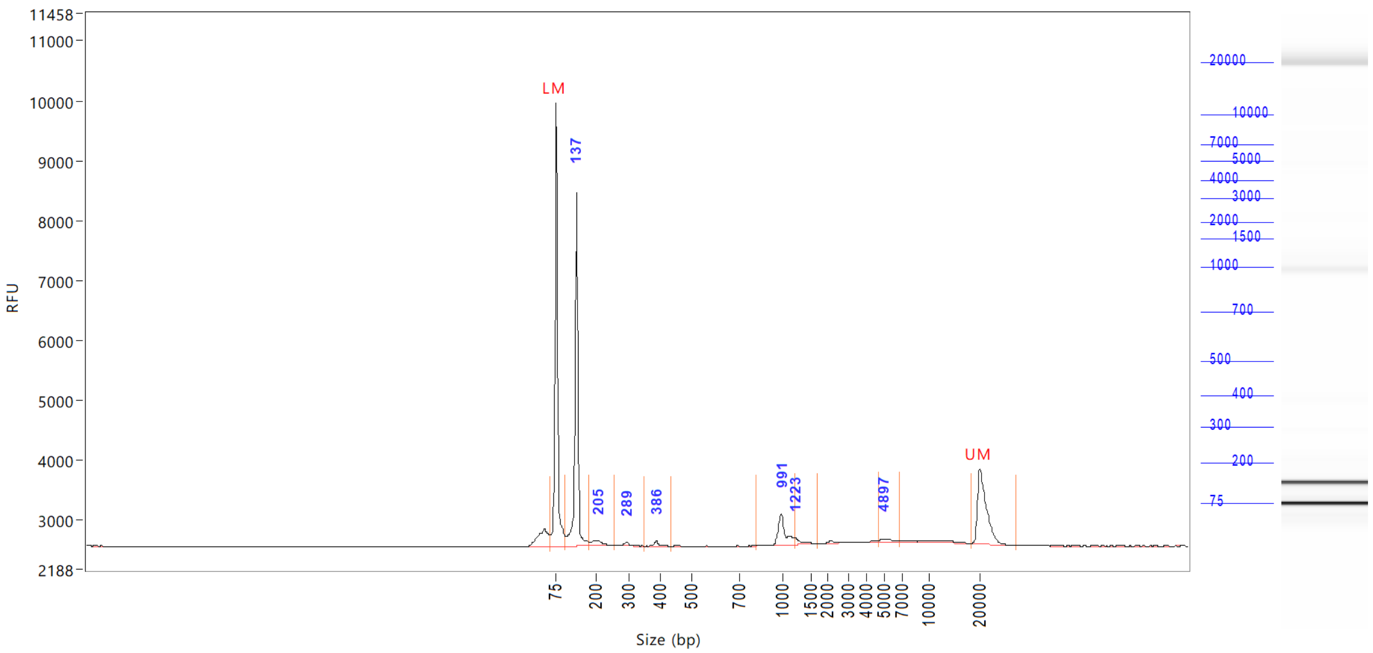

Peak	Size<br>(bp)	Concentration<br>(ng/uL)	From<br>(bp)	To<br>(bp)	Average size<br>(bp)	CV%	RFU	Corrected peak area
1	75 (LM)	0.5000	56	103	74	8.87	7405	48.712
2	137	5.2770	103	179	134	6.90	5892	42.843
3	205	0.2690	179	253	201	7.32	85	2.184
4	289	0.0968	253	345	288	5.17	67	0.786
5	386	0.1220	345	431	387	3.75	83	0.991
6	991	0.8028	814	1223	1024	7.28	517	6.518
7	1223	0.1277	1223	1693	1331	7.55	110	1.037
8	4897	0.1147	4621	6778	5394	11.18	55	0.931
9	20000 (UM)	0.1508	18293	27375	20652	5.22	1261	14.692

TIC:	6.8101	ng/uL
TIM:	69.4652	nmole/L
Total concentration:	7.1575	ng/uL

Sample peak width (sec): 5   Sample min peak height: 50   Sample baseline V to V?: Y   Sample baseline V to V points: 3
Sample filter: Binomial   Number of points for filter: 3 Sample start region (min): 0 Sample end region (min): 30
Marker peak width (sec): 5   Marker min peak height: 200   Marker baseline V to V?: Y   Marker baseline V to V points: 3
Lower marker selection: First peak > 500 RFU   Upper marker selection: Last peak > 200 RFU
Ladder size (bp)75, 200, 300, 400, 500, 700, 1000, 1500, 2000, 3000, 4000, 5000, 7000, 10000, 20000
Quantification using: Lower Marker   Final concentration (ng/uL): 0.5000   Dilution factor: 12.0

Sample: SampG2
Well location: G2
Created: Tuesday, August 10, 2021 11:20:27 PM

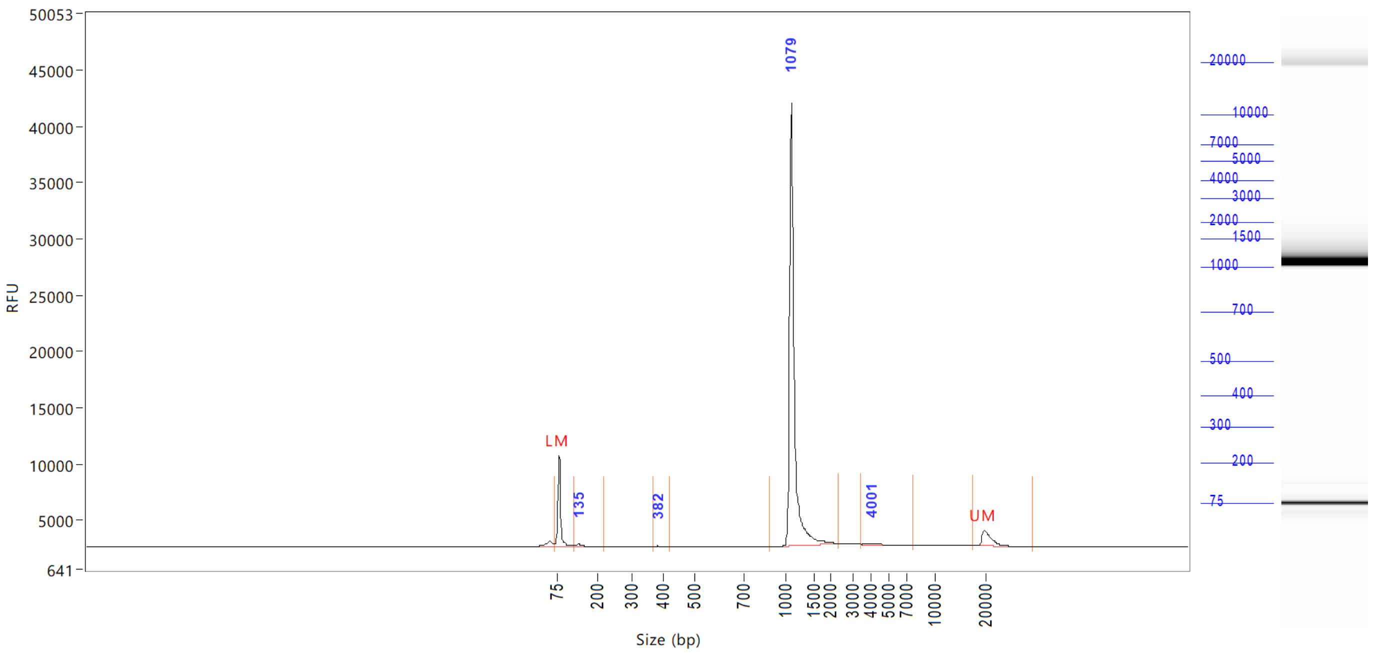

Peak	Size (bp)	Concentration (ng/uL)	From (bp)	To (bp)	Average size (bp)	CV%	RFU	Corrected peak area
1	75 (LM)	0.5000	64	124	76	11.51	8170	55.245
2	135	0.4971	124	215	155	16.63	240	4.577
3	382	0.1014	370	420	388	3.04	79	0.933
4	1079	33.2053	883	2315	1118	11.13	39437	305.736
5	4001	0.1523	3465	7667	4654	21.84	77	1.402
6	20000 (UM)	0.1439	17561	29430	20588	5.32	1376	15.895

TIC:	33.9560	ng/uL
TIM:	54.6418	nmole/L
Total concentration:	34.2140	ng/uL

Sample peak width (sec): 5 Sample min peak height: 50 Sample baseline V to V?: Y Sample baseline V to V points: 3
Sample filter: Binomial Number of points for filter: 3 Sample start region (min): 0 Sample end region (min): 30
Marker peak width (sec): 5 Marker min peak height: 200 Marker baseline V to V?: Y Marker baseline V to V points: 3
Lower marker selection: First peak > 500 RFU Upper marker selection: Last peak > 200 RFU
Ladder size (bp)75, 200, 300, 400, 500, 700, 1000, 1500, 2000, 3000, 4000, 5000, 7000, 10000, 20000
Quantification using: Lower Marker Final concentration (ng/uL): 0.5000 Dilution factor: 12.0

Sample: SampH2
Well location: H2
Created: Tuesday, August 10, 2021 11:20:27 PM

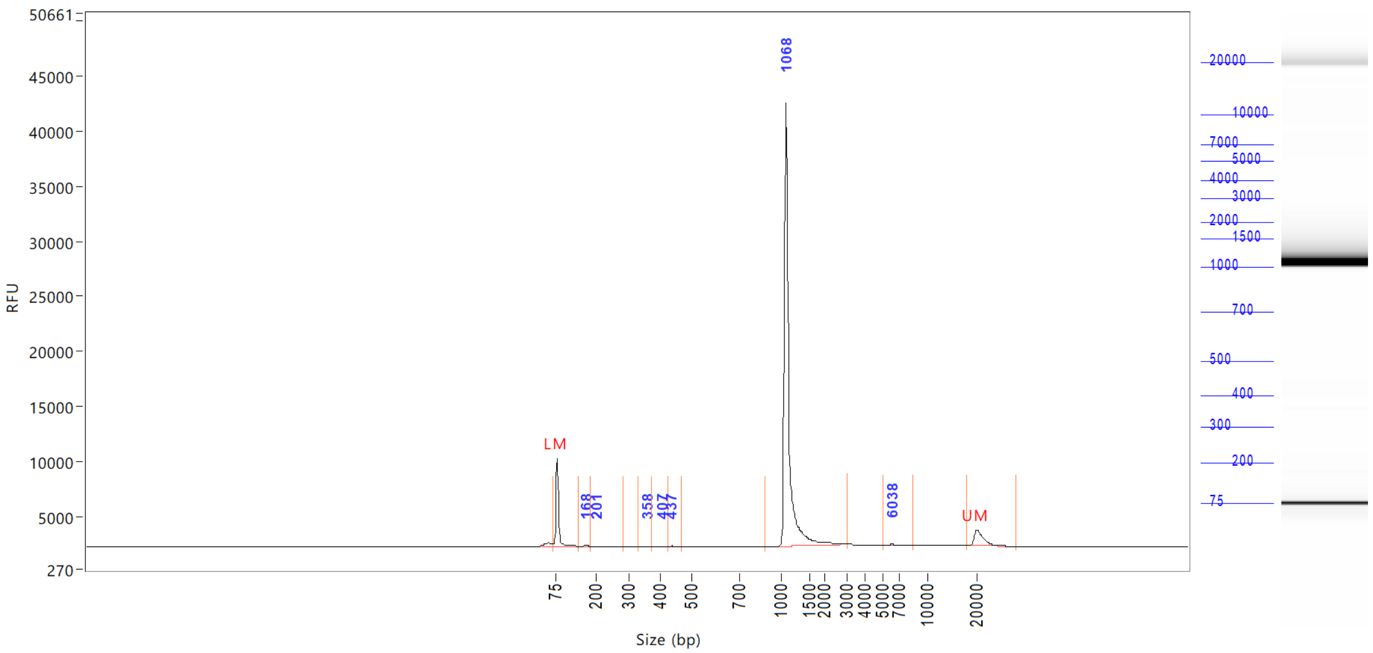

Peak	Size (bp)	Concentration (ng/uL)	From (bp)	To (bp)	Average size (bp)	CV%	RFU	Corrected peak area
1	75 (LM)	0.5000	64	145	77	15.24	8037	51.697
2	168	0.1856	145	183	164	6.21	103	1.599
3	201	0.1743	183	278	211	10.13	59	1.501
4	358	0.0355	327	374	354	1.51	55	0.306
5	407	0.0896	374	426	406	2.47	57	0.772
6	437	0.0564	426	468	437	1.91	74	0.486
7	1068	36.0206	883	3000	1122	14.86	40244	310.360
8	6038	0.1149	5001	8534	6560	12.44	52	0.990
9	20000 (UM)	0.1643	18049	27979	20657	5.38	1471	16.984

TIC:	36.6770	ng/uL
TIM:	56.8303	nmole/L
Total concentration:	36.8850	ng/uL

```
Sample peak width (sec): 5    Sample min peak height: 50    Sample baseline V to V?: Y    Sample baseline V to V points: 3
Sample filter: Binomial       Number of points for filter: 3  Sample start region (min): 0  Sample end region (min): 30
Marker peak width (sec): 5    Marker min peak height: 200   Marker baseline V to V?: Y    Marker baseline V to V points: 3
Lower marker selection: First peak > 500 RFU                Upper marker selection: Last peak > 200 RFU
Ladder size (bp)75, 200, 300, 400, 500, 700, 1000, 1500, 2000, 3000, 4000, 5000, 7000, 10000, 20000
Quantification using: Lower Marker          Final concentration (ng/uL): 0.5000          Dilution factor: 12.0
```

Sample: SampA3
Well location: A3
Created: Tuesday, August 10, 2021 11:20:27 PM

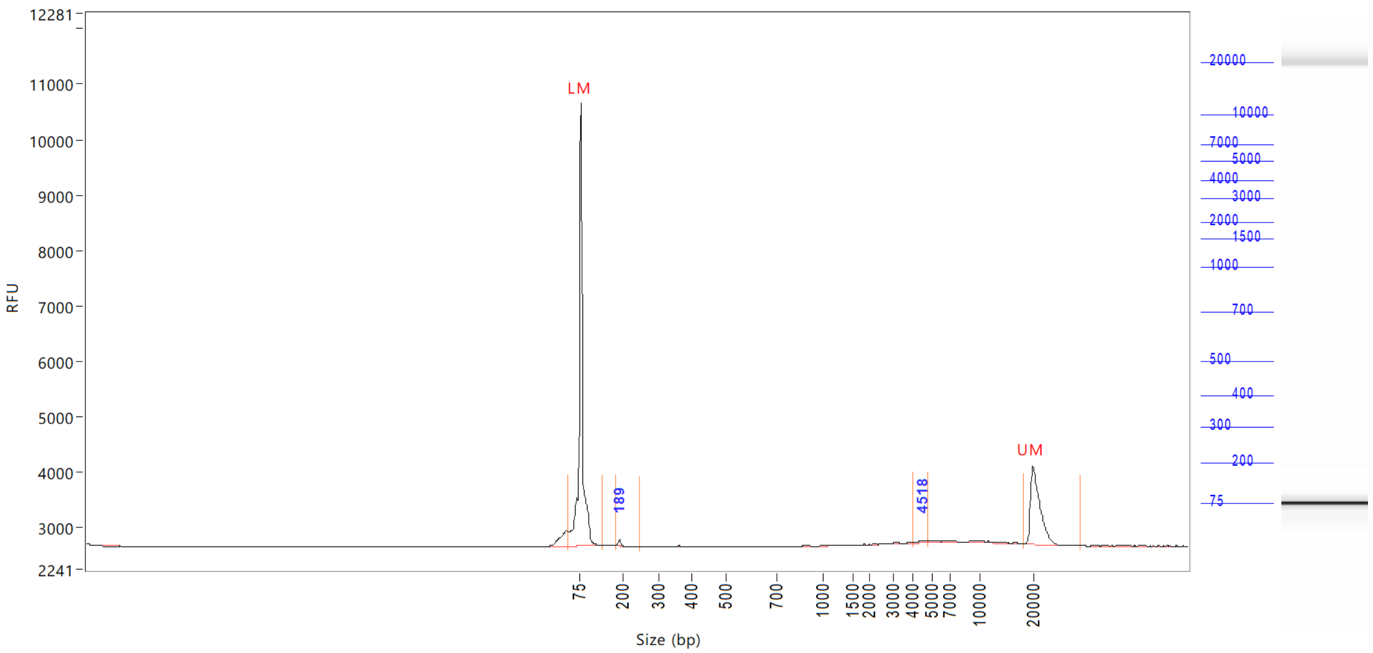

Peak	Size (bp)	Concentration (ng/uL)	From (bp)	To (bp)	Average size (bp)	CV%	RFU	Corrected peak area
1	75 (LM)	0.5000	39	139	73	14.37	8014	64.304
2	189	0.0621	179	248	190	6.52	116	0.666
3	4518	0.0463	4070	4793	4518	3.71	51	0.496
4	20000 (UM)	0.1277	18293	28947	20613	5.15	1424	16.420

TIC:	0.1084	ng/uL
TIM:	0.5545	nmole/L
Total concentration:	0.4798	ng/uL

Sample peak width (sec): 5 Sample min peak height: 50 Sample baseline V to V?: Y Sample baseline V to V points: 3
Sample filter: Binomial Number of points for filter: 3 Sample start region (min): 0 Sample end region (min): 30
Marker peak width (sec): 5 Marker min peak height: 200 Marker baseline V to V?: Y Marker baseline V to V points: 3
Lower marker selection: First peak > 500 RFU Upper marker selection: Last peak > 200 RFU
Ladder size (bp)75, 200, 300, 400, 500, 700, 1000, 1500, 2000, 3000, 4000, 5000, 7000, 10000, 20000
Quantification using: Lower Marker Final concentration (ng/uL): 0.5000 Dilution factor: 12.0

Sample: SampB3
Well location: B3
Created: Tuesday, August 10, 2021 11:20:27 PM

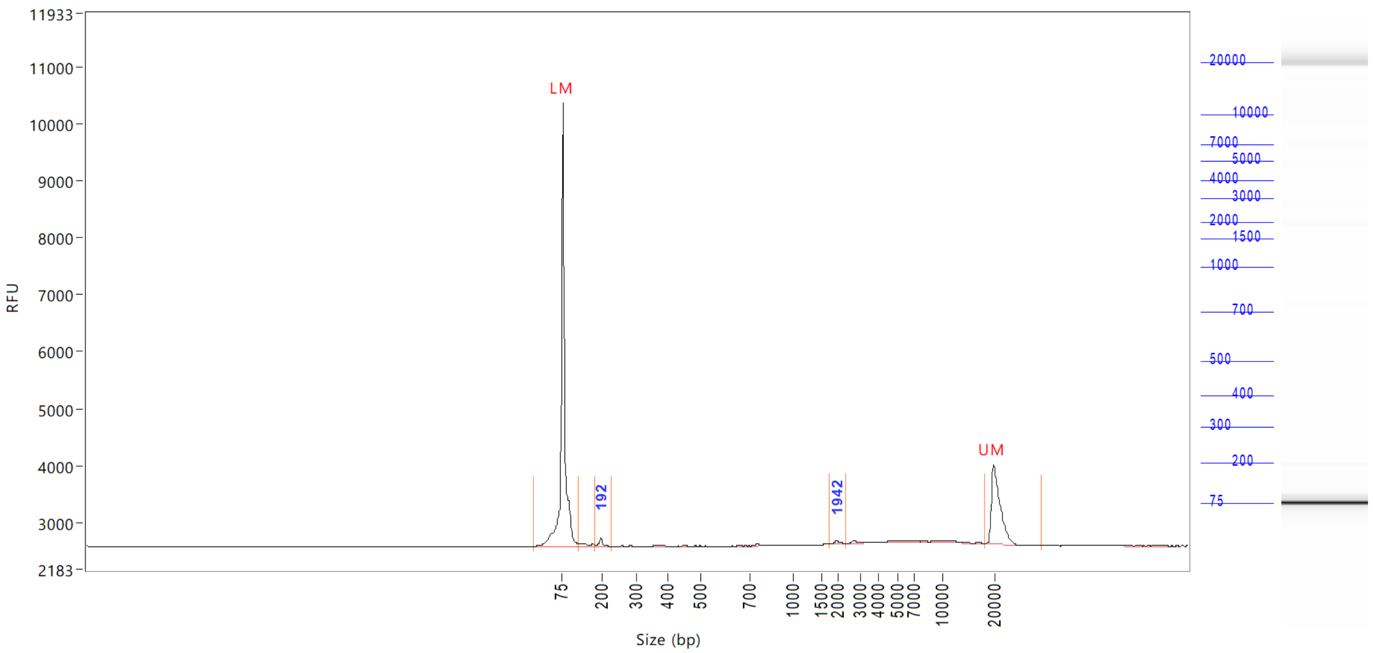

Peak	Size (bp)	Concentration (ng/uL)	From (bp)	To (bp)	Average size (bp)	CV%	RFU	Corrected peak area
1	75 (LM)	0.5000	0	128	73	20.02	7789	67.071
2	192	0.1120	177	224	191	3.90	161	1.252
3	1942	0.0446	1712	2344	1964	5.57	61	0.498
4	20000 (UM)	0.1157	18293	29189	20608	5.11	1391	15.514

TIC:	0.1566	ng/uL
TIM:	1.0015	nmole/L
Total concentration:	0.6490	ng/uL

Sample peak width (sec): 5 Sample min peak height: 50 Sample baseline V to V?: Y Sample baseline V to V points: 3
Sample filter: Binomial Number of points for filter: 3 Sample start region (min): 0 Sample end region (min): 30
Marker peak width (sec): 5 Marker min peak height: 200 Marker baseline V to V?: Y Marker baseline V to V points: 3
Lower marker selection: First peak > 500 RFU Upper marker selection: Last peak > 200 RFU
Ladder size (bp)75, 200, 300, 400, 500, 700, 1000, 1500, 2000, 3000, 4000, 5000, 7000, 10000, 20000
Quantification using: Lower Marker Final concentration (ng/uL): 0.5000 Dilution factor: 12.0

Sample: SampC3
Well location: C3
Created: Tuesday, August 10, 2021 11:20:27 PM

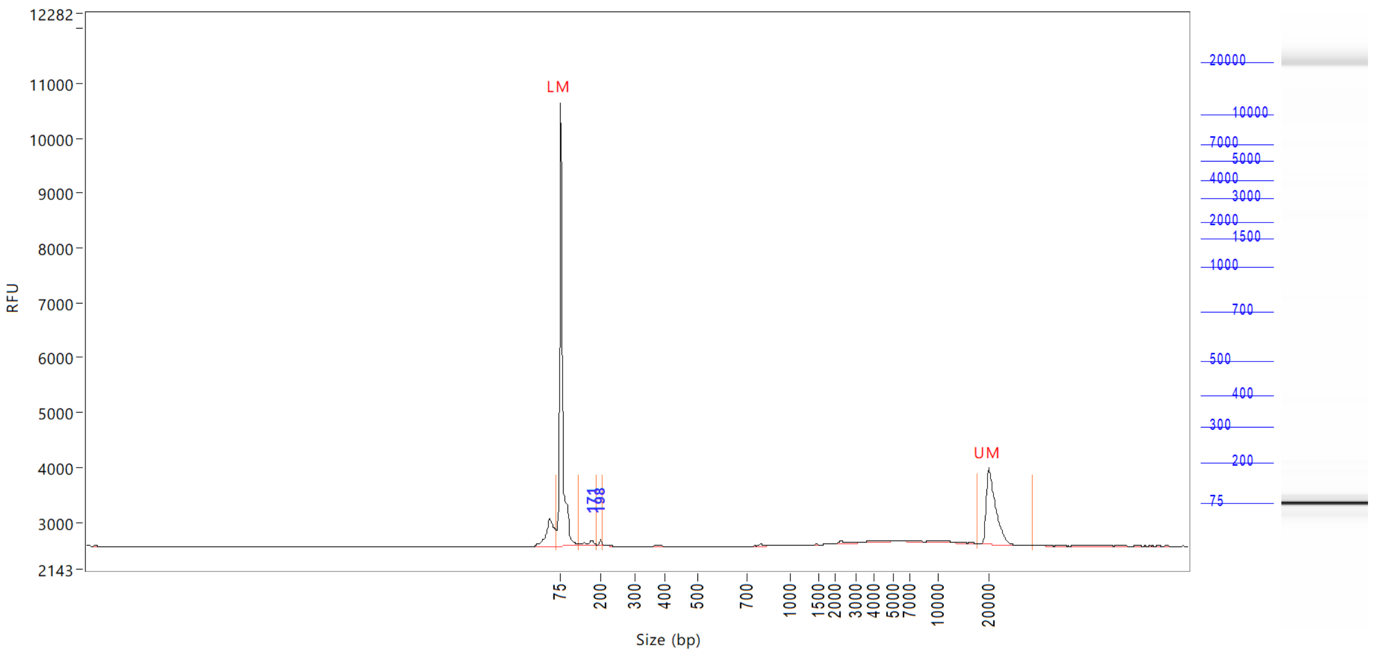

Peak	Size (bp)	Concentration (ng/uL)	From (bp)	To (bp)	Average size (bp)	CV%	RFU	Corrected peak area
1	75 (LM)	0.5000	62	133	76	12.13	8094	57.764
2	171	0.1889	133	189	161	8.93	100	1.818
3	198	0.0668	189	206	196	1.68	115	0.643
4	20000 (UM)	0.1431	17805	28584	20634	5.26	1393	16.538

TIC:	0.2557	ng/uL
TIM:	2.4886	nmole/L
Total concentration:	0.7758	ng/uL

Sample peak width (sec): 5 Sample min peak height: 50 Sample baseline V to V?: Y Sample baseline V to V points: 3
Sample filter: Binomial Number of points for filter: 3 Sample start region (min): 0 Sample end region (min): 30
Marker peak width (sec): 5 Marker min peak height: 200 Marker baseline V to V?: Y Marker baseline V to V points: 3
Lower marker selection: First peak > 500 RFU Upper marker selection: Last peak > 200 RFU
Ladder size (bp)75, 200, 300, 400, 500, 700, 1000, 1500, 2000, 3000, 4000, 5000, 7000, 10000, 20000
Quantification using: Lower Marker Final concentration (ng/uL): 0.5000 Dilution factor: 12.0

Sample: SampD3
Well location: D3
Created: Tuesday, August 10, 2021 11:20:27 PM

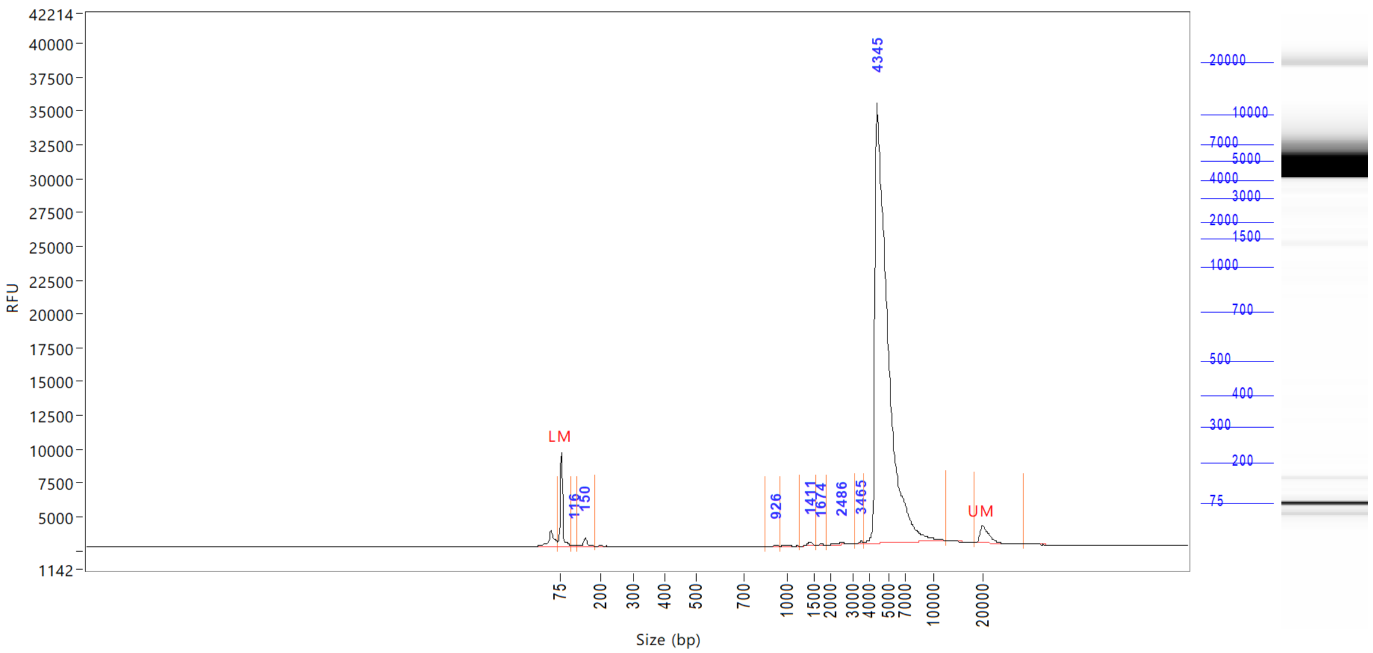

Peak	Size (bp)	Concentration (ng/uL)	From (bp)	To (bp)	Average size (bp)	CV%	RFU	Corrected peak area
1	75 (LM)	0.5000	64	107	74	7.83	6884	42.740
2	116	0.1394	107	124	115	4.30	114	0.993
3	150	0.7011	124	183	146	6.27	588	4.994
4	926	0.0780	848	952	917	2.13	59	0.555
5	1411	0.2998	1245	1520	1408	2.81	298	2.135
6	1674	0.0832	1520	1846	1654	3.92	76	0.592
7	2486	0.3249	1846	3108	2488	11.25	135	2.314
8	3465	0.1758	3108	3643	3449	3.58	176	1.253
9	4345	84.7033	3643	12684	4928	18.64	32577	603.374
10	20000 (UM)	0.1637	18415	28221	20516	4.95	1251	13.995

TIC:	86.5055	ng/uL
TIM:	39.0522	nmole/L
Total concentration:	86.9266	ng/uL

```
Sample peak width (sec): 5     Sample min peak height: 50    Sample baseline V to V?: Y    Sample baseline V to V points: 3
Sample filter: Binomial        Number of points for filter: 3  Sample start region (min): 0  Sample end region (min): 30
Marker peak width (sec): 5     Marker min peak height: 200   Marker baseline V to V?: Y    Marker baseline V to V points: 3
Lower marker selection: First peak > 500 RFU                 Upper marker selection: Last peak > 200 RFU
Ladder size (bp)75, 200, 300, 400, 500, 700, 1000, 1500, 2000, 3000, 4000, 5000, 7000, 10000, 20000
Quantification using: Lower Marker          Final concentration (ng/uL): 0.5000          Dilution factor: 12.0
```

Sample: SampE3
Well location: E3
Created: Tuesday, August 10, 2021 11:20:27 PM

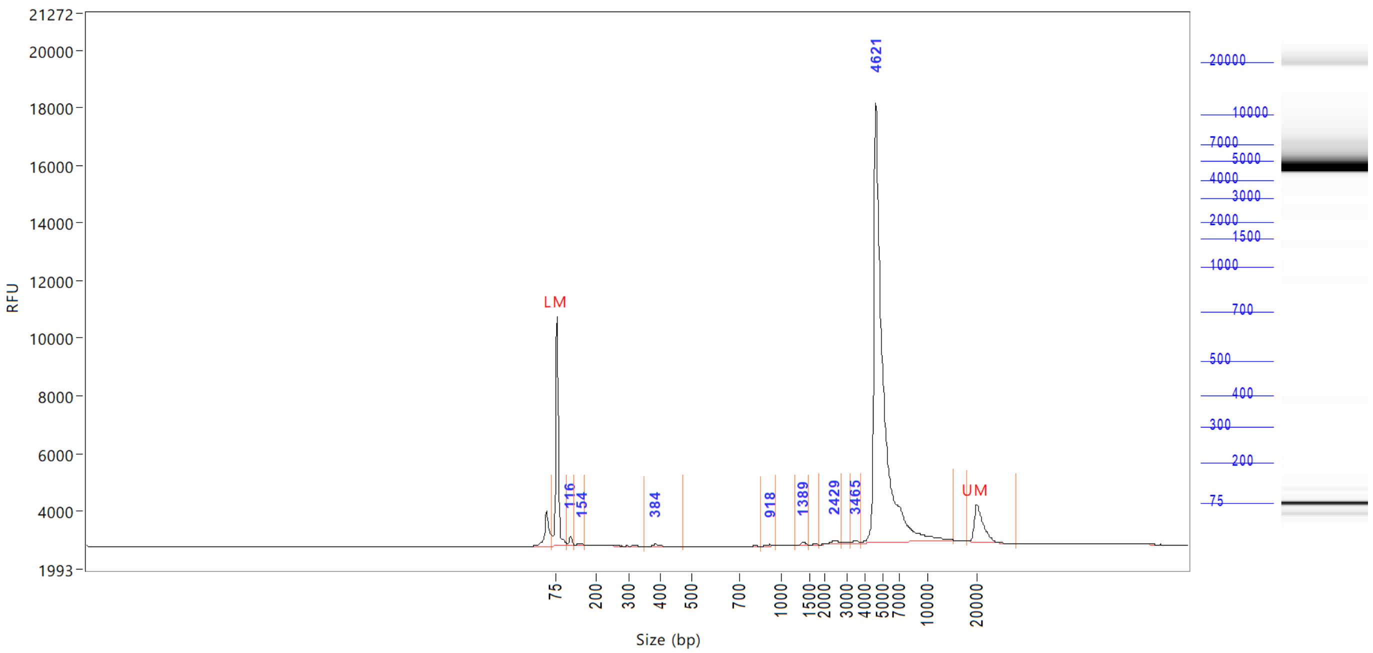

Peak	Size (bp)	Concentration (ng/uL)	From (bp)	To (bp)	Average size (bp)	CV%	RFU	Corrected peak area
1	75 (LM)	0.5000	62	107	74	7.92	7936	50.346
2	116	0.2920	107	131	115	4.35	347	2.451
3	154	0.1032	131	162	145	5.09	73	0.866
4	384	0.1127	347	473	391	5.61	69	0.946
5	918	0.0537	848	961	909	2.22	54	0.450
6	1389	0.1022	1245	1500	1386	2.91	120	0.857
7	2429	0.1951	1808	2772	2381	8.45	118	1.637
8	3465	0.0982	3144	3750	3451	4.64	85	0.824
9	4621	24.2943	3750	15122	5248	23.28	15276	203.854
10	20000 (UM)	0.1498	18049	27979	20626	5.28	1306	15.084

TIC:	25.2513	ng/uL
TIM:	13.8363	nmole/L
Total concentration:	25.5570	ng/uL

```
Sample peak width (sec): 5    Sample min peak height: 50    Sample baseline V to V?: Y    Sample baseline V to V points: 3
Sample filter: Binomial       Number of points for filter: 3  Sample start region (min): 0  Sample end region (min): 30
Marker peak width (sec): 5    Marker min peak height: 200   Marker baseline V to V?: Y    Marker baseline V to V points: 3
Lower marker selection: First peak > 500 RFU                Upper marker selection: Last peak > 200 RFU
Ladder size (bp)75, 200, 300, 400, 500, 700, 1000, 1500, 2000, 3000, 4000, 5000, 7000, 10000, 20000
Quantification using: Lower Marker          Final concentration (ng/uL): 0.5000          Dilution factor: 12.0
```

Sample: SampF3
Well location: F3
Created: Tuesday, August 10, 2021 11:20:27 PM

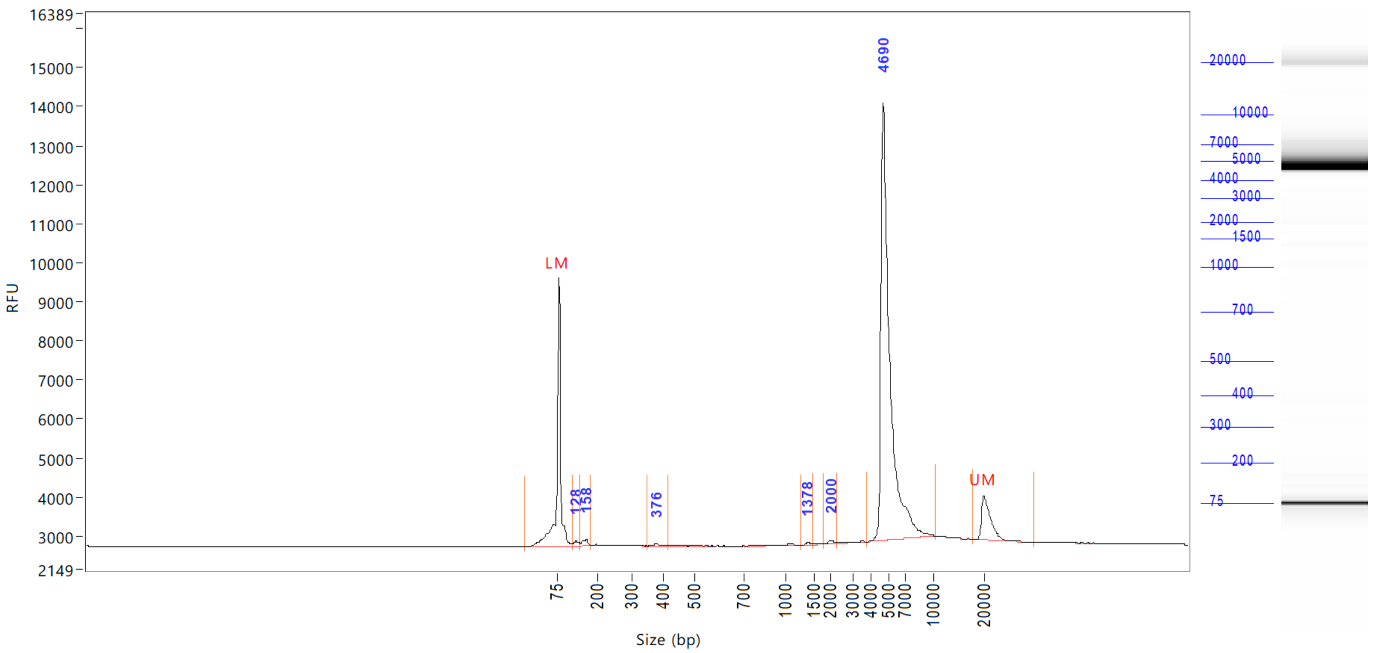

Peak	Size (bp)	Concentration (ng/uL)	From (bp)	To (bp)	Average size (bp)	CV%	RFU	Corrected peak area
1	75 (LM)	0.5000	0	116	67	27.04	6874	59.097
2	128	0.1609	116	141	128	5.44	134	1.585
3	158	0.2210	141	175	154	5.34	155	2.176
4	376	0.0967	349	416	381	3.80	71	0.953
5	1378	0.0545	1267	1478	1374	2.55	81	0.536
6	2000	0.0903	1770	2315	2039	5.59	99	0.889
7	4690	14.3698	3786	10489	5136	16.13	11225	141.535
8	20000 (UM)	0.1108	17683	29793	20640	5.69	1134	13.097

TIC:	14.9931	ng/uL
TIM:	9.5854	nmole/L
Total concentration:	15.3666	ng/uL

```
Sample peak width (sec): 5    Sample min peak height: 50    Sample baseline V to V?: Y    Sample baseline V to V points: 3
Sample filter: Binomial       Number of points for filter: 3  Sample start region (min): 0  Sample end region (min): 30
Marker peak width (sec): 5    Marker min peak height: 200   Marker baseline V to V?: Y    Marker baseline V to V points: 3
Lower marker selection: First peak > 500 RFU                Upper marker selection: Last peak > 200 RFU
Ladder size (bp)75, 200, 300, 400, 500, 700, 1000, 1500, 2000, 3000, 4000, 5000, 7000, 10000, 20000
Quantification using: Lower Marker          Final concentration (ng/uL): 0.5000          Dilution factor: 12.0
```

Sample: SampG3
Well location: G3
Created: Tuesday, August 10, 2021 11:20:27 PM

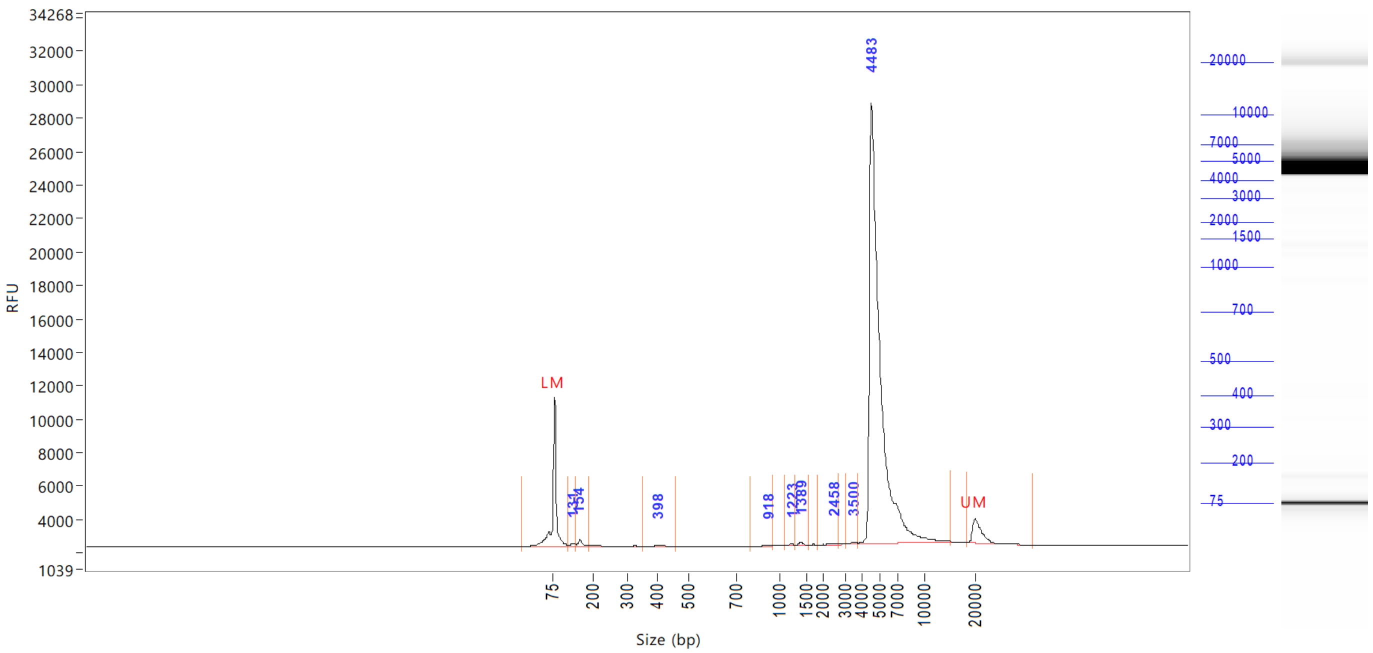

Peak	Size (bp)	Concentration (ng/uL)	From (bp)	To (bp)	Average size (bp)	CV%	RFU	Corrected peak area
1	75 (LM)	0.5000	0	116	69	23.64	8941	76.903
2	131	0.1655	116	145	131	6.42	132	2.121
3	154	0.3033	145	183	156	4.82	405	3.888
4	398	0.1041	352	456	408	5.53	55	1.335
5	918	0.0562	792	957	906	2.26	79	0.720
6	1223	0.0573	1101	1289	1211	2.70	117	0.734
7	1389	0.1311	1289	1520	1383	3.15	222	1.680
8	2458	0.0941	1846	2715	2342	9.49	92	1.206
9	3500	0.1055	3001	3750	3417	5.56	133	1.352
10	4483	31.4532	3750	15122	5099	21.92	26392	403.142
11	20000 (UM)	0.1087	18415	31607	20661	5.59	1438	16.726

TIC:	32.4703	ng/uL
TIM:	16.2993	nmole/L
Total concentration:	32.6844	ng/uL

Sample peak width (sec): 5 Sample min peak height: 50 Sample baseline V to V?: Y Sample baseline V to V points: 3
Sample filter: Binomial Number of points for filter: 3 Sample start region (min): 0 Sample end region (min): 30
Marker peak width (sec): 5 Marker min peak height: 200 Marker baseline V to V?: Y Marker baseline V to V points: 3
Lower marker selection: First peak > 500 RFU Upper marker selection: Last peak > 200 RFU
Ladder size (bp)75, 200, 300, 400, 500, 700, 1000, 1500, 2000, 3000, 4000, 5000, 7000, 10000, 20000
Quantification using: Lower Marker Final concentration (ng/uL): 0.5000 Dilution factor: 12.0

Sample: SampH3
Well location: H3
Created: Tuesday, August 10, 2021 11:20:27 PM

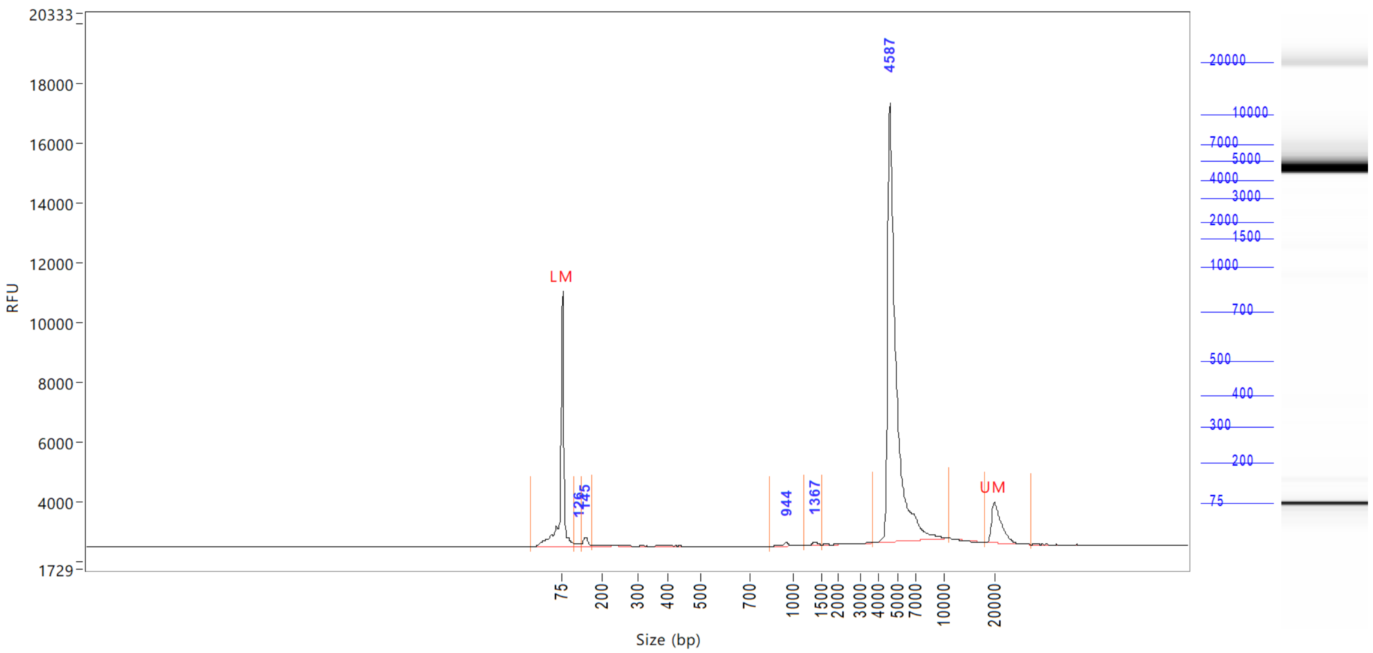

Peak	Size (bp)	Concentration (ng/uL)	From (bp)	To (bp)	Average size (bp)	CV%	RFU	Corrected peak area
1	75 (LM)	0.5000	0	112	66	26.33	8537	70.221
2	126	0.1163	112	133	123	5.27	100	1.361
3	145	0.2813	133	169	145	5.08	307	3.292
4	944	0.1186	835	1178	947	4.62	131	1.389
5	1367	0.0881	1178	1500	1368	3.55	122	1.031
6	4587	15.2368	3679	10977	4979	16.54	14714	178.324
7	20000 (UM)	0.1190	18049	27133	20612	5.37	1354	16.715

TIC:	15.8412	ng/uL
TIM:	10.0920	nmole/L
Total concentration:	16.1920	ng/uL

Sample peak width (sec): 5 Sample min peak height: 50 Sample baseline V to V?: Y Sample baseline V to V points: 3
Sample filter: Binomial Number of points for filter: 3 Sample start region (min): 0 Sample end region (min): 30
Marker peak width (sec): 5 Marker min peak height: 200 Marker baseline V to V?: Y Marker baseline V to V points: 3
Lower marker selection: First peak > 500 RFU Upper marker selection: Last peak > 200 RFU
Ladder size (bp)75, 200, 300, 400, 500, 700, 1000, 1500, 2000, 3000, 4000, 5000, 7000, 10000, 20000
Quantification using: Lower Marker Final concentration (ng/uL): 0.5000 Dilution factor: 12.0

Sample: SampA4
Well location: A4
Created: Tuesday, August 10, 2021 11:20:27 PM

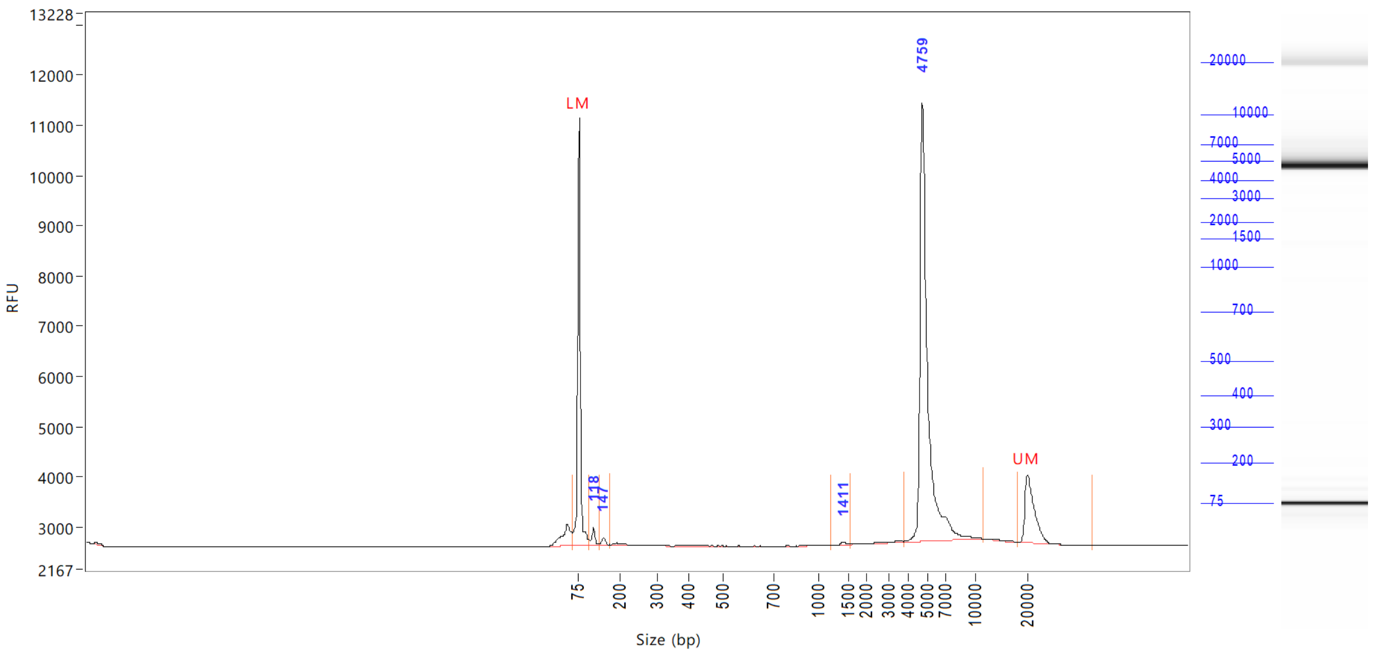

Peak	Size (bp)	Concentration (ng/uL)	From (bp)	To (bp)	Average size (bp)	CV%	RFU	Corrected peak area
1	75 (LM)	0.5000	56	107	73	8.45	8506	55.030
2	118	0.2907	107	135	116	4.98	352	2.667
3	147	0.1227	135	169	145	3.51	140	1.125
4	1411	0.0546	1212	1539	1395	3.40	63	0.501
5	4759	9.8890	3786	11464	5129	16.26	8739	90.698
6	20000 (UM)	0.1409	18171	32332	20650	5.87	1357	15.512

TIC:	10.3570	ng/uL
TIM:	8.7463	nmole/L
Total concentration:	10.7551	ng/uL

```
Sample peak width (sec): 5    Sample min peak height: 50    Sample baseline V to V?: Y    Sample baseline V to V points: 3
Sample filter: Binomial       Number of points for filter: 3  Sample start region (min): 0  Sample end region (min): 30
Marker peak width (sec): 5    Marker min peak height: 200   Marker baseline V to V?: Y    Marker baseline V to V points: 3
Lower marker selection: First peak > 500 RFU                Upper marker selection: Last peak > 200 RFU
Ladder size (bp)75, 200, 300, 400, 500, 700, 1000, 1500, 2000, 3000, 4000, 5000, 7000, 10000, 20000
Quantification using: Lower Marker          Final concentration (ng/uL): 0.5000          Dilution factor: 12.0
```

Sample: SampB4
Well location: B4
Created: Tuesday, August 10, 2021 11:20:27 PM

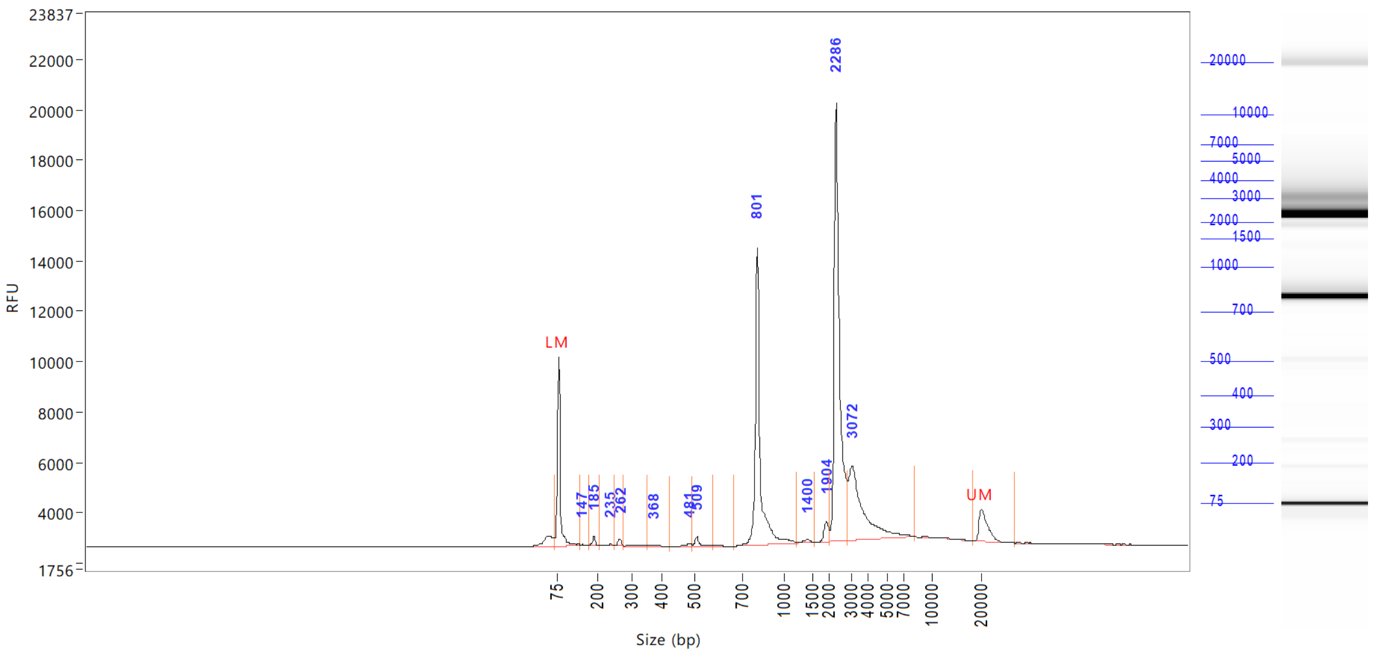

Peak	Size (bp)	Concentration (ng/uL)	From (bp)	To (bp)	Average size (bp)	CV%	RFU	Corrected peak area
1	75 (LM)	0.5000	64	141	76	14.24	7484	50.391
2	147	0.0909	141	169	154	5.53	51	0.763
3	185	0.2910	169	203	183	2.63	370	2.444
4	235	0.0789	203	248	231	4.27	62	0.663
5	262	0.2474	248	275	259	1.75	261	2.078
6	368	0.0687	352	420	371	3.62	52	0.577
7	481	0.1265	420	492	475	2.92	96	1.062
8	509	0.3866	492	575	512	3.07	372	3.247
9	801	11.9266	664	1223	818	6.80	11810	100.166
10	1400	0.1811	1223	1539	1358	4.85	121	1.521
11	1904	0.7517	1539	2001	1896	3.67	832	6.313
12	2286	17.9054	2001	2829	2354	7.39	17437	150.379
13	3072	7.9052	2829	8134	3605	23.69	2974	66.393
14	20000 (UM)	0.1420	18171	26770	20525	4.74	1282	14.315

TIC:	39.9600	ng/uL
TIM:	48.7025	nmole/L
Total concentration:	39.8418	ng/uL

Sample peak width (sec): 5 Sample min peak height: 50 Sample baseline V to V?: Y Sample baseline V to V points: 3
Sample filter: Binomial Number of points for filter: 3 Sample start region (min): 0 Sample end region (min): 30
Marker peak width (sec): 5 Marker min peak height: 200 Marker baseline V to V?: Y Marker baseline V to V points: 3
Lower marker selection: First peak > 500 RFU Upper marker selection: Last peak > 200 RFU
Ladder size (bp)75, 200, 300, 400, 500, 700, 1000, 1500, 2000, 3000, 4000, 5000, 7000, 10000, 20000
Quantification using: Lower Marker Final concentration (ng/uL): 0.5000 Dilution factor: 12.0

Sample: SampC4
Well location: C4
Created: Tuesday, August 10, 2021 11:20:27 PM

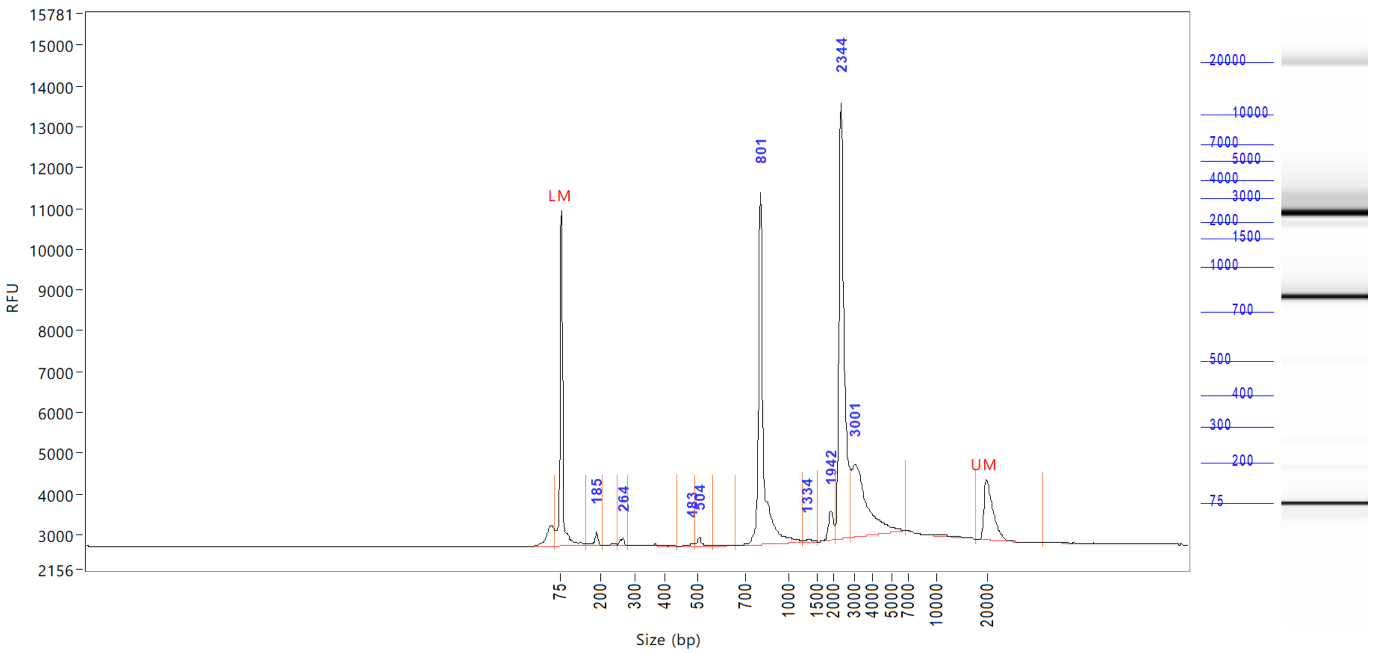

Peak	Size (bp)	Concentration (ng/uL)	From (bp)	To (bp)	Average size (bp)	CV%	RFU	Corrected peak area
1	75 (LM)	0.5000	56	152	75	16.48	8231	56.180
2	185	0.3056	152	203	178	5.38	348	2.861
3	264	0.1740	248	280	259	2.02	185	1.629
4	483	0.0674	433	489	475	2.30	58	0.631
5	504	0.2119	489	562	507	2.93	216	1.984
6	801	8.0569	654	1234	817	7.28	8620	75.440
7	1334	0.0950	1234	1520	1341	4.87	61	0.890
8	1942	0.6215	1520	2087	1928	4.30	710	5.820
9	2344	9.5927	2087	2829	2402	6.65	10701	89.820
10	3001	4.4380	2829	6704	3476	17.67	1782	41.555
11	20000 (UM)	0.1498	17805	31365	20626	5.37	1476	16.833

TIC:	23.5629	ng/uL
TIM:	30.4017	nmole/L
Total concentration:	23.7375	ng/uL

```
Sample peak width (sec): 5    Sample min peak height: 50    Sample baseline V to V?: Y    Sample baseline V to V points: 3
Sample filter: Binomial       Number of points for filter: 3  Sample start region (min): 0  Sample end region (min): 30
Marker peak width (sec): 5    Marker min peak height: 200   Marker baseline V to V?: Y    Marker baseline V to V points: 3
Lower marker selection: First peak > 500 RFU                Upper marker selection: Last peak > 200 RFU
Ladder size (bp)75, 200, 300, 400, 500, 700, 1000, 1500, 2000, 3000, 4000, 5000, 7000, 10000, 20000
Quantification using: Lower Marker          Final concentration (ng/uL): 0.5000          Dilution factor: 12.0
```

Sample: SampD4
Well location: D4
Created: Tuesday, August 10, 2021 11:20:27 PM

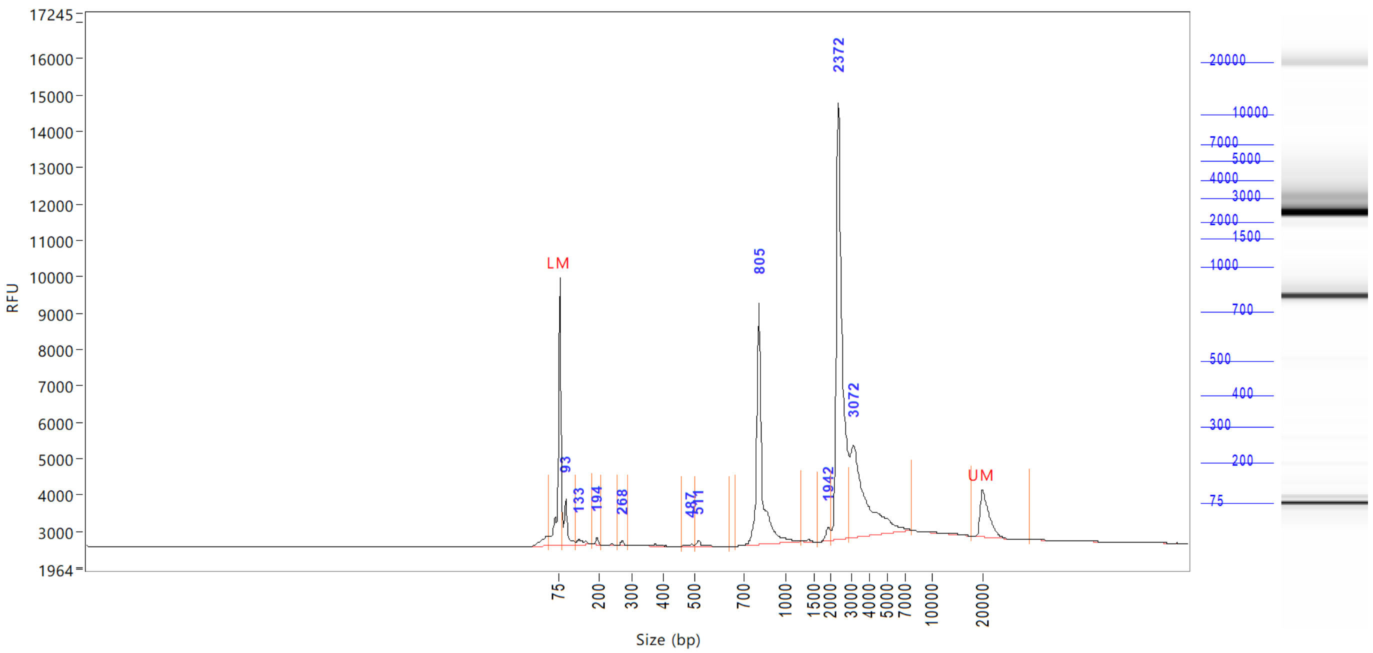

Peak	Size (bp)	Concentration (ng/uL)	From (bp)	To (bp)	Average size (bp)	CV%	RFU	Corrected peak area
1	75 (LM)	0.5000	45	86	72	10.01	7344	50.960
2	93	1.2573	86	126	96	9.33	1258	10.678
3	133	0.2908	126	181	143	8.54	149	2.470
4	194	0.1356	181	206	191	1.86	199	1.152
5	268	0.1110	255	286	265	1.54	137	0.943
6	487	0.0729	454	496	481	2.17	54	0.619
7	511	0.1798	496	641	522	5.49	154	1.527
8	805	7.4681	669	1278	821	6.65	6638	63.429
9	1942	0.3209	1597	2001	1913	3.45	358	2.726
10	2372	13.5477	2001	2886	2440	7.39	12019	115.064
11	3072	7.2081	2886	7734	3697	22.01	2540	61.221
12	20000 (UM)	0.1447	17805	29309	20549	5.15	1291	14.743

TIC:	30.5922	ng/uL
TIM:	55.1150	nmole/L
Total concentration:	30.6934	ng/uL

Sample peak width (sec): 5 Sample min peak height: 50 Sample baseline V to V?: Y Sample baseline V to V points: 3
Sample filter: Binomial Number of points for filter: 3 Sample start region (min): 0 Sample end region (min): 30
Marker peak width (sec): 5 Marker min peak height: 200 Marker baseline V to V?: Y Marker baseline V to V points: 3
Lower marker selection: First peak > 500 RFU Upper marker selection: Last peak > 200 RFU
Ladder size (bp)75, 200, 300, 400, 500, 700, 1000, 1500, 2000, 3000, 4000, 5000, 7000, 10000, 20000
Quantification using: Lower Marker Final concentration (ng/uL): 0.5000 Dilution factor: 12.0

Sample: SampE4
Well location: E4
Created: Tuesday, August 10, 2021 11:20:27 PM

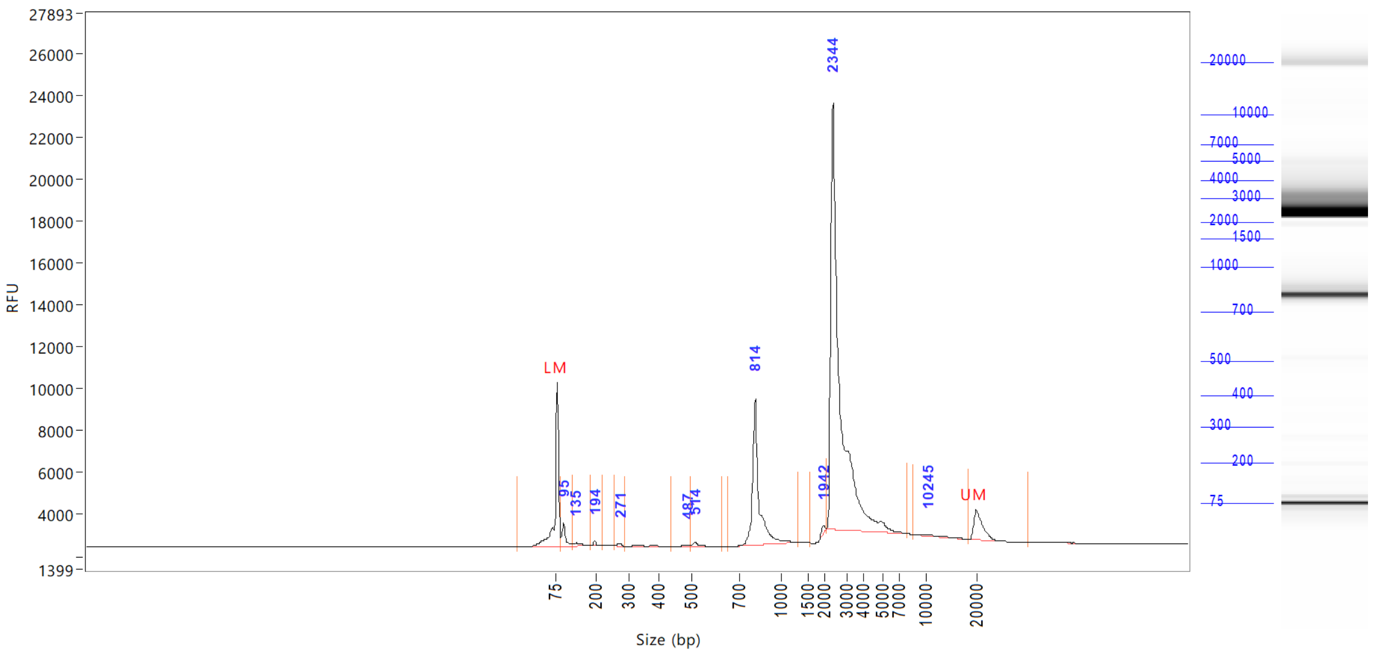

Peak	Size (bp)	Concentration (ng/uL)	From (bp)	To (bp)	Average size (bp)	CV%	RFU	Corrected peak area
1	75 (LM)	0.5000	0	86	66	24.91	7799	63.385
2	95	0.9728	86	128	97	10.23	1117	10.276
3	135	0.2664	128	183	147	9.78	144	2.814
4	194	0.1400	183	219	193	2.84	224	1.479
5	271	0.1174	253	284	265	1.97	139	1.240
6	487	0.0847	435	494	479	2.57	75	0.895
7	514	0.2164	494	623	520	4.68	224	2.286
8	814	7.1046	657	1311	830	6.97	6970	75.054
9	1942	0.2030	1501	2030	1912	2.38	400	2.145
10	2344	26.8630	2030	7867	2751	24.11	20361	283.784
11	10245	0.1873	8534	18415	11771	18.04	62	1.978
12	20000 (UM)	0.1280	18415	30519	20643	5.35	1413	16.226

TIC:	36.1555	ng/uL
TIM:	52.7049	nmole/L
Total concentration:	36.2844	ng/uL

```
Sample peak width (sec): 5    Sample min peak height: 50    Sample baseline V to V?: Y    Sample baseline V to V points: 3
Sample filter: Binomial       Number of points for filter: 3  Sample start region (min): 0  Sample end region (min): 30
Marker peak width (sec): 5    Marker min peak height: 200   Marker baseline V to V?: Y    Marker baseline V to V points: 3
Lower marker selection: First peak > 500 RFU                Upper marker selection: Last peak > 200 RFU
Ladder size (bp)75, 200, 300, 400, 500, 700, 1000, 1500, 2000, 3000, 4000, 5000, 7000, 10000, 20000
Quantification using: Lower Marker          Final concentration (ng/uL): 0.5000          Dilution factor: 12.0
```

Sample: SampF4
Well location: F4
Created: Tuesday, August 10, 2021 11:20:27 PM

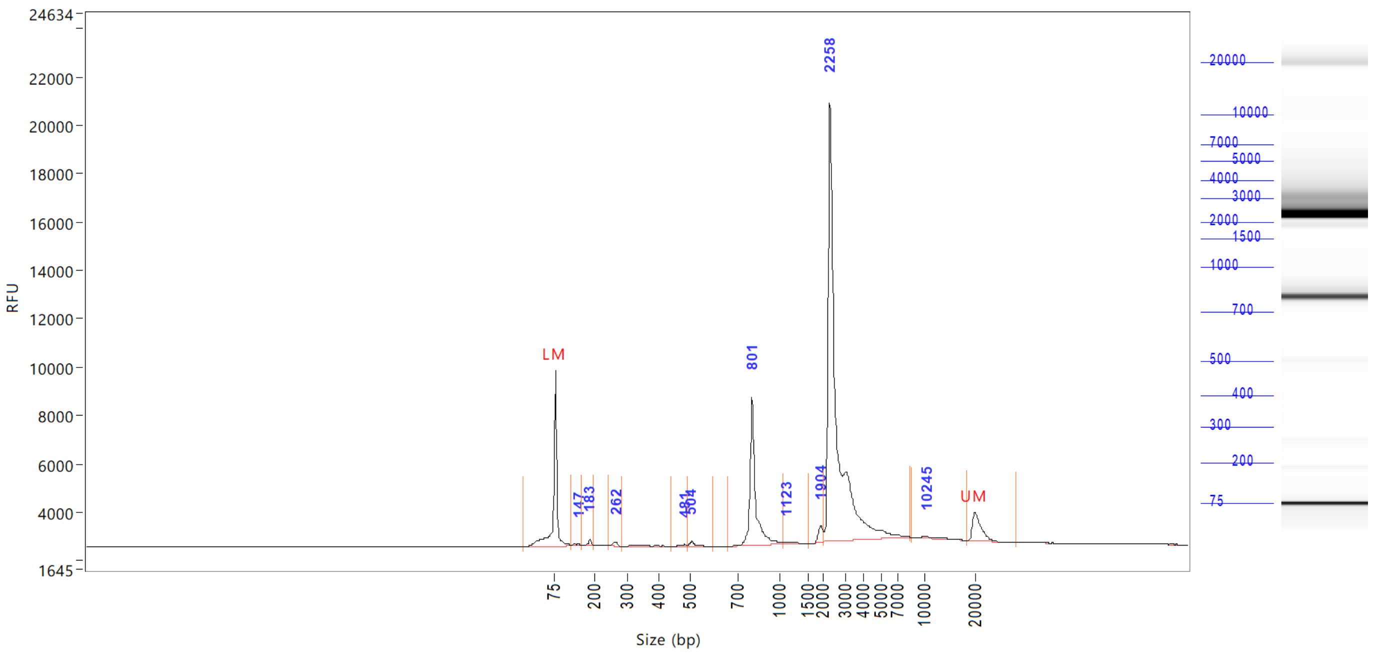

Peak	Size (bp)	Concentration (ng/uL)	From (bp)	To (bp)	Average size (bp)	CV%	RFU	Corrected peak area
1	75 (LM)	0.5000	0	124	65	30.78	7241	61.286
2	147	0.1490	124	158	140	6.64	104	1.522
3	183	0.2166	158	194	178	4.08	288	2.213
4	262	0.1764	241	280	256	2.40	183	1.802
5	481	0.0782	441	489	474	2.20	75	0.798
6	504	0.2025	489	593	510	3.87	213	2.069
7	801	5.6265	657	1056	812	5.73	6100	57.471
8	1123	0.1462	1056	1501	1208	8.97	72	1.493
9	1904	0.5123	1501	2001	1895	3.66	688	5.232
10	2258	22.3784	2001	8334	2714	28.27	18154	228.580
11	10245	0.1935	8534	18537	11758	17.07	61	1.976
12	20000 (UM)	0.1071	18537	28221	20594	5.01	1169	13.129

TIC:	29.6796	ng/uL
TIM:	31.4580	nmole/L
Total concentration:	29.7945	ng/uL

```
Sample peak width (sec): 5    Sample min peak height: 50    Sample baseline V to V?: Y    Sample baseline V to V points: 3
Sample filter: Binomial       Number of points for filter: 3  Sample start region (min): 0  Sample end region (min): 30
Marker peak width (sec): 5    Marker min peak height: 200   Marker baseline V to V?: Y    Marker baseline V to V points: 3
Lower marker selection: First peak > 500 RFU                Upper marker selection: Last peak > 200 RFU
Ladder size (bp)75, 200, 300, 400, 500, 700, 1000, 1500, 2000, 3000, 4000, 5000, 7000, 10000, 20000
Quantification using: Lower Marker          Final concentration (ng/uL): 0.5000          Dilution factor: 12.0
```

Sample: SampG4
Well location: G4
Created: Tuesday, August 10, 2021 11:20:27 PM

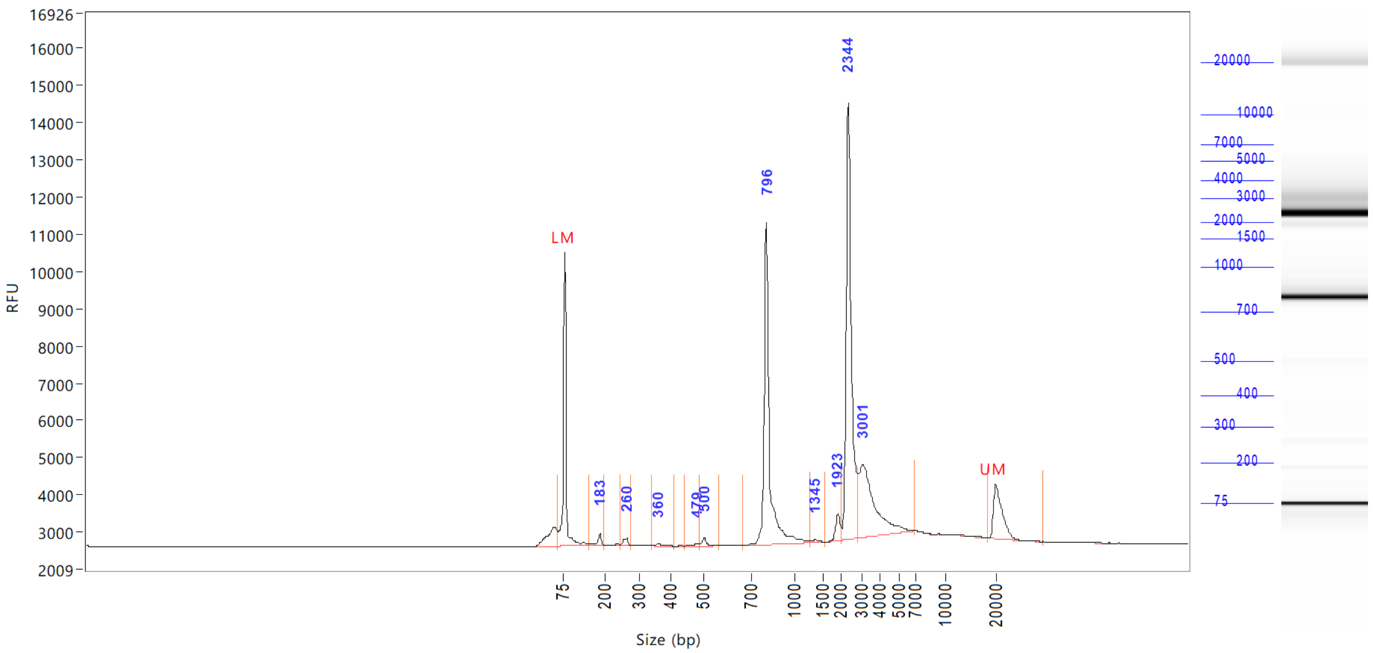

Peak	Size (bp)	Concentration (ng/uL)	From (bp)	To (bp)	Average size (bp)	CV%	RFU	Corrected peak area
1	75 (LM)	0.5000	54	152	74	16.39	7881	54.393
2	183	0.2780	152	198	177	4.86	335	2.521
3	260	0.2150	241	273	254	2.30	199	1.949
4	360	0.1062	339	409	363	3.65	67	0.963
5	479	0.0747	441	487	474	2.05	63	0.677
6	500	0.2093	487	562	503	2.64	216	1.897
7	796	8.3220	664	1267	816	7.82	8631	75.444
8	1345	0.0949	1267	1539	1354	4.20	71	0.861
9	1923	0.6090	1539	2058	1916	3.98	705	5.521
10	2344	10.7649	2058	2829	2391	6.75	11729	97.590
11	3001	5.0061	2829	6852	3485	18.08	1944	45.383
12	20000 (UM)	0.1492	18293	29189	20639	5.10	1450	16.231

TIC:	25.6801	ng/uL
TIM:	32.5997	nmole/L
Total concentration:	25.7252	ng/uL

```
Sample peak width (sec): 5    Sample min peak height: 50    Sample baseline V to V?: Y    Sample baseline V to V points: 3
Sample filter: Binomial       Number of points for filter: 3  Sample start region (min): 0  Sample end region (min): 30
Marker peak width (sec): 5    Marker min peak height: 200   Marker baseline V to V?: Y    Marker baseline V to V points: 3
Lower marker selection: First peak > 500 RFU                Upper marker selection: Last peak > 200 RFU
Ladder size (bp)75, 200, 300, 400, 500, 700, 1000, 1500, 2000, 3000, 4000, 5000, 7000, 10000, 20000
Quantification using: Lower Marker          Final concentration (ng/uL): 0.5000          Dilution factor: 12.0
```

Sample: SampH4
Well location: H4
Created: Tuesday, August 10, 2021 11:20:27 PM

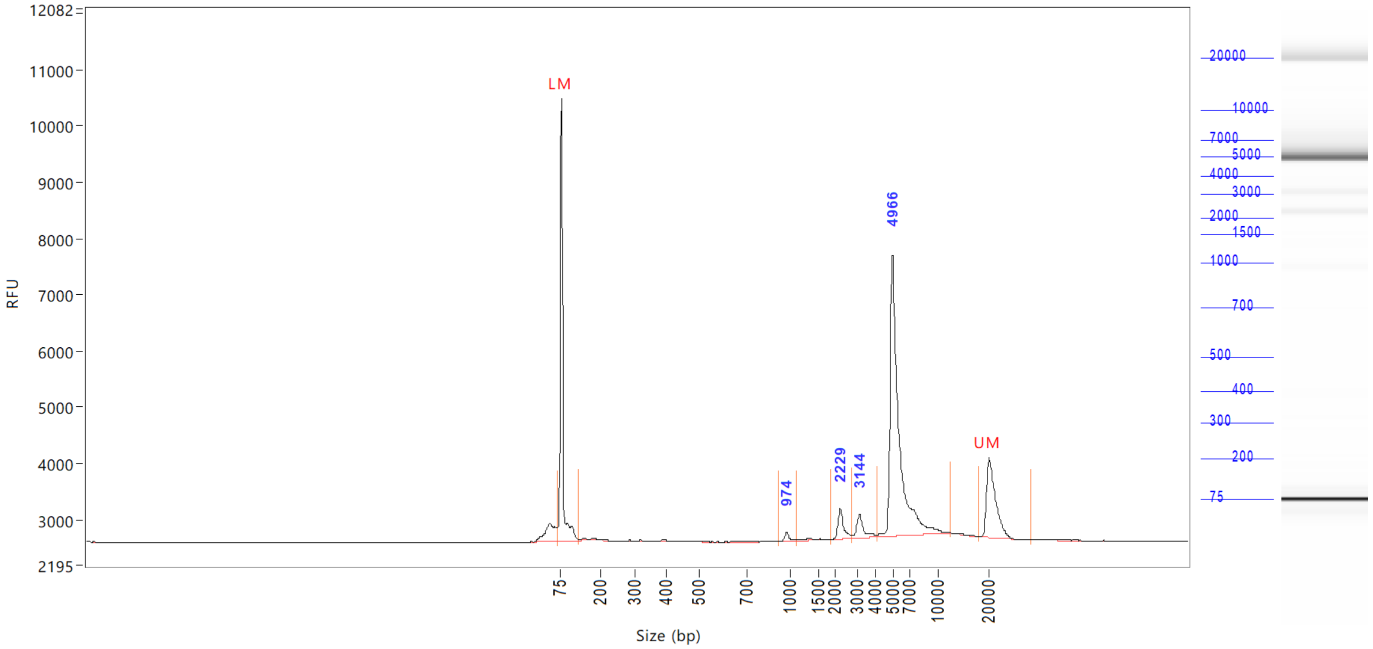

Peak	Size (bp)	Concentration (ng/uL)	From (bp)	To (bp)	Average size (bp)	CV%	RFU	Corrected peak area
1	75 (LM)	0.5000	64	128	76	12.33	7878	49.913
2	974	0.1501	922	1112	979	2.88	155	1.249
3	2229	0.5760	1904	2800	2276	7.69	549	4.791
4	3144	0.5221	2800	4104	3242	9.35	412	4.343
5	4966	7.5017	4104	12318	5606	19.32	4990	62.405
6	20000 (UM)	0.1620	18049	28463	20639	5.10	1409	16.172

TIC:	8.7498	ng/uL
TIM:	3.1370	nmole/L
Total concentration:	9.2546	ng/uL

```
Sample peak width (sec): 5    Sample min peak height: 50    Sample baseline V to V?: Y    Sample baseline V to V points: 3
Sample filter: Binomial       Number of points for filter: 3  Sample start region (min): 0  Sample end region (min): 30
Marker peak width (sec): 5    Marker min peak height: 200   Marker baseline V to V?: Y    Marker baseline V to V points: 3
Lower marker selection: First peak > 500 RFU                Upper marker selection: Last peak > 200 RFU
Ladder size (bp)75, 200, 300, 400, 500, 700, 1000, 1500, 2000, 3000, 4000, 5000, 7000, 10000, 20000
Quantification using: Lower Marker          Final concentration (ng/uL): 0.5000          Dilution factor: 12.0
```

Sample: SampA5
Well location: A5
Created: Tuesday, August 10, 2021 11:20:27 PM

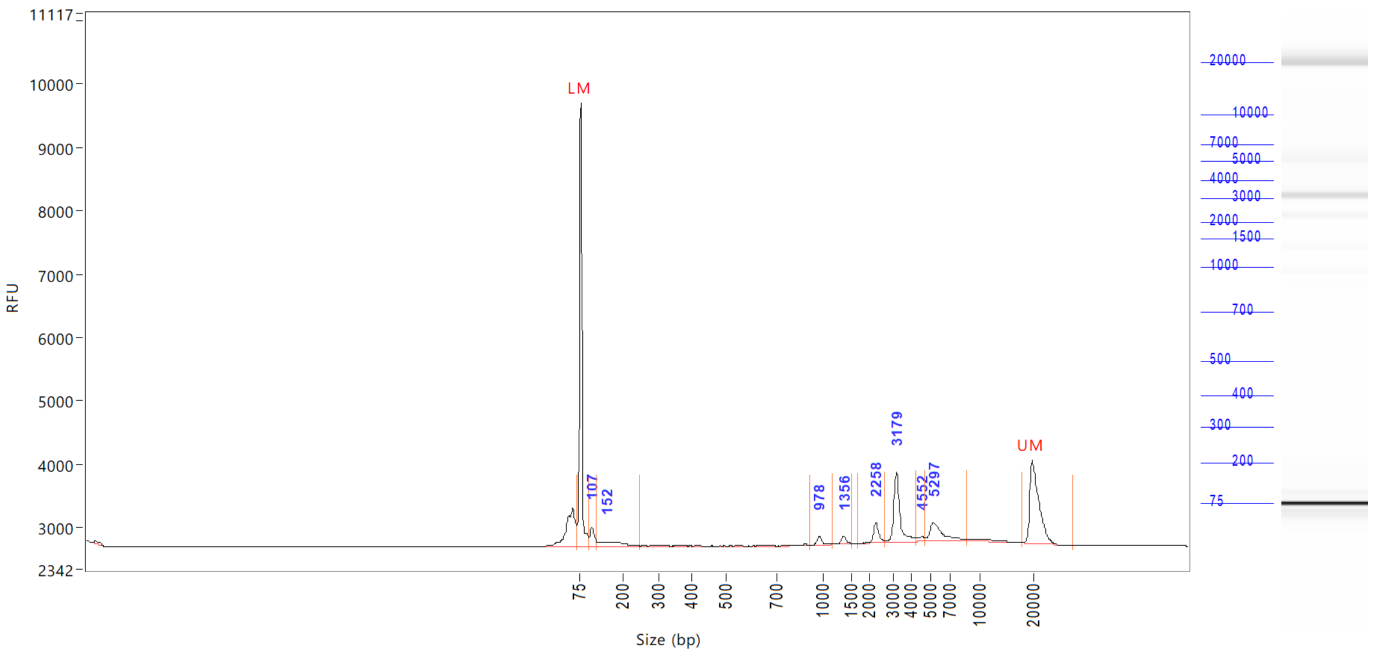

Peak	Size (bp)	Concentration (ng/uL)	From (bp)	To (bp)	Average size (bp)	CV%	RFU	Corrected peak area
1	75 (LM)	0.5000	64	97	73	6.60	7008	41.161
2	107	0.4823	97	124	107	6.26	306	3.309
3	152	0.5215	124	246	165	15.64	72	3.578
4	978	0.1472	913	1167	980	2.78	137	1.010
5	1356	0.1378	1167	1501	1348	3.70	122	0.946
6	2258	0.3780	1655	2686	2262	6.69	321	2.593
7	3179	1.4340	2686	4242	3236	8.76	1097	9.837
8	4552	0.0977	4242	4690	4464	2.98	74	0.670
9	5297	0.7344	4690	8734	5826	15.92	289	5.038
10	20000 (UM)	0.1782	18049	27617	20539	4.90	1306	14.673

TIC:	3.9328	ng/uL
TIM:	14.2617	nmole/L
Total concentration:	4.2847	ng/uL

Sample peak width (sec): 5 Sample min peak height: 50 Sample baseline V to V?: Y Sample baseline V to V points: 3
Sample filter: Binomial Number of points for filter: 3 Sample start region (min): 0 Sample end region (min): 30
Marker peak width (sec): 5 Marker min peak height: 200 Marker baseline V to V?: Y Marker baseline V to V points: 3
Lower marker selection: First peak > 500 RFU Upper marker selection: Last peak > 200 RFU
Ladder size (bp)75, 200, 300, 400, 500, 700, 1000, 1500, 2000, 3000, 4000, 5000, 7000, 10000, 20000
Quantification using: Lower Marker Final concentration (ng/uL): 0.5000 Dilution factor: 12.0

Sample: SampB5
Well location: B5
Created: Tuesday, August 10, 2021 11:20:27 PM

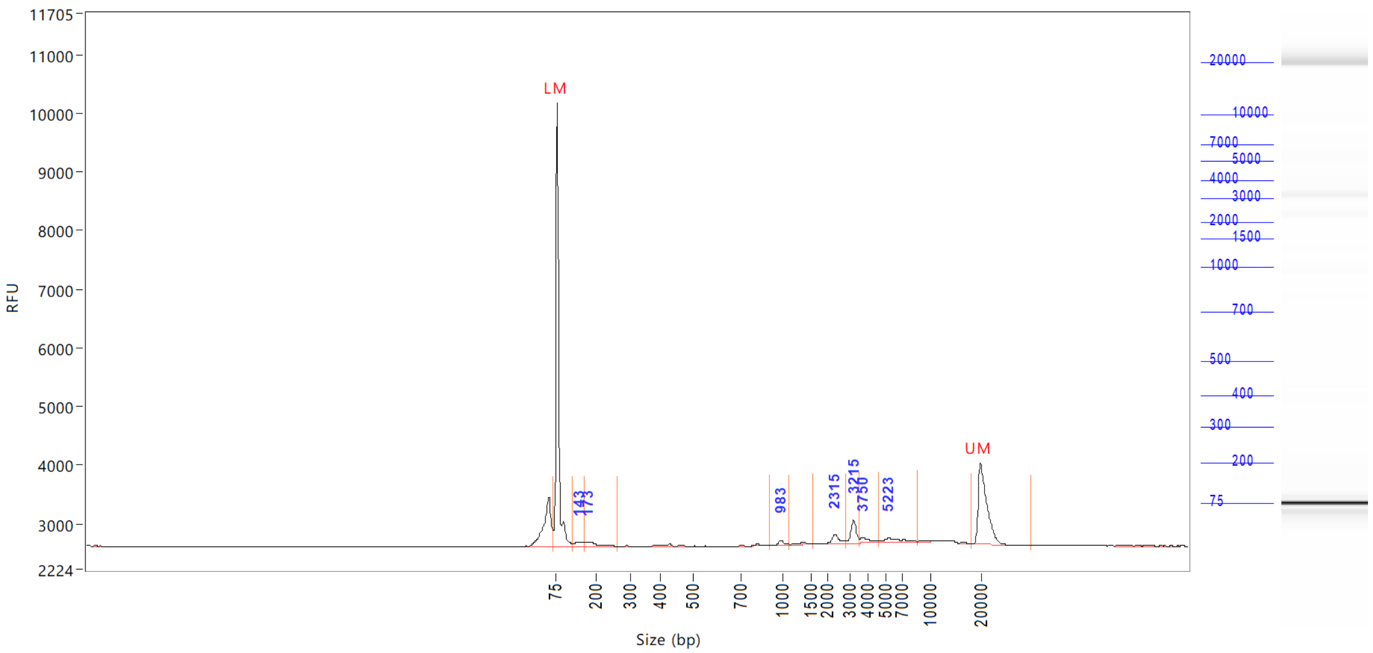

Peak	Size (bp)	Concentration (ng/uL)	From (bp)	To (bp)	Average size (bp)	CV%	RFU	Corrected peak area
1	75 (LM)	0.5000	64	124	76	10.66	7574	48.637
2	143	0.1894	124	162	143	7.87	64	1.536
3	173	0.2289	162	260	189	11.07	65	1.856
4	983	0.1001	909	1112	989	3.37	84	0.811
5	2315	0.2278	1539	2829	2344	9.47	158	1.847
6	3215	0.4178	2829	3572	3188	4.96	401	3.387
7	3750	0.1722	3572	4621	3989	7.39	88	1.396
8	5223	0.2138	4621	8600	5991	16.51	70	1.733
9	20000 (UM)	0.1602	18171	30035	20675	5.43	1381	15.587

TIC:	1.5501	ng/uL
TIM:	4.8407	nmole/L
Total concentration:	1.8797	ng/uL

Sample peak width (sec): 5   Sample min peak height: 50   Sample baseline V to V?: Y   Sample baseline V to V points: 3
Sample filter: Binomial   Number of points for filter: 3   Sample start region (min): 0   Sample end region (min): 30
Marker peak width (sec): 5   Marker min peak height: 200   Marker baseline V to V?: Y   Marker baseline V to V points: 3
Lower marker selection: First peak > 500 RFU   Upper marker selection: Last peak > 200 RFU
Ladder size (bp)75, 200, 300, 400, 500, 700, 1000, 1500, 2000, 3000, 4000, 5000, 7000, 10000, 20000
Quantification using: Lower Marker   Final concentration (ng/uL): 0.5000   Dilution factor: 12.0

Sample: SampC5
Well location: C5
Created: Tuesday, August 10, 2021 11:20:27 PM

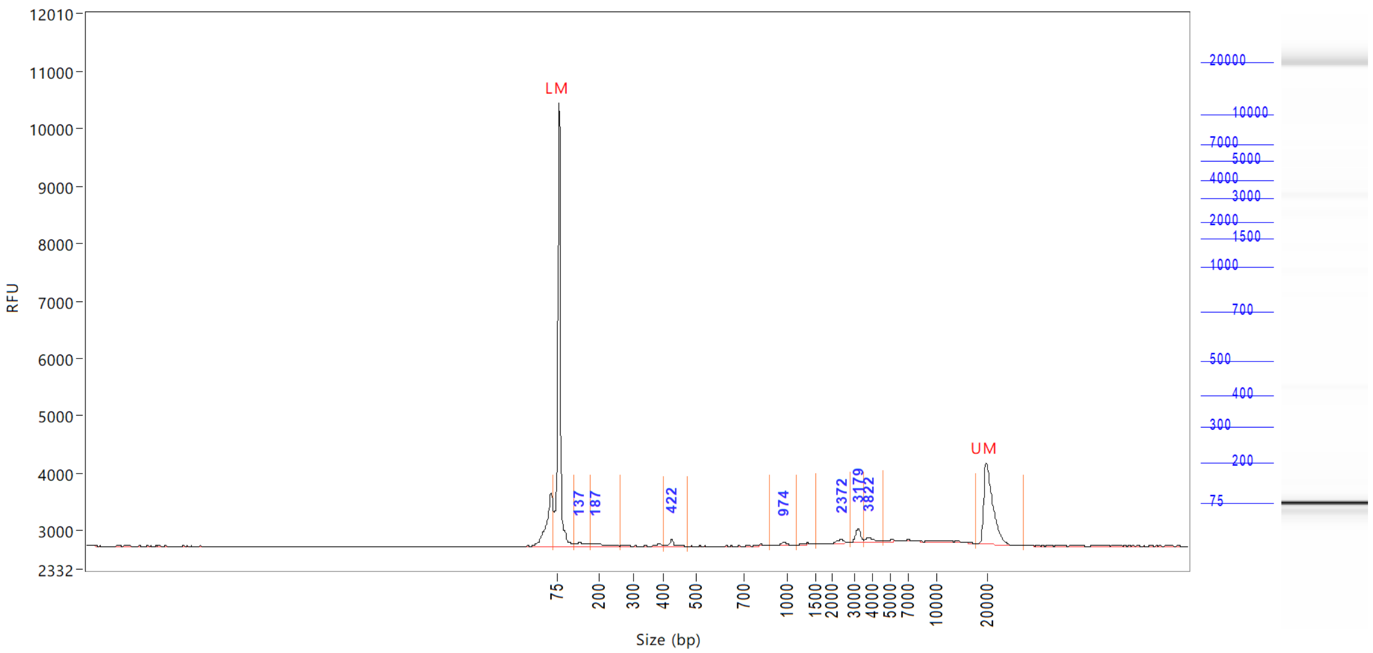

Peak	Size (bp)	Concentration (ng/uL)	From (bp)	To (bp)	Average size (bp)	CV%	RFU	Corrected peak area
1	75 (LM)	0.5000	60	122	74	9.74	7728	51.479
2	137	0.2012	122	175	147	10.67	54	1.726
3	187	0.1463	175	260	200	9.59	52	1.256
4	422	0.1376	398	471	422	3.21	123	1.181
5	974	0.0692	874	1156	976	3.28	60	0.594
6	2372	0.1016	1500	2829	2313	10.18	62	0.872
7	3179	0.2223	2829	3536	3166	4.63	235	1.907
8	3822	0.1274	3536	4621	3953	7.06	78	1.093
9	20000 (UM)	0.1586	17927	27254	20585	5.13	1415	16.327

TIC:	1.0056	ng/uL
TIM:	4.3462	nmole/L
Total concentration:	1.3944	ng/uL

Sample peak width (sec): 5   Sample min peak height: 50   Sample baseline V to V?: Y   Sample baseline V to V points: 3
Sample filter: Binomial   Number of points for filter: 3 Sample start region (min): 0 Sample end region (min): 30
Marker peak width (sec): 5   Marker min peak height: 200   Marker baseline V to V?: Y   Marker baseline V to V points: 3
Lower marker selection: First peak > 500 RFU   Upper marker selection: Last peak > 200 RFU
Ladder size (bp)75, 200, 300, 400, 500, 700, 1000, 1500, 2000, 3000, 4000, 5000, 7000, 10000, 20000
Quantification using: Lower Marker   Final concentration (ng/uL): 0.5000   Dilution factor: 12.0

Sample: SampD5
Well location: D5
Created: Tuesday, August 10, 2021 11:20:27 PM

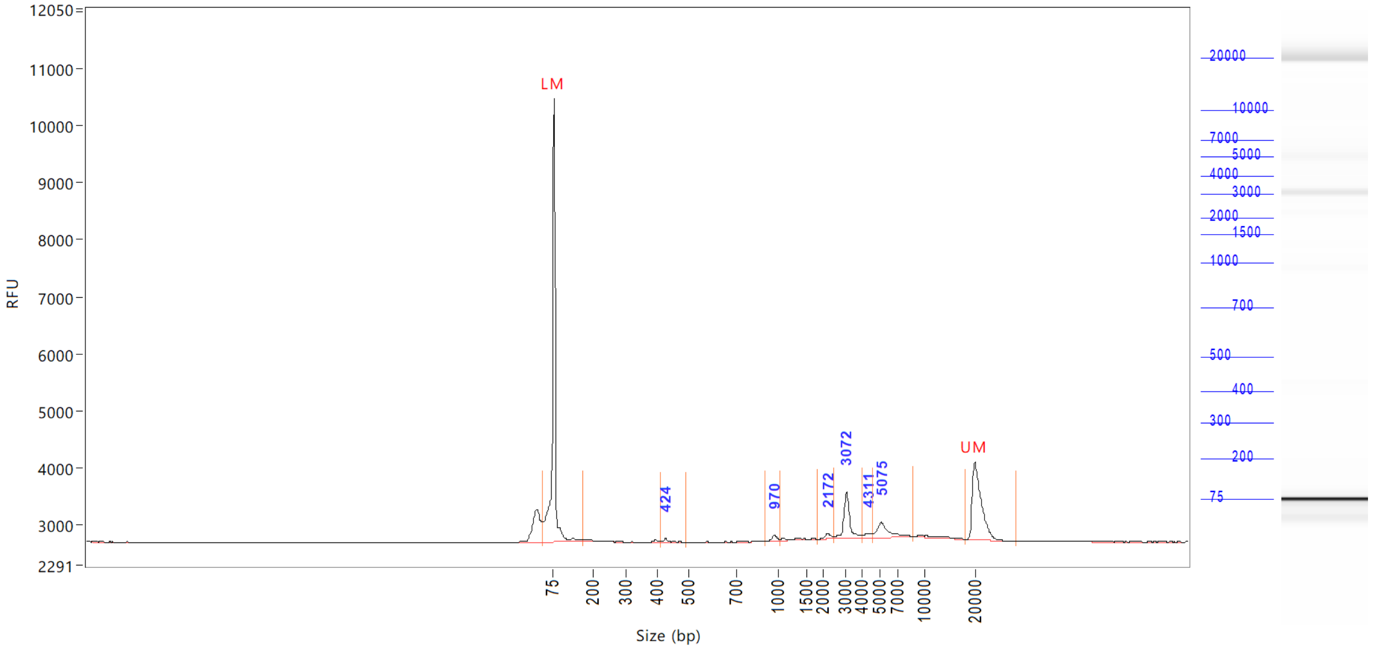

Peak	Size (bp)	Concentration (ng/uL)	From (bp)	To (bp)	Average size (bp)	CV%	RFU	Corrected peak area
1	75 (LM)	0.5000	43	166	73	19.57	7790	58.574
2	424	0.0611	412	489	432	4.14	62	0.596
3	970	0.0915	900	1001	965	1.88	103	0.894
4	2172	0.0906	1827	2486	2199	5.99	99	0.885
5	3072	0.7701	2486	4001	3119	8.63	813	7.518
6	4311	0.0935	4001	4552	4289	3.77	79	0.912
7	5075	0.4743	4552	8734	5572	16.13	268	4.630
8	20000 (UM)	0.1363	18171	28221	20623	5.35	1354	15.969

TIC:	1.5810	ng/uL
TIM:	1.0390	nmole/L
Total concentration:	1.9835	ng/uL

```
Sample peak width (sec): 5    Sample min peak height: 50    Sample baseline V to V?: Y    Sample baseline V to V points: 3
Sample filter: Binomial       Number of points for filter: 3  Sample start region (min): 0  Sample end region (min): 30
Marker peak width (sec): 5    Marker min peak height: 200   Marker baseline V to V?: Y    Marker baseline V to V points: 3
Lower marker selection: First peak > 500 RFU                Upper marker selection: Last peak > 200 RFU
Ladder size (bp)75, 200, 300, 400, 500, 700, 1000, 1500, 2000, 3000, 4000, 5000, 7000, 10000, 20000
Quantification using: Lower Marker          Final concentration (ng/uL): 0.5000          Dilution factor: 12.0
```

Sample: SampE5
Well location: E5
Created: Tuesday, August 10, 2021 11:20:27 PM

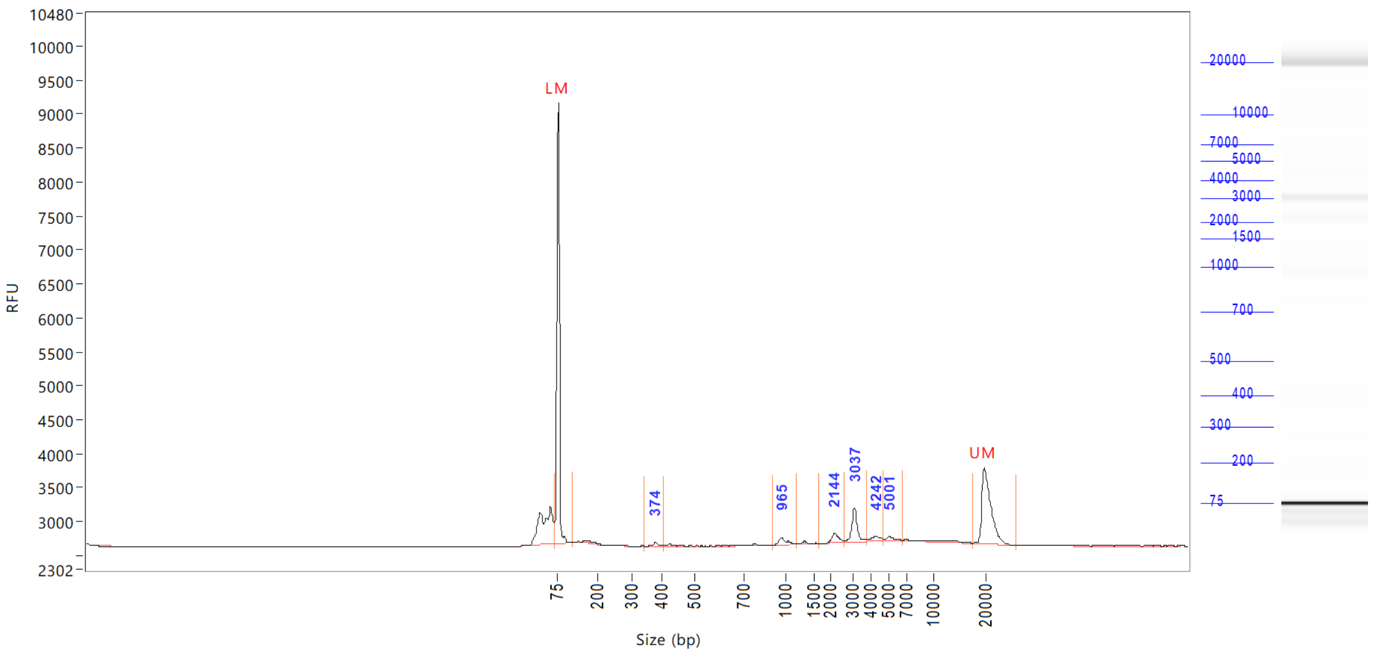

Peak	Size (bp)	Concentration (ng/uL)	From (bp)	To (bp)	Average size (bp)	CV%	RFU	Corrected peak area
1	75 (LM)	0.5000	64	122	73	6.25	6490	37.707
2	374	0.0996	339	400	374	3.13	57	0.626
3	965	0.2241	905	1178	984	4.91	115	1.409
4	2144	0.2492	1635	2600	2179	7.73	135	1.566
5	3037	0.6849	2600	3750	3061	6.28	503	4.304
6	4242	0.1682	3750	4724	4228	5.93	72	1.057
7	5001	0.1285	4724	6556	5312	9.23	63	0.808
8	20000 (UM)	0.1733	17561	26166	20530	5.12	1130	13.067

TIC:	1.5545	ng/uL
TIM:	1.4750	nmole/L
Total concentration:	2.1254	ng/uL

Sample peak width (sec): 5   Sample min peak height: 50   Sample baseline V to V?: Y   Sample baseline V to V points: 3
Sample filter: Binomial   Number of points for filter: 3   Sample start region (min): 0   Sample end region (min): 30
Marker peak width (sec): 5   Marker min peak height: 200   Marker baseline V to V?: Y   Marker baseline V to V points: 3
Lower marker selection: First peak > 500 RFU   Upper marker selection: Last peak > 200 RFU
Ladder size (bp)75, 200, 300, 400, 500, 700, 1000, 1500, 2000, 3000, 4000, 5000, 7000, 10000, 20000
Quantification using: Lower Marker   Final concentration (ng/uL): 0.5000   Dilution factor: 12.0

Sample: SampF5
Well location: F5
Created: Tuesday, August 10, 2021 11:20:27 PM

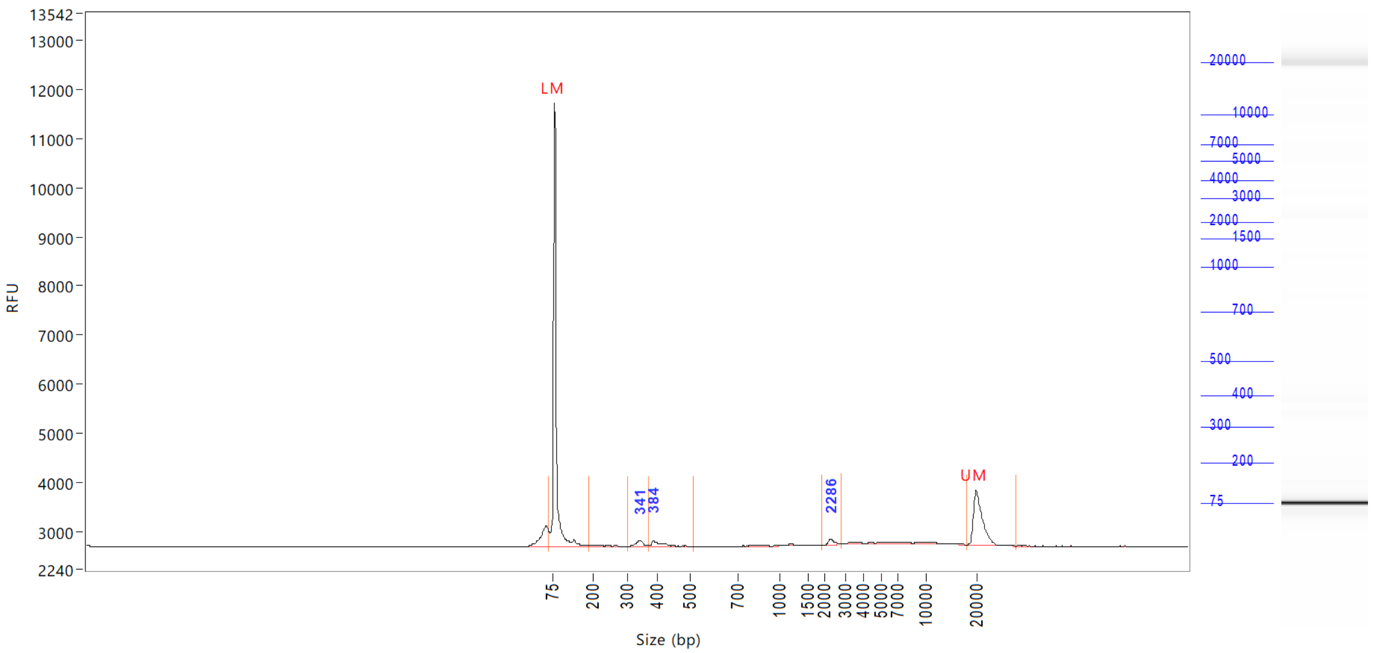

Peak	Size (bp)	Concentration (ng/uL)	From (bp)	To (bp)	Average size (bp)	CV%	RFU	Corrected peak area
1	75 (LM)	0.5000	60	185	78	19.46	9030	63.047
2	341	0.1679	301	368	336	3.66	111	1.765
3	384	0.2166	368	514	404	7.07	128	2.276
4	2286	0.1082	1942	2829	2343	7.65	113	1.137
5	20000 (UM)	0.1011	18293	27979	20569	5.17	1116	12.751

TIC:	0.4928	ng/uL
TIM:	1.7806	nmole/L
Total concentration:	0.9285	ng/uL

Sample peak width (sec): 5 Sample min peak height: 50 Sample baseline V to V?: Y Sample baseline V to V points: 3
Sample filter: Binomial Number of points for filter: 3 Sample start region (min): 0 Sample end region (min): 30
Marker peak width (sec): 5 Marker min peak height: 200 Marker baseline V to V?: Y Marker baseline V to V points: 3
Lower marker selection: First peak > 500 RFU Upper marker selection: Last peak > 200 RFU
Ladder size (bp)75, 200, 300, 400, 500, 700, 1000, 1500, 2000, 3000, 4000, 5000, 7000, 10000, 20000
Quantification using: Lower Marker Final concentration (ng/uL): 0.5000 Dilution factor: 12.0

Sample: SampG5
Well location: G5
Created: Tuesday, August 10, 2021 11:20:27 PM

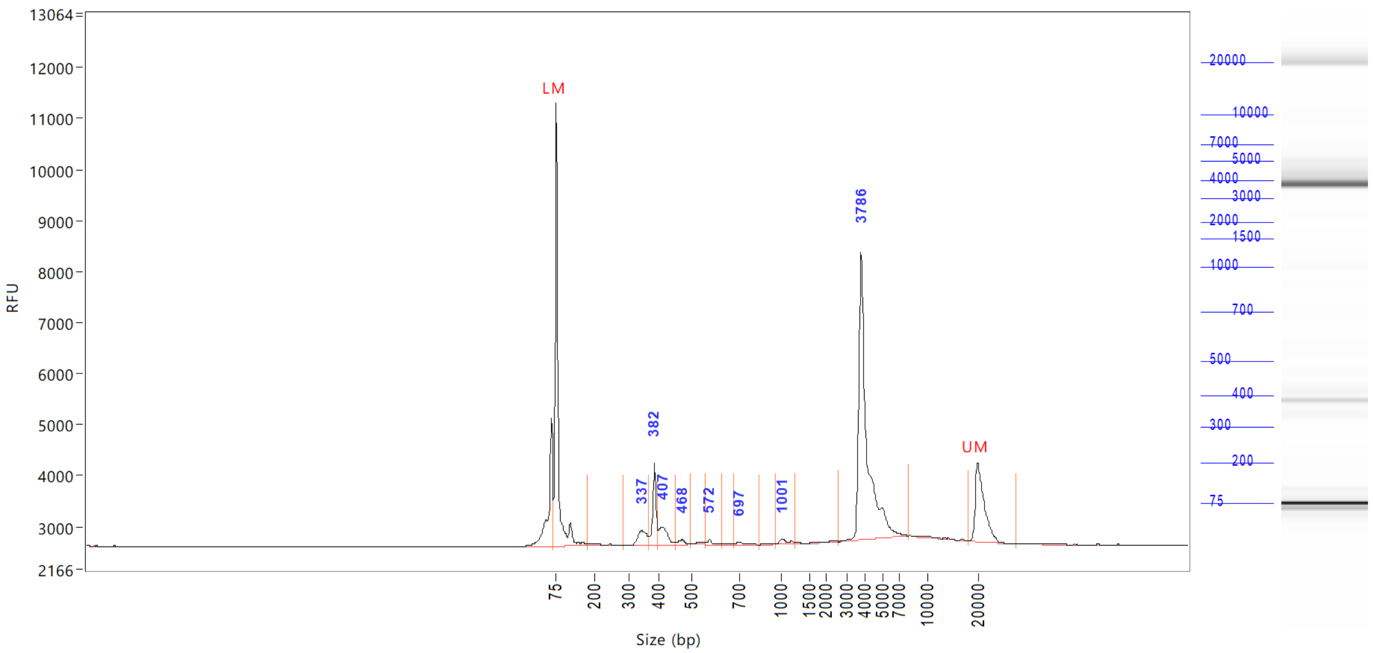

Peak	Size (bp)	Concentration (ng/uL)	From (bp)	To (bp)	Average size (bp)	CV%	RFU	Corrected peak area
1	75 (LM)	0.5000	66	173	79	21.12	8701	62.085
2	337	0.5623	282	364	339	4.04	293	5.819
3	382	1.0905	364	396	380	1.82	1597	11.284
4	407	0.5875	396	449	412	2.92	357	6.079
5	468	0.1208	449	498	465	1.93	99	1.250
6	572	0.0800	560	623	579	2.64	90	0.828
7	697	0.0803	677	835	719	4.78	55	0.831
8	1001	0.1459	957	1234	1062	7.38	103	1.510
9	3786	6.6054	2600	7867	4081	13.66	5640	68.350
10	20000 (UM)	0.1460	18171	27738	20720	5.26	1551	18.128

TIC:	9.2728	ng/uL
TIM:	13.5261	nmole/L
Total concentration:	9.6475	ng/uL

Sample peak width (sec): 5 Sample min peak height: 50 Sample baseline V to V?: Y Sample baseline V to V points: 3
Sample filter: Binomial Number of points for filter: 3 Sample start region (min): 0 Sample end region (min): 30
Marker peak width (sec): 5 Marker min peak height: 200 Marker baseline V to V?: Y Marker baseline V to V points: 3
Lower marker selection: First peak > 500 RFU Upper marker selection: Last peak > 200 RFU
Ladder size (bp)75, 200, 300, 400, 500, 700, 1000, 1500, 2000, 3000, 4000, 5000, 7000, 10000, 20000
Quantification using: Lower Marker Final concentration (ng/uL): 0.5000 Dilution factor: 12.0

Sample: SampH5
Well location: H5
Created: Tuesday, August 10, 2021 11:20:27 PM

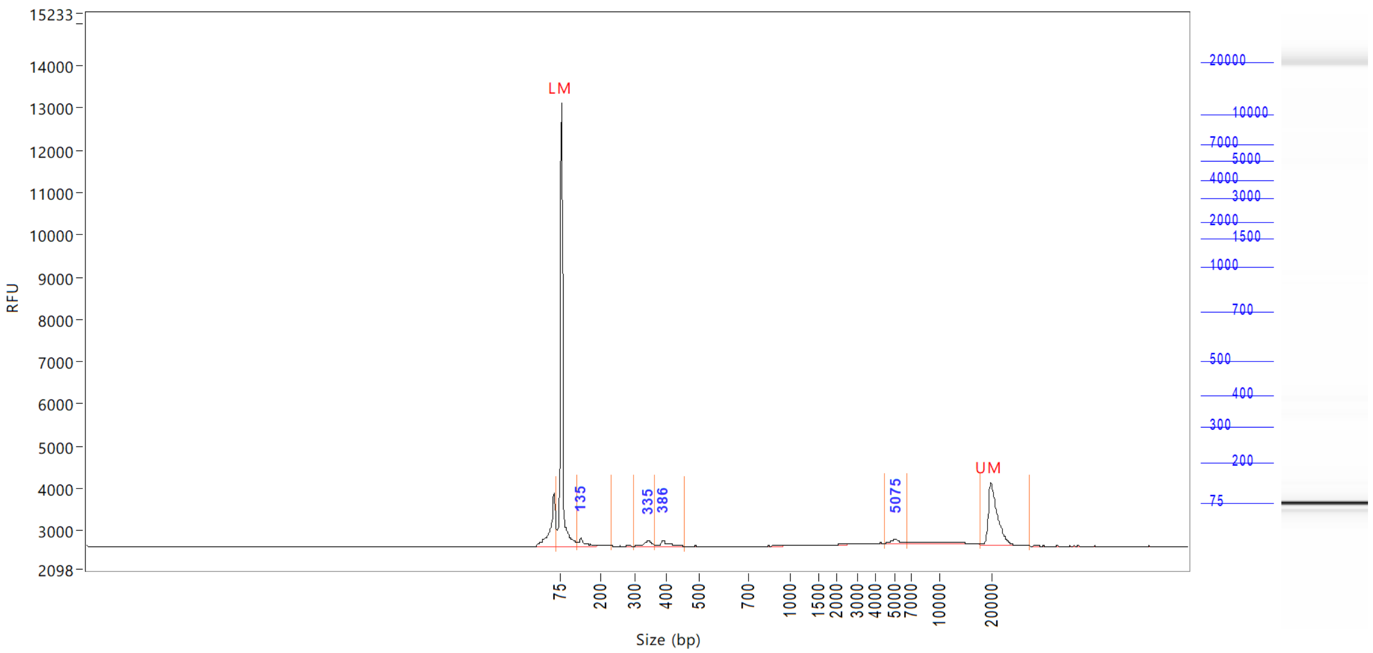

Peak	Size (bp)	Concentration (ng/uL)	From (bp)	To (bp)	Average size (bp)	CV%	RFU	Corrected peak area
1	75 (LM)	0.5000	62	126	75	11.45	10493	66.430
2	135	0.2599	126	228	151	15.75	183	2.877
3	335	0.1757	293	362	335	3.82	120	1.945
4	386	0.1969	362	452	395	4.65	143	2.180
5	5075	0.1249	4483	6556	5271	10.15	91	1.383
6	20000 (UM)	0.1281	17927	27738	20637	5.17	1461	17.026

TIC:	0.7574	ng/uL
TIM:	4.5504	nmole/L
Total concentration:	1.1338	ng/uL

Sample peak width (sec): 5 Sample min peak height: 50 Sample baseline V to V?: Y Sample baseline V to V points: 3
Sample filter: Binomial Number of points for filter: 3 Sample start region (min): 0 Sample end region (min): 30
Marker peak width (sec): 5 Marker min peak height: 200 Marker baseline V to V?: Y Marker baseline V to V points: 3
Lower marker selection: First peak > 500 RFU Upper marker selection: Last peak > 200 RFU
Ladder size (bp)75, 200, 300, 400, 500, 700, 1000, 1500, 2000, 3000, 4000, 5000, 7000, 10000, 20000
Quantification using: Lower Marker Final concentration (ng/uL): 0.5000 Dilution factor: 12.0

Sample: SampA6
Well location: A6
Created: Tuesday, August 10, 2021 11:20:27 PM

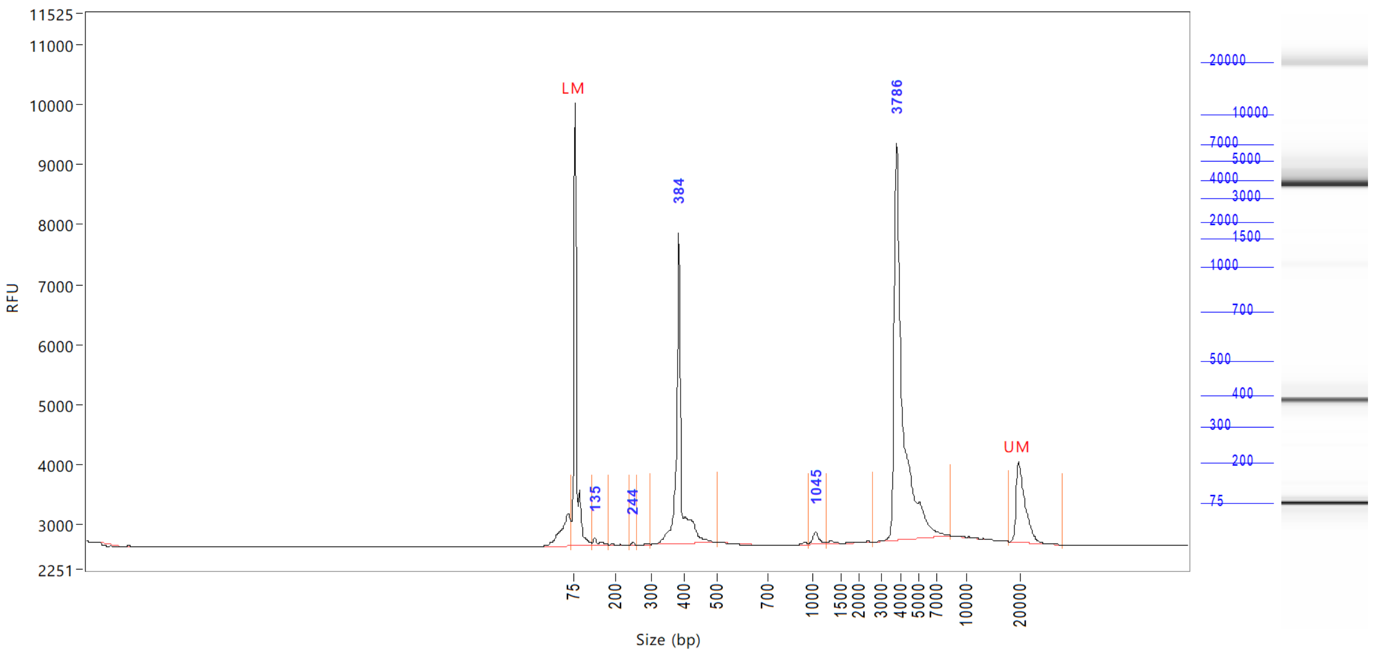

Peak	Size (bp)	Concentration (ng/uL)	From (bp)	To (bp)	Average size (bp)	CV%	RFU	Corrected peak area
1	75 (LM)	0.5000	66	126	76	10.94	7402	49.787
2	135	0.1943	126	177	143	9.12	123	1.612
3	244	0.0340	235	255	244	1.14	56	0.282
4	384	5.7750	295	498	385	5.82	5176	47.920
5	1045	0.2523	978	1234	1054	5.34	220	2.093
6	3786	9.6122	2600	8334	4136	14.95	6613	79.762
7	20000 (UM)	0.1524	18171	28221	20527	5.14	1349	15.177

TIC:	15.8677	ng/uL
TIM:	31.3599	nmole/L
Total concentration:	16.1125	ng/uL

Sample peak width (sec): 5   Sample min peak height: 50   Sample baseline V to V?: Y   Sample baseline V to V points: 3
Sample filter: Binomial   Number of points for filter: 3 Sample start region (min): 0 Sample end region (min): 30
Marker peak width (sec): 5   Marker min peak height: 200   Marker baseline V to V?: Y   Marker baseline V to V points: 3
Lower marker selection: First peak > 500 RFU   Upper marker selection: Last peak > 200 RFU
Ladder size (bp)75, 200, 300, 400, 500, 700, 1000, 1500, 2000, 3000, 4000, 5000, 7000, 10000, 20000
Quantification using: Lower Marker   Final concentration (ng/uL): 0.5000   Dilution factor: 12.0

Sample: SampB6
Well location: B6
Created: Tuesday, August 10, 2021 11:20:27 PM

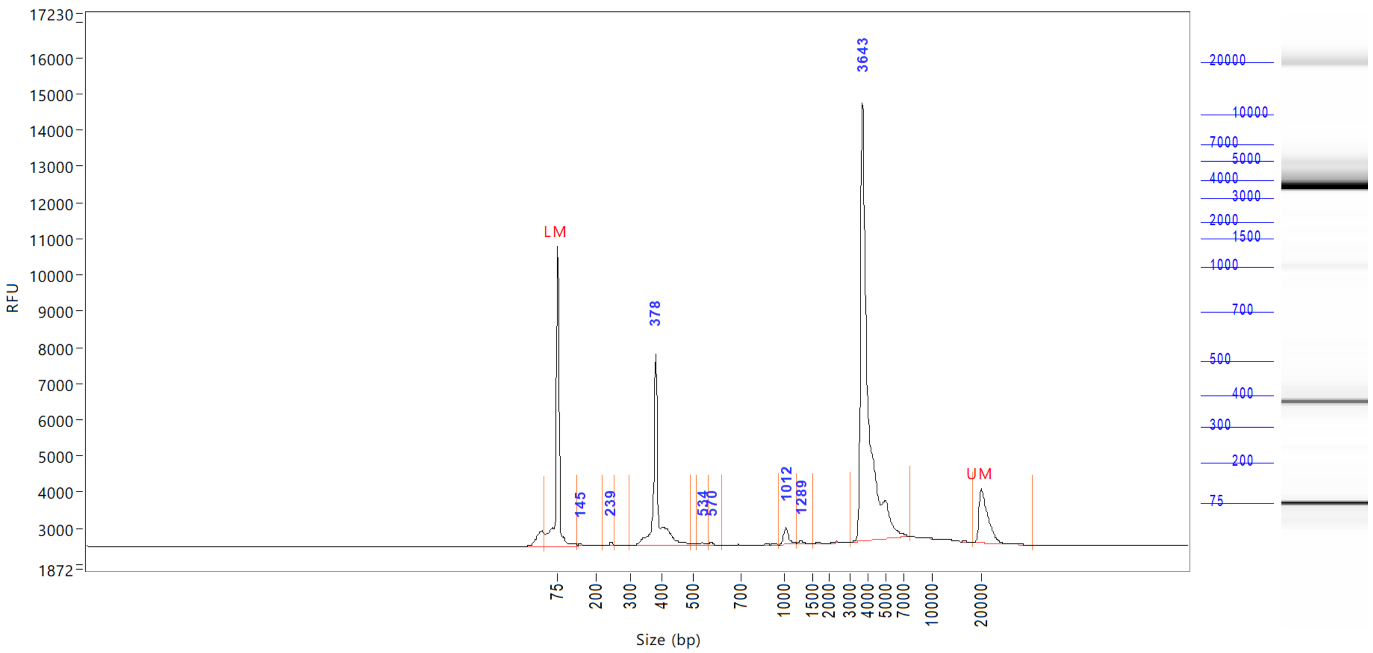

Peak	Size (bp)	Concentration (ng/uL)	From (bp)	To (bp)	Average size (bp)	CV%	RFU	Corrected peak area
1	75 (LM)	0.5000	35	137	72	17.39	8298	59.942
2	145	0.1401	137	214	166	13.51	69	1.400
3	239	0.0786	214	250	234	3.23	104	0.785
4	378	5.1041	295	489	383	6.65	5270	50.992
5	534	0.1001	509	560	534	2.82	56	1.000
6	570	0.0551	560	616	570	1.61	78	0.551
7	1012	0.4024	957	1201	1029	4.74	464	4.020
8	1289	0.0955	1201	1539	1311	5.89	91	0.954
9	3643	13.6947	3000	7734	3959	13.66	12123	136.814
10	20000 (UM)	0.1433	18171	30156	20586	5.46	1492	17.177

TIC:	19.6707	ng/uL
TIM:	30.7914	nmole/L
Total concentration:	19.9327	ng/uL

Sample peak width (sec): 5   Sample min peak height: 50   Sample baseline V to V?: Y   Sample baseline V to V points: 3
Sample filter: Binomial   Number of points for filter: 3 Sample start region (min): 0 Sample end region (min): 30
Marker peak width (sec): 5   Marker min peak height: 200   Marker baseline V to V?: Y   Marker baseline V to V points: 3
Lower marker selection: First peak > 500 RFU   Upper marker selection: Last peak > 200 RFU
Ladder size (bp)75, 200, 300, 400, 500, 700, 1000, 1500, 2000, 3000, 4000, 5000, 7000, 10000, 20000
Quantification using: Lower Marker   Final concentration (ng/uL): 0.5000   Dilution factor: 12.0

Sample: SampC6
Well location: C6
Created: Tuesday, August 10, 2021 11:20:27 PM

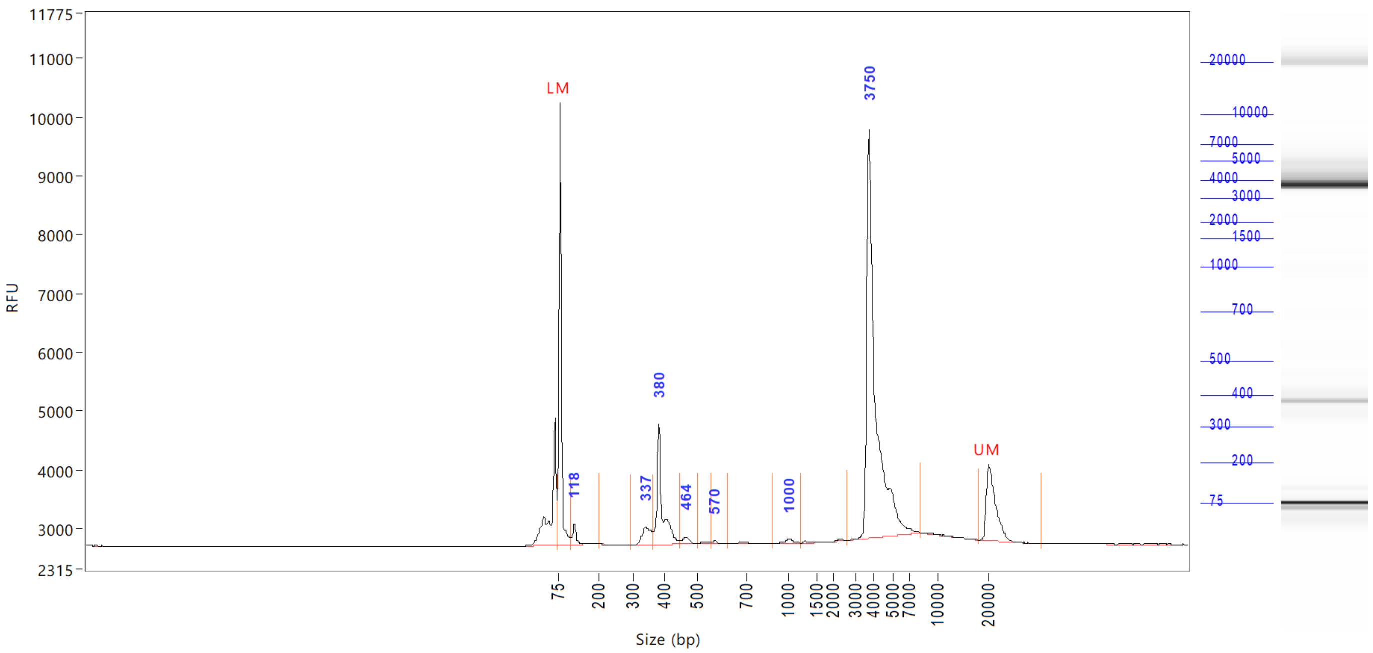

Peak	Size (bp)	Concentration (ng/uL)	From (bp)	To (bp)	Average size (bp)	CV%	RFU	Corrected peak area
1	75 (LM)	0.5000	67	108	75	8.38	7539	46.502
2	118	0.4375	108	200	123	12.87	353	3.391
3	337	0.7556	291	360	337	3.97	306	5.856
4	380	2.8830	360	445	387	4.35	2041	22.344
5	464	0.2032	445	498	461	2.06	119	1.575
6	570	0.0626	552	621	571	2.36	55	0.485
7	1000	0.1533	887	1201	998	5.45	82	1.188
8	3750	11.5804	2600	8134	4056	14.56	6954	89.752
9	20000 (UM)	0.1667	18049	30277	20684	5.49	1293	15.506

TIC:	16.0756	ng/uL
TIM:	27.6477	nmole/L
Total concentration:	16.3413	ng/uL

Sample peak width (sec): 5   Sample min peak height: 50   Sample baseline V to V?: Y   Sample baseline V to V points: 3
Sample filter: Binomial   Number of points for filter: 3 Sample start region (min): 0 Sample end region (min): 30
Marker peak width (sec): 5   Marker min peak height: 200   Marker baseline V to V?: Y   Marker baseline V to V points: 3
Lower marker selection: First peak > 500 RFU   Upper marker selection: Last peak > 200 RFU
Ladder size (bp)75, 200, 300, 400, 500, 700, 1000, 1500, 2000, 3000, 4000, 5000, 7000, 10000, 20000
Quantification using: Lower Marker   Final concentration (ng/uL): 0.5000   Dilution factor: 12.0

Sample: SampD6
Well location: D6
Created: Tuesday, August 10, 2021 11:20:27 PM

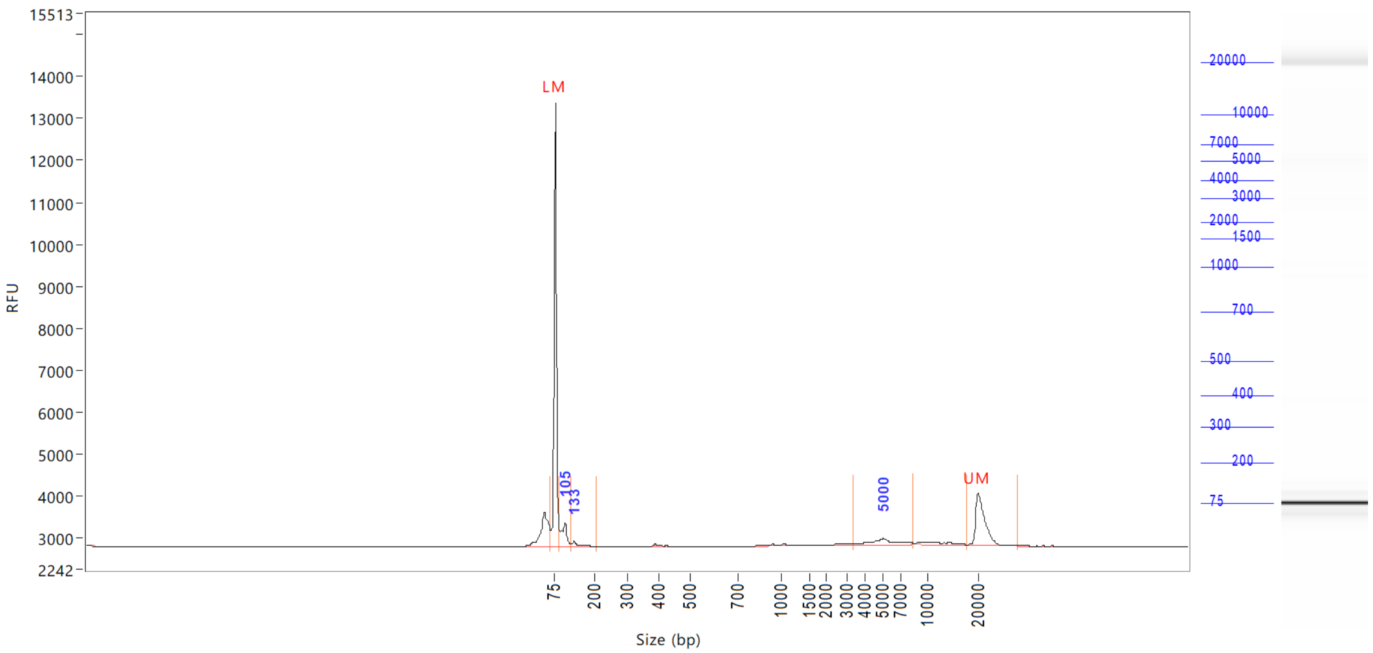

Peak	Size (bp)	Concentration (ng/uL)	From (bp)	To (bp)	Average size (bp)	CV%	RFU	Corrected peak area
1	75 (LM)	0.5000	62	89	74	5.42	10600	64.951
2	105	0.6528	89	124	102	7.83	547	7.066
3	133	0.1399	124	206	142	12.76	133	1.514
4	5000	0.4622	3322	8467	5350	23.73	149	5.003
5	20000 (UM)	0.1135	17805	27859	20665	5.28	1258	14.738

TIC:	1.2548	ng/uL
TIM:	12.2703	nmole/L
Total concentration:	1.7524	ng/uL

Sample peak width (sec): 5   Sample min peak height: 50   Sample baseline V to V?: Y   Sample baseline V to V points: 3
Sample filter: Binomial   Number of points for filter: 3   Sample start region (min): 0   Sample end region (min): 30
Marker peak width (sec): 5   Marker min peak height: 200   Marker baseline V to V?: Y   Marker baseline V to V points: 3
Lower marker selection: First peak > 500 RFU   Upper marker selection: Last peak > 200 RFU
Ladder size (bp)75, 200, 300, 400, 500, 700, 1000, 1500, 2000, 3000, 4000, 5000, 7000, 10000, 20000
Quantification using: Lower Marker   Final concentration (ng/uL): 0.5000   Dilution factor: 12.0

Sample: SampE6
Well location: E6
Created: Tuesday, August 10, 2021 11:20:27 PM

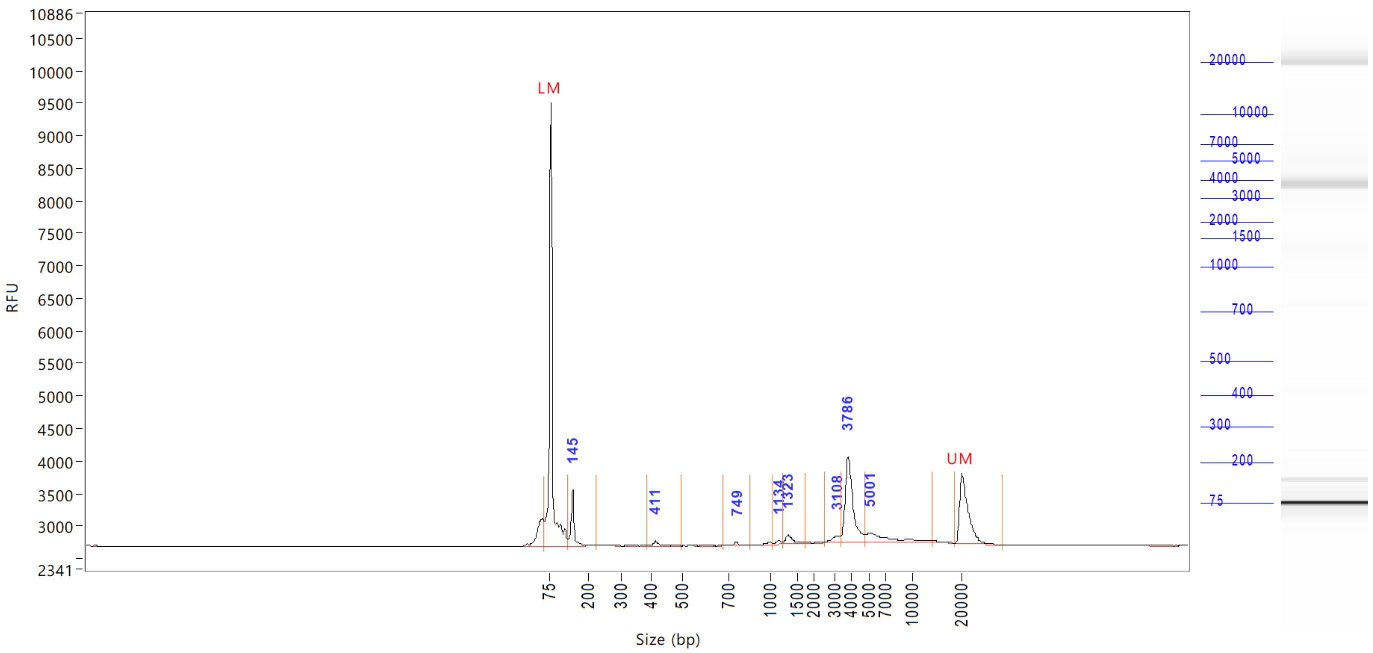

Peak	Size (bp)	Concentration (ng/uL)	From (bp)	To (bp)	Average size (bp)	CV%	RFU	Corrected peak area
1	75 (LM)	0.5000	56	129	77	16.69	6826	53.271
2	145	0.7590	129	223	148	10.74	860	6.739
3	411	0.1076	386	498	419	4.96	71	0.955
4	749	0.0611	672	853	749	4.31	58	0.543
5	1134	0.0569	1045	1212	1137	3.74	56	0.505
6	1323	0.1954	1212	1750	1361	7.73	135	1.735
7	3108	0.1553	2515	3322	3013	6.57	106	1.379
8	3786	1.8067	3322	4793	3870	7.43	1305	16.040
9	5001	0.5439	4793	14025	7302	32.13	142	4.829
10	20000 (UM)	0.1130	18537	28463	20736	5.64	1065	12.036

TIC:	3.6858	ng/uL
TIM:	10.2793	nmole/L
Total concentration:	3.8415	ng/uL

Sample peak width (sec): 5 Sample min peak height: 50 Sample baseline V to V?: Y Sample baseline V to V points: 3
Sample filter: Binomial Number of points for filter: 3 Sample start region (min): 0 Sample end region (min): 30
Marker peak width (sec): 5 Marker min peak height: 200 Marker baseline V to V?: Y Marker baseline V to V points: 3
Lower marker selection: First peak > 500 RFU Upper marker selection: Last peak > 200 RFU
Ladder size (bp)75, 200, 300, 400, 500, 700, 1000, 1500, 2000, 3000, 4000, 5000, 7000, 10000, 20000
Quantification using: Lower Marker Final concentration (ng/uL): 0.5000 Dilution factor: 12.0

Sample: SampF6
Well location: F6
Created: Tuesday, August 10, 2021 11:20:27 PM

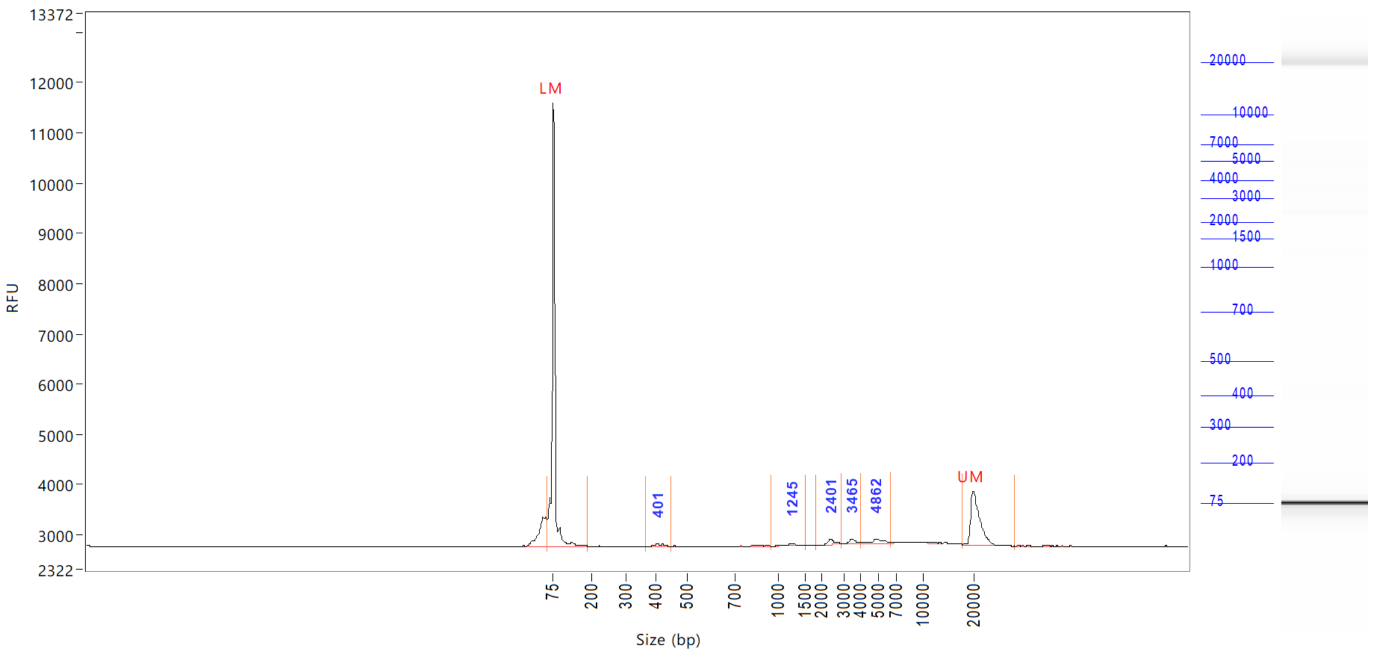

Peak	Size (bp)	Concentration (ng/uL)	From (bp)	To (bp)	Average size (bp)	CV%	RFU	Corrected peak area
1	75 (LM)	0.5000	56	185	75	16.84	8834	63.869
2	401	0.0887	364	445	407	3.68	51	0.944
3	1245	0.0604	957	1501	1229	8.49	50	0.643
4	2401	0.1060	1827	2914	2450	7.90	108	1.128
5	3465	0.1112	2914	4035	3484	7.64	100	1.184
6	4862	0.1436	4035	6408	4998	11.18	90	1.528
7	20000 (UM)	0.0973	17805	28463	20667	5.31	1076	12.429

TIC:	0.5099	ng/uL
TIM:	0.6105	nmole/L
Total concentration:	0.7097	ng/uL

Sample peak width (sec): 5 Sample min peak height: 50 Sample baseline V to V?: Y Sample baseline V to V points: 3
Sample filter: Binomial Number of points for filter: 3 Sample start region (min): 0 Sample end region (min): 30
Marker peak width (sec): 5 Marker min peak height: 200 Marker baseline V to V?: Y Marker baseline V to V points: 3
Lower marker selection: First peak > 500 RFU Upper marker selection: Last peak > 200 RFU
Ladder size (bp)75, 200, 300, 400, 500, 700, 1000, 1500, 2000, 3000, 4000, 5000, 7000, 10000, 20000
Quantification using: Lower Marker Final concentration (ng/uL): 0.5000 Dilution factor: 12.0

Sample: SampG6
Well location: G6
Created: Tuesday, August 10, 2021 11:20:27 PM

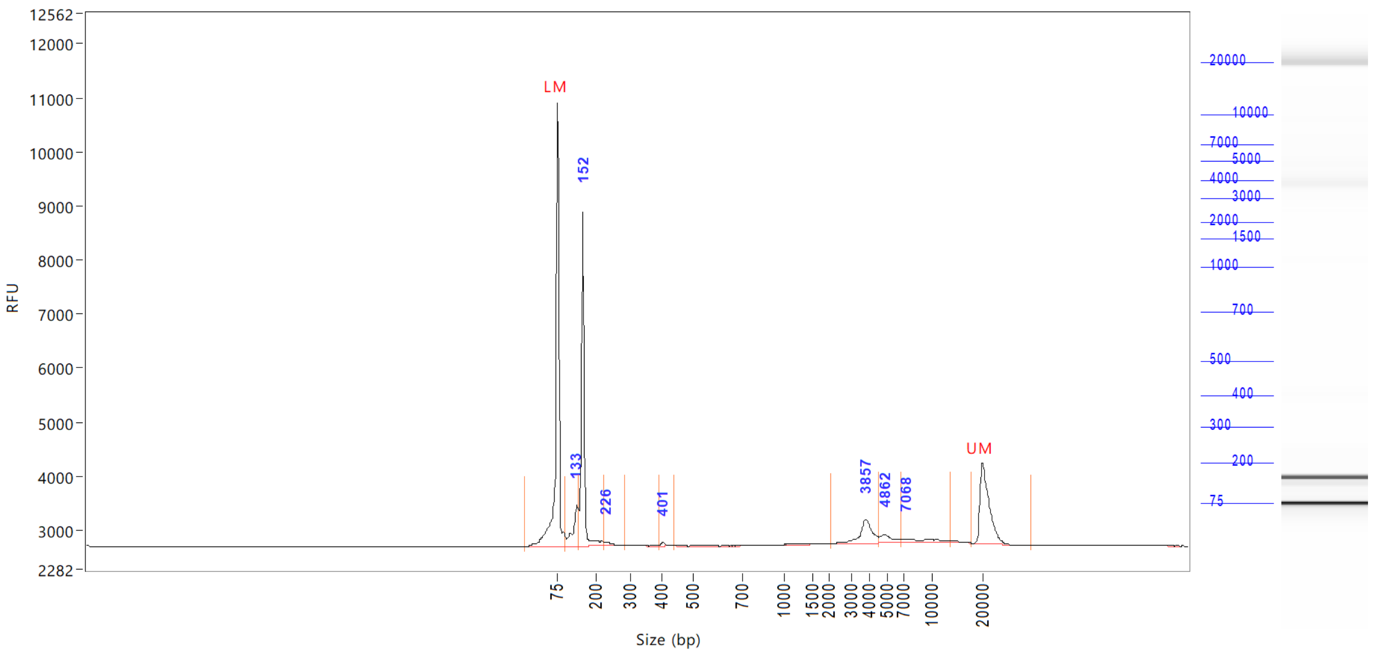

Peak	Size (bp)	Concentration (ng/uL)	From (bp)	To (bp)	Average size (bp)	CV%	RFU	Corrected peak area
1	75 (LM)	0.5000	0	101	68	22.53	8213	63.928
2	133	0.9634	101	141	127	8.46	751	10.265
3	152	3.8672	141	219	154	8.06	6179	41.204
4	226	0.1126	219	280	232	4.55	77	1.199
5	401	0.0684	390	435	405	2.42	67	0.729
6	3857	0.7519	2030	4552	3745	12.56	437	8.011
7	4862	0.2826	4552	6704	5277	11.82	165	3.011
8	7068	0.2914	6704	13781	9378	20.73	78	3.105
9	20000 (UM)	0.1394	17927	29793	20701	5.49	1491	17.826

TIC:	6.3375	ng/uL
TIM:	55.2833	nmole/L
Total concentration:	6.4597	ng/uL

Sample peak width (sec): 5   Sample min peak height: 50   Sample baseline V to V?: Y   Sample baseline V to V points: 3
Sample filter: Binomial   Number of points for filter: 3 Sample start region (min): 0 Sample end region (min): 30
Marker peak width (sec): 5   Marker min peak height: 200   Marker baseline V to V?: Y   Marker baseline V to V points: 3
Lower marker selection: First peak > 500 RFU   Upper marker selection: Last peak > 200 RFU
Ladder size (bp)75, 200, 300, 400, 500, 700, 1000, 1500, 2000, 3000, 4000, 5000, 7000, 10000, 20000
Quantification using: Lower Marker   Final concentration (ng/uL): 0.5000   Dilution factor: 12.0

Sample: SampH6
Well location: H6
Created: Tuesday, August 10, 2021 11:20:27 PM

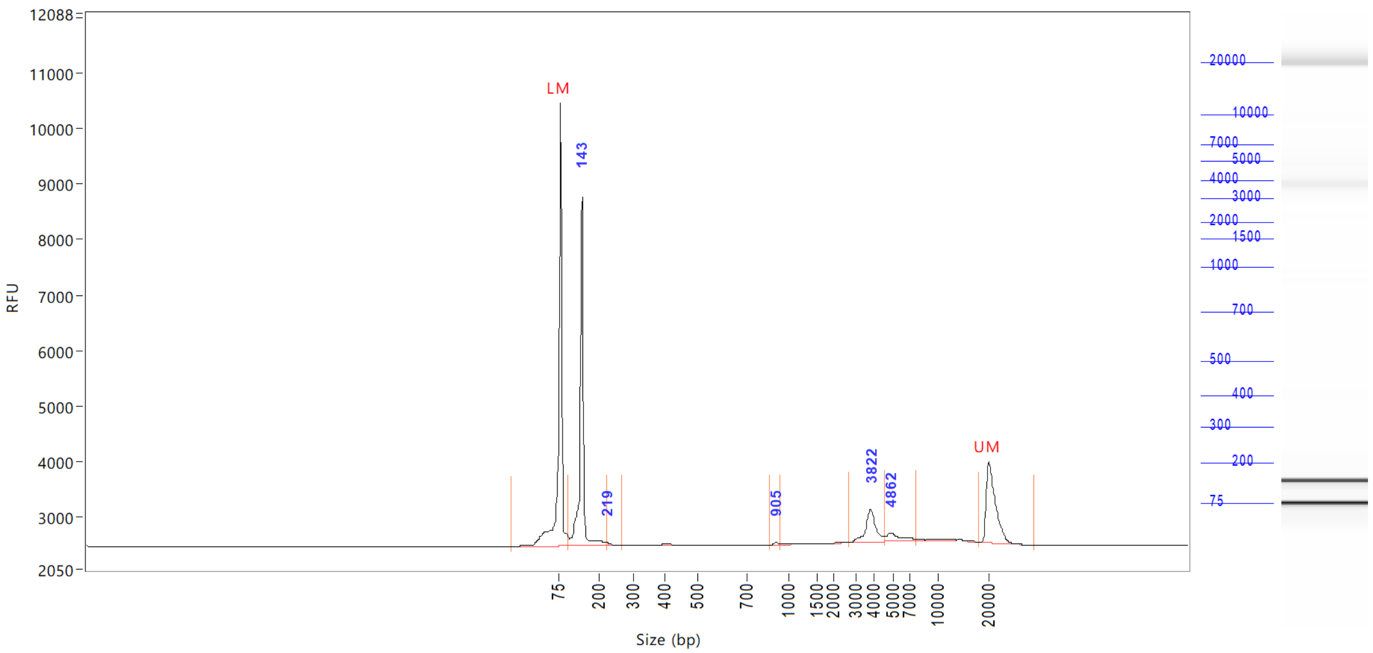

Peak	Size<br>(bp)	Concentration<br>(ng/uL)	From<br>(bp)	To<br>(bp)	Average size<br>(bp)	CV%	RFU	Corrected peak area
1	75 (LM)	0.5000	0	103	67	28.27	8014	64.965
2	143	4.5784	103	219	140	10.98	6288	49.572
3	219	0.0562	219	264	233	5.39	60	0.609
4	905	0.0325	861	939	901	1.66	50	0.352
5	3822	0.8240	2657	4552	3758	9.39	583	8.921
6	4862	0.2814	4552	7534	5475	15.34	150	3.047
7	20000 (UM)	0.1327	18049	28947	20608	5.22	1476	17.246

TIC:	5.7724	ng/uL
TIM:	54.6422	nmole/L
Total concentration:	6.0607	ng/uL

```
Sample peak width (sec): 5    Sample min peak height: 50    Sample baseline V to V?: Y    Sample baseline V to V points: 3
Sample filter: Binomial       Number of points for filter: 3  Sample start region (min): 0  Sample end region (min): 30
Marker peak width (sec): 5    Marker min peak height: 200   Marker baseline V to V?: Y    Marker baseline V to V points: 3
Lower marker selection: First peak > 500 RFU                Upper marker selection: Last peak > 200 RFU
Ladder size (bp)75, 200, 300, 400, 500, 700, 1000, 1500, 2000, 3000, 4000, 5000, 7000, 10000, 20000
Quantification using: Lower Marker          Final concentration (ng/uL): 0.5000          Dilution factor: 12.0
```

Sample: SampA7
Well location: A7
Created: Tuesday, August 10, 2021 11:20:27 PM

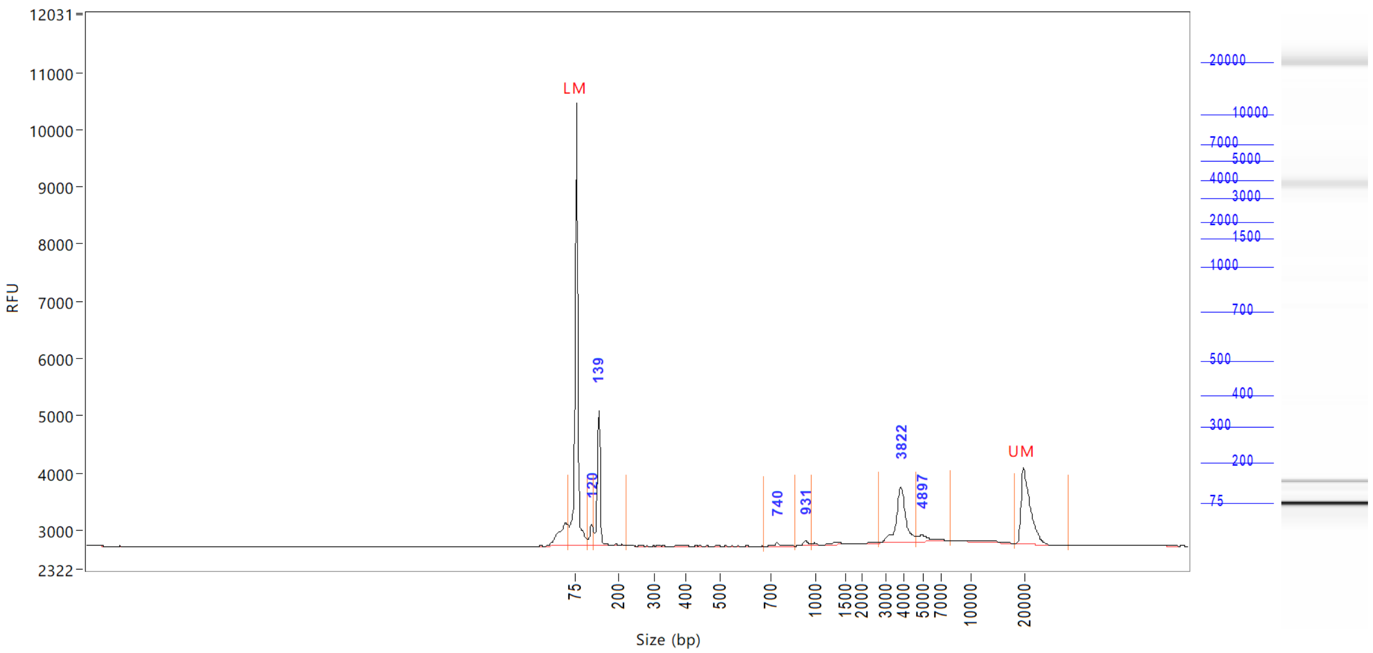

Peak	Size (bp)	Concentration (ng/uL)	From (bp)	To (bp)	Average size (bp)	CV%	RFU	Corrected peak area
1	75 (LM)	0.5000	54	107	73	9.59	7744	53.787
2	120	0.3754	107	128	118	4.83	358	3.365
3	139	1.6849	128	219	139	5.74	2342	15.104
4	740	0.0650	674	857	742	4.90	62	0.583
5	931	0.0832	857	970	929	2.20	86	0.746
6	3822	1.5023	2715	4656	3777	8.98	976	13.467
7	4897	0.2154	4656	7934	5368	13.58	111	1.931
8	20000 (UM)	0.1395	18293	28463	20568	5.13	1323	15.007

TIC:	3.9262	ng/uL
TIM:	26.1577	nmole/L
Total concentration:	4.2791	ng/uL

Sample peak width (sec): 5   Sample min peak height: 50   Sample baseline V to V?: Y   Sample baseline V to V points: 3
Sample filter: Binomial   Number of points for filter: 3 Sample start region (min): 0 Sample end region (min): 30
Marker peak width (sec): 5   Marker min peak height: 200   Marker baseline V to V?: Y   Marker baseline V to V points: 3
Lower marker selection: First peak > 500 RFU   Upper marker selection: Last peak > 200 RFU
Ladder size (bp)75, 200, 300, 400, 500, 700, 1000, 1500, 2000, 3000, 4000, 5000, 7000, 10000, 20000
Quantification using: Lower Marker   Final concentration (ng/uL): 0.5000   Dilution factor: 12.0

Sample: SampB7
Well location: B7
Created: Tuesday, August 10, 2021 11:20:27 PM

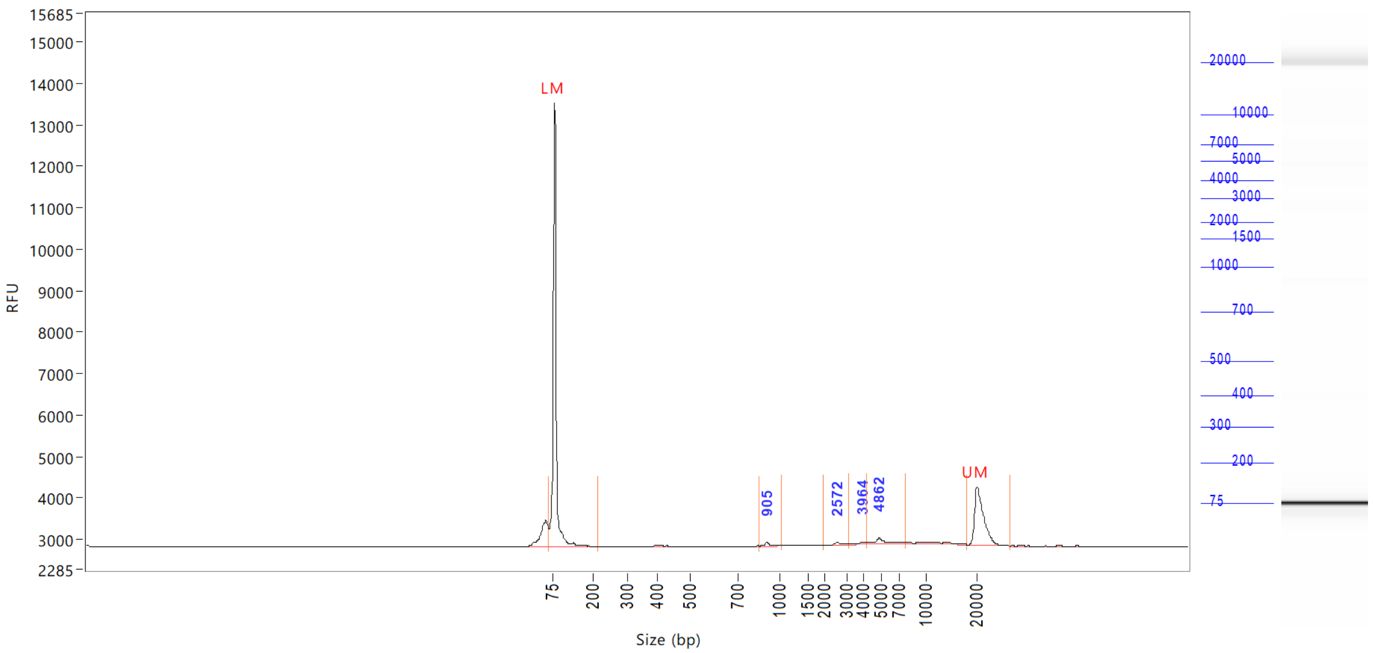

Peak	Size (bp)	Concentration (ng/uL)	From (bp)	To (bp)	Average size (bp)	CV%	RFU	Corrected peak area
1	75 (LM)	0.5000	58	212	76	17.82	10711	80.030
2	905	0.0614	853	1023	910	3.43	87	0.819
3	2572	0.0574	1962	3108	2614	9.00	59	0.766
4	3964	0.0541	3108	4139	3780	7.08	67	0.721
5	4862	0.1897	4139	7667	5367	17.36	136	2.530
6	20000 (UM)	0.1037	18171	26770	20673	5.09	1414	16.591

TIC:	0.3625	ng/uL
TIM:	0.2289	nmole/L
Total concentration:	0.6140	ng/uL

```
Sample peak width (sec): 5    Sample min peak height: 50    Sample baseline V to V?: Y    Sample baseline V to V points: 3
Sample filter: Binomial       Number of points for filter: 3  Sample start region (min): 0  Sample end region (min): 30
Marker peak width (sec): 5    Marker min peak height: 200   Marker baseline V to V?: Y    Marker baseline V to V points: 3
Lower marker selection: First peak > 500 RFU                Upper marker selection: Last peak > 200 RFU
Ladder size (bp)75, 200, 300, 400, 500, 700, 1000, 1500, 2000, 3000, 4000, 5000, 7000, 10000, 20000
Quantification using: Lower Marker          Final concentration (ng/uL): 0.5000          Dilution factor: 12.0
```

Sample: SampC7
Well location: C7
Created: Tuesday, August 10, 2021 11:20:27 PM

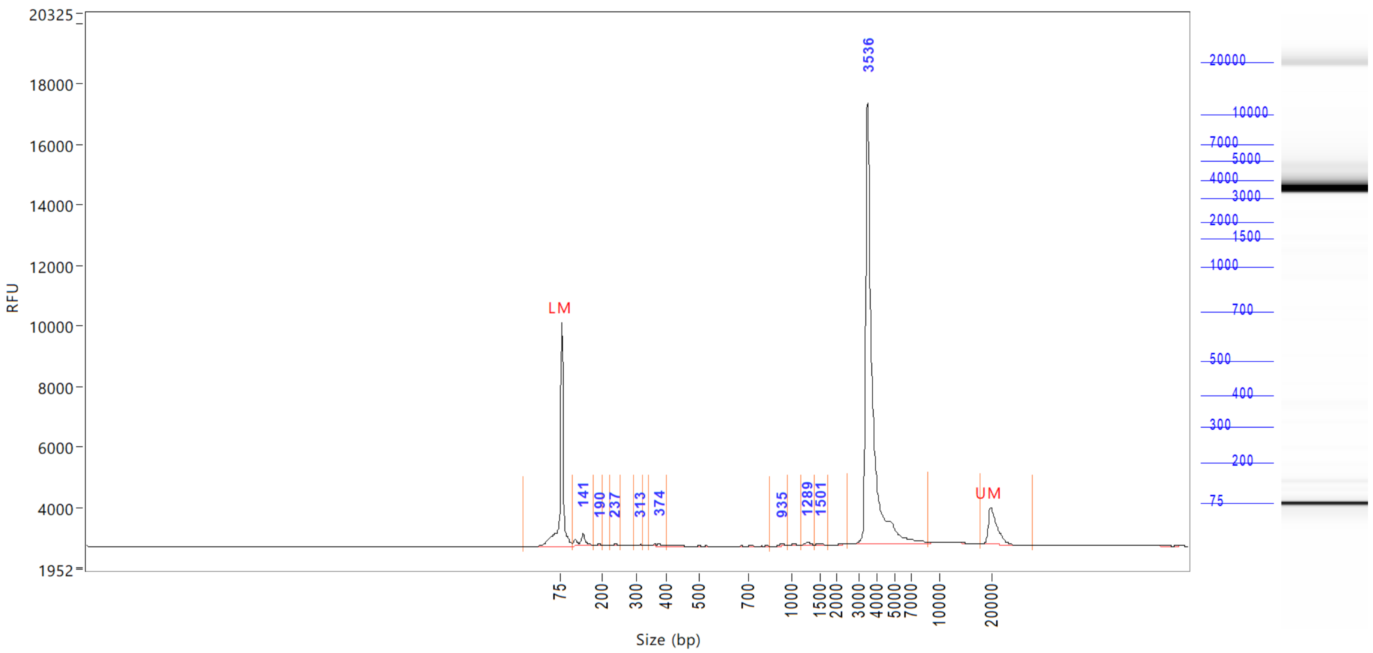

Peak	Size (bp)	Concentration (ng/uL)	From (bp)	To (bp)	Average size (bp)	CV%	RFU	Corrected peak area
1	75 (LM)	0.5000	0	107	68	21.18	7409	59.479
2	141	0.6316	107	173	133	11.12	428	6.261
3	190	0.0541	173	200	186	3.14	64	0.536
4	237	0.0509	224	253	236	1.84	79	0.505
5	313	0.0326	291	321	310	1.78	55	0.324
6	374	0.1286	339	400	367	3.36	95	1.275
7	935	0.0616	853	978	934	1.91	80	0.610
8	1289	0.0923	1167	1411	1272	3.85	97	0.915
9	1501	0.0483	1411	1750	1512	4.09	61	0.479
10	3536	14.7902	2543	8667	3791	16.55	14604	146.618
11	20000 (UM)	0.1171	17805	28342	20606	5.37	1220	13.929

TIC:	15.8902	ng/uL
TIM:	16.0888	nmole/L
Total concentration:	16.2536	ng/uL

```
Sample peak width (sec): 5    Sample min peak height: 50    Sample baseline V to V?: Y    Sample baseline V to V points: 3
Sample filter: Binomial       Number of points for filter: 3  Sample start region (min): 0  Sample end region (min): 30
Marker peak width (sec): 5    Marker min peak height: 200   Marker baseline V to V?: Y    Marker baseline V to V points: 3
Lower marker selection: First peak > 500 RFU                Upper marker selection: Last peak > 200 RFU
Ladder size (bp)75, 200, 300, 400, 500, 700, 1000, 1500, 2000, 3000, 4000, 5000, 7000, 10000, 20000
Quantification using: Lower Marker          Final concentration (ng/uL): 0.5000          Dilution factor: 12.0
```

Sample: SampD7
Well location: D7
Created: Tuesday, August 10, 2021 11:20:27 PM

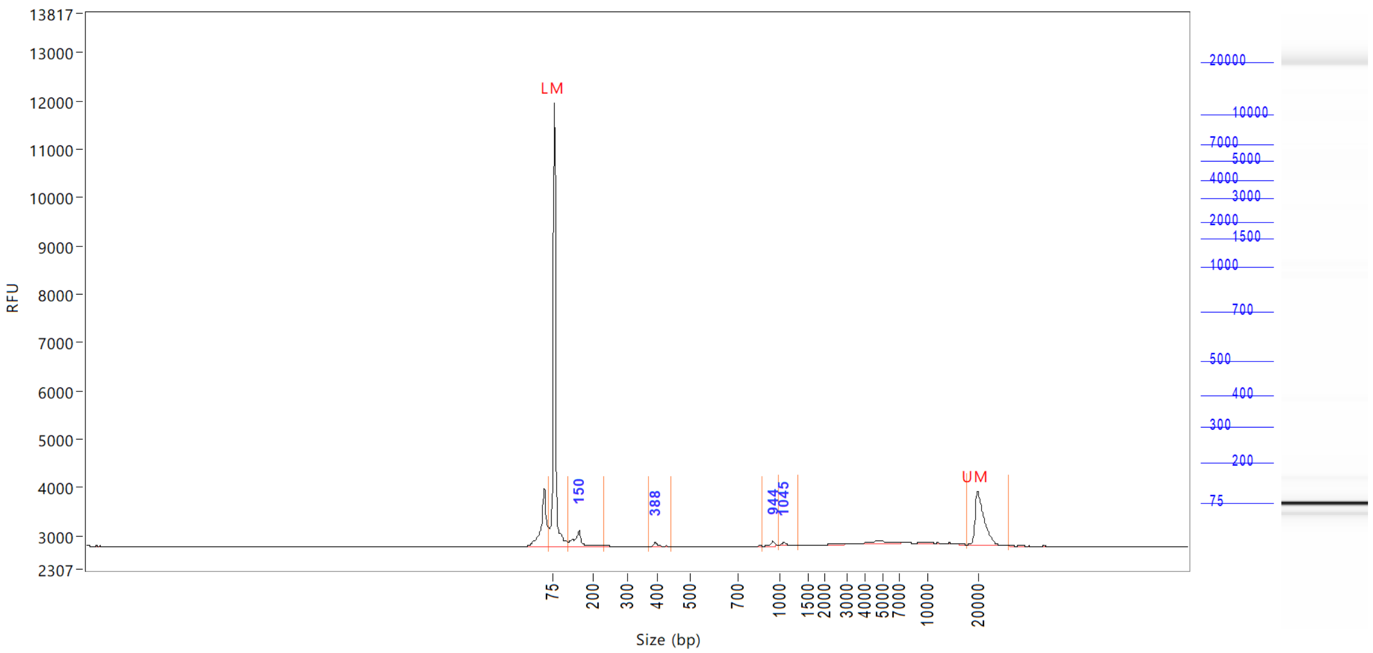

Peak	Size (bp)	Concentration (ng/uL)	From (bp)	To (bp)	Average size (bp)	CV%	RFU	Corrected peak area
1	75 (LM)	0.5000	58	116	74	10.03	9197	61.935
2	150	0.5819	116	230	149	15.45	341	6.007
3	388	0.0752	368	437	393	3.65	74	0.777
4	944	0.0729	874	983	939	2.00	99	0.753
5	1045	0.0743	983	1323	1073	7.23	72	0.767
6	20000 (UM)	0.1047	17927	26287	20661	5.07	1104	12.969

TIC:	0.8044	ng/uL
TIM:	6.9754	nmole/L
Total concentration:	1.2222	ng/uL

Sample peak width (sec): 5   Sample min peak height: 50   Sample baseline V to V?: Y   Sample baseline V to V points: 3
Sample filter: Binomial   Number of points for filter: 3 Sample start region (min): 0 Sample end region (min): 30
Marker peak width (sec): 5   Marker min peak height: 200   Marker baseline V to V?: Y   Marker baseline V to V points: 3
Lower marker selection: First peak > 500 RFU   Upper marker selection: Last peak > 200 RFU
Ladder size (bp)75, 200, 300, 400, 500, 700, 1000, 1500, 2000, 3000, 4000, 5000, 7000, 10000, 20000
Quantification using: Lower Marker   Final concentration (ng/uL): 0.5000   Dilution factor: 12.0

Sample: SampE7
Well location: E7
Created: Tuesday, August 10, 2021 11:20:27 PM

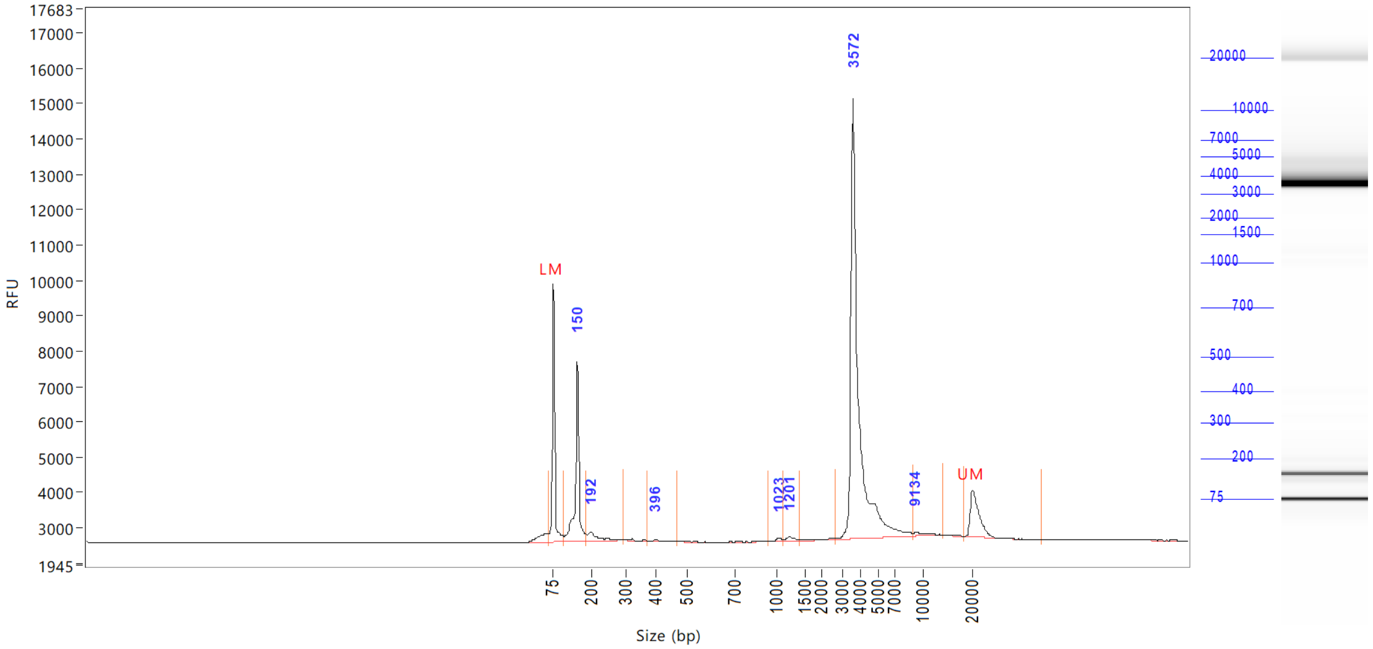

Peak	Size (bp)	Concentration (ng/uL)	From (bp)	To (bp)	Average size (bp)	CV%	RFU	Corrected peak area
1	75 (LM)	0.5000	60	107	75	8.97	7311	46.340
2	150	5.7574	107	181	146	8.38	5103	44.467
3	192	0.7863	181	289	209	11.44	269	6.073
4	396	0.0953	372	464	402	4.12	63	0.736
5	1023	0.1106	931	1101	1019	3.41	104	0.854
6	1201	0.2124	1101	1400	1228	5.73	117	1.641
7	3572	18.5598	2629	8934	3950	20.92	12476	143.345
8	9134	0.2128	8934	14147	10127	10.62	92	1.643
9	20000 (UM)	0.1635	18171	33783	20756	6.46	1307	15.155

TIC:	25.7346	ng/uL
TIM:	79.6168	nmole/L
Total concentration:	26.0020	ng/uL

```
Sample peak width (sec): 5    Sample min peak height: 50    Sample baseline V to V?: Y    Sample baseline V to V points: 3
Sample filter: Binomial       Number of points for filter: 3  Sample start region (min): 0  Sample end region (min): 30
Marker peak width (sec): 5    Marker min peak height: 200   Marker baseline V to V?: Y    Marker baseline V to V points: 3
Lower marker selection: First peak > 500 RFU                Upper marker selection: Last peak > 200 RFU
Ladder size (bp)75, 200, 300, 400, 500, 700, 1000, 1500, 2000, 3000, 4000, 5000, 7000, 10000, 20000
Quantification using: Lower Marker          Final concentration (ng/uL): 0.5000          Dilution factor: 12.0
```

Sample: SampF7
Well location: F7
Created: Tuesday, August 10, 2021 11:20:27 PM

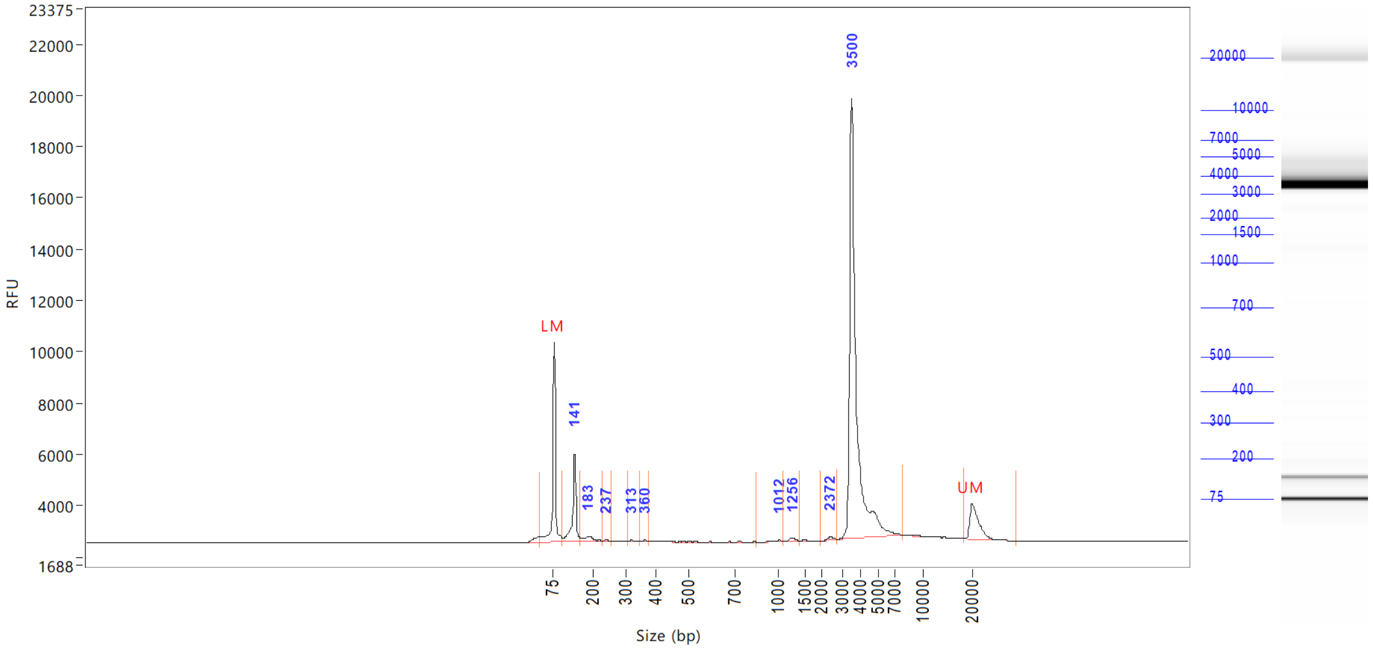

Peak	Size (bp)	Concentration (ng/uL)	From (bp)	To (bp)	Average size (bp)	CV%	RFU	Corrected peak area
1	75 (LM)	0.5000	33	103	72	13.11	7788	55.608
2	141	3.0954	103	160	135	7.31	3418	28.688
3	183	0.4050	160	228	182	7.40	184	3.754
4	237	0.0444	228	253	237	1.66	66	0.411
5	313	0.0439	303	341	313	1.73	71	0.407
6	360	0.0363	341	372	358	1.27	60	0.336
7	1012	0.0536	848	1079	1007	4.20	52	0.497
8	1256	0.1748	1079	1389	1239	5.50	140	1.620
9	2372	0.1127	2000	2743	2380	4.94	133	1.044
10	3500	18.5759	2743	7867	3724	14.00	17172	172.162
11	20000 (UM)	0.1422	18415	28826	20604	5.19	1379	15.815

TIC:	22.5420	ng/uL
TIM:	50.6499	nmole/L
Total concentration:	22.8212	ng/uL

```
Sample peak width (sec): 5    Sample min peak height: 50    Sample baseline V to V?: Y    Sample baseline V to V points: 3
Sample filter: Binomial       Number of points for filter: 3  Sample start region (min): 0  Sample end region (min): 30
Marker peak width (sec): 5    Marker min peak height: 200   Marker baseline V to V?: Y    Marker baseline V to V points: 3
Lower marker selection: First peak > 500 RFU                Upper marker selection: Last peak > 200 RFU
Ladder size (bp)75, 200, 300, 400, 500, 700, 1000, 1500, 2000, 3000, 4000, 5000, 7000, 10000, 20000
Quantification using: Lower Marker          Final concentration (ng/uL): 0.5000          Dilution factor: 12.0
```

Sample: SampG7
Well location: G7
Created: Tuesday, August 10, 2021 11:20:27 PM

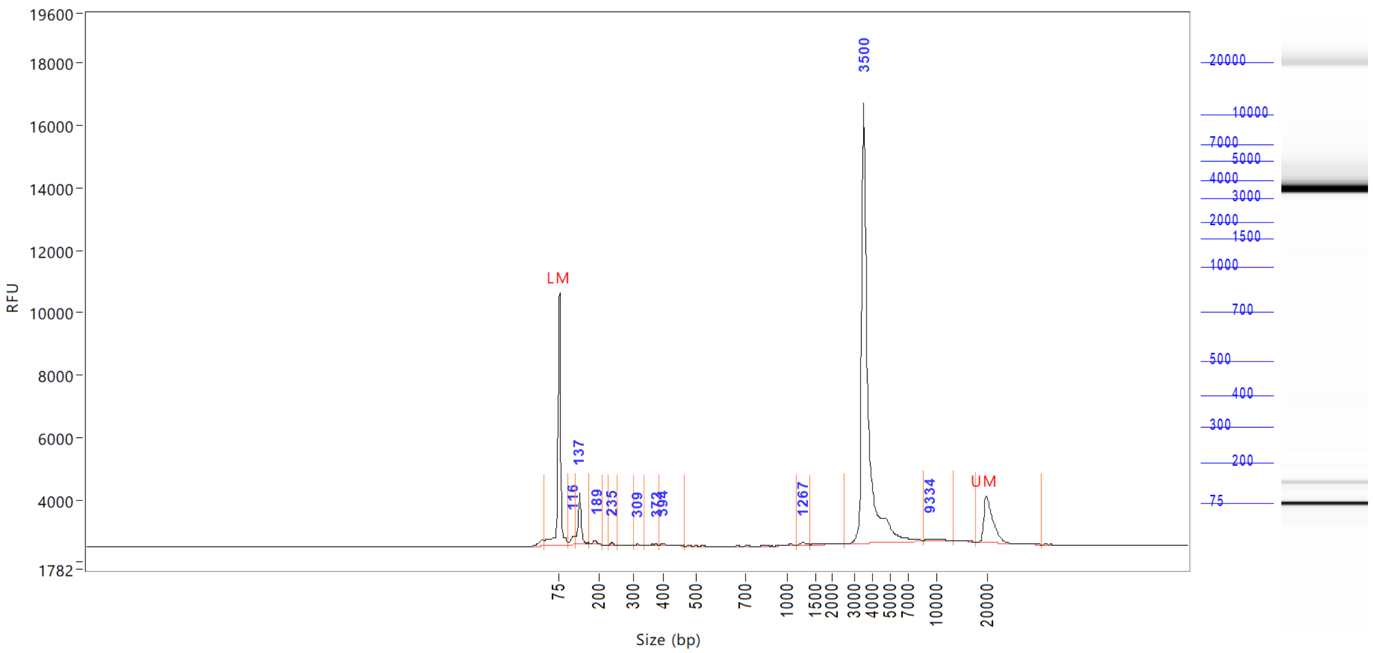

Peak	Size (bp)	Concentration (ng/uL)	From (bp)	To (bp)	Average size (bp)	CV%	RFU	Corrected peak area
1	75 (LM)	0.5000	28	103	71	14.14	8114	55.416
2	116	0.3103	103	124	115	5.09	270	2.866
3	137	1.3217	124	169	136	4.50	1653	12.207
4	189	0.1134	169	206	184	3.37	98	1.047
5	235	0.0468	226	251	234	1.77	69	0.432
6	309	0.0351	300	333	310	2.22	54	0.324
7	372	0.0842	333	382	363	3.10	60	0.778
8	394	0.1175	382	464	404	4.48	87	1.085
9	1267	0.0968	1156	1400	1262	4.40	82	0.894
10	3500	14.9533	2515	8734	3766	17.05	14136	138.108
11	9334	0.1155	8734	13415	9950	9.87	60	1.067
12	20000 (UM)	0.1523	17805	31002	20610	5.64	1469	16.880

TIC:	17.1946	ng/uL
TIM:	29.4722	nmole/L
Total concentration:	17.4085	ng/uL

```
Sample peak width (sec): 5    Sample min peak height: 50    Sample baseline V to V?: Y    Sample baseline V to V points: 3
Sample filter: Binomial       Number of points for filter: 3  Sample start region (min): 0  Sample end region (min): 30
Marker peak width (sec): 5    Marker min peak height: 200   Marker baseline V to V?: Y    Marker baseline V to V points: 3
Lower marker selection: First peak > 500 RFU                Upper marker selection: Last peak > 200 RFU
Ladder size (bp)75, 200, 300, 400, 500, 700, 1000, 1500, 2000, 3000, 4000, 5000, 7000, 10000, 20000
Quantification using: Lower Marker          Final concentration (ng/uL): 0.5000          Dilution factor: 12.0
```

Sample: SampH7
Well location: H7
Created: Tuesday, August 10, 2021 11:20:27 PM

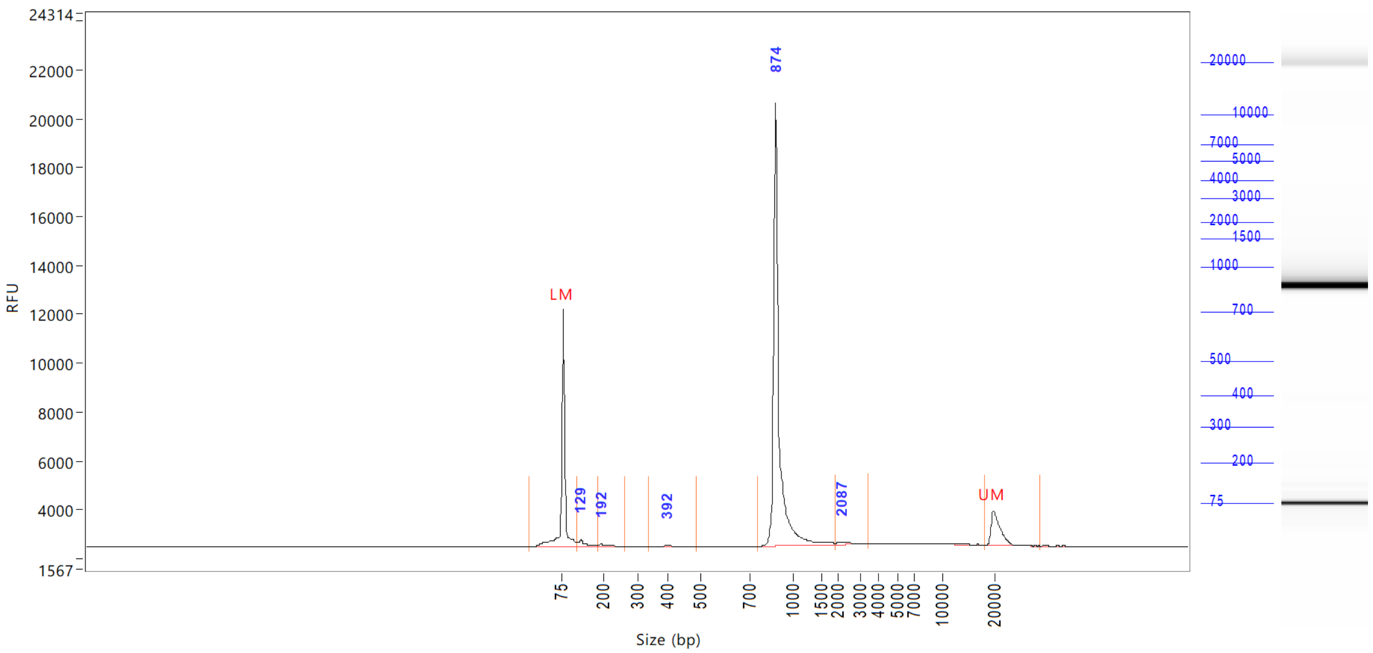

Peak	Size (bp)	Concentration (ng/uL)	From (bp)	To (bp)	Average size (bp)	CV%	RFU	Corrected peak area
1	75 (LM)	0.5000	0	120	70	25.83	9738	77.762
2	129	0.3448	120	183	141	12.48	282	4.469
3	192	0.1421	183	260	205	8.36	106	1.841
4	392	0.0962	335	485	398	6.33	66	1.247
5	874	12.7845	749	1904	908	12.34	18160	165.692
6	2087	0.2013	1904	3393	2416	16.61	109	2.608
7	20000 (UM)	0.1049	18171	29068	20581	5.45	1413	16.317

TIC:	13.5689	ng/uL
TIM:	28.8669	nmole/L
Total concentration:	13.7440	ng/uL

```
Sample peak width (sec): 5    Sample min peak height: 50    Sample baseline V to V?: Y    Sample baseline V to V points: 3
Sample filter: Binomial       Number of points for filter: 3  Sample start region (min): 0  Sample end region (min): 30
Marker peak width (sec): 5    Marker min peak height: 200   Marker baseline V to V?: Y    Marker baseline V to V points: 3
Lower marker selection: First peak > 500 RFU                Upper marker selection: Last peak > 200 RFU
Ladder size (bp)75, 200, 300, 400, 500, 700, 1000, 1500, 2000, 3000, 4000, 5000, 7000, 10000, 20000
Quantification using: Lower Marker          Final concentration (ng/uL): 0.5000          Dilution factor: 12.0
```

Sample: SampA8
Well location: A8
Created: Tuesday, August 10, 2021 11:20:27 PM

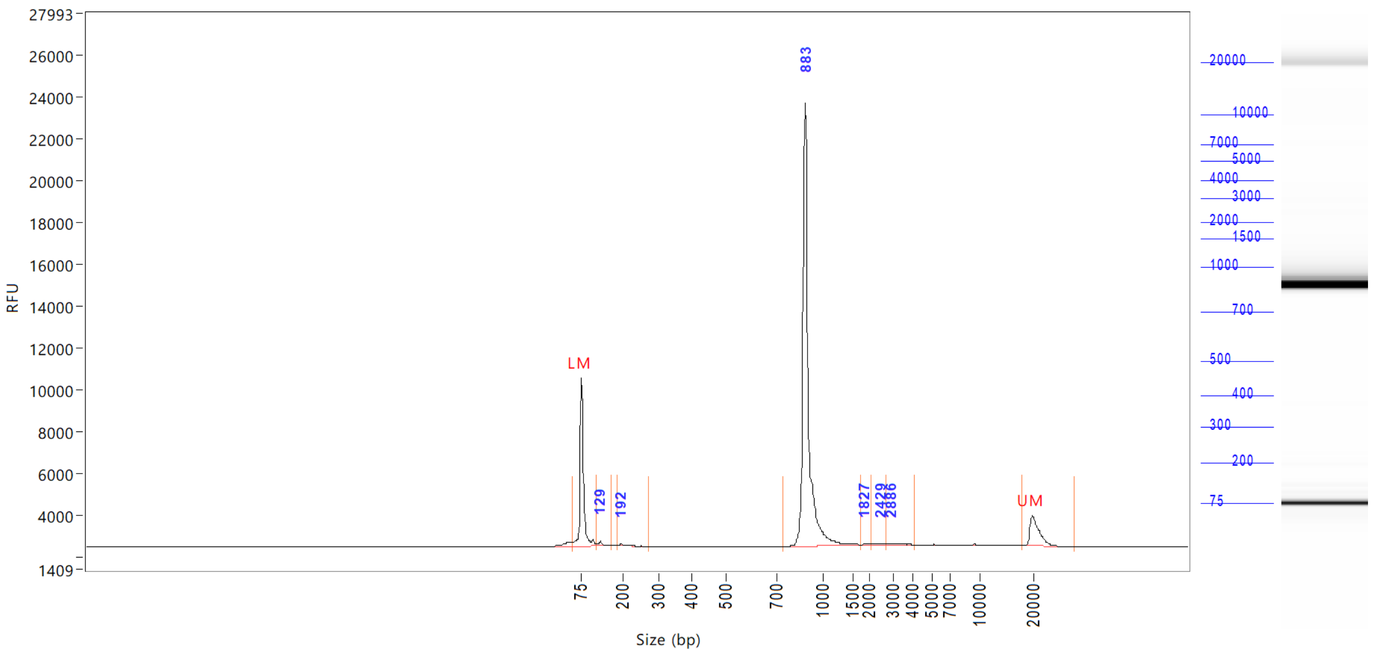

Peak	Size (bp)	Concentration (ng/uL)	From (bp)	To (bp)	Average size (bp)	CV%	RFU	Corrected peak area
1	75 (LM)	0.5000	50	120	75	11.93	8056	65.435
2	129	0.1939	120	164	131	6.71	215	2.115
3	192	0.1049	183	269	205	8.65	102	1.144
4	883	16.9722	740	1712	900	8.43	21231	185.095
5	1827	0.0603	1712	2058	1878	5.34	53	0.658
6	2429	0.0869	2058	2686	2367	7.56	57	0.948
7	2886	0.1073	2686	4104	3126	10.89	67	1.170
8	20000 (UM)	0.1226	18049	27859	20565	5.01	1436	16.050

TIC:	17.5255	ng/uL
TIM:	34.4810	nmole/L
Total concentration:	17.7872	ng/uL

```
Sample peak width (sec): 5    Sample min peak height: 50    Sample baseline V to V?: Y    Sample baseline V to V points: 3
Sample filter: Binomial       Number of points for filter: 3  Sample start region (min): 0  Sample end region (min): 30
Marker peak width (sec): 5    Marker min peak height: 200   Marker baseline V to V?: Y    Marker baseline V to V points: 3
Lower marker selection: First peak > 500 RFU                Upper marker selection: Last peak > 200 RFU
Ladder size (bp)75, 200, 300, 400, 500, 700, 1000, 1500, 2000, 3000, 4000, 5000, 7000, 10000, 20000
Quantification using: Lower Marker          Final concentration (ng/uL): 0.5000          Dilution factor: 12.0
```

Sample: SampB8
Well location: B8
Created: Tuesday, August 10, 2021 11:20:27 PM

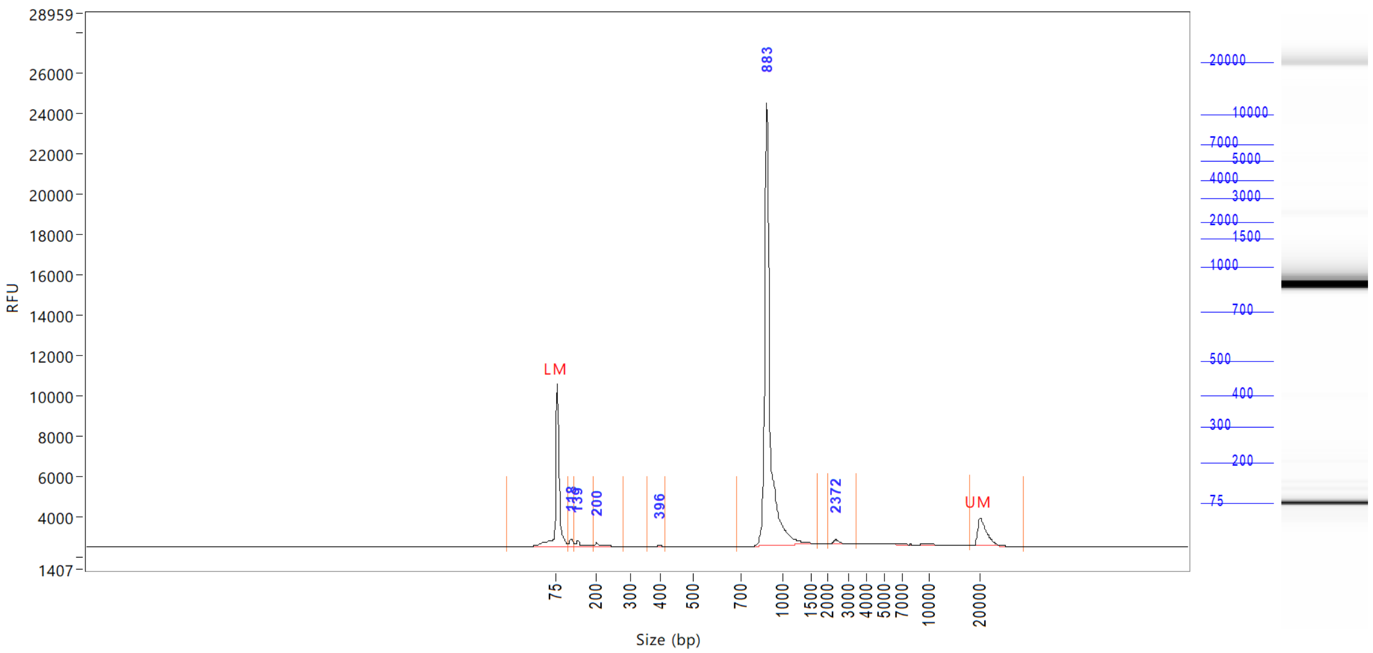

Peak	Size (bp)	Concentration (ng/uL)	From (bp)	To (bp)	Average size (bp)	CV%	RFU	Corrected peak area
1	75 (LM)	0.5000	0	108	71	23.48	8100	69.985
2	118	0.2888	108	128	117	4.44	398	3.368
3	139	0.4159	128	192	151	12.59	317	4.851
4	200	0.2322	192	278	214	8.53	181	2.708
5	396	0.0583	358	412	391	2.67	71	0.680
6	883	16.8936	685	1712	904	7.82	21979	197.051
7	2372	0.1765	2000	3393	2425	8.51	211	2.059
8	20000 (UM)	0.1119	18171	28705	20656	5.03	1370	15.668

TIC:	18.0652	ng/uL
TIM:	41.4878	nmole/L
Total concentration:	18.2787	ng/uL

Sample peak width (sec): 5   Sample min peak height: 50   Sample baseline V to V?: Y   Sample baseline V to V points: 3
Sample filter: Binomial   Number of points for filter: 3 Sample start region (min): 0 Sample end region (min): 30
Marker peak width (sec): 5   Marker min peak height: 200   Marker baseline V to V?: Y   Marker baseline V to V points: 3
Lower marker selection: First peak > 500 RFU   Upper marker selection: Last peak > 200 RFU
Ladder size (bp)75, 200, 300, 400, 500, 700, 1000, 1500, 2000, 3000, 4000, 5000, 7000, 10000, 20000
Quantification using: Lower Marker   Final concentration (ng/uL): 0.5000   Dilution factor: 12.0

Sample: SampC8
Well location: C8
Created: Tuesday, August 10, 2021 11:20:27 PM

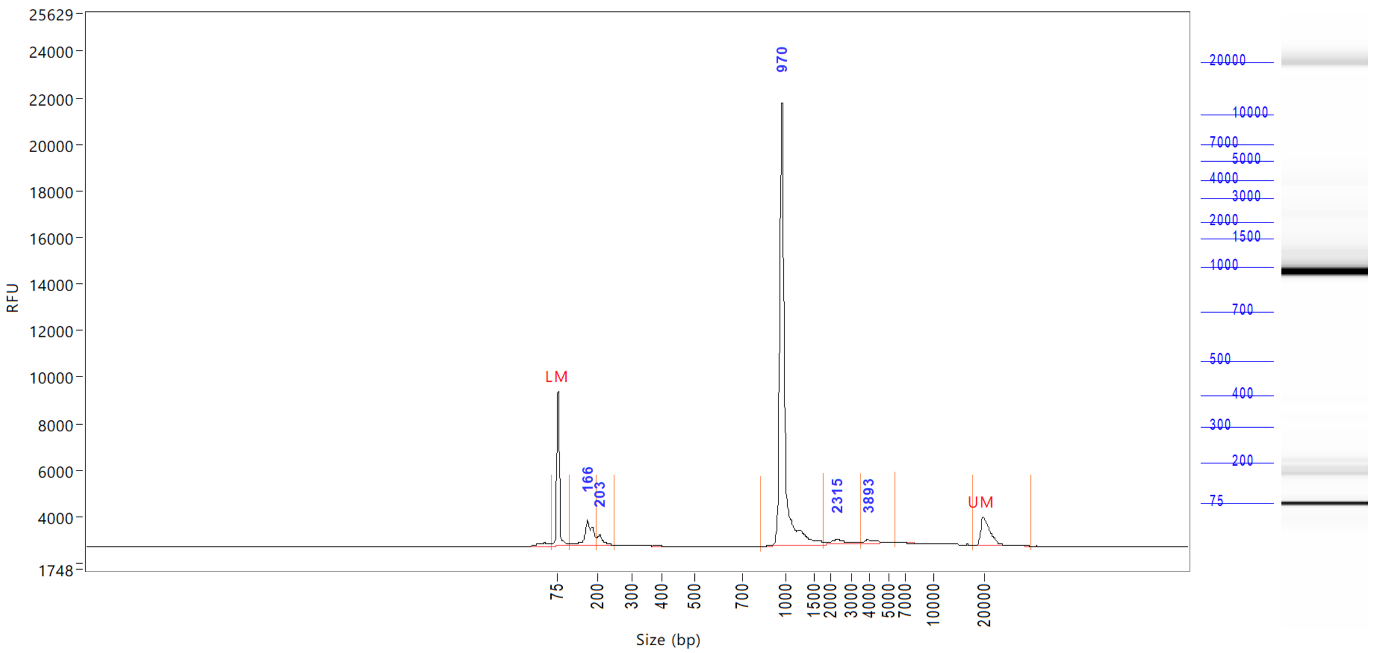

Peak	Size (bp)	Concentration (ng/uL)	From (bp)	To (bp)	Average size (bp)	CV%	RFU	Corrected peak area
1	75 (LM)	0.5000	54	112	73	8.82	6634	41.622
2	166	2.9531	112	194	166	9.73	1114	20.486
3	203	0.8764	194	250	208	5.74	470	6.079
4	970	22.6458	827	1827	1009	11.76	19053	157.094
5	2315	0.8081	1827	3465	2444	17.55	210	5.606
6	3893	0.4305	3465	5889	4149	10.41	149	2.987
7	20000 (UM)	0.1713	17805	29551	20613	5.46	1217	14.258

TIC:	27.7138	ng/uL
TIM:	73.8266	nmole/L
Total concentration:	27.9115	ng/uL

Sample peak width (sec): 5   Sample min peak height: 50   Sample baseline V to V?: Y   Sample baseline V to V points: 3
Sample filter: Binomial   Number of points for filter: 3 Sample start region (min): 0 Sample end region (min): 30
Marker peak width (sec): 5   Marker min peak height: 200   Marker baseline V to V?: Y   Marker baseline V to V points: 3
Lower marker selection: First peak > 500 RFU   Upper marker selection: Last peak > 200 RFU
Ladder size (bp)75, 200, 300, 400, 500, 700, 1000, 1500, 2000, 3000, 4000, 5000, 7000, 10000, 20000
Quantification using: Lower Marker   Final concentration (ng/uL): 0.5000   Dilution factor: 12.0

Sample: SampD8
Well location: D8
Created: Tuesday, August 10, 2021 11:20:27 PM

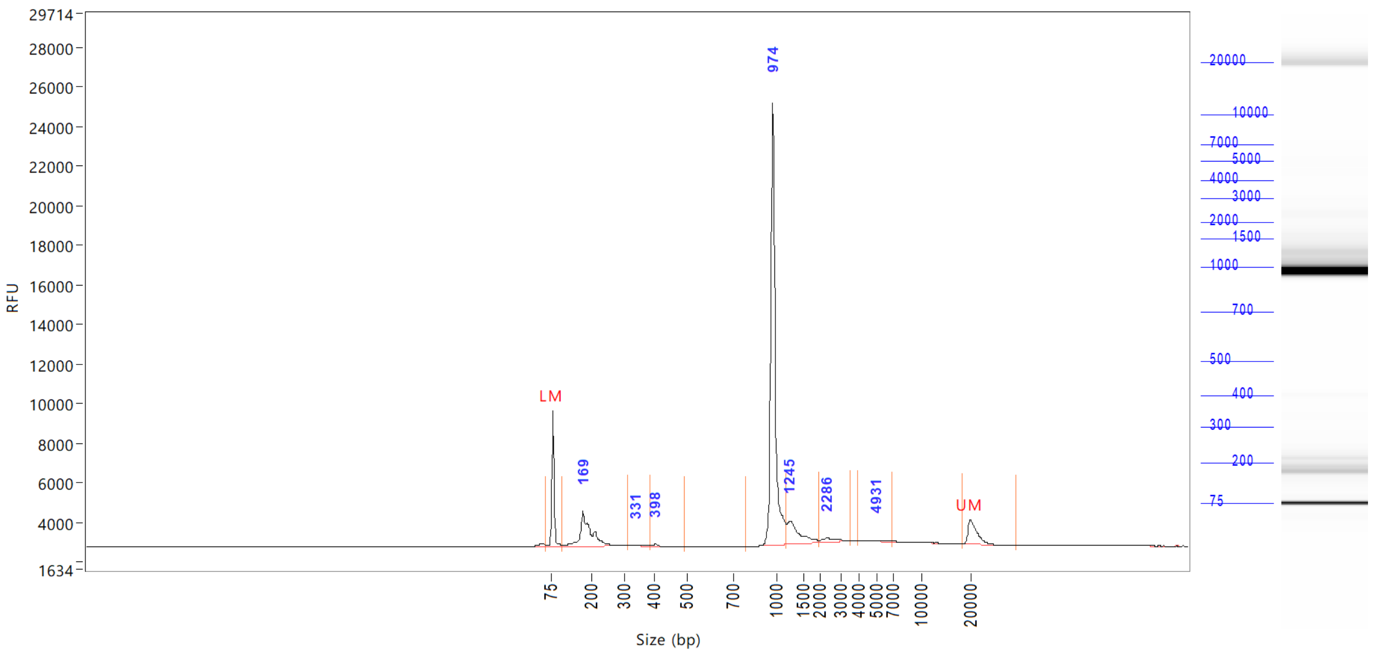

Peak	Size (bp)	Concentration (ng/uL)	From (bp)	To (bp)	Average size (bp)	CV%	RFU	Corrected peak area
1	75 (LM)	0.5000	56	105	74	7.12	6838	40.500
2	169	6.5405	105	307	185	16.81	1764	44.148
3	331	0.2447	307	386	342	6.24	62	1.652
4	398	0.1541	386	492	404	4.90	108	1.040
5	974	26.2093	779	1178	983	4.25	22375	176.911
6	1245	3.6250	1178	1981	1377	13.38	1182	24.469
7	2286	0.6653	1981	3465	2472	14.15	221	4.490
8	4931	0.2715	4000	6852	5016	14.51	62	1.833
9	20000 (UM)	0.1776	18293	29189	20647	5.72	1225	14.382

TIC:	37.7104	ng/uL
TIM:	108.6786	nmole/L
Total concentration:	37.6973	ng/uL

Sample peak width (sec): 5 Sample min peak height: 50 Sample baseline V to V?: Y Sample baseline V to V points: 3
Sample filter: Binomial Number of points for filter: 3 Sample start region (min): 0 Sample end region (min): 30
Marker peak width (sec): 5 Marker min peak height: 200 Marker baseline V to V?: Y Marker baseline V to V points: 3
Lower marker selection: First peak > 500 RFU Upper marker selection: Last peak > 200 RFU
Ladder size (bp)75, 200, 300, 400, 500, 700, 1000, 1500, 2000, 3000, 4000, 5000, 7000, 10000, 20000
Quantification using: Lower Marker Final concentration (ng/uL): 0.5000 Dilution factor: 12.0

Sample: SampE8
Well location: E8
Created: Tuesday, August 10, 2021 11:20:27 PM

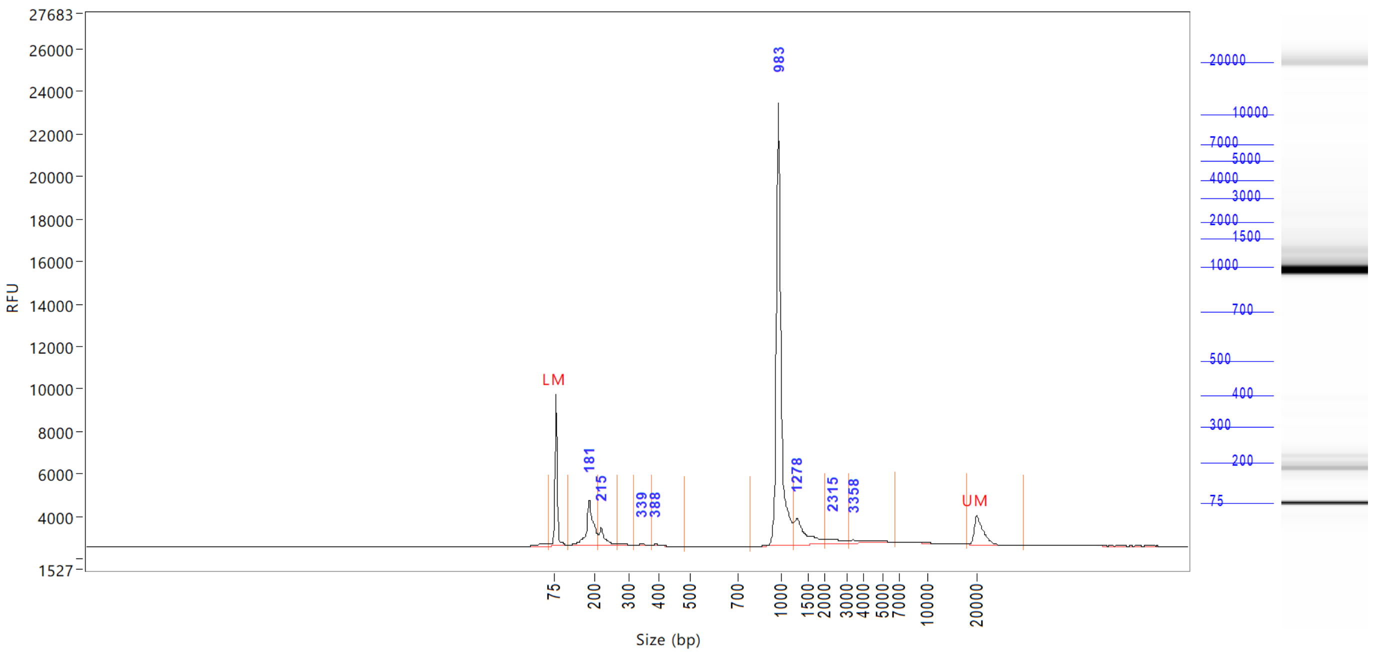

Peak	Size (bp)	Concentration (ng/uL)	From (bp)	To (bp)	Average size (bp)	CV%	RFU	Corrected peak area
1	75 (LM)	0.5000	54	112	74	7.72	7102	42.858
2	181	4.7555	112	206	179	9.19	2126	33.969
3	215	1.5029	206	268	221	5.93	823	10.735
4	339	0.1185	313	376	343	3.98	51	0.846
5	388	0.1224	376	479	394	4.79	93	0.874
6	983	23.7309	783	1212	996	5.06	20861	169.511
7	1278	3.5366	1212	2001	1428	14.22	1219	25.262
8	2315	0.8045	2001	3108	2493	12.72	248	5.747
9	3358	0.7112	3108	6630	4107	18.01	151	5.080
10	20000 (UM)	0.1815	18049	29430	20610	5.14	1351	15.562

TIC:	35.2825	ng/uL
TIM:	100.0468	nmole/L
Total concentration:	35.2763	ng/uL

Sample peak width (sec): 5 Sample min peak height: 50 Sample baseline V to V?: Y Sample baseline V to V points: 3
Sample filter: Binomial Number of points for filter: 3 Sample start region (min): 0 Sample end region (min): 30
Marker peak width (sec): 5 Marker min peak height: 200 Marker baseline V to V?: Y Marker baseline V to V points: 3
Lower marker selection: First peak > 500 RFU Upper marker selection: Last peak > 200 RFU
Ladder size (bp)75, 200, 300, 400, 500, 700, 1000, 1500, 2000, 3000, 4000, 5000, 7000, 10000, 20000
Quantification using: Lower Marker Final concentration (ng/uL): 0.5000 Dilution factor: 12.0

Sample: SampF8
Well location: F8
Created: Tuesday, August 10, 2021 11:20:27 PM

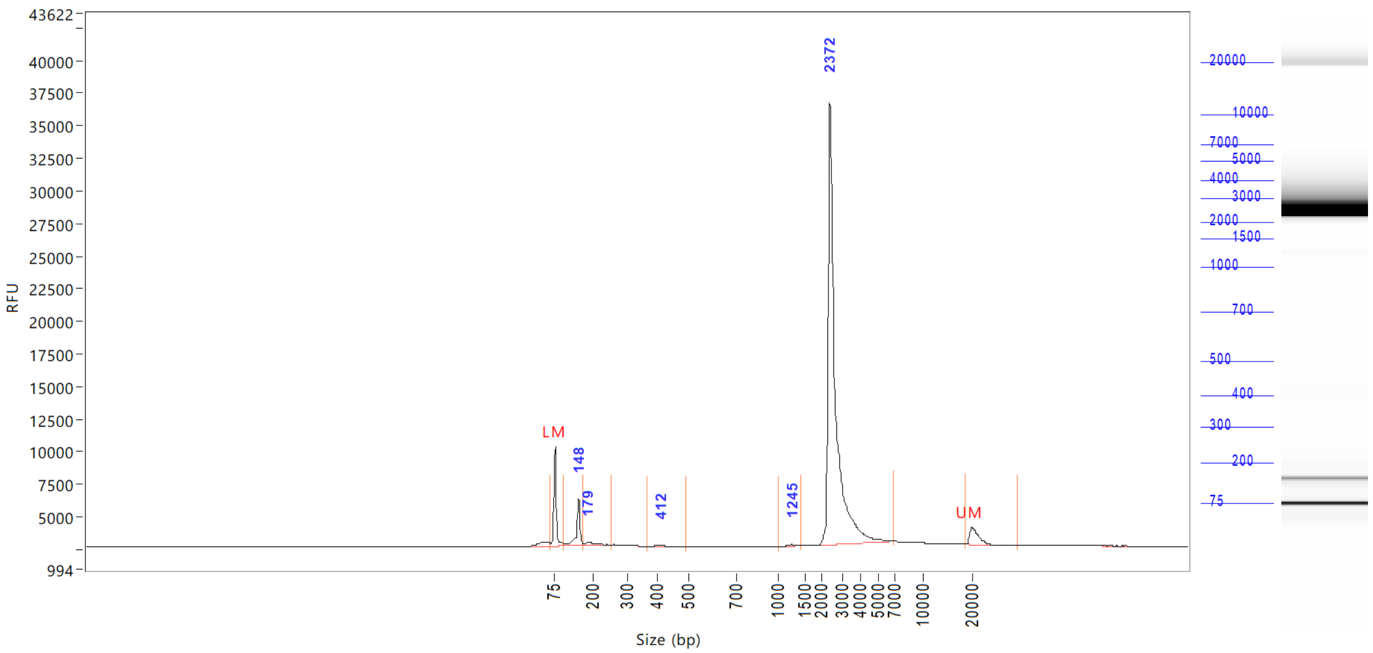

Peak	Size (bp)	Concentration (ng/uL)	From (bp)	To (bp)	Average size (bp)	CV%	RFU	Corrected peak area
1	75 (LM)	0.5000	60	101	74	7.81	7609	47.793
2	148	3.8236	101	166	142	8.38	3572	30.457
3	179	0.7343	166	251	193	9.75	214	5.849
4	412	0.1865	368	492	406	3.82	82	1.485
5	1245	0.1410	1012	1433	1236	5.24	123	1.123
6	2372	47.1654	1433	6852	2640	18.76	33945	375.693
7	20000 (UM)	0.1598	18659	29189	20690	5.28	1328	15.274

TIC:	52.0508	ng/uL
TIM:	80.8602	nmole/L
Total concentration:	52.1920	ng/uL

Sample peak width (sec): 5   Sample min peak height: 50   Sample baseline V to V?: Y   Sample baseline V to V points: 3
Sample filter: Binomial   Number of points for filter: 3 Sample start region (min): 0 Sample end region (min): 30
Marker peak width (sec): 5   Marker min peak height: 200   Marker baseline V to V?: Y   Marker baseline V to V points: 3
Lower marker selection: First peak > 500 RFU   Upper marker selection: Last peak > 200 RFU
Ladder size (bp)75, 200, 300, 400, 500, 700, 1000, 1500, 2000, 3000, 4000, 5000, 7000, 10000, 20000
Quantification using: Lower Marker   Final concentration (ng/uL): 0.5000   Dilution factor: 12.0

Sample: SampG8
Well location: G8
Created: Tuesday, August 10, 2021 11:20:27 PM

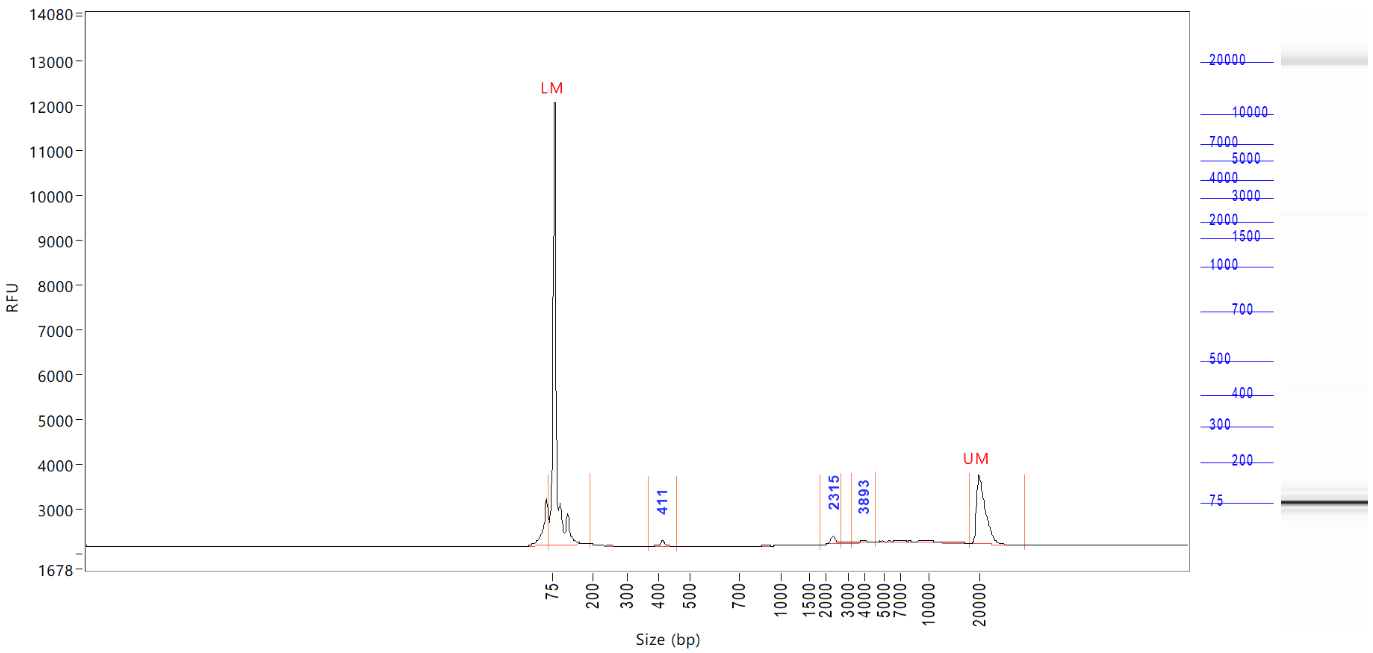

Peak	Size (bp)	Concentration (ng/uL)	From (bp)	To (bp)	Average size (bp)	CV%	RFU	Corrected peak area
1	75 (LM)	0.5000	60	187	79	19.15	9885	87.334
2	411	0.0850	366	454	409	3.39	115	1.238
3	2315	0.0979	1808	2657	2300	5.86	175	1.425
4	3893	0.0579	3215	4587	3908	8.26	58	0.843
5	20000 (UM)	0.1021	18049	29189	20601	5.14	1554	17.826

TIC:	0.2408	ng/uL
TIM:	0.4365	nmole/L
Total concentration:	0.5003	ng/uL

```
Sample peak width (sec): 5    Sample min peak height: 50    Sample baseline V to V?: Y    Sample baseline V to V points: 3
Sample filter: Binomial       Number of points for filter: 3  Sample start region (min): 0  Sample end region (min): 30
Marker peak width (sec): 5    Marker min peak height: 200   Marker baseline V to V?: Y    Marker baseline V to V points: 3
Lower marker selection: First peak > 500 RFU                Upper marker selection: Last peak > 200 RFU
Ladder size (bp)75, 200, 300, 400, 500, 700, 1000, 1500, 2000, 3000, 4000, 5000, 7000, 10000, 20000
Quantification using: Lower Marker          Final concentration (ng/uL): 0.5000          Dilution factor: 12.0
```

Sample: SampH8
Well location: H8
Created: Tuesday, August 10, 2021 11:20:27 PM

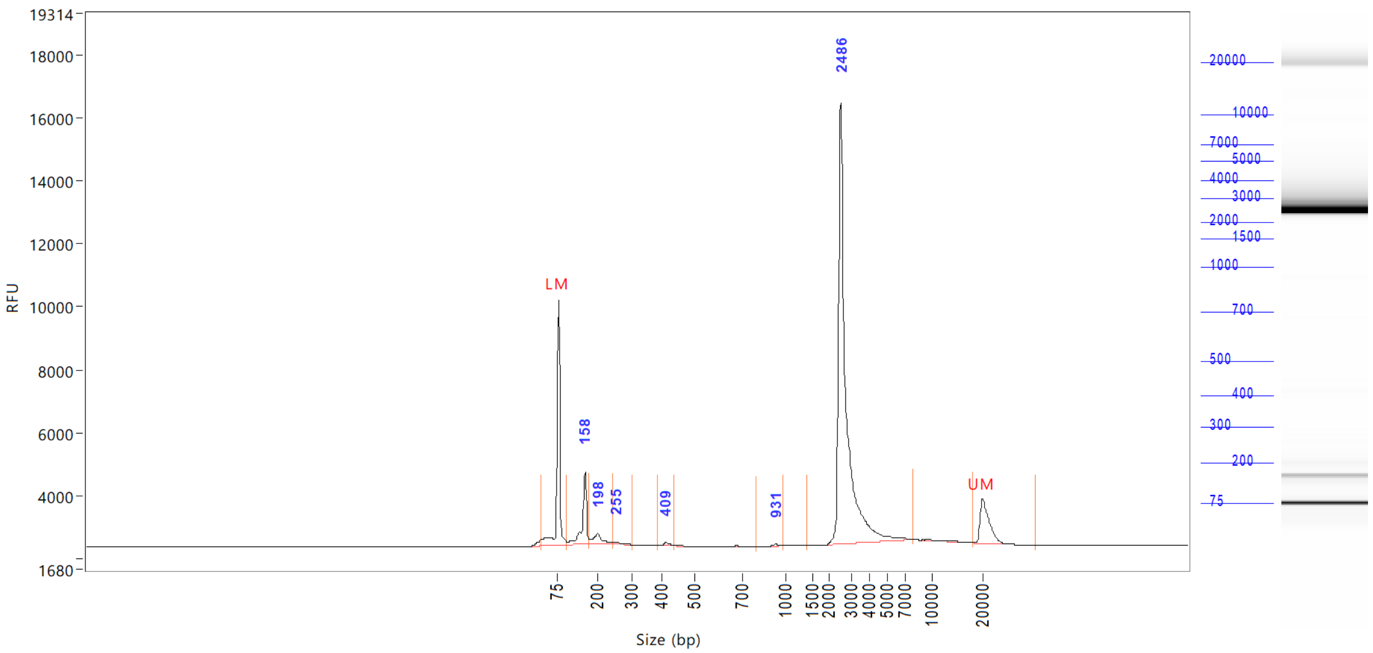

Peak	Size (bp)	Concentration (ng/uL)	From (bp)	To (bp)	Average size (bp)	CV%	RFU	Corrected peak area
1	75 (LM)	0.5000	24	103	71	17.20	7788	53.937
2	158	2.3324	103	171	149	9.10	2303	20.967
3	198	0.7430	171	242	199	8.40	335	6.679
4	255	0.1271	242	301	261	5.93	79	1.143
5	409	0.1348	384	433	409	2.50	108	1.212
6	931	0.0524	796	983	926	2.79	60	0.471
7	2486	18.1071	1378	7867	2767	21.74	14014	162.774
8	20000 (UM)	0.1552	18049	30639	20577	5.36	1424	16.744

TIC:	21.4969	ng/uL
TIM:	44.0750	nmole/L
Total concentration:	21.7022	ng/uL

Sample peak width (sec): 5   Sample min peak height: 50   Sample baseline V to V?: Y   Sample baseline V to V points: 3
Sample filter: Binomial   Number of points for filter: 3 Sample start region (min): 0 Sample end region (min): 30
Marker peak width (sec): 5   Marker min peak height: 200   Marker baseline V to V?: Y   Marker baseline V to V points: 3
Lower marker selection: First peak > 500 RFU   Upper marker selection: Last peak > 200 RFU
Ladder size (bp)75, 200, 300, 400, 500, 700, 1000, 1500, 2000, 3000, 4000, 5000, 7000, 10000, 20000
Quantification using: Lower Marker   Final concentration (ng/uL): 0.5000   Dilution factor: 12.0

Sample: SampA9
Well location: A9
Created: Tuesday, August 10, 2021 11:20:27 PM

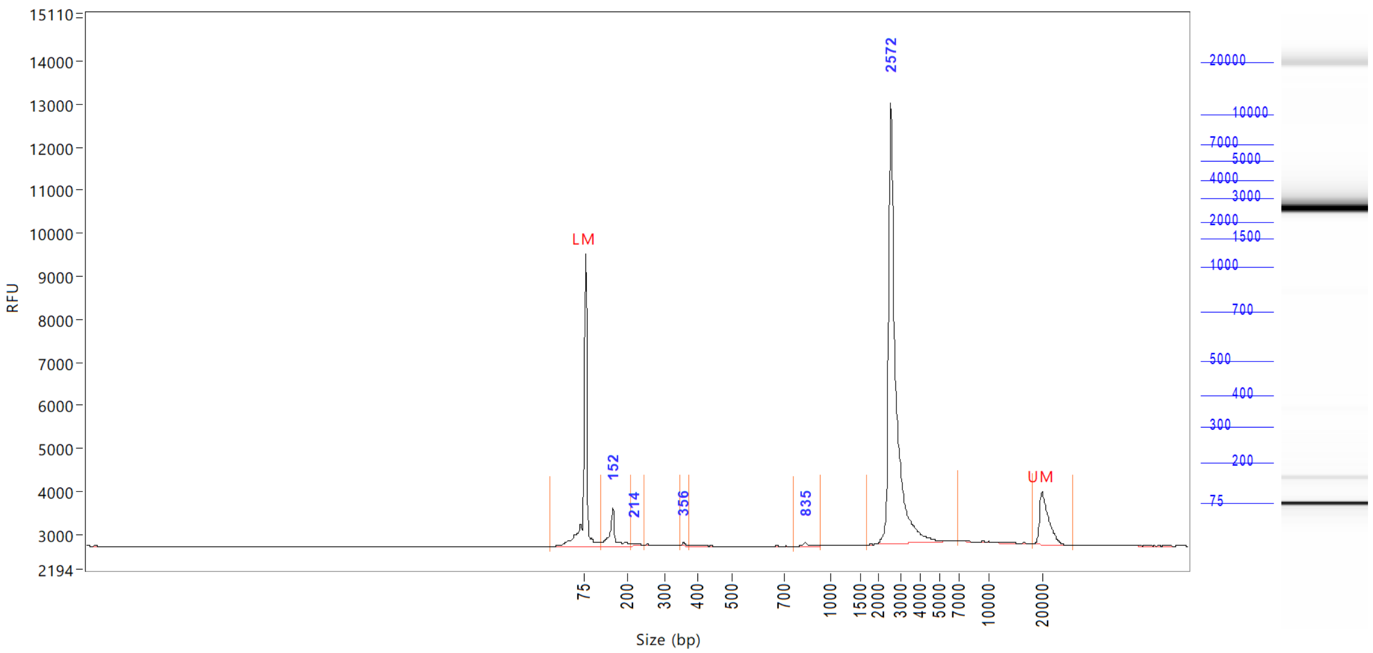

Peak	Size (bp)	Concentration (ng/uL)	From (bp)	To (bp)	Average size (bp)	CV%	RFU	Corrected peak area
1	75 (LM)	0.5000	0	118	68	23.87	6801	49.957
2	152	1.2389	118	206	153	12.08	896	10.316
3	214	0.1169	206	241	221	4.47	52	0.973
4	356	0.0642	343	374	355	1.65	78	0.535
5	835	0.0835	766	939	841	3.37	76	0.695
6	2572	13.0968	1674	7001	2727	15.19	10259	109.046
7	20000 (UM)	0.1369	18171	25561	20528	4.82	1218	13.676

TIC:	14.6003	ng/uL
TIM:	22.5459	nmole/L
Total concentration:	14.9691	ng/uL

Sample peak width (sec): 5   Sample min peak height: 50   Sample baseline V to V?: Y   Sample baseline V to V points: 3
Sample filter: Binomial   Number of points for filter: 3 Sample start region (min): 0 Sample end region (min): 30
Marker peak width (sec): 5   Marker min peak height: 200   Marker baseline V to V?: Y   Marker baseline V to V points: 3
Lower marker selection: First peak > 500 RFU   Upper marker selection: Last peak > 200 RFU
Ladder size (bp)75, 200, 300, 400, 500, 700, 1000, 1500, 2000, 3000, 4000, 5000, 7000, 10000, 20000
Quantification using: Lower Marker   Final concentration (ng/uL): 0.5000   Dilution factor: 12.0

Sample: SampB9
Well location: B9
Created: Tuesday, August 10, 2021 11:20:27 PM

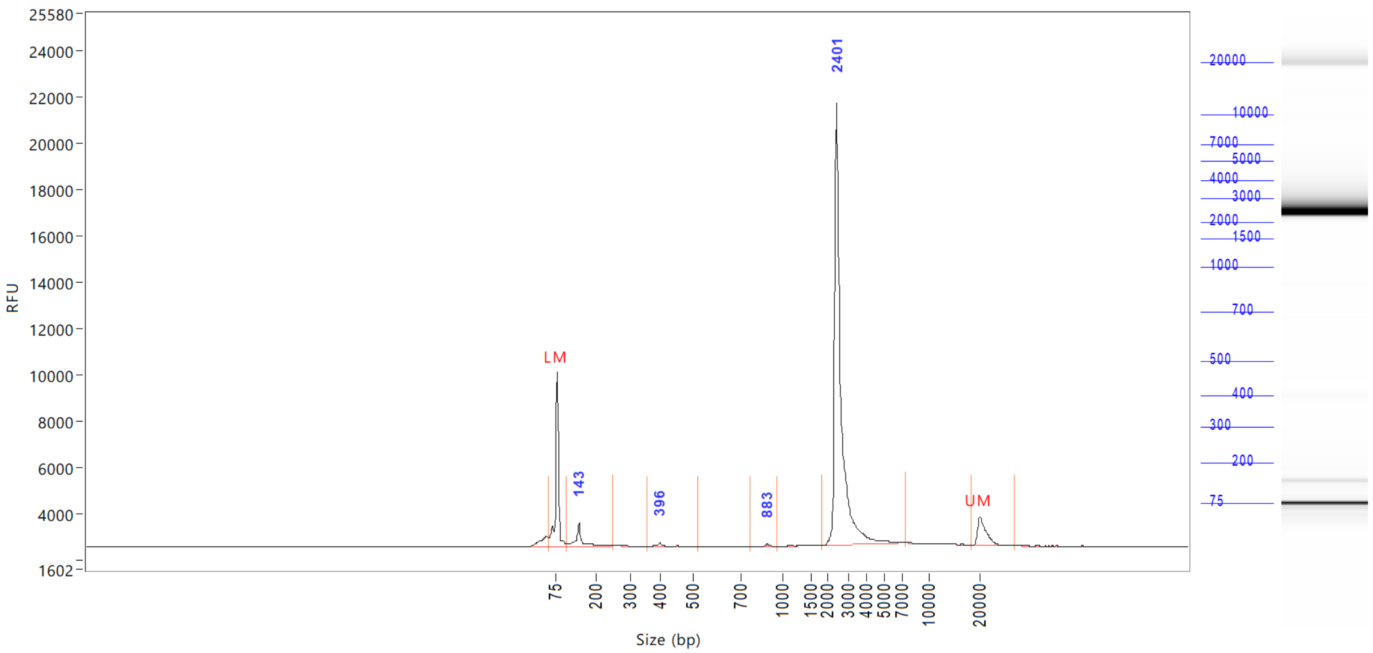

Peak	Size (bp)	Concentration (ng/uL)	From (bp)	To (bp)	Average size (bp)	CV%	RFU	Corrected peak area
1	75 (LM)	0.5000	50	105	72	10.73	7502	52.813
2	143	1.5049	105	246	150	19.63	981	13.247
3	396	0.2033	358	521	406	6.44	149	1.790
4	883	0.0863	766	961	879	3.60	84	0.760
5	2401	21.4160	1827	7334	2595	18.23	19100	188.507
6	20000 (UM)	0.1276	18415	27133	20638	5.08	1205	13.474

TIC:	23.2106	ng/uL
TIM:	31.0607	nmole/L
Total concentration:	23.4279	ng/uL

Sample peak width (sec): 5 Sample min peak height: 50 Sample baseline V to V?: Y Sample baseline V to V points: 3
Sample filter: Binomial Number of points for filter: 3 Sample start region (min): 0 Sample end region (min): 30
Marker peak width (sec): 5 Marker min peak height: 200 Marker baseline V to V?: Y Marker baseline V to V points: 3
Lower marker selection: First peak > 500 RFU Upper marker selection: Last peak > 200 RFU
Ladder size (bp)75, 200, 300, 400, 500, 700, 1000, 1500, 2000, 3000, 4000, 5000, 7000, 10000, 20000
Quantification using: Lower Marker Final concentration (ng/uL): 0.5000 Dilution factor: 12.0

Sample: SampC9
Well location: C9
Created: Tuesday, August 10, 2021 11:20:27 PM

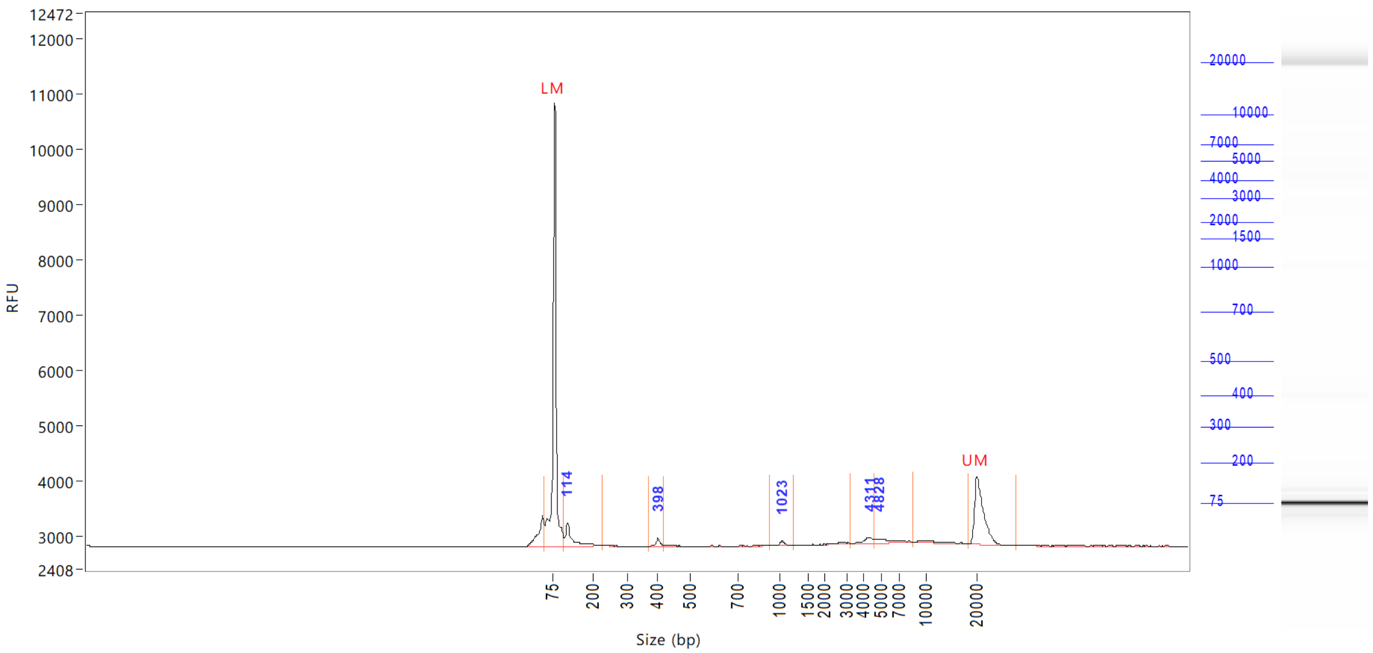

Peak	Size (bp)	Concentration (ng/uL)	From (bp)	To (bp)	Average size (bp)	CV%	RFU	Corrected peak area
1	75 (LM)	0.5000	45	105	73	12.17	8041	63.910
2	114	0.6153	105	223	132	20.01	421	6.555
3	398	0.1166	368	416	396	2.26	149	1.242
4	1023	0.0652	922	1234	1037	5.47	80	0.695
5	4311	0.1496	3215	4587	4130	7.90	101	1.593
6	4828	0.2035	4587	8534	5753	18.15	83	2.167
7	20000 (UM)	0.1125	18415	27979	20688	5.32	1222	14.383

TIC:	1.1502	ng/uL
TIM:	8.3654	nmole/L
Total concentration:	1.4690	ng/uL

Sample peak width (sec): 5 Sample min peak height: 50 Sample baseline V to V?: Y Sample baseline V to V points: 3
Sample filter: Binomial Number of points for filter: 3 Sample start region (min): 0 Sample end region (min): 30
Marker peak width (sec): 5 Marker min peak height: 200 Marker baseline V to V?: Y Marker baseline V to V points: 3
Lower marker selection: First peak > 500 RFU Upper marker selection: Last peak > 200 RFU
Ladder size (bp)75, 200, 300, 400, 500, 700, 1000, 1500, 2000, 3000, 4000, 5000, 7000, 10000, 20000
Quantification using: Lower Marker Final concentration (ng/uL): 0.5000 Dilution factor: 12.0

Sample: SampD9
Well location: D9
Created: Tuesday, August 10, 2021 11:20:27 PM

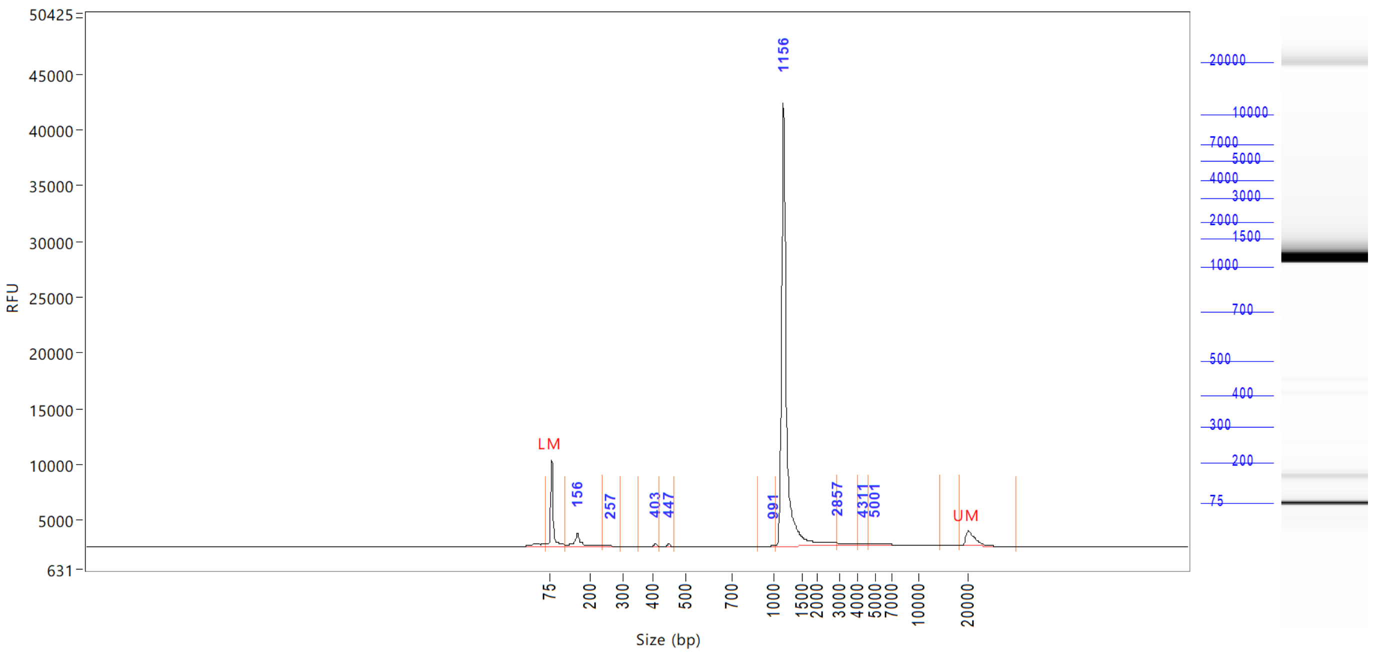

Peak	Size (bp)	Concentration (ng/uL)	From (bp)	To (bp)	Average size (bp)	CV%	RFU	Corrected peak area
1	75 (LM)	0.5000	60	122	77	12.60	7730	52.980
2	156	2.0455	122	237	160	13.43	1188	18.062
3	257	0.1102	237	291	255	3.92	88	0.973
4	403	0.2141	349	418	399	2.56	227	1.891
5	447	0.1952	418	464	441	1.90	224	1.724
6	991	0.0457	879	1012	984	1.90	61	0.404
7	1156	37.0613	1012	2857	1221	17.80	39766	327.252
8	2857	0.5432	2857	4035	3374	10.95	247	4.797
9	4311	0.1931	4035	4621	4324	4.11	131	1.705
10	5001	0.7415	4621	14391	7300	31.89	140	6.547
11	20000 (UM)	0.1513	18171	29672	20712	5.65	1339	16.035

TIC:	41.1500	ng/uL
TIM:	73.8799	nmole/L
Total concentration:	41.2127	ng/uL

Sample peak width (sec): 5 Sample min peak height: 50 Sample baseline V to V?: Y Sample baseline V to V points: 3
Sample filter: Binomial Number of points for filter: 3 Sample start region (min): 0 Sample end region (min): 30
Marker peak width (sec): 5 Marker min peak height: 200 Marker baseline V to V?: Y Marker baseline V to V points: 3
Lower marker selection: First peak > 500 RFU Upper marker selection: Last peak > 200 RFU
Ladder size (bp)75, 200, 300, 400, 500, 700, 1000, 1500, 2000, 3000, 4000, 5000, 7000, 10000, 20000
Quantification using: Lower Marker Final concentration (ng/uL): 0.5000 Dilution factor: 12.0

Sample: SampE9
Well location: E9
Created: Tuesday, August 10, 2021 11:20:27 PM

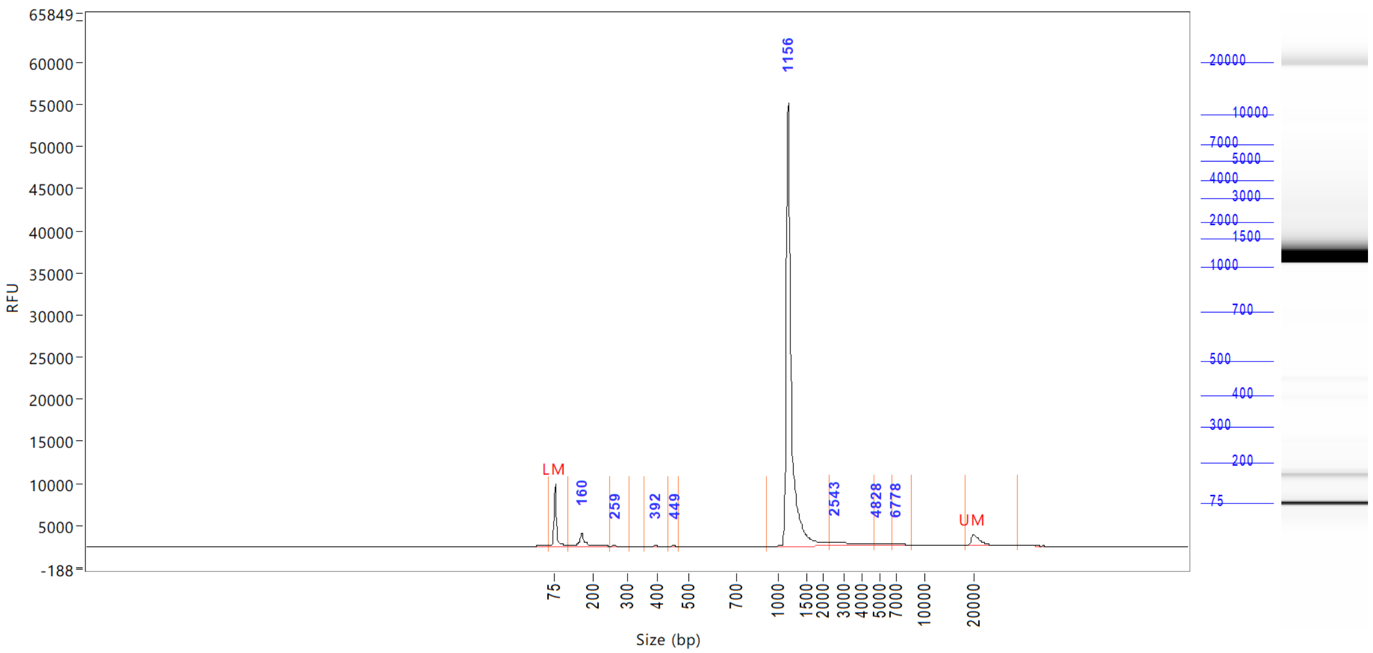

Peak	Size (bp)	Concentration (ng/uL)	From (bp)	To (bp)	Average size (bp)	CV%	RFU	Corrected peak area
1	75 (LM)	0.5000	58	120	76	12.90	7435	50.636
2	160	3.0158	120	244	164	14.98	1658	25.451
3	259	0.1996	244	305	261	4.72	118	1.685
4	392	0.2427	354	431	394	3.83	184	2.048
5	449	0.1867	431	466	446	1.28	229	1.576
6	1156	53.4039	909	2315	1207	12.52	52762	450.694
7	2543	1.9031	2315	4690	3208	20.47	406	16.061
8	4828	0.2816	4690	6408	5308	9.72	133	2.377
9	6778	0.1363	6408	8667	7125	7.03	81	1.151
10	20000 (UM)	0.1423	18415	29068	20679	5.65	1235	14.409

TIC:	59.3699	ng/uL
TIM:	107.1109	nmole/L
Total concentration:	59.5156	ng/uL

Sample peak width (sec): 5 Sample min peak height: 50 Sample baseline V to V?: Y Sample baseline V to V points: 3
Sample filter: Binomial Number of points for filter: 3 Sample start region (min): 0 Sample end region (min): 30
Marker peak width (sec): 5 Marker min peak height: 200 Marker baseline V to V?: Y Marker baseline V to V points: 3
Lower marker selection: First peak > 500 RFU Upper marker selection: Last peak > 200 RFU
Ladder size (bp)75, 200, 300, 400, 500, 700, 1000, 1500, 2000, 3000, 4000, 5000, 7000, 10000, 20000
Quantification using: Lower Marker Final concentration (ng/uL): 0.5000 Dilution factor: 12.0

Sample: SampF9
Well location: F9
Created: Tuesday, August 10, 2021 11:20:27 PM

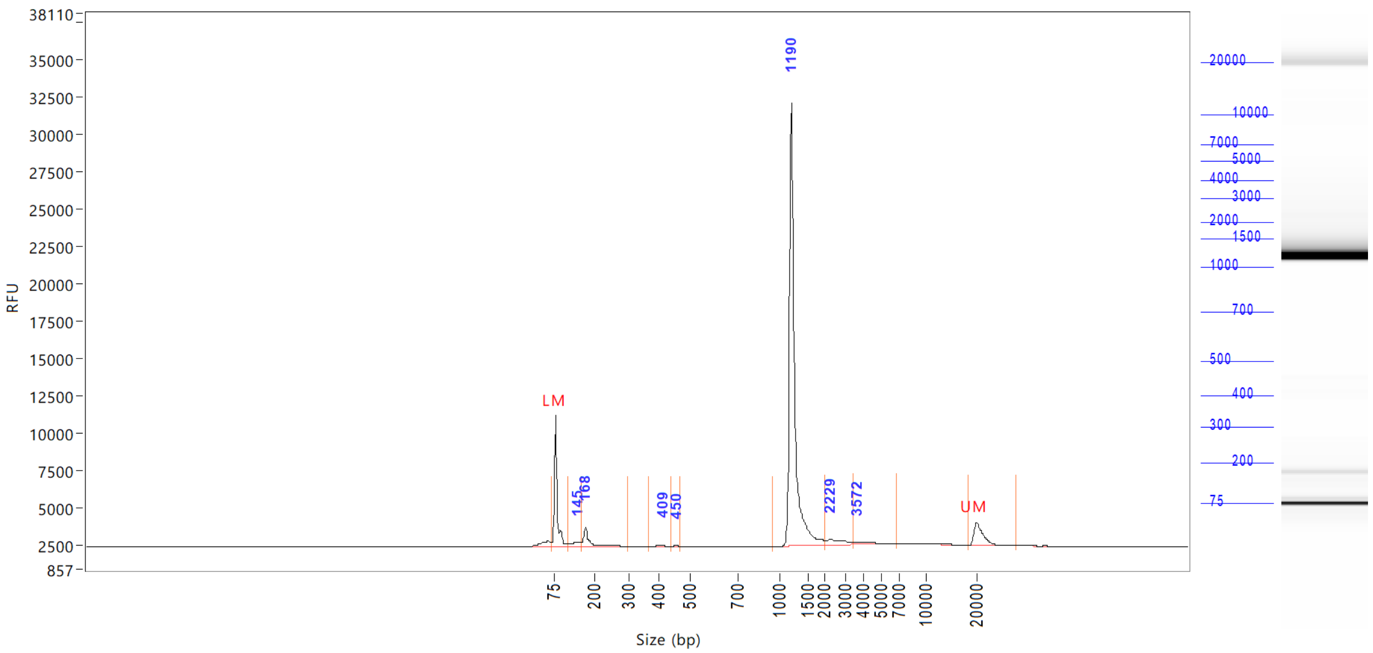

Peak	Size (bp)	Concentration (ng/uL)	From (bp)	To (bp)	Average size (bp)	CV%	RFU	Corrected peak area
1	75 (LM)	0.5000	62	114	76	10.97	8829	62.235
2	145	0.6407	114	156	137	8.86	300	6.646
3	168	1.5966	156	296	185	15.68	1247	16.561
4	409	0.1649	366	437	404	3.39	128	1.711
5	450	0.0673	437	468	449	1.20	113	0.698
6	1190	24.5981	939	2030	1236	10.71	29737	255.143
7	2229	0.9288	2030	3358	2576	14.41	375	9.634
8	3572	0.3988	3358	6852	4091	14.28	166	4.137
9	20000 (UM)	0.1426	18415	28100	20670	5.09	1517	17.746

TIC:	28.3952	ng/uL
TIM:	56.3039	nmole/L
Total concentration:	28.5191	ng/uL

```
Sample peak width (sec): 5    Sample min peak height: 50    Sample baseline V to V?: Y    Sample baseline V to V points: 3
Sample filter: Binomial       Number of points for filter: 3  Sample start region (min): 0  Sample end region (min): 30
Marker peak width (sec): 5    Marker min peak height: 200   Marker baseline V to V?: Y    Marker baseline V to V points: 3
Lower marker selection: First peak > 500 RFU                Upper marker selection: Last peak > 200 RFU
Ladder size (bp)75, 200, 300, 400, 500, 700, 1000, 1500, 2000, 3000, 4000, 5000, 7000, 10000, 20000
Quantification using: Lower Marker          Final concentration (ng/uL): 0.5000          Dilution factor: 12.0
```

Sample: SampG9
Well location: G9
Created: Tuesday, August 10, 2021 11:20:27 PM

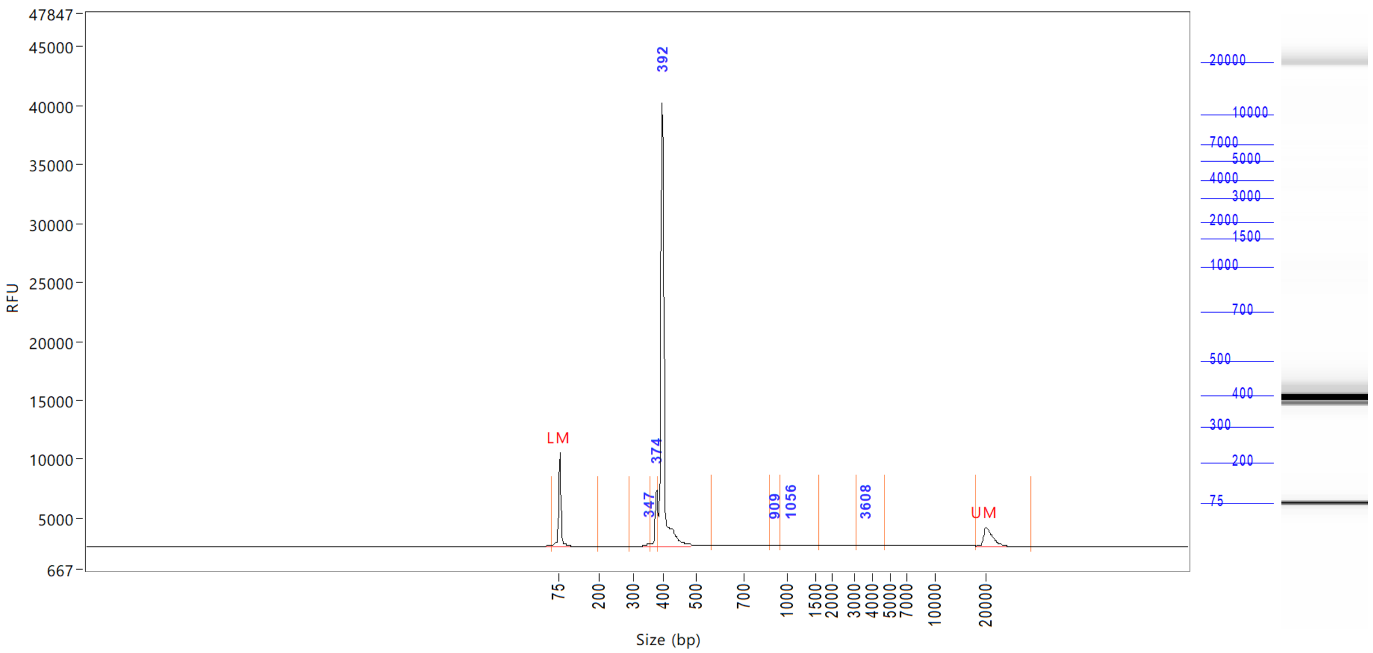

Peak	Size (bp)	Concentration (ng/uL)	From (bp)	To (bp)	Average size (bp)	CV%	RFU	Corrected peak area
1	75 (LM)	0.5000	54	194	75	15.22	8036	51.837
2	347	0.2974	286	356	342	2.91	204	2.570
3	374	3.3150	356	380	372	1.36	4794	28.640
4	392	28.3976	380	560	396	4.18	37697	245.341
5	909	0.0807	874	944	909	2.19	58	0.697
6	1056	0.1749	944	1578	1068	11.05	63	1.511
7	3608	0.1126	3108	4724	3905	9.25	56	0.973
8	20000 (UM)	0.1725	18049	28826	20694	5.32	1552	17.880

TIC:	32.3782	ng/uL
TIM:	134.5397	nmole/L
Total concentration:	32.4222	ng/uL

Sample peak width (sec): 5   Sample min peak height: 50   Sample baseline V to V?: Y   Sample baseline V to V points: 3
Sample filter: Binomial   Number of points for filter: 3 Sample start region (min): 0 Sample end region (min): 30
Marker peak width (sec): 5   Marker min peak height: 200   Marker baseline V to V?: Y   Marker baseline V to V points: 3
Lower marker selection: First peak > 500 RFU   Upper marker selection: Last peak > 200 RFU
Ladder size (bp)75, 200, 300, 400, 500, 700, 1000, 1500, 2000, 3000, 4000, 5000, 7000, 10000, 20000
Quantification using: Lower Marker   Final concentration (ng/uL): 0.5000   Dilution factor: 12.0

Sample: SampH9
Well location: H9
Created: Tuesday, August 10, 2021 11:20:27 PM

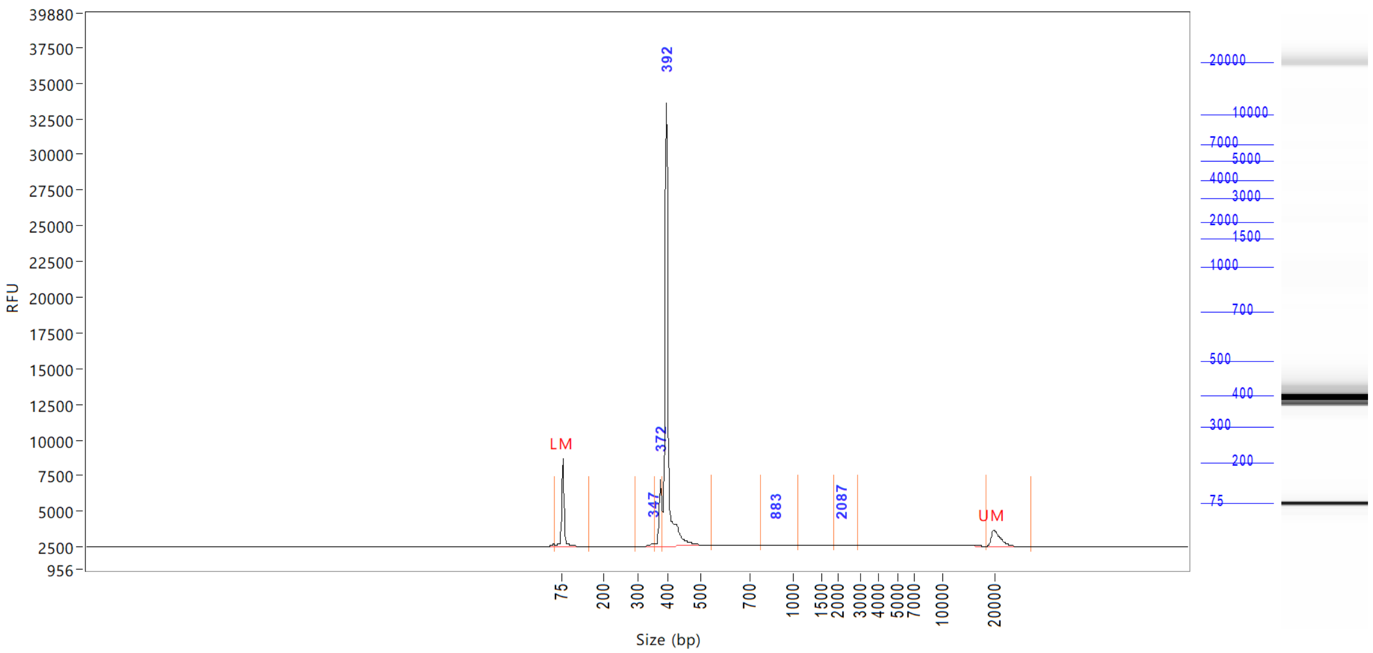

Peak	Size (bp)	Concentration (ng/uL)	From (bp)	To (bp)	Average size (bp)	CV%	RFU	Corrected peak area
1	75 (LM)	0.5000	52	158	73	11.50	6162	39.045
2	347	0.3650	291	356	341	2.85	179	2.375
3	372	4.4118	356	380	371	1.43	4691	28.709
4	392	32.7701	380	539	395	4.14	31086	213.250
5	883	0.3707	775	1079	931	7.13	67	2.412
6	2087	0.1249	1866	2857	2252	10.63	65	0.813
7	20000 (UM)	0.1655	18415	27254	20592	4.99	1144	12.924

TIC:	38.0424	ng/uL
TIM:	158.5676	nmole/L
Total concentration:	38.0553	ng/uL

```
Sample peak width (sec): 5    Sample min peak height: 50    Sample baseline V to V?: Y    Sample baseline V to V points: 3
Sample filter: Binomial       Number of points for filter: 3  Sample start region (min): 0  Sample end region (min): 30
Marker peak width (sec): 5    Marker min peak height: 200   Marker baseline V to V?: Y    Marker baseline V to V points: 3
Lower marker selection: First peak > 500 RFU                Upper marker selection: Last peak > 200 RFU
Ladder size (bp)75, 200, 300, 400, 500, 700, 1000, 1500, 2000, 3000, 4000, 5000, 7000, 10000, 20000
Quantification using: Lower Marker          Final concentration (ng/uL): 0.5000          Dilution factor: 12.0
```

Sample: SampA10
Well location: A10
Created: Tuesday, August 10, 2021 11:20:27 PM

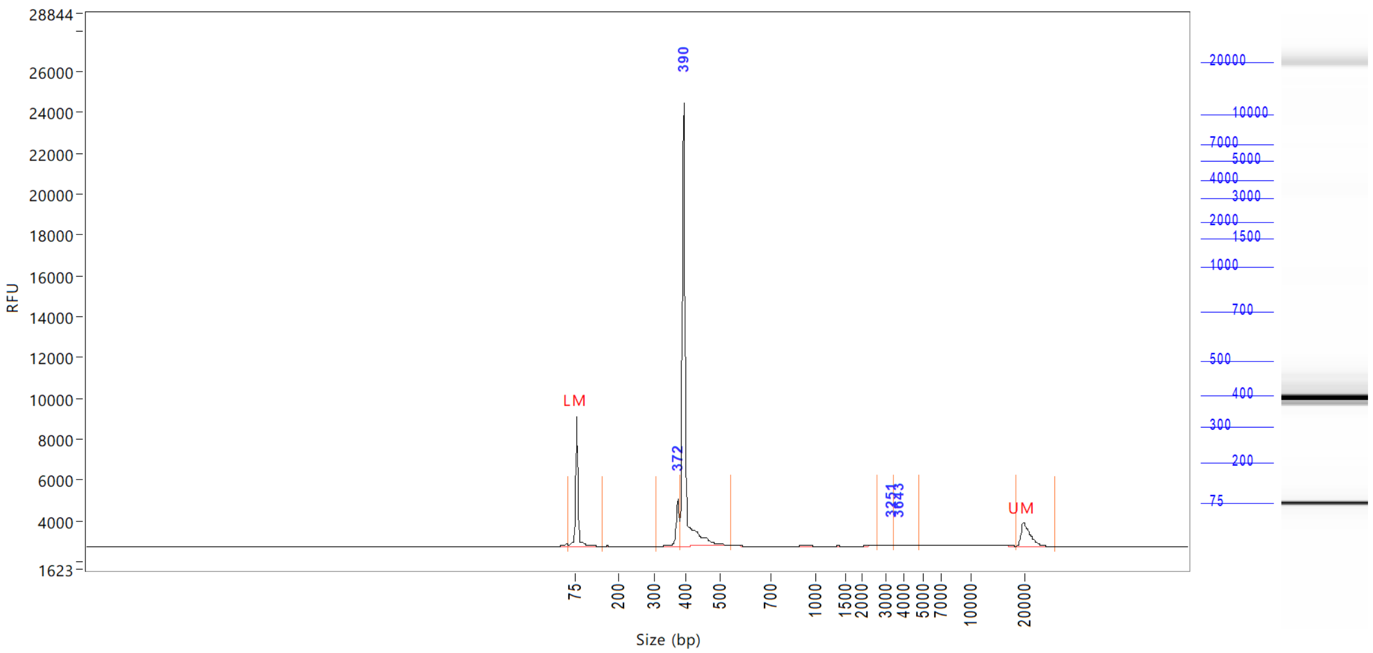

Peak	Size (bp)	Concentration (ng/uL)	From (bp)	To (bp)	Average size (bp)	CV%	RFU	Corrected peak area
1	75 (LM)	0.5000	54	150	76	14.89	6339	39.611
2	372	2.2933	303	380	370	2.57	2324	15.140
3	390	21.7694	380	539	396	5.01	21736	143.718
4	3251	0.0671	2629	3429	3206	5.57	53	0.443
5	3643	0.1159	3429	4793	3818	7.96	56	0.765
6	20000 (UM)	0.1625	18537	25924	20539	4.97	1148	12.870

TIC:	24.2458	ng/uL
TIM:	100.7283	nmole/L
Total concentration:	24.6814	ng/uL

Sample peak width (sec): 5 Sample min peak height: 50 Sample baseline V to V?: Y Sample baseline V to V points: 3
Sample filter: Binomial Number of points for filter: 3 Sample start region (min): 0 Sample end region (min): 30
Marker peak width (sec): 5 Marker min peak height: 200 Marker baseline V to V?: Y Marker baseline V to V points: 3
Lower marker selection: First peak > 500 RFU Upper marker selection: Last peak > 200 RFU
Ladder size (bp)75, 200, 300, 400, 500, 700, 1000, 1500, 2000, 3000, 4000, 5000, 7000, 10000, 20000
Quantification using: Lower Marker Final concentration (ng/uL): 0.5000 Dilution factor: 12.0

Sample: SampB10
Well location: B10
Created: Tuesday, August 10, 2021 11:20:27 PM

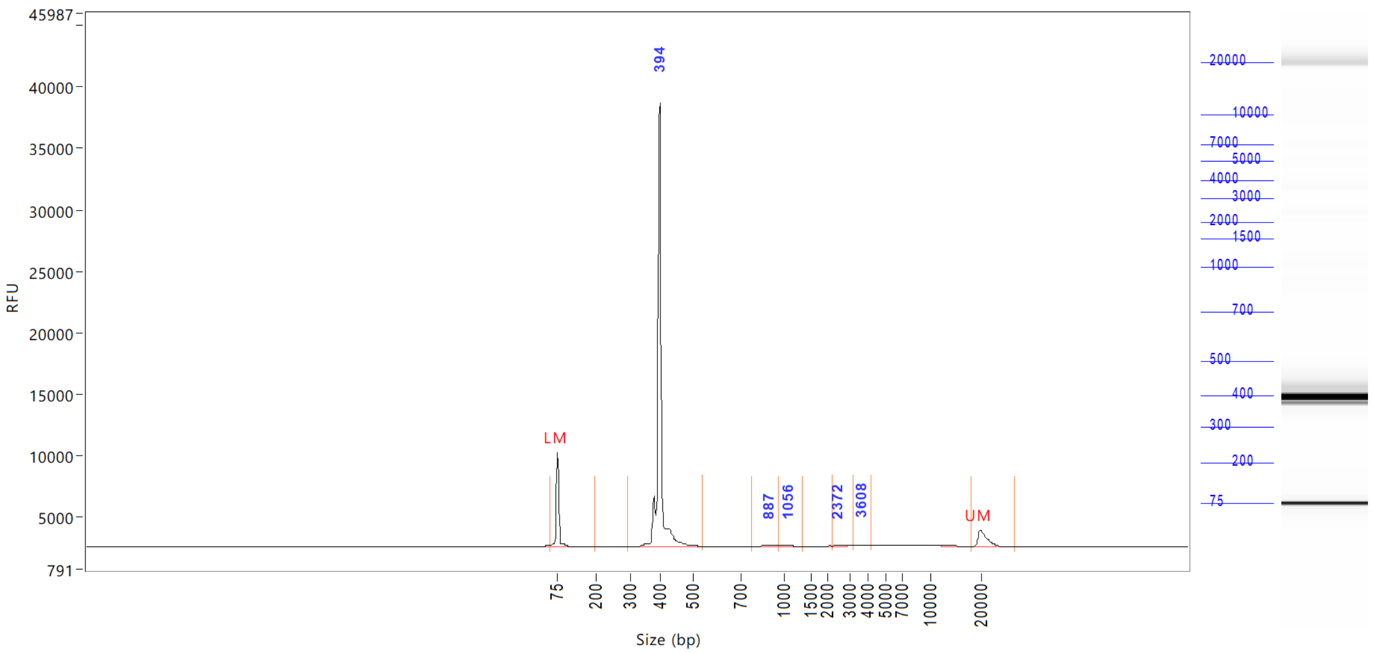

Peak	Size (bp)	Concentration (ng/uL)	From (bp)	To (bp)	Average size (bp)	CV%	RFU	Corrected peak area
1	75 (LM)	0.5000	52	192	75	18.93	7715	49.453
2	394	31.9983	289	537	393	4.33	36122	263.736
3	887	0.1899	770	965	891	4.88	69	1.566
4	1056	0.1456	965	1334	1070	8.23	59	1.200
5	2372	0.0950	2172	3179	2444	7.33	88	0.783
6	3608	0.1224	3179	4173	3670	5.54	99	1.009
7	20000 (UM)	0.1515	18171	27012	20610	4.98	1317	14.981

TIC:	32.5514	ng/uL
TIM:	134.6532	nmole/L
Total concentration:	32.8925	ng/uL

```
Sample peak width (sec): 5    Sample min peak height: 50    Sample baseline V to V?: Y    Sample baseline V to V points: 3
Sample filter: Binomial       Number of points for filter: 3  Sample start region (min): 0  Sample end region (min): 30
Marker peak width (sec): 5    Marker min peak height: 200   Marker baseline V to V?: Y    Marker baseline V to V points: 3
Lower marker selection: First peak > 500 RFU                Upper marker selection: Last peak > 200 RFU
Ladder size (bp)75, 200, 300, 400, 500, 700, 1000, 1500, 2000, 3000, 4000, 5000, 7000, 10000, 20000
Quantification using: Lower Marker          Final concentration (ng/uL): 0.5000          Dilution factor: 12.0
```

Sample: SampC10
Well location: C10
Created: Tuesday, August 10, 2021 11:20:27 PM

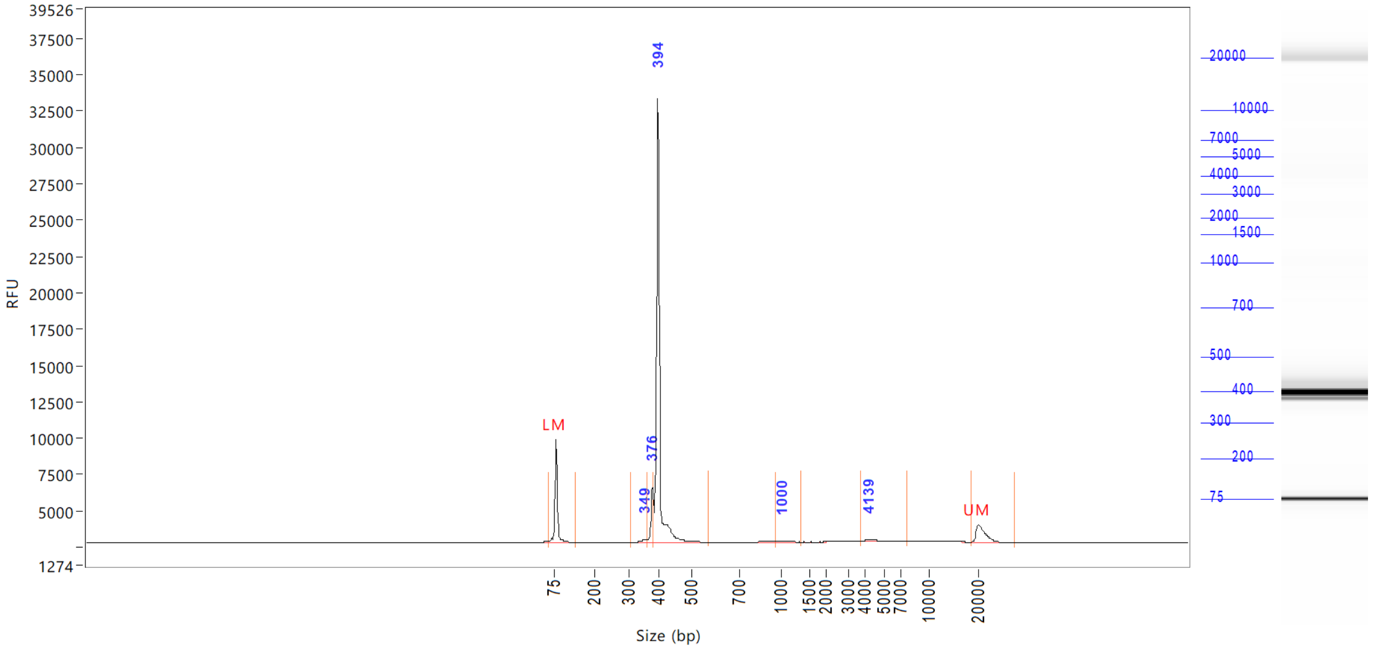

Peak	Size (bp)	Concentration (ng/uL)	From (bp)	To (bp)	Average size (bp)	CV%	RFU	Corrected peak area
1	75 (LM)	0.5000	54	139	75	11.30	7068	45.905
2	349	0.2891	303	358	344	2.98	171	2.212
3	376	3.0130	358	382	374	1.37	3765	23.052
4	394	26.0355	382	567	397	3.93	30573	199.192
5	1000	0.1669	961	1356	1060	8.28	68	1.277
6	4139	0.4403	3786	7734	4952	20.42	103	3.369
7	20000 (UM)	0.1551	18537	27254	20609	5.16	1224	14.238

TIC:	29.9449	ng/uL
TIM:	122.9412	nmole/L
Total concentration:	30.2198	ng/uL

Sample peak width (sec): 5 Sample min peak height: 50 Sample baseline V to V?: Y Sample baseline V to V points: 3
Sample filter: Binomial Number of points for filter: 3 Sample start region (min): 0 Sample end region (min): 30
Marker peak width (sec): 5 Marker min peak height: 200 Marker baseline V to V?: Y Marker baseline V to V points: 3
Lower marker selection: First peak > 500 RFU Upper marker selection: Last peak > 200 RFU
Ladder size (bp)75, 200, 300, 400, 500, 700, 1000, 1500, 2000, 3000, 4000, 5000, 7000, 10000, 20000
Quantification using: Lower Marker Final concentration (ng/uL): 0.5000 Dilution factor: 12.0

Sample: SampD10
Well location: D10
Created: Tuesday, August 10, 2021 11:20:27 PM

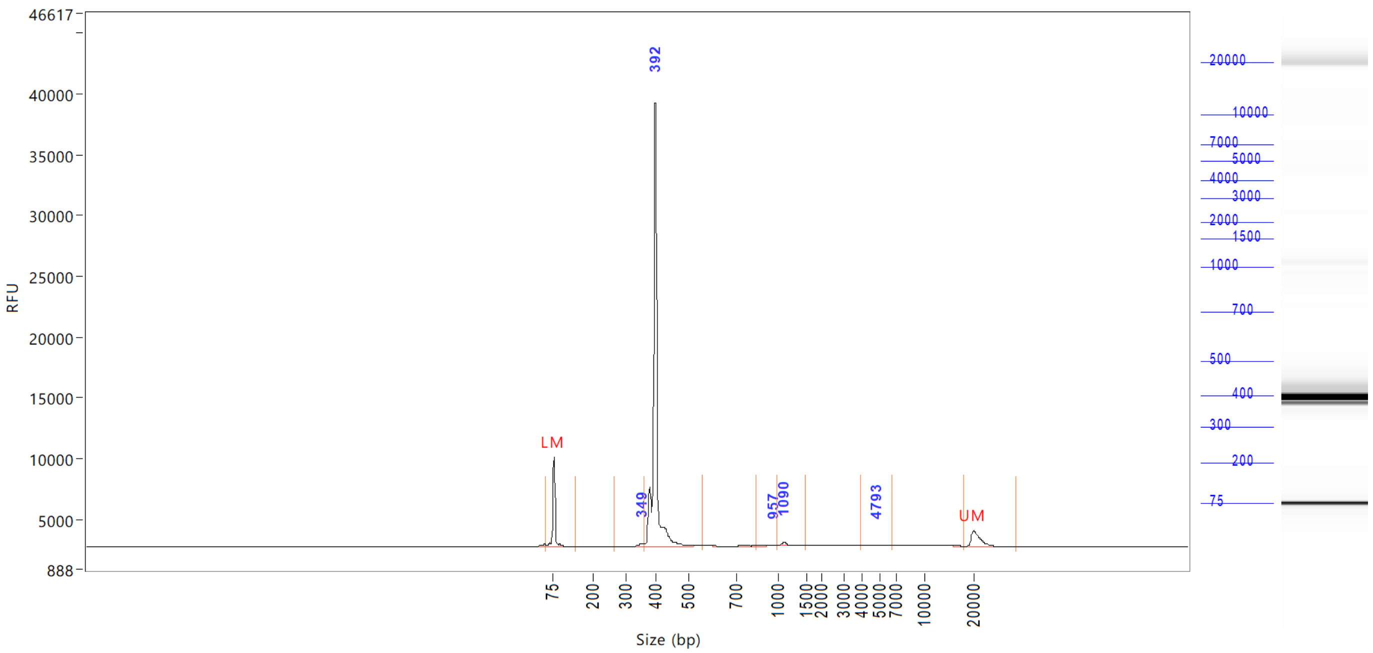

Peak	Size (bp)	Concentration (ng/uL)	From (bp)	To (bp)	Average size (bp)	CV%	RFU	Corrected peak area
1	75 (LM)	0.5000	52	147	74	13.22	7344	46.164
2	349	0.3691	262	358	343	3.23	219	2.840
3	392	35.6980	358	560	393	4.24	36537	274.658
4	957	0.2367	844	991	926	4.44	86	1.821
5	1090	0.4325	991	1500	1103	7.59	327	3.327
6	4793	0.1729	3929	6556	5040	12.75	51	1.331
7	20000 (UM)	0.1566	18171	28463	20658	5.34	1252	14.457

TIC:	36.9092	ng/uL
TIM:	152.3403	nmole/L
Total concentration:	37.3028	ng/uL

```
Sample peak width (sec): 5    Sample min peak height: 50    Sample baseline V to V?: Y    Sample baseline V to V points: 3
Sample filter: Binomial       Number of points for filter: 3  Sample start region (min): 0  Sample end region (min): 30
Marker peak width (sec): 5    Marker min peak height: 200   Marker baseline V to V?: Y    Marker baseline V to V points: 3
Lower marker selection: First peak > 500 RFU                Upper marker selection: Last peak > 200 RFU
Ladder size (bp)75, 200, 300, 400, 500, 700, 1000, 1500, 2000, 3000, 4000, 5000, 7000, 10000, 20000
Quantification using: Lower Marker          Final concentration (ng/uL): 0.5000          Dilution factor: 12.0
```

Sample: SampE10
Well location: E10
Created: Tuesday, August 10, 2021 11:20:27 PM

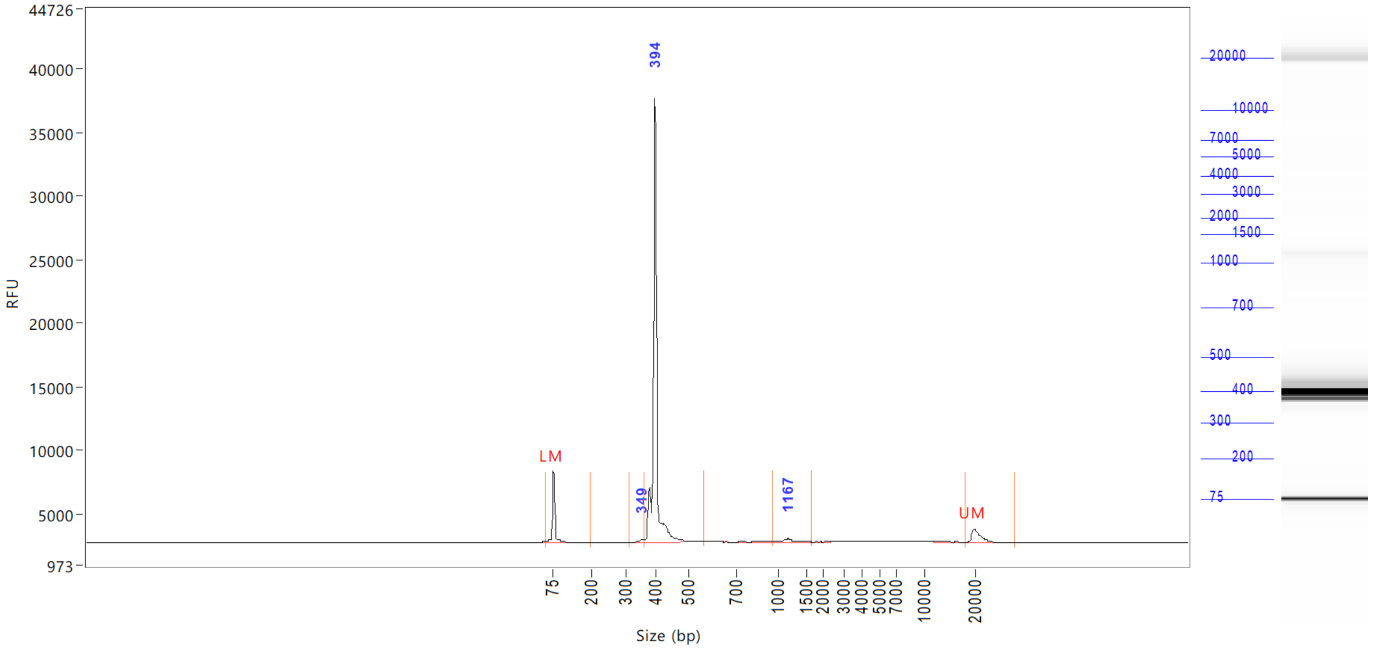

Peak	Size (bp)	Concentration (ng/uL)	From (bp)	To (bp)	Average size (bp)	CV%	RFU	Corrected peak area
1	75 (LM)	0.5000	54	194	76	16.99	5650	37.851
2	349	0.3606	307	356	343	2.64	187	2.275
3	394	40.4761	356	562	395	4.33	34955	255.341
4	1167	0.6366	961	1674	1144	10.19	272	4.016
5	20000 (UM)	0.1530	18171	28221	20601	5.29	992	11.580

TIC:	41.4734	ng/uL
TIM:	171.2392	nmole/L
Total concentration:	42.0869	ng/uL

Sample peak width (sec): 5 Sample min peak height: 50 Sample baseline V to V?: Y Sample baseline V to V points: 3
Sample filter: Binomial Number of points for filter: 3 Sample start region (min): 0 Sample end region (min): 30
Marker peak width (sec): 5 Marker min peak height: 200 Marker baseline V to V?: Y Marker baseline V to V points: 3
Lower marker selection: First peak > 500 RFU Upper marker selection: Last peak > 200 RFU
Ladder size (bp)75, 200, 300, 400, 500, 700, 1000, 1500, 2000, 3000, 4000, 5000, 7000, 10000, 20000
Quantification using: Lower Marker Final concentration (ng/uL): 0.5000 Dilution factor: 12.0

Sample: SampF10
Well location: F10
Created: Tuesday, August 10, 2021 11:20:27 PM

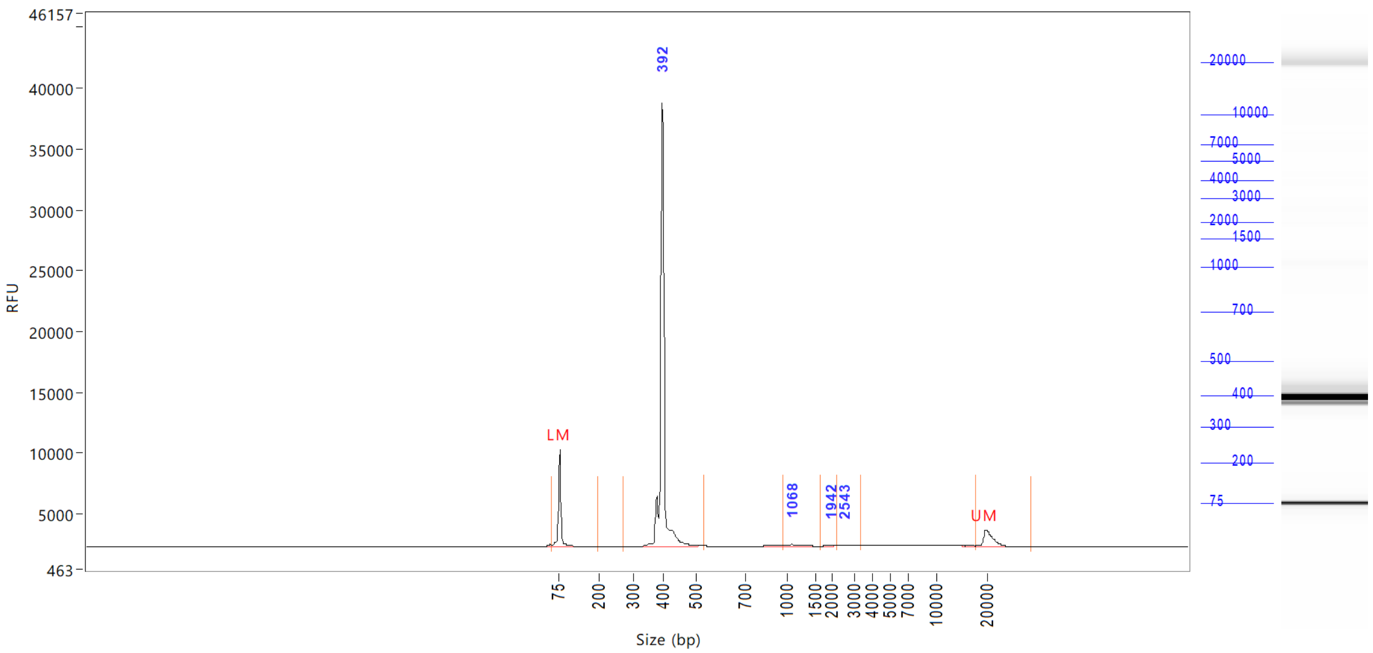

Peak	Size (bp)	Concentration (ng/uL)	From (bp)	To (bp)	Average size (bp)	CV%	RFU	Corrected peak area
1	75 (LM)	0.5000	52	196	75	16.62	7965	50.855
2	392	30.5219	269	527	392	4.14	36511	258.697
3	1068	0.2456	965	1655	1102	11.27	145	2.082
4	1942	0.0618	1655	2172	1951	5.24	54	0.523
5	2543	0.1217	2172	3358	2623	10.09	75	1.032
6	20000 (UM)	0.1516	17805	28705	20550	5.24	1325	15.419

TIC:	30.9511	ng/uL
TIM:	128.5995	nmole/L
Total concentration:	31.4026	ng/uL

Sample peak width (sec): 5   Sample min peak height: 50   Sample baseline V to V?: Y   Sample baseline V to V points: 3
Sample filter: Binomial   Number of points for filter: 3   Sample start region (min): 0   Sample end region (min): 30
Marker peak width (sec): 5   Marker min peak height: 200   Marker baseline V to V?: Y   Marker baseline V to V points: 3
Lower marker selection: First peak > 500 RFU   Upper marker selection: Last peak > 200 RFU
Ladder size (bp)75, 200, 300, 400, 500, 700, 1000, 1500, 2000, 3000, 4000, 5000, 7000, 10000, 20000
Quantification using: Lower Marker   Final concentration (ng/uL): 0.5000   Dilution factor: 12.0

Sample: SampG10
Well location: G10
Created: Tuesday, August 10, 2021 11:20:27 PM

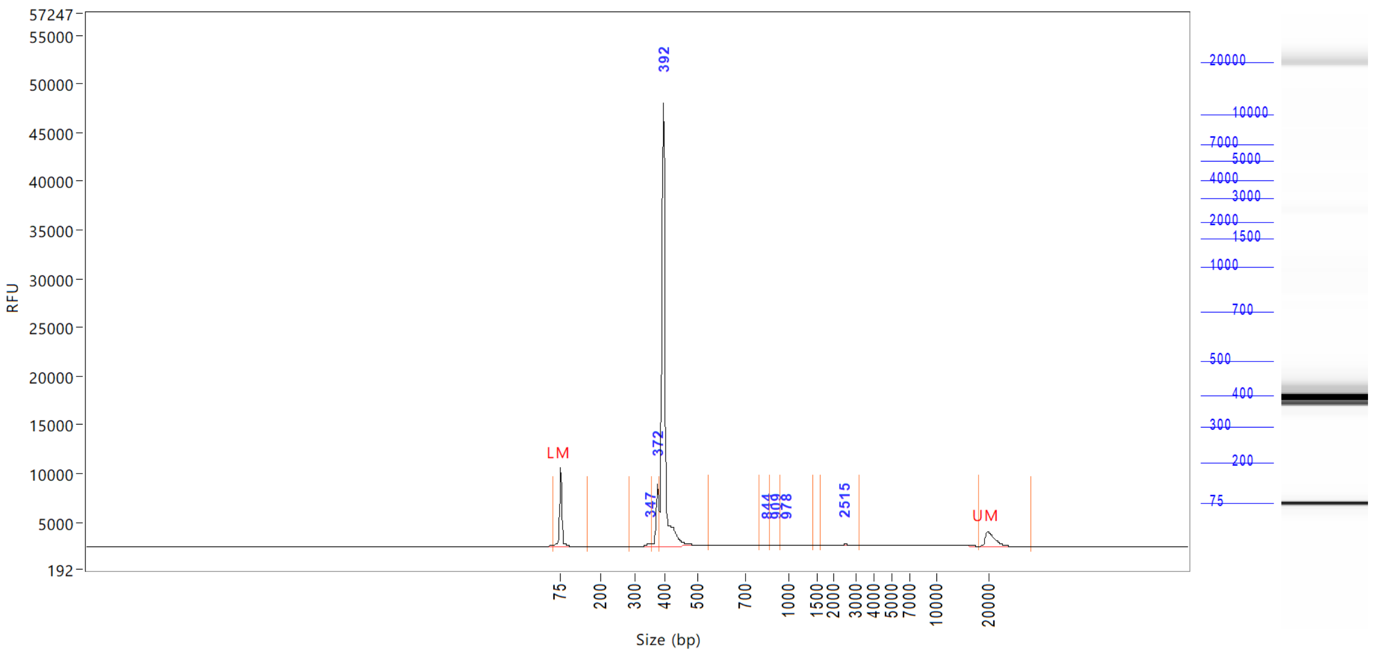

Peak	Size (bp)	Concentration (ng/uL)	From (bp)	To (bp)	Average size (bp)	CV%	RFU	Corrected peak area
1	75 (LM)	0.5000	52	160	73	10.64	8193	51.131
2	347	0.3740	282	354	341	2.79	272	3.187
3	372	4.7561	354	380	371	1.47	6468	40.530
4	392	36.0867	380	544	394	3.80	45578	307.522
5	844	0.0760	783	866	836	2.42	62	0.647
6	909	0.1368	866	944	906	2.49	87	1.166
7	978	0.2834	944	1445	1056	9.70	98	2.415
8	2515	0.2052	1616	3215	2528	8.97	159	1.748
9	20000 (UM)	0.1759	18293	28584	20676	5.20	1525	17.990

TIC:	41.9182	ng/uL
TIM:	174.5607	nmole/L
Total concentration:	41.8884	ng/uL

Sample peak width (sec): 5 Sample min peak height: 50 Sample baseline V to V?: Y Sample baseline V to V points: 3
Sample filter: Binomial Number of points for filter: 3 Sample start region (min): 0 Sample end region (min): 30
Marker peak width (sec): 5 Marker min peak height: 200 Marker baseline V to V?: Y Marker baseline V to V points: 3
Lower marker selection: First peak > 500 RFU Upper marker selection: Last peak > 200 RFU
Ladder size (bp)75, 200, 300, 400, 500, 700, 1000, 1500, 2000, 3000, 4000, 5000, 7000, 10000, 20000
Quantification using: Lower Marker Final concentration (ng/uL): 0.5000 Dilution factor: 12.0

Sample: SampH10
Well location: H10
Created: Tuesday, August 10, 2021 11:20:27 PM

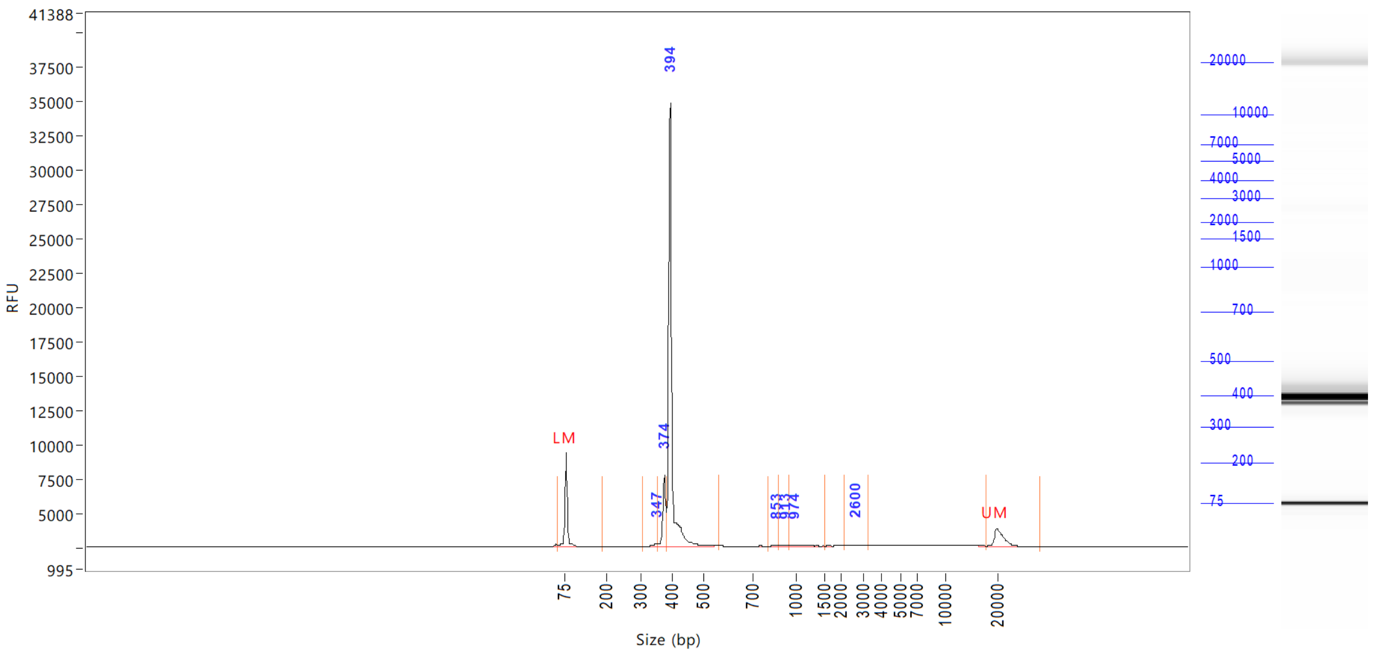

Peak	Size (bp)	Concentration (ng/uL)	From (bp)	To (bp)	Average size (bp)	CV%	RFU	Corrected peak area
1	75 (LM)	0.5000	50	187	75	17.32	6887	43.326
2	347	0.3626	305	356	342	2.83	207	2.619
3	374	4.3770	356	382	373	1.45	5190	31.606
4	394	29.7801	382	560	397	4.04	32283	215.040
5	853	0.0789	801	879	850	2.24	58	0.570
6	913	0.1490	879	952	916	2.31	76	1.076
7	974	0.2271	952	1500	1060	10.05	69	1.640
8	2600	0.1359	2087	3215	2588	8.19	90	0.981
9	20000 (UM)	0.1676	17927	28463	20668	5.35	1271	14.524

TIC:	35.1107	ng/uL
TIM:	145.3276	nmole/L
Total concentration:	35.1123	ng/uL

Sample peak width (sec): 5 Sample min peak height: 50 Sample baseline V to V?: Y Sample baseline V to V points: 3
Sample filter: Binomial Number of points for filter: 3 Sample start region (min): 0 Sample end region (min): 30
Marker peak width (sec): 5 Marker min peak height: 200 Marker baseline V to V?: Y Marker baseline V to V points: 3
Lower marker selection: First peak > 500 RFU Upper marker selection: Last peak > 200 RFU
Ladder size (bp)75, 200, 300, 400, 500, 700, 1000, 1500, 2000, 3000, 4000, 5000, 7000, 10000, 20000
Quantification using: Lower Marker Final concentration (ng/uL): 0.5000 Dilution factor: 12.0

Sample: SampA11
Well location: A11
Created: Tuesday, August 10, 2021 11:20:27 PM

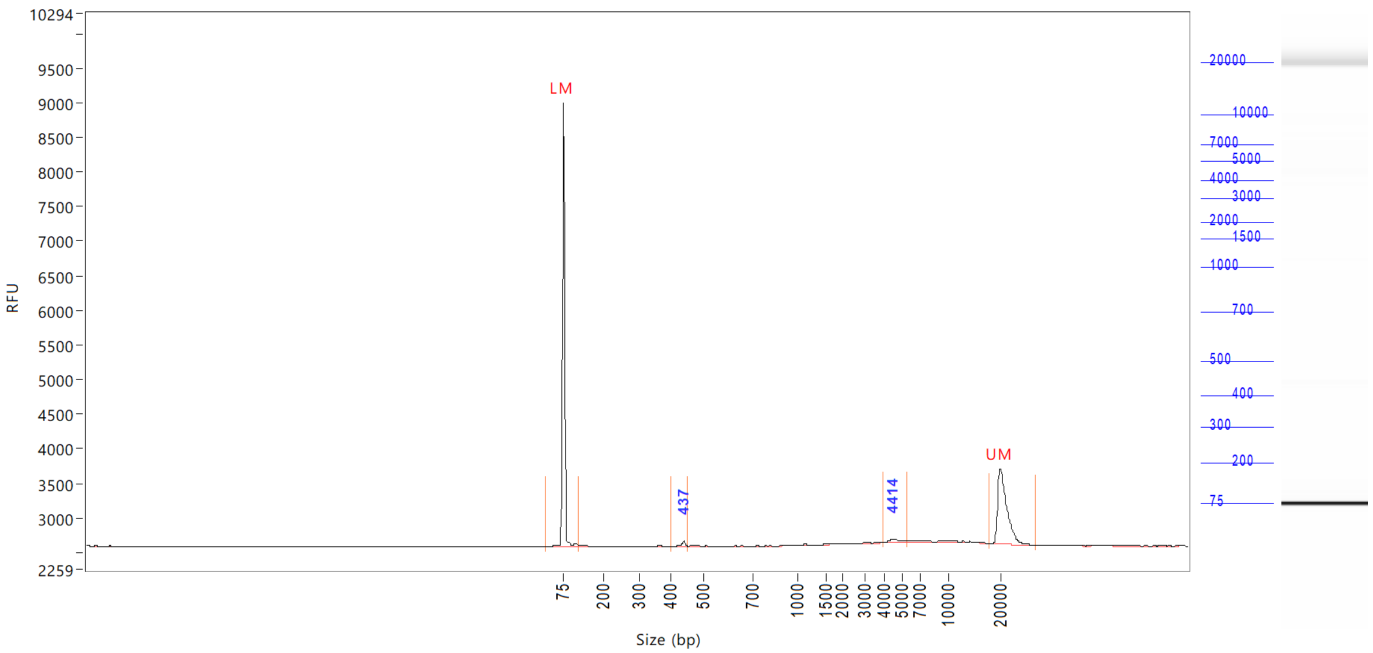

Peak	Size (bp)	Concentration (ng/uL)	From (bp)	To (bp)	Average size (bp)	CV%	RFU	Corrected peak area
1	75 (LM)	0.5000	24	120	73	8.36	6425	37.208
2	437	0.0896	400	452	433	2.16	72	0.555
3	4414	0.1413	3964	5519	4590	7.69	51	0.876
4	20000 (UM)	0.1698	17683	26891	20564	5.36	1084	12.633

TIC:	0.2309	ng/uL
TIM:	0.3911	nmole/L
Total concentration:	0.7605	ng/uL

```
Sample peak width (sec): 5    Sample min peak height: 50    Sample baseline V to V?: Y    Sample baseline V to V points: 3
Sample filter: Binomial       Number of points for filter: 3  Sample start region (min): 0  Sample end region (min): 30
Marker peak width (sec): 5    Marker min peak height: 200   Marker baseline V to V?: Y    Marker baseline V to V points: 3
Lower marker selection: First peak > 500 RFU                Upper marker selection: Last peak > 200 RFU
Ladder size (bp)75, 200, 300, 400, 500, 700, 1000, 1500, 2000, 3000, 4000, 5000, 7000, 10000, 20000
Quantification using: Lower Marker          Final concentration (ng/uL): 0.5000          Dilution factor: 12.0
```

Sample: SampB11
Well location: B11
Created: Tuesday, August 10, 2021 11:20:27 PM

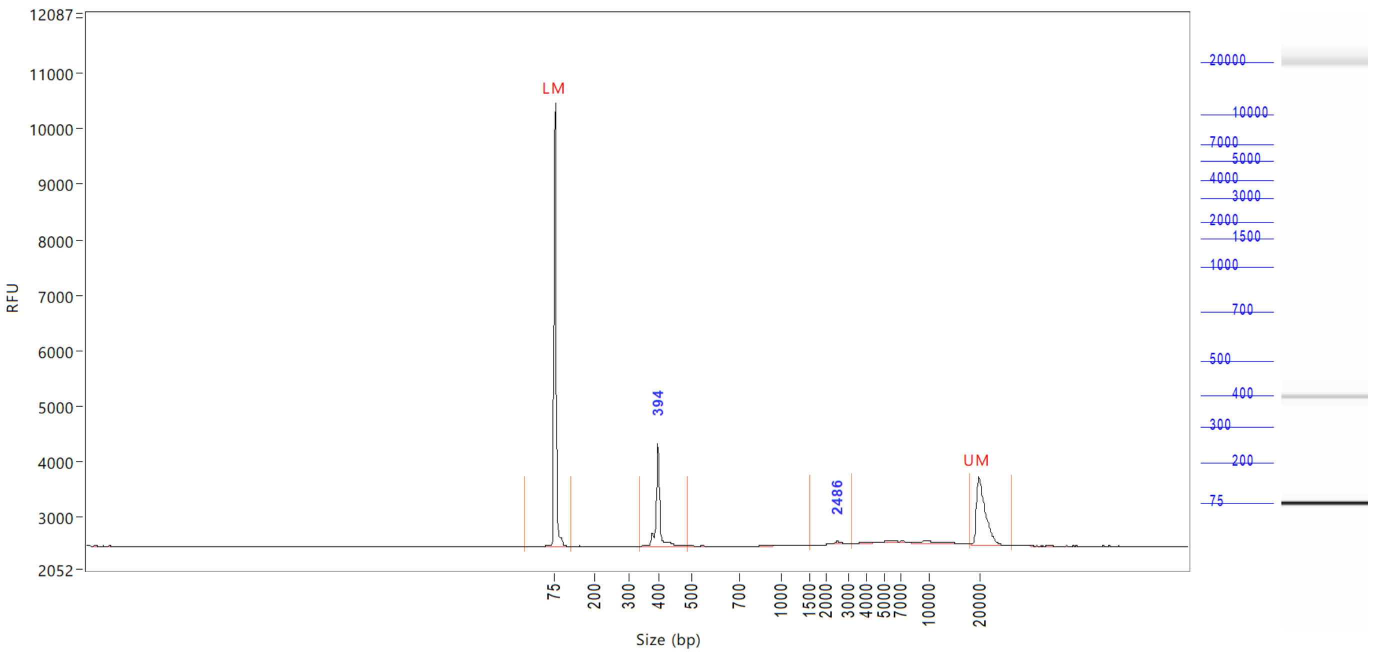

Peak	Size (bp)	Concentration (ng/uL)	From (bp)	To (bp)	Average size (bp)	CV%	RFU	Corrected peak area
1	75 (LM)	0.5000	0	126	73	9.61	8025	46.817
2	394	1.8907	333	489	395	4.71	1858	14.753
3	2486	0.0691	1500	3179	2487	12.06	52	0.539
4	20000 (UM)	0.1520	18293	26287	20540	5.00	1224	14.230

TIC:	1.9598	ng/uL
TIM:	7.9210	nmole/L
Total concentration:	2.5010	ng/uL

Sample peak width (sec): 5 Sample min peak height: 50 Sample baseline V to V?: Y Sample baseline V to V points: 3
Sample filter: Binomial Number of points for filter: 3 Sample start region (min): 0 Sample end region (min): 30
Marker peak width (sec): 5 Marker min peak height: 200 Marker baseline V to V?: Y Marker baseline V to V points: 3
Lower marker selection: First peak > 500 RFU Upper marker selection: Last peak > 200 RFU
Ladder size (bp)75, 200, 300, 400, 500, 700, 1000, 1500, 2000, 3000, 4000, 5000, 7000, 10000, 20000
Quantification using: Lower Marker Final concentration (ng/uL): 0.5000 Dilution factor: 12.0

Sample: SampC11
Well location: C11
Created: Tuesday, August 10, 2021 11:20:27 PM

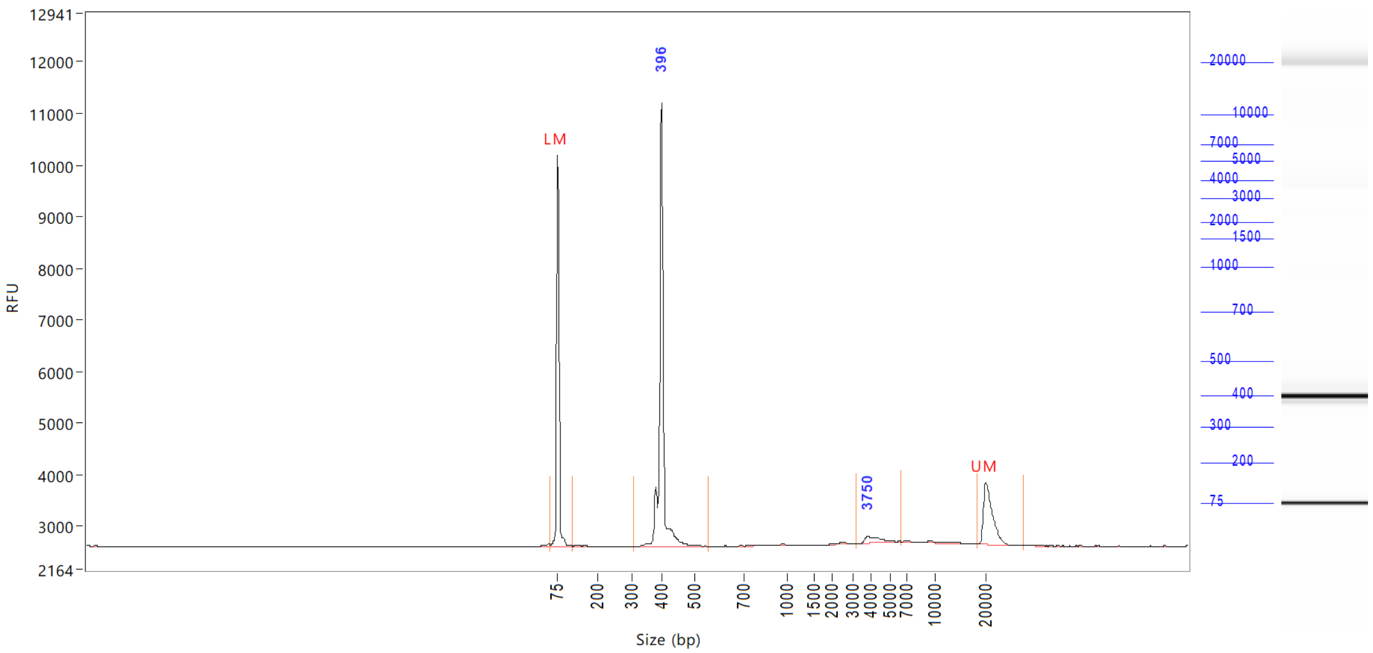

Peak	Size (bp)	Concentration (ng/uL)	From (bp)	To (bp)	Average size (bp)	CV%	RFU	Corrected peak area
1	75 (LM)	0.5000	52	122	74	7.17	7579	44.106
2	396	8.8040	307	555	395	4.55	8612	64.719
3	3750	0.3623	3144	6334	4122	10.75	121	2.663
4	20000 (UM)	0.1566	18415	27375	20623	5.03	1198	13.814

TIC:	9.1663	ng/uL
TIM:	36.8157	nmole/L
Total concentration:	9.6421	ng/uL

Sample peak width (sec): 5   Sample min peak height: 50   Sample baseline V to V?: Y   Sample baseline V to V points: 3
Sample filter: Binomial   Number of points for filter: 3 Sample start region (min): 0 Sample end region (min): 30
Marker peak width (sec): 5   Marker min peak height: 200   Marker baseline V to V?: Y   Marker baseline V to V points: 3
Lower marker selection: First peak > 500 RFU   Upper marker selection: Last peak > 200 RFU
Ladder size (bp)75, 200, 300, 400, 500, 700, 1000, 1500, 2000, 3000, 4000, 5000, 7000, 10000, 20000
Quantification using: Lower Marker   Final concentration (ng/uL): 0.5000   Dilution factor: 12.0

Sample: SampD11
Well location: D11
Created: Tuesday, August 10, 2021 11:20:27 PM

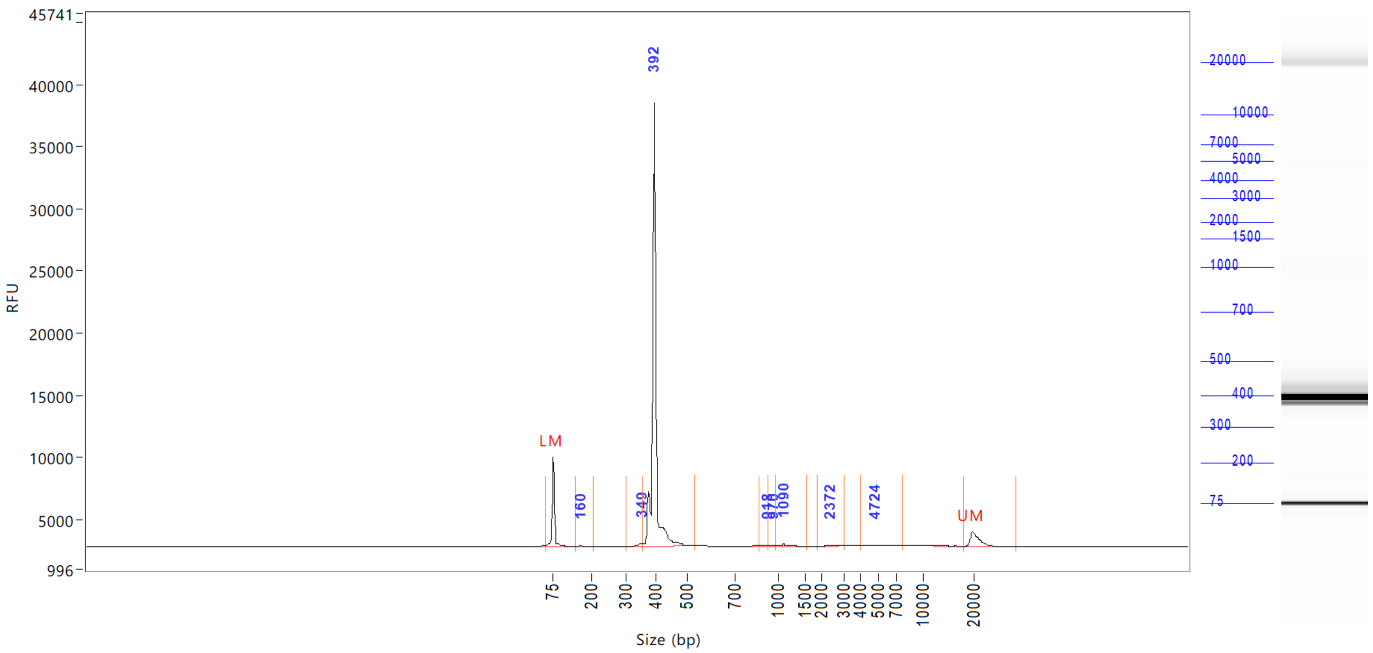

Peak	Size (bp)	Concentration (ng/uL)	From (bp)	To (bp)	Average size (bp)	CV%	RFU	Corrected peak area
1	75 (LM)	0.5000	52	145	74	13.72	7234	45.455
2	160	0.1717	145	201	163	8.14	93	1.301
3	349	0.3363	298	356	342	2.82	205	2.547
4	392	34.6600	356	527	393	4.23	35742	262.579
5	918	0.0956	861	935	900	2.36	56	0.725
6	970	0.0950	935	991	963	1.73	69	0.719
7	1090	0.2527	991	1539	1113	8.69	138	1.914
8	2372	0.1029	1846	3037	2481	9.55	66	0.780
9	4724	0.2988	4001	7667	5391	17.64	80	2.264
10	20000 (UM)	0.1495	18171	28705	20623	5.72	1139	13.588

TIC:	36.0131	ng/uL
TIM:	149.3221	nmole/L
Total concentration:	36.3013	ng/uL

Sample peak width (sec): 5 Sample min peak height: 50 Sample baseline V to V?: Y Sample baseline V to V points: 3
Sample filter: Binomial Number of points for filter: 3 Sample start region (min): 0 Sample end region (min): 30
Marker peak width (sec): 5 Marker min peak height: 200 Marker baseline V to V?: Y Marker baseline V to V points: 3
Lower marker selection: First peak > 500 RFU Upper marker selection: Last peak > 200 RFU
Ladder size (bp)75, 200, 300, 400, 500, 700, 1000, 1500, 2000, 3000, 4000, 5000, 7000, 10000, 20000
Quantification using: Lower Marker Final concentration (ng/uL): 0.5000 Dilution factor: 12.0

Sample: SampE11
Well location: E11
Created: Tuesday, August 10, 2021 11:20:27 PM

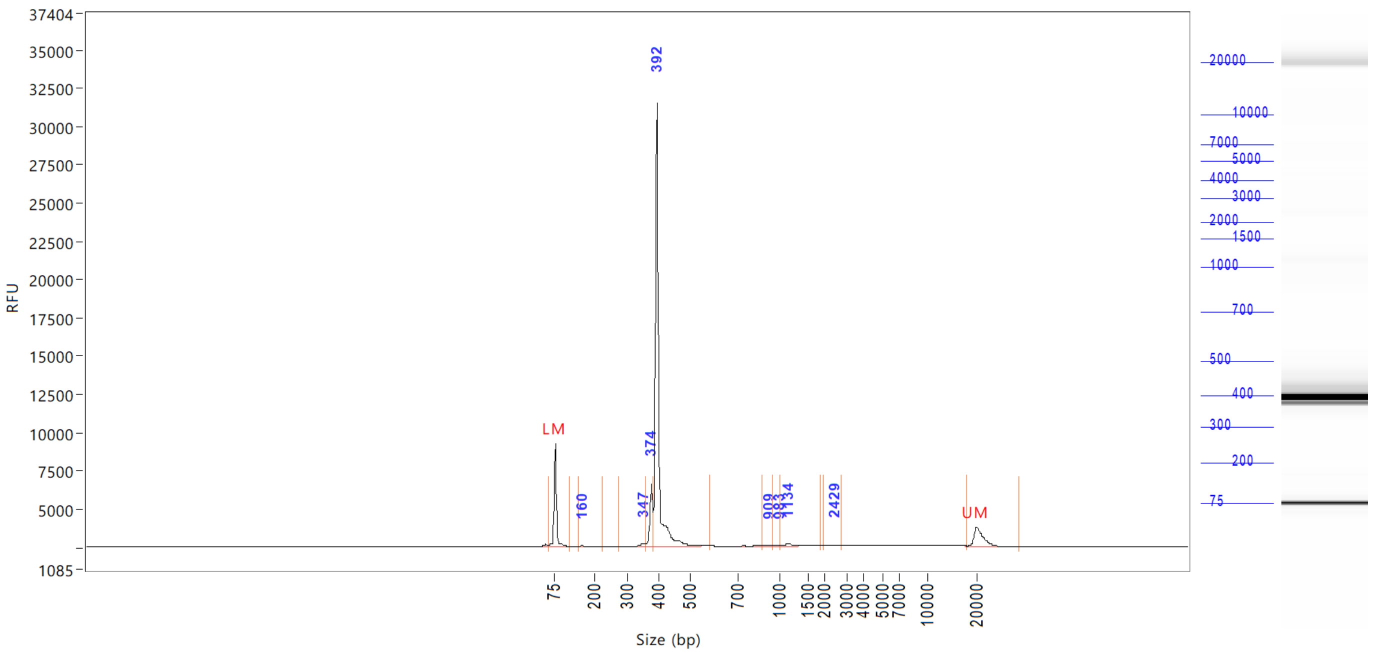

Peak	Size (bp)	Concentration (ng/uL)	From (bp)	To (bp)	Average size (bp)	CV%	RFU	Corrected peak area
1	75 (LM)	0.5000	54	122	74	9.15	6736	41.719
2	160	0.1383	150	223	168	9.57	77	0.961
3	347	0.3233	273	356	342	3.42	180	2.248
4	374	3.5054	356	380	372	1.34	4097	24.373
5	392	28.8428	380	580	397	4.64	29022	200.547
6	909	0.1190	866	948	909	2.61	54	0.828
7	983	0.0968	948	1000	975	1.60	67	0.673
8	1134	0.3132	1000	1866	1159	10.50	148	2.178
9	2429	0.1071	1962	2743	2405	6.28	79	0.744
10	20000 (UM)	0.1722	18171	28463	20572	5.24	1235	14.367

TIC:	33.4458	ng/uL
TIM:	138.8410	nmole/L
Total concentration:	33.5946	ng/uL

Sample peak width (sec): 5 Sample min peak height: 50 Sample baseline V to V?: Y Sample baseline V to V points: 3
Sample filter: Binomial Number of points for filter: 3 Sample start region (min): 0 Sample end region (min): 30
Marker peak width (sec): 5 Marker min peak height: 200 Marker baseline V to V?: Y Marker baseline V to V points: 3
Lower marker selection: First peak > 500 RFU Upper marker selection: Last peak > 200 RFU
Ladder size (bp)75, 200, 300, 400, 500, 700, 1000, 1500, 2000, 3000, 4000, 5000, 7000, 10000, 20000
Quantification using: Lower Marker Final concentration (ng/uL): 0.5000 Dilution factor: 12.0

Sample: SampF11
Well location: F11
Created: Tuesday, August 10, 2021 11:20:27 PM

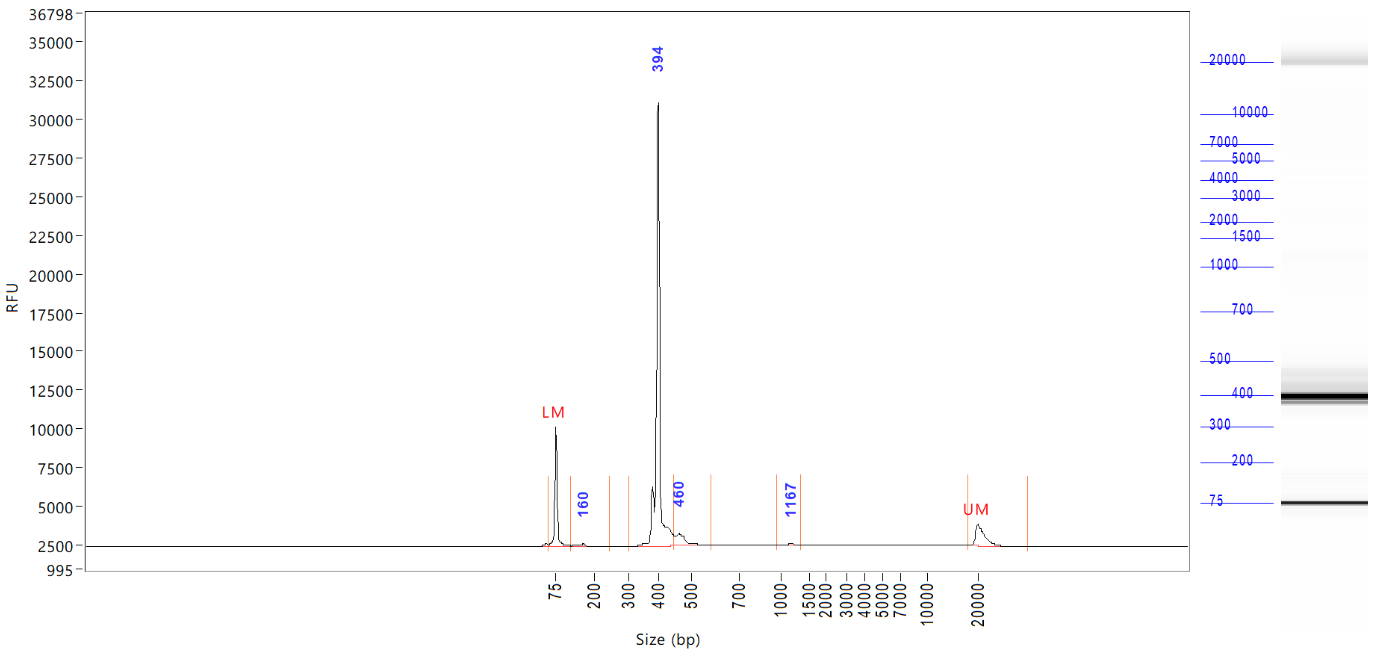

Peak	Size (bp)	Concentration (ng/uL)	From (bp)	To (bp)	Average size (bp)	CV%	RFU	Corrected peak area
1	75 (LM)	0.5000	52	122	73	10.42	7745	49.061
2	160	0.4109	122	242	154	14.07	169	3.360
3	394	25.9702	298	447	392	3.69	28595	212.353
4	460	1.8174	447	580	470	4.54	766	14.861
5	1167	0.2291	965	1356	1119	8.50	115	1.873
6	20000 (UM)	0.1622	18171	29914	20716	5.64	1360	15.920

TIC:	28.4276	ng/uL
TIM:	120.0850	nmole/L
Total concentration:	29.0146	ng/uL

Sample peak width (sec): 5 Sample min peak height: 50 Sample baseline V to V?: Y Sample baseline V to V points: 3
Sample filter: Binomial Number of points for filter: 3 Sample start region (min): 0 Sample end region (min): 30
Marker peak width (sec): 5 Marker min peak height: 200 Marker baseline V to V?: Y Marker baseline V to V points: 3
Lower marker selection: First peak > 500 RFU Upper marker selection: Last peak > 200 RFU
Ladder size (bp)75, 200, 300, 400, 500, 700, 1000, 1500, 2000, 3000, 4000, 5000, 7000, 10000, 20000
Quantification using: Lower Marker Final concentration (ng/uL): 0.5000 Dilution factor: 12.0

Sample: SampG11
Well location: G11
Created: Tuesday, August 10, 2021 11:20:27 PM

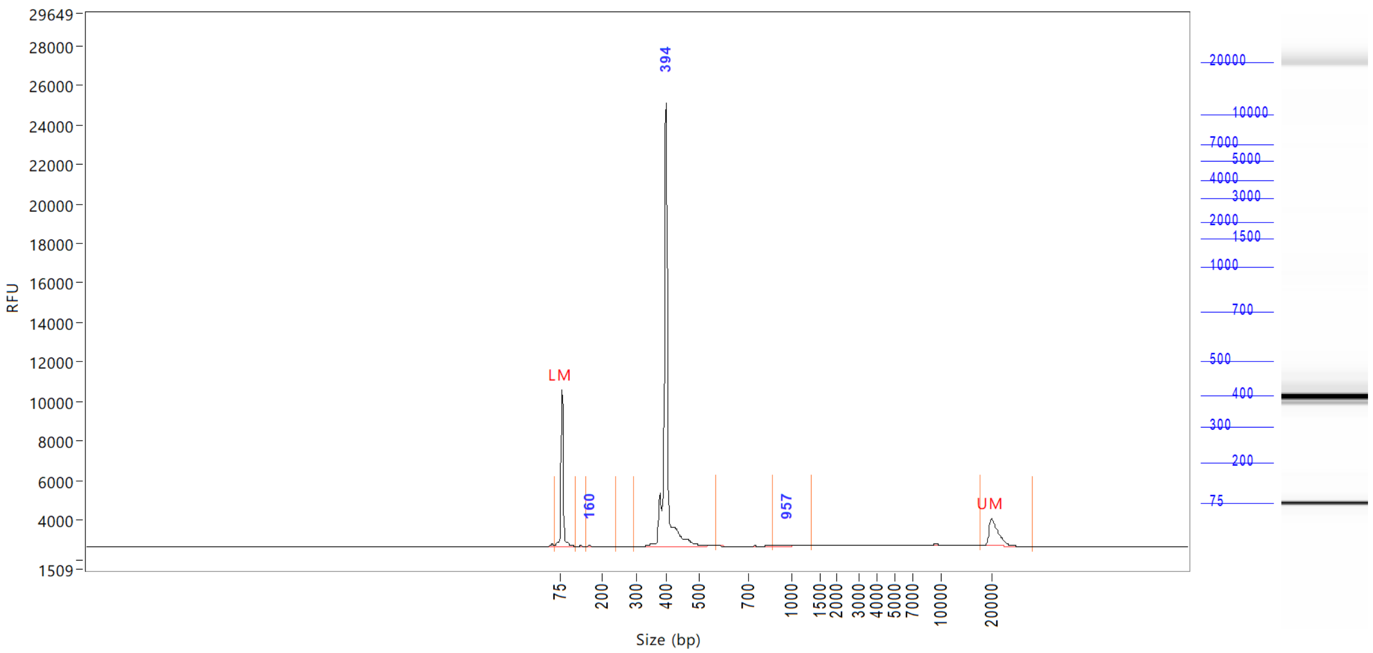

Peak	Size (bp)	Concentration (ng/uL)	From (bp)	To (bp)	Average size (bp)	CV%	RFU	Corrected peak area
1	75 (LM)	0.5000	52	118	74	8.73	7980	47.900
2	160	0.1095	148	239	169	10.91	62	0.874
3	394	21.3236	291	565	395	5.16	22487	170.233
4	957	0.2058	870	1356	1014	10.40	62	1.643
5	20000 (UM)	0.1702	17927	28221	20653	5.25	1395	16.308

TIC:	21.6388	ng/uL
TIM:	90.2172	nmole/L
Total concentration:	22.0684	ng/uL

```
Sample peak width (sec): 5    Sample min peak height: 50    Sample baseline V to V?: Y    Sample baseline V to V points: 3
Sample filter: Binomial       Number of points for filter: 3  Sample start region (min): 0  Sample end region (min): 30
Marker peak width (sec): 5    Marker min peak height: 200   Marker baseline V to V?: Y    Marker baseline V to V points: 3
Lower marker selection: First peak > 500 RFU                Upper marker selection: Last peak > 200 RFU
Ladder size (bp)75, 200, 300, 400, 500, 700, 1000, 1500, 2000, 3000, 4000, 5000, 7000, 10000, 20000
Quantification using: Lower Marker          Final concentration (ng/uL): 0.5000          Dilution factor: 12.0
```

Sample: SampH11
Well location: H11
Created: Tuesday, August 10, 2021 11:20:27 PM

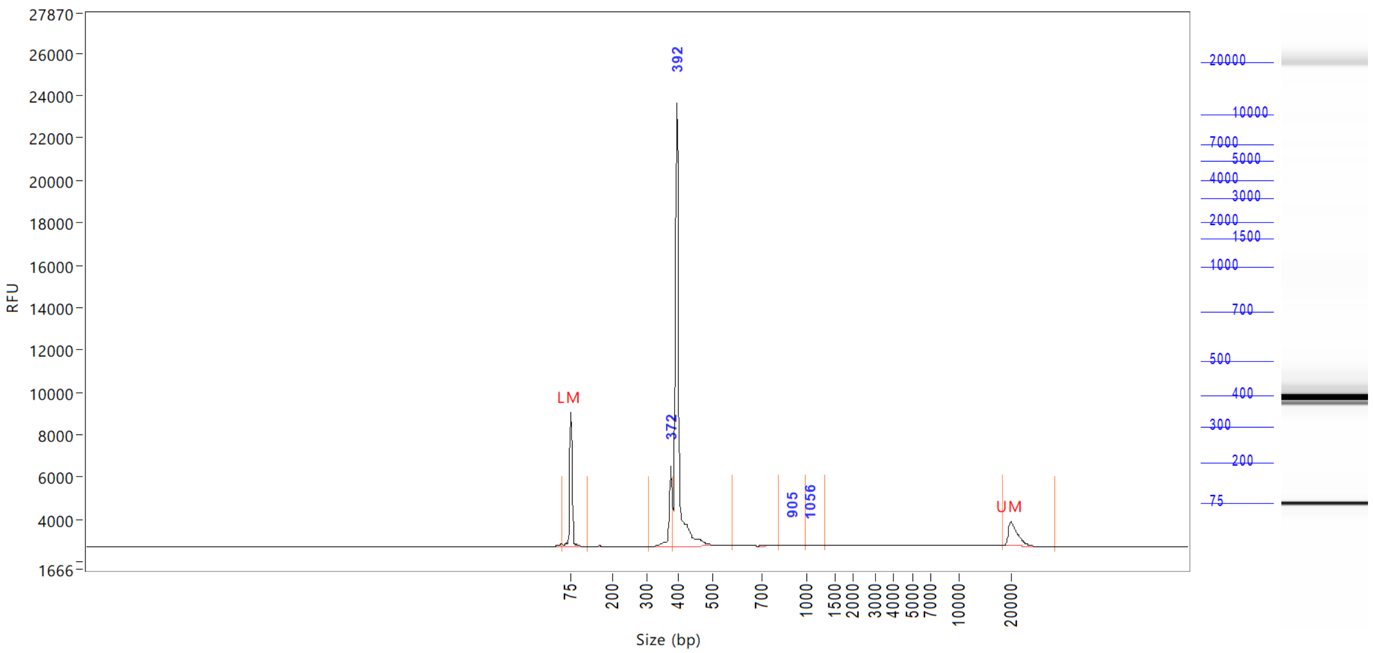

Peak	Size (bp)	Concentration (ng/uL)	From (bp)	To (bp)	Average size (bp)	CV%	RFU	Corrected peak area
1	75 (LM)	0.5000	50	124	73	8.90	6343	37.464
2	372	4.0447	301	380	369	2.83	3781	25.255
3	392	23.2116	380	580	397	4.91	20933	144.932
4	905	0.2794	809	996	911	5.49	61	1.745
5	1056	0.1266	996	1323	1090	6.98	50	0.790
6	20000 (UM)	0.1715	18293	28463	20569	4.89	1142	12.850

TIC:	27.6623	ng/uL
TIM:	114.9251	nmole/L
Total concentration:	27.9301	ng/uL

Sample peak width (sec): 5   Sample min peak height: 50   Sample baseline V to V?: Y   Sample baseline V to V points: 3
Sample filter: Binomial   Number of points for filter: 3   Sample start region (min): 0   Sample end region (min): 30
Marker peak width (sec): 5   Marker min peak height: 200   Marker baseline V to V?: Y   Marker baseline V to V points: 3
Lower marker selection: First peak > 500 RFU   Upper marker selection: Last peak > 200 RFU
Ladder size (bp)75, 200, 300, 400, 500, 700, 1000, 1500, 2000, 3000, 4000, 5000, 7000, 10000, 20000
Quantification using: Lower Marker   Final concentration (ng/uL): 0.5000   Dilution factor: 12.0

Sample: SampA12
Well location: A12
Created: Tuesday, August 10, 2021 11:20:27 PM

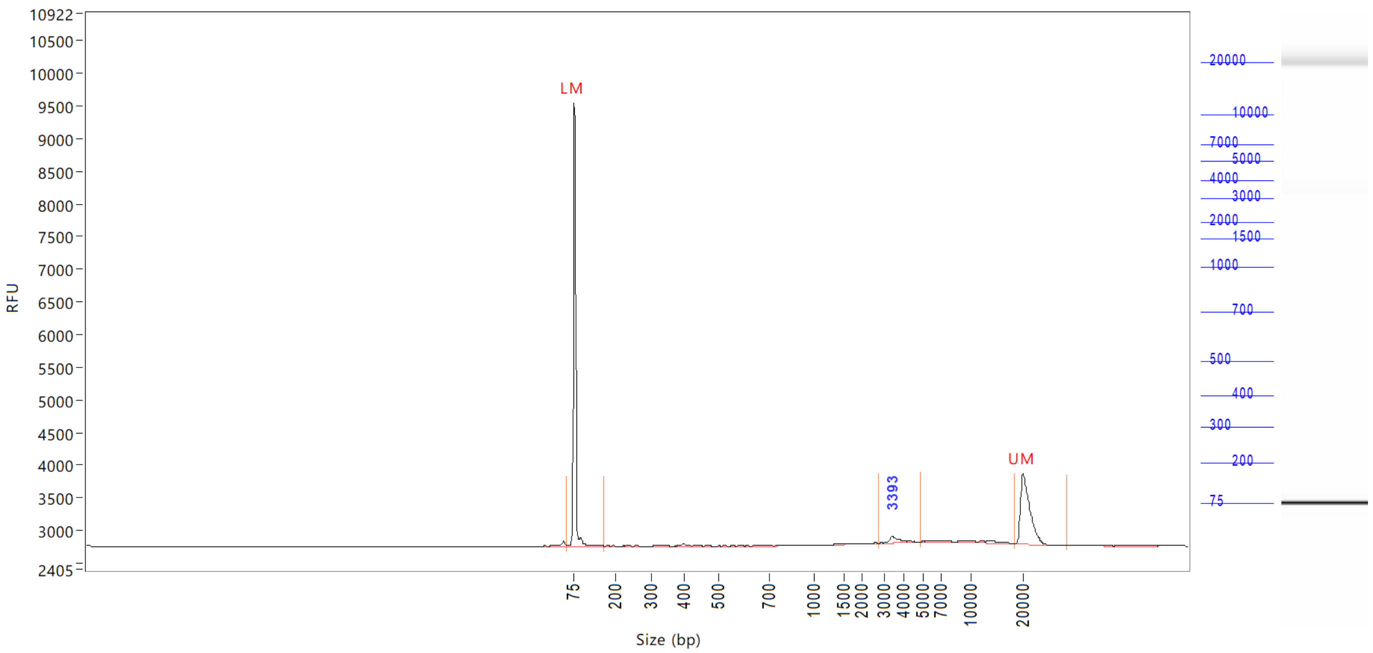

Peak	Size (bp)	Concentration (ng/uL)	From (bp)	To (bp)	Average size (bp)	CV%	RFU	Corrected peak area
1	75 (LM)	0.5000	54	166	75	9.42	6803	38.845
2	3393	0.2449	2743	4897	3677	12.00	95	1.586
3	20000 (UM)	0.1618	18537	28342	20562	5.00	1087	12.569

TIC:	0.2449	ng/uL
TIM:	0.1097	nmole/L
Total concentration:	0.8570	ng/uL

Sample peak width (sec): 5   Sample min peak height: 50   Sample baseline V to V?: Y   Sample baseline V to V points: 3
Sample filter: Binomial   Number of points for filter: 3   Sample start region (min): 0   Sample end region (min): 30
Marker peak width (sec): 5   Marker min peak height: 200   Marker baseline V to V?: Y   Marker baseline V to V points: 3
Lower marker selection: First peak > 500 RFU   Upper marker selection: Last peak > 200 RFU
Ladder size (bp)75, 200, 300, 400, 500, 700, 1000, 1500, 2000, 3000, 4000, 5000, 7000, 10000, 20000
Quantification using: Lower Marker   Final concentration (ng/uL): 0.5000   Dilution factor: 12.0

Sample: SampB12
Well location: B12
Created: Tuesday, August 10, 2021 11:20:27 PM

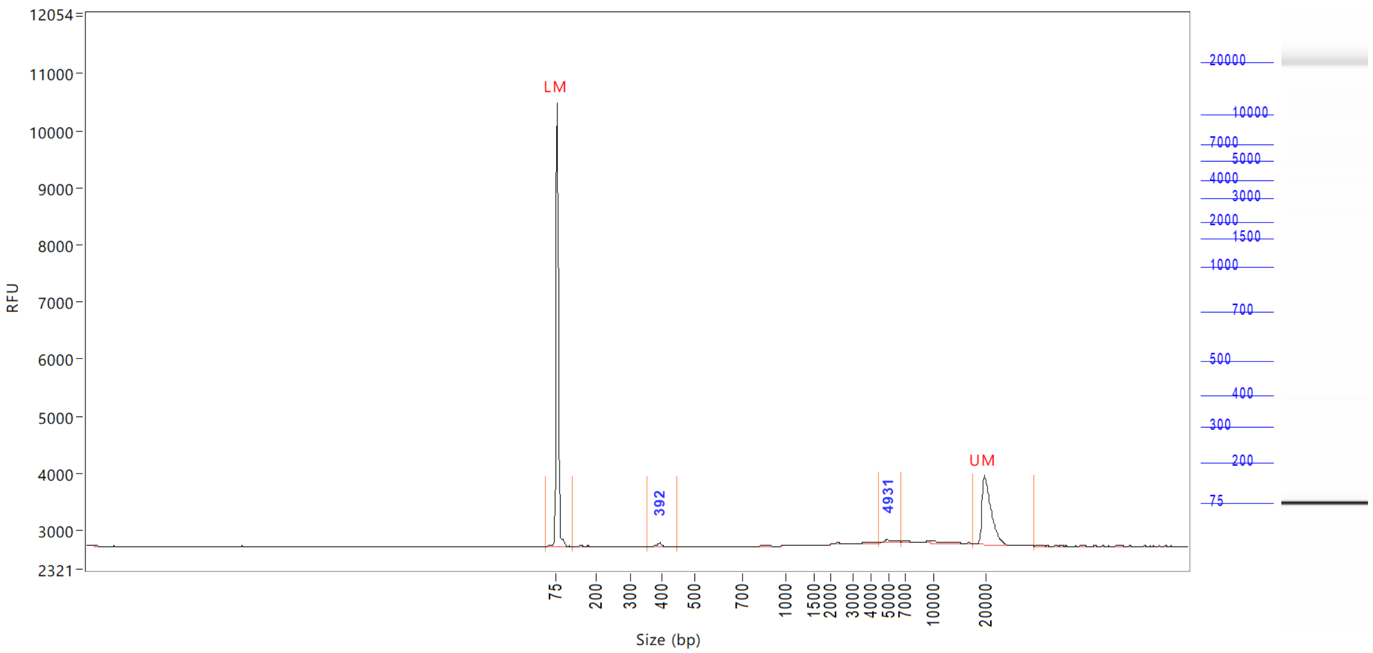

Peak	Size (bp)	Concentration (ng/uL)	From (bp)	To (bp)	Average size (bp)	CV%	RFU	Corrected peak area
1	75 (LM)	0.5000	39	126	74	6.40	7781	44.102
2	392	0.1024	354	445	392	4.15	65	0.753
3	4931	0.1247	4449	6482	5225	10.31	53	0.916
4	20000 (UM)	0.1600	17683	29793	20595	5.26	1226	14.115

TIC:	0.2271	ng/uL
TIM:	0.4693	nmole/L
Total concentration:	0.7531	ng/uL

Sample peak width (sec): 5   Sample min peak height: 50   Sample baseline V to V?: Y   Sample baseline V to V points: 3
Sample filter: Binomial   Number of points for filter: 3   Sample start region (min): 0   Sample end region (min): 30
Marker peak width (sec): 5   Marker min peak height: 200   Marker baseline V to V?: Y   Marker baseline V to V points: 3
Lower marker selection: First peak > 500 RFU   Upper marker selection: Last peak > 200 RFU
Ladder size (bp)75, 200, 300, 400, 500, 700, 1000, 1500, 2000, 3000, 4000, 5000, 7000, 10000, 20000
Quantification using: Lower Marker   Final concentration (ng/uL): 0.5000   Dilution factor: 12.0

Sample: SampC12
Well location: C12
Created: Tuesday, August 10, 2021 11:20:27 PM

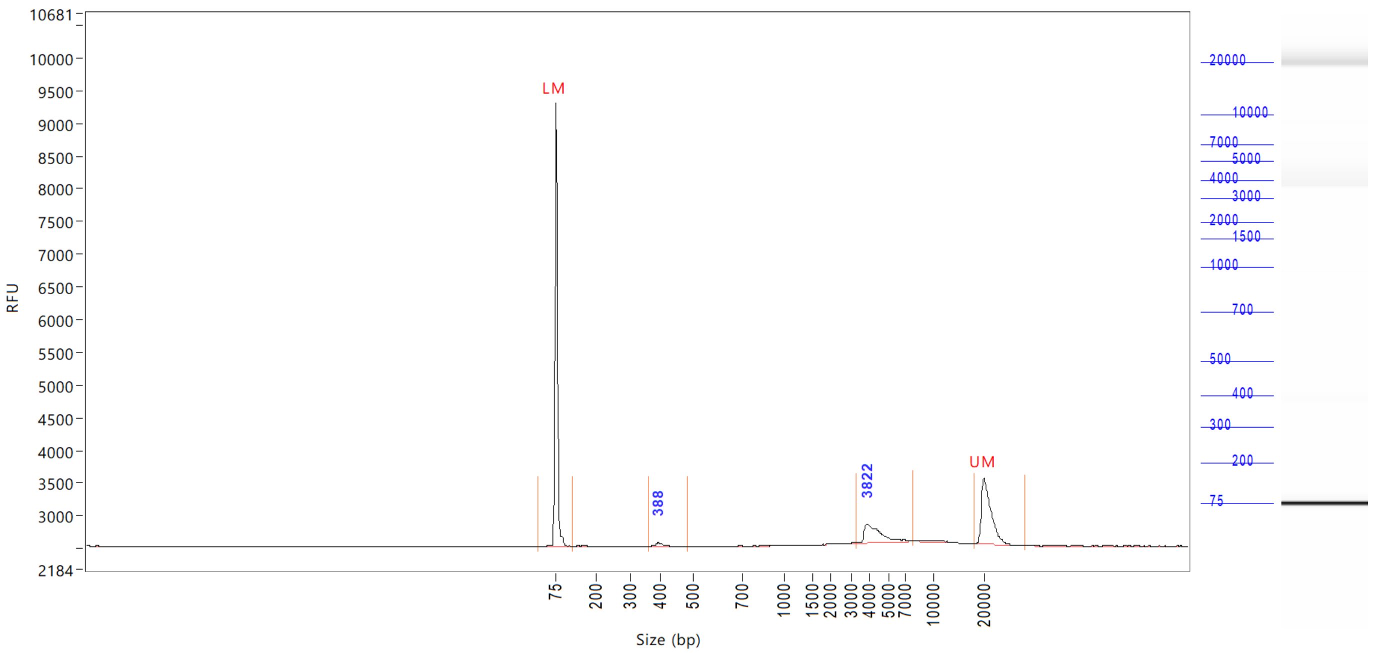

Peak	Size (bp)	Concentration (ng/uL)	From (bp)	To (bp)	Average size (bp)	CV%	RFU	Corrected peak area
1	75 (LM)	0.5000	20	124	73	7.01	6791	42.012
2	388	0.1335	360	479	396	5.33	66	0.935
3	3822	0.9838	3251	7867	4380	16.99	286	6.889
4	20000 (UM)	0.1488	18171	28100	20685	5.47	1019	12.499

TIC:	1.1173	ng/uL
TIM:	0.9244	nmole/L
Total concentration:	1.4073	ng/uL

Sample peak width (sec): 5   Sample min peak height: 50   Sample baseline V to V?: Y   Sample baseline V to V points: 3
Sample filter: Binomial   Number of points for filter: 3 Sample start region (min): 0 Sample end region (min): 30
Marker peak width (sec): 5   Marker min peak height: 200   Marker baseline V to V?: Y   Marker baseline V to V points: 3
Lower marker selection: First peak > 500 RFU   Upper marker selection: Last peak > 200 RFU
Ladder size (bp)75, 200, 300, 400, 500, 700, 1000, 1500, 2000, 3000, 4000, 5000, 7000, 10000, 20000
Quantification using: Lower Marker   Final concentration (ng/uL): 0.5000   Dilution factor: 12.0

Sample: SampD12
Well location: D12
Created: Tuesday, August 10, 2021 11:20:27 PM

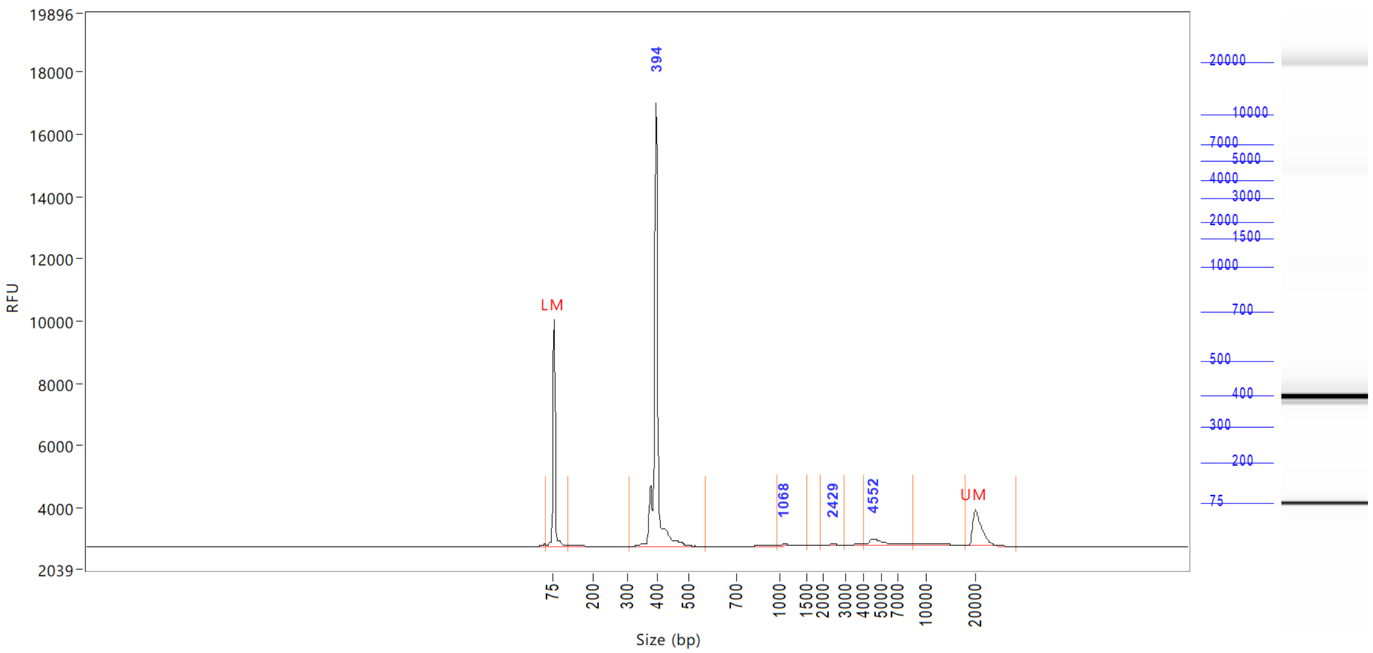

Peak	Size (bp)	Concentration (ng/uL)	From (bp)	To (bp)	Average size (bp)	CV%	RFU	Corrected peak area
1	75 (LM)	0.5000	52	122	74	8.03	7311	42.981
2	394	15.1174	309	565	394	4.75	14271	108.293
3	1068	0.1486	983	1489	1119	9.72	64	1.064
4	2429	0.0903	1885	2943	2457	9.36	51	0.647
5	4552	0.6611	4001	8600	5377	21.45	198	4.736
6	20000 (UM)	0.1543	18049	28100	20559	5.19	1138	13.260

TIC:	16.0174	ng/uL
TIM:	63.6090	nmole/L
Total concentration:	16.6159	ng/uL

```
Sample peak width (sec): 5    Sample min peak height: 50    Sample baseline V to V?: Y    Sample baseline V to V points: 3
Sample filter: Binomial       Number of points for filter: 3  Sample start region (min): 0  Sample end region (min): 30
Marker peak width (sec): 5    Marker min peak height: 200   Marker baseline V to V?: Y    Marker baseline V to V points: 3
Lower marker selection: First peak > 500 RFU                Upper marker selection: Last peak > 200 RFU
Ladder size (bp)75, 200, 300, 400, 500, 700, 1000, 1500, 2000, 3000, 4000, 5000, 7000, 10000, 20000
Quantification using: Lower Marker          Final concentration (ng/uL): 0.5000          Dilution factor: 12.0
```

Sample: SampE12
Well location: E12
Created: Tuesday, August 10, 2021 11:20:27 PM

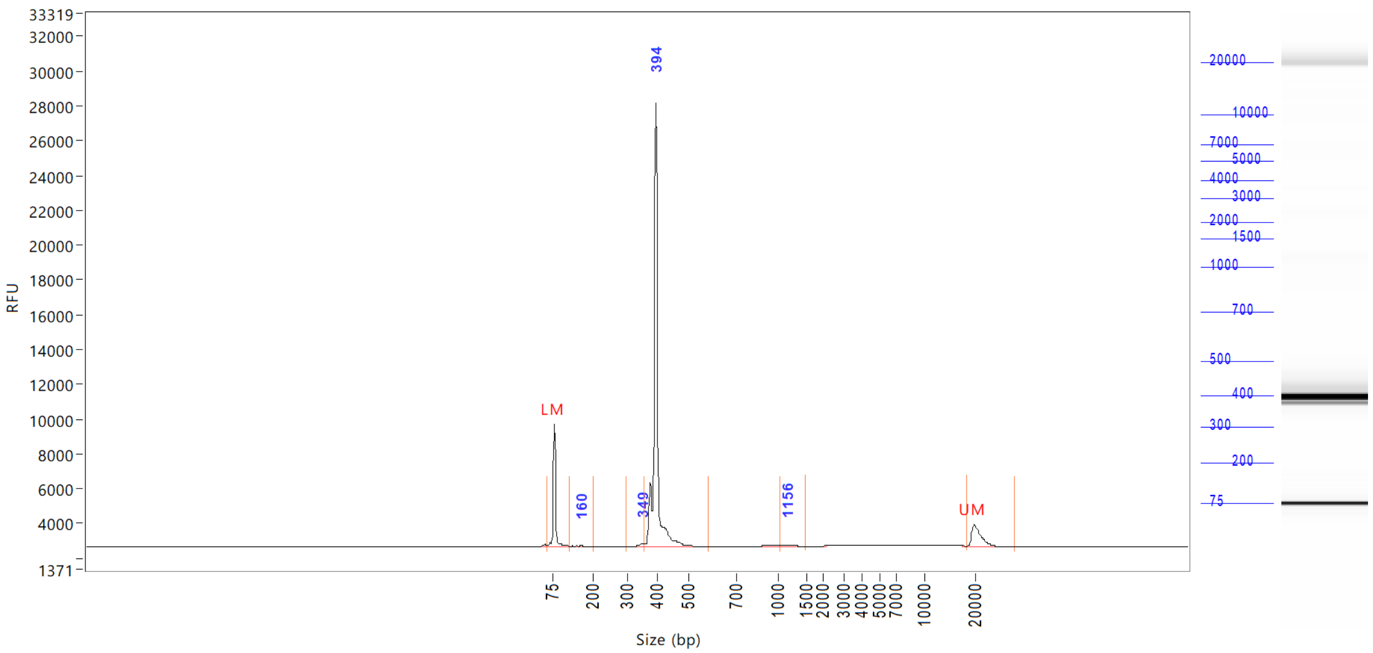

Peak	Size (bp)	Concentration (ng/uL)	From (bp)	To (bp)	Average size (bp)	CV%	RFU	Corrected peak area
1	75 (LM)	0.5000	52	124	74	10.55	7007	43.818
2	160	0.2168	124	198	150	11.51	67	1.583
3	349	0.2728	298	356	342	3.08	162	1.992
4	394	26.8650	356	578	394	4.55	25540	196.197
5	1156	0.2009	1012	1489	1161	7.64	96	1.467
6	20000 (UM)	0.1632	18415	28221	20623	5.22	1229	14.307

TIC:	27.5555	ng/uL
TIM:	116.1562	nmole/L
Total concentration:	28.1158	ng/uL

Sample peak width (sec): 5 Sample min peak height: 50 Sample baseline V to V?: Y Sample baseline V to V points: 3
Sample filter: Binomial Number of points for filter: 3 Sample start region (min): 0 Sample end region (min): 30
Marker peak width (sec): 5 Marker min peak height: 200 Marker baseline V to V?: Y Marker baseline V to V points: 3
Lower marker selection: First peak > 500 RFU Upper marker selection: Last peak > 200 RFU
Ladder size (bp)75, 200, 300, 400, 500, 700, 1000, 1500, 2000, 3000, 4000, 5000, 7000, 10000, 20000
Quantification using: Lower Marker Final concentration (ng/uL): 0.5000 Dilution factor: 12.0

Sample: SampF12
Well location: F12
Created: Tuesday, August 10, 2021 11:20:27 PM

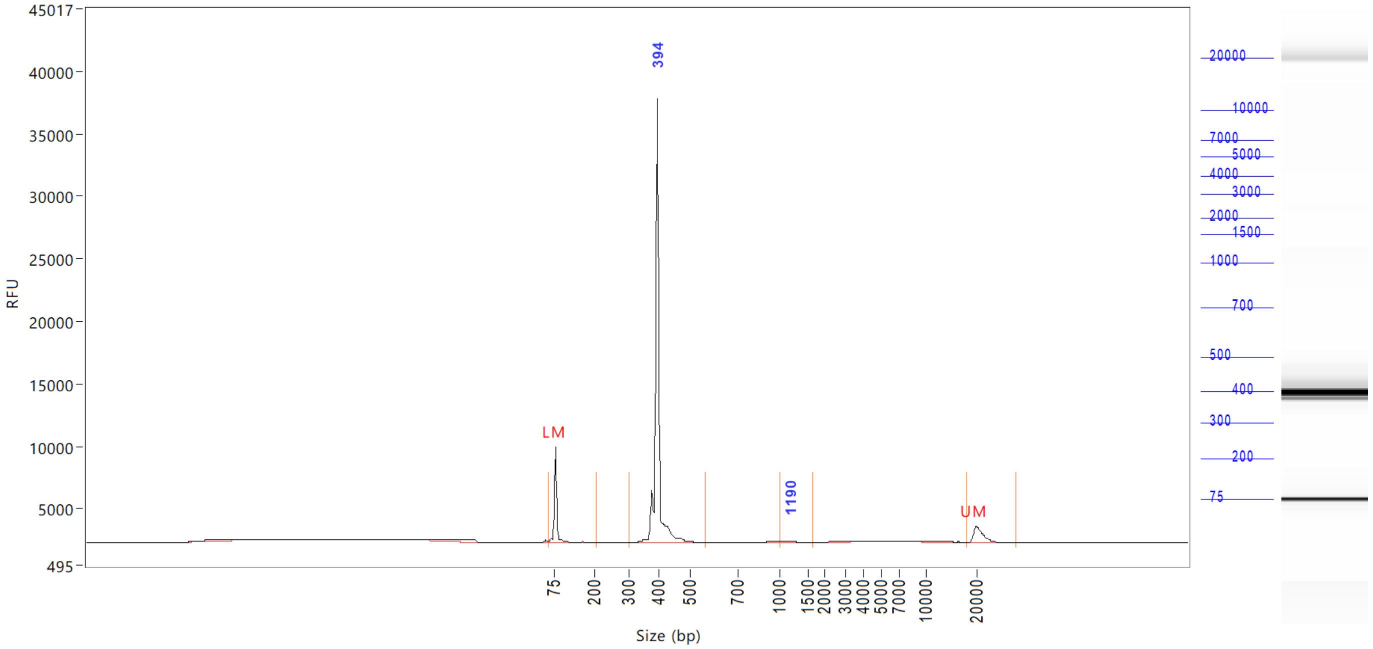

Peak	Size (bp)	Concentration (ng/uL)	From (bp)	To (bp)	Average size (bp)	CV%	RFU	Corrected peak area
1	75 (LM)	0.5000	52	205	76	20.68	7659	50.365
2	394	30.9420	301	560	394	4.55	35594	259.732
3	1190	0.1995	991	1655	1158	9.70	105	1.675
4	20000 (UM)	0.1508	18293	27979	20657	5.37	1306	15.190

TIC:	31.1415	ng/uL
TIM:	129.4921	nmole/L
Total concentration:	31.7651	ng/uL

Sample peak width (sec): 5 Sample min peak height: 50 Sample baseline V to V?: Y Sample baseline V to V points: 3
Sample filter: Binomial Number of points for filter: 3 Sample start region (min): 0 Sample end region (min): 30
Marker peak width (sec): 5 Marker min peak height: 200 Marker baseline V to V?: Y Marker baseline V to V points: 3
Lower marker selection: First peak > 500 RFU Upper marker selection: Last peak > 200 RFU
Ladder size (bp)75, 200, 300, 400, 500, 700, 1000, 1500, 2000, 3000, 4000, 5000, 7000, 10000, 20000
Quantification using: Lower Marker Final concentration (ng/uL): 0.5000 Dilution factor: 12.0

Sample: SampG12
Well location: G12
Created: Tuesday, August 10, 2021 11:20:27 PM

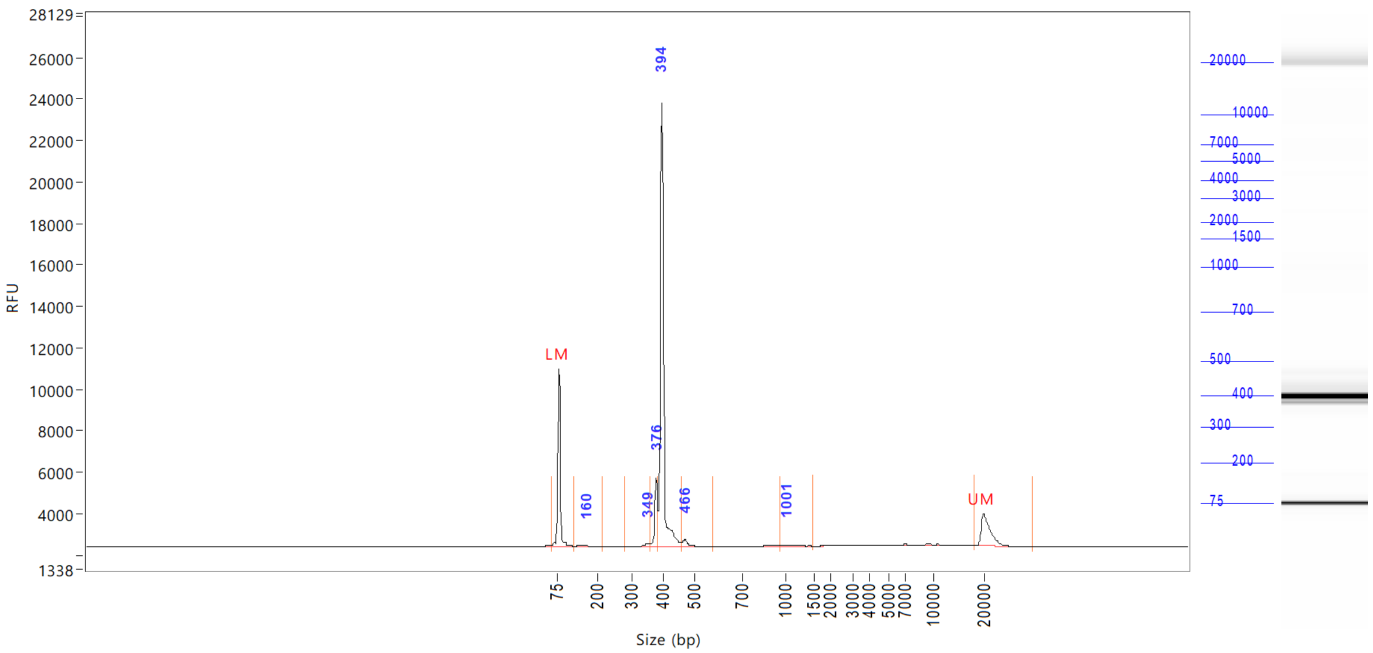

Peak	Size (bp)	Concentration (ng/uL)	From (bp)	To (bp)	Average size (bp)	CV%	RFU	Corrected peak area
1	75 (LM)	0.5000	52	124	74	9.02	8555	50.939
2	160	0.2037	124	212	153	11.96	87	1.729
3	349	0.2262	278	358	343	3.79	147	1.920
4	376	2.2575	358	382	374	1.31	3320	19.166
5	394	16.5698	382	454	396	2.83	21421	140.676
6	466	0.4676	454	575	471	3.59	336	3.970
7	1001	0.1642	957	1489	1078	10.08	78	1.394
8	20000 (UM)	0.1721	18171	29793	20689	5.34	1513	17.538

TIC:	19.8890	ng/uL
TIM:	83.9311	nmole/L
Total concentration:	20.1447	ng/uL

```
Sample peak width (sec): 5    Sample min peak height: 50    Sample baseline V to V?: Y    Sample baseline V to V points: 3
Sample filter: Binomial       Number of points for filter: 3  Sample start region (min): 0  Sample end region (min): 30
Marker peak width (sec): 5    Marker min peak height: 200   Marker baseline V to V?: Y    Marker baseline V to V points: 3
Lower marker selection: First peak > 500 RFU                Upper marker selection: Last peak > 200 RFU
Ladder size (bp)75, 200, 300, 400, 500, 700, 1000, 1500, 2000, 3000, 4000, 5000, 7000, 10000, 20000
Quantification using: Lower Marker          Final concentration (ng/uL): 0.5000          Dilution factor: 12.0
```

Sample: SampH12
Well location: H12
Created: Tuesday, August 10, 2021 11:20:27 PM

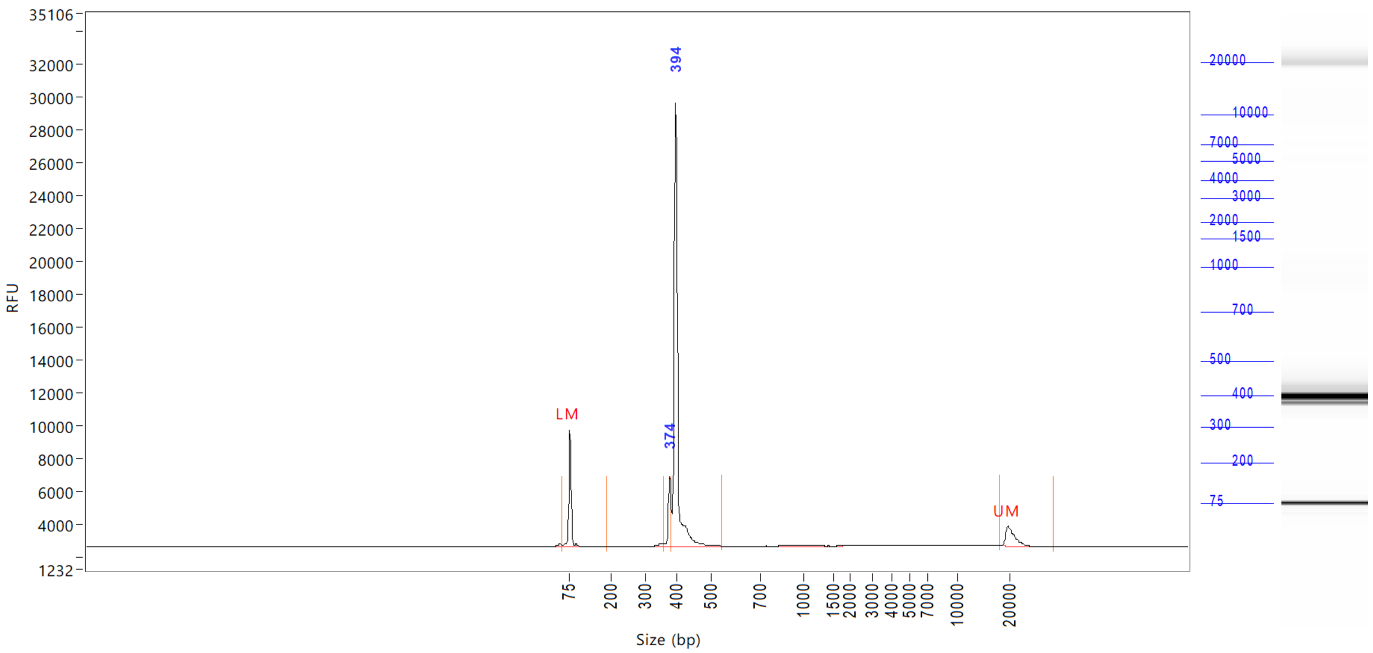

Peak	Size (bp)	Concentration (ng/uL)	From (bp)	To (bp)	Average size (bp)	CV%	RFU	Corrected peak area
1	75 (LM)	0.5000	52	187	75	12.79	7125	41.980
2	374	3.6874	356	382	373	1.43	4267	25.799
3	394	25.8169	382	542	397	3.90	27071	180.632
4	20000 (UM)	0.1612	18171	28705	20500	4.96	1192	13.534

TIC:	29.5043	ng/uL
TIM:	123.2569	nmole/L
Total concentration:	30.2290	ng/uL

Sample peak width (sec): 5 Sample min peak height: 200 Sample baseline V to V?: Y Sample baseline V to V points: 3
Sample filter: Binomial Number of points for filter: 3 Sample start region (min): 0 Sample end region (min): 30
Marker peak width (sec): 5 Marker min peak height: 200 Marker baseline V to V?: Y Marker baseline V to V points: 3
Lower marker selection: First peak > 500 RFU Upper marker selection: Last peak > 200 RFU
Ladder size (bp)75, 200, 300, 400, 500, 700, 1000, 1500, 2000, 3000, 4000, 5000, 7000, 10000, 20000
Quantification using: Lower Marker Final concentration (ng/uL): 0.5000 Dilution factor: 12.0

Sample: DNA size ladder
Well location: A7
Created: Tuesday, August 10, 2021 11:20:27 PM
Import from: C:\AATI\Data\2021 08 10\22-31-23\210811_alberto_ladder.SCAL
Fit type: Point to point

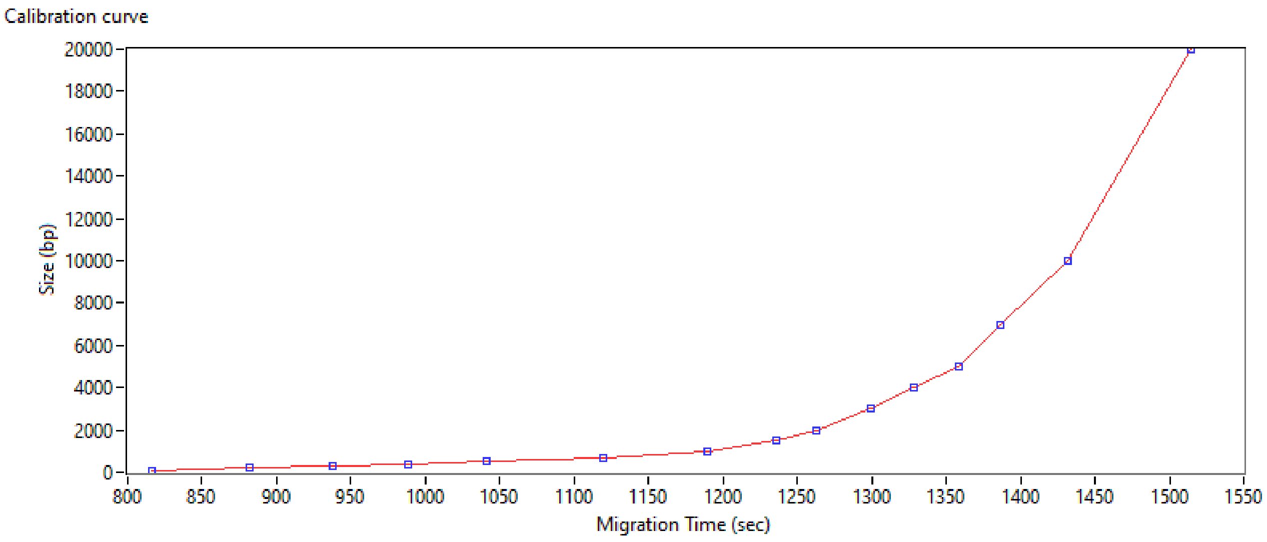

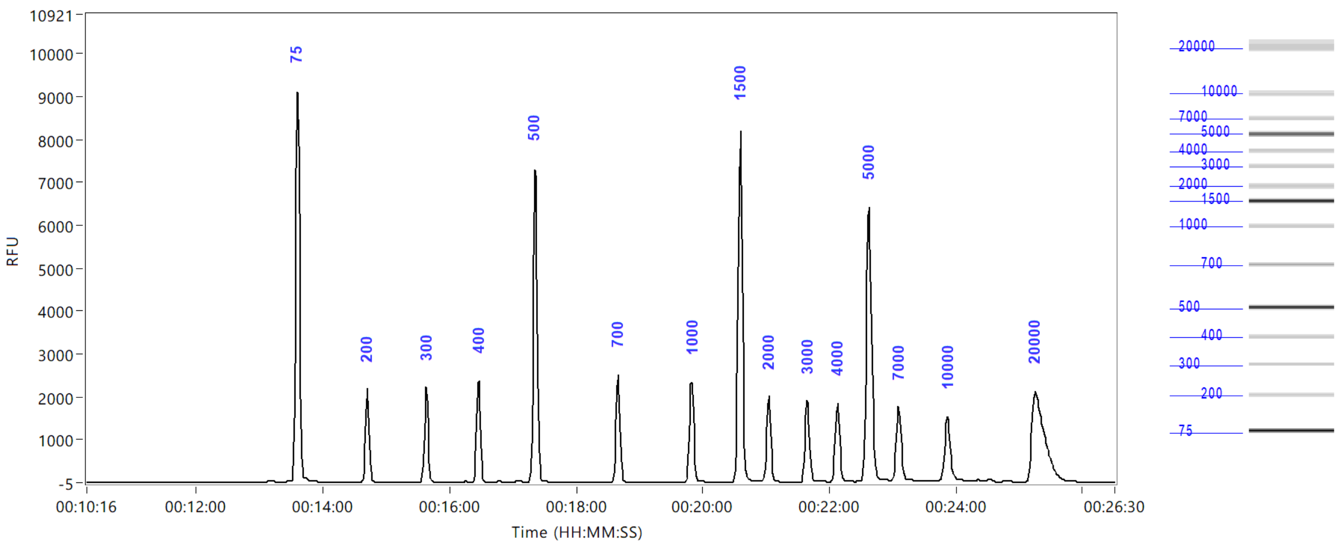

Supplement: Supplementary file 2 — sb3c00292_si_002.zip [file sb3c00292_si_002.zip › dnada_supplementary_material_pks_library_build/ZAG_trials/ZAG_Trial_1/23-11-21_plate8/plate8_report.pdf]
