# Supplementary material for: Automated Platform for the Plasmid Construction Process
Source: ACS Synth Biol. 2023 Nov 10;12(12):3506–13. doi: 10.1021/acssynbio.3c00292 (PMC10729297; doi:10.1021/acssynbio.3c00292)

Instrument controller software run summary:

Filename and data path: C:\AATI\Data\2021 08 10\19-51-25\2021 08 10 19H 51M..raw
Created: Tuesday, August 10, 2021 8:01:08 PM
Number of capillaries: 96
Array serial number: 111920-09SFS
Effect length: 33 cm
Array usage count: 166
Instrument type: ZAG DNA Analyzer
Instrument controller software version: 1.0.1.9
Device serial number: Z7597

Method Information

Method name: ZAG130GP33 - DNA 75-20000bp - Gel Prime Only.mthds
Gel prime: Yes
Full conditioning: No
Gel prime to buffer: No
Gel selection: Gel 1
Perform prerun: 8.0 kV, 30 sec.
Rinse: No
Marker 1: Row: A, 5.0 kV, 5 sec.
Rinse: No
Sample injection: 5.0 kV, 5 sec.
Separation: 8.0 kV, 30.0 min.
Tray name: Sample Tray-3

Analysis mode: DNA

Notes

Gel Image

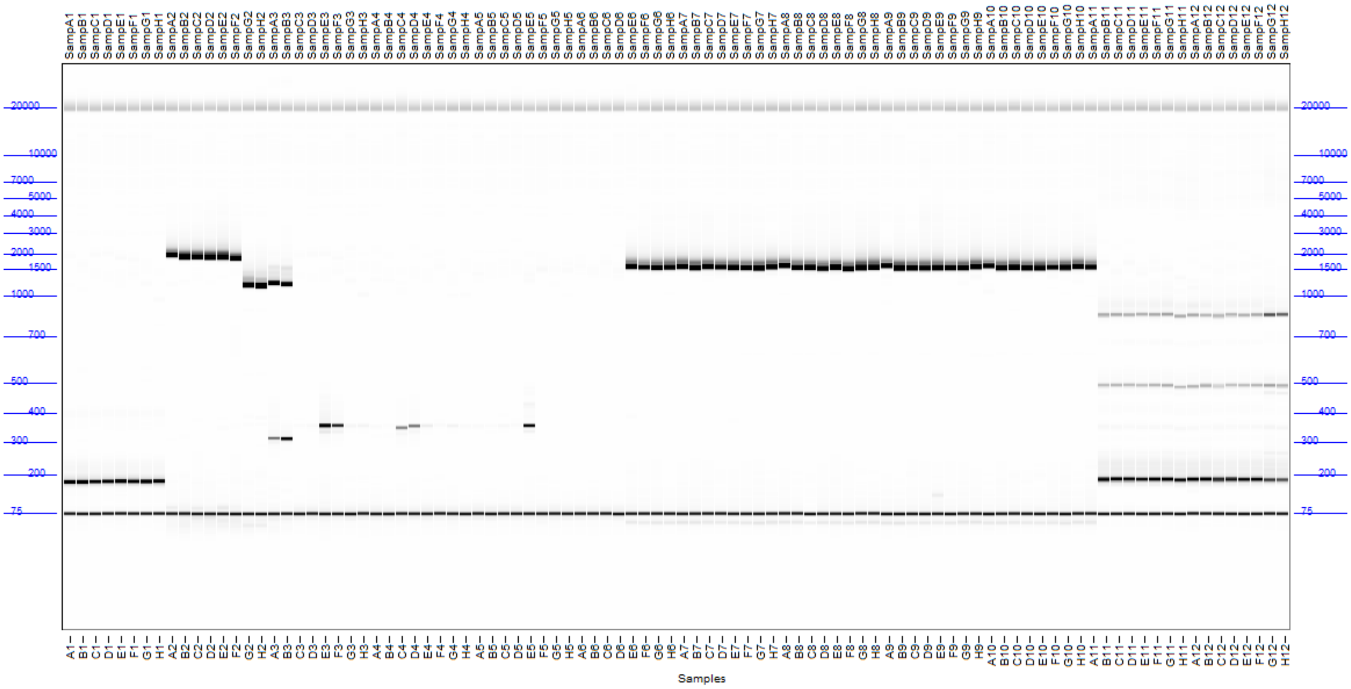
20000
10000
7000
5000
4000
3000
2000
1500
1000
700
500
400
300
200
75
Samples

Filename and data path: C:\AATI\Data\2021 08 10\19-51-25\2021 08 10 19H 51M..raw

A1: SampA1

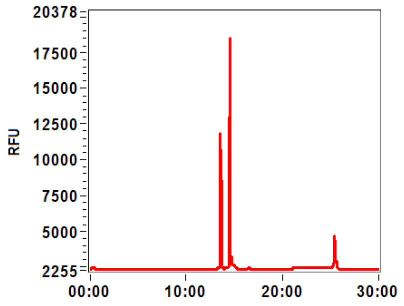

B1: SampB1

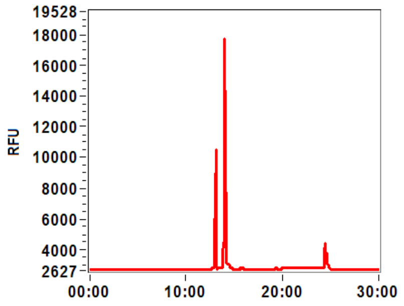

C1: SampC1

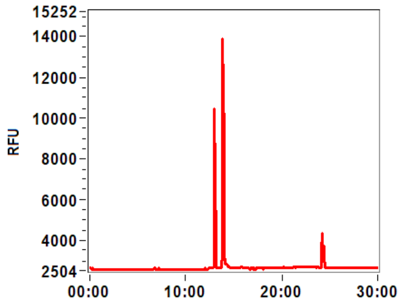

D1: SampD1

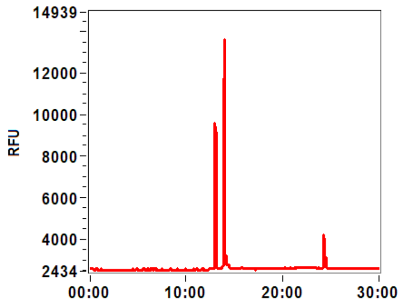

E1: SampE1

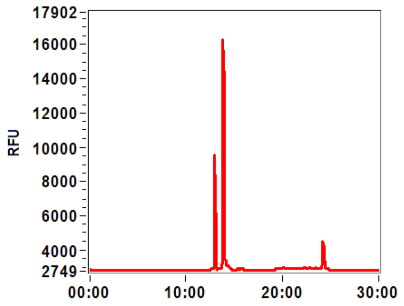

F1: SampF1

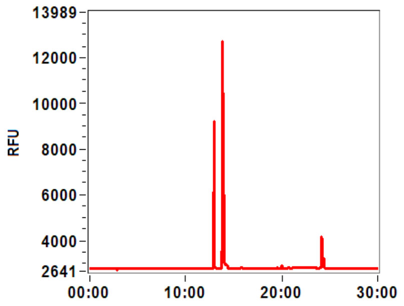

G1: SampG1

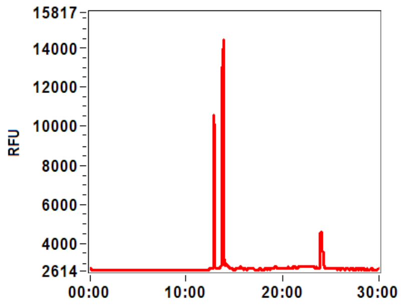

H1: SampH1

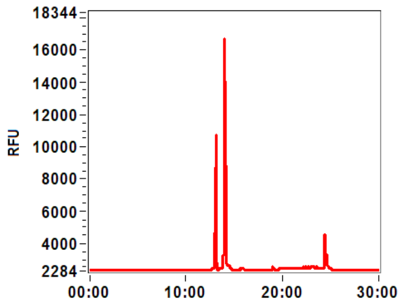

A2: SampA2

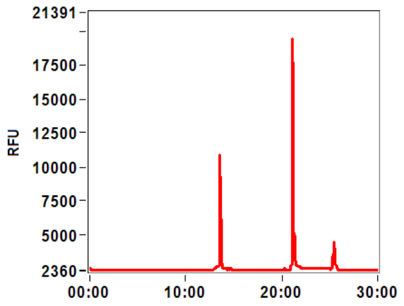

B2: SampB2

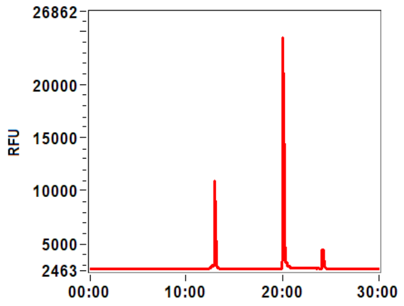

C2: SampC2

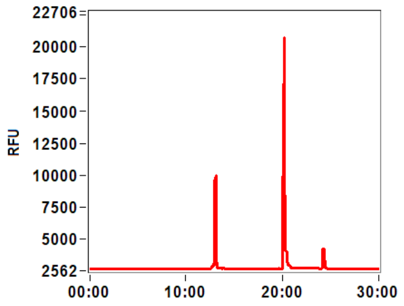

D2: SampD2

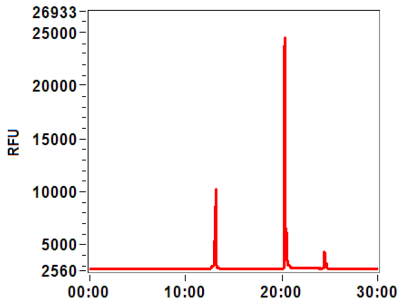

Filename and data path: C:\AATI\Data\2021 08 10\19-51-25\2021 08 10 19H 51M..raw

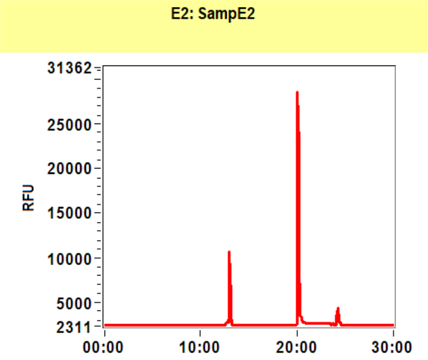

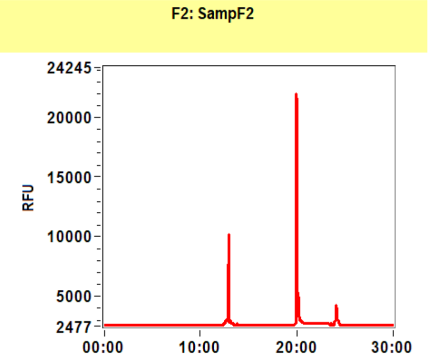

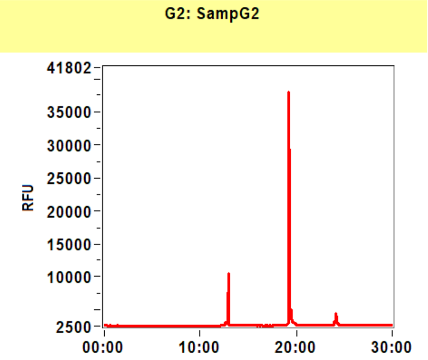

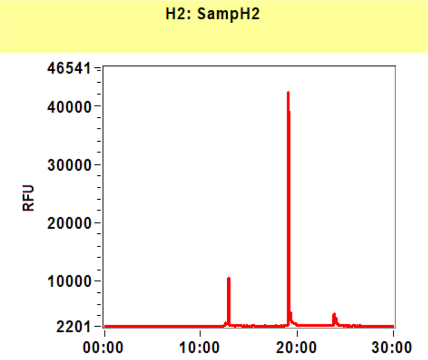

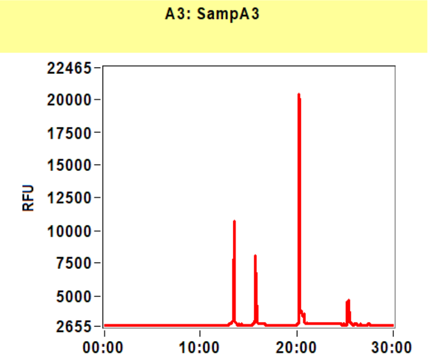

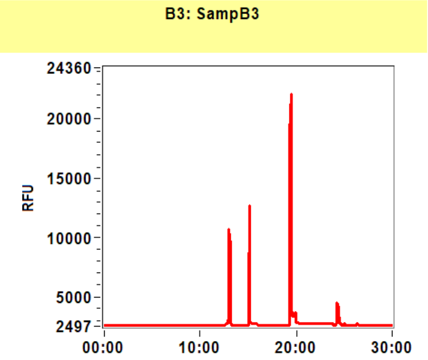

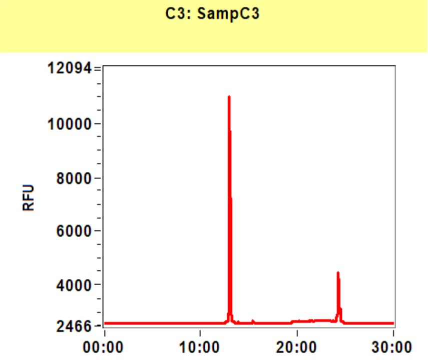

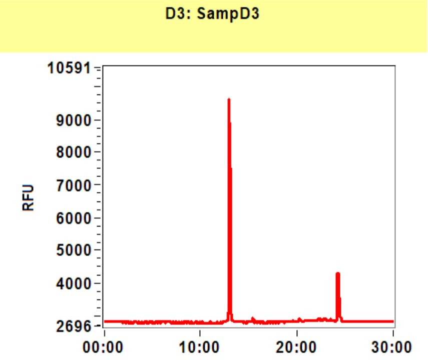

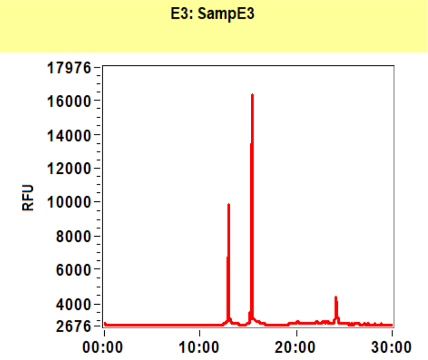

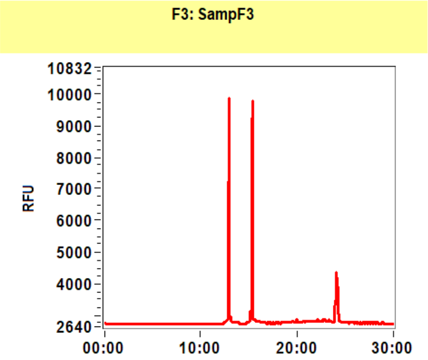

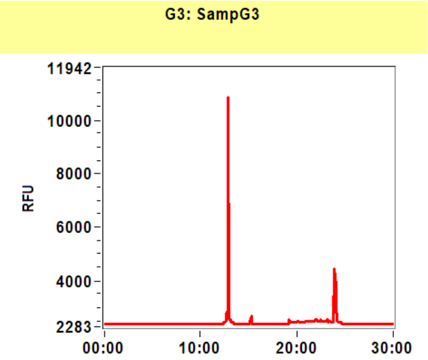

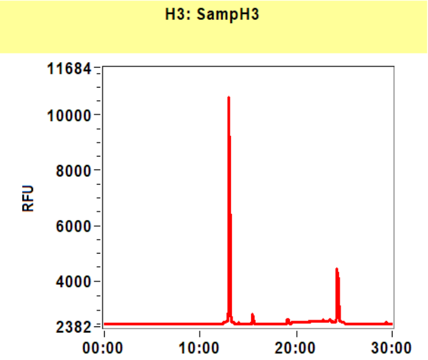

Filename and data path: C:\AATI\Data\2021 08 10\19-51-25\2021 08 10 19H 51M..raw

A4: SampA4

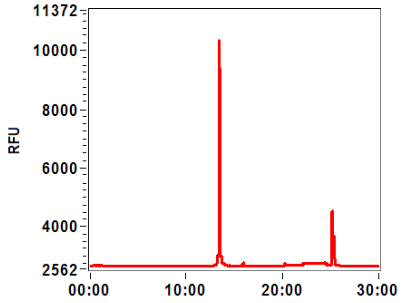

B4: SampB4

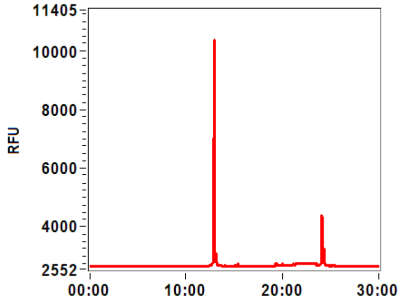

C4: SampC4

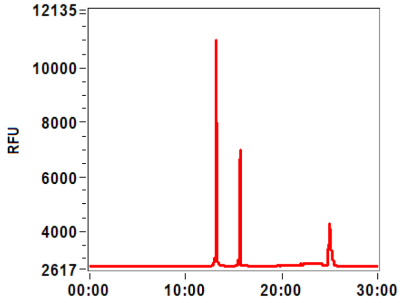

D4: SampD4

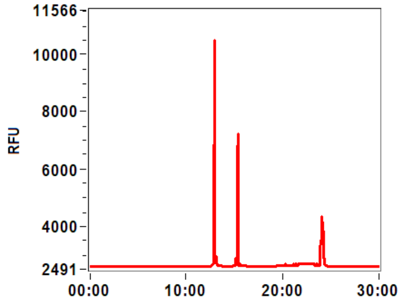

E4: SampE4

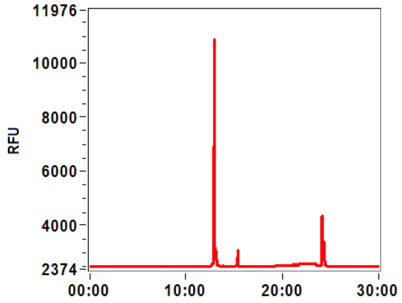

F4: SampF4

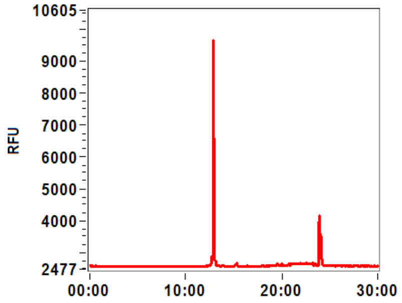

G4: SampG4

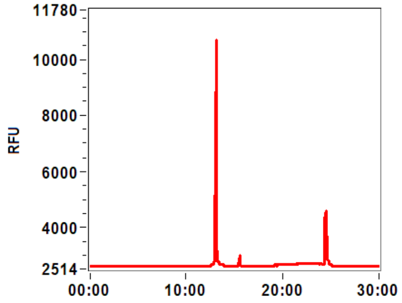

H4: SampH4

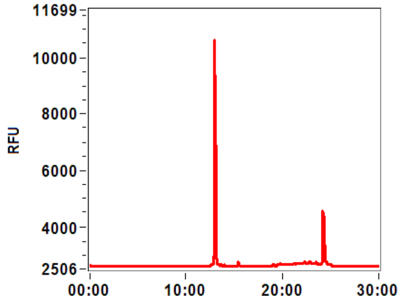

A5: SampA5

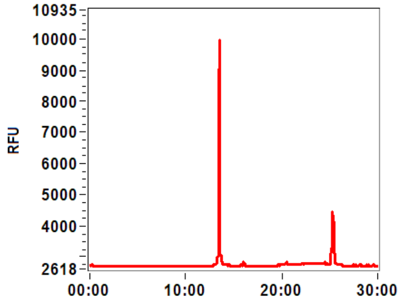

B5: SampB5

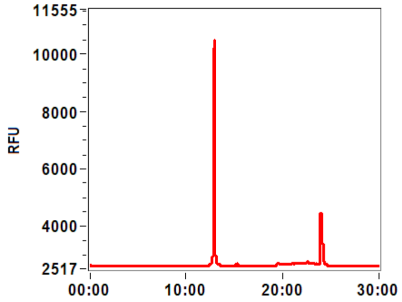

C5: SampC5

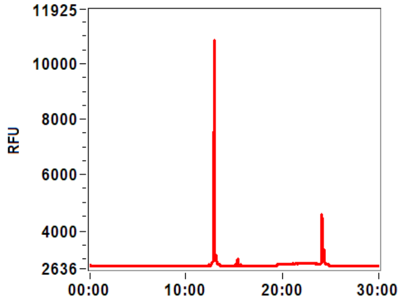

D5: SampD5

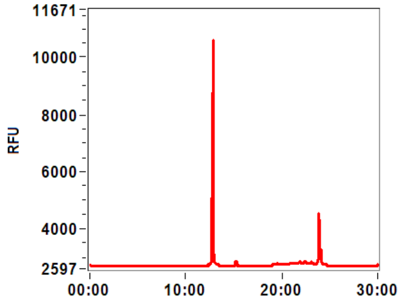

Filename and data path: C:\AATI\Data\2021 08 10\19-51-25\2021 08 10 19H 51M..raw

E5: SampE5

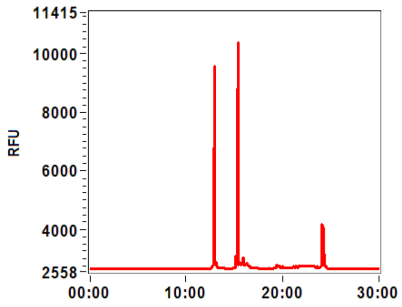

F5: SampF5

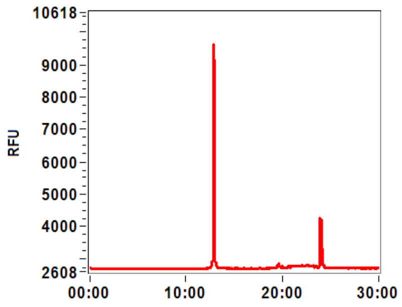

G5: SampG5

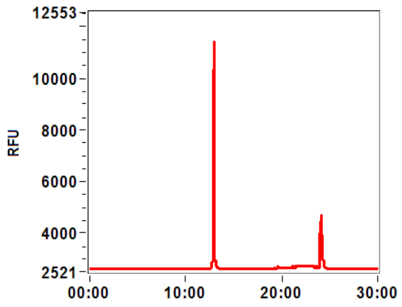

H5: SampH5

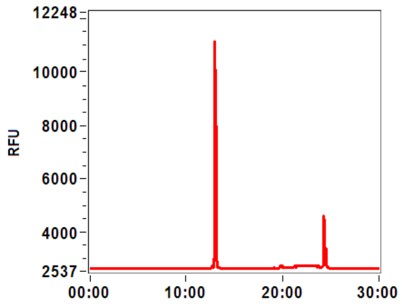

A6: SampA6

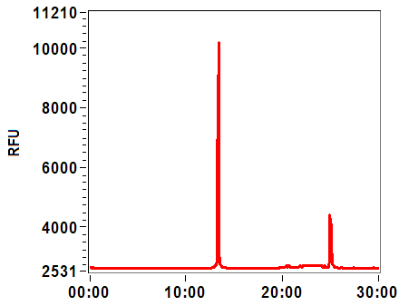

B6: SampB6

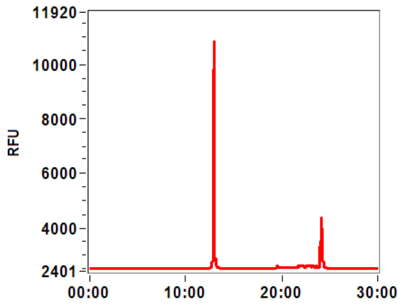

C6: SampC6

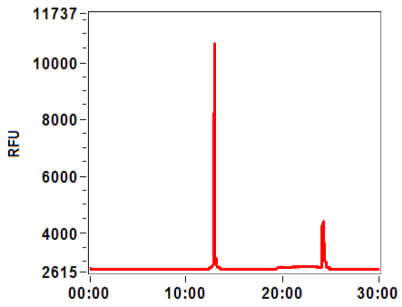

D6: SampD6

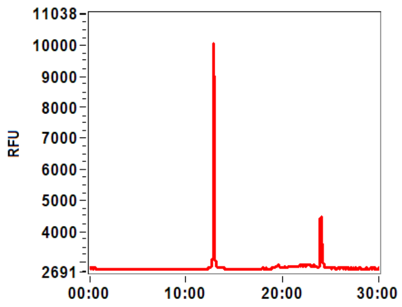

E6: SampE6

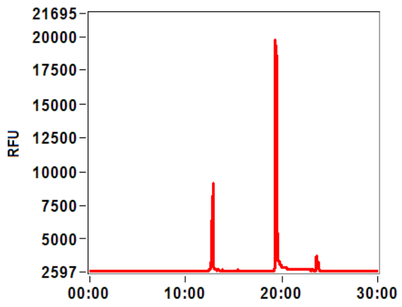

F6: SampF6

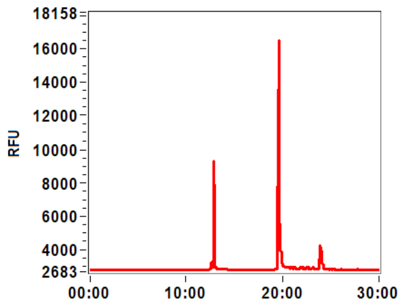

G6: SampG6

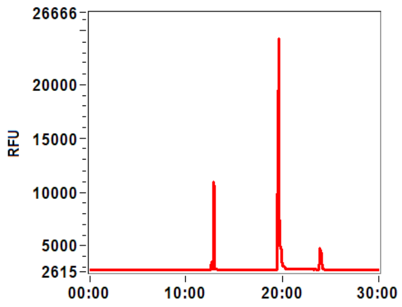

H6: SampH6

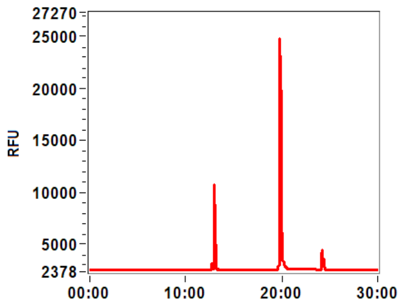

Filename and data path: C:\AATI\Data\2021 08 10\19-51-25\2021 08 10 19H 51M..raw

A7: SampA7

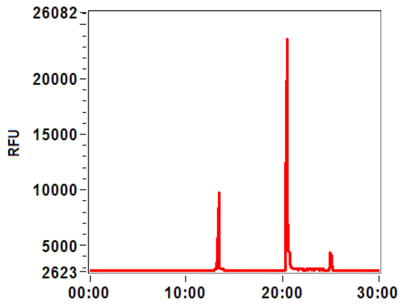

B7: SampB7

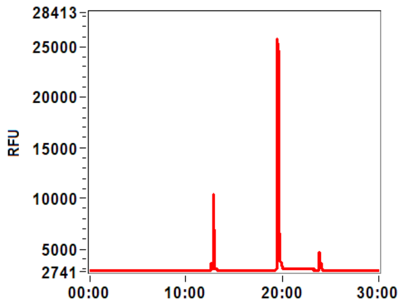

C7: SampC7

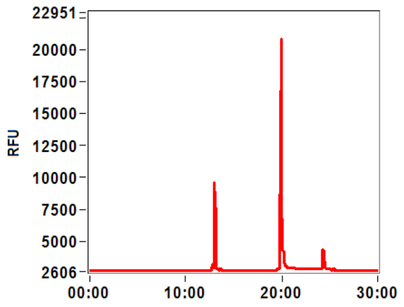

D7: SampD7

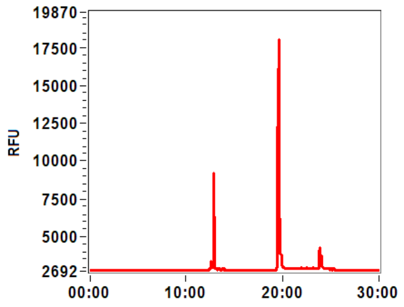

E7: SampE7

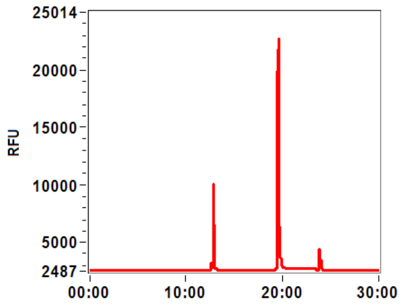

F7: SampF7

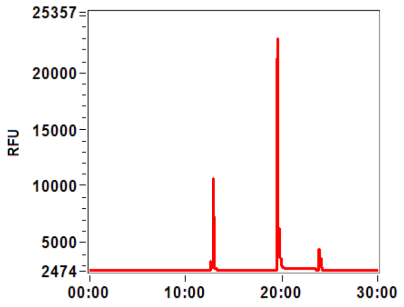

G7: SampG7

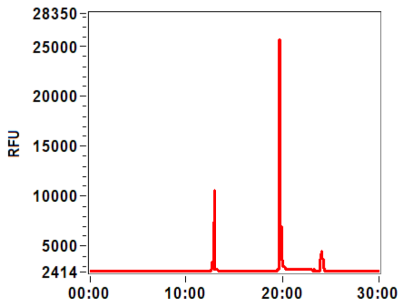

H7: SampH7

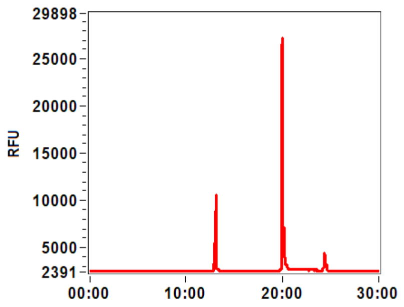

A8: SampA8

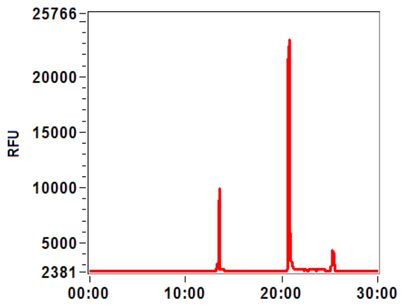

B8: SampB8

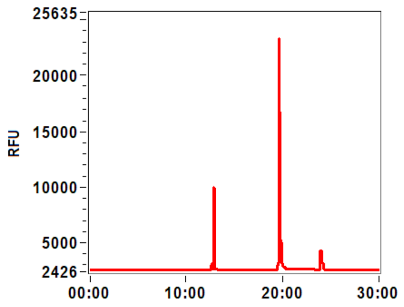

C8: SampC8

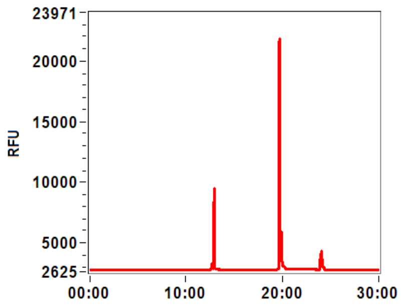

D8: SampD8

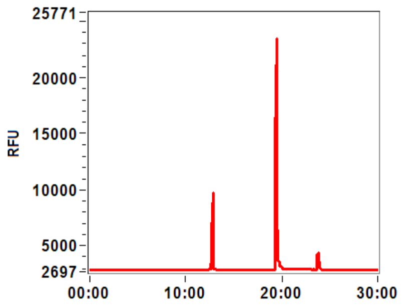

Filename and data path: C:\AATI\Data\2021 08 10\19-51-25\2021 08 10 19H 51M..raw

E8: SampE8

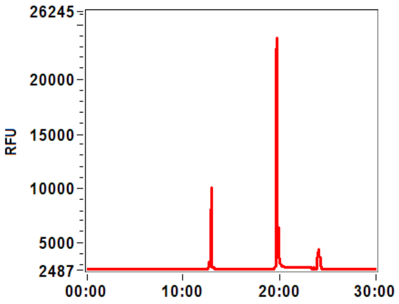

F8: SampF8

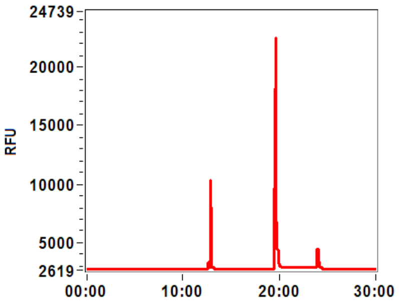

G8: SampG8

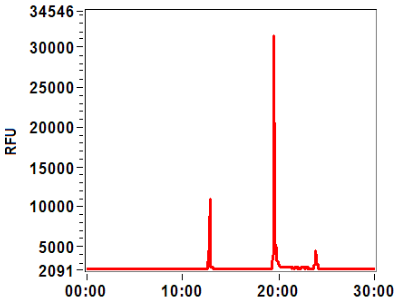

H8: SampH8

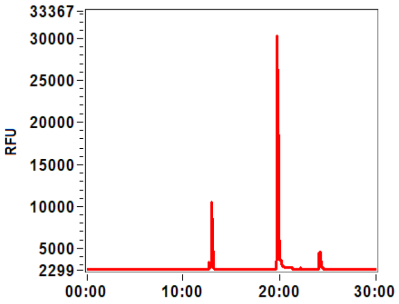

A9: SampA9

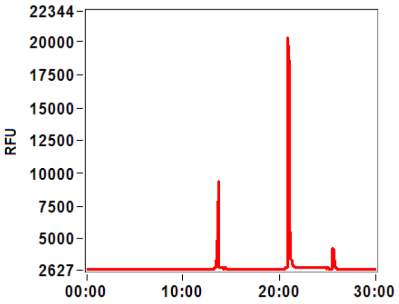

B9: SampB9

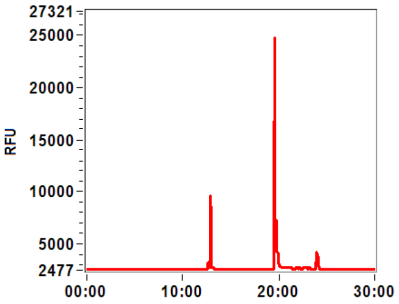

C9: SampC9

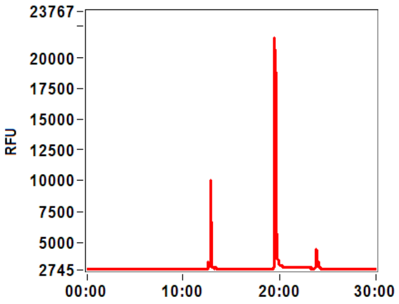

D9: SampD9

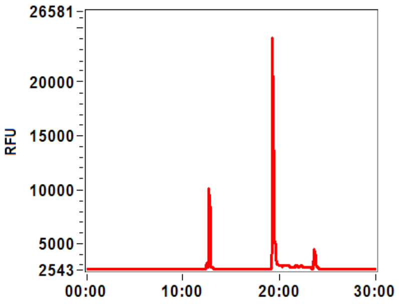

E9: SampE9

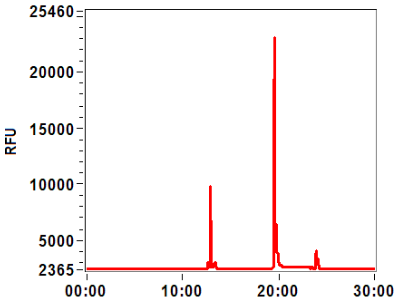

F9: SampF9

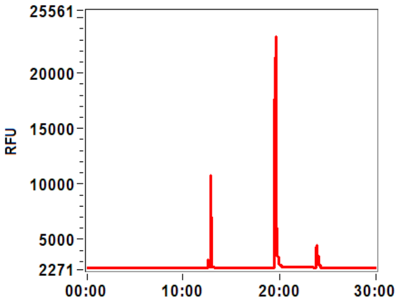

G9: SampG9

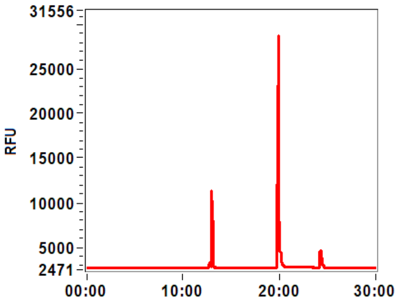

H9: SampH9

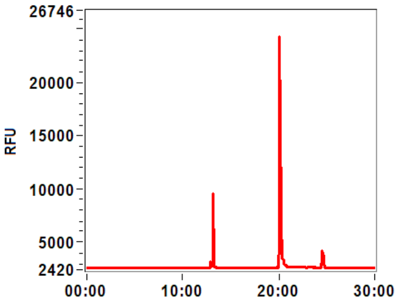

Filename and data path: C:\AATI\Data\2021 08 10\19-51-25\2021 08 10 19H 51M..raw

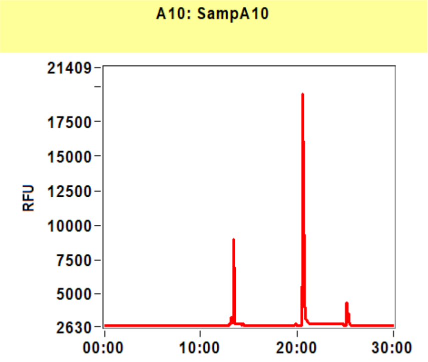

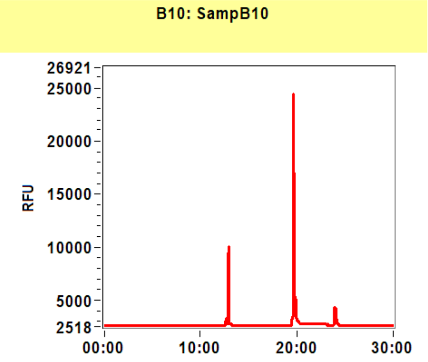

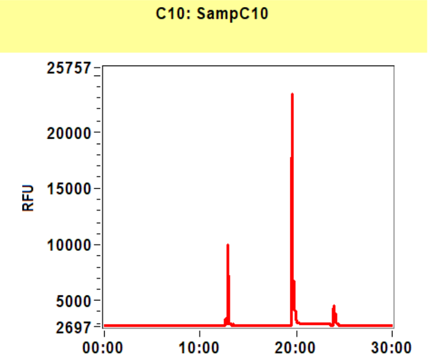

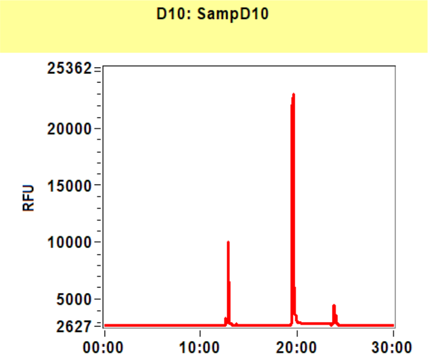

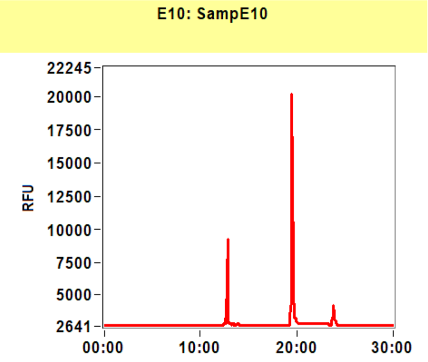

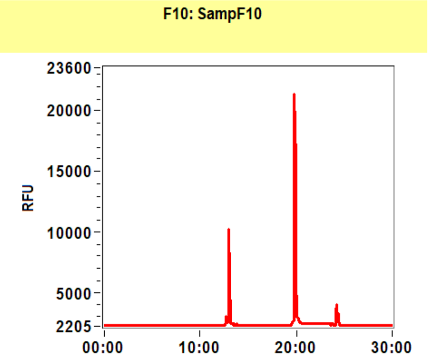

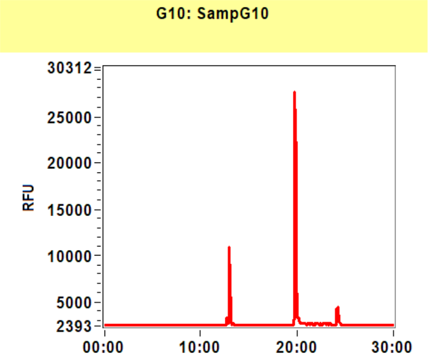

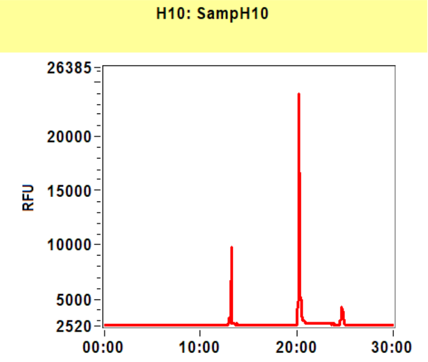

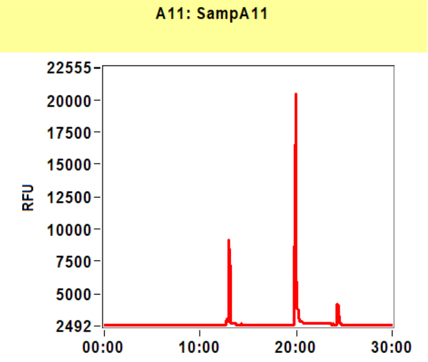

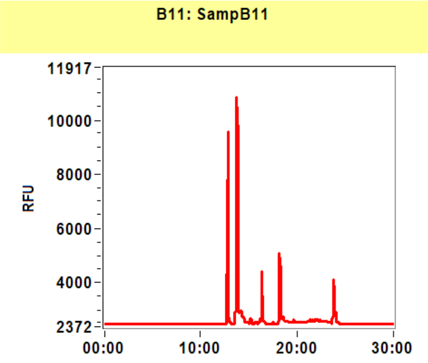

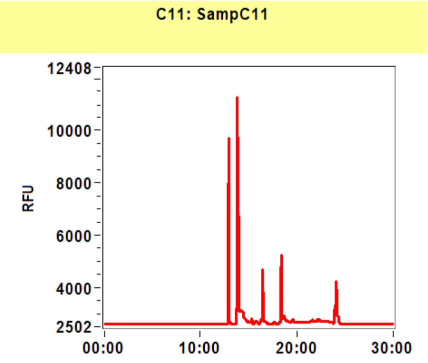

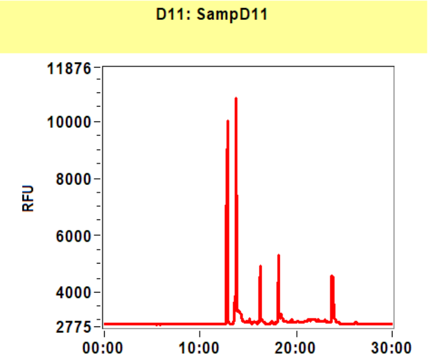

Filename and data path: C:\AATI\Data\2021 08 10\19-51-25\2021 08 10 19H 51M..raw

E11: SampE11

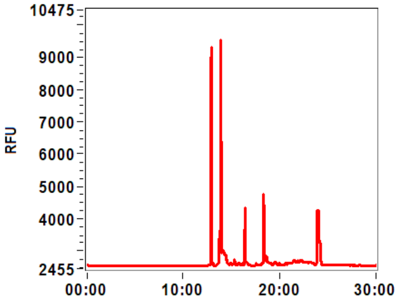

F11: SampF11

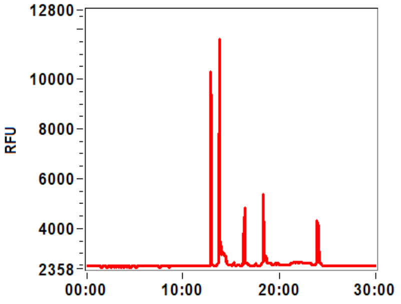

G11: SampG11

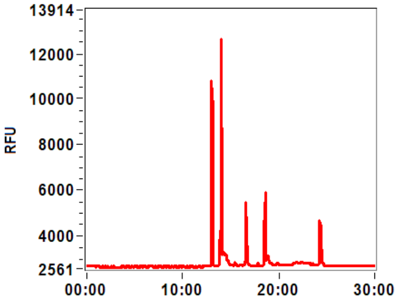

H11: SampH11

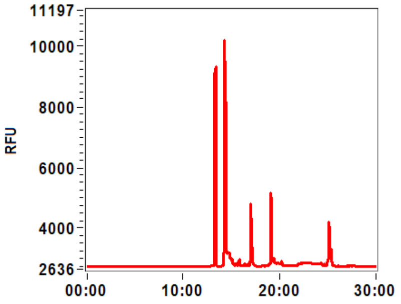

A12: SampA12

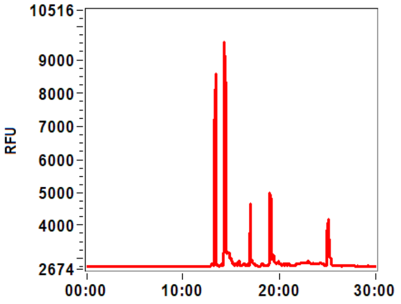

B12: SampB12

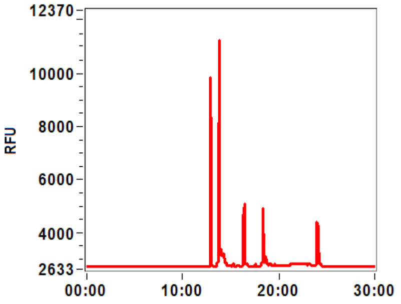

C12: SampC12

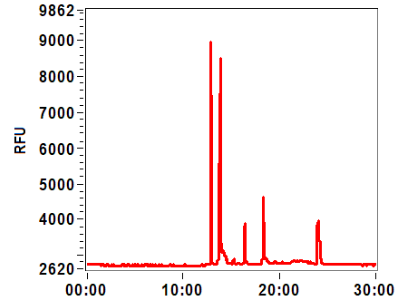

D12: SampD12

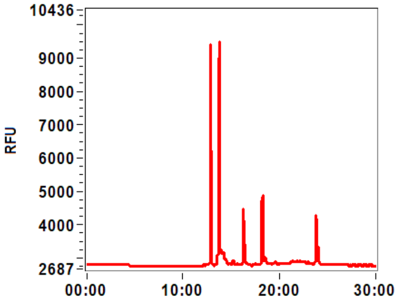

E12: SampE12

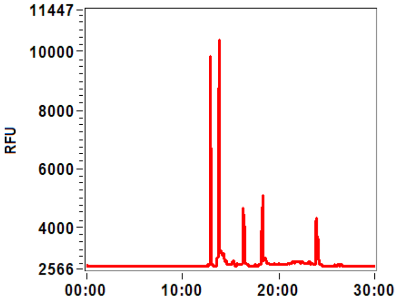

F12: SampF12

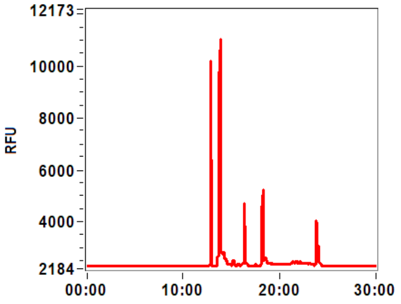

G12: SampG12

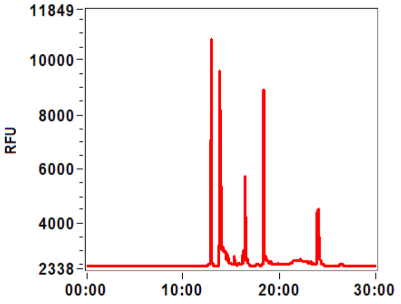

H12: SampH12

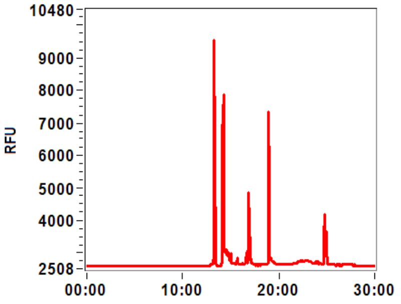

Sample: SampA1
Well location: A1
Created: Tuesday, August 10, 2021 8:01:08 PM

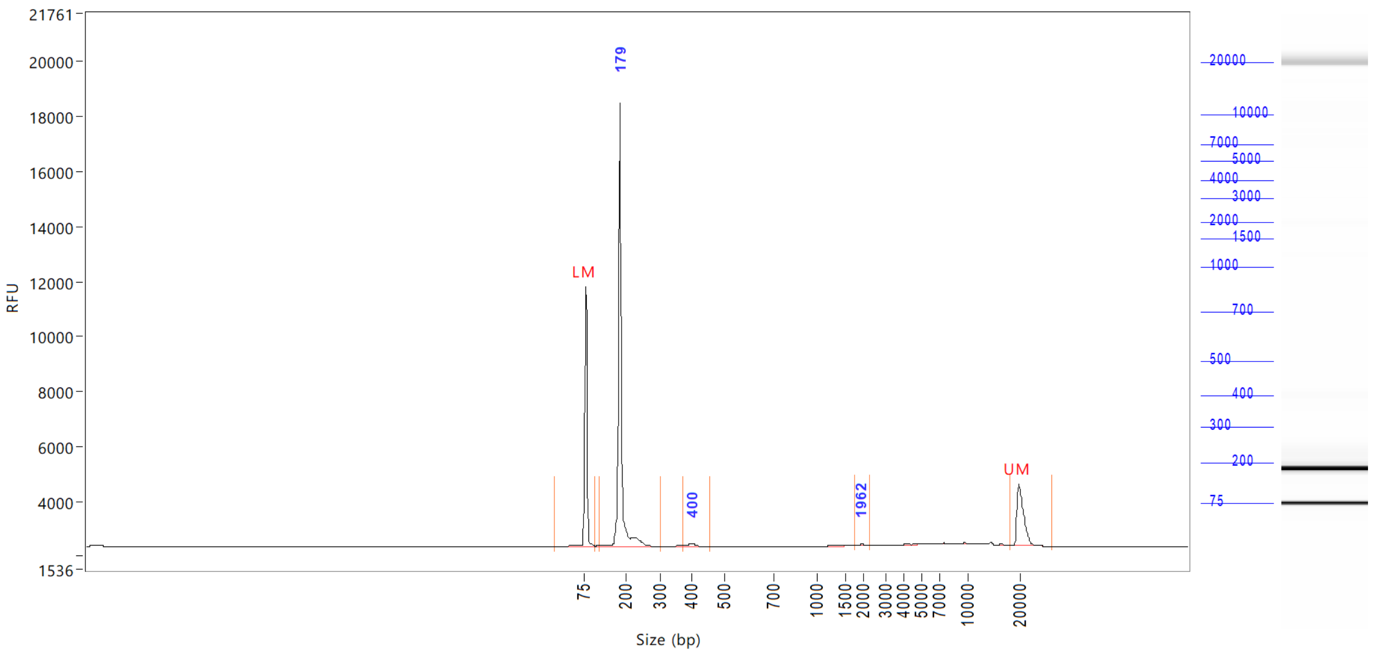

Peak	Size (bp)	Concentration (ng/uL)	From (bp)	To (bp)	Average size (bp)	CV%	RFU	Corrected peak area
1	75 (LM)	0.5000	0	105	72	11.37	9471	50.763
2	179	12.4968	120	300	180	8.61	16166	105.729
3	400	0.1942	372	454	397	3.11	114	1.643
4	1962	0.0613	1731	2286	1971	4.86	73	0.518
5	20000 (UM)	0.1848	18293	26529	20367	3.68	2246	18.762

TIC:	12.7523	ng/uL
TIM:	114.9926	nmole/L
Total concentration:	13.1985	ng/uL

Sample peak width (sec): 5 Sample min peak height: 50 Sample baseline V to V?: Y Sample baseline V to V points: 3
Sample filter: Binomial Number of points for filter: 3 Sample start region (min): 0 Sample end region (min): 30
Marker peak width (sec): 5 Marker min peak height: 200 Marker baseline V to V?: Y Marker baseline V to V points: 3
Lower marker selection: First peak > 500 RFU Upper marker selection: Last peak > 200 RFU
Ladder size (bp)75, 200, 300, 400, 500, 700, 1000, 1500, 2000, 3000, 4000, 5000, 7000, 10000, 20000
Quantification using: Lower Marker Final concentration (ng/uL): 0.5000 Dilution factor: 12.0

Sample: SampB1
Well location: B1
Created: Tuesday, August 10, 2021 8:01:08 PM

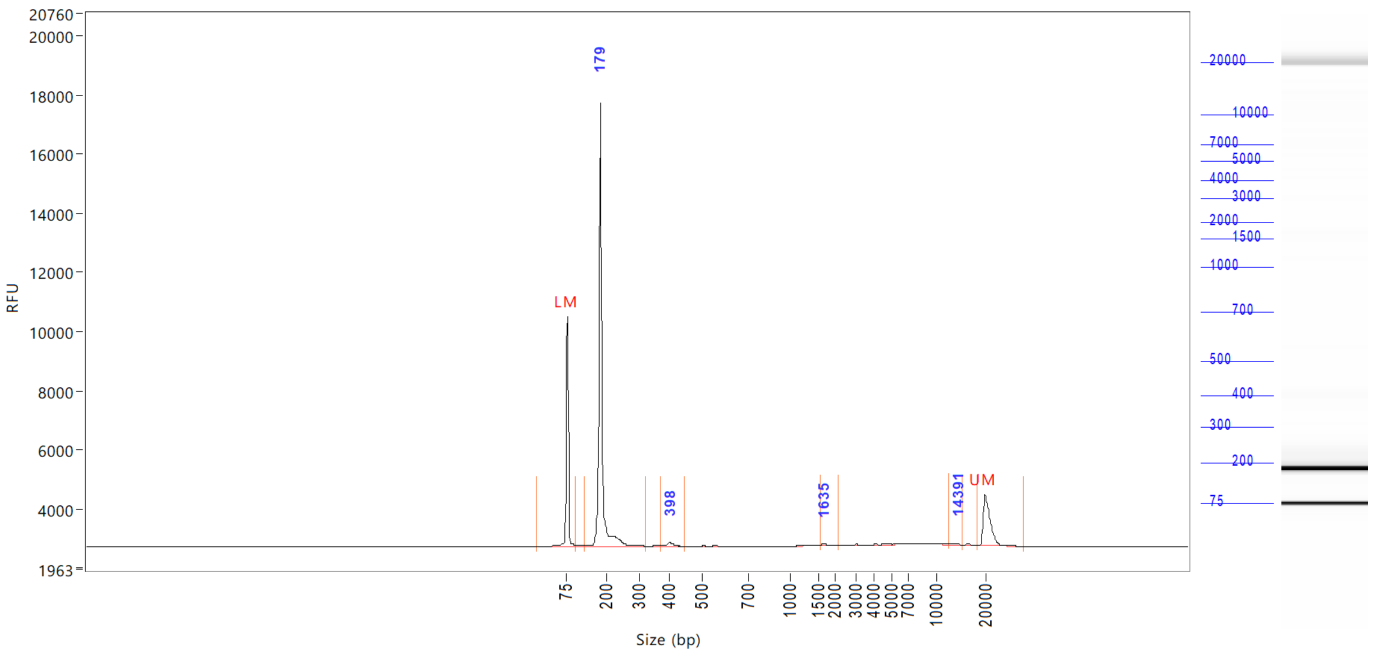

Peak	Size (bp)	Concentration (ng/uL)	From (bp)	To (bp)	Average size (bp)	CV%	RFU	Corrected peak area
1	75 (LM)	0.5000	0	99	72	11.71	7758	43.413
2	179	14.2031	129	319	181	9.16	15020	102.768
3	398	0.2436	372	445	398	3.16	117	1.762
4	1635	0.0857	1539	2172	1700	7.47	78	0.620
5	14391	0.0600	12684	15366	13827	4.29	61	0.434
6	20000 (UM)	0.1738	18293	27738	20442	4.35	1714	15.088

TIC:	14.5924	ng/uL
TIM:	130.1024	nmole/L
Total concentration:	15.2228	ng/uL

Sample peak width (sec): 5   Sample min peak height: 50   Sample baseline V to V?: Y   Sample baseline V to V points: 3
Sample filter: Binomial   Number of points for filter: 3 Sample start region (min): 0 Sample end region (min): 30
Marker peak width (sec): 5   Marker min peak height: 200   Marker baseline V to V?: Y   Marker baseline V to V points: 3
Lower marker selection: First peak > 500 RFU   Upper marker selection: Last peak > 200 RFU
Ladder size (bp)75, 200, 300, 400, 500, 700, 1000, 1500, 2000, 3000, 4000, 5000, 7000, 10000, 20000
Quantification using: Lower Marker   Final concentration (ng/uL): 0.5000   Dilution factor: 12.0

Sample: SampC1
Well location: C1
Created: Tuesday, August 10, 2021 8:01:08 PM

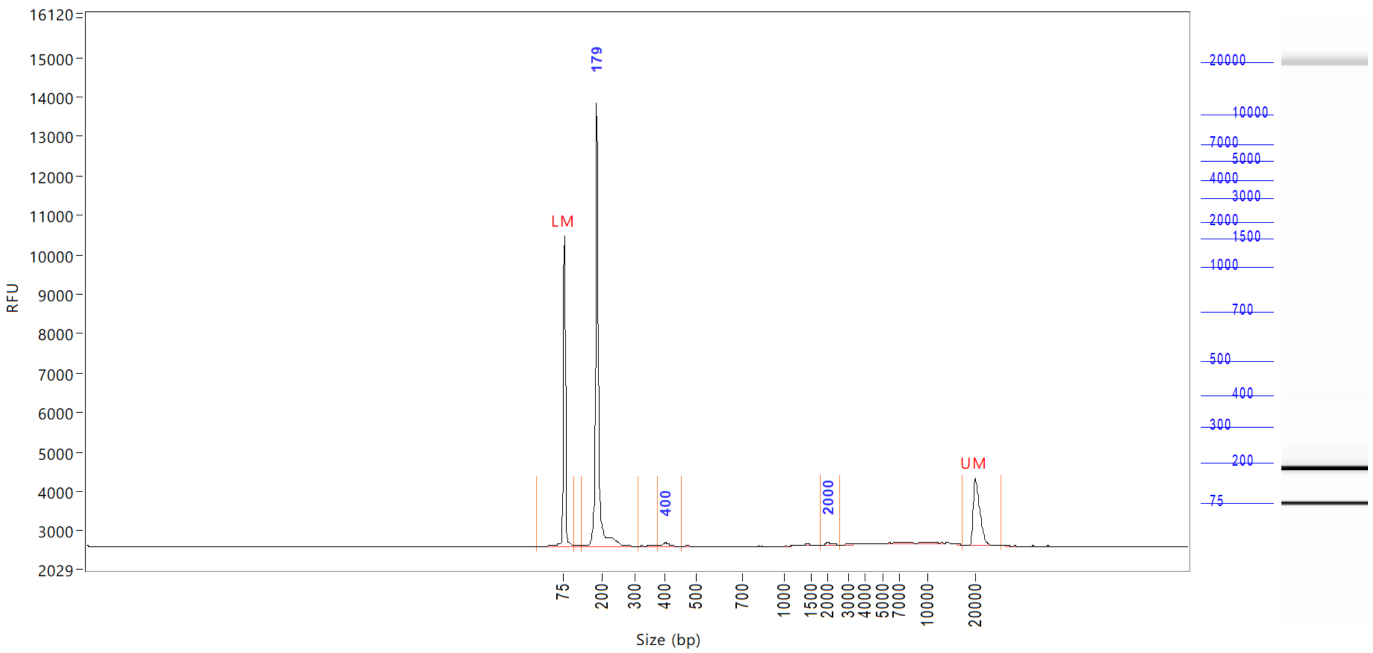

Peak	Size (bp)	Concentration (ng/uL)	From (bp)	To (bp)	Average size (bp)	CV%	RFU	Corrected peak area
1	75 (LM)	0.5000	0	107	72	10.18	7856	43.716
2	179	10.3428	129	307	182	8.46	11259	75.358
3	400	0.1827	374	454	400	3.61	87	1.331
4	2000	0.0930	1789	2572	2056	7.07	86	0.677
5	20000 (UM)	0.1662	17195	25199	20313	3.72	1709	14.527

TIC:	10.6185	ng/uL
TIM:	94.2530	nmole/L
Total concentration:	11.1533	ng/uL

```
Sample peak width (sec): 5    Sample min peak height: 50    Sample baseline V to V?: Y    Sample baseline V to V points: 3
Sample filter: Binomial       Number of points for filter: 3  Sample start region (min): 0  Sample end region (min): 30
Marker peak width (sec): 5    Marker min peak height: 200   Marker baseline V to V?: Y    Marker baseline V to V points: 3
Lower marker selection: First peak > 500 RFU                Upper marker selection: Last peak > 200 RFU
Ladder size (bp)75, 200, 300, 400, 500, 700, 1000, 1500, 2000, 3000, 4000, 5000, 7000, 10000, 20000
Quantification using: Lower Marker          Final concentration (ng/uL): 0.5000          Dilution factor: 12.0
```

Sample: SampD1
Well location: D1
Created: Tuesday, August 10, 2021 8:01:08 PM

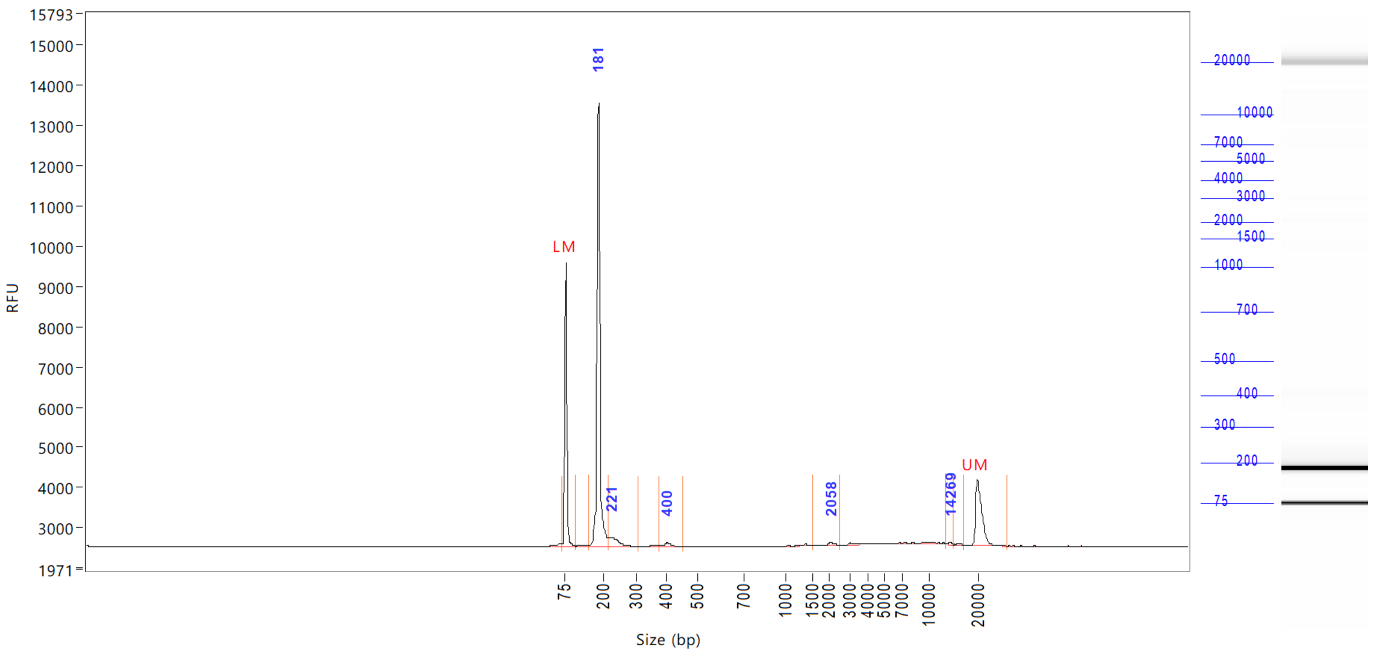

Peak	Size (bp)	Concentration (ng/uL)	From (bp)	To (bp)	Average size (bp)	CV%	RFU	Corrected peak area
1	75 (LM)	0.5000	64	107	74	4.68	7043	38.022
2	181	11.0061	152	212	180	4.06	11038	69.746
3	221	0.7226	212	303	231	6.62	212	4.579
4	400	0.2030	376	450	401	3.06	88	1.287
5	2058	0.0990	1501	2486	2020	9.44	79	0.627
6	14269	0.0414	13659	15244	14191	2.35	52	0.262
7	20000 (UM)	0.1775	17317	26166	20405	3.95	1632	13.501

TIC:	12.0720	ng/uL
TIM:	106.5842	nmole/L
Total concentration:	12.6994	ng/uL

```
Sample peak width (sec): 5    Sample min peak height: 50    Sample baseline V to V?: Y    Sample baseline V to V points: 3
Sample filter: Binomial       Number of points for filter: 3  Sample start region (min): 0  Sample end region (min): 30
Marker peak width (sec): 5    Marker min peak height: 200   Marker baseline V to V?: Y    Marker baseline V to V points: 3
Lower marker selection: First peak > 500 RFU                Upper marker selection: Last peak > 200 RFU
Ladder size (bp)75, 200, 300, 400, 500, 700, 1000, 1500, 2000, 3000, 4000, 5000, 7000, 10000, 20000
Quantification using: Lower Marker          Final concentration (ng/uL): 0.5000          Dilution factor: 12.0
```

Sample: SampE1
Well location: E1
Created: Tuesday, August 10, 2021 8:01:08 PM

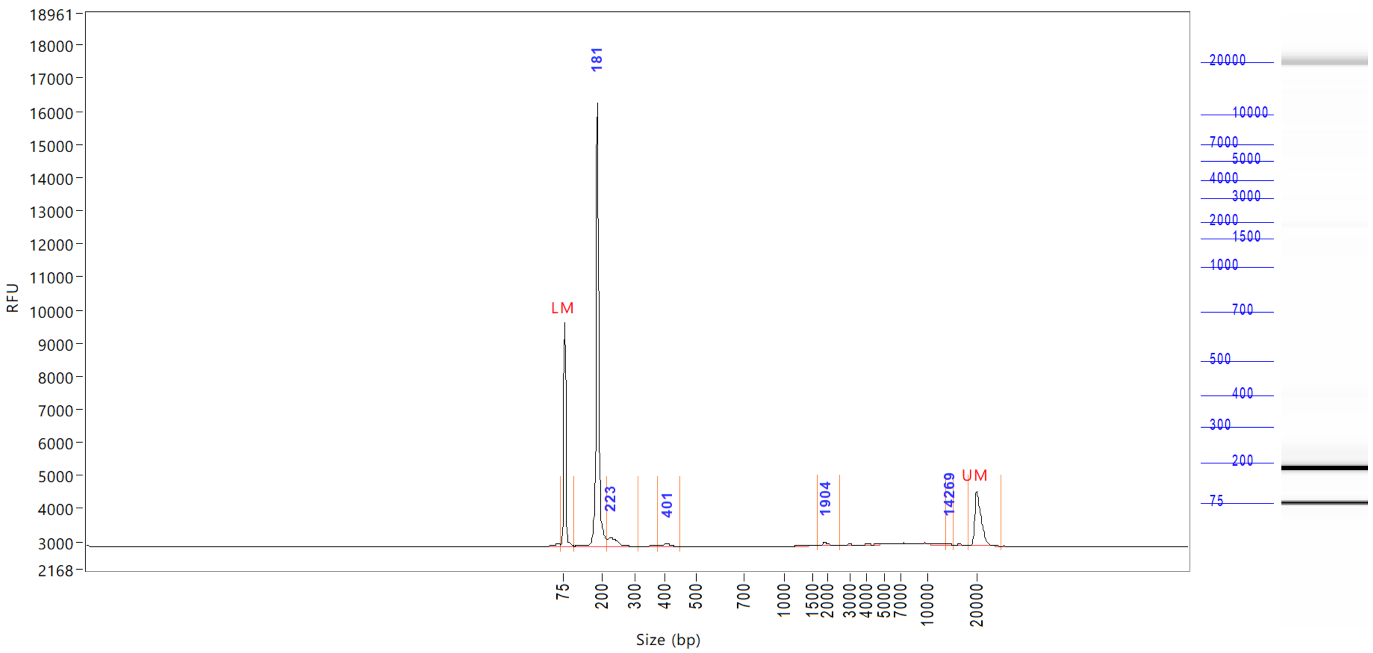

Peak	Size (bp)	Concentration (ng/uL)	From (bp)	To (bp)	Average size (bp)	CV%	RFU	Corrected peak area
1	75 (LM)	0.5000	64	107	74	5.19	6739	37.989
2	181	13.1199	107	212	179	4.95	13420	83.069
3	223	0.8992	212	305	231	6.60	251	5.693
4	401	0.2247	374	447	401	3.14	96	1.423
5	1904	0.1359	1635	2543	1942	7.39	108	0.860
6	14269	0.0401	13659	15244	14206	2.48	55	0.254
7	20000 (UM)	0.1813	18171	24957	20347	3.65	1633	13.777

TIC:	14.4198	ng/uL
TIM:	127.9388	nmole/L
Total concentration:	15.0419	ng/uL

Sample peak width (sec): 5 Sample min peak height: 50 Sample baseline V to V?: Y Sample baseline V to V points: 3
Sample filter: Binomial Number of points for filter: 3 Sample start region (min): 0 Sample end region (min): 30
Marker peak width (sec): 5 Marker min peak height: 200 Marker baseline V to V?: Y Marker baseline V to V points: 3
Lower marker selection: First peak > 500 RFU Upper marker selection: Last peak > 200 RFU
Ladder size (bp)75, 200, 300, 400, 500, 700, 1000, 1500, 2000, 3000, 4000, 5000, 7000, 10000, 20000
Quantification using: Lower Marker Final concentration (ng/uL): 0.5000 Dilution factor: 12.0

Sample: SampF1
Well location: F1
Created: Tuesday, August 10, 2021 8:01:08 PM

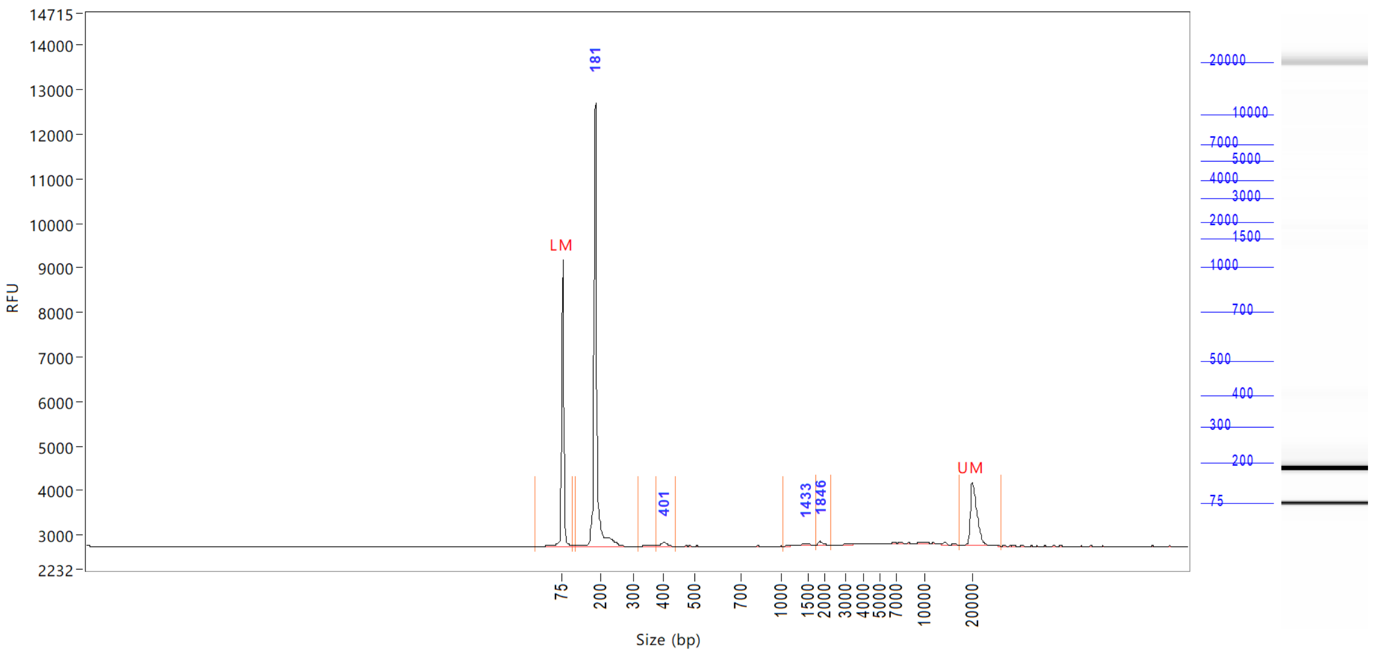

Peak	Size (bp)	Concentration (ng/uL)	From (bp)	To (bp)	Average size (bp)	CV%	RFU	Corrected peak area
1	75 (LM)	0.5000	0	107	72	11.18	6432	36.387
2	181	11.3004	120	313	182	8.26	9976	68.531
3	401	0.1921	376	441	400	2.95	80	1.165
4	1433	0.1188	1012	1712	1420	8.71	51	0.720
5	1846	0.1107	1712	2258	1871	5.38	95	0.671
6	20000 (UM)	0.1664	17317	25924	20385	4.09	1415	12.113

TIC:	11.7219	ng/uL
TIM:	103.1022	nmole/L
Total concentration:	12.3091	ng/uL

```
Sample peak width (sec): 5    Sample min peak height: 50    Sample baseline V to V?: Y    Sample baseline V to V points: 3
Sample filter: Binomial       Number of points for filter: 3  Sample start region (min): 0  Sample end region (min): 30
Marker peak width (sec): 5    Marker min peak height: 200   Marker baseline V to V?: Y    Marker baseline V to V points: 3
Lower marker selection: First peak > 500 RFU                Upper marker selection: Last peak > 200 RFU
Ladder size (bp)75, 200, 300, 400, 500, 700, 1000, 1500, 2000, 3000, 4000, 5000, 7000, 10000, 20000
Quantification using: Lower Marker          Final concentration (ng/uL): 0.5000          Dilution factor: 12.0
```

Sample: SampG1
Well location: G1
Created: Tuesday, August 10, 2021 8:01:08 PM

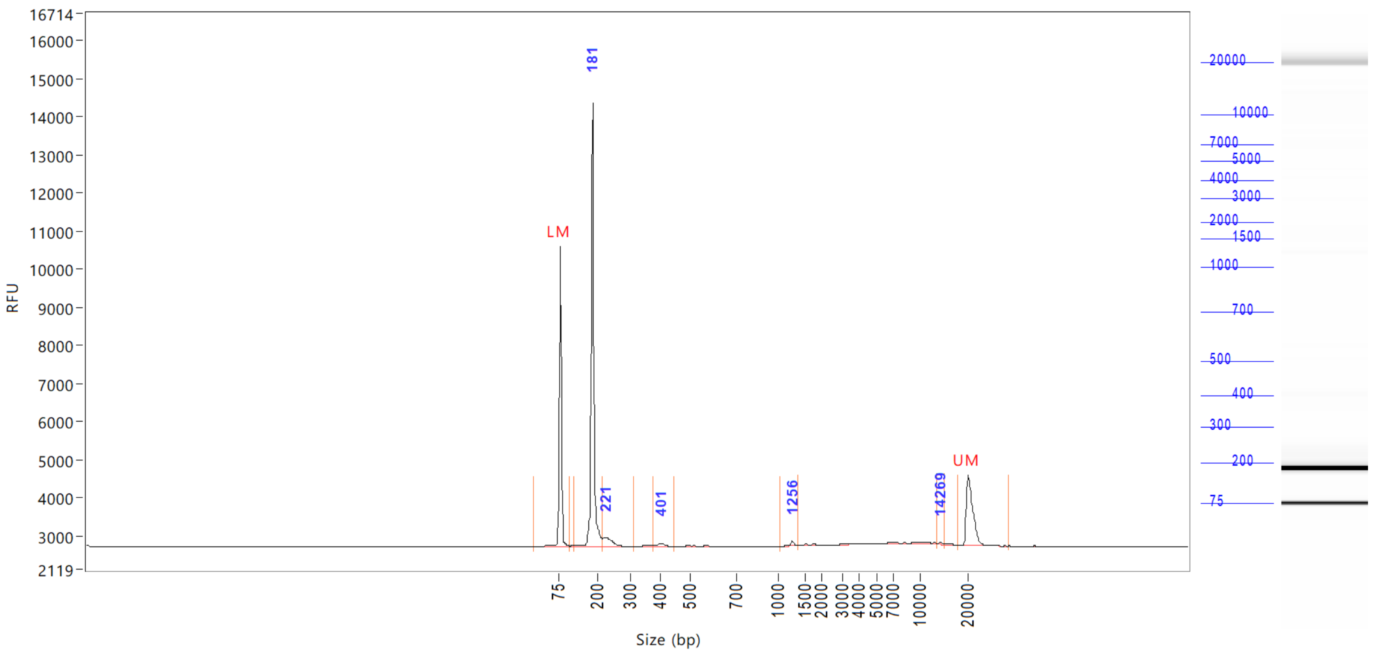

Peak	Size (bp)	Concentration (ng/uL)	From (bp)	To (bp)	Average size (bp)	CV%	RFU	Corrected peak area
1	75 (LM)	0.5000	0	107	73	8.93	7867	43.600
2	181	9.9899	120	210	179	4.58	11664	72.594
3	221	0.7043	210	307	230	7.02	229	5.118
4	401	0.1594	378	443	400	2.57	88	1.158
5	1256	0.0918	1012	1378	1251	3.76	118	0.667
6	14269	0.0446	13659	15122	14233	2.51	59	0.324
7	20000 (UM)	0.1816	17927	28342	20425	4.00	1844	15.833

TIC:	10.9899	ng/uL
TIM:	97.5664	nmole/L
Total concentration:	11.5875	ng/uL

Sample peak width (sec): 5 Sample min peak height: 50 Sample baseline V to V?: Y Sample baseline V to V points: 3
Sample filter: Binomial Number of points for filter: 3 Sample start region (min): 0 Sample end region (min): 30
Marker peak width (sec): 5 Marker min peak height: 200 Marker baseline V to V?: Y Marker baseline V to V points: 3
Lower marker selection: First peak > 500 RFU Upper marker selection: Last peak > 200 RFU
Ladder size (bp)75, 200, 300, 400, 500, 700, 1000, 1500, 2000, 3000, 4000, 5000, 7000, 10000, 20000
Quantification using: Lower Marker Final concentration (ng/uL): 0.5000 Dilution factor: 12.0

Sample: SampH1
Well location: H1
Created: Tuesday, August 10, 2021 8:01:08 PM

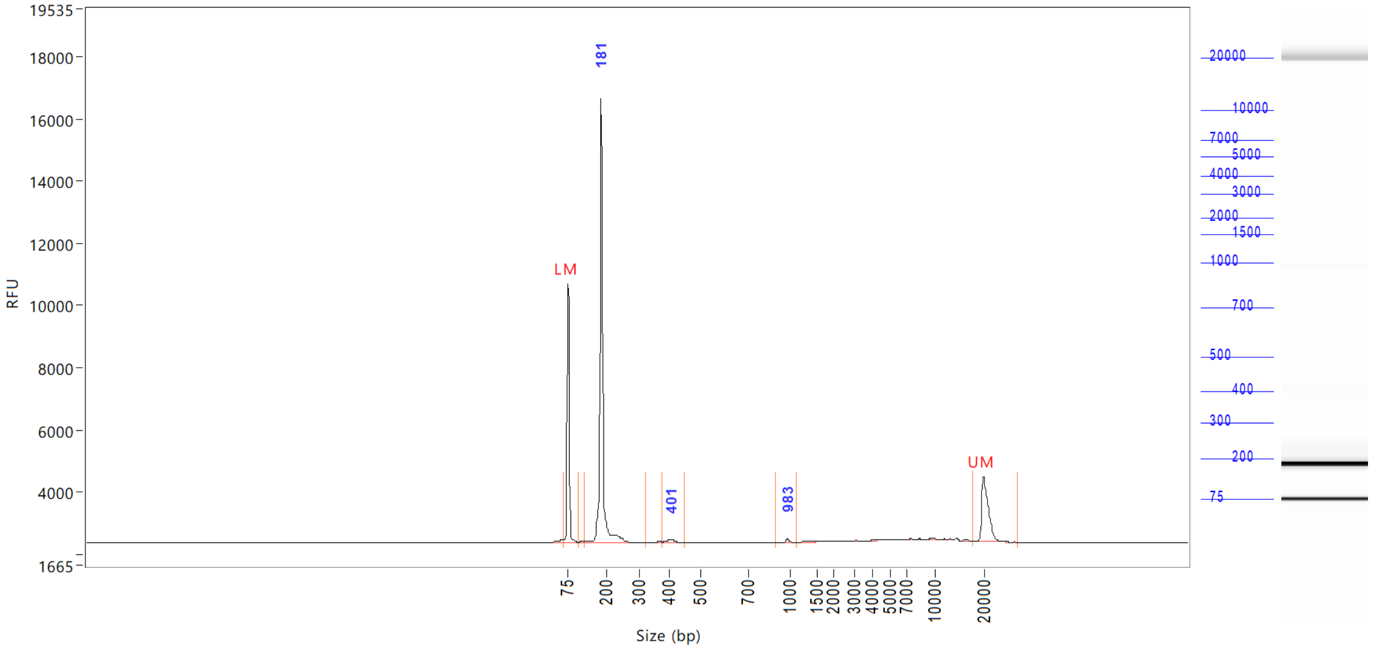

Peak	Size (bp)	Concentration (ng/uL)	From (bp)	To (bp)	Average size (bp)	CV%	RFU	Corrected peak area
1	75 (LM)	0.5000	62	108	74	5.11	8323	45.133
2	181	12.3306	129	319	182	7.82	14287	92.753
3	401	0.2103	374	447	400	2.93	110	1.582
4	983	0.0832	900	1123	983	2.39	114	0.626
5	20000 (UM)	0.1972	18049	27012	20449	3.95	2077	17.804

TIC:	12.6241	ng/uL
TIM:	112.3872	nmole/L
Total concentration:	13.1757	ng/uL

Sample peak width (sec): 5   Sample min peak height: 50   Sample baseline V to V?: Y   Sample baseline V to V points: 3
Sample filter: Binomial   Number of points for filter: 3 Sample start region (min): 0 Sample end region (min): 30
Marker peak width (sec): 5   Marker min peak height: 200   Marker baseline V to V?: Y   Marker baseline V to V points: 3
Lower marker selection: First peak > 500 RFU   Upper marker selection: Last peak > 200 RFU
Ladder size (bp)75, 200, 300, 400, 500, 700, 1000, 1500, 2000, 3000, 4000, 5000, 7000, 10000, 20000
Quantification using: Lower Marker   Final concentration (ng/uL): 0.5000   Dilution factor: 12.0

Sample: SampA2
Well location: A2
Created: Tuesday, August 10, 2021 8:01:08 PM

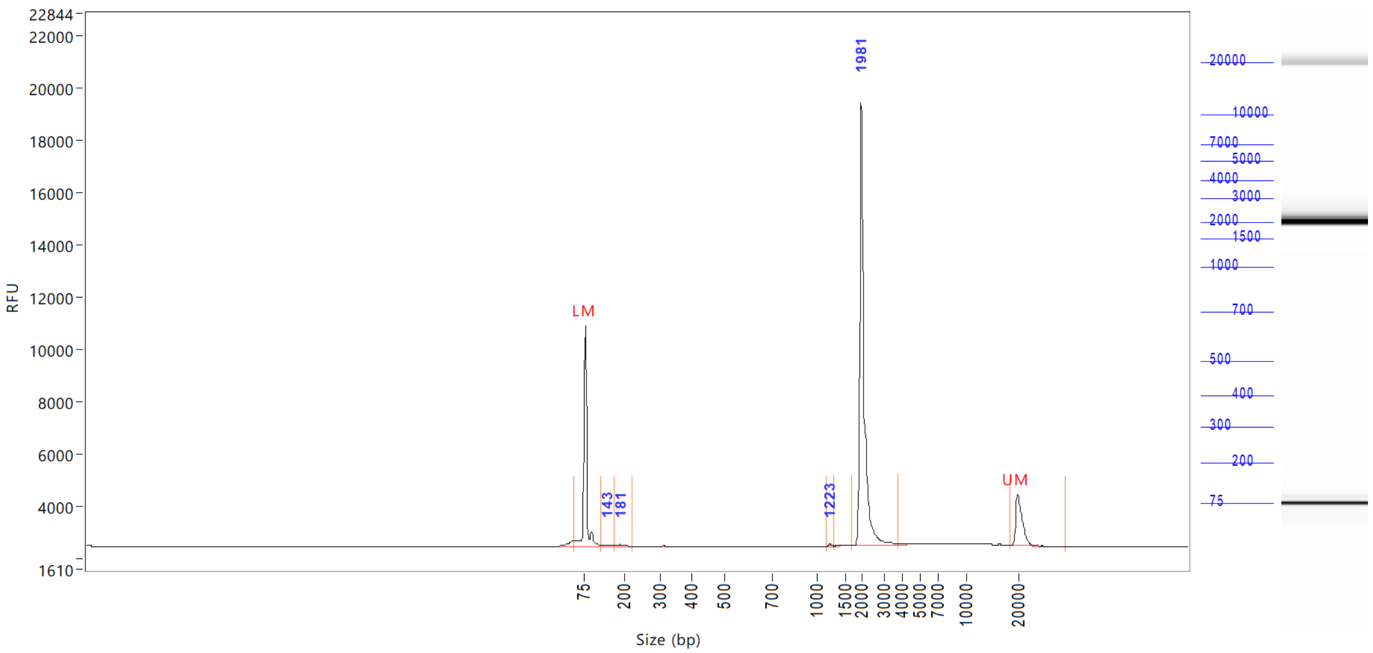

Peak	Size (bp)	Concentration (ng/uL)	From (bp)	To (bp)	Average size (bp)	CV%	RFU	Corrected peak area
1	75 (LM)	0.5000	43	126	74	13.55	8418	54.091
2	143	0.1442	126	164	141	7.31	66	1.300
3	181	0.1073	164	219	184	6.26	75	0.968
4	1223	0.0393	1167	1300	1222	1.99	76	0.354
5	1981	12.0457	1693	3822	2061	10.48	16935	108.594
6	20000 (UM)	0.1489	18415	29189	20377	4.15	1957	16.105

TIC:	12.3365	ng/uL
TIM:	12.3138	nmole/L
Total concentration:	12.7469	ng/uL

Sample peak width (sec): 5 Sample min peak height: 50 Sample baseline V to V?: Y Sample baseline V to V points: 3
Sample filter: Binomial Number of points for filter: 3 Sample start region (min): 0 Sample end region (min): 30
Marker peak width (sec): 5 Marker min peak height: 200 Marker baseline V to V?: Y Marker baseline V to V points: 3
Lower marker selection: First peak > 500 RFU Upper marker selection: Last peak > 200 RFU
Ladder size (bp)75, 200, 300, 400, 500, 700, 1000, 1500, 2000, 3000, 4000, 5000, 7000, 10000, 20000
Quantification using: Lower Marker Final concentration (ng/uL): 0.5000 Dilution factor: 12.0

Sample: SampB2
Well location: B2
Created: Tuesday, August 10, 2021 8:01:08 PM

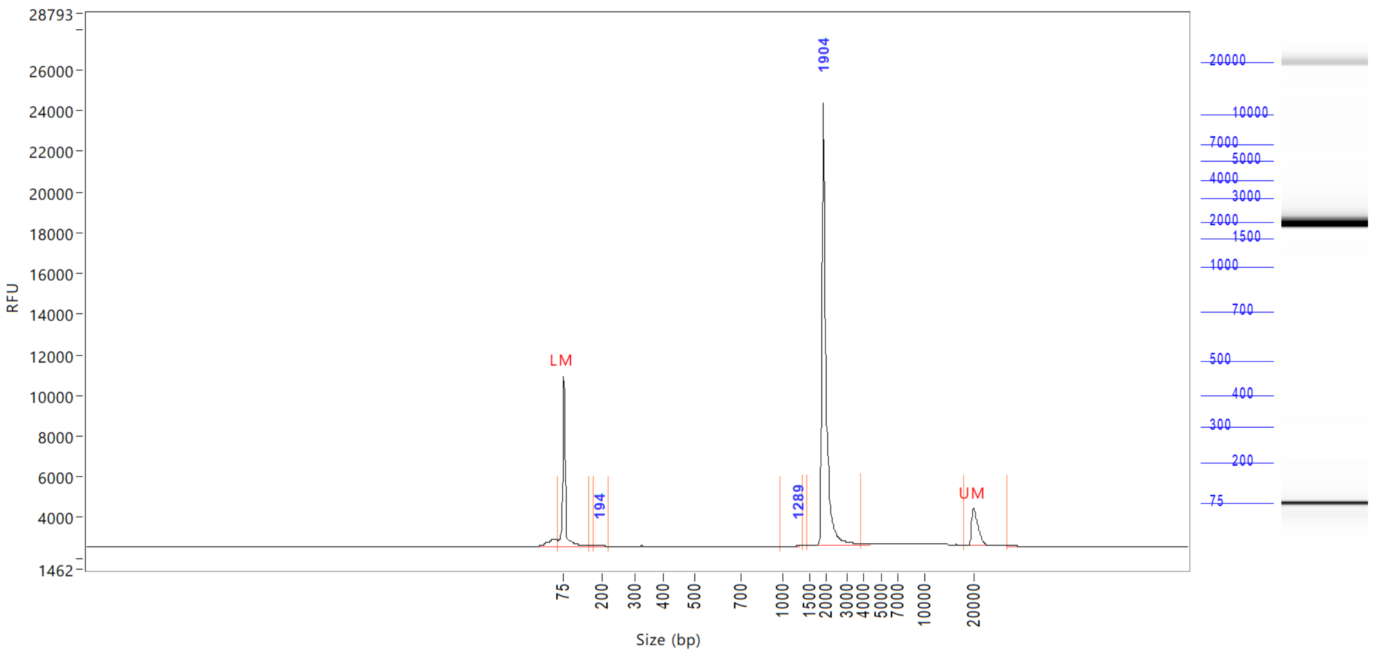

Peak	Size (bp)	Concentration (ng/uL)	From (bp)	To (bp)	Average size (bp)	CV%	RFU	Corrected peak area
1	75 (LM)	0.5000	58	160	77	17.53	8361	56.578
2	194	0.1001	173	217	191	4.74	73	0.944
3	1289	0.0295	974	1356	1264	5.38	58	0.278
4	1904	15.0521	1456	3857	1981	10.80	21791	141.935
5	20000 (UM)	0.1355	18049	26770	20376	3.89	1842	15.337

TIC:	15.1816	ng/uL
TIM:	13.4075	nmole/L
Total concentration:	15.5863	ng/uL

Sample peak width (sec): 5 Sample min peak height: 50 Sample baseline V to V?: Y Sample baseline V to V points: 3
Sample filter: Binomial Number of points for filter: 3 Sample start region (min): 0 Sample end region (min): 30
Marker peak width (sec): 5 Marker min peak height: 200 Marker baseline V to V?: Y Marker baseline V to V points: 3
Lower marker selection: First peak > 500 RFU Upper marker selection: Last peak > 200 RFU
Ladder size (bp)75, 200, 300, 400, 500, 700, 1000, 1500, 2000, 3000, 4000, 5000, 7000, 10000, 20000
Quantification using: Lower Marker Final concentration (ng/uL): 0.5000 Dilution factor: 12.0

Sample: SampC2
Well location: C2
Created: Tuesday, August 10, 2021 8:01:08 PM

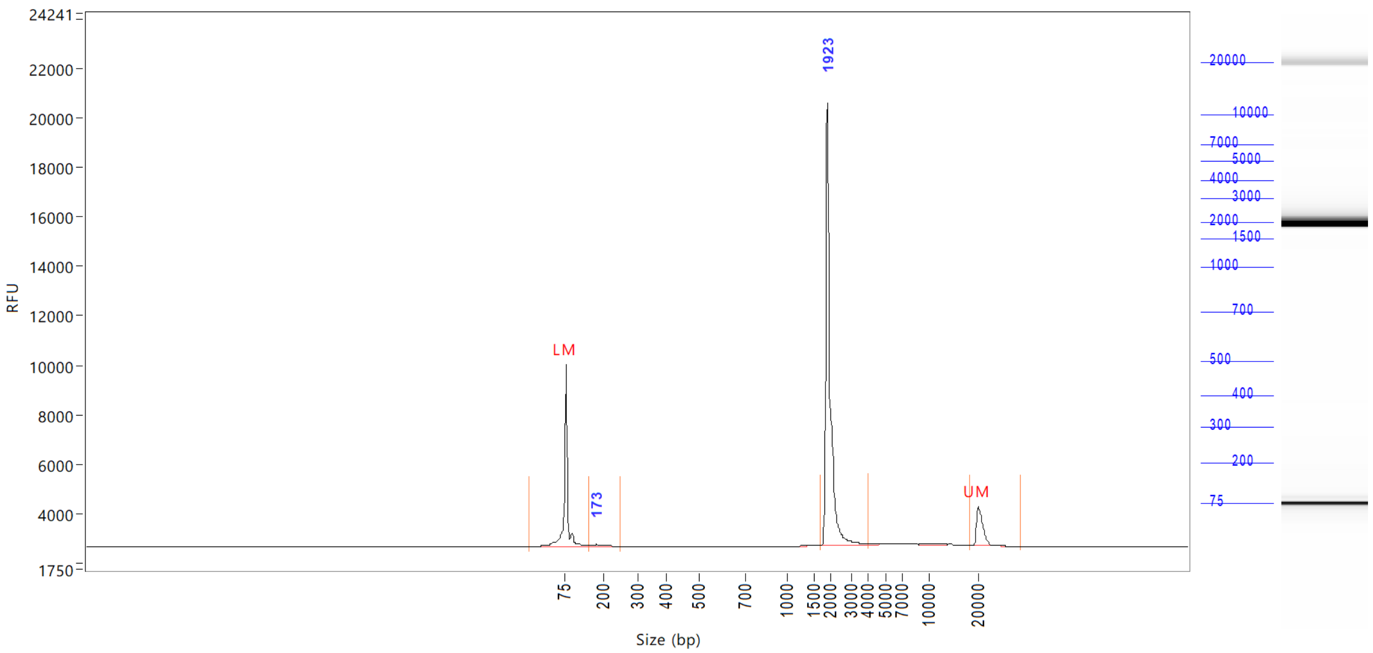

Peak	Size (bp)	Concentration (ng/uL)	From (bp)	To (bp)	Average size (bp)	CV%	RFU	Corrected peak area
1	75 (LM)	0.5000	0	148	71	26.68	7342	56.809
2	173	0.2617	148	248	186	13.99	85	2.477
3	1923	12.7810	1674	4000	2001	11.81	17936	121.012
4	20000 (UM)	0.1150	18415	28705	20329	3.95	1612	13.068

TIC:	13.0426	ng/uL
TIM:	12.8272	nmole/L
Total concentration:	13.4621	ng/uL

```
Sample peak width (sec): 5    Sample min peak height: 50    Sample baseline V to V?: Y    Sample baseline V to V points: 3
Sample filter: Binomial       Number of points for filter: 3  Sample start region (min): 0  Sample end region (min): 30
Marker peak width (sec): 5    Marker min peak height: 200   Marker baseline V to V?: Y    Marker baseline V to V points: 3
Lower marker selection: First peak > 500 RFU                Upper marker selection: Last peak > 200 RFU
Ladder size (bp)75, 200, 300, 400, 500, 700, 1000, 1500, 2000, 3000, 4000, 5000, 7000, 10000, 20000
Quantification using: Lower Marker          Final concentration (ng/uL): 0.5000          Dilution factor: 12.0
```

Sample: SampD2
Well location: D2
Created: Tuesday, August 10, 2021 8:01:08 PM

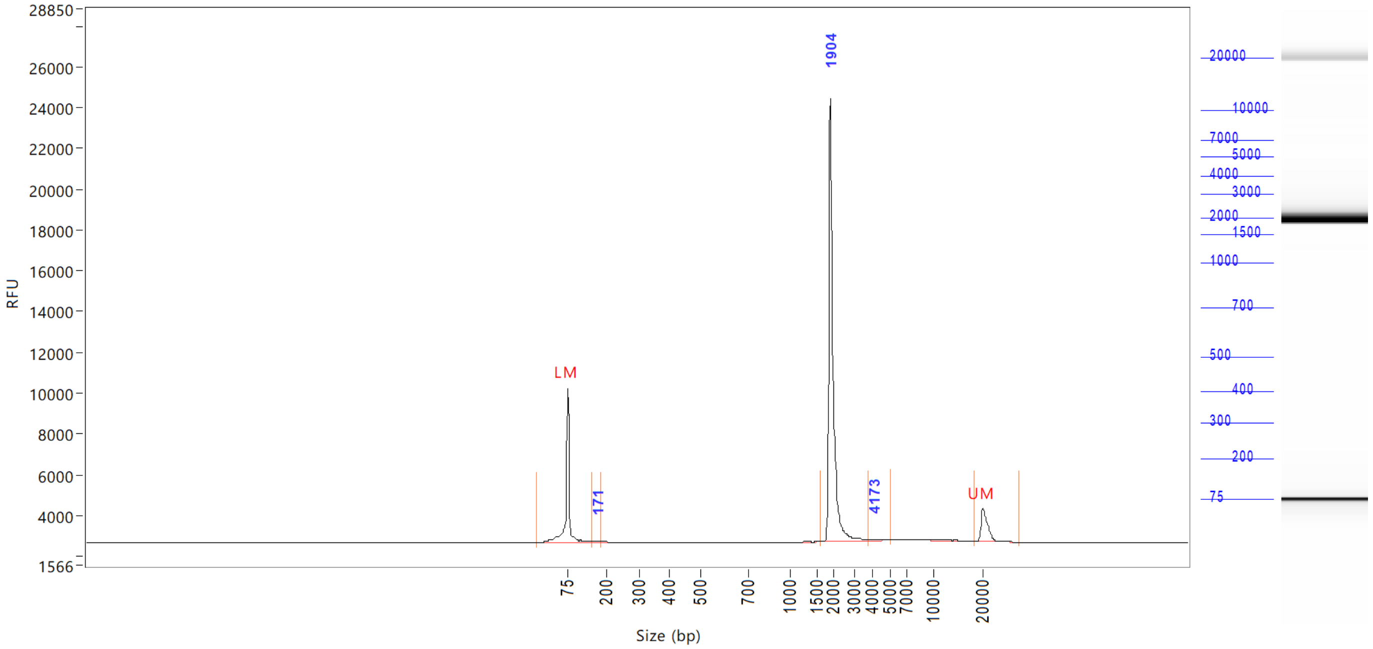

Peak	Size (bp)	Concentration (ng/uL)	From (bp)	To (bp)	Average size (bp)	CV%	RFU	Corrected peak area
1	75 (LM)	0.5000	0	154	69	28.65	7573	60.667
2	171	0.0995	154	183	168	4.71	79	1.006
3	1904	13.6588	1616	3822	1976	11.42	21767	138.106
4	4173	0.1098	3822	5075	4297	7.28	57	1.111
5	20000 (UM)	0.1113	18415	27375	20413	3.76	1614	13.500

TIC:	13.8681	ng/uL
TIM:	12.3941	nmole/L
Total concentration:	14.2614	ng/uL

```
Sample peak width (sec): 5    Sample min peak height: 50    Sample baseline V to V?: Y    Sample baseline V to V points: 3
Sample filter: Binomial       Number of points for filter: 3  Sample start region (min): 0  Sample end region (min): 30
Marker peak width (sec): 5    Marker min peak height: 200   Marker baseline V to V?: Y    Marker baseline V to V points: 3
Lower marker selection: First peak > 500 RFU                Upper marker selection: Last peak > 200 RFU
Ladder size (bp)75, 200, 300, 400, 500, 700, 1000, 1500, 2000, 3000, 4000, 5000, 7000, 10000, 20000
Quantification using: Lower Marker          Final concentration (ng/uL): 0.5000          Dilution factor: 12.0
```

Sample: SampE2
Well location: E2
Created: Tuesday, August 10, 2021 8:01:08 PM

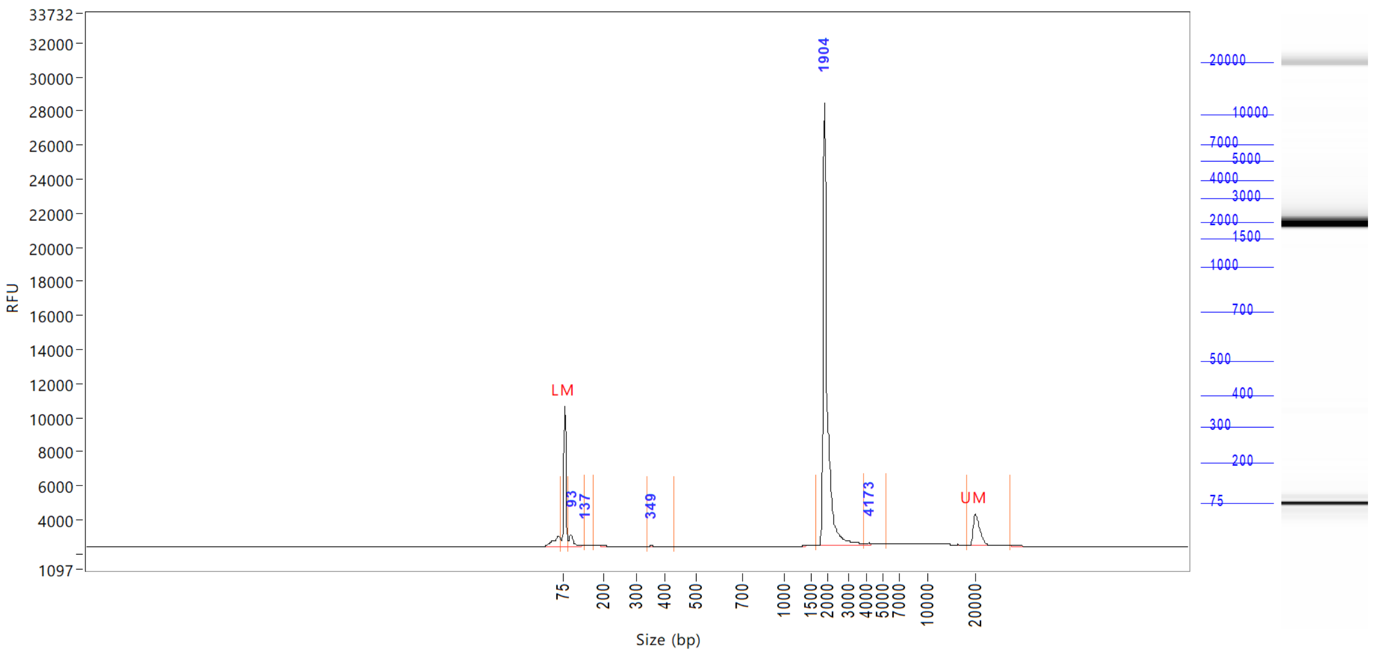

Peak	Size (bp)	Concentration (ng/uL)	From (bp)	To (bp)	Average size (bp)	CV%	RFU	Corrected peak area
1	75 (LM)	0.5000	64	84	73	4.63	8250	46.224
2	93	0.9918	84	137	97	12.60	698	7.641
3	137	0.0631	137	169	147	5.56	56	0.486
4	349	0.1039	337	424	371	6.73	62	0.800
5	1904	22.3215	1616	3893	1994	12.57	26032	171.966
6	4173	0.2131	3893	5297	4363	7.71	95	1.642
7	20000 (UM)	0.1721	18171	27012	20330	3.75	1899	15.913

TIC:	23.6933	ng/uL
TIM:	36.4617	nmole/L
Total concentration:	24.0273	ng/uL

```
Sample peak width (sec): 5    Sample min peak height: 50    Sample baseline V to V?: Y    Sample baseline V to V points: 3
Sample filter: Binomial       Number of points for filter: 3  Sample start region (min): 0  Sample end region (min): 30
Marker peak width (sec): 5    Marker min peak height: 200   Marker baseline V to V?: Y    Marker baseline V to V points: 3
Lower marker selection: First peak > 500 RFU                Upper marker selection: Last peak > 200 RFU
Ladder size (bp)75, 200, 300, 400, 500, 700, 1000, 1500, 2000, 3000, 4000, 5000, 7000, 10000, 20000
Quantification using: Lower Marker          Final concentration (ng/uL): 0.5000          Dilution factor: 12.0
```

Sample: SampF2
Well location: F2
Created: Tuesday, August 10, 2021 8:01:08 PM

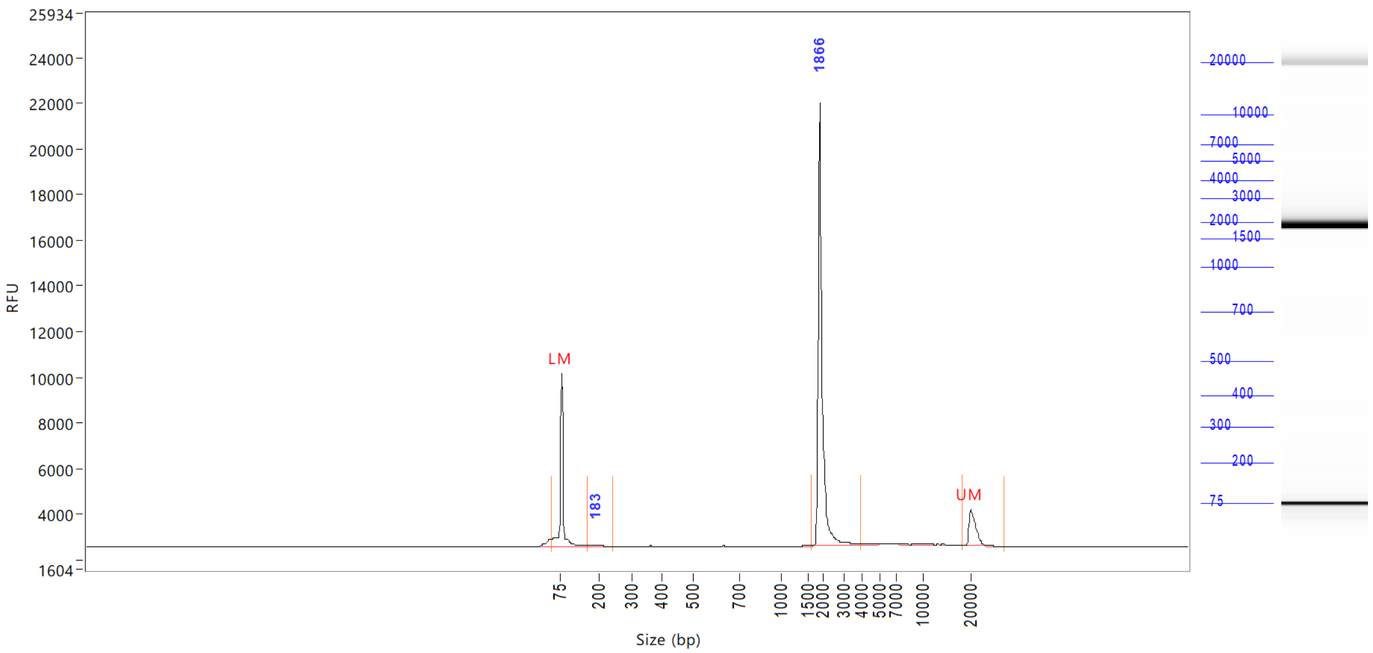

Peak	Size (bp)	Concentration (ng/uL)	From (bp)	To (bp)	Average size (bp)	CV%	RFU	Corrected peak area
1	75 (LM)	0.5000	43	160	74	19.94	7594	55.210
2	183	0.2046	160	239	191	10.58	82	1.883
3	1866	12.9342	1616	3929	1942	12.14	19417	119.017
4	20000 (UM)	0.1268	18049	26649	20403	4.09	1592	14.003

TIC:	13.1389	ng/uL
TIM:	12.7251	nmole/L
Total concentration:	13.7052	ng/uL

Sample peak width (sec): 5 Sample min peak height: 50 Sample baseline V to V?: Y Sample baseline V to V points: 3
Sample filter: Binomial Number of points for filter: 3 Sample start region (min): 0 Sample end region (min): 30
Marker peak width (sec): 5 Marker min peak height: 200 Marker baseline V to V?: Y Marker baseline V to V points: 3
Lower marker selection: First peak > 500 RFU Upper marker selection: Last peak > 200 RFU
Ladder size (bp)75, 200, 300, 400, 500, 700, 1000, 1500, 2000, 3000, 4000, 5000, 7000, 10000, 20000
Quantification using: Lower Marker Final concentration (ng/uL): 0.5000 Dilution factor: 12.0

Sample: SampG2
Well location: G2
Created: Tuesday, August 10, 2021 8:01:08 PM

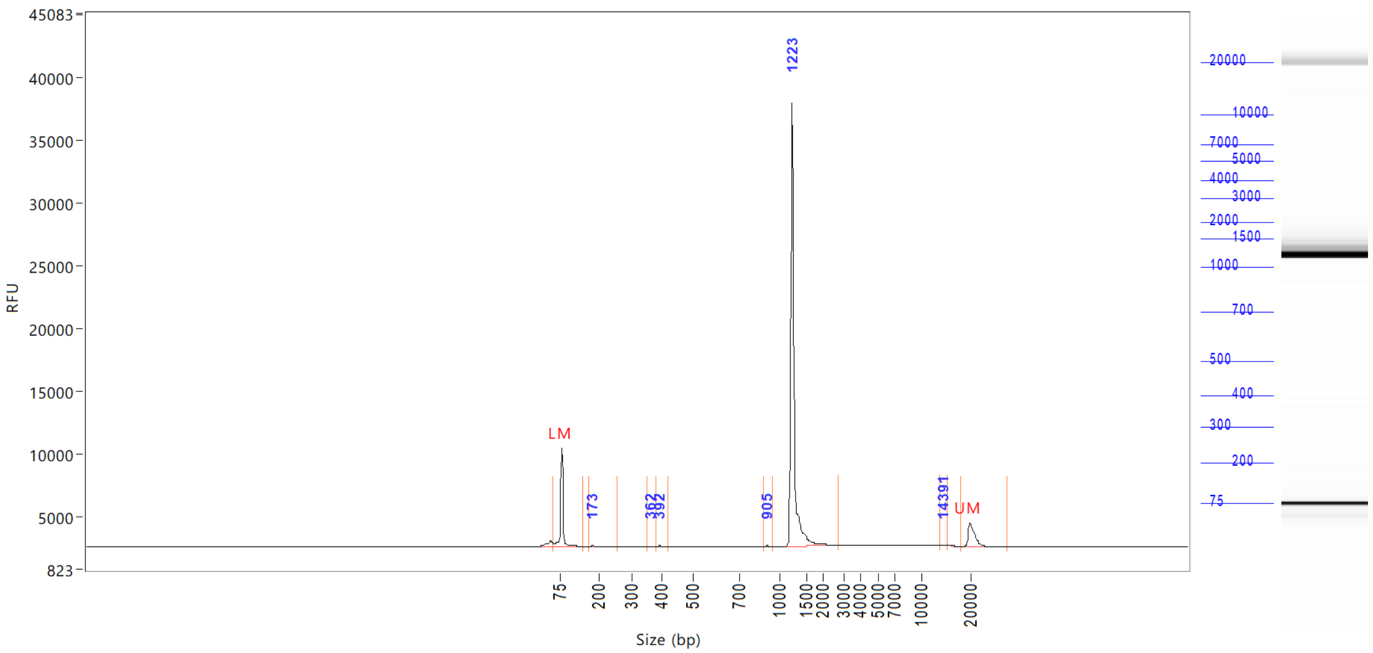

Peak	Size (bp)	Concentration (ng/uL)	From (bp)	To (bp)	Average size (bp)	CV%	RFU	Corrected peak area
1	75 (LM)	0.5000	47	147	73	14.70	7843	50.257
2	173	0.1615	164	251	189	10.21	75	1.353
3	362	0.0356	352	378	361	1.45	54	0.298
4	392	0.0576	378	420	391	1.87	84	0.482
5	905	0.0329	879	948	901	1.14	59	0.276
6	1223	22.9702	948	2743	1245	9.31	35358	192.400
7	14391	0.0306	13659	15366	14168	1.91	55	0.257
8	20000 (UM)	0.1550	18171	27617	20429	4.03	1836	15.575

TIC:	23.2884	ng/uL
TIM:	32.2419	nmole/L
Total concentration:	23.6943	ng/uL

Sample peak width (sec): 5 Sample min peak height: 50 Sample baseline V to V?: Y Sample baseline V to V points: 3
Sample filter: Binomial Number of points for filter: 3 Sample start region (min): 0 Sample end region (min): 30
Marker peak width (sec): 5 Marker min peak height: 200 Marker baseline V to V?: Y Marker baseline V to V points: 3
Lower marker selection: First peak > 500 RFU Upper marker selection: Last peak > 200 RFU
Ladder size (bp)75, 200, 300, 400, 500, 700, 1000, 1500, 2000, 3000, 4000, 5000, 7000, 10000, 20000
Quantification using: Lower Marker Final concentration (ng/uL): 0.5000 Dilution factor: 12.0

Sample: SampH2
Well location: H2
Created: Tuesday, August 10, 2021 8:01:08 PM

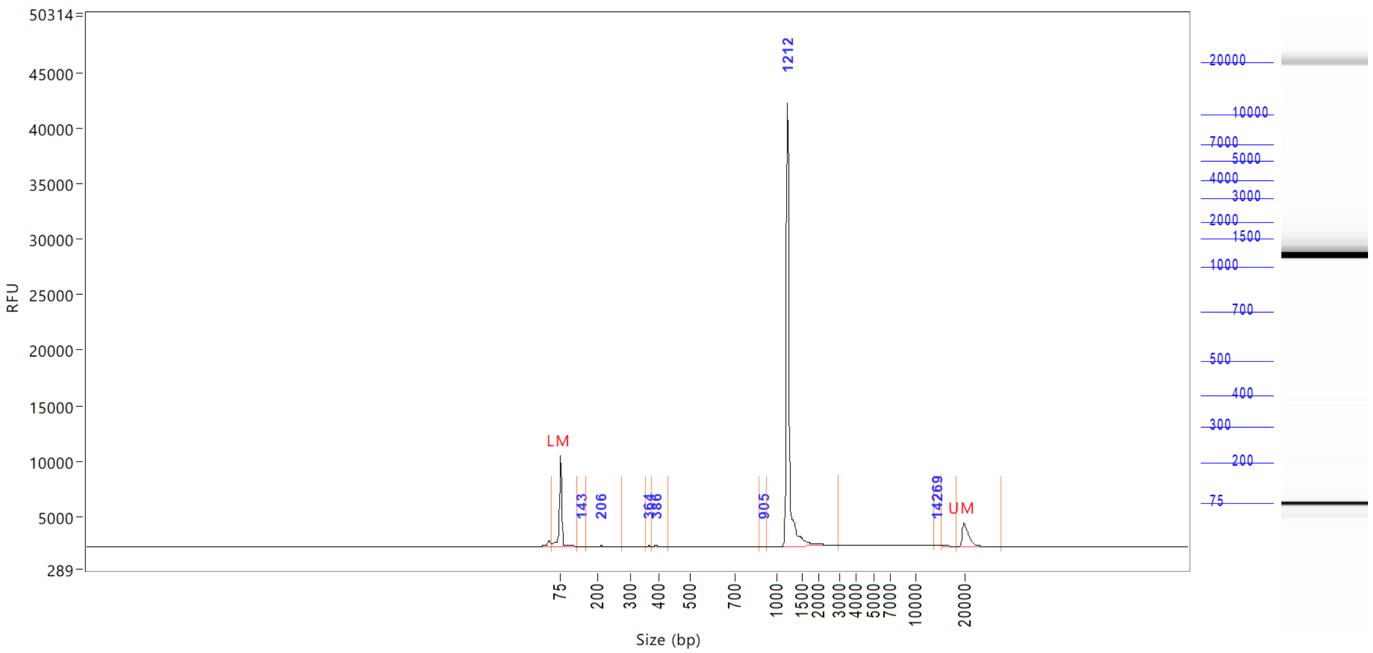

Peak	Size (bp)	Concentration (ng/uL)	From (bp)	To (bp)	Average size (bp)	CV%	RFU	Corrected peak area
1	75 (LM)	0.5000	45	131	72	13.45	8179	53.766
2	143	0.0597	131	156	142	4.22	58	0.535
3	206	0.1826	156	271	198	11.39	67	1.636
4	364	0.0398	352	372	361	0.99	79	0.356
5	386	0.0686	372	430	386	2.07	122	0.615
6	905	0.0273	870	926	898	1.01	52	0.245
7	1212	23.3088	926	3000	1229	10.40	39969	208.871
8	14269	0.0364	13659	15366	14219	2.31	63	0.327
9	20000 (UM)	0.1662	18415	27738	20503	4.36	2064	17.875

TIC:	23.7232	ng/uL
TIM:	33.9524	nmole/L
Total concentration:	24.0454	ng/uL

Sample peak width (sec): 5 Sample min peak height: 50 Sample baseline V to V?: Y Sample baseline V to V points: 3
Sample filter: Binomial Number of points for filter: 3 Sample start region (min): 0 Sample end region (min): 30
Marker peak width (sec): 5 Marker min peak height: 200 Marker baseline V to V?: Y Marker baseline V to V points: 3
Lower marker selection: First peak > 500 RFU Upper marker selection: Last peak > 200 RFU
Ladder size (bp)75, 200, 300, 400, 500, 700, 1000, 1500, 2000, 3000, 4000, 5000, 7000, 10000, 20000
Quantification using: Lower Marker Final concentration (ng/uL): 0.5000 Dilution factor: 12.0

Sample: SampA3
Well location: A3
Created: Tuesday, August 10, 2021 8:01:08 PM

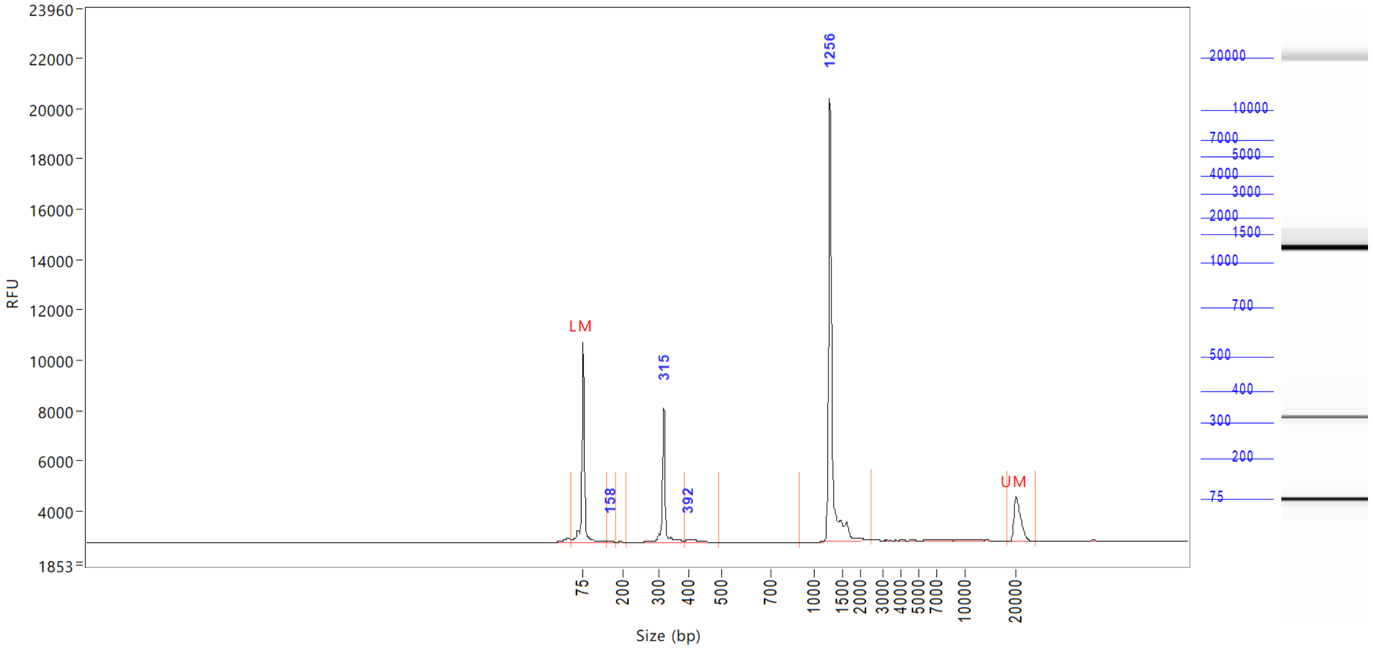

Peak	Size (bp)	Concentration (ng/uL)	From (bp)	To (bp)	Average size (bp)	CV%	RFU	Corrected peak area
1	75 (LM)	0.5000	39	148	73	15.35	7938	50.825
2	158	0.0643	148	173	157	3.91	75	0.544
3	315	3.7421	208	384	315	5.57	5351	31.699
4	392	0.3026	384	490	416	5.61	90	2.564
5	1256	11.1332	896	2543	1297	9.71	17637	94.307
6	20000 (UM)	0.1508	18293	23990	20390	3.50	1824	15.327

TIC:	15.2422	ng/uL
TIM:	35.5411	nmole/L
Total concentration:	15.5382	ng/uL

```
Sample peak width (sec): 5    Sample min peak height: 50    Sample baseline V to V?: Y    Sample baseline V to V points: 3
Sample filter: Binomial       Number of points for filter: 3  Sample start region (min): 0  Sample end region (min): 30
Marker peak width (sec): 5    Marker min peak height: 200   Marker baseline V to V?: Y    Marker baseline V to V points: 3
Lower marker selection: First peak > 500 RFU                Upper marker selection: Last peak > 200 RFU
Ladder size (bp)75, 200, 300, 400, 500, 700, 1000, 1500, 2000, 3000, 4000, 5000, 7000, 10000, 20000
Quantification using: Lower Marker          Final concentration (ng/uL): 0.5000          Dilution factor: 12.0
```

Sample: SampB3
Well location: B3
Created: Tuesday, August 10, 2021 8:01:08 PM

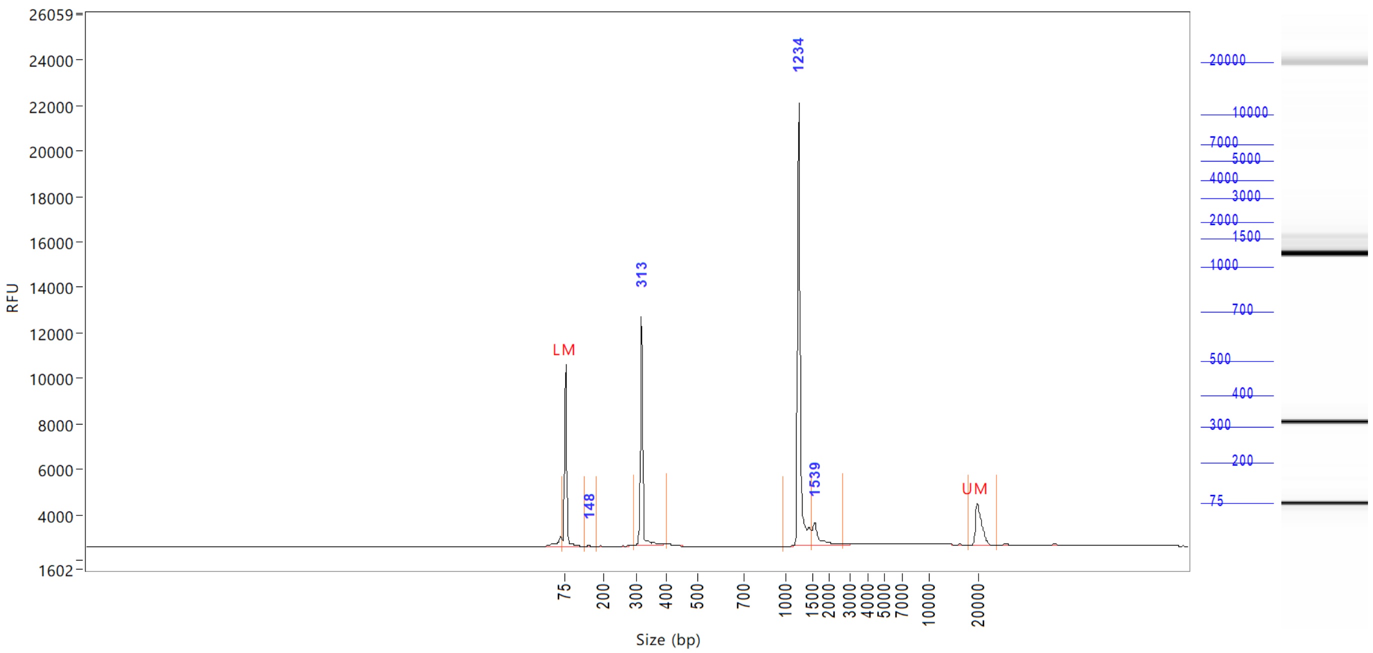

Peak	Size (bp)	Concentration (ng/uL)	From (bp)	To (bp)	Average size (bp)	CV%	RFU	Corrected peak area
1	75 (LM)	0.5000	66	137	74	8.96	8027	44.962
2	148	0.0597	137	177	149	4.96	64	0.447
3	313	6.7222	291	398	313	2.82	10068	50.374
4	1234	12.4807	978	1478	1246	4.67	19518	93.527
5	1539	1.4600	1478	2686	1692	15.69	991	10.941
6	20000 (UM)	0.1698	18171	23869	20338	3.42	1853	15.272

TIC:	20.7225	ng/uL
TIM:	53.8949	nmole/L
Total concentration:	21.0929	ng/uL

Sample peak width (sec): 5 Sample min peak height: 50 Sample baseline V to V?: Y Sample baseline V to V points: 3
Sample filter: Binomial Number of points for filter: 3 Sample start region (min): 0 Sample end region (min): 30
Marker peak width (sec): 5 Marker min peak height: 200 Marker baseline V to V?: Y Marker baseline V to V points: 3
Lower marker selection: First peak > 500 RFU Upper marker selection: Last peak > 200 RFU
Ladder size (bp)75, 200, 300, 400, 500, 700, 1000, 1500, 2000, 3000, 4000, 5000, 7000, 10000, 20000
Quantification using: Lower Marker Final concentration (ng/uL): 0.5000 Dilution factor: 12.0

Sample: SampC3
Well location: C3
Created: Tuesday, August 10, 2021 8:01:08 PM

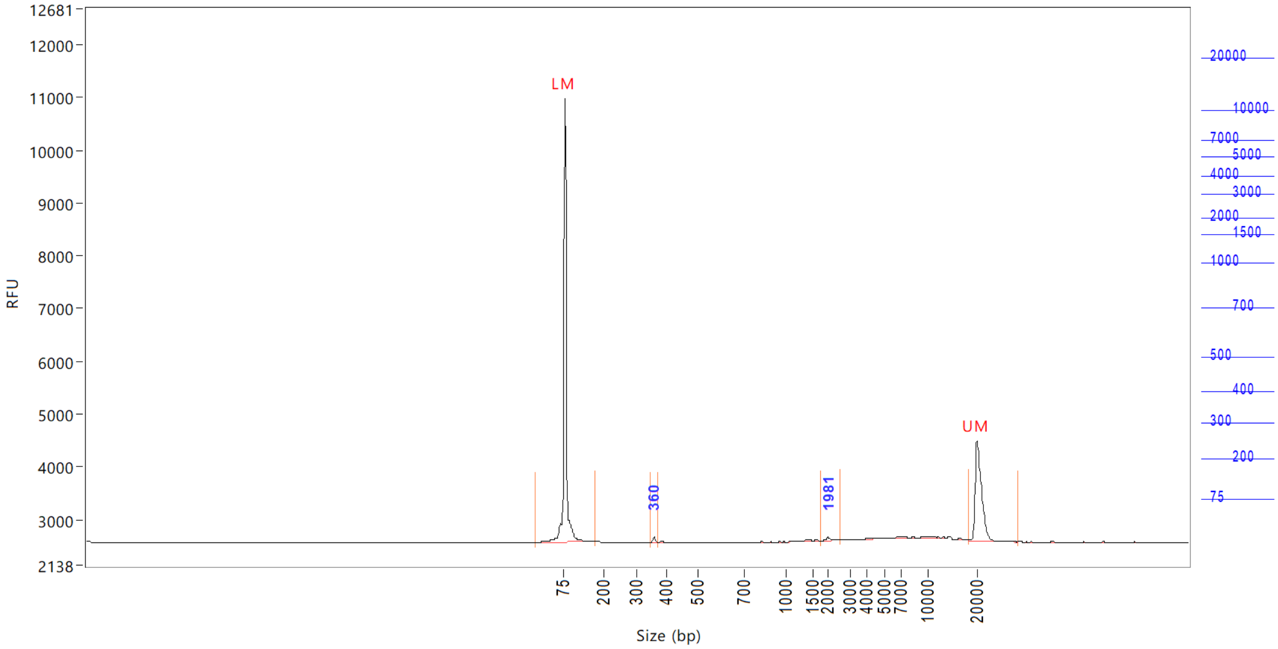

Peak	Size (bp)	Concentration (ng/uL)	From (bp)	To (bp)	Average size (bp)	CV%	RFU	Corrected peak area
1	75 (LM)	0.5000	0	171	73	15.73	8417	55.278
2	360	0.0592	343	370	356	1.23	109	0.545
3	1981	0.0530	1770	2515	2015	6.76	59	0.489
4	20000 (UM)	0.1441	18171	28584	20364	3.90	1891	15.929

TIC:	0.1122	ng/uL
TIM:	0.3167	nmole/L
Total concentration:	0.4901	ng/uL

Sample peak width (sec): 5   Sample min peak height: 50   Sample baseline V to V?: Y   Sample baseline V to V points: 3
Sample filter: Binomial   Number of points for filter: 3 Sample start region (min): 0 Sample end region (min): 30
Marker peak width (sec): 5   Marker min peak height: 200   Marker baseline V to V?: Y   Marker baseline V to V points: 3
Lower marker selection: First peak > 500 RFU   Upper marker selection: Last peak > 200 RFU
Ladder size (bp)75, 200, 300, 400, 500, 700, 1000, 1500, 2000, 3000, 4000, 5000, 7000, 10000, 20000
Quantification using: Lower Marker   Final concentration (ng/uL): 0.5000   Dilution factor: 12.0

Sample: SampD3
Well location: D3
Created: Tuesday, August 10, 2021 8:01:08 PM

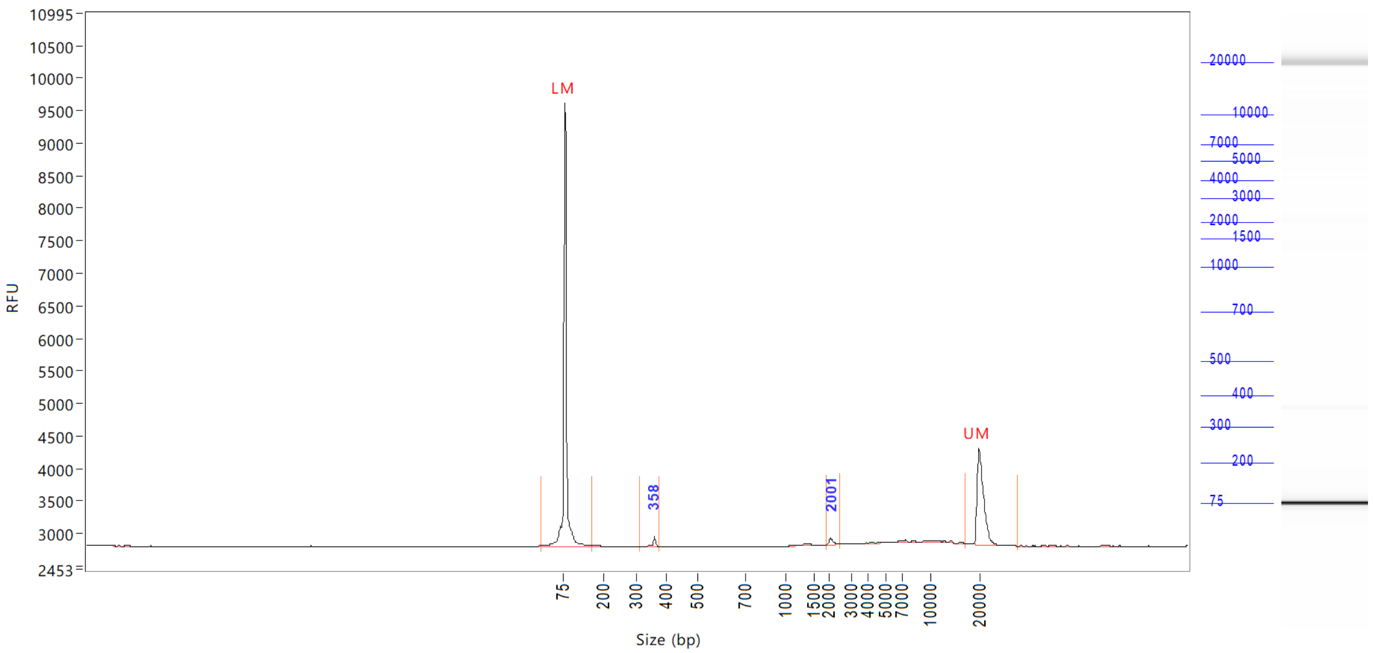

Peak	Size (bp)	Concentration (ng/uL)	From (bp)	To (bp)	Average size (bp)	CV%	RFU	Corrected peak area
1	75 (LM)	0.5000	0	162	74	18.09	6830	46.476
2	358	0.1334	307	374	349	3.90	148	1.034
3	2001	0.0844	1885	2486	2058	5.55	98	0.654
4	20000 (UM)	0.1362	17195	27979	20339	4.14	1479	12.661

TIC:	0.2178	ng/uL
TIM:	0.6965	nmole/L
Total concentration:	0.7361	ng/uL

Sample peak width (sec): 5   Sample min peak height: 50   Sample baseline V to V?: Y   Sample baseline V to V points: 3
Sample filter: Binomial   Number of points for filter: 3   Sample start region (min): 0   Sample end region (min): 30
Marker peak width (sec): 5   Marker min peak height: 200   Marker baseline V to V?: Y   Marker baseline V to V points: 3
Lower marker selection: First peak > 500 RFU   Upper marker selection: Last peak > 200 RFU
Ladder size (bp)75, 200, 300, 400, 500, 700, 1000, 1500, 2000, 3000, 4000, 5000, 7000, 10000, 20000
Quantification using: Lower Marker   Final concentration (ng/uL): 0.5000   Dilution factor: 12.0

Sample: SampE3
Well location: E3
Created: Tuesday, August 10, 2021 8:01:08 PM

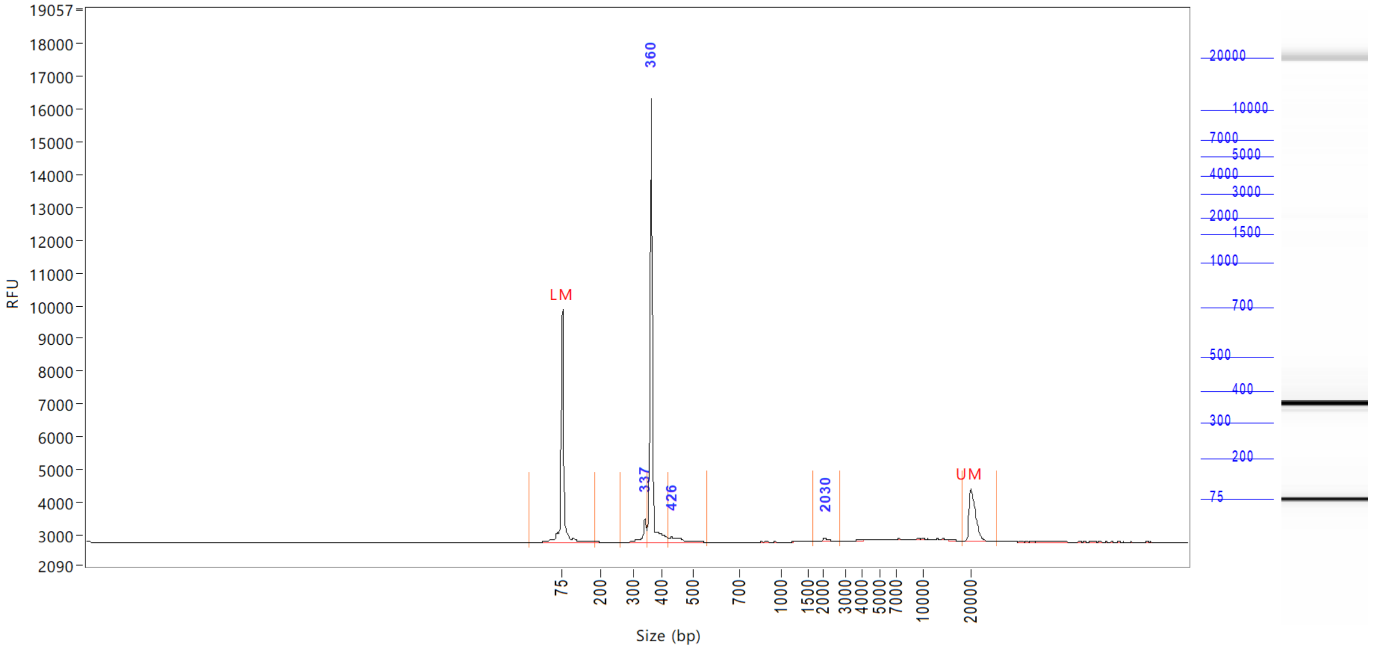

Peak	Size<br>(bp)	Concentration<br>(ng/uL)	From<br>(bp)	To<br>(bp)	Average size<br>(bp)	CV%	RFU	Corrected peak area
1	75 (LM)	0.5000	0	179	74	27.18	7123	51.463
2	337	0.6672	259	343	328	4.45	726	5.723
3	360	8.2151	343	418	360	3.05	13559	70.463
4	426	0.4134	418	555	448	5.49	152	3.546
5	2030	0.0902	1674	2743	2090	7.98	104	0.774
6	20000 (UM)	0.1324	18049	25440	20358	3.89	1608	13.631

TIC:	9.3859	ng/uL
TIM:	42.4781	nmole/L
Total concentration:	9.7040	ng/uL

Sample peak width (sec): 5   Sample min peak height: 50   Sample baseline V to V?: Y   Sample baseline V to V points: 3
Sample filter: Binomial   Number of points for filter: 3   Sample start region (min): 0   Sample end region (min): 30
Marker peak width (sec): 5   Marker min peak height: 200   Marker baseline V to V?: Y   Marker baseline V to V points: 3
Lower marker selection: First peak > 500 RFU   Upper marker selection: Last peak > 200 RFU
Ladder size (bp)75, 200, 300, 400, 500, 700, 1000, 1500, 2000, 3000, 4000, 5000, 7000, 10000, 20000
Quantification using: Lower Marker   Final concentration (ng/uL): 0.5000   Dilution factor: 12.0

Sample: SampF3
Well location: F3
Created: Tuesday, August 10, 2021 8:01:08 PM

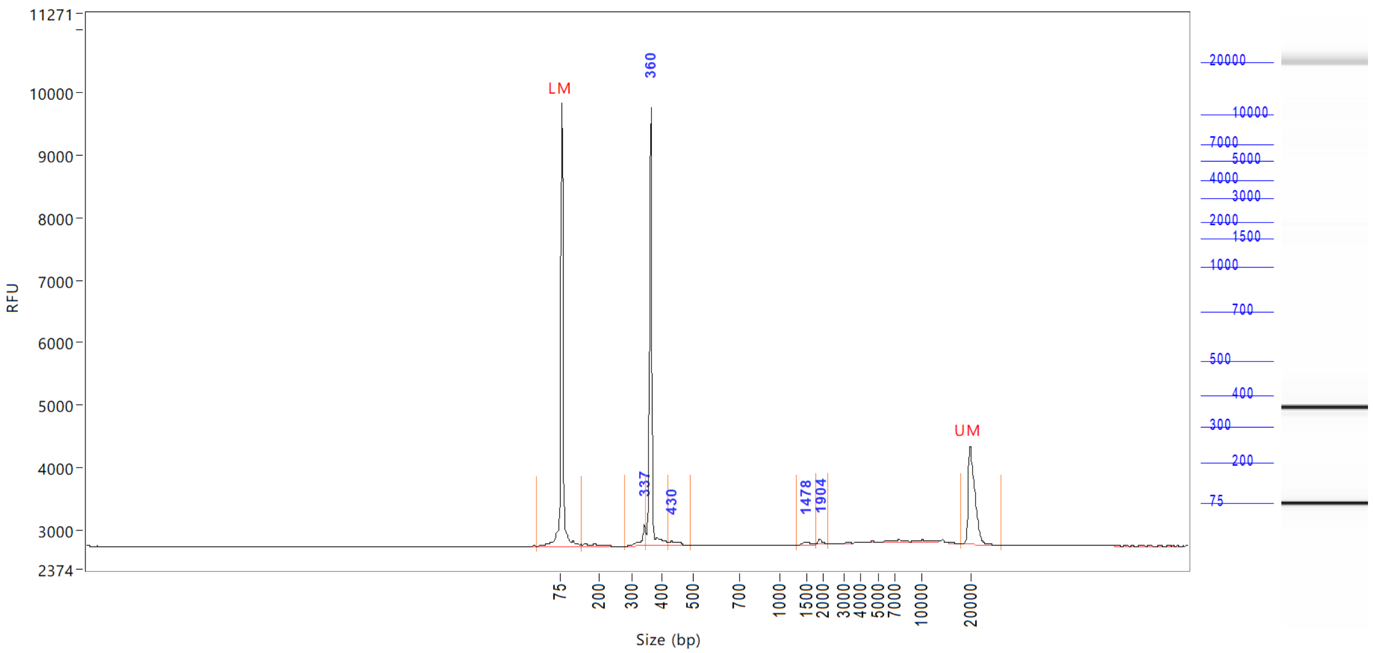

Peak	Size (bp)	Concentration (ng/uL)	From (bp)	To (bp)	Average size (bp)	CV%	RFU	Corrected peak area
1	75 (LM)	0.5000	0	141	72	16.76	7109	45.727
2	337	0.3971	275	345	329	4.30	357	3.026
3	360	4.6257	345	418	360	2.80	7014	35.253
4	430	0.1601	418	494	439	3.31	68	1.220
5	1478	0.0823	1300	1770	1504	6.88	53	0.627
6	1904	0.0780	1770	2229	1919	4.70	87	0.595
7	20000 (UM)	0.1491	18171	26287	20458	4.05	1574	13.640

TIC:	5.3432	ng/uL
TIM:	23.8816	nmole/L
Total concentration:	5.7331	ng/uL

Sample peak width (sec): 5   Sample min peak height: 50   Sample baseline V to V?: Y   Sample baseline V to V points: 3
Sample filter: Binomial   Number of points for filter: 3   Sample start region (min): 0   Sample end region (min): 30
Marker peak width (sec): 5   Marker min peak height: 200   Marker baseline V to V?: Y   Marker baseline V to V points: 3
Lower marker selection: First peak > 500 RFU   Upper marker selection: Last peak > 200 RFU
Ladder size (bp)75, 200, 300, 400, 500, 700, 1000, 1500, 2000, 3000, 4000, 5000, 7000, 10000, 20000
Quantification using: Lower Marker   Final concentration (ng/uL): 0.5000   Dilution factor: 12.0

Sample: SampG3
Well location: G3
Created: Tuesday, August 10, 2021 8:01:08 PM

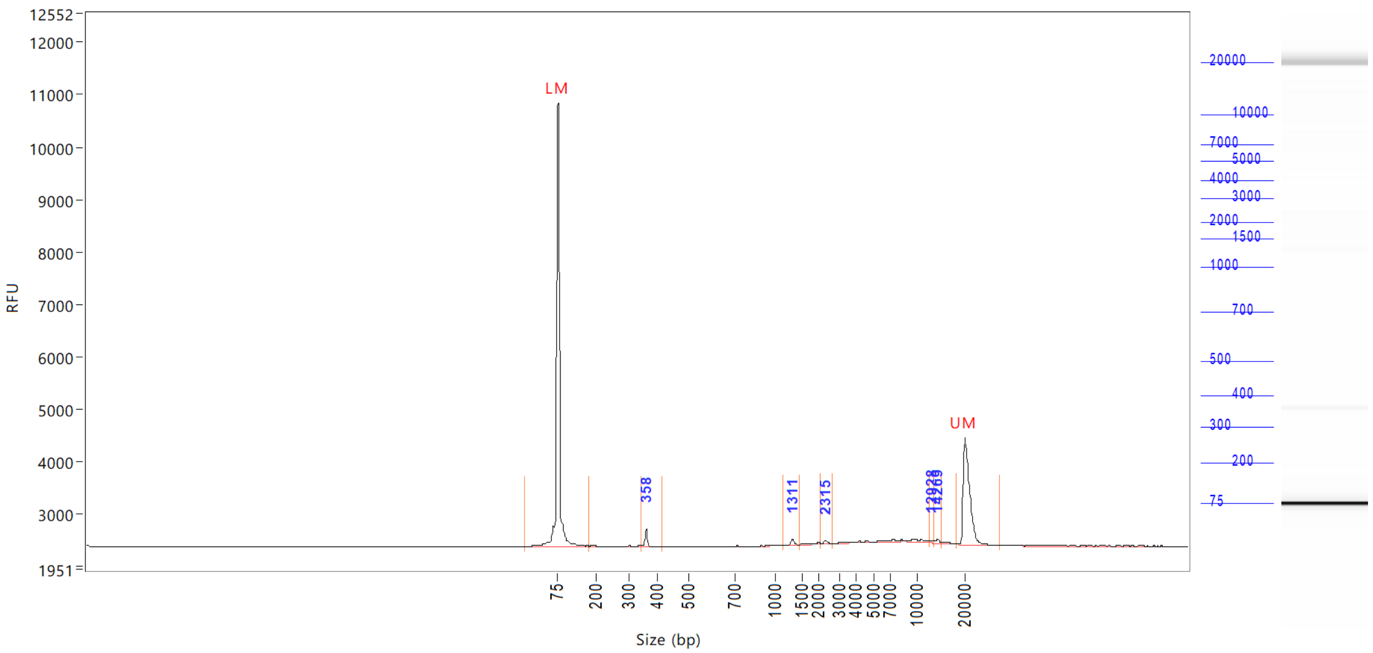

Peak	Size (bp)	Concentration (ng/uL)	From (bp)	To (bp)	Average size (bp)	CV%	RFU	Corrected peak area
1	75 (LM)	0.5000	0	177	73	19.78	8474	59.086
2	358	0.1719	343	414	358	2.85	331	1.693
3	1311	0.0697	1123	1433	1300	3.48	117	0.686
4	2315	0.0426	2115	2686	2306	4.55	67	0.420
5	12928	0.0299	12440	13537	12974	2.53	52	0.294
6	14269	0.0426	13537	15122	14199	2.75	76	0.420
7	20000 (UM)	0.1493	18293	27254	20427	4.23	2032	17.644

TIC:	0.3567	ng/uL
TIM:	0.9175	nmole/L
Total concentration:	0.7887	ng/uL

```
Sample peak width (sec): 5    Sample min peak height: 50    Sample baseline V to V?: Y    Sample baseline V to V points: 3
Sample filter: Binomial       Number of points for filter: 3  Sample start region (min): 0  Sample end region (min): 30
Marker peak width (sec): 5    Marker min peak height: 200   Marker baseline V to V?: Y    Marker baseline V to V points: 3
Lower marker selection: First peak > 500 RFU                Upper marker selection: Last peak > 200 RFU
Ladder size (bp)75, 200, 300, 400, 500, 700, 1000, 1500, 2000, 3000, 4000, 5000, 7000, 10000, 20000
Quantification using: Lower Marker          Final concentration (ng/uL): 0.5000          Dilution factor: 12.0
```

Sample: SampH3
Well location: H3
Created: Tuesday, August 10, 2021 8:01:08 PM

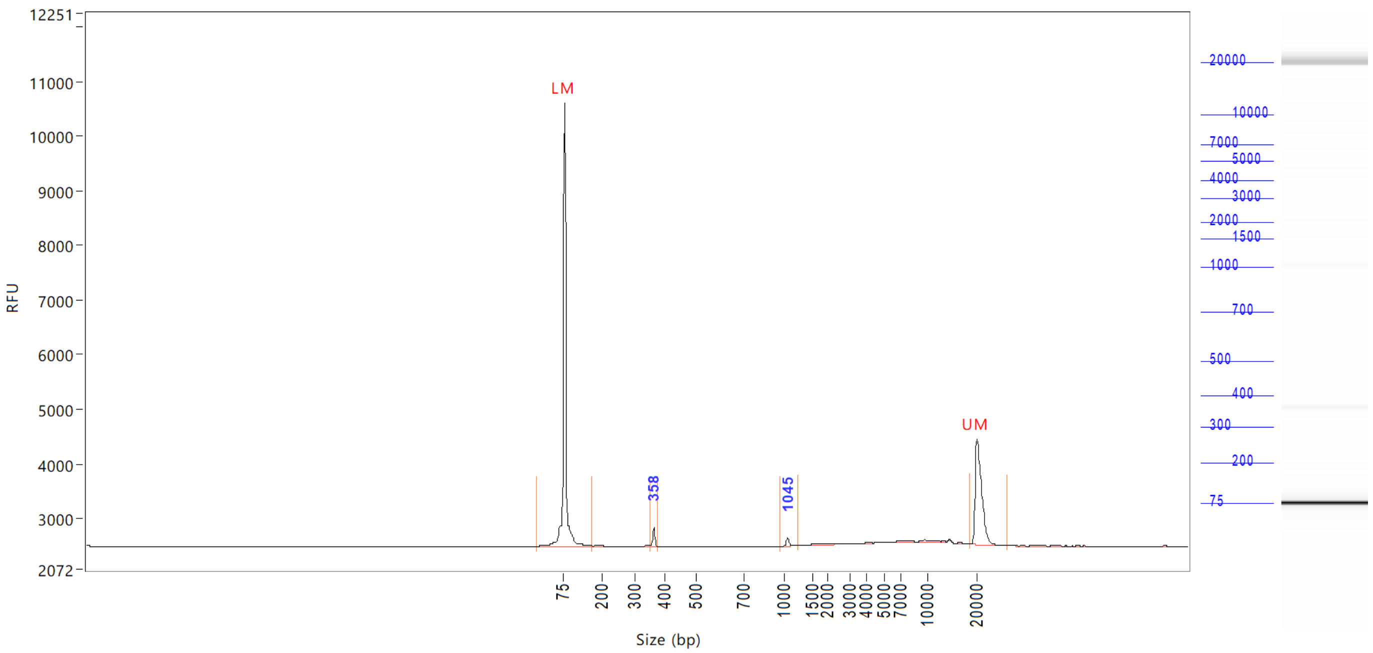

Peak	Size	Concentration	From	To	Average size	CV%	RFU	Corrected peak area
	(bp)	(ng/uL)	(bp)	(bp)	(bp)			
1	75 (LM)	0.5000	0	168	74	18.78	8138	55.766
2	358	0.1786	345	376	357	1.18	346	1.660
3	1045	0.0885	961	1234	1042	3.96	152	0.822
4	20000 (UM)	0.1491	18537	26408	20464	3.93	1938	16.633

TIC:	0.2671	ng/uL
TIM:	0.9628	nmole/L
Total concentration:	0.7053	ng/uL

Sample peak width (sec): 5   Sample min peak height: 50   Sample baseline V to V?: Y   Sample baseline V to V points: 3
Sample filter: Binomial   Number of points for filter: 3   Sample start region (min): 0   Sample end region (min): 30
Marker peak width (sec): 5   Marker min peak height: 200   Marker baseline V to V?: Y   Marker baseline V to V points: 3
Lower marker selection: First peak > 500 RFU   Upper marker selection: Last peak > 200 RFU
Ladder size (bp)75, 200, 300, 400, 500, 700, 1000, 1500, 2000, 3000, 4000, 5000, 7000, 10000, 20000
Quantification using: Lower Marker   Final concentration (ng/uL): 0.5000   Dilution factor: 12.0

Sample: SampA4
Well location: A4
Created: Tuesday, August 10, 2021 8:01:08 PM

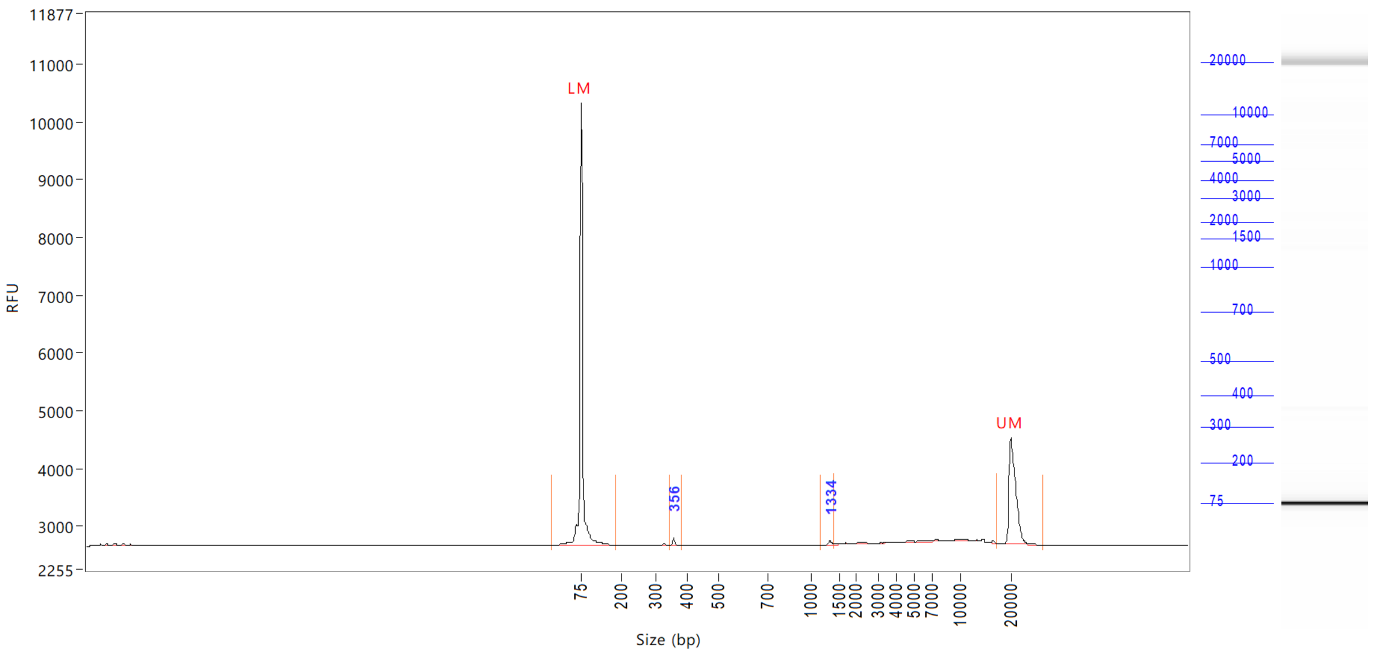

Peak	Size (bp)	Concentration (ng/uL)	From (bp)	To (bp)	Average size (bp)	CV%	RFU	Corrected peak area
1	75 (LM)	0.5000	0	183	74	21.99	7676	52.583
2	356	0.0697	343	380	354	1.47	123	0.611
3	1334	0.0364	1167	1400	1322	2.58	62	0.319
4	20000 (UM)	0.1477	17317	26408	20394	3.86	1837	15.529

TIC:	0.1061	ng/uL
TIM:	0.3692	nmole/L
Total concentration:	0.5557	ng/uL

```
Sample peak width (sec): 5    Sample min peak height: 50    Sample baseline V to V?: Y    Sample baseline V to V points: 3
Sample filter: Binomial       Number of points for filter: 3  Sample start region (min): 0  Sample end region (min): 30
Marker peak width (sec): 5    Marker min peak height: 200   Marker baseline V to V?: Y    Marker baseline V to V points: 3
Lower marker selection: First peak > 500 RFU                Upper marker selection: Last peak > 200 RFU
Ladder size (bp)75, 200, 300, 400, 500, 700, 1000, 1500, 2000, 3000, 4000, 5000, 7000, 10000, 20000
Quantification using: Lower Marker          Final concentration (ng/uL): 0.5000          Dilution factor: 12.0
```

Sample: SampB4
Well location: B4
Created: Tuesday, August 10, 2021 8:01:08 PM

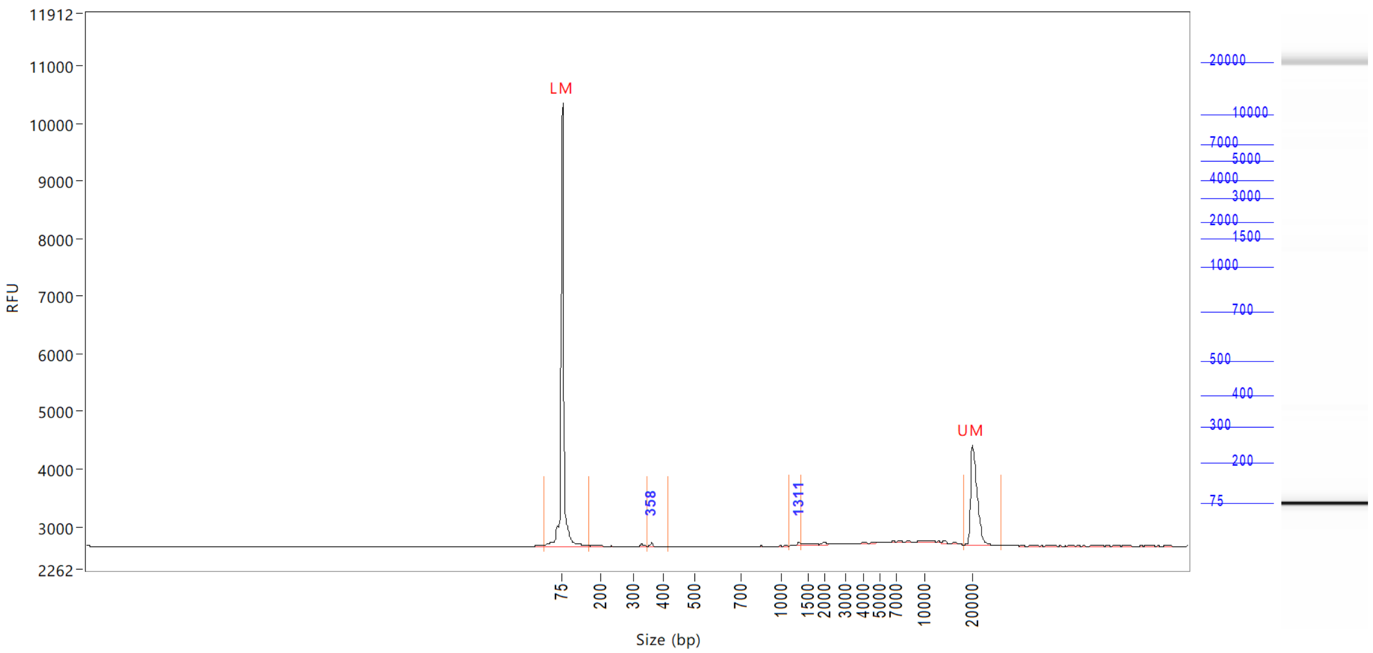

Peak	Size (bp)	Concentration (ng/uL)	From (bp)	To (bp)	Average size (bp)	CV%	RFU	Corrected peak area
1	75 (LM)	0.5000	18	164	73	16.77	7712	53.244
2	358	0.0503	345	412	362	4.06	70	0.447
3	1311	0.0375	1123	1367	1299	3.31	60	0.333
4	20000 (UM)	0.1373	18171	25803	20419	3.89	1733	14.619

TIC:	0.0878	ng/uL
TIM:	0.2762	nmole/L
Total concentration:	0.5511	ng/uL

Sample peak width (sec): 5 Sample min peak height: 50 Sample baseline V to V?: Y Sample baseline V to V points: 3
Sample filter: Binomial Number of points for filter: 3 Sample start region (min): 0 Sample end region (min): 30
Marker peak width (sec): 5 Marker min peak height: 200 Marker baseline V to V?: Y Marker baseline V to V points: 3
Lower marker selection: First peak > 500 RFU Upper marker selection: Last peak > 200 RFU
Ladder size (bp)75, 200, 300, 400, 500, 700, 1000, 1500, 2000, 3000, 4000, 5000, 7000, 10000, 20000
Quantification using: Lower Marker Final concentration (ng/uL): 0.5000 Dilution factor: 12.0

Sample: SampC4
Well location: C4
Created: Tuesday, August 10, 2021 8:01:08 PM

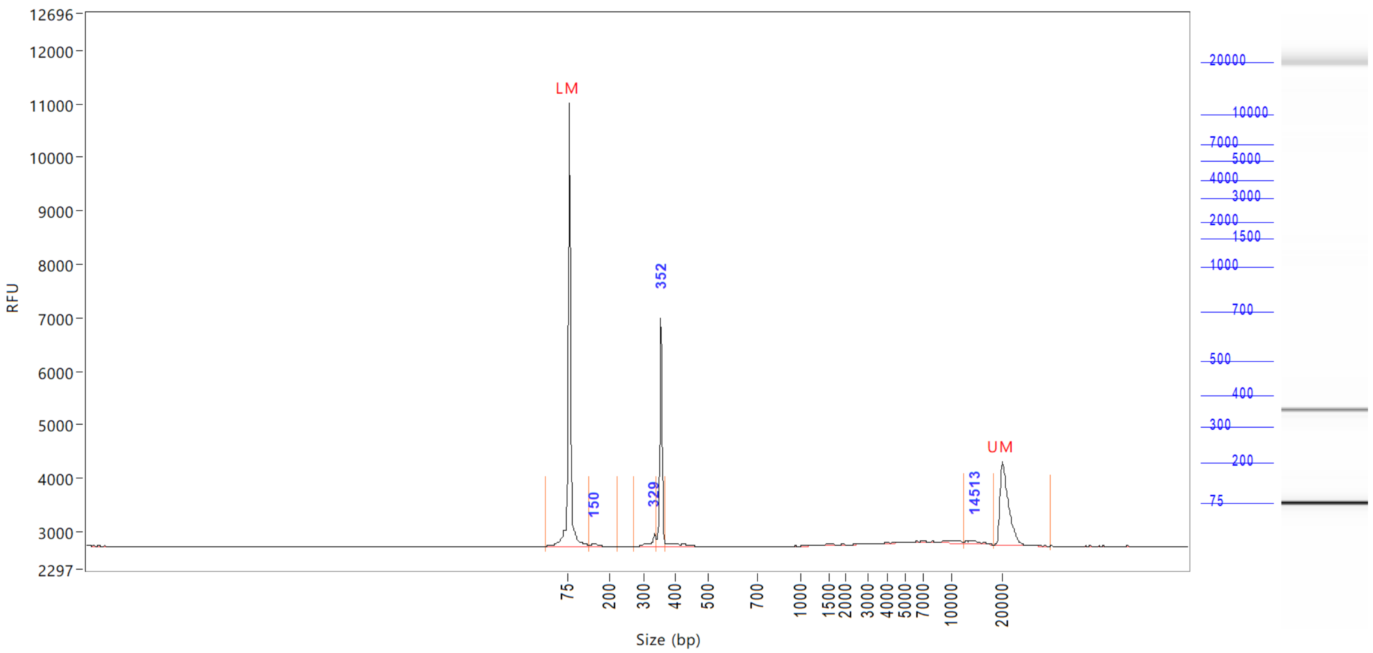

Peak	Size (bp)	Concentration (ng/uL)	From (bp)	To (bp)	Average size (bp)	CV%	RFU	Corrected peak area
1	75 (LM)	0.5000	3	137	73	15.85	8314	54.243
2	150	0.1161	137	223	158	10.83	57	1.050
3	329	0.2942	271	337	319	4.66	251	2.660
4	352	2.2620	337	368	349	1.15	4280	20.449
5	14513	0.1151	12318	18171	14440	9.06	58	1.041
6	20000 (UM)	0.1541	18171	29309	20583	5.07	1562	16.718

TIC:	2.7874	ng/uL
TIM:	13.4005	nmole/L
Total concentration:	3.3619	ng/uL

Sample peak width (sec): 5 Sample min peak height: 50 Sample baseline V to V?: Y Sample baseline V to V points: 3
Sample filter: Binomial Number of points for filter: 3 Sample start region (min): 0 Sample end region (min): 30
Marker peak width (sec): 5 Marker min peak height: 200 Marker baseline V to V?: Y Marker baseline V to V points: 3
Lower marker selection: First peak > 500 RFU Upper marker selection: Last peak > 200 RFU
Ladder size (bp)75, 200, 300, 400, 500, 700, 1000, 1500, 2000, 3000, 4000, 5000, 7000, 10000, 20000
Quantification using: Lower Marker Final concentration (ng/uL): 0.5000 Dilution factor: 12.0

Sample: SampD4
Well location: D4
Created: Tuesday, August 10, 2021 8:01:08 PM

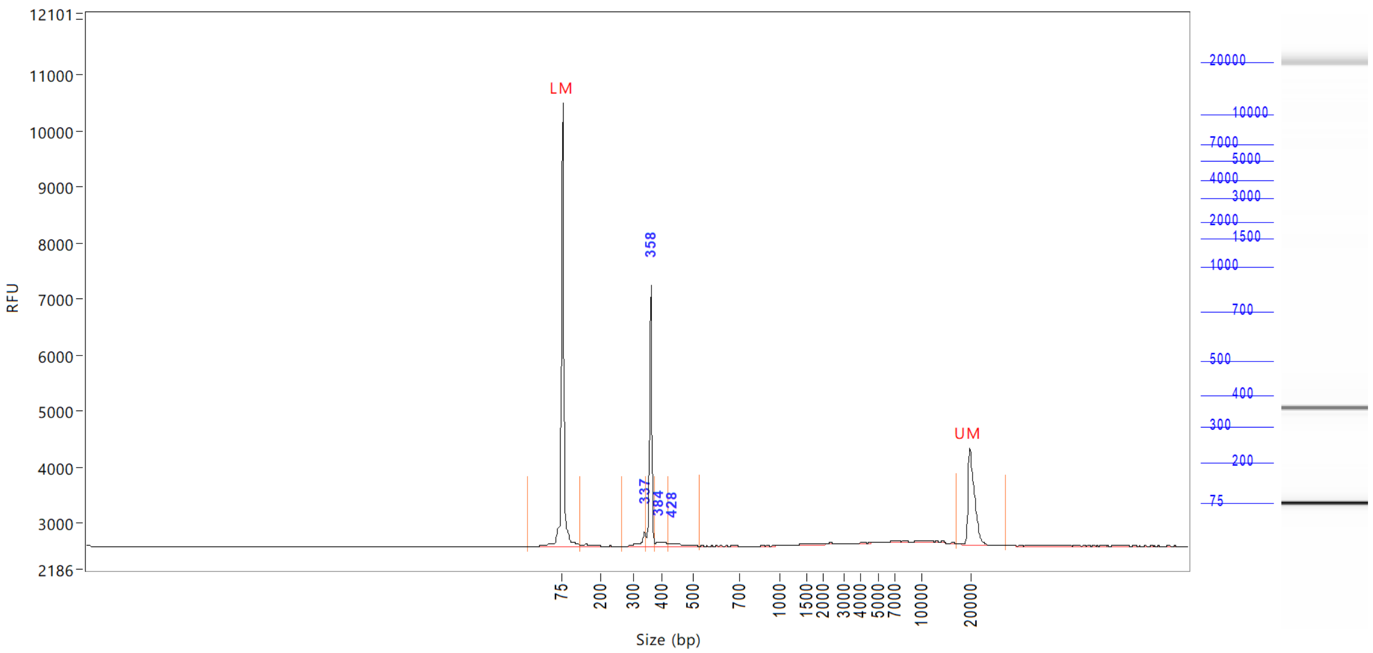

Peak	Size (bp)	Concentration (ng/uL)	From (bp)	To (bp)	Average size (bp)	CV%	RFU	Corrected peak area
1	75 (LM)	0.5000	0	131	73	17.35	7927	50.822
2	337	0.3045	266	343	325	4.91	271	2.579
3	358	2.5927	343	372	356	1.09	4649	21.961
4	384	0.1698	372	418	394	3.36	67	1.438
5	428	0.1393	418	524	446	4.99	51	1.180
6	20000 (UM)	0.1425	17195	27496	20399	4.05	1719	14.487

TIC:	3.2063	ng/uL
TIM:	14.7457	nmole/L
Total concentration:	3.6747	ng/uL

Sample peak width (sec): 5   Sample min peak height: 50   Sample baseline V to V?: Y   Sample baseline V to V points: 3
Sample filter: Binomial   Number of points for filter: 3   Sample start region (min): 0   Sample end region (min): 30
Marker peak width (sec): 5   Marker min peak height: 200   Marker baseline V to V?: Y   Marker baseline V to V points: 3
Lower marker selection: First peak > 500 RFU   Upper marker selection: Last peak > 200 RFU
Ladder size (bp)75, 200, 300, 400, 500, 700, 1000, 1500, 2000, 3000, 4000, 5000, 7000, 10000, 20000
Quantification using: Lower Marker   Final concentration (ng/uL): 0.5000   Dilution factor: 12.0

Sample: SampE4
Well location: E4
Created: Tuesday, August 10, 2021 8:01:08 PM

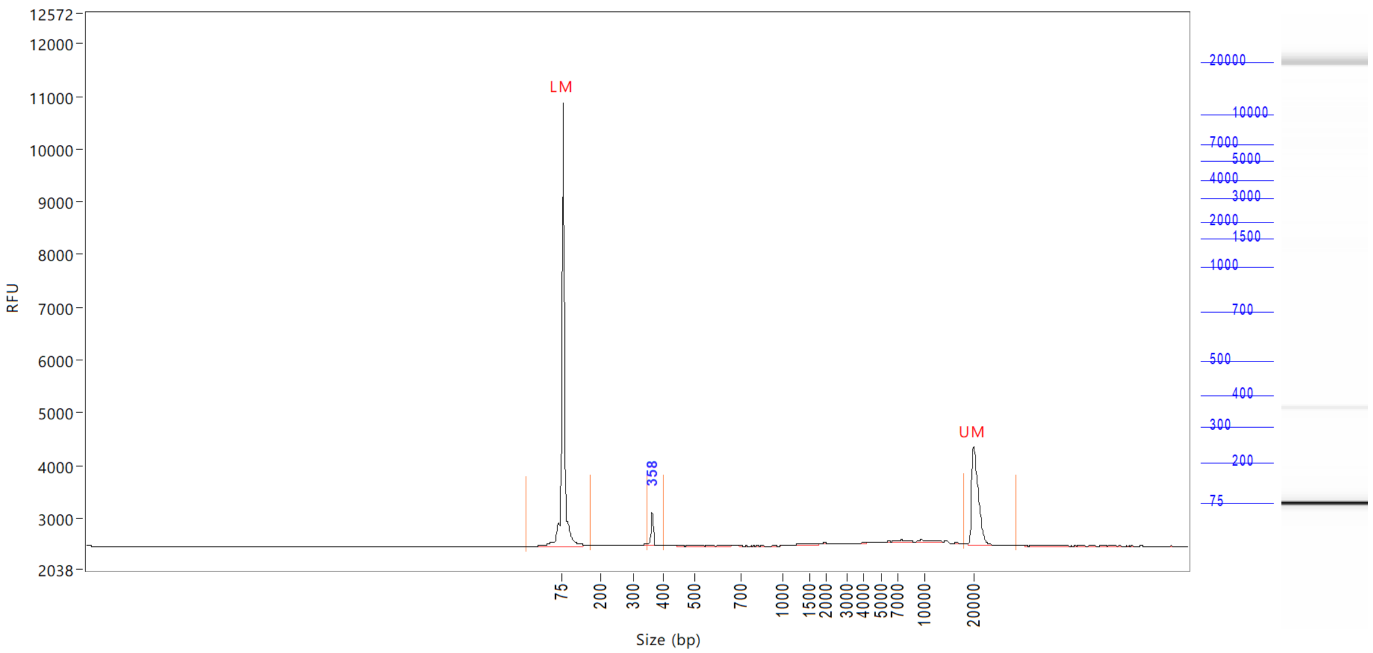

Peak	Size (bp)	Concentration (ng/uL)	From (bp)	To (bp)	Average size (bp)	CV%	RFU	Corrected peak area
1	75 (LM)	0.5000	0	166	73	19.37	8415	58.157
2	358	0.3386	343	398	357	1.60	626	3.282
3	20000 (UM)	0.1384	18049	28705	20410	4.08	1861	16.093

TIC:	0.3386	ng/uL
TIM:	1.5605	nmole/L
Total concentration:	0.7675	ng/uL

Sample peak width (sec): 5 Sample min peak height: 50 Sample baseline V to V?: Y Sample baseline V to V points: 3
Sample filter: Binomial Number of points for filter: 3 Sample start region (min): 0 Sample end region (min): 30
Marker peak width (sec): 5 Marker min peak height: 200 Marker baseline V to V?: Y Marker baseline V to V points: 3
Lower marker selection: First peak > 500 RFU Upper marker selection: Last peak > 200 RFU
Ladder size (bp)75, 200, 300, 400, 500, 700, 1000, 1500, 2000, 3000, 4000, 5000, 7000, 10000, 20000
Quantification using: Lower Marker Final concentration (ng/uL): 0.5000 Dilution factor: 12.0

Sample: SampF4
Well location: F4
Created: Tuesday, August 10, 2021 8:01:08 PM

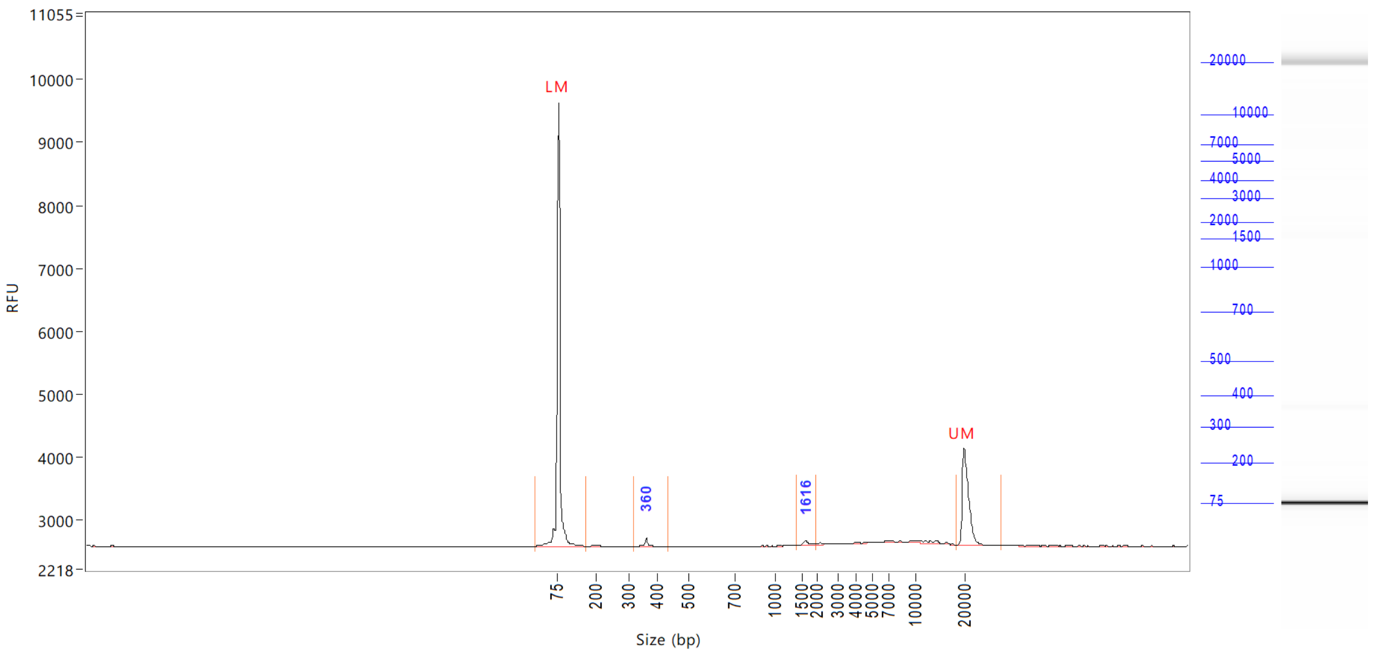

Peak	Size (bp)	Concentration (ng/uL)	From (bp)	To (bp)	Average size (bp)	CV%	RFU	Corrected peak area
1	75 (LM)	0.5000	3	164	74	16.53	7064	48.407
2	360	0.1306	315	435	362	5.43	133	1.053
3	1616	0.0859	1400	1962	1646	7.95	69	0.693
4	20000 (UM)	0.1360	18537	27859	20457	4.03	1548	13.162

TIC:	0.2165	ng/uL
TIM:	0.6793	nmole/L
Total concentration:	0.6860	ng/uL

Sample peak width (sec): 5   Sample min peak height: 50   Sample baseline V to V?: Y   Sample baseline V to V points: 3
Sample filter: Binomial   Number of points for filter: 3 Sample start region (min): 0 Sample end region (min): 30
Marker peak width (sec): 5   Marker min peak height: 200   Marker baseline V to V?: Y   Marker baseline V to V points: 3
Lower marker selection: First peak > 500 RFU   Upper marker selection: Last peak > 200 RFU
Ladder size (bp)75, 200, 300, 400, 500, 700, 1000, 1500, 2000, 3000, 4000, 5000, 7000, 10000, 20000
Quantification using: Lower Marker   Final concentration (ng/uL): 0.5000   Dilution factor: 12.0

Sample: SampG4
Well location: G4
Created: Tuesday, August 10, 2021 8:01:08 PM

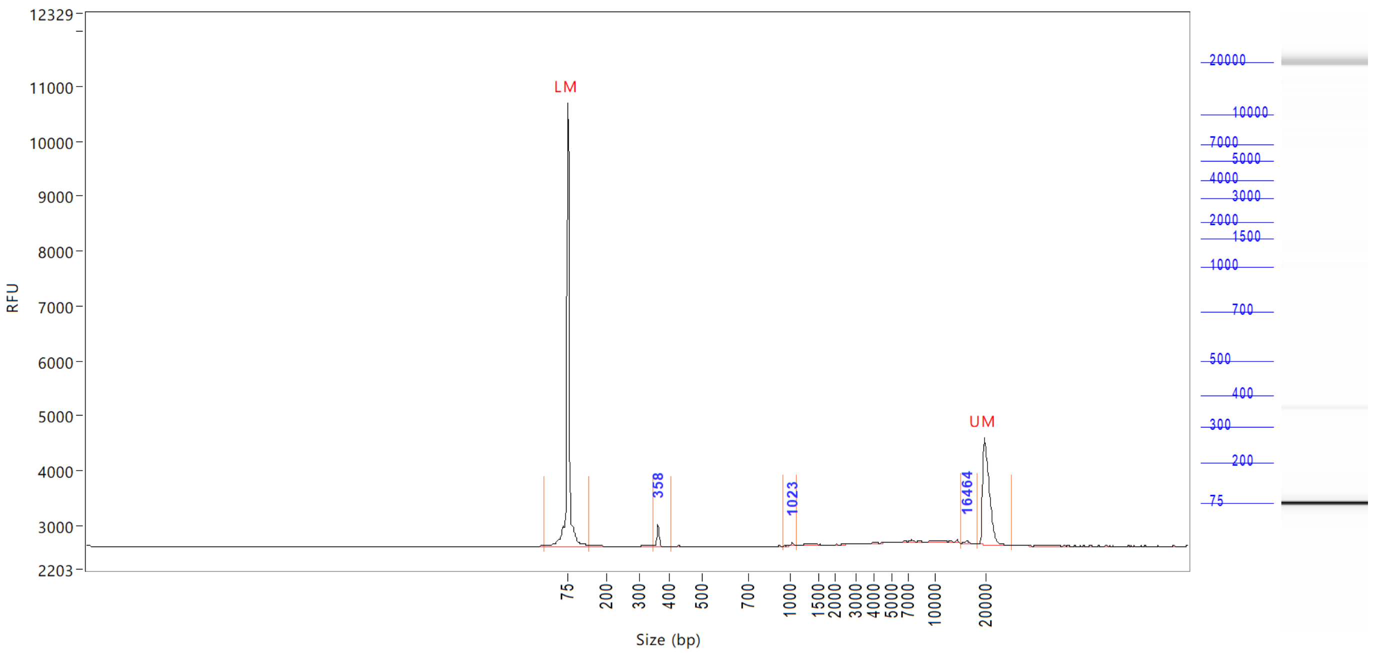

Peak	Size (bp)	Concentration (ng/uL)	From (bp)	To (bp)	Average size (bp)	CV%	RFU	Corrected peak area
1	75 (LM)	0.5000	1	141	73	17.17	8096	53.928
2	358	0.2129	343	400	356	1.60	392	1.913
3	1023	0.0351	939	1112	1017	3.04	53	0.315
4	16464	0.0390	15122	18415	16386	3.67	54	0.351
5	20000 (UM)	0.1520	18415	25440	20356	3.66	1952	16.393

TIC:	0.2869	ng/uL
TIM:	1.0445	nmole/L
Total concentration:	0.8127	ng/uL

```
Sample peak width (sec): 5    Sample min peak height: 50    Sample baseline V to V?: Y    Sample baseline V to V points: 3
Sample filter: Binomial       Number of points for filter: 3  Sample start region (min): 0  Sample end region (min): 30
Marker peak width (sec): 5    Marker min peak height: 200   Marker baseline V to V?: Y    Marker baseline V to V points: 3
Lower marker selection: First peak > 500 RFU                Upper marker selection: Last peak > 200 RFU
Ladder size (bp)75, 200, 300, 400, 500, 700, 1000, 1500, 2000, 3000, 4000, 5000, 7000, 10000, 20000
Quantification using: Lower Marker          Final concentration (ng/uL): 0.5000          Dilution factor: 12.0
```

Sample: SampH4
Well location: H4
Created: Tuesday, August 10, 2021 8:01:08 PM

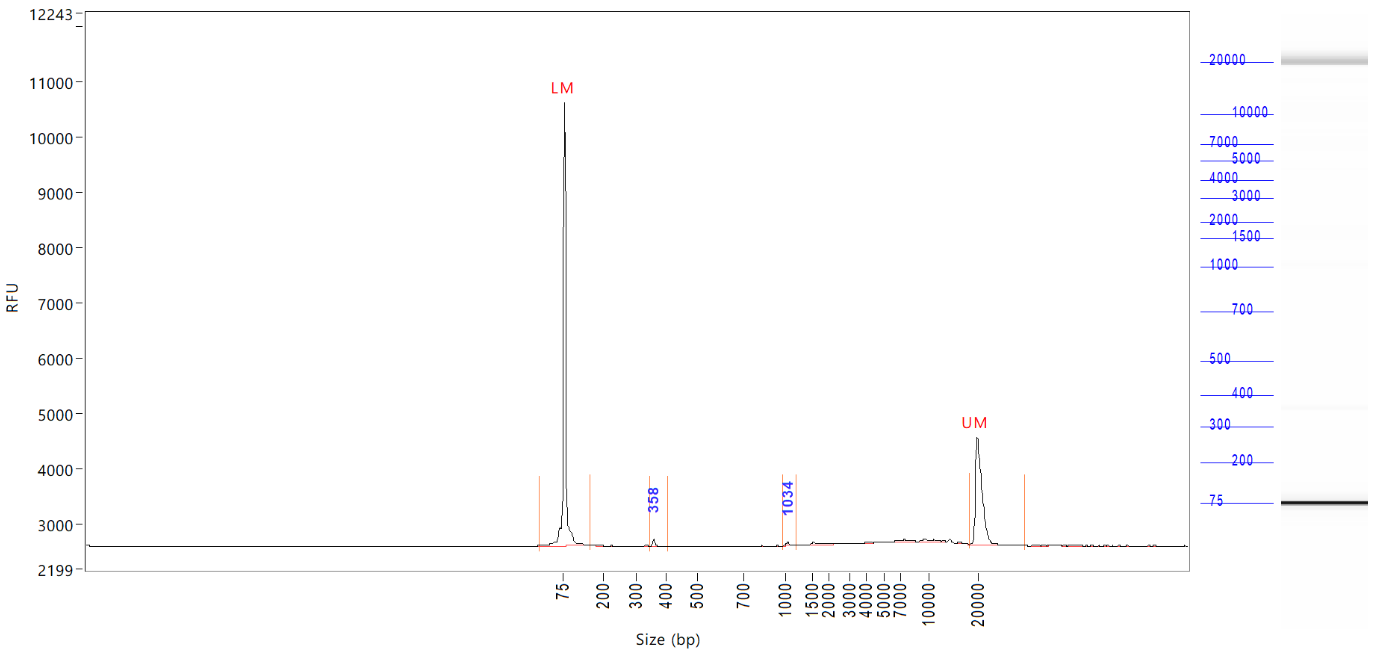

Peak	Size (bp)	Concentration (ng/uL)	From (bp)	To (bp)	Average size (bp)	CV%	RFU	Corrected peak area
1	75 (LM)	0.5000	0	158	72	15.29	8019	52.768
2	358	0.0857	345	405	359	2.89	128	0.753
3	1034	0.0384	978	1212	1034	3.96	57	0.338
4	20000 (UM)	0.1590	18415	29793	20486	4.48	1939	16.784

TIC:	0.1241	ng/uL
TIM:	0.4537	nmole/L
Total concentration:	0.5538	ng/uL

Sample peak width (sec): 5 Sample min peak height: 50 Sample baseline V to V?: Y Sample baseline V to V points: 3
Sample filter: Binomial Number of points for filter: 3 Sample start region (min): 0 Sample end region (min): 30
Marker peak width (sec): 5 Marker min peak height: 200 Marker baseline V to V?: Y Marker baseline V to V points: 3
Lower marker selection: First peak > 500 RFU Upper marker selection: Last peak > 200 RFU
Ladder size (bp)75, 200, 300, 400, 500, 700, 1000, 1500, 2000, 3000, 4000, 5000, 7000, 10000, 20000
Quantification using: Lower Marker Final concentration (ng/uL): 0.5000 Dilution factor: 12.0

Sample: SampA5
Well location: A5
Created: Tuesday, August 10, 2021 8:01:08 PM

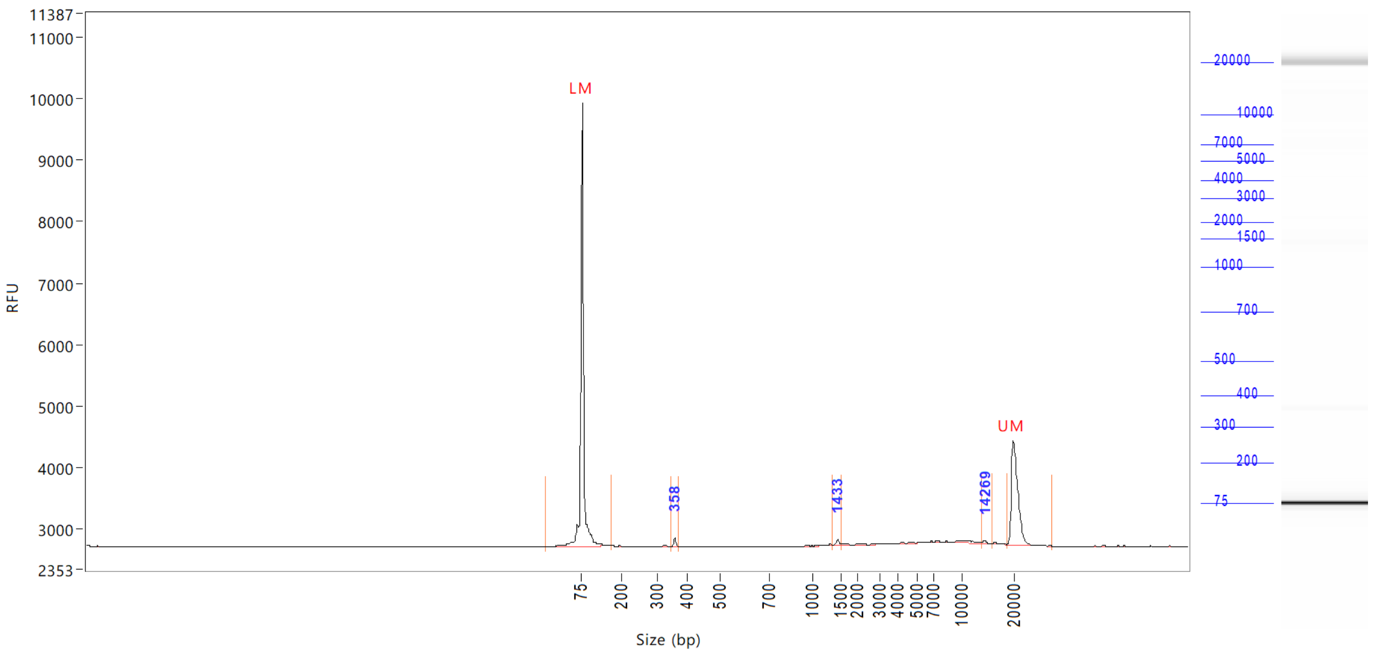

Peak	Size (bp)	Concentration (ng/uL)	From (bp)	To (bp)	Average size (bp)	CV%	RFU	Corrected peak area
1	75 (LM)	0.5000	0	168	74	17.75	7216	48.364
2	358	0.0787	343	370	355	1.09	133	0.635
3	1433	0.0667	1356	1500	1424	2.37	90	0.537
4	14269	0.0354	13659	15732	14370	3.72	52	0.285
5	20000 (UM)	0.1399	18659	27617	20348	3.75	1689	13.533

TIC:	0.1808	ng/uL
TIM:	0.4460	nmole/L
Total concentration:	0.6306	ng/uL

```
Sample peak width (sec): 5    Sample min peak height: 50    Sample baseline V to V?: Y    Sample baseline V to V points: 3
Sample filter: Binomial       Number of points for filter: 3  Sample start region (min): 0  Sample end region (min): 30
Marker peak width (sec): 5    Marker min peak height: 200   Marker baseline V to V?: Y    Marker baseline V to V points: 3
Lower marker selection: First peak > 500 RFU                Upper marker selection: Last peak > 200 RFU
Ladder size (bp)75, 200, 300, 400, 500, 700, 1000, 1500, 2000, 3000, 4000, 5000, 7000, 10000, 20000
Quantification using: Lower Marker          Final concentration (ng/uL): 0.5000          Dilution factor: 12.0
```

Sample: SampB5
Well location: B5
Created: Tuesday, August 10, 2021 8:01:08 PM

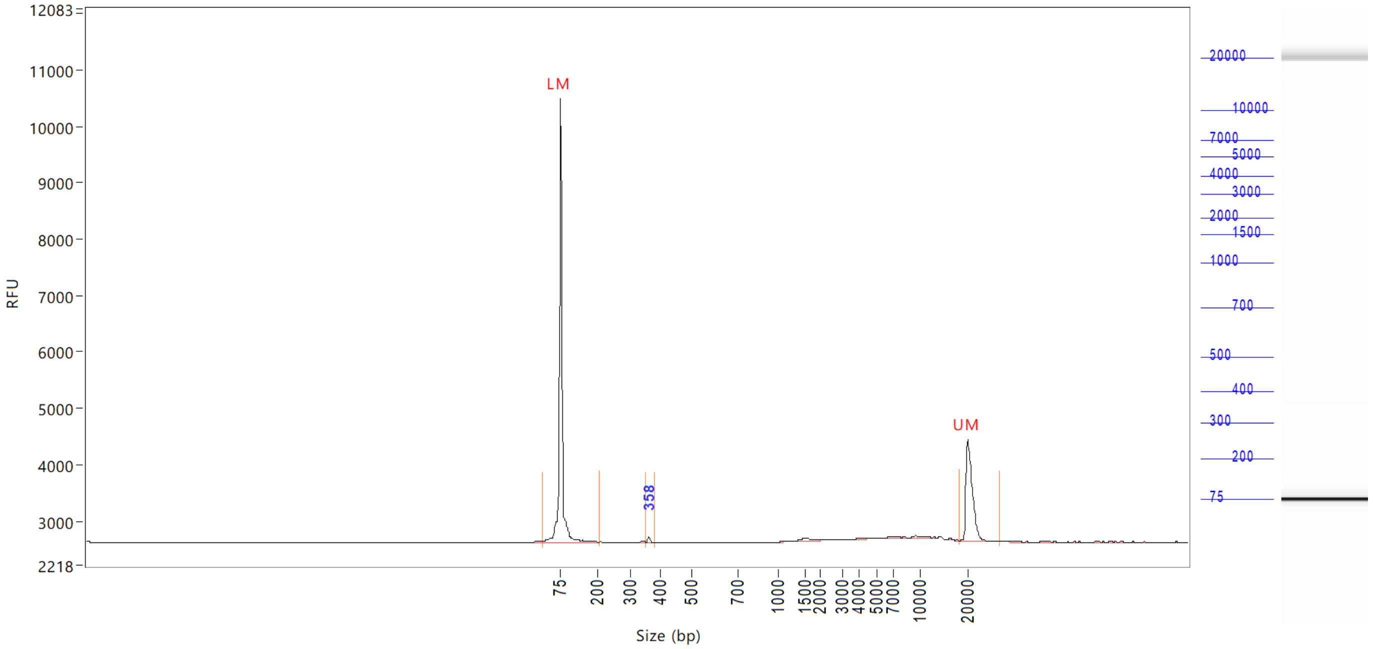

Peak	Size (bp)	Concentration (ng/uL)	From (bp)	To (bp)	Average size (bp)	CV%	RFU	Corrected peak area
1	75 (LM)	0.5000	18	201	74	18.00	7882	54.172
2	358	0.0493	349	380	357	1.30	91	0.445
3	20000 (UM)	0.1411	18171	26770	20459	3.92	1804	15.285

TIC:	0.0493	ng/uL
TIM:	0.2272	nmole/L
Total concentration:	0.5023	ng/uL

Sample peak width (sec): 5   Sample min peak height: 50   Sample baseline V to V?: Y   Sample baseline V to V points: 3
Sample filter: Binomial   Number of points for filter: 3   Sample start region (min): 0   Sample end region (min): 30
Marker peak width (sec): 5   Marker min peak height: 200   Marker baseline V to V?: Y   Marker baseline V to V points: 3
Lower marker selection: First peak > 500 RFU   Upper marker selection: Last peak > 200 RFU
Ladder size (bp)75, 200, 300, 400, 500, 700, 1000, 1500, 2000, 3000, 4000, 5000, 7000, 10000, 20000
Quantification using: Lower Marker   Final concentration (ng/uL): 0.5000   Dilution factor: 12.0

Sample: SampC5
Well location: C5
Created: Tuesday, August 10, 2021 8:01:08 PM

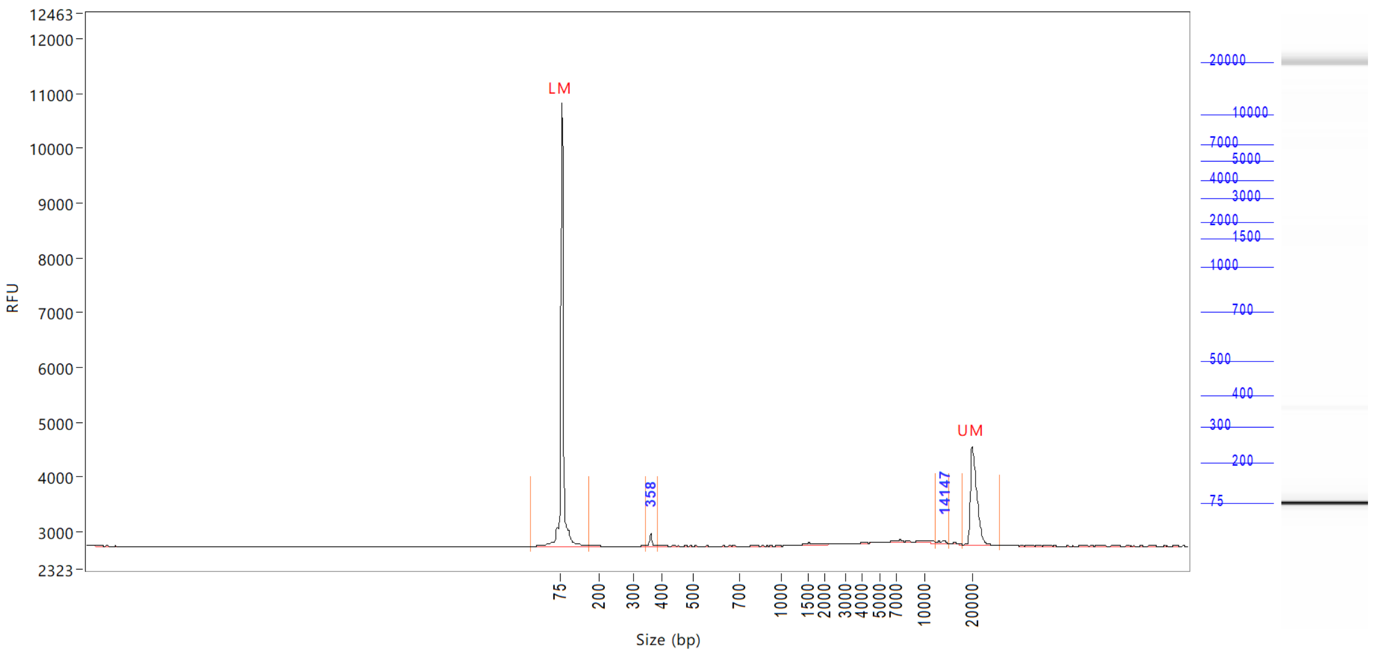

Peak	Size (bp)	Concentration (ng/uL)	From (bp)	To (bp)	Average size (bp)	CV%	RFU	Corrected peak area
1	75 (LM)	0.5000	0	164	74	18.73	8104	54.404
2	358	0.1304	343	384	357	1.79	226	1.183
3	14147	0.0592	12562	15000	13680	4.56	63	0.537
4	20000 (UM)	0.1375	17927	25682	20369	3.80	1796	14.960

TIC:	0.1896	ng/uL
TIM:	0.6081	nmole/L
Total concentration:	0.6987	ng/uL

Sample peak width (sec): 5 Sample min peak height: 50 Sample baseline V to V?: Y Sample baseline V to V points: 3
Sample filter: Binomial Number of points for filter: 3 Sample start region (min): 0 Sample end region (min): 30
Marker peak width (sec): 5 Marker min peak height: 200 Marker baseline V to V?: Y Marker baseline V to V points: 3
Lower marker selection: First peak > 500 RFU Upper marker selection: Last peak > 200 RFU
Ladder size (bp)75, 200, 300, 400, 500, 700, 1000, 1500, 2000, 3000, 4000, 5000, 7000, 10000, 20000
Quantification using: Lower Marker Final concentration (ng/uL): 0.5000 Dilution factor: 12.0

Sample: SampD5
Well location: D5
Created: Tuesday, August 10, 2021 8:01:08 PM

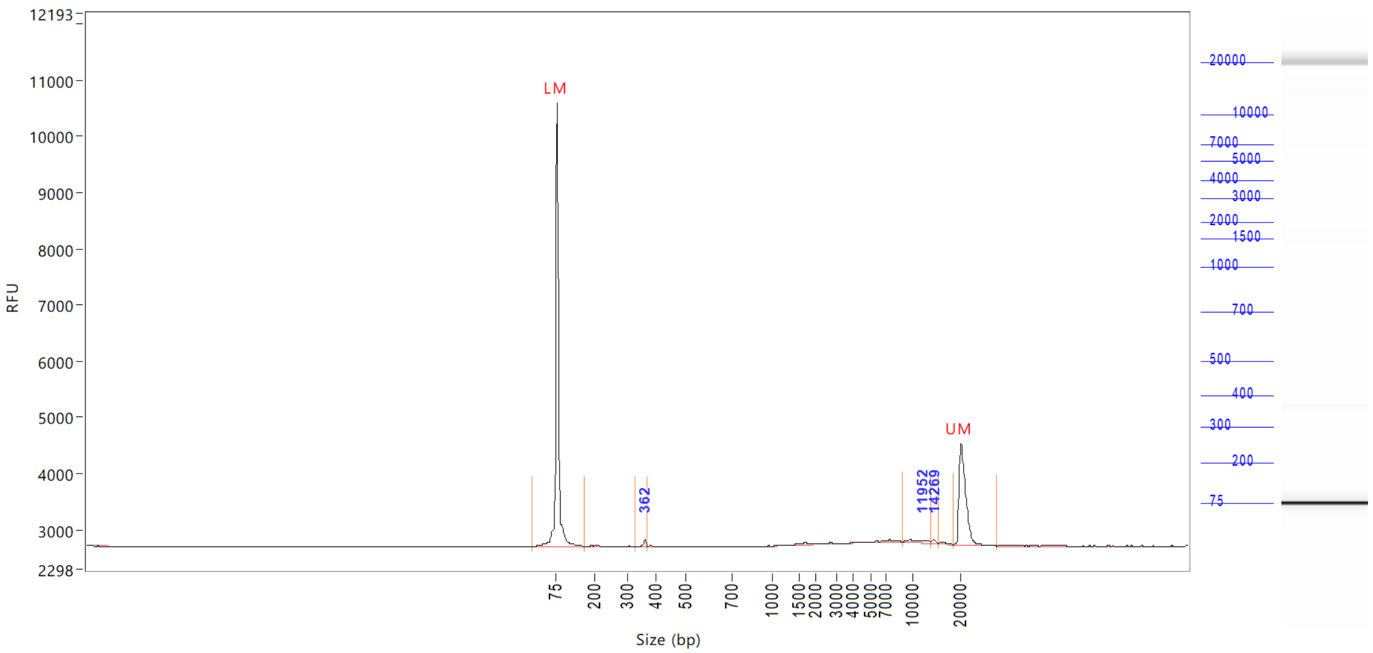

Peak	Size (bp)	Concentration (ng/uL)	From (bp)	To (bp)	Average size (bp)	CV%	RFU	Corrected peak area
1	75 (LM)	0.5000	0	168	74	17.21	7912	52.614
2	362	0.0806	327	372	356	2.07	124	0.707
3	11952	0.1429	8734	13659	11089	12.23	51	1.253
4	14269	0.0447	13659	15244	14298	2.74	73	0.392
5	20000 (UM)	0.1499	18415	27496	20510	4.47	1790	15.769

TIC:	0.2683	ng/uL
TIM:	0.3989	nmole/L
Total concentration:	0.6514	ng/uL

```
Sample peak width (sec): 5    Sample min peak height: 50    Sample baseline V to V?: Y    Sample baseline V to V points: 3
Sample filter: Binomial       Number of points for filter: 3  Sample start region (min): 0  Sample end region (min): 30
Marker peak width (sec): 5    Marker min peak height: 200   Marker baseline V to V?: Y    Marker baseline V to V points: 3
Lower marker selection: First peak > 500 RFU                Upper marker selection: Last peak > 200 RFU
Ladder size (bp)75, 200, 300, 400, 500, 700, 1000, 1500, 2000, 3000, 4000, 5000, 7000, 10000, 20000
Quantification using: Lower Marker          Final concentration (ng/uL): 0.5000          Dilution factor: 12.0
```

Sample: SampE5
Well location: E5
Created: Tuesday, August 10, 2021 8:01:08 PM

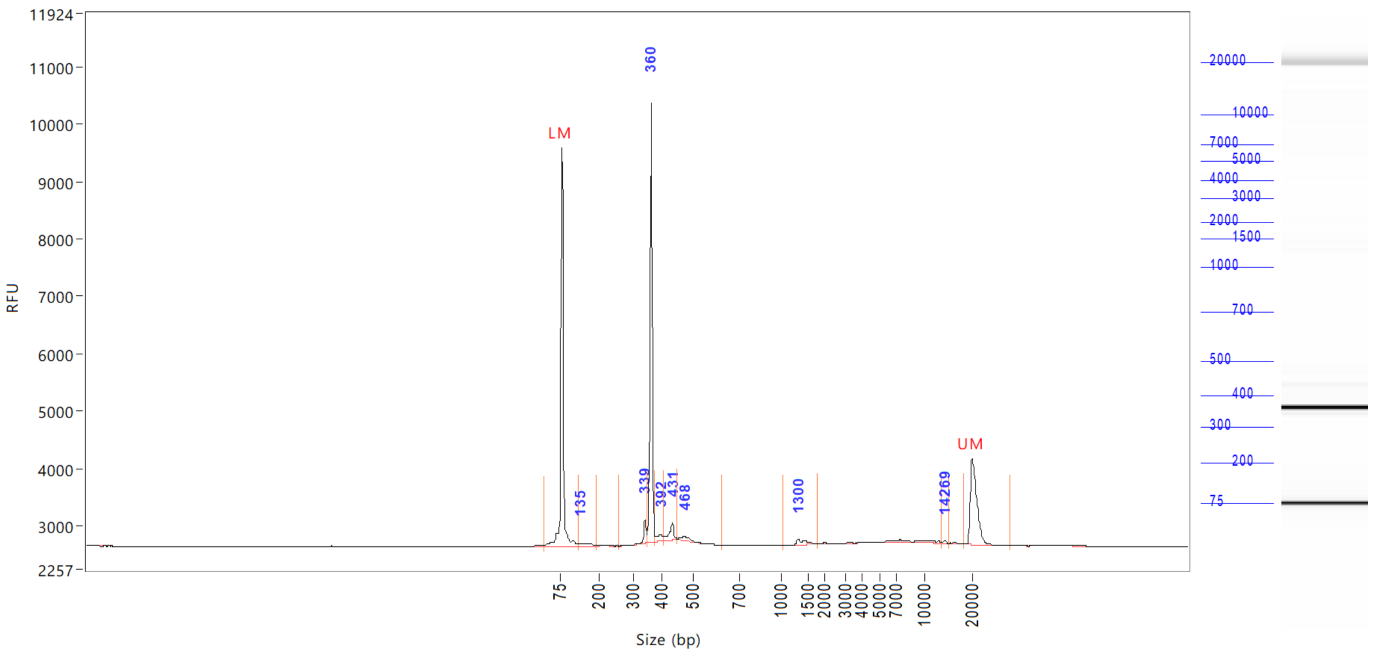

Peak	Size (bp)	Concentration (ng/uL)	From (bp)	To (bp)	Average size (bp)	CV%	RFU	Corrected peak area
1	75 (LM)	0.5000	20	129	73	14.61	6928	43.963
2	135	0.1496	129	185	153	10.75	52	1.096
3	339	0.3045	257	345	334	3.63	391	2.231
4	360	4.9104	345	374	357	1.06	7652	35.980
5	392	0.1858	374	401	387	2.10	121	1.361
6	431	0.3739	401	447	423	2.30	288	2.740
7	468	0.1952	447	618	477	5.59	82	1.430
8	1300	0.1987	1012	1789	1397	8.71	96	1.456
9	14269	0.0330	13659	15000	14173	2.10	52	0.242
10	20000 (UM)	0.1439	18171	27738	20368	4.04	1483	12.656

TIC:	6.3510	ng/uL
TIM:	28.8907	nmole/L
Total concentration:	6.7222	ng/uL

```
Sample peak width (sec): 5    Sample min peak height: 50    Sample baseline V to V?: Y    Sample baseline V to V points: 3
Sample filter: Binomial       Number of points for filter: 3  Sample start region (min): 0  Sample end region (min): 30
Marker peak width (sec): 5    Marker min peak height: 200   Marker baseline V to V?: Y    Marker baseline V to V points: 3
Lower marker selection: First peak > 500 RFU                Upper marker selection: Last peak > 200 RFU
Ladder size (bp)75, 200, 300, 400, 500, 700, 1000, 1500, 2000, 3000, 4000, 5000, 7000, 10000, 20000
Quantification using: Lower Marker          Final concentration (ng/uL): 0.5000          Dilution factor: 12.0
```

Sample: SampF5
Well location: F5
Created: Tuesday, August 10, 2021 8:01:08 PM

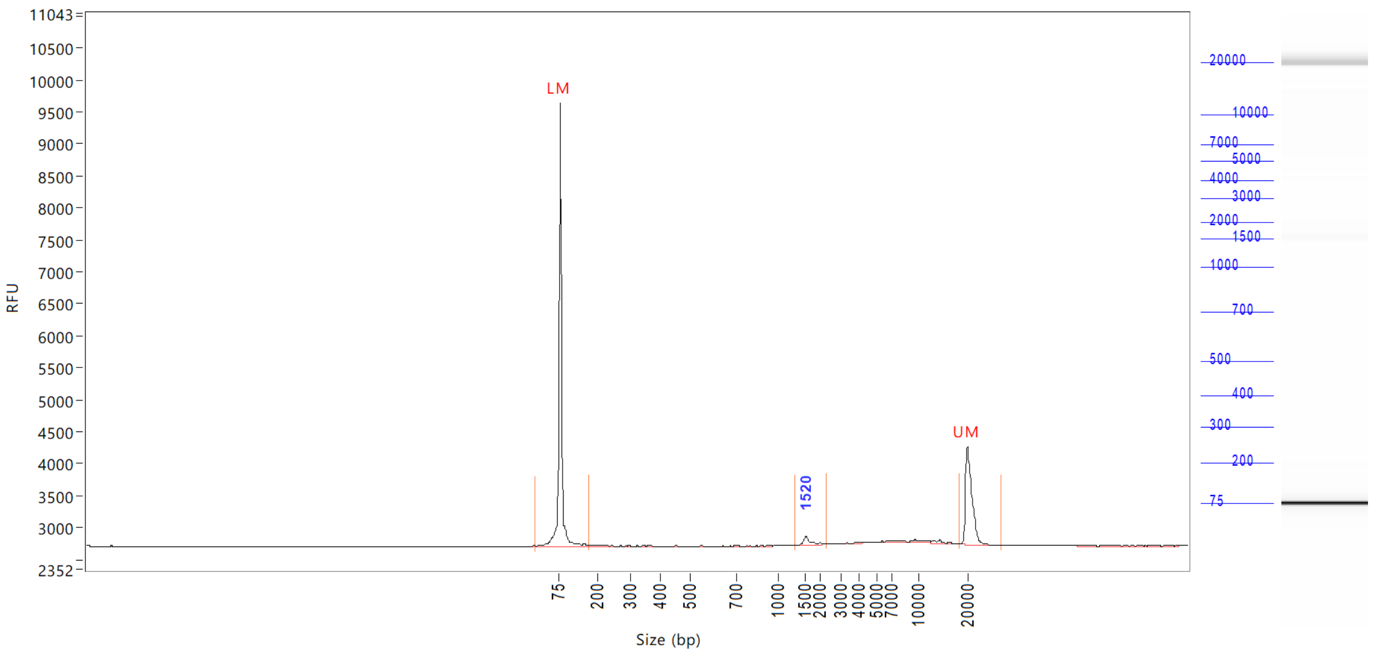

Peak	Size (bp)	Concentration (ng/uL)	From (bp)	To (bp)	Average size (bp)	CV%	RFU	Corrected peak area
1	75 (LM)	0.5000	0	168	74	19.43	6943	46.556
2	1520	0.1923	1311	2286	1621	11.60	142	1.492
3	20000 (UM)	0.1423	18171	26891	20371	4.05	1525	13.245

TIC:	0.1923	ng/uL
TIM:	0.1952	nmole/L
Total concentration:	0.7588	ng/uL

Sample peak width (sec): 5   Sample min peak height: 50   Sample baseline V to V?: Y   Sample baseline V to V points: 3
Sample filter: Binomial   Number of points for filter: 3 Sample start region (min): 0 Sample end region (min): 30
Marker peak width (sec): 5   Marker min peak height: 200   Marker baseline V to V?: Y   Marker baseline V to V points: 3
Lower marker selection: First peak > 500 RFU   Upper marker selection: Last peak > 200 RFU
Ladder size (bp)75, 200, 300, 400, 500, 700, 1000, 1500, 2000, 3000, 4000, 5000, 7000, 10000, 20000
Quantification using: Lower Marker   Final concentration (ng/uL): 0.5000   Dilution factor: 12.0

Sample: SampG5
Well location: G5
Created: Tuesday, August 10, 2021 8:01:08 PM

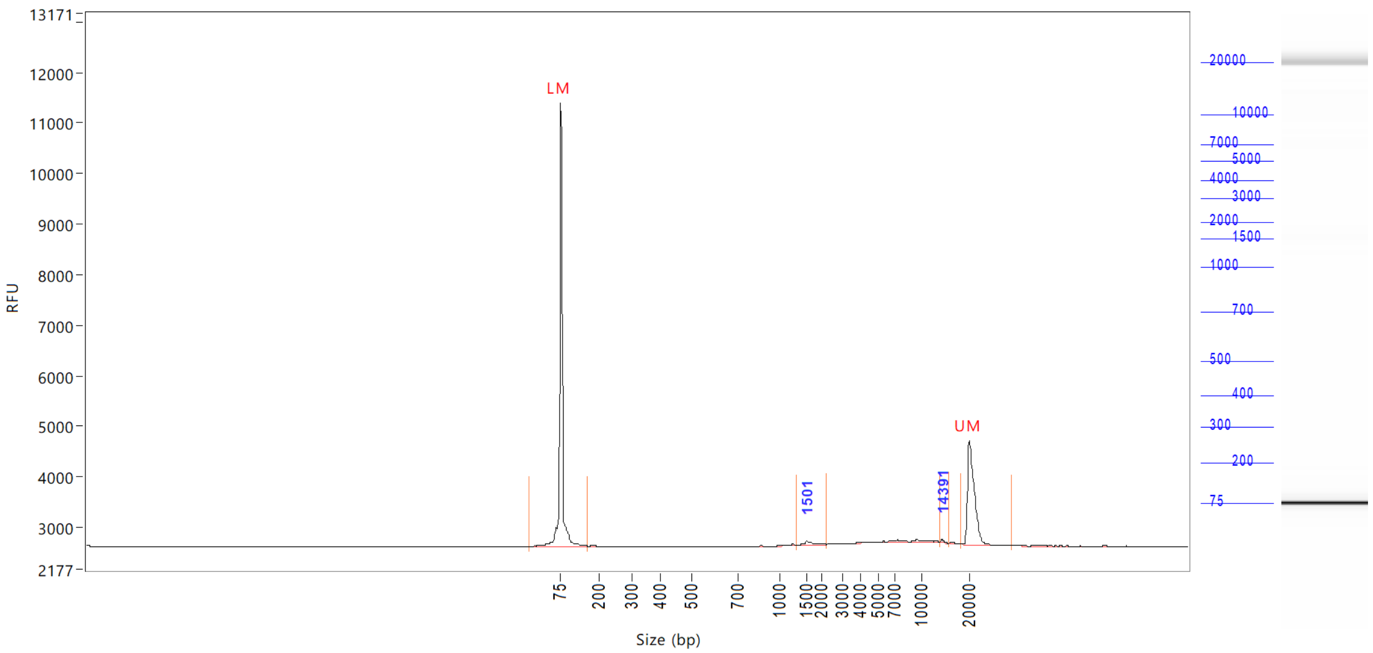

Peak	Size (bp)	Concentration (ng/uL)	From (bp)	To (bp)	Average size (bp)	CV%	RFU	Corrected peak area
1	75 (LM)	0.5000	0	164	74	19.28	8791	59.828
2	1501	0.1132	1334	2201	1608	10.44	83	1.128
3	14391	0.0273	13781	15610	14262	2.00	64	0.272
4	20000 (UM)	0.1531	18293	28584	20472	4.27	2064	18.321

TIC:	0.1404	ng/uL
TIM:	0.1190	nmole/L
Total concentration:	0.5573	ng/uL

Sample peak width (sec): 5   Sample min peak height: 50   Sample baseline V to V?: Y   Sample baseline V to V points: 3
Sample filter: Binomial   Number of points for filter: 3 Sample start region (min): 0 Sample end region (min): 30
Marker peak width (sec): 5   Marker min peak height: 200   Marker baseline V to V?: Y   Marker baseline V to V points: 3
Lower marker selection: First peak > 500 RFU   Upper marker selection: Last peak > 200 RFU
Ladder size (bp)75, 200, 300, 400, 500, 700, 1000, 1500, 2000, 3000, 4000, 5000, 7000, 10000, 20000
Quantification using: Lower Marker   Final concentration (ng/uL): 0.5000   Dilution factor: 12.0

Sample: SampH5
Well location: H5
Created: Tuesday, August 10, 2021 8:01:08 PM

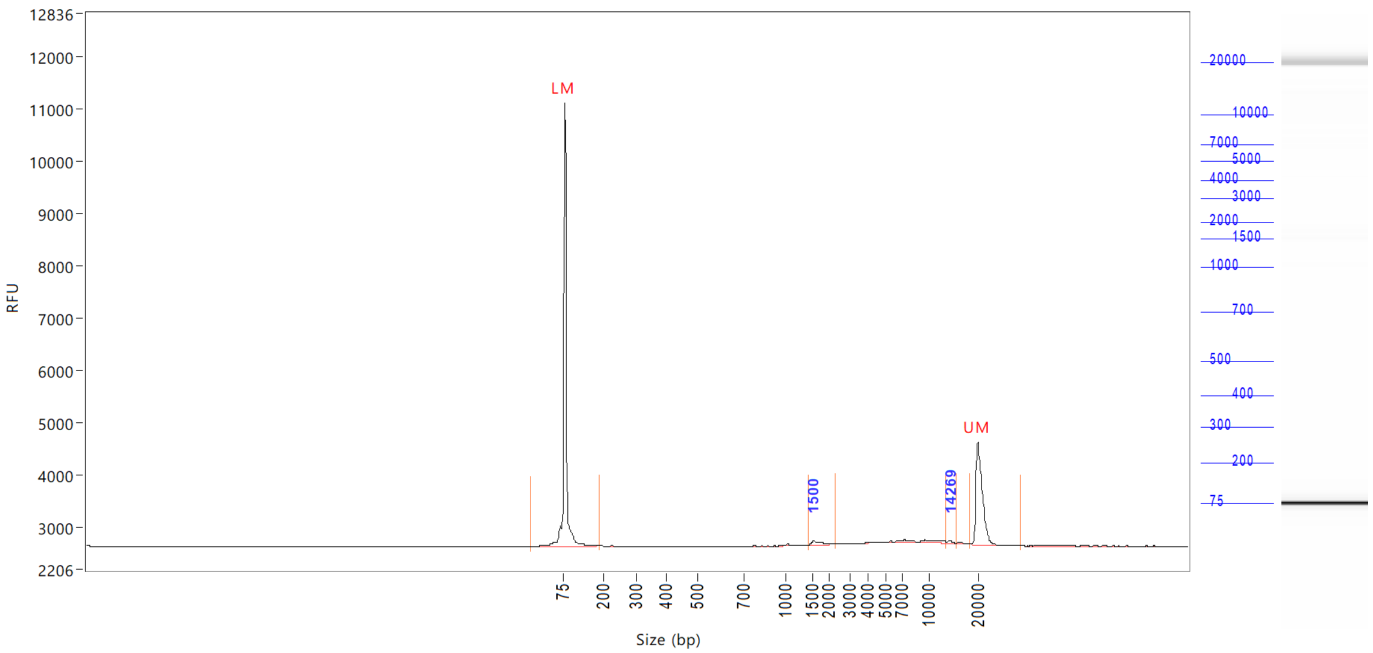

Peak	Size (bp)	Concentration (ng/uL)	From (bp)	To (bp)	Average size (bp)	CV%	RFU	Corrected peak area
1	75 (LM)	0.5000	0	187	73	17.99	8491	55.996
2	1500	0.1335	1411	2315	1647	11.07	98	1.246
3	14269	0.0398	13537	15488	14273	3.35	65	0.372
4	20000 (UM)	0.1494	18415	28947	20363	4.16	1955	16.733

TIC:	0.1734	ng/uL
TIM:	0.1381	nmole/L
Total concentration:	0.5929	ng/uL

```
Sample peak width (sec): 5    Sample min peak height: 50    Sample baseline V to V?: Y    Sample baseline V to V points: 3
Sample filter: Binomial       Number of points for filter: 3  Sample start region (min): 0  Sample end region (min): 30
Marker peak width (sec): 5    Marker min peak height: 200   Marker baseline V to V?: Y    Marker baseline V to V points: 3
Lower marker selection: First peak > 500 RFU                Upper marker selection: Last peak > 200 RFU
Ladder size (bp)75, 200, 300, 400, 500, 700, 1000, 1500, 2000, 3000, 4000, 5000, 7000, 10000, 20000
Quantification using: Lower Marker          Final concentration (ng/uL): 0.5000          Dilution factor: 12.0
```

Sample: SampA6
Well location: A6
Created: Tuesday, August 10, 2021 8:01:08 PM

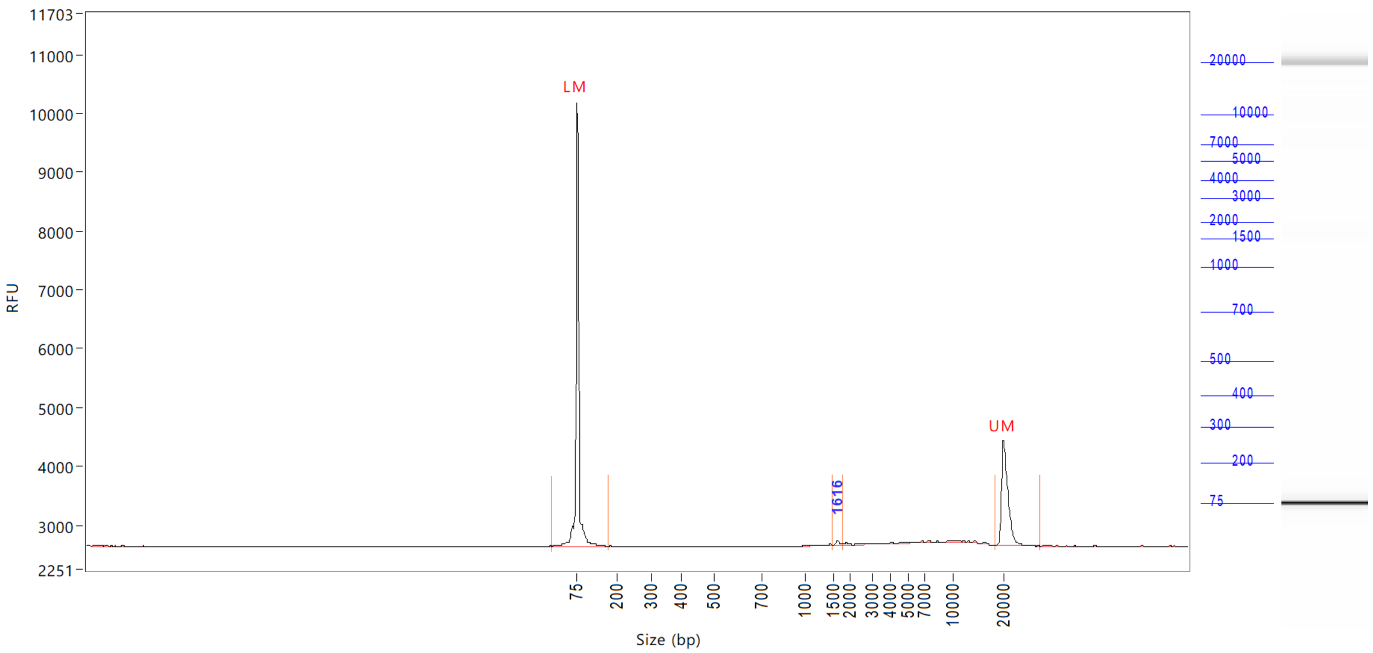

Peak	Size<br>(bp)	Concentration<br>(ng/uL)	From<br>(bp)	To<br>(bp)	Average size<br>(bp)	CV%	RFU	Corrected peak area
1	75 (LM)	0.5000	0	173	74	16.91	7552	49.821
2	1616	0.0595	1489	1770	1609	4.29	81	0.494
3	20000 (UM)	0.1457	18415	27254	20325	3.95	1774	14.523

TIC:	0.0595	ng/uL
TIM:	0.0609	nmole/L
Total concentration:	0.4838	ng/uL

Sample peak width (sec): 5 Sample min peak height: 50 Sample baseline V to V?: Y Sample baseline V to V points: 3
Sample filter: Binomial Number of points for filter: 3 Sample start region (min): 0 Sample end region (min): 30
Marker peak width (sec): 5 Marker min peak height: 200 Marker baseline V to V?: Y Marker baseline V to V points: 3
Lower marker selection: First peak > 500 RFU Upper marker selection: Last peak > 200 RFU
Ladder size (bp)75, 200, 300, 400, 500, 700, 1000, 1500, 2000, 3000, 4000, 5000, 7000, 10000, 20000
Quantification using: Lower Marker Final concentration (ng/uL): 0.5000 Dilution factor: 12.0

Sample: SampB6
Well location: B6
Created: Tuesday, August 10, 2021 8:01:08 PM

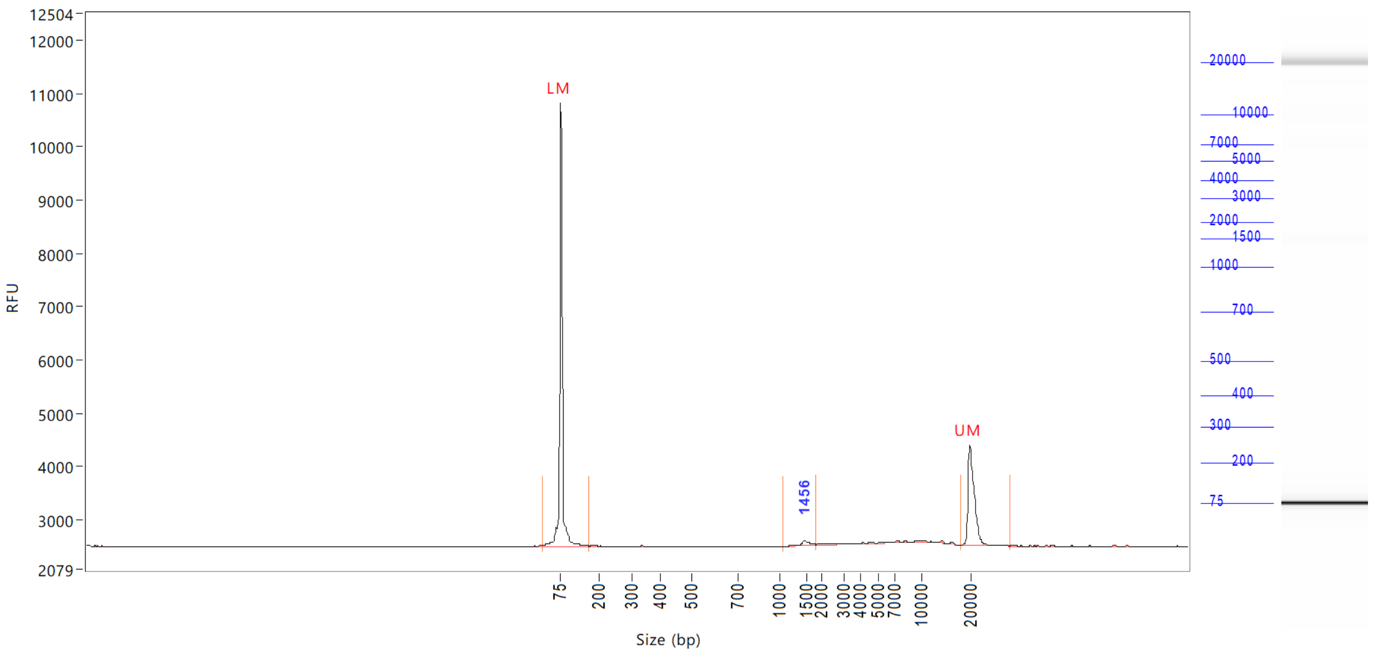

Peak	Size (bp)	Concentration (ng/uL)	From (bp)	To (bp)	Average size (bp)	CV%	RFU	Corrected peak area
1	75 (LM)	0.5000	20	168	75	17.34	8336	55.936
2	1456	0.1263	1056	1808	1474	8.58	88	1.178
3	20000 (UM)	0.1360	18171	28221	20380	4.31	1861	15.212

TIC:	0.1263	ng/uL
TIM:	0.1411	nmole/L
Total concentration:	0.5089	ng/uL

Sample peak width (sec): 5   Sample min peak height: 50   Sample baseline V to V?: Y   Sample baseline V to V points: 3
Sample filter: Binomial   Number of points for filter: 3   Sample start region (min): 0   Sample end region (min): 30
Marker peak width (sec): 5   Marker min peak height: 200   Marker baseline V to V?: Y   Marker baseline V to V points: 3
Lower marker selection: First peak > 500 RFU   Upper marker selection: Last peak > 200 RFU
Ladder size (bp)75, 200, 300, 400, 500, 700, 1000, 1500, 2000, 3000, 4000, 5000, 7000, 10000, 20000
Quantification using: Lower Marker   Final concentration (ng/uL): 0.5000   Dilution factor: 12.0

Sample: SampC6
Well location: C6
Created: Tuesday, August 10, 2021 8:01:08 PM

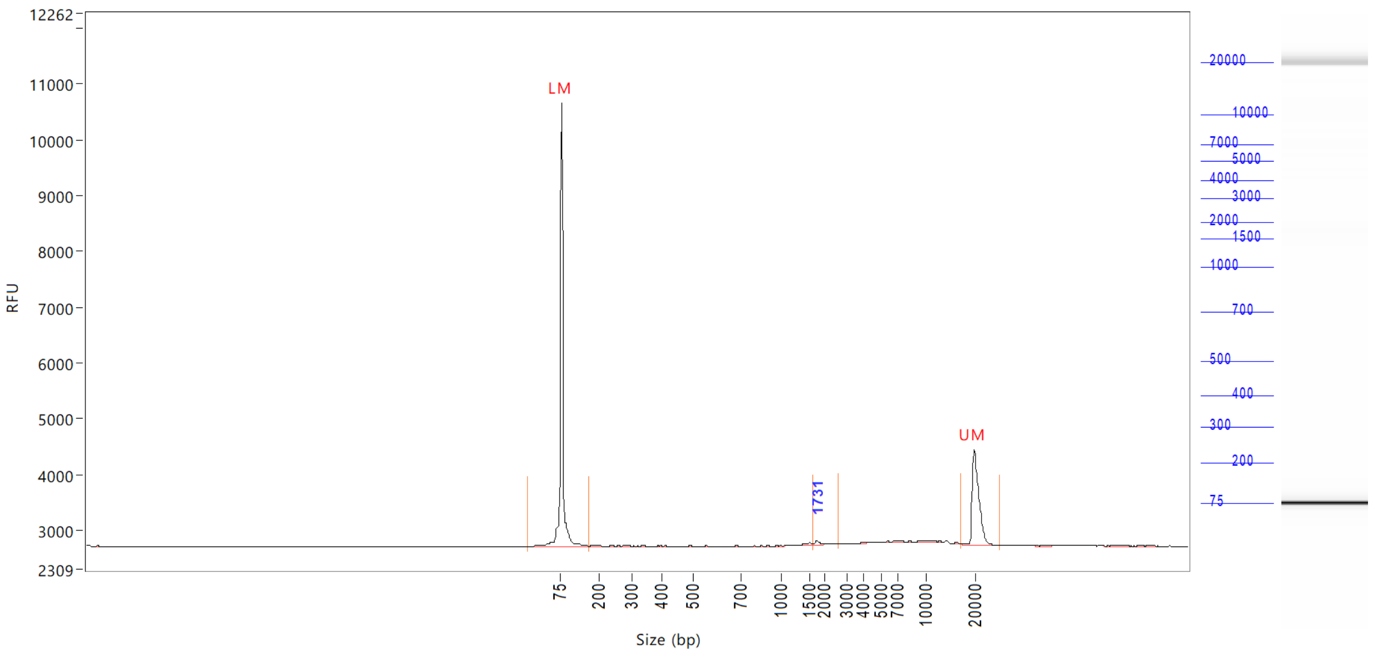

Peak	Size	Concentration	From	To	Average size	CV%	RFU	Corrected peak area
	(bp)	(ng/uL)	(bp)	(bp)	(bp)			
1	75 (LM)	0.5000	0	164	74	18.33	7954	54.207
2	1731	0.0869	1616	2600	1873	12.09	77	0.785
3	20000 (UM)	0.1350	17317	25199	20383	3.87	1698	14.638

TIC:	0.0869	ng/uL
TIM:	0.0763	nmole/L
Total concentration:	0.5158	ng/uL

Sample peak width (sec): 5   Sample min peak height: 50   Sample baseline V to V?: Y   Sample baseline V to V points: 3
Sample filter: Binomial   Number of points for filter: 3 Sample start region (min): 0 Sample end region (min): 30
Marker peak width (sec): 5   Marker min peak height: 200   Marker baseline V to V?: Y   Marker baseline V to V points: 3
Lower marker selection: First peak > 500 RFU   Upper marker selection: Last peak > 200 RFU
Ladder size (bp)75, 200, 300, 400, 500, 700, 1000, 1500, 2000, 3000, 4000, 5000, 7000, 10000, 20000
Quantification using: Lower Marker   Final concentration (ng/uL): 0.5000   Dilution factor: 12.0

Sample: SampD6
Well location: D6
Created: Tuesday, August 10, 2021 8:01:08 PM

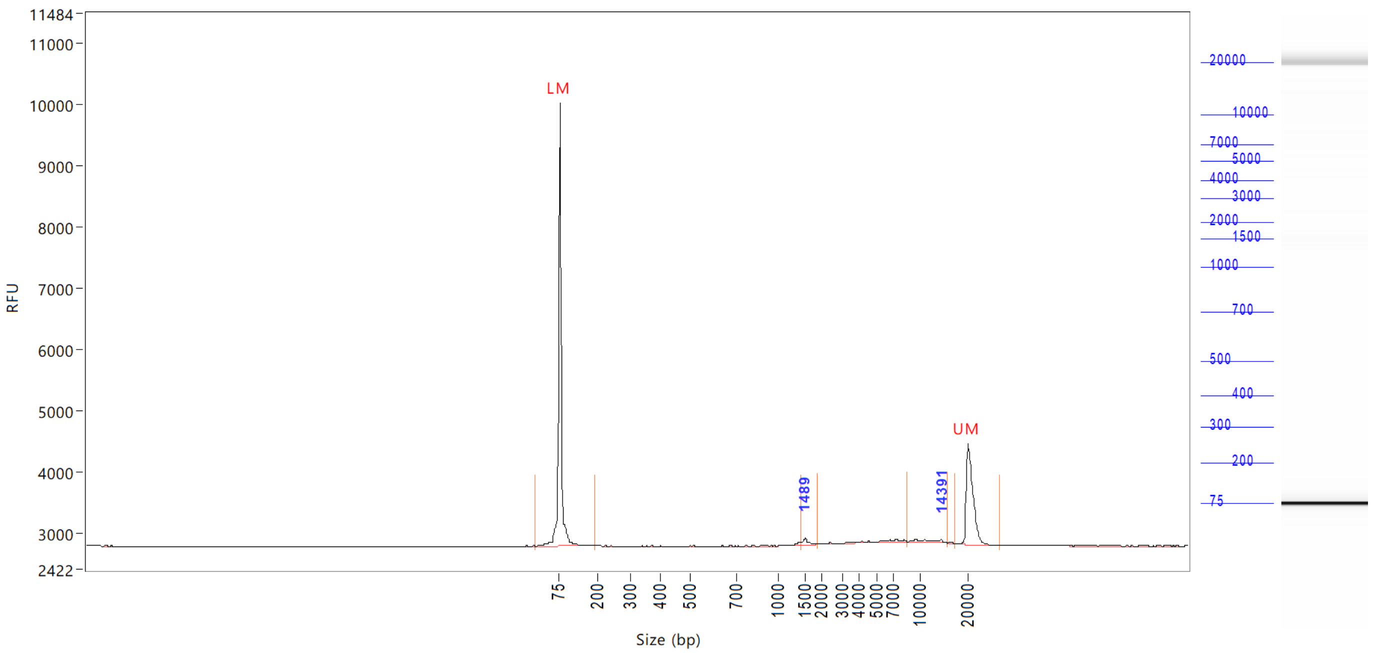

Peak	Size (bp)	Concentration (ng/uL)	From (bp)	To (bp)	Average size (bp)	CV%	RFU	Corrected peak area
1	75 (LM)	0.5000	0	190	73	16.30	7235	49.285
2	1489	0.1079	1433	1904	1543	6.75	104	0.887
3	14391	0.1553	8600	15610	11540	15.68	55	1.275
4	20000 (UM)	0.1490	17195	26529	20447	4.31	1657	14.689

TIC:	0.2632	ng/uL
TIM:	0.1373	nmole/L
Total concentration:	0.6230	ng/uL

Sample peak width (sec): 5 Sample min peak height: 50 Sample baseline V to V?: Y Sample baseline V to V points: 3
Sample filter: Binomial Number of points for filter: 3 Sample start region (min): 0 Sample end region (min): 30
Marker peak width (sec): 5 Marker min peak height: 200 Marker baseline V to V?: Y Marker baseline V to V points: 3
Lower marker selection: First peak > 500 RFU Upper marker selection: Last peak > 200 RFU
Ladder size (bp)75, 200, 300, 400, 500, 700, 1000, 1500, 2000, 3000, 4000, 5000, 7000, 10000, 20000
Quantification using: Lower Marker Final concentration (ng/uL): 0.5000 Dilution factor: 12.0

Sample: SampE6
Well location: E6
Created: Tuesday, August 10, 2021 8:01:08 PM

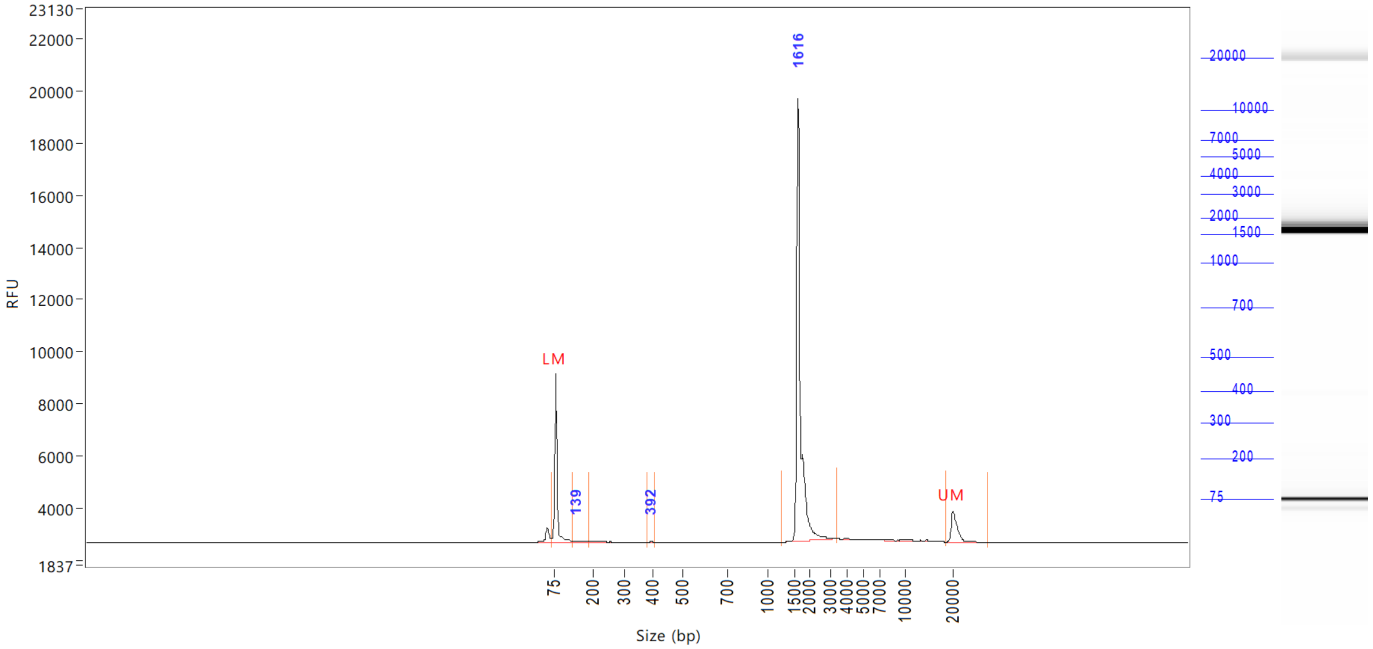

Peak	Size (bp)	Concentration (ng/uL)	From (bp)	To (bp)	Average size (bp)	CV%	RFU	Corrected peak area
1	75 (LM)	0.5000	64	131	76	12.53	6458	39.372
2	139	0.2340	131	187	155	10.27	62	1.536
3	392	0.0419	380	403	390	1.03	50	0.275
4	1616	16.2335	1245	3358	1666	10.85	16971	106.525
5	20000 (UM)	0.1225	18415	27496	20502	4.68	1153	9.643

TIC:	16.5094	ng/uL
TIM:	18.6982	nmole/L
Total concentration:	17.0795	ng/uL

Sample peak width (sec): 5 Sample min peak height: 50 Sample baseline V to V?: Y Sample baseline V to V points: 3
Sample filter: Binomial Number of points for filter: 3 Sample start region (min): 0 Sample end region (min): 30
Marker peak width (sec): 5 Marker min peak height: 200 Marker baseline V to V?: Y Marker baseline V to V points: 3
Lower marker selection: First peak > 500 RFU Upper marker selection: Last peak > 200 RFU
Ladder size (bp)75, 200, 300, 400, 500, 700, 1000, 1500, 2000, 3000, 4000, 5000, 7000, 10000, 20000
Quantification using: Lower Marker Final concentration (ng/uL): 0.5000 Dilution factor: 12.0

Sample: SampF6
Well location: F6
Created: Tuesday, August 10, 2021 8:01:08 PM

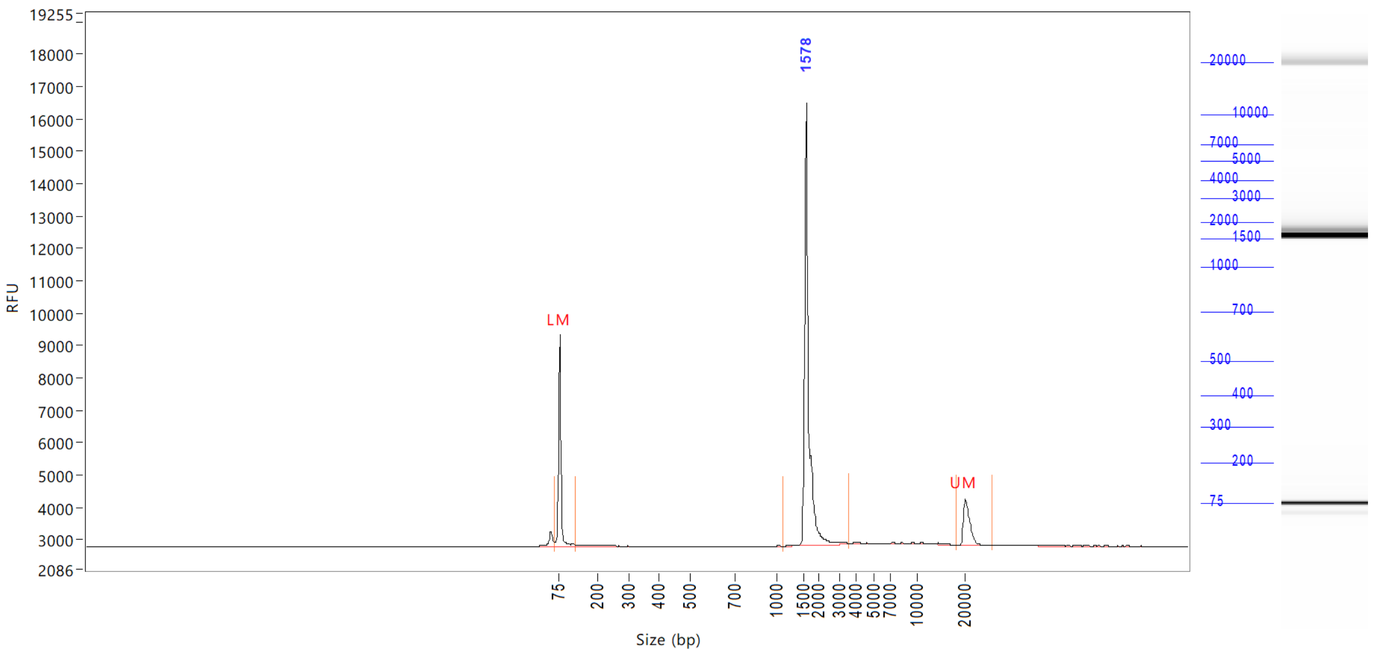

Peak	Size (bp)	Concentration (ng/uL)	From (bp)	To (bp)	Average size (bp)	CV%	RFU	Corrected peak area
1	75 (LM)	0.5000	64	131	75	11.16	6551	38.887
2	1578	13.6430	1112	3572	1657	13.81	13693	88.422
3	20000 (UM)	0.1574	18049	25682	20427	3.75	1435	12.245

TIC:	13.6430	ng/uL
TIM:	13.5533	nmole/L
Total concentration:	14.5571	ng/uL

Sample peak width (sec): 5 Sample min peak height: 50 Sample baseline V to V?: Y Sample baseline V to V points: 3
Sample filter: Binomial Number of points for filter: 3 Sample start region (min): 0 Sample end region (min): 30
Marker peak width (sec): 5 Marker min peak height: 200 Marker baseline V to V?: Y Marker baseline V to V points: 3
Lower marker selection: First peak > 500 RFU Upper marker selection: Last peak > 200 RFU
Ladder size (bp)75, 200, 300, 400, 500, 700, 1000, 1500, 2000, 3000, 4000, 5000, 7000, 10000, 20000
Quantification using: Lower Marker Final concentration (ng/uL): 0.5000 Dilution factor: 12.0

Sample: SampG6
Well location: G6
Created: Tuesday, August 10, 2021 8:01:08 PM

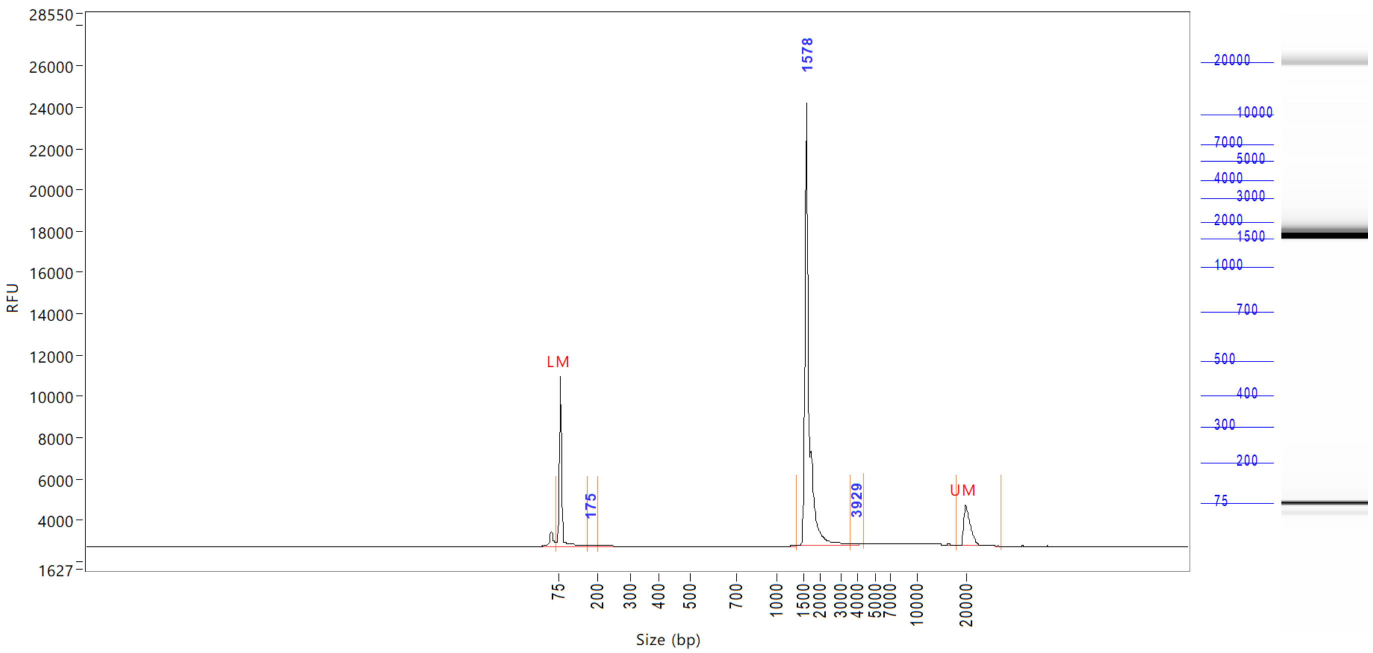

Peak	Size (bp)	Concentration (ng/uL)	From (bp)	To (bp)	Average size (bp)	CV%	RFU	Corrected peak area
1	75 (LM)	0.5000	64	166	78	19.04	8277	51.438
2	175	0.1067	166	198	181	5.20	52	0.915
3	1578	16.5112	1356	3572	1652	13.99	21481	141.551
4	3929	0.1061	3572	4380	3933	5.44	61	0.910
5	20000 (UM)	0.1689	18171	27254	20430	4.19	1983	17.374

TIC:	16.7241	ng/uL
TIM:	17.4661	nmole/L
Total concentration:	17.1818	ng/uL

```
Sample peak width (sec): 5    Sample min peak height: 50    Sample baseline V to V?: Y    Sample baseline V to V points: 3
Sample filter: Binomial       Number of points for filter: 3  Sample start region (min): 0  Sample end region (min): 30
Marker peak width (sec): 5    Marker min peak height: 200   Marker baseline V to V?: Y    Marker baseline V to V points: 3
Lower marker selection: First peak > 500 RFU                Upper marker selection: Last peak > 200 RFU
Ladder size (bp)75, 200, 300, 400, 500, 700, 1000, 1500, 2000, 3000, 4000, 5000, 7000, 10000, 20000
Quantification using: Lower Marker          Final concentration (ng/uL): 0.5000          Dilution factor: 12.0
```

Sample: SampH6
Well location: H6
Created: Tuesday, August 10, 2021 8:01:08 PM

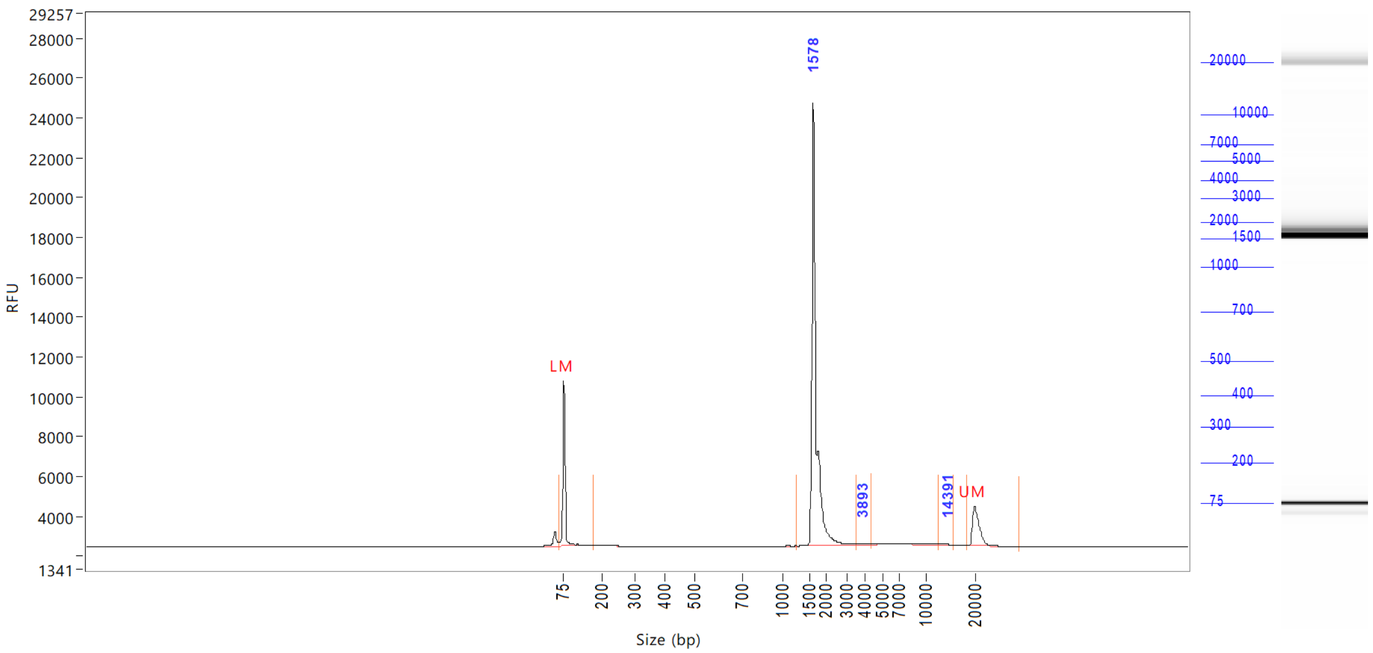

Peak	Size (bp)	Concentration (ng/uL)	From (bp)	To (bp)	Average size (bp)	CV%	RFU	Corrected peak area
1	75 (LM)	0.5000	64	173	76	13.68	8309	48.353
2	1578	18.0384	1256	3536	1661	13.18	22273	145.368
3	3893	0.1103	3536	4380	3909	5.48	60	0.889
4	14391	0.0663	12684	15610	13745	4.46	66	0.535
5	20000 (UM)	0.1783	18293	29189	20488	4.28	1993	17.241

TIC:	18.2150	ng/uL
TIM:	17.9310	nmole/L
Total concentration:	18.6091	ng/uL

Sample peak width (sec): 5   Sample min peak height: 50   Sample baseline V to V?: Y   Sample baseline V to V points: 3
Sample filter: Binomial   Number of points for filter: 3   Sample start region (min): 0   Sample end region (min): 30
Marker peak width (sec): 5   Marker min peak height: 200   Marker baseline V to V?: Y   Marker baseline V to V points: 3
Lower marker selection: First peak > 500 RFU   Upper marker selection: Last peak > 200 RFU
Ladder size (bp)75, 200, 300, 400, 500, 700, 1000, 1500, 2000, 3000, 4000, 5000, 7000, 10000, 20000
Quantification using: Lower Marker   Final concentration (ng/uL): 0.5000   Dilution factor: 12.0

Sample: SampA7
Well location: A7
Created: Tuesday, August 10, 2021 8:01:08 PM

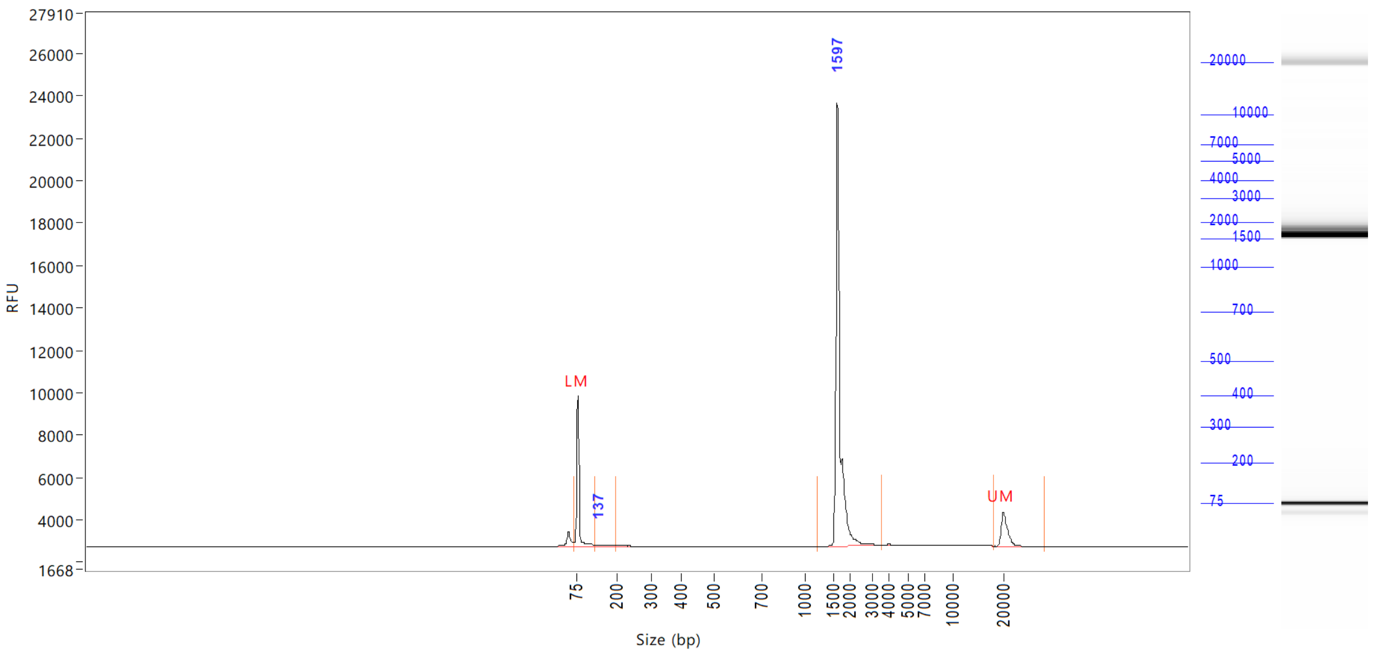

Peak	Size (bp)	Concentration (ng/uL)	From (bp)	To (bp)	Average size (bp)	CV%	RFU	Corrected peak area
1	75 (LM)	0.5000	64	129	75	12.33	7106	42.118
2	137	0.2909	129	194	157	11.67	73	2.042
3	1597	18.6802	1212	3608	1670	11.96	20956	131.129
4	20000 (UM)	0.1540	18171	28100	20389	4.04	1625	12.975

TIC:	18.9711	ng/uL
TIM:	21.4579	nmole/L
Total concentration:	19.4980	ng/uL

Sample peak width (sec): 5 Sample min peak height: 50 Sample baseline V to V?: Y Sample baseline V to V points: 3
Sample filter: Binomial Number of points for filter: 3 Sample start region (min): 0 Sample end region (min): 30
Marker peak width (sec): 5 Marker min peak height: 200 Marker baseline V to V?: Y Marker baseline V to V points: 3
Lower marker selection: First peak > 500 RFU Upper marker selection: Last peak > 200 RFU
Ladder size (bp)75, 200, 300, 400, 500, 700, 1000, 1500, 2000, 3000, 4000, 5000, 7000, 10000, 20000
Quantification using: Lower Marker Final concentration (ng/uL): 0.5000 Dilution factor: 12.0

Sample: SampB7
Well location: B7
Created: Tuesday, August 10, 2021 8:01:08 PM

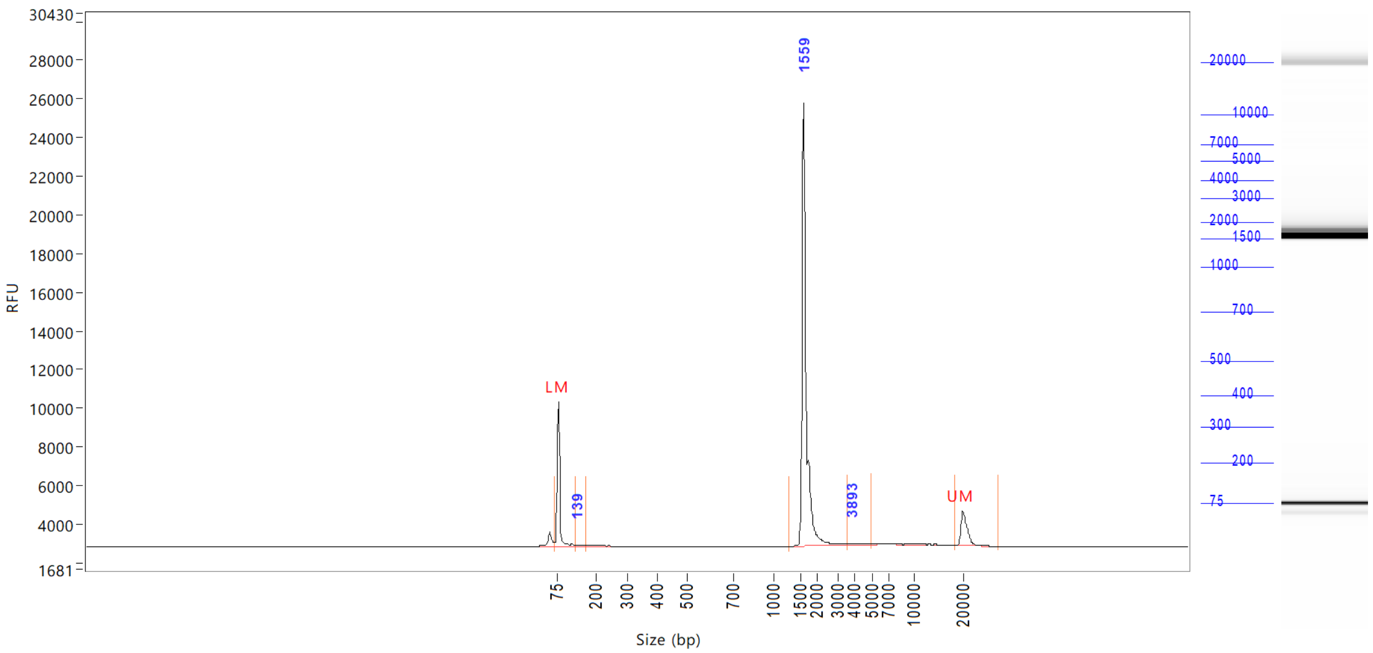

Peak	Size (bp)	Concentration (ng/uL)	From (bp)	To (bp)	Average size (bp)	CV%	RFU	Corrected peak area
1	75 (LM)	0.5000	64	131	76	12.38	7518	45.853
2	139	0.1808	131	168	148	6.95	75	1.382
3	1559	18.9740	1278	3536	1633	13.38	22953	145.004
4	3893	0.1767	3536	4931	4049	8.56	70	1.351
5	20000 (UM)	0.1640	18293	27496	20431	4.16	1790	15.043

TIC:	19.3315	ng/uL
TIM:	21.2059	nmole/L
Total concentration:	19.8547	ng/uL

Sample peak width (sec): 5 Sample min peak height: 50 Sample baseline V to V?: Y Sample baseline V to V points: 3
Sample filter: Binomial Number of points for filter: 3 Sample start region (min): 0 Sample end region (min): 30
Marker peak width (sec): 5 Marker min peak height: 200 Marker baseline V to V?: Y Marker baseline V to V points: 3
Lower marker selection: First peak > 500 RFU Upper marker selection: Last peak > 200 RFU
Ladder size (bp)75, 200, 300, 400, 500, 700, 1000, 1500, 2000, 3000, 4000, 5000, 7000, 10000, 20000
Quantification using: Lower Marker Final concentration (ng/uL): 0.5000 Dilution factor: 12.0

Sample: SampC7
Well location: C7
Created: Tuesday, August 10, 2021 8:01:08 PM

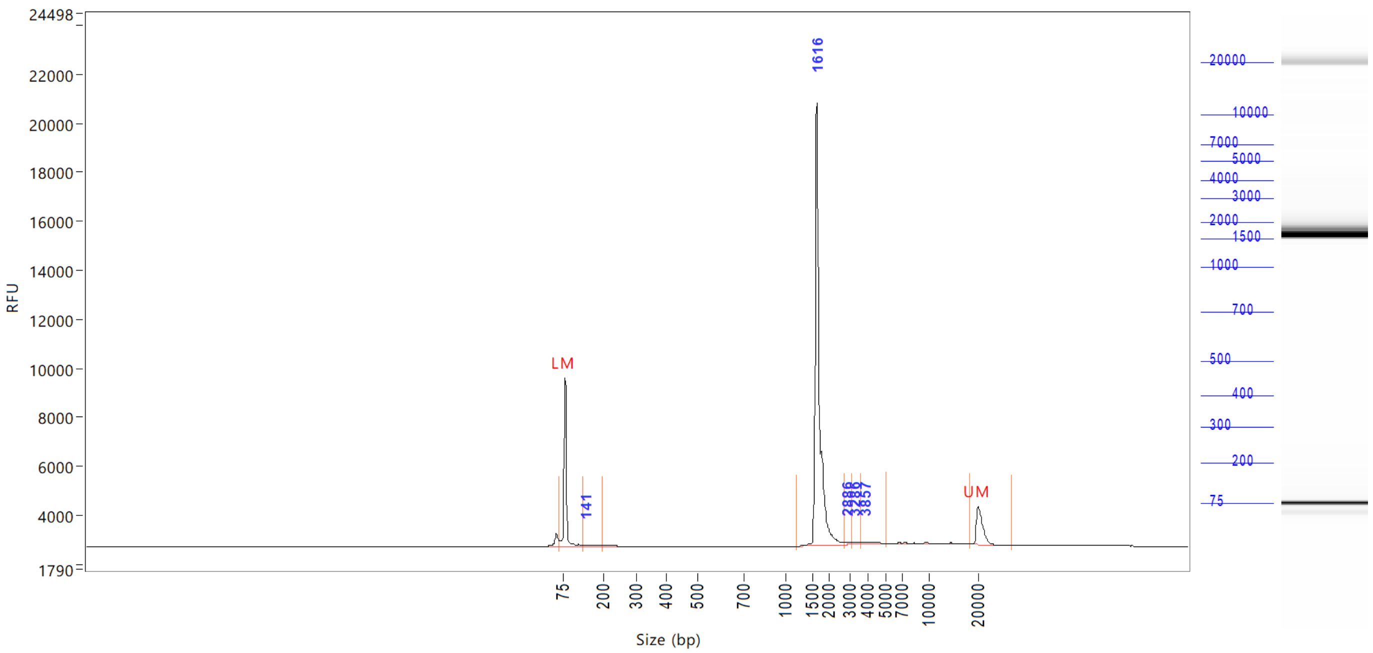

Peak	Size (bp)	Concentration (ng/uL)	From (bp)	To (bp)	Average size (bp)	CV%	RFU	Corrected peak area
1	75 (LM)	0.5000	58	131	75	11.58	6899	41.881
2	141	0.2612	131	196	161	11.56	63	1.823
3	1616	16.8151	1212	2715	1658	9.47	18109	117.373
4	2886	0.1387	2715	3108	2907	4.02	92	0.968
5	3286	0.1357	3108	3608	3351	4.55	82	0.947
6	3857	0.1956	3608	5075	4037	7.54	78	1.365
7	20000 (UM)	0.1534	18415	26891	20419	3.74	1551	12.846

TIC:	17.5462	ng/uL
TIM:	19.5858	nmole/L
Total concentration:	17.9521	ng/uL

Sample peak width (sec): 5 Sample min peak height: 50 Sample baseline V to V?: Y Sample baseline V to V points: 3
Sample filter: Binomial Number of points for filter: 3 Sample start region (min): 0 Sample end region (min): 30
Marker peak width (sec): 5 Marker min peak height: 200 Marker baseline V to V?: Y Marker baseline V to V points: 3
Lower marker selection: First peak > 500 RFU Upper marker selection: Last peak > 200 RFU
Ladder size (bp)75, 200, 300, 400, 500, 700, 1000, 1500, 2000, 3000, 4000, 5000, 7000, 10000, 20000
Quantification using: Lower Marker Final concentration (ng/uL): 0.5000 Dilution factor: 12.0

Sample: SampD7
Well location: D7
Created: Tuesday, August 10, 2021 8:01:08 PM

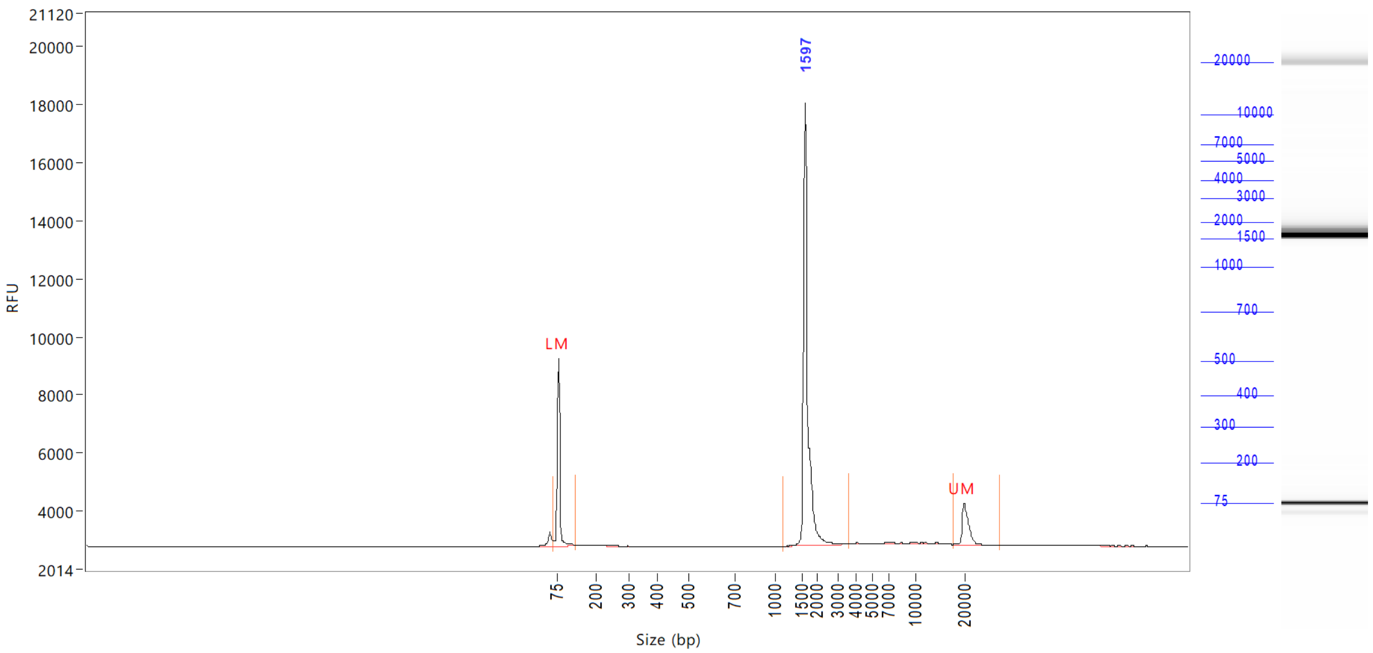

Peak	Size (bp)	Concentration (ng/uL)	From (bp)	To (bp)	Average size (bp)	CV%	RFU	Corrected peak area
1	75 (LM)	0.5000	62	131	75	10.16	6427	39.027
2	1597	15.5215	1134	3608	1649	12.01	15240	100.960
3	20000 (UM)	0.1570	17683	27496	20464	4.30	1425	12.255

TIC:	15.5215	ng/uL
TIM:	15.4943	nmole/L
Total concentration:	16.2727	ng/uL

```
Sample peak width (sec): 5    Sample min peak height: 50    Sample baseline V to V?: Y    Sample baseline V to V points: 3
Sample filter: Binomial       Number of points for filter: 3  Sample start region (min): 0  Sample end region (min): 30
Marker peak width (sec): 5    Marker min peak height: 200   Marker baseline V to V?: Y    Marker baseline V to V points: 3
Lower marker selection: First peak > 500 RFU                Upper marker selection: Last peak > 200 RFU
Ladder size (bp)75, 200, 300, 400, 500, 700, 1000, 1500, 2000, 3000, 4000, 5000, 7000, 10000, 20000
Quantification using: Lower Marker          Final concentration (ng/uL): 0.5000          Dilution factor: 12.0
```

Sample: SampE7
Well location: E7
Created: Tuesday, August 10, 2021 8:01:08 PM

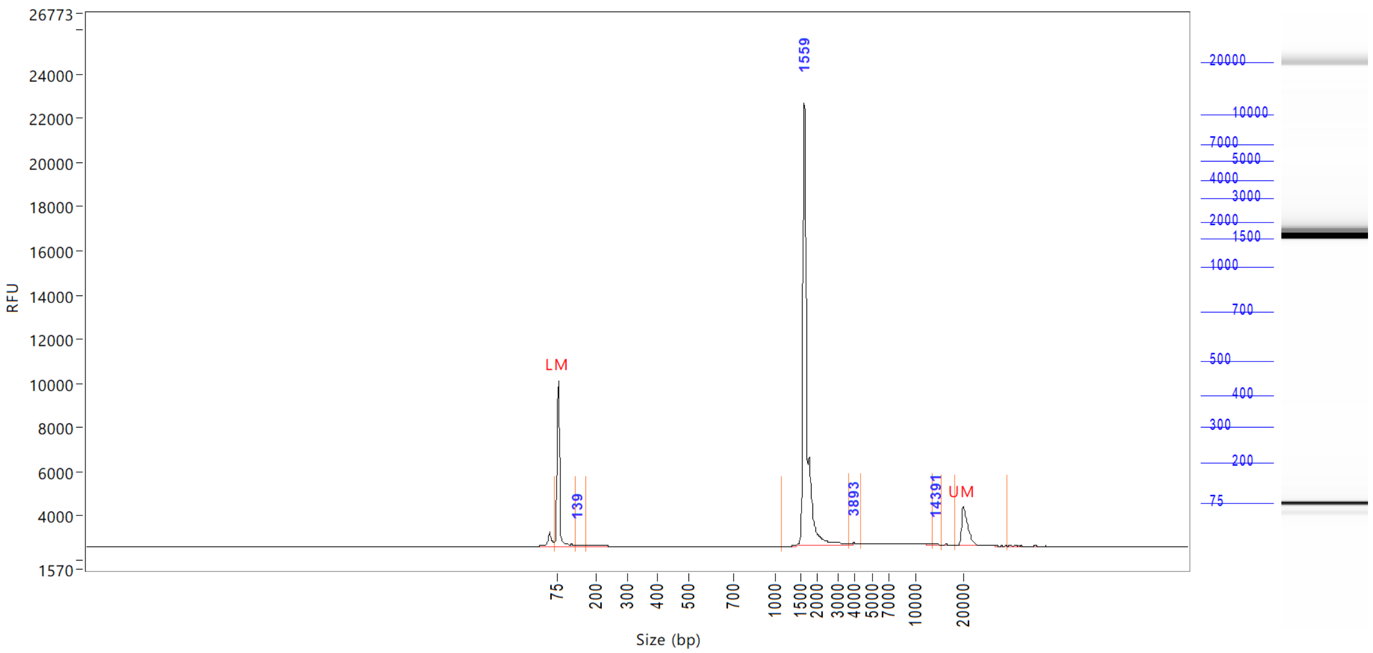

Peak	Size (bp)	Concentration (ng/uL)	From (bp)	To (bp)	Average size (bp)	CV%	RFU	Corrected peak area
1	75 (LM)	0.5000	64	131	75	11.49	7500	44.422
2	139	0.1372	131	166	147	6.60	61	1.016
3	1559	17.5993	1123	3679	1645	14.43	20112	130.298
4	3893	0.1274	3679	4380	3988	4.75	69	0.943
5	14391	0.0372	13537	15366	14155	2.31	56	0.275
6	20000 (UM)	0.1659	18171	29068	20455	4.34	1736	14.741

TIC:	17.9011	ng/uL
TIM:	19.2019	nmole/L
Total concentration:	18.4739	ng/uL

```
Sample peak width (sec): 5    Sample min peak height: 50    Sample baseline V to V?: Y    Sample baseline V to V points: 3
Sample filter: Binomial       Number of points for filter: 3  Sample start region (min): 0  Sample end region (min): 30
Marker peak width (sec): 5    Marker min peak height: 200   Marker baseline V to V?: Y    Marker baseline V to V points: 3
Lower marker selection: First peak > 500 RFU                Upper marker selection: Last peak > 200 RFU
Ladder size (bp)75, 200, 300, 400, 500, 700, 1000, 1500, 2000, 3000, 4000, 5000, 7000, 10000, 20000
Quantification using: Lower Marker          Final concentration (ng/uL): 0.5000          Dilution factor: 12.0
```

Sample: SampF7
Well location: F7
Created: Tuesday, August 10, 2021 8:01:08 PM

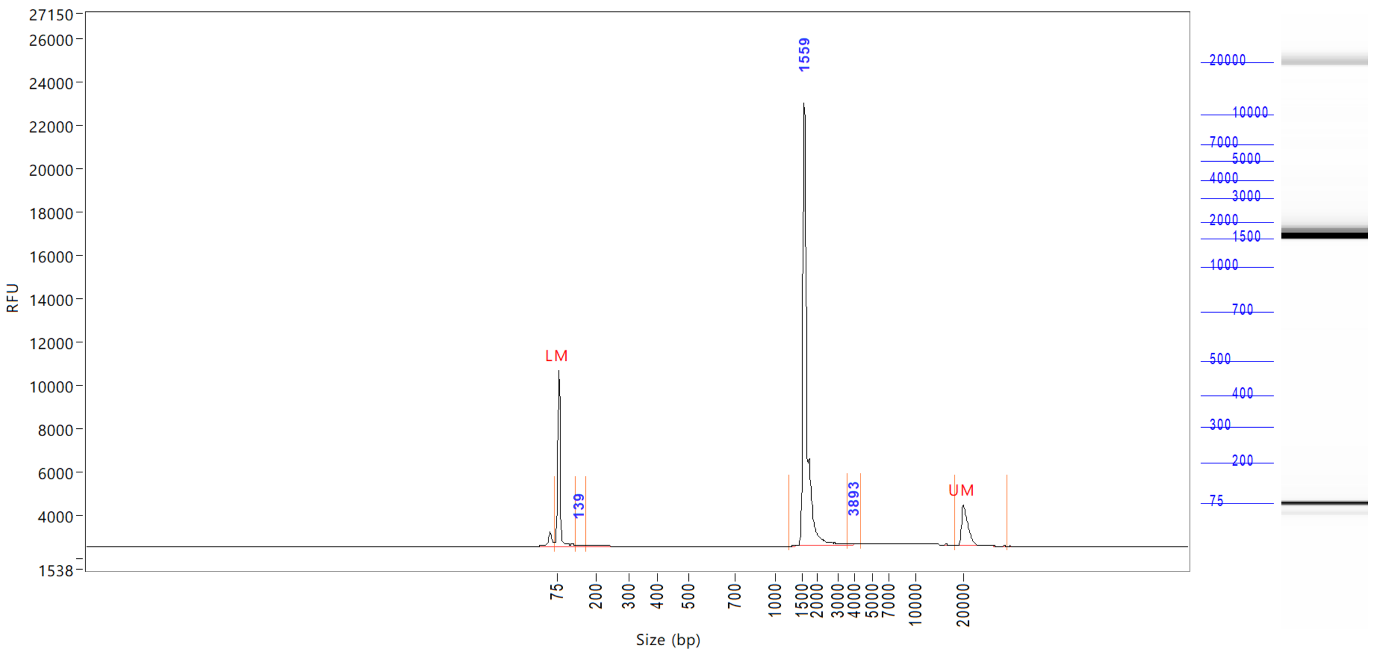

Peak	Size<br>(bp)	Concentration<br>(ng/uL)	From<br>(bp)	To<br>(bp)	Average size<br>(bp)	CV%	RFU	Corrected peak area
1	75 (LM)	0.5000	64	131	75	11.78	8144	48.088
2	139	0.1555	131	168	148	6.95	68	1.246
3	1559	16.2651	1278	3500	1636	13.23	20436	130.358
4	3893	0.1182	3500	4345	3884	5.66	61	0.948
5	20000 (UM)	0.1677	18293	29068	20459	4.22	1846	16.133

TIC:	16.5388	ng/uL
TIM:	18.1421	nmole/L
Total concentration:	17.0646	ng/uL

Sample peak width (sec): 5   Sample min peak height: 50   Sample baseline V to V?: Y   Sample baseline V to V points: 3
Sample filter: Binomial   Number of points for filter: 3 Sample start region (min): 0 Sample end region (min): 30
Marker peak width (sec): 5   Marker min peak height: 200   Marker baseline V to V?: Y   Marker baseline V to V points: 3
Lower marker selection: First peak > 500 RFU   Upper marker selection: Last peak > 200 RFU
Ladder size (bp)75, 200, 300, 400, 500, 700, 1000, 1500, 2000, 3000, 4000, 5000, 7000, 10000, 20000
Quantification using: Lower Marker   Final concentration (ng/uL): 0.5000   Dilution factor: 12.0

Sample: SampG7
Well location: G7
Created: Tuesday, August 10, 2021 8:01:08 PM

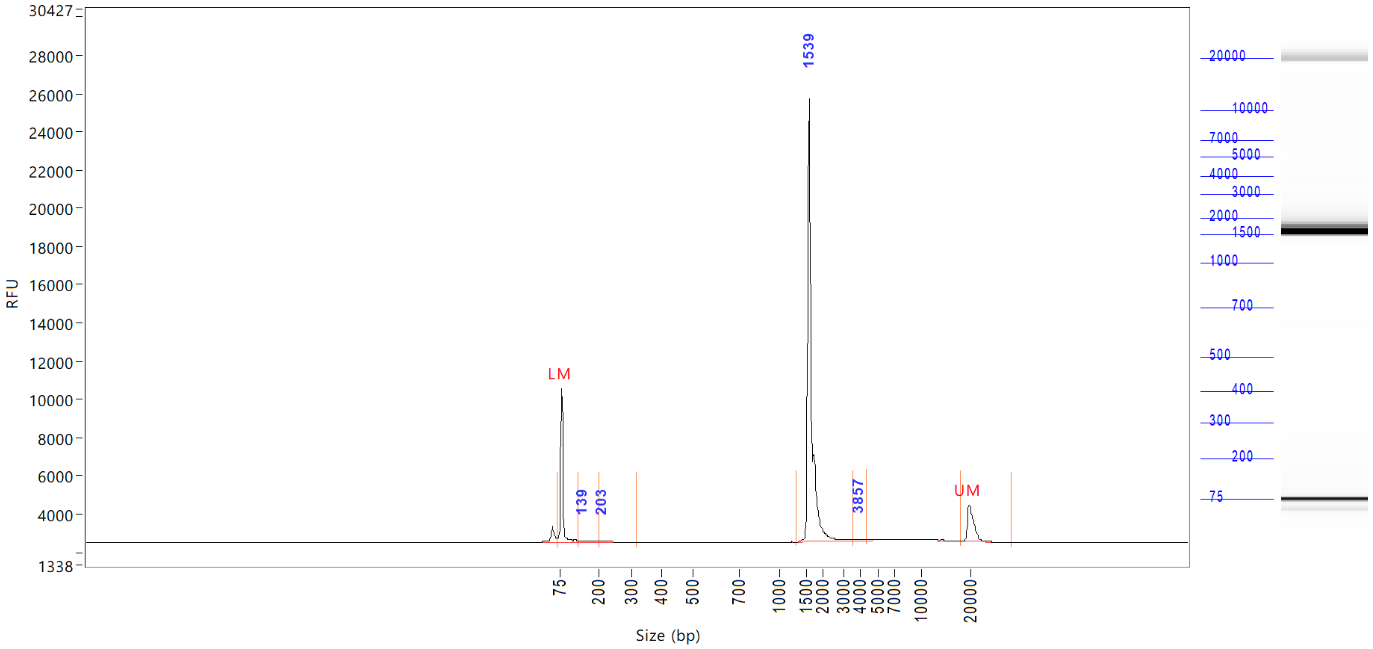

Peak	Size (bp)	Concentration (ng/uL)	From (bp)	To (bp)	Average size (bp)	CV%	RFU	Corrected peak area
1	75 (LM)	0.5000	64	129	75	12.36	8017	48.298
2	139	0.3038	129	196	158	12.22	89	2.445
3	203	0.1854	196	311	228	11.37	55	1.492
4	1539	18.5519	1289	3536	1619	12.46	23215	149.335
5	3857	0.0989	3536	4311	3885	5.15	58	0.796
6	20000 (UM)	0.1654	18171	28584	20306	3.87	1935	15.973

TIC:	19.1400	ng/uL
TIM:	23.4018	nmole/L
Total concentration:	19.4484	ng/uL

Sample peak width (sec): 5   Sample min peak height: 50   Sample baseline V to V?: Y   Sample baseline V to V points: 3
Sample filter: Binomial   Number of points for filter: 3 Sample start region (min): 0 Sample end region (min): 30
Marker peak width (sec): 5   Marker min peak height: 200   Marker baseline V to V?: Y   Marker baseline V to V points: 3
Lower marker selection: First peak > 500 RFU   Upper marker selection: Last peak > 200 RFU
Ladder size (bp)75, 200, 300, 400, 500, 700, 1000, 1500, 2000, 3000, 4000, 5000, 7000, 10000, 20000
Quantification using: Lower Marker   Final concentration (ng/uL): 0.5000   Dilution factor: 12.0

Sample: SampH7
Well location: H7
Created: Tuesday, August 10, 2021 8:01:08 PM

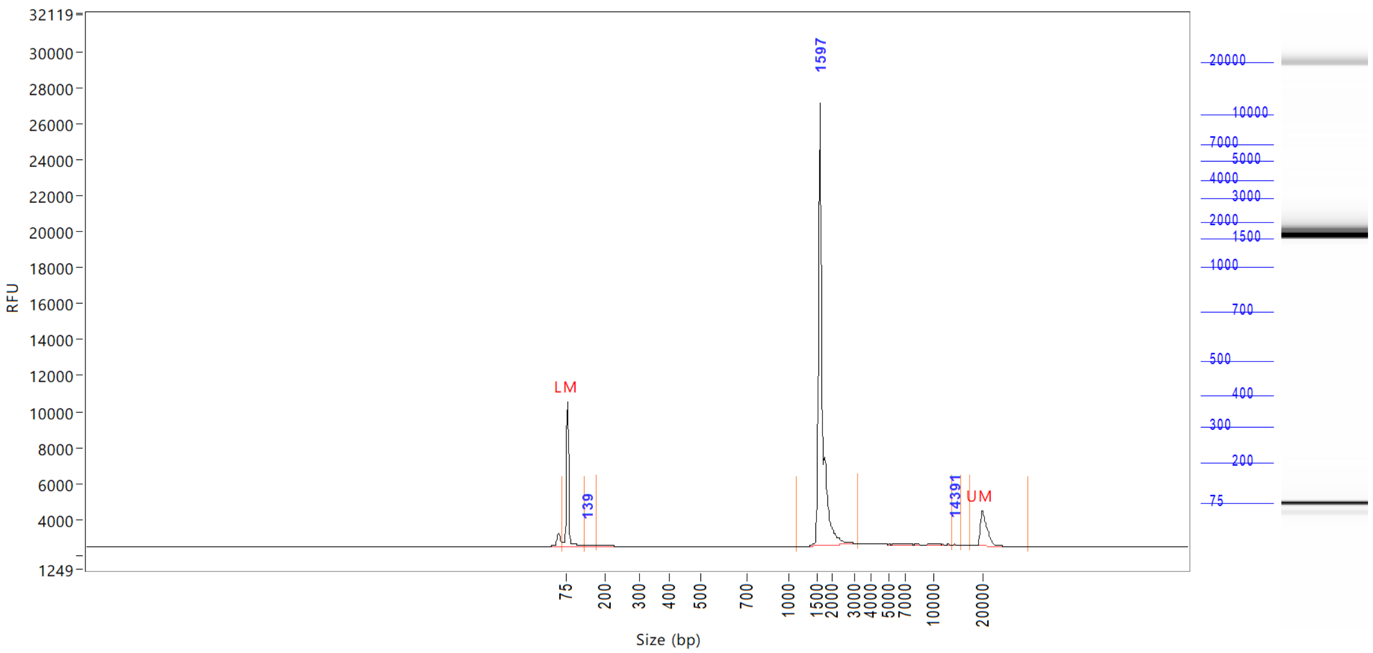

Peak	Size (bp)	Concentration (ng/uL)	From (bp)	To (bp)	Average size (bp)	CV%	RFU	Corrected peak area
1	75 (LM)	0.5000	58	131	75	11.01	8050	47.596
2	139	0.1078	131	166	145	6.23	57	0.855
3	1597	19.7449	1134	3251	1651	10.60	24622	156.631
4	14391	0.0314	13781	15610	14296	2.06	57	0.249
5	20000 (UM)	0.1771	17317	29430	20407	4.30	1983	16.860

TIC:	19.8841	ng/uL
TIM:	20.9117	nmole/L
Total concentration:	20.3442	ng/uL

Sample peak width (sec): 5   Sample min peak height: 50   Sample baseline V to V?: Y   Sample baseline V to V points: 3
Sample filter: Binomial   Number of points for filter: 3   Sample start region (min): 0   Sample end region (min): 30
Marker peak width (sec): 5   Marker min peak height: 200   Marker baseline V to V?: Y   Marker baseline V to V points: 3
Lower marker selection: First peak > 500 RFU   Upper marker selection: Last peak > 200 RFU
Ladder size (bp)75, 200, 300, 400, 500, 700, 1000, 1500, 2000, 3000, 4000, 5000, 7000, 10000, 20000
Quantification using: Lower Marker   Final concentration (ng/uL): 0.5000   Dilution factor: 12.0

Sample: SampA8
Well location: A8
Created: Tuesday, August 10, 2021 8:01:08 PM

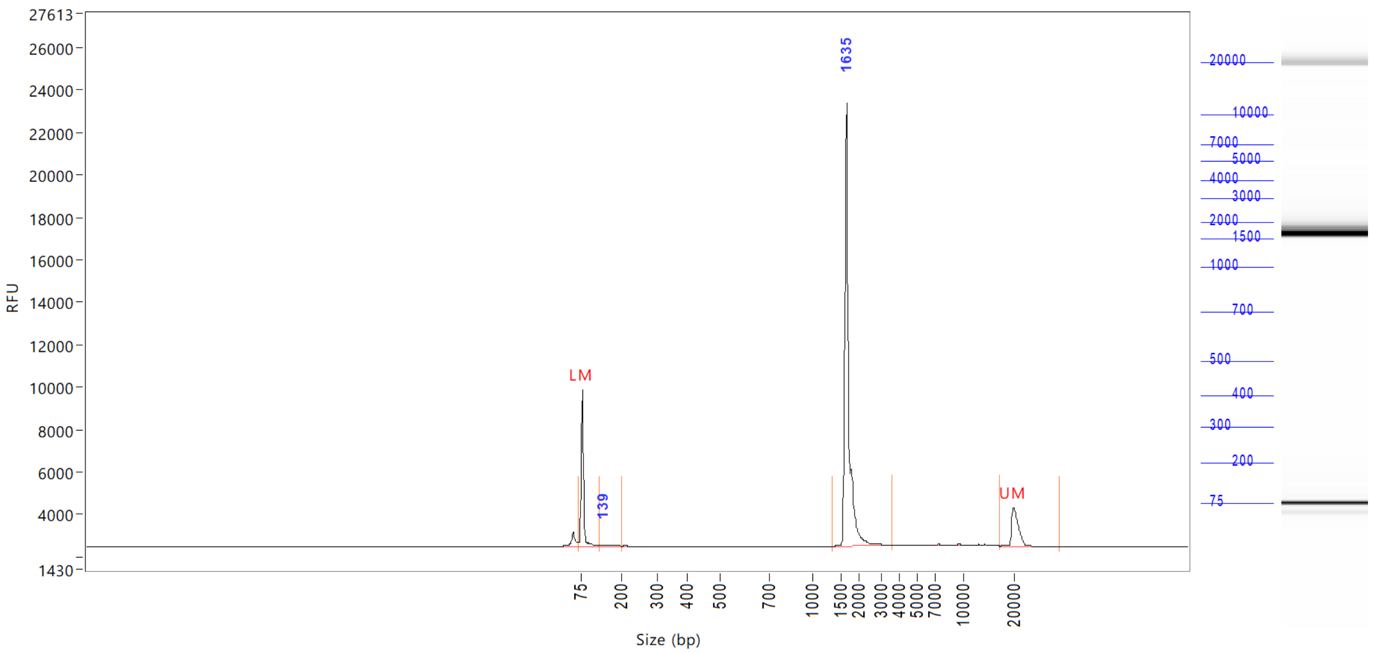

Peak	Size (bp)	Concentration (ng/uL)	From (bp)	To (bp)	Average size (bp)	CV%	RFU	Corrected peak area
1	75 (LM)	0.5000	66	131	76	11.54	7397	43.937
2	139	0.2494	131	198	158	11.62	67	1.826
3	1635	16.8656	1334	3643	1687	11.25	20906	123.503
4	20000 (UM)	0.1728	17195	28826	20314	4.09	1834	15.183

TIC:	17.1150	ng/uL
TIM:	19.0508	nmole/L
Total concentration:	17.5522	ng/uL

```
Sample peak width (sec): 5    Sample min peak height: 50    Sample baseline V to V?: Y    Sample baseline V to V points: 3
Sample filter: Binomial       Number of points for filter: 3  Sample start region (min): 0  Sample end region (min): 30
Marker peak width (sec): 5    Marker min peak height: 200   Marker baseline V to V?: Y    Marker baseline V to V points: 3
Lower marker selection: First peak > 500 RFU                Upper marker selection: Last peak > 200 RFU
Ladder size (bp)75, 200, 300, 400, 500, 700, 1000, 1500, 2000, 3000, 4000, 5000, 7000, 10000, 20000
Quantification using: Lower Marker          Final concentration (ng/uL): 0.5000          Dilution factor: 12.0
```

Sample: SampB8
Well location: B8
Created: Tuesday, August 10, 2021 8:01:08 PM

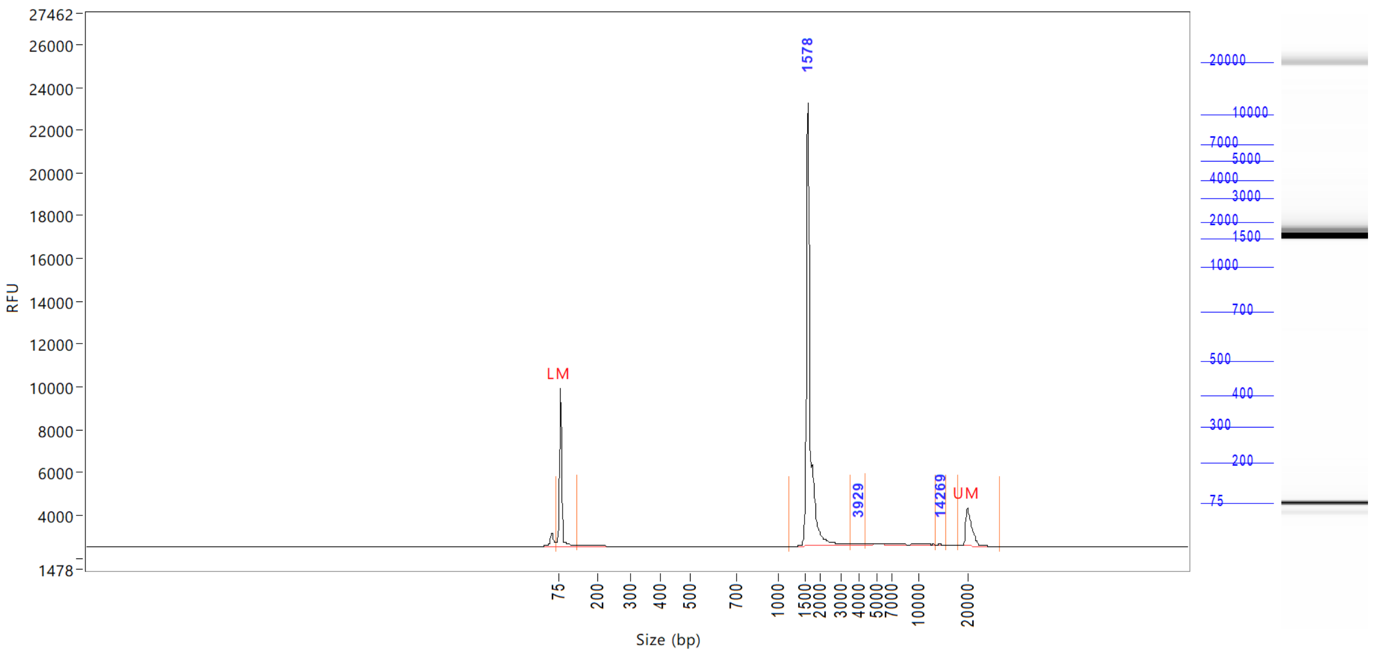

Peak	Size (bp)	Concentration (ng/uL)	From (bp)	To (bp)	Average size (bp)	CV%	RFU	Corrected peak area
1	75 (LM)	0.5000	64	131	75	10.42	7404	45.050
2	1578	17.0966	1223	3465	1645	13.09	20740	128.367
3	3929	0.1104	3465	4345	3862	5.96	53	0.829
4	14269	0.0478	13415	15610	14196	3.50	60	0.359
5	20000 (UM)	0.1613	18049	26770	20398	4.05	1760	14.536

TIC:	17.2548	ng/uL
TIM:	17.1606	nmole/L
Total concentration:	17.8291	ng/uL

```
Sample peak width (sec): 5    Sample min peak height: 50    Sample baseline V to V?: Y    Sample baseline V to V points: 3
Sample filter: Binomial       Number of points for filter: 3  Sample start region (min): 0  Sample end region (min): 30
Marker peak width (sec): 5    Marker min peak height: 200   Marker baseline V to V?: Y    Marker baseline V to V points: 3
Lower marker selection: First peak > 500 RFU                Upper marker selection: Last peak > 200 RFU
Ladder size (bp)75, 200, 300, 400, 500, 700, 1000, 1500, 2000, 3000, 4000, 5000, 7000, 10000, 20000
Quantification using: Lower Marker          Final concentration (ng/uL): 0.5000          Dilution factor: 12.0
```

Sample: SampC8
Well location: C8
Created: Tuesday, August 10, 2021 8:01:08 PM

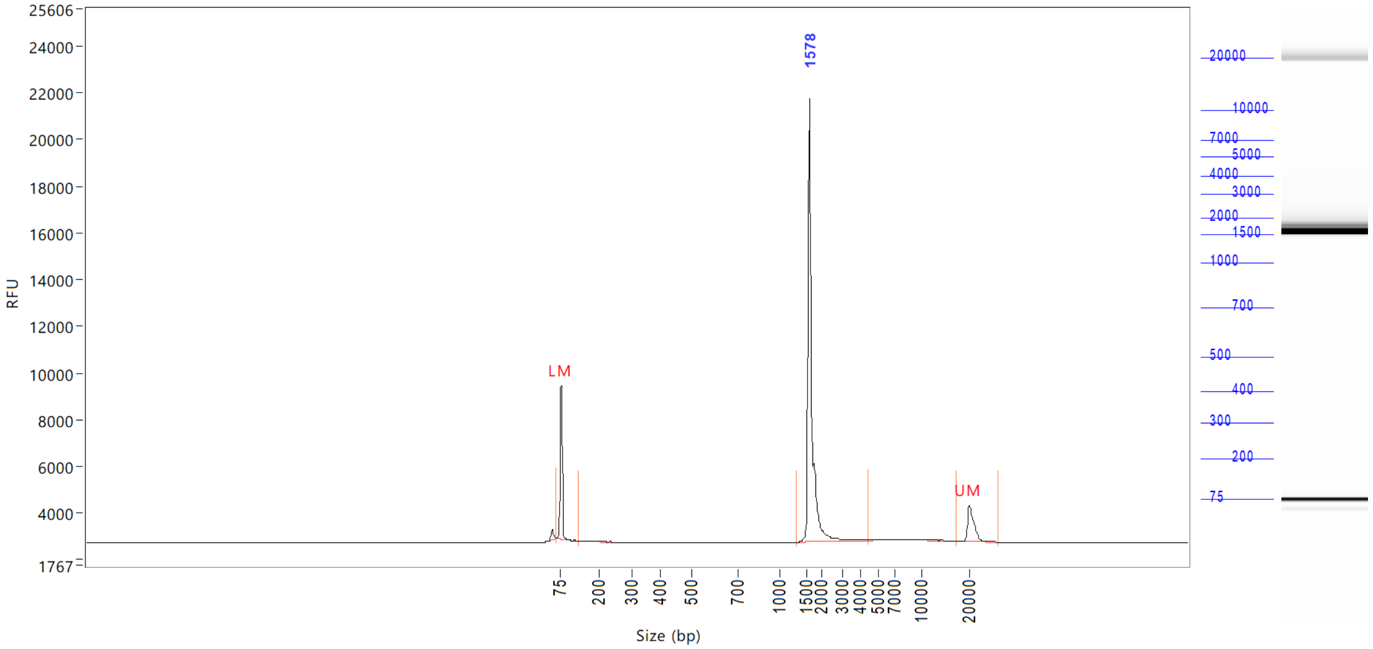

Peak	Size (bp)	Concentration (ng/uL)	From (bp)	To (bp)	Average size (bp)	CV%	RFU	Corrected peak area
1	75 (LM)	0.5000	60	129	73	6.39	6580	36.689
2	1578	19.5399	1334	4414	1654	17.39	19027	119.483
3	20000 (UM)	0.1774	17317	25924	20417	3.97	1553	13.015

TIC:	19.5399	ng/uL
TIM:	19.4466	nmole/L
Total concentration:	20.0701	ng/uL

Sample peak width (sec): 5 Sample min peak height: 50 Sample baseline V to V?: Y Sample baseline V to V points: 3
Sample filter: Binomial Number of points for filter: 3 Sample start region (min): 0 Sample end region (min): 30
Marker peak width (sec): 5 Marker min peak height: 200 Marker baseline V to V?: Y Marker baseline V to V points: 3
Lower marker selection: First peak > 500 RFU Upper marker selection: Last peak > 200 RFU
Ladder size (bp)75, 200, 300, 400, 500, 700, 1000, 1500, 2000, 3000, 4000, 5000, 7000, 10000, 20000
Quantification using: Lower Marker Final concentration (ng/uL): 0.5000 Dilution factor: 12.0

Sample: SampD8
Well location: D8
Created: Tuesday, August 10, 2021 8:01:08 PM

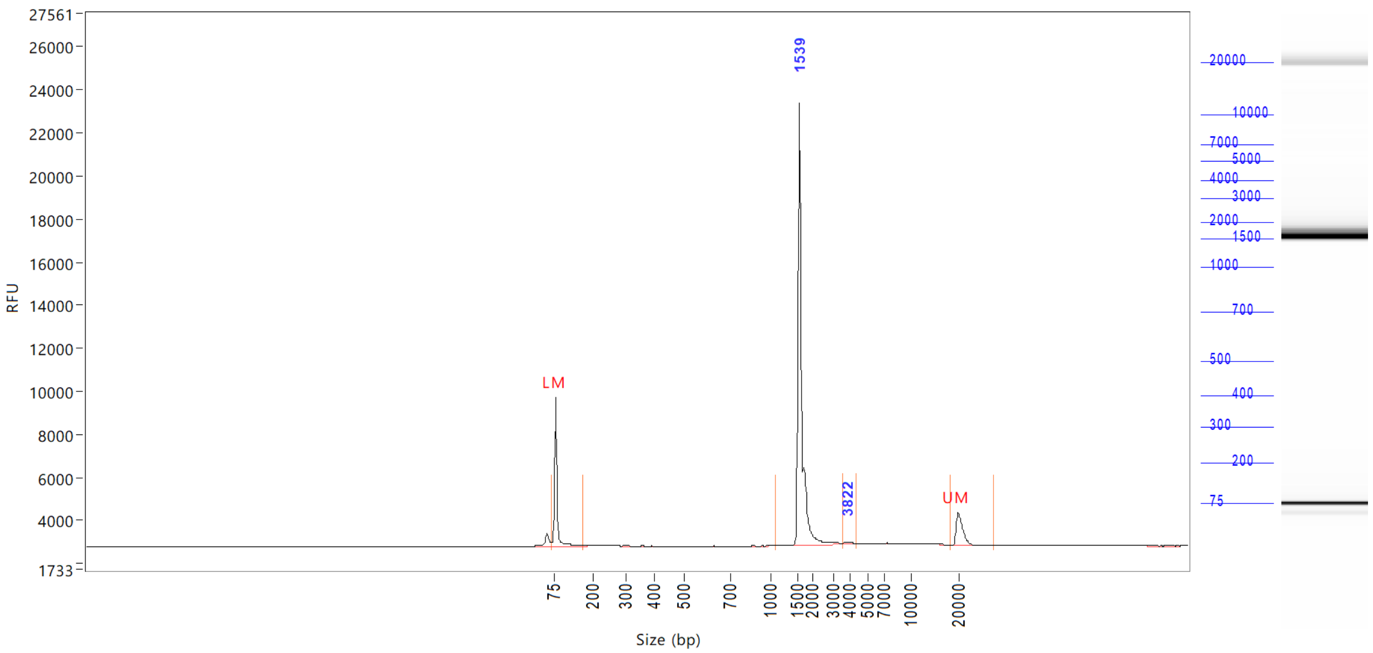

Peak	Size (bp)	Concentration (ng/uL)	From (bp)	To (bp)	Average size (bp)	CV%	RFU	Corrected peak area
1	75 (LM)	0.5000	64	168	77	19.45	6893	42.851
2	1539	17.7490	1079	3500	1615	13.92	20590	126.761
3	3822	0.1376	3500	4311	3868	5.79	65	0.983
4	20000 (UM)	0.1501	18171	27375	20387	4.18	1511	12.868

TIC:	17.8865	ng/uL
TIM:	18.1493	nmole/L
Total concentration:	18.4822	ng/uL

Sample peak width (sec): 5 Sample min peak height: 50 Sample baseline V to V?: Y Sample baseline V to V points: 3
Sample filter: Binomial Number of points for filter: 3 Sample start region (min): 0 Sample end region (min): 30
Marker peak width (sec): 5 Marker min peak height: 200 Marker baseline V to V?: Y Marker baseline V to V points: 3
Lower marker selection: First peak > 500 RFU Upper marker selection: Last peak > 200 RFU
Ladder size (bp)75, 200, 300, 400, 500, 700, 1000, 1500, 2000, 3000, 4000, 5000, 7000, 10000, 20000
Quantification using: Lower Marker Final concentration (ng/uL): 0.5000 Dilution factor: 12.0

Sample: SampE8
Well location: E8
Created: Tuesday, August 10, 2021 8:01:08 PM

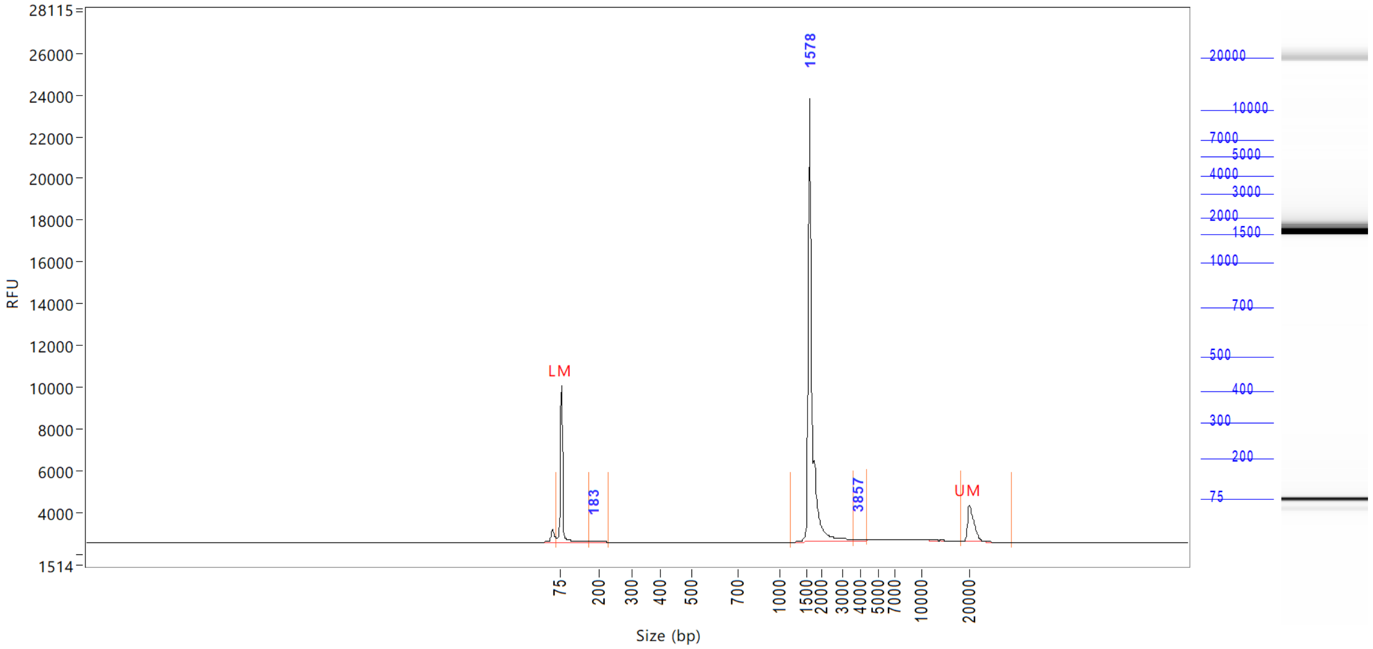

Peak	Size<br>(bp)	Concentration<br>(ng/uL)	From<br>(bp)	To<br>(bp)	Average size<br>(bp)	CV%	RFU	Corrected peak area
1	75 (LM)	0.5000	60	166	77	19.81	7522	46.812
2	183	0.2054	166	226	192	8.90	59	1.603
3	1578	17.1742	1201	3608	1654	14.43	21231	133.995
4	3857	0.1415	3608	4380	3952	5.25	75	1.104
5	20000 (UM)	0.1589	18171	28826	20427	4.04	1753	14.877

TIC:	17.5211	ng/uL
TIM:	18.9100	nmole/L
Total concentration:	17.9428	ng/uL

Sample peak width (sec): 5 Sample min peak height: 50 Sample baseline V to V?: Y Sample baseline V to V points: 3
Sample filter: Binomial Number of points for filter: 3 Sample start region (min): 0 Sample end region (min): 30
Marker peak width (sec): 5 Marker min peak height: 200 Marker baseline V to V?: Y Marker baseline V to V points: 3
Lower marker selection: First peak > 500 RFU Upper marker selection: Last peak > 200 RFU
Ladder size (bp)75, 200, 300, 400, 500, 700, 1000, 1500, 2000, 3000, 4000, 5000, 7000, 10000, 20000
Quantification using: Lower Marker Final concentration (ng/uL): 0.5000 Dilution factor: 12.0

Sample: SampF8
Well location: F8
Created: Tuesday, August 10, 2021 8:01:08 PM

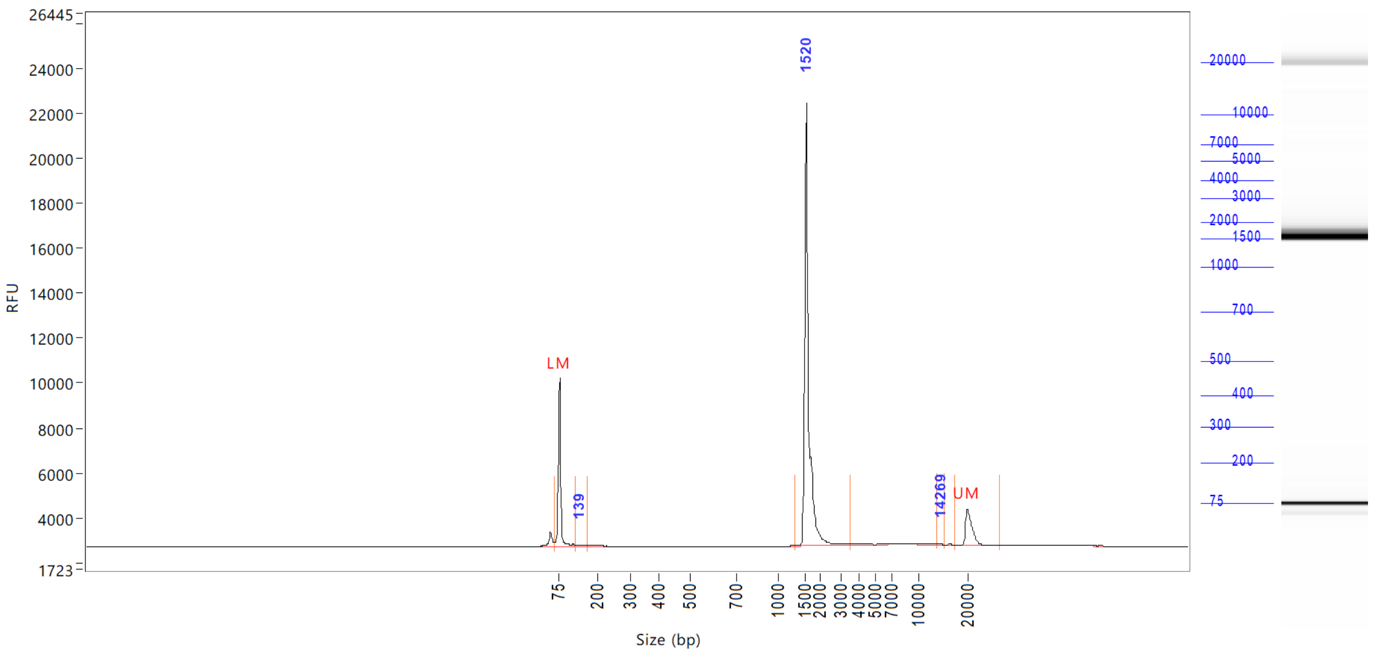

Peak	Size (bp)	Concentration (ng/uL)	From (bp)	To (bp)	Average size (bp)	CV%	RFU	Corrected peak area
1	75 (LM)	0.5000	64	129	75	11.62	7525	44.661
2	139	0.1629	129	166	146	7.00	64	1.213
3	1520	16.7671	1311	3536	1601	12.72	19733	124.807
4	14269	0.0356	13659	15244	14154	2.06	53	0.265
5	20000 (UM)	0.1541	17317	26770	20403	4.19	1620	13.763

TIC:	16.9656	ng/uL
TIM:	19.0772	nmole/L
Total concentration:	17.6542	ng/uL

Sample peak width (sec): 5   Sample min peak height: 50   Sample baseline V to V?: Y   Sample baseline V to V points: 3
Sample filter: Binomial   Number of points for filter: 3   Sample start region (min): 0   Sample end region (min): 30
Marker peak width (sec): 5   Marker min peak height: 200   Marker baseline V to V?: Y   Marker baseline V to V points: 3
Lower marker selection: First peak > 500 RFU   Upper marker selection: Last peak > 200 RFU
Ladder size (bp)75, 200, 300, 400, 500, 700, 1000, 1500, 2000, 3000, 4000, 5000, 7000, 10000, 20000
Quantification using: Lower Marker   Final concentration (ng/uL): 0.5000   Dilution factor: 12.0

Sample: SampG8
Well location: G8
Created: Tuesday, August 10, 2021 8:01:08 PM

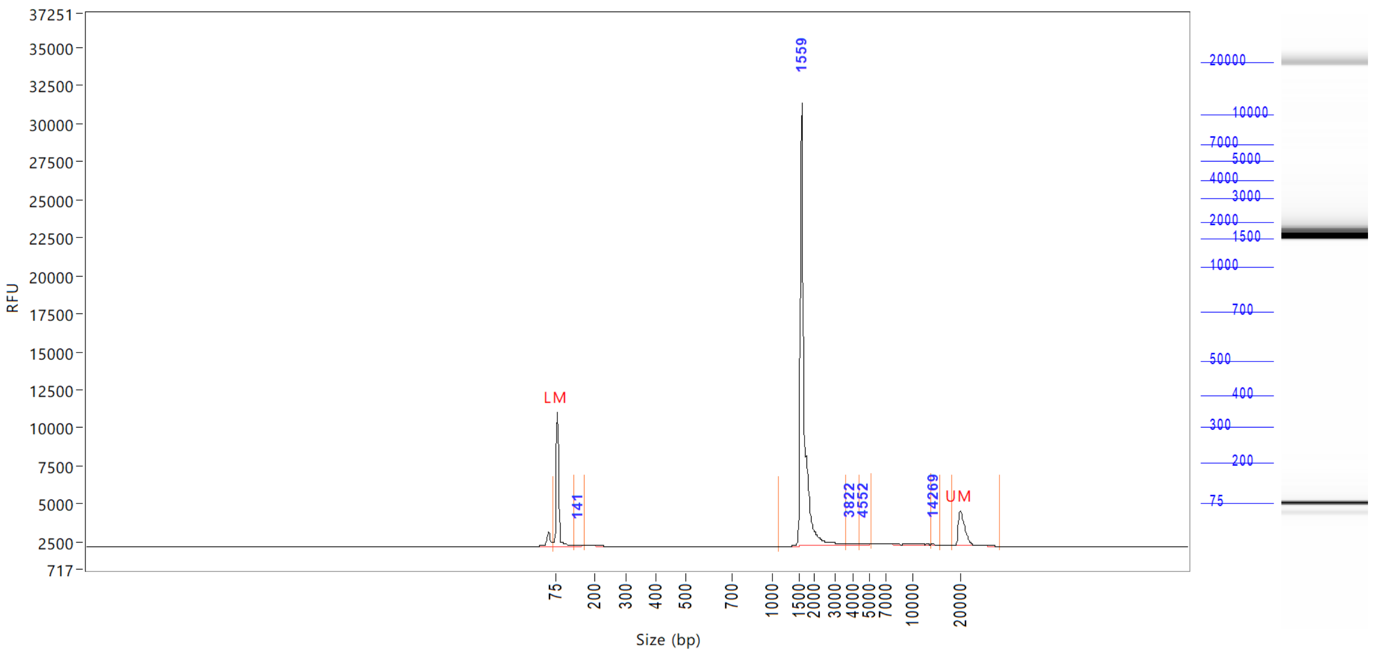

Peak	Size (bp)	Concentration (ng/uL)	From (bp)	To (bp)	Average size (bp)	CV%	RFU	Corrected peak area
1	75 (LM)	0.5000	64	131	76	11.16	8783	54.615
2	141	0.1047	131	168	146	6.36	63	0.953
3	1559	21.0268	1101	3608	1647	14.78	29162	191.397
4	3822	0.1581	3608	4380	3950	5.33	96	1.439
5	4552	0.0584	4380	5223	4645	4.29	53	0.532
6	14269	0.0189	13903	15732	14309	2.08	52	0.172
7	20000 (UM)	0.1808	18171	28342	20473	4.29	2299	19.749

TIC:	21.3669	ng/uL
TIM:	22.2826	nmole/L
Total concentration:	21.6969	ng/uL

Sample peak width (sec): 5 Sample min peak height: 50 Sample baseline V to V?: Y Sample baseline V to V points: 3
Sample filter: Binomial Number of points for filter: 3 Sample start region (min): 0 Sample end region (min): 30
Marker peak width (sec): 5 Marker min peak height: 200 Marker baseline V to V?: Y Marker baseline V to V points: 3
Lower marker selection: First peak > 500 RFU Upper marker selection: Last peak > 200 RFU
Ladder size (bp)75, 200, 300, 400, 500, 700, 1000, 1500, 2000, 3000, 4000, 5000, 7000, 10000, 20000
Quantification using: Lower Marker Final concentration (ng/uL): 0.5000 Dilution factor: 12.0

Sample: SampH8
Well location: H8
Created: Tuesday, August 10, 2021 8:01:08 PM

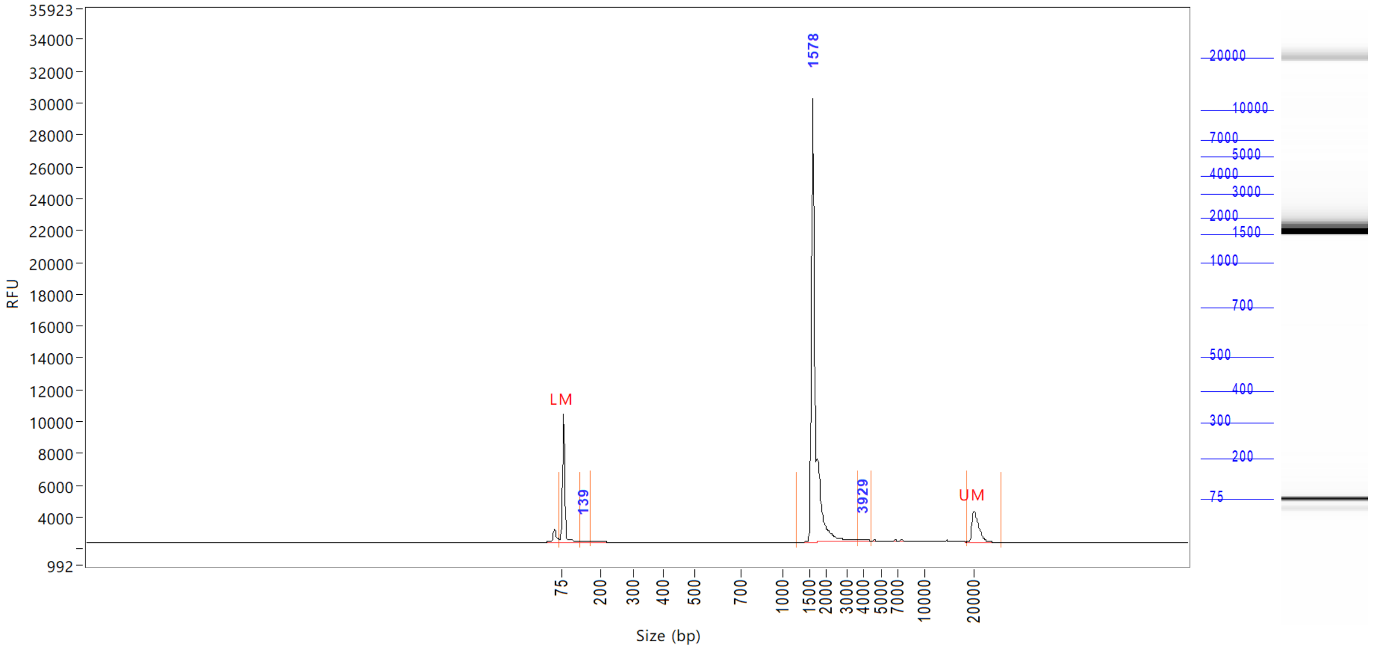

Peak	Size (bp)	Concentration (ng/uL)	From (bp)	To (bp)	Average size (bp)	CV%	RFU	Corrected peak area
1	75 (LM)	0.5000	64	129	76	10.59	8085	49.240
2	139	0.1063	129	166	145	6.50	64	0.872
3	1578	21.7716	1267	3643	1667	14.66	27892	178.674
4	3929	0.1326	3643	4380	3960	4.93	79	1.088
5	20000 (UM)	0.1714	18415	25561	20486	3.95	1950	16.883

TIC:	22.0105	ng/uL
TIM:	22.7585	nmole/L
Total concentration:	22.4043	ng/uL

Sample peak width (sec): 5 Sample min peak height: 50 Sample baseline V to V?: Y Sample baseline V to V points: 3
Sample filter: Binomial Number of points for filter: 3 Sample start region (min): 0 Sample end region (min): 30
Marker peak width (sec): 5 Marker min peak height: 200 Marker baseline V to V?: Y Marker baseline V to V points: 3
Lower marker selection: First peak > 500 RFU Upper marker selection: Last peak > 200 RFU
Ladder size (bp)75, 200, 300, 400, 500, 700, 1000, 1500, 2000, 3000, 4000, 5000, 7000, 10000, 20000
Quantification using: Lower Marker Final concentration (ng/uL): 0.5000 Dilution factor: 12.0

Sample: SampA9
Well location: A9
Created: Tuesday, August 10, 2021 8:01:08 PM

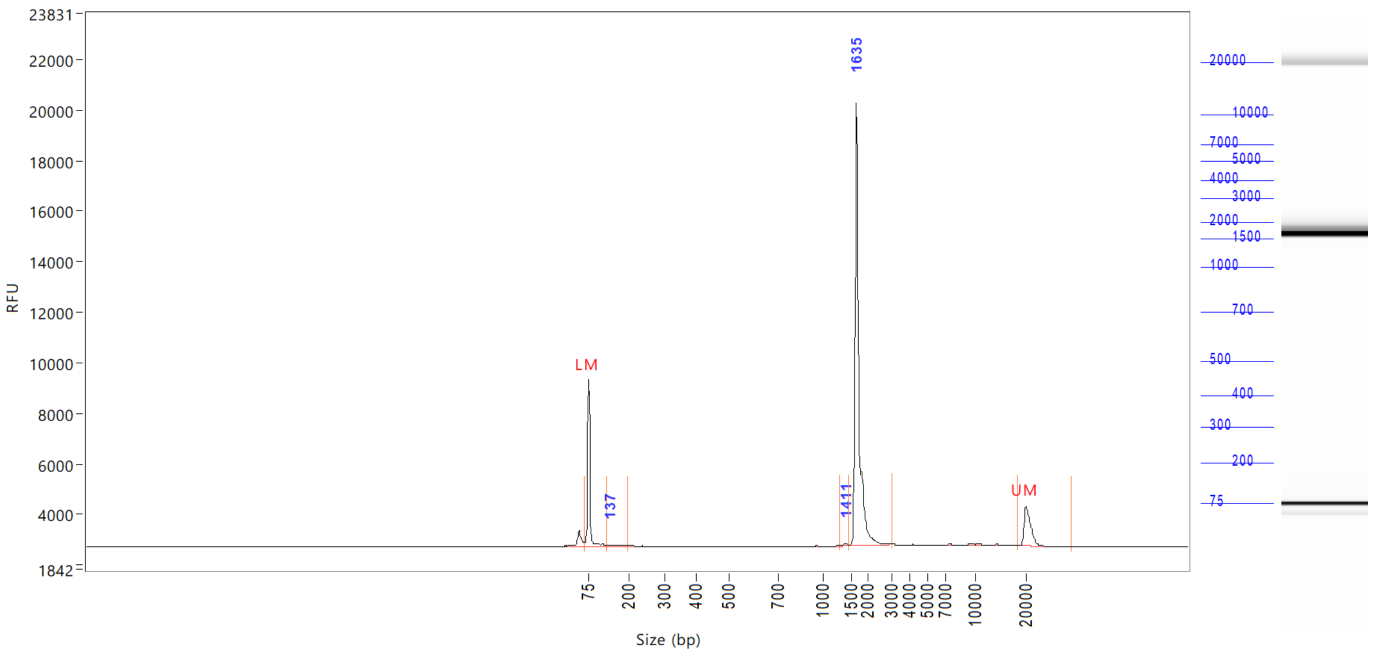

Peak	Size (bp)	Concentration (ng/uL)	From (bp)	To (bp)	Average size (bp)	CV%	RFU	Corrected peak area
1	75 (LM)	0.5000	62	129	74	11.17	6603	38.701
2	137	0.2045	129	196	154	11.57	59	1.319
3	1411	0.0951	1300	1456	1386	2.61	80	0.613
4	1635	15.4036	1456	3037	1669	8.11	17536	99.356
5	20000 (UM)	0.1607	18415	28584	20337	3.89	1551	12.441

TIC:	15.7032	ng/uL
TIM:	17.4878	nmole/L
Total concentration:	16.2706	ng/uL

```
Sample peak width (sec): 5    Sample min peak height: 50    Sample baseline V to V?: Y    Sample baseline V to V points: 3
Sample filter: Binomial       Number of points for filter: 3  Sample start region (min): 0  Sample end region (min): 30
Marker peak width (sec): 5    Marker min peak height: 200   Marker baseline V to V?: Y    Marker baseline V to V points: 3
Lower marker selection: First peak > 500 RFU                Upper marker selection: Last peak > 200 RFU
Ladder size (bp)75, 200, 300, 400, 500, 700, 1000, 1500, 2000, 3000, 4000, 5000, 7000, 10000, 20000
Quantification using: Lower Marker          Final concentration (ng/uL): 0.5000          Dilution factor: 12.0
```

Sample: SampB9
Well location: B9
Created: Tuesday, August 10, 2021 8:01:08 PM

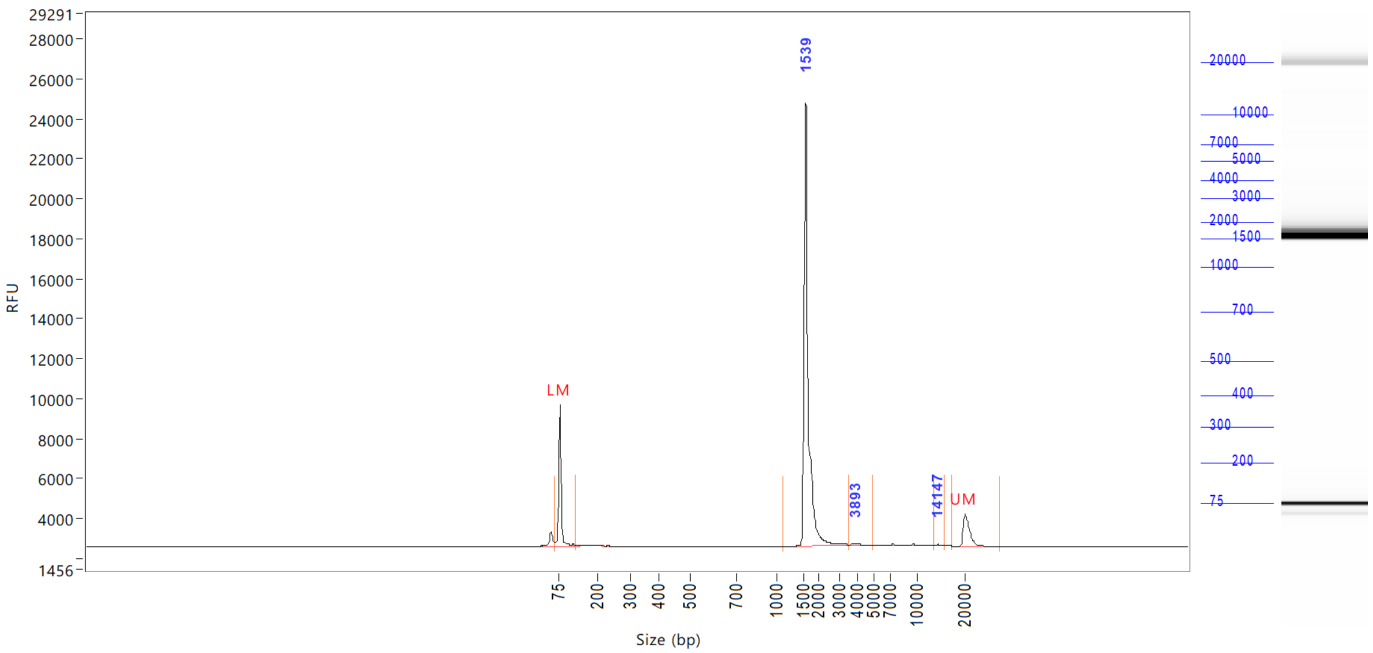

Peak	Size (bp)	Concentration (ng/uL)	From (bp)	To (bp)	Average size (bp)	CV%	RFU	Corrected peak area
1	75 (LM)	0.5000	62	129	74	11.07	7102	43.818
2	1539	19.9971	1112	3536	1618	12.33	22226	146.039
3	3893	0.1379	3536	4966	4046	8.55	56	1.007
4	14147	0.0312	13537	15610	14114	2.16	50	0.228
5	20000 (UM)	0.1564	17317	27254	20419	4.31	1636	13.710

TIC:	20.1662	ng/uL
TIM:	20.4041	nmole/L
Total concentration:	20.6384	ng/uL

Sample peak width (sec): 5 Sample min peak height: 50 Sample baseline V to V?: Y Sample baseline V to V points: 3
Sample filter: Binomial Number of points for filter: 3 Sample start region (min): 0 Sample end region (min): 30
Marker peak width (sec): 5 Marker min peak height: 200 Marker baseline V to V?: Y Marker baseline V to V points: 3
Lower marker selection: First peak > 500 RFU Upper marker selection: Last peak > 200 RFU
Ladder size (bp)75, 200, 300, 400, 500, 700, 1000, 1500, 2000, 3000, 4000, 5000, 7000, 10000, 20000
Quantification using: Lower Marker Final concentration (ng/uL): 0.5000 Dilution factor: 12.0

Sample: SampC9
Well location: C9
Created: Tuesday, August 10, 2021 8:01:08 PM

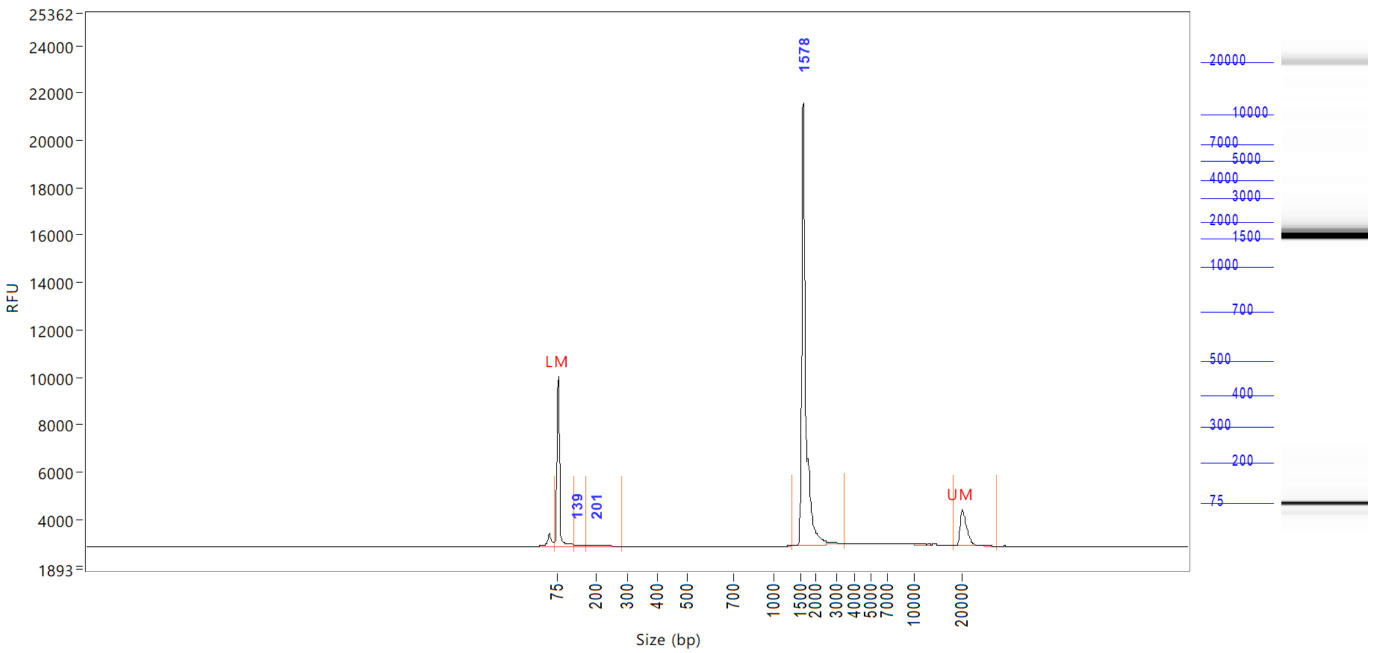

Peak	Size (bp)	Concentration (ng/uL)	From (bp)	To (bp)	Average size (bp)	CV%	RFU	Corrected peak area
1	75 (LM)	0.5000	64	129	75	12.12	7158	43.376
2	139	0.1816	129	168	147	7.36	72	1.313
3	201	0.2586	168	278	206	12.26	55	1.870
4	1578	16.6091	1345	3465	1624	11.24	18714	120.074
5	20000 (UM)	0.1534	18171	27254	20433	4.19	1565	13.308

TIC:	17.0493	ng/uL
TIM:	20.9296	nmole/L
Total concentration:	17.3621	ng/uL

```
Sample peak width (sec): 5    Sample min peak height: 50    Sample baseline V to V?: Y    Sample baseline V to V points: 3
Sample filter: Binomial       Number of points for filter: 3  Sample start region (min): 0  Sample end region (min): 30
Marker peak width (sec): 5    Marker min peak height: 200   Marker baseline V to V?: Y    Marker baseline V to V points: 3
Lower marker selection: First peak > 500 RFU                Upper marker selection: Last peak > 200 RFU
Ladder size (bp)75, 200, 300, 400, 500, 700, 1000, 1500, 2000, 3000, 4000, 5000, 7000, 10000, 20000
Quantification using: Lower Marker          Final concentration (ng/uL): 0.5000          Dilution factor: 12.0
```

Sample: SampD9
Well location: D9
Created: Tuesday, August 10, 2021 8:01:08 PM

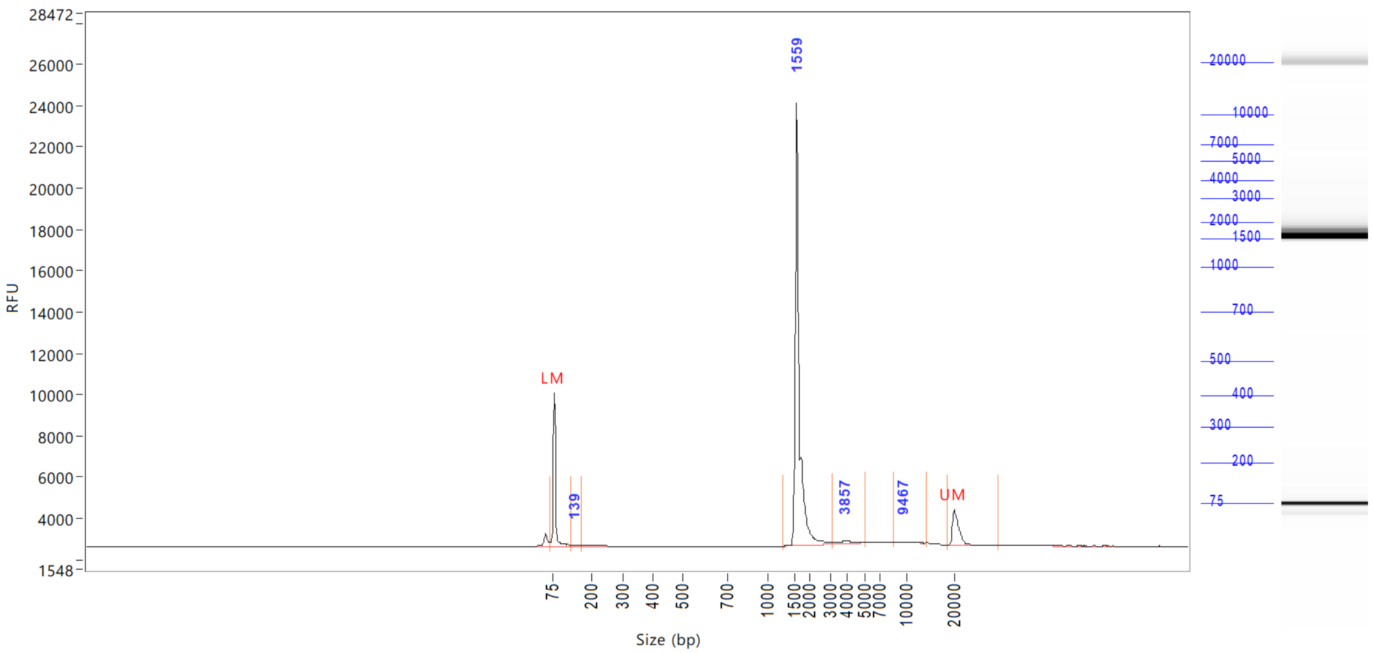

Peak	Size (bp)	Concentration (ng/uL)	From (bp)	To (bp)	Average size (bp)	CV%	RFU	Corrected peak area
1	75 (LM)	0.5000	62	129	75	12.33	7444	46.495
2	139	0.1741	129	166	146	6.97	72	1.350
3	1559	17.9223	1267	3108	1630	12.91	21475	138.884
4	3857	0.4511	3108	5149	3898	12.75	117	3.495
5	9467	0.1370	8534	14025	10173	10.97	54	1.062
6	20000 (UM)	0.1546	18537	29430	20505	4.60	1695	14.380

TIC:	18.6846	ng/uL
TIM:	20.2723	nmole/L
Total concentration:	19.1325	ng/uL

```
Sample peak width (sec): 5    Sample min peak height: 50    Sample baseline V to V?: Y    Sample baseline V to V points: 3
Sample filter: Binomial       Number of points for filter: 3  Sample start region (min): 0  Sample end region (min): 30
Marker peak width (sec): 5    Marker min peak height: 200   Marker baseline V to V?: Y    Marker baseline V to V points: 3
Lower marker selection: First peak > 500 RFU                Upper marker selection: Last peak > 200 RFU
Ladder size (bp)75, 200, 300, 400, 500, 700, 1000, 1500, 2000, 3000, 4000, 5000, 7000, 10000, 20000
Quantification using: Lower Marker          Final concentration (ng/uL): 0.5000          Dilution factor: 12.0
```

Sample: SampE9
Well location: E9
Created: Tuesday, August 10, 2021 8:01:08 PM

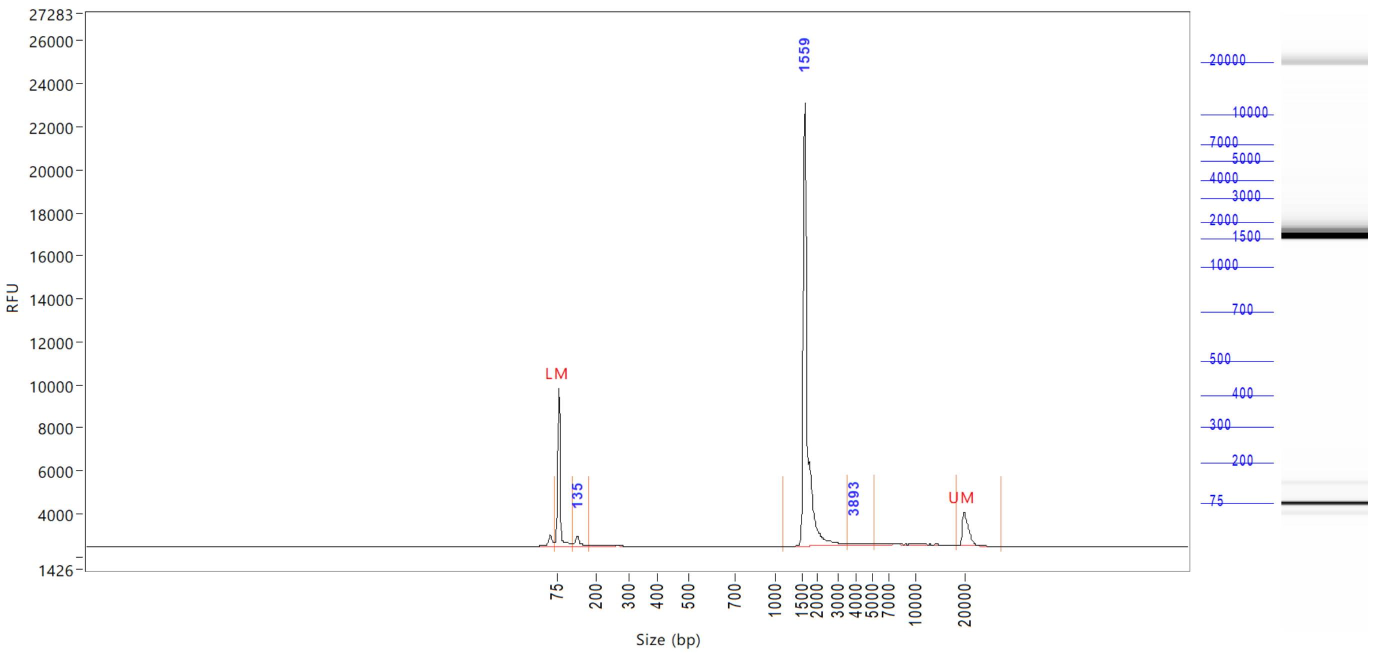

Peak	Size (bp)	Concentration (ng/uL)	From (bp)	To (bp)	Average size (bp)	CV%	RFU	Corrected peak area
1	75 (LM)	0.5000	62	122	75	12.62	7339	44.753
2	135	0.7438	122	177	139	8.96	495	5.548
3	1559	18.2353	1156	3536	1647	14.71	20647	136.013
4	3893	0.2919	3536	5223	4157	9.98	97	2.177
5	20000 (UM)	0.1466	18293	27738	20460	3.97	1564	13.125

TIC:	19.2709	ng/uL
TIM:	27.1338	nmole/L
Total concentration:	19.7364	ng/uL

Sample peak width (sec): 5 Sample min peak height: 50 Sample baseline V to V?: Y Sample baseline V to V points: 3
Sample filter: Binomial Number of points for filter: 3 Sample start region (min): 0 Sample end region (min): 30
Marker peak width (sec): 5 Marker min peak height: 200 Marker baseline V to V?: Y Marker baseline V to V points: 3
Lower marker selection: First peak > 500 RFU Upper marker selection: Last peak > 200 RFU
Ladder size (bp)75, 200, 300, 400, 500, 700, 1000, 1500, 2000, 3000, 4000, 5000, 7000, 10000, 20000
Quantification using: Lower Marker Final concentration (ng/uL): 0.5000 Dilution factor: 12.0

Sample: SampF9
Well location: F9
Created: Tuesday, August 10, 2021 8:01:08 PM

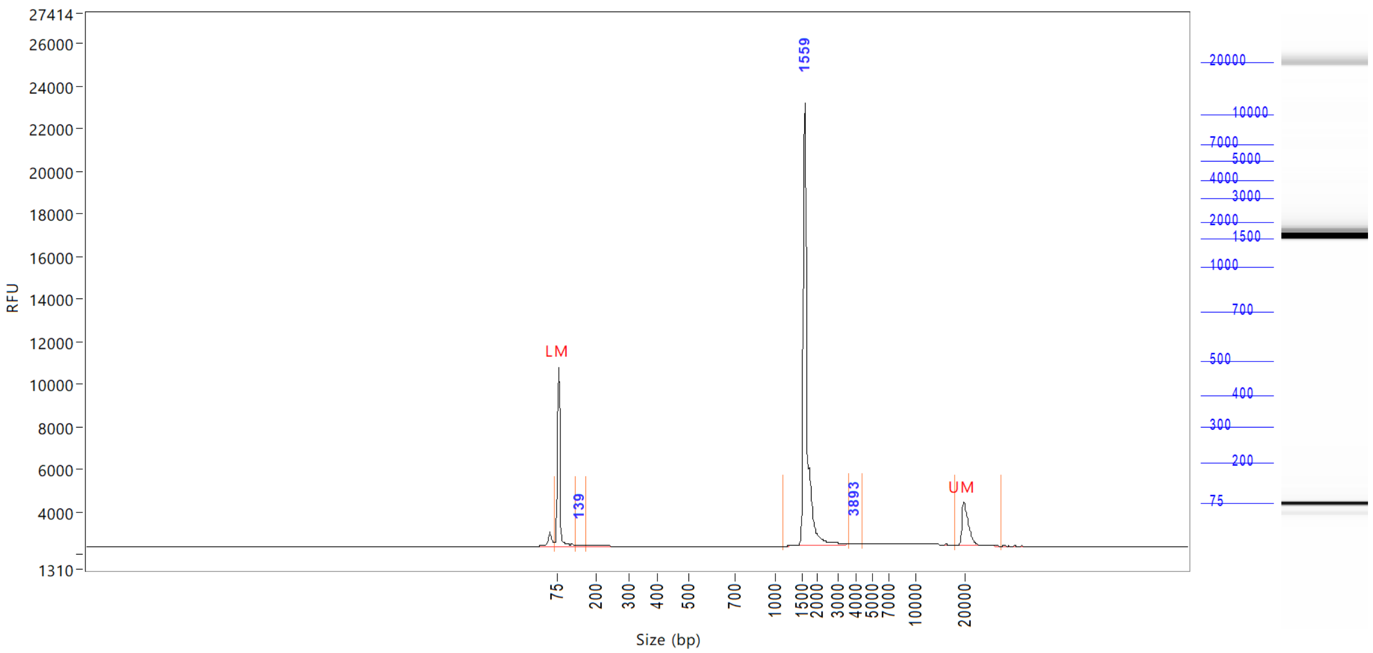

Peak	Size (bp)	Concentration (ng/uL)	From (bp)	To (bp)	Average size (bp)	CV%	RFU	Corrected peak area
1	75 (LM)	0.5000	62	129	74	11.42	8391	51.182
2	139	0.1456	129	166	146	7.03	67	1.242
3	1559	14.8626	1123	3643	1626	13.19	20821	126.783
4	3893	0.0853	3643	4380	3954	4.74	55	0.728
5	20000 (UM)	0.1730	18171	27617	20422	4.23	2057	17.712

TIC:	15.0935	ng/uL
TIM:	16.7203	nmole/L
Total concentration:	15.5882	ng/uL

Sample peak width (sec): 5   Sample min peak height: 50   Sample baseline V to V?: Y   Sample baseline V to V points: 3
Sample filter: Binomial   Number of points for filter: 3 Sample start region (min): 0 Sample end region (min): 30
Marker peak width (sec): 5   Marker min peak height: 200   Marker baseline V to V?: Y   Marker baseline V to V points: 3
Lower marker selection: First peak > 500 RFU   Upper marker selection: Last peak > 200 RFU
Ladder size (bp)75, 200, 300, 400, 500, 700, 1000, 1500, 2000, 3000, 4000, 5000, 7000, 10000, 20000
Quantification using: Lower Marker   Final concentration (ng/uL): 0.5000   Dilution factor: 12.0

Sample: SampG9
Well location: G9
Created: Tuesday, August 10, 2021 8:01:08 PM

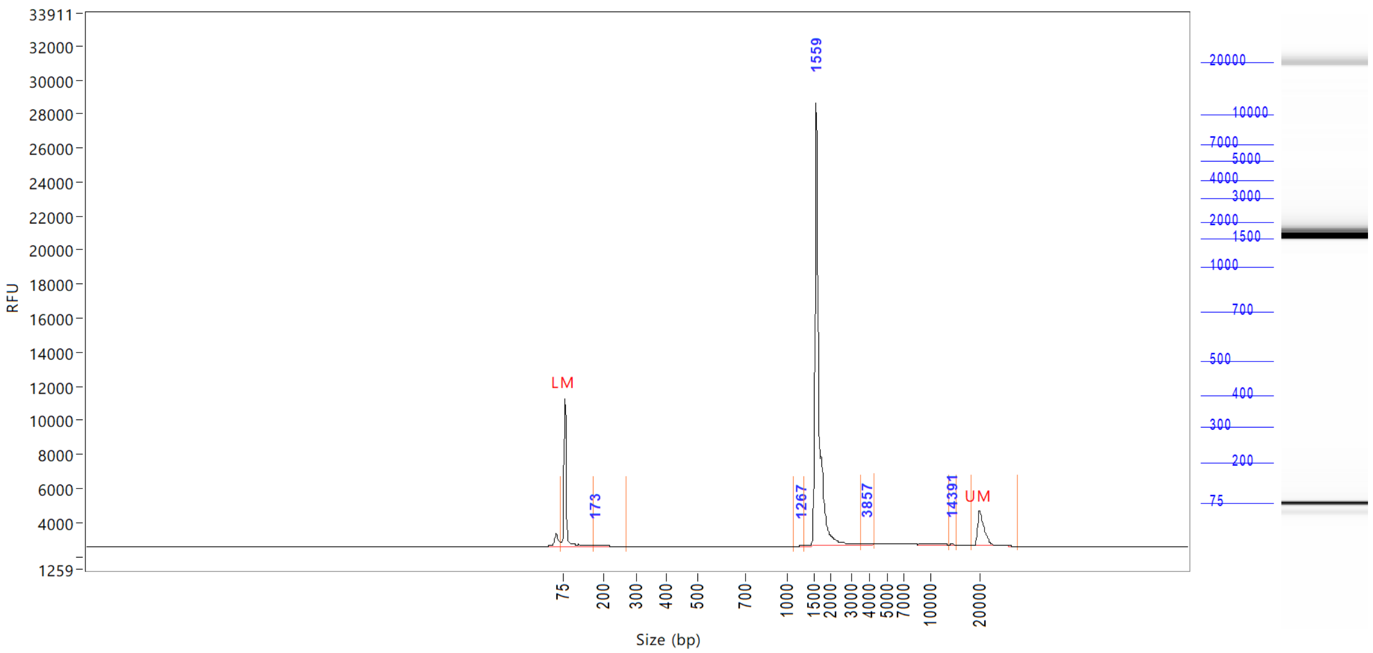

Peak	Size (bp)	Concentration (ng/uL)	From (bp)	To (bp)	Average size (bp)	CV%	RFU	Corrected peak area
1	75 (LM)	0.5000	64	166	78	19.94	8719	53.714
2	173	0.2249	166	269	198	11.71	64	2.014
3	1267	0.0375	1134	1311	1261	2.43	67	0.335
4	1559	18.3907	1311	3572	1632	13.41	26061	164.639
5	3857	0.0950	3572	4345	3906	5.32	61	0.850
6	14391	0.0288	13537	15366	14171	2.19	58	0.257
7	20000 (UM)	0.1633	18293	27738	20369	4.15	2005	17.548

TIC:	18.7768	ng/uL
TIM:	20.5097	nmole/L
Total concentration:	19.1177	ng/uL

```
Sample peak width (sec): 5     Sample min peak height: 50     Sample baseline V to V?: Y     Sample baseline V to V points: 3
Sample filter: Binomial        Number of points for filter: 3  Sample start region (min): 0  Sample end region (min): 30
Marker peak width (sec): 5     Marker min peak height: 200    Marker baseline V to V?: Y     Marker baseline V to V points: 3
Lower marker selection: First peak > 500 RFU                  Upper marker selection: Last peak > 200 RFU
Ladder size (bp)75, 200, 300, 400, 500, 700, 1000, 1500, 2000, 3000, 4000, 5000, 7000, 10000, 20000
Quantification using: Lower Marker          Final concentration (ng/uL): 0.5000          Dilution factor: 12.0
```

Sample: SampH9
Well location: H9
Created: Tuesday, August 10, 2021 8:01:08 PM

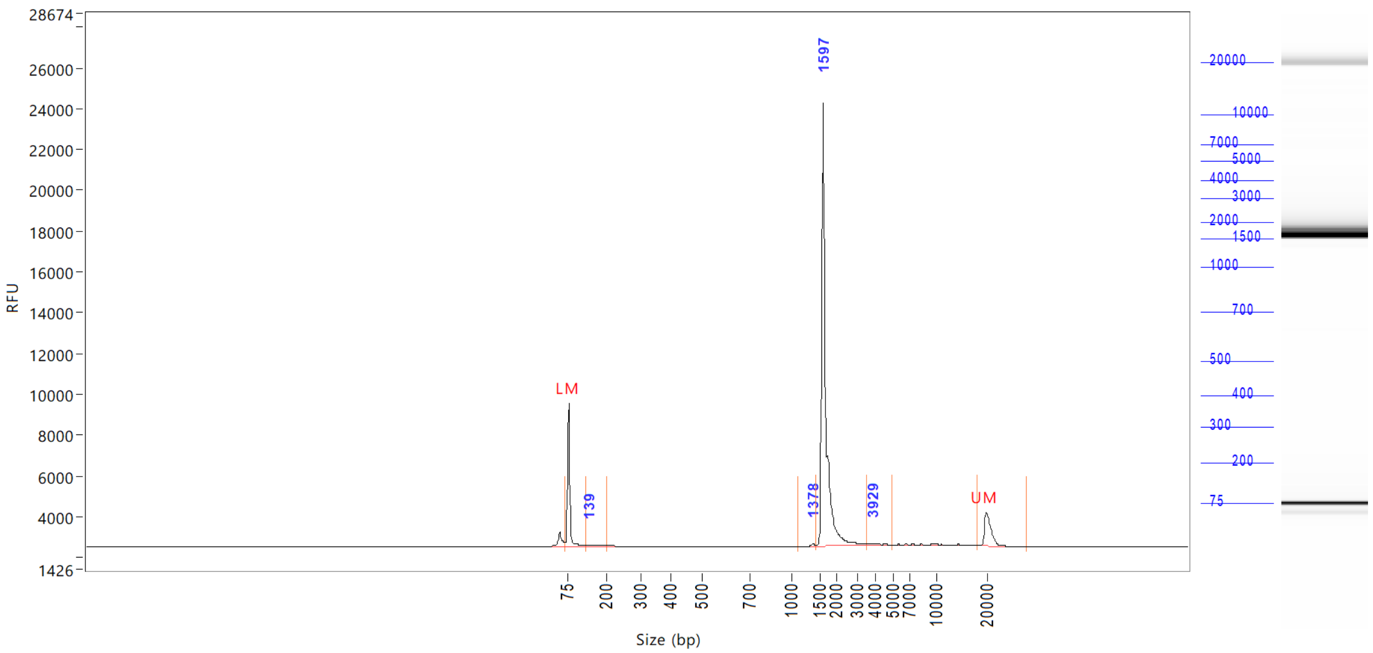

Peak	Size (bp)	Concentration (ng/uL)	From (bp)	To (bp)	Average size (bp)	CV%	RFU	Corrected peak area
1	75 (LM)	0.5000	64	129	75	11.66	7062	41.318
2	139	0.2730	129	196	157	11.70	69	1.880
3	1378	0.1058	1112	1433	1367	3.13	121	0.728
4	1597	20.5632	1433	3536	1679	14.20	21754	141.606
5	3929	0.1773	3536	5000	4033	8.55	64	1.221
6	20000 (UM)	0.1653	18293	28100	20379	4.01	1647	13.662

TIC:	21.1193	ng/uL
TIM:	23.2181	nmole/L
Total concentration:	21.5111	ng/uL

Sample peak width (sec): 5 Sample min peak height: 50 Sample baseline V to V?: Y Sample baseline V to V points: 3
Sample filter: Binomial Number of points for filter: 3 Sample start region (min): 0 Sample end region (min): 30
Marker peak width (sec): 5 Marker min peak height: 200 Marker baseline V to V?: Y Marker baseline V to V points: 3
Lower marker selection: First peak > 500 RFU Upper marker selection: Last peak > 200 RFU
Ladder size (bp)75, 200, 300, 400, 500, 700, 1000, 1500, 2000, 3000, 4000, 5000, 7000, 10000, 20000
Quantification using: Lower Marker Final concentration (ng/uL): 0.5000 Dilution factor: 12.0

Sample: SampA10
Well location: A10
Created: Tuesday, August 10, 2021 8:01:08 PM

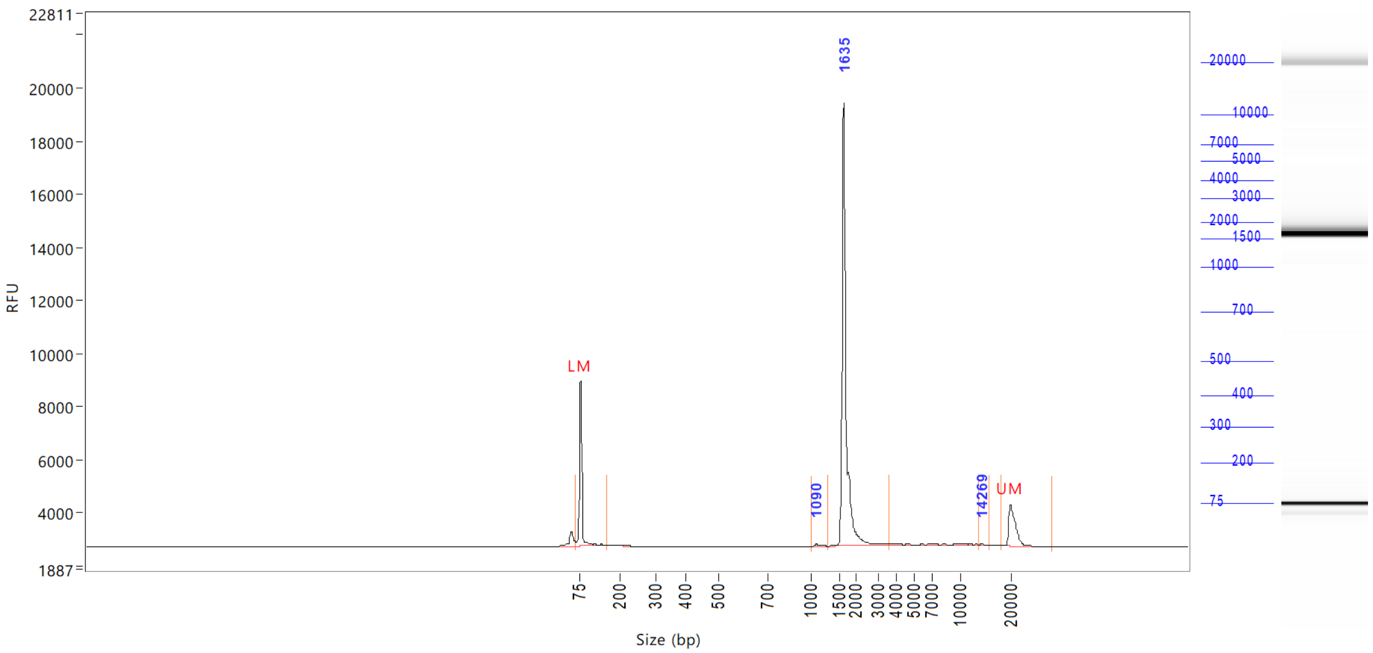

Peak	Size (bp)	Concentration (ng/uL)	From (bp)	To (bp)	Average size (bp)	CV%	RFU	Corrected peak area
1	75 (LM)	0.5000	60	158	74	13.31	6228	37.241
2	1090	0.0826	1012	1289	1112	5.02	77	0.513
3	1635	15.6885	1289	3572	1683	12.24	16699	97.376
4	14269	0.0643	13537	15732	14334	3.85	61	0.399
5	20000 (UM)	0.1764	17927	28100	20369	4.17	1561	13.141

TIC:	15.8354	ng/uL
TIM:	15.4742	nmole/L
Total concentration:	16.4286	ng/uL

Sample peak width (sec): 5 Sample min peak height: 50 Sample baseline V to V?: Y Sample baseline V to V points: 3
Sample filter: Binomial Number of points for filter: 3 Sample start region (min): 0 Sample end region (min): 30
Marker peak width (sec): 5 Marker min peak height: 200 Marker baseline V to V?: Y Marker baseline V to V points: 3
Lower marker selection: First peak > 500 RFU Upper marker selection: Last peak > 200 RFU
Ladder size (bp)75, 200, 300, 400, 500, 700, 1000, 1500, 2000, 3000, 4000, 5000, 7000, 10000, 20000
Quantification using: Lower Marker Final concentration (ng/uL): 0.5000 Dilution factor: 12.0

Sample: SampB10
Well location: B10
Created: Tuesday, August 10, 2021 8:01:08 PM

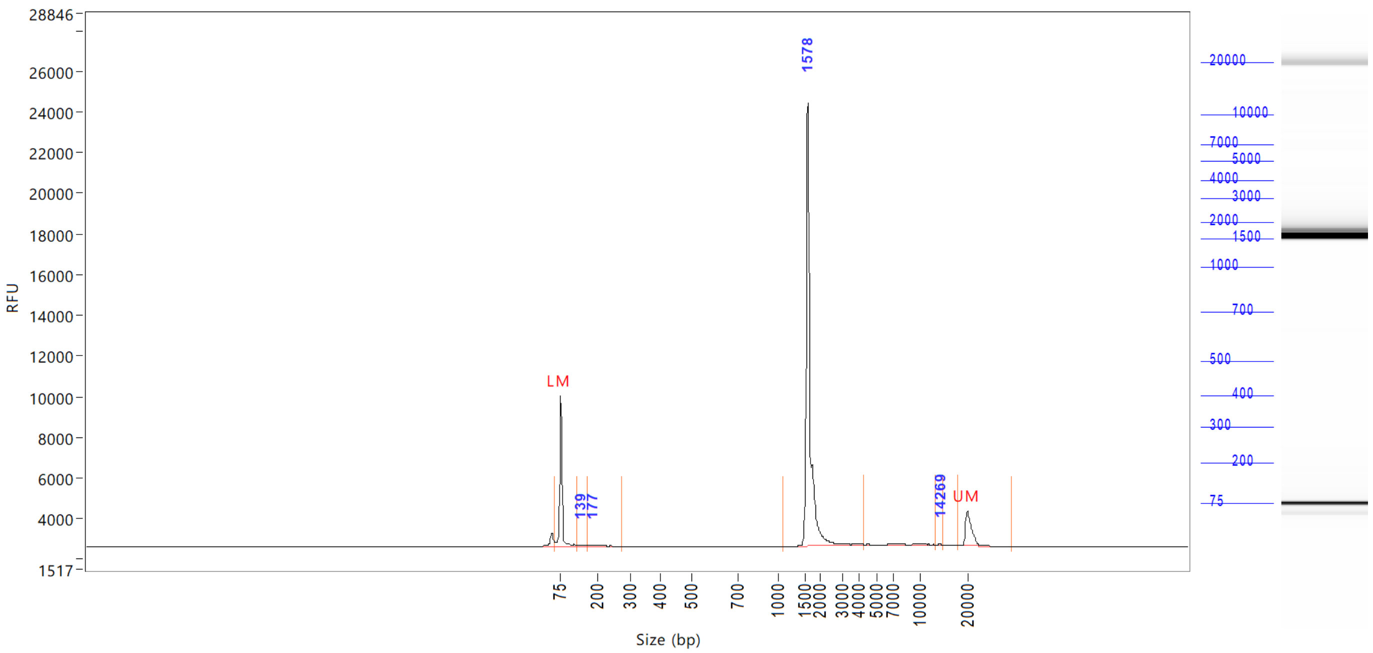

Peak	Size (bp)	Concentration (ng/uL)	From (bp)	To (bp)	Average size (bp)	CV%	RFU	Corrected peak area
1	75 (LM)	0.5000	60	129	75	11.96	7400	44.702
2	139	0.1598	129	164	146	6.62	65	1.190
3	177	0.2525	164	271	196	12.19	77	1.881
4	1578	18.5222	1090	4277	1646	15.99	21818	137.997
5	14269	0.0431	13415	15000	14080	2.67	59	0.321
6	20000 (UM)	0.1580	18049	29189	20374	4.47	1689	14.129

TIC:	18.9776	ng/uL
TIM:	22.4448	nmole/L
Total concentration:	19.3933	ng/uL

```
Sample peak width (sec): 5    Sample min peak height: 50    Sample baseline V to V?: Y    Sample baseline V to V points: 3
Sample filter: Binomial       Number of points for filter: 3  Sample start region (min): 0  Sample end region (min): 30
Marker peak width (sec): 5    Marker min peak height: 200   Marker baseline V to V?: Y    Marker baseline V to V points: 3
Lower marker selection: First peak > 500 RFU                Upper marker selection: Last peak > 200 RFU
Ladder size (bp)75, 200, 300, 400, 500, 700, 1000, 1500, 2000, 3000, 4000, 5000, 7000, 10000, 20000
Quantification using: Lower Marker          Final concentration (ng/uL): 0.5000          Dilution factor: 12.0
```

Sample: SampC10
Well location: C10
Created: Tuesday, August 10, 2021 8:01:08 PM

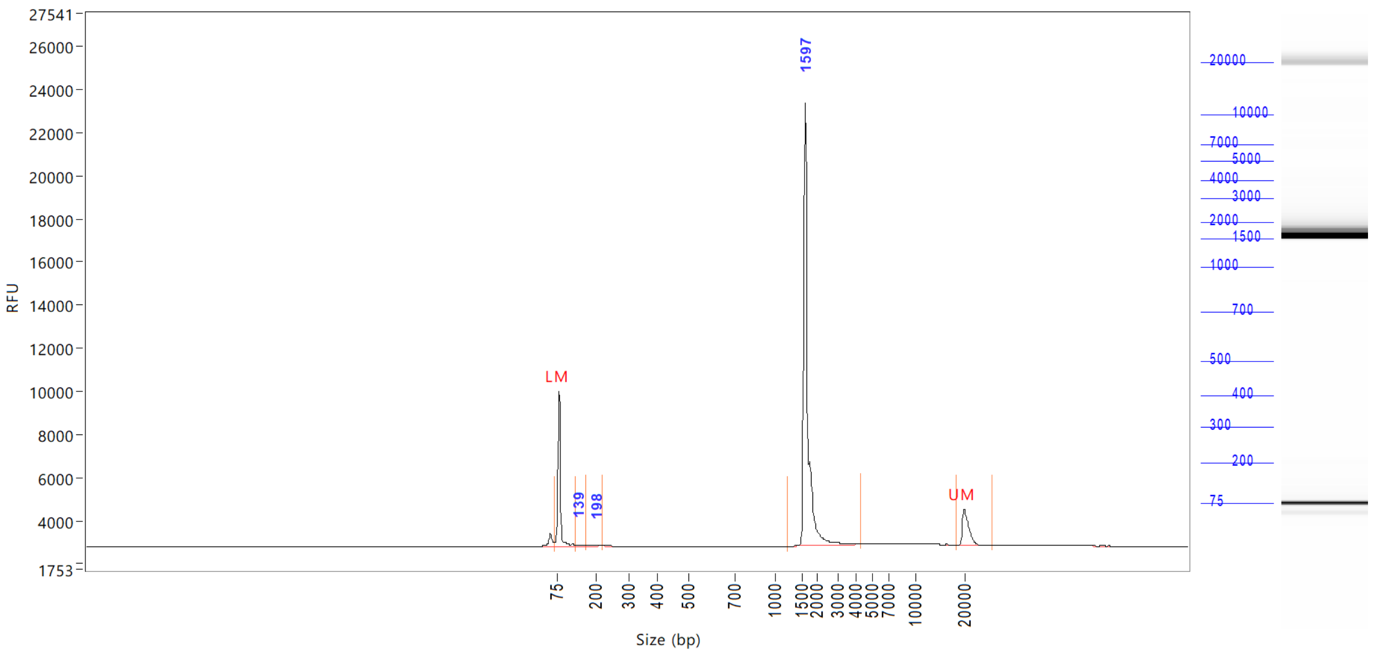

Peak	Size (bp)	Concentration (ng/uL)	From (bp)	To (bp)	Average size (bp)	CV%	RFU	Corrected peak area
1	75 (LM)	0.5000	64	131	76	11.52	7216	43.294
2	139	0.1437	131	168	146	6.68	73	1.037
3	198	0.1334	168	217	192	6.12	55	0.963
4	1597	18.3468	1223	4345	1662	16.82	20576	132.384
5	20000 (UM)	0.1653	18293	26045	20487	3.98	1663	14.313

TIC:	18.6240	ng/uL
TIM:	20.9316	nmole/L
Total concentration:	19.0061	ng/uL

Sample peak width (sec): 5   Sample min peak height: 50   Sample baseline V to V?: Y   Sample baseline V to V points: 3
Sample filter: Binomial   Number of points for filter: 3 Sample start region (min): 0 Sample end region (min): 30
Marker peak width (sec): 5   Marker min peak height: 200   Marker baseline V to V?: Y   Marker baseline V to V points: 3
Lower marker selection: First peak > 500 RFU   Upper marker selection: Last peak > 200 RFU
Ladder size (bp)75, 200, 300, 400, 500, 700, 1000, 1500, 2000, 3000, 4000, 5000, 7000, 10000, 20000
Quantification using: Lower Marker   Final concentration (ng/uL): 0.5000   Dilution factor: 12.0

Sample: SampD10
Well location: D10
Created: Tuesday, August 10, 2021 8:01:08 PM

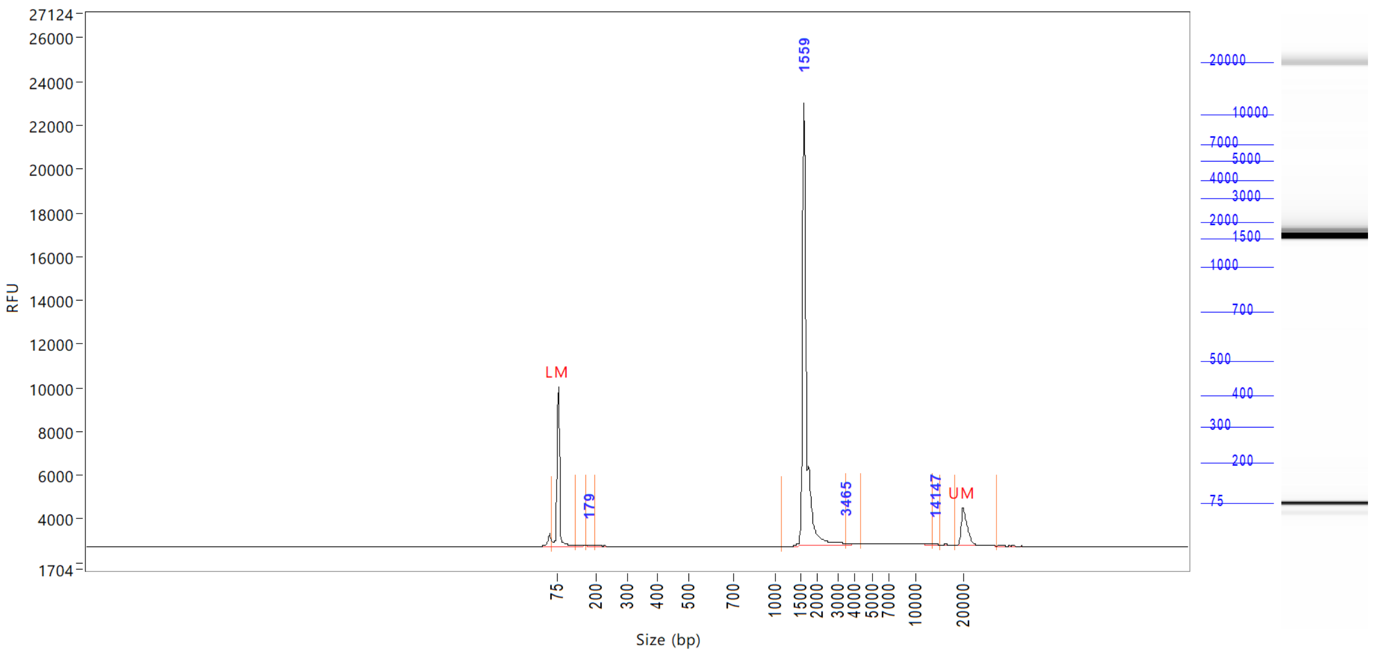

Peak	Size (bp)	Concentration (ng/uL)	From (bp)	To (bp)	Average size (bp)	CV%	RFU	Corrected peak area
1	75 (LM)	0.5000	56	131	74	10.46	7323	43.856
2	179	0.0764	166	194	179	3.95	51	0.558
3	1559	17.3885	1112	3465	1631	14.17	20286	127.100
4	3465	0.1565	3465	4345	3861	6.30	66	1.144
5	14147	0.0419	13537	15122	14164	2.60	59	0.306
6	20000 (UM)	0.1631	18171	27012	20347	4.09	1688	14.302

TIC:	17.6633	ng/uL
TIM:	18.3224	nmole/L
Total concentration:	18.2296	ng/uL

Sample peak width (sec): 5 Sample min peak height: 50 Sample baseline V to V?: Y Sample baseline V to V points: 3
Sample filter: Binomial Number of points for filter: 3 Sample start region (min): 0 Sample end region (min): 30
Marker peak width (sec): 5 Marker min peak height: 200 Marker baseline V to V?: Y Marker baseline V to V points: 3
Lower marker selection: First peak > 500 RFU Upper marker selection: Last peak > 200 RFU
Ladder size (bp)75, 200, 300, 400, 500, 700, 1000, 1500, 2000, 3000, 4000, 5000, 7000, 10000, 20000
Quantification using: Lower Marker Final concentration (ng/uL): 0.5000 Dilution factor: 12.0

Sample: SampE10
Well location: E10
Created: Tuesday, August 10, 2021 8:01:08 PM

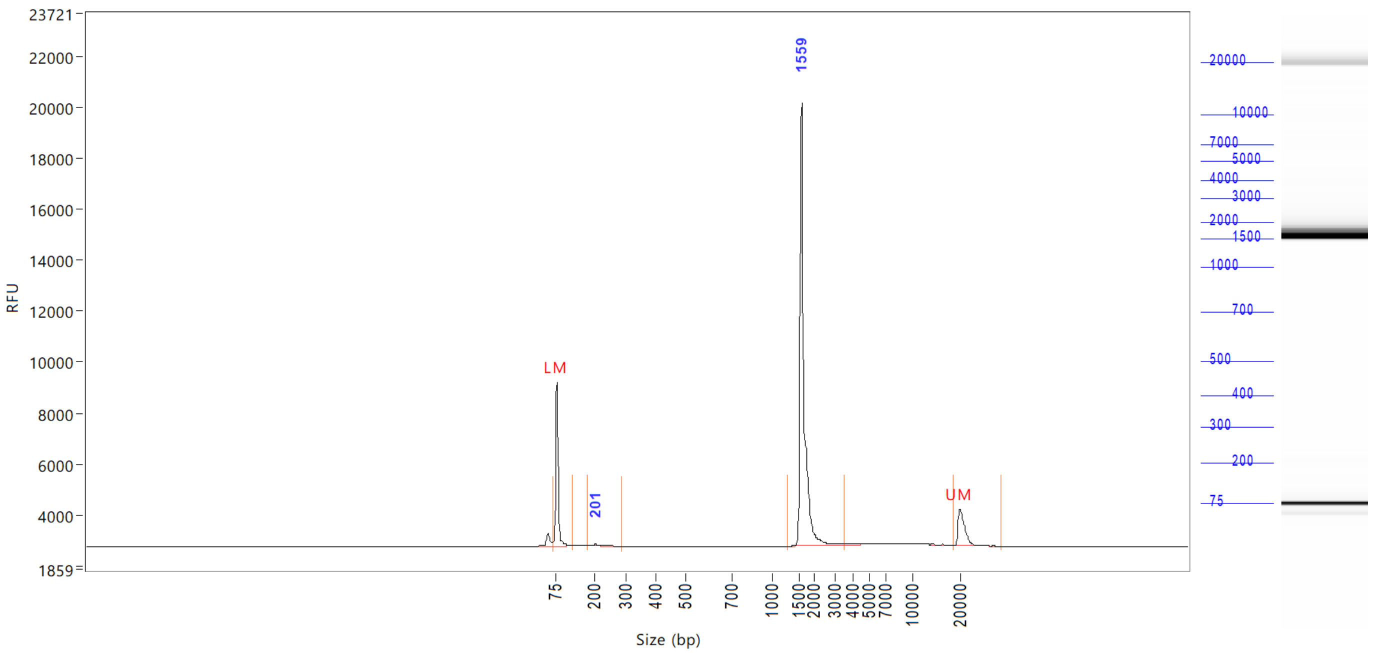

Peak	Size (bp)	Concentration (ng/uL)	From (bp)	To (bp)	Average size (bp)	CV%	RFU	Corrected peak area
1	75 (LM)	0.5000	64	129	74	10.00	6435	38.527
2	201	0.1603	177	282	208	9.41	67	1.030
3	1559	17.9716	1278	3465	1626	12.62	17448	115.400
4	20000 (UM)	0.1603	18415	28463	20417	4.30	1415	12.353

TIC:	18.1320	ng/uL
TIM:	19.4613	nmole/L
Total concentration:	18.7023	ng/uL

Sample peak width (sec): 5 Sample min peak height: 50 Sample baseline V to V?: Y Sample baseline V to V points: 3
Sample filter: Binomial Number of points for filter: 3 Sample start region (min): 0 Sample end region (min): 30
Marker peak width (sec): 5 Marker min peak height: 200 Marker baseline V to V?: Y Marker baseline V to V points: 3
Lower marker selection: First peak > 500 RFU Upper marker selection: Last peak > 200 RFU
Ladder size (bp)75, 200, 300, 400, 500, 700, 1000, 1500, 2000, 3000, 4000, 5000, 7000, 10000, 20000
Quantification using: Lower Marker Final concentration (ng/uL): 0.5000 Dilution factor: 12.0

Sample: SampF10
Well location: F10
Created: Tuesday, August 10, 2021 8:01:08 PM

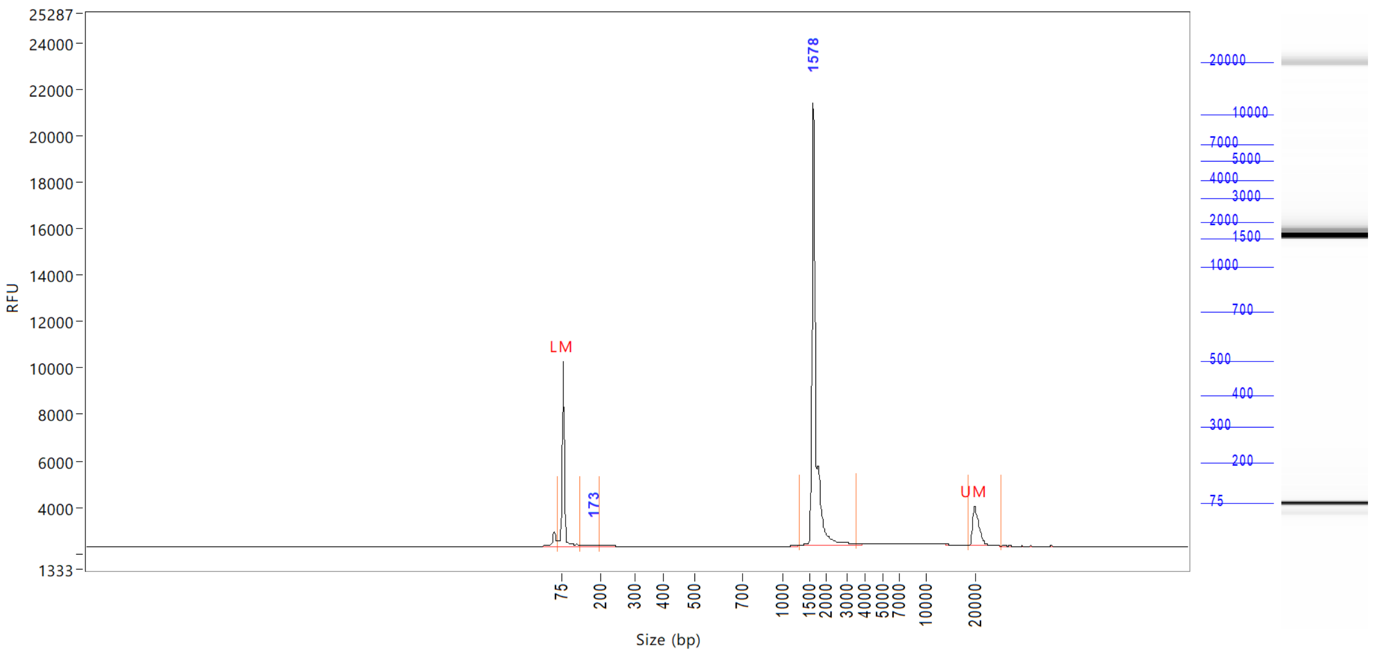

Peak	Size (bp)	Concentration (ng/uL)	From (bp)	To (bp)	Average size (bp)	CV%	RFU	Corrected peak area
1	75 (LM)	0.5000	58	129	75	11.36	7958	47.167
2	173	0.2767	129	192	160	10.98	80	2.175
3	1578	14.9291	1289	3536	1649	13.51	19108	117.360
4	20000 (UM)	0.1477	18659	25320	20318	3.79	1682	13.928

TIC:	15.2058	ng/uL
TIM:	17.7451	nmole/L
Total concentration:	15.7453	ng/uL

```
Sample peak width (sec): 5    Sample min peak height: 50    Sample baseline V to V?: Y    Sample baseline V to V points: 3
Sample filter: Binomial       Number of points for filter: 3  Sample start region (min): 0  Sample end region (min): 30
Marker peak width (sec): 5    Marker min peak height: 200   Marker baseline V to V?: Y    Marker baseline V to V points: 3
Lower marker selection: First peak > 500 RFU                Upper marker selection: Last peak > 200 RFU
Ladder size (bp)75, 200, 300, 400, 500, 700, 1000, 1500, 2000, 3000, 4000, 5000, 7000, 10000, 20000
Quantification using: Lower Marker          Final concentration (ng/uL): 0.5000          Dilution factor: 12.0
```

Sample: SampG10
Well location: G10
Created: Tuesday, August 10, 2021 8:01:08 PM

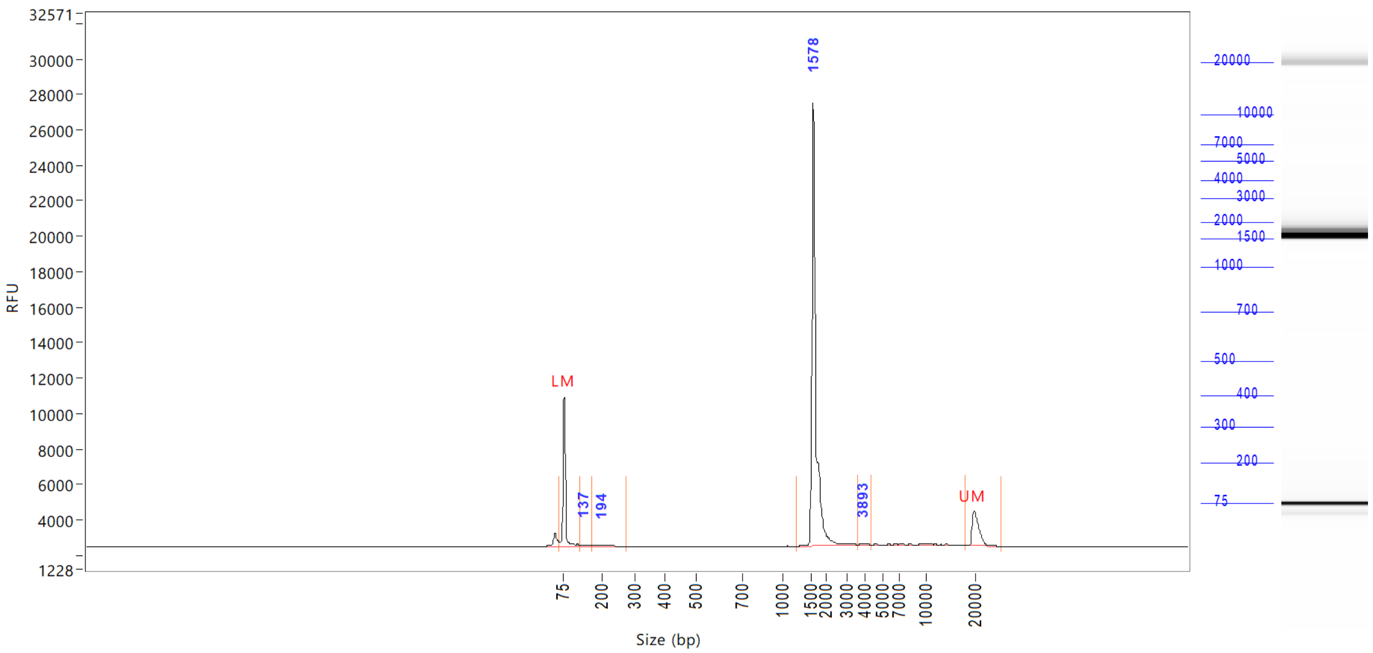

Peak	Size (bp)	Concentration (ng/uL)	From (bp)	To (bp)	Average size (bp)	CV%	RFU	Corrected peak area
1	75 (LM)	0.5000	62	128	74	12.20	8393	52.179
2	137	0.2100	128	166	145	7.40	109	1.826
3	194	0.2457	166	269	198	11.57	68	2.137
4	1578	17.9334	1245	3608	1634	11.84	25020	155.957
5	3893	0.0822	3608	4345	3943	4.85	54	0.714
6	20000 (UM)	0.1627	18049	25682	20381	3.82	2004	16.979

TIC:	18.4713	ng/uL
TIM:	22.5207	nmole/L
Total concentration:	18.7807	ng/uL

Sample peak width (sec): 5 Sample min peak height: 50 Sample baseline V to V?: Y Sample baseline V to V points: 3
Sample filter: Binomial Number of points for filter: 3 Sample start region (min): 0 Sample end region (min): 30
Marker peak width (sec): 5 Marker min peak height: 200 Marker baseline V to V?: Y Marker baseline V to V points: 3
Lower marker selection: First peak > 500 RFU Upper marker selection: Last peak > 200 RFU
Ladder size (bp)75, 200, 300, 400, 500, 700, 1000, 1500, 2000, 3000, 4000, 5000, 7000, 10000, 20000
Quantification using: Lower Marker Final concentration (ng/uL): 0.5000 Dilution factor: 12.0

Sample: SampH10
Well location: H10
Created: Tuesday, August 10, 2021 8:01:08 PM

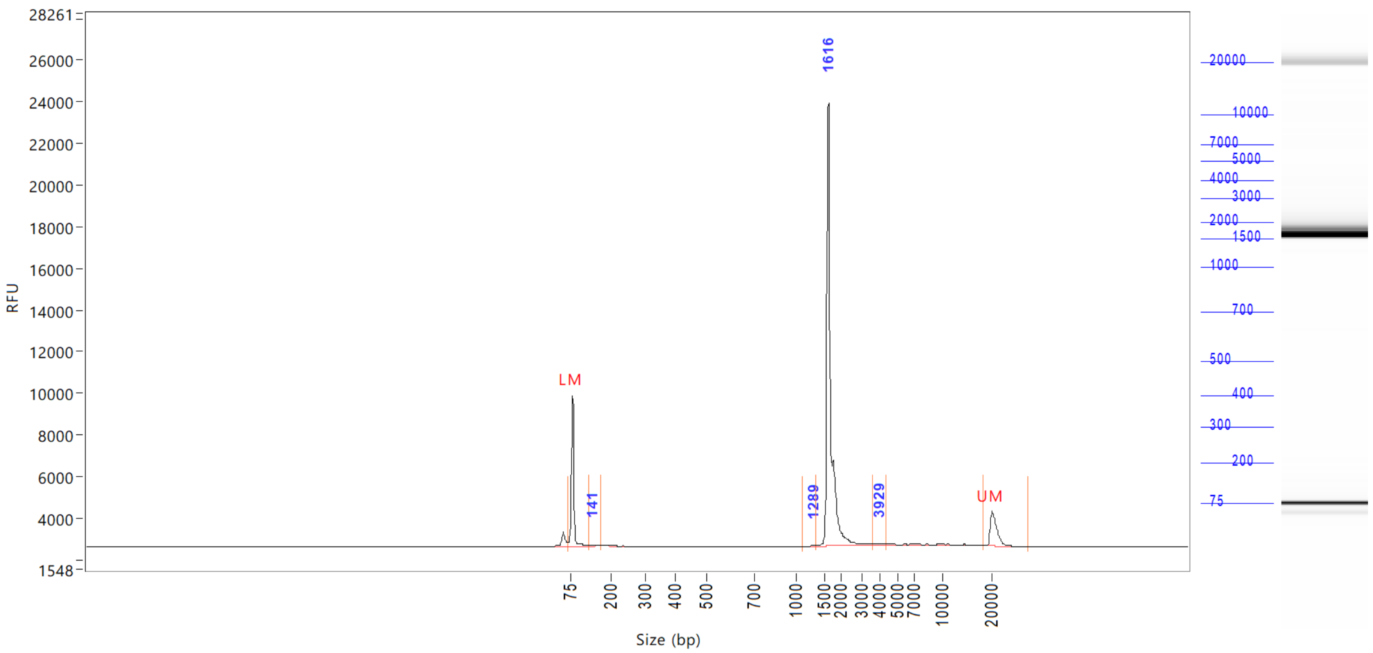

Peak	Size (bp)	Concentration (ng/uL)	From (bp)	To (bp)	Average size (bp)	CV%	RFU	Corrected peak area
1	75 (LM)	0.5000	64	131	75	10.13	7208	41.945
2	141	0.1135	131	166	144	5.76	72	0.794
3	1289	0.0457	1101	1356	1277	3.73	55	0.320
4	1616	19.6007	1356	3608	1677	13.26	21324	137.026
5	3929	0.1182	3608	4380	3953	5.10	61	0.827
6	20000 (UM)	0.1690	18415	27375	20421	3.98	1677	14.177

TIC:	19.8782	ng/uL
TIM:	20.6433	nmole/L
Total concentration:	20.3639	ng/uL

Sample peak width (sec): 5 Sample min peak height: 50 Sample baseline V to V?: Y Sample baseline V to V points: 3
Sample filter: Binomial Number of points for filter: 3 Sample start region (min): 0 Sample end region (min): 30
Marker peak width (sec): 5 Marker min peak height: 200 Marker baseline V to V?: Y Marker baseline V to V points: 3
Lower marker selection: First peak > 500 RFU Upper marker selection: Last peak > 200 RFU
Ladder size (bp)75, 200, 300, 400, 500, 700, 1000, 1500, 2000, 3000, 4000, 5000, 7000, 10000, 20000
Quantification using: Lower Marker Final concentration (ng/uL): 0.5000 Dilution factor: 12.0

Sample: SampA11
Well location: A11
Created: Tuesday, August 10, 2021 8:01:08 PM

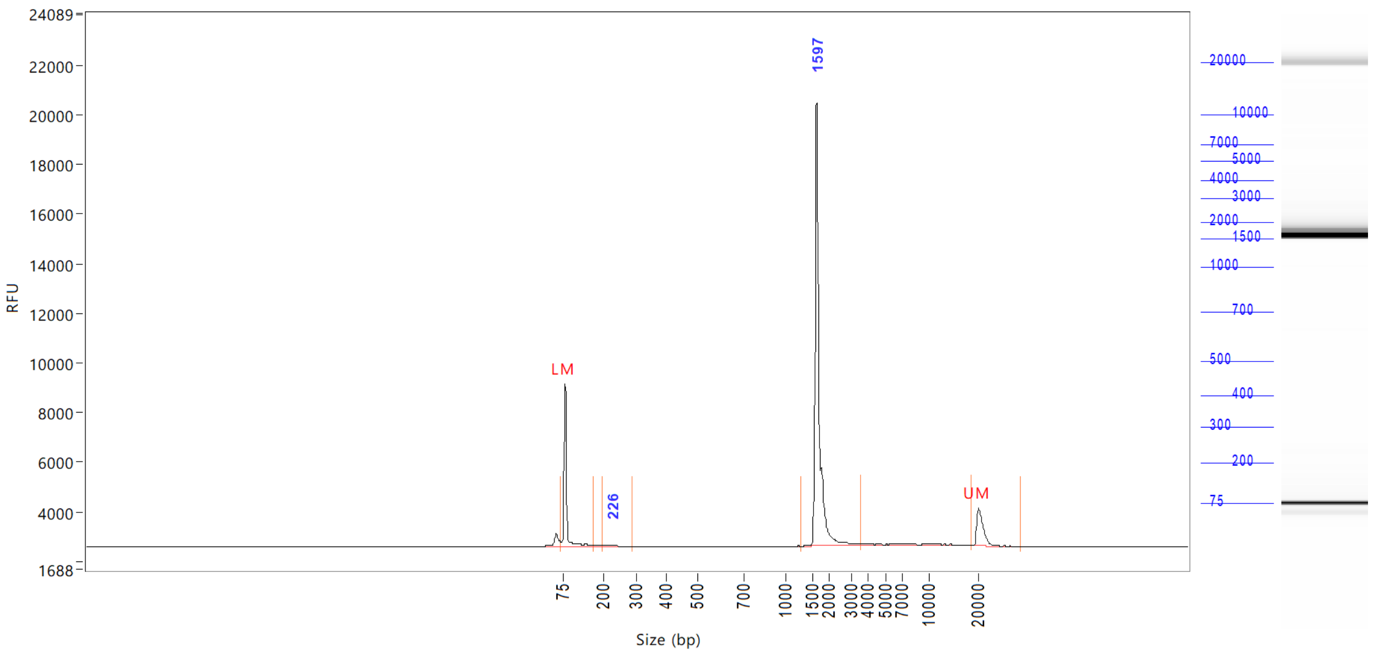

Peak	Size (bp)	Concentration (ng/uL)	From (bp)	To (bp)	Average size (bp)	CV%	RFU	Corrected peak area
1	75 (LM)	0.5000	64	168	79	21.14	6546	41.886
2	226	0.1725	196	286	225	8.64	53	1.204
3	1597	15.8780	1278	3572	1656	13.84	17879	110.844
4	20000 (UM)	0.1557	18537	28705	20435	4.55	1545	13.047

TIC:	16.0505	ng/uL
TIM:	17.0439	nmole/L
Total concentration:	16.6786	ng/uL

Sample peak width (sec): 5 Sample min peak height: 50 Sample baseline V to V?: Y Sample baseline V to V points: 3
Sample filter: Binomial Number of points for filter: 3 Sample start region (min): 0 Sample end region (min): 30
Marker peak width (sec): 5 Marker min peak height: 200 Marker baseline V to V?: Y Marker baseline V to V points: 3
Lower marker selection: First peak > 500 RFU Upper marker selection: Last peak > 200 RFU
Ladder size (bp)75, 200, 300, 400, 500, 700, 1000, 1500, 2000, 3000, 4000, 5000, 7000, 10000, 20000
Quantification using: Lower Marker Final concentration (ng/uL): 0.5000 Dilution factor: 12.0

Sample: SampB11
Well location: B11
Created: Tuesday, August 10, 2021 8:01:08 PM

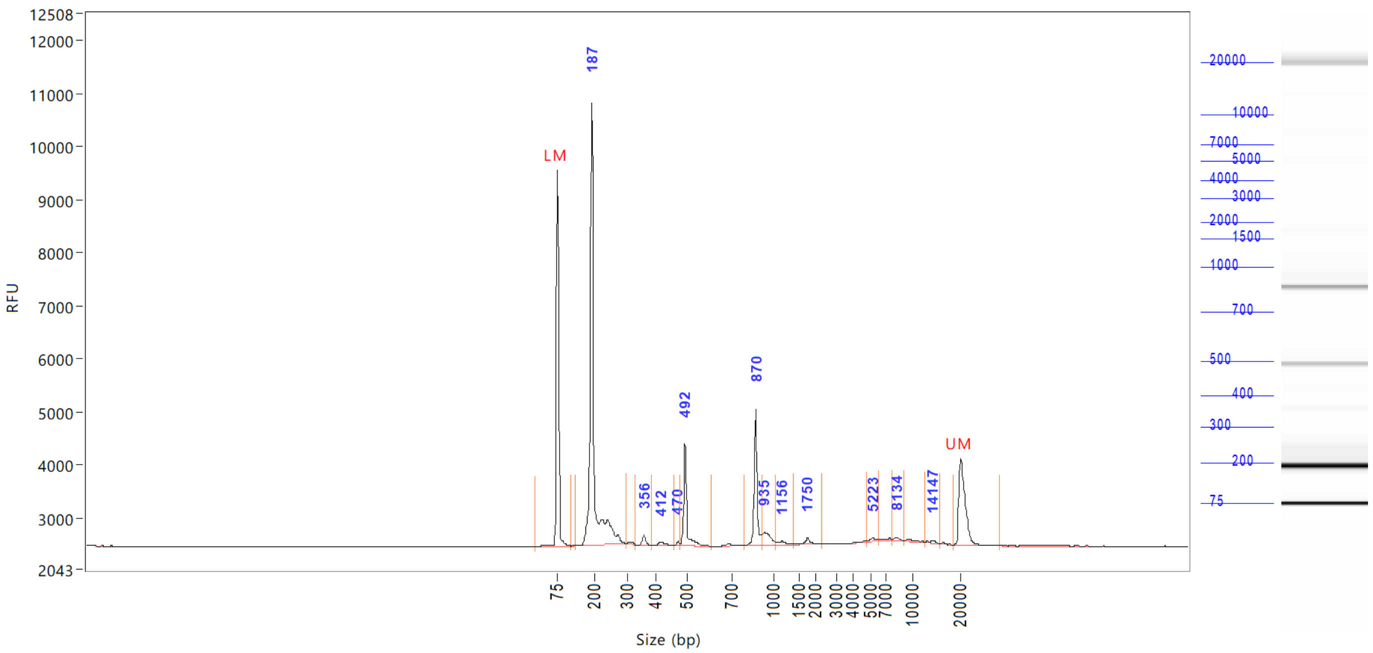

Peak	Size (bp)	Concentration (ng/uL)	From (bp)	To (bp)	Average size (bp)	CV%	RFU	Corrected peak area
1	75 (LM)	0.5000	3	118	72	8.78	7085	39.898
2	187	10.9967	137	295	193	10.90	8339	73.125
3	356	0.2233	327	384	353	2.13	203	1.485
4	412	0.1564	384	458	420	3.65	59	1.040
5	470	0.0686	458	475	468	0.94	84	0.456
6	492	1.7493	475	608	496	3.06	1915	11.632
7	870	1.9974	788	913	864	1.85	2566	13.282
8	935	0.5287	913	1056	956	3.32	250	3.516
9	1156	0.1399	1056	1389	1160	6.25	65	0.930
10	1750	0.1504	1389	2286	1738	7.65	114	1.000
11	5223	0.0859	4828	5963	5258	5.85	57	0.571
12	8134	0.0895	7534	8934	8132	4.14	68	0.595
13	14147	0.0694	12318	15610	13850	5.29	58	0.462
14	20000 (UM)	0.1706	18293	28221	20410	4.24	1617	13.614

TIC:	16.2556	ng/uL
TIM:	106.4873	nmole/L
Total concentration:	16.5609	ng/uL

```
Sample peak width (sec): 5    Sample min peak height: 50    Sample baseline V to V?: Y    Sample baseline V to V points: 3
Sample filter: Binomial       Number of points for filter: 3  Sample start region (min): 0  Sample end region (min): 30
Marker peak width (sec): 5    Marker min peak height: 200   Marker baseline V to V?: Y    Marker baseline V to V points: 3
Lower marker selection: First peak > 500 RFU                Upper marker selection: Last peak > 200 RFU
Ladder size (bp)75, 200, 300, 400, 500, 700, 1000, 1500, 2000, 3000, 4000, 5000, 7000, 10000, 20000
Quantification using: Lower Marker          Final concentration (ng/uL): 0.5000          Dilution factor: 12.0
```

Sample: SampC11
Well location: C11
Created: Tuesday, August 10, 2021 8:01:08 PM

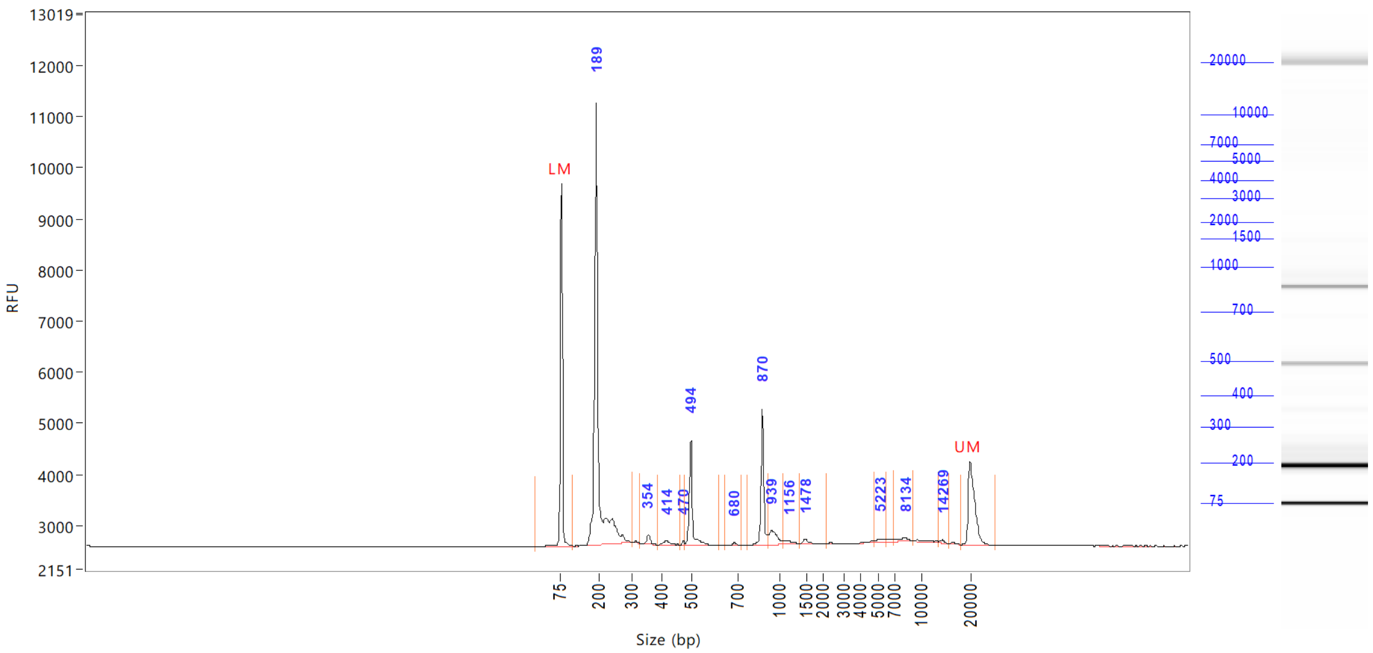

Peak	Size (bp)	Concentration (ng/uL)	From (bp)	To (bp)	Average size (bp)	CV%	RFU	Corrected peak area
1	75 (LM)	0.5000	0	114	73	7.89	7092	38.534
2	189	11.6463	114	298	195	10.95	8654	74.797
3	354	0.2154	327	386	352	2.14	191	1.384
4	414	0.1672	386	458	417	3.43	67	1.074
5	470	0.0590	458	475	468	0.94	72	0.379
6	494	1.9081	475	616	496	3.09	2048	12.254
7	680	0.0628	639	714	677	1.41	57	0.403
8	870	2.0658	762	918	867	1.95	2652	13.267
9	939	0.5921	918	1056	958	3.21	285	3.803
10	1156	0.1603	1056	1367	1168	6.82	68	1.030
11	1478	0.1590	1367	2172	1526	9.16	103	1.021
12	5223	0.1224	4828	6038	5332	6.69	68	0.786
13	8134	0.1640	6926	9134	7879	7.03	73	1.053
14	14269	0.0562	13537	15610	14241	3.30	62	0.361
15	20000 (UM)	0.1802	18171	25320	20440	3.94	1640	13.888

TIC:	17.3787	ng/uL
TIM:	111.9694	nmole/L
Total concentration:	17.6380	ng/uL

```
Sample peak width (sec): 5     Sample min peak height: 50     Sample baseline V to V?: Y     Sample baseline V to V points: 3
Sample filter: Binomial        Number of points for filter: 3  Sample start region (min): 0  Sample end region (min): 30
Marker peak width (sec): 5     Marker min peak height: 200    Marker baseline V to V?: Y     Marker baseline V to V points: 3
Lower marker selection: First peak > 500 RFU                  Upper marker selection: Last peak > 200 RFU
Ladder size (bp)75, 200, 300, 400, 500, 700, 1000, 1500, 2000, 3000, 4000, 5000, 7000, 10000, 20000
Quantification using: Lower Marker          Final concentration (ng/uL): 0.5000          Dilution factor: 12.0
```

Sample: SampD11
Well location: D11
Created: Tuesday, August 10, 2021 8:01:08 PM

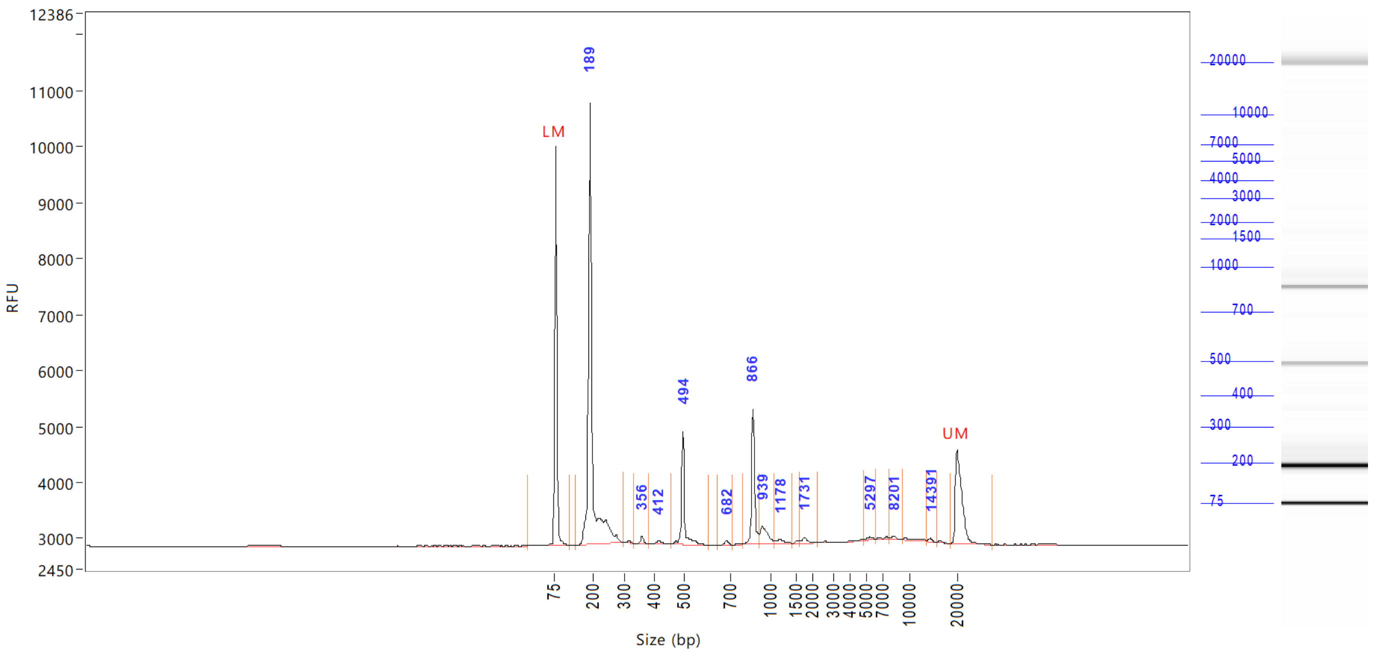

Peak	Size (bp)	Concentration (ng/uL)	From (bp)	To (bp)	Average size (bp)	CV%	RFU	Corrected peak area
1	75 (LM)	0.5000	0	124	73	8.21	7143	39.441
2	189	10.6603	141	295	194	10.84	7902	70.076
3	356	0.1637	329	382	354	2.04	149	1.076
4	412	0.1224	382	456	415	2.71	58	0.804
5	494	1.8585	456	600	496	3.28	2012	12.217
6	682	0.0815	641	714	679	1.36	78	0.536
7	866	1.9005	792	913	866	1.84	2413	12.493
8	939	0.6350	913	1056	955	3.13	322	4.174
9	1178	0.1473	1056	1411	1171	6.53	69	0.969
10	1731	0.1351	1597	2258	1769	6.97	109	0.888
11	5297	0.0721	4828	6038	5279	5.86	52	0.474
12	8201	0.0750	7667	9134	8250	4.39	61	0.493
13	14391	0.0573	13537	15732	14293	3.33	62	0.377
14	20000 (UM)	0.1857	18537	27375	20494	4.56	1683	14.647

TIC:	15.9088	ng/uL
TIM:	103.0390	nmole/L
Total concentration:	16.2765	ng/uL

```
Sample peak width (sec): 5    Sample min peak height: 50    Sample baseline V to V?: Y    Sample baseline V to V points: 3
Sample filter: Binomial       Number of points for filter: 3  Sample start region (min): 0  Sample end region (min): 30
Marker peak width (sec): 5    Marker min peak height: 200   Marker baseline V to V?: Y    Marker baseline V to V points: 3
Lower marker selection: First peak > 500 RFU                Upper marker selection: Last peak > 200 RFU
Ladder size (bp)75, 200, 300, 400, 500, 700, 1000, 1500, 2000, 3000, 4000, 5000, 7000, 10000, 20000
Quantification using: Lower Marker          Final concentration (ng/uL): 0.5000          Dilution factor: 12.0
```

Sample: SampE11
Well location: E11
Created: Tuesday, August 10, 2021 8:01:08 PM

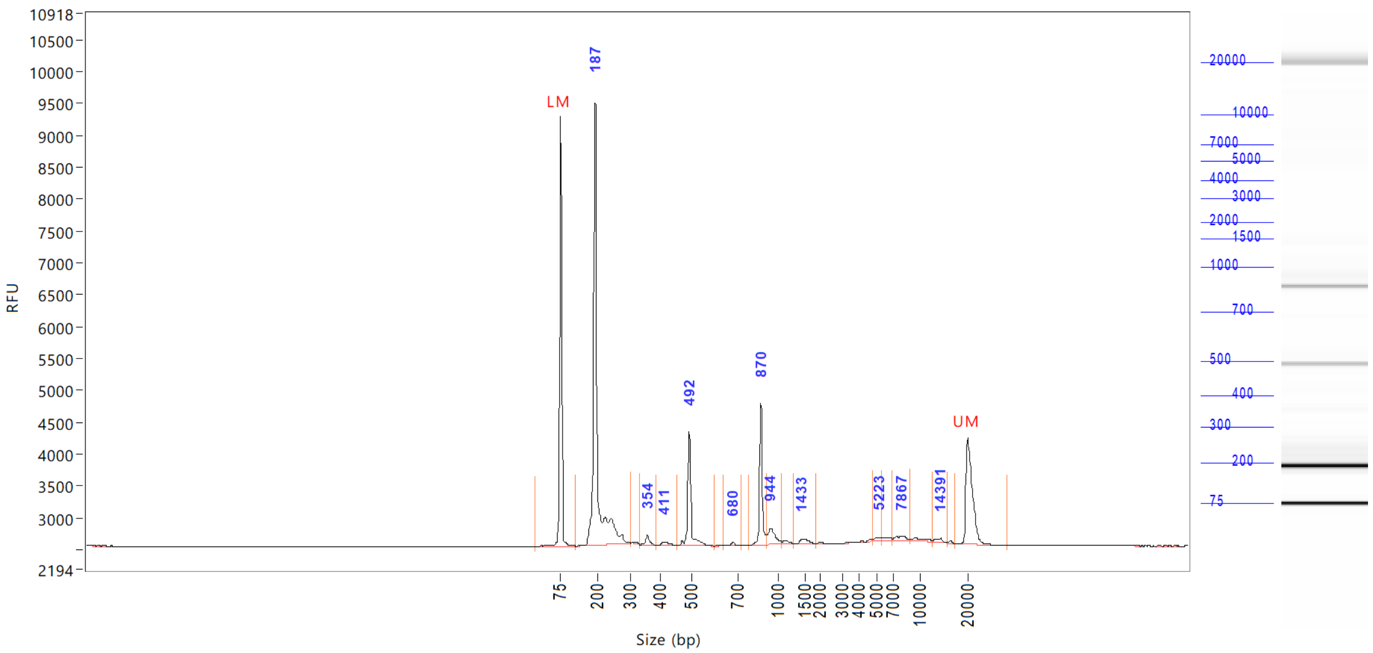

Peak	Size (bp)	Concentration (ng/uL)	From (bp)	To (bp)	Average size (bp)	CV%	RFU	Corrected peak area
1	75 (LM)	0.5000	0	126	72	9.15	6754	37.462
2	187	9.9150	126	300	194	11.15	6951	61.906
3	354	0.1920	329	386	353	2.13	159	1.199
4	411	0.1348	386	456	416	3.28	56	0.842
5	492	1.7230	456	598	495	3.29	1779	10.758
6	680	0.0611	641	723	679	1.66	50	0.381
7	870	1.7641	788	913	868	1.72	2210	11.015
8	944	0.5211	913	1068	955	3.33	253	3.254
9	1433	0.1533	1278	1827	1479	5.90	78	0.957
10	5223	0.1006	4793	5741	5189	5.42	60	0.628
11	7867	0.1449	7001	8934	7837	6.01	65	0.905
12	14391	0.0971	12684	15732	13941	5.28	67	0.606
13	20000 (UM)	0.1908	17439	28221	20474	4.33	1664	14.297

TIC:	14.8070	ng/uL
TIM:	95.8212	nmole/L
Total concentration:	15.2151	ng/uL

```
Sample peak width (sec): 5     Sample min peak height: 50    Sample baseline V to V?: Y    Sample baseline V to V points: 3
Sample filter: Binomial        Number of points for filter: 3  Sample start region (min): 0  Sample end region (min): 30
Marker peak width (sec): 5     Marker min peak height: 200   Marker baseline V to V?: Y    Marker baseline V to V points: 3
Lower marker selection: First peak > 500 RFU                 Upper marker selection: Last peak > 200 RFU
Ladder size (bp)75, 200, 300, 400, 500, 700, 1000, 1500, 2000, 3000, 4000, 5000, 7000, 10000, 20000
Quantification using: Lower Marker          Final concentration (ng/uL): 0.5000          Dilution factor: 12.0
```

Sample: SampF11
Well location: F11
Created: Tuesday, August 10, 2021 8:01:08 PM

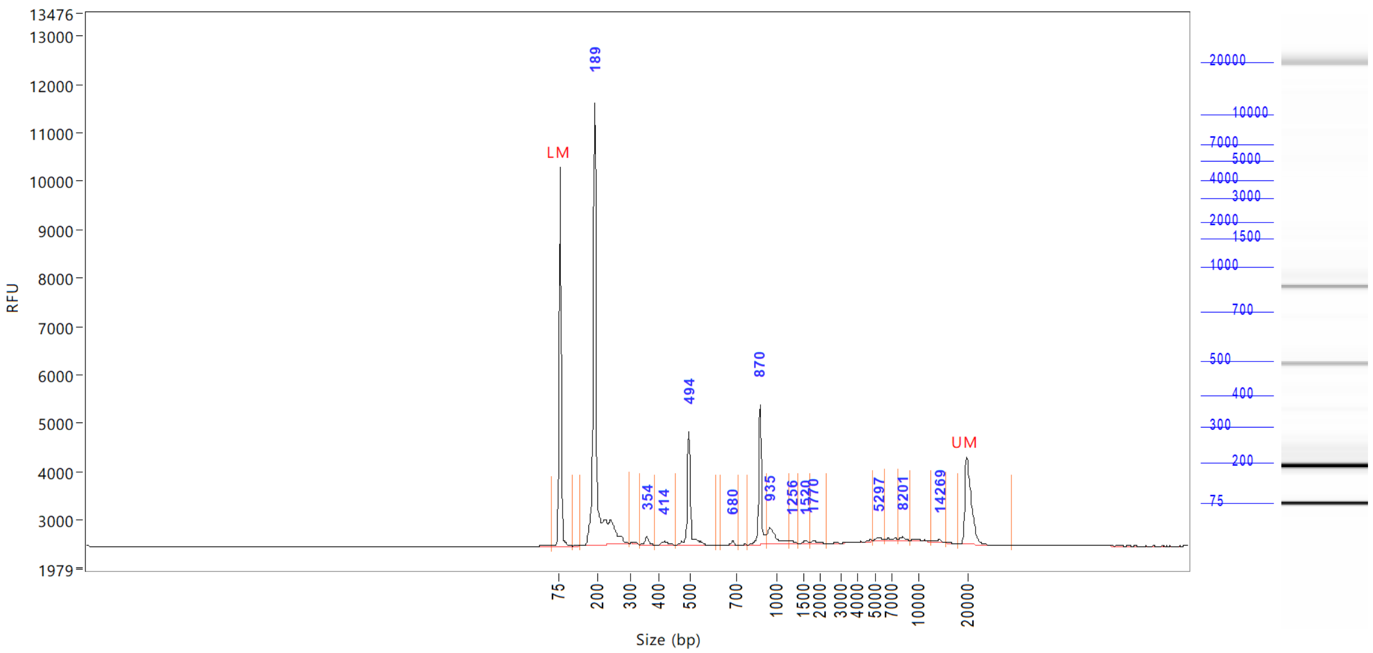

Peak	Size (bp)	Concentration (ng/uL)	From (bp)	To (bp)	Average size (bp)	CV%	RFU	Corrected peak area
1	75 (LM)	0.5000	47	114	73	5.60	7854	42.747
2	189	11.2949	141	296	194	10.90	9161	80.470
3	354	0.1889	329	382	352	2.17	182	1.346
4	414	0.1314	382	450	413	2.54	70	0.936
5	494	2.0030	450	608	495	3.22	2347	14.270
6	680	0.0815	626	710	677	1.45	87	0.581
7	870	1.9988	779	913	866	1.84	2879	14.240
8	935	0.7403	913	1223	984	7.73	347	5.274
9	1256	0.0579	1223	1389	1268	2.83	64	0.412
10	1520	0.0674	1389	1674	1526	4.07	65	0.480
11	1770	0.0781	1674	2258	1827	6.24	69	0.556
12	5297	0.0945	4828	6038	5295	6.08	68	0.673
13	8201	0.0740	7601	9000	8103	3.64	68	0.527
14	14269	0.0696	12440	15610	13882	5.27	64	0.496
15	20000 (UM)	0.1808	18171	29189	20371	4.05	1817	15.460

TIC:	16.8803	ng/uL
TIM:	109.2955	nmole/L
Total concentration:	17.1302	ng/uL

Sample peak width (sec): 5   Sample min peak height: 50   Sample baseline V to V?: Y   Sample baseline V to V points: 3
Sample filter: Binomial   Number of points for filter: 3 Sample start region (min): 0 Sample end region (min): 30
Marker peak width (sec): 5   Marker min peak height: 200   Marker baseline V to V?: Y   Marker baseline V to V points: 3
Lower marker selection: First peak > 500 RFU   Upper marker selection: Last peak > 200 RFU
Ladder size (bp)75, 200, 300, 400, 500, 700, 1000, 1500, 2000, 3000, 4000, 5000, 7000, 10000, 20000
Quantification using: Lower Marker   Final concentration (ng/uL): 0.5000   Dilution factor: 12.0

Sample: SampG11
Well location: G11
Created: Tuesday, August 10, 2021 8:01:08 PM

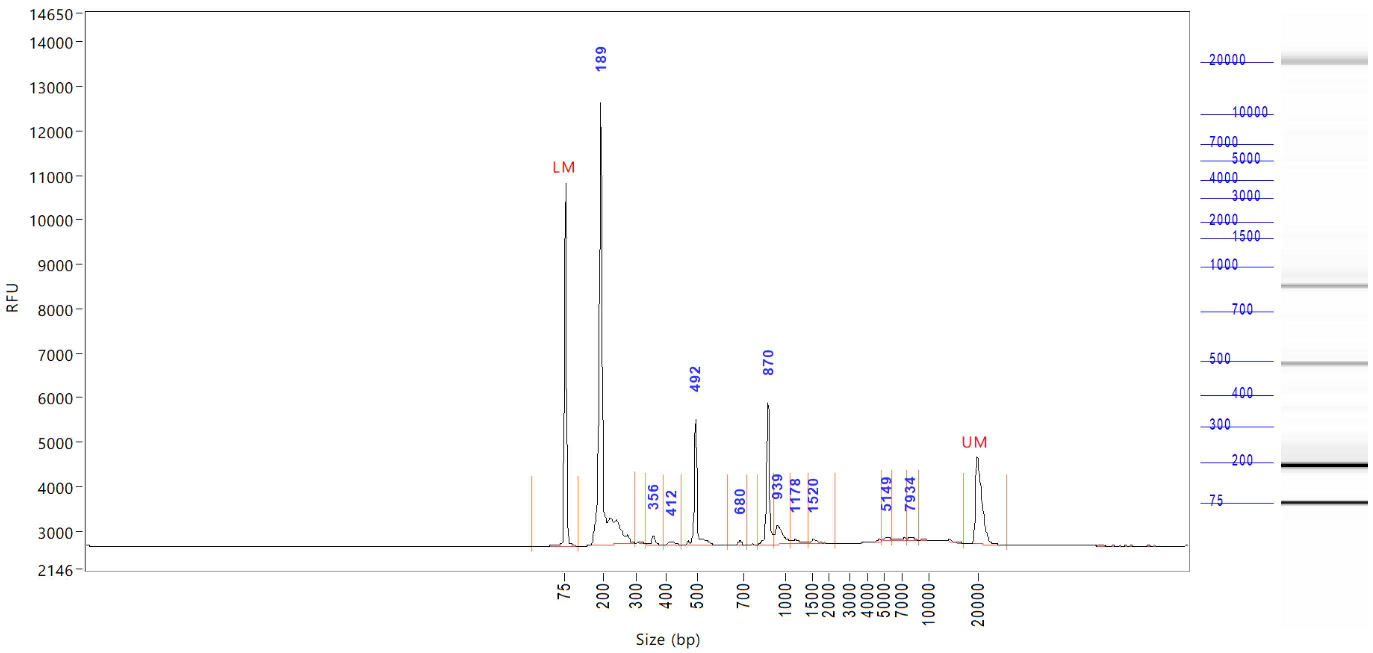

Peak	Size (bp)	Concentration (ng/uL)	From (bp)	To (bp)	Average size (bp)	CV%	RFU	Corrected peak area
1	75 (LM)	0.5000	0	118	72	10.42	8144	44.247
2	189	11.9346	118	295	195	11.09	9965	88.012
3	356	0.1851	329	388	353	2.04	197	1.365
4	412	0.1569	388	445	415	2.74	77	1.157
5	492	2.2585	445	626	495	3.28	2823	16.655
6	680	0.0909	626	723	677	1.52	95	0.670
7	870	2.1447	796	913	868	1.77	3190	15.816
8	939	0.7804	913	1079	960	3.75	416	5.755
9	1178	0.2169	1079	1411	1206	7.56	99	1.600
10	1520	0.1682	1411	2315	1603	10.40	105	1.241
11	5149	0.0983	4828	5963	5235	5.84	77	0.725
12	7934	0.0735	7667	8867	8070	3.20	63	0.542
13	20000 (UM)	0.1877	17317	26166	20437	3.86	1954	16.613

TIC:	18.1080	ng/uL
TIM:	115.7609	nmole/L
Total concentration:	18.3477	ng/uL

Sample peak width (sec): 5 Sample min peak height: 50 Sample baseline V to V?: Y Sample baseline V to V points: 3
Sample filter: Binomial Number of points for filter: 3 Sample start region (min): 0 Sample end region (min): 30
Marker peak width (sec): 5 Marker min peak height: 200 Marker baseline V to V?: Y Marker baseline V to V points: 3
Lower marker selection: First peak > 500 RFU Upper marker selection: Last peak > 200 RFU
Ladder size (bp)75, 200, 300, 400, 500, 700, 1000, 1500, 2000, 3000, 4000, 5000, 7000, 10000, 20000
Quantification using: Lower Marker Final concentration (ng/uL): 0.5000 Dilution factor: 12.0

Sample: SampH11
Well location: H11
Created: Tuesday, August 10, 2021 8:01:08 PM

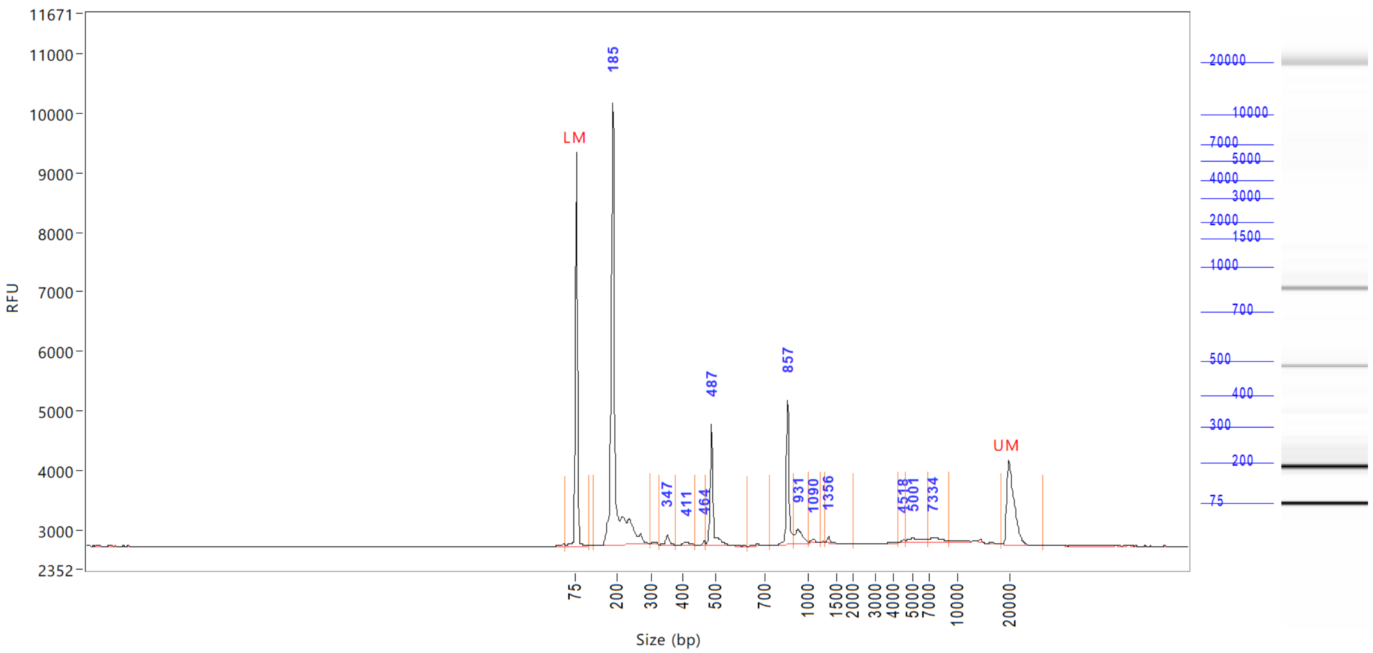

Peak	Size (bp)	Concentration (ng/uL)	From (bp)	To (bp)	Average size (bp)	CV%	RFU	Corrected peak area
1	75 (LM)	0.5000	43	112	72	5.69	6596	35.537
2	185	10.7860	129	295	193	11.33	7425	63.884
3	347	0.2200	323	378	347	2.19	180	1.303
4	411	0.1361	378	437	410	2.69	55	0.806
5	464	0.0810	437	471	461	1.75	76	0.480
6	487	1.9950	471	631	491	3.55	2030	11.816
7	857	1.9618	731	905	855	1.93	2408	11.620
8	931	0.4716	905	1001	938	2.45	238	2.793
9	1090	0.0836	1001	1212	1081	4.23	58	0.495
10	1356	0.0987	1300	2001	1389	8.19	101	0.584
11	4518	0.0545	4242	4656	4493	2.42	51	0.323
12	5001	0.2573	4656	6852	5515	11.89	76	1.524
13	7334	0.2010	6852	9000	7683	6.96	75	1.190
14	20000 (UM)	0.1834	18293	26529	20397	4.14	1412	13.038

TIC:	16.3466	ng/uL
TIM:	105.4375	nmole/L
Total concentration:	16.5988	ng/uL

```
Sample peak width (sec): 5    Sample min peak height: 50    Sample baseline V to V?: Y    Sample baseline V to V points: 3
Sample filter: Binomial       Number of points for filter: 3  Sample start region (min): 0  Sample end region (min): 30
Marker peak width (sec): 5    Marker min peak height: 200   Marker baseline V to V?: Y    Marker baseline V to V points: 3
Lower marker selection: First peak > 500 RFU                Upper marker selection: Last peak > 200 RFU
Ladder size (bp)75, 200, 300, 400, 500, 700, 1000, 1500, 2000, 3000, 4000, 5000, 7000, 10000, 20000
Quantification using: Lower Marker          Final concentration (ng/uL): 0.5000          Dilution factor: 12.0
```

Sample: SampA12
Well location: A12
Created: Tuesday, August 10, 2021 8:01:08 PM

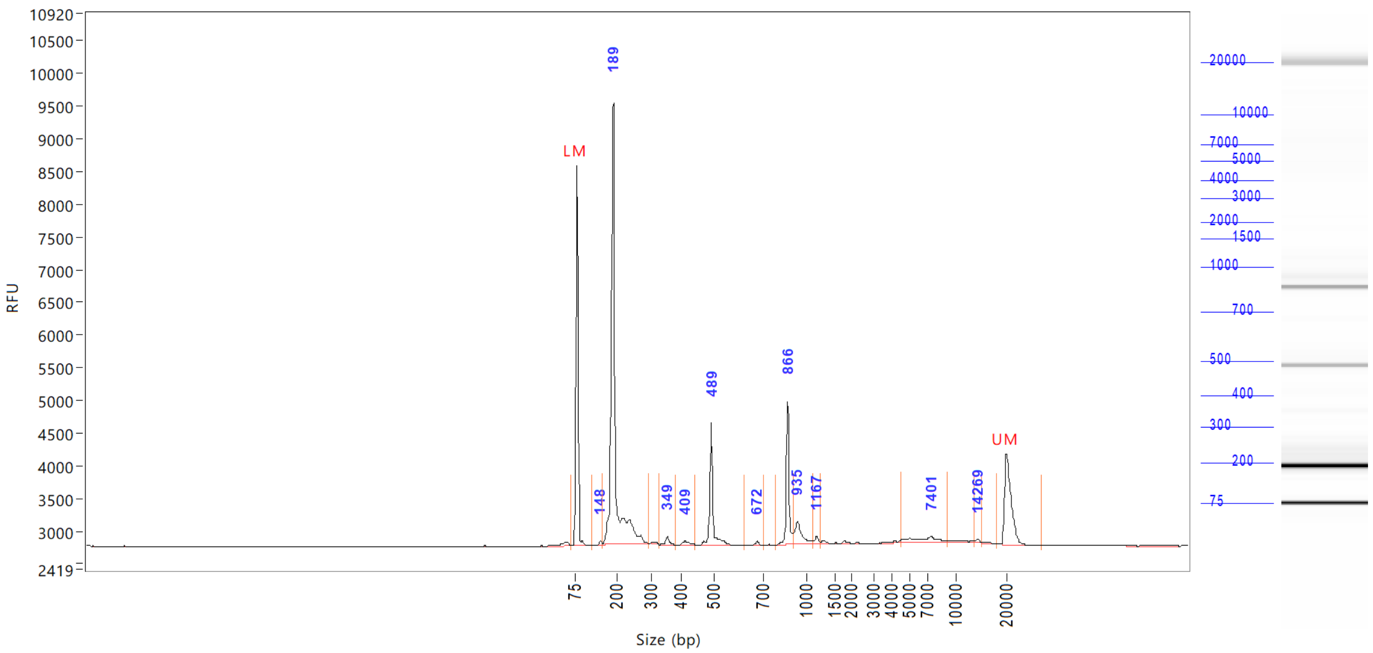

Peak	Size (bp)	Concentration (ng/uL)	From (bp)	To (bp)	Average size (bp)	CV%	RFU	Corrected peak area
1	75 (LM)	0.5000	58	126	74	5.37	5816	31.850
2	148	0.0764	126	156	147	3.57	68	0.405
3	189	11.1934	156	293	194	10.51	6761	59.418
4	349	0.1859	323	380	349	2.21	135	0.987
5	409	0.1444	380	439	410	2.61	54	0.767
6	489	2.1014	439	618	492	3.59	1867	11.155
7	672	0.0861	618	701	668	1.74	61	0.457
8	866	1.9425	779	905	863	1.67	2176	10.311
9	935	0.8029	905	1123	955	4.88	348	4.262
10	1167	0.1112	1123	1245	1170	2.63	104	0.590
11	7401	0.5071	4518	9200	6361	20.35	86	2.692
12	14269	0.0539	13659	15122	14193	2.30	59	0.286
13	20000 (UM)	0.1840	18171	27012	20339	3.90	1393	11.723

TIC:	17.2052	ng/uL
TIM:	109.7969	nmole/L
Total concentration:	17.5726	ng/uL

```
Sample peak width (sec): 5     Sample min peak height: 50     Sample baseline V to V?: Y     Sample baseline V to V points: 3
Sample filter: Binomial        Number of points for filter: 3  Sample start region (min): 0  Sample end region (min): 30
Marker peak width (sec): 5     Marker min peak height: 200    Marker baseline V to V?: Y     Marker baseline V to V points: 3
Lower marker selection: First peak > 500 RFU                  Upper marker selection: Last peak > 200 RFU
Ladder size (bp)75, 200, 300, 400, 500, 700, 1000, 1500, 2000, 3000, 4000, 5000, 7000, 10000, 20000
Quantification using: Lower Marker          Final concentration (ng/uL): 0.5000          Dilution factor: 12.0
```

Sample: SampB12
Well location: B12
Created: Tuesday, August 10, 2021 8:01:08 PM

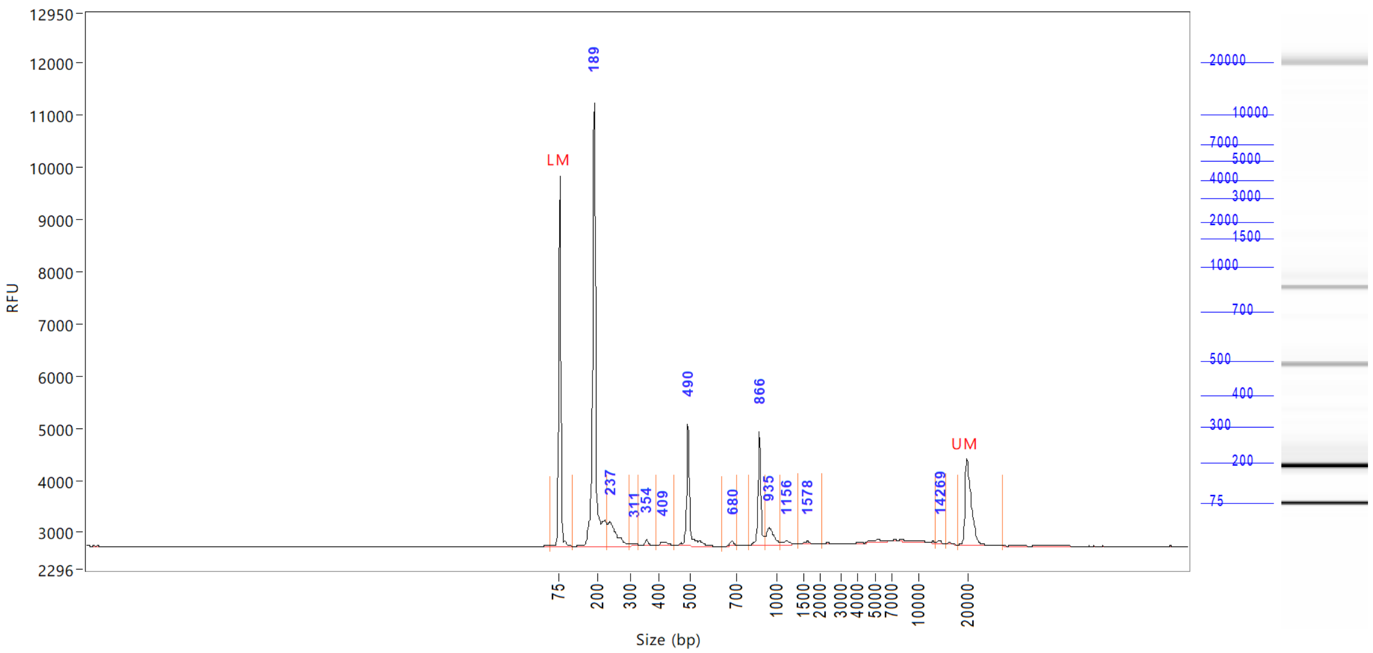

Peak	Size (bp)	Concentration (ng/uL)	From (bp)	To (bp)	Average size (bp)	CV%	RFU	Corrected peak area
1	75 (LM)	0.5000	47	118	74	5.66	7098	38.835
2	189	10.3837	118	228	189	6.36	8507	67.208
3	237	1.4314	228	296	248	6.54	467	9.264
4	311	0.1128	296	327	309	2.61	50	0.730
5	354	0.1643	327	388	352	2.89	116	1.064
6	409	0.1803	388	445	414	2.83	72	1.167
7	490	2.2144	445	634	495	3.61	2340	14.333
8	680	0.1055	634	700	676	1.18	106	0.683
9	866	1.6704	783	909	862	1.82	2170	10.811
10	935	0.6309	909	1034	949	2.84	330	4.084
11	1156	0.1613	1034	1378	1152	6.54	76	1.044
12	1578	0.0974	1378	2087	1604	8.39	58	0.631
13	14269	0.0577	13415	15732	14205	3.71	62	0.374
14	20000 (UM)	0.1766	18049	27375	20397	4.16	1644	13.716

TIC:	17.2102	ng/uL
TIM:	114.1434	nmole/L
Total concentration:	17.5199	ng/uL

```
Sample peak width (sec): 5    Sample min peak height: 50    Sample baseline V to V?: Y    Sample baseline V to V points: 3
Sample filter: Binomial       Number of points for filter: 3  Sample start region (min): 0  Sample end region (min): 30
Marker peak width (sec): 5    Marker min peak height: 200   Marker baseline V to V?: Y    Marker baseline V to V points: 3
Lower marker selection: First peak > 500 RFU                Upper marker selection: Last peak > 200 RFU
Ladder size (bp)75, 200, 300, 400, 500, 700, 1000, 1500, 2000, 3000, 4000, 5000, 7000, 10000, 20000
Quantification using: Lower Marker          Final concentration (ng/uL): 0.5000          Dilution factor: 12.0
```

Sample: SampC12
Well location: C12
Created: Tuesday, August 10, 2021 8:01:08 PM

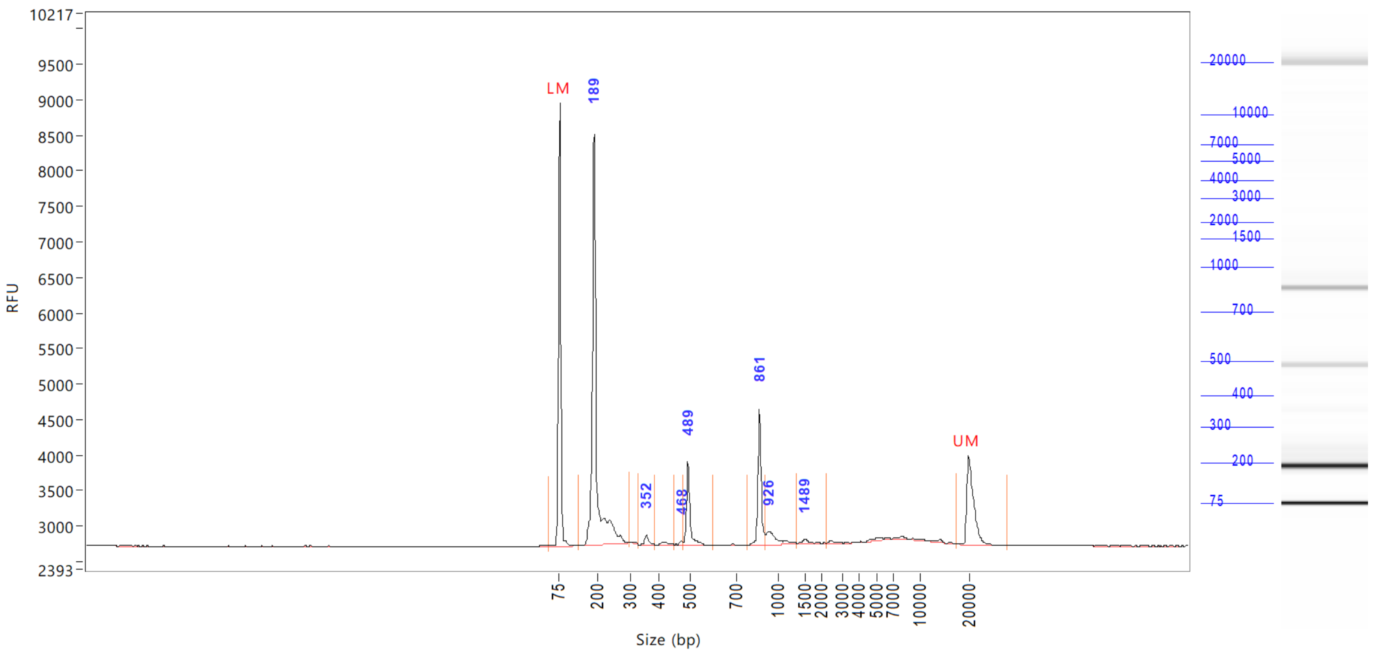

Peak	Size (bp)	Concentration (ng/uL)	From (bp)	To (bp)	Average size (bp)	CV%	RFU	Corrected peak area
1	75 (LM)	0.5000	43	137	73	6.48	6248	36.085
2	189	9.2962	137	298	193	10.66	5787	55.908
3	352	0.2012	327	382	352	2.33	144	1.210
4	468	0.0783	445	473	464	1.52	65	0.471
5	489	1.3586	473	593	493	2.94	1171	8.171
6	861	1.8869	775	905	857	1.94	1913	11.348
7	926	0.5929	905	1345	995	10.20	188	3.566
8	1489	0.1479	1345	2258	1590	12.50	65	0.889
9	20000 (UM)	0.1583	17317	27979	20514	5.15	1249	11.422

TIC:	13.5619	ng/uL
TIM:	89.7032	nmole/L
Total concentration:	14.1921	ng/uL

Sample peak width (sec): 5 Sample min peak height: 50 Sample baseline V to V?: Y Sample baseline V to V points: 3
Sample filter: Binomial Number of points for filter: 3 Sample start region (min): 0 Sample end region (min): 30
Marker peak width (sec): 5 Marker min peak height: 200 Marker baseline V to V?: Y Marker baseline V to V points: 3
Lower marker selection: First peak > 500 RFU Upper marker selection: Last peak > 200 RFU
Ladder size (bp)75, 200, 300, 400, 500, 700, 1000, 1500, 2000, 3000, 4000, 5000, 7000, 10000, 20000
Quantification using: Lower Marker Final concentration (ng/uL): 0.5000 Dilution factor: 12.0

Sample: SampD12
Well location: D12
Created: Tuesday, August 10, 2021 8:01:08 PM

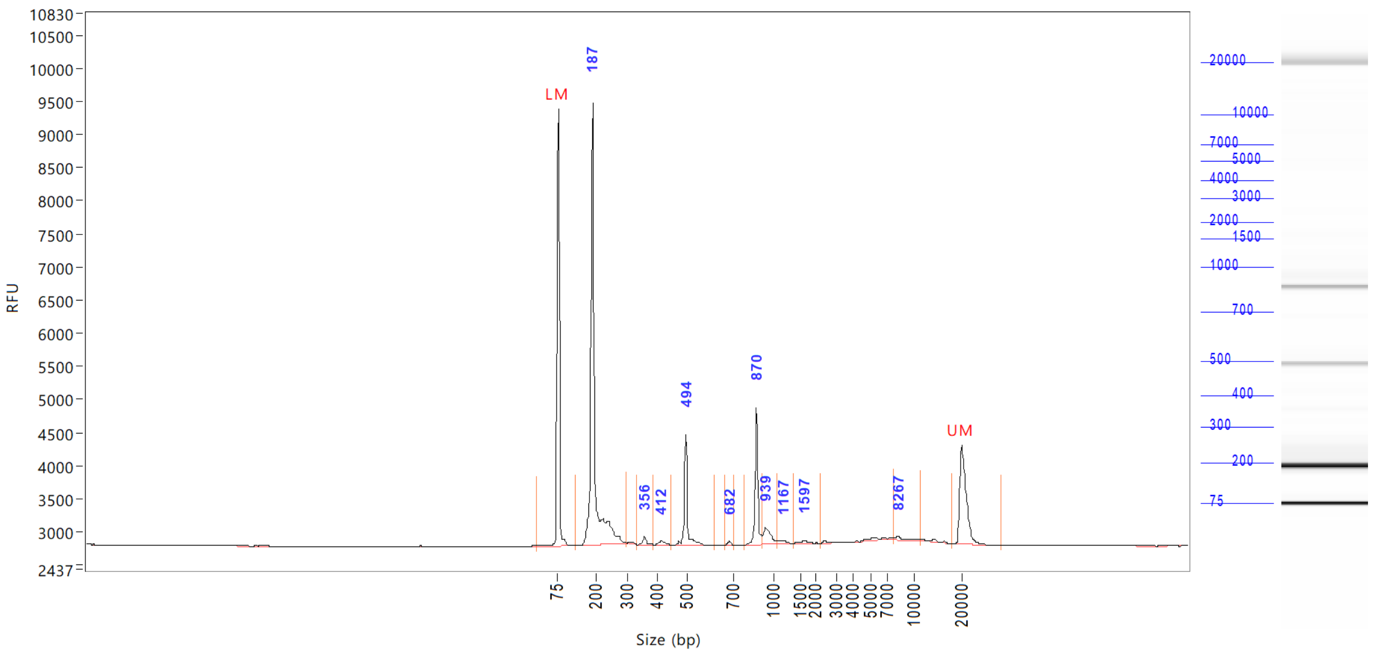

Peak	Size (bp)	Concentration (ng/uL)	From (bp)	To (bp)	Average size (bp)	CV%	RFU	Corrected peak area
1	75 (LM)	0.5000	5	131	73	9.25	6606	36.779
2	187	9.6350	131	295	193	10.45	6684	59.060
3	356	0.1757	329	390	354	2.71	132	1.077
4	412	0.1224	390	445	413	2.44	56	0.750
5	494	1.6632	445	618	496	3.45	1663	10.195
6	682	0.0651	662	701	678	1.02	61	0.399
7	870	1.6818	775	913	865	1.83	2075	10.309
8	939	0.5136	913	1056	954	3.15	248	3.148
9	1167	0.1282	1056	1367	1169	6.41	55	0.786
10	1597	0.1157	1367	2172	1594	9.50	52	0.709
11	8267	0.1383	7667	11464	9019	11.37	55	0.848
12	20000 (UM)	0.1738	18049	28221	20486	4.36	1490	12.785

TIC:	14.2390	ng/uL
TIM:	93.4709	nmole/L
Total concentration:	14.5832	ng/uL

Sample peak width (sec): 5 Sample min peak height: 50 Sample baseline V to V?: Y Sample baseline V to V points: 3
Sample filter: Binomial Number of points for filter: 3 Sample start region (min): 0 Sample end region (min): 30
Marker peak width (sec): 5 Marker min peak height: 200 Marker baseline V to V?: Y Marker baseline V to V points: 3
Lower marker selection: First peak > 500 RFU Upper marker selection: Last peak > 200 RFU
Ladder size (bp)75, 200, 300, 400, 500, 700, 1000, 1500, 2000, 3000, 4000, 5000, 7000, 10000, 20000
Quantification using: Lower Marker Final concentration (ng/uL): 0.5000 Dilution factor: 12.0

Sample: SampE12
Well location: E12
Created: Tuesday, August 10, 2021 8:01:08 PM

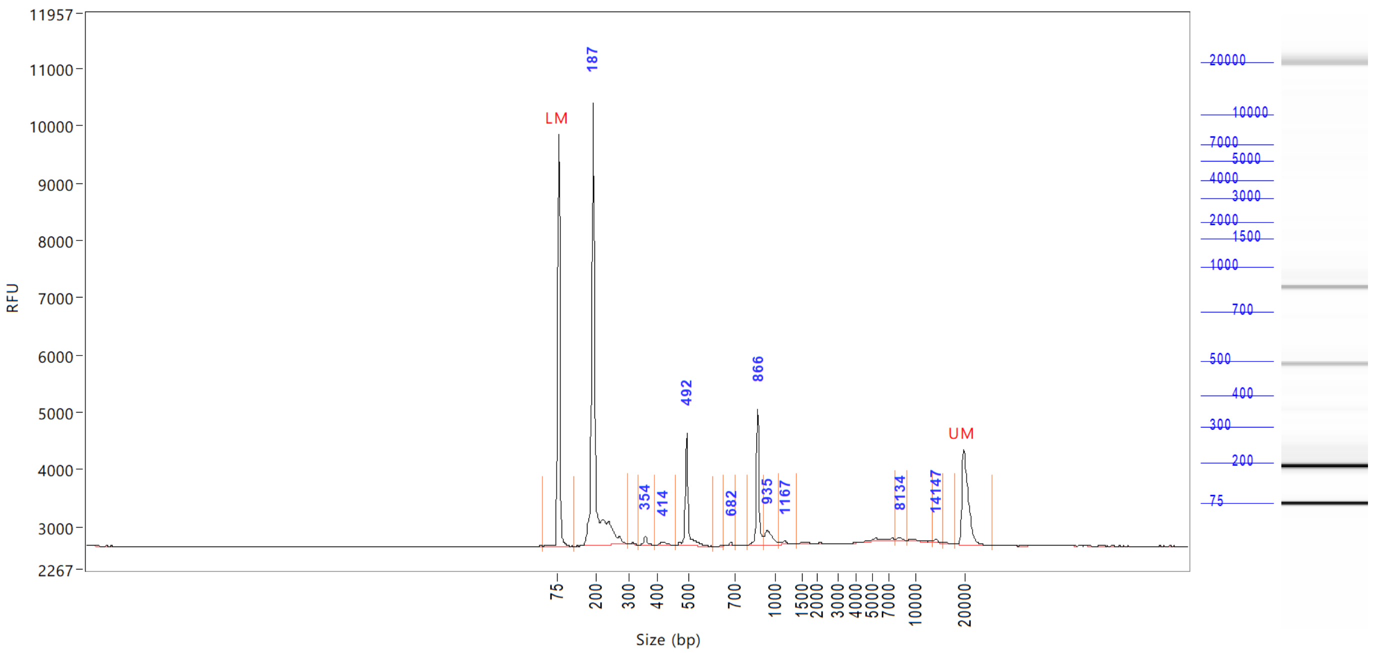

Peak	Size (bp)	Concentration (ng/uL)	From (bp)	To (bp)	Average size (bp)	CV%	RFU	Corrected peak area
1	75 (LM)	0.5000	24	124	72	7.29	7183	39.975
2	187	10.0452	124	296	194	10.91	7724	66.926
3	354	0.1909	329	388	354	2.50	164	1.272
4	414	0.1586	388	456	417	3.31	66	1.056
5	492	1.7647	456	606	495	3.23	1960	11.757
6	682	0.0496	652	705	677	1.07	55	0.331
7	866	1.7436	792	913	863	1.79	2379	11.617
8	935	0.4838	913	1056	955	3.19	249	3.224
9	1167	0.1077	1056	1411	1162	6.89	56	0.717
10	8134	0.0753	7601	9067	8166	4.33	59	0.502
11	14147	0.0532	13415	15732	14190	3.72	58	0.354
12	20000 (UM)	0.1774	18171	26045	20381	4.00	1649	14.179

TIC:	14.6726	ng/uL
TIM:	96.9661	nmole/L
Total concentration:	15.0949	ng/uL

```
Sample peak width (sec): 5    Sample min peak height: 50    Sample baseline V to V?: Y    Sample baseline V to V points: 3
Sample filter: Binomial       Number of points for filter: 3  Sample start region (min): 0  Sample end region (min): 30
Marker peak width (sec): 5    Marker min peak height: 200   Marker baseline V to V?: Y    Marker baseline V to V points: 3
Lower marker selection: First peak > 500 RFU                Upper marker selection: Last peak > 200 RFU
Ladder size (bp)75, 200, 300, 400, 500, 700, 1000, 1500, 2000, 3000, 4000, 5000, 7000, 10000, 20000
Quantification using: Lower Marker          Final concentration (ng/uL): 0.5000          Dilution factor: 12.0
```

Sample: SampF12
Well location: F12
Created: Tuesday, August 10, 2021 8:01:08 PM

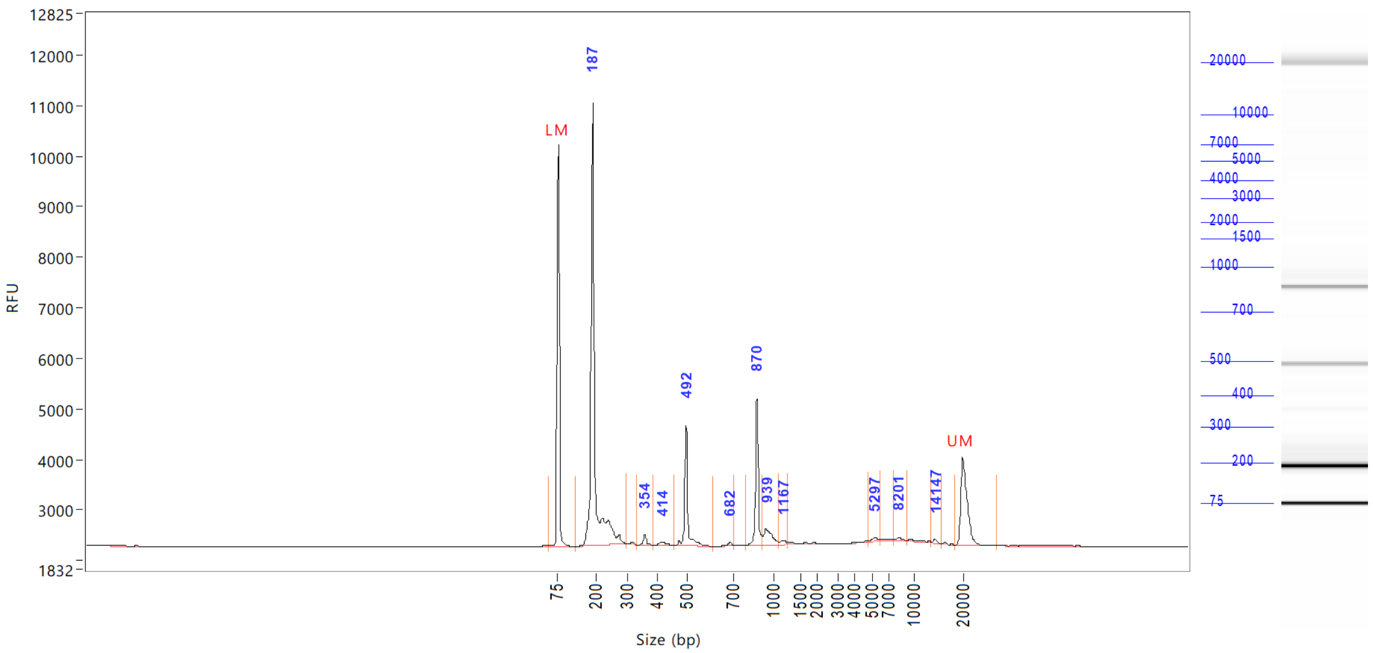

Peak	Size (bp)	Concentration (ng/uL)	From (bp)	To (bp)	Average size (bp)	CV%	RFU	Corrected peak area
1	75 (LM)	0.5000	45	131	73	6.05	7931	43.209
2	187	10.7689	131	295	194	10.95	8765	77.553
3	354	0.2138	329	386	354	2.23	206	1.540
4	414	0.1598	386	456	417	3.37	71	1.151
5	492	1.9976	456	611	495	3.20	2385	14.386
6	682	0.0593	611	701	675	1.92	64	0.427
7	870	2.0299	792	913	865	1.76	2906	14.619
8	939	0.5965	913	1079	959	3.68	312	4.296
9	1167	0.1068	1079	1245	1153	4.06	74	0.769
10	5297	0.0923	4828	6038	5299	5.91	69	0.665
11	8201	0.0805	7601	9134	8236	4.48	73	0.580
12	14147	0.0517	13415	15488	14164	2.92	67	0.372
13	20000 (UM)	0.1705	18293	27133	20407	4.04	1741	14.737

TIC:	16.1573	ng/uL
TIM:	104.7658	nmole/L
Total concentration:	16.5367	ng/uL

```
Sample peak width (sec): 5     Sample min peak height: 50    Sample baseline V to V?: Y    Sample baseline V to V points: 3
Sample filter: Binomial        Number of points for filter: 3  Sample start region (min): 0  Sample end region (min): 30
Marker peak width (sec): 5     Marker min peak height: 200   Marker baseline V to V?: Y    Marker baseline V to V points: 3
Lower marker selection: First peak > 500 RFU                 Upper marker selection: Last peak > 200 RFU
Ladder size (bp)75, 200, 300, 400, 500, 700, 1000, 1500, 2000, 3000, 4000, 5000, 7000, 10000, 20000
Quantification using: Lower Marker          Final concentration (ng/uL): 0.5000          Dilution factor: 12.0
```

Sample: SampG12
Well location: G12
Created: Tuesday, August 10, 2021 8:01:08 PM

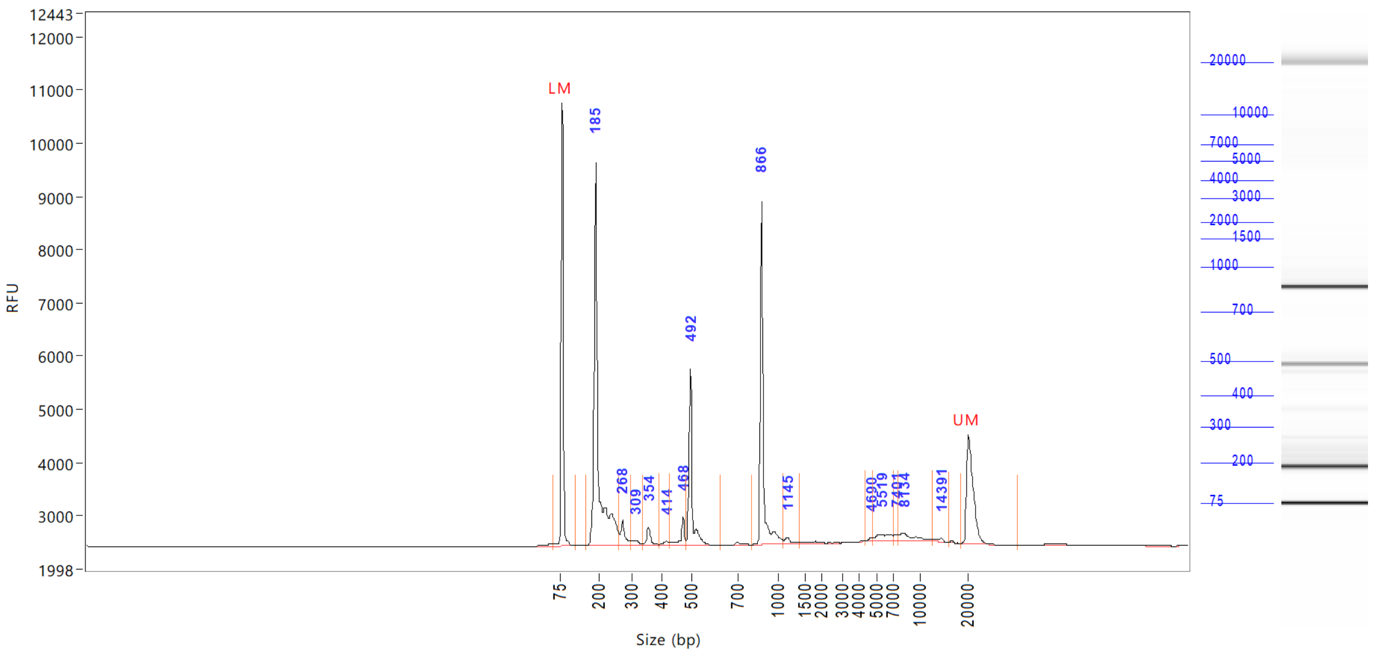

Peak	Size (bp)	Concentration (ng/uL)	From (bp)	To (bp)	Average size (bp)	CV%	RFU	Corrected peak area
1	75 (LM)	0.5000	48	122	74	6.01	8338	45.859
2	185	9.1813	154	259	195	10.74	7181	70.175
3	268	0.5832	259	296	272	3.61	461	4.457
4	309	0.1652	296	335	310	3.26	87	1.263
5	354	0.3150	335	390	353	2.06	318	2.407
6	414	0.0927	390	426	412	1.96	60	0.709
7	468	0.4440	426	479	462	2.61	520	3.394
8	492	2.5430	479	623	496	2.95	3295	19.437
9	866	4.4820	796	1068	877	4.06	6451	34.257
10	1145	0.1777	1068	1389	1173	6.97	126	1.358
11	4690	0.0498	4311	4793	4627	2.47	63	0.380
12	5519	0.3524	4793	7001	5830	11.65	127	2.693
13	7401	0.1086	7001	7601	7309	2.55	122	0.830
14	8134	0.3862	7601	12562	9117	13.81	140	2.952
15	14391	0.0823	12562	15854	13720	5.03	67	0.629
16	20000 (UM)	0.1953	18415	30398	20506	4.40	2064	17.911

TIC:	18.9633	ng/uL
TIM:	102.5549	nmole/L
Total concentration:	19.0998	ng/uL

```
Sample peak width (sec): 5    Sample min peak height: 50    Sample baseline V to V?: Y    Sample baseline V to V points: 3
Sample filter: Binomial       Number of points for filter: 3  Sample start region (min): 0  Sample end region (min): 30
Marker peak width (sec): 5    Marker min peak height: 200   Marker baseline V to V?: Y    Marker baseline V to V points: 3
Lower marker selection: First peak > 500 RFU                Upper marker selection: Last peak > 200 RFU
Ladder size (bp)75, 200, 300, 400, 500, 700, 1000, 1500, 2000, 3000, 4000, 5000, 7000, 10000, 20000
Quantification using: Lower Marker          Final concentration (ng/uL): 0.5000          Dilution factor: 12.0
```

Sample: SampH12
Well location: H12
Created: Tuesday, August 10, 2021 8:01:08 PM

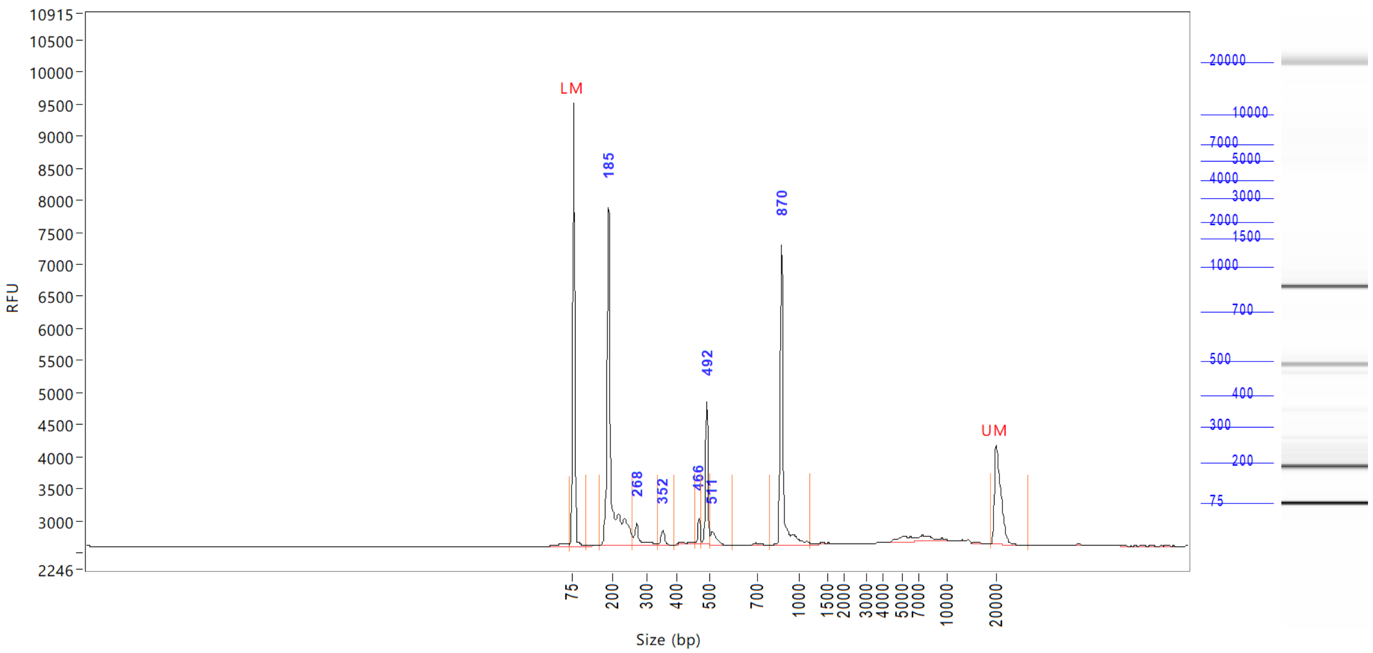

Peak	Size (bp)	Concentration (ng/uL)	From (bp)	To (bp)	Average size (bp)	CV%	RFU	Corrected peak area
1	75 (LM)	0.5000	64	114	73	5.08	6924	36.110
2	185	8.1245	154	257	194	10.34	5265	48.896
3	268	0.6522	257	335	278	6.78	354	3.925
4	352	0.2805	335	388	350	1.96	225	1.688
5	466	0.3125	456	475	465	0.79	410	1.881
6	492	1.7699	475	501	489	0.93	2214	10.652
7	511	0.3861	501	595	519	2.89	210	2.324
8	870	3.9872	792	1178	881	4.92	4693	23.997
9	20000 (UM)	0.1768	18781	26287	20381	3.77	1553	12.770

TIC:	15.5129	ng/uL
TIM:	89.7671	nmole/L
Total concentration:	16.5393	ng/uL

Sample peak width (sec): 5   Sample min peak height: 200   Sample baseline V to V?: Y   Sample baseline V to V points: 3
Sample filter: Binomial   Number of points for filter: 3   Sample start region (min): 0   Sample end region (min): 30
Marker peak width (sec): 5   Marker min peak height: 200   Marker baseline V to V?: Y   Marker baseline V to V points: 3
Lower marker selection: First peak > 500 RFU   Upper marker selection: Last peak > 200 RFU
Ladder size (bp)75, 200, 300, 400, 500, 700, 1000, 1500, 2000, 3000, 4000, 5000, 7000, 10000, 20000
Quantification using: Lower Marker   Final concentration (ng/uL): 0.5000   Dilution factor: 12.0

Sample: DNA size ladder
Well location: A7
Created: Tuesday, August 10, 2021 8:01:08 PM
Import from: C:\Users\admin\Desktop\210811_alberto_ladder.SCAL
Fit type: Point to point

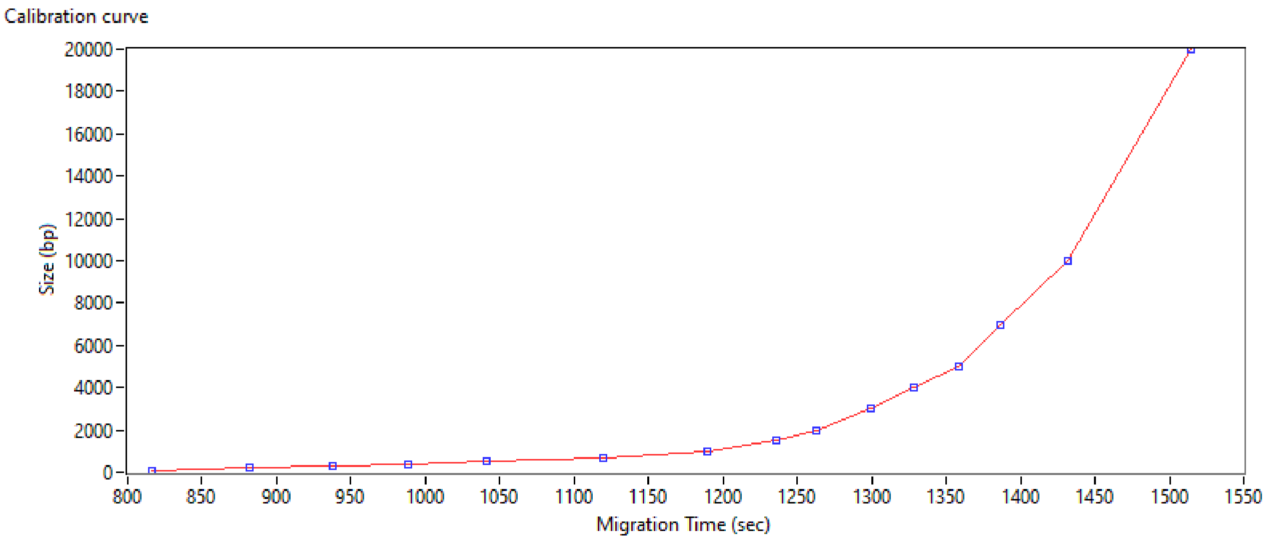

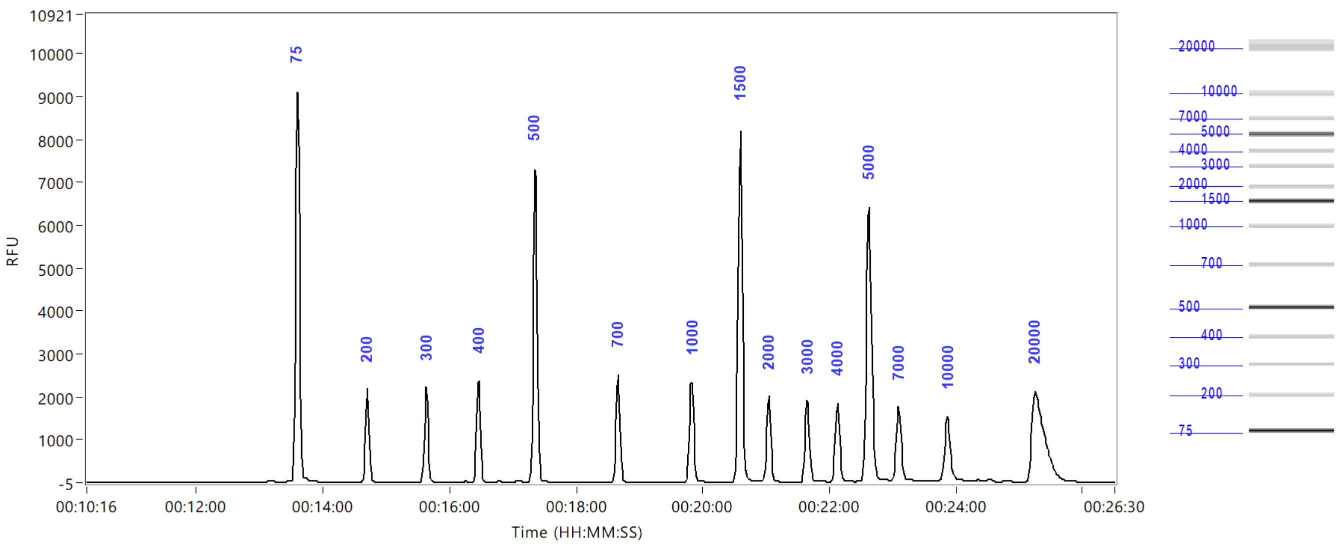

Supplement: Supplementary file 2 — sb3c00292_si_002.zip [file sb3c00292_si_002.zip › dnada_supplementary_material_pks_library_build/ZAG_trials/ZAG_Trial_1/19-51-25_plate3/plate3_report.pdf]
