# Supplementary material for: Automated Platform for the Plasmid Construction Process
Source: ACS Synth Biol. 2023 Nov 10;12(12):3506–13. doi: 10.1021/acssynbio.3c00292 (PMC10729297; doi:10.1021/acssynbio.3c00292)

Instrument controller software run summary:

Filename and data path: C:\AATI\Data\2021 08 10\20-31-19\2021 08 10 20H 31M..raw
Created: Tuesday, August 10, 2021 8:41:15 PM
Number of capillaries: 96
Array serial number: 111920-09SFS
Effect length: 33 cm
Array usage count: 167
Instrument type: ZAG DNA Analyzer
Instrument controller software version: 1.0.1.9
Device serial number: Z7597

Method Information

Method name: ZAG130GP33 - DNA 75-20000bp - Gel Prime Only.mthds
Gel prime: Yes
Full conditioning: No
Gel prime to buffer: No
Gel selection: Gel 1
Perform prerun: 8.0 kV, 30 sec.
Rinse: No
Marker 1: Row: A, 5.0 kV, 5 sec.
Rinse: No
Sample injection: 5.0 kV, 5 sec.
Separation: 8.0 kV, 30.0 min.
Tray name: Sample Tray-4

Analysis mode: DNA

Notes

Gel Image

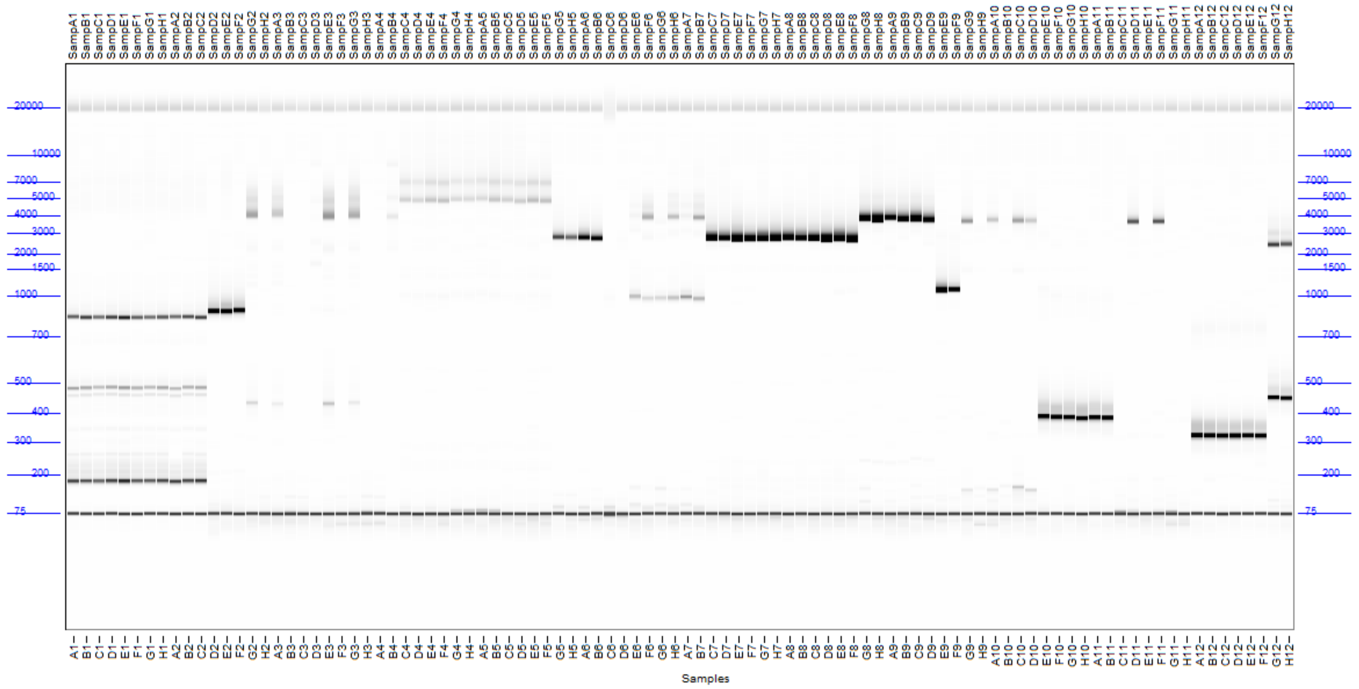

Filename and data path: C:\AATI\Data\2021 08 10\20-31-19\2021 08 10 20H 31M..raw

A1: SampA1

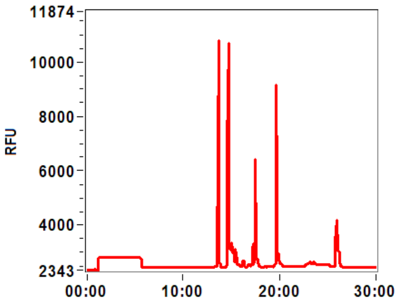

B1: SampB1

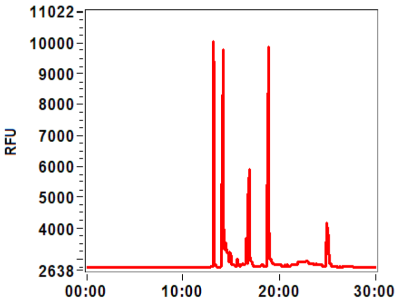

C1: SampC1

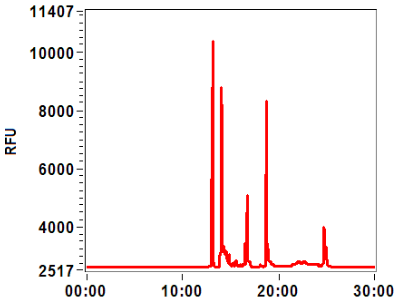

D1: SampD1

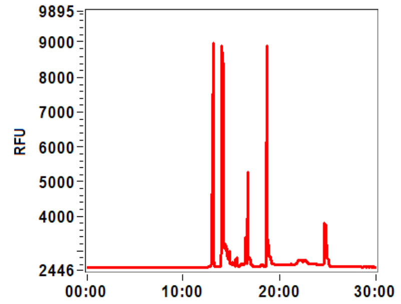

E1: SampE1

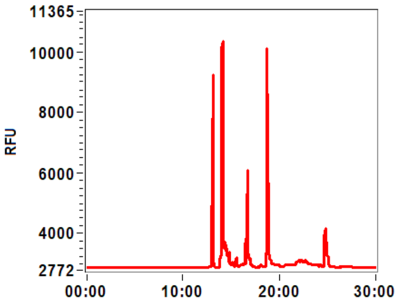

F1: SampF1

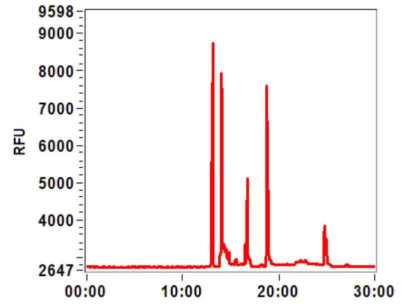

G1: SampG1

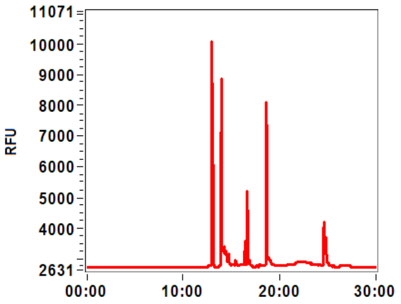

H1: SampH1

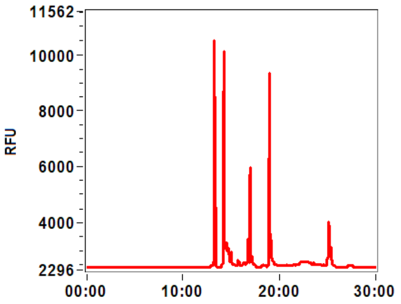

A2: SampA2

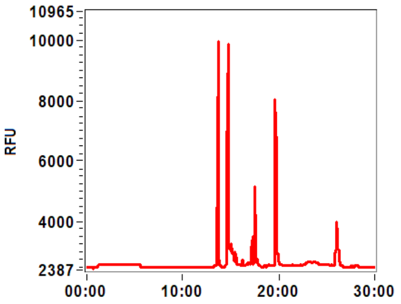

B2: SampB2

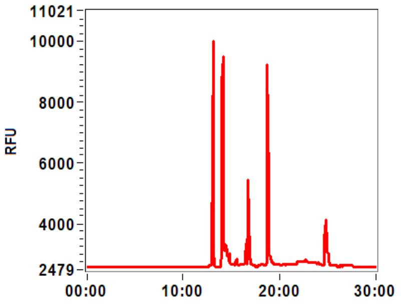

C2: SampC2

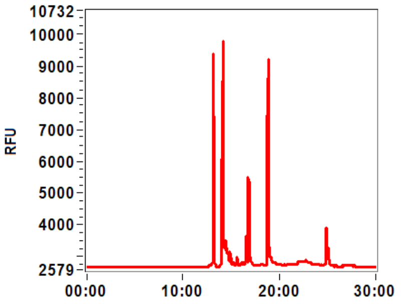

D2: SampD2

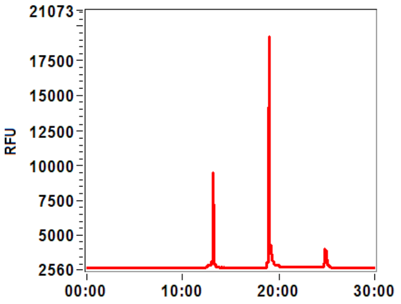

Filename and data path: C:\AATI\Data\2021 08 10\20-31-19\2021 08 10 20H 31M..raw

E2: SampE2

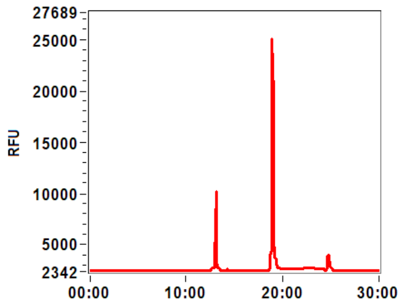

F2: SampF2

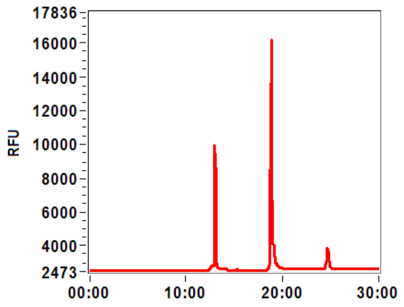

G2: SampG2

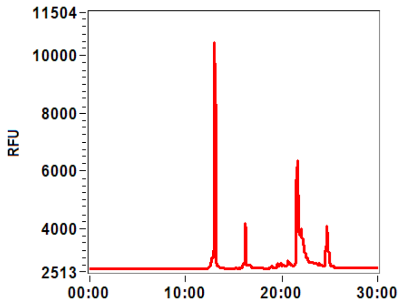

H2: SampH2

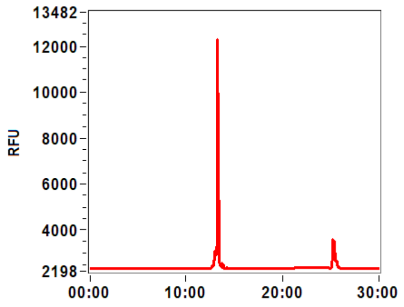

A3: SampA3

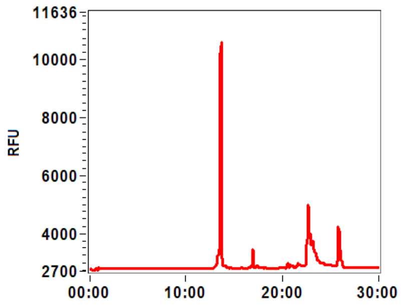

B3: SampB3

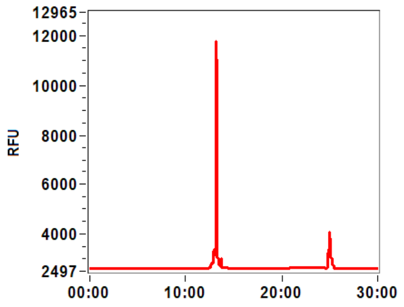

C3: SampC3

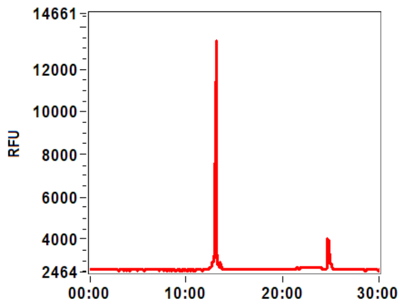

D3: SampD3

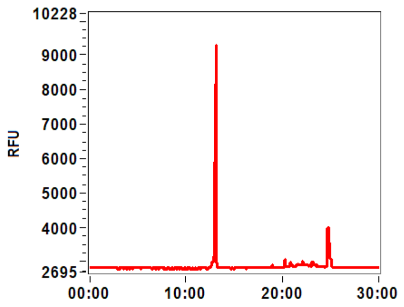

E3: SampE3

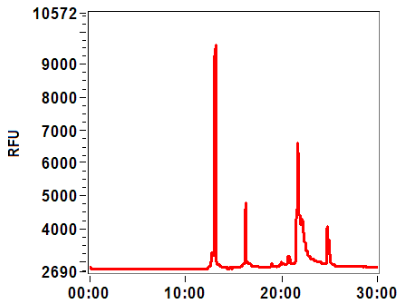

F3: SampF3

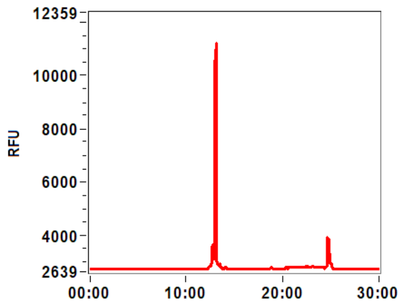

G3: SampG3

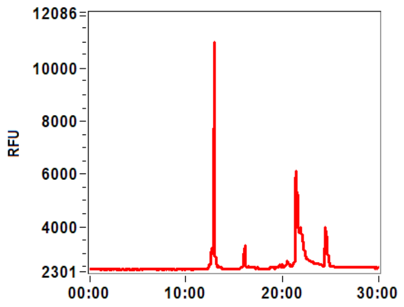

H3: SampH3

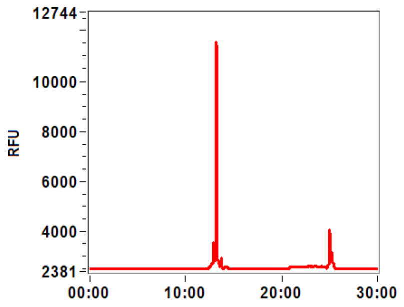

Filename and data path: C:\AATI\Data\2021 08 10\20-31-19\2021 08 10 20H 31M..raw

A4: SampA4

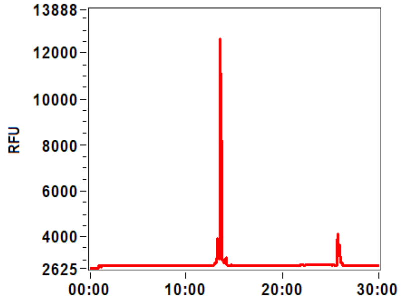

B4: SampB4

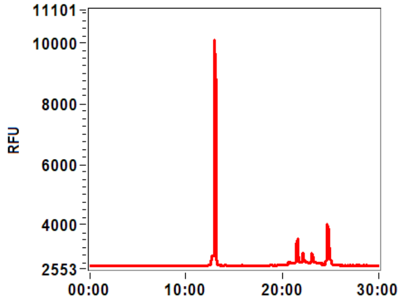

C4: SampC4

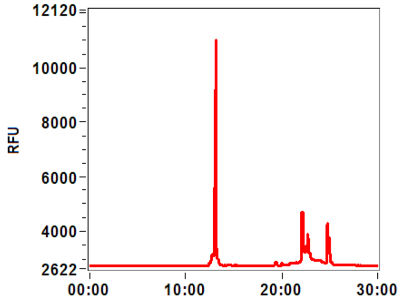

D4: SampD4

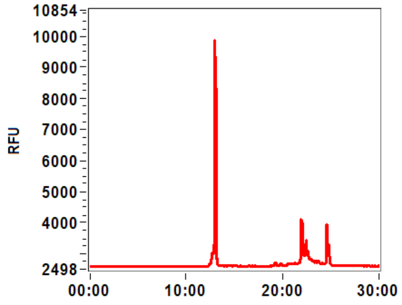

E4: SampE4

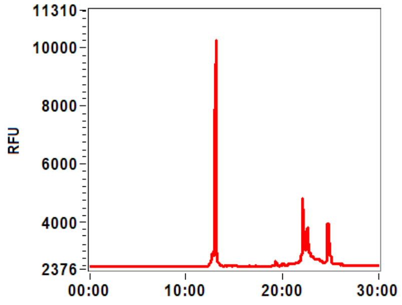

F4: SampF4

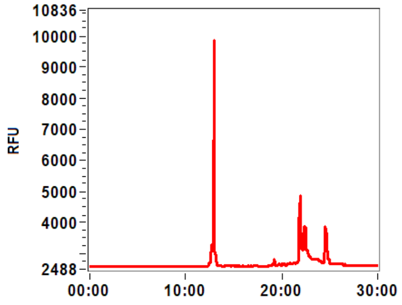

G4: SampG4

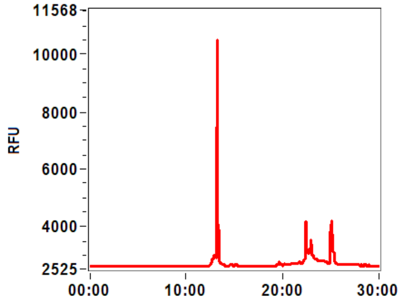

H4: SampH4

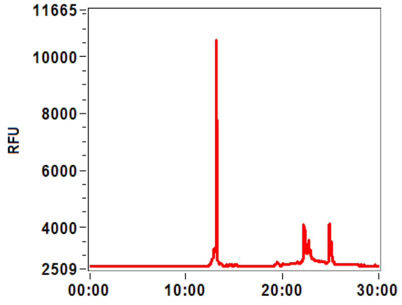

A5: SampA5

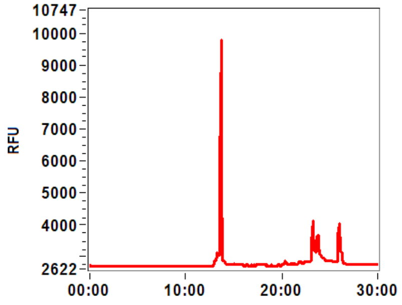

B5: SampB5

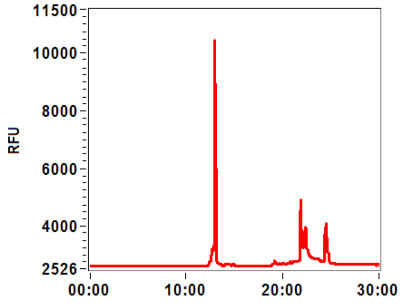

C5: SampC5

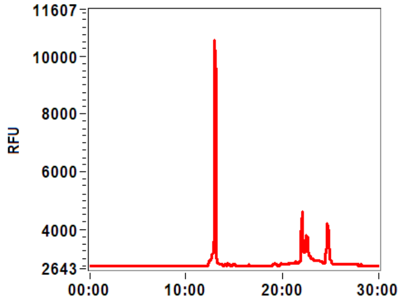

D5: SampD5

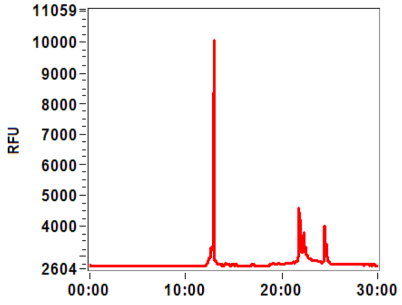

Filename and data path: C:\AATI\Data\2021 08 10\20-31-19\2021 08 10 20H 31M..raw

E5: SampE5

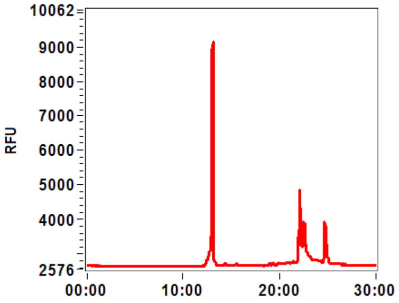

F5: SampF5

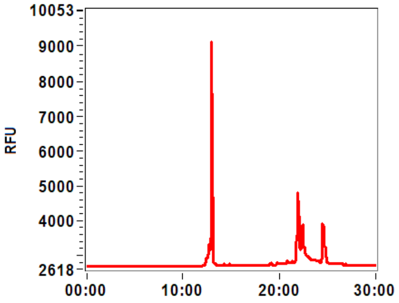

G5: SampG5

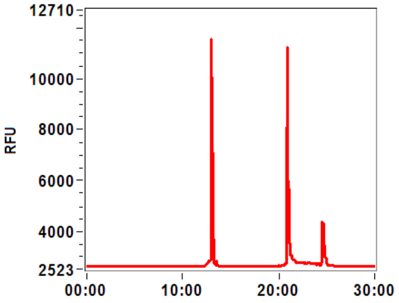

H5: SampH5

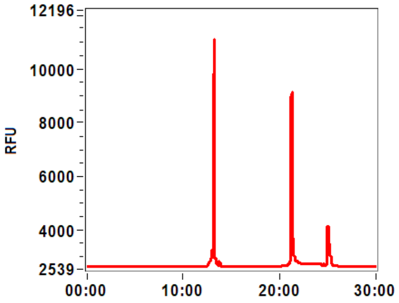

A6: SampA6

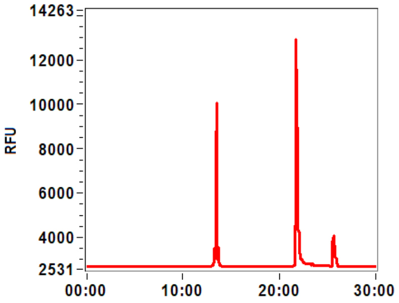

B6: SampB6

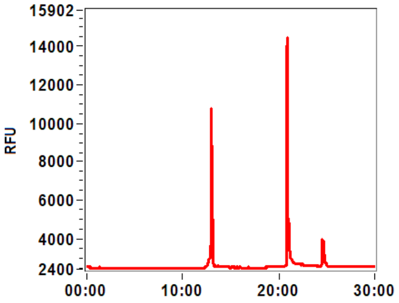

C6: SampC6

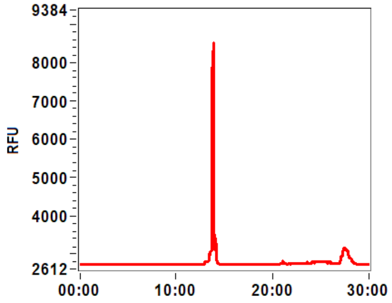

D6: SampD6

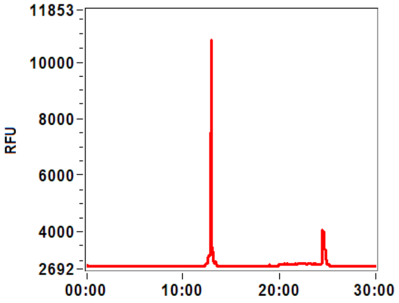

E6: SampE6

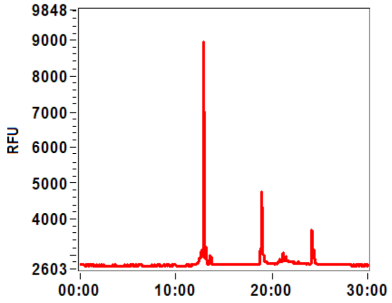

F6: SampF6

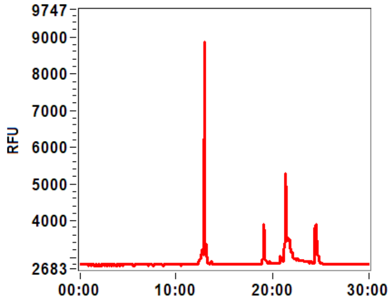

G6: SampG6

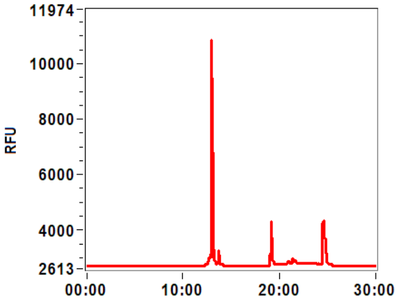

H6: SampH6

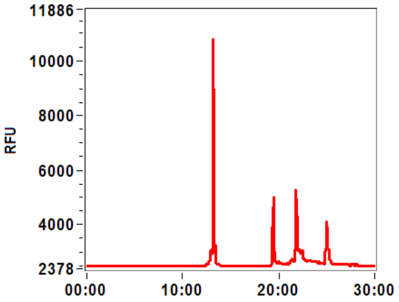

Filename and data path: C:\AATI\Data\2021 08 10\20-31-19\2021 08 10 20H 31M..raw

A7: SampA7

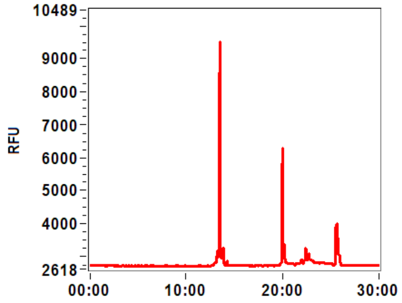

B7: SampB7

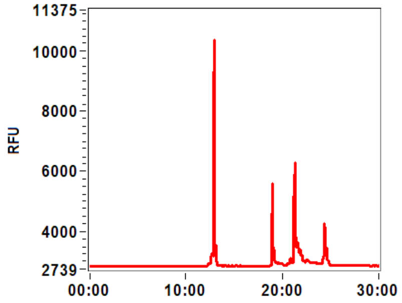

C7: SampC7

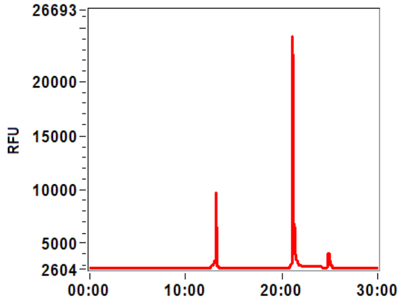

D7: SampD7

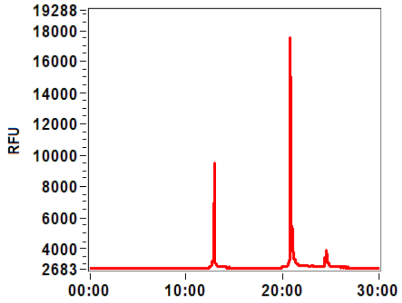

E7: SampE7

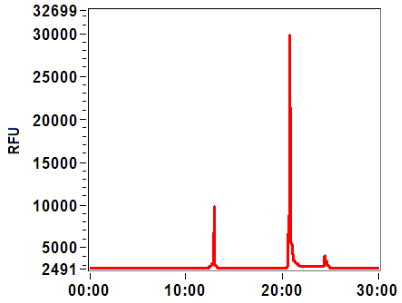

F7: SampF7

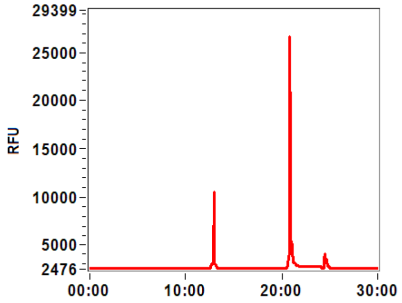

G7: SampG7

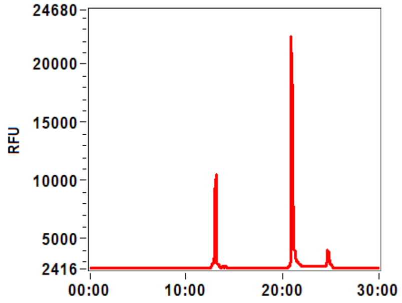

H7: SampH7

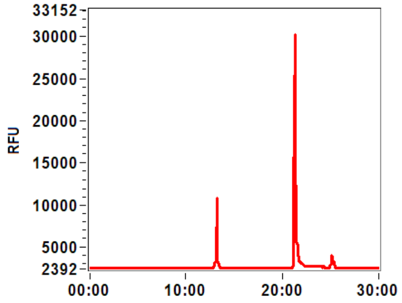

A8: SampA8

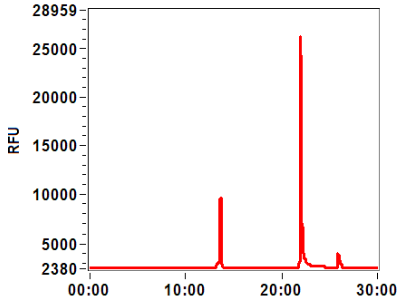

B8: SampB8

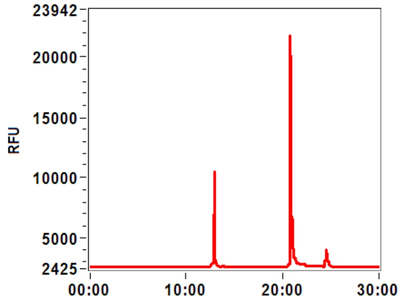

C8: SampC8

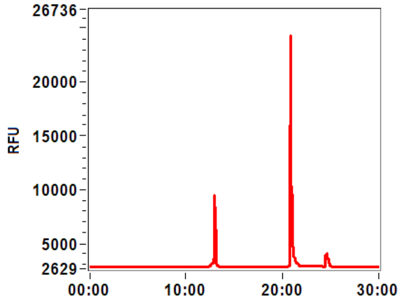

D8: SampD8

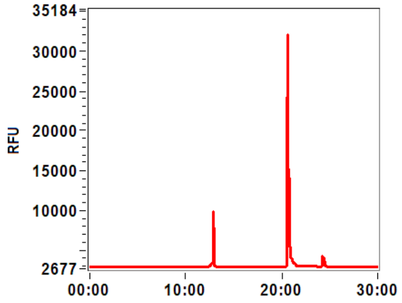

Filename and data path: C:\AATI\Data\2021 08 10\20-31-19\2021 08 10 20H 31M..raw

E8: SampE8

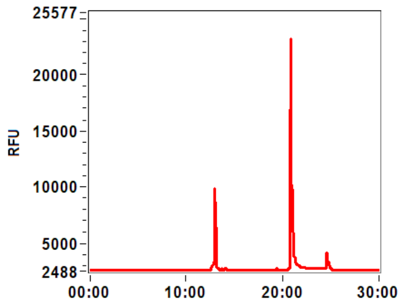

F8: SampF8

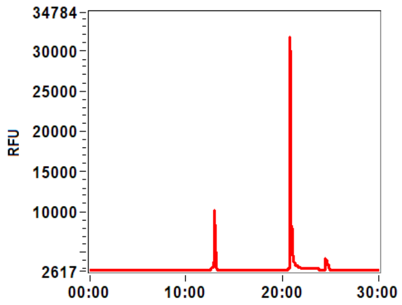

G8: SampG8

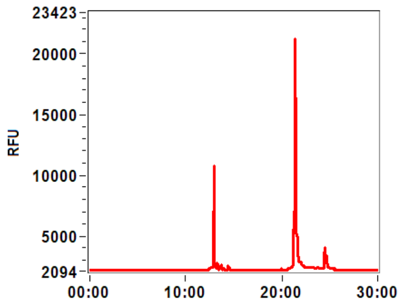

H8: SampH8

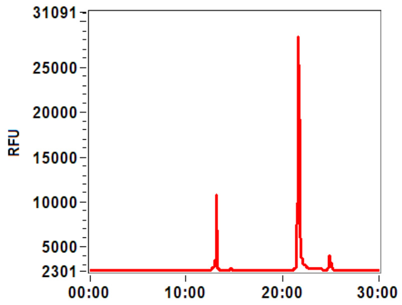

A9: SampA9

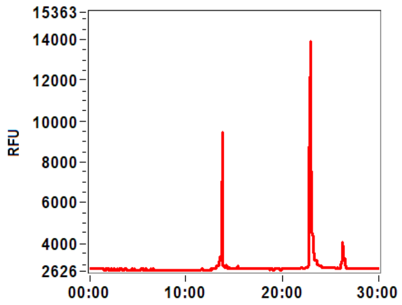

B9: SampB9

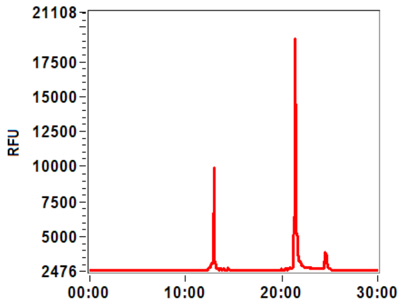

C9: SampC9

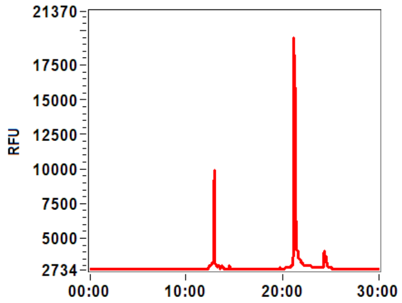

D9: SampD9

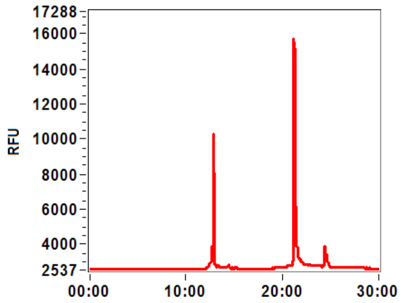

E9: SampE9

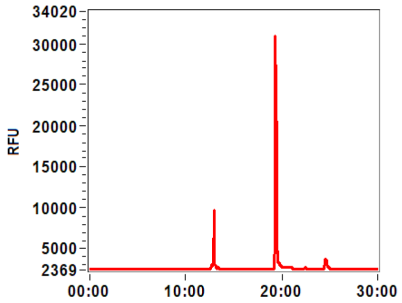

F9: SampF9

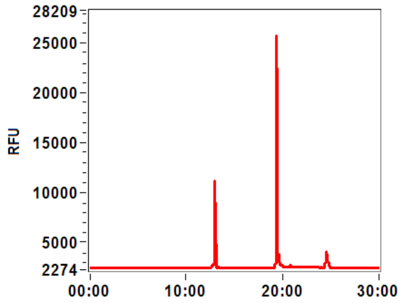

G9: SampG9

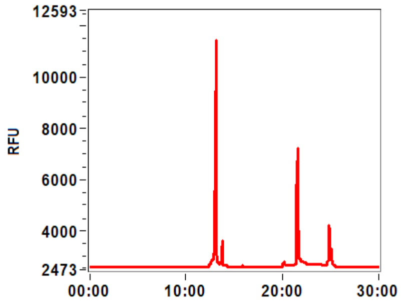

H9: SampH9

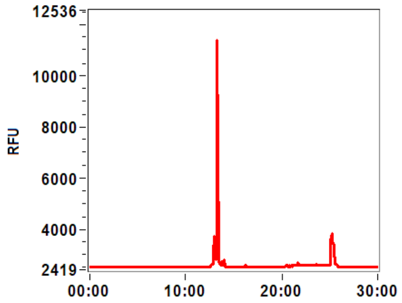

Filename and data path: C:\AATI\Data\2021 08 10\20-31-19\2021 08 10 20H 31M..raw

A10: SampA10

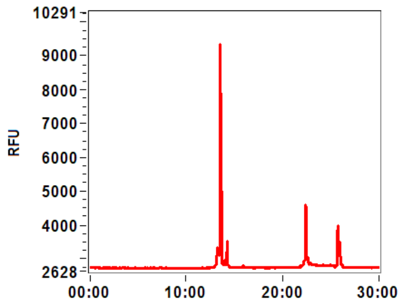

B10: SampB10

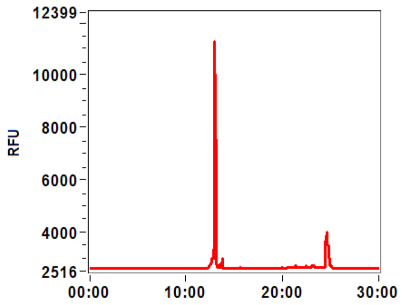

C10: SampC10

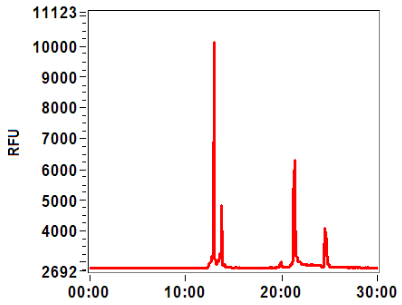

D10: SampD10

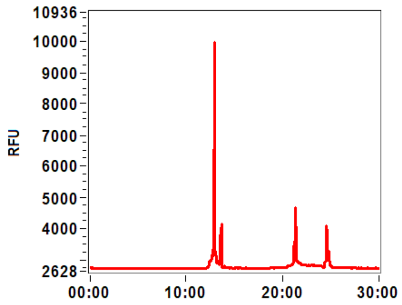

E10: SampE10

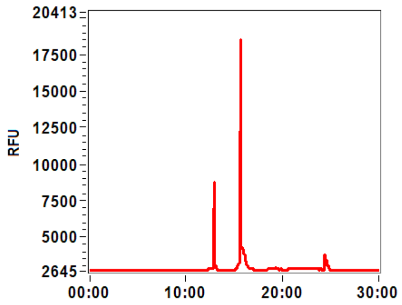

F10: SampF10

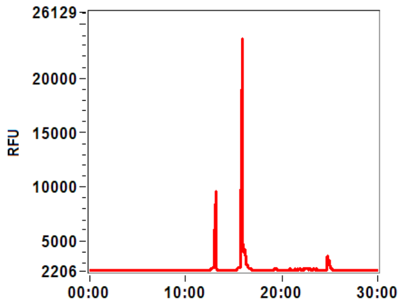

G10: SampG10

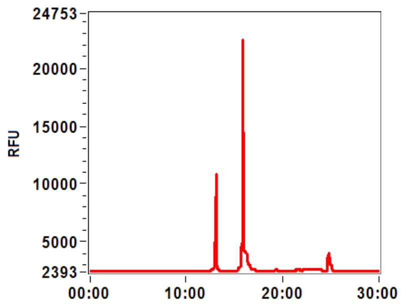

H10: SampH10

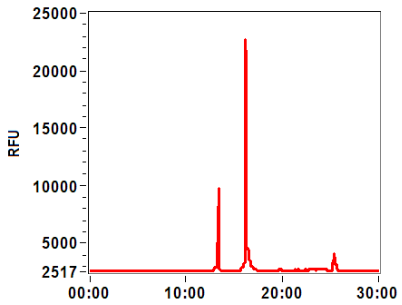

A11: SampA11

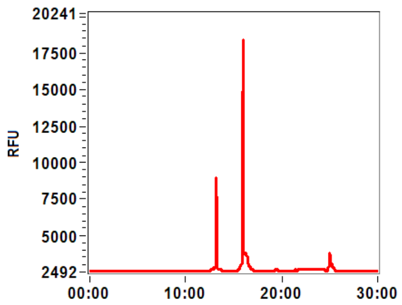

B11: SampB11

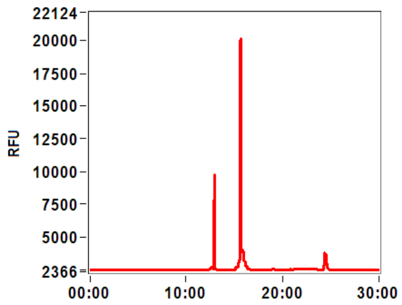

C11: SampC11

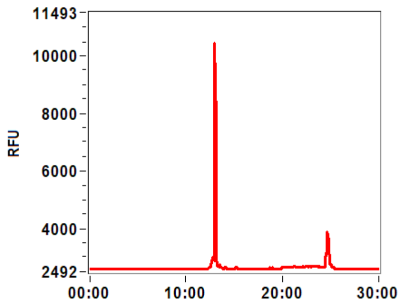

D11: SampD11

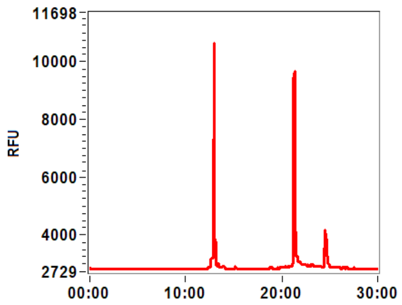

Filename and data path: C:\AATI\Data\2021 08 10\20-31-19\2021 08 10 20H 31M..raw

E11: SampE11

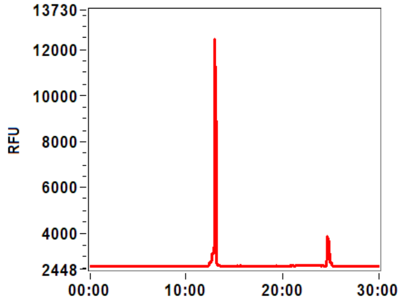

F11: SampF11

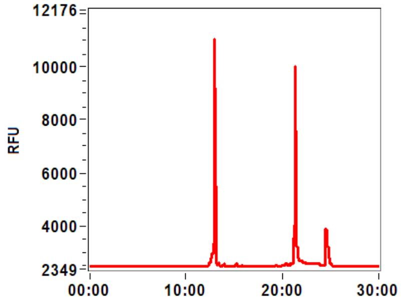

G11: SampG11

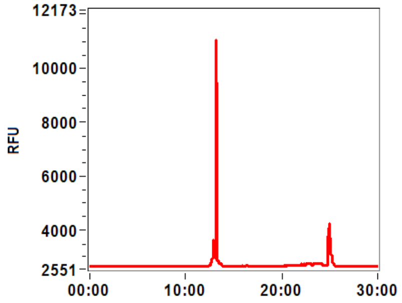

H11: SampH11

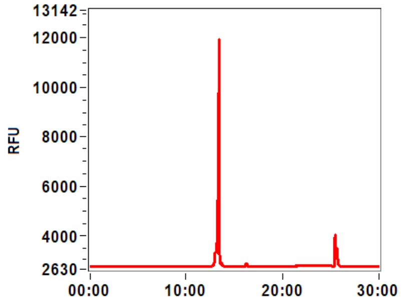

A12: SampA12

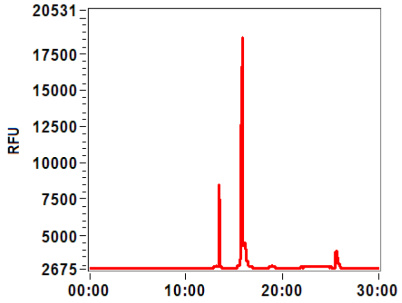

B12: SampB12

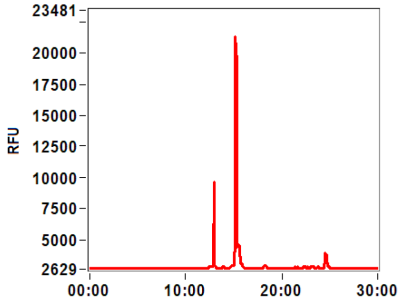

C12: SampC12

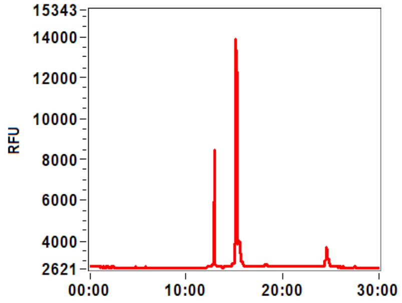

D12: SampD12

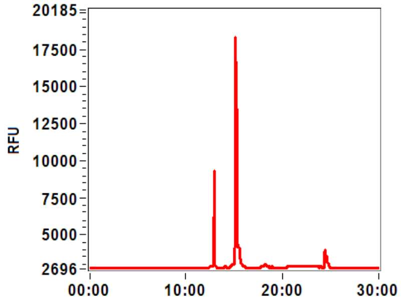

E12: SampE12

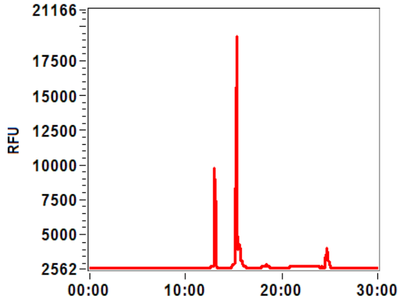

F12: SampF12

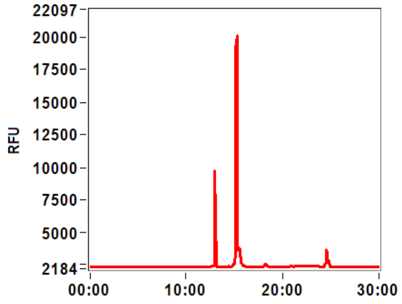

G12: SampG12

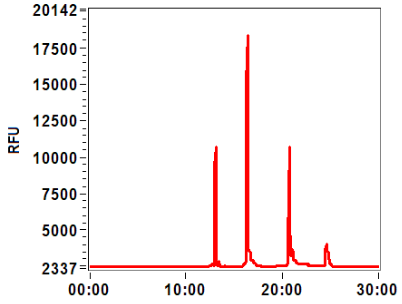

H12: SampH12

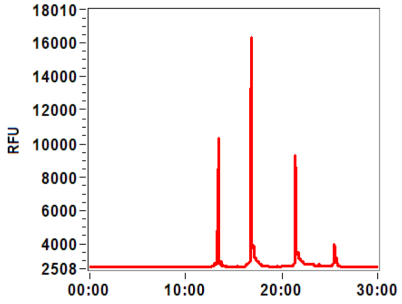

Sample: SampA1
Well location: A1
Created: Tuesday, August 10, 2021 8:41:15 PM

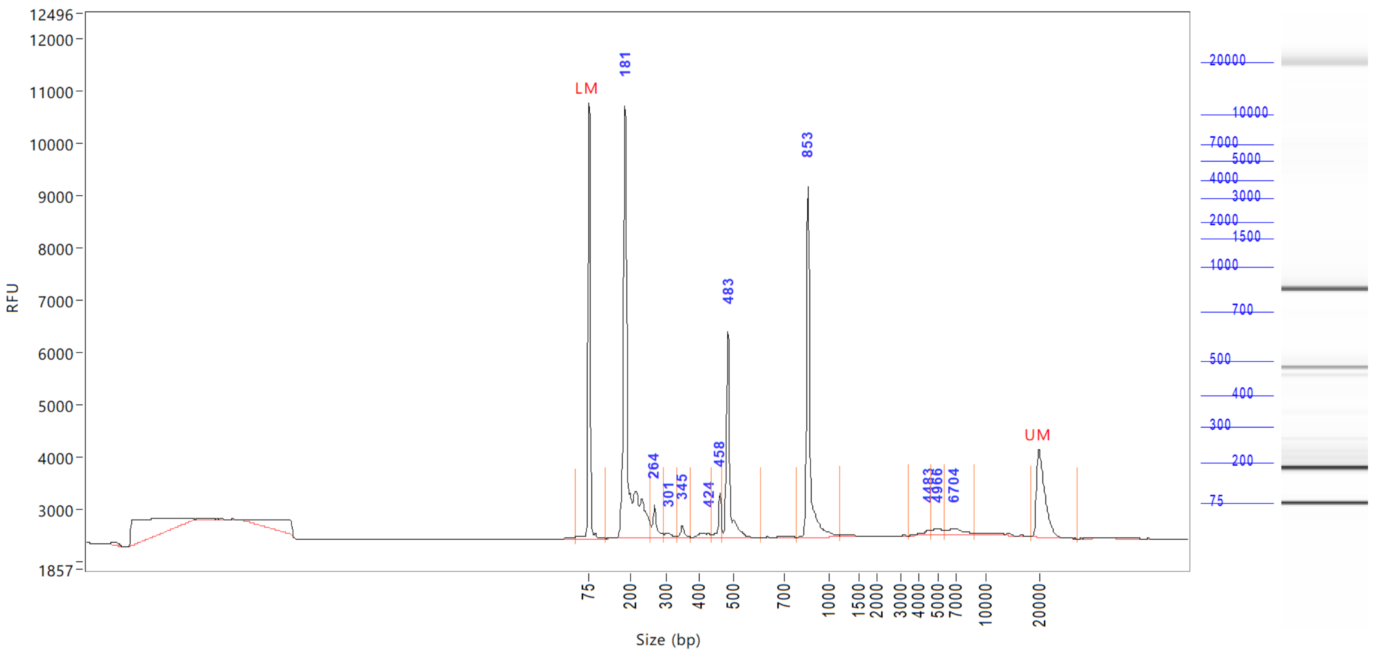

Peak	Size (bp)	Concentration (ng/uL)	From (bp)	To (bp)	Average size (bp)	CV%	RFU	Corrected peak area
1	75 (LM)	0.5000	37	126	74	6.85	8355	45.486
2	181	10.6585	126	255	191	10.83	8262	80.801
3	264	0.6893	255	291	266	3.35	633	5.225
4	301	0.1671	291	331	305	3.51	83	1.267
5	345	0.2191	331	376	345	2.05	217	1.661
6	424	0.2291	376	433	410	3.25	85	1.737
7	458	0.6212	433	468	455	1.43	840	4.710
8	483	3.2436	468	606	487	3.13	3934	24.589
9	853	5.1765	779	1156	862	4.32	6700	39.243
10	4483	0.1513	3465	4621	4298	5.94	109	1.147
11	4966	0.2405	4621	5741	5056	6.34	119	1.823
12	6704	0.3405	5741	8867	6935	10.89	120	2.581
13	20000 (UM)	0.1842	18415	27254	20446	4.47	1688	16.758

TIC:	21.7367	ng/uL
TIM:	122.1827	nmole/L
Total concentration:	22.0600	ng/uL

Sample peak width (sec): 5 Sample min peak height: 50 Sample baseline V to V?: Y Sample baseline V to V points: 3
Sample filter: Binomial Number of points for filter: 3 Sample start region (min): 0 Sample end region (min): 30
Marker peak width (sec): 5 Marker min peak height: 200 Marker baseline V to V?: Y Marker baseline V to V points: 3
Lower marker selection: First peak > 500 RFU Upper marker selection: Last peak > 200 RFU
Ladder size (bp)75, 200, 300, 400, 500, 700, 1000, 1500, 2000, 3000, 4000, 5000, 7000, 10000, 20000
Quantification using: Lower Marker Final concentration (ng/uL): 0.5000 Dilution factor: 12.0

Sample: SampB1
Well location: B1
Created: Tuesday, August 10, 2021 8:41:15 PM

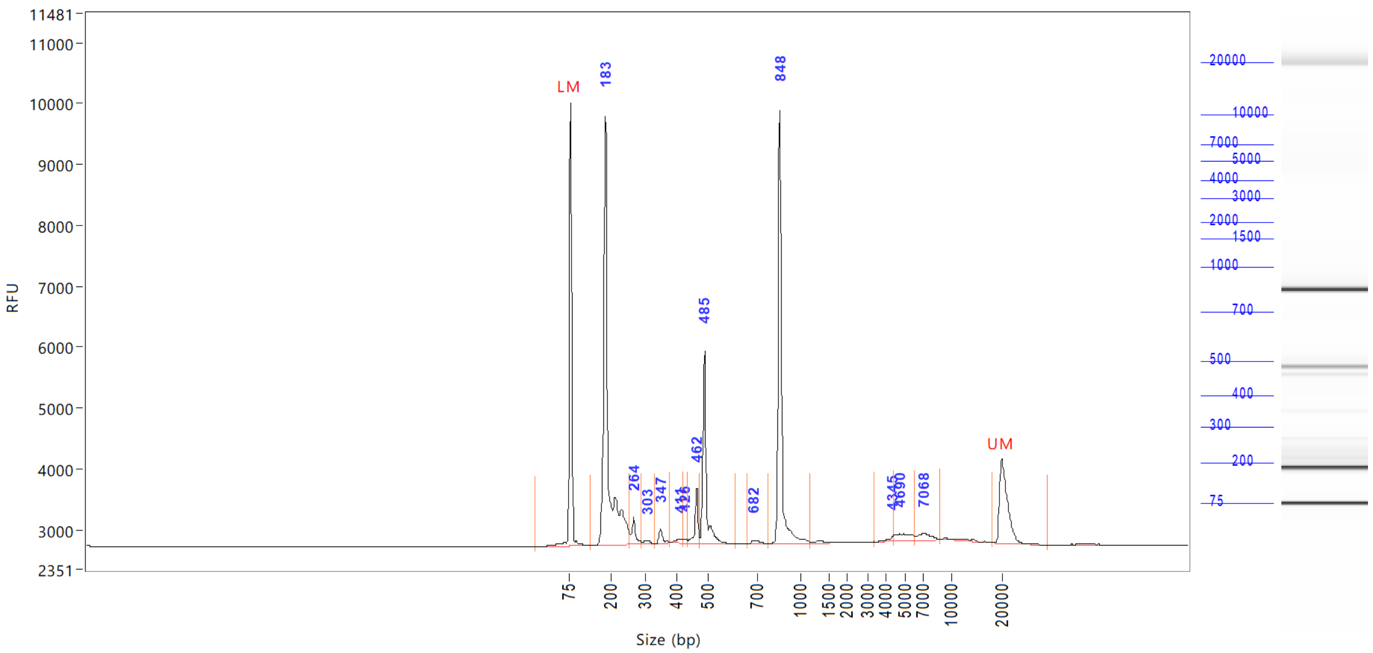

Peak	Size (bp)	Concentration (ng/uL)	From (bp)	To (bp)	Average size (bp)	CV%	RFU	Corrected peak area
1	75 (LM)	0.5000	0	139	72	12.68	7289	40.405
2	183	10.6573	139	255	192	10.70	7035	71.767
3	264	0.5256	255	289	265	2.89	448	3.539
4	303	0.1182	289	329	302	2.54	59	0.796
5	347	0.2799	329	380	349	2.49	246	1.885
6	411	0.1439	380	420	405	2.36	66	0.969
7	426	0.0722	420	433	426	0.99	74	0.486
8	462	0.7963	433	470	457	1.55	896	5.362
9	485	3.0141	470	611	488	3.03	3158	20.297
10	682	0.1441	657	766	693	3.11	68	0.971
11	848	5.9261	766	1167	856	4.48	7107	39.907
12	4345	0.0796	3358	4380	4149	5.23	84	0.536
13	4690	0.4050	4380	6112	5046	9.61	115	2.727
14	7068	0.3228	6112	8800	7201	8.93	118	2.174
15	20000 (UM)	0.1720	18049	28826	20535	4.93	1392	13.895

TIC:	22.4851	ng/uL
TIM:	122.3534	nmole/L
Total concentration:	22.6760	ng/uL

Sample peak width (sec): 5   Sample min peak height: 50   Sample baseline V to V?: Y   Sample baseline V to V points: 3
Sample filter: Binomial   Number of points for filter: 3 Sample start region (min): 0 Sample end region (min): 30
Marker peak width (sec): 5   Marker min peak height: 200   Marker baseline V to V?: Y   Marker baseline V to V points: 3
Lower marker selection: First peak > 500 RFU   Upper marker selection: Last peak > 200 RFU
Ladder size (bp)75, 200, 300, 400, 500, 700, 1000, 1500, 2000, 3000, 4000, 5000, 7000, 10000, 20000
Quantification using: Lower Marker   Final concentration (ng/uL): 0.5000   Dilution factor: 12.0

Sample: SampC1
Well location: C1
Created: Tuesday, August 10, 2021 8:41:15 PM

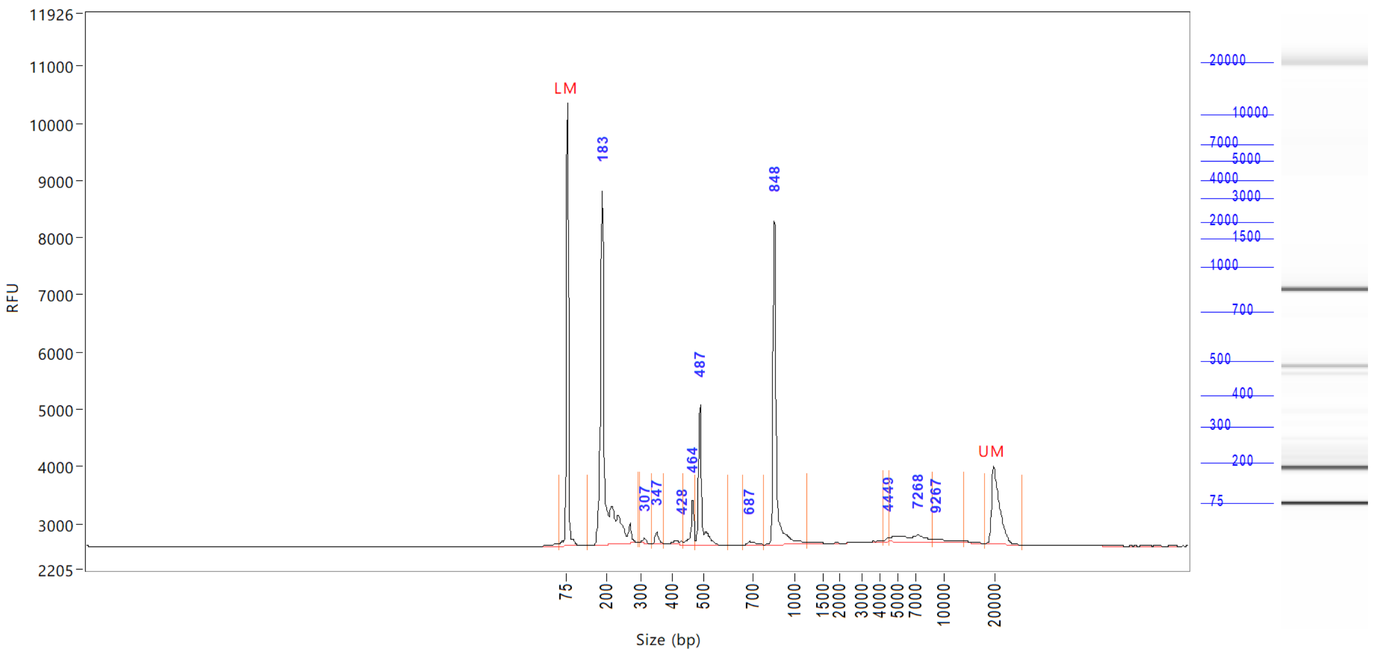

Peak	Size (bp)	Concentration (ng/uL)	From (bp)	To (bp)	Average size (bp)	CV%	RFU	Corrected peak area
1	75 (LM)	0.5000	48	137	73	6.78	7759	42.816
2	183	9.1123	137	289	193	12.30	6183	65.026
3	307	0.0861	293	331	305	1.78	84	0.614
4	347	0.2281	331	374	347	1.96	214	1.628
5	428	0.1742	374	435	413	3.24	65	1.243
6	464	0.6585	435	471	459	1.55	785	4.699
7	487	2.2267	471	600	489	2.84	2453	15.890
8	687	0.1276	662	779	700	3.34	67	0.911
9	848	4.6708	779	1190	860	4.73	5667	33.331
10	4449	0.0549	4139	4552	4392	2.44	68	0.392
11	7268	0.6403	4552	8934	6430	19.80	117	4.569
12	9267	0.2082	8934	13903	10931	13.84	54	1.486
13	20000 (UM)	0.1614	18171	25682	20516	4.50	1352	13.819

TIC:	18.1878	ng/uL
TIM:	99.1750	nmole/L
Total concentration:	18.3448	ng/uL

```
Sample peak width (sec): 5    Sample min peak height: 50    Sample baseline V to V?: Y    Sample baseline V to V points: 3
Sample filter: Binomial       Number of points for filter: 3  Sample start region (min): 0  Sample end region (min): 30
Marker peak width (sec): 5    Marker min peak height: 200   Marker baseline V to V?: Y    Marker baseline V to V points: 3
Lower marker selection: First peak > 500 RFU                Upper marker selection: Last peak > 200 RFU
Ladder size (bp)75, 200, 300, 400, 500, 700, 1000, 1500, 2000, 3000, 4000, 5000, 7000, 10000, 20000
Quantification using: Lower Marker          Final concentration (ng/uL): 0.5000          Dilution factor: 12.0
```

Sample: SampD1
Well location: D1
Created: Tuesday, August 10, 2021 8:41:15 PM

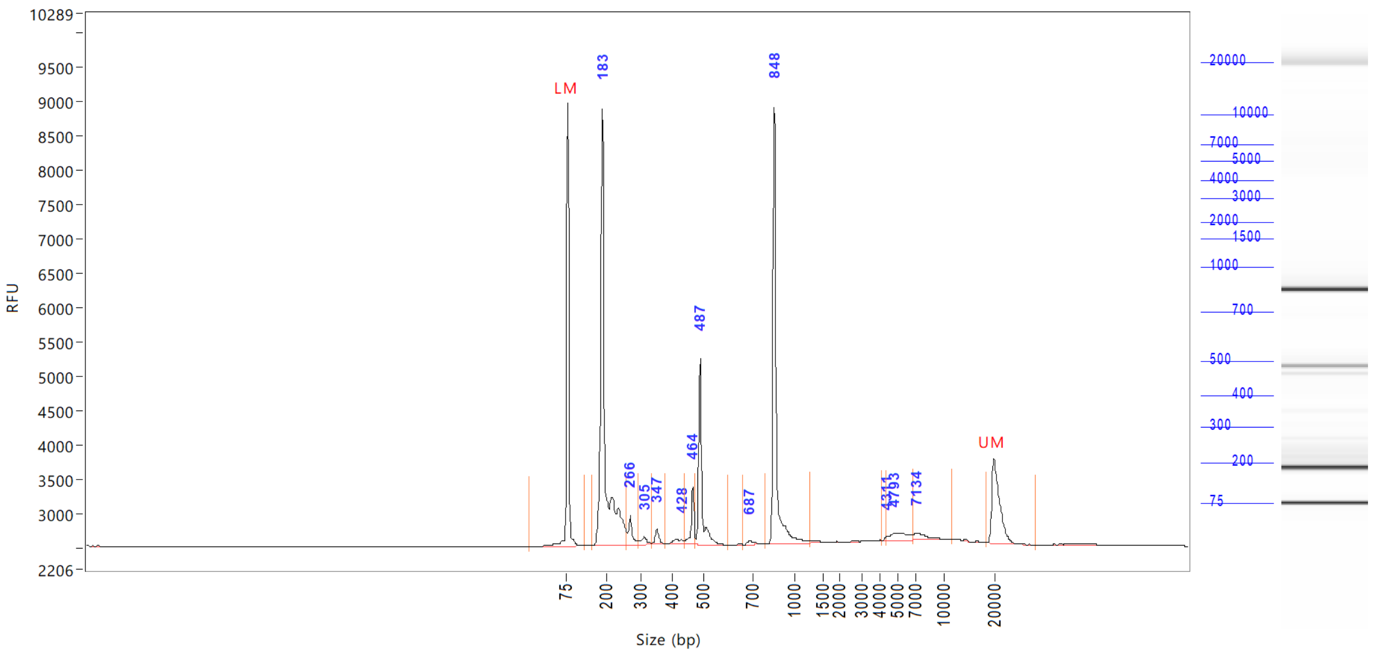

Peak	Size (bp)	Concentration (ng/uL)	From (bp)	To (bp)	Average size (bp)	CV%	RFU	Corrected peak area
1	75 (LM)	0.5000	0	128	73	13.58	6456	38.257
2	183	10.1897	152	257	192	10.78	6344	64.972
3	266	0.5651	257	291	268	3.19	436	3.603
4	305	0.2142	291	331	306	3.15	109	1.366
5	347	0.2806	331	378	347	1.96	230	1.789
6	428	0.2169	378	437	414	3.28	71	1.383
7	464	0.7725	437	473	460	1.53	845	4.925
8	487	2.6937	473	595	490	2.80	2713	17.175
9	687	0.1286	662	783	698	3.11	61	0.820
10	848	5.8572	783	1256	862	5.57	6343	37.347
11	4311	0.0285	4104	4345	4270	1.49	55	0.182
12	4793	0.4395	4345	6778	5329	13.21	100	2.803
13	7134	0.3172	6778	11708	7953	12.31	106	2.023
14	20000 (UM)	0.1604	18415	28100	20563	4.74	1227	12.272

TIC:	21.7037	ng/uL
TIM:	117.5766	nmole/L
Total concentration:	21.8128	ng/uL

Sample peak width (sec): 5 Sample min peak height: 50 Sample baseline V to V?: Y Sample baseline V to V points: 3
Sample filter: Binomial Number of points for filter: 3 Sample start region (min): 0 Sample end region (min): 30
Marker peak width (sec): 5 Marker min peak height: 200 Marker baseline V to V?: Y Marker baseline V to V points: 3
Lower marker selection: First peak > 500 RFU Upper marker selection: Last peak > 200 RFU
Ladder size (bp)75, 200, 300, 400, 500, 700, 1000, 1500, 2000, 3000, 4000, 5000, 7000, 10000, 20000
Quantification using: Lower Marker Final concentration (ng/uL): 0.5000 Dilution factor: 12.0

Sample: SampE1
Well location: E1
Created: Tuesday, August 10, 2021 8:41:15 PM

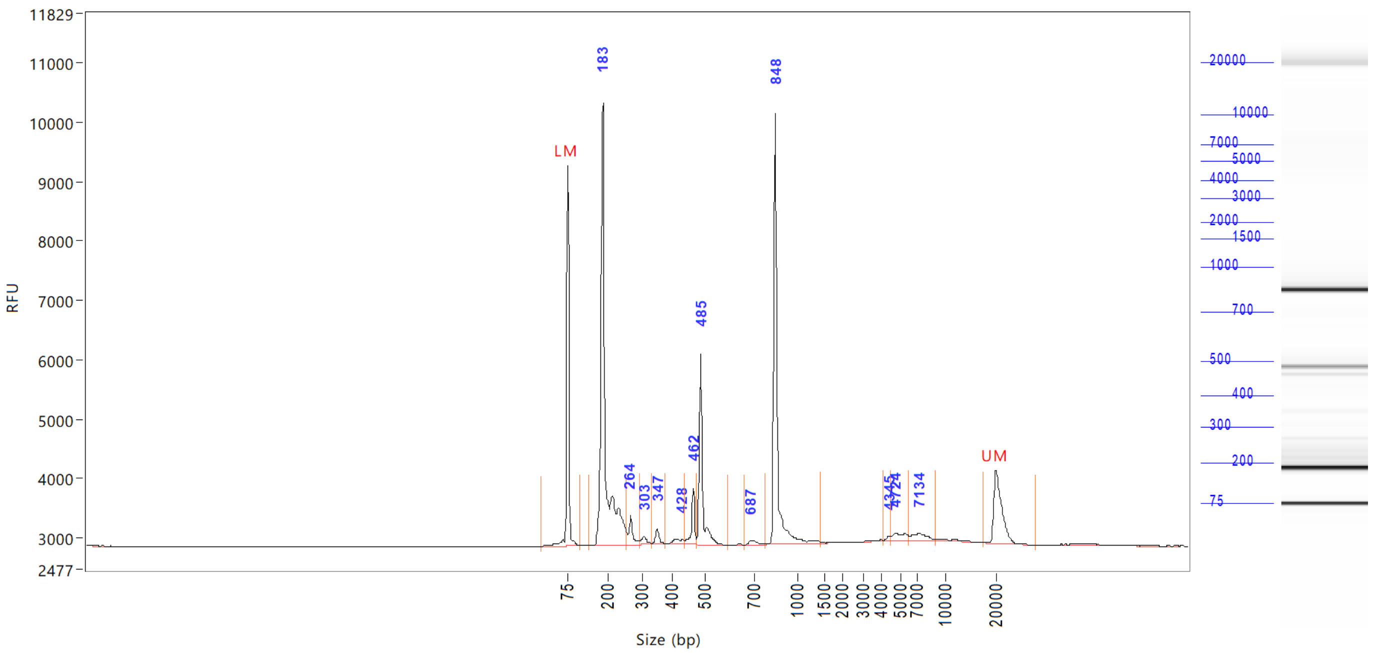

Peak	Size (bp)	Concentration (ng/uL)	From (bp)	To (bp)	Average size (bp)	CV%	RFU	Corrected peak area
1	75 (LM)	0.5000	0	114	71	11.81	6395	37.281
2	183	12.3878	141	255	190	10.62	7455	76.970
3	264	0.6729	255	291	267	3.28	487	4.181
4	303	0.2486	291	329	304	2.90	142	1.544
5	347	0.3368	329	376	346	1.97	271	2.093
6	428	0.2585	376	433	412	3.10	85	1.606
7	462	0.9213	433	471	457	1.65	950	5.725
8	485	3.3127	471	593	488	2.90	3213	20.583
9	687	0.1646	662	779	699	3.36	68	1.023
10	848	7.0334	779	1400	863	7.50	7239	43.702
11	4345	0.0777	4035	4449	4298	2.47	81	0.483
12	4724	0.3881	4449	6038	5079	8.79	115	2.411
13	7134	0.4005	6038	8867	7240	9.61	123	2.489
14	20000 (UM)	0.1671	17561	27738	20469	4.64	1225	12.460

TIC:	26.2027	ng/uL
TIM:	143.8527	nmole/L
Total concentration:	26.4060	ng/uL

```
Sample peak width (sec): 5     Sample min peak height: 50     Sample baseline V to V?: Y     Sample baseline V to V points: 3
Sample filter: Binomial        Number of points for filter: 3  Sample start region (min): 0  Sample end region (min): 30
Marker peak width (sec): 5     Marker min peak height: 200    Marker baseline V to V?: Y     Marker baseline V to V points: 3
Lower marker selection: First peak > 500 RFU                  Upper marker selection: Last peak > 200 RFU
Ladder size (bp)75, 200, 300, 400, 500, 700, 1000, 1500, 2000, 3000, 4000, 5000, 7000, 10000, 20000
Quantification using: Lower Marker          Final concentration (ng/uL): 0.5000          Dilution factor: 12.0
```

Sample: SampF1
Well location: F1
Created: Tuesday, August 10, 2021 8:41:15 PM

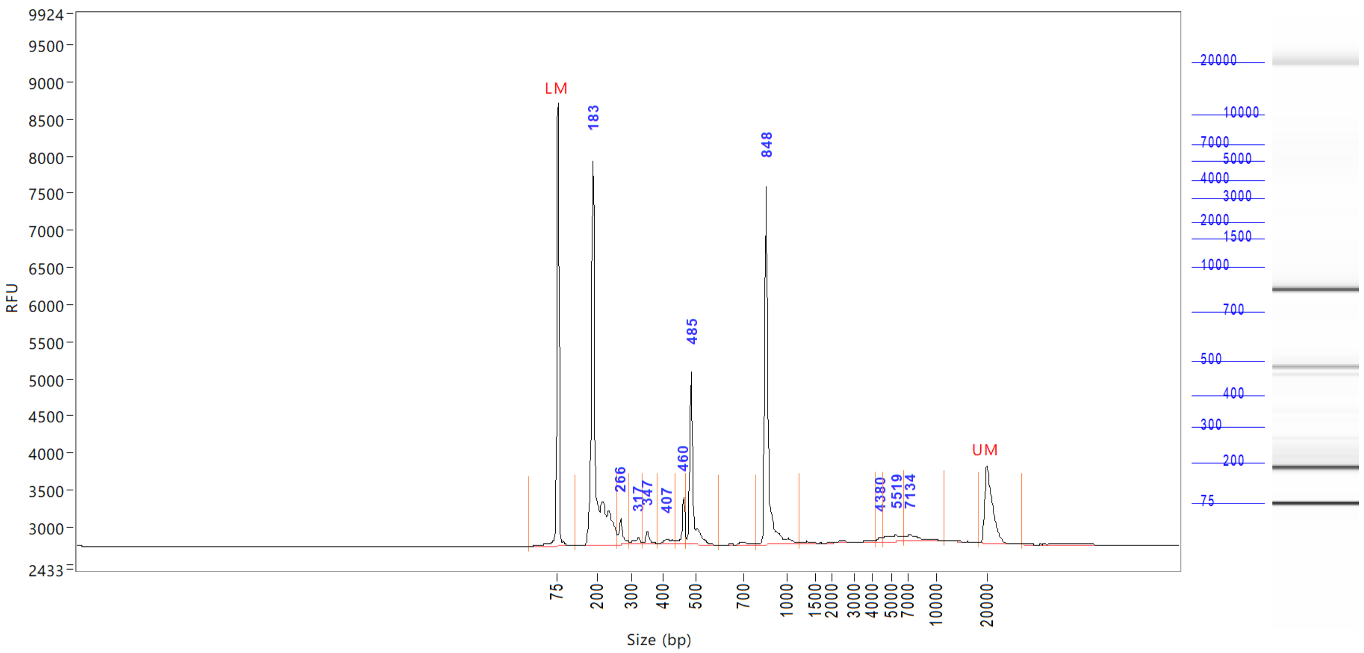

Peak	Size (bp)	Concentration (ng/uL)	From (bp)	To (bp)	Average size (bp)	CV%	RFU	Corrected peak area
1	75 (LM)	0.5000	0	129	71	10.98	5981	34.475
2	183	9.2037	129	257	192	10.80	5185	52.883
3	266	0.5031	257	291	267	3.04	357	2.891
4	317	0.1859	291	331	309	3.29	79	1.068
5	347	0.2253	331	380	348	2.37	158	1.295
6	407	0.1734	380	433	413	2.87	53	0.996
7	460	0.6231	433	470	457	1.53	619	3.580
8	485	2.6870	470	595	487	2.85	2325	15.439
9	848	5.1401	775	1201	859	4.97	4816	29.534
10	4380	0.0789	4139	4518	4362	2.44	62	0.453
11	5519	0.3539	4518	6556	5334	11.34	84	2.034
12	7134	0.2979	6556	11342	7743	11.89	89	1.712
13	20000 (UM)	0.1484	18171	27012	20452	4.44	1039	10.230

TIC:	19.4723	ng/uL
TIM:	106.0314	nmole/L
Total concentration:	19.7810	ng/uL

```
Sample peak width (sec): 5    Sample min peak height: 50    Sample baseline V to V?: Y    Sample baseline V to V points: 3
Sample filter: Binomial       Number of points for filter: 3  Sample start region (min): 0  Sample end region (min): 30
Marker peak width (sec): 5    Marker min peak height: 200   Marker baseline V to V?: Y    Marker baseline V to V points: 3
Lower marker selection: First peak > 500 RFU                Upper marker selection: Last peak > 200 RFU
Ladder size (bp)75, 200, 300, 400, 500, 700, 1000, 1500, 2000, 3000, 4000, 5000, 7000, 10000, 20000
Quantification using: Lower Marker          Final concentration (ng/uL): 0.5000          Dilution factor: 12.0
```

Sample: SampG1
Well location: G1
Created: Tuesday, August 10, 2021 8:41:15 PM

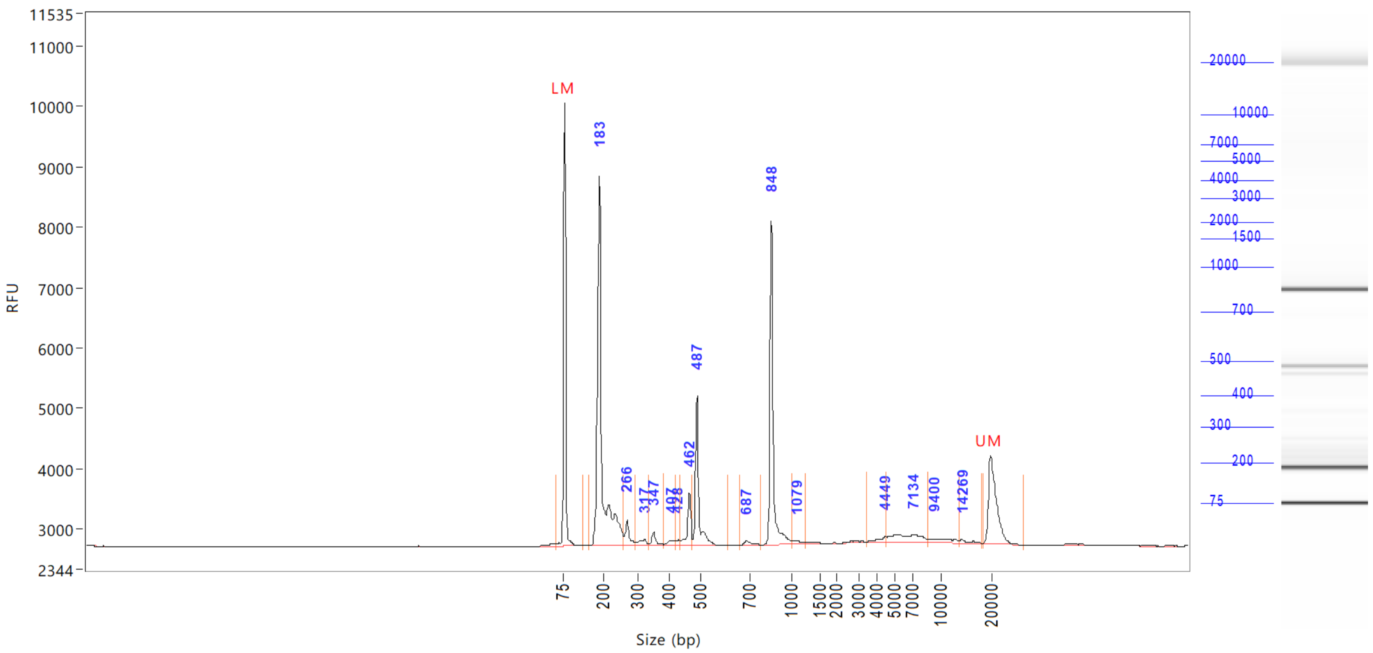

Peak	Size (bp)	Concentration (ng/uL)	From (bp)	To (bp)	Average size (bp)	CV%	RFU	Corrected peak area
1	75 (LM)	0.5000	50	131	74	6.17	7338	40.942
2	183	9.0326	154	257	192	10.76	6116	61.635
3	266	0.5058	257	291	267	3.08	423	3.451
4	317	0.1932	291	331	309	3.41	80	1.318
5	347	0.2416	331	380	346	2.21	201	1.649
6	407	0.1601	380	422	406	2.36	74	1.092
7	428	0.0837	422	435	428	0.99	83	0.571
8	462	0.7829	435	473	459	1.70	852	5.342
9	487	2.3361	473	613	490	3.02	2455	15.941
10	687	0.1182	662	779	696	3.06	63	0.807
11	848	4.5048	779	996	855	3.33	5348	30.740
12	1079	0.1273	996	1234	1077	6.19	63	0.868
13	4449	0.1738	3393	4552	4131	7.67	91	1.186
14	7134	0.7819	4552	8667	6329	19.17	135	5.336
15	9400	0.3105	8667	13781	10715	14.13	69	2.119
16	14269	0.0931	13781	18293	15205	7.39	57	0.635
17	20000 (UM)	0.1810	18537	26529	20491	4.56	1455	14.825

TIC:	19.4454	ng/uL
TIM:	103.7384	nmole/L
Total concentration:	19.5237	ng/uL

```
Sample peak width (sec): 5    Sample min peak height: 50    Sample baseline V to V?: Y    Sample baseline V to V points: 3
Sample filter: Binomial       Number of points for filter: 3  Sample start region (min): 0  Sample end region (min): 30
Marker peak width (sec): 5    Marker min peak height: 200   Marker baseline V to V?: Y    Marker baseline V to V points: 3
Lower marker selection: First peak > 500 RFU                Upper marker selection: Last peak > 200 RFU
Ladder size (bp)75, 200, 300, 400, 500, 700, 1000, 1500, 2000, 3000, 4000, 5000, 7000, 10000, 20000
Quantification using: Lower Marker          Final concentration (ng/uL): 0.5000          Dilution factor: 12.0
```

Sample: SampH1
Well location: H1
Created: Tuesday, August 10, 2021 8:41:15 PM

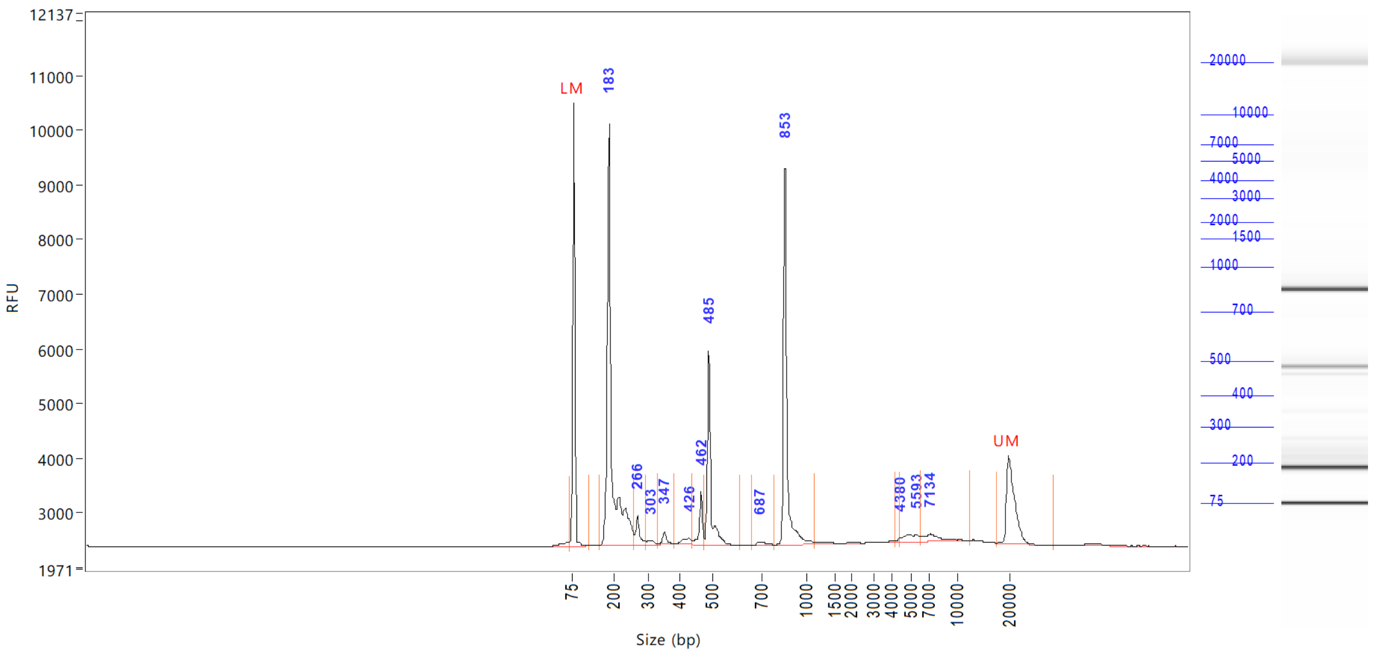

Peak	Size (bp)	Concentration (ng/uL)	From (bp)	To (bp)	Average size (bp)	CV%	RFU	Corrected peak area
1	75 (LM)	0.5000	64	126	74	5.21	8119	43.339
2	183	10.8093	154	255	192	10.56	7713	78.078
3	266	0.6453	255	291	266	3.20	546	4.661
4	303	0.1534	291	329	305	3.13	78	1.108
5	347	0.2736	329	380	347	2.21	243	1.976
6	426	0.2634	380	435	414	3.00	115	1.902
7	462	0.8240	435	471	458	1.62	970	5.952
8	485	3.2570	471	608	489	3.04	3547	23.526
9	687	0.1466	657	775	700	3.48	67	1.059
10	853	5.8312	775	1134	859	4.30	6892	42.120
11	4380	0.0453	4139	4414	4313	1.86	77	0.327
12	5593	0.3915	4414	6112	5114	9.42	127	2.828
13	7134	0.4582	6112	12562	7761	16.77	137	3.310
14	20000 (UM)	0.1884	17683	28826	20592	4.76	1605	16.329

TIC:	23.0987	ng/uL
TIM:	125.4013	nmole/L
Total concentration:	23.2598	ng/uL

```
Sample peak width (sec): 5    Sample min peak height: 50    Sample baseline V to V?: Y    Sample baseline V to V points: 3
Sample filter: Binomial       Number of points for filter: 3  Sample start region (min): 0  Sample end region (min): 30
Marker peak width (sec): 5    Marker min peak height: 200   Marker baseline V to V?: Y    Marker baseline V to V points: 3
Lower marker selection: First peak > 500 RFU                Upper marker selection: Last peak > 200 RFU
Ladder size (bp)75, 200, 300, 400, 500, 700, 1000, 1500, 2000, 3000, 4000, 5000, 7000, 10000, 20000
Quantification using: Lower Marker          Final concentration (ng/uL): 0.5000          Dilution factor: 12.0
```

Sample: SampA2
Well location: A2
Created: Tuesday, August 10, 2021 8:41:15 PM

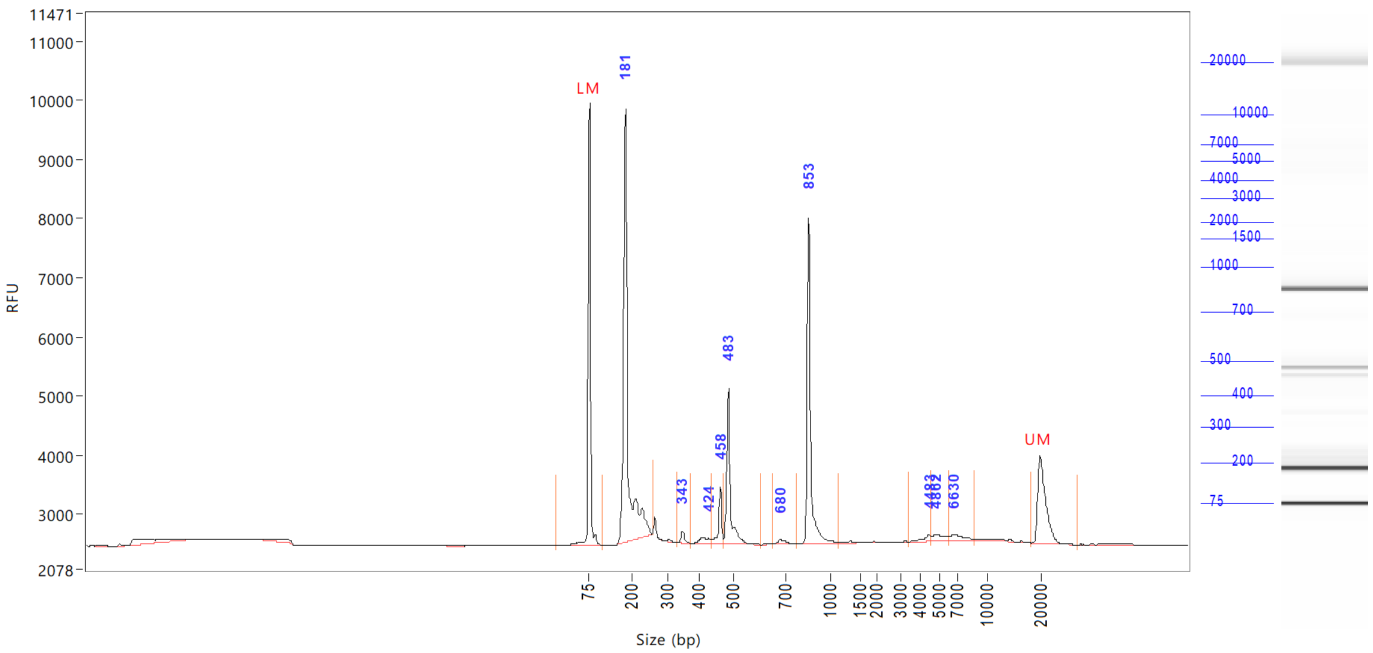

Peak	Size (bp)	Concentration (ng/uL)	From (bp)	To (bp)	Average size (bp)	CV%	RFU	Corrected peak area
1	75 (LM)	0.5000	0	116	72	11.29	7484	42.220
2	181	9.3935	116	259	186	9.36	7311	66.099
3	343	0.2020	329	374	344	1.82	202	1.421
4	424	0.2539	374	431	410	3.16	90	1.787
5	458	0.7910	431	468	454	1.58	967	5.566
6	483	2.4326	468	606	486	2.99	2631	17.117
7	680	0.1504	654	779	691	3.00	75	1.058
8	853	4.4778	779	1134	860	3.53	5506	31.508
9	4483	0.1629	3393	4621	4283	6.15	103	1.146
10	4862	0.2733	4621	6038	5170	8.17	102	1.923
11	6630	0.2854	6038	8800	7078	9.72	108	2.008
12	20000 (UM)	0.1707	18415	27133	20508	4.37	1492	14.410

TIC:	18.4227	ng/uL
TIM:	105.2613	nmole/L
Total concentration:	18.7436	ng/uL

```
Sample peak width (sec): 5     Sample min peak height: 50    Sample baseline V to V?: Y    Sample baseline V to V points: 3
Sample filter: Binomial        Number of points for filter: 3  Sample start region (min): 0  Sample end region (min): 30
Marker peak width (sec): 5     Marker min peak height: 200   Marker baseline V to V?: Y    Marker baseline V to V points: 3
Lower marker selection: First peak > 500 RFU                 Upper marker selection: Last peak > 200 RFU
Ladder size (bp)75, 200, 300, 400, 500, 700, 1000, 1500, 2000, 3000, 4000, 5000, 7000, 10000, 20000
Quantification using: Lower Marker          Final concentration (ng/uL): 0.5000          Dilution factor: 12.0
```

Sample: SampB2
Well location: B2
Created: Tuesday, August 10, 2021 8:41:15 PM

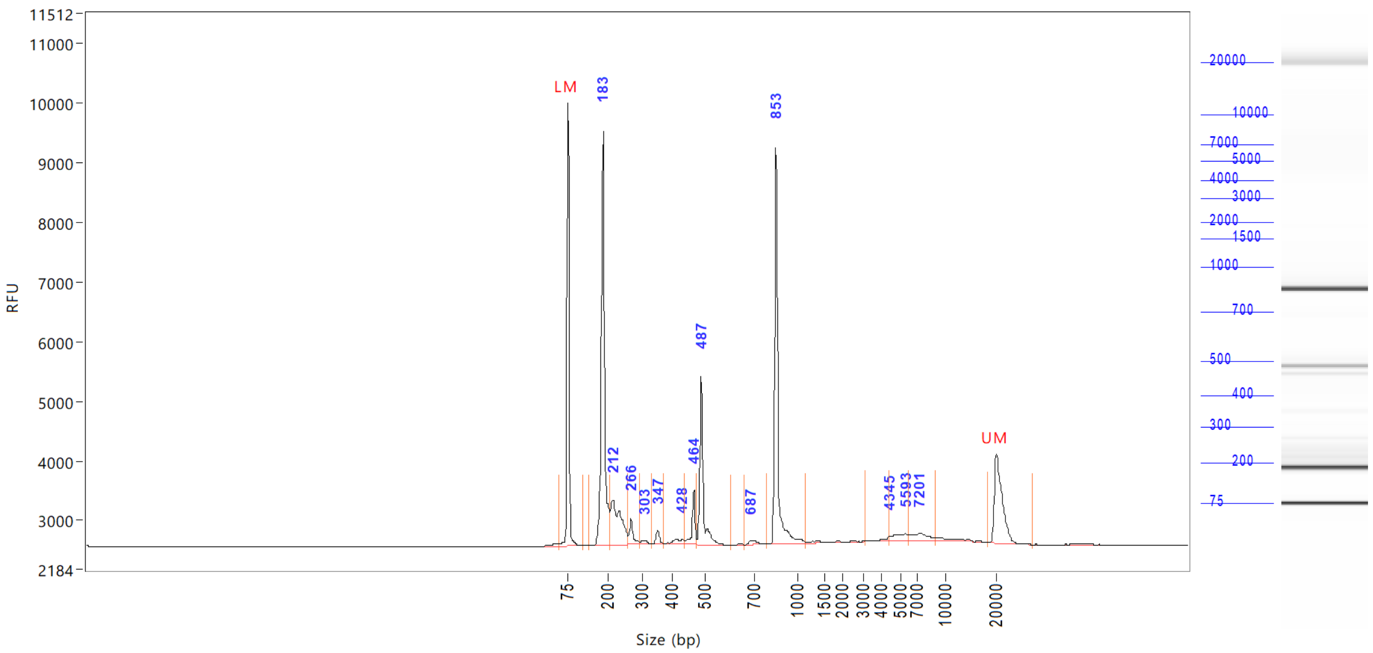

Peak	Size (bp)	Concentration (ng/uL)	From (bp)	To (bp)	Average size (bp)	CV%	RFU	Corrected peak area
1	75 (LM)	0.5000	48	124	73	6.13	7447	40.578
2	183	7.9545	141	203	182	3.98	6942	53.796
3	212	2.5296	203	257	224	6.43	752	17.107
4	266	0.5152	257	291	267	2.99	447	3.485
5	303	0.1084	291	329	302	2.35	57	0.733
6	347	0.2475	329	372	347	1.79	235	1.674
7	428	0.2534	372	435	411	3.67	83	1.713
8	464	0.8081	435	473	459	1.66	904	5.465
9	487	2.6541	473	606	489	2.76	2832	17.950
10	687	0.1425	659	783	700	3.16	67	0.964
11	853	5.5727	783	1145	861	4.23	6649	37.688
12	4345	0.0832	3001	4380	4098	6.42	76	0.563
13	5593	0.3819	4380	5963	5032	8.89	118	2.583
14	7201	0.4280	5963	8934	7261	10.56	130	2.894
15	20000 (UM)	0.1826	18293	27133	20569	4.48	1489	14.818

TIC:	21.6790	ng/uL
TIM:	119.4470	nmole/L
Total concentration:	21.8920	ng/uL

```
Sample peak width (sec): 5     Sample min peak height: 50     Sample baseline V to V?: Y     Sample baseline V to V points: 3
Sample filter: Binomial        Number of points for filter: 3  Sample start region (min): 0  Sample end region (min): 30
Marker peak width (sec): 5     Marker min peak height: 200    Marker baseline V to V?: Y     Marker baseline V to V points: 3
Lower marker selection: First peak > 500 RFU                  Upper marker selection: Last peak > 200 RFU
Ladder size (bp)75, 200, 300, 400, 500, 700, 1000, 1500, 2000, 3000, 4000, 5000, 7000, 10000, 20000
Quantification using: Lower Marker          Final concentration (ng/uL): 0.5000          Dilution factor: 12.0
```

Sample: SampC2
Well location: C2
Created: Tuesday, August 10, 2021 8:41:15 PM

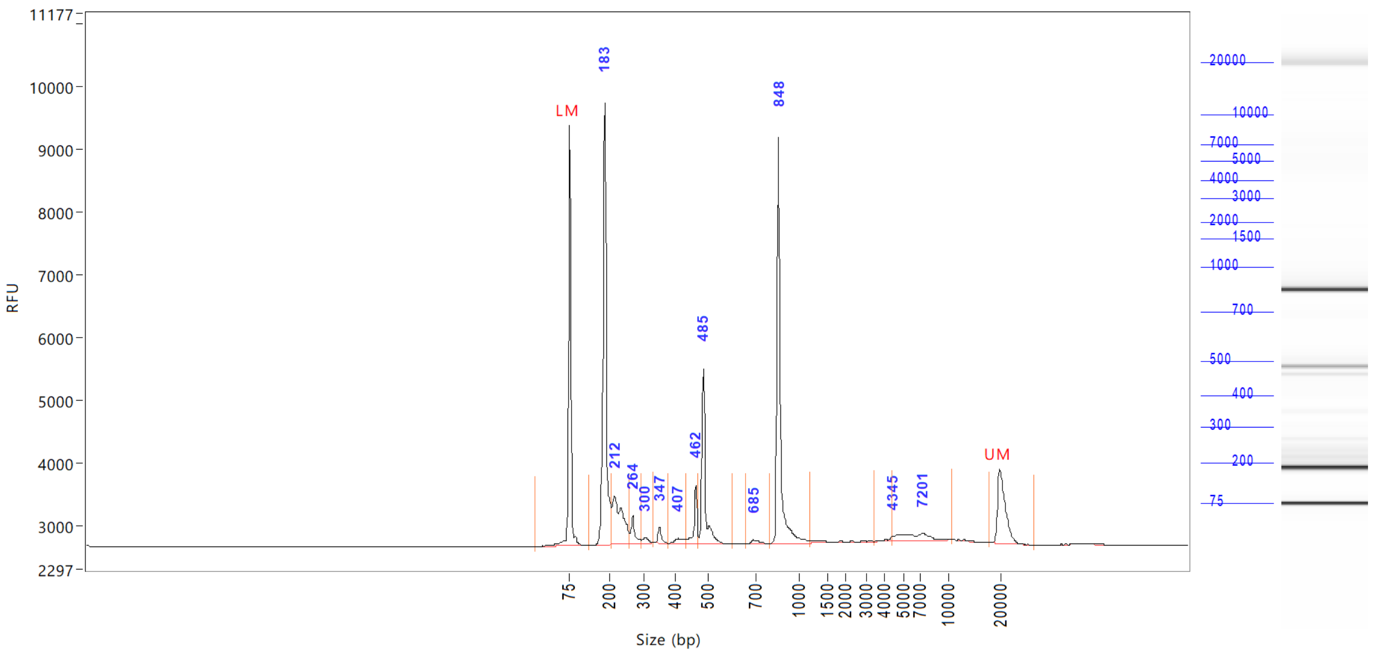

Peak	Size (bp)	Concentration (ng/uL)	From (bp)	To (bp)	Average size (bp)	CV%	RFU	Corrected peak area
1	75 (LM)	0.5000	0	137	72	12.72	6717	39.089
2	183	8.3964	137	203	182	4.05	7069	54.701
3	212	2.6818	203	255	223	6.25	782	17.471
4	264	0.5621	255	289	266	3.15	453	3.662
5	300	0.1670	289	329	301	2.56	90	1.088
6	347	0.2740	329	378	346	1.78	248	1.785
7	407	0.2431	378	433	412	3.17	78	1.584
8	462	0.8523	433	471	457	1.69	935	5.553
9	485	2.8177	471	600	489	3.01	2783	18.357
10	685	0.1517	657	783	696	3.20	68	0.989
11	848	5.8831	783	1178	860	4.93	6500	38.328
12	4345	0.0637	3358	4380	4167	4.90	71	0.415
13	7201	0.7202	4380	10367	6178	21.68	112	4.692
14	20000 (UM)	0.1461	17927	26649	20447	4.36	1181	11.421

TIC:	22.8131	ng/uL
TIM:	126.6679	nmole/L
Total concentration:	22.8648	ng/uL

```
Sample peak width (sec): 5     Sample min peak height: 50    Sample baseline V to V?: Y    Sample baseline V to V points: 3
Sample filter: Binomial        Number of points for filter: 3  Sample start region (min): 0  Sample end region (min): 30
Marker peak width (sec): 5     Marker min peak height: 200   Marker baseline V to V?: Y    Marker baseline V to V points: 3
Lower marker selection: First peak > 500 RFU                 Upper marker selection: Last peak > 200 RFU
Ladder size (bp)75, 200, 300, 400, 500, 700, 1000, 1500, 2000, 3000, 4000, 5000, 7000, 10000, 20000
Quantification using: Lower Marker          Final concentration (ng/uL): 0.5000          Dilution factor: 12.0
```

Sample: SampD2
Well location: D2
Created: Tuesday, August 10, 2021 8:41:15 PM

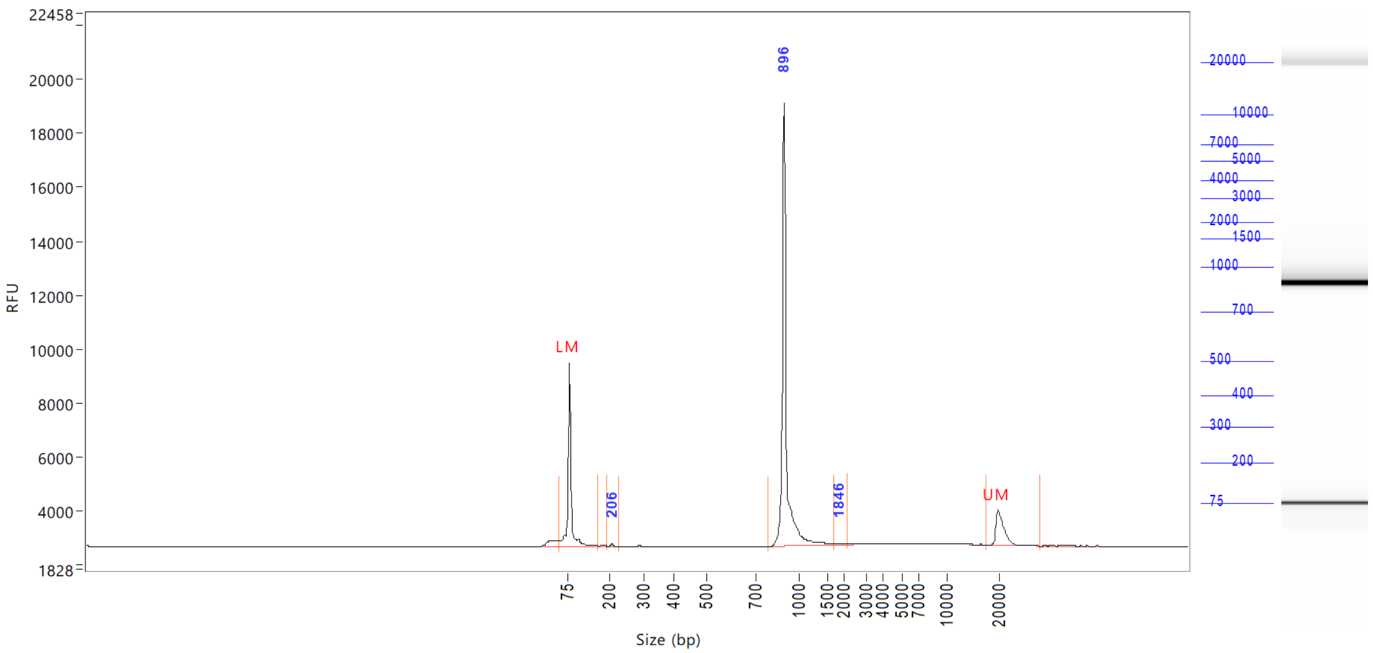

Peak	Size (bp)	Concentration (ng/uL)	From (bp)	To (bp)	Average size (bp)	CV%	RFU	Corrected peak area
1	75 (LM)	0.5000	43	166	76	18.79	6819	50.967
2	206	0.0439	194	228	205	1.85	70	0.373
3	896	13.0767	788	1731	919	9.76	16469	111.081
4	1846	0.0908	1731	2172	1919	6.26	60	0.771
5	20000 (UM)	0.1284	17561	28342	20528	4.69	1315	13.087

TIC:	13.2114	ng/uL
TIM:	23.8497	nmole/L
Total concentration:	13.5923	ng/uL

Sample peak width (sec): 5   Sample min peak height: 50   Sample baseline V to V?: Y   Sample baseline V to V points: 3
Sample filter: Binomial   Number of points for filter: 3   Sample start region (min): 0   Sample end region (min): 30
Marker peak width (sec): 5   Marker min peak height: 200   Marker baseline V to V?: Y   Marker baseline V to V points: 3
Lower marker selection: First peak > 500 RFU   Upper marker selection: Last peak > 200 RFU
Ladder size (bp)75, 200, 300, 400, 500, 700, 1000, 1500, 2000, 3000, 4000, 5000, 7000, 10000, 20000
Quantification using: Lower Marker   Final concentration (ng/uL): 0.5000   Dilution factor: 12.0

Sample: SampE2
Well location: E2
Created: Tuesday, August 10, 2021 8:41:15 PM

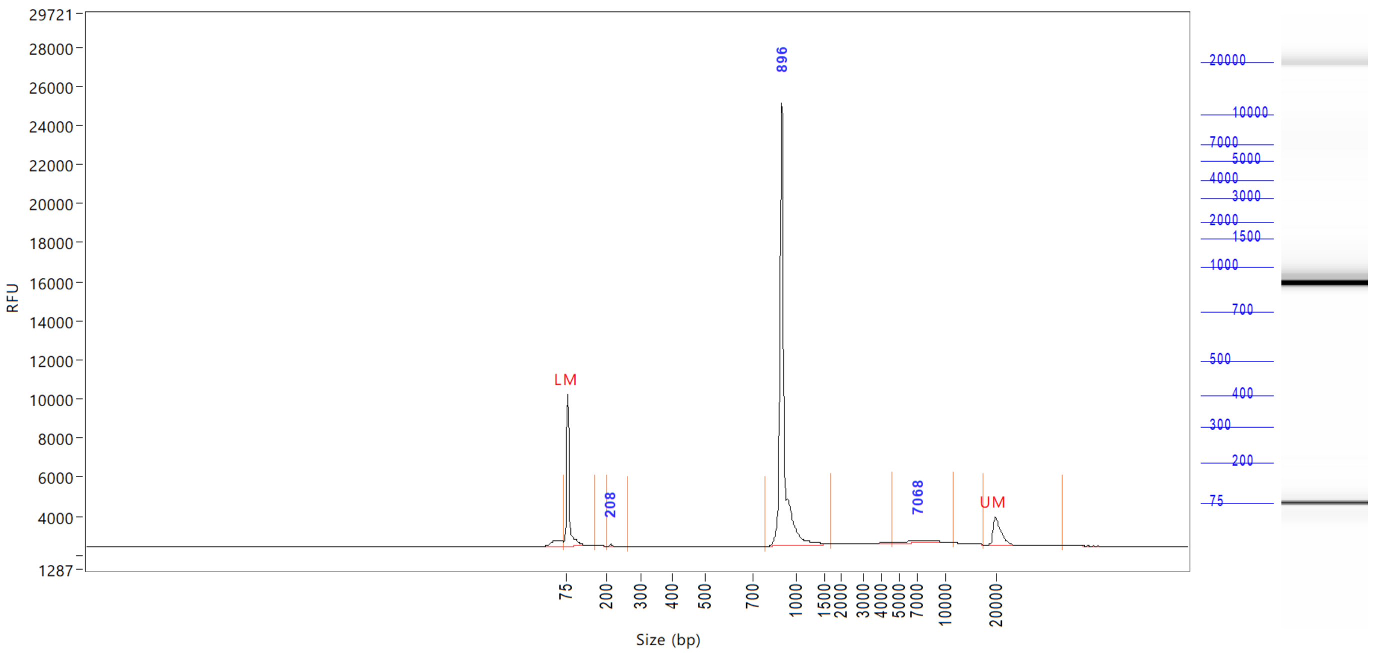

Peak	Size (bp)	Concentration (ng/uL)	From (bp)	To (bp)	Average size (bp)	CV%	RFU	Corrected peak area
1	75 (LM)	0.5000	64	162	77	14.10	7771	54.184
2	208	0.0571	200	259	208	3.50	100	0.516
3	896	17.6974	783	1693	915	7.98	22687	159.819
4	7068	0.5626	4587	11464	7274	19.52	131	5.081
5	20000 (UM)	0.1297	17683	33058	20508	6.04	1399	14.055

TIC:	18.3171	ng/uL
TIM:	32.4130	nmole/L
Total concentration:	18.5911	ng/uL

Sample peak width (sec): 5   Sample min peak height: 50   Sample baseline V to V?: Y   Sample baseline V to V points: 3
Sample filter: Binomial   Number of points for filter: 3 Sample start region (min): 0 Sample end region (min): 30
Marker peak width (sec): 5   Marker min peak height: 200   Marker baseline V to V?: Y   Marker baseline V to V points: 3
Lower marker selection: First peak > 500 RFU   Upper marker selection: Last peak > 200 RFU
Ladder size (bp)75, 200, 300, 400, 500, 700, 1000, 1500, 2000, 3000, 4000, 5000, 7000, 10000, 20000
Quantification using: Lower Marker   Final concentration (ng/uL): 0.5000   Dilution factor: 12.0

Sample: SampF2
Well location: F2
Created: Tuesday, August 10, 2021 8:41:15 PM

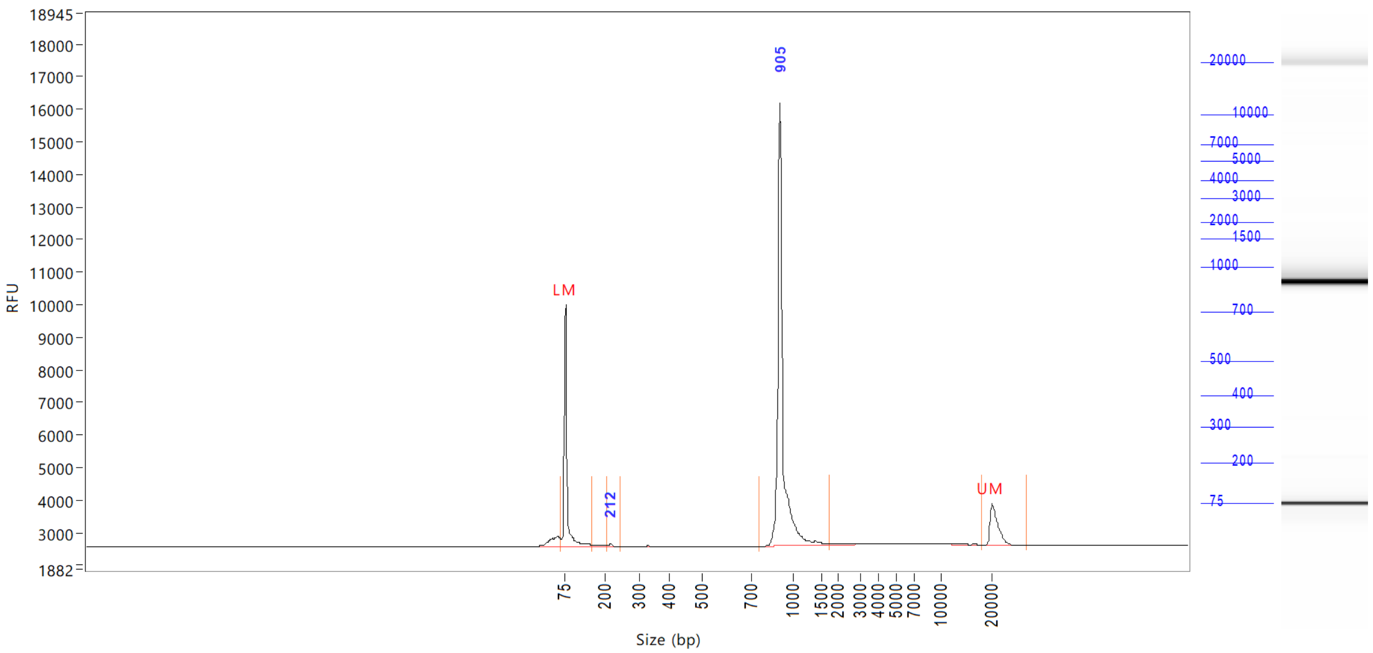

Peak	Size (bp)	Concentration (ng/uL)	From (bp)	To (bp)	Average size (bp)	CV%	RFU	Corrected peak area
1	75 (LM)	0.5000	62	160	77	18.11	7413	50.368
2	212	0.0827	203	244	213	3.32	99	0.694
3	905	13.1292	753	1712	927	9.43	13619	110.217
4	20000 (UM)	0.1284	18049	26770	20571	4.64	1264	12.937

TIC:	13.2119	ng/uL
TIM:	23.9492	nmole/L
Total concentration:	13.8180	ng/uL

```
Sample peak width (sec): 5    Sample min peak height: 50    Sample baseline V to V?: Y    Sample baseline V to V points: 3
Sample filter: Binomial       Number of points for filter: 3  Sample start region (min): 0  Sample end region (min): 30
Marker peak width (sec): 5    Marker min peak height: 200   Marker baseline V to V?: Y    Marker baseline V to V points: 3
Lower marker selection: First peak > 500 RFU                Upper marker selection: Last peak > 200 RFU
Ladder size (bp)75, 200, 300, 400, 500, 700, 1000, 1500, 2000, 3000, 4000, 5000, 7000, 10000, 20000
Quantification using: Lower Marker          Final concentration (ng/uL): 0.5000          Dilution factor: 12.0
```

Sample: SampG2
Well location: G2
Created: Tuesday, August 10, 2021 8:41:15 PM

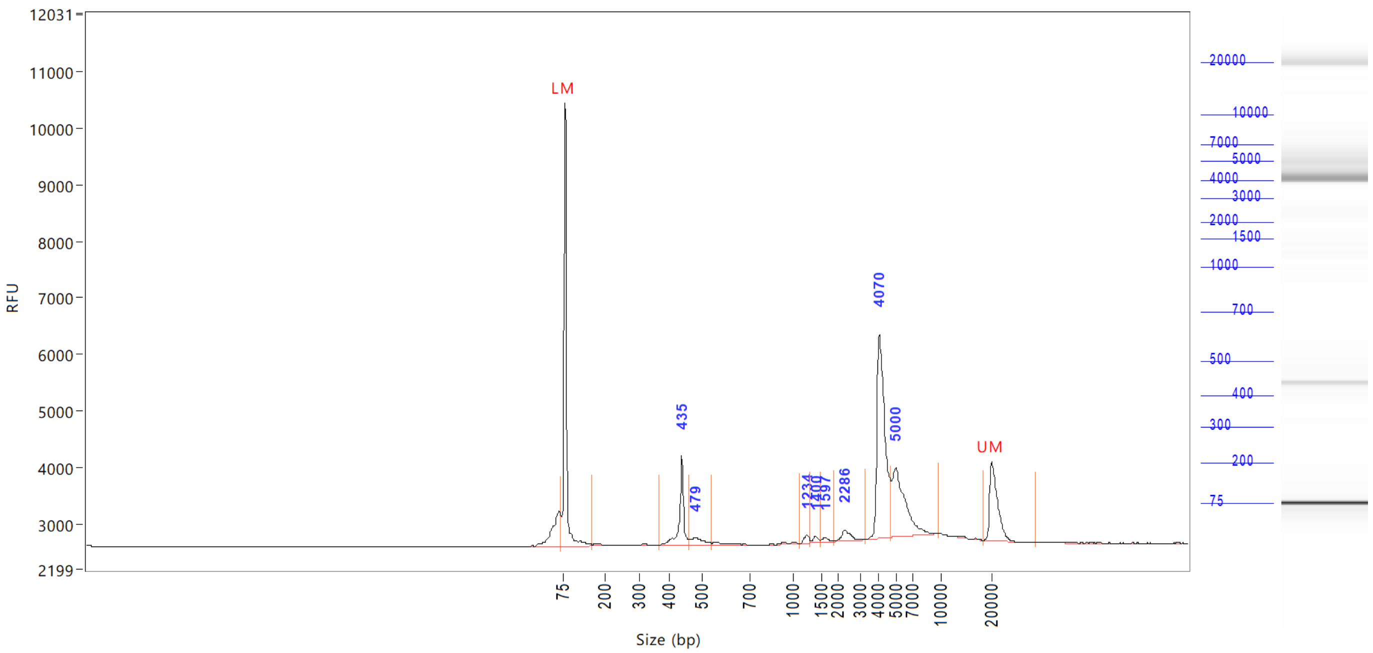

Peak	Size (bp)	Concentration (ng/uL)	From (bp)	To (bp)	Average size (bp)	CV%	RFU	Corrected peak area
1	75 (LM)	0.5000	64	160	77	16.10	7854	51.092
2	435	1.5189	366	458	429	3.50	1573	12.934
3	479	0.3701	458	537	486	4.08	124	3.151
4	1234	0.1356	1112	1311	1223	3.28	148	1.155
5	1400	0.1074	1311	1478	1391	2.99	114	0.914
6	1597	0.1107	1478	1904	1610	6.23	90	0.942
7	2286	0.3109	1904	3286	2376	9.86	198	2.647
8	4070	5.0292	3286	4724	4193	5.49	3601	42.825
9	5000	2.7585	4724	9733	5633	15.88	1211	23.489
10	20000 (UM)	0.1405	18293	28463	20472	4.56	1408	14.360

TIC:	10.3412	ng/uL
TIM:	10.4974	nmole/L
Total concentration:	10.6577	ng/uL

Sample peak width (sec): 5   Sample min peak height: 50   Sample baseline V to V?: Y   Sample baseline V to V points: 3
Sample filter: Binomial   Number of points for filter: 3   Sample start region (min): 0   Sample end region (min): 30
Marker peak width (sec): 5   Marker min peak height: 200   Marker baseline V to V?: Y   Marker baseline V to V points: 3
Lower marker selection: First peak > 500 RFU   Upper marker selection: Last peak > 200 RFU
Ladder size (bp)75, 200, 300, 400, 500, 700, 1000, 1500, 2000, 3000, 4000, 5000, 7000, 10000, 20000
Quantification using: Lower Marker   Final concentration (ng/uL): 0.5000   Dilution factor: 12.0

Sample: SampH2
Well location: H2
Created: Tuesday, August 10, 2021 8:41:15 PM

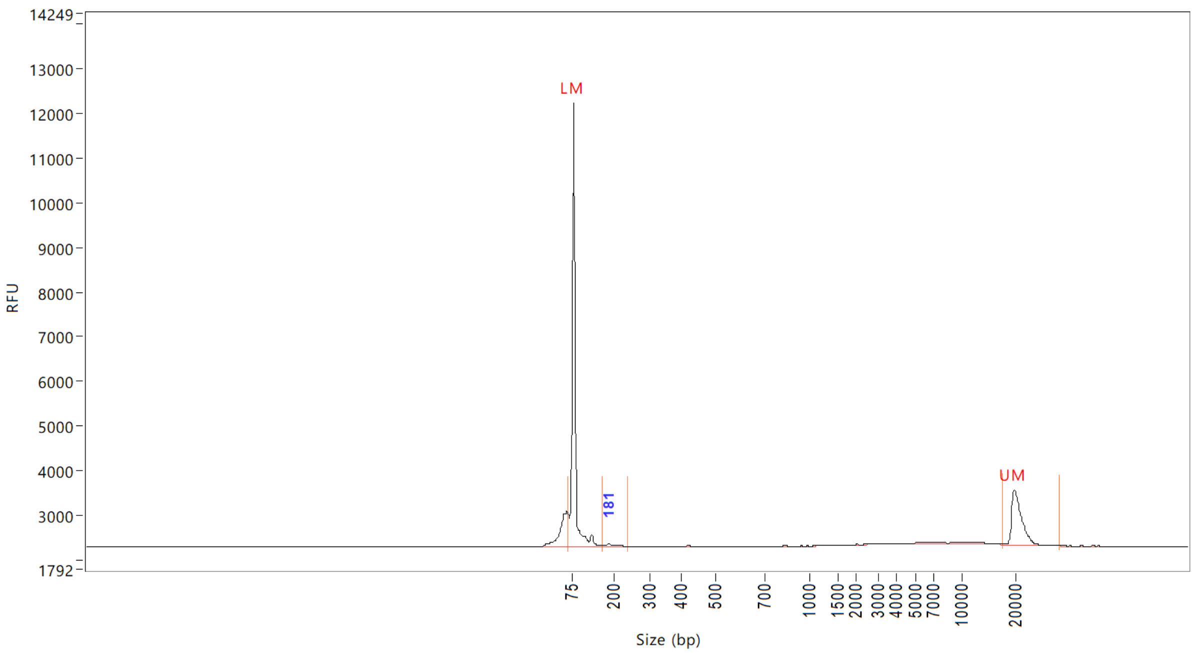

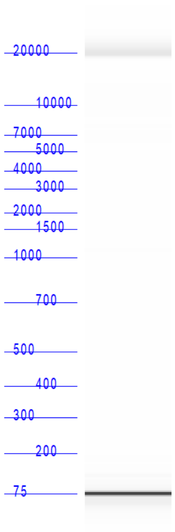

Peak	Size (bp)	Concentration (ng/uL)	From (bp)	To (bp)	Average size (bp)	CV%	RFU	Corrected peak area
1	75 (LM)	0.5000	62	166	77	17.60	9956	71.167
2	181	0.0832	166	237	188	8.09	51	0.987
3	20000 (UM)	0.1058	17805	28584	20599	5.40	1241	15.065

TIC:	0.0832	ng/uL
TIM:	0.7278	nmole/L
Total concentration:	0.4681	ng/uL

Sample peak width (sec): 5 Sample min peak height: 50 Sample baseline V to V?: Y Sample baseline V to V points: 3
Sample filter: Binomial Number of points for filter: 3 Sample start region (min): 0 Sample end region (min): 30
Marker peak width (sec): 5 Marker min peak height: 200 Marker baseline V to V?: Y Marker baseline V to V points: 3
Lower marker selection: First peak > 500 RFU Upper marker selection: Last peak > 200 RFU
Ladder size (bp)75, 200, 300, 400, 500, 700, 1000, 1500, 2000, 3000, 4000, 5000, 7000, 10000, 20000
Quantification using: Lower Marker Final concentration (ng/uL): 0.5000 Dilution factor: 12.0

Sample: SampA3
Well location: A3
Created: Tuesday, August 10, 2021 8:41:15 PM

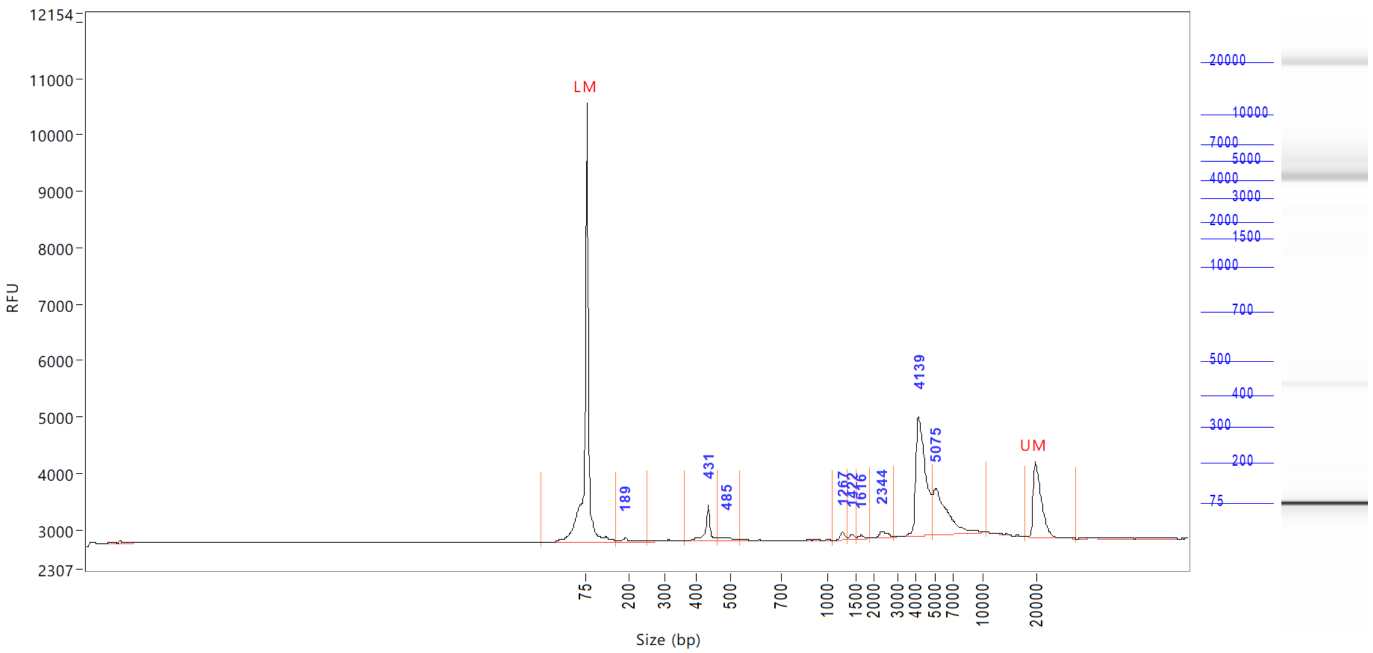

Peak	Size (bp)	Concentration (ng/uL)	From (bp)	To (bp)	Average size (bp)	CV%	RFU	Corrected peak area
1	75 (LM)	0.5000	0	162	68	29.64	7792	67.902
2	189	0.1182	162	250	194	10.69	66	1.338
3	431	0.5177	362	460	424	3.95	641	5.858
4	485	0.1445	460	532	487	3.70	67	1.636
5	1267	0.0934	1090	1345	1254	3.38	140	1.057
6	1422	0.0644	1345	1501	1422	2.98	90	0.729
7	1616	0.0494	1501	1885	1619	4.79	66	0.560
8	2344	0.1267	1885	2886	2398	7.68	115	1.433
9	4139	2.5844	2886	4862	4280	6.40	2108	29.248
10	5075	1.4593	4862	10611	5973	17.62	820	16.515
11	20000 (UM)	0.0976	18171	27738	20483	4.48	1343	13.258

TIC:	5.1580	ng/uL
TIM:	5.2297	nmole/L
Total concentration:	5.3174	ng/uL

Sample peak width (sec): 5   Sample min peak height: 50   Sample baseline V to V?: Y   Sample baseline V to V points: 3
Sample filter: Binomial   Number of points for filter: 3 Sample start region (min): 0 Sample end region (min): 30
Marker peak width (sec): 5   Marker min peak height: 200   Marker baseline V to V?: Y   Marker baseline V to V points: 3
Lower marker selection: First peak > 500 RFU   Upper marker selection: Last peak > 200 RFU
Ladder size (bp)75, 200, 300, 400, 500, 700, 1000, 1500, 2000, 3000, 4000, 5000, 7000, 10000, 20000
Quantification using: Lower Marker   Final concentration (ng/uL): 0.5000   Dilution factor: 12.0

Sample: SampB3
Well location: B3
Created: Tuesday, August 10, 2021 8:41:15 PM

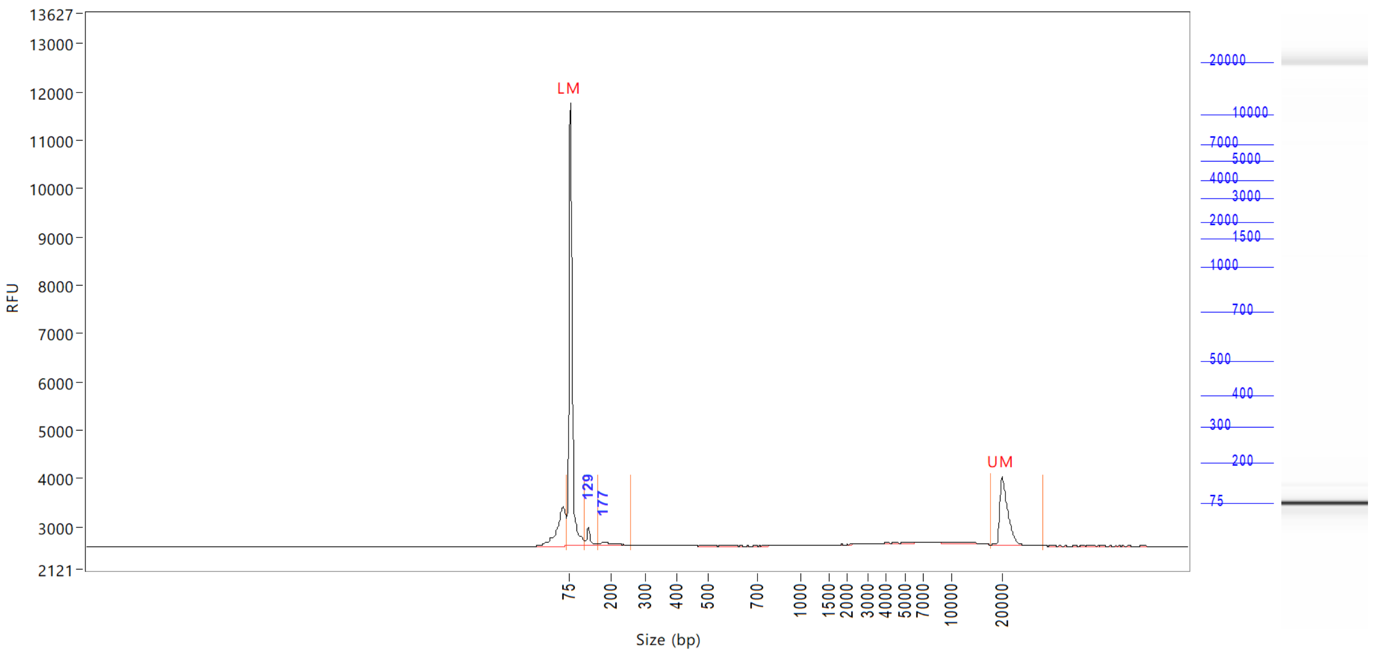

Peak	Size (bp)	Concentration (ng/uL)	From (bp)	To (bp)	Average size (bp)	CV%	RFU	Corrected peak area
1	75 (LM)	0.5000	64	120	75	10.77	9184	68.095
2	129	0.2794	120	160	131	6.95	390	3.171
3	177	0.1560	160	259	190	10.46	61	1.770
4	20000 (UM)	0.1036	17927	27979	20561	4.48	1423	14.109

TIC:	0.4354	ng/uL
TIM:	4.8542	nmole/L
Total concentration:	0.7780	ng/uL

Sample peak width (sec): 5   Sample min peak height: 50   Sample baseline V to V?: Y   Sample baseline V to V points: 3
Sample filter: Binomial   Number of points for filter: 3 Sample start region (min): 0 Sample end region (min): 30
Marker peak width (sec): 5   Marker min peak height: 200   Marker baseline V to V?: Y   Marker baseline V to V points: 3
Lower marker selection: First peak > 500 RFU   Upper marker selection: Last peak > 200 RFU
Ladder size (bp)75, 200, 300, 400, 500, 700, 1000, 1500, 2000, 3000, 4000, 5000, 7000, 10000, 20000
Quantification using: Lower Marker   Final concentration (ng/uL): 0.5000   Dilution factor: 12.0

Sample: SampC3
Well location: C3
Created: Tuesday, August 10, 2021 8:41:15 PM

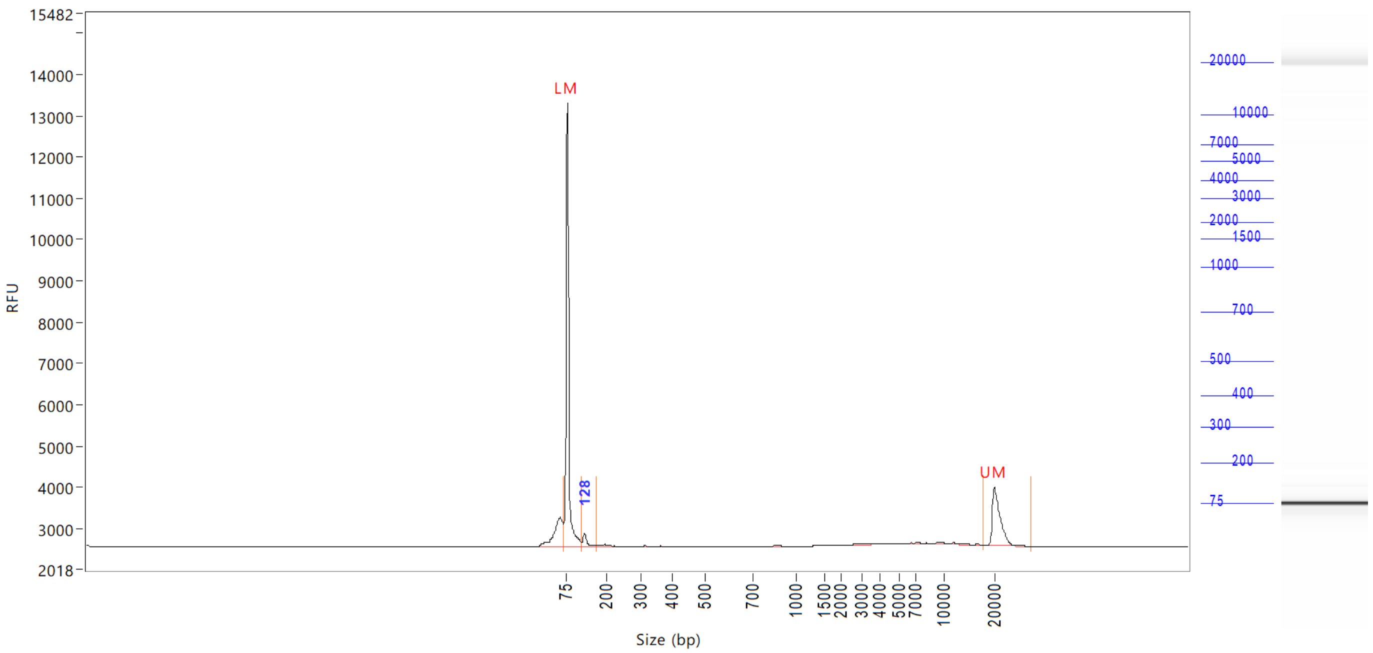

Peak	Size (bp)	Concentration (ng/uL)	From (bp)	To (bp)	Average size (bp)	CV%	RFU	Corrected peak area
1	75 (LM)	0.5000	64	118	75	10.34	10758	70.491
2	128	0.2474	118	168	131	8.70	328	2.906
3	20000 (UM)	0.0999	17805	27375	20538	4.65	1415	14.088

TIC:	0.2474	ng/uL
TIM:	3.1026	nmole/L
Total concentration:	0.6554	ng/uL

Sample peak width (sec): 5 Sample min peak height: 50 Sample baseline V to V?: Y Sample baseline V to V points: 3
Sample filter: Binomial Number of points for filter: 3 Sample start region (min): 0 Sample end region (min): 30
Marker peak width (sec): 5 Marker min peak height: 200 Marker baseline V to V?: Y Marker baseline V to V points: 3
Lower marker selection: First peak > 500 RFU Upper marker selection: Last peak > 200 RFU
Ladder size (bp)75, 200, 300, 400, 500, 700, 1000, 1500, 2000, 3000, 4000, 5000, 7000, 10000, 20000
Quantification using: Lower Marker Final concentration (ng/uL): 0.5000 Dilution factor: 12.0

Sample: SampD3
Well location: D3
Created: Tuesday, August 10, 2021 8:41:15 PM

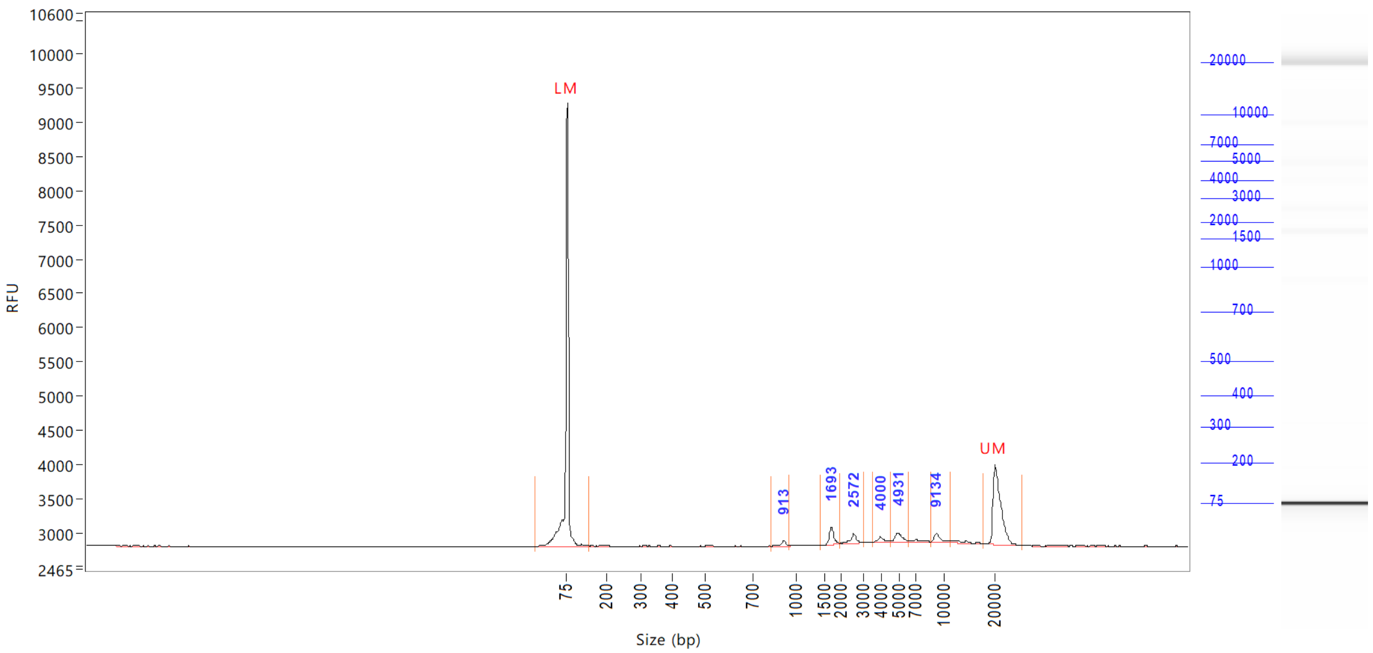

Peak	Size (bp)	Concentration (ng/uL)	From (bp)	To (bp)	Average size (bp)	CV%	RFU	Corrected peak area
1	75 (LM)	0.5000	0	143	68	22.01	6503	46.115
2	913	0.0839	822	957	898	2.83	75	0.645
3	1693	0.2298	1422	1962	1699	5.11	244	1.767
4	2572	0.1756	1962	3037	2485	8.63	139	1.350
5	4000	0.0933	3536	4518	4034	5.40	77	0.717
6	4931	0.1805	4518	6038	5039	6.37	138	1.388
7	9134	0.1427	8534	10977	9393	5.55	126	1.097
8	20000 (UM)	0.1247	17561	25320	20506	4.32	1163	11.502

TIC:	0.9060	ng/uL
TIM:	0.6149	nmole/L
Total concentration:	1.3011	ng/uL

Sample peak width (sec): 5   Sample min peak height: 50   Sample baseline V to V?: Y   Sample baseline V to V points: 3
Sample filter: Binomial   Number of points for filter: 3 Sample start region (min): 0 Sample end region (min): 30
Marker peak width (sec): 5   Marker min peak height: 200   Marker baseline V to V?: Y   Marker baseline V to V points: 3
Lower marker selection: First peak > 500 RFU   Upper marker selection: Last peak > 200 RFU
Ladder size (bp)75, 200, 300, 400, 500, 700, 1000, 1500, 2000, 3000, 4000, 5000, 7000, 10000, 20000
Quantification using: Lower Marker   Final concentration (ng/uL): 0.5000   Dilution factor: 12.0

Sample: SampE3
Well location: E3
Created: Tuesday, August 10, 2021 8:41:15 PM

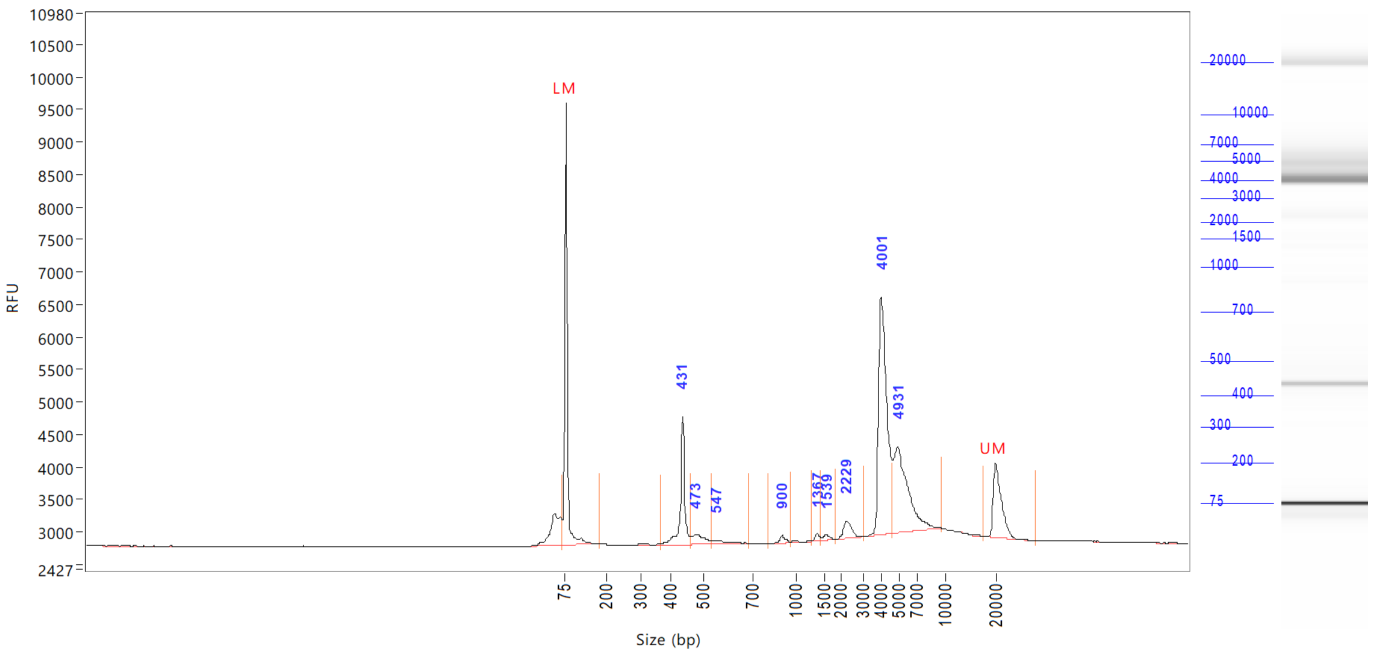

Peak	Size (bp)	Concentration (ng/uL)	From (bp)	To (bp)	Average size (bp)	CV%	RFU	Corrected peak area
1	75 (LM)	0.5000	64	181	75	15.35	6810	43.504
2	431	2.2894	362	458	427	3.42	1977	16.599
3	473	0.5179	458	532	485	3.93	153	3.755
4	547	0.2382	532	685	583	6.41	62	1.727
5	900	0.1688	805	961	900	3.00	124	1.224
6	1367	0.1084	1278	1445	1365	2.80	112	0.786
7	1539	0.1182	1445	1827	1552	5.05	84	0.857
8	2229	0.4657	1827	3001	2290	8.32	273	3.376
9	4001	6.3254	3001	4656	4126	5.71	3646	45.863
10	4931	3.7688	4656	9600	5562	16.22	1331	27.327
11	20000 (UM)	0.1389	17683	27979	20489	4.72	1151	12.081

TIC:	14.0007	ng/uL
TIM:	15.7899	nmole/L
Total concentration:	14.0226	ng/uL

```
Sample peak width (sec): 5    Sample min peak height: 50    Sample baseline V to V?: Y    Sample baseline V to V points: 3
Sample filter: Binomial       Number of points for filter: 3  Sample start region (min): 0  Sample end region (min): 30
Marker peak width (sec): 5    Marker min peak height: 200   Marker baseline V to V?: Y    Marker baseline V to V points: 3
Lower marker selection: First peak > 500 RFU                Upper marker selection: Last peak > 200 RFU
Ladder size (bp)75, 200, 300, 400, 500, 700, 1000, 1500, 2000, 3000, 4000, 5000, 7000, 10000, 20000
Quantification using: Lower Marker          Final concentration (ng/uL): 0.5000          Dilution factor: 12.0
```

Sample: SampF3
Well location: F3
Created: Tuesday, August 10, 2021 8:41:15 PM

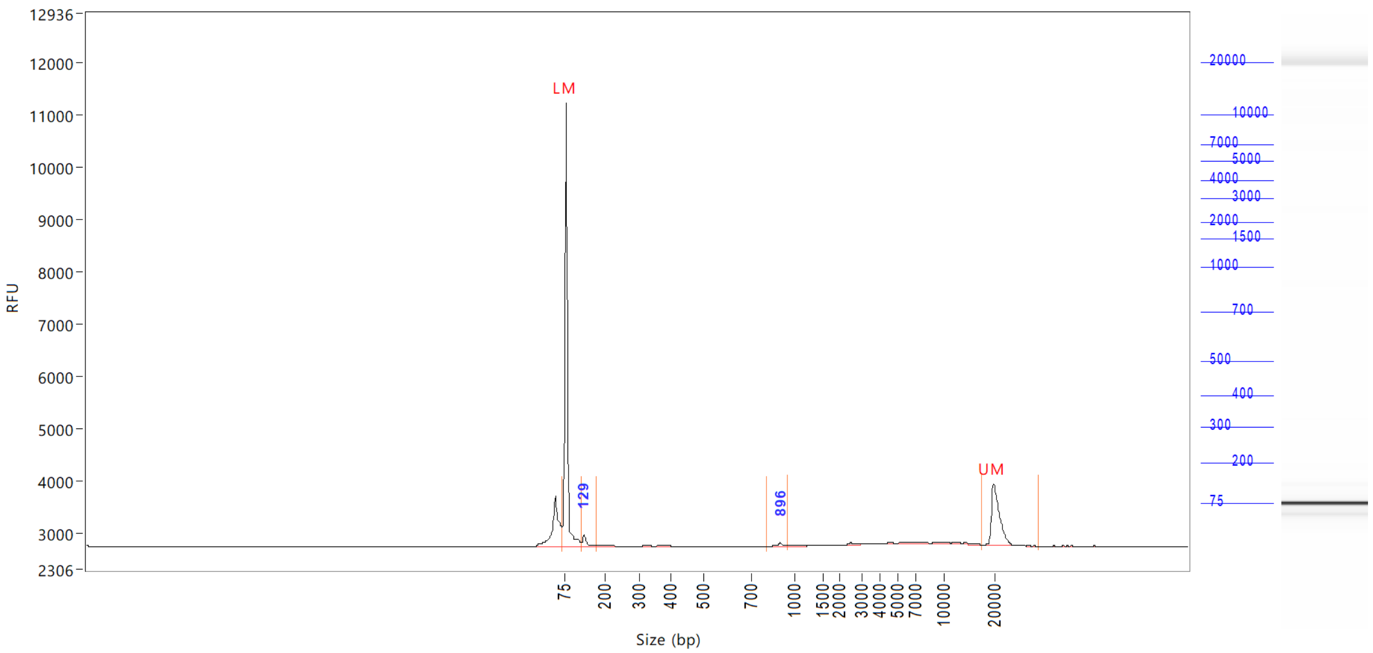

Peak	Size (bp)	Concentration (ng/uL)	From (bp)	To (bp)	Average size (bp)	CV%	RFU	Corrected peak area
1	75 (LM)	0.5000	62	122	75	11.03	8493	54.205
2	129	0.2215	122	171	134	8.97	212	2.001
3	896	0.0603	796	952	886	3.12	62	0.545
4	20000 (UM)	0.1125	17683	28826	20515	4.93	1184	12.201

TIC:	0.2818	ng/uL
TIM:	2.8285	nmole/L
Total concentration:	0.7979	ng/uL

```
Sample peak width (sec): 5    Sample min peak height: 50    Sample baseline V to V?: Y    Sample baseline V to V points: 3
Sample filter: Binomial       Number of points for filter: 3  Sample start region (min): 0  Sample end region (min): 30
Marker peak width (sec): 5    Marker min peak height: 200   Marker baseline V to V?: Y    Marker baseline V to V points: 3
Lower marker selection: First peak > 500 RFU                Upper marker selection: Last peak > 200 RFU
Ladder size (bp)75, 200, 300, 400, 500, 700, 1000, 1500, 2000, 3000, 4000, 5000, 7000, 10000, 20000
Quantification using: Lower Marker          Final concentration (ng/uL): 0.5000          Dilution factor: 12.0
```

Sample: SampG3
Well location: G3
Created: Tuesday, August 10, 2021 8:41:15 PM

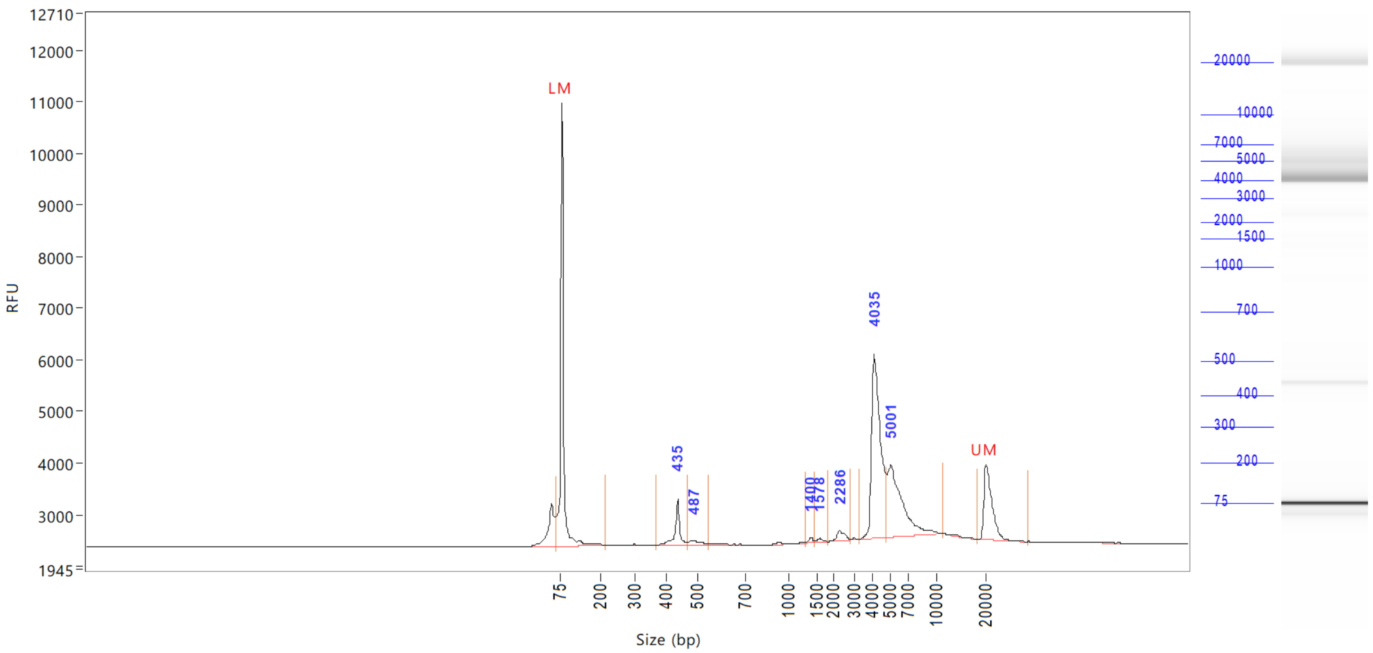

Peak	Size (bp)	Concentration (ng/uL)	From (bp)	To (bp)	Average size (bp)	CV%	RFU	Corrected peak area
1	75 (LM)	0.5000	58	210	78	23.51	8598	62.000
2	435	0.7838	366	466	429	3.83	902	8.099
3	487	0.1888	466	539	493	3.87	80	1.951
4	1400	0.0625	1300	1467	1384	2.64	100	0.646
5	1578	0.0578	1467	1866	1570	4.36	73	0.597
6	2286	0.2261	1866	2800	2334	7.68	204	2.336
7	4035	4.6578	3251	4793	4220	6.31	3576	48.131
8	5001	2.9280	4793	11220	5957	19.48	1394	30.256
9	20000 (UM)	0.1195	18415	28342	20587	4.63	1465	14.815

TIC:	8.9048	ng/uL
TIM:	6.5570	nmole/L
Total concentration:	9.1115	ng/uL

Sample peak width (sec): 5   Sample min peak height: 50   Sample baseline V to V?: Y   Sample baseline V to V points: 3
Sample filter: Binomial   Number of points for filter: 3   Sample start region (min): 0   Sample end region (min): 30
Marker peak width (sec): 5   Marker min peak height: 200   Marker baseline V to V?: Y   Marker baseline V to V points: 3
Lower marker selection: First peak > 500 RFU   Upper marker selection: Last peak > 200 RFU
Ladder size (bp)75, 200, 300, 400, 500, 700, 1000, 1500, 2000, 3000, 4000, 5000, 7000, 10000, 20000
Quantification using: Lower Marker   Final concentration (ng/uL): 0.5000   Dilution factor: 12.0

Sample: SampH3
Well location: H3
Created: Tuesday, August 10, 2021 8:41:15 PM

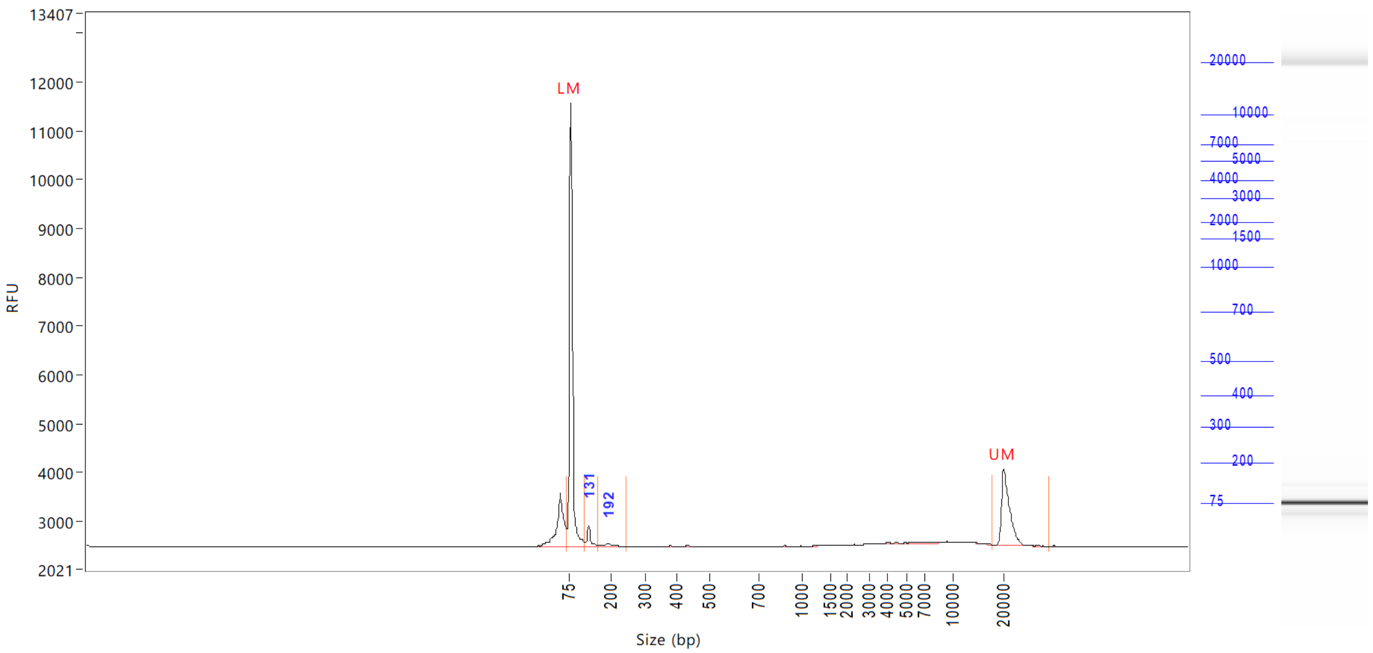

Peak	Size (bp)	Concentration (ng/uL)	From (bp)	To (bp)	Average size (bp)	CV%	RFU	Corrected peak area
1	75 (LM)	0.5000	64	120	76	9.93	9103	65.731
2	131	0.3258	120	158	132	6.25	436	3.569
3	192	0.1370	158	246	188	9.60	50	1.501
4	20000 (UM)	0.1211	17805	28826	20585	5.01	1555	15.921

TIC:	0.4628	ng/uL
TIM:	5.2535	nmole/L
Total concentration:	0.7406	ng/uL

Sample peak width (sec): 5 Sample min peak height: 50 Sample baseline V to V?: Y Sample baseline V to V points: 3
Sample filter: Binomial Number of points for filter: 3 Sample start region (min): 0 Sample end region (min): 30
Marker peak width (sec): 5 Marker min peak height: 200 Marker baseline V to V?: Y Marker baseline V to V points: 3
Lower marker selection: First peak > 500 RFU Upper marker selection: Last peak > 200 RFU
Ladder size (bp)75, 200, 300, 400, 500, 700, 1000, 1500, 2000, 3000, 4000, 5000, 7000, 10000, 20000
Quantification using: Lower Marker Final concentration (ng/uL): 0.5000 Dilution factor: 12.0

Sample: SampA4
Well location: A4
Created: Tuesday, August 10, 2021 8:41:15 PM

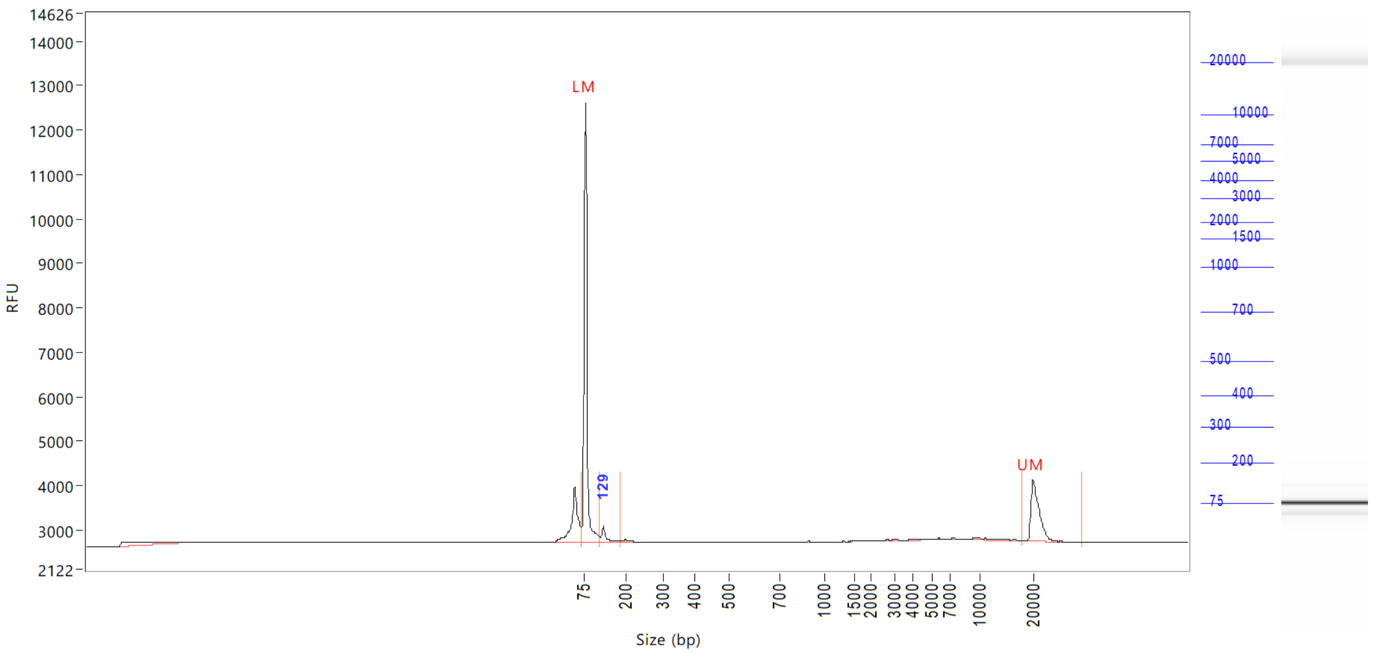

Peak	Size (bp)	Concentration (ng/uL)	From (bp)	To (bp)	Average size (bp)	CV%	RFU	Corrected peak area
1	75 (LM)	0.5000	64	120	76	10.34	9891	64.412
2	129	0.3131	120	181	135	10.94	360	3.362
3	20000 (UM)	0.1079	18049	29068	20564	4.83	1376	13.901

TIC:	0.3131	ng/uL
TIM:	3.8116	nmole/L
Total concentration:	0.8025	ng/uL

Sample peak width (sec): 5 Sample min peak height: 50 Sample baseline V to V?: Y Sample baseline V to V points: 3
Sample filter: Binomial Number of points for filter: 3 Sample start region (min): 0 Sample end region (min): 30
Marker peak width (sec): 5 Marker min peak height: 200 Marker baseline V to V?: Y Marker baseline V to V points: 3
Lower marker selection: First peak > 500 RFU Upper marker selection: Last peak > 200 RFU
Ladder size (bp)75, 200, 300, 400, 500, 700, 1000, 1500, 2000, 3000, 4000, 5000, 7000, 10000, 20000
Quantification using: Lower Marker Final concentration (ng/uL): 0.5000 Dilution factor: 12.0

Sample: SampB4
Well location: B4
Created: Tuesday, August 10, 2021 8:41:15 PM

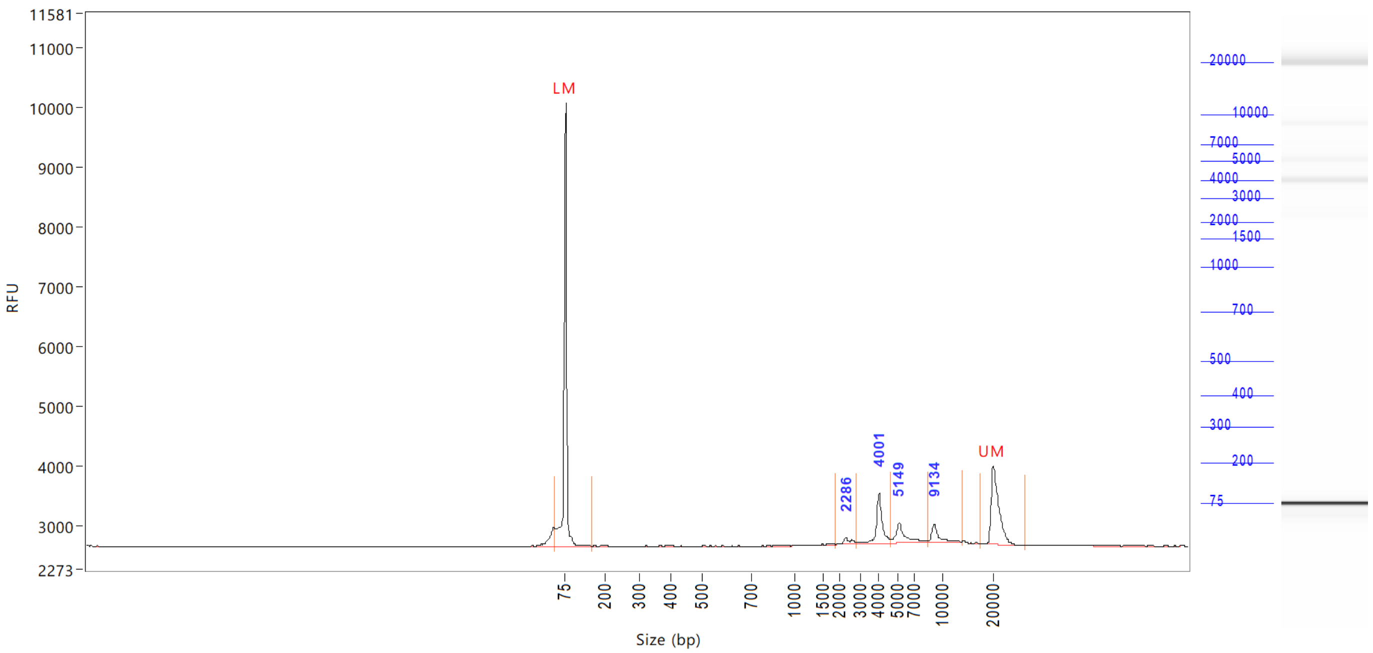

Peak	Size (bp)	Concentration (ng/uL)	From (bp)	To (bp)	Average size (bp)	CV%	RFU	Corrected peak area
1	75 (LM)	0.5000	45	156	72	12.57	7436	48.108
2	2286	0.1470	1885	2800	2389	8.24	105	1.179
3	4001	0.9168	2800	4621	3962	7.41	834	7.351
4	5149	0.5664	4621	8467	5677	16.24	341	4.542
5	9134	0.3351	8467	13903	9460	9.11	309	2.687
6	20000 (UM)	0.1351	17561	26408	20509	4.43	1316	12.995

TIC:	1.9653	ng/uL
TIM:	0.7048	nmole/L
Total concentration:	2.1806	ng/uL

```
Sample peak width (sec): 5    Sample min peak height: 50    Sample baseline V to V?: Y    Sample baseline V to V points: 3
Sample filter: Binomial       Number of points for filter: 3  Sample start region (min): 0  Sample end region (min): 30
Marker peak width (sec): 5    Marker min peak height: 200   Marker baseline V to V?: Y    Marker baseline V to V points: 3
Lower marker selection: First peak > 500 RFU                Upper marker selection: Last peak > 200 RFU
Ladder size (bp)75, 200, 300, 400, 500, 700, 1000, 1500, 2000, 3000, 4000, 5000, 7000, 10000, 20000
Quantification using: Lower Marker          Final concentration (ng/uL): 0.5000          Dilution factor: 12.0
```

Sample: SampC4
Well location: C4
Created: Tuesday, August 10, 2021 8:41:15 PM

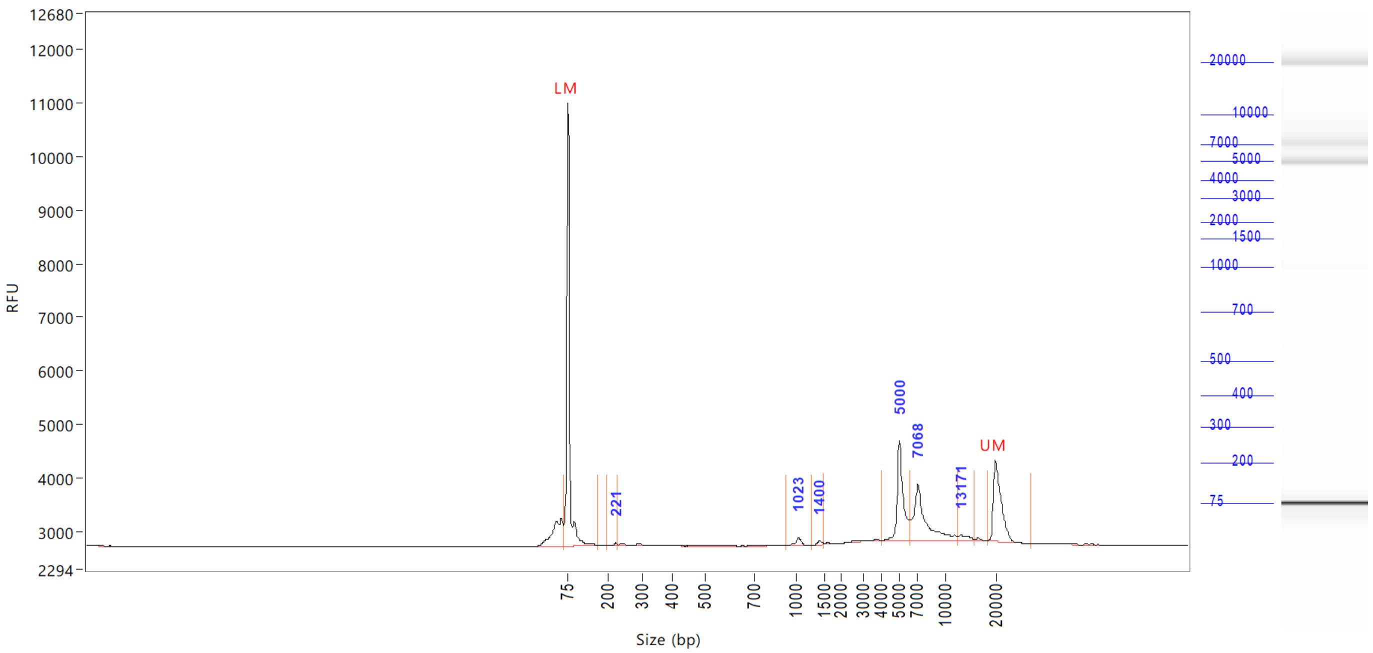

Peak	Size (bp)	Concentration (ng/uL)	From (bp)	To (bp)	Average size (bp)	CV%	RFU	Corrected peak area
1	75 (LM)	0.5000	62	168	76	15.16	8293	56.614
2	221	0.0400	198	228	218	2.86	52	0.377
3	1023	0.1409	931	1267	1023	4.74	134	1.330
4	1400	0.0557	1267	1489	1396	3.09	69	0.526
5	5000	1.7768	4000	6260	5164	8.02	1854	16.765
6	7068	1.8240	6260	12562	7857	17.47	1043	17.211
7	13171	0.1519	12562	15732	13875	6.20	102	1.433
8	20000 (UM)	0.1324	18293	27012	20465	4.39	1505	14.994

TIC:	3.9894	ng/uL
TIM:	1.5609	nmole/L
Total concentration:	4.3961	ng/uL

Sample peak width (sec): 5 Sample min peak height: 50 Sample baseline V to V?: Y Sample baseline V to V points: 3
Sample filter: Binomial Number of points for filter: 3 Sample start region (min): 0 Sample end region (min): 30
Marker peak width (sec): 5 Marker min peak height: 200 Marker baseline V to V?: Y Marker baseline V to V points: 3
Lower marker selection: First peak > 500 RFU Upper marker selection: Last peak > 200 RFU
Ladder size (bp)75, 200, 300, 400, 500, 700, 1000, 1500, 2000, 3000, 4000, 5000, 7000, 10000, 20000
Quantification using: Lower Marker Final concentration (ng/uL): 0.5000 Dilution factor: 12.0

Sample: SampD4
Well location: D4
Created: Tuesday, August 10, 2021 8:41:15 PM

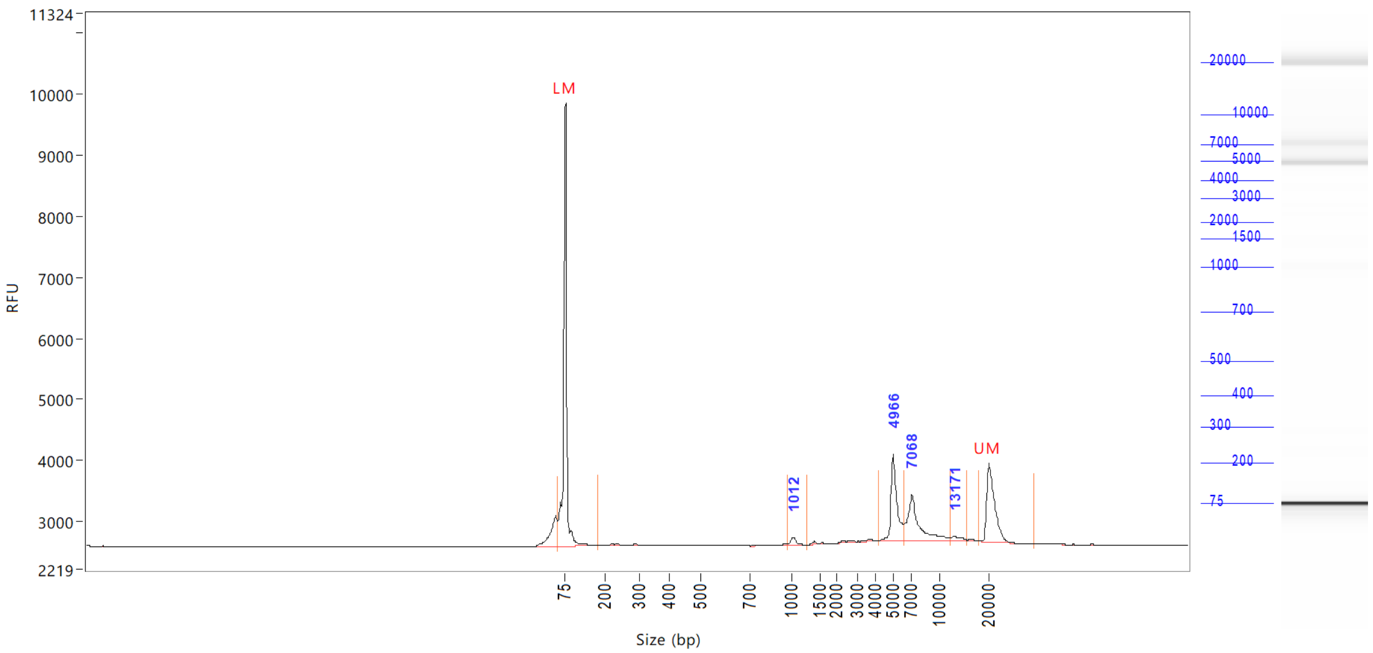

Peak	Size (bp)	Concentration (ng/uL)	From (bp)	To (bp)	Average size (bp)	CV%	RFU	Corrected peak area
1	75 (LM)	0.5000	50	177	72	14.71	7271	54.140
2	1012	0.1290	970	1256	1023	4.45	126	1.164
3	4966	1.3798	4139	6186	5110	7.67	1416	12.451
4	7068	1.3758	6186	12196	7757	16.75	752	12.414
5	13171	0.1290	12196	15732	13671	6.79	76	1.164
6	20000 (UM)	0.1201	18049	29068	20528	4.77	1281	13.001

TIC:	3.0136	ng/uL
TIM:	0.9596	nmole/L
Total concentration:	3.4604	ng/uL

Sample peak width (sec): 5 Sample min peak height: 50 Sample baseline V to V?: Y Sample baseline V to V points: 3
Sample filter: Binomial Number of points for filter: 3 Sample start region (min): 0 Sample end region (min): 30
Marker peak width (sec): 5 Marker min peak height: 200 Marker baseline V to V?: Y Marker baseline V to V points: 3
Lower marker selection: First peak > 500 RFU Upper marker selection: Last peak > 200 RFU
Ladder size (bp)75, 200, 300, 400, 500, 700, 1000, 1500, 2000, 3000, 4000, 5000, 7000, 10000, 20000
Quantification using: Lower Marker Final concentration (ng/uL): 0.5000 Dilution factor: 12.0

Sample: SampE4
Well location: E4
Created: Tuesday, August 10, 2021 8:41:15 PM

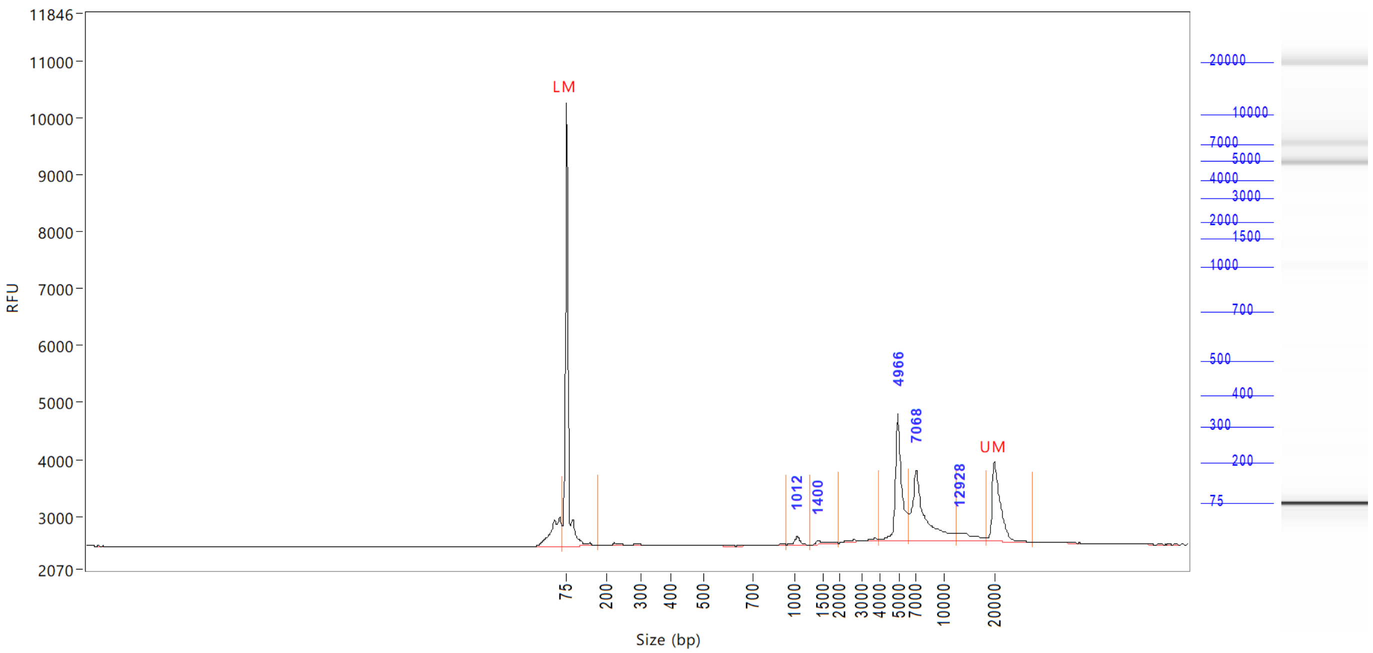

Peak	Size (bp)	Concentration (ng/uL)	From (bp)	To (bp)	Average size (bp)	CV%	RFU	Corrected peak area
1	75 (LM)	0.5000	60	171	76	15.80	7808	54.652
2	1012	0.1726	935	1245	1020	4.81	148	1.572
3	1400	0.0993	1245	1962	1477	9.18	74	0.905
4	4966	2.2504	3893	6186	5113	8.03	2228	20.498
5	7068	2.3635	6186	12318	7835	17.41	1236	21.529
6	12928	0.3642	12318	18415	14778	11.49	134	3.317
7	20000 (UM)	0.1338	18415	27496	20534	5.09	1416	14.630

TIC:	5.2500	ng/uL
TIM:	1.6509	nmole/L
Total concentration:	5.6919	ng/uL

```
Sample peak width (sec): 5    Sample min peak height: 50    Sample baseline V to V?: Y    Sample baseline V to V points: 3
Sample filter: Binomial       Number of points for filter: 3  Sample start region (min): 0  Sample end region (min): 30
Marker peak width (sec): 5    Marker min peak height: 200   Marker baseline V to V?: Y    Marker baseline V to V points: 3
Lower marker selection: First peak > 500 RFU                Upper marker selection: Last peak > 200 RFU
Ladder size (bp)75, 200, 300, 400, 500, 700, 1000, 1500, 2000, 3000, 4000, 5000, 7000, 10000, 20000
Quantification using: Lower Marker          Final concentration (ng/uL): 0.5000          Dilution factor: 12.0
```

Sample: SampF4
Well location: F4
Created: Tuesday, August 10, 2021 8:41:15 PM

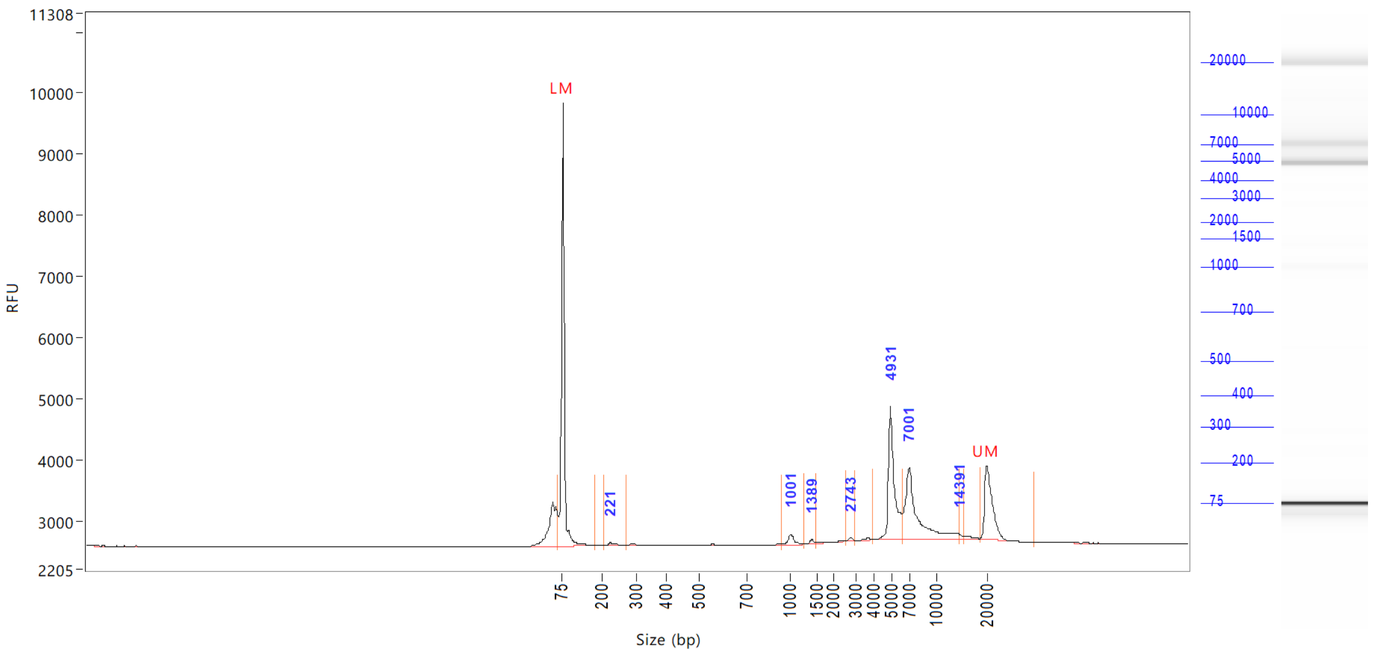

Peak	Size (bp)	Concentration (ng/uL)	From (bp)	To (bp)	Average size (bp)	CV%	RFU	Corrected peak area
1	75 (LM)	0.5000	60	173	74	13.89	7268	50.246
2	221	0.1038	205	268	230	6.15	53	0.869
3	1001	0.2144	944	1245	1017	4.56	175	1.796
4	1389	0.0558	1245	1467	1379	2.84	69	0.467
5	2743	0.0380	2515	2971	2723	3.22	51	0.318
6	4931	2.2795	3929	6186	5100	7.75	2173	19.090
7	7001	2.3646	6186	14391	7998	22.49	1167	19.802
8	14391	0.0434	14391	15488	14836	2.32	79	0.363
9	20000 (UM)	0.1169	18537	29309	20457	4.72	1185	11.750

TIC:	5.0995	ng/uL
TIM:	2.4063	nmole/L
Total concentration:	5.4151	ng/uL

Sample peak width (sec): 5   Sample min peak height: 50   Sample baseline V to V?: Y   Sample baseline V to V points: 3
Sample filter: Binomial   Number of points for filter: 3   Sample start region (min): 0   Sample end region (min): 30
Marker peak width (sec): 5   Marker min peak height: 200   Marker baseline V to V?: Y   Marker baseline V to V points: 3
Lower marker selection: First peak > 500 RFU   Upper marker selection: Last peak > 200 RFU
Ladder size (bp)75, 200, 300, 400, 500, 700, 1000, 1500, 2000, 3000, 4000, 5000, 7000, 10000, 20000
Quantification using: Lower Marker   Final concentration (ng/uL): 0.5000   Dilution factor: 12.0

Sample: SampG4
Well location: G4
Created: Tuesday, August 10, 2021 8:41:15 PM

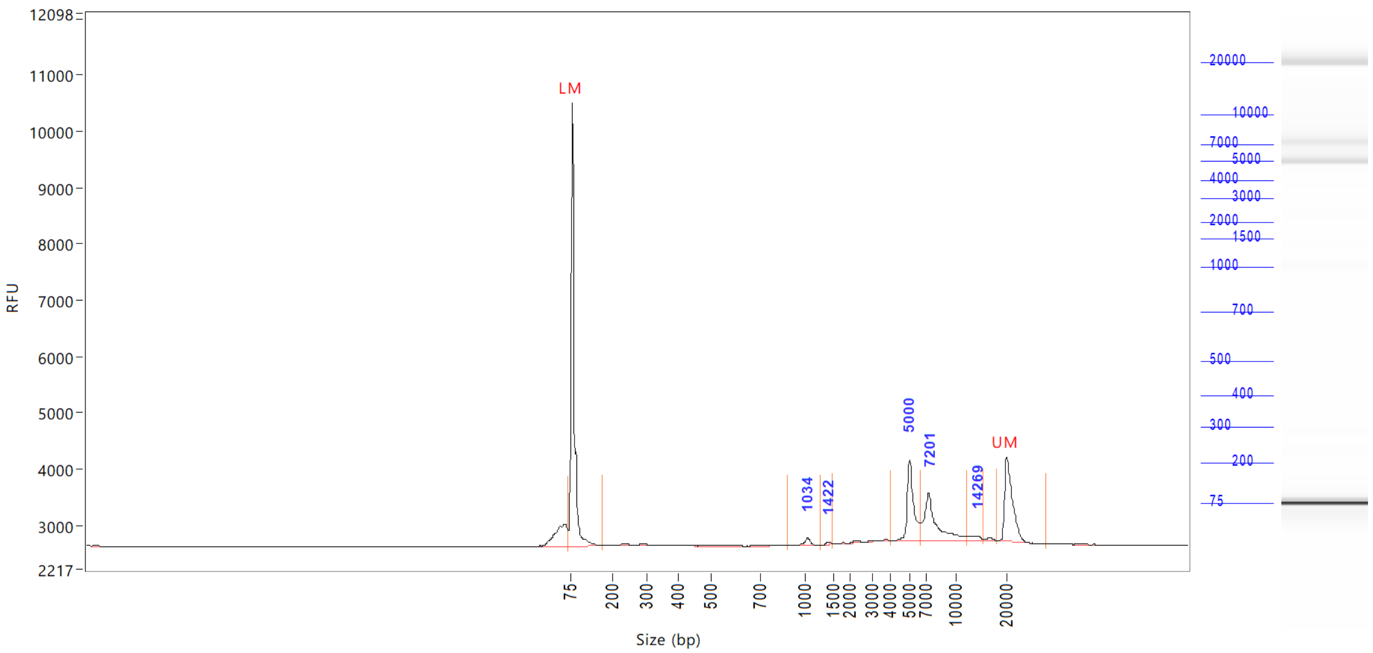

Peak	Size (bp)	Concentration (ng/uL)	From (bp)	To (bp)	Average size (bp)	CV%	RFU	Corrected peak area
1	75 (LM)	0.5000	64	169	77	14.71	7891	56.842
2	1034	0.1465	883	1278	1029	5.43	133	1.388
3	1422	0.0394	1278	1489	1408	2.64	54	0.373
4	5000	1.3938	4000	6334	5232	8.21	1441	13.204
5	7201	1.4374	6334	12074	7811	15.55	833	13.617
6	14269	0.1289	12074	15488	13615	6.76	80	1.221
7	20000 (UM)	0.1307	18049	27617	20529	4.43	1490	14.854

TIC:	3.1460	ng/uL
TIM:	1.0376	nmole/L
Total concentration:	3.5536	ng/uL

```
Sample peak width (sec): 5    Sample min peak height: 50    Sample baseline V to V?: Y    Sample baseline V to V points: 3
Sample filter: Binomial       Number of points for filter: 3  Sample start region (min): 0  Sample end region (min): 30
Marker peak width (sec): 5    Marker min peak height: 200   Marker baseline V to V?: Y    Marker baseline V to V points: 3
Lower marker selection: First peak > 500 RFU                Upper marker selection: Last peak > 200 RFU
Ladder size (bp)75, 200, 300, 400, 500, 700, 1000, 1500, 2000, 3000, 4000, 5000, 7000, 10000, 20000
Quantification using: Lower Marker          Final concentration (ng/uL): 0.5000         Dilution factor: 12.0
```

Sample: SampH4
Well location: H4
Created: Tuesday, August 10, 2021 8:41:15 PM

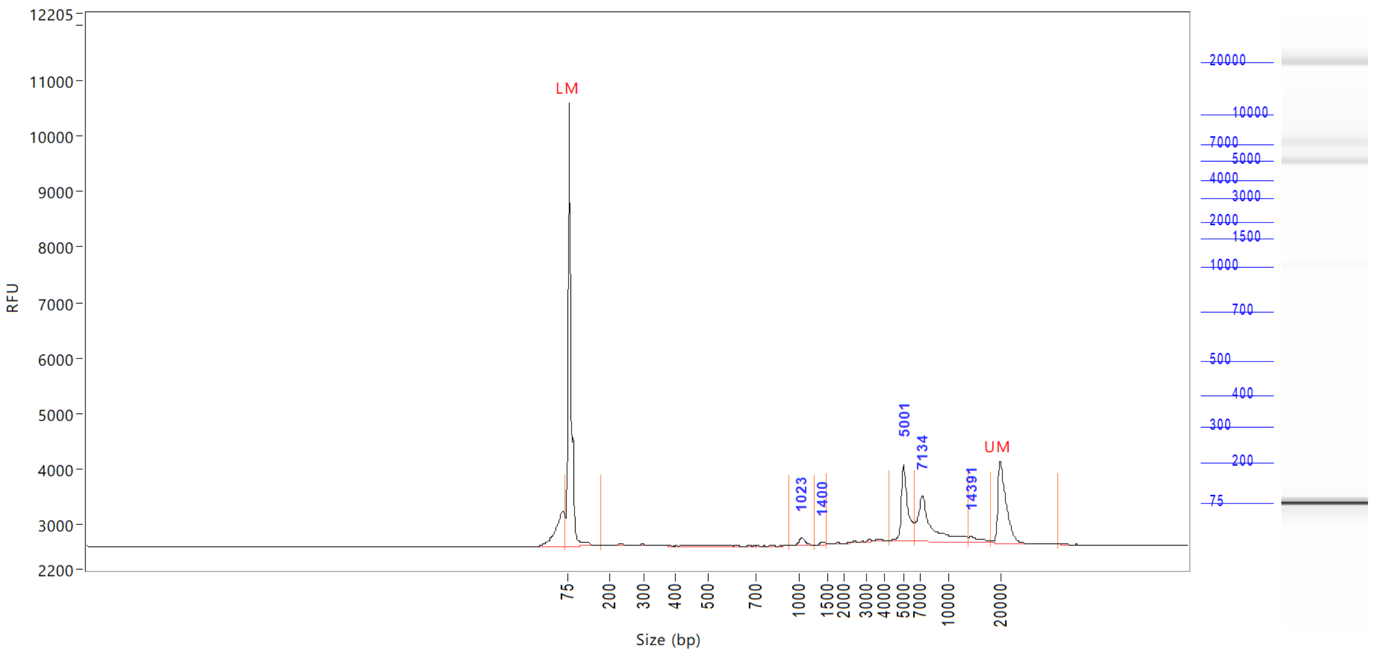

Peak	Size (bp)	Concentration (ng/uL)	From (bp)	To (bp)	Average size (bp)	CV%	RFU	Corrected peak area
1	75 (LM)	0.5000	64	173	77	13.36	7990	58.605
2	1023	0.1632	931	1256	1032	5.26	148	1.594
3	1400	0.0399	1256	1478	1391	2.92	54	0.390
4	5001	1.3165	4242	6260	5222	7.79	1384	12.859
5	7134	1.6303	6260	13659	8178	21.27	820	15.924
6	14391	0.1807	13659	18293	15528	8.54	103	1.765
7	20000 (UM)	0.1313	18293	31244	20648	5.72	1469	15.388

TIC:	3.3305	ng/uL
TIM:	1.0699	nmole/L
Total concentration:	3.7220	ng/uL

```
Sample peak width (sec): 5    Sample min peak height: 50    Sample baseline V to V?: Y    Sample baseline V to V points: 3
Sample filter: Binomial       Number of points for filter: 3  Sample start region (min): 0  Sample end region (min): 30
Marker peak width (sec): 5    Marker min peak height: 200   Marker baseline V to V?: Y    Marker baseline V to V points: 3
Lower marker selection: First peak > 500 RFU                Upper marker selection: Last peak > 200 RFU
Ladder size (bp)75, 200, 300, 400, 500, 700, 1000, 1500, 2000, 3000, 4000, 5000, 7000, 10000, 20000
Quantification using: Lower Marker          Final concentration (ng/uL): 0.5000          Dilution factor: 12.0
```

Sample: SampA5
Well location: A5
Created: Tuesday, August 10, 2021 8:41:15 PM

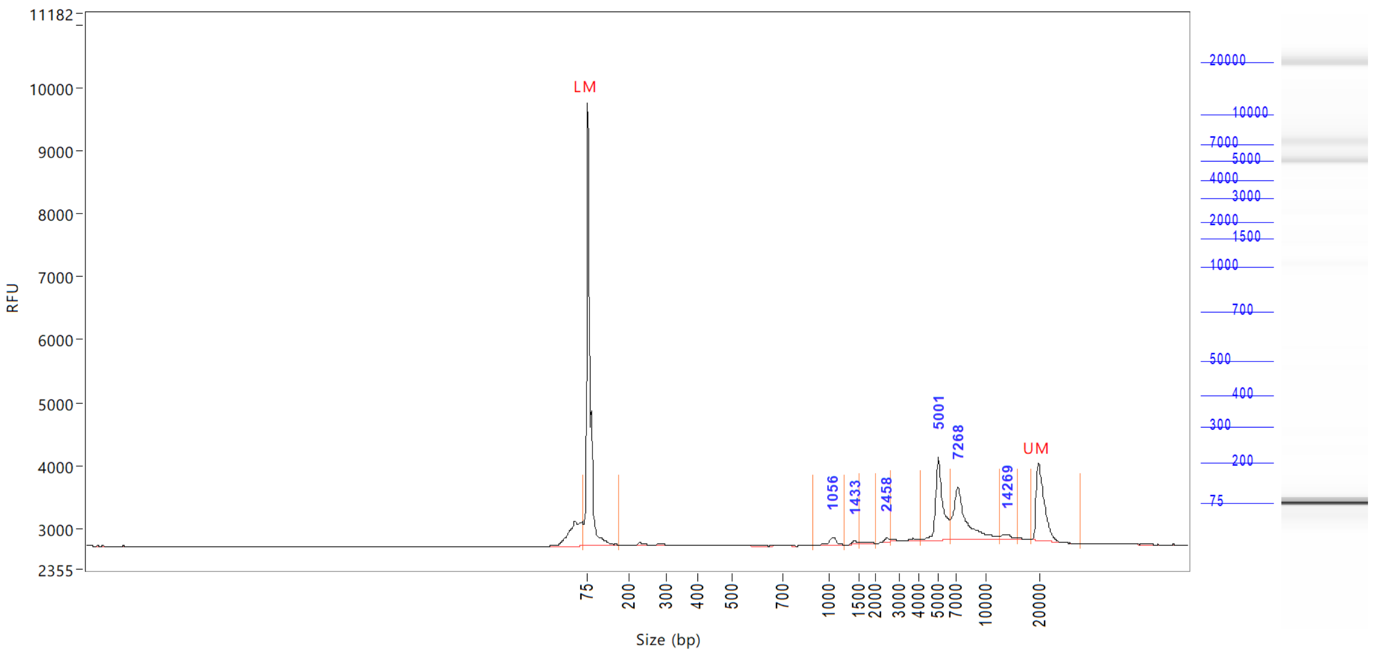

Peak	Size (bp)	Concentration (ng/uL)	From (bp)	To (bp)	Average size (bp)	CV%	RFU	Corrected peak area
1	75 (LM)	0.5000	64	168	78	14.29	7046	55.297
2	1056	0.1612	900	1267	1043	5.65	129	1.485
3	1433	0.0472	1267	1501	1425	3.03	57	0.435
4	2458	0.0509	2000	2600	2416	4.41	68	0.469
5	5001	1.4065	4104	6334	5273	8.44	1321	12.963
6	7268	1.5801	6334	12562	7865	16.19	845	14.563
7	14269	0.1191	12562	15976	13961	6.38	79	1.098
8	20000 (UM)	0.1077	18659	27738	20460	4.36	1237	11.906

TIC:	3.3651	ng/uL
TIM:	1.1275	nmole/L
Total concentration:	3.7947	ng/uL

```
Sample peak width (sec): 5    Sample min peak height: 50    Sample baseline V to V?: Y    Sample baseline V to V points: 3
Sample filter: Binomial       Number of points for filter: 3  Sample start region (min): 0  Sample end region (min): 30
Marker peak width (sec): 5    Marker min peak height: 200   Marker baseline V to V?: Y    Marker baseline V to V points: 3
Lower marker selection: First peak > 500 RFU                Upper marker selection: Last peak > 200 RFU
Ladder size (bp)75, 200, 300, 400, 500, 700, 1000, 1500, 2000, 3000, 4000, 5000, 7000, 10000, 20000
Quantification using: Lower Marker          Final concentration (ng/uL): 0.5000          Dilution factor: 12.0
```

Sample: SampB5
Well location: B5
Created: Tuesday, August 10, 2021 8:41:15 PM

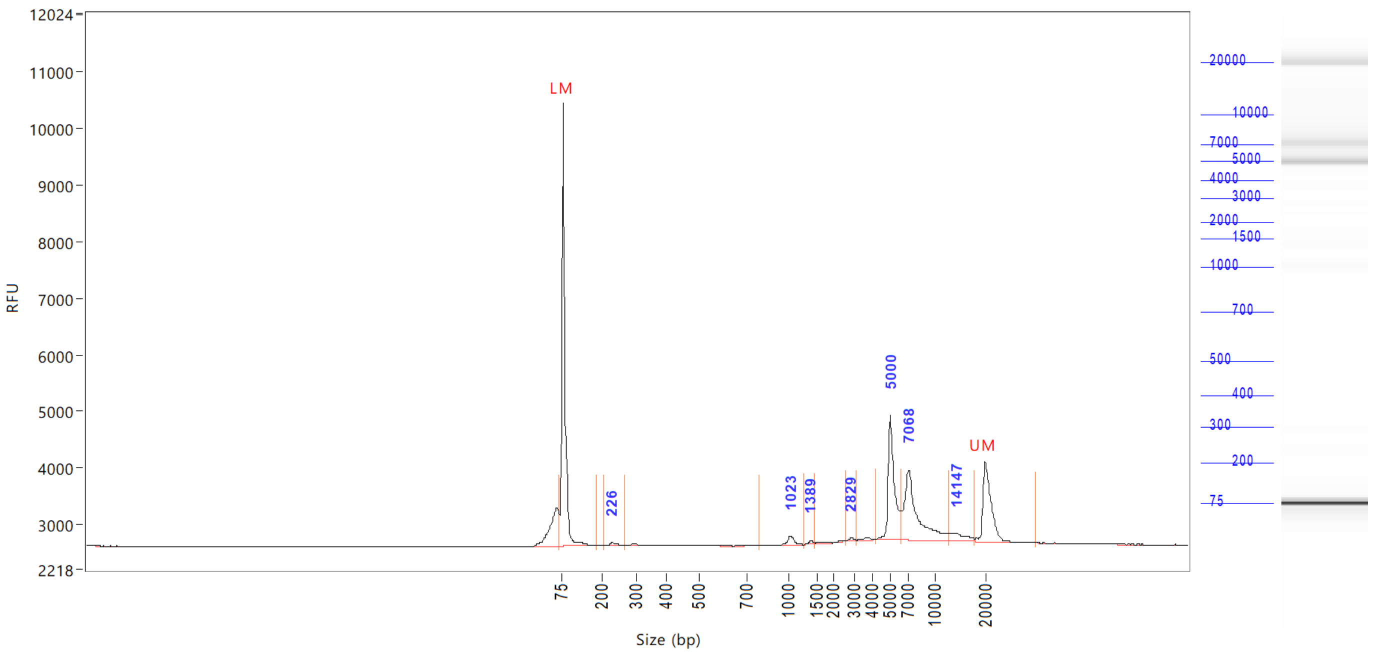

Peak	Size (bp)	Concentration (ng/uL)	From (bp)	To (bp)	Average size (bp)	CV%	RFU	Corrected peak area
1	75 (LM)	0.5000	64	183	77	14.40	7828	58.781
2	226	0.0969	205	266	232	6.23	56	0.949
3	1023	0.1839	783	1256	1020	5.76	162	1.802
4	1389	0.0512	1256	1467	1380	2.84	70	0.502
5	2829	0.0361	2572	3072	2803	3.51	53	0.354
6	5000	2.1028	4173	6260	5195	7.94	2187	20.600
7	7068	2.3020	6260	12562	7915	17.85	1234	22.553
8	14147	0.3155	12562	17927	14753	10.14	138	3.091
9	20000 (UM)	0.1262	17927	29914	20551	5.64	1419	14.840

TIC:	5.0884	ng/uL
TIM:	2.2460	nmole/L
Total concentration:	5.3454	ng/uL

```
Sample peak width (sec): 5    Sample min peak height: 50    Sample baseline V to V?: Y    Sample baseline V to V points: 3
Sample filter: Binomial       Number of points for filter: 3  Sample start region (min): 0  Sample end region (min): 30
Marker peak width (sec): 5    Marker min peak height: 200    Marker baseline V to V?: Y    Marker baseline V to V points: 3
Lower marker selection: First peak > 500 RFU                  Upper marker selection: Last peak > 200 RFU
Ladder size (bp)75, 200, 300, 400, 500, 700, 1000, 1500, 2000, 3000, 4000, 5000, 7000, 10000, 20000
Quantification using: Lower Marker          Final concentration (ng/uL): 0.5000          Dilution factor: 12.0
```

Sample: SampC5
Well location: C5
Created: Tuesday, August 10, 2021 8:41:15 PM

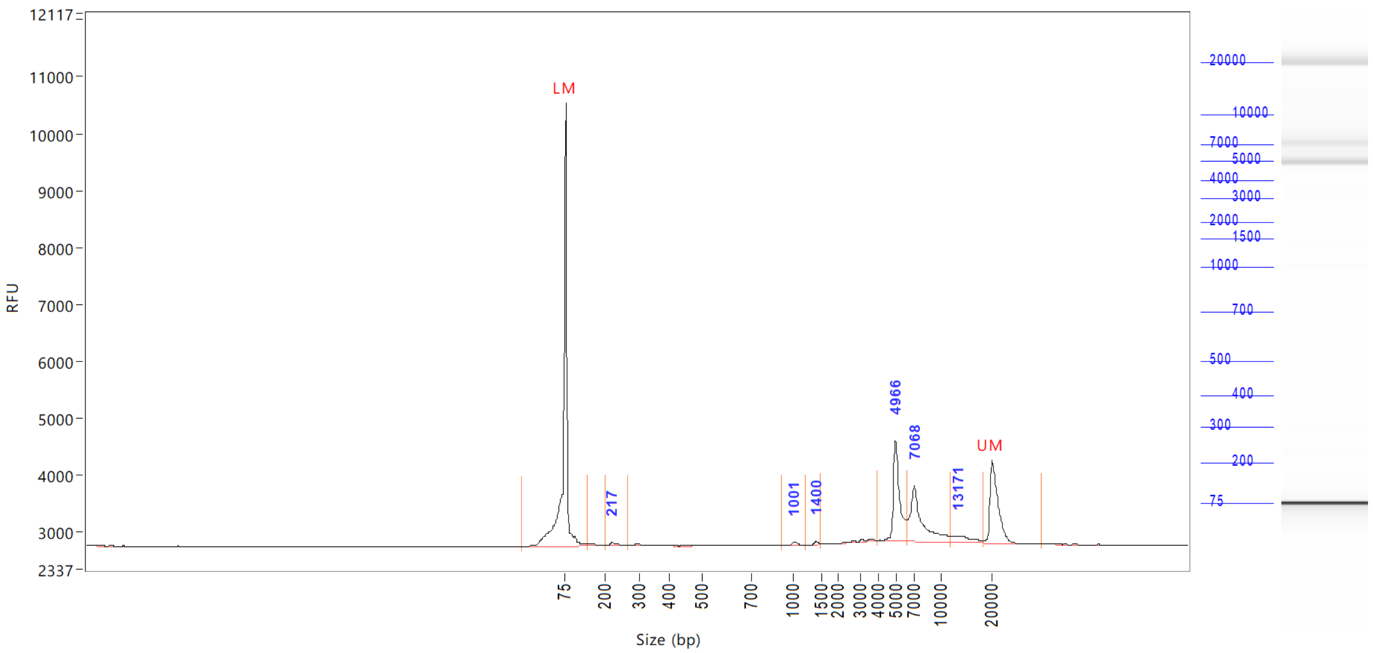

Peak	Size (bp)	Concentration (ng/uL)	From (bp)	To (bp)	Average size (bp)	CV%	RFU	Corrected peak area
1	75 (LM)	0.5000	0	143	65	33.57	7810	67.112
2	217	0.0837	201	266	225	6.28	65	0.936
3	1001	0.0602	913	1212	1011	4.64	64	0.673
4	1400	0.0323	1212	1467	1384	2.71	55	0.361
5	4966	1.4584	3929	6334	5189	8.44	1759	16.312
6	7068	1.4541	6334	11708	7823	15.74	982	16.265
7	13171	0.2856	11708	18415	14457	12.62	117	3.195
8	20000 (UM)	0.1142	18415	29793	20632	5.67	1450	15.331

TIC:	3.3743	ng/uL
TIM:	1.5492	nmole/L
Total concentration:	3.6764	ng/uL

```
Sample peak width (sec): 5    Sample min peak height: 50    Sample baseline V to V?: Y    Sample baseline V to V points: 3
Sample filter: Binomial       Number of points for filter: 3  Sample start region (min): 0  Sample end region (min): 30
Marker peak width (sec): 5    Marker min peak height: 200   Marker baseline V to V?: Y    Marker baseline V to V points: 3
Lower marker selection: First peak > 500 RFU                Upper marker selection: Last peak > 200 RFU
Ladder size (bp)75, 200, 300, 400, 500, 700, 1000, 1500, 2000, 3000, 4000, 5000, 7000, 10000, 20000
Quantification using: Lower Marker          Final concentration (ng/uL): 0.5000          Dilution factor: 12.0
```

Sample: SampD5
Well location: D5
Created: Tuesday, August 10, 2021 8:41:15 PM

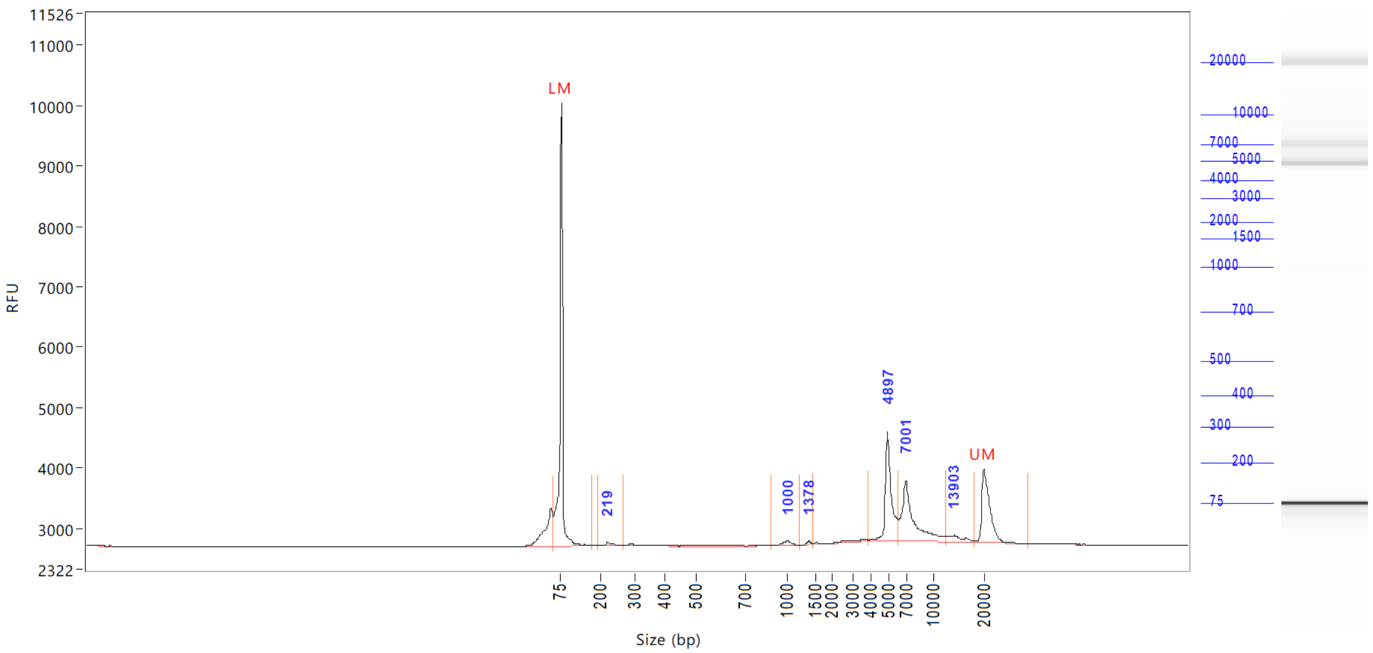

Peak	Size (bp)	Concentration (ng/uL)	From (bp)	To (bp)	Average size (bp)	CV%	RFU	Corrected peak area
1	75 (LM)	0.5000	50	173	73	15.25	7349	51.960
2	219	0.1138	190	264	225	7.30	59	0.986
3	1000	0.1188	883	1234	995	4.99	80	1.029
4	1378	0.0431	1234	1456	1372	2.84	54	0.373
5	4897	1.8942	3893	6112	5050	7.68	1814	16.403
6	7001	1.9009	6112	12318	7723	17.67	994	16.462
7	13903	0.2565	12318	18049	14581	10.56	105	2.222
8	20000 (UM)	0.1204	18049	28705	20511	5.01	1222	12.510

TIC:	4.3273	ng/uL
TIM:	2.1319	nmole/L
Total concentration:	4.6572	ng/uL

```
Sample peak width (sec): 5    Sample min peak height: 50    Sample baseline V to V?: Y    Sample baseline V to V points: 3
Sample filter: Binomial       Number of points for filter: 3  Sample start region (min): 0  Sample end region (min): 30
Marker peak width (sec): 5    Marker min peak height: 200   Marker baseline V to V?: Y    Marker baseline V to V points: 3
Lower marker selection: First peak > 500 RFU                Upper marker selection: Last peak > 200 RFU
Ladder size (bp)75, 200, 300, 400, 500, 700, 1000, 1500, 2000, 3000, 4000, 5000, 7000, 10000, 20000
Quantification using: Lower Marker          Final concentration (ng/uL): 0.5000          Dilution factor: 12.0
```

Sample: SampE5
Well location: E5
Created: Tuesday, August 10, 2021 8:41:15 PM

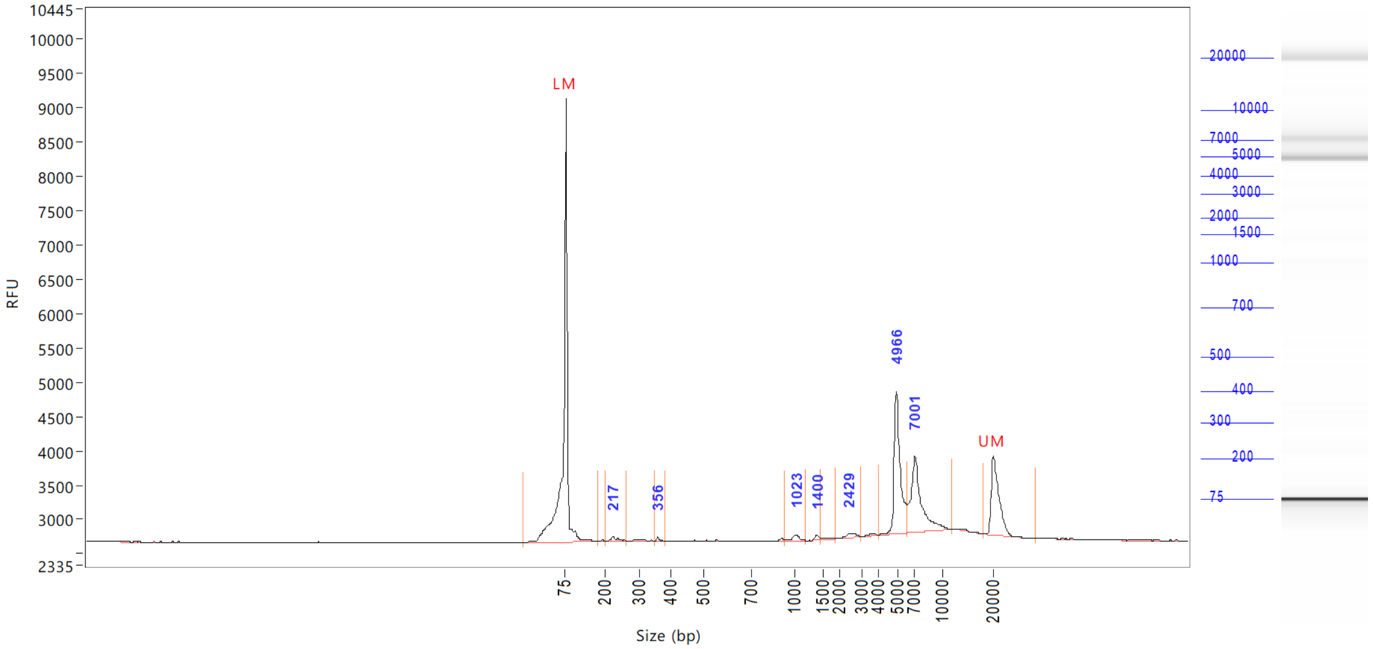

Peak	Size (bp)	Concentration (ng/uL)	From (bp)	To (bp)	Average size (bp)	CV%	RFU	Corrected peak area
1	75 (LM)	0.5000	0	173	63	37.59	6476	62.193
2	217	0.1002	198	259	223	6.12	75	1.039
3	356	0.0382	345	382	356	2.03	58	0.396
4	1023	0.0957	931	1201	1013	4.38	88	0.992
5	1400	0.0455	1201	1467	1386	3.05	69	0.471
6	2429	0.0832	1904	2971	2501	8.16	58	0.862
7	4966	1.8005	3964	6186	5125	7.54	2074	18.663
8	7001	1.5483	6186	11830	7389	11.75	1111	16.048
9	20000 (UM)	0.0927	17927	28221	20527	4.53	1152	11.529

TIC:	3.7115	ng/uL
TIM:	2.1030	nmole/L
Total concentration:	4.0110	ng/uL

Sample peak width (sec): 5   Sample min peak height: 50   Sample baseline V to V?: Y   Sample baseline V to V points: 3
Sample filter: Binomial   Number of points for filter: 3   Sample start region (min): 0   Sample end region (min): 30
Marker peak width (sec): 5   Marker min peak height: 200   Marker baseline V to V?: Y   Marker baseline V to V points: 3
Lower marker selection: First peak > 500 RFU   Upper marker selection: Last peak > 200 RFU
Ladder size (bp)75, 200, 300, 400, 500, 700, 1000, 1500, 2000, 3000, 4000, 5000, 7000, 10000, 20000
Quantification using: Lower Marker   Final concentration (ng/uL): 0.5000   Dilution factor: 12.0

Sample: SampF5
Well location: F5
Created: Tuesday, August 10, 2021 8:41:15 PM

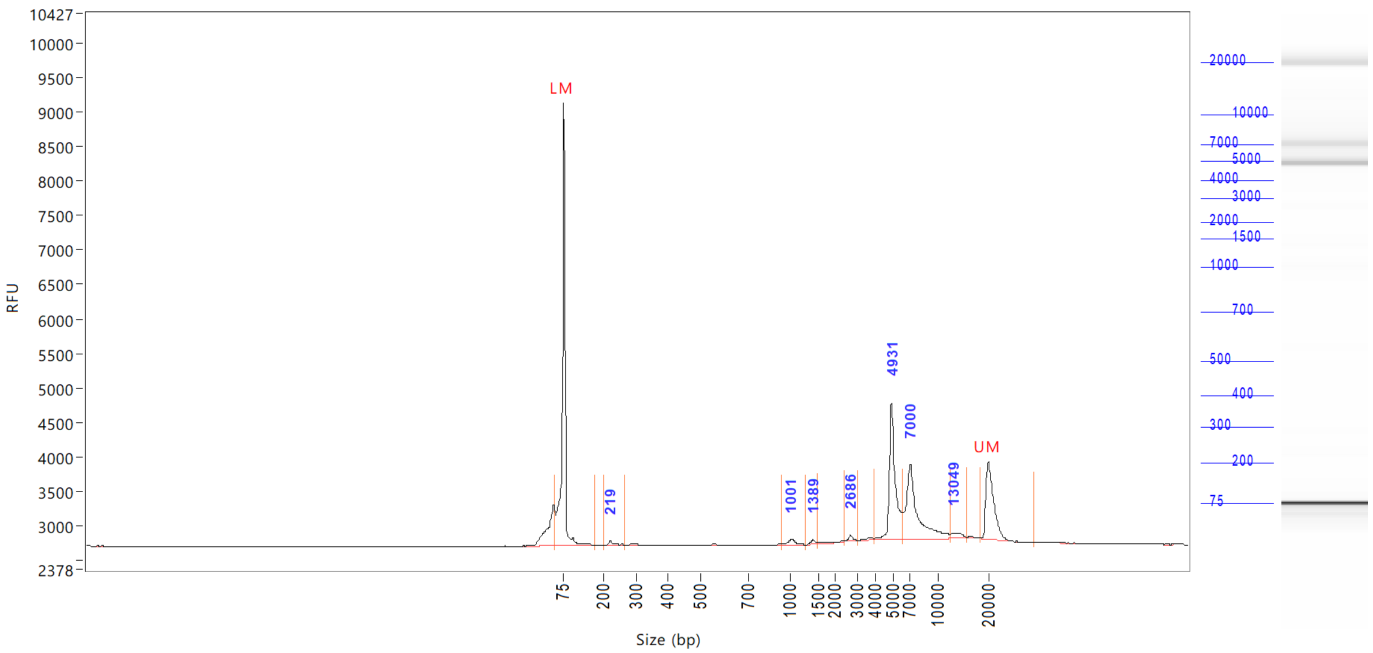

Peak	Size (bp)	Concentration (ng/uL)	From (bp)	To (bp)	Average size (bp)	CV%	RFU	Corrected peak area
1	75 (LM)	0.5000	50	173	73	15.73	6420	45.880
2	219	0.0995	201	264	227	5.96	60	0.761
3	1001	0.1479	939	1267	1023	5.95	84	1.131
4	1389	0.0629	1267	1467	1382	2.89	67	0.481
5	2686	0.0734	2401	3000	2688	3.96	81	0.561
6	4931	2.3144	3964	6186	5084	7.83	1979	17.697
7	7000	2.1679	6186	12318	7670	16.63	1071	16.577
8	13049	0.1638	12318	15732	13702	6.39	85	1.253
9	20000 (UM)	0.1227	18415	29068	20481	4.92	1115	11.256

TIC:	5.0297	ng/uL
TIM:	2.3127	nmole/L
Total concentration:	5.4101	ng/uL

```
Sample peak width (sec): 5    Sample min peak height: 50    Sample baseline V to V?: Y    Sample baseline V to V points: 3
Sample filter: Binomial       Number of points for filter: 3  Sample start region (min): 0  Sample end region (min): 30
Marker peak width (sec): 5    Marker min peak height: 200    Marker baseline V to V?: Y    Marker baseline V to V points: 3
Lower marker selection: First peak > 500 RFU                 Upper marker selection: Last peak > 200 RFU
Ladder size (bp)75, 200, 300, 400, 500, 700, 1000, 1500, 2000, 3000, 4000, 5000, 7000, 10000, 20000
Quantification using: Lower Marker          Final concentration (ng/uL): 0.5000          Dilution factor: 12.0
```

Sample: SampG5
Well location: G5
Created: Tuesday, August 10, 2021 8:41:15 PM

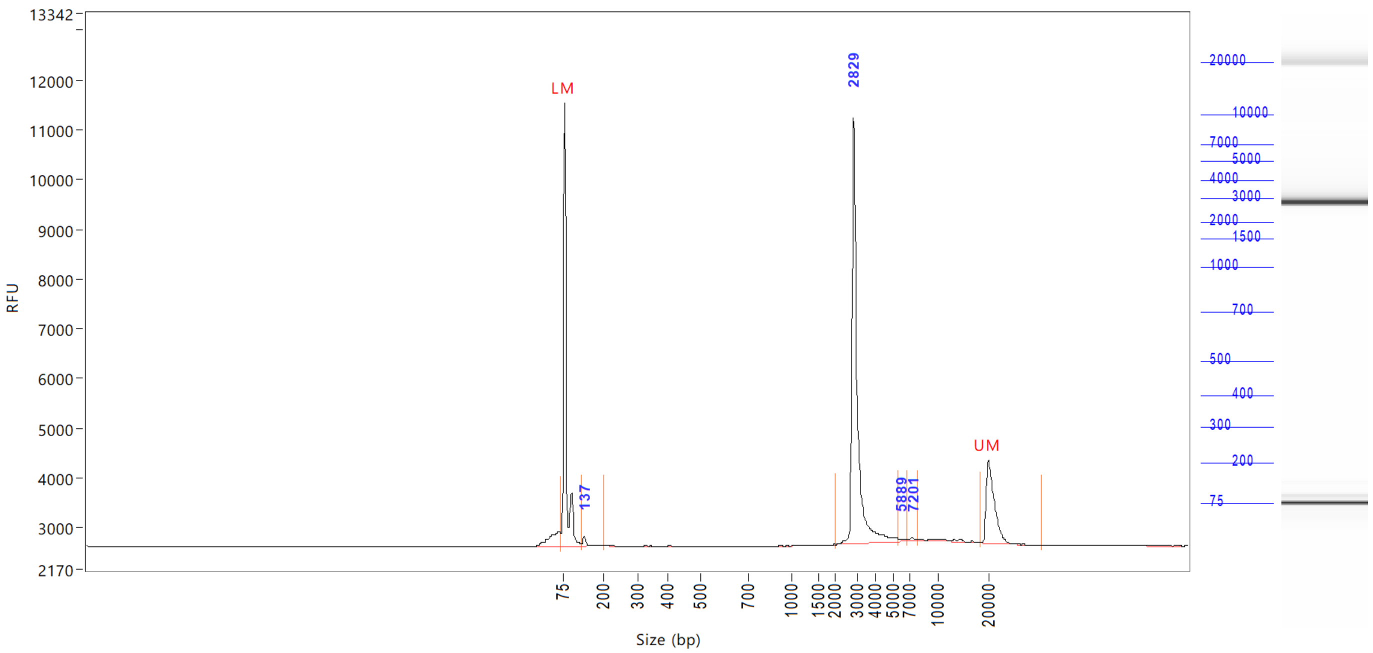

Peak	Size (bp)	Concentration (ng/uL)	From (bp)	To (bp)	Average size (bp)	CV%	RFU	Corrected peak area
1	75 (LM)	0.5000	64	128	77	12.21	8922	61.612
2	137	0.1666	128	201	142	10.80	196	1.711
3	2829	6.7205	2001	5667	2993	14.50	8571	69.010
4	5889	0.0580	5667	6704	6165	5.11	57	0.596
5	7201	0.0513	6704	7801	7149	4.12	54	0.527
6	20000 (UM)	0.1418	18415	30519	20630	5.37	1674	17.475

TIC:	6.9965	ng/uL
TIM:	5.6523	nmole/L
Total concentration:	7.2616	ng/uL

```
Sample peak width (sec): 5    Sample min peak height: 50    Sample baseline V to V?: Y    Sample baseline V to V points: 3
Sample filter: Binomial       Number of points for filter: 3  Sample start region (min): 0  Sample end region (min): 30
Marker peak width (sec): 5    Marker min peak height: 200   Marker baseline V to V?: Y    Marker baseline V to V points: 3
Lower marker selection: First peak > 500 RFU                Upper marker selection: Last peak > 200 RFU
Ladder size (bp)75, 200, 300, 400, 500, 700, 1000, 1500, 2000, 3000, 4000, 5000, 7000, 10000, 20000
Quantification using: Lower Marker          Final concentration (ng/uL): 0.5000          Dilution factor: 12.0
```

Sample: SampH5
Well location: H5
Created: Tuesday, August 10, 2021 8:41:15 PM

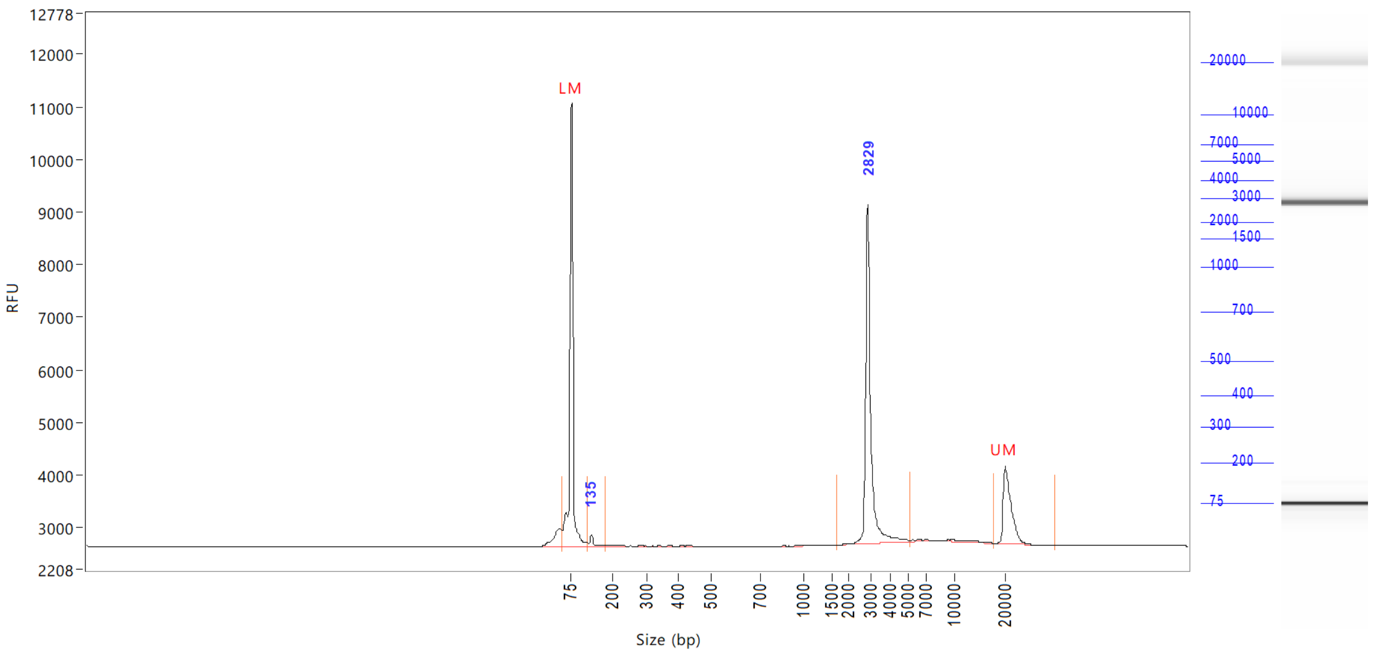

Peak	Size (bp)	Concentration (ng/uL)	From (bp)	To (bp)	Average size (bp)	CV%	RFU	Corrected peak area
1	75 (LM)	0.5000	45	126	72	14.14	8447	61.478
2	135	0.1943	126	179	140	9.58	228	1.991
3	2829	4.7778	1616	5149	2924	12.17	6459	48.955
4	20000 (UM)	0.1225	17805	29793	20527	4.98	1477	15.068

TIC:	4.9722	ng/uL
TIM:	4.9712	nmole/L
Total concentration:	5.3282	ng/uL

Sample peak width (sec): 5   Sample min peak height: 50   Sample baseline V to V?: Y   Sample baseline V to V points: 3
Sample filter: Binomial   Number of points for filter: 3 Sample start region (min): 0 Sample end region (min): 30
Marker peak width (sec): 5   Marker min peak height: 200   Marker baseline V to V?: Y   Marker baseline V to V points: 3
Lower marker selection: First peak > 500 RFU   Upper marker selection: Last peak > 200 RFU
Ladder size (bp)75, 200, 300, 400, 500, 700, 1000, 1500, 2000, 3000, 4000, 5000, 7000, 10000, 20000
Quantification using: Lower Marker   Final concentration (ng/uL): 0.5000   Dilution factor: 12.0

Sample: SampA6
Well location: A6
Created: Tuesday, August 10, 2021 8:41:15 PM

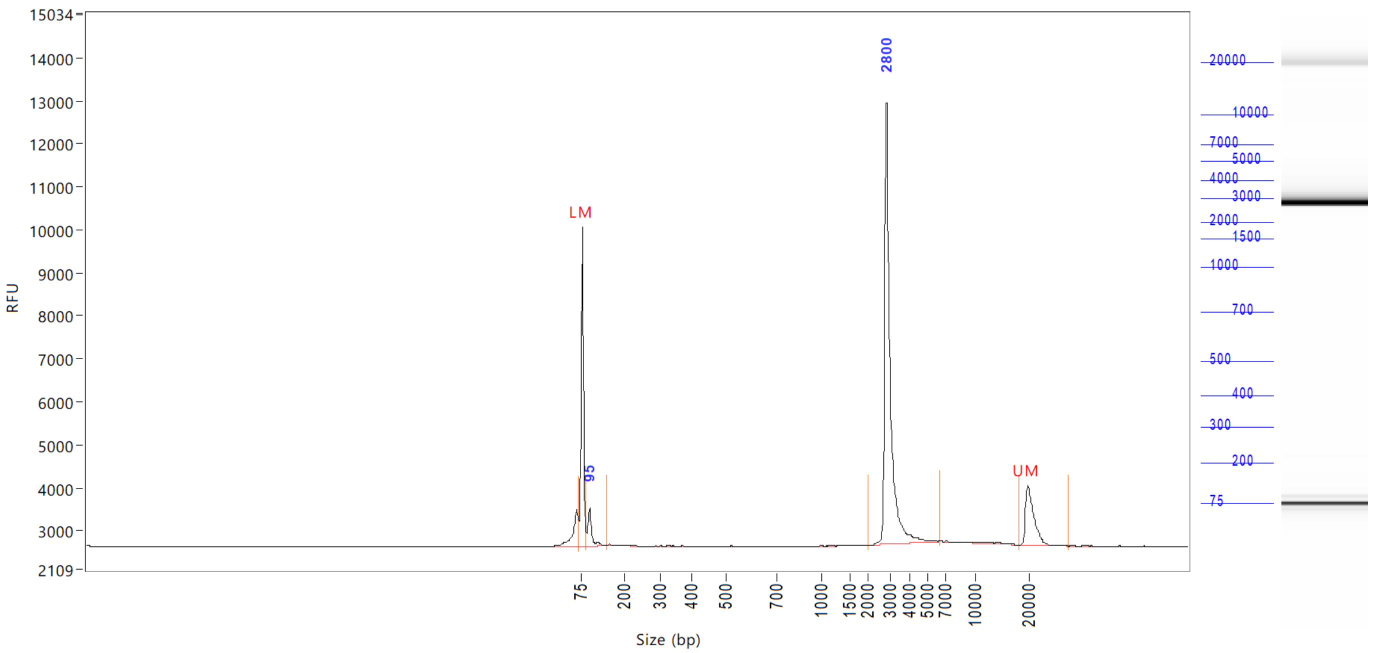

Peak	Size (bp)	Concentration (ng/uL)	From (bp)	To (bp)	Average size (bp)	CV%	RFU	Corrected peak area
1	75 (LM)	0.5000	64	86	72	5.23	7429	43.806
2	95	1.1536	86	148	98	13.49	871	8.423
3	2800	11.4768	2030	6260	2940	11.53	10277	83.792
4	20000 (UM)	0.1603	18415	27617	20487	4.76	1376	14.042

TIC:	12.6304	ng/uL
TIM:	25.7554	nmole/L
Total concentration:	13.1102	ng/uL

Sample peak width (sec): 5 Sample min peak height: 50 Sample baseline V to V?: Y Sample baseline V to V points: 3
Sample filter: Binomial Number of points for filter: 3 Sample start region (min): 0 Sample end region (min): 30
Marker peak width (sec): 5 Marker min peak height: 200 Marker baseline V to V?: Y Marker baseline V to V points: 3
Lower marker selection: First peak > 500 RFU Upper marker selection: Last peak > 200 RFU
Ladder size (bp)75, 200, 300, 400, 500, 700, 1000, 1500, 2000, 3000, 4000, 5000, 7000, 10000, 20000
Quantification using: Lower Marker Final concentration (ng/uL): 0.5000 Dilution factor: 12.0

Sample: SampB6
Well location: B6
Created: Tuesday, August 10, 2021 8:41:15 PM

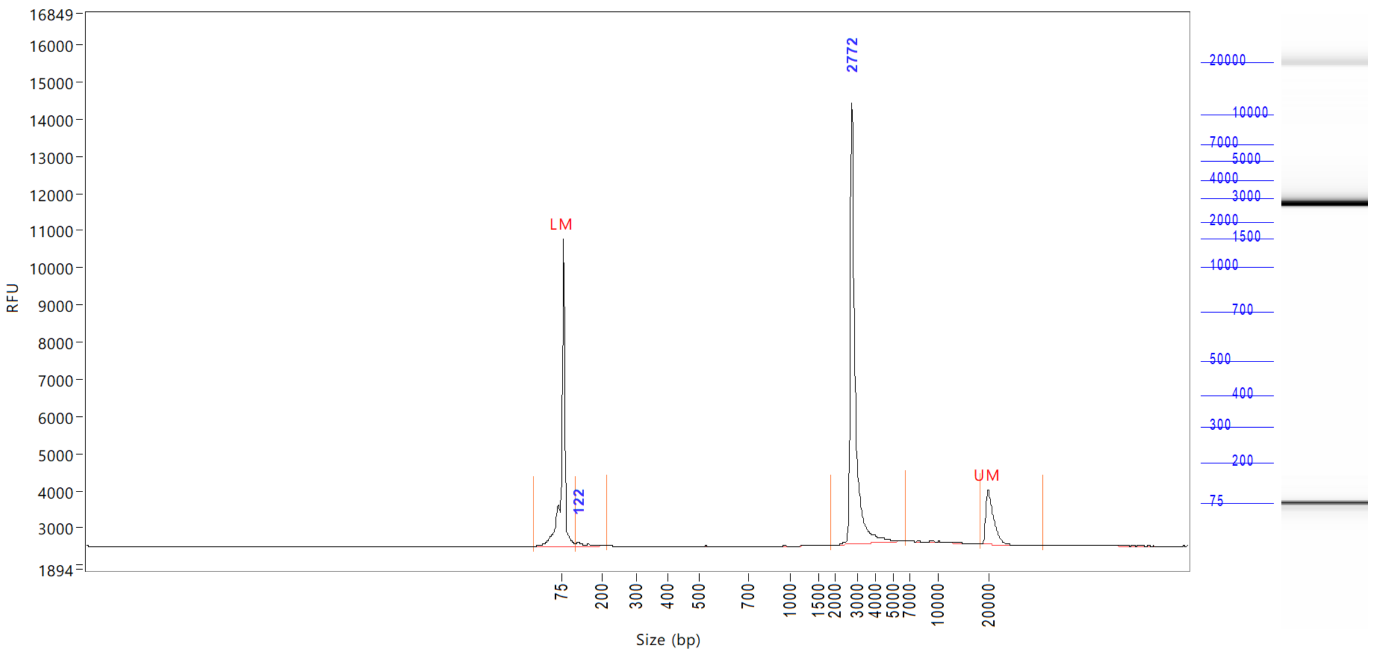

Peak	Size (bp)	Concentration (ng/uL)	From (bp)	To (bp)	Average size (bp)	CV%	RFU	Corrected peak area
1	75 (LM)	0.5000	0	112	68	21.25	8273	70.598
2	122	0.1886	112	210	137	14.93	122	2.219
3	2772	7.7554	1904	6556	2897	12.32	11888	91.253
4	20000 (UM)	0.1029	18415	30881	20498	5.28	1465	14.531

TIC:	7.9440	ng/uL
TIM:	6.6691	nmole/L
Total concentration:	8.1595	ng/uL

```
Sample peak width (sec): 5    Sample min peak height: 50    Sample baseline V to V?: Y    Sample baseline V to V points: 3
Sample filter: Binomial       Number of points for filter: 3  Sample start region (min): 0  Sample end region (min): 30
Marker peak width (sec): 5    Marker min peak height: 200   Marker baseline V to V?: Y    Marker baseline V to V points: 3
Lower marker selection: First peak > 500 RFU                Upper marker selection: Last peak > 200 RFU
Ladder size (bp)75, 200, 300, 400, 500, 700, 1000, 1500, 2000, 3000, 4000, 5000, 7000, 10000, 20000
Quantification using: Lower Marker          Final concentration (ng/uL): 0.5000          Dilution factor: 12.0
```

Sample: SampC6
Well location: C6
Created: Tuesday, August 10, 2021 8:41:15 PM

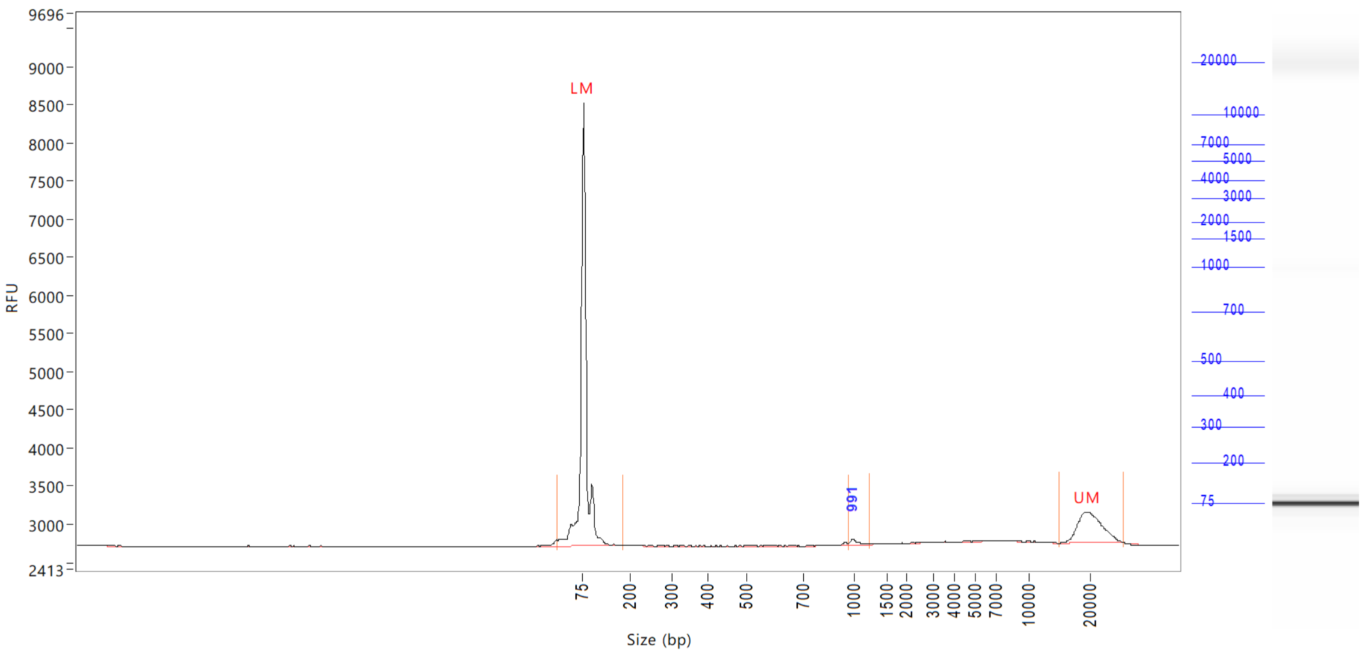

Peak	Size (bp)	Concentration (ng/uL)	From (bp)	To (bp)	Average size (bp)	CV%	RFU	Corrected peak area
1	75 (LM)	0.5000	7	177	72	22.16	5812	72.485
2	991	0.0774	965	1234	1021	5.50	86	0.935
3	20000 (UM)	0.0778	15000	25682	20232	9.08	405	11.281

TIC:	0.0774	ng/uL
TIM:	0.1247	nmole/L
Total concentration:	0.2890	ng/uL

```
Sample peak width (sec): 5    Sample min peak height: 50    Sample baseline V to V?: Y    Sample baseline V to V points: 3
Sample filter: Binomial       Number of points for filter: 3  Sample start region (min): 0  Sample end region (min): 30
Marker peak width (sec): 5    Marker min peak height: 200   Marker baseline V to V?: Y    Marker baseline V to V points: 3
Lower marker selection: First peak > 500 RFU                Upper marker selection: Last peak > 200 RFU
Ladder size (bp)75, 200, 300, 400, 500, 700, 1000, 1500, 2000, 3000, 4000, 5000, 7000, 10000, 20000
Quantification using: Lower Marker          Final concentration (ng/uL): 0.5000          Dilution factor: 12.0
```

Sample: SampD6
Well location: D6
Created: Tuesday, August 10, 2021 8:41:15 PM

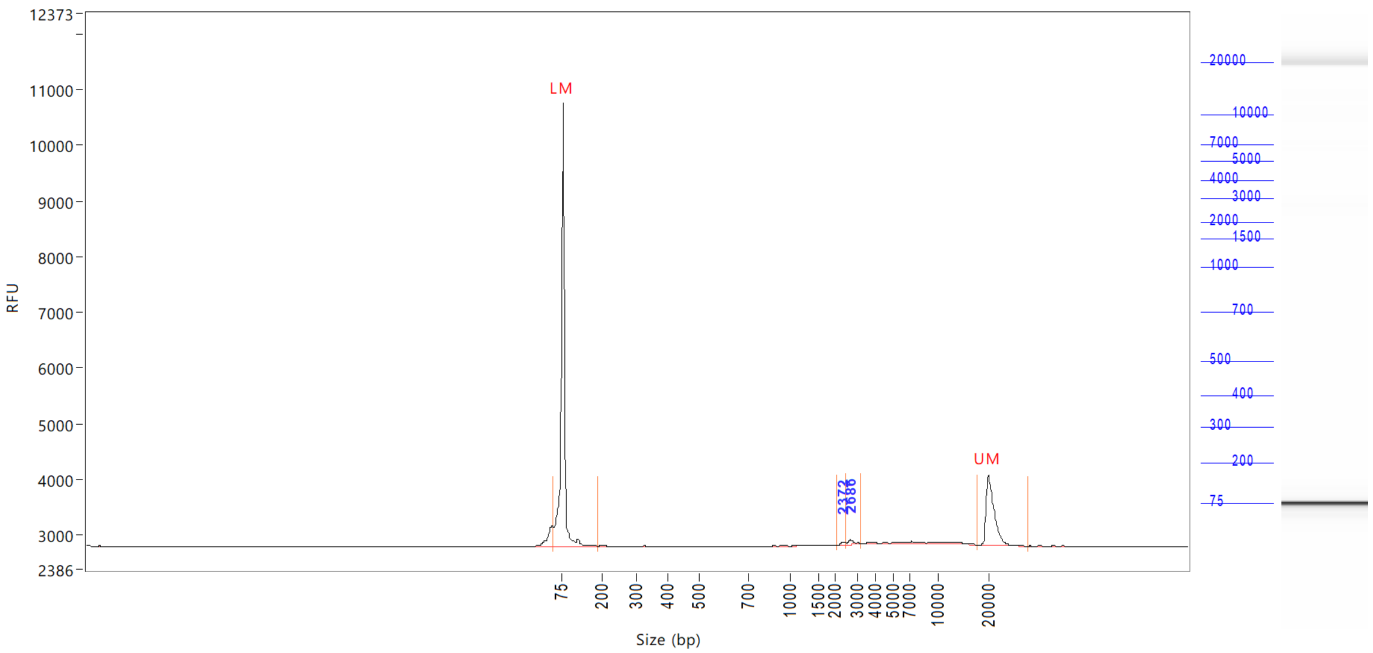

Peak	Size (bp)	Concentration (ng/uL)	From (bp)	To (bp)	Average size (bp)	CV%	RFU	Corrected peak area
1	75 (LM)	0.5000	45	183	73	17.47	7983	65.500
2	2372	0.0328	2058	2515	2355	3.87	56	0.358
3	2686	0.0718	2515	3215	2767	6.07	83	0.784
4	20000 (UM)	0.0979	17927	27859	20562	4.91	1248	12.824

TIC:	0.1046	ng/uL
TIM:	0.0656	nmole/L
Total concentration:	0.4265	ng/uL

Sample peak width (sec): 5   Sample min peak height: 50   Sample baseline V to V?: Y   Sample baseline V to V points: 3
Sample filter: Binomial   Number of points for filter: 3 Sample start region (min): 0 Sample end region (min): 30
Marker peak width (sec): 5   Marker min peak height: 200   Marker baseline V to V?: Y   Marker baseline V to V points: 3
Lower marker selection: First peak > 500 RFU   Upper marker selection: Last peak > 200 RFU
Ladder size (bp)75, 200, 300, 400, 500, 700, 1000, 1500, 2000, 3000, 4000, 5000, 7000, 10000, 20000
Quantification using: Lower Marker   Final concentration (ng/uL): 0.5000   Dilution factor: 12.0

Sample: SampE6
Well location: E6
Created: Tuesday, August 10, 2021 8:41:15 PM

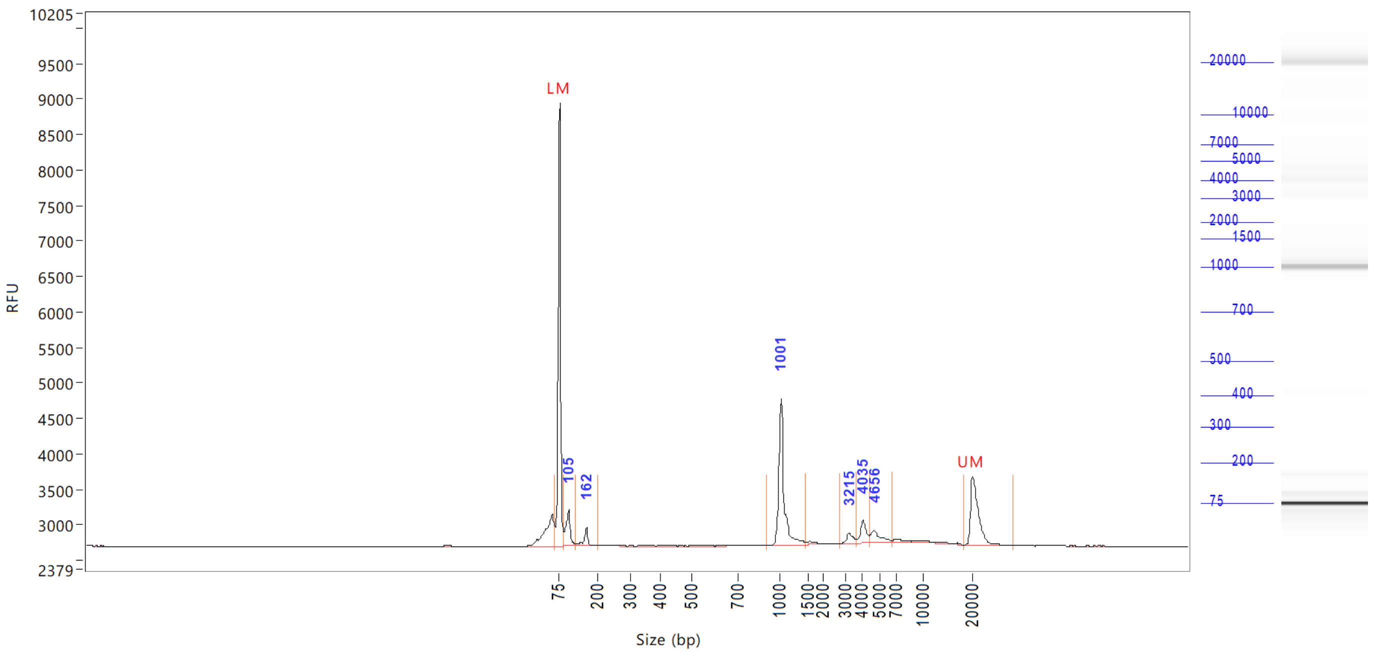

Peak	Size (bp)	Concentration (ng/uL)	From (bp)	To (bp)	Average size (bp)	CV%	RFU	Corrected peak area
1	75 (LM)	0.5000	64	87	73	5.10	6248	36.250
2	105	0.9239	87	128	101	7.87	513	5.582
3	162	0.3761	128	198	155	6.69	268	2.272
4	1001	2.8655	896	1467	1043	8.78	2066	17.312
5	3215	0.2424	2743	3679	3275	5.93	149	1.464
6	4035	0.4670	3679	4414	4056	4.10	327	2.821
7	4656	0.3989	4414	6408	4932	9.72	181	2.410
8	20000 (UM)	0.1345	18293	28342	20690	5.52	968	9.749

TIC:	5.2737	ng/uL
TIM:	23.9762	nmole/L
Total concentration:	5.7815	ng/uL

Sample peak width (sec): 5 Sample min peak height: 50 Sample baseline V to V?: Y Sample baseline V to V points: 3
Sample filter: Binomial Number of points for filter: 3 Sample start region (min): 0 Sample end region (min): 30
Marker peak width (sec): 5 Marker min peak height: 200 Marker baseline V to V?: Y Marker baseline V to V points: 3
Lower marker selection: First peak > 500 RFU Upper marker selection: Last peak > 200 RFU
Ladder size (bp)75, 200, 300, 400, 500, 700, 1000, 1500, 2000, 3000, 4000, 5000, 7000, 10000, 20000
Quantification using: Lower Marker Final concentration (ng/uL): 0.5000 Dilution factor: 12.0

Sample: SampF6
Well location: F6
Created: Tuesday, August 10, 2021 8:41:15 PM

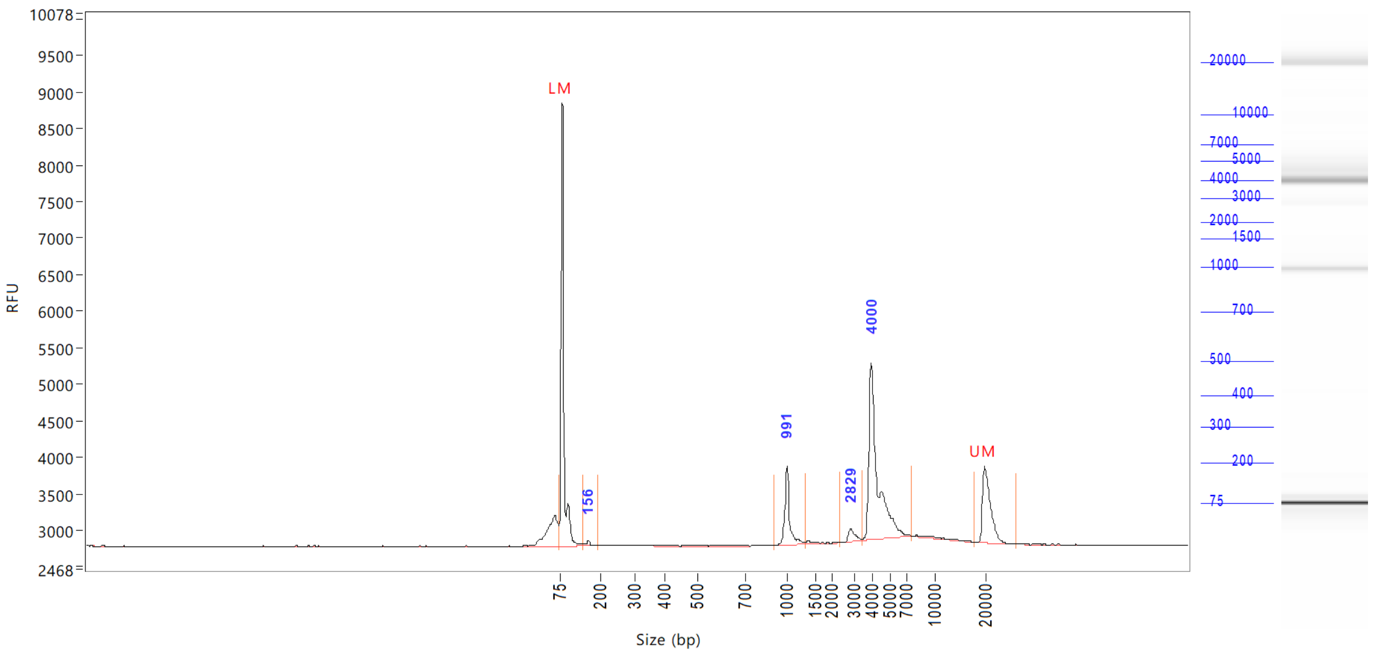

Peak	Size (bp)	Concentration (ng/uL)	From (bp)	To (bp)	Average size (bp)	CV%	RFU	Corrected peak area
1	75 (LM)	0.5000	66	143	77	12.41	6076	42.297
2	156	0.0974	143	189	157	6.17	72	0.686
3	991	1.2056	900	1311	1010	6.45	1080	8.499
4	2829	0.2502	2315	3465	2904	6.28	179	1.763
5	4000	4.3054	3465	7468	4286	12.45	2427	30.351
6	20000 (UM)	0.1274	17927	26408	20537	4.71	1056	10.777

TIC:	5.8585	ng/uL
TIM:	4.7795	nmole/L
Total concentration:	6.2782	ng/uL

Sample peak width (sec): 5 Sample min peak height: 50 Sample baseline V to V?: Y Sample baseline V to V points: 3
Sample filter: Binomial Number of points for filter: 3 Sample start region (min): 0 Sample end region (min): 30
Marker peak width (sec): 5 Marker min peak height: 200 Marker baseline V to V?: Y Marker baseline V to V points: 3
Lower marker selection: First peak > 500 RFU Upper marker selection: Last peak > 200 RFU
Ladder size (bp)75, 200, 300, 400, 500, 700, 1000, 1500, 2000, 3000, 4000, 5000, 7000, 10000, 20000
Quantification using: Lower Marker Final concentration (ng/uL): 0.5000 Dilution factor: 12.0

Sample: SampG6
Well location: G6
Created: Tuesday, August 10, 2021 8:41:15 PM

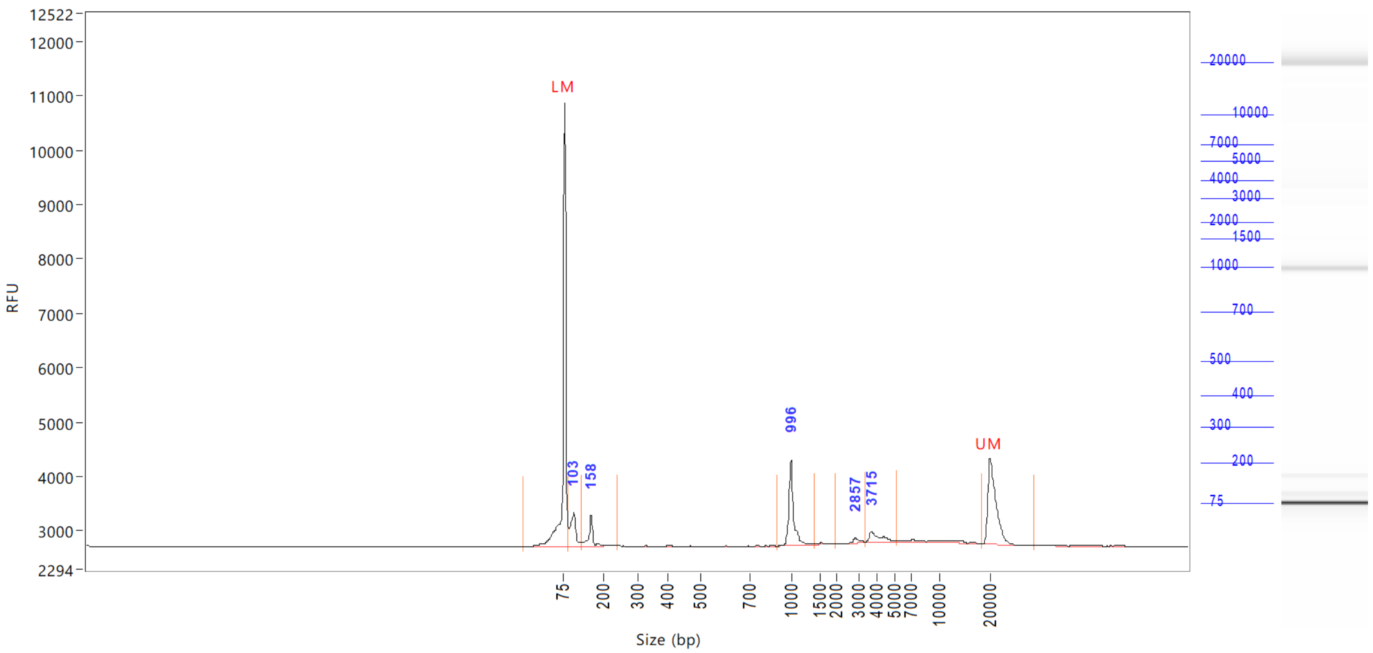

Peak	Size (bp)	Concentration (ng/uL)	From (bp)	To (bp)	Average size (bp)	CV%	RFU	Corrected peak area
1	75 (LM)	0.5000	0	89	69	22.21	8171	58.654
2	103	0.7782	89	129	102	8.72	622	7.608
3	158	0.5525	129	237	156	9.74	566	5.401
4	996	1.2508	896	1411	1010	5.91	1579	12.227
5	2857	0.0941	1962	3322	2869	7.21	93	0.920
6	3715	0.3312	3322	5223	4046	10.18	198	3.237
7	20000 (UM)	0.1420	18293	28705	20537	4.91	1590	16.662

TIC:	3.0068	ng/uL
TIM:	20.5774	nmole/L
Total concentration:	3.3445	ng/uL

Sample peak width (sec): 5 Sample min peak height: 50 Sample baseline V to V?: Y Sample baseline V to V points: 3
Sample filter: Binomial Number of points for filter: 3 Sample start region (min): 0 Sample end region (min): 30
Marker peak width (sec): 5 Marker min peak height: 200 Marker baseline V to V?: Y Marker baseline V to V points: 3
Lower marker selection: First peak > 500 RFU Upper marker selection: Last peak > 200 RFU
Ladder size (bp)75, 200, 300, 400, 500, 700, 1000, 1500, 2000, 3000, 4000, 5000, 7000, 10000, 20000
Quantification using: Lower Marker Final concentration (ng/uL): 0.5000 Dilution factor: 12.0

Sample: SampH6
Well location: H6
Created: Tuesday, August 10, 2021 8:41:15 PM

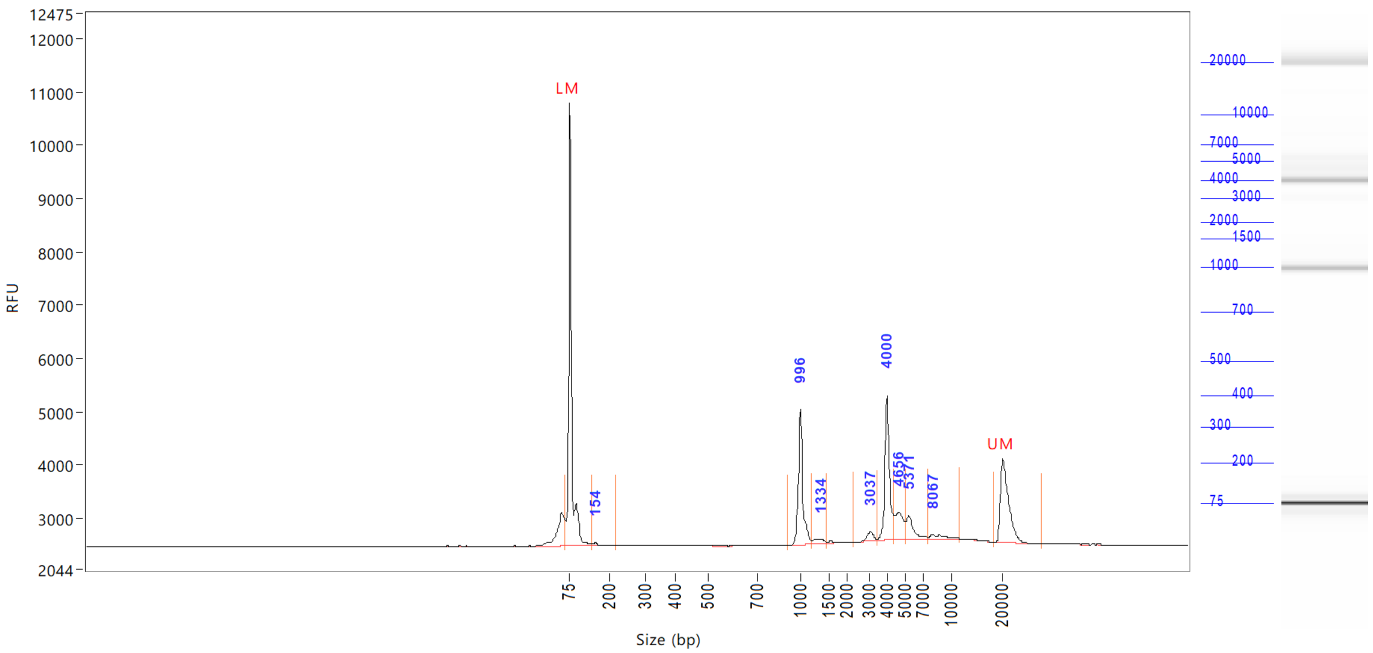

Peak	Size (bp)	Concentration (ng/uL)	From (bp)	To (bp)	Average size (bp)	CV%	RFU	Corrected peak area
1	75 (LM)	0.5000	64	143	77	13.50	8327	57.595
2	154	0.0929	143	217	165	12.09	70	0.892
3	996	1.9152	905	1178	1002	3.78	2532	18.384
4	1334	0.1582	1178	1445	1295	5.50	92	1.518
5	3037	0.1737	2286	3429	3042	5.04	173	1.667
6	4000	1.9653	3429	4380	3997	3.81	2704	18.865
7	4656	0.6460	4380	5000	4668	3.87	495	6.201
8	5371	0.4966	5000	7468	5645	10.59	428	4.767
9	8067	0.1428	7468	11708	8679	9.30	70	1.371
10	20000 (UM)	0.1423	18415	27617	20605	4.79	1585	16.392

TIC:	5.5907	ng/uL
TIM:	5.5759	nmole/L
Total concentration:	5.7271	ng/uL

Sample peak width (sec): 5   Sample min peak height: 50   Sample baseline V to V?: Y   Sample baseline V to V points: 3
Sample filter: Binomial   Number of points for filter: 3 Sample start region (min): 0 Sample end region (min): 30
Marker peak width (sec): 5   Marker min peak height: 200   Marker baseline V to V?: Y   Marker baseline V to V points: 3
Lower marker selection: First peak > 500 RFU   Upper marker selection: Last peak > 200 RFU
Ladder size (bp)75, 200, 300, 400, 500, 700, 1000, 1500, 2000, 3000, 4000, 5000, 7000, 10000, 20000
Quantification using: Lower Marker   Final concentration (ng/uL): 0.5000   Dilution factor: 12.0

Sample: SampA7
Well location: A7
Created: Tuesday, August 10, 2021 8:41:15 PM

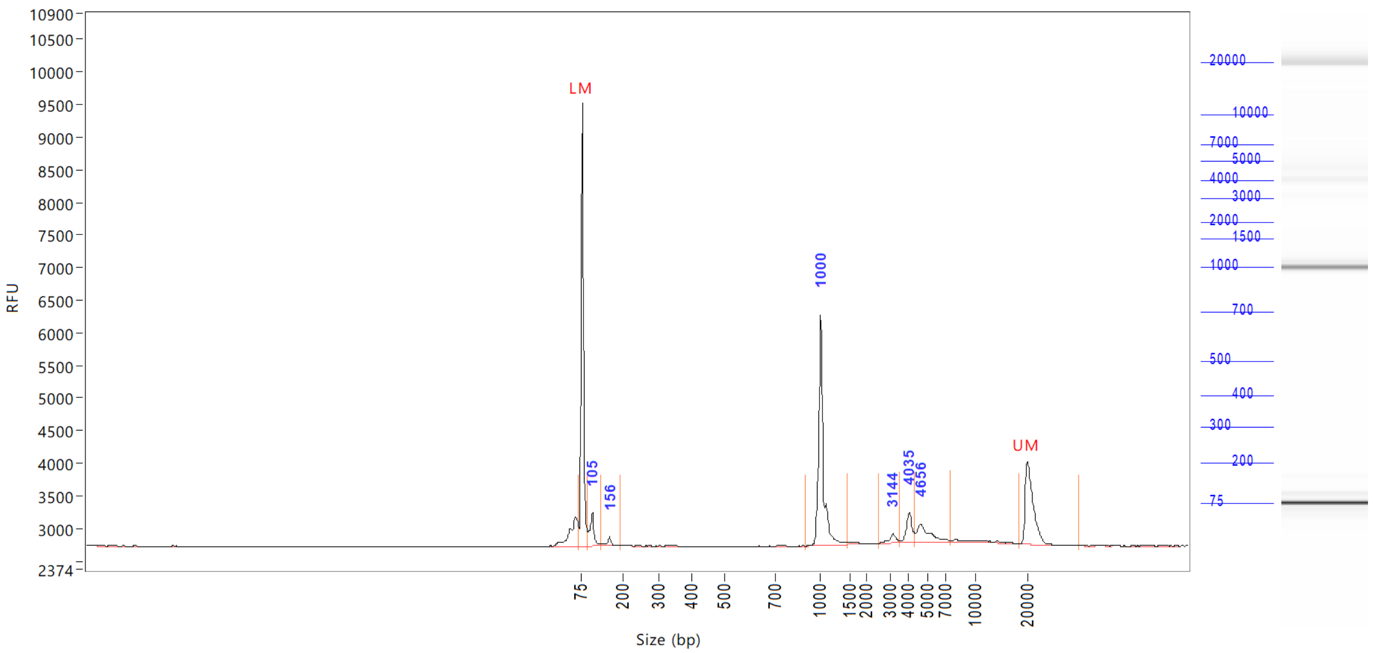

Peak	Size (bp)	Concentration (ng/uL)	From (bp)	To (bp)	Average size (bp)	CV%	RFU	Corrected peak area
1	75 (LM)	0.5000	66	91	74	5.38	6807	39.915
2	105	0.7769	91	133	103	8.06	520	5.168
3	156	0.1876	133	190	152	5.84	139	1.248
4	1000	3.8563	896	1467	1030	7.53	3536	25.654
5	3144	0.2055	2486	3536	3088	6.54	139	1.367
6	4035	0.5506	3536	4311	4005	3.76	467	3.663
7	4656	0.7209	4311	7534	5052	14.02	281	4.796
8	20000 (UM)	0.1552	18537	29672	20532	5.08	1262	12.391

TIC:	6.2978	ng/uL
TIM:	21.1473	nmole/L
Total concentration:	6.8032	ng/uL

Sample peak width (sec): 5 Sample min peak height: 50 Sample baseline V to V?: Y Sample baseline V to V points: 3
Sample filter: Binomial Number of points for filter: 3 Sample start region (min): 0 Sample end region (min): 30
Marker peak width (sec): 5 Marker min peak height: 200 Marker baseline V to V?: Y Marker baseline V to V points: 3
Lower marker selection: First peak > 500 RFU Upper marker selection: Last peak > 200 RFU
Ladder size (bp)75, 200, 300, 400, 500, 700, 1000, 1500, 2000, 3000, 4000, 5000, 7000, 10000, 20000
Quantification using: Lower Marker Final concentration (ng/uL): 0.5000 Dilution factor: 12.0

Sample: SampB7
Well location: B7
Created: Tuesday, August 10, 2021 8:41:15 PM

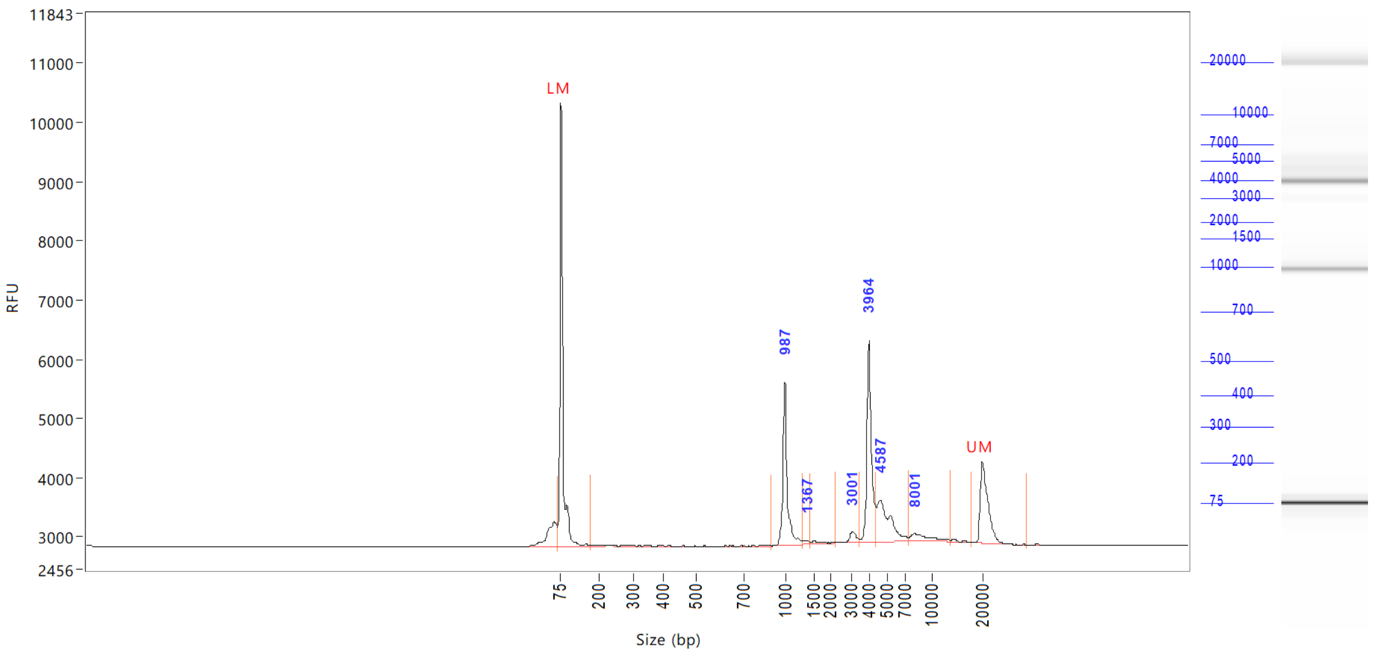

Peak	Size (bp)	Concentration (ng/uL)	From (bp)	To (bp)	Average size (bp)	CV%	RFU	Corrected peak area
1	75 (LM)	0.5000	66	169	78	14.30	7499	54.276
2	987	2.2345	896	1289	1006	5.81	2743	20.213
3	1367	0.0825	1289	1445	1358	3.29	72	0.747
4	3001	0.2084	2258	3429	3036	5.74	180	1.886
5	3964	2.6236	3429	4345	3965	3.86	3402	23.733
6	4587	1.5034	4345	7401	5054	13.64	708	13.600
7	8001	0.3687	7401	13537	9243	16.22	128	3.336
8	20000 (UM)	0.1271	17805	28947	20549	4.73	1379	13.799

TIC:	7.0211	ng/uL
TIM:	5.5136	nmole/L
Total concentration:	7.2259	ng/uL

Sample peak width (sec): 5   Sample min peak height: 50   Sample baseline V to V?: Y   Sample baseline V to V points: 3
Sample filter: Binomial   Number of points for filter: 3   Sample start region (min): 0   Sample end region (min): 30
Marker peak width (sec): 5   Marker min peak height: 200   Marker baseline V to V?: Y   Marker baseline V to V points: 3
Lower marker selection: First peak > 500 RFU   Upper marker selection: Last peak > 200 RFU
Ladder size (bp)75, 200, 300, 400, 500, 700, 1000, 1500, 2000, 3000, 4000, 5000, 7000, 10000, 20000
Quantification using: Lower Marker   Final concentration (ng/uL): 0.5000   Dilution factor: 12.0

Sample: SampC7
Well location: C7
Created: Tuesday, August 10, 2021 8:41:15 PM

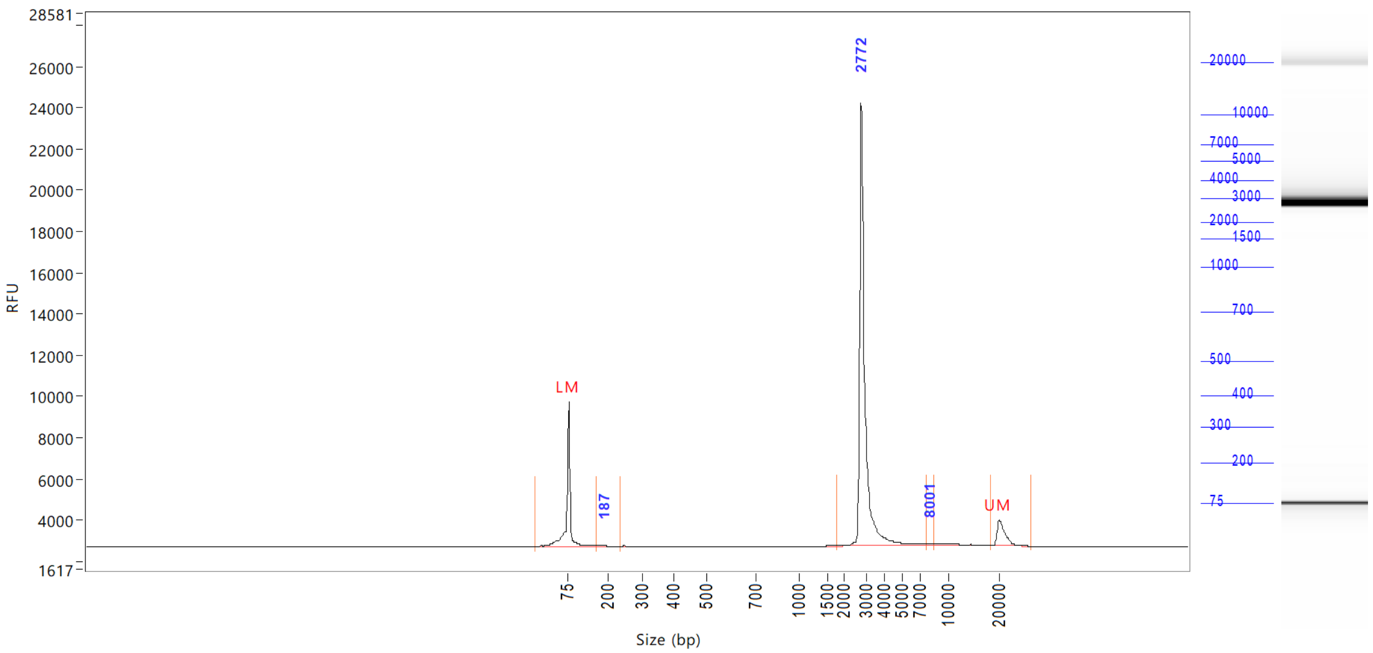

Peak	Size (bp)	Concentration (ng/uL)	From (bp)	To (bp)	Average size (bp)	CV%	RFU	Corrected peak area
1	75 (LM)	0.5000	0	160	69	29.80	7008	58.379
2	187	0.1182	160	232	183	8.59	74	1.150
3	2772	17.3466	1770	7734	2954	17.71	21517	168.780
4	8001	0.0470	7734	8534	8097	2.96	57	0.457
5	20000 (UM)	0.1033	18171	26287	20536	4.56	1229	12.057

TIC:	17.5118	ng/uL
TIM:	10.7387	nmole/L
Total concentration:	17.8408	ng/uL

Sample peak width (sec): 5   Sample min peak height: 50   Sample baseline V to V?: Y   Sample baseline V to V points: 3
Sample filter: Binomial   Number of points for filter: 3 Sample start region (min): 0 Sample end region (min): 30
Marker peak width (sec): 5   Marker min peak height: 200   Marker baseline V to V?: Y   Marker baseline V to V points: 3
Lower marker selection: First peak > 500 RFU   Upper marker selection: Last peak > 200 RFU
Ladder size (bp)75, 200, 300, 400, 500, 700, 1000, 1500, 2000, 3000, 4000, 5000, 7000, 10000, 20000
Quantification using: Lower Marker   Final concentration (ng/uL): 0.5000   Dilution factor: 12.0

Sample: SampD7
Well location: D7
Created: Tuesday, August 10, 2021 8:41:15 PM

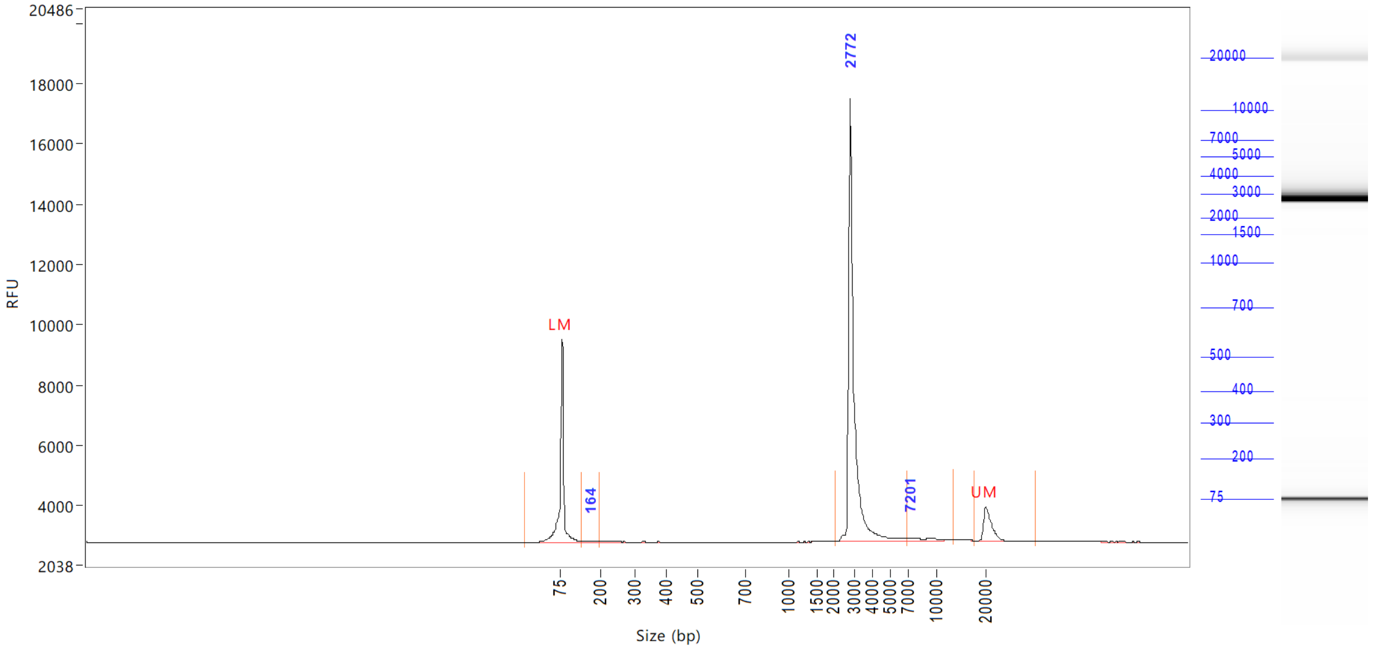

Peak	Size (bp)	Concentration (ng/uL)	From (bp)	To (bp)	Average size (bp)	CV%	RFU	Corrected peak area
1	75 (LM)	0.5000	0	137	70	23.26	6727	57.440
2	164	0.1795	137	194	165	9.92	64	1.719
3	2772	12.6607	2087	6926	2974	17.47	14709	121.206
4	7201	0.3119	6926	13659	9261	19.31	81	2.986
5	20000 (UM)	0.0982	17683	29793	20547	5.17	1107	11.283

TIC:	13.1521	ng/uL
TIM:	8.8519	nmole/L
Total concentration:	13.5034	ng/uL

Sample peak width (sec): 5   Sample min peak height: 50   Sample baseline V to V?: Y   Sample baseline V to V points: 3
Sample filter: Binomial   Number of points for filter: 3 Sample start region (min): 0 Sample end region (min): 30
Marker peak width (sec): 5   Marker min peak height: 200   Marker baseline V to V?: Y   Marker baseline V to V points: 3
Lower marker selection: First peak > 500 RFU   Upper marker selection: Last peak > 200 RFU
Ladder size (bp)75, 200, 300, 400, 500, 700, 1000, 1500, 2000, 3000, 4000, 5000, 7000, 10000, 20000
Quantification using: Lower Marker   Final concentration (ng/uL): 0.5000   Dilution factor: 12.0

Sample: SampE7
Well location: E7
Created: Tuesday, August 10, 2021 8:41:15 PM

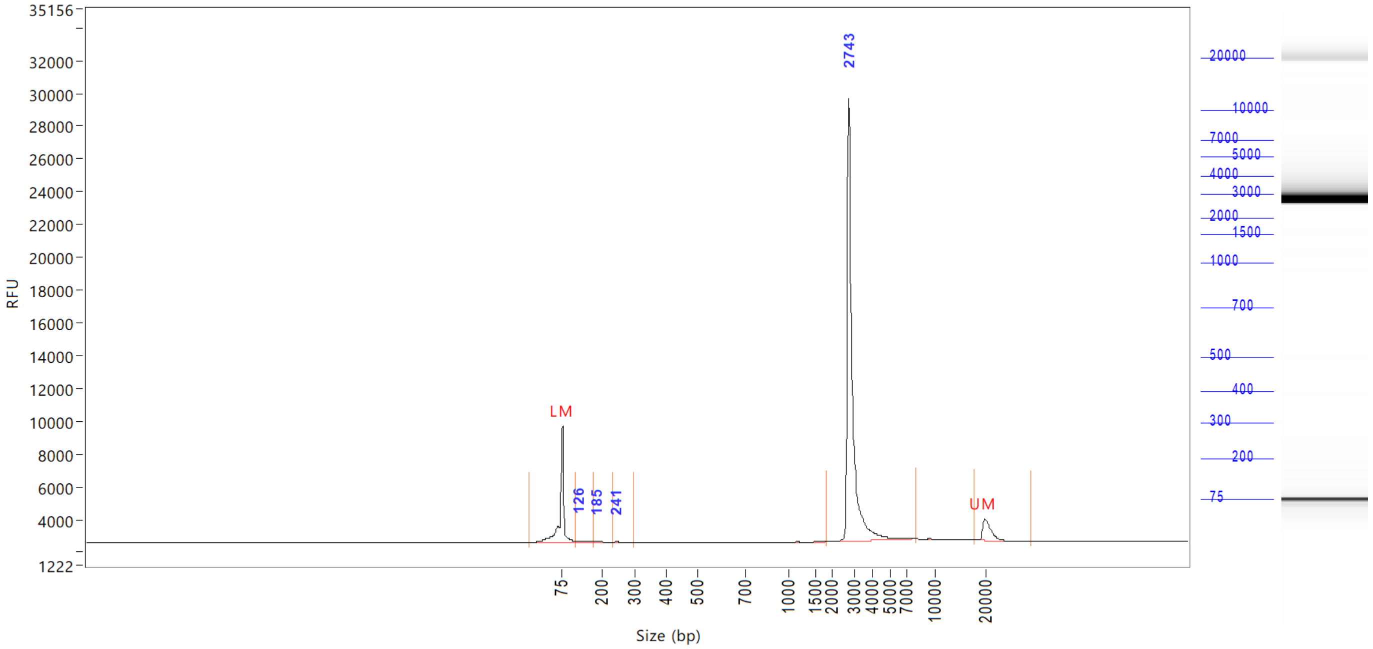

Peak	Size<br>(bp)	Concentration<br>(ng/uL)	From<br>(bp)	To<br>(bp)	Average size<br>(bp)	CV%	RFU	Corrected peak area
1	75 (LM)	0.5000	0	118	66	28.10	7124	63.573
2	126	0.2621	118	175	144	11.89	118	2.777
3	185	0.1324	175	232	197	8.12	87	1.403
4	241	0.0769	232	296	248	5.35	82	0.814
5	2743	20.3215	1827	7934	2913	15.67	27048	215.317
6	20000 (UM)	0.1070	18049	29430	20606	4.97	1337	13.604

TIC:	20.7928	ng/uL
TIM:	16.0898	nmole/L
Total concentration:	21.0869	ng/uL

```
Sample peak width (sec): 5     Sample min peak height: 50     Sample baseline V to V?: Y     Sample baseline V to V points: 3
Sample filter: Binomial        Number of points for filter: 3  Sample start region (min): 0  Sample end region (min): 30
Marker peak width (sec): 5     Marker min peak height: 200    Marker baseline V to V?: Y     Marker baseline V to V points: 3
Lower marker selection: First peak > 500 RFU                  Upper marker selection: Last peak > 200 RFU
Ladder size (bp)75, 200, 300, 400, 500, 700, 1000, 1500, 2000, 3000, 4000, 5000, 7000, 10000, 20000
Quantification using: Lower Marker          Final concentration (ng/uL): 0.5000          Dilution factor: 12.0
```

Sample: SampF7
Well location: F7
Created: Tuesday, August 10, 2021 8:41:15 PM

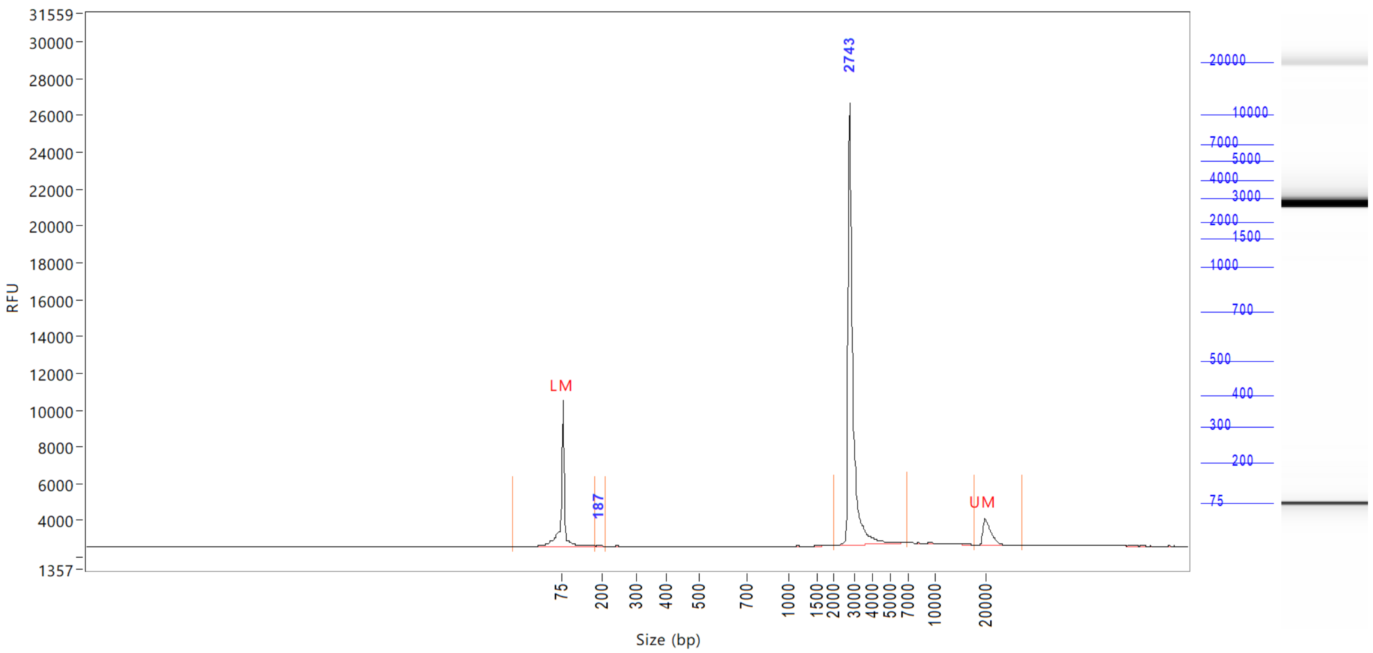

Peak	Size (bp)	Concentration (ng/uL)	From (bp)	To (bp)	Average size (bp)	CV%	RFU	Corrected peak area
1	75 (LM)	0.5000	0	177	70	31.53	7965	67.002
2	187	0.0620	177	210	189	4.45	69	0.692
3	2743	15.9466	2001	7000	2871	11.83	24065	178.076
4	20000 (UM)	0.1091	17805	27496	20535	4.84	1423	14.621

TIC:	16.0085	ng/uL
TIM:	9.6828	nmole/L
Total concentration:	16.3518	ng/uL

```
Sample peak width (sec): 5    Sample min peak height: 50    Sample baseline V to V?: Y    Sample baseline V to V points: 3
Sample filter: Binomial       Number of points for filter: 3  Sample start region (min): 0  Sample end region (min): 30
Marker peak width (sec): 5    Marker min peak height: 200   Marker baseline V to V?: Y    Marker baseline V to V points: 3
Lower marker selection: First peak > 500 RFU                Upper marker selection: Last peak > 200 RFU
Ladder size (bp)75, 200, 300, 400, 500, 700, 1000, 1500, 2000, 3000, 4000, 5000, 7000, 10000, 20000
Quantification using: Lower Marker          Final concentration (ng/uL): 0.5000          Dilution factor: 12.0
```

Sample: SampG7
Well location: G7
Created: Tuesday, August 10, 2021 8:41:15 PM

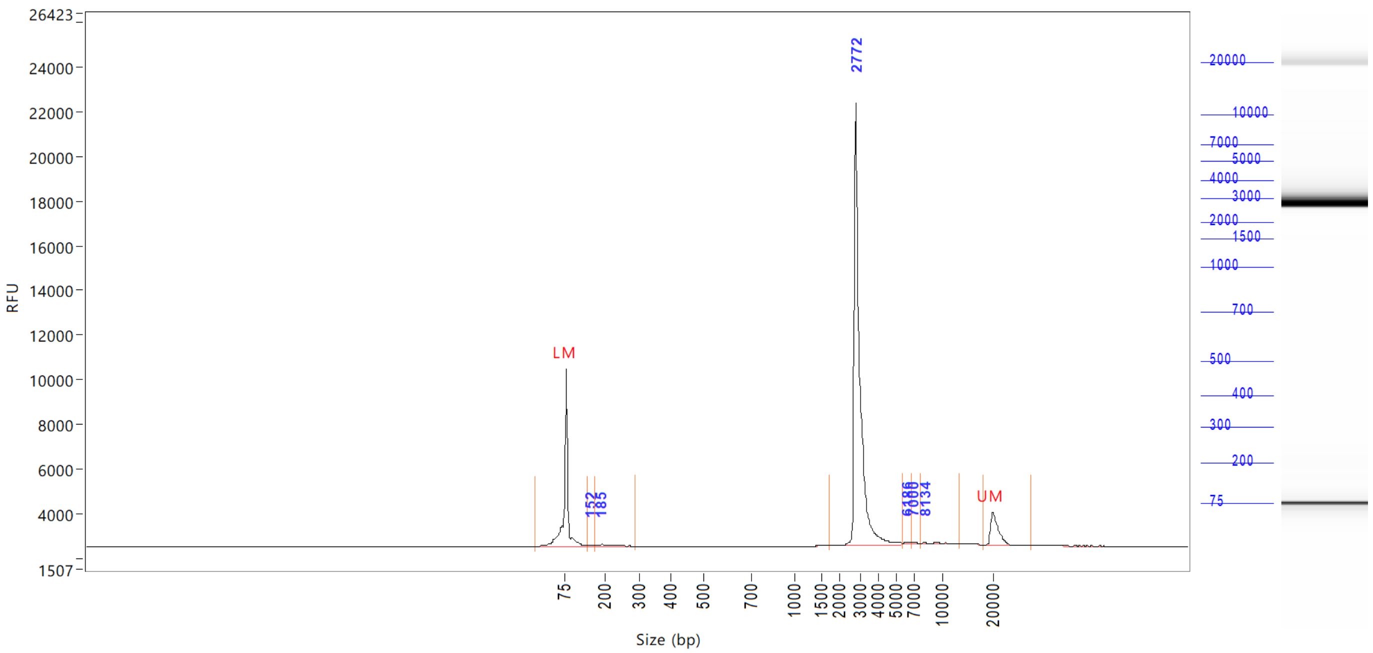

Peak	Size (bp)	Concentration (ng/uL)	From (bp)	To (bp)	Average size (bp)	CV%	RFU	Corrected peak area
1	75 (LM)	0.5000	0	143	69	26.21	7963	67.935
2	152	0.0833	143	166	153	4.27	83	0.944
3	185	0.2702	166	284	204	11.69	91	3.060
4	2772	14.8158	1693	5667	2912	12.34	19858	167.752
5	6186	0.0699	5667	6704	6186	5.05	78	0.792
6	7000	0.0696	6704	7734	7167	4.20	75	0.788
7	8134	0.1821	7734	13537	9826	15.59	63	2.062
8	20000 (UM)	0.1088	18049	27738	20548	4.82	1484	14.789

TIC:	15.4910	ng/uL
TIM:	11.5140	nmole/L
Total concentration:	15.6075	ng/uL

Sample peak width (sec): 5 Sample min peak height: 50 Sample baseline V to V?: Y Sample baseline V to V points: 3
Sample filter: Binomial Number of points for filter: 3 Sample start region (min): 0 Sample end region (min): 30
Marker peak width (sec): 5 Marker min peak height: 200 Marker baseline V to V?: Y Marker baseline V to V points: 3
Lower marker selection: First peak > 500 RFU Upper marker selection: Last peak > 200 RFU
Ladder size (bp)75, 200, 300, 400, 500, 700, 1000, 1500, 2000, 3000, 4000, 5000, 7000, 10000, 20000
Quantification using: Lower Marker Final concentration (ng/uL): 0.5000 Dilution factor: 12.0

Sample: SampH7
Well location: H7
Created: Tuesday, August 10, 2021 8:41:15 PM

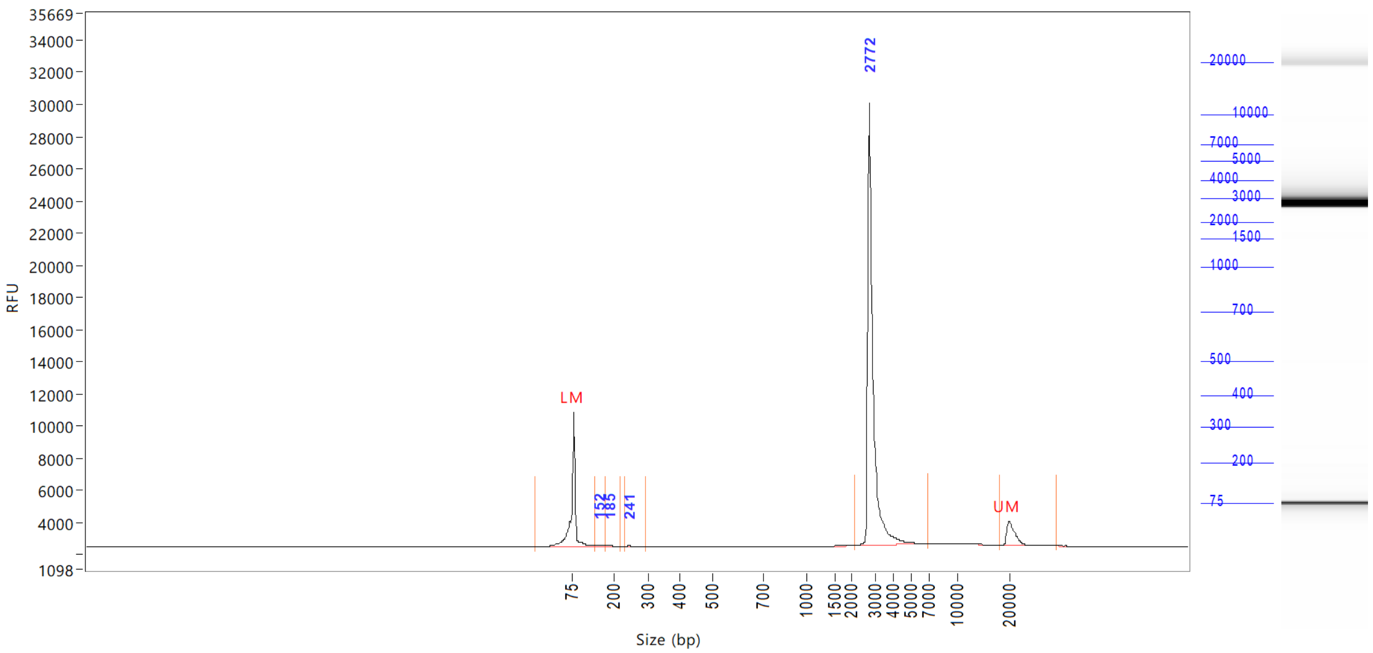

Peak	Size (bp)	Concentration (ng/uL)	From (bp)	To (bp)	Average size (bp)	CV%	RFU	Corrected peak area
1	75 (LM)	0.5000	0	139	68	25.22	8313	73.646
2	152	0.0970	139	171	154	5.69	73	1.190
3	185	0.0684	171	217	185	4.79	86	0.840
4	241	0.0389	230	291	247	4.88	54	0.478
5	2772	17.5073	2087	6926	2905	12.39	27561	214.891
6	20000 (UM)	0.1059	18049	29189	20516	4.71	1529	15.604

TIC:	17.7116	ng/uL
TIM:	11.8234	nmole/L
Total concentration:	17.9124	ng/uL

```
Sample peak width (sec): 5    Sample min peak height: 50    Sample baseline V to V?: Y    Sample baseline V to V points: 3
Sample filter: Binomial       Number of points for filter: 3  Sample start region (min): 0  Sample end region (min): 30
Marker peak width (sec): 5    Marker min peak height: 200   Marker baseline V to V?: Y    Marker baseline V to V points: 3
Lower marker selection: First peak > 500 RFU                Upper marker selection: Last peak > 200 RFU
Ladder size (bp)75, 200, 300, 400, 500, 700, 1000, 1500, 2000, 3000, 4000, 5000, 7000, 10000, 20000
Quantification using: Lower Marker          Final concentration (ng/uL): 0.5000          Dilution factor: 12.0
```

Sample: SampA8
Well location: A8
Created: Tuesday, August 10, 2021 8:41:15 PM

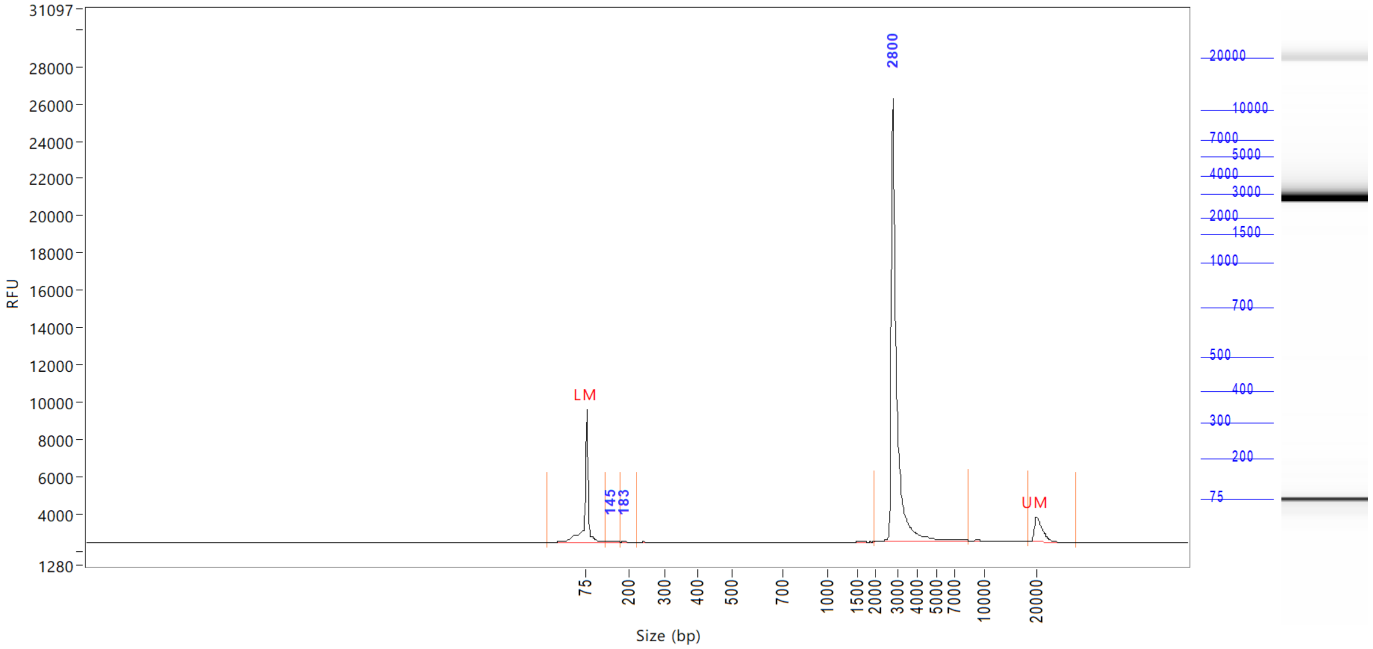

Peak	Size (bp)	Concentration (ng/uL)	From (bp)	To (bp)	Average size (bp)	CV%	RFU	Corrected peak area
1	75 (LM)	0.5000	0	129	66	29.30	7128	59.295
2	145	0.1239	129	173	148	8.06	65	1.224
3	183	0.0687	173	221	186	5.30	86	0.679
4	2800	18.2577	2001	8467	2978	16.76	23790	180.432
5	20000 (UM)	0.1096	18415	27375	20484	4.46	1352	12.996

TIC:	18.4502	ng/uL
TIM:	12.0755	nmole/L
Total concentration:	18.7471	ng/uL

```
Sample peak width (sec): 5    Sample min peak height: 50    Sample baseline V to V?: Y    Sample baseline V to V points: 3
Sample filter: Binomial       Number of points for filter: 3  Sample start region (min): 0  Sample end region (min): 30
Marker peak width (sec): 5    Marker min peak height: 200   Marker baseline V to V?: Y    Marker baseline V to V points: 3
Lower marker selection: First peak > 500 RFU                Upper marker selection: Last peak > 200 RFU
Ladder size (bp)75, 200, 300, 400, 500, 700, 1000, 1500, 2000, 3000, 4000, 5000, 7000, 10000, 20000
Quantification using: Lower Marker          Final concentration (ng/uL): 0.5000          Dilution factor: 12.0
```

Sample: SampB8
Well location: B8
Created: Tuesday, August 10, 2021 8:41:15 PM

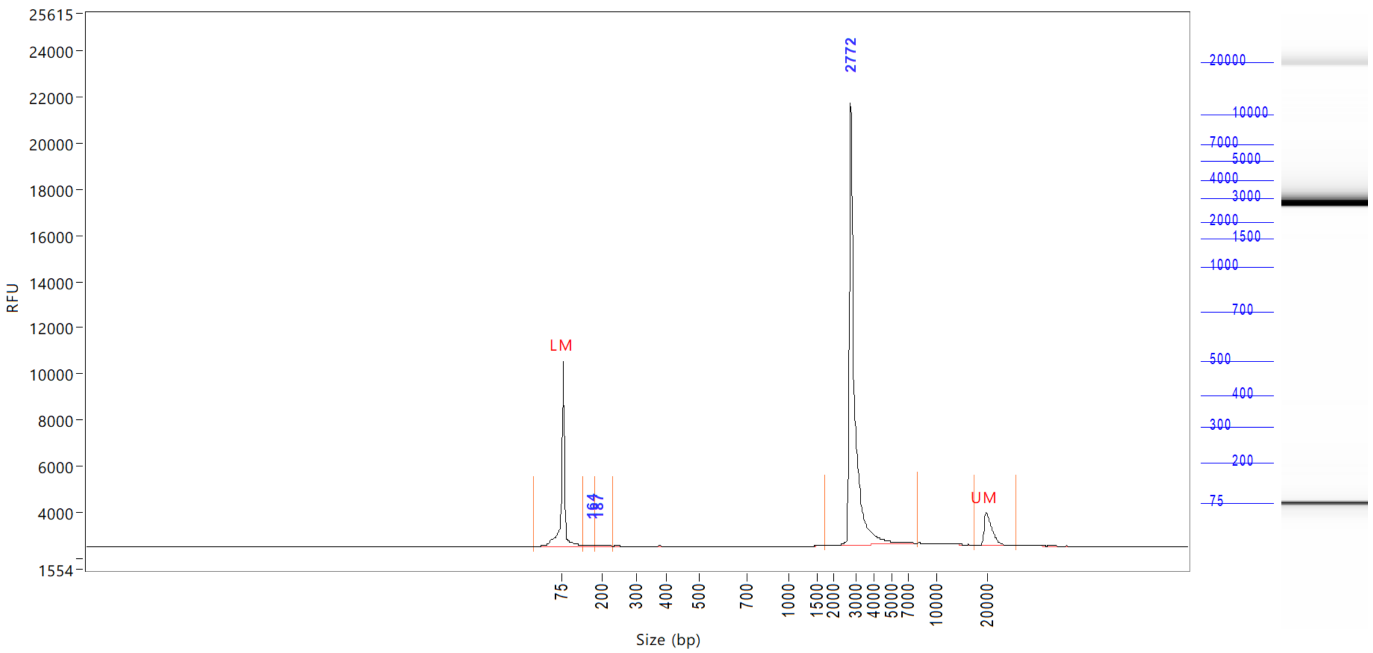

Peak	Size (bp)	Concentration (ng/uL)	From (bp)	To (bp)	Average size (bp)	CV%	RFU	Corrected peak area
1	75 (LM)	0.5000	0	137	70	22.91	8001	63.569
2	164	0.1289	137	177	158	7.14	75	1.365
3	187	0.1234	177	228	198	7.28	73	1.307
4	2772	14.7300	1712	7934	2969	15.82	19172	156.061
5	20000 (UM)	0.1117	17683	25924	20508	4.58	1419	14.206

TIC:	14.9822	ng/uL
TIM:	10.5324	nmole/L
Total concentration:	15.2942	ng/uL

```
Sample peak width (sec): 5    Sample min peak height: 50    Sample baseline V to V?: Y    Sample baseline V to V points: 3
Sample filter: Binomial       Number of points for filter: 3  Sample start region (min): 0  Sample end region (min): 30
Marker peak width (sec): 5    Marker min peak height: 200   Marker baseline V to V?: Y    Marker baseline V to V points: 3
Lower marker selection: First peak > 500 RFU                Upper marker selection: Last peak > 200 RFU
Ladder size (bp)75, 200, 300, 400, 500, 700, 1000, 1500, 2000, 3000, 4000, 5000, 7000, 10000, 20000
Quantification using: Lower Marker          Final concentration (ng/uL): 0.5000          Dilution factor: 12.0
```

Sample: SampC8
Well location: C8
Created: Tuesday, August 10, 2021 8:41:15 PM

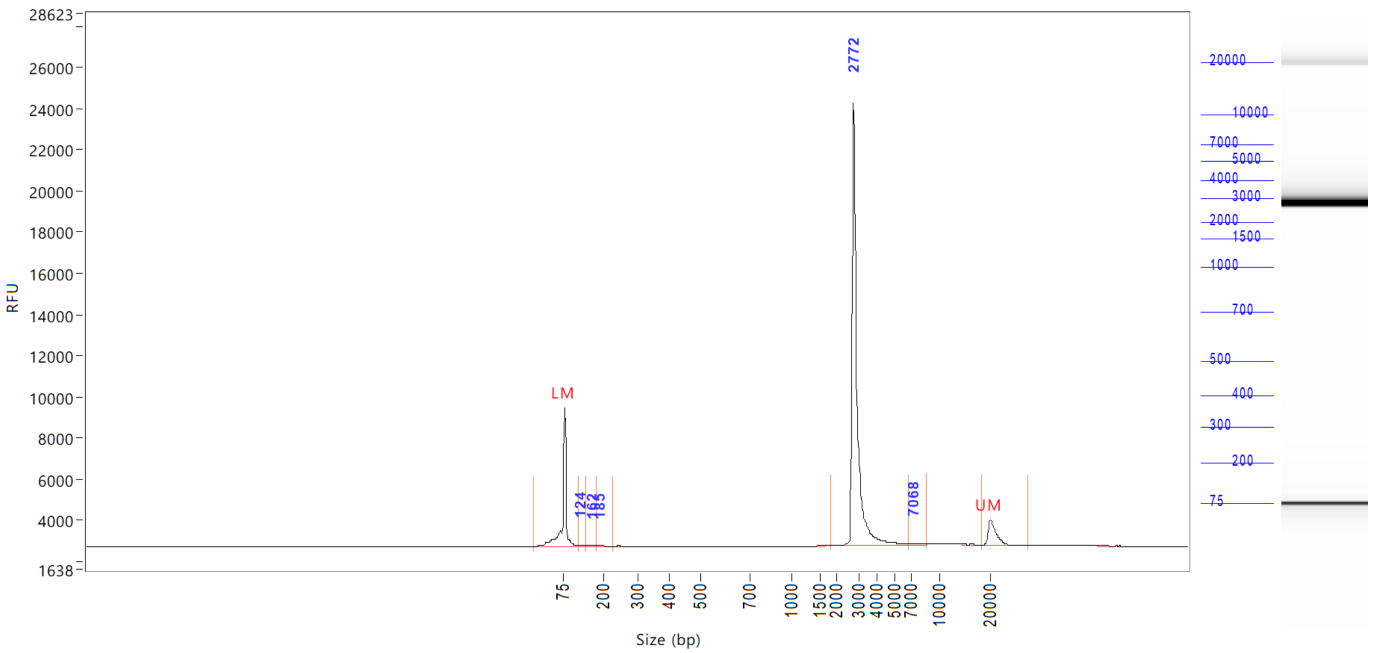

Peak	Size (bp)	Concentration (ng/uL)	From (bp)	To (bp)	Average size (bp)	CV%	RFU	Corrected peak area
1	75 (LM)	0.5000	0	120	66	28.42	6764	57.927
2	124	0.0864	120	139	127	4.54	89	0.834
3	162	0.1221	139	175	156	5.91	77	1.179
4	185	0.0709	175	221	188	5.16	69	0.684
5	2772	16.6496	1846	6704	2924	15.10	21523	160.742
6	7068	0.1310	6704	8534	7484	7.05	79	1.264
7	20000 (UM)	0.1062	18293	27617	20575	4.79	1222	12.299

TIC:	17.0599	ng/uL
TIM:	12.4271	nmole/L
Total concentration:	17.4176	ng/uL

```
Sample peak width (sec): 5    Sample min peak height: 50    Sample baseline V to V?: Y    Sample baseline V to V points: 3
Sample filter: Binomial       Number of points for filter: 3  Sample start region (min): 0  Sample end region (min): 30
Marker peak width (sec): 5    Marker min peak height: 200   Marker baseline V to V?: Y    Marker baseline V to V points: 3
Lower marker selection: First peak > 500 RFU                Upper marker selection: Last peak > 200 RFU
Ladder size (bp)75, 200, 300, 400, 500, 700, 1000, 1500, 2000, 3000, 4000, 5000, 7000, 10000, 20000
Quantification using: Lower Marker          Final concentration (ng/uL): 0.5000          Dilution factor: 12.0
```

Sample: SampD8
Well location: D8
Created: Tuesday, August 10, 2021 8:41:15 PM

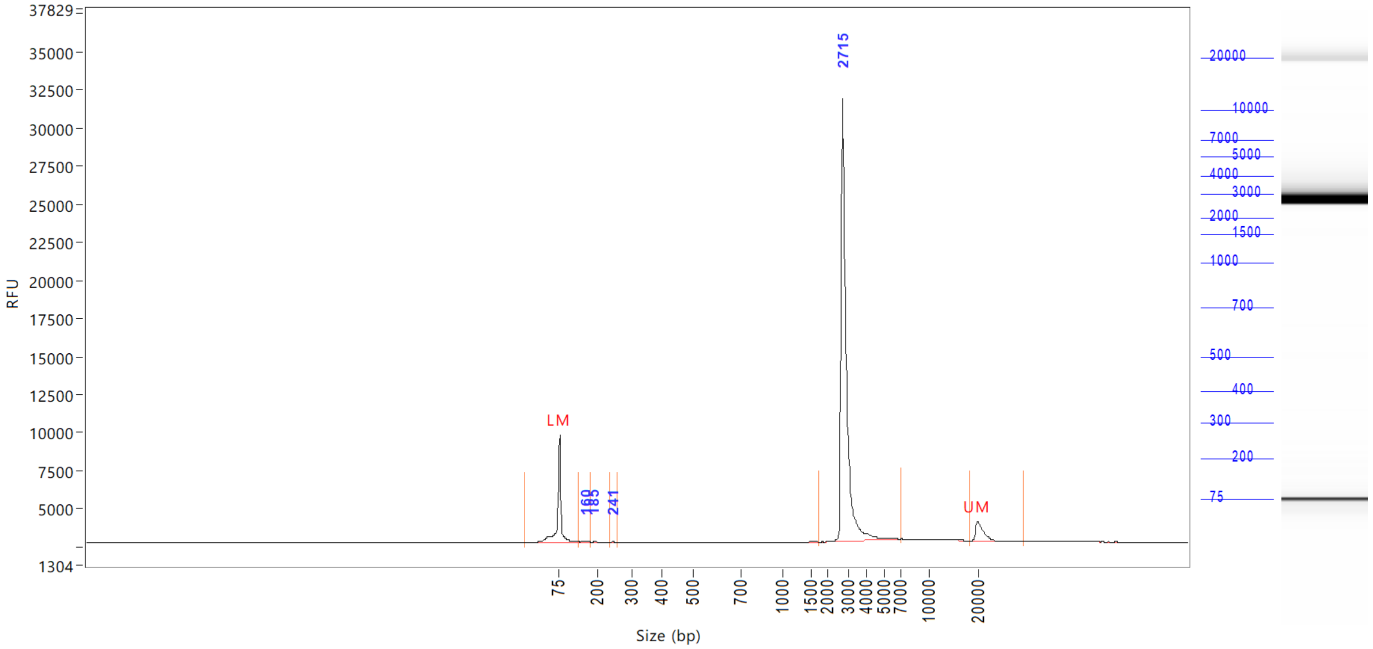

Peak	Size (bp)	Concentration (ng/uL)	From (bp)	To (bp)	Average size (bp)	CV%	RFU	Corrected peak area
1	75 (LM)	0.5000	0	139	69	28.06	7073	58.832
2	160	0.1373	139	175	156	5.94	82	1.346
3	185	0.1038	175	232	193	7.36	89	1.018
4	241	0.0293	232	255	240	1.51	50	0.287
5	2715	23.6208	1750	7000	2851	12.65	29119	231.608
6	20000 (UM)	0.1112	18537	29309	20509	4.87	1289	13.079

TIC:	23.8912	ng/uL
TIM:	16.1707	nmole/L
Total concentration:	24.1505	ng/uL

```
Sample peak width (sec): 5    Sample min peak height: 50    Sample baseline V to V?: Y    Sample baseline V to V points: 3
Sample filter: Binomial       Number of points for filter: 3  Sample start region (min): 0  Sample end region (min): 30
Marker peak width (sec): 5    Marker min peak height: 200   Marker baseline V to V?: Y    Marker baseline V to V points: 3
Lower marker selection: First peak > 500 RFU                Upper marker selection: Last peak > 200 RFU
Ladder size (bp)75, 200, 300, 400, 500, 700, 1000, 1500, 2000, 3000, 4000, 5000, 7000, 10000, 20000
Quantification using: Lower Marker          Final concentration (ng/uL): 0.5000          Dilution factor: 12.0
```

Sample: SampE8
Well location: E8
Created: Tuesday, August 10, 2021 8:41:15 PM

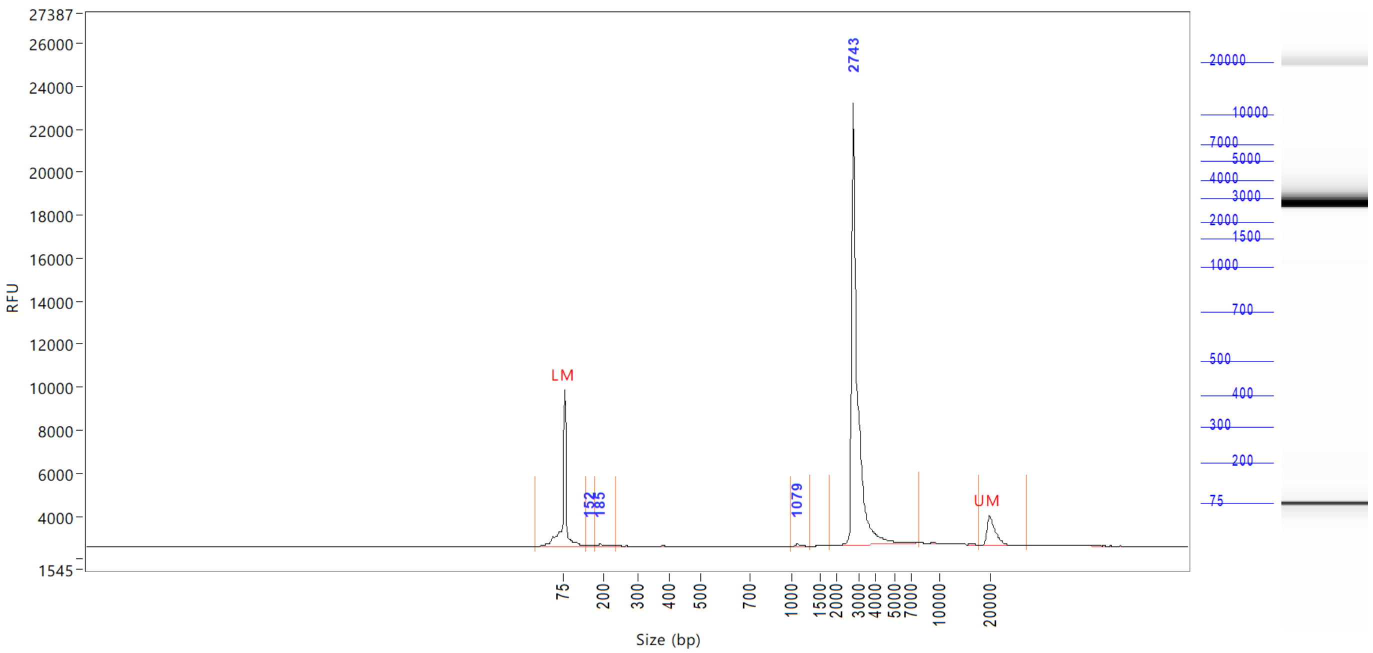

Peak	Size (bp)	Concentration (ng/uL)	From (bp)	To (bp)	Average size (bp)	CV%	RFU	Corrected peak area
1	75 (LM)	0.5000	0	139	68	27.31	7319	63.701
2	152	0.1194	139	169	154	5.60	87	1.268
3	185	0.2540	169	235	199	9.08	105	2.696
4	1079	0.0710	991	1334	1095	5.04	102	0.754
5	2743	16.8953	1789	7867	2945	15.22	20587	179.376
6	20000 (UM)	0.1121	17805	27375	20507	4.80	1382	14.277

TIC:	17.3396	ng/uL
TIM:	12.9236	nmole/L
Total concentration:	17.6352	ng/uL

Sample peak width (sec): 5 Sample min peak height: 50 Sample baseline V to V?: Y Sample baseline V to V points: 3
Sample filter: Binomial Number of points for filter: 3 Sample start region (min): 0 Sample end region (min): 30
Marker peak width (sec): 5 Marker min peak height: 200 Marker baseline V to V?: Y Marker baseline V to V points: 3
Lower marker selection: First peak > 500 RFU Upper marker selection: Last peak > 200 RFU
Ladder size (bp)75, 200, 300, 400, 500, 700, 1000, 1500, 2000, 3000, 4000, 5000, 7000, 10000, 20000
Quantification using: Lower Marker Final concentration (ng/uL): 0.5000 Dilution factor: 12.0

Sample: SampF8
Well location: F8
Created: Tuesday, August 10, 2021 8:41:15 PM

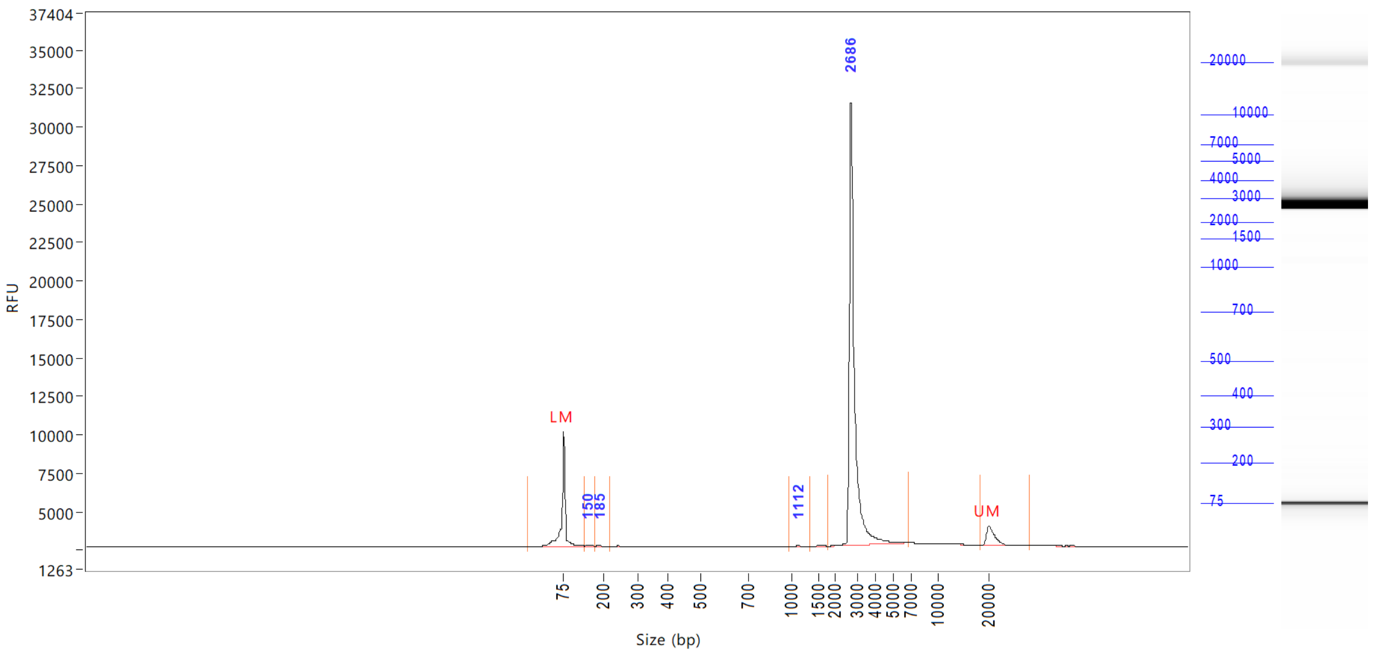

Peak	Size (bp)	Concentration (ng/uL)	From (bp)	To (bp)	Average size (bp)	CV%	RFU	Corrected peak area
1	75 (LM)	0.5000	0	139	68	25.72	7504	64.066
2	150	0.1174	139	171	153	5.54	92	1.253
3	185	0.0917	171	219	187	5.73	94	0.980
4	1112	0.0591	991	1345	1120	5.23	87	0.631
5	2686	21.3866	1731	6852	2838	12.91	28810	228.361
6	20000 (UM)	0.1018	18415	28100	20523	4.67	1272	13.042

TIC:	21.6549	ng/uL
TIM:	14.5599	nmole/L
Total concentration:	21.9356	ng/uL

```
Sample peak width (sec): 5    Sample min peak height: 50    Sample baseline V to V?: Y    Sample baseline V to V points: 3
Sample filter: Binomial       Number of points for filter: 3  Sample start region (min): 0  Sample end region (min): 30
Marker peak width (sec): 5    Marker min peak height: 200   Marker baseline V to V?: Y    Marker baseline V to V points: 3
Lower marker selection: First peak > 500 RFU                Upper marker selection: Last peak > 200 RFU
Ladder size (bp)75, 200, 300, 400, 500, 700, 1000, 1500, 2000, 3000, 4000, 5000, 7000, 10000, 20000
Quantification using: Lower Marker          Final concentration (ng/uL): 0.5000          Dilution factor: 12.0
```

Sample: SampG8
Well location: G8
Created: Tuesday, August 10, 2021 8:41:15 PM

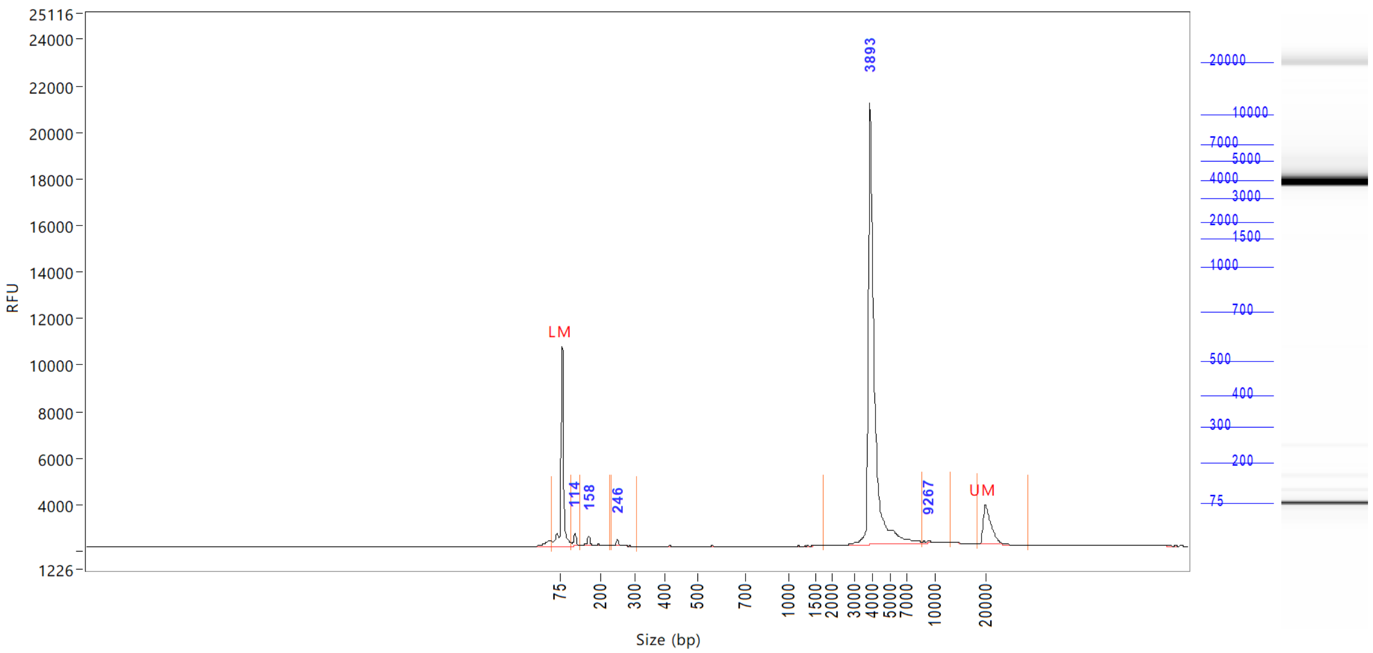

Peak	Size (bp)	Concentration (ng/uL)	From (bp)	To (bp)	Average size (bp)	CV%	RFU	Corrected peak area
1	75 (LM)	0.5000	47	105	74	10.25	8557	57.571
2	114	0.3884	105	131	114	4.79	529	3.726
3	158	0.3876	131	224	158	9.19	403	3.719
4	246	0.1618	228	301	245	3.44	288	1.553
5	3893	17.3896	1712	8534	4095	14.91	19007	166.856
6	9267	0.1502	8534	12928	9646	8.98	73	1.441
7	20000 (UM)	0.1459	18537	28584	20563	4.54	1678	16.803

TIC:	18.4776	ng/uL
TIM:	17.7305	nmole/L
Total concentration:	18.6448	ng/uL

Sample peak width (sec): 5 Sample min peak height: 50 Sample baseline V to V?: Y Sample baseline V to V points: 3
Sample filter: Binomial Number of points for filter: 3 Sample start region (min): 0 Sample end region (min): 30
Marker peak width (sec): 5 Marker min peak height: 200 Marker baseline V to V?: Y Marker baseline V to V points: 3
Lower marker selection: First peak > 500 RFU Upper marker selection: Last peak > 200 RFU
Ladder size (bp)75, 200, 300, 400, 500, 700, 1000, 1500, 2000, 3000, 4000, 5000, 7000, 10000, 20000
Quantification using: Lower Marker Final concentration (ng/uL): 0.5000 Dilution factor: 12.0

Sample: SampH8
Well location: H8
Created: Tuesday, August 10, 2021 8:41:15 PM

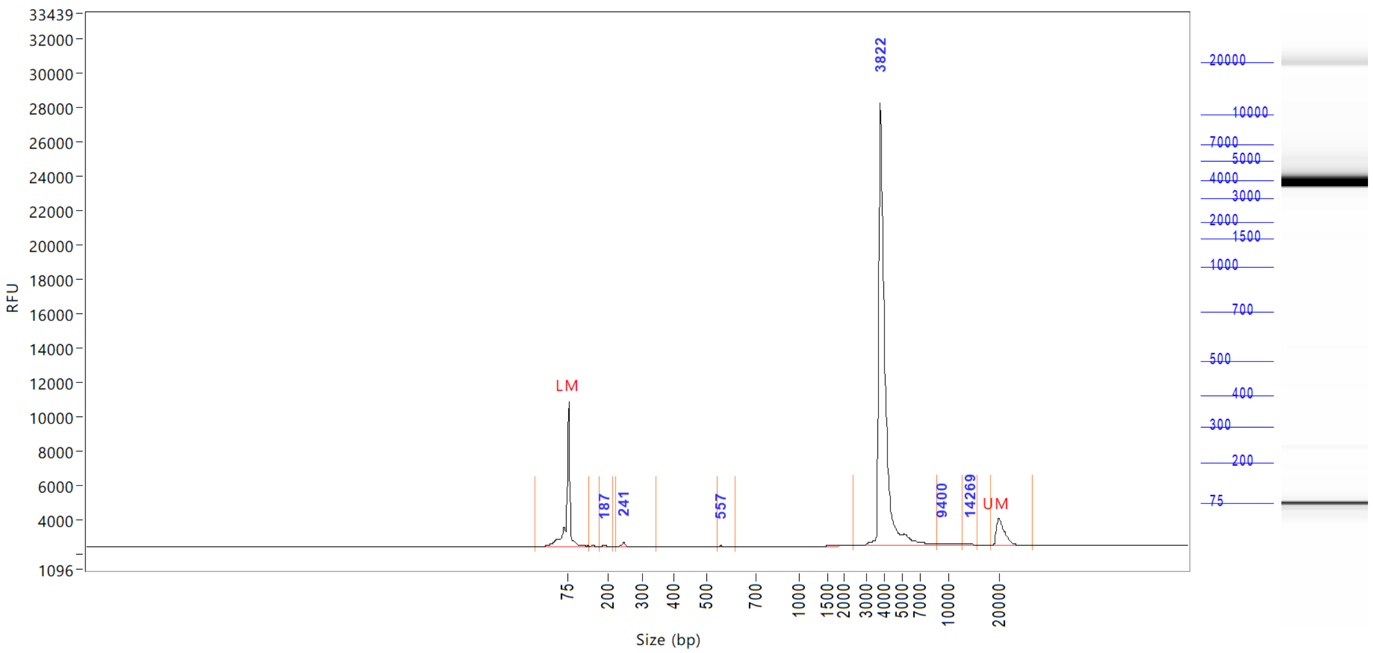

Peak	Size (bp)	Concentration (ng/uL)	From (bp)	To (bp)	Average size (bp)	CV%	RFU	Corrected peak area
1	75 (LM)	0.5000	0	141	67	24.80	8406	68.134
2	187	0.0534	171	210	188	4.67	77	0.607
3	241	0.1554	221	341	246	8.48	250	1.764
4	557	0.0314	544	613	561	2.45	51	0.357
5	3822	20.4228	2401	8934	4022	14.88	25801	231.913
6	9400	0.2035	8934	12684	10354	10.44	104	2.311
7	14269	0.0763	12684	15854	13997	6.13	60	0.867
8	20000 (UM)	0.1194	18415	26649	20529	4.67	1571	16.264

TIC:	20.9428	ng/uL
TIM:	9.9988	nmole/L
Total concentration:	21.1531	ng/uL

```
Sample peak width (sec): 5    Sample min peak height: 50    Sample baseline V to V?: Y    Sample baseline V to V points: 3
Sample filter: Binomial       Number of points for filter: 3  Sample start region (min): 0  Sample end region (min): 30
Marker peak width (sec): 5    Marker min peak height: 200   Marker baseline V to V?: Y    Marker baseline V to V points: 3
Lower marker selection: First peak > 500 RFU                Upper marker selection: Last peak > 200 RFU
Ladder size (bp)75, 200, 300, 400, 500, 700, 1000, 1500, 2000, 3000, 4000, 5000, 7000, 10000, 20000
Quantification using: Lower Marker          Final concentration (ng/uL): 0.5000          Dilution factor: 12.0
```

Sample: SampA9
Well location: A9
Created: Tuesday, August 10, 2021 8:41:15 PM

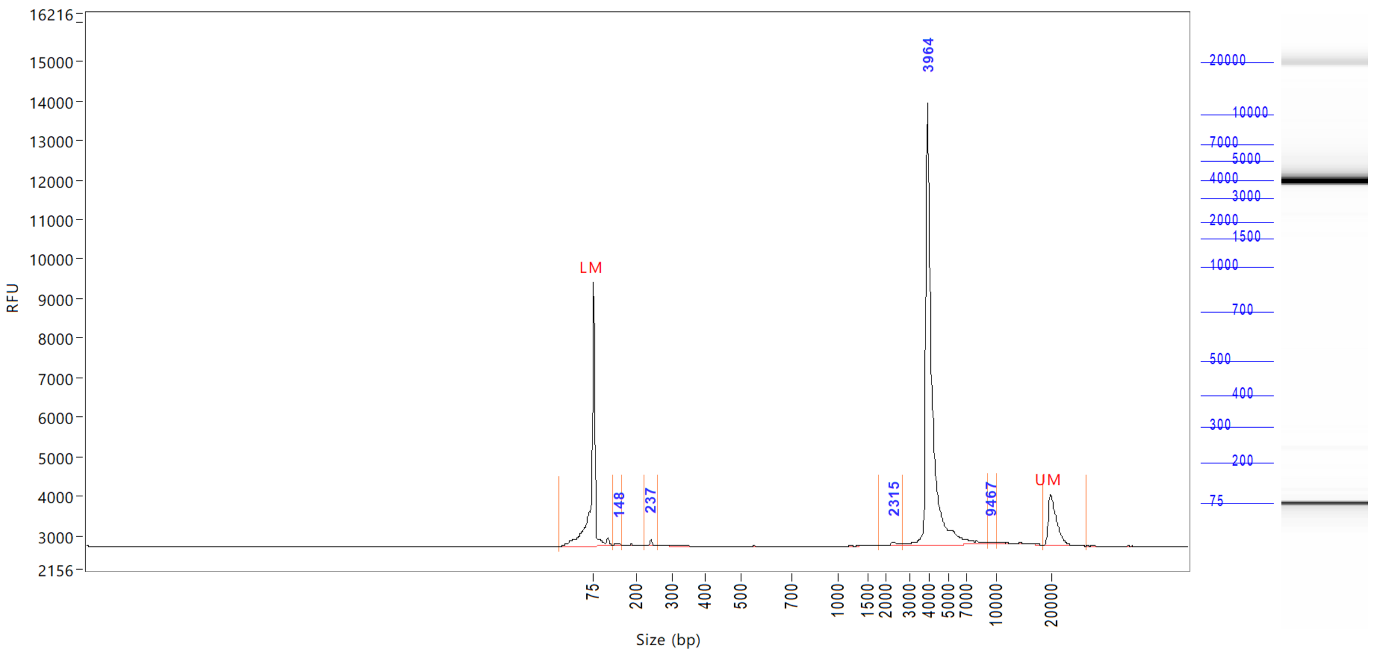

Peak	Size (bp)	Concentration (ng/uL)	From (bp)	To (bp)	Average size (bp)	CV%	RFU	Corrected peak area
1	75 (LM)	0.5000	0	129	65	30.71	6669	56.843
2	148	0.0738	129	158	141	4.82	51	0.699
3	237	0.0984	221	255	235	2.07	159	0.932
4	2315	0.0957	1770	2715	2304	9.03	89	0.907
5	3964	10.3920	2715	9067	4179	15.84	11198	98.452
6	9467	0.0534	9067	10000	9515	2.93	54	0.506
7	20000 (UM)	0.1087	18537	26408	20476	4.36	1269	12.358

TIC:	10.7133	ng/uL
TIM:	5.7201	nmole/L
Total concentration:	11.0597	ng/uL

Sample peak width (sec): 5 Sample min peak height: 50 Sample baseline V to V?: Y Sample baseline V to V points: 3
Sample filter: Binomial Number of points for filter: 3 Sample start region (min): 0 Sample end region (min): 30
Marker peak width (sec): 5 Marker min peak height: 200 Marker baseline V to V?: Y Marker baseline V to V points: 3
Lower marker selection: First peak > 500 RFU Upper marker selection: Last peak > 200 RFU
Ladder size (bp)75, 200, 300, 400, 500, 700, 1000, 1500, 2000, 3000, 4000, 5000, 7000, 10000, 20000
Quantification using: Lower Marker Final concentration (ng/uL): 0.5000 Dilution factor: 12.0

Sample: SampB9
Well location: B9
Created: Tuesday, August 10, 2021 8:41:15 PM

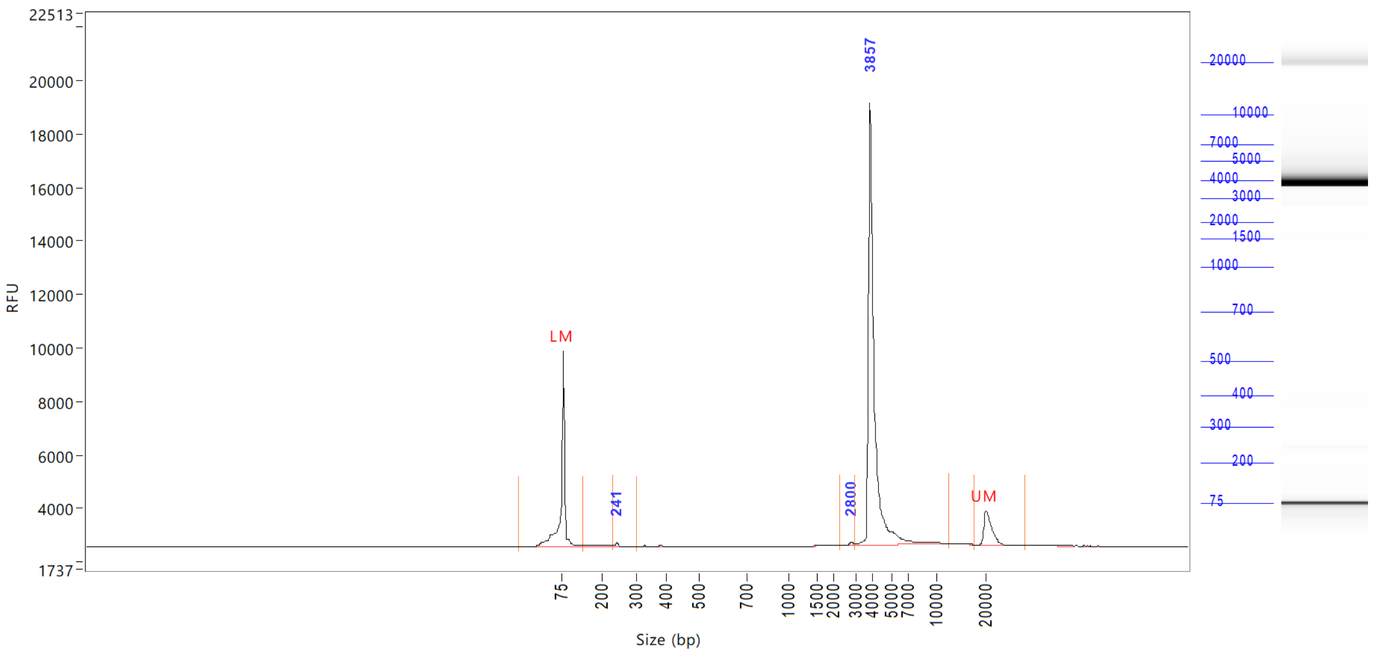

Peak	Size (bp)	Concentration (ng/uL)	From (bp)	To (bp)	Average size (bp)	CV%	RFU	Corrected peak area
1	75 (LM)	0.5000	0	135	65	31.21	7318	62.676
2	241	0.0806	228	300	241	4.27	141	0.842
3	2800	0.0748	2286	3000	2792	5.04	103	0.782
4	3857	13.3727	3000	12440	4114	18.58	16548	139.691
5	20000 (UM)	0.1032	17805	27738	20562	4.58	1276	12.940

TIC:	13.5282	ng/uL
TIM:	5.9454	nmole/L
Total concentration:	13.7984	ng/uL

```
Sample peak width (sec): 5    Sample min peak height: 50    Sample baseline V to V?: Y    Sample baseline V to V points: 3
Sample filter: Binomial       Number of points for filter: 3  Sample start region (min): 0  Sample end region (min): 30
Marker peak width (sec): 5    Marker min peak height: 200   Marker baseline V to V?: Y    Marker baseline V to V points: 3
Lower marker selection: First peak > 500 RFU                Upper marker selection: Last peak > 200 RFU
Ladder size (bp)75, 200, 300, 400, 500, 700, 1000, 1500, 2000, 3000, 4000, 5000, 7000, 10000, 20000
Quantification using: Lower Marker          Final concentration (ng/uL): 0.5000          Dilution factor: 12.0
```

Sample: SampC9
Well location: C9
Created: Tuesday, August 10, 2021 8:41:15 PM

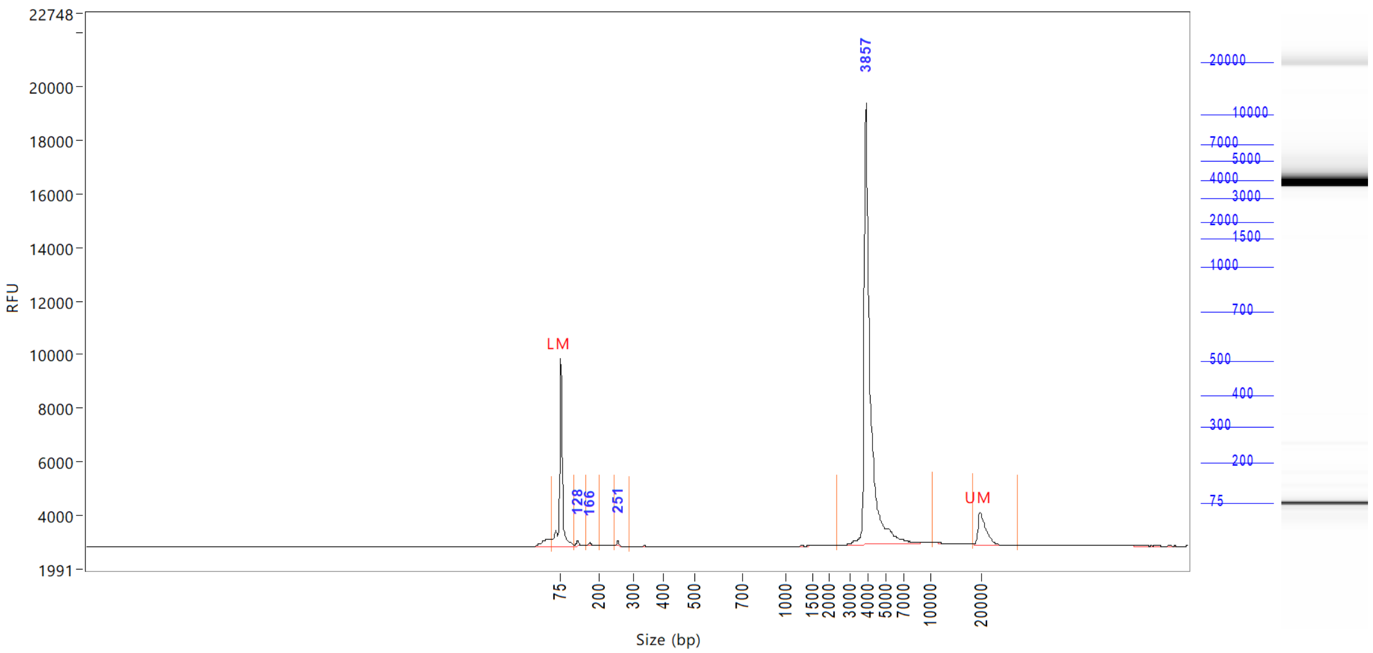

Peak	Size (bp)	Concentration (ng/uL)	From (bp)	To (bp)	Average size (bp)	CV%	RFU	Corrected peak area
1	75 (LM)	0.5000	50	116	74	11.60	7035	49.088
2	128	0.1956	116	154	129	7.19	182	1.600
3	166	0.1473	154	198	169	5.62	144	1.205
4	251	0.1319	242	289	250	2.38	213	1.079
5	3857	18.3924	2344	10245	4082	14.53	16513	150.476
6	20000 (UM)	0.1262	18659	27617	20535	4.96	1217	12.390

TIC:	18.8672	ng/uL
TIM:	12.2094	nmole/L
Total concentration:	19.1259	ng/uL

Sample peak width (sec): 5 Sample min peak height: 50 Sample baseline V to V?: Y Sample baseline V to V points: 3
Sample filter: Binomial Number of points for filter: 3 Sample start region (min): 0 Sample end region (min): 30
Marker peak width (sec): 5 Marker min peak height: 200 Marker baseline V to V?: Y Marker baseline V to V points: 3
Lower marker selection: First peak > 500 RFU Upper marker selection: Last peak > 200 RFU
Ladder size (bp)75, 200, 300, 400, 500, 700, 1000, 1500, 2000, 3000, 4000, 5000, 7000, 10000, 20000
Quantification using: Lower Marker Final concentration (ng/uL): 0.5000 Dilution factor: 12.0

Sample: SampD9
Well location: D9
Created: Tuesday, August 10, 2021 8:41:15 PM

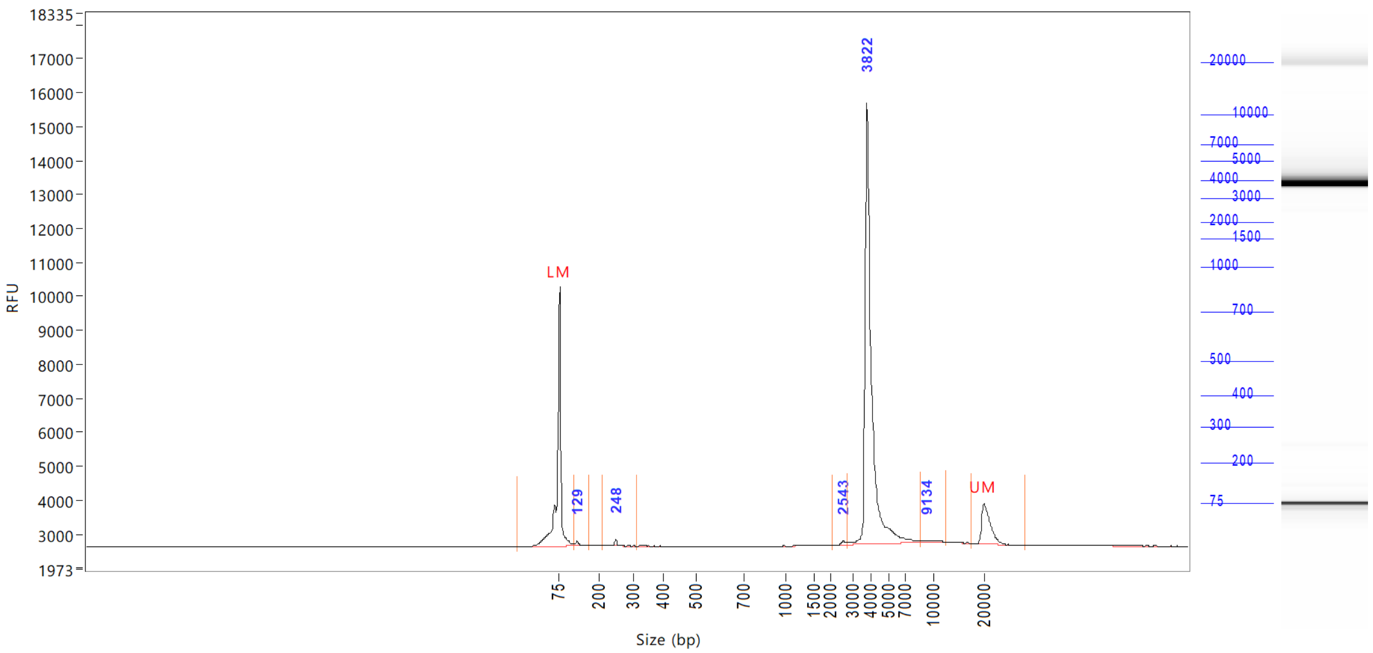

Peak	Size (bp)	Concentration (ng/uL)	From (bp)	To (bp)	Average size (bp)	CV%	RFU	Corrected peak area
1	75 (LM)	0.5000	0	120	66	25.25	7615	66.202
2	129	0.1343	120	169	135	9.45	149	1.482
3	248	0.1247	208	309	242	5.91	174	1.376
4	2543	0.0730	2030	2743	2536	4.90	104	0.805
5	3822	11.2401	2743	8600	4031	14.83	13005	124.019
6	9134	0.0787	8600	12318	9507	7.18	55	0.869
7	20000 (UM)	0.0993	17561	28221	20555	4.85	1205	13.146

TIC:	11.6508	ng/uL
TIM:	7.1337	nmole/L
Total concentration:	11.8019	ng/uL

Sample peak width (sec): 5 Sample min peak height: 50 Sample baseline V to V?: Y Sample baseline V to V points: 3
Sample filter: Binomial Number of points for filter: 3 Sample start region (min): 0 Sample end region (min): 30
Marker peak width (sec): 5 Marker min peak height: 200 Marker baseline V to V?: Y Marker baseline V to V points: 3
Lower marker selection: First peak > 500 RFU Upper marker selection: Last peak > 200 RFU
Ladder size (bp)75, 200, 300, 400, 500, 700, 1000, 1500, 2000, 3000, 4000, 5000, 7000, 10000, 20000
Quantification using: Lower Marker Final concentration (ng/uL): 0.5000 Dilution factor: 12.0

Sample: SampE9
Well location: E9
Created: Tuesday, August 10, 2021 8:41:15 PM

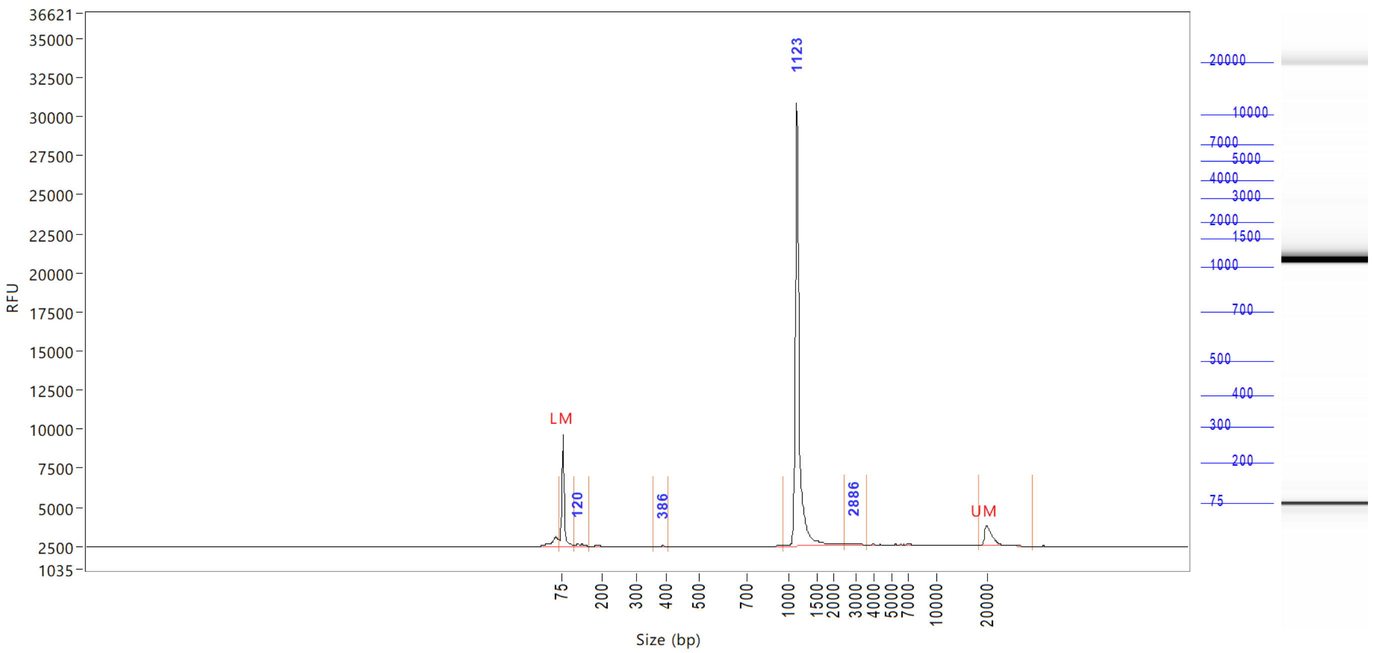

Peak	Size (bp)	Concentration (ng/uL)	From (bp)	To (bp)	Average size (bp)	CV%	RFU	Corrected peak area
1	75 (LM)	0.5000	64	108	74	8.22	7125	44.283
2	120	0.3567	108	156	125	8.90	181	2.633
3	386	0.0636	358	405	385	2.05	65	0.469
4	1123	25.0809	961	2515	1156	11.49	28410	185.112
5	2886	0.2724	2515	3643	2929	9.04	133	2.010
6	20000 (UM)	0.1406	18415	29189	20579	4.67	1248	12.453

TIC:	25.7736	ng/uL
TIM:	40.8250	nmole/L
Total concentration:	26.4052	ng/uL

Sample peak width (sec): 5 Sample min peak height: 50 Sample baseline V to V?: Y Sample baseline V to V points: 3
Sample filter: Binomial Number of points for filter: 3 Sample start region (min): 0 Sample end region (min): 30
Marker peak width (sec): 5 Marker min peak height: 200 Marker baseline V to V?: Y Marker baseline V to V points: 3
Lower marker selection: First peak > 500 RFU Upper marker selection: Last peak > 200 RFU
Ladder size (bp)75, 200, 300, 400, 500, 700, 1000, 1500, 2000, 3000, 4000, 5000, 7000, 10000, 20000
Quantification using: Lower Marker Final concentration (ng/uL): 0.5000 Dilution factor: 12.0

Sample: SampF9
Well location: F9
Created: Tuesday, August 10, 2021 8:41:15 PM

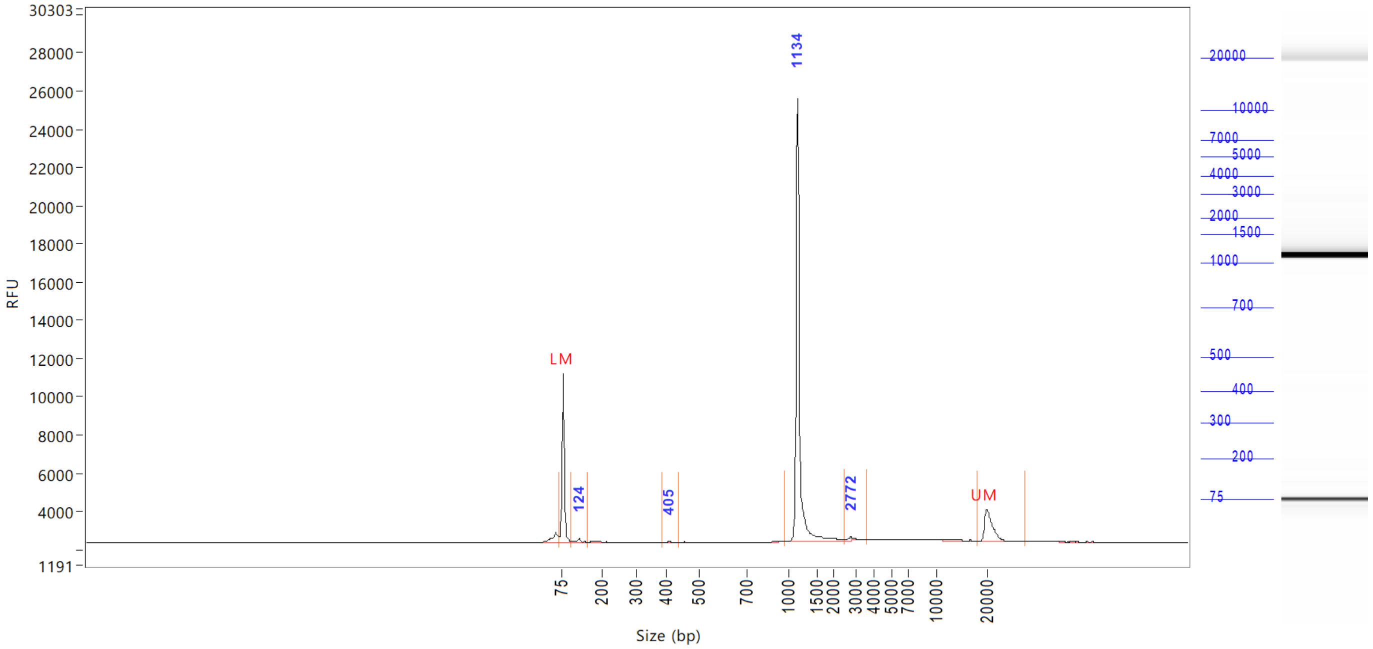

Peak	Size (bp)	Concentration (ng/uL)	From (bp)	To (bp)	Average size (bp)	CV%	RFU	Corrected peak area
1	75 (LM)	0.5000	62	101	73	6.10	8802	51.902
2	124	0.2390	101	150	118	7.72	188	2.067
3	405	0.0558	384	437	406	2.38	62	0.483
4	1134	15.5296	965	2486	1171	12.89	23221	134.336
5	2772	0.2393	2486	3608	2863	8.55	158	2.070
6	20000 (UM)	0.1615	18293	27617	20609	4.69	1626	16.766

TIC:	16.0638	ng/uL
TIM:	25.5198	nmole/L
Total concentration:	16.6448	ng/uL

Sample peak width (sec): 5   Sample min peak height: 50   Sample baseline V to V?: Y   Sample baseline V to V points: 3
Sample filter: Binomial   Number of points for filter: 3   Sample start region (min): 0   Sample end region (min): 30
Marker peak width (sec): 5   Marker min peak height: 200   Marker baseline V to V?: Y   Marker baseline V to V points: 3
Lower marker selection: First peak > 500 RFU   Upper marker selection: Last peak > 200 RFU
Ladder size (bp)75, 200, 300, 400, 500, 700, 1000, 1500, 2000, 3000, 4000, 5000, 7000, 10000, 20000
Quantification using: Lower Marker   Final concentration (ng/uL): 0.5000   Dilution factor: 12.0

Sample: SampG9
Well location: G9
Created: Tuesday, August 10, 2021 8:41:15 PM

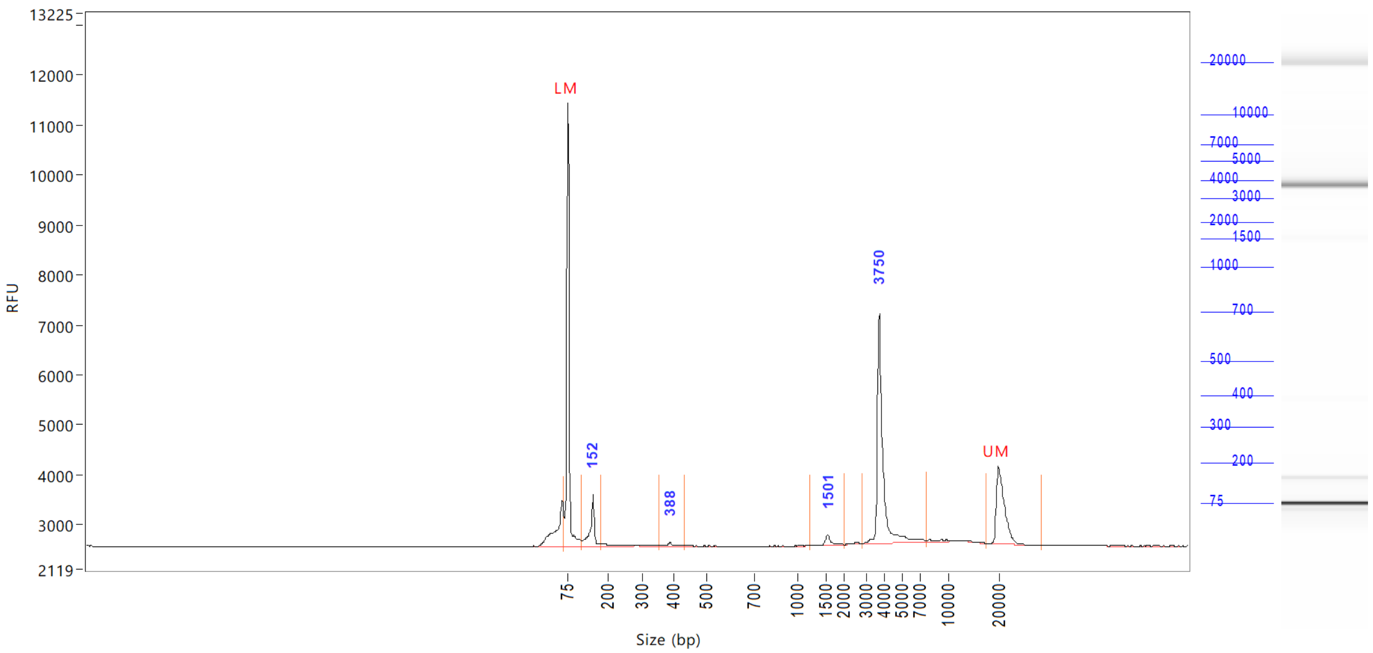

Peak	Size (bp)	Concentration (ng/uL)	From (bp)	To (bp)	Average size (bp)	CV%	RFU	Corrected peak area
1	75 (LM)	0.5000	64	118	74	10.47	8871	54.456
2	152	1.0521	118	177	145	7.56	1033	9.549
3	388	0.0791	354	433	390	4.10	71	0.718
4	1501	0.1856	1212	2030	1534	6.89	205	1.685
5	3750	4.0927	2886	7734	3946	15.48	4595	37.145
6	20000 (UM)	0.1446	17561	28463	20511	4.69	1559	15.751

TIC:	5.4095	ng/uL
TIM:	14.1646	nmole/L
Total concentration:	5.8186	ng/uL

```
Sample peak width (sec): 5    Sample min peak height: 50    Sample baseline V to V?: Y    Sample baseline V to V points: 3
Sample filter: Binomial       Number of points for filter: 3  Sample start region (min): 0  Sample end region (min): 30
Marker peak width (sec): 5    Marker min peak height: 200   Marker baseline V to V?: Y    Marker baseline V to V points: 3
Lower marker selection: First peak > 500 RFU                Upper marker selection: Last peak > 200 RFU
Ladder size (bp)75, 200, 300, 400, 500, 700, 1000, 1500, 2000, 3000, 4000, 5000, 7000, 10000, 20000
Quantification using: Lower Marker          Final concentration (ng/uL): 0.5000         Dilution factor: 12.0
```

Sample: SampH9
Well location: H9
Created: Tuesday, August 10, 2021 8:41:15 PM

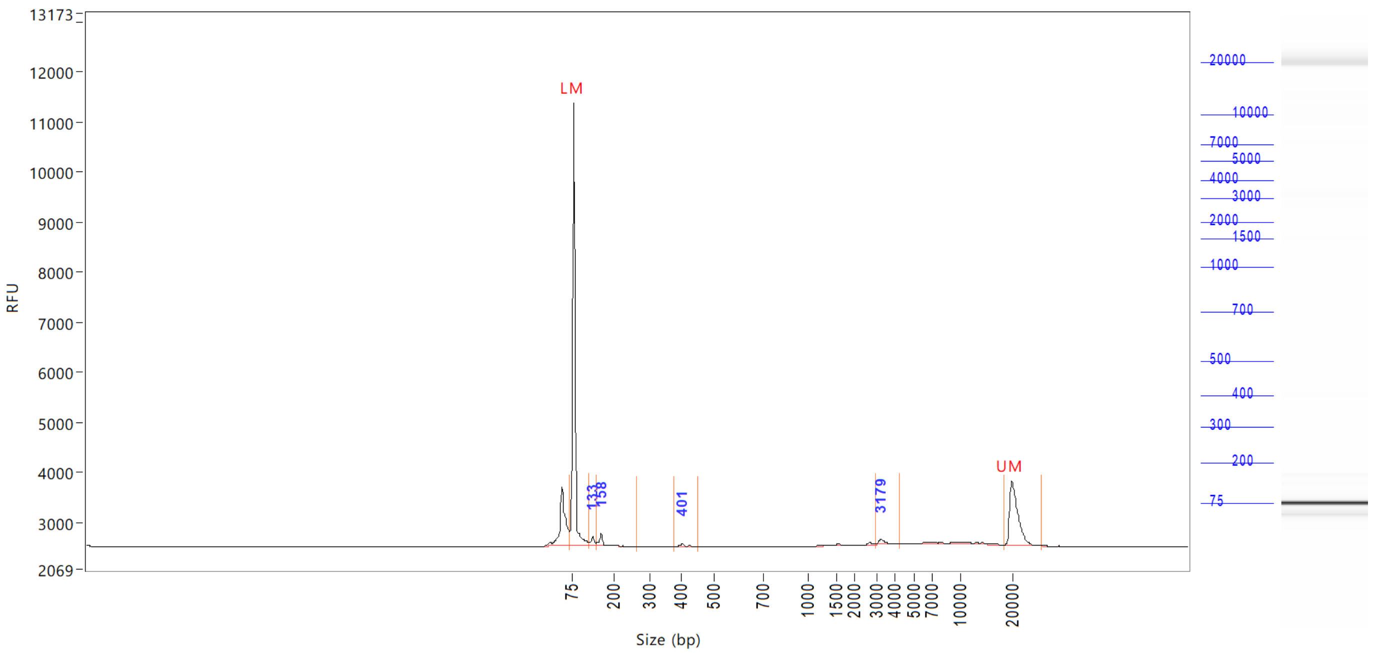

Peak	Size (bp)	Concentration (ng/uL)	From (bp)	To (bp)	Average size (bp)	CV%	RFU	Corrected peak area
1	75 (LM)	0.5000	64	124	75	9.25	8839	54.107
2	133	0.1258	124	143	132	3.60	168	1.135
3	158	0.1898	143	262	158	10.39	240	1.711
4	401	0.0672	378	450	406	3.68	67	0.606
5	3179	0.1129	3000	4242	3329	8.02	106	1.018
6	20000 (UM)	0.1217	18415	25682	20482	4.66	1288	13.167

TIC:	0.4957	ng/uL
TIM:	3.8686	nmole/L
Total concentration:	0.8757	ng/uL

Sample peak width (sec): 5 Sample min peak height: 50 Sample baseline V to V?: Y Sample baseline V to V points: 3
Sample filter: Binomial Number of points for filter: 3 Sample start region (min): 0 Sample end region (min): 30
Marker peak width (sec): 5 Marker min peak height: 200 Marker baseline V to V?: Y Marker baseline V to V points: 3
Lower marker selection: First peak > 500 RFU Upper marker selection: Last peak > 200 RFU
Ladder size (bp)75, 200, 300, 400, 500, 700, 1000, 1500, 2000, 3000, 4000, 5000, 7000, 10000, 20000
Quantification using: Lower Marker Final concentration (ng/uL): 0.5000 Dilution factor: 12.0

Sample: SampA10
Well location: A10
Created: Tuesday, August 10, 2021 8:41:15 PM

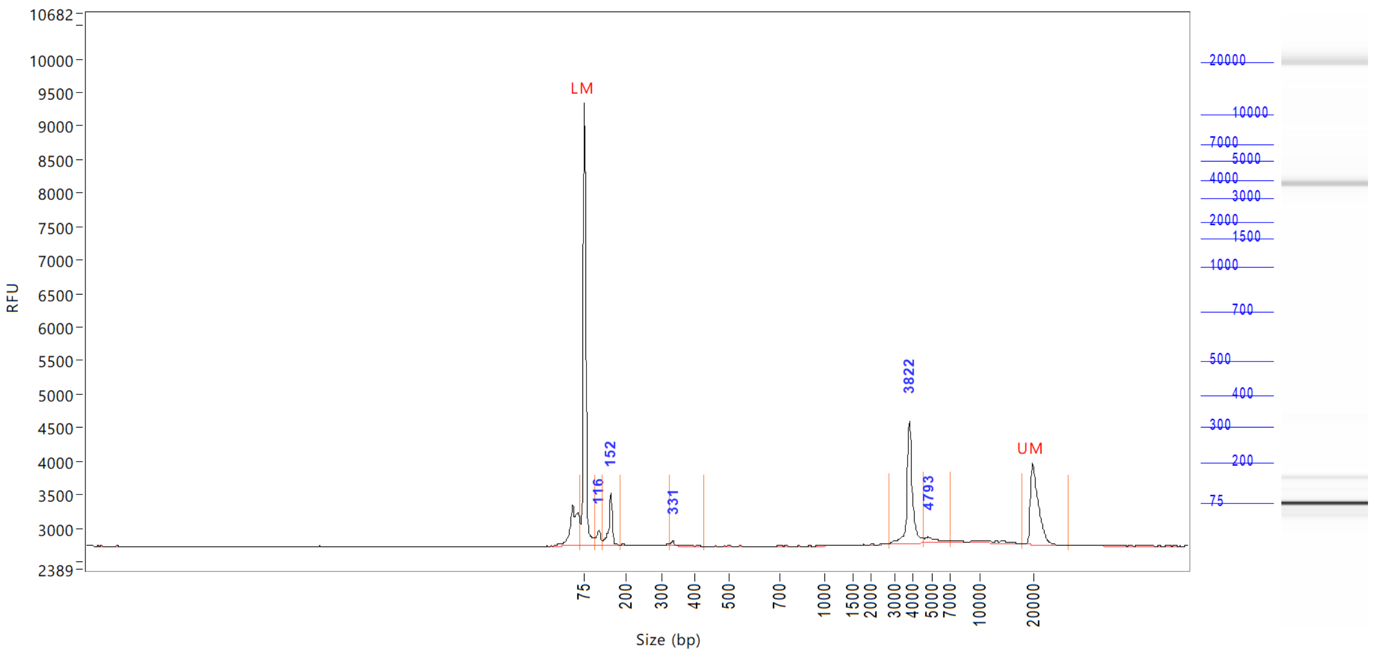

Peak	Size (bp)	Concentration (ng/uL)	From (bp)	To (bp)	Average size (bp)	CV%	RFU	Corrected peak area
1	75 (LM)	0.5000	64	107	74	8.10	6613	40.549
2	116	0.2919	107	128	116	4.81	227	1.972
3	152	0.8600	128	181	148	5.00	773	5.812
4	331	0.1098	321	426	345	7.12	75	0.742
5	3822	2.0381	2800	4587	3806	6.64	1822	13.774
6	4793	0.2262	4587	7000	5308	12.50	89	1.529
7	20000 (UM)	0.1472	18171	26891	20538	4.56	1216	11.938

TIC:	3.5260	ng/uL
TIM:	15.1579	nmole/L
Total concentration:	3.9605	ng/uL

Sample peak width (sec): 5   Sample min peak height: 50   Sample baseline V to V?: Y   Sample baseline V to V points: 3
Sample filter: Binomial   Number of points for filter: 3   Sample start region (min): 0   Sample end region (min): 30
Marker peak width (sec): 5   Marker min peak height: 200   Marker baseline V to V?: Y   Marker baseline V to V points: 3
Lower marker selection: First peak > 500 RFU   Upper marker selection: Last peak > 200 RFU
Ladder size (bp)75, 200, 300, 400, 500, 700, 1000, 1500, 2000, 3000, 4000, 5000, 7000, 10000, 20000
Quantification using: Lower Marker   Final concentration (ng/uL): 0.5000   Dilution factor: 12.0

Sample: SampB10
Well location: B10
Created: Tuesday, August 10, 2021 8:41:15 PM

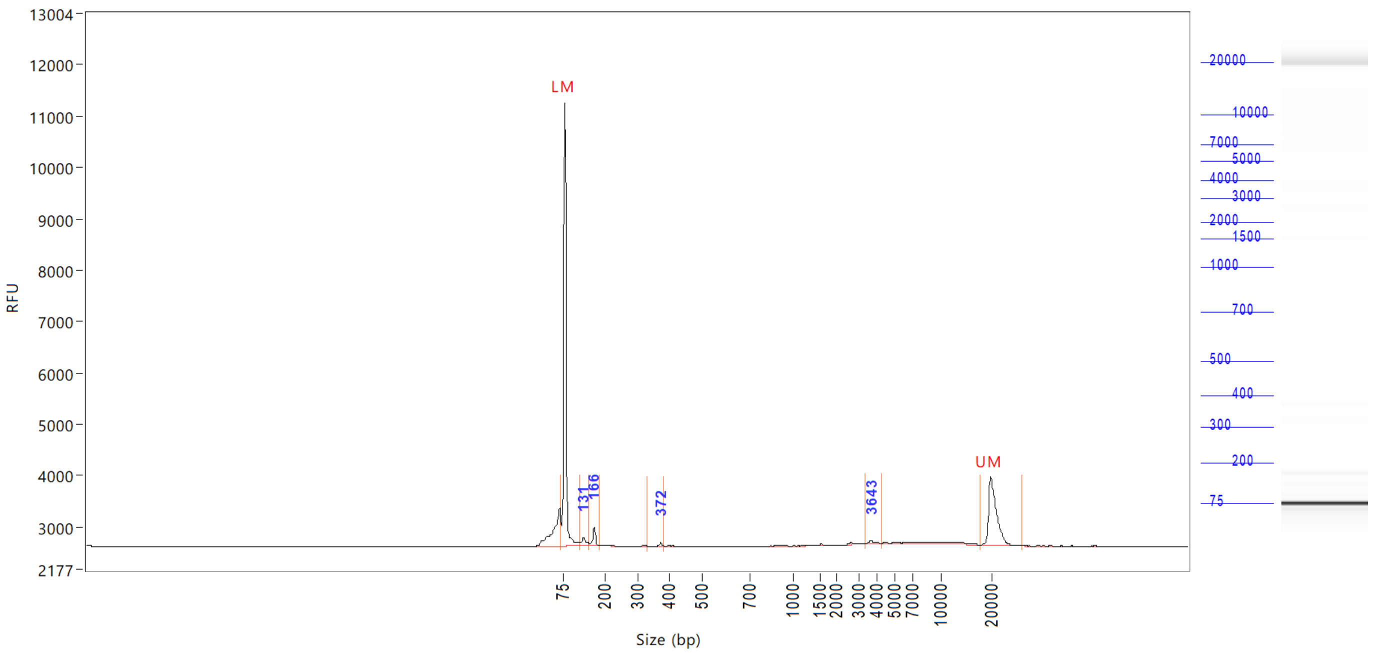

Peak	Size (bp)	Concentration (ng/uL)	From (bp)	To (bp)	Average size (bp)	CV%	RFU	Corrected peak area
1	75 (LM)	0.5000	64	122	74	9.45	8644	53.638
2	131	0.1915	122	152	134	6.29	156	1.712
3	166	0.2492	152	183	163	2.60	371	2.227
4	372	0.0647	329	384	363	3.39	71	0.578
5	3643	0.0620	3322	4242	3733	5.55	59	0.554
6	20000 (UM)	0.1248	17927	26287	20505	4.47	1327	13.385

TIC:	0.5673	ng/uL
TIM:	5.1815	nmole/L
Total concentration:	1.0558	ng/uL

Sample peak width (sec): 5 Sample min peak height: 50 Sample baseline V to V?: Y Sample baseline V to V points: 3
Sample filter: Binomial Number of points for filter: 3 Sample start region (min): 0 Sample end region (min): 30
Marker peak width (sec): 5 Marker min peak height: 200 Marker baseline V to V?: Y Marker baseline V to V points: 3
Lower marker selection: First peak > 500 RFU Upper marker selection: Last peak > 200 RFU
Ladder size (bp)75, 200, 300, 400, 500, 700, 1000, 1500, 2000, 3000, 4000, 5000, 7000, 10000, 20000
Quantification using: Lower Marker Final concentration (ng/uL): 0.5000 Dilution factor: 12.0

Sample: SampC10
Well location: C10
Created: Tuesday, August 10, 2021 8:41:15 PM

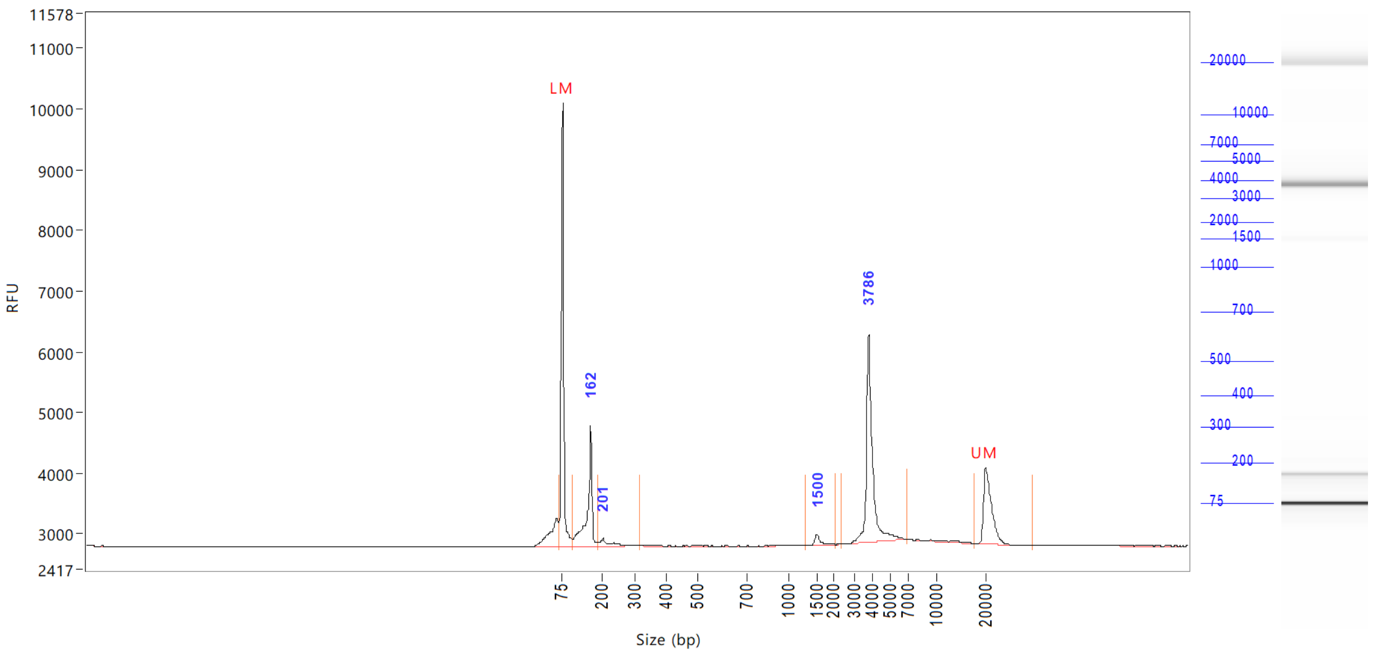

Peak	Size (bp)	Concentration (ng/uL)	From (bp)	To (bp)	Average size (bp)	CV%	RFU	Corrected peak area
1	75 (LM)	0.5000	64	105	74	7.96	7316	45.144
2	162	2.7633	105	187	149	11.17	1992	20.791
3	201	0.3516	187	309	217	10.63	124	2.646
4	1500	0.1934	1300	2115	1531	7.47	181	1.455
5	3786	4.0636	2344	7000	3871	10.36	3428	30.575
6	20000 (UM)	0.1431	17683	29430	20533	5.15	1260	12.922

TIC:	7.3720	ng/uL
TIM:	35.0806	nmole/L
Total concentration:	7.6830	ng/uL

```
Sample peak width (sec): 5    Sample min peak height: 50    Sample baseline V to V?: Y    Sample baseline V to V points: 3
Sample filter: Binomial       Number of points for filter: 3  Sample start region (min): 0  Sample end region (min): 30
Marker peak width (sec): 5    Marker min peak height: 200   Marker baseline V to V?: Y    Marker baseline V to V points: 3
Lower marker selection: First peak > 500 RFU                Upper marker selection: Last peak > 200 RFU
Ladder size (bp)75, 200, 300, 400, 500, 700, 1000, 1500, 2000, 3000, 4000, 5000, 7000, 10000, 20000
Quantification using: Lower Marker          Final concentration (ng/uL): 0.5000          Dilution factor: 12.0
```

Sample: SampD10
Well location: D10
Created: Tuesday, August 10, 2021 8:41:15 PM

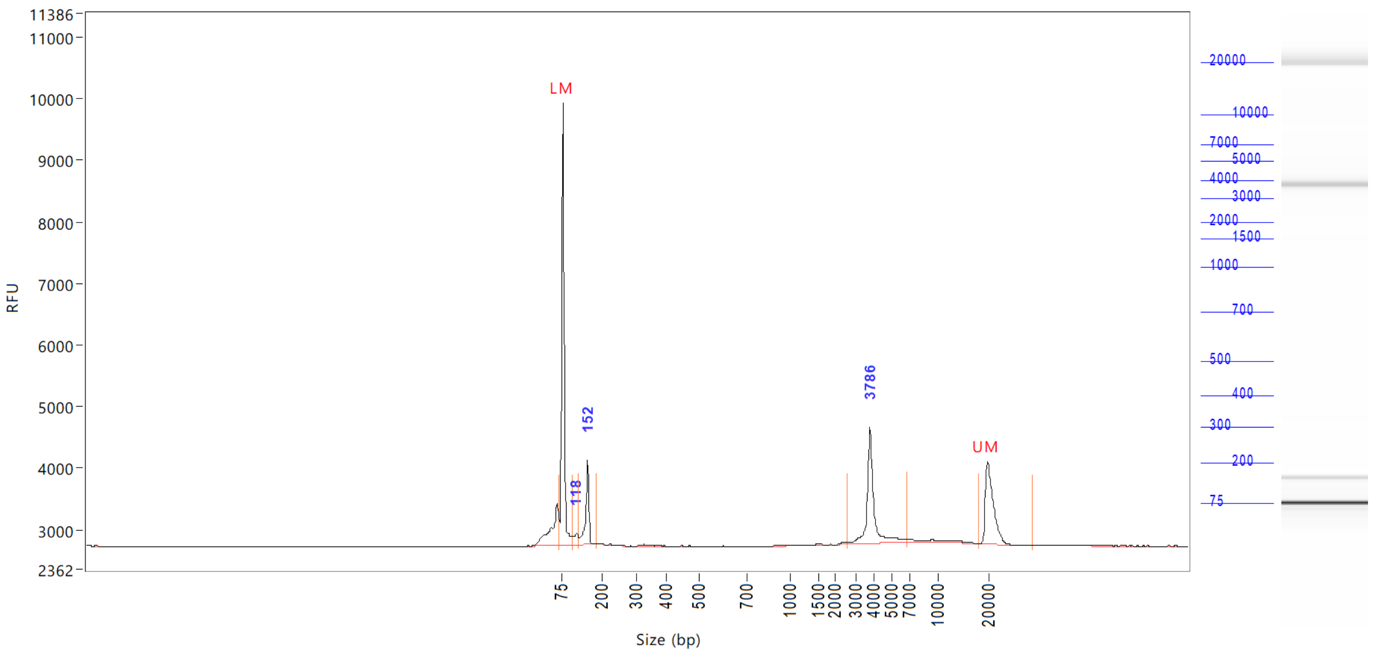

Peak	Size (bp)	Concentration (ng/uL)	From (bp)	To (bp)	Average size (bp)	CV%	RFU	Corrected peak area
1	75 (LM)	0.5000	66	108	75	8.23	7190	45.479
2	118	0.2477	108	126	117	4.34	196	1.877
3	152	1.3437	126	183	147	5.01	1375	10.185
4	3786	2.3711	2600	6778	3896	14.83	1890	17.973
5	20000 (UM)	0.1495	18293	28947	20518	4.78	1332	13.601

TIC:	3.9624	ng/uL
TIM:	19.5014	nmole/L
Total concentration:	4.4625	ng/uL

```
Sample peak width (sec): 5    Sample min peak height: 50    Sample baseline V to V?: Y    Sample baseline V to V points: 3
Sample filter: Binomial       Number of points for filter: 3  Sample start region (min): 0  Sample end region (min): 30
Marker peak width (sec): 5    Marker min peak height: 200   Marker baseline V to V?: Y    Marker baseline V to V points: 3
Lower marker selection: First peak > 500 RFU                Upper marker selection: Last peak > 200 RFU
Ladder size (bp)75, 200, 300, 400, 500, 700, 1000, 1500, 2000, 3000, 4000, 5000, 7000, 10000, 20000
Quantification using: Lower Marker          Final concentration (ng/uL): 0.5000          Dilution factor: 12.0
```

Sample: SampE10
Well location: E10
Created: Tuesday, August 10, 2021 8:41:15 PM

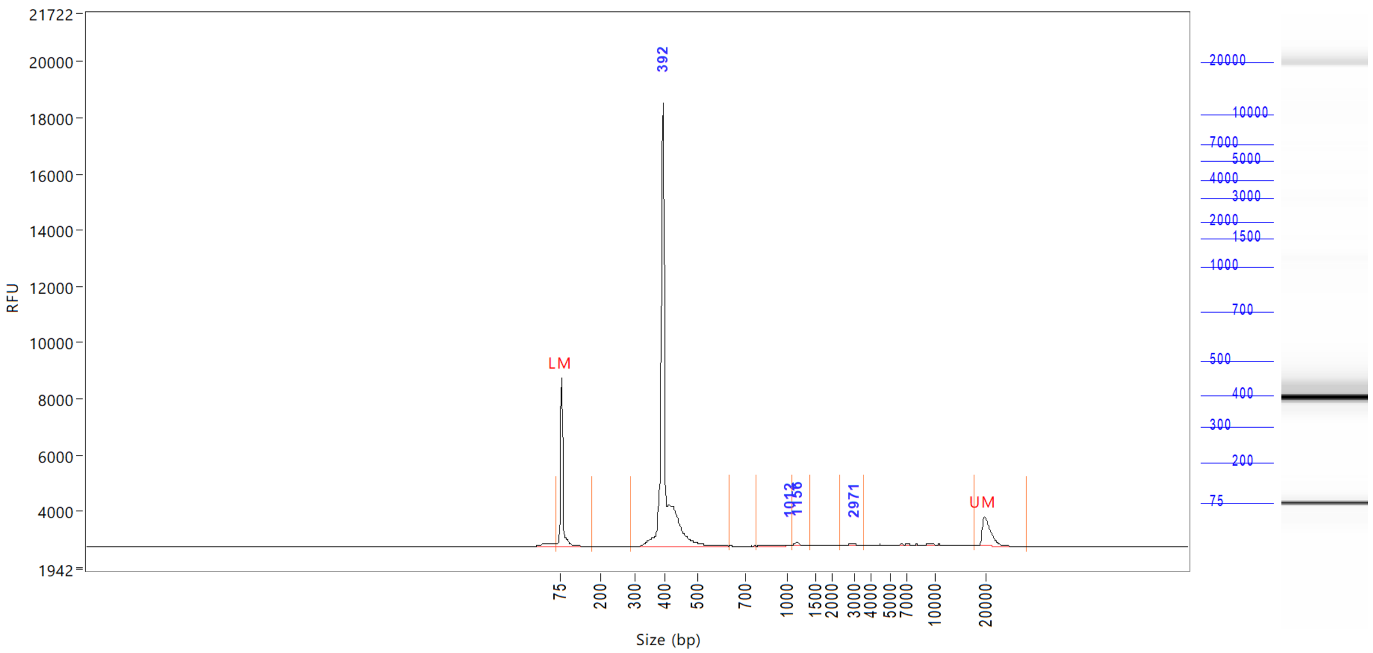

Peak	Size<br>(bp)	Concentration<br>(ng/uL)	From<br>(bp)	To<br>(bp)	Average size<br>(bp)	CV%	RFU	Corrected peak area
1	75 (LM)	0.5000	62	171	76	13.89	6018	37.508
2	392	22.2436	289	636	400	7.06	15804	139.054
3	1012	0.2424	775	1068	938	7.36	51	1.515
4	1156	0.2046	1068	1411	1168	5.65	138	1.279
5	2971	0.1290	2372	3572	2957	8.04	60	0.807
6	20000 (UM)	0.1471	17927	28463	20579	5.03	1055	11.034

TIC:	22.8196	ng/uL
TIM:	92.2785	nmole/L
Total concentration:	23.1815	ng/uL

Sample peak width (sec): 5 Sample min peak height: 50 Sample baseline V to V?: Y Sample baseline V to V points: 3
Sample filter: Binomial Number of points for filter: 3 Sample start region (min): 0 Sample end region (min): 30
Marker peak width (sec): 5 Marker min peak height: 200 Marker baseline V to V?: Y Marker baseline V to V points: 3
Lower marker selection: First peak > 500 RFU Upper marker selection: Last peak > 200 RFU
Ladder size (bp)75, 200, 300, 400, 500, 700, 1000, 1500, 2000, 3000, 4000, 5000, 7000, 10000, 20000
Quantification using: Lower Marker Final concentration (ng/uL): 0.5000 Dilution factor: 12.0

Sample: SampF10
Well location: F10
Created: Tuesday, August 10, 2021 8:41:15 PM

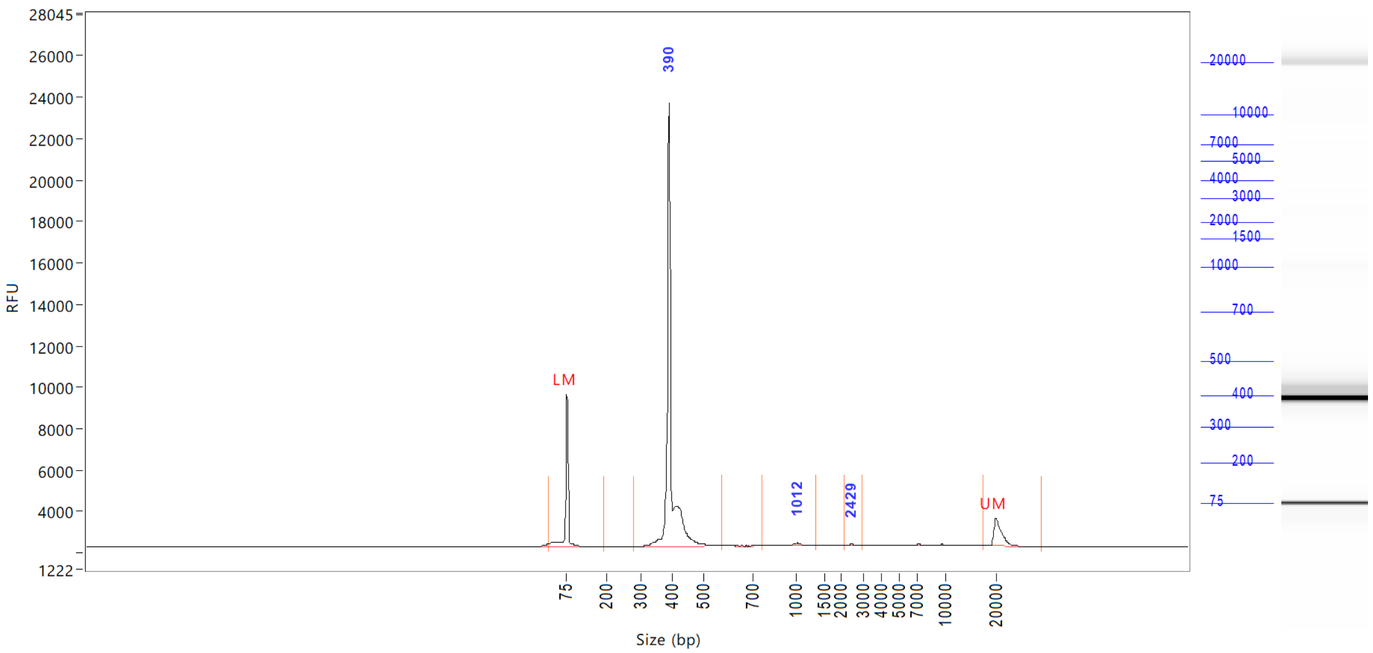

Peak	Size (bp)	Concentration (ng/uL)	From (bp)	To (bp)	Average size (bp)	CV%	RFU	Corrected peak area
1	75 (LM)	0.5000	20	192	71	20.76	7355	49.152
2	390	20.8144	277	567	395	5.81	21429	170.512
3	1012	0.3246	766	1345	995	10.25	137	2.659
4	2429	0.0673	2115	2971	2492	6.33	62	0.552
5	20000 (UM)	0.1330	17683	28947	20596	4.80	1313	13.077

TIC:	21.2063	ng/uL
TIM:	87.2788	nmole/L
Total concentration:	21.5219	ng/uL

```
Sample peak width (sec): 5    Sample min peak height: 50    Sample baseline V to V?: Y    Sample baseline V to V points: 3
Sample filter: Binomial       Number of points for filter: 3  Sample start region (min): 0  Sample end region (min): 30
Marker peak width (sec): 5    Marker min peak height: 200   Marker baseline V to V?: Y    Marker baseline V to V points: 3
Lower marker selection: First peak > 500 RFU                Upper marker selection: Last peak > 200 RFU
Ladder size (bp)75, 200, 300, 400, 500, 700, 1000, 1500, 2000, 3000, 4000, 5000, 7000, 10000, 20000
Quantification using: Lower Marker          Final concentration (ng/uL): 0.5000          Dilution factor: 12.0
```

Sample: SampG10
Well location: G10
Created: Tuesday, August 10, 2021 8:41:15 PM

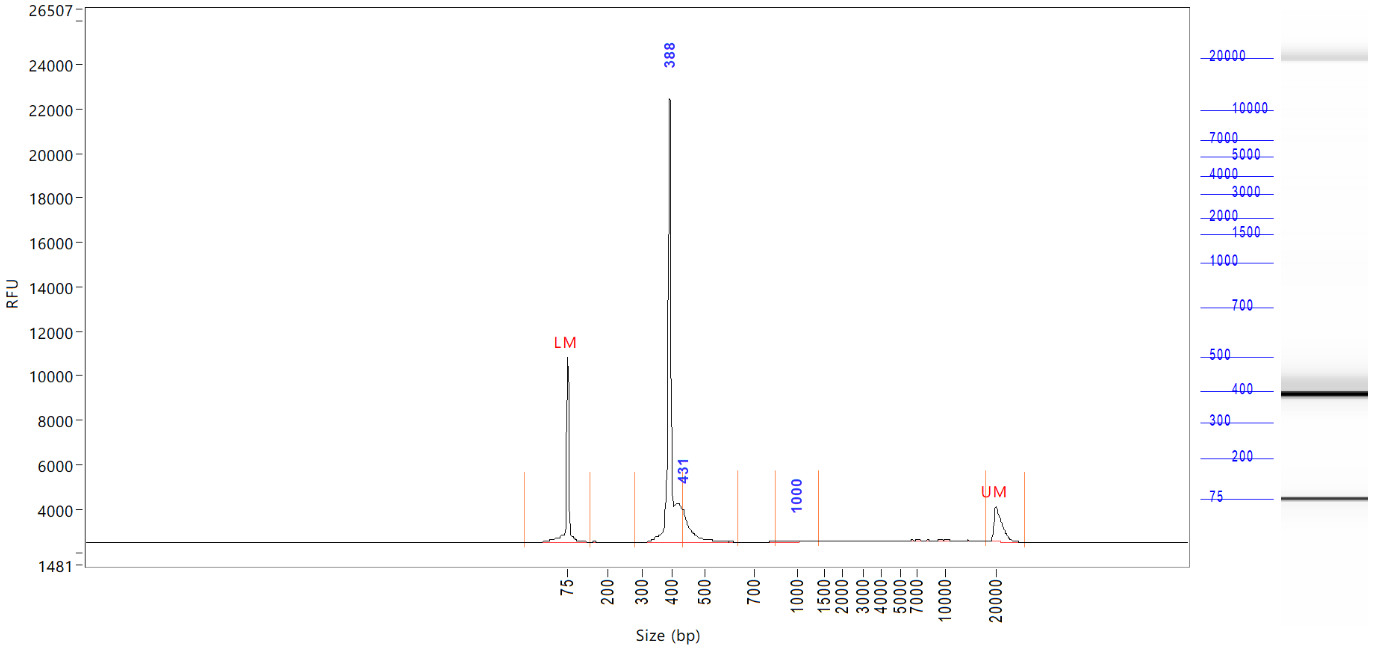

Peak	Size (bp)	Concentration (ng/uL)	From (bp)	To (bp)	Average size (bp)	CV%	RFU	Corrected peak area
1	75 (LM)	0.5000	0	145	70	25.43	8347	57.561
2	388	15.9391	278	431	390	4.14	20000	152.913
3	431	2.1035	431	639	459	6.66	1442	20.180
4	1000	0.1993	853	1378	1008	10.21	65	1.912
5	20000 (UM)	0.1386	18049	25682	20569	4.59	1580	15.953

TIC:	18.2419	ng/uL
TIM:	75.1071	nmole/L
Total concentration:	18.4562	ng/uL

Sample peak width (sec): 5 Sample min peak height: 50 Sample baseline V to V?: Y Sample baseline V to V points: 3
Sample filter: Binomial Number of points for filter: 3 Sample start region (min): 0 Sample end region (min): 30
Marker peak width (sec): 5 Marker min peak height: 200 Marker baseline V to V?: Y Marker baseline V to V points: 3
Lower marker selection: First peak > 500 RFU Upper marker selection: Last peak > 200 RFU
Ladder size (bp)75, 200, 300, 400, 500, 700, 1000, 1500, 2000, 3000, 4000, 5000, 7000, 10000, 20000
Quantification using: Lower Marker Final concentration (ng/uL): 0.5000 Dilution factor: 12.0

Sample: SampH10
Well location: H10
Created: Tuesday, August 10, 2021 8:41:15 PM

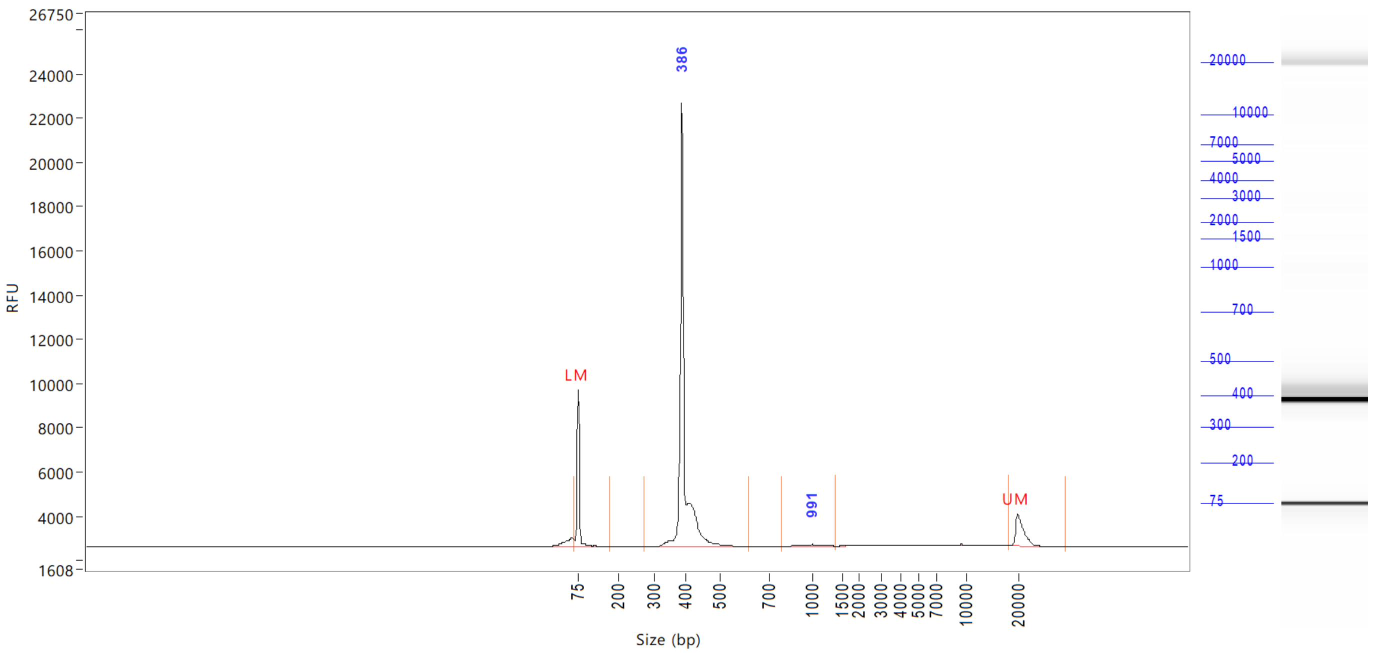

Peak	Size (bp)	Concentration (ng/uL)	From (bp)	To (bp)	Average size (bp)	CV%	RFU	Corrected peak area
1	75 (LM)	0.5000	64	173	74	11.59	7059	41.346
2	386	24.1760	269	616	393	6.66	20097	166.597
3	991	0.3157	779	1389	992	10.07	76	2.176
4	20000 (UM)	0.1741	18293	29068	20567	4.69	1429	14.399

TIC:	24.4917	ng/uL
TIM:	101.7354	nmole/L
Total concentration:	24.9693	ng/uL

Sample peak width (sec): 5   Sample min peak height: 50   Sample baseline V to V?: Y   Sample baseline V to V points: 3
Sample filter: Binomial   Number of points for filter: 3   Sample start region (min): 0   Sample end region (min): 30
Marker peak width (sec): 5   Marker min peak height: 200   Marker baseline V to V?: Y   Marker baseline V to V points: 3
Lower marker selection: First peak > 500 RFU   Upper marker selection: Last peak > 200 RFU
Ladder size (bp)75, 200, 300, 400, 500, 700, 1000, 1500, 2000, 3000, 4000, 5000, 7000, 10000, 20000
Quantification using: Lower Marker   Final concentration (ng/uL): 0.5000   Dilution factor: 12.0

Sample: SampA11
Well location: A11
Created: Tuesday, August 10, 2021 8:41:15 PM

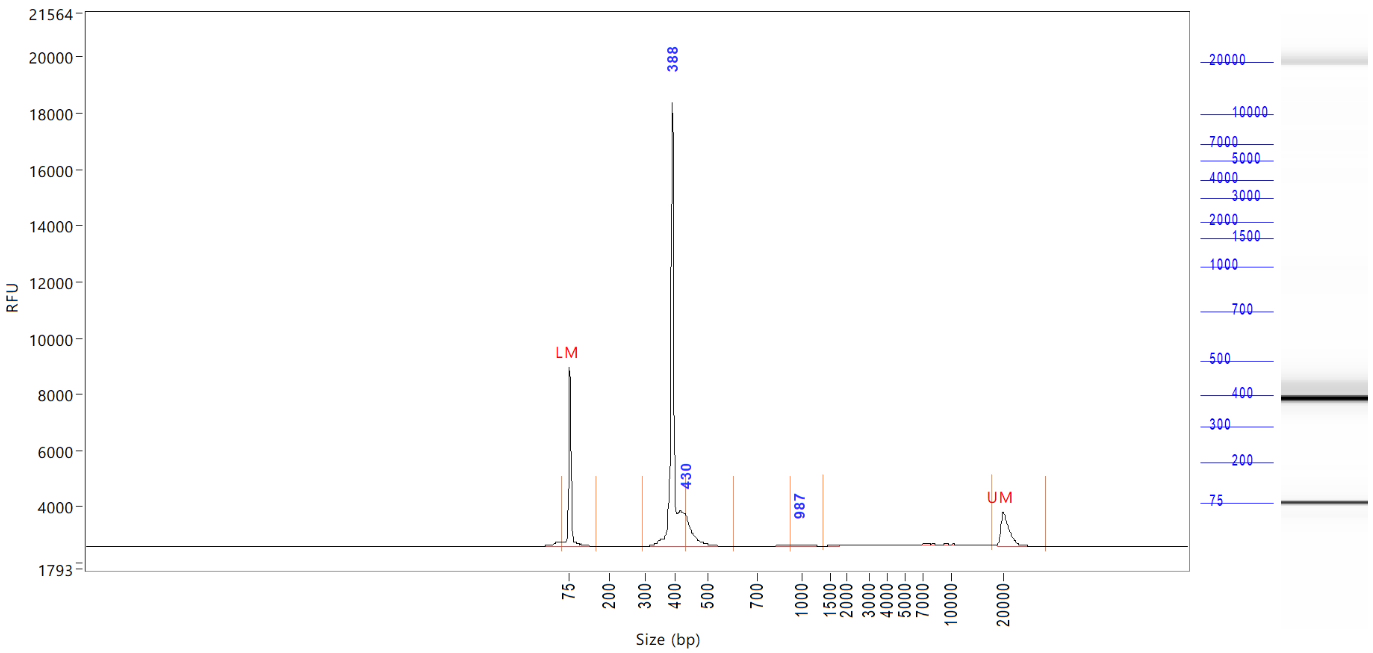

Peak	Size (bp)	Concentration (ng/uL)	From (bp)	To (bp)	Average size (bp)	CV%	RFU	Corrected peak area
1	75 (LM)	0.5000	54	158	75	13.63	6359	38.843
2	388	18.0466	293	430	389	4.11	15808	116.830
3	430	2.3727	430	603	454	5.94	1119	15.360
4	987	0.1718	926	1400	1032	9.02	52	1.112
5	20000 (UM)	0.1595	17805	28342	20647	5.23	1200	12.393

TIC:	20.5911	ng/uL
TIM:	85.2006	nmole/L
Total concentration:	21.0686	ng/uL

```
Sample peak width (sec): 5    Sample min peak height: 50    Sample baseline V to V?: Y    Sample baseline V to V points: 3
Sample filter: Binomial       Number of points for filter: 3  Sample start region (min): 0  Sample end region (min): 30
Marker peak width (sec): 5    Marker min peak height: 200   Marker baseline V to V?: Y    Marker baseline V to V points: 3
Lower marker selection: First peak > 500 RFU                Upper marker selection: Last peak > 200 RFU
Ladder size (bp)75, 200, 300, 400, 500, 700, 1000, 1500, 2000, 3000, 4000, 5000, 7000, 10000, 20000
Quantification using: Lower Marker          Final concentration (ng/uL): 0.5000          Dilution factor: 12.0
```

Sample: SampB11
Well location: B11
Created: Tuesday, August 10, 2021 8:41:15 PM

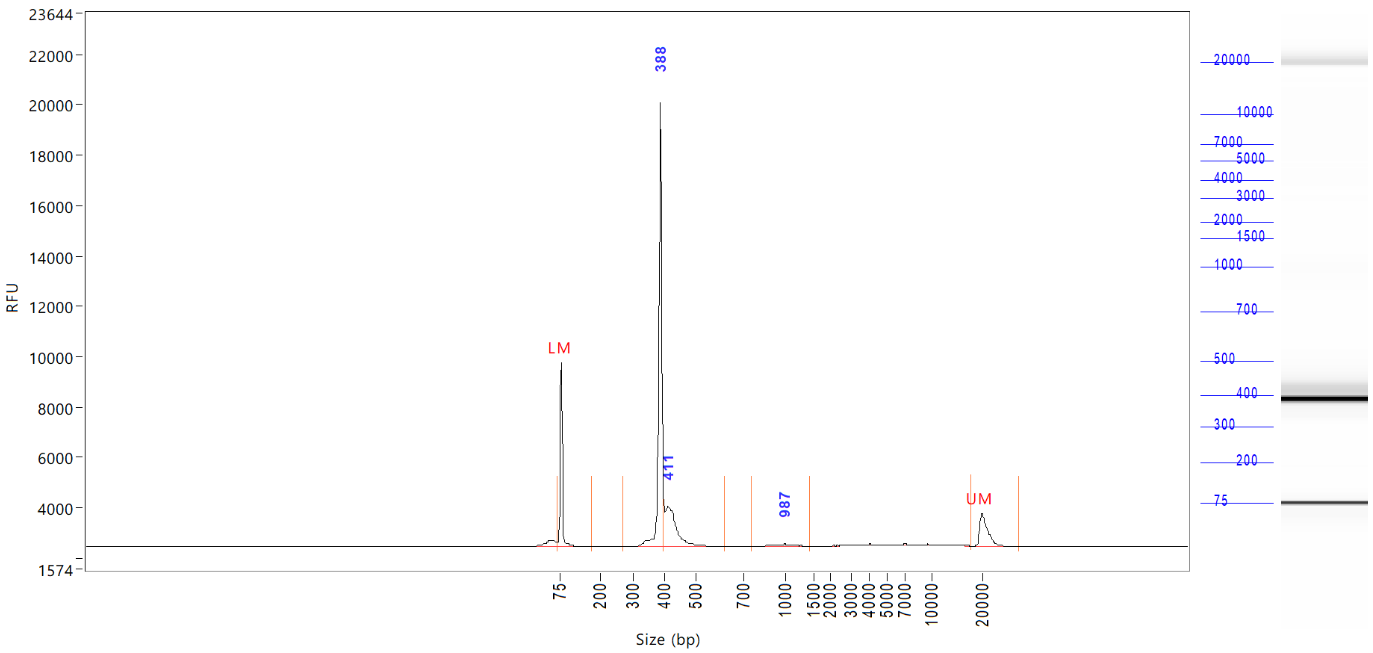

Peak	Size (bp)	Concentration (ng/uL)	From (bp)	To (bp)	Average size (bp)	CV%	RFU	Corrected peak area
1	75 (LM)	0.5000	64	171	75	12.36	7301	41.811
2	388	16.1154	268	400	382	3.06	17645	112.299
3	411	5.2519	400	616	426	6.26	1571	36.598
4	987	0.3343	757	1445	986	11.09	75	2.329
5	20000 (UM)	0.1585	17927	27496	20550	4.98	1312	13.253

TIC:	21.7016	ng/uL
TIM:	90.2504	nmole/L
Total concentration:	22.1574	ng/uL

Sample peak width (sec): 5 Sample min peak height: 50 Sample baseline V to V?: Y Sample baseline V to V points: 3
Sample filter: Binomial Number of points for filter: 3 Sample start region (min): 0 Sample end region (min): 30
Marker peak width (sec): 5 Marker min peak height: 200 Marker baseline V to V?: Y Marker baseline V to V points: 3
Lower marker selection: First peak > 500 RFU Upper marker selection: Last peak > 200 RFU
Ladder size (bp)75, 200, 300, 400, 500, 700, 1000, 1500, 2000, 3000, 4000, 5000, 7000, 10000, 20000
Quantification using: Lower Marker Final concentration (ng/uL): 0.5000 Dilution factor: 12.0

Sample: SampC11
Well location: C11
Created: Tuesday, August 10, 2021 8:41:15 PM

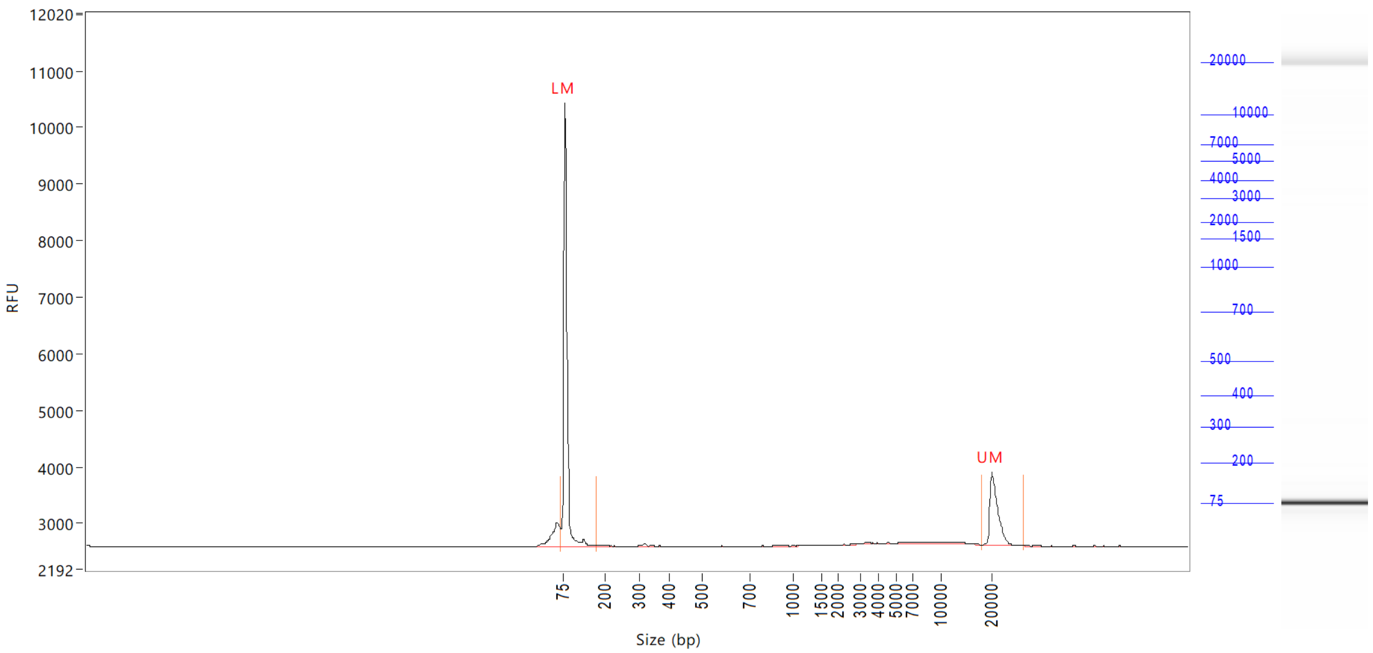

Peak	Size (bp)	Concentration (ng/uL)	From (bp)	To (bp)	Average size (bp)	CV%	RFU	Corrected peak area
1	75 (LM)	0.5000	62	175	77	15.62	7856	63.997
2	20000 (UM)	0.1021	17927	26166	20526	4.59	1278	13.062

TIC:	0.0000	ng/uL
TIM:	0.0000	nmole/L
Total concentration:	0.4532	ng/uL

```
Sample peak width (sec): 5    Sample min peak height: 50    Sample baseline V to V?: Y    Sample baseline V to V points: 3
Sample filter: Binomial       Number of points for filter: 3  Sample start region (min): 0  Sample end region (min): 30
Marker peak width (sec): 5    Marker min peak height: 200   Marker baseline V to V?: Y    Marker baseline V to V points: 3
Lower marker selection: First peak > 500 RFU                Upper marker selection: Last peak > 200 RFU
Ladder size (bp)75, 200, 300, 400, 500, 700, 1000, 1500, 2000, 3000, 4000, 5000, 7000, 10000, 20000
Quantification using: Lower Marker          Final concentration (ng/uL): 0.5000          Dilution factor: 12.0
```

Sample: SampD11
Well location: D11
Created: Tuesday, August 10, 2021 8:41:15 PM

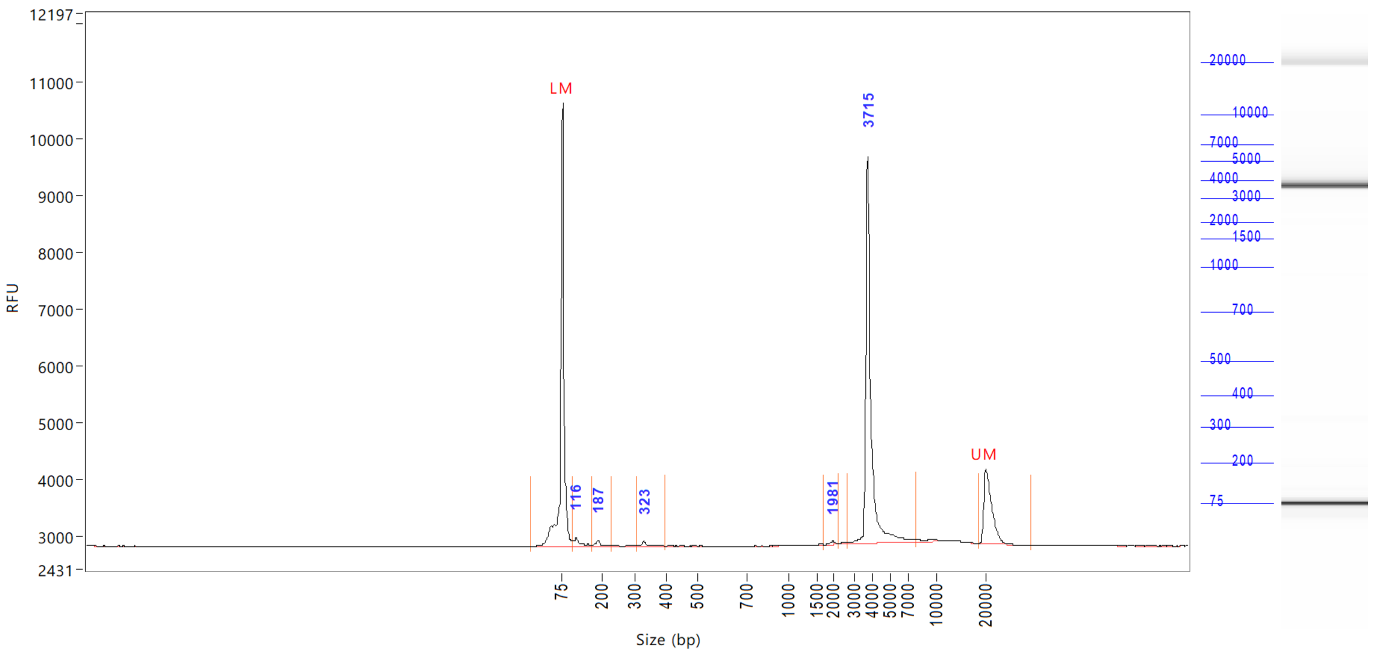

Peak	Size	Concentration	From	To	Average size	CV%	RFU	Corrected peak area
	(bp)	(ng/uL)	(bp)	(bp)	(bp)			
1	75 (LM)	0.5000	0	108	69	20.58	7804	63.515
2	116	0.2135	108	168	128	13.43	164	2.260
3	187	0.1205	168	226	189	7.03	109	1.275
4	323	0.0917	300	398	333	6.31	91	0.971
5	1981	0.0408	1712	2258	1979	5.13	56	0.432
6	3715	4.8801	2657	7867	3888	14.87	6790	51.659
7	20000 (UM)	0.1047	18537	29189	20591	4.86	1299	13.300

TIC:	5.3466	ng/uL
TIM:	6.3424	nmole/L
Total concentration:	5.5978	ng/uL

```
Sample peak width (sec): 5    Sample min peak height: 50    Sample baseline V to V?: Y    Sample baseline V to V points: 3
Sample filter: Binomial       Number of points for filter: 3  Sample start region (min): 0  Sample end region (min): 30
Marker peak width (sec): 5    Marker min peak height: 200   Marker baseline V to V?: Y    Marker baseline V to V points: 3
Lower marker selection: First peak > 500 RFU                Upper marker selection: Last peak > 200 RFU
Ladder size (bp)75, 200, 300, 400, 500, 700, 1000, 1500, 2000, 3000, 4000, 5000, 7000, 10000, 20000
Quantification using: Lower Marker          Final concentration (ng/uL): 0.5000          Dilution factor: 12.0
```

Sample: SampE11
Well location: E11
Created: Tuesday, August 10, 2021 8:41:15 PM

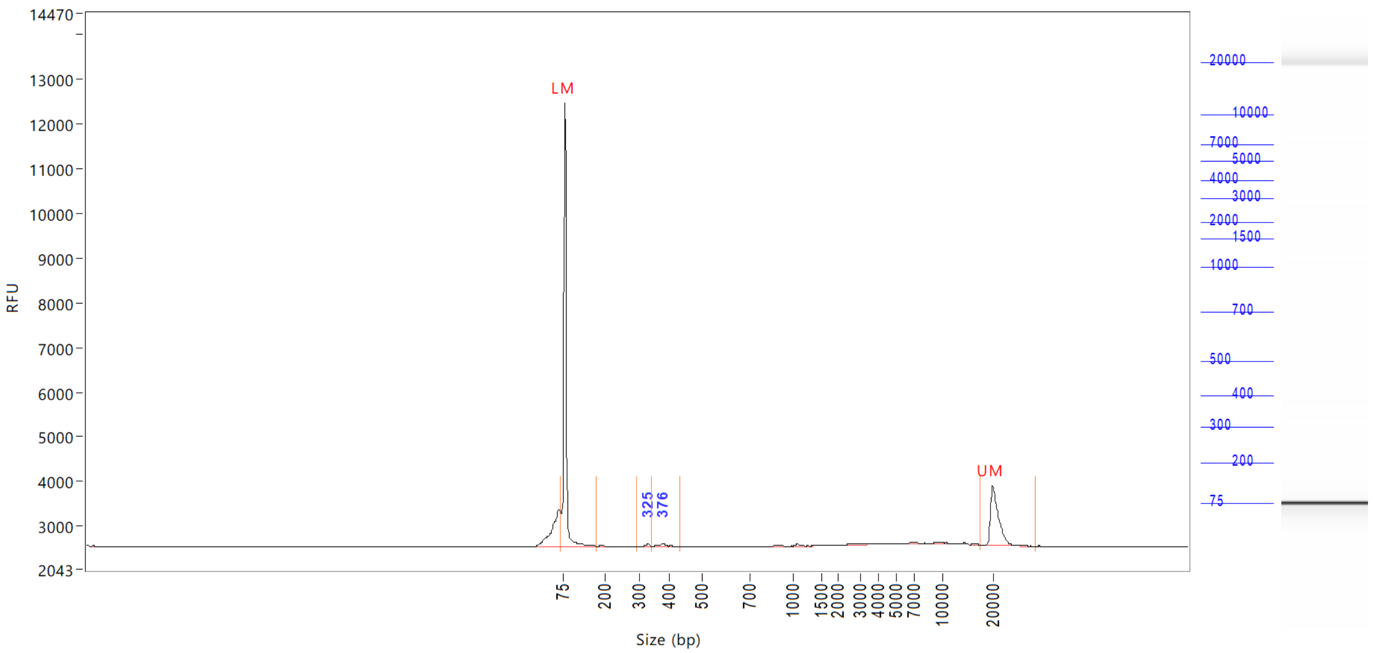

Peak	Size (bp)	Concentration (ng/uL)	From (bp)	To (bp)	Average size (bp)	CV%	RFU	Corrected peak area
1	75 (LM)	0.5000	64	175	75	12.81	9932	61.234
2	325	0.0473	291	341	323	2.70	63	0.483
3	376	0.0844	341	431	378	5.00	73	0.861
4	20000 (UM)	0.1114	17683	28342	20566	4.92	1323	13.642

TIC:	0.1317	ng/uL
TIM:	0.6082	nmole/L
Total concentration:	0.4690	ng/uL

```
Sample peak width (sec): 5    Sample min peak height: 50    Sample baseline V to V?: Y    Sample baseline V to V points: 3
Sample filter: Binomial       Number of points for filter: 3  Sample start region (min): 0  Sample end region (min): 30
Marker peak width (sec): 5    Marker min peak height: 200   Marker baseline V to V?: Y    Marker baseline V to V points: 3
Lower marker selection: First peak > 500 RFU                Upper marker selection: Last peak > 200 RFU
Ladder size (bp)75, 200, 300, 400, 500, 700, 1000, 1500, 2000, 3000, 4000, 5000, 7000, 10000, 20000
Quantification using: Lower Marker          Final concentration (ng/uL): 0.5000          Dilution factor: 12.0
```

Sample: SampF11
Well location: F11
Created: Tuesday, August 10, 2021 8:41:15 PM

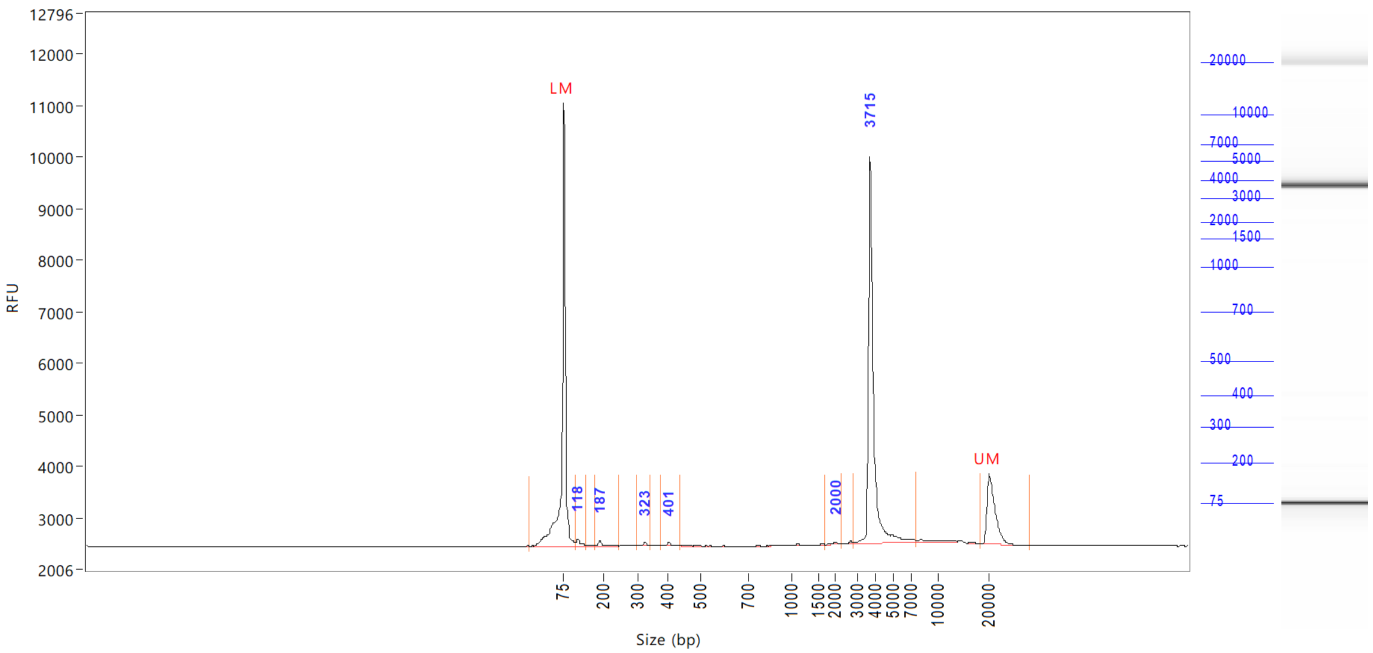

Peak	Size (bp)	Concentration (ng/uL)	From (bp)	To (bp)	Average size (bp)	CV%	RFU	Corrected peak area
1	75 (LM)	0.5000	0	110	66	27.42	8616	71.721
2	118	0.1269	110	147	122	7.97	144	1.517
3	187	0.0847	171	244	190	6.83	113	1.012
4	323	0.0478	298	343	322	2.33	81	0.572
5	401	0.0477	378	435	402	2.70	75	0.570
6	2000	0.0266	1674	2286	1980	4.20	52	0.319
7	3715	4.5481	2829	7601	3879	13.68	7499	54.366
8	20000 (UM)	0.0974	18293	28100	20599	4.72	1357	13.970

TIC:	4.8819	ng/uL
TIM:	4.8336	nmole/L
Total concentration:	5.1418	ng/uL

```
Sample peak width (sec): 5    Sample min peak height: 50    Sample baseline V to V?: Y    Sample baseline V to V points: 3
Sample filter: Binomial       Number of points for filter: 3  Sample start region (min): 0  Sample end region (min): 30
Marker peak width (sec): 5    Marker min peak height: 200   Marker baseline V to V?: Y    Marker baseline V to V points: 3
Lower marker selection: First peak > 500 RFU                Upper marker selection: Last peak > 200 RFU
Ladder size (bp)75, 200, 300, 400, 500, 700, 1000, 1500, 2000, 3000, 4000, 5000, 7000, 10000, 20000
Quantification using: Lower Marker          Final concentration (ng/uL): 0.5000          Dilution factor: 12.0
```

Sample: SampG11
Well location: G11
Created: Tuesday, August 10, 2021 8:41:15 PM

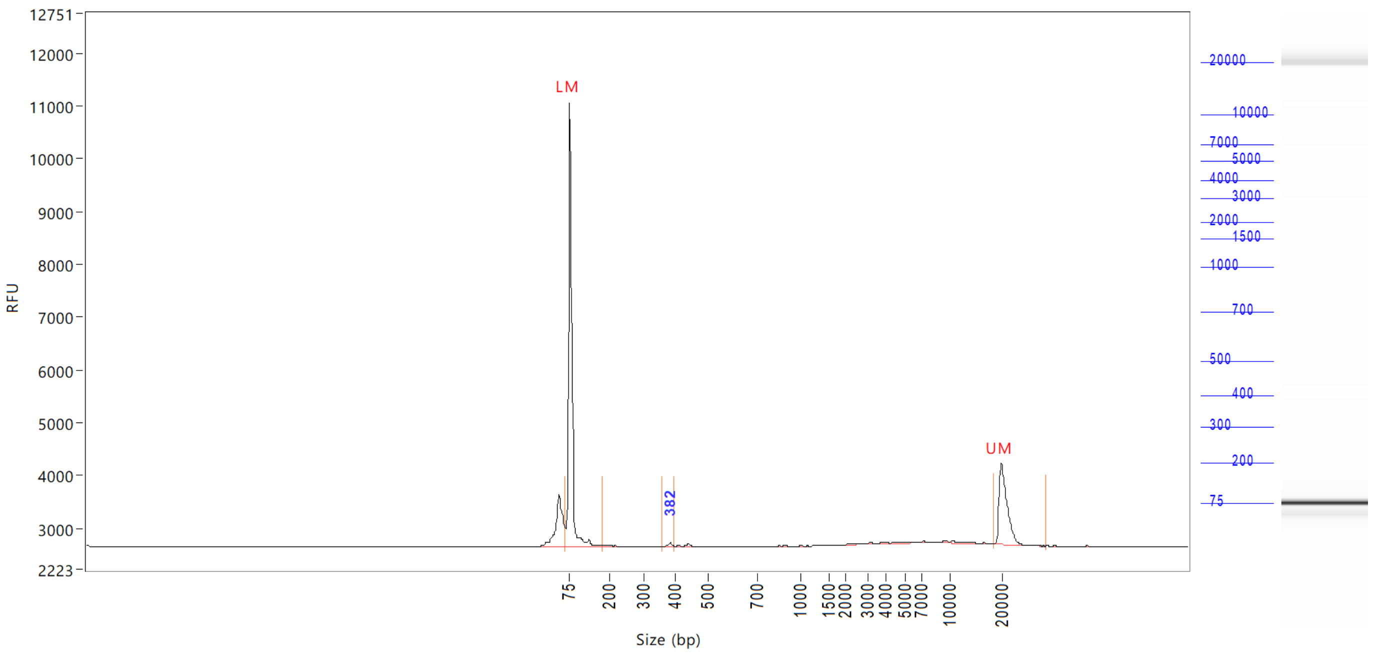

Peak	Size (bp)	Concentration (ng/uL)	From (bp)	To (bp)	Average size (bp)	CV%	RFU	Corrected peak area
1	75 (LM)	0.5000	64	177	77	16.35	8417	64.737
2	382	0.0391	358	396	378	1.61	64	0.421
3	20000 (UM)	0.1193	18537	28826	20569	4.60	1537	15.446

TIC:	0.0391	ng/uL
TIM:	0.1700	nmole/L
Total concentration:	0.4462	ng/uL

Sample peak width (sec): 5 Sample min peak height: 50 Sample baseline V to V?: Y Sample baseline V to V points: 3
Sample filter: Binomial Number of points for filter: 3 Sample start region (min): 0 Sample end region (min): 30
Marker peak width (sec): 5 Marker min peak height: 200 Marker baseline V to V?: Y Marker baseline V to V points: 3
Lower marker selection: First peak > 500 RFU Upper marker selection: Last peak > 200 RFU
Ladder size (bp)75, 200, 300, 400, 500, 700, 1000, 1500, 2000, 3000, 4000, 5000, 7000, 10000, 20000
Quantification using: Lower Marker Final concentration (ng/uL): 0.5000 Dilution factor: 12.0

Sample: SampH11
Well location: H11
Created: Tuesday, August 10, 2021 8:41:15 PM

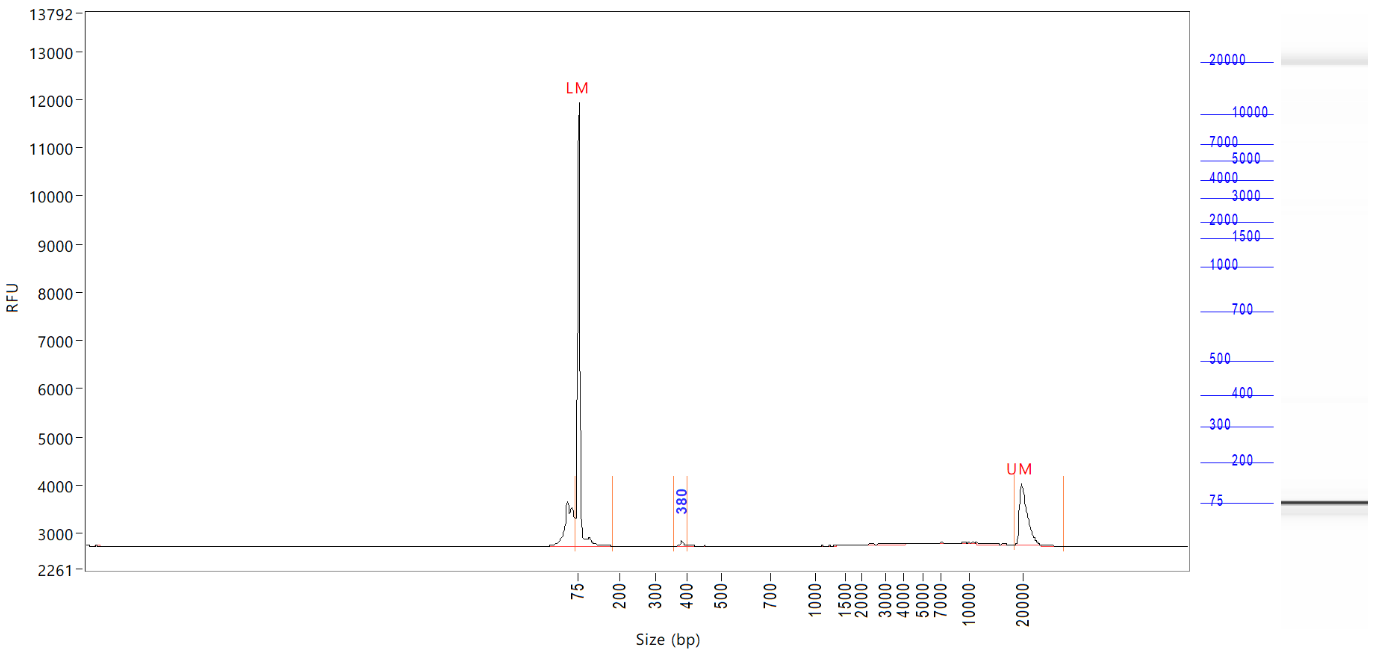

Peak	Size (bp)	Concentration (ng/uL)	From (bp)	To (bp)	Average size (bp)	CV%	RFU	Corrected peak area
1	75 (LM)	0.5000	64	175	75	14.42	9217	55.650
2	380	0.0806	356	400	379	2.11	106	0.748
3	20000 (UM)	0.1153	18415	28100	20529	4.99	1269	12.838

TIC:	0.0806	ng/uL
TIM:	0.3499	nmole/L
Total concentration:	0.5289	ng/uL

Sample peak width (sec): 5 Sample min peak height: 50 Sample baseline V to V?: Y Sample baseline V to V points: 3
Sample filter: Binomial Number of points for filter: 3 Sample start region (min): 0 Sample end region (min): 30
Marker peak width (sec): 5 Marker min peak height: 200 Marker baseline V to V?: Y Marker baseline V to V points: 3
Lower marker selection: First peak > 500 RFU Upper marker selection: Last peak > 200 RFU
Ladder size (bp)75, 200, 300, 400, 500, 700, 1000, 1500, 2000, 3000, 4000, 5000, 7000, 10000, 20000
Quantification using: Lower Marker Final concentration (ng/uL): 0.5000 Dilution factor: 12.0

Sample: SampA12
Well location: A12
Created: Tuesday, August 10, 2021 8:41:15 PM

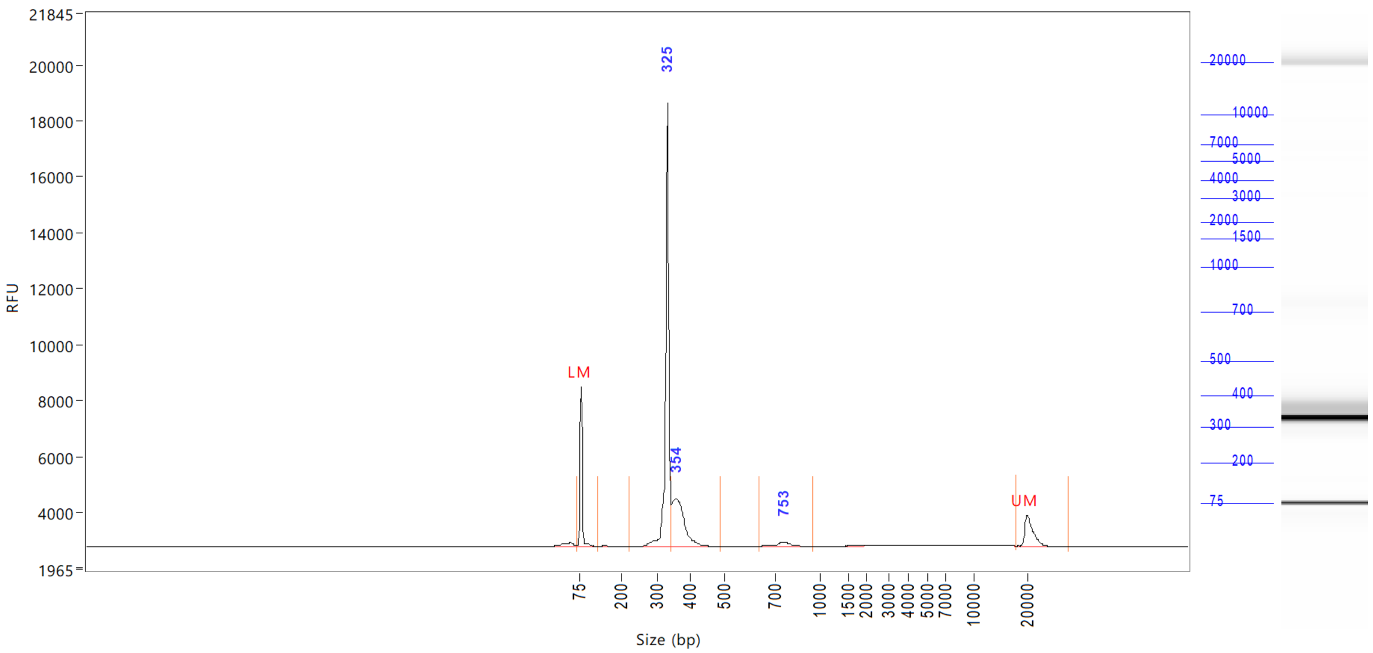

Peak	Size (bp)	Concentration (ng/uL)	From (bp)	To (bp)	Average size (bp)	CV%	RFU	Corrected peak area
1	75 (LM)	0.5000	64	126	75	6.86	5694	32.052
2	325	19.4036	221	339	322	3.39	15888	103.654
3	354	7.9089	339	490	366	5.85	1682	42.250
4	753	0.7504	641	961	749	7.44	140	4.009
5	20000 (UM)	0.1697	17927	27859	20584	4.82	1082	10.881

TIC:	28.0629	ng/uL
TIM:	136.3289	nmole/L
Total concentration:	28.2486	ng/uL

```
Sample peak width (sec): 5    Sample min peak height: 50    Sample baseline V to V?: Y    Sample baseline V to V points: 3
Sample filter: Binomial       Number of points for filter: 3  Sample start region (min): 0  Sample end region (min): 30
Marker peak width (sec): 5    Marker min peak height: 200   Marker baseline V to V?: Y    Marker baseline V to V points: 3
Lower marker selection: First peak > 500 RFU                Upper marker selection: Last peak > 200 RFU
Ladder size (bp)75, 200, 300, 400, 500, 700, 1000, 1500, 2000, 3000, 4000, 5000, 7000, 10000, 20000
Quantification using: Lower Marker          Final concentration (ng/uL): 0.5000          Dilution factor: 12.0
```

Sample: SampB12
Well location: B12
Created: Tuesday, August 10, 2021 8:41:15 PM

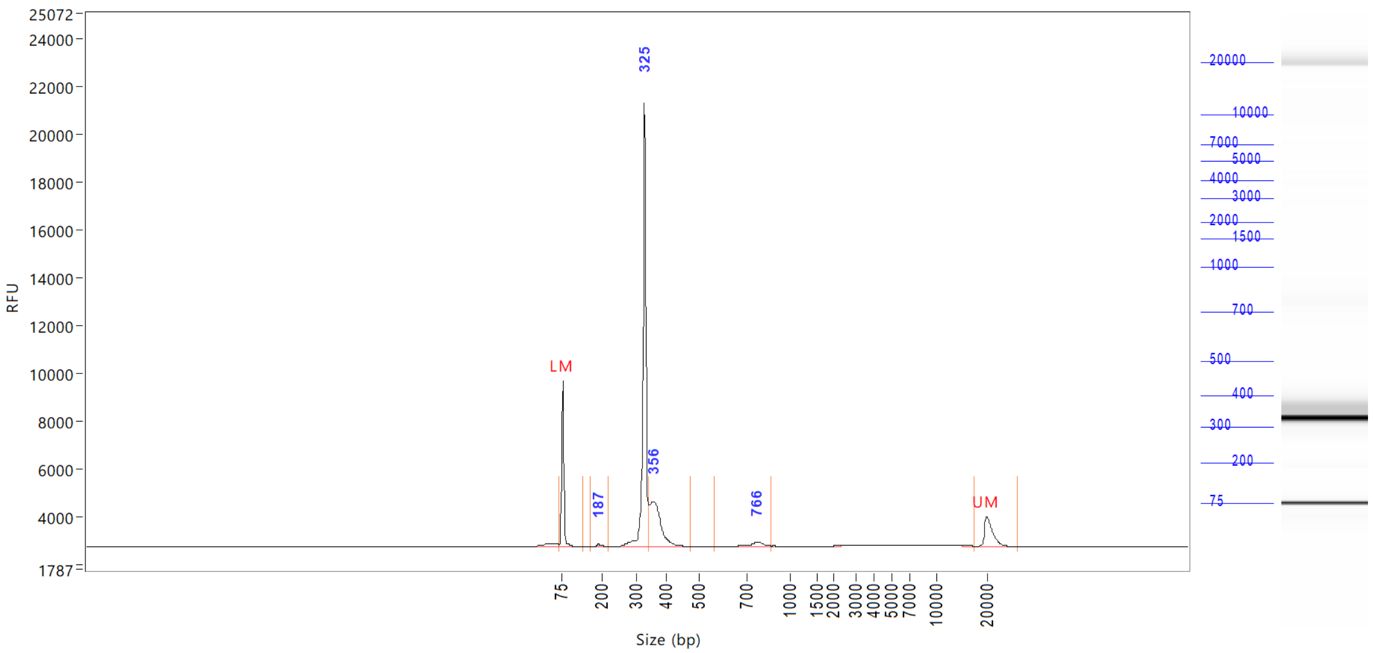

Peak	Size (bp)	Concentration (ng/uL)	From (bp)	To (bp)	Average size (bp)	CV%	RFU	Corrected peak area
1	75 (LM)	0.5000	64	137	74	6.94	6958	39.176
2	187	0.1348	164	217	189	5.27	97	0.880
3	325	18.8657	217	341	322	3.47	18611	123.179
4	356	6.6238	341	475	366	5.53	1888	43.249
5	766	0.7081	562	870	750	7.37	183	4.624
6	20000 (UM)	0.1629	17561	26045	20466	4.72	1252	12.763

TIC:	26.3324	ng/uL
TIM:	128.8814	nmole/L
Total concentration:	26.5645	ng/uL

```
Sample peak width (sec): 5    Sample min peak height: 50    Sample baseline V to V?: Y    Sample baseline V to V points: 3
Sample filter: Binomial       Number of points for filter: 3  Sample start region (min): 0  Sample end region (min): 30
Marker peak width (sec): 5    Marker min peak height: 200   Marker baseline V to V?: Y    Marker baseline V to V points: 3
Lower marker selection: First peak > 500 RFU                Upper marker selection: Last peak > 200 RFU
Ladder size (bp)75, 200, 300, 400, 500, 700, 1000, 1500, 2000, 3000, 4000, 5000, 7000, 10000, 20000
Quantification using: Lower Marker          Final concentration (ng/uL): 0.5000          Dilution factor: 12.0
```

Sample: SampC12
Well location: C12
Created: Tuesday, August 10, 2021 8:41:15 PM

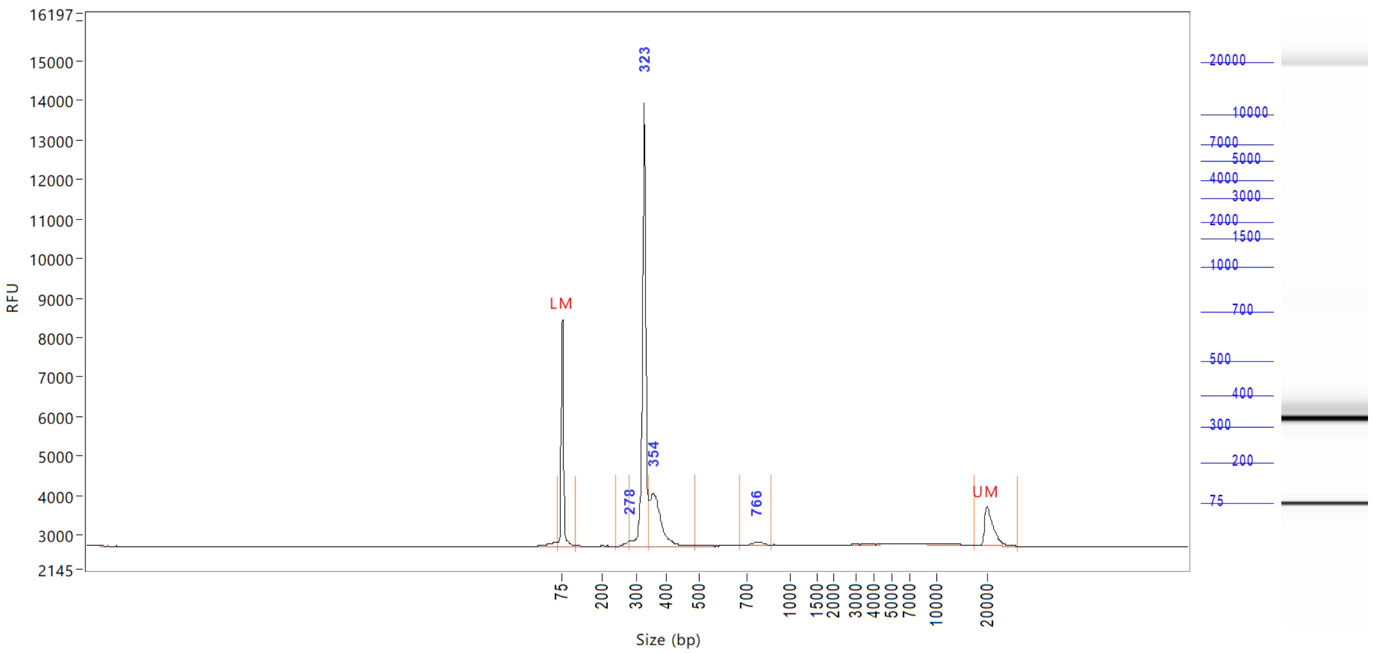

Peak	Size (bp)	Concentration (ng/uL)	From (bp)	To (bp)	Average size (bp)	CV%	RFU	Corrected peak area
1	75 (LM)	0.5000	62	114	73	7.22	5732	35.564
2	278	0.2034	242	280	271	2.74	131	1.205
3	323	14.3435	280	341	322	2.68	11220	85.020
4	354	5.0999	341	490	367	6.01	1328	30.229
5	766	0.4341	669	870	762	6.40	104	2.573
6	20000 (UM)	0.1518	17561	25924	20582	5.03	991	10.801

TIC:	20.0808	ng/uL
TIM:	98.3118	nmole/L
Total concentration:	20.4367	ng/uL

```
Sample peak width (sec): 5    Sample min peak height: 50    Sample baseline V to V?: Y    Sample baseline V to V points: 3
Sample filter: Binomial       Number of points for filter: 3  Sample start region (min): 0  Sample end region (min): 30
Marker peak width (sec): 5    Marker min peak height: 200   Marker baseline V to V?: Y    Marker baseline V to V points: 3
Lower marker selection: First peak > 500 RFU                Upper marker selection: Last peak > 200 RFU
Ladder size (bp)75, 200, 300, 400, 500, 700, 1000, 1500, 2000, 3000, 4000, 5000, 7000, 10000, 20000
Quantification using: Lower Marker          Final concentration (ng/uL): 0.5000         Dilution factor: 12.0
```

Sample: SampD12
Well location: D12
Created: Tuesday, August 10, 2021 8:41:15 PM

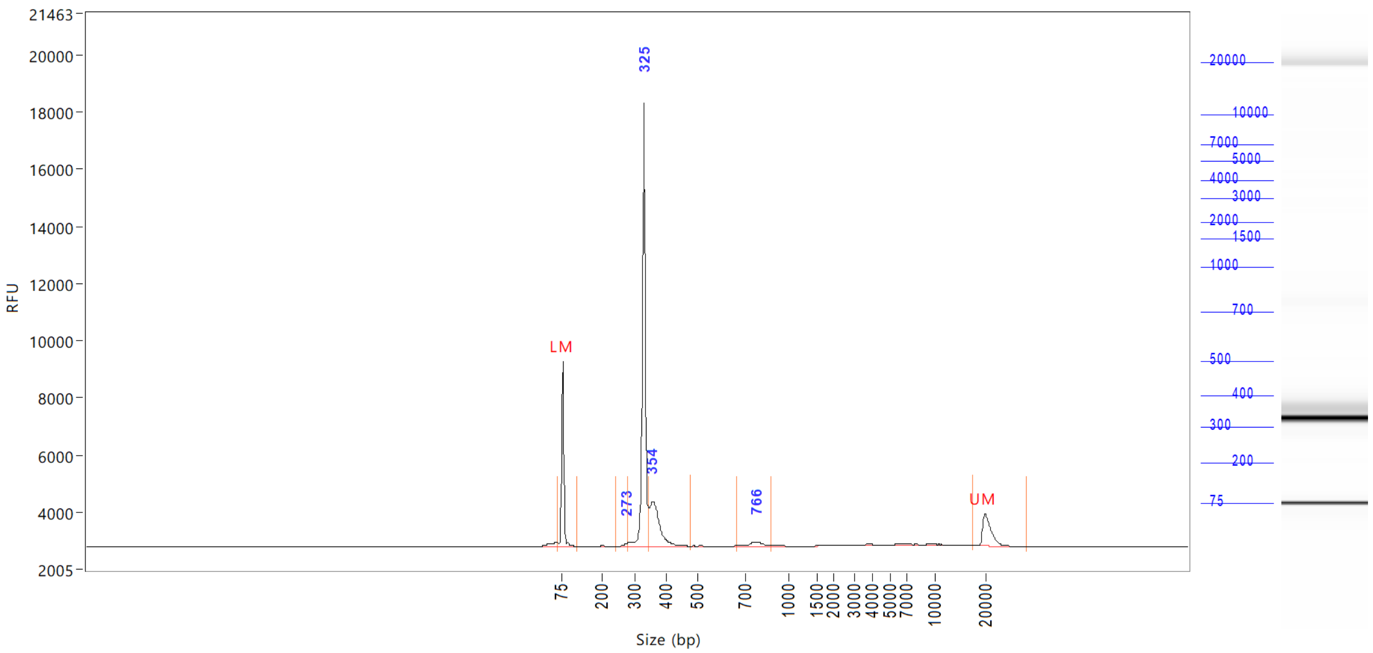

Peak	Size (bp)	Concentration (ng/uL)	From (bp)	To (bp)	Average size (bp)	CV%	RFU	Corrected peak area
1	75 (LM)	0.5000	62	120	74	6.67	6496	36.939
2	273	0.1590	239	275	267	2.92	124	0.979
3	325	16.4500	275	341	322	2.73	15541	101.274
4	354	5.3807	341	473	365	5.41	1560	33.126
5	766	0.6265	662	879	758	6.35	166	3.857
6	20000 (UM)	0.1621	17683	28221	20500	4.86	1146	11.973

TIC:	22.6161	ng/uL
TIM:	110.6317	nmole/L
Total concentration:	23.1733	ng/uL

```
Sample peak width (sec): 5    Sample min peak height: 50    Sample baseline V to V?: Y    Sample baseline V to V points: 3
Sample filter: Binomial       Number of points for filter: 3  Sample start region (min): 0  Sample end region (min): 30
Marker peak width (sec): 5    Marker min peak height: 200   Marker baseline V to V?: Y    Marker baseline V to V points: 3
Lower marker selection: First peak > 500 RFU                Upper marker selection: Last peak > 200 RFU
Ladder size (bp)75, 200, 300, 400, 500, 700, 1000, 1500, 2000, 3000, 4000, 5000, 7000, 10000, 20000
Quantification using: Lower Marker          Final concentration (ng/uL): 0.5000          Dilution factor: 12.0
```

Sample: SampE12
Well location: E12
Created: Tuesday, August 10, 2021 8:41:15 PM

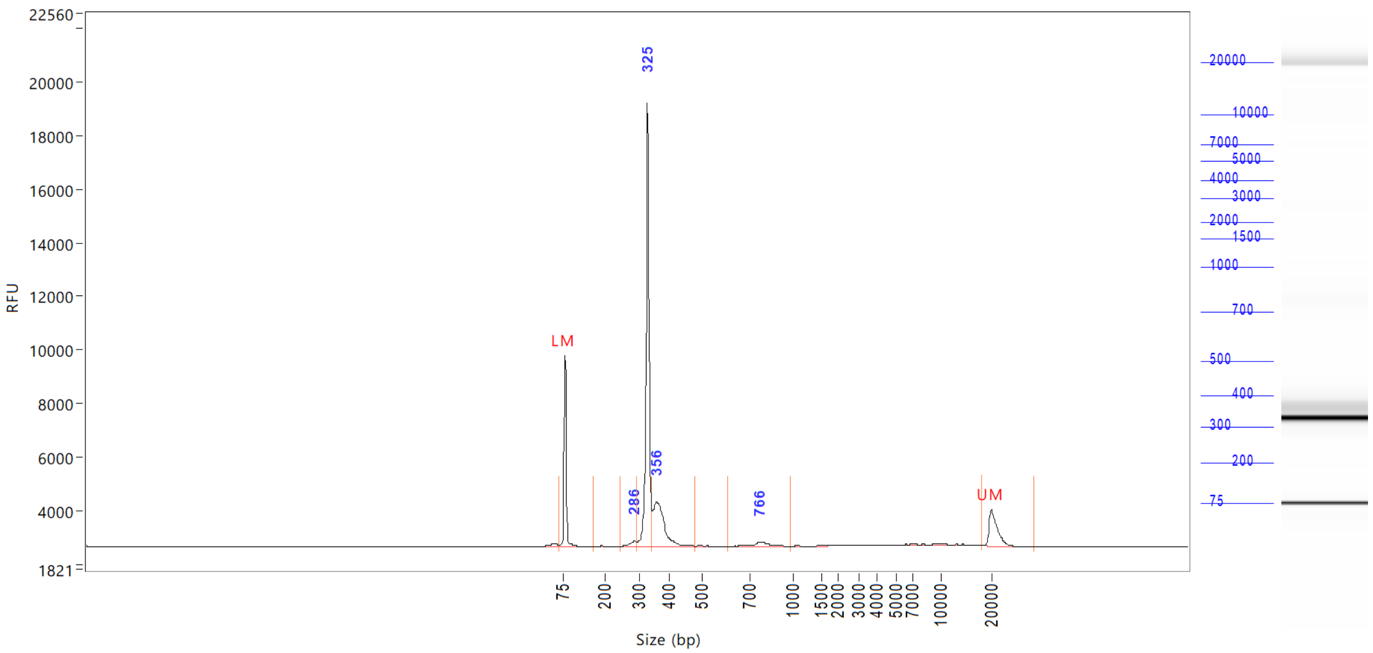

Peak	Size (bp)	Concentration (ng/uL)	From (bp)	To (bp)	Average size (bp)	CV%	RFU	Corrected peak area
1	75 (LM)	0.5000	56	166	74	9.00	7140	40.510
2	286	0.4214	242	293	278	3.84	189	2.845
3	325	15.2537	293	339	323	2.11	16570	102.989
4	356	5.7226	339	479	366	5.85	1636	38.637
5	766	0.6543	608	987	768	8.94	155	4.418
6	20000 (UM)	0.1760	18049	28463	20548	5.05	1355	14.262

TIC:	22.0520	ng/uL
TIM:	107.3056	nmole/L
Total concentration:	22.4136	ng/uL

```
Sample peak width (sec): 5    Sample min peak height: 50    Sample baseline V to V?: Y    Sample baseline V to V points: 3
Sample filter: Binomial       Number of points for filter: 3  Sample start region (min): 0  Sample end region (min): 30
Marker peak width (sec): 5    Marker min peak height: 200   Marker baseline V to V?: Y    Marker baseline V to V points: 3
Lower marker selection: First peak > 500 RFU                Upper marker selection: Last peak > 200 RFU
Ladder size (bp)75, 200, 300, 400, 500, 700, 1000, 1500, 2000, 3000, 4000, 5000, 7000, 10000, 20000
Quantification using: Lower Marker          Final concentration (ng/uL): 0.5000          Dilution factor: 12.0
```

Sample: SampF12
Well location: F12
Created: Tuesday, August 10, 2021 8:41:15 PM

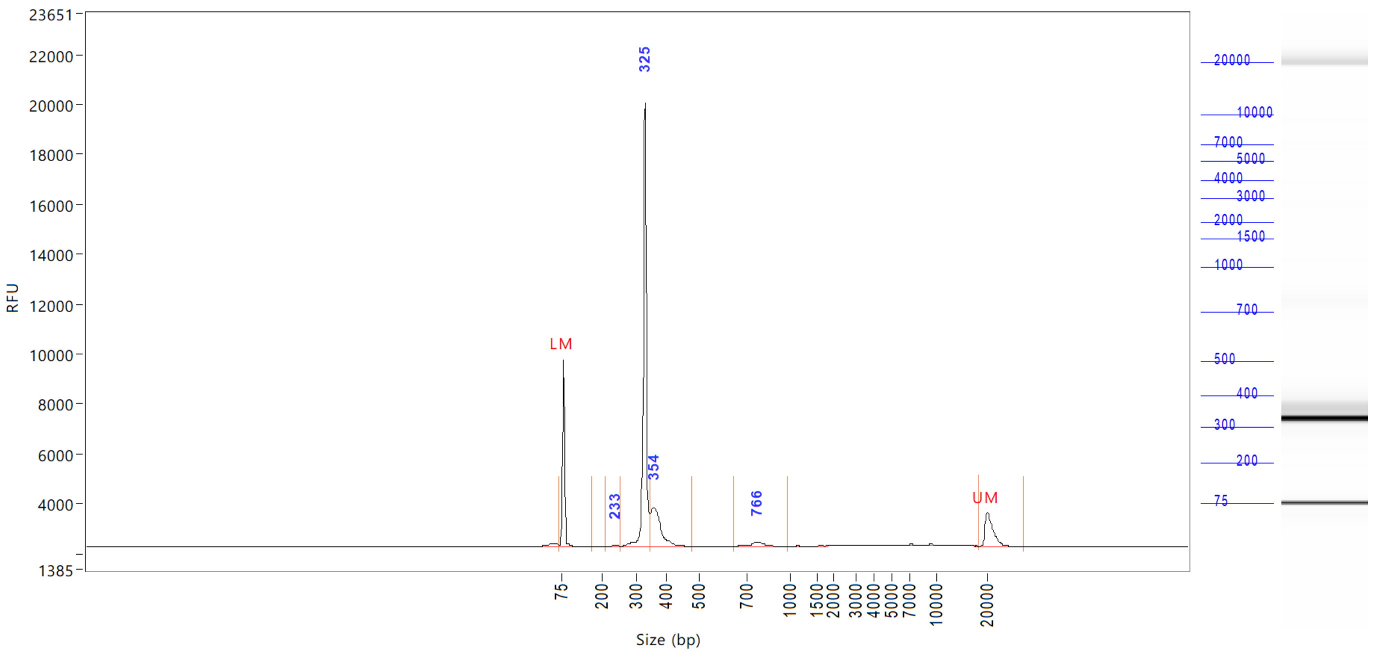

Peak	Size (bp)	Concentration (ng/uL)	From (bp)	To (bp)	Average size (bp)	CV%	RFU	Corrected peak area
1	75 (LM)	0.5000	64	164	74	7.77	7482	42.814
2	233	0.1198	206	253	234	4.57	59	0.855
3	325	16.4207	253	341	321	3.01	17799	117.173
4	354	4.7013	341	477	366	5.74	1578	33.547
5	766	0.6391	641	983	763	7.42	187	4.561
6	20000 (UM)	0.1597	18415	27375	20582	4.67	1338	13.676

TIC:	21.8809	ng/uL
TIM:	107.5043	nmole/L
Total concentration:	22.0804	ng/uL

Sample peak width (sec): 5 Sample min peak height: 50 Sample baseline V to V?: Y Sample baseline V to V points: 3
Sample filter: Binomial Number of points for filter: 3 Sample start region (min): 0 Sample end region (min): 30
Marker peak width (sec): 5 Marker min peak height: 200 Marker baseline V to V?: Y Marker baseline V to V points: 3
Lower marker selection: First peak > 500 RFU Upper marker selection: Last peak > 200 RFU
Ladder size (bp)75, 200, 300, 400, 500, 700, 1000, 1500, 2000, 3000, 4000, 5000, 7000, 10000, 20000
Quantification using: Lower Marker Final concentration (ng/uL): 0.5000 Dilution factor: 12.0

Sample: SampG12
Well location: G12
Created: Tuesday, August 10, 2021 8:41:15 PM

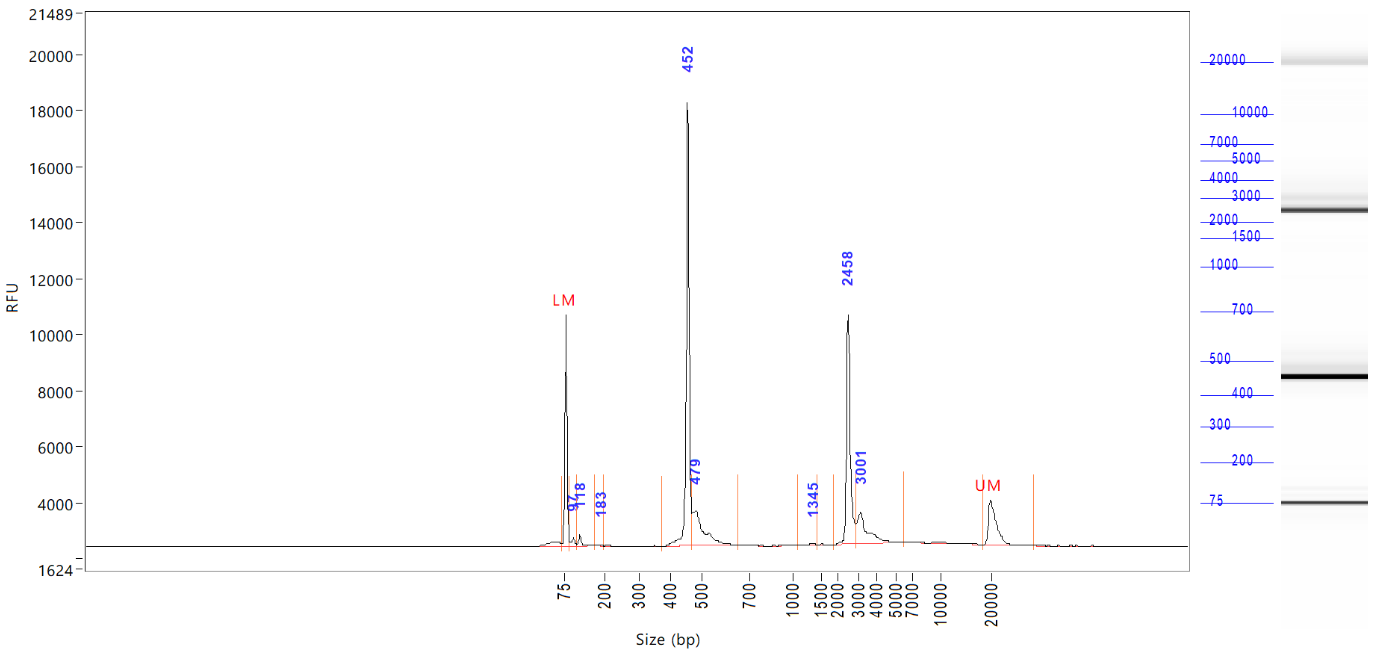

Peak	Size (bp)	Concentration (ng/uL)	From (bp)	To (bp)	Average size (bp)	CV%	RFU	Corrected peak area
1	75 (LM)	0.5000	62	87	73	4.57	8257	44.596
2	97	0.3285	87	108	95	5.48	294	2.442
3	118	0.3706	108	166	120	8.38	381	2.755
4	183	0.0435	166	192	179	2.30	52	0.323
5	452	12.1049	374	466	449	2.06	15854	89.973
6	479	3.6977	466	649	499	6.39	1226	27.484
7	1345	0.0951	1079	1445	1288	7.31	62	0.707
8	2458	6.6552	1904	2829	2471	4.93	8195	49.467
9	3001	2.3367	2829	6038	3343	14.42	1138	17.368
10	20000 (UM)	0.1836	18537	28584	20562	4.79	1607	16.374

TIC:	25.6323	ng/uL
TIM:	73.4101	nmole/L
Total concentration:	25.7035	ng/uL

```
Sample peak width (sec): 5    Sample min peak height: 50    Sample baseline V to V?: Y    Sample baseline V to V points: 3
Sample filter: Binomial       Number of points for filter: 3  Sample start region (min): 0  Sample end region (min): 30
Marker peak width (sec): 5    Marker min peak height: 200   Marker baseline V to V?: Y    Marker baseline V to V points: 3
Lower marker selection: First peak > 500 RFU                Upper marker selection: Last peak > 200 RFU
Ladder size (bp)75, 200, 300, 400, 500, 700, 1000, 1500, 2000, 3000, 4000, 5000, 7000, 10000, 20000
Quantification using: Lower Marker          Final concentration (ng/uL): 0.5000          Dilution factor: 12.0
```

Sample: SampH12
Well location: H12
Created: Tuesday, August 10, 2021 8:41:15 PM

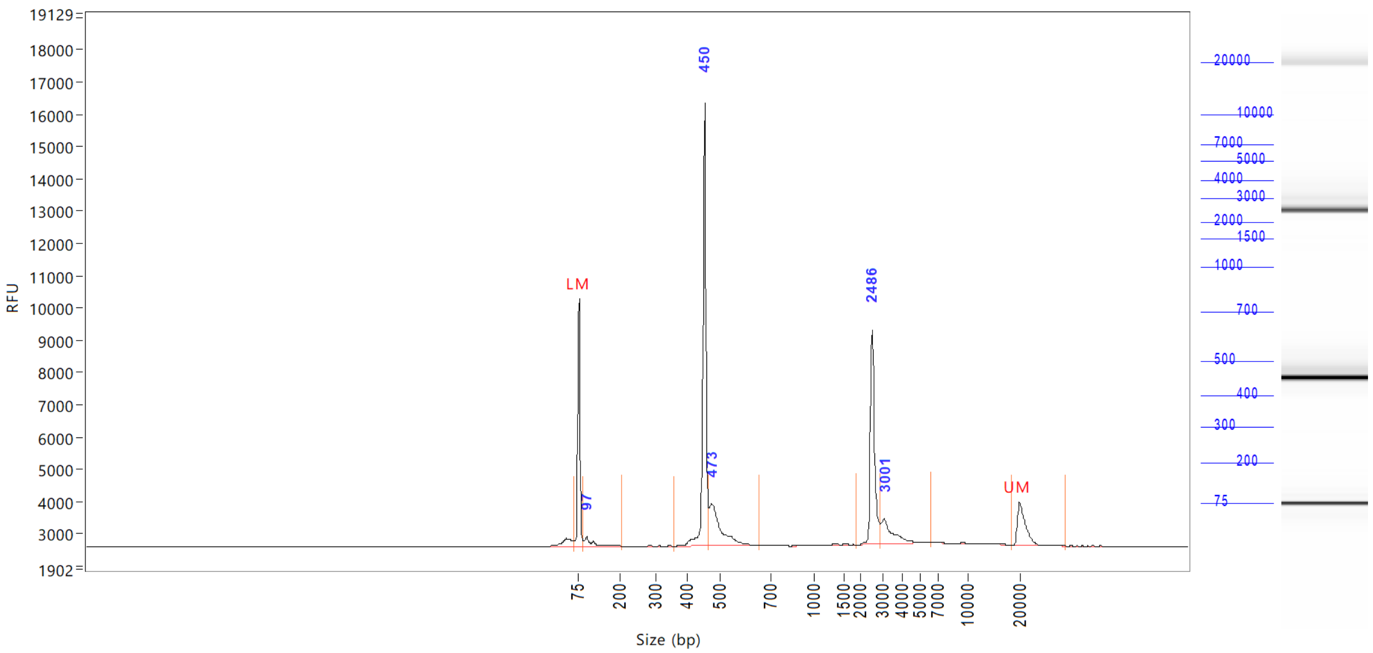

Peak	Size (bp)	Concentration (ng/uL)	From (bp)	To (bp)	Average size (bp)	CV%	RFU	Corrected peak area
1	75 (LM)	0.5000	60	87	73	5.10	7667	42.949
2	97	0.7207	87	203	112	21.29	311	5.159
3	450	11.1701	358	464	447	2.25	13747	79.957
4	473	3.9134	464	657	495	6.47	1290	28.013
5	2486	5.7273	1885	2857	2501	4.94	6626	40.997
6	3001	1.8683	2857	6112	3382	14.41	771	13.374
7	20000 (UM)	0.1551	18537	28947	20546	4.82	1334	13.325

TIC:	23.3997	ng/uL
TIM:	69.3742	nmole/L
Total concentration:	23.4791	ng/uL

Sample peak width (sec): 5 Sample min peak height: 200 Sample baseline V to V?: Y Sample baseline V to V points: 3
Sample filter: Binomial Number of points for filter: 3 Sample start region (min): 0 Sample end region (min): 30
Marker peak width (sec): 5 Marker min peak height: 200 Marker baseline V to V?: Y Marker baseline V to V points: 3
Lower marker selection: First peak > 500 RFU Upper marker selection: Last peak > 200 RFU
Ladder size (bp)75, 200, 300, 400, 500, 700, 1000, 1500, 2000, 3000, 4000, 5000, 7000, 10000, 20000
Quantification using: Lower Marker Final concentration (ng/uL): 0.5000 Dilution factor: 12.0

Sample: DNA size ladder
Well location: A7
Created: Tuesday, August 10, 2021 8:41:15 PM
Import from: C:\Users\admin\Desktop\210811_alberto_ladder.SCAL
Fit type: Point to point

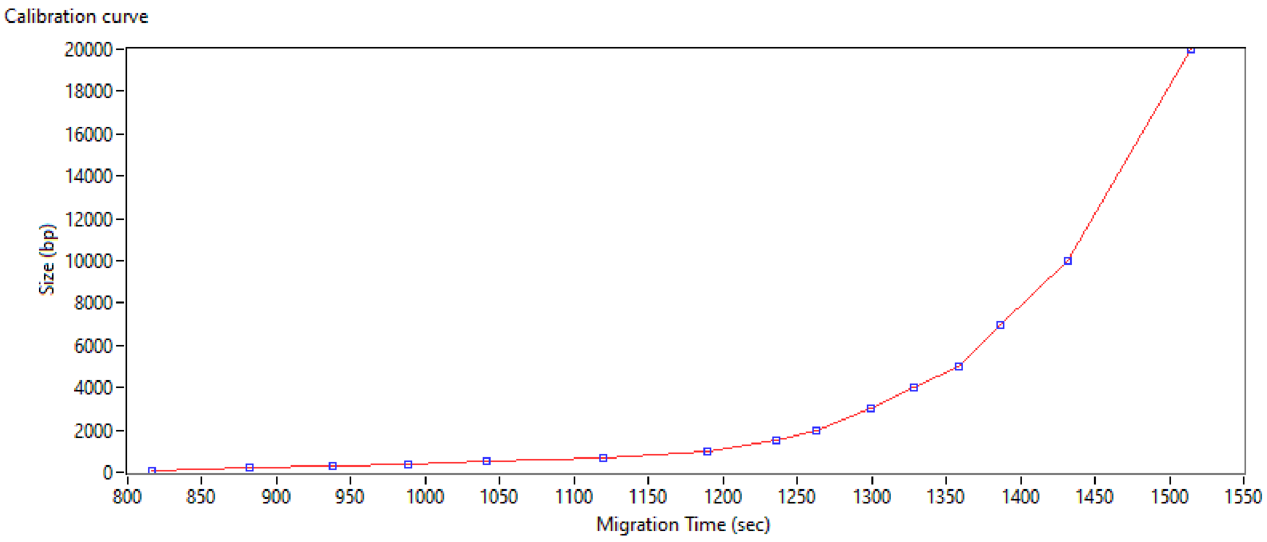

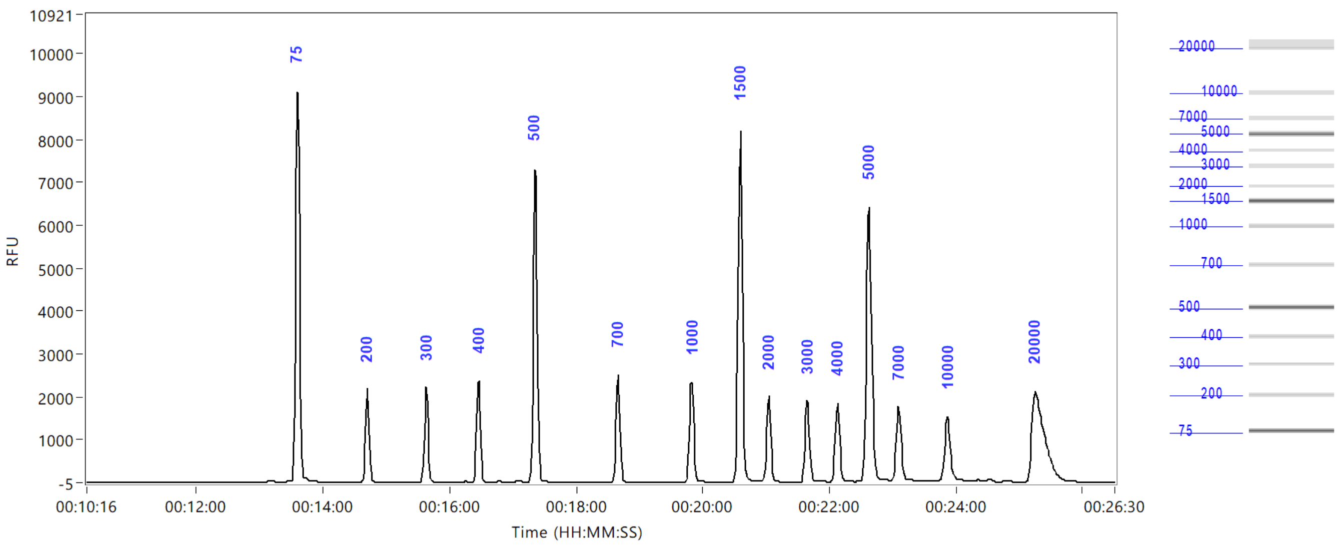

Supplement: Supplementary file 2 — sb3c00292_si_002.zip [file sb3c00292_si_002.zip › dnada_supplementary_material_pks_library_build/ZAG_trials/ZAG_Trial_1/20-31-19_plate4/plate4_report.pdf]
