# Supplementary material for: Automated Platform for the Plasmid Construction Process
Source: ACS Synth Biol. 2023 Nov 10;12(12):3506–13. doi: 10.1021/acssynbio.3c00292 (PMC10729297; doi:10.1021/acssynbio.3c00292)

Instrument controller software run summary:

Filename and data path: C:\AATI\Data\2021 09 20\19-10-17\2021 09 20 19H 10M..raw
Created: Monday, September 20, 2021 7:19:57 PM
Number of capillaries: 96
Array serial number: 111920-09SFS
Effect length: 33 cm
Array usage count: 225
Instrument type: ZAG DNA Analyzer
Instrument controller software version: 1.0.1.9
Device serial number: Z7597

Method Information

Method name: ZAG130GP33 - DNA 75-20000bp - Gel Prime Only.mthds
Gel prime: Yes
Full conditioning: No
Gel prime to buffer: No
Gel selection: Gel 1
Perform prerun: 8.0 kV, 30 sec.
Rinse: No
Marker 1: Row: A, 5.0 kV, 5 sec.
Rinse: No
Sample injection: 5.0 kV, 5 sec.
Separation: 8.0 kV, 30.0 min.
Tray name: Sample Tray-3

Analysis mode: DNA

Notes

Gel Image

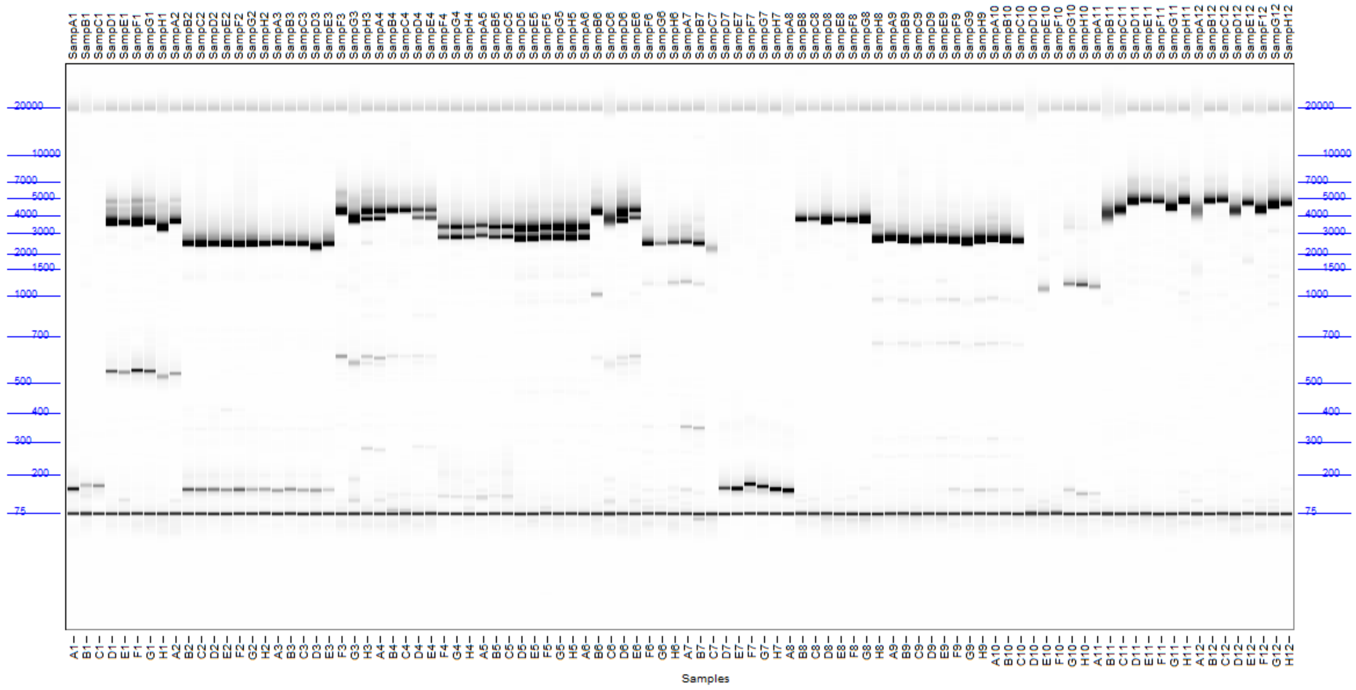
20000
10000
7000
5000
4000
3000
2000
1500
1000
700
500
400
300
200
75
Samples

Filename and data path: C:\AATI\Data\2021 09 20\19-10-17\2021 09 20 19H 10M..raw

A1: SampA1

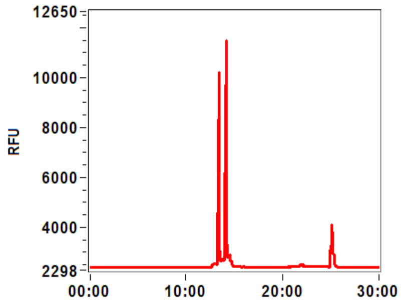

B1: SampB1

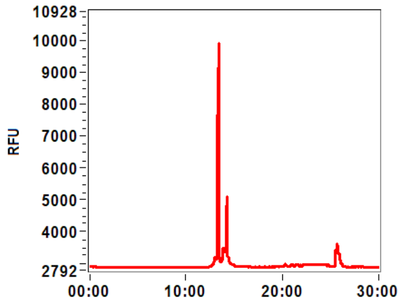

C1: SampC1

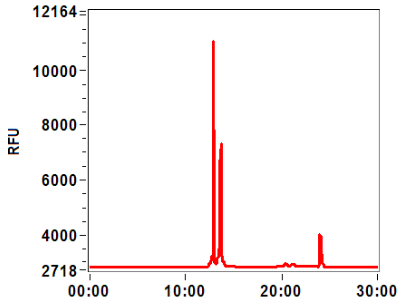

D1: SampD1

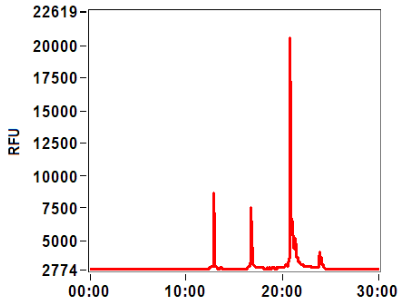

E1: SampE1

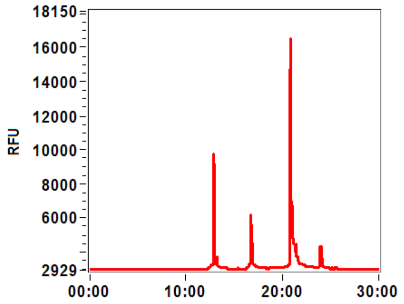

F1: SampF1

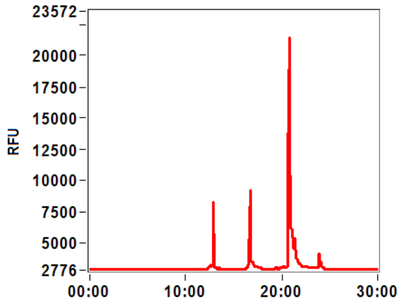

G1: SampG1

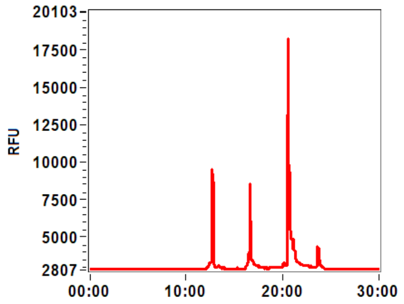

H1: SampH1

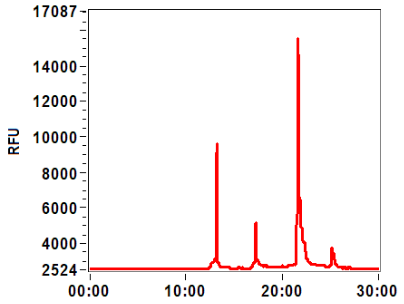

A2: SampA2

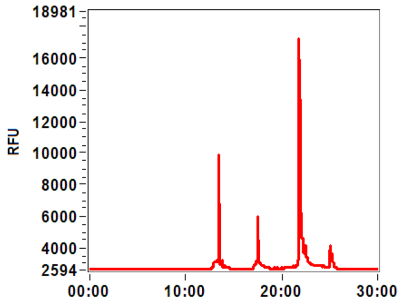

B2: SampB2

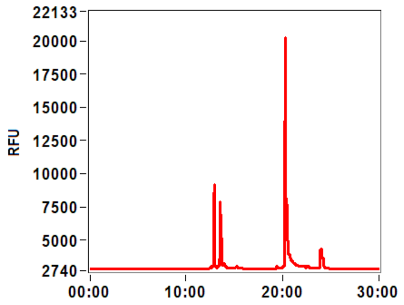

C2: SampC2

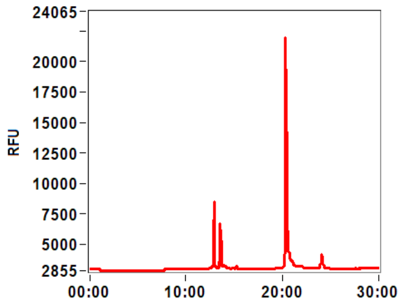

D2: SampD2

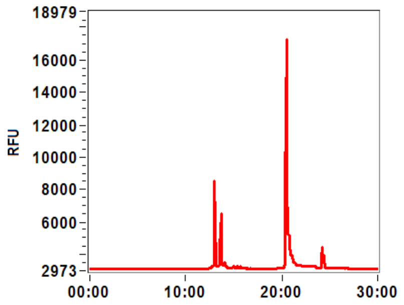

Filename and data path: C:\AATI\Data\2021 09 20\19-10-17\2021 09 20 19H 10M..raw

E2: SampE2

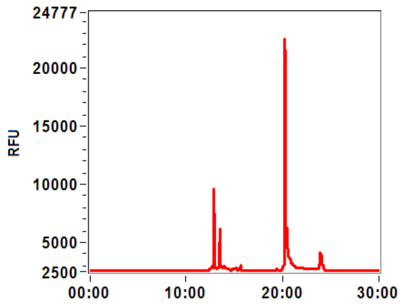

F2: SampF2

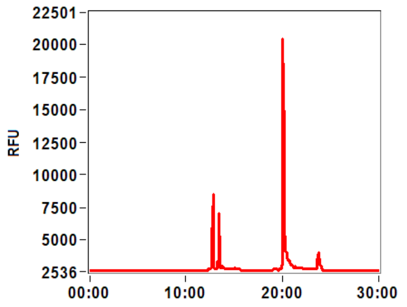

G2: SampG2

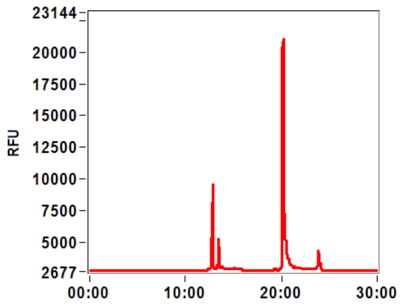

H2: SampH2

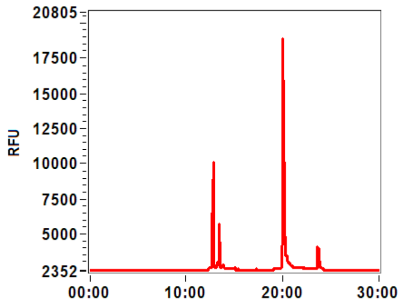

A3: SampA3

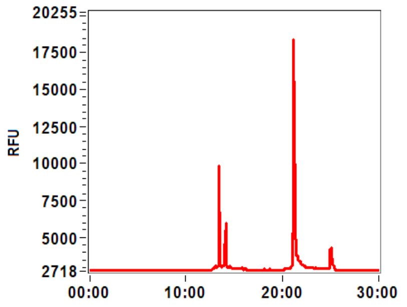

B3: SampB3

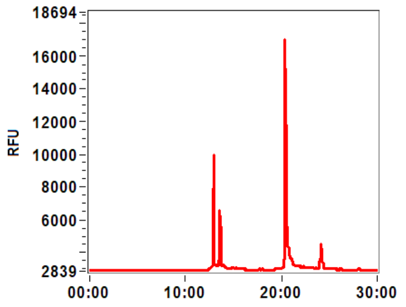

C3: SampC3

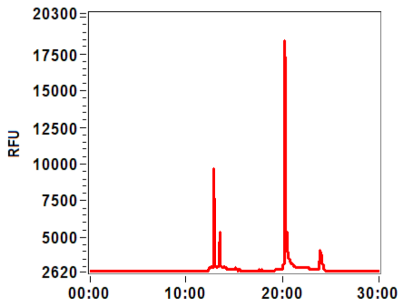

D3: SampD3

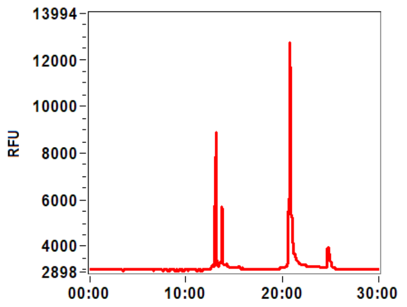

E3: SampE3

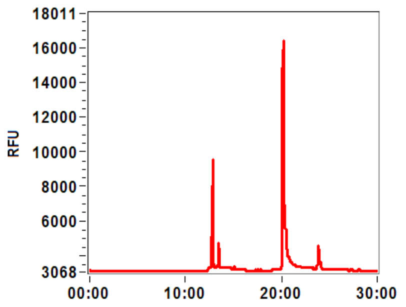

F3: SampF3

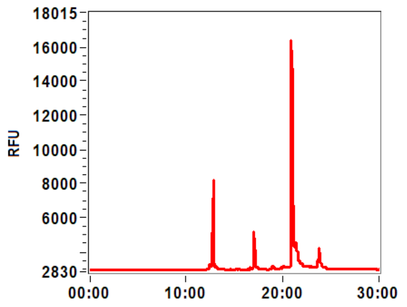

G3: SampG3

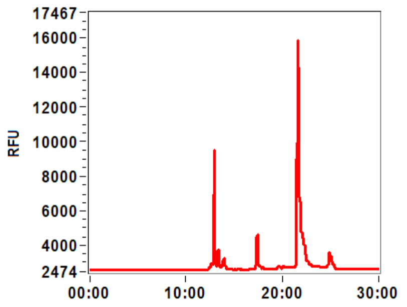

H3: SampH3

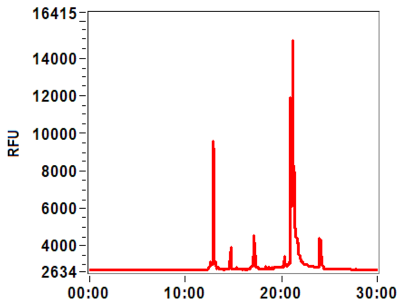

Filename and data path: C:\AATI\Data\2021 09 20\19-10-17\2021 09 20 19H 10M..raw

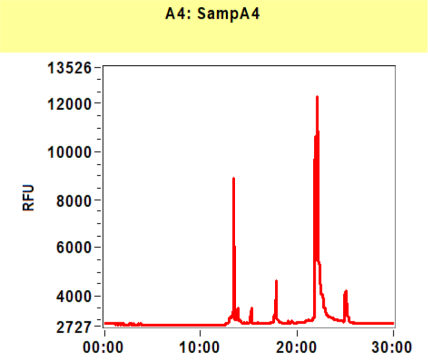

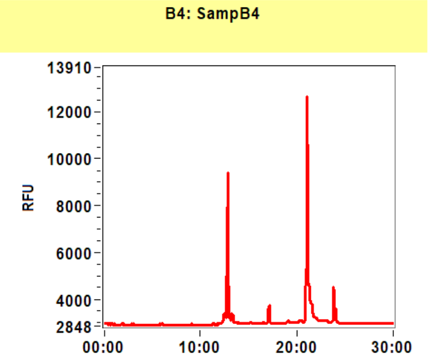

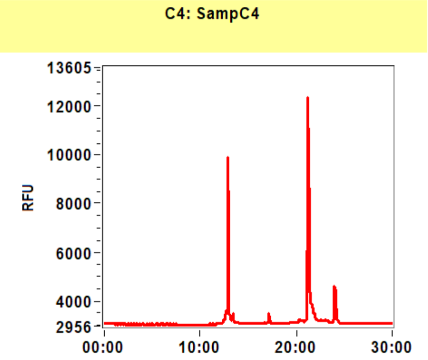

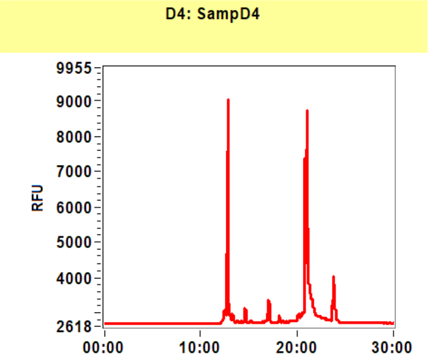

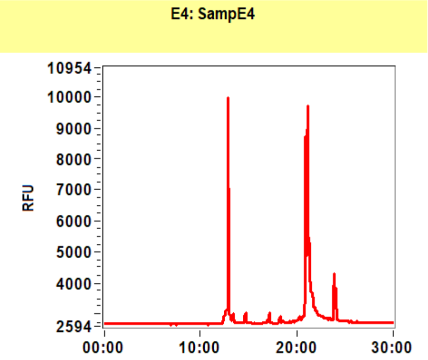

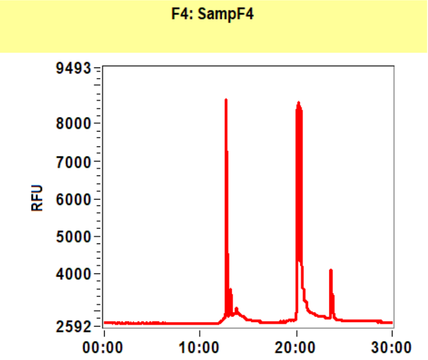

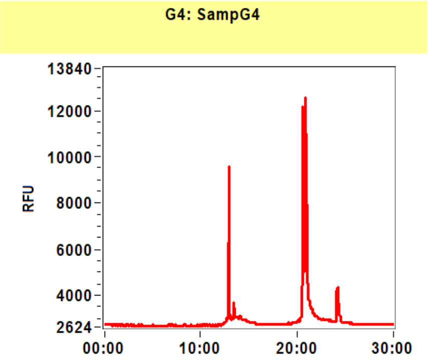

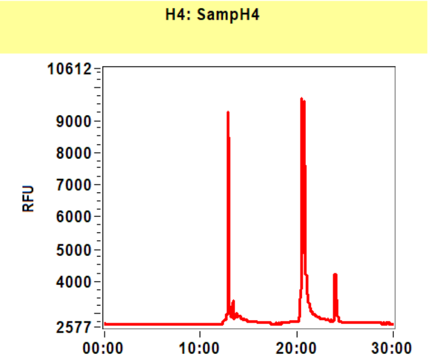

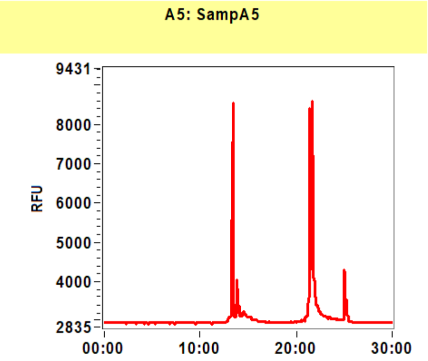

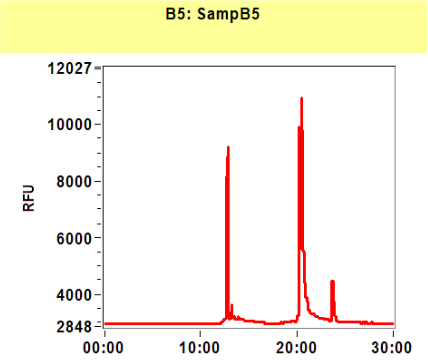

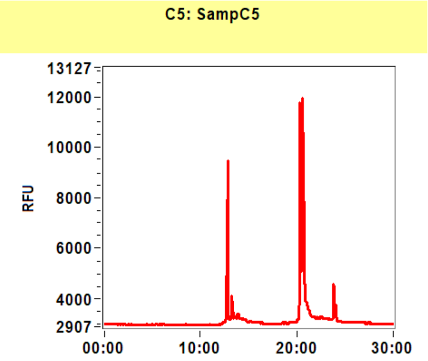

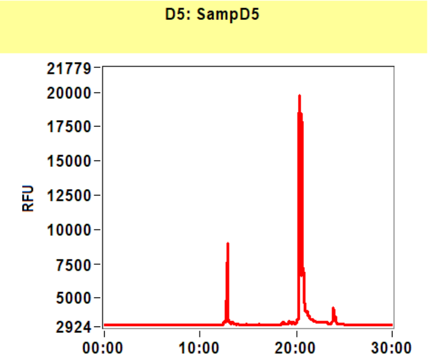

Filename and data path: C:\AATI\Data\2021 09 20\19-10-17\2021 09 20 19H 10M..raw

E5: SampE5

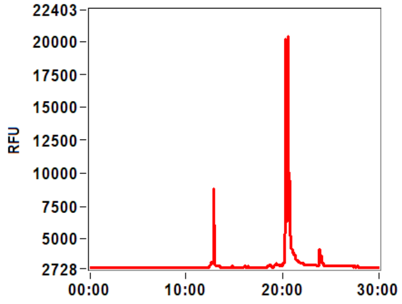

F5: SampF5

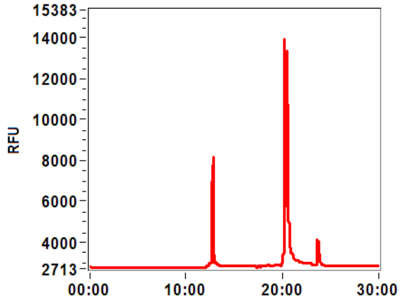

G5: SampG5

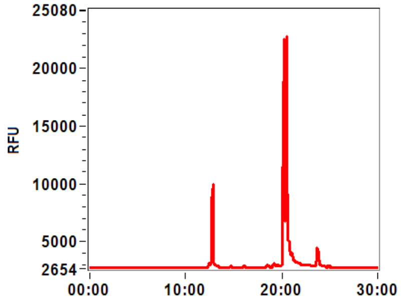

H5: SampH5

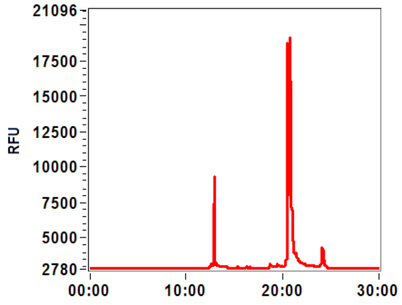

A6: SampA6

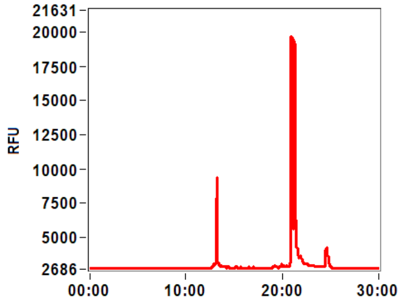

B6: SampB6

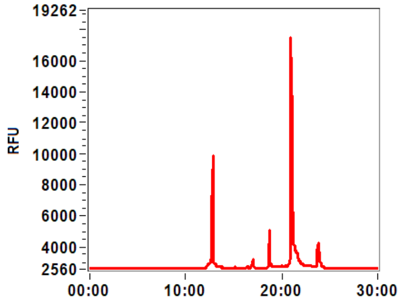

C6: SampC6

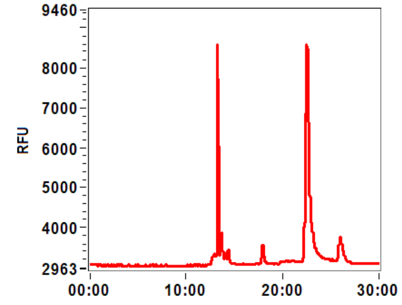

D6: SampD6

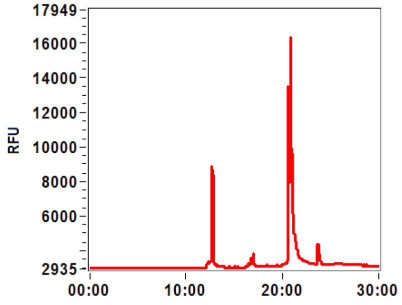

E6: SampE6

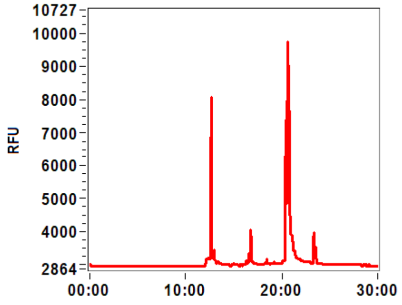

F6: SampF6

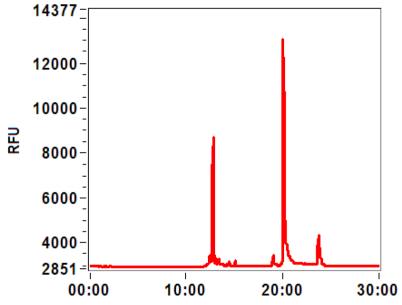

G6: SampG6

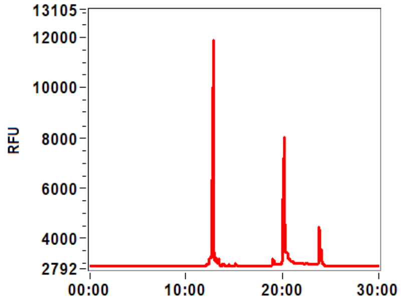

H6: SampH6

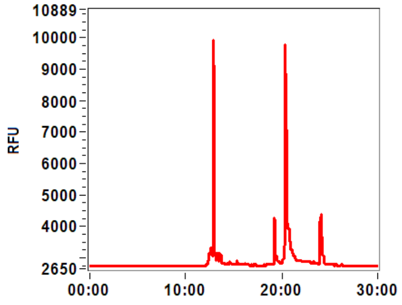

Filename and data path: C:\AATI\Data\2021 09 20\19-10-17\2021 09 20 19H 10M..raw

A7: SampA7

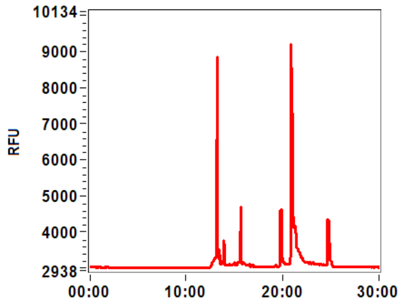

B7: SampB7

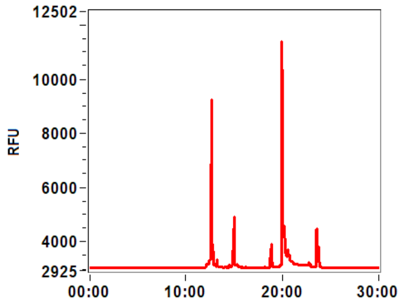

C7: SampC7

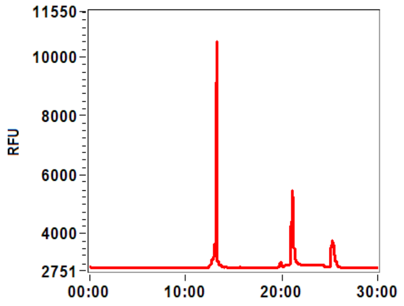

D7: SampD7

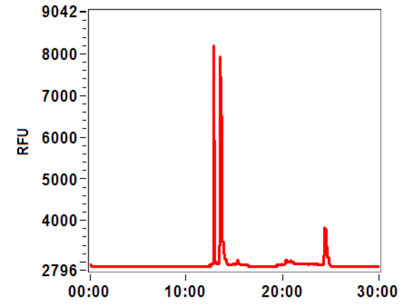

E7: SampE7

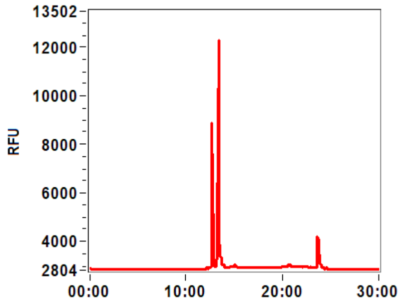

F7: SampF7

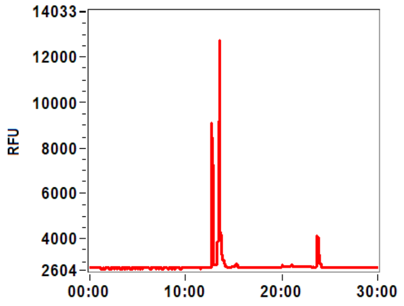

G7: SampG7

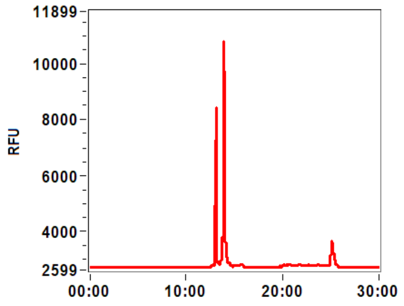

H7: SampH7

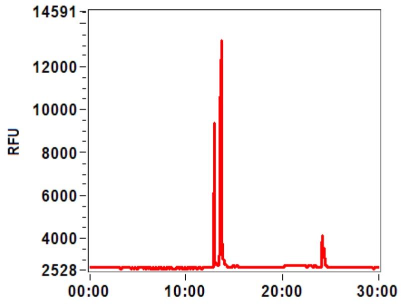

A8: SampA8

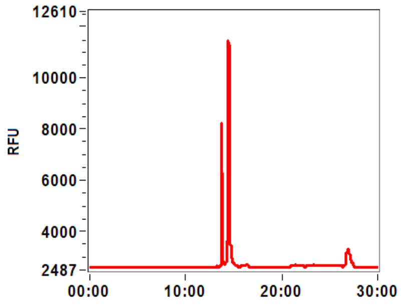

B8: SampB8

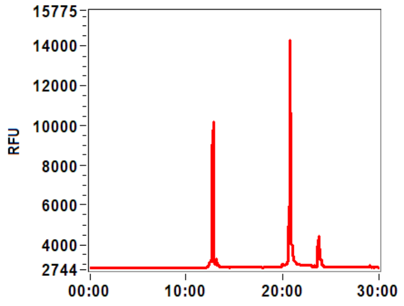

C8: SampC8

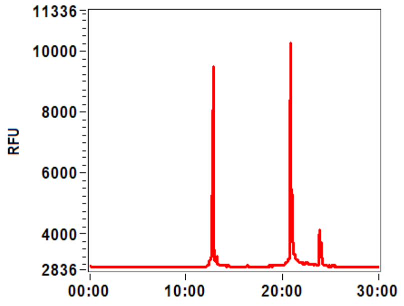

D8: SampD8

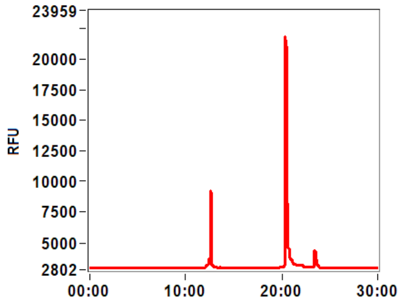

Filename and data path: C:\AATI\Data\2021 09 20\19-10-17\2021 09 20 19H 10M..raw

E8: SampE8

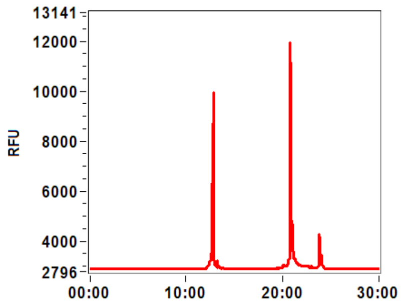

F8: SampF8

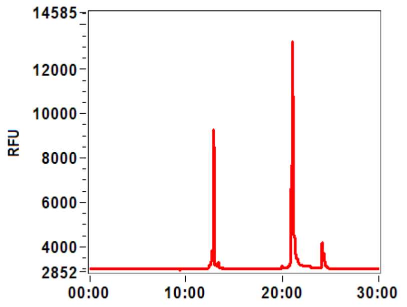

G8: SampG8

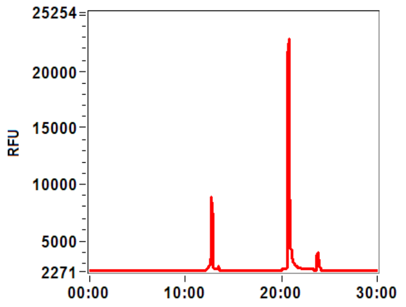

H8: SampH8

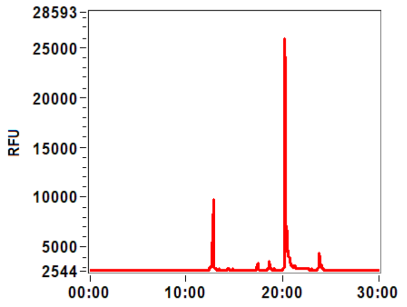

A9: SampA9

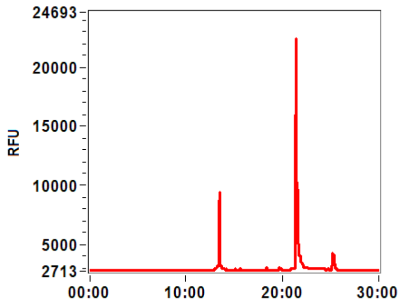

B9: SampB9

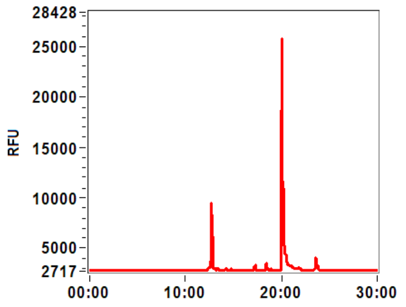

C9: SampC9

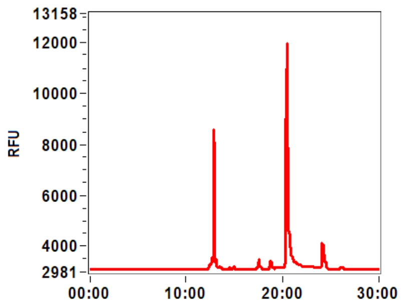

D9: SampD9

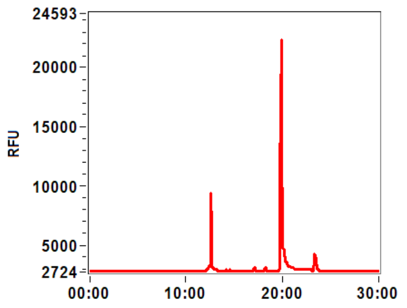

E9: SampE9

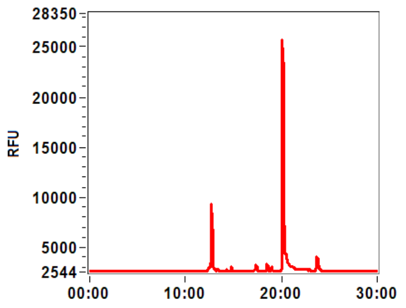

F9: SampF9

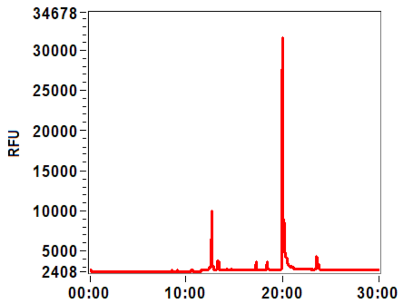

G9: SampG9

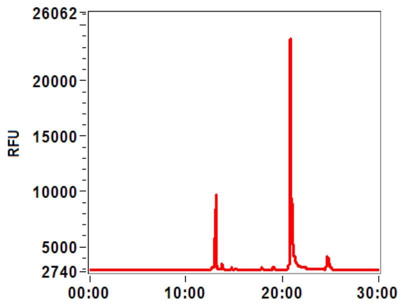

H9: SampH9

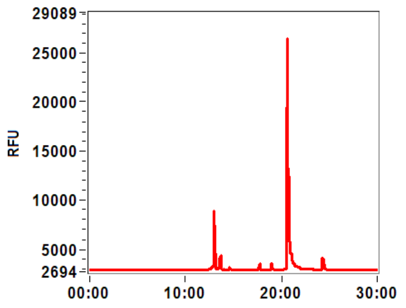

Filename and data path: C:\AATI\Data\2021 09 20\19-10-17\2021 09 20 19H 10M..raw

A10: SampA10

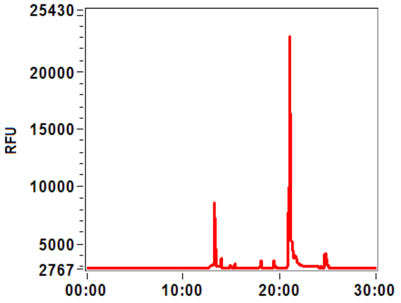

B10: SampB10

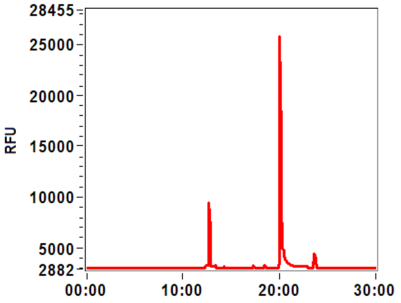

C10: SampC10

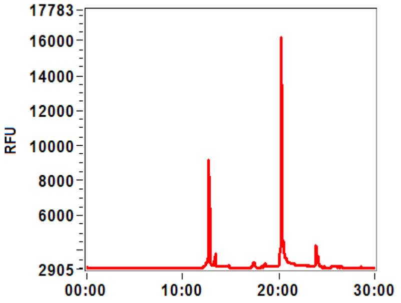

D10: SampD10

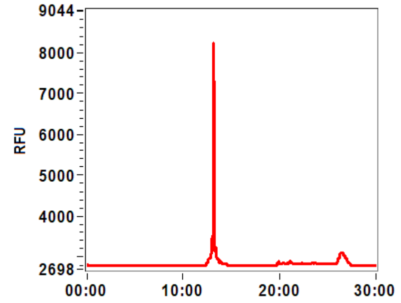

E10: SampE10

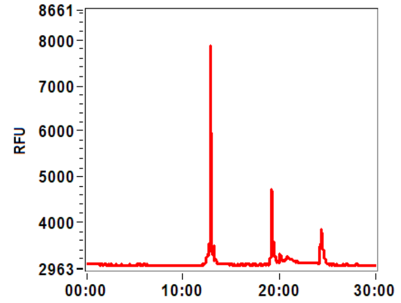

F10: SampF10

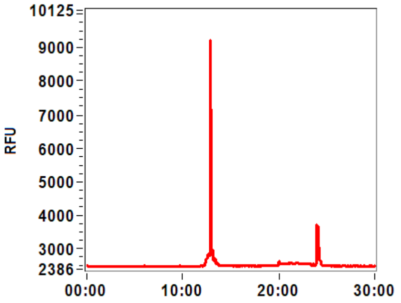

G10: SampG10

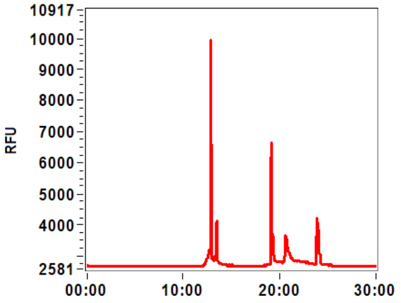

H10: SampH10

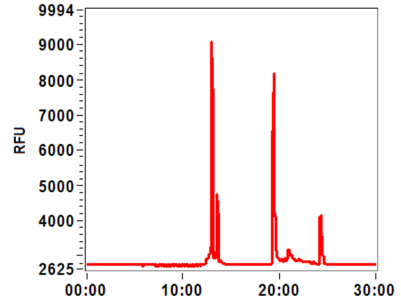

A11: SampA11

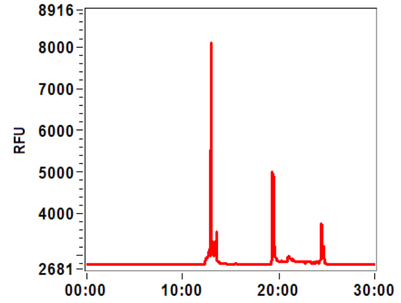

B11: SampB11

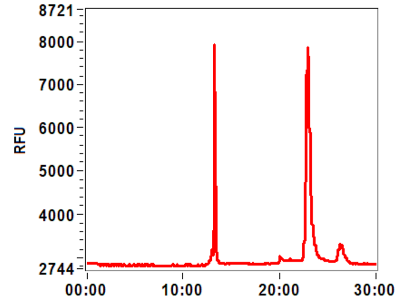

C11: SampC11

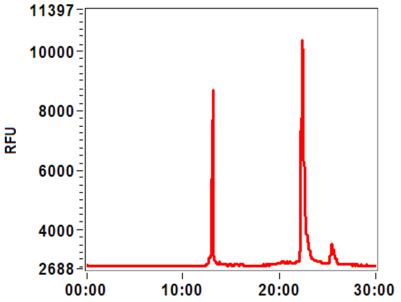

D11: SampD11

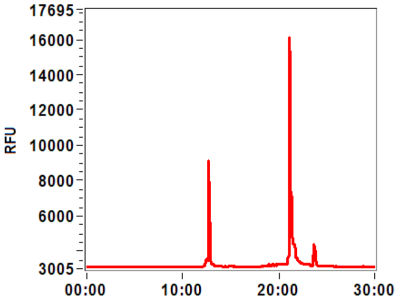

Filename and data path: C:\AATI\Data\2021 09 20\19-10-17\2021 09 20 19H 10M..raw

E11: SampE11

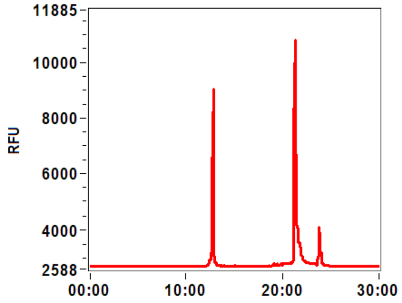

F11: SampF11

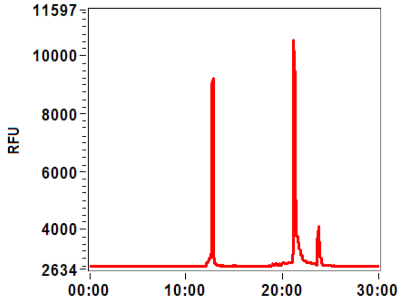

G11: SampG11

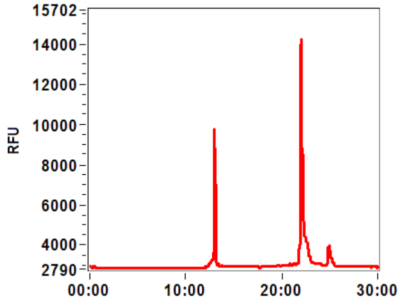

H11: SampH11

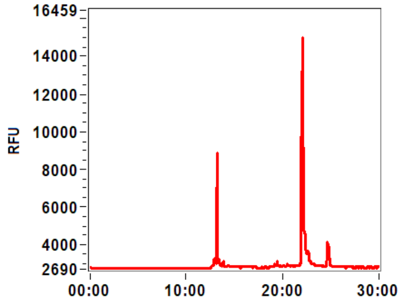

A12: SampA12

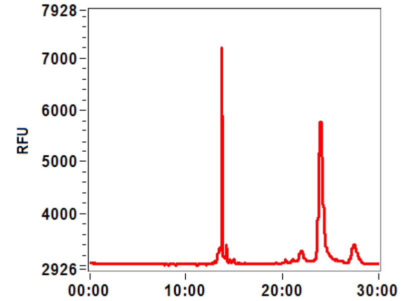

B12: SampB12

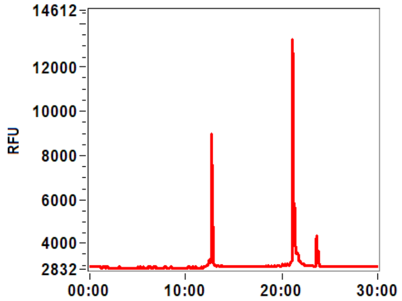

C12: SampC12

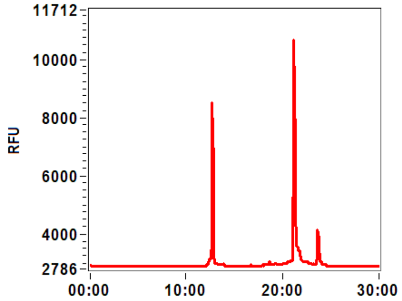

D12: SampD12

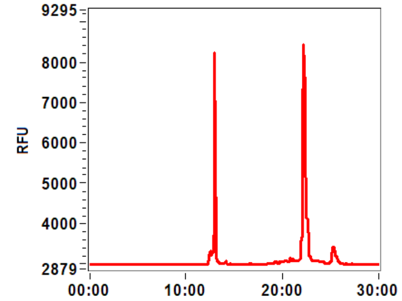

E12: SampE12

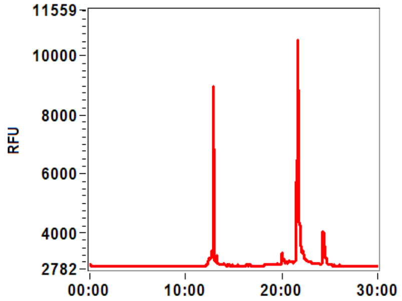

F12: SampF12

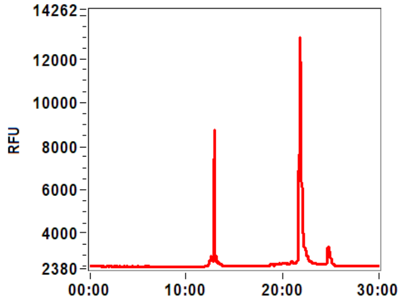

G12: SampG12

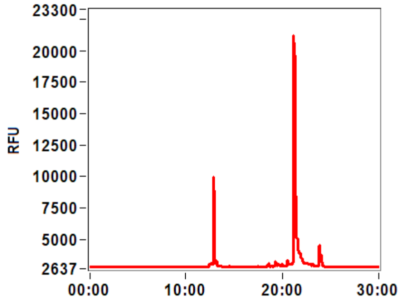

H12: SampH12

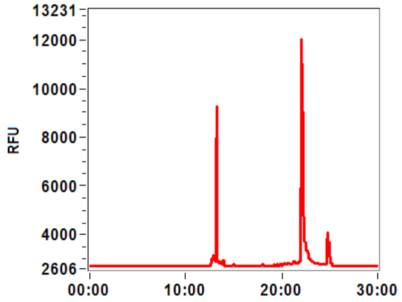

Sample: SampA1
Well location: A1
Created: Monday, September 20, 2021 7:19:57 PM

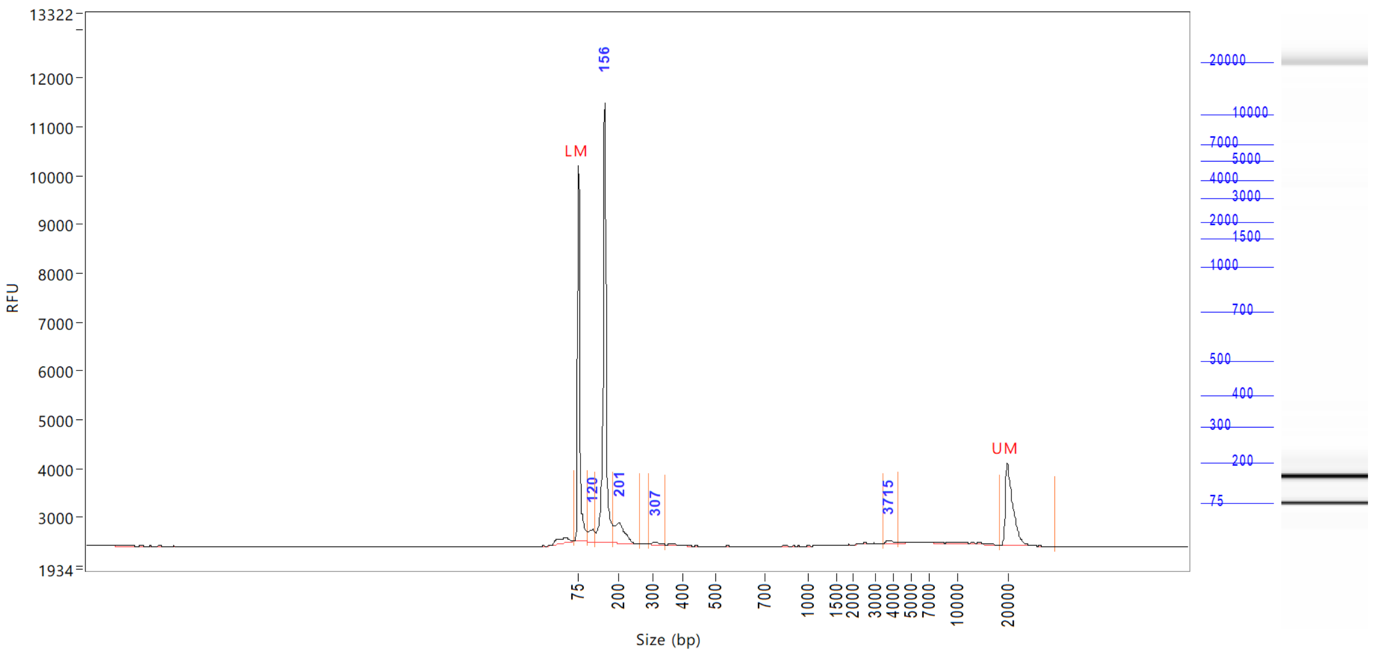

Peak	Size (bp)	Concentration (ng/uL)	From (bp)	To (bp)	Average size (bp)	CV%	RFU	Corrected peak area
1	75 (LM)	0.5000	64	103	75	7.98	7694	44.917
2	120	0.5034	103	128	115	6.43	267	3.769
3	156	8.9151	128	185	154	5.52	9018	66.740
4	201	1.2226	185	260	204	6.44	418	9.153
5	307	0.1056	289	341	310	3.93	50	0.790
6	3715	0.0856	3465	4277	3848	4.66	62	0.640
7	20000 (UM)	0.1768	18415	29309	20556	4.60	1678	15.883

TIC:	10.8322	ng/uL
TIM:	112.7896	nmole/L
Total concentration:	11.2686	ng/uL

Sample peak width (sec): 5 Sample min peak height: 50 Sample baseline V to V?: Y Sample baseline V to V points: 3
Sample filter: Binomial Number of points for filter: 3 Sample start region (min): 0 Sample end region (min): 30
Marker peak width (sec): 5 Marker min peak height: 200 Marker baseline V to V?: Y Marker baseline V to V points: 3
Lower marker selection: First peak > 500 RFU Upper marker selection: Last peak > 200 RFU
Ladder size (bp)75, 200, 300, 400, 500, 700, 1000, 1500, 2000, 3000, 4000, 5000, 7000, 10000, 20000
Quantification using: Lower Marker Final concentration (ng/uL): 0.5000 Dilution factor: 12.0

Sample: SampB1
Well location: B1
Created: Monday, September 20, 2021 7:19:57 PM

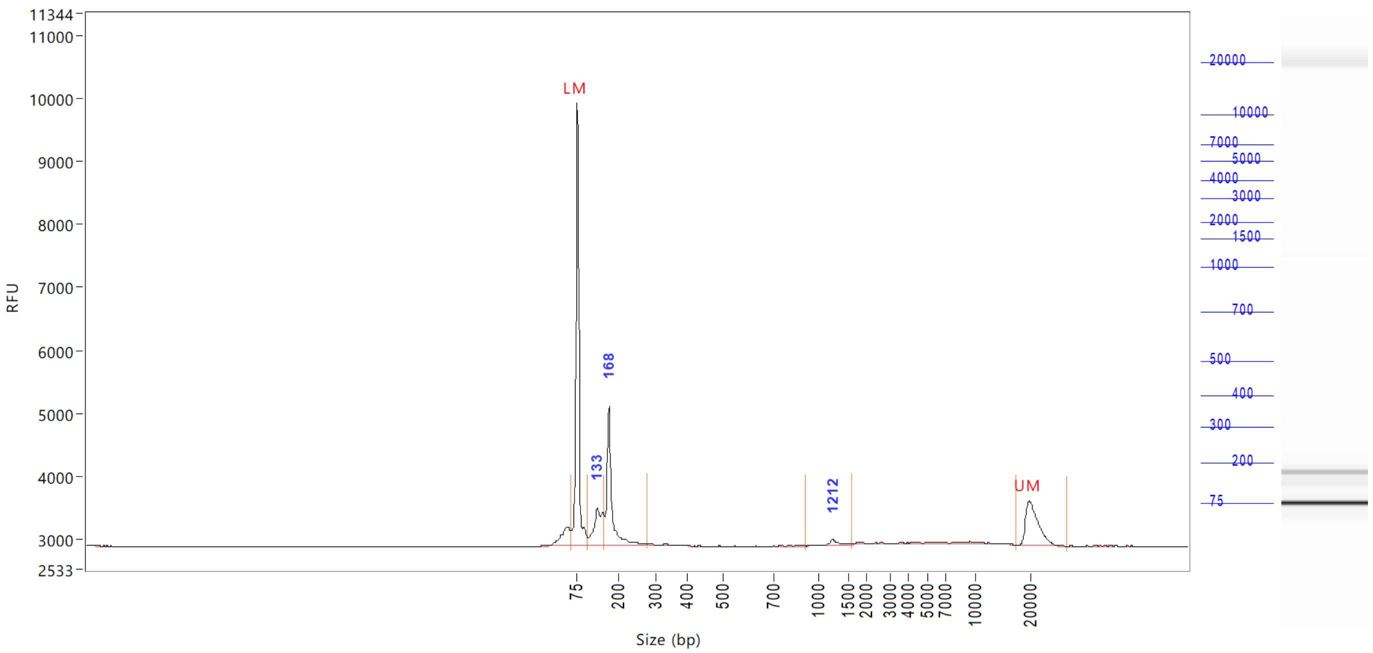

Peak	Size (bp)	Concentration (ng/uL)	From (bp)	To (bp)	Average size (bp)	CV%	RFU	Corrected peak area
1	75 (LM)	0.5000	58	107	74	8.95	7027	52.128
2	133	1.4396	107	156	136	9.50	596	12.508
3	168	2.6605	156	277	173	10.25	2194	23.114
4	1212	0.1526	913	1597	1249	10.61	96	1.326
5	20000 (UM)	0.1026	17439	27133	20711	6.18	701	10.701

TIC:	4.2528	ng/uL
TIM:	42.8764	nmole/L
Total concentration:	4.5394	ng/uL

```
Sample peak width (sec): 5    Sample min peak height: 50    Sample baseline V to V?: Y    Sample baseline V to V points: 3
Sample filter: Binomial       Number of points for filter: 3  Sample start region (min): 0  Sample end region (min): 30
Marker peak width (sec): 5    Marker min peak height: 200   Marker baseline V to V?: Y    Marker baseline V to V points: 3
Lower marker selection: First peak > 500 RFU                Upper marker selection: Last peak > 200 RFU
Ladder size (bp)75, 200, 300, 400, 500, 700, 1000, 1500, 2000, 3000, 4000, 5000, 7000, 10000, 20000
Quantification using: Lower Marker          Final concentration (ng/uL): 0.5000          Dilution factor: 12.0
```

Sample: SampC1
Well location: C1
Created: Monday, September 20, 2021 7:19:57 PM

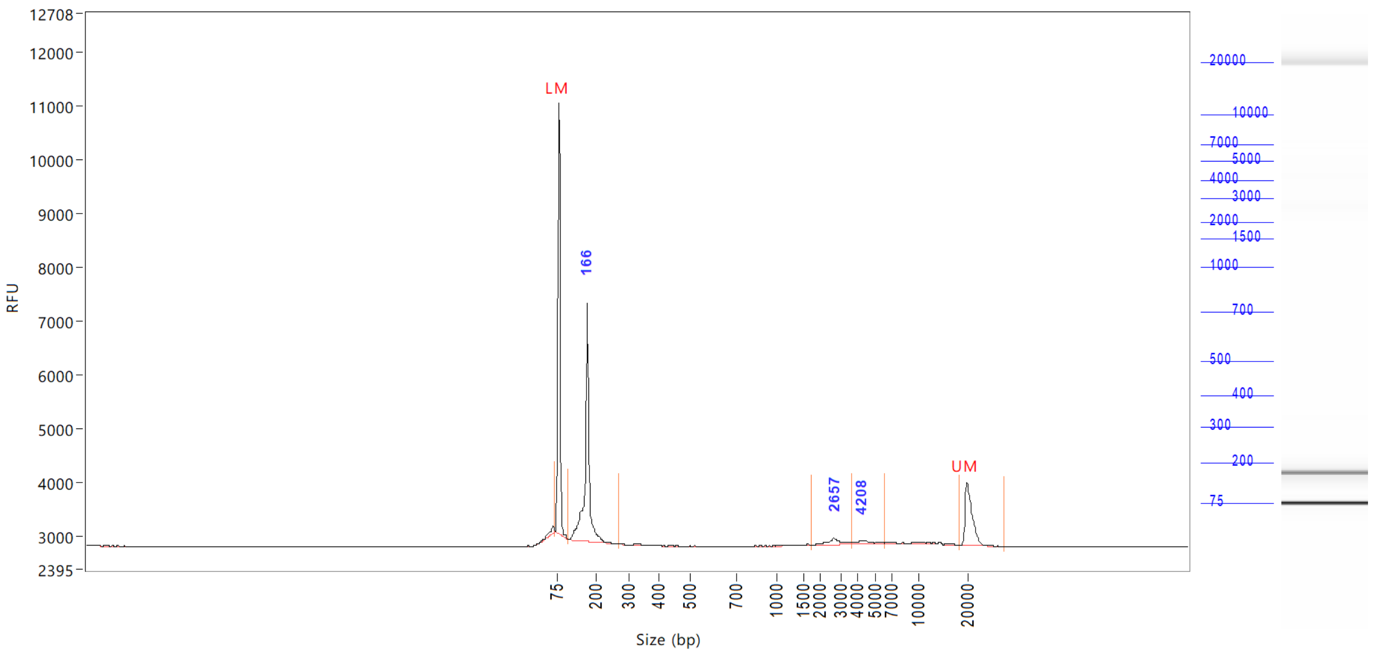

Peak	Size (bp)	Concentration (ng/uL)	From (bp)	To (bp)	Average size (bp)	CV%	RFU	Corrected peak area
1	75 (LM)	0.5000	64	107	73	4.73	8011	43.016
2	166	5.7193	107	266	162	10.19	4416	41.003
3	2657	0.3262	1731	3679	2700	15.68	121	2.339
4	4208	0.2039	3679	6112	4463	12.35	72	1.462
5	20000 (UM)	0.1255	18537	27738	20514	4.34	1176	10.793

TIC:	6.2494	ng/uL
TIM:	58.3048	nmole/L
Total concentration:	6.5151	ng/uL

Sample peak width (sec): 5 Sample min peak height: 50 Sample baseline V to V?: Y Sample baseline V to V points: 3
Sample filter: Binomial Number of points for filter: 3 Sample start region (min): 0 Sample end region (min): 30
Marker peak width (sec): 5 Marker min peak height: 200 Marker baseline V to V?: Y Marker baseline V to V points: 3
Lower marker selection: First peak > 500 RFU Upper marker selection: Last peak > 200 RFU
Ladder size (bp)75, 200, 300, 400, 500, 700, 1000, 1500, 2000, 3000, 4000, 5000, 7000, 10000, 20000
Quantification using: Lower Marker Final concentration (ng/uL): 0.5000 Dilution factor: 12.0

Sample: SampD1
Well location: D1
Created: Monday, September 20, 2021 7:19:57 PM

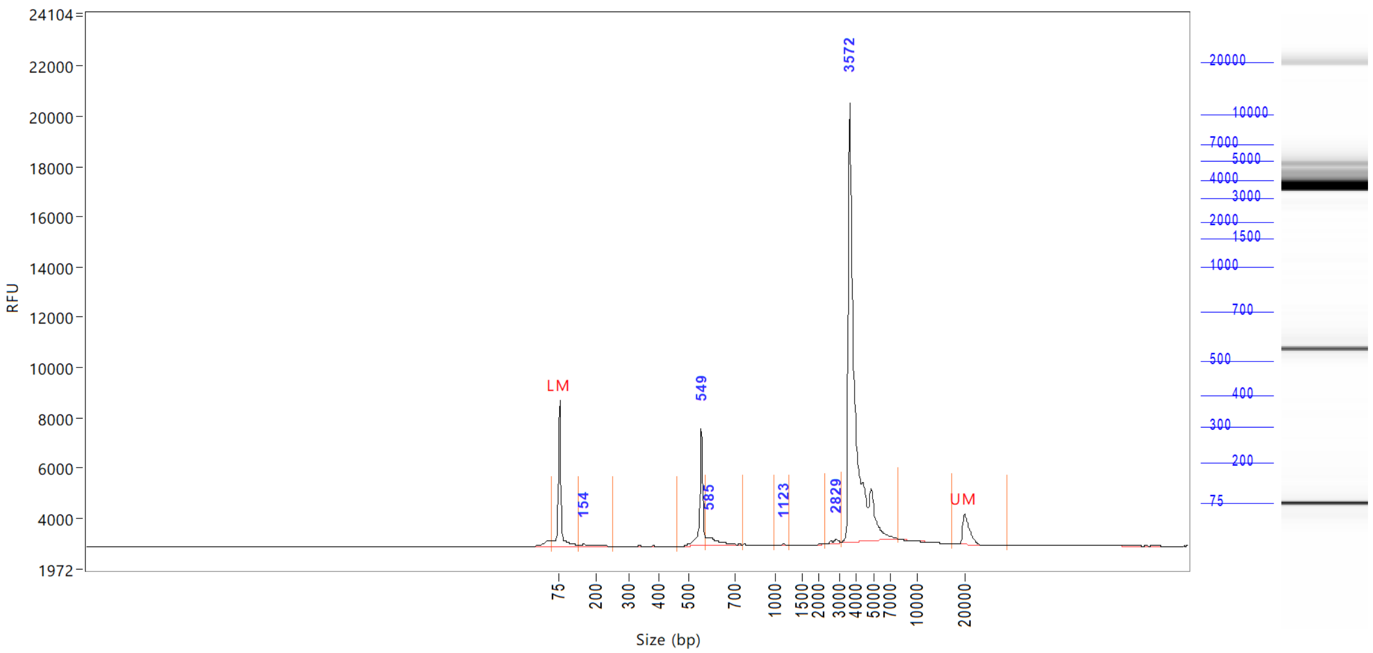

Peak	Size (bp)	Concentration (ng/uL)	From (bp)	To (bp)	Average size (bp)	CV%	RFU	Corrected peak area
1	75 (LM)	0.5000	50	139	76	16.14	5846	39.747
2	154	0.3605	139	250	175	14.20	86	2.388
3	549	4.1037	462	570	543	2.53	4670	27.185
4	585	1.1306	570	749	613	6.11	335	7.490
5	1123	0.0696	991	1245	1127	4.40	64	0.461
6	2829	0.2596	2286	3108	2774	5.99	179	1.720
7	3572	27.2441	3108	7867	3938	14.34	17518	180.477
8	20000 (UM)	0.1411	17439	29068	20507	4.75	1230	11.214

TIC:	33.1681	ng/uL
TIM:	30.5033	nmole/L
Total concentration:	33.3889	ng/uL

```
Sample peak width (sec): 5    Sample min peak height: 50    Sample baseline V to V?: Y    Sample baseline V to V points: 3
Sample filter: Binomial       Number of points for filter: 3  Sample start region (min): 0  Sample end region (min): 30
Marker peak width (sec): 5    Marker min peak height: 200   Marker baseline V to V?: Y    Marker baseline V to V points: 3
Lower marker selection: First peak > 500 RFU                Upper marker selection: Last peak > 200 RFU
Ladder size (bp)75, 200, 300, 400, 500, 700, 1000, 1500, 2000, 3000, 4000, 5000, 7000, 10000, 20000
Quantification using: Lower Marker          Final concentration (ng/uL): 0.5000          Dilution factor: 12.0
```

Sample: SampE1
Well location: E1
Created: Monday, September 20, 2021 7:19:57 PM

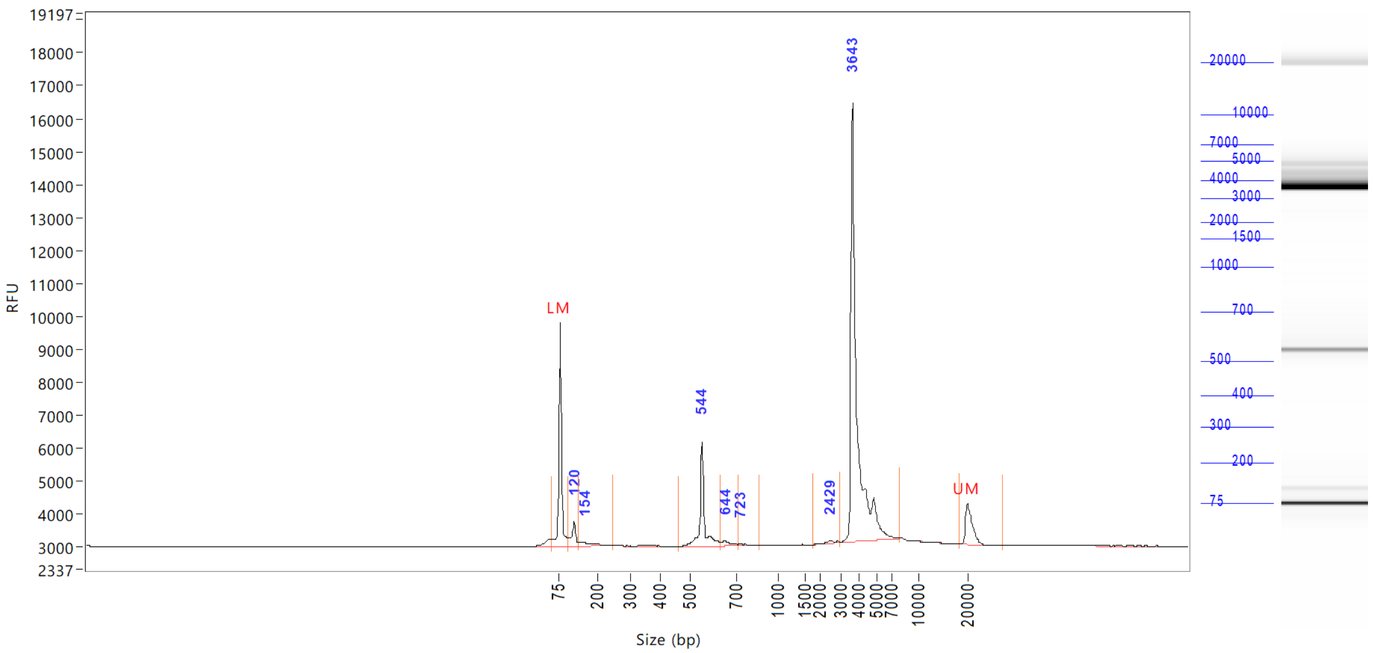

Peak	Size (bp)	Concentration (ng/uL)	From (bp)	To (bp)	Average size (bp)	CV%	RFU	Corrected peak area
1	75 (LM)	0.5000	47	101	73	10.48	6775	46.028
2	120	1.0370	101	139	116	7.42	742	7.955
3	154	0.4304	139	246	171	14.34	117	3.302
4	544	3.2010	460	629	548	4.65	3166	24.556
5	644	0.3436	629	701	658	3.18	142	2.636
6	723	0.1229	701	861	747	4.82	71	0.943
7	2429	0.1421	1731	2943	2447	10.71	85	1.090
8	3643	15.7209	2943	7867	3928	13.69	13330	120.600
9	20000 (UM)	0.1218	18293	27375	20424	4.10	1246	11.212

TIC:	20.9980	ng/uL
TIM:	36.2497	nmole/L
Total concentration:	21.1199	ng/uL

Sample peak width (sec): 5   Sample min peak height: 50   Sample baseline V to V?: Y   Sample baseline V to V points: 3
Sample filter: Binomial   Number of points for filter: 3 Sample start region (min): 0 Sample end region (min): 30
Marker peak width (sec): 5   Marker min peak height: 200   Marker baseline V to V?: Y   Marker baseline V to V points: 3
Lower marker selection: First peak > 500 RFU   Upper marker selection: Last peak > 200 RFU
Ladder size (bp)75, 200, 300, 400, 500, 700, 1000, 1500, 2000, 3000, 4000, 5000, 7000, 10000, 20000
Quantification using: Lower Marker   Final concentration (ng/uL): 0.5000   Dilution factor: 12.0

Sample: SampF1
Well location: F1
Created: Monday, September 20, 2021 7:19:57 PM

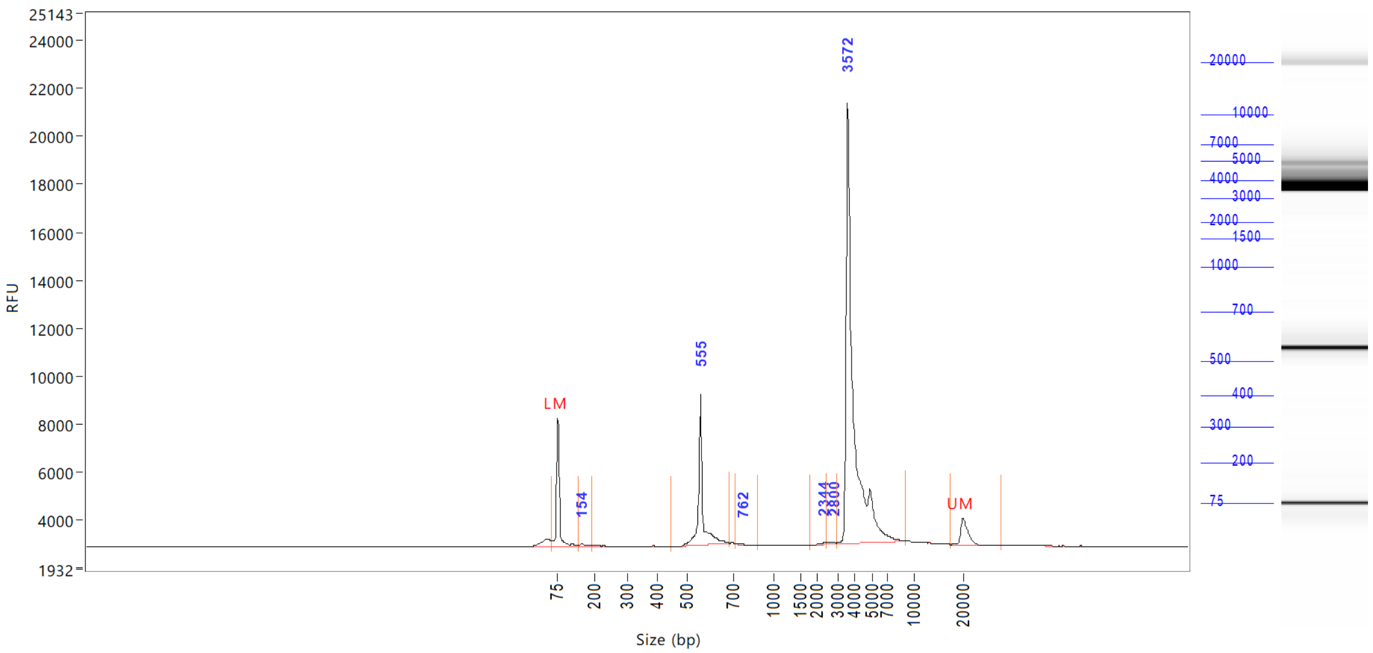

Peak	Size (bp)	Concentration (ng/uL)	From (bp)	To (bp)	Average size (bp)	CV%	RFU	Corrected peak area
1	75 (LM)	0.5000	60	143	77	15.88	5366	36.175
2	154	0.2602	143	189	161	7.55	87	1.569
3	555	7.9657	447	685	557	5.02	6290	48.027
4	762	0.0704	714	874	754	4.16	55	0.424
5	2344	0.1273	1770	2486	2273	6.41	99	0.768
6	2800	0.1495	2486	2943	2700	4.79	87	0.901
7	3572	33.5005	2943	9000	3987	17.18	18426	201.980
8	20000 (UM)	0.1473	17439	28100	20492	4.55	1137	10.654

TIC:	42.0736	ng/uL
TIM:	40.3602	nmole/L
Total concentration:	42.4063	ng/uL

Sample peak width (sec): 5 Sample min peak height: 50 Sample baseline V to V?: Y Sample baseline V to V points: 3
Sample filter: Binomial Number of points for filter: 3 Sample start region (min): 0 Sample end region (min): 30
Marker peak width (sec): 5 Marker min peak height: 200 Marker baseline V to V?: Y Marker baseline V to V points: 3
Lower marker selection: First peak > 500 RFU Upper marker selection: Last peak > 200 RFU
Ladder size (bp)75, 200, 300, 400, 500, 700, 1000, 1500, 2000, 3000, 4000, 5000, 7000, 10000, 20000
Quantification using: Lower Marker Final concentration (ng/uL): 0.5000 Dilution factor: 12.0

Sample: SampG1
Well location: G1
Created: Monday, September 20, 2021 7:19:57 PM

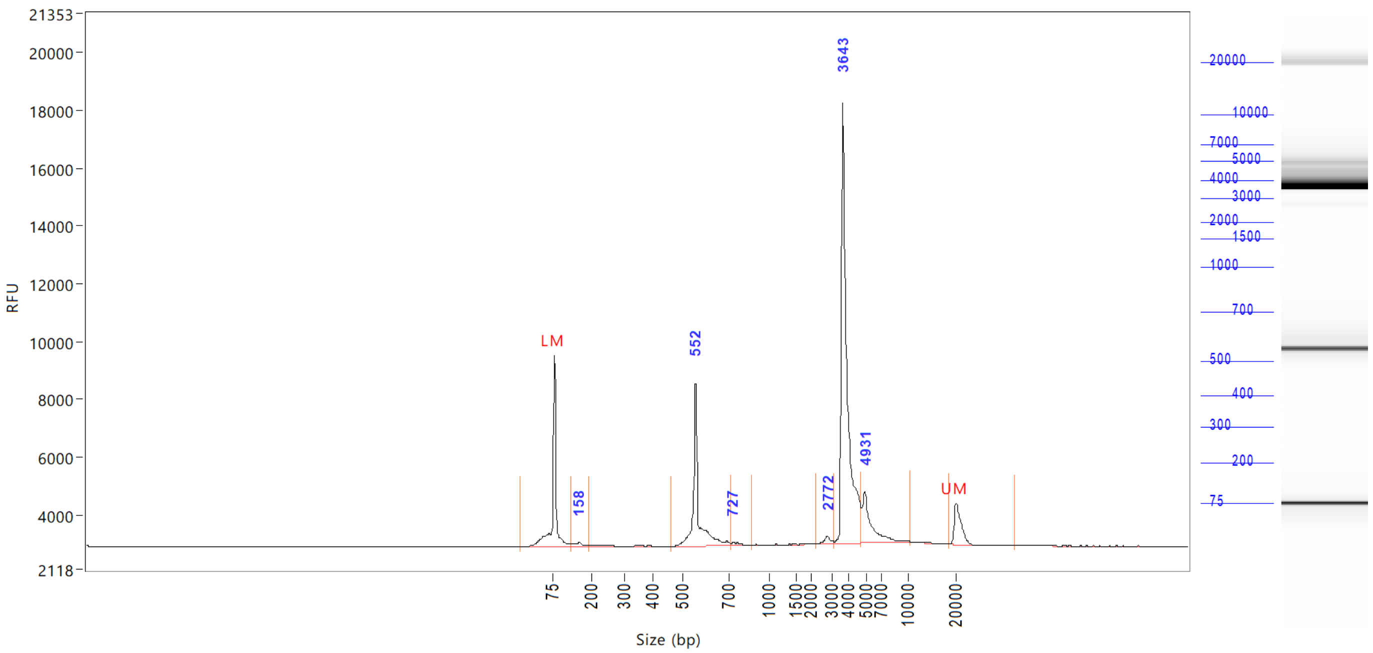

Peak	Size (bp)	Concentration (ng/uL)	From (bp)	To (bp)	Average size (bp)	CV%	RFU	Corrected peak area
1	75 (LM)	0.5000	0	129	68	29.58	6613	55.045
2	158	0.3304	129	192	155	10.49	141	3.031
3	552	5.7856	462	710	564	7.20	5634	53.078
4	727	0.1998	710	870	759	5.00	118	1.833
5	2772	0.2940	2229	3108	2755	6.39	276	2.698
6	3643	14.7692	3108	4759	3849	8.26	15270	135.495
7	4931	2.5621	4759	10367	5662	18.85	1773	23.505
8	20000 (UM)	0.1223	18537	32332	20632	6.13	1446	13.461

TIC:	23.9412	ng/uL
TIM:	28.0550	nmole/L
Total concentration:	24.1928	ng/uL

Sample peak width (sec): 5   Sample min peak height: 50   Sample baseline V to V?: Y   Sample baseline V to V points: 3
Sample filter: Binomial   Number of points for filter: 3 Sample start region (min): 0 Sample end region (min): 30
Marker peak width (sec): 5   Marker min peak height: 200   Marker baseline V to V?: Y   Marker baseline V to V points: 3
Lower marker selection: First peak > 500 RFU   Upper marker selection: Last peak > 200 RFU
Ladder size (bp)75, 200, 300, 400, 500, 700, 1000, 1500, 2000, 3000, 4000, 5000, 7000, 10000, 20000
Quantification using: Lower Marker   Final concentration (ng/uL): 0.5000   Dilution factor: 12.0

Sample: SampH1
Well location: H1
Created: Monday, September 20, 2021 7:19:57 PM

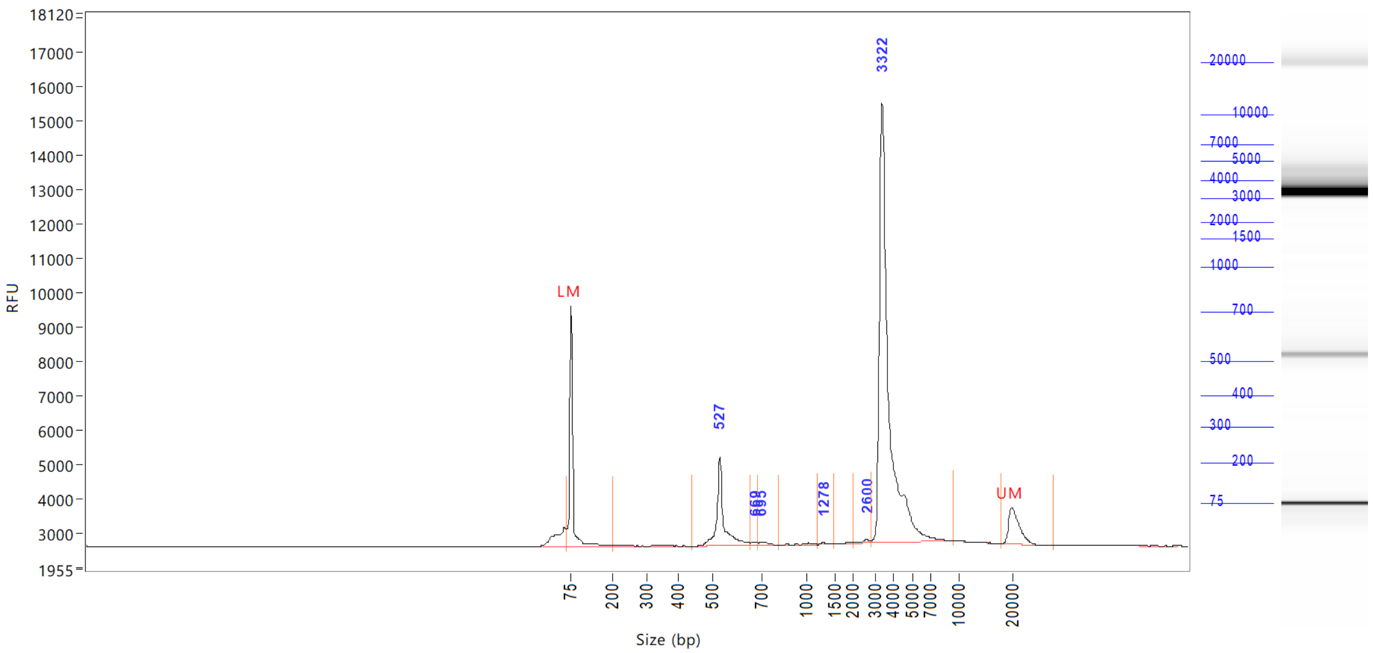

Peak	Size (bp)	Concentration (ng/uL)	From (bp)	To (bp)	Average size (bp)	CV%	RFU	Corrected peak area
1	75 (LM)	0.5000	62	203	80	26.60	6994	50.148
2	527	3.8570	437	649	535	6.13	2577	32.237
3	669	0.1249	649	682	665	1.47	95	1.044
4	695	0.1741	682	809	715	3.81	81	1.455
5	1278	0.0612	1178	1478	1310	4.78	54	0.512
6	2600	0.1281	2030	2800	2530	7.34	100	1.070
7	3322	20.5665	2800	9400	3668	17.91	12803	171.894
8	20000 (UM)	0.1411	17927	27979	20642	5.59	1070	14.153

TIC:	24.9118	ng/uL
TIM:	21.9640	nmole/L
Total concentration:	25.2277	ng/uL

Sample peak width (sec): 5 Sample min peak height: 50 Sample baseline V to V?: Y Sample baseline V to V points: 3
Sample filter: Binomial Number of points for filter: 3 Sample start region (min): 0 Sample end region (min): 30
Marker peak width (sec): 5 Marker min peak height: 200 Marker baseline V to V?: Y Marker baseline V to V points: 3
Lower marker selection: First peak > 500 RFU Upper marker selection: Last peak > 200 RFU
Ladder size (bp)75, 200, 300, 400, 500, 700, 1000, 1500, 2000, 3000, 4000, 5000, 7000, 10000, 20000
Quantification using: Lower Marker Final concentration (ng/uL): 0.5000 Dilution factor: 12.0

Sample: SampA2
Well location: A2
Created: Monday, September 20, 2021 7:19:57 PM

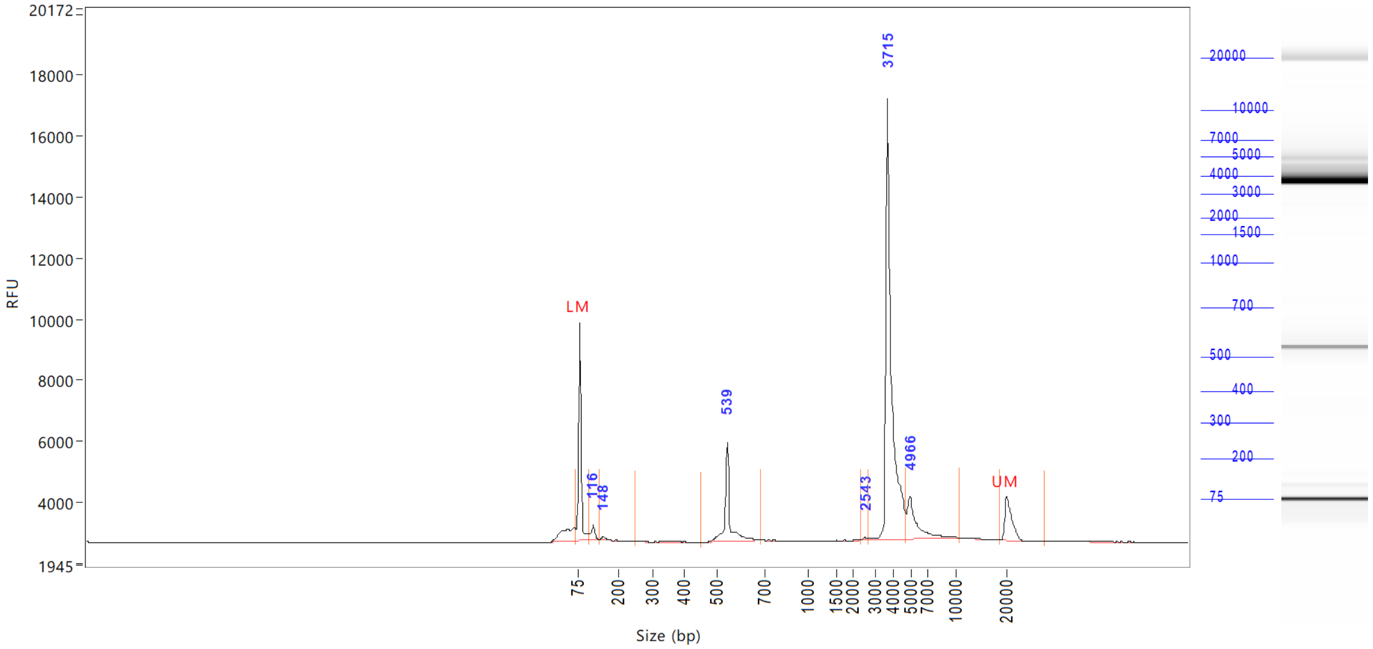

Peak	Size (bp)	Concentration (ng/uL)	From (bp)	To (bp)	Average size (bp)	CV%	RFU	Corrected peak area
1	75 (LM)	0.5000	64	107	75	8.96	7127	44.261
2	116	0.4972	107	137	115	4.19	464	3.668
3	148	0.1399	137	246	158	13.69	101	1.032
4	539	3.4991	450	680	544	5.48	3246	25.812
5	2543	0.0846	2372	2743	2559	3.73	94	0.624
6	3715	16.3990	2743	4759	3871	7.63	14476	120.973
7	4966	2.4803	4759	10489	5649	18.62	1399	18.297
8	20000 (UM)	0.1425	18659	27617	20451	4.00	1429	12.617

TIC:	23.1001	ng/uL
TIM:	26.8942	nmole/L
Total concentration:	23.3496	ng/uL

Sample peak width (sec): 5   Sample min peak height: 50   Sample baseline V to V?: Y   Sample baseline V to V points: 3
Sample filter: Binomial   Number of points for filter: 3 Sample start region (min): 0 Sample end region (min): 30
Marker peak width (sec): 5   Marker min peak height: 200   Marker baseline V to V?: Y   Marker baseline V to V points: 3
Lower marker selection: First peak > 500 RFU   Upper marker selection: Last peak > 200 RFU
Ladder size (bp)75, 200, 300, 400, 500, 700, 1000, 1500, 2000, 3000, 4000, 5000, 7000, 10000, 20000
Quantification using: Lower Marker   Final concentration (ng/uL): 0.5000   Dilution factor: 12.0

Sample: SampB2
Well location: B2
Created: Monday, September 20, 2021 7:19:57 PM

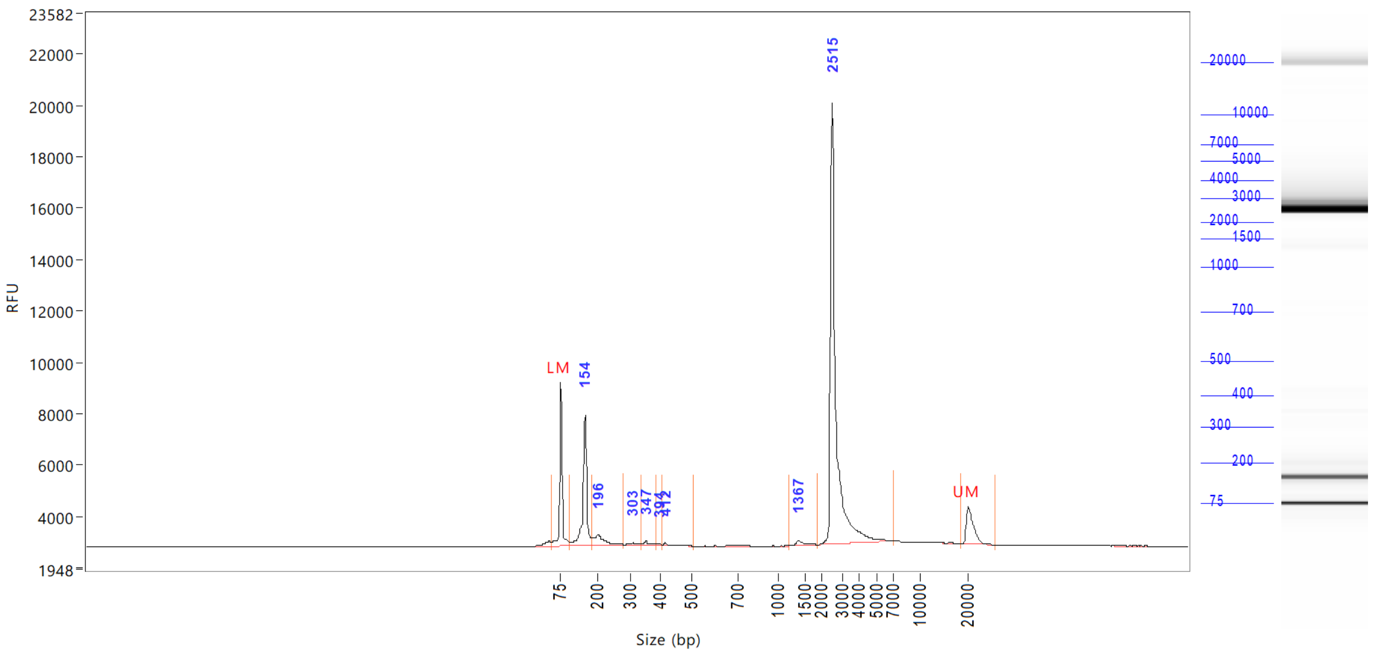

Peak	Size (bp)	Concentration (ng/uL)	From (bp)	To (bp)	Average size (bp)	CV%	RFU	Corrected peak area
1	75 (LM)	0.5000	45	107	73	11.38	6346	40.236
2	154	6.5910	107	181	150	8.09	5054	44.199
3	196	1.2921	181	277	205	8.94	397	8.665
4	303	0.2011	277	333	307	4.40	73	1.349
5	347	0.2373	333	386	355	4.01	157	1.591
6	394	0.0659	386	403	394	1.28	58	0.442
7	412	0.1131	403	509	420	4.72	99	0.758
8	1367	0.3546	1201	1885	1415	7.59	207	2.378
9	2515	21.3766	1885	7001	2742	18.73	17191	143.351
10	20000 (UM)	0.1689	18415	25682	20515	4.17	1472	13.590

TIC:	30.2317	ng/uL
TIM:	98.7211	nmole/L
Total concentration:	30.3961	ng/uL

Sample peak width (sec): 5   Sample min peak height: 50   Sample baseline V to V?: Y   Sample baseline V to V points: 3
Sample filter: Binomial   Number of points for filter: 3 Sample start region (min): 0 Sample end region (min): 30
Marker peak width (sec): 5   Marker min peak height: 200   Marker baseline V to V?: Y   Marker baseline V to V points: 3
Lower marker selection: First peak > 500 RFU   Upper marker selection: Last peak > 200 RFU
Ladder size (bp)75, 200, 300, 400, 500, 700, 1000, 1500, 2000, 3000, 4000, 5000, 7000, 10000, 20000
Quantification using: Lower Marker   Final concentration (ng/uL): 0.5000   Dilution factor: 12.0

Sample: SampC2
Well location: C2
Created: Monday, September 20, 2021 7:19:57 PM

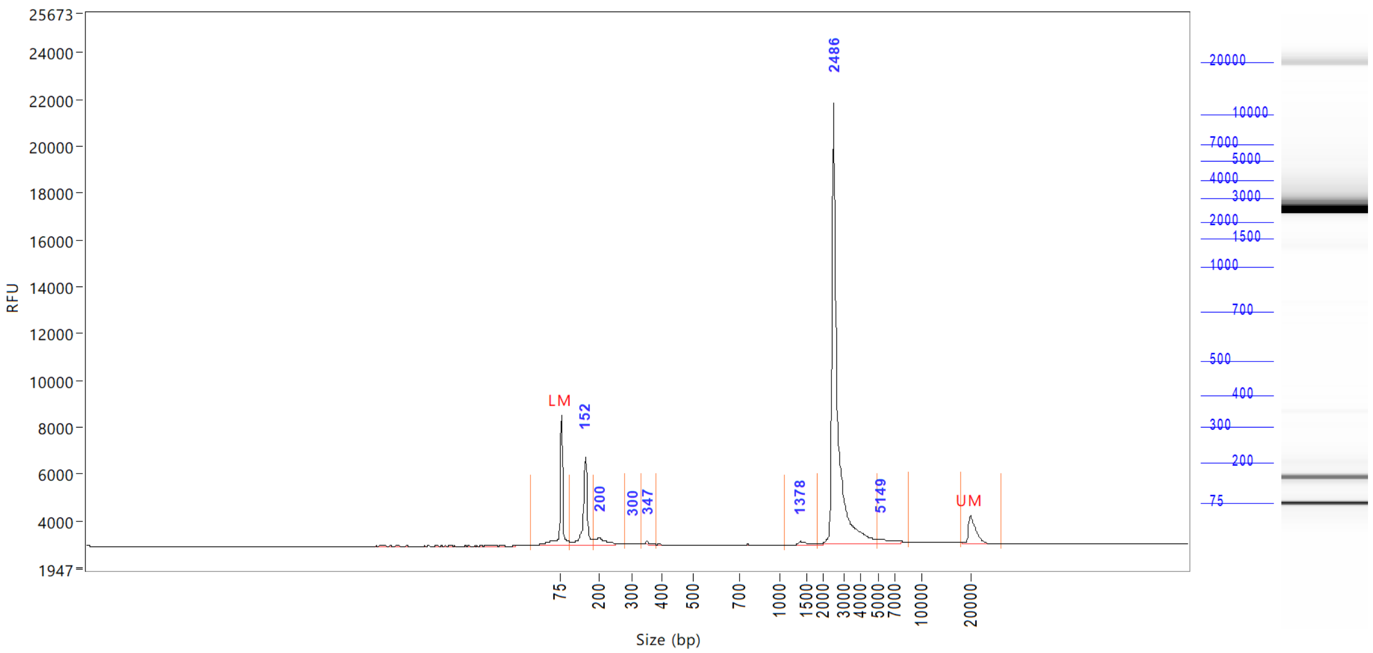

Peak	Size (bp)	Concentration (ng/uL)	From (bp)	To (bp)	Average size (bp)	CV%	RFU	Corrected peak area
1	75 (LM)	0.5000	0	105	68	24.94	5550	40.065
2	152	5.2153	105	179	149	8.36	3761	34.825
3	200	1.1237	179	277	208	10.59	289	7.503
4	300	0.1504	277	331	302	4.38	57	1.005
5	347	0.1796	331	382	348	2.78	180	1.200
6	1378	0.3355	1079	1808	1443	9.41	149	2.241
7	2486	24.2971	1808	4931	2707	17.02	18870	162.243
8	5149	0.6247	4931	8401	6225	15.94	175	4.171
9	20000 (UM)	0.1372	18049	26408	20506	4.40	1177	10.993

TIC:	31.9264	ng/uL
TIM:	83.4007	nmole/L
Total concentration:	32.2594	ng/uL

```
Sample peak width (sec): 5    Sample min peak height: 50    Sample baseline V to V?: Y    Sample baseline V to V points: 3
Sample filter: Binomial       Number of points for filter: 3  Sample start region (min): 0  Sample end region (min): 30
Marker peak width (sec): 5    Marker min peak height: 200   Marker baseline V to V?: Y    Marker baseline V to V points: 3
Lower marker selection: First peak > 500 RFU                Upper marker selection: Last peak > 200 RFU
Ladder size (bp)75, 200, 300, 400, 500, 700, 1000, 1500, 2000, 3000, 4000, 5000, 7000, 10000, 20000
Quantification using: Lower Marker          Final concentration (ng/uL): 0.5000          Dilution factor: 12.0
```

Sample: SampD2
Well location: D2
Created: Monday, September 20, 2021 7:19:57 PM

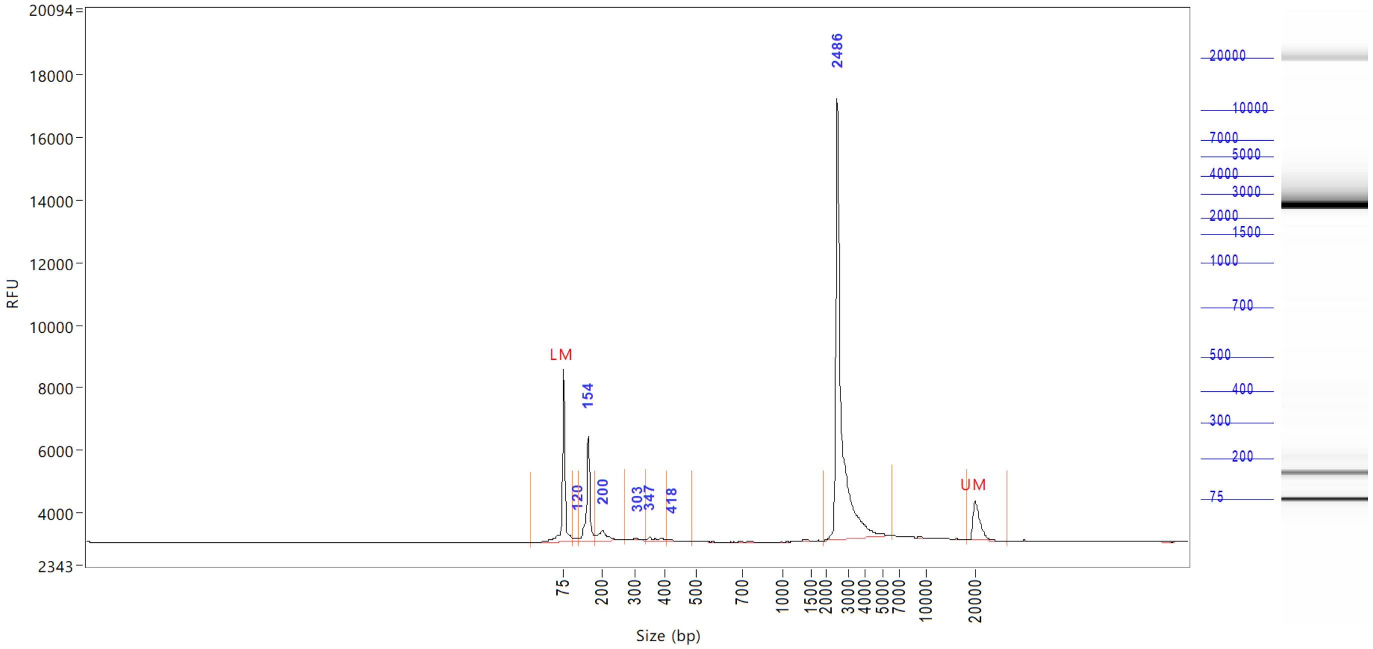

Peak	Size (bp)	Concentration (ng/uL)	From (bp)	To (bp)	Average size (bp)	CV%	RFU	Corrected peak area
1	75 (LM)	0.5000	0	107	70	19.07	5485	37.651
2	120	0.2392	107	126	116	5.06	125	1.501
3	154	4.3235	126	177	151	5.61	3331	27.131
4	200	1.1196	177	269	203	8.70	326	7.026
5	303	0.1756	269	335	306	5.03	54	1.102
6	347	0.3434	335	407	369	5.51	115	2.155
7	418	0.0984	407	487	425	4.19	53	0.617
8	2486	19.2846	1885	6186	2733	16.94	14098	121.016
9	20000 (UM)	0.1483	18537	26649	20479	4.08	1250	11.166

TIC:	25.5844	ng/uL
TIM:	73.9870	nmole/L
Total concentration:	25.8318	ng/uL

```
Sample peak width (sec): 5    Sample min peak height: 50    Sample baseline V to V?: Y    Sample baseline V to V points: 3
Sample filter: Binomial       Number of points for filter: 3  Sample start region (min): 0  Sample end region (min): 30
Marker peak width (sec): 5    Marker min peak height: 200   Marker baseline V to V?: Y    Marker baseline V to V points: 3
Lower marker selection: First peak > 500 RFU                Upper marker selection: Last peak > 200 RFU
Ladder size (bp)75, 200, 300, 400, 500, 700, 1000, 1500, 2000, 3000, 4000, 5000, 7000, 10000, 20000
Quantification using: Lower Marker          Final concentration (ng/uL): 0.5000          Dilution factor: 12.0
```

Sample: SampE2
Well location: E2
Created: Monday, September 20, 2021 7:19:57 PM

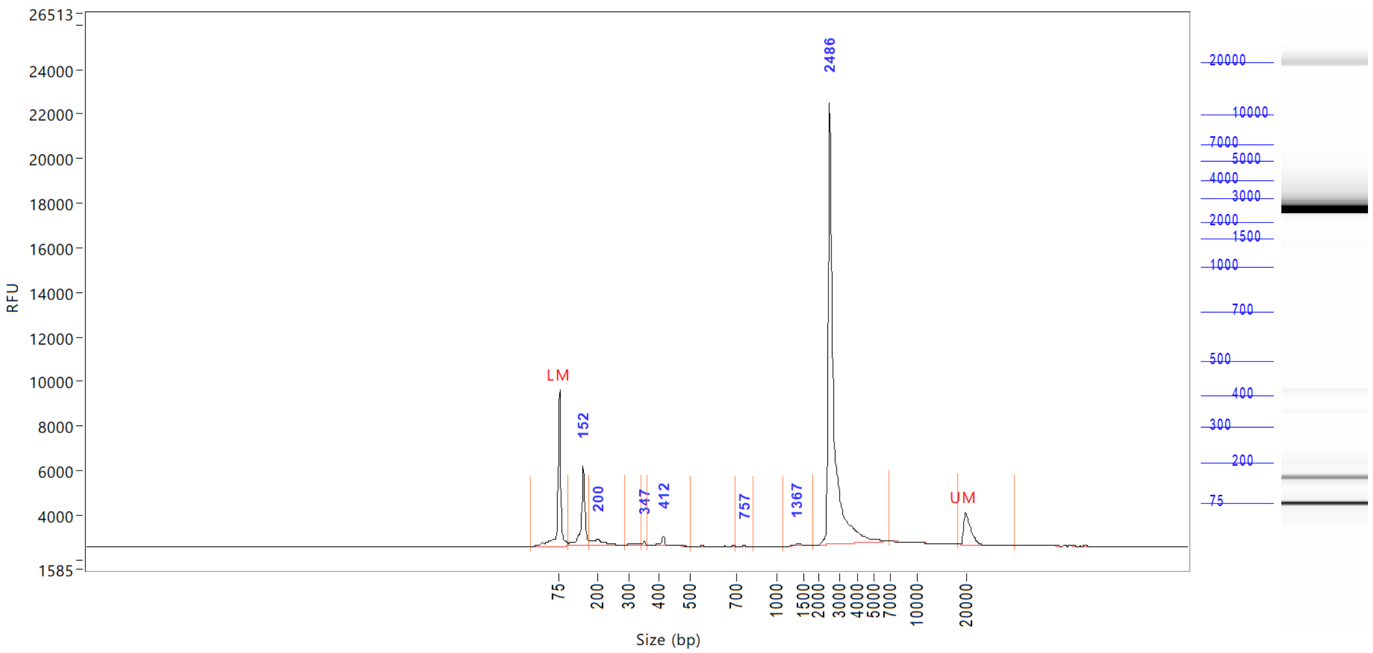

Peak	Size (bp)	Concentration (ng/uL)	From (bp)	To (bp)	Average size (bp)	CV%	RFU	Corrected peak area
1	75 (LM)	0.5000	0	105	68	24.46	7024	51.449
2	152	3.6033	105	175	147	8.11	3586	30.897
3	200	0.8678	175	284	203	9.66	307	7.441
4	347	0.1039	337	360	345	1.30	172	0.891
5	412	0.3803	360	504	407	4.16	448	3.261
6	757	0.0467	700	831	749	3.03	53	0.401
7	1367	0.1249	1123	1827	1423	6.98	81	1.071
8	2486	19.4170	1827	6704	2723	18.08	19827	166.496
9	20000 (UM)	0.1327	18293	30277	20521	5.00	1465	13.658

TIC:	24.5439	ng/uL
TIM:	61.3320	nmole/L
Total concentration:	24.7454	ng/uL

Sample peak width (sec): 5   Sample min peak height: 50   Sample baseline V to V?: Y   Sample baseline V to V points: 3
Sample filter: Binomial   Number of points for filter: 3 Sample start region (min): 0 Sample end region (min): 30
Marker peak width (sec): 5   Marker min peak height: 200   Marker baseline V to V?: Y   Marker baseline V to V points: 3
Lower marker selection: First peak > 500 RFU   Upper marker selection: Last peak > 200 RFU
Ladder size (bp)75, 200, 300, 400, 500, 700, 1000, 1500, 2000, 3000, 4000, 5000, 7000, 10000, 20000
Quantification using: Lower Marker   Final concentration (ng/uL): 0.5000   Dilution factor: 12.0

Sample: SampF2
Well location: F2
Created: Monday, September 20, 2021 7:19:57 PM

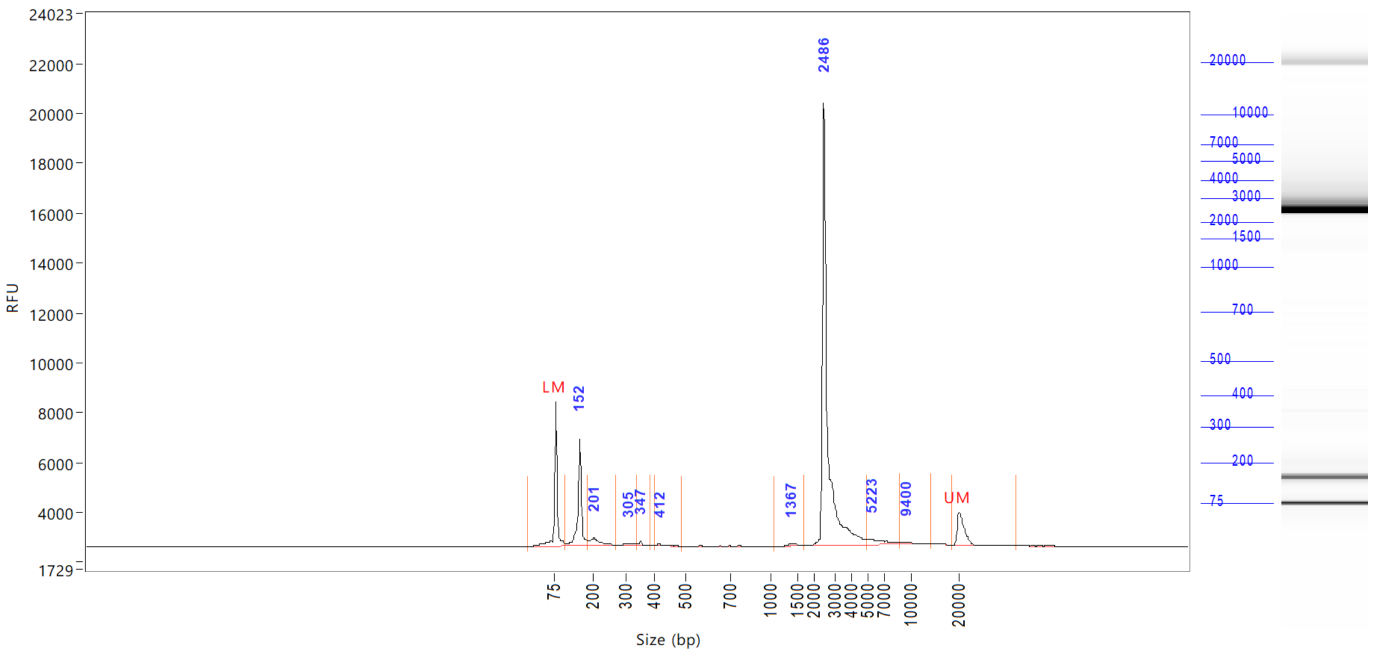

Peak	Size<br>(bp)	Concentration<br>(ng/uL)	From<br>(bp)	To<br>(bp)	Average size<br>(bp)	CV%	RFU	Corrected peak area
1	75 (LM)	0.5000	0	105	70	20.67	5814	39.108
2	152	5.6057	105	179	150	7.49	4298	36.538
3	201	1.0310	179	271	204	8.78	292	6.720
4	305	0.2196	271	337	311	4.57	70	1.431
5	347	0.2140	337	382	353	3.36	177	1.395
6	412	0.1210	401	489	418	4.44	100	0.789
7	1367	0.2313	1056	1693	1423	7.72	95	1.508
8	2486	22.0732	1693	4897	2711	18.05	17775	143.873
9	5223	0.8463	4897	8667	6301	16.92	206	5.516
10	9400	0.1778	8667	14147	10038	11.69	56	1.159
11	20000 (UM)	0.1520	18415	31969	20552	4.88	1299	11.889

TIC:	30.5199	ng/uL
TIM:	86.2873	nmole/L
Total concentration:	30.6668	ng/uL

Sample peak width (sec): 5 Sample min peak height: 50 Sample baseline V to V?: Y Sample baseline V to V points: 3
Sample filter: Binomial Number of points for filter: 3 Sample start region (min): 0 Sample end region (min): 30
Marker peak width (sec): 5 Marker min peak height: 200 Marker baseline V to V?: Y Marker baseline V to V points: 3
Lower marker selection: First peak > 500 RFU Upper marker selection: Last peak > 200 RFU
Ladder size (bp)75, 200, 300, 400, 500, 700, 1000, 1500, 2000, 3000, 4000, 5000, 7000, 10000, 20000
Quantification using: Lower Marker Final concentration (ng/uL): 0.5000 Dilution factor: 12.0

Sample: SampG2
Well location: G2
Created: Monday, September 20, 2021 7:19:57 PM

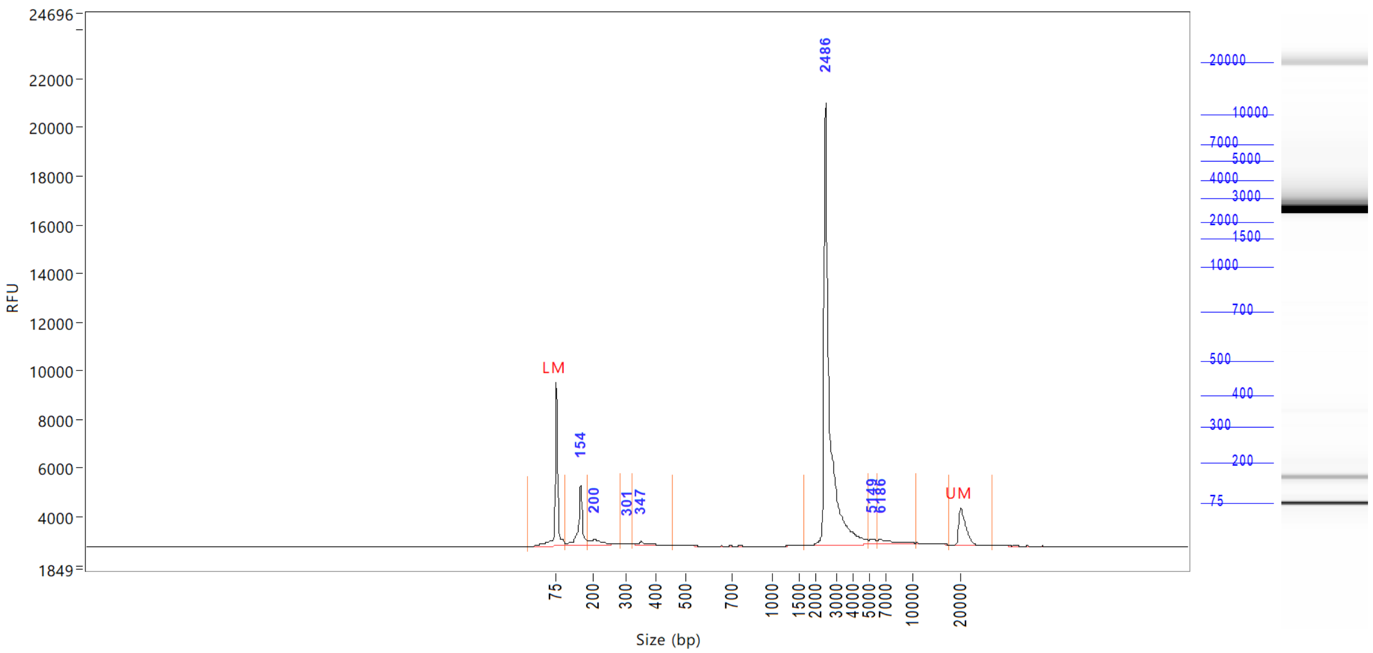

Peak	Size (bp)	Concentration (ng/uL)	From (bp)	To (bp)	Average size (bp)	CV%	RFU	Corrected peak area
1	75 (LM)	0.5000	0	107	70	20.30	6724	45.072
2	154	3.1703	107	177	149	8.37	2468	23.815
3	200	0.9075	177	280	208	11.43	229	6.817
4	301	0.1022	280	319	301	3.43	50	0.768
5	347	0.3074	319	454	358	6.90	154	2.309
6	2486	21.7562	1635	4931	2727	17.07	18208	163.431
7	5149	0.2667	4931	5815	5306	5.37	197	2.003
8	6186	0.6372	5815	10489	7432	16.25	186	4.787
9	20000 (UM)	0.1560	17561	26649	20464	4.13	1526	14.063

TIC:	27.1475	ng/uL
TIM:	57.4708	nmole/L
Total concentration:	27.4105	ng/uL

Sample peak width (sec): 5   Sample min peak height: 50   Sample baseline V to V?: Y   Sample baseline V to V points: 3
Sample filter: Binomial   Number of points for filter: 3 Sample start region (min): 0 Sample end region (min): 30
Marker peak width (sec): 5   Marker min peak height: 200   Marker baseline V to V?: Y   Marker baseline V to V points: 3
Lower marker selection: First peak > 500 RFU   Upper marker selection: Last peak > 200 RFU
Ladder size (bp)75, 200, 300, 400, 500, 700, 1000, 1500, 2000, 3000, 4000, 5000, 7000, 10000, 20000
Quantification using: Lower Marker   Final concentration (ng/uL): 0.5000   Dilution factor: 12.0

Sample: SampH2
Well location: H2
Created: Monday, September 20, 2021 7:19:57 PM

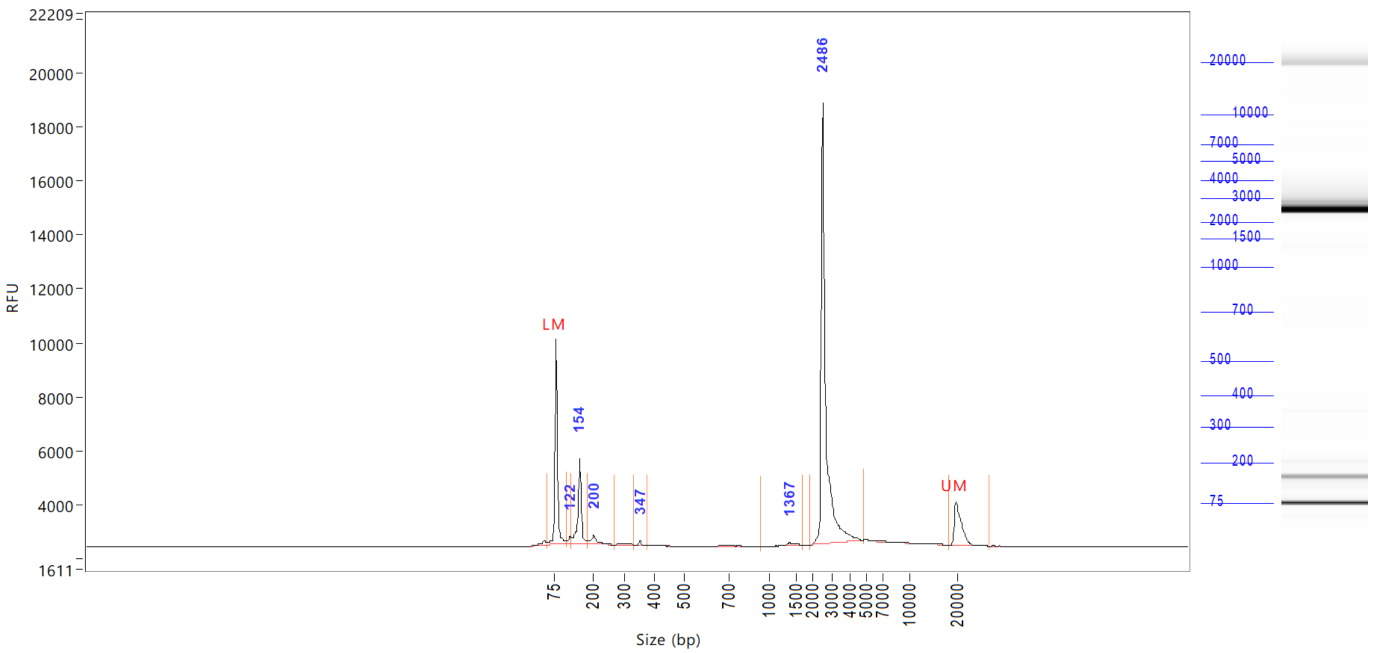

Peak	Size (bp)	Concentration (ng/uL)	From (bp)	To (bp)	Average size (bp)	CV%	RFU	Corrected peak area
1	75 (LM)	0.5000	47	110	75	9.12	7594	48.512
2	122	0.2480	110	128	120	4.05	262	2.005
3	154	3.1415	128	179	151	5.65	3121	25.401
4	200	0.5504	179	264	201	6.79	318	4.450
5	347	0.1192	331	376	347	2.29	150	0.963
6	1367	0.1275	935	1674	1383	7.29	101	1.031
7	2486	14.6361	1923	4862	2645	13.09	16358	118.338
8	20000 (UM)	0.1548	18415	26891	20478	4.35	1592	15.021

TIC:	18.8227	ng/uL
TIM:	51.9175	nmole/L
Total concentration:	19.2452	ng/uL

Sample peak width (sec): 5 Sample min peak height: 50 Sample baseline V to V?: Y Sample baseline V to V points: 3
Sample filter: Binomial Number of points for filter: 3 Sample start region (min): 0 Sample end region (min): 30
Marker peak width (sec): 5 Marker min peak height: 200 Marker baseline V to V?: Y Marker baseline V to V points: 3
Lower marker selection: First peak > 500 RFU Upper marker selection: Last peak > 200 RFU
Ladder size (bp)75, 200, 300, 400, 500, 700, 1000, 1500, 2000, 3000, 4000, 5000, 7000, 10000, 20000
Quantification using: Lower Marker Final concentration (ng/uL): 0.5000 Dilution factor: 12.0

Sample: SampA3
Well location: A3
Created: Monday, September 20, 2021 7:19:57 PM

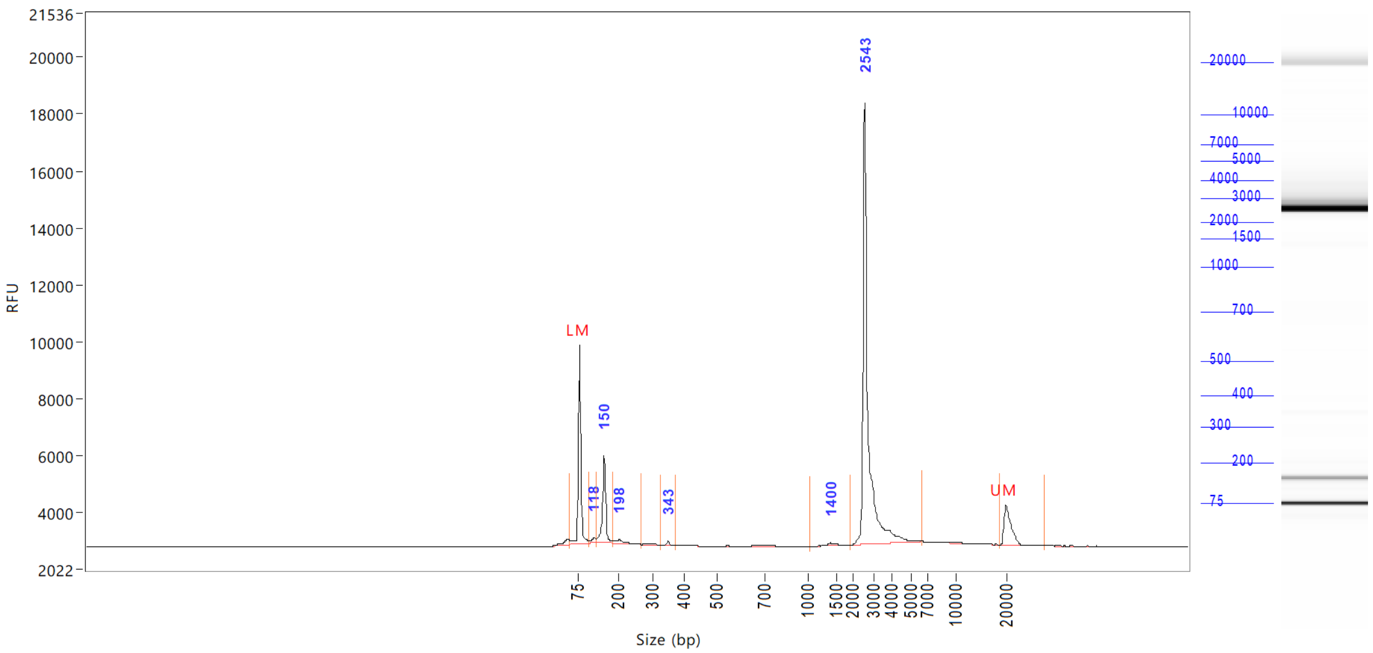

Peak	Size (bp)	Concentration (ng/uL)	From (bp)	To (bp)	Average size (bp)	CV%	RFU	Corrected peak area
1	75 (LM)	0.5000	47	103	73	8.60	6978	42.767
2	118	0.2707	103	126	115	5.67	186	1.930
3	150	3.3003	126	181	148	5.36	3053	23.523
4	198	0.3289	181	262	204	8.09	140	2.344
5	343	0.1164	323	370	342	2.09	148	0.829
6	1400	0.1498	1034	1923	1446	8.53	94	1.068
7	2543	16.1481	1923	6408	2736	16.68	15519	115.100
8	20000 (UM)	0.1453	18781	27738	20412	4.03	1393	12.432

TIC:	20.3141	ng/uL
TIM:	53.6123	nmole/L
Total concentration:	20.7663	ng/uL

```
Sample peak width (sec): 5     Sample min peak height: 50    Sample baseline V to V?: Y    Sample baseline V to V points: 3
Sample filter: Binomial        Number of points for filter: 3  Sample start region (min): 0  Sample end region (min): 30
Marker peak width (sec): 5     Marker min peak height: 200   Marker baseline V to V?: Y    Marker baseline V to V points: 3
Lower marker selection: First peak > 500 RFU                 Upper marker selection: Last peak > 200 RFU
Ladder size (bp)75, 200, 300, 400, 500, 700, 1000, 1500, 2000, 3000, 4000, 5000, 7000, 10000, 20000
Quantification using: Lower Marker          Final concentration (ng/uL): 0.5000         Dilution factor: 12.0
```

Sample: SampB3
Well location: B3
Created: Monday, September 20, 2021 7:19:57 PM

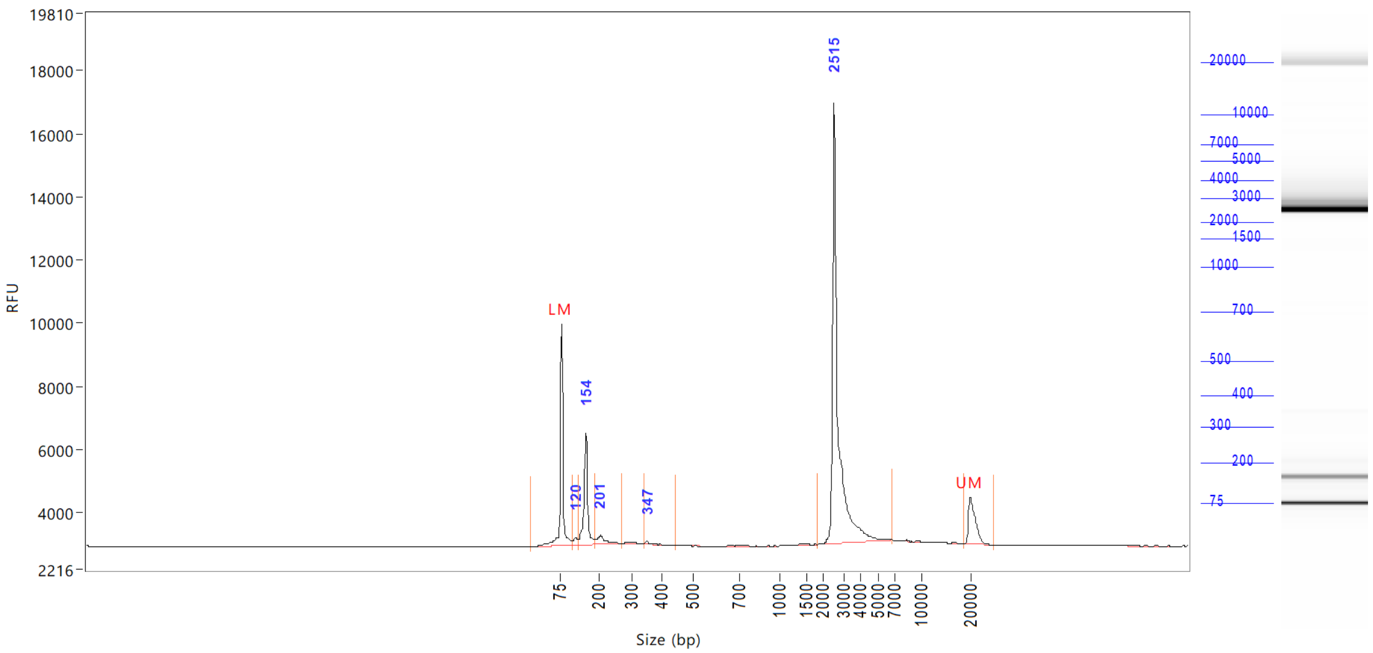

Peak	Size (bp)	Concentration (ng/uL)	From (bp)	To (bp)	Average size (bp)	CV%	RFU	Corrected peak area
1	75 (LM)	0.5000	0	110	72	18.92	7022	48.758
2	120	0.3430	110	129	120	4.78	248	2.787
3	154	3.6043	129	183	152	5.96	3554	29.290
4	201	0.6372	183	266	205	7.94	285	5.178
5	347	0.1166	337	443	359	6.38	127	0.948
6	2515	14.2956	1808	6556	2753	17.55	13966	116.171
7	20000 (UM)	0.1370	18659	24715	20413	3.85	1482	13.355

TIC:	18.9967	ng/uL
TIM:	57.8619	nmole/L
Total concentration:	19.3307	ng/uL

```
Sample peak width (sec): 5    Sample min peak height: 50    Sample baseline V to V?: Y    Sample baseline V to V points: 3
Sample filter: Binomial       Number of points for filter: 3  Sample start region (min): 0  Sample end region (min): 30
Marker peak width (sec): 5    Marker min peak height: 200   Marker baseline V to V?: Y    Marker baseline V to V points: 3
Lower marker selection: First peak > 500 RFU                Upper marker selection: Last peak > 200 RFU
Ladder size (bp)75, 200, 300, 400, 500, 700, 1000, 1500, 2000, 3000, 4000, 5000, 7000, 10000, 20000
Quantification using: Lower Marker          Final concentration (ng/uL): 0.5000          Dilution factor: 12.0
```

Sample: SampC3
Well location: C3
Created: Monday, September 20, 2021 7:19:57 PM

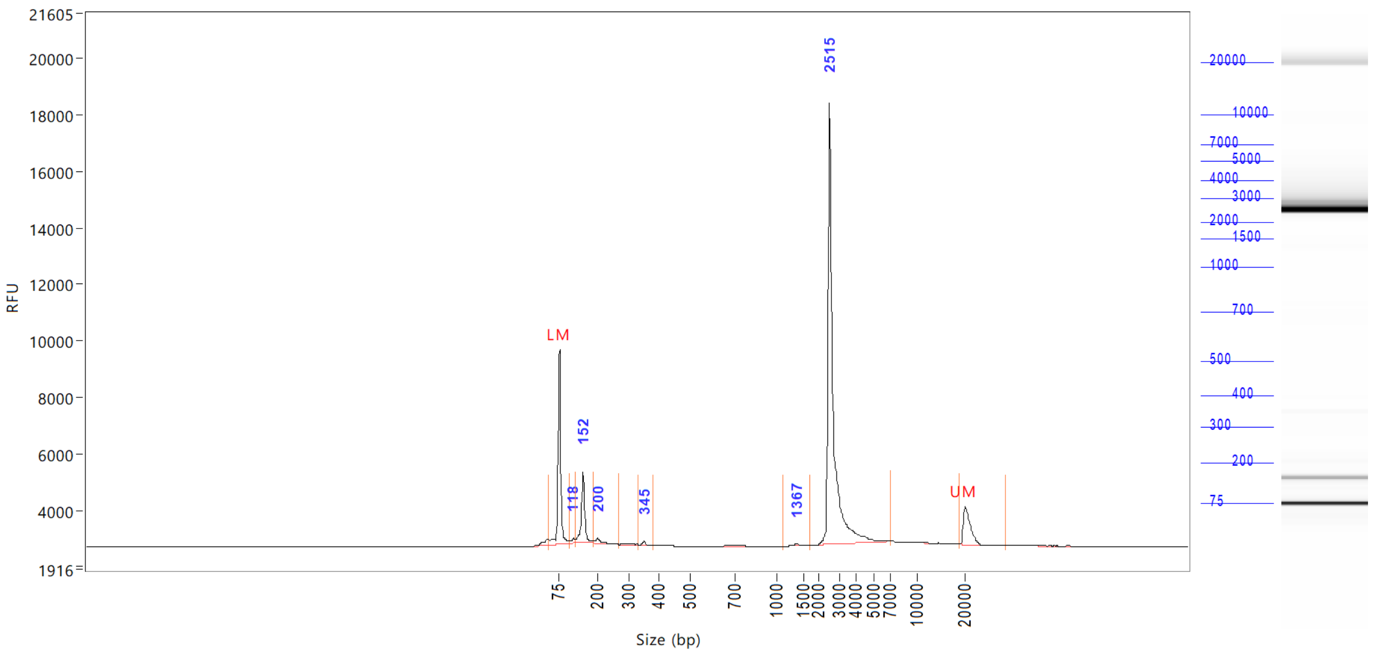

Peak	Size (bp)	Concentration (ng/uL)	From (bp)	To (bp)	Average size (bp)	CV%	RFU	Corrected peak area
1	75 (LM)	0.5000	43	108	73	10.62	6890	45.854
2	118	0.1894	108	126	117	4.27	161	1.447
3	152	2.6866	126	187	150	6.07	2535	20.532
4	200	0.3096	187	264	206	7.20	167	2.366
5	345	0.1118	329	378	344	1.81	157	0.854
6	1367	0.0857	1134	1712	1414	6.11	58	0.655
7	2515	16.2408	1712	7000	2721	17.83	15655	124.117
8	20000 (UM)	0.1372	18903	28584	20528	4.43	1375	12.578

TIC:	19.6238	ng/uL
TIM:	45.0263	nmole/L
Total concentration:	19.9691	ng/uL

```
Sample peak width (sec): 5    Sample min peak height: 50    Sample baseline V to V?: Y    Sample baseline V to V points: 3
Sample filter: Binomial       Number of points for filter: 3  Sample start region (min): 0  Sample end region (min): 30
Marker peak width (sec): 5    Marker min peak height: 200   Marker baseline V to V?: Y    Marker baseline V to V points: 3
Lower marker selection: First peak > 500 RFU                Upper marker selection: Last peak > 200 RFU
Ladder size (bp)75, 200, 300, 400, 500, 700, 1000, 1500, 2000, 3000, 4000, 5000, 7000, 10000, 20000
Quantification using: Lower Marker          Final concentration (ng/uL): 0.5000          Dilution factor: 12.0
```

Sample: SampD3
Well location: D3
Created: Monday, September 20, 2021 7:19:57 PM

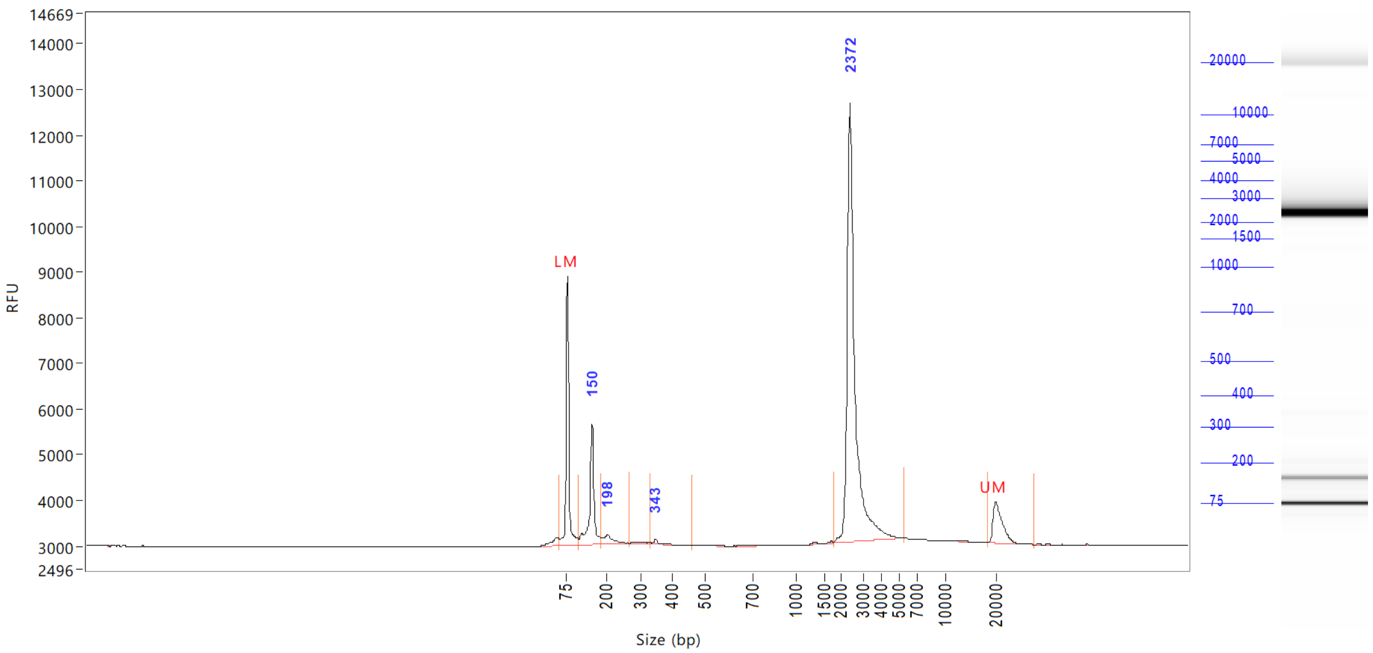

Peak	Size (bp)	Concentration (ng/uL)	From (bp)	To (bp)	Average size (bp)	CV%	RFU	Corrected peak area
1	75 (LM)	0.5000	50	110	74	11.00	5898	40.833
2	150	3.9486	110	181	147	8.64	2646	26.872
3	198	0.6234	181	262	202	7.62	216	4.243
4	343	0.1754	329	462	360	8.45	112	1.194
5	2372	16.7584	1750	5445	2522	16.01	9626	114.049
6	20000 (UM)	0.1302	18293	27617	20586	5.15	915	10.630

TIC:	21.5059	ng/uL
TIM:	60.9604	nmole/L
Total concentration:	21.9304	ng/uL

```
Sample peak width (sec): 5    Sample min peak height: 50    Sample baseline V to V?: Y    Sample baseline V to V points: 3
Sample filter: Binomial       Number of points for filter: 3  Sample start region (min): 0  Sample end region (min): 30
Marker peak width (sec): 5    Marker min peak height: 200   Marker baseline V to V?: Y    Marker baseline V to V points: 3
Lower marker selection: First peak > 500 RFU                Upper marker selection: Last peak > 200 RFU
Ladder size (bp)75, 200, 300, 400, 500, 700, 1000, 1500, 2000, 3000, 4000, 5000, 7000, 10000, 20000
Quantification using: Lower Marker          Final concentration (ng/uL): 0.5000          Dilution factor: 12.0
```

Sample: SampE3
Well location: E3
Created: Monday, September 20, 2021 7:19:57 PM

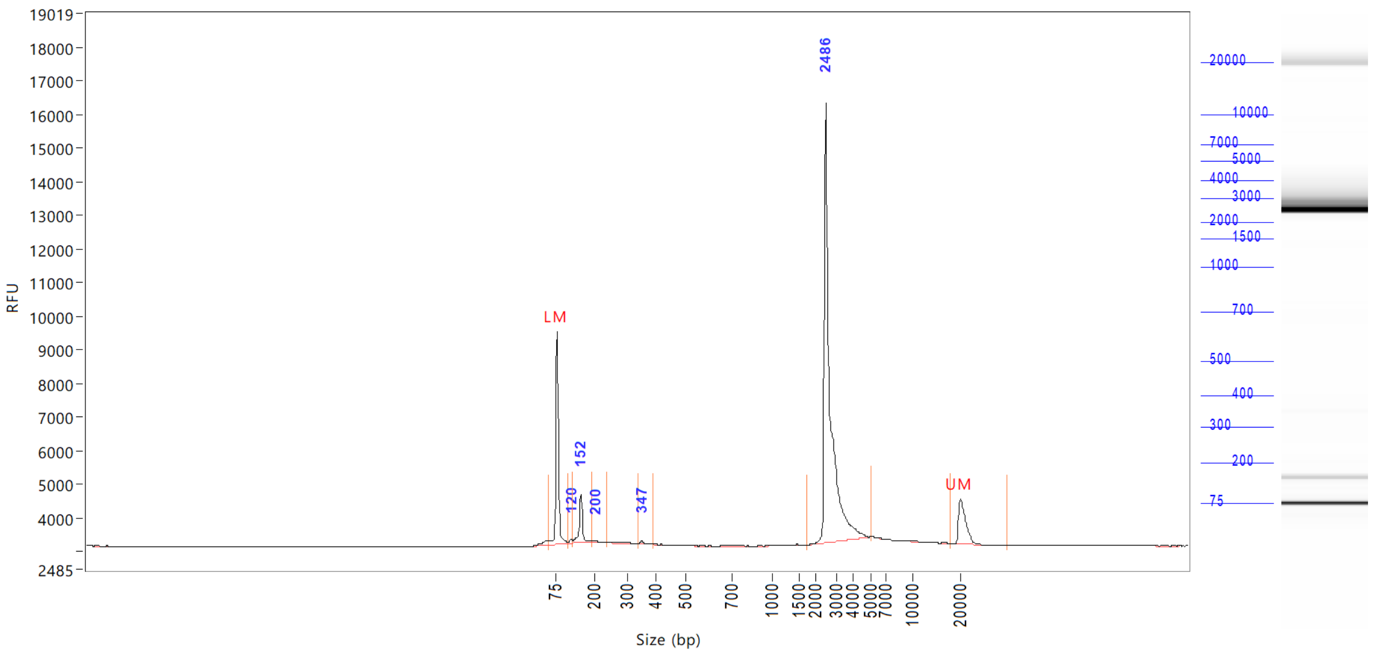

Peak	Size (bp)	Concentration (ng/uL)	From (bp)	To (bp)	Average size (bp)	CV%	RFU	Corrected peak area
1	75 (LM)	0.5000	50	110	74	8.80	6345	39.321
2	120	0.1312	110	128	119	4.20	107	0.860
3	152	1.7619	128	189	151	5.89	1455	11.547
4	200	0.1177	189	237	203	4.83	63	0.772
5	347	0.1571	337	392	358	4.53	116	1.030
6	2486	18.3386	1712	5001	2737	15.25	13104	120.182
7	20000 (UM)	0.1604	17805	29914	20464	4.74	1338	12.616

TIC:	20.5065	ng/uL
TIM:	33.6941	nmole/L
Total concentration:	21.0085	ng/uL

Sample peak width (sec): 5   Sample min peak height: 50   Sample baseline V to V?: Y   Sample baseline V to V points: 3
Sample filter: Binomial   Number of points for filter: 3 Sample start region (min): 0 Sample end region (min): 30
Marker peak width (sec): 5   Marker min peak height: 200   Marker baseline V to V?: Y   Marker baseline V to V points: 3
Lower marker selection: First peak > 500 RFU   Upper marker selection: Last peak > 200 RFU
Ladder size (bp)75, 200, 300, 400, 500, 700, 1000, 1500, 2000, 3000, 4000, 5000, 7000, 10000, 20000
Quantification using: Lower Marker   Final concentration (ng/uL): 0.5000   Dilution factor: 12.0

Sample: SampF3
Well location: F3
Created: Monday, September 20, 2021 7:19:57 PM

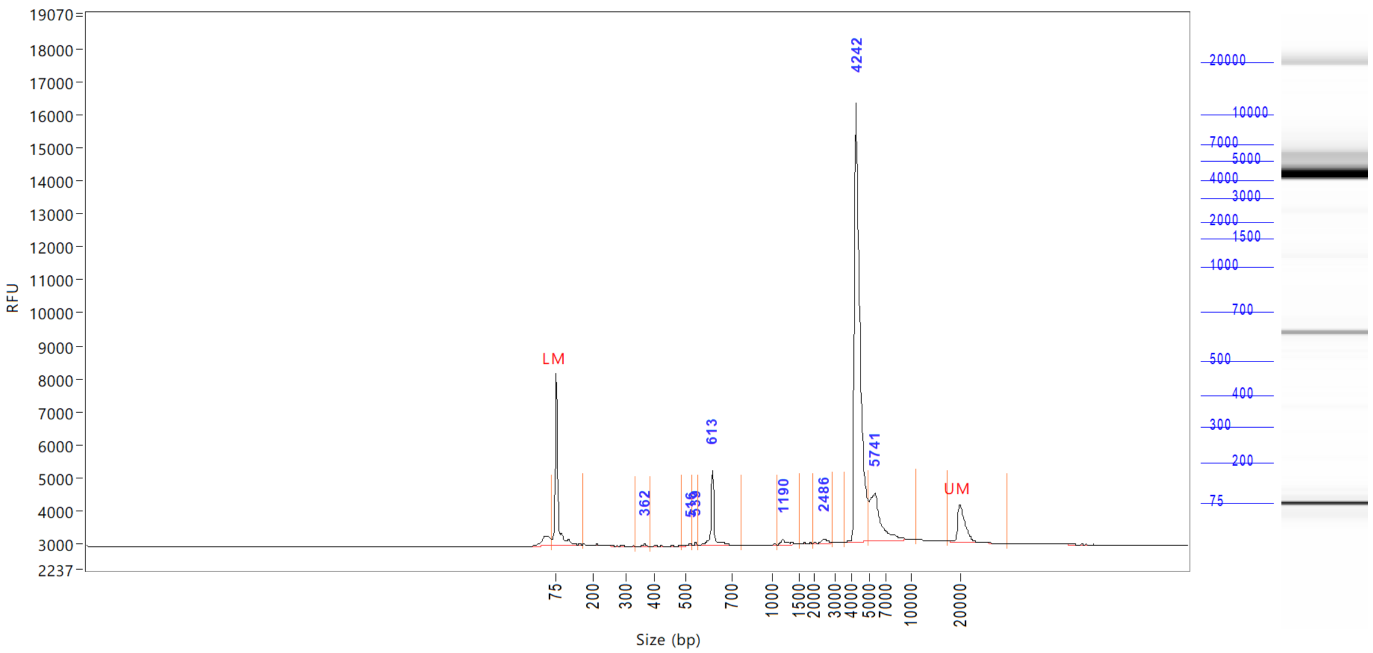

Peak	Size (bp)	Concentration (ng/uL)	From (bp)	To (bp)	Average size (bp)	CV%	RFU	Corrected peak area
1	75 (LM)	0.5000	60	164	77	15.76	5199	34.951
2	362	0.0976	331	380	358	2.23	95	0.568
3	516	0.0769	485	529	510	1.86	76	0.448
4	539	0.0739	529	552	538	0.93	97	0.431
5	613	2.5291	552	766	618	4.09	2275	14.733
6	1190	0.3104	1101	1520	1245	7.08	177	1.808
7	2486	0.2577	1942	2886	2445	8.40	144	1.501
8	4242	19.4320	3536	5000	4342	5.08	13315	113.195
9	5741	4.0861	5000	10977	6034	15.84	1436	23.802
10	20000 (UM)	0.1537	17561	29914	20585	4.85	1127	10.746

TIC:	26.8637	ng/uL
TIM:	16.7236	nmole/L
Total concentration:	27.1165	ng/uL

```
Sample peak width (sec): 5    Sample min peak height: 50    Sample baseline V to V?: Y    Sample baseline V to V points: 3
Sample filter: Binomial       Number of points for filter: 3  Sample start region (min): 0  Sample end region (min): 30
Marker peak width (sec): 5    Marker min peak height: 200   Marker baseline V to V?: Y    Marker baseline V to V points: 3
Lower marker selection: First peak > 500 RFU                Upper marker selection: Last peak > 200 RFU
Ladder size (bp)75, 200, 300, 400, 500, 700, 1000, 1500, 2000, 3000, 4000, 5000, 7000, 10000, 20000
Quantification using: Lower Marker          Final concentration (ng/uL): 0.5000          Dilution factor: 12.0
```

Sample: SampG3
Well location: G3
Created: Monday, September 20, 2021 7:19:57 PM

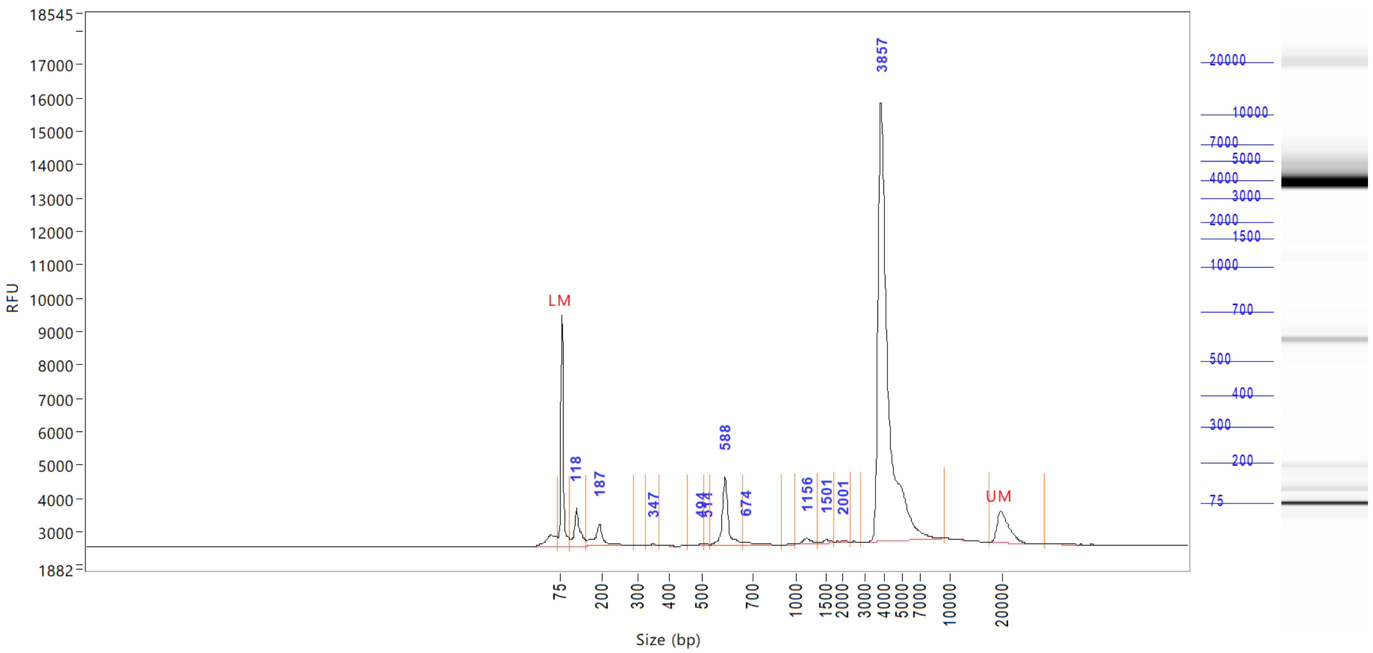

Peak	Size (bp)	Concentration (ng/uL)	From (bp)	To (bp)	Average size (bp)	CV%	RFU	Corrected peak area
1	75 (LM)	0.5000	64	101	74	8.03	6934	45.202
2	118	2.0106	101	148	121	9.12	1145	15.147
3	187	1.7683	148	289	188	13.28	658	13.322
4	347	0.0688	321	366	344	1.74	71	0.518
5	494	0.0685	452	501	488	2.12	52	0.516
6	514	0.0683	501	529	513	1.55	61	0.514
7	588	2.9834	529	662	588	3.52	2053	22.476
8	674	0.2986	662	896	720	7.64	95	2.249
9	1156	0.3158	987	1345	1157	6.53	169	2.379
10	1501	0.1790	1345	1750	1520	6.44	102	1.348
11	2001	0.1190	1750	2344	1993	7.54	69	0.896
12	3857	25.4996	2829	9533	4155	15.82	13162	192.104
13	20000 (UM)	0.1580	17683	28221	20585	6.14	943	14.284

TIC:	33.3797	ng/uL
TIM:	63.4182	nmole/L
Total concentration:	33.4742	ng/uL

```
Sample peak width (sec): 5     Sample min peak height: 50     Sample baseline V to V?: Y     Sample baseline V to V points: 3
Sample filter: Binomial        Number of points for filter: 3  Sample start region (min): 0  Sample end region (min): 30
Marker peak width (sec): 5     Marker min peak height: 200    Marker baseline V to V?: Y     Marker baseline V to V points: 3
Lower marker selection: First peak > 500 RFU                  Upper marker selection: Last peak > 200 RFU
Ladder size (bp)75, 200, 300, 400, 500, 700, 1000, 1500, 2000, 3000, 4000, 5000, 7000, 10000, 20000
Quantification using: Lower Marker          Final concentration (ng/uL): 0.5000          Dilution factor: 12.0
```

Sample: SampH3
Well location: H3
Created: Monday, September 20, 2021 7:19:57 PM

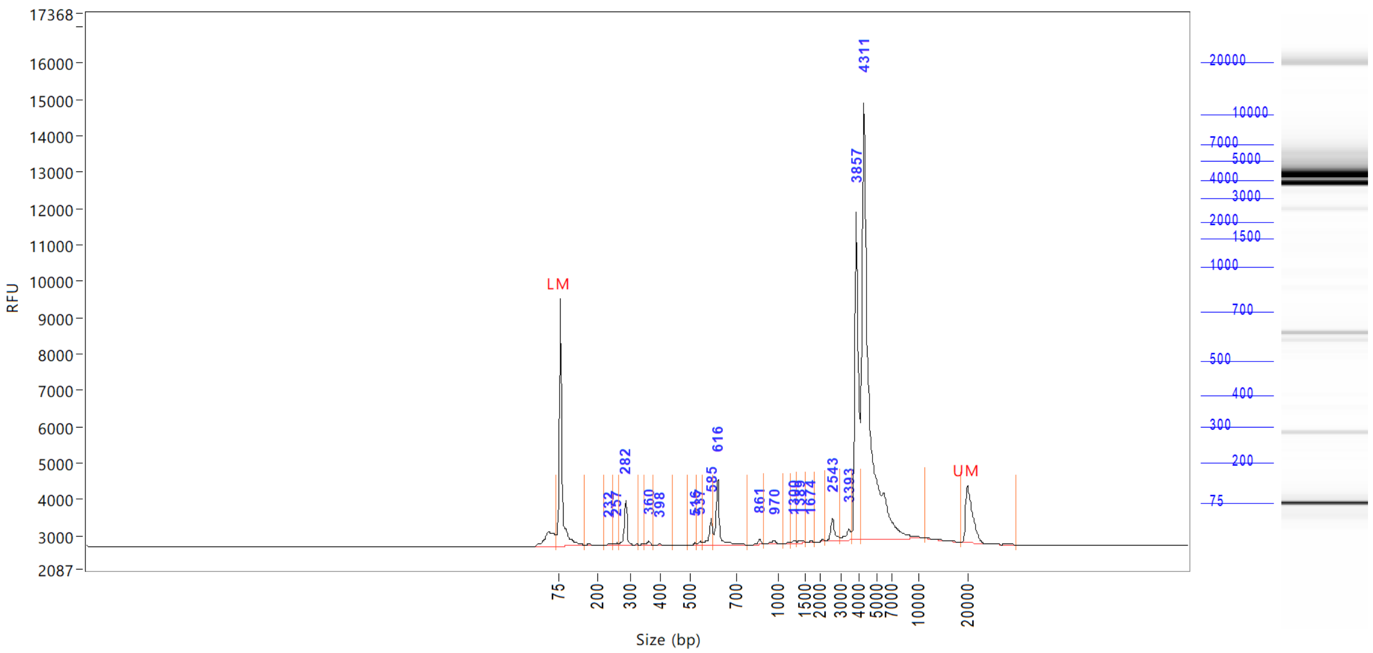

Peak	Size (bp)	Concentration (ng/uL)	From (bp)	To (bp)	Average size (bp)	CV%	RFU	Corrected peak area
1	75 (LM)	0.5000	64	154	78	16.58	6814	47.731
2	232	0.0558	217	244	233	2.45	53	0.444
3	257	0.0612	244	262	254	2.09	54	0.487
4	282	0.8916	262	323	281	2.17	1224	7.093
5	360	0.0737	345	380	357	1.44	98	0.586
6	398	0.0494	380	441	404	3.02	53	0.393
7	516	0.0491	487	524	512	1.53	70	0.390
8	537	0.0935	524	549	538	1.32	114	0.744
9	585	0.6442	549	595	579	1.86	732	5.125
10	616	1.5009	595	775	620	3.81	1820	11.940
11	861	0.1088	775	900	852	1.99	146	0.865
12	970	0.1221	900	1079	959	2.35	101	0.971
13	1300	0.0630	1234	1356	1299	2.54	75	0.501
14	1389	0.0668	1356	1520	1426	3.27	54	0.532
15	1674	0.0433	1520	1808	1639	3.57	56	0.344
16	2543	0.4667	2229	2943	2576	5.24	601	3.713
17	3393	0.3290	2943	3608	3332	5.56	294	2.617
18	3857	6.3435	3608	4070	3873	2.72	9027	50.464
19	4311	16.5306	4070	11220	4736	18.46	12025	131.504
20	20000 (UM)	0.1519	18537	30035	20518	5.08	1559	14.498

TIC:	27.4932	ng/uL
TIM:	22.3321	nmole/L
Total concentration:	27.2902	ng/uL

Sample peak width (sec): 5   Sample min peak height: 50   Sample baseline V to V?: Y   Sample baseline V to V points: 3
Sample filter: Binomial   Number of points for filter: 3 Sample start region (min): 0 Sample end region (min): 30
Marker peak width (sec): 5   Marker min peak height: 200   Marker baseline V to V?: Y   Marker baseline V to V points: 3
Lower marker selection: First peak > 500 RFU   Upper marker selection: Last peak > 200 RFU
Ladder size (bp)75, 200, 300, 400, 500, 700, 1000, 1500, 2000, 3000, 4000, 5000, 7000, 10000, 20000
Quantification using: Lower Marker   Final concentration (ng/uL): 0.5000   Dilution factor: 12.0

Sample: SampA4
Well location: A4
Created: Monday, September 20, 2021 7:19:57 PM

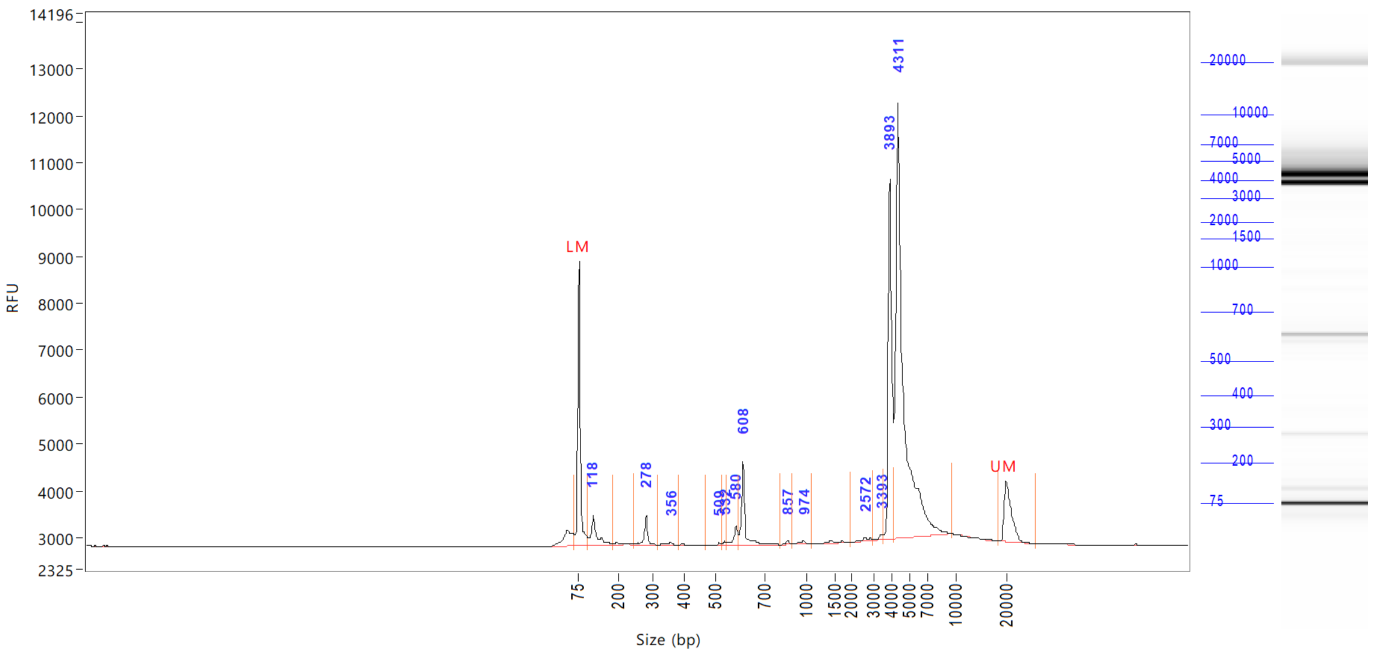

Peak	Size (bp)	Concentration (ng/uL)	From (bp)	To (bp)	Average size (bp)	CV%	RFU	Corrected peak area
1	75 (LM)	0.5000	62	103	74	8.56	6075	37.819
2	118	1.3301	103	179	125	13.07	647	8.384
3	278	0.6741	242	311	274	3.25	637	4.249
4	356	0.1207	311	382	349	4.50	71	0.761
5	509	0.0572	466	519	503	2.13	55	0.361
6	532	0.0835	519	542	531	1.21	86	0.527
7	580	0.5133	542	590	573	2.08	404	3.235
8	608	1.8428	590	801	616	4.55	1797	11.615
9	857	0.0713	801	896	850	1.61	77	0.449
10	974	0.1240	896	1079	959	2.59	79	0.781
11	2572	0.1322	1981	2971	2580	8.68	66	0.834
12	3393	0.1267	2971	3536	3329	4.58	115	0.799
13	3893	6.7857	3536	4104	3892	2.79	7687	42.771
14	4311	16.3357	4104	9600	4774	17.16	9314	102.966
15	20000 (UM)	0.1587	18415	25924	20465	4.06	1299	12.004

TIC:	28.1972	ng/uL
TIM:	37.9415	nmole/L
Total concentration:	28.1876	ng/uL

```
Sample peak width (sec): 5     Sample min peak height: 50    Sample baseline V to V?: Y    Sample baseline V to V points: 3
Sample filter: Binomial        Number of points for filter: 3  Sample start region (min): 0  Sample end region (min): 30
Marker peak width (sec): 5     Marker min peak height: 200   Marker baseline V to V?: Y    Marker baseline V to V points: 3
Lower marker selection: First peak > 500 RFU                  Upper marker selection: Last peak > 200 RFU
Ladder size (bp)75, 200, 300, 400, 500, 700, 1000, 1500, 2000, 3000, 4000, 5000, 7000, 10000, 20000
Quantification using: Lower Marker          Final concentration (ng/uL): 0.5000          Dilution factor: 12.0
```

Sample: SampB4
Well location: B4
Created: Monday, September 20, 2021 7:19:57 PM

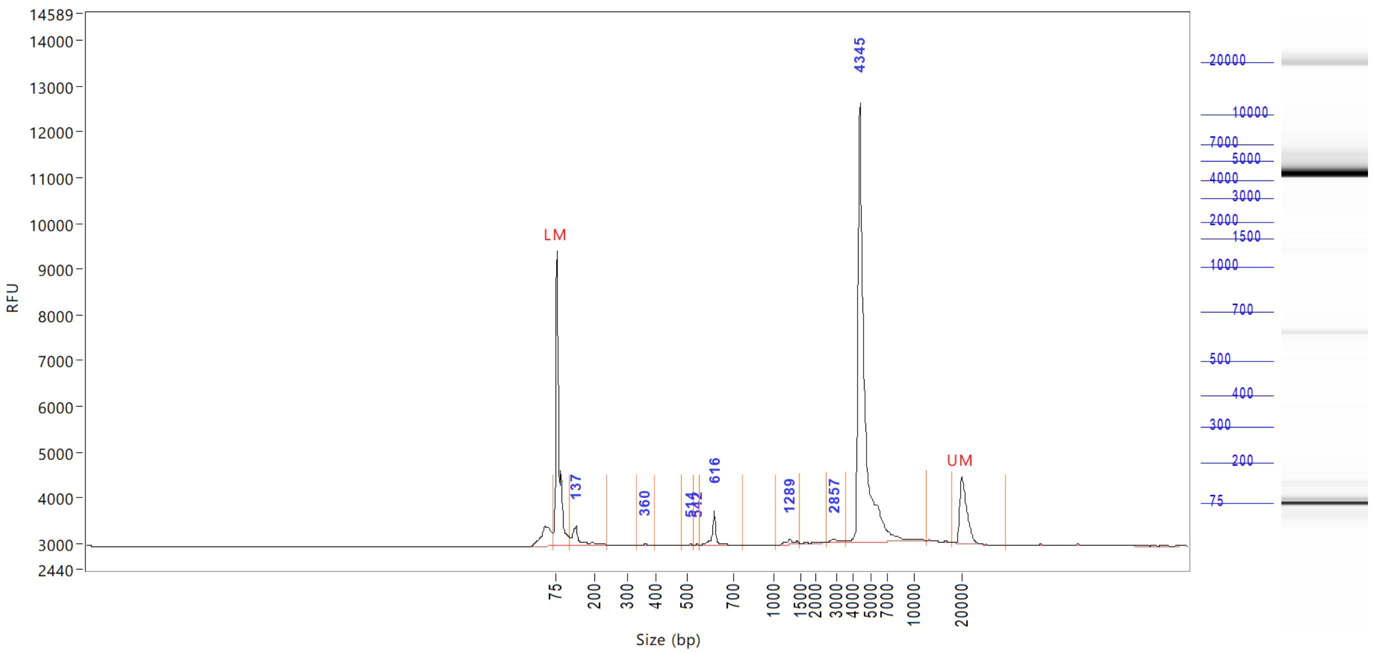

Peak	Size (bp)	Concentration (ng/uL)	From (bp)	To (bp)	Average size (bp)	CV%	RFU	Corrected peak area
1	75 (LM)	0.5000	64	118	78	12.79	6443	50.468
2	137	0.8743	118	239	146	17.91	426	7.354
3	360	0.0560	333	394	359	3.69	52	0.471
4	514	0.0365	483	524	508	1.66	50	0.307
5	542	0.0380	524	555	540	1.32	57	0.320
6	616	0.7215	555	775	618	4.74	773	6.069
7	1289	0.1738	1023	1489	1302	7.04	112	1.462
8	2857	0.1450	2486	3500	2949	8.54	76	1.220
9	4345	11.0386	3500	12440	4717	18.81	9615	92.849
10	20000 (UM)	0.1356	17927	29068	20433	4.52	1472	13.685

TIC:	13.0838	ng/uL
TIM:	16.4066	nmole/L
Total concentration:	13.2484	ng/uL

```
Sample peak width (sec): 5    Sample min peak height: 50    Sample baseline V to V?: Y    Sample baseline V to V points: 3
Sample filter: Binomial       Number of points for filter: 3  Sample start region (min): 0  Sample end region (min): 30
Marker peak width (sec): 5    Marker min peak height: 200   Marker baseline V to V?: Y    Marker baseline V to V points: 3
Lower marker selection: First peak > 500 RFU                Upper marker selection: Last peak > 200 RFU
Ladder size (bp)75, 200, 300, 400, 500, 700, 1000, 1500, 2000, 3000, 4000, 5000, 7000, 10000, 20000
Quantification using: Lower Marker          Final concentration (ng/uL): 0.5000          Dilution factor: 12.0
```

Sample: SampC4
Well location: C4
Created: Monday, September 20, 2021 7:19:57 PM

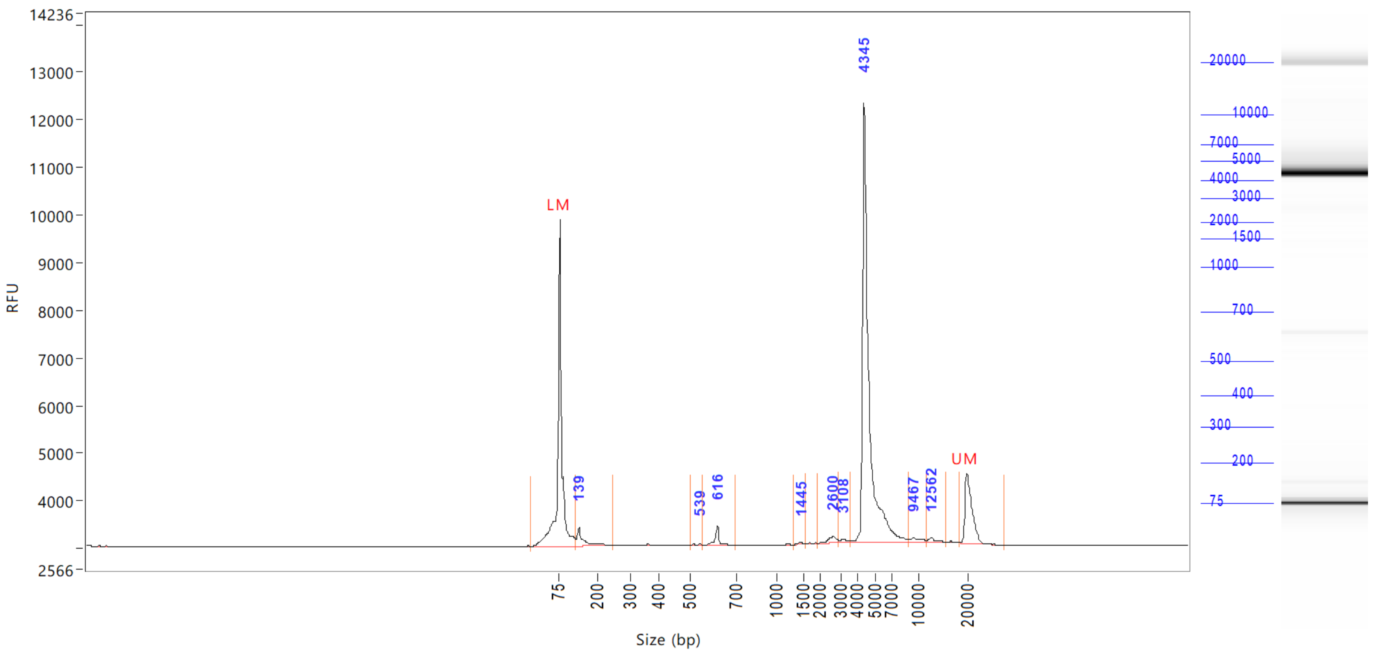

Peak	Size (bp)	Concentration (ng/uL)	From (bp)	To (bp)	Average size (bp)	CV%	RFU	Corrected peak area
1	75 (LM)	0.5000	0	128	71	27.35	6871	66.153
2	139	0.5575	128	248	159	18.81	397	6.146
3	539	0.0425	504	552	527	2.63	54	0.469
4	616	0.3088	552	695	611	3.65	413	3.405
5	1445	0.0374	1300	1539	1417	3.44	50	0.413
6	2600	0.1608	1923	2914	2517	8.90	131	1.773
7	3108	0.0694	2914	3572	3166	5.69	73	0.765
8	4345	7.8874	3572	8867	4686	15.70	9237	86.962
9	9467	0.1365	8867	11464	9881	7.13	83	1.504
10	12562	0.1101	11464	15732	13080	8.36	100	1.214
11	20000 (UM)	0.1028	18537	27738	20463	4.00	1477	13.606

TIC:	9.3104	ng/uL
TIM:	9.7198	nmole/L
Total concentration:	9.4548	ng/uL

Sample peak width (sec): 5   Sample min peak height: 50   Sample baseline V to V?: Y   Sample baseline V to V points: 3
Sample filter: Binomial   Number of points for filter: 3 Sample start region (min): 0 Sample end region (min): 30
Marker peak width (sec): 5   Marker min peak height: 200   Marker baseline V to V?: Y   Marker baseline V to V points: 3
Lower marker selection: First peak > 500 RFU   Upper marker selection: Last peak > 200 RFU
Ladder size (bp)75, 200, 300, 400, 500, 700, 1000, 1500, 2000, 3000, 4000, 5000, 7000, 10000, 20000
Quantification using: Lower Marker   Final concentration (ng/uL): 0.5000   Dilution factor: 12.0

Sample: SampD4
Well location: D4
Created: Monday, September 20, 2021 7:19:57 PM

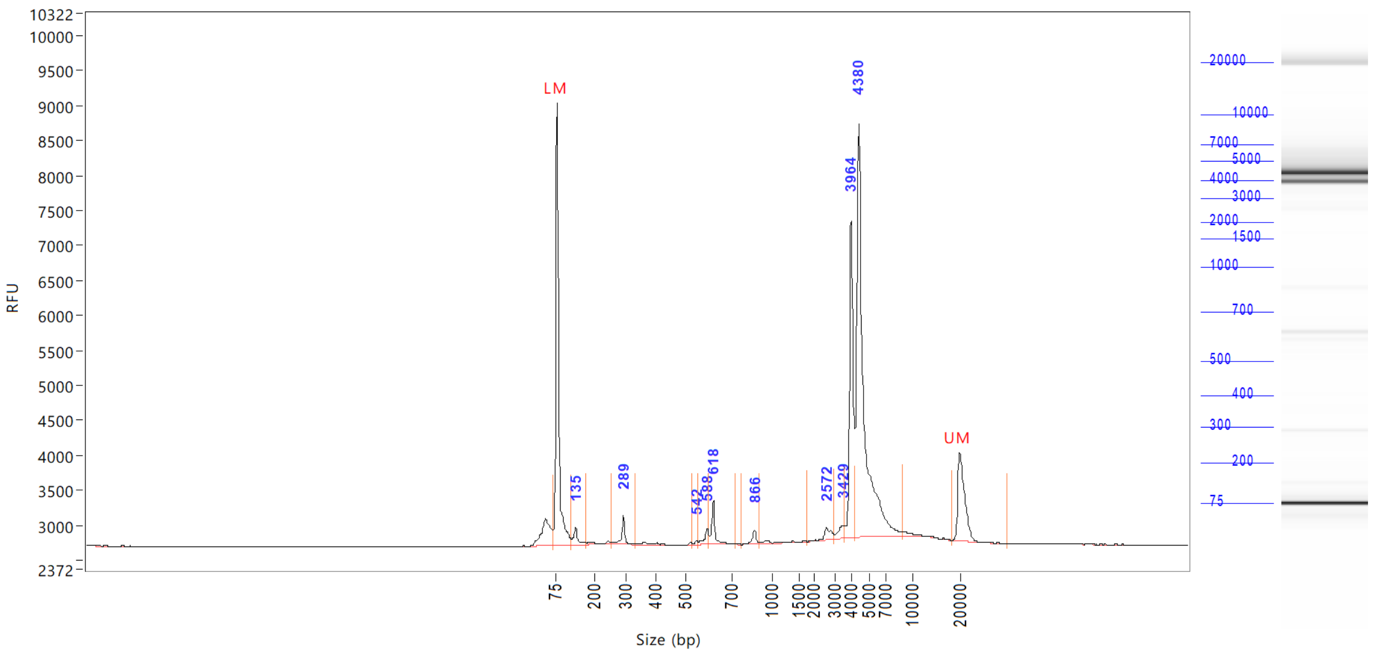

Peak	Size (bp)	Concentration (ng/uL)	From (bp)	To (bp)	Average size (bp)	CV%	RFU	Corrected peak area
1	75 (LM)	0.5000	64	120	76	12.31	6338	44.283
2	135	0.3608	120	171	137	8.81	243	2.663
3	289	0.3976	253	329	285	3.90	420	2.934
4	542	0.0483	524	549	539	1.28	53	0.357
5	588	0.2538	549	600	582	2.24	231	1.873
6	618	0.5503	600	718	622	3.17	629	4.061
7	866	0.1750	770	905	856	2.43	202	1.292
8	2572	0.2587	1750	2943	2596	8.33	179	1.909
9	3429	0.2726	2943	3572	3298	5.55	185	2.012
10	3964	3.5042	3572	4139	3938	2.70	4531	25.863
11	4380	8.3789	4139	9000	4825	17.18	5900	61.840
12	20000 (UM)	0.1293	18293	30035	20523	4.62	1253	11.448

TIC:	14.2001	ng/uL
TIM:	13.9031	nmole/L
Total concentration:	14.4801	ng/uL

```
Sample peak width (sec): 5    Sample min peak height: 50    Sample baseline V to V?: Y    Sample baseline V to V points: 3
Sample filter: Binomial       Number of points for filter: 3  Sample start region (min): 0  Sample end region (min): 30
Marker peak width (sec): 5    Marker min peak height: 200   Marker baseline V to V?: Y    Marker baseline V to V points: 3
Lower marker selection: First peak > 500 RFU                Upper marker selection: Last peak > 200 RFU
Ladder size (bp)75, 200, 300, 400, 500, 700, 1000, 1500, 2000, 3000, 4000, 5000, 7000, 10000, 20000
Quantification using: Lower Marker          Final concentration (ng/uL): 0.5000          Dilution factor: 12.0
```

Sample: SampE4
Well location: E4
Created: Monday, September 20, 2021 7:19:57 PM

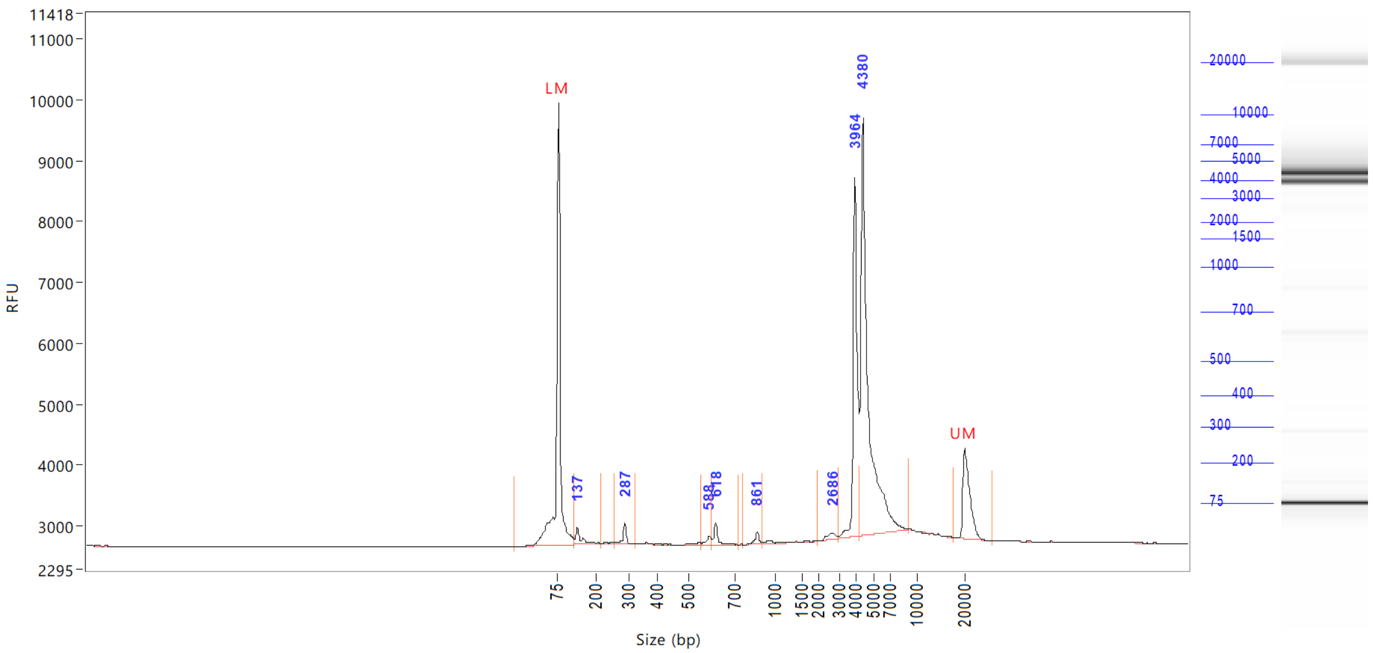

Peak	Size (bp)	Concentration (ng/uL)	From (bp)	To (bp)	Average size (bp)	CV%	RFU	Corrected peak area
1	75 (LM)	0.5000	0	126	70	27.56	7268	63.710
2	137	0.3243	126	215	146	12.44	284	3.444
3	287	0.2134	255	321	282	3.09	341	2.266
4	588	0.1204	552	598	581	2.10	143	1.279
5	618	0.2479	598	727	620	3.18	367	2.632
6	861	0.1377	762	909	856	2.46	202	1.462
7	2686	0.1403	1962	3000	2580	8.19	108	1.490
8	3964	3.4349	3000	4173	3923	4.08	5879	36.472
9	4380	7.0982	4173	9200	4810	15.93	6845	75.370
10	20000 (UM)	0.1082	17683	25803	20508	4.00	1481	13.788

TIC:	11.7171	ng/uL
TIM:	10.1197	nmole/L
Total concentration:	11.8112	ng/uL

```
Sample peak width (sec): 5    Sample min peak height: 50    Sample baseline V to V?: Y    Sample baseline V to V points: 3
Sample filter: Binomial       Number of points for filter: 3  Sample start region (min): 0  Sample end region (min): 30
Marker peak width (sec): 5    Marker min peak height: 200   Marker baseline V to V?: Y    Marker baseline V to V points: 3
Lower marker selection: First peak > 500 RFU                Upper marker selection: Last peak > 200 RFU
Ladder size (bp)75, 200, 300, 400, 500, 700, 1000, 1500, 2000, 3000, 4000, 5000, 7000, 10000, 20000
Quantification using: Lower Marker          Final concentration (ng/uL): 0.5000          Dilution factor: 12.0
```

Sample: SampF4
Well location: F4
Created: Monday, September 20, 2021 7:19:57 PM

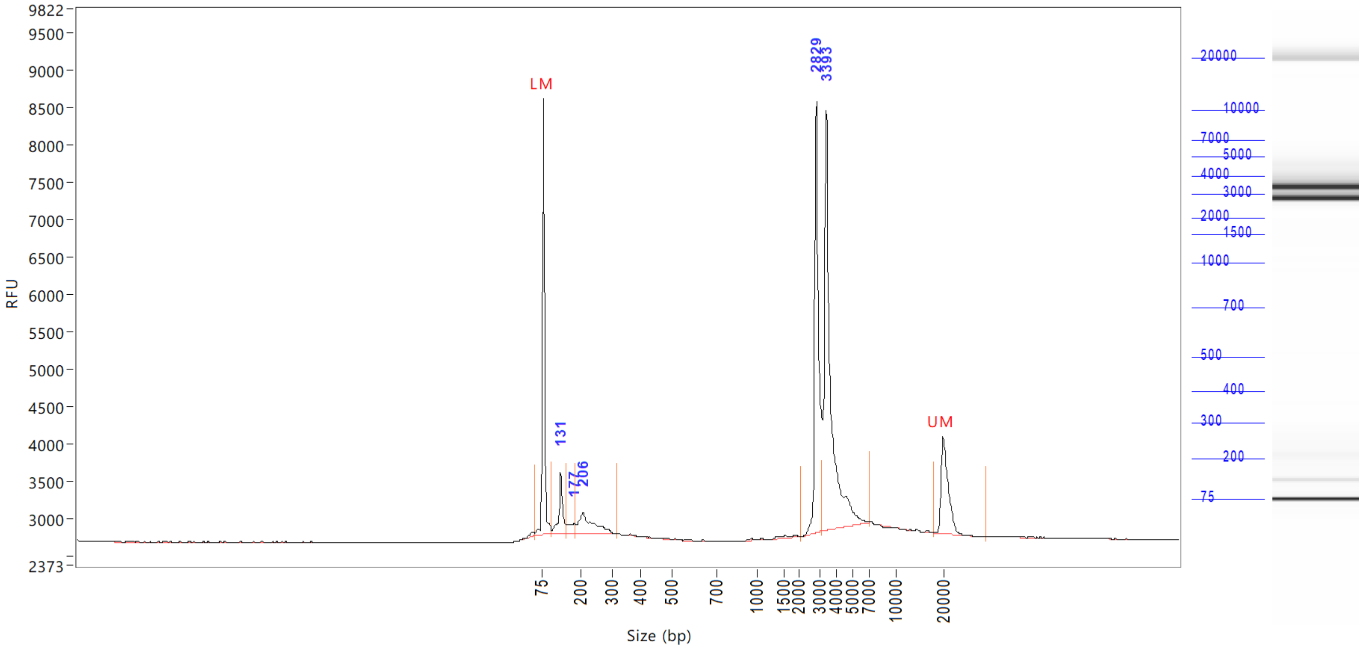

Peak	Size (bp)	Concentration (ng/uL)	From (bp)	To (bp)	Average size (bp)	CV%	RFU	Corrected peak area
1	75 (LM)	0.5000	47	103	73	7.61	5845	33.352
2	131	1.3137	103	152	129	7.95	806	7.302
3	177	0.4070	152	183	168	5.53	130	2.262
4	206	1.6506	183	311	225	12.58	276	9.175
5	2829	6.9413	2115	3144	2855	4.63	5764	38.585
6	3393	9.9551	3144	7068	3647	13.64	5617	55.338
7	20000 (UM)	0.1872	17927	29189	20504	4.56	1311	12.486

TIC:	20.2677	ng/uL
TIM:	41.2741	nmole/L
Total concentration:	20.3858	ng/uL

```
Sample peak width (sec): 5    Sample min peak height: 50    Sample baseline V to V?: Y    Sample baseline V to V points: 3
Sample filter: Binomial       Number of points for filter: 3  Sample start region (min): 0  Sample end region (min): 30
Marker peak width (sec): 5    Marker min peak height: 200   Marker baseline V to V?: Y    Marker baseline V to V points: 3
Lower marker selection: First peak > 500 RFU                Upper marker selection: Last peak > 200 RFU
Ladder size (bp)75, 200, 300, 400, 500, 700, 1000, 1500, 2000, 3000, 4000, 5000, 7000, 10000, 20000
Quantification using: Lower Marker          Final concentration (ng/uL): 0.5000          Dilution factor: 12.0
```

Sample: SampG4
Well location: G4
Created: Monday, September 20, 2021 7:19:57 PM

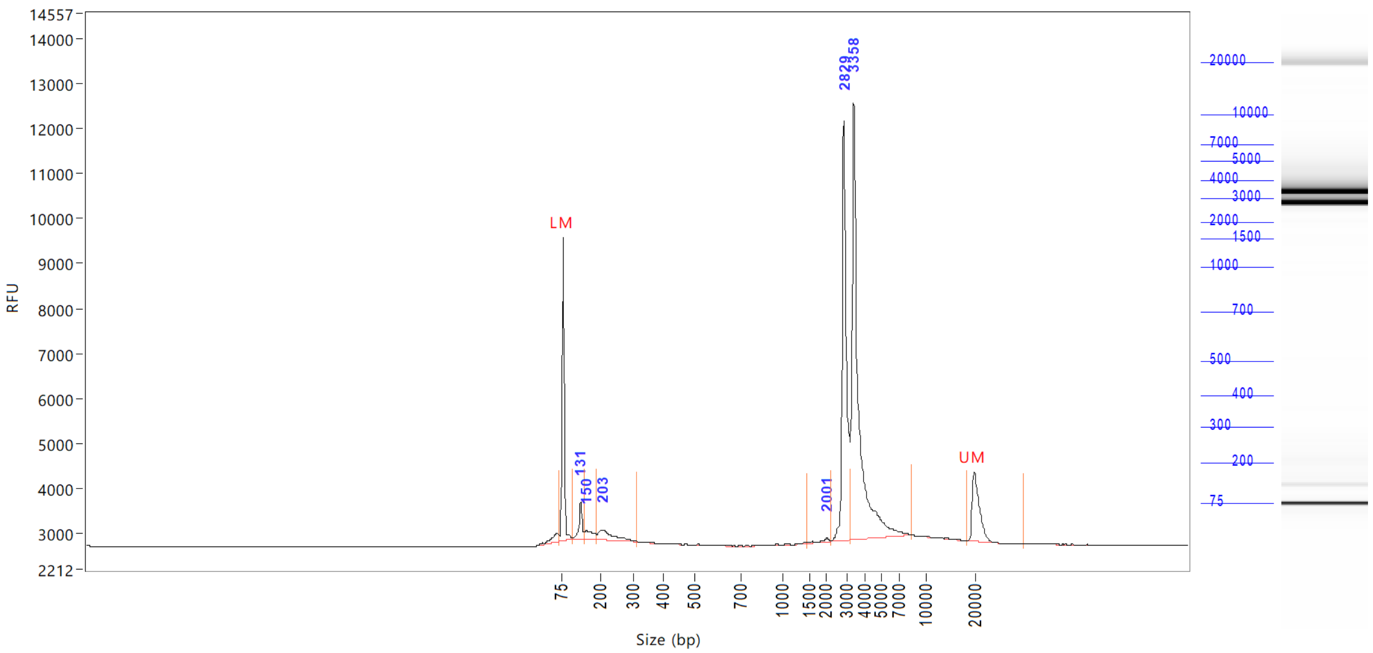

Peak	Size (bp)	Concentration (ng/uL)	From (bp)	To (bp)	Average size (bp)	CV%	RFU	Corrected peak area
1	75 (LM)	0.5000	64	105	73	5.90	6740	37.174
2	131	1.0208	105	143	128	5.43	820	6.325
3	150	0.6293	143	183	161	7.40	193	3.899
4	203	1.2251	183	309	222	13.04	224	7.590
5	2001	0.1617	1433	2229	1847	12.54	83	1.002
6	2829	9.6330	2229	3144	2851	4.60	9328	59.684
7	3358	15.4191	3144	8401	3725	18.42	9714	95.532
8	20000 (UM)	0.1925	18537	30035	20508	4.68	1540	14.309

TIC:	28.0890	ng/uL
TIM:	41.1237	nmole/L
Total concentration:	28.0684	ng/uL

Sample peak width (sec): 5   Sample min peak height: 50   Sample baseline V to V?: Y   Sample baseline V to V points: 3
Sample filter: Binomial   Number of points for filter: 3   Sample start region (min): 0   Sample end region (min): 30
Marker peak width (sec): 5   Marker min peak height: 200   Marker baseline V to V?: Y   Marker baseline V to V points: 3
Lower marker selection: First peak > 500 RFU   Upper marker selection: Last peak > 200 RFU
Ladder size (bp)75, 200, 300, 400, 500, 700, 1000, 1500, 2000, 3000, 4000, 5000, 7000, 10000, 20000
Quantification using: Lower Marker   Final concentration (ng/uL): 0.5000   Dilution factor: 12.0

Sample: SampH4
Well location: H4
Created: Monday, September 20, 2021 7:19:57 PM

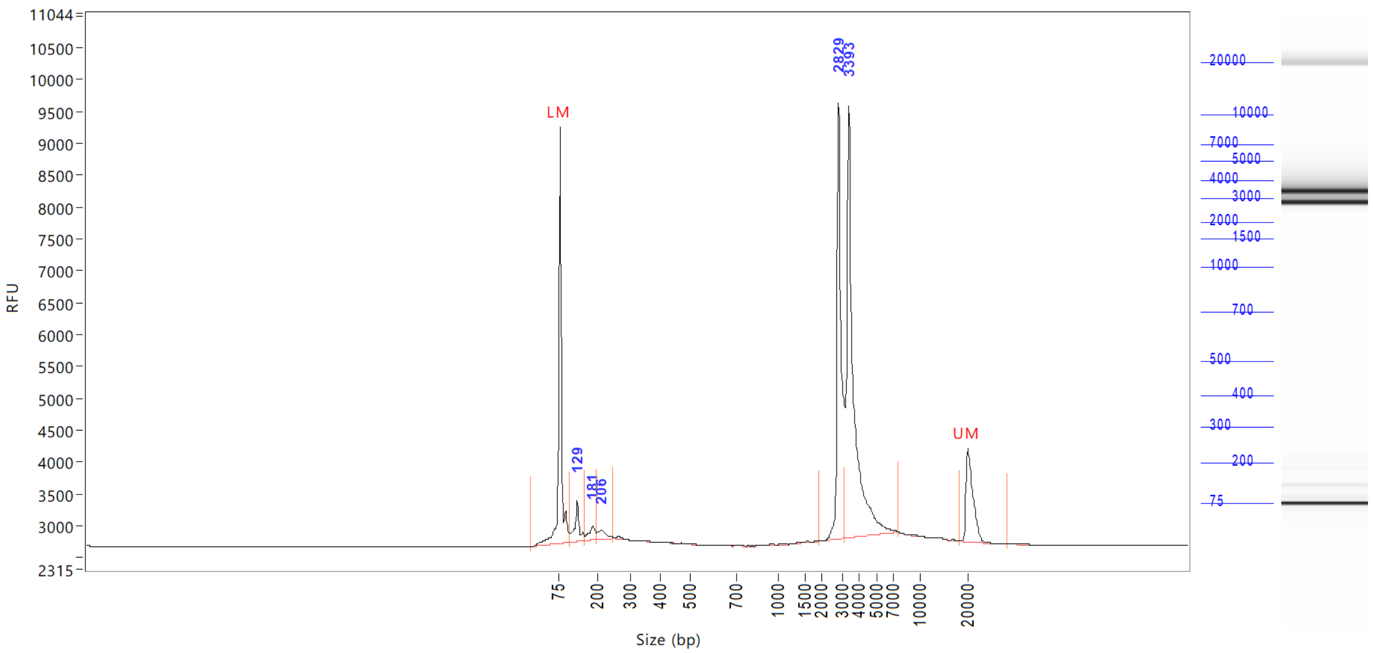

Peak	Size (bp)	Concentration (ng/uL)	From (bp)	To (bp)	Average size (bp)	CV%	RFU	Corrected peak area
1	75 (LM)	0.5000	0	107	71	19.94	6532	45.835
2	129	0.9225	107	156	128	8.95	656	7.048
3	181	0.4133	156	192	175	5.60	224	3.157
4	206	0.3678	192	246	210	6.00	140	2.809
5	2829	6.1831	1942	3144	2864	4.85	6858	47.234
6	3393	9.1679	3144	7601	3688	15.34	6771	70.036
7	20000 (UM)	0.1472	18293	28100	20439	4.29	1466	13.490

TIC:	17.0546	ng/uL
TIM:	26.2503	nmole/L
Total concentration:	17.0991	ng/uL

```
Sample peak width (sec): 5    Sample min peak height: 50    Sample baseline V to V?: Y    Sample baseline V to V points: 3
Sample filter: Binomial       Number of points for filter: 3  Sample start region (min): 0  Sample end region (min): 30
Marker peak width (sec): 5    Marker min peak height: 200   Marker baseline V to V?: Y    Marker baseline V to V points: 3
Lower marker selection: First peak > 500 RFU                Upper marker selection: Last peak > 200 RFU
Ladder size (bp)75, 200, 300, 400, 500, 700, 1000, 1500, 2000, 3000, 4000, 5000, 7000, 10000, 20000
Quantification using: Lower Marker          Final concentration (ng/uL): 0.5000          Dilution factor: 12.0
```

Sample: SampA5
Well location: A5
Created: Monday, September 20, 2021 7:19:57 PM

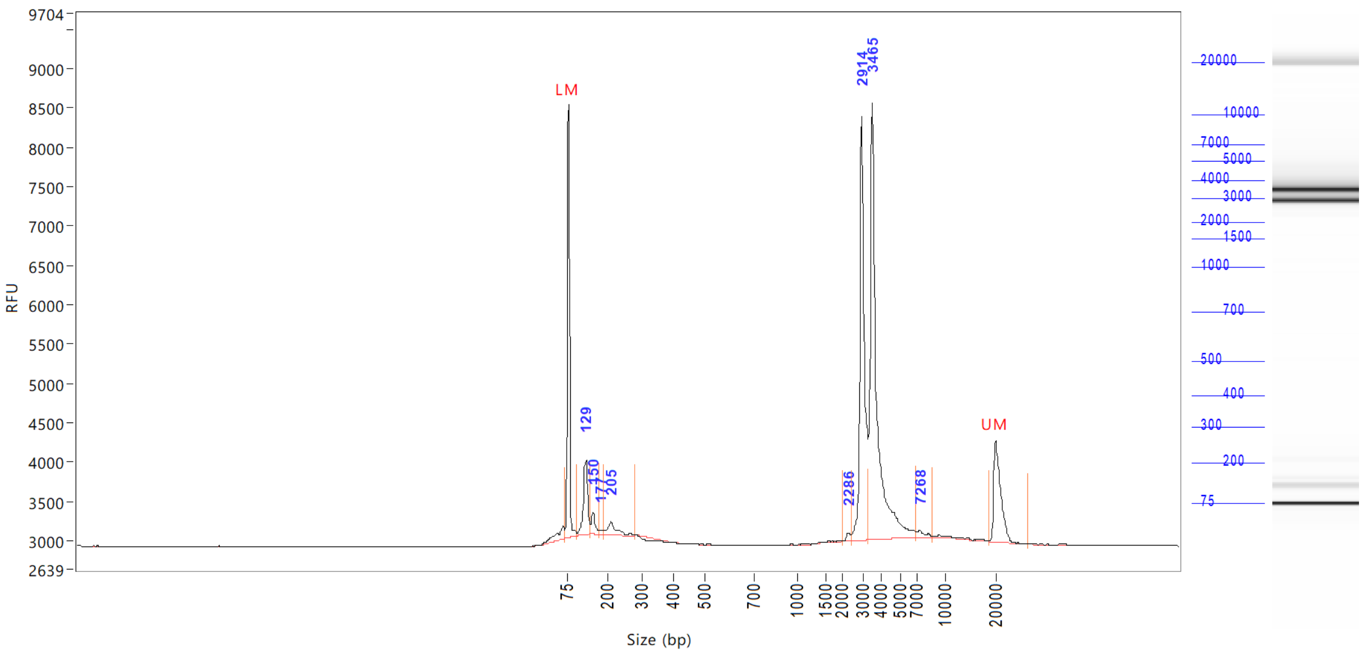

Peak	Size (bp)	Concentration (ng/uL)	From (bp)	To (bp)	Average size (bp)	CV%	RFU	Corrected peak area
1	75 (LM)	0.5000	64	101	73	5.69	5508	30.214
2	129	1.9881	101	141	125	5.81	951	10.011
3	150	0.5354	141	171	152	5.01	284	2.696
4	177	0.0963	171	185	178	2.43	57	0.485
5	205	0.6700	185	275	215	9.53	163	3.374
6	2286	0.1295	2058	2429	2292	3.72	105	0.652
7	2914	7.0555	2429	3251	2934	4.78	5392	35.529
8	3465	10.2908	3251	6926	3772	15.33	5560	51.821
9	7268	0.2017	6926	8600	7554	6.31	84	1.016
10	20000 (UM)	0.1917	18659	26408	20426	4.00	1290	11.586

TIC:	20.9673	ng/uL
TIM:	46.5209	nmole/L
Total concentration:	21.1258	ng/uL

```
Sample peak width (sec): 5    Sample min peak height: 50    Sample baseline V to V?: Y    Sample baseline V to V points: 3
Sample filter: Binomial       Number of points for filter: 3  Sample start region (min): 0  Sample end region (min): 30
Marker peak width (sec): 5    Marker min peak height: 200   Marker baseline V to V?: Y    Marker baseline V to V points: 3
Lower marker selection: First peak > 500 RFU                Upper marker selection: Last peak > 200 RFU
Ladder size (bp)75, 200, 300, 400, 500, 700, 1000, 1500, 2000, 3000, 4000, 5000, 7000, 10000, 20000
Quantification using: Lower Marker          Final concentration (ng/uL): 0.5000          Dilution factor: 12.0
```

Sample: SampB5
Well location: B5
Created: Monday, September 20, 2021 7:19:57 PM

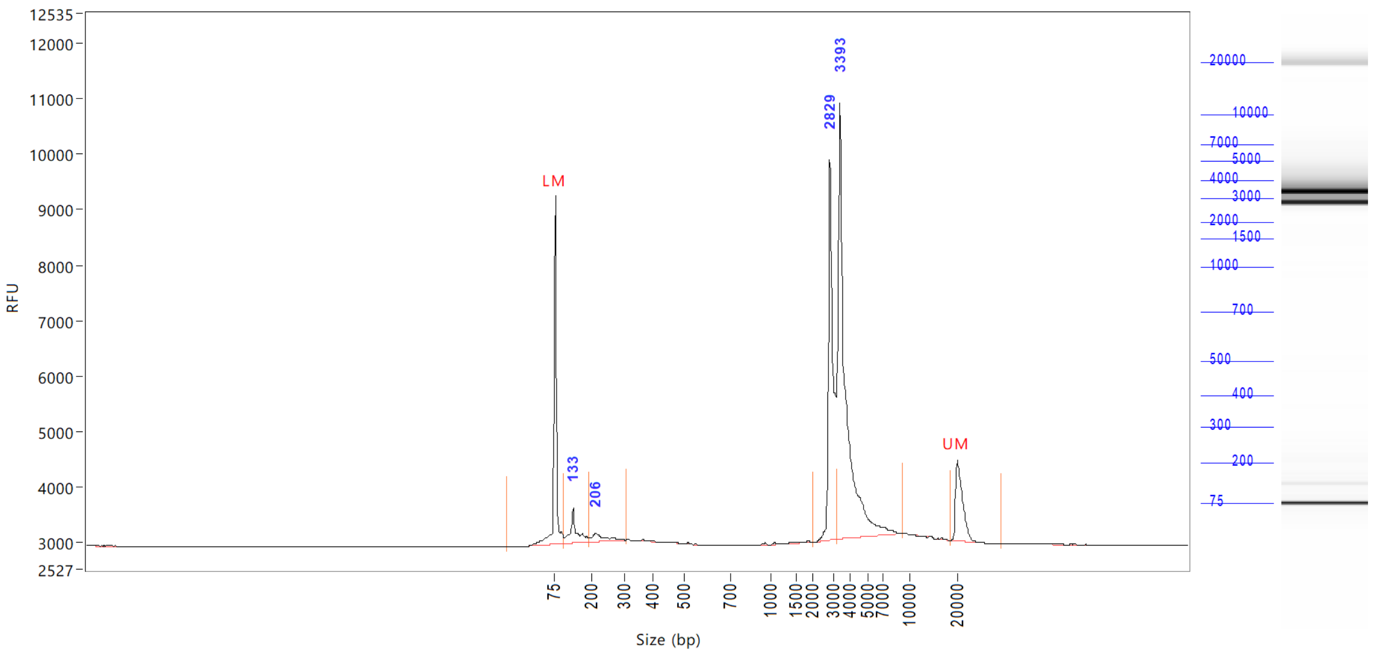

Peak	Size (bp)	Concentration (ng/uL)	From (bp)	To (bp)	Average size (bp)	CV%	RFU	Corrected peak area
1	75 (LM)	0.5000	0	105	71	20.71	6262	41.100
2	133	1.3388	105	185	139	14.08	623	9.170
3	206	0.6458	185	303	222	11.70	148	4.424
4	2829	7.2224	2000	3179	2889	5.13	6857	49.473
5	3393	13.2812	3179	9134	3825	20.33	7860	90.976
6	20000 (UM)	0.1655	18415	29068	20459	4.51	1453	13.603

TIC:	22.4882	ng/uL
TIM:	30.4427	nmole/L
Total concentration:	22.4793	ng/uL

Sample peak width (sec): 5   Sample min peak height: 50   Sample baseline V to V?: Y   Sample baseline V to V points: 3
Sample filter: Binomial   Number of points for filter: 3 Sample start region (min): 0 Sample end region (min): 30
Marker peak width (sec): 5   Marker min peak height: 200   Marker baseline V to V?: Y   Marker baseline V to V points: 3
Lower marker selection: First peak > 500 RFU   Upper marker selection: Last peak > 200 RFU
Ladder size (bp)75, 200, 300, 400, 500, 700, 1000, 1500, 2000, 3000, 4000, 5000, 7000, 10000, 20000
Quantification using: Lower Marker   Final concentration (ng/uL): 0.5000   Dilution factor: 12.0

Sample: SampC5
Well location: C5
Created: Monday, September 20, 2021 7:19:57 PM

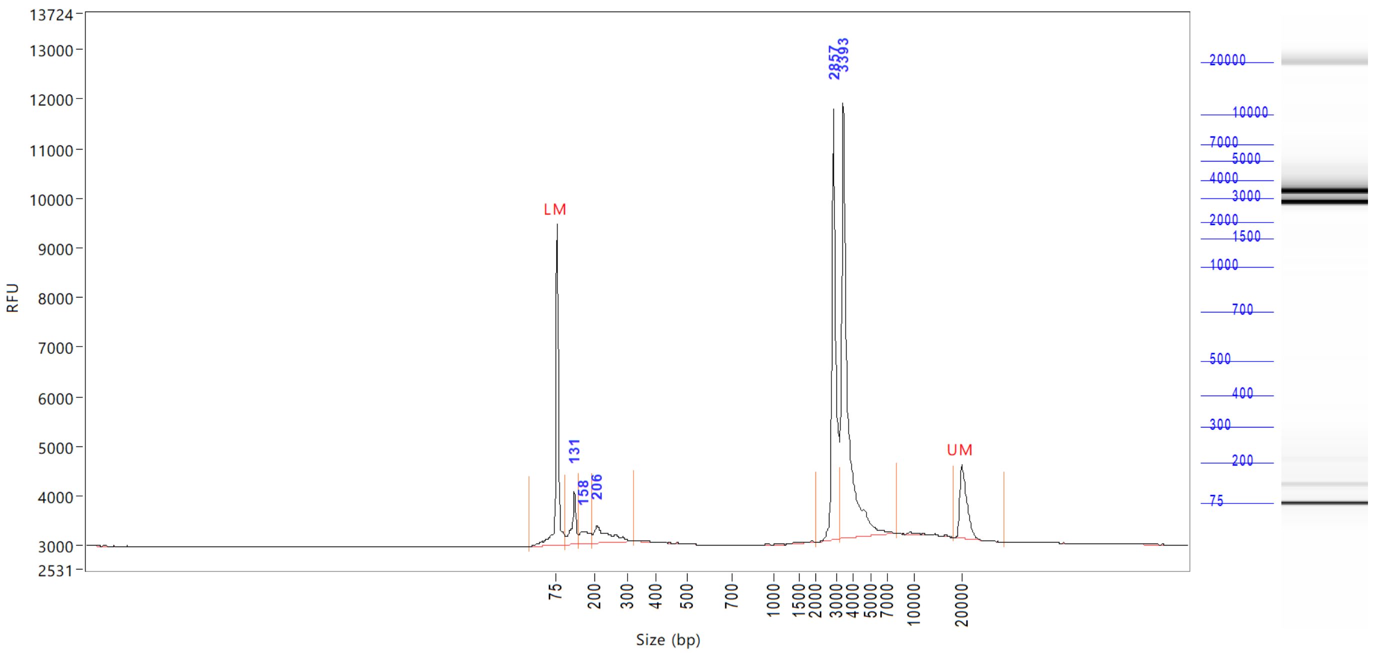

Peak	Size (bp)	Concentration (ng/uL)	From (bp)	To (bp)	Average size (bp)	CV%	RFU	Corrected peak area
1	75 (LM)	0.5000	0	103	71	20.19	6469	43.397
2	131	1.4755	103	145	126	7.63	1078	10.672
3	158	0.8870	145	190	167	8.04	248	6.415
4	206	1.5305	190	319	231	12.74	342	11.070
5	2857	7.5455	2001	3179	2876	4.65	8661	54.575
6	3393	11.2782	3179	8067	3694	15.49	8780	81.574
7	20000 (UM)	0.1584	18293	29068	20550	4.20	1469	13.752

TIC:	22.7166	ng/uL
TIM:	48.2111	nmole/L
Total concentration:	22.8414	ng/uL

```
Sample peak width (sec): 5     Sample min peak height: 50     Sample baseline V to V?: Y     Sample baseline V to V points: 3
Sample filter: Binomial        Number of points for filter: 3  Sample start region (min): 0  Sample end region (min): 30
Marker peak width (sec): 5     Marker min peak height: 200    Marker baseline V to V?: Y     Marker baseline V to V points: 3
Lower marker selection: First peak > 500 RFU                  Upper marker selection: Last peak > 200 RFU
Ladder size (bp)75, 200, 300, 400, 500, 700, 1000, 1500, 2000, 3000, 4000, 5000, 7000, 10000, 20000
Quantification using: Lower Marker          Final concentration (ng/uL): 0.5000          Dilution factor: 12.0
```

Sample: SampD5
Well location: D5
Created: Monday, September 20, 2021 7:19:57 PM

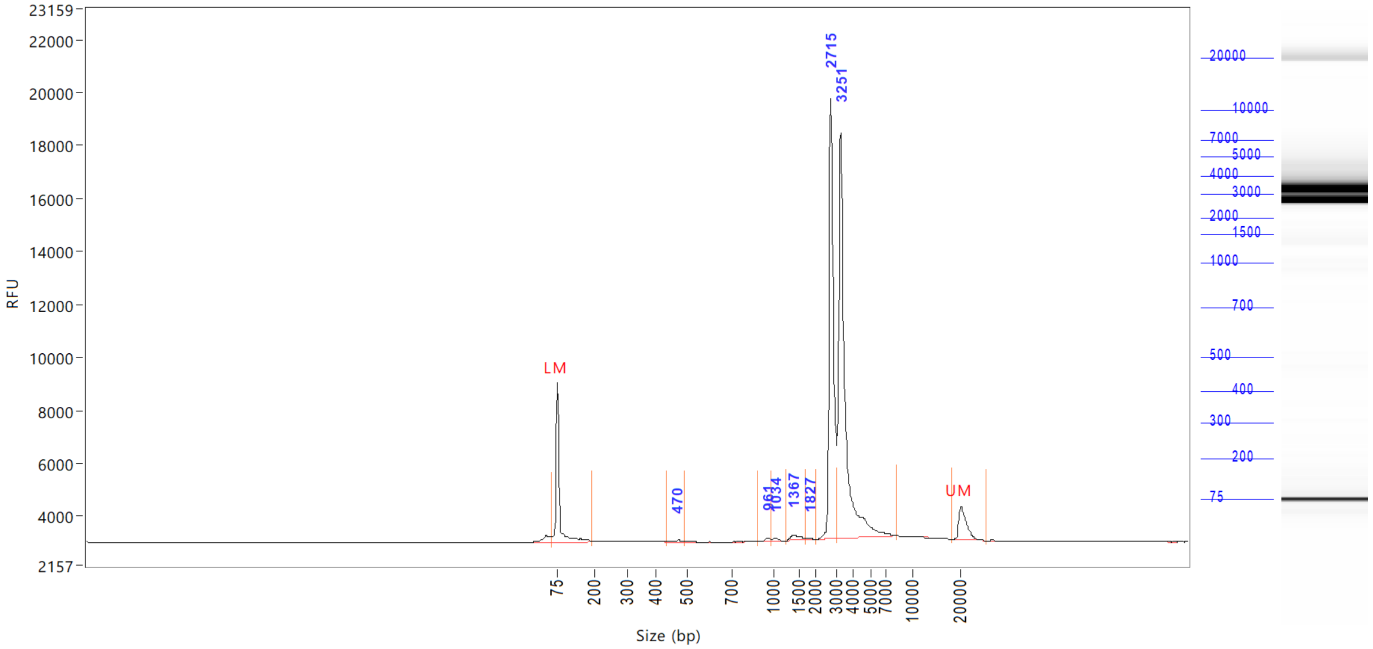

Peak	Size (bp)	Concentration (ng/uL)	From (bp)	To (bp)	Average size (bp)	CV%	RFU	Corrected peak area
1	75 (LM)	0.5000	54	192	82	29.16	6003	46.794
2	470	0.1173	433	494	465	3.02	80	0.915
3	961	0.1462	883	987	955	1.93	134	1.140
4	1034	0.1294	987	1234	1033	4.12	114	1.009
5	1367	0.3889	1234	1693	1421	6.58	214	3.033
6	1827	0.0684	1693	2001	1796	3.36	69	0.533
7	2715	14.1659	2001	3001	2751	4.41	16643	110.480
8	3251	19.6521	3001	8201	3527	17.32	15314	153.268
9	20000 (UM)	0.1327	18049	25440	20557	4.32	1252	12.417

TIC:	34.6682	ng/uL
TIM:	19.0360	nmole/L
Total concentration:	34.6692	ng/uL

Sample peak width (sec): 5 Sample min peak height: 50 Sample baseline V to V?: Y Sample baseline V to V points: 3
Sample filter: Binomial Number of points for filter: 3 Sample start region (min): 0 Sample end region (min): 30
Marker peak width (sec): 5 Marker min peak height: 200 Marker baseline V to V?: Y Marker baseline V to V points: 3
Lower marker selection: First peak > 500 RFU Upper marker selection: Last peak > 200 RFU
Ladder size (bp)75, 200, 300, 400, 500, 700, 1000, 1500, 2000, 3000, 4000, 5000, 7000, 10000, 20000
Quantification using: Lower Marker Final concentration (ng/uL): 0.5000 Dilution factor: 12.0

Sample: SampE5
Well location: E5
Created: Monday, September 20, 2021 7:19:57 PM

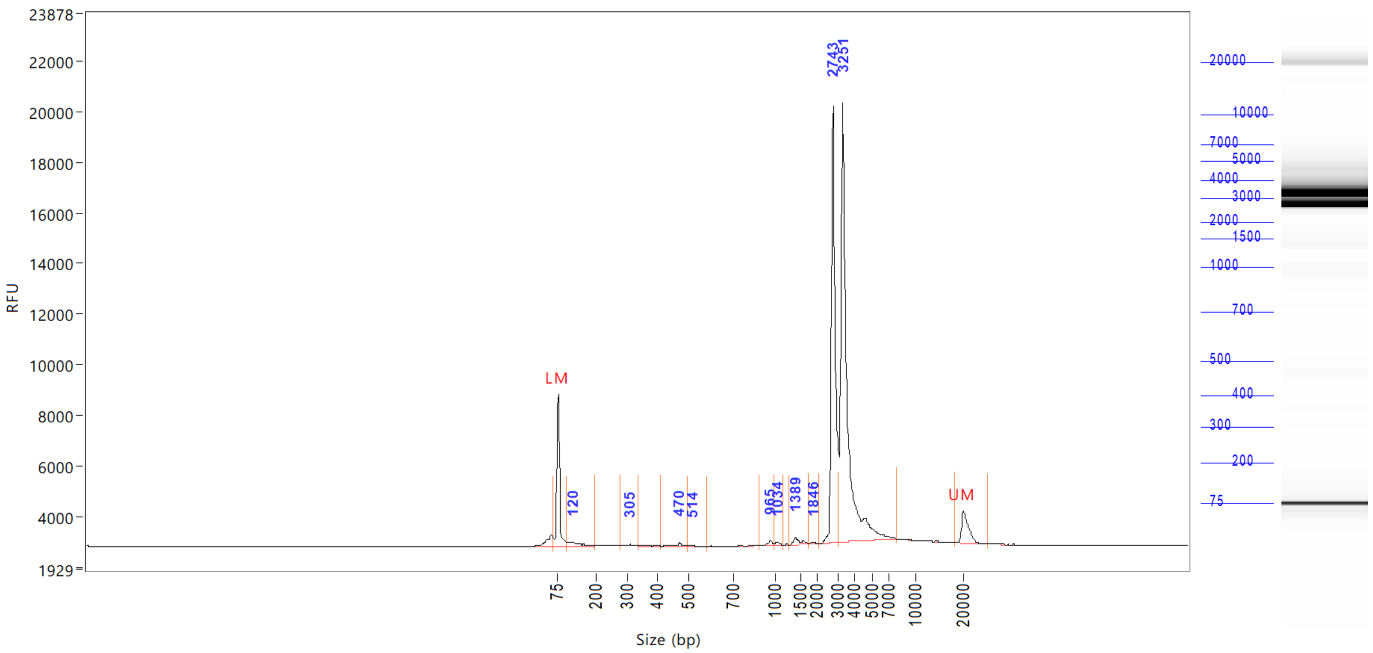

Peak	Size (bp)	Concentration (ng/uL)	From (bp)	To (bp)	Average size (bp)	CV%	RFU	Corrected peak area
1	75 (LM)	0.5000	62	105	74	9.14	6017	38.749
2	120	0.6817	105	196	132	16.25	157	4.403
3	305	0.0693	275	331	299	2.81	61	0.447
4	470	0.1963	411	496	463	3.62	133	1.268
5	514	0.0777	496	580	515	3.28	60	0.502
6	965	0.2145	883	996	958	2.09	183	1.385
7	1034	0.1358	996	1145	1034	2.91	125	0.877
8	1389	0.4744	1278	1731	1440	6.59	258	3.064
9	1846	0.0805	1731	2087	1840	3.23	80	0.520
10	2743	16.5974	2087	3037	2767	4.50	17218	107.188
11	3251	24.8726	3037	7934	3549	16.60	17359	160.631
12	20000 (UM)	0.1494	18171	24957	20504	3.97	1257	11.582

TIC:	43.4001	ng/uL
TIM:	32.4241	nmole/L
Total concentration:	43.2432	ng/uL

Sample peak width (sec): 5   Sample min peak height: 50   Sample baseline V to V?: Y   Sample baseline V to V points: 3
Sample filter: Binomial   Number of points for filter: 3 Sample start region (min): 0 Sample end region (min): 30
Marker peak width (sec): 5   Marker min peak height: 200   Marker baseline V to V?: Y   Marker baseline V to V points: 3
Lower marker selection: First peak > 500 RFU   Upper marker selection: Last peak > 200 RFU
Ladder size (bp)75, 200, 300, 400, 500, 700, 1000, 1500, 2000, 3000, 4000, 5000, 7000, 10000, 20000
Quantification using: Lower Marker   Final concentration (ng/uL): 0.5000   Dilution factor: 12.0

Sample: SampF5
Well location: F5
Created: Monday, September 20, 2021 7:19:57 PM

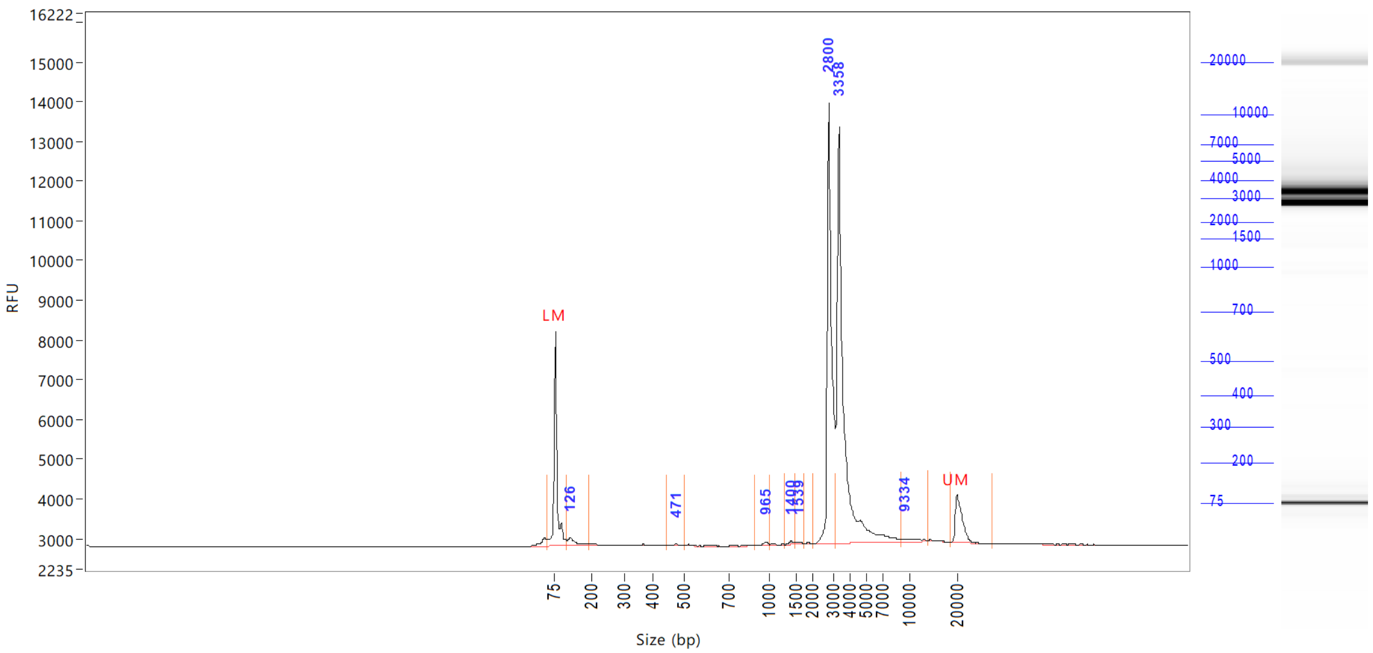

Peak	Size (bp)	Concentration (ng/uL)	From (bp)	To (bp)	Average size (bp)	CV%	RFU	Corrected peak area
1	75 (LM)	0.5000	50	112	76	12.36	5385	39.872
2	126	0.5946	112	190	136	14.46	200	3.952
3	471	0.0742	443	501	470	2.86	51	0.493
4	965	0.1023	883	996	958	1.89	94	0.680
5	1400	0.0935	1278	1467	1390	3.02	85	0.621
6	1539	0.0892	1467	1750	1563	4.92	63	0.593
7	2800	11.5649	2058	3108	2831	4.73	11102	76.852
8	3358	15.9303	3108	9000	3765	24.11	10494	105.862
9	9334	0.2835	9000	13781	10658	12.70	82	1.884
10	20000 (UM)	0.1366	18537	27496	20502	4.18	1199	10.889

TIC:	28.7325	ng/uL
TIM:	21.5590	nmole/L
Total concentration:	28.7356	ng/uL

Sample peak width (sec): 5 Sample min peak height: 50 Sample baseline V to V?: Y Sample baseline V to V points: 3
Sample filter: Binomial Number of points for filter: 3 Sample start region (min): 0 Sample end region (min): 30
Marker peak width (sec): 5 Marker min peak height: 200 Marker baseline V to V?: Y Marker baseline V to V points: 3
Lower marker selection: First peak > 500 RFU Upper marker selection: Last peak > 200 RFU
Ladder size (bp)75, 200, 300, 400, 500, 700, 1000, 1500, 2000, 3000, 4000, 5000, 7000, 10000, 20000
Quantification using: Lower Marker Final concentration (ng/uL): 0.5000 Dilution factor: 12.0

Sample: SampG5
Well location: G5
Created: Monday, September 20, 2021 7:19:57 PM

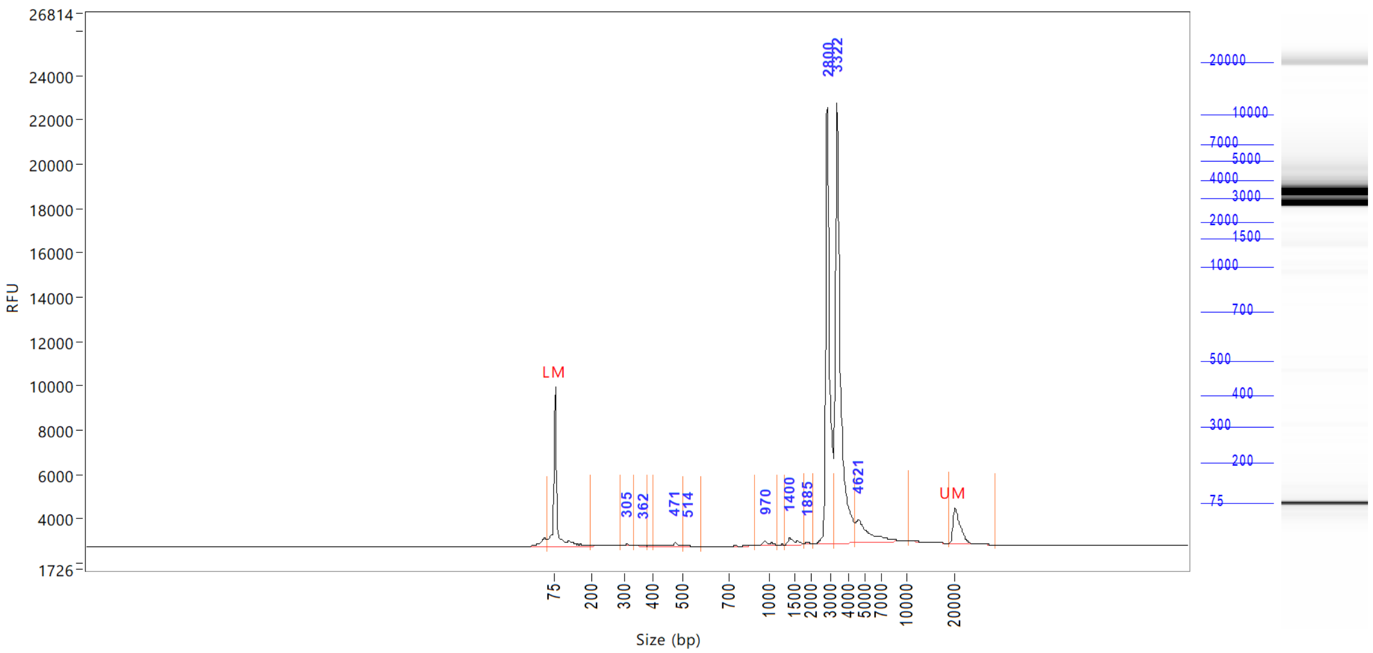

Peak	Size (bp)	Concentration (ng/uL)	From (bp)	To (bp)	Average size (bp)	CV%	RFU	Corrected peak area
1	75 (LM)	0.5000	50	194	80	26.00	7229	55.474
2	305	0.0752	287	333	303	1.85	118	0.696
3	362	0.0314	333	378	358	1.87	51	0.290
4	471	0.1766	401	498	462	4.59	165	1.633
5	514	0.0633	498	580	515	2.86	75	0.585
6	970	0.2647	892	1167	991	4.65	219	2.448
7	1400	0.4045	1289	1770	1463	7.00	291	3.740
8	1885	0.0847	1770	2144	1874	3.20	115	0.783
9	2800	13.5798	2144	3108	2822	4.37	19703	125.555
10	3322	17.8927	3108	4449	3455	8.14	19911	165.431
11	4621	2.2591	4449	10367	5569	21.87	1020	20.887
12	20000 (UM)	0.1370	18659	28705	20563	4.24	1609	15.202

TIC:	34.8320	ng/uL
TIM:	19.4684	nmole/L
Total concentration:	34.6458	ng/uL

```
Sample peak width (sec): 5    Sample min peak height: 50    Sample baseline V to V?: Y    Sample baseline V to V points: 3
Sample filter: Binomial       Number of points for filter: 3  Sample start region (min): 0  Sample end region (min): 30
Marker peak width (sec): 5    Marker min peak height: 200   Marker baseline V to V?: Y    Marker baseline V to V points: 3
Lower marker selection: First peak > 500 RFU                Upper marker selection: Last peak > 200 RFU
Ladder size (bp)75, 200, 300, 400, 500, 700, 1000, 1500, 2000, 3000, 4000, 5000, 7000, 10000, 20000
Quantification using: Lower Marker          Final concentration (ng/uL): 0.5000          Dilution factor: 12.0
```

Sample: SampH5
Well location: H5
Created: Monday, September 20, 2021 7:19:57 PM

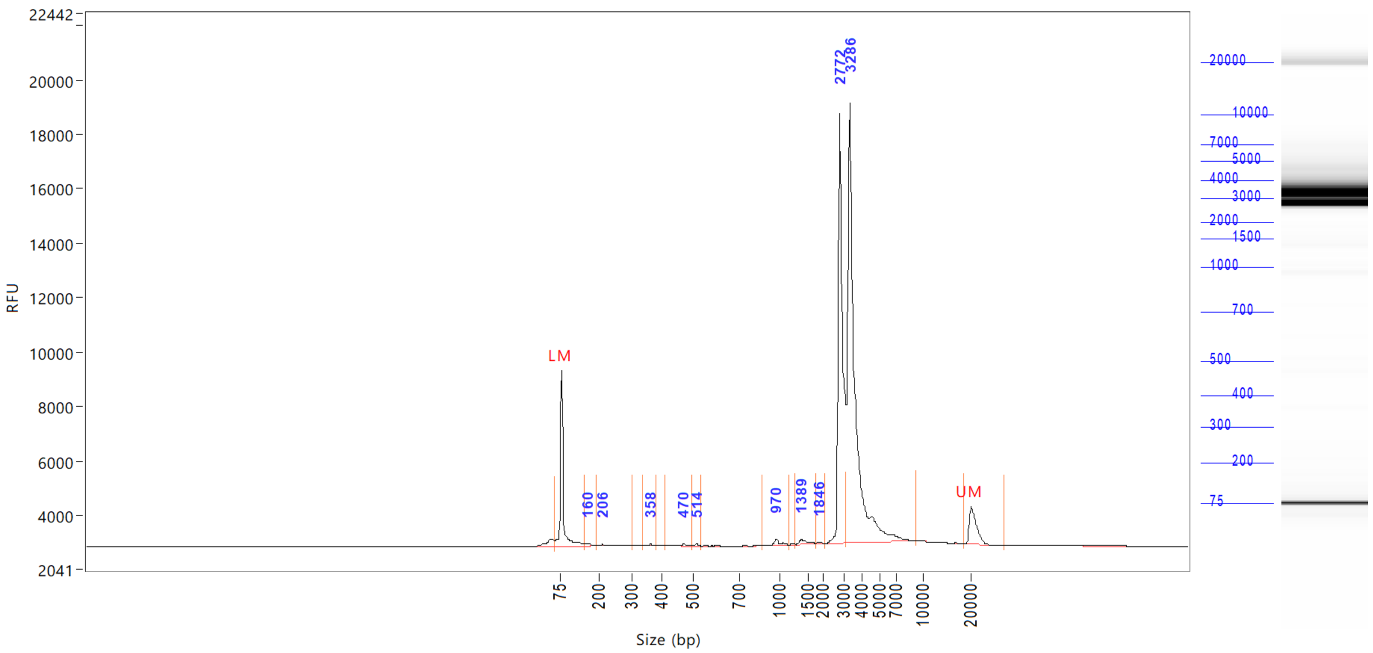

Peak	Size (bp)	Concentration (ng/uL)	From (bp)	To (bp)	Average size (bp)	CV%	RFU	Corrected peak area
1	75 (LM)	0.5000	54	152	79	19.99	6448	46.591
2	160	0.1663	152	187	167	6.08	79	1.291
3	206	0.2076	187	298	221	10.95	54	1.612
4	358	0.0419	335	380	356	1.65	56	0.325
5	470	0.1256	411	496	464	3.75	98	0.975
6	514	0.0575	496	537	509	1.46	69	0.446
7	970	0.2774	870	1145	981	4.24	225	2.154
8	1389	0.3083	1278	1750	1452	7.15	180	2.394
9	1846	0.0580	1750	2087	1860	3.76	60	0.451
10	2772	14.4598	2087	3072	2815	4.71	15756	112.282
11	3286	22.4418	3072	9334	3630	19.40	16170	174.263
12	20000 (UM)	0.1373	18293	26770	20497	4.10	1376	12.791

TIC:	38.1441	ng/uL
TIM:	23.5059	nmole/L
Total concentration:	37.7292	ng/uL

```
Sample peak width (sec): 5    Sample min peak height: 50    Sample baseline V to V?: Y    Sample baseline V to V points: 3
Sample filter: Binomial       Number of points for filter: 3  Sample start region (min): 0  Sample end region (min): 30
Marker peak width (sec): 5    Marker min peak height: 200   Marker baseline V to V?: Y    Marker baseline V to V points: 3
Lower marker selection: First peak > 500 RFU                Upper marker selection: Last peak > 200 RFU
Ladder size (bp)75, 200, 300, 400, 500, 700, 1000, 1500, 2000, 3000, 4000, 5000, 7000, 10000, 20000
Quantification using: Lower Marker          Final concentration (ng/uL): 0.5000          Dilution factor: 12.0
```

Sample: SampA6
Well location: A6
Created: Monday, September 20, 2021 7:19:57 PM

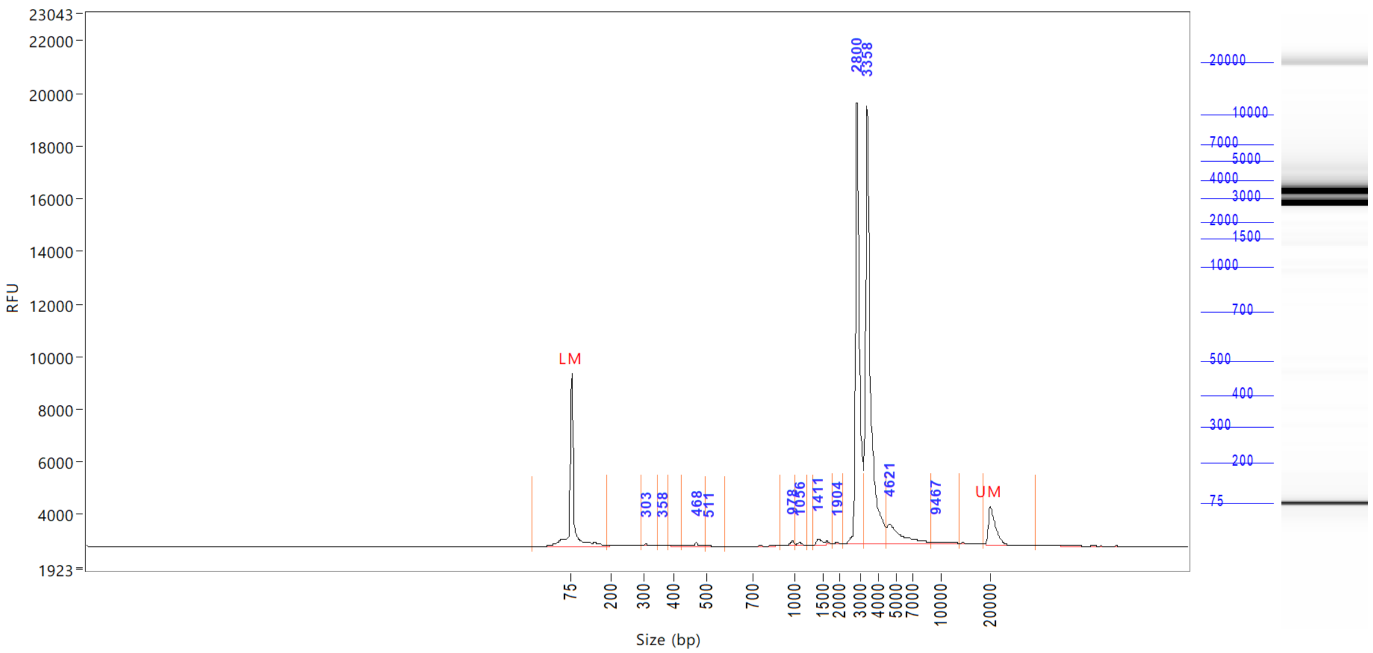

Peak	Size (bp)	Concentration (ng/uL)	From (bp)	To (bp)	Average size (bp)	CV%	RFU	Corrected peak area
1	75 (LM)	0.5000	0	189	76	34.83	6560	55.836
2	303	0.0451	289	341	302	2.44	67	0.420
3	358	0.0349	341	376	356	1.68	54	0.325
4	468	0.1215	420	496	462	3.29	125	1.131
5	511	0.0461	496	575	516	3.87	61	0.429
6	978	0.1279	883	1000	967	1.84	171	1.190
7	1056	0.0831	1000	1201	1056	3.32	104	0.773
8	1411	0.2856	1311	1789	1487	7.11	229	2.658
9	1904	0.0555	1789	2144	1897	2.99	84	0.517
10	2800	10.8557	2144	3108	2830	4.07	16786	101.024
11	3358	13.8062	3108	4414	3472	7.67	16628	128.481
12	4621	1.7085	4414	9000	5485	20.72	763	15.899
13	9467	0.1264	9000	13781	10265	10.95	66	1.176
14	20000 (UM)	0.1181	18781	29068	20514	4.50	1453	13.192

TIC:	27.2965	ng/uL
TIM:	15.0923	nmole/L
Total concentration:	27.2257	ng/uL

```
Sample peak width (sec): 5     Sample min peak height: 50    Sample baseline V to V?: Y    Sample baseline V to V points: 3
Sample filter: Binomial        Number of points for filter: 3  Sample start region (min): 0  Sample end region (min): 30
Marker peak width (sec): 5     Marker min peak height: 200   Marker baseline V to V?: Y    Marker baseline V to V points: 3
Lower marker selection: First peak > 500 RFU                 Upper marker selection: Last peak > 200 RFU
Ladder size (bp)75, 200, 300, 400, 500, 700, 1000, 1500, 2000, 3000, 4000, 5000, 7000, 10000, 20000
Quantification using: Lower Marker          Final concentration (ng/uL): 0.5000          Dilution factor: 12.0
```

Sample: SampB6
Well location: B6
Created: Monday, September 20, 2021 7:19:57 PM

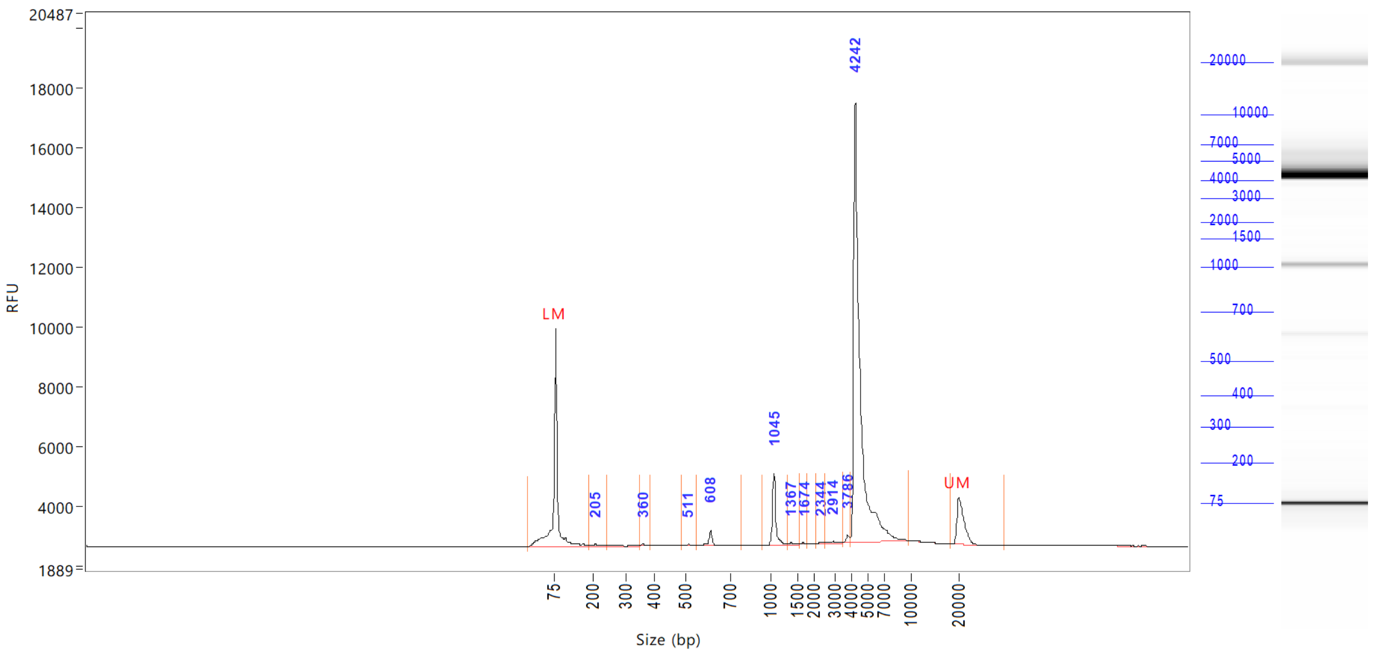

Peak	Size (bp)	Concentration (ng/uL)	From (bp)	To (bp)	Average size (bp)	CV%	RFU	Corrected peak area
1	75 (LM)	0.5000	0	185	70	37.27	7271	65.517
2	205	0.1015	185	241	208	6.89	64	1.109
3	360	0.0462	347	386	357	1.68	91	0.504
4	511	0.0462	485	547	514	2.77	60	0.505
5	608	0.3762	547	770	609	4.08	546	4.108
6	1045	1.3056	939	1311	1043	5.03	2404	14.256
7	1367	0.0463	1311	1520	1376	3.28	66	0.505
8	1674	0.0221	1520	1770	1631	2.86	51	0.241
9	2344	0.0491	2087	2572	2346	4.80	69	0.536
10	2914	0.0764	2572	3536	2971	8.01	66	0.834
11	3786	0.1179	3536	3929	3774	2.49	244	1.287
12	4242	13.0664	3929	9733	4576	15.95	14730	142.679
13	20000 (UM)	0.1107	18293	29309	20484	4.47	1575	14.508

TIC:	15.2538	ng/uL
TIM:	9.1470	nmole/L
Total concentration:	15.4025	ng/uL

Sample peak width (sec): 5   Sample min peak height: 50   Sample baseline V to V?: Y   Sample baseline V to V points: 3
Sample filter: Binomial   Number of points for filter: 3 Sample start region (min): 0 Sample end region (min): 30
Marker peak width (sec): 5   Marker min peak height: 200   Marker baseline V to V?: Y   Marker baseline V to V points: 3
Lower marker selection: First peak > 500 RFU   Upper marker selection: Last peak > 200 RFU
Ladder size (bp)75, 200, 300, 400, 500, 700, 1000, 1500, 2000, 3000, 4000, 5000, 7000, 10000, 20000
Quantification using: Lower Marker   Final concentration (ng/uL): 0.5000   Dilution factor: 12.0

Sample: SampC6
Well location: C6
Created: Monday, September 20, 2021 7:19:57 PM

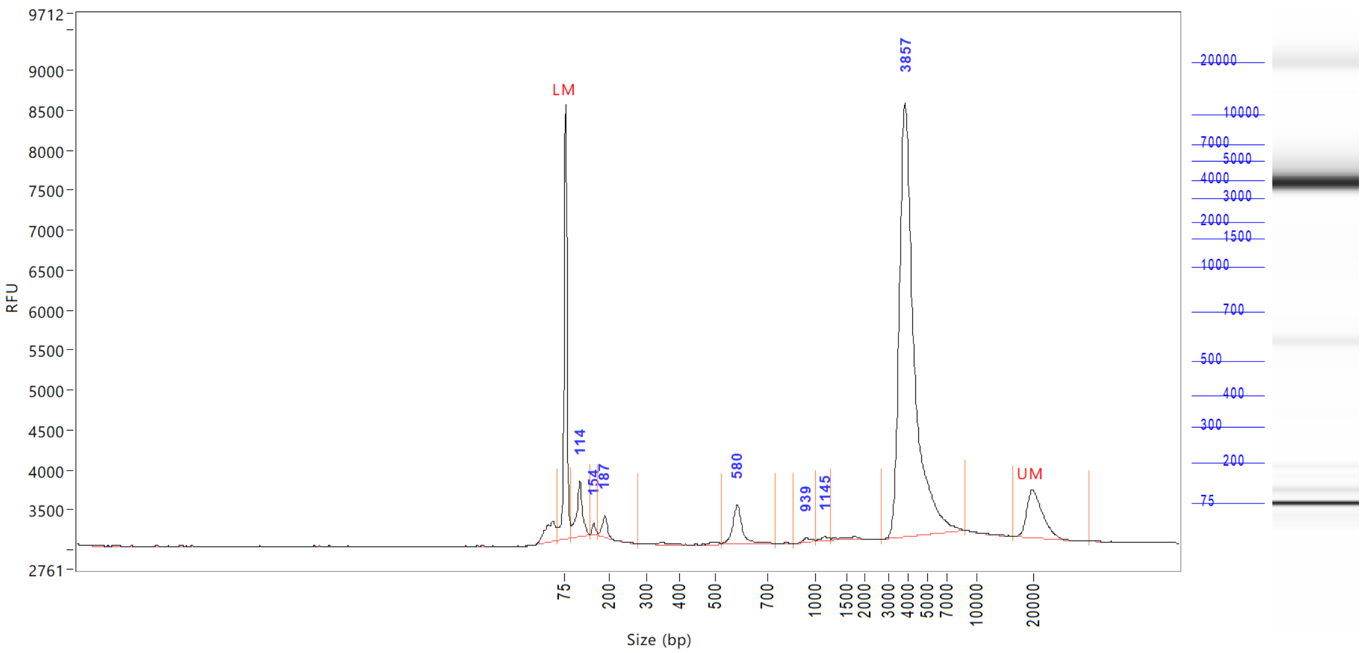

Peak	Size (bp)	Concentration (ng/uL)	From (bp)	To (bp)	Average size (bp)	CV%	RFU	Corrected peak area
1	75 (LM)	0.5000	54	89	73	6.67	5438	39.749
2	114	1.4792	89	145	111	9.21	691	9.799
3	154	0.1808	145	168	154	3.45	159	1.198
4	187	0.4823	168	277	186	7.05	276	3.195
5	580	1.2732	521	744	584	5.02	487	8.435
6	939	0.1037	861	1012	946	2.91	50	0.687
7	1145	0.0857	1012	1256	1141	5.34	54	0.568
8	3857	16.9889	2743	8867	4097	16.16	5434	112.549
9	20000 (UM)	0.1464	16586	30277	20646	7.64	603	11.637

TIC:	20.5938	ng/uL
TIM:	38.7991	nmole/L
Total concentration:	20.9958	ng/uL

```
Sample peak width (sec): 5    Sample min peak height: 50    Sample baseline V to V?: Y    Sample baseline V to V points: 3
Sample filter: Binomial       Number of points for filter: 3  Sample start region (min): 0  Sample end region (min): 30
Marker peak width (sec): 5    Marker min peak height: 200   Marker baseline V to V?: Y    Marker baseline V to V points: 3
Lower marker selection: First peak > 500 RFU                Upper marker selection: Last peak > 200 RFU
Ladder size (bp)75, 200, 300, 400, 500, 700, 1000, 1500, 2000, 3000, 4000, 5000, 7000, 10000, 20000
Quantification using: Lower Marker          Final concentration (ng/uL): 0.5000          Dilution factor: 12.0
```

Sample: SampD6
Well location: D6
Created: Monday, September 20, 2021 7:19:57 PM

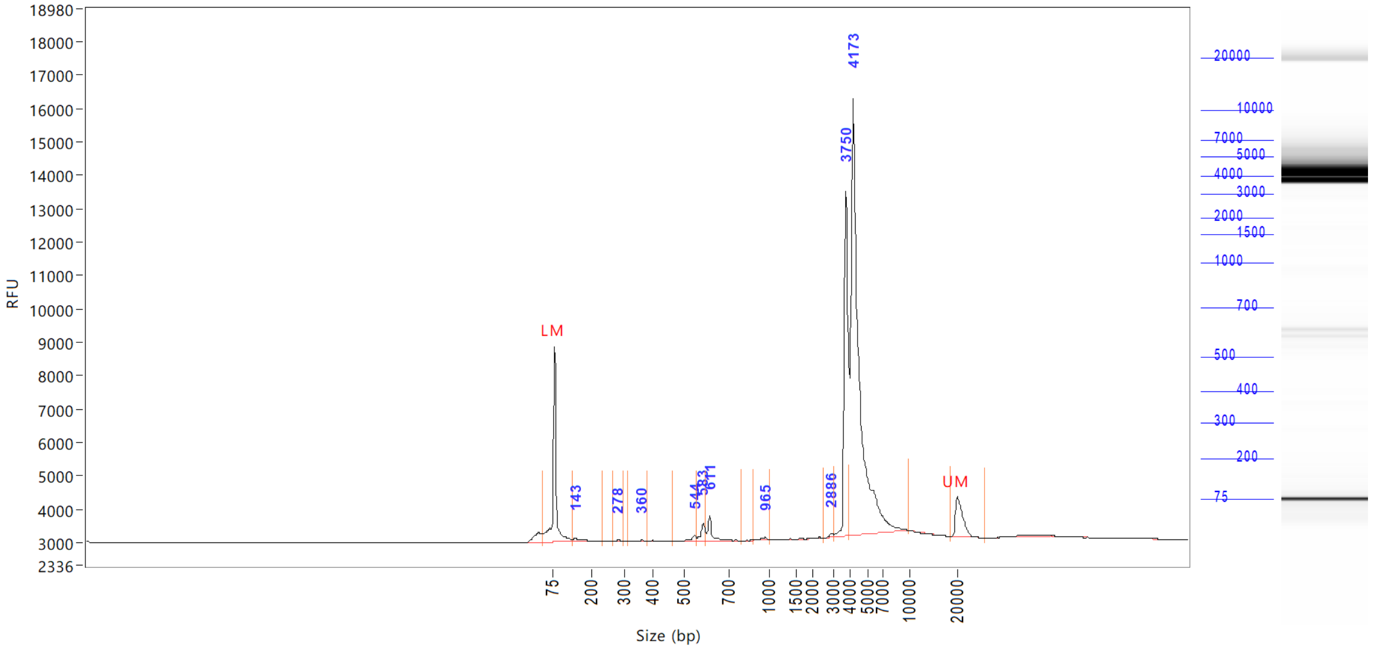

Peak	Size (bp)	Concentration (ng/uL)	From (bp)	To (bp)	Average size (bp)	CV%	RFU	Corrected peak area
1	75 (LM)	0.5000	37	133	73	19.21	5831	45.343
2	143	0.3195	133	232	160	13.47	119	2.414
3	278	0.0490	264	296	278	1.73	65	0.370
4	360	0.0961	311	378	349	4.14	65	0.727
5	544	0.2829	462	555	530	3.59	180	2.138
6	583	0.5652	555	595	578	1.78	549	4.271
7	611	0.8079	595	783	620	4.28	744	6.105
8	965	0.1177	879	1000	944	2.39	89	0.889
9	2886	0.0877	2543	3037	2896	3.10	102	0.663
10	3750	8.1612	3037	3964	3752	3.42	10318	61.675
11	4173	21.4112	3964	9800	4575	17.11	13087	161.807
12	20000 (UM)	0.1260	18415	25803	20424	3.99	1222	11.425

TIC:	31.8982	ng/uL
TIM:	20.1967	nmole/L
Total concentration:	31.5687	ng/uL

```
Sample peak width (sec): 5    Sample min peak height: 50    Sample baseline V to V?: Y    Sample baseline V to V points: 3
Sample filter: Binomial       Number of points for filter: 3  Sample start region (min): 0  Sample end region (min): 30
Marker peak width (sec): 5    Marker min peak height: 200   Marker baseline V to V?: Y    Marker baseline V to V points: 3
Lower marker selection: First peak > 500 RFU                Upper marker selection: Last peak > 200 RFU
Ladder size (bp)75, 200, 300, 400, 500, 700, 1000, 1500, 2000, 3000, 4000, 5000, 7000, 10000, 20000
Quantification using: Lower Marker          Final concentration (ng/uL): 0.5000          Dilution factor: 12.0
```

Sample: SampE6
Well location: E6
Created: Monday, September 20, 2021 7:19:57 PM

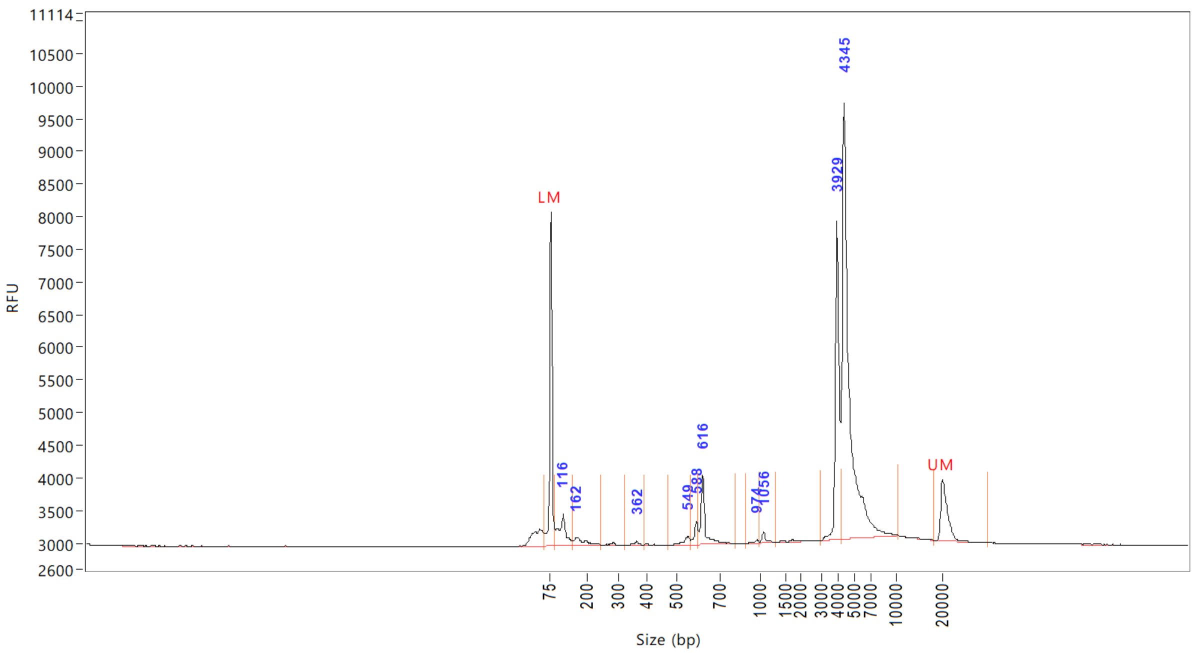

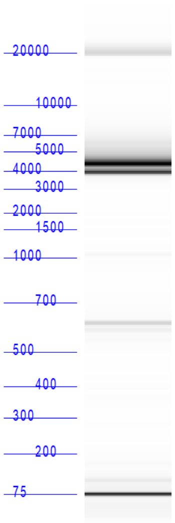

Peak	Size (bp)	Concentration (ng/uL)	From (bp)	To (bp)	Average size (bp)	CV%	RFU	Corrected peak area
1	75 (LM)	0.5000	54	91	73	7.54	5116	31.882
2	116	1.5059	91	148	113	12.36	485	8.002
3	162	0.5238	148	242	176	11.60	118	2.783
4	362	0.1254	319	388	356	4.10	58	0.666
5	549	0.2847	471	560	535	3.48	131	1.513
6	588	0.5238	560	598	583	1.69	364	2.783
7	616	1.4785	598	809	627	4.89	1057	7.856
8	974	0.0826	896	991	956	2.42	51	0.439
9	1056	0.2151	991	1311	1069	5.47	165	1.143
10	3929	5.4482	2971	4173	3938	3.83	4884	28.950
11	4345	13.1665	4173	10367	4806	17.36	6689	69.962
12	20000 (UM)	0.1365	18293	29672	20602	5.33	940	8.707

TIC:	23.3546	ng/uL
TIM:	40.8582	nmole/L
Total concentration:	23.2963	ng/uL

```
Sample peak width (sec): 5     Sample min peak height: 50    Sample baseline V to V?: Y    Sample baseline V to V points: 3
Sample filter: Binomial        Number of points for filter: 3  Sample start region (min): 0  Sample end region (min): 30
Marker peak width (sec): 5     Marker min peak height: 200   Marker baseline V to V?: Y    Marker baseline V to V points: 3
Lower marker selection: First peak > 500 RFU                 Upper marker selection: Last peak > 200 RFU
Ladder size (bp)75, 200, 300, 400, 500, 700, 1000, 1500, 2000, 3000, 4000, 5000, 7000, 10000, 20000
Quantification using: Lower Marker          Final concentration (ng/uL): 0.5000          Dilution factor: 12.0
```

Sample: SampF6
Well location: F6
Created: Monday, September 20, 2021 7:19:57 PM

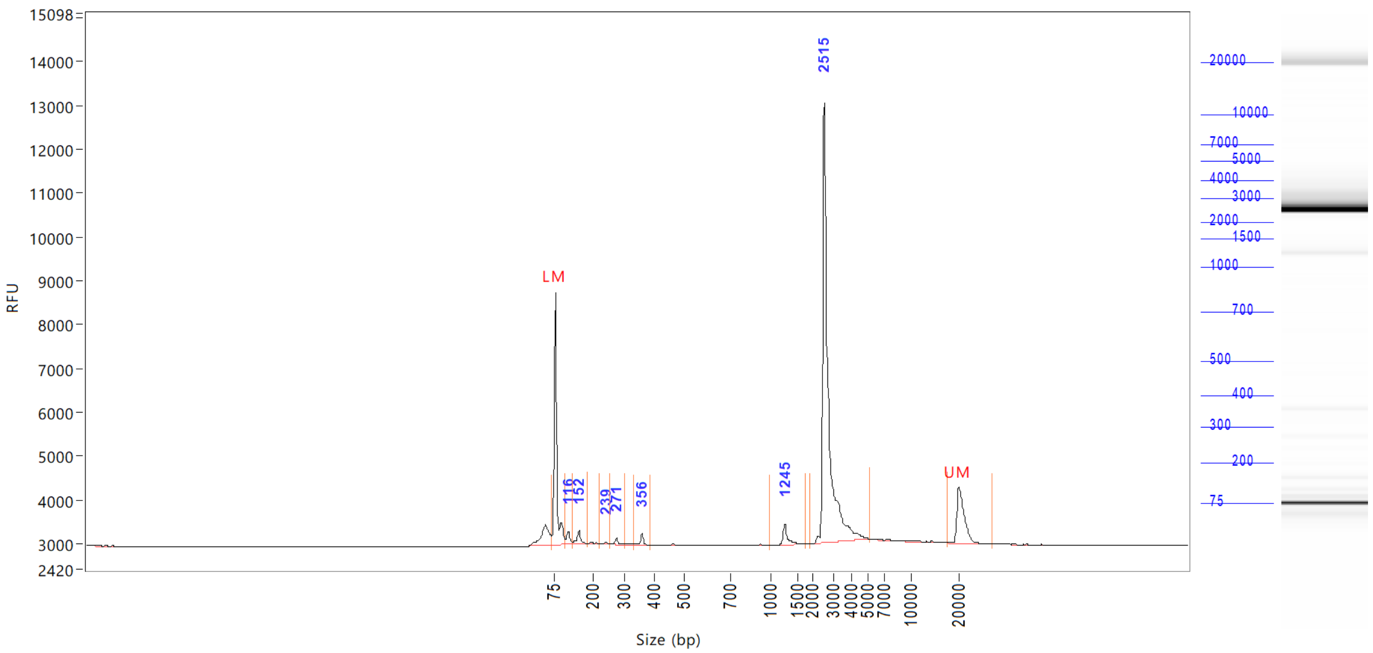

Peak	Size (bp)	Concentration (ng/uL)	From (bp)	To (bp)	Average size (bp)	CV%	RFU	Corrected peak area
1	75 (LM)	0.5000	64	108	77	10.35	5744	38.362
2	116	0.3726	108	131	117	4.87	294	2.382
3	152	0.4146	131	183	149	5.58	296	2.651
4	239	0.0567	221	251	237	1.90	63	0.362
5	271	0.1591	251	298	271	2.68	148	1.017
6	356	0.2580	329	384	352	2.28	266	1.649
7	1245	0.5834	996	1750	1280	6.57	481	3.730
8	2515	13.6376	1885	5149	2724	15.20	10046	87.195
9	20000 (UM)	0.1607	17683	27012	20521	4.52	1291	12.329

TIC:	15.4820	ng/uL
TIM:	21.3608	nmole/L
Total concentration:	15.9326	ng/uL

Sample peak width (sec): 5   Sample min peak height: 50   Sample baseline V to V?: Y   Sample baseline V to V points: 3
Sample filter: Binomial   Number of points for filter: 3   Sample start region (min): 0   Sample end region (min): 30
Marker peak width (sec): 5   Marker min peak height: 200   Marker baseline V to V?: Y   Marker baseline V to V points: 3
Lower marker selection: First peak > 500 RFU   Upper marker selection: Last peak > 200 RFU
Ladder size (bp)75, 200, 300, 400, 500, 700, 1000, 1500, 2000, 3000, 4000, 5000, 7000, 10000, 20000
Quantification using: Lower Marker   Final concentration (ng/uL): 0.5000   Dilution factor: 12.0

Sample: SampG6
Well location: G6
Created: Monday, September 20, 2021 7:19:57 PM

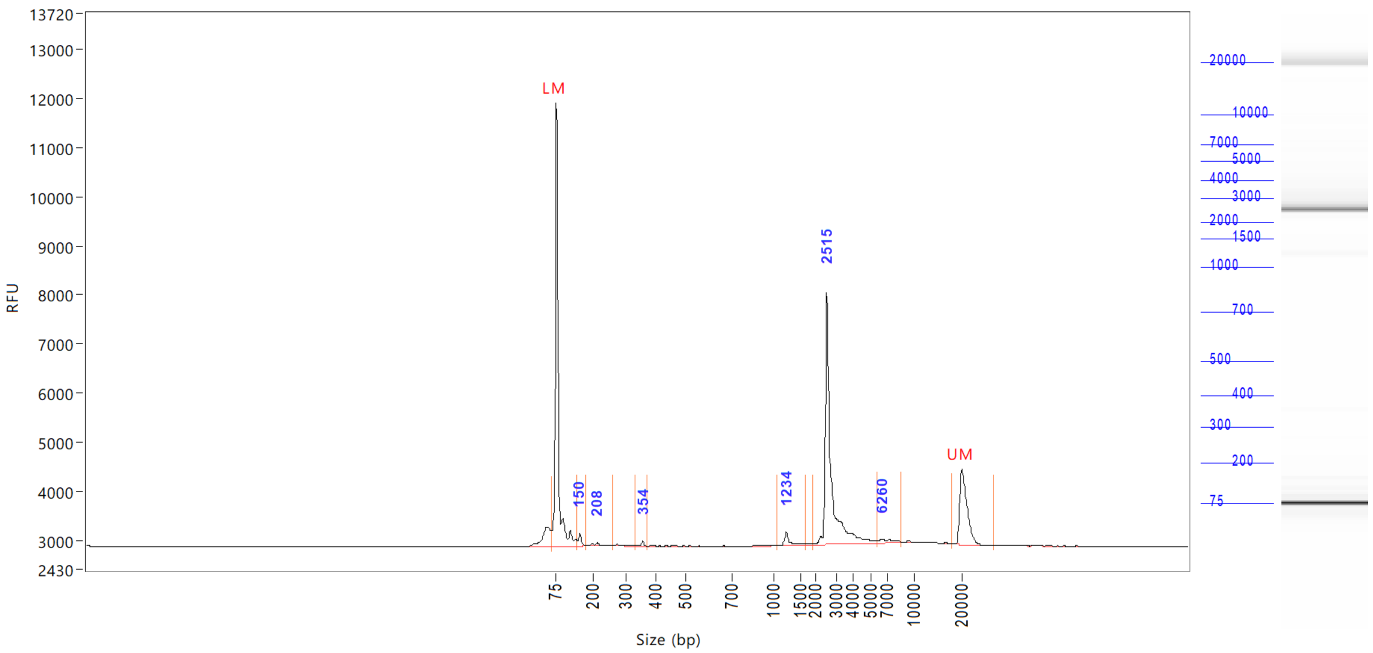

Peak	Size (bp)	Concentration (ng/uL)	From (bp)	To (bp)	Average size (bp)	CV%	RFU	Corrected peak area
1	75 (LM)	0.5000	60	143	78	17.46	9019	64.745
2	150	0.1823	143	173	151	4.48	255	1.967
3	208	0.1034	173	255	204	9.11	58	1.116
4	354	0.0520	327	370	348	2.08	95	0.561
5	1234	0.2246	1079	1674	1287	8.66	280	2.424
6	2515	4.1498	1923	5815	2817	20.91	5119	44.780
7	6260	0.1371	5815	8600	6983	10.51	69	1.479
8	20000 (UM)	0.1139	17805	26649	20444	4.33	1545	14.750

TIC:	4.8492	ng/uL
TIM:	5.8078	nmole/L
Total concentration:	5.0443	ng/uL

Sample peak width (sec): 5   Sample min peak height: 50   Sample baseline V to V?: Y   Sample baseline V to V points: 3
Sample filter: Binomial   Number of points for filter: 3   Sample start region (min): 0   Sample end region (min): 30
Marker peak width (sec): 5   Marker min peak height: 200   Marker baseline V to V?: Y   Marker baseline V to V points: 3
Lower marker selection: First peak > 500 RFU   Upper marker selection: Last peak > 200 RFU
Ladder size (bp)75, 200, 300, 400, 500, 700, 1000, 1500, 2000, 3000, 4000, 5000, 7000, 10000, 20000
Quantification using: Lower Marker   Final concentration (ng/uL): 0.5000   Dilution factor: 12.0

Sample: SampH6
Well location: H6
Created: Monday, September 20, 2021 7:19:57 PM

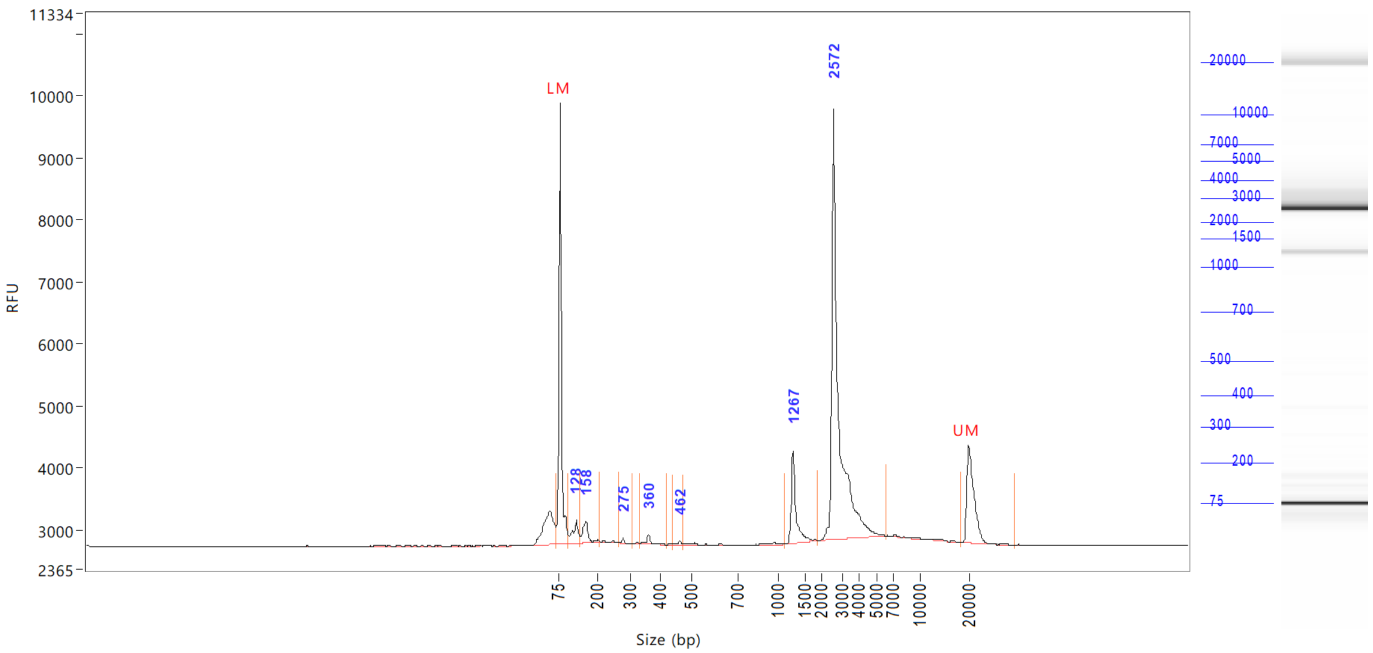

Peak	Size (bp)	Concentration (ng/uL)	From (bp)	To (bp)	Average size (bp)	CV%	RFU	Corrected peak area
1	75 (LM)	0.5000	64	105	75	8.71	7128	44.378
2	128	0.6453	105	143	123	8.06	371	4.773
3	158	0.5800	143	205	158	7.84	346	4.290
4	275	0.0598	260	301	274	2.10	79	0.442
5	360	0.1428	327	420	356	3.80	154	1.056
6	462	0.0520	441	471	458	1.35	65	0.385
7	1267	1.4872	1101	1885	1295	7.38	1500	11.000
8	2572	9.6717	1885	6186	2874	17.63	6950	71.536
9	20000 (UM)	0.1671	18415	29430	20507	4.45	1580	14.832

TIC:	12.6388	ng/uL
TIM:	23.2893	nmole/L
Total concentration:	12.9895	ng/uL

Sample peak width (sec): 5   Sample min peak height: 50   Sample baseline V to V?: Y   Sample baseline V to V points: 3
Sample filter: Binomial   Number of points for filter: 3 Sample start region (min): 0 Sample end region (min): 30
Marker peak width (sec): 5   Marker min peak height: 200   Marker baseline V to V?: Y   Marker baseline V to V points: 3
Lower marker selection: First peak > 500 RFU   Upper marker selection: Last peak > 200 RFU
Ladder size (bp)75, 200, 300, 400, 500, 700, 1000, 1500, 2000, 3000, 4000, 5000, 7000, 10000, 20000
Quantification using: Lower Marker   Final concentration (ng/uL): 0.5000   Dilution factor: 12.0

Sample: SampA7
Well location: A7
Created: Monday, September 20, 2021 7:19:57 PM

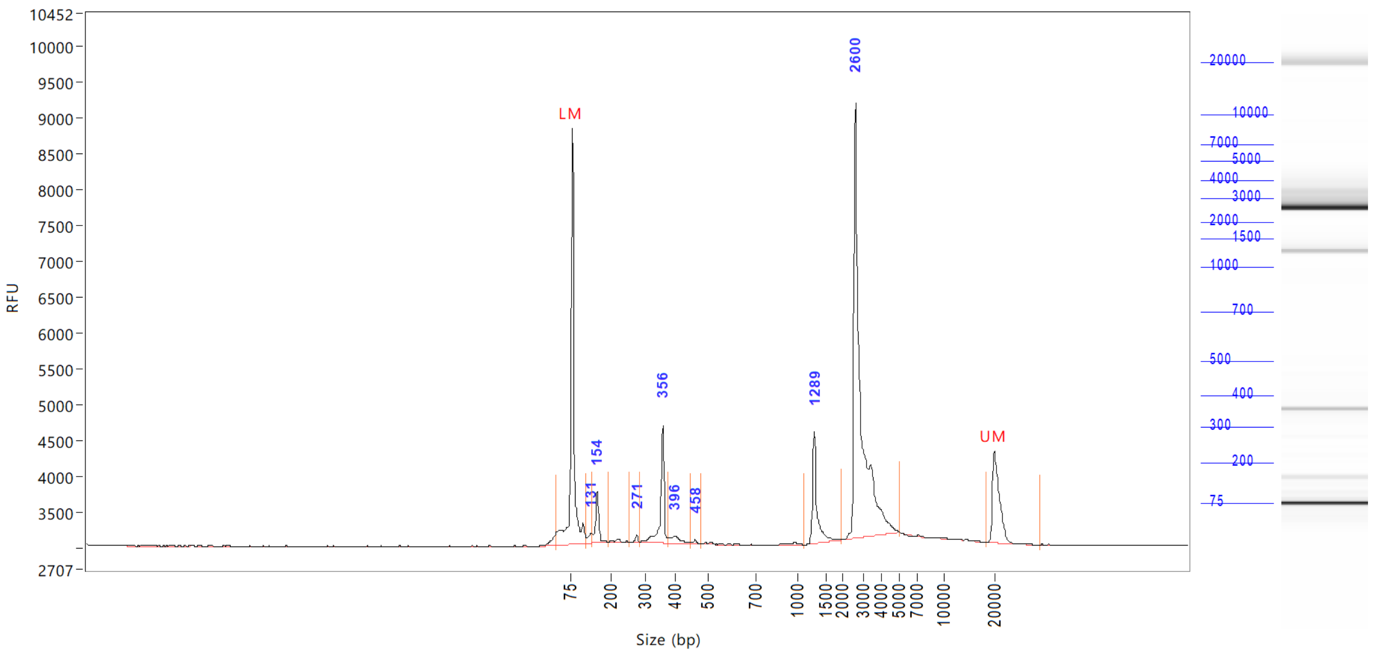

Peak	Size (bp)	Concentration (ng/uL)	From (bp)	To (bp)	Average size (bp)	CV%	RFU	Corrected peak area
1	75 (LM)	0.5000	26	120	73	20.00	5793	44.025
2	131	0.1993	120	139	130	4.39	134	1.462
3	154	0.6999	139	189	151	4.13	706	5.136
4	271	0.0754	250	280	269	1.93	99	0.553
5	356	1.5143	280	372	346	4.66	1630	11.111
6	396	0.2992	372	449	398	4.75	95	2.195
7	458	0.0541	449	481	459	1.43	59	0.397
8	1289	1.4695	1101	2000	1319	7.94	1552	10.782
9	2600	8.4002	2000	5000	2903	16.07	6073	61.636
10	20000 (UM)	0.1302	18415	29068	20447	4.49	1276	11.462

TIC:	12.7119	ng/uL
TIM:	25.8266	nmole/L
Total concentration:	13.0139	ng/uL

Sample peak width (sec): 5 Sample min peak height: 50 Sample baseline V to V?: Y Sample baseline V to V points: 3
Sample filter: Binomial Number of points for filter: 3 Sample start region (min): 0 Sample end region (min): 30
Marker peak width (sec): 5 Marker min peak height: 200 Marker baseline V to V?: Y Marker baseline V to V points: 3
Lower marker selection: First peak > 500 RFU Upper marker selection: Last peak > 200 RFU
Ladder size (bp)75, 200, 300, 400, 500, 700, 1000, 1500, 2000, 3000, 4000, 5000, 7000, 10000, 20000
Quantification using: Lower Marker Final concentration (ng/uL): 0.5000 Dilution factor: 12.0

Sample: SampB7
Well location: B7
Created: Monday, September 20, 2021 7:19:57 PM

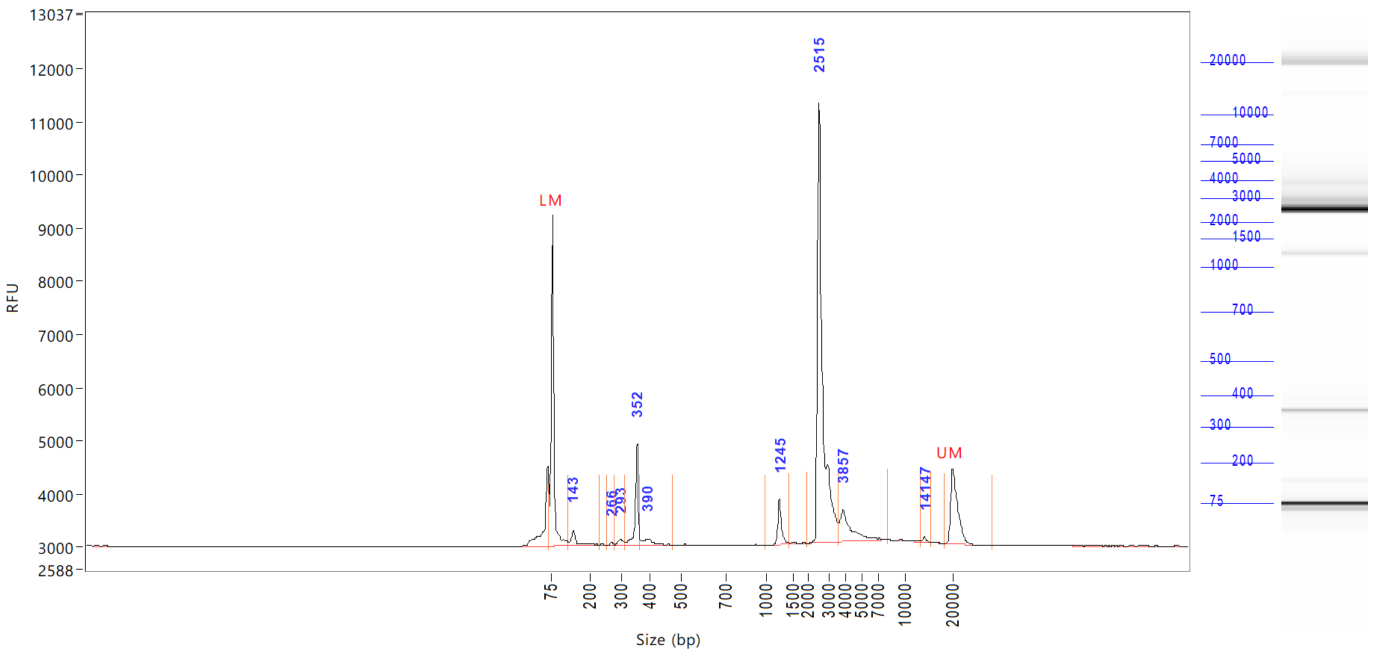

Peak	Size (bp)	Concentration (ng/uL)	From (bp)	To (bp)	Average size (bp)	CV%	RFU	Corrected peak area
1	75 (LM)	0.5000	66	128	75	13.16	6225	42.434
2	143	0.5623	128	228	155	16.13	299	3.977
3	266	0.0482	251	275	264	1.63	60	0.341
4	293	0.2115	275	309	293	2.86	110	1.496
5	352	1.6564	309	364	345	2.77	1896	11.715
6	390	0.3991	364	473	396	6.32	125	2.822
7	1245	0.7322	987	1445	1246	4.02	846	5.179
8	2515	9.4631	1962	3572	2652	9.90	8279	66.926
9	3857	1.4592	3572	8001	4288	17.15	601	10.320
10	14147	0.0674	13293	15610	14056	2.45	111	0.476
11	20000 (UM)	0.1585	18415	28463	20583	4.55	1401	13.452

TIC:	14.5994	ng/uL
TIM:	24.4169	nmole/L
Total concentration:	14.8048	ng/uL

```
Sample peak width (sec): 5     Sample min peak height: 50     Sample baseline V to V?: Y     Sample baseline V to V points: 3
Sample filter: Binomial        Number of points for filter: 3  Sample start region (min): 0  Sample end region (min): 30
Marker peak width (sec): 5     Marker min peak height: 200    Marker baseline V to V?: Y     Marker baseline V to V points: 3
Lower marker selection: First peak > 500 RFU                  Upper marker selection: Last peak > 200 RFU
Ladder size (bp)75, 200, 300, 400, 500, 700, 1000, 1500, 2000, 3000, 4000, 5000, 7000, 10000, 20000
Quantification using: Lower Marker          Final concentration (ng/uL): 0.5000          Dilution factor: 12.0
```

Sample: SampC7
Well location: C7
Created: Monday, September 20, 2021 7:19:57 PM

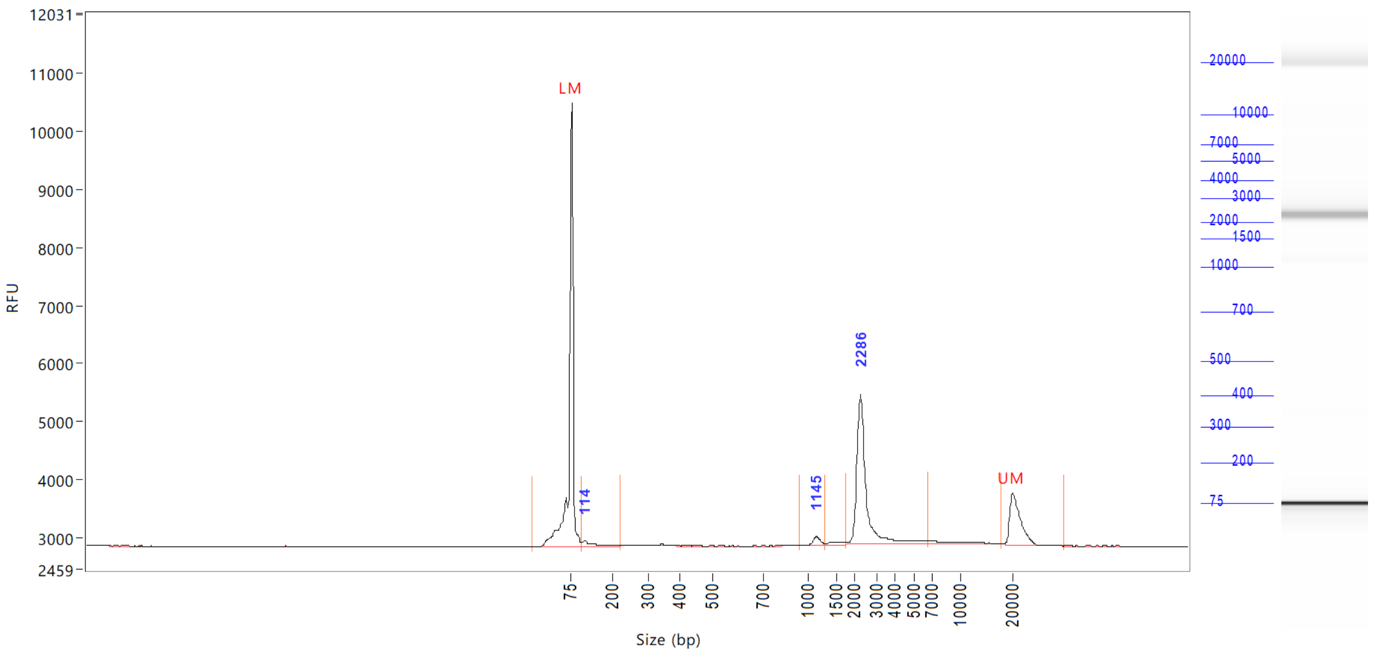

Peak	Size (bp)	Concentration (ng/uL)	From (bp)	To (bp)	Average size (bp)	CV%	RFU	Corrected peak area
1	75 (LM)	0.5000	0	105	65	28.93	7647	66.993
2	114	0.2021	105	221	136	20.86	95	2.257
3	1145	0.1480	944	1300	1144	5.50	147	1.653
4	2286	3.1888	1770	6630	2513	27.37	2575	35.605
5	20000 (UM)	0.0881	17927	29672	20677	5.89	886	11.800

TIC:	3.5390	ng/uL
TIM:	4.7442	nmole/L
Total concentration:	3.8723	ng/uL

Sample peak width (sec): 5   Sample min peak height: 50   Sample baseline V to V?: Y   Sample baseline V to V points: 3
Sample filter: Binomial   Number of points for filter: 3 Sample start region (min): 0 Sample end region (min): 30
Marker peak width (sec): 5   Marker min peak height: 200   Marker baseline V to V?: Y   Marker baseline V to V points: 3
Lower marker selection: First peak > 500 RFU   Upper marker selection: Last peak > 200 RFU
Ladder size (bp)75, 200, 300, 400, 500, 700, 1000, 1500, 2000, 3000, 4000, 5000, 7000, 10000, 20000
Quantification using: Lower Marker   Final concentration (ng/uL): 0.5000   Dilution factor: 12.0

Sample: SampD7
Well location: D7
Created: Monday, September 20, 2021 7:19:57 PM

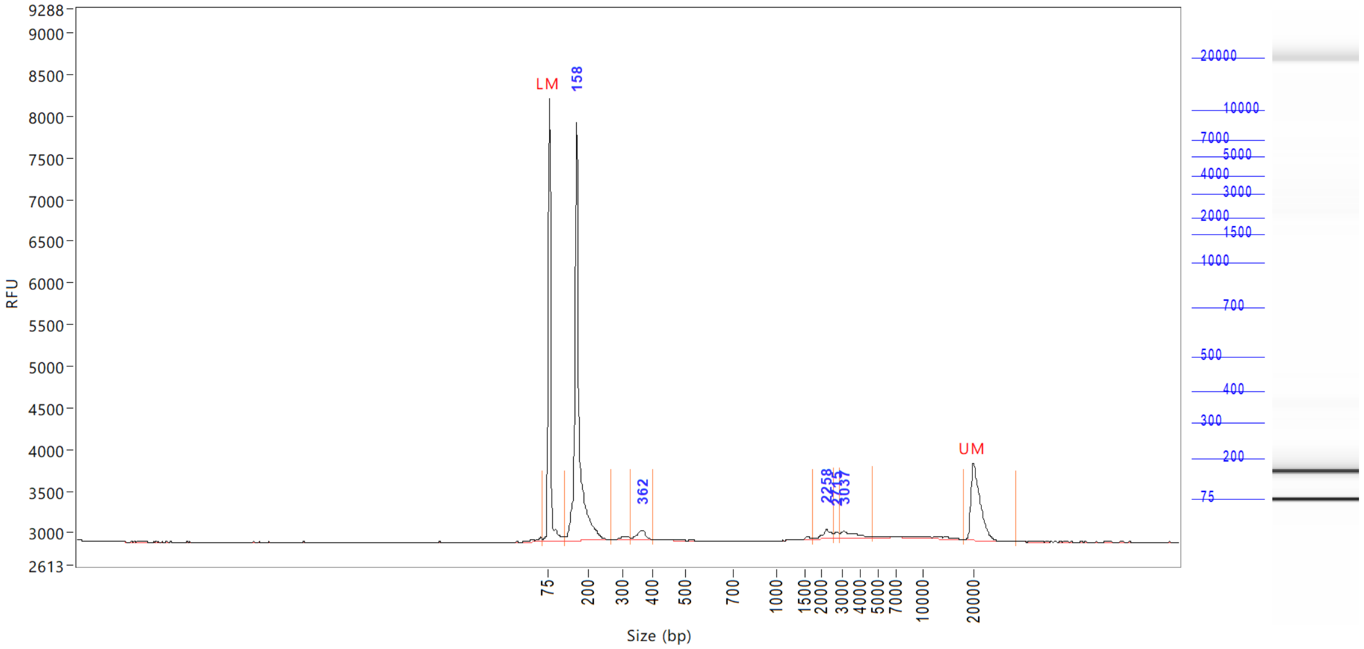

Peak	Size (bp)	Concentration (ng/uL)	From (bp)	To (bp)	Average size (bp)	CV%	RFU	Corrected peak area
1	75 (LM)	0.5000	54	122	74	10.76	5322	32.821
2	158	8.9934	122	264	163	10.90	5020	49.195
3	362	0.3882	323	398	355	4.10	115	2.124
4	2258	0.2729	1750	2600	2243	9.06	109	1.493
5	2715	0.1078	2600	2886	2738	3.20	78	0.590
6	3037	0.3130	2886	4690	3469	13.36	86	1.712
7	20000 (UM)	0.1688	18049	28463	20572	5.22	930	11.079

TIC:	10.0753	ng/uL
TIM:	92.9049	nmole/L
Total concentration:	10.6592	ng/uL

```
Sample peak width (sec): 5    Sample min peak height: 50    Sample baseline V to V?: Y    Sample baseline V to V points: 3
Sample filter: Binomial       Number of points for filter: 3  Sample start region (min): 0  Sample end region (min): 30
Marker peak width (sec): 5    Marker min peak height: 200   Marker baseline V to V?: Y    Marker baseline V to V points: 3
Lower marker selection: First peak > 500 RFU                Upper marker selection: Last peak > 200 RFU
Ladder size (bp)75, 200, 300, 400, 500, 700, 1000, 1500, 2000, 3000, 4000, 5000, 7000, 10000, 20000
Quantification using: Lower Marker          Final concentration (ng/uL): 0.5000          Dilution factor: 12.0
```

Sample: SampE7
Well location: E7
Created: Monday, September 20, 2021 7:19:57 PM

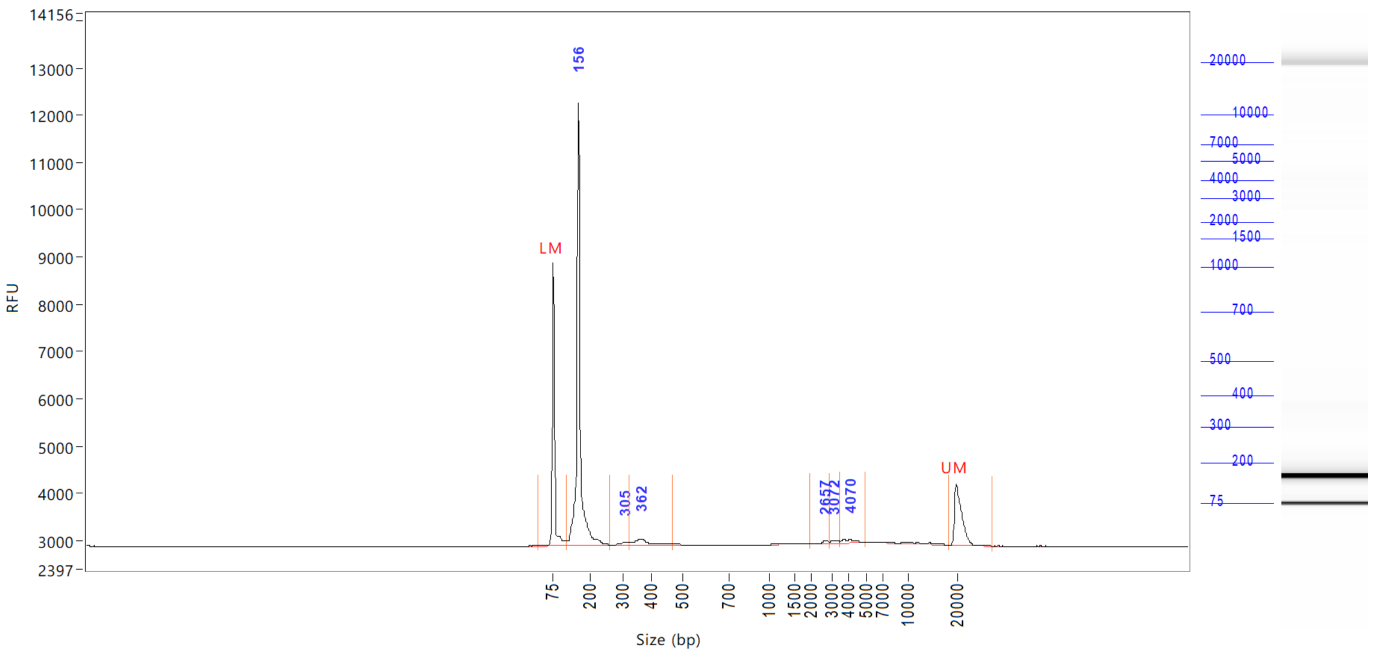

Peak	Size (bp)	Concentration (ng/uL)	From (bp)	To (bp)	Average size (bp)	CV%	RFU	Corrected peak area
1	75 (LM)	0.5000	24	118	75	12.09	5985	35.902
2	156	12.9680	118	259	158	9.46	9376	77.595
3	305	0.1581	259	321	301	4.54	50	0.946
4	362	0.6290	321	468	373	9.60	129	3.764
5	2657	0.1004	1942	2886	2593	8.62	62	0.601
6	3072	0.1074	2886	3465	3149	5.32	51	0.643
7	4070	0.2501	3465	4966	4053	8.82	73	1.496
8	20000 (UM)	0.1734	18415	27496	20548	4.57	1298	12.452

TIC:	14.2130	ng/uL
TIM:	138.7642	nmole/L
Total concentration:	14.6327	ng/uL

Sample peak width (sec): 5 Sample min peak height: 50 Sample baseline V to V?: Y Sample baseline V to V points: 3
Sample filter: Binomial Number of points for filter: 3 Sample start region (min): 0 Sample end region (min): 30
Marker peak width (sec): 5 Marker min peak height: 200 Marker baseline V to V?: Y Marker baseline V to V points: 3
Lower marker selection: First peak > 500 RFU Upper marker selection: Last peak > 200 RFU
Ladder size (bp)75, 200, 300, 400, 500, 700, 1000, 1500, 2000, 3000, 4000, 5000, 7000, 10000, 20000
Quantification using: Lower Marker Final concentration (ng/uL): 0.5000 Dilution factor: 12.0

Sample: SampF7
Well location: F7
Created: Monday, September 20, 2021 7:19:57 PM

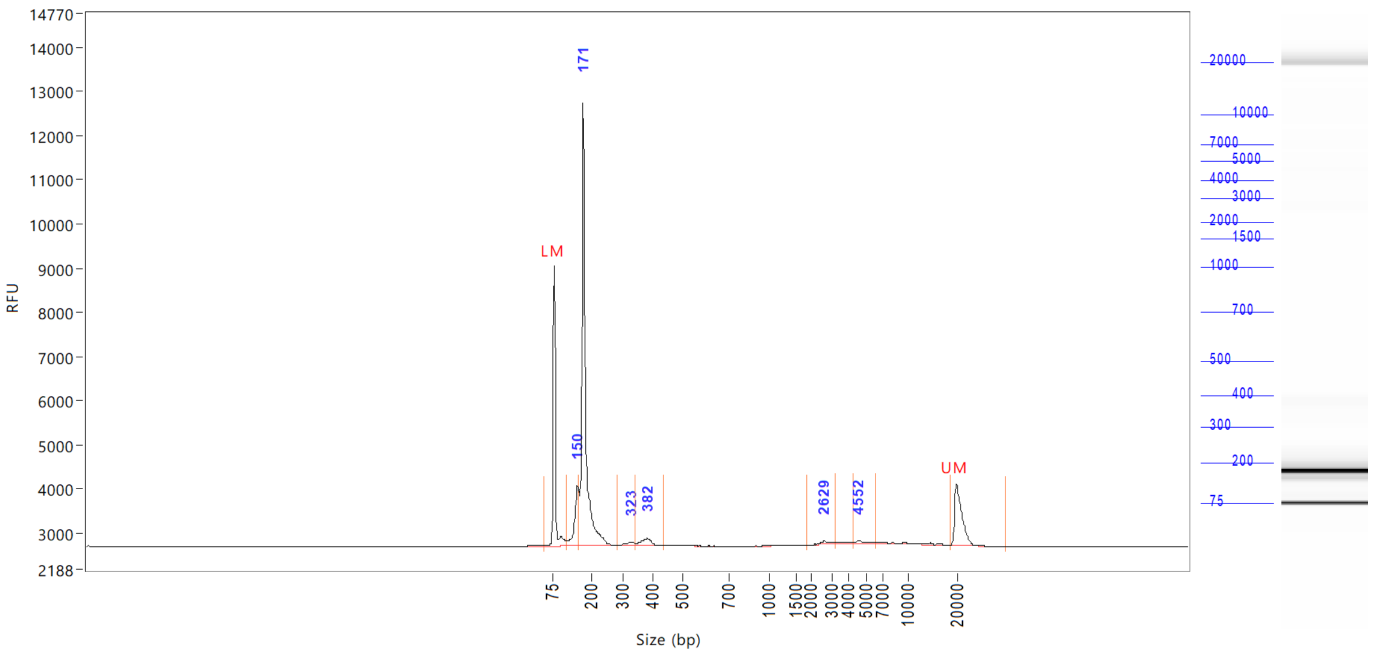

Peak	Size (bp)	Concentration (ng/uL)	From (bp)	To (bp)	Average size (bp)	CV%	RFU	Corrected peak area
1	75 (LM)	0.5000	43	118	76	11.81	6345	38.938
2	150	2.4136	118	160	148	6.30	1352	15.664
3	171	12.9270	160	282	177	8.76	10037	83.892
4	323	0.1851	282	339	318	4.23	62	1.201
5	382	0.5041	339	435	373	4.39	157	3.271
6	2629	0.1558	1846	3179	2679	9.02	69	1.011
7	4552	0.1316	4208	6186	4834	9.73	59	0.854
8	20000 (UM)	0.1728	18537	30277	20593	4.85	1393	13.459

TIC:	16.3171	ng/uL
TIM:	150.1867	nmole/L
Total concentration:	16.5486	ng/uL

```
Sample peak width (sec): 5    Sample min peak height: 50    Sample baseline V to V?: Y    Sample baseline V to V points: 3
Sample filter: Binomial       Number of points for filter: 3  Sample start region (min): 0  Sample end region (min): 30
Marker peak width (sec): 5    Marker min peak height: 200   Marker baseline V to V?: Y    Marker baseline V to V points: 3
Lower marker selection: First peak > 500 RFU                Upper marker selection: Last peak > 200 RFU
Ladder size (bp)75, 200, 300, 400, 500, 700, 1000, 1500, 2000, 3000, 4000, 5000, 7000, 10000, 20000
Quantification using: Lower Marker          Final concentration (ng/uL): 0.5000          Dilution factor: 12.0
```

Sample: SampG7
Well location: G7
Created: Monday, September 20, 2021 7:19:57 PM

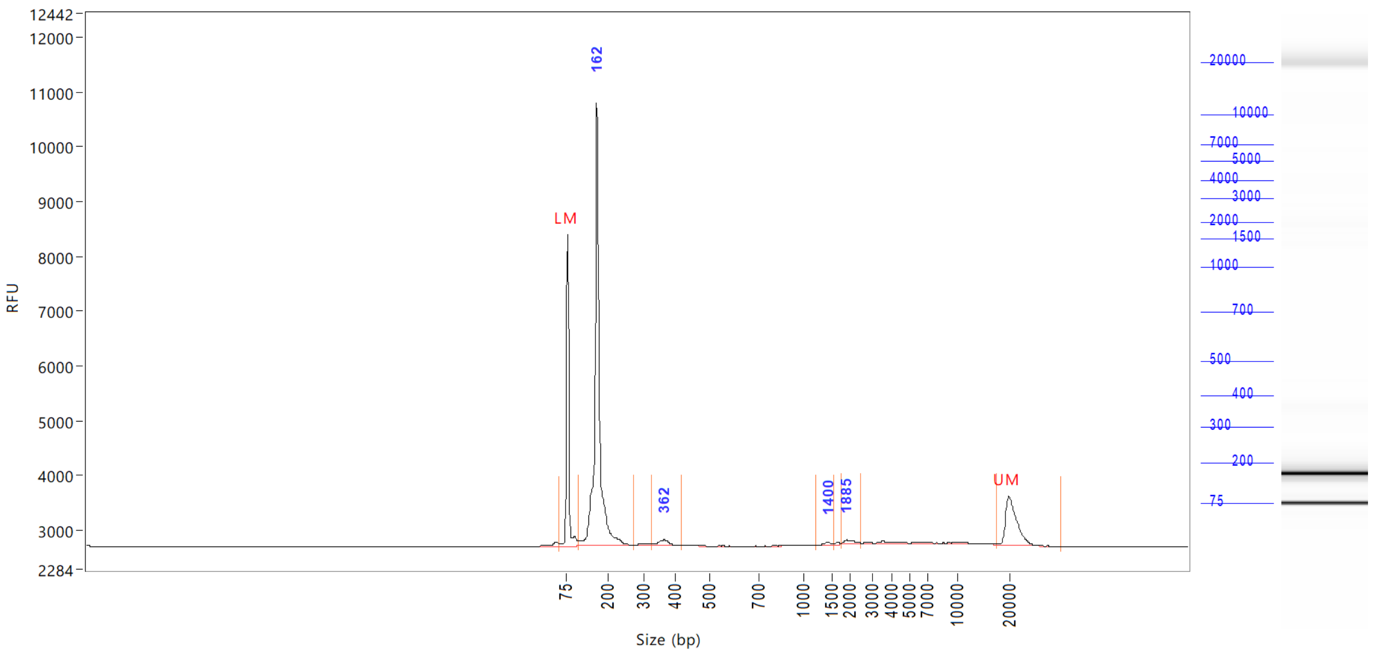

Peak	Size (bp)	Concentration (ng/uL)	From (bp)	To (bp)	Average size (bp)	CV%	RFU	Corrected peak area
1	75 (LM)	0.5000	52	108	75	8.77	5690	35.396
2	162	14.1301	108	269	164	10.72	8104	83.357
3	362	0.3929	323	416	358	4.99	103	2.318
4	1400	0.0966	1212	1501	1396	4.38	61	0.570
5	1885	0.2128	1731	2515	2004	8.79	82	1.255
6	20000 (UM)	0.1763	17561	29914	20600	6.04	896	12.484

TIC:	14.8323	ng/uL
TIM:	143.7187	nmole/L
Total concentration:	15.4999	ng/uL

```
Sample peak width (sec): 5    Sample min peak height: 50    Sample baseline V to V?: Y    Sample baseline V to V points: 3
Sample filter: Binomial       Number of points for filter: 3  Sample start region (min): 0  Sample end region (min): 30
Marker peak width (sec): 5    Marker min peak height: 200   Marker baseline V to V?: Y    Marker baseline V to V points: 3
Lower marker selection: First peak > 500 RFU                 Upper marker selection: Last peak > 200 RFU
Ladder size (bp)75, 200, 300, 400, 500, 700, 1000, 1500, 2000, 3000, 4000, 5000, 7000, 10000, 20000
Quantification using: Lower Marker          Final concentration (ng/uL): 0.5000          Dilution factor: 12.0
```

Sample: SampH7
Well location: H7
Created: Monday, September 20, 2021 7:19:57 PM

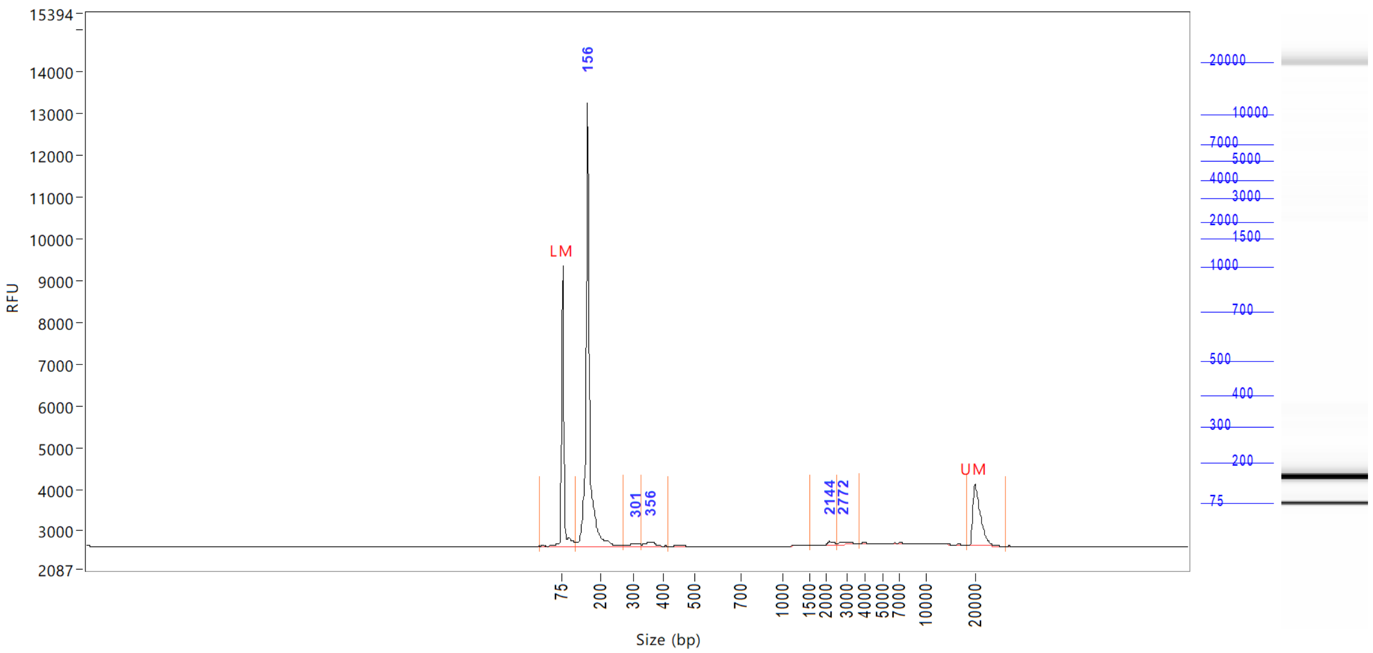

Peak	Size (bp)	Concentration (ng/uL)	From (bp)	To (bp)	Average size (bp)	CV%	RFU	Corrected peak area
1	75 (LM)	0.5000	3	118	75	12.96	6700	39.674
2	156	13.6364	118	269	156	9.40	10628	90.169
3	301	0.1892	269	323	299	4.53	59	1.251
4	356	0.3201	323	411	352	4.39	106	2.117
5	2144	0.1266	1501	2486	2189	7.43	83	0.837
6	2772	0.1849	2486	3715	2951	10.24	69	1.223
7	20000 (UM)	0.1824	18537	26287	20555	4.39	1478	14.470

TIC:	14.4573	ng/uL
TIM:	146.4097	nmole/L
Total concentration:	14.8331	ng/uL

Sample peak width (sec): 5   Sample min peak height: 50   Sample baseline V to V?: Y   Sample baseline V to V points: 3
Sample filter: Binomial   Number of points for filter: 3   Sample start region (min): 0   Sample end region (min): 30
Marker peak width (sec): 5   Marker min peak height: 200   Marker baseline V to V?: Y   Marker baseline V to V points: 3
Lower marker selection: First peak > 500 RFU   Upper marker selection: Last peak > 200 RFU
Ladder size (bp)75, 200, 300, 400, 500, 700, 1000, 1500, 2000, 3000, 4000, 5000, 7000, 10000, 20000
Quantification using: Lower Marker   Final concentration (ng/uL): 0.5000   Dilution factor: 12.0

Sample: SampA8
Well location: A8
Created: Monday, September 20, 2021 7:19:57 PM

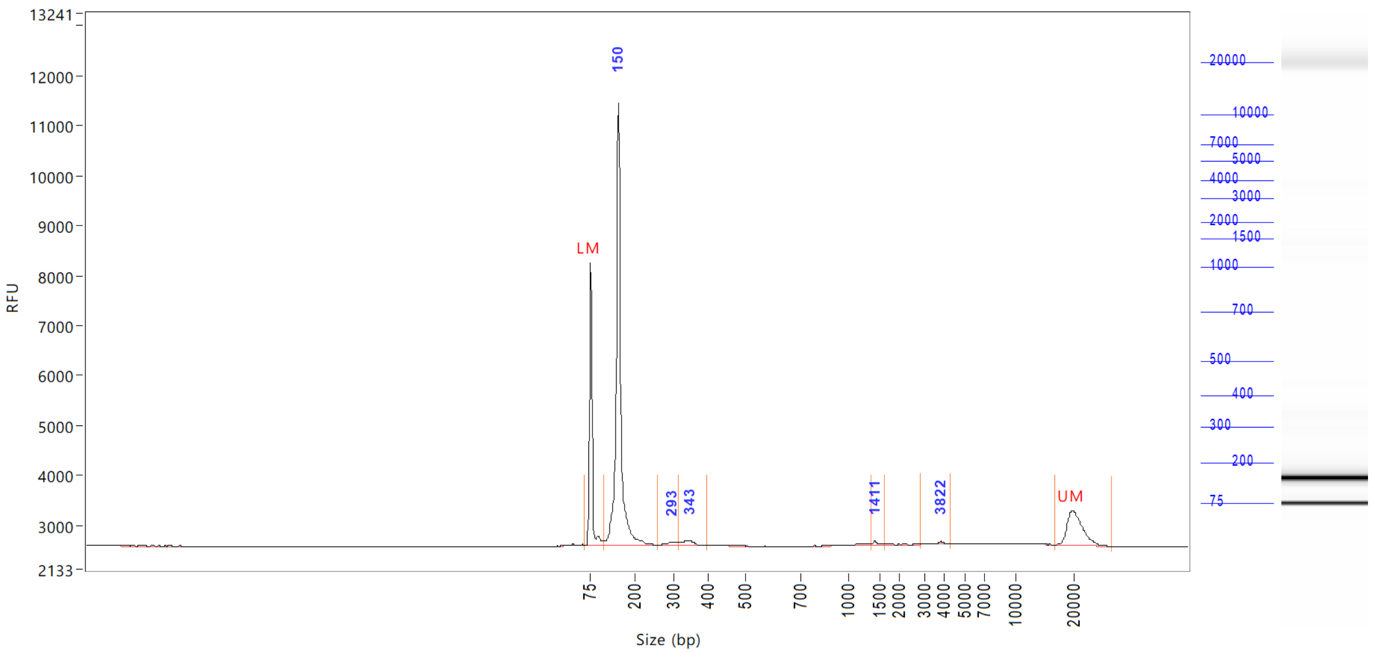

Peak	Size (bp)	Concentration (ng/uL)	From (bp)	To (bp)	Average size (bp)	CV%	RFU	Corrected peak area
1	75 (LM)	0.5000	60	112	74	9.16	5637	36.156
2	150	15.1780	112	255	151	9.85	8857	91.464
3	293	0.2985	255	313	290	5.06	74	1.799
4	343	0.3996	313	394	337	4.31	105	2.408
5	1411	0.1051	1345	1616	1436	4.30	74	0.633
6	3822	0.0931	2886	4277	3682	8.32	52	0.561
7	20000 (UM)	0.1728	16708	26891	20513	6.71	690	12.499

TIC:	16.0744	ng/uL
TIM:	169.0087	nmole/L
Total concentration:	16.4668	ng/uL

```
Sample peak width (sec): 5    Sample min peak height: 50    Sample baseline V to V?: Y    Sample baseline V to V points: 3
Sample filter: Binomial       Number of points for filter: 3  Sample start region (min): 0  Sample end region (min): 30
Marker peak width (sec): 5    Marker min peak height: 200   Marker baseline V to V?: Y    Marker baseline V to V points: 3
Lower marker selection: First peak > 500 RFU                Upper marker selection: Last peak > 200 RFU
Ladder size (bp)75, 200, 300, 400, 500, 700, 1000, 1500, 2000, 3000, 4000, 5000, 7000, 10000, 20000
Quantification using: Lower Marker          Final concentration (ng/uL): 0.5000          Dilution factor: 12.0
```

Sample: SampB8
Well location: B8
Created: Monday, September 20, 2021 7:19:57 PM

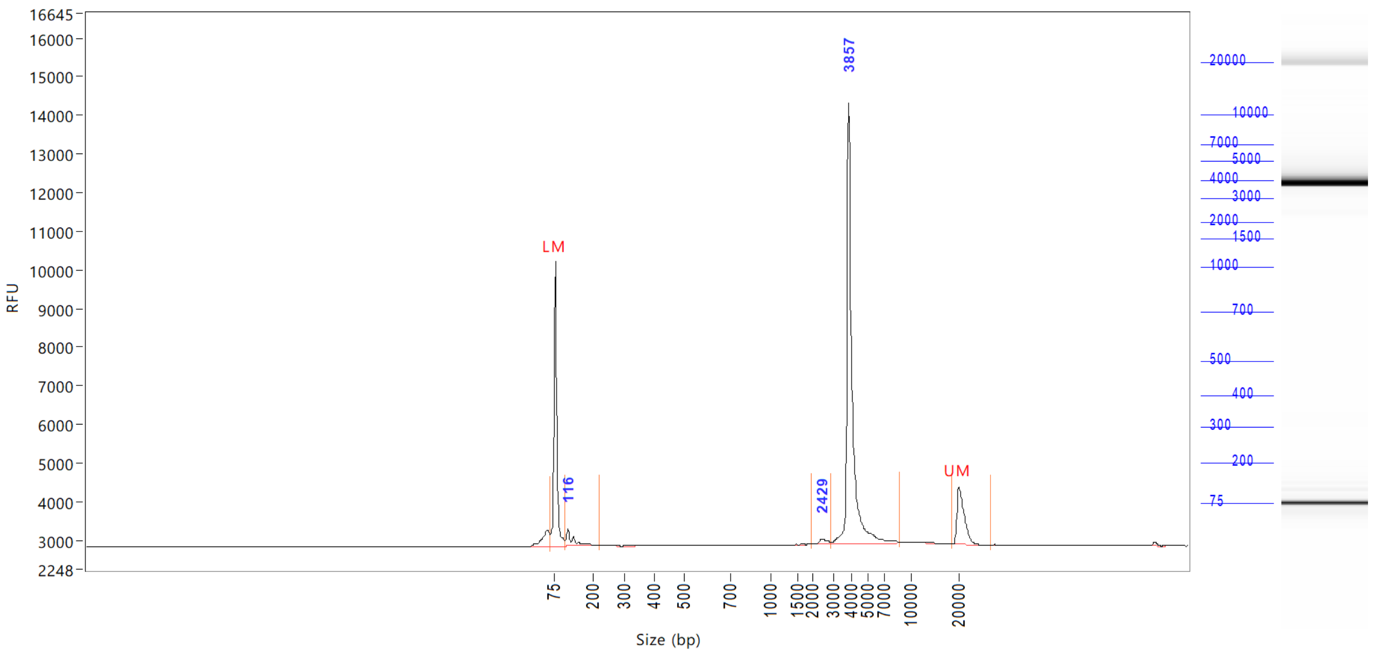

Peak	Size (bp)	Concentration (ng/uL)	From (bp)	To (bp)	Average size (bp)	CV%	RFU	Corrected peak area
1	75 (LM)	0.5000	60	107	75	8.86	7385	52.037
2	116	0.8045	107	219	135	18.35	427	6.978
3	2429	0.1941	1962	2857	2468	8.03	135	1.683
4	3857	10.6167	2857	8734	4032	14.52	11435	92.077
5	20000 (UM)	0.1353	18415	26649	20508	4.15	1490	14.082

TIC:	11.6153	ng/uL
TIM:	14.2568	nmole/L
Total concentration:	11.9848	ng/uL

Sample peak width (sec): 5   Sample min peak height: 50   Sample baseline V to V?: Y   Sample baseline V to V points: 3
Sample filter: Binomial   Number of points for filter: 3   Sample start region (min): 0   Sample end region (min): 30
Marker peak width (sec): 5   Marker min peak height: 200   Marker baseline V to V?: Y   Marker baseline V to V points: 3
Lower marker selection: First peak > 500 RFU   Upper marker selection: Last peak > 200 RFU
Ladder size (bp)75, 200, 300, 400, 500, 700, 1000, 1500, 2000, 3000, 4000, 5000, 7000, 10000, 20000
Quantification using: Lower Marker   Final concentration (ng/uL): 0.5000   Dilution factor: 12.0

Sample: SampC8
Well location: C8
Created: Monday, September 20, 2021 7:19:57 PM

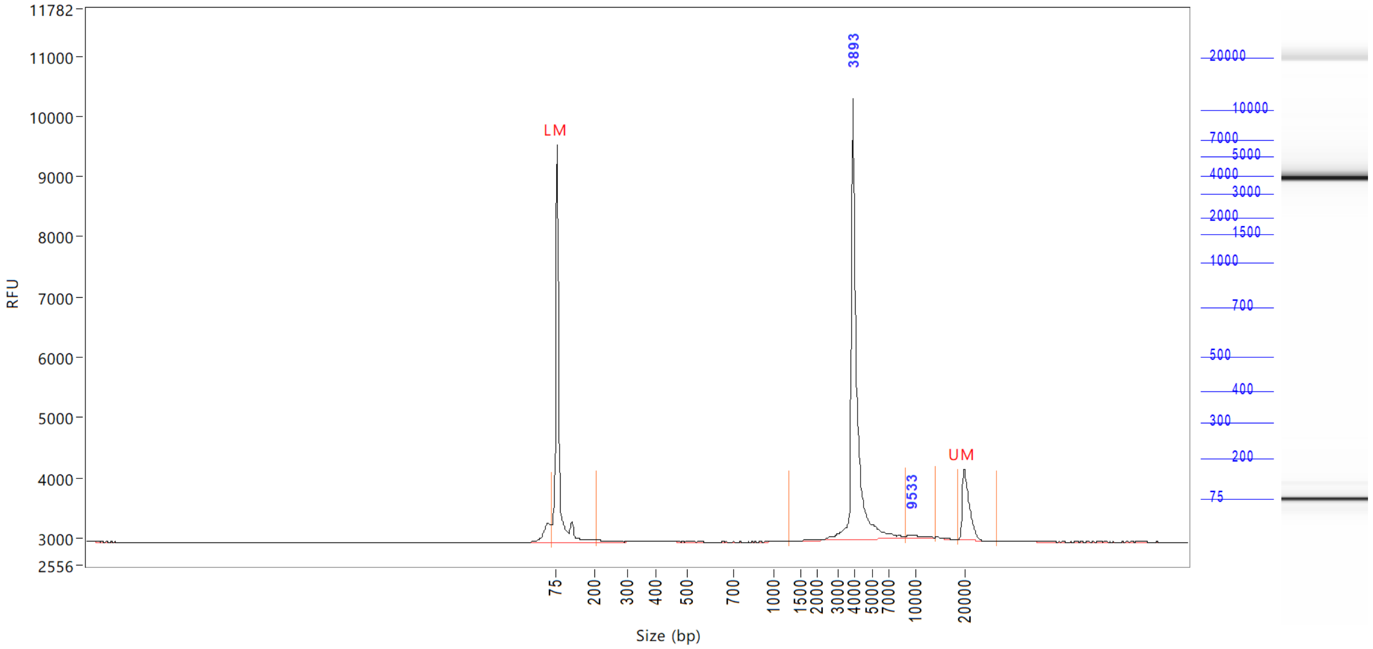

Peak	Size (bp)	Concentration (ng/uL)	From (bp)	To (bp)	Average size (bp)	CV%	RFU	Corrected peak area
1	75 (LM)	0.5000	58	203	81	26.66	6595	52.153
2	3893	7.3593	1256	8800	4066	17.71	7326	63.969
3	9533	0.1082	8800	14147	10110	10.76	51	0.941
4	20000 (UM)	0.1059	18903	26770	20553	4.14	1178	11.042

TIC:	7.4675	ng/uL
TIM:	2.9973	nmole/L
Total concentration:	7.6721	ng/uL

Sample peak width (sec): 5 Sample min peak height: 50 Sample baseline V to V?: Y Sample baseline V to V points: 3
Sample filter: Binomial Number of points for filter: 3 Sample start region (min): 0 Sample end region (min): 30
Marker peak width (sec): 5 Marker min peak height: 200 Marker baseline V to V?: Y Marker baseline V to V points: 3
Lower marker selection: First peak > 500 RFU Upper marker selection: Last peak > 200 RFU
Ladder size (bp)75, 200, 300, 400, 500, 700, 1000, 1500, 2000, 3000, 4000, 5000, 7000, 10000, 20000
Quantification using: Lower Marker Final concentration (ng/uL): 0.5000 Dilution factor: 12.0

Sample: SampD8
Well location: D8
Created: Monday, September 20, 2021 7:19:57 PM

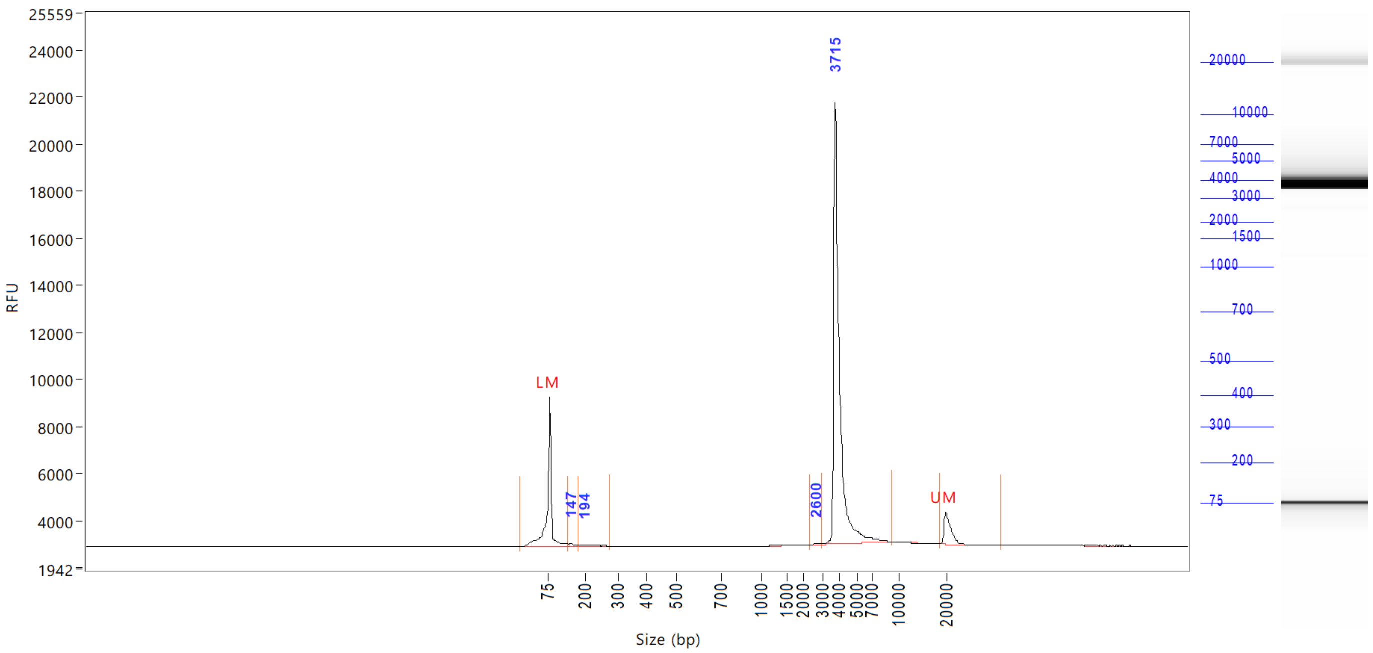

Peak	Size (bp)	Concentration (ng/uL)	From (bp)	To (bp)	Average size (bp)	CV%	RFU	Corrected peak area
1	75 (LM)	0.5000	0	141	67	32.39	6352	61.981
2	147	0.1444	141	175	155	6.47	101	1.492
3	194	0.1615	175	275	204	10.32	66	1.668
4	2600	0.0579	2372	2943	2673	4.93	77	0.598
5	3715	16.3401	2943	9200	3950	14.66	18774	168.795
6	20000 (UM)	0.1020	18537	31607	20639	6.29	1353	12.646

TIC:	16.7038	ng/uL
TIM:	9.6783	nmole/L
Total concentration:	16.8607	ng/uL

Sample peak width (sec): 5 Sample min peak height: 50 Sample baseline V to V?: Y Sample baseline V to V points: 3
Sample filter: Binomial Number of points for filter: 3 Sample start region (min): 0 Sample end region (min): 30
Marker peak width (sec): 5 Marker min peak height: 200 Marker baseline V to V?: Y Marker baseline V to V points: 3
Lower marker selection: First peak > 500 RFU Upper marker selection: Last peak > 200 RFU
Ladder size (bp)75, 200, 300, 400, 500, 700, 1000, 1500, 2000, 3000, 4000, 5000, 7000, 10000, 20000
Quantification using: Lower Marker Final concentration (ng/uL): 0.5000 Dilution factor: 12.0

Sample: SampE8
Well location: E8
Created: Monday, September 20, 2021 7:19:57 PM

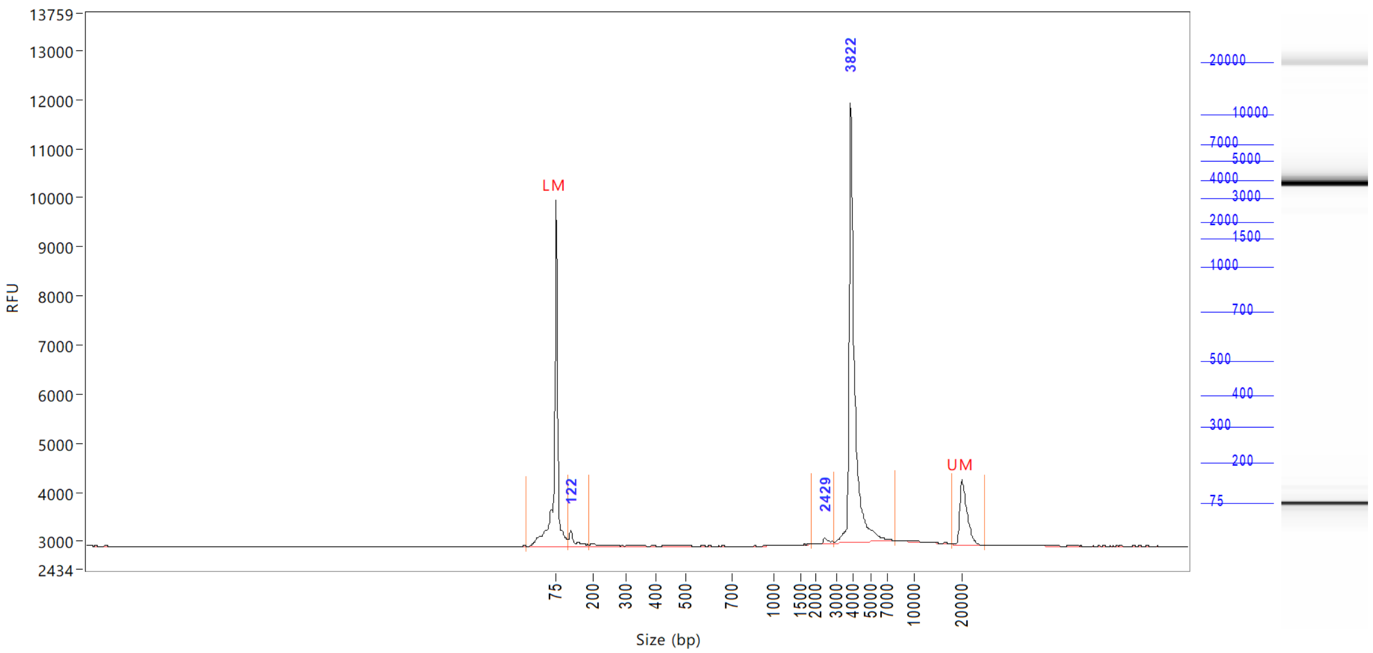

Peak	Size<br>(bp)	Concentration<br>(ng/uL)	From<br>(bp)	To<br>(bp)	Average size<br>(bp)	CV%	RFU	Corrected peak area
1	75 (LM)	0.5000	0	112	66	28.77	7047	61.027
2	122	0.3967	112	181	133	13.89	310	4.034
3	2429	0.1029	1866	2914	2522	7.45	114	1.047
4	3822	7.3204	2914	7934	3989	10.94	8969	74.457
5	20000 (UM)	0.1029	17805	24715	20420	4.10	1339	12.560

TIC:	7.8200	ng/uL
TIM:	7.9888	nmole/L
Total concentration:	8.1085	ng/uL

Sample peak width (sec): 5   Sample min peak height: 50   Sample baseline V to V?: Y   Sample baseline V to V points: 3
Sample filter: Binomial   Number of points for filter: 3 Sample start region (min): 0 Sample end region (min): 30
Marker peak width (sec): 5   Marker min peak height: 200   Marker baseline V to V?: Y   Marker baseline V to V points: 3
Lower marker selection: First peak > 500 RFU   Upper marker selection: Last peak > 200 RFU
Ladder size (bp)75, 200, 300, 400, 500, 700, 1000, 1500, 2000, 3000, 4000, 5000, 7000, 10000, 20000
Quantification using: Lower Marker   Final concentration (ng/uL): 0.5000   Dilution factor: 12.0

Sample: SampF8
Well location: F8
Created: Monday, September 20, 2021 7:19:57 PM

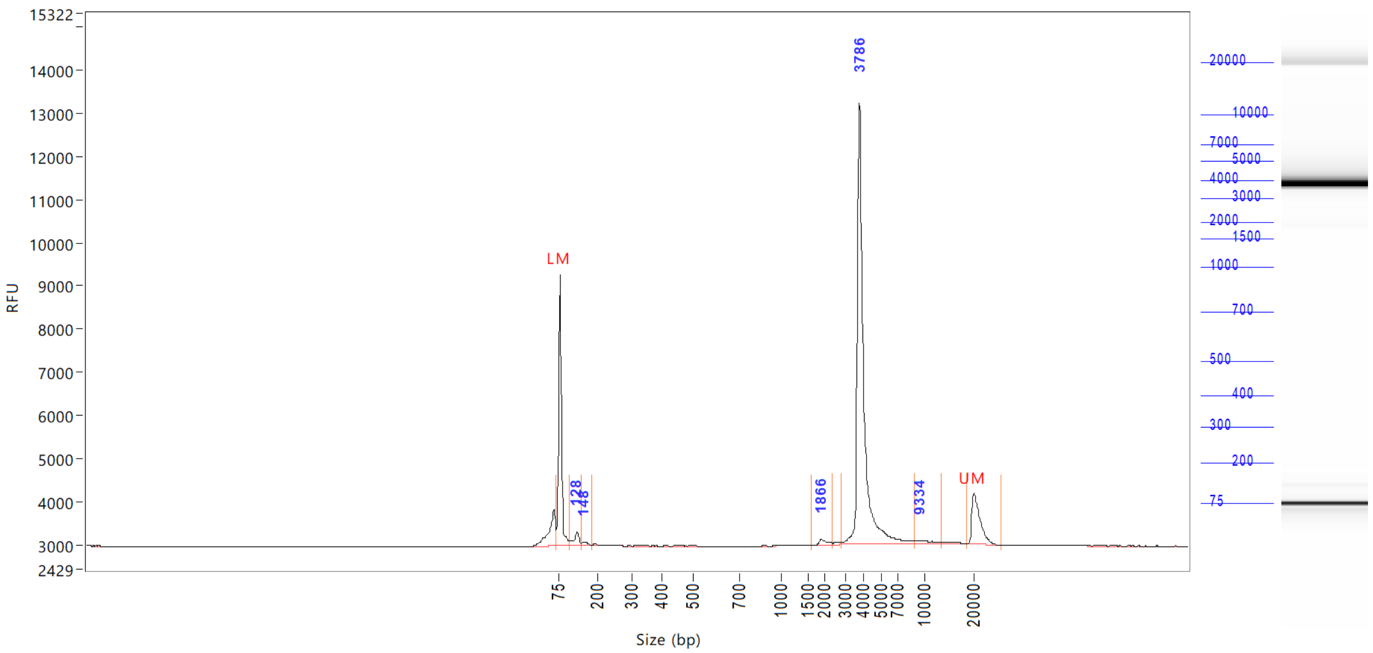

Peak	Size (bp)	Concentration (ng/uL)	From (bp)	To (bp)	Average size (bp)	CV%	RFU	Corrected peak area
1	75 (LM)	0.5000	64	108	74	8.82	6290	44.358
2	128	0.4938	108	143	125	6.51	309	3.650
3	148	0.1191	143	177	153	4.57	76	0.881
4	1866	0.2167	1578	2372	1975	8.31	128	1.602
5	3786	13.1015	2772	8934	4015	16.72	10251	96.860
6	9334	0.1993	8934	13415	10657	12.35	58	1.473
7	20000 (UM)	0.1403	18781	25803	20698	4.79	1176	12.445

TIC:	14.1304	ng/uL
TIM:	13.3529	nmole/L
Total concentration:	14.4861	ng/uL

Sample peak width (sec): 5 Sample min peak height: 50 Sample baseline V to V?: Y Sample baseline V to V points: 3
Sample filter: Binomial Number of points for filter: 3 Sample start region (min): 0 Sample end region (min): 30
Marker peak width (sec): 5 Marker min peak height: 200 Marker baseline V to V?: Y Marker baseline V to V points: 3
Lower marker selection: First peak > 500 RFU Upper marker selection: Last peak > 200 RFU
Ladder size (bp)75, 200, 300, 400, 500, 700, 1000, 1500, 2000, 3000, 4000, 5000, 7000, 10000, 20000
Quantification using: Lower Marker Final concentration (ng/uL): 0.5000 Dilution factor: 12.0

Sample: SampG8
Well location: G8
Created: Monday, September 20, 2021 7:19:57 PM

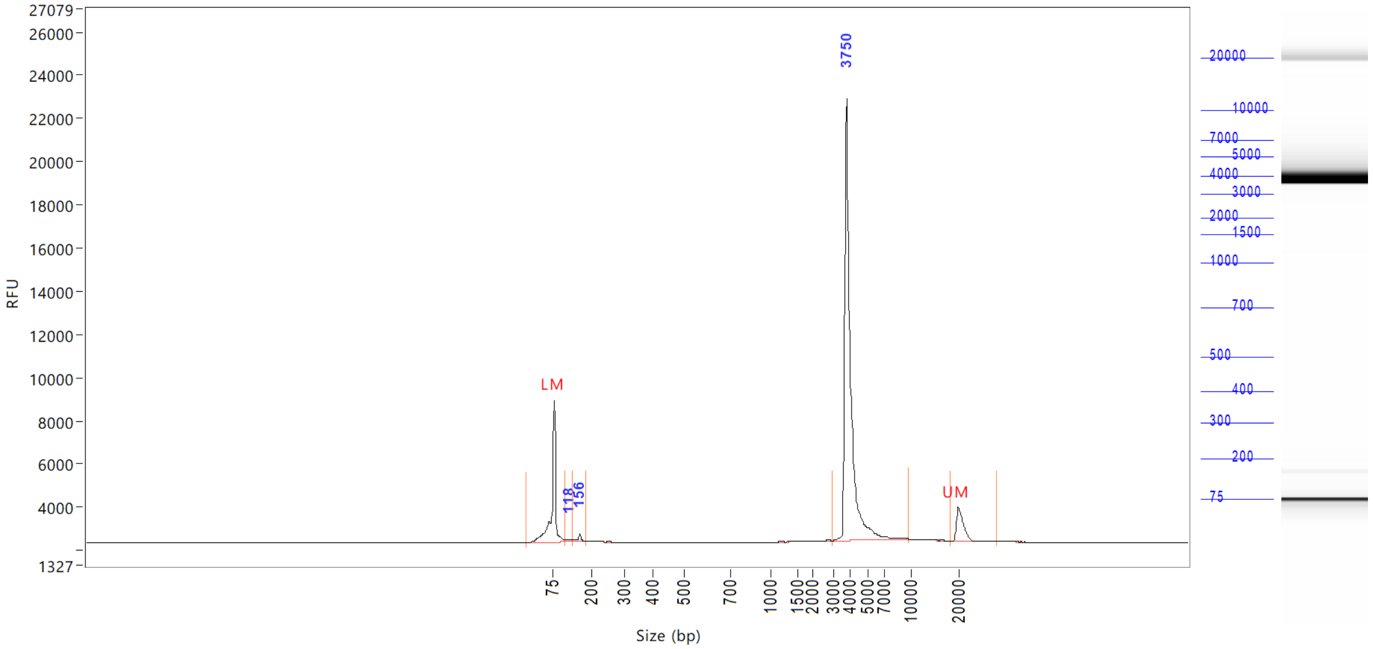

Peak	Size (bp)	Concentration (ng/uL)	From (bp)	To (bp)	Average size (bp)	CV%	RFU	Corrected peak area
1	75 (LM)	0.5000	0	110	65	26.30	6566	61.016
2	118	0.1204	110	133	121	5.67	93	1.225
3	156	0.2994	133	177	153	5.24	376	3.045
4	3750	17.4132	2943	9733	3993	14.60	20494	177.079
5	20000 (UM)	0.1158	18293	28221	20479	4.15	1576	14.127

TIC:	17.8330	ng/uL
TIM:	12.0308	nmole/L
Total concentration:	18.0585	ng/uL

```
Sample peak width (sec): 5    Sample min peak height: 50    Sample baseline V to V?: Y    Sample baseline V to V points: 3
Sample filter: Binomial       Number of points for filter: 3  Sample start region (min): 0  Sample end region (min): 30
Marker peak width (sec): 5    Marker min peak height: 200   Marker baseline V to V?: Y    Marker baseline V to V points: 3
Lower marker selection: First peak > 500 RFU                Upper marker selection: Last peak > 200 RFU
Ladder size (bp)75, 200, 300, 400, 500, 700, 1000, 1500, 2000, 3000, 4000, 5000, 7000, 10000, 20000
Quantification using: Lower Marker          Final concentration (ng/uL): 0.5000          Dilution factor: 12.0
```

Sample: SampH8
Well location: H8
Created: Monday, September 20, 2021 7:19:57 PM

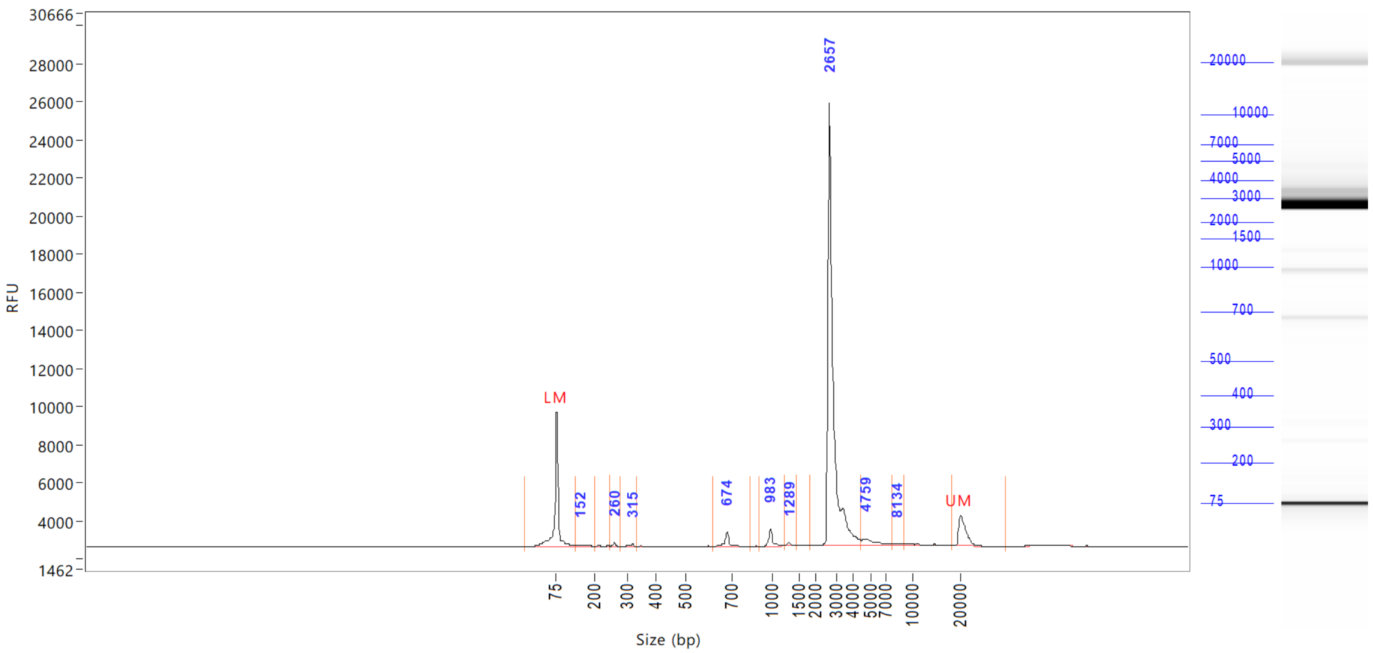

Peak	Size (bp)	Concentration (ng/uL)	From (bp)	To (bp)	Average size (bp)	CV%	RFU	Corrected peak area
1	75 (LM)	0.5000	0	137	68	27.11	7071	59.849
2	152	0.1759	137	201	159	9.27	93	1.755
3	260	0.0985	246	278	257	1.86	179	0.982
4	315	0.1023	278	333	308	3.14	115	1.020
5	674	0.6026	613	831	675	3.73	786	6.011
6	983	0.6089	905	1212	991	4.49	887	6.074
7	1289	0.1269	1212	1445	1298	3.47	192	1.266
8	2657	18.8337	1866	4449	2846	12.19	23286	187.862
9	4759	0.7073	4449	7667	5352	16.41	348	7.055
10	8134	0.0813	7667	9067	8260	4.96	73	0.811
11	20000 (UM)	0.1266	18171	29430	20500	4.49	1614	15.160

TIC:	21.3373	ng/uL
TIM:	16.7643	nmole/L
Total concentration:	21.5558	ng/uL

```
Sample peak width (sec): 5    Sample min peak height: 50    Sample baseline V to V?: Y    Sample baseline V to V points: 3
Sample filter: Binomial       Number of points for filter: 3  Sample start region (min): 0  Sample end region (min): 30
Marker peak width (sec): 5    Marker min peak height: 200   Marker baseline V to V?: Y    Marker baseline V to V points: 3
Lower marker selection: First peak > 500 RFU                Upper marker selection: Last peak > 200 RFU
Ladder size (bp)75, 200, 300, 400, 500, 700, 1000, 1500, 2000, 3000, 4000, 5000, 7000, 10000, 20000
Quantification using: Lower Marker          Final concentration (ng/uL): 0.5000          Dilution factor: 12.0
```

Sample: SampA9
Well location: A9
Created: Monday, September 20, 2021 7:19:57 PM

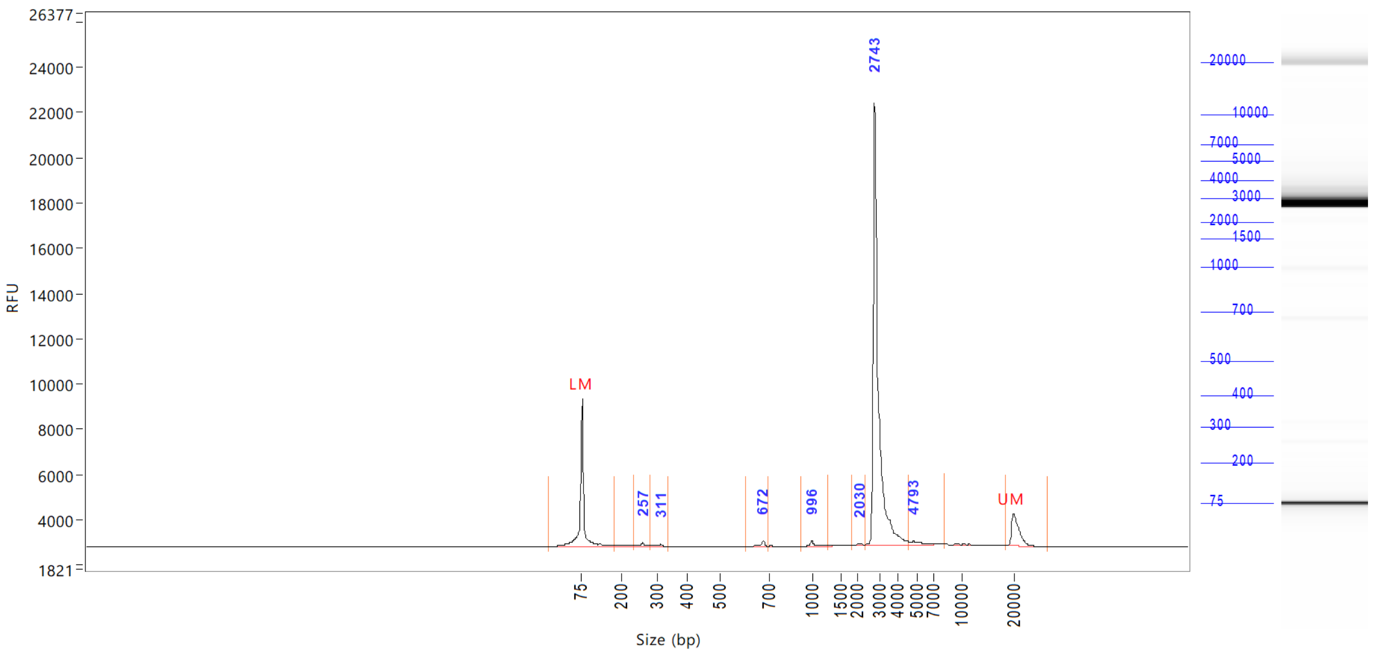

Peak	Size (bp)	Concentration (ng/uL)	From (bp)	To (bp)	Average size (bp)	CV%	RFU	Corrected peak area
1	75 (LM)	0.5000	0	175	73	29.32	6533	53.315
2	257	0.1091	232	280	254	3.30	156	0.969
3	311	0.0800	280	333	303	3.10	79	0.711
4	672	0.2175	603	695	664	2.30	265	1.933
5	996	0.1989	922	1256	1015	5.78	236	1.768
6	2030	0.0830	1827	2286	2060	5.03	97	0.737
7	2743	16.7799	2286	4587	2902	11.21	19575	149.103
8	4793	0.3072	4587	8134	5339	14.13	145	2.730
9	20000 (UM)	0.1209	18537	26770	20529	4.09	1402	12.890

TIC:	17.7755	ng/uL
TIM:	11.6817	nmole/L
Total concentration:	18.1228	ng/uL

Sample peak width (sec): 5   Sample min peak height: 50   Sample baseline V to V?: Y   Sample baseline V to V points: 3
Sample filter: Binomial   Number of points for filter: 3 Sample start region (min): 0 Sample end region (min): 30
Marker peak width (sec): 5   Marker min peak height: 200   Marker baseline V to V?: Y   Marker baseline V to V points: 3
Lower marker selection: First peak > 500 RFU   Upper marker selection: Last peak > 200 RFU
Ladder size (bp)75, 200, 300, 400, 500, 700, 1000, 1500, 2000, 3000, 4000, 5000, 7000, 10000, 20000
Quantification using: Lower Marker   Final concentration (ng/uL): 0.5000   Dilution factor: 12.0

Sample: SampB9
Well location: B9
Created: Monday, September 20, 2021 7:19:57 PM

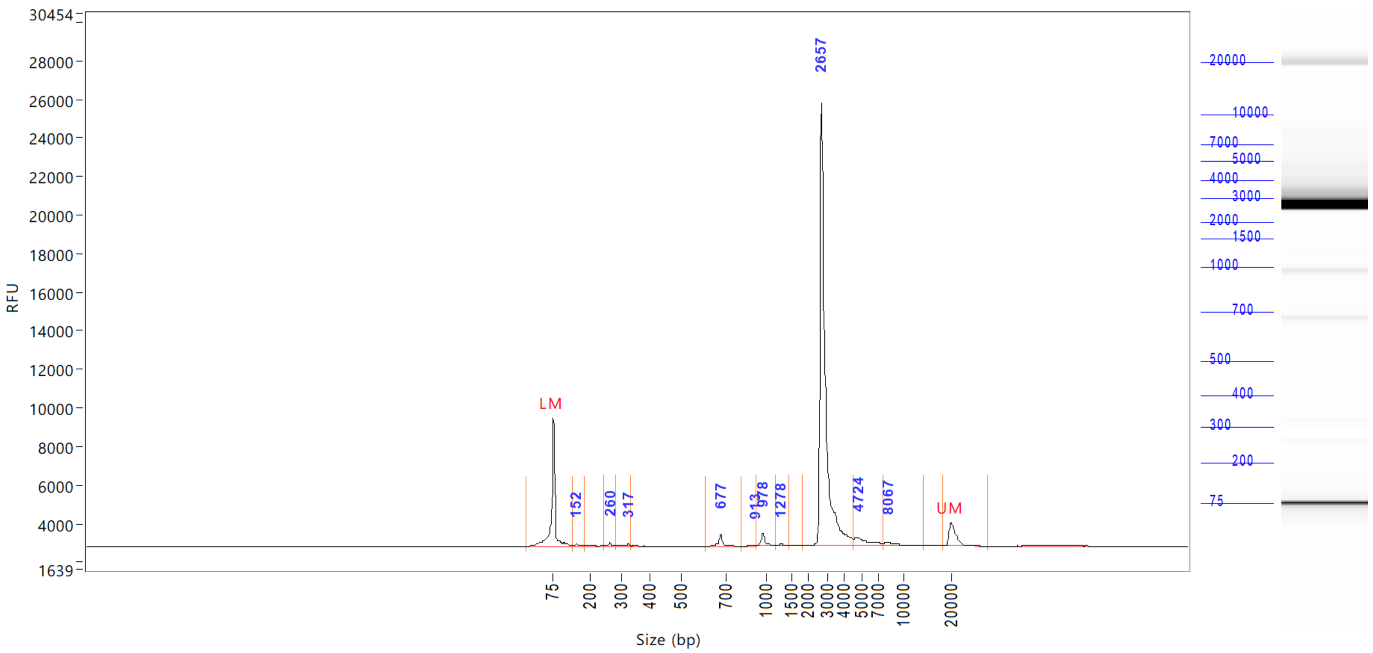

Peak	Size (bp)	Concentration (ng/uL)	From (bp)	To (bp)	Average size (bp)	CV%	RFU	Corrected peak area
1	75 (LM)	0.5000	0	141	70	28.13	6608	59.414
2	152	0.1368	141	179	154	6.12	113	1.355
3	260	0.1055	242	278	258	2.06	182	1.045
4	317	0.1075	278	331	310	3.27	125	1.065
5	677	0.5036	608	809	673	3.26	605	4.987
6	913	0.0487	809	931	896	2.42	65	0.482
7	978	0.5008	931	1178	985	3.86	675	4.959
8	1278	0.0645	1178	1433	1286	3.78	80	0.639
9	2657	19.7725	1827	4483	2843	12.68	22979	195.795
10	4724	0.8951	4483	7601	5469	16.62	395	8.864
11	8067	0.2952	7601	14269	8750	12.75	201	2.924
12	20000 (UM)	0.0911	18171	27859	20491	4.53	1213	10.825

TIC:	22.4303	ng/uL
TIM:	16.7181	nmole/L
Total concentration:	22.5336	ng/uL

Sample peak width (sec): 5   Sample min peak height: 50   Sample baseline V to V?: Y   Sample baseline V to V points: 3
Sample filter: Binomial   Number of points for filter: 3 Sample start region (min): 0 Sample end region (min): 30
Marker peak width (sec): 5   Marker min peak height: 200   Marker baseline V to V?: Y   Marker baseline V to V points: 3
Lower marker selection: First peak > 500 RFU   Upper marker selection: Last peak > 200 RFU
Ladder size (bp)75, 200, 300, 400, 500, 700, 1000, 1500, 2000, 3000, 4000, 5000, 7000, 10000, 20000
Quantification using: Lower Marker   Final concentration (ng/uL): 0.5000   Dilution factor: 12.0

Sample: SampC9
Well location: C9
Created: Monday, September 20, 2021 7:19:57 PM

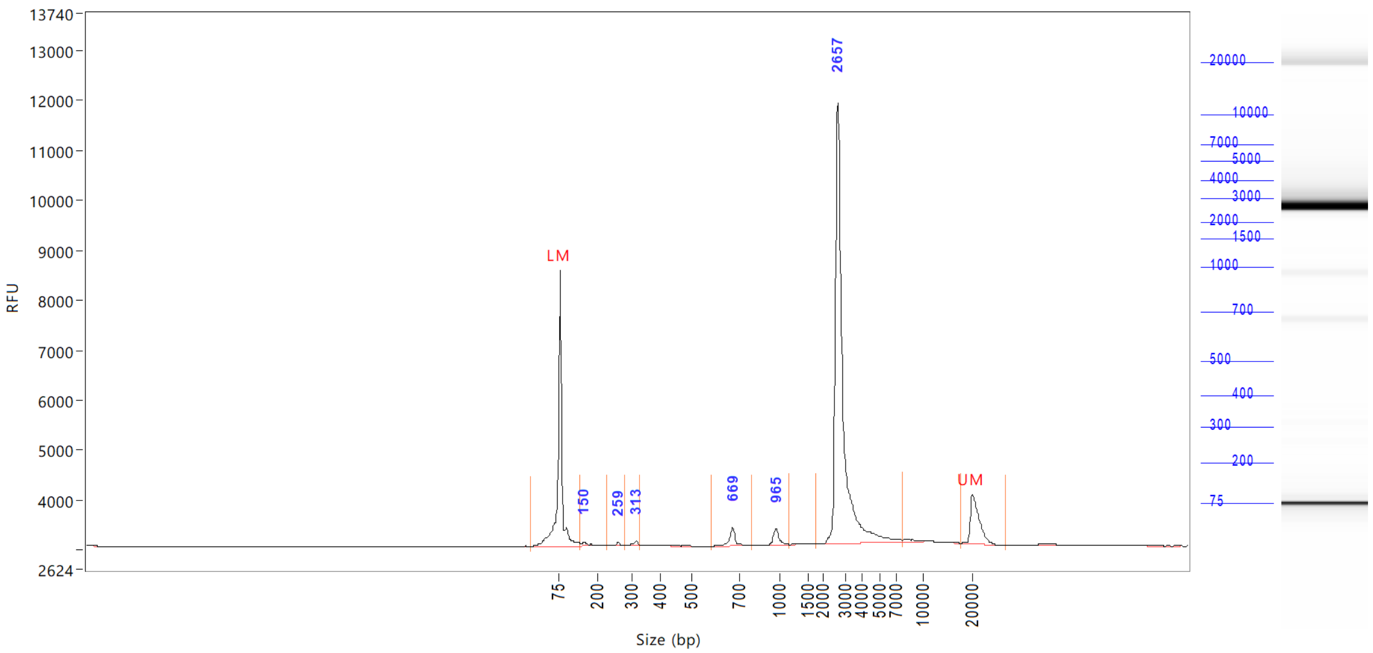

Peak	Size (bp)	Concentration (ng/uL)	From (bp)	To (bp)	Average size (bp)	CV%	RFU	Corrected peak area
1	75 (LM)	0.5000	0	141	70	27.14	5523	51.046
2	150	0.1776	141	226	166	13.06	86	1.511
3	259	0.0745	226	280	255	4.23	62	0.634
4	313	0.1185	280	329	306	3.40	92	1.008
5	669	0.4604	583	792	668	3.41	359	3.917
6	965	0.3976	792	1167	962	4.35	325	3.383
7	2657	12.1872	1750	7601	2914	22.33	8826	103.684
8	20000 (UM)	0.1101	17561	26891	20732	5.17	982	11.237

TIC:	13.4158	ng/uL
TIM:	11.5752	nmole/L
Total concentration:	13.6329	ng/uL

Sample peak width (sec): 5   Sample min peak height: 50   Sample baseline V to V?: Y   Sample baseline V to V points: 3
Sample filter: Binomial   Number of points for filter: 3 Sample start region (min): 0 Sample end region (min): 30
Marker peak width (sec): 5   Marker min peak height: 200   Marker baseline V to V?: Y   Marker baseline V to V points: 3
Lower marker selection: First peak > 500 RFU   Upper marker selection: Last peak > 200 RFU
Ladder size (bp)75, 200, 300, 400, 500, 700, 1000, 1500, 2000, 3000, 4000, 5000, 7000, 10000, 20000
Quantification using: Lower Marker   Final concentration (ng/uL): 0.5000   Dilution factor: 12.0

Sample: SampD9
Well location: D9
Created: Monday, September 20, 2021 7:19:57 PM

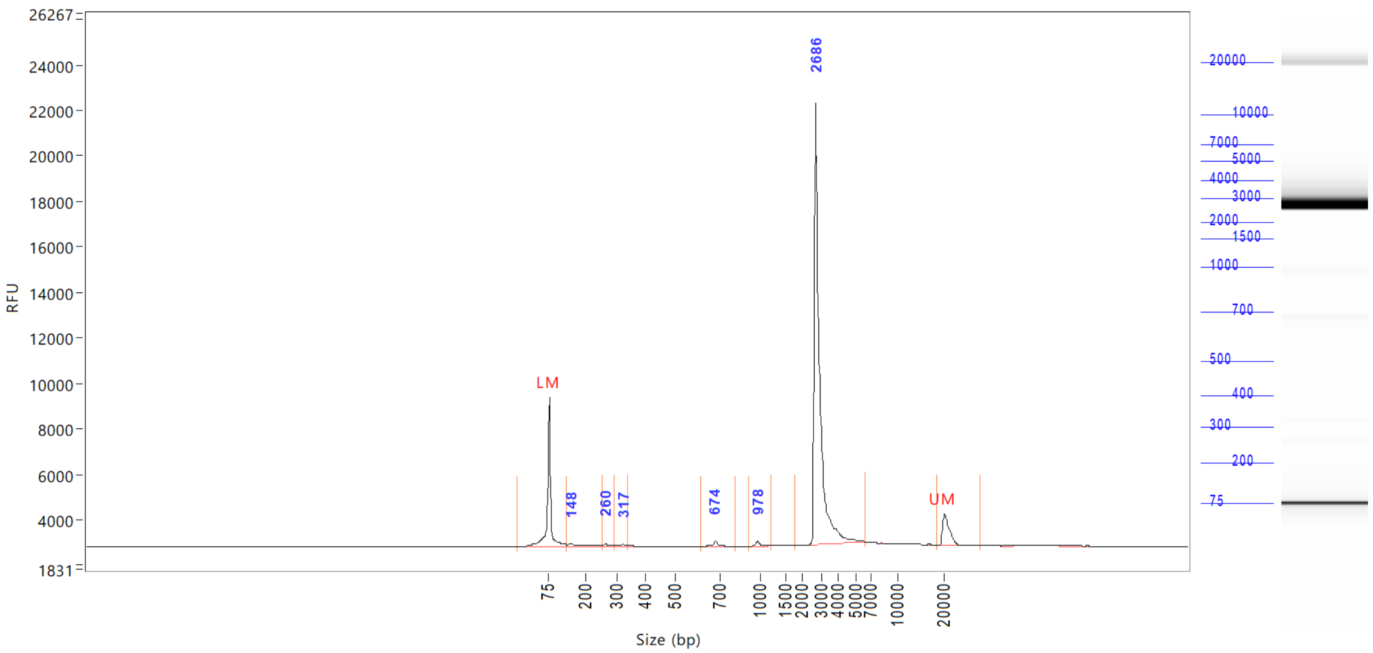

Peak	Size (bp)	Concentration (ng/uL)	From (bp)	To (bp)	Average size (bp)	CV%	RFU	Corrected peak area
1	75 (LM)	0.5000	0	135	71	25.24	6527	52.502
2	148	0.3441	135	250	176	18.24	91	3.011
3	260	0.1002	250	291	261	3.40	130	0.877
4	317	0.0885	291	337	310	3.07	93	0.774
5	674	0.2729	611	809	675	4.01	260	2.388
6	978	0.2021	909	1201	991	4.49	209	1.769
7	2686	18.5879	1770	6186	2871	14.15	19448	162.650
8	20000 (UM)	0.1200	18537	27617	20521	4.11	1378	12.604

TIC:	19.5956	ng/uL
TIM:	15.9741	nmole/L
Total concentration:	19.7514	ng/uL

```
Sample peak width (sec): 5    Sample min peak height: 50    Sample baseline V to V?: Y    Sample baseline V to V points: 3
Sample filter: Binomial       Number of points for filter: 3  Sample start region (min): 0  Sample end region (min): 30
Marker peak width (sec): 5    Marker min peak height: 200   Marker baseline V to V?: Y    Marker baseline V to V points: 3
Lower marker selection: First peak > 500 RFU                Upper marker selection: Last peak > 200 RFU
Ladder size (bp)75, 200, 300, 400, 500, 700, 1000, 1500, 2000, 3000, 4000, 5000, 7000, 10000, 20000
Quantification using: Lower Marker          Final concentration (ng/uL): 0.5000          Dilution factor: 12.0
```

Sample: SampE9
Well location: E9
Created: Monday, September 20, 2021 7:19:57 PM

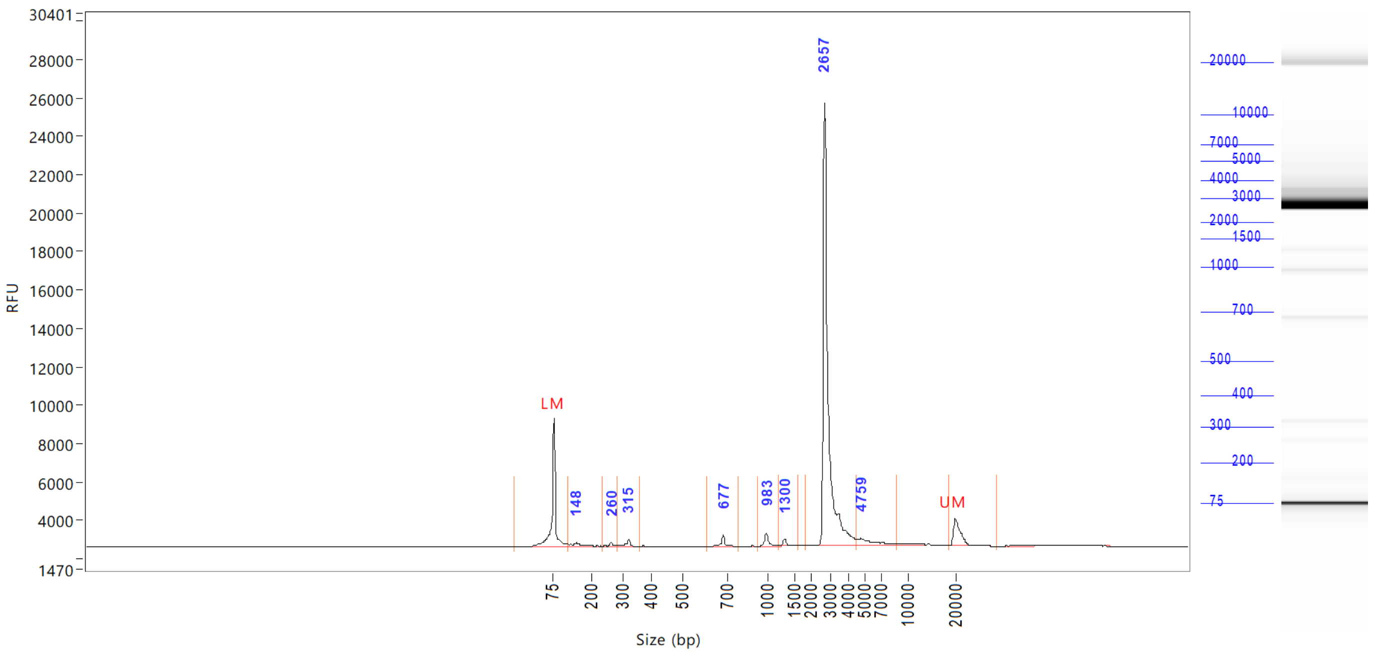

Peak	Size (bp)	Concentration (ng/uL)	From (bp)	To (bp)	Average size (bp)	CV%	RFU	Corrected peak area
1	75 (LM)	0.5000	0	126	69	25.94	6673	56.695
2	148	0.4474	126	233	158	16.28	188	4.227
3	260	0.1551	233	282	258	3.87	199	1.466
4	315	0.3056	282	354	310	3.46	386	2.888
5	677	0.4665	606	779	673	3.04	596	4.408
6	983	0.4981	922	1190	991	4.18	698	4.706
7	1300	0.2449	1190	1597	1313	5.36	365	2.314
8	2657	19.3985	1827	4483	2851	12.92	23074	183.299
9	4759	0.8709	4483	8800	5674	20.53	347	8.229
10	20000 (UM)	0.1145	18659	28584	20514	4.22	1393	12.979

TIC:	22.3869	ng/uL
TIM:	20.9932	nmole/L
Total concentration:	22.5487	ng/uL

Sample peak width (sec): 5   Sample min peak height: 50   Sample baseline V to V?: Y   Sample baseline V to V points: 3
Sample filter: Binomial   Number of points for filter: 3 Sample start region (min): 0 Sample end region (min): 30
Marker peak width (sec): 5   Marker min peak height: 200   Marker baseline V to V?: Y   Marker baseline V to V points: 3
Lower marker selection: First peak > 500 RFU   Upper marker selection: Last peak > 200 RFU
Ladder size (bp)75, 200, 300, 400, 500, 700, 1000, 1500, 2000, 3000, 4000, 5000, 7000, 10000, 20000
Quantification using: Lower Marker   Final concentration (ng/uL): 0.5000   Dilution factor: 12.0

Sample: SampF9
Well location: F9
Created: Monday, September 20, 2021 7:19:57 PM

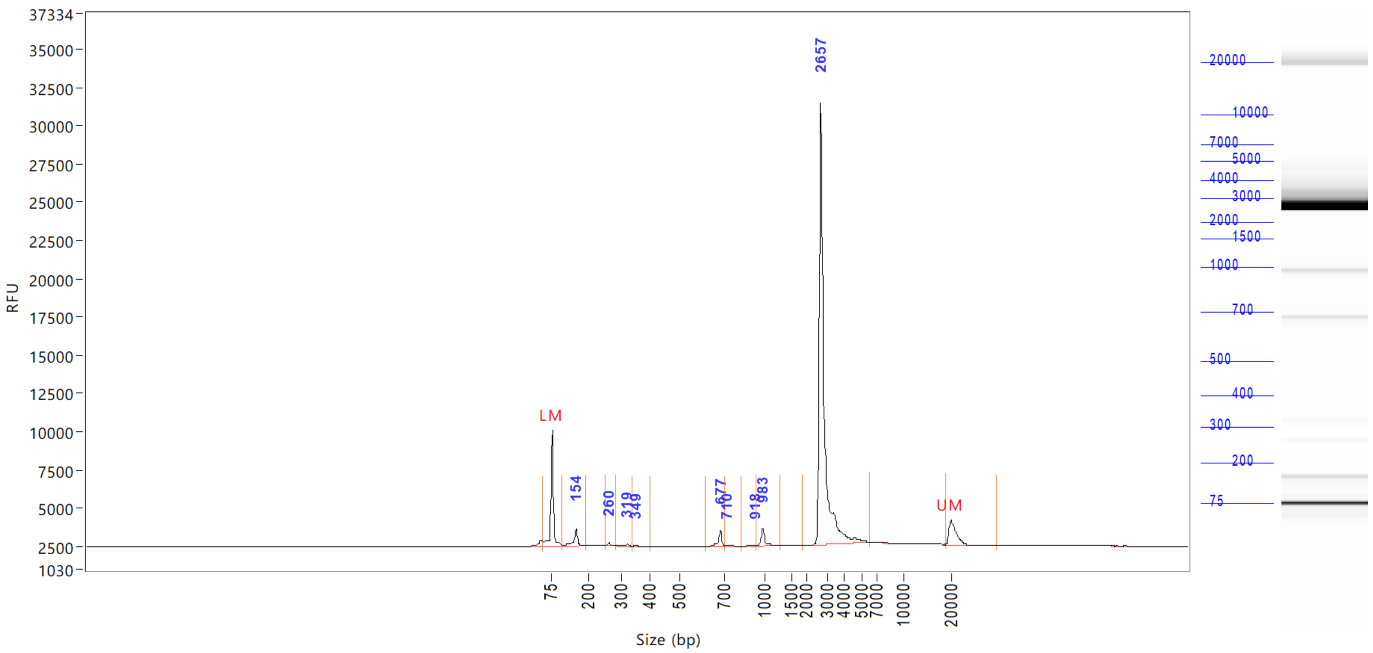

Peak	Size	Concentration	From	To	Average size	CV%	RFU	Corrected peak area
	(bp)	(ng/uL)	(bp)	(bp)	(bp)			
1	75 (LM)	0.5000	47	110	73	11.57	7556	50.102
2	154	1.3100	110	189	148	9.22	1169	10.939
3	260	0.1359	250	286	259	1.63	228	1.135
4	319	0.1677	286	335	311	2.92	183	1.400
5	349	0.0478	335	401	352	3.68	62	0.399
6	677	0.7694	611	695	669	1.94	1015	6.424
7	710	0.1143	695	818	720	3.43	99	0.955
8	918	0.0877	818	935	899	2.66	90	0.732
9	983	0.9022	935	1267	992	4.94	1105	7.534
10	2657	26.5125	1827	6038	2819	14.66	28905	221.389
11	20000 (UM)	0.1534	18781	29793	20536	4.70	1603	15.368

TIC:	30.0475	ng/uL
TIM:	35.8143	nmole/L
Total concentration:	30.3135	ng/uL

Sample peak width (sec): 5 Sample min peak height: 50 Sample baseline V to V?: Y Sample baseline V to V points: 3
Sample filter: Binomial Number of points for filter: 3 Sample start region (min): 0 Sample end region (min): 30
Marker peak width (sec): 5 Marker min peak height: 200 Marker baseline V to V?: Y Marker baseline V to V points: 3
Lower marker selection: First peak > 500 RFU Upper marker selection: Last peak > 200 RFU
Ladder size (bp)75, 200, 300, 400, 500, 700, 1000, 1500, 2000, 3000, 4000, 5000, 7000, 10000, 20000
Quantification using: Lower Marker Final concentration (ng/uL): 0.5000 Dilution factor: 12.0

Sample: SampG9
Well location: G9
Created: Monday, September 20, 2021 7:19:57 PM

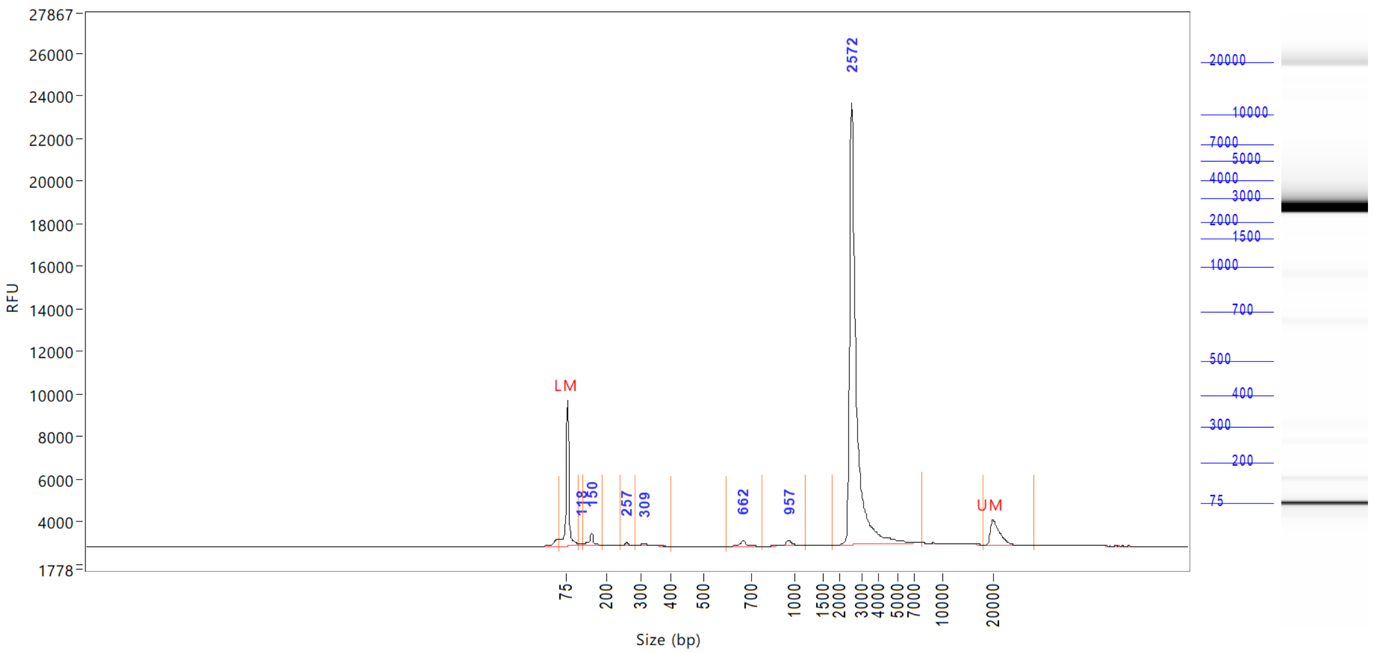

Peak	Size (bp)	Concentration (ng/uL)	From (bp)	To (bp)	Average size (bp)	CV%	RFU	Corrected peak area
1	75 (LM)	0.5000	50	108	74	10.48	6827	49.182
2	118	0.1608	108	126	117	4.60	116	1.318
3	150	0.6977	126	185	146	6.47	566	5.719
4	257	0.1543	241	284	257	3.10	163	1.265
5	309	0.1728	284	400	311	6.35	109	1.417
6	662	0.3897	595	775	660	3.06	276	3.195
7	957	0.3741	775	1190	954	4.77	257	3.066
8	2572	25.8565	1789	7801	2759	17.75	20759	211.948
9	20000 (UM)	0.1426	18171	28100	20698	5.22	1191	14.023

TIC:	27.8059	ng/uL
TIM:	29.0575	nmole/L
Total concentration:	28.0006	ng/uL

```
Sample peak width (sec): 5    Sample min peak height: 50    Sample baseline V to V?: Y    Sample baseline V to V points: 3
Sample filter: Binomial       Number of points for filter: 3  Sample start region (min): 0  Sample end region (min): 30
Marker peak width (sec): 5    Marker min peak height: 200   Marker baseline V to V?: Y    Marker baseline V to V points: 3
Lower marker selection: First peak > 500 RFU                Upper marker selection: Last peak > 200 RFU
Ladder size (bp)75, 200, 300, 400, 500, 700, 1000, 1500, 2000, 3000, 4000, 5000, 7000, 10000, 20000
Quantification using: Lower Marker          Final concentration (ng/uL): 0.5000          Dilution factor: 12.0
```

Sample: SampH9
Well location: H9
Created: Monday, September 20, 2021 7:19:57 PM

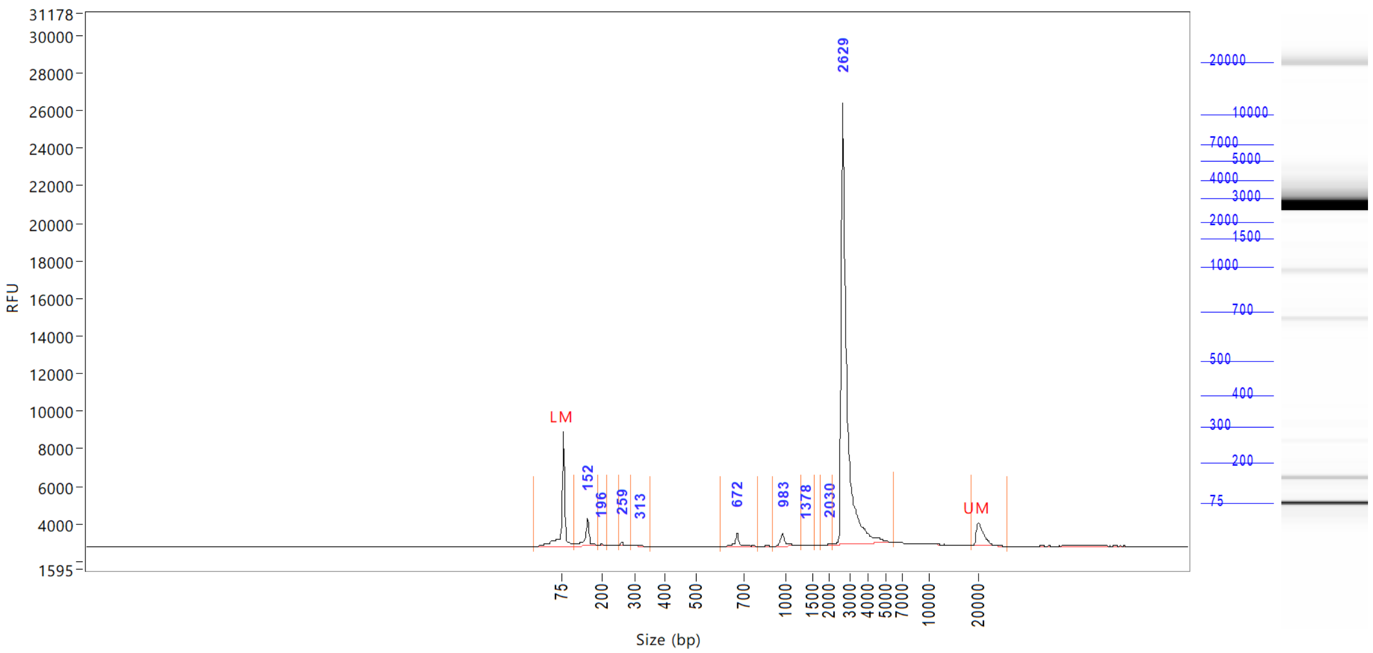

Peak	Size (bp)	Concentration (ng/uL)	From (bp)	To (bp)	Average size (bp)	CV%	RFU	Corrected peak area
1	75 (LM)	0.5000	0	110	67	26.41	6063	47.464
2	152	1.7985	110	187	146	9.17	1500	14.228
3	196	0.1198	187	215	198	3.62	75	0.948
4	259	0.1296	250	287	257	1.62	209	1.025
5	313	0.0994	287	349	308	3.92	71	0.786
6	672	0.7110	603	801	672	3.82	700	5.625
7	983	0.6935	905	1289	1002	6.69	674	5.486
8	1378	0.0577	1289	1559	1366	3.51	52	0.456
9	2030	0.0612	1731	2172	2030	3.46	83	0.484
10	2629	25.4495	2172	6038	2823	13.89	23531	201.324
11	20000 (UM)	0.1246	18537	25803	20459	4.36	1242	11.824

TIC:	29.1203	ng/uL
TIM:	40.4404	nmole/L
Total concentration:	29.2949	ng/uL

```
Sample peak width (sec): 5    Sample min peak height: 50    Sample baseline V to V?: Y    Sample baseline V to V points: 3
Sample filter: Binomial       Number of points for filter: 3  Sample start region (min): 0  Sample end region (min): 30
Marker peak width (sec): 5    Marker min peak height: 200   Marker baseline V to V?: Y    Marker baseline V to V points: 3
Lower marker selection: First peak > 500 RFU                Upper marker selection: Last peak > 200 RFU
Ladder size (bp)75, 200, 300, 400, 500, 700, 1000, 1500, 2000, 3000, 4000, 5000, 7000, 10000, 20000
Quantification using: Lower Marker          Final concentration (ng/uL): 0.5000          Dilution factor: 12.0
```

Sample: SampA10
Well location: A10
Created: Monday, September 20, 2021 7:19:57 PM

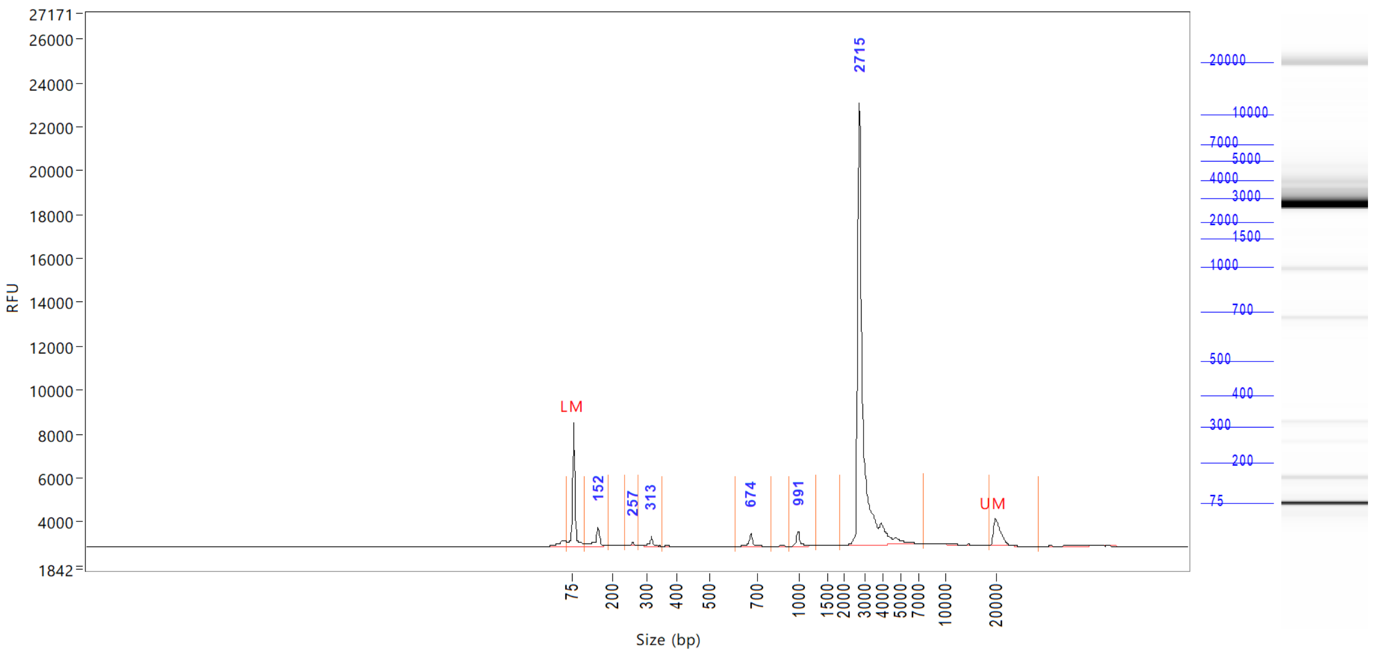

Peak	Size (bp)	Concentration (ng/uL)	From (bp)	To (bp)	Average size (bp)	CV%	RFU	Corrected peak area
1	75 (LM)	0.5000	56	110	74	10.04	5659	35.443
2	152	1.3504	110	185	145	9.21	829	7.977
3	257	0.1795	233	273	256	2.37	185	1.060
4	313	0.5026	273	352	307	3.64	418	2.969
5	674	0.7303	606	796	674	3.60	570	4.314
6	991	0.7590	926	1289	1007	5.07	664	4.484
7	2715	26.9423	1904	7534	2955	17.71	20182	159.154
8	20000 (UM)	0.1586	18781	28705	20433	4.36	1242	11.240

TIC:	30.4642	ng/uL
TIM:	37.1853	nmole/L
Total concentration:	30.9499	ng/uL

```
Sample peak width (sec): 5    Sample min peak height: 50    Sample baseline V to V?: Y    Sample baseline V to V points: 3
Sample filter: Binomial       Number of points for filter: 3  Sample start region (min): 0  Sample end region (min): 30
Marker peak width (sec): 5    Marker min peak height: 200   Marker baseline V to V?: Y    Marker baseline V to V points: 3
Lower marker selection: First peak > 500 RFU                Upper marker selection: Last peak > 200 RFU
Ladder size (bp)75, 200, 300, 400, 500, 700, 1000, 1500, 2000, 3000, 4000, 5000, 7000, 10000, 20000
Quantification using: Lower Marker          Final concentration (ng/uL): 0.5000          Dilution factor: 12.0
```

Sample: SampB10
Well location: B10
Created: Monday, September 20, 2021 7:19:57 PM

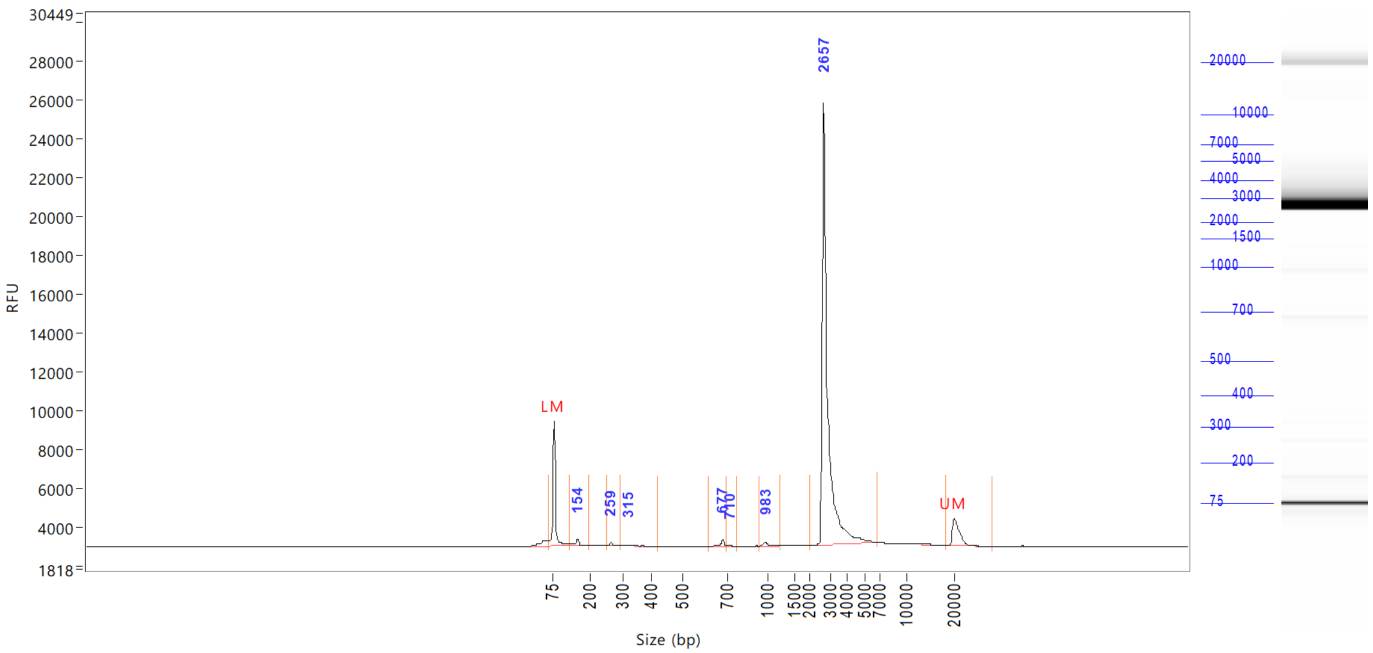

Peak	Size (bp)	Concentration (ng/uL)	From (bp)	To (bp)	Average size (bp)	CV%	RFU	Corrected peak area
1	75 (LM)	0.5000	58	128	76	13.12	6396	42.567
2	154	0.5384	128	190	150	8.85	326	3.820
3	259	0.1209	248	293	259	2.45	152	0.858
4	315	0.1012	293	418	325	8.48	61	0.718
5	677	0.3287	611	692	666	2.21	332	2.332
6	710	0.0570	692	770	711	2.31	55	0.404
7	983	0.2674	931	1212	996	4.82	231	1.897
8	2657	26.9288	2001	6556	2877	15.73	22803	191.048
9	20000 (UM)	0.1516	18415	27979	20518	4.14	1402	12.903

TIC:	28.3424	ng/uL
TIM:	23.9738	nmole/L
Total concentration:	28.6515	ng/uL

Sample peak width (sec): 5   Sample min peak height: 50   Sample baseline V to V?: Y   Sample baseline V to V points: 3
Sample filter: Binomial   Number of points for filter: 3   Sample start region (min): 0   Sample end region (min): 30
Marker peak width (sec): 5   Marker min peak height: 200   Marker baseline V to V?: Y   Marker baseline V to V points: 3
Lower marker selection: First peak > 500 RFU   Upper marker selection: Last peak > 200 RFU
Ladder size (bp)75, 200, 300, 400, 500, 700, 1000, 1500, 2000, 3000, 4000, 5000, 7000, 10000, 20000
Quantification using: Lower Marker   Final concentration (ng/uL): 0.5000   Dilution factor: 12.0

Sample: SampC10
Well location: C10
Created: Monday, September 20, 2021 7:19:57 PM

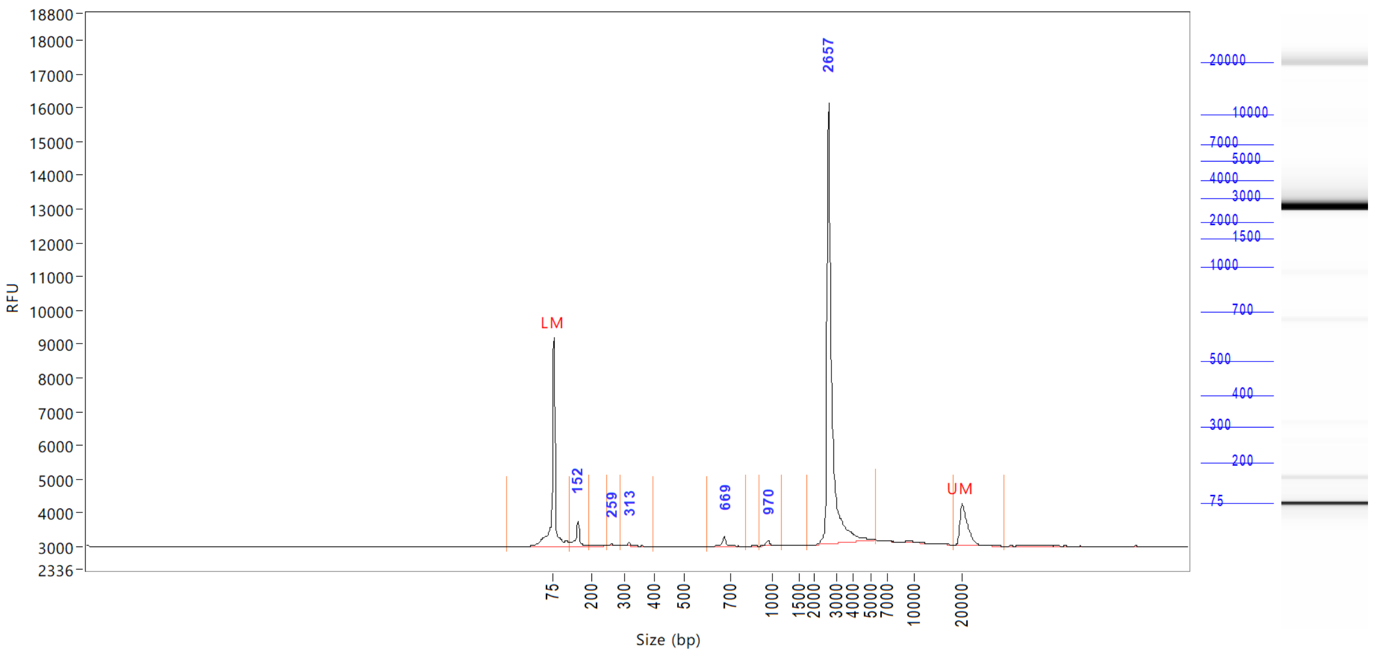

Peak	Size (bp)	Concentration (ng/uL)	From (bp)	To (bp)	Average size (bp)	CV%	RFU	Corrected peak area
1	75 (LM)	0.5000	0	126	69	28.11	6191	50.804
2	152	0.8314	126	190	150	8.11	749	7.039
3	259	0.0530	244	284	256	2.29	72	0.449
4	313	0.1036	284	396	312	5.88	110	0.877
5	669	0.2649	595	809	667	3.11	309	2.243
6	970	0.1565	900	1178	981	4.47	163	1.325
7	2657	11.7062	1731	5593	2774	13.17	13078	99.120
8	20000 (UM)	0.1213	18049	28826	20545	4.79	1228	12.324

TIC:	13.1155	ng/uL
TIM:	17.8587	nmole/L
Total concentration:	13.3964	ng/uL

Sample peak width (sec): 5   Sample min peak height: 50   Sample baseline V to V?: Y   Sample baseline V to V points: 3
Sample filter: Binomial   Number of points for filter: 3 Sample start region (min): 0 Sample end region (min): 30
Marker peak width (sec): 5   Marker min peak height: 200   Marker baseline V to V?: Y   Marker baseline V to V points: 3
Lower marker selection: First peak > 500 RFU   Upper marker selection: Last peak > 200 RFU
Ladder size (bp)75, 200, 300, 400, 500, 700, 1000, 1500, 2000, 3000, 4000, 5000, 7000, 10000, 20000
Quantification using: Lower Marker   Final concentration (ng/uL): 0.5000   Dilution factor: 12.0

Sample: SampD10
Well location: D10
Created: Monday, September 20, 2021 7:19:57 PM

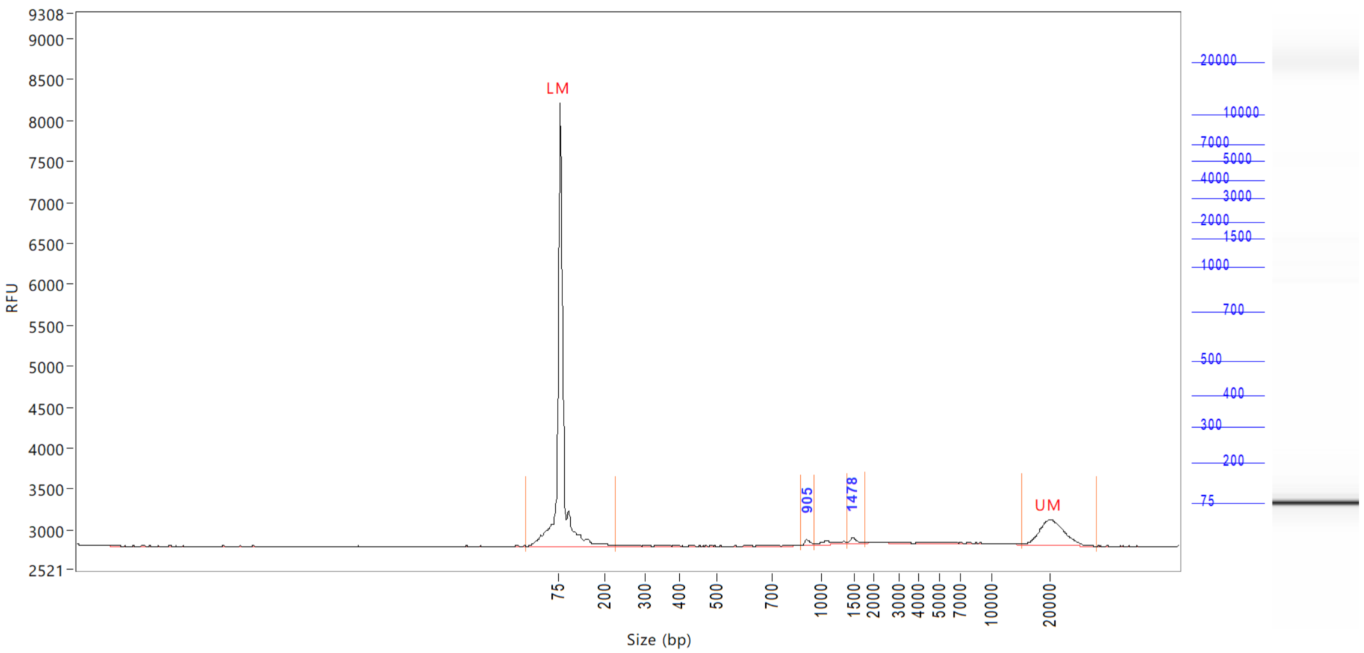

Peak	Size (bp)	Concentration (ng/uL)	From (bp)	To (bp)	Average size (bp)	CV%	RFU	Corrected peak area
1	75 (LM)	0.5000	0	224	76	32.85	5423	68.210
2	905	0.0561	874	952	912	1.92	80	0.638
3	1478	0.0635	1389	1789	1494	5.03	65	0.722
4	20000 (UM)	0.0675	15244	27979	20501	10.08	310	9.208

TIC:	0.1196	ng/uL
TIM:	0.1711	nmole/L
Total concentration:	0.4479	ng/uL

Sample peak width (sec): 5 Sample min peak height: 50 Sample baseline V to V?: Y Sample baseline V to V points: 3
Sample filter: Binomial Number of points for filter: 3 Sample start region (min): 0 Sample end region (min): 30
Marker peak width (sec): 5 Marker min peak height: 200 Marker baseline V to V?: Y Marker baseline V to V points: 3
Lower marker selection: First peak > 500 RFU Upper marker selection: Last peak > 200 RFU
Ladder size (bp)75, 200, 300, 400, 500, 700, 1000, 1500, 2000, 3000, 4000, 5000, 7000, 10000, 20000
Quantification using: Lower Marker Final concentration (ng/uL): 0.5000 Dilution factor: 12.0

Sample: SampE10
Well location: E10
Created: Monday, September 20, 2021 7:19:57 PM

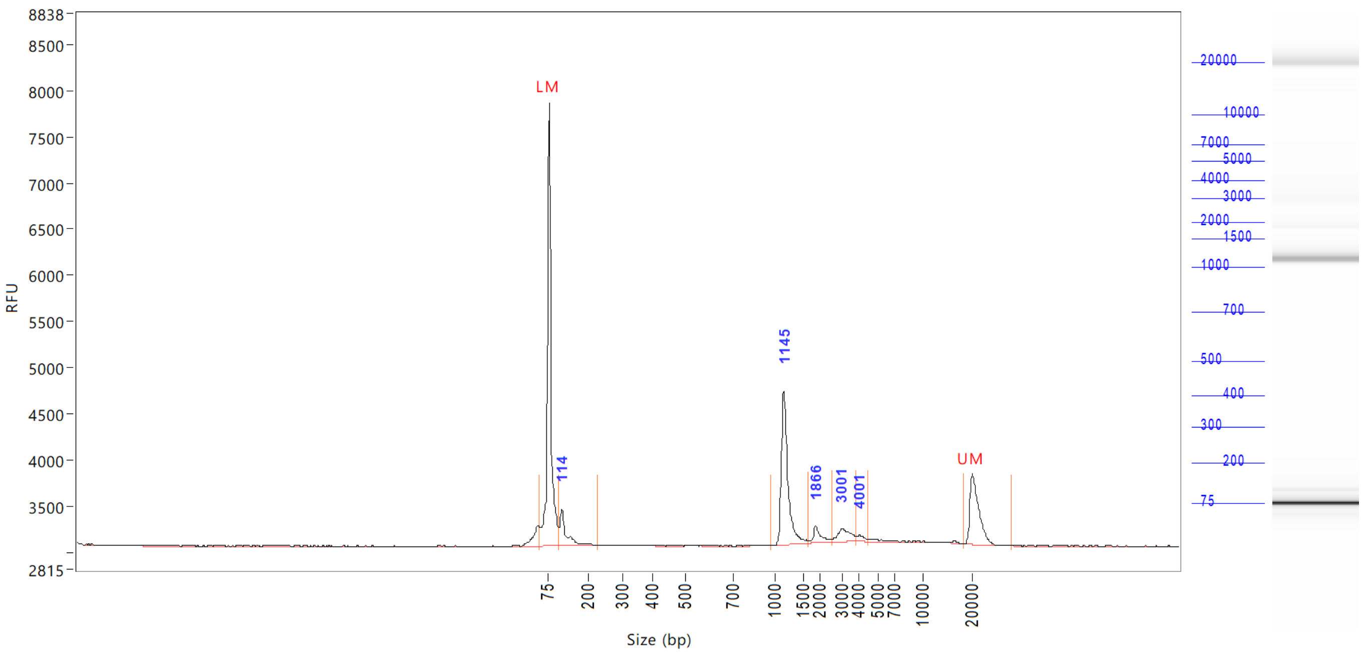

Peak	Size (bp)	Concentration (ng/uL)	From (bp)	To (bp)	Average size (bp)	CV%	RFU	Corrected peak area
1	75 (LM)	0.5000	47	105	74	12.67	4807	40.427
2	114	0.8727	105	228	128	18.17	399	5.880
3	1145	2.6358	970	1635	1173	7.80	1664	17.760
4	1866	0.3033	1635	2543	1961	10.26	182	2.043
5	3001	0.4127	2543	3857	3170	10.31	138	2.780
6	4001	0.1066	3857	4449	4096	4.00	66	0.718
7	20000 (UM)	0.1102	18415	27859	20580	5.20	773	8.913

TIC:	4.3311	ng/uL
TIM:	15.4129	nmole/L
Total concentration:	4.6257	ng/uL

Sample peak width (sec): 5 Sample min peak height: 50 Sample baseline V to V?: Y Sample baseline V to V points: 3
Sample filter: Binomial Number of points for filter: 3 Sample start region (min): 0 Sample end region (min): 30
Marker peak width (sec): 5 Marker min peak height: 200 Marker baseline V to V?: Y Marker baseline V to V points: 3
Lower marker selection: First peak > 500 RFU Upper marker selection: Last peak > 200 RFU
Ladder size (bp)75, 200, 300, 400, 500, 700, 1000, 1500, 2000, 3000, 4000, 5000, 7000, 10000, 20000
Quantification using: Lower Marker Final concentration (ng/uL): 0.5000 Dilution factor: 12.0

Sample: SampF10
Well location: F10
Created: Monday, September 20, 2021 7:19:57 PM

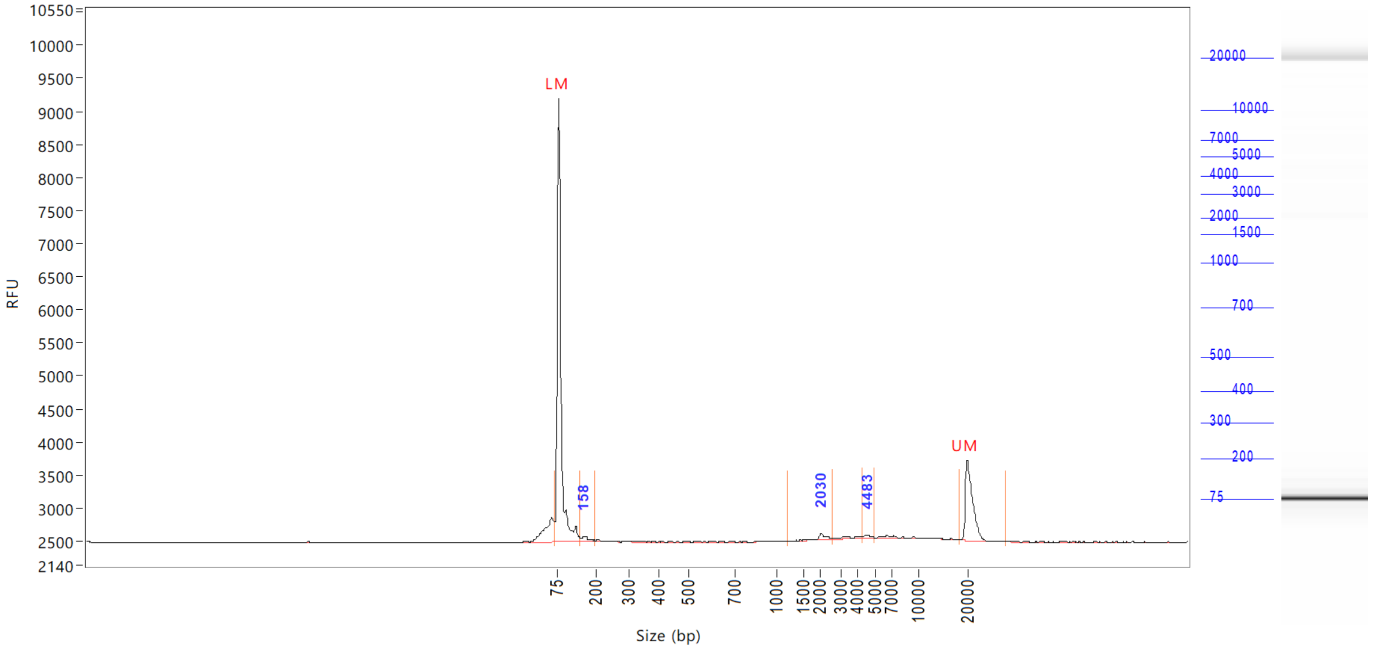

Peak	Size (bp)	Concentration (ng/uL)	From (bp)	To (bp)	Average size (bp)	CV%	RFU	Corrected peak area
1	75 (LM)	0.5000	64	148	80	17.74	6713	58.281
2	158	0.1401	148	194	165	7.42	84	1.360
3	2030	0.1197	1201	2629	2090	14.07	93	1.163
4	4483	0.0450	4277	4966	4536	3.49	51	0.438
5	20000 (UM)	0.0993	18537	28100	20603	4.38	1213	11.569

TIC:	0.3048	ng/uL
TIM:	1.5059	nmole/L
Total concentration:	0.6829	ng/uL

```
Sample peak width (sec): 5    Sample min peak height: 50    Sample baseline V to V?: Y    Sample baseline V to V points: 3
Sample filter: Binomial       Number of points for filter: 3  Sample start region (min): 0  Sample end region (min): 30
Marker peak width (sec): 5    Marker min peak height: 200   Marker baseline V to V?: Y    Marker baseline V to V points: 3
Lower marker selection: First peak > 500 RFU                Upper marker selection: Last peak > 200 RFU
Ladder size (bp)75, 200, 300, 400, 500, 700, 1000, 1500, 2000, 3000, 4000, 5000, 7000, 10000, 20000
Quantification using: Lower Marker          Final concentration (ng/uL): 0.5000          Dilution factor: 12.0
```

Sample: SampG10
Well location: G10
Created: Monday, September 20, 2021 7:19:57 PM

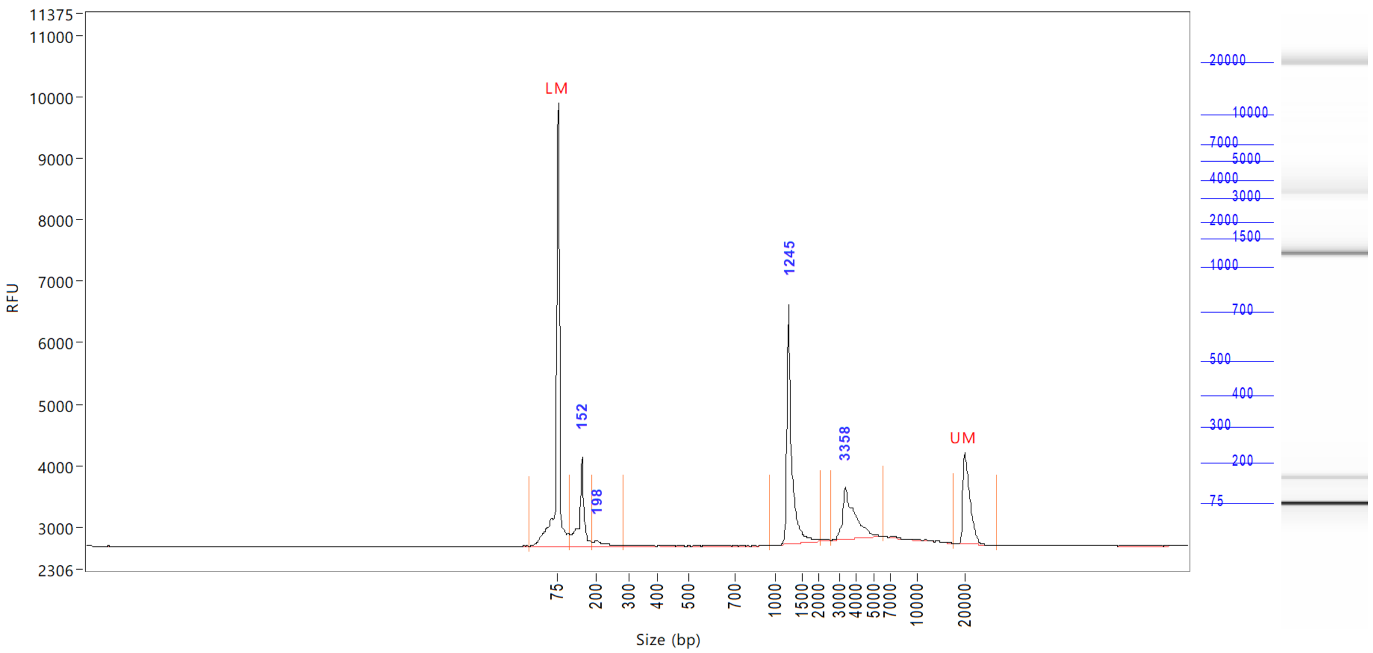

Peak	Size (bp)	Concentration (ng/uL)	From (bp)	To (bp)	Average size (bp)	CV%	RFU	Corrected peak area
1	75 (LM)	0.5000	0	114	68	27.01	7243	58.431
2	152	1.6516	114	189	147	9.87	1460	16.084
3	198	0.2296	189	286	218	10.76	84	2.236
4	1245	3.1586	952	2087	1287	9.77	3912	30.760
5	3358	1.5352	2629	6186	3712	13.16	843	14.951
6	20000 (UM)	0.1197	17683	26649	20523	4.02	1491	13.987

TIC:	6.5751	ng/uL
TIM:	24.9177	nmole/L
Total concentration:	6.7957	ng/uL

```
Sample peak width (sec): 5    Sample min peak height: 50    Sample baseline V to V?: Y    Sample baseline V to V points: 3
Sample filter: Binomial       Number of points for filter: 3  Sample start region (min): 0  Sample end region (min): 30
Marker peak width (sec): 5    Marker min peak height: 200   Marker baseline V to V?: Y    Marker baseline V to V points: 3
Lower marker selection: First peak > 500 RFU                Upper marker selection: Last peak > 200 RFU
Ladder size (bp)75, 200, 300, 400, 500, 700, 1000, 1500, 2000, 3000, 4000, 5000, 7000, 10000, 20000
Quantification using: Lower Marker          Final concentration (ng/uL): 0.5000          Dilution factor: 12.0
```

Sample: SampH10
Well location: H10
Created: Monday, September 20, 2021 7:19:57 PM

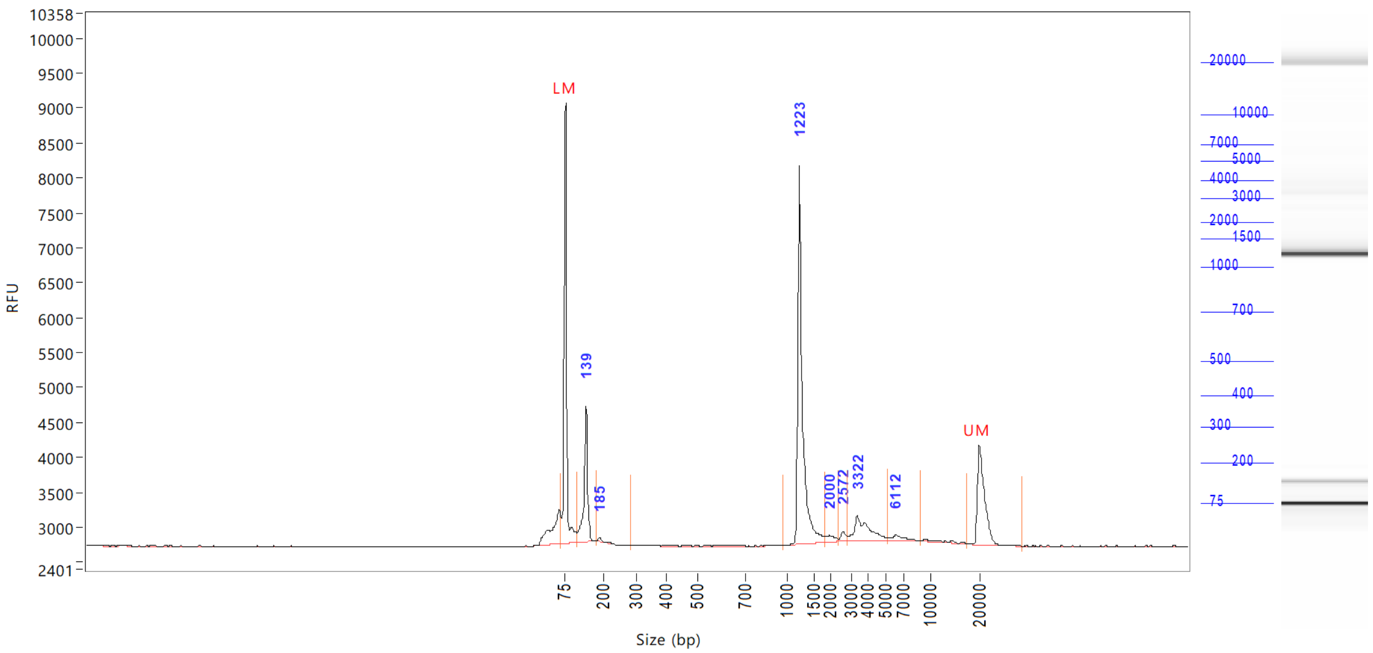

Peak	Size (bp)	Concentration (ng/uL)	From (bp)	To (bp)	Average size (bp)	CV%	RFU	Corrected peak area
1	75 (LM)	0.5000	62	112	74	10.98	6313	40.427
2	139	2.3093	112	173	135	6.05	1944	15.560
3	185	0.0943	173	282	199	13.15	61	0.636
4	1223	6.0031	970	1846	1268	8.53	5419	40.448
5	2000	0.1885	1846	2372	2044	7.09	94	1.270
6	2572	0.1712	2372	2857	2609	4.79	142	1.153
7	3322	1.1024	2857	5149	3730	13.74	351	7.428
8	6112	0.1981	5149	9000	6548	13.37	73	1.335
9	20000 (UM)	0.1653	17561	28826	20557	4.80	1417	13.367

TIC:	10.0668	ng/uL
TIM:	37.4764	nmole/L
Total concentration:	10.3384	ng/uL

```
Sample peak width (sec): 5     Sample min peak height: 50    Sample baseline V to V?: Y    Sample baseline V to V points: 3
Sample filter: Binomial        Number of points for filter: 3  Sample start region (min): 0  Sample end region (min): 30
Marker peak width (sec): 5     Marker min peak height: 200   Marker baseline V to V?: Y    Marker baseline V to V points: 3
Lower marker selection: First peak > 500 RFU                 Upper marker selection: Last peak > 200 RFU
Ladder size (bp)75, 200, 300, 400, 500, 700, 1000, 1500, 2000, 3000, 4000, 5000, 7000, 10000, 20000
Quantification using: Lower Marker          Final concentration (ng/uL): 0.5000          Dilution factor: 12.0
```

Sample: SampA11
Well location: A11
Created: Monday, September 20, 2021 7:19:57 PM

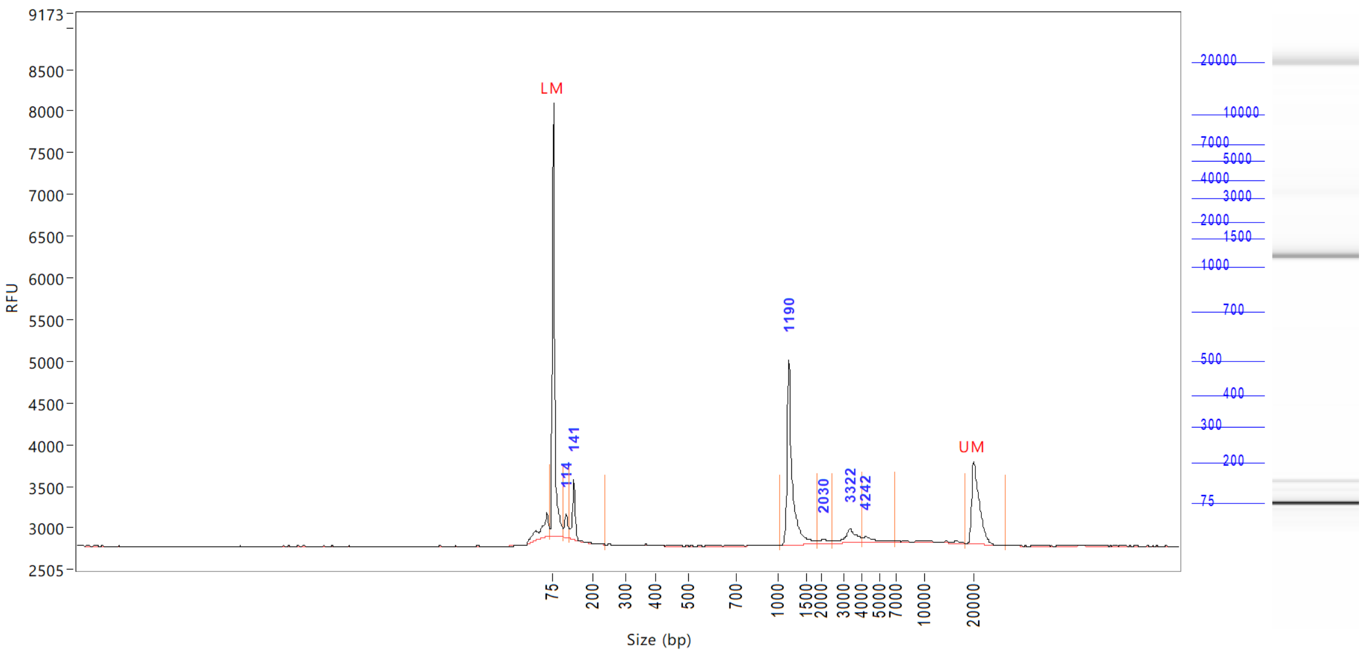

Peak	Size (bp)	Concentration (ng/uL)	From (bp)	To (bp)	Average size (bp)	CV%	RFU	Corrected peak area
1	75 (LM)	0.5000	64	105	75	8.71	5204	32.321
2	114	0.5008	105	128	115	5.49	283	2.698
3	141	0.8874	128	239	141	9.05	728	4.780
4	1190	3.4809	1012	1846	1237	9.51	2224	18.751
5	2030	0.1661	1846	2486	2111	8.69	57	0.895
6	3322	0.5361	2486	4070	3352	11.42	170	2.888
7	4242	0.2370	4070	6926	4869	15.79	69	1.277
8	20000 (UM)	0.1504	18415	26529	20516	4.27	986	9.719

TIC:	5.8083	ng/uL
TIM:	22.6005	nmole/L
Total concentration:	6.1040	ng/uL

Sample peak width (sec): 5   Sample min peak height: 50   Sample baseline V to V?: Y   Sample baseline V to V points: 3
Sample filter: Binomial   Number of points for filter: 3 Sample start region (min): 0 Sample end region (min): 30
Marker peak width (sec): 5   Marker min peak height: 200   Marker baseline V to V?: Y   Marker baseline V to V points: 3
Lower marker selection: First peak > 500 RFU   Upper marker selection: Last peak > 200 RFU
Ladder size (bp)75, 200, 300, 400, 500, 700, 1000, 1500, 2000, 3000, 4000, 5000, 7000, 10000, 20000
Quantification using: Lower Marker   Final concentration (ng/uL): 0.5000   Dilution factor: 12.0

Sample: SampB11
Well location: B11
Created: Monday, September 20, 2021 7:19:57 PM

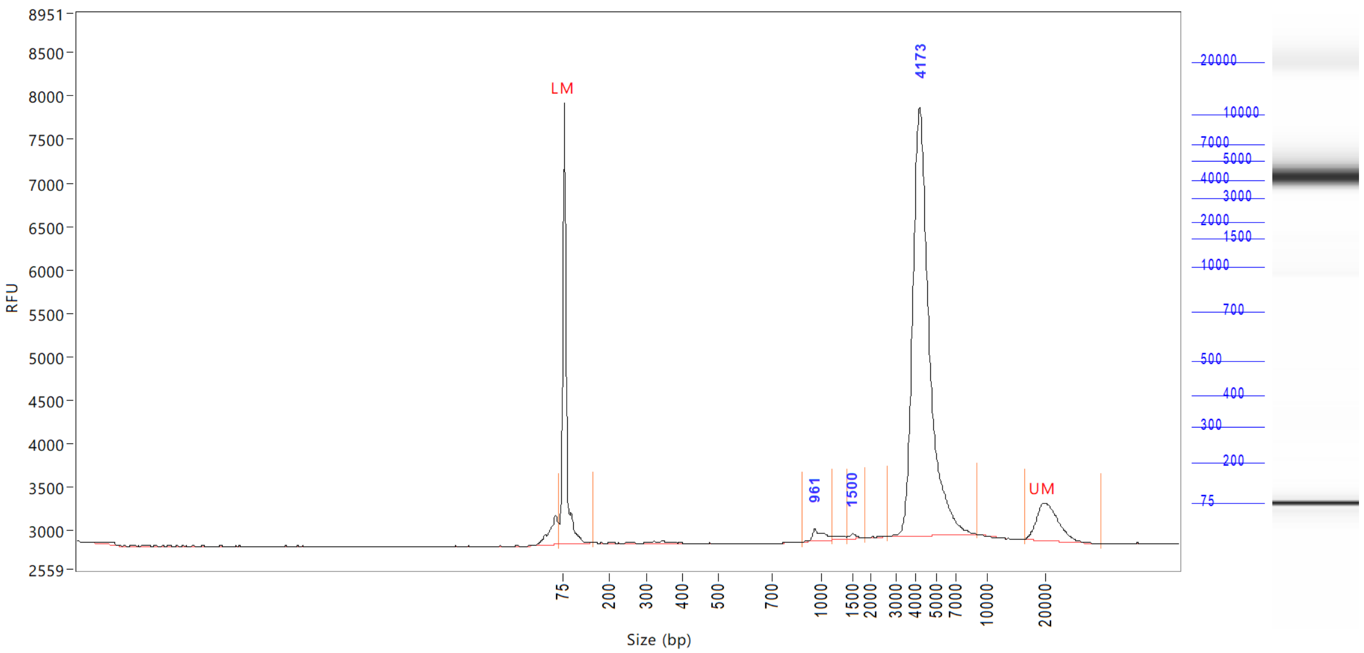

Peak	Size (bp)	Concentration (ng/uL)	From (bp)	To (bp)	Average size (bp)	CV%	RFU	Corrected peak area
1	75 (LM)	0.5000	62	158	76	12.62	5079	42.955
2	961	0.3342	883	1178	1005	6.43	140	2.393
3	1500	0.0900	1411	1846	1532	6.25	55	0.644
4	4173	15.6413	2686	9000	4355	14.78	4919	111.979
5	20000 (UM)	0.1312	16464	30156	20804	9.14	435	11.271

TIC:	16.0655	ng/uL
TIM:	6.5567	nmole/L
Total concentration:	16.5172	ng/uL

```
Sample peak width (sec): 5    Sample min peak height: 50    Sample baseline V to V?: Y    Sample baseline V to V points: 3
Sample filter: Binomial       Number of points for filter: 3  Sample start region (min): 0  Sample end region (min): 30
Marker peak width (sec): 5    Marker min peak height: 200   Marker baseline V to V?: Y    Marker baseline V to V points: 3
Lower marker selection: First peak > 500 RFU                Upper marker selection: Last peak > 200 RFU
Ladder size (bp)75, 200, 300, 400, 500, 700, 1000, 1500, 2000, 3000, 4000, 5000, 7000, 10000, 20000
Quantification using: Lower Marker          Final concentration (ng/uL): 0.5000          Dilution factor: 12.0
```

Sample: SampC11
Well location: C11
Created: Monday, September 20, 2021 7:19:57 PM

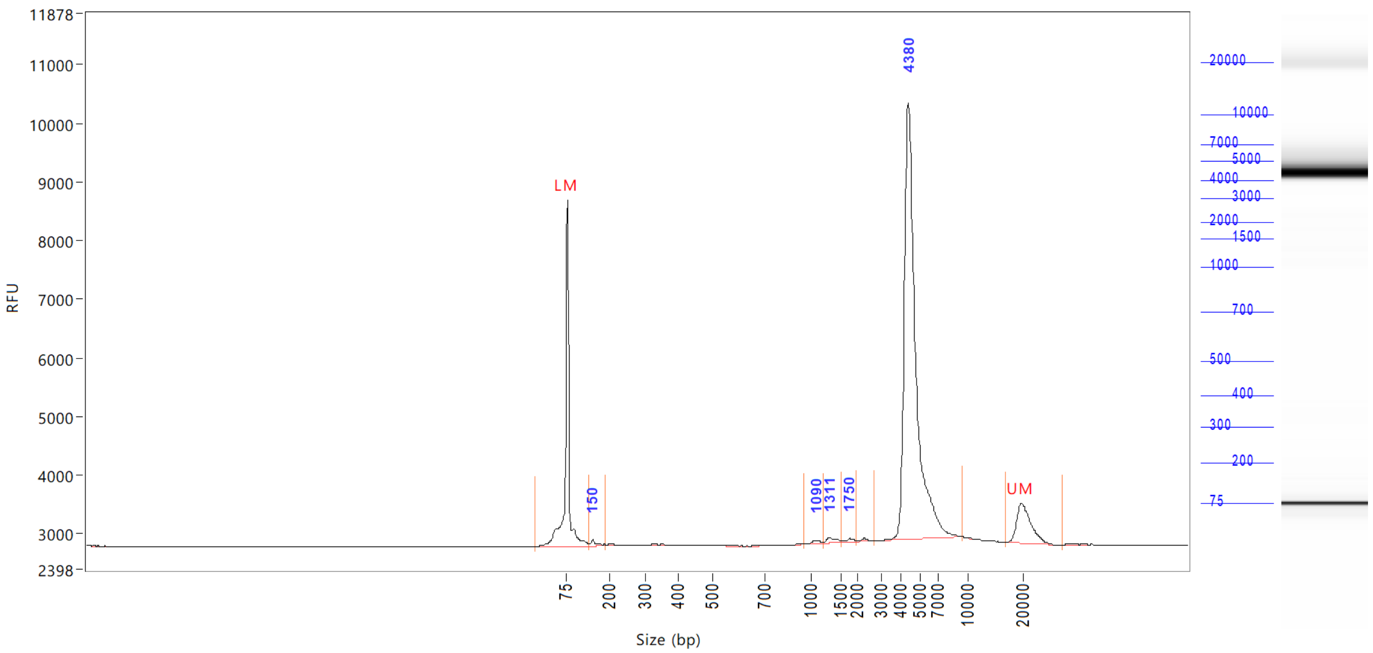

Peak	Size (bp)	Concentration (ng/uL)	From (bp)	To (bp)	Average size (bp)	CV%	RFU	Corrected peak area
1	75 (LM)	0.5000	0	139	70	24.47	5909	51.143
2	150	0.1833	139	189	157	8.58	111	1.562
3	1090	0.0894	952	1212	1084	5.51	59	0.762
4	1311	0.1570	1212	1520	1353	5.84	93	1.338
5	1750	0.0816	1520	1942	1733	6.35	60	0.695
6	4380	14.0630	2715	9400	4651	14.28	7452	119.870
7	20000 (UM)	0.1172	16952	27617	20525	6.78	678	11.991

TIC:	14.5742	ng/uL
TIM:	7.3007	nmole/L
Total concentration:	14.9581	ng/uL

Sample peak width (sec): 5 Sample min peak height: 50 Sample baseline V to V?: Y Sample baseline V to V points: 3
Sample filter: Binomial Number of points for filter: 3 Sample start region (min): 0 Sample end region (min): 30
Marker peak width (sec): 5 Marker min peak height: 200 Marker baseline V to V?: Y Marker baseline V to V points: 3
Lower marker selection: First peak > 500 RFU Upper marker selection: Last peak > 200 RFU
Ladder size (bp)75, 200, 300, 400, 500, 700, 1000, 1500, 2000, 3000, 4000, 5000, 7000, 10000, 20000
Quantification using: Lower Marker Final concentration (ng/uL): 0.5000 Dilution factor: 12.0

Sample: SampD11
Well location: D11
Created: Monday, September 20, 2021 7:19:57 PM

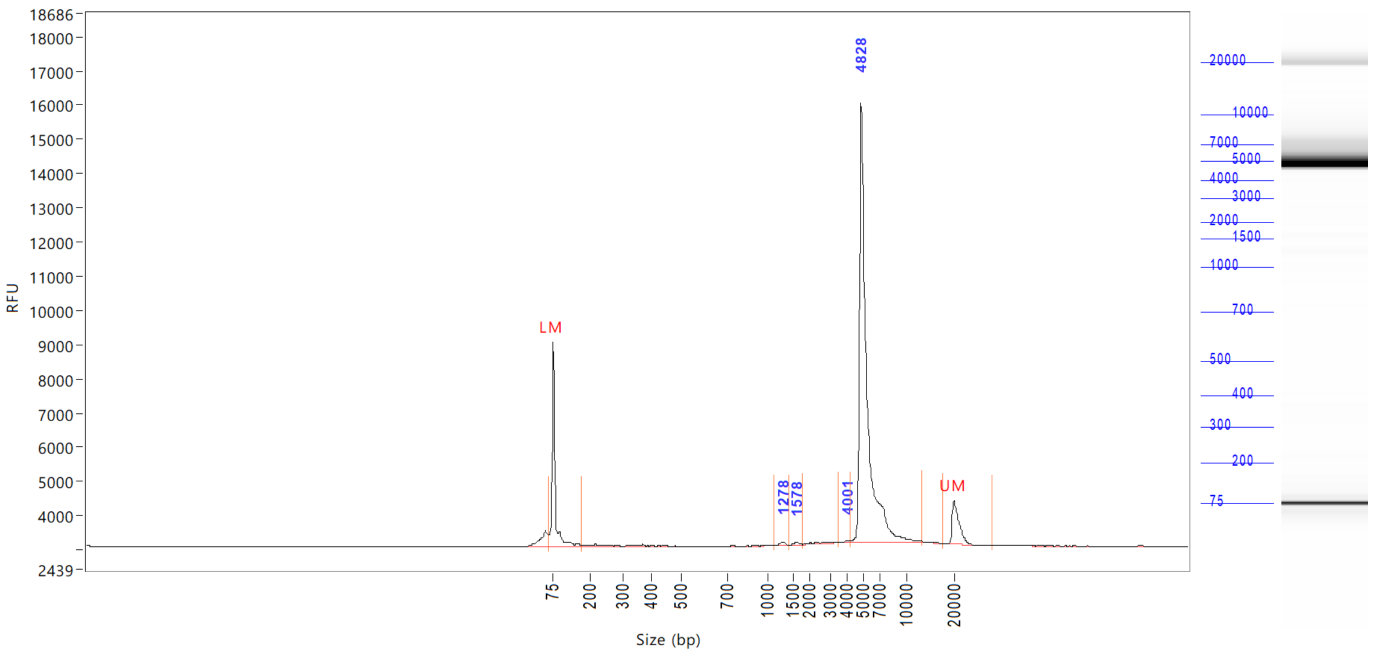

Peak	Size (bp)	Concentration (ng/uL)	From (bp)	To (bp)	Average size (bp)	CV%	RFU	Corrected peak area
1	75 (LM)	0.5000	64	169	80	21.70	5994	43.875
2	1278	0.1297	1123	1400	1266	3.84	108	0.949
3	1578	0.0913	1400	1770	1542	4.35	80	0.667
4	4001	0.0712	3500	4173	3906	4.45	54	0.521
5	4828	19.2719	4173	13415	5392	18.92	12868	140.927
6	20000 (UM)	0.1351	17561	27979	20481	4.28	1274	11.857

TIC:	19.5641	ng/uL
TIM:	6.1802	nmole/L
Total concentration:	20.2022	ng/uL

Sample peak width (sec): 5 Sample min peak height: 50 Sample baseline V to V?: Y Sample baseline V to V points: 3
Sample filter: Binomial Number of points for filter: 3 Sample start region (min): 0 Sample end region (min): 30
Marker peak width (sec): 5 Marker min peak height: 200 Marker baseline V to V?: Y Marker baseline V to V points: 3
Lower marker selection: First peak > 500 RFU Upper marker selection: Last peak > 200 RFU
Ladder size (bp)75, 200, 300, 400, 500, 700, 1000, 1500, 2000, 3000, 4000, 5000, 7000, 10000, 20000
Quantification using: Lower Marker Final concentration (ng/uL): 0.5000 Dilution factor: 12.0

Sample: SampE11
Well location: E11
Created: Monday, September 20, 2021 7:19:57 PM

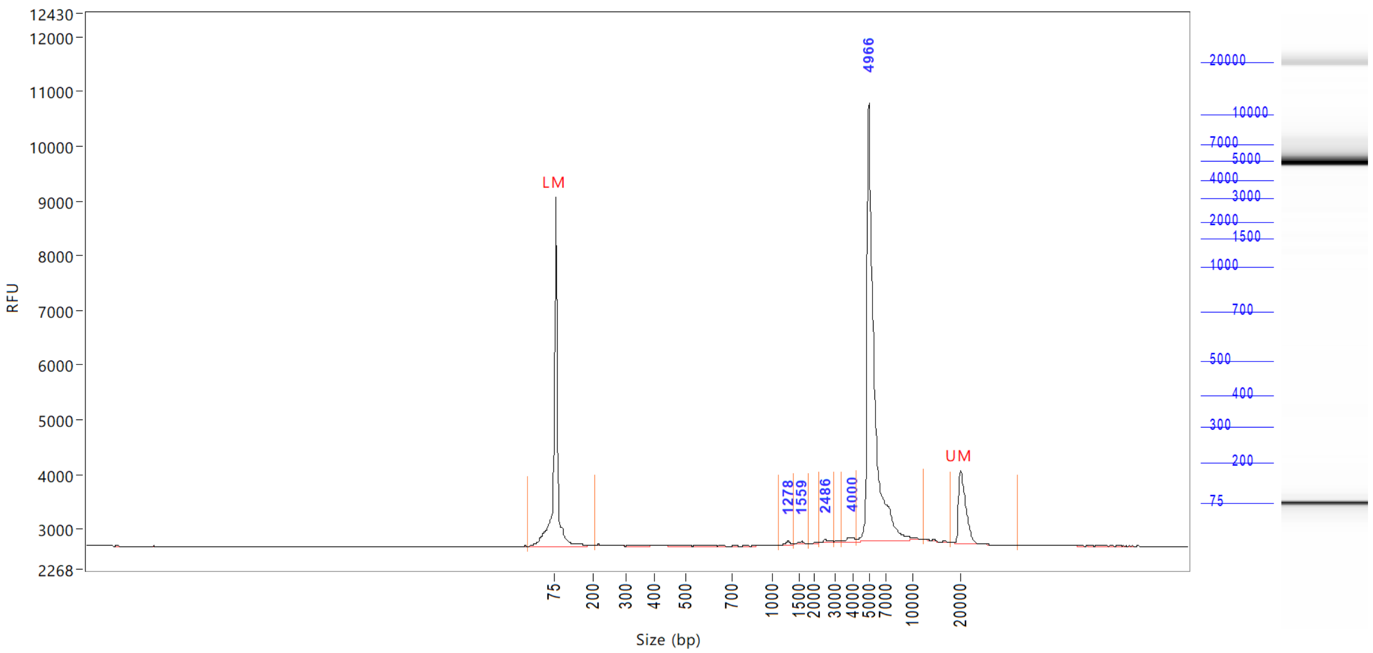

Peak	Size (bp)	Concentration (ng/uL)	From (bp)	To (bp)	Average size (bp)	CV%	RFU	Corrected peak area
1	75 (LM)	0.5000	0	205	71	30.62	6386	58.009
2	1278	0.0567	1112	1389	1273	4.01	64	0.548
3	1559	0.0523	1389	1808	1533	5.14	51	0.506
4	2486	0.0649	2201	2886	2561	5.95	59	0.628
5	4000	0.1032	3358	4208	3847	5.54	72	0.998
6	4966	8.7682	4208	12318	5450	17.06	8026	84.773
7	20000 (UM)	0.1096	17683	31728	20461	4.93	1342	12.710

TIC:	9.0454	ng/uL
TIM:	2.8640	nmole/L
Total concentration:	9.3068	ng/uL

```
Sample peak width (sec): 5    Sample min peak height: 50    Sample baseline V to V?: Y    Sample baseline V to V points: 3
Sample filter: Binomial       Number of points for filter: 3  Sample start region (min): 0  Sample end region (min): 30
Marker peak width (sec): 5    Marker min peak height: 200   Marker baseline V to V?: Y    Marker baseline V to V points: 3
Lower marker selection: First peak > 500 RFU                Upper marker selection: Last peak > 200 RFU
Ladder size (bp)75, 200, 300, 400, 500, 700, 1000, 1500, 2000, 3000, 4000, 5000, 7000, 10000, 20000
Quantification using: Lower Marker          Final concentration (ng/uL): 0.5000          Dilution factor: 12.0
```

Sample: SampF11
Well location: F11
Created: Monday, September 20, 2021 7:19:57 PM

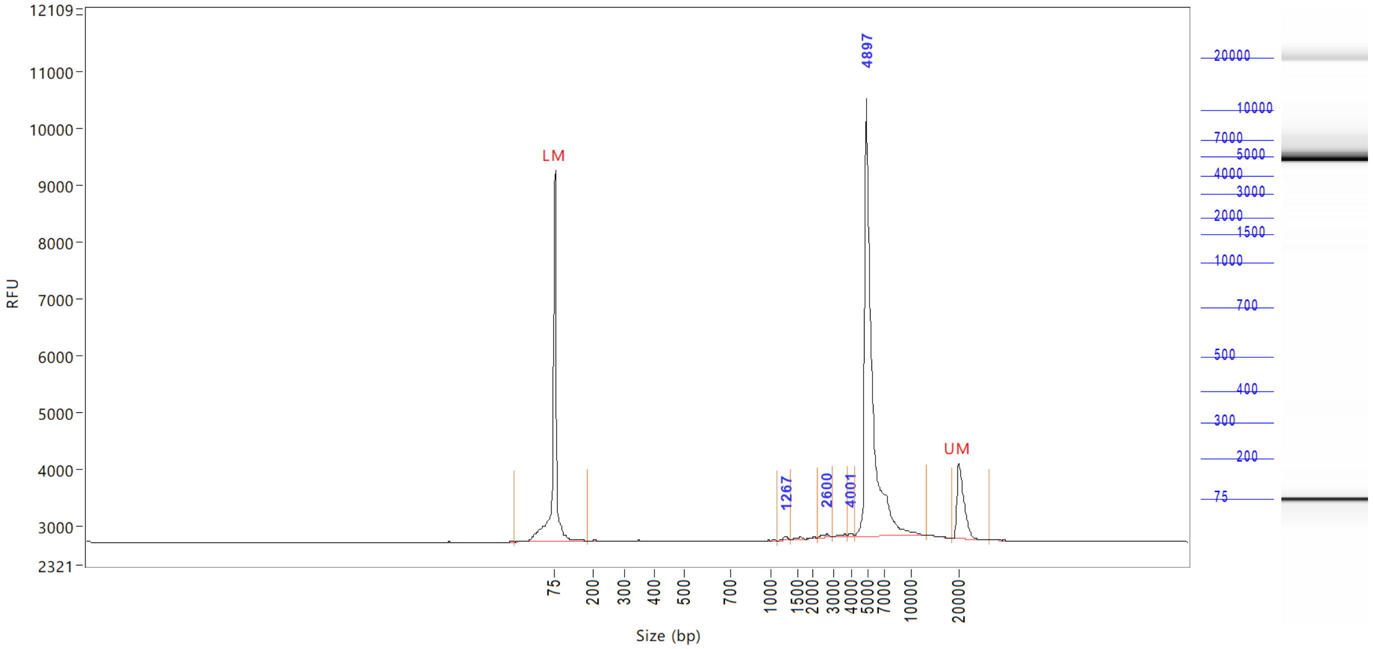

Peak	Size (bp)	Concentration (ng/uL)	From (bp)	To (bp)	Average size (bp)	CV%	RFU	Corrected peak area
1	75 (LM)	0.5000	0	181	67	31.72	6524	57.139
2	1267	0.0588	1123	1378	1252	3.65	68	0.560
3	2600	0.0697	2144	2914	2492	6.54	51	0.663
4	4001	0.0400	3786	4173	3973	2.68	52	0.381
5	4897	8.8889	4173	13293	5472	18.98	7713	84.649
6	20000 (UM)	0.1060	18415	26287	20463	4.00	1313	12.116

TIC:	9.0573	ng/uL
TIM:	2.8142	nmole/L
Total concentration:	9.3638	ng/uL

Sample peak width (sec): 5   Sample min peak height: 50   Sample baseline V to V?: Y   Sample baseline V to V points: 3
Sample filter: Binomial   Number of points for filter: 3   Sample start region (min): 0   Sample end region (min): 30
Marker peak width (sec): 5   Marker min peak height: 200   Marker baseline V to V?: Y   Marker baseline V to V points: 3
Lower marker selection: First peak > 500 RFU   Upper marker selection: Last peak > 200 RFU
Ladder size (bp)75, 200, 300, 400, 500, 700, 1000, 1500, 2000, 3000, 4000, 5000, 7000, 10000, 20000
Quantification using: Lower Marker   Final concentration (ng/uL): 0.5000   Dilution factor: 12.0

Sample: SampG11
Well location: G11
Created: Monday, September 20, 2021 7:19:57 PM

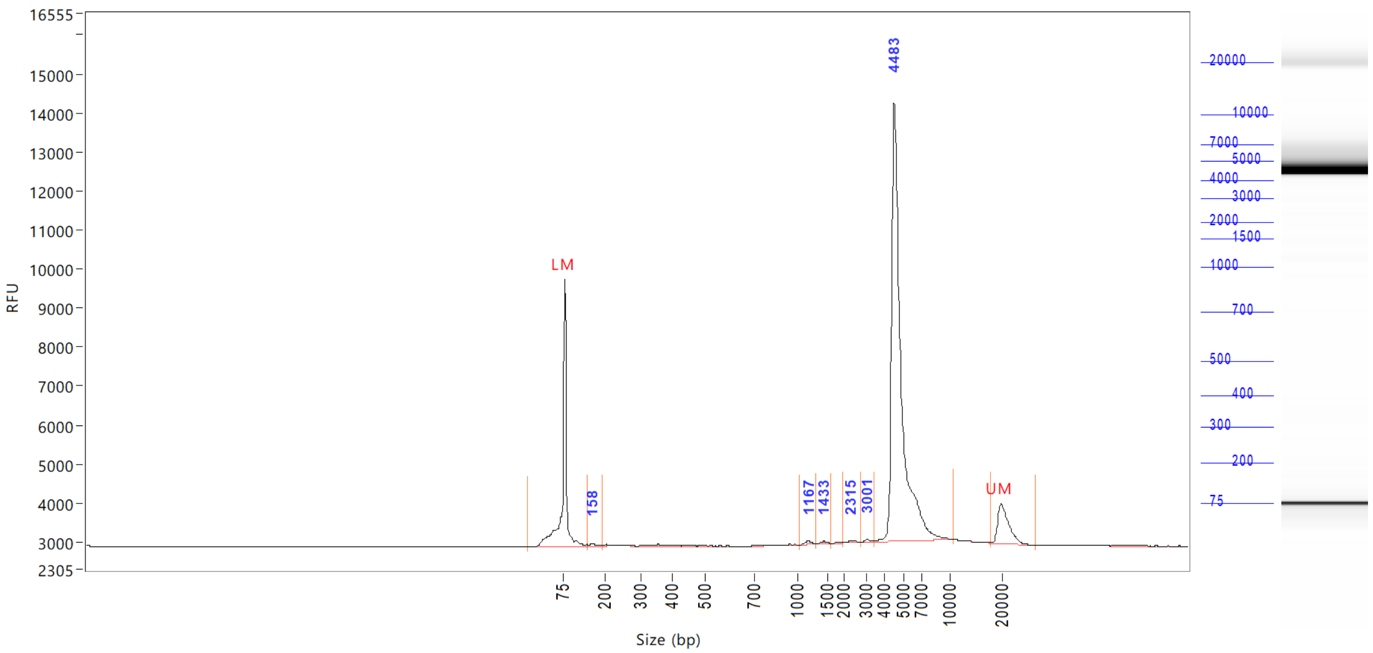

Peak	Size (bp)	Concentration (ng/uL)	From (bp)	To (bp)	Average size (bp)	CV%	RFU	Corrected peak area
1	75 (LM)	0.5000	0	145	66	32.81	6860	63.494
2	158	0.1044	145	192	162	7.70	77	1.104
3	1167	0.0859	1001	1300	1155	4.76	89	0.909
4	1433	0.0607	1300	1597	1429	3.70	61	0.643
5	2315	0.0665	1981	2743	2323	7.84	56	0.704
6	3001	0.0658	2743	3429	3065	5.56	70	0.697
7	4483	14.2941	3429	10733	4910	16.69	11257	151.265
8	20000 (UM)	0.1115	17805	26649	20614	5.79	1014	14.155

TIC:	14.6775	ng/uL
TIM:	6.1265	nmole/L
Total concentration:	15.0293	ng/uL

Sample peak width (sec): 5   Sample min peak height: 50   Sample baseline V to V?: Y   Sample baseline V to V points: 3
Sample filter: Binomial   Number of points for filter: 3 Sample start region (min): 0 Sample end region (min): 30
Marker peak width (sec): 5   Marker min peak height: 200   Marker baseline V to V?: Y   Marker baseline V to V points: 3
Lower marker selection: First peak > 500 RFU   Upper marker selection: Last peak > 200 RFU
Ladder size (bp)75, 200, 300, 400, 500, 700, 1000, 1500, 2000, 3000, 4000, 5000, 7000, 10000, 20000
Quantification using: Lower Marker   Final concentration (ng/uL): 0.5000   Dilution factor: 12.0

Sample: SampH11
Well location: H11
Created: Monday, September 20, 2021 7:19:57 PM

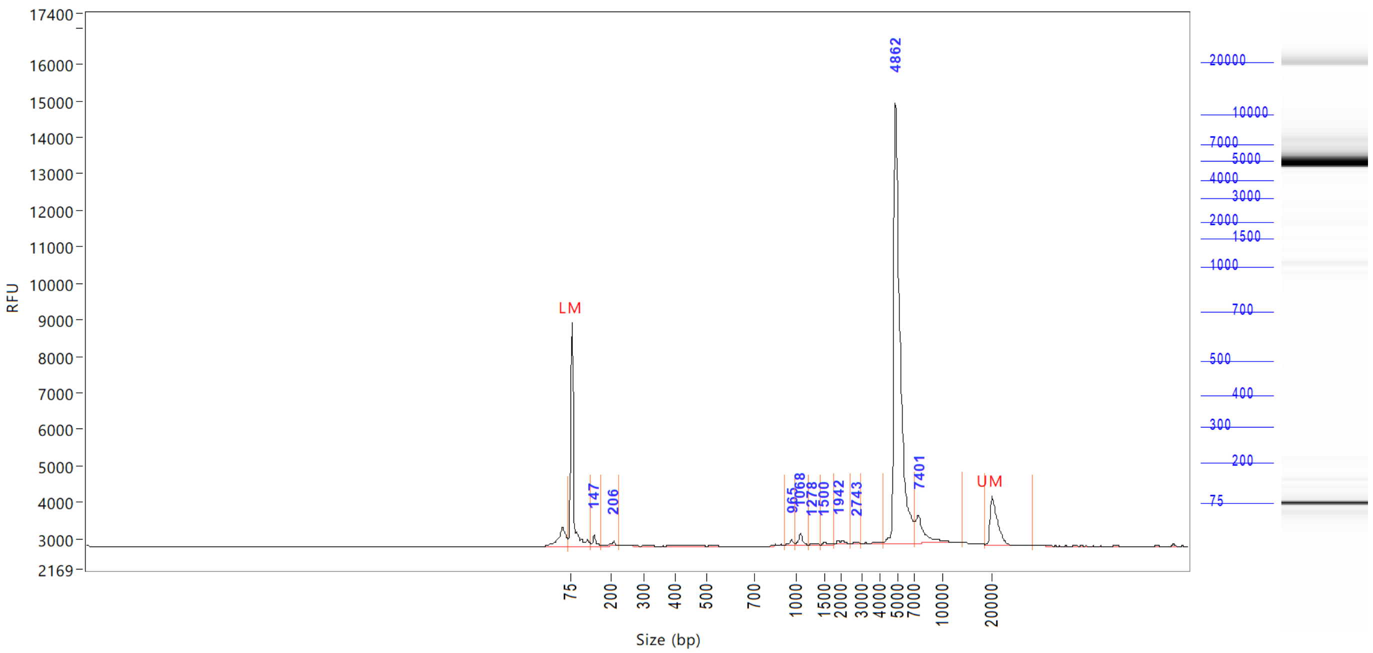

Peak	Size (bp)	Concentration (ng/uL)	From (bp)	To (bp)	Average size (bp)	CV%	RFU	Corrected peak area
1	75 (LM)	0.5000	64	135	78	15.71	6112	40.316
2	147	0.3538	135	168	146	4.70	305	2.377
3	206	0.2126	168	223	196	5.76	125	1.429
4	965	0.1615	913	987	956	1.81	158	1.085
5	1068	0.3165	987	1212	1056	3.99	321	2.126
6	1278	0.0645	1212	1433	1299	3.63	51	0.434
7	1500	0.0858	1433	1789	1554	6.19	75	0.577
8	1942	0.1924	1789	2486	2041	7.54	112	1.293
9	2743	0.0665	2486	2971	2686	3.69	56	0.447
10	4862	16.2639	4208	7000	5118	9.25	12085	109.283
11	7401	1.5377	7000	14025	7993	13.89	742	10.332
12	20000 (UM)	0.1536	18659	28221	20513	4.39	1334	12.386

TIC:	19.2553	ng/uL
TIM:	12.4546	nmole/L
Total concentration:	19.6018	ng/uL

```
Sample peak width (sec): 5    Sample min peak height: 50    Sample baseline V to V?: Y    Sample baseline V to V points: 3
Sample filter: Binomial       Number of points for filter: 3  Sample start region (min): 0  Sample end region (min): 30
Marker peak width (sec): 5    Marker min peak height: 200    Marker baseline V to V?: Y    Marker baseline V to V points: 3
Lower marker selection: First peak > 500 RFU                 Upper marker selection: Last peak > 200 RFU
Ladder size (bp)75, 200, 300, 400, 500, 700, 1000, 1500, 2000, 3000, 4000, 5000, 7000, 10000, 20000
Quantification using: Lower Marker          Final concentration (ng/uL): 0.5000          Dilution factor: 12.0
```

Sample: SampA12
Well location: A12
Created: Monday, September 20, 2021 7:19:57 PM

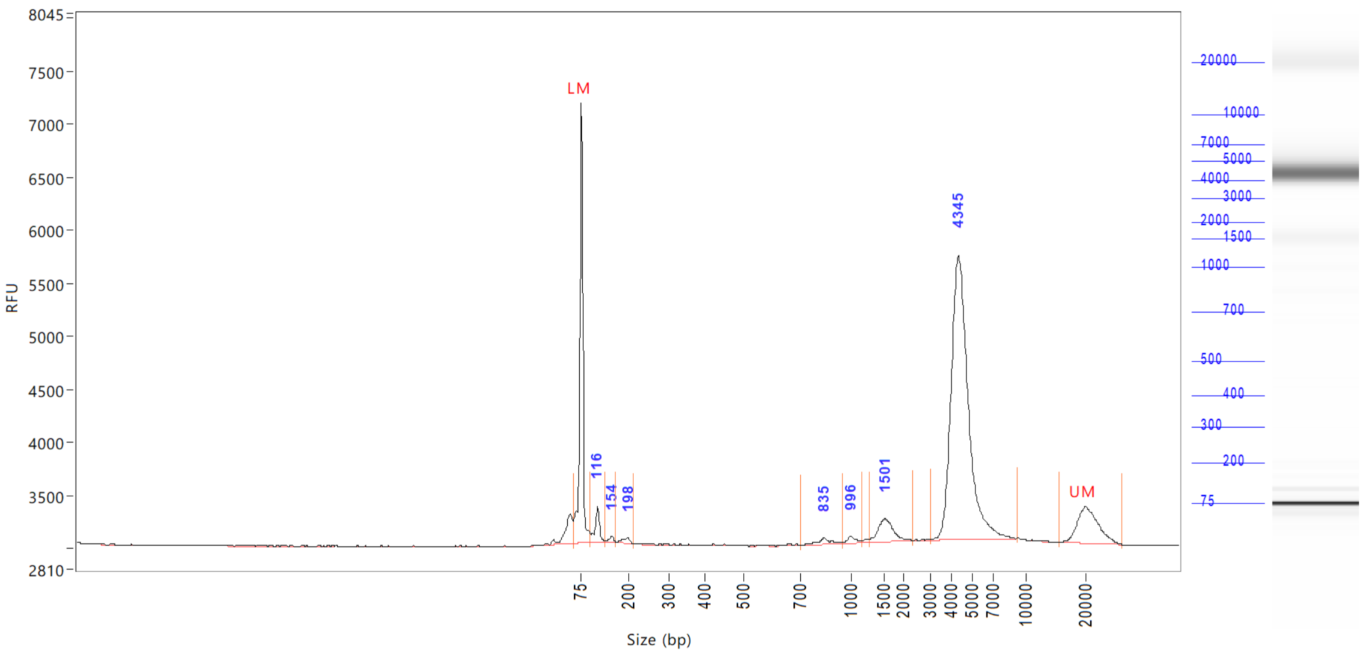

Peak	Size (bp)	Concentration (ng/uL)	From (bp)	To (bp)	Average size (bp)	CV%	RFU	Corrected peak area
1	75 (LM)	0.5000	54	99	72	8.36	4149	31.464
2	116	0.6575	99	137	114	6.08	342	3.448
3	154	0.1059	137	166	151	3.84	56	0.555
4	198	0.1548	166	212	189	4.69	54	0.812
5	835	0.2445	705	957	842	6.73	62	1.282
6	996	0.1648	957	1178	1032	5.93	66	0.864
7	1501	0.8983	1267	2344	1559	11.10	214	4.711
8	4345	11.4604	3072	9267	4518	14.18	2672	60.099
9	20000 (UM)	0.1315	15610	26287	20569	8.44	345	8.274

TIC:	13.6862	ng/uL
TIM:	17.8373	nmole/L
Total concentration:	14.0055	ng/uL

Sample peak width (sec): 5   Sample min peak height: 50   Sample baseline V to V?: Y   Sample baseline V to V points: 3
Sample filter: Binomial   Number of points for filter: 3 Sample start region (min): 0 Sample end region (min): 30
Marker peak width (sec): 5   Marker min peak height: 200   Marker baseline V to V?: Y   Marker baseline V to V points: 3
Lower marker selection: First peak > 500 RFU   Upper marker selection: Last peak > 200 RFU
Ladder size (bp)75, 200, 300, 400, 500, 700, 1000, 1500, 2000, 3000, 4000, 5000, 7000, 10000, 20000
Quantification using: Lower Marker   Final concentration (ng/uL): 0.5000   Dilution factor: 12.0

Sample: SampB12
Well location: B12
Created: Monday, September 20, 2021 7:19:57 PM

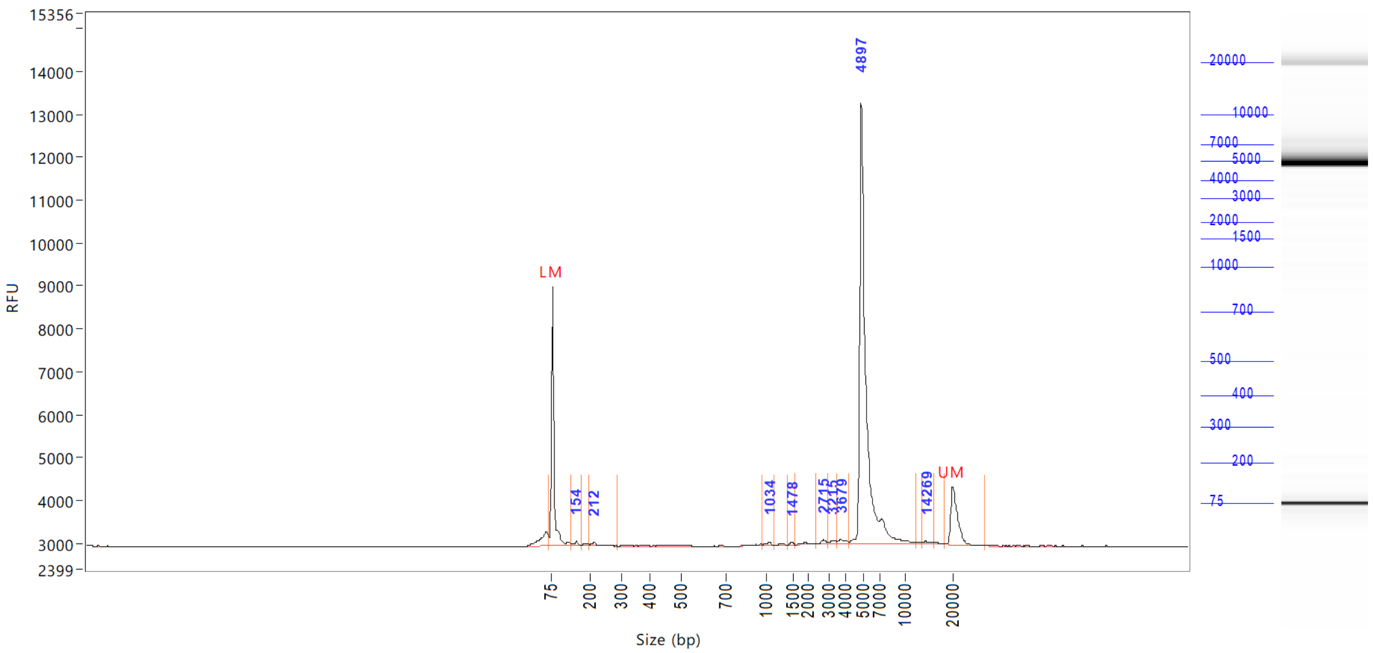

Peak	Size (bp)	Concentration (ng/uL)	From (bp)	To (bp)	Average size (bp)	CV%	RFU	Corrected peak area
1	75 (LM)	0.5000	64	135	76	13.27	6044	39.045
2	154	0.1145	135	169	148	4.35	87	0.745
3	212	0.1100	196	284	217	9.20	79	0.716
4	1034	0.0796	974	1145	1022	2.88	82	0.518
5	1478	0.0489	1389	1559	1465	1.74	65	0.318
6	2715	0.1099	2344	2914	2692	4.30	91	0.715
7	3215	0.1192	2914	3465	3180	4.93	76	0.775
8	3679	0.1664	3465	4173	3787	5.19	98	1.083
9	4897	14.7833	4173	12196	5372	18.18	10286	96.201
10	14269	0.0878	13293	15976	14488	5.32	57	0.571
11	20000 (UM)	0.1593	18293	26770	20421	4.21	1359	12.436

TIC:	15.6195	ng/uL
TIM:	7.0295	nmole/L
Total concentration:	16.0398	ng/uL

Sample peak width (sec): 5 Sample min peak height: 50 Sample baseline V to V?: Y Sample baseline V to V points: 3
Sample filter: Binomial Number of points for filter: 3 Sample start region (min): 0 Sample end region (min): 30
Marker peak width (sec): 5 Marker min peak height: 200 Marker baseline V to V?: Y Marker baseline V to V points: 3
Lower marker selection: First peak > 500 RFU Upper marker selection: Last peak > 200 RFU
Ladder size (bp)75, 200, 300, 400, 500, 700, 1000, 1500, 2000, 3000, 4000, 5000, 7000, 10000, 20000
Quantification using: Lower Marker Final concentration (ng/uL): 0.5000 Dilution factor: 12.0

Sample: SampC12
Well location: C12
Created: Monday, September 20, 2021 7:19:57 PM

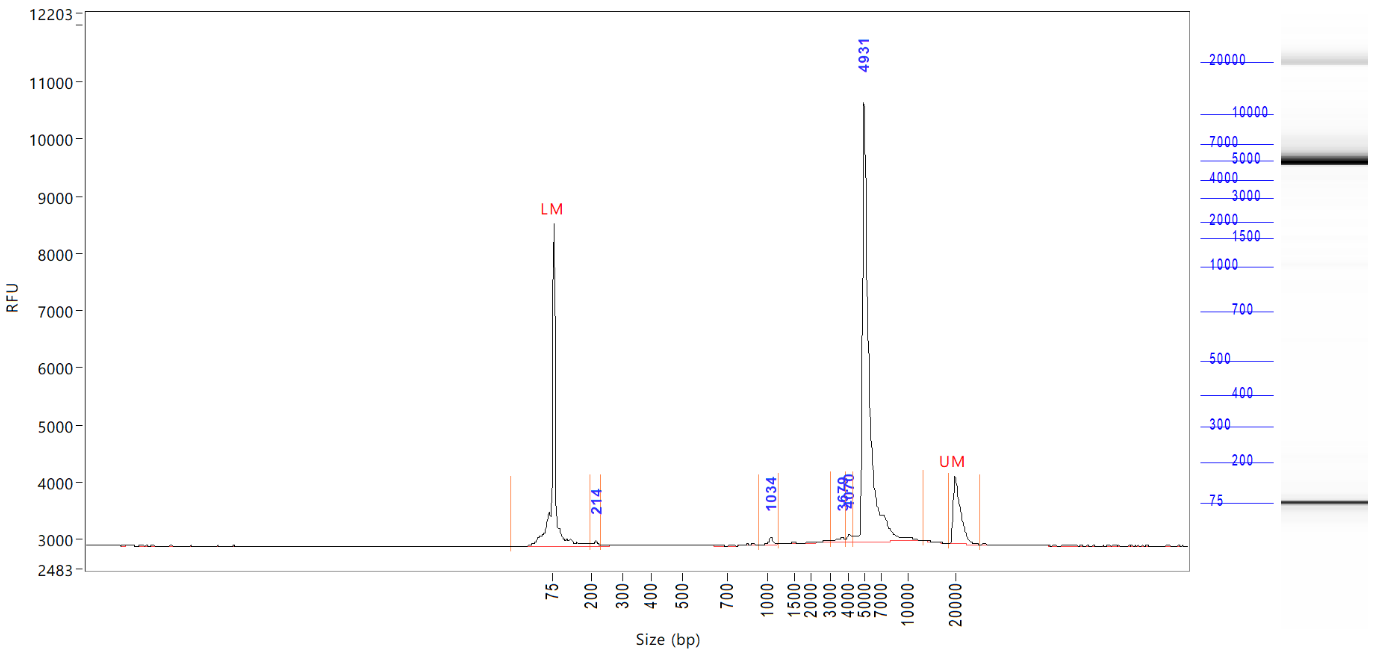

Peak	Size (bp)	Concentration (ng/uL)	From (bp)	To (bp)	Average size (bp)	CV%	RFU	Corrected peak area
1	75 (LM)	0.5000	0	196	73	35.91	5635	51.357
2	214	0.1138	196	230	210	3.82	80	0.974
3	1034	0.1085	935	1190	1034	4.29	122	0.929
4	3679	0.1322	3000	3857	3459	7.26	81	1.131
5	4070	0.1203	3857	4277	4074	2.86	132	1.030
6	4931	9.0435	4277	13415	5461	18.22	7690	77.408
7	20000 (UM)	0.1125	18537	25440	20579	4.51	1179	11.559

TIC:	9.5184	ng/uL
TIM:	3.9020	nmole/L
Total concentration:	9.8414	ng/uL

```
Sample peak width (sec): 5    Sample min peak height: 50    Sample baseline V to V?: Y    Sample baseline V to V points: 3
Sample filter: Binomial       Number of points for filter: 3  Sample start region (min): 0  Sample end region (min): 30
Marker peak width (sec): 5    Marker min peak height: 200   Marker baseline V to V?: Y    Marker baseline V to V points: 3
Lower marker selection: First peak > 500 RFU                Upper marker selection: Last peak > 200 RFU
Ladder size (bp)75, 200, 300, 400, 500, 700, 1000, 1500, 2000, 3000, 4000, 5000, 7000, 10000, 20000
Quantification using: Lower Marker          Final concentration (ng/uL): 0.5000          Dilution factor: 12.0
```

Sample: SampD12
Well location: D12
Created: Monday, September 20, 2021 7:19:57 PM

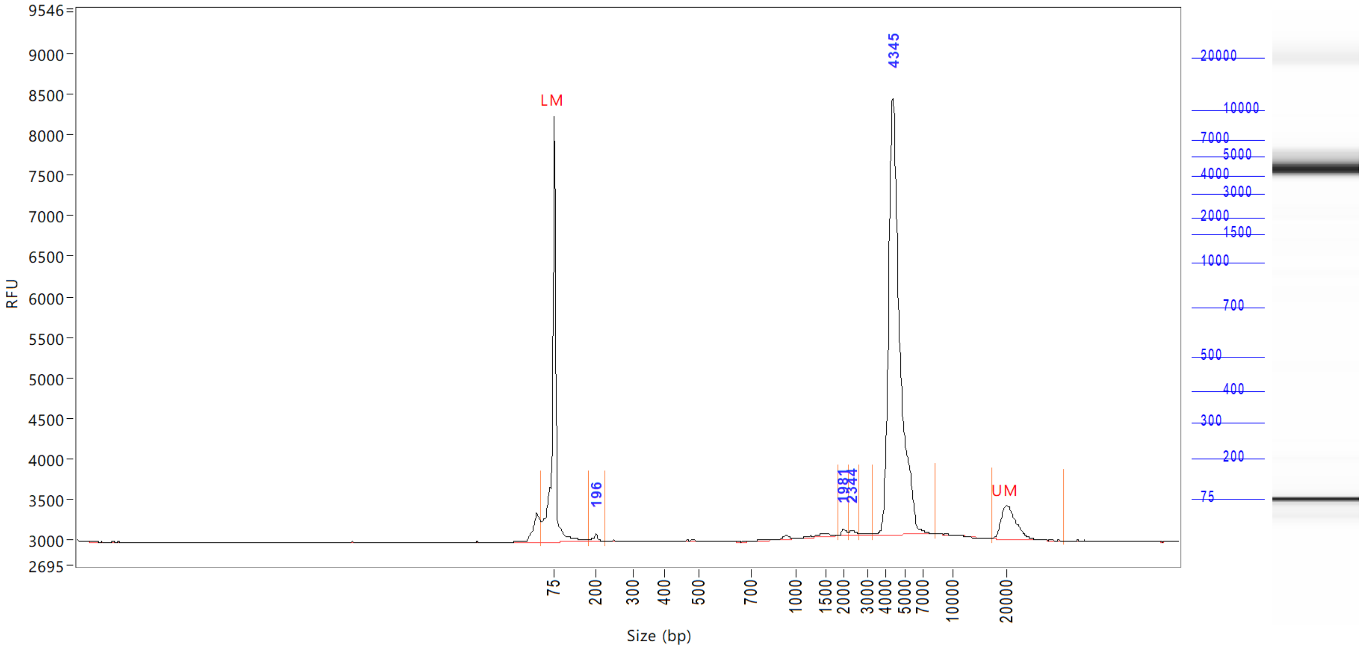

Peak	Size (bp)	Concentration (ng/uL)	From (bp)	To (bp)	Average size (bp)	CV%	RFU	Corrected peak area
1	75 (LM)	0.5000	37	175	72	23.07	5237	46.670
2	196	0.1477	175	223	193	5.17	88	1.149
3	1981	0.0834	1846	2201	2027	4.16	83	0.649
4	2344	0.0817	2201	2686	2388	5.37	65	0.635
5	4345	11.1326	3286	8201	4496	9.66	5379	86.592
6	20000 (UM)	0.0841	17195	30639	20619	7.76	415	7.847

TIC:	11.4454	ng/uL
TIM:	5.4589	nmole/L
Total concentration:	11.9485	ng/uL

```
Sample peak width (sec): 5    Sample min peak height: 50    Sample baseline V to V?: Y    Sample baseline V to V points: 3
Sample filter: Binomial       Number of points for filter: 3  Sample start region (min): 0  Sample end region (min): 30
Marker peak width (sec): 5    Marker min peak height: 200   Marker baseline V to V?: Y    Marker baseline V to V points: 3
Lower marker selection: First peak > 500 RFU                Upper marker selection: Last peak > 200 RFU
Ladder size (bp)75, 200, 300, 400, 500, 700, 1000, 1500, 2000, 3000, 4000, 5000, 7000, 10000, 20000
Quantification using: Lower Marker          Final concentration (ng/uL): 0.5000         Dilution factor: 12.0
```

Sample: SampE12
Well location: E12
Created: Monday, September 20, 2021 7:19:57 PM

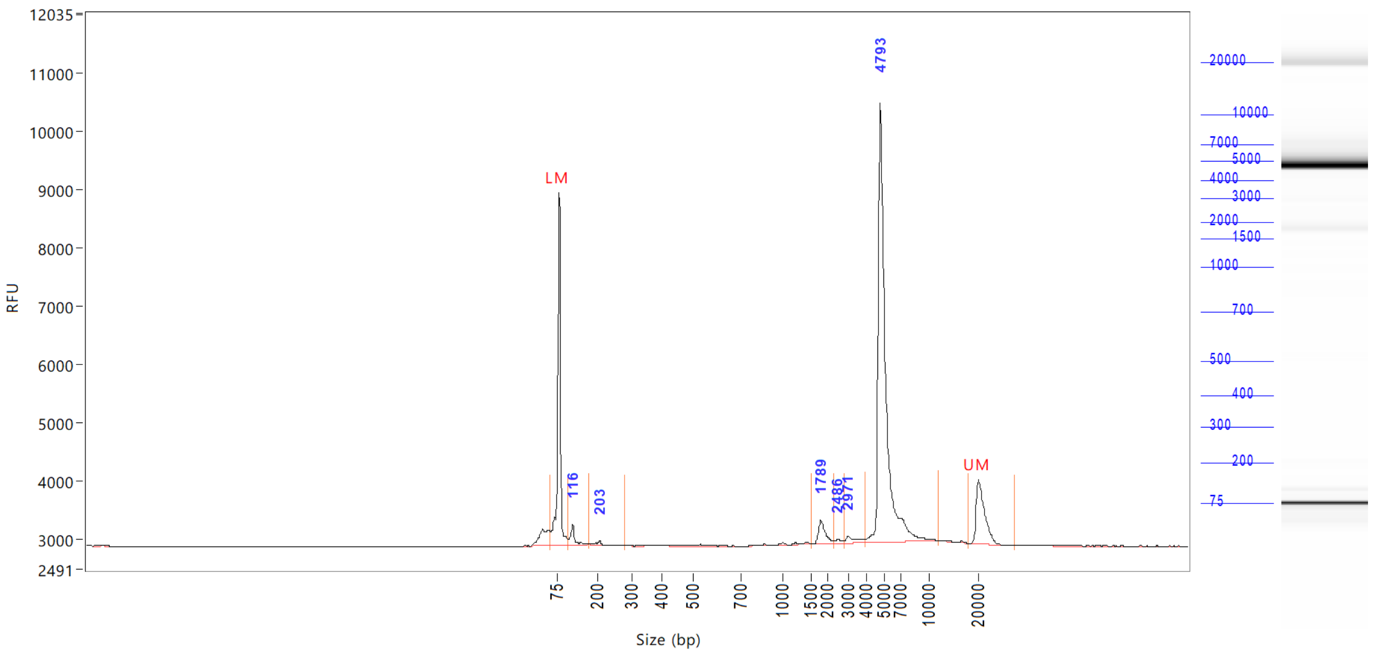

Peak	Size (bp)	Concentration (ng/uL)	From (bp)	To (bp)	Average size (bp)	CV%	RFU	Corrected peak area
1	75 (LM)	0.5000	48	103	73	10.29	6056	41.272
2	116	0.5061	103	171	118	10.53	341	3.481
3	203	0.1203	171	278	202	10.38	67	0.828
4	1789	0.6502	1501	2315	1849	7.74	405	4.472
5	2486	0.0986	2315	2772	2522	5.18	64	0.678
6	2971	0.2753	2772	3929	3256	10.24	114	1.894
7	4793	11.6449	3929	11952	5150	16.48	7555	80.101
8	20000 (UM)	0.1480	17927	27254	20546	4.84	1107	12.213

TIC:	13.2954	ng/uL
TIM:	12.5295	nmole/L
Total concentration:	13.7040	ng/uL

```
Sample peak width (sec): 5    Sample min peak height: 50    Sample baseline V to V?: Y    Sample baseline V to V points: 3
Sample filter: Binomial       Number of points for filter: 3  Sample start region (min): 0  Sample end region (min): 30
Marker peak width (sec): 5    Marker min peak height: 200   Marker baseline V to V?: Y    Marker baseline V to V points: 3
Lower marker selection: First peak > 500 RFU                Upper marker selection: Last peak > 200 RFU
Ladder size (bp)75, 200, 300, 400, 500, 700, 1000, 1500, 2000, 3000, 4000, 5000, 7000, 10000, 20000
Quantification using: Lower Marker          Final concentration (ng/uL): 0.5000          Dilution factor: 12.0
```

Sample: SampF12
Well location: F12
Created: Monday, September 20, 2021 7:19:57 PM

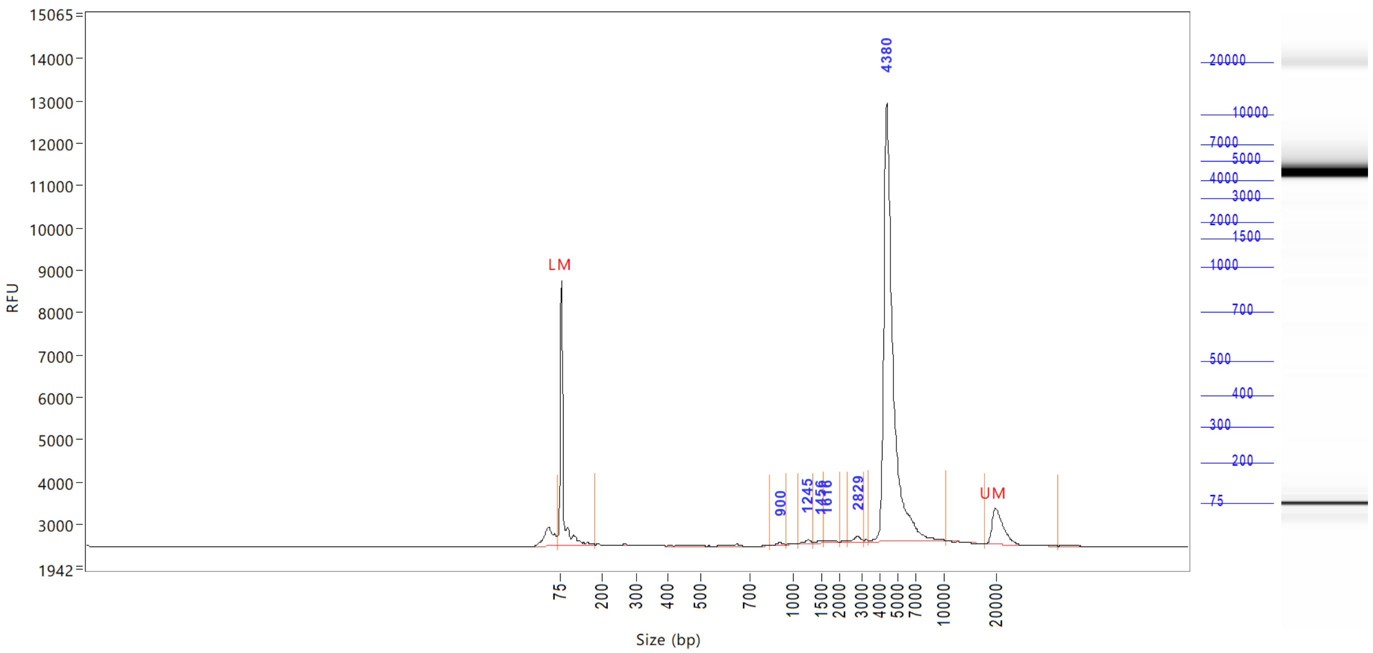

Peak	Size (bp)	Concentration (ng/uL)	From (bp)	To (bp)	Average size (bp)	CV%	RFU	Corrected peak area
1	75 (LM)	0.5000	64	177	79	19.67	6261	45.482
2	900	0.0755	831	944	895	2.05	59	0.573
3	1245	0.1357	1068	1356	1222	5.35	90	1.029
4	1456	0.0729	1356	1501	1440	2.90	56	0.553
5	1616	0.1035	1501	2001	1686	7.55	55	0.785
6	2829	0.1872	2344	3108	2770	6.20	119	1.419
7	4380	18.3173	3358	10245	4655	14.90	10368	138.852
8	20000 (UM)	0.1329	17805	32211	20624	6.41	848	12.089

TIC:	18.8921	ng/uL
TIM:	7.0954	nmole/L
Total concentration:	19.2705	ng/uL

```
Sample peak width (sec): 5     Sample min peak height: 50     Sample baseline V to V?: Y     Sample baseline V to V points: 3
Sample filter: Binomial        Number of points for filter: 3  Sample start region (min): 0  Sample end region (min): 30
Marker peak width (sec): 5     Marker min peak height: 200    Marker baseline V to V?: Y     Marker baseline V to V points: 3
Lower marker selection: First peak > 500 RFU                  Upper marker selection: Last peak > 200 RFU
Ladder size (bp)75, 200, 300, 400, 500, 700, 1000, 1500, 2000, 3000, 4000, 5000, 7000, 10000, 20000
Quantification using: Lower Marker          Final concentration (ng/uL): 0.5000          Dilution factor: 12.0
```

Sample: SampG12
Well location: G12
Created: Monday, September 20, 2021 7:19:57 PM

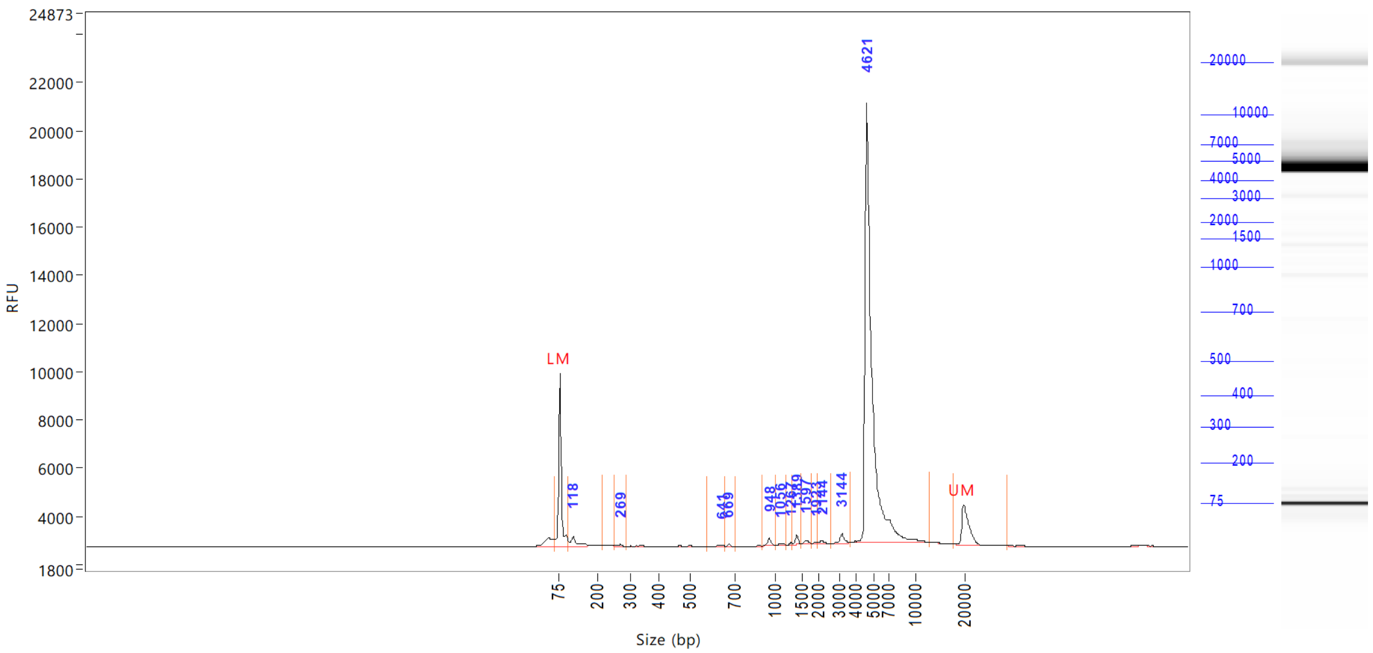

Peak	Size (bp)	Concentration (ng/uL)	From (bp)	To (bp)	Average size (bp)	CV%	RFU	Corrected peak area
1	75 (LM)	0.5000	62	105	74	9.85	7205	48.206
2	118	0.7890	105	212	130	16.99	431	6.339
3	269	0.0457	251	287	267	1.86	71	0.367
4	641	0.0788	572	654	623	3.16	51	0.633
5	669	0.0740	654	700	668	1.12	115	0.595
6	948	0.2555	896	1001	945	1.91	337	2.053
7	1056	0.0856	1001	1190	1079	4.05	88	0.688
8	1267	0.0515	1190	1300	1260	1.91	89	0.414
9	1389	0.2551	1300	1478	1375	2.20	395	2.049
10	1597	0.1325	1478	1789	1601	4.05	157	1.064
11	1923	0.0359	1789	1981	1897	2.67	56	0.288
12	2144	0.1099	1981	2600	2156	5.66	112	0.883
13	3144	0.3529	2600	3643	3144	5.78	401	2.835
14	4621	23.4281	3643	12806	5040	18.36	18287	188.227
15	20000 (UM)	0.1621	17805	29068	20485	4.58	1657	15.631

TIC:	25.6945	ng/uL
TIM:	19.6810	nmole/L
Total concentration:	25.9051	ng/uL

```
Sample peak width (sec): 5     Sample min peak height: 50     Sample baseline V to V?: Y     Sample baseline V to V points: 3
Sample filter: Binomial        Number of points for filter: 3  Sample start region (min): 0  Sample end region (min): 30
Marker peak width (sec): 5     Marker min peak height: 200    Marker baseline V to V?: Y     Marker baseline V to V points: 3
Lower marker selection: First peak > 500 RFU                  Upper marker selection: Last peak > 200 RFU
Ladder size (bp)75, 200, 300, 400, 500, 700, 1000, 1500, 2000, 3000, 4000, 5000, 7000, 10000, 20000
Quantification using: Lower Marker          Final concentration (ng/uL): 0.5000          Dilution factor: 12.0
```

Sample: SampH12
Well location: H12
Created: Monday, September 20, 2021 7:19:57 PM

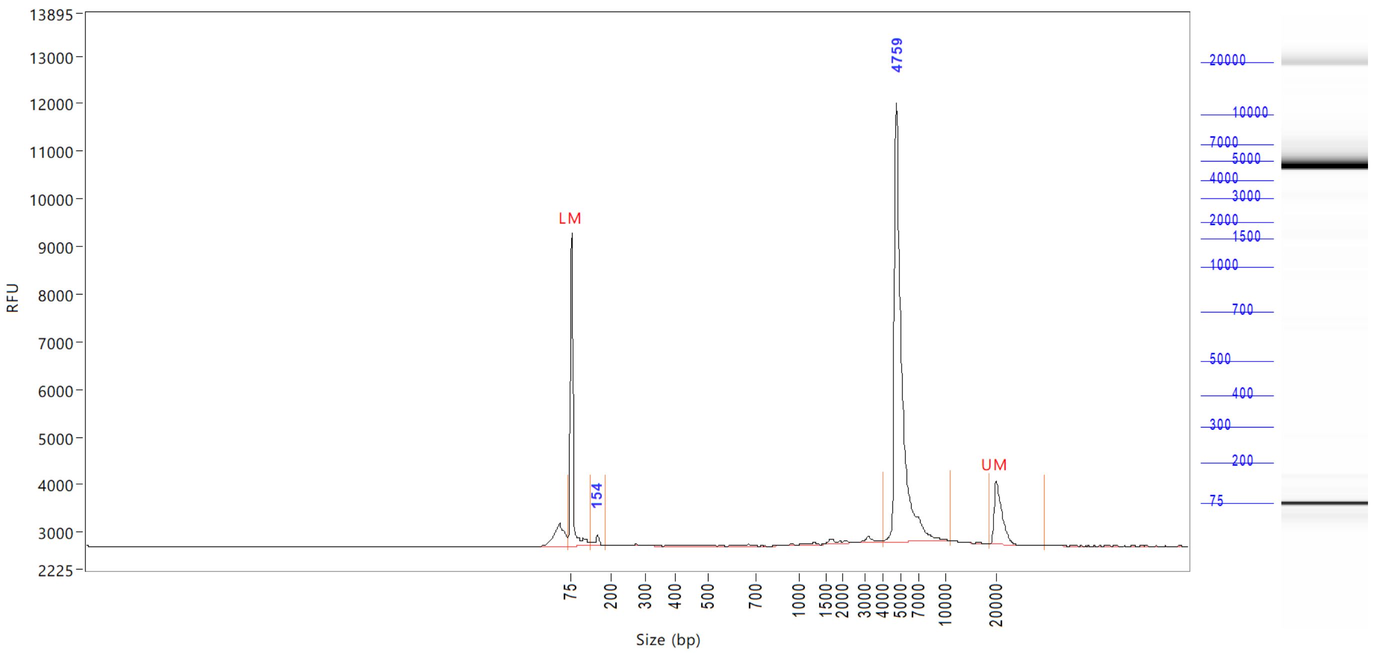

Peak	Size (bp)	Concentration (ng/uL)	From (bp)	To (bp)	Average size (bp)	CV%	RFU	Corrected peak area
1	75 (LM)	0.5000	64	135	76	14.90	6567	40.718
2	154	0.3097	135	179	151	5.47	218	2.102
3	4759	14.2577	4001	10733	5125	15.26	9228	96.758
4	20000 (UM)	0.1603	18537	29551	20535	4.70	1313	13.054

TIC:	14.5674	ng/uL
TIM:	7.9510	nmole/L
Total concentration:	15.5503	ng/uL

```
Sample peak width (sec): 5    Sample min peak height: 200   Sample baseline V to V?: Y    Sample baseline V to V points: 3
Sample filter: Binomial       Number of points for filter: 3  Sample start region (min): 0  Sample end region (min): 30
Marker peak width (sec): 5    Marker min peak height: 200   Marker baseline V to V?: Y    Marker baseline V to V points: 3
Lower marker selection: First peak > 500 RFU                Upper marker selection: Last peak > 200 RFU
Ladder size (bp)75, 200, 300, 400, 500, 700, 1000, 1500, 2000, 3000, 4000, 5000, 7000, 10000, 20000
Quantification using: Lower Marker          Final concentration (ng/uL): 0.5000         Dilution factor: 12.0
```

Sample: DNA size ladder
Well location: A7
Created: Monday, September 20, 2021 7:19:57 PM
Import from: C:\Users\admin\Desktop\210810_alberto_ladder.SCAL
Fit type: Point to point

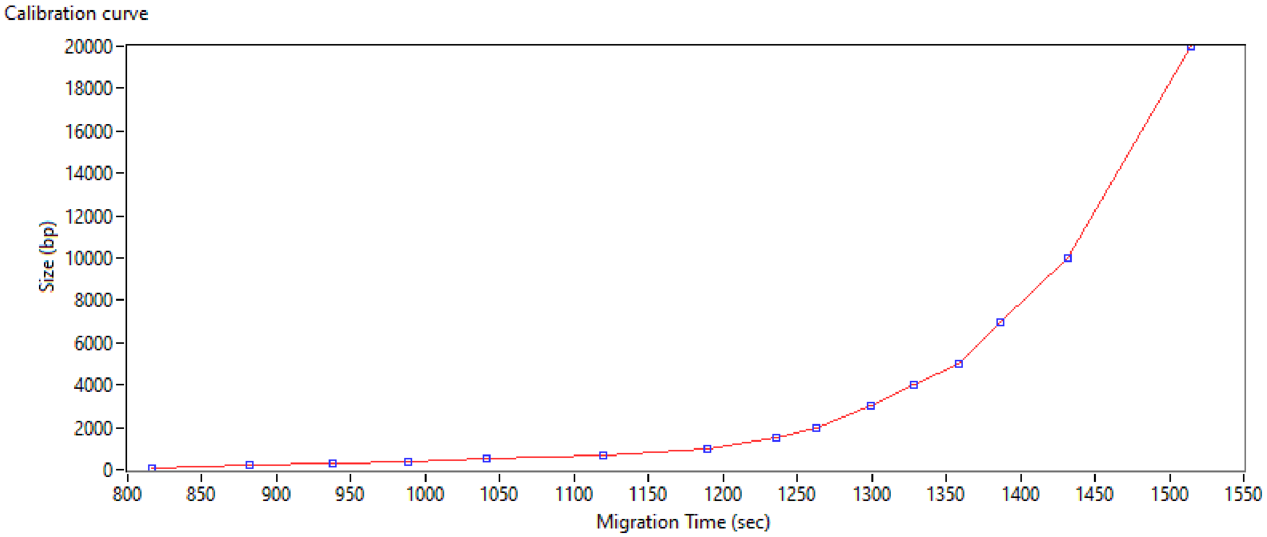

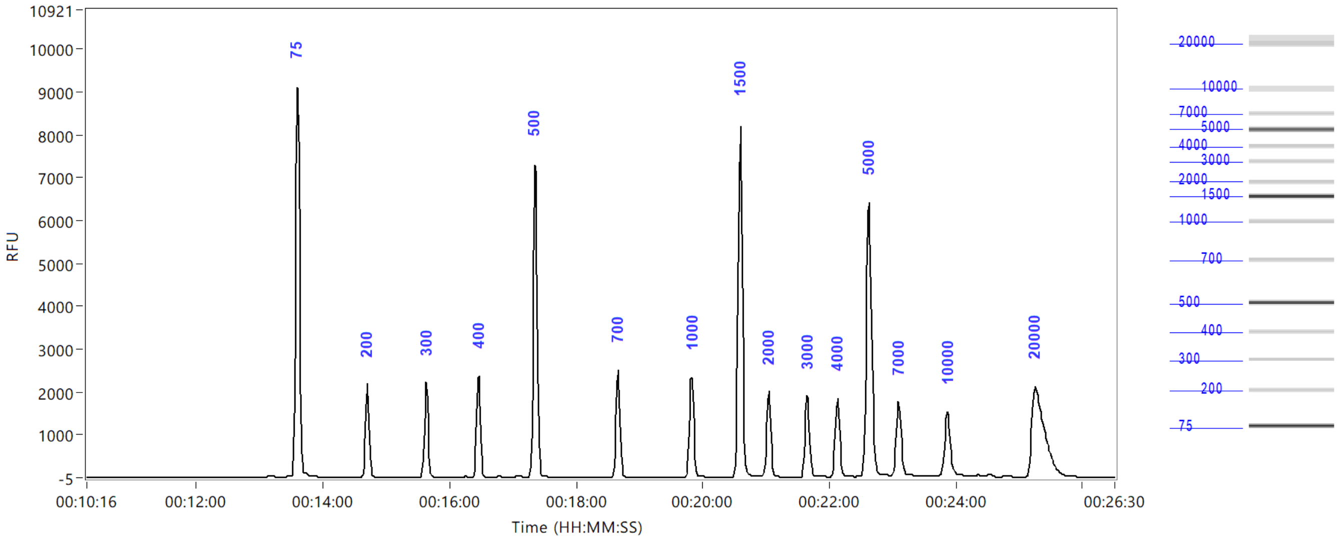

Supplement: Supplementary file 2 — sb3c00292_si_002.zip [file sb3c00292_si_002.zip › dnada_supplementary_material_pks_library_build/ZAG_trials/ZAG_Trial_6/19-10-17_plate3/plate3-t2.pdf]
