# Supplementary material for: Automated Platform for the Plasmid Construction Process
Source: ACS Synth Biol. 2023 Nov 10;12(12):3506–13. doi: 10.1021/acssynbio.3c00292 (PMC10729297; doi:10.1021/acssynbio.3c00292)

Instrument controller software run summary:

Filename and data path: C:\AATI\Data\2021 09 20\18-30-13\2021 09 20 18H 30M..raw
Created: Monday, September 20, 2021 6:40:06 PM
Number of capillaries: 96
Array serial number: 111920-09SFS
Effect length: 33 cm
Array usage count: 224
Instrument type: ZAG DNA Analyzer
Instrument controller software version: 1.0.1.9
Device serial number: Z7597

Method Information

Method name: ZAG130GP33 - DNA 75-20000bp - Gel Prime Only.mthds
Gel prime: Yes
Full conditioning: No
Gel prime to buffer: No
Gel selection: Gel 1
Perform prerun: 8.0 kV, 30 sec.
Rinse: No
Marker 1: Row: A, 5.0 kV, 5 sec.
Rinse: No
Sample injection: 5.0 kV, 5 sec.
Separation: 8.0 kV, 30.0 min.
Tray name: Sample Tray-2

Analysis mode: DNA

Notes

Gel Image

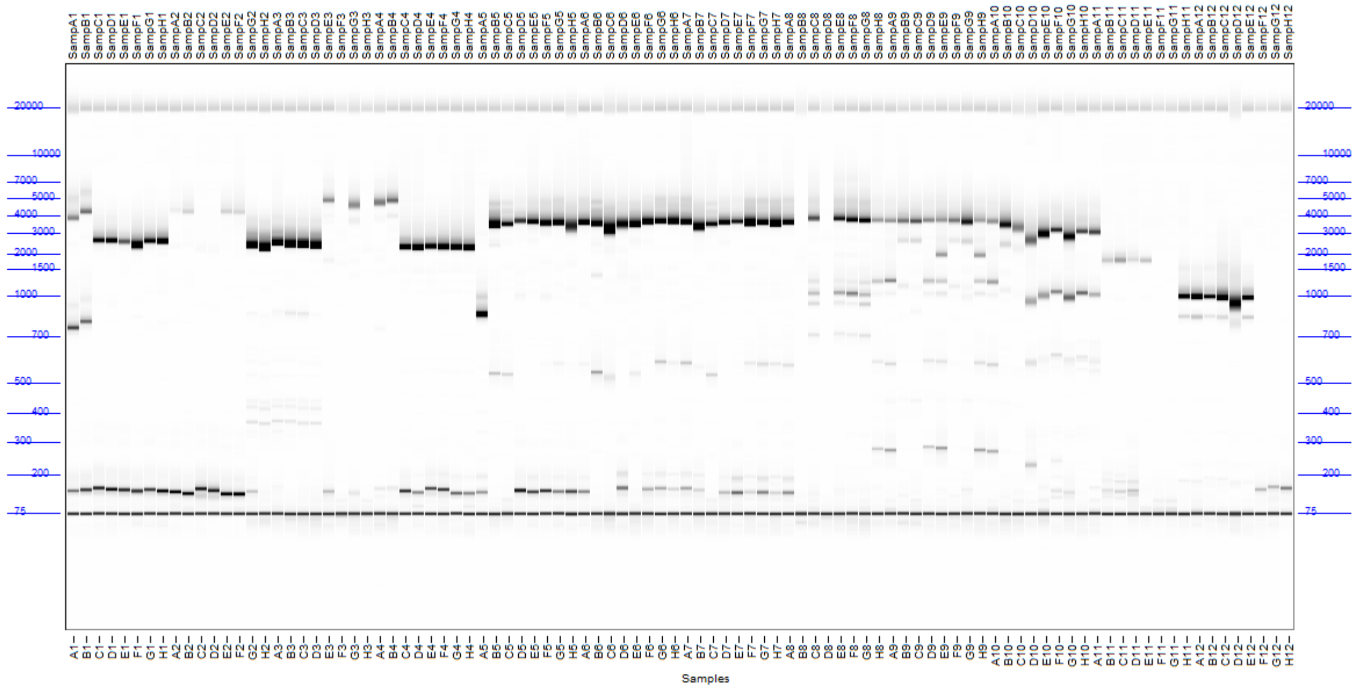

Filename and data path: C:\AATI\Data\2021 09 20\18-30-13\2021 09 20 18H 30M..raw

A1: SampA1

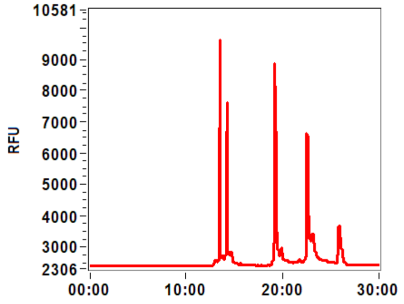

B1: SampB1

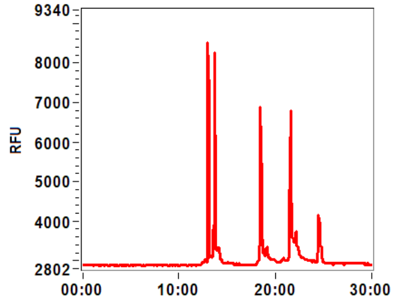

C1: SampC1

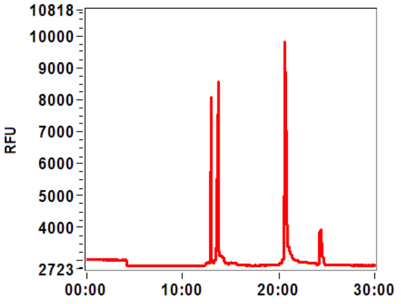

D1: SampD1

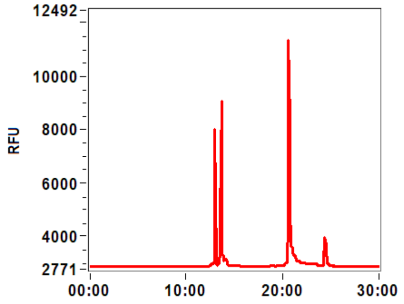

E1: SampE1

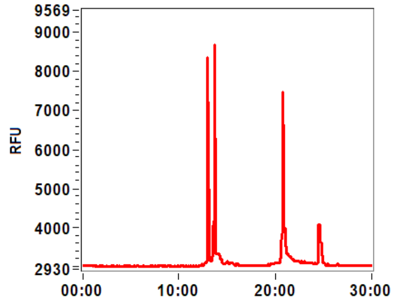

F1: SampF1

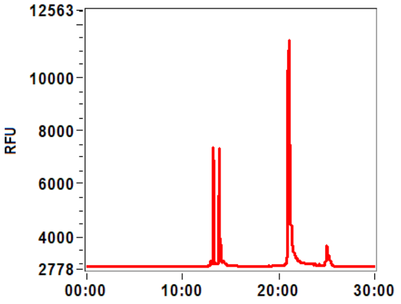

G1: SampG1

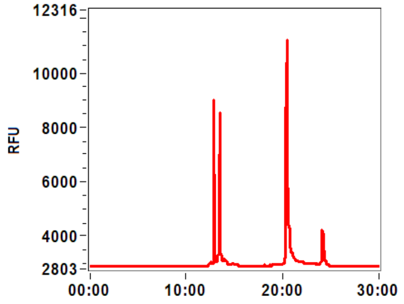

H1: SampH1

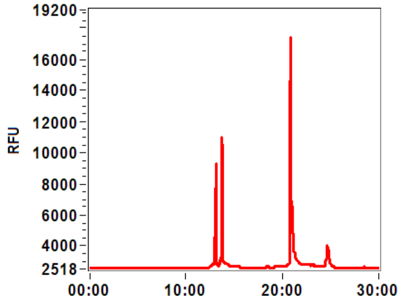

A2: SampA2

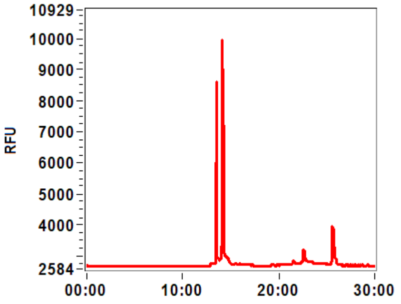

B2: SampB2

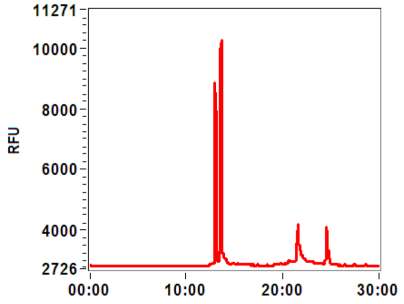

C2: SampC2

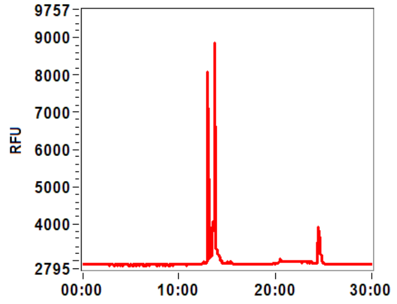

D2: SampD2

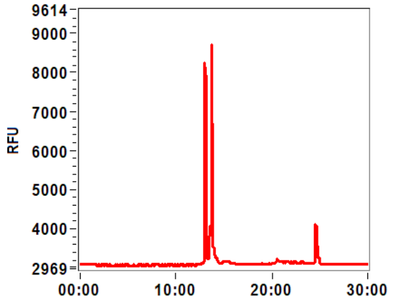

Filename and data path: C:\AATI\Data\2021 09 20\18-30-13\2021 09 20 18H 30M..raw

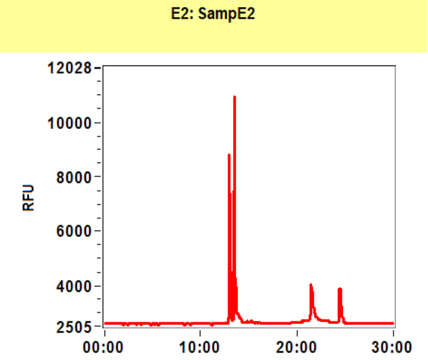

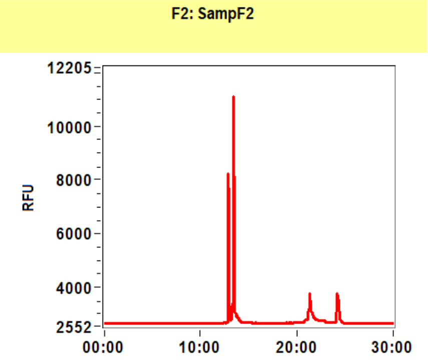

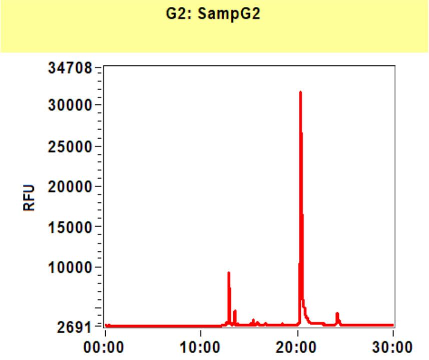

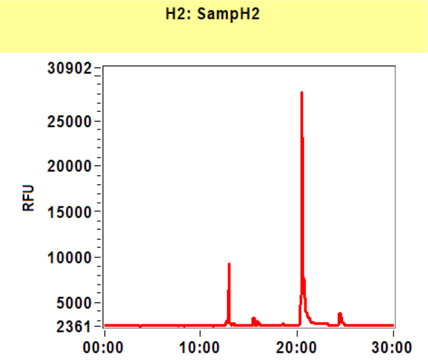

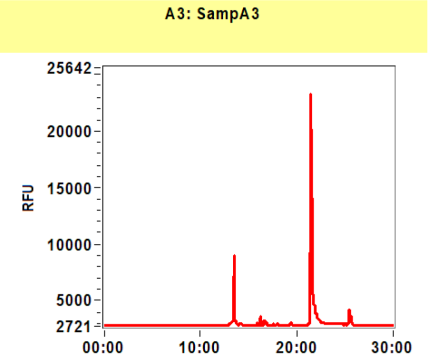

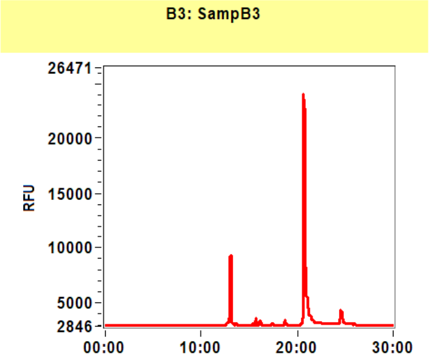

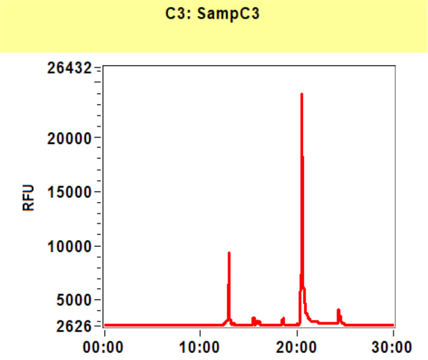

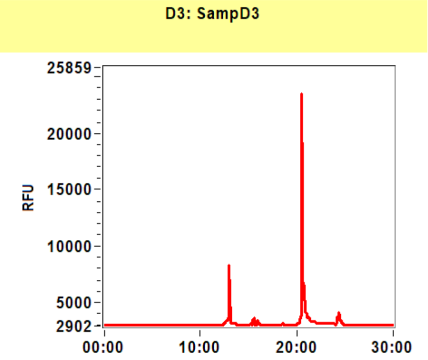

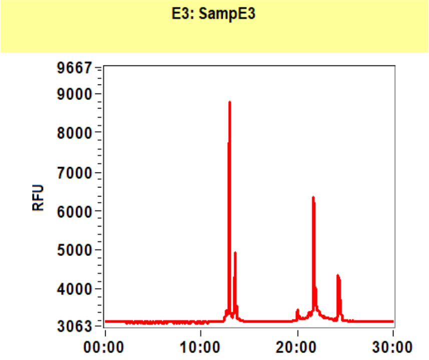

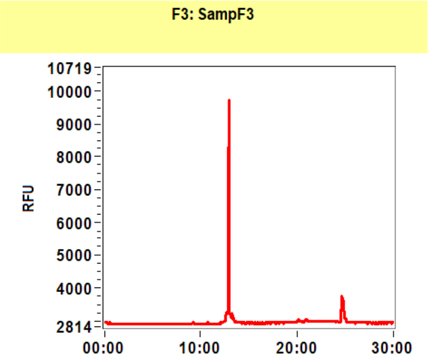

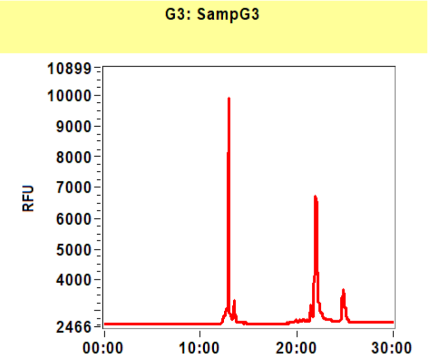

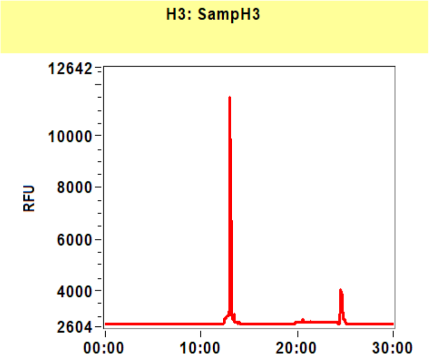

Filename and data path: C:\AATI\Data\2021 09 20\18-30-13\2021 09 20 18H 30M..raw

A4: SampA4

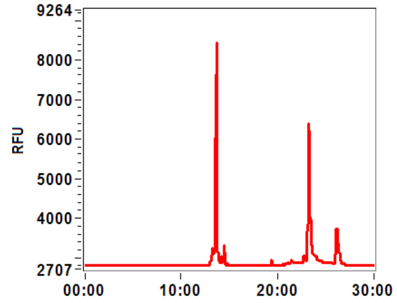

B4: SampB4

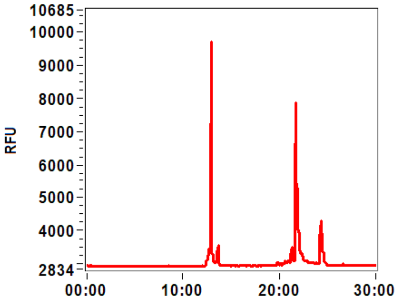

C4: SampC4

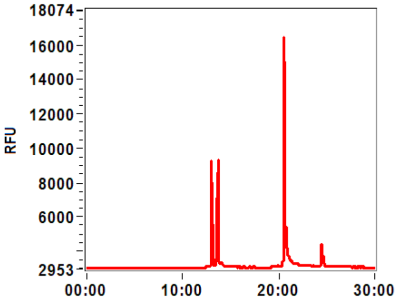

D4: SampD4

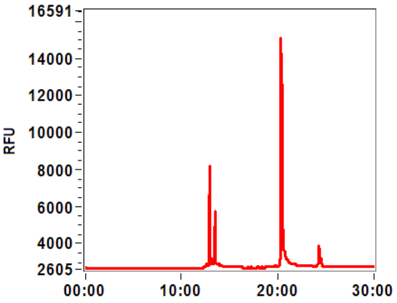

E4: SampE4

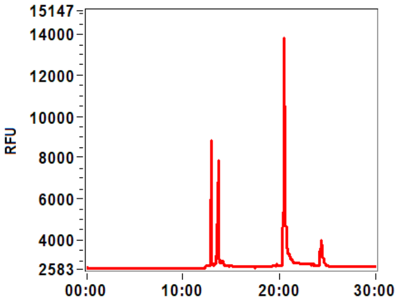

F4: SampF4

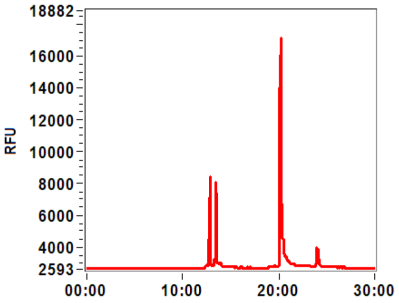

G4: SampG4

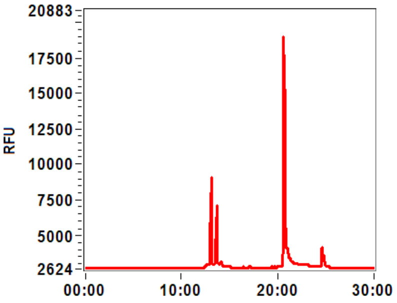

H4: SampH4

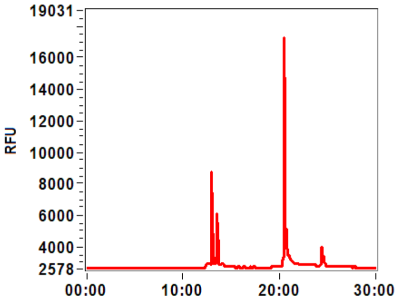

A5: SampA5

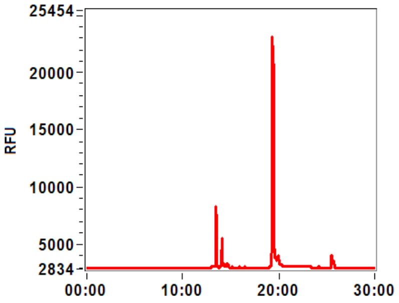

B5: SampB5

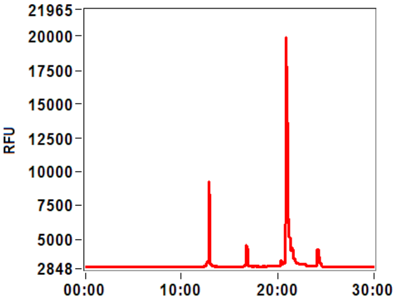

C5: SampC5

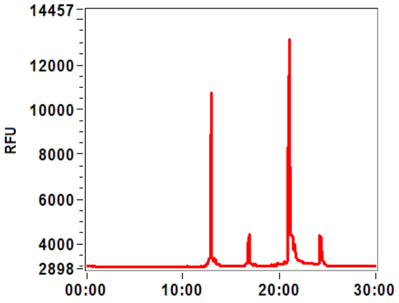

D5: SampD5

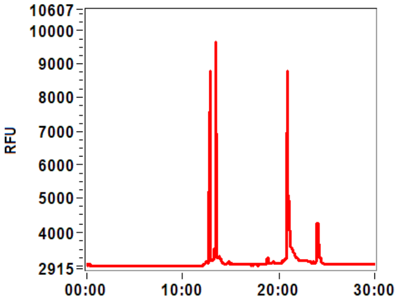

Filename and data path: C:\AATI\Data\2021 09 20\18-30-13\2021 09 20 18H 30M..raw

E5: SampE5

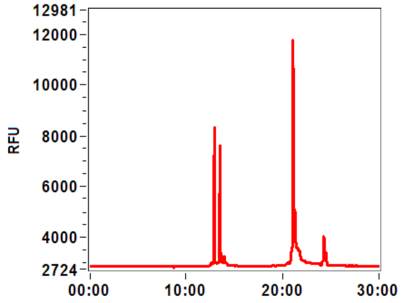

F5: SampF5

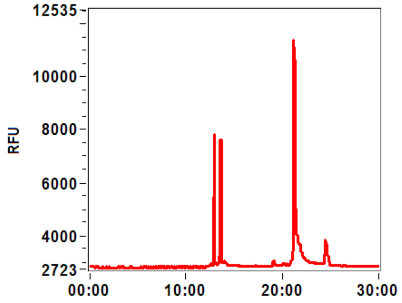

G5: SampG5

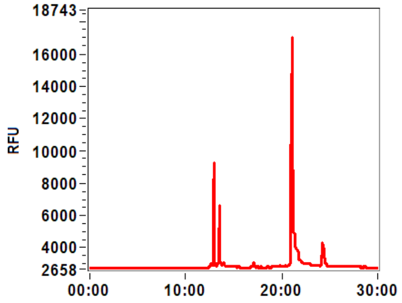

H5: SampH5

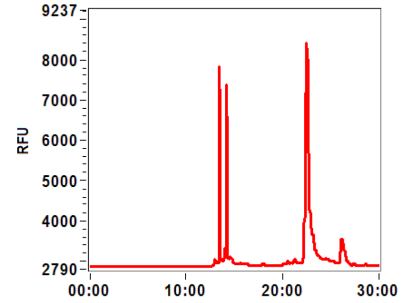

A6: SampA6

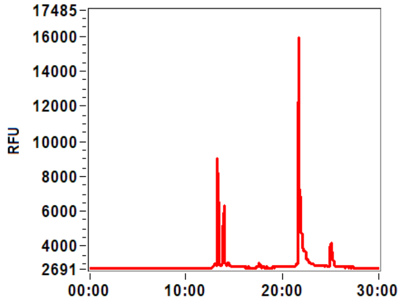

B6: SampB6

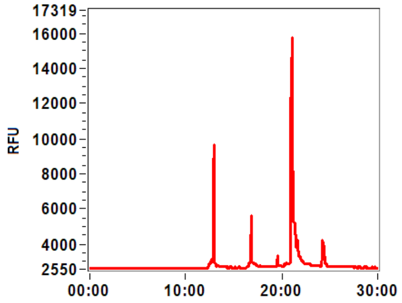

C6: SampC6

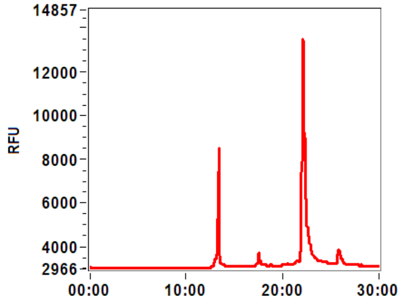

D6: SampD6

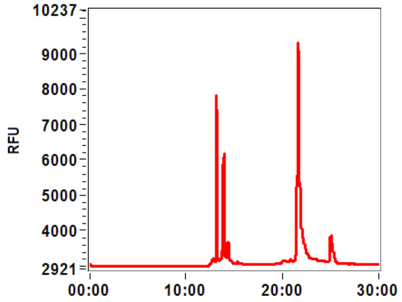

E6: SampE6

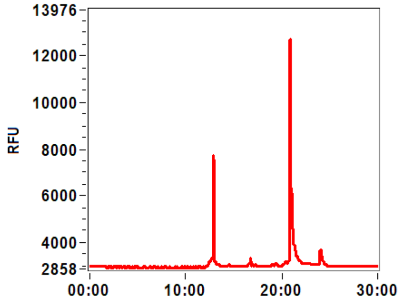

F6: SampF6

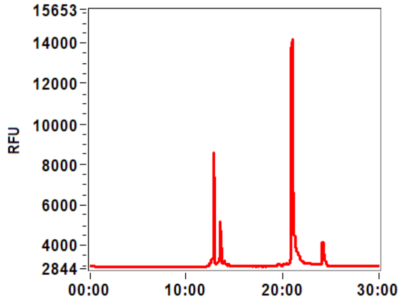

G6: SampG6

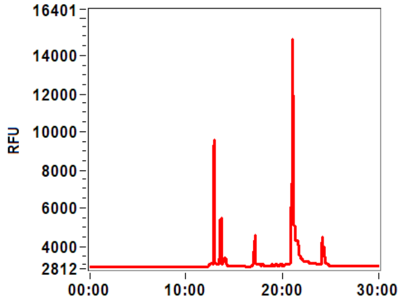

H6: SampH6

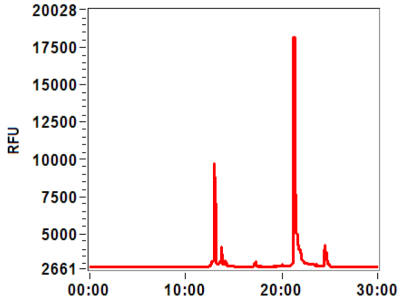

Filename and data path: C:\AATI\Data\2021 09 20\18-30-13\2021 09 20 18H 30M..raw

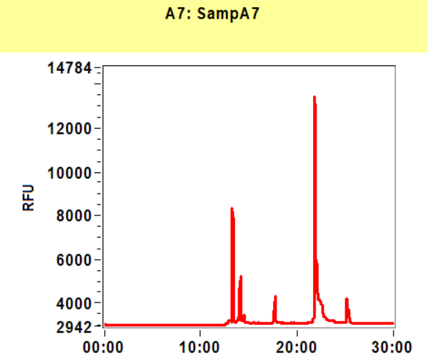

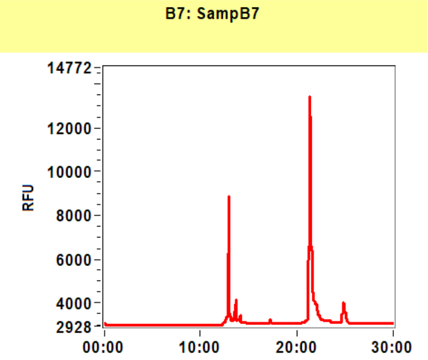

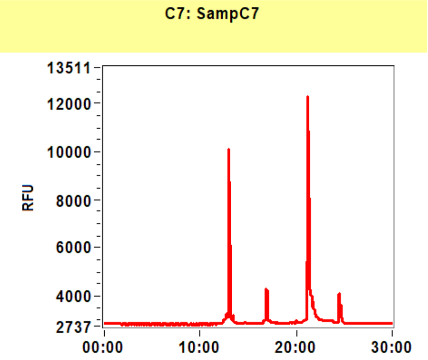

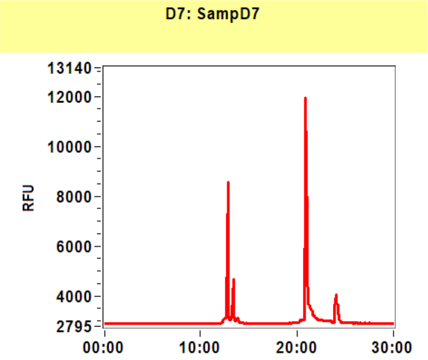

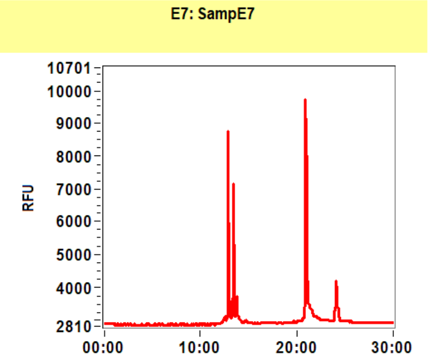

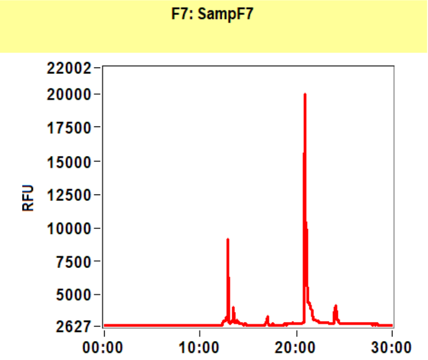

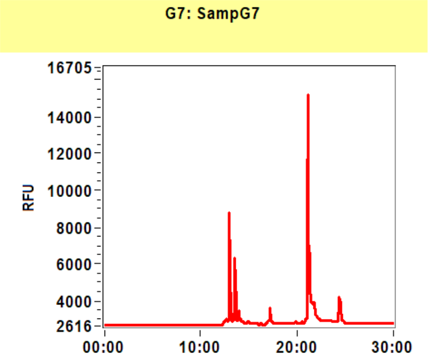

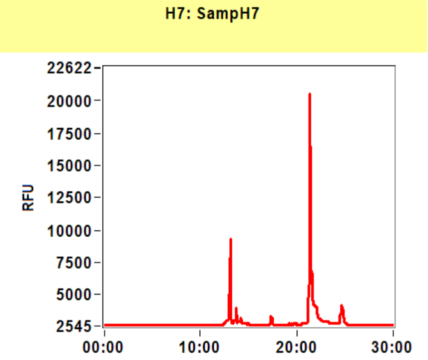

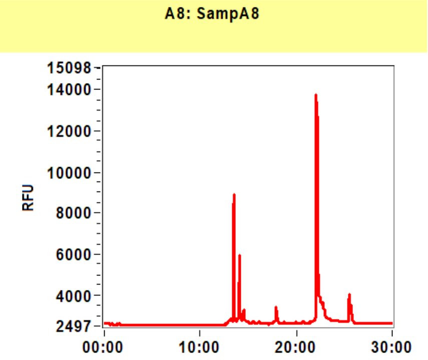

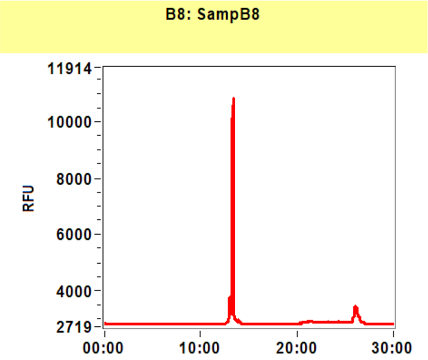

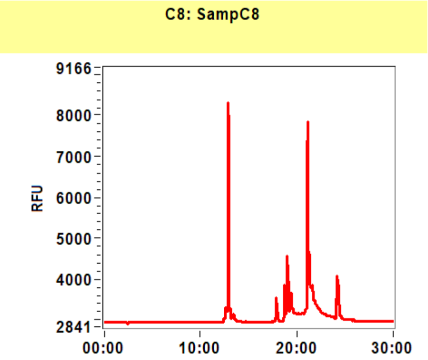

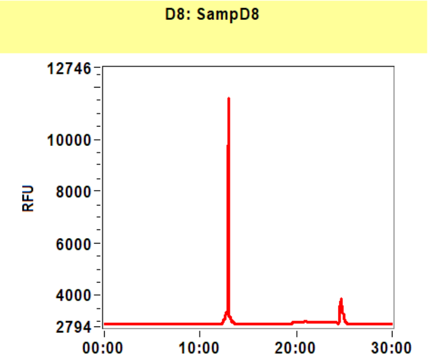

Filename and data path: C:\AATI\Data\2021 09 20\18-30-13\2021 09 20 18H 30M..raw

E8: SampE8

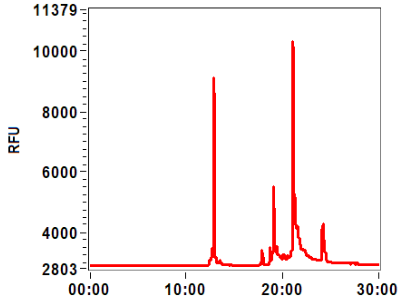

F8: SampF8

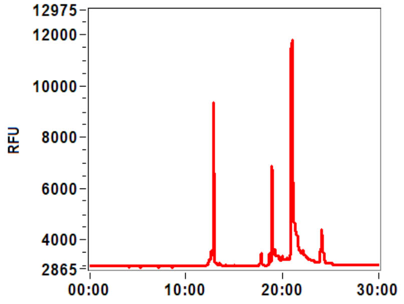

G8: SampG8

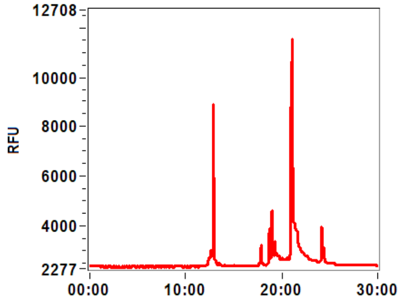

H8: SampH8

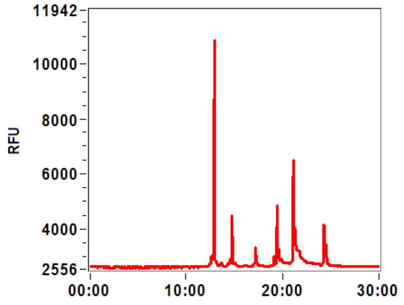

A9: SampA9

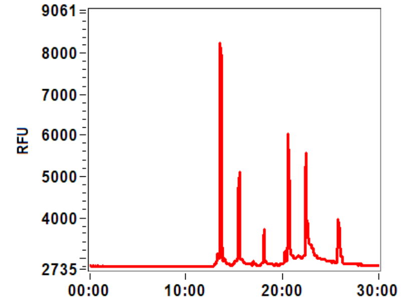

B9: SampB9

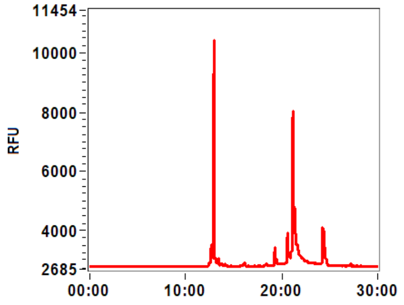

C9: SampC9

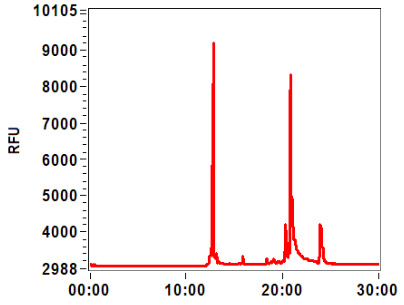

D9: SampD9

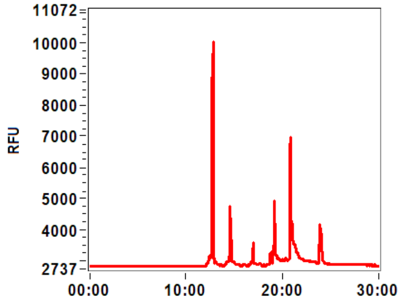

E9: SampE9

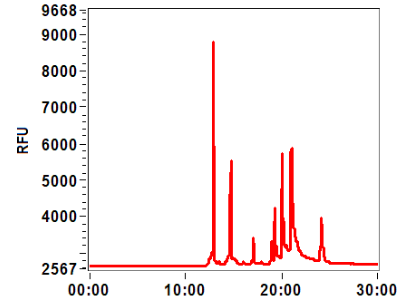

F9: SampF9

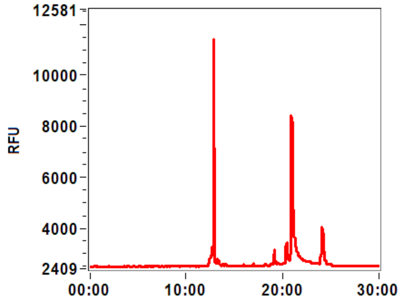

G9: SampG9

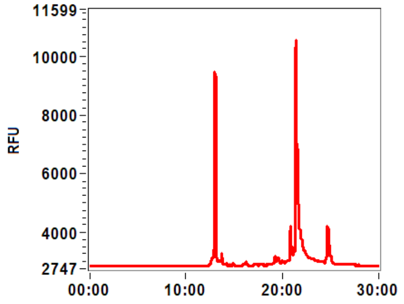

H9: SampH9

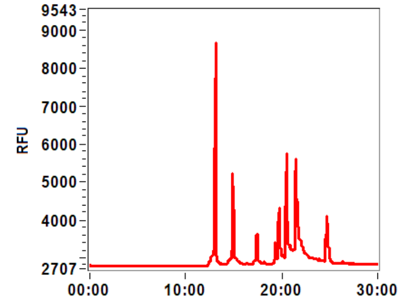

Filename and data path: C:\AATI\Data\2021 09 20\18-30-13\2021 09 20 18H 30M..raw

A10: SampA10

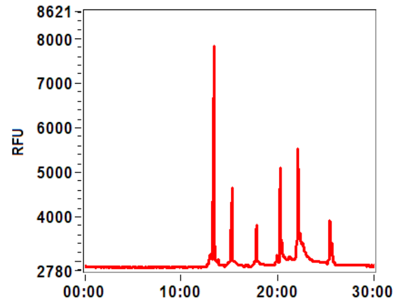

B10: SampB10

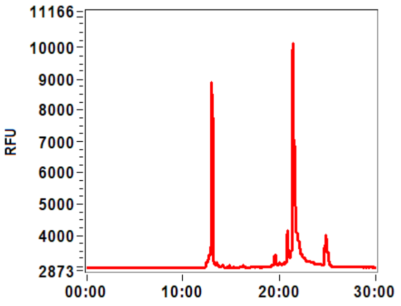

C10: SampC10

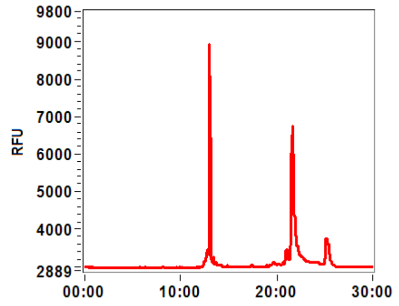

D10: SampD10

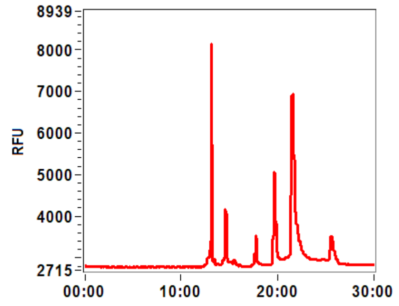

E10: SampE10

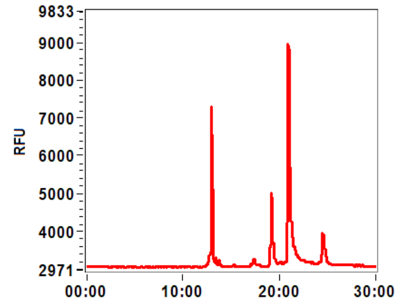

F10: SampF10

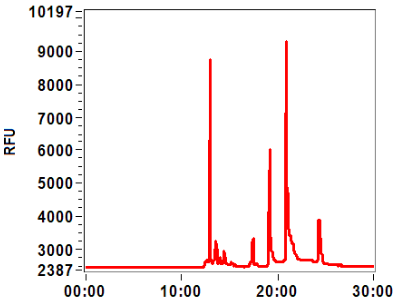

G10: SampG10

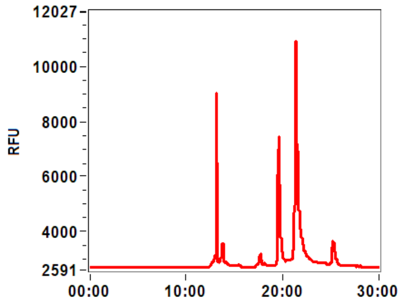

H10: SampH10

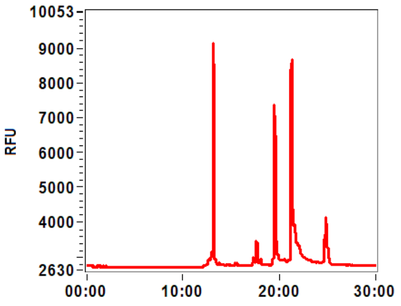

A11: SampA11

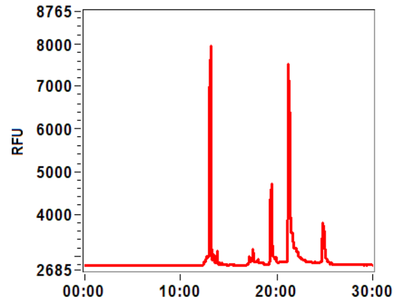

B11: SampB11

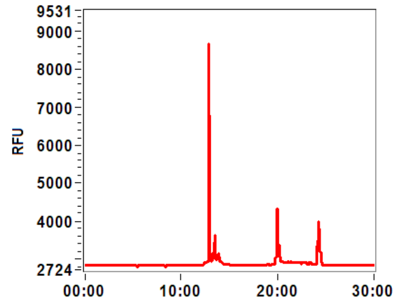

C11: SampC11

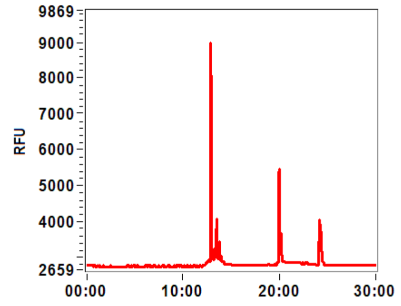

D11: SampD11

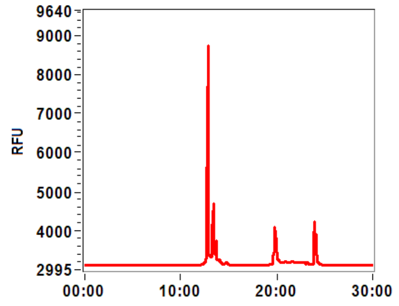

Filename and data path: C:\AATI\Data\2021 09 20\18-30-13\2021 09 20 18H 30M..raw

E11: SampE11

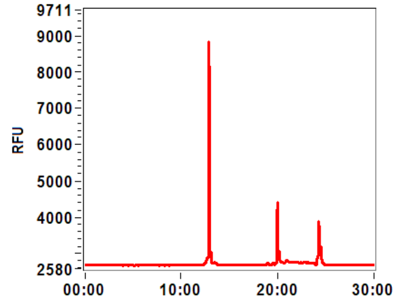

F11: SampF11

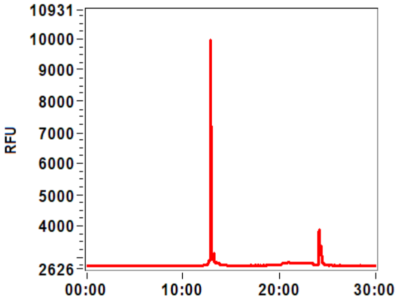

G11: SampG11

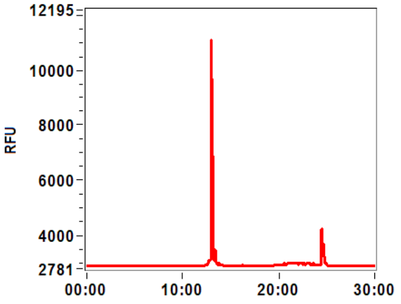

H11: SampH11

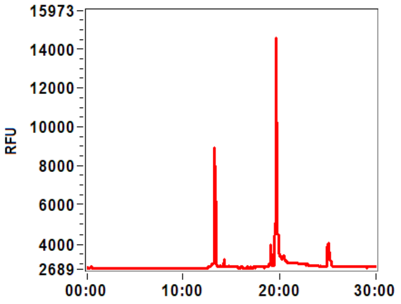

A12: SampA12

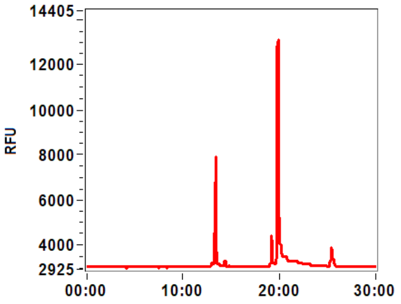

B12: SampB12

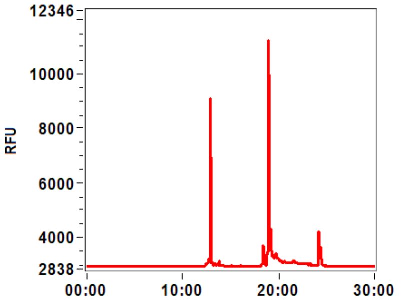

C12: SampC12

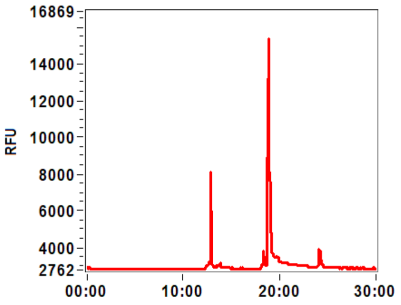

D12: SampD12

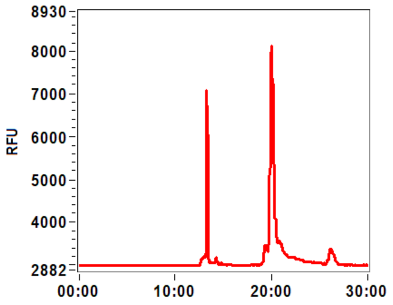

E12: SampE12

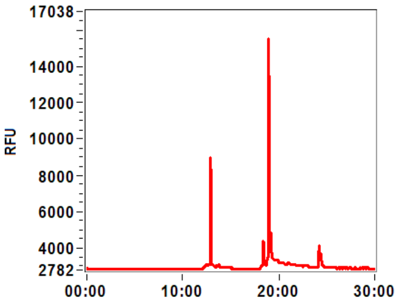

F12: SampF12

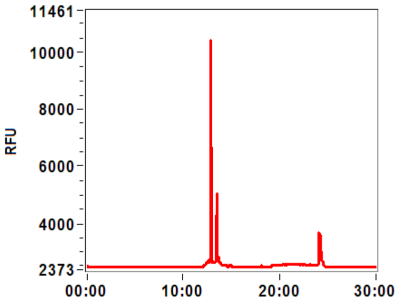

G12: SampG12

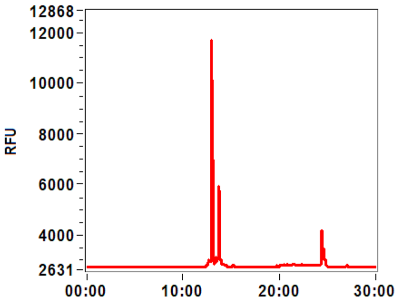

H12: SampH12

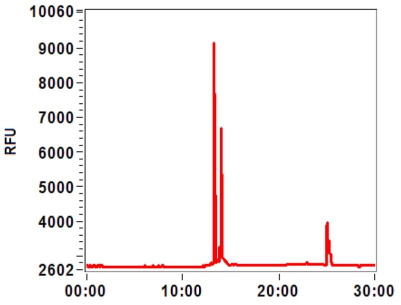

Sample: SampA1
Well location: A1
Created: Monday, September 20, 2021 6:40:06 PM

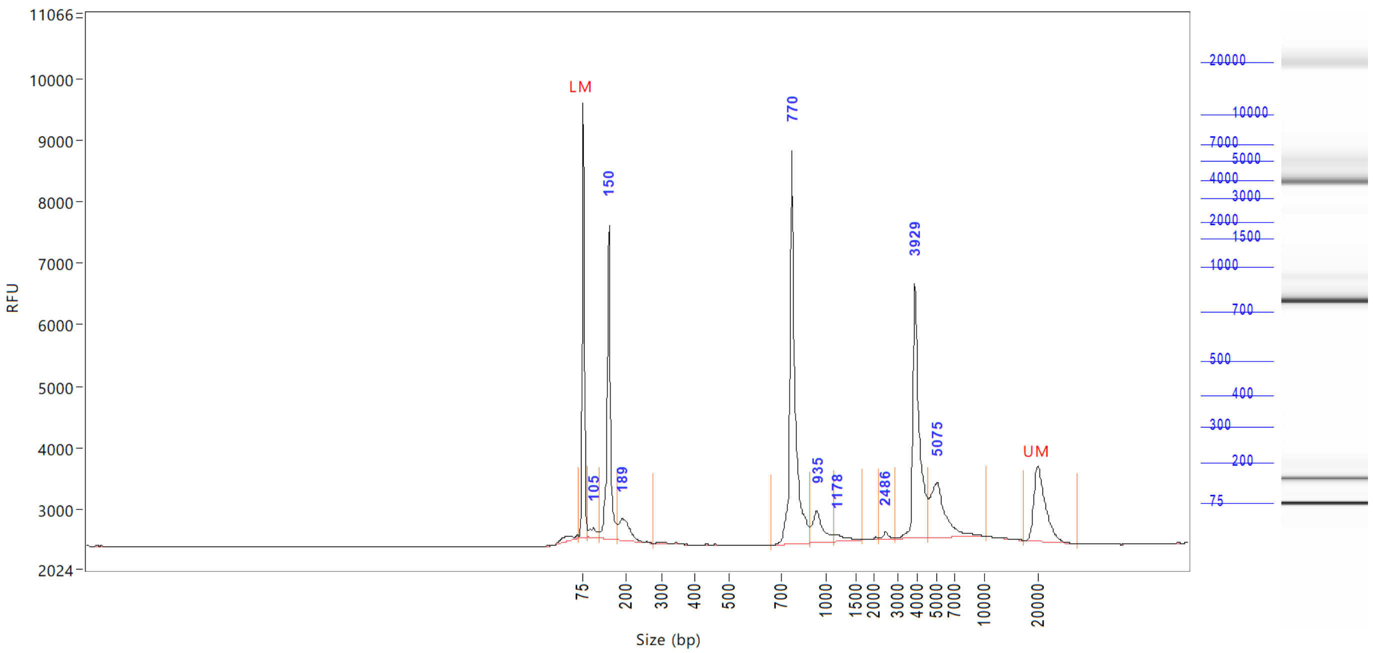

Peak	Size (bp)	Concentration (ng/uL)	From (bp)	To (bp)	Average size (bp)	CV%	RFU	Corrected peak area
1	75 (LM)	0.5000	62	87	73	4.43	7079	36.736
2	105	0.4808	87	122	104	9.83	157	2.944
3	150	6.4026	122	175	148	5.87	5111	39.201
4	189	1.3940	175	273	197	8.36	356	8.535
5	770	8.9670	662	887	777	4.17	6402	54.901
6	935	1.4034	887	1112	950	5.13	524	8.592
7	1178	0.3328	1112	1674	1259	9.25	111	2.038
8	2486	0.1594	2201	2943	2491	5.61	122	0.976
9	3929	6.2915	2943	4621	4010	6.04	4132	38.520
10	5075	2.9944	4621	10245	5550	17.68	890	18.334
11	20000 (UM)	0.2324	17317	27254	20514	5.97	1215	17.076

TIC:	28.4258	ng/uL
TIM:	115.7605	nmole/L
Total concentration:	28.5267	ng/uL

```
Sample peak width (sec): 5     Sample min peak height: 50     Sample baseline V to V?: Y     Sample baseline V to V points: 3
Sample filter: Binomial        Number of points for filter: 3  Sample start region (min): 0  Sample end region (min): 30
Marker peak width (sec): 5     Marker min peak height: 200    Marker baseline V to V?: Y     Marker baseline V to V points: 3
Lower marker selection: First peak > 500 RFU                  Upper marker selection: Last peak > 200 RFU
Ladder size (bp)75, 200, 300, 400, 500, 700, 1000, 1500, 2000, 3000, 4000, 5000, 7000, 10000, 20000
Quantification using: Lower Marker          Final concentration (ng/uL): 0.5000          Dilution factor: 12.0
```

Sample: SampB1
Well location: B1
Created: Monday, September 20, 2021 6:40:06 PM

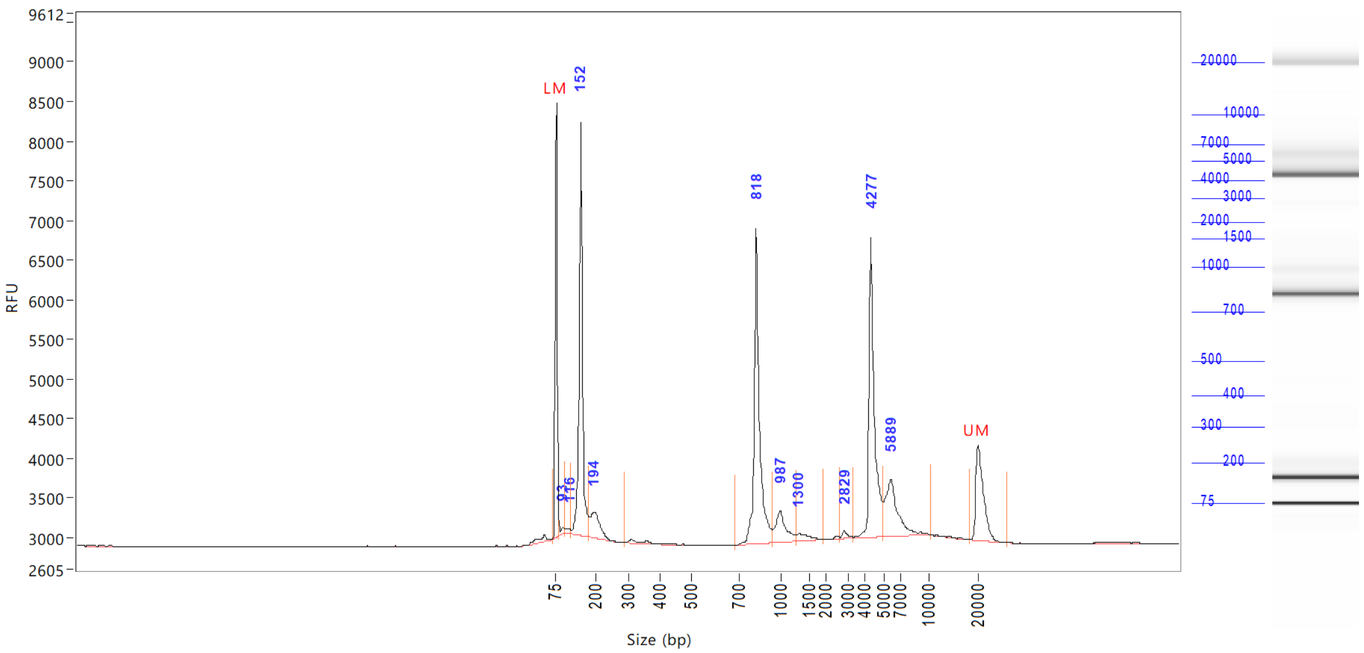

Peak	Size (bp)	Concentration (ng/uL)	From (bp)	To (bp)	Average size (bp)	CV%	RFU	Corrected peak area
1	75 (LM)	0.5000	64	86	73	4.02	5485	28.671
2	93	0.1365	86	101	93	4.79	84	0.652
3	116	0.1708	101	122	111	5.83	64	0.816
4	152	8.6366	122	179	151	5.74	5216	41.269
5	194	1.5100	179	284	199	7.06	324	7.216
6	818	7.2775	682	931	823	4.03	3980	34.775
7	987	1.4330	931	1256	1021	7.95	402	6.848
8	1300	0.3893	1256	1923	1412	8.66	92	1.860
9	2829	0.1693	2657	3322	2883	5.10	102	0.809
10	4277	6.2425	3322	5000	4369	5.43	3781	29.830
11	5889	2.6849	5000	10367	6168	15.34	720	12.830
12	20000 (UM)	0.2032	18415	25924	20491	4.24	1196	11.653

TIC:	28.6505	ng/uL
TIM:	131.8994	nmole/L
Total concentration:	28.8983	ng/uL

```
Sample peak width (sec): 5    Sample min peak height: 50    Sample baseline V to V?: Y    Sample baseline V to V points: 3
Sample filter: Binomial       Number of points for filter: 3  Sample start region (min): 0  Sample end region (min): 30
Marker peak width (sec): 5    Marker min peak height: 200   Marker baseline V to V?: Y    Marker baseline V to V points: 3
Lower marker selection: First peak > 500 RFU                Upper marker selection: Last peak > 200 RFU
Ladder size (bp)75, 200, 300, 400, 500, 700, 1000, 1500, 2000, 3000, 4000, 5000, 7000, 10000, 20000
Quantification using: Lower Marker          Final concentration (ng/uL): 0.5000          Dilution factor: 12.0
```

Sample: SampC1
Well location: C1
Created: Monday, September 20, 2021 6:40:06 PM

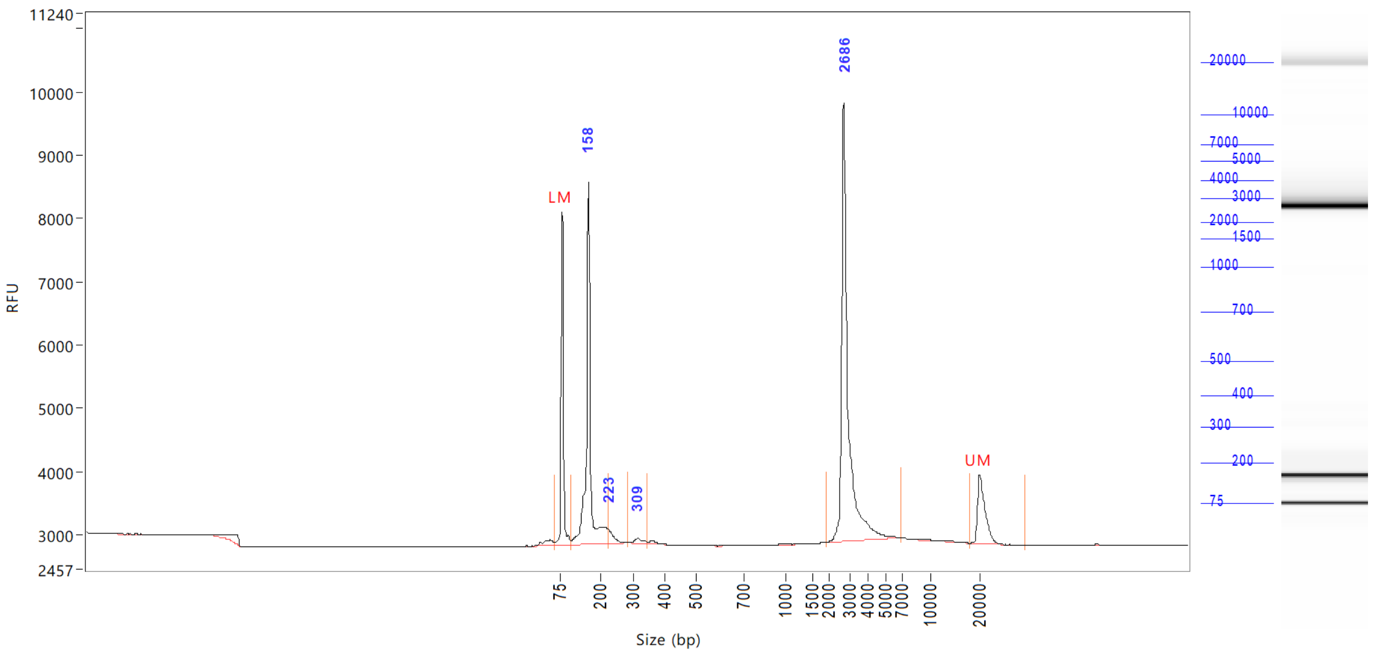

Peak	Size (bp)	Concentration (ng/uL)	From (bp)	To (bp)	Average size (bp)	CV%	RFU	Corrected peak area
1	75 (LM)	0.5000	52	103	74	7.48	5266	31.456
2	158	10.2949	103	223	159	12.43	5710	53.973
3	223	0.4377	223	280	234	4.58	217	2.294
4	309	0.2592	280	343	314	4.63	82	1.359
5	2686	12.4654	1904	6778	2876	16.17	6938	65.353
6	20000 (UM)	0.1710	18049	29309	20487	4.96	1093	10.756

TIC:	23.4571	ng/uL
TIM:	117.9928	nmole/L
Total concentration:	23.8408	ng/uL

```
Sample peak width (sec): 5    Sample min peak height: 50    Sample baseline V to V?: Y    Sample baseline V to V points: 3
Sample filter: Binomial       Number of points for filter: 3  Sample start region (min): 0  Sample end region (min): 30
Marker peak width (sec): 5    Marker min peak height: 200   Marker baseline V to V?: Y    Marker baseline V to V points: 3
Lower marker selection: First peak > 500 RFU                Upper marker selection: Last peak > 200 RFU
Ladder size (bp)75, 200, 300, 400, 500, 700, 1000, 1500, 2000, 3000, 4000, 5000, 7000, 10000, 20000
Quantification using: Lower Marker          Final concentration (ng/uL): 0.5000          Dilution factor: 12.0
```

Sample: SampD1
Well location: D1
Created: Monday, September 20, 2021 6:40:06 PM

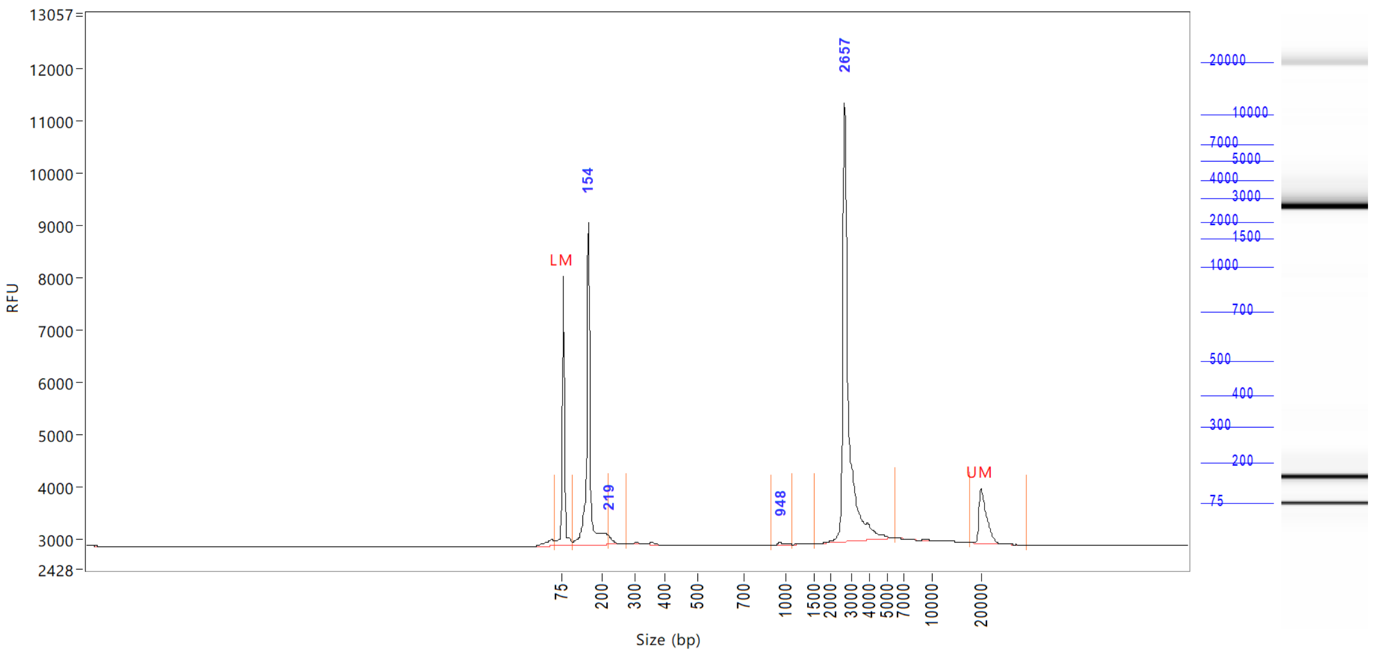

Peak	Size (bp)	Concentration (ng/uL)	From (bp)	To (bp)	Average size (bp)	CV%	RFU	Corrected peak area
1	75 (LM)	0.5000	52	103	74	8.04	5162	30.489
2	154	10.4076	103	219	156	11.61	6174	52.885
3	219	0.2687	219	269	229	4.34	162	1.365
4	948	0.1120	892	1101	964	3.49	58	0.569
5	2657	14.3953	1520	5889	2847	15.14	8402	73.149
6	20000 (UM)	0.1718	17805	29189	20541	4.91	1055	10.474

TIC:	25.1836	ng/uL
TIM:	120.0994	nmole/L
Total concentration:	25.6248	ng/uL

```
Sample peak width (sec): 5    Sample min peak height: 50    Sample baseline V to V?: Y    Sample baseline V to V points: 3
Sample filter: Binomial       Number of points for filter: 3  Sample start region (min): 0  Sample end region (min): 30
Marker peak width (sec): 5    Marker min peak height: 200   Marker baseline V to V?: Y    Marker baseline V to V points: 3
Lower marker selection: First peak > 500 RFU                Upper marker selection: Last peak > 200 RFU
Ladder size (bp)75, 200, 300, 400, 500, 700, 1000, 1500, 2000, 3000, 4000, 5000, 7000, 10000, 20000
Quantification using: Lower Marker          Final concentration (ng/uL): 0.5000          Dilution factor: 12.0
```

Sample: SampE1
Well location: E1
Created: Monday, September 20, 2021 6:40:06 PM

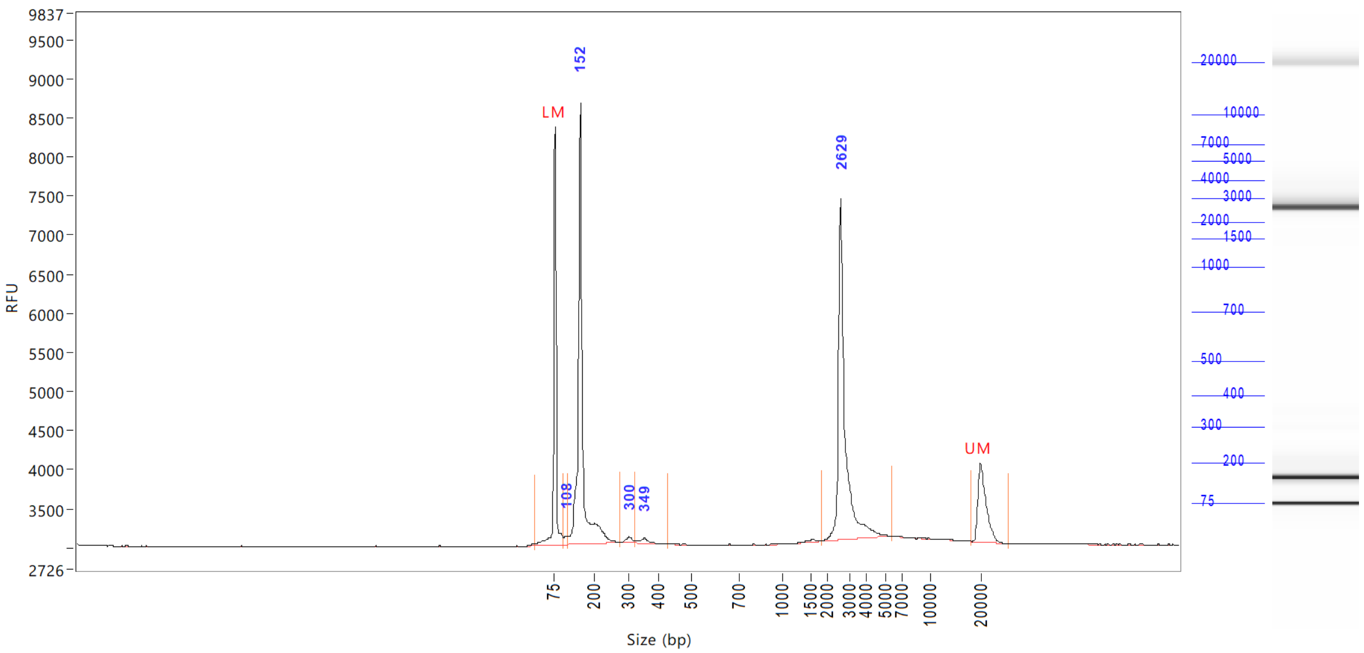

Peak	Size (bp)	Concentration (ng/uL)	From (bp)	To (bp)	Average size (bp)	CV%	RFU	Corrected peak area
1	75 (LM)	0.5000	14	101	71	13.60	5344	32.902
2	108	0.2191	101	116	108	4.46	116	1.201
3	152	10.0544	116	275	157	13.73	5652	55.135
4	300	0.1911	275	321	299	3.33	74	1.048
5	349	0.2213	321	424	352	6.01	63	1.213
6	2629	8.0170	1866	5741	2787	15.25	4365	43.962
7	20000 (UM)	0.1610	18293	25682	20456	4.48	1008	10.592

TIC:	18.7029	ng/uL
TIM:	115.4128	nmole/L
Total concentration:	18.9636	ng/uL

```
Sample peak width (sec): 5    Sample min peak height: 50    Sample baseline V to V?: Y    Sample baseline V to V points: 3
Sample filter: Binomial       Number of points for filter: 3  Sample start region (min): 0  Sample end region (min): 30
Marker peak width (sec): 5    Marker min peak height: 200   Marker baseline V to V?: Y    Marker baseline V to V points: 3
Lower marker selection: First peak > 500 RFU               Upper marker selection: Last peak > 200 RFU
Ladder size (bp)75, 200, 300, 400, 500, 700, 1000, 1500, 2000, 3000, 4000, 5000, 7000, 10000, 20000
Quantification using: Lower Marker          Final concentration (ng/uL): 0.5000          Dilution factor: 12.0
```

Sample: SampF1
Well location: F1
Created: Monday, September 20, 2021 6:40:06 PM

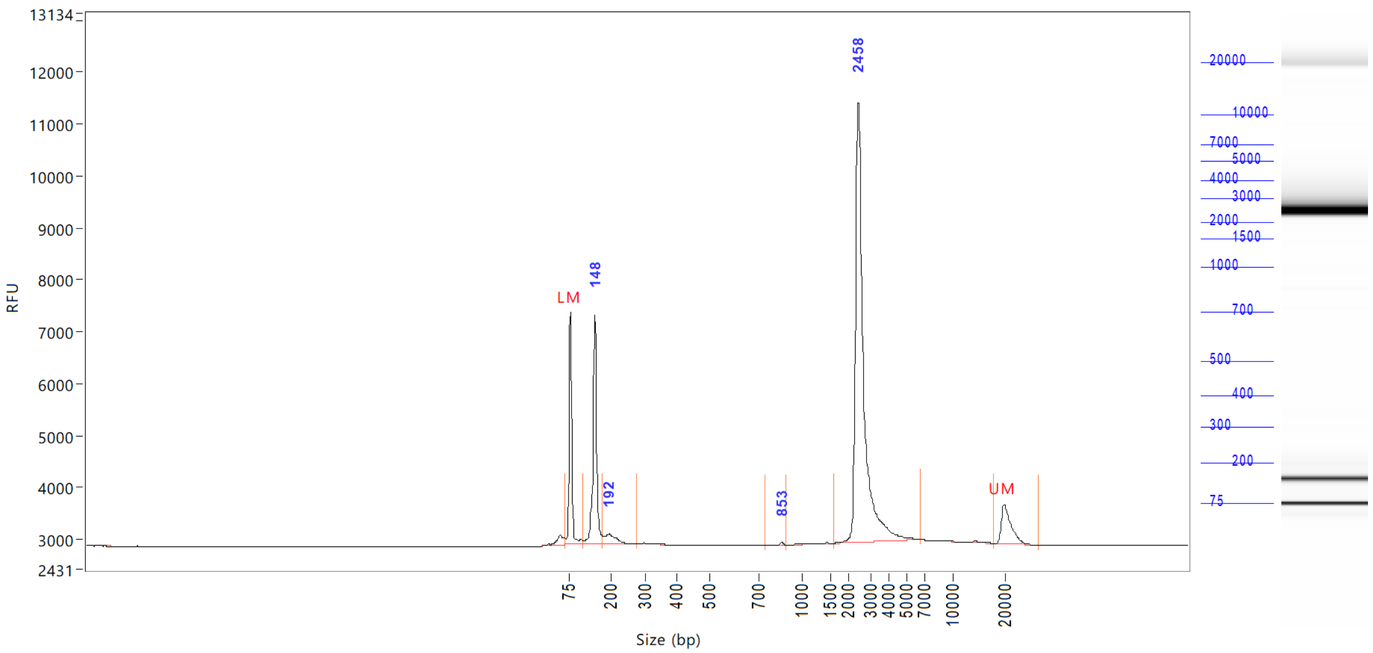

Peak	Size (bp)	Concentration (ng/uL)	From (bp)	To (bp)	Average size (bp)	CV%	RFU	Corrected peak area
1	75 (LM)	0.5000	62	114	74	10.08	4481	27.975
2	148	8.0840	114	175	146	5.60	4405	37.692
3	192	1.0663	175	271	199	8.39	196	4.972
4	853	0.0852	744	892	836	4.08	60	0.397
5	2458	22.0020	1559	6630	2628	17.13	8471	102.586
6	20000 (UM)	0.1655	18049	26770	20603	5.35	751	9.259

TIC:	31.2375	ng/uL
TIM:	113.7567	nmole/L
Total concentration:	31.6314	ng/uL

```
Sample peak width (sec): 5    Sample min peak height: 50    Sample baseline V to V?: Y    Sample baseline V to V points: 3
Sample filter: Binomial       Number of points for filter: 3  Sample start region (min): 0  Sample end region (min): 30
Marker peak width (sec): 5    Marker min peak height: 200   Marker baseline V to V?: Y    Marker baseline V to V points: 3
Lower marker selection: First peak > 500 RFU                Upper marker selection: Last peak > 200 RFU
Ladder size (bp)75, 200, 300, 400, 500, 700, 1000, 1500, 2000, 3000, 4000, 5000, 7000, 10000, 20000
Quantification using: Lower Marker          Final concentration (ng/uL): 0.5000          Dilution factor: 12.0
```

Sample: SampG1
Well location: G1
Created: Monday, September 20, 2021 6:40:06 PM

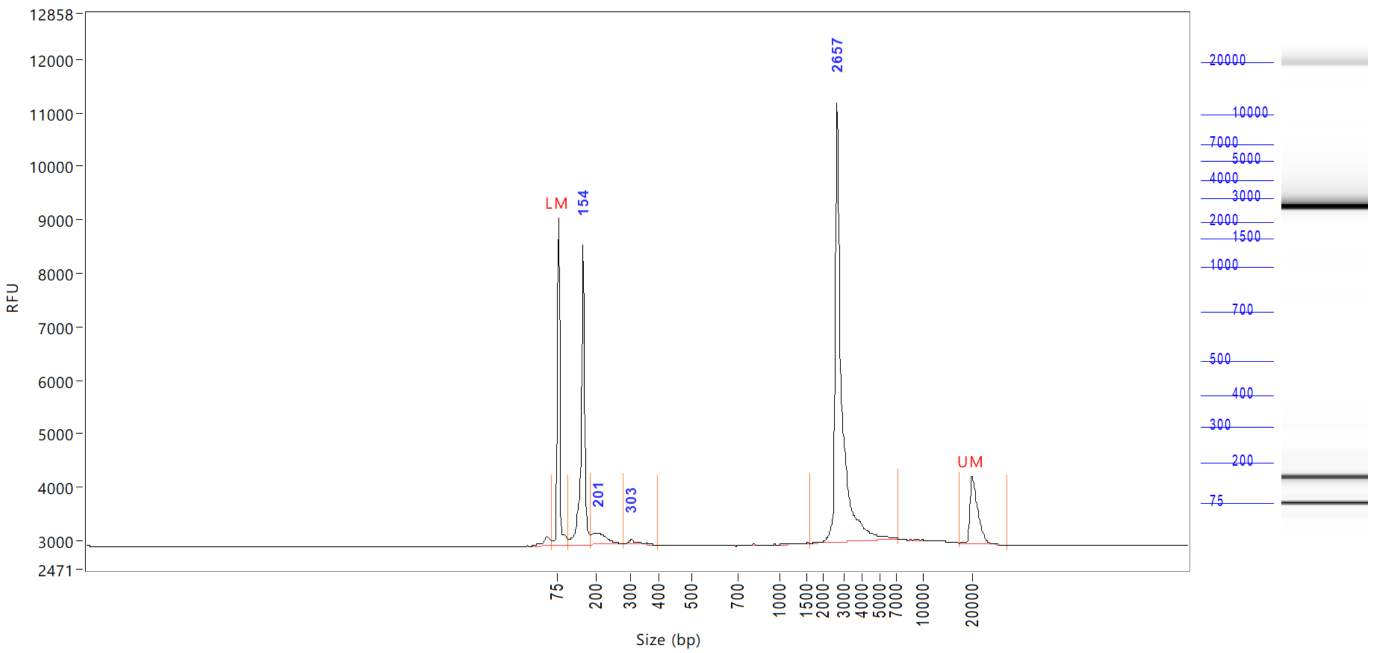

Peak	Size (bp)	Concentration (ng/uL)	From (bp)	To (bp)	Average size (bp)	CV%	RFU	Corrected peak area
1	75 (LM)	0.5000	58	107	75	8.56	6108	36.615
2	154	7.8119	107	179	149	7.13	5605	47.673
3	201	1.1556	179	277	208	9.31	208	7.052
4	303	0.2590	277	392	319	7.44	79	1.580
5	2657	13.7521	1559	7268	2887	18.65	8218	83.923
6	20000 (UM)	0.1734	17317	27254	20471	4.57	1261	12.697

TIC:	22.9786	ng/uL
TIM:	104.4794	nmole/L
Total concentration:	23.2552	ng/uL

Sample peak width (sec): 5 Sample min peak height: 50 Sample baseline V to V?: Y Sample baseline V to V points: 3
Sample filter: Binomial Number of points for filter: 3 Sample start region (min): 0 Sample end region (min): 30
Marker peak width (sec): 5 Marker min peak height: 200 Marker baseline V to V?: Y Marker baseline V to V points: 3
Lower marker selection: First peak > 500 RFU Upper marker selection: Last peak > 200 RFU
Ladder size (bp)75, 200, 300, 400, 500, 700, 1000, 1500, 2000, 3000, 4000, 5000, 7000, 10000, 20000
Quantification using: Lower Marker Final concentration (ng/uL): 0.5000 Dilution factor: 12.0

Sample: SampH1
Well location: H1
Created: Monday, September 20, 2021 6:40:06 PM

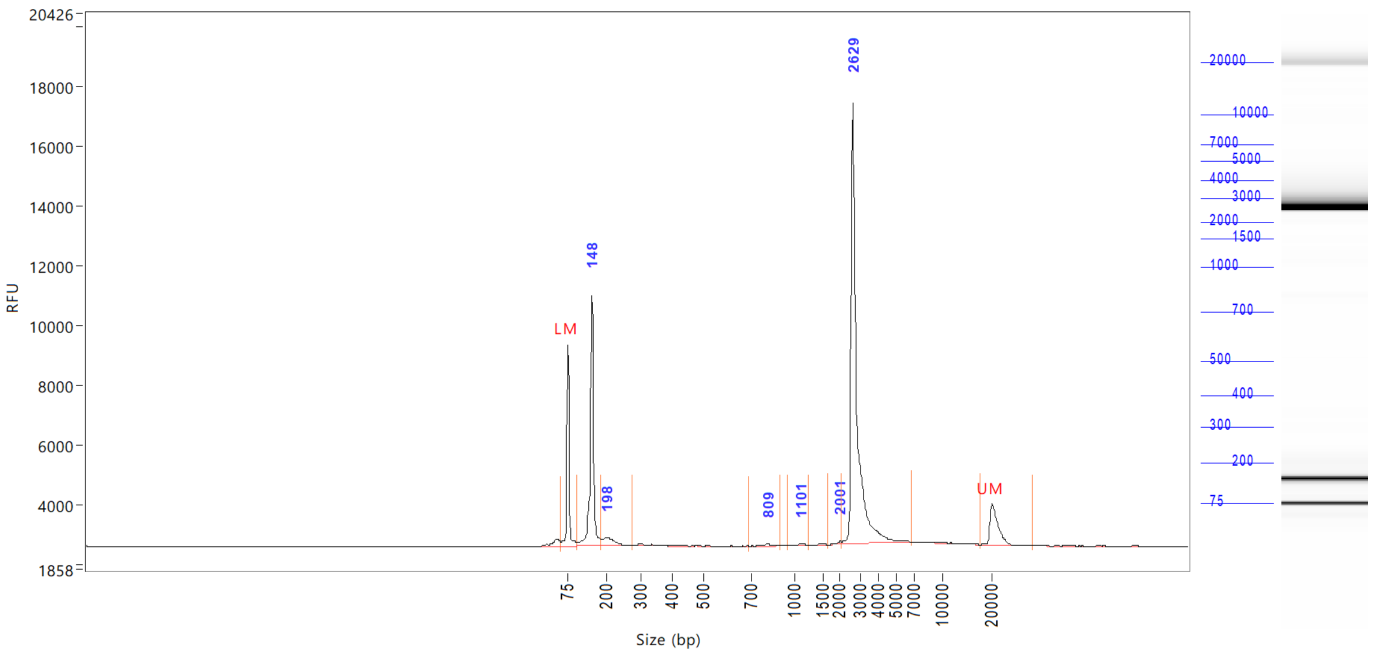

Peak	Size (bp)	Concentration (ng/uL)	From (bp)	To (bp)	Average size (bp)	CV%	RFU	Corrected peak area
1	75 (LM)	0.5000	52	103	73	8.45	6696	39.501
2	148	9.8376	103	177	146	6.63	8338	64.766
3	198	1.0565	177	271	201	8.11	253	6.956
4	809	0.1482	690	896	791	4.26	91	0.976
5	1101	0.1139	948	1245	1091	6.90	52	0.750
6	2001	0.0883	1674	2115	1998	3.80	84	0.581
7	2629	19.5296	2115	6778	2797	14.90	14739	128.573
8	20000 (UM)	0.1812	17561	28100	20562	4.67	1393	14.315

TIC:	30.7743	ng/uL
TIM:	131.4266	nmole/L
Total concentration:	31.2312	ng/uL

```
Sample peak width (sec): 5    Sample min peak height: 50    Sample baseline V to V?: Y    Sample baseline V to V points: 3
Sample filter: Binomial       Number of points for filter: 3  Sample start region (min): 0  Sample end region (min): 30
Marker peak width (sec): 5    Marker min peak height: 200   Marker baseline V to V?: Y    Marker baseline V to V points: 3
Lower marker selection: First peak > 500 RFU                Upper marker selection: Last peak > 200 RFU
Ladder size (bp)75, 200, 300, 400, 500, 700, 1000, 1500, 2000, 3000, 4000, 5000, 7000, 10000, 20000
Quantification using: Lower Marker          Final concentration (ng/uL): 0.5000          Dilution factor: 12.0
```

Sample: SampA2
Well location: A2
Created: Monday, September 20, 2021 6:40:06 PM

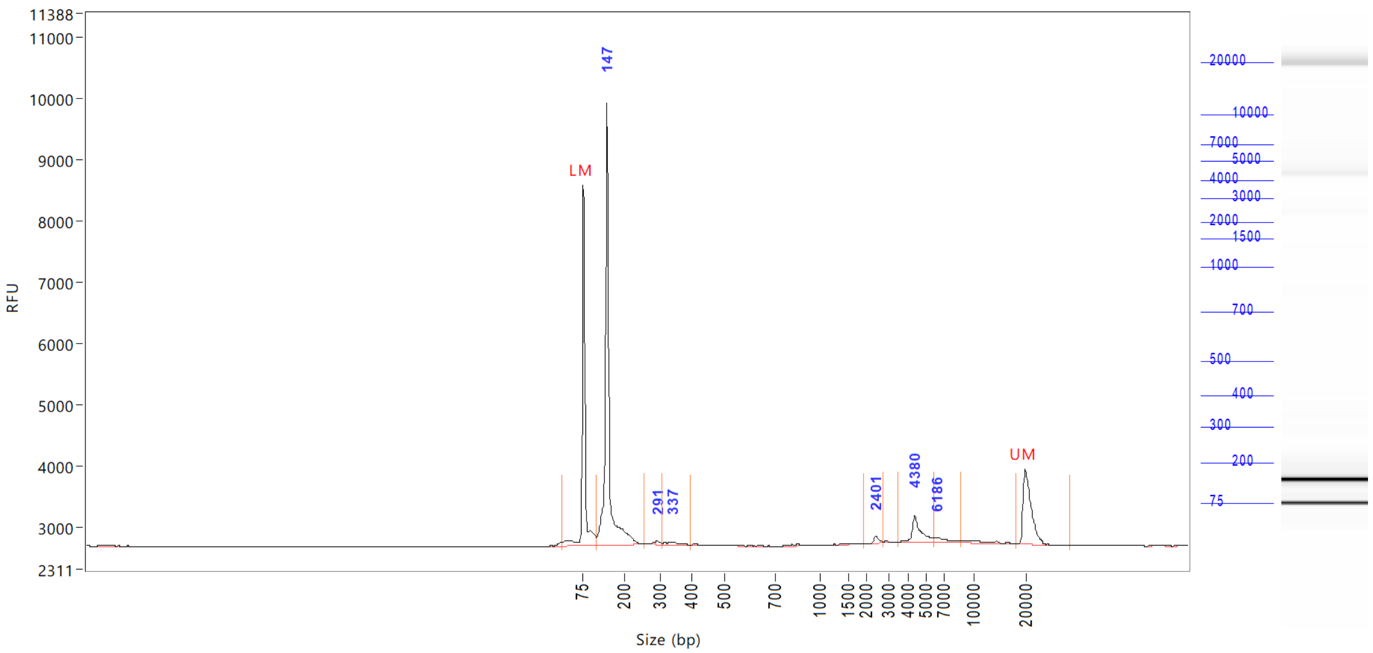

Peak	Size (bp)	Concentration (ng/uL)	From (bp)	To (bp)	Average size (bp)	CV%	RFU	Corrected peak area
1	75 (LM)	0.5000	12	114	74	18.42	5892	38.446
2	147	9.6108	114	253	150	12.61	7232	61.583
3	291	0.1304	253	305	286	4.04	56	0.835
4	337	0.2142	305	396	341	6.38	53	1.373
5	2401	0.1368	1923	2743	2411	5.60	114	0.877
6	4380	0.8292	3536	5815	4573	8.84	444	5.313
7	6186	0.2384	5815	8734	6947	11.66	81	1.528
8	20000 (UM)	0.1593	18171	28584	20458	4.74	1231	12.250

TIC:	11.1597	ng/uL
TIM:	107.5342	nmole/L
Total concentration:	11.5943	ng/uL

```
Sample peak width (sec): 5    Sample min peak height: 50    Sample baseline V to V?: Y    Sample baseline V to V points: 3
Sample filter: Binomial       Number of points for filter: 3  Sample start region (min): 0  Sample end region (min): 30
Marker peak width (sec): 5    Marker min peak height: 200   Marker baseline V to V?: Y    Marker baseline V to V points: 3
Lower marker selection: First peak > 500 RFU                Upper marker selection: Last peak > 200 RFU
Ladder size (bp)75, 200, 300, 400, 500, 700, 1000, 1500, 2000, 3000, 4000, 5000, 7000, 10000, 20000
Quantification using: Lower Marker          Final concentration (ng/uL): 0.5000          Dilution factor: 12.0
```

Sample: SampB2
Well location: B2
Created: Monday, September 20, 2021 6:40:06 PM

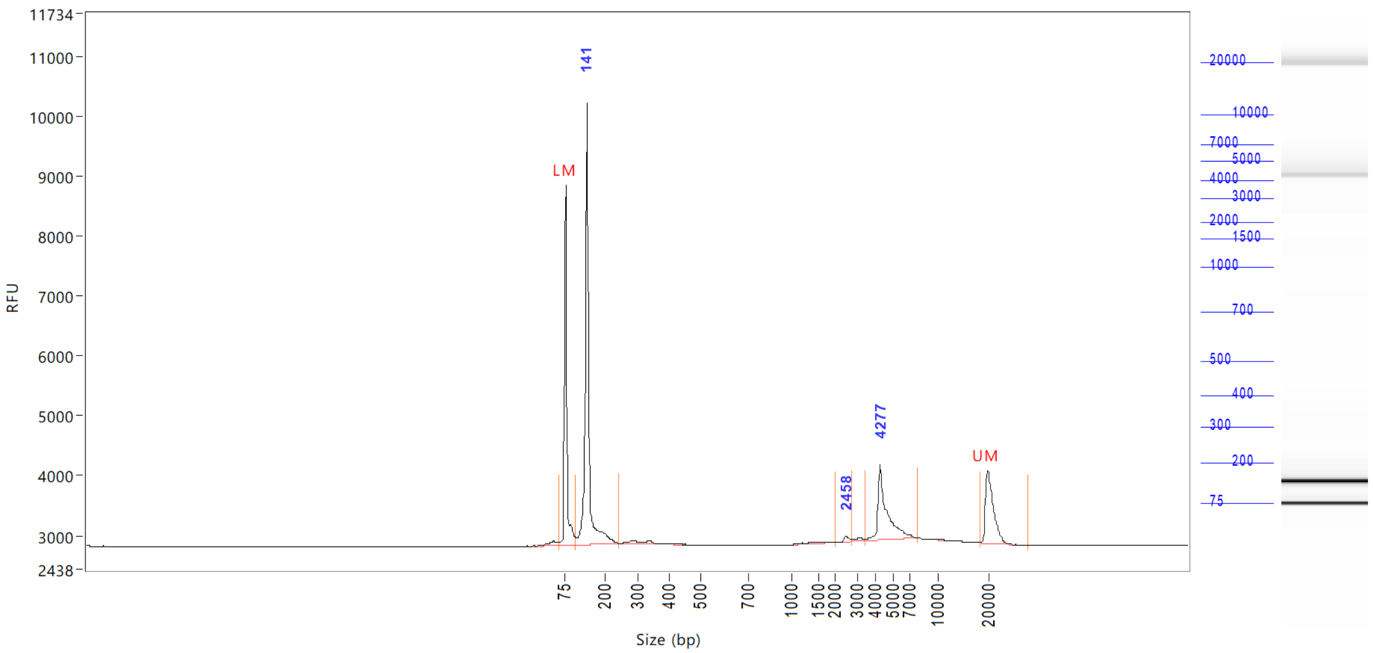

Peak	Size (bp)	Concentration (ng/uL)	From (bp)	To (bp)	Average size (bp)	CV%	RFU	Corrected peak area
1	75 (LM)	0.5000	54	108	75	9.86	6021	36.729
2	141	9.9190	108	241	144	12.27	7398	60.718
3	2458	0.1296	2000	2743	2497	5.35	94	0.793
4	4277	2.6165	3429	7734	4636	13.64	1253	16.016
5	20000 (UM)	0.1679	18415	28100	20537	4.73	1222	12.332

TIC:	12.6650	ng/uL
TIM:	114.2148	nmole/L
Total concentration:	13.4262	ng/uL

```
Sample peak width (sec): 5    Sample min peak height: 50    Sample baseline V to V?: Y    Sample baseline V to V points: 3
Sample filter: Binomial       Number of points for filter: 3  Sample start region (min): 0  Sample end region (min): 30
Marker peak width (sec): 5    Marker min peak height: 200   Marker baseline V to V?: Y    Marker baseline V to V points: 3
Lower marker selection: First peak > 500 RFU                Upper marker selection: Last peak > 200 RFU
Ladder size (bp)75, 200, 300, 400, 500, 700, 1000, 1500, 2000, 3000, 4000, 5000, 7000, 10000, 20000
Quantification using: Lower Marker          Final concentration (ng/uL): 0.5000          Dilution factor: 12.0
```

Sample: SampC2
Well location: C2
Created: Monday, September 20, 2021 6:40:06 PM

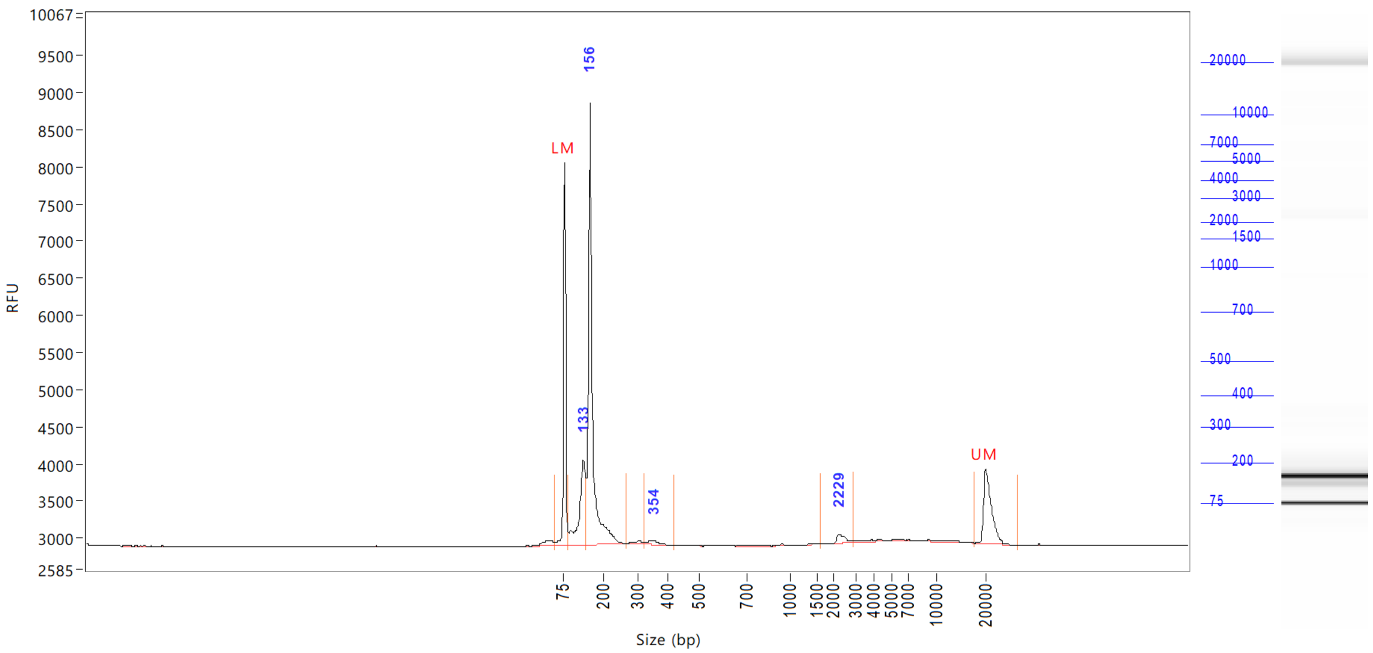

Peak	Size (bp)	Concentration (ng/uL)	From (bp)	To (bp)	Average size (bp)	CV%	RFU	Corrected peak area
1	75 (LM)	0.5000	47	87	73	6.25	5160	29.449
2	133	3.6466	87	145	127	11.28	1146	17.898
3	156	11.0904	145	266	162	11.02	5956	54.433
4	354	0.2531	323	418	353	5.99	54	1.242
5	2229	0.3239	1578	2914	2338	9.51	122	1.590
6	20000 (UM)	0.1693	17683	26408	20489	4.55	999	9.970

TIC:	15.3139	ng/uL
TIM:	161.1112	nmole/L
Total concentration:	15.8828	ng/uL

Sample peak width (sec): 5   Sample min peak height: 50   Sample baseline V to V?: Y   Sample baseline V to V points: 3
Sample filter: Binomial   Number of points for filter: 3 Sample start region (min): 0 Sample end region (min): 30
Marker peak width (sec): 5   Marker min peak height: 200   Marker baseline V to V?: Y   Marker baseline V to V points: 3
Lower marker selection: First peak > 500 RFU   Upper marker selection: Last peak > 200 RFU
Ladder size (bp)75, 200, 300, 400, 500, 700, 1000, 1500, 2000, 3000, 4000, 5000, 7000, 10000, 20000
Quantification using: Lower Marker   Final concentration (ng/uL): 0.5000   Dilution factor: 12.0

Sample: SampD2
Well location: D2
Created: Monday, September 20, 2021 6:40:06 PM

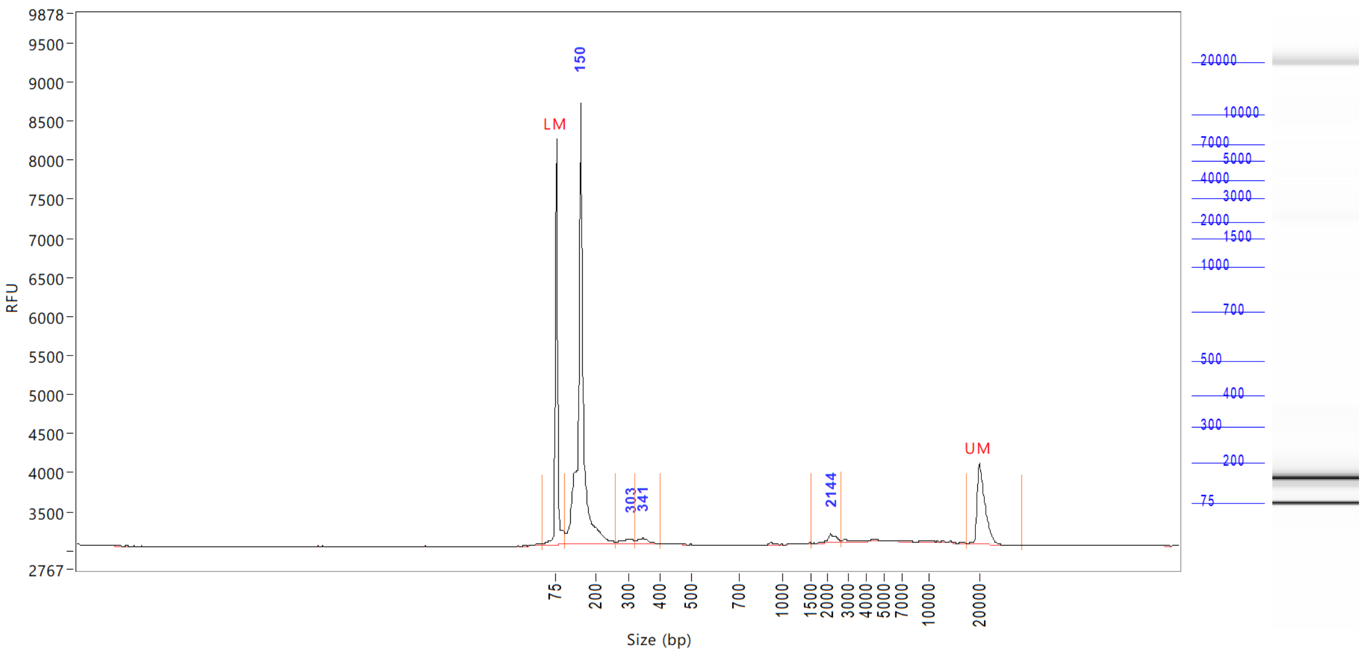

Peak	Size	Concentration	From	To	Average size	CV%	RFU	Corrected peak area
	(bp)	(ng/uL)	(bp)	(bp)	(bp)			
1	75 (LM)	0.5000	33	103	74	9.79	5189	31.470
2	150	12.5537	103	259	151	13.96	5651	65.844
3	303	0.2700	259	315	289	5.35	54	1.416
4	341	0.2666	315	400	341	4.62	68	1.398
5	2144	0.2729	1539	2629	2198	9.38	112	1.431
6	20000 (UM)	0.1637	17439	28584	20548	4.78	1031	10.304

TIC:	13.3633	ng/uL
TIM:	139.6664	nmole/L
Total concentration:	13.8990	ng/uL

```
Sample peak width (sec): 5    Sample min peak height: 50    Sample baseline V to V?: Y    Sample baseline V to V points: 3
Sample filter: Binomial       Number of points for filter: 3  Sample start region (min): 0  Sample end region (min): 30
Marker peak width (sec): 5    Marker min peak height: 200   Marker baseline V to V?: Y    Marker baseline V to V points: 3
Lower marker selection: First peak > 500 RFU                Upper marker selection: Last peak > 200 RFU
Ladder size (bp)75, 200, 300, 400, 500, 700, 1000, 1500, 2000, 3000, 4000, 5000, 7000, 10000, 20000
Quantification using: Lower Marker          Final concentration (ng/uL): 0.5000          Dilution factor: 12.0
```

Sample: SampE2
Well location: E2
Created: Monday, September 20, 2021 6:40:06 PM

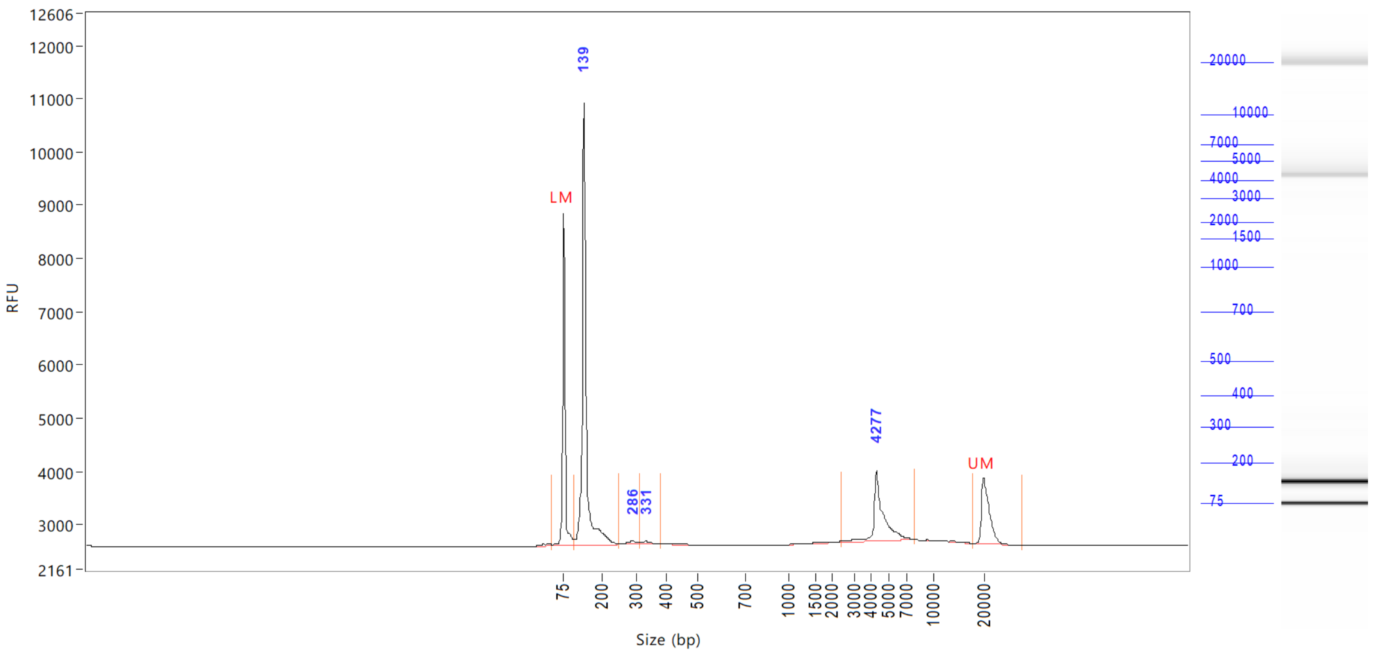

Peak	Size (bp)	Concentration (ng/uL)	From (bp)	To (bp)	Average size (bp)	CV%	RFU	Corrected peak area
1	75 (LM)	0.5000	39	107	74	9.32	6228	37.796
2	139	11.5187	107	246	143	13.34	8318	72.559
3	286	0.2354	246	307	280	5.33	77	1.483
4	331	0.1778	307	380	333	4.85	59	1.120
5	4277	2.8215	2458	7934	4537	15.92	1324	17.773
6	20000 (UM)	0.1659	18049	27859	20553	4.56	1250	12.541

TIC:	14.7533	ng/uL
TIM:	135.6590	nmole/L
Total concentration:	15.0575	ng/uL

```
Sample peak width (sec): 5    Sample min peak height: 50    Sample baseline V to V?: Y    Sample baseline V to V points: 3
Sample filter: Binomial       Number of points for filter: 3  Sample start region (min): 0  Sample end region (min): 30
Marker peak width (sec): 5    Marker min peak height: 200   Marker baseline V to V?: Y    Marker baseline V to V points: 3
Lower marker selection: First peak > 500 RFU                Upper marker selection: Last peak > 200 RFU
Ladder size (bp)75, 200, 300, 400, 500, 700, 1000, 1500, 2000, 3000, 4000, 5000, 7000, 10000, 20000
Quantification using: Lower Marker          Final concentration (ng/uL): 0.5000          Dilution factor: 12.0
```

Sample: SampF2
Well location: F2
Created: Monday, September 20, 2021 6:40:06 PM

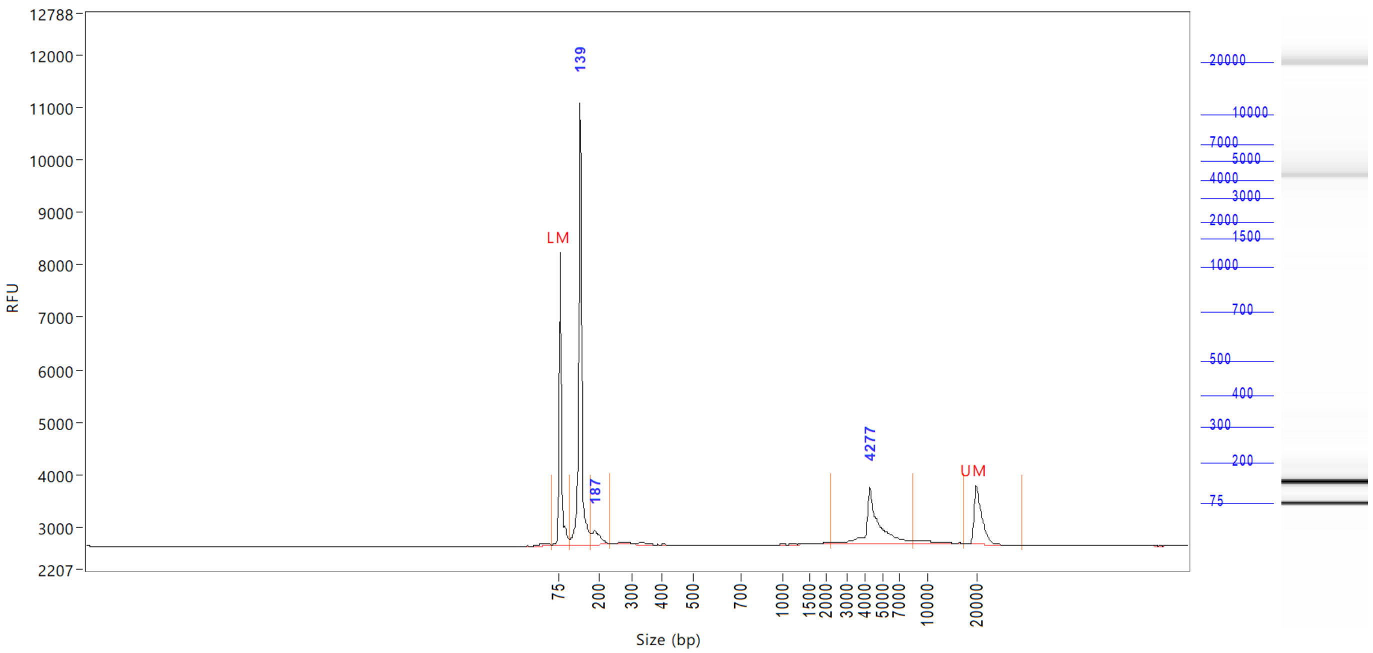

Peak	Size (bp)	Concentration (ng/uL)	From (bp)	To (bp)	Average size (bp)	CV%	RFU	Corrected peak area
1	75 (LM)	0.5000	50	105	75	8.95	5574	34.244
2	139	11.1397	105	171	137	6.52	8429	63.577
3	187	0.9464	171	233	190	6.75	269	5.401
4	4277	3.2758	2229	8467	4685	22.39	1071	18.696
5	20000 (UM)	0.1645	17561	29430	20605	5.30	1115	11.267

TIC:	15.3619	ng/uL
TIM:	142.9556	nmole/L
Total concentration:	16.1676	ng/uL

```
Sample peak width (sec): 5    Sample min peak height: 50    Sample baseline V to V?: Y    Sample baseline V to V points: 3
Sample filter: Binomial       Number of points for filter: 3  Sample start region (min): 0  Sample end region (min): 30
Marker peak width (sec): 5    Marker min peak height: 200   Marker baseline V to V?: Y    Marker baseline V to V points: 3
Lower marker selection: First peak > 500 RFU                Upper marker selection: Last peak > 200 RFU
Ladder size (bp)75, 200, 300, 400, 500, 700, 1000, 1500, 2000, 3000, 4000, 5000, 7000, 10000, 20000
Quantification using: Lower Marker          Final concentration (ng/uL): 0.5000          Dilution factor: 12.0
```

Sample: SampG2
Well location: G2
Created: Monday, September 20, 2021 6:40:06 PM

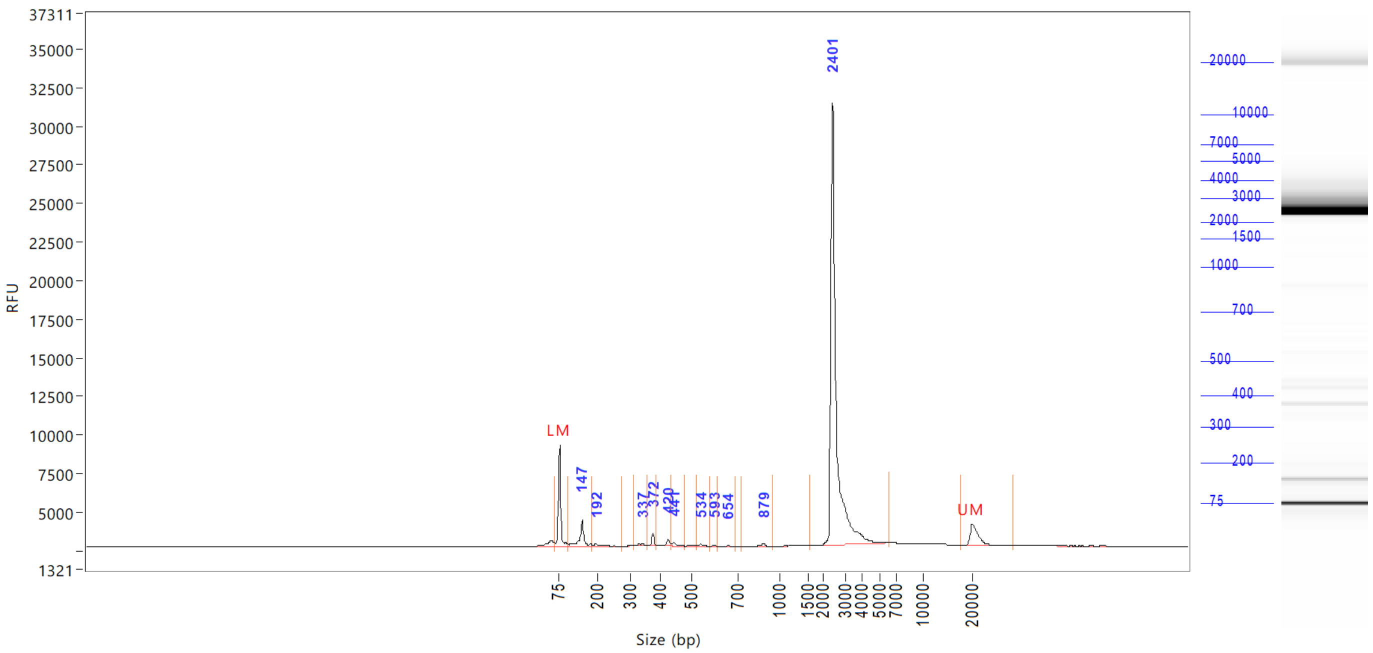

Peak	Size (bp)	Concentration (ng/uL)	From (bp)	To (bp)	Average size (bp)	CV%	RFU	Corrected peak area
1	75 (LM)	0.5000	64	105	74	8.39	6528	39.080
2	147	2.7328	105	183	143	10.47	1765	17.800
3	192	0.4351	183	271	205	9.47	175	2.834
4	337	0.2659	307	356	333	3.32	120	1.732
5	372	0.6364	356	386	369	1.34	774	4.145
6	420	0.5314	386	435	418	2.60	417	3.461
7	441	0.2687	435	477	443	1.67	267	1.750
8	534	0.1288	519	578	535	1.97	144	0.839
9	593	0.0748	578	606	590	0.83	104	0.487
10	654	0.0700	606	687	642	2.75	54	0.456
11	879	0.2087	718	939	859	3.71	179	1.359
12	2401	36.1651	1559	6112	2599	15.84	28666	235.555
13	20000 (UM)	0.1773	17683	28342	20569	4.83	1362	13.857

TIC:	41.5177	ng/uL
TIM:	66.2272	nmole/L
Total concentration:	41.6386	ng/uL

```
Sample peak width (sec): 5     Sample min peak height: 50     Sample baseline V to V?: Y     Sample baseline V to V points: 3
Sample filter: Binomial        Number of points for filter: 3  Sample start region (min): 0  Sample end region (min): 30
Marker peak width (sec): 5     Marker min peak height: 200    Marker baseline V to V?: Y     Marker baseline V to V points: 3
Lower marker selection: First peak > 500 RFU                  Upper marker selection: Last peak > 200 RFU
Ladder size (bp)75, 200, 300, 400, 500, 700, 1000, 1500, 2000, 3000, 4000, 5000, 7000, 10000, 20000
Quantification using: Lower Marker          Final concentration (ng/uL): 0.5000          Dilution factor: 12.0
```

Sample: SampH2
Well location: H2
Created: Monday, September 20, 2021 6:40:06 PM

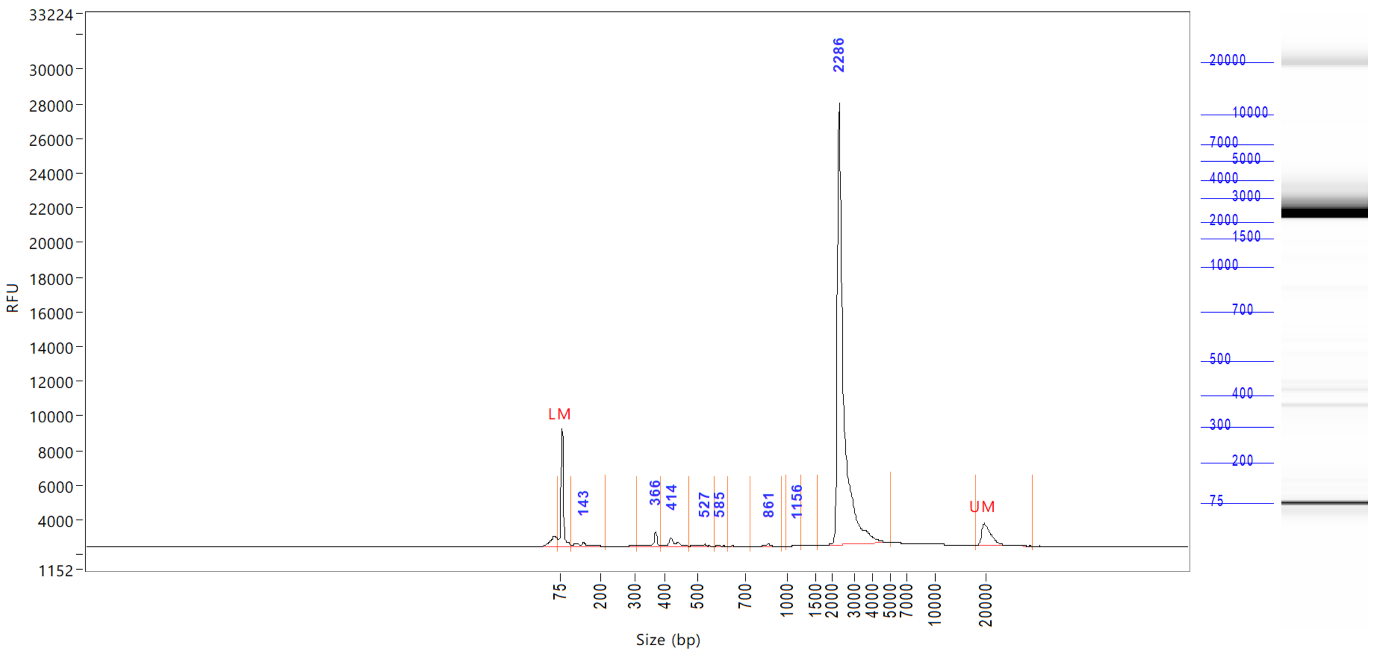

Peak	Size (bp)	Concentration (ng/uL)	From (bp)	To (bp)	Average size (bp)	CV%	RFU	Corrected peak area
1	75 (LM)	0.5000	64	105	75	7.71	6758	42.040
2	143	0.7626	105	212	138	16.26	205	5.343
3	366	1.0083	301	382	354	5.07	859	7.065
4	414	0.9887	382	471	419	3.97	533	6.928
5	527	0.1798	471	567	513	4.02	111	1.260
6	585	0.0867	567	623	587	1.85	94	0.607
7	861	0.1874	736	952	846	3.49	143	1.313
8	1156	0.0566	987	1234	1145	3.46	59	0.397
9	2286	35.8247	1559	5075	2488	15.81	25529	251.014
10	20000 (UM)	0.1798	18171	29672	20591	5.80	1255	15.118

TIC:	39.0948	ng/uL
TIM:	42.6184	nmole/L
Total concentration:	39.2926	ng/uL

Sample peak width (sec): 5   Sample min peak height: 50   Sample baseline V to V?: Y   Sample baseline V to V points: 3
Sample filter: Binomial   Number of points for filter: 3 Sample start region (min): 0 Sample end region (min): 30
Marker peak width (sec): 5   Marker min peak height: 200   Marker baseline V to V?: Y   Marker baseline V to V points: 3
Lower marker selection: First peak > 500 RFU   Upper marker selection: Last peak > 200 RFU
Ladder size (bp)75, 200, 300, 400, 500, 700, 1000, 1500, 2000, 3000, 4000, 5000, 7000, 10000, 20000
Quantification using: Lower Marker   Final concentration (ng/uL): 0.5000   Dilution factor: 12.0

Sample: SampA3
Well location: A3
Created: Monday, September 20, 2021 6:40:06 PM

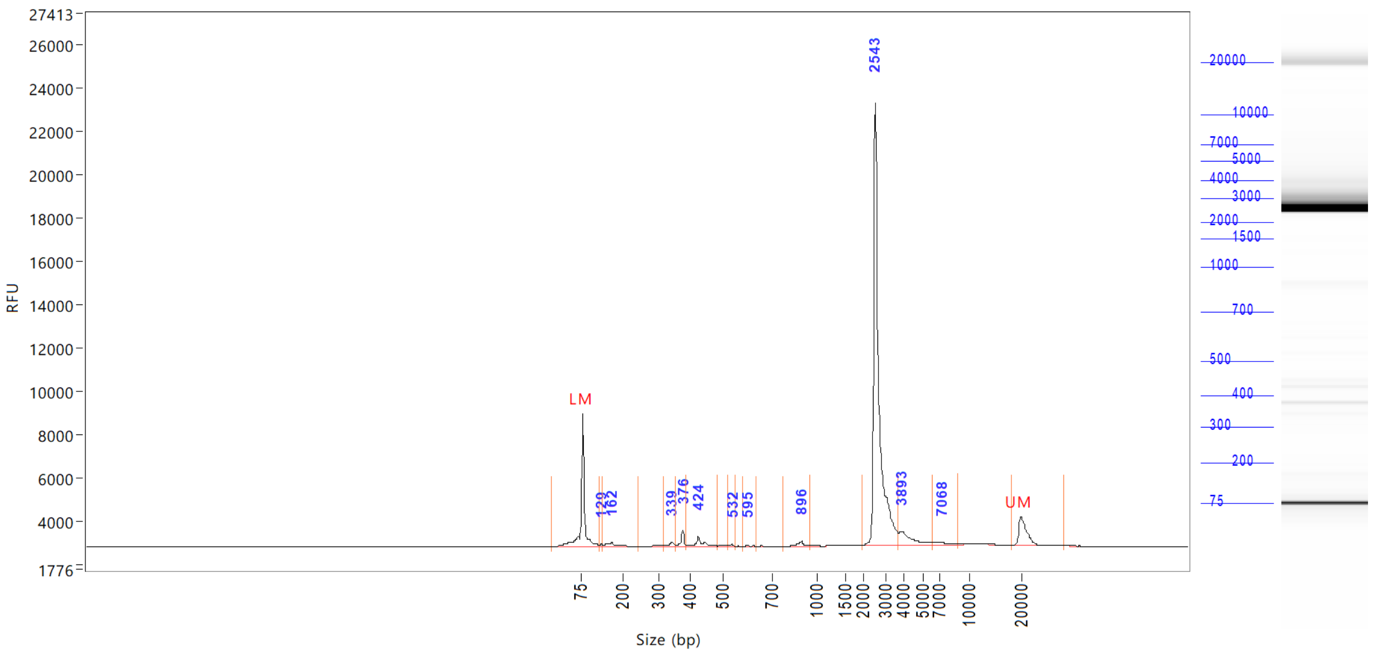

Peak	Size (bp)	Concentration (ng/uL)	From (bp)	To (bp)	Average size (bp)	CV%	RFU	Corrected peak area
1	75 (LM)	0.5000	0	124	70	25.63	6146	48.271
2	129	0.1090	124	137	130	3.31	101	0.877
3	162	0.4542	137	242	166	13.37	181	3.654
4	339	0.2510	317	354	337	2.70	198	2.019
5	376	0.5306	354	388	372	1.71	733	4.269
6	424	0.6106	388	481	425	3.91	430	4.912
7	532	0.0473	519	547	529	0.85	87	0.381
8	595	0.0509	578	634	597	1.77	76	0.410
9	896	0.2453	762	952	875	3.10	221	1.973
10	2543	20.1291	2000	3750	2674	10.10	20432	161.941
11	3893	1.3963	3750	6112	4348	12.77	617	11.233
12	7068	0.2675	6112	8867	7222	10.07	104	2.152
13	20000 (UM)	0.1322	18049	28221	20542	4.59	1301	12.764

TIC:	24.0918	ng/uL
TIM:	25.5419	nmole/L
Total concentration:	24.3627	ng/uL

```
Sample peak width (sec): 5    Sample min peak height: 50    Sample baseline V to V?: Y    Sample baseline V to V points: 3
Sample filter: Binomial       Number of points for filter: 3  Sample start region (min): 0  Sample end region (min): 30
Marker peak width (sec): 5    Marker min peak height: 200   Marker baseline V to V?: Y    Marker baseline V to V points: 3
Lower marker selection: First peak > 500 RFU               Upper marker selection: Last peak > 200 RFU
Ladder size (bp)75, 200, 300, 400, 500, 700, 1000, 1500, 2000, 3000, 4000, 5000, 7000, 10000, 20000
Quantification using: Lower Marker          Final concentration (ng/uL): 0.5000          Dilution factor: 12.0
```

Sample: SampB3
Well location: B3
Created: Monday, September 20, 2021 6:40:06 PM

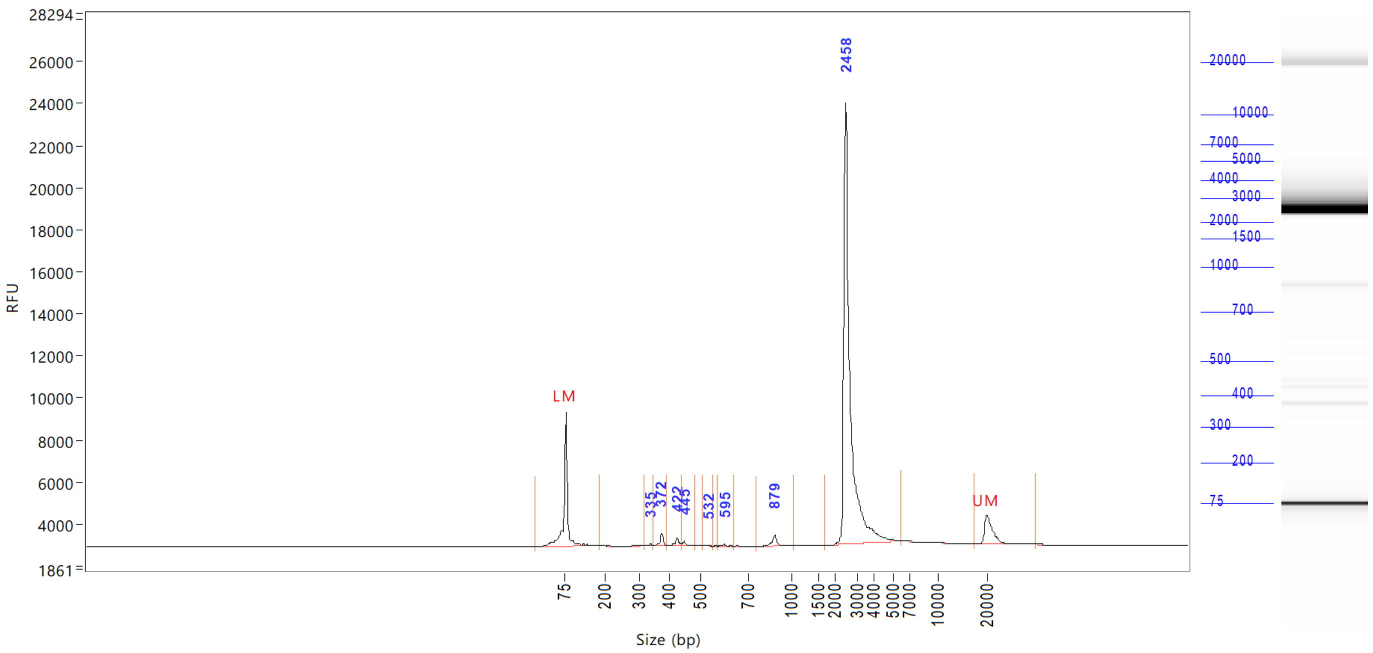

Peak	Size (bp)	Concentration (ng/uL)	From (bp)	To (bp)	Average size (bp)	CV%	RFU	Corrected peak area
1	75 (LM)	0.5000	0	179	69	27.12	6365	55.070
2	335	0.0831	313	345	332	2.41	68	0.762
3	372	0.4162	345	392	370	2.17	599	3.820
4	422	0.3417	392	437	419	2.38	353	3.136
5	445	0.1504	437	479	445	1.68	210	1.380
6	532	0.0339	506	547	526	1.37	60	0.311
7	595	0.0556	565	634	595	2.01	85	0.510
8	879	0.5088	749	1023	879	3.93	538	4.670
9	2458	21.5269	1693	6038	2670	16.18	21010	197.580
10	20000 (UM)	0.1253	17439	29551	20511	5.18	1331	13.801

TIC:	23.1166	ng/uL
TIM:	18.6452	nmole/L
Total concentration:	23.3339	ng/uL

```
Sample peak width (sec): 5    Sample min peak height: 50    Sample baseline V to V?: Y    Sample baseline V to V points: 3
Sample filter: Binomial       Number of points for filter: 3  Sample start region (min): 0  Sample end region (min): 30
Marker peak width (sec): 5    Marker min peak height: 200   Marker baseline V to V?: Y    Marker baseline V to V points: 3
Lower marker selection: First peak > 500 RFU                Upper marker selection: Last peak > 200 RFU
Ladder size (bp)75, 200, 300, 400, 500, 700, 1000, 1500, 2000, 3000, 4000, 5000, 7000, 10000, 20000
Quantification using: Lower Marker          Final concentration (ng/uL): 0.5000          Dilution factor: 12.0
```

Sample: SampC3
Well location: C3
Created: Monday, September 20, 2021 6:40:06 PM

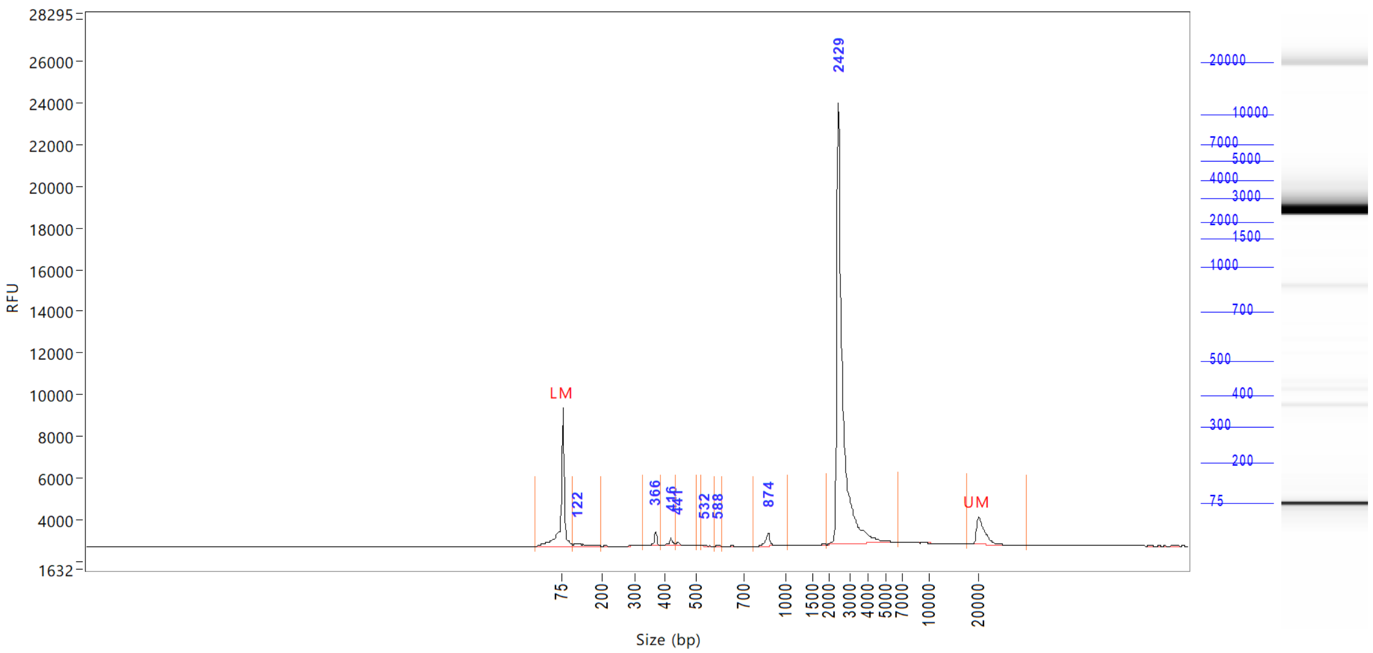

Peak	Size (bp)	Concentration (ng/uL)	From (bp)	To (bp)	Average size (bp)	CV%	RFU	Corrected peak area
1	75 (LM)	0.5000	0	103	65	27.41	6662	57.222
2	122	0.4831	103	196	138	17.66	136	4.607
3	366	0.4504	325	384	362	2.91	651	4.295
4	416	0.3250	384	431	414	2.48	354	3.099
5	441	0.1488	431	498	442	2.44	202	1.420
6	532	0.0316	521	572	533	1.91	60	0.302
7	588	0.0355	572	606	587	0.94	71	0.339
8	874	0.5211	766	1045	874	3.56	616	4.969
9	2429	20.1994	1942	6482	2636	16.01	21196	192.641
10	20000 (UM)	0.1213	17439	29793	20660	5.36	1309	13.878

TIC:	22.1948	ng/uL
TIM:	23.4375	nmole/L
Total concentration:	22.3802	ng/uL

Sample peak width (sec): 5   Sample min peak height: 50   Sample baseline V to V?: Y   Sample baseline V to V points: 3
Sample filter: Binomial   Number of points for filter: 3 Sample start region (min): 0 Sample end region (min): 30
Marker peak width (sec): 5   Marker min peak height: 200   Marker baseline V to V?: Y   Marker baseline V to V points: 3
Lower marker selection: First peak > 500 RFU   Upper marker selection: Last peak > 200 RFU
Ladder size (bp)75, 200, 300, 400, 500, 700, 1000, 1500, 2000, 3000, 4000, 5000, 7000, 10000, 20000
Quantification using: Lower Marker   Final concentration (ng/uL): 0.5000   Dilution factor: 12.0

Sample: SampD3
Well location: D3
Created: Monday, September 20, 2021 6:40:06 PM

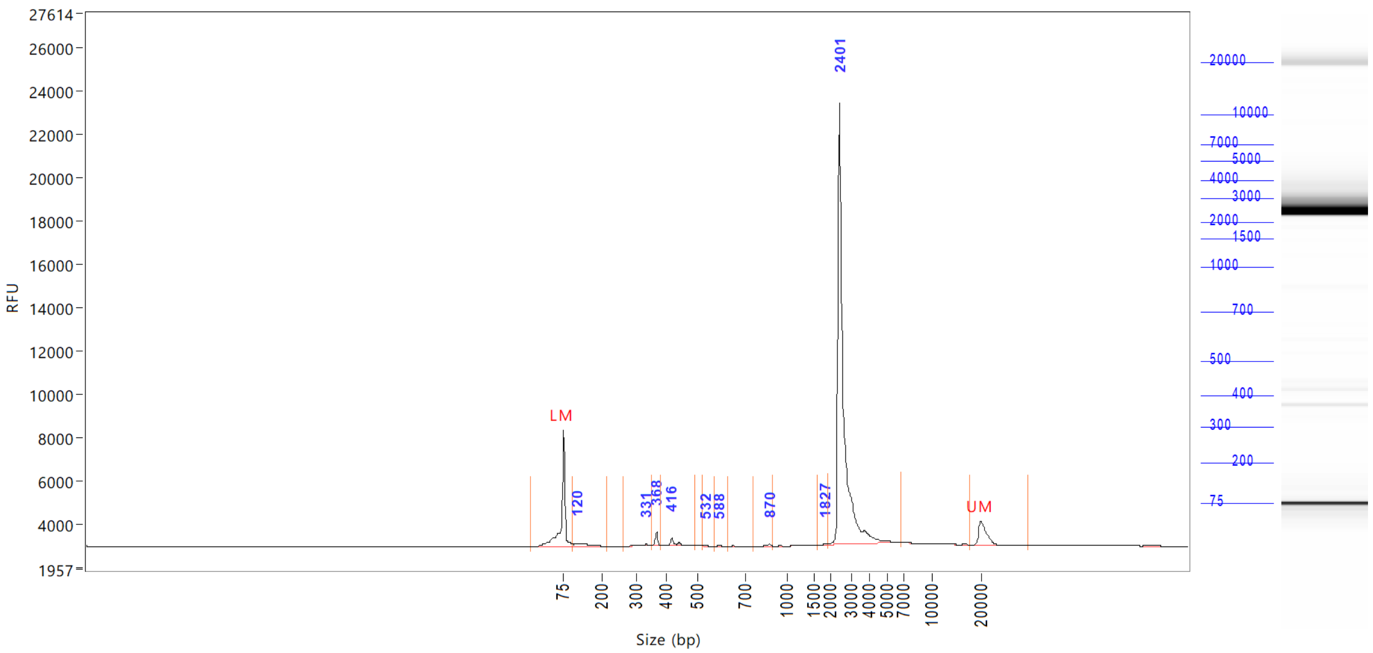

Peak	Size (bp)	Concentration (ng/uL)	From (bp)	To (bp)	Average size (bp)	CV%	RFU	Corrected peak area
1	75 (LM)	0.5000	0	105	63	30.35	5342	48.093
2	120	0.5560	105	214	135	16.47	150	4.456
3	331	0.0751	259	354	322	6.02	62	0.602
4	368	0.3608	354	380	365	1.01	591	2.892
5	416	0.4304	380	492	420	3.55	339	3.450
6	532	0.0361	521	570	533	1.93	56	0.289
7	588	0.0472	570	629	590	1.95	66	0.378
8	870	0.0863	757	900	851	3.07	100	0.691
9	1827	0.0526	1559	1942	1814	3.13	77	0.422
10	2401	21.9579	1942	6630	2604	16.73	20404	176.004
11	20000 (UM)	0.1159	17683	29672	20504	5.09	1108	11.146

TIC:	23.6023	ng/uL
TIM:	24.8018	nmole/L
Total concentration:	23.7590	ng/uL

```
Sample peak width (sec): 5    Sample min peak height: 50    Sample baseline V to V?: Y    Sample baseline V to V points: 3
Sample filter: Binomial       Number of points for filter: 3  Sample start region (min): 0  Sample end region (min): 30
Marker peak width (sec): 5    Marker min peak height: 200   Marker baseline V to V?: Y    Marker baseline V to V points: 3
Lower marker selection: First peak > 500 RFU                Upper marker selection: Last peak > 200 RFU
Ladder size (bp)75, 200, 300, 400, 500, 700, 1000, 1500, 2000, 3000, 4000, 5000, 7000, 10000, 20000
Quantification using: Lower Marker          Final concentration (ng/uL): 0.5000          Dilution factor: 12.0
```

Sample: SampE3
Well location: E3
Created: Monday, September 20, 2021 6:40:06 PM

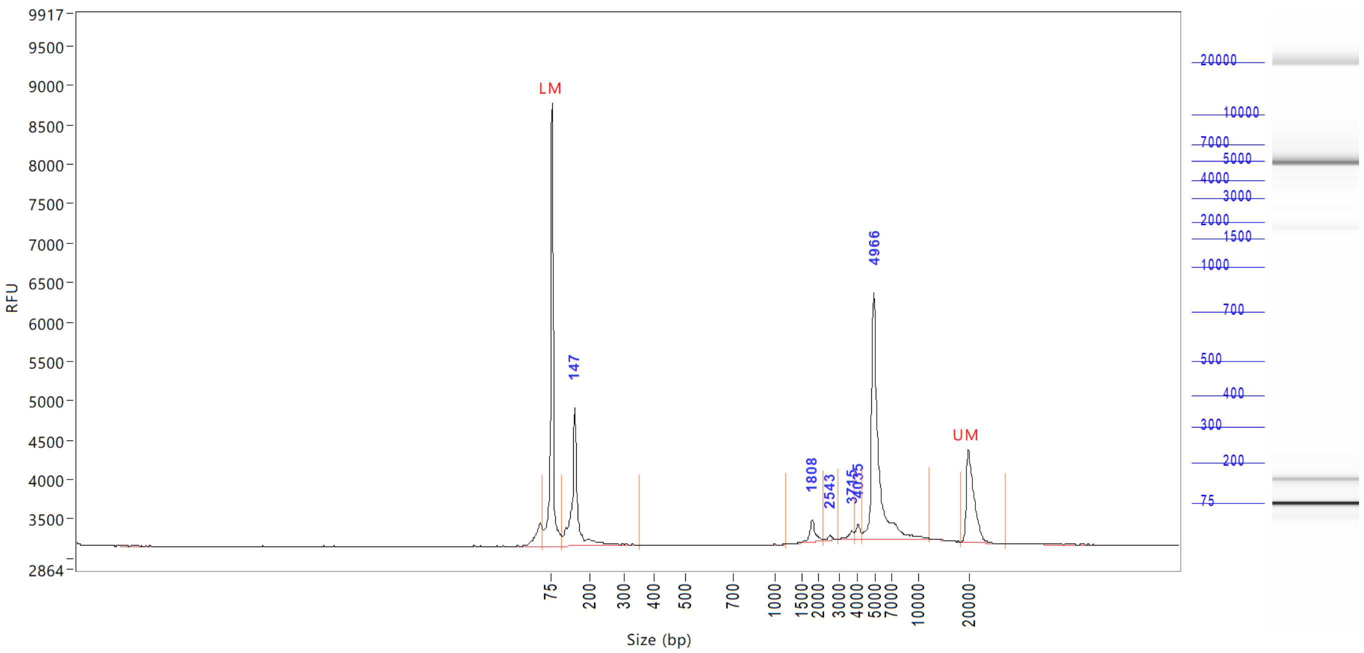

Peak	Size (bp)	Concentration (ng/uL)	From (bp)	To (bp)	Average size (bp)	CV%	RFU	Corrected peak area
1	75 (LM)	0.5000	48	107	73	11.26	5632	39.545
2	147	3.4082	107	349	154	23.75	1760	22.463
3	1808	0.3913	1201	2201	1799	7.97	284	2.579
4	2543	0.0805	2201	2914	2497	5.80	60	0.530
5	3715	0.1507	2914	3857	3554	6.64	109	0.993
6	4035	0.2073	3857	4277	4050	2.76	185	1.366
7	4966	4.7090	4277	12074	5441	18.92	3119	31.036
8	20000 (UM)	0.1486	18537	27617	20580	4.61	1165	11.757

TIC:	8.9469	ng/uL
TIM:	38.3647	nmole/L
Total concentration:	9.0795	ng/uL

Sample peak width (sec): 5 Sample min peak height: 50 Sample baseline V to V?: Y Sample baseline V to V points: 3
Sample filter: Binomial Number of points for filter: 3 Sample start region (min): 0 Sample end region (min): 30
Marker peak width (sec): 5 Marker min peak height: 200 Marker baseline V to V?: Y Marker baseline V to V points: 3
Lower marker selection: First peak > 500 RFU Upper marker selection: Last peak > 200 RFU
Ladder size (bp)75, 200, 300, 400, 500, 700, 1000, 1500, 2000, 3000, 4000, 5000, 7000, 10000, 20000
Quantification using: Lower Marker Final concentration (ng/uL): 0.5000 Dilution factor: 12.0

Sample: SampF3
Well location: F3
Created: Monday, September 20, 2021 6:40:06 PM

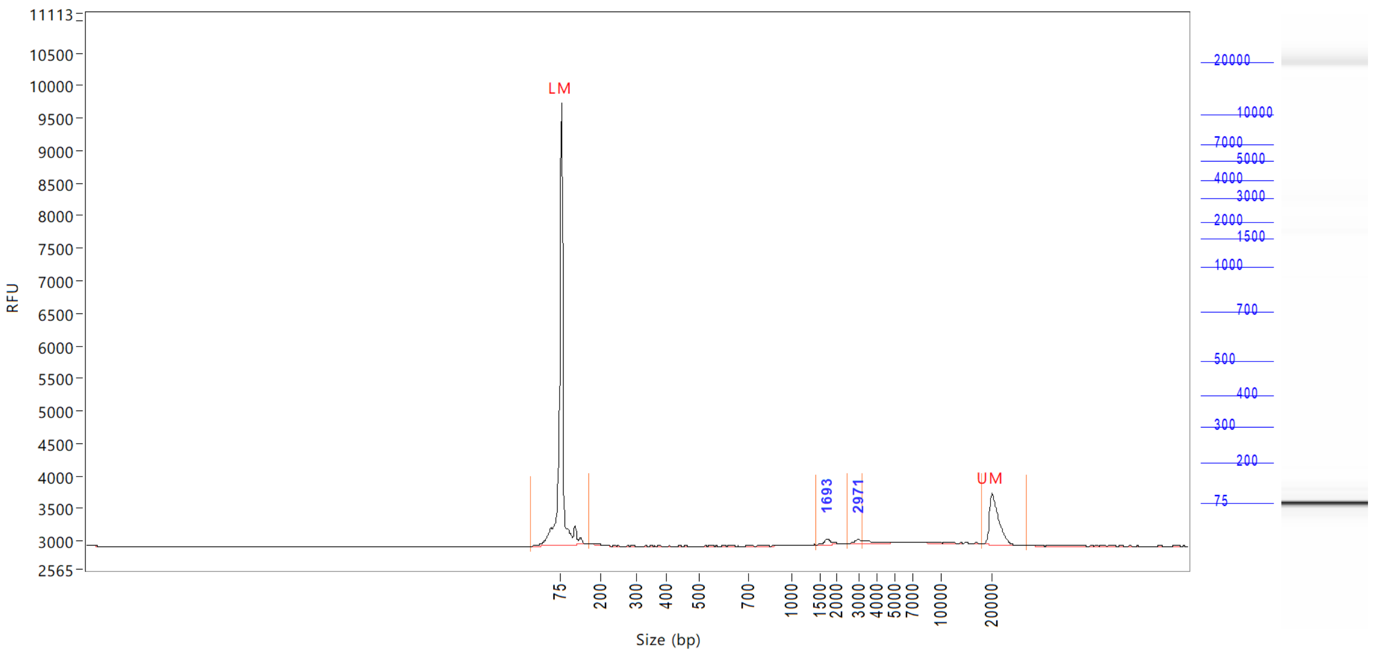

Peak	Size (bp)	Concentration (ng/uL)	From (bp)	To (bp)	Average size (bp)	CV%	RFU	Corrected peak area
1	75 (LM)	0.5000	0	162	72	23.62	6811	61.175
2	1693	0.1212	1433	2486	1807	12.24	88	1.236
3	2971	0.0729	2486	3179	2902	5.61	64	0.743
4	20000 (UM)	0.0777	17927	26770	20611	5.32	796	9.511

TIC:	0.1941	ng/uL
TIM:	0.1518	nmole/L
Total concentration:	0.5027	ng/uL

Sample peak width (sec): 5 Sample min peak height: 50 Sample baseline V to V?: Y Sample baseline V to V points: 3
Sample filter: Binomial Number of points for filter: 3 Sample start region (min): 0 Sample end region (min): 30
Marker peak width (sec): 5 Marker min peak height: 200 Marker baseline V to V?: Y Marker baseline V to V points: 3
Lower marker selection: First peak > 500 RFU Upper marker selection: Last peak > 200 RFU
Ladder size (bp)75, 200, 300, 400, 500, 700, 1000, 1500, 2000, 3000, 4000, 5000, 7000, 10000, 20000
Quantification using: Lower Marker Final concentration (ng/uL): 0.5000 Dilution factor: 12.0

Sample: SampG3
Well location: G3
Created: Monday, September 20, 2021 6:40:06 PM

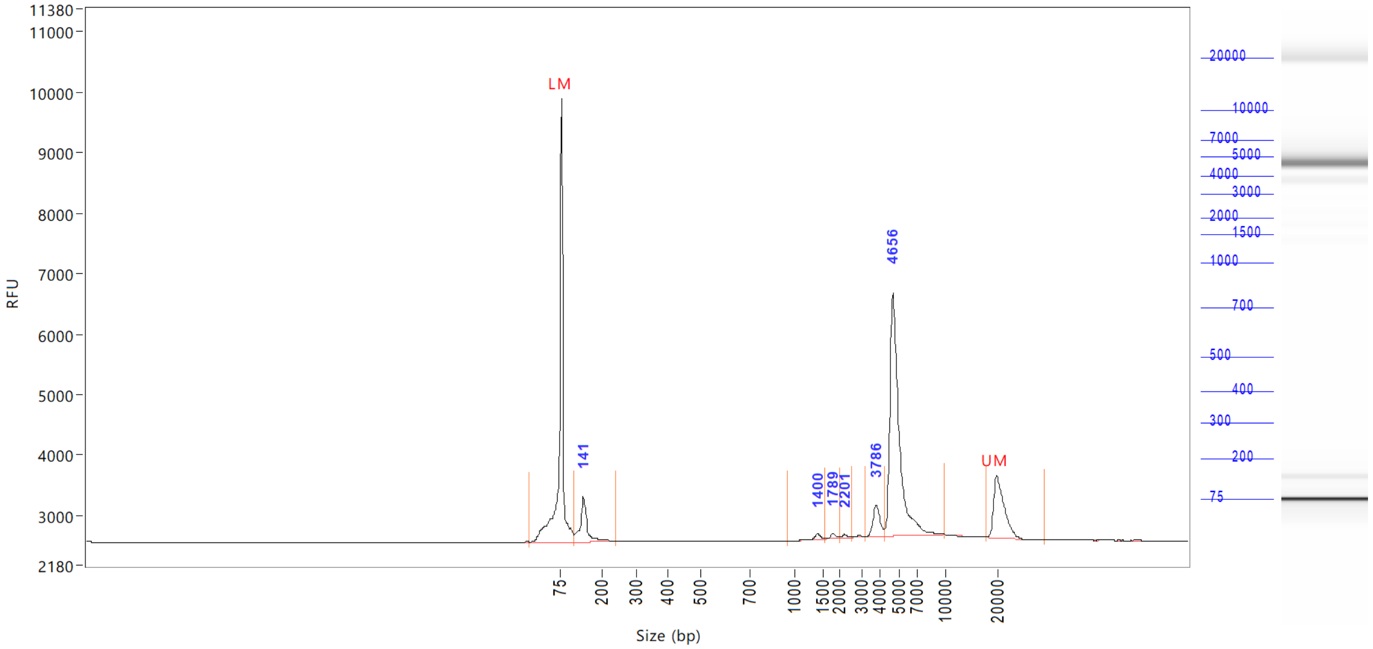

Peak	Size (bp)	Concentration (ng/uL)	From (bp)	To (bp)	Average size (bp)	CV%	RFU	Corrected peak area
1	75 (LM)	0.5000	0	114	66	29.18	7348	66.661
2	141	1.0720	114	241	146	14.96	766	11.910
3	1400	0.0759	957	1559	1370	6.63	90	0.843
4	1789	0.0619	1559	2000	1802	4.62	91	0.688
5	2201	0.0409	2000	2515	2195	5.31	58	0.455
6	3786	0.4892	3144	4173	3769	4.80	527	5.435
7	4656	4.7831	4173	9933	4954	14.27	4021	53.140
8	20000 (UM)	0.1037	17927	29189	20607	5.68	1032	13.829

TIC:	6.5230	ng/uL
TIM:	14.0485	nmole/L
Total concentration:	6.6334	ng/uL

Sample peak width (sec): 5 Sample min peak height: 50 Sample baseline V to V?: Y Sample baseline V to V points: 3
Sample filter: Binomial Number of points for filter: 3 Sample start region (min): 0 Sample end region (min): 30
Marker peak width (sec): 5 Marker min peak height: 200 Marker baseline V to V?: Y Marker baseline V to V points: 3
Lower marker selection: First peak > 500 RFU Upper marker selection: Last peak > 200 RFU
Ladder size (bp)75, 200, 300, 400, 500, 700, 1000, 1500, 2000, 3000, 4000, 5000, 7000, 10000, 20000
Quantification using: Lower Marker Final concentration (ng/uL): 0.5000 Dilution factor: 12.0

Sample: SampH3
Well location: H3
Created: Monday, September 20, 2021 6:40:06 PM

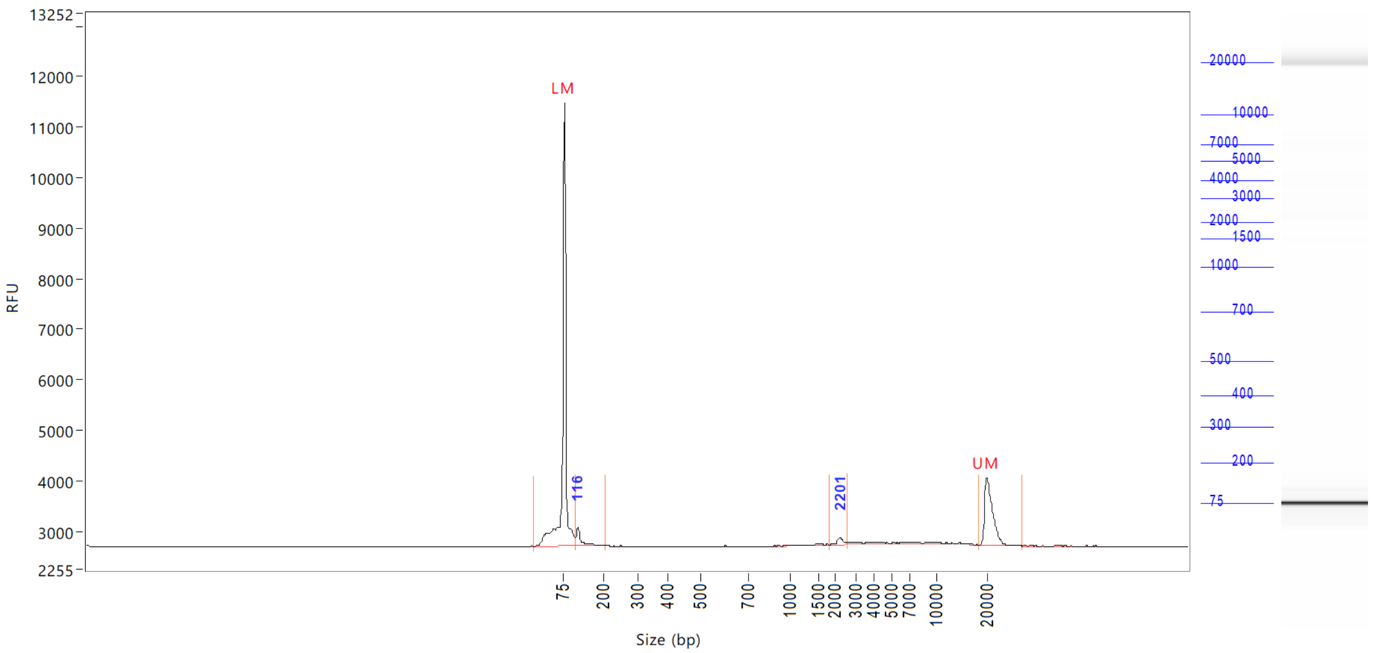

Peak	Size (bp)	Concentration (ng/uL)	From (bp)	To (bp)	Average size (bp)	CV%	RFU	Corrected peak area
1	75 (LM)	0.5000	0	108	68	26.87	8777	71.274
2	116	0.3441	108	206	126	16.11	368	4.088
3	2201	0.1108	1846	2572	2228	6.76	146	1.316
4	20000 (UM)	0.0952	18415	27133	20465	4.49	1334	13.571

TIC:	0.4549	ng/uL
TIM:	4.5693	nmole/L
Total concentration:	0.7846	ng/uL

```
Sample peak width (sec): 5    Sample min peak height: 50    Sample baseline V to V?: Y    Sample baseline V to V points: 3
Sample filter: Binomial       Number of points for filter: 3  Sample start region (min): 0  Sample end region (min): 30
Marker peak width (sec): 5    Marker min peak height: 200   Marker baseline V to V?: Y    Marker baseline V to V points: 3
Lower marker selection: First peak > 500 RFU                Upper marker selection: Last peak > 200 RFU
Ladder size (bp)75, 200, 300, 400, 500, 700, 1000, 1500, 2000, 3000, 4000, 5000, 7000, 10000, 20000
Quantification using: Lower Marker          Final concentration (ng/uL): 0.5000          Dilution factor: 12.0
```

Sample: SampA4
Well location: A4
Created: Monday, September 20, 2021 6:40:06 PM

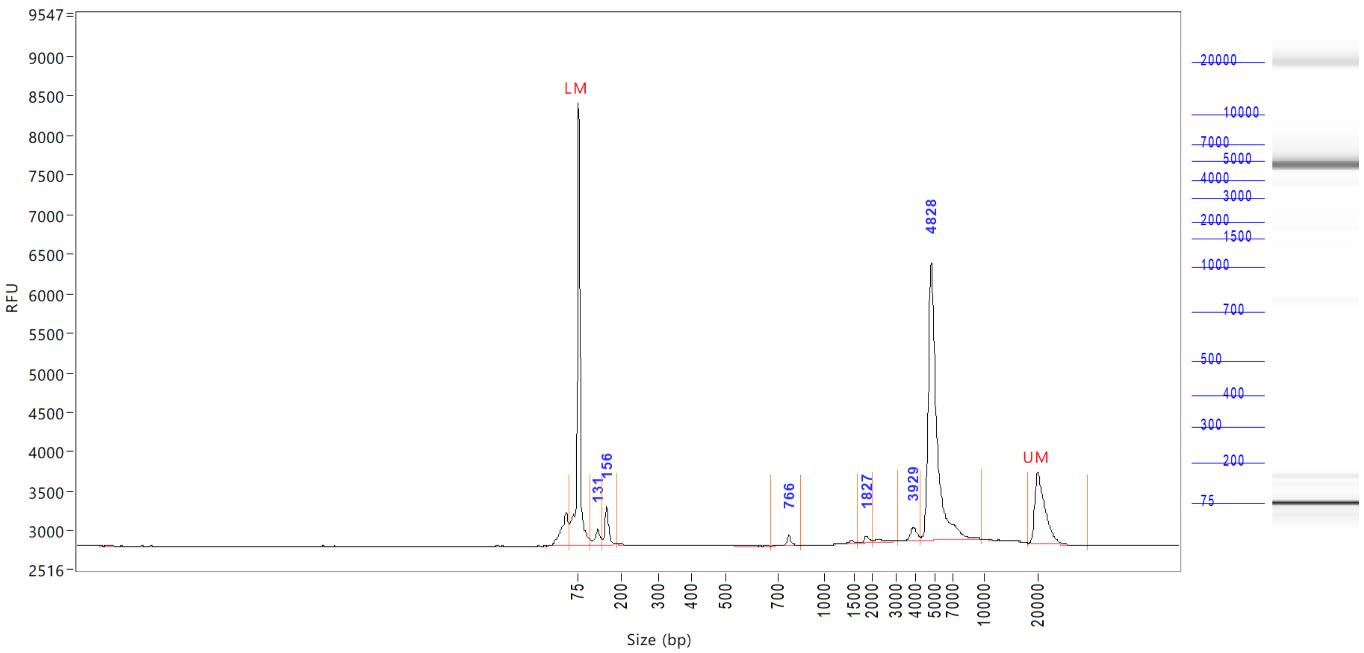

Peak	Size (bp)	Concentration (ng/uL)	From (bp)	To (bp)	Average size (bp)	CV%	RFU	Corrected peak area
1	75 (LM)	0.5000	48	112	73	11.48	5604	39.650
2	131	0.3730	112	145	129	6.38	203	2.465
3	156	0.6014	145	187	156	3.85	492	3.974
4	766	0.1622	672	844	763	3.69	131	1.072
5	1827	0.1047	1559	2001	1830	5.00	90	0.692
6	3929	0.2391	3072	4277	3901	4.38	164	1.580
7	4828	6.1612	4277	9800	5106	13.75	3515	40.715
8	20000 (UM)	0.1403	18171	29189	20573	5.56	901	11.125

TIC:	7.6416	ng/uL
TIM:	13.6189	nmole/L
Total concentration:	7.9804	ng/uL

Sample peak width (sec): 5 Sample min peak height: 50 Sample baseline V to V?: Y Sample baseline V to V points: 3
Sample filter: Binomial Number of points for filter: 3 Sample start region (min): 0 Sample end region (min): 30
Marker peak width (sec): 5 Marker min peak height: 200 Marker baseline V to V?: Y Marker baseline V to V points: 3
Lower marker selection: First peak > 500 RFU Upper marker selection: Last peak > 200 RFU
Ladder size (bp)75, 200, 300, 400, 500, 700, 1000, 1500, 2000, 3000, 4000, 5000, 7000, 10000, 20000
Quantification using: Lower Marker Final concentration (ng/uL): 0.5000 Dilution factor: 12.0

Sample: SampB4
Well location: B4
Created: Monday, September 20, 2021 6:40:06 PM

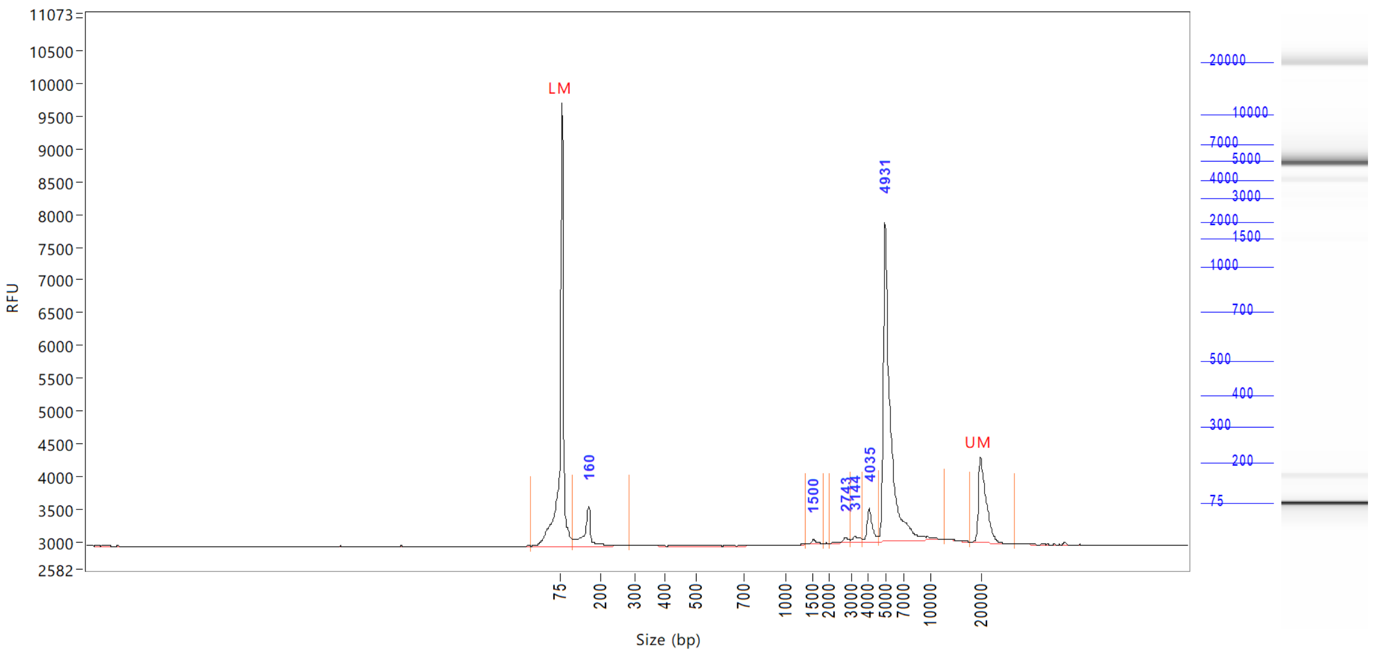

Peak	Size (bp)	Concentration (ng/uL)	From (bp)	To (bp)	Average size (bp)	CV%	RFU	Corrected peak area
1	75 (LM)	0.5000	0	110	67	25.61	6781	59.898
2	160	0.9756	110	286	157	19.28	605	9.739
3	1500	0.0637	1367	1827	1526	6.00	77	0.636
4	2743	0.0792	2001	2943	2654	7.84	82	0.791
5	3144	0.1110	2943	3679	3279	6.33	89	1.108
6	4035	0.4105	3679	4518	4063	4.18	519	4.098
7	4931	5.0168	4518	12806	5416	17.31	4872	50.083
8	20000 (UM)	0.1091	17927	26891	20562	4.54	1296	13.067

TIC:	6.6569	ng/uL
TIM:	12.0781	nmole/L
Total concentration:	6.8249	ng/uL

```
Sample peak width (sec): 5    Sample min peak height: 50    Sample baseline V to V?: Y    Sample baseline V to V points: 3
Sample filter: Binomial       Number of points for filter: 3  Sample start region (min): 0  Sample end region (min): 30
Marker peak width (sec): 5    Marker min peak height: 200   Marker baseline V to V?: Y    Marker baseline V to V points: 3
Lower marker selection: First peak > 500 RFU                Upper marker selection: Last peak > 200 RFU
Ladder size (bp)75, 200, 300, 400, 500, 700, 1000, 1500, 2000, 3000, 4000, 5000, 7000, 10000, 20000
Quantification using: Lower Marker          Final concentration (ng/uL): 0.5000          Dilution factor: 12.0
```

Sample: SampC4
Well location: C4
Created: Monday, September 20, 2021 6:40:06 PM

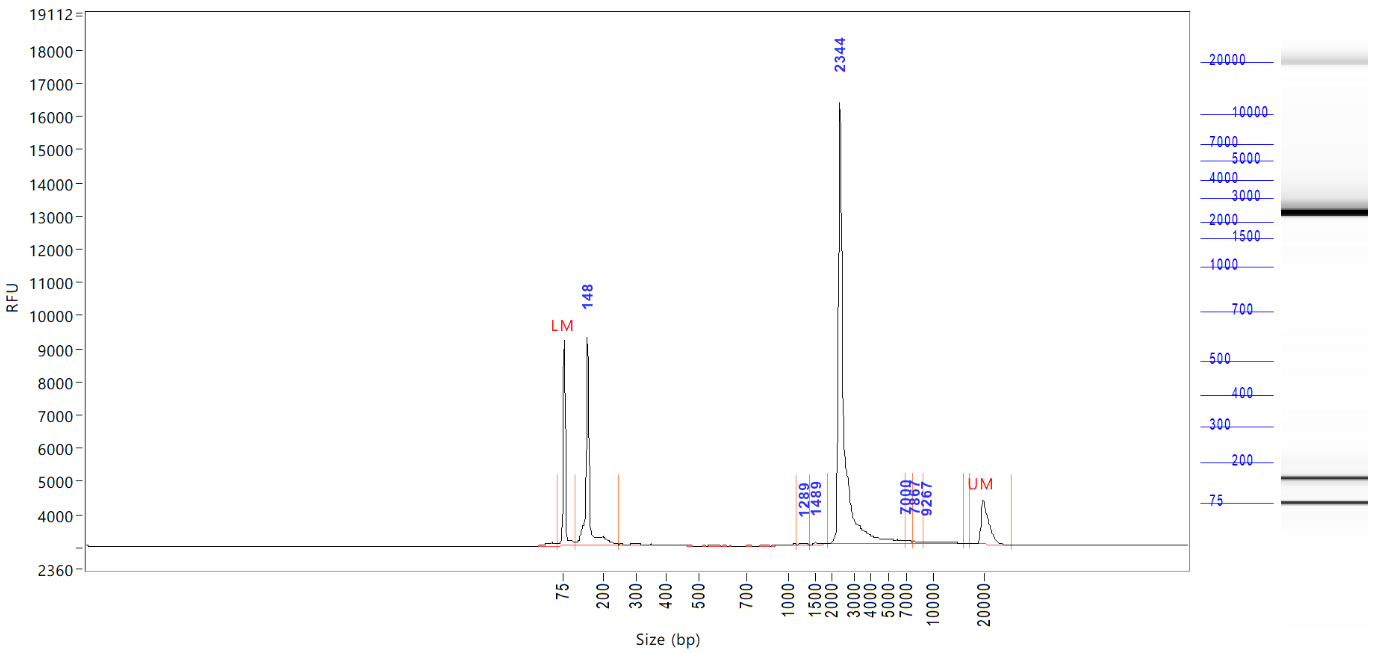

Peak	Size (bp)	Concentration (ng/uL)	From (bp)	To (bp)	Average size (bp)	CV%	RFU	Corrected peak area
1	75 (LM)	0.5000	54	112	75	10.21	6189	36.522
2	148	8.4645	112	246	152	12.90	6264	51.524
3	1289	0.0928	1156	1389	1251	3.93	51	0.565
4	1489	0.1248	1389	1866	1594	7.74	62	0.760
5	2344	18.8569	1866	6926	2629	25.42	13322	114.783
6	7000	0.1126	6926	7667	7246	3.25	91	0.685
7	7867	0.1301	7667	8867	8218	4.46	69	0.792
8	9267	0.2650	8867	15976	11100	16.87	57	1.613
9	20000 (UM)	0.1804	17439	25561	20516	4.51	1312	13.178

TIC:	28.0466	ng/uL
TIM:	103.6748	nmole/L
Total concentration:	28.4368	ng/uL

```
Sample peak width (sec): 5     Sample min peak height: 50     Sample baseline V to V?: Y     Sample baseline V to V points: 3
Sample filter: Binomial        Number of points for filter: 3  Sample start region (min): 0  Sample end region (min): 30
Marker peak width (sec): 5     Marker min peak height: 200    Marker baseline V to V?: Y     Marker baseline V to V points: 3
Lower marker selection: First peak > 500 RFU                  Upper marker selection: Last peak > 200 RFU
Ladder size (bp)75, 200, 300, 400, 500, 700, 1000, 1500, 2000, 3000, 4000, 5000, 7000, 10000, 20000
Quantification using: Lower Marker          Final concentration (ng/uL): 0.5000          Dilution factor: 12.0
```

Sample: SampD4
Well location: D4
Created: Monday, September 20, 2021 6:40:06 PM

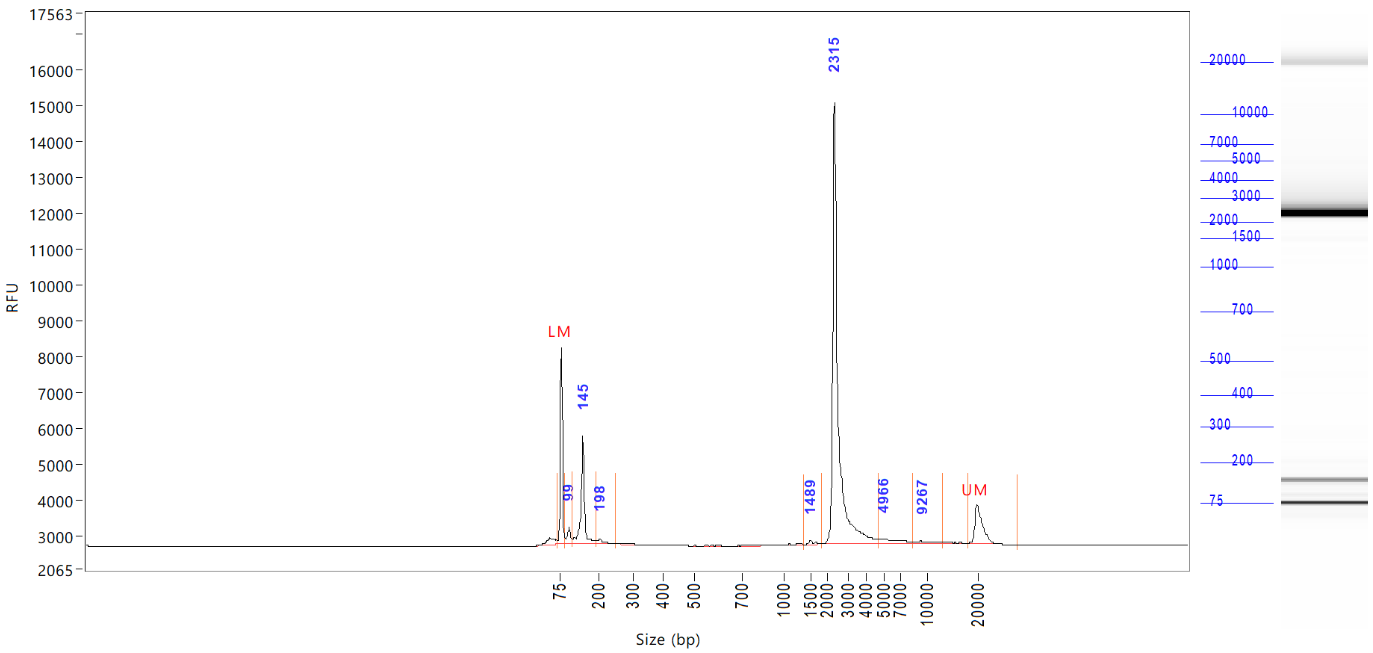

Peak	Size (bp)	Concentration (ng/uL)	From (bp)	To (bp)	Average size (bp)	CV%	RFU	Corrected peak area
1	75 (LM)	0.5000	64	87	74	4.65	5463	30.329
2	99	0.7035	87	110	98	5.84	428	3.556
3	145	4.6892	110	187	141	7.73	2971	23.703
4	198	0.3141	187	244	202	5.64	132	1.588
5	1489	0.2086	1378	1846	1556	7.37	115	1.054
6	2315	20.5108	1846	4724	2502	17.16	12323	103.678
7	4966	0.6842	4724	8334	6081	17.85	121	3.458
8	9267	0.3001	8334	13049	10140	12.82	56	1.517
9	20000 (UM)	0.1785	18171	28342	20499	4.66	1073	10.830

TIC:	27.4105	ng/uL
TIM:	82.9455	nmole/L
Total concentration:	27.8620	ng/uL

Sample peak width (sec): 5   Sample min peak height: 50   Sample baseline V to V?: Y   Sample baseline V to V points: 3
Sample filter: Binomial   Number of points for filter: 3   Sample start region (min): 0   Sample end region (min): 30
Marker peak width (sec): 5   Marker min peak height: 200   Marker baseline V to V?: Y   Marker baseline V to V points: 3
Lower marker selection: First peak > 500 RFU   Upper marker selection: Last peak > 200 RFU
Ladder size (bp)75, 200, 300, 400, 500, 700, 1000, 1500, 2000, 3000, 4000, 5000, 7000, 10000, 20000
Quantification using: Lower Marker   Final concentration (ng/uL): 0.5000   Dilution factor: 12.0

Sample: SampE4
Well location: E4
Created: Monday, September 20, 2021 6:40:06 PM

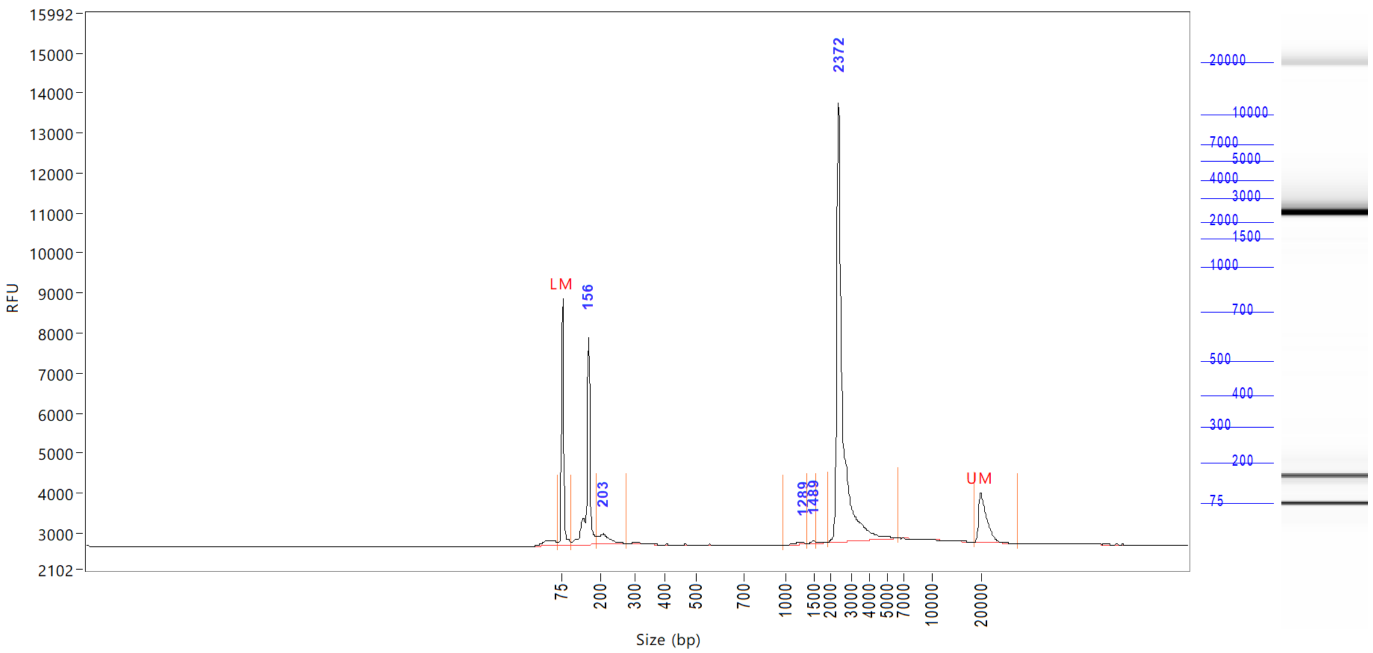

Peak	Size (bp)	Concentration (ng/uL)	From (bp)	To (bp)	Average size (bp)	CV%	RFU	Corrected peak area
1	75 (LM)	0.5000	62	103	74	7.05	6162	35.191
2	156	7.2057	103	183	152	8.33	5156	42.263
3	203	1.1589	183	273	209	9.20	276	6.797
4	1289	0.1608	983	1367	1196	7.60	56	0.943
5	1489	0.0893	1367	1559	1468	2.87	81	0.524
6	2372	17.0203	1904	6334	2598	18.41	10989	99.828
7	20000 (UM)	0.1830	18659	27496	20547	4.82	1236	12.881

TIC:	25.6351	ng/uL
TIM:	98.1383	nmole/L
Total concentration:	25.9871	ng/uL

Sample peak width (sec): 5 Sample min peak height: 50 Sample baseline V to V?: Y Sample baseline V to V points: 3
Sample filter: Binomial Number of points for filter: 3 Sample start region (min): 0 Sample end region (min): 30
Marker peak width (sec): 5 Marker min peak height: 200 Marker baseline V to V?: Y Marker baseline V to V points: 3
Lower marker selection: First peak > 500 RFU Upper marker selection: Last peak > 200 RFU
Ladder size (bp)75, 200, 300, 400, 500, 700, 1000, 1500, 2000, 3000, 4000, 5000, 7000, 10000, 20000
Quantification using: Lower Marker Final concentration (ng/uL): 0.5000 Dilution factor: 12.0

Sample: SampF4
Well location: F4
Created: Monday, September 20, 2021 6:40:06 PM

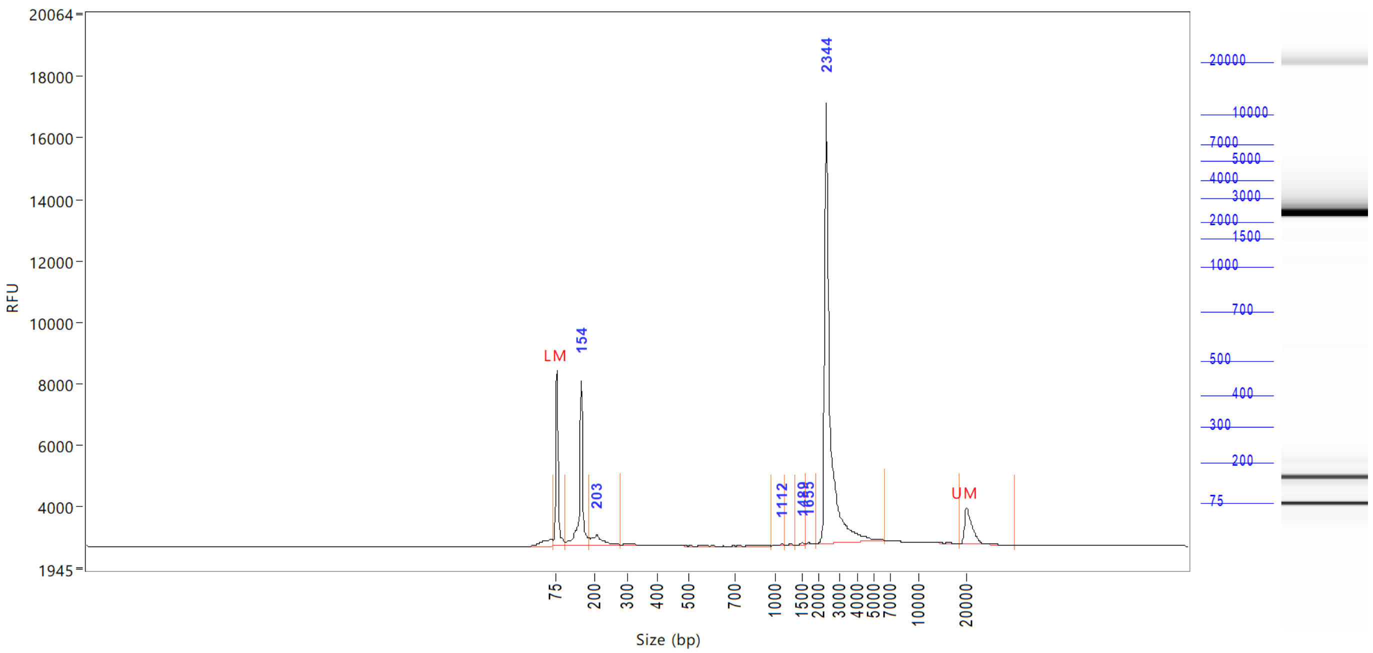

Peak	Size (bp)	Concentration (ng/uL)	From (bp)	To (bp)	Average size (bp)	CV%	RFU	Corrected peak area
1	75 (LM)	0.5000	64	103	74	8.11	5706	34.666
2	154	7.6161	103	181	149	8.52	5346	44.003
3	203	1.3122	181	278	207	9.03	318	7.582
4	1112	0.0609	965	1167	1084	3.88	51	0.352
5	1489	0.0664	1356	1559	1480	2.25	71	0.383
6	1655	0.0673	1559	1904	1668	4.19	58	0.389
7	2344	21.4415	1904	6186	2554	18.20	14382	123.881
8	20000 (UM)	0.1734	18415	29793	20647	5.24	1188	12.024

TIC:	30.5643	ng/uL
TIM:	108.4832	nmole/L
Total concentration:	31.0955	ng/uL

```
Sample peak width (sec): 5    Sample min peak height: 50    Sample baseline V to V?: Y    Sample baseline V to V points: 3
Sample filter: Binomial       Number of points for filter: 3  Sample start region (min): 0  Sample end region (min): 30
Marker peak width (sec): 5    Marker min peak height: 200   Marker baseline V to V?: Y    Marker baseline V to V points: 3
Lower marker selection: First peak > 500 RFU                Upper marker selection: Last peak > 200 RFU
Ladder size (bp)75, 200, 300, 400, 500, 700, 1000, 1500, 2000, 3000, 4000, 5000, 7000, 10000, 20000
Quantification using: Lower Marker          Final concentration (ng/uL): 0.5000          Dilution factor: 12.0
```

Sample: SampG4
Well location: G4
Created: Monday, September 20, 2021 6:40:06 PM

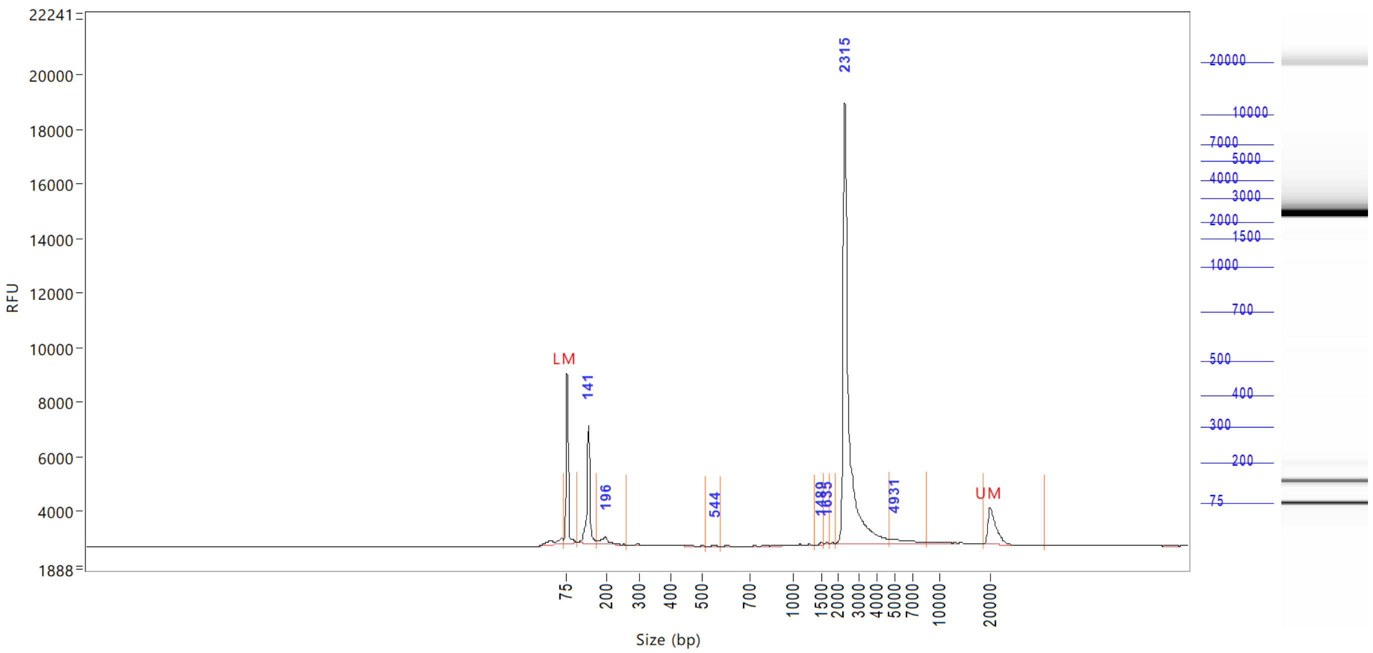

Peak	Size (bp)	Concentration (ng/uL)	From (bp)	To (bp)	Average size (bp)	CV%	RFU	Corrected peak area
1	75 (LM)	0.5000	66	108	75	6.19	6217	35.793
2	141	5.1698	108	168	139	5.45	4288	30.840
3	196	0.7813	168	259	193	8.56	258	4.661
4	544	0.0593	509	575	542	1.75	59	0.354
5	1489	0.0751	1389	1559	1488	2.07	86	0.448
6	1635	0.0687	1559	1750	1640	3.20	69	0.410
7	2315	23.1130	1923	4690	2537	17.34	16158	137.880
8	4931	0.6412	4690	8467	5928	18.03	151	3.825
9	20000 (UM)	0.1960	18537	31002	20568	5.34	1347	14.032

TIC:	29.9083	ng/uL
TIM:	83.2812	nmole/L
Total concentration:	30.4152	ng/uL

```
Sample peak width (sec): 5    Sample min peak height: 50    Sample baseline V to V?: Y    Sample baseline V to V points: 3
Sample filter: Binomial       Number of points for filter: 3  Sample start region (min): 0  Sample end region (min): 30
Marker peak width (sec): 5    Marker min peak height: 200   Marker baseline V to V?: Y    Marker baseline V to V points: 3
Lower marker selection: First peak > 500 RFU                Upper marker selection: Last peak > 200 RFU
Ladder size (bp)75, 200, 300, 400, 500, 700, 1000, 1500, 2000, 3000, 4000, 5000, 7000, 10000, 20000
Quantification using: Lower Marker          Final concentration (ng/uL): 0.5000          Dilution factor: 12.0
```

Sample: SampH4
Well location: H4
Created: Monday, September 20, 2021 6:40:06 PM

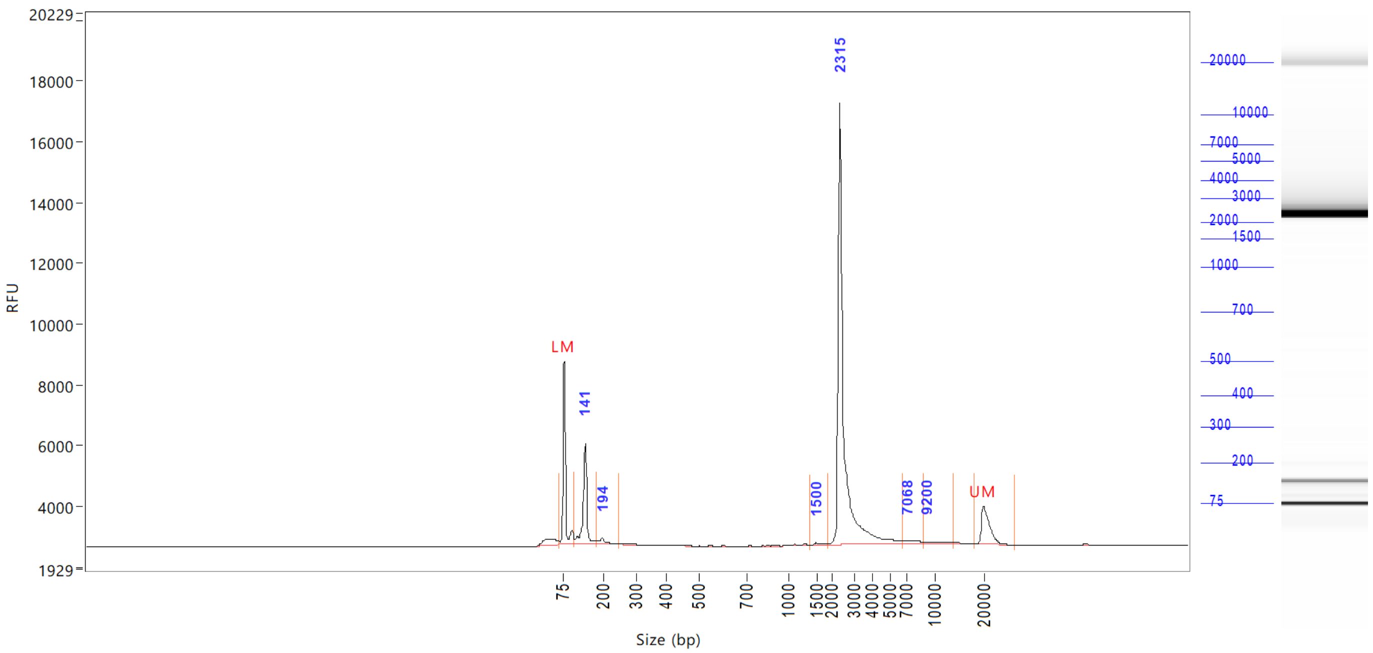

Peak	Size (bp)	Concentration (ng/uL)	From (bp)	To (bp)	Average size (bp)	CV%	RFU	Corrected peak area
1	75 (LM)	0.5000	62	107	75	10.49	6009	37.712
2	141	4.1156	107	175	137	7.19	3273	25.868
3	194	0.3681	175	244	195	6.08	184	2.313
4	1500	0.1151	1378	1866	1590	7.34	62	0.723
5	2315	19.8381	1866	6556	2579	24.32	14561	124.689
6	7068	0.3632	6556	8867	7598	8.65	107	2.283
7	9200	0.2583	8867	13781	10580	13.32	67	1.623
8	20000 (UM)	0.1709	18171	26166	20450	4.47	1256	12.889

TIC:	25.0583	ng/uL
TIM:	65.3686	nmole/L
Total concentration:	25.4888	ng/uL

Sample peak width (sec): 5 Sample min peak height: 50 Sample baseline V to V?: Y Sample baseline V to V points: 3
Sample filter: Binomial Number of points for filter: 3 Sample start region (min): 0 Sample end region (min): 30
Marker peak width (sec): 5 Marker min peak height: 200 Marker baseline V to V?: Y Marker baseline V to V points: 3
Lower marker selection: First peak > 500 RFU Upper marker selection: Last peak > 200 RFU
Ladder size (bp)75, 200, 300, 400, 500, 700, 1000, 1500, 2000, 3000, 4000, 5000, 7000, 10000, 20000
Quantification using: Lower Marker Final concentration (ng/uL): 0.5000 Dilution factor: 12.0

Sample: SampA5
Well location: A5
Created: Monday, September 20, 2021 6:40:06 PM

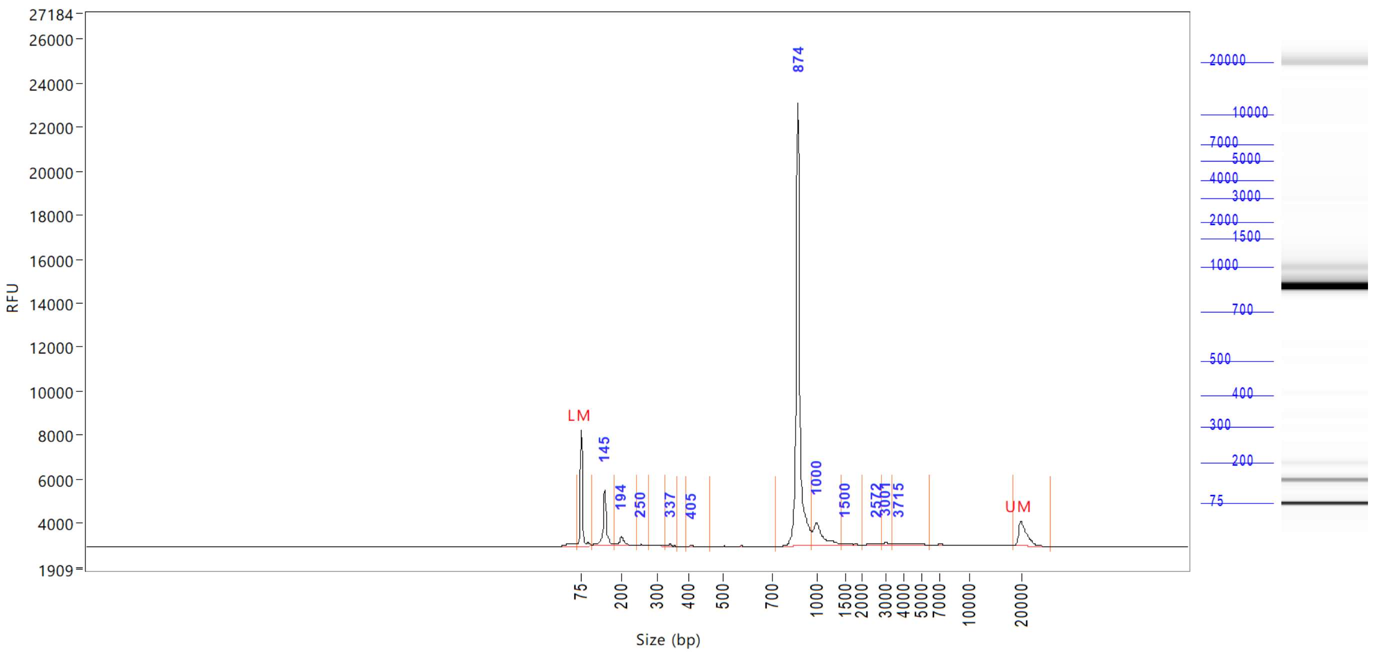

Peak	Size (bp)	Concentration (ng/uL)	From (bp)	To (bp)	Average size (bp)	CV%	RFU	Corrected peak area
1	75 (LM)	0.5000	64	108	74	8.19	5305	29.872
2	145	4.0493	108	175	142	6.95	2564	20.160
3	194	0.9181	175	239	197	6.08	394	4.571
4	250	0.0867	239	273	248	1.94	58	0.431
5	337	0.0935	321	364	336	1.83	88	0.465
6	405	0.0916	390	462	412	4.57	74	0.456
7	874	26.2372	727	970	874	2.84	20175	130.628
8	1000	3.1646	970	1445	1071	10.54	1062	15.756
9	1500	0.1919	1445	2001	1582	8.68	81	0.956
10	2572	0.1341	2001	2829	2530	7.14	62	0.668
11	3001	0.2028	2829	3429	3096	5.31	97	1.010
12	3715	0.3247	3429	5889	4169	13.18	64	1.616
13	20000 (UM)	0.1862	18415	25803	20511	4.34	1134	11.125

TIC:	35.4944	ng/uL
TIM:	110.7176	nmole/L
Total concentration:	35.7668	ng/uL

```
Sample peak width (sec): 5    Sample min peak height: 50    Sample baseline V to V?: Y    Sample baseline V to V points: 3
Sample filter: Binomial       Number of points for filter: 3  Sample start region (min): 0  Sample end region (min): 30
Marker peak width (sec): 5    Marker min peak height: 200   Marker baseline V to V?: Y    Marker baseline V to V points: 3
Lower marker selection: First peak > 500 RFU                Upper marker selection: Last peak > 200 RFU
Ladder size (bp)75, 200, 300, 400, 500, 700, 1000, 1500, 2000, 3000, 4000, 5000, 7000, 10000, 20000
Quantification using: Lower Marker          Final concentration (ng/uL): 0.5000          Dilution factor: 12.0
```

Sample: SampB5
Well location: B5
Created: Monday, September 20, 2021 6:40:06 PM

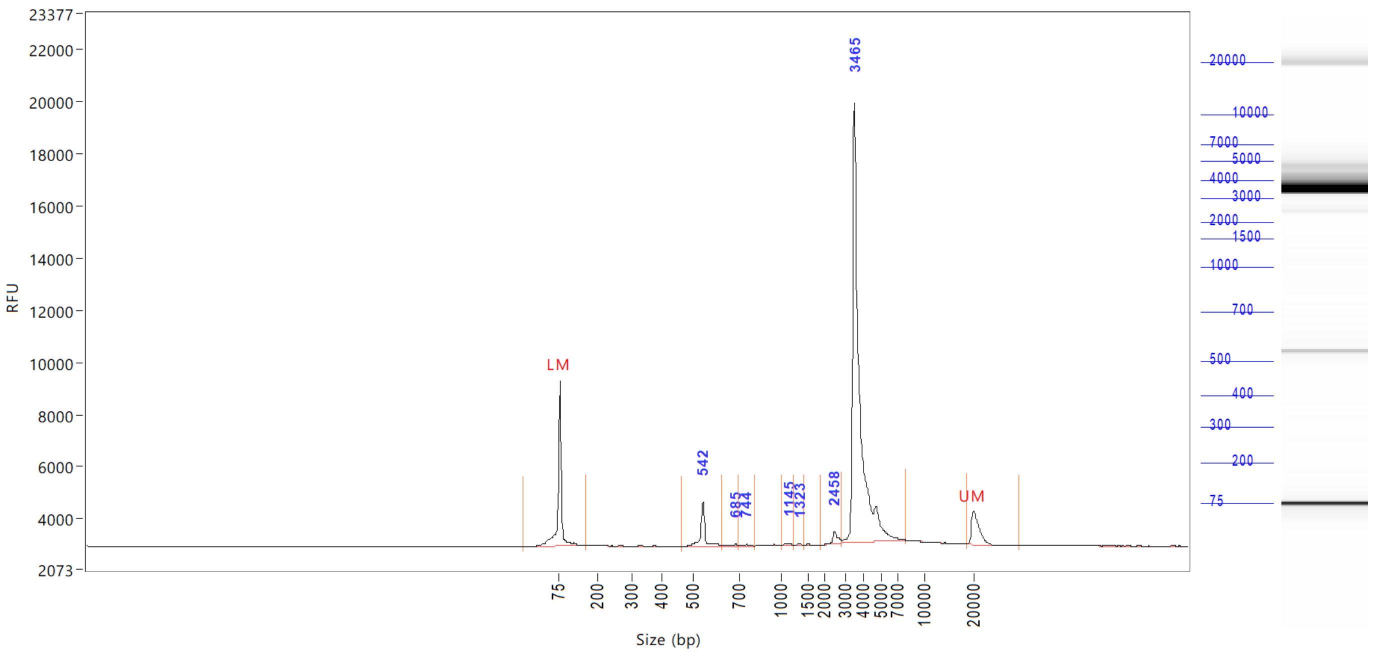

Peak	Size (bp)	Concentration (ng/uL)	From (bp)	To (bp)	Average size (bp)	CV%	RFU	Corrected peak area
1	75 (LM)	0.5000	0	156	68	26.40	6310	51.644
2	542	1.5384	466	626	541	4.66	1673	13.242
3	685	0.1172	626	695	660	3.15	65	1.008
4	744	0.0782	695	805	727	3.13	61	0.673
5	1145	0.0963	1000	1212	1103	4.66	84	0.829
6	1323	0.0498	1212	1411	1306	3.88	52	0.429
7	2458	0.4262	1866	2829	2503	6.07	447	3.668
8	3465	20.0790	2829	7867	3765	14.06	16871	172.828
9	20000 (UM)	0.1239	18537	29430	20531	4.60	1292	12.800

TIC:	22.3850	ng/uL
TIM:	14.4148	nmole/L
Total concentration:	22.6417	ng/uL

Sample peak width (sec): 5 Sample min peak height: 50 Sample baseline V to V?: Y Sample baseline V to V points: 3
Sample filter: Binomial Number of points for filter: 3 Sample start region (min): 0 Sample end region (min): 30
Marker peak width (sec): 5 Marker min peak height: 200 Marker baseline V to V?: Y Marker baseline V to V points: 3
Lower marker selection: First peak > 500 RFU Upper marker selection: Last peak > 200 RFU
Ladder size (bp)75, 200, 300, 400, 500, 700, 1000, 1500, 2000, 3000, 4000, 5000, 7000, 10000, 20000
Quantification using: Lower Marker Final concentration (ng/uL): 0.5000 Dilution factor: 12.0

Sample: SampC5
Well location: C5
Created: Monday, September 20, 2021 6:40:06 PM

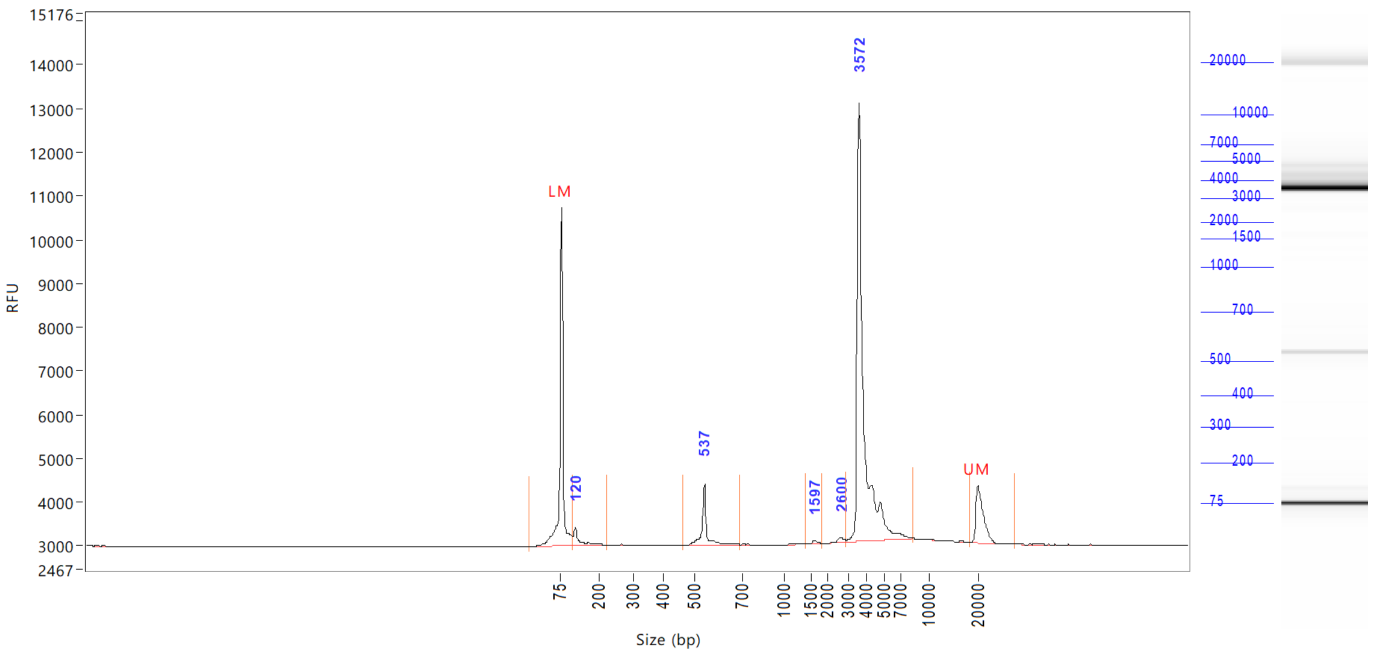

Peak	Size (bp)	Concentration (ng/uL)	From (bp)	To (bp)	Average size (bp)	CV%	RFU	Corrected peak area
1	75 (LM)	0.5000	0	108	71	19.41	7745	61.153
2	120	0.5790	108	219	134	18.77	401	5.902
3	537	1.2982	460	692	546	7.49	1418	13.231
4	1597	0.0546	1400	1808	1581	4.15	79	0.557
5	2600	0.1084	1808	2857	2576	6.18	111	1.105
6	3572	9.4492	2857	8334	3877	16.41	10041	96.308
7	20000 (UM)	0.1065	18415	27496	20513	4.57	1323	13.023

TIC:	11.4894	ng/uL
TIM:	15.1514	nmole/L
Total concentration:	11.6812	ng/uL

Sample peak width (sec): 5   Sample min peak height: 50   Sample baseline V to V?: Y   Sample baseline V to V points: 3
Sample filter: Binomial   Number of points for filter: 3 Sample start region (min): 0 Sample end region (min): 30
Marker peak width (sec): 5   Marker min peak height: 200   Marker baseline V to V?: Y   Marker baseline V to V points: 3
Lower marker selection: First peak > 500 RFU   Upper marker selection: Last peak > 200 RFU
Ladder size (bp)75, 200, 300, 400, 500, 700, 1000, 1500, 2000, 3000, 4000, 5000, 7000, 10000, 20000
Quantification using: Lower Marker   Final concentration (ng/uL): 0.5000   Dilution factor: 12.0

Sample: SampD5
Well location: D5
Created: Monday, September 20, 2021 6:40:06 PM

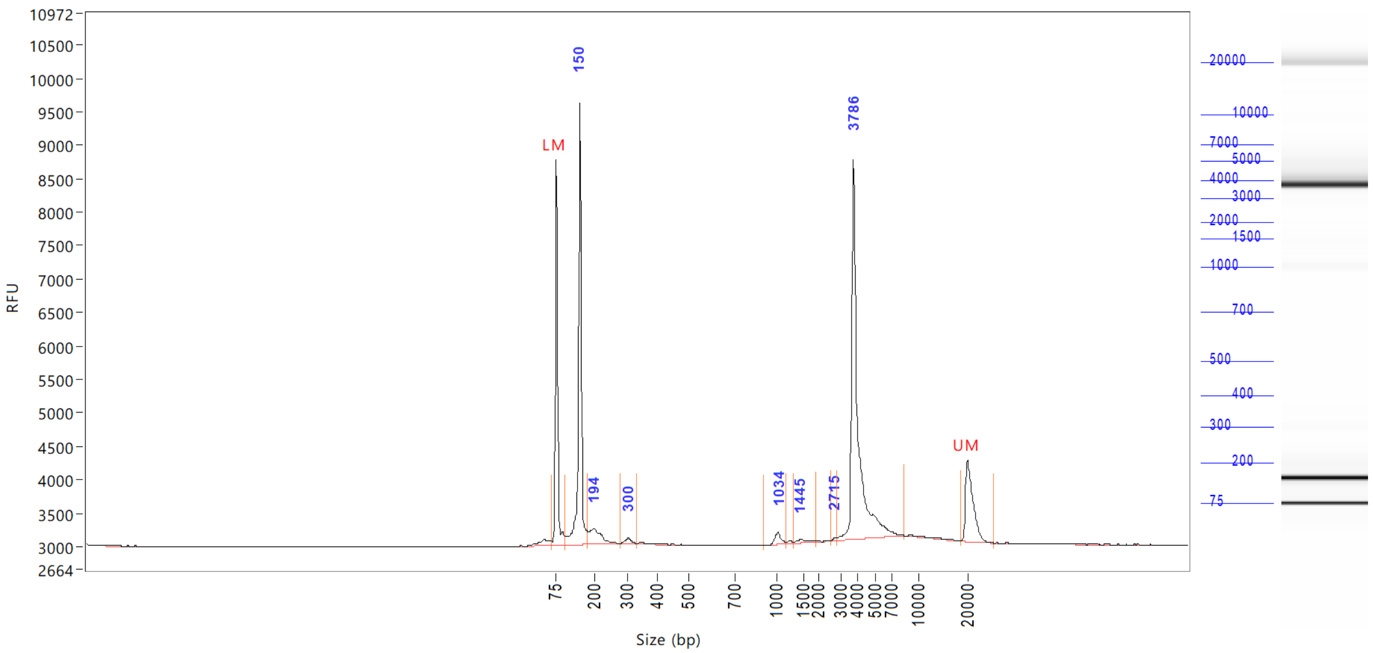

Peak	Size (bp)	Concentration (ng/uL)	From (bp)	To (bp)	Average size (bp)	CV%	RFU	Corrected peak area
1	75 (LM)	0.5000	64	103	74	7.79	5769	32.974
2	150	8.6947	103	177	147	6.76	6612	47.783
3	194	1.1349	177	275	202	8.72	232	6.237
4	300	0.1985	275	329	299	3.43	79	1.091
5	1034	0.3626	913	1190	1032	5.20	194	1.993
6	1445	0.1773	1323	1904	1509	9.32	55	0.975
7	2715	0.0591	2515	2829	2705	2.95	55	0.325
8	3786	9.8007	2829	8334	4021	15.45	5678	53.861
9	20000 (UM)	0.1837	18781	25561	20541	4.48	1212	12.115

TIC:	20.4279	ng/uL
TIM:	112.3575	nmole/L
Total concentration:	20.7917	ng/uL

Sample peak width (sec): 5 Sample min peak height: 50 Sample baseline V to V?: Y Sample baseline V to V points: 3
Sample filter: Binomial Number of points for filter: 3 Sample start region (min): 0 Sample end region (min): 30
Marker peak width (sec): 5 Marker min peak height: 200 Marker baseline V to V?: Y Marker baseline V to V points: 3
Lower marker selection: First peak > 500 RFU Upper marker selection: Last peak > 200 RFU
Ladder size (bp)75, 200, 300, 400, 500, 700, 1000, 1500, 2000, 3000, 4000, 5000, 7000, 10000, 20000
Quantification using: Lower Marker Final concentration (ng/uL): 0.5000 Dilution factor: 12.0

Sample: SampE5
Well location: E5
Created: Monday, September 20, 2021 6:40:06 PM

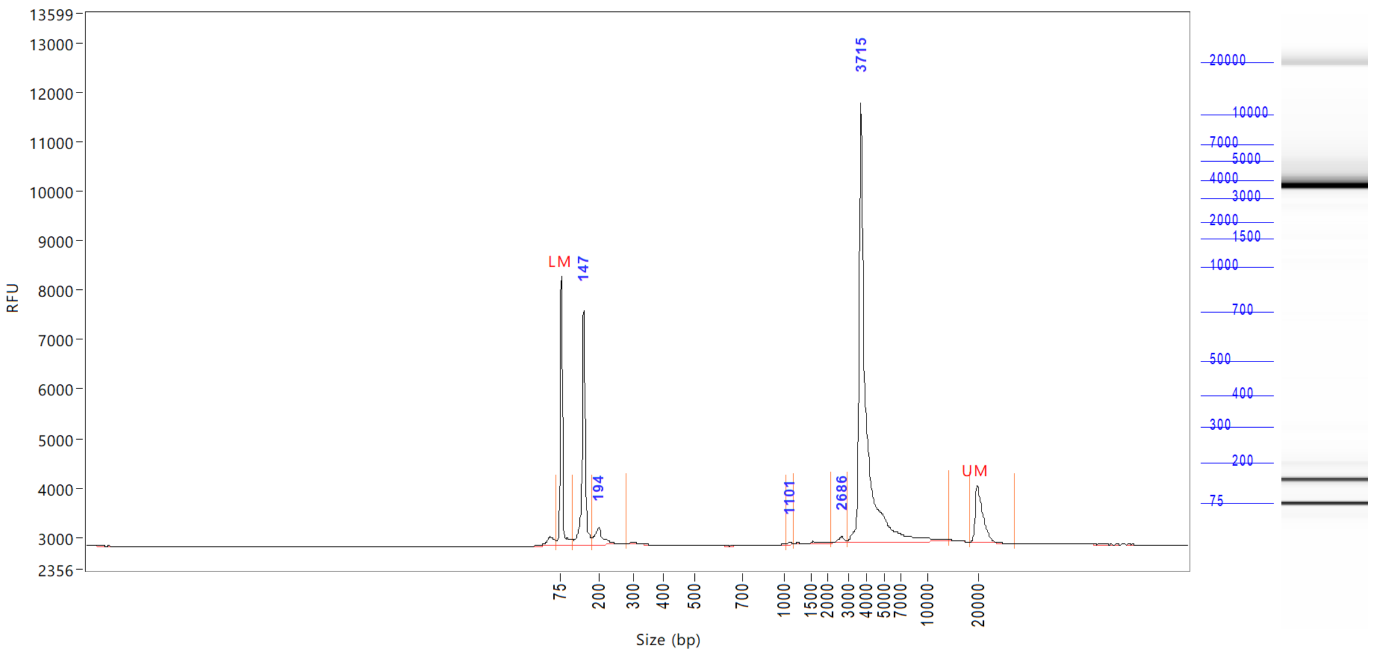

Peak	Size (bp)	Concentration (ng/uL)	From (bp)	To (bp)	Average size (bp)	CV%	RFU	Corrected peak area
1	75 (LM)	0.5000	60	114	74	10.74	5458	32.937
2	147	6.4527	114	171	143	5.69	4745	35.422
3	194	1.2215	171	277	198	9.38	358	6.705
4	1101	0.0628	1023	1178	1084	2.91	58	0.345
5	2686	0.2315	2115	2914	2601	6.34	125	1.271
6	3715	16.8790	2914	14269	4241	31.08	8904	92.657
7	20000 (UM)	0.1739	18537	27496	20519	4.62	1148	11.452

TIC:	24.8476	ng/uL
TIM:	91.0931	nmole/L
Total concentration:	25.3120	ng/uL

Sample peak width (sec): 5   Sample min peak height: 50   Sample baseline V to V?: Y   Sample baseline V to V points: 3
Sample filter: Binomial   Number of points for filter: 3 Sample start region (min): 0 Sample end region (min): 30
Marker peak width (sec): 5   Marker min peak height: 200   Marker baseline V to V?: Y   Marker baseline V to V points: 3
Lower marker selection: First peak > 500 RFU   Upper marker selection: Last peak > 200 RFU
Ladder size (bp)75, 200, 300, 400, 500, 700, 1000, 1500, 2000, 3000, 4000, 5000, 7000, 10000, 20000
Quantification using: Lower Marker   Final concentration (ng/uL): 0.5000   Dilution factor: 12.0

Sample: SampF5
Well location: F5
Created: Monday, September 20, 2021 6:40:06 PM

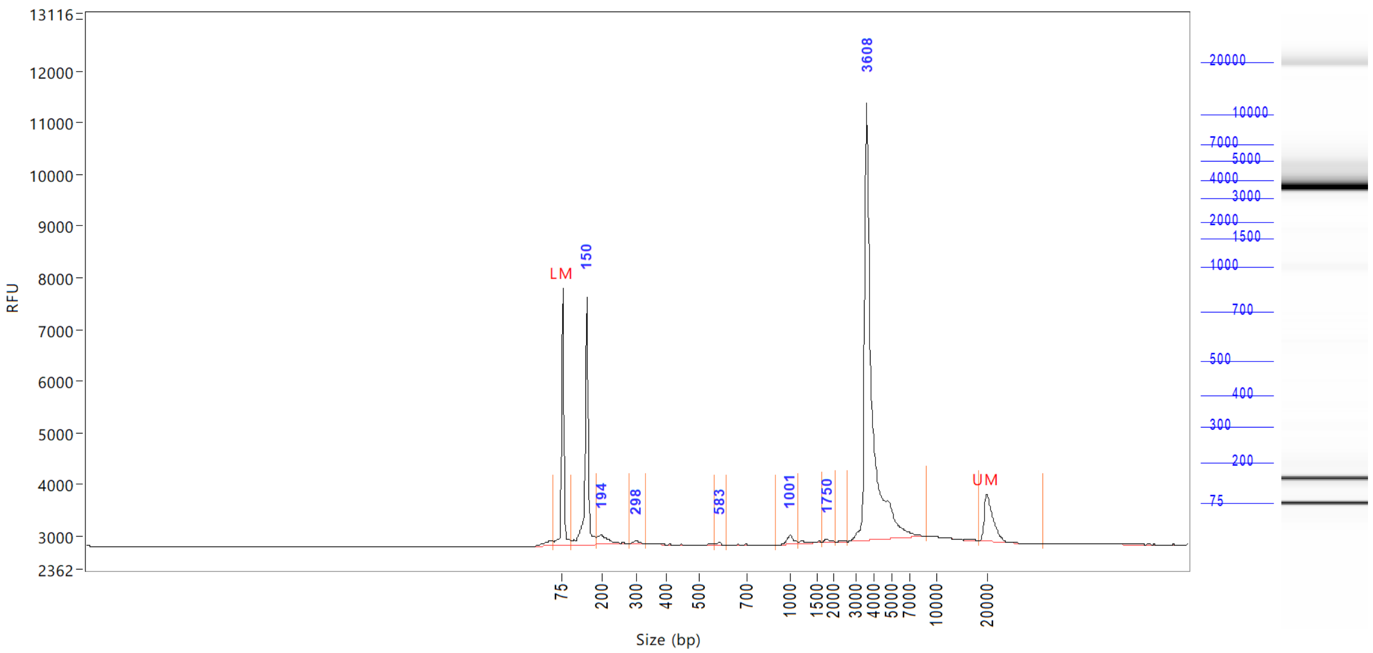

Peak	Size (bp)	Concentration (ng/uL)	From (bp)	To (bp)	Average size (bp)	CV%	RFU	Corrected peak area
1	75 (LM)	0.5000	48	101	74	8.26	4975	29.969
2	150	7.2816	101	179	146	7.49	4781	36.371
3	194	0.9312	179	278	205	9.64	178	4.651
4	298	0.1528	278	329	300	3.45	50	0.763
5	583	0.1061	565	616	581	1.69	68	0.530
6	1001	0.3990	905	1156	1018	4.81	178	1.993
7	1750	0.1041	1616	2058	1772	4.92	63	0.520
8	3608	19.0247	2600	8934	3923	16.51	8474	95.026
9	20000 (UM)	0.1673	18293	31244	20554	6.02	905	10.029

TIC:	27.9995	ng/uL
TIM:	99.2971	nmole/L
Total concentration:	28.4370	ng/uL

```
Sample peak width (sec): 5     Sample min peak height: 50    Sample baseline V to V?: Y    Sample baseline V to V points: 3
Sample filter: Binomial        Number of points for filter: 3  Sample start region (min): 0  Sample end region (min): 30
Marker peak width (sec): 5     Marker min peak height: 200   Marker baseline V to V?: Y    Marker baseline V to V points: 3
Lower marker selection: First peak > 500 RFU                 Upper marker selection: Last peak > 200 RFU
Ladder size (bp)75, 200, 300, 400, 500, 700, 1000, 1500, 2000, 3000, 4000, 5000, 7000, 10000, 20000
Quantification using: Lower Marker          Final concentration (ng/uL): 0.5000          Dilution factor: 12.0
```

Sample: SampG5
Well location: G5
Created: Monday, September 20, 2021 6:40:06 PM

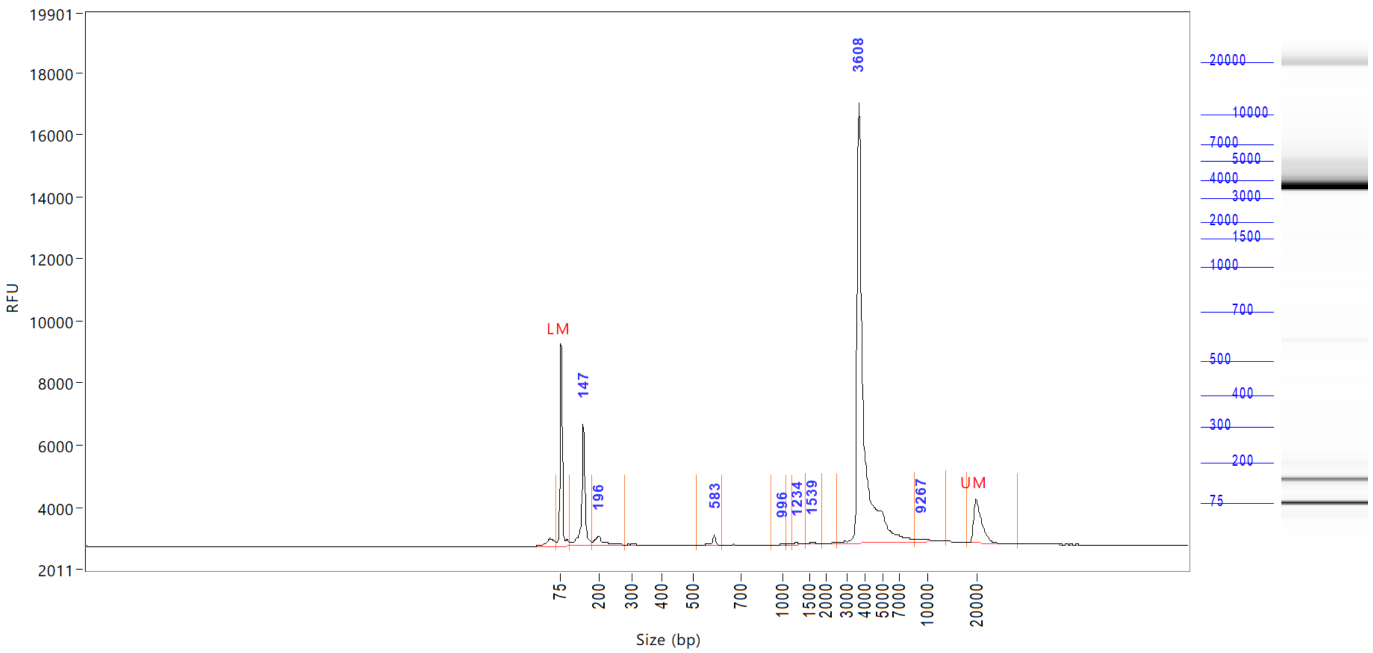

Peak	Size (bp)	Concentration (ng/uL)	From (bp)	To (bp)	Average size (bp)	CV%	RFU	Corrected peak area
1	75 (LM)	0.5000	64	105	75	7.50	6506	39.327
2	147	4.6895	105	175	143	7.40	3889	30.737
3	196	0.9653	175	277	203	10.41	310	6.327
4	583	0.4350	511	618	575	3.08	344	2.851
5	996	0.0553	913	1045	993	2.05	60	0.362
6	1234	0.1066	1156	1411	1259	4.98	69	0.699
7	1539	0.1173	1411	1866	1574	6.30	78	0.769
8	3608	20.7550	2486	8667	4000	19.11	14188	136.037
9	9267	0.2376	8667	14025	9843	10.11	77	1.557
10	20000 (UM)	0.1867	18049	28584	20520	5.00	1426	14.681

TIC:	27.3616	ng/uL
TIM:	71.8918	nmole/L
Total concentration:	27.7712	ng/uL

```
Sample peak width (sec): 5    Sample min peak height: 50    Sample baseline V to V?: Y    Sample baseline V to V points: 3
Sample filter: Binomial       Number of points for filter: 3  Sample start region (min): 0  Sample end region (min): 30
Marker peak width (sec): 5    Marker min peak height: 200   Marker baseline V to V?: Y    Marker baseline V to V points: 3
Lower marker selection: First peak > 500 RFU                Upper marker selection: Last peak > 200 RFU
Ladder size (bp)75, 200, 300, 400, 500, 700, 1000, 1500, 2000, 3000, 4000, 5000, 7000, 10000, 20000
Quantification using: Lower Marker          Final concentration (ng/uL): 0.5000          Dilution factor: 12.0
```

Sample: SampH5
Well location: H5
Created: Monday, September 20, 2021 6:40:06 PM

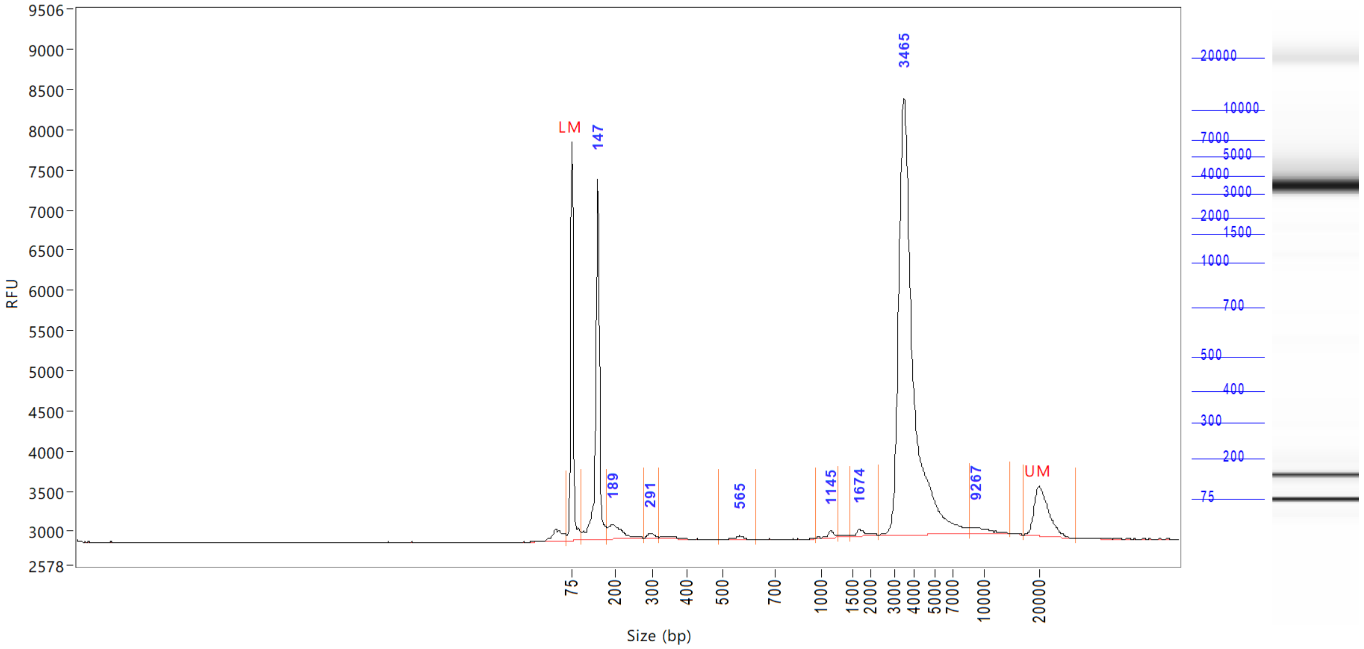

Peak	Size (bp)	Concentration (ng/uL)	From (bp)	To (bp)	Average size (bp)	CV%	RFU	Corrected peak area
1	75 (LM)	0.5000	62	101	73	7.39	4956	33.238
2	147	7.2995	101	173	143	7.37	4492	40.437
3	189	0.9448	173	273	199	9.69	182	5.234
4	291	0.1516	273	315	293	3.07	53	0.840
5	565	0.1810	492	626	558	4.85	51	1.002
6	1145	0.1759	965	1267	1121	6.59	93	0.975
7	1674	0.2046	1467	2315	1756	10.13	82	1.133
8	3465	19.9401	2315	8600	3775	21.32	5443	110.462
9	9267	0.3197	8600	14635	10020	12.54	80	1.771
10	20000 (UM)	0.1622	17073	26649	20571	6.65	616	10.780

TIC:	29.2173	ng/uL
TIM:	102.2765	nmole/L
Total concentration:	29.4797	ng/uL

Sample peak width (sec): 5 Sample min peak height: 50 Sample baseline V to V?: Y Sample baseline V to V points: 3
Sample filter: Binomial Number of points for filter: 3 Sample start region (min): 0 Sample end region (min): 30
Marker peak width (sec): 5 Marker min peak height: 200 Marker baseline V to V?: Y Marker baseline V to V points: 3
Lower marker selection: First peak > 500 RFU Upper marker selection: Last peak > 200 RFU
Ladder size (bp)75, 200, 300, 400, 500, 700, 1000, 1500, 2000, 3000, 4000, 5000, 7000, 10000, 20000
Quantification using: Lower Marker Final concentration (ng/uL): 0.5000 Dilution factor: 12.0

Sample: SampA6
Well location: A6
Created: Monday, September 20, 2021 6:40:06 PM

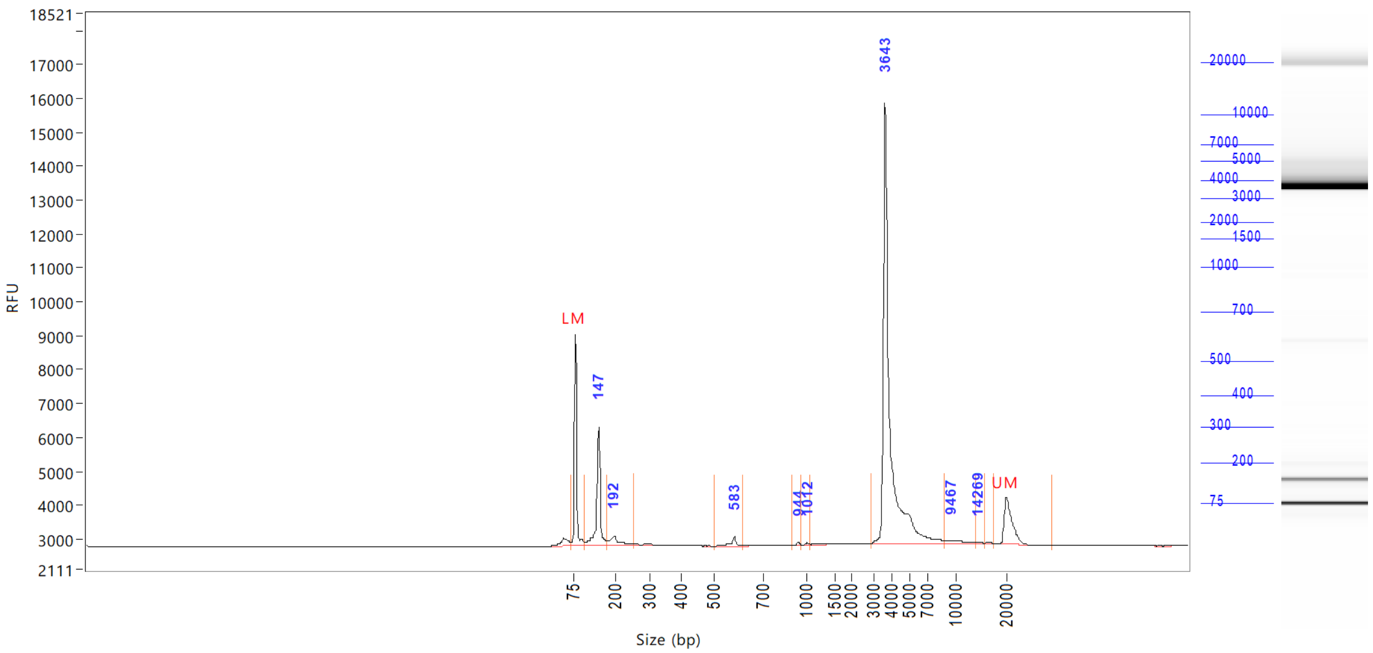

Peak	Size (bp)	Concentration (ng/uL)	From (bp)	To (bp)	Average size (bp)	CV%	RFU	Corrected peak area
1	75 (LM)	0.5000	64	105	74	7.89	6243	35.381
2	147	4.5656	105	173	143	7.46	3475	26.922
3	192	0.8110	173	250	197	8.16	268	4.782
4	583	0.4057	500	616	572	3.45	275	2.392
5	944	0.0672	905	970	940	1.19	72	0.396
6	1012	0.0579	970	1079	1009	2.19	60	0.342
7	3643	20.6961	2886	8867	4037	20.29	13039	122.040
8	9467	0.4135	8867	13781	10588	13.23	97	2.439
9	14269	0.0639	13781	15610	14482	3.58	53	0.377
10	20000 (UM)	0.1948	17561	28826	20441	4.59	1381	13.784

TIC:	27.0808	ng/uL
TIM:	69.1271	nmole/L
Total concentration:	27.6090	ng/uL

```
Sample peak width (sec): 5    Sample min peak height: 50    Sample baseline V to V?: Y    Sample baseline V to V points: 3
Sample filter: Binomial       Number of points for filter: 3  Sample start region (min): 0  Sample end region (min): 30
Marker peak width (sec): 5    Marker min peak height: 200   Marker baseline V to V?: Y    Marker baseline V to V points: 3
Lower marker selection: First peak > 500 RFU                Upper marker selection: Last peak > 200 RFU
Ladder size (bp)75, 200, 300, 400, 500, 700, 1000, 1500, 2000, 3000, 4000, 5000, 7000, 10000, 20000
Quantification using: Lower Marker          Final concentration (ng/uL): 0.5000          Dilution factor: 12.0
```

Sample: SampB6
Well location: B6
Created: Monday, September 20, 2021 6:40:06 PM

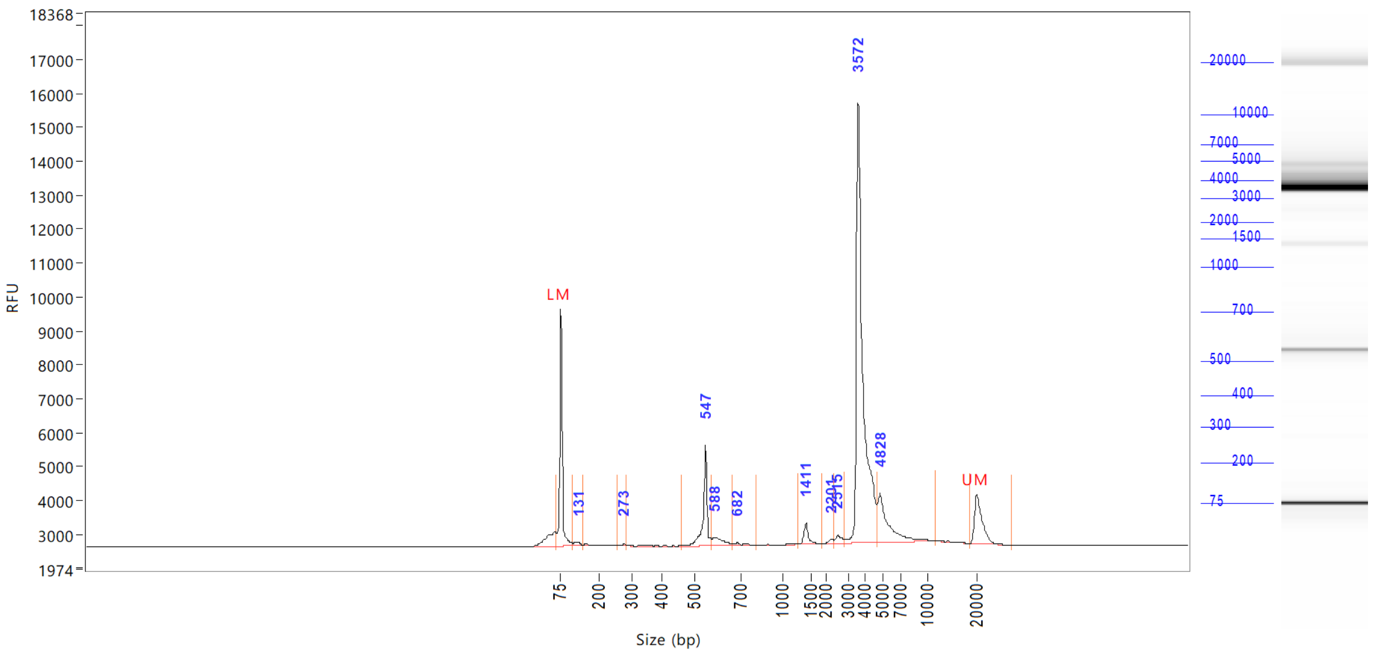

Peak	Size (bp)	Concentration (ng/uL)	From (bp)	To (bp)	Average size (bp)	CV%	RFU	Corrected peak area
1	75 (LM)	0.5000	64	116	75	9.67	6985	43.740
2	131	0.2043	116	145	127	5.82	100	1.489
3	273	0.0629	255	282	269	1.72	80	0.459
4	547	2.9220	460	575	538	3.16	2958	21.301
5	588	0.6710	575	659	606	3.73	251	4.892
6	682	0.1992	659	805	699	4.32	84	1.452
7	1411	0.6092	1267	1846	1419	5.06	627	4.441
8	2201	0.1330	1846	2315	2147	4.86	116	0.970
9	2515	0.3201	2315	2857	2565	5.69	235	2.333
10	3572	17.3293	2857	4621	3748	8.36	12995	126.332
11	4828	3.1087	4621	11586	5648	21.81	1444	22.663
12	20000 (UM)	0.1656	18659	27133	20501	4.70	1442	14.490

TIC:	25.5598	ng/uL
TIM:	23.7883	nmole/L
Total concentration:	25.7214	ng/uL

```
Sample peak width (sec): 5    Sample min peak height: 50    Sample baseline V to V?: Y    Sample baseline V to V points: 3
Sample filter: Binomial       Number of points for filter: 3  Sample start region (min): 0  Sample end region (min): 30
Marker peak width (sec): 5    Marker min peak height: 200   Marker baseline V to V?: Y    Marker baseline V to V points: 3
Lower marker selection: First peak > 500 RFU                Upper marker selection: Last peak > 200 RFU
Ladder size (bp)75, 200, 300, 400, 500, 700, 1000, 1500, 2000, 3000, 4000, 5000, 7000, 10000, 20000
Quantification using: Lower Marker          Final concentration (ng/uL): 0.5000          Dilution factor: 12.0
```

Sample: SampC6
Well location: C6
Created: Monday, September 20, 2021 6:40:06 PM

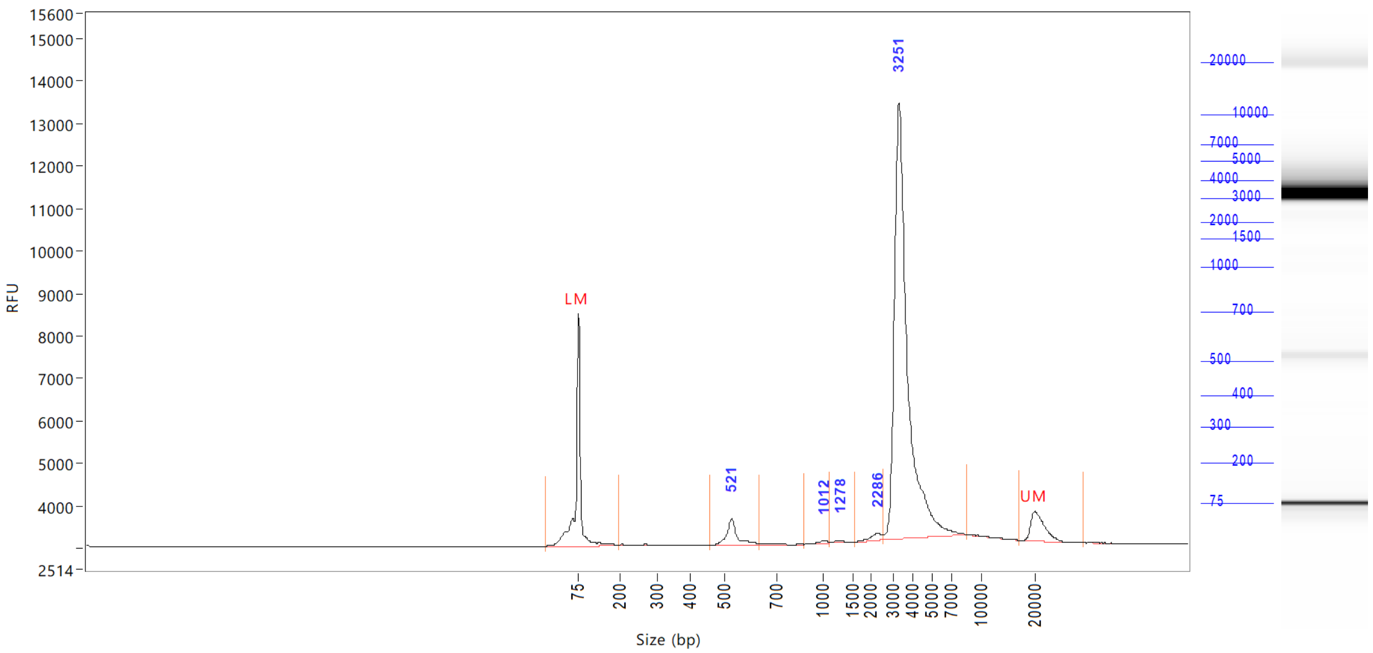

Peak	Size (bp)	Concentration (ng/uL)	From (bp)	To (bp)	Average size (bp)	CV%	RFU	Corrected peak area
1	75 (LM)	0.5000	0	192	70	34.52	5471	59.379
2	521	1.2469	454	634	530	6.32	633	12.340
3	1012	0.0871	879	1112	1002	4.55	53	0.862
4	1278	0.0913	1112	1559	1258	6.14	66	0.904
5	2286	0.2973	1559	2543	2142	11.20	163	2.943
6	3251	19.3411	2543	8600	3531	18.21	10277	191.410
7	20000 (UM)	0.0989	17195	28947	20770	6.72	694	11.741

TIC:	21.0637	ng/uL
TIM:	13.3797	nmole/L
Total concentration:	21.4306	ng/uL

```
Sample peak width (sec): 5    Sample min peak height: 50    Sample baseline V to V?: Y    Sample baseline V to V points: 3
Sample filter: Binomial       Number of points for filter: 3  Sample start region (min): 0  Sample end region (min): 30
Marker peak width (sec): 5    Marker min peak height: 200   Marker baseline V to V?: Y    Marker baseline V to V points: 3
Lower marker selection: First peak > 500 RFU                Upper marker selection: Last peak > 200 RFU
Ladder size (bp)75, 200, 300, 400, 500, 700, 1000, 1500, 2000, 3000, 4000, 5000, 7000, 10000, 20000
Quantification using: Lower Marker          Final concentration (ng/uL): 0.5000          Dilution factor: 12.0
```

Sample: SampD6
Well location: D6
Created: Monday, September 20, 2021 6:40:06 PM

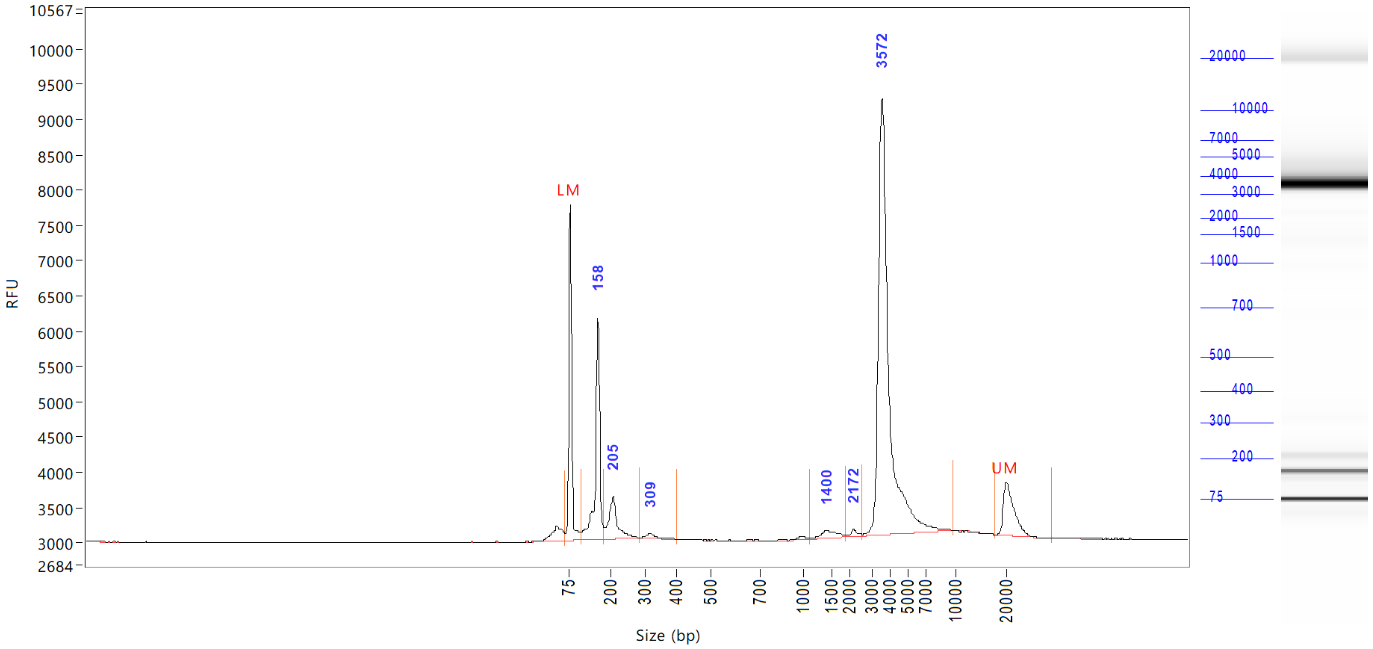

Peak	Size (bp)	Concentration (ng/uL)	From (bp)	To (bp)	Average size (bp)	CV%	RFU	Corrected peak area
1	75 (LM)	0.5000	62	108	74	9.41	4760	31.582
2	158	5.7886	108	179	152	8.26	3125	30.469
3	205	2.1154	179	282	207	8.42	607	11.135
4	309	0.2675	282	400	322	7.72	65	1.408
5	1400	0.5400	1090	1904	1453	12.12	112	2.842
6	2172	0.1928	1904	2600	2207	7.64	101	1.015
7	3572	17.6680	2600	9800	3850	19.33	6194	92.998
8	20000 (UM)	0.1629	17683	28826	20655	5.85	750	10.287

TIC:	26.5722	ng/uL
TIM:	89.0712	nmole/L
Total concentration:	26.8516	ng/uL

```
Sample peak width (sec): 5     Sample min peak height: 50     Sample baseline V to V?: Y     Sample baseline V to V points: 3
Sample filter: Binomial        Number of points for filter: 3  Sample start region (min): 0  Sample end region (min): 30
Marker peak width (sec): 5     Marker min peak height: 200    Marker baseline V to V?: Y     Marker baseline V to V points: 3
Lower marker selection: First peak > 500 RFU                  Upper marker selection: Last peak > 200 RFU
Ladder size (bp)75, 200, 300, 400, 500, 700, 1000, 1500, 2000, 3000, 4000, 5000, 7000, 10000, 20000
Quantification using: Lower Marker          Final concentration (ng/uL): 0.5000          Dilution factor: 12.0
```

Sample: SampE6
Well location: E6
Created: Monday, September 20, 2021 6:40:06 PM

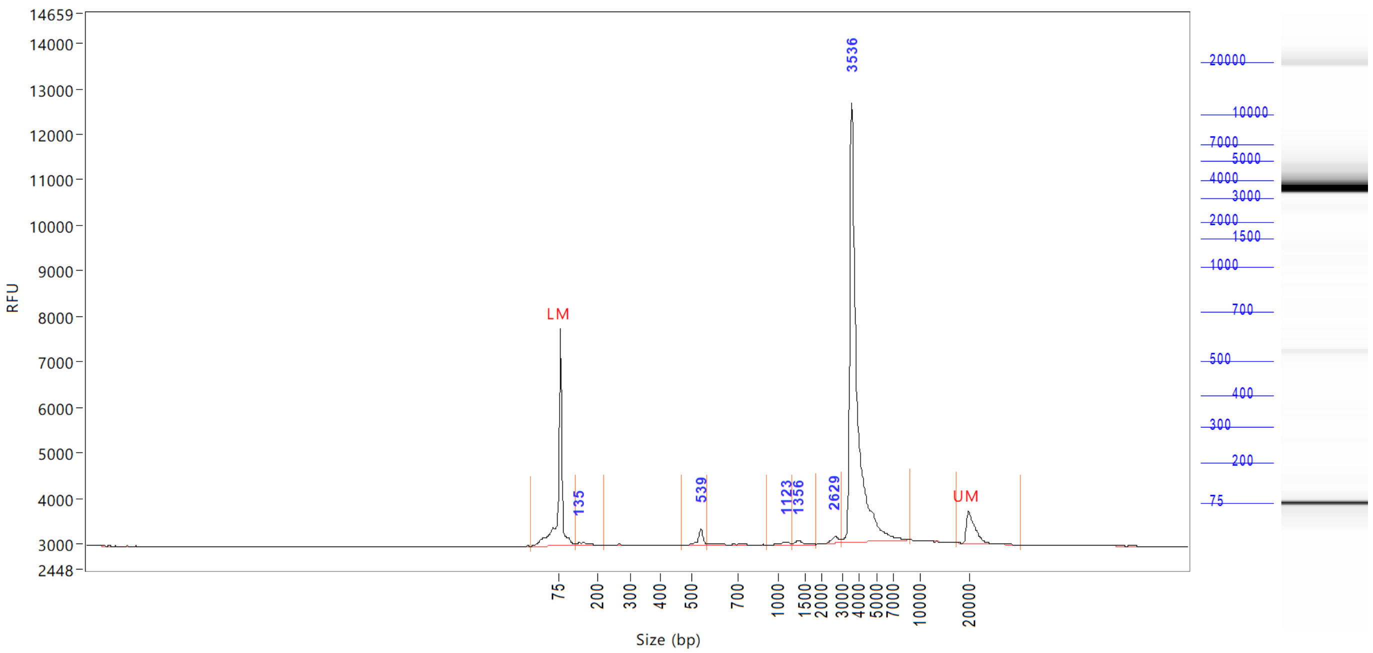

Peak	Size (bp)	Concentration (ng/uL)	From (bp)	To (bp)	Average size (bp)	CV%	RFU	Corrected peak area
1	75 (LM)	0.5000	0	128	66	31.34	4775	44.001
2	135	0.2010	128	215	150	11.12	68	1.474
3	539	0.5136	470	567	531	3.23	373	3.766
4	1123	0.1545	918	1245	1087	7.33	77	1.133
5	1356	0.1728	1245	1827	1391	6.81	90	1.267
6	2629	0.2354	1827	2971	2607	8.04	139	1.726
7	3536	14.5410	2971	8934	3823	15.43	9662	106.638
8	20000 (UM)	0.0900	17317	30639	20749	6.36	697	7.916

TIC:	15.8183	ng/uL
TIM:	10.6425	nmole/L
Total concentration:	16.1590	ng/uL

Sample peak width (sec): 5 Sample min peak height: 50 Sample baseline V to V?: Y Sample baseline V to V points: 3
Sample filter: Binomial Number of points for filter: 3 Sample start region (min): 0 Sample end region (min): 30
Marker peak width (sec): 5 Marker min peak height: 200 Marker baseline V to V?: Y Marker baseline V to V points: 3
Lower marker selection: First peak > 500 RFU Upper marker selection: Last peak > 200 RFU
Ladder size (bp)75, 200, 300, 400, 500, 700, 1000, 1500, 2000, 3000, 4000, 5000, 7000, 10000, 20000
Quantification using: Lower Marker Final concentration (ng/uL): 0.5000 Dilution factor: 12.0

Sample: SampF6
Well location: F6
Created: Monday, September 20, 2021 6:40:06 PM

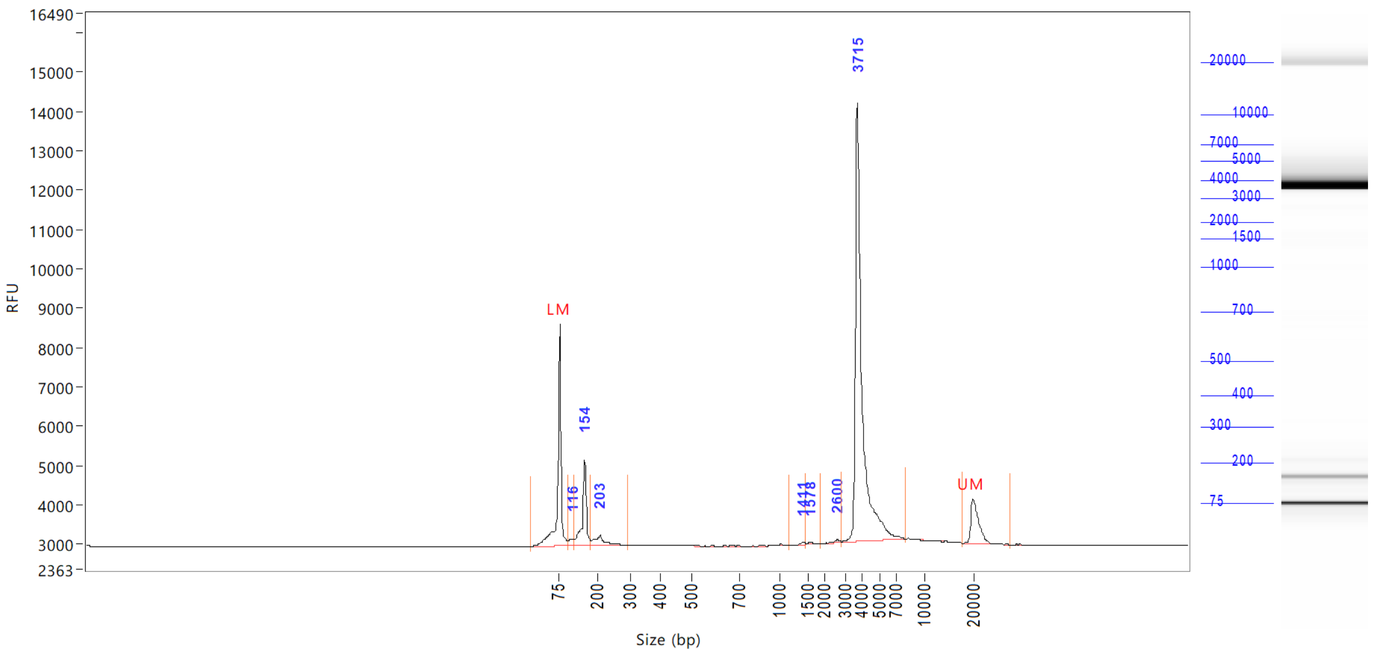

Peak	Size (bp)	Concentration (ng/uL)	From (bp)	To (bp)	Average size (bp)	CV%	RFU	Corrected peak area
1	75 (LM)	0.5000	0	101	67	24.73	5654	45.588
2	116	0.3193	101	124	113	6.13	179	2.426
3	154	2.5673	124	177	150	6.93	2182	19.506
4	203	0.7863	177	289	208	10.26	276	5.974
5	1411	0.0713	1156	1467	1379	4.33	68	0.542
6	1578	0.0679	1467	1923	1574	5.62	53	0.516
7	2600	0.0953	1923	2857	2506	8.48	69	0.724
8	3715	14.0660	2857	8001	3935	13.24	11162	106.872
9	20000 (UM)	0.1251	17805	27496	20559	4.82	1126	11.403

TIC:	17.9734	ng/uL
TIM:	45.0897	nmole/L
Total concentration:	18.1004	ng/uL

Sample peak width (sec): 5   Sample min peak height: 50   Sample baseline V to V?: Y   Sample baseline V to V points: 3
Sample filter: Binomial   Number of points for filter: 3 Sample start region (min): 0 Sample end region (min): 30
Marker peak width (sec): 5   Marker min peak height: 200   Marker baseline V to V?: Y   Marker baseline V to V points: 3
Lower marker selection: First peak > 500 RFU   Upper marker selection: Last peak > 200 RFU
Ladder size (bp)75, 200, 300, 400, 500, 700, 1000, 1500, 2000, 3000, 4000, 5000, 7000, 10000, 20000
Quantification using: Lower Marker   Final concentration (ng/uL): 0.5000   Dilution factor: 12.0

Sample: SampG6
Well location: G6
Created: Monday, September 20, 2021 6:40:06 PM

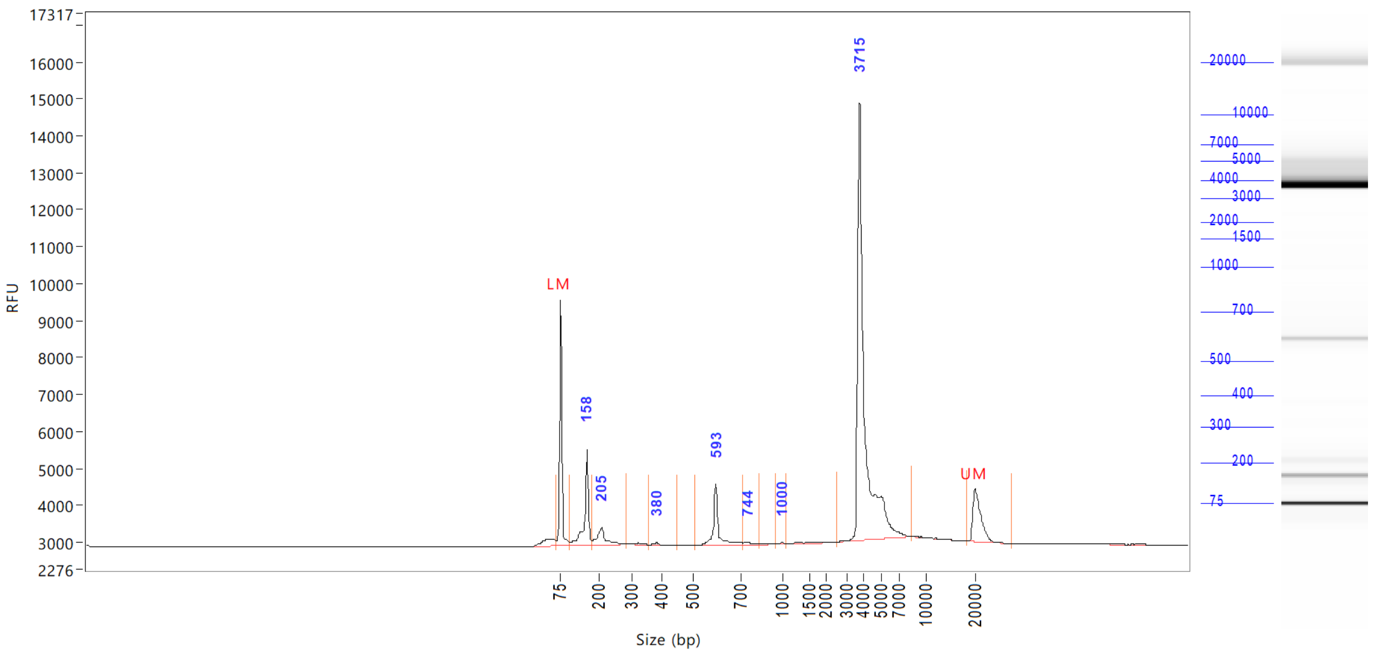

Peak	Size (bp)	Concentration (ng/uL)	From (bp)	To (bp)	Average size (bp)	CV%	RFU	Corrected peak area
1	75 (LM)	0.5000	62	103	73	7.21	6648	38.948
2	158	3.5502	103	177	150	9.36	2594	23.045
3	205	1.3440	177	278	207	9.14	464	8.724
4	380	0.1010	356	449	384	5.05	54	0.655
5	593	2.2939	504	714	599	5.60	1646	14.890
6	744	0.1634	714	835	756	4.09	77	1.061
7	1000	0.0486	952	1056	999	1.88	52	0.315
8	3715	18.4311	2515	8467	4084	15.90	11856	119.641
9	20000 (UM)	0.1894	18537	27496	20604	4.75	1436	14.755

TIC:	25.9320	ng/uL
TIM:	64.1742	nmole/L
Total concentration:	26.2729	ng/uL

Sample peak width (sec): 5 Sample min peak height: 50 Sample baseline V to V?: Y Sample baseline V to V points: 3
Sample filter: Binomial Number of points for filter: 3 Sample start region (min): 0 Sample end region (min): 30
Marker peak width (sec): 5 Marker min peak height: 200 Marker baseline V to V?: Y Marker baseline V to V points: 3
Lower marker selection: First peak > 500 RFU Upper marker selection: Last peak > 200 RFU
Ladder size (bp)75, 200, 300, 400, 500, 700, 1000, 1500, 2000, 3000, 4000, 5000, 7000, 10000, 20000
Quantification using: Lower Marker Final concentration (ng/uL): 0.5000 Dilution factor: 12.0

Sample: SampH6
Well location: H6
Created: Monday, September 20, 2021 6:40:06 PM

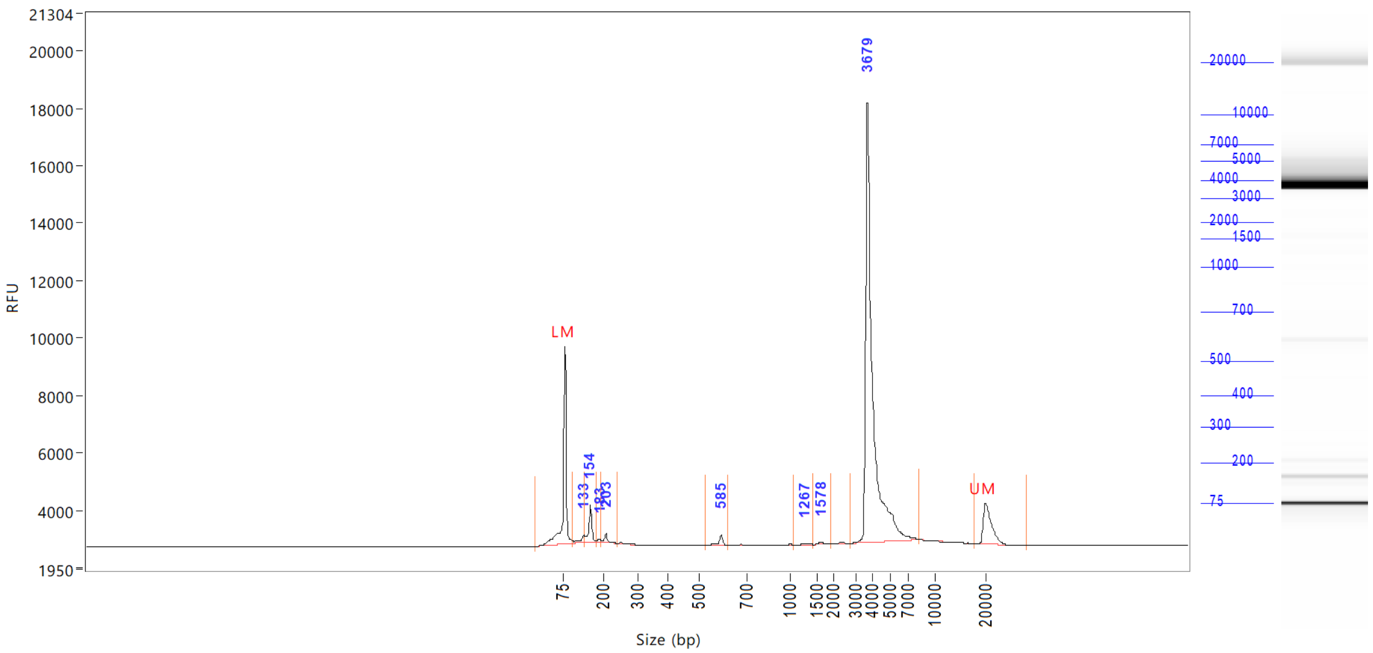

Peak	Size (bp)	Concentration (ng/uL)	From (bp)	To (bp)	Average size (bp)	CV%	RFU	Corrected peak area
1	75 (LM)	0.5000	0	101	67	22.31	6883	50.159
2	133	0.3061	101	139	126	8.65	234	2.559
3	154	1.1215	139	173	153	3.98	1285	9.376
4	183	0.1089	173	190	182	2.74	108	0.911
5	203	0.2877	190	241	203	3.69	322	2.405
6	585	0.3954	519	616	578	2.93	396	3.305
7	1267	0.0666	1079	1422	1260	5.09	54	0.557
8	1578	0.0903	1422	1923	1606	6.33	89	0.755
9	3679	18.7869	2743	8267	3996	14.45	15326	157.056
10	20000 (UM)	0.1483	17927	28342	20590	4.65	1438	14.878

TIC:	21.1634	ng/uL
TIM:	28.3981	nmole/L
Total concentration:	21.4679	ng/uL

```
Sample peak width (sec): 5     Sample min peak height: 50     Sample baseline V to V?: Y     Sample baseline V to V points: 3
Sample filter: Binomial        Number of points for filter: 3  Sample start region (min): 0  Sample end region (min): 30
Marker peak width (sec): 5     Marker min peak height: 200    Marker baseline V to V?: Y     Marker baseline V to V points: 3
Lower marker selection: First peak > 500 RFU                  Upper marker selection: Last peak > 200 RFU
Ladder size (bp)75, 200, 300, 400, 500, 700, 1000, 1500, 2000, 3000, 4000, 5000, 7000, 10000, 20000
Quantification using: Lower Marker          Final concentration (ng/uL): 0.5000          Dilution factor: 12.0
```

Sample: SampA7
Well location: A7
Created: Monday, September 20, 2021 6:40:06 PM

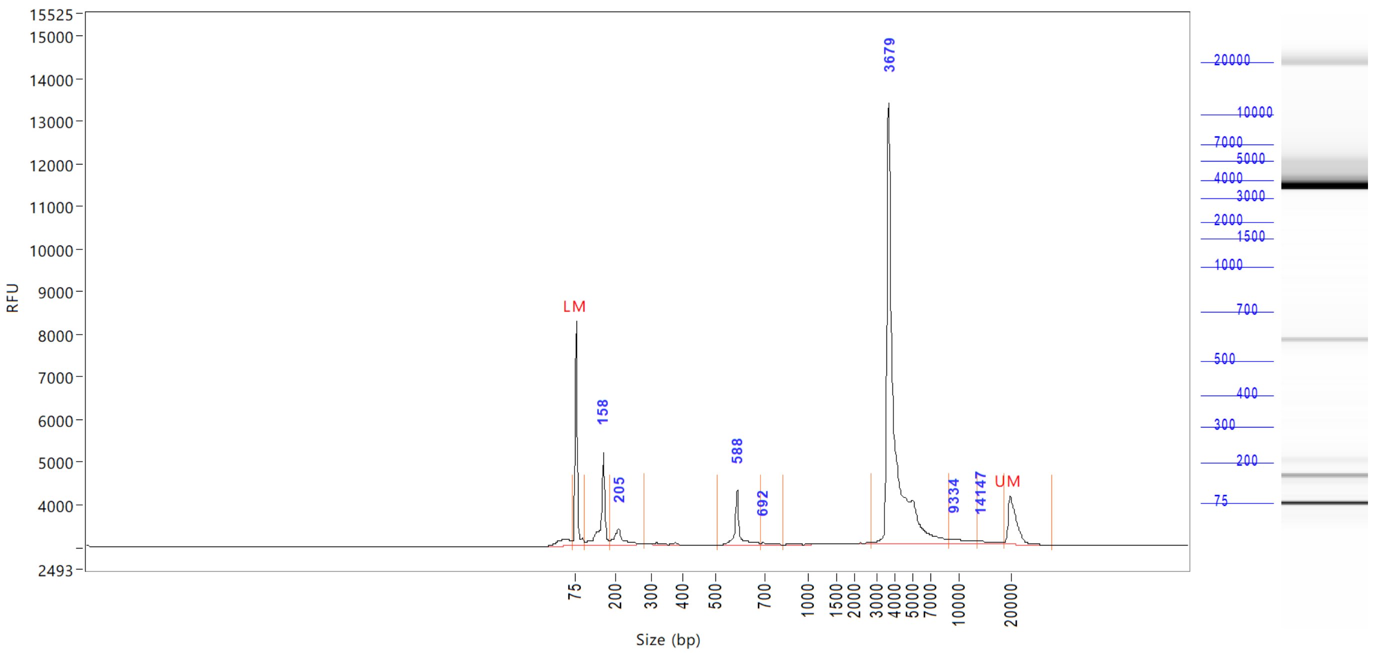

Peak	Size (bp)	Concentration (ng/uL)	From (bp)	To (bp)	Average size (bp)	CV%	RFU	Corrected peak area
1	75 (LM)	0.5000	64	103	74	7.34	5252	29.796
2	158	4.0001	103	177	149	9.52	2167	19.864
3	205	1.4282	177	278	207	9.23	379	7.093
4	588	2.2433	504	682	590	4.79	1297	11.140
5	692	0.2043	682	831	726	5.05	54	1.014
6	3679	22.2594	2772	9000	4155	21.78	10346	110.539
7	9334	0.5665	9000	13659	10791	12.94	109	2.813
8	14147	0.3032	13659	18659	15843	9.34	75	1.506
9	20000 (UM)	0.1950	18659	28100	20589	5.05	1123	11.620

TIC:	31.0051	ng/uL
TIM:	71.1245	nmole/L
Total concentration:	31.5882	ng/uL

```
Sample peak width (sec): 5    Sample min peak height: 50    Sample baseline V to V?: Y    Sample baseline V to V points: 3
Sample filter: Binomial       Number of points for filter: 3  Sample start region (min): 0  Sample end region (min): 30
Marker peak width (sec): 5    Marker min peak height: 200   Marker baseline V to V?: Y    Marker baseline V to V points: 3
Lower marker selection: First peak > 500 RFU                Upper marker selection: Last peak > 200 RFU
Ladder size (bp)75, 200, 300, 400, 500, 700, 1000, 1500, 2000, 3000, 4000, 5000, 7000, 10000, 20000
Quantification using: Lower Marker          Final concentration (ng/uL): 0.5000          Dilution factor: 12.0
```

Sample: SampB7
Well location: B7
Created: Monday, September 20, 2021 6:40:06 PM

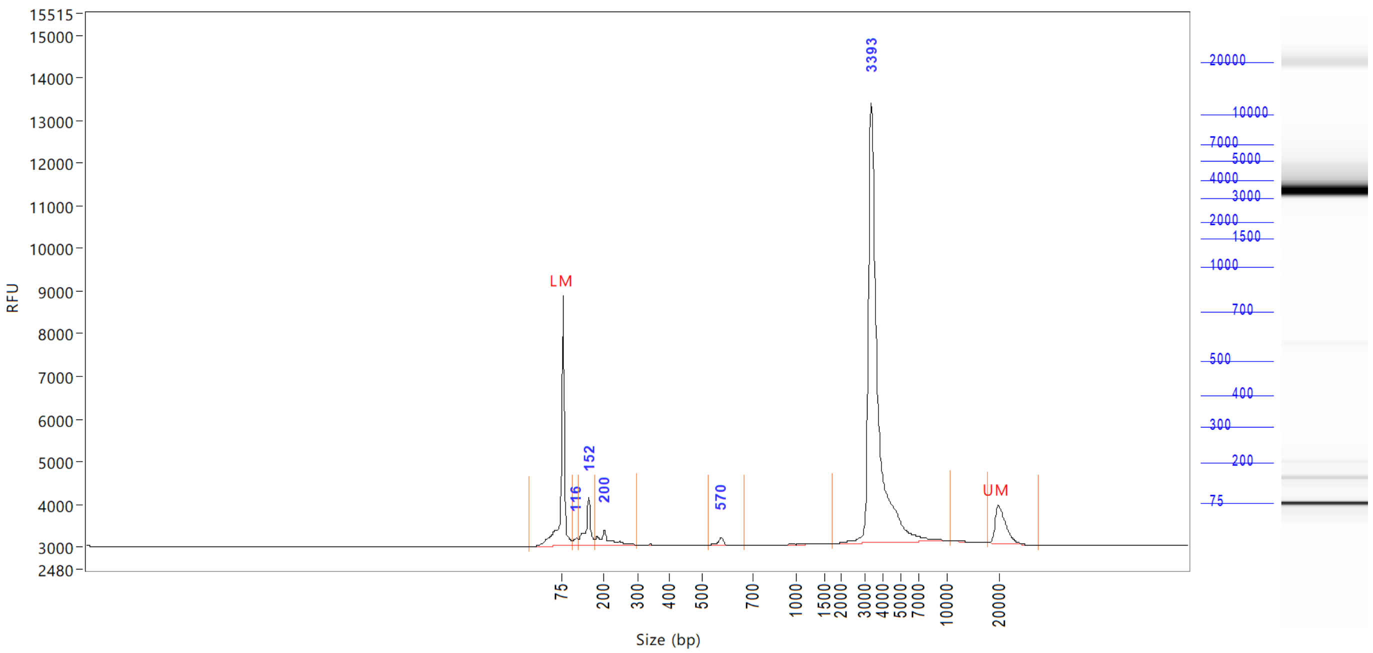

Peak	Size (bp)	Concentration (ng/uL)	From (bp)	To (bp)	Average size (bp)	CV%	RFU	Corrected peak area
1	75 (LM)	0.5000	0	103	67	23.31	5860	47.454
2	116	0.2601	103	122	113	5.19	172	2.057
3	152	1.6188	122	171	147	7.64	1110	12.803
4	200	0.9838	171	293	207	12.41	363	7.781
5	570	0.3490	519	669	578	5.81	200	2.760
6	3393	17.9691	1712	10489	3694	20.41	10330	142.117
7	20000 (UM)	0.1247	17683	27738	20529	5.56	890	11.837

TIC:	21.1809	ng/uL
TIM:	38.6961	nmole/L
Total concentration:	21.3469	ng/uL

Sample peak width (sec): 5   Sample min peak height: 50   Sample baseline V to V?: Y   Sample baseline V to V points: 3
Sample filter: Binomial   Number of points for filter: 3 Sample start region (min): 0 Sample end region (min): 30
Marker peak width (sec): 5   Marker min peak height: 200   Marker baseline V to V?: Y   Marker baseline V to V points: 3
Lower marker selection: First peak > 500 RFU   Upper marker selection: Last peak > 200 RFU
Ladder size (bp)75, 200, 300, 400, 500, 700, 1000, 1500, 2000, 3000, 4000, 5000, 7000, 10000, 20000
Quantification using: Lower Marker   Final concentration (ng/uL): 0.5000   Dilution factor: 12.0

Sample: SampC7
Well location: C7
Created: Monday, September 20, 2021 6:40:06 PM

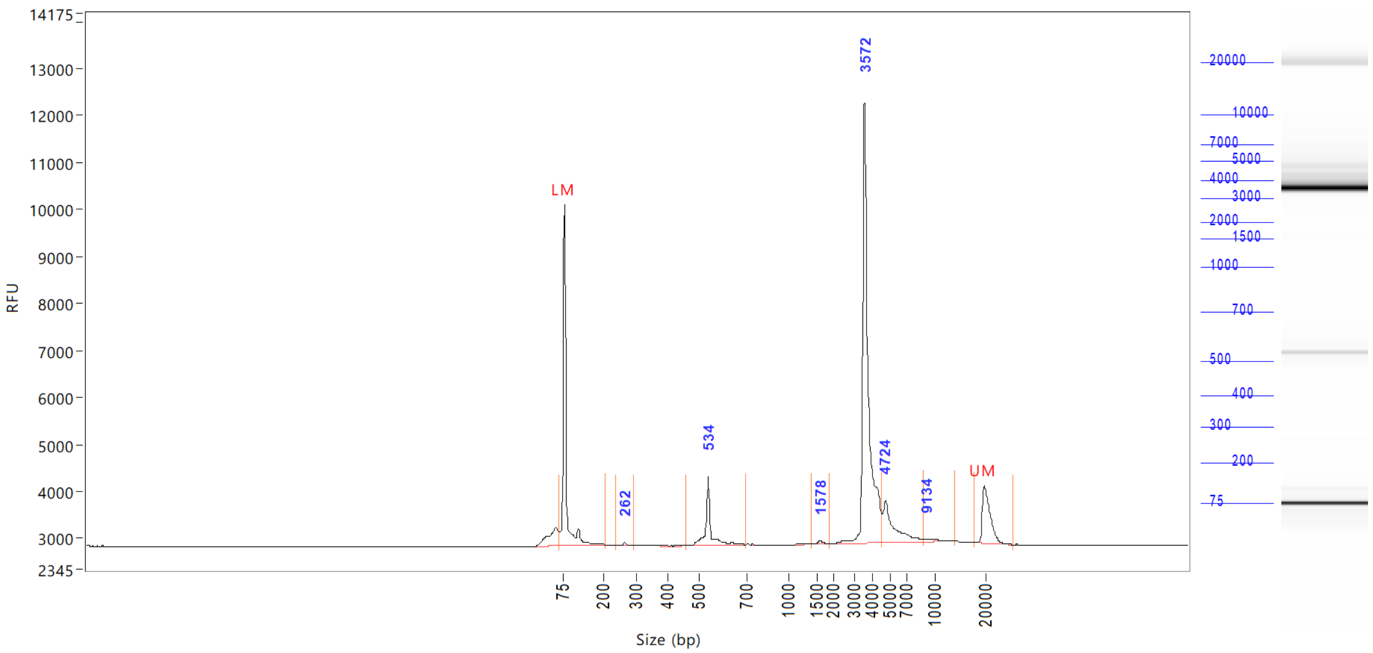

Peak	Size (bp)	Concentration (ng/uL)	From (bp)	To (bp)	Average size (bp)	CV%	RFU	Corrected peak area
1	75 (LM)	0.5000	58	203	79	21.74	7260	52.706
2	262	0.0519	233	287	259	3.21	58	0.456
3	534	1.5061	458	697	540	6.37	1461	13.230
4	1578	0.0669	1400	1904	1604	6.19	73	0.587
5	3572	9.2978	1904	4552	3671	8.75	9382	81.675
6	4724	1.5972	4552	8734	5530	19.25	890	14.030
7	9134	0.1211	8734	13903	9858	10.07	60	1.064
8	20000 (UM)	0.1158	18049	25803	20469	4.36	1238	12.212

TIC:	12.6410	ng/uL
TIM:	9.6532	nmole/L
Total concentration:	12.7070	ng/uL

Sample peak width (sec): 5   Sample min peak height: 50   Sample baseline V to V?: Y   Sample baseline V to V points: 3
Sample filter: Binomial   Number of points for filter: 3 Sample start region (min): 0 Sample end region (min): 30
Marker peak width (sec): 5   Marker min peak height: 200   Marker baseline V to V?: Y   Marker baseline V to V points: 3
Lower marker selection: First peak > 500 RFU   Upper marker selection: Last peak > 200 RFU
Ladder size (bp)75, 200, 300, 400, 500, 700, 1000, 1500, 2000, 3000, 4000, 5000, 7000, 10000, 20000
Quantification using: Lower Marker   Final concentration (ng/uL): 0.5000   Dilution factor: 12.0

Sample: SampD7
Well location: D7
Created: Monday, September 20, 2021 6:40:06 PM

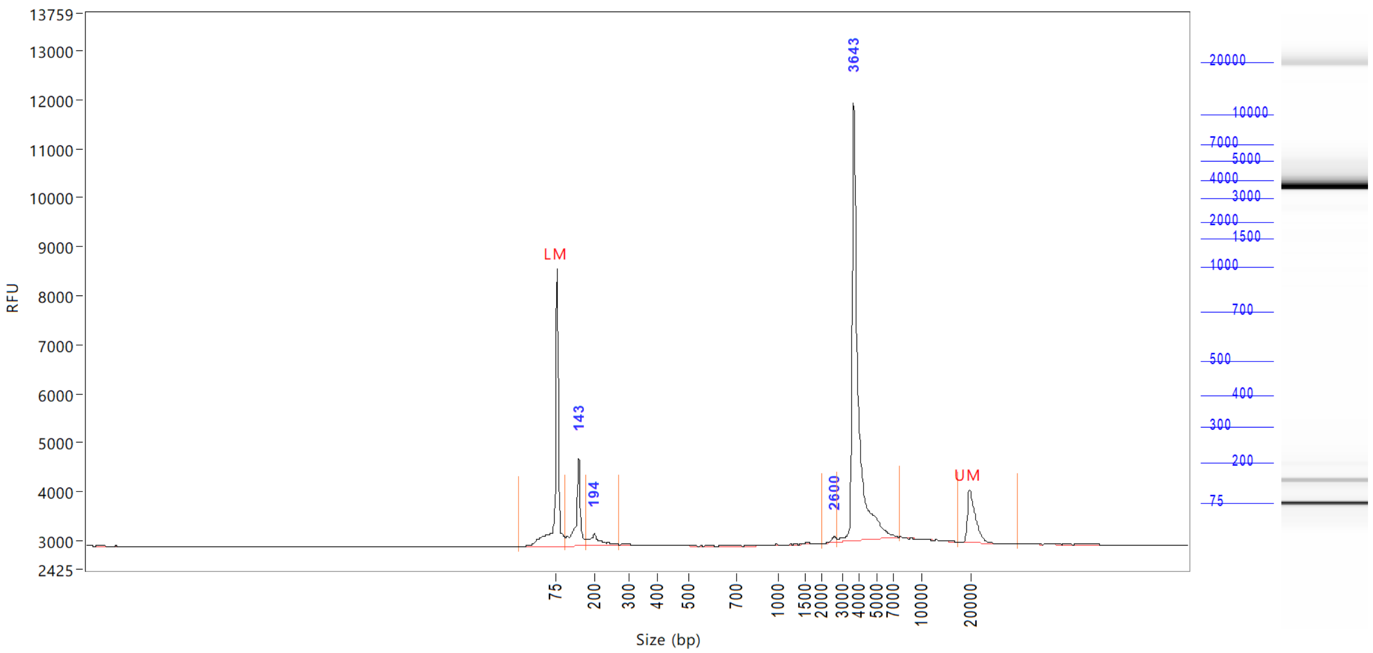

Peak	Size (bp)	Concentration (ng/uL)	From (bp)	To (bp)	Average size (bp)	CV%	RFU	Corrected peak area
1	75 (LM)	0.5000	0	101	66	30.30	5657	44.399
2	143	2.5412	101	168	137	10.03	1783	18.804
3	194	0.8684	168	271	202	12.09	241	6.426
4	2600	0.1337	2001	2772	2550	5.51	121	0.990
5	3643	11.1458	2772	7667	3889	13.04	8939	82.477
6	20000 (UM)	0.1273	17561	29793	20542	5.36	1087	11.305

TIC:	14.6890	ng/uL
TIM:	42.3528	nmole/L
Total concentration:	15.0077	ng/uL

```
Sample peak width (sec): 5    Sample min peak height: 50    Sample baseline V to V?: Y    Sample baseline V to V points: 3
Sample filter: Binomial       Number of points for filter: 3  Sample start region (min): 0  Sample end region (min): 30
Marker peak width (sec): 5    Marker min peak height: 200   Marker baseline V to V?: Y    Marker baseline V to V points: 3
Lower marker selection: First peak > 500 RFU                Upper marker selection: Last peak > 200 RFU
Ladder size (bp)75, 200, 300, 400, 500, 700, 1000, 1500, 2000, 3000, 4000, 5000, 7000, 10000, 20000
Quantification using: Lower Marker          Final concentration (ng/uL): 0.5000          Dilution factor: 12.0
```

Sample: SampE7
Well location: E7
Created: Monday, September 20, 2021 6:40:06 PM

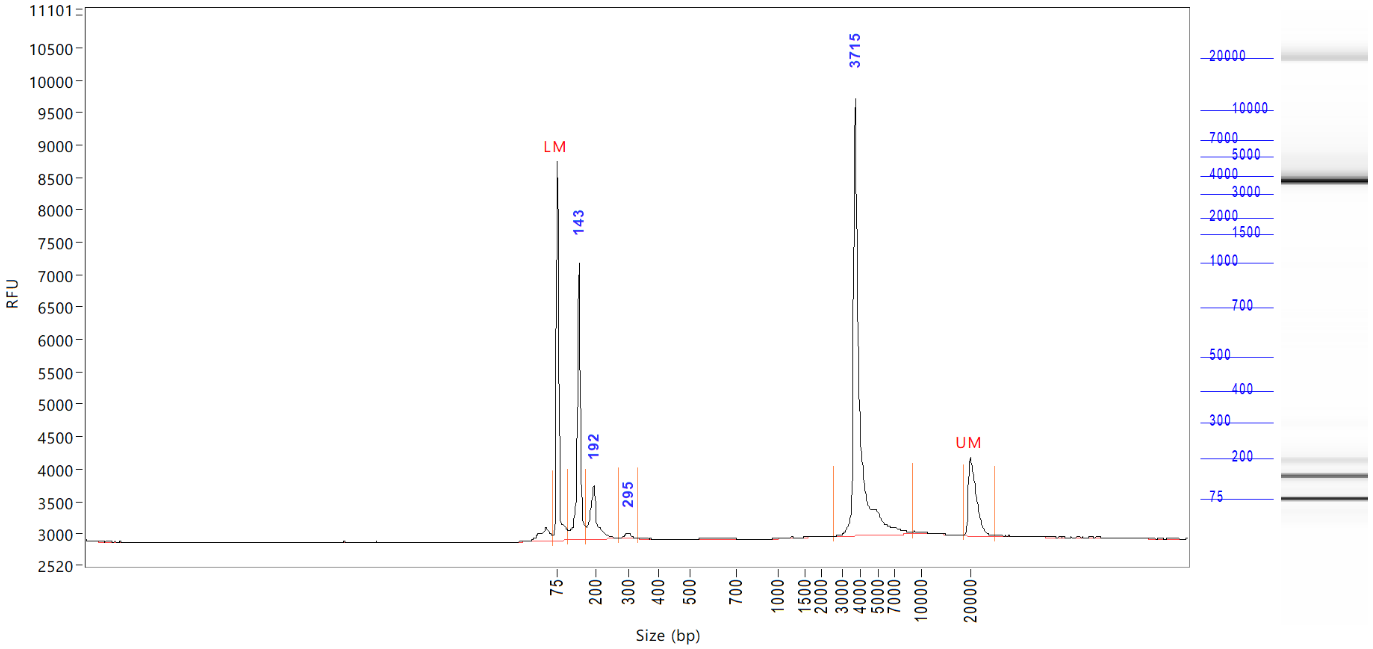

Peak	Size (bp)	Concentration (ng/uL)	From (bp)	To (bp)	Average size (bp)	CV%	RFU	Corrected peak area
1	75 (LM)	0.5000	64	112	76	10.99	5856	35.721
2	143	5.6997	112	168	141	6.10	4268	33.934
3	192	2.1846	168	269	193	8.42	817	13.006
4	295	0.2066	269	329	295	3.74	79	1.230
5	3715	10.4354	2543	9000	4058	19.83	6758	62.128
6	20000 (UM)	0.1741	18659	24957	20601	4.59	1220	12.437

TIC:	18.5263	ng/uL
TIM:	90.4243	nmole/L
Total concentration:	18.9968	ng/uL

```
Sample peak width (sec): 5    Sample min peak height: 50    Sample baseline V to V?: Y    Sample baseline V to V points: 3
Sample filter: Binomial       Number of points for filter: 3  Sample start region (min): 0  Sample end region (min): 30
Marker peak width (sec): 5    Marker min peak height: 200   Marker baseline V to V?: Y    Marker baseline V to V points: 3
Lower marker selection: First peak > 500 RFU                Upper marker selection: Last peak > 200 RFU
Ladder size (bp)75, 200, 300, 400, 500, 700, 1000, 1500, 2000, 3000, 4000, 5000, 7000, 10000, 20000
Quantification using: Lower Marker          Final concentration (ng/uL): 0.5000          Dilution factor: 12.0
```

Sample: SampF7
Well location: F7
Created: Monday, September 20, 2021 6:40:06 PM

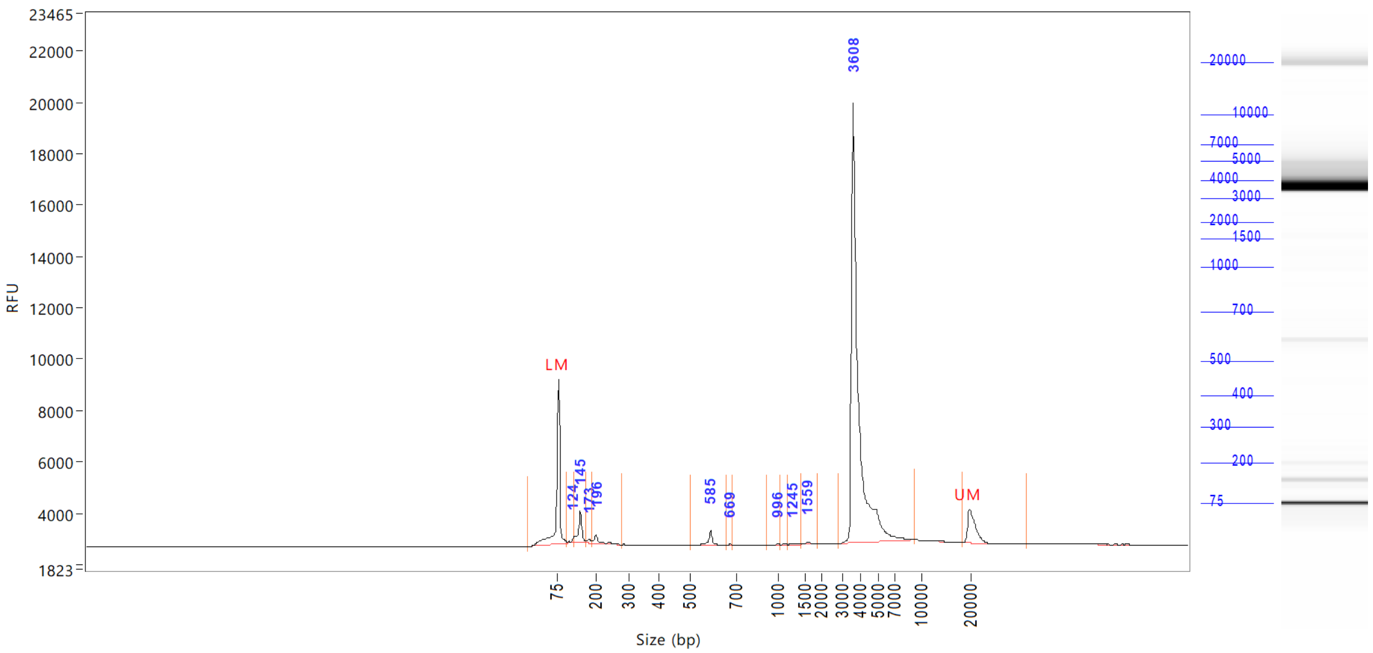

Peak	Size (bp)	Concentration (ng/uL)	From (bp)	To (bp)	Average size (bp)	CV%	RFU	Corrected peak area
1	75 (LM)	0.5000	0	103	66	28.08	6379	49.153
2	124	0.2270	103	128	120	5.63	225	1.860
3	145	1.2242	128	166	143	5.09	1238	10.029
4	173	0.1590	166	185	175	3.29	130	1.303
5	196	0.4756	185	277	205	8.86	363	3.897
6	585	0.6651	501	657	583	4.20	596	5.449
7	669	0.0448	657	682	668	1.05	54	0.367
8	996	0.0398	913	1034	991	2.06	53	0.326
9	1245	0.0676	1156	1422	1254	4.31	59	0.553
10	1559	0.1022	1422	1866	1576	5.35	104	0.837
11	3608	21.4873	2829	9334	3929	16.44	17151	176.029
12	20000 (UM)	0.1355	18537	31728	20530	5.30	1332	13.324

TIC:	24.4926	ng/uL
TIM:	33.7379	nmole/L
Total concentration:	24.6710	ng/uL

```
Sample peak width (sec): 5    Sample min peak height: 50    Sample baseline V to V?: Y    Sample baseline V to V points: 3
Sample filter: Binomial       Number of points for filter: 3  Sample start region (min): 0  Sample end region (min): 30
Marker peak width (sec): 5    Marker min peak height: 200   Marker baseline V to V?: Y    Marker baseline V to V points: 3
Lower marker selection: First peak > 500 RFU                Upper marker selection: Last peak > 200 RFU
Ladder size (bp)75, 200, 300, 400, 500, 700, 1000, 1500, 2000, 3000, 4000, 5000, 7000, 10000, 20000
Quantification using: Lower Marker          Final concentration (ng/uL): 0.5000          Dilution factor: 12.0
```

Sample: SampG7
Well location: G7
Created: Monday, September 20, 2021 6:40:06 PM

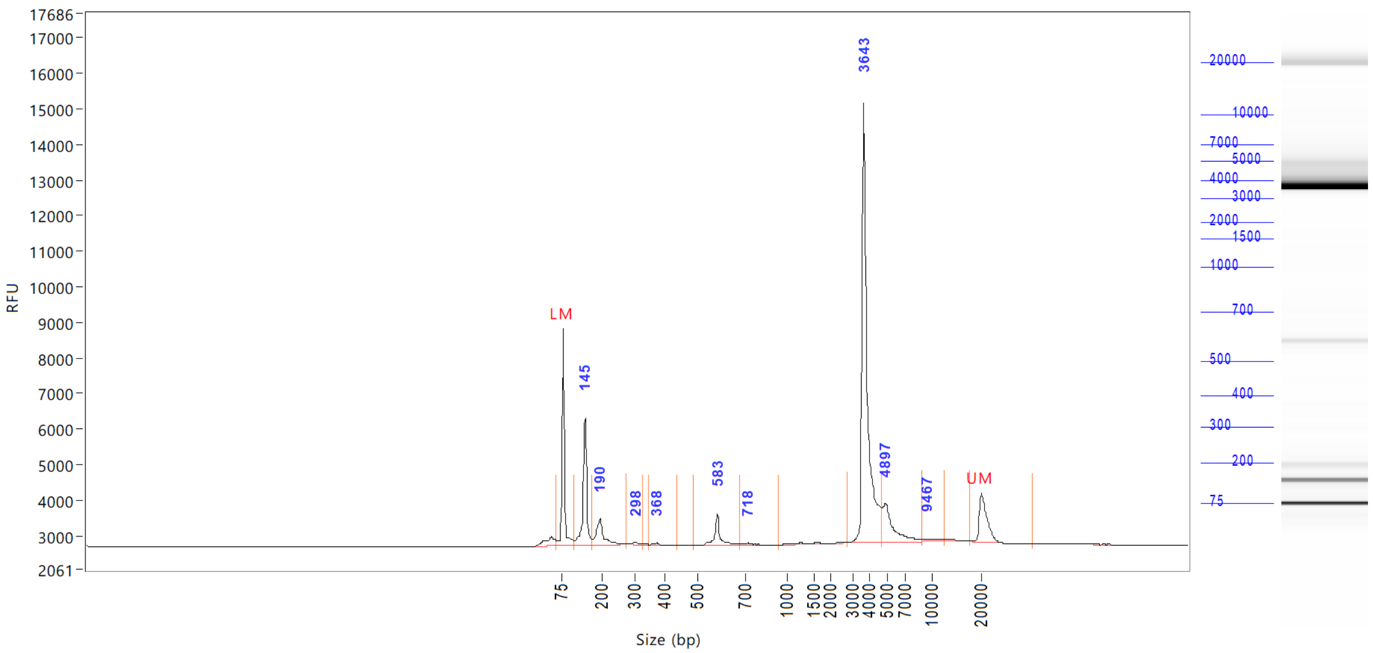

Peak	Size (bp)	Concentration (ng/uL)	From (bp)	To (bp)	Average size (bp)	CV%	RFU	Corrected peak area
1	75 (LM)	0.5000	56	108	74	10.08	6099	37.075
2	145	4.8889	108	168	141	6.94	3578	30.209
3	190	1.9369	168	271	194	9.45	733	11.968
4	298	0.1393	271	323	296	3.76	54	0.861
5	368	0.1178	343	437	370	4.90	58	0.728
6	583	1.4848	487	677	584	5.08	903	9.175
7	718	0.2156	677	944	743	8.31	63	1.332
8	3643	16.4928	2772	4656	3783	7.62	12379	101.912
9	4897	2.8461	4656	9000	5553	18.89	1084	17.587
10	9467	0.2328	9000	12440	10227	9.60	75	1.439
11	20000 (UM)	0.1831	17683	30398	20471	4.88	1364	13.575

TIC:	28.3549	ng/uL
TIM:	87.4119	nmole/L
Total concentration:	28.6163	ng/uL

```
Sample peak width (sec): 5     Sample min peak height: 50     Sample baseline V to V?: Y     Sample baseline V to V points: 3
Sample filter: Binomial        Number of points for filter: 3  Sample start region (min): 0  Sample end region (min): 30
Marker peak width (sec): 5     Marker min peak height: 200    Marker baseline V to V?: Y     Marker baseline V to V points: 3
Lower marker selection: First peak > 500 RFU                  Upper marker selection: Last peak > 200 RFU
Ladder size (bp)75, 200, 300, 400, 500, 700, 1000, 1500, 2000, 3000, 4000, 5000, 7000, 10000, 20000
Quantification using: Lower Marker          Final concentration (ng/uL): 0.5000          Dilution factor: 12.0
```

Sample: SampH7
Well location: H7
Created: Monday, September 20, 2021 6:40:06 PM

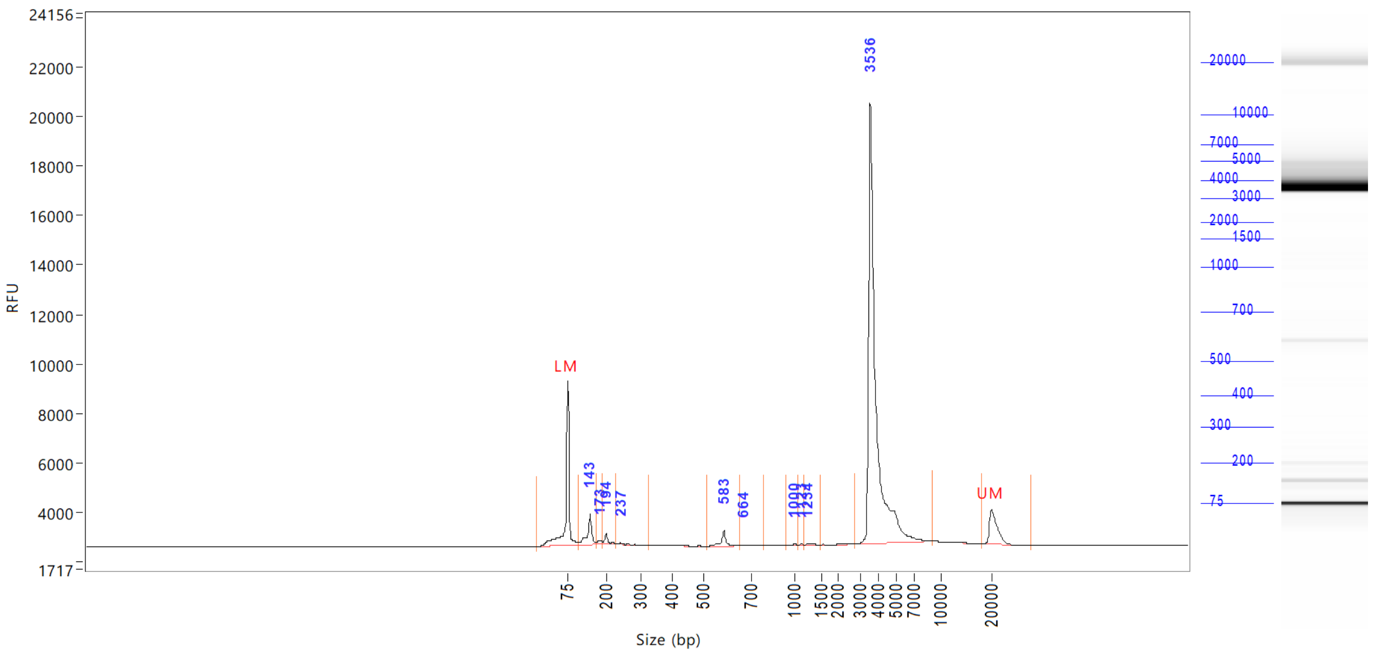

Peak	Size (bp)	Concentration (ng/uL)	From (bp)	To (bp)	Average size (bp)	CV%	RFU	Corrected peak area
1	75 (LM)	0.5000	0	110	65	29.73	6630	53.170
2	143	1.4019	110	162	137	7.88	1249	12.423
3	173	0.1923	162	185	173	3.87	160	1.704
4	194	0.3717	185	223	196	4.16	414	3.293
5	237	0.0934	223	321	246	7.93	61	0.828
6	583	0.6294	511	649	579	4.03	641	5.577
7	664	0.0798	649	783	680	3.69	51	0.707
8	1000	0.0441	948	1056	999	2.08	61	0.391
9	1123	0.0408	1056	1178	1118	3.00	52	0.362
10	1234	0.0825	1178	1456	1294	5.83	51	0.731
11	3536	20.6440	2772	9134	3886	16.48	17818	182.939
12	20000 (UM)	0.1317	18049	27859	20517	4.35	1408	14.010

TIC:	23.5798	ng/uL
TIM:	33.3494	nmole/L
Total concentration:	23.7378	ng/uL

```
Sample peak width (sec): 5     Sample min peak height: 50     Sample baseline V to V?: Y     Sample baseline V to V points: 3
Sample filter: Binomial        Number of points for filter: 3  Sample start region (min): 0  Sample end region (min): 30
Marker peak width (sec): 5     Marker min peak height: 200    Marker baseline V to V?: Y     Marker baseline V to V points: 3
Lower marker selection: First peak > 500 RFU                  Upper marker selection: Last peak > 200 RFU
Ladder size (bp)75, 200, 300, 400, 500, 700, 1000, 1500, 2000, 3000, 4000, 5000, 7000, 10000, 20000
Quantification using: Lower Marker          Final concentration (ng/uL): 0.5000          Dilution factor: 12.0
```

Sample: SampA8
Well location: A8
Created: Monday, September 20, 2021 6:40:06 PM

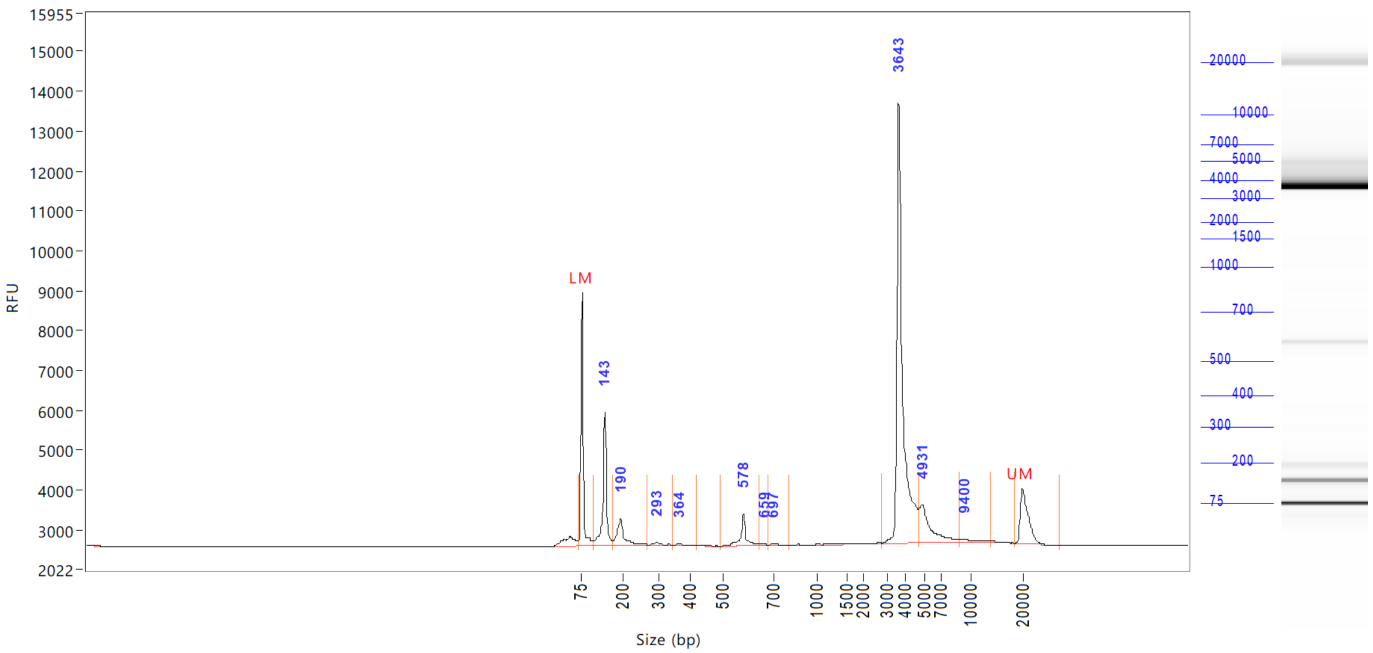

Peak	Size (bp)	Concentration (ng/uL)	From (bp)	To (bp)	Average size (bp)	CV%	RFU	Corrected peak area
1	75 (LM)	0.5000	64	108	75	9.18	6327	36.235
2	143	4.4819	108	168	140	6.81	3325	27.067
3	190	1.6955	168	269	193	9.27	661	10.239
4	293	0.1720	269	343	301	6.04	57	1.039
5	364	0.1106	343	418	367	4.40	53	0.668
6	578	1.2564	489	644	575	4.21	811	7.588
7	659	0.1006	644	680	659	1.55	57	0.608
8	697	0.1520	680	805	717	4.51	58	0.918
9	3643	15.2948	2829	4724	3805	8.05	11051	92.367
10	4931	2.6116	4724	8800	5670	18.52	952	15.772
11	9400	0.3176	8800	13781	10474	13.51	81	1.918
12	20000 (UM)	0.1847	18537	27133	20468	4.28	1380	13.384

TIC:	26.1929	ng/uL
TIM:	80.1089	nmole/L
Total concentration:	26.4126	ng/uL

```
Sample peak width (sec): 5    Sample min peak height: 50    Sample baseline V to V?: Y    Sample baseline V to V points: 3
Sample filter: Binomial       Number of points for filter: 3  Sample start region (min): 0  Sample end region (min): 30
Marker peak width (sec): 5    Marker min peak height: 200   Marker baseline V to V?: Y    Marker baseline V to V points: 3
Lower marker selection: First peak > 500 RFU                Upper marker selection: Last peak > 200 RFU
Ladder size (bp)75, 200, 300, 400, 500, 700, 1000, 1500, 2000, 3000, 4000, 5000, 7000, 10000, 20000
Quantification using: Lower Marker          Final concentration (ng/uL): 0.5000          Dilution factor: 12.0
```

Sample: SampB8
Well location: B8
Created: Monday, September 20, 2021 6:40:06 PM

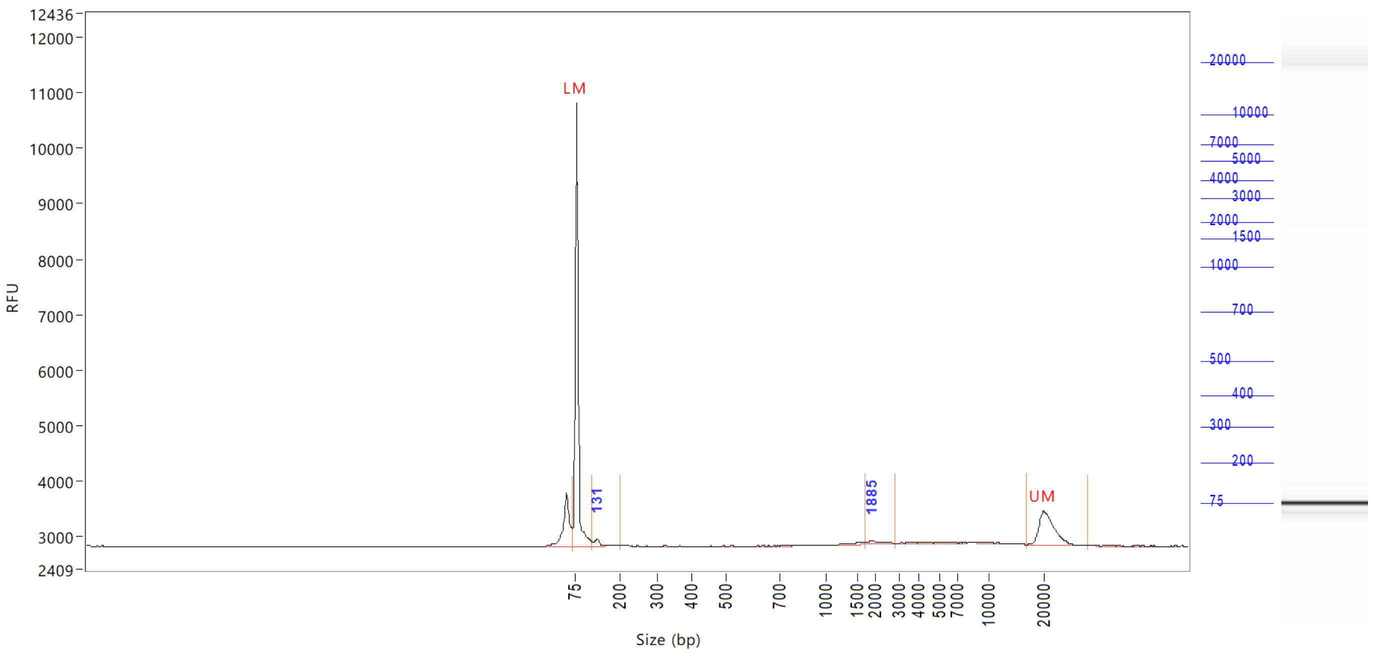

Peak	Size (bp)	Concentration (ng/uL)	From (bp)	To (bp)	Average size (bp)	CV%	RFU	Corrected peak area
1	75 (LM)	0.5000	64	122	75	10.60	8008	57.688
2	131	0.1709	122	201	135	9.11	129	1.643
3	1885	0.1090	1731	2800	2035	12.19	63	1.048
4	20000 (UM)	0.0970	16830	27979	20691	7.14	603	11.195

TIC:	0.2799	ng/uL
TIM:	2.1682	nmole/L
Total concentration:	0.5774	ng/uL

Sample peak width (sec): 5   Sample min peak height: 50   Sample baseline V to V?: Y   Sample baseline V to V points: 3
Sample filter: Binomial   Number of points for filter: 3 Sample start region (min): 0 Sample end region (min): 30
Marker peak width (sec): 5   Marker min peak height: 200   Marker baseline V to V?: Y   Marker baseline V to V points: 3
Lower marker selection: First peak > 500 RFU   Upper marker selection: Last peak > 200 RFU
Ladder size (bp)75, 200, 300, 400, 500, 700, 1000, 1500, 2000, 3000, 4000, 5000, 7000, 10000, 20000
Quantification using: Lower Marker   Final concentration (ng/uL): 0.5000   Dilution factor: 12.0

Sample: SampC8
Well location: C8
Created: Monday, September 20, 2021 6:40:06 PM

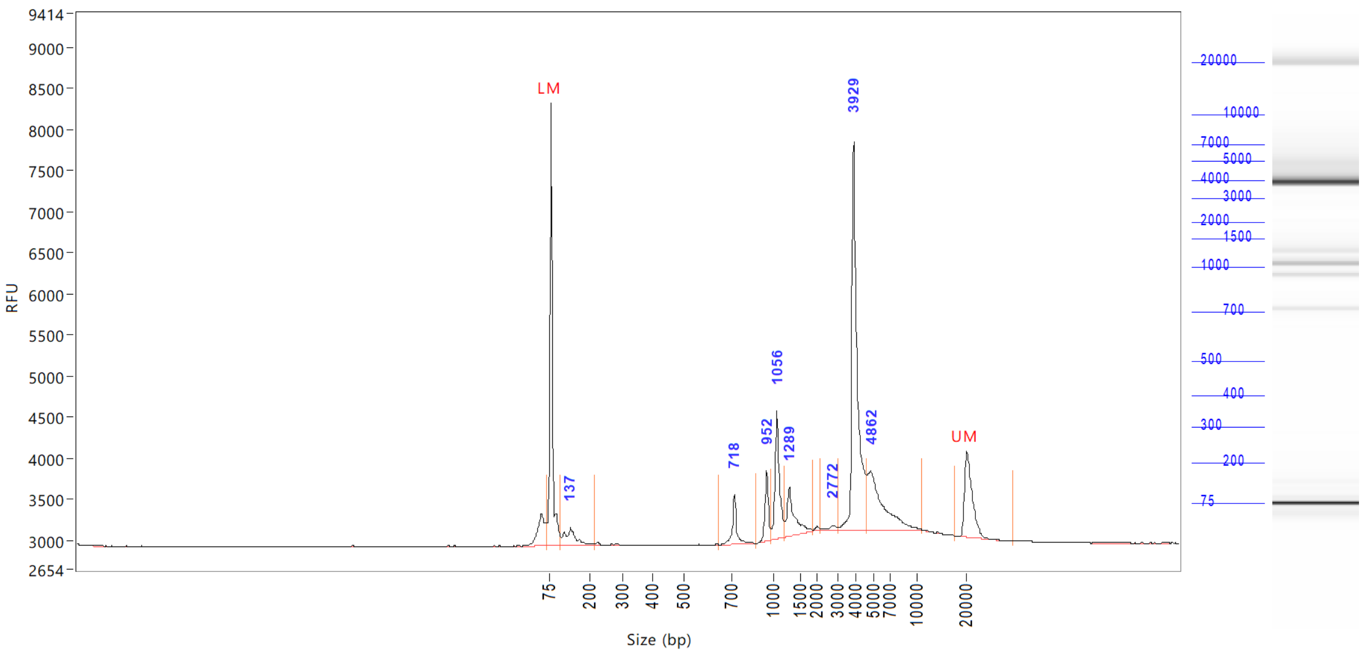

Peak	Size (bp)	Concentration (ng/uL)	From (bp)	To (bp)	Average size (bp)	CV%	RFU	Corrected peak area
1	75 (LM)	0.5000	64	108	76	9.70	5394	35.037
2	137	0.9520	108	214	141	16.75	217	5.559
3	718	0.7916	649	874	720	4.16	605	4.622
4	952	0.8771	874	987	950	1.62	853	5.122
5	1056	2.0533	987	1212	1070	4.79	1550	11.990
6	1289	1.2236	1212	1904	1360	9.41	598	7.145
7	2772	0.1080	2172	3037	2723	7.47	57	0.631
8	3929	6.9432	3037	4621	3989	6.12	4715	40.545
9	4862	2.8216	4621	10855	5849	21.48	713	16.477
10	20000 (UM)	0.1504	17683	29551	20587	4.87	1052	10.536

TIC:	15.7702	ng/uL
TIM:	22.7884	nmole/L
Total concentration:	15.9268	ng/uL

```
Sample peak width (sec): 5    Sample min peak height: 50    Sample baseline V to V?: Y    Sample baseline V to V points: 3
Sample filter: Binomial       Number of points for filter: 3  Sample start region (min): 0  Sample end region (min): 30
Marker peak width (sec): 5    Marker min peak height: 200   Marker baseline V to V?: Y    Marker baseline V to V points: 3
Lower marker selection: First peak > 500 RFU                Upper marker selection: Last peak > 200 RFU
Ladder size (bp)75, 200, 300, 400, 500, 700, 1000, 1500, 2000, 3000, 4000, 5000, 7000, 10000, 20000
Quantification using: Lower Marker          Final concentration (ng/uL): 0.5000          Dilution factor: 12.0
```

Sample: SampD8
Well location: D8
Created: Monday, September 20, 2021 6:40:06 PM

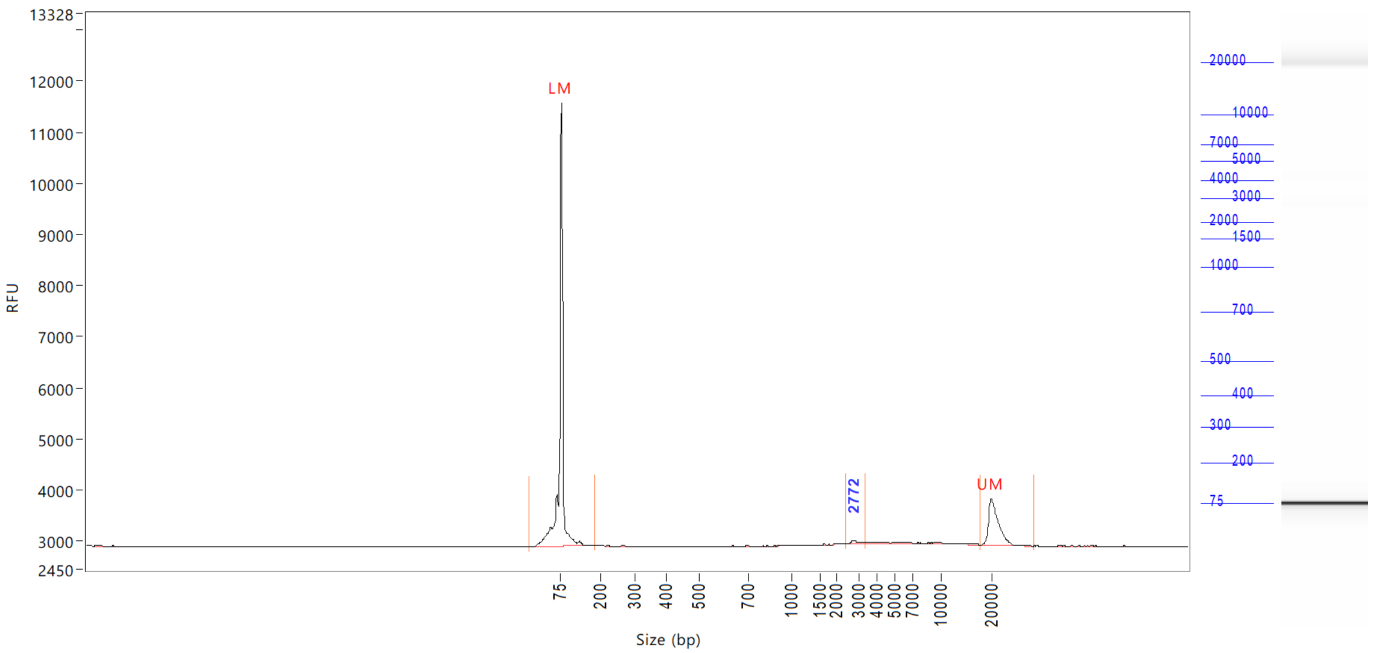

Peak	Size (bp)	Concentration (ng/uL)	From (bp)	To (bp)	Average size (bp)	CV%	RFU	Corrected peak area
1	75 (LM)	0.5000	0	179	69	25.34	8684	70.637
2	2772	0.0707	2429	3358	2840	7.12	76	0.833
3	20000 (UM)	0.0810	17927	28463	20655	5.77	912	11.450

TIC:	0.0707	ng/uL
TIM:	0.0410	nmole/L
Total concentration:	0.3419	ng/uL

Sample peak width (sec): 5 Sample min peak height: 50 Sample baseline V to V?: Y Sample baseline V to V points: 3
Sample filter: Binomial Number of points for filter: 3 Sample start region (min): 0 Sample end region (min): 30
Marker peak width (sec): 5 Marker min peak height: 200 Marker baseline V to V?: Y Marker baseline V to V points: 3
Lower marker selection: First peak > 500 RFU Upper marker selection: Last peak > 200 RFU
Ladder size (bp)75, 200, 300, 400, 500, 700, 1000, 1500, 2000, 3000, 4000, 5000, 7000, 10000, 20000
Quantification using: Lower Marker Final concentration (ng/uL): 0.5000 Dilution factor: 12.0

Sample: SampE8
Well location: E8
Created: Monday, September 20, 2021 6:40:06 PM

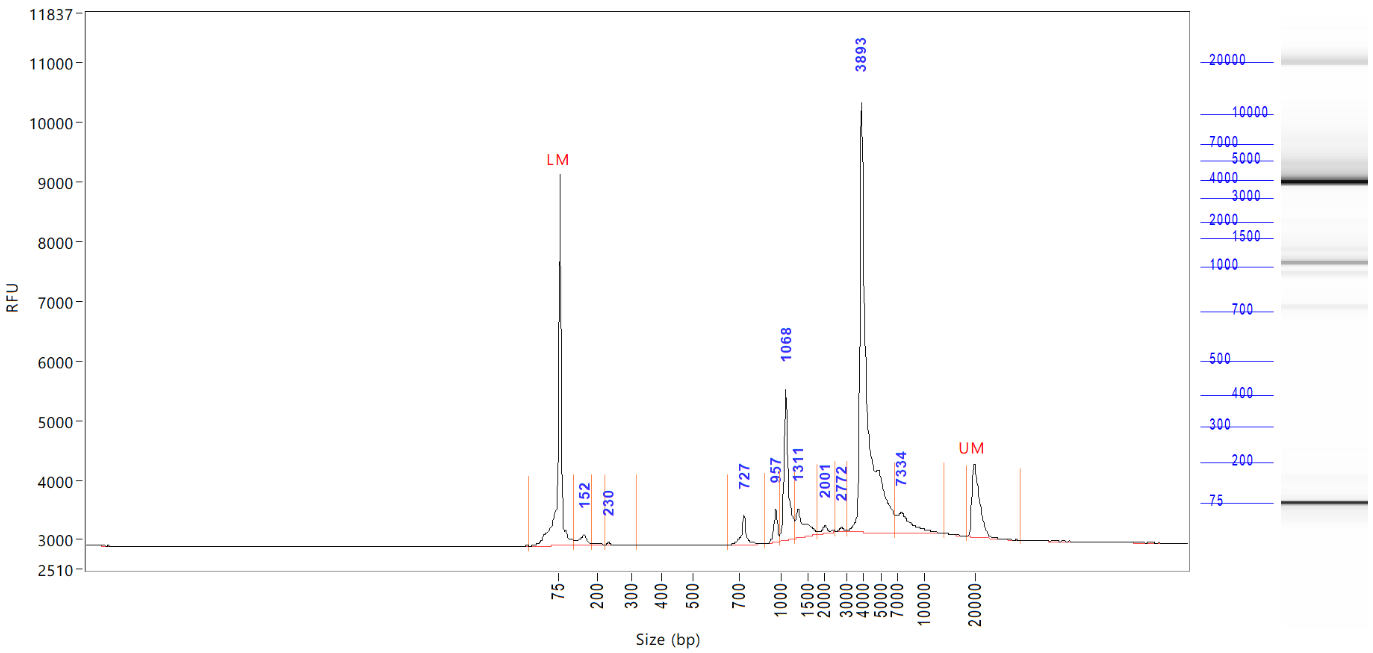

Peak	Size (bp)	Concentration (ng/uL)	From (bp)	To (bp)	Average size (bp)	CV%	RFU	Corrected peak area
1	75 (LM)	0.5000	0	122	68	25.70	6239	51.210
2	152	0.4097	122	179	146	9.38	178	3.497
3	230	0.0775	219	313	248	9.93	51	0.662
4	727	0.5187	646	879	725	4.72	490	4.427
5	957	0.4133	879	987	953	1.67	553	3.527
6	1068	2.2531	987	1234	1082	5.09	2539	19.230
7	1311	0.8529	1234	1770	1388	8.85	494	7.280
8	2001	0.1742	1770	2515	2027	8.36	140	1.487
9	2772	0.0730	2515	3001	2779	4.36	72	0.623
10	3893	9.5532	3001	6778	4274	14.78	7220	81.536
11	7334	0.8657	6778	13903	8142	14.78	341	7.389
12	20000 (UM)	0.1196	18415	29672	20631	5.69	1232	12.245

TIC:	15.1914	ng/uL
TIM:	15.4957	nmole/L
Total concentration:	15.2904	ng/uL

```
Sample peak width (sec): 5    Sample min peak height: 50    Sample baseline V to V?: Y    Sample baseline V to V points: 3
Sample filter: Binomial       Number of points for filter: 3  Sample start region (min): 0  Sample end region (min): 30
Marker peak width (sec): 5    Marker min peak height: 200   Marker baseline V to V?: Y    Marker baseline V to V points: 3
Lower marker selection: First peak > 500 RFU                Upper marker selection: Last peak > 200 RFU
Ladder size (bp)75, 200, 300, 400, 500, 700, 1000, 1500, 2000, 3000, 4000, 5000, 7000, 10000, 20000
Quantification using: Lower Marker          Final concentration (ng/uL): 0.5000          Dilution factor: 12.0
```

Sample: SampF8
Well location: F8
Created: Monday, September 20, 2021 6:40:06 PM

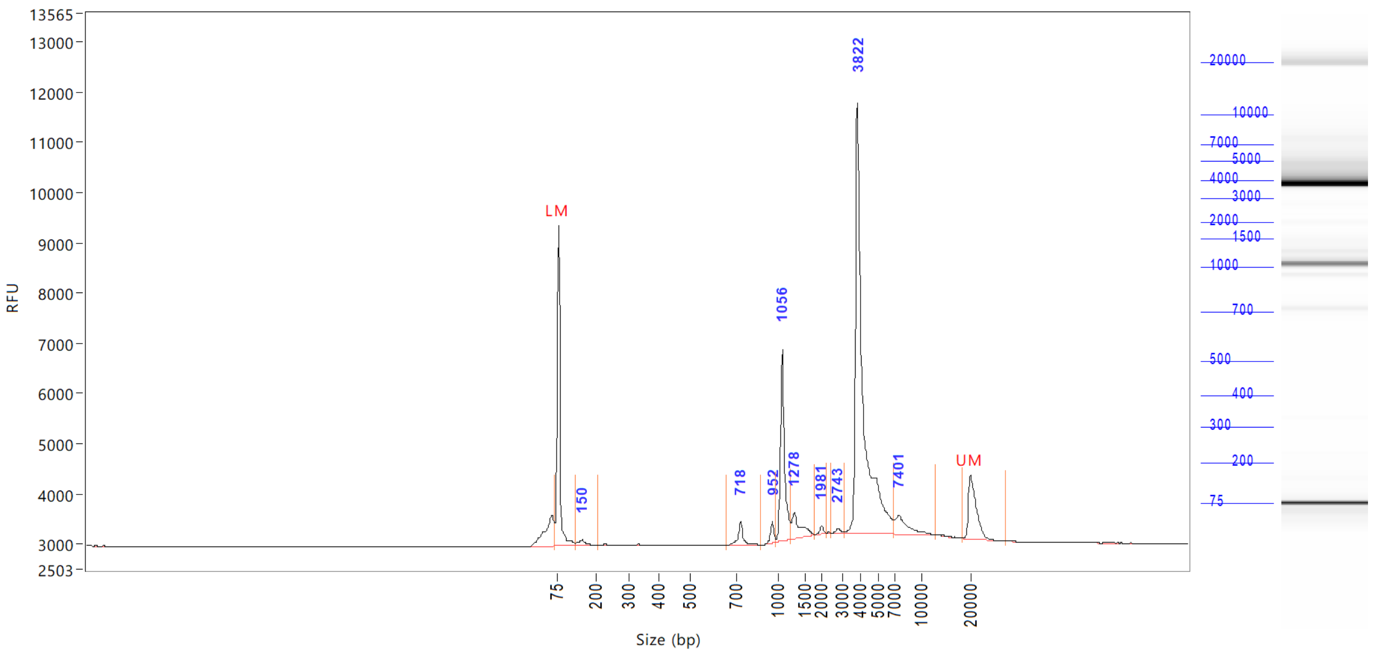

Peak	Size (bp)	Concentration (ng/uL)	From (bp)	To (bp)	Average size (bp)	CV%	RFU	Corrected peak area
1	75 (LM)	0.5000	64	129	76	12.10	6371	43.168
2	150	0.3316	129	203	155	11.40	117	2.386
3	718	0.5654	654	866	719	4.18	468	4.068
4	952	0.3564	866	978	946	1.52	430	2.564
5	1056	3.6480	978	1223	1063	4.89	3801	26.246
6	1278	0.9753	1223	1789	1362	8.29	518	7.017
7	1981	0.1354	1789	2172	1964	3.21	158	0.974
8	2743	0.0971	2429	3072	2791	5.11	78	0.698
9	3822	13.2541	3072	6852	4199	14.98	8580	95.359
10	7401	0.9779	6852	12928	8043	12.90	382	7.036
11	20000 (UM)	0.1517	18171	27133	20558	4.73	1285	13.096

TIC:	20.3412	ng/uL
TIM:	17.8249	nmole/L
Total concentration:	20.5211	ng/uL

Sample peak width (sec): 5   Sample min peak height: 50   Sample baseline V to V?: Y   Sample baseline V to V points: 3
Sample filter: Binomial   Number of points for filter: 3 Sample start region (min): 0 Sample end region (min): 30
Marker peak width (sec): 5   Marker min peak height: 200   Marker baseline V to V?: Y   Marker baseline V to V points: 3
Lower marker selection: First peak > 500 RFU   Upper marker selection: Last peak > 200 RFU
Ladder size (bp)75, 200, 300, 400, 500, 700, 1000, 1500, 2000, 3000, 4000, 5000, 7000, 10000, 20000
Quantification using: Lower Marker   Final concentration (ng/uL): 0.5000   Dilution factor: 12.0

Sample: SampG8
Well location: G8
Created: Monday, September 20, 2021 6:40:06 PM

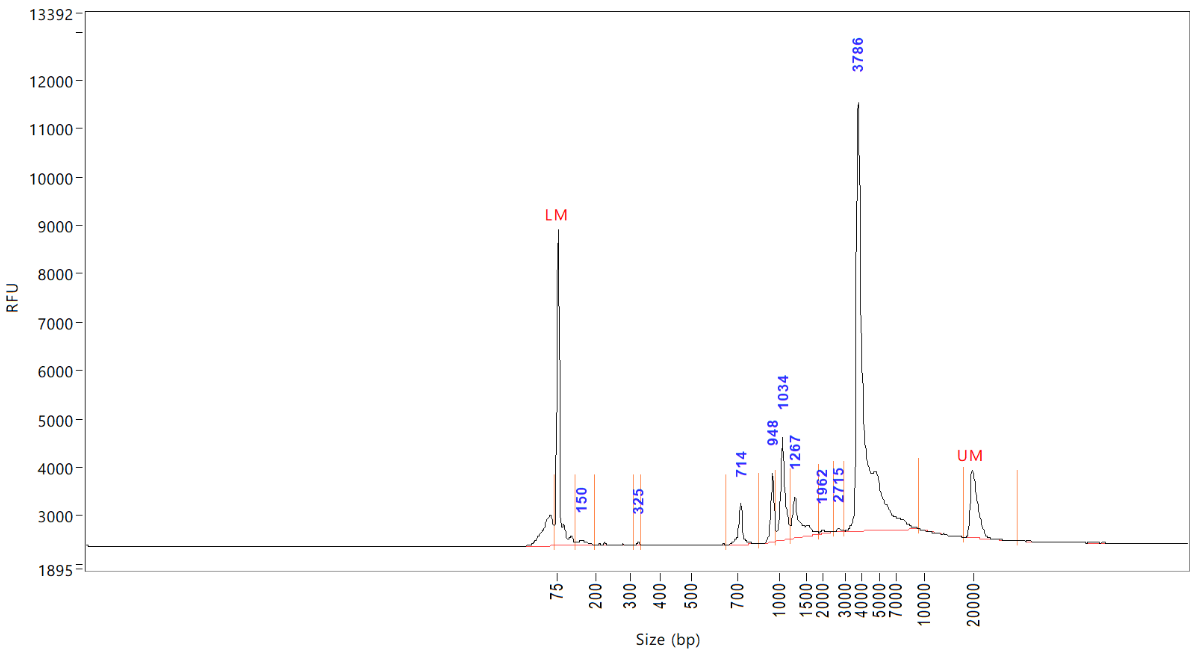

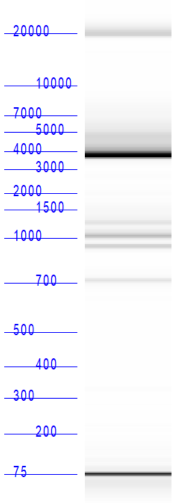

Peak	Size (bp)	Concentration (ng/uL)	From (bp)	To (bp)	Average size (bp)	CV%	RFU	Corrected peak area
1	75 (LM)	0.5000	64	129	77	14.48	6529	44.569
2	150	0.3345	129	194	152	10.00	112	2.485
3	325	0.0633	309	337	322	1.62	78	0.470
4	714	0.8697	652	853	712	3.58	842	6.460
5	948	1.1483	853	974	942	1.49	1426	8.530
6	1034	2.2475	974	1190	1048	4.88	2137	16.695
7	1267	1.4984	1190	1846	1357	10.20	852	11.131
8	1962	0.1030	1846	2458	2015	6.20	83	0.765
9	2715	0.0609	2458	3001	2732	4.43	66	0.453
10	3786	13.5381	3001	9533	4295	21.34	8873	100.564
11	20000 (UM)	0.1521	18171	29189	20424	4.73	1410	13.558

TIC:	19.8637	ng/uL
TIM:	18.6143	nmole/L
Total concentration:	20.0136	ng/uL

```
Sample peak width (sec): 5    Sample min peak height: 50    Sample baseline V to V?: Y    Sample baseline V to V points: 3
Sample filter: Binomial       Number of points for filter: 3  Sample start region (min): 0  Sample end region (min): 30
Marker peak width (sec): 5    Marker min peak height: 200   Marker baseline V to V?: Y    Marker baseline V to V points: 3
Lower marker selection: First peak > 500 RFU                Upper marker selection: Last peak > 200 RFU
Ladder size (bp)75, 200, 300, 400, 500, 700, 1000, 1500, 2000, 3000, 4000, 5000, 7000, 10000, 20000
Quantification using: Lower Marker          Final concentration (ng/uL): 0.5000          Dilution factor: 12.0
```

Sample: SampH8
Well location: H8
Created: Monday, September 20, 2021 6:40:06 PM

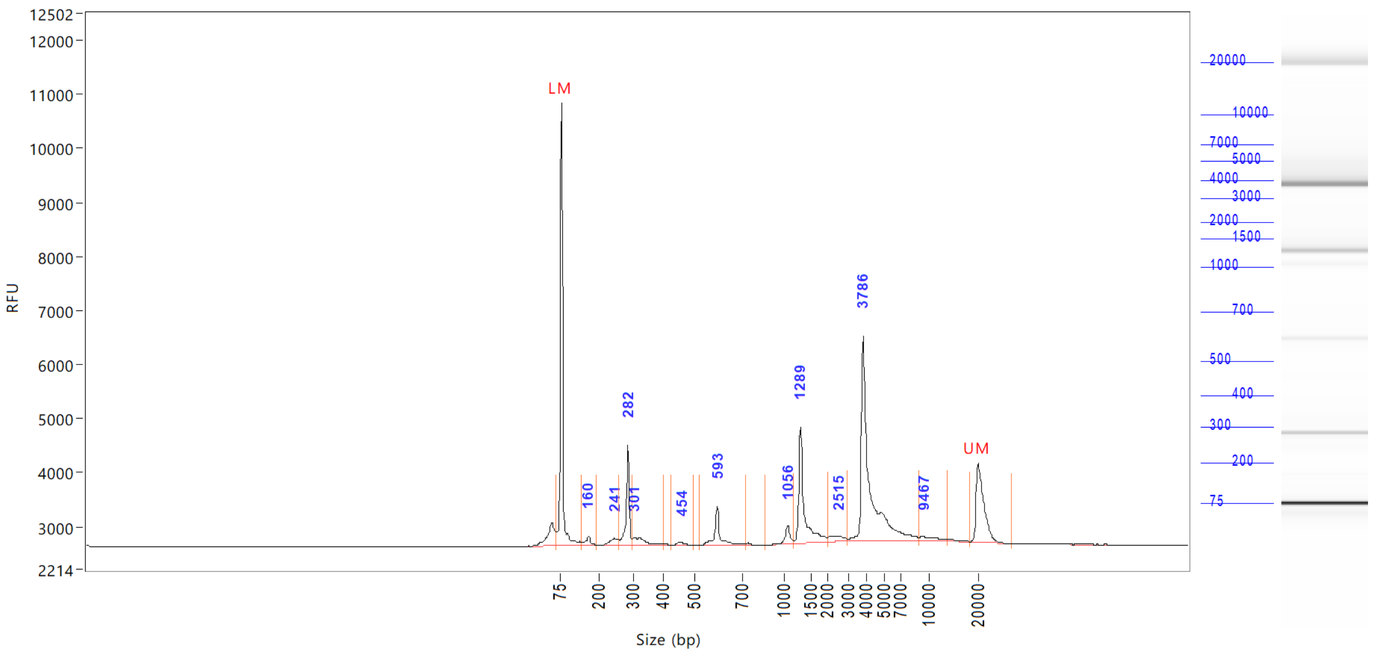

Peak	Size (bp)	Concentration (ng/uL)	From (bp)	To (bp)	Average size (bp)	CV%	RFU	Corrected peak area
1	75 (LM)	0.5000	60	141	75	13.58	8206	50.507
2	160	0.2145	141	187	157	5.17	171	1.806
3	241	0.2688	187	257	238	5.29	120	2.263
4	282	1.4615	257	295	278	2.40	1853	12.303
5	301	0.4417	295	401	325	7.98	135	3.718
6	454	0.0981	426	494	453	2.75	57	0.826
7	593	0.8406	519	718	600	6.08	726	7.076
8	1056	0.3408	866	1178	1050	5.02	338	2.869
9	1289	2.2661	1178	1981	1368	11.72	2155	19.076
10	2515	0.2691	1981	2914	2373	10.99	99	2.266
11	3786	5.5029	2914	8934	4369	23.16	3788	46.322
12	9467	0.2112	8934	13781	10737	13.24	71	1.778
13	20000 (UM)	0.1477	18537	26770	20619	4.78	1456	14.920

TIC:	11.9154	ng/uL
TIM:	23.2019	nmole/L
Total concentration:	11.9994	ng/uL

```
Sample peak width (sec): 5     Sample min peak height: 50     Sample baseline V to V?: Y     Sample baseline V to V points: 3
Sample filter: Binomial        Number of points for filter: 3  Sample start region (min): 0  Sample end region (min): 30
Marker peak width (sec): 5     Marker min peak height: 200    Marker baseline V to V?: Y     Marker baseline V to V points: 3
Lower marker selection: First peak > 500 RFU                  Upper marker selection: Last peak > 200 RFU
Ladder size (bp)75, 200, 300, 400, 500, 700, 1000, 1500, 2000, 3000, 4000, 5000, 7000, 10000, 20000
Quantification using: Lower Marker          Final concentration (ng/uL): 0.5000          Dilution factor: 12.0
```

Sample: SampA9
Well location: A9
Created: Monday, September 20, 2021 6:40:06 PM

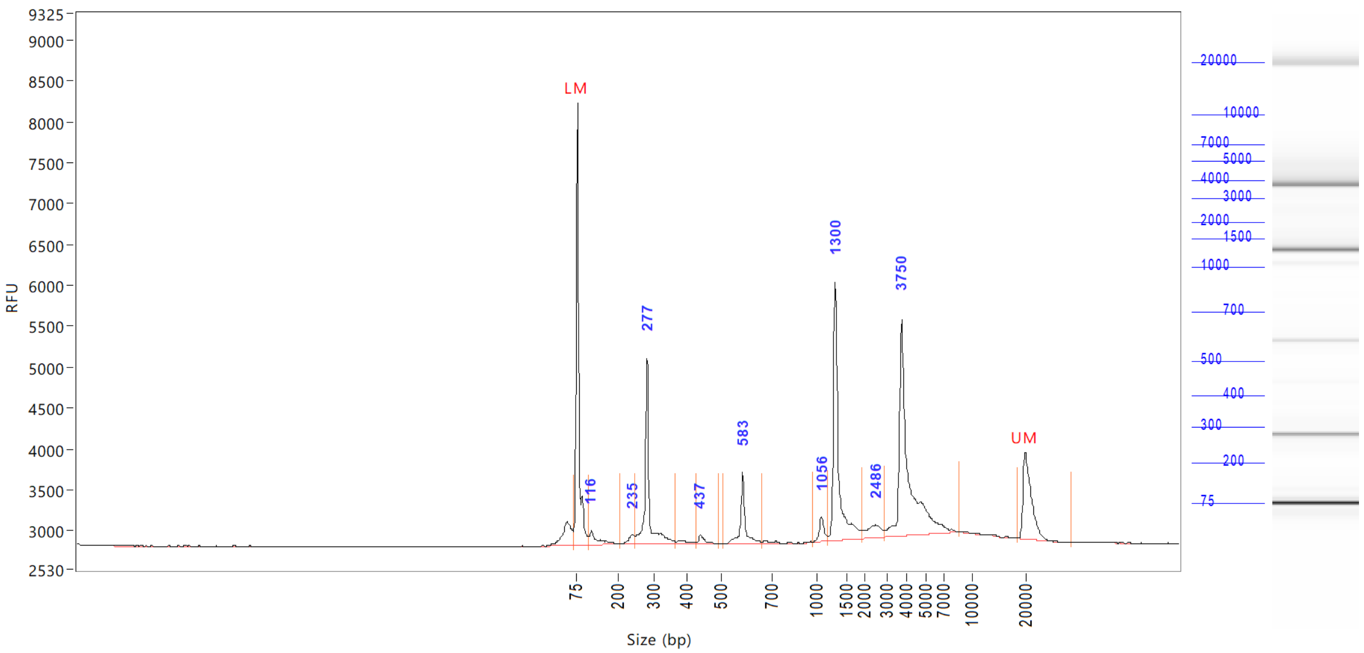

Peak	Size (bp)	Concentration (ng/uL)	From (bp)	To (bp)	Average size (bp)	CV%	RFU	Corrected peak area
1	75 (LM)	0.5000	64	108	76	9.34	5415	34.900
2	116	0.5224	108	205	133	15.87	175	3.039
3	235	0.2643	205	244	231	3.80	116	1.537
4	277	3.2966	244	364	281	7.27	2262	19.175
5	437	0.2362	424	492	445	3.13	118	1.374
6	583	1.2316	506	659	583	4.16	874	7.164
7	1056	0.3826	970	1178	1066	4.29	302	2.226
8	1300	4.1832	1178	1923	1363	10.18	3156	24.332
9	2486	0.6058	1923	2914	2382	11.60	155	3.524
10	3750	5.7769	2914	8667	4227	19.49	2651	33.602
11	20000 (UM)	0.1557	18537	28705	20483	5.02	1065	10.865

TIC:	16.4996	ng/uL
TIM:	40.2931	nmole/L
Total concentration:	16.8654	ng/uL

Sample peak width (sec): 5 Sample min peak height: 50 Sample baseline V to V?: Y Sample baseline V to V points: 3
Sample filter: Binomial Number of points for filter: 3 Sample start region (min): 0 Sample end region (min): 30
Marker peak width (sec): 5 Marker min peak height: 200 Marker baseline V to V?: Y Marker baseline V to V points: 3
Lower marker selection: First peak > 500 RFU Upper marker selection: Last peak > 200 RFU
Ladder size (bp)75, 200, 300, 400, 500, 700, 1000, 1500, 2000, 3000, 4000, 5000, 7000, 10000, 20000
Quantification using: Lower Marker Final concentration (ng/uL): 0.5000 Dilution factor: 12.0

Sample: SampB9
Well location: B9
Created: Monday, September 20, 2021 6:40:06 PM

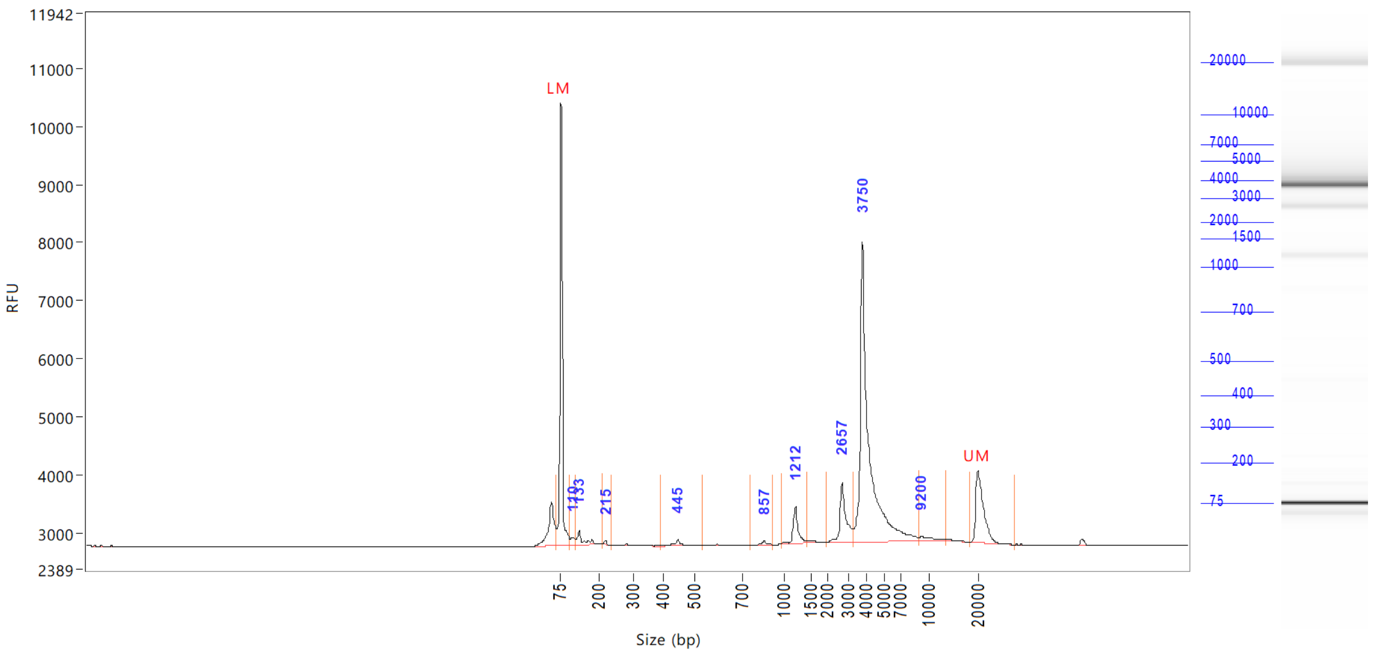

Peak	Size (bp)	Concentration (ng/uL)	From (bp)	To (bp)	Average size (bp)	CV%	RFU	Corrected peak area
1	75 (LM)	0.5000	64	103	75	7.09	7617	46.830
2	110	0.2043	103	124	113	5.76	125	1.594
3	133	0.4248	124	208	146	13.36	253	3.315
4	215	0.0408	208	235	214	1.56	66	0.319
5	445	0.2220	390	534	444	6.44	116	1.733
6	857	0.0767	762	926	851	2.74	67	0.599
7	1212	0.6322	983	1411	1205	5.65	623	4.935
8	2657	1.2237	1962	3215	2734	7.66	1025	9.551
9	3750	8.3491	3215	8934	4248	21.98	5158	65.166
10	9200	0.2013	8934	13537	10434	12.22	74	1.571
11	20000 (UM)	0.1342	18415	27375	20525	4.79	1246	12.569

TIC:	11.3749	ng/uL
TIM:	13.9031	nmole/L
Total concentration:	11.5679	ng/uL

```
Sample peak width (sec): 5    Sample min peak height: 50    Sample baseline V to V?: Y    Sample baseline V to V points: 3
Sample filter: Binomial       Number of points for filter: 3  Sample start region (min): 0  Sample end region (min): 30
Marker peak width (sec): 5    Marker min peak height: 200   Marker baseline V to V?: Y    Marker baseline V to V points: 3
Lower marker selection: First peak > 500 RFU                Upper marker selection: Last peak > 200 RFU
Ladder size (bp)75, 200, 300, 400, 500, 700, 1000, 1500, 2000, 3000, 4000, 5000, 7000, 10000, 20000
Quantification using: Lower Marker          Final concentration (ng/uL): 0.5000          Dilution factor: 12.0
```

Sample: SampC9
Well location: C9
Created: Monday, September 20, 2021 6:40:06 PM

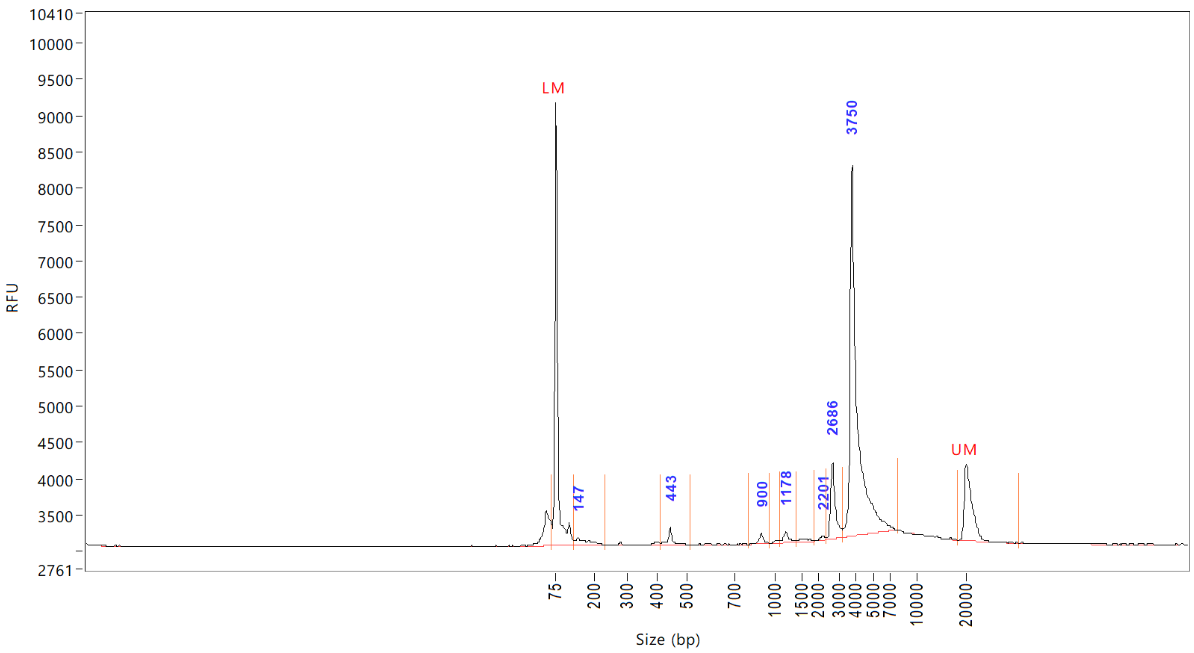

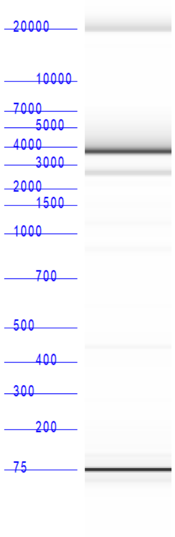

Peak	Size (bp)	Concentration (ng/uL)	From (bp)	To (bp)	Average size (bp)	CV%	RFU	Corrected peak area
1	75 (LM)	0.5000	64	131	78	16.04	6104	43.085
2	147	0.4109	131	230	166	14.39	97	2.951
3	443	0.3218	409	514	442	4.01	241	2.311
4	900	0.1615	805	957	890	2.46	152	1.160
5	1178	0.1984	1090	1378	1203	5.72	148	1.425
6	2201	0.0614	1885	2372	2162	5.19	55	0.441
7	2686	1.0817	2372	3144	2699	5.52	1046	7.767
8	3750	7.6882	3144	7734	4047	14.40	5106	55.207
9	20000 (UM)	0.1263	18171	31002	20664	5.50	1059	10.885

TIC:	9.9239	ng/uL
TIM:	9.6710	nmole/L
Total concentration:	10.2844	ng/uL

```
Sample peak width (sec): 5    Sample min peak height: 50    Sample baseline V to V?: Y    Sample baseline V to V points: 3
Sample filter: Binomial       Number of points for filter: 3  Sample start region (min): 0  Sample end region (min): 30
Marker peak width (sec): 5    Marker min peak height: 200   Marker baseline V to V?: Y    Marker baseline V to V points: 3
Lower marker selection: First peak > 500 RFU                Upper marker selection: Last peak > 200 RFU
Ladder size (bp)75, 200, 300, 400, 500, 700, 1000, 1500, 2000, 3000, 4000, 5000, 7000, 10000, 20000
Quantification using: Lower Marker          Final concentration (ng/uL): 0.5000          Dilution factor: 12.0
```

Sample: SampD9
Well location: D9
Created: Monday, September 20, 2021 6:40:06 PM

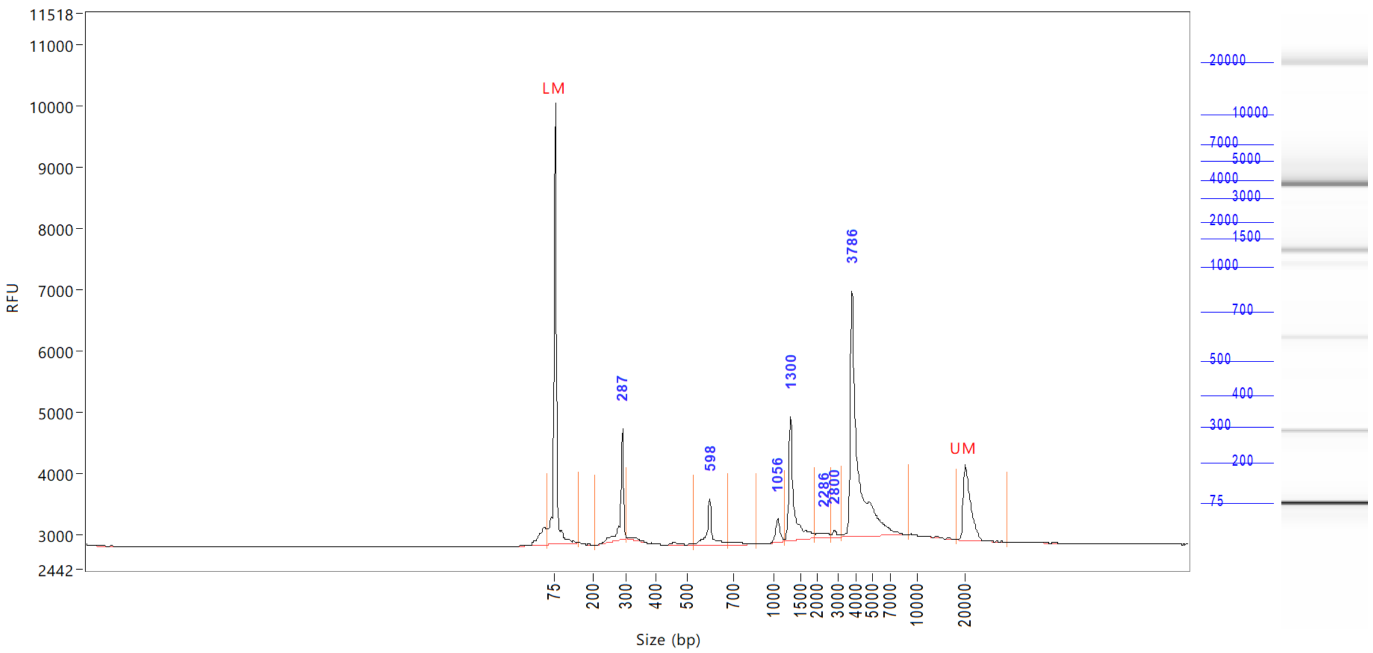

Peak	Size (bp)	Concentration (ng/uL)	From (bp)	To (bp)	Average size (bp)	CV%	RFU	Corrected peak area
1	75 (LM)	0.5000	48	152	74	13.50	7215	47.991
2	287	1.5574	203	300	278	5.36	1812	12.457
3	598	0.8956	527	680	597	4.60	752	7.163
4	1056	0.3710	861	1178	1049	4.35	390	2.967
5	1300	2.1511	1178	1923	1354	10.21	2028	17.205
6	2286	0.1826	1923	2657	2244	9.65	74	1.460
7	2800	0.1106	2657	3108	2840	4.16	114	0.884
8	3786	5.7951	3108	9134	4255	18.44	3996	46.352
9	20000 (UM)	0.1264	18293	28826	20542	4.63	1236	12.136

TIC:	11.0633	ng/uL
TIM:	17.3205	nmole/L
Total concentration:	11.3869	ng/uL

```
Sample peak width (sec): 5    Sample min peak height: 50    Sample baseline V to V?: Y    Sample baseline V to V points: 3
Sample filter: Binomial       Number of points for filter: 3  Sample start region (min): 0  Sample end region (min): 30
Marker peak width (sec): 5    Marker min peak height: 200   Marker baseline V to V?: Y    Marker baseline V to V points: 3
Lower marker selection: First peak > 500 RFU                Upper marker selection: Last peak > 200 RFU
Ladder size (bp)75, 200, 300, 400, 500, 700, 1000, 1500, 2000, 3000, 4000, 5000, 7000, 10000, 20000
Quantification using: Lower Marker          Final concentration (ng/uL): 0.5000          Dilution factor: 12.0
```

Sample: SampE9
Well location: E9
Created: Monday, September 20, 2021 6:40:06 PM

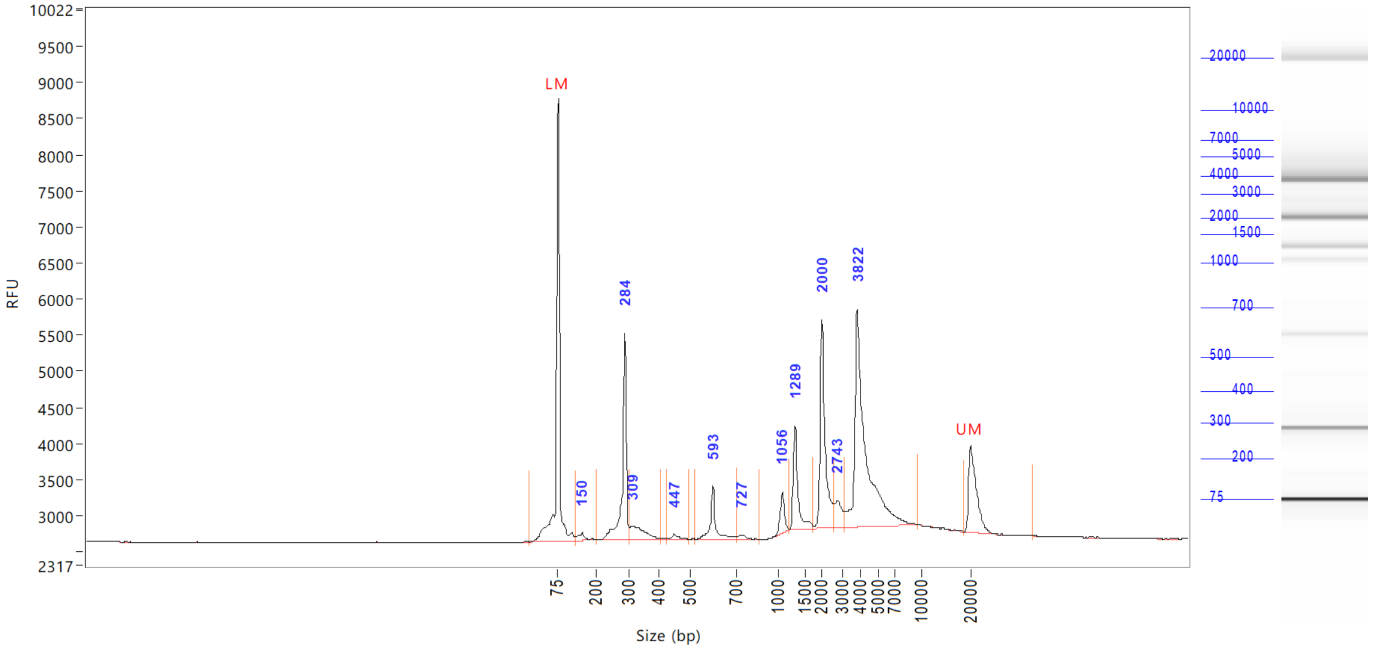

Peak	Size (bp)	Concentration (ng/uL)	From (bp)	To (bp)	Average size (bp)	CV%	RFU	Corrected peak area
1	75 (LM)	0.5000	0	129	68	26.21	6140	47.849
2	150	0.2219	129	198	150	9.59	107	1.769
3	284	2.8753	198	298	275	5.66	2857	22.930
4	309	0.7310	298	403	332	7.67	188	5.830
5	447	0.1212	424	494	448	3.01	77	0.966
6	593	0.8922	519	697	599	5.28	741	7.115
7	727	0.1093	697	861	737	4.57	63	0.872
8	1056	0.5184	861	1190	1047	3.91	583	4.134
9	1289	1.3808	1190	1750	1327	7.08	1434	11.012
10	2000	2.9511	1750	2543	2062	7.69	2881	23.535
11	2743	0.6178	2543	3072	2782	5.52	383	4.927
12	3822	6.0613	3072	9600	4334	20.96	3012	48.338
13	20000 (UM)	0.1288	18659	32816	20699	6.24	1193	12.327

TIC:	16.4805	ng/uL
TIM:	33.9431	nmole/L
Total concentration:	16.4315	ng/uL

```
Sample peak width (sec): 5     Sample min peak height: 50    Sample baseline V to V?: Y    Sample baseline V to V points: 3
Sample filter: Binomial        Number of points for filter: 3  Sample start region (min): 0  Sample end region (min): 30
Marker peak width (sec): 5     Marker min peak height: 200   Marker baseline V to V?: Y    Marker baseline V to V points: 3
Lower marker selection: First peak > 500 RFU                 Upper marker selection: Last peak > 200 RFU
Ladder size (bp)75, 200, 300, 400, 500, 700, 1000, 1500, 2000, 3000, 4000, 5000, 7000, 10000, 20000
Quantification using: Lower Marker          Final concentration (ng/uL): 0.5000         Dilution factor: 12.0
```

Sample: SampF9
Well location: F9
Created: Monday, September 20, 2021 6:40:06 PM

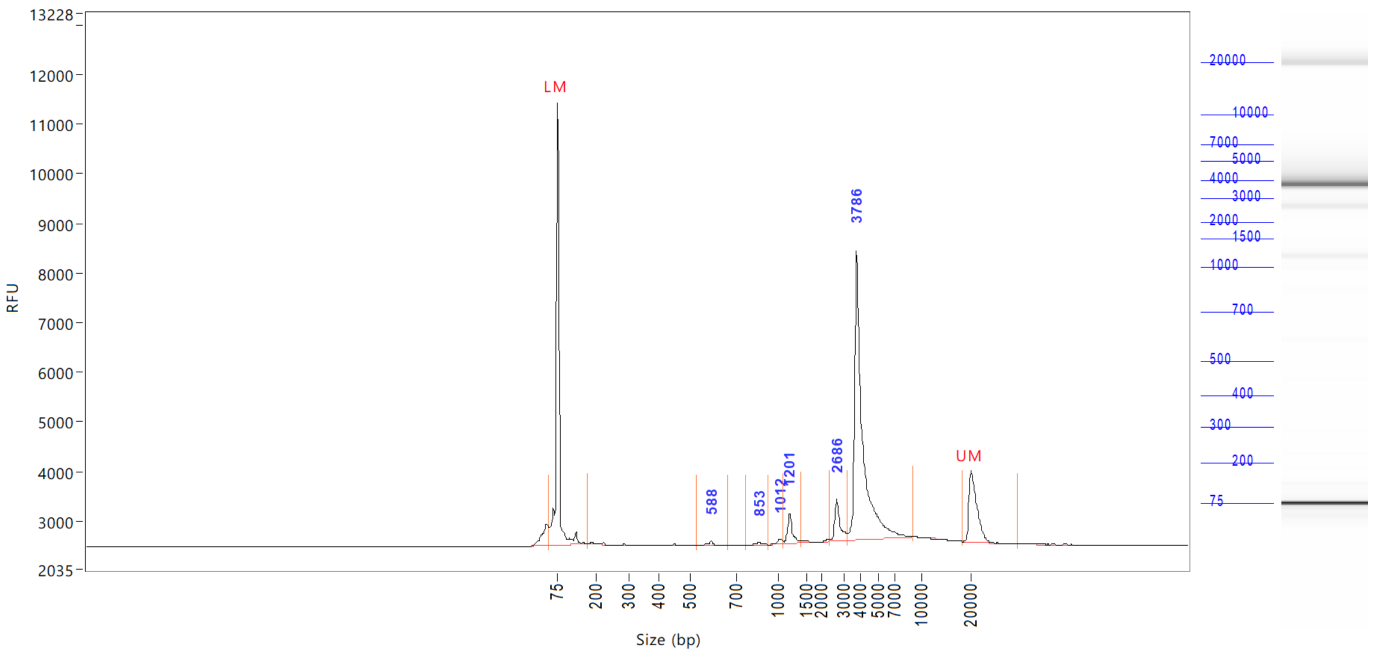

Peak	Size (bp)	Concentration (ng/uL)	From (bp)	To (bp)	Average size (bp)	CV%	RFU	Corrected peak area
1	75 (LM)	0.5000	48	173	75	19.16	8915	62.251
2	588	0.0854	527	657	582	3.90	98	0.886
3	853	0.0575	757	922	847	3.17	60	0.597
4	1012	0.0815	922	1090	1015	3.34	101	0.845
5	1201	0.4665	1090	1411	1203	5.04	608	4.840
6	2686	0.6927	2344	3215	2732	6.60	838	7.186
7	3786	6.5569	3215	9000	4120	16.66	5809	68.028
8	20000 (UM)	0.1212	18293	29672	20569	5.14	1449	15.087

TIC:	7.9404	ng/uL
TIM:	4.1610	nmole/L
Total concentration:	8.1395	ng/uL

```
Sample peak width (sec): 5    Sample min peak height: 50    Sample baseline V to V?: Y    Sample baseline V to V points: 3
Sample filter: Binomial       Number of points for filter: 3  Sample start region (min): 0  Sample end region (min): 30
Marker peak width (sec): 5    Marker min peak height: 200   Marker baseline V to V?: Y    Marker baseline V to V points: 3
Lower marker selection: First peak > 500 RFU                Upper marker selection: Last peak > 200 RFU
Ladder size (bp)75, 200, 300, 400, 500, 700, 1000, 1500, 2000, 3000, 4000, 5000, 7000, 10000, 20000
Quantification using: Lower Marker          Final concentration (ng/uL): 0.5000          Dilution factor: 12.0
```

Sample: SampG9
Well location: G9
Created: Monday, September 20, 2021 6:40:06 PM

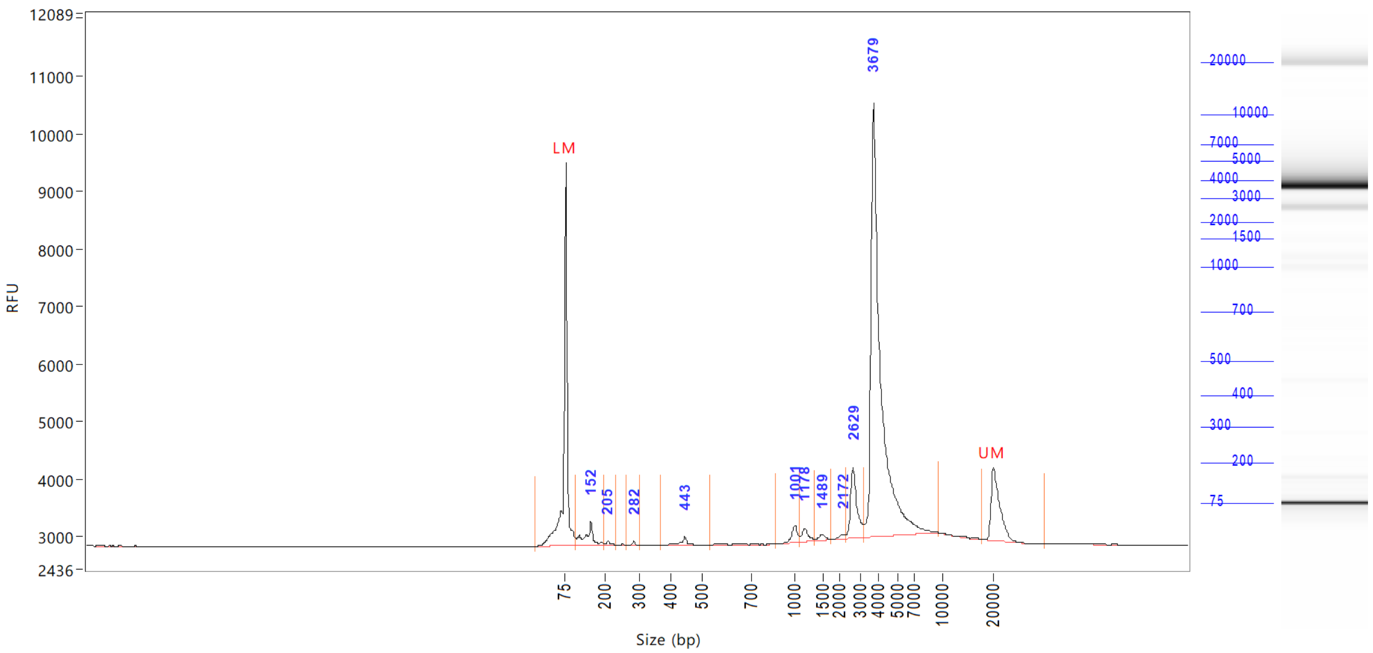

Peak	Size (bp)	Concentration (ng/uL)	From (bp)	To (bp)	Average size (bp)	CV%	RFU	Corrected peak area
1	75 (LM)	0.5000	0	108	68	23.93	6647	55.136
2	152	0.7855	108	196	143	14.14	407	7.218
3	205	0.0689	196	228	206	3.28	68	0.633
4	282	0.0471	259	298	278	2.06	71	0.433
5	443	0.2227	368	527	434	5.75	145	2.046
6	1001	0.3018	874	1079	1000	3.21	289	2.774
7	1178	0.2766	1079	1367	1184	5.51	233	2.542
8	1489	0.1207	1367	1750	1487	4.48	102	1.109
9	2172	0.0752	1750	2286	2089	5.69	66	0.691
10	2629	1.3477	2286	3144	2680	6.74	1237	12.384
11	3679	11.4963	3144	9533	4077	18.46	7548	105.643
12	20000 (UM)	0.1290	17683	30035	20685	5.68	1262	14.229

TIC:	14.7424	ng/uL
TIM:	17.2441	nmole/L
Total concentration:	14.9109	ng/uL

```
Sample peak width (sec): 5     Sample min peak height: 50     Sample baseline V to V?: Y     Sample baseline V to V points: 3
Sample filter: Binomial        Number of points for filter: 3  Sample start region (min): 0  Sample end region (min): 30
Marker peak width (sec): 5     Marker min peak height: 200    Marker baseline V to V?: Y     Marker baseline V to V points: 3
Lower marker selection: First peak > 500 RFU                  Upper marker selection: Last peak > 200 RFU
Ladder size (bp)75, 200, 300, 400, 500, 700, 1000, 1500, 2000, 3000, 4000, 5000, 7000, 10000, 20000
Quantification using: Lower Marker          Final concentration (ng/uL): 0.5000          Dilution factor: 12.0
```

Sample: SampH9
Well location: H9
Created: Monday, September 20, 2021 6:40:06 PM

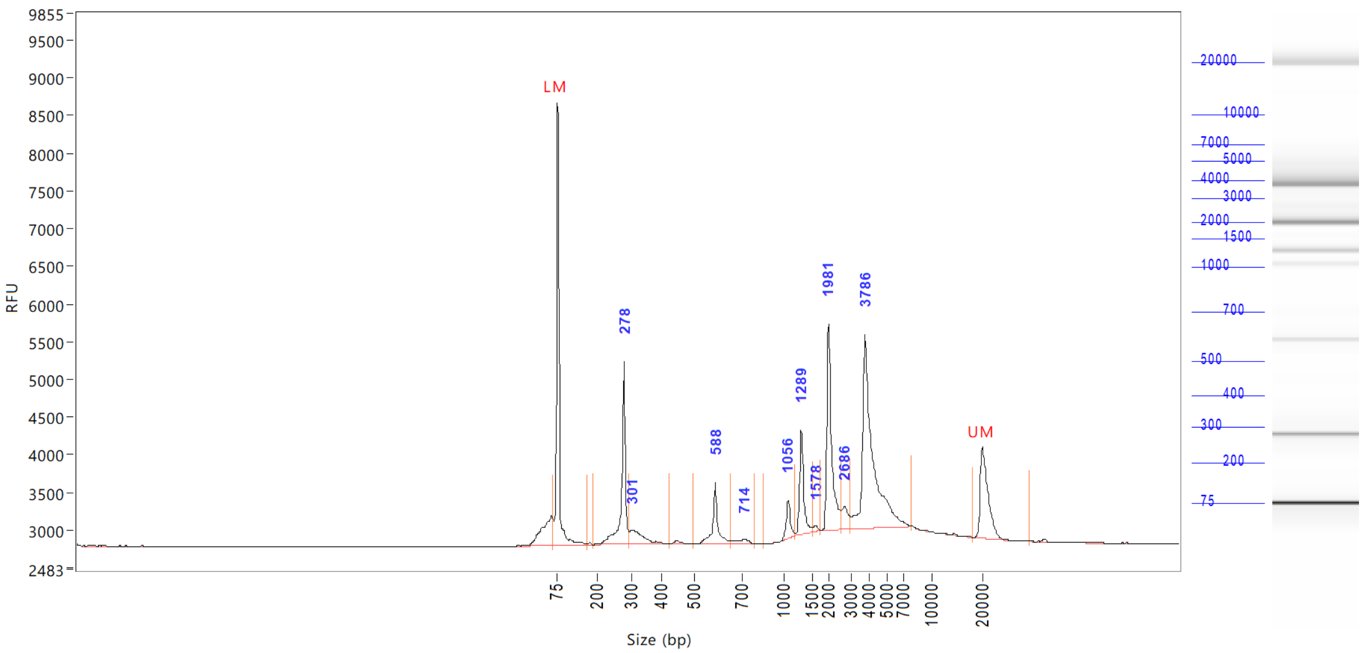

Peak	Size (bp)	Concentration (ng/uL)	From (bp)	To (bp)	Average size (bp)	CV%	RFU	Corrected peak area
1	75 (LM)	0.5000	64	168	77	15.87	5877	38.076
2	278	3.0838	187	293	269	5.87	2417	19.569
3	301	0.7907	293	422	322	7.55	179	5.018
4	588	1.1756	498	654	585	4.07	814	7.460
5	714	0.2035	654	783	698	3.89	61	1.292
6	1056	0.5667	848	1190	1051	4.23	510	3.596
7	1289	1.4698	1190	1500	1301	4.12	1378	9.327
8	1578	0.0860	1500	1731	1563	3.16	74	0.546
9	1981	3.3034	1731	2515	2035	7.20	2722	20.963
10	2686	0.5167	2515	2971	2716	4.85	306	3.279
11	3786	6.1292	2971	7801	4154	16.05	2573	38.896
12	20000 (UM)	0.1607	18171	29309	20542	4.76	1205	12.237

TIC:	17.3254	ng/uL
TIM:	34.9337	nmole/L
Total concentration:	17.3932	ng/uL

Sample peak width (sec): 5   Sample min peak height: 50   Sample baseline V to V?: Y   Sample baseline V to V points: 3
Sample filter: Binomial   Number of points for filter: 3 Sample start region (min): 0 Sample end region (min): 30
Marker peak width (sec): 5   Marker min peak height: 200   Marker baseline V to V?: Y   Marker baseline V to V points: 3
Lower marker selection: First peak > 500 RFU   Upper marker selection: Last peak > 200 RFU
Ladder size (bp)75, 200, 300, 400, 500, 700, 1000, 1500, 2000, 3000, 4000, 5000, 7000, 10000, 20000
Quantification using: Lower Marker   Final concentration (ng/uL): 0.5000   Dilution factor: 12.0

Sample: SampA10
Well location: A10
Created: Monday, September 20, 2021 6:40:06 PM

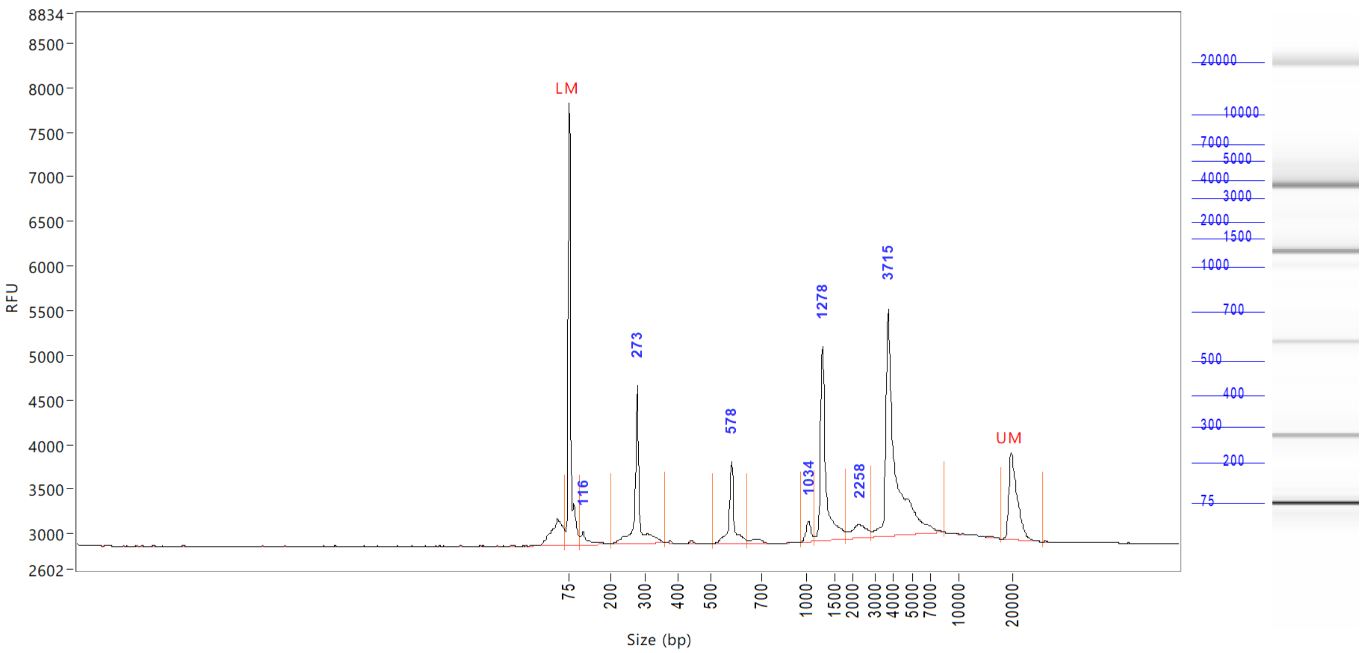

Peak	Size (bp)	Concentration (ng/uL)	From (bp)	To (bp)	Average size (bp)	CV%	RFU	Corrected peak area
1	75 (LM)	0.5000	64	107	76	9.19	4966	31.453
2	116	0.4584	107	200	131	16.70	143	2.403
3	273	3.1003	200	362	273	8.18	1766	16.252
4	578	1.5562	504	641	577	3.87	925	8.158
5	1034	0.3654	961	1156	1039	4.40	228	1.915
6	1278	3.4747	1156	1827	1327	9.12	2177	18.215
7	2258	0.6651	1827	2857	2286	12.17	156	3.486
8	3715	6.4872	2857	8467	4155	18.78	2546	34.007
9	20000 (UM)	0.1610	17927	25924	20409	4.50	976	10.127

TIC:	16.1073	ng/uL
TIM:	36.8053	nmole/L
Total concentration:	16.4875	ng/uL

```
Sample peak width (sec): 5     Sample min peak height: 50     Sample baseline V to V?: Y     Sample baseline V to V points: 3
Sample filter: Binomial        Number of points for filter: 3  Sample start region (min): 0  Sample end region (min): 30
Marker peak width (sec): 5     Marker min peak height: 200    Marker baseline V to V?: Y     Marker baseline V to V points: 3
Lower marker selection: First peak > 500 RFU                  Upper marker selection: Last peak > 200 RFU
Ladder size (bp)75, 200, 300, 400, 500, 700, 1000, 1500, 2000, 3000, 4000, 5000, 7000, 10000, 20000
Quantification using: Lower Marker          Final concentration (ng/uL): 0.5000          Dilution factor: 12.0
```

Sample: SampB10
Well location: B10
Created: Monday, September 20, 2021 6:40:06 PM

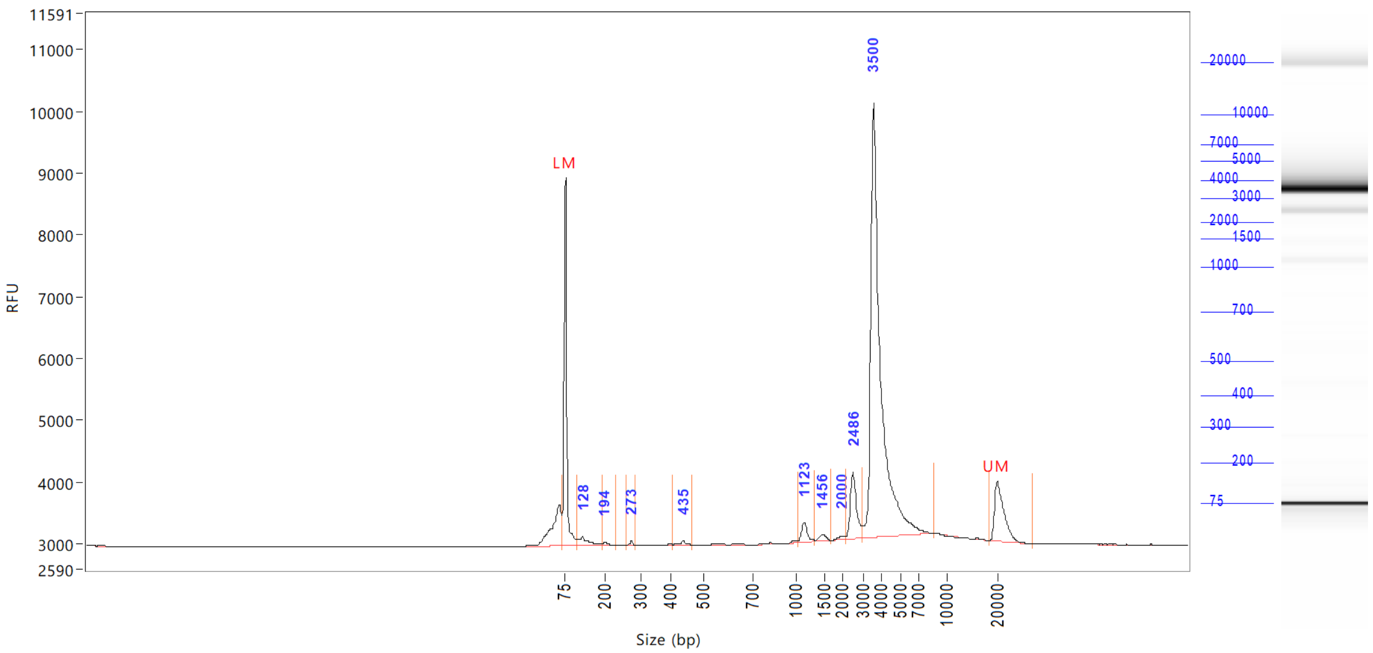

Peak	Size (bp)	Concentration (ng/uL)	From (bp)	To (bp)	Average size (bp)	CV%	RFU	Corrected peak area
1	75 (LM)	0.5000	64	112	74	9.86	5955	39.783
2	128	0.4770	112	187	138	14.09	155	3.163
3	194	0.0781	187	226	196	3.59	57	0.518
4	273	0.0657	259	284	270	1.48	73	0.436
5	435	0.1563	401	462	430	3.19	78	1.037
6	1123	0.4448	1012	1323	1127	5.18	315	2.949
7	1456	0.1662	1323	1655	1443	3.83	104	1.102
8	2000	0.0958	1655	2144	1972	4.76	63	0.635
9	2486	1.6019	2144	2943	2514	6.70	1070	10.621
10	3500	15.1929	2943	8667	3837	16.31	7045	100.737
11	20000 (UM)	0.1453	18293	26891	20555	5.21	964	11.564

TIC:	18.2789	ng/uL
TIM:	15.8212	nmole/L
Total concentration:	18.5996	ng/uL

```
Sample peak width (sec): 5    Sample min peak height: 50    Sample baseline V to V?: Y    Sample baseline V to V points: 3
Sample filter: Binomial       Number of points for filter: 3  Sample start region (min): 0  Sample end region (min): 30
Marker peak width (sec): 5    Marker min peak height: 200   Marker baseline V to V?: Y    Marker baseline V to V points: 3
Lower marker selection: First peak > 500 RFU                Upper marker selection: Last peak > 200 RFU
Ladder size (bp)75, 200, 300, 400, 500, 700, 1000, 1500, 2000, 3000, 4000, 5000, 7000, 10000, 20000
Quantification using: Lower Marker          Final concentration (ng/uL): 0.5000          Dilution factor: 12.0
```

Sample: SampC10
Well location: C10
Created: Monday, September 20, 2021 6:40:06 PM

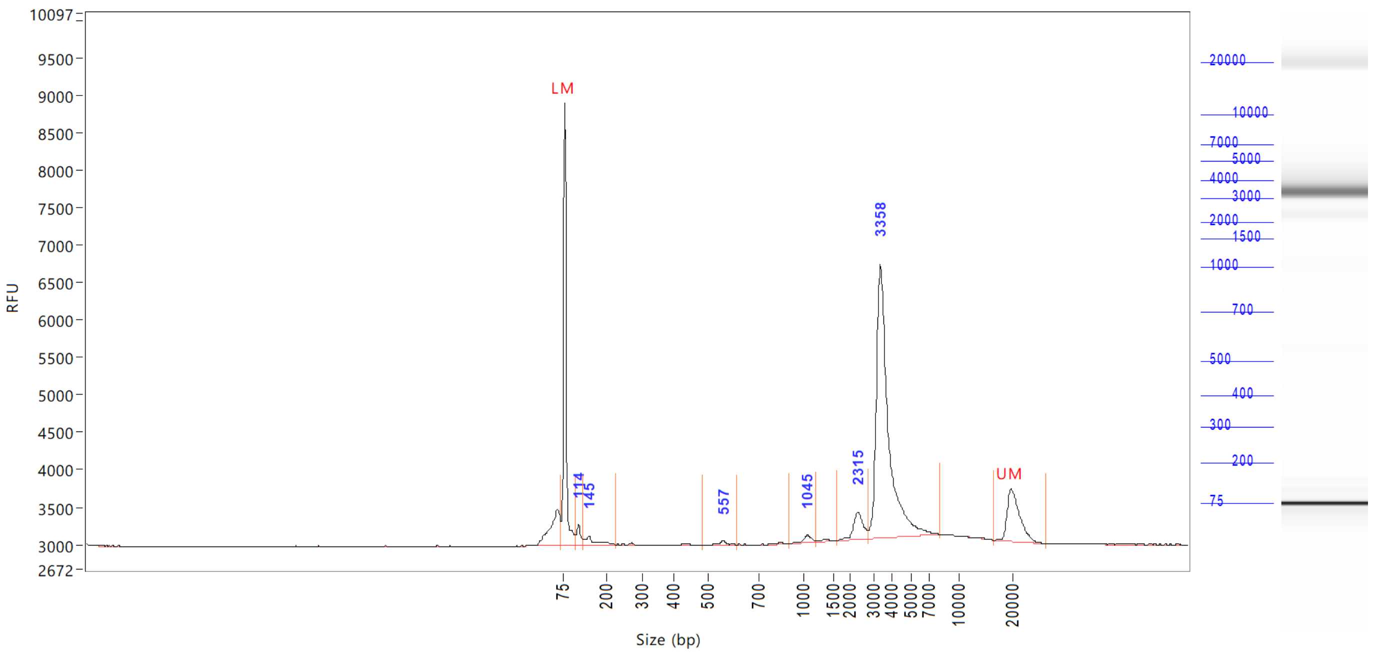

Peak	Size (bp)	Concentration (ng/uL)	From (bp)	To (bp)	Average size (bp)	CV%	RFU	Corrected peak area
1	75 (LM)	0.5000	64	105	74	8.56	5918	40.373
2	114	0.4162	105	131	115	6.09	270	2.801
3	145	0.3818	131	224	159	14.20	124	2.569
4	557	0.1489	483	611	551	4.62	62	1.002
5	1045	0.1938	900	1212	1047	6.16	98	1.304
6	2315	0.8149	1578	2743	2287	10.00	367	5.484
7	3358	9.5106	2743	8134	3615	16.98	3649	63.996
8	20000 (UM)	0.1360	16708	26529	20526	6.26	702	10.985

TIC:	11.4663	ng/uL
TIM:	15.5591	nmole/L
Total concentration:	11.7803	ng/uL

Sample peak width (sec): 5 Sample min peak height: 50 Sample baseline V to V?: Y Sample baseline V to V points: 3
Sample filter: Binomial Number of points for filter: 3 Sample start region (min): 0 Sample end region (min): 30
Marker peak width (sec): 5 Marker min peak height: 200 Marker baseline V to V?: Y Marker baseline V to V points: 3
Lower marker selection: First peak > 500 RFU Upper marker selection: Last peak > 200 RFU
Ladder size (bp)75, 200, 300, 400, 500, 700, 1000, 1500, 2000, 3000, 4000, 5000, 7000, 10000, 20000
Quantification using: Lower Marker Final concentration (ng/uL): 0.5000 Dilution factor: 12.0

Sample: SampD10
Well location: D10
Created: Monday, September 20, 2021 6:40:06 PM

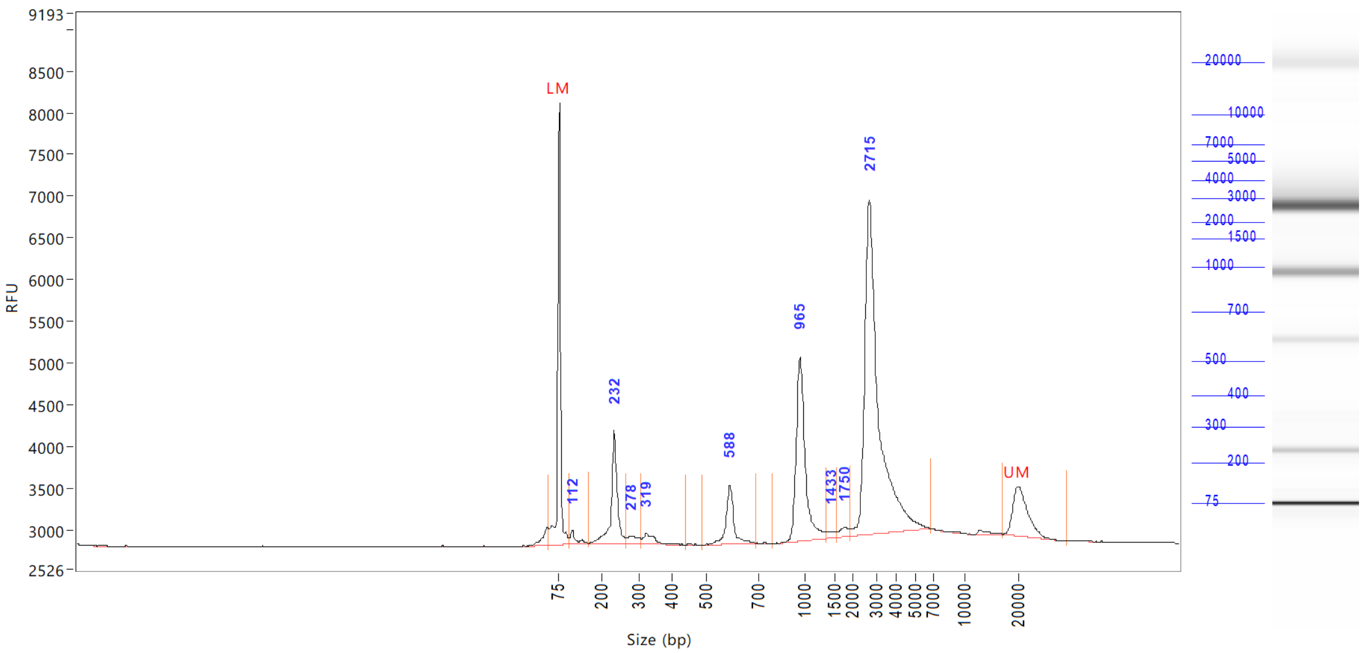

Peak	Size (bp)	Concentration (ng/uL)	From (bp)	To (bp)	Average size (bp)	CV%	RFU	Corrected peak area
1	75 (LM)	0.5000	45	103	72	10.57	5306	36.804
2	112	0.3270	103	160	117	10.54	166	2.006
3	232	2.8183	160	264	227	6.67	1351	17.287
4	278	0.3741	264	305	284	4.22	90	2.295
5	319	0.4949	305	439	336	7.67	132	3.036
6	588	1.6066	489	687	587	4.36	707	9.855
7	965	5.2501	788	1356	988	7.40	2202	32.204
8	1433	0.1805	1356	1539	1442	3.66	83	1.107
9	1750	0.2701	1539	1923	1740	6.33	112	1.657
10	2715	13.1527	1923	6778	2970	19.73	4009	80.679
11	20000 (UM)	0.1472	17195	28826	20546	7.05	590	10.833

TIC:	24.4743	ng/uL
TIM:	50.6000	nmole/L
Total concentration:	24.6197	ng/uL

```
Sample peak width (sec): 5     Sample min peak height: 50    Sample baseline V to V?: Y    Sample baseline V to V points: 3
Sample filter: Binomial        Number of points for filter: 3  Sample start region (min): 0  Sample end region (min): 30
Marker peak width (sec): 5     Marker min peak height: 200   Marker baseline V to V?: Y    Marker baseline V to V points: 3
Lower marker selection: First peak > 500 RFU                Upper marker selection: Last peak > 200 RFU
Ladder size (bp)75, 200, 300, 400, 500, 700, 1000, 1500, 2000, 3000, 4000, 5000, 7000, 10000, 20000
Quantification using: Lower Marker          Final concentration (ng/uL): 0.5000          Dilution factor: 12.0
```

Sample: SampE10
Well location: E10
Created: Monday, September 20, 2021 6:40:06 PM

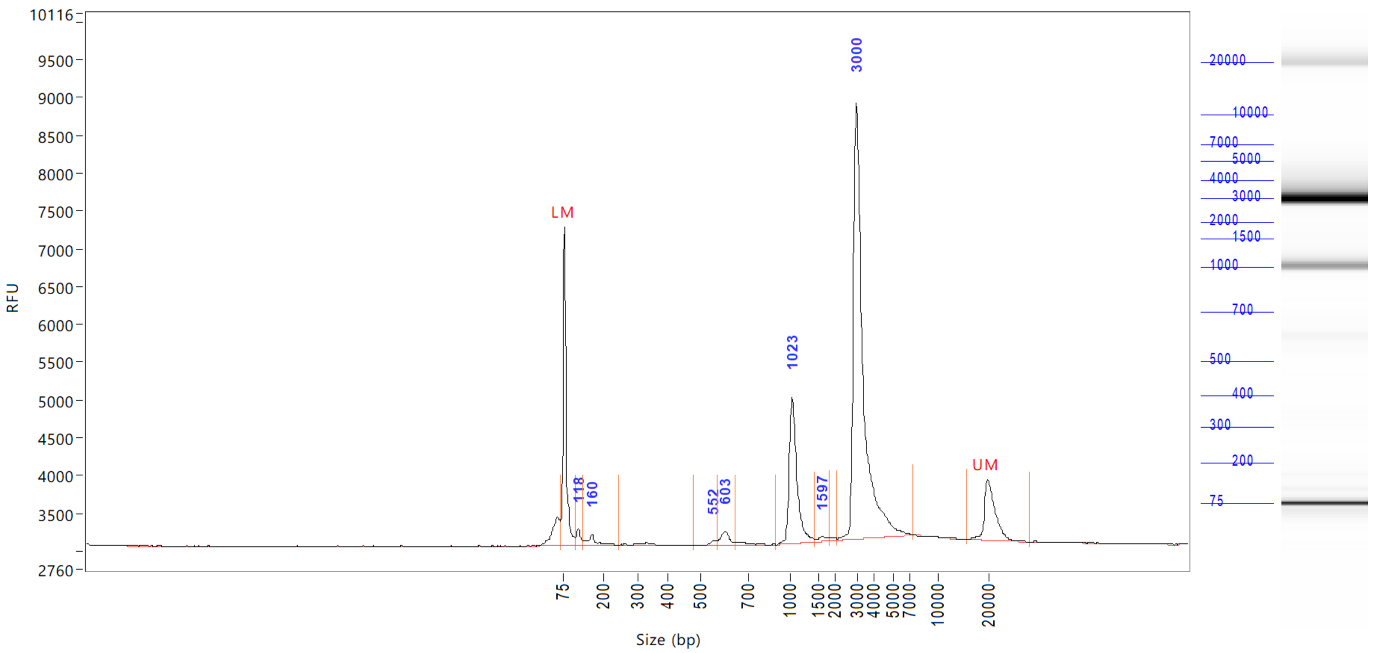

Peak	Size (bp)	Concentration (ng/uL)	From (bp)	To (bp)	Average size (bp)	CV%	RFU	Corrected peak area
1	75 (LM)	0.5000	64	108	75	10.02	4217	31.930
2	118	0.4421	108	135	119	5.97	219	2.353
3	160	0.4948	135	242	165	13.20	145	2.633
4	552	0.1683	477	570	546	3.55	68	0.895
5	603	0.5575	570	649	602	2.95	186	2.967
6	1023	4.6094	900	1433	1053	7.97	1942	24.530
7	1597	0.1809	1433	1808	1590	6.72	64	0.963
8	3000	15.9032	2087	7334	3244	16.88	5780	84.631
9	20000 (UM)	0.1744	15732	28463	20582	6.96	804	11.137

TIC:	22.3562	ng/uL
TIM:	28.5264	nmole/L
Total concentration:	22.7983	ng/uL

Sample peak width (sec): 5   Sample min peak height: 50   Sample baseline V to V?: Y   Sample baseline V to V points: 3
Sample filter: Binomial   Number of points for filter: 3   Sample start region (min): 0   Sample end region (min): 30
Marker peak width (sec): 5   Marker min peak height: 200   Marker baseline V to V?: Y   Marker baseline V to V points: 3
Lower marker selection: First peak > 500 RFU   Upper marker selection: Last peak > 200 RFU
Ladder size (bp)75, 200, 300, 400, 500, 700, 1000, 1500, 2000, 3000, 4000, 5000, 7000, 10000, 20000
Quantification using: Lower Marker   Final concentration (ng/uL): 0.5000   Dilution factor: 12.0

Sample: SampF10
Well location: F10
Created: Monday, September 20, 2021 6:40:06 PM

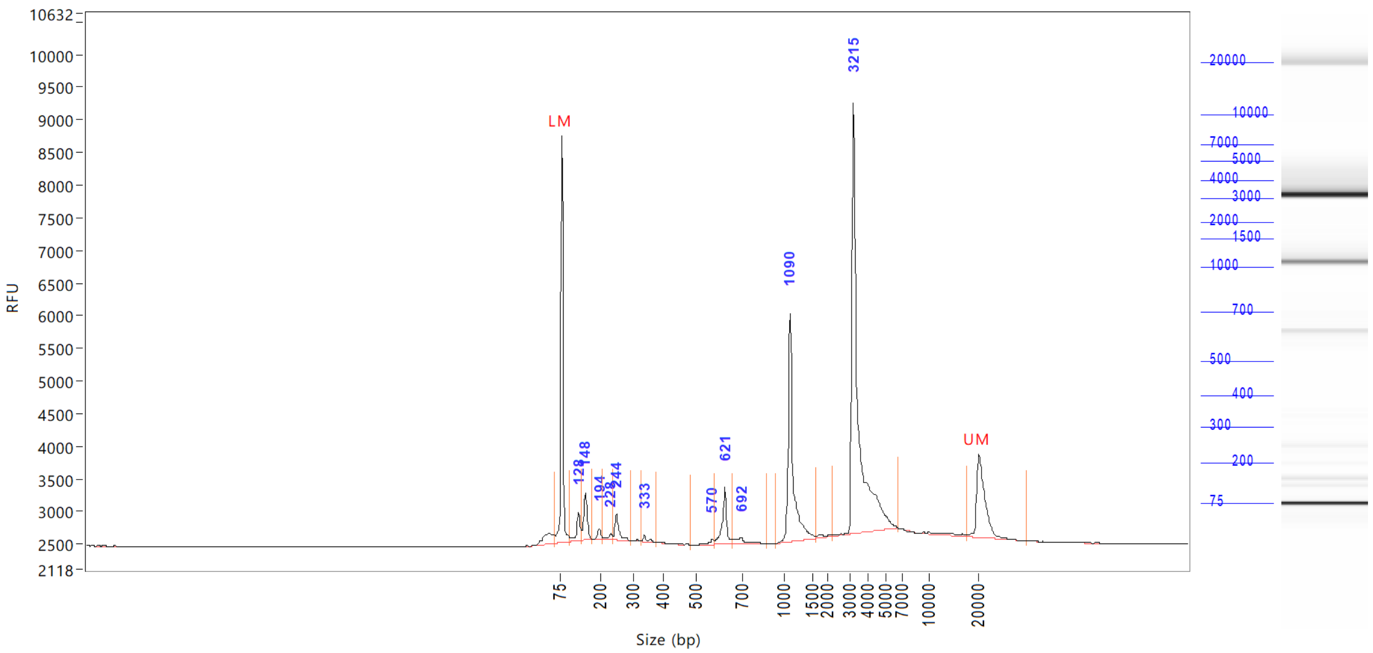

Peak	Size (bp)	Concentration (ng/uL)	From (bp)	To (bp)	Average size (bp)	CV%	RFU	Corrected peak area
1	75 (LM)	0.5000	56	101	73	6.73	6221	35.841
2	128	0.6616	101	139	125	6.78	438	3.952
3	148	0.9297	139	171	148	3.32	712	5.554
4	194	0.2026	171	206	193	3.09	170	1.210
5	228	0.1601	206	235	224	3.66	89	0.956
6	244	0.5988	235	289	247	3.93	397	3.577
7	333	0.1296	321	372	338	3.40	97	0.774
8	570	0.1376	485	580	558	3.37	83	0.822
9	621	1.1588	580	652	616	2.20	869	6.922
10	692	0.3485	652	870	702	6.63	93	2.082
11	1090	4.7098	935	1597	1127	8.98	3491	28.134
12	3215	10.7404	2201	6556	3525	15.71	6606	64.158
13	20000 (UM)	0.1929	17439	29672	20630	5.63	1286	13.829

TIC:	19.7776	ng/uL
TIM:	42.7522	nmole/L
Total concentration:	20.1456	ng/uL

```
Sample peak width (sec): 5    Sample min peak height: 50    Sample baseline V to V?: Y    Sample baseline V to V points: 3
Sample filter: Binomial       Number of points for filter: 3  Sample start region (min): 0  Sample end region (min): 30
Marker peak width (sec): 5    Marker min peak height: 200   Marker baseline V to V?: Y    Marker baseline V to V points: 3
Lower marker selection: First peak > 500 RFU                Upper marker selection: Last peak > 200 RFU
Ladder size (bp)75, 200, 300, 400, 500, 700, 1000, 1500, 2000, 3000, 4000, 5000, 7000, 10000, 20000
Quantification using: Lower Marker          Final concentration (ng/uL): 0.5000          Dilution factor: 12.0
```

Sample: SampG10
Well location: G10
Created: Monday, September 20, 2021 6:40:06 PM

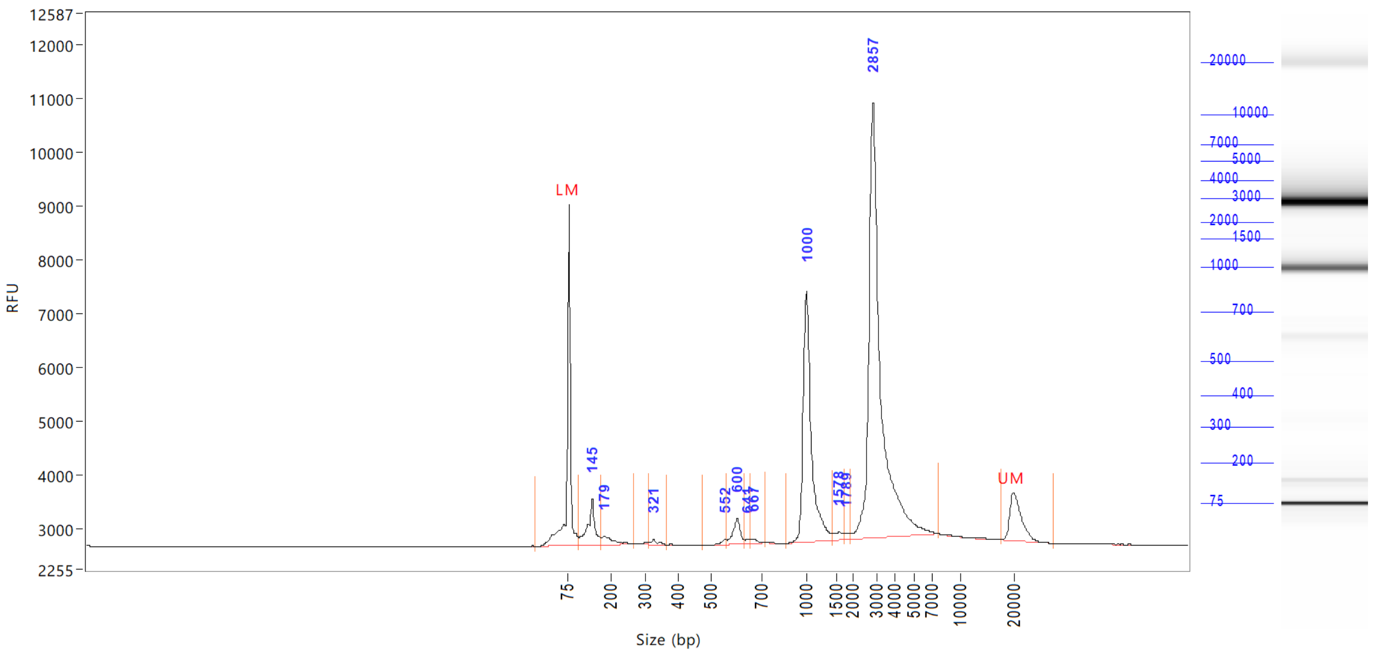

Peak	Size (bp)	Concentration (ng/uL)	From (bp)	To (bp)	Average size (bp)	CV%	RFU	Corrected peak area
1	75 (LM)	0.5000	0	101	67	25.21	6337	50.836
2	145	1.6809	101	169	136	11.60	855	14.242
3	179	0.5051	169	266	196	10.68	167	4.279
4	321	0.1443	305	364	325	3.67	96	1.223
5	552	0.1338	473	557	537	3.21	95	1.133
6	600	0.7469	557	629	593	2.61	481	6.328
7	641	0.0902	629	654	641	1.24	79	0.764
8	667	0.1059	654	718	668	1.58	87	0.897
9	1000	7.2730	866	1445	1037	8.97	4673	61.621
10	1578	0.2396	1445	1712	1560	5.28	142	2.030
11	1789	0.1339	1712	1923	1815	3.64	122	1.134
12	2857	16.4371	1923	7667	3132	20.28	8098	139.265
13	20000 (UM)	0.1273	17805	27617	20606	5.96	890	12.938

TIC:	27.4906	ng/uL
TIM:	48.8095	nmole/L
Total concentration:	27.5712	ng/uL

```
Sample peak width (sec): 5     Sample min peak height: 50    Sample baseline V to V?: Y    Sample baseline V to V points: 3
Sample filter: Binomial        Number of points for filter: 3  Sample start region (min): 0  Sample end region (min): 30
Marker peak width (sec): 5     Marker min peak height: 200   Marker baseline V to V?: Y    Marker baseline V to V points: 3
Lower marker selection: First peak > 500 RFU                 Upper marker selection: Last peak > 200 RFU
Ladder size (bp)75, 200, 300, 400, 500, 700, 1000, 1500, 2000, 3000, 4000, 5000, 7000, 10000, 20000
Quantification using: Lower Marker          Final concentration (ng/uL): 0.5000          Dilution factor: 12.0
```

Sample: SampH10
Well location: H10
Created: Monday, September 20, 2021 6:40:06 PM

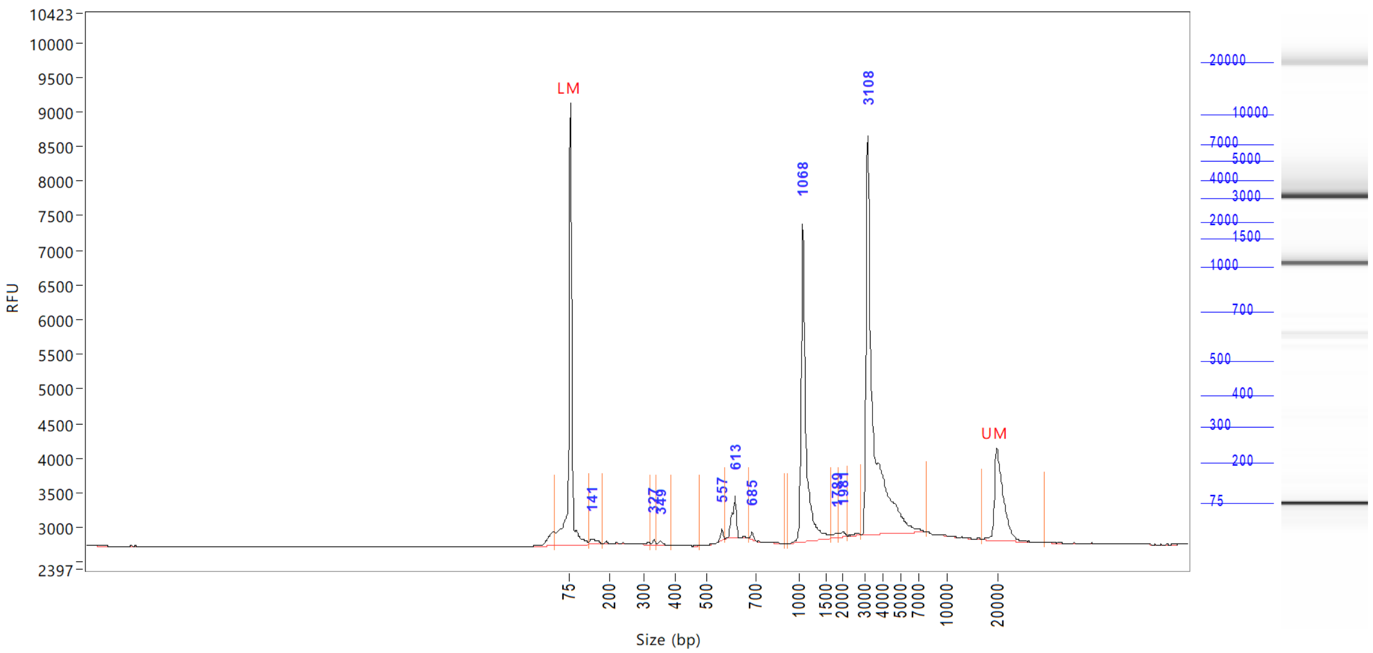

Peak	Size (bp)	Concentration (ng/uL)	From (bp)	To (bp)	Average size (bp)	CV%	RFU	Corrected peak area
1	75 (LM)	0.5000	30	131	71	18.42	6395	45.787
2	141	0.1744	131	177	147	7.37	84	1.331
3	327	0.0615	319	337	327	1.41	80	0.469
4	349	0.0767	337	388	350	2.45	67	0.586
5	557	0.1164	475	572	551	2.73	167	0.888
6	613	0.6452	572	669	606	1.96	611	4.924
7	685	0.0840	669	900	704	7.08	107	0.641
8	1068	4.5481	926	1635	1103	9.15	4582	34.707
9	1789	0.0786	1635	1866	1752	3.95	64	0.600
10	1981	0.0771	1866	2172	1969	3.56	70	0.588
11	3108	8.2117	2829	7867	3561	19.44	5762	62.664
12	20000 (UM)	0.1634	17073	29551	20610	5.84	1342	14.959

TIC:	14.0738	ng/uL
TIM:	15.6378	nmole/L
Total concentration:	14.2923	ng/uL

```
Sample peak width (sec): 5    Sample min peak height: 50    Sample baseline V to V?: Y    Sample baseline V to V points: 3
Sample filter: Binomial       Number of points for filter: 3  Sample start region (min): 0  Sample end region (min): 30
Marker peak width (sec): 5    Marker min peak height: 200   Marker baseline V to V?: Y    Marker baseline V to V points: 3
Lower marker selection: First peak > 500 RFU                Upper marker selection: Last peak > 200 RFU
Ladder size (bp)75, 200, 300, 400, 500, 700, 1000, 1500, 2000, 3000, 4000, 5000, 7000, 10000, 20000
Quantification using: Lower Marker          Final concentration (ng/uL): 0.5000          Dilution factor: 12.0
```

Sample: SampA11
Well location: A11
Created: Monday, September 20, 2021 6:40:06 PM

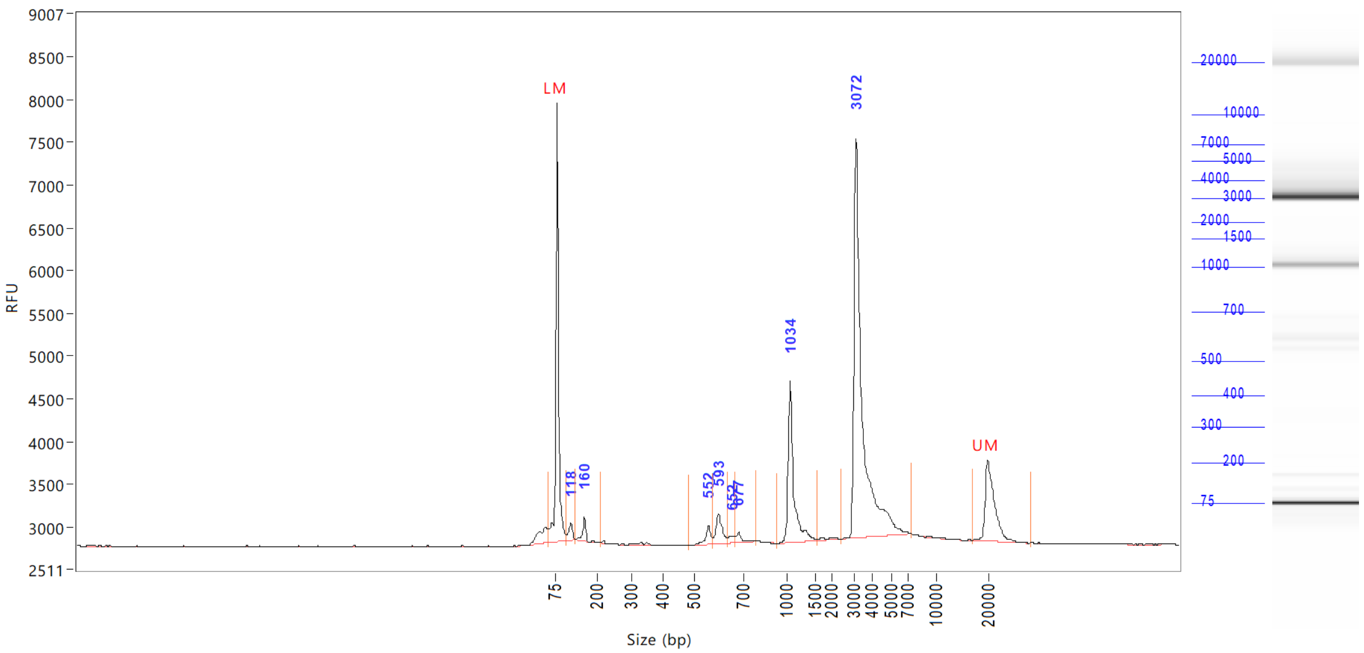

Peak	Size (bp)	Concentration (ng/uL)	From (bp)	To (bp)	Average size (bp)	CV%	RFU	Corrected peak area
1	75 (LM)	0.5000	48	103	74	9.60	5136	34.551
2	118	0.3331	103	131	114	4.97	200	1.918
3	160	0.3758	131	208	157	6.44	289	2.164
4	552	0.3668	477	567	543	3.16	226	2.112
5	593	0.6969	567	631	595	2.45	357	4.013
6	652	0.1522	631	662	646	1.45	76	0.876
7	677	0.1871	662	779	679	2.33	118	1.078
8	1034	2.9930	926	1539	1078	9.26	1894	17.235
9	3072	9.7976	2401	7268	3435	17.73	4664	56.420
10	20000 (UM)	0.1606	16952	28463	20685	6.21	940	11.098

TIC:	14.9024	ng/uL
TIM:	21.8794	nmole/L
Total concentration:	15.1758	ng/uL

```
Sample peak width (sec): 5    Sample min peak height: 50    Sample baseline V to V?: Y    Sample baseline V to V points: 3
Sample filter: Binomial       Number of points for filter: 3  Sample start region (min): 0  Sample end region (min): 30
Marker peak width (sec): 5    Marker min peak height: 200   Marker baseline V to V?: Y    Marker baseline V to V points: 3
Lower marker selection: First peak > 500 RFU                Upper marker selection: Last peak > 200 RFU
Ladder size (bp)75, 200, 300, 400, 500, 700, 1000, 1500, 2000, 3000, 4000, 5000, 7000, 10000, 20000
Quantification using: Lower Marker          Final concentration (ng/uL): 0.5000          Dilution factor: 12.0
```

Sample: SampB11
Well location: B11
Created: Monday, September 20, 2021 6:40:06 PM

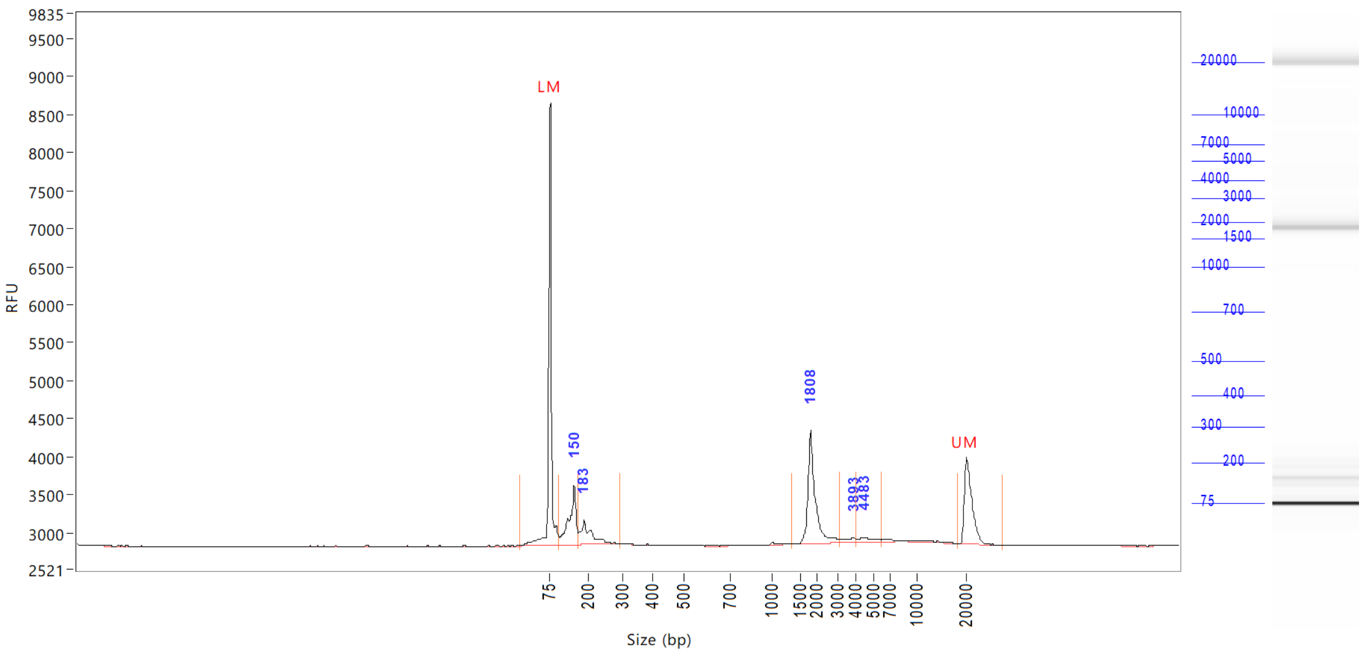

Peak	Size (bp)	Concentration (ng/uL)	From (bp)	To (bp)	Average size (bp)	CV%	RFU	Corrected peak area
1	75 (LM)	0.5000	0	103	70	20.22	5830	38.998
2	150	1.9000	103	164	139	10.51	788	12.349
3	183	1.0995	164	293	196	11.99	317	7.146
4	1808	2.6173	1356	3108	1923	14.37	1485	17.011
5	3893	0.1572	3108	4070	3633	7.88	58	1.022
6	4483	0.2139	4070	5963	4731	10.18	66	1.391
7	20000 (UM)	0.1448	18415	27496	20557	4.79	1138	11.295

TIC:	5.9878	ng/uL
TIM:	34.0708	nmole/L
Total concentration:	6.3349	ng/uL

```
Sample peak width (sec): 5    Sample min peak height: 50    Sample baseline V to V?: Y    Sample baseline V to V points: 3
Sample filter: Binomial       Number of points for filter: 3  Sample start region (min): 0  Sample end region (min): 30
Marker peak width (sec): 5    Marker min peak height: 200   Marker baseline V to V?: Y    Marker baseline V to V points: 3
Lower marker selection: First peak > 500 RFU                Upper marker selection: Last peak > 200 RFU
Ladder size (bp)75, 200, 300, 400, 500, 700, 1000, 1500, 2000, 3000, 4000, 5000, 7000, 10000, 20000
Quantification using: Lower Marker          Final concentration (ng/uL): 0.5000          Dilution factor: 12.0
```

Sample: SampC11
Well location: C11
Created: Monday, September 20, 2021 6:40:06 PM

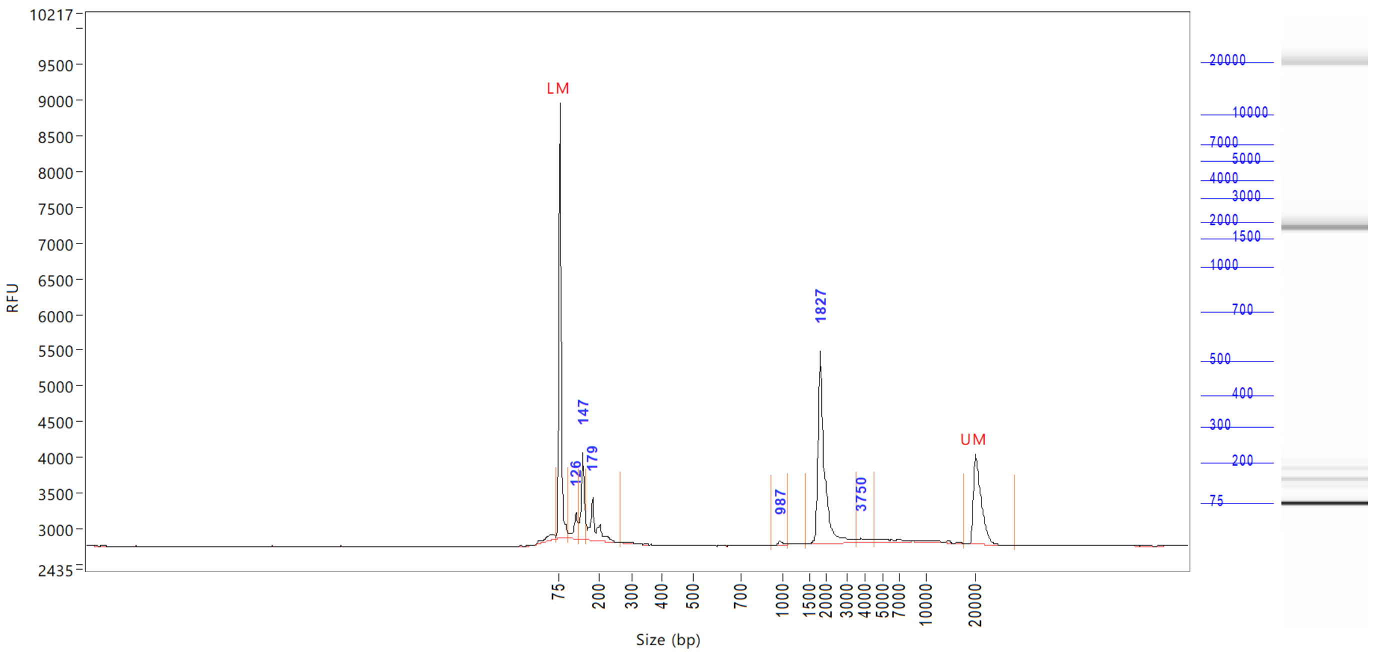

Peak	Size (bp)	Concentration (ng/uL)	From (bp)	To (bp)	Average size (bp)	CV%	RFU	Corrected peak area
1	75 (LM)	0.5000	64	101	73	6.82	6107	36.688
2	126	0.5901	101	133	122	6.85	371	3.608
3	147	1.5599	133	160	145	3.94	1219	9.538
4	179	1.3376	160	264	187	9.99	596	8.179
5	987	0.0696	922	1090	987	2.80	53	0.425
6	1827	4.3964	1422	3500	1928	14.85	2686	26.883
7	3750	0.1818	3500	4518	3977	7.19	58	1.112
8	20000 (UM)	0.1725	17561	28100	20554	4.69	1256	12.660

TIC:	8.1353	ng/uL
TIM:	41.3308	nmole/L
Total concentration:	8.6273	ng/uL

Sample peak width (sec): 5 Sample min peak height: 50 Sample baseline V to V?: Y Sample baseline V to V points: 3
Sample filter: Binomial Number of points for filter: 3 Sample start region (min): 0 Sample end region (min): 30
Marker peak width (sec): 5 Marker min peak height: 200 Marker baseline V to V?: Y Marker baseline V to V points: 3
Lower marker selection: First peak > 500 RFU Upper marker selection: Last peak > 200 RFU
Ladder size (bp)75, 200, 300, 400, 500, 700, 1000, 1500, 2000, 3000, 4000, 5000, 7000, 10000, 20000
Quantification using: Lower Marker Final concentration (ng/uL): 0.5000 Dilution factor: 12.0

Sample: SampD11
Well location: D11
Created: Monday, September 20, 2021 6:40:06 PM

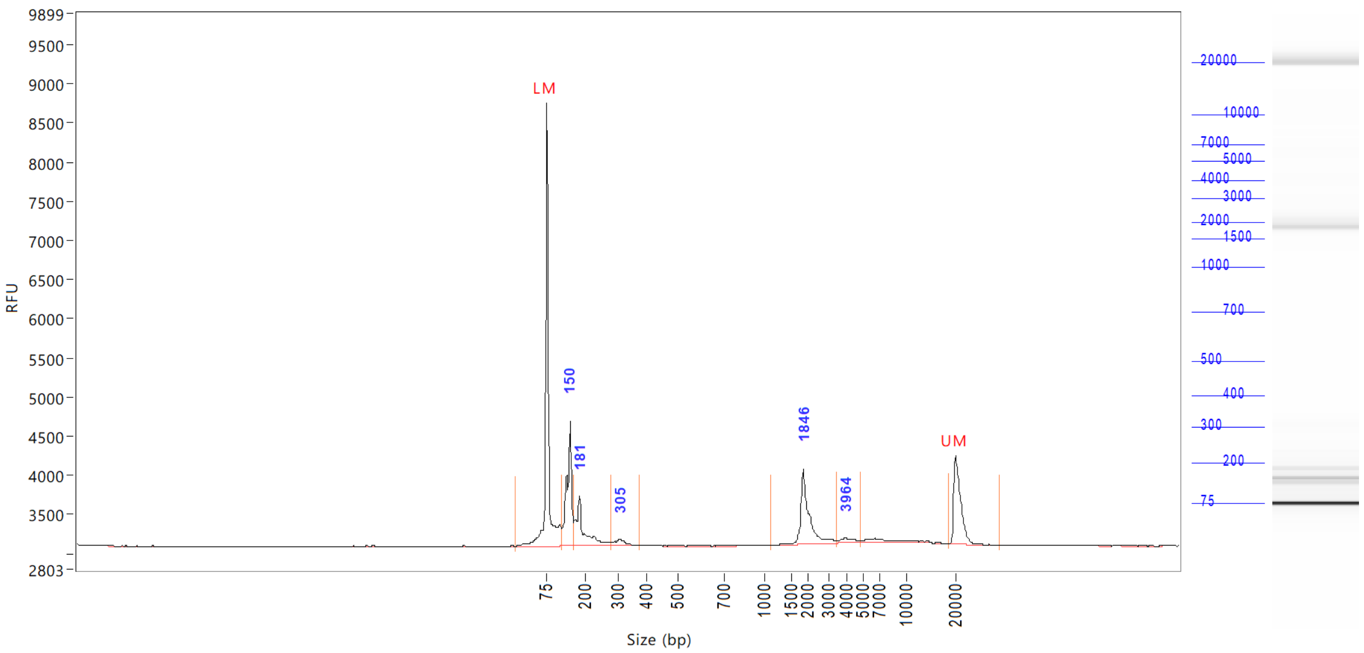

Peak	Size (bp)	Concentration (ng/uL)	From (bp)	To (bp)	Average size (bp)	CV%	RFU	Corrected peak area
1	75 (LM)	0.5000	0	122	74	24.27	5667	42.739
2	150	2.5055	122	160	142	6.35	1591	17.847
3	181	1.6023	160	277	192	14.39	630	11.413
4	305	0.2418	277	374	305	5.82	76	1.722
5	1846	1.7679	1101	3465	1997	18.22	961	12.593
6	3964	0.1777	3465	4862	4086	8.78	61	1.266
7	20000 (UM)	0.1241	18537	29068	20609	5.24	1133	10.605

TIC:	6.2952	ng/uL
TIM:	45.5500	nmole/L
Total concentration:	6.6385	ng/uL

```
Sample peak width (sec): 5    Sample min peak height: 50    Sample baseline V to V?: Y    Sample baseline V to V points: 3
Sample filter: Binomial       Number of points for filter: 3  Sample start region (min): 0  Sample end region (min): 30
Marker peak width (sec): 5    Marker min peak height: 200   Marker baseline V to V?: Y    Marker baseline V to V points: 3
Lower marker selection: First peak > 500 RFU                Upper marker selection: Last peak > 200 RFU
Ladder size (bp)75, 200, 300, 400, 500, 700, 1000, 1500, 2000, 3000, 4000, 5000, 7000, 10000, 20000
Quantification using: Lower Marker          Final concentration (ng/uL): 0.5000          Dilution factor: 12.0
```

Sample: SampE11
Well location: E11
Created: Monday, September 20, 2021 6:40:06 PM

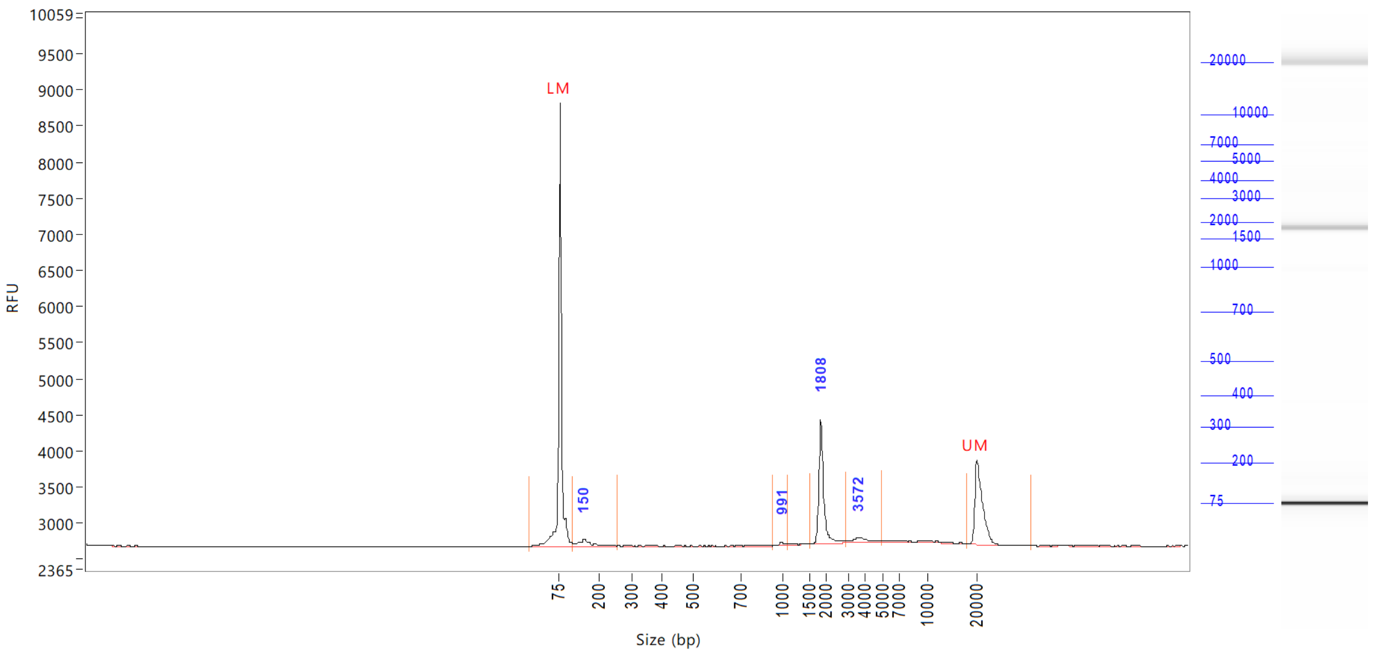

Peak	Size (bp)	Concentration (ng/uL)	From (bp)	To (bp)	Average size (bp)	CV%	RFU	Corrected peak area
1	75 (LM)	0.5000	0	118	71	18.35	6147	43.543
2	150	0.3576	118	257	157	16.94	103	2.595
3	991	0.0471	922	1079	995	2.69	53	0.341
4	1808	1.7988	1501	2914	1859	9.86	1713	13.054
5	3572	0.2127	2914	4931	3742	12.48	70	1.544
6	20000 (UM)	0.1351	18049	31123	20618	5.19	1162	11.766

TIC:	2.4162	ng/uL
TIM:	5.5084	nmole/L
Total concentration:	2.7780	ng/uL

Sample peak width (sec): 5 Sample min peak height: 50 Sample baseline V to V?: Y Sample baseline V to V points: 3
Sample filter: Binomial Number of points for filter: 3 Sample start region (min): 0 Sample end region (min): 30
Marker peak width (sec): 5 Marker min peak height: 200 Marker baseline V to V?: Y Marker baseline V to V points: 3
Lower marker selection: First peak > 500 RFU Upper marker selection: Last peak > 200 RFU
Ladder size (bp)75, 200, 300, 400, 500, 700, 1000, 1500, 2000, 3000, 4000, 5000, 7000, 10000, 20000
Quantification using: Lower Marker Final concentration (ng/uL): 0.5000 Dilution factor: 12.0

Sample: SampF11
Well location: F11
Created: Monday, September 20, 2021 6:40:06 PM

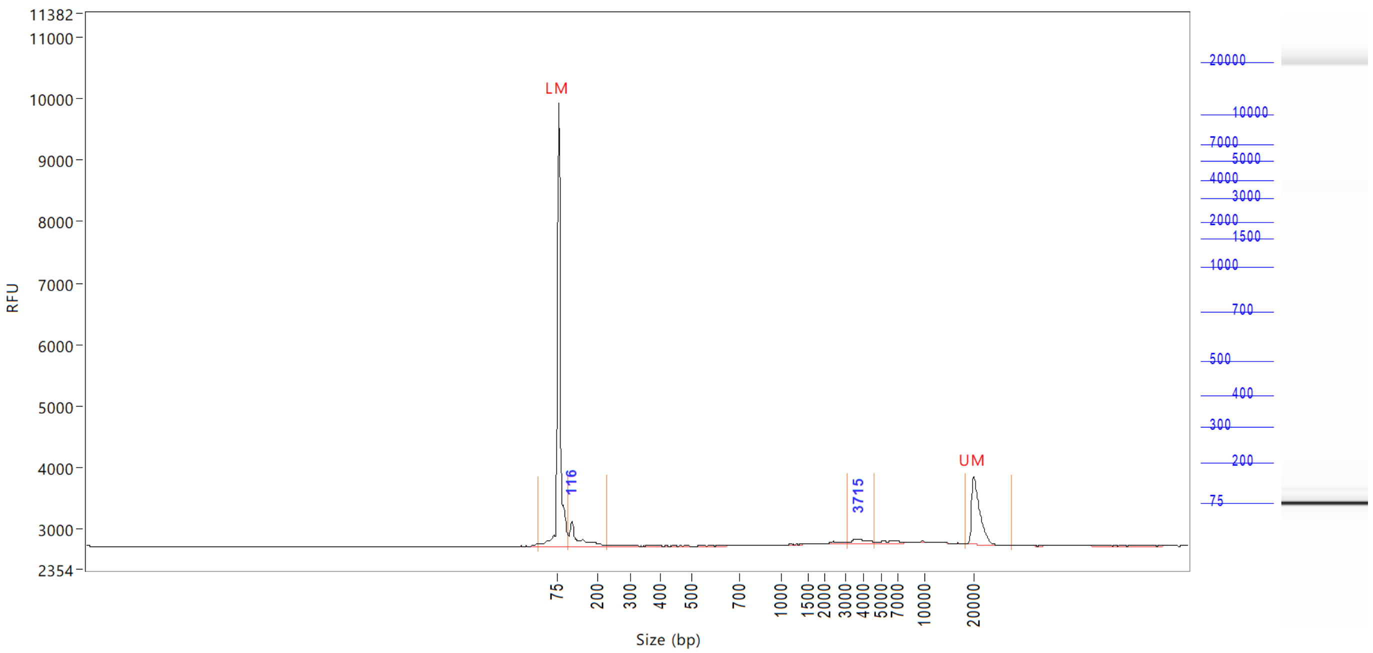

Peak	Size (bp)	Concentration (ng/uL)	From (bp)	To (bp)	Average size (bp)	CV%	RFU	Corrected peak area
1	75 (LM)	0.5000	11	105	73	14.51	7210	53.746
2	116	0.8065	105	226	139	21.73	406	7.225
3	3715	0.1849	3072	4621	3820	10.13	78	1.656
4	20000 (UM)	0.1078	18415	27617	20656	4.91	1107	11.593

TIC:	0.9914	ng/uL
TIM:	9.6146	nmole/L
Total concentration:	1.4552	ng/uL

Sample peak width (sec): 5 Sample min peak height: 50 Sample baseline V to V?: Y Sample baseline V to V points: 3
Sample filter: Binomial Number of points for filter: 3 Sample start region (min): 0 Sample end region (min): 30
Marker peak width (sec): 5 Marker min peak height: 200 Marker baseline V to V?: Y Marker baseline V to V points: 3
Lower marker selection: First peak > 500 RFU Upper marker selection: Last peak > 200 RFU
Ladder size (bp)75, 200, 300, 400, 500, 700, 1000, 1500, 2000, 3000, 4000, 5000, 7000, 10000, 20000
Quantification using: Lower Marker Final concentration (ng/uL): 0.5000 Dilution factor: 12.0

Sample: SampG11
Well location: G11
Created: Monday, September 20, 2021 6:40:06 PM

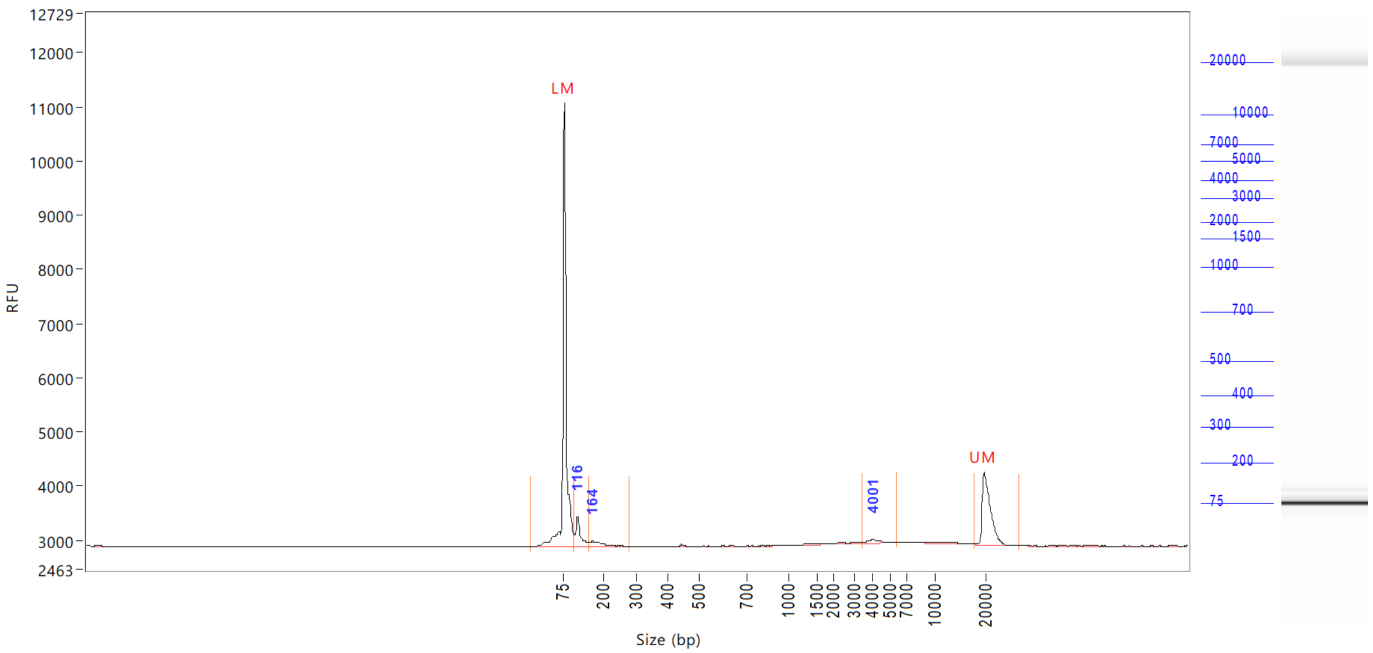

Peak	Size (bp)	Concentration (ng/uL)	From (bp)	To (bp)	Average size (bp)	CV%	RFU	Corrected peak area
1	75 (LM)	0.5000	0	107	72	20.39	8204	67.150
2	116	0.5736	107	156	122	10.72	545	6.420
3	164	0.2234	156	277	185	13.49	94	2.501
4	4001	0.1186	3465	5667	4176	10.50	64	1.327
5	20000 (UM)	0.1043	17927	27012	20534	4.77	1336	14.002

TIC:	0.9157	ng/uL
TIM:	9.7571	nmole/L
Total concentration:	1.2322	ng/uL

```
Sample peak width (sec): 5    Sample min peak height: 50    Sample baseline V to V?: Y    Sample baseline V to V points: 3
Sample filter: Binomial       Number of points for filter: 3  Sample start region (min): 0  Sample end region (min): 30
Marker peak width (sec): 5    Marker min peak height: 200   Marker baseline V to V?: Y    Marker baseline V to V points: 3
Lower marker selection: First peak > 500 RFU                Upper marker selection: Last peak > 200 RFU
Ladder size (bp)75, 200, 300, 400, 500, 700, 1000, 1500, 2000, 3000, 4000, 5000, 7000, 10000, 20000
Quantification using: Lower Marker          Final concentration (ng/uL): 0.5000          Dilution factor: 12.0
```

Sample: SampH11
Well location: H11
Created: Monday, September 20, 2021 6:40:06 PM

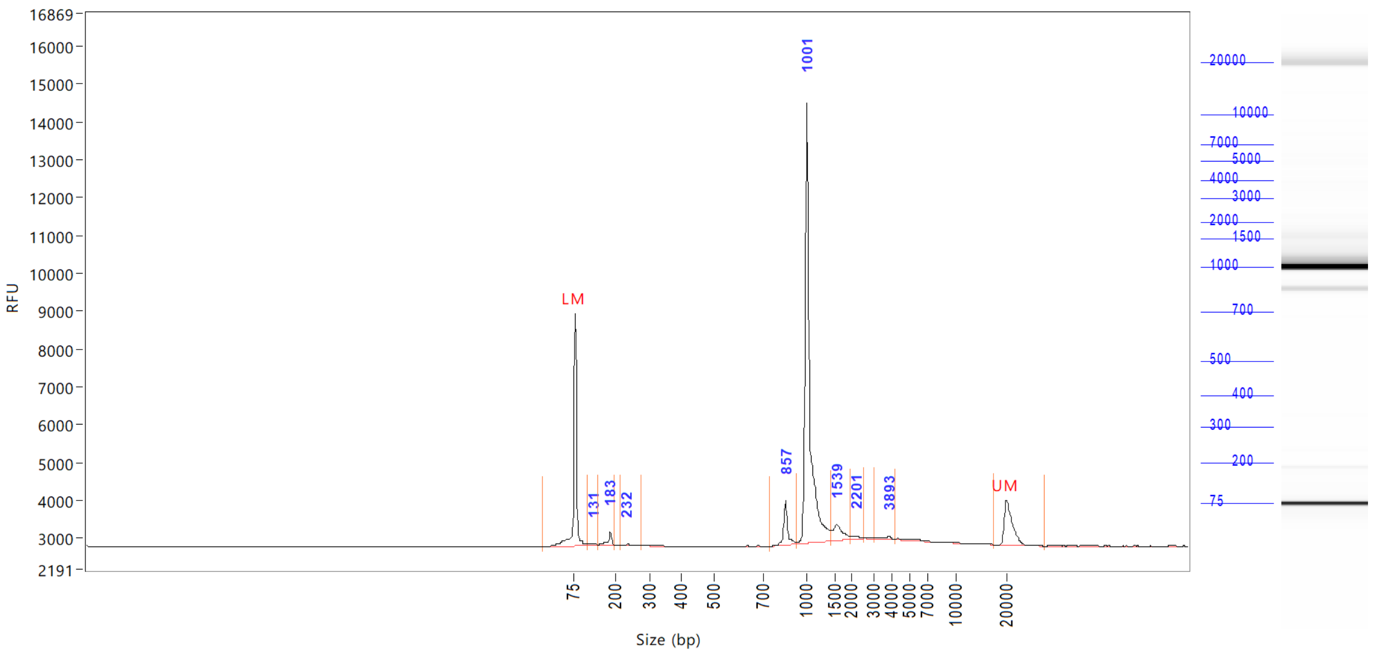

Peak	Size (bp)	Concentration (ng/uL)	From (bp)	To (bp)	Average size (bp)	CV%	RFU	Corrected peak area
1	75 (LM)	0.5000	0	114	69	19.90	6146	43.261
2	131	0.1232	114	145	128	6.62	62	0.888
3	183	0.4058	145	196	173	6.37	341	2.926
4	232	0.0770	210	275	234	5.06	52	0.555
5	857	1.0753	749	931	855	2.68	1162	7.753
6	1001	12.3157	931	1445	1052	8.99	11642	88.799
7	1539	0.8777	1445	2000	1616	8.81	432	6.328
8	2201	0.1273	2000	2572	2184	6.39	84	0.918
9	3893	0.1488	3072	4173	3638	7.58	81	1.073
10	20000 (UM)	0.1412	17561	27254	20498	4.76	1197	12.220

TIC:	15.1508	ng/uL
TIM:	28.3746	nmole/L
Total concentration:	15.5555	ng/uL

```
Sample peak width (sec): 5    Sample min peak height: 50    Sample baseline V to V?: Y    Sample baseline V to V points: 3
Sample filter: Binomial       Number of points for filter: 3  Sample start region (min): 0  Sample end region (min): 30
Marker peak width (sec): 5    Marker min peak height: 200   Marker baseline V to V?: Y    Marker baseline V to V points: 3
Lower marker selection: First peak > 500 RFU                Upper marker selection: Last peak > 200 RFU
Ladder size (bp)75, 200, 300, 400, 500, 700, 1000, 1500, 2000, 3000, 4000, 5000, 7000, 10000, 20000
Quantification using: Lower Marker          Final concentration (ng/uL): 0.5000          Dilution factor: 12.0
```

Sample: SampA12
Well location: A12
Created: Monday, September 20, 2021 6:40:06 PM

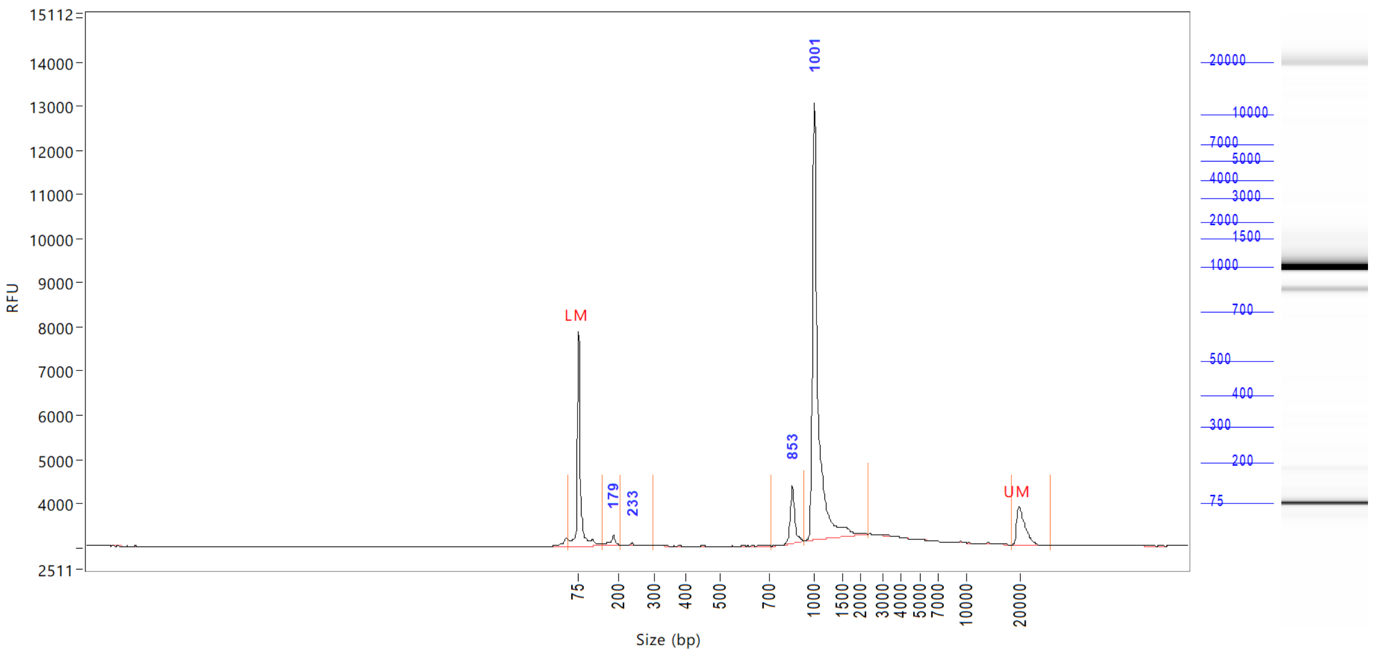

Peak	Size (bp)	Concentration (ng/uL)	From (bp)	To (bp)	Average size (bp)	CV%	RFU	Corrected peak area
1	75 (LM)	0.5000	47	150	78	19.06	4853	36.582
2	179	0.4743	150	203	174	6.64	241	2.892
3	233	0.1685	203	293	231	8.06	62	1.028
4	853	1.5722	710	931	849	2.36	1311	9.586
5	1001	14.6824	931	2372	1067	13.45	9919	89.520
6	20000 (UM)	0.1262	18537	26045	20560	4.81	879	9.237

TIC:	16.8975	ng/uL
TIM:	31.3782	nmole/L
Total concentration:	17.2582	ng/uL

Sample peak width (sec): 5 Sample min peak height: 50 Sample baseline V to V?: Y Sample baseline V to V points: 3
Sample filter: Binomial Number of points for filter: 3 Sample start region (min): 0 Sample end region (min): 30
Marker peak width (sec): 5 Marker min peak height: 200 Marker baseline V to V?: Y Marker baseline V to V points: 3
Lower marker selection: First peak > 500 RFU Upper marker selection: Last peak > 200 RFU
Ladder size (bp)75, 200, 300, 400, 500, 700, 1000, 1500, 2000, 3000, 4000, 5000, 7000, 10000, 20000
Quantification using: Lower Marker Final concentration (ng/uL): 0.5000 Dilution factor: 12.0

Sample: SampB12
Well location: B12
Created: Monday, September 20, 2021 6:40:06 PM

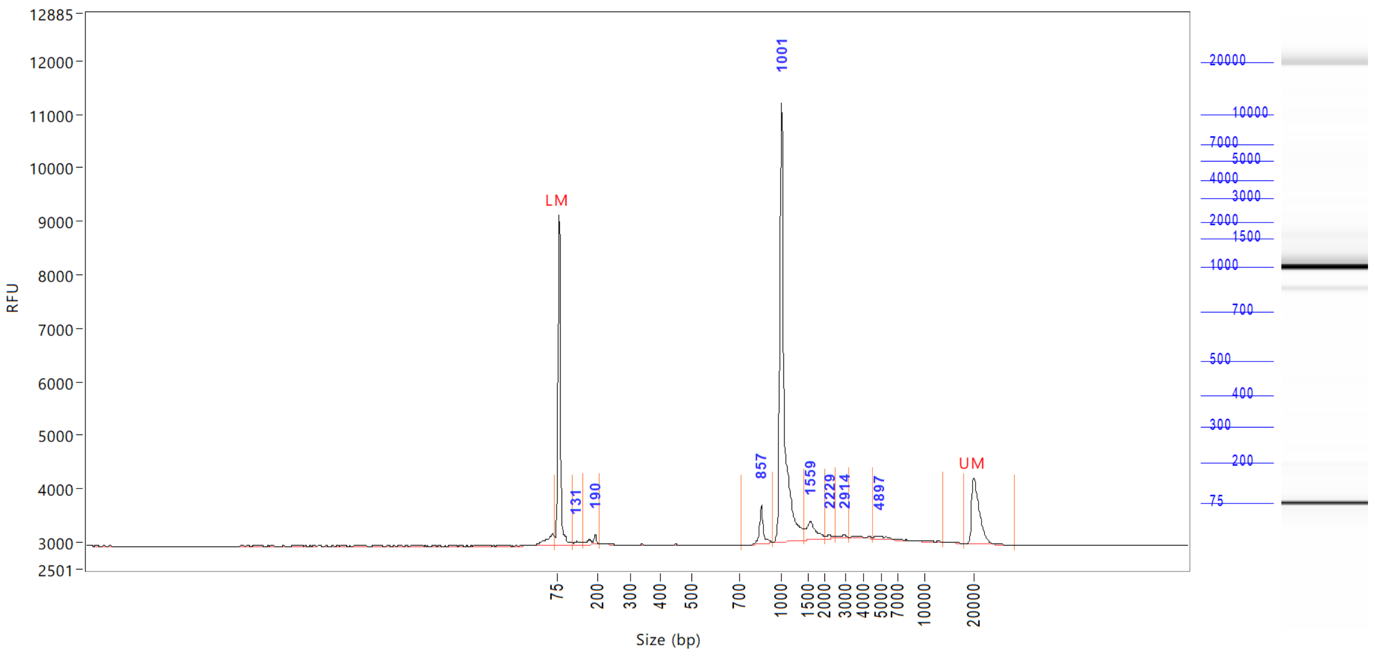

Peak	Size (bp)	Concentration (ng/uL)	From (bp)	To (bp)	Average size (bp)	CV%	RFU	Corrected peak area
1	75 (LM)	0.5000	64	122	75	9.09	6164	37.862
2	131	0.1461	122	152	136	6.59	63	0.922
3	190	0.3260	152	205	178	6.96	169	2.057
4	857	0.7540	710	944	856	2.70	721	4.758
5	1001	9.8312	944	1422	1051	8.71	8213	62.038
6	1559	0.8500	1422	2001	1613	9.32	345	5.364
7	2229	0.1172	2001	2543	2228	6.73	63	0.739
8	2914	0.0889	2543	3215	2821	5.42	52	0.561
9	4897	0.2849	4552	13537	6973	35.12	59	1.798
10	20000 (UM)	0.1640	18171	28342	20566	4.68	1242	12.420

TIC:	12.3983	ng/uL
TIM:	22.6953	nmole/L
Total concentration:	12.6995	ng/uL

Sample peak width (sec): 5 Sample min peak height: 50 Sample baseline V to V?: Y Sample baseline V to V points: 3
Sample filter: Binomial Number of points for filter: 3 Sample start region (min): 0 Sample end region (min): 30
Marker peak width (sec): 5 Marker min peak height: 200 Marker baseline V to V?: Y Marker baseline V to V points: 3
Lower marker selection: First peak > 500 RFU Upper marker selection: Last peak > 200 RFU
Ladder size (bp)75, 200, 300, 400, 500, 700, 1000, 1500, 2000, 3000, 4000, 5000, 7000, 10000, 20000
Quantification using: Lower Marker Final concentration (ng/uL): 0.5000 Dilution factor: 12.0

Sample: SampC12
Well location: C12
Created: Monday, September 20, 2021 6:40:06 PM

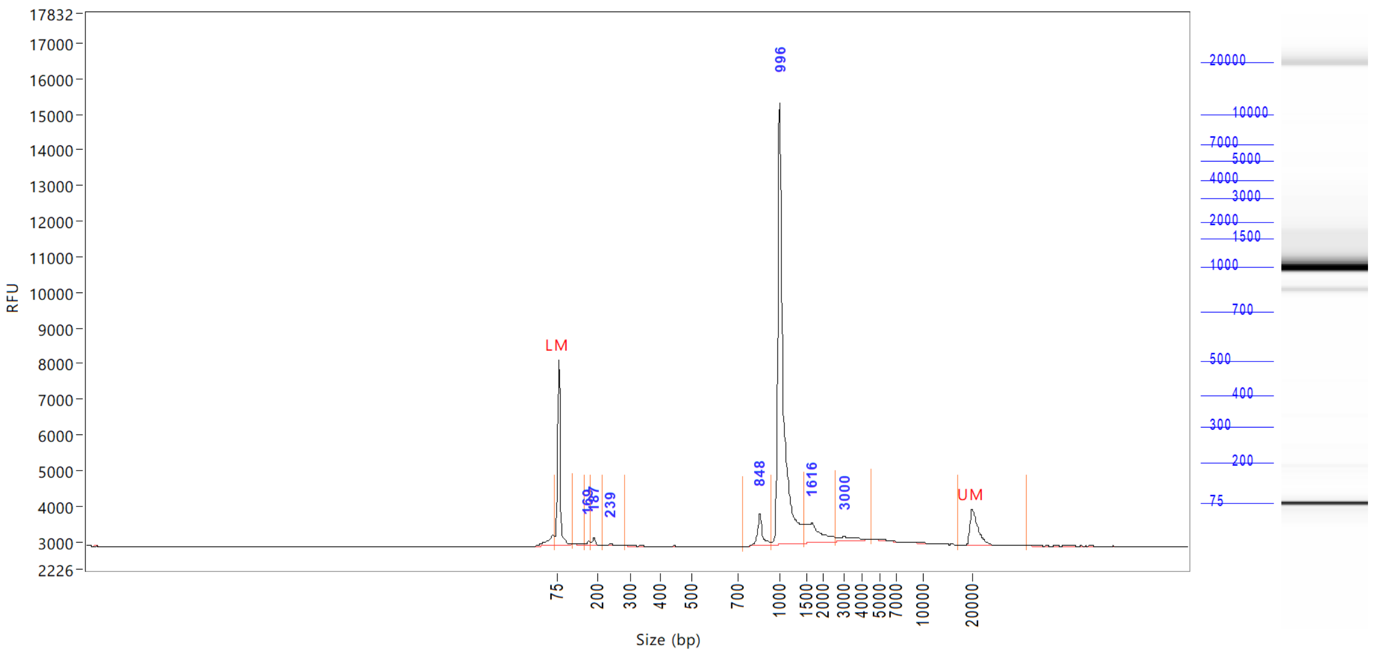

Peak	Size (bp)	Concentration (ng/uL)	From (bp)	To (bp)	Average size (bp)	CV%	RFU	Corrected peak area
1	75 (LM)	0.5000	64	122	74	7.83	5184	32.526
2	169	0.1615	158	179	169	3.38	99	0.876
3	187	0.2698	179	214	188	3.93	203	1.462
4	239	0.1236	214	284	237	6.04	56	0.670
5	848	1.4254	727	939	854	3.34	906	7.727
6	996	20.8558	939	1433	1049	9.65	12415	113.061
7	1616	2.6456	1433	2572	1755	16.54	543	14.342
8	3000	0.6141	2572	4552	3191	14.55	122	3.329
9	20000 (UM)	0.1646	17073	31002	20623	6.14	1019	10.707

TIC:	26.0958	ng/uL
TIM:	43.0579	nmole/L
Total concentration:	26.4451	ng/uL

```
Sample peak width (sec): 5    Sample min peak height: 50    Sample baseline V to V?: Y    Sample baseline V to V points: 3
Sample filter: Binomial       Number of points for filter: 3  Sample start region (min): 0  Sample end region (min): 30
Marker peak width (sec): 5    Marker min peak height: 200   Marker baseline V to V?: Y    Marker baseline V to V points: 3
Lower marker selection: First peak > 500 RFU                Upper marker selection: Last peak > 200 RFU
Ladder size (bp)75, 200, 300, 400, 500, 700, 1000, 1500, 2000, 3000, 4000, 5000, 7000, 10000, 20000
Quantification using: Lower Marker          Final concentration (ng/uL): 0.5000          Dilution factor: 12.0
```

Sample: SampD12
Well location: D12
Created: Monday, September 20, 2021 6:40:06 PM

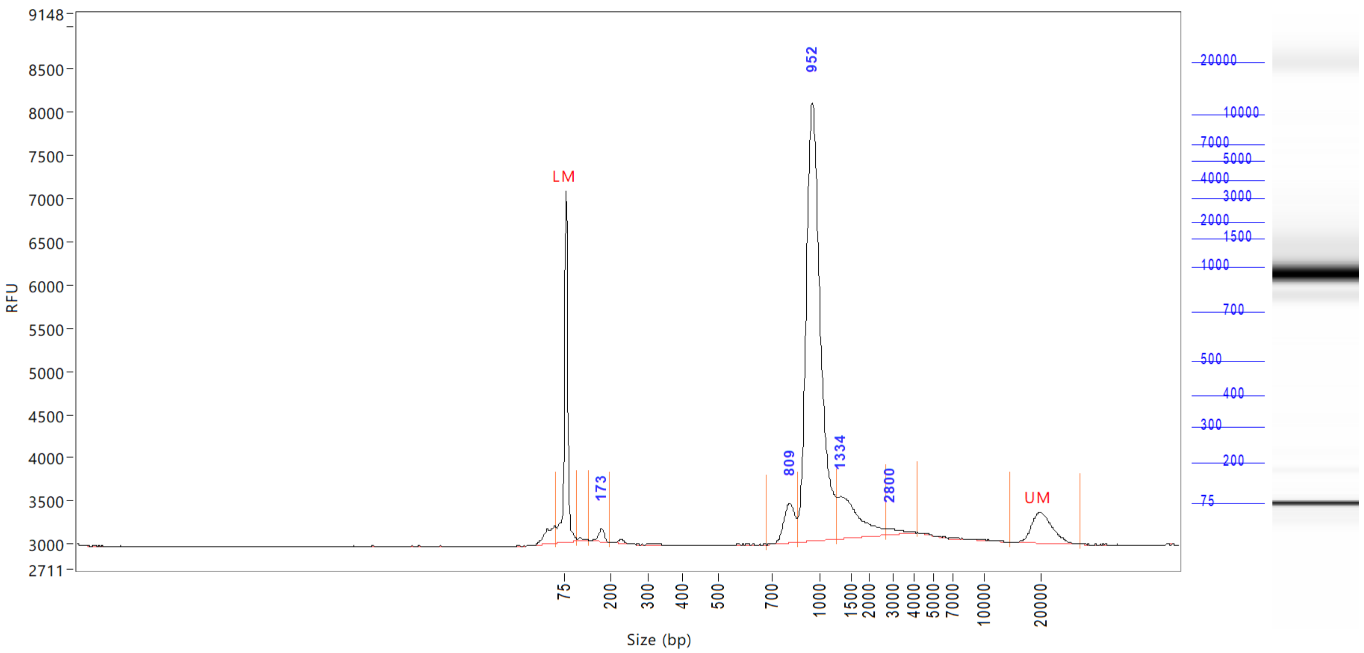

Peak	Size (bp)	Concentration (ng/uL)	From (bp)	To (bp)	Average size (bp)	CV%	RFU	Corrected peak area
1	75 (LM)	0.5000	48	105	72	9.53	4061	34.155
2	173	0.3440	139	196	170	5.25	158	1.958
3	809	1.4873	680	857	803	3.98	461	8.466
4	952	20.2543	857	1278	976	7.68	5082	115.298
5	1334	2.8243	1278	2715	1615	20.74	489	16.077
6	2800	0.2380	2715	4139	3123	10.42	72	1.355
7	20000 (UM)	0.1287	14635	27375	20474	8.80	354	8.794

TIC:	25.1480	ng/uL
TIM:	43.5361	nmole/L
Total concentration:	25.5328	ng/uL

Sample peak width (sec): 5 Sample min peak height: 50 Sample baseline V to V?: Y Sample baseline V to V points: 3
Sample filter: Binomial Number of points for filter: 3 Sample start region (min): 0 Sample end region (min): 30
Marker peak width (sec): 5 Marker min peak height: 200 Marker baseline V to V?: Y Marker baseline V to V points: 3
Lower marker selection: First peak > 500 RFU Upper marker selection: Last peak > 200 RFU
Ladder size (bp)75, 200, 300, 400, 500, 700, 1000, 1500, 2000, 3000, 4000, 5000, 7000, 10000, 20000
Quantification using: Lower Marker Final concentration (ng/uL): 0.5000 Dilution factor: 12.0

Sample: SampE12
Well location: E12
Created: Monday, September 20, 2021 6:40:06 PM

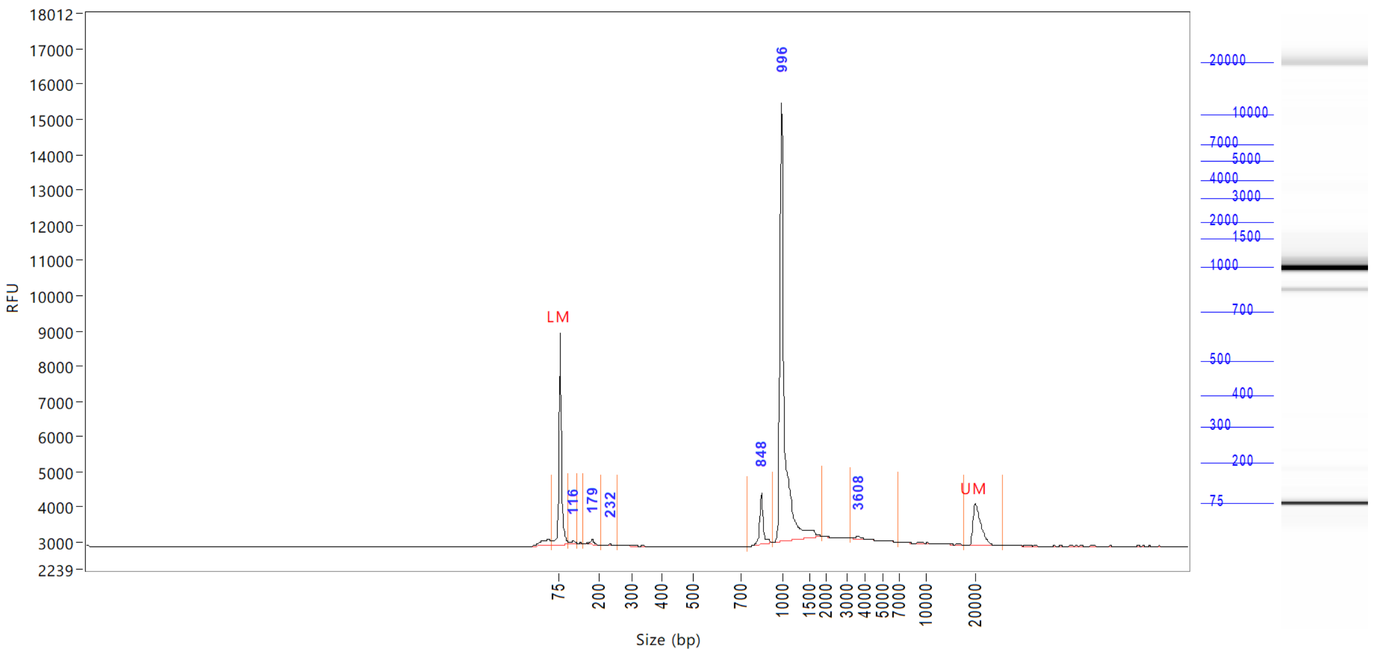

Peak	Size (bp)	Concentration (ng/uL)	From (bp)	To (bp)	Average size (bp)	CV%	RFU	Corrected peak area
1	75 (LM)	0.5000	50	101	73	8.14	6017	38.302
2	116	0.1714	101	131	113	6.56	91	1.094
3	179	0.2181	150	205	173	5.03	149	1.392
4	232	0.0589	205	257	228	3.32	51	0.376
5	848	1.4153	744	926	846	2.24	1459	9.035
6	996	14.5414	926	1904	1049	11.82	12459	92.829
7	3608	0.1499	3179	6852	3952	17.22	66	0.957
8	20000 (UM)	0.1602	17683	25561	20485	4.56	1209	12.276

TIC:	16.5551	ng/uL
TIM:	30.6212	nmole/L
Total concentration:	16.9365	ng/uL

Sample peak width (sec): 5 Sample min peak height: 50 Sample baseline V to V?: Y Sample baseline V to V points: 3
Sample filter: Binomial Number of points for filter: 3 Sample start region (min): 0 Sample end region (min): 30
Marker peak width (sec): 5 Marker min peak height: 200 Marker baseline V to V?: Y Marker baseline V to V points: 3
Lower marker selection: First peak > 500 RFU Upper marker selection: Last peak > 200 RFU
Ladder size (bp)75, 200, 300, 400, 500, 700, 1000, 1500, 2000, 3000, 4000, 5000, 7000, 10000, 20000
Quantification using: Lower Marker Final concentration (ng/uL): 0.5000 Dilution factor: 12.0

Sample: SampF12
Well location: F12
Created: Monday, September 20, 2021 6:40:06 PM

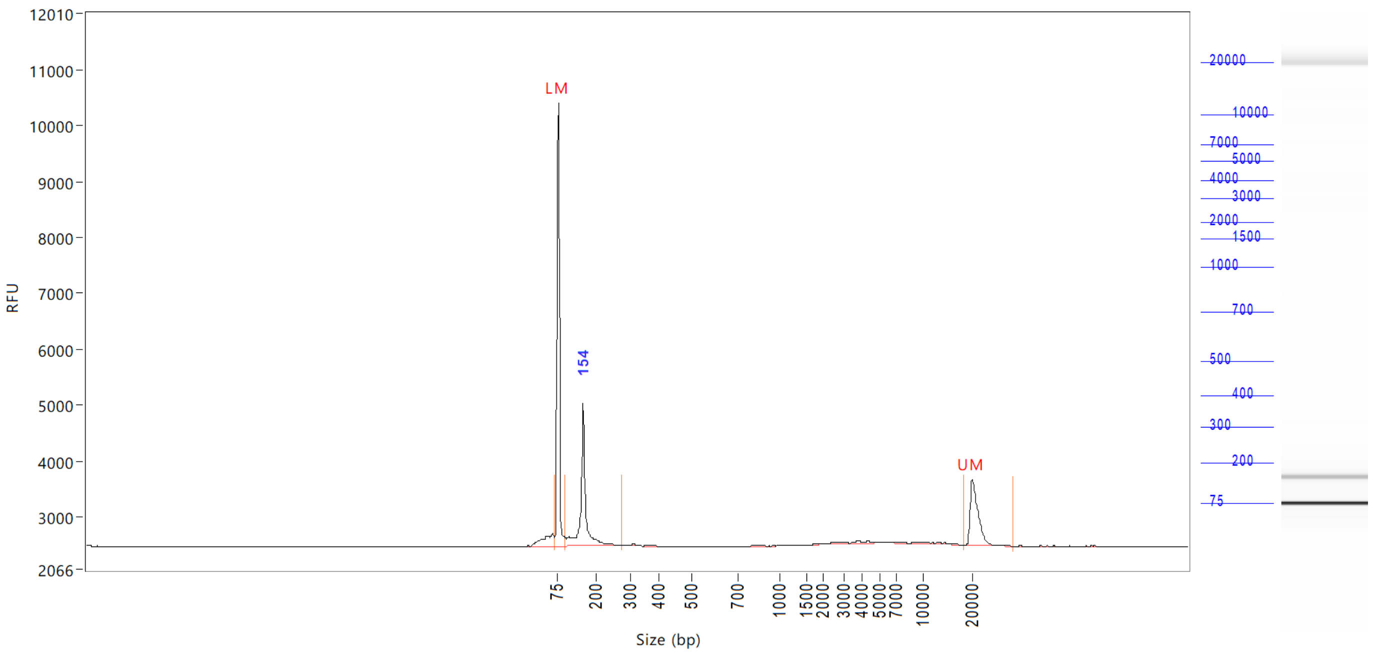

Peak	Size (bp)	Concentration (ng/uL)	From (bp)	To (bp)	Average size (bp)	CV%	RFU	Corrected peak area
1	75 (LM)	0.5000	64	99	73	6.44	7940	45.383
2	154	3.5830	99	275	153	15.18	2554	27.101
3	20000 (UM)	0.1314	18293	28342	20553	4.86	1177	11.930

TIC:	3.5830	ng/uL
TIM:	38.4895	nmole/L
Total concentration:	4.1661	ng/uL

Sample peak width (sec): 5 Sample min peak height: 50 Sample baseline V to V?: Y Sample baseline V to V points: 3
Sample filter: Binomial Number of points for filter: 3 Sample start region (min): 0 Sample end region (min): 30
Marker peak width (sec): 5 Marker min peak height: 200 Marker baseline V to V?: Y Marker baseline V to V points: 3
Lower marker selection: First peak > 500 RFU Upper marker selection: Last peak > 200 RFU
Ladder size (bp)75, 200, 300, 400, 500, 700, 1000, 1500, 2000, 3000, 4000, 5000, 7000, 10000, 20000
Quantification using: Lower Marker Final concentration (ng/uL): 0.5000 Dilution factor: 12.0

Sample: SampG12
Well location: G12
Created: Monday, September 20, 2021 6:40:06 PM

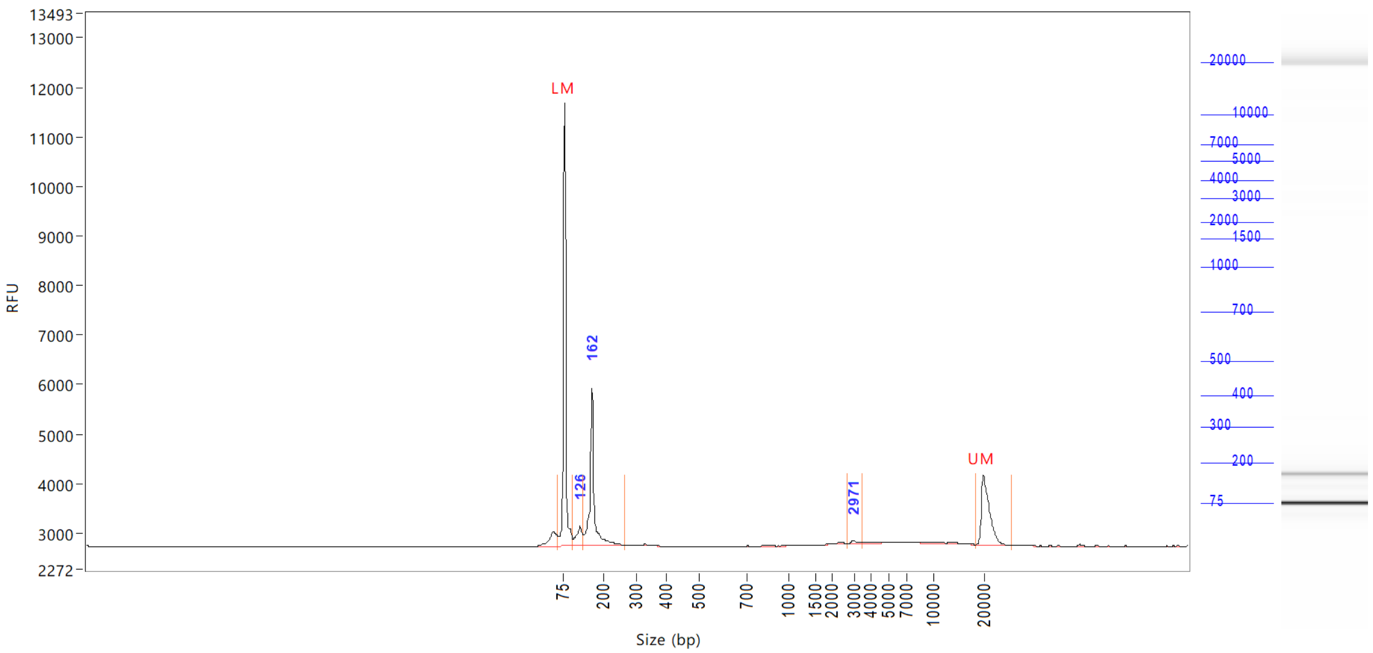

Peak	Size (bp)	Concentration (ng/uL)	From (bp)	To (bp)	Average size (bp)	CV%	RFU	Corrected peak area
1	75 (LM)	0.5000	56	101	74	7.60	8957	56.921
2	126	0.6029	101	137	120	8.11	393	5.720
3	162	3.0980	137	264	165	10.72	3168	29.390
4	2971	0.0632	2715	3500	3070	6.30	55	0.599
5	20000 (UM)	0.1269	18415	25561	20596	4.66	1401	14.448

TIC:	3.7641	ng/uL
TIM:	39.1510	nmole/L
Total concentration:	4.1692	ng/uL

Sample peak width (sec): 5 Sample min peak height: 50 Sample baseline V to V?: Y Sample baseline V to V points: 3
Sample filter: Binomial Number of points for filter: 3 Sample start region (min): 0 Sample end region (min): 30
Marker peak width (sec): 5 Marker min peak height: 200 Marker baseline V to V?: Y Marker baseline V to V points: 3
Lower marker selection: First peak > 500 RFU Upper marker selection: Last peak > 200 RFU
Ladder size (bp)75, 200, 300, 400, 500, 700, 1000, 1500, 2000, 3000, 4000, 5000, 7000, 10000, 20000
Quantification using: Lower Marker Final concentration (ng/uL): 0.5000 Dilution factor: 12.0

Sample: SampH12
Well location: H12
Created: Monday, September 20, 2021 6:40:06 PM

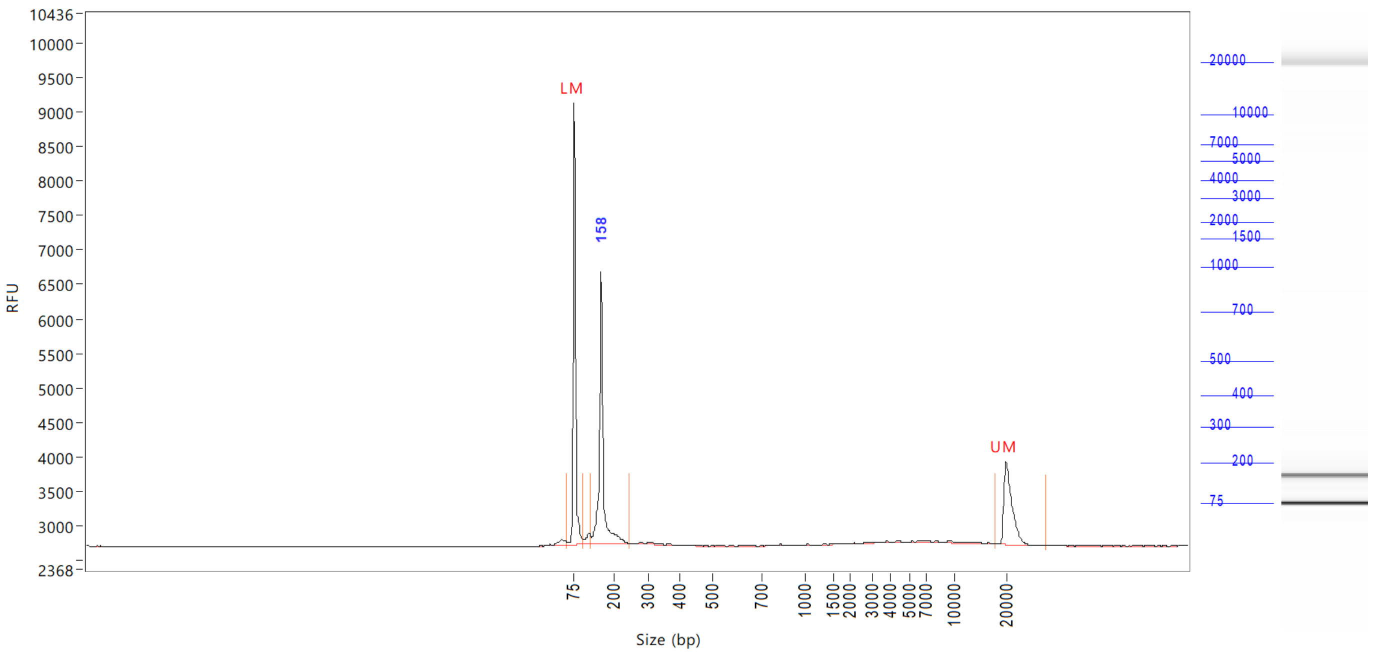

Peak	Size (bp)	Concentration (ng/uL)	From (bp)	To (bp)	Average size (bp)	CV%	RFU	Corrected peak area
1	75 (LM)	0.5000	54	105	74	7.47	6420	36.994
2	158	5.5605	128	244	160	10.03	3953	34.285
3	20000 (UM)	0.1691	17927	27859	20562	4.87	1213	12.510

TIC:	5.5605	ng/uL
TIM:	57.1235	nmole/L
Total concentration:	6.4973	ng/uL

Sample peak width (sec): 5   Sample min peak height: 200   Sample baseline V to V?: Y   Sample baseline V to V points: 3
Sample filter: Binomial   Number of points for filter: 3 Sample start region (min): 0 Sample end region (min): 30
Marker peak width (sec): 5   Marker min peak height: 200   Marker baseline V to V?: Y   Marker baseline V to V points: 3
Lower marker selection: First peak > 500 RFU   Upper marker selection: Last peak > 200 RFU
Ladder size (bp)75, 200, 300, 400, 500, 700, 1000, 1500, 2000, 3000, 4000, 5000, 7000, 10000, 20000
Quantification using: Lower Marker   Final concentration (ng/uL): 0.5000   Dilution factor: 12.0

Sample: DNA size ladder
Well location: A7
Created: Monday, September 20, 2021 6:40:06 PM
Import from: C:\Users\admin\Desktop\210810_alberto_ladder.SCAL
Fit type: Point to point

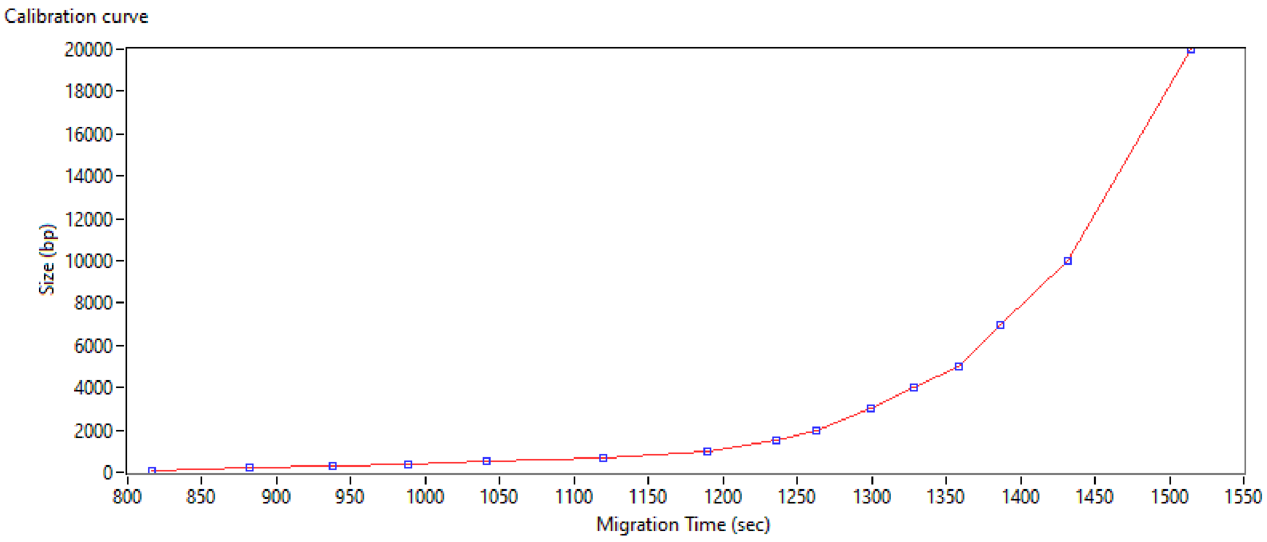

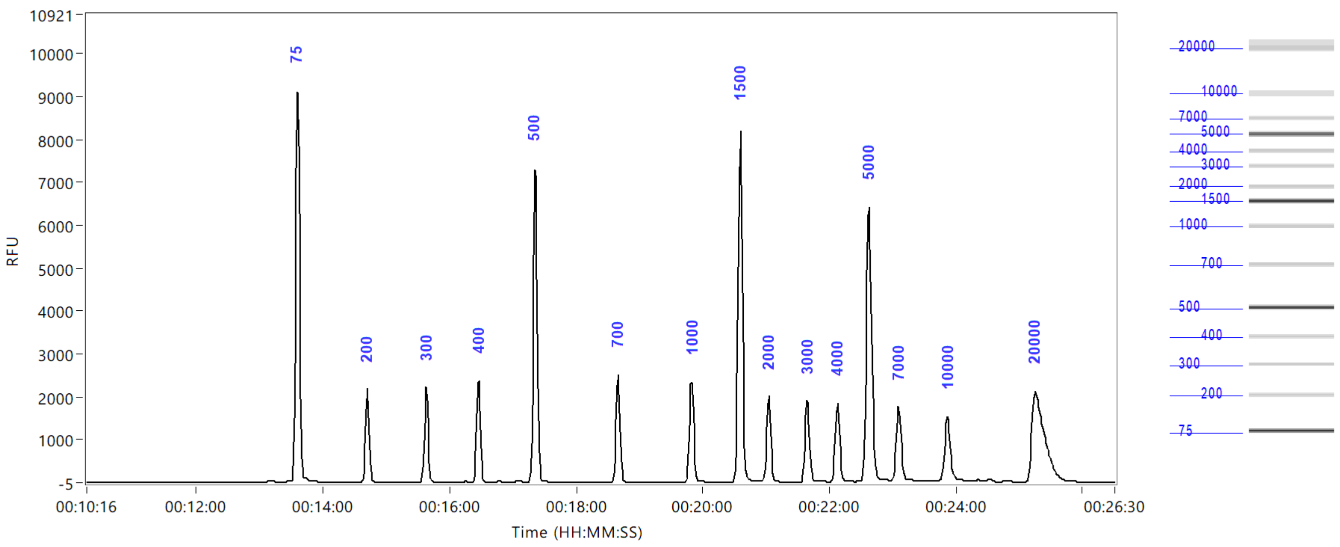

Supplement: Supplementary file 2 — sb3c00292_si_002.zip [file sb3c00292_si_002.zip › dnada_supplementary_material_pks_library_build/ZAG_trials/ZAG_Trial_6/18-30-13_plate2/plate2-t2.pdf]
