# Supplementary material for: Automated Platform for the Plasmid Construction Process
Source: ACS Synth Biol. 2023 Nov 10;12(12):3506–13. doi: 10.1021/acssynbio.3c00292 (PMC10729297; doi:10.1021/acssynbio.3c00292)

Instrument controller software run summary:

Filename and data path: C:\AATI\Data\2021 09 20\20-30-11\2021 09 20 20H 30M..raw
Created: Monday, September 20, 2021 8:39:56 PM
Number of capillaries: 96
Array serial number: 111920-09SFS
Effect length: 33 cm
Array usage count: 227
Instrument type: ZAG DNA Analyzer
Instrument controller software version: 1.0.1.9
Device serial number: Z7597

Method Information

Method name: ZAG130GP33 - DNA 75-20000bp - Gel Prime Only.mthds
Gel prime: Yes
Full conditioning: No
Gel prime to buffer: No
Gel selection: Gel 1
Perform prerun: 8.0 kV, 30 sec.
Rinse: No
Marker 1: Row: A, 5.0 kV, 5 sec.
Rinse: No
Sample injection: 5.0 kV, 5 sec.
Separation: 8.0 kV, 30.0 min.
Tray name: Sample Tray-5

Analysis mode: DNA

Notes

Gel Image

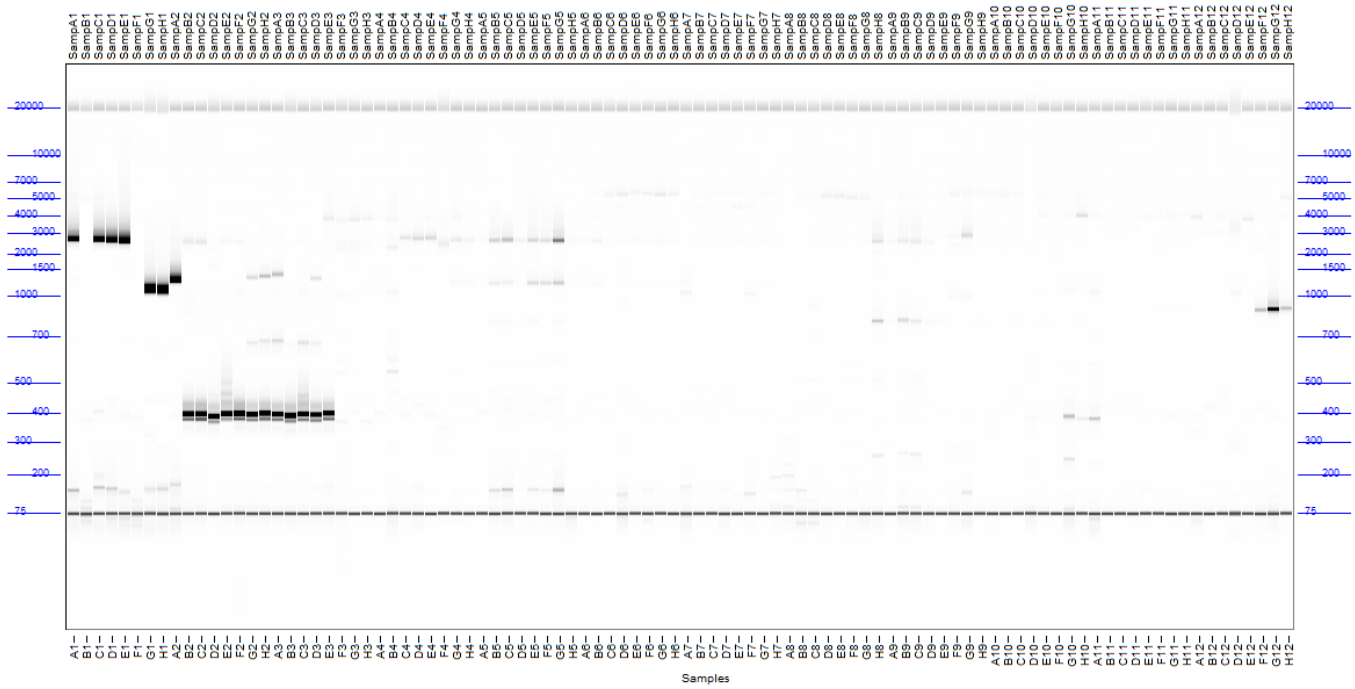

Filename and data path: C:\AATI\Data\2021 09 20\20-30-11\2021 09 20 20H 30M..raw

A1: SampA1

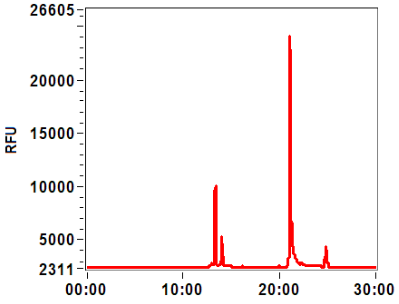

B1: SampB1

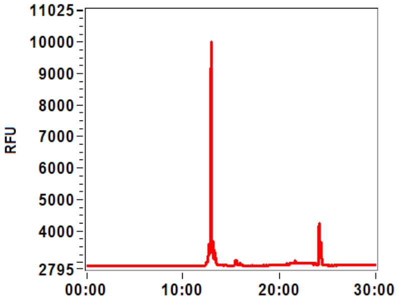

C1: SampC1

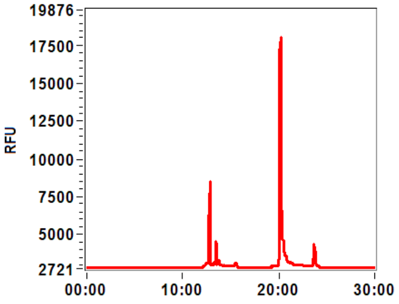

D1: SampD1

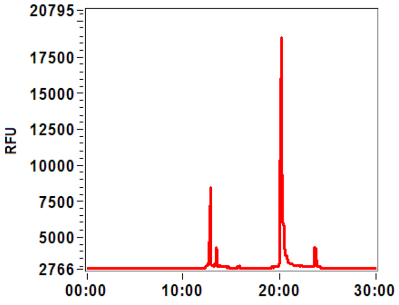

E1: SampE1

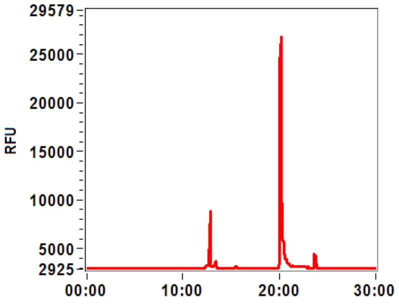

F1: SampF1

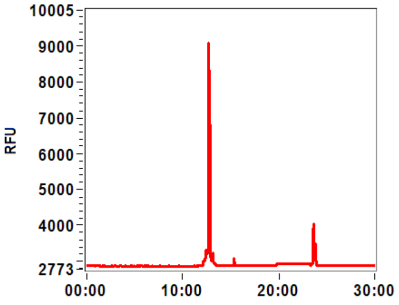

G1: SampG1

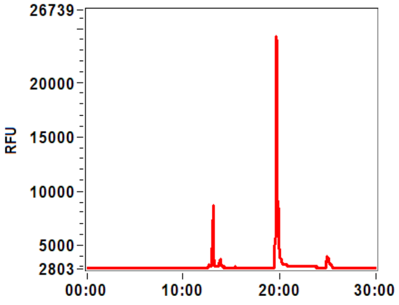

H1: SampH1

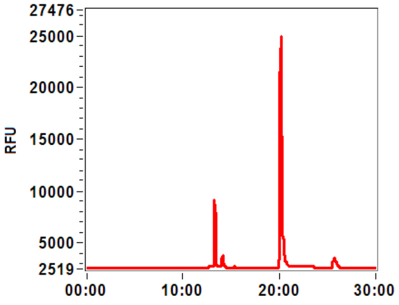

A2: SampA2

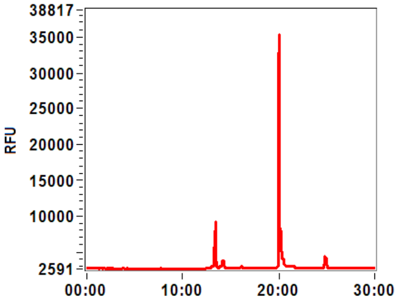

B2: SampB2

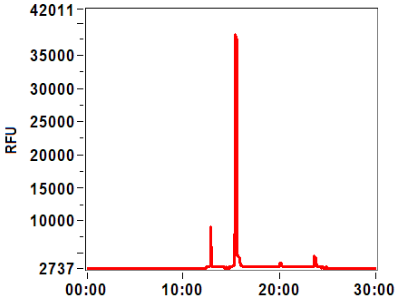

C2: SampC2

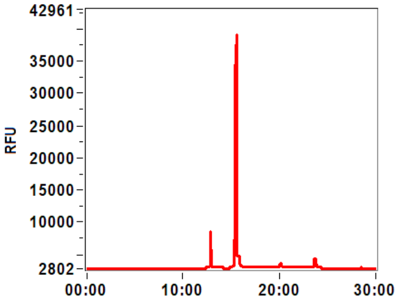

D2: SampD2

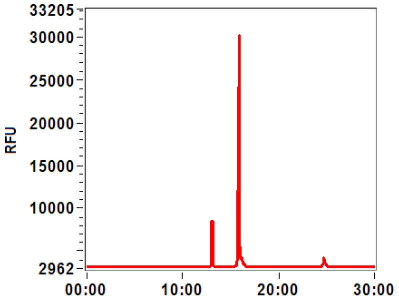

Filename and data path: C:\AATI\Data\2021 09 20\20-30-11\2021 09 20 20H 30M..raw

E2: SampE2

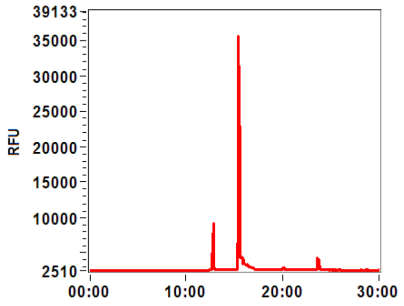

F2: SampF2

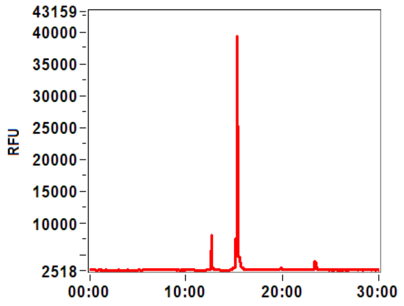

G2: SampG2

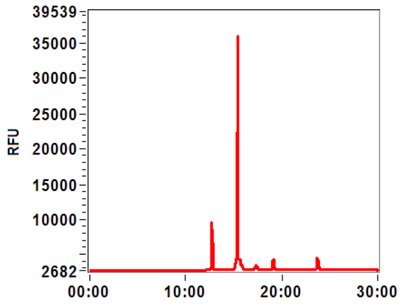

H2: SampH2

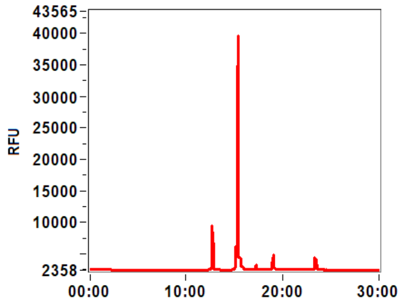

A3: SampA3

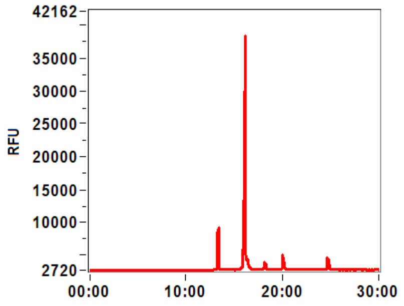

B3: SampB3

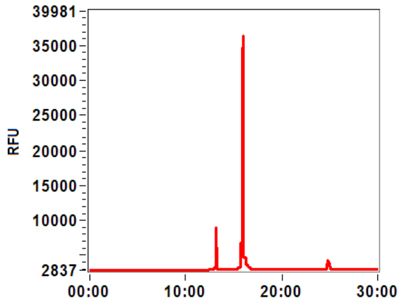

C3: SampC3

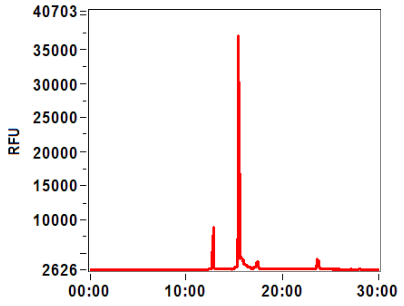

D3: SampD3

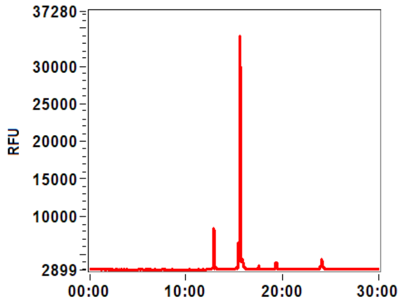

E3: SampE3

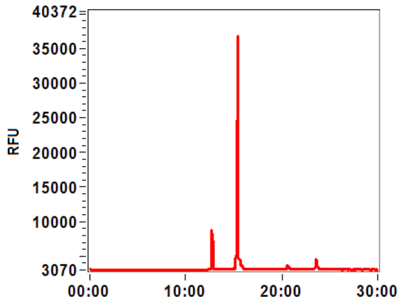

F3: SampF3

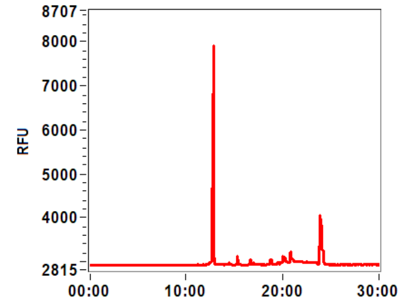

G3: SampG3

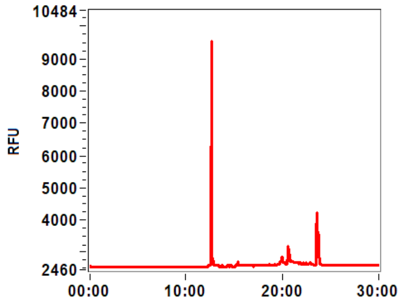

H3: SampH3

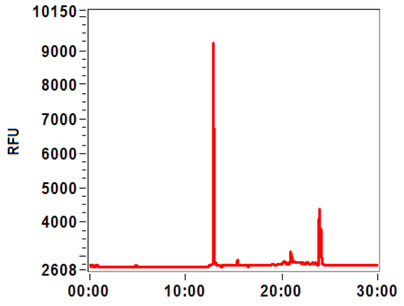

Filename and data path: C:\AATI\Data\2021 09 20\20-30-11\2021 09 20 20H 30M..raw

A4: SampA4

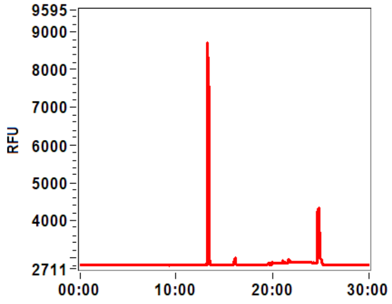

B4: SampB4

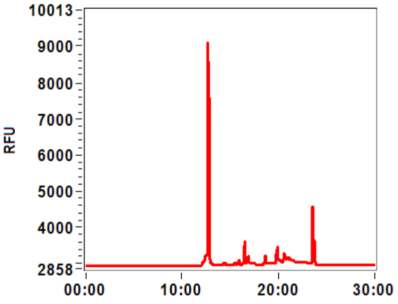

C4: SampC4

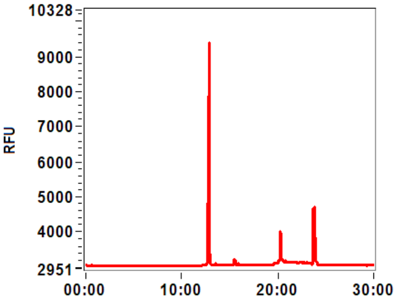

D4: SampD4

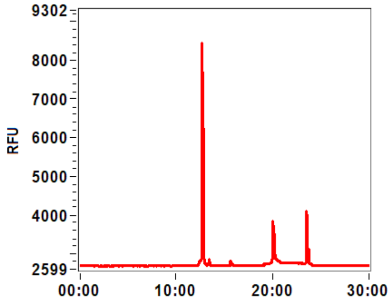

E4: SampE4

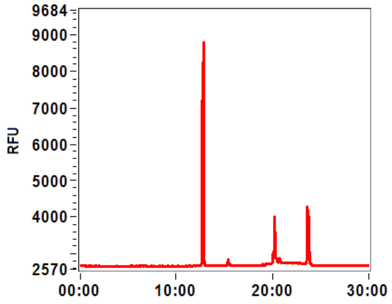

F4: SampF4

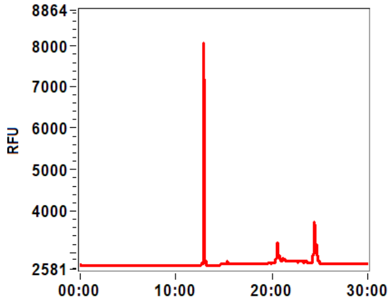

G4: SampG4

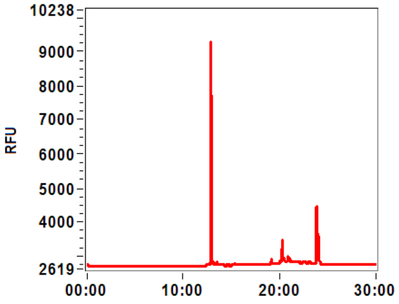

H4: SampH4

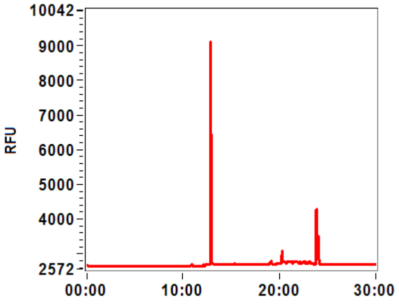

A5: SampA5

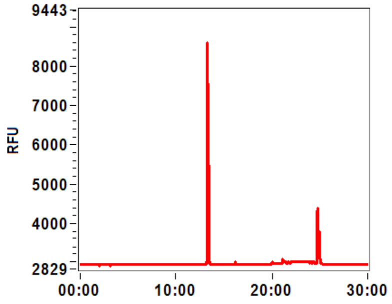

B5: SampB5

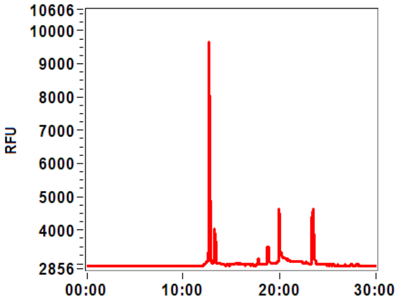

C5: SampC5

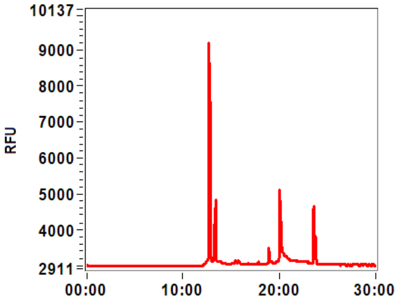

D5: SampD5

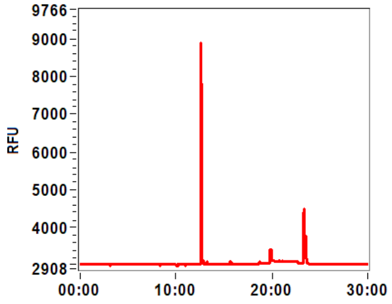

Filename and data path: C:\AATI\Data\2021 09 20\20-30-11\2021 09 20 20H 30M..raw

E5: SampE5

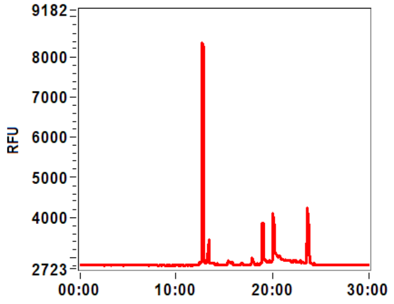

F5: SampF5

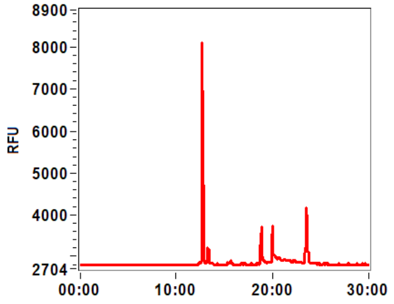

G5: SampG5

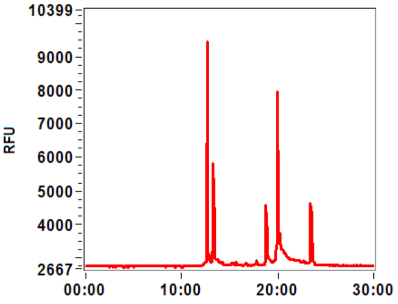

H5: SampH5

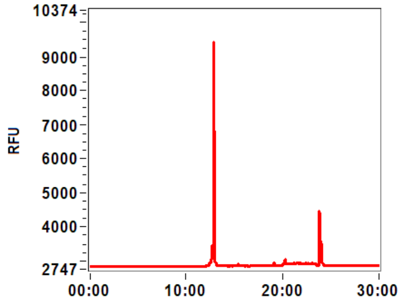

A6: SampA6

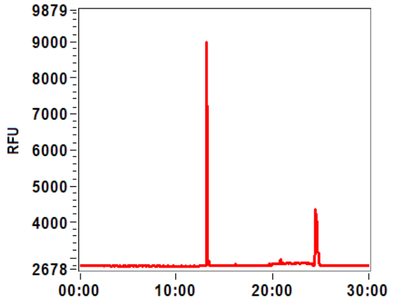

B6: SampB6

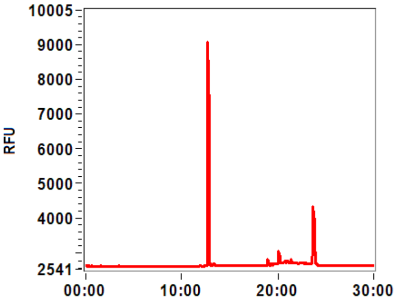

C6: SampC6

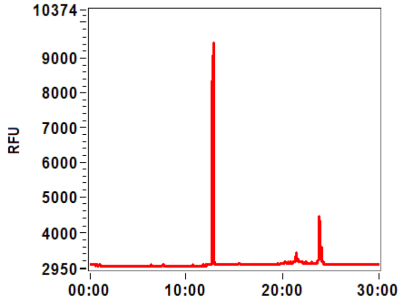

D6: SampD6

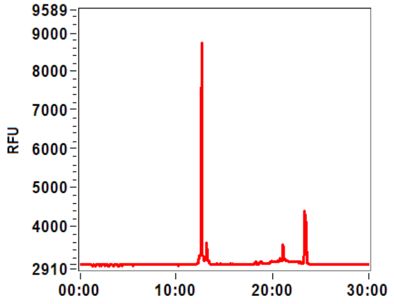

E6: SampE6

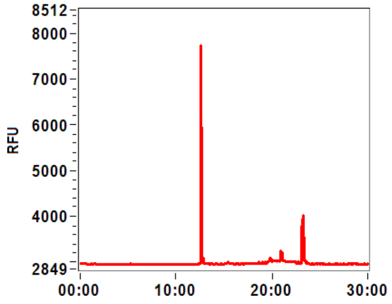

F6: SampF6

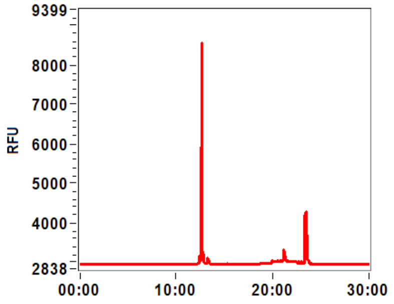

G6: SampG6

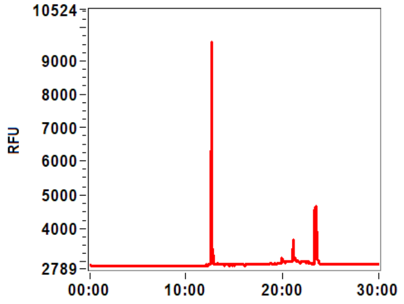

H6: SampH6

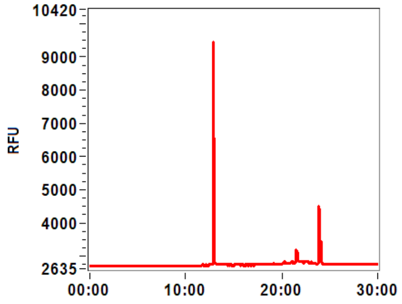

Filename and data path: C:\AATI\Data\2021 09 20\20-30-11\2021 09 20 20H 30M..raw

A7: SampA7

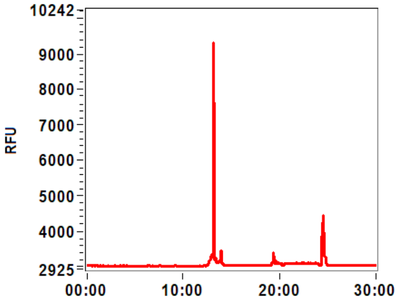

B7: SampB7

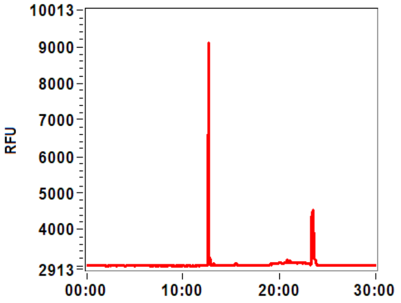

C7: SampC7

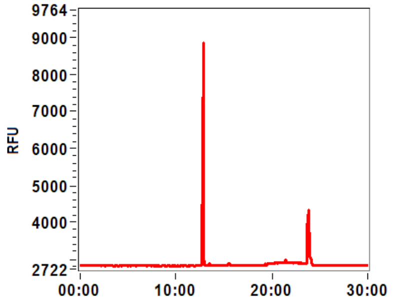

D7: SampD7

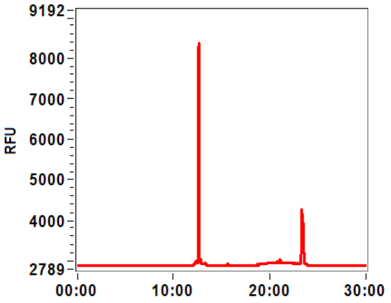

E7: SampE7

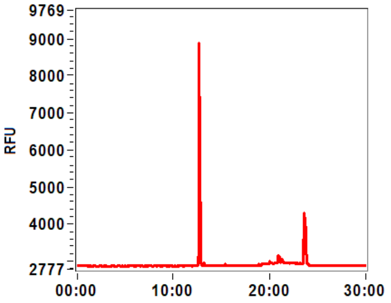

F7: SampF7

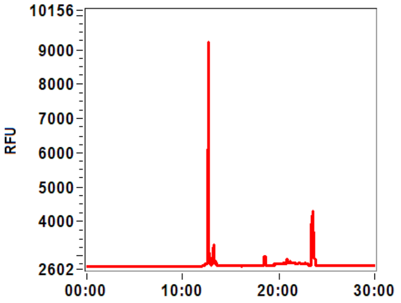

G7: SampG7

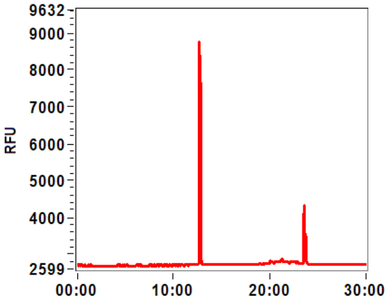

H7: SampH7

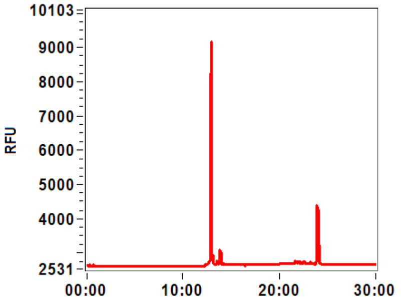

A8: SampA8

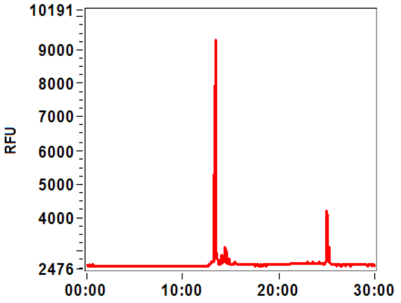

B8: SampB8

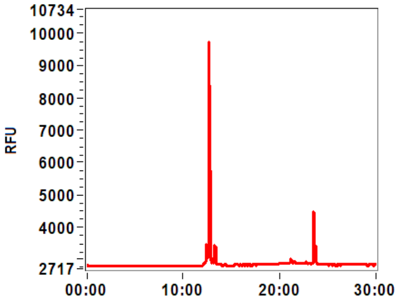

C8: SampC8

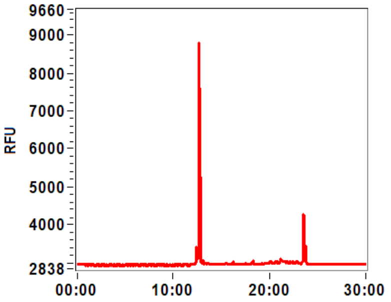

D8: SampD8

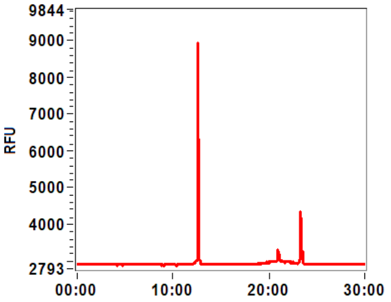

Filename and data path: C:\AATI\Data\2021 09 20\20-30-11\2021 09 20 20H 30M..raw

E8: SampE8

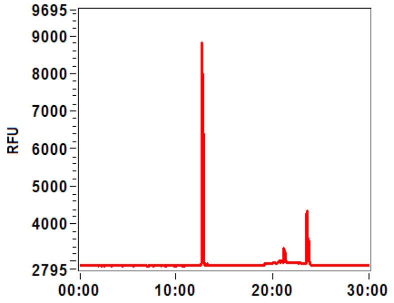

F8: SampF8

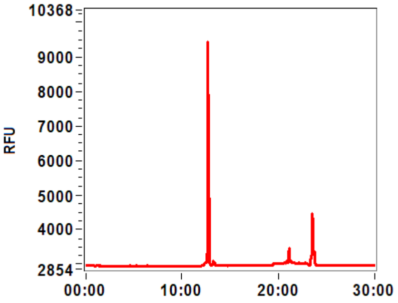

G8: SampG8

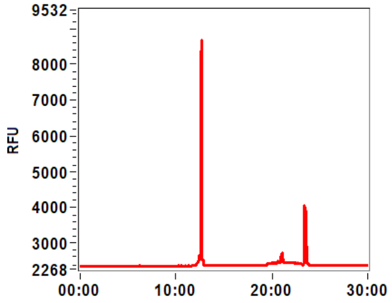

H8: SampH8

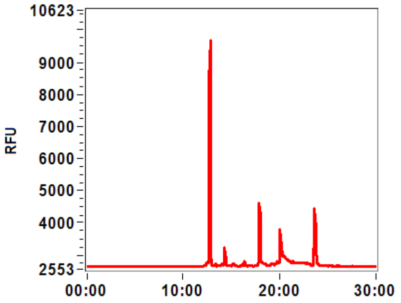

A9: SampA9

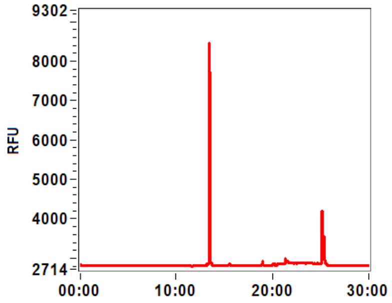

B9: SampB9

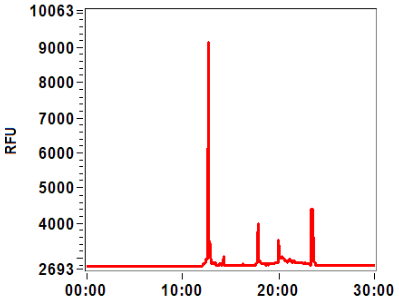

C9: SampC9

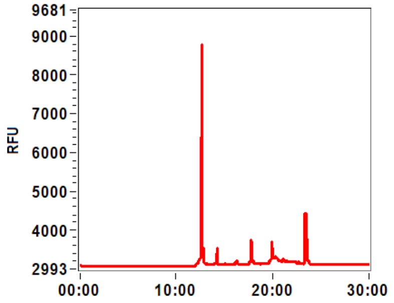

D9: SampD9

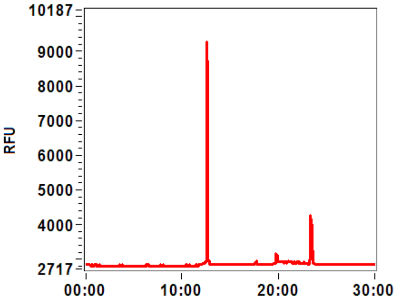

E9: SampE9

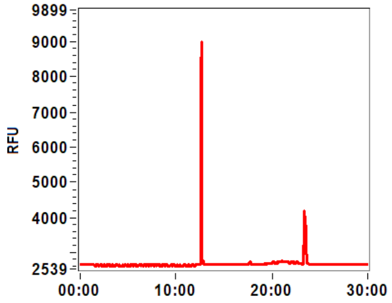

F9: SampF9

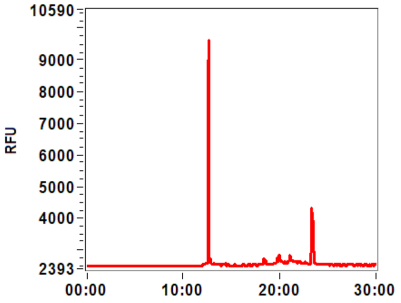

G9: SampG9

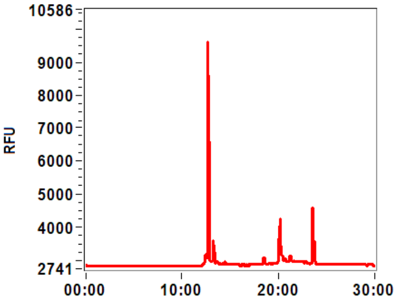

H9: SampH9

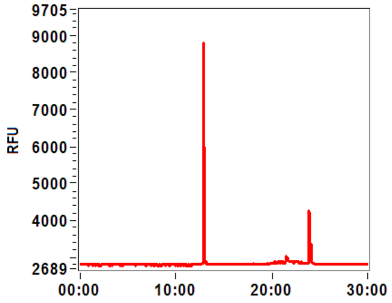

Filename and data path: C:\AATI\Data\2021 09 20\20-30-11\2021 09 20 20H 30M..raw

A10: SampA10

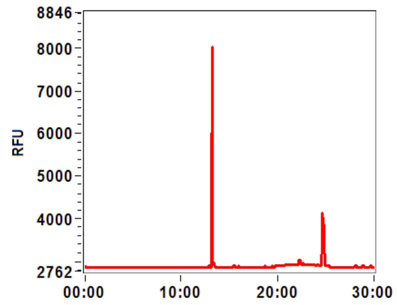

B10: SampB10

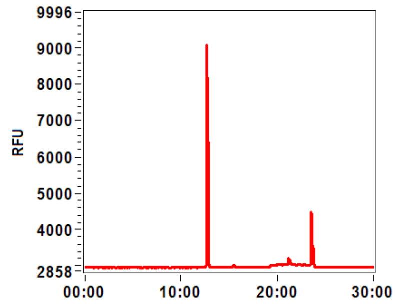

C10: SampC10

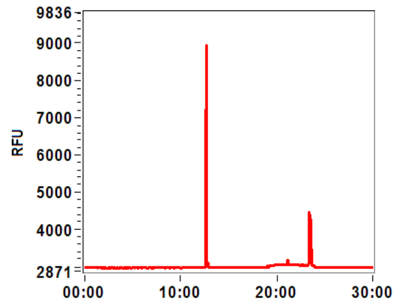

D10: SampD10

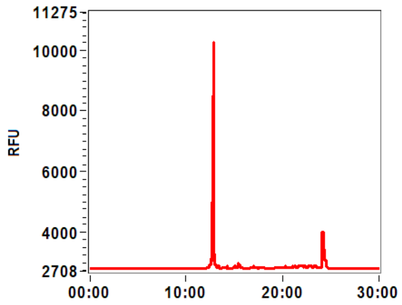

E10: SampE10

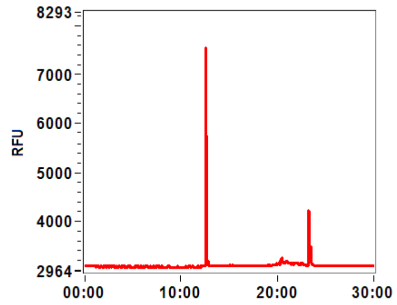

F10: SampF10

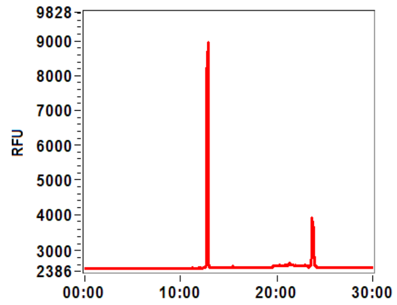

G10: SampG10

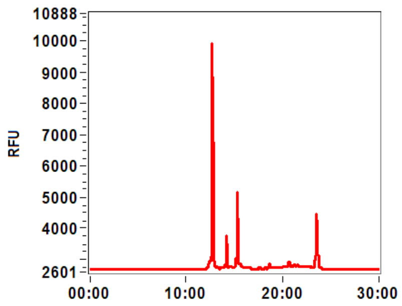

H10: SampH10

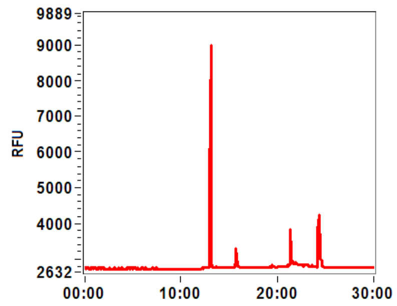

A11: SampA11

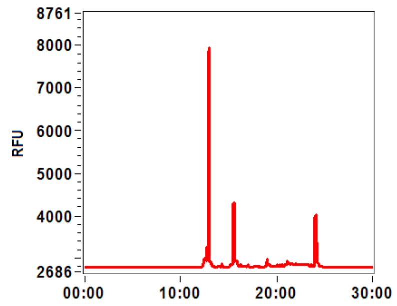

B11: SampB11

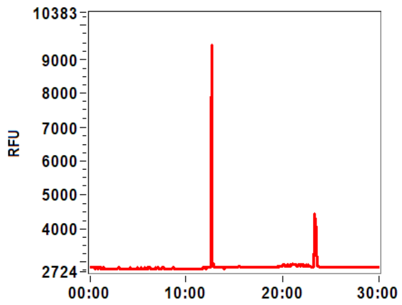

C11: SampC11

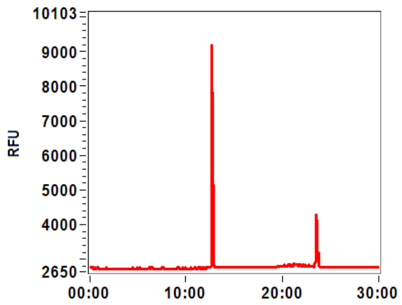

D11: SampD11

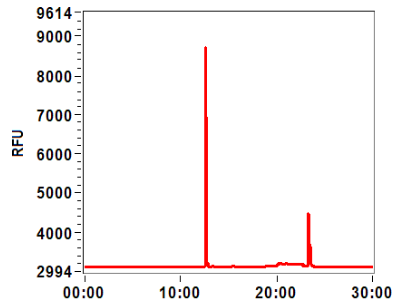

Filename and data path: C:\AATI\Data\2021 09 20\20-30-11\2021 09 20 20H 30M..raw

E11: SampE11

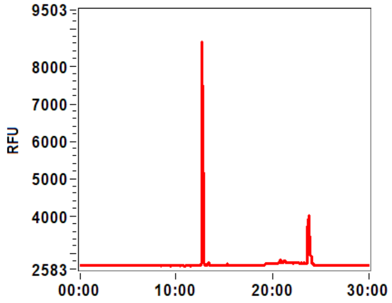

F11: SampF11

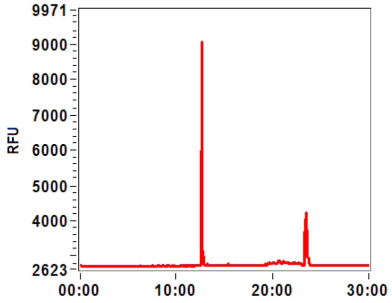

G11: SampG11

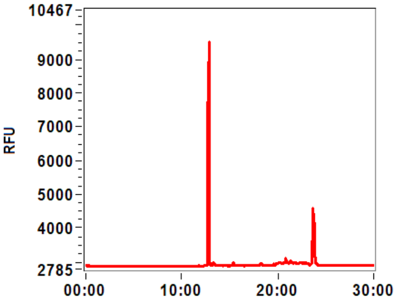

H11: SampH11

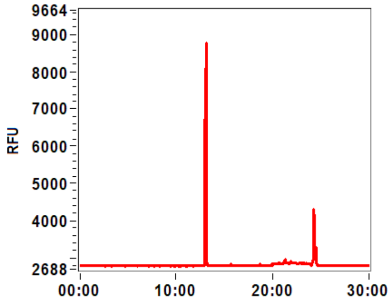

A12: SampA12

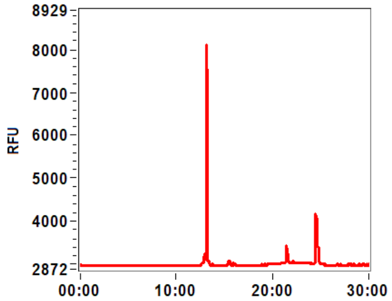

B12: SampB12

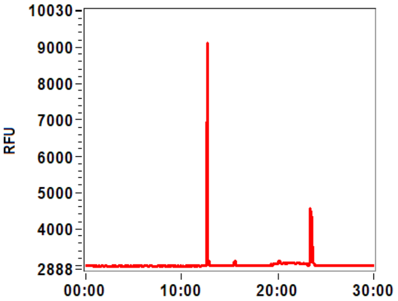

C12: SampC12

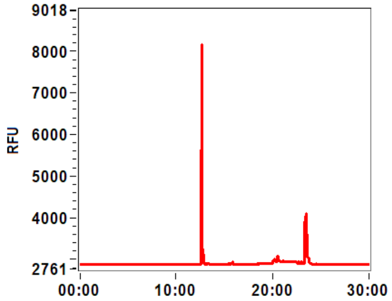

D12: SampD12

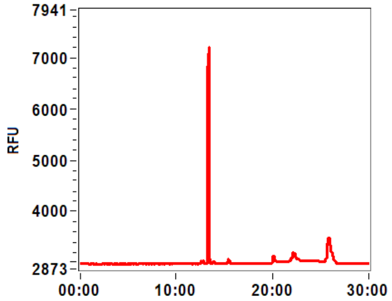

E12: SampE12

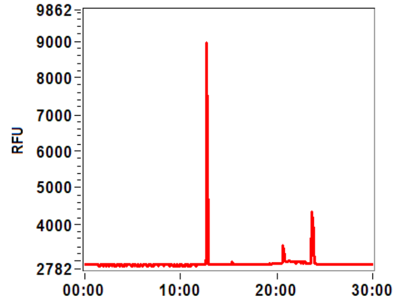

F12: SampF12

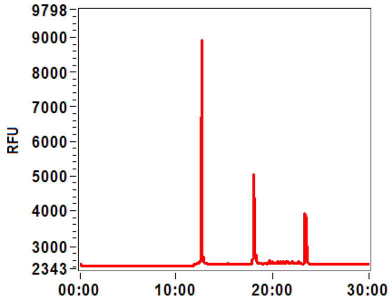

G12: SampG12

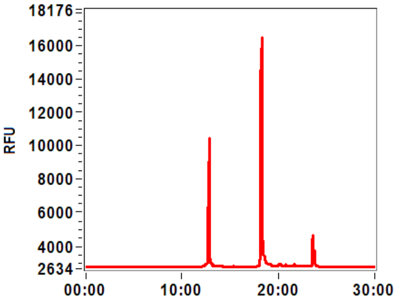

H12: SampH12

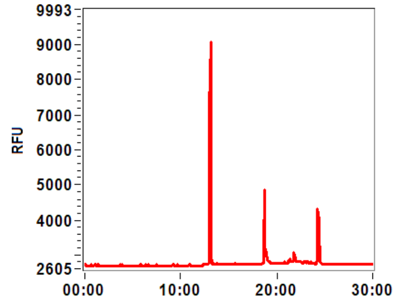

Sample: SampA1
Well location: A1
Created: Monday, September 20, 2021 8:39:56 PM

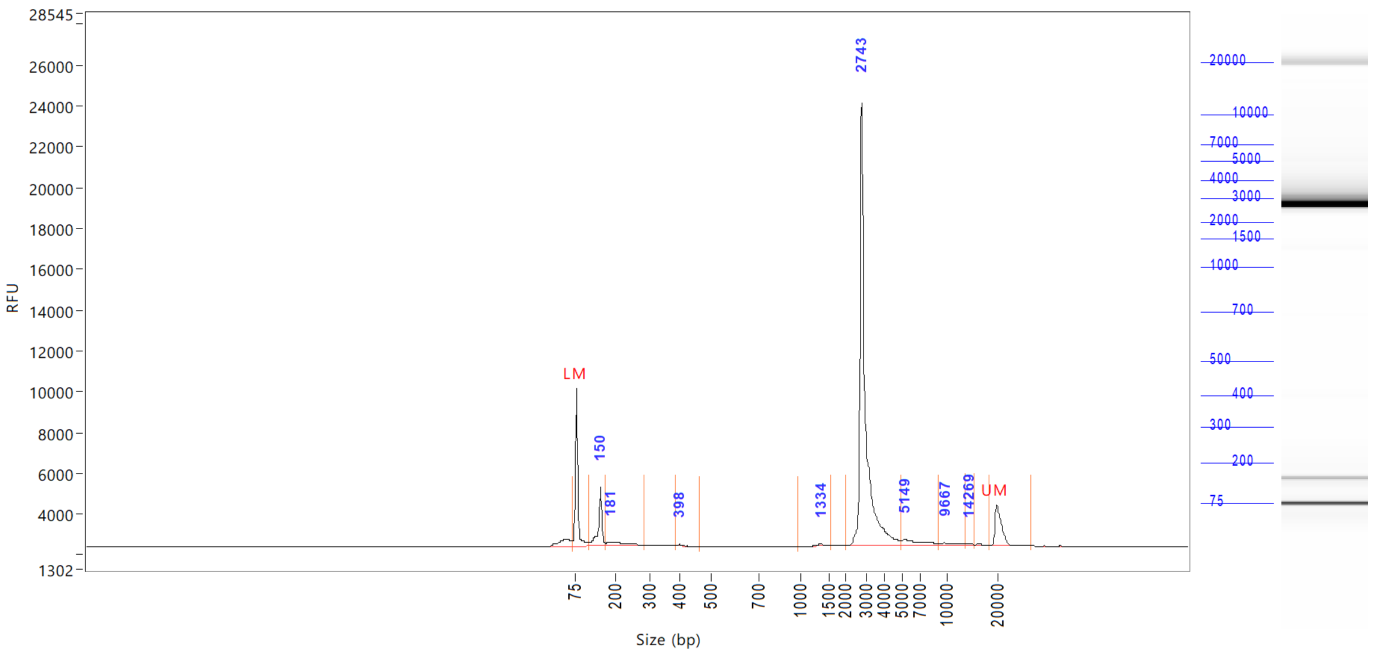

Peak	Size (bp)	Concentration (ng/uL)	From (bp)	To (bp)	Average size (bp)	CV%	RFU	Corrected peak area
1	75 (LM)	0.5000	62	114	75	11.16	7711	48.014
2	150	2.8874	114	168	144	7.12	2880	23.106
3	181	0.7948	168	286	204	12.55	182	6.360
4	398	0.0711	386	462	403	3.67	70	0.569
5	1334	0.1202	974	1520	1342	6.25	123	0.962
6	2743	22.1939	2030	4897	2938	14.35	21713	177.604
7	5149	0.8555	4897	9067	6427	18.79	301	6.846
8	9667	0.2533	9067	13659	10767	12.30	90	2.027
9	14269	0.0438	13659	15488	14193	2.43	64	0.350
10	20000 (UM)	0.1653	18537	27012	20369	3.58	1949	15.873

TIC:	27.2200	ng/uL
TIM:	52.4947	nmole/L
Total concentration:	27.2741	ng/uL

```
Sample peak width (sec): 5    Sample min peak height: 50    Sample baseline V to V?: Y    Sample baseline V to V points: 3
Sample filter: Binomial       Number of points for filter: 3  Sample start region (min): 0  Sample end region (min): 30
Marker peak width (sec): 5    Marker min peak height: 200   Marker baseline V to V?: Y    Marker baseline V to V points: 3
Lower marker selection: First peak > 500 RFU                Upper marker selection: Last peak > 200 RFU
Ladder size (bp)75, 200, 300, 400, 500, 700, 1000, 1500, 2000, 3000, 4000, 5000, 7000, 10000, 20000
Quantification using: Lower Marker          Final concentration (ng/uL): 0.5000         Dilution factor: 12.0
```

Sample: SampB1
Well location: B1
Created: Monday, September 20, 2021 8:39:56 PM

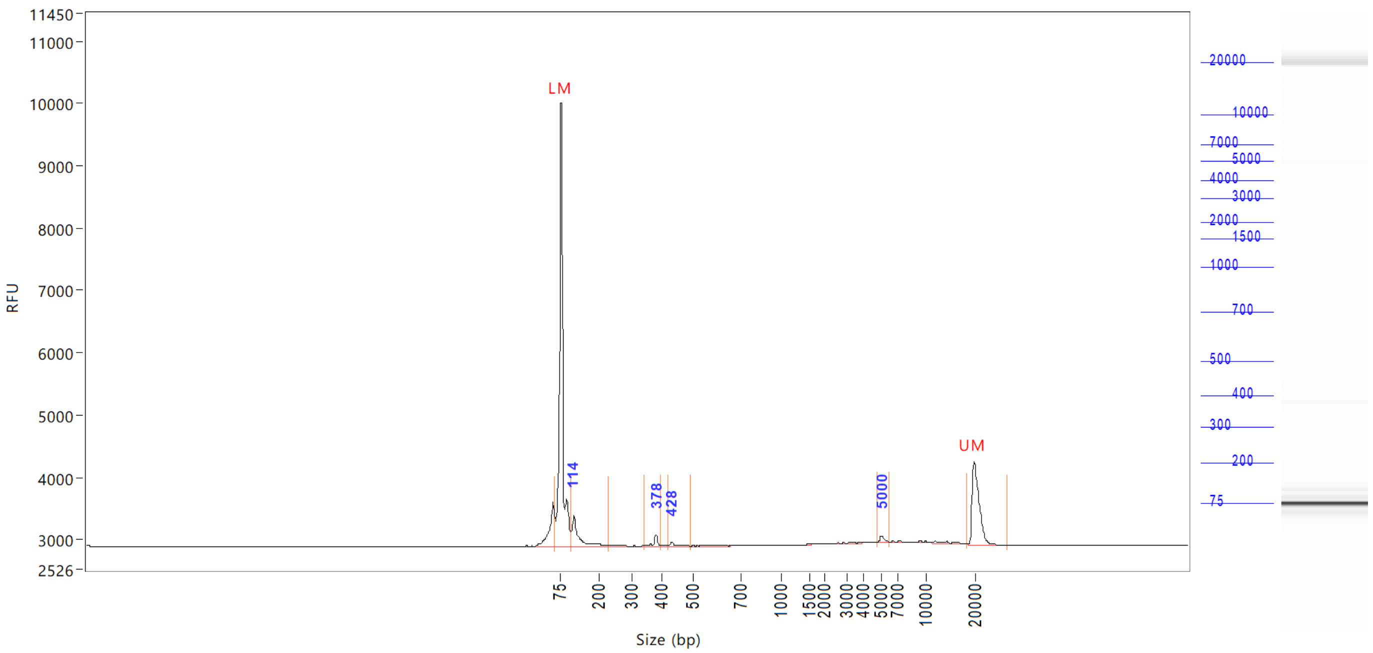

Peak	Size (bp)	Concentration (ng/uL)	From (bp)	To (bp)	Average size (bp)	CV%	RFU	Corrected peak area
1	75 (LM)	0.5000	58	107	74	11.56	7128	58.147
2	114	0.7620	107	226	131	20.18	505	7.385
3	378	0.1354	339	392	372	2.48	183	1.312
4	428	0.0660	416	489	434	3.36	64	0.640
5	5000	0.0802	4724	5963	5106	5.40	114	0.777
6	20000 (UM)	0.1046	18415	26649	20447	4.40	1344	12.162

TIC:	1.0436	ng/uL
TIM:	10.4324	nmole/L
Total concentration:	1.3877	ng/uL

```
Sample peak width (sec): 5    Sample min peak height: 50    Sample baseline V to V?: Y    Sample baseline V to V points: 3
Sample filter: Binomial       Number of points for filter: 3  Sample start region (min): 0  Sample end region (min): 30
Marker peak width (sec): 5    Marker min peak height: 200   Marker baseline V to V?: Y    Marker baseline V to V points: 3
Lower marker selection: First peak > 500 RFU                Upper marker selection: Last peak > 200 RFU
Ladder size (bp)75, 200, 300, 400, 500, 700, 1000, 1500, 2000, 3000, 4000, 5000, 7000, 10000, 20000
Quantification using: Lower Marker          Final concentration (ng/uL): 0.5000          Dilution factor: 12.0
```

Sample: SampC1
Well location: C1
Created: Monday, September 20, 2021 8:39:56 PM

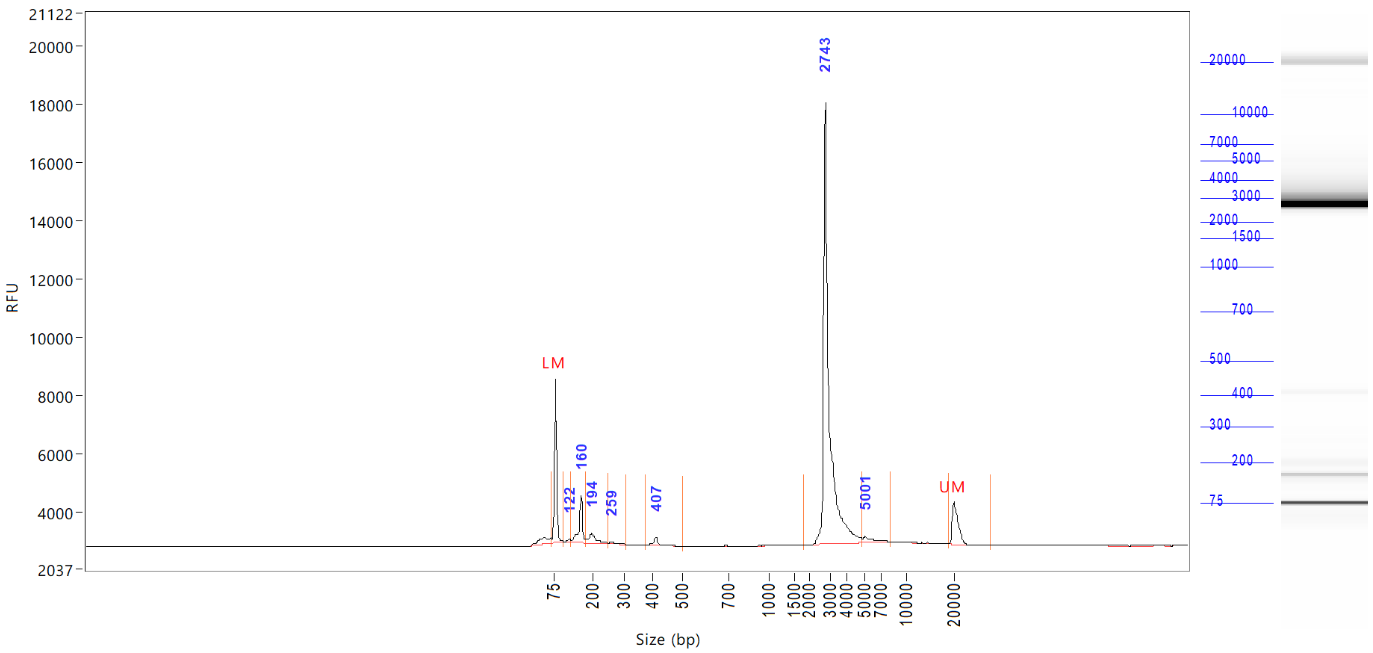

Peak	Size (bp)	Concentration (ng/uL)	From (bp)	To (bp)	Average size (bp)	CV%	RFU	Corrected peak area
1	75 (LM)	0.5000	64	103	73	5.40	5593	31.409
2	122	0.1731	103	128	118	5.19	108	0.906
3	160	2.5491	128	175	154	6.15	1615	13.344
4	194	1.1206	175	246	200	7.98	328	5.866
5	259	0.1737	246	307	265	5.41	62	0.909
6	407	0.4738	374	498	408	4.26	281	2.480
7	2743	25.0449	1789	4862	2921	14.21	15177	131.105
8	5001	0.5612	4862	8134	5723	13.66	201	2.938
9	20000 (UM)	0.1849	18781	27617	20393	3.59	1446	11.614

TIC:	30.0965	ng/uL
TIM:	56.0936	nmole/L
Total concentration:	30.2790	ng/uL

Sample peak width (sec): 5   Sample min peak height: 50   Sample baseline V to V?: Y   Sample baseline V to V points: 3
Sample filter: Binomial   Number of points for filter: 3   Sample start region (min): 0   Sample end region (min): 30
Marker peak width (sec): 5   Marker min peak height: 200   Marker baseline V to V?: Y   Marker baseline V to V points: 3
Lower marker selection: First peak > 500 RFU   Upper marker selection: Last peak > 200 RFU
Ladder size (bp)75, 200, 300, 400, 500, 700, 1000, 1500, 2000, 3000, 4000, 5000, 7000, 10000, 20000
Quantification using: Lower Marker   Final concentration (ng/uL): 0.5000   Dilution factor: 12.0

Sample: SampD1
Well location: D1
Created: Monday, September 20, 2021 8:39:56 PM

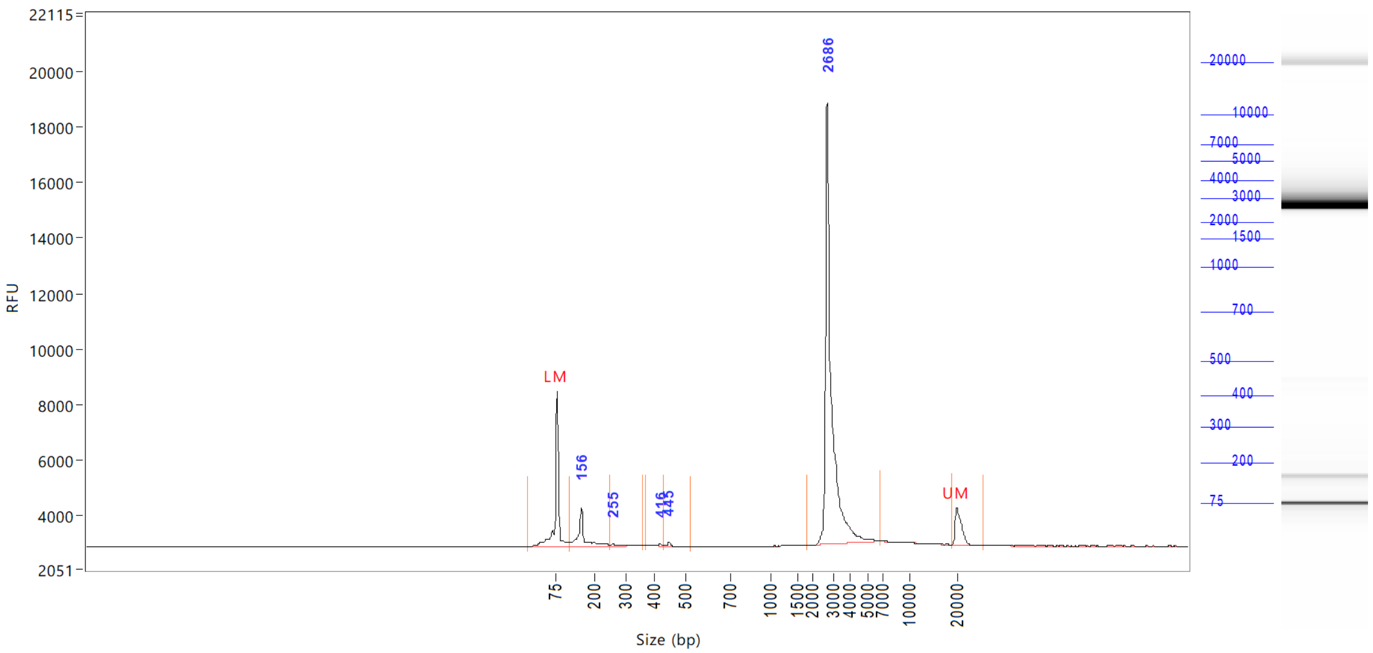

Peak	Size (bp)	Concentration (ng/uL)	From (bp)	To (bp)	Average size (bp)	CV%	RFU	Corrected peak area
1	75 (LM)	0.5000	0	120	68	27.19	5622	44.820
2	156	2.4583	120	250	162	17.30	1412	18.363
3	255	0.1949	250	358	279	8.96	68	1.456
4	416	0.0914	368	430	410	2.54	69	0.683
5	445	0.1174	430	521	445	2.99	133	0.877
6	2686	19.4864	1789	6630	2900	15.28	15950	145.564
7	20000 (UM)	0.1225	18781	25561	20348	3.47	1353	10.984

TIC:	22.3483	ng/uL
TIM:	37.9541	nmole/L
Total concentration:	22.4851	ng/uL

Sample peak width (sec): 5 Sample min peak height: 50 Sample baseline V to V?: Y Sample baseline V to V points: 3
Sample filter: Binomial Number of points for filter: 3 Sample start region (min): 0 Sample end region (min): 30
Marker peak width (sec): 5 Marker min peak height: 200 Marker baseline V to V?: Y Marker baseline V to V points: 3
Lower marker selection: First peak > 500 RFU Upper marker selection: Last peak > 200 RFU
Ladder size (bp)75, 200, 300, 400, 500, 700, 1000, 1500, 2000, 3000, 4000, 5000, 7000, 10000, 20000
Quantification using: Lower Marker Final concentration (ng/uL): 0.5000 Dilution factor: 12.0

Sample: SampE1
Well location: E1
Created: Monday, September 20, 2021 8:39:56 PM

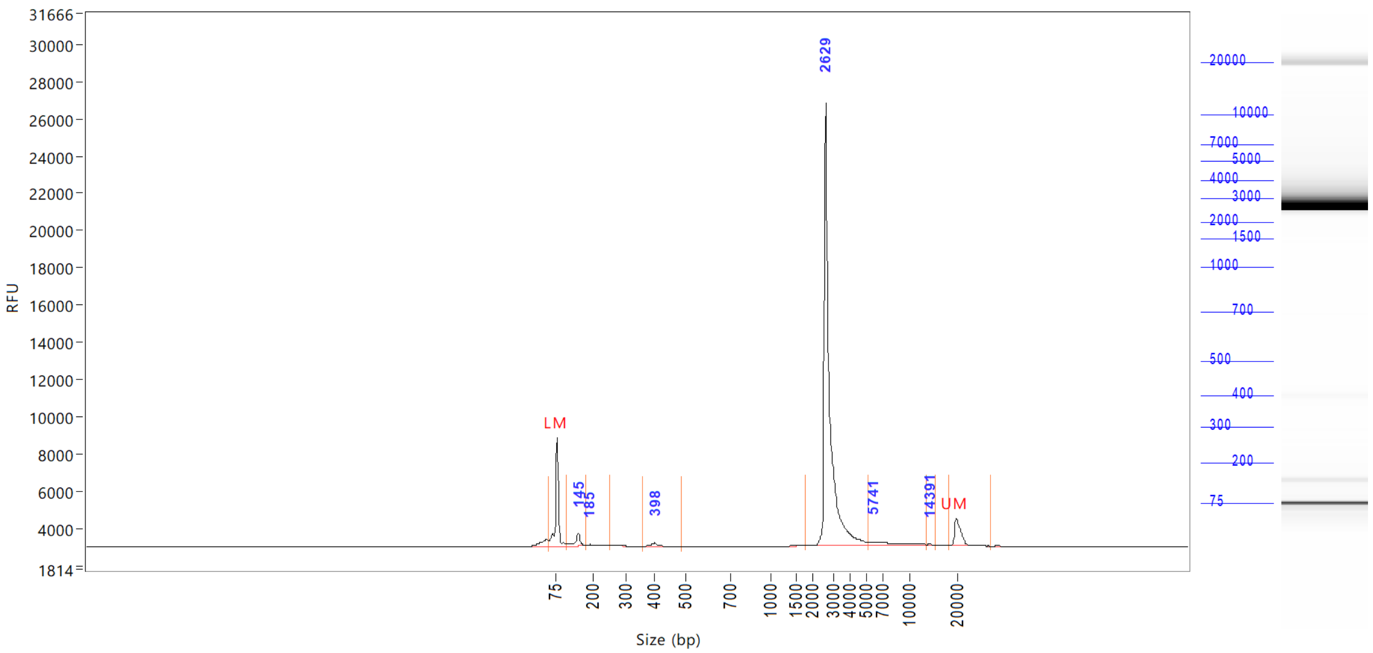

Peak	Size (bp)	Concentration (ng/uL)	From (bp)	To (bp)	Average size (bp)	CV%	RFU	Corrected peak area
1	75 (LM)	0.5000	47	107	72	12.81	5843	41.254
2	145	1.2423	107	173	139	10.13	689	8.542
3	185	0.2491	173	250	200	9.99	72	1.712
4	398	0.3232	356	489	398	4.69	184	2.222
5	2629	29.4695	1770	5075	2855	15.54	23820	202.621
6	5741	1.1717	5075	13781	7860	27.47	209	8.056
7	14391	0.0662	13781	15732	14560	3.84	60	0.455
8	20000 (UM)	0.1421	18415	27375	20340	3.50	1461	11.726

TIC:	32.5219	ng/uL
TIM:	35.3159	nmole/L
Total concentration:	32.7524	ng/uL

```
Sample peak width (sec): 5    Sample min peak height: 50    Sample baseline V to V?: Y    Sample baseline V to V points: 3
Sample filter: Binomial       Number of points for filter: 3  Sample start region (min): 0  Sample end region (min): 30
Marker peak width (sec): 5    Marker min peak height: 200   Marker baseline V to V?: Y    Marker baseline V to V points: 3
Lower marker selection: First peak > 500 RFU                Upper marker selection: Last peak > 200 RFU
Ladder size (bp)75, 200, 300, 400, 500, 700, 1000, 1500, 2000, 3000, 4000, 5000, 7000, 10000, 20000
Quantification using: Lower Marker         Final concentration (ng/uL): 0.5000         Dilution factor: 12.0
```

Sample: SampF1
Well location: F1
Created: Monday, September 20, 2021 8:39:56 PM

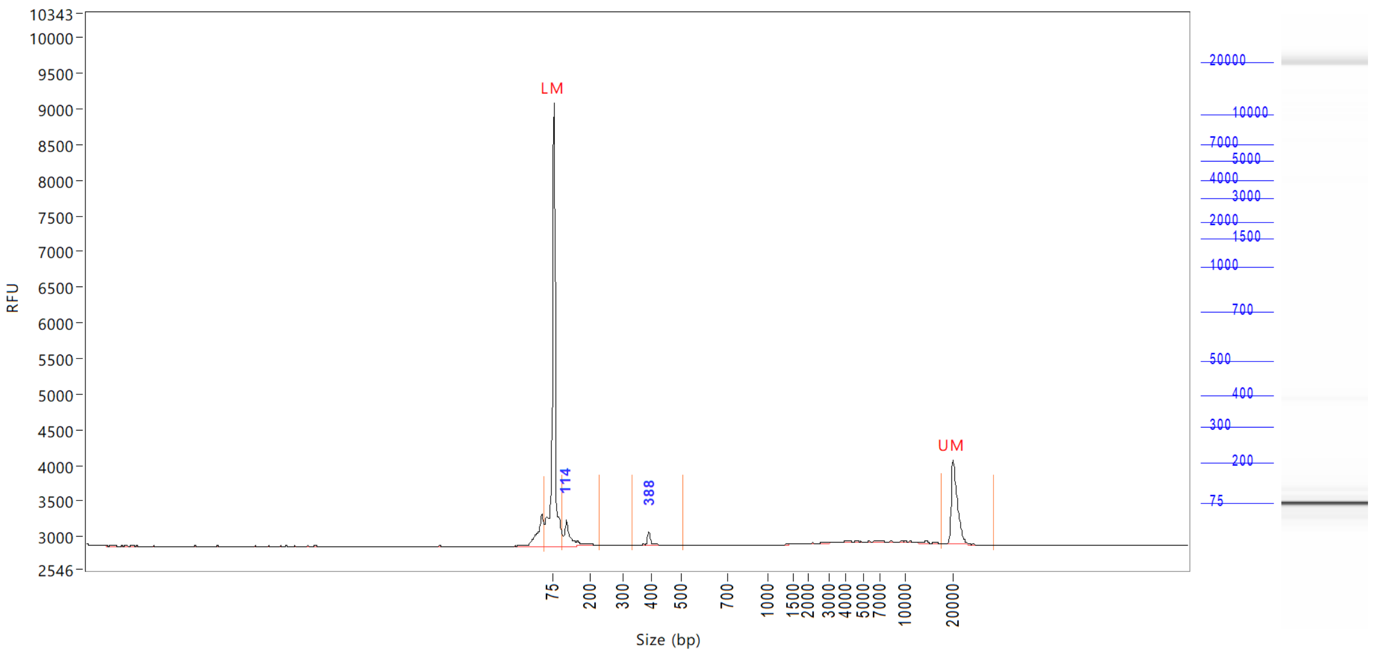

Peak	Size (bp)	Concentration (ng/uL)	From (bp)	To (bp)	Average size (bp)	CV%	RFU	Corrected peak area
1	75 (LM)	0.5000	43	105	72	13.56	6227	49.482
2	114	0.6967	105	226	133	20.39	363	5.746
3	388	0.2098	329	504	395	6.44	191	1.731
4	20000 (UM)	0.0994	17683	28705	20451	4.36	1170	9.837

TIC:	0.9066	ng/uL
TIM:	9.4818	nmole/L
Total concentration:	1.2205	ng/uL

Sample peak width (sec): 5   Sample min peak height: 50   Sample baseline V to V?: Y   Sample baseline V to V points: 3
Sample filter: Binomial   Number of points for filter: 3 Sample start region (min): 0 Sample end region (min): 30
Marker peak width (sec): 5   Marker min peak height: 200   Marker baseline V to V?: Y   Marker baseline V to V points: 3
Lower marker selection: First peak > 500 RFU   Upper marker selection: Last peak > 200 RFU
Ladder size (bp)75, 200, 300, 400, 500, 700, 1000, 1500, 2000, 3000, 4000, 5000, 7000, 10000, 20000
Quantification using: Lower Marker   Final concentration (ng/uL): 0.5000   Dilution factor: 12.0

Sample: SampG1
Well location: G1
Created: Monday, September 20, 2021 8:39:56 PM

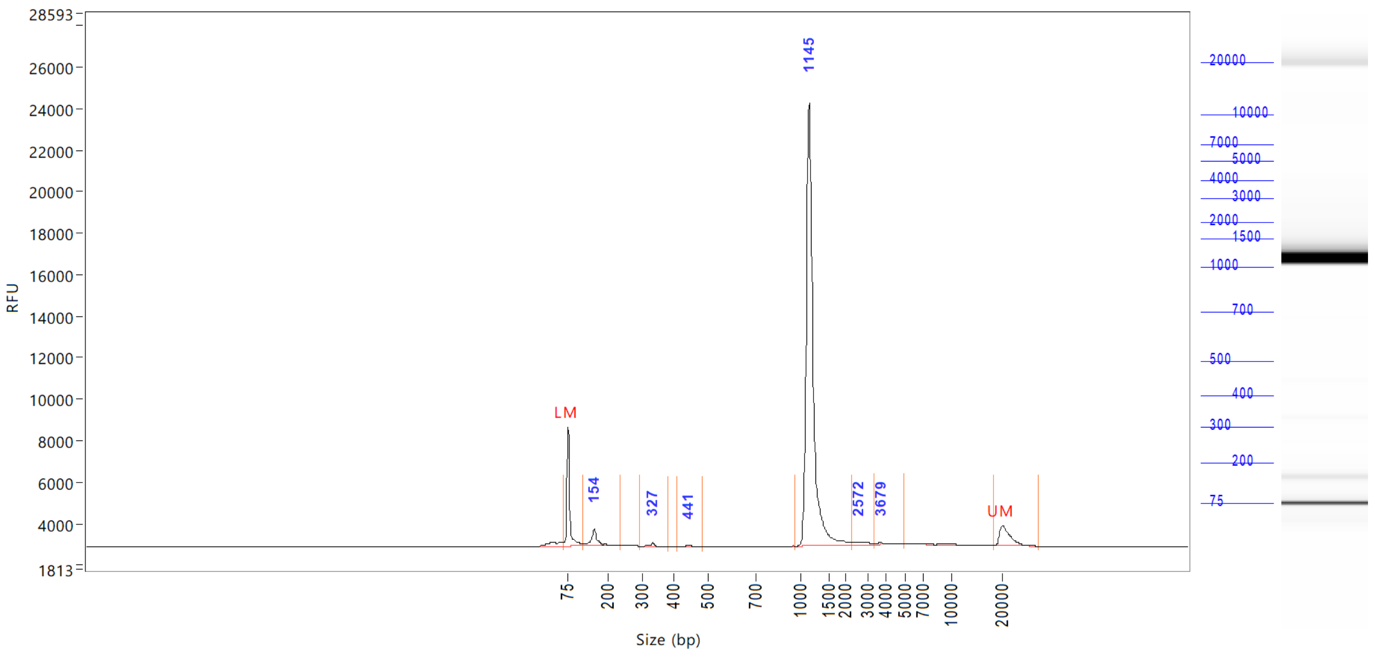

Peak	Size (bp)	Concentration (ng/uL)	From (bp)	To (bp)	Average size (bp)	CV%	RFU	Corrected peak area
1	75 (LM)	0.5000	62	124	76	12.49	5742	40.335
2	154	1.6185	124	232	155	10.74	774	10.880
3	327	0.1754	291	378	325	3.73	155	1.179
4	441	0.0883	407	485	440	2.73	64	0.594
5	1145	35.1716	965	2258	1179	11.30	21373	236.440
6	2572	0.4902	2258	3322	2696	10.71	140	3.295
7	3679	0.2134	3322	4931	3805	9.03	80	1.434
8	20000 (UM)	0.1563	18171	27133	20720	5.70	972	12.608

TIC:	37.7574	ng/uL
TIM:	67.8753	nmole/L
Total concentration:	38.0537	ng/uL

```
Sample peak width (sec): 5     Sample min peak height: 50     Sample baseline V to V?: Y     Sample baseline V to V points: 3
Sample filter: Binomial        Number of points for filter: 3  Sample start region (min): 0  Sample end region (min): 30
Marker peak width (sec): 5     Marker min peak height: 200    Marker baseline V to V?: Y     Marker baseline V to V points: 3
Lower marker selection: First peak > 500 RFU                  Upper marker selection: Last peak > 200 RFU
Ladder size (bp)75, 200, 300, 400, 500, 700, 1000, 1500, 2000, 3000, 4000, 5000, 7000, 10000, 20000
Quantification using: Lower Marker          Final concentration (ng/uL): 0.5000          Dilution factor: 12.0
```

Sample: SampH1
Well location: H1
Created: Monday, September 20, 2021 8:39:56 PM

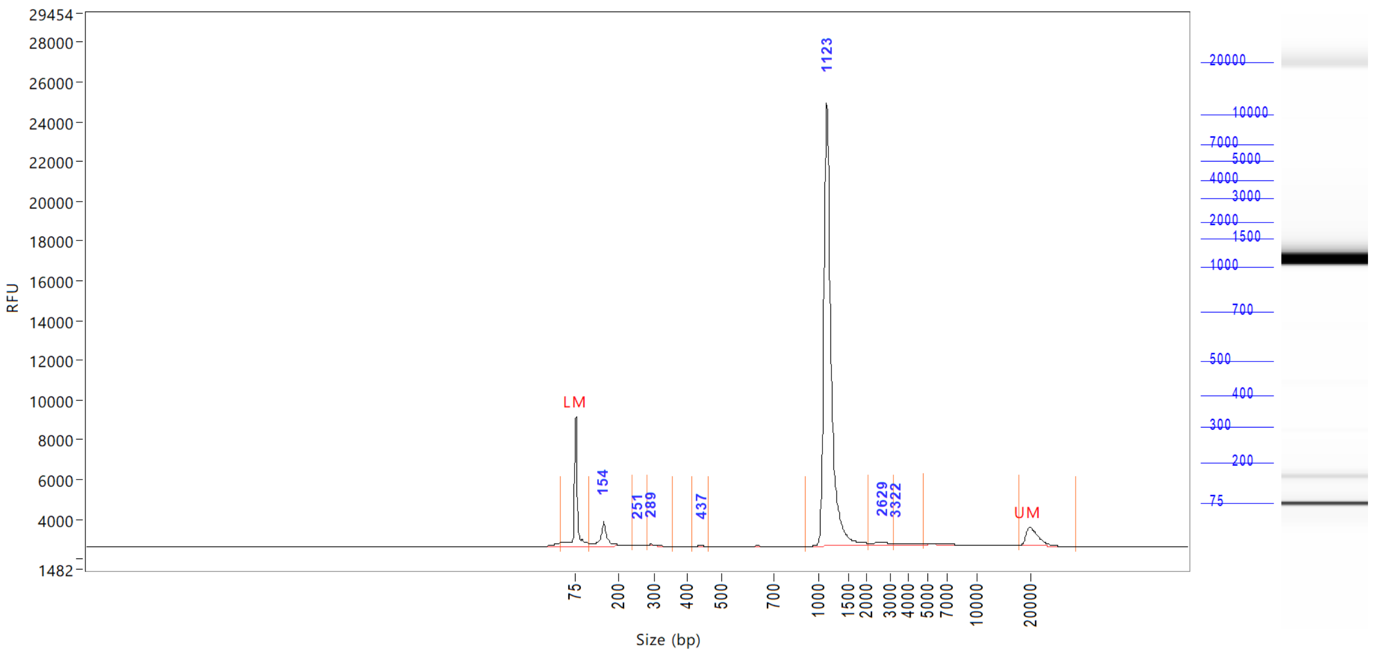

Peak	Size (bp)	Concentration (ng/uL)	From (bp)	To (bp)	Average size (bp)	CV%	RFU	Corrected peak area
1	75 (LM)	0.5000	31	116	72	16.84	6507	50.861
2	154	2.2210	116	239	158	14.16	1219	18.827
3	251	0.0801	239	278	252	3.62	52	0.679
4	289	0.0925	278	356	292	4.02	127	0.784
5	437	0.0698	412	458	435	1.70	73	0.592
6	1123	30.4095	909	2058	1156	9.80	22329	257.776
7	2629	0.4154	2058	3215	2588	12.37	123	3.521
8	3322	0.1874	3215	4793	3761	10.73	76	1.589
9	20000 (UM)	0.1363	17927	28463	20601	5.91	960	13.860

TIC:	33.4758	ng/uL
TIM:	68.0583	nmole/L
Total concentration:	33.7071	ng/uL

```
Sample peak width (sec): 5    Sample min peak height: 50    Sample baseline V to V?: Y    Sample baseline V to V points: 3
Sample filter: Binomial       Number of points for filter: 3  Sample start region (min): 0  Sample end region (min): 30
Marker peak width (sec): 5    Marker min peak height: 200   Marker baseline V to V?: Y    Marker baseline V to V points: 3
Lower marker selection: First peak > 500 RFU                Upper marker selection: Last peak > 200 RFU
Ladder size (bp)75, 200, 300, 400, 500, 700, 1000, 1500, 2000, 3000, 4000, 5000, 7000, 10000, 20000
Quantification using: Lower Marker          Final concentration (ng/uL): 0.5000          Dilution factor: 12.0
```

Sample: SampA2
Well location: A2
Created: Monday, September 20, 2021 8:39:56 PM

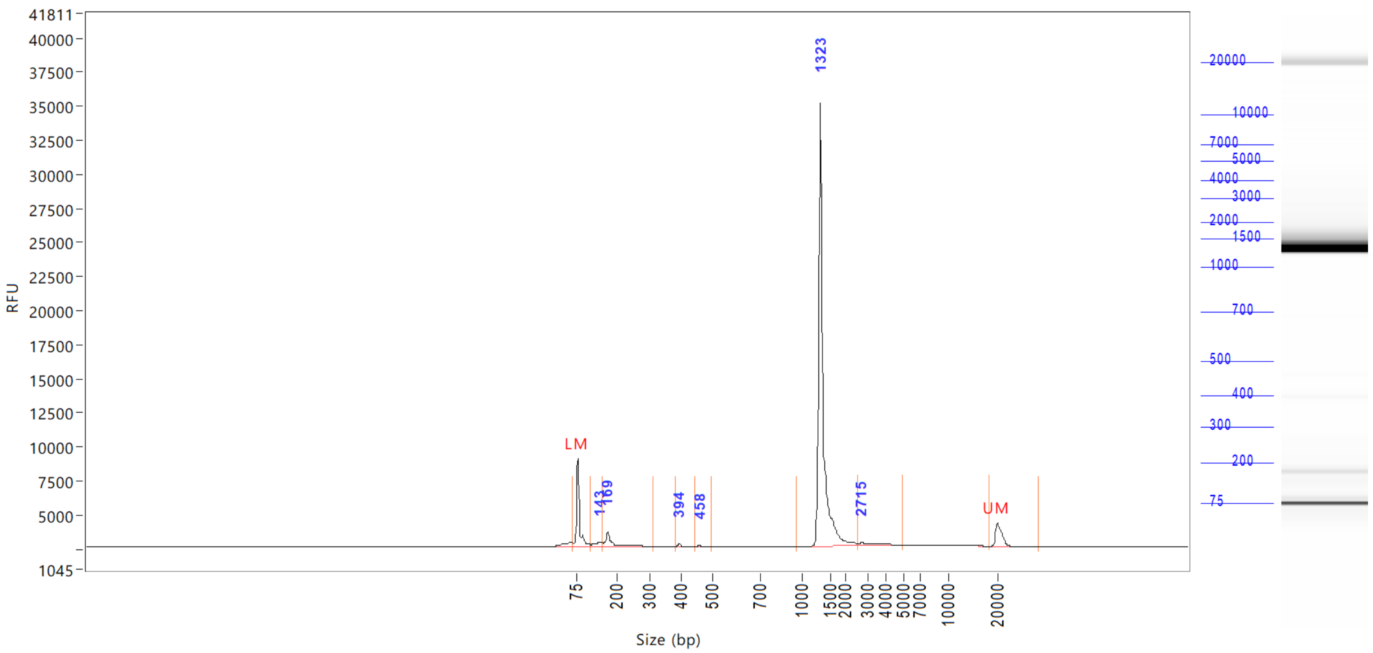

Peak	Size (bp)	Concentration (ng/uL)	From (bp)	To (bp)	Average size (bp)	CV%	RFU	Corrected peak area
1	75 (LM)	0.5000	62	114	76	11.72	6456	44.736
2	143	0.7907	114	154	136	8.48	300	5.896
3	169	2.0100	154	307	186	16.33	1085	14.986
4	394	0.1849	382	445	396	2.85	217	1.378
5	458	0.0489	445	498	458	1.79	73	0.365
6	1323	30.3867	957	2543	1370	11.30	32560	226.561
7	2715	0.7623	2543	5000	3269	17.40	218	5.684
8	20000 (UM)	0.1471	18415	28342	20320	3.78	1670	13.161

TIC:	34.1836	ng/uL
TIM:	65.1577	nmole/L
Total concentration:	34.4157	ng/uL

```
Sample peak width (sec): 5    Sample min peak height: 50    Sample baseline V to V?: Y    Sample baseline V to V points: 3
Sample filter: Binomial       Number of points for filter: 3  Sample start region (min): 0  Sample end region (min): 30
Marker peak width (sec): 5    Marker min peak height: 200   Marker baseline V to V?: Y    Marker baseline V to V points: 3
Lower marker selection: First peak > 500 RFU                Upper marker selection: Last peak > 200 RFU
Ladder size (bp)75, 200, 300, 400, 500, 700, 1000, 1500, 2000, 3000, 4000, 5000, 7000, 10000, 20000
Quantification using: Lower Marker          Final concentration (ng/uL): 0.5000          Dilution factor: 12.0
```

Sample: SampB2
Well location: B2
Created: Monday, September 20, 2021 8:39:56 PM

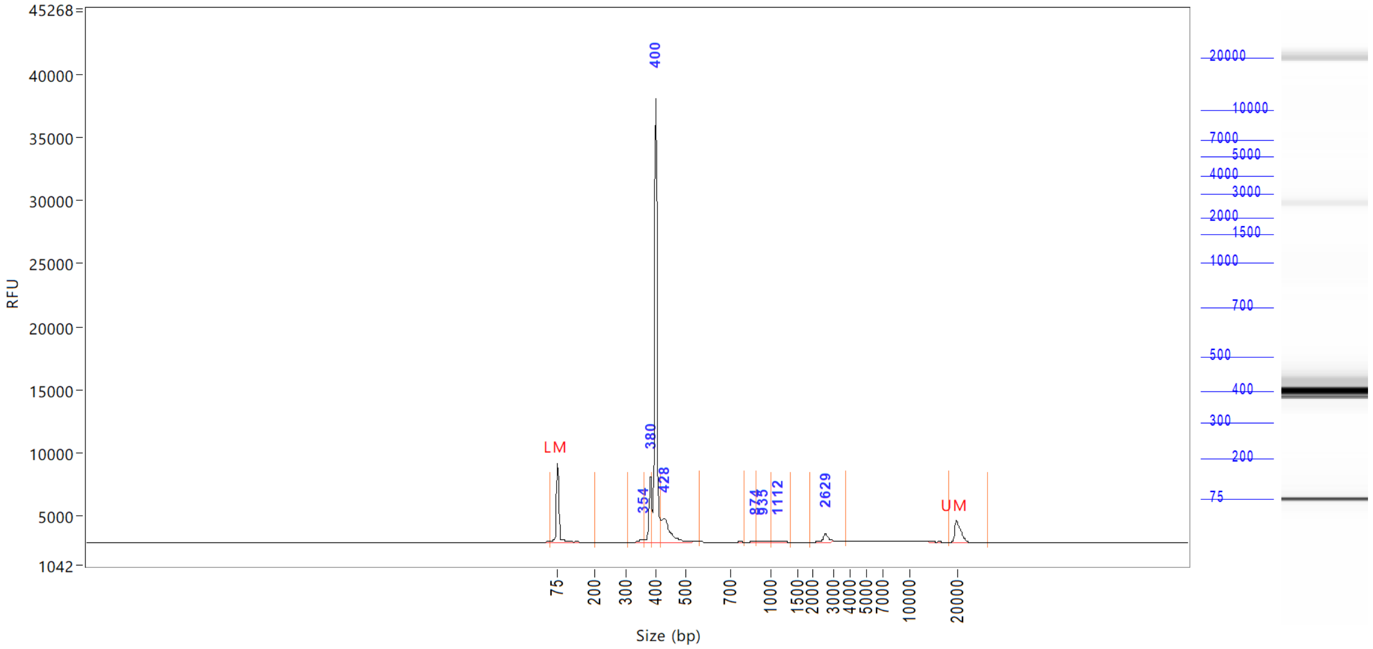

Peak	Size (bp)	Concentration (ng/uL)	From (bp)	To (bp)	Average size (bp)	CV%	RFU	Corrected peak area
1	75 (LM)	0.5000	52	200	78	24.32	6269	40.950
2	354	0.4837	303	362	346	2.97	247	3.301
3	380	4.3939	362	388	380	1.38	5232	29.988
4	400	28.1484	388	416	398	1.23	35332	192.113
5	428	4.9578	416	560	438	4.68	1869	33.837
6	874	0.0753	801	896	866	2.45	58	0.514
7	935	0.1972	896	1000	946	3.17	77	1.346
8	1112	0.2112	1000	1389	1124	7.65	86	1.441
9	2629	0.9715	1904	3786	2678	10.26	663	6.631
10	20000 (UM)	0.1663	18293	26649	20352	3.51	1690	13.616

TIC:	39.4390	ng/uL
TIM:	157.7030	nmole/L
Total concentration:	39.1972	ng/uL

```
Sample peak width (sec): 5    Sample min peak height: 50    Sample baseline V to V?: Y    Sample baseline V to V points: 3
Sample filter: Binomial       Number of points for filter: 3  Sample start region (min): 0  Sample end region (min): 30
Marker peak width (sec): 5    Marker min peak height: 200   Marker baseline V to V?: Y    Marker baseline V to V points: 3
Lower marker selection: First peak > 500 RFU                Upper marker selection: Last peak > 200 RFU
Ladder size (bp)75, 200, 300, 400, 500, 700, 1000, 1500, 2000, 3000, 4000, 5000, 7000, 10000, 20000
Quantification using: Lower Marker          Final concentration (ng/uL): 0.5000          Dilution factor: 12.0
```

Sample: SampC2
Well location: C2
Created: Monday, September 20, 2021 8:39:56 PM

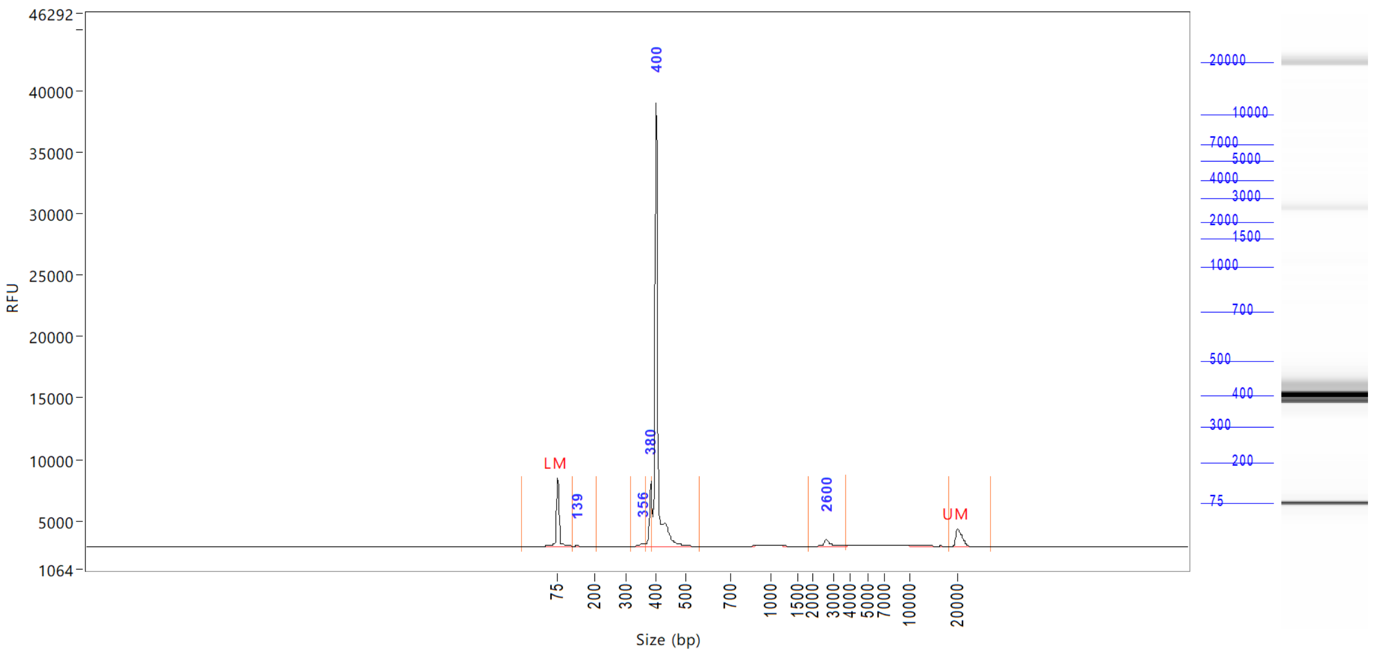

Peak	Size (bp)	Concentration (ng/uL)	From (bp)	To (bp)	Average size (bp)	CV%	RFU	Corrected peak area
1	75 (LM)	0.5000	0	128	73	19.76	5617	37.612
2	139	0.2971	128	201	157	13.27	69	1.862
3	356	0.5609	313	364	348	3.04	252	3.516
4	380	4.4450	364	388	379	1.30	5269	27.864
5	400	35.9078	388	560	404	4.03	36146	225.093
6	2600	0.9250	1827	3715	2671	9.70	619	5.798
7	20000 (UM)	0.1589	18049	26891	20369	3.98	1464	11.952

TIC:	42.1358	ng/uL
TIM:	171.8635	nmole/L
Total concentration:	42.3118	ng/uL

```
Sample peak width (sec): 5    Sample min peak height: 50    Sample baseline V to V?: Y    Sample baseline V to V points: 3
Sample filter: Binomial       Number of points for filter: 3  Sample start region (min): 0  Sample end region (min): 30
Marker peak width (sec): 5    Marker min peak height: 200   Marker baseline V to V?: Y    Marker baseline V to V points: 3
Lower marker selection: First peak > 500 RFU                Upper marker selection: Last peak > 200 RFU
Ladder size (bp)75, 200, 300, 400, 500, 700, 1000, 1500, 2000, 3000, 4000, 5000, 7000, 10000, 20000
Quantification using: Lower Marker          Final concentration (ng/uL): 0.5000          Dilution factor: 12.0
```

Sample: SampD2
Well location: D2
Created: Monday, September 20, 2021 8:39:56 PM

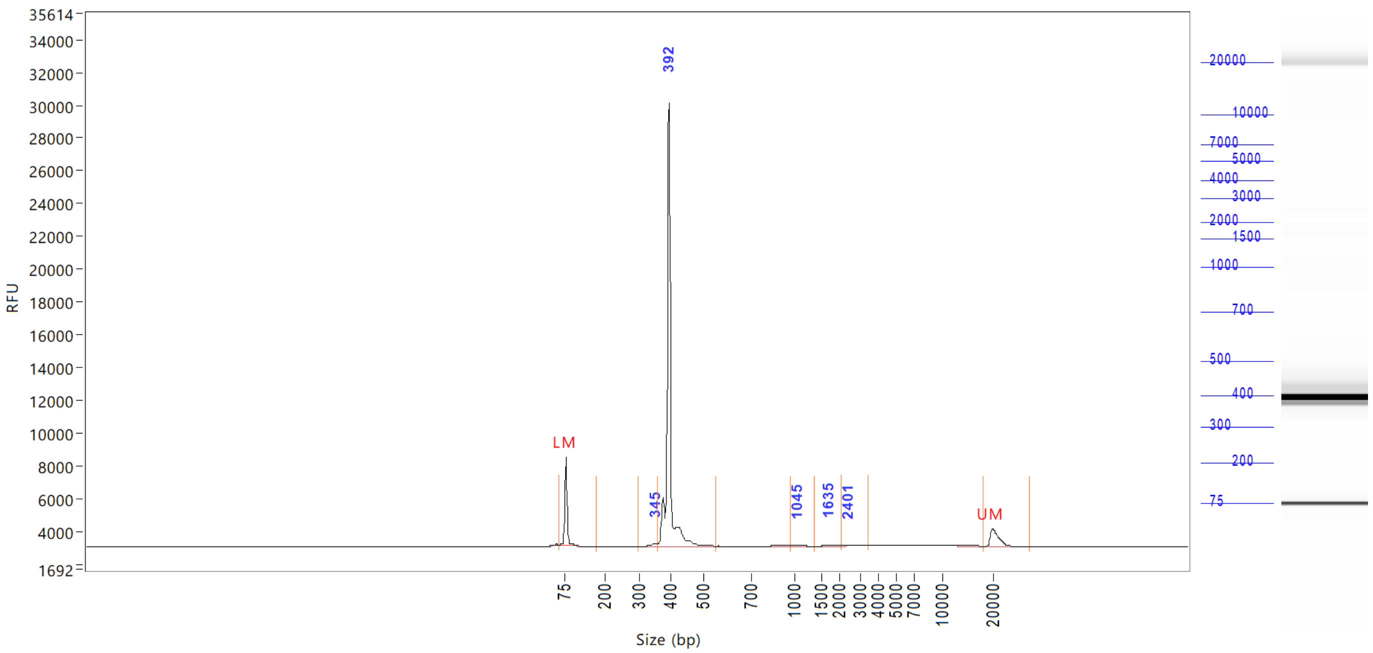

Peak	Size (bp)	Concentration (ng/uL)	From (bp)	To (bp)	Average size (bp)	CV%	RFU	Corrected peak area
1	75 (LM)	0.5000	52	171	73	8.54	5388	31.694
2	345	0.5068	293	354	338	3.49	199	2.677
3	392	36.8242	354	552	394	5.15	27117	194.519
4	1045	0.1800	974	1367	1067	7.75	56	0.951
5	1635	0.1765	1367	2087	1682	8.94	88	0.932
6	2401	0.1472	2087	3393	2527	11.13	65	0.777
7	20000 (UM)	0.1821	18049	27254	20520	4.61	1107	11.546

TIC:	37.8346	ng/uL
TIM:	156.7845	nmole/L
Total concentration:	38.5495	ng/uL

Sample peak width (sec): 5   Sample min peak height: 50   Sample baseline V to V?: Y   Sample baseline V to V points: 3
Sample filter: Binomial   Number of points for filter: 3   Sample start region (min): 0   Sample end region (min): 30
Marker peak width (sec): 5   Marker min peak height: 200   Marker baseline V to V?: Y   Marker baseline V to V points: 3
Lower marker selection: First peak > 500 RFU   Upper marker selection: Last peak > 200 RFU
Ladder size (bp)75, 200, 300, 400, 500, 700, 1000, 1500, 2000, 3000, 4000, 5000, 7000, 10000, 20000
Quantification using: Lower Marker   Final concentration (ng/uL): 0.5000   Dilution factor: 12.0

Sample: SampE2
Well location: E2
Created: Monday, September 20, 2021 8:39:56 PM

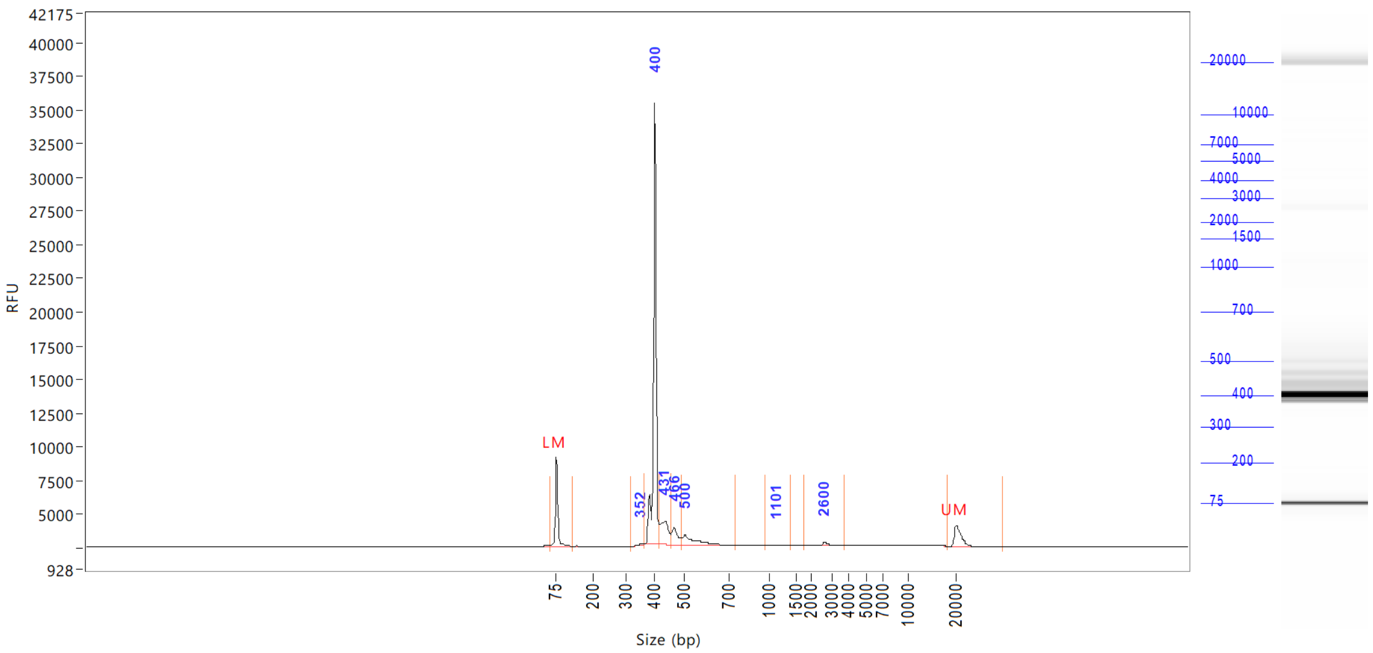

Peak	Size (bp)	Concentration (ng/uL)	From (bp)	To (bp)	Average size (bp)	CV%	RFU	Corrected peak area
1	75 (LM)	0.5000	54	129	75	11.42	6598	40.805
2	352	0.1079	317	364	346	2.60	65	0.734
3	400	27.7499	364	416	397	1.92	32809	188.722
4	431	4.1334	416	452	432	2.33	1738	28.111
5	466	2.2472	452	487	467	2.02	1273	15.283
6	500	2.7822	487	744	540	8.52	750	18.921
7	1101	0.1746	961	1400	1102	9.17	60	1.188
8	2600	0.3450	1770	3750	2743	11.10	235	2.347
9	20000 (UM)	0.1668	18293	29793	20500	4.02	1561	13.611

TIC:	37.5403	ng/uL
TIM:	148.1239	nmole/L
Total concentration:	37.5676	ng/uL

```
Sample peak width (sec): 5    Sample min peak height: 50    Sample baseline V to V?: Y    Sample baseline V to V points: 3
Sample filter: Binomial       Number of points for filter: 3  Sample start region (min): 0  Sample end region (min): 30
Marker peak width (sec): 5    Marker min peak height: 200   Marker baseline V to V?: Y    Marker baseline V to V points: 3
Lower marker selection: First peak > 500 RFU                Upper marker selection: Last peak > 200 RFU
Ladder size (bp)75, 200, 300, 400, 500, 700, 1000, 1500, 2000, 3000, 4000, 5000, 7000, 10000, 20000
Quantification using: Lower Marker          Final concentration (ng/uL): 0.5000          Dilution factor: 12.0
```

Sample: SampF2
Well location: F2
Created: Monday, September 20, 2021 8:39:56 PM

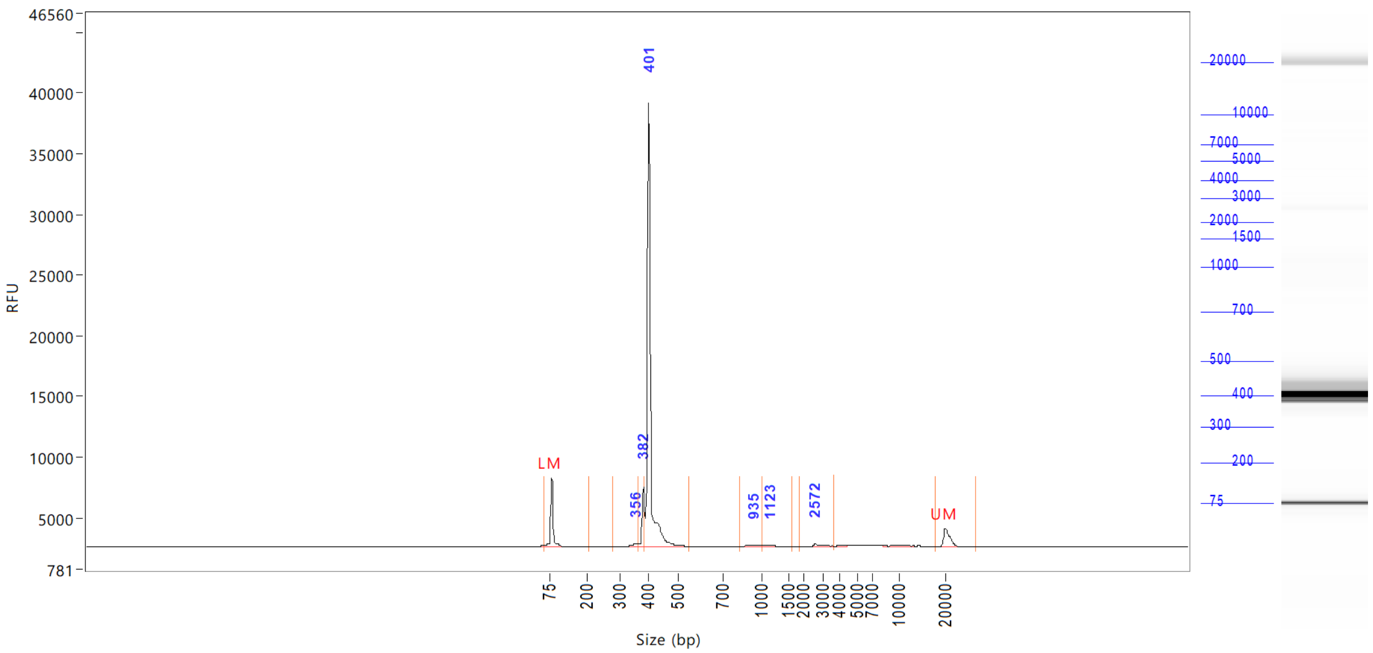

Peak	Size (bp)	Concentration (ng/uL)	From (bp)	To (bp)	Average size (bp)	CV%	RFU	Corrected peak area
1	75 (LM)	0.5000	52	200	77	17.74	5621	37.334
2	356	0.5255	278	364	348	3.28	243	3.270
3	382	4.2553	364	388	380	1.28	4927	26.478
4	401	37.3647	388	549	404	3.99	36591	232.495
5	935	0.2819	831	1000	927	4.70	79	1.754
6	1123	0.2500	1000	1616	1153	9.94	81	1.556
7	2572	0.2540	1885	3572	2659	9.81	203	1.580
8	20000 (UM)	0.1659	18049	26529	20399	3.92	1494	12.389

TIC:	42.9314	ng/uL
TIM:	174.0916	nmole/L
Total concentration:	42.7799	ng/uL

```
Sample peak width (sec): 5    Sample min peak height: 50    Sample baseline V to V?: Y    Sample baseline V to V points: 3
Sample filter: Binomial       Number of points for filter: 3  Sample start region (min): 0  Sample end region (min): 30
Marker peak width (sec): 5    Marker min peak height: 200   Marker baseline V to V?: Y    Marker baseline V to V points: 3
Lower marker selection: First peak > 500 RFU                Upper marker selection: Last peak > 200 RFU
Ladder size (bp)75, 200, 300, 400, 500, 700, 1000, 1500, 2000, 3000, 4000, 5000, 7000, 10000, 20000
Quantification using: Lower Marker          Final concentration (ng/uL): 0.5000          Dilution factor: 12.0
```

Sample: SampG2
Well location: G2
Created: Monday, September 20, 2021 8:39:56 PM

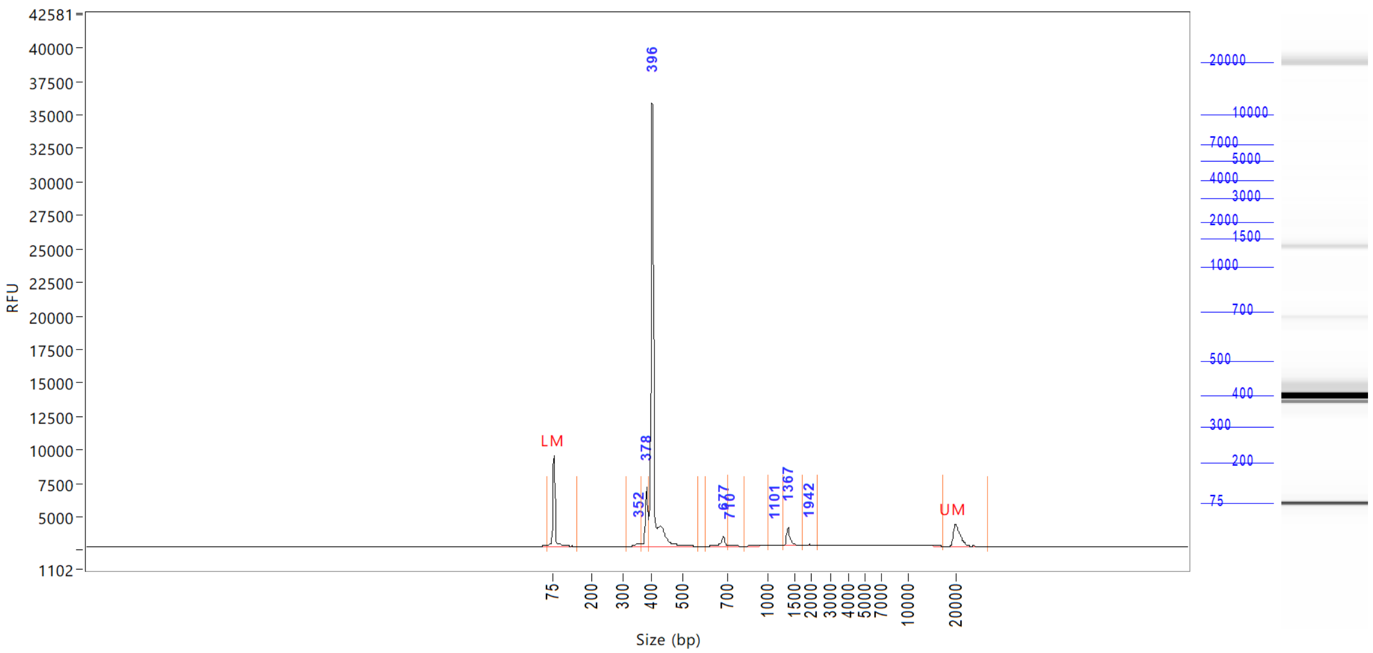

Peak	Size (bp)	Concentration (ng/uL)	From (bp)	To (bp)	Average size (bp)	CV%	RFU	Corrected peak area
1	75 (LM)	0.5000	52	150	75	15.32	6843	42.796
2	352	0.4634	307	362	346	3.05	230	3.305
3	378	3.5494	362	386	378	1.33	4411	25.316
4	396	28.3999	386	562	401	3.77	33148	202.566
5	677	0.7852	598	697	665	2.69	705	5.600
6	710	0.1356	697	814	724	3.34	85	0.967
7	1101	0.0800	1000	1267	1088	5.06	51	0.570
8	1367	1.2606	1267	1750	1385	5.34	1341	8.992
9	1942	0.1602	1750	2344	1973	7.01	96	1.142
10	20000 (UM)	0.1716	17317	26770	20419	4.12	1668	14.690

TIC:	34.8342	ng/uL
TIM:	138.1802	nmole/L
Total concentration:	35.0363	ng/uL

```
Sample peak width (sec): 5    Sample min peak height: 50    Sample baseline V to V?: Y    Sample baseline V to V points: 3
Sample filter: Binomial       Number of points for filter: 3  Sample start region (min): 0  Sample end region (min): 30
Marker peak width (sec): 5    Marker min peak height: 200   Marker baseline V to V?: Y    Marker baseline V to V points: 3
Lower marker selection: First peak > 500 RFU                Upper marker selection: Last peak > 200 RFU
Ladder size (bp)75, 200, 300, 400, 500, 700, 1000, 1500, 2000, 3000, 4000, 5000, 7000, 10000, 20000
Quantification using: Lower Marker          Final concentration (ng/uL): 0.5000          Dilution factor: 12.0
```

Sample: SampH2
Well location: H2
Created: Monday, September 20, 2021 8:39:56 PM

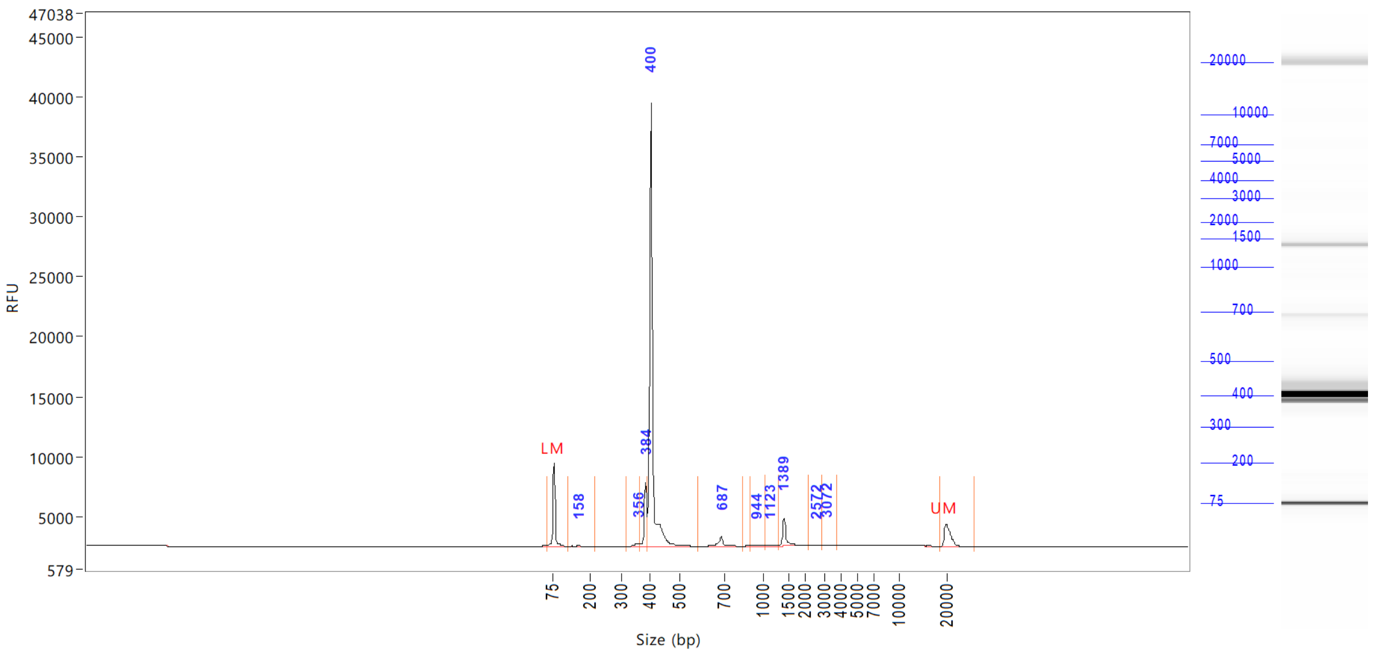

Peak	Size (bp)	Concentration (ng/uL)	From (bp)	To (bp)	Average size (bp)	CV%	RFU	Corrected peak area
1	75 (LM)	0.5000	52	126	74	11.37	6966	43.057
2	158	0.2826	126	214	153	11.42	113	2.028
3	356	0.5270	315	366	350	3.06	277	3.782
4	384	4.2325	366	390	381	1.37	5361	30.374
5	400	32.9155	390	583	405	4.02	37137	236.210
6	687	1.2152	583	840	682	4.50	847	8.721
7	944	0.1698	900	1012	953	3.14	79	1.218
8	1123	0.1306	1012	1278	1124	6.00	65	0.937
9	1389	2.0275	1278	2172	1418	6.77	2364	14.550
10	2572	0.0798	2172	2857	2591	5.93	55	0.573
11	3072	0.1498	2857	3750	3143	6.09	97	1.075
12	20000 (UM)	0.1798	18659	26166	20403	3.79	1887	15.485

TIC:	41.7303	ng/uL
TIM:	163.4077	nmole/L
Total concentration:	41.6105	ng/uL

```
Sample peak width (sec): 5     Sample min peak height: 50     Sample baseline V to V?: Y     Sample baseline V to V points: 3
Sample filter: Binomial        Number of points for filter: 3  Sample start region (min): 0  Sample end region (min): 30
Marker peak width (sec): 5     Marker min peak height: 200    Marker baseline V to V?: Y     Marker baseline V to V points: 3
Lower marker selection: First peak > 500 RFU                  Upper marker selection: Last peak > 200 RFU
Ladder size (bp)75, 200, 300, 400, 500, 700, 1000, 1500, 2000, 3000, 4000, 5000, 7000, 10000, 20000
Quantification using: Lower Marker          Final concentration (ng/uL): 0.5000          Dilution factor: 12.0
```

Sample: SampA3
Well location: A3
Created: Monday, September 20, 2021 8:39:56 PM

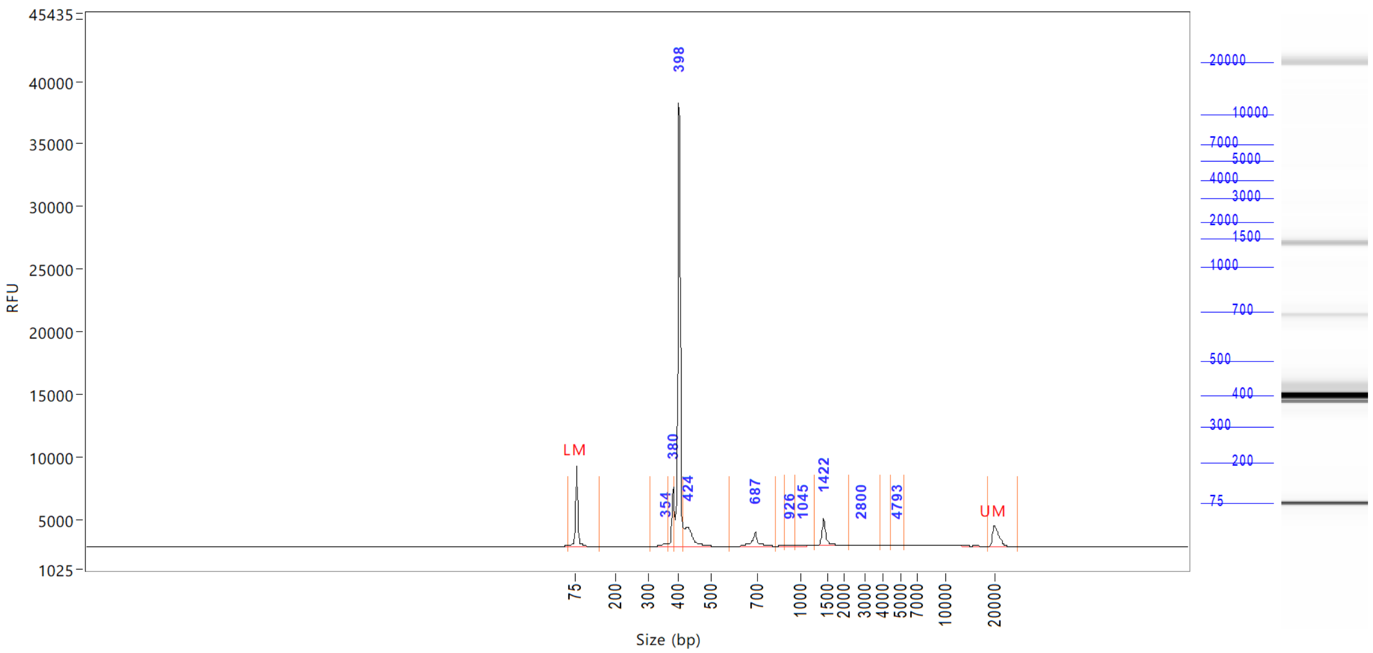

Peak	Size (bp)	Concentration (ng/uL)	From (bp)	To (bp)	Average size (bp)	CV%	RFU	Corrected peak area
1	75 (LM)	0.5000	52	147	75	13.97	6437	38.920
2	354	0.5213	303	364	347	3.20	240	3.382
3	380	3.8127	364	386	378	1.20	4808	24.732
4	398	28.4331	386	412	397	1.10	35495	184.438
5	424	4.1105	412	578	434	4.97	1517	26.664
6	687	1.8035	578	831	684	4.37	1198	11.699
7	926	0.1032	892	965	926	2.31	53	0.670
8	1045	0.1522	965	1267	1048	5.95	65	0.987
9	1422	2.2748	1267	2229	1454	7.90	2174	14.756
10	2800	0.1524	2229	3857	2834	12.06	58	0.988
11	4793	0.0847	4414	5371	4879	4.39	55	0.550
12	20000 (UM)	0.1715	18537	24715	20316	3.28	1665	13.352

TIC:	41.4486	ng/uL
TIM:	159.9397	nmole/L
Total concentration:	41.4483	ng/uL

Sample peak width (sec): 5   Sample min peak height: 50   Sample baseline V to V?: Y   Sample baseline V to V points: 3
Sample filter: Binomial   Number of points for filter: 3 Sample start region (min): 0 Sample end region (min): 30
Marker peak width (sec): 5   Marker min peak height: 200   Marker baseline V to V?: Y   Marker baseline V to V points: 3
Lower marker selection: First peak > 500 RFU   Upper marker selection: Last peak > 200 RFU
Ladder size (bp)75, 200, 300, 400, 500, 700, 1000, 1500, 2000, 3000, 4000, 5000, 7000, 10000, 20000
Quantification using: Lower Marker   Final concentration (ng/uL): 0.5000   Dilution factor: 12.0

Sample: SampB3
Well location: B3
Created: Monday, September 20, 2021 8:39:56 PM

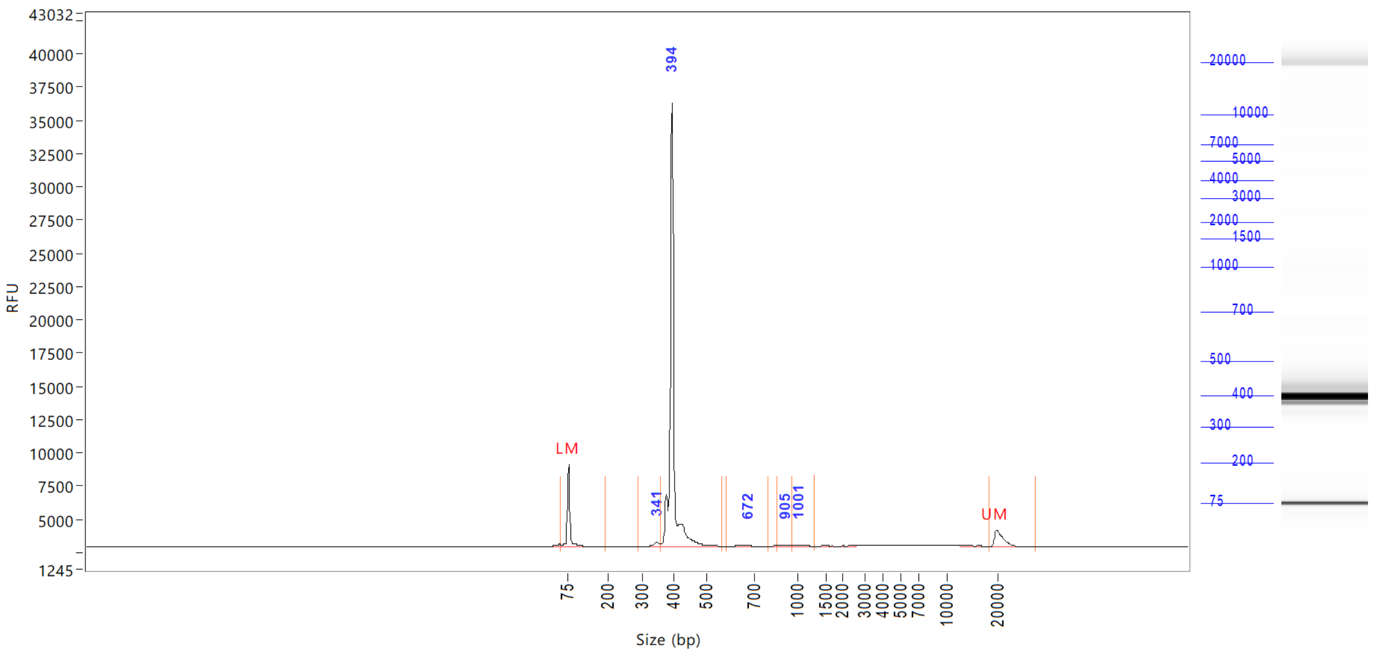

Peak	Size (bp)	Concentration (ng/uL)	From (bp)	To (bp)	Average size (bp)	CV%	RFU	Corrected peak area
1	75 (LM)	0.5000	52	189	75	16.47	6140	39.061
2	341	0.6685	287	358	341	3.22	349	4.352
3	394	39.7139	358	567	396	4.79	33394	258.541
4	672	0.2519	583	796	662	4.79	103	1.640
5	905	0.1597	861	957	909	3.00	60	1.040
6	1001	0.1985	957	1289	1041	7.14	69	1.292
7	20000 (UM)	0.1666	18537	27496	20562	4.81	1215	13.013

TIC:	40.9925	ng/uL
TIM:	169.4557	nmole/L
Total concentration:	41.4694	ng/uL

```
Sample peak width (sec): 5    Sample min peak height: 50    Sample baseline V to V?: Y    Sample baseline V to V points: 3
Sample filter: Binomial       Number of points for filter: 3  Sample start region (min): 0  Sample end region (min): 30
Marker peak width (sec): 5    Marker min peak height: 200   Marker baseline V to V?: Y    Marker baseline V to V points: 3
Lower marker selection: First peak > 500 RFU                Upper marker selection: Last peak > 200 RFU
Ladder size (bp)75, 200, 300, 400, 500, 700, 1000, 1500, 2000, 3000, 4000, 5000, 7000, 10000, 20000
Quantification using: Lower Marker          Final concentration (ng/uL): 0.5000          Dilution factor: 12.0
```

Sample: SampC3
Well location: C3
Created: Monday, September 20, 2021 8:39:56 PM

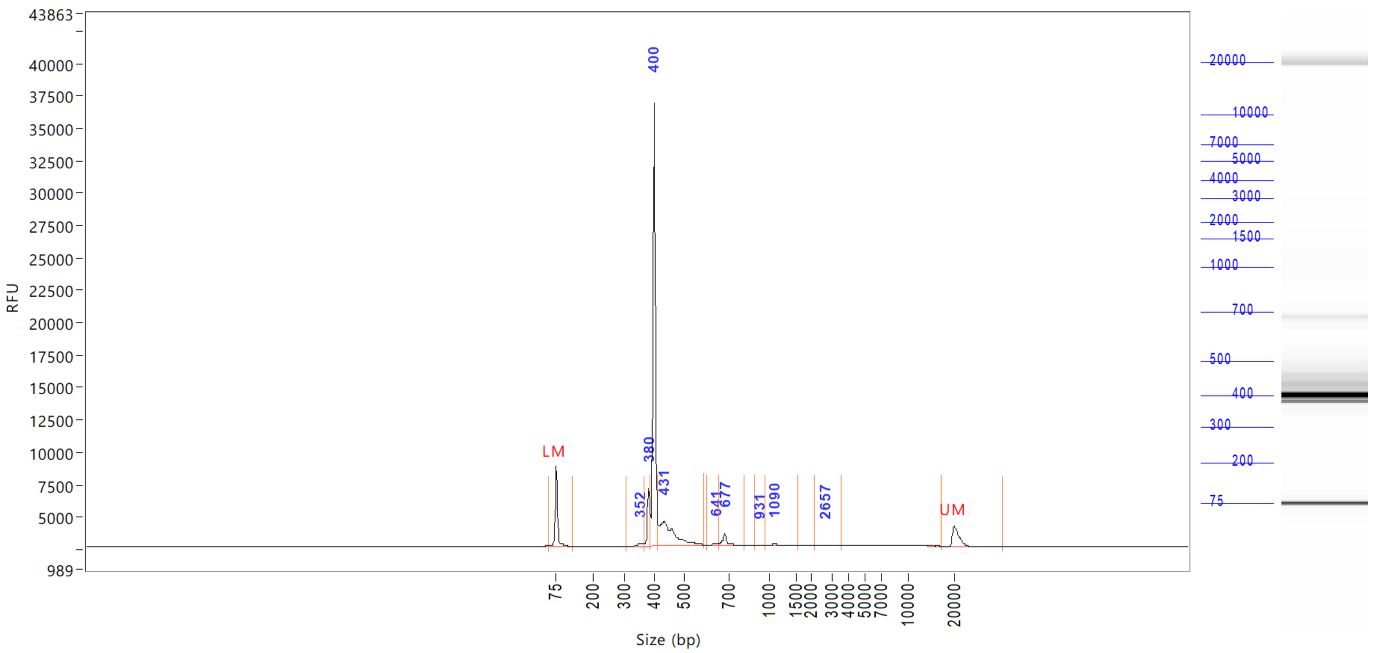

Peak	Size (bp)	Concentration (ng/uL)	From (bp)	To (bp)	Average size (bp)	CV%	RFU	Corrected peak area
1	75 (LM)	0.5000	52	128	74	11.08	6178	37.826
2	352	0.4704	305	366	349	3.16	198	2.965
3	380	3.8109	366	386	378	1.18	4394	24.025
4	400	28.6027	386	412	397	1.11	34223	180.323
5	431	8.5679	412	588	450	6.92	1869	54.015
6	641	0.1873	600	652	637	1.42	139	1.181
7	677	1.1471	652	814	681	3.02	936	7.232
8	931	0.1376	887	965	929	2.42	65	0.868
9	1090	0.4436	965	1559	1175	14.03	101	2.797
10	2657	0.1141	2172	3572	2775	8.35	56	0.719
11	20000 (UM)	0.1772	17195	30277	20352	4.18	1580	13.406

TIC:	43.4815	ng/uL
TIM:	172.8588	nmole/L
Total concentration:	43.3438	ng/uL

```
Sample peak width (sec): 5    Sample min peak height: 50    Sample baseline V to V?: Y    Sample baseline V to V points: 3
Sample filter: Binomial       Number of points for filter: 3  Sample start region (min): 0  Sample end region (min): 30
Marker peak width (sec): 5    Marker min peak height: 200   Marker baseline V to V?: Y    Marker baseline V to V points: 3
Lower marker selection: First peak > 500 RFU                Upper marker selection: Last peak > 200 RFU
Ladder size (bp)75, 200, 300, 400, 500, 700, 1000, 1500, 2000, 3000, 4000, 5000, 7000, 10000, 20000
Quantification using: Lower Marker          Final concentration (ng/uL): 0.5000          Dilution factor: 12.0
```

Sample: SampD3
Well location: D3
Created: Monday, September 20, 2021 8:39:56 PM

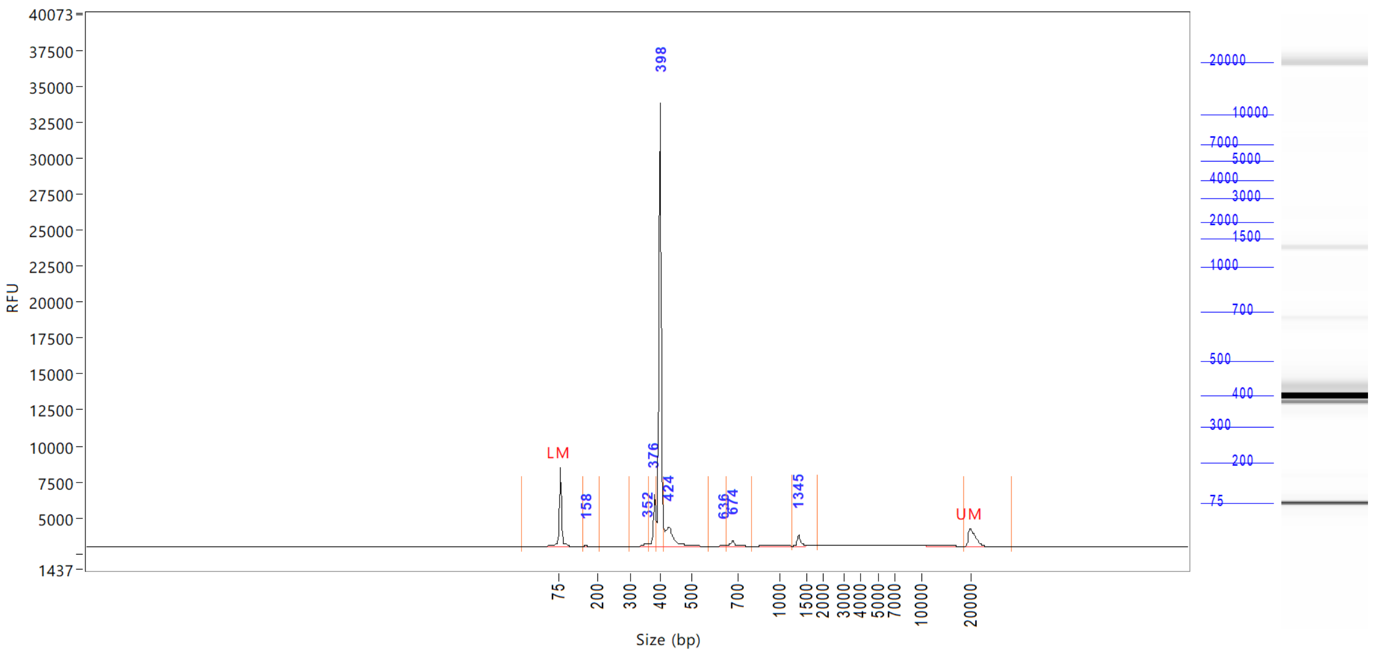

Peak	Size (bp)	Concentration (ng/uL)	From (bp)	To (bp)	Average size (bp)	CV%	RFU	Corrected peak area
1	75 (LM)	0.5000	0	148	73	21.57	5481	36.581
2	158	0.1612	148	205	164	8.18	85	0.983
3	352	0.4399	296	360	344	3.28	190	2.682
4	376	3.4030	360	386	377	1.47	3599	20.747
5	398	25.8610	386	411	395	1.05	30883	157.669
6	424	3.8293	411	567	434	5.31	1315	23.347
7	636	0.1302	567	646	630	2.15	79	0.794
8	674	0.5655	646	796	678	3.39	409	3.448
9	1345	0.8300	1234	1827	1365	5.37	808	5.060
10	20000 (UM)	0.1577	18659	28342	20433	4.33	1266	11.538

TIC:	35.2199	ng/uL
TIM:	143.5183	nmole/L
Total concentration:	35.4065	ng/uL

Sample peak width (sec): 5   Sample min peak height: 50   Sample baseline V to V?: Y   Sample baseline V to V points: 3
Sample filter: Binomial   Number of points for filter: 3   Sample start region (min): 0   Sample end region (min): 30
Marker peak width (sec): 5   Marker min peak height: 200   Marker baseline V to V?: Y   Marker baseline V to V points: 3
Lower marker selection: First peak > 500 RFU   Upper marker selection: Last peak > 200 RFU
Ladder size (bp)75, 200, 300, 400, 500, 700, 1000, 1500, 2000, 3000, 4000, 5000, 7000, 10000, 20000
Quantification using: Lower Marker   Final concentration (ng/uL): 0.5000   Dilution factor: 12.0

Sample: SampE3
Well location: E3
Created: Monday, September 20, 2021 8:39:56 PM

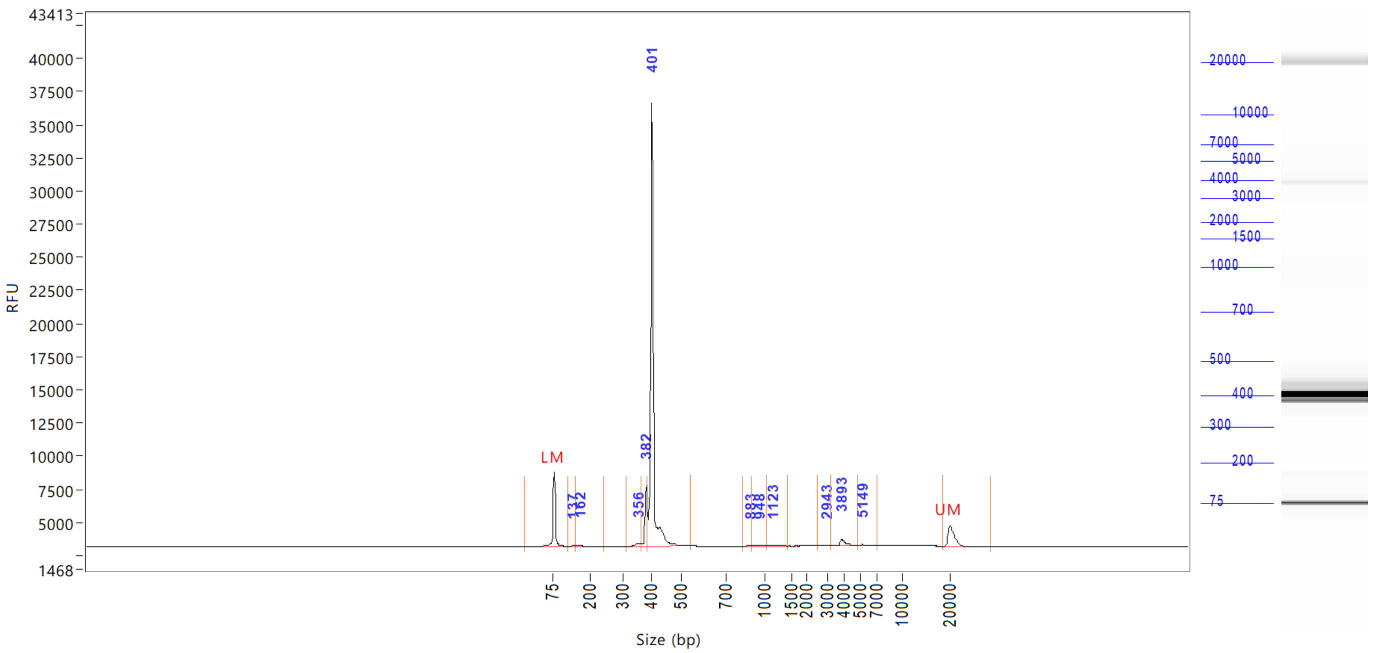

Peak	Size (bp)	Concentration (ng/uL)	From (bp)	To (bp)	Average size (bp)	CV%	RFU	Corrected peak area
1	75 (LM)	0.5000	0	126	73	16.29	5682	37.412
2	137	0.1179	126	147	137	4.48	66	0.735
3	162	0.3660	147	242	173	13.17	165	2.282
4	356	0.4928	313	368	350	3.19	209	3.073
5	382	3.8779	368	388	380	1.15	4581	24.180
6	401	31.4619	388	542	404	3.39	33504	196.176
7	883	0.0789	827	900	873	2.03	52	0.492
8	948	0.1869	900	1001	950	3.11	73	1.166
9	1123	0.1961	1001	1422	1142	8.25	73	1.223
10	2943	0.1153	2515	3144	2865	5.72	64	0.719
11	3893	0.8199	3144	4862	3992	8.86	528	5.112
12	5149	0.2048	4862	7068	5681	11.85	84	1.277
13	20000 (UM)	0.1679	18415	28584	20456	3.99	1498	12.564

TIC:	37.9184	ng/uL
TIM:	153.3462	nmole/L
Total concentration:	37.8119	ng/uL

Sample peak width (sec): 5   Sample min peak height: 50   Sample baseline V to V?: Y   Sample baseline V to V points: 3
Sample filter: Binomial   Number of points for filter: 3 Sample start region (min): 0 Sample end region (min): 30
Marker peak width (sec): 5   Marker min peak height: 200   Marker baseline V to V?: Y   Marker baseline V to V points: 3
Lower marker selection: First peak > 500 RFU   Upper marker selection: Last peak > 200 RFU
Ladder size (bp)75, 200, 300, 400, 500, 700, 1000, 1500, 2000, 3000, 4000, 5000, 7000, 10000, 20000
Quantification using: Lower Marker   Final concentration (ng/uL): 0.5000   Dilution factor: 12.0

Sample: SampF3
Well location: F3
Created: Monday, September 20, 2021 8:39:56 PM

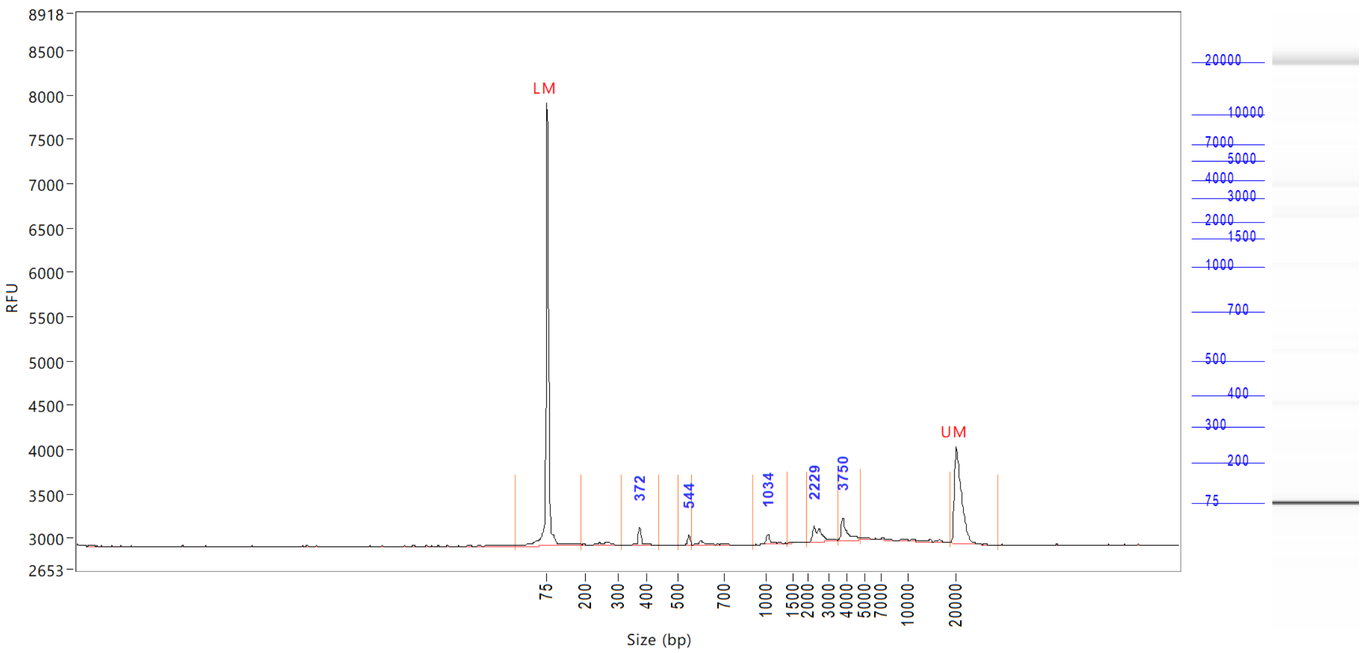

Peak	Size (bp)	Concentration (ng/uL)	From (bp)	To (bp)	Average size (bp)	CV%	RFU	Corrected peak area
1	75 (LM)	0.5000	0	189	74	26.09	5001	33.426
2	372	0.3098	313	433	373	4.42	212	1.726
3	544	0.1213	498	560	539	1.84	124	0.676
4	1034	0.1472	905	1389	1056	7.71	111	0.820
5	2229	0.4230	1942	3465	2467	11.92	177	2.356
6	3750	0.4284	3465	4793	3957	7.80	258	2.387
7	20000 (UM)	0.1535	18781	28826	20583	4.54	1086	10.261

TIC:	1.4297	ng/uL
TIM:	2.4267	nmole/L
Total concentration:	2.2014	ng/uL

```
Sample peak width (sec): 5     Sample min peak height: 50     Sample baseline V to V?: Y     Sample baseline V to V points: 3
Sample filter: Binomial        Number of points for filter: 3  Sample start region (min): 0  Sample end region (min): 30
Marker peak width (sec): 5     Marker min peak height: 200    Marker baseline V to V?: Y     Marker baseline V to V points: 3
Lower marker selection: First peak > 500 RFU                  Upper marker selection: Last peak > 200 RFU
Ladder size (bp)75, 200, 300, 400, 500, 700, 1000, 1500, 2000, 3000, 4000, 5000, 7000, 10000, 20000
Quantification using: Lower Marker          Final concentration (ng/uL): 0.5000          Dilution factor: 12.0
```

Sample: SampG3
Well location: G3
Created: Monday, September 20, 2021 8:39:56 PM

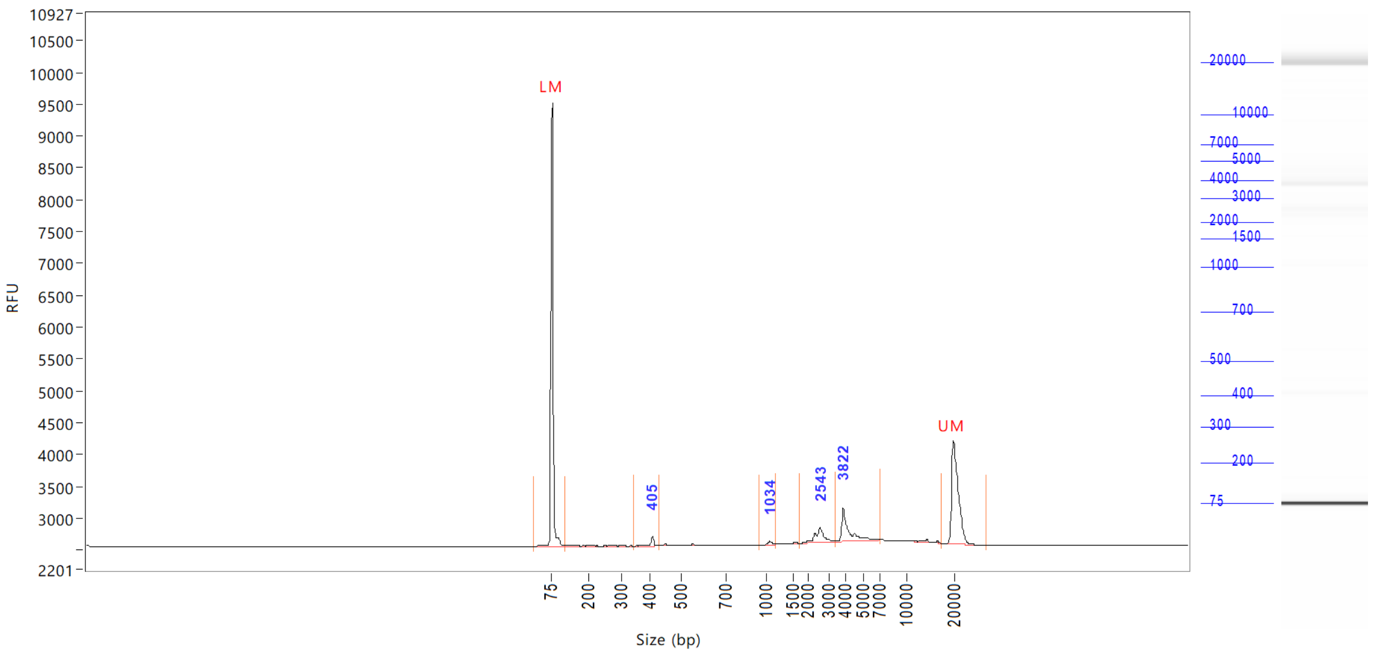

Peak	Size (bp)	Concentration (ng/uL)	From (bp)	To (bp)	Average size (bp)	CV%	RFU	Corrected peak area
1	75 (LM)	0.5000	16	116	73	8.45	6972	40.281
2	405	0.1582	341	426	399	3.34	144	1.062
3	1034	0.0664	948	1178	1050	4.52	59	0.446
4	2543	0.4868	1731	3358	2488	12.00	241	3.268
5	3822	0.8159	3358	7068	4177	13.43	538	5.477
6	20000 (UM)	0.1817	17561	27012	20425	3.86	1625	14.639

TIC:	1.5273	ng/uL
TIM:	1.4000	nmole/L
Total concentration:	1.9233	ng/uL

Sample peak width (sec): 5   Sample min peak height: 50   Sample baseline V to V?: Y   Sample baseline V to V points: 3
Sample filter: Binomial   Number of points for filter: 3   Sample start region (min): 0   Sample end region (min): 30
Marker peak width (sec): 5   Marker min peak height: 200   Marker baseline V to V?: Y   Marker baseline V to V points: 3
Lower marker selection: First peak > 500 RFU   Upper marker selection: Last peak > 200 RFU
Ladder size (bp)75, 200, 300, 400, 500, 700, 1000, 1500, 2000, 3000, 4000, 5000, 7000, 10000, 20000
Quantification using: Lower Marker   Final concentration (ng/uL): 0.5000   Dilution factor: 12.0

Sample: SampH3
Well location: H3
Created: Monday, September 20, 2021 8:39:56 PM

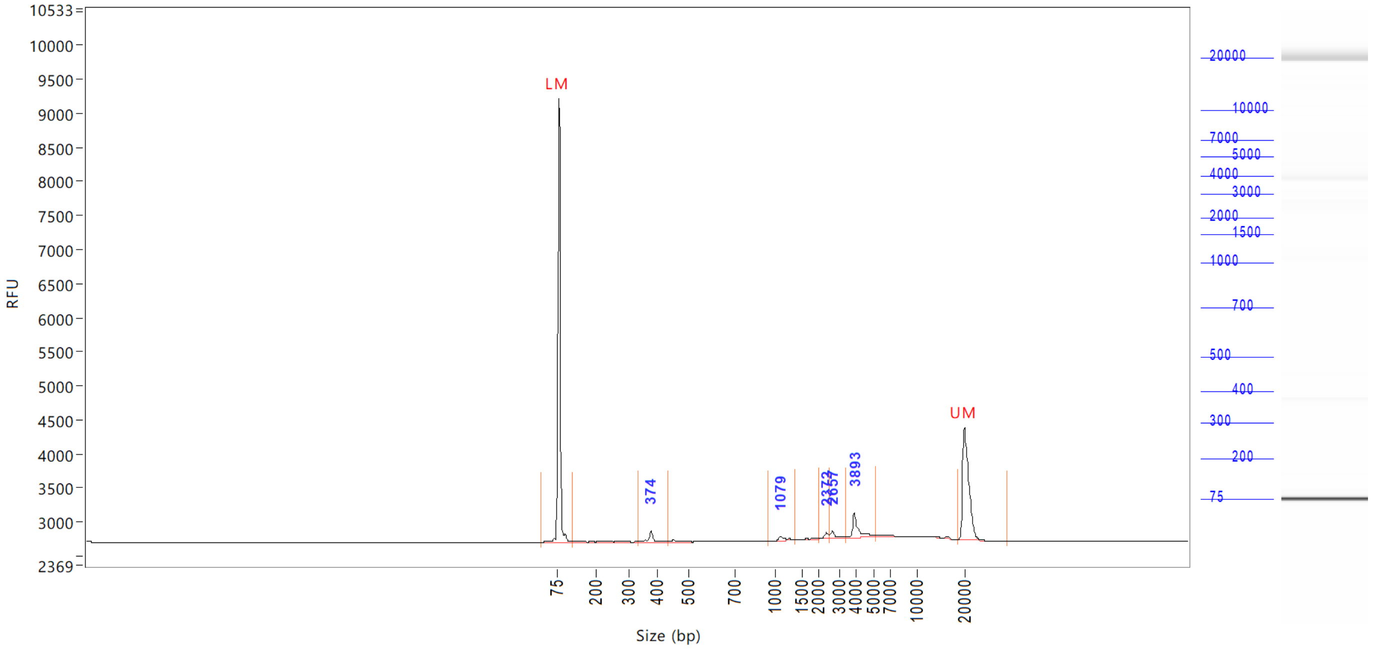

Peak	Size (bp)	Concentration (ng/uL)	From (bp)	To (bp)	Average size (bp)	CV%	RFU	Corrected peak area
1	75 (LM)	0.5000	22	118	74	7.48	6522	36.441
2	374	0.2370	329	430	374	4.66	173	1.440
3	1079	0.1203	948	1367	1130	8.12	62	0.731
4	2372	0.0934	2030	2515	2348	4.54	83	0.567
5	2657	0.1456	2515	3358	2769	7.57	103	0.884
6	3893	0.5356	3358	5297	4119	9.33	356	3.253
7	20000 (UM)	0.1900	18537	29068	20370	4.12	1638	13.848

TIC:	1.1319	ng/uL
TIM:	1.5840	nmole/L
Total concentration:	1.6280	ng/uL

Sample peak width (sec): 5 Sample min peak height: 50 Sample baseline V to V?: Y Sample baseline V to V points: 3
Sample filter: Binomial Number of points for filter: 3 Sample start region (min): 0 Sample end region (min): 30
Marker peak width (sec): 5 Marker min peak height: 200 Marker baseline V to V?: Y Marker baseline V to V points: 3
Lower marker selection: First peak > 500 RFU Upper marker selection: Last peak > 200 RFU
Ladder size (bp)75, 200, 300, 400, 500, 700, 1000, 1500, 2000, 3000, 4000, 5000, 7000, 10000, 20000
Quantification using: Lower Marker Final concentration (ng/uL): 0.5000 Dilution factor: 12.0

Sample: SampA4
Well location: A4
Created: Monday, September 20, 2021 8:39:56 PM

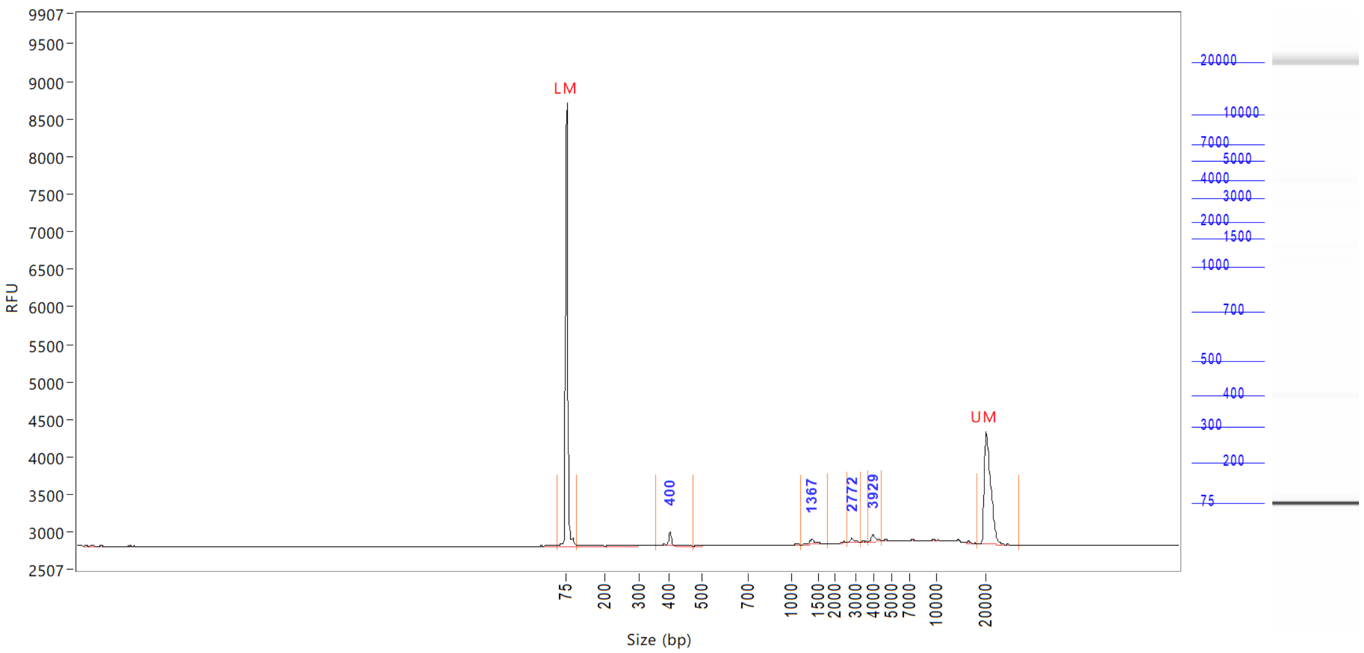

Peak	Size (bp)	Concentration (ng/uL)	From (bp)	To (bp)	Average size (bp)	CV%	RFU	Corrected peak area
1	75 (LM)	0.5000	47	108	73	6.26	5910	32.670
2	400	0.2678	354	473	400	4.21	181	1.458
3	1367	0.1338	1167	1770	1392	7.28	73	0.728
4	2772	0.0673	2572	3251	2813	4.71	54	0.366
5	3929	0.1167	3643	4449	4009	4.12	92	0.635
6	20000 (UM)	0.1907	18293	26529	20410	3.77	1501	12.459

TIC:	0.5856	ng/uL
TIM:	1.3471	nmole/L
Total concentration:	1.1369	ng/uL

Sample peak width (sec): 5   Sample min peak height: 50   Sample baseline V to V?: Y   Sample baseline V to V points: 3
Sample filter: Binomial   Number of points for filter: 3   Sample start region (min): 0   Sample end region (min): 30
Marker peak width (sec): 5   Marker min peak height: 200   Marker baseline V to V?: Y   Marker baseline V to V points: 3
Lower marker selection: First peak > 500 RFU   Upper marker selection: Last peak > 200 RFU
Ladder size (bp)75, 200, 300, 400, 500, 700, 1000, 1500, 2000, 3000, 4000, 5000, 7000, 10000, 20000
Quantification using: Lower Marker   Final concentration (ng/uL): 0.5000   Dilution factor: 12.0

Sample: SampB4
Well location: B4
Created: Monday, September 20, 2021 8:39:56 PM

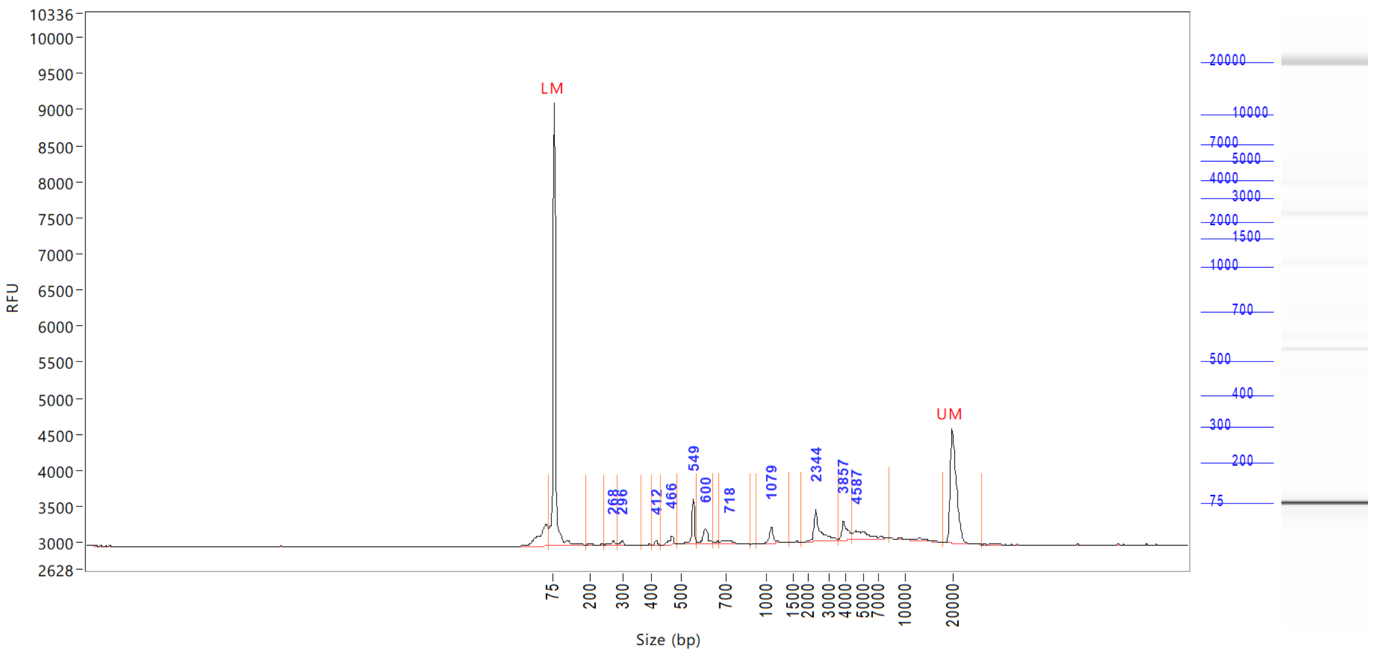

Peak	Size (bp)	Concentration (ng/uL)	From (bp)	To (bp)	Average size (bp)	CV%	RFU	Corrected peak area
1	75 (LM)	0.5000	56	183	77	17.55	6152	41.168
2	268	0.0877	239	282	263	3.89	58	0.602
3	296	0.0792	282	364	298	4.68	68	0.544
4	412	0.0526	400	430	411	1.12	62	0.361
5	466	0.1557	430	483	460	1.95	121	1.068
6	549	0.5163	483	565	543	2.20	639	3.542
7	600	0.3421	565	639	601	2.53	214	2.348
8	718	0.2023	667	874	713	5.32	60	1.388
9	1079	0.2560	913	1411	1085	6.79	230	1.757
10	2344	0.6389	1770	3572	2514	13.22	454	4.384
11	3857	0.3507	3572	4380	3980	4.85	268	2.407
12	4587	0.4004	4380	8067	5257	15.65	122	2.747
13	20000 (UM)	0.1594	18171	26408	20317	3.65	1585	13.128

TIC:	3.0820	ng/uL
TIM:	5.7992	nmole/L
Total concentration:	3.3094	ng/uL

```
Sample peak width (sec): 5    Sample min peak height: 50    Sample baseline V to V?: Y    Sample baseline V to V points: 3
Sample filter: Binomial       Number of points for filter: 3  Sample start region (min): 0  Sample end region (min): 30
Marker peak width (sec): 5    Marker min peak height: 200   Marker baseline V to V?: Y    Marker baseline V to V points: 3
Lower marker selection: First peak > 500 RFU                Upper marker selection: Last peak > 200 RFU
Ladder size (bp)75, 200, 300, 400, 500, 700, 1000, 1500, 2000, 3000, 4000, 5000, 7000, 10000, 20000
Quantification using: Lower Marker          Final concentration (ng/uL): 0.5000          Dilution factor: 12.0
```

Sample: SampC4
Well location: C4
Created: Monday, September 20, 2021 8:39:56 PM

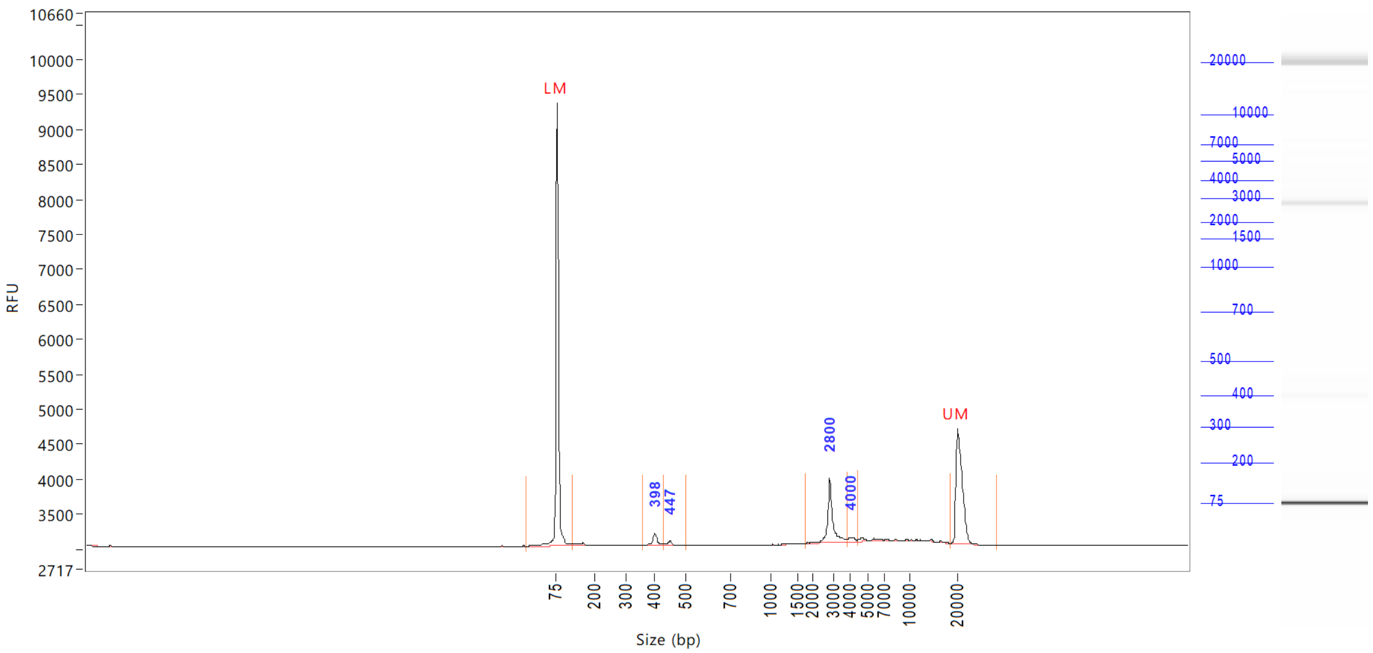

Peak	Size (bp)	Concentration (ng/uL)	From (bp)	To (bp)	Average size (bp)	CV%	RFU	Corrected peak area
1	75 (LM)	0.5000	0	128	73	13.83	6341	38.253
2	398	0.2964	354	428	396	3.19	183	1.890
3	447	0.1220	428	501	451	3.74	68	0.778
4	2800	1.1014	1731	3786	2842	9.95	920	7.022
5	4000	0.1040	3786	4380	4040	3.84	66	0.663
6	20000 (UM)	0.1789	18415	28100	20388	3.79	1638	13.686

TIC:	1.6238	ng/uL
TIM:	2.3568	nmole/L
Total concentration:	2.1639	ng/uL

```
Sample peak width (sec): 5    Sample min peak height: 50    Sample baseline V to V?: Y    Sample baseline V to V points: 3
Sample filter: Binomial       Number of points for filter: 3  Sample start region (min): 0  Sample end region (min): 30
Marker peak width (sec): 5    Marker min peak height: 200   Marker baseline V to V?: Y    Marker baseline V to V points: 3
Lower marker selection: First peak > 500 RFU                Upper marker selection: Last peak > 200 RFU
Ladder size (bp)75, 200, 300, 400, 500, 700, 1000, 1500, 2000, 3000, 4000, 5000, 7000, 10000, 20000
Quantification using: Lower Marker          Final concentration (ng/uL): 0.5000          Dilution factor: 12.0
```

Sample: SampD4
Well location: D4
Created: Monday, September 20, 2021 8:39:56 PM

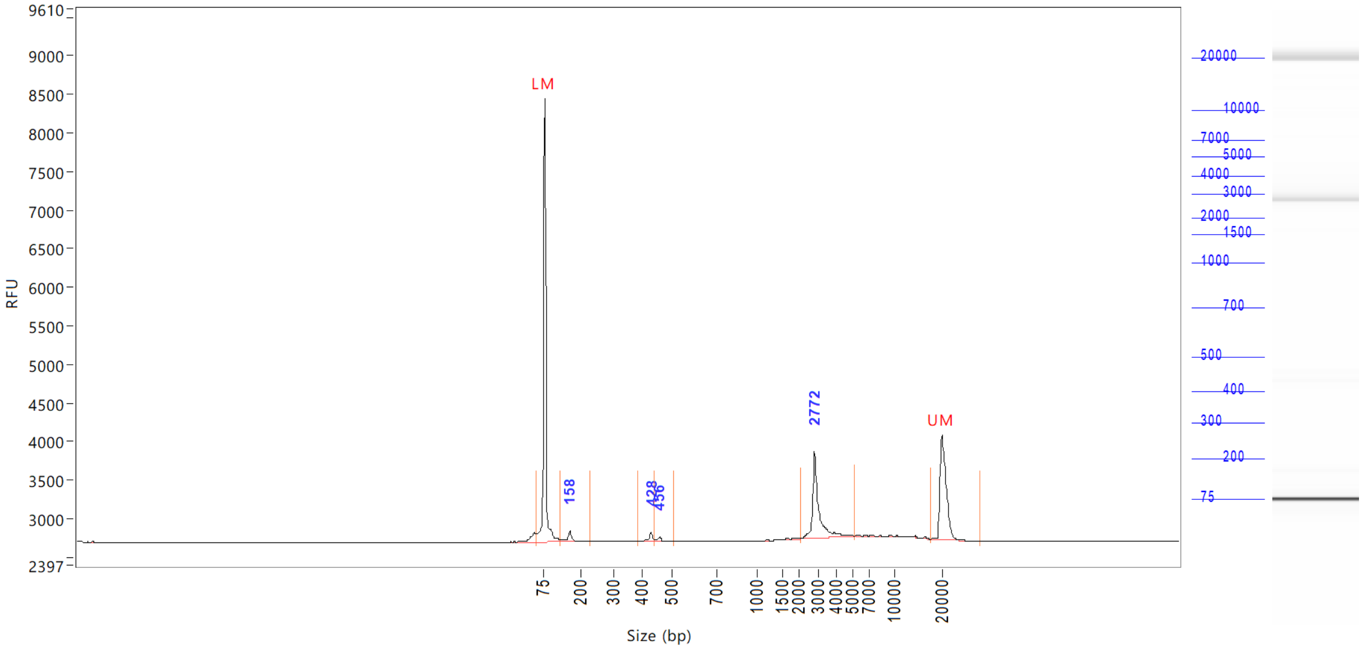

Peak	Size (bp)	Concentration (ng/uL)	From (bp)	To (bp)	Average size (bp)	CV%	RFU	Corrected peak area
1	75 (LM)	0.5000	47	129	74	11.80	5759	35.831
2	158	0.2616	129	224	158	10.62	136	1.562
3	428	0.1662	384	441	423	2.71	127	0.993
4	456	0.1032	441	504	458	2.86	57	0.616
5	2772	1.7867	2058	5149	2961	14.77	1119	10.670
6	20000 (UM)	0.1562	17561	28221	20391	4.03	1361	11.194

TIC:	2.3178	ng/uL
TIM:	4.7322	nmole/L
Total concentration:	2.6227	ng/uL

```
Sample peak width (sec): 5    Sample min peak height: 50    Sample baseline V to V?: Y    Sample baseline V to V points: 3
Sample filter: Binomial       Number of points for filter: 3  Sample start region (min): 0  Sample end region (min): 30
Marker peak width (sec): 5    Marker min peak height: 200   Marker baseline V to V?: Y    Marker baseline V to V points: 3
Lower marker selection: First peak > 500 RFU                Upper marker selection: Last peak > 200 RFU
Ladder size (bp)75, 200, 300, 400, 500, 700, 1000, 1500, 2000, 3000, 4000, 5000, 7000, 10000, 20000
Quantification using: Lower Marker          Final concentration (ng/uL): 0.5000          Dilution factor: 12.0
```

Sample: SampE4
Well location: E4
Created: Monday, September 20, 2021 8:39:56 PM

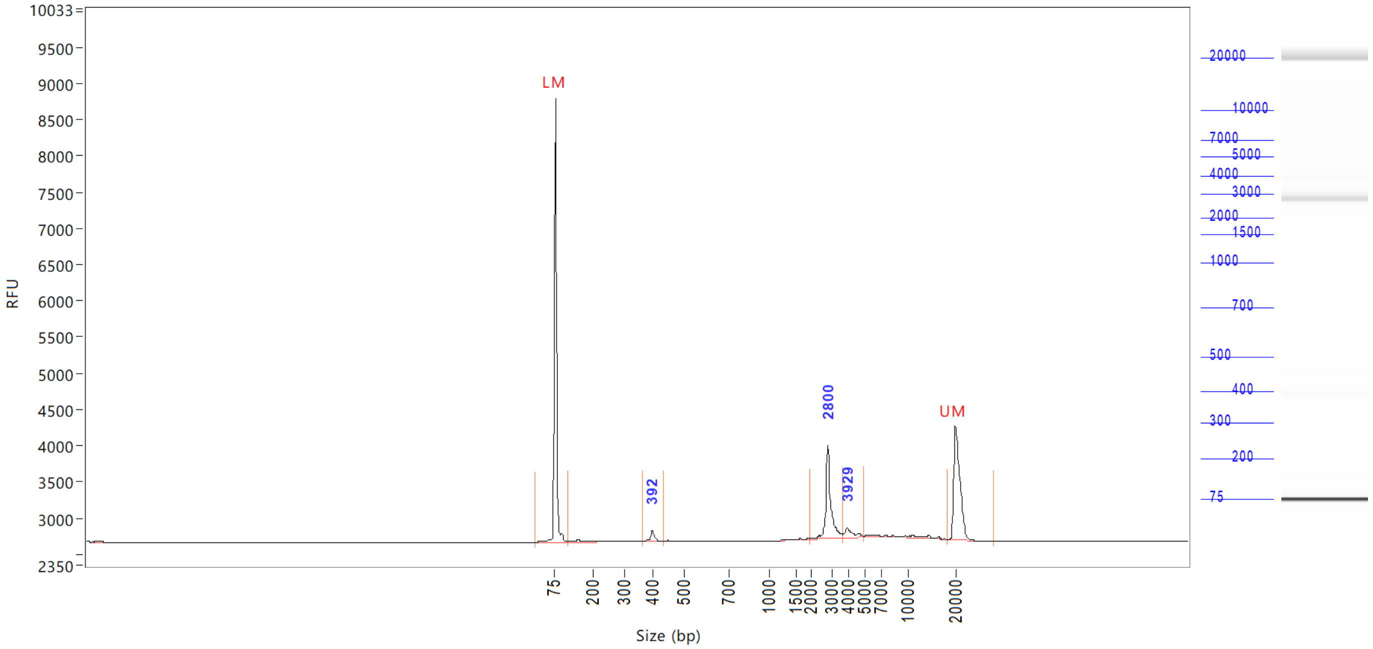

Peak	Size (bp)	Concentration (ng/uL)	From (bp)	To (bp)	Average size (bp)	CV%	RFU	Corrected peak area
1	75 (LM)	0.5000	11	120	72	8.89	6137	35.797
2	392	0.2618	360	435	395	3.48	155	1.562
3	2800	1.6454	1942	3643	2839	8.05	1280	9.817
4	3929	0.2923	3643	4931	4109	7.74	138	1.744
5	20000 (UM)	0.1828	18293	28342	20424	3.88	1573	13.091

TIC:	2.1994	ng/uL
TIM:	2.1615	nmole/L
Total concentration:	2.7602	ng/uL

Sample peak width (sec): 5   Sample min peak height: 50   Sample baseline V to V?: Y   Sample baseline V to V points: 3
Sample filter: Binomial   Number of points for filter: 3 Sample start region (min): 0 Sample end region (min): 30
Marker peak width (sec): 5   Marker min peak height: 200   Marker baseline V to V?: Y   Marker baseline V to V points: 3
Lower marker selection: First peak > 500 RFU   Upper marker selection: Last peak > 200 RFU
Ladder size (bp)75, 200, 300, 400, 500, 700, 1000, 1500, 2000, 3000, 4000, 5000, 7000, 10000, 20000
Quantification using: Lower Marker   Final concentration (ng/uL): 0.5000   Dilution factor: 12.0

Sample: SampF4
Well location: F4
Created: Monday, September 20, 2021 8:39:56 PM

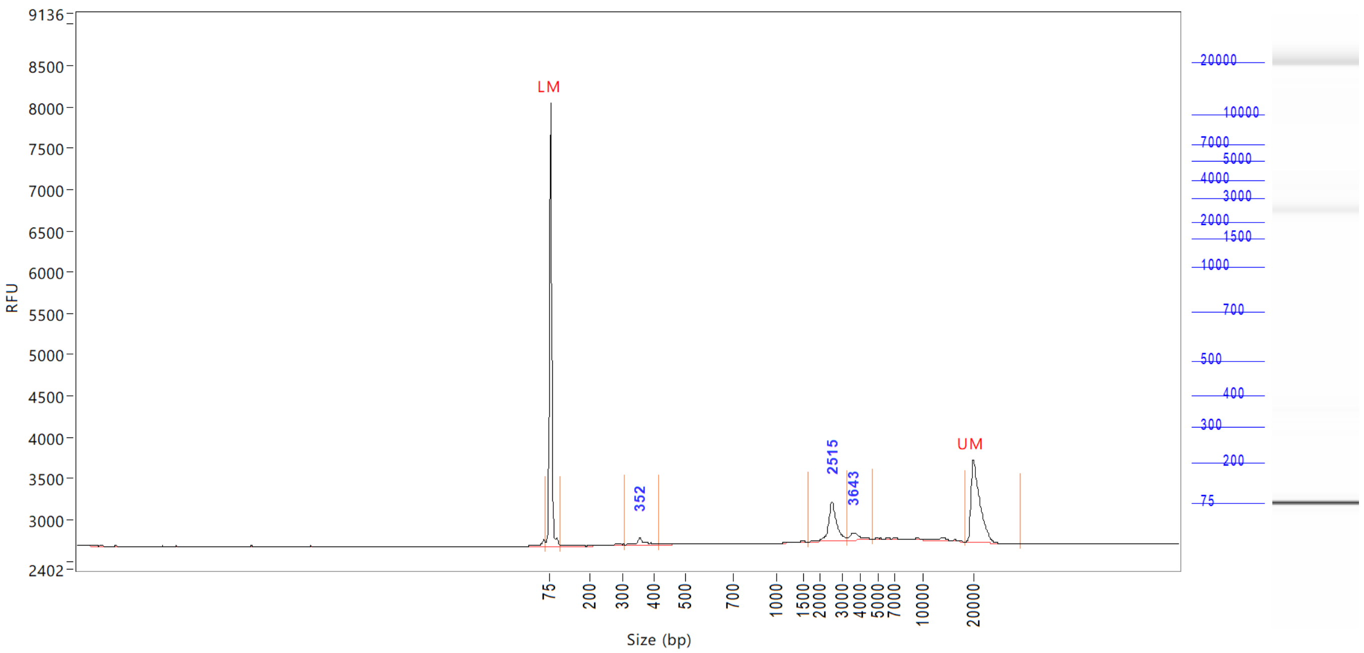

Peak	Size (bp)	Concentration (ng/uL)	From (bp)	To (bp)	Average size (bp)	CV%	RFU	Corrected peak area
1	75 (LM)	0.5000	62	108	74	6.01	5380	31.648
2	352	0.2619	303	412	353	6.42	86	1.381
3	2515	1.1306	1597	3286	2523	9.91	479	5.963
4	3643	0.2640	3286	4724	3793	8.58	92	1.393
5	20000 (UM)	0.1809	18415	29430	20708	5.36	991	11.452

TIC:	1.6565	ng/uL
TIM:	2.0727	nmole/L
Total concentration:	2.2457	ng/uL

```
Sample peak width (sec): 5    Sample min peak height: 50    Sample baseline V to V?: Y    Sample baseline V to V points: 3
Sample filter: Binomial       Number of points for filter: 3  Sample start region (min): 0  Sample end region (min): 30
Marker peak width (sec): 5    Marker min peak height: 200   Marker baseline V to V?: Y    Marker baseline V to V points: 3
Lower marker selection: First peak > 500 RFU                Upper marker selection: Last peak > 200 RFU
Ladder size (bp)75, 200, 300, 400, 500, 700, 1000, 1500, 2000, 3000, 4000, 5000, 7000, 10000, 20000
Quantification using: Lower Marker          Final concentration (ng/uL): 0.5000          Dilution factor: 12.0
```

Sample: SampG4
Well location: G4
Created: Monday, September 20, 2021 8:39:56 PM

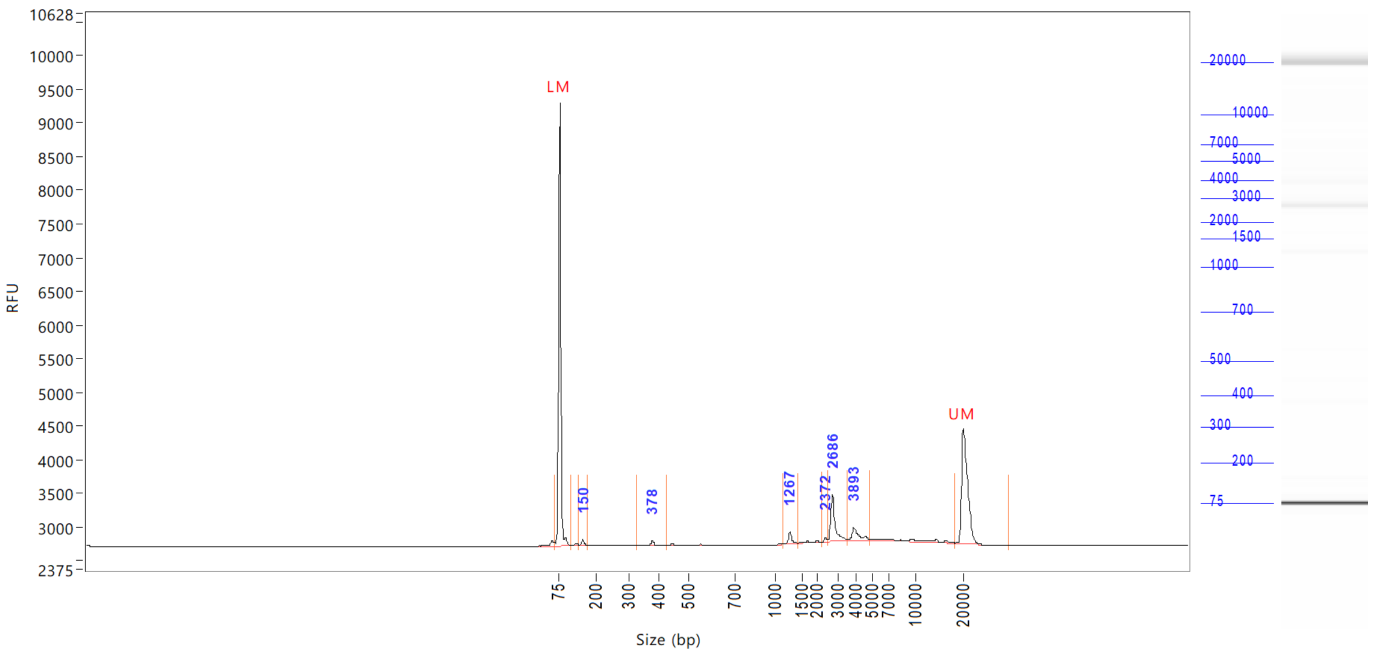

Peak	Size (bp)	Concentration (ng/uL)	From (bp)	To (bp)	Average size (bp)	CV%	RFU	Corrected peak area
1	75 (LM)	0.5000	64	114	74	6.42	6589	36.128
2	150	0.0974	141	169	150	3.08	93	0.586
3	378	0.1153	327	426	379	4.54	72	0.694
4	1267	0.1657	1156	1422	1268	3.32	174	0.998
5	2372	0.0497	2172	2486	2356	3.09	67	0.299
6	2686	0.7821	2486	3500	2771	7.11	692	4.709
7	3893	0.3704	3500	4862	4082	7.70	191	2.230
8	20000 (UM)	0.1946	18293	29551	20384	4.01	1701	14.062

TIC:	1.5806	ng/uL
TIM:	2.4314	nmole/L
Total concentration:	2.1967	ng/uL

```
Sample peak width (sec): 5    Sample min peak height: 50    Sample baseline V to V?: Y    Sample baseline V to V points: 3
Sample filter: Binomial       Number of points for filter: 3  Sample start region (min): 0  Sample end region (min): 30
Marker peak width (sec): 5    Marker min peak height: 200   Marker baseline V to V?: Y    Marker baseline V to V points: 3
Lower marker selection: First peak > 500 RFU                Upper marker selection: Last peak > 200 RFU
Ladder size (bp)75, 200, 300, 400, 500, 700, 1000, 1500, 2000, 3000, 4000, 5000, 7000, 10000, 20000
Quantification using: Lower Marker          Final concentration (ng/uL): 0.5000          Dilution factor: 12.0
```

Sample: SampH4
Well location: H4
Created: Monday, September 20, 2021 8:39:56 PM

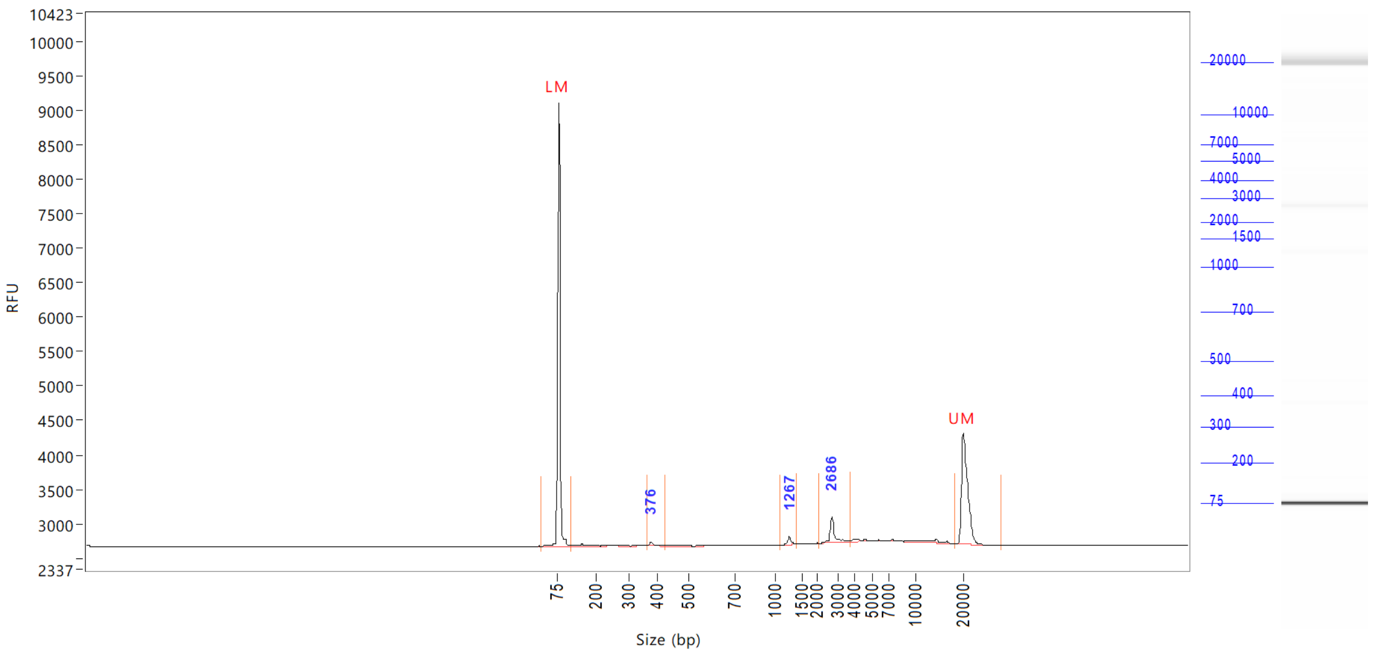

Peak	Size (bp)	Concentration (ng/uL)	From (bp)	To (bp)	Average size (bp)	CV%	RFU	Corrected peak area
1	75 (LM)	0.5000	20	116	73	7.72	6459	35.698
2	376	0.0975	360	424	382	4.04	64	0.580
3	1267	0.1171	1079	1400	1258	4.09	112	0.697
4	2686	0.4415	2058	3679	2769	9.64	370	2.627
5	20000 (UM)	0.1885	18293	28100	20382	3.83	1609	13.460

TIC:	0.6561	ng/uL
TIM:	0.8355	nmole/L
Total concentration:	1.3161	ng/uL

Sample peak width (sec): 5 Sample min peak height: 50 Sample baseline V to V?: Y Sample baseline V to V points: 3
Sample filter: Binomial Number of points for filter: 3 Sample start region (min): 0 Sample end region (min): 30
Marker peak width (sec): 5 Marker min peak height: 200 Marker baseline V to V?: Y Marker baseline V to V points: 3
Lower marker selection: First peak > 500 RFU Upper marker selection: Last peak > 200 RFU
Ladder size (bp)75, 200, 300, 400, 500, 700, 1000, 1500, 2000, 3000, 4000, 5000, 7000, 10000, 20000
Quantification using: Lower Marker Final concentration (ng/uL): 0.5000 Dilution factor: 12.0

Sample: SampA5
Well location: A5
Created: Monday, September 20, 2021 8:39:56 PM

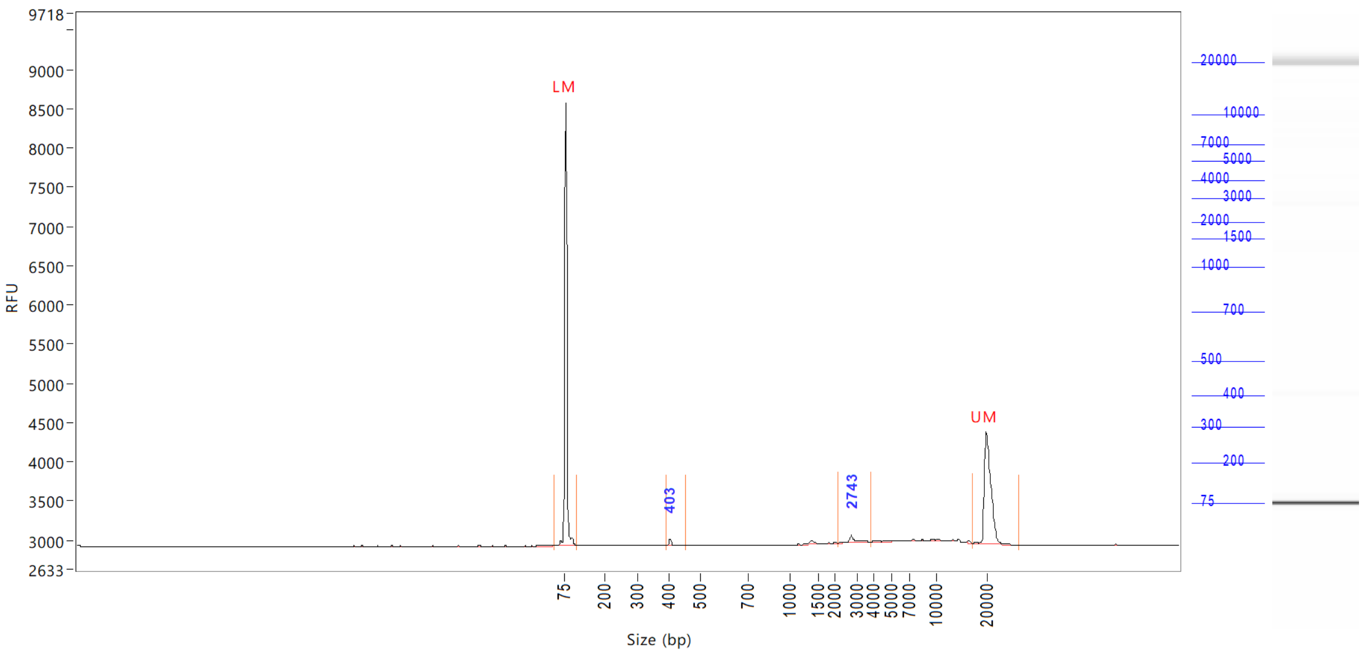

Peak	Size (bp)	Concentration (ng/uL)	From (bp)	To (bp)	Average size (bp)	CV%	RFU	Corrected peak area
1	75 (LM)	0.5000	43	110	74	6.36	5654	31.275
2	403	0.1182	392	454	407	3.26	88	0.616
3	2743	0.1673	2115	3822	2875	10.67	97	0.872
4	20000 (UM)	0.1917	17195	26529	20281	3.77	1437	11.993

TIC:	0.2855	ng/uL
TIM:	0.5737	nmole/L
Total concentration:	0.9575	ng/uL

```
Sample peak width (sec): 5    Sample min peak height: 50    Sample baseline V to V?: Y    Sample baseline V to V points: 3
Sample filter: Binomial       Number of points for filter: 3  Sample start region (min): 0  Sample end region (min): 30
Marker peak width (sec): 5    Marker min peak height: 200   Marker baseline V to V?: Y    Marker baseline V to V points: 3
Lower marker selection: First peak > 500 RFU                Upper marker selection: Last peak > 200 RFU
Ladder size (bp)75, 200, 300, 400, 500, 700, 1000, 1500, 2000, 3000, 4000, 5000, 7000, 10000, 20000
Quantification using: Lower Marker          Final concentration (ng/uL): 0.5000          Dilution factor: 12.0
```

Sample: SampB5
Well location: B5
Created: Monday, September 20, 2021 8:39:56 PM

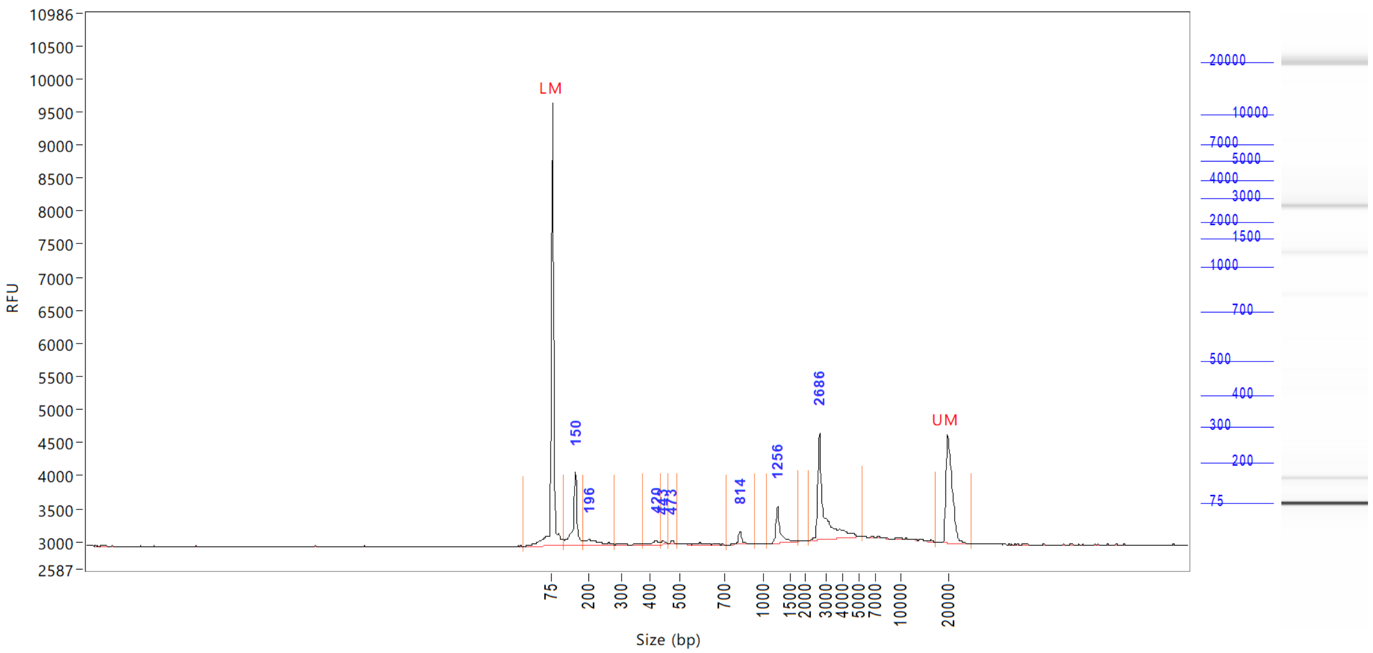

Peak	Size (bp)	Concentration (ng/uL)	From (bp)	To (bp)	Average size (bp)	CV%	RFU	Corrected peak area
1	75 (LM)	0.5000	0	114	71	18.64	6703	43.985
2	150	1.4078	114	177	146	8.24	1108	10.320
3	196	0.3816	177	277	211	11.38	95	2.797
4	420	0.1212	374	433	411	3.53	66	0.889
5	443	0.0660	433	462	445	1.72	55	0.484
6	473	0.0601	462	492	472	1.18	57	0.441
7	814	0.1791	710	931	818	3.71	199	1.313
8	1256	0.5756	1034	1750	1292	7.68	561	4.219
9	2686	2.0781	2144	5371	3016	18.31	1610	15.234
10	20000 (UM)	0.1543	17317	25078	20343	3.70	1648	13.577

TIC:	4.8694	ng/uL
TIM:	21.9866	nmole/L
Total concentration:	5.0985	ng/uL

Sample peak width (sec): 5 Sample min peak height: 50 Sample baseline V to V?: Y Sample baseline V to V points: 3
Sample filter: Binomial Number of points for filter: 3 Sample start region (min): 0 Sample end region (min): 30
Marker peak width (sec): 5 Marker min peak height: 200 Marker baseline V to V?: Y Marker baseline V to V points: 3
Lower marker selection: First peak > 500 RFU Upper marker selection: Last peak > 200 RFU
Ladder size (bp)75, 200, 300, 400, 500, 700, 1000, 1500, 2000, 3000, 4000, 5000, 7000, 10000, 20000
Quantification using: Lower Marker Final concentration (ng/uL): 0.5000 Dilution factor: 12.0

Sample: SampC5
Well location: C5
Created: Monday, September 20, 2021 8:39:56 PM

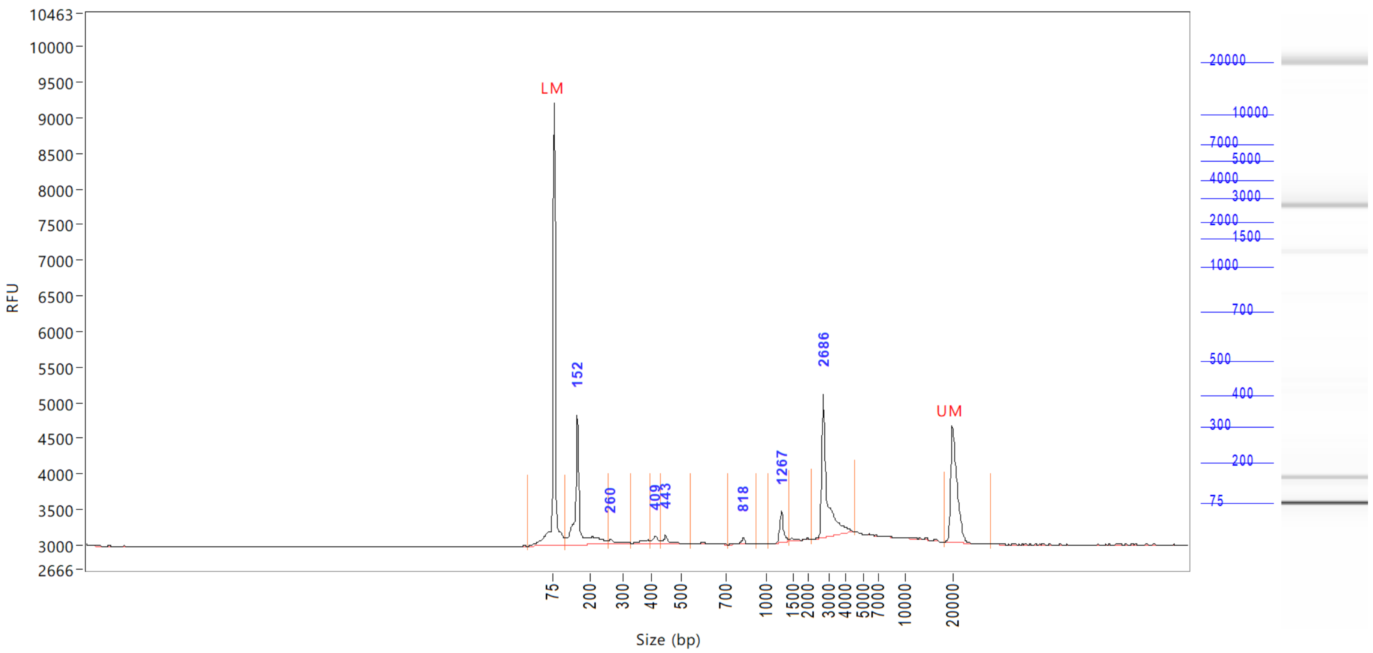

Peak	Size (bp)	Concentration (ng/uL)	From (bp)	To (bp)	Average size (bp)	CV%	RFU	Corrected peak area
1	75 (LM)	0.5000	0	110	72	18.41	6216	42.202
2	152	2.7993	110	253	159	18.07	1819	19.689
3	260	0.1277	253	327	274	6.84	61	0.898
4	409	0.1559	398	428	411	1.89	103	1.097
5	443	0.1922	428	534	452	4.06	114	1.352
6	818	0.1069	701	913	804	4.16	90	0.752
7	1267	0.3886	1012	1411	1272	3.69	435	2.733
8	2686	2.4255	2115	4518	2894	12.39	2016	17.060
9	20000 (UM)	0.1652	18293	28100	20464	3.94	1625	13.940

TIC:	6.1961	ng/uL
TIM:	33.1297	nmole/L
Total concentration:	6.5604	ng/uL

Sample peak width (sec): 5   Sample min peak height: 50   Sample baseline V to V?: Y   Sample baseline V to V points: 3
Sample filter: Binomial   Number of points for filter: 3 Sample start region (min): 0 Sample end region (min): 30
Marker peak width (sec): 5   Marker min peak height: 200   Marker baseline V to V?: Y   Marker baseline V to V points: 3
Lower marker selection: First peak > 500 RFU   Upper marker selection: Last peak > 200 RFU
Ladder size (bp)75, 200, 300, 400, 500, 700, 1000, 1500, 2000, 3000, 4000, 5000, 7000, 10000, 20000
Quantification using: Lower Marker   Final concentration (ng/uL): 0.5000   Dilution factor: 12.0

Sample: SampD5
Well location: D5
Created: Monday, September 20, 2021 8:39:56 PM

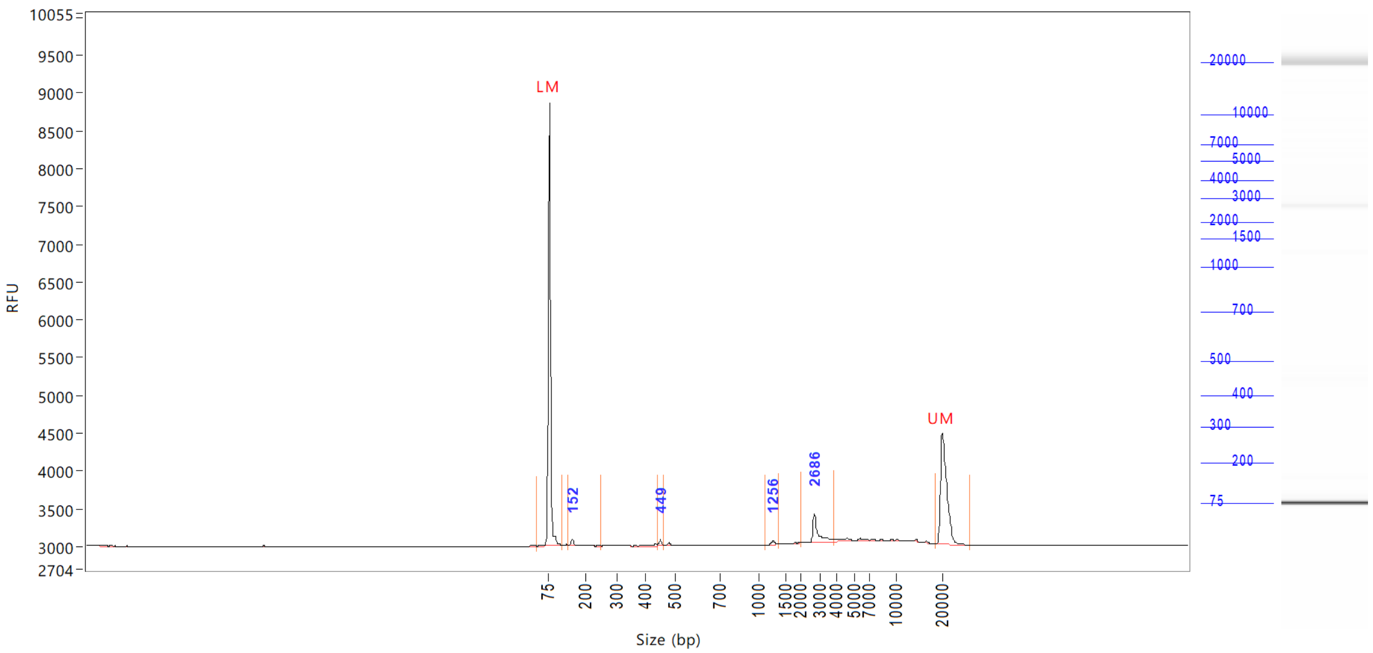

Peak	Size (bp)	Concentration (ng/uL)	From (bp)	To (bp)	Average size (bp)	CV%	RFU	Corrected peak area
1	75 (LM)	0.5000	33	122	75	6.96	5867	33.985
2	152	0.1021	141	248	163	15.19	74	0.578
3	449	0.0913	437	462	447	1.21	82	0.517
4	1256	0.0696	1112	1367	1248	3.89	60	0.394
5	2686	0.5595	2030	3750	2852	10.81	381	3.169
6	20000 (UM)	0.1868	18415	26045	20380	3.80	1470	12.694

TIC:	0.8225	ng/uL
TIM:	1.7805	nmole/L
Total concentration:	1.3913	ng/uL

Sample peak width (sec): 5   Sample min peak height: 50   Sample baseline V to V?: Y   Sample baseline V to V points: 3
Sample filter: Binomial   Number of points for filter: 3 Sample start region (min): 0 Sample end region (min): 30
Marker peak width (sec): 5   Marker min peak height: 200   Marker baseline V to V?: Y   Marker baseline V to V points: 3
Lower marker selection: First peak > 500 RFU   Upper marker selection: Last peak > 200 RFU
Ladder size (bp)75, 200, 300, 400, 500, 700, 1000, 1500, 2000, 3000, 4000, 5000, 7000, 10000, 20000
Quantification using: Lower Marker   Final concentration (ng/uL): 0.5000   Dilution factor: 12.0

Sample: SampE5
Well location: E5
Created: Monday, September 20, 2021 8:39:56 PM

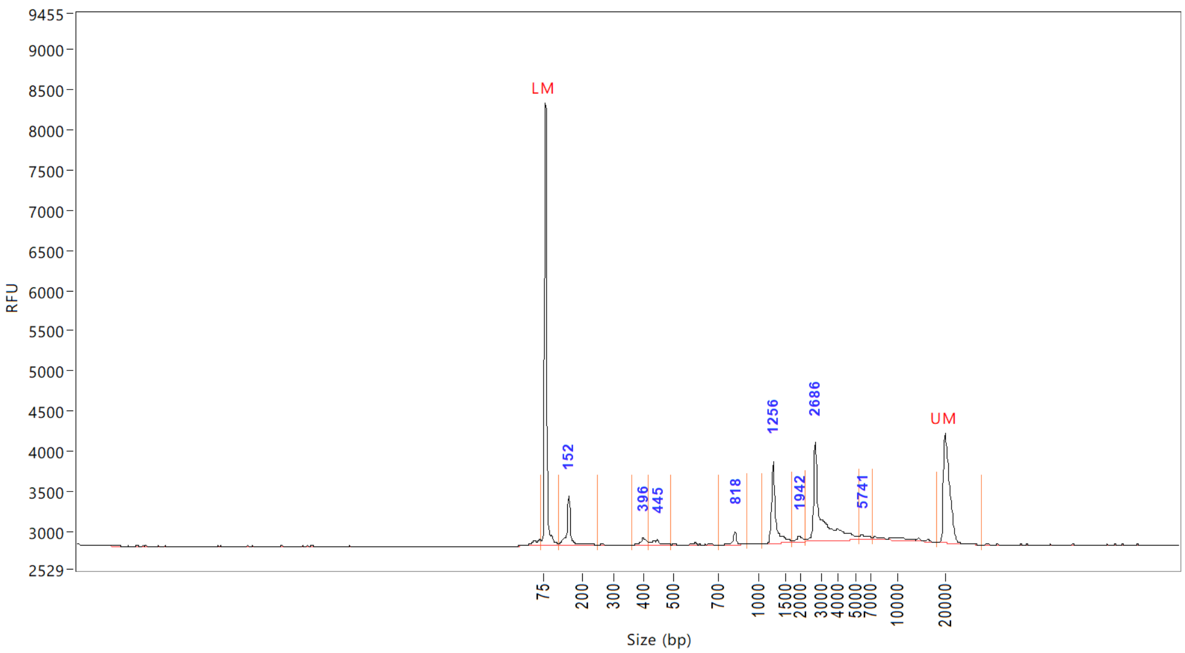

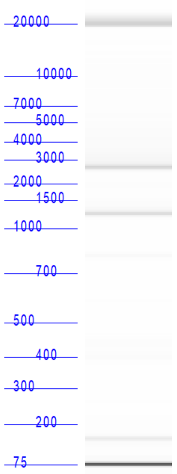

Peak	Size (bp)	Concentration (ng/uL)	From (bp)	To (bp)	Average size (bp)	CV%	RFU	Corrected peak area
1	75 (LM)	0.5000	64	120	75	8.06	5524	32.744
2	152	1.0766	120	246	155	13.48	611	5.876
3	396	0.2263	362	416	396	3.09	91	1.235
4	445	0.2131	416	492	442	4.12	66	1.163
5	818	0.2048	700	909	813	3.48	167	1.117
6	1256	1.2858	1045	1693	1292	7.04	1029	7.017
7	1942	0.1439	1693	2229	1940	6.59	70	0.786
8	2686	2.5301	2229	5371	3176	21.39	1228	13.807
9	5741	0.1381	5371	7068	6159	8.26	59	0.754
10	20000 (UM)	0.1746	18293	27617	20420	3.72	1372	11.432

TIC:	5.8188	ng/uL
TIM:	16.6732	nmole/L
Total concentration:	6.2492	ng/uL

```
Sample peak width (sec): 5    Sample min peak height: 50    Sample baseline V to V?: Y    Sample baseline V to V points: 3
Sample filter: Binomial       Number of points for filter: 3  Sample start region (min): 0  Sample end region (min): 30
Marker peak width (sec): 5    Marker min peak height: 200   Marker baseline V to V?: Y    Marker baseline V to V points: 3
Lower marker selection: First peak > 500 RFU                Upper marker selection: Last peak > 200 RFU
Ladder size (bp)75, 200, 300, 400, 500, 700, 1000, 1500, 2000, 3000, 4000, 5000, 7000, 10000, 20000
Quantification using: Lower Marker          Final concentration (ng/uL): 0.5000          Dilution factor: 12.0
```

Sample: SampF5
Well location: F5
Created: Monday, September 20, 2021 8:39:56 PM

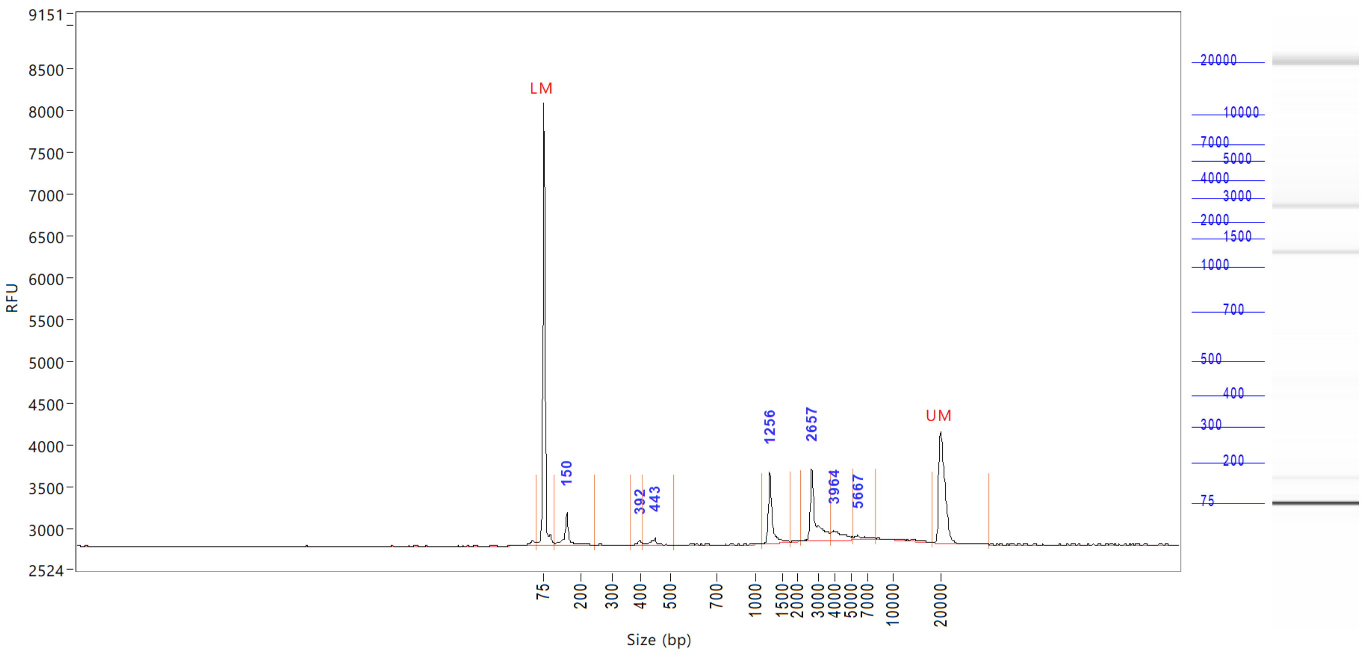

Peak	Size (bp)	Concentration (ng/uL)	From (bp)	To (bp)	Average size (bp)	CV%	RFU	Corrected peak area
1	75 (LM)	0.5000	48	108	73	7.93	5285	30.955
2	150	0.8099	108	244	152	15.18	392	4.178
3	392	0.1032	360	403	388	2.57	60	0.533
4	443	0.2727	403	511	440	5.34	86	1.407
5	1256	1.0721	1101	1750	1283	6.72	862	5.531
6	2657	1.4106	2172	3750	2861	11.16	866	7.278
7	3964	0.4210	3750	5223	4287	8.97	114	2.172
8	5667	0.1664	5223	8001	6349	12.14	54	0.859
9	20000 (UM)	0.1775	18293	30398	20427	4.84	1321	10.988

TIC:	4.2560	ng/uL
TIM:	12.6065	nmole/L
Total concentration:	4.5974	ng/uL

Sample peak width (sec): 5 Sample min peak height: 50 Sample baseline V to V?: Y Sample baseline V to V points: 3
Sample filter: Binomial Number of points for filter: 3 Sample start region (min): 0 Sample end region (min): 30
Marker peak width (sec): 5 Marker min peak height: 200 Marker baseline V to V?: Y Marker baseline V to V points: 3
Lower marker selection: First peak > 500 RFU Upper marker selection: Last peak > 200 RFU
Ladder size (bp)75, 200, 300, 400, 500, 700, 1000, 1500, 2000, 3000, 4000, 5000, 7000, 10000, 20000
Quantification using: Lower Marker Final concentration (ng/uL): 0.5000 Dilution factor: 12.0

Sample: SampG5
Well location: G5
Created: Monday, September 20, 2021 8:39:56 PM

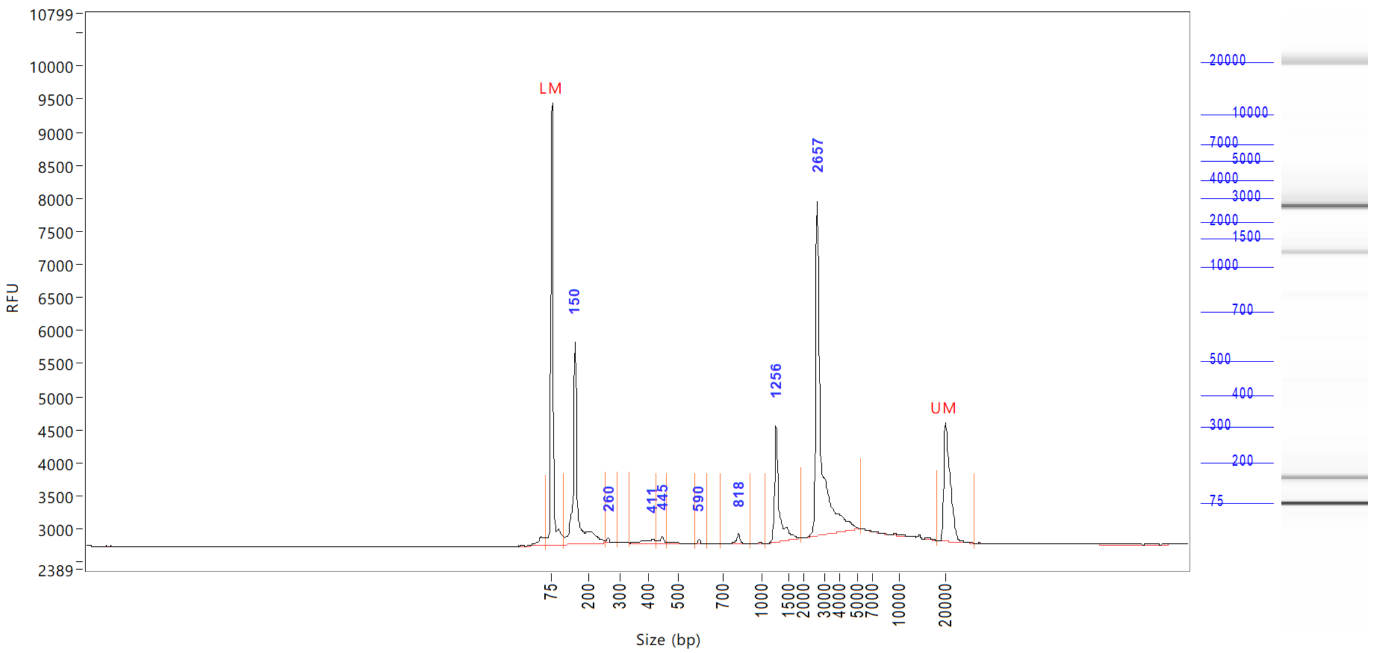

Peak	Size (bp)	Concentration (ng/uL)	From (bp)	To (bp)	Average size (bp)	CV%	RFU	Corrected peak area
1	75 (LM)	0.5000	52	116	74	11.77	6701	42.773
2	150	4.4028	116	251	159	15.14	3050	31.387
3	260	0.0807	251	291	260	2.60	80	0.575
4	411	0.1958	331	424	390	5.82	66	1.396
5	445	0.1180	424	458	440	1.83	104	0.841
6	590	0.0565	575	626	590	1.20	80	0.403
7	818	0.1715	687	905	804	3.83	162	1.223
8	1256	1.8860	1045	1904	1305	8.13	1762	13.445
9	2657	6.0737	1904	5445	2953	17.12	5048	43.298
10	20000 (UM)	0.1809	18171	26287	20477	3.92	1785	15.478

TIC:	12.9850	ng/uL
TIM:	53.5654	nmole/L
Total concentration:	13.2250	ng/uL

Sample peak width (sec): 5   Sample min peak height: 50   Sample baseline V to V?: Y   Sample baseline V to V points: 3
Sample filter: Binomial   Number of points for filter: 3 Sample start region (min): 0 Sample end region (min): 30
Marker peak width (sec): 5   Marker min peak height: 200   Marker baseline V to V?: Y   Marker baseline V to V points: 3
Lower marker selection: First peak > 500 RFU   Upper marker selection: Last peak > 200 RFU
Ladder size (bp)75, 200, 300, 400, 500, 700, 1000, 1500, 2000, 3000, 4000, 5000, 7000, 10000, 20000
Quantification using: Lower Marker   Final concentration (ng/uL): 0.5000   Dilution factor: 12.0

Sample: SampH5
Well location: H5
Created: Monday, September 20, 2021 8:39:56 PM

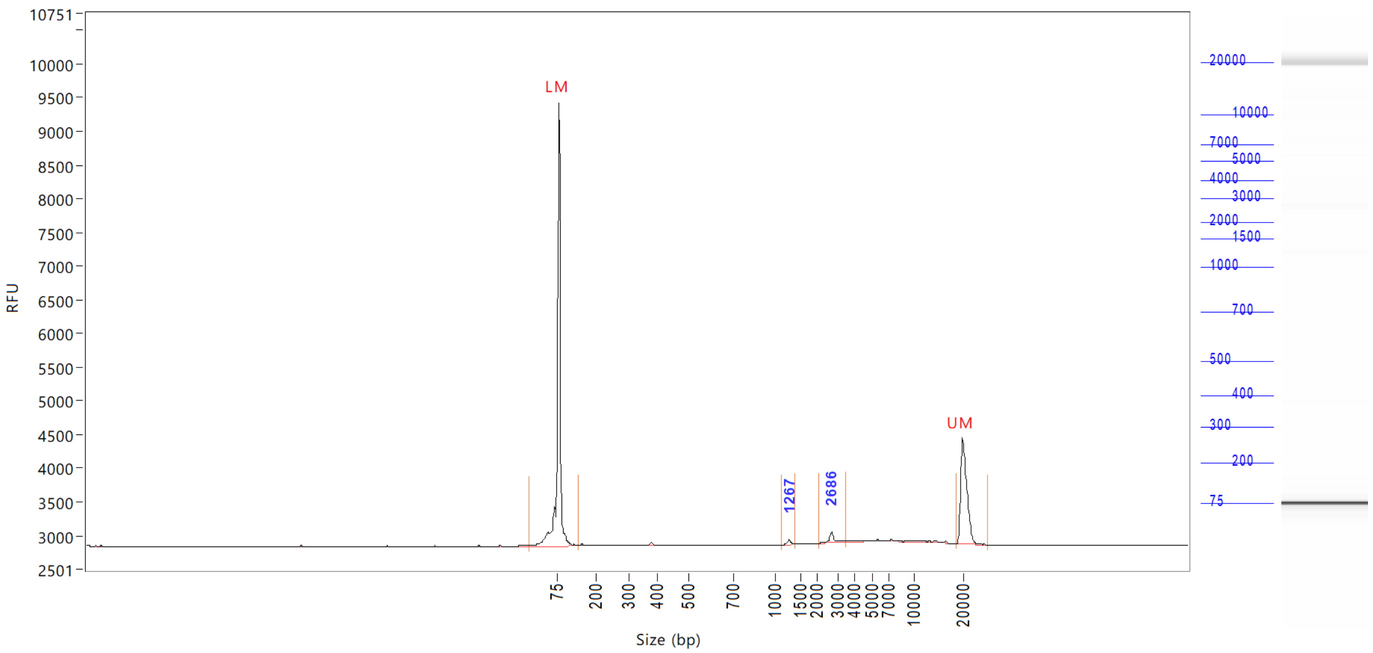

Peak	Size (bp)	Concentration (ng/uL)	From (bp)	To (bp)	Average size (bp)	CV%	RFU	Corrected peak area
1	75 (LM)	0.5000	0	143	69	22.51	6588	46.972
2	1267	0.0620	1123	1389	1259	3.85	77	0.486
3	2686	0.1700	2058	3465	2752	9.77	158	1.331
4	20000 (UM)	0.1391	18781	25078	20321	3.59	1570	13.065

TIC:	0.2320	ng/uL
TIM:	0.1828	nmole/L
Total concentration:	0.7283	ng/uL

```
Sample peak width (sec): 5    Sample min peak height: 50    Sample baseline V to V?: Y    Sample baseline V to V points: 3
Sample filter: Binomial       Number of points for filter: 3  Sample start region (min): 0  Sample end region (min): 30
Marker peak width (sec): 5    Marker min peak height: 200   Marker baseline V to V?: Y    Marker baseline V to V points: 3
Lower marker selection: First peak > 500 RFU                Upper marker selection: Last peak > 200 RFU
Ladder size (bp)75, 200, 300, 400, 500, 700, 1000, 1500, 2000, 3000, 4000, 5000, 7000, 10000, 20000
Quantification using: Lower Marker          Final concentration (ng/uL): 0.5000          Dilution factor: 12.0
```

Sample: SampA6
Well location: A6
Created: Monday, September 20, 2021 8:39:56 PM

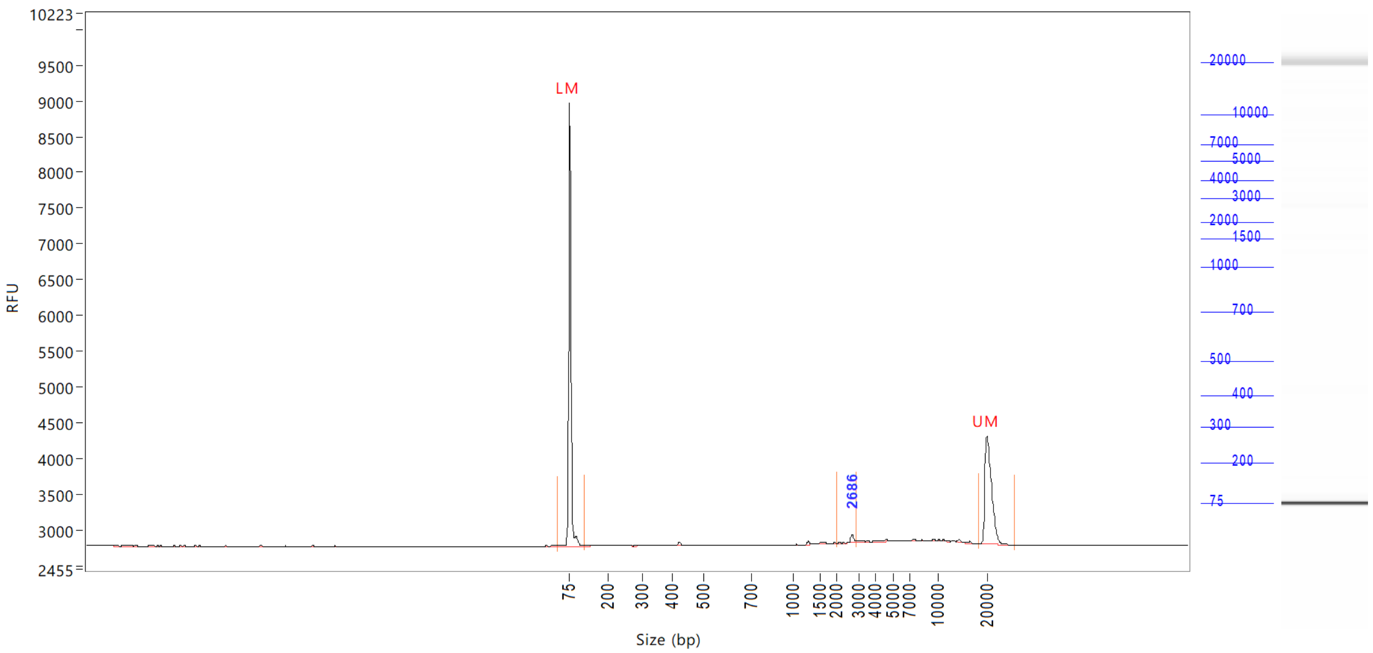

Peak	Size (bp)	Concentration (ng/uL)	From (bp)	To (bp)	Average size (bp)	CV%	RFU	Corrected peak area
1	75 (LM)	0.5000	37	124	74	7.61	6204	34.109
2	2686	0.1047	2001	2914	2673	5.19	110	0.595
3	20000 (UM)	0.1874	18293	25440	20310	3.56	1508	12.785

TIC:	0.1047	ng/uL
TIM:	0.0645	nmole/L
Total concentration:	0.8378	ng/uL

```
Sample peak width (sec): 5    Sample min peak height: 50    Sample baseline V to V?: Y    Sample baseline V to V points: 3
Sample filter: Binomial       Number of points for filter: 3  Sample start region (min): 0  Sample end region (min): 30
Marker peak width (sec): 5    Marker min peak height: 200   Marker baseline V to V?: Y    Marker baseline V to V points: 3
Lower marker selection: First peak > 500 RFU                Upper marker selection: Last peak > 200 RFU
Ladder size (bp)75, 200, 300, 400, 500, 700, 1000, 1500, 2000, 3000, 4000, 5000, 7000, 10000, 20000
Quantification using: Lower Marker          Final concentration (ng/uL): 0.5000          Dilution factor: 12.0
```

Sample: SampB6
Well location: B6
Created: Monday, September 20, 2021 8:39:56 PM

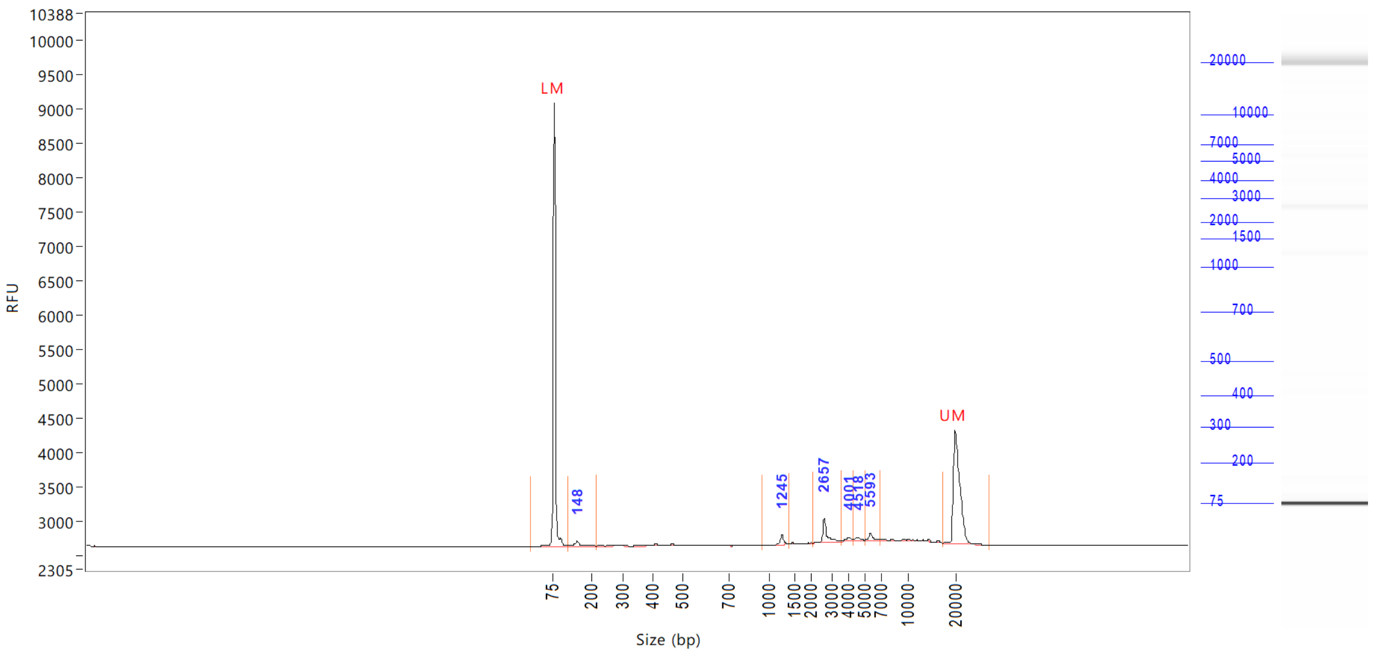

Peak	Size (bp)	Concentration (ng/uL)	From (bp)	To (bp)	Average size (bp)	CV%	RFU	Corrected peak area
1	75 (LM)	0.5000	0	124	73	8.91	6457	37.655
2	148	0.1516	124	212	150	11.43	72	0.951
3	1245	0.1436	948	1389	1230	5.86	142	0.901
4	2657	0.3900	2058	3643	2744	9.38	351	2.448
5	4001	0.0864	3643	4277	3976	3.79	60	0.542
6	4518	0.0654	4277	4966	4532	3.46	54	0.410
7	5593	0.1127	4966	6852	5734	7.56	100	0.707
8	20000 (UM)	0.1912	17317	27133	20383	3.90	1658	14.402

TIC:	0.9497	ng/uL
TIM:	2.1787	nmole/L
Total concentration:	1.3836	ng/uL

Sample peak width (sec): 5 Sample min peak height: 50 Sample baseline V to V?: Y Sample baseline V to V points: 3
Sample filter: Binomial Number of points for filter: 3 Sample start region (min): 0 Sample end region (min): 30
Marker peak width (sec): 5 Marker min peak height: 200 Marker baseline V to V?: Y Marker baseline V to V points: 3
Lower marker selection: First peak > 500 RFU Upper marker selection: Last peak > 200 RFU
Ladder size (bp)75, 200, 300, 400, 500, 700, 1000, 1500, 2000, 3000, 4000, 5000, 7000, 10000, 20000
Quantification using: Lower Marker Final concentration (ng/uL): 0.5000 Dilution factor: 12.0

Sample: SampC6
Well location: C6
Created: Monday, September 20, 2021 8:39:56 PM

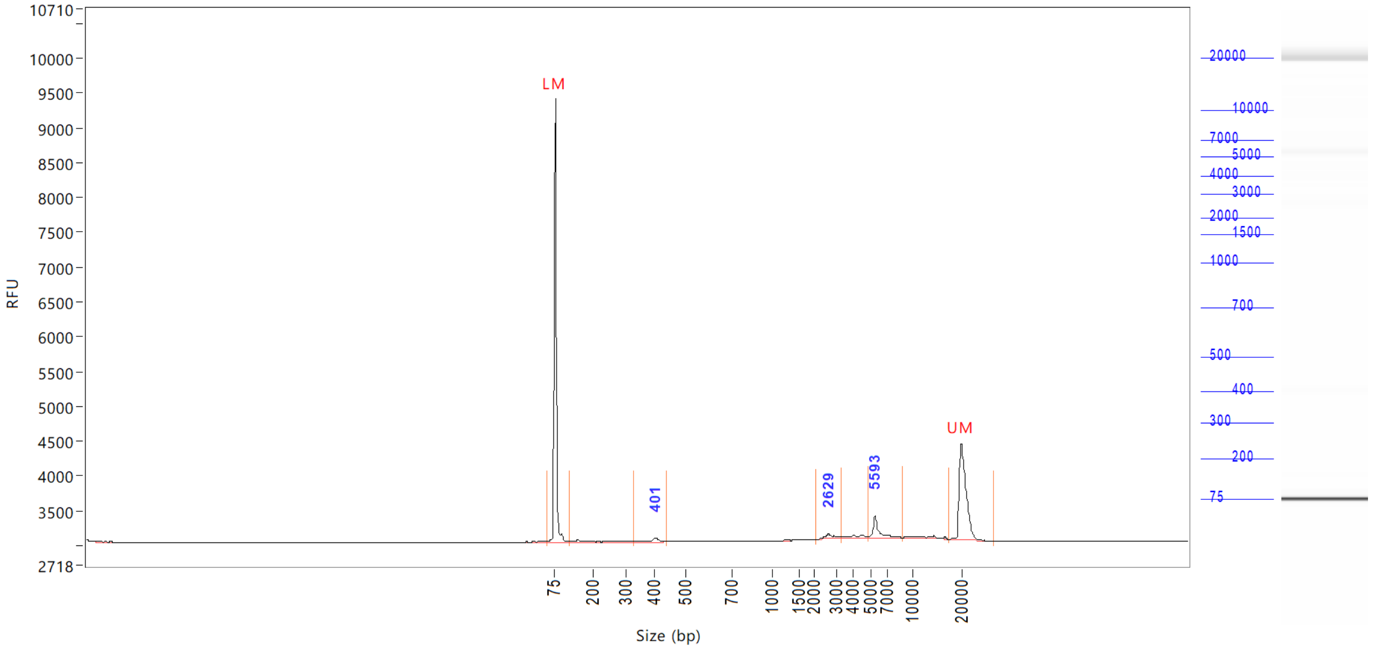

Peak	Size (bp)	Concentration (ng/uL)	From (bp)	To (bp)	Average size (bp)	CV%	RFU	Corrected peak area
1	75 (LM)	0.5000	50	124	74	7.08	6382	35.847
2	401	0.1496	327	439	396	5.22	58	0.894
3	2629	0.1376	2001	3322	2685	8.92	65	0.822
4	5593	0.4149	4931	8800	5916	13.14	304	2.479
5	20000 (UM)	0.1744	17439	26770	20395	4.10	1381	12.502

TIC:	0.7021	ng/uL
TIM:	0.8215	nmole/L
Total concentration:	1.2607	ng/uL

Sample peak width (sec): 5   Sample min peak height: 50   Sample baseline V to V?: Y   Sample baseline V to V points: 3
Sample filter: Binomial   Number of points for filter: 3 Sample start region (min): 0 Sample end region (min): 30
Marker peak width (sec): 5   Marker min peak height: 200   Marker baseline V to V?: Y   Marker baseline V to V points: 3
Lower marker selection: First peak > 500 RFU   Upper marker selection: Last peak > 200 RFU
Ladder size (bp)75, 200, 300, 400, 500, 700, 1000, 1500, 2000, 3000, 4000, 5000, 7000, 10000, 20000
Quantification using: Lower Marker   Final concentration (ng/uL): 0.5000   Dilution factor: 12.0

Sample: SampD6
Well location: D6
Created: Monday, September 20, 2021 8:39:56 PM

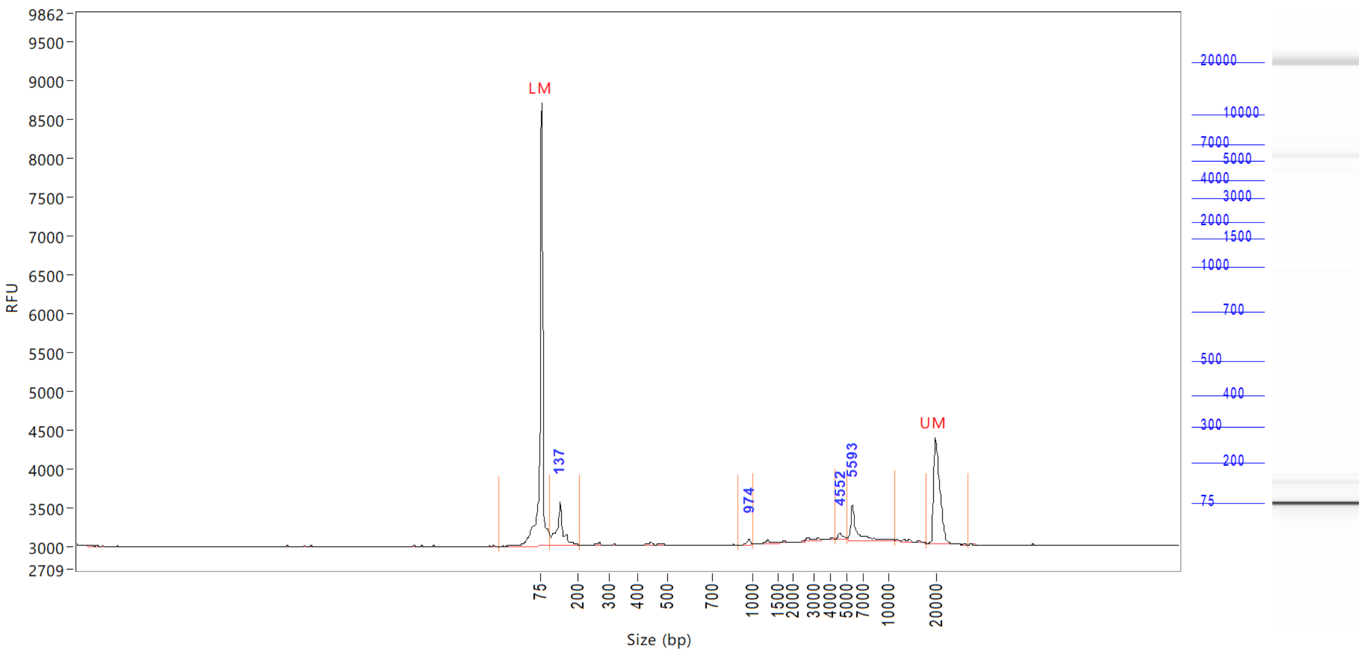

Peak	Size	Concentration	From	To	Average size	CV%	RFU	Corrected peak area
	(bp)	(ng/uL)	(bp)	(bp)	(bp)			
1	75 (LM)	0.5000	0	105	68	23.91	5710	43.402
2	137	1.1597	105	205	136	13.33	572	8.389
3	974	0.0588	887	1012	963	1.67	68	0.425
4	4552	0.1087	4311	5000	4614	3.94	95	0.786
5	5593	0.5808	5000	11098	6389	20.95	461	4.201
6	20000 (UM)	0.1328	18171	27133	20316	3.58	1362	11.530

TIC:	1.9080	ng/uL
TIM:	14.3014	nmole/L
Total concentration:	2.5380	ng/uL

Sample peak width (sec): 5 Sample min peak height: 50 Sample baseline V to V?: Y Sample baseline V to V points: 3
Sample filter: Binomial Number of points for filter: 3 Sample start region (min): 0 Sample end region (min): 30
Marker peak width (sec): 5 Marker min peak height: 200 Marker baseline V to V?: Y Marker baseline V to V points: 3
Lower marker selection: First peak > 500 RFU Upper marker selection: Last peak > 200 RFU
Ladder size (bp)75, 200, 300, 400, 500, 700, 1000, 1500, 2000, 3000, 4000, 5000, 7000, 10000, 20000
Quantification using: Lower Marker Final concentration (ng/uL): 0.5000 Dilution factor: 12.0

Sample: SampE6
Well location: E6
Created: Monday, September 20, 2021 8:39:56 PM

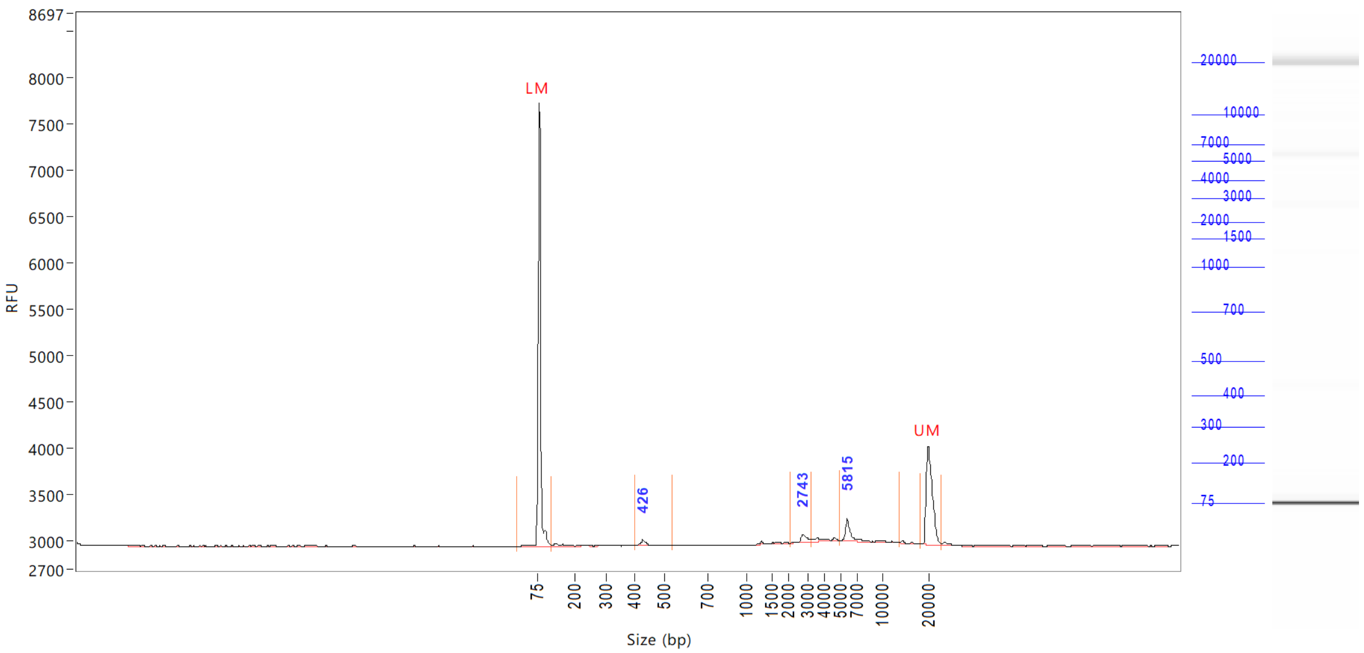

Peak	Size (bp)	Concentration (ng/uL)	From (bp)	To (bp)	Average size (bp)	CV%	RFU	Corrected peak area
1	75 (LM)	0.5000	3	116	74	10.06	4792	28.355
2	426	0.1612	398	539	437	6.00	59	0.762
3	2743	0.1854	2087	3144	2753	6.99	89	0.876
4	5815	0.4907	4966	13659	6858	27.55	252	2.319
5	20000 (UM)	0.1548	18415	22901	20268	3.32	1059	8.779

TIC:	0.8373	ng/uL
TIM:	0.8355	nmole/L
Total concentration:	1.4202	ng/uL

Sample peak width (sec): 5   Sample min peak height: 50   Sample baseline V to V?: Y   Sample baseline V to V points: 3
Sample filter: Binomial   Number of points for filter: 3   Sample start region (min): 0   Sample end region (min): 30
Marker peak width (sec): 5   Marker min peak height: 200   Marker baseline V to V?: Y   Marker baseline V to V points: 3
Lower marker selection: First peak > 500 RFU   Upper marker selection: Last peak > 200 RFU
Ladder size (bp)75, 200, 300, 400, 500, 700, 1000, 1500, 2000, 3000, 4000, 5000, 7000, 10000, 20000
Quantification using: Lower Marker   Final concentration (ng/uL): 0.5000   Dilution factor: 12.0

Sample: SampF6
Well location: F6
Created: Monday, September 20, 2021 8:39:56 PM

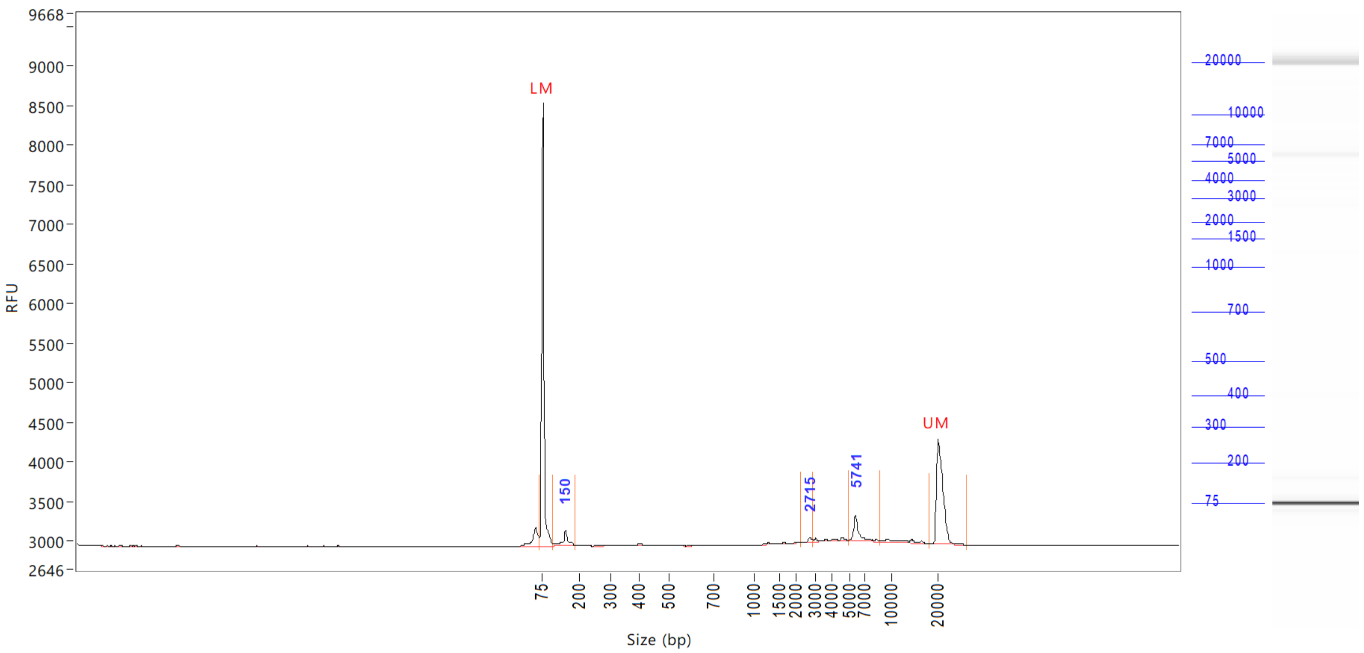

Peak	Size (bp)	Concentration (ng/uL)	From (bp)	To (bp)	Average size (bp)	CV%	RFU	Corrected peak area
1	75 (LM)	0.5000	64	110	74	7.62	5608	32.831
2	150	0.4295	110	183	145	10.50	189	2.350
3	2715	0.0598	2258	2857	2679	4.12	53	0.327
4	5741	0.4934	4931	8667	6105	12.78	334	2.700
5	20000 (UM)	0.1700	18171	26166	20429	3.63	1324	11.164

TIC:	0.9827	ng/uL
TIM:	5.0376	nmole/L
Total concentration:	1.6556	ng/uL

```
Sample peak width (sec): 5    Sample min peak height: 50    Sample baseline V to V?: Y    Sample baseline V to V points: 3
Sample filter: Binomial       Number of points for filter: 3  Sample start region (min): 0  Sample end region (min): 30
Marker peak width (sec): 5    Marker min peak height: 200   Marker baseline V to V?: Y    Marker baseline V to V points: 3
Lower marker selection: First peak > 500 RFU                Upper marker selection: Last peak > 200 RFU
Ladder size (bp)75, 200, 300, 400, 500, 700, 1000, 1500, 2000, 3000, 4000, 5000, 7000, 10000, 20000
Quantification using: Lower Marker          Final concentration (ng/uL): 0.5000          Dilution factor: 12.0
```

Sample: SampG6
Well location: G6
Created: Monday, September 20, 2021 8:39:56 PM

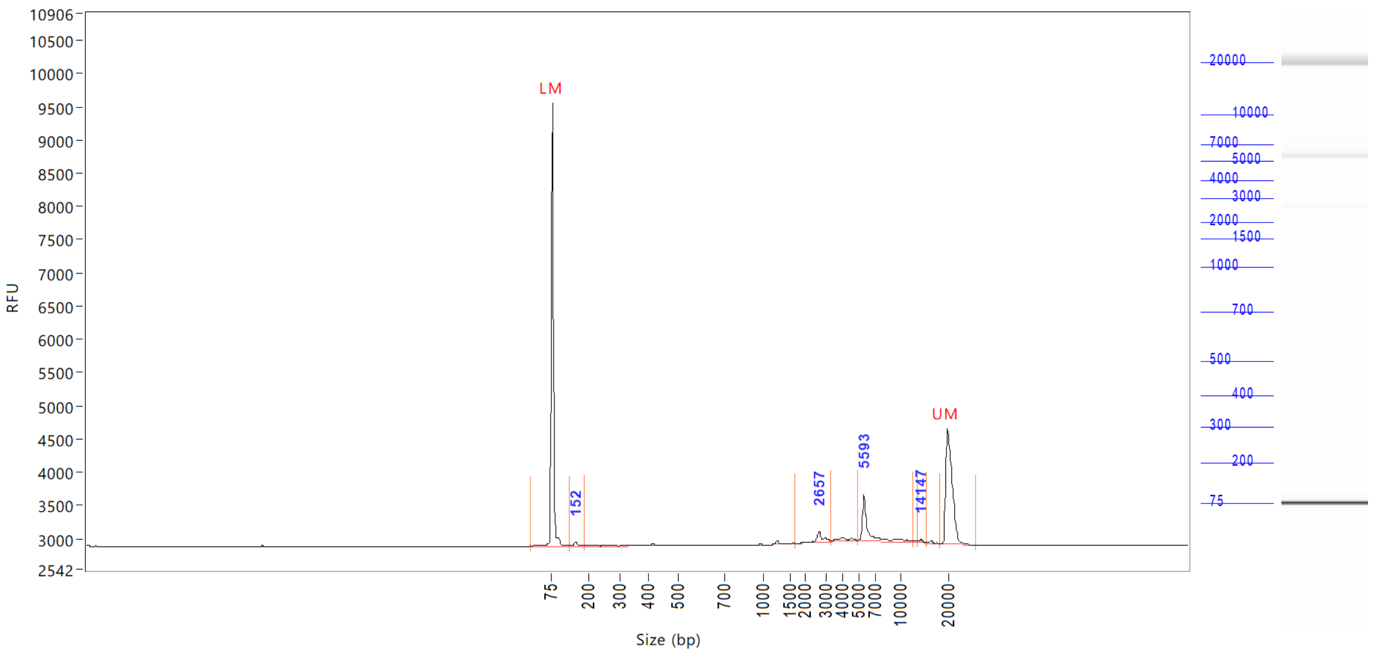

Peak	Size (bp)	Concentration (ng/uL)	From (bp)	To (bp)	Average size (bp)	CV%	RFU	Corrected peak area
1	75 (LM)	0.5000	7	133	74	9.97	6683	38.650
2	152	0.1040	133	183	154	7.45	56	0.670
3	2657	0.2272	1674	3322	2723	9.25	159	1.463
4	5593	0.9428	4897	12440	6666	26.43	701	6.073
5	14147	0.0432	13537	15244	14190	2.83	52	0.279
6	20000 (UM)	0.1916	18293	25924	20319	3.65	1725	14.812

TIC:	1.3172	ng/uL
TIM:	1.4852	nmole/L
Total concentration:	1.8412	ng/uL

```
Sample peak width (sec): 5    Sample min peak height: 50    Sample baseline V to V?: Y    Sample baseline V to V points: 3
Sample filter: Binomial       Number of points for filter: 3  Sample start region (min): 0  Sample end region (min): 30
Marker peak width (sec): 5    Marker min peak height: 200   Marker baseline V to V?: Y    Marker baseline V to V points: 3
Lower marker selection: First peak > 500 RFU                Upper marker selection: Last peak > 200 RFU
Ladder size (bp)75, 200, 300, 400, 500, 700, 1000, 1500, 2000, 3000, 4000, 5000, 7000, 10000, 20000
Quantification using: Lower Marker          Final concentration (ng/uL): 0.5000          Dilution factor: 12.0
```

Sample: SampH6
Well location: H6
Created: Monday, September 20, 2021 8:39:56 PM

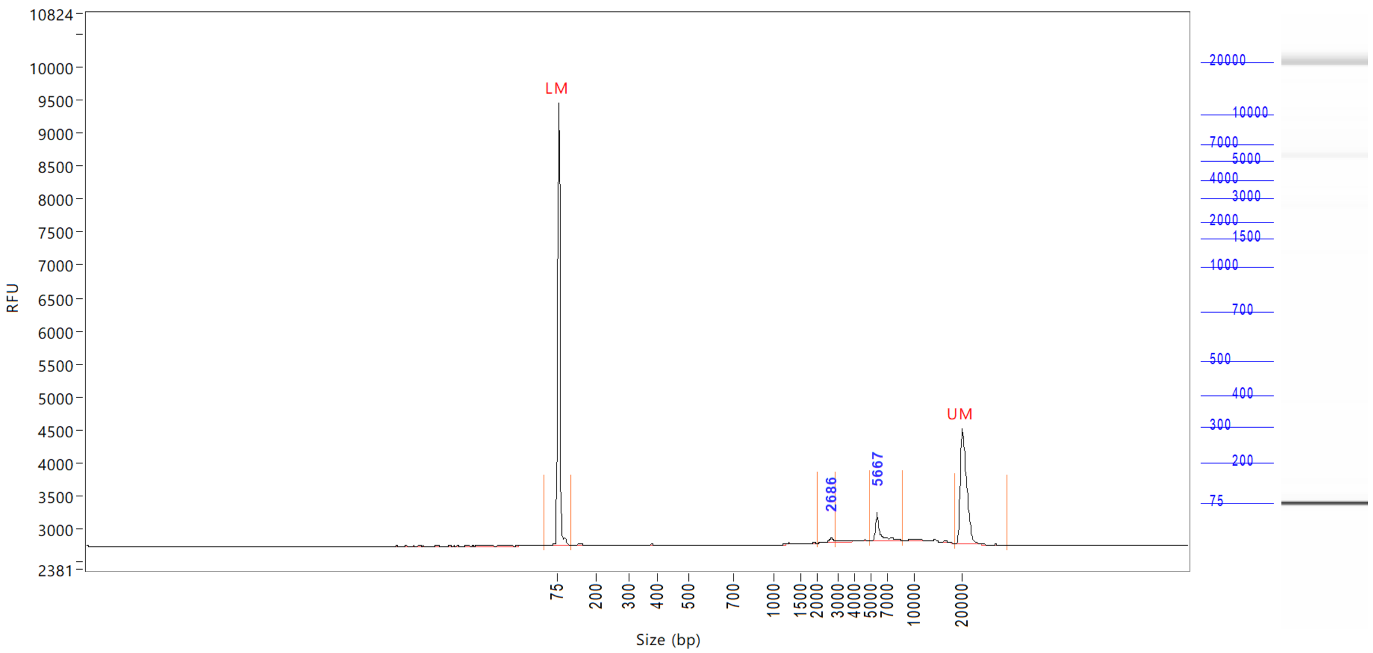

Peak	Size (bp)	Concentration (ng/uL)	From (bp)	To (bp)	Average size (bp)	CV%	RFU	Corrected peak area
1	75 (LM)	0.5000	30	114	73	7.47	6738	37.683
2	2686	0.0783	2001	2886	2611	6.41	65	0.492
3	5667	0.4990	4897	8734	6082	13.53	430	3.134
4	20000 (UM)	0.1970	18415	29672	20423	4.25	1755	14.848

TIC:	0.5773	ng/uL
TIM:	0.1845	nmole/L
Total concentration:	1.1563	ng/uL

```
Sample peak width (sec): 5    Sample min peak height: 50    Sample baseline V to V?: Y    Sample baseline V to V points: 3
Sample filter: Binomial       Number of points for filter: 3  Sample start region (min): 0  Sample end region (min): 30
Marker peak width (sec): 5    Marker min peak height: 200   Marker baseline V to V?: Y    Marker baseline V to V points: 3
Lower marker selection: First peak > 500 RFU                Upper marker selection: Last peak > 200 RFU
Ladder size (bp)75, 200, 300, 400, 500, 700, 1000, 1500, 2000, 3000, 4000, 5000, 7000, 10000, 20000
Quantification using: Lower Marker          Final concentration (ng/uL): 0.5000          Dilution factor: 12.0
```

Sample: SampA7
Well location: A7
Created: Monday, September 20, 2021 8:39:56 PM

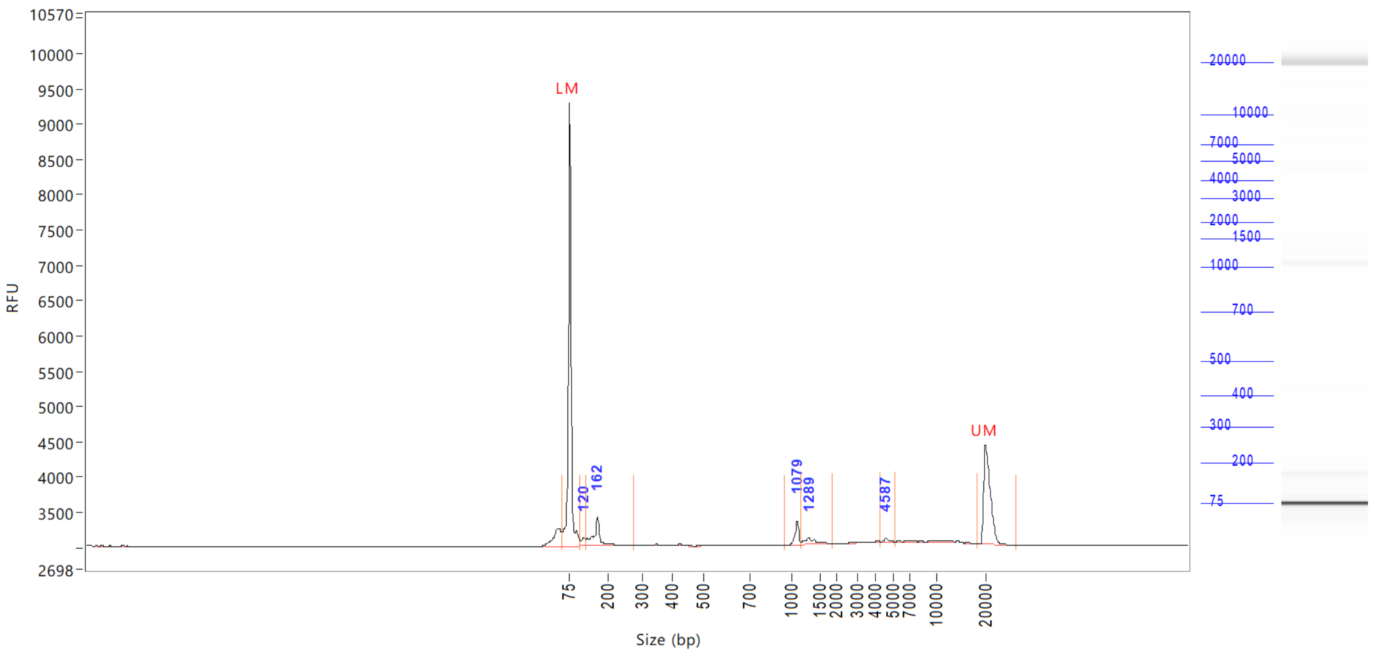

Peak	Size (bp)	Concentration (ng/uL)	From (bp)	To (bp)	Average size (bp)	CV%	RFU	Corrected peak area
1	75 (LM)	0.5000	50	110	73	10.54	6286	41.569
2	120	0.1574	110	126	118	4.00	117	1.090
3	162	0.8377	126	277	160	14.74	405	5.804
4	1079	0.2970	948	1156	1060	3.55	338	2.058
5	1289	0.2257	1156	1904	1345	9.75	92	1.564
6	4587	0.0556	4311	5297	4655	3.87	55	0.385
7	20000 (UM)	0.1438	18293	26045	20324	3.66	1407	11.958

TIC:	1.5734	ng/uL
TIM:	11.5541	nmole/L
Total concentration:	1.9460	ng/uL

Sample peak width (sec): 5 Sample min peak height: 50 Sample baseline V to V?: Y Sample baseline V to V points: 3
Sample filter: Binomial Number of points for filter: 3 Sample start region (min): 0 Sample end region (min): 30
Marker peak width (sec): 5 Marker min peak height: 200 Marker baseline V to V?: Y Marker baseline V to V points: 3
Lower marker selection: First peak > 500 RFU Upper marker selection: Last peak > 200 RFU
Ladder size (bp)75, 200, 300, 400, 500, 700, 1000, 1500, 2000, 3000, 4000, 5000, 7000, 10000, 20000
Quantification using: Lower Marker Final concentration (ng/uL): 0.5000 Dilution factor: 12.0

Sample: SampB7
Well location: B7
Created: Monday, September 20, 2021 8:39:56 PM

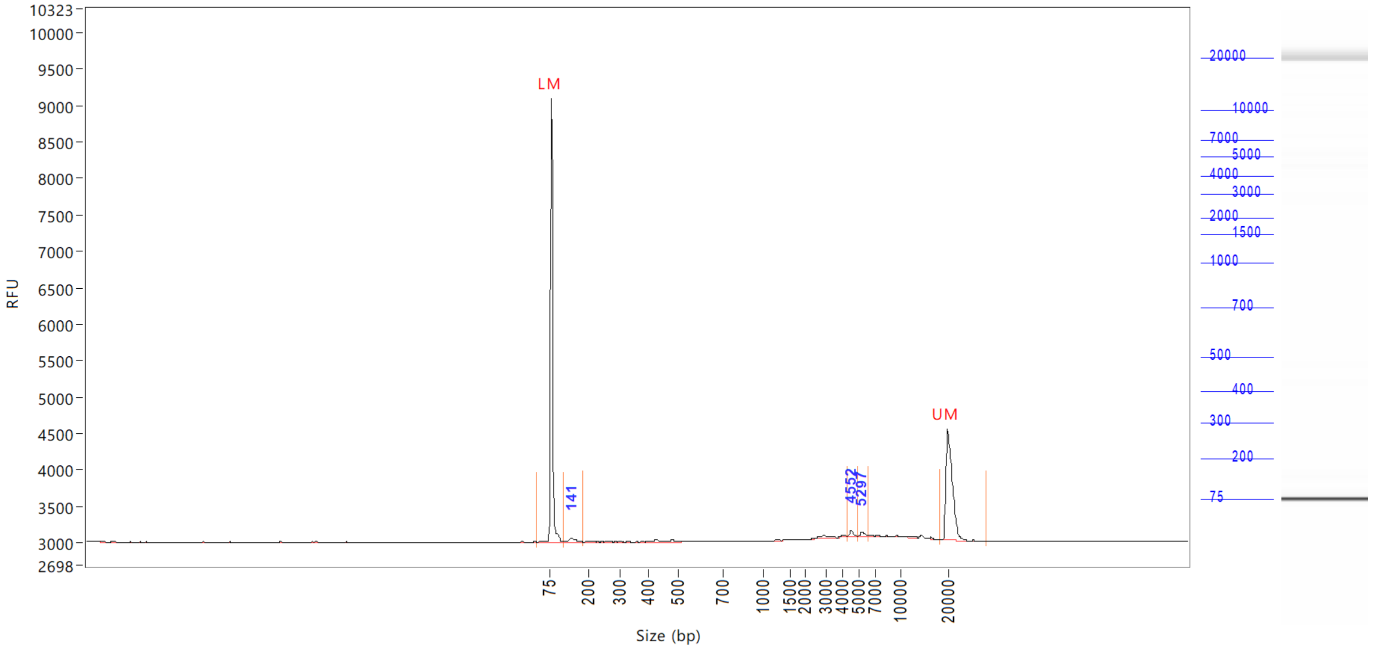

Peak	Size (bp)	Concentration (ng/uL)	From (bp)	To (bp)	Average size (bp)	CV%	RFU	Corrected peak area
1	75 (LM)	0.5000	28	118	74	8.00	6092	34.671
2	141	0.1648	118	183	143	8.95	63	0.952
3	4552	0.0995	4277	4931	4541	3.00	96	0.575
4	5297	0.0769	4931	6112	5404	5.61	57	0.444
5	20000 (UM)	0.1873	18415	28221	20402	4.10	1515	12.989

TIC:	0.3412	ng/uL
TIM:	1.9535	nmole/L
Total concentration:	0.9791	ng/uL

Sample peak width (sec): 5   Sample min peak height: 50   Sample baseline V to V?: Y   Sample baseline V to V points: 3
Sample filter: Binomial   Number of points for filter: 3   Sample start region (min): 0   Sample end region (min): 30
Marker peak width (sec): 5   Marker min peak height: 200   Marker baseline V to V?: Y   Marker baseline V to V points: 3
Lower marker selection: First peak > 500 RFU   Upper marker selection: Last peak > 200 RFU
Ladder size (bp)75, 200, 300, 400, 500, 700, 1000, 1500, 2000, 3000, 4000, 5000, 7000, 10000, 20000
Quantification using: Lower Marker   Final concentration (ng/uL): 0.5000   Dilution factor: 12.0

Sample: SampC7
Well location: C7
Created: Monday, September 20, 2021 8:39:56 PM

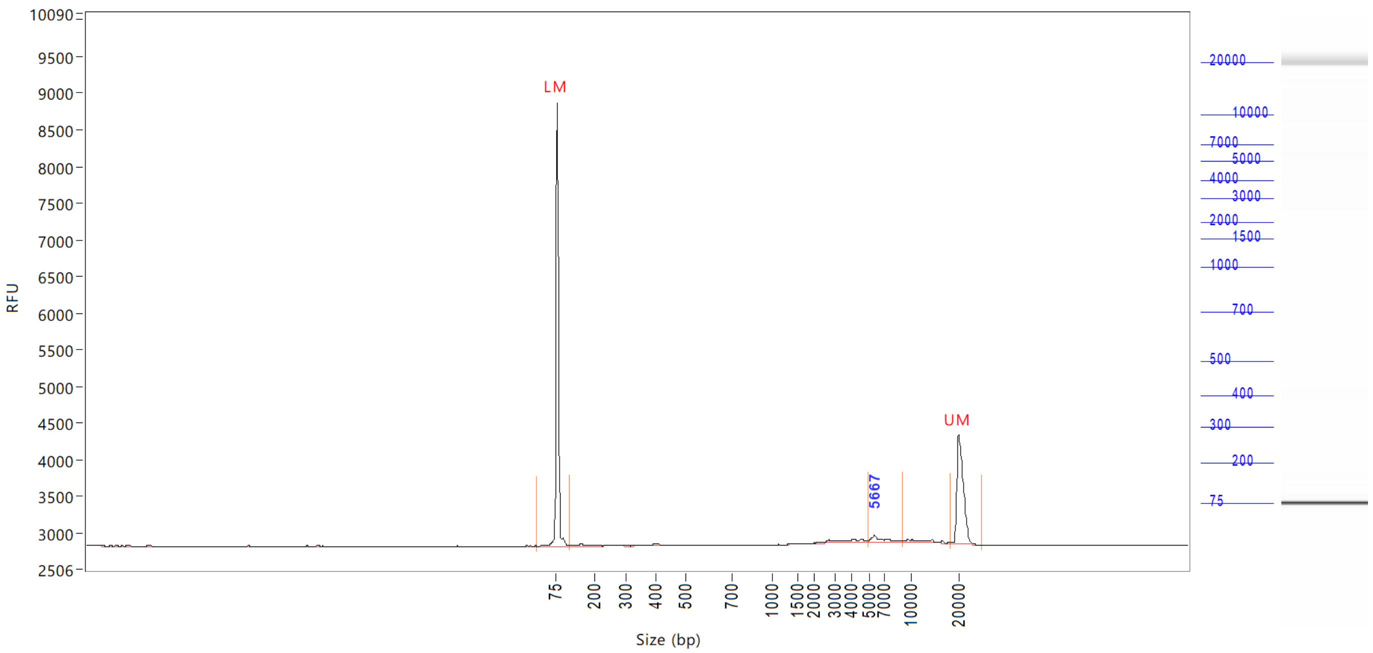

Peak	Size (bp)	Concentration (ng/uL)	From (bp)	To (bp)	Average size (bp)	CV%	RFU	Corrected peak area
1	75 (LM)	0.5000	11	118	74	8.39	6057	34.359
2	5667	0.2579	4931	9067	6486	16.92	91	1.477
3	20000 (UM)	0.1825	18293	24836	20323	3.40	1482	12.543

TIC:	0.2579	ng/uL
TIM:	0.0655	nmole/L
Total concentration:	1.1420	ng/uL

Sample peak width (sec): 5 Sample min peak height: 50 Sample baseline V to V?: Y Sample baseline V to V points: 3
Sample filter: Binomial Number of points for filter: 3 Sample start region (min): 0 Sample end region (min): 30
Marker peak width (sec): 5 Marker min peak height: 200 Marker baseline V to V?: Y Marker baseline V to V points: 3
Lower marker selection: First peak > 500 RFU Upper marker selection: Last peak > 200 RFU
Ladder size (bp)75, 200, 300, 400, 500, 700, 1000, 1500, 2000, 3000, 4000, 5000, 7000, 10000, 20000
Quantification using: Lower Marker Final concentration (ng/uL): 0.5000 Dilution factor: 12.0

Sample: SampD7
Well location: D7
Created: Monday, September 20, 2021 8:39:56 PM

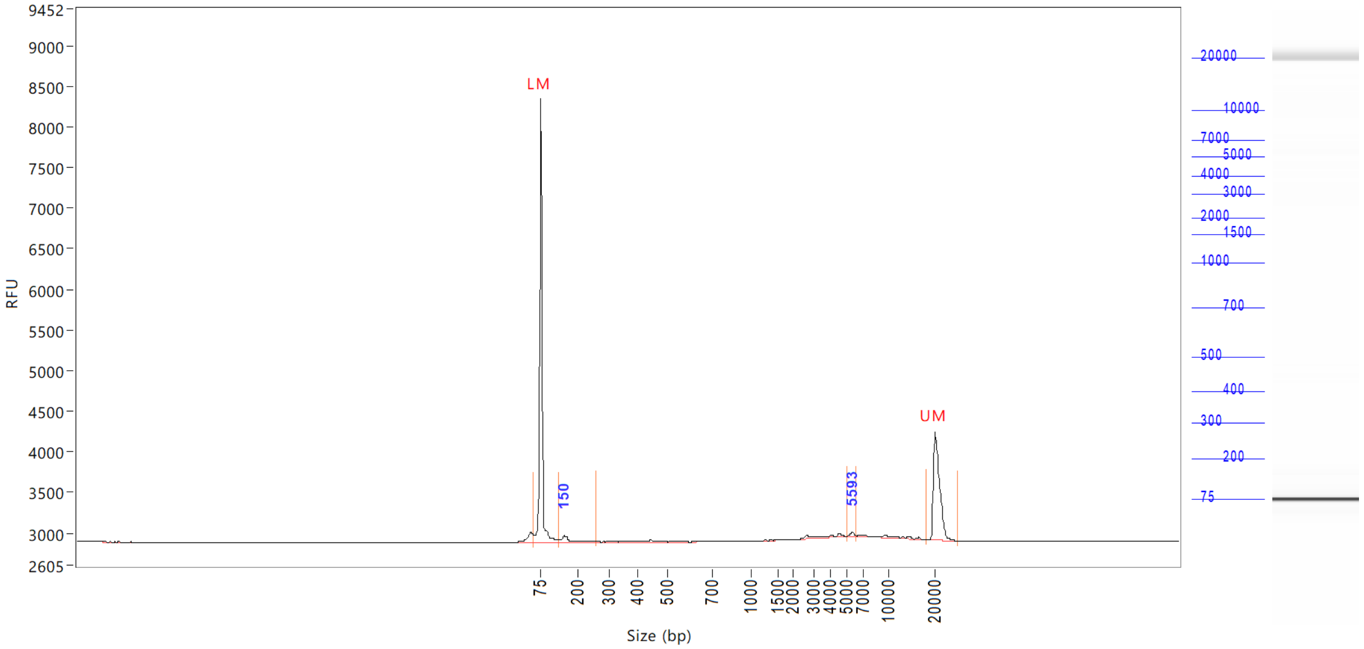

Peak	Size (bp)	Concentration (ng/uL)	From (bp)	To (bp)	Average size (bp)	CV%	RFU	Corrected peak area
1	75 (LM)	0.5000	50	133	74	11.97	5472	34.694
2	150	0.2463	133	257	164	15.41	80	1.424
3	5593	0.0723	5001	6260	5600	5.04	64	0.418
4	20000 (UM)	0.1611	18171	24957	20406	3.57	1337	11.180

TIC:	0.3187	ng/uL
TIM:	2.4904	nmole/L
Total concentration:	0.9995	ng/uL

```
Sample peak width (sec): 5    Sample min peak height: 50    Sample baseline V to V?: Y    Sample baseline V to V points: 3
Sample filter: Binomial       Number of points for filter: 3  Sample start region (min): 0  Sample end region (min): 30
Marker peak width (sec): 5    Marker min peak height: 200   Marker baseline V to V?: Y    Marker baseline V to V points: 3
Lower marker selection: First peak > 500 RFU                Upper marker selection: Last peak > 200 RFU
Ladder size (bp)75, 200, 300, 400, 500, 700, 1000, 1500, 2000, 3000, 4000, 5000, 7000, 10000, 20000
Quantification using: Lower Marker          Final concentration (ng/uL): 0.5000          Dilution factor: 12.0
```

Sample: SampE7
Well location: E7
Created: Monday, September 20, 2021 8:39:56 PM

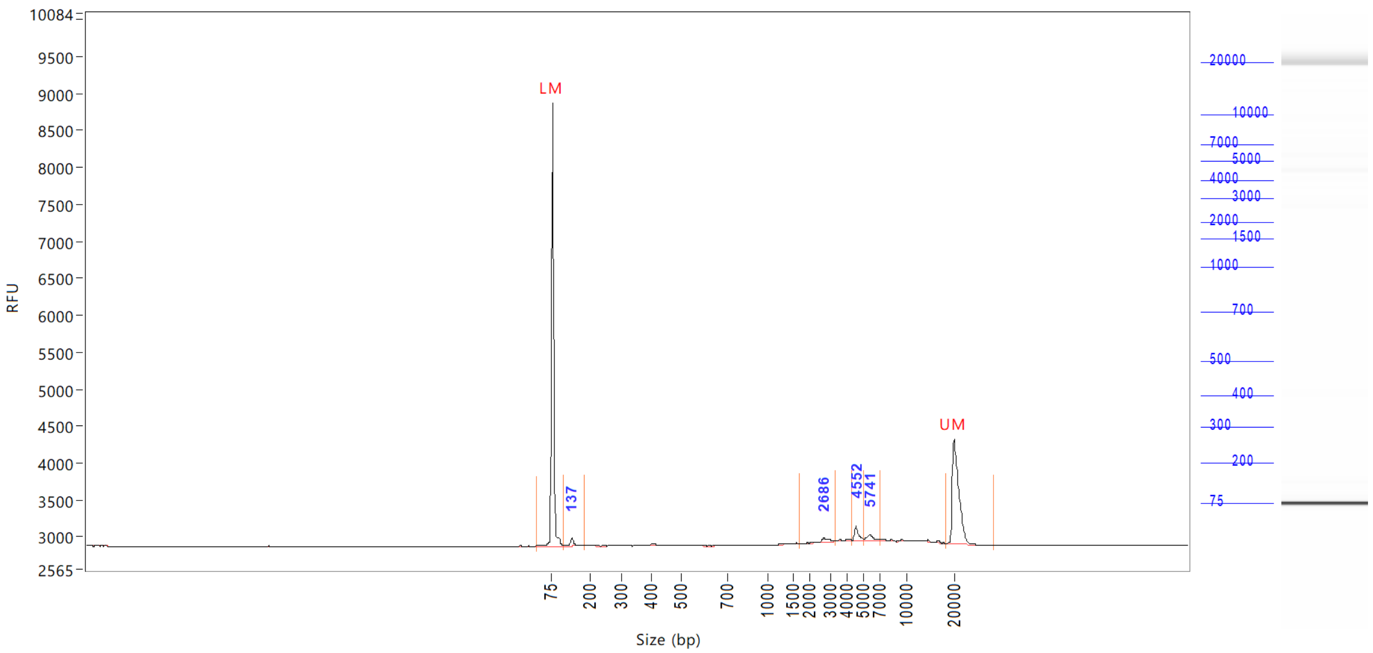

Peak	Size (bp)	Concentration (ng/uL)	From (bp)	To (bp)	Average size (bp)	CV%	RFU	Corrected peak area
1	75 (LM)	0.5000	22	112	73	7.81	6005	34.457
2	137	0.1703	112	179	136	8.56	103	0.978
3	2686	0.1173	1674	3322	2742	9.64	61	0.673
4	4552	0.2138	4311	5000	4565	3.25	207	1.228
5	5741	0.1530	5000	7001	5795	8.87	89	0.879
6	20000 (UM)	0.1820	18293	28342	20456	4.23	1412	12.544

TIC:	0.6544	ng/uL
TIM:	2.2487	nmole/L
Total concentration:	1.1445	ng/uL

```
Sample peak width (sec): 5    Sample min peak height: 50    Sample baseline V to V?: Y    Sample baseline V to V points: 3
Sample filter: Binomial       Number of points for filter: 3  Sample start region (min): 0  Sample end region (min): 30
Marker peak width (sec): 5    Marker min peak height: 200   Marker baseline V to V?: Y    Marker baseline V to V points: 3
Lower marker selection: First peak > 500 RFU                Upper marker selection: Last peak > 200 RFU
Ladder size (bp)75, 200, 300, 400, 500, 700, 1000, 1500, 2000, 3000, 4000, 5000, 7000, 10000, 20000
Quantification using: Lower Marker          Final concentration (ng/uL): 0.5000          Dilution factor: 12.0
```

Sample: SampF7
Well location: F7
Created: Monday, September 20, 2021 8:39:56 PM

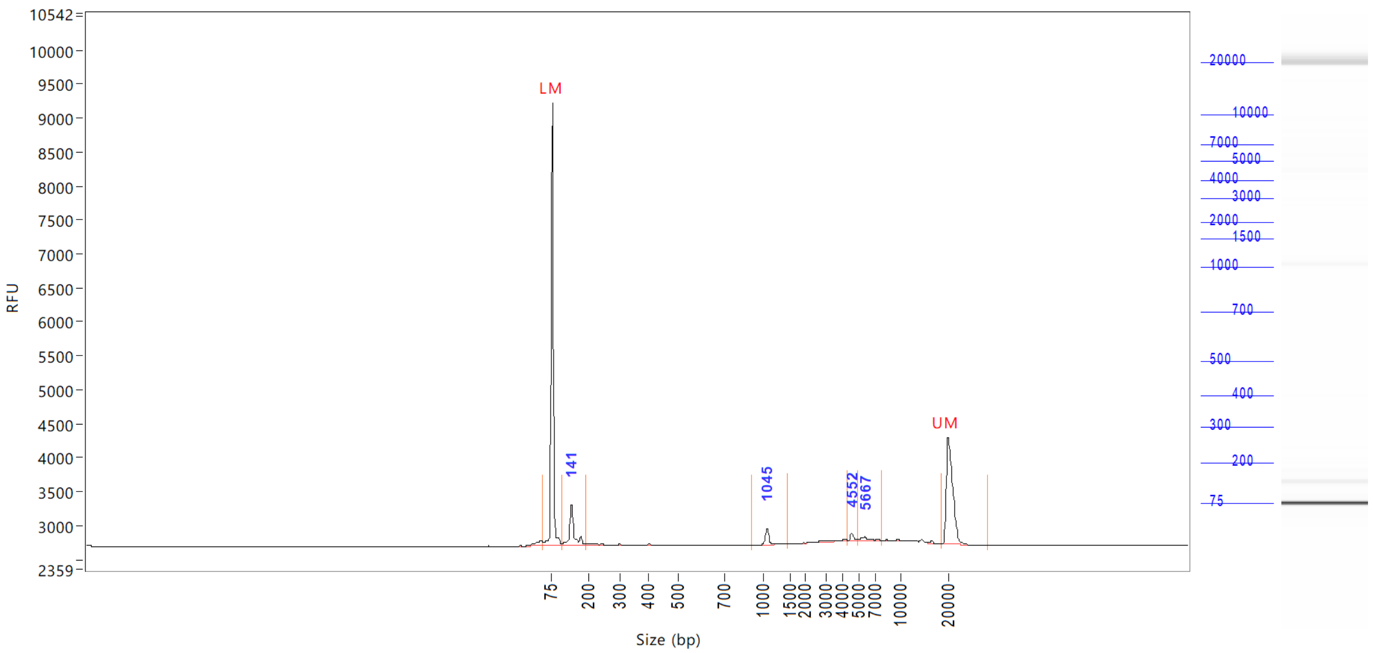

Peak	Size (bp)	Concentration (ng/uL)	From (bp)	To (bp)	Average size (bp)	CV%	RFU	Corrected peak area
1	75 (LM)	0.5000	47	107	73	7.38	6526	37.540
2	141	0.9522	107	187	141	10.15	600	5.957
3	1045	0.2590	909	1456	1071	8.01	243	1.620
4	4552	0.1174	4345	4931	4593	3.12	116	0.735
5	5667	0.1541	4931	7734	6037	13.42	62	0.964
6	20000 (UM)	0.1771	18537	28342	20463	4.07	1573	13.297

TIC:	1.4827	ng/uL
TIM:	11.5794	nmole/L
Total concentration:	1.9651	ng/uL

Sample peak width (sec): 5 Sample min peak height: 50 Sample baseline V to V?: Y Sample baseline V to V points: 3
Sample filter: Binomial Number of points for filter: 3 Sample start region (min): 0 Sample end region (min): 30
Marker peak width (sec): 5 Marker min peak height: 200 Marker baseline V to V?: Y Marker baseline V to V points: 3
Lower marker selection: First peak > 500 RFU Upper marker selection: Last peak > 200 RFU
Ladder size (bp)75, 200, 300, 400, 500, 700, 1000, 1500, 2000, 3000, 4000, 5000, 7000, 10000, 20000
Quantification using: Lower Marker Final concentration (ng/uL): 0.5000 Dilution factor: 12.0

Sample: SampG7
Well location: G7
Created: Monday, September 20, 2021 8:39:56 PM

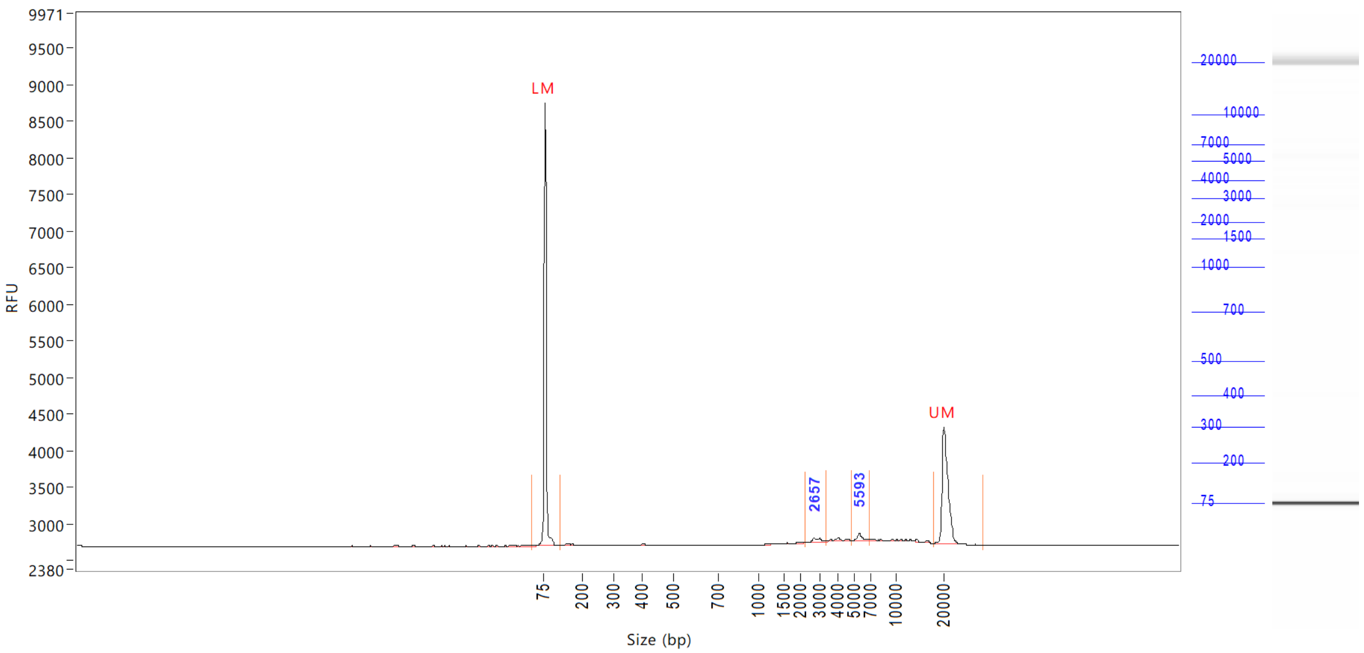

Peak	Size (bp)	Concentration (ng/uL)	From (bp)	To (bp)	Average size (bp)	CV%	RFU	Corrected peak area
1	75 (LM)	0.5000	31	128	73	8.19	6060	34.384
2	2657	0.1359	2258	3358	2797	8.20	64	0.779
3	5593	0.1336	4897	6926	5677	7.79	104	0.765
4	20000 (UM)	0.1843	18049	28463	20371	3.62	1585	12.676

TIC:	0.2694	ng/uL
TIM:	0.1187	nmole/L
Total concentration:	0.8946	ng/uL

Sample peak width (sec): 5   Sample min peak height: 50   Sample baseline V to V?: Y   Sample baseline V to V points: 3
Sample filter: Binomial   Number of points for filter: 3   Sample start region (min): 0   Sample end region (min): 30
Marker peak width (sec): 5   Marker min peak height: 200   Marker baseline V to V?: Y   Marker baseline V to V points: 3
Lower marker selection: First peak > 500 RFU   Upper marker selection: Last peak > 200 RFU
Ladder size (bp)75, 200, 300, 400, 500, 700, 1000, 1500, 2000, 3000, 4000, 5000, 7000, 10000, 20000
Quantification using: Lower Marker   Final concentration (ng/uL): 0.5000   Dilution factor: 12.0

Sample: SampH7
Well location: H7
Created: Monday, September 20, 2021 8:39:56 PM

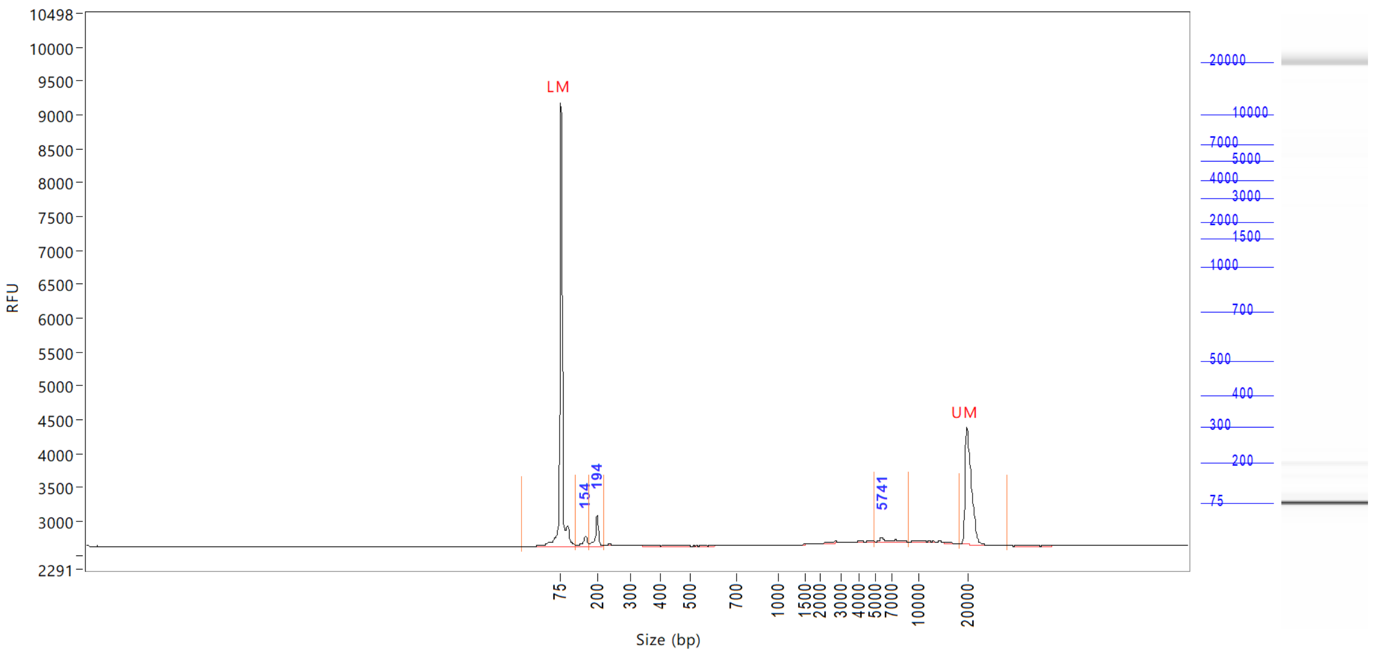

Peak	Size (bp)	Concentration (ng/uL)	From (bp)	To (bp)	Average size (bp)	CV%	RFU	Corrected peak area
1	75 (LM)	0.5000	0	124	74	16.14	6556	42.908
2	154	0.2051	124	166	150	6.31	155	1.466
3	194	0.4587	166	217	189	4.68	449	3.281
4	5741	0.1275	4966	8867	6523	14.88	72	0.912
5	20000 (UM)	0.1688	18293	28342	20379	3.76	1728	14.488

TIC:	0.7914	ng/uL
TIM:	6.2696	nmole/L
Total concentration:	1.1798	ng/uL

```
Sample peak width (sec): 5    Sample min peak height: 50    Sample baseline V to V?: Y    Sample baseline V to V points: 3
Sample filter: Binomial       Number of points for filter: 3  Sample start region (min): 0  Sample end region (min): 30
Marker peak width (sec): 5    Marker min peak height: 200   Marker baseline V to V?: Y    Marker baseline V to V points: 3
Lower marker selection: First peak > 500 RFU                Upper marker selection: Last peak > 200 RFU
Ladder size (bp)75, 200, 300, 400, 500, 700, 1000, 1500, 2000, 3000, 4000, 5000, 7000, 10000, 20000
Quantification using: Lower Marker          Final concentration (ng/uL): 0.5000          Dilution factor: 12.0
```

Sample: SampA8
Well location: A8
Created: Monday, September 20, 2021 8:39:56 PM

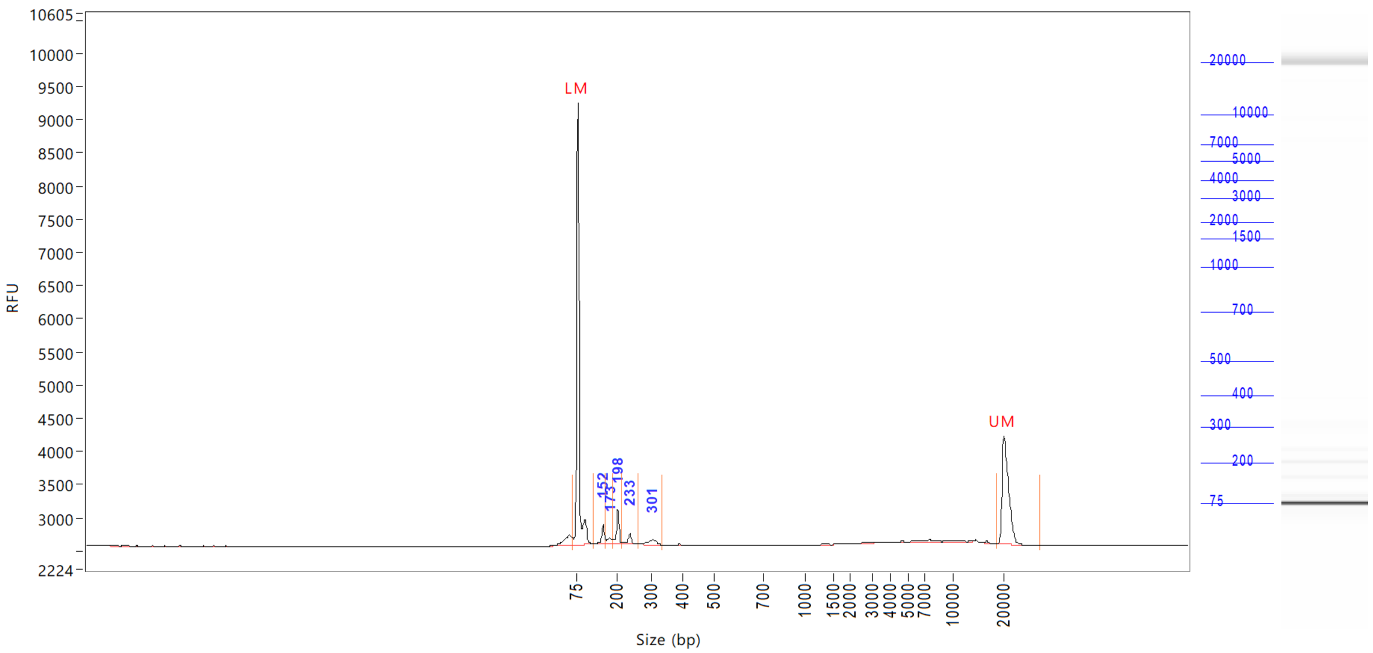

Peak	Size (bp)	Concentration (ng/uL)	From (bp)	To (bp)	Average size (bp)	CV%	RFU	Corrected peak area
1	75 (LM)	0.5000	62	124	75	9.35	6671	39.057
2	152	0.3184	124	162	150	4.14	299	2.073
3	173	0.1988	162	187	174	4.22	94	1.294
4	198	0.4849	187	210	197	2.23	519	3.157
5	233	0.2359	210	259	229	3.55	177	1.536
6	301	0.2034	259	335	297	4.57	71	1.324
7	20000 (UM)	0.1755	18659	27254	20432	3.77	1624	13.712

TIC:	1.4415	ng/uL
TIM:	12.2351	nmole/L
Total concentration:	1.7986	ng/uL

Sample peak width (sec): 5 Sample min peak height: 50 Sample baseline V to V?: Y Sample baseline V to V points: 3
Sample filter: Binomial Number of points for filter: 3 Sample start region (min): 0 Sample end region (min): 30
Marker peak width (sec): 5 Marker min peak height: 200 Marker baseline V to V?: Y Marker baseline V to V points: 3
Lower marker selection: First peak > 500 RFU Upper marker selection: Last peak > 200 RFU
Ladder size (bp)75, 200, 300, 400, 500, 700, 1000, 1500, 2000, 3000, 4000, 5000, 7000, 10000, 20000
Quantification using: Lower Marker Final concentration (ng/uL): 0.5000 Dilution factor: 12.0

Sample: SampB8
Well location: B8
Created: Monday, September 20, 2021 8:39:56 PM

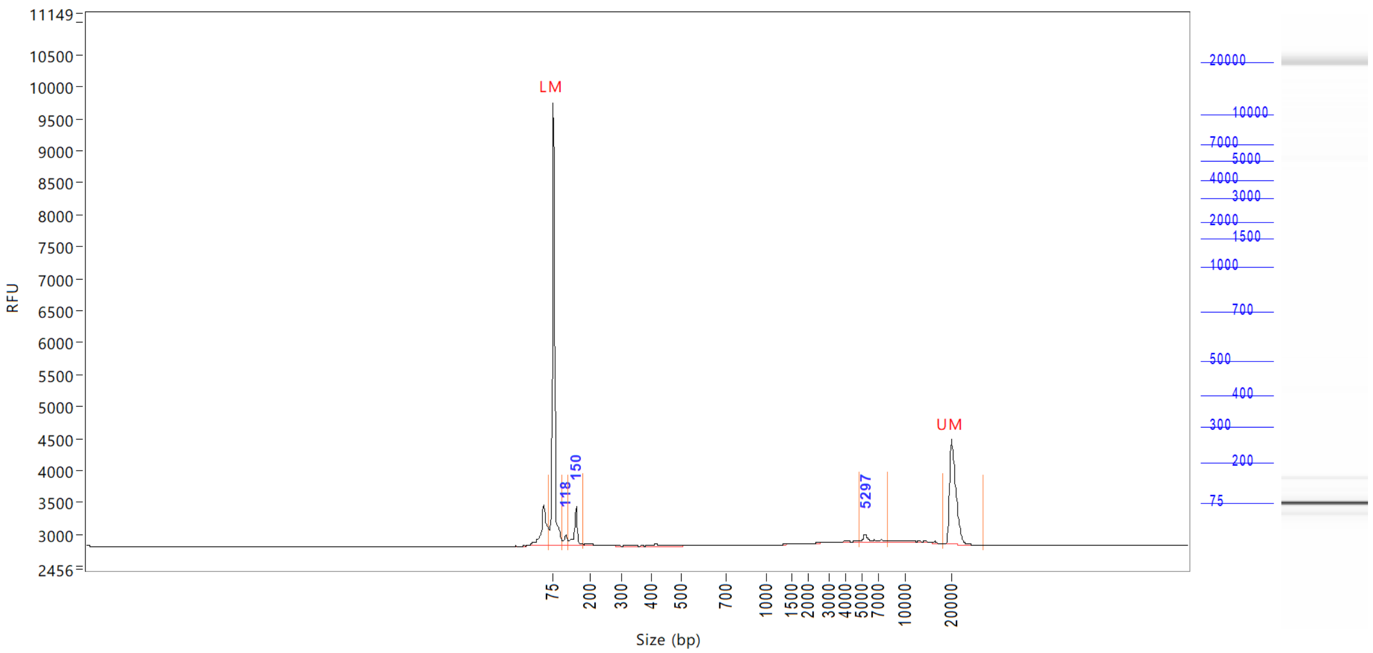

Peak	Size (bp)	Concentration (ng/uL)	From (bp)	To (bp)	Average size (bp)	CV%	RFU	Corrected peak area
1	75 (LM)	0.5000	64	107	74	7.77	6928	41.679
2	118	0.1868	107	126	115	4.44	167	1.298
3	150	0.6030	126	177	146	5.38	591	4.189
4	5297	0.1829	4862	8001	5932	14.80	116	1.271
5	20000 (UM)	0.1671	18293	26649	20372	4.00	1637	13.929

TIC:	0.9727	ng/uL
TIM:	9.5067	nmole/L
Total concentration:	1.5151	ng/uL

Sample peak width (sec): 5 Sample min peak height: 50 Sample baseline V to V?: Y Sample baseline V to V points: 3
Sample filter: Binomial Number of points for filter: 3 Sample start region (min): 0 Sample end region (min): 30
Marker peak width (sec): 5 Marker min peak height: 200 Marker baseline V to V?: Y Marker baseline V to V points: 3
Lower marker selection: First peak > 500 RFU Upper marker selection: Last peak > 200 RFU
Ladder size (bp)75, 200, 300, 400, 500, 700, 1000, 1500, 2000, 3000, 4000, 5000, 7000, 10000, 20000
Quantification using: Lower Marker Final concentration (ng/uL): 0.5000 Dilution factor: 12.0

Sample: SampC8
Well location: C8
Created: Monday, September 20, 2021 8:39:56 PM

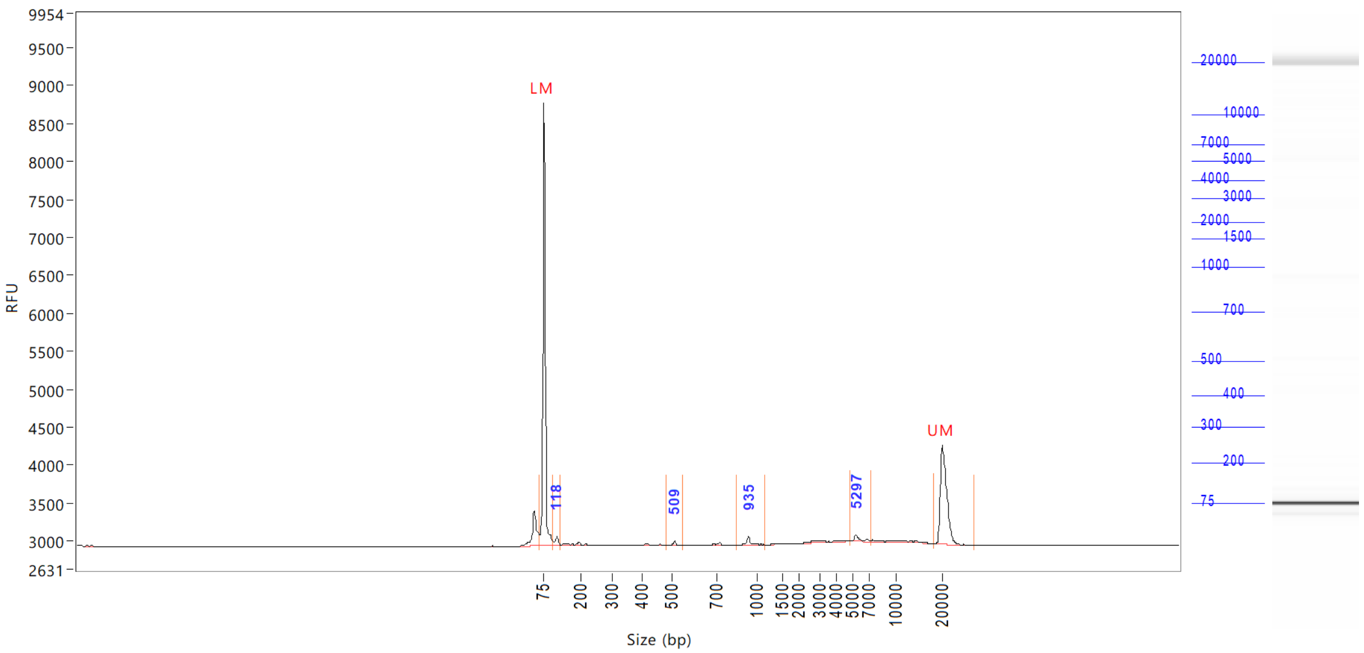

Peak	Size (bp)	Concentration (ng/uL)	From (bp)	To (bp)	Average size (bp)	CV%	RFU	Corrected peak area
1	75 (LM)	0.5000	60	107	74	7.60	5841	34.873
2	118	0.1595	107	131	115	4.44	117	0.927
3	509	0.0903	481	549	507	2.34	72	0.525
4	935	0.1416	848	1156	938	4.60	107	0.823
5	5297	0.1356	4862	7134	5698	11.20	82	0.788
6	20000 (UM)	0.1609	18049	26649	20346	3.85	1314	11.226

TIC:	0.5271	ng/uL
TIM:	2.8591	nmole/L
Total concentration:	1.2767	ng/uL

```
Sample peak width (sec): 5    Sample min peak height: 50    Sample baseline V to V?: Y    Sample baseline V to V points: 3
Sample filter: Binomial       Number of points for filter: 3  Sample start region (min): 0  Sample end region (min): 30
Marker peak width (sec): 5    Marker min peak height: 200   Marker baseline V to V?: Y    Marker baseline V to V points: 3
Lower marker selection: First peak > 500 RFU                Upper marker selection: Last peak > 200 RFU
Ladder size (bp)75, 200, 300, 400, 500, 700, 1000, 1500, 2000, 3000, 4000, 5000, 7000, 10000, 20000
Quantification using: Lower Marker          Final concentration (ng/uL): 0.5000          Dilution factor: 12.0
```

Sample: SampD8
Well location: D8
Created: Monday, September 20, 2021 8:39:56 PM

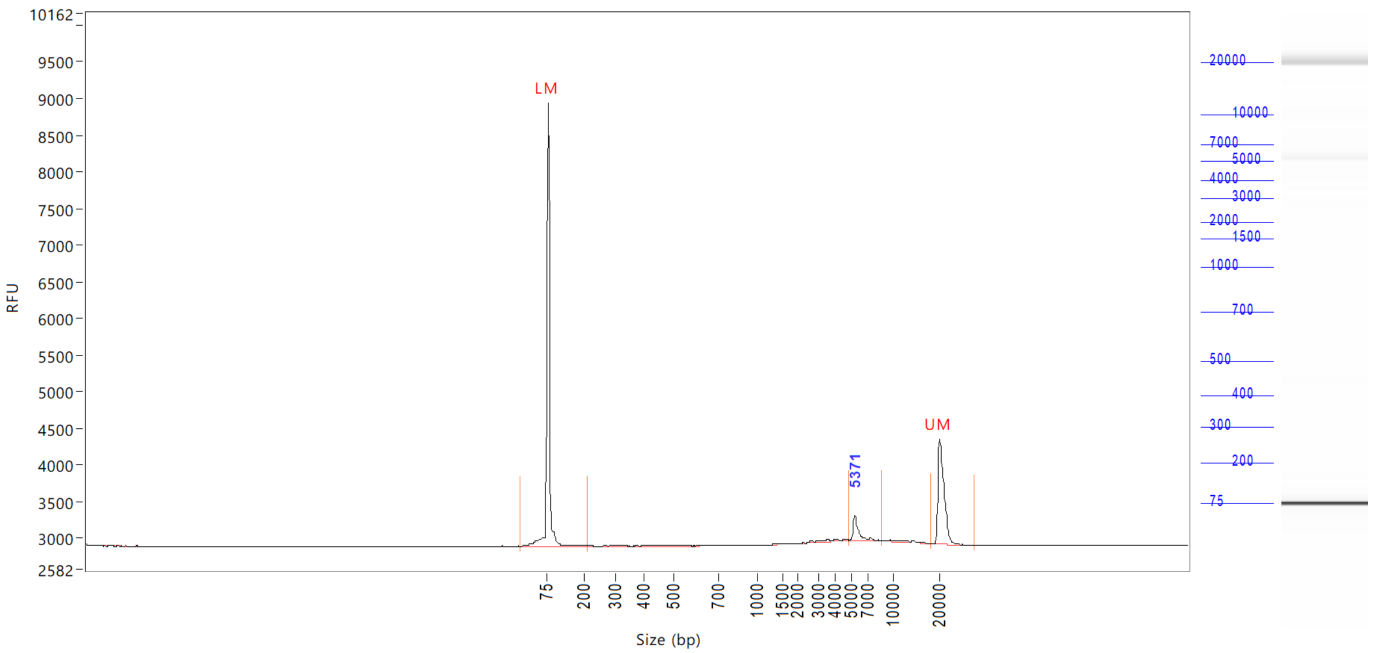

Peak	Size (bp)	Concentration (ng/uL)	From (bp)	To (bp)	Average size (bp)	CV%	RFU	Corrected peak area
1	75 (LM)	0.5000	0	206	72	25.28	6059	40.786
2	5371	0.4778	4862	8734	5827	13.73	361	3.248
3	20000 (UM)	0.1486	18171	27496	20471	3.85	1426	12.122

TIC:	0.4778	ng/uL
TIM:	0.1350	nmole/L
Total concentration:	0.9855	ng/uL

Sample peak width (sec): 5   Sample min peak height: 50   Sample baseline V to V?: Y   Sample baseline V to V points: 3
Sample filter: Binomial   Number of points for filter: 3   Sample start region (min): 0   Sample end region (min): 30
Marker peak width (sec): 5   Marker min peak height: 200   Marker baseline V to V?: Y   Marker baseline V to V points: 3
Lower marker selection: First peak > 500 RFU   Upper marker selection: Last peak > 200 RFU
Ladder size (bp)75, 200, 300, 400, 500, 700, 1000, 1500, 2000, 3000, 4000, 5000, 7000, 10000, 20000
Quantification using: Lower Marker   Final concentration (ng/uL): 0.5000   Dilution factor: 12.0

Sample: SampE8
Well location: E8
Created: Monday, September 20, 2021 8:39:56 PM

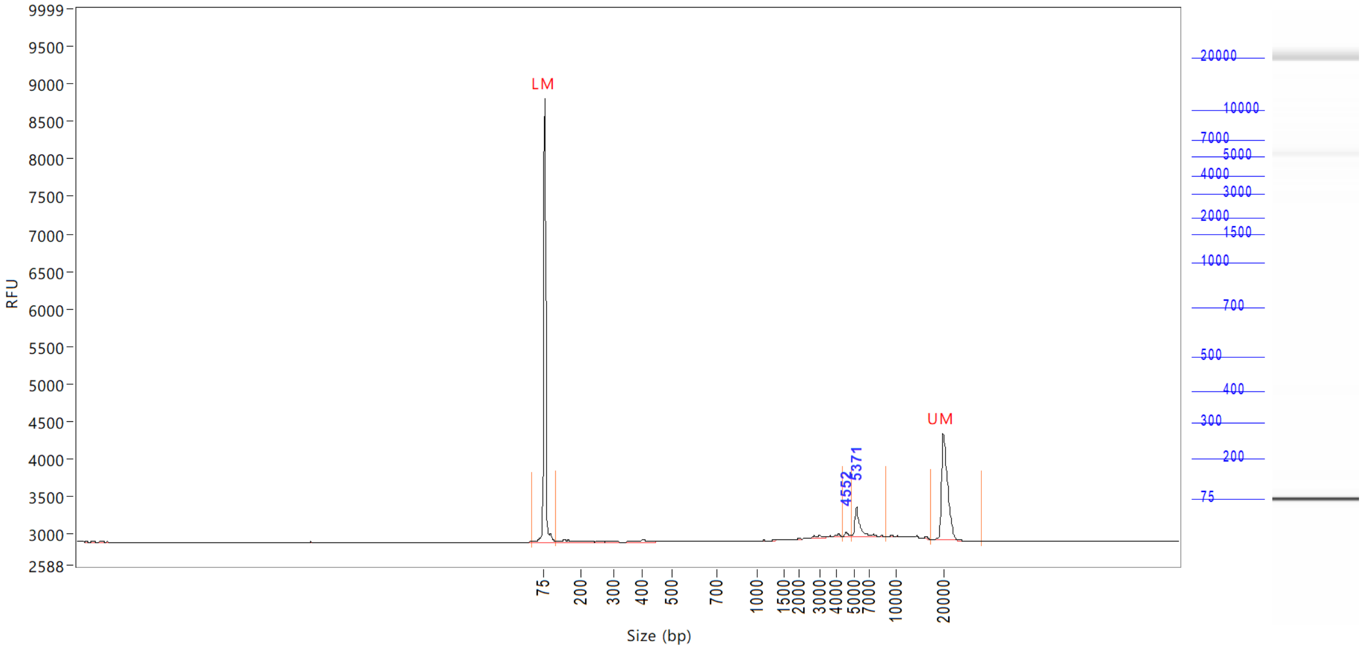

Peak	Size (bp)	Concentration (ng/uL)	From (bp)	To (bp)	Average size (bp)	CV%	RFU	Corrected peak area
1	75 (LM)	0.5000	31	110	73	7.28	5920	33.188
2	4552	0.0771	4345	4897	4593	3.04	63	0.426
3	5371	0.5910	4897	8800	5742	14.07	401	3.269
4	20000 (UM)	0.1760	17195	28100	20403	3.70	1420	11.683

TIC:	0.6681	ng/uL
TIM:	0.1971	nmole/L
Total concentration:	1.2873	ng/uL

Sample peak width (sec): 5   Sample min peak height: 50   Sample baseline V to V?: Y   Sample baseline V to V points: 3
Sample filter: Binomial   Number of points for filter: 3 Sample start region (min): 0 Sample end region (min): 30
Marker peak width (sec): 5   Marker min peak height: 200   Marker baseline V to V?: Y   Marker baseline V to V points: 3
Lower marker selection: First peak > 500 RFU   Upper marker selection: Last peak > 200 RFU
Ladder size (bp)75, 200, 300, 400, 500, 700, 1000, 1500, 2000, 3000, 4000, 5000, 7000, 10000, 20000
Quantification using: Lower Marker   Final concentration (ng/uL): 0.5000   Dilution factor: 12.0

Sample: SampF8
Well location: F8
Created: Monday, September 20, 2021 8:39:56 PM

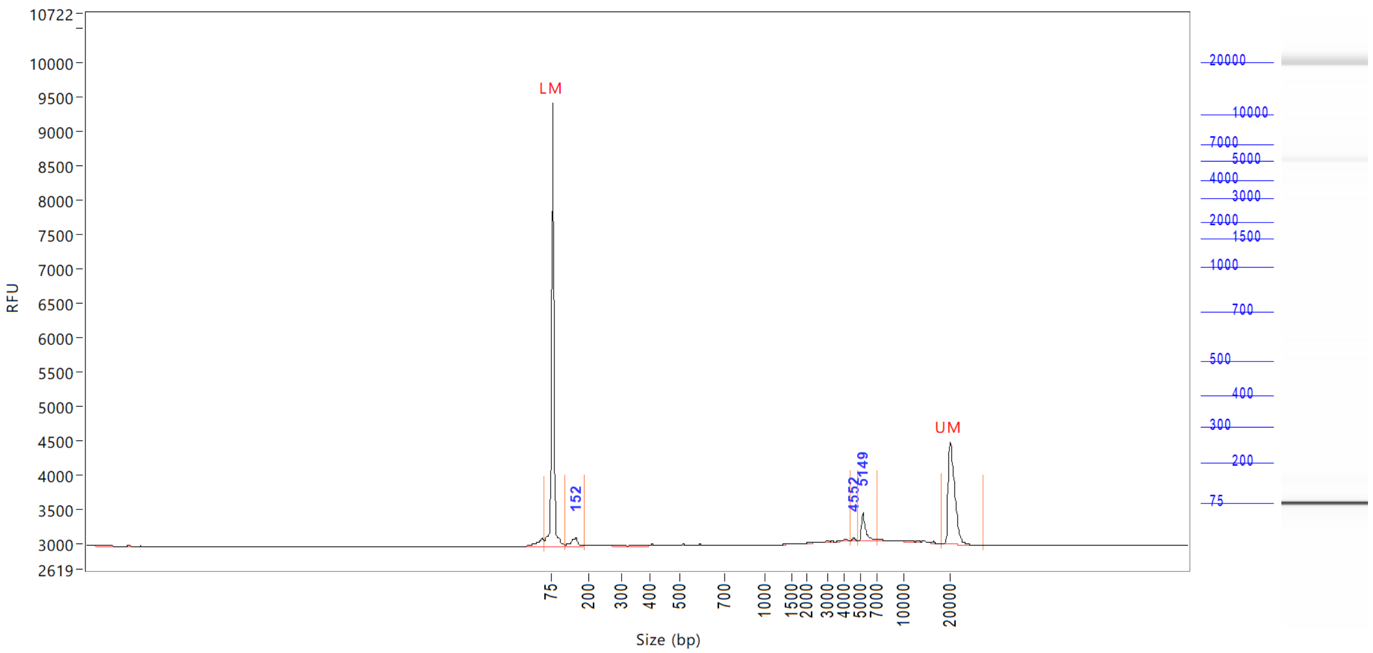

Peak	Size (bp)	Concentration (ng/uL)	From (bp)	To (bp)	Average size (bp)	CV%	RFU	Corrected peak area
1	75 (LM)	0.5000	47	116	73	9.14	6472	39.070
2	152	0.2897	116	181	144	8.46	120	1.886
3	4552	0.0467	4345	4828	4562	2.64	51	0.304
4	5149	0.4276	4828	7000	5398	8.84	416	2.784
5	20000 (UM)	0.1641	18171	26891	20349	3.72	1495	12.825

TIC:	0.7639	ng/uL
TIM:	3.4531	nmole/L
Total concentration:	1.3042	ng/uL

Sample peak width (sec): 5   Sample min peak height: 50   Sample baseline V to V?: Y   Sample baseline V to V points: 3
Sample filter: Binomial   Number of points for filter: 3 Sample start region (min): 0 Sample end region (min): 30
Marker peak width (sec): 5   Marker min peak height: 200   Marker baseline V to V?: Y   Marker baseline V to V points: 3
Lower marker selection: First peak > 500 RFU   Upper marker selection: Last peak > 200 RFU
Ladder size (bp)75, 200, 300, 400, 500, 700, 1000, 1500, 2000, 3000, 4000, 5000, 7000, 10000, 20000
Quantification using: Lower Marker   Final concentration (ng/uL): 0.5000   Dilution factor: 12.0

Sample: SampG8
Well location: G8
Created: Monday, September 20, 2021 8:39:56 PM

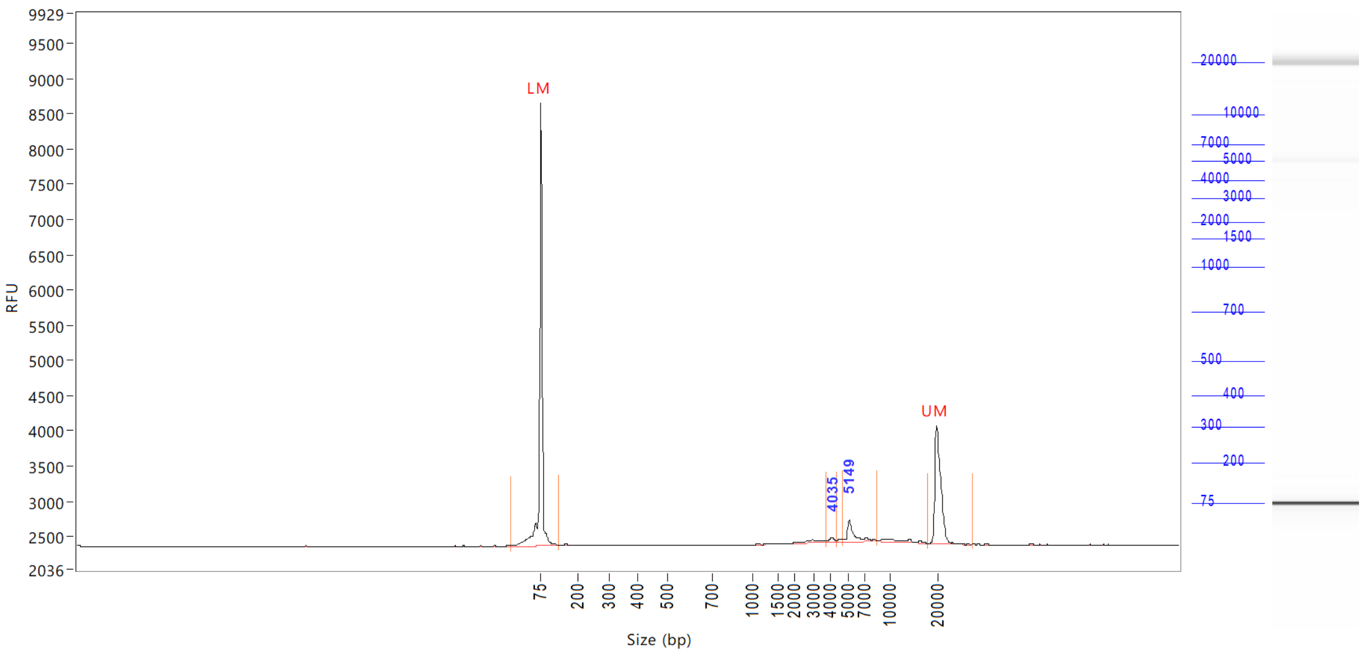

Peak	Size (bp)	Concentration (ng/uL)	From (bp)	To (bp)	Average size (bp)	CV%	RFU	Corrected peak area
1	75 (LM)	0.5000	0	133	69	22.40	6300	43.102
2	4035	0.0720	3750	4311	4026	3.45	63	0.517
3	5149	0.4226	4724	8534	5749	16.57	299	3.036
4	20000 (UM)	0.1549	18049	27617	20286	3.70	1671	13.352

TIC:	0.4946	ng/uL
TIM:	0.1505	nmole/L
Total concentration:	1.1412	ng/uL

Sample peak width (sec): 5 Sample min peak height: 50 Sample baseline V to V?: Y Sample baseline V to V points: 3
Sample filter: Binomial Number of points for filter: 3 Sample start region (min): 0 Sample end region (min): 30
Marker peak width (sec): 5 Marker min peak height: 200 Marker baseline V to V?: Y Marker baseline V to V points: 3
Lower marker selection: First peak > 500 RFU Upper marker selection: Last peak > 200 RFU
Ladder size (bp)75, 200, 300, 400, 500, 700, 1000, 1500, 2000, 3000, 4000, 5000, 7000, 10000, 20000
Quantification using: Lower Marker Final concentration (ng/uL): 0.5000 Dilution factor: 12.0

Sample: SampH8
Well location: H8
Created: Monday, September 20, 2021 8:39:56 PM

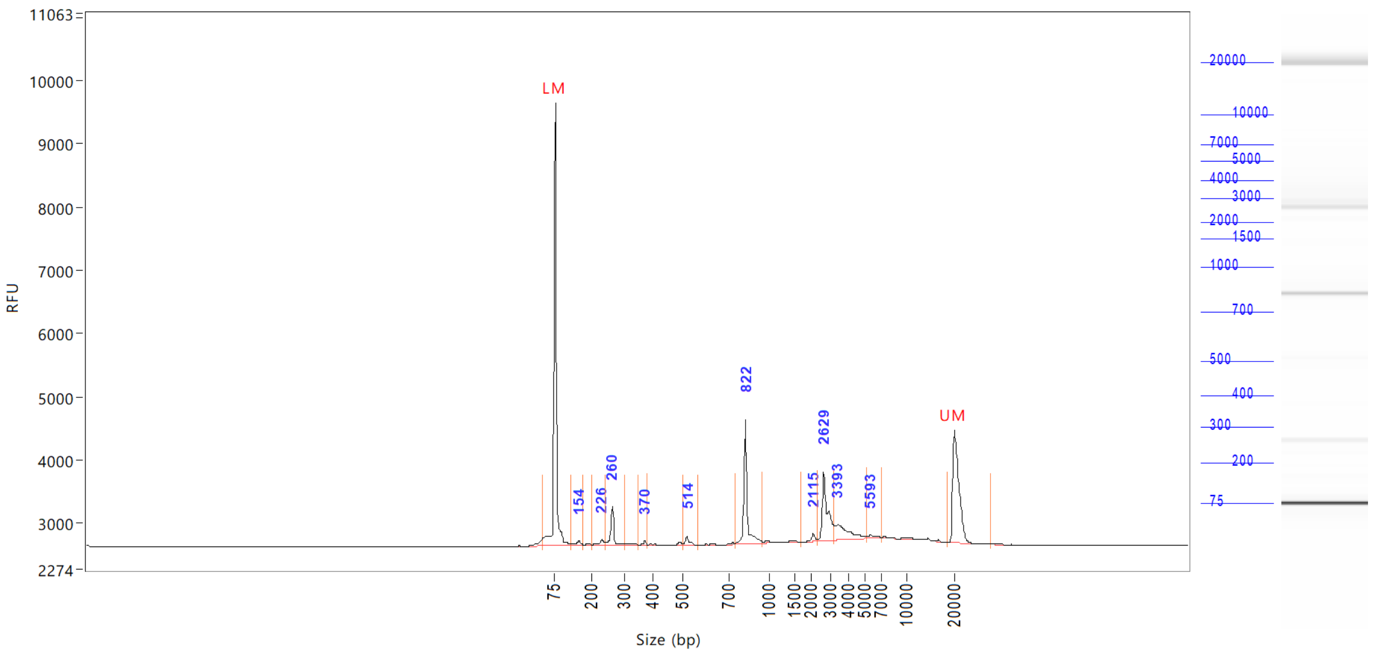

Peak	Size (bp)	Concentration (ng/uL)	From (bp)	To (bp)	Average size (bp)	CV%	RFU	Corrected peak area
1	75 (LM)	0.5000	37	128	73	12.40	7009	43.504
2	154	0.1015	128	169	147	6.26	69	0.736
3	226	0.1202	196	241	225	4.41	82	0.871
4	260	0.5211	241	300	259	2.90	605	3.778
5	370	0.0408	347	382	366	1.35	57	0.295
6	514	0.1530	498	562	515	2.15	145	1.109
7	822	1.5958	744	957	826	3.84	1961	11.571
8	2115	0.1067	1674	2315	2086	5.28	121	0.774
9	2629	1.3110	2315	3179	2742	6.89	1096	9.506
10	3393	0.7147	3179	5223	3816	12.77	237	5.182
11	5593	0.0764	5223	7001	5941	8.55	56	0.554
12	20000 (UM)	0.1706	18415	27496	20365	3.86	1778	14.839

TIC:	4.7412	ng/uL
TIM:	10.3747	nmole/L
Total concentration:	5.0909	ng/uL

```
Sample peak width (sec): 5    Sample min peak height: 50    Sample baseline V to V?: Y    Sample baseline V to V points: 3
Sample filter: Binomial       Number of points for filter: 3  Sample start region (min): 0  Sample end region (min): 30
Marker peak width (sec): 5    Marker min peak height: 200   Marker baseline V to V?: Y    Marker baseline V to V points: 3
Lower marker selection: First peak > 500 RFU                Upper marker selection: Last peak > 200 RFU
Ladder size (bp)75, 200, 300, 400, 500, 700, 1000, 1500, 2000, 3000, 4000, 5000, 7000, 10000, 20000
Quantification using: Lower Marker          Final concentration (ng/uL): 0.5000          Dilution factor: 12.0
```

Sample: SampA9
Well location: A9
Created: Monday, September 20, 2021 8:39:56 PM

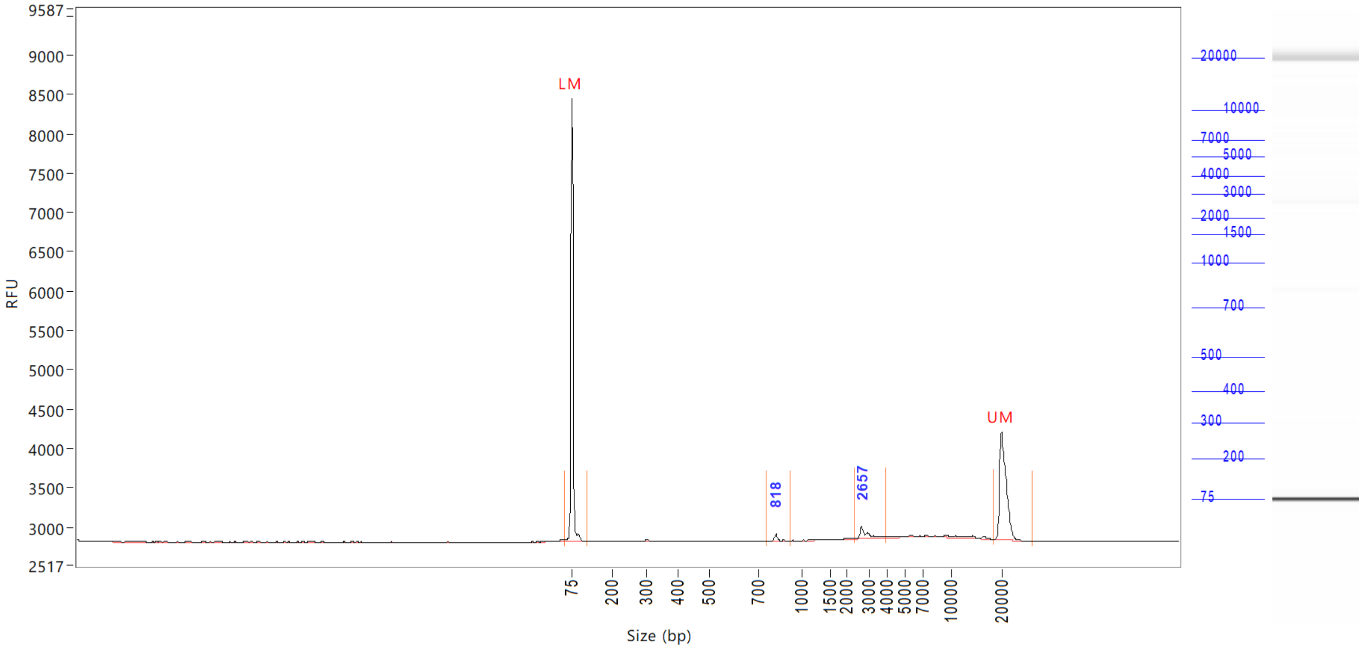

Peak	Size (bp)	Concentration (ng/uL)	From (bp)	To (bp)	Average size (bp)	CV%	RFU	Corrected peak area
1	75 (LM)	0.5000	52	120	73	6.50	5642	30.914
2	818	0.1064	757	913	821	2.96	92	0.548
3	2657	0.3070	2344	3929	2906	12.03	151	1.582
4	20000 (UM)	0.1842	18293	25924	20310	3.68	1364	11.388

TIC:	0.4134	ng/uL
TIM:	0.3871	nmole/L
Total concentration:	1.1137	ng/uL

Sample peak width (sec): 5   Sample min peak height: 50   Sample baseline V to V?: Y   Sample baseline V to V points: 3
Sample filter: Binomial   Number of points for filter: 3 Sample start region (min): 0 Sample end region (min): 30
Marker peak width (sec): 5   Marker min peak height: 200   Marker baseline V to V?: Y   Marker baseline V to V points: 3
Lower marker selection: First peak > 500 RFU   Upper marker selection: Last peak > 200 RFU
Ladder size (bp)75, 200, 300, 400, 500, 700, 1000, 1500, 2000, 3000, 4000, 5000, 7000, 10000, 20000
Quantification using: Lower Marker   Final concentration (ng/uL): 0.5000   Dilution factor: 12.0

Sample: SampB9
Well location: B9
Created: Monday, September 20, 2021 8:39:56 PM

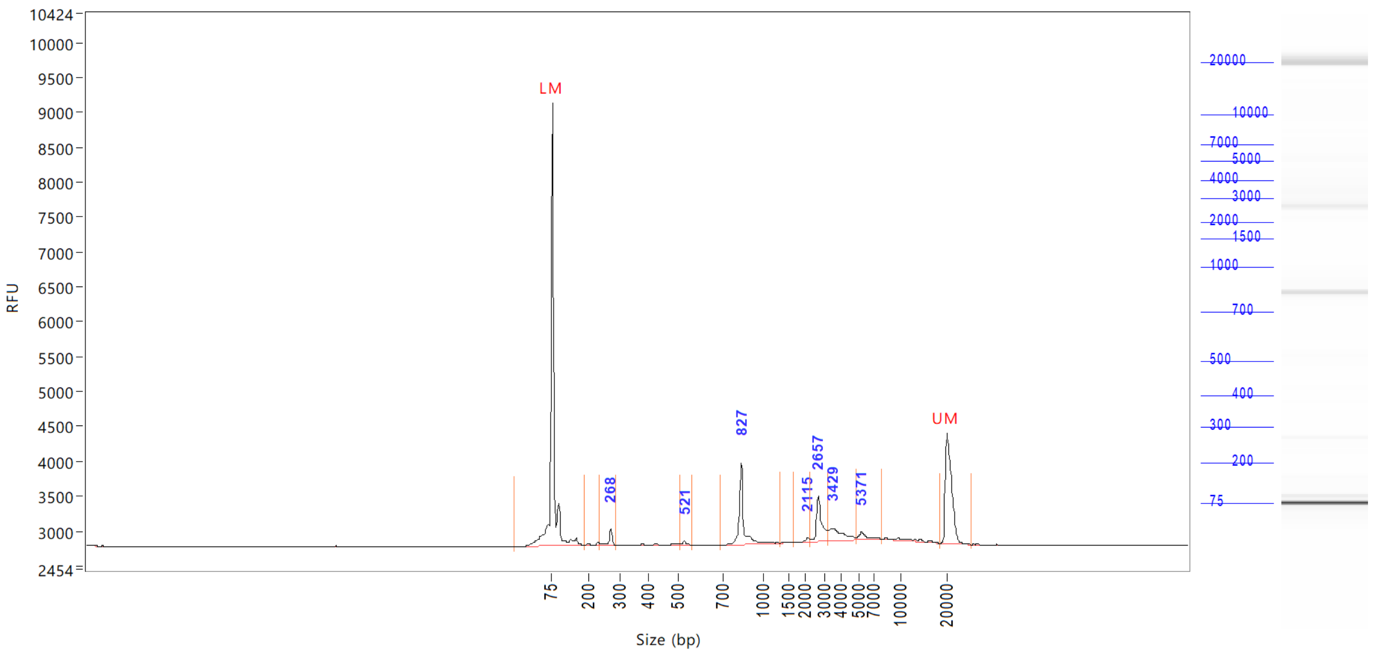

Peak	Size<br>(bp)	Concentration<br>(ng/uL)	From<br>(bp)	To<br>(bp)	Average size<br>(bp)	CV%	RFU	Corrected peak area
1	75 (LM)	0.5000	0	183	74	28.07	6358	49.266
2	268	0.1931	233	287	262	3.67	234	1.586
3	521	0.0656	501	560	521	2.09	71	0.538
4	827	1.0833	687	1323	842	7.31	1179	8.895
5	2115	0.0442	1635	2258	2091	4.90	60	0.363
6	2657	0.6480	2258	3108	2714	6.46	652	5.321
7	3429	0.4884	3108	4862	3754	12.29	180	4.010
8	5371	0.1870	4862	7734	5851	13.82	111	1.535
9	20000 (UM)	0.1352	18415	25078	20339	3.56	1581	13.324

TIC:	2.7096	ng/uL
TIM:	4.2317	nmole/L
Total concentration:	3.0277	ng/uL

```
Sample peak width (sec): 5    Sample min peak height: 50    Sample baseline V to V?: Y    Sample baseline V to V points: 3
Sample filter: Binomial       Number of points for filter: 3  Sample start region (min): 0  Sample end region (min): 30
Marker peak width (sec): 5    Marker min peak height: 200   Marker baseline V to V?: Y    Marker baseline V to V points: 3
Lower marker selection: First peak > 500 RFU                Upper marker selection: Last peak > 200 RFU
Ladder size (bp)75, 200, 300, 400, 500, 700, 1000, 1500, 2000, 3000, 4000, 5000, 7000, 10000, 20000
Quantification using: Lower Marker          Final concentration (ng/uL): 0.5000          Dilution factor: 12.0
```

Sample: SampC9
Well location: C9
Created: Monday, September 20, 2021 8:39:56 PM

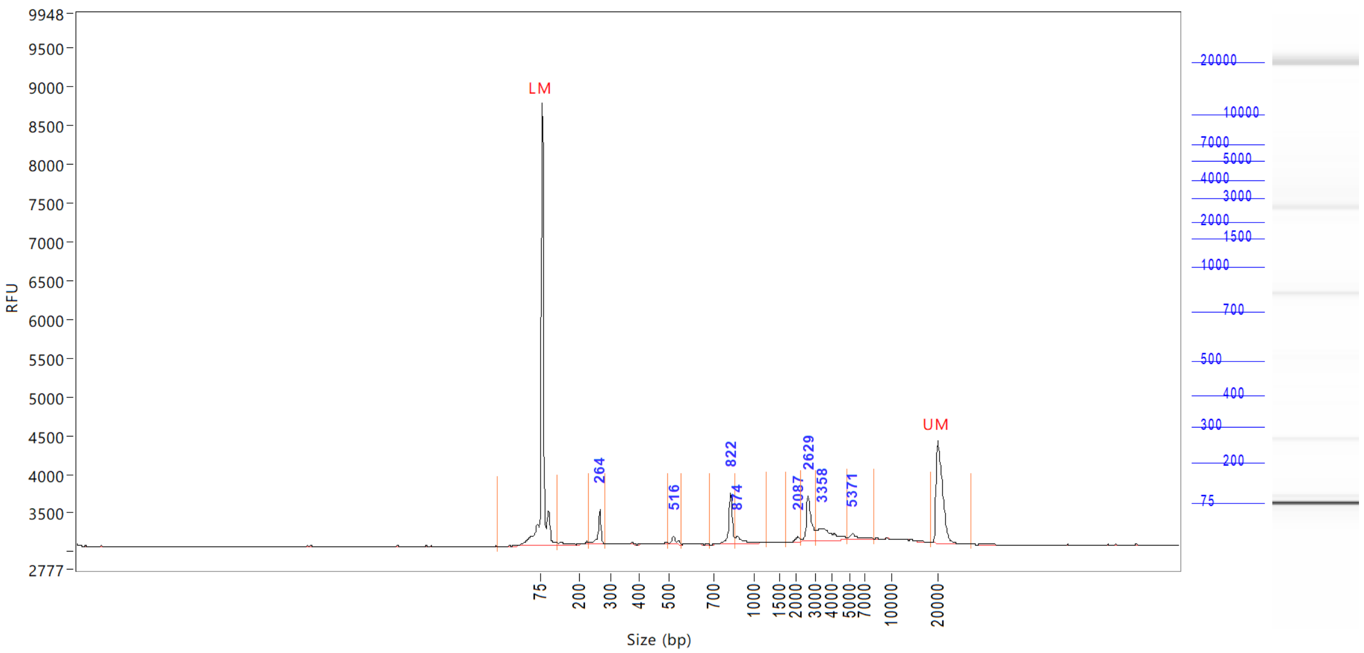

Peak	Size (bp)	Concentration (ng/uL)	From (bp)	To (bp)	Average size (bp)	CV%	RFU	Corrected peak area
1	75 (LM)	0.5000	0	128	71	22.44	5717	40.785
2	264	0.4514	230	280	259	3.24	445	3.069
3	516	0.1438	498	552	519	2.25	109	0.978
4	822	0.5991	682	853	810	3.27	648	4.073
5	874	0.1979	853	1223	918	7.70	88	1.345
6	2087	0.0667	1693	2258	2071	4.78	65	0.454
7	2629	0.6950	2258	3001	2668	6.01	583	4.724
8	3358	0.5701	3001	4897	3696	13.41	154	3.875
9	5371	0.1694	4897	8001	5962	14.22	74	1.151
10	20000 (UM)	0.1390	18293	27133	20372	3.85	1321	11.335

TIC:	2.8936	ng/uL
TIM:	5.6776	nmole/L
Total concentration:	3.2452	ng/uL

Sample peak width (sec): 5   Sample min peak height: 50   Sample baseline V to V?: Y   Sample baseline V to V points: 3
Sample filter: Binomial   Number of points for filter: 3   Sample start region (min): 0   Sample end region (min): 30
Marker peak width (sec): 5   Marker min peak height: 200   Marker baseline V to V?: Y   Marker baseline V to V points: 3
Lower marker selection: First peak > 500 RFU   Upper marker selection: Last peak > 200 RFU
Ladder size (bp)75, 200, 300, 400, 500, 700, 1000, 1500, 2000, 3000, 4000, 5000, 7000, 10000, 20000
Quantification using: Lower Marker   Final concentration (ng/uL): 0.5000   Dilution factor: 12.0

Sample: SampD9
Well location: D9
Created: Monday, September 20, 2021 8:39:56 PM

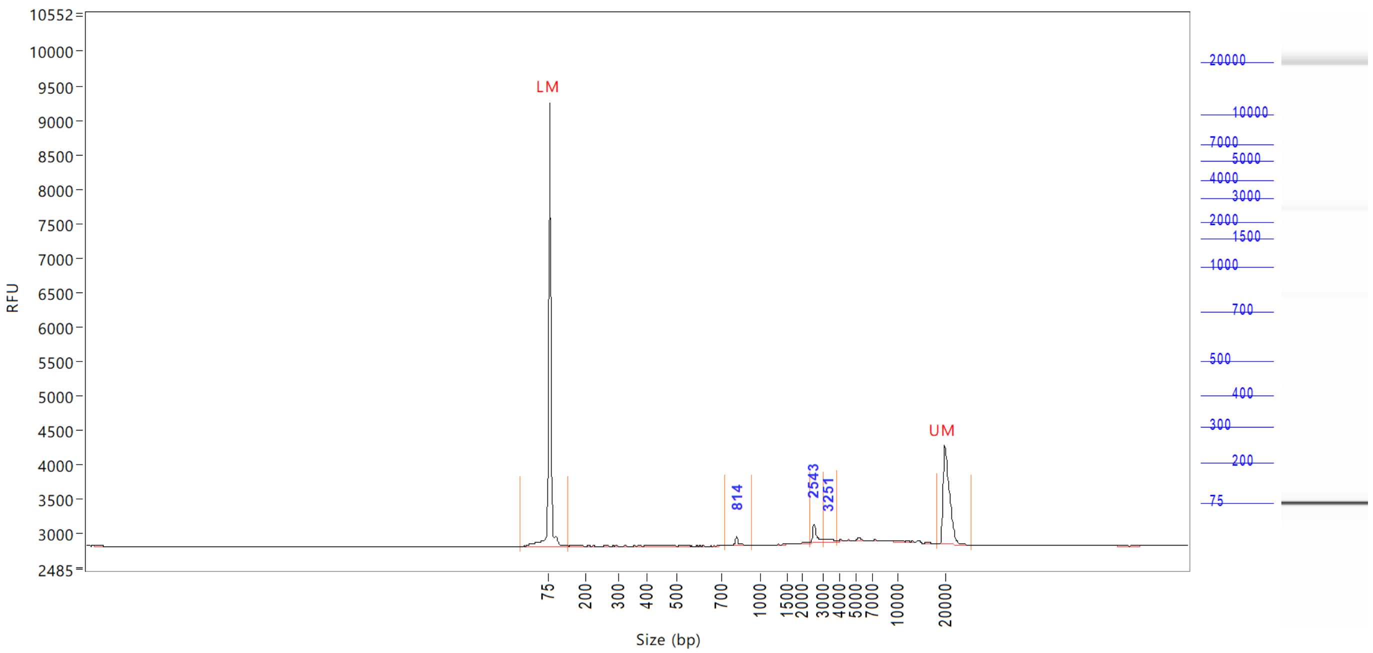

Peak	Size (bp)	Concentration (ng/uL)	From (bp)	To (bp)	Average size (bp)	CV%	RFU	Corrected peak area
1	75 (LM)	0.5000	0	139	72	17.92	6447	39.662
2	814	0.1401	718	935	817	4.03	127	0.926
3	2543	0.2748	2344	3037	2626	6.05	271	1.816
4	3251	0.0962	3037	3822	3350	6.27	50	0.636
5	20000 (UM)	0.1557	18415	25682	20427	3.87	1442	12.347

TIC:	0.5111	ng/uL
TIM:	0.5018	nmole/L
Total concentration:	1.0018	ng/uL

Sample peak width (sec): 5 Sample min peak height: 50 Sample baseline V to V?: Y Sample baseline V to V points: 3
Sample filter: Binomial Number of points for filter: 3 Sample start region (min): 0 Sample end region (min): 30
Marker peak width (sec): 5 Marker min peak height: 200 Marker baseline V to V?: Y Marker baseline V to V points: 3
Lower marker selection: First peak > 500 RFU Upper marker selection: Last peak > 200 RFU
Ladder size (bp)75, 200, 300, 400, 500, 700, 1000, 1500, 2000, 3000, 4000, 5000, 7000, 10000, 20000
Quantification using: Lower Marker Final concentration (ng/uL): 0.5000 Dilution factor: 12.0

Sample: SampE9
Well location: E9
Created: Monday, September 20, 2021 8:39:56 PM

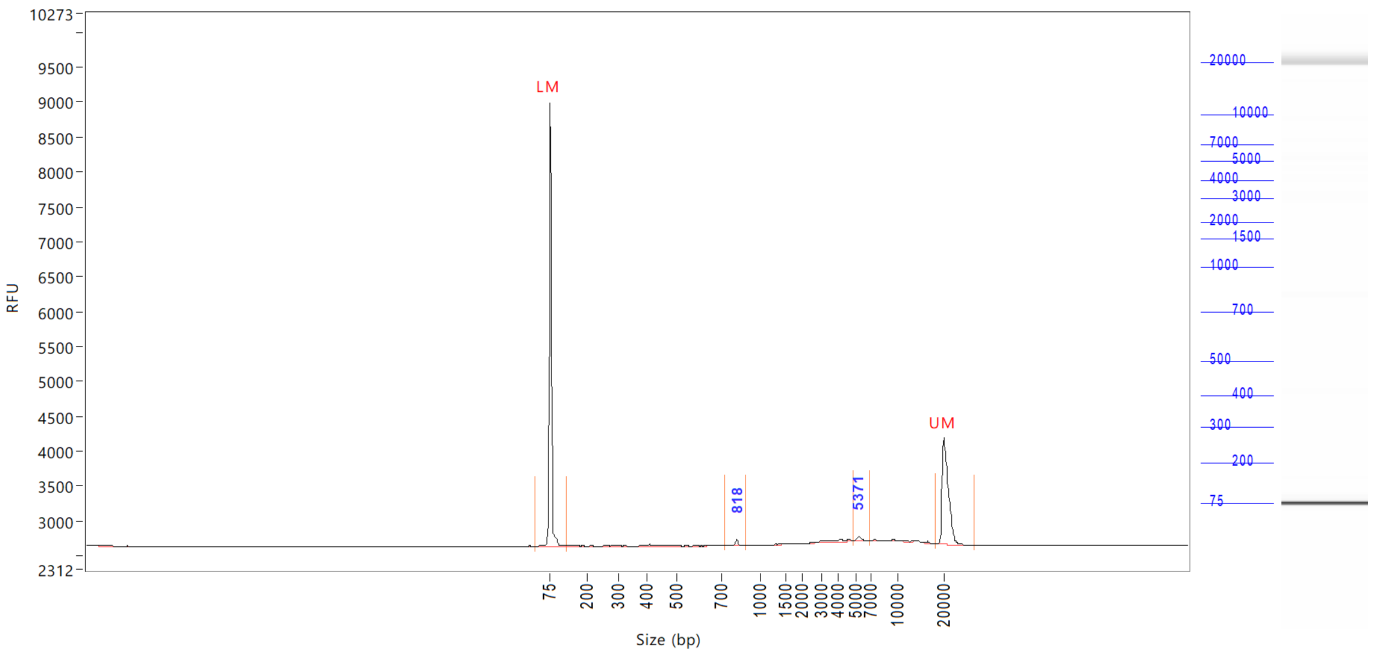

Peak	Size<br>(bp)	Concentration<br>(ng/uL)	From<br>(bp)	To<br>(bp)	Average size<br>(bp)	CV%	RFU	Corrected peak area
1	75 (LM)	0.5000	28	131	74	8.58	6363	36.027
2	818	0.0966	727	892	813	3.19	89	0.580
3	5371	0.1072	4862	6926	5613	9.58	65	0.644
4	20000 (UM)	0.1812	18415	26649	20457	3.72	1523	13.053

TIC:	0.2037	ng/uL
TIM:	0.2269	nmole/L
Total concentration:	0.8345	ng/uL

```
Sample peak width (sec): 5    Sample min peak height: 50    Sample baseline V to V?: Y    Sample baseline V to V points: 3
Sample filter: Binomial       Number of points for filter: 3  Sample start region (min): 0  Sample end region (min): 30
Marker peak width (sec): 5    Marker min peak height: 200   Marker baseline V to V?: Y    Marker baseline V to V points: 3
Lower marker selection: First peak > 500 RFU                Upper marker selection: Last peak > 200 RFU
Ladder size (bp)75, 200, 300, 400, 500, 700, 1000, 1500, 2000, 3000, 4000, 5000, 7000, 10000, 20000
Quantification using: Lower Marker          Final concentration (ng/uL): 0.5000          Dilution factor: 12.0
```

Sample: SampF9
Well location: F9
Created: Monday, September 20, 2021 8:39:56 PM

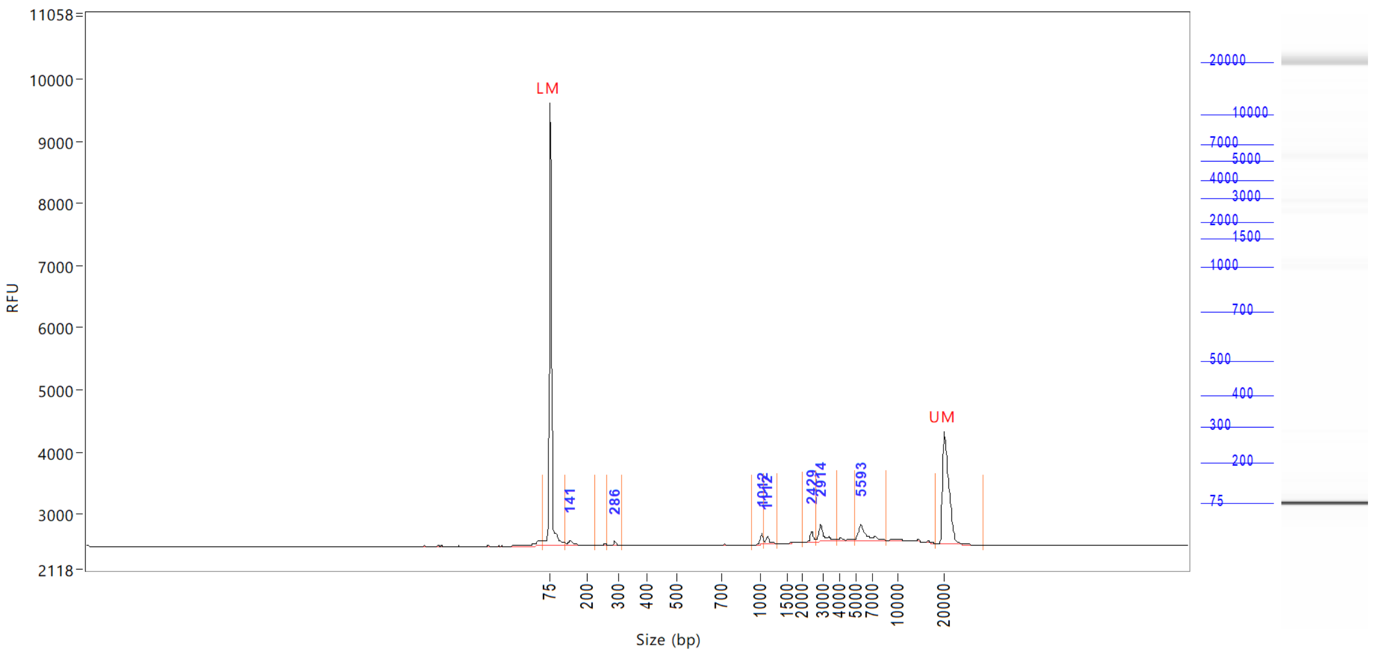

Peak	Size (bp)	Concentration (ng/uL)	From (bp)	To (bp)	Average size (bp)	CV%	RFU	Corrected peak area
1	75 (LM)	0.5000	50	128	75	11.17	7134	42.321
2	141	0.2186	128	224	154	14.58	89	1.542
3	286	0.0615	264	311	283	2.65	69	0.434
4	1012	0.1321	931	1056	1004	1.99	178	0.932
5	1112	0.1079	1056	1323	1125	5.01	118	0.761
6	2429	0.1329	2000	2657	2439	4.05	170	0.938
7	2914	0.3386	2657	3857	3021	8.67	282	2.389
8	5593	0.4505	4897	8734	6171	14.67	265	3.178
9	20000 (UM)	0.1821	18171	28221	20439	3.94	1806	15.416

TIC:	1.4422	ng/uL
TIM:	3.4599	nmole/L
Total concentration:	1.7872	ng/uL

Sample peak width (sec): 5 Sample min peak height: 50 Sample baseline V to V?: Y Sample baseline V to V points: 3
Sample filter: Binomial Number of points for filter: 3 Sample start region (min): 0 Sample end region (min): 30
Marker peak width (sec): 5 Marker min peak height: 200 Marker baseline V to V?: Y Marker baseline V to V points: 3
Lower marker selection: First peak > 500 RFU Upper marker selection: Last peak > 200 RFU
Ladder size (bp)75, 200, 300, 400, 500, 700, 1000, 1500, 2000, 3000, 4000, 5000, 7000, 10000, 20000
Quantification using: Lower Marker Final concentration (ng/uL): 0.5000 Dilution factor: 12.0

Sample: SampG9
Well location: G9
Created: Monday, September 20, 2021 8:39:56 PM

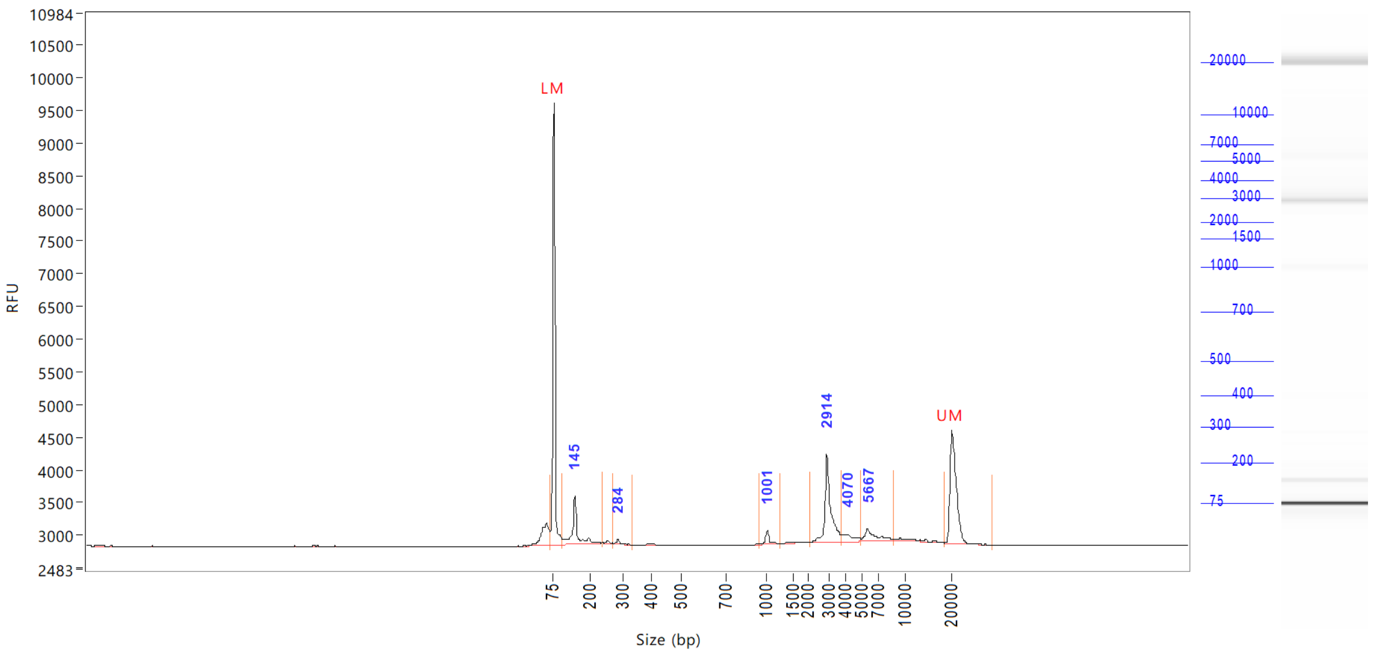

Peak	Size (bp)	Concentration (ng/uL)	From (bp)	To (bp)	Average size (bp)	CV%	RFU	Corrected peak area
1	75 (LM)	0.5000	64	103	74	7.07	6771	39.238
2	145	1.2900	103	235	145	14.91	730	8.436
3	284	0.0730	269	333	285	4.34	70	0.477
4	1001	0.2139	944	1245	1023	5.28	207	1.399
5	2914	1.8771	2087	3822	2998	9.29	1341	12.275
6	4070	0.3006	3822	4931	4273	7.43	113	1.966
7	5667	0.4816	4931	8734	6353	15.58	196	3.150
8	20000 (UM)	0.1847	18415	28705	20427	3.80	1721	14.492

TIC:	4.2362	ng/uL
TIM:	16.6577	nmole/L
Total concentration:	4.6643	ng/uL

```
Sample peak width (sec): 5    Sample min peak height: 50    Sample baseline V to V?: Y    Sample baseline V to V points: 3
Sample filter: Binomial       Number of points for filter: 3  Sample start region (min): 0  Sample end region (min): 30
Marker peak width (sec): 5    Marker min peak height: 200   Marker baseline V to V?: Y    Marker baseline V to V points: 3
Lower marker selection: First peak > 500 RFU                Upper marker selection: Last peak > 200 RFU
Ladder size (bp)75, 200, 300, 400, 500, 700, 1000, 1500, 2000, 3000, 4000, 5000, 7000, 10000, 20000
Quantification using: Lower Marker          Final concentration (ng/uL): 0.5000          Dilution factor: 12.0
```

Sample: SampH9
Well location: H9
Created: Monday, September 20, 2021 8:39:56 PM

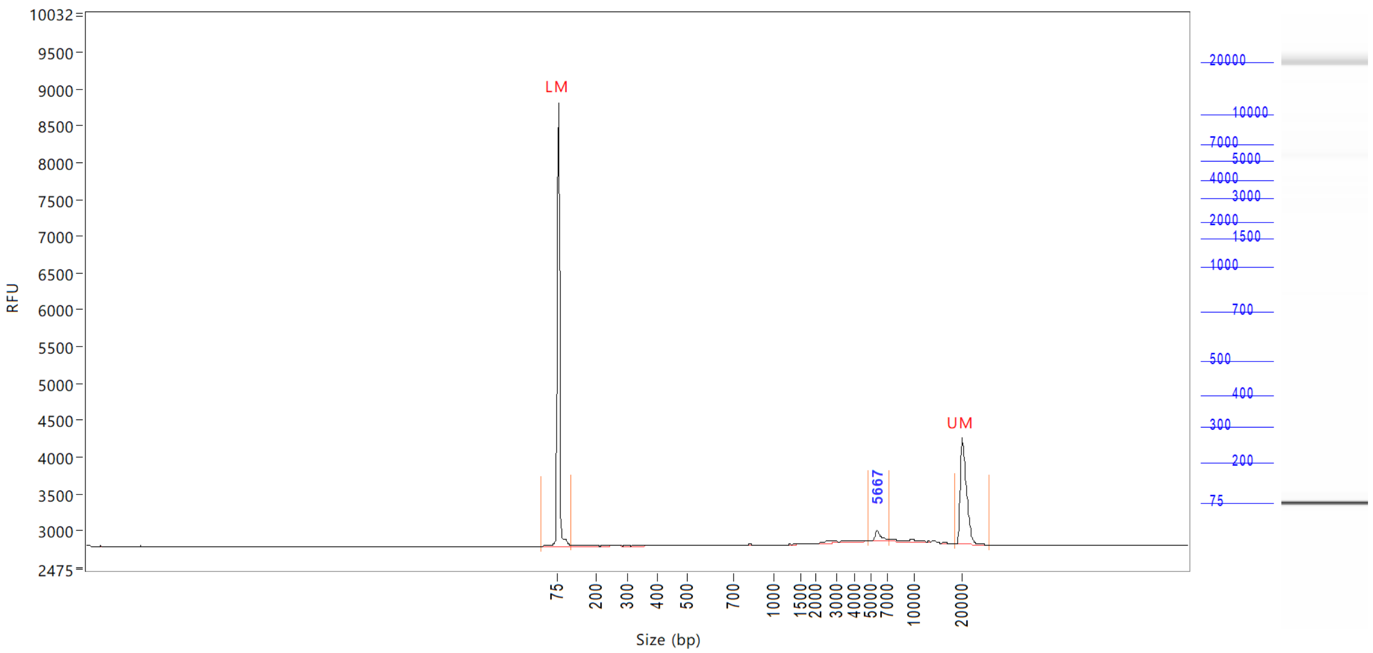

Peak	Size (bp)	Concentration (ng/uL)	From (bp)	To (bp)	Average size (bp)	CV%	RFU	Corrected peak area
1	75 (LM)	0.5000	22	118	74	7.85	6035	33.782
2	5667	0.2456	4793	7068	5885	8.83	156	1.383
3	20000 (UM)	0.1771	18415	25682	20389	3.79	1454	11.968

TIC:	0.2456	ng/uL
TIM:	0.0687	nmole/L
Total concentration:	0.9697	ng/uL

Sample peak width (sec): 5 Sample min peak height: 50 Sample baseline V to V?: Y Sample baseline V to V points: 3
Sample filter: Binomial Number of points for filter: 3 Sample start region (min): 0 Sample end region (min): 30
Marker peak width (sec): 5 Marker min peak height: 200 Marker baseline V to V?: Y Marker baseline V to V points: 3
Lower marker selection: First peak > 500 RFU Upper marker selection: Last peak > 200 RFU
Ladder size (bp)75, 200, 300, 400, 500, 700, 1000, 1500, 2000, 3000, 4000, 5000, 7000, 10000, 20000
Quantification using: Lower Marker Final concentration (ng/uL): 0.5000 Dilution factor: 12.0

Sample: SampA10
Well location: A10
Created: Monday, September 20, 2021 8:39:56 PM

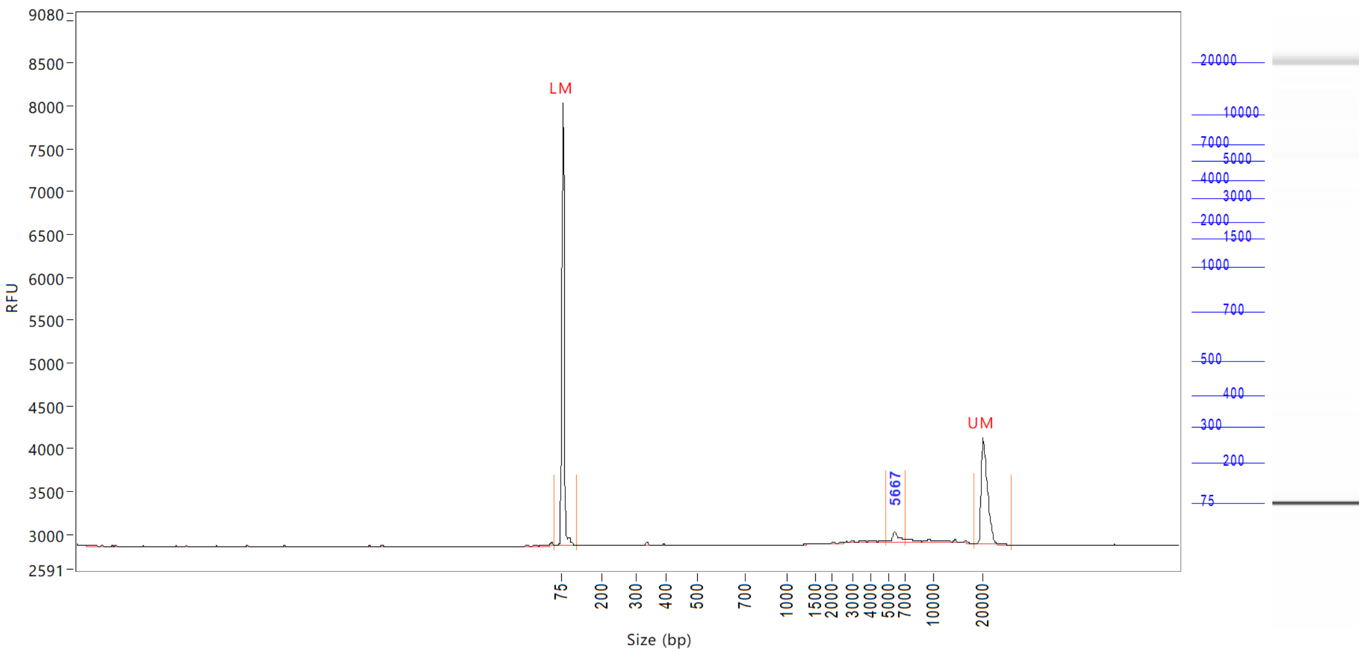

Peak	Size (bp)	Concentration (ng/uL)	From (bp)	To (bp)	Average size (bp)	CV%	RFU	Corrected peak area
1	75 (LM)	0.5000	50	116	73	6.63	5179	28.299
2	5667	0.2129	4862	7000	5858	8.17	116	1.004
3	20000 (UM)	0.1771	18293	25924	20367	3.55	1239	10.022

TIC:	0.2129	ng/uL
TIM:	0.0598	nmole/L
Total concentration:	1.0300	ng/uL

```
Sample peak width (sec): 5    Sample min peak height: 50    Sample baseline V to V?: Y    Sample baseline V to V points: 3
Sample filter: Binomial       Number of points for filter: 3  Sample start region (min): 0  Sample end region (min): 30
Marker peak width (sec): 5    Marker min peak height: 200   Marker baseline V to V?: Y    Marker baseline V to V points: 3
Lower marker selection: First peak > 500 RFU                Upper marker selection: Last peak > 200 RFU
Ladder size (bp)75, 200, 300, 400, 500, 700, 1000, 1500, 2000, 3000, 4000, 5000, 7000, 10000, 20000
Quantification using: Lower Marker          Final concentration (ng/uL): 0.5000          Dilution factor: 12.0
```

Sample: SampB10
Well location: B10
Created: Monday, September 20, 2021 8:39:56 PM

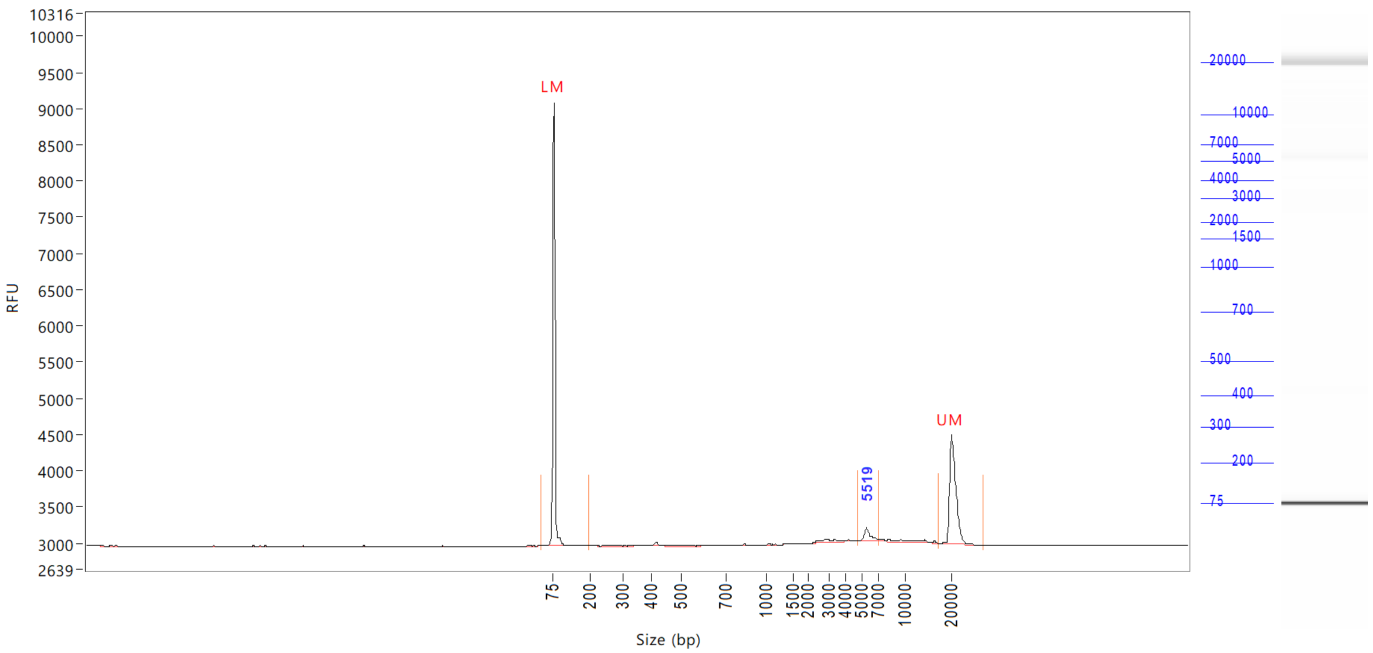

Peak	Size (bp)	Concentration (ng/uL)	From (bp)	To (bp)	Average size (bp)	CV%	RFU	Corrected peak area
1	75 (LM)	0.5000	31	190	74	10.45	6126	34.708
2	5519	0.3050	4690	7001	5690	9.34	189	1.764
3	20000 (UM)	0.1813	17073	26649	20350	3.91	1506	12.586

TIC:	0.3050	ng/uL
TIM:	0.0882	nmole/L
Total concentration:	1.1081	ng/uL

```
Sample peak width (sec): 5    Sample min peak height: 50    Sample baseline V to V?: Y    Sample baseline V to V points: 3
Sample filter: Binomial       Number of points for filter: 3  Sample start region (min): 0  Sample end region (min): 30
Marker peak width (sec): 5    Marker min peak height: 200   Marker baseline V to V?: Y    Marker baseline V to V points: 3
Lower marker selection: First peak > 500 RFU                Upper marker selection: Last peak > 200 RFU
Ladder size (bp)75, 200, 300, 400, 500, 700, 1000, 1500, 2000, 3000, 4000, 5000, 7000, 10000, 20000
Quantification using: Lower Marker          Final concentration (ng/uL): 0.5000          Dilution factor: 12.0
```

Sample: SampC10
Well location: C10
Created: Monday, September 20, 2021 8:39:56 PM

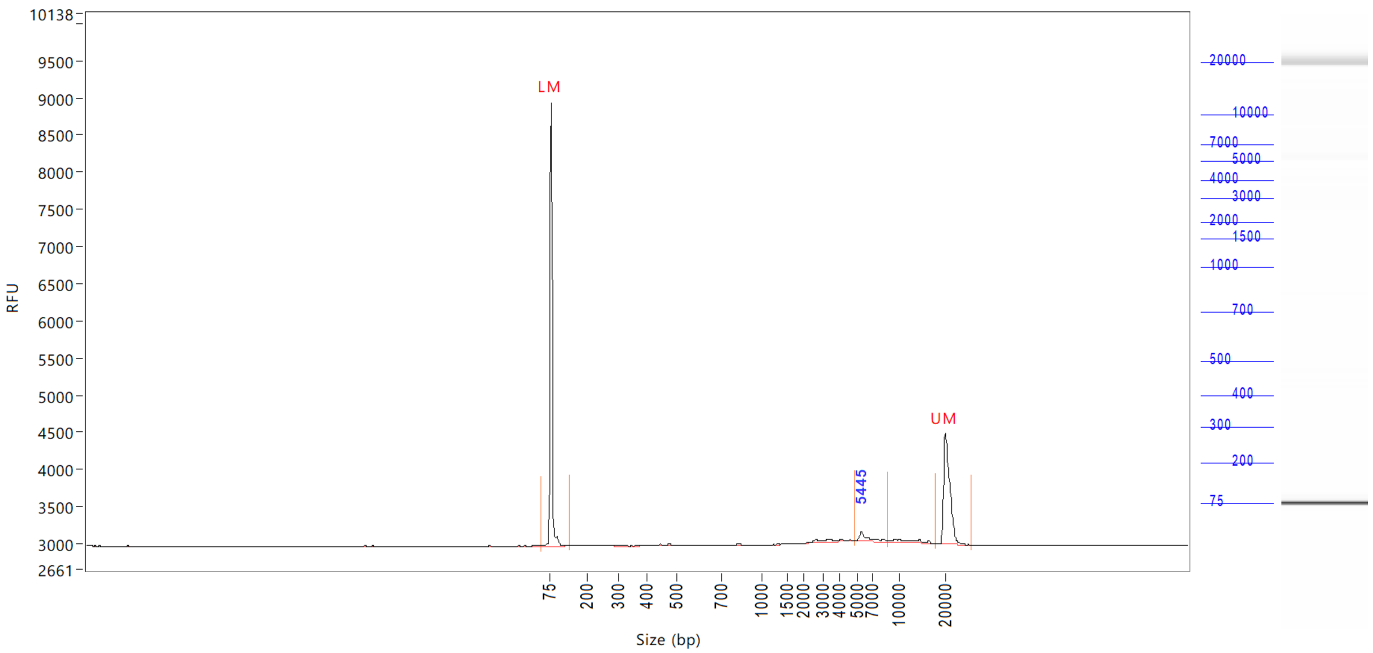

Peak	Size (bp)	Concentration (ng/uL)	From (bp)	To (bp)	Average size (bp)	CV%	RFU	Corrected peak area
1	75 (LM)	0.5000	47	141	74	8.27	5971	33.648
2	5445	0.2979	4931	8734	6296	16.16	135	1.671
3	20000 (UM)	0.1851	18049	25682	20383	3.74	1480	12.459

TIC:	0.2979	ng/uL
TIM:	0.0779	nmole/L
Total concentration:	1.0956	ng/uL

```
Sample peak width (sec): 5    Sample min peak height: 50    Sample baseline V to V?: Y    Sample baseline V to V points: 3
Sample filter: Binomial       Number of points for filter: 3  Sample start region (min): 0  Sample end region (min): 30
Marker peak width (sec): 5    Marker min peak height: 200   Marker baseline V to V?: Y    Marker baseline V to V points: 3
Lower marker selection: First peak > 500 RFU                Upper marker selection: Last peak > 200 RFU
Ladder size (bp)75, 200, 300, 400, 500, 700, 1000, 1500, 2000, 3000, 4000, 5000, 7000, 10000, 20000
Quantification using: Lower Marker          Final concentration (ng/uL): 0.5000          Dilution factor: 12.0
```

Sample: SampD10
Well location: D10
Created: Monday, September 20, 2021 8:39:56 PM

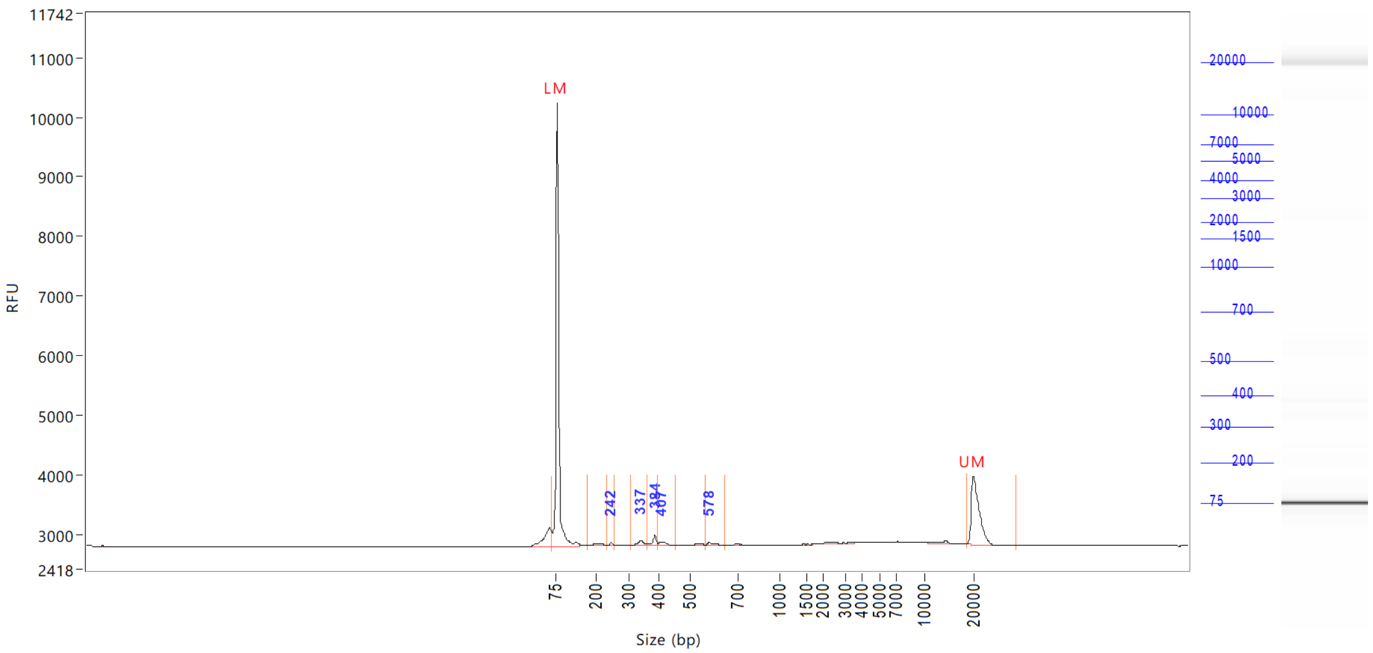

Peak	Size (bp)	Concentration (ng/uL)	From (bp)	To (bp)	Average size (bp)	CV%	RFU	Corrected peak area
1	75 (LM)	0.5000	60	171	77	15.30	7448	49.290
2	242	0.0424	233	253	241	1.41	60	0.349
3	337	0.1591	303	362	335	3.72	81	1.307
4	384	0.1724	362	394	380	1.86	191	1.416
5	407	0.1210	394	452	411	2.94	58	0.994
6	578	0.0727	560	646	587	3.28	58	0.597
7	20000 (UM)	0.1197	18659	28584	20530	4.84	1153	11.796

TIC:	0.5676	ng/uL
TIM:	2.5052	nmole/L
Total concentration:	1.0982	ng/uL

Sample peak width (sec): 5 Sample min peak height: 50 Sample baseline V to V?: Y Sample baseline V to V points: 3
Sample filter: Binomial Number of points for filter: 3 Sample start region (min): 0 Sample end region (min): 30
Marker peak width (sec): 5 Marker min peak height: 200 Marker baseline V to V?: Y Marker baseline V to V points: 3
Lower marker selection: First peak > 500 RFU Upper marker selection: Last peak > 200 RFU
Ladder size (bp)75, 200, 300, 400, 500, 700, 1000, 1500, 2000, 3000, 4000, 5000, 7000, 10000, 20000
Quantification using: Lower Marker Final concentration (ng/uL): 0.5000 Dilution factor: 12.0

Sample: SampE10
Well location: E10
Created: Monday, September 20, 2021 8:39:56 PM

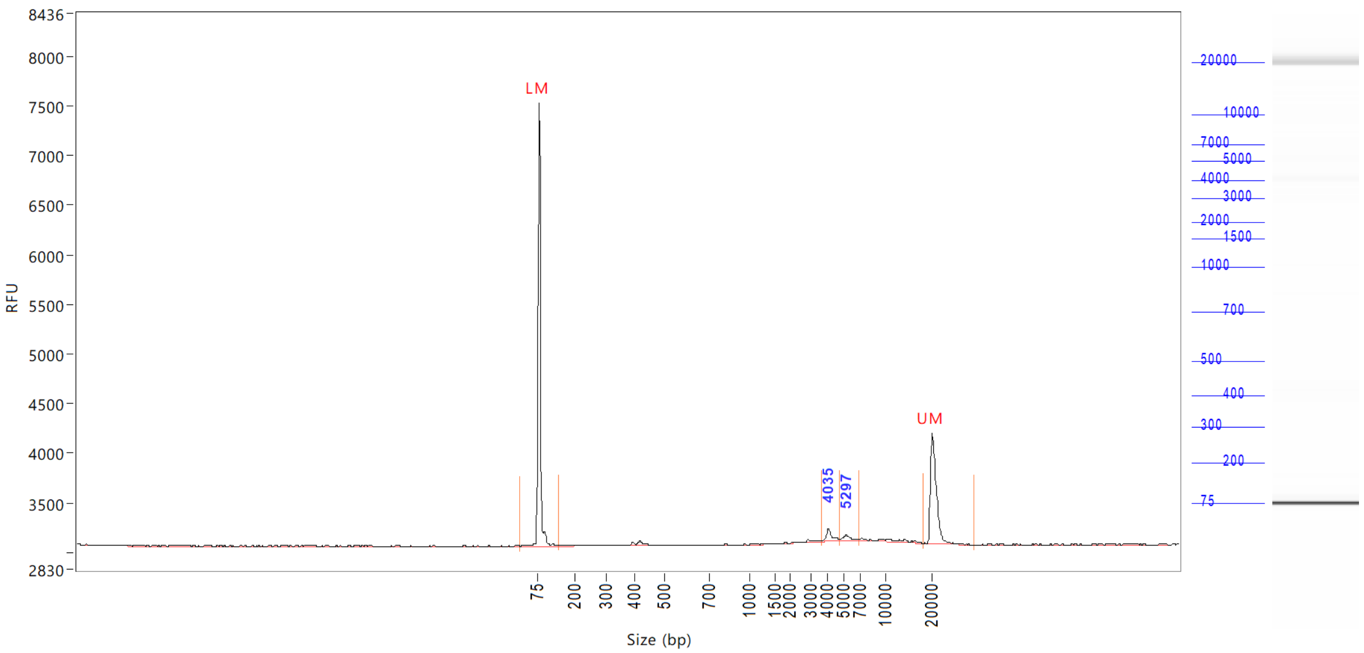

Peak	Size (bp)	Concentration (ng/uL)	From (bp)	To (bp)	Average size (bp)	CV%	RFU	Corrected peak area
1	75 (LM)	0.5000	11	143	74	10.94	4477	26.217
2	4035	0.2703	3643	4793	4168	6.16	129	1.181
3	5297	0.1683	4793	6852	5493	10.06	55	0.735
4	20000 (UM)	0.1729	18049	29068	20453	4.69	1117	9.067

TIC:	0.4386	ng/uL
TIM:	0.1572	nmole/L
Total concentration:	1.3517	ng/uL

```
Sample peak width (sec): 5    Sample min peak height: 50    Sample baseline V to V?: Y    Sample baseline V to V points: 3
Sample filter: Binomial       Number of points for filter: 3  Sample start region (min): 0  Sample end region (min): 30
Marker peak width (sec): 5    Marker min peak height: 200   Marker baseline V to V?: Y    Marker baseline V to V points: 3
Lower marker selection: First peak > 500 RFU                Upper marker selection: Last peak > 200 RFU
Ladder size (bp)75, 200, 300, 400, 500, 700, 1000, 1500, 2000, 3000, 4000, 5000, 7000, 10000, 20000
Quantification using: Lower Marker          Final concentration (ng/uL): 0.5000          Dilution factor: 12.0
```

Sample: SampF10
Well location: F10
Created: Monday, September 20, 2021 8:39:56 PM

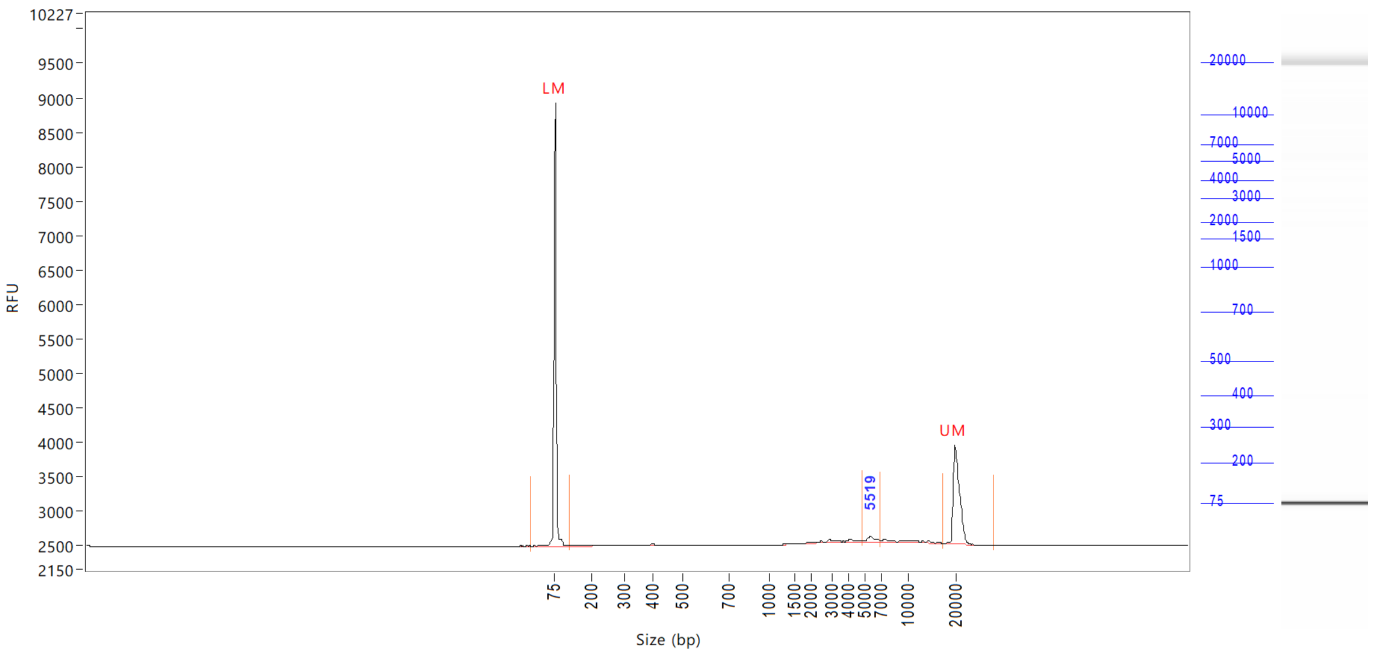

Peak	Size (bp)	Concentration (ng/uL)	From (bp)	To (bp)	Average size (bp)	CV%	RFU	Corrected peak area
1	75 (LM)	0.5000	0	126	73	9.25	6450	36.106
2	5519	0.1471	4862	6926	5726	9.10	79	0.885
3	20000 (UM)	0.1686	17317	28100	20327	4.04	1429	12.172

TIC:	0.1471	ng/uL
TIM:	0.0423	nmole/L
Total concentration:	0.8463	ng/uL

```
Sample peak width (sec): 5    Sample min peak height: 50    Sample baseline V to V?: Y    Sample baseline V to V points: 3
Sample filter: Binomial       Number of points for filter: 3  Sample start region (min): 0  Sample end region (min): 30
Marker peak width (sec): 5    Marker min peak height: 200   Marker baseline V to V?: Y    Marker baseline V to V points: 3
Lower marker selection: First peak > 500 RFU                Upper marker selection: Last peak > 200 RFU
Ladder size (bp)75, 200, 300, 400, 500, 700, 1000, 1500, 2000, 3000, 4000, 5000, 7000, 10000, 20000
Quantification using: Lower Marker          Final concentration (ng/uL): 0.5000          Dilution factor: 12.0
```

Sample: SampG10
Well location: G10
Created: Monday, September 20, 2021 8:39:56 PM

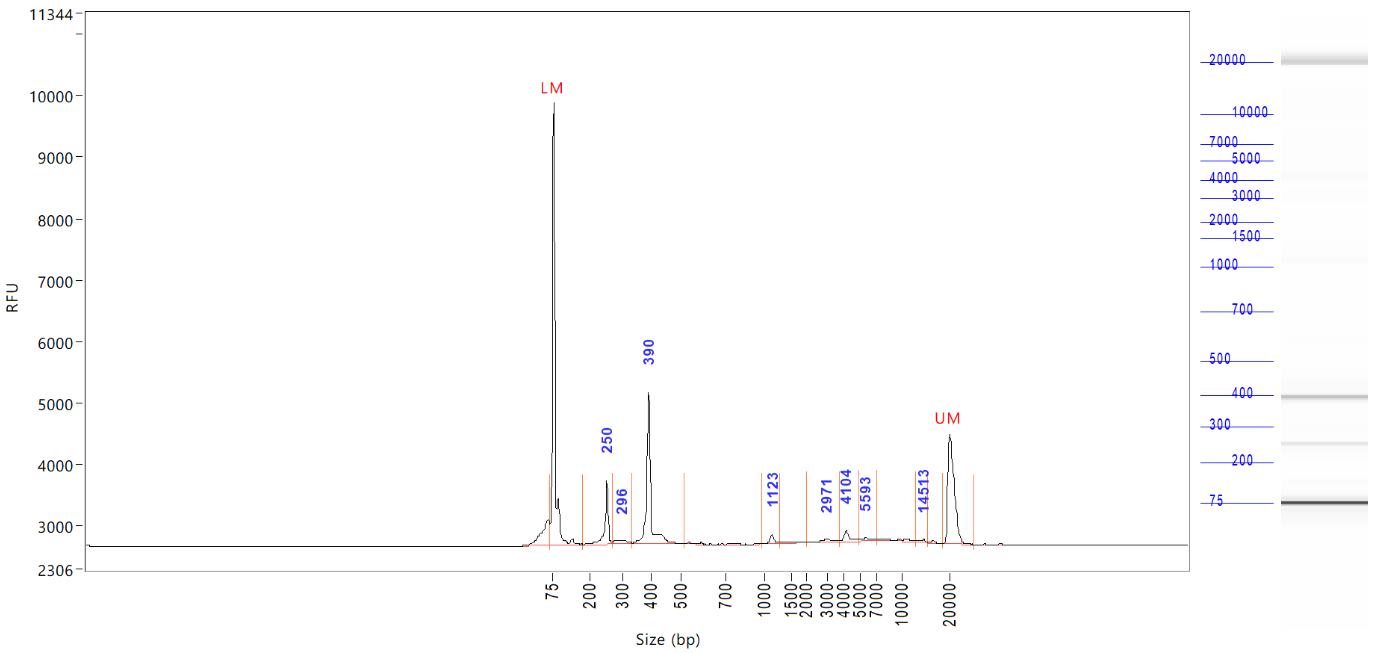

Peak	Size (bp)	Concentration (ng/uL)	From (bp)	To (bp)	Average size (bp)	CV%	RFU	Corrected peak area
1	75 (LM)	0.5000	64	175	76	15.36	7204	47.790
2	250	0.9703	175	268	242	5.63	1044	7.728
3	296	0.2272	268	333	295	5.68	65	1.810
4	390	2.5310	333	516	394	5.56	2471	20.159
5	1123	0.1537	970	1278	1117	5.50	159	1.224
6	2971	0.1118	2000	3750	3004	12.13	52	0.891
7	4104	0.2065	3750	4897	4243	6.39	176	1.645
8	5593	0.1084	4897	7068	5853	11.01	57	0.863
9	14513	0.0579	12562	15244	13812	5.03	50	0.461
10	20000 (UM)	0.1579	18415	25320	20440	3.63	1770	15.096

TIC:	4.3667	ng/uL
TIM:	18.8348	nmole/L
Total concentration:	4.6894	ng/uL

```
Sample peak width (sec): 5    Sample min peak height: 50    Sample baseline V to V?: Y    Sample baseline V to V points: 3
Sample filter: Binomial       Number of points for filter: 3  Sample start region (min): 0  Sample end region (min): 30
Marker peak width (sec): 5    Marker min peak height: 200    Marker baseline V to V?: Y    Marker baseline V to V points: 3
Lower marker selection: First peak > 500 RFU                 Upper marker selection: Last peak > 200 RFU
Ladder size (bp)75, 200, 300, 400, 500, 700, 1000, 1500, 2000, 3000, 4000, 5000, 7000, 10000, 20000
Quantification using: Lower Marker          Final concentration (ng/uL): 0.5000          Dilution factor: 12.0
```

Sample: SampH10
Well location: H10
Created: Monday, September 20, 2021 8:39:56 PM

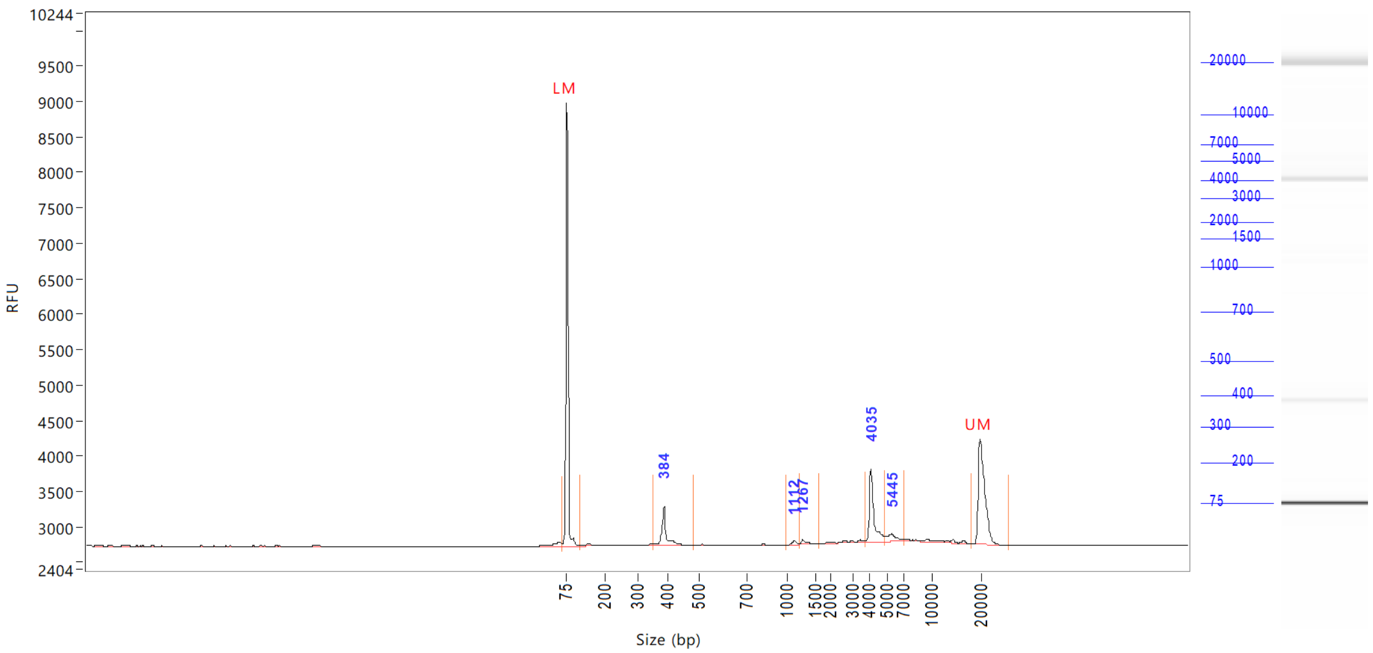

Peak	Size (bp)	Concentration (ng/uL)	From (bp)	To (bp)	Average size (bp)	CV%	RFU	Corrected peak area
1	75 (LM)	0.5000	64	118	74	6.44	6262	34.329
2	384	0.8529	352	479	389	4.79	560	4.880
3	1112	0.0732	987	1201	1101	4.33	58	0.419
4	1267	0.0912	1201	1616	1307	6.23	66	0.522
5	4035	1.1469	3786	4828	4147	5.32	1033	6.562
6	5445	0.2848	4828	7001	5566	10.71	106	1.629
7	20000 (UM)	0.1854	18293	25803	20326	3.71	1479	12.729

TIC:	2.4490	ng/uL
TIM:	4.3712	nmole/L
Total concentration:	3.1256	ng/uL

Sample peak width (sec): 5   Sample min peak height: 50   Sample baseline V to V?: Y   Sample baseline V to V points: 3
Sample filter: Binomial   Number of points for filter: 3   Sample start region (min): 0   Sample end region (min): 30
Marker peak width (sec): 5   Marker min peak height: 200   Marker baseline V to V?: Y   Marker baseline V to V points: 3
Lower marker selection: First peak > 500 RFU   Upper marker selection: Last peak > 200 RFU
Ladder size (bp)75, 200, 300, 400, 500, 700, 1000, 1500, 2000, 3000, 4000, 5000, 7000, 10000, 20000
Quantification using: Lower Marker   Final concentration (ng/uL): 0.5000   Dilution factor: 12.0

Sample: SampA11
Well location: A11
Created: Monday, September 20, 2021 8:39:56 PM

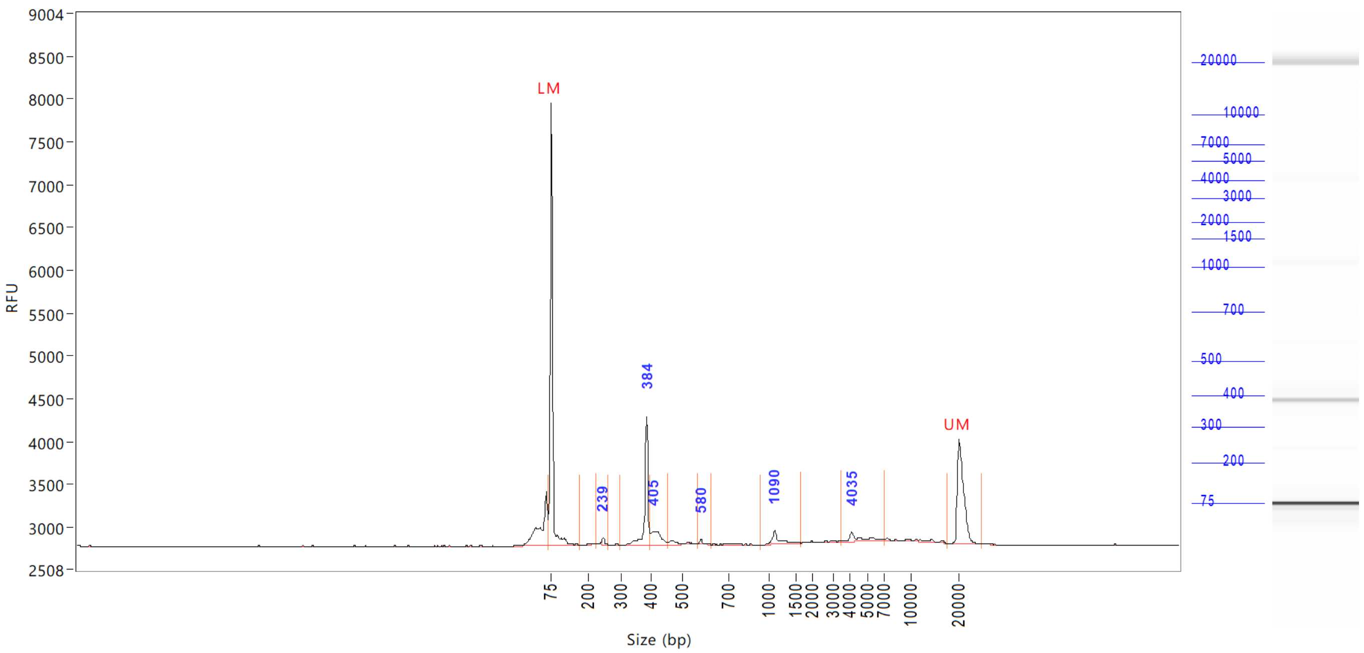

Peak	Size (bp)	Concentration (ng/uL)	From (bp)	To (bp)	Average size (bp)	CV%	RFU	Corrected peak area
1	75 (LM)	0.5000	66	168	75	13.82	5180	31.581
2	239	0.0935	219	259	237	2.23	80	0.492
3	384	1.9221	296	396	376	3.90	1507	10.117
4	405	0.6038	396	450	416	3.27	154	3.178
5	580	0.0544	565	626	580	1.86	56	0.286
6	1090	0.3222	931	1616	1139	10.16	164	1.696
7	4035	0.3631	3429	7001	4836	18.53	116	1.911
8	20000 (UM)	0.1605	17439	24715	20389	3.56	1218	10.139

TIC:	3.3589	ng/uL
TIM:	12.1901	nmole/L
Total concentration:	3.9073	ng/uL

Sample peak width (sec): 5   Sample min peak height: 50   Sample baseline V to V?: Y   Sample baseline V to V points: 3
Sample filter: Binomial   Number of points for filter: 3 Sample start region (min): 0 Sample end region (min): 30
Marker peak width (sec): 5   Marker min peak height: 200   Marker baseline V to V?: Y   Marker baseline V to V points: 3
Lower marker selection: First peak > 500 RFU   Upper marker selection: Last peak > 200 RFU
Ladder size (bp)75, 200, 300, 400, 500, 700, 1000, 1500, 2000, 3000, 4000, 5000, 7000, 10000, 20000
Quantification using: Lower Marker   Final concentration (ng/uL): 0.5000   Dilution factor: 12.0

Sample: SampB11
Well location: B11
Created: Monday, September 20, 2021 8:39:56 PM

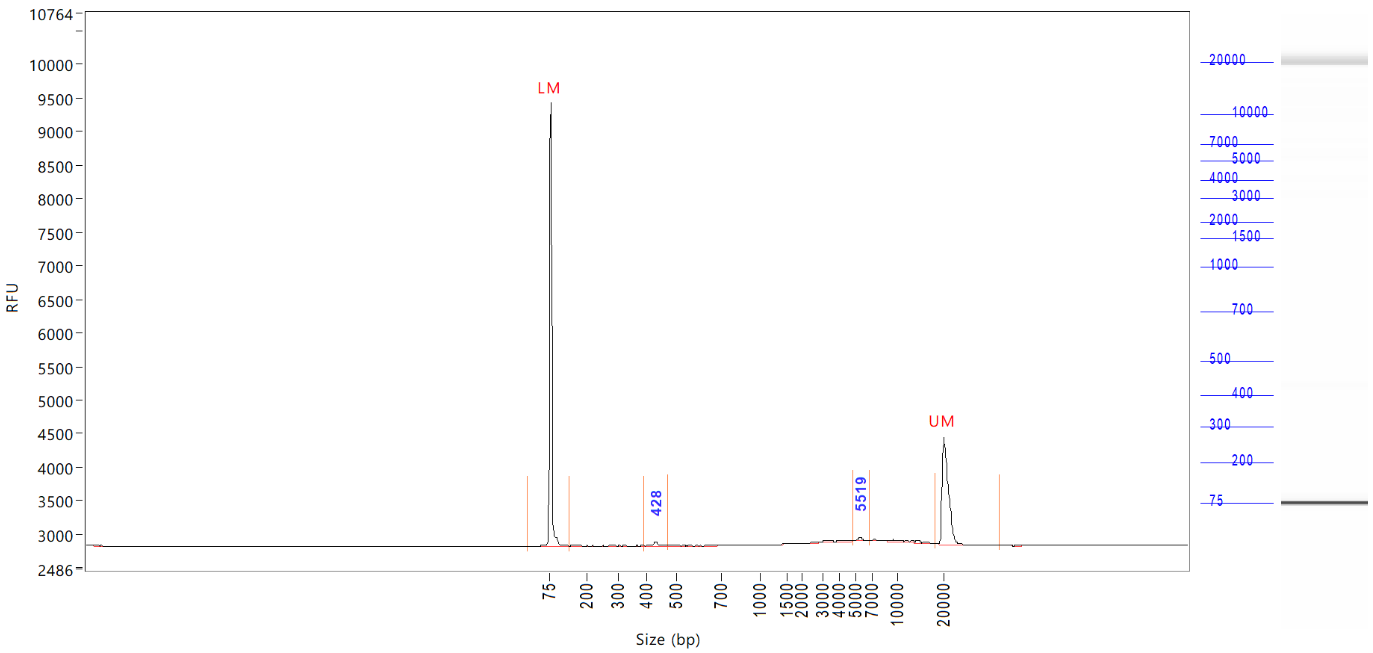

Peak	Size (bp)	Concentration (ng/uL)	From (bp)	To (bp)	Average size (bp)	CV%	RFU	Corrected peak area
1	75 (LM)	0.5000	1	139	73	9.63	6617	37.624
2	428	0.1166	390	468	427	3.65	63	0.731
3	5519	0.0889	4828	6778	5638	8.65	59	0.558
4	20000 (UM)	0.1801	18171	31849	20423	4.64	1585	13.552

TIC:	0.2056	ng/uL
TIM:	0.4754	nmole/L
Total concentration:	0.8040	ng/uL

Sample peak width (sec): 5   Sample min peak height: 50   Sample baseline V to V?: Y   Sample baseline V to V points: 3
Sample filter: Binomial   Number of points for filter: 3   Sample start region (min): 0   Sample end region (min): 30
Marker peak width (sec): 5   Marker min peak height: 200   Marker baseline V to V?: Y   Marker baseline V to V points: 3
Lower marker selection: First peak > 500 RFU   Upper marker selection: Last peak > 200 RFU
Ladder size (bp)75, 200, 300, 400, 500, 700, 1000, 1500, 2000, 3000, 4000, 5000, 7000, 10000, 20000
Quantification using: Lower Marker   Final concentration (ng/uL): 0.5000   Dilution factor: 12.0

Sample: SampC11
Well location: C11
Created: Monday, September 20, 2021 8:39:56 PM

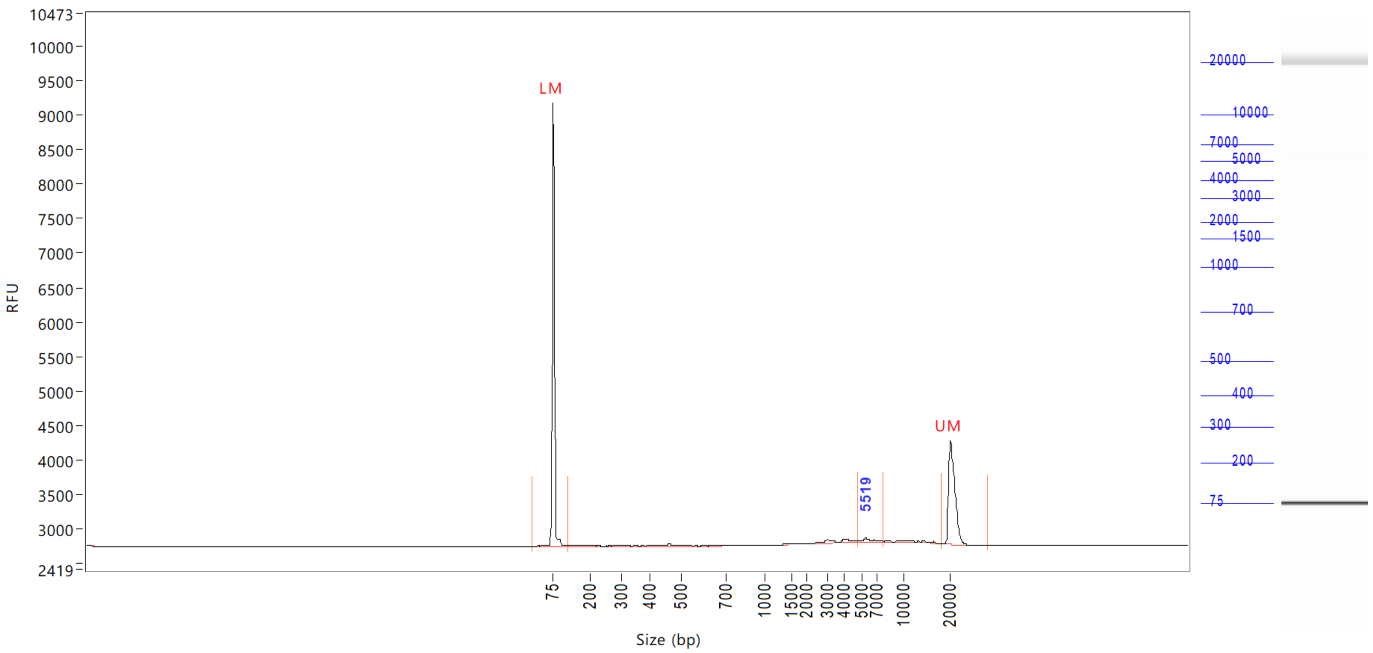

Peak	Size (bp)	Concentration (ng/uL)	From (bp)	To (bp)	Average size (bp)	CV%	RFU	Corrected peak area
1	75 (LM)	0.5000	5	128	73	8.82	6436	36.004
2	5519	0.1529	4793	7734	6028	13.26	60	0.918
3	20000 (UM)	0.1764	18049	27859	20418	3.88	1501	12.706

TIC:	0.1529	ng/uL
TIM:	0.0418	nmole/L
Total concentration:	0.9065	ng/uL

Sample peak width (sec): 5 Sample min peak height: 50 Sample baseline V to V?: Y Sample baseline V to V points: 3
Sample filter: Binomial Number of points for filter: 3 Sample start region (min): 0 Sample end region (min): 30
Marker peak width (sec): 5 Marker min peak height: 200 Marker baseline V to V?: Y Marker baseline V to V points: 3
Lower marker selection: First peak > 500 RFU Upper marker selection: Last peak > 200 RFU
Ladder size (bp)75, 200, 300, 400, 500, 700, 1000, 1500, 2000, 3000, 4000, 5000, 7000, 10000, 20000
Quantification using: Lower Marker Final concentration (ng/uL): 0.5000 Dilution factor: 12.0

Sample: SampD11
Well location: D11
Created: Monday, September 20, 2021 8:39:56 PM

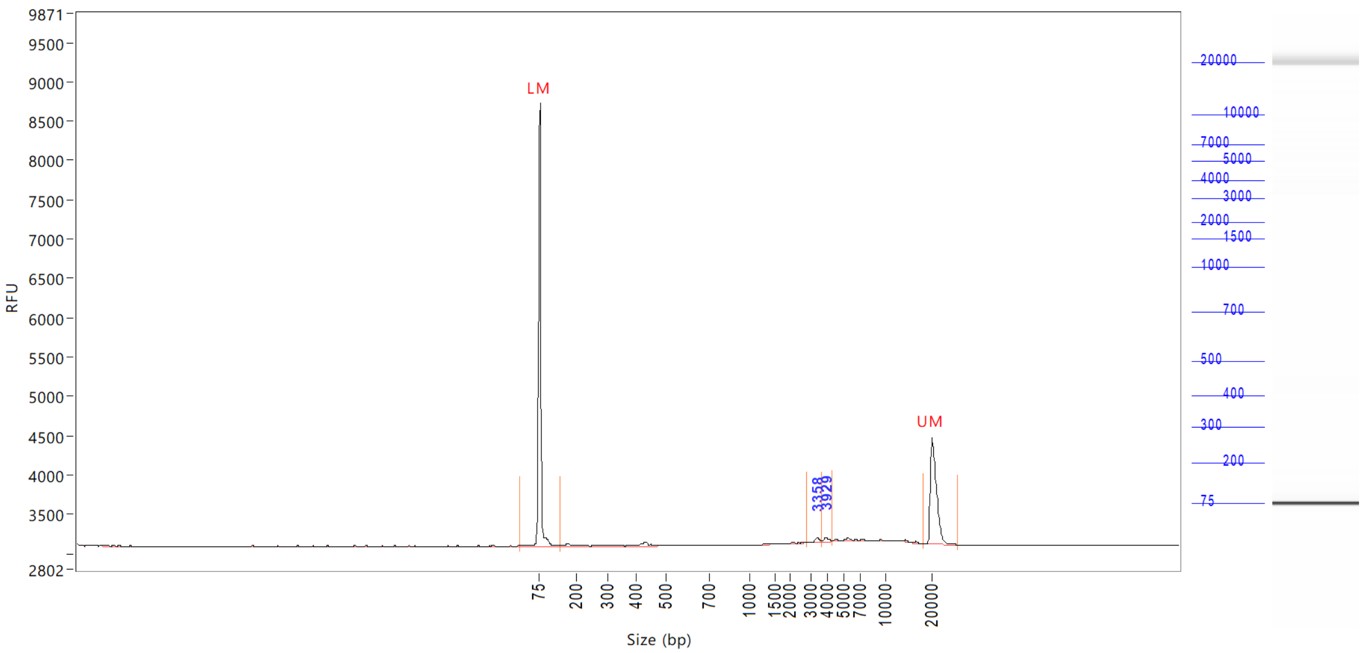

Peak	Size (bp)	Concentration (ng/uL)	From (bp)	To (bp)	Average size (bp)	CV%	RFU	Corrected peak area
1	75 (LM)	0.5000	9	147	74	12.30	5647	33.284
2	3358	0.0752	2772	3679	3336	5.68	53	0.417
3	3929	0.0836	3679	4311	3951	3.61	59	0.464
4	20000 (UM)	0.1715	18049	25682	20409	3.86	1363	11.418

TIC:	0.1588	ng/uL
TIM:	0.0719	nmole/L
Total concentration:	0.9545	ng/uL

Sample peak width (sec): 5   Sample min peak height: 50   Sample baseline V to V?: Y   Sample baseline V to V points: 3
Sample filter: Binomial   Number of points for filter: 3 Sample start region (min): 0 Sample end region (min): 30
Marker peak width (sec): 5   Marker min peak height: 200   Marker baseline V to V?: Y   Marker baseline V to V points: 3
Lower marker selection: First peak > 500 RFU   Upper marker selection: Last peak > 200 RFU
Ladder size (bp)75, 200, 300, 400, 500, 700, 1000, 1500, 2000, 3000, 4000, 5000, 7000, 10000, 20000
Quantification using: Lower Marker   Final concentration (ng/uL): 0.5000   Dilution factor: 12.0

Sample: SampE11
Well location: E11
Created: Monday, September 20, 2021 8:39:56 PM

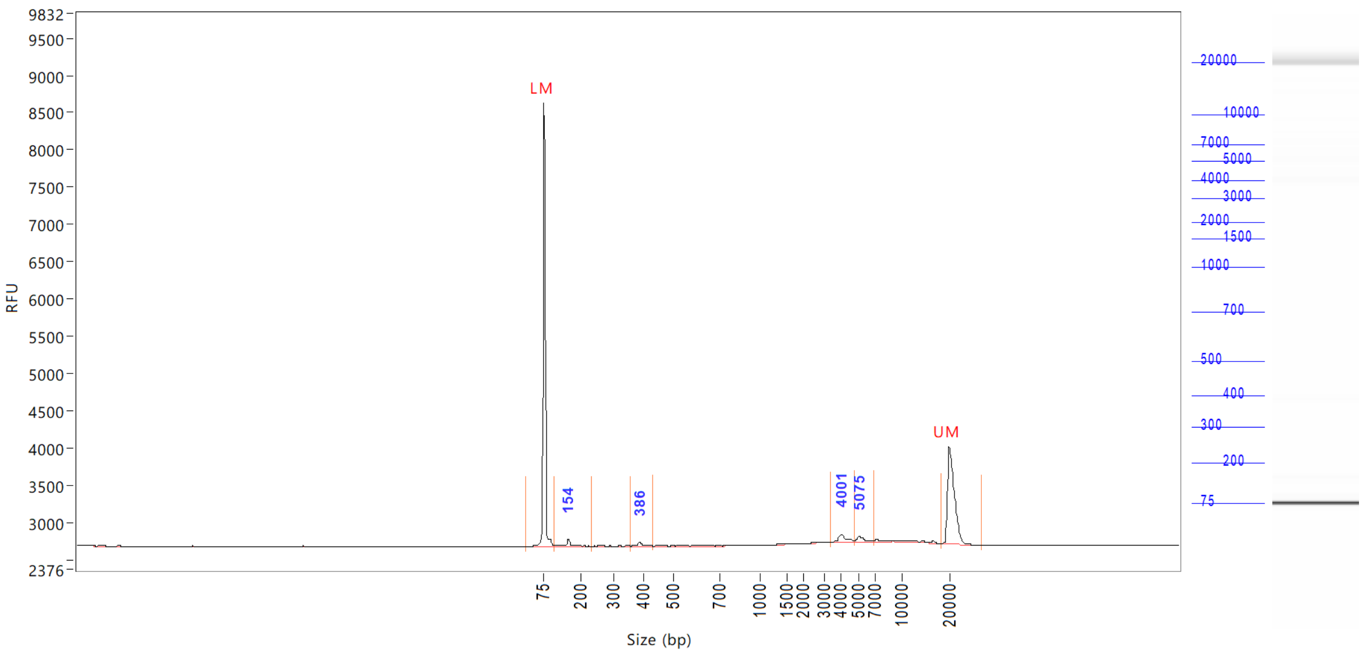

Peak	Size (bp)	Concentration (ng/uL)	From (bp)	To (bp)	Average size (bp)	CV%	RFU	Corrected peak area
1	75 (LM)	0.5000	18	110	74	7.66	5961	33.599
2	154	0.1982	110	230	153	14.87	92	1.110
3	386	0.1034	354	431	389	3.95	50	0.579
4	4001	0.2148	3358	4793	4074	7.04	104	1.203
5	5075	0.1385	4793	6926	5469	10.53	63	0.775
6	20000 (UM)	0.1743	18293	26770	20506	3.99	1298	11.712

TIC:	0.6549	ng/uL
TIM:	2.6949	nmole/L
Total concentration:	1.0241	ng/uL

Sample peak width (sec): 5 Sample min peak height: 50 Sample baseline V to V?: Y Sample baseline V to V points: 3
Sample filter: Binomial Number of points for filter: 3 Sample start region (min): 0 Sample end region (min): 30
Marker peak width (sec): 5 Marker min peak height: 200 Marker baseline V to V?: Y Marker baseline V to V points: 3
Lower marker selection: First peak > 500 RFU Upper marker selection: Last peak > 200 RFU
Ladder size (bp)75, 200, 300, 400, 500, 700, 1000, 1500, 2000, 3000, 4000, 5000, 7000, 10000, 20000
Quantification using: Lower Marker Final concentration (ng/uL): 0.5000 Dilution factor: 12.0

Sample: SampF11
Well location: F11
Created: Monday, September 20, 2021 8:39:56 PM

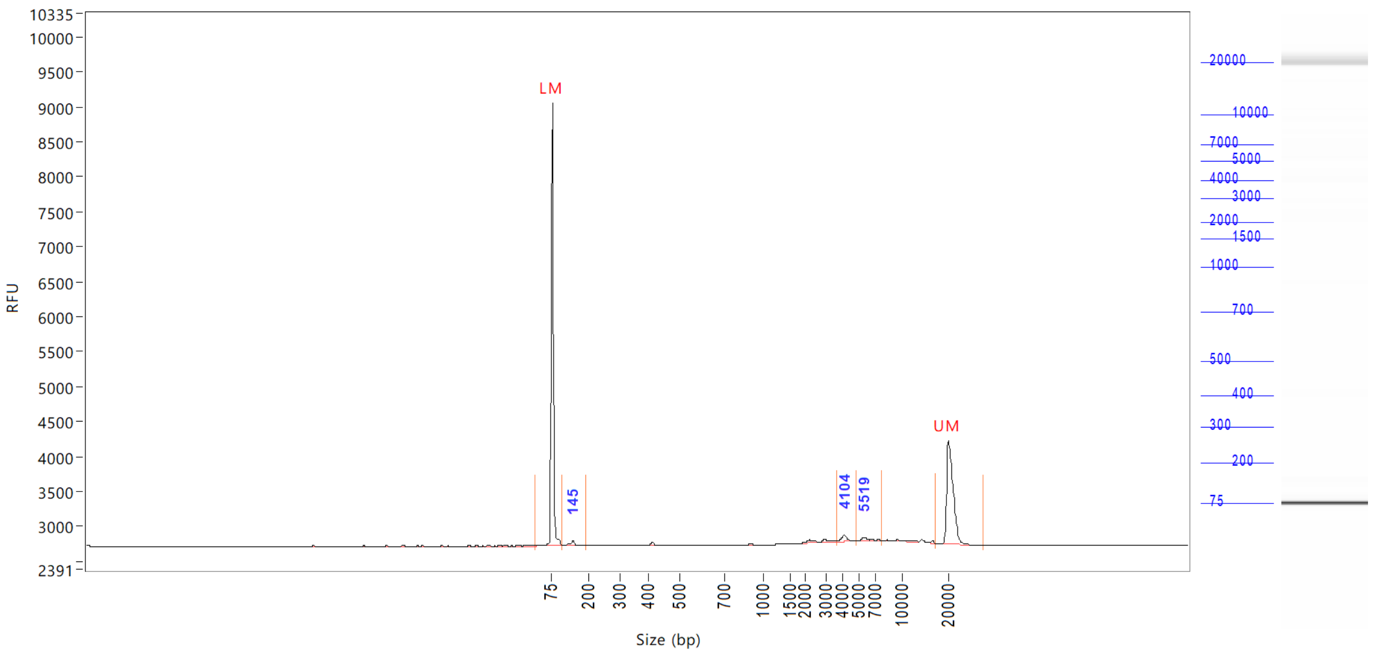

Peak	Size (bp)	Concentration (ng/uL)	From (bp)	To (bp)	Average size (bp)	CV%	RFU	Corrected peak area
1	75 (LM)	0.5000	22	108	74	6.99	6341	35.604
2	145	0.1609	108	189	140	11.40	76	0.955
3	4104	0.1461	3643	4793	4130	5.64	86	0.867
4	5519	0.1418	4793	7734	6020	13.49	54	0.842
5	20000 (UM)	0.1776	17317	27254	20410	3.94	1483	12.643

TIC:	0.4488	ng/uL
TIM:	1.9856	nmole/L
Total concentration:	1.0280	ng/uL

Sample peak width (sec): 5   Sample min peak height: 50   Sample baseline V to V?: Y   Sample baseline V to V points: 3
Sample filter: Binomial   Number of points for filter: 3   Sample start region (min): 0   Sample end region (min): 30
Marker peak width (sec): 5   Marker min peak height: 200   Marker baseline V to V?: Y   Marker baseline V to V points: 3
Lower marker selection: First peak > 500 RFU   Upper marker selection: Last peak > 200 RFU
Ladder size (bp)75, 200, 300, 400, 500, 700, 1000, 1500, 2000, 3000, 4000, 5000, 7000, 10000, 20000
Quantification using: Lower Marker   Final concentration (ng/uL): 0.5000   Dilution factor: 12.0

Sample: SampG11
Well location: G11
Created: Monday, September 20, 2021 8:39:56 PM

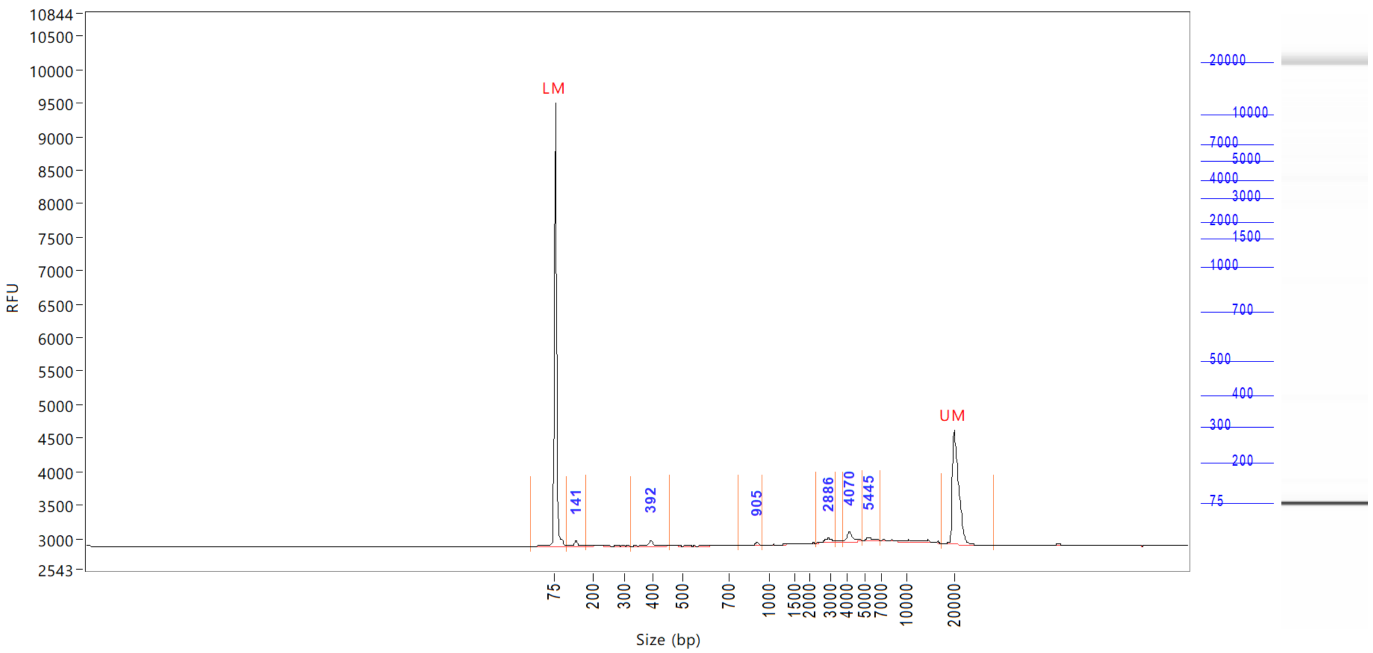

Peak	Size (bp)	Concentration (ng/uL)	From (bp)	To (bp)	Average size (bp)	CV%	RFU	Corrected peak area
1	75 (LM)	0.5000	0	112	72	9.10	6631	38.474
2	141	0.1316	112	179	139	8.91	85	0.844
3	392	0.1582	321	452	388	5.12	90	1.014
4	905	0.0525	766	948	895	3.71	54	0.337
5	2886	0.1257	2229	3251	2831	7.00	66	0.806
6	4070	0.2371	3750	4897	4180	5.97	154	1.520
7	5445	0.1135	4897	7000	5721	9.66	61	0.728
8	20000 (UM)	0.1894	17439	28584	20446	4.27	1693	14.572

TIC:	0.8186	ng/uL
TIM:	2.5223	nmole/L
Total concentration:	1.1735	ng/uL

Sample peak width (sec): 5 Sample min peak height: 50 Sample baseline V to V?: Y Sample baseline V to V points: 3
Sample filter: Binomial Number of points for filter: 3 Sample start region (min): 0 Sample end region (min): 30
Marker peak width (sec): 5 Marker min peak height: 200 Marker baseline V to V?: Y Marker baseline V to V points: 3
Lower marker selection: First peak > 500 RFU Upper marker selection: Last peak > 200 RFU
Ladder size (bp)75, 200, 300, 400, 500, 700, 1000, 1500, 2000, 3000, 4000, 5000, 7000, 10000, 20000
Quantification using: Lower Marker Final concentration (ng/uL): 0.5000 Dilution factor: 12.0

Sample: SampH11
Well location: H11
Created: Monday, September 20, 2021 8:39:56 PM

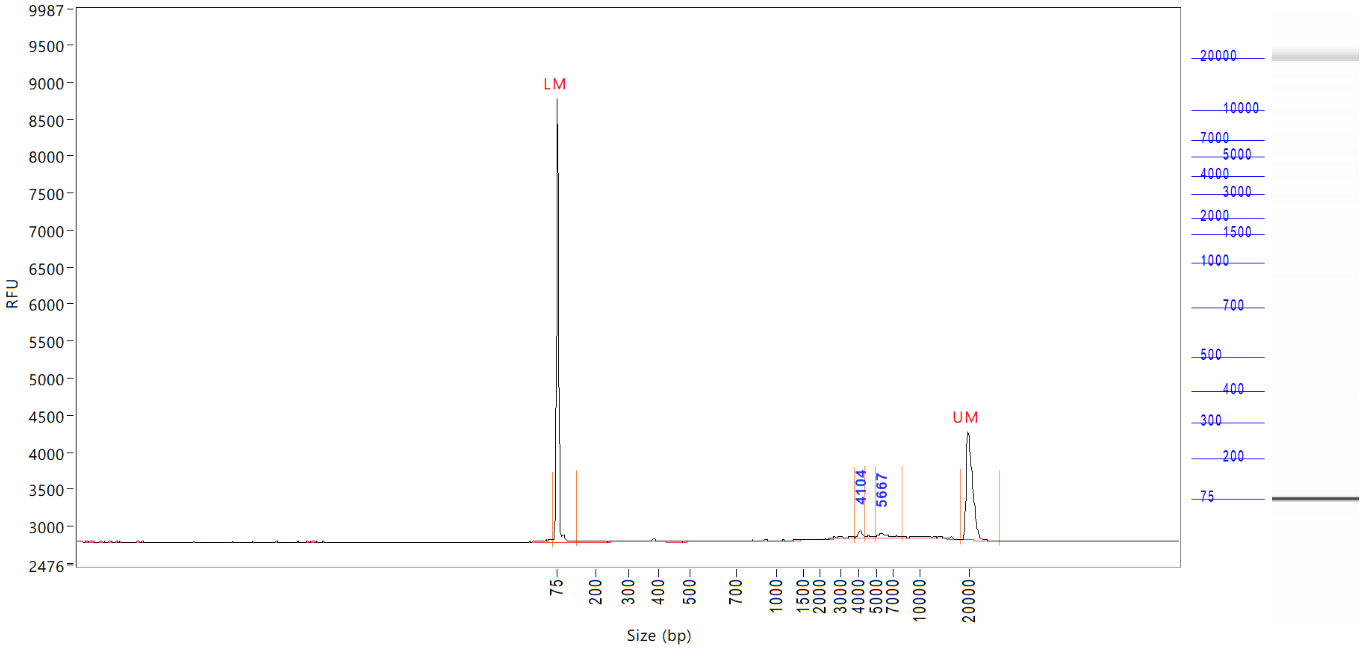

Peak	Size (bp)	Concentration (ng/uL)	From (bp)	To (bp)	Average size (bp)	CV%	RFU	Corrected peak area
1	75 (LM)	0.5000	62	139	73	7.51	6000	32.730
2	4104	0.1007	3786	4380	4080	2.75	94	0.549
3	5667	0.1550	4966	8001	6139	13.56	56	0.846
4	20000 (UM)	0.1886	18415	26529	20358	3.77	1467	12.348

TIC:	0.2557	ng/uL
TIM:	0.0822	nmole/L
Total concentration:	0.8911	ng/uL

```
Sample peak width (sec): 5    Sample min peak height: 50    Sample baseline V to V?: Y    Sample baseline V to V points: 3
Sample filter: Binomial       Number of points for filter: 3  Sample start region (min): 0  Sample end region (min): 30
Marker peak width (sec): 5    Marker min peak height: 200   Marker baseline V to V?: Y    Marker baseline V to V points: 3
Lower marker selection: First peak > 500 RFU                Upper marker selection: Last peak > 200 RFU
Ladder size (bp)75, 200, 300, 400, 500, 700, 1000, 1500, 2000, 3000, 4000, 5000, 7000, 10000, 20000
Quantification using: Lower Marker          Final concentration (ng/uL): 0.5000          Dilution factor: 12.0
```

Sample: SampA12
Well location: A12
Created: Monday, September 20, 2021 8:39:56 PM

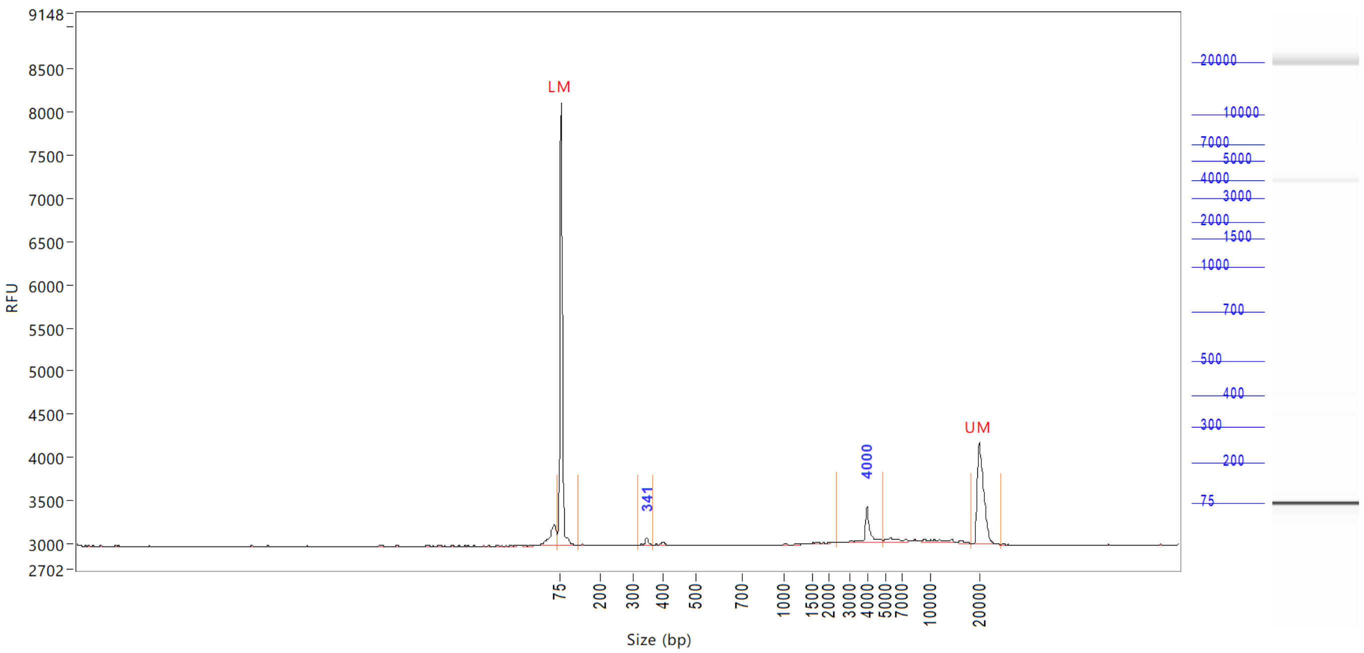

Peak	Size<br>(bp)	Concentration<br>(ng/uL)	From<br>(bp)	To<br>(bp)	Average size<br>(bp)	CV%	RFU	Corrected peak area
1	75 (LM)	0.5000	64	131	74	7.79	5143	28.970
2	341	0.1449	313	364	338	2.80	92	0.700
3	4000	0.6152	2372	4862	3971	9.73	421	2.971
4	20000 (UM)	0.1662	18415	24473	20331	3.36	1175	9.628

TIC:	0.7602	ng/uL
TIM:	0.9604	nmole/L
Total concentration:	1.5648	ng/uL

Sample peak width (sec): 5   Sample min peak height: 50   Sample baseline V to V?: Y   Sample baseline V to V points: 3
Sample filter: Binomial   Number of points for filter: 3   Sample start region (min): 0   Sample end region (min): 30
Marker peak width (sec): 5   Marker min peak height: 200   Marker baseline V to V?: Y   Marker baseline V to V points: 3
Lower marker selection: First peak > 500 RFU   Upper marker selection: Last peak > 200 RFU
Ladder size (bp)75, 200, 300, 400, 500, 700, 1000, 1500, 2000, 3000, 4000, 5000, 7000, 10000, 20000
Quantification using: Lower Marker   Final concentration (ng/uL): 0.5000   Dilution factor: 12.0

Sample: SampB12
Well location: B12
Created: Monday, September 20, 2021 8:39:56 PM

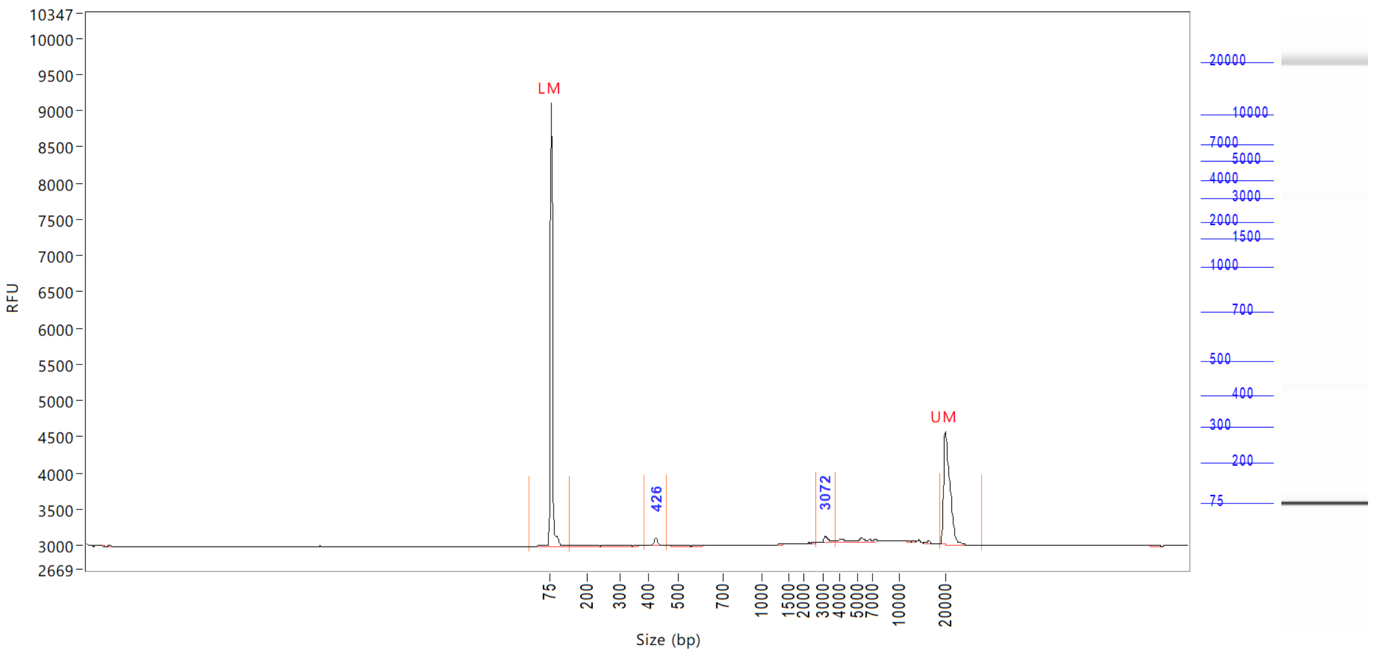

Peak	Size (bp)	Concentration (ng/uL)	From (bp)	To (bp)	Average size (bp)	CV%	RFU	Corrected peak area
1	75 (LM)	0.5000	1	137	73	9.55	6134	36.009
2	426	0.1845	388	462	424	3.10	111	1.107
3	3072	0.1528	2600	3750	3195	7.74	90	0.917
4	20000 (UM)	0.1838	18659	27859	20431	4.24	1549	13.238

TIC:	0.3373	ng/uL
TIM:	0.7948	nmole/L
Total concentration:	0.9542	ng/uL

Sample peak width (sec): 5   Sample min peak height: 50   Sample baseline V to V?: Y   Sample baseline V to V points: 3
Sample filter: Binomial   Number of points for filter: 3 Sample start region (min): 0 Sample end region (min): 30
Marker peak width (sec): 5   Marker min peak height: 200   Marker baseline V to V?: Y   Marker baseline V to V points: 3
Lower marker selection: First peak > 500 RFU   Upper marker selection: Last peak > 200 RFU
Ladder size (bp)75, 200, 300, 400, 500, 700, 1000, 1500, 2000, 3000, 4000, 5000, 7000, 10000, 20000
Quantification using: Lower Marker   Final concentration (ng/uL): 0.5000   Dilution factor: 12.0

Sample: SampC12
Well location: C12
Created: Monday, September 20, 2021 8:39:56 PM

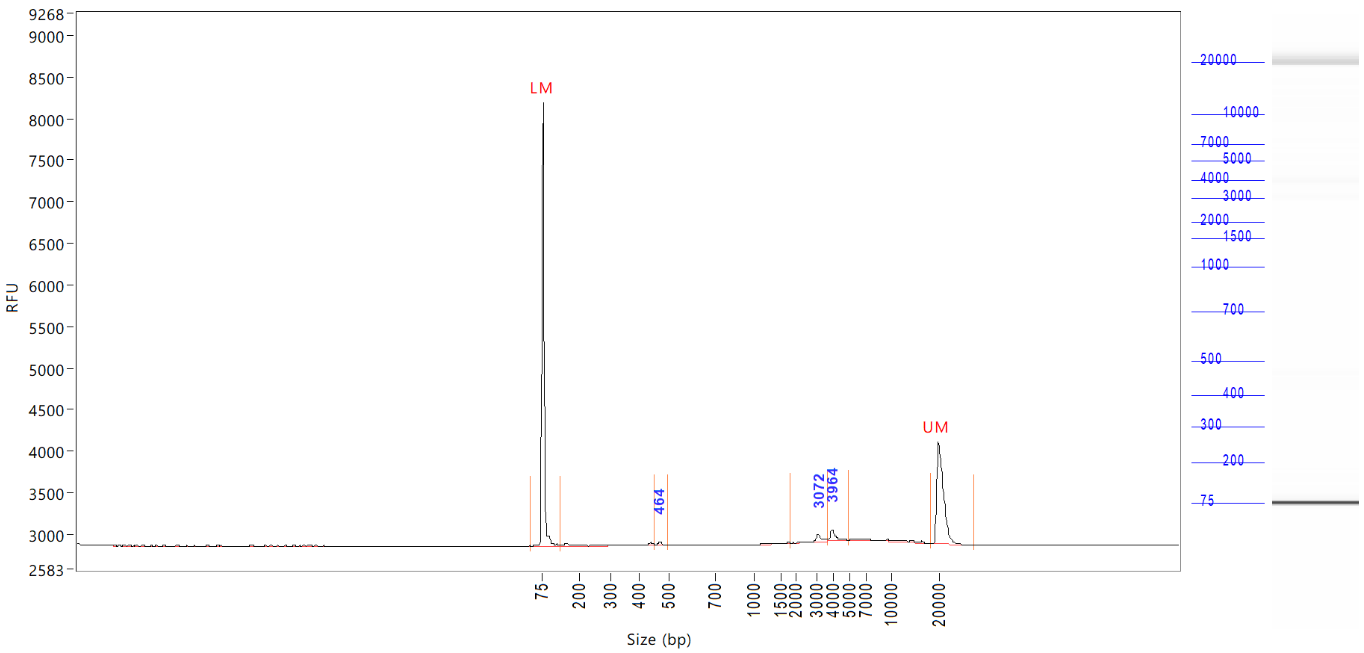

Peak	Size (bp)	Concentration (ng/uL)	From (bp)	To (bp)	Average size (bp)	CV%	RFU	Corrected peak area
1	75 (LM)	0.5000	35	133	74	9.29	5342	31.658
2	464	0.0827	450	490	465	1.82	51	0.436
3	3072	0.1672	1827	3643	3067	11.82	90	0.882
4	3964	0.2374	3643	4897	4063	6.35	141	1.253
5	20000 (UM)	0.1713	18293	27496	20468	4.22	1225	10.847

TIC:	0.4872	ng/uL
TIM:	0.4785	nmole/L
Total concentration:	1.1484	ng/uL

Sample peak width (sec): 5 Sample min peak height: 50 Sample baseline V to V?: Y Sample baseline V to V points: 3
Sample filter: Binomial Number of points for filter: 3 Sample start region (min): 0 Sample end region (min): 30
Marker peak width (sec): 5 Marker min peak height: 200 Marker baseline V to V?: Y Marker baseline V to V points: 3
Lower marker selection: First peak > 500 RFU Upper marker selection: Last peak > 200 RFU
Ladder size (bp)75, 200, 300, 400, 500, 700, 1000, 1500, 2000, 3000, 4000, 5000, 7000, 10000, 20000
Quantification using: Lower Marker Final concentration (ng/uL): 0.5000 Dilution factor: 12.0

Sample: SampD12
Well location: D12
Created: Monday, September 20, 2021 8:39:56 PM

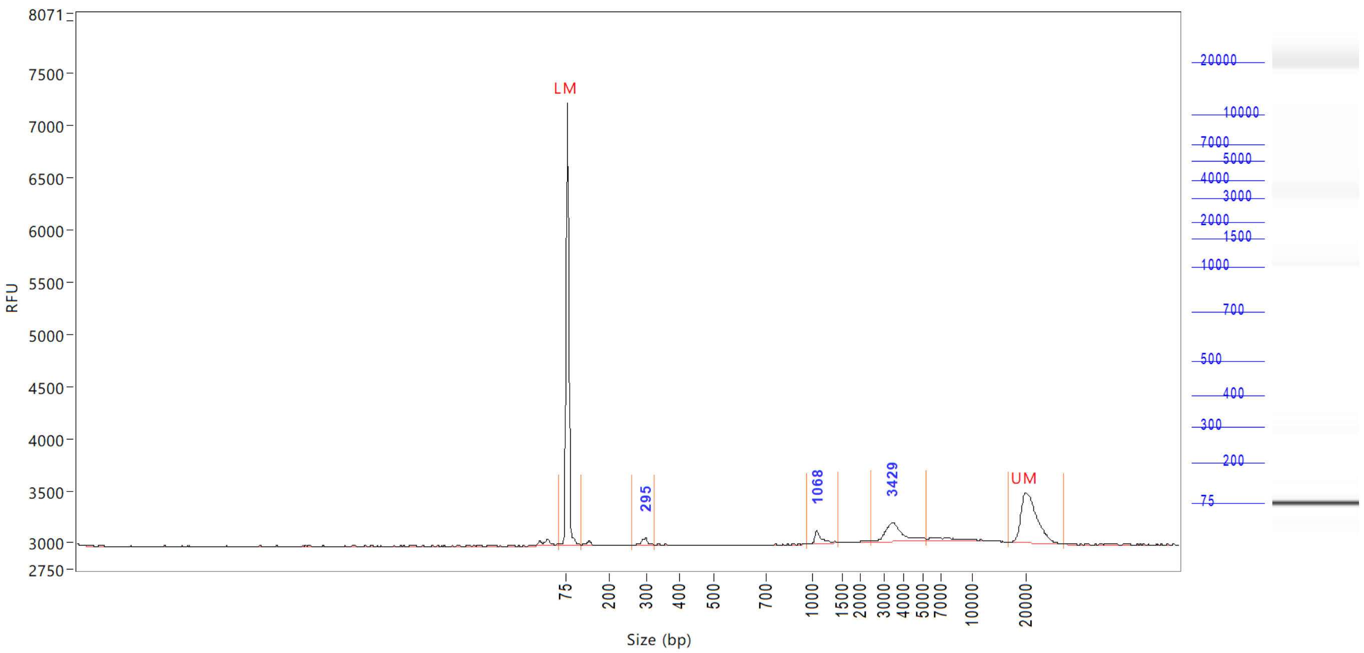

Peak	Size (bp)	Concentration (ng/uL)	From (bp)	To (bp)	Average size (bp)	CV%	RFU	Corrected peak area
1	75 (LM)	0.5000	52	114	73	6.58	4241	29.957
2	295	0.2032	259	323	291	3.99	76	1.014
3	1068	0.2815	965	1411	1116	8.99	132	1.406
4	3429	0.8927	2458	5445	3635	16.39	178	4.457
5	20000 (UM)	0.1553	16952	26891	20812	7.10	481	9.305

TIC:	1.3774	ng/uL
TIM:	1.9679	nmole/L
Total concentration:	2.0809	ng/uL

Sample peak width (sec): 5   Sample min peak height: 50   Sample baseline V to V?: Y   Sample baseline V to V points: 3
Sample filter: Binomial   Number of points for filter: 3 Sample start region (min): 0 Sample end region (min): 30
Marker peak width (sec): 5   Marker min peak height: 200   Marker baseline V to V?: Y   Marker baseline V to V points: 3
Lower marker selection: First peak > 500 RFU   Upper marker selection: Last peak > 200 RFU
Ladder size (bp)75, 200, 300, 400, 500, 700, 1000, 1500, 2000, 3000, 4000, 5000, 7000, 10000, 20000
Quantification using: Lower Marker   Final concentration (ng/uL): 0.5000   Dilution factor: 12.0

Sample: SampE12
Well location: E12
Created: Monday, September 20, 2021 8:39:56 PM

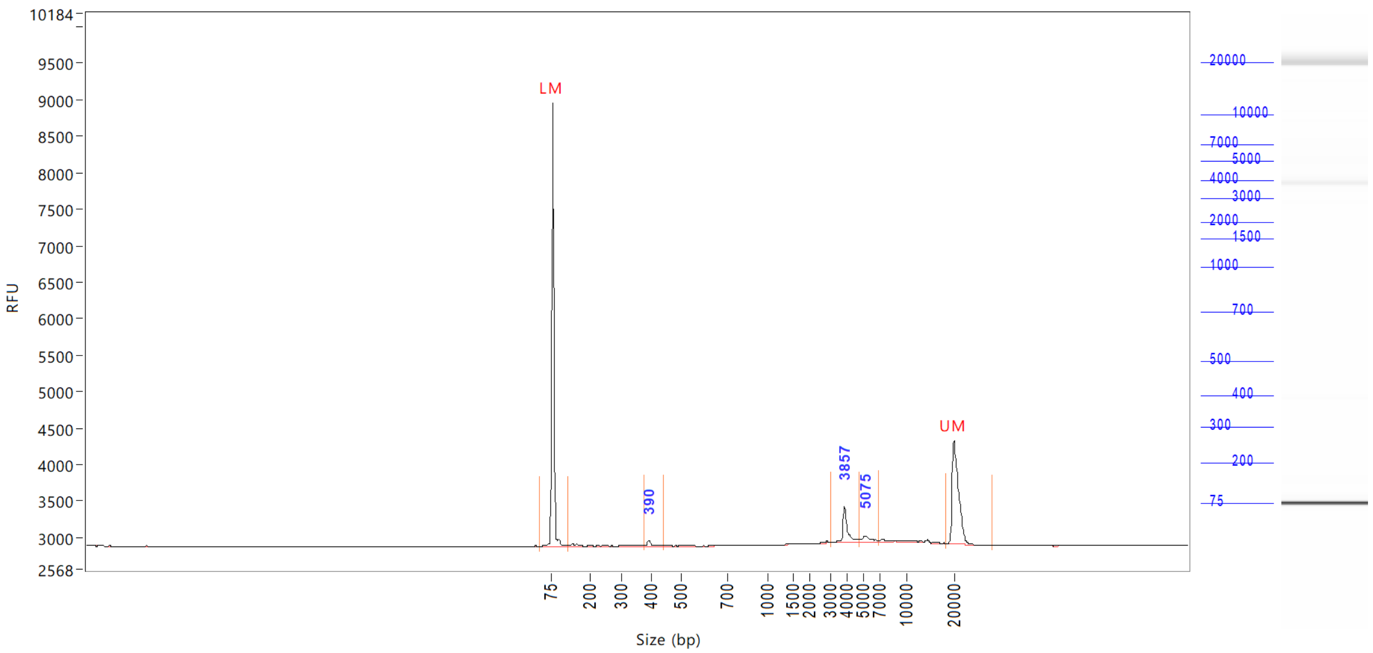

Peak	Size<br>(bp)	Concentration<br>(ng/uL)	From<br>(bp)	To<br>(bp)	Average size<br>(bp)	CV%	RFU	Corrected peak area
1	75 (LM)	0.5000	35	126	74	7.55	6086	33.944
2	390	0.1170	378	441	398	3.84	72	0.662
3	3857	0.5915	3001	4724	3933	7.09	496	3.346
4	5075	0.1857	4724	6778	5384	10.03	84	1.051
5	20000 (UM)	0.1810	18415	27979	20394	3.95	1413	12.287

TIC:	0.8942	ng/uL
TIM:	0.7881	nmole/L
Total concentration:	1.3709	ng/uL

```
Sample peak width (sec): 5    Sample min peak height: 50    Sample baseline V to V?: Y    Sample baseline V to V points: 3
Sample filter: Binomial       Number of points for filter: 3  Sample start region (min): 0  Sample end region (min): 30
Marker peak width (sec): 5    Marker min peak height: 200   Marker baseline V to V?: Y    Marker baseline V to V points: 3
Lower marker selection: First peak > 500 RFU                Upper marker selection: Last peak > 200 RFU
Ladder size (bp)75, 200, 300, 400, 500, 700, 1000, 1500, 2000, 3000, 4000, 5000, 7000, 10000, 20000
Quantification using: Lower Marker          Final concentration (ng/uL): 0.5000          Dilution factor: 12.0
```

Sample: SampF12
Well location: F12
Created: Monday, September 20, 2021 8:39:56 PM

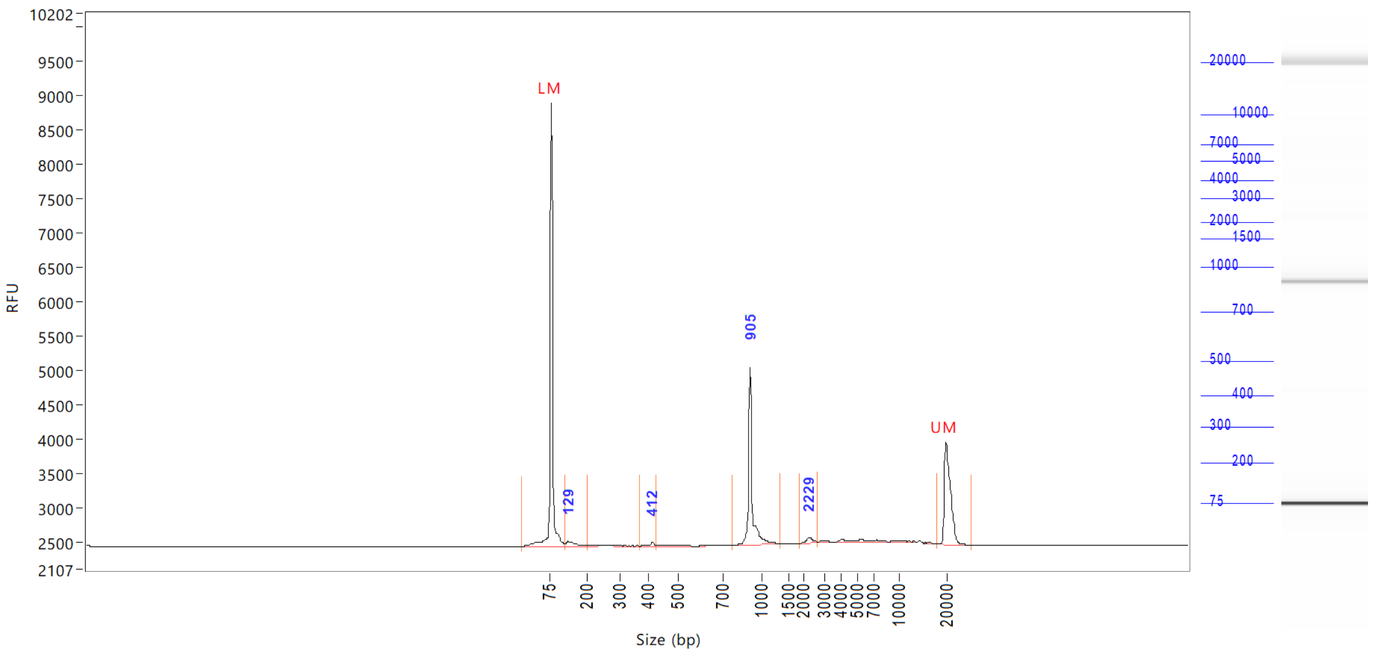

Peak	Size (bp)	Concentration (ng/uL)	From (bp)	To (bp)	Average size (bp)	CV%	RFU	Corrected peak area
1	75 (LM)	0.5000	0	120	71	19.64	6467	43.451
2	129	0.1955	120	198	145	13.75	68	1.416
3	412	0.0642	368	426	406	2.62	57	0.465
4	905	2.2709	766	1323	915	5.41	2578	16.445
5	2229	0.1283	1846	2715	2261	8.07	81	0.929
6	20000 (UM)	0.1432	18049	25320	20416	3.80	1485	12.445

TIC:	2.6588	ng/uL
TIM:	6.6537	nmole/L
Total concentration:	3.0977	ng/uL

```
Sample peak width (sec): 5    Sample min peak height: 50    Sample baseline V to V?: Y    Sample baseline V to V points: 3
Sample filter: Binomial       Number of points for filter: 3  Sample start region (min): 0  Sample end region (min): 30
Marker peak width (sec): 5    Marker min peak height: 200   Marker baseline V to V?: Y    Marker baseline V to V points: 3
Lower marker selection: First peak > 500 RFU                Upper marker selection: Last peak > 200 RFU
Ladder size (bp)75, 200, 300, 400, 500, 700, 1000, 1500, 2000, 3000, 4000, 5000, 7000, 10000, 20000
Quantification using: Lower Marker          Final concentration (ng/uL): 0.5000          Dilution factor: 12.0
```

Sample: SampG12
Well location: G12
Created: Monday, September 20, 2021 8:39:56 PM

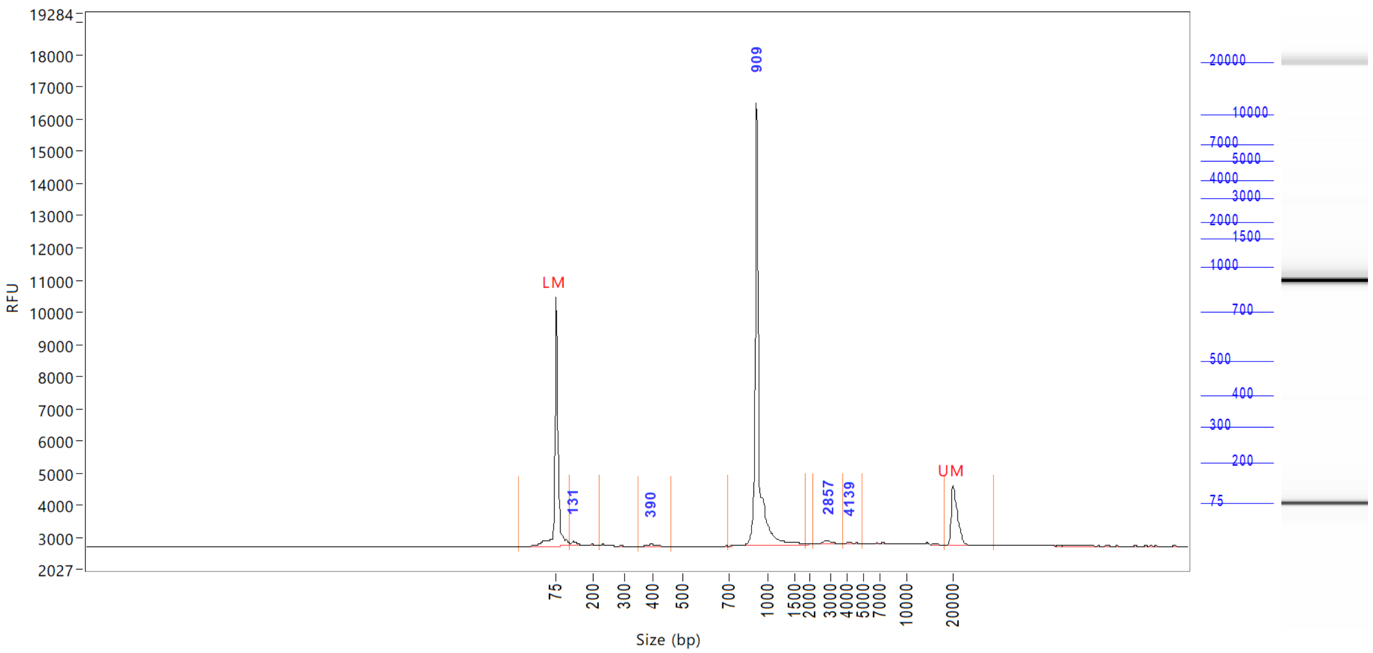

Peak	Size (bp)	Concentration (ng/uL)	From (bp)	To (bp)	Average size (bp)	CV%	RFU	Corrected peak area
1	75 (LM)	0.5000	0	122	72	21.63	7709	60.557
2	131	0.2567	122	219	149	16.67	144	2.591
3	390	0.0996	345	460	395	5.93	84	1.005
4	909	9.1066	700	1846	936	10.63	13763	91.910
5	2857	0.1903	2172	3750	2832	11.32	113	1.920
6	4139	0.0631	3750	4931	4222	6.68	57	0.637
7	20000 (UM)	0.1310	18293	28584	20449	4.15	1853	15.866

TIC:	9.7162	ng/uL
TIM:	19.3951	nmole/L
Total concentration:	9.9064	ng/uL

Sample peak width (sec): 5 Sample min peak height: 50 Sample baseline V to V?: Y Sample baseline V to V points: 3
Sample filter: Binomial Number of points for filter: 3 Sample start region (min): 0 Sample end region (min): 30
Marker peak width (sec): 5 Marker min peak height: 200 Marker baseline V to V?: Y Marker baseline V to V points: 3
Lower marker selection: First peak > 500 RFU Upper marker selection: Last peak > 200 RFU
Ladder size (bp)75, 200, 300, 400, 500, 700, 1000, 1500, 2000, 3000, 4000, 5000, 7000, 10000, 20000
Quantification using: Lower Marker Final concentration (ng/uL): 0.5000 Dilution factor: 12.0

Sample: SampH12
Well location: H12
Created: Monday, September 20, 2021 8:39:56 PM

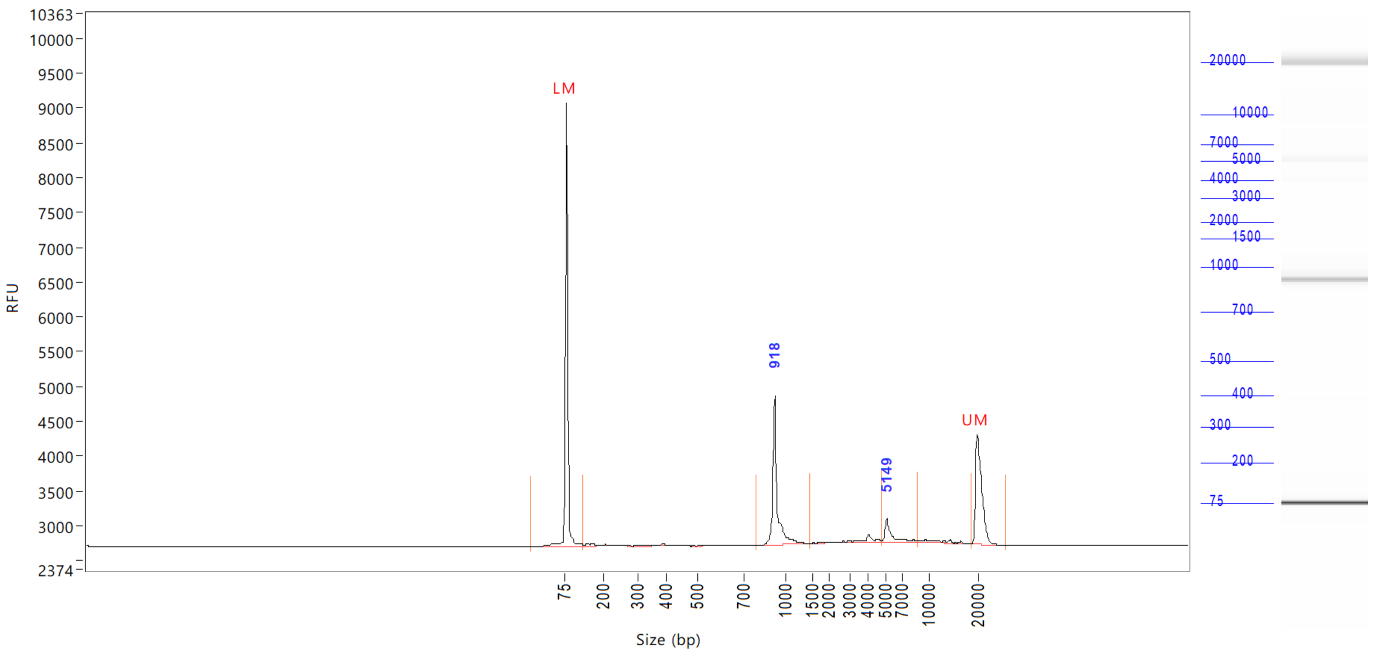

Peak	Size (bp)	Concentration (ng/uL)	From (bp)	To (bp)	Average size (bp)	CV%	RFU	Corrected peak area
1	75 (LM)	0.5000	0	131	73	16.42	6382	38.953
2	918	2.4362	779	1456	933	6.75	2136	15.817
3	5149	0.4654	4793	8734	5789	16.79	323	3.022
4	20000 (UM)	0.1684	18781	25803	20373	3.70	1574	13.119

TIC:	2.9017	ng/uL
TIM:	4.4301	nmole/L
Total concentration:	3.5906	ng/uL

```
Sample peak width (sec): 5    Sample min peak height: 200   Sample baseline V to V?: Y    Sample baseline V to V points: 3
Sample filter: Binomial       Number of points for filter: 3  Sample start region (min): 0  Sample end region (min): 30
Marker peak width (sec): 5    Marker min peak height: 200   Marker baseline V to V?: Y    Marker baseline V to V points: 3
Lower marker selection: First peak > 500 RFU                Upper marker selection: Last peak > 200 RFU
Ladder size (bp)75, 200, 300, 400, 500, 700, 1000, 1500, 2000, 3000, 4000, 5000, 7000, 10000, 20000
Quantification using: Lower Marker          Final concentration (ng/uL): 0.5000          Dilution factor: 12.0
```

Sample: DNA size ladder
Well location: A7
Created: Monday, September 20, 2021 8:39:56 PM
Import from: C:\Users\admin\Desktop\210810_alberto_ladder.SCAL
Fit type: Point to point

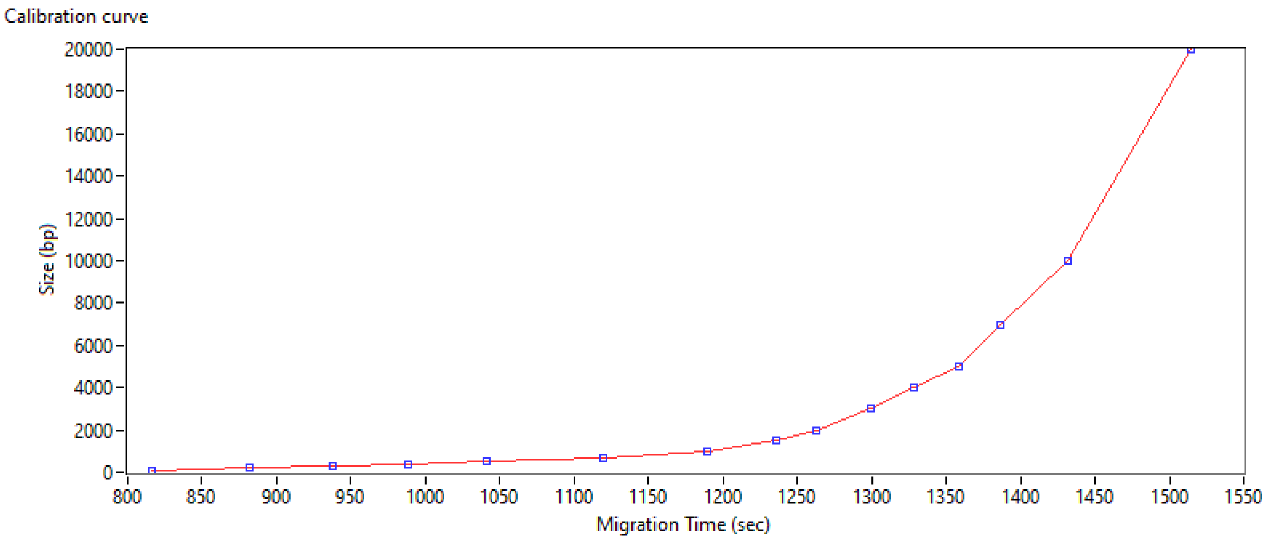

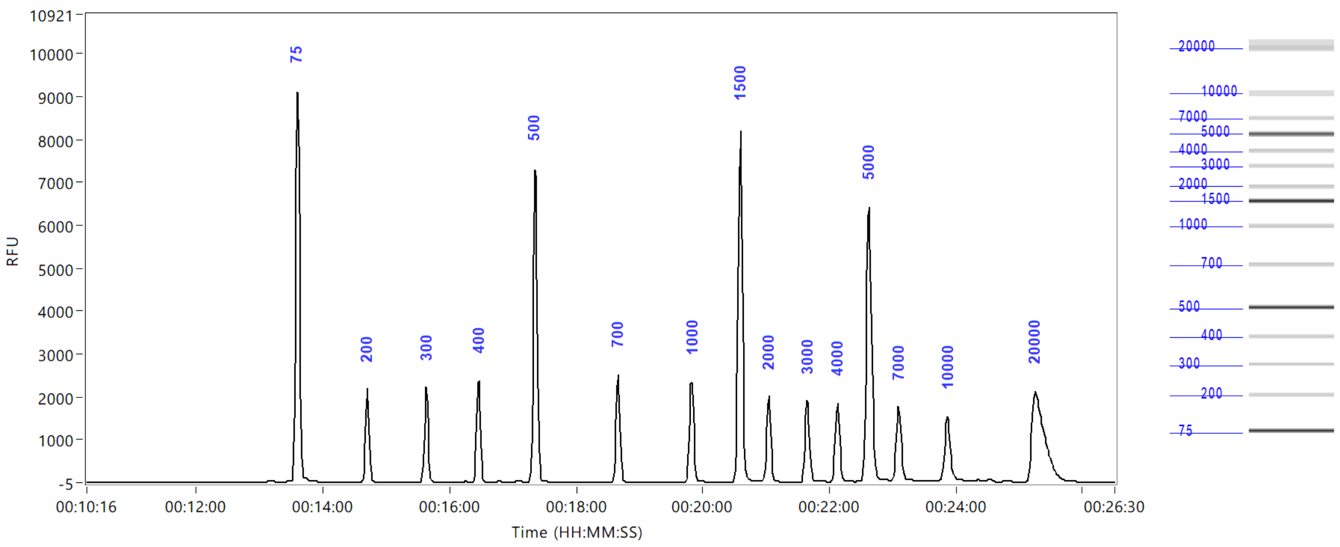

Supplement: Supplementary file 2 — sb3c00292_si_002.zip [file sb3c00292_si_002.zip › dnada_supplementary_material_pks_library_build/ZAG_trials/ZAG_Trial_6/20-30-11_plate5/plate5-t2.pdf]
