# Supplementary material for: Automated Platform for the Plasmid Construction Process
Source: ACS Synth Biol. 2023 Nov 10;12(12):3506–13. doi: 10.1021/acssynbio.3c00292 (PMC10729297; doi:10.1021/acssynbio.3c00292)

Instrument controller software run summary:

Filename and data path: C:\AATI\Data\2021 09 20\17-39-27\2021 09 20 17H 39M..raw
Created: Monday, September 20, 2021 6:00:03 PM
Number of capillaries: 96
Array serial number: 111920-09SFS
Effect length: 33 cm
Array usage count: 223
Instrument type: ZAG DNA Analyzer
Instrument controller software version: 1.0.1.9
Device serial number: Z7597

Method Information

Method name: ZAG130FC33 - DNA 75-20000bp - Full Conditioning.mthds
Gel prime: No
Full conditioning: Yes
Gel prime to buffer: No
Gel selection: Gel 1
Perform prerun: 8.0 kV, 30 sec.
Rinse: No
Marker 1: Row: A, 5.0 kV, 5 sec.
Rinse: No
Sample injection: 5.0 kV, 5 sec.
Separation: 8.0 kV, 30.0 min.
Tray name: Sample Tray-1

Analysis mode: DNA

Notes

Gel Image

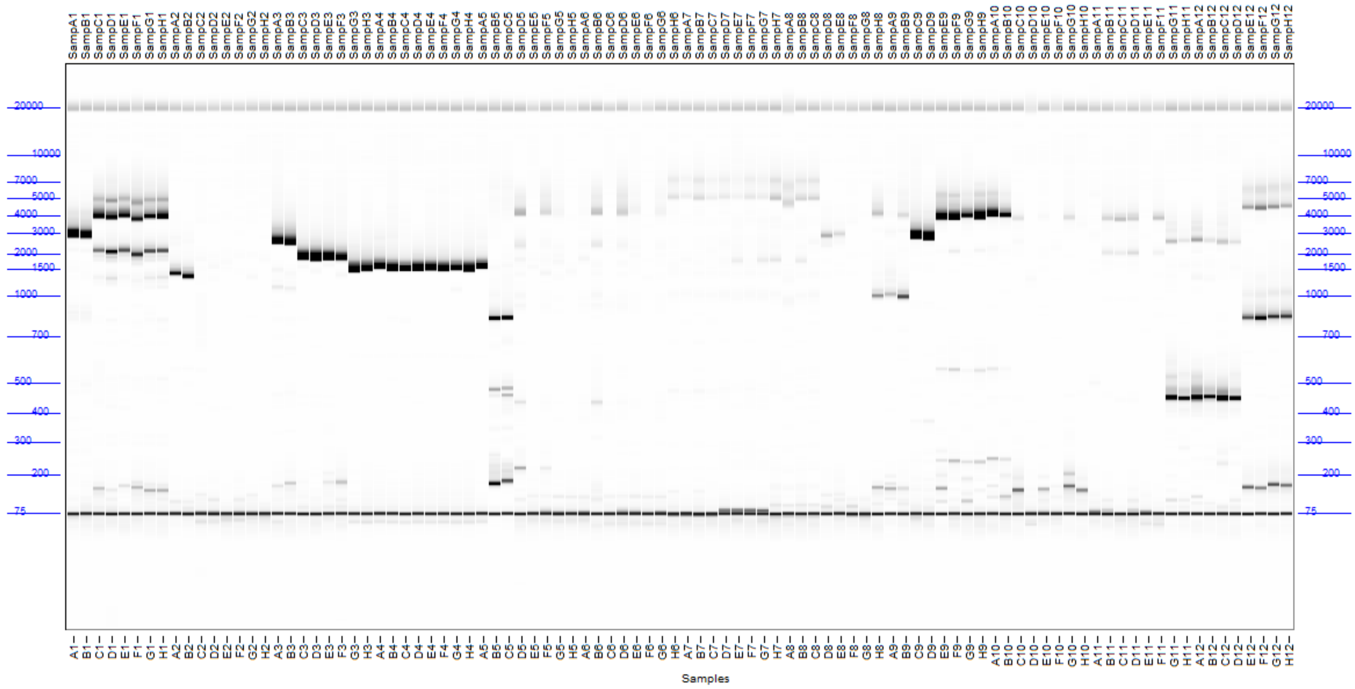

Filename and data path: C:\AATI\Data\2021 09 20\17-39-27\2021 09 20 17H 39M..raw

A1: SampA1

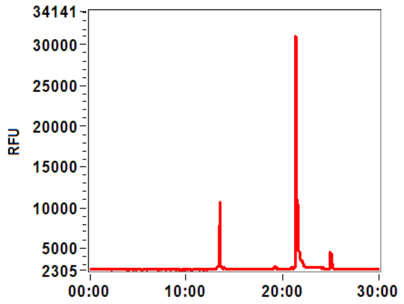

B1: SampB1

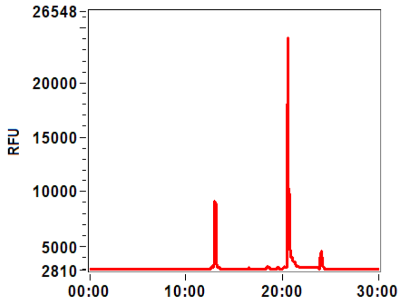

C1: SampC1

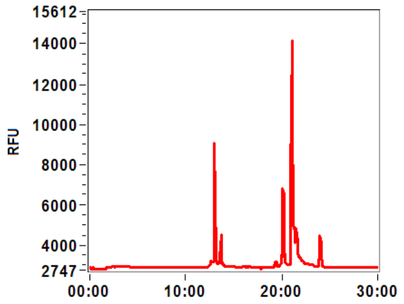

D1: SampD1

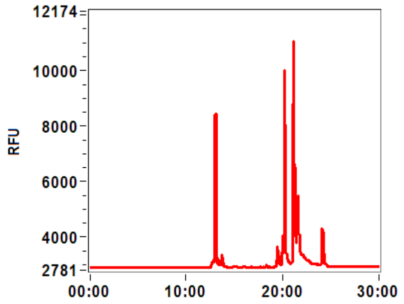

E1: SampE1

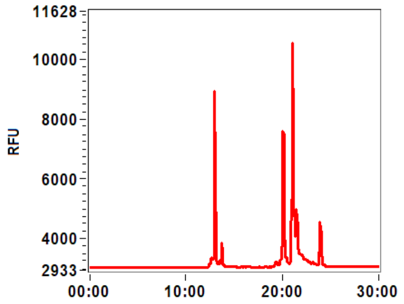

F1: SampF1

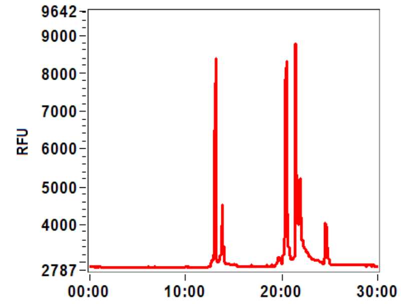

G1: SampG1

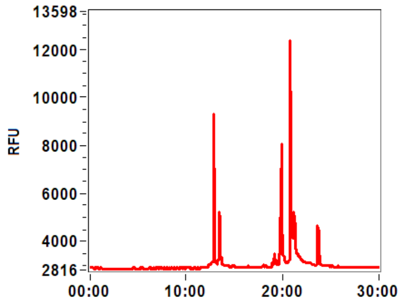

H1: SampH1

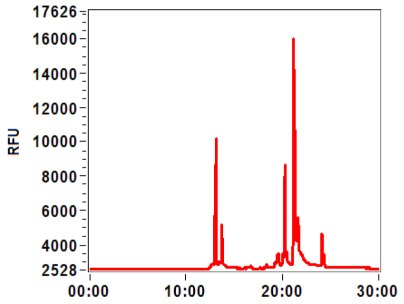

A2: SampA2

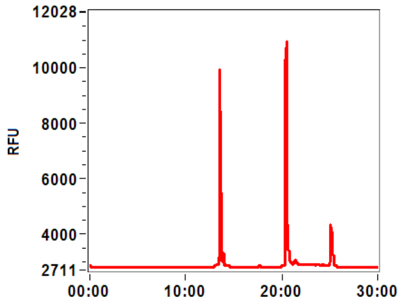

B2: SampB2

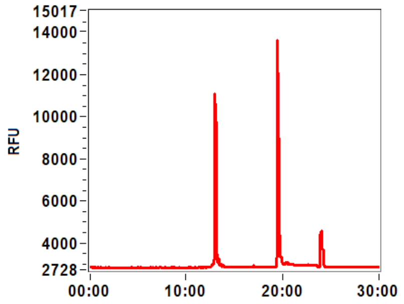

C2: SampC2

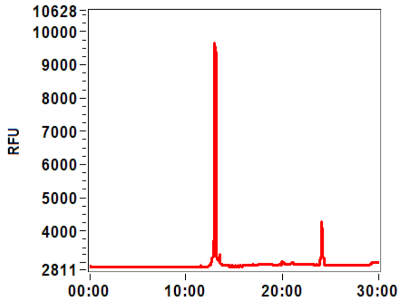

D2: SampD2

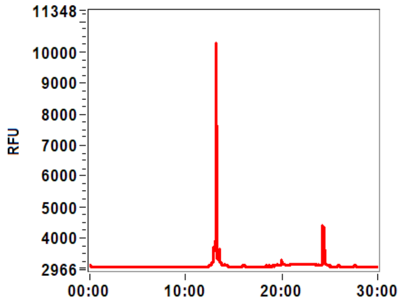

Filename and data path: C:\AATI\Data\2021 09 20\17-39-27\2021 09 20 17H 39M..raw

E2: SampE2

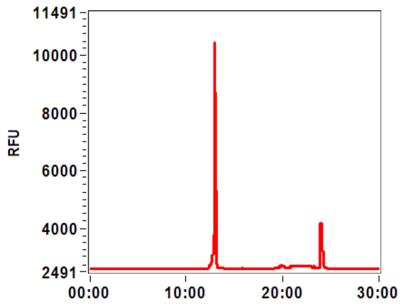

F2: SampF2

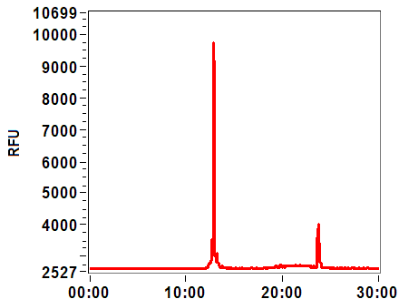

G2: SampG2

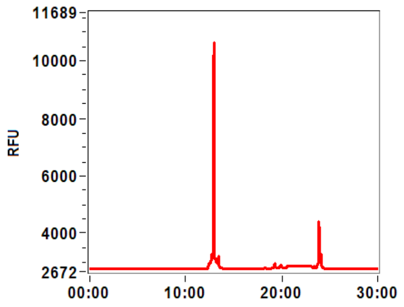

H2: SampH2

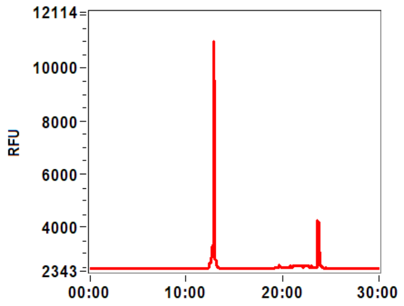

A3: SampA3

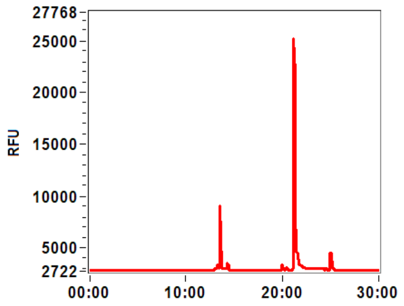

B3: SampB3

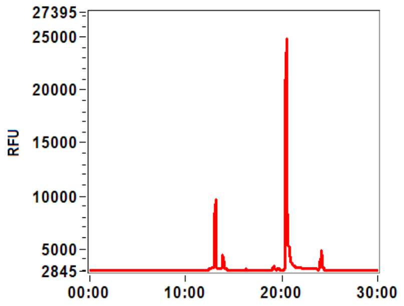

C3: SampC3

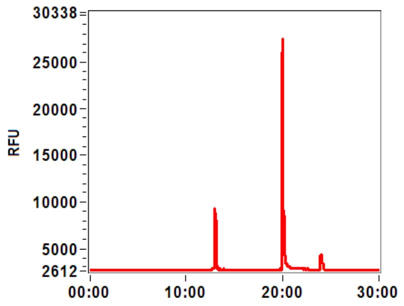

D3: SampD3

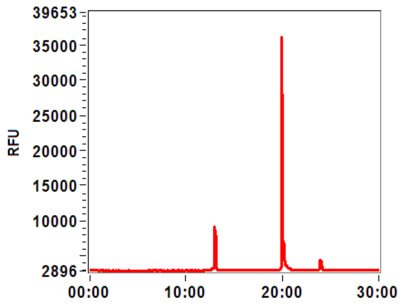

E3: SampE3

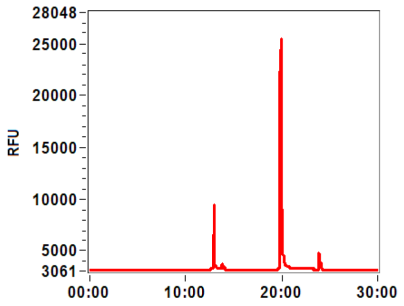

F3: SampF3

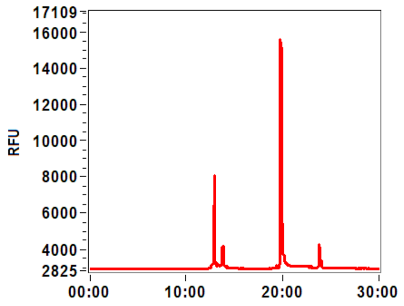

G3: SampG3

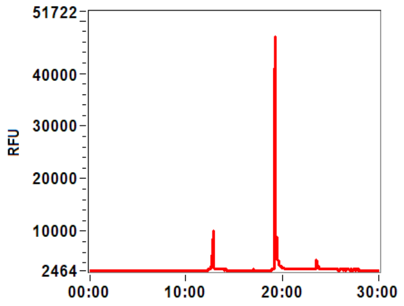

H3: SampH3

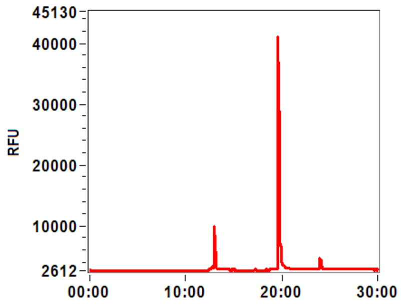

Filename and data path: C:\AATI\Data\2021 09 20\17-39-27\2021 09 20 17H 39M..raw

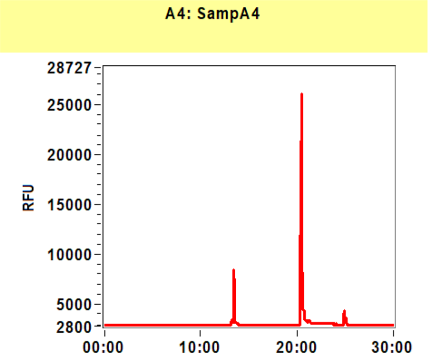

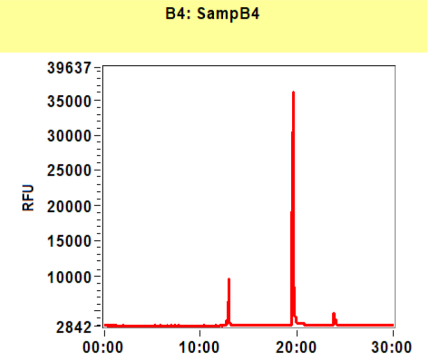

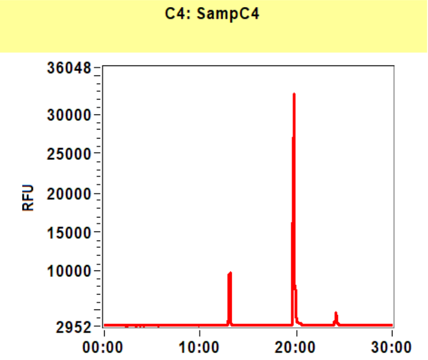

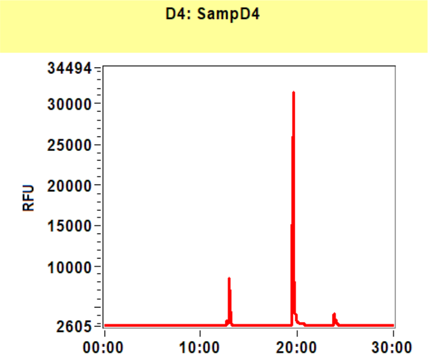

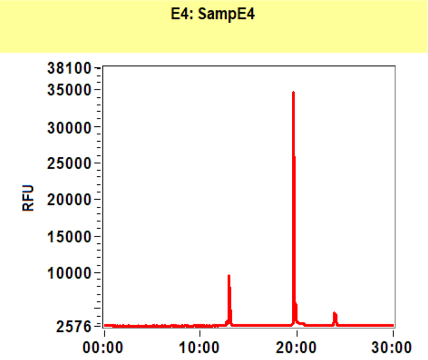

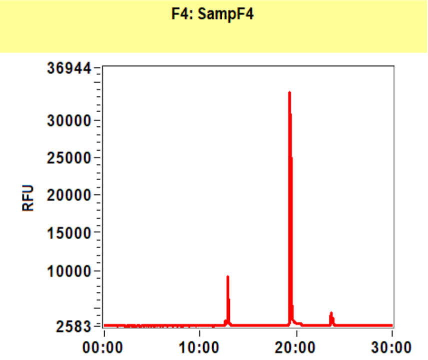

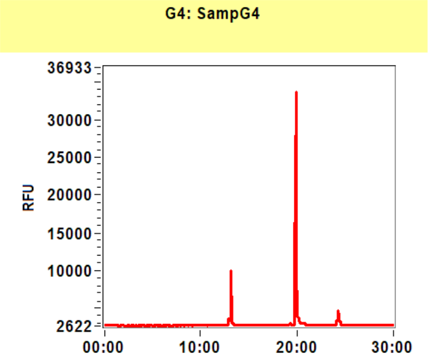

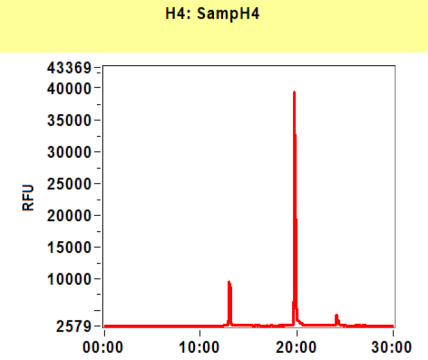

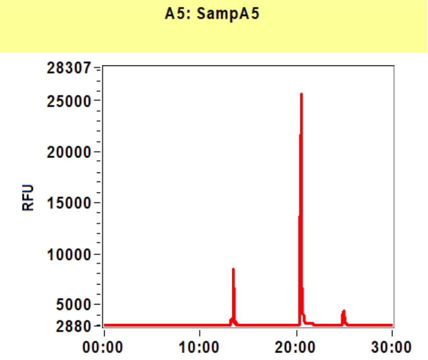

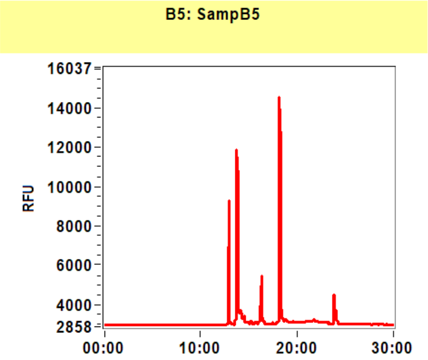

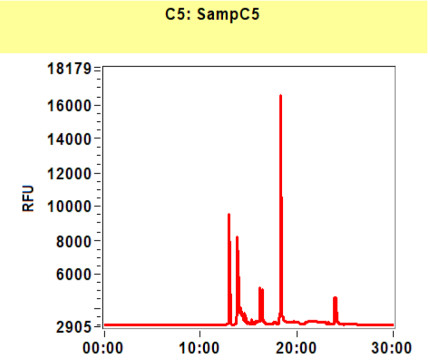

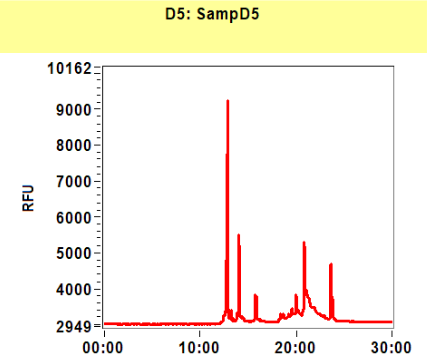

Filename and data path: C:\AATI\Data\2021 09 20\17-39-27\2021 09 20 17H 39M..raw

E5: SampE5

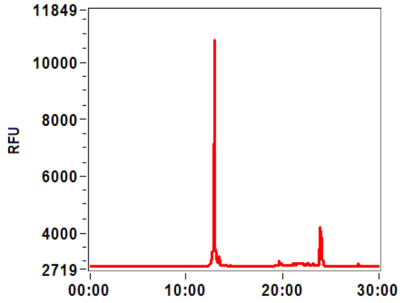

F5: SampF5

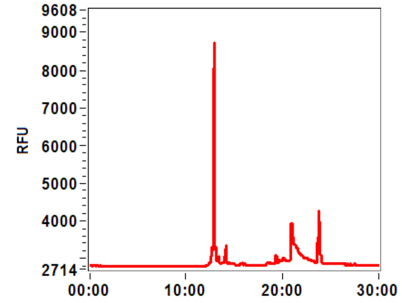

G5: SampG5

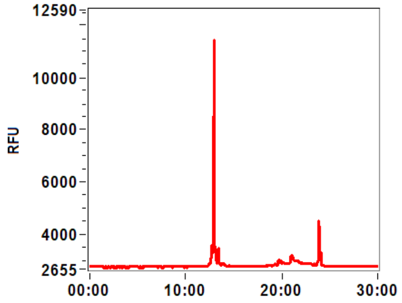

H5: SampH5

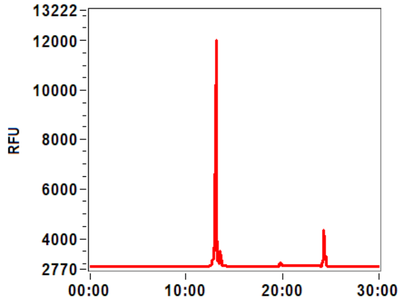

A6: SampA6

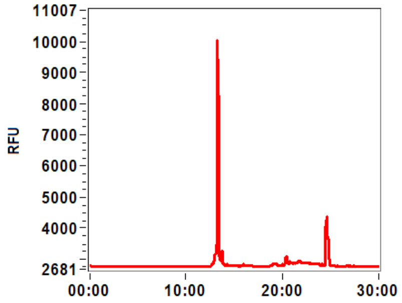

B6: SampB6

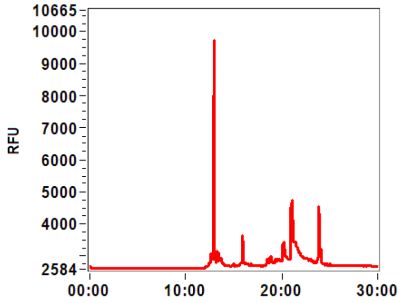

C6: SampC6

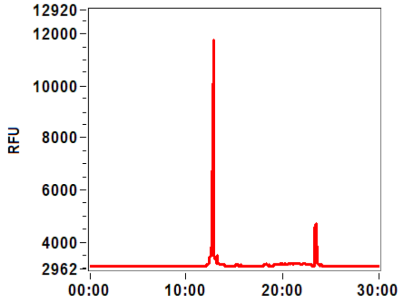

D6: SampD6

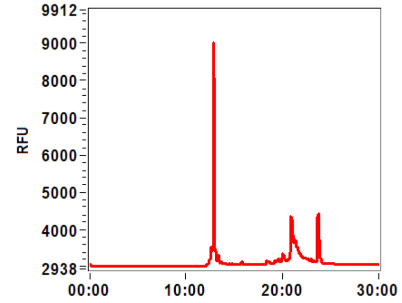

E6: SampE6

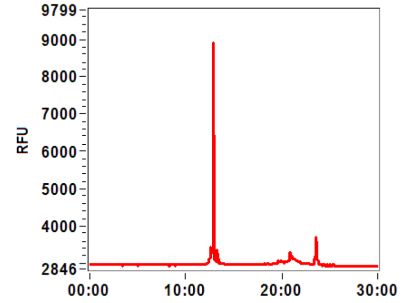

F6: SampF6

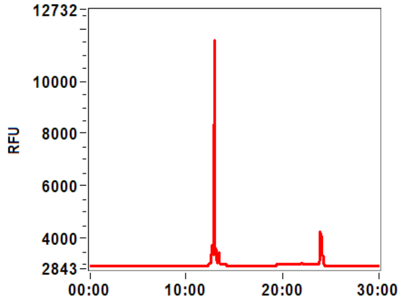

G6: SampG6

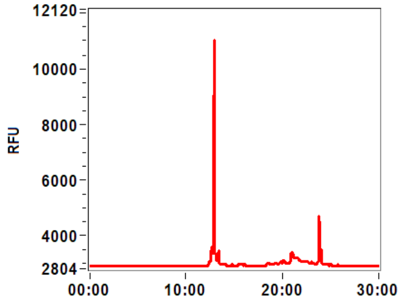

H6: SampH6

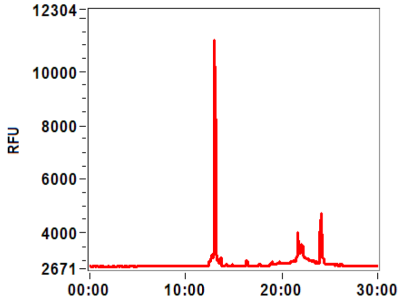

Filename and data path: C:\AATI\Data\2021 09 20\17-39-27\2021 09 20 17H 39M..raw

A7: SampA7

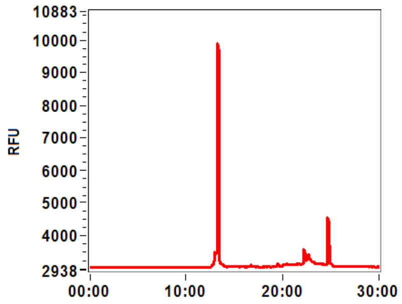

B7: SampB7

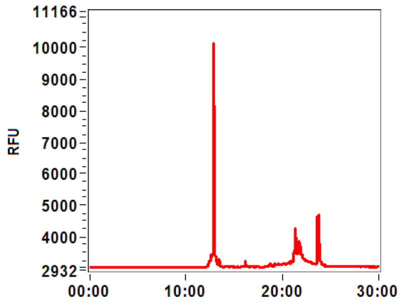

C7: SampC7

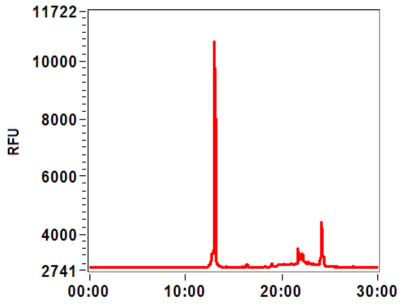

D7: SampD7

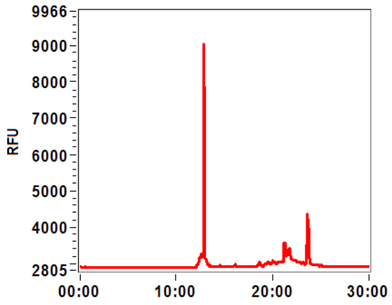

E7: SampE7

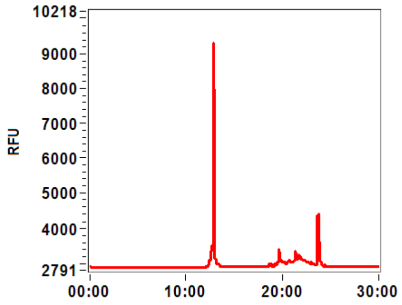

F7: SampF7

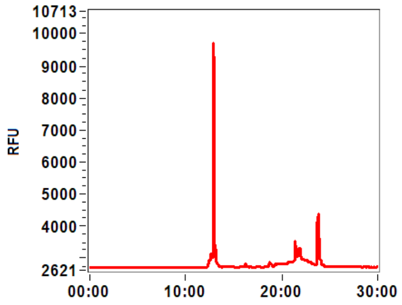

G7: SampG7

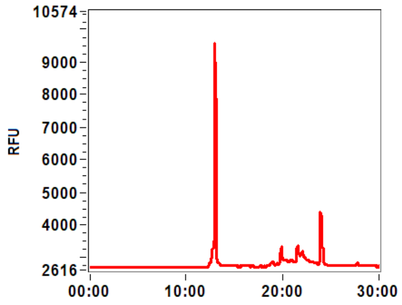

H7: SampH7

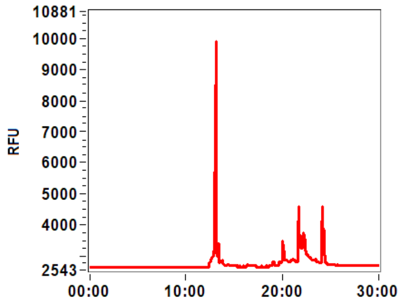

A8: SampA8

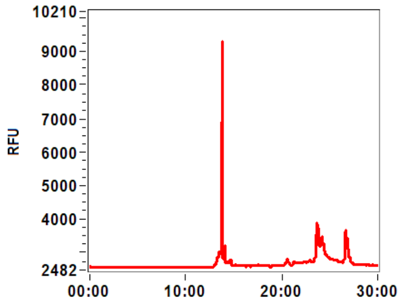

B8: SampB8

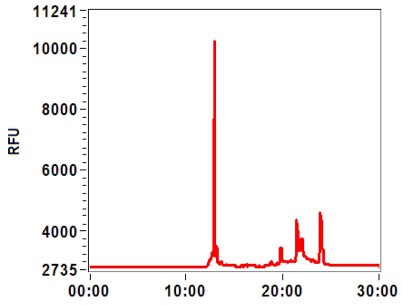

C8: SampC8

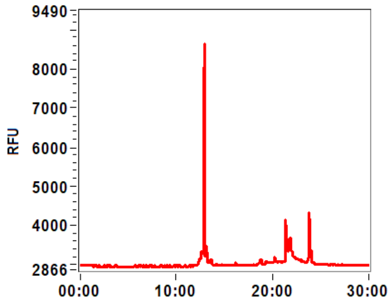

D8: SampD8

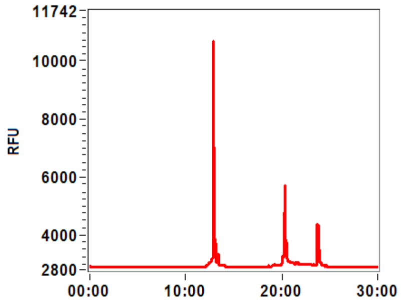

Filename and data path: C:\AATI\Data\2021 09 20\17-39-27\2021 09 20 17H 39M..raw

E8: SampE8

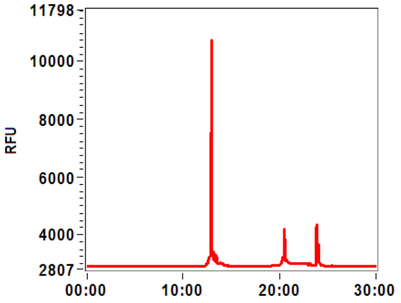

F8: SampF8

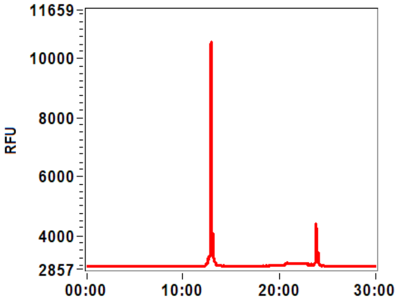

G8: SampG8

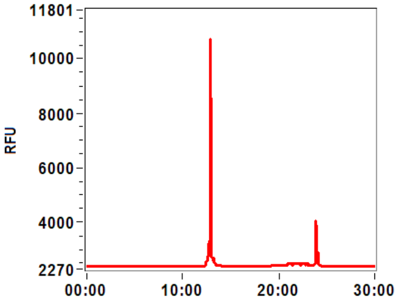

H8: SampH8

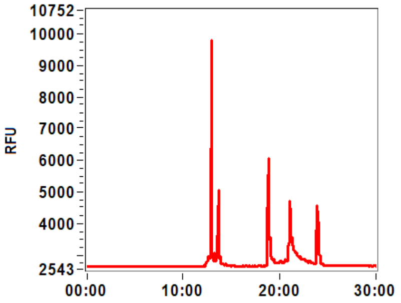

A9: SampA9

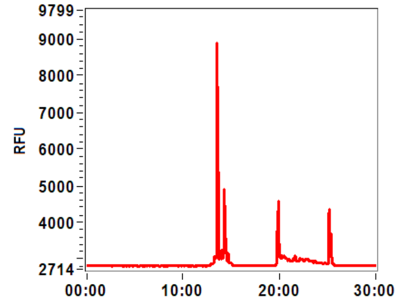

B9: SampB9

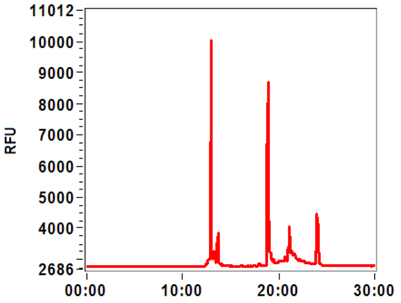

C9: SampC9

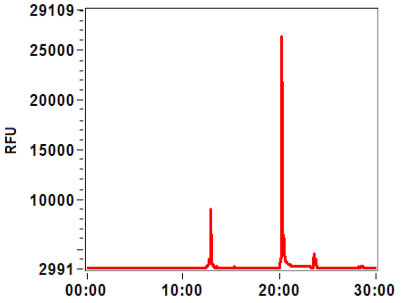

D9: SampD9

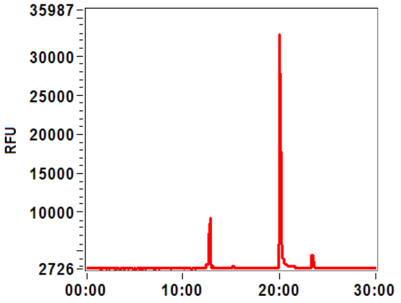

E9: SampE9

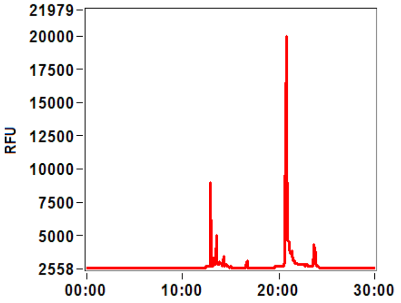

F9: SampF9

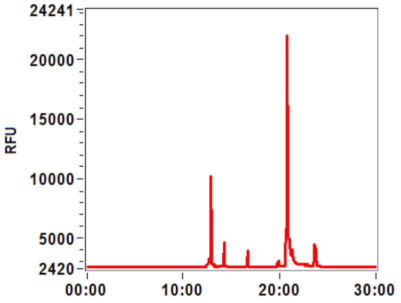

G9: SampG9

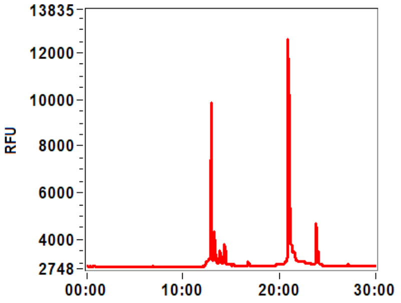

H9: SampH9

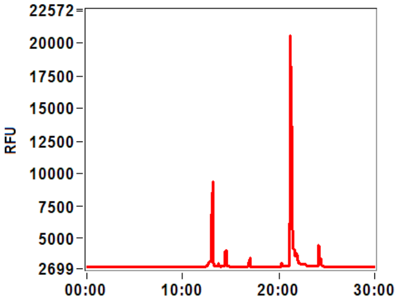

Filename and data path: C:\AATI\Data\2021 09 20\17-39-27\2021 09 20 17H 39M..raw

A10: SampA10

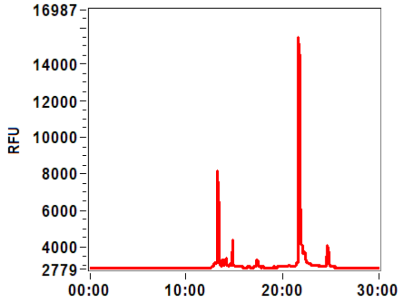

B10: SampB10

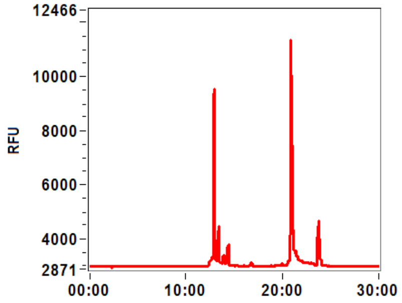

C10: SampC10

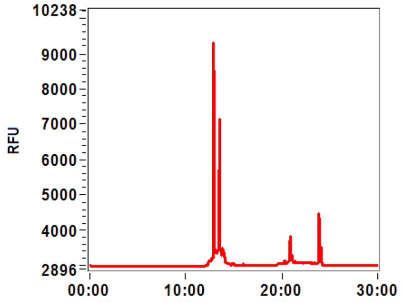

D10: SampD10

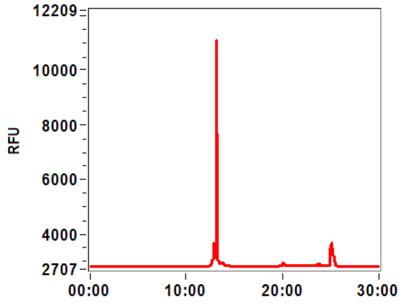

E10: SampE10

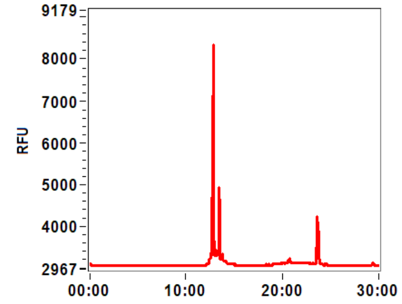

F10: SampF10

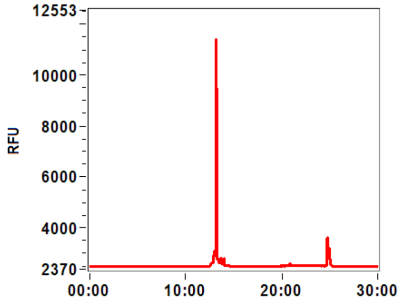

G10: SampG10

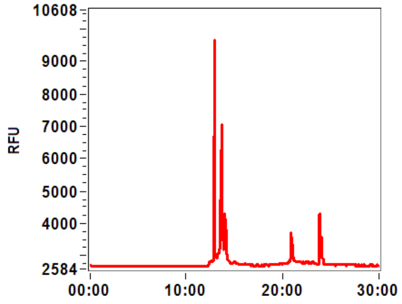

H10: SampH10

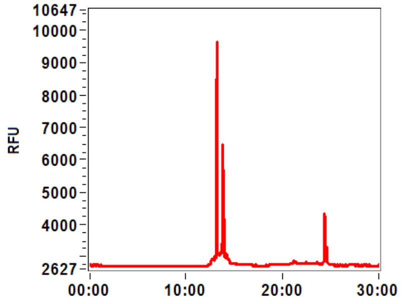

A11: SampA11

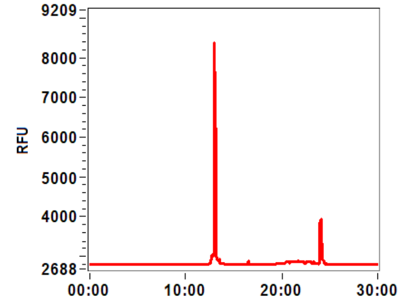

B11: SampB11

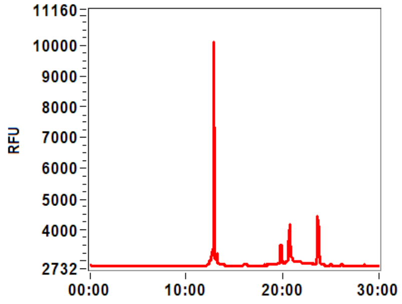

C11: SampC11

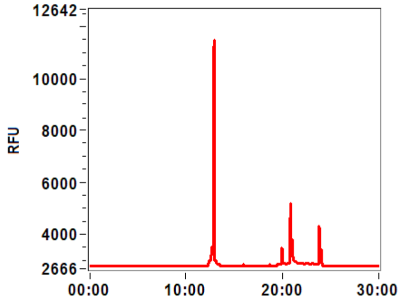

D11: SampD11

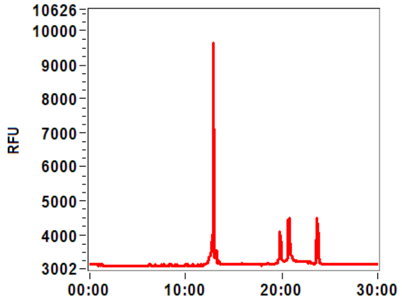

Filename and data path: C:\AATI\Data\2021 09 20\17-39-27\2021 09 20 17H 39M..raw

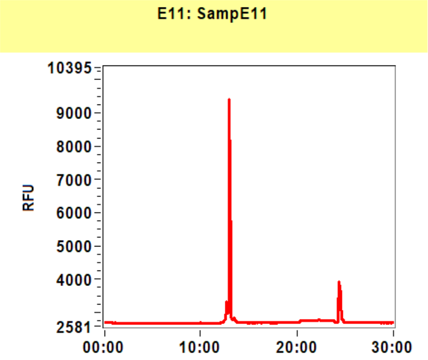

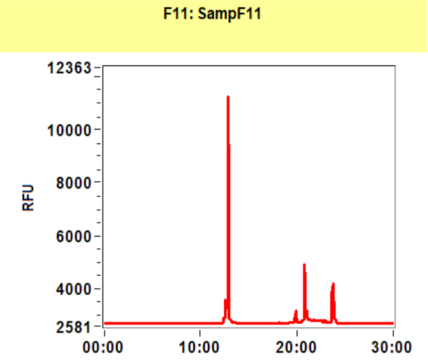

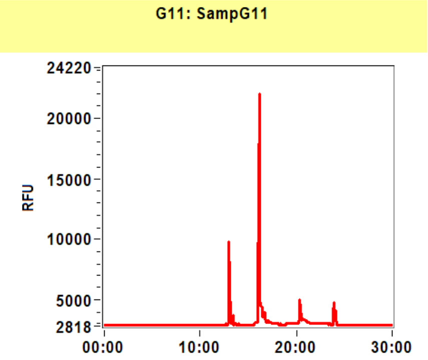

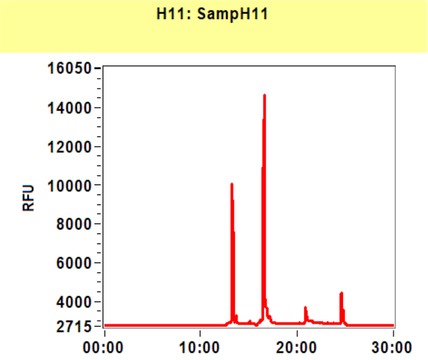

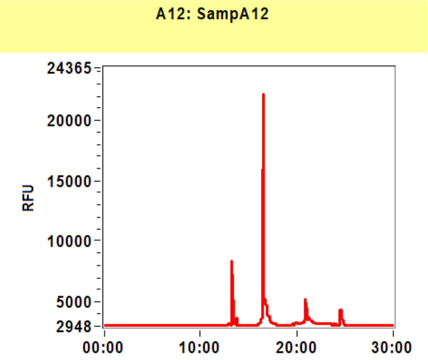

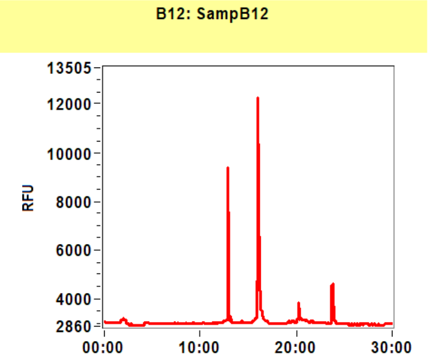

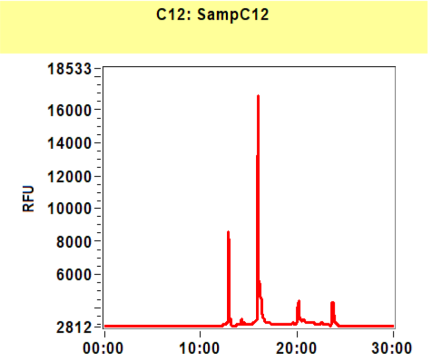

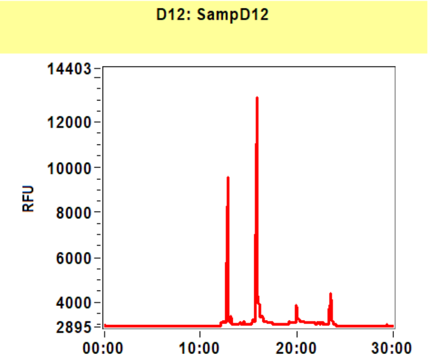

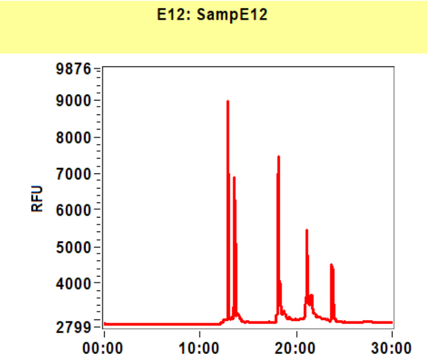

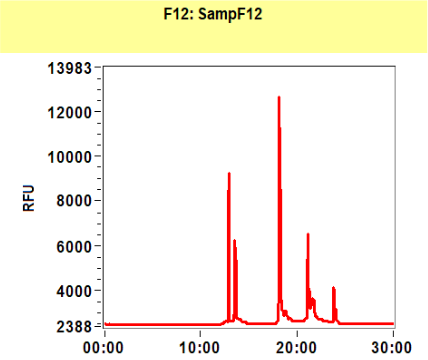

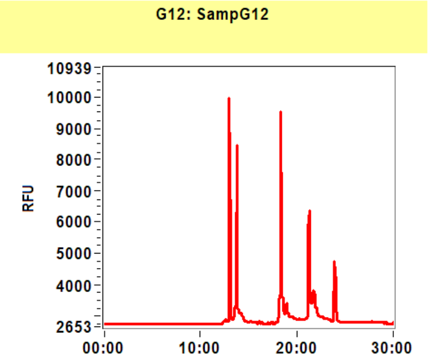

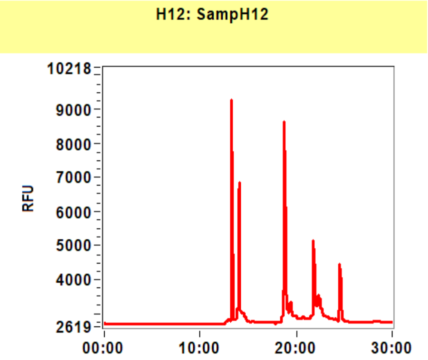

Sample: SampA1
Well location: A1
Created: Monday, September 20, 2021 6:00:03 PM

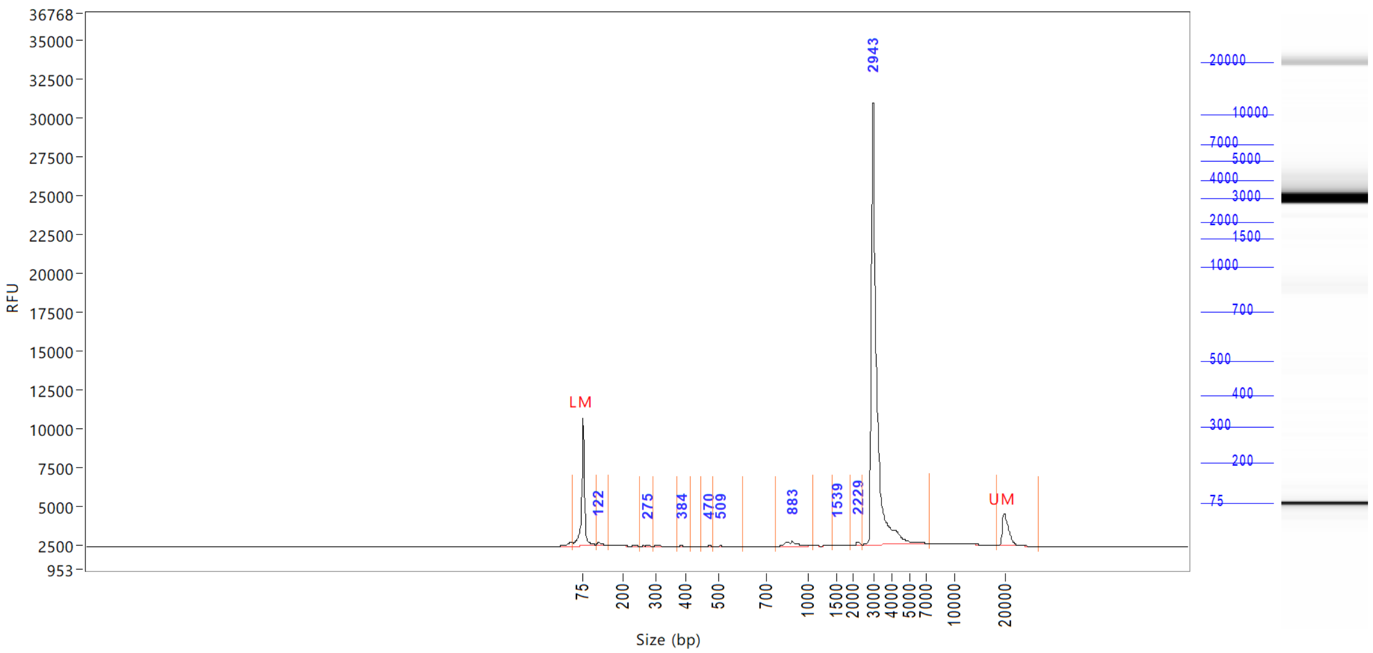

Peak	Size (bp)	Concentration (ng/uL)	From (bp)	To (bp)	Average size (bp)	CV%	RFU	Corrected peak area
1	75 (LM)	0.5000	43	114	72	12.23	8212	56.809
2	122	0.1999	114	154	126	6.67	198	1.892
3	275	0.0567	250	289	270	2.92	71	0.537
4	384	0.0357	368	414	381	2.03	59	0.338
5	470	0.0569	445	481	466	1.65	77	0.539
6	509	0.0675	481	600	512	4.70	56	0.639
7	883	0.5764	762	1068	876	4.96	310	5.457
8	1539	0.0769	1433	1923	1589	6.32	72	0.728
9	2229	0.1088	1923	2429	2215	3.51	227	1.031
10	2943	23.3104	2429	7268	3122	12.94	28512	220.709
11	20000 (UM)	0.1461	18415	27012	20327	3.45	2081	16.594

TIC:	24.4892	ng/uL
TIM:	17.0584	nmole/L
Total concentration:	24.7994	ng/uL

Sample peak width (sec): 5 Sample min peak height: 50 Sample baseline V to V?: Y Sample baseline V to V points: 3
Sample filter: Binomial Number of points for filter: 3 Sample start region (min): 0 Sample end region (min): 30
Marker peak width (sec): 5 Marker min peak height: 200 Marker baseline V to V?: Y Marker baseline V to V points: 3
Lower marker selection: First peak > 500 RFU Upper marker selection: Last peak > 200 RFU
Ladder size (bp)75, 200, 300, 400, 500, 700, 1000, 1500, 2000, 3000, 4000, 5000, 7000, 10000, 20000
Quantification using: Lower Marker Final concentration (ng/uL): 0.5000 Dilution factor: 12.0

Sample: SampB1
Well location: B1
Created: Monday, September 20, 2021 6:00:03 PM

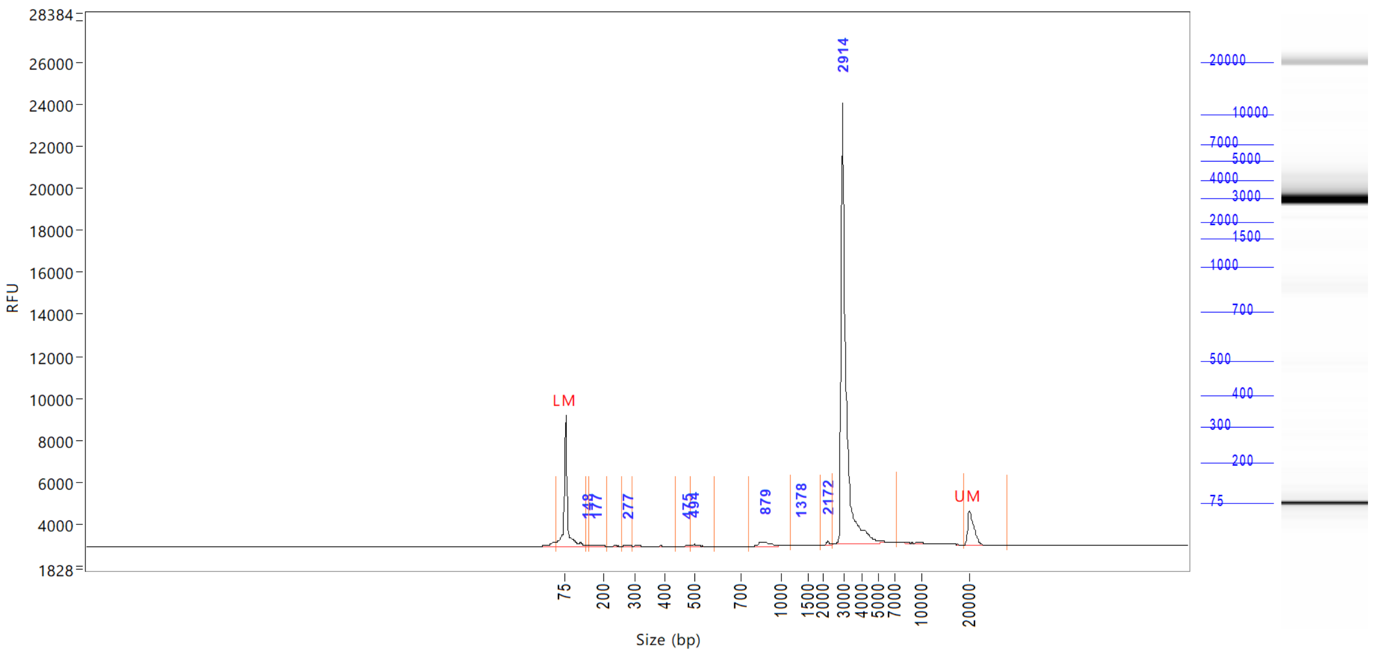

Peak	Size (bp)	Concentration (ng/uL)	From (bp)	To (bp)	Average size (bp)	CV%	RFU	Corrected peak area
1	75 (LM)	0.5000	45	143	76	19.09	6305	49.085
2	148	0.0601	143	154	148	2.53	66	0.491
3	177	0.1664	154	212	172	7.28	73	1.361
4	277	0.0652	255	289	273	2.89	59	0.533
5	475	0.0612	437	483	467	2.50	58	0.500
6	494	0.1278	483	580	505	3.51	98	1.046
7	879	0.5223	753	1167	878	6.16	220	4.273
8	1378	0.1097	1167	1885	1461	8.69	53	0.898
9	2172	0.0871	1885	2401	2160	3.56	160	0.713
10	2914	19.2872	2401	7134	3088	13.65	21114	157.787
11	20000 (UM)	0.1319	18781	27979	20409	3.80	1607	12.947

TIC:	20.4870	ng/uL
TIM:	14.7332	nmole/L
Total concentration:	20.7223	ng/uL

```
Sample peak width (sec): 5    Sample min peak height: 50    Sample baseline V to V?: Y    Sample baseline V to V points: 3
Sample filter: Binomial       Number of points for filter: 3  Sample start region (min): 0  Sample end region (min): 30
Marker peak width (sec): 5    Marker min peak height: 200   Marker baseline V to V?: Y    Marker baseline V to V points: 3
Lower marker selection: First peak > 500 RFU                Upper marker selection: Last peak > 200 RFU
Ladder size (bp)75, 200, 300, 400, 500, 700, 1000, 1500, 2000, 3000, 4000, 5000, 7000, 10000, 20000
Quantification using: Lower Marker          Final concentration (ng/uL): 0.5000          Dilution factor: 12.0
```

Sample: SampC1
Well location: C1
Created: Monday, September 20, 2021 6:00:03 PM

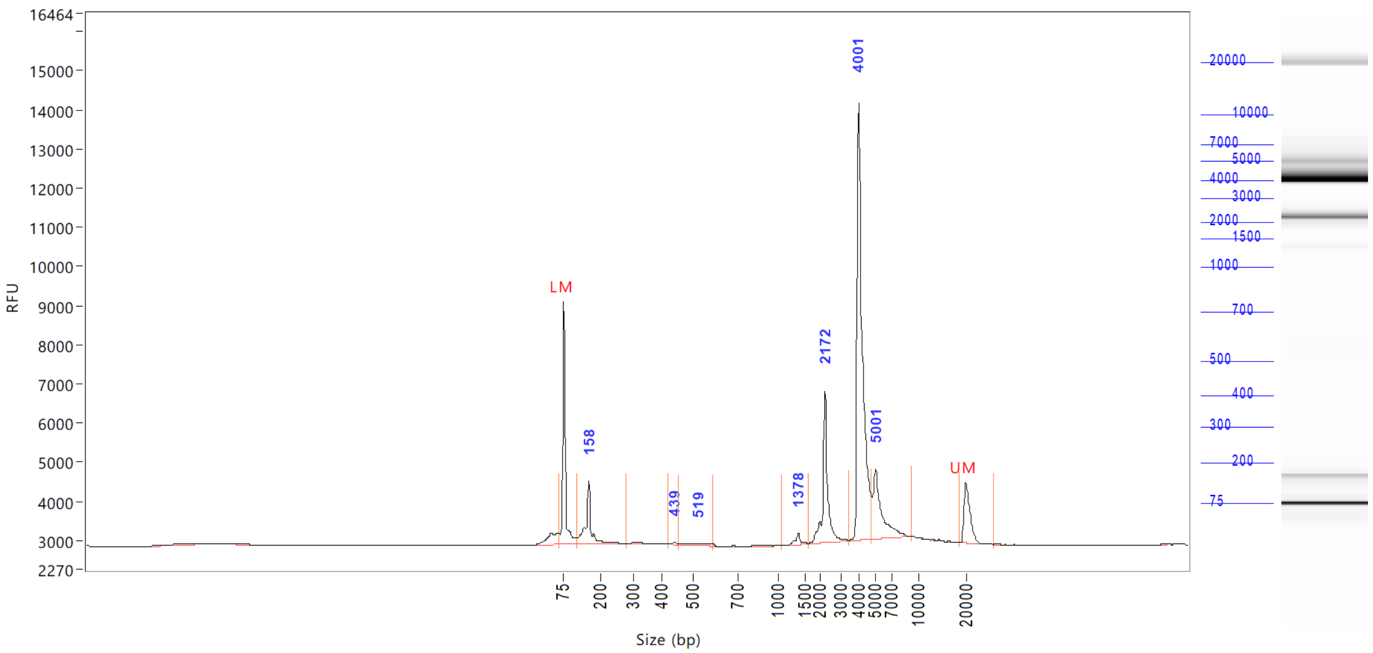

Peak	Size (bp)	Concentration (ng/uL)	From (bp)	To (bp)	Average size (bp)	CV%	RFU	Corrected peak area
1	75 (LM)	0.5000	64	118	76	12.59	6196	39.883
2	158	2.8543	118	278	157	14.80	1590	18.973
3	439	0.0870	420	452	437	1.86	60	0.578
4	519	0.3441	452	588	519	7.53	52	2.288
5	1378	0.3790	1045	1597	1345	5.43	282	2.520
6	2172	4.3039	1597	3429	2197	10.26	3874	28.609
7	4001	13.5470	3429	4793	4147	5.46	11183	90.049
8	5001	3.4207	4793	9200	5613	15.80	1781	22.738
9	20000 (UM)	0.1562	18537	25803	20374	3.62	1543	12.463

TIC:	24.9361	ng/uL
TIM:	41.3707	nmole/L
Total concentration:	25.0496	ng/uL

```
Sample peak width (sec): 5     Sample min peak height: 50     Sample baseline V to V?: Y     Sample baseline V to V points: 3
Sample filter: Binomial        Number of points for filter: 3  Sample start region (min): 0  Sample end region (min): 30
Marker peak width (sec): 5     Marker min peak height: 200    Marker baseline V to V?: Y     Marker baseline V to V points: 3
Lower marker selection: First peak > 500 RFU                  Upper marker selection: Last peak > 200 RFU
Ladder size (bp)75, 200, 300, 400, 500, 700, 1000, 1500, 2000, 3000, 4000, 5000, 7000, 10000, 20000
Quantification using: Lower Marker          Final concentration (ng/uL): 0.5000          Dilution factor: 12.0
```

Sample: SampD1
Well location: D1
Created: Monday, September 20, 2021 6:00:03 PM

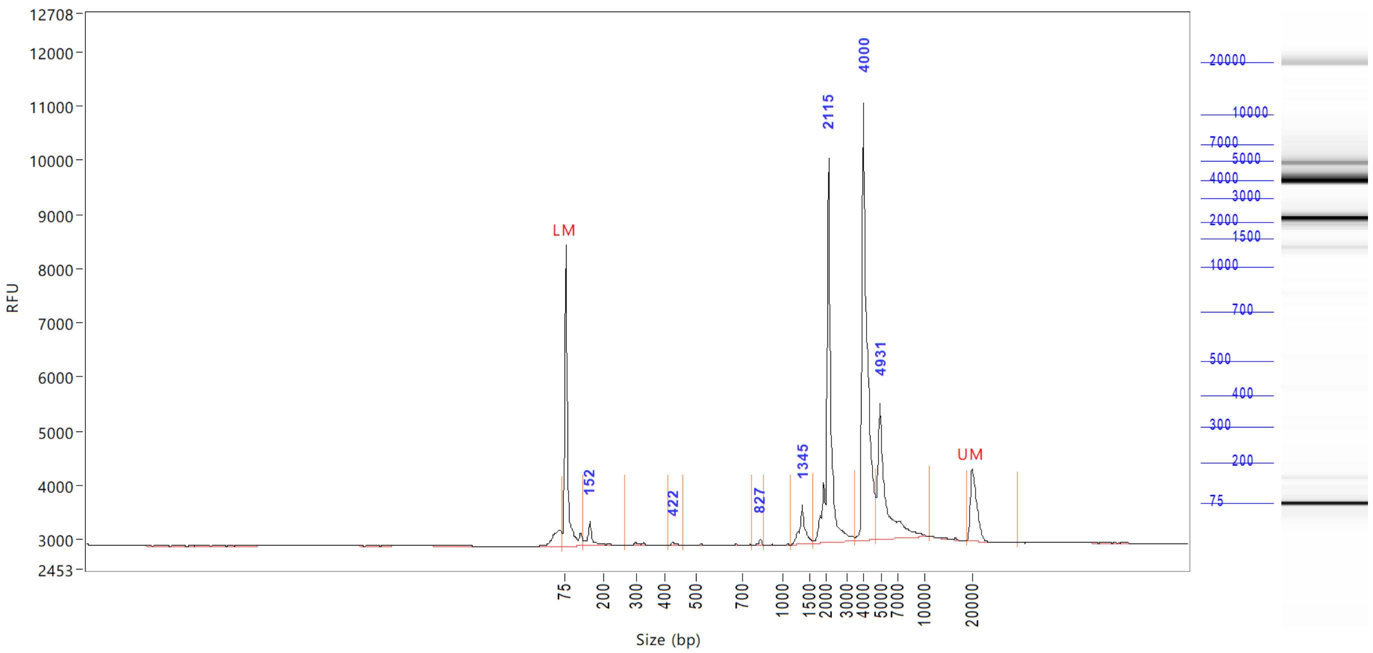

Peak	Size (bp)	Concentration (ng/uL)	From (bp)	To (bp)	Average size (bp)	CV%	RFU	Corrected peak area
1	75 (LM)	0.5000	64	133	77	16.86	5566	40.351
2	152	0.7982	133	262	162	15.92	454	5.368
3	422	0.1003	409	456	428	2.67	70	0.675
4	827	0.0932	770	853	819	1.81	112	0.626
5	1345	0.9398	1123	1597	1342	5.81	713	6.320
6	2115	7.1528	1597	3500	2139	11.93	7095	48.104
7	4000	9.6006	3500	4724	4084	5.39	8084	64.565
8	4931	4.1903	4724	11098	5736	20.96	2514	28.180
9	20000 (UM)	0.1391	18659	29309	20372	4.39	1330	11.226

TIC:	22.8752	ng/uL
TIM:	20.4019	nmole/L
Total concentration:	23.1580	ng/uL

```
Sample peak width (sec): 5    Sample min peak height: 50    Sample baseline V to V?: Y    Sample baseline V to V points: 3
Sample filter: Binomial       Number of points for filter: 3  Sample start region (min): 0  Sample end region (min): 30
Marker peak width (sec): 5    Marker min peak height: 200   Marker baseline V to V?: Y    Marker baseline V to V points: 3
Lower marker selection: First peak > 500 RFU                Upper marker selection: Last peak > 200 RFU
Ladder size (bp)75, 200, 300, 400, 500, 700, 1000, 1500, 2000, 3000, 4000, 5000, 7000, 10000, 20000
Quantification using: Lower Marker          Final concentration (ng/uL): 0.5000          Dilution factor: 12.0
```

Sample: SampE1
Well location: E1
Created: Monday, September 20, 2021 6:00:03 PM

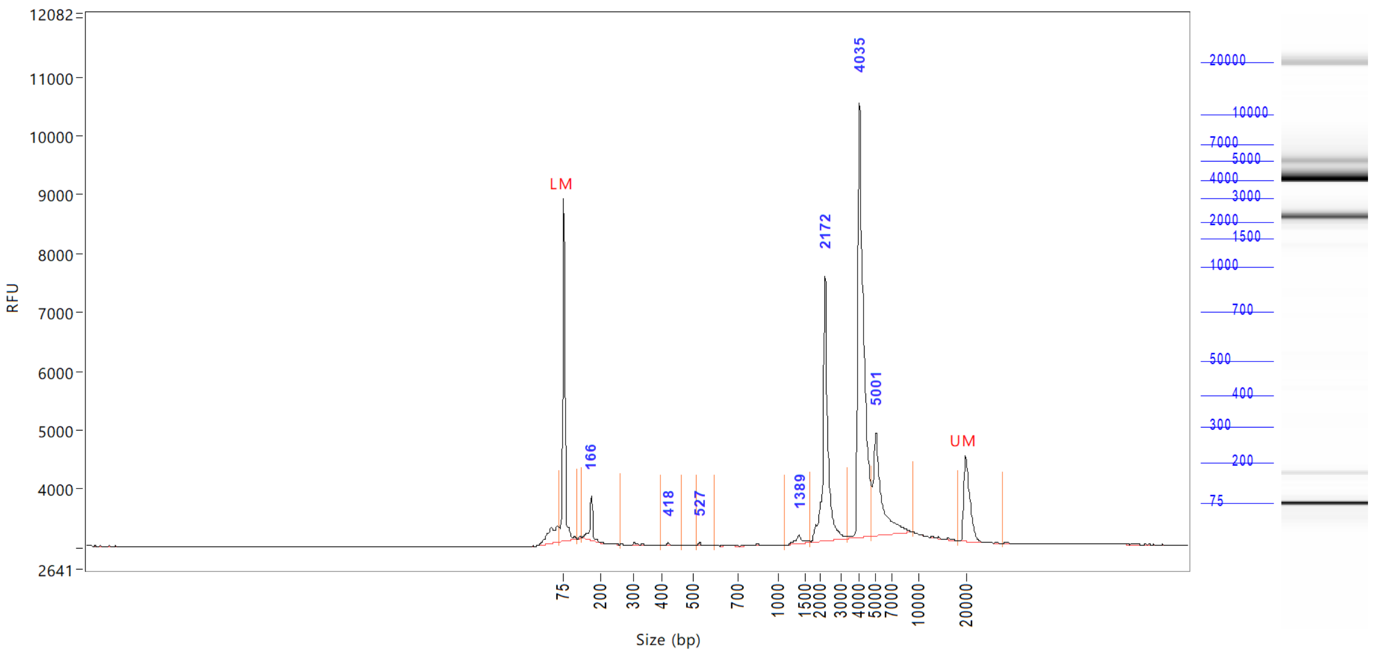

Peak	Size (bp)	Concentration (ng/uL)	From (bp)	To (bp)	Average size (bp)	CV%	RFU	Corrected peak area
1	75 (LM)	0.5000	64	118	74	8.03	5817	34.285
2	166	0.9781	135	259	164	8.62	752	5.589
3	418	0.0851	396	464	426	3.88	51	0.486
4	527	0.0545	516	598	530	2.69	53	0.311
5	1389	0.2889	1101	1674	1380	7.32	144	1.651
6	2172	6.1636	1674	3358	2209	9.84	4484	35.220
7	4035	11.3126	3358	4793	4188	5.24	7392	64.642
8	5001	3.7714	4793	9467	5644	16.76	1754	21.551
9	20000 (UM)	0.1742	18293	27859	20351	3.88	1453	11.942

TIC:	22.6543	ng/uL
TIM:	20.7862	nmole/L
Total concentration:	22.9233	ng/uL

Sample peak width (sec): 5   Sample min peak height: 50   Sample baseline V to V?: Y   Sample baseline V to V points: 3
Sample filter: Binomial   Number of points for filter: 3   Sample start region (min): 0   Sample end region (min): 30
Marker peak width (sec): 5   Marker min peak height: 200   Marker baseline V to V?: Y   Marker baseline V to V points: 3
Lower marker selection: First peak > 500 RFU   Upper marker selection: Last peak > 200 RFU
Ladder size (bp)75, 200, 300, 400, 500, 700, 1000, 1500, 2000, 3000, 4000, 5000, 7000, 10000, 20000
Quantification using: Lower Marker   Final concentration (ng/uL): 0.5000   Dilution factor: 12.0

Sample: SampF1
Well location: F1
Created: Monday, September 20, 2021 6:00:03 PM

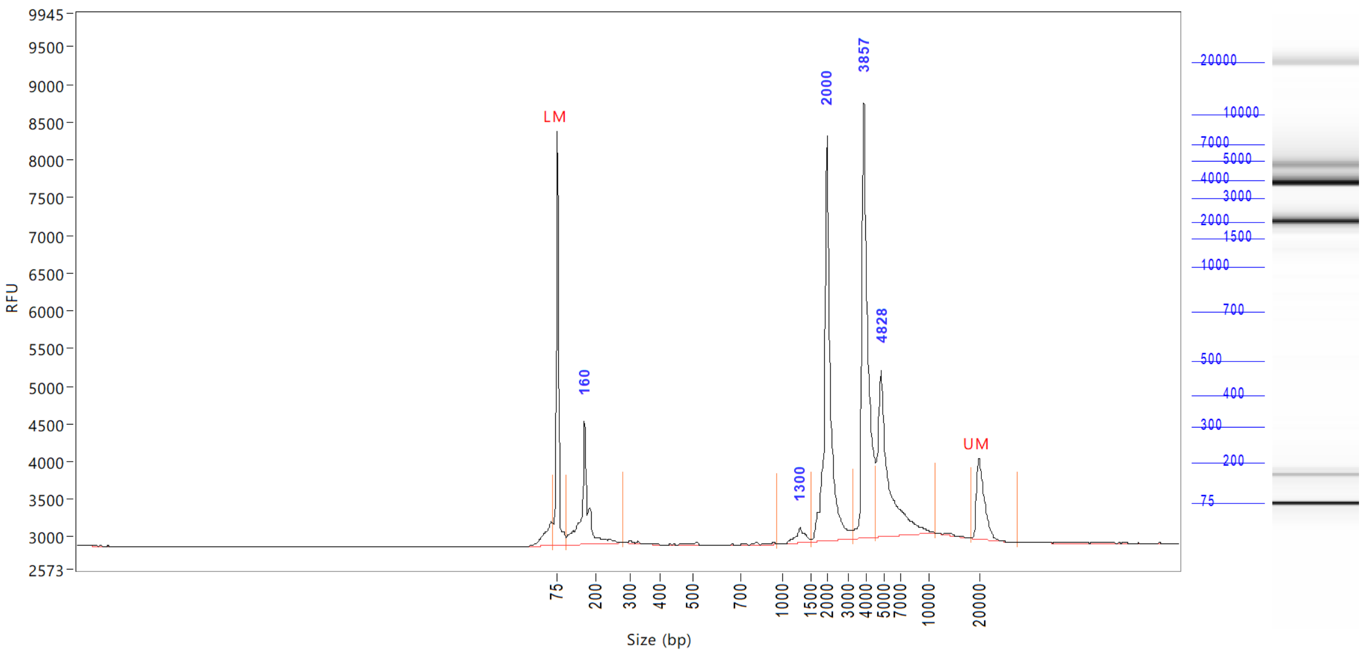

Peak	Size (bp)	Concentration (ng/uL)	From (bp)	To (bp)	Average size (bp)	CV%	RFU	Corrected peak area
1	75 (LM)	0.5000	64	101	74	7.46	5496	32.672
2	160	4.2681	101	277	159	17.28	1623	23.241
3	1300	0.5565	965	1539	1297	8.24	198	3.030
4	2000	9.3127	1539	3251	2067	12.35	5380	50.711
5	3857	9.7211	3251	4518	3967	5.63	5782	52.935
6	4828	6.3394	4518	11220	5573	21.71	2200	34.520
7	20000 (UM)	0.1640	18659	27738	20538	4.57	1070	10.718

TIC:	30.1978	ng/uL
TIM:	58.1511	nmole/L
Total concentration:	30.5092	ng/uL

Sample peak width (sec): 5 Sample min peak height: 50 Sample baseline V to V?: Y Sample baseline V to V points: 3
Sample filter: Binomial Number of points for filter: 3 Sample start region (min): 0 Sample end region (min): 30
Marker peak width (sec): 5 Marker min peak height: 200 Marker baseline V to V?: Y Marker baseline V to V points: 3
Lower marker selection: First peak > 500 RFU Upper marker selection: Last peak > 200 RFU
Ladder size (bp)75, 200, 300, 400, 500, 700, 1000, 1500, 2000, 3000, 4000, 5000, 7000, 10000, 20000
Quantification using: Lower Marker Final concentration (ng/uL): 0.5000 Dilution factor: 12.0

Sample: SampG1
Well location: G1
Created: Monday, September 20, 2021 6:00:03 PM

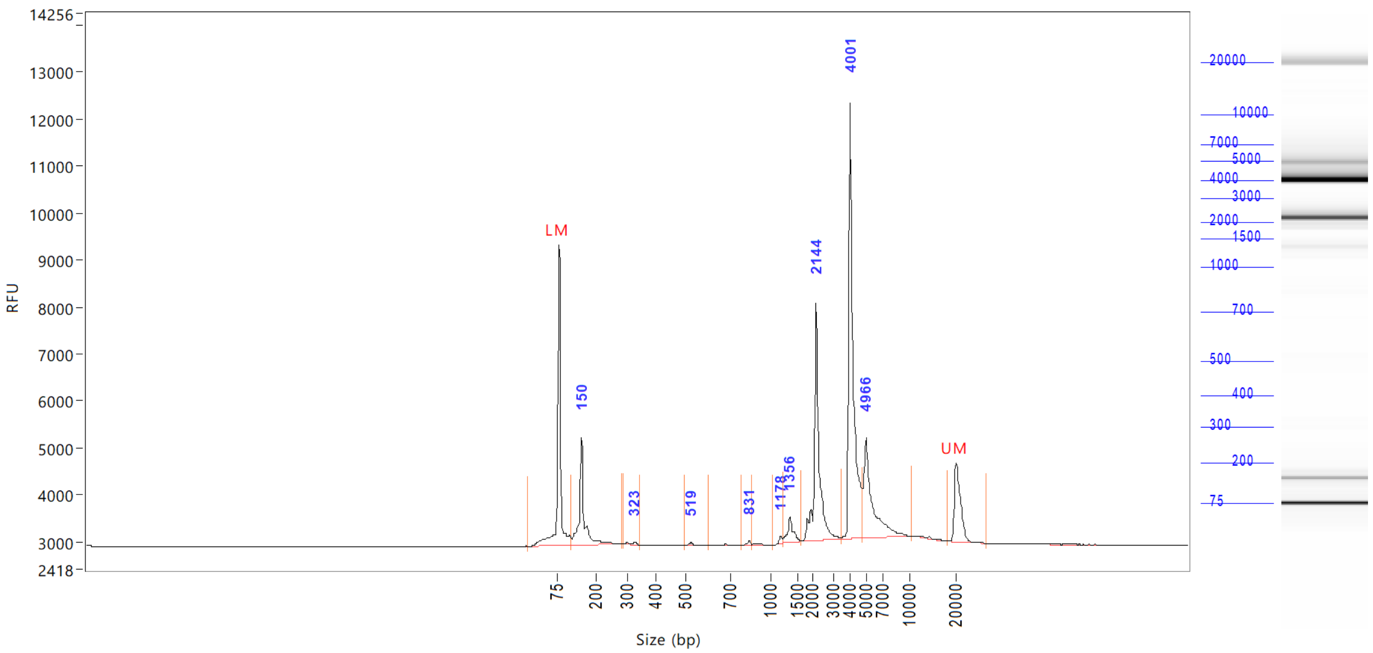

Peak	Size (bp)	Concentration (ng/uL)	From (bp)	To (bp)	Average size (bp)	CV%	RFU	Corrected peak area
1	75 (LM)	0.5000	0	118	70	27.42	6402	47.577
2	150	3.1090	118	280	155	15.08	2298	24.653
3	323	0.0912	286	341	310	3.93	62	0.724
4	519	0.0779	496	595	524	4.12	69	0.618
5	831	0.0778	770	853	821	1.98	97	0.617
6	1178	0.0867	1034	1212	1171	2.11	144	0.687
7	1356	0.6311	1212	1616	1359	5.34	544	5.004
8	2144	4.8043	1616	3500	2190	11.02	5056	38.096
9	4001	8.2303	3500	4724	4118	5.29	9293	65.263
10	4966	3.4709	4724	10489	5691	18.91	2126	27.523
11	20000 (UM)	0.1459	18293	26408	20432	3.76	1675	13.887

TIC:	20.5792	ng/uL
TIM:	42.6446	nmole/L
Total concentration:	20.6819	ng/uL

Sample peak width (sec): 5 Sample min peak height: 50 Sample baseline V to V?: Y Sample baseline V to V points: 3
Sample filter: Binomial Number of points for filter: 3 Sample start region (min): 0 Sample end region (min): 30
Marker peak width (sec): 5 Marker min peak height: 200 Marker baseline V to V?: Y Marker baseline V to V points: 3
Lower marker selection: First peak > 500 RFU Upper marker selection: Last peak > 200 RFU
Ladder size (bp)75, 200, 300, 400, 500, 700, 1000, 1500, 2000, 3000, 4000, 5000, 7000, 10000, 20000
Quantification using: Lower Marker Final concentration (ng/uL): 0.5000 Dilution factor: 12.0

Sample: SampH1
Well location: H1
Created: Monday, September 20, 2021 6:00:03 PM

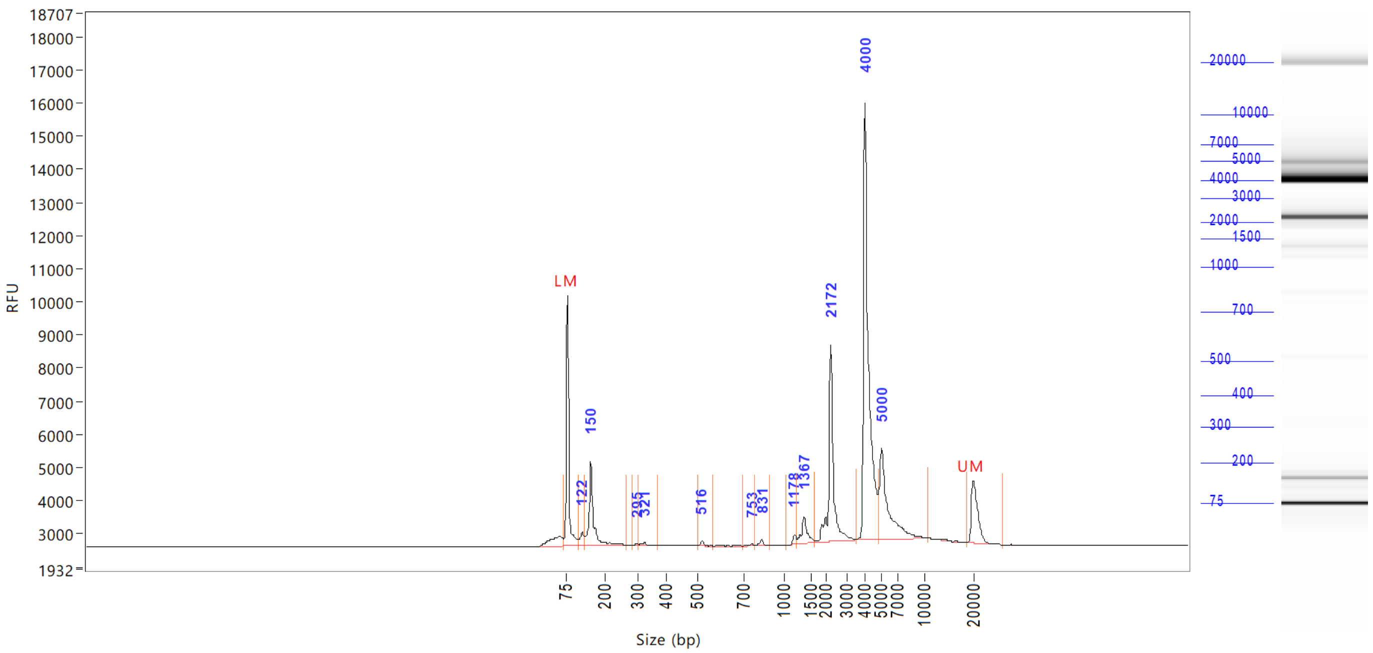

Peak	Size (bp)	Concentration (ng/uL)	From (bp)	To (bp)	Average size (bp)	CV%	RFU	Corrected peak area
1	75 (LM)	0.5000	64	112	76	10.80	7550	47.487
2	122	0.4580	112	131	122	4.52	405	3.625
3	150	3.2459	131	262	157	13.18	2534	25.690
4	295	0.0411	282	301	294	1.48	55	0.325
5	321	0.0764	301	368	318	3.79	77	0.604
6	516	0.1277	500	567	516	1.88	170	1.010
7	753	0.0513	695	779	745	2.51	68	0.406
8	831	0.1651	779	892	824	2.29	203	1.307
9	1178	0.1811	1012	1223	1177	2.22	304	1.433
10	1367	0.9242	1223	1616	1370	5.58	819	7.315
11	2172	5.1852	1616	3500	2190	10.61	5916	41.038
12	4000	12.9020	3500	4793	4127	5.49	13203	102.113
13	5000	4.3784	4793	10489	5744	18.49	2735	34.653
14	20000 (UM)	0.1624	18659	26287	20419	3.56	1879	15.421

TIC:	27.7364	ng/uL
TIM:	53.2870	nmole/L
Total concentration:	27.7540	ng/uL

```
Sample peak width (sec): 5     Sample min peak height: 50     Sample baseline V to V?: Y     Sample baseline V to V points: 3
Sample filter: Binomial        Number of points for filter: 3  Sample start region (min): 0  Sample end region (min): 30
Marker peak width (sec): 5     Marker min peak height: 200    Marker baseline V to V?: Y     Marker baseline V to V points: 3
Lower marker selection: First peak > 500 RFU                  Upper marker selection: Last peak > 200 RFU
Ladder size (bp)75, 200, 300, 400, 500, 700, 1000, 1500, 2000, 3000, 4000, 5000, 7000, 10000, 20000
Quantification using: Lower Marker          Final concentration (ng/uL): 0.5000          Dilution factor: 12.0
```

Sample: SampA2
Well location: A2
Created: Monday, September 20, 2021 6:00:03 PM

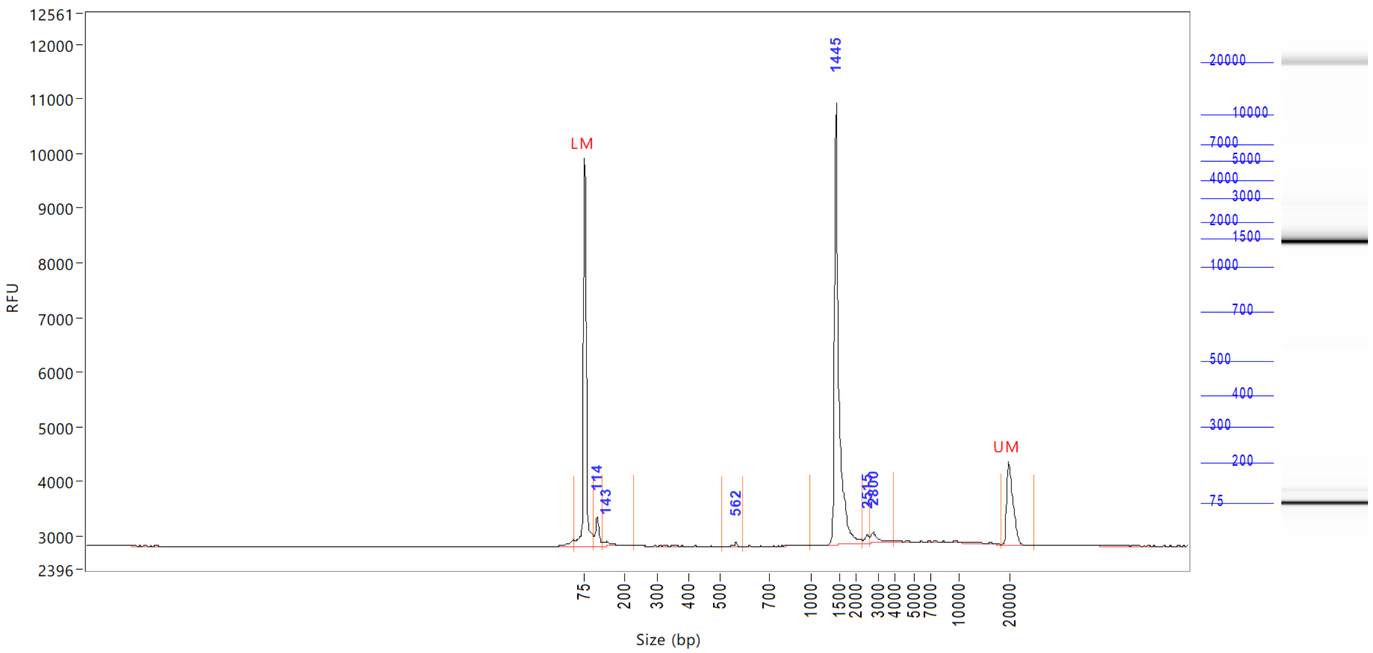

Peak	Size (bp)	Concentration (ng/uL)	From (bp)	To (bp)	Average size (bp)	CV%	RFU	Corrected peak area
1	75 (LM)	0.5000	45	103	74	9.65	7099	45.949
2	114	0.6116	103	133	113	5.98	540	4.684
3	143	0.2060	133	226	159	13.55	84	1.577
4	562	0.0570	506	588	552	2.78	64	0.437
5	1445	7.2141	991	2315	1488	8.38	8090	55.246
6	2515	0.1365	2315	2629	2483	3.51	160	1.045
7	2800	0.2943	2629	3929	2991	10.87	192	2.254
8	20000 (UM)	0.1324	18415	24957	20337	3.40	1534	12.170

TIC:	8.5194	ng/uL
TIM:	19.4219	nmole/L
Total concentration:	8.8076	ng/uL

```
Sample peak width (sec): 5    Sample min peak height: 50    Sample baseline V to V?: Y    Sample baseline V to V points: 3
Sample filter: Binomial       Number of points for filter: 3  Sample start region (min): 0  Sample end region (min): 30
Marker peak width (sec): 5    Marker min peak height: 200    Marker baseline V to V?: Y    Marker baseline V to V points: 3
Lower marker selection: First peak > 500 RFU                 Upper marker selection: Last peak > 200 RFU
Ladder size (bp)75, 200, 300, 400, 500, 700, 1000, 1500, 2000, 3000, 4000, 5000, 7000, 10000, 20000
Quantification using: Lower Marker          Final concentration (ng/uL): 0.5000          Dilution factor: 12.0
```

Sample: SampB2
Well location: B2
Created: Monday, September 20, 2021 6:00:03 PM

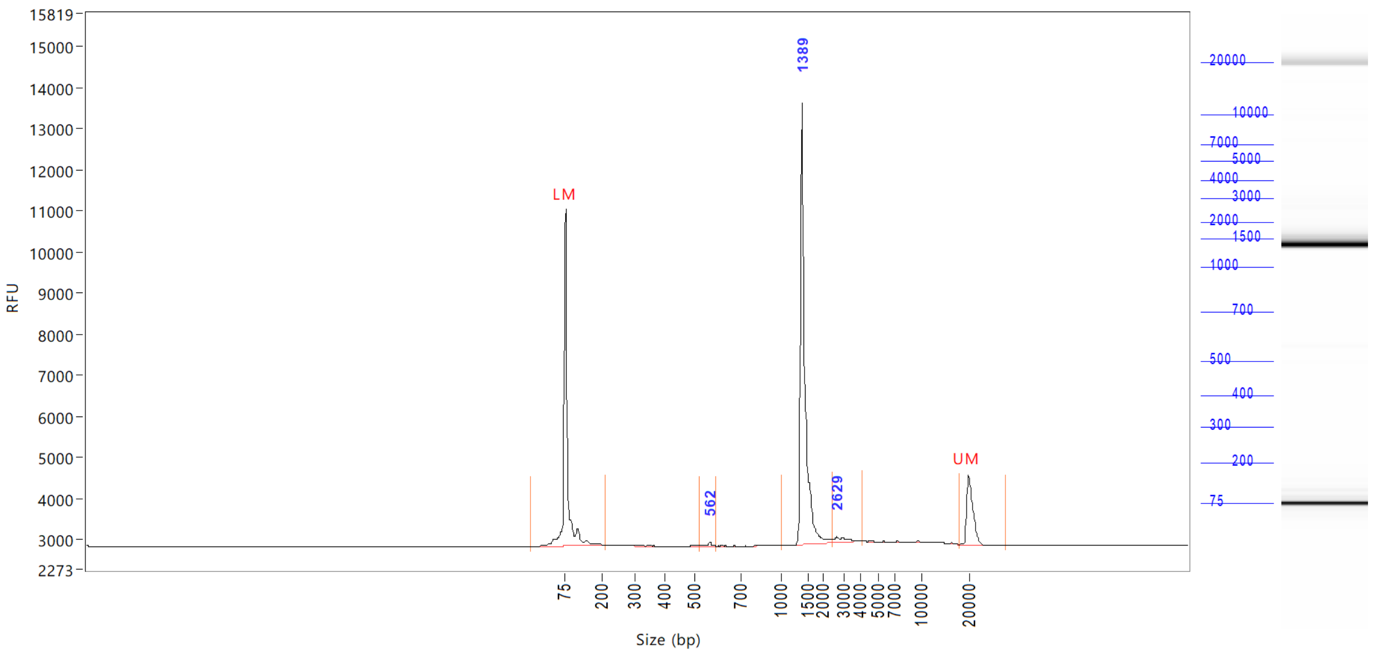

Peak	Size (bp)	Concentration (ng/uL)	From (bp)	To (bp)	Average size (bp)	CV%	RFU	Corrected peak area
1	75 (LM)	0.5000	0	205	76	27.71	8215	69.609
2	562	0.0555	519	593	557	2.58	94	0.644
3	1389	6.7267	1000	2458	1433	9.19	10779	78.040
4	2629	0.1984	2458	4070	2919	11.76	134	2.301
5	20000 (UM)	0.0996	17927	27738	20307	3.66	1698	13.872

TIC:	6.9806	ng/uL
TIM:	8.0027	nmole/L
Total concentration:	7.1555	ng/uL

Sample peak width (sec): 5 Sample min peak height: 50 Sample baseline V to V?: Y Sample baseline V to V points: 3
Sample filter: Binomial Number of points for filter: 3 Sample start region (min): 0 Sample end region (min): 30
Marker peak width (sec): 5 Marker min peak height: 200 Marker baseline V to V?: Y Marker baseline V to V points: 3
Lower marker selection: First peak > 500 RFU Upper marker selection: Last peak > 200 RFU
Ladder size (bp)75, 200, 300, 400, 500, 700, 1000, 1500, 2000, 3000, 4000, 5000, 7000, 10000, 20000
Quantification using: Lower Marker Final concentration (ng/uL): 0.5000 Dilution factor: 12.0

Sample: SampC2
Well location: C2
Created: Monday, September 20, 2021 6:00:03 PM

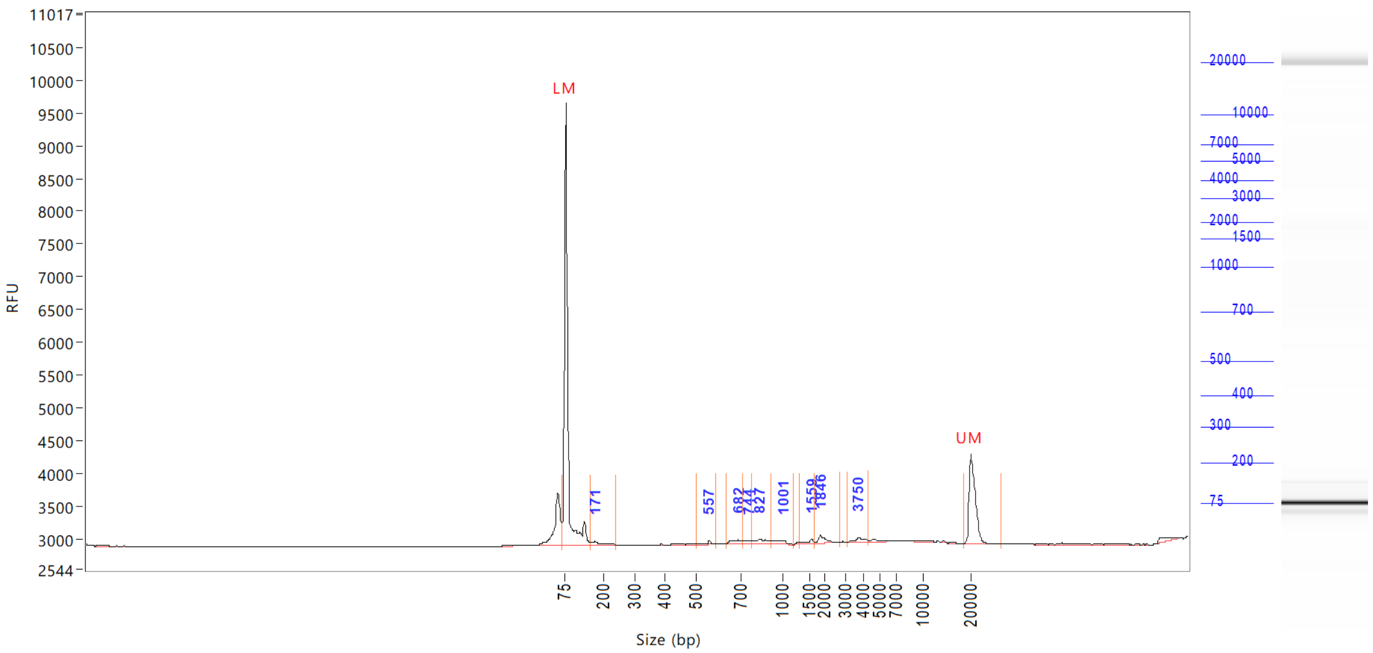

Peak	Size (bp)	Concentration (ng/uL)	From (bp)	To (bp)	Average size (bp)	CV%	RFU	Corrected peak area
1	75 (LM)	0.5000	64	158	81	21.17	6752	53.953
2	171	0.1175	158	239	183	11.27	54	1.056
3	557	0.0467	504	585	546	3.04	55	0.419
4	682	0.1225	631	705	672	2.90	62	1.101
5	744	0.0749	705	770	738	2.65	53	0.674
6	827	0.2051	770	918	845	4.97	73	1.844
7	1001	0.1401	918	1201	977	4.13	63	1.260
8	1559	0.0720	1334	1655	1484	5.76	62	0.648
9	1846	0.1549	1655	2686	1938	9.60	126	1.393
10	3750	0.0849	3037	4345	3847	6.93	66	0.764
11	20000 (UM)	0.1021	18293	26287	20301	3.62	1358	11.013

TIC:	1.0185	ng/uL
TIM:	2.5461	nmole/L
Total concentration:	1.2716	ng/uL

```
Sample peak width (sec): 5    Sample min peak height: 50    Sample baseline V to V?: Y    Sample baseline V to V points: 3
Sample filter: Binomial       Number of points for filter: 3  Sample start region (min): 0  Sample end region (min): 30
Marker peak width (sec): 5    Marker min peak height: 200   Marker baseline V to V?: Y    Marker baseline V to V points: 3
Lower marker selection: First peak > 500 RFU                Upper marker selection: Last peak > 200 RFU
Ladder size (bp)75, 200, 300, 400, 500, 700, 1000, 1500, 2000, 3000, 4000, 5000, 7000, 10000, 20000
Quantification using: Lower Marker          Final concentration (ng/uL): 0.5000          Dilution factor: 12.0
```

Sample: SampD2
Well location: D2
Created: Monday, September 20, 2021 6:00:03 PM

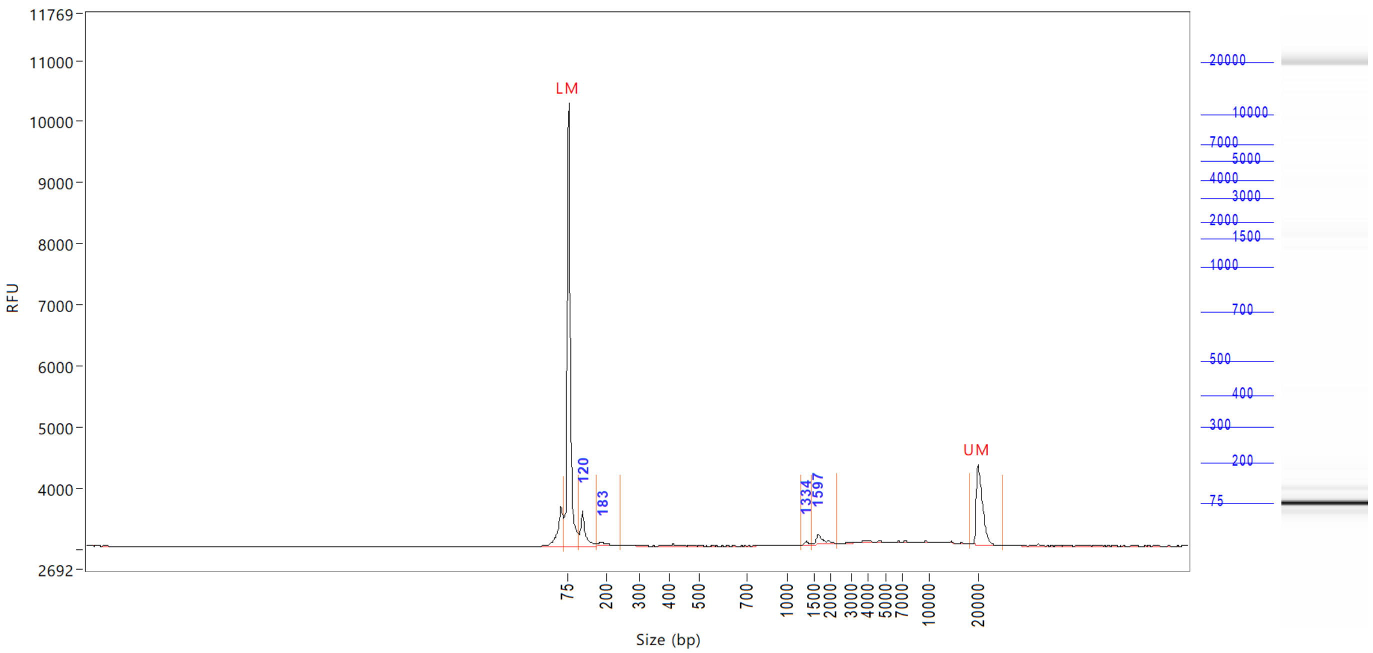

Peak	Size (bp)	Concentration (ng/uL)	From (bp)	To (bp)	Average size (bp)	CV%	RFU	Corrected peak area
1	75 (LM)	0.5000	60	108	75	10.20	7249	56.221
2	120	0.6858	108	166	124	10.52	585	6.426
3	183	0.1361	166	241	187	7.54	56	1.276
4	1334	0.0414	1245	1445	1344	3.05	59	0.388
5	1597	0.1932	1445	2315	1706	9.87	169	1.811
6	20000 (UM)	0.0958	18293	25199	20410	3.66	1312	10.769

TIC:	1.0566	ng/uL
TIM:	10.5206	nmole/L
Total concentration:	1.3620	ng/uL

Sample peak width (sec): 5 Sample min peak height: 50 Sample baseline V to V?: Y Sample baseline V to V points: 3
Sample filter: Binomial Number of points for filter: 3 Sample start region (min): 0 Sample end region (min): 30
Marker peak width (sec): 5 Marker min peak height: 200 Marker baseline V to V?: Y Marker baseline V to V points: 3
Lower marker selection: First peak > 500 RFU Upper marker selection: Last peak > 200 RFU
Ladder size (bp)75, 200, 300, 400, 500, 700, 1000, 1500, 2000, 3000, 4000, 5000, 7000, 10000, 20000
Quantification using: Lower Marker Final concentration (ng/uL): 0.5000 Dilution factor: 12.0

Sample: SampE2
Well location: E2
Created: Monday, September 20, 2021 6:00:03 PM

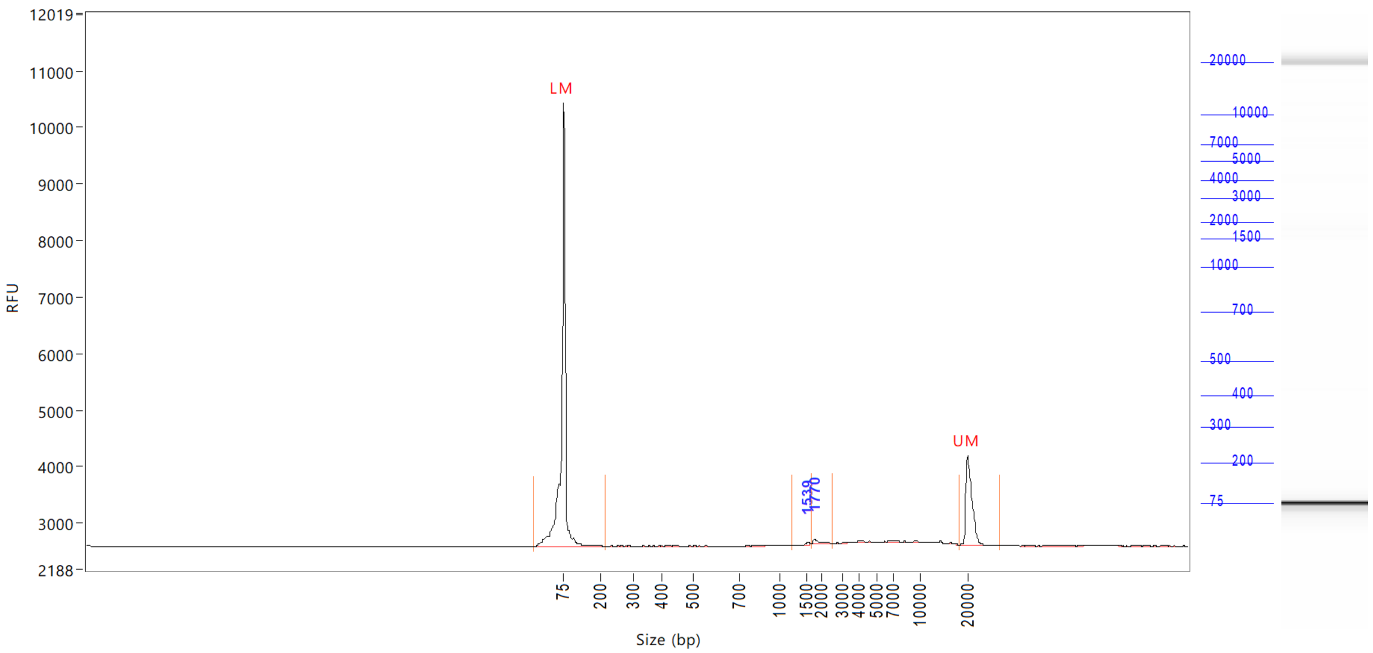

Peak	Size (bp)	Concentration (ng/uL)	From (bp)	To (bp)	Average size (bp)	CV%	RFU	Corrected peak area
1	75 (LM)	0.5000	0	210	67	29.38	7853	73.368
2	1539	0.0307	1223	1655	1512	5.54	55	0.375
3	1770	0.0881	1655	2515	1899	10.08	99	1.077
4	20000 (UM)	0.0882	18293	26770	20398	3.71	1579	12.943

TIC:	0.1188	ng/uL
TIM:	0.1098	nmole/L
Total concentration:	0.3541	ng/uL

Sample peak width (sec): 5 Sample min peak height: 50 Sample baseline V to V?: Y Sample baseline V to V points: 3
Sample filter: Binomial Number of points for filter: 3 Sample start region (min): 0 Sample end region (min): 30
Marker peak width (sec): 5 Marker min peak height: 200 Marker baseline V to V?: Y Marker baseline V to V points: 3
Lower marker selection: First peak > 500 RFU Upper marker selection: Last peak > 200 RFU
Ladder size (bp)75, 200, 300, 400, 500, 700, 1000, 1500, 2000, 3000, 4000, 5000, 7000, 10000, 20000
Quantification using: Lower Marker Final concentration (ng/uL): 0.5000 Dilution factor: 12.0

Sample: SampF2
Well location: F2
Created: Monday, September 20, 2021 6:00:03 PM

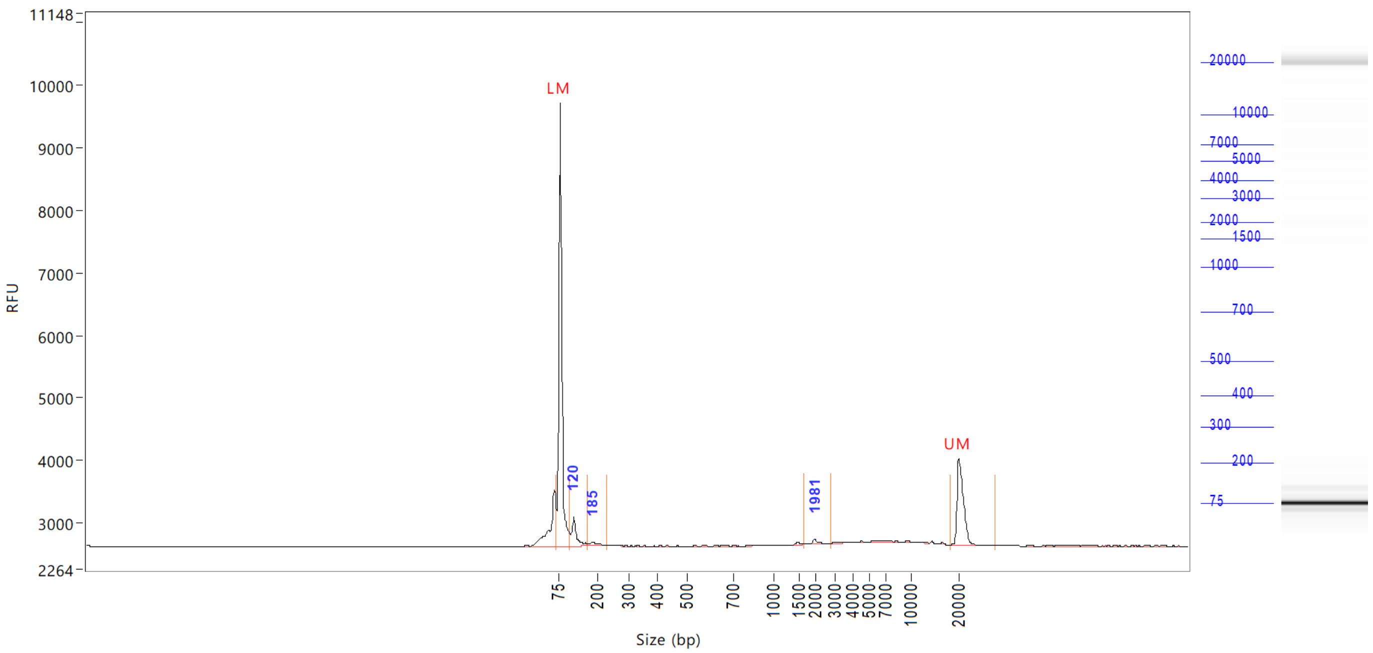

Peak	Size (bp)	Concentration (ng/uL)	From (bp)	To (bp)	Average size (bp)	CV%	RFU	Corrected peak area
1	75 (LM)	0.5000	66	108	76	9.87	7093	53.538
2	120	0.5971	108	166	124	10.94	468	5.328
3	185	0.1394	166	232	187	7.19	55	1.244
4	1981	0.0792	1655	2743	2038	10.05	72	0.707
5	20000 (UM)	0.1065	18049	27738	20375	3.86	1382	11.408

TIC:	0.8158	ng/uL
TIM:	9.2016	nmole/L
Total concentration:	1.1775	ng/uL

```
Sample peak width (sec): 5    Sample min peak height: 50    Sample baseline V to V?: Y    Sample baseline V to V points: 3
Sample filter: Binomial       Number of points for filter: 3  Sample start region (min): 0  Sample end region (min): 30
Marker peak width (sec): 5    Marker min peak height: 200   Marker baseline V to V?: Y    Marker baseline V to V points: 3
Lower marker selection: First peak > 500 RFU                Upper marker selection: Last peak > 200 RFU
Ladder size (bp)75, 200, 300, 400, 500, 700, 1000, 1500, 2000, 3000, 4000, 5000, 7000, 10000, 20000
Quantification using: Lower Marker          Final concentration (ng/uL): 0.5000          Dilution factor: 12.0
```

Sample: SampG2
Well location: G2
Created: Monday, September 20, 2021 6:00:03 PM

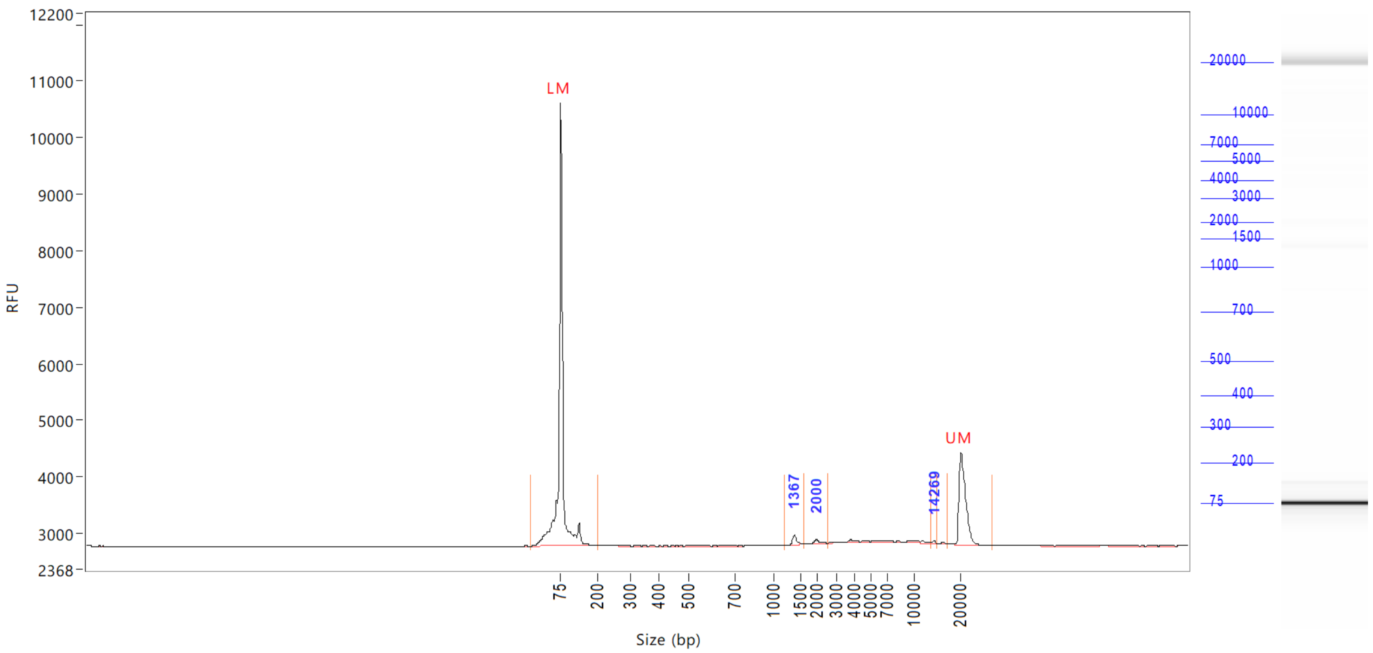

Peak	Size<br>(bp)	Concentration<br>(ng/uL)	From<br>(bp)	To<br>(bp)	Average size<br>(bp)	CV%	RFU	Corrected peak area
1	75 (LM)	0.5000	0	200	72	31.67	7845	73.809
2	1367	0.0924	1190	1597	1383	4.06	172	1.137
3	2000	0.0557	1597	2515	1985	8.92	77	0.685
4	14269	0.0205	13659	15000	14174	2.19	53	0.252
5	20000 (UM)	0.0953	17317	26649	20444	3.89	1631	14.069

TIC:	0.1686	ng/uL
TIM:	0.1586	nmole/L
Total concentration:	0.4971	ng/uL

```
Sample peak width (sec): 5    Sample min peak height: 50    Sample baseline V to V?: Y    Sample baseline V to V points: 3
Sample filter: Binomial       Number of points for filter: 3  Sample start region (min): 0  Sample end region (min): 30
Marker peak width (sec): 5    Marker min peak height: 200   Marker baseline V to V?: Y    Marker baseline V to V points: 3
Lower marker selection: First peak > 500 RFU                Upper marker selection: Last peak > 200 RFU
Ladder size (bp)75, 200, 300, 400, 500, 700, 1000, 1500, 2000, 3000, 4000, 5000, 7000, 10000, 20000
Quantification using: Lower Marker          Final concentration (ng/uL): 0.5000          Dilution factor: 12.0
```

Sample: SampH2
Well location: H2
Created: Monday, September 20, 2021 6:00:03 PM

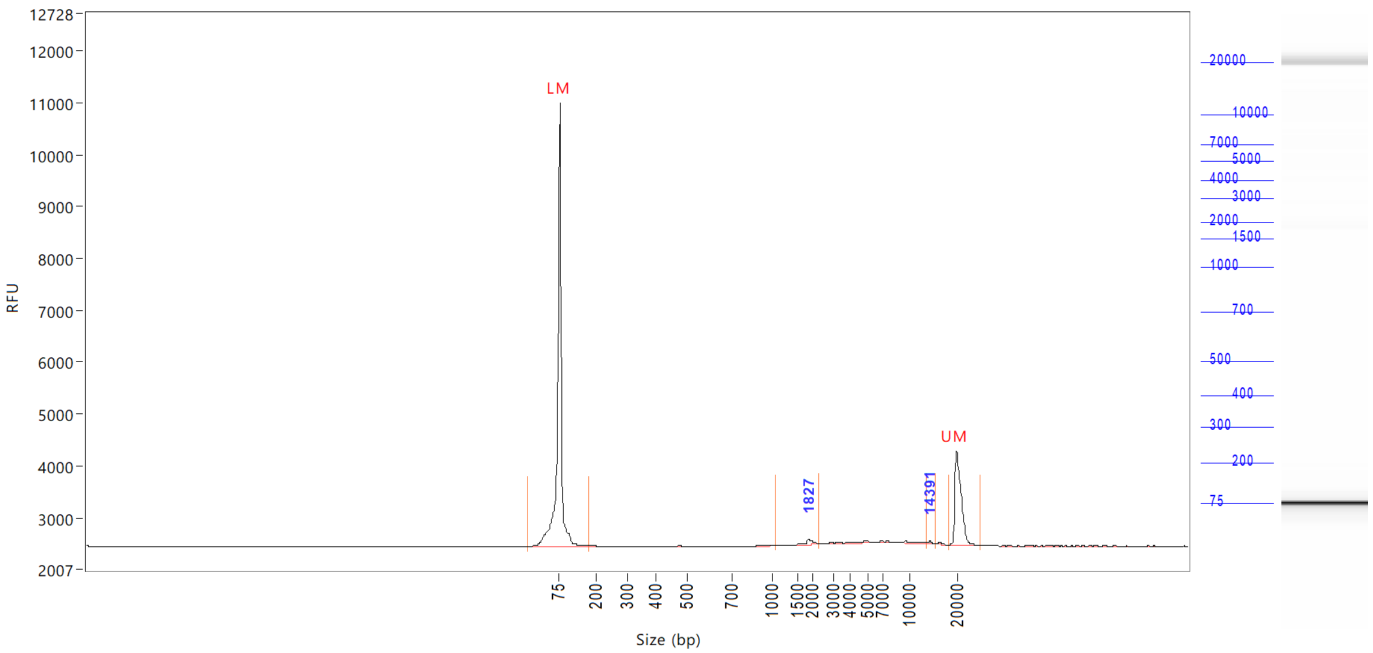

Peak	Size (bp)	Concentration (ng/uL)	From (bp)	To (bp)	Average size (bp)	CV%	RFU	Corrected peak area
1	75 (LM)	0.5000	0	177	70	26.45	8569	78.564
2	1827	0.0954	1079	2286	1818	11.97	109	1.249
3	14391	0.0221	13659	15488	14296	2.72	61	0.289
4	20000 (UM)	0.0959	18293	25199	20442	3.75	1801	15.076

TIC:	0.1174	ng/uL
TIM:	0.0889	nmole/L
Total concentration:	0.4616	ng/uL

Sample peak width (sec): 5   Sample min peak height: 50   Sample baseline V to V?: Y   Sample baseline V to V points: 3
Sample filter: Binomial   Number of points for filter: 3   Sample start region (min): 0   Sample end region (min): 30
Marker peak width (sec): 5   Marker min peak height: 200   Marker baseline V to V?: Y   Marker baseline V to V points: 3
Lower marker selection: First peak > 500 RFU   Upper marker selection: Last peak > 200 RFU
Ladder size (bp)75, 200, 300, 400, 500, 700, 1000, 1500, 2000, 3000, 4000, 5000, 7000, 10000, 20000
Quantification using: Lower Marker   Final concentration (ng/uL): 0.5000   Dilution factor: 12.0

Sample: SampA3
Well location: A3
Created: Monday, September 20, 2021 6:00:03 PM

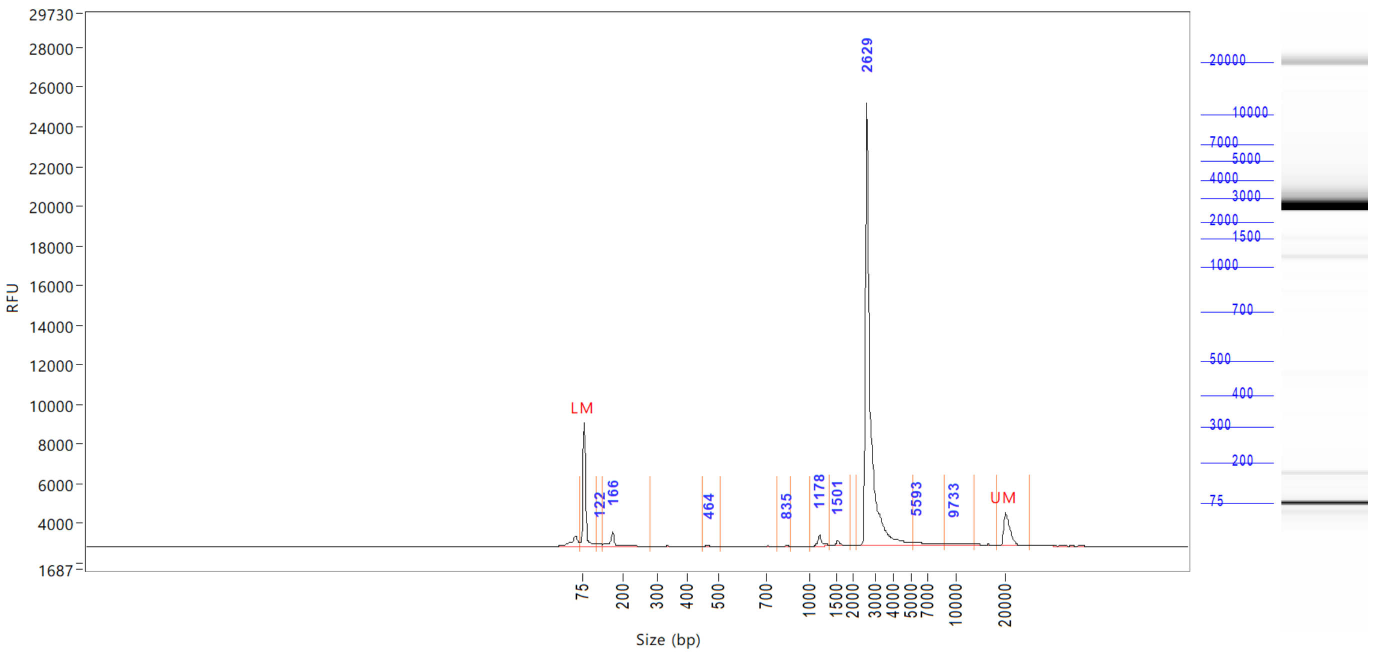

Peak	Size (bp)	Concentration (ng/uL)	From (bp)	To (bp)	Average size (bp)	CV%	RFU	Corrected peak area
1	75 (LM)	0.5000	64	112	76	9.47	6249	38.224
2	122	0.2538	112	131	121	4.68	156	1.617
3	166	1.2979	131	282	168	14.50	719	8.268
4	464	0.1156	447	501	463	2.18	95	0.736
5	835	0.0626	770	870	828	2.05	69	0.399
6	1178	0.5824	1001	1367	1190	5.08	569	3.710
7	1501	0.3161	1367	1904	1517	5.72	240	2.014
8	2629	25.8753	2087	5149	2796	14.43	22357	164.844
9	5593	0.5682	5149	8800	6709	15.43	139	3.620
10	9733	0.2574	8800	13781	10655	13.16	67	1.640
11	20000 (UM)	0.1693	18415	24836	20342	3.31	1628	12.943

TIC:	29.3291	ng/uL
TIM:	33.2419	nmole/L
Total concentration:	29.5491	ng/uL

```
Sample peak width (sec): 5    Sample min peak height: 50    Sample baseline V to V?: Y    Sample baseline V to V points: 3
Sample filter: Binomial       Number of points for filter: 3  Sample start region (min): 0  Sample end region (min): 30
Marker peak width (sec): 5    Marker min peak height: 200   Marker baseline V to V?: Y    Marker baseline V to V points: 3
Lower marker selection: First peak > 500 RFU                Upper marker selection: Last peak > 200 RFU
Ladder size (bp)75, 200, 300, 400, 500, 700, 1000, 1500, 2000, 3000, 4000, 5000, 7000, 10000, 20000
Quantification using: Lower Marker          Final concentration (ng/uL): 0.5000          Dilution factor: 12.0
```

Sample: SampB3
Well location: B3
Created: Monday, September 20, 2021 6:00:03 PM

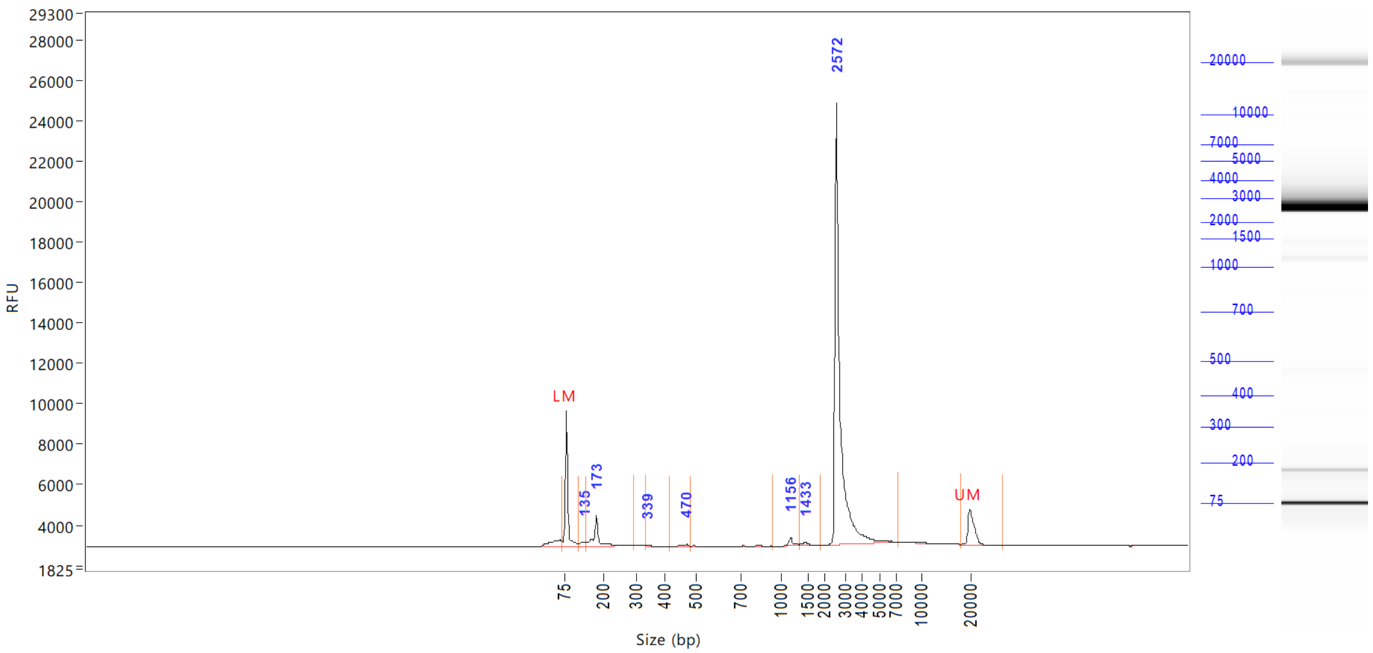

Peak	Size (bp)	Concentration (ng/uL)	From (bp)	To (bp)	Average size (bp)	CV%	RFU	Corrected peak area
1	75 (LM)	0.5000	64	114	76	10.58	6700	41.742
2	135	0.4102	114	141	128	6.20	189	2.854
3	173	2.4098	141	295	177	13.79	1503	16.765
4	339	0.0566	331	412	352	6.38	57	0.394
5	470	0.1577	412	481	456	3.62	104	1.097
6	1156	0.4314	935	1323	1160	5.57	410	3.001
7	1433	0.2413	1323	1885	1440	5.70	162	1.679
8	2572	24.8374	1885	7201	2779	16.80	21866	172.792
9	20000 (UM)	0.1700	18049	26891	20334	3.67	1751	14.189

TIC:	28.5443	ng/uL
TIM:	44.0815	nmole/L
Total concentration:	28.8059	ng/uL

Sample peak width (sec): 5   Sample min peak height: 50   Sample baseline V to V?: Y   Sample baseline V to V points: 3
Sample filter: Binomial   Number of points for filter: 3 Sample start region (min): 0 Sample end region (min): 30
Marker peak width (sec): 5   Marker min peak height: 200   Marker baseline V to V?: Y   Marker baseline V to V points: 3
Lower marker selection: First peak > 500 RFU   Upper marker selection: Last peak > 200 RFU
Ladder size (bp)75, 200, 300, 400, 500, 700, 1000, 1500, 2000, 3000, 4000, 5000, 7000, 10000, 20000
Quantification using: Lower Marker   Final concentration (ng/uL): 0.5000   Dilution factor: 12.0

Sample: SampC3
Well location: C3
Created: Monday, September 20, 2021 6:00:03 PM

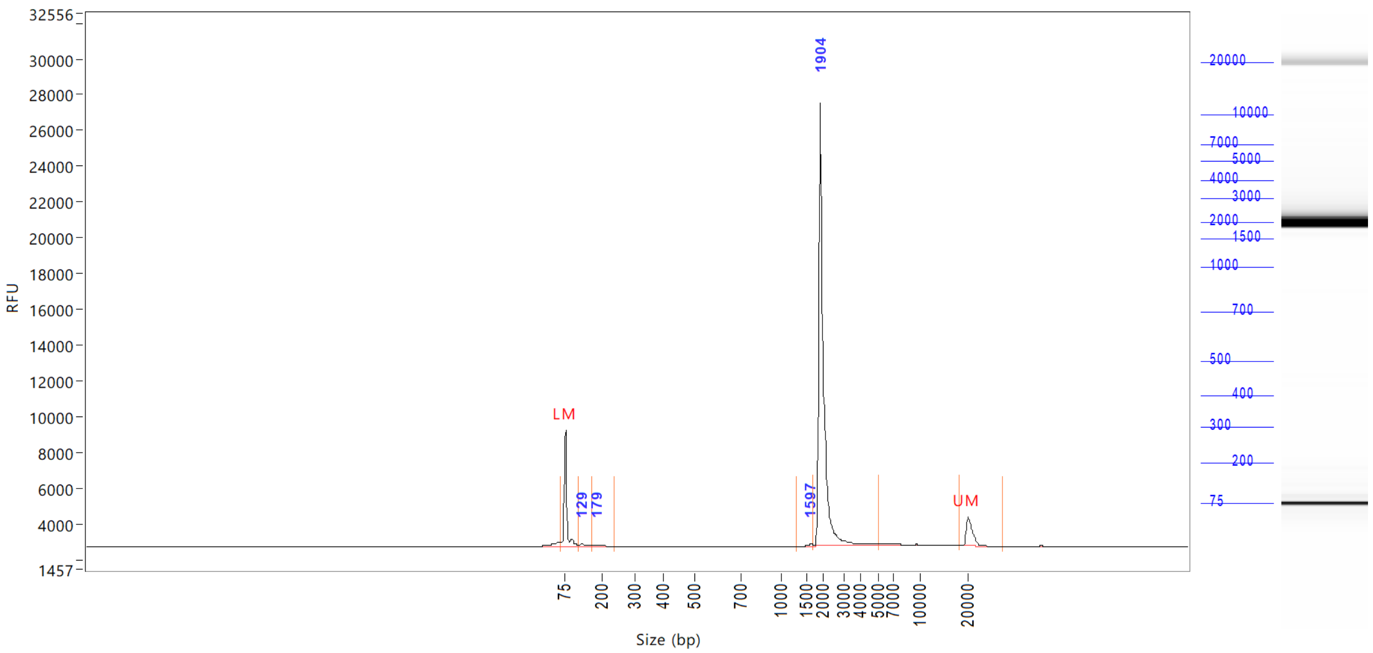

Peak	Size (bp)	Concentration (ng/uL)	From (bp)	To (bp)	Average size (bp)	CV%	RFU	Corrected peak area
1	75 (LM)	0.5000	62	120	76	12.36	6522	42.262
2	129	0.3463	120	166	138	9.08	145	2.439
3	179	0.1981	166	233	186	8.15	106	1.396
4	1597	0.1226	1311	1693	1575	4.85	137	0.864
5	1904	25.9500	1693	5149	2038	18.16	24824	182.782
6	20000 (UM)	0.1562	18049	27133	20357	3.74	1611	13.202

TIC:	26.6170	ng/uL
TIM:	26.9631	nmole/L
Total concentration:	27.1050	ng/uL

Sample peak width (sec): 5 Sample min peak height: 50 Sample baseline V to V?: Y Sample baseline V to V points: 3
Sample filter: Binomial Number of points for filter: 3 Sample start region (min): 0 Sample end region (min): 30
Marker peak width (sec): 5 Marker min peak height: 200 Marker baseline V to V?: Y Marker baseline V to V points: 3
Lower marker selection: First peak > 500 RFU Upper marker selection: Last peak > 200 RFU
Ladder size (bp)75, 200, 300, 400, 500, 700, 1000, 1500, 2000, 3000, 4000, 5000, 7000, 10000, 20000
Quantification using: Lower Marker Final concentration (ng/uL): 0.5000 Dilution factor: 12.0

Sample: SampD3
Well location: D3
Created: Monday, September 20, 2021 6:00:03 PM

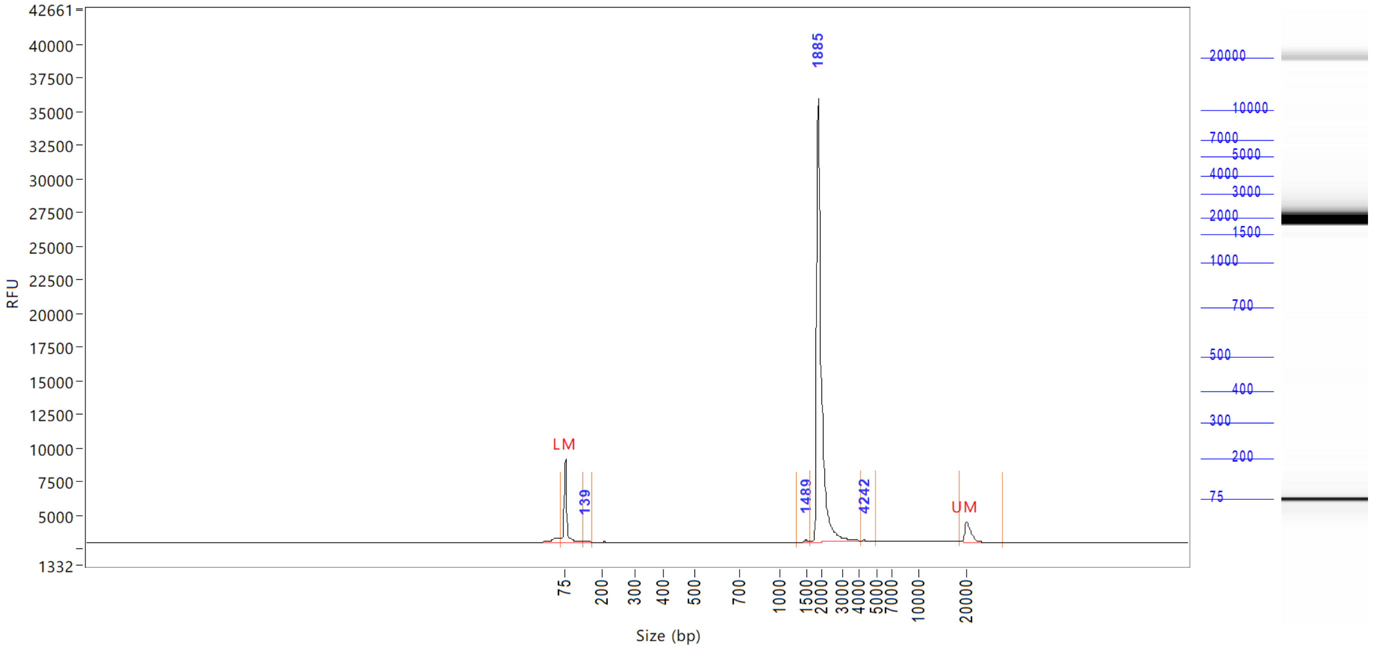

Peak	Size (bp)	Concentration (ng/uL)	From (bp)	To (bp)	Average size (bp)	CV%	RFU	Corrected peak area
1	75 (LM)	0.5000	62	133	76	14.92	6191	41.819
2	139	0.1254	133	162	144	5.04	64	0.874
3	1489	0.1332	1323	1635	1498	3.43	147	0.929
4	1885	33.8293	1635	4035	1973	12.71	32998	235.785
5	4242	0.1429	4035	4966	4398	5.58	68	0.996
6	20000 (UM)	0.1421	18537	27738	20431	3.81	1465	11.882

TIC:	34.2309	ng/uL
TIM:	29.8564	nmole/L
Total concentration:	34.6562	ng/uL

```
Sample peak width (sec): 5    Sample min peak height: 50    Sample baseline V to V?: Y    Sample baseline V to V points: 3
Sample filter: Binomial       Number of points for filter: 3  Sample start region (min): 0  Sample end region (min): 30
Marker peak width (sec): 5    Marker min peak height: 200   Marker baseline V to V?: Y    Marker baseline V to V points: 3
Lower marker selection: First peak > 500 RFU                Upper marker selection: Last peak > 200 RFU
Ladder size (bp)75, 200, 300, 400, 500, 700, 1000, 1500, 2000, 3000, 4000, 5000, 7000, 10000, 20000
Quantification using: Lower Marker          Final concentration (ng/uL): 0.5000          Dilution factor: 12.0
```

Sample: SampE3
Well location: E3
Created: Monday, September 20, 2021 6:00:03 PM

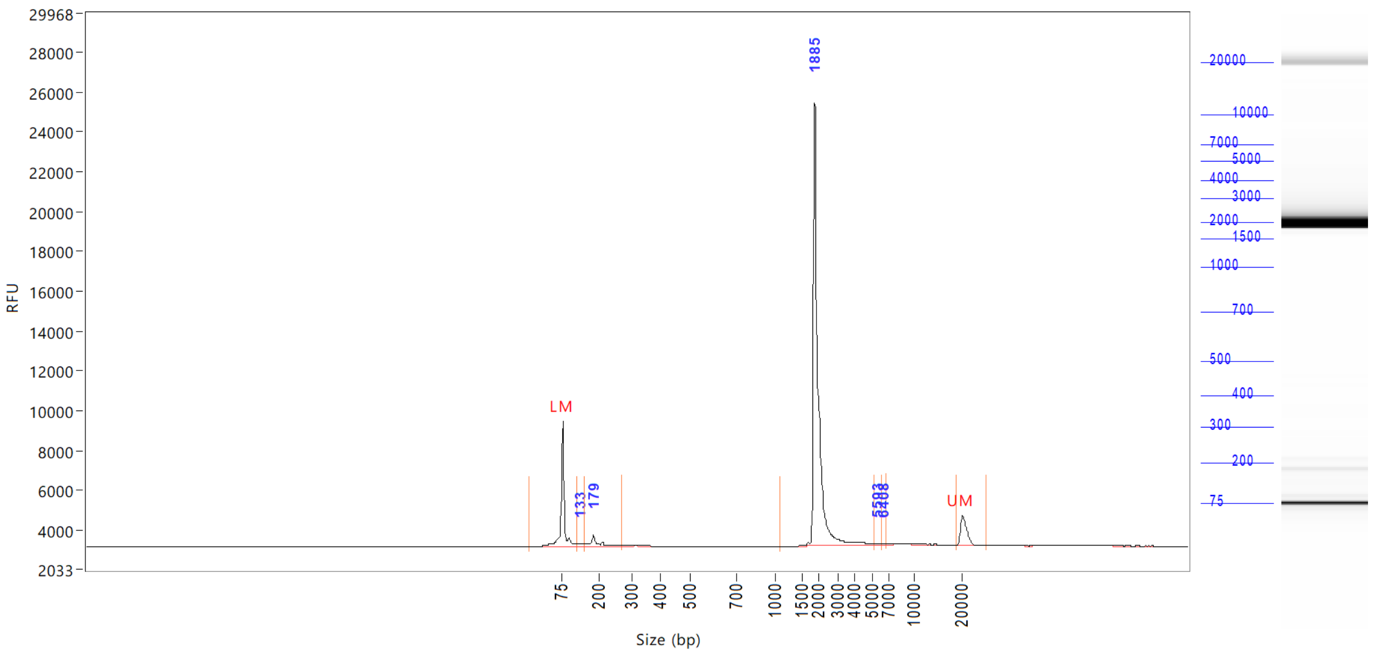

Peak	Size (bp)	Concentration (ng/uL)	From (bp)	To (bp)	Average size (bp)	CV%	RFU	Corrected peak area
1	75 (LM)	0.5000	0	124	72	22.97	6271	47.991
2	133	0.2712	124	150	137	5.79	148	2.169
3	179	1.0495	150	269	186	12.97	582	8.394
4	1885	21.0413	1079	5297	2046	20.26	22294	168.297
5	5593	0.0633	5297	6186	5715	4.77	62	0.506
6	6408	0.0368	6186	6704	6431	2.56	54	0.295
7	20000 (UM)	0.1325	18903	25078	20419	3.42	1545	12.715

TIC:	22.4621	ng/uL
TIM:	29.4865	nmole/L
Total concentration:	22.8309	ng/uL

```
Sample peak width (sec): 5     Sample min peak height: 50    Sample baseline V to V?: Y    Sample baseline V to V points: 3
Sample filter: Binomial        Number of points for filter: 3  Sample start region (min): 0  Sample end region (min): 30
Marker peak width (sec): 5     Marker min peak height: 200   Marker baseline V to V?: Y    Marker baseline V to V points: 3
Lower marker selection: First peak > 500 RFU                 Upper marker selection: Last peak > 200 RFU
Ladder size (bp)75, 200, 300, 400, 500, 700, 1000, 1500, 2000, 3000, 4000, 5000, 7000, 10000, 20000
Quantification using: Lower Marker          Final concentration (ng/uL): 0.5000          Dilution factor: 12.0
```

Sample: SampF3
Well location: F3
Created: Monday, September 20, 2021 6:00:03 PM

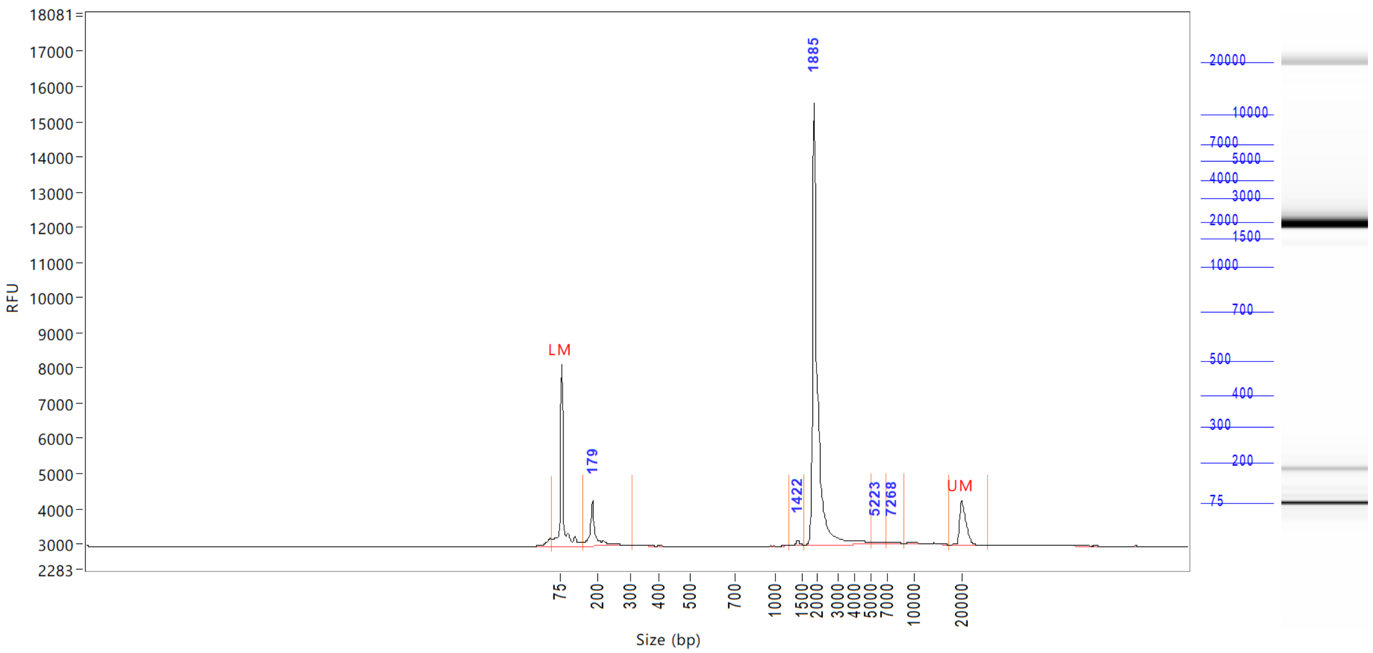

Peak	Size (bp)	Concentration (ng/uL)	From (bp)	To (bp)	Average size (bp)	CV%	RFU	Corrected peak area
1	75 (LM)	0.5000	47	147	79	21.52	5165	40.467
2	179	2.3388	147	303	186	13.55	1288	15.774
3	1422	0.1603	1267	1578	1424	3.46	161	1.081
4	1885	14.4351	1578	4966	2057	21.31	12593	97.357
5	5223	0.1628	4966	6852	5784	10.05	66	1.098
6	7268	0.1202	6852	8867	7626	7.25	55	0.811
7	20000 (UM)	0.1315	17317	25440	20393	3.78	1282	10.647

TIC:	17.2172	ng/uL
TIM:	32.4824	nmole/L
Total concentration:	17.4270	ng/uL

Sample peak width (sec): 5 Sample min peak height: 50 Sample baseline V to V?: Y Sample baseline V to V points: 3
Sample filter: Binomial Number of points for filter: 3 Sample start region (min): 0 Sample end region (min): 30
Marker peak width (sec): 5 Marker min peak height: 200 Marker baseline V to V?: Y Marker baseline V to V points: 3
Lower marker selection: First peak > 500 RFU Upper marker selection: Last peak > 200 RFU
Ladder size (bp)75, 200, 300, 400, 500, 700, 1000, 1500, 2000, 3000, 4000, 5000, 7000, 10000, 20000
Quantification using: Lower Marker Final concentration (ng/uL): 0.5000 Dilution factor: 12.0

Sample: SampG3
Well location: G3
Created: Monday, September 20, 2021 6:00:03 PM

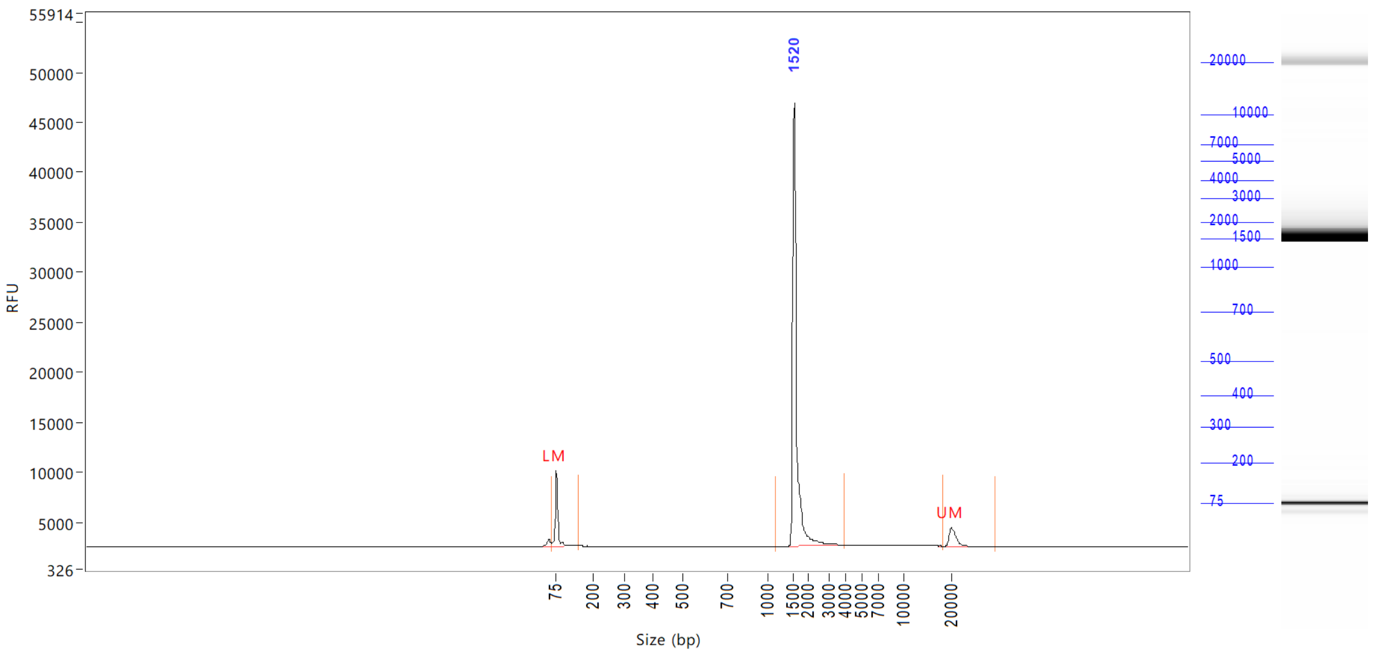

Peak	Size (bp)	Concentration (ng/uL)	From (bp)	To (bp)	Average size (bp)	CV%	RFU	Corrected peak area
1	75 (LM)	0.5000	58	152	75	12.87	7508	48.299
2	1520	33.9706	1156	4000	1578	13.12	44388	273.455
3	20000 (UM)	0.1687	18415	29551	20461	4.33	1936	16.299

TIC:	33.9706	ng/uL
TIM:	35.4364	nmole/L
Total concentration:	34.2732	ng/uL

Sample peak width (sec): 5 Sample min peak height: 50 Sample baseline V to V?: Y Sample baseline V to V points: 3
Sample filter: Binomial Number of points for filter: 3 Sample start region (min): 0 Sample end region (min): 30
Marker peak width (sec): 5 Marker min peak height: 200 Marker baseline V to V?: Y Marker baseline V to V points: 3
Lower marker selection: First peak > 500 RFU Upper marker selection: Last peak > 200 RFU
Ladder size (bp)75, 200, 300, 400, 500, 700, 1000, 1500, 2000, 3000, 4000, 5000, 7000, 10000, 20000
Quantification using: Lower Marker Final concentration (ng/uL): 0.5000 Dilution factor: 12.0

Sample: SampH3
Well location: H3
Created: Monday, September 20, 2021 6:00:03 PM

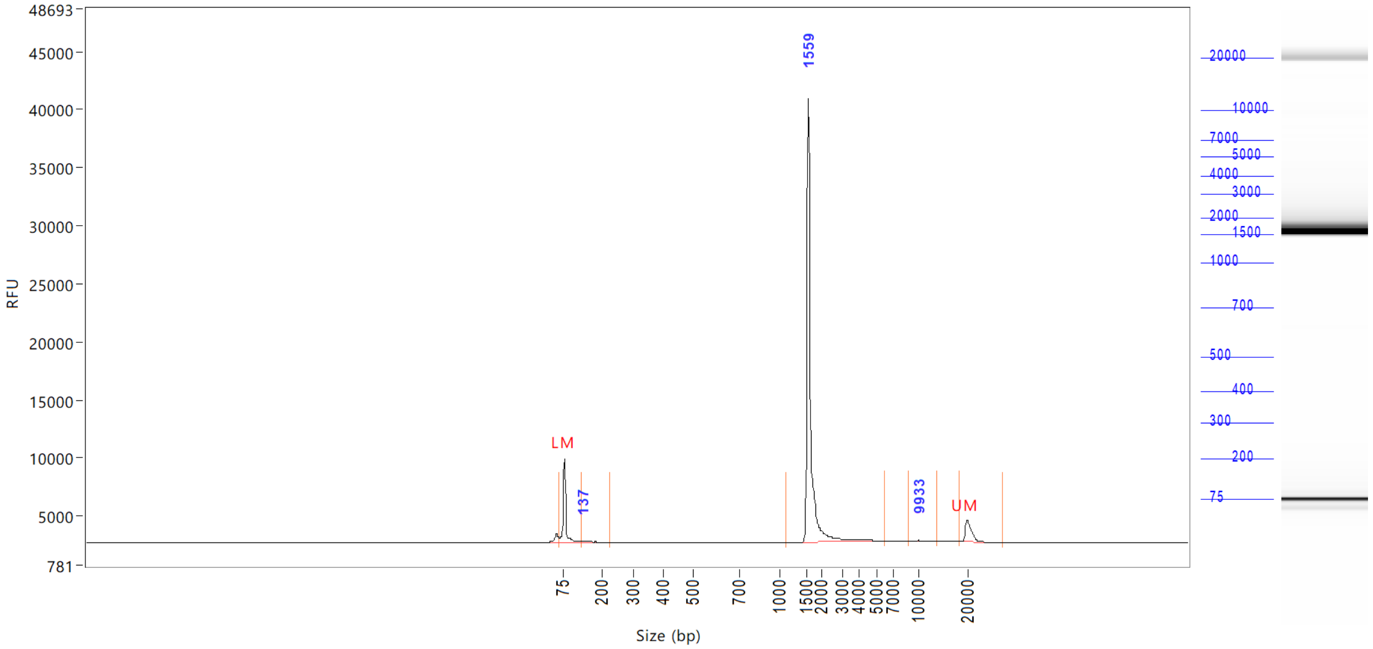

Peak	Size (bp)	Concentration (ng/uL)	From (bp)	To (bp)	Average size (bp)	CV%	RFU	Corrected peak area
1	75 (LM)	0.5000	60	131	76	15.05	7232	48.845
2	137	0.4546	131	224	164	15.36	137	3.701
3	1559	28.6265	1112	6112	1666	23.70	38264	233.045
4	9933	0.0701	8800	13781	10418	10.54	59	0.571
5	20000 (UM)	0.1547	18415	27617	20409	3.74	1845	15.114

TIC:	29.1512	ng/uL
TIM:	32.8524	nmole/L
Total concentration:	29.3829	ng/uL

```
Sample peak width (sec): 5    Sample min peak height: 50    Sample baseline V to V?: Y    Sample baseline V to V points: 3
Sample filter: Binomial       Number of points for filter: 3  Sample start region (min): 0  Sample end region (min): 30
Marker peak width (sec): 5    Marker min peak height: 200   Marker baseline V to V?: Y    Marker baseline V to V points: 3
Lower marker selection: First peak > 500 RFU                Upper marker selection: Last peak > 200 RFU
Ladder size (bp)75, 200, 300, 400, 500, 700, 1000, 1500, 2000, 3000, 4000, 5000, 7000, 10000, 20000
Quantification using: Lower Marker          Final concentration (ng/uL): 0.5000          Dilution factor: 12.0
```

Sample: SampA4
Well location: A4
Created: Monday, September 20, 2021 6:00:03 PM

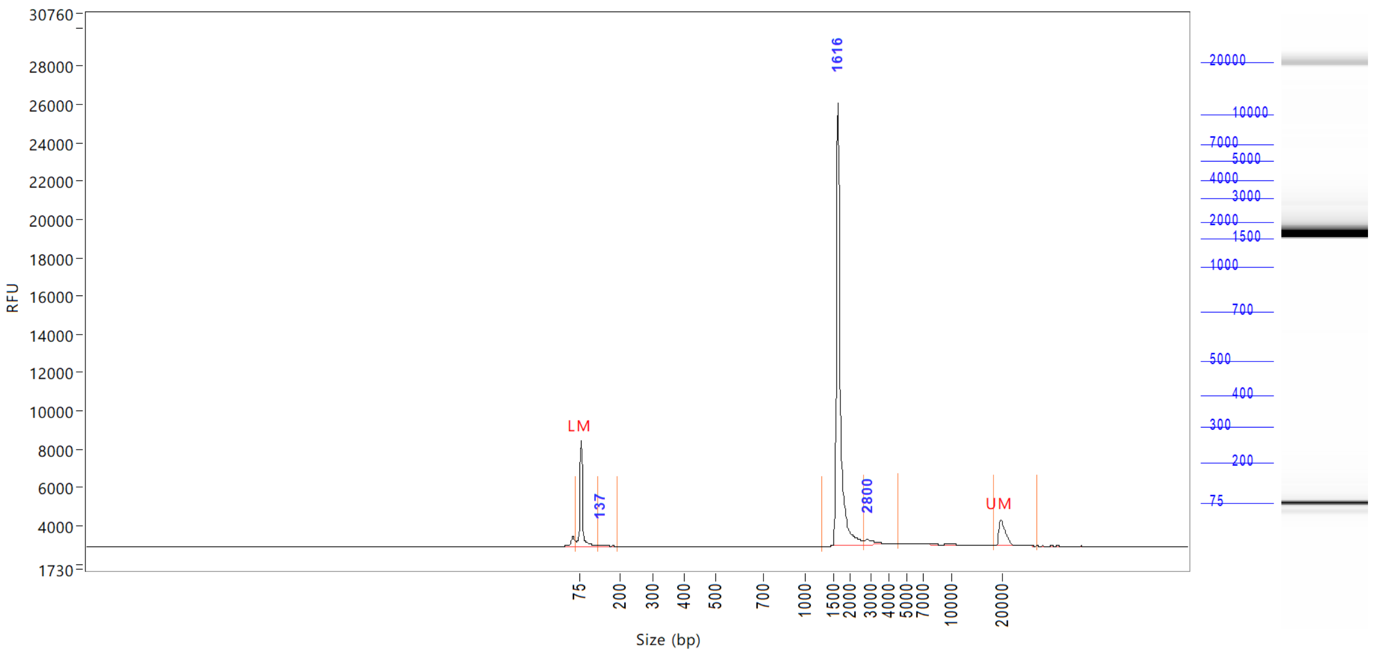

Peak	Size<br>(bp)	Concentration<br>(ng/uL)	From<br>(bp)	To<br>(bp)	Average size<br>(bp)	CV%	RFU	Corrected peak area
1	75 (LM)	0.5000	60	131	76	13.60	5555	37.708
2	137	0.2833	131	190	153	10.57	86	1.780
3	1616	23.1211	1300	2629	1668	9.60	23163	145.310
4	2800	0.7350	2629	4483	3063	12.13	294	4.619
5	20000 (UM)	0.1474	18537	27133	20311	3.60	1354	11.118

TIC:	24.1394	ng/uL
TIM:	26.2555	nmole/L
Total concentration:	24.5862	ng/uL

Sample peak width (sec): 5   Sample min peak height: 50   Sample baseline V to V?: Y   Sample baseline V to V points: 3
Sample filter: Binomial   Number of points for filter: 3   Sample start region (min): 0   Sample end region (min): 30
Marker peak width (sec): 5   Marker min peak height: 200   Marker baseline V to V?: Y   Marker baseline V to V points: 3
Lower marker selection: First peak > 500 RFU   Upper marker selection: Last peak > 200 RFU
Ladder size (bp)75, 200, 300, 400, 500, 700, 1000, 1500, 2000, 3000, 4000, 5000, 7000, 10000, 20000
Quantification using: Lower Marker   Final concentration (ng/uL): 0.5000   Dilution factor: 12.0

Sample: SampB4
Well location: B4
Created: Monday, September 20, 2021 6:00:03 PM

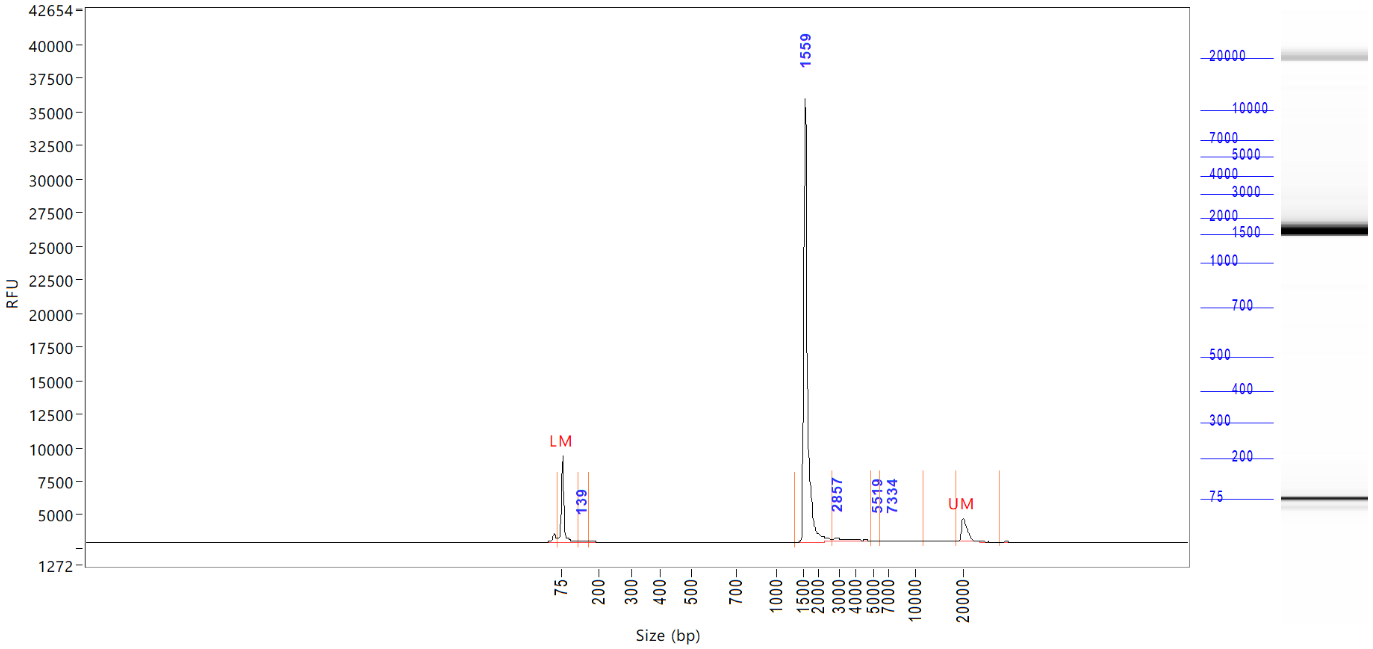

Peak	Size (bp)	Concentration (ng/uL)	From (bp)	To (bp)	Average size (bp)	CV%	RFU	Corrected peak area
1	75 (LM)	0.5000	58	131	76	13.66	6480	43.780
2	139	0.1841	131	166	146	6.59	96	1.343
3	1559	26.7444	1356	2686	1604	9.72	33052	195.147
4	2857	0.9413	2686	4931	3511	18.49	256	6.868
5	5519	0.0905	4931	5815	5315	5.36	65	0.660
6	7334	0.3348	5815	11586	8049	18.75	66	2.443
7	20000 (UM)	0.1590	18537	27738	20416	3.88	1701	13.925

TIC:	28.2951	ng/uL
TIM:	30.0565	nmole/L
Total concentration:	28.6363	ng/uL

Sample peak width (sec): 5   Sample min peak height: 50   Sample baseline V to V?: Y   Sample baseline V to V points: 3
Sample filter: Binomial   Number of points for filter: 3 Sample start region (min): 0 Sample end region (min): 30
Marker peak width (sec): 5   Marker min peak height: 200   Marker baseline V to V?: Y   Marker baseline V to V points: 3
Lower marker selection: First peak > 500 RFU   Upper marker selection: Last peak > 200 RFU
Ladder size (bp)75, 200, 300, 400, 500, 700, 1000, 1500, 2000, 3000, 4000, 5000, 7000, 10000, 20000
Quantification using: Lower Marker   Final concentration (ng/uL): 0.5000   Dilution factor: 12.0

Sample: SampC4
Well location: C4
Created: Monday, September 20, 2021 6:00:03 PM

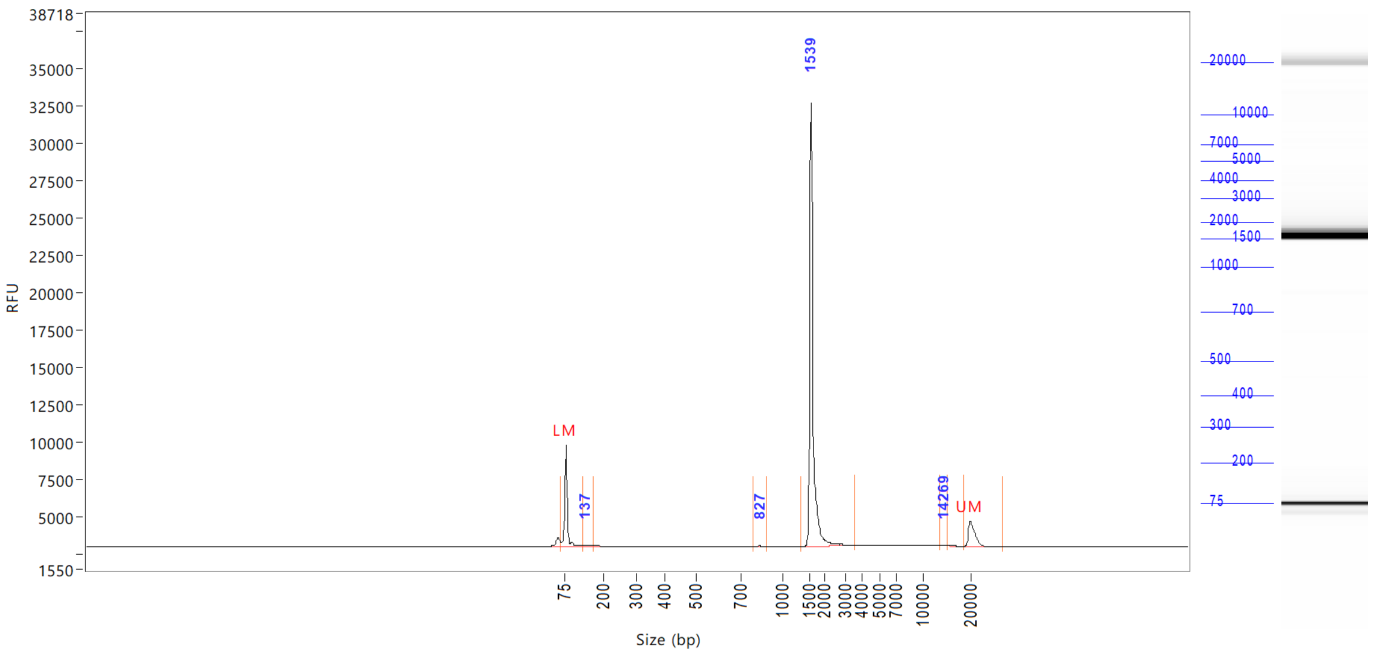

Peak	Size (bp)	Concentration (ng/uL)	From (bp)	To (bp)	Average size (bp)	CV%	RFU	Corrected peak area
1	75 (LM)	0.5000	56	129	74	13.10	6777	43.888
2	137	0.1715	129	166	145	7.01	81	1.255
3	827	0.0547	788	883	824	1.68	71	0.400
4	1539	22.3001	1345	3500	1594	11.79	29687	163.119
5	14269	0.0356	13537	15122	14166	2.56	50	0.260
6	20000 (UM)	0.1574	18415	26649	20335	3.78	1671	13.818

TIC:	22.5620	ng/uL
TIM:	25.0866	nmole/L
Total concentration:	23.1234	ng/uL

Sample peak width (sec): 5 Sample min peak height: 50 Sample baseline V to V?: Y Sample baseline V to V points: 3
Sample filter: Binomial Number of points for filter: 3 Sample start region (min): 0 Sample end region (min): 30
Marker peak width (sec): 5 Marker min peak height: 200 Marker baseline V to V?: Y Marker baseline V to V points: 3
Lower marker selection: First peak > 500 RFU Upper marker selection: Last peak > 200 RFU
Ladder size (bp)75, 200, 300, 400, 500, 700, 1000, 1500, 2000, 3000, 4000, 5000, 7000, 10000, 20000
Quantification using: Lower Marker Final concentration (ng/uL): 0.5000 Dilution factor: 12.0

Sample: SampD4
Well location: D4
Created: Monday, September 20, 2021 6:00:03 PM

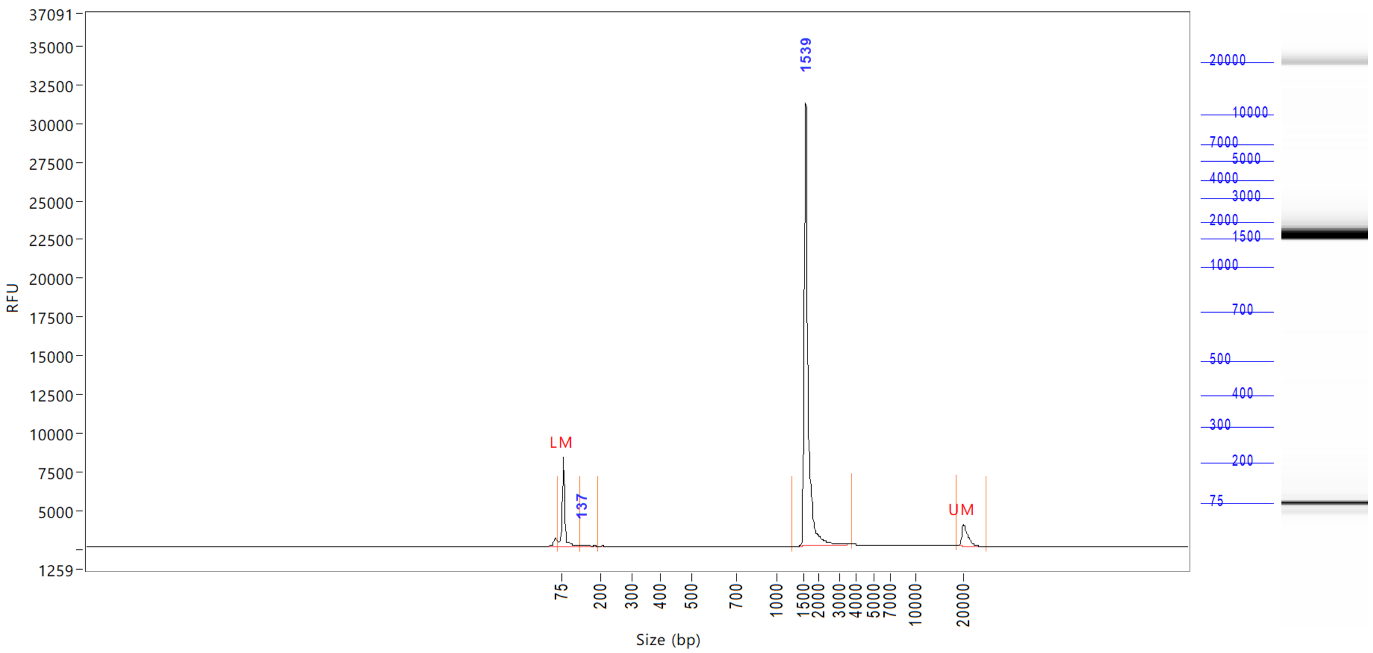

Peak	Size (bp)	Concentration (ng/uL)	From (bp)	To (bp)	Average size (bp)	CV%	RFU	Corrected peak area
1	75 (LM)	0.5000	58	131	76	14.74	5821	39.706
2	137	0.3427	131	192	155	11.07	105	2.268
3	1539	26.3772	1278	3715	1606	11.20	28600	174.557
4	20000 (UM)	0.1410	18537	24957	20437	3.57	1356	11.198

TIC:	26.7199	ng/uL
TIM:	30.6699	nmole/L
Total concentration:	27.1972	ng/uL

```
Sample peak width (sec): 5    Sample min peak height: 50    Sample baseline V to V?: Y    Sample baseline V to V points: 3
Sample filter: Binomial       Number of points for filter: 3  Sample start region (min): 0  Sample end region (min): 30
Marker peak width (sec): 5    Marker min peak height: 200   Marker baseline V to V?: Y    Marker baseline V to V points: 3
Lower marker selection: First peak > 500 RFU                Upper marker selection: Last peak > 200 RFU
Ladder size (bp)75, 200, 300, 400, 500, 700, 1000, 1500, 2000, 3000, 4000, 5000, 7000, 10000, 20000
Quantification using: Lower Marker          Final concentration (ng/uL): 0.5000          Dilution factor: 12.0
```

Sample: SampE4
Well location: E4
Created: Monday, September 20, 2021 6:00:03 PM

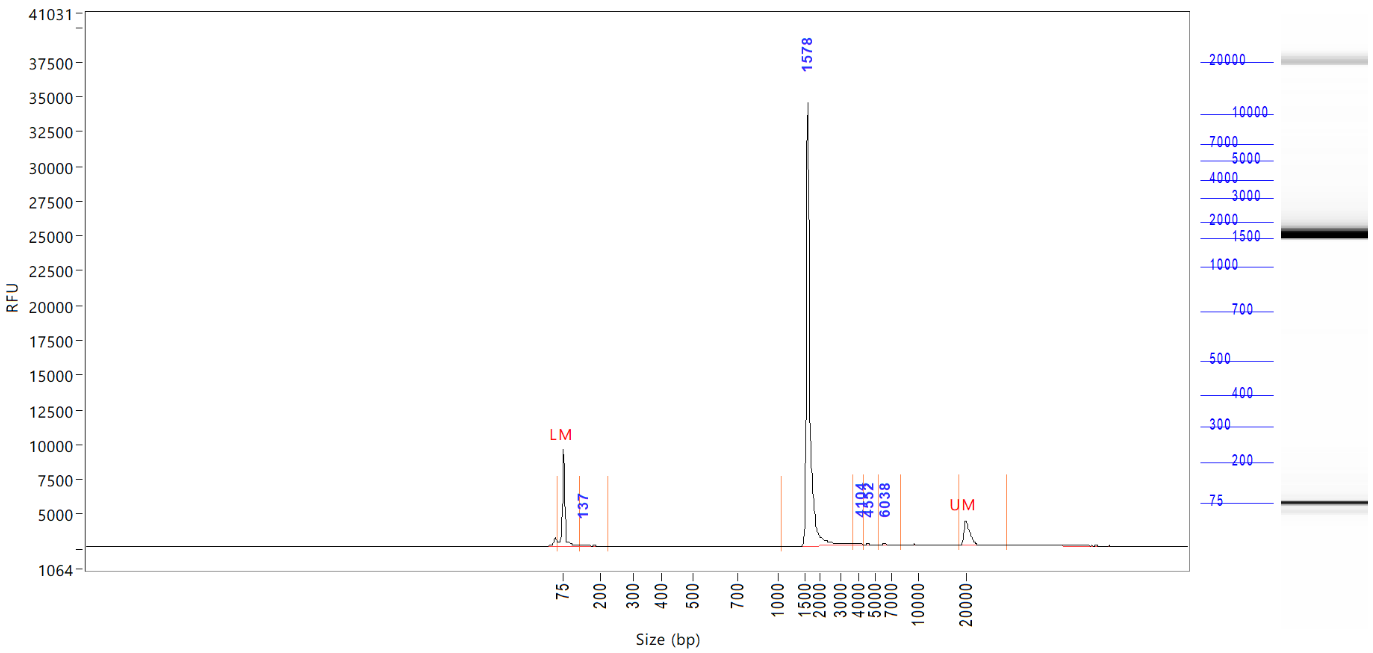

Peak	Size<br>(bp)	Concentration<br>(ng/uL)	From<br>(bp)	To<br>(bp)	Average size<br>(bp)	CV%	RFU	Corrected peak area
1	75 (LM)	0.5000	56	131	76	14.90	6932	45.913
2	137	0.3841	131	223	163	15.08	113	2.940
3	1578	25.0083	1056	3679	1632	14.19	31919	191.368
4	4104	0.1383	3679	4311	3979	4.53	76	1.058
5	4552	0.1117	4311	5371	4705	5.85	58	0.855
6	6038	0.1097	5371	8001	6418	10.45	63	0.839
7	20000 (UM)	0.1510	18537	28584	20427	3.76	1713	13.865

TIC:	25.7521	ng/uL
TIM:	29.2226	nmole/L
Total concentration:	25.9963	ng/uL

Sample peak width (sec): 5 Sample min peak height: 50 Sample baseline V to V?: Y Sample baseline V to V points: 3
Sample filter: Binomial Number of points for filter: 3 Sample start region (min): 0 Sample end region (min): 30
Marker peak width (sec): 5 Marker min peak height: 200 Marker baseline V to V?: Y Marker baseline V to V points: 3
Lower marker selection: First peak > 500 RFU Upper marker selection: Last peak > 200 RFU
Ladder size (bp)75, 200, 300, 400, 500, 700, 1000, 1500, 2000, 3000, 4000, 5000, 7000, 10000, 20000
Quantification using: Lower Marker Final concentration (ng/uL): 0.5000 Dilution factor: 12.0

Sample: SampF4
Well location: F4
Created: Monday, September 20, 2021 6:00:03 PM

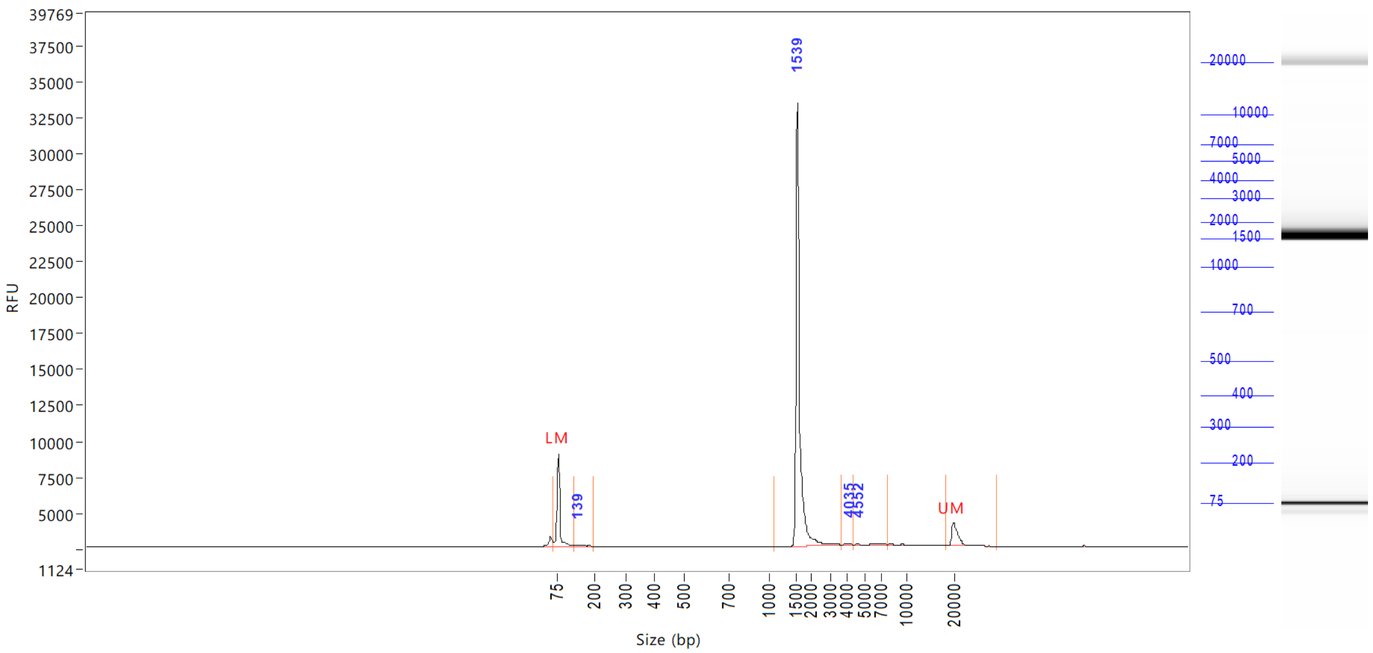

Peak	Size (bp)	Concentration (ng/uL)	From (bp)	To (bp)	Average size (bp)	CV%	RFU	Corrected peak area
1	75 (LM)	0.5000	58	129	76	14.60	6475	44.119
2	139	0.3742	129	196	155	12.08	119	2.752
3	1539	25.2465	1079	3643	1611	14.45	30861	185.643
4	4035	0.1401	3643	4345	3980	5.00	72	1.031
5	4552	0.2447	4345	7734	5688	17.98	58	1.800
6	20000 (UM)	0.1503	18537	29309	20415	4.07	1598	13.258

TIC:	26.0056	ng/uL
TIM:	29.8936	nmole/L
Total concentration:	26.3337	ng/uL

Sample peak width (sec): 5   Sample min peak height: 50   Sample baseline V to V?: Y   Sample baseline V to V points: 3
Sample filter: Binomial   Number of points for filter: 3   Sample start region (min): 0   Sample end region (min): 30
Marker peak width (sec): 5   Marker min peak height: 200   Marker baseline V to V?: Y   Marker baseline V to V points: 3
Lower marker selection: First peak > 500 RFU   Upper marker selection: Last peak > 200 RFU
Ladder size (bp)75, 200, 300, 400, 500, 700, 1000, 1500, 2000, 3000, 4000, 5000, 7000, 10000, 20000
Quantification using: Lower Marker   Final concentration (ng/uL): 0.5000   Dilution factor: 12.0

Sample: SampG4
Well location: G4
Created: Monday, September 20, 2021 6:00:03 PM

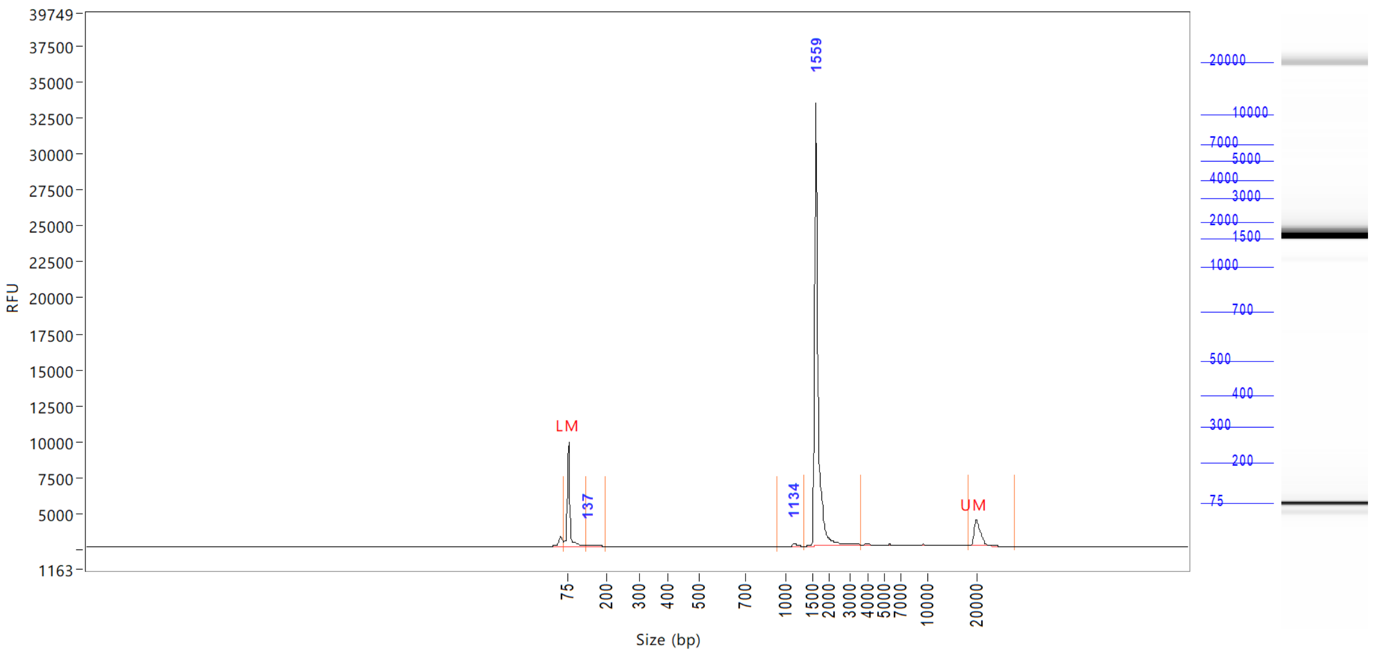

Peak	Size (bp)	Concentration (ng/uL)	From (bp)	To (bp)	Average size (bp)	CV%	RFU	Corrected peak area
1	75 (LM)	0.5000	60	129	76	14.07	7269	47.195
2	137	0.3414	129	194	154	11.73	124	2.685
3	1134	0.1535	935	1311	1126	5.20	193	1.207
4	1559	22.1791	1311	3679	1624	13.41	30803	174.456
5	20000 (UM)	0.1607	18293	27979	20349	3.98	1846	15.166

TIC:	22.6739	ng/uL
TIM:	26.3487	nmole/L
Total concentration:	23.2197	ng/uL

Sample peak width (sec): 5 Sample min peak height: 50 Sample baseline V to V?: Y Sample baseline V to V points: 3
Sample filter: Binomial Number of points for filter: 3 Sample start region (min): 0 Sample end region (min): 30
Marker peak width (sec): 5 Marker min peak height: 200 Marker baseline V to V?: Y Marker baseline V to V points: 3
Lower marker selection: First peak > 500 RFU Upper marker selection: Last peak > 200 RFU
Ladder size (bp)75, 200, 300, 400, 500, 700, 1000, 1500, 2000, 3000, 4000, 5000, 7000, 10000, 20000
Quantification using: Lower Marker Final concentration (ng/uL): 0.5000 Dilution factor: 12.0

Sample: SampH4
Well location: H4
Created: Monday, September 20, 2021 6:00:03 PM

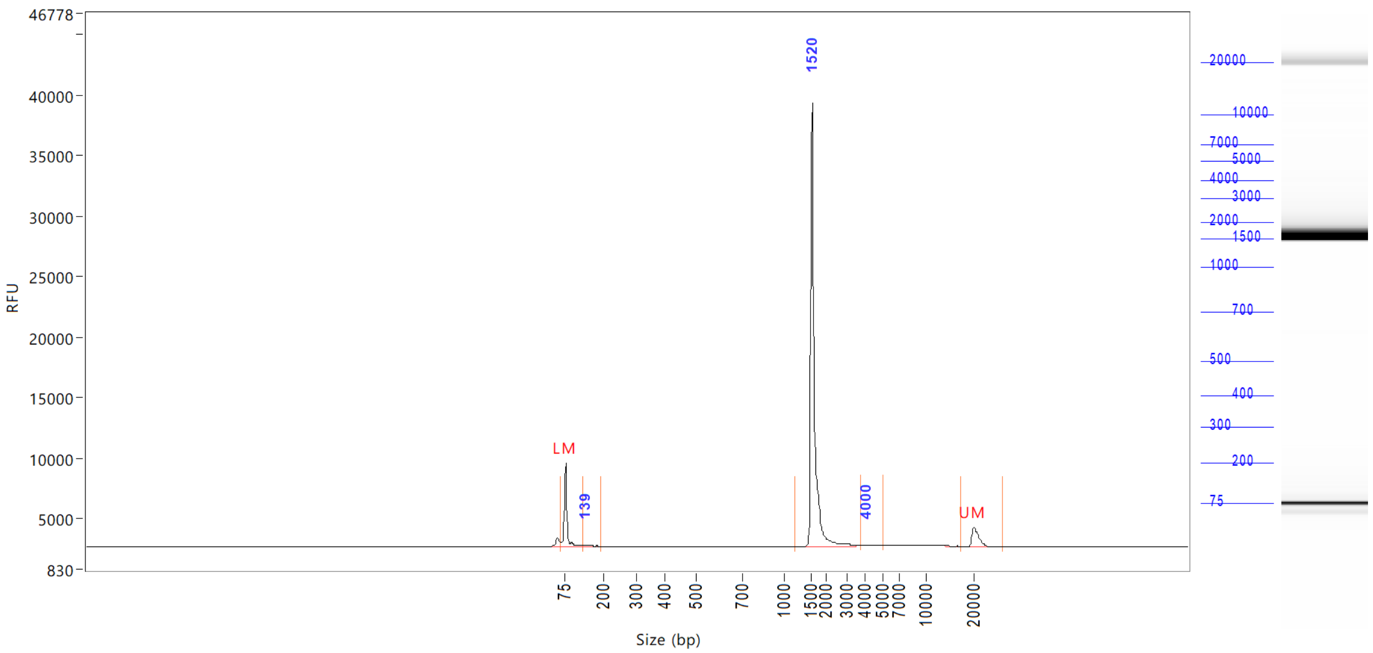

Peak	Size (bp)	Concentration (ng/uL)	From (bp)	To (bp)	Average size (bp)	CV%	RFU	Corrected peak area
1	75 (LM)	0.5000	60	129	76	14.36	6894	46.108
2	139	0.3696	129	192	154	11.45	125	2.840
3	1520	28.8402	1212	3822	1600	14.93	36711	221.629
4	4000	0.1879	3822	5075	4292	7.99	85	1.444
5	20000 (UM)	0.1483	17317	25924	20478	3.89	1582	13.672

TIC:	29.3977	ng/uL
TIM:	33.6873	nmole/L
Total concentration:	29.8596	ng/uL

```
Sample peak width (sec): 5    Sample min peak height: 50    Sample baseline V to V?: Y    Sample baseline V to V points: 3
Sample filter: Binomial       Number of points for filter: 3  Sample start region (min): 0  Sample end region (min): 30
Marker peak width (sec): 5    Marker min peak height: 200   Marker baseline V to V?: Y    Marker baseline V to V points: 3
Lower marker selection: First peak > 500 RFU                Upper marker selection: Last peak > 200 RFU
Ladder size (bp)75, 200, 300, 400, 500, 700, 1000, 1500, 2000, 3000, 4000, 5000, 7000, 10000, 20000
Quantification using: Lower Marker          Final concentration (ng/uL): 0.5000          Dilution factor: 12.0
```

Sample: SampA5
Well location: A5
Created: Monday, September 20, 2021 6:00:03 PM

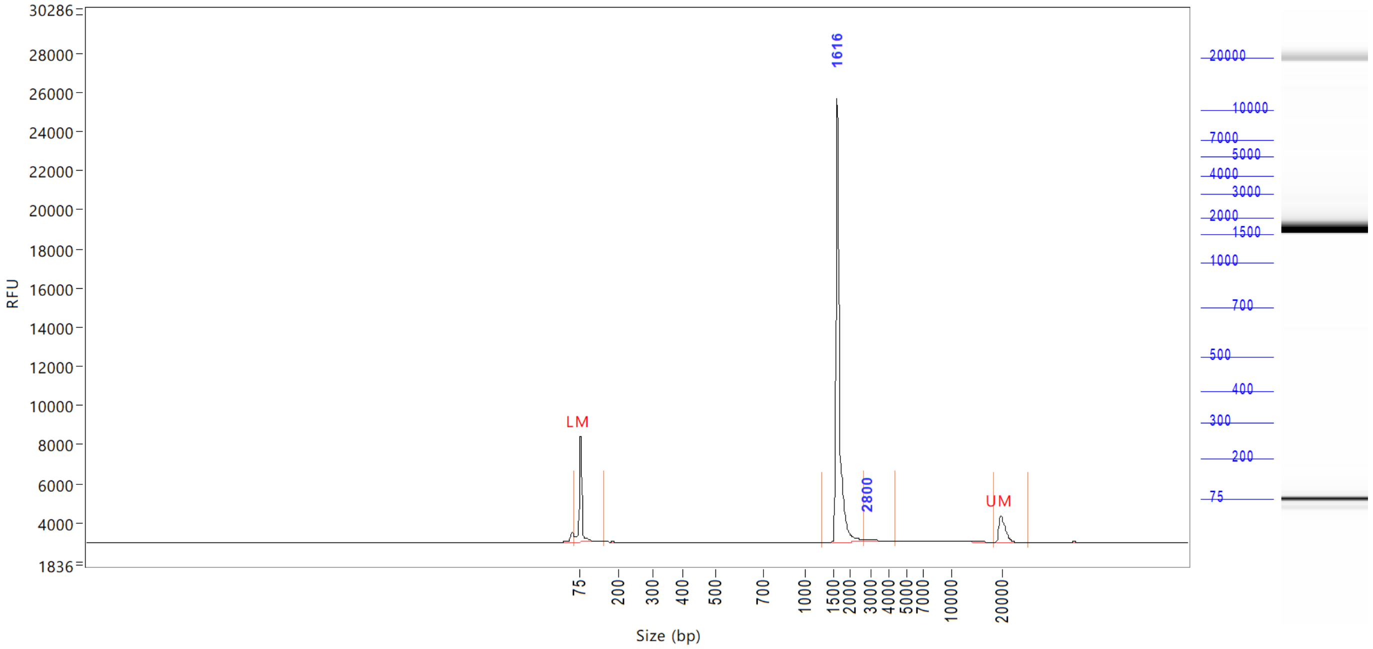

Peak	Size (bp)	Concentration (ng/uL)	From (bp)	To (bp)	Average size (bp)	CV%	RFU	Corrected peak area
1	75 (LM)	0.5000	58	150	75	11.77	5382	34.992
2	1616	21.7659	1289	2629	1654	8.05	22721	126.940
3	2800	0.4781	2629	4380	3202	14.42	136	2.788
4	20000 (UM)	0.1545	18537	25561	20274	3.49	1351	10.809

TIC:	22.2440	ng/uL
TIM:	21.9078	nmole/L
Total concentration:	22.7818	ng/uL

```
Sample peak width (sec): 5    Sample min peak height: 50    Sample baseline V to V?: Y    Sample baseline V to V points: 3
Sample filter: Binomial       Number of points for filter: 3  Sample start region (min): 0  Sample end region (min): 30
Marker peak width (sec): 5    Marker min peak height: 200   Marker baseline V to V?: Y    Marker baseline V to V points: 3
Lower marker selection: First peak > 500 RFU                Upper marker selection: Last peak > 200 RFU
Ladder size (bp)75, 200, 300, 400, 500, 700, 1000, 1500, 2000, 3000, 4000, 5000, 7000, 10000, 20000
Quantification using: Lower Marker          Final concentration (ng/uL): 0.5000          Dilution factor: 12.0
```

Sample: SampB5
Well location: B5
Created: Monday, September 20, 2021 6:00:03 PM

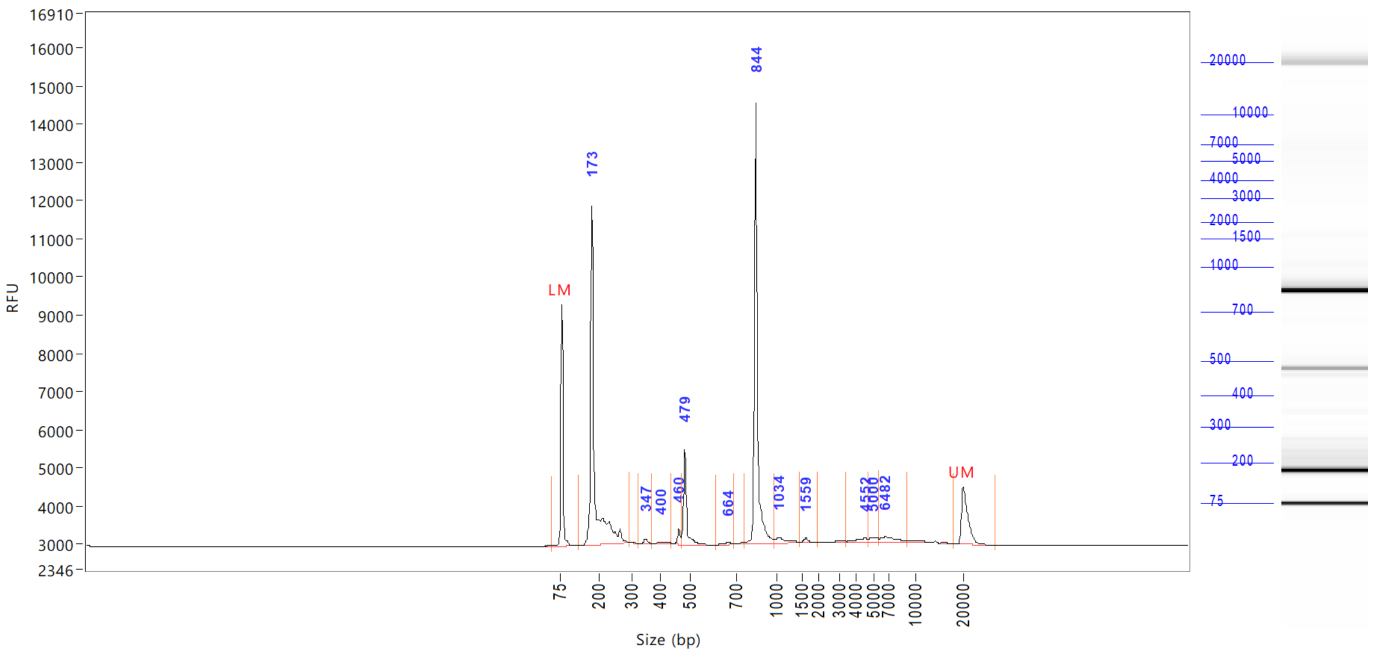

Peak	Size (bp)	Concentration (ng/uL)	From (bp)	To (bp)	Average size (bp)	CV%	RFU	Corrected peak area
1	75 (LM)	0.5000	45	133	73	7.36	6314	36.038
2	173	13.4697	133	293	186	13.89	8873	80.904
3	347	0.2030	323	372	347	2.28	146	1.219
4	400	0.2505	372	437	406	4.02	69	1.505
5	460	0.4164	437	468	457	1.44	420	2.501
6	479	2.5759	468	611	482	2.91	2506	15.472
7	664	0.1120	611	687	657	2.32	69	0.673
8	844	10.4118	757	987	848	3.20	11567	62.537
9	1034	0.4641	987	1456	1129	11.20	158	2.787
10	1559	0.1597	1456	1962	1590	5.90	124	0.959
11	4552	0.2414	3393	4690	4237	7.65	97	1.450
12	5000	0.2394	4690	5741	5105	5.99	110	1.438
13	6482	0.4308	5741	9000	6981	11.27	135	2.587
14	20000 (UM)	0.1801	18049	26770	20453	4.21	1493	12.980

TIC:	28.9748	ng/uL
TIM:	152.9331	nmole/L
Total concentration:	29.1823	ng/uL

```
Sample peak width (sec): 5     Sample min peak height: 50    Sample baseline V to V?: Y    Sample baseline V to V points: 3
Sample filter: Binomial        Number of points for filter: 3  Sample start region (min): 0  Sample end region (min): 30
Marker peak width (sec): 5     Marker min peak height: 200   Marker baseline V to V?: Y    Marker baseline V to V points: 3
Lower marker selection: First peak > 500 RFU                 Upper marker selection: Last peak > 200 RFU
Ladder size (bp)75, 200, 300, 400, 500, 700, 1000, 1500, 2000, 3000, 4000, 5000, 7000, 10000, 20000
Quantification using: Lower Marker          Final concentration (ng/uL): 0.5000         Dilution factor: 12.0
```

Sample: SampC5
Well location: C5
Created: Monday, September 20, 2021 6:00:03 PM

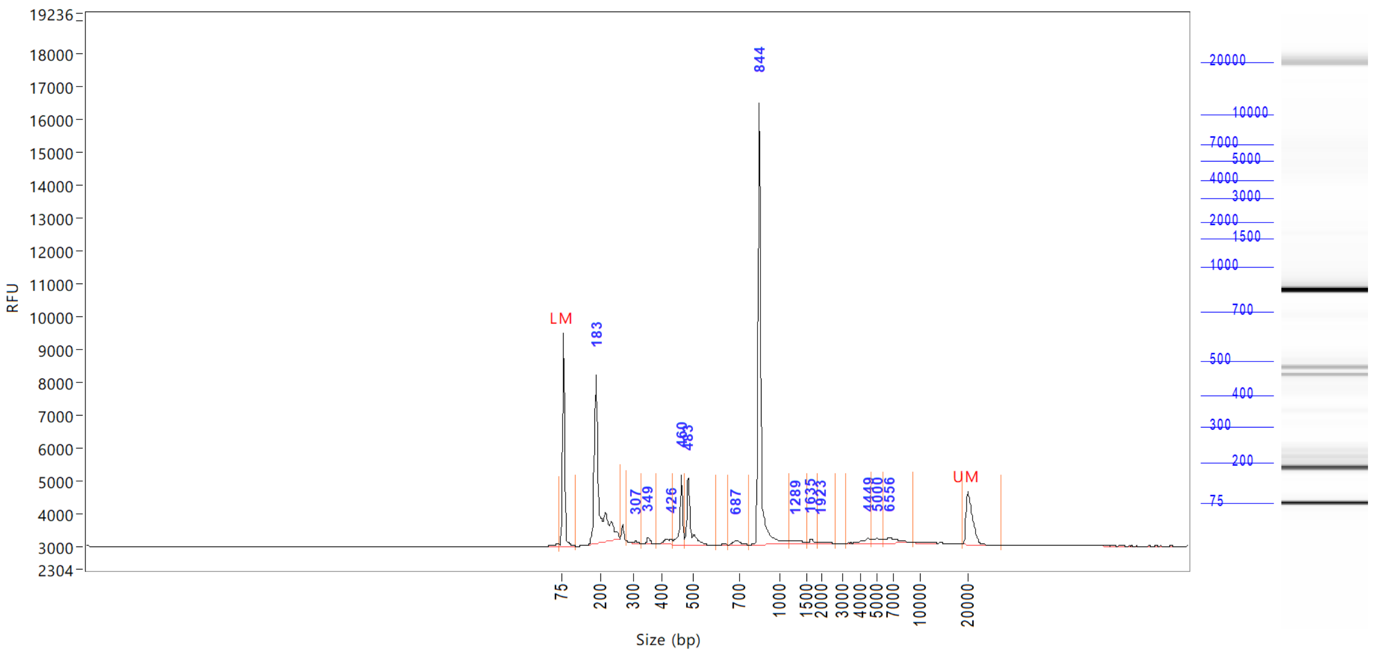

Peak	Size (bp)	Concentration (ng/uL)	From (bp)	To (bp)	Average size (bp)	CV%	RFU	Corrected peak area
1	75 (LM)	0.5000	64	118	74	5.92	6498	36.894
2	183	9.6746	118	260	192	10.18	5112	59.489
3	307	0.0955	278	325	306	2.18	61	0.587
4	349	0.2448	325	376	349	1.68	209	1.505
5	426	0.4874	376	433	414	2.92	174	2.997
6	460	1.9317	433	470	456	1.56	2134	11.878
7	483	2.3787	470	598	488	3.22	2054	14.627
8	687	0.2798	654	770	690	2.65	132	1.720
9	844	11.6088	770	1167	856	4.97	13475	71.383
10	1289	0.3416	1167	1501	1315	7.20	97	2.101
11	1635	0.1644	1501	1827	1647	5.02	133	1.011
12	1923	0.1279	1827	2629	2051	8.68	61	0.787
13	4449	0.2928	3179	4621	4237	6.98	144	1.801
14	5000	0.3372	4621	5667	5032	6.02	141	2.073
15	6556	0.5453	5667	9134	6947	11.89	160	3.353
16	20000 (UM)	0.1881	18659	26891	20397	4.13	1600	13.879

TIC:	28.5105	ng/uL
TIM:	125.4768	nmole/L
Total concentration:	28.5850	ng/uL

Sample peak width (sec): 5   Sample min peak height: 50   Sample baseline V to V?: Y   Sample baseline V to V points: 3
Sample filter: Binomial   Number of points for filter: 3 Sample start region (min): 0 Sample end region (min): 30
Marker peak width (sec): 5   Marker min peak height: 200   Marker baseline V to V?: Y   Marker baseline V to V points: 3
Lower marker selection: First peak > 500 RFU   Upper marker selection: Last peak > 200 RFU
Ladder size (bp)75, 200, 300, 400, 500, 700, 1000, 1500, 2000, 3000, 4000, 5000, 7000, 10000, 20000
Quantification using: Lower Marker   Final concentration (ng/uL): 0.5000   Dilution factor: 12.0

Sample: SampD5
Well location: D5
Created: Monday, September 20, 2021 6:00:03 PM

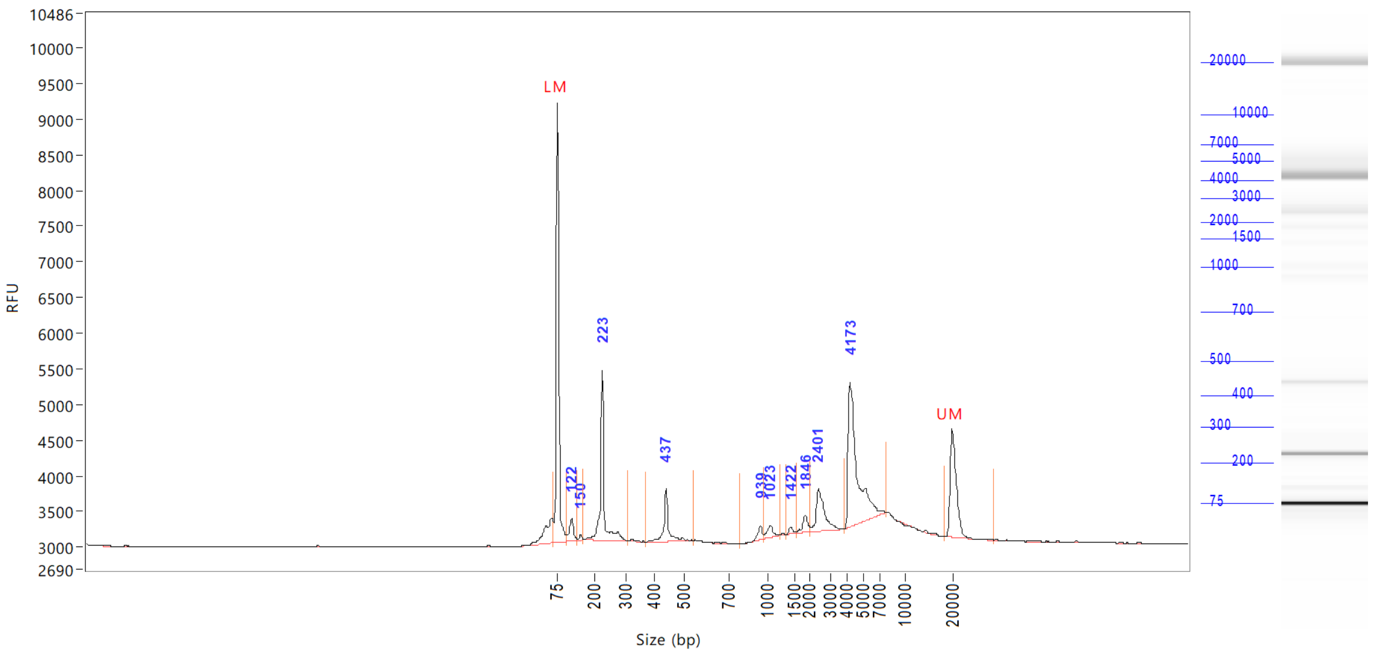

Peak	Size (bp)	Concentration (ng/uL)	From (bp)	To (bp)	Average size (bp)	CV%	RFU	Corrected peak area
1	75 (LM)	0.5000	62	105	74	8.03	6171	38.229
2	122	0.4639	105	141	119	6.21	307	2.955
3	150	0.0787	141	164	149	2.94	75	0.501
4	223	2.9917	164	305	225	8.35	2389	19.062
5	437	1.0830	368	542	438	5.20	740	6.900
6	939	0.2405	779	961	925	2.36	200	1.532
7	1023	0.2908	961	1201	1025	5.00	166	1.853
8	1422	0.0989	1323	1539	1428	2.84	98	0.630
9	1846	0.3290	1539	2030	1826	5.83	241	2.096
10	2401	1.1969	2030	3822	2544	10.67	595	7.626
11	4173	4.4314	3822	7734	4572	13.50	2025	28.235
12	20000 (UM)	0.1644	18415	28947	20318	3.90	1530	12.573

TIC:	11.2047	ng/uL
TIM:	36.8808	nmole/L
Total concentration:	11.3353	ng/uL

```
Sample peak width (sec): 5     Sample min peak height: 50     Sample baseline V to V?: Y     Sample baseline V to V points: 3
Sample filter: Binomial        Number of points for filter: 3  Sample start region (min): 0  Sample end region (min): 30
Marker peak width (sec): 5     Marker min peak height: 200    Marker baseline V to V?: Y     Marker baseline V to V points: 3
Lower marker selection: First peak > 500 RFU                  Upper marker selection: Last peak > 200 RFU
Ladder size (bp)75, 200, 300, 400, 500, 700, 1000, 1500, 2000, 3000, 4000, 5000, 7000, 10000, 20000
Quantification using: Lower Marker          Final concentration (ng/uL): 0.5000          Dilution factor: 12.0
```

Sample: SampE5
Well location: E5
Created: Monday, September 20, 2021 6:00:03 PM

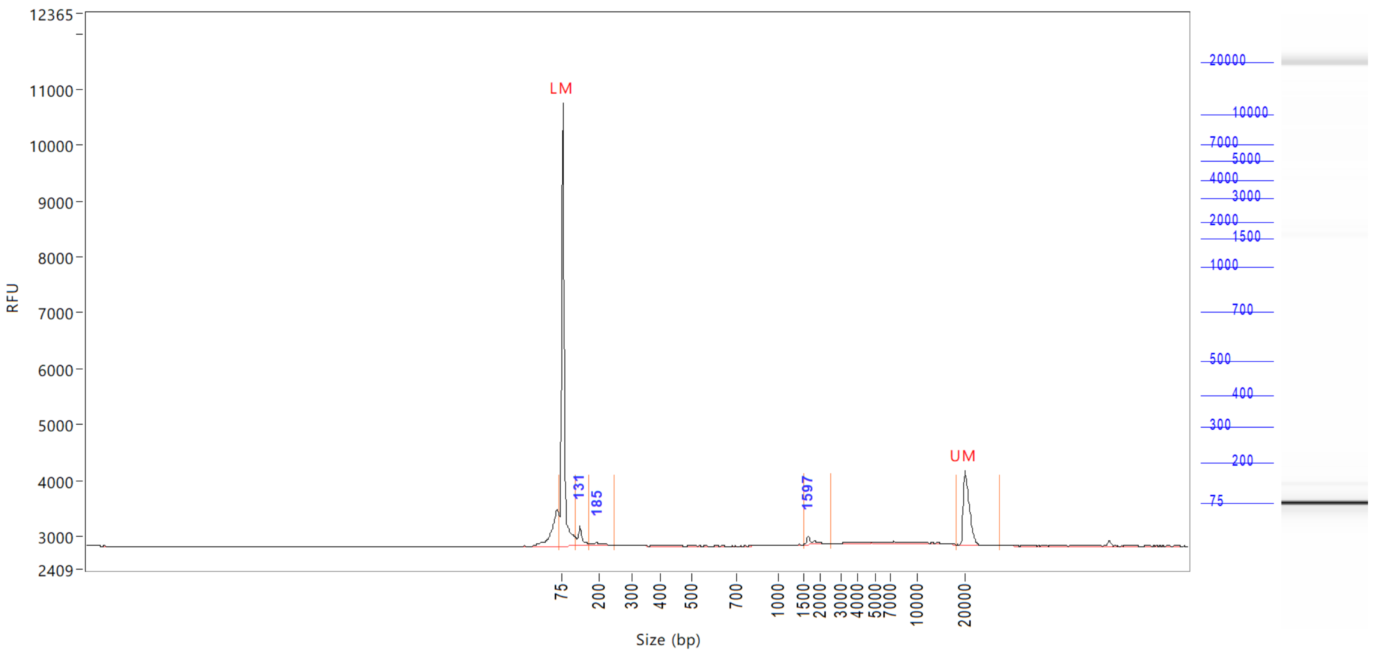

Peak	Size (bp)	Concentration (ng/uL)	From (bp)	To (bp)	Average size (bp)	CV%	RFU	Corrected peak area
1	75 (LM)	0.5000	66	120	76	11.68	7949	54.022
2	131	0.4040	120	166	135	8.26	353	3.638
3	185	0.1836	166	242	193	8.84	54	1.653
4	1597	0.1668	1489	2486	1748	11.98	150	1.502
5	20000 (UM)	0.0999	18171	27375	20360	3.66	1329	10.799

TIC:	0.7544	ng/uL
TIM:	6.6389	nmole/L
Total concentration:	1.1823	ng/uL

Sample peak width (sec): 5 Sample min peak height: 50 Sample baseline V to V?: Y Sample baseline V to V points: 3
Sample filter: Binomial Number of points for filter: 3 Sample start region (min): 0 Sample end region (min): 30
Marker peak width (sec): 5 Marker min peak height: 200 Marker baseline V to V?: Y Marker baseline V to V points: 3
Lower marker selection: First peak > 500 RFU Upper marker selection: Last peak > 200 RFU
Ladder size (bp)75, 200, 300, 400, 500, 700, 1000, 1500, 2000, 3000, 4000, 5000, 7000, 10000, 20000
Quantification using: Lower Marker Final concentration (ng/uL): 0.5000 Dilution factor: 12.0

Sample: SampF5
Well location: F5
Created: Monday, September 20, 2021 6:00:03 PM

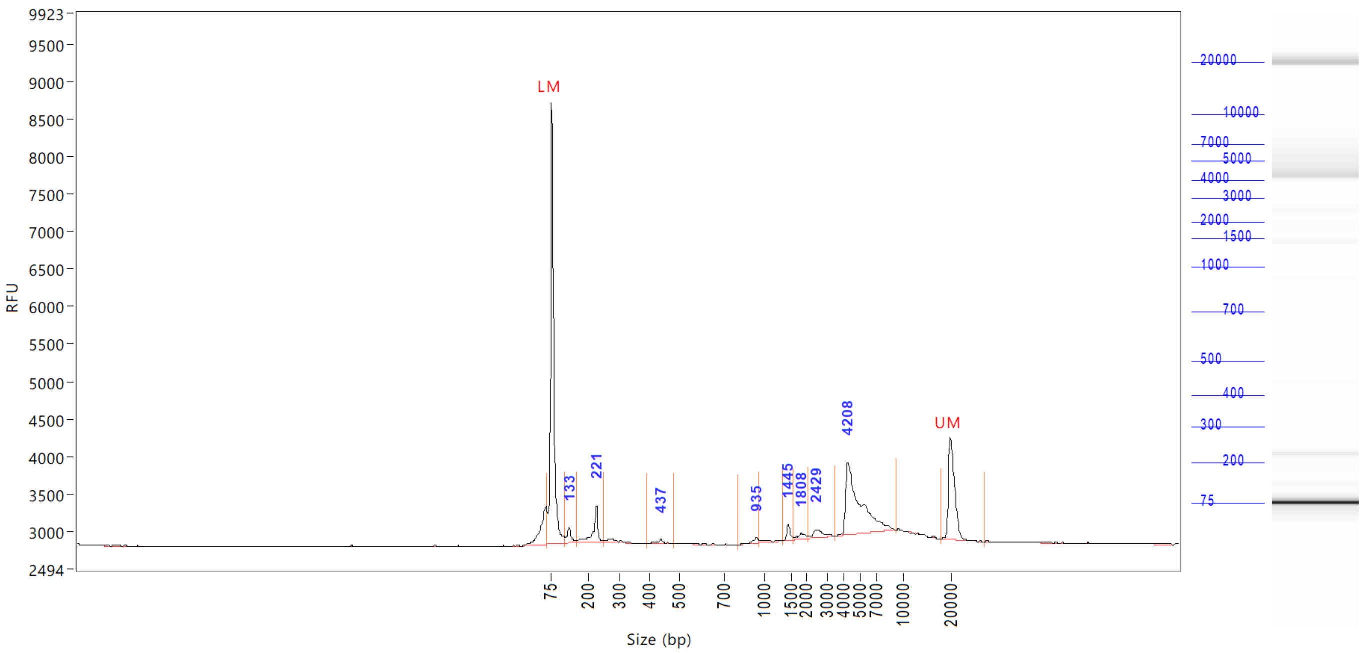

Peak	Size (bp)	Concentration (ng/uL)	From (bp)	To (bp)	Average size (bp)	CV%	RFU	Corrected peak area
1	75 (LM)	0.5000	64	122	77	11.23	5906	45.950
2	133	0.2697	122	160	134	6.64	199	2.065
3	221	0.6363	160	246	211	8.33	482	4.873
4	437	0.1122	392	481	434	4.46	60	0.859
5	935	0.0843	801	961	918	3.03	64	0.645
6	1445	0.1650	1345	1578	1447	2.91	213	1.264
7	1808	0.1246	1578	2030	1810	6.68	78	0.954
8	2429	0.2278	2030	3465	2546	10.92	110	1.745
9	4208	2.6545	3465	9334	5046	20.69	969	20.329
10	20000 (UM)	0.1217	17927	27133	20361	3.88	1362	11.181

TIC:	4.2744	ng/uL
TIM:	10.1566	nmole/L
Total concentration:	4.5583	ng/uL

```
Sample peak width (sec): 5    Sample min peak height: 50    Sample baseline V to V?: Y    Sample baseline V to V points: 3
Sample filter: Binomial       Number of points for filter: 3  Sample start region (min): 0  Sample end region (min): 30
Marker peak width (sec): 5    Marker min peak height: 200   Marker baseline V to V?: Y    Marker baseline V to V points: 3
Lower marker selection: First peak > 500 RFU                Upper marker selection: Last peak > 200 RFU
Ladder size (bp)75, 200, 300, 400, 500, 700, 1000, 1500, 2000, 3000, 4000, 5000, 7000, 10000, 20000
Quantification using: Lower Marker          Final concentration (ng/uL): 0.5000          Dilution factor: 12.0
```

Sample: SampG5
Well location: G5
Created: Monday, September 20, 2021 6:00:03 PM

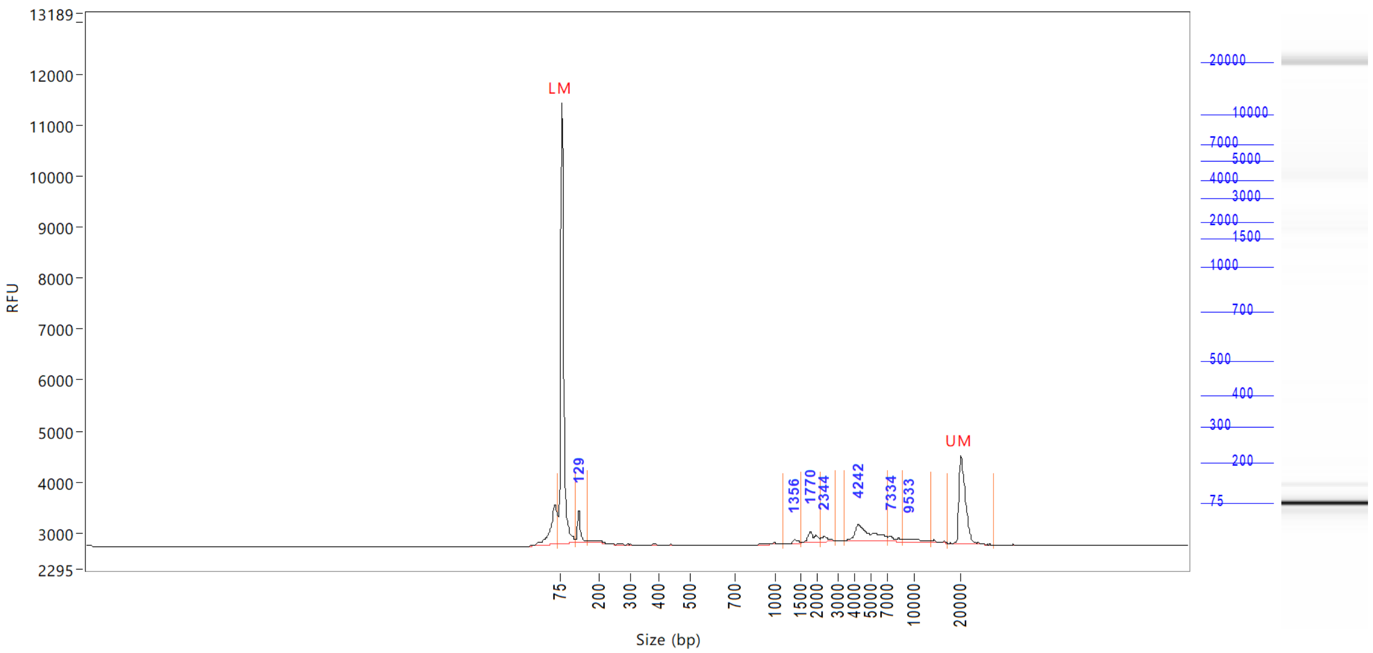

Peak	Size (bp)	Concentration (ng/uL)	From (bp)	To (bp)	Average size (bp)	CV%	RFU	Corrected peak area
1	75 (LM)	0.5000	64	120	75	10.08	8649	66.306
2	129	0.3995	120	162	130	5.07	630	4.415
3	1356	0.0533	1134	1500	1376	3.38	76	0.589
4	1770	0.2328	1500	2115	1826	7.46	219	2.573
5	2344	0.1051	2115	2886	2377	7.31	102	1.162
6	4242	0.7320	3358	7001	4870	17.70	316	8.090
7	7334	0.1106	7001	8734	7714	6.53	100	1.222
8	9533	0.1219	8734	13537	10477	12.35	54	1.347
9	20000 (UM)	0.1097	17195	27012	20426	3.97	1717	14.549

TIC:	1.7553	ng/uL
TIM:	5.6860	nmole/L
Total concentration:	2.0355	ng/uL

```
Sample peak width (sec): 5     Sample min peak height: 50    Sample baseline V to V?: Y    Sample baseline V to V points: 3
Sample filter: Binomial        Number of points for filter: 3  Sample start region (min): 0  Sample end region (min): 30
Marker peak width (sec): 5     Marker min peak height: 200   Marker baseline V to V?: Y    Marker baseline V to V points: 3
Lower marker selection: First peak > 500 RFU                 Upper marker selection: Last peak > 200 RFU
Ladder size (bp)75, 200, 300, 400, 500, 700, 1000, 1500, 2000, 3000, 4000, 5000, 7000, 10000, 20000
Quantification using: Lower Marker          Final concentration (ng/uL): 0.5000          Dilution factor: 12.0
```

Sample: SampH5
Well location: H5
Created: Monday, September 20, 2021 6:00:03 PM

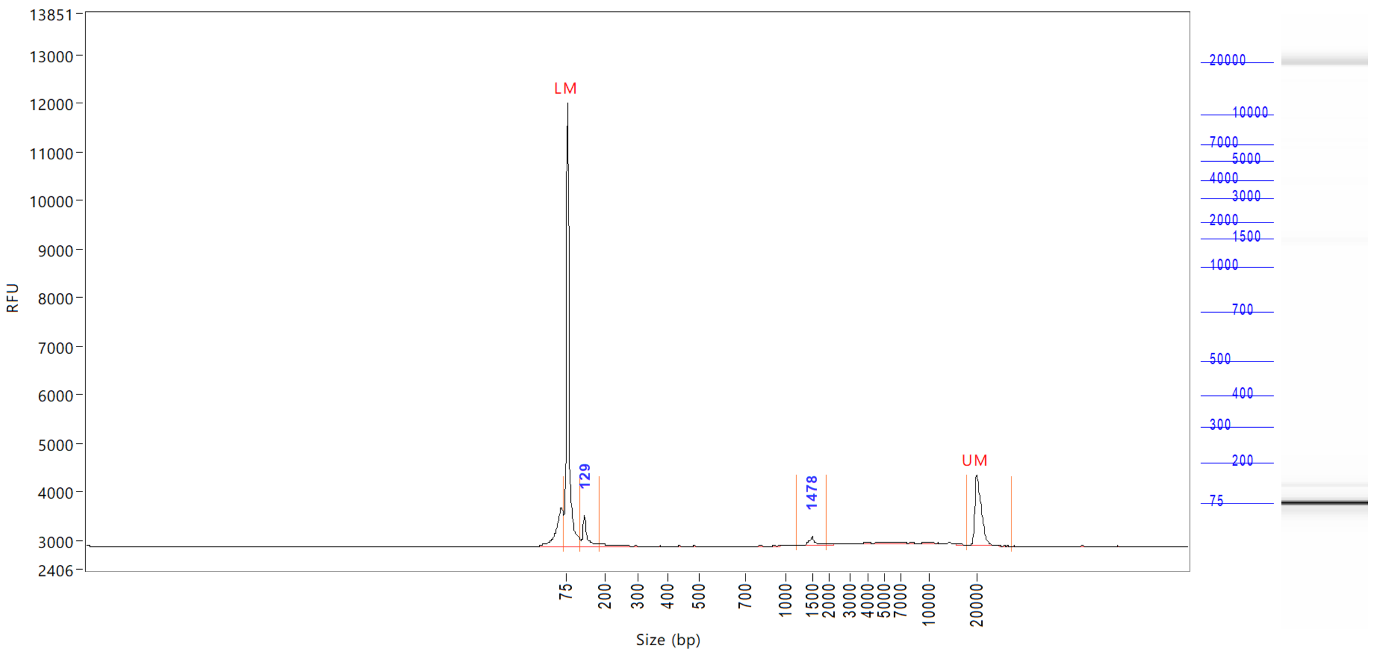

Peak	Size (bp)	Concentration (ng/uL)	From (bp)	To (bp)	Average size (bp)	CV%	RFU	Corrected peak area
1	75 (LM)	0.5000	64	118	76	11.06	9142	64.991
2	129	0.5522	118	177	135	10.10	627	5.982
3	1478	0.1504	1190	1904	1492	7.51	171	1.629
4	20000 (UM)	0.0945	17927	27133	20393	3.93	1437	12.280

TIC:	0.7026	ng/uL
TIM:	6.8874	nmole/L
Total concentration:	1.0765	ng/uL

Sample peak width (sec): 5 Sample min peak height: 50 Sample baseline V to V?: Y Sample baseline V to V points: 3
Sample filter: Binomial Number of points for filter: 3 Sample start region (min): 0 Sample end region (min): 30
Marker peak width (sec): 5 Marker min peak height: 200 Marker baseline V to V?: Y Marker baseline V to V points: 3
Lower marker selection: First peak > 500 RFU Upper marker selection: Last peak > 200 RFU
Ladder size (bp)75, 200, 300, 400, 500, 700, 1000, 1500, 2000, 3000, 4000, 5000, 7000, 10000, 20000
Quantification using: Lower Marker Final concentration (ng/uL): 0.5000 Dilution factor: 12.0

Sample: SampA6
Well location: A6
Created: Monday, September 20, 2021 6:00:03 PM

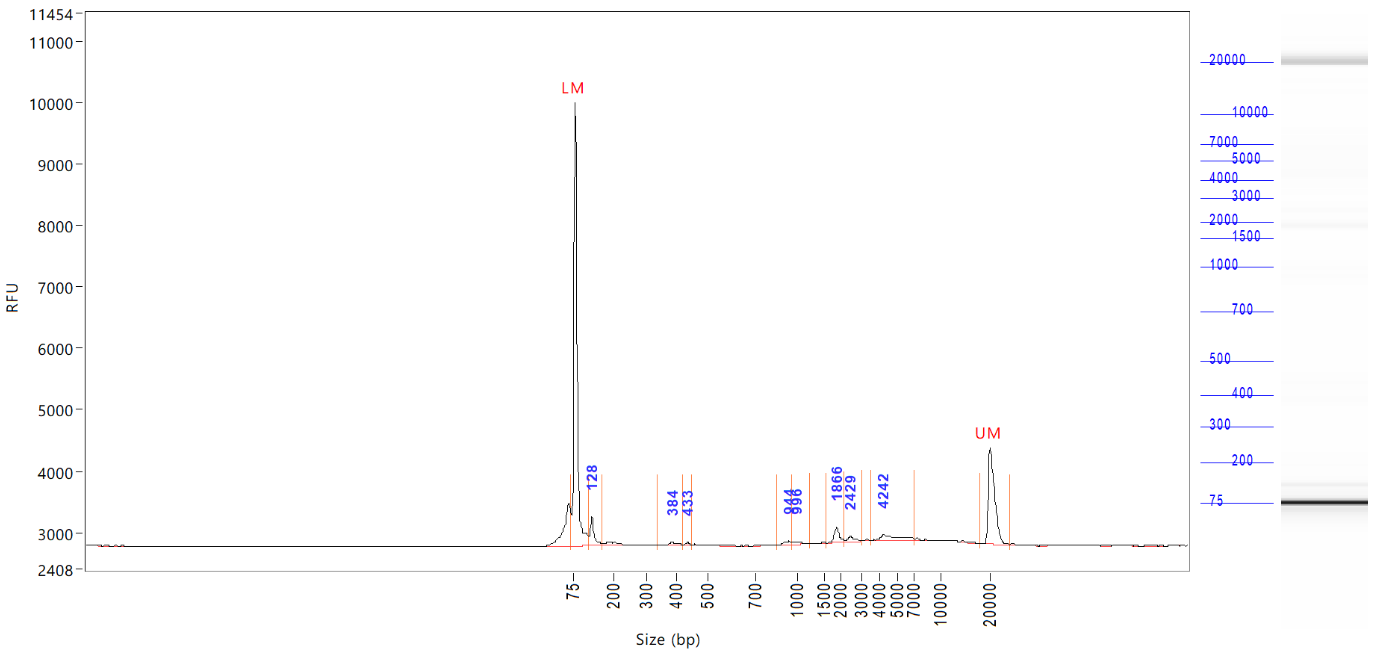

Peak	Size (bp)	Concentration (ng/uL)	From (bp)	To (bp)	Average size (bp)	CV%	RFU	Corrected peak area
1	75 (LM)	0.5000	64	118	76	11.59	7223	57.143
2	128	0.4839	118	164	131	7.99	463	4.609
3	384	0.1096	333	418	387	4.40	55	1.043
4	433	0.0465	418	447	432	1.73	50	0.443
5	944	0.0710	853	961	921	2.74	59	0.676
6	996	0.0743	961	1223	1021	5.80	50	0.708
7	1866	0.2131	1559	2201	1884	5.66	249	2.029
8	2429	0.1022	2201	3001	2491	6.88	87	0.973
9	4242	0.3069	3500	7068	4982	18.62	104	2.923
10	20000 (UM)	0.1133	18171	23990	20398	3.52	1561	12.954

TIC:	1.4074	ng/uL
TIM:	7.3145	nmole/L
Total concentration:	1.7648	ng/uL

Sample peak width (sec): 5 Sample min peak height: 50 Sample baseline V to V?: Y Sample baseline V to V points: 3
Sample filter: Binomial Number of points for filter: 3 Sample start region (min): 0 Sample end region (min): 30
Marker peak width (sec): 5 Marker min peak height: 200 Marker baseline V to V?: Y Marker baseline V to V points: 3
Lower marker selection: First peak > 500 RFU Upper marker selection: Last peak > 200 RFU
Ladder size (bp)75, 200, 300, 400, 500, 700, 1000, 1500, 2000, 3000, 4000, 5000, 7000, 10000, 20000
Quantification using: Lower Marker Final concentration (ng/uL): 0.5000 Dilution factor: 12.0

Sample: SampB6
Well location: B6
Created: Monday, September 20, 2021 6:00:03 PM

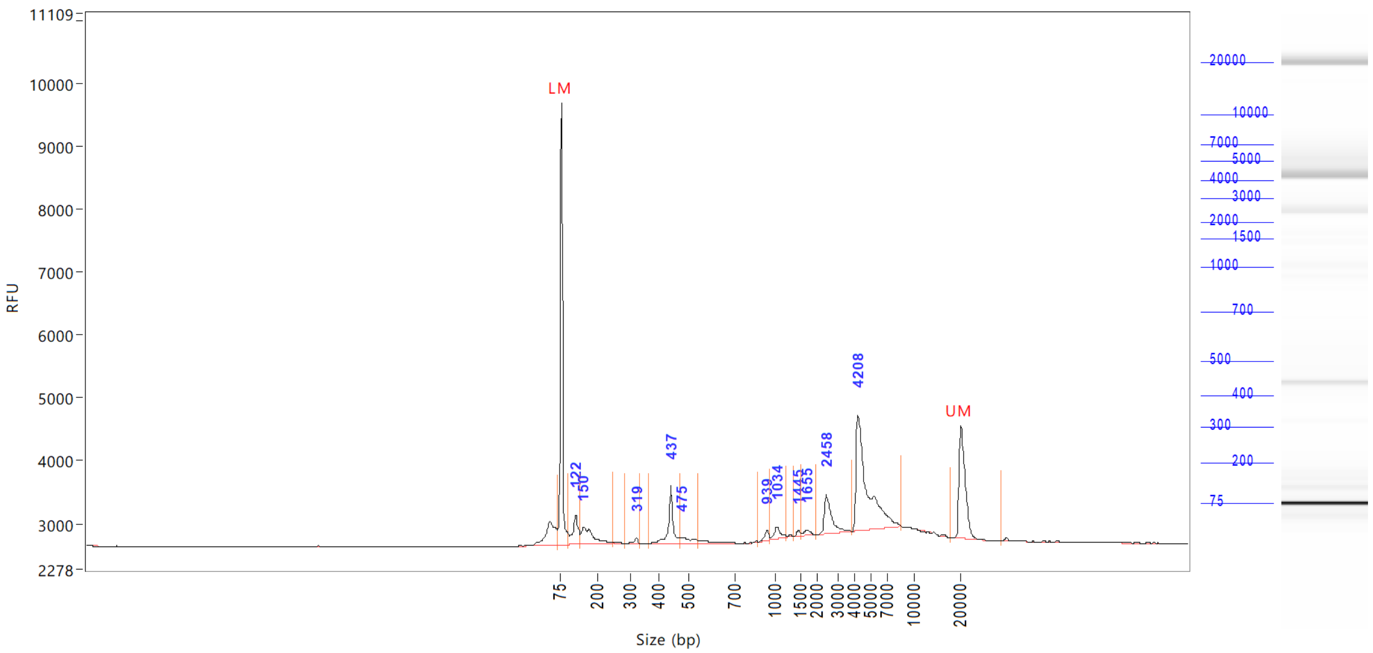

Peak	Size (bp)	Concentration (ng/uL)	From (bp)	To (bp)	Average size (bp)	CV%	RFU	Corrected peak area
1	75 (LM)	0.5000	64	101	74	7.40	7031	41.439
2	122	0.8718	101	137	120	7.64	467	6.021
3	150	1.0078	137	244	166	13.26	265	6.960
4	319	0.0916	280	331	311	3.05	85	0.633
5	437	1.1944	364	468	432	3.89	919	8.249
6	475	0.3008	468	537	496	4.07	90	2.077
7	939	0.1620	874	965	930	1.94	156	1.119
8	1034	0.3088	965	1223	1045	5.78	188	2.133
9	1445	0.0798	1356	1501	1440	2.39	90	0.551
10	1655	0.1269	1501	1962	1667	5.40	88	0.876
11	2458	1.0337	1962	3857	2588	10.63	630	7.139
12	4208	4.3702	3857	8600	4864	18.44	1835	30.183
13	20000 (UM)	0.1738	17927	28584	20395	3.88	1779	14.408

TIC:	9.5477	ng/uL
TIM:	31.0715	nmole/L
Total concentration:	9.7864	ng/uL

Sample peak width (sec): 5 Sample min peak height: 50 Sample baseline V to V?: Y Sample baseline V to V points: 3
Sample filter: Binomial Number of points for filter: 3 Sample start region (min): 0 Sample end region (min): 30
Marker peak width (sec): 5 Marker min peak height: 200 Marker baseline V to V?: Y Marker baseline V to V points: 3
Lower marker selection: First peak > 500 RFU Upper marker selection: Last peak > 200 RFU
Ladder size (bp)75, 200, 300, 400, 500, 700, 1000, 1500, 2000, 3000, 4000, 5000, 7000, 10000, 20000
Quantification using: Lower Marker Final concentration (ng/uL): 0.5000 Dilution factor: 12.0

Sample: SampC6
Well location: C6
Created: Monday, September 20, 2021 6:00:03 PM

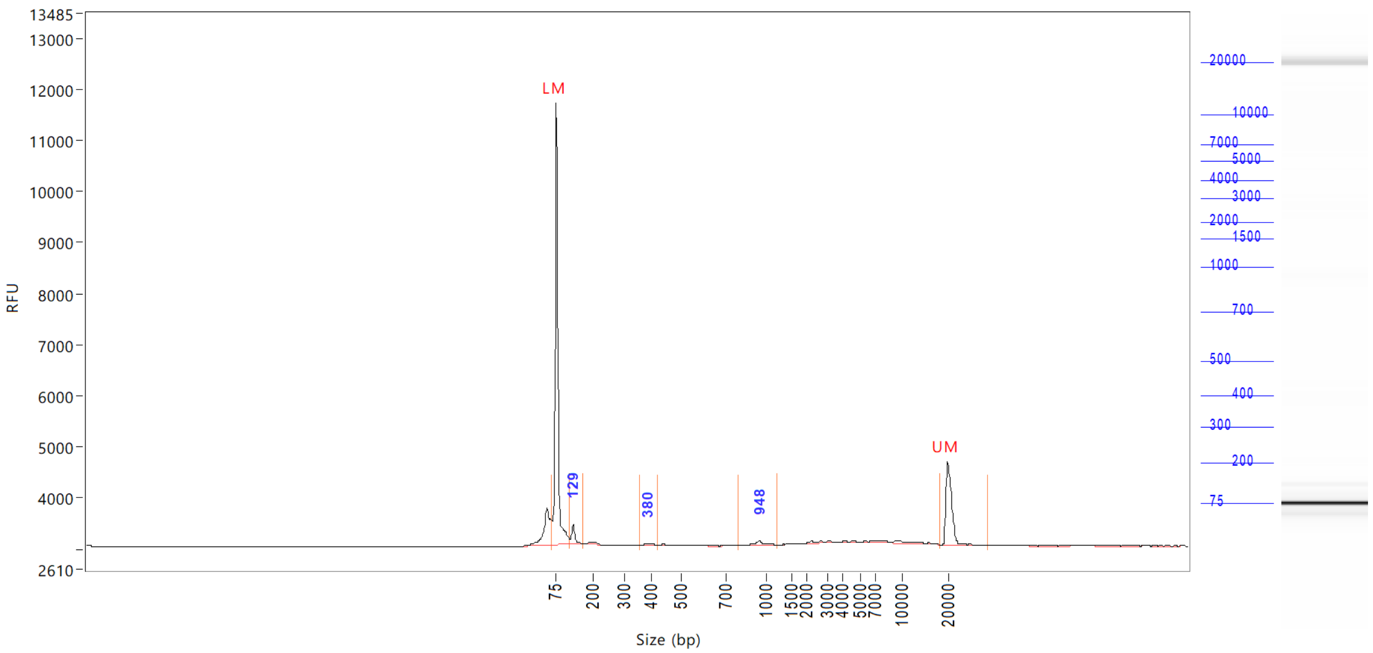

Peak	Size (bp)	Concentration (ng/uL)	From (bp)	To (bp)	Average size (bp)	CV%	RFU	Corrected peak area
1	75 (LM)	0.5000	62	120	76	11.68	8654	58.097
2	129	0.3126	120	164	131	5.28	378	3.027
3	380	0.0893	358	414	385	3.35	50	0.864
4	948	0.1401	796	1223	946	5.99	95	1.356
5	20000 (UM)	0.1074	18415	28463	20327	4.59	1639	12.477

TIC:	0.5419	ng/uL
TIM:	4.5457	nmole/L
Total concentration:	1.1226	ng/uL

Sample peak width (sec): 5 Sample min peak height: 50 Sample baseline V to V?: Y Sample baseline V to V points: 3
Sample filter: Binomial Number of points for filter: 3 Sample start region (min): 0 Sample end region (min): 30
Marker peak width (sec): 5 Marker min peak height: 200 Marker baseline V to V?: Y Marker baseline V to V points: 3
Lower marker selection: First peak > 500 RFU Upper marker selection: Last peak > 200 RFU
Ladder size (bp)75, 200, 300, 400, 500, 700, 1000, 1500, 2000, 3000, 4000, 5000, 7000, 10000, 20000
Quantification using: Lower Marker Final concentration (ng/uL): 0.5000 Dilution factor: 12.0

Sample: SampD6
Well location: D6
Created: Monday, September 20, 2021 6:00:03 PM

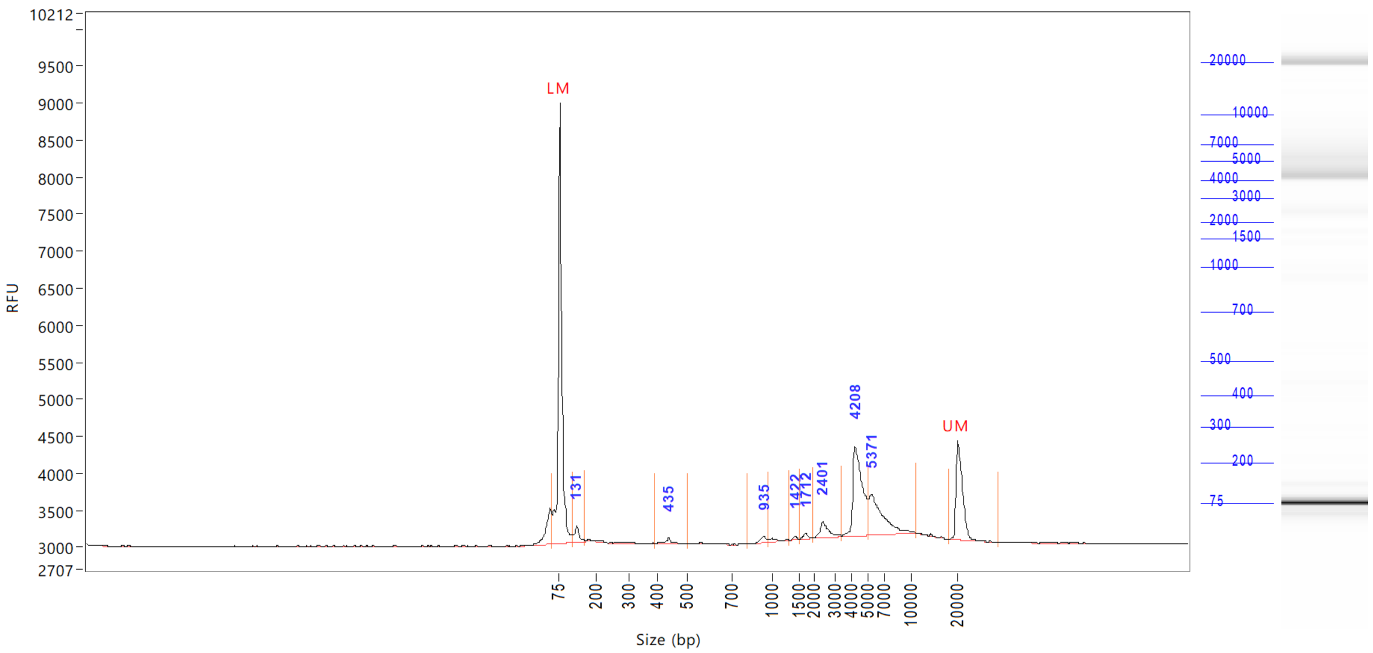

Peak	Size (bp)	Concentration (ng/uL)	From (bp)	To (bp)	Average size (bp)	CV%	RFU	Corrected peak area
1	75 (LM)	0.5000	50	120	76	13.32	5958	51.315
2	131	0.2834	120	160	133	6.67	226	2.423
3	435	0.1582	394	498	436	5.42	83	1.353
4	935	0.1179	809	970	922	3.16	89	1.009
5	1422	0.0356	1334	1501	1421	2.19	55	0.304
6	1712	0.0617	1501	2000	1689	4.51	74	0.528
7	2401	0.3347	2000	3429	2527	11.03	216	2.863
8	4208	2.0959	3429	5000	4400	6.82	1208	17.925
9	5371	1.3348	5000	11220	6387	18.66	546	11.416
10	20000 (UM)	0.1098	18171	28584	20451	4.11	1336	11.267

TIC:	4.4223	ng/uL
TIM:	5.7561	nmole/L
Total concentration:	4.7105	ng/uL

```
Sample peak width (sec): 5    Sample min peak height: 50    Sample baseline V to V?: Y    Sample baseline V to V points: 3
Sample filter: Binomial       Number of points for filter: 3  Sample start region (min): 0  Sample end region (min): 30
Marker peak width (sec): 5    Marker min peak height: 200   Marker baseline V to V?: Y    Marker baseline V to V points: 3
Lower marker selection: First peak > 500 RFU                Upper marker selection: Last peak > 200 RFU
Ladder size (bp)75, 200, 300, 400, 500, 700, 1000, 1500, 2000, 3000, 4000, 5000, 7000, 10000, 20000
Quantification using: Lower Marker          Final concentration (ng/uL): 0.5000          Dilution factor: 12.0
```

Sample: SampE6
Well location: E6
Created: Monday, September 20, 2021 6:00:03 PM

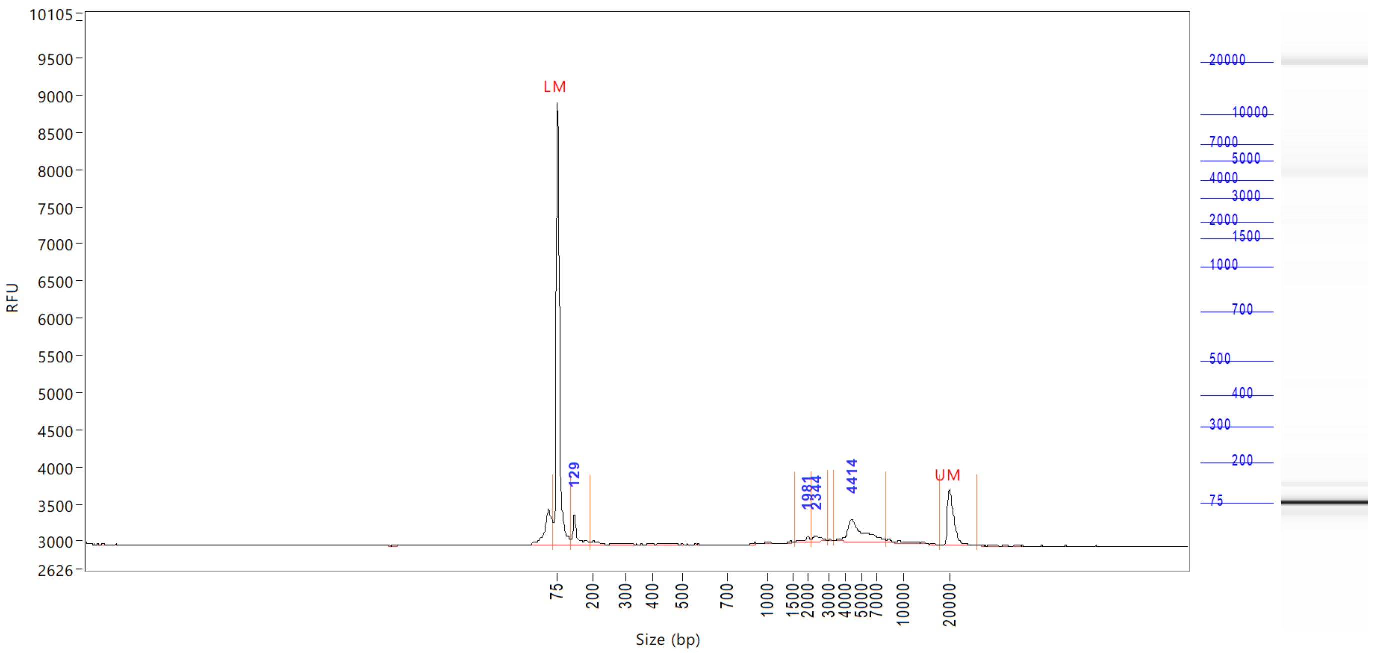

Peak	Size (bp)	Concentration (ng/uL)	From (bp)	To (bp)	Average size (bp)	CV%	RFU	Corrected peak area
1	75 (LM)	0.5000	62	120	76	10.96	5956	45.829
2	129	0.5682	120	187	140	12.49	406	4.340
3	1981	0.0892	1539	2144	1890	8.61	70	0.682
4	2344	0.1294	2144	2943	2466	8.13	71	0.988
5	4414	0.9608	3286	8067	5068	20.70	289	7.339
6	20000 (UM)	0.0697	18049	25924	20438	4.48	749	6.391

TIC:	1.7477	ng/uL
TIM:	7.1459	nmole/L
Total concentration:	2.3150	ng/uL

```
Sample peak width (sec): 5    Sample min peak height: 50    Sample baseline V to V?: Y    Sample baseline V to V points: 3
Sample filter: Binomial       Number of points for filter: 3  Sample start region (min): 0  Sample end region (min): 30
Marker peak width (sec): 5    Marker min peak height: 200   Marker baseline V to V?: Y    Marker baseline V to V points: 3
Lower marker selection: First peak > 500 RFU                Upper marker selection: Last peak > 200 RFU
Ladder size (bp)75, 200, 300, 400, 500, 700, 1000, 1500, 2000, 3000, 4000, 5000, 7000, 10000, 20000
Quantification using: Lower Marker          Final concentration (ng/uL): 0.5000          Dilution factor: 12.0
```

Sample: SampF6
Well location: F6
Created: Monday, September 20, 2021 6:00:03 PM

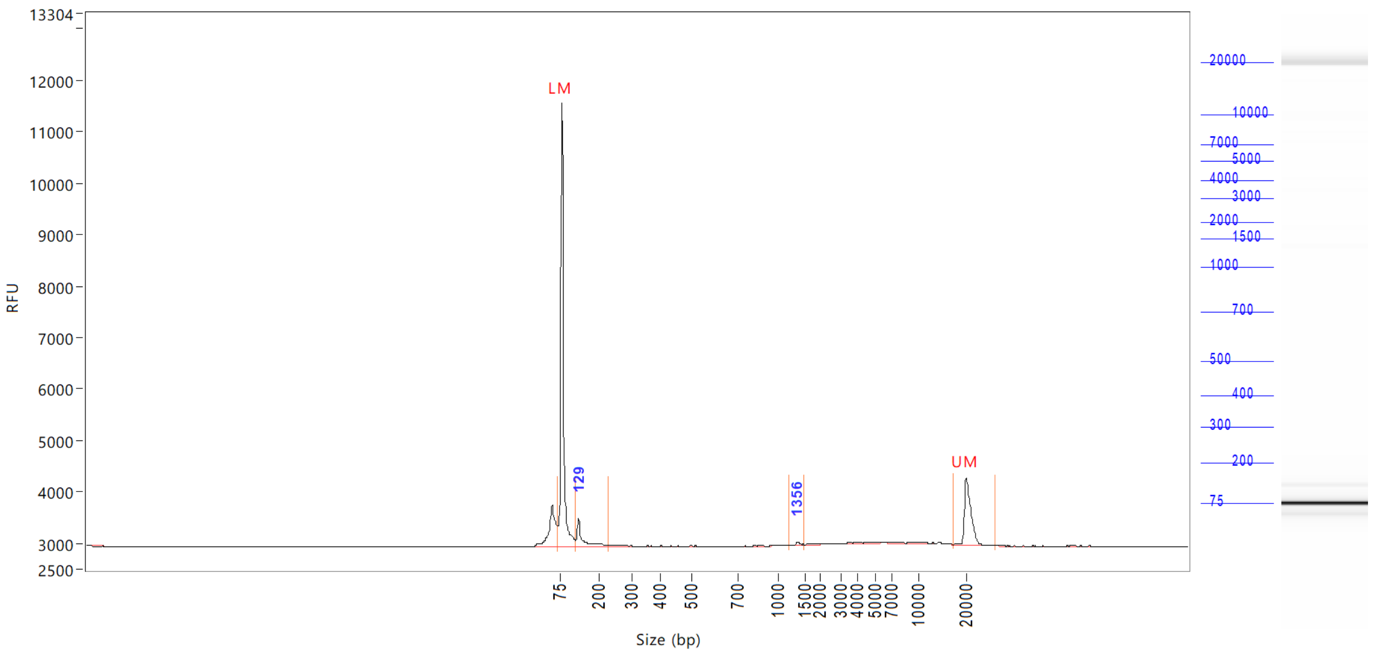

Peak	Size (bp)	Concentration (ng/uL)	From (bp)	To (bp)	Average size (bp)	CV%	RFU	Corrected peak area
1	75 (LM)	0.5000	62	118	76	11.41	8633	59.909
2	129	0.6523	118	226	144	18.28	548	6.513
3	1356	0.0479	1201	1500	1362	4.25	59	0.478
4	20000 (UM)	0.0942	17317	26166	20400	4.03	1295	11.281

TIC:	0.7002	ng/uL
TIM:	7.5023	nmole/L
Total concentration:	1.0203	ng/uL

Sample peak width (sec): 5 Sample min peak height: 50 Sample baseline V to V?: Y Sample baseline V to V points: 3
Sample filter: Binomial Number of points for filter: 3 Sample start region (min): 0 Sample end region (min): 30
Marker peak width (sec): 5 Marker min peak height: 200 Marker baseline V to V?: Y Marker baseline V to V points: 3
Lower marker selection: First peak > 500 RFU Upper marker selection: Last peak > 200 RFU
Ladder size (bp)75, 200, 300, 400, 500, 700, 1000, 1500, 2000, 3000, 4000, 5000, 7000, 10000, 20000
Quantification using: Lower Marker Final concentration (ng/uL): 0.5000 Dilution factor: 12.0

Sample: SampG6
Well location: G6
Created: Monday, September 20, 2021 6:00:03 PM

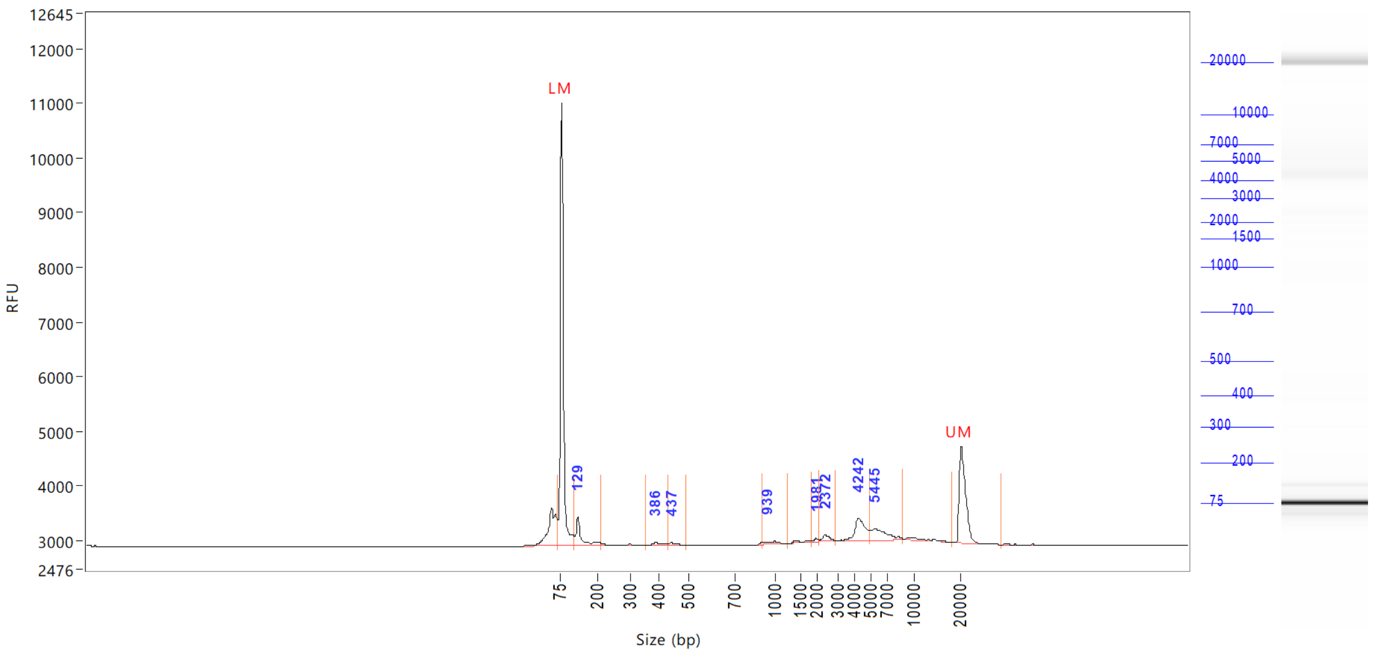

Peak	Size (bp)	Concentration (ng/uL)	From (bp)	To (bp)	Average size (bp)	CV%	RFU	Corrected peak area
1	75 (LM)	0.5000	64	120	76	11.75	8114	63.705
2	129	0.6026	120	208	144	17.12	528	6.398
3	386	0.1120	354	428	396	4.26	55	1.190
4	437	0.0723	428	489	449	3.60	63	0.767
5	939	0.1006	900	1234	971	5.60	50	1.068
6	1981	0.0354	1846	2115	1976	3.25	61	0.376
7	2372	0.1163	2115	2943	2417	6.44	120	1.235
8	4242	0.6015	2943	4966	4355	7.64	396	6.386
9	5445	0.4824	4966	8800	6292	15.80	209	5.122
10	20000 (UM)	0.1164	18171	28463	20462	3.91	1762	14.829

TIC:	2.1231	ng/uL
TIM:	8.2405	nmole/L
Total concentration:	2.3694	ng/uL

Sample peak width (sec): 5 Sample min peak height: 50 Sample baseline V to V?: Y Sample baseline V to V points: 3
Sample filter: Binomial Number of points for filter: 3 Sample start region (min): 0 Sample end region (min): 30
Marker peak width (sec): 5 Marker min peak height: 200 Marker baseline V to V?: Y Marker baseline V to V points: 3
Lower marker selection: First peak > 500 RFU Upper marker selection: Last peak > 200 RFU
Ladder size (bp)75, 200, 300, 400, 500, 700, 1000, 1500, 2000, 3000, 4000, 5000, 7000, 10000, 20000
Quantification using: Lower Marker Final concentration (ng/uL): 0.5000 Dilution factor: 12.0

Sample: SampH6
Well location: H6
Created: Monday, September 20, 2021 6:00:03 PM

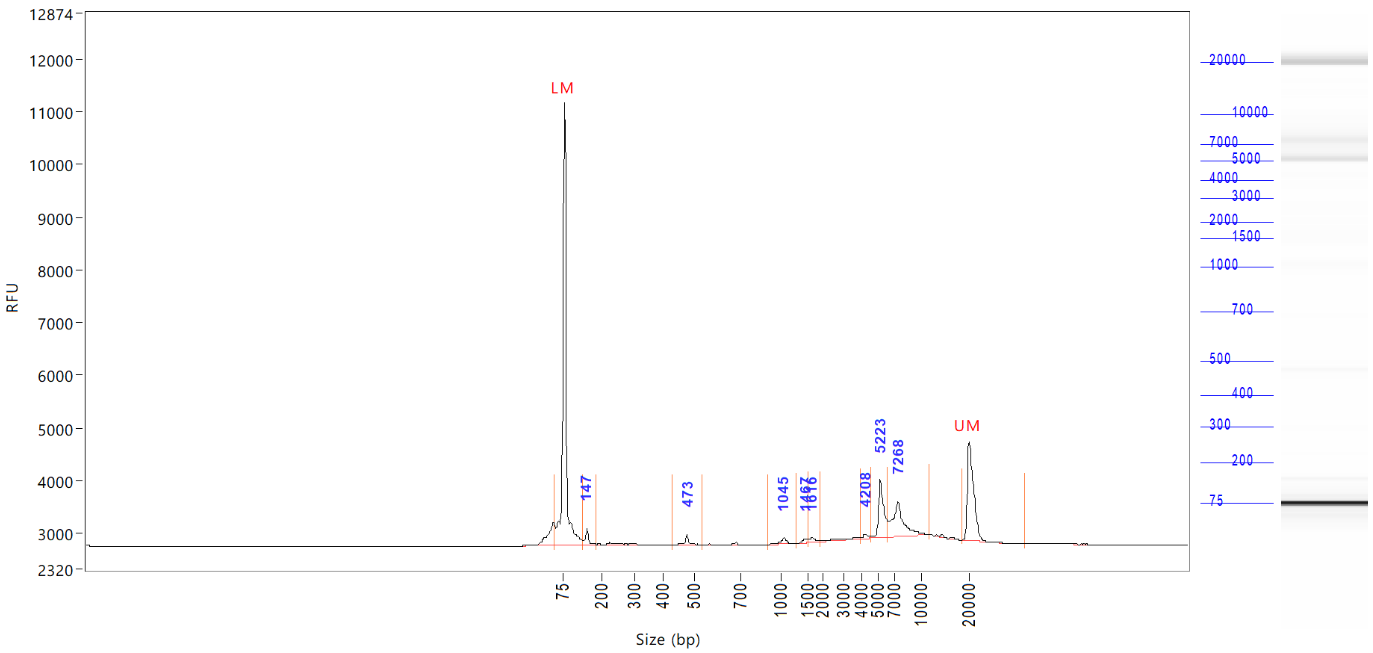

Peak	Size (bp)	Concentration (ng/uL)	From (bp)	To (bp)	Average size (bp)	CV%	RFU	Corrected peak area
1	75 (LM)	0.5000	45	137	74	16.94	8414	71.720
2	147	0.1877	137	179	147	4.84	319	2.244
3	473	0.1431	428	534	473	3.82	214	1.710
4	1045	0.1033	905	1300	1029	5.75	113	1.234
5	1467	0.0593	1300	1520	1446	3.18	83	0.708
6	1616	0.0711	1520	1904	1660	5.97	91	0.850
7	4208	0.0527	4001	4552	4257	3.39	88	0.630
8	5223	0.7002	4552	6112	5332	6.45	1108	8.369
9	7268	0.8953	6112	11586	7499	12.21	672	10.702
10	20000 (UM)	0.1076	18415	31486	20443	4.86	1871	15.428

TIC:	2.2127	ng/uL
TIM:	3.3329	nmole/L
Total concentration:	2.4945	ng/uL

```
Sample peak width (sec): 5    Sample min peak height: 50    Sample baseline V to V?: Y    Sample baseline V to V points: 3
Sample filter: Binomial       Number of points for filter: 3  Sample start region (min): 0  Sample end region (min): 30
Marker peak width (sec): 5    Marker min peak height: 200   Marker baseline V to V?: Y    Marker baseline V to V points: 3
Lower marker selection: First peak > 500 RFU               Upper marker selection: Last peak > 200 RFU
Ladder size (bp)75, 200, 300, 400, 500, 700, 1000, 1500, 2000, 3000, 4000, 5000, 7000, 10000, 20000
Quantification using: Lower Marker          Final concentration (ng/uL): 0.5000          Dilution factor: 12.0
```

Sample: SampA7
Well location: A7
Created: Monday, September 20, 2021 6:00:03 PM

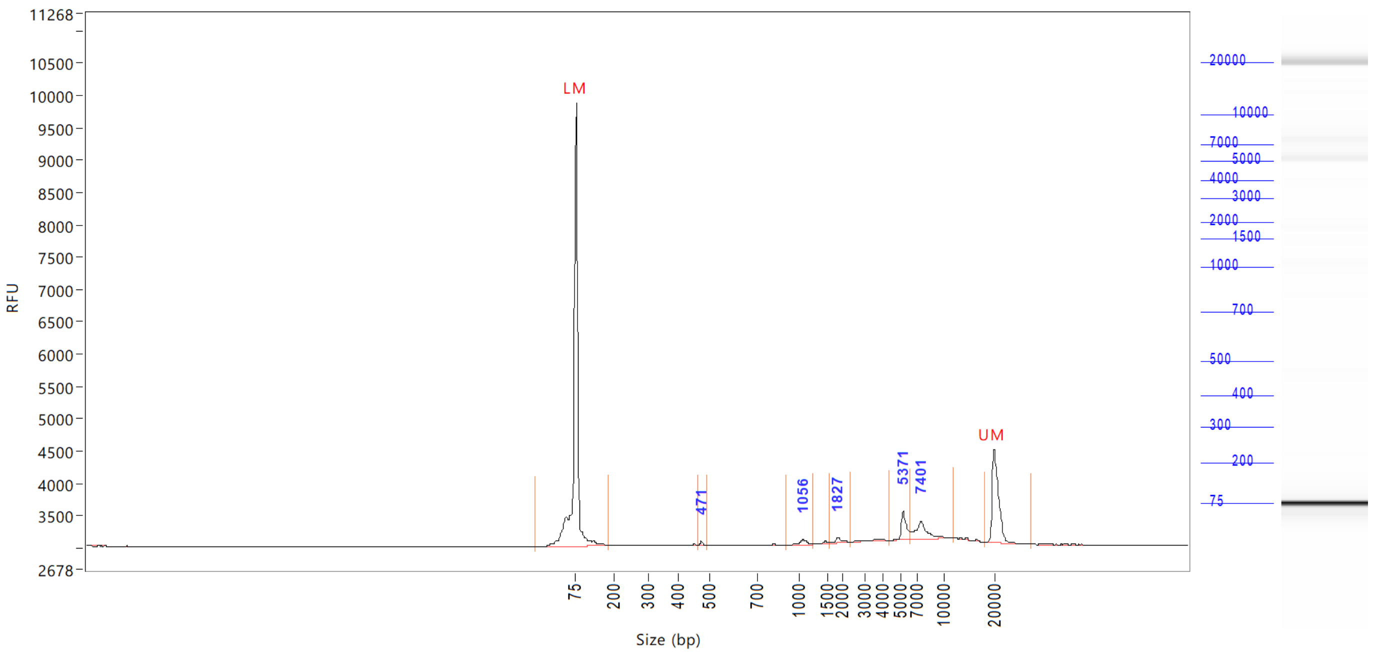

Peak	Size (bp)	Concentration (ng/uL)	From (bp)	To (bp)	Average size (bp)	CV%	RFU	Corrected peak area
1	75 (LM)	0.5000	0	179	69	28.54	6861	66.926
2	471	0.0329	460	489	470	1.08	66	0.367
3	1056	0.0878	909	1256	1046	5.87	80	0.979
4	1827	0.0737	1578	2286	1850	7.07	86	0.822
5	5371	0.2922	4380	6260	5438	6.55	448	3.259
6	7401	0.4023	6260	11708	7620	11.80	283	4.487
7	20000 (UM)	0.0889	18293	27375	20409	4.01	1447	11.894

TIC:	0.8888	ng/uL
TIM:	0.4942	nmole/L
Total concentration:	1.1018	ng/uL

Sample peak width (sec): 5   Sample min peak height: 50   Sample baseline V to V?: Y   Sample baseline V to V points: 3
Sample filter: Binomial   Number of points for filter: 3   Sample start region (min): 0   Sample end region (min): 30
Marker peak width (sec): 5   Marker min peak height: 200   Marker baseline V to V?: Y   Marker baseline V to V points: 3
Lower marker selection: First peak > 500 RFU   Upper marker selection: Last peak > 200 RFU
Ladder size (bp)75, 200, 300, 400, 500, 700, 1000, 1500, 2000, 3000, 4000, 5000, 7000, 10000, 20000
Quantification using: Lower Marker   Final concentration (ng/uL): 0.5000   Dilution factor: 12.0

Sample: SampB7
Well location: B7
Created: Monday, September 20, 2021 6:00:03 PM

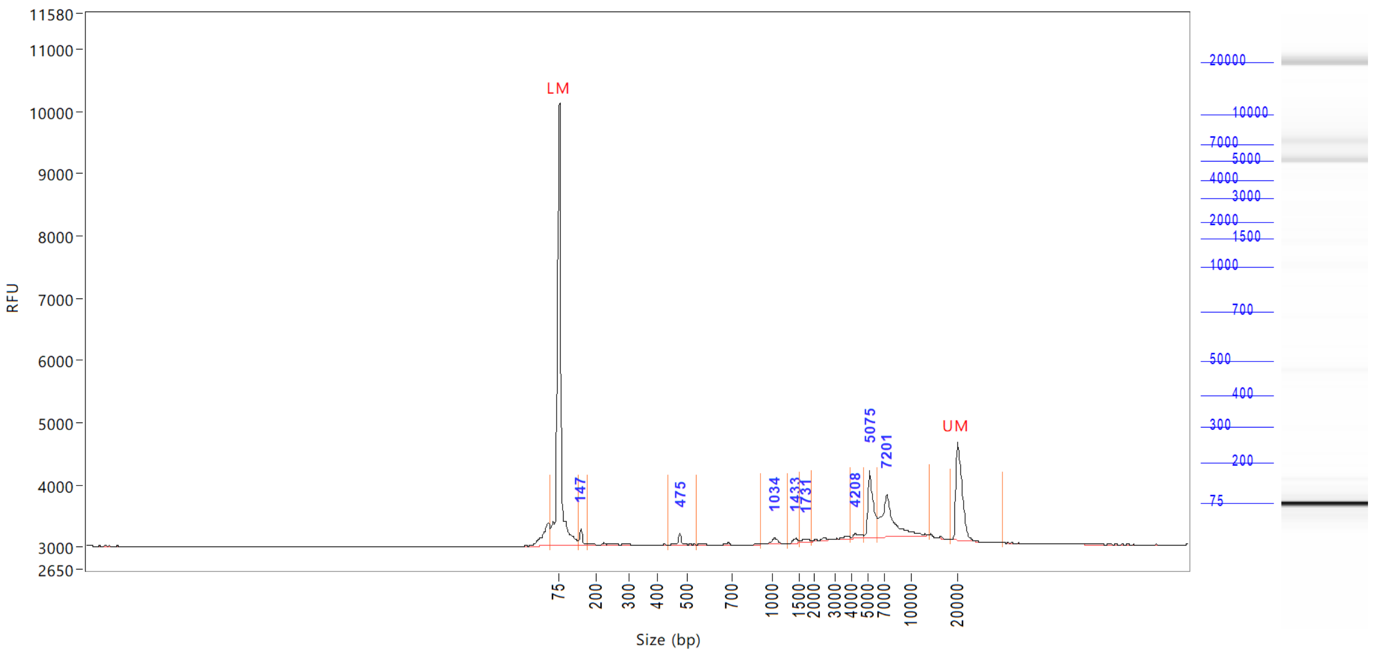

Peak	Size (bp)	Concentration (ng/uL)	From (bp)	To (bp)	Average size (bp)	CV%	RFU	Corrected peak area
1	75 (LM)	0.5000	45	137	74	17.39	7123	63.386
2	147	0.1699	137	169	146	4.24	254	1.795
3	475	0.1608	433	542	475	4.24	193	1.699
4	1034	0.1081	909	1289	1038	5.67	108	1.142
5	1433	0.0636	1289	1520	1430	3.25	81	0.671
6	1731	0.0598	1520	1904	1695	5.99	50	0.632
7	4208	0.0770	4000	4724	4334	4.82	75	0.813
8	5075	0.8161	4724	6112	5300	6.48	1091	8.622
9	7201	1.1181	6112	14025	7703	16.10	685	11.812
10	20000 (UM)	0.1029	18293	29430	20411	4.30	1580	13.051

TIC:	2.5735	ng/uL
TIM:	3.2945	nmole/L
Total concentration:	2.8761	ng/uL

Sample peak width (sec): 5   Sample min peak height: 50   Sample baseline V to V?: Y   Sample baseline V to V points: 3
Sample filter: Binomial   Number of points for filter: 3 Sample start region (min): 0 Sample end region (min): 30
Marker peak width (sec): 5   Marker min peak height: 200   Marker baseline V to V?: Y   Marker baseline V to V points: 3
Lower marker selection: First peak > 500 RFU   Upper marker selection: Last peak > 200 RFU
Ladder size (bp)75, 200, 300, 400, 500, 700, 1000, 1500, 2000, 3000, 4000, 5000, 7000, 10000, 20000
Quantification using: Lower Marker   Final concentration (ng/uL): 0.5000   Dilution factor: 12.0

Sample: SampC7
Well location: C7
Created: Monday, September 20, 2021 6:00:03 PM

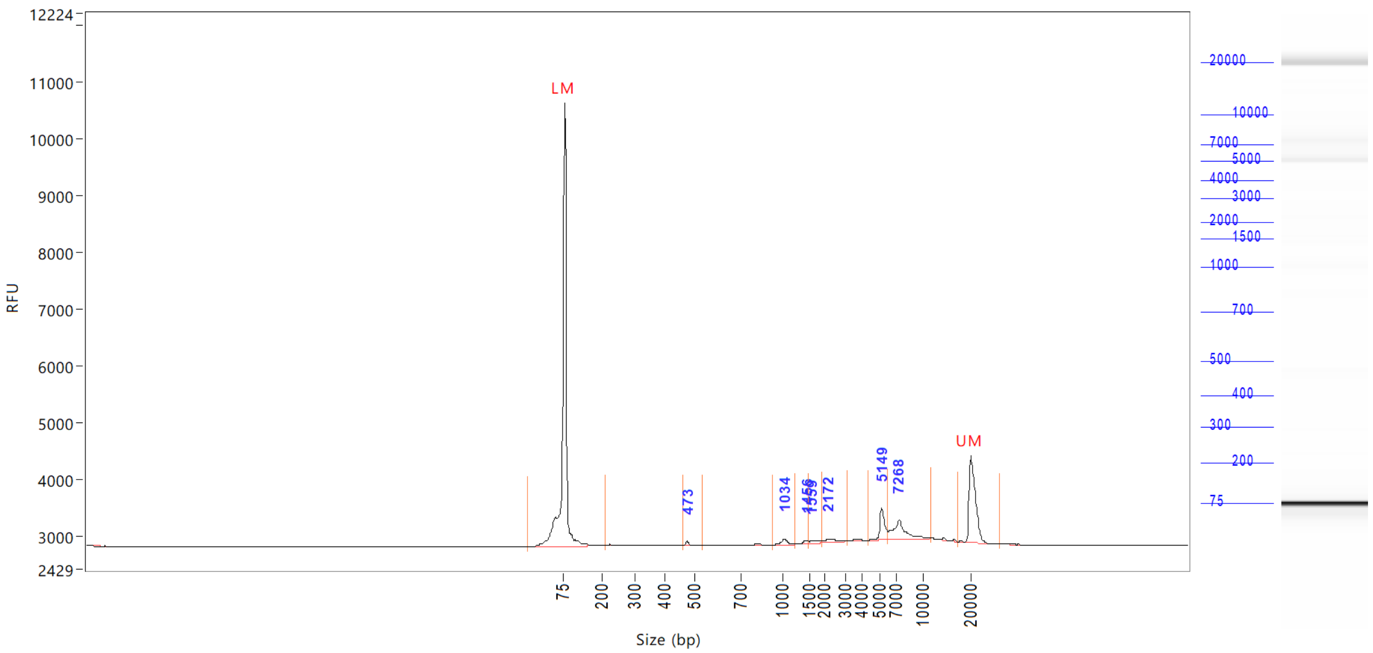

Peak	Size (bp)	Concentration (ng/uL)	From (bp)	To (bp)	Average size (bp)	CV%	RFU	Corrected peak area
1	75 (LM)	0.5000	0	210	68	29.81	7822	74.823
2	473	0.0509	460	532	478	3.03	81	0.635
3	1034	0.0897	931	1245	1032	5.27	107	1.118
4	1456	0.0411	1245	1500	1425	3.28	64	0.513
5	1559	0.0585	1500	1904	1681	7.67	53	0.729
6	2172	0.0926	1904	3108	2325	13.00	67	1.155
7	5149	0.3209	4277	5963	5240	6.08	560	4.002
8	7268	0.4798	5963	11708	7473	13.48	344	5.983
9	20000 (UM)	0.0852	17317	25924	20321	3.78	1531	12.756

TIC:	1.1335	ng/uL
TIM:	0.6951	nmole/L
Total concentration:	1.3448	ng/uL

```
Sample peak width (sec): 5    Sample min peak height: 50    Sample baseline V to V?: Y    Sample baseline V to V points: 3
Sample filter: Binomial       Number of points for filter: 3  Sample start region (min): 0  Sample end region (min): 30
Marker peak width (sec): 5    Marker min peak height: 200   Marker baseline V to V?: Y    Marker baseline V to V points: 3
Lower marker selection: First peak > 500 RFU                Upper marker selection: Last peak > 200 RFU
Ladder size (bp)75, 200, 300, 400, 500, 700, 1000, 1500, 2000, 3000, 4000, 5000, 7000, 10000, 20000
Quantification using: Lower Marker          Final concentration (ng/uL): 0.5000          Dilution factor: 12.0
```

Sample: SampD7
Well location: D7
Created: Monday, September 20, 2021 6:00:03 PM

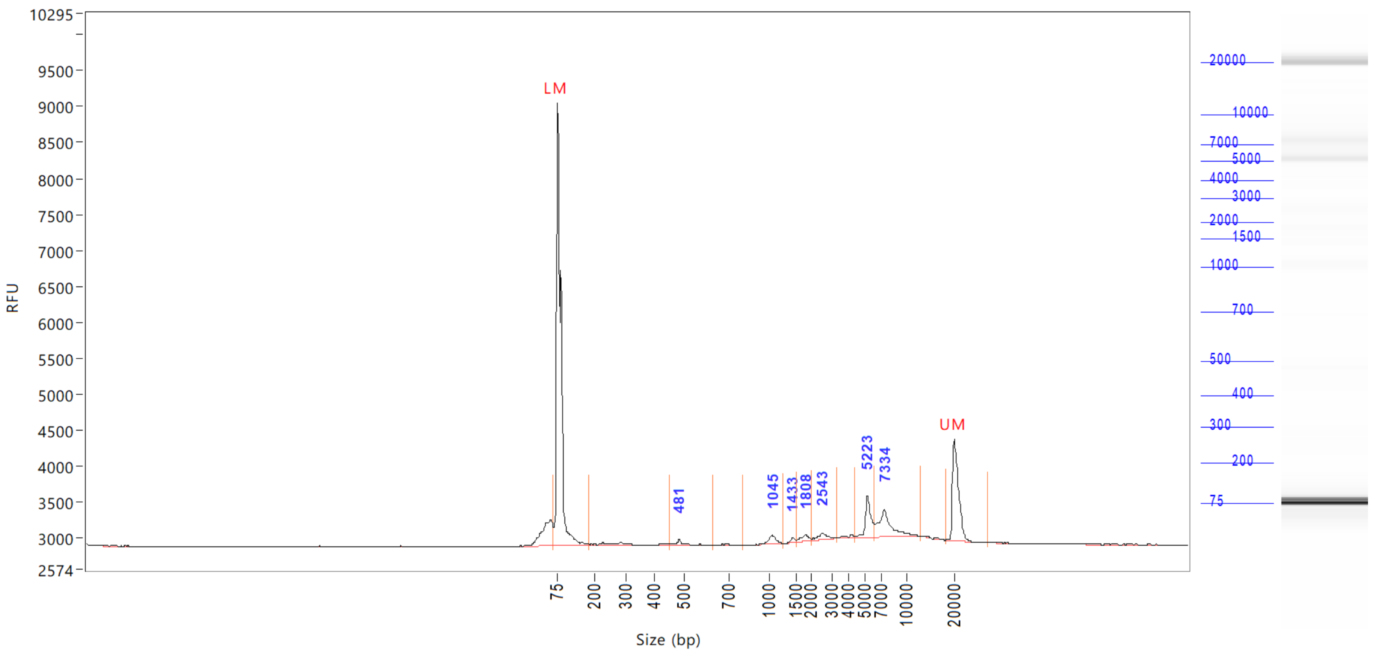

Peak	Size (bp)	Concentration (ng/uL)	From (bp)	To (bp)	Average size (bp)	CV%	RFU	Corrected peak area
1	75 (LM)	0.5000	64	181	81	16.04	6159	61.783
2	481	0.1229	447	623	493	7.95	88	1.266
3	1045	0.1530	801	1256	1032	6.12	120	1.575
4	1433	0.0445	1256	1501	1432	2.93	59	0.459
5	1808	0.1101	1501	2001	1759	7.37	87	1.134
6	2543	0.1342	2001	3322	2527	10.47	95	1.382
7	5223	0.4755	4380	6186	5355	7.35	574	4.896
8	7334	0.6710	6186	12928	7686	14.19	387	6.910
9	20000 (UM)	0.0941	18171	27012	20409	3.86	1417	11.630

TIC:	1.7113	ng/uL
TIM:	1.1859	nmole/L
Total concentration:	1.9642	ng/uL

Sample peak width (sec): 5 Sample min peak height: 50 Sample baseline V to V?: Y Sample baseline V to V points: 3
Sample filter: Binomial Number of points for filter: 3 Sample start region (min): 0 Sample end region (min): 30
Marker peak width (sec): 5 Marker min peak height: 200 Marker baseline V to V?: Y Marker baseline V to V points: 3
Lower marker selection: First peak > 500 RFU Upper marker selection: Last peak > 200 RFU
Ladder size (bp)75, 200, 300, 400, 500, 700, 1000, 1500, 2000, 3000, 4000, 5000, 7000, 10000, 20000
Quantification using: Lower Marker Final concentration (ng/uL): 0.5000 Dilution factor: 12.0

Sample: SampE7
Well location: E7
Created: Monday, September 20, 2021 6:00:03 PM

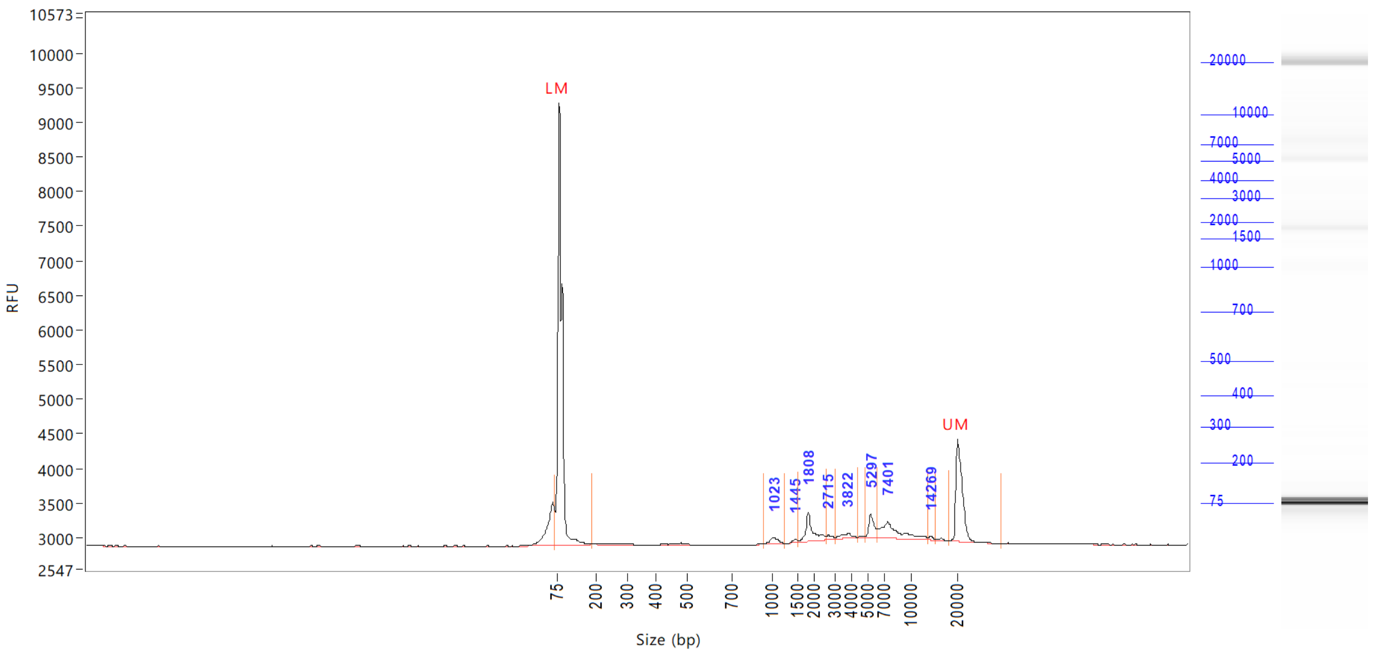

Peak	Size (bp)	Concentration (ng/uL)	From (bp)	To (bp)	Average size (bp)	CV%	RFU	Corrected peak area
1	75 (LM)	0.5000	66	185	80	14.86	6396	62.243
2	1023	0.1239	935	1234	1032	5.31	96	1.285
3	1445	0.0396	1234	1500	1419	3.63	51	0.411
4	1808	0.4671	1500	2600	1916	12.67	431	4.845
5	2715	0.0607	2600	3037	2788	4.57	61	0.630
6	3822	0.1063	3037	4345	3613	8.41	67	1.103
7	5297	0.2623	4793	6186	5416	6.60	332	2.722
8	7401	0.5830	6186	13537	8368	20.83	235	6.048
9	14269	0.0329	13537	15122	14198	2.84	58	0.342
10	20000 (UM)	0.0994	18171	29309	20401	4.11	1486	12.377

TIC:	1.6759	ng/uL
TIM:	0.9274	nmole/L
Total concentration:	1.8833	ng/uL

```
Sample peak width (sec): 5    Sample min peak height: 50    Sample baseline V to V?: Y    Sample baseline V to V points: 3
Sample filter: Binomial       Number of points for filter: 3  Sample start region (min): 0  Sample end region (min): 30
Marker peak width (sec): 5    Marker min peak height: 200   Marker baseline V to V?: Y    Marker baseline V to V points: 3
Lower marker selection: First peak > 500 RFU                Upper marker selection: Last peak > 200 RFU
Ladder size (bp)75, 200, 300, 400, 500, 700, 1000, 1500, 2000, 3000, 4000, 5000, 7000, 10000, 20000
Quantification using: Lower Marker          Final concentration (ng/uL): 0.5000          Dilution factor: 12.0
```

Sample: SampF7
Well location: F7
Created: Monday, September 20, 2021 6:00:03 PM

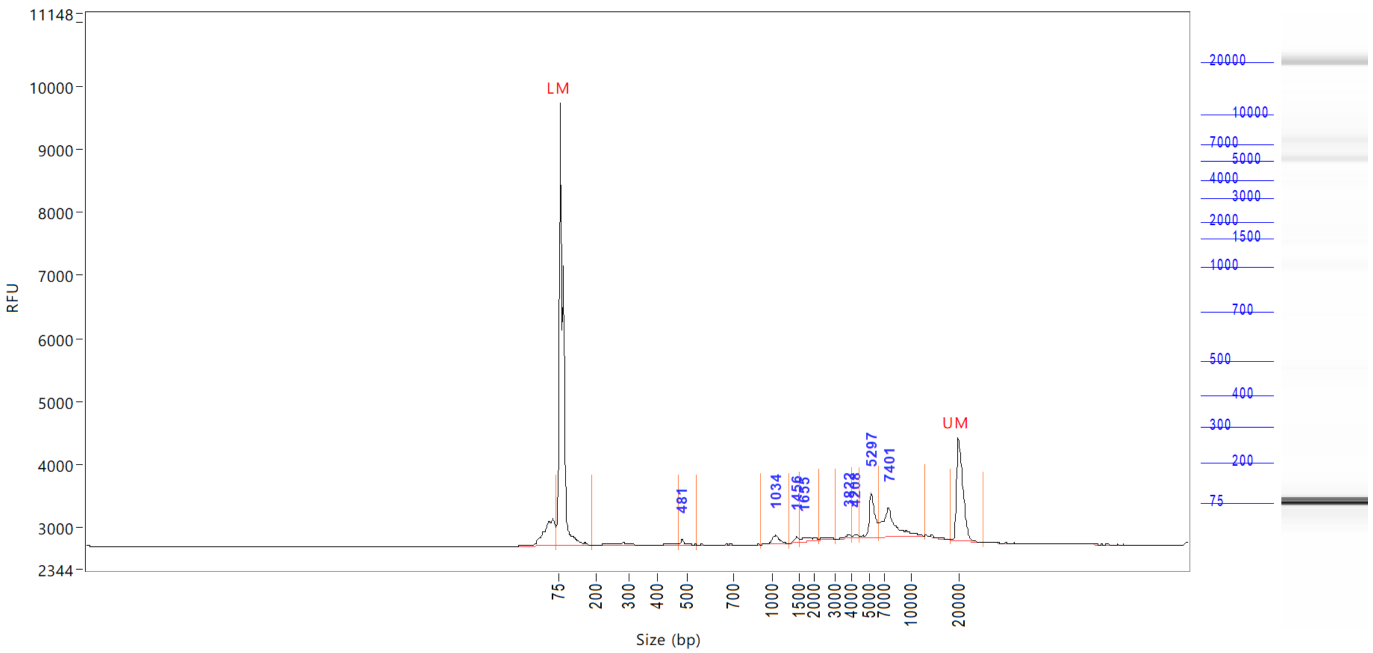

Peak	Size (bp)	Concentration (ng/uL)	From (bp)	To (bp)	Average size (bp)	CV%	RFU	Corrected peak area
1	75 (LM)	0.5000	64	185	80	14.87	7021	69.310
2	481	0.0722	468	539	488	3.57	97	0.834
3	1034	0.1507	905	1311	1045	6.20	135	1.741
4	1456	0.0611	1311	1520	1437	3.05	89	0.706
5	1655	0.1051	1520	2172	1781	9.37	67	1.214
6	3822	0.0504	3001	4001	3728	5.34	67	0.582
7	4208	0.0389	4001	4449	4209	3.01	60	0.449
8	5297	0.5165	4449	6186	5357	7.14	705	5.966
9	7401	0.7333	6186	12928	7729	14.72	463	8.471
10	20000 (UM)	0.0969	18171	25320	20386	3.57	1621	13.432

TIC:	1.7282	ng/uL
TIM:	1.0003	nmole/L
Total concentration:	2.0236	ng/uL

Sample peak width (sec): 5 Sample min peak height: 50 Sample baseline V to V?: Y Sample baseline V to V points: 3
Sample filter: Binomial Number of points for filter: 3 Sample start region (min): 0 Sample end region (min): 30
Marker peak width (sec): 5 Marker min peak height: 200 Marker baseline V to V?: Y Marker baseline V to V points: 3
Lower marker selection: First peak > 500 RFU Upper marker selection: Last peak > 200 RFU
Ladder size (bp)75, 200, 300, 400, 500, 700, 1000, 1500, 2000, 3000, 4000, 5000, 7000, 10000, 20000
Quantification using: Lower Marker Final concentration (ng/uL): 0.5000 Dilution factor: 12.0

Sample: SampG7
Well location: G7
Created: Monday, September 20, 2021 6:00:03 PM

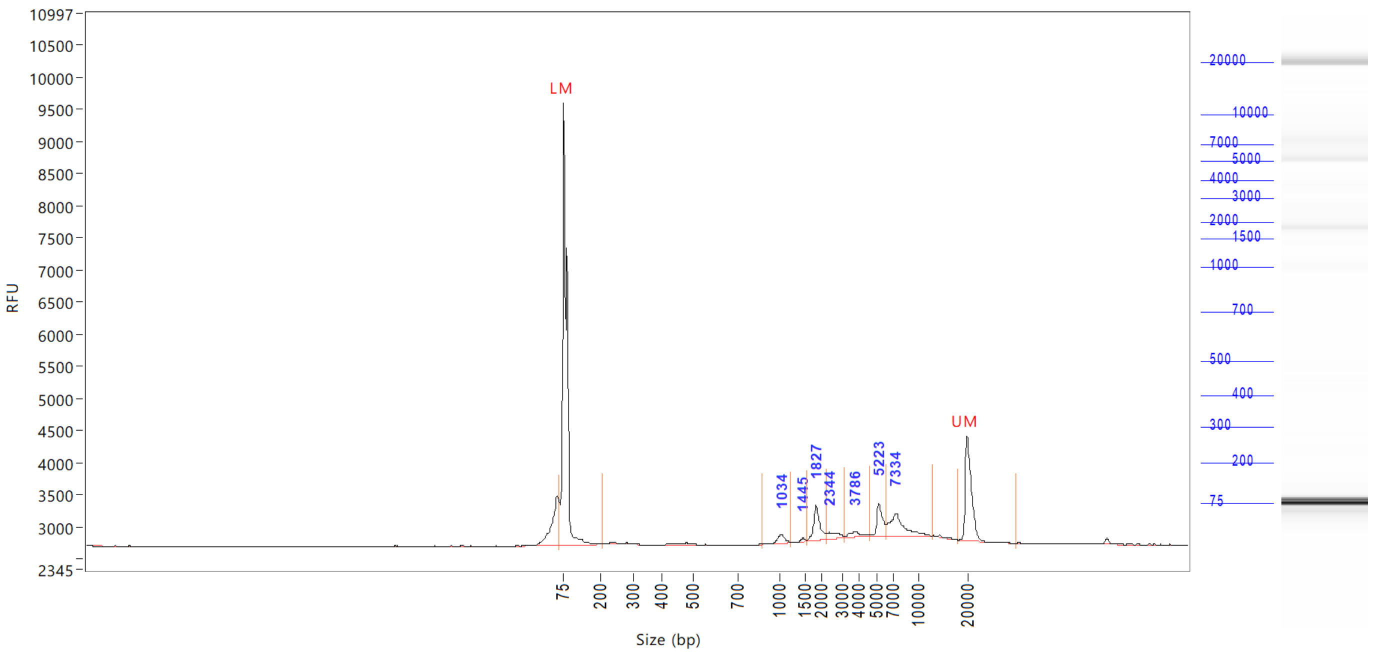

Peak	Size (bp)	Concentration (ng/uL)	From (bp)	To (bp)	Average size (bp)	CV%	RFU	Corrected peak area
1	75 (LM)	0.5000	64	201	79	16.59	6895	69.944
2	1034	0.1426	879	1223	1026	5.05	131	1.662
3	1445	0.0344	1223	1501	1414	4.34	52	0.401
4	1827	0.4720	1501	2229	1848	7.59	544	5.502
5	2344	0.1603	2229	3144	2594	9.48	104	1.869
6	3786	0.1233	3144	4621	3700	8.92	82	1.437
7	5223	0.3685	4621	6186	5406	6.63	514	4.295
8	7334	0.6093	6186	12806	7742	14.57	349	7.103
9	20000 (UM)	0.0947	18171	30398	20401	4.20	1626	13.245

TIC:	1.9103	ng/uL
TIM:	1.0876	nmole/L
Total concentration:	2.0927	ng/uL

Sample peak width (sec): 5 Sample min peak height: 50 Sample baseline V to V?: Y Sample baseline V to V points: 3
Sample filter: Binomial Number of points for filter: 3 Sample start region (min): 0 Sample end region (min): 30
Marker peak width (sec): 5 Marker min peak height: 200 Marker baseline V to V?: Y Marker baseline V to V points: 3
Lower marker selection: First peak > 500 RFU Upper marker selection: Last peak > 200 RFU
Ladder size (bp)75, 200, 300, 400, 500, 700, 1000, 1500, 2000, 3000, 4000, 5000, 7000, 10000, 20000
Quantification using: Lower Marker Final concentration (ng/uL): 0.5000 Dilution factor: 12.0

Sample: SampH7
Well location: H7
Created: Monday, September 20, 2021 6:00:03 PM

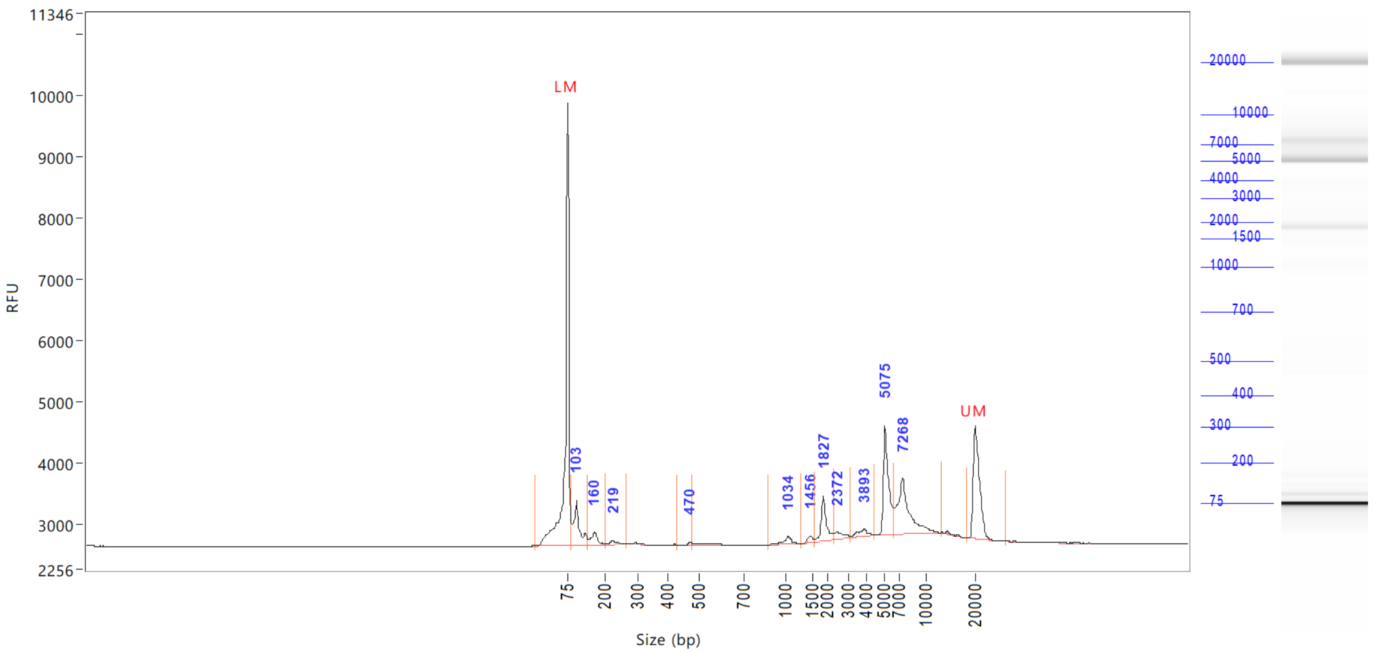

Peak	Size (bp)	Concentration (ng/uL)	From (bp)	To (bp)	Average size (bp)	CV%	RFU	Corrected peak area
1	75 (LM)	0.5000	0	86	62	32.02	7244	63.148
2	103	0.9588	86	141	105	13.46	739	10.091
3	160	0.3113	141	198	160	7.78	201	3.276
4	219	0.1011	198	260	222	6.40	72	1.064
5	470	0.0394	431	479	464	2.21	58	0.414
6	1034	0.1512	870	1300	1024	6.34	131	1.592
7	1456	0.0936	1300	1559	1450	3.44	111	0.985
8	1827	0.5850	1559	2229	1858	6.78	747	6.157
9	2372	0.1735	2229	3072	2554	8.83	125	1.826
10	3893	0.1627	3072	4449	3692	7.97	114	1.712
11	5075	1.3417	4449	6260	5326	7.10	1793	14.121
12	7268	1.4478	6260	12928	7659	13.80	914	15.238
13	20000 (UM)	0.1183	18293	26529	20404	3.53	1831	14.941

TIC:	5.3660	ng/uL
TIM:	20.8606	nmole/L
Total concentration:	5.4949	ng/uL

```
Sample peak width (sec): 5     Sample min peak height: 50    Sample baseline V to V?: Y    Sample baseline V to V points: 3
Sample filter: Binomial        Number of points for filter: 3  Sample start region (min): 0  Sample end region (min): 30
Marker peak width (sec): 5     Marker min peak height: 200   Marker baseline V to V?: Y    Marker baseline V to V points: 3
Lower marker selection: First peak > 500 RFU                 Upper marker selection: Last peak > 200 RFU
Ladder size (bp)75, 200, 300, 400, 500, 700, 1000, 1500, 2000, 3000, 4000, 5000, 7000, 10000, 20000
Quantification using: Lower Marker          Final concentration (ng/uL): 0.5000          Dilution factor: 12.0
```

Sample: SampA8
Well location: A8
Created: Monday, September 20, 2021 6:00:03 PM

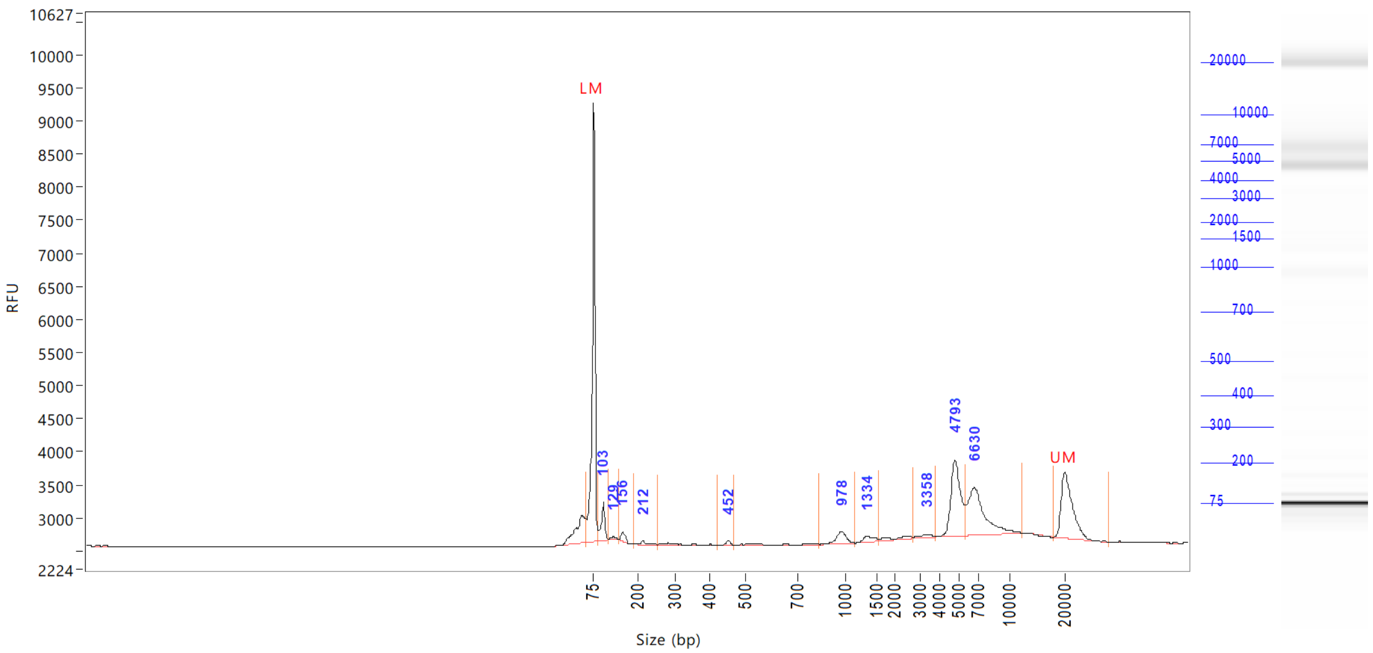

Peak	Size (bp)	Concentration (ng/uL)	From (bp)	To (bp)	Average size (bp)	CV%	RFU	Corrected peak area
1	75 (LM)	0.5000	54	87	72	7.44	6640	45.252
2	103	0.6143	87	116	98	5.61	582	4.633
3	129	0.0795	116	145	129	5.78	51	0.600
4	156	0.1903	145	190	157	3.86	144	1.435
5	212	0.0869	190	250	218	5.44	56	0.655
6	452	0.0785	420	468	449	1.82	70	0.592
7	978	0.3762	831	1156	965	3.94	183	2.837
8	1334	0.1883	1156	1539	1367	5.97	93	1.420
9	3358	0.1136	2743	3857	3263	8.27	53	0.857
10	4793	2.0530	3857	5667	4875	6.41	1159	15.483
11	6630	2.0931	5667	12196	7103	15.66	719	15.786
12	20000 (UM)	0.1473	17927	27738	20556	5.58	998	13.328

TIC:	5.8735	ng/uL
TIM:	16.3433	nmole/L
Total concentration:	6.2882	ng/uL

```
Sample peak width (sec): 5     Sample min peak height: 50     Sample baseline V to V?: Y     Sample baseline V to V points: 3
Sample filter: Binomial        Number of points for filter: 3  Sample start region (min): 0  Sample end region (min): 30
Marker peak width (sec): 5     Marker min peak height: 200    Marker baseline V to V?: Y     Marker baseline V to V points: 3
Lower marker selection: First peak > 500 RFU                  Upper marker selection: Last peak > 200 RFU
Ladder size (bp)75, 200, 300, 400, 500, 700, 1000, 1500, 2000, 3000, 4000, 5000, 7000, 10000, 20000
Quantification using: Lower Marker          Final concentration (ng/uL): 0.5000          Dilution factor: 12.0
```

Sample: SampB8
Well location: B8
Created: Monday, September 20, 2021 6:00:03 PM

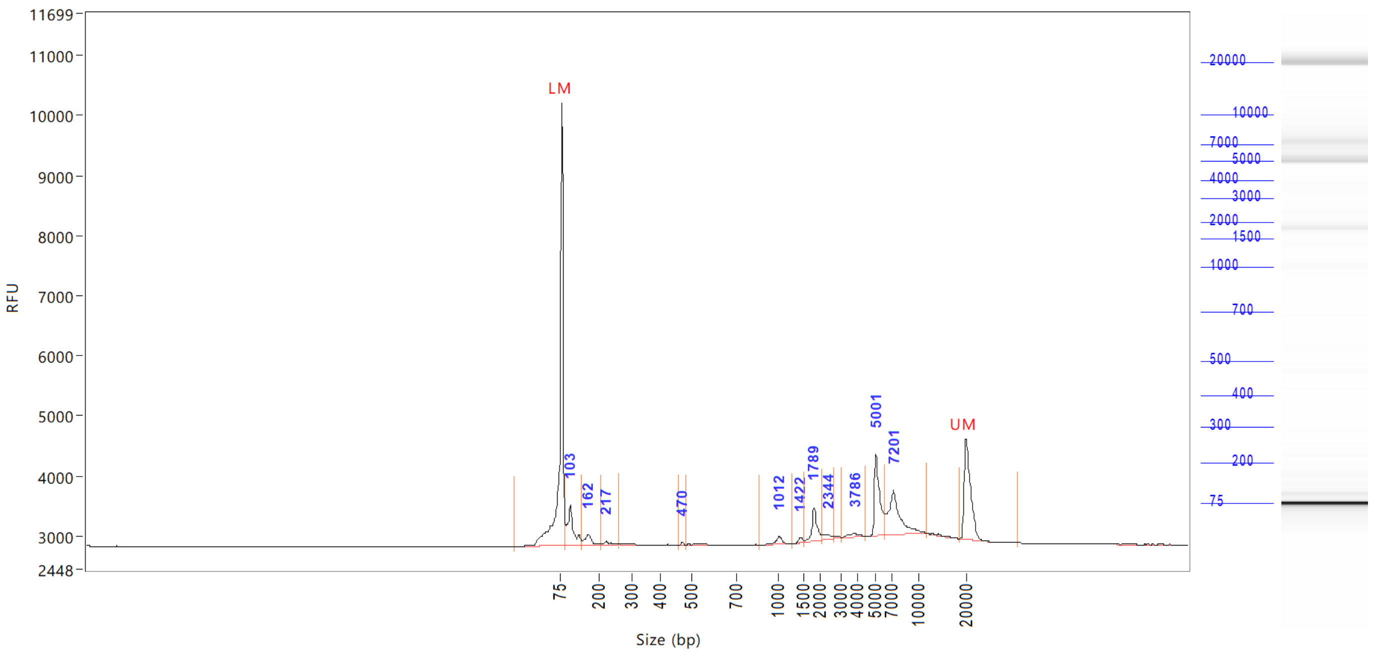

Peak	Size (bp)	Concentration (ng/uL)	From (bp)	To (bp)	Average size (bp)	CV%	RFU	Corrected peak area
1	75 (LM)	0.5000	0	87	63	32.10	7379	60.825
2	103	0.8870	87	141	106	12.93	671	8.992
3	162	0.2838	141	203	161	8.35	172	2.877
4	217	0.0809	203	260	224	6.08	58	0.820
5	470	0.0266	456	481	467	1.04	51	0.270
6	1012	0.1336	866	1267	1015	5.36	130	1.355
7	1422	0.0584	1267	1489	1414	2.87	78	0.593
8	1789	0.4688	1489	2087	1805	6.31	562	4.753
9	2344	0.1009	2087	2629	2324	6.59	83	1.023
10	3786	0.0981	3037	4414	3633	7.82	60	0.995
11	5001	1.0868	4414	6186	5297	6.92	1352	11.017
12	7201	1.1637	6186	11586	7486	12.22	735	11.797
13	20000 (UM)	0.1188	18537	30760	20489	4.58	1676	14.451

TIC:	4.3887	ng/uL
TIM:	18.7495	nmole/L
Total concentration:	4.5769	ng/uL

```
Sample peak width (sec): 5    Sample min peak height: 50    Sample baseline V to V?: Y    Sample baseline V to V points: 3
Sample filter: Binomial       Number of points for filter: 3  Sample start region (min): 0  Sample end region (min): 30
Marker peak width (sec): 5    Marker min peak height: 200   Marker baseline V to V?: Y    Marker baseline V to V points: 3
Lower marker selection: First peak > 500 RFU                Upper marker selection: Last peak > 200 RFU
Ladder size (bp)75, 200, 300, 400, 500, 700, 1000, 1500, 2000, 3000, 4000, 5000, 7000, 10000, 20000
Quantification using: Lower Marker          Final concentration (ng/uL): 0.5000          Dilution factor: 12.0
```

Sample: SampC8
Well location: C8
Created: Monday, September 20, 2021 6:00:03 PM

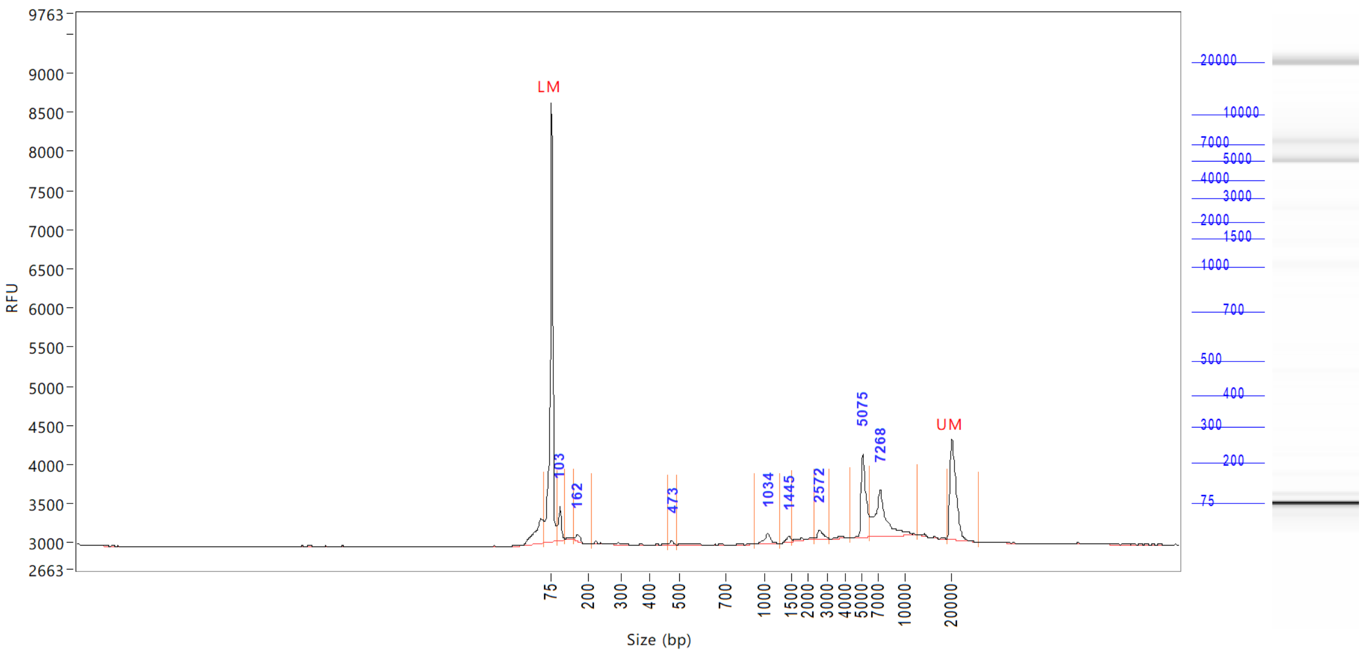

Peak	Size (bp)	Concentration (ng/uL)	From (bp)	To (bp)	Average size (bp)	CV%	RFU	Corrected peak area
1	75 (LM)	0.5000	52	95	72	9.23	5614	38.730
2	103	0.4844	95	122	102	4.72	442	3.127
3	162	0.1429	150	208	163	5.41	89	0.922
4	473	0.0603	460	489	471	1.26	63	0.389
5	1034	0.2330	918	1267	1025	5.27	132	1.504
6	1445	0.0849	1267	1501	1422	3.06	69	0.548
7	2572	0.1539	2344	3072	2621	5.24	122	0.994
8	5075	1.1817	4345	6038	5253	6.17	1068	7.628
9	7268	1.5149	6038	12806	7555	14.45	600	9.779
10	20000 (UM)	0.1344	18903	25561	20388	3.53	1288	10.413

TIC:	3.8559	ng/uL
TIM:	10.7189	nmole/L
Total concentration:	4.3902	ng/uL

Sample peak width (sec): 5 Sample min peak height: 50 Sample baseline V to V?: Y Sample baseline V to V points: 3
Sample filter: Binomial Number of points for filter: 3 Sample start region (min): 0 Sample end region (min): 30
Marker peak width (sec): 5 Marker min peak height: 200 Marker baseline V to V?: Y Marker baseline V to V points: 3
Lower marker selection: First peak > 500 RFU Upper marker selection: Last peak > 200 RFU
Ladder size (bp)75, 200, 300, 400, 500, 700, 1000, 1500, 2000, 3000, 4000, 5000, 7000, 10000, 20000
Quantification using: Lower Marker Final concentration (ng/uL): 0.5000 Dilution factor: 12.0

Sample: SampD8
Well location: D8
Created: Monday, September 20, 2021 6:00:03 PM

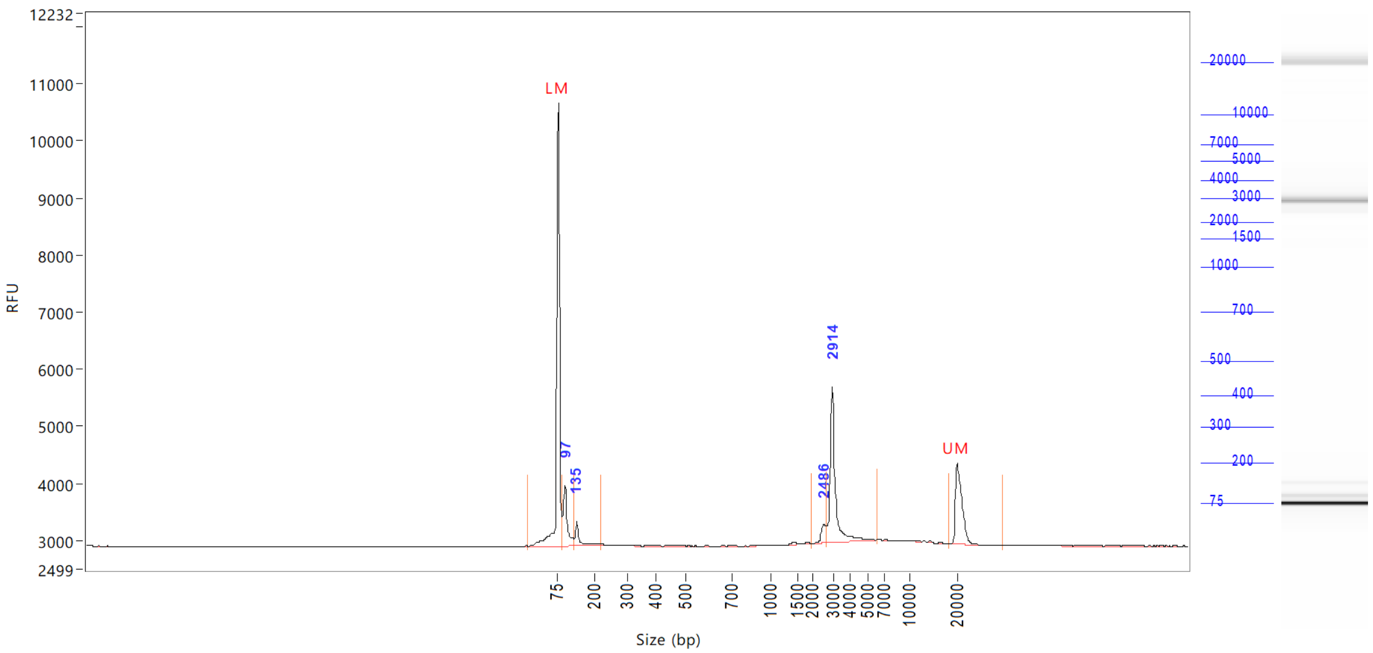

Peak	Size (bp)	Concentration (ng/uL)	From (bp)	To (bp)	Average size (bp)	CV%	RFU	Corrected peak area
1	75 (LM)	0.5000	0	86	69	20.98	7771	52.747
2	97	1.2822	86	128	99	10.32	1063	11.272
3	135	0.5357	128	217	153	17.19	439	4.709
4	2486	0.2850	1942	2629	2460	4.94	324	2.506
5	2914	2.4238	2629	6260	3058	13.48	2732	21.308
6	20000 (UM)	0.1167	18293	29551	20496	4.55	1428	12.312

TIC:	4.5267	ng/uL
TIM:	28.5171	nmole/L
Total concentration:	4.7694	ng/uL

Sample peak width (sec): 5   Sample min peak height: 50   Sample baseline V to V?: Y   Sample baseline V to V points: 3
Sample filter: Binomial   Number of points for filter: 3 Sample start region (min): 0 Sample end region (min): 30
Marker peak width (sec): 5   Marker min peak height: 200   Marker baseline V to V?: Y   Marker baseline V to V points: 3
Lower marker selection: First peak > 500 RFU   Upper marker selection: Last peak > 200 RFU
Ladder size (bp)75, 200, 300, 400, 500, 700, 1000, 1500, 2000, 3000, 4000, 5000, 7000, 10000, 20000
Quantification using: Lower Marker   Final concentration (ng/uL): 0.5000   Dilution factor: 12.0

Sample: SampE8
Well location: E8
Created: Monday, September 20, 2021 6:00:03 PM

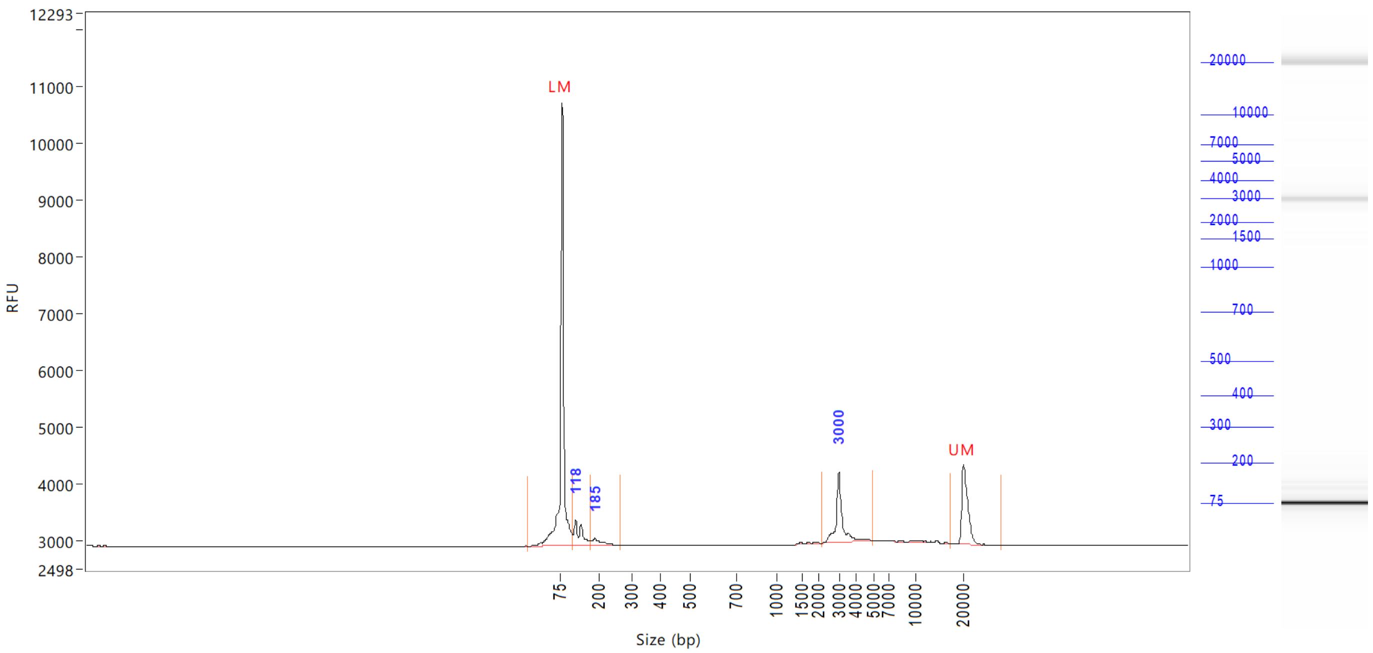

Peak	Size<br>(bp)	Concentration<br>(ng/uL)	From<br>(bp)	To<br>(bp)	Average size<br>(bp)	CV%	RFU	Corrected peak area
1	75 (LM)	0.5000	0	110	71	22.95	7814	62.978
2	118	0.7095	110	169	132	11.65	441	7.447
3	185	0.2452	169	264	195	9.37	118	2.573
4	3000	1.1127	2144	5000	3054	12.83	1246	11.679
5	20000 (UM)	0.0915	17317	27979	20417	4.11	1402	11.528

TIC:	2.0674	ng/uL
TIM:	11.4984	nmole/L
Total concentration:	2.2717	ng/uL

```
Sample peak width (sec): 5    Sample min peak height: 50    Sample baseline V to V?: Y    Sample baseline V to V points: 3
Sample filter: Binomial       Number of points for filter: 3  Sample start region (min): 0  Sample end region (min): 30
Marker peak width (sec): 5    Marker min peak height: 200   Marker baseline V to V?: Y    Marker baseline V to V points: 3
Lower marker selection: First peak > 500 RFU                Upper marker selection: Last peak > 200 RFU
Ladder size (bp)75, 200, 300, 400, 500, 700, 1000, 1500, 2000, 3000, 4000, 5000, 7000, 10000, 20000
Quantification using: Lower Marker          Final concentration (ng/uL): 0.5000          Dilution factor: 12.0
```

Sample: SampF8
Well location: F8
Created: Monday, September 20, 2021 6:00:03 PM

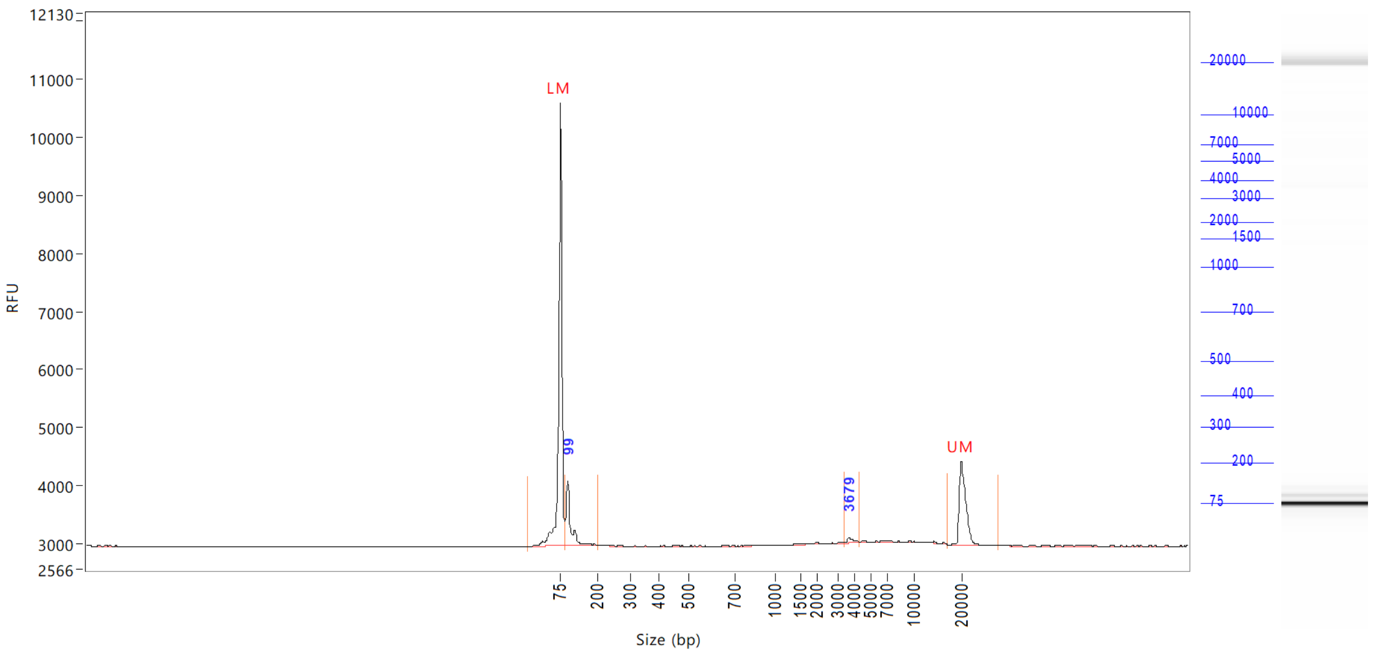

Peak	Size (bp)	Concentration (ng/uL)	From (bp)	To (bp)	Average size (bp)	CV%	RFU	Corrected peak area
1	75 (LM)	0.5000	0	87	68	18.96	7629	60.938
2	99	1.1959	87	200	104	15.70	1101	12.146
3	3679	0.0838	3429	4277	3809	5.38	89	0.851
4	20000 (UM)	0.1024	17195	27859	20392	4.42	1437	12.483

TIC:	1.2797	ng/uL
TIM:	18.9209	nmole/L
Total concentration:	1.6227	ng/uL

Sample peak width (sec): 5   Sample min peak height: 50   Sample baseline V to V?: Y   Sample baseline V to V points: 3
Sample filter: Binomial   Number of points for filter: 3 Sample start region (min): 0 Sample end region (min): 30
Marker peak width (sec): 5   Marker min peak height: 200   Marker baseline V to V?: Y   Marker baseline V to V points: 3
Lower marker selection: First peak > 500 RFU   Upper marker selection: Last peak > 200 RFU
Ladder size (bp)75, 200, 300, 400, 500, 700, 1000, 1500, 2000, 3000, 4000, 5000, 7000, 10000, 20000
Quantification using: Lower Marker   Final concentration (ng/uL): 0.5000   Dilution factor: 12.0

Sample: SampG8
Well location: G8
Created: Monday, September 20, 2021 6:00:03 PM

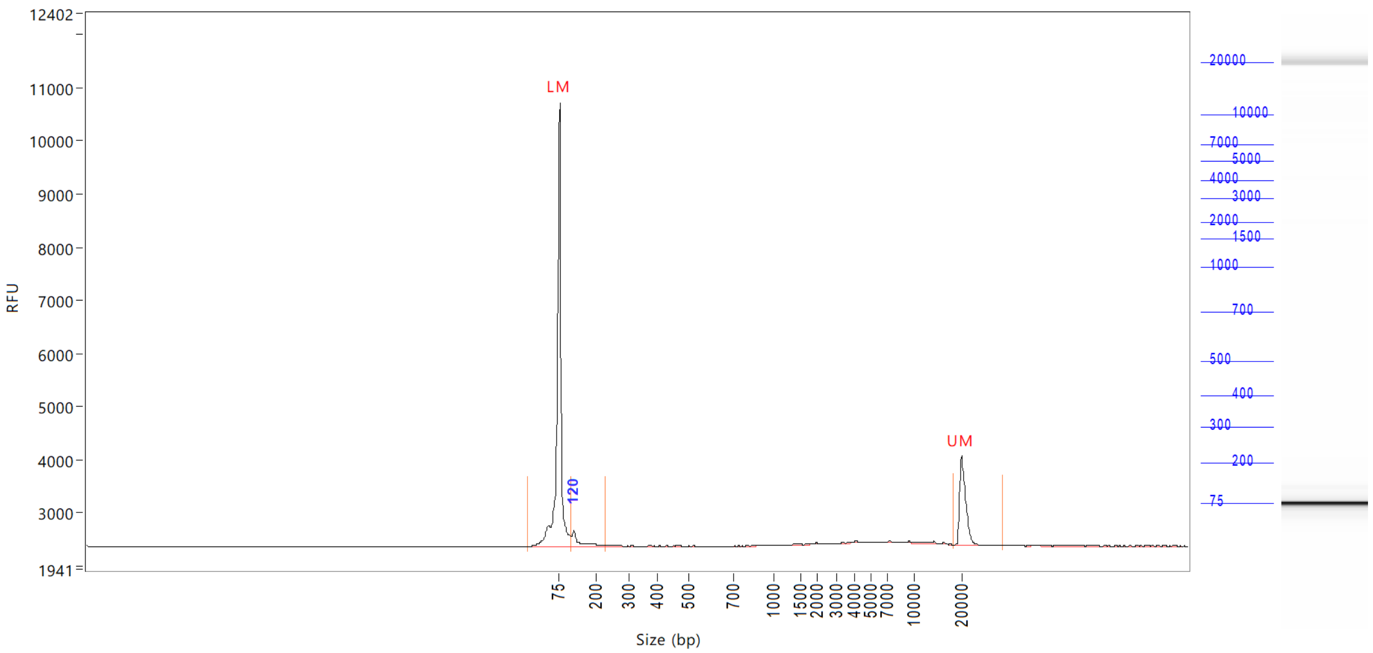

Peak	Size<br>(bp)	Concentration<br>(ng/uL)	From<br>(bp)	To<br>(bp)	Average size<br>(bp)	CV%	RFU	Corrected peak area
1	75 (LM)	0.5000	0	112	68	23.11	8357	77.229
2	120	0.3778	112	228	141	20.71	300	4.863
3	20000 (UM)	0.0863	18415	28705	20393	4.07	1677	13.326

TIC:	0.3778	ng/uL
TIM:	4.4034	nmole/L
Total concentration:	0.6695	ng/uL

```
Sample peak width (sec): 5    Sample min peak height: 50    Sample baseline V to V?: Y    Sample baseline V to V points: 3
Sample filter: Binomial       Number of points for filter: 3  Sample start region (min): 0  Sample end region (min): 30
Marker peak width (sec): 5    Marker min peak height: 200   Marker baseline V to V?: Y    Marker baseline V to V points: 3
Lower marker selection: First peak > 500 RFU                Upper marker selection: Last peak > 200 RFU
Ladder size (bp)75, 200, 300, 400, 500, 700, 1000, 1500, 2000, 3000, 4000, 5000, 7000, 10000, 20000
Quantification using: Lower Marker          Final concentration (ng/uL): 0.5000          Dilution factor: 12.0
```

Sample: SampH8
Well location: H8
Created: Monday, September 20, 2021 6:00:03 PM

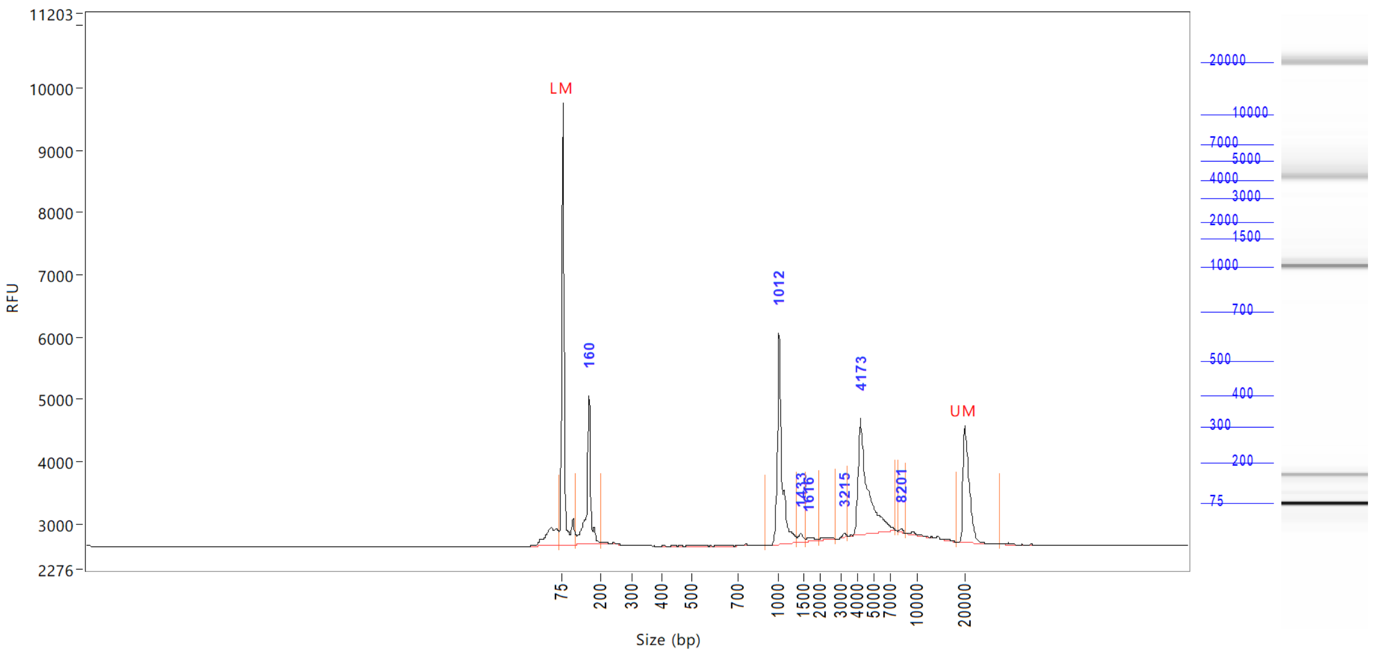

Peak	Size (bp)	Concentration (ng/uL)	From (bp)	To (bp)	Average size (bp)	CV%	RFU	Corrected peak area
1	75 (LM)	0.5000	62	118	75	13.51	7112	46.484
2	160	2.8112	118	200	155	8.53	2385	21.779
3	1012	3.3050	905	1356	1050	7.33	3389	25.605
4	1433	0.1555	1356	1520	1428	3.05	140	1.205
5	1616	0.1045	1520	1962	1707	7.17	53	0.810
6	3215	0.0451	2772	3465	3154	3.23	61	0.350
7	4173	3.3982	3465	7601	4591	14.43	1865	26.327
8	8201	0.0369	7801	8800	8172	2.25	57	0.286
9	20000 (UM)	0.1677	18415	27375	20458	3.84	1869	15.593

TIC:	9.8566	ng/uL
TIM:	36.5205	nmole/L
Total concentration:	10.2120	ng/uL

```
Sample peak width (sec): 5    Sample min peak height: 50    Sample baseline V to V?: Y    Sample baseline V to V points: 3
Sample filter: Binomial       Number of points for filter: 3  Sample start region (min): 0  Sample end region (min): 30
Marker peak width (sec): 5    Marker min peak height: 200   Marker baseline V to V?: Y    Marker baseline V to V points: 3
Lower marker selection: First peak > 500 RFU                Upper marker selection: Last peak > 200 RFU
Ladder size (bp)75, 200, 300, 400, 500, 700, 1000, 1500, 2000, 3000, 4000, 5000, 7000, 10000, 20000
Quantification using: Lower Marker          Final concentration (ng/uL): 0.5000          Dilution factor: 12.0
```

Sample: SampA9
Well location: A9
Created: Monday, September 20, 2021 6:00:03 PM

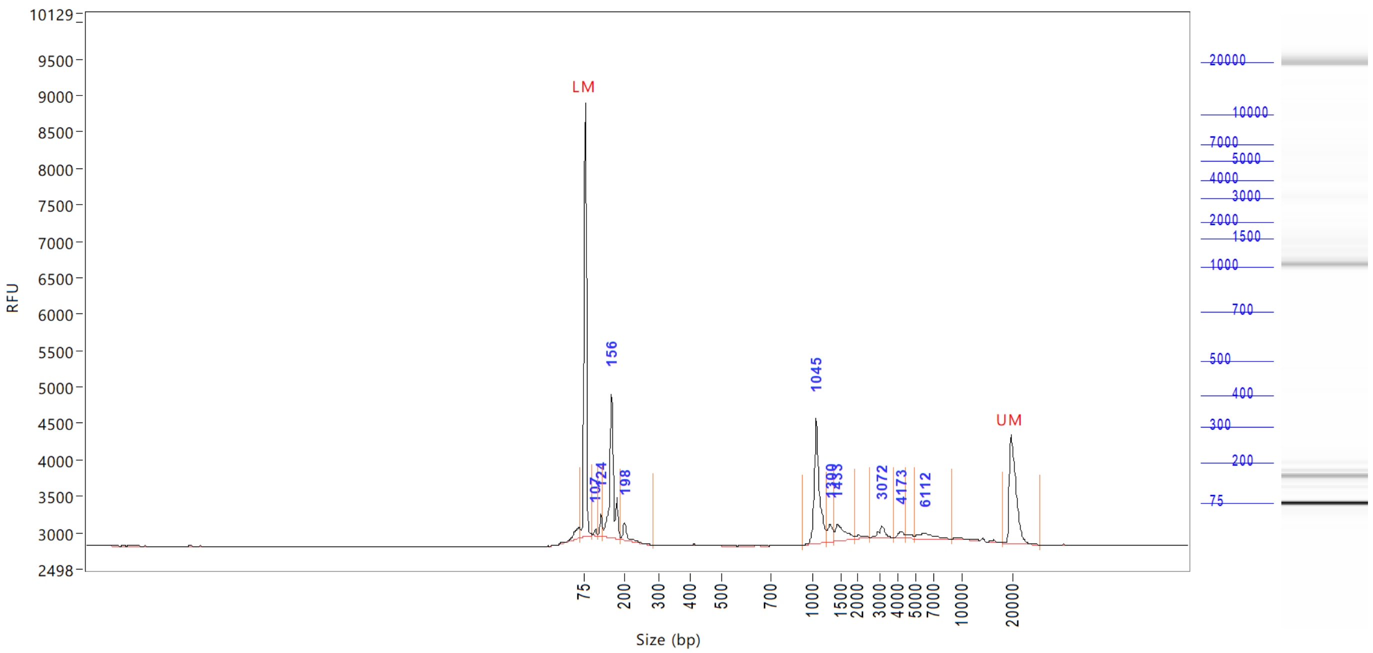

Peak	Size (bp)	Concentration (ng/uL)	From (bp)	To (bp)	Average size (bp)	CV%	RFU	Corrected peak area
1	75 (LM)	0.5000	62	97	73	4.59	5965	31.420
2	107	0.0916	97	114	105	4.10	84	0.480
3	124	0.3317	114	131	122	3.44	300	1.737
4	156	3.3001	131	187	156	6.28	1974	17.282
5	198	0.4216	187	284	204	7.35	245	2.208
6	1045	2.6568	922	1234	1060	5.56	1712	13.913
7	1300	0.3818	1234	1367	1296	2.98	236	1.999
8	1433	0.6672	1367	1904	1526	8.86	221	3.494
9	3072	0.2780	2515	3750	3067	6.33	155	1.456
10	4173	0.1633	3750	4449	4145	3.71	100	0.855
11	6112	0.3288	4931	9000	6426	15.44	72	1.722
12	20000 (UM)	0.1966	18171	25561	20326	3.80	1491	12.354

TIC:	8.6209	ng/uL
TIM:	49.6960	nmole/L
Total concentration:	9.0637	ng/uL

Sample peak width (sec): 5   Sample min peak height: 50   Sample baseline V to V?: Y   Sample baseline V to V points: 3
Sample filter: Binomial   Number of points for filter: 3   Sample start region (min): 0   Sample end region (min): 30
Marker peak width (sec): 5   Marker min peak height: 200   Marker baseline V to V?: Y   Marker baseline V to V points: 3
Lower marker selection: First peak > 500 RFU   Upper marker selection: Last peak > 200 RFU
Ladder size (bp)75, 200, 300, 400, 500, 700, 1000, 1500, 2000, 3000, 4000, 5000, 7000, 10000, 20000
Quantification using: Lower Marker   Final concentration (ng/uL): 0.5000   Dilution factor: 12.0

Sample: SampB9
Well location: B9
Created: Monday, September 20, 2021 6:00:03 PM

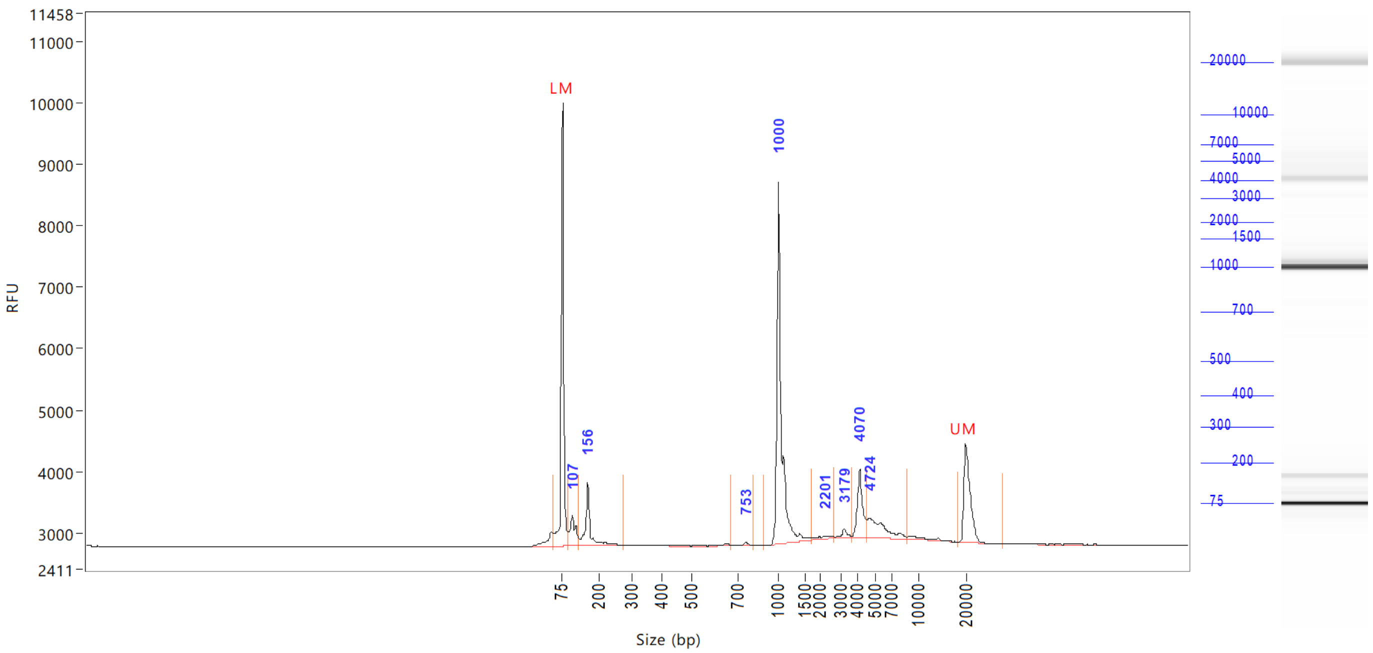

Peak	Size (bp)	Concentration (ng/uL)	From (bp)	To (bp)	Average size (bp)	CV%	RFU	Corrected peak area
1	75 (LM)	0.5000	45	97	72	9.88	7223	46.368
2	107	0.8017	97	131	111	8.17	489	6.196
3	156	1.4093	131	271	163	14.17	1033	10.891
4	753	0.0616	672	814	749	3.34	62	0.476
5	1000	5.5992	896	1731	1047	9.71	5880	43.270
6	2201	0.1427	1731	2686	2163	11.87	52	1.103
7	3179	0.1596	2686	3643	3167	6.67	151	1.234
8	4070	1.1411	3643	4483	4099	4.11	1132	8.819
9	4724	1.0894	4483	8734	5660	19.46	327	8.419
10	20000 (UM)	0.1463	18293	27738	20392	3.96	1610	13.563

TIC:	10.4046	ng/uL
TIM:	35.9798	nmole/L
Total concentration:	10.6937	ng/uL

```
Sample peak width (sec): 5    Sample min peak height: 50    Sample baseline V to V?: Y    Sample baseline V to V points: 3
Sample filter: Binomial       Number of points for filter: 3  Sample start region (min): 0  Sample end region (min): 30
Marker peak width (sec): 5    Marker min peak height: 200   Marker baseline V to V?: Y    Marker baseline V to V points: 3
Lower marker selection: First peak > 500 RFU                Upper marker selection: Last peak > 200 RFU
Ladder size (bp)75, 200, 300, 400, 500, 700, 1000, 1500, 2000, 3000, 4000, 5000, 7000, 10000, 20000
Quantification using: Lower Marker          Final concentration (ng/uL): 0.5000          Dilution factor: 12.0
```

Sample: SampC9
Well location: C9
Created: Monday, September 20, 2021 6:00:03 PM

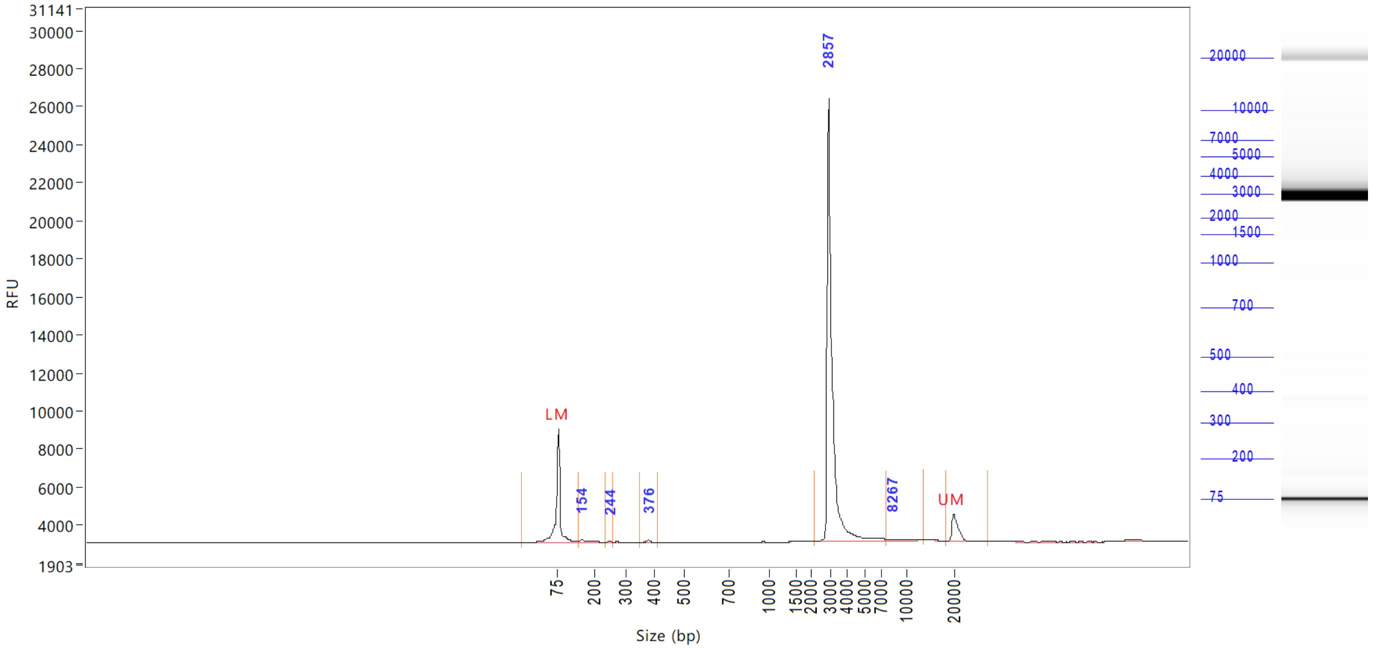

Peak	Size (bp)	Concentration (ng/uL)	From (bp)	To (bp)	Average size (bp)	CV%	RFU	Corrected peak area
1	75 (LM)	0.5000	0	143	70	27.81	5957	53.881
2	154	0.3463	143	233	173	13.21	146	3.110
3	244	0.0444	233	255	243	2.26	52	0.399
4	376	0.0916	343	405	370	2.71	120	0.823
5	2857	20.0205	2144	7601	3069	18.01	23313	179.787
6	8267	0.3179	7601	13659	9724	16.35	83	2.855
7	20000 (UM)	0.1105	18293	27496	20499	4.23	1387	11.913

TIC:	20.8207	ng/uL
TIM:	14.7913	nmole/L
Total concentration:	21.0725	ng/uL

```
Sample peak width (sec): 5    Sample min peak height: 50    Sample baseline V to V?: Y    Sample baseline V to V points: 3
Sample filter: Binomial       Number of points for filter: 3  Sample start region (min): 0  Sample end region (min): 30
Marker peak width (sec): 5    Marker min peak height: 200   Marker baseline V to V?: Y    Marker baseline V to V points: 3
Lower marker selection: First peak > 500 RFU                Upper marker selection: Last peak > 200 RFU
Ladder size (bp)75, 200, 300, 400, 500, 700, 1000, 1500, 2000, 3000, 4000, 5000, 7000, 10000, 20000
Quantification using: Lower Marker          Final concentration (ng/uL): 0.5000          Dilution factor: 12.0
```

Sample: SampD9
Well location: D9
Created: Monday, September 20, 2021 6:00:03 PM

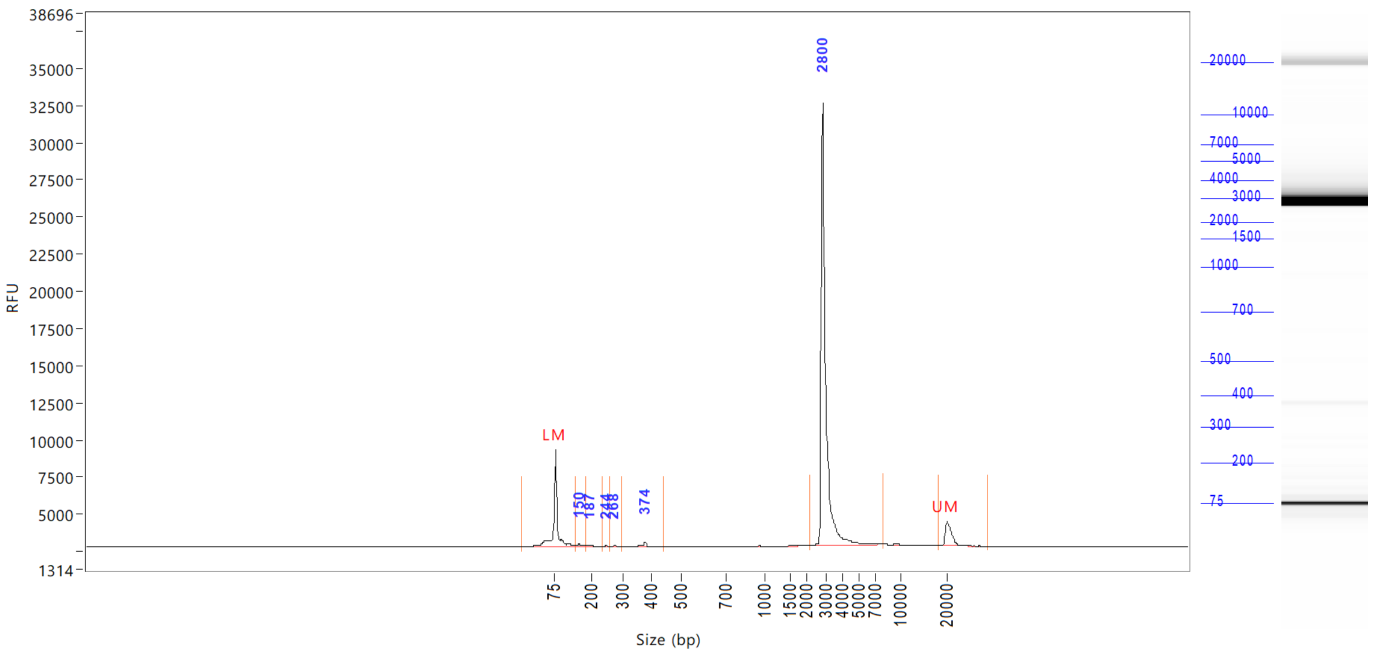

Peak	Size (bp)	Concentration (ng/uL)	From (bp)	To (bp)	Average size (bp)	CV%	RFU	Corrected peak area
1	75 (LM)	0.5000	0	141	70	30.40	6485	55.438
2	150	0.2153	141	179	157	6.47	152	1.989
3	187	0.1168	179	232	192	6.11	112	1.079
4	244	0.0326	232	257	241	1.79	54	0.301
5	268	0.0442	257	295	270	2.62	52	0.408
6	374	0.2868	295	441	368	5.90	325	2.650
7	2800	23.2708	2172	7934	2958	14.06	29811	215.013
8	20000 (UM)	0.1209	18171	28584	20394	4.54	1619	13.409

TIC:	23.9666	ng/uL
TIM:	17.9792	nmole/L
Total concentration:	24.2632	ng/uL

Sample peak width (sec): 5 Sample min peak height: 50 Sample baseline V to V?: Y Sample baseline V to V points: 3
Sample filter: Binomial Number of points for filter: 3 Sample start region (min): 0 Sample end region (min): 30
Marker peak width (sec): 5 Marker min peak height: 200 Marker baseline V to V?: Y Marker baseline V to V points: 3
Lower marker selection: First peak > 500 RFU Upper marker selection: Last peak > 200 RFU
Ladder size (bp)75, 200, 300, 400, 500, 700, 1000, 1500, 2000, 3000, 4000, 5000, 7000, 10000, 20000
Quantification using: Lower Marker Final concentration (ng/uL): 0.5000 Dilution factor: 12.0

Sample: SampE9
Well location: E9
Created: Monday, September 20, 2021 6:00:03 PM

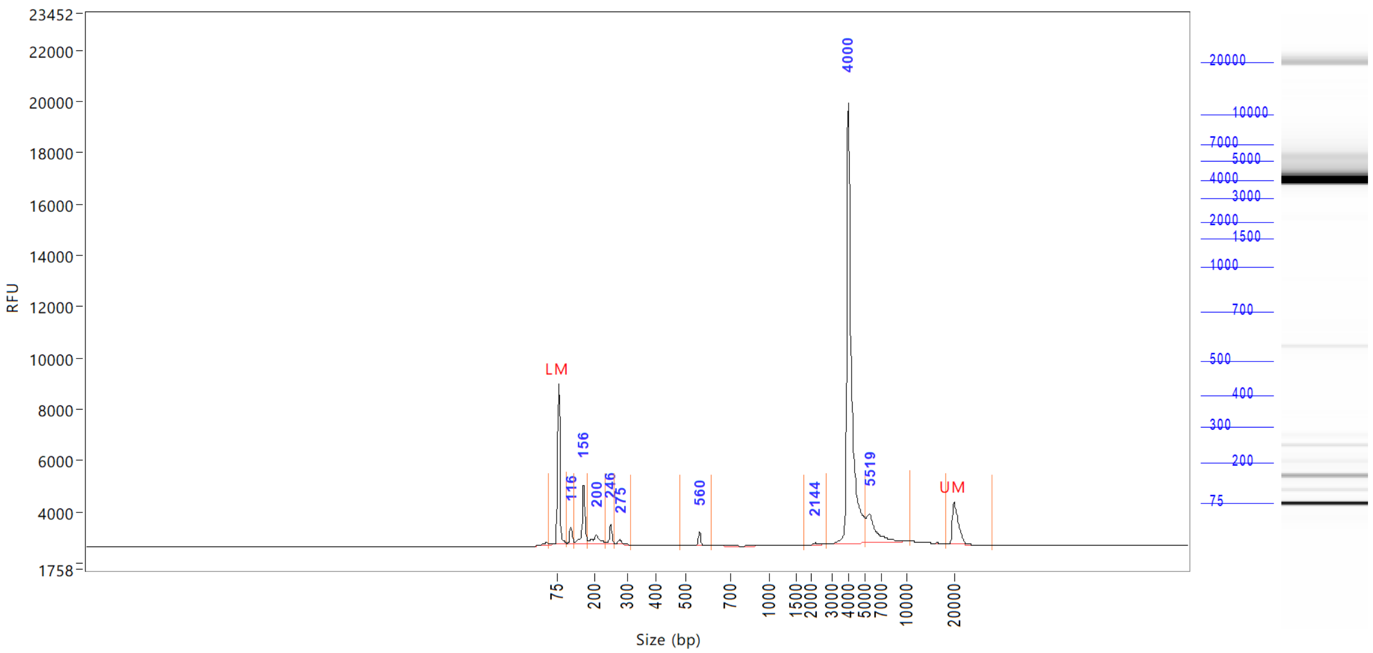

Peak	Size (bp)	Concentration (ng/uL)	From (bp)	To (bp)	Average size (bp)	CV%	RFU	Corrected peak area
1	75 (LM)	0.5000	45	103	73	6.48	6266	35.426
2	116	0.6316	103	128	113	4.14	612	3.729
3	156	2.5554	128	173	155	4.63	2273	15.088
4	200	0.8960	173	230	199	6.81	360	5.290
5	246	0.6909	230	260	243	1.95	737	4.080
6	275	0.2499	260	311	274	2.77	183	1.476
7	560	0.5791	481	618	555	3.44	542	3.419
8	2144	0.0989	1750	2743	2158	8.08	69	0.584
9	4000	22.3446	2743	5001	4102	6.85	17221	131.931
10	5519	2.7701	5001	10489	6079	16.27	1148	16.356
11	20000 (UM)	0.1865	18415	28100	20403	3.83	1648	13.214

TIC:	30.8166	ng/uL
TIM:	61.3682	nmole/L
Total concentration:	30.9336	ng/uL

```
Sample peak width (sec): 5    Sample min peak height: 50    Sample baseline V to V?: Y    Sample baseline V to V points: 3
Sample filter: Binomial       Number of points for filter: 3  Sample start region (min): 0  Sample end region (min): 30
Marker peak width (sec): 5    Marker min peak height: 200   Marker baseline V to V?: Y    Marker baseline V to V points: 3
Lower marker selection: First peak > 500 RFU                Upper marker selection: Last peak > 200 RFU
Ladder size (bp)75, 200, 300, 400, 500, 700, 1000, 1500, 2000, 3000, 4000, 5000, 7000, 10000, 20000
Quantification using: Lower Marker          Final concentration (ng/uL): 0.5000          Dilution factor: 12.0
```

Sample: SampF9
Well location: F9
Created: Monday, September 20, 2021 6:00:03 PM

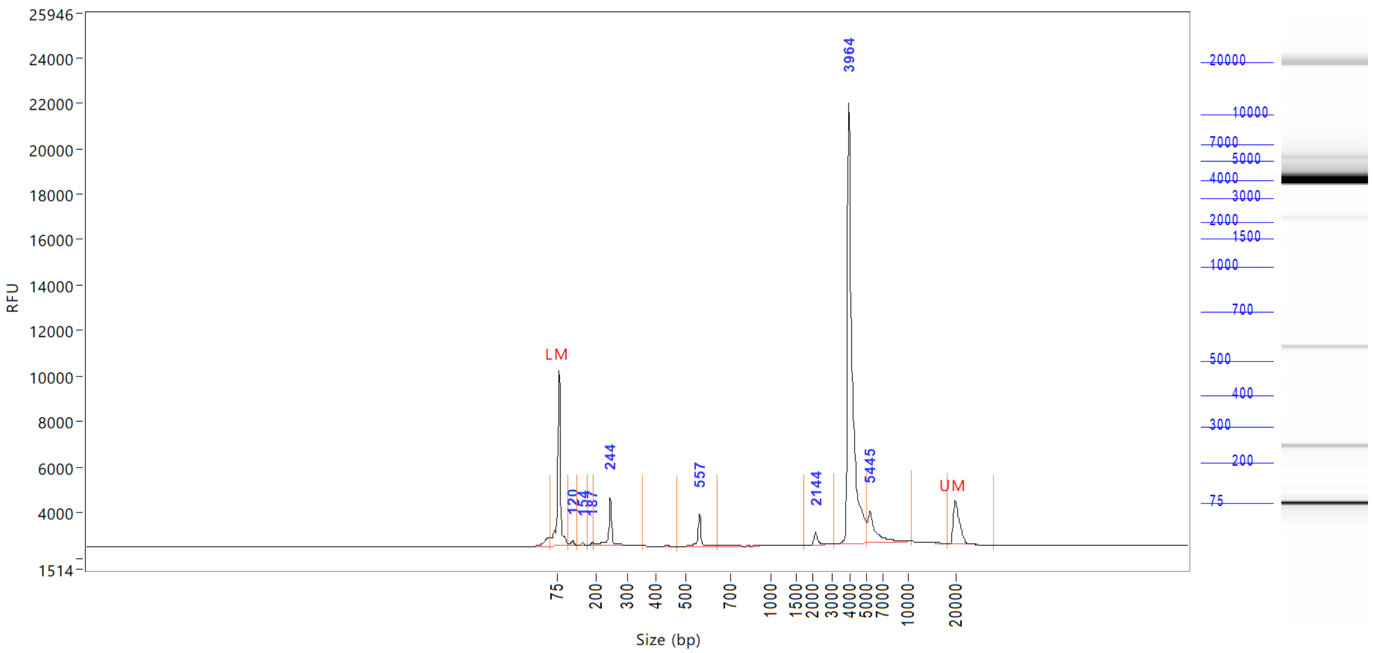

Peak	Size (bp)	Concentration (ng/uL)	From (bp)	To (bp)	Average size (bp)	CV%	RFU	Corrected peak area
1	75 (LM)	0.5000	47	107	73	11.87	7703	53.582
2	120	0.1969	107	137	117	5.27	190	1.759
3	154	0.0963	137	171	153	4.19	122	0.860
4	187	0.0898	171	194	185	2.97	111	0.802
5	244	1.8105	194	354	243	8.25	2131	16.168
6	557	1.0129	470	639	557	4.01	1411	9.046
7	2144	0.3675	1750	3001	2173	7.82	576	3.282
8	3964	16.7448	3001	5075	4097	6.74	19400	149.538
9	5445	1.8244	5075	10489	6094	16.44	1433	16.292
10	20000 (UM)	0.1489	18293	28100	20328	3.75	1931	15.957

TIC:	22.1431	ng/uL
TIM:	27.3426	nmole/L
Total concentration:	22.2840	ng/uL

```
Sample peak width (sec): 5    Sample min peak height: 50    Sample baseline V to V?: Y    Sample baseline V to V points: 3
Sample filter: Binomial       Number of points for filter: 3  Sample start region (min): 0  Sample end region (min): 30
Marker peak width (sec): 5    Marker min peak height: 200   Marker baseline V to V?: Y    Marker baseline V to V points: 3
Lower marker selection: First peak > 500 RFU                Upper marker selection: Last peak > 200 RFU
Ladder size (bp)75, 200, 300, 400, 500, 700, 1000, 1500, 2000, 3000, 4000, 5000, 7000, 10000, 20000
Quantification using: Lower Marker          Final concentration (ng/uL): 0.5000          Dilution factor: 12.0
```

Sample: SampG9
Well location: G9
Created: Monday, September 20, 2021 6:00:03 PM

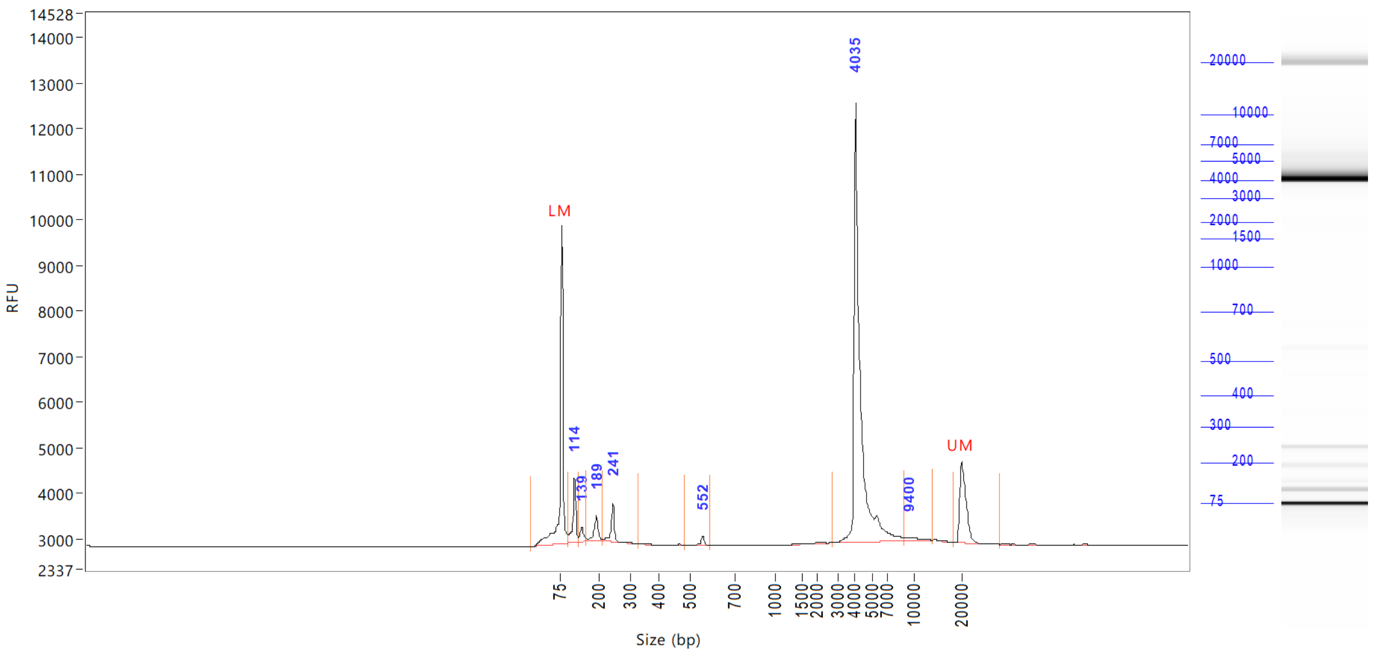

Peak	Size (bp)	Concentration (ng/uL)	From (bp)	To (bp)	Average size (bp)	CV%	RFU	Corrected peak area
1	75 (LM)	0.5000	0	97	66	27.90	6974	51.323
2	114	1.2380	97	129	113	5.56	1406	10.590
3	139	0.3364	129	158	140	4.72	301	2.877
4	189	0.5998	158	208	185	4.83	548	5.131
5	241	0.7150	208	325	240	6.05	850	6.116
6	552	0.1777	479	585	542	3.76	196	1.520
7	4035	10.3624	2743	8800	4398	17.35	9651	88.638
8	9400	0.1601	8800	14025	10099	10.92	68	1.369
9	20000 (UM)	0.1443	18293	28100	20429	3.79	1783	14.815

TIC:	13.5892	ng/uL
TIM:	36.6177	nmole/L
Total concentration:	13.8138	ng/uL

Sample peak width (sec): 5 Sample min peak height: 50 Sample baseline V to V?: Y Sample baseline V to V points: 3
Sample filter: Binomial Number of points for filter: 3 Sample start region (min): 0 Sample end region (min): 30
Marker peak width (sec): 5 Marker min peak height: 200 Marker baseline V to V?: Y Marker baseline V to V points: 3
Lower marker selection: First peak > 500 RFU Upper marker selection: Last peak > 200 RFU
Ladder size (bp)75, 200, 300, 400, 500, 700, 1000, 1500, 2000, 3000, 4000, 5000, 7000, 10000, 20000
Quantification using: Lower Marker Final concentration (ng/uL): 0.5000 Dilution factor: 12.0

Sample: SampH9
Well location: H9
Created: Monday, September 20, 2021 6:00:03 PM

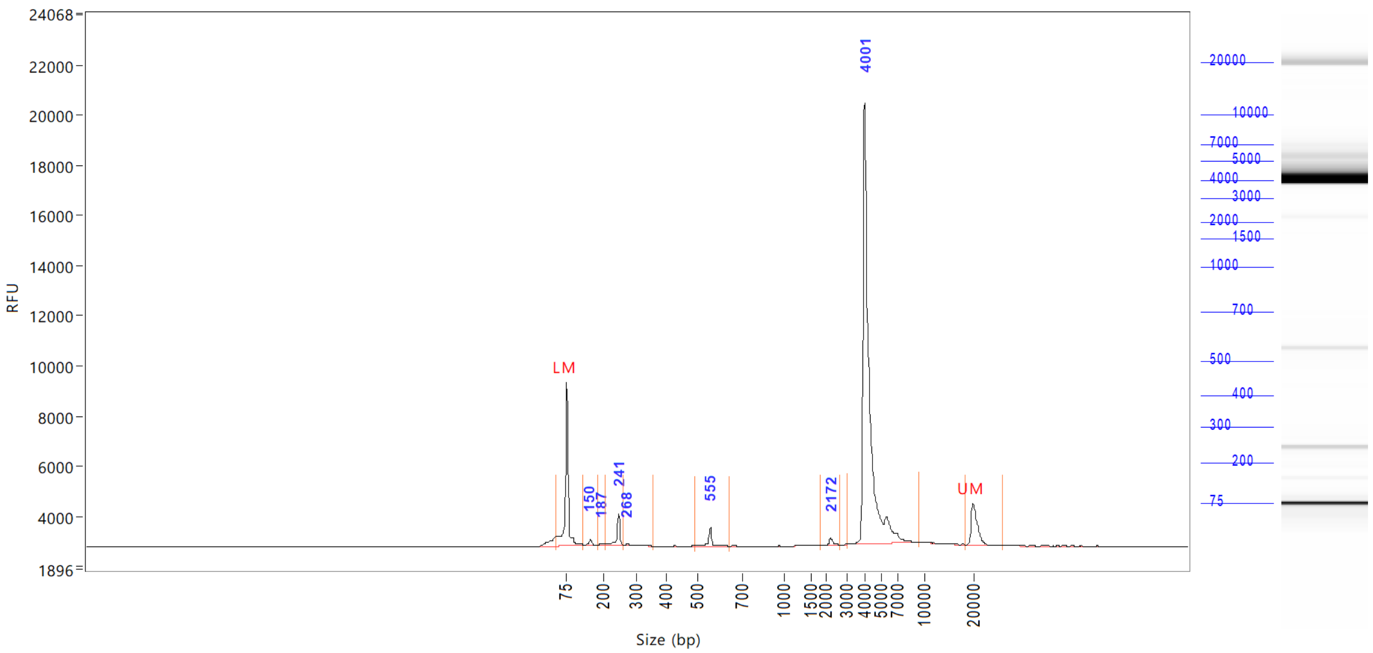

Peak	Size (bp)	Concentration (ng/uL)	From (bp)	To (bp)	Average size (bp)	CV%	RFU	Corrected peak area
1	75 (LM)	0.5000	43	129	72	15.06	6543	47.003
2	150	0.2453	129	177	148	4.96	265	1.922
3	187	0.0905	177	203	190	3.69	73	0.709
4	241	1.0671	203	259	237	3.68	1279	8.360
5	268	0.1331	259	354	282	6.93	69	1.043
6	555	0.6877	494	641	553	4.49	743	5.387
7	2172	0.1840	1808	2629	2182	5.10	263	1.442
8	4001	20.7916	3001	9334	4295	14.57	17617	162.878
9	20000 (UM)	0.1416	18415	26287	20378	3.77	1637	13.314

TIC:	23.1994	ng/uL
TIM:	21.8431	nmole/L
Total concentration:	23.4734	ng/uL

```
Sample peak width (sec): 5    Sample min peak height: 50    Sample baseline V to V?: Y    Sample baseline V to V points: 3
Sample filter: Binomial       Number of points for filter: 3  Sample start region (min): 0  Sample end region (min): 30
Marker peak width (sec): 5    Marker min peak height: 200   Marker baseline V to V?: Y    Marker baseline V to V points: 3
Lower marker selection: First peak > 500 RFU                Upper marker selection: Last peak > 200 RFU
Ladder size (bp)75, 200, 300, 400, 500, 700, 1000, 1500, 2000, 3000, 4000, 5000, 7000, 10000, 20000
Quantification using: Lower Marker          Final concentration (ng/uL): 0.5000          Dilution factor: 12.0
```

Sample: SampA10
Well location: A10
Created: Monday, September 20, 2021 6:00:03 PM

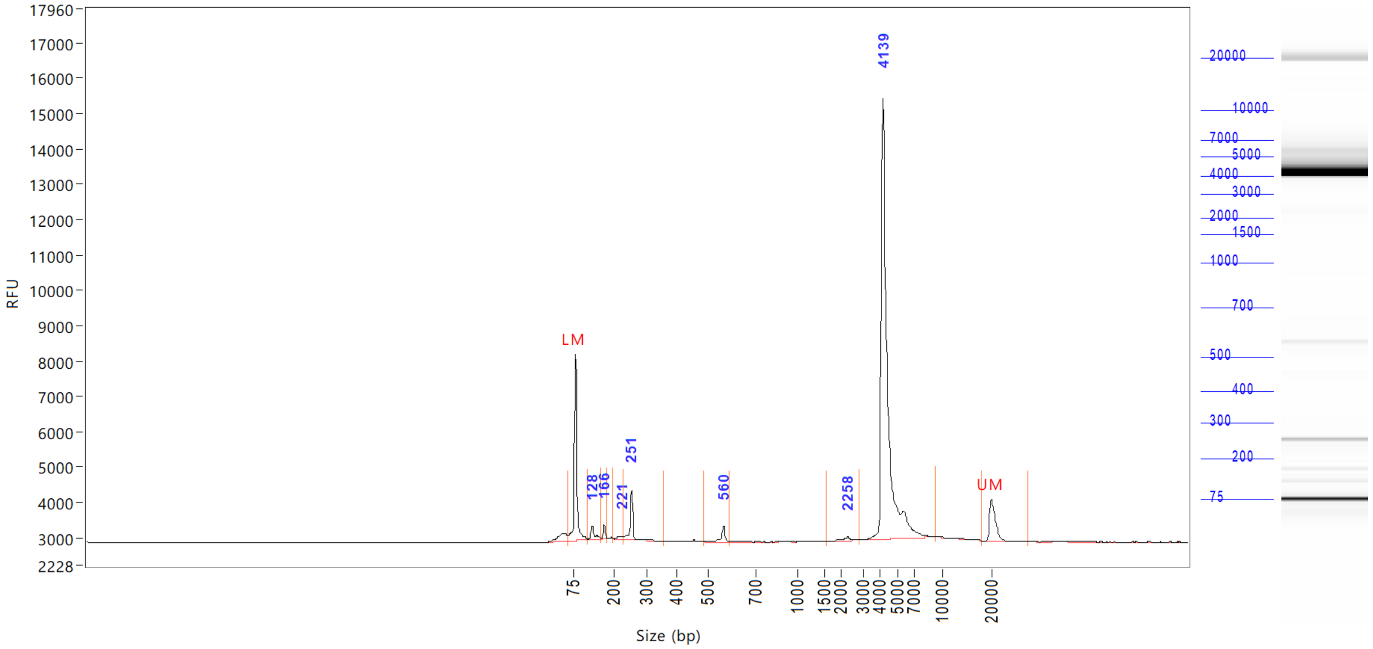

Peak	Size (bp)	Concentration (ng/uL)	From (bp)	To (bp)	Average size (bp)	CV%	RFU	Corrected peak area
1	75 (LM)	0.5000	52	114	74	9.78	5248	33.269
2	128	0.6015	114	156	131	6.66	383	3.335
3	166	0.3590	156	175	164	1.91	399	1.991
4	221	0.2177	198	228	216	3.46	93	1.207
5	251	1.5475	228	352	250	5.00	1413	8.581
6	560	0.5694	483	588	552	3.03	453	3.157
7	2258	0.2146	1520	2857	2154	10.82	102	1.190
8	4139	20.1420	2857	9267	4420	14.48	12483	111.684
9	20000 (UM)	0.1383	18171	27375	20315	3.96	1192	9.205

TIC:	23.6518	ng/uL
TIM:	32.3444	nmole/L
Total concentration:	23.9696	ng/uL

Sample peak width (sec): 5 Sample min peak height: 50 Sample baseline V to V?: Y Sample baseline V to V points: 3
Sample filter: Binomial Number of points for filter: 3 Sample start region (min): 0 Sample end region (min): 30
Marker peak width (sec): 5 Marker min peak height: 200 Marker baseline V to V?: Y Marker baseline V to V points: 3
Lower marker selection: First peak > 500 RFU Upper marker selection: Last peak > 200 RFU
Ladder size (bp)75, 200, 300, 400, 500, 700, 1000, 1500, 2000, 3000, 4000, 5000, 7000, 10000, 20000
Quantification using: Lower Marker Final concentration (ng/uL): 0.5000 Dilution factor: 12.0

Sample: SampB10
Well location: B10
Created: Monday, September 20, 2021 6:00:03 PM

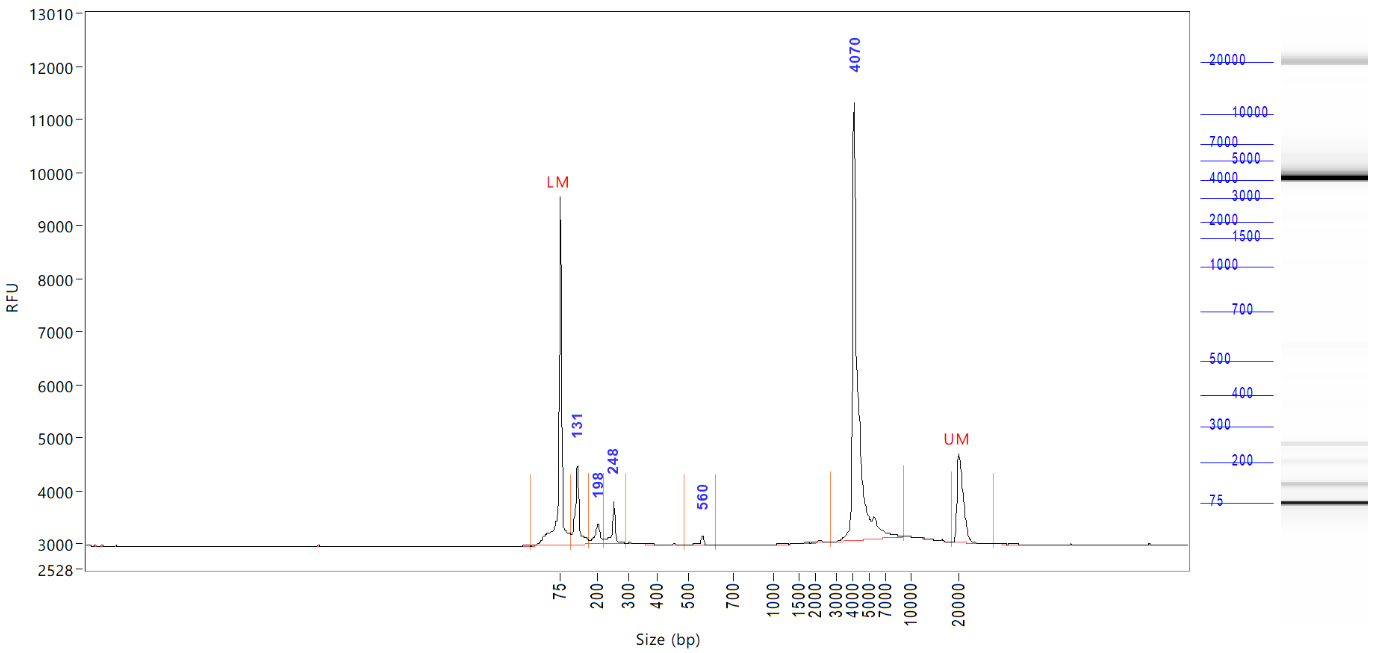

Peak	Size (bp)	Concentration (ng/uL)	From (bp)	To (bp)	Average size (bp)	CV%	RFU	Corrected peak area
1	75 (LM)	0.5000	0	110	68	26.64	6574	51.417
2	131	1.7618	110	173	131	9.59	1486	15.098
3	198	0.5464	173	219	196	5.38	365	4.683
4	248	0.6714	219	287	244	3.94	798	5.754
5	560	0.1426	489	623	553	4.01	158	1.222
6	4070	7.9361	2743	9134	4347	14.53	8268	68.008
7	20000 (UM)	0.1322	18415	27375	20417	3.75	1657	13.594

TIC:	11.0583	ng/uL
TIM:	34.6369	nmole/L
Total concentration:	11.2905	ng/uL

Sample peak width (sec): 5 Sample min peak height: 50 Sample baseline V to V?: Y Sample baseline V to V points: 3
Sample filter: Binomial Number of points for filter: 3 Sample start region (min): 0 Sample end region (min): 30
Marker peak width (sec): 5 Marker min peak height: 200 Marker baseline V to V?: Y Marker baseline V to V points: 3
Lower marker selection: First peak > 500 RFU Upper marker selection: Last peak > 200 RFU
Ladder size (bp)75, 200, 300, 400, 500, 700, 1000, 1500, 2000, 3000, 4000, 5000, 7000, 10000, 20000
Quantification using: Lower Marker Final concentration (ng/uL): 0.5000 Dilution factor: 12.0

Sample: SampC10
Well location: C10
Created: Monday, September 20, 2021 6:00:03 PM

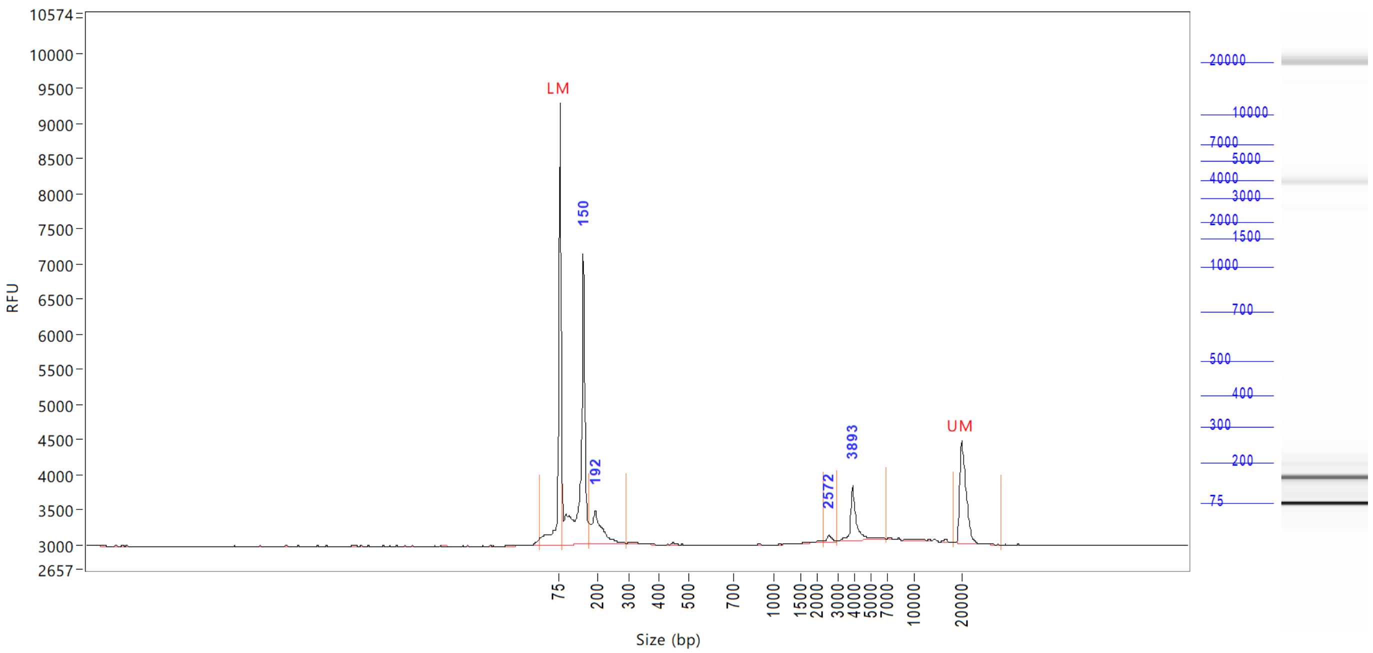

Peak	Size (bp)	Concentration (ng/uL)	From (bp)	To (bp)	Average size (bp)	CV%	RFU	Corrected peak area
1	75 (LM)	0.5000	9	84	68	20.36	6304	41.722
2	150	6.2706	84	173	139	15.09	4130	43.603
3	192	1.5457	173	289	202	10.78	472	10.748
4	2572	0.1184	2286	3000	2601	5.65	101	0.823
5	3893	1.0003	3000	6926	4027	10.74	784	6.956
6	20000 (UM)	0.1473	18293	28221	20452	4.22	1453	12.293

TIC:	8.9349	ng/uL
TIM:	87.1976	nmole/L
Total concentration:	9.3097	ng/uL

```
Sample peak width (sec): 5    Sample min peak height: 50    Sample baseline V to V?: Y    Sample baseline V to V points: 3
Sample filter: Binomial       Number of points for filter: 3  Sample start region (min): 0  Sample end region (min): 30
Marker peak width (sec): 5    Marker min peak height: 200   Marker baseline V to V?: Y    Marker baseline V to V points: 3
Lower marker selection: First peak > 500 RFU                Upper marker selection: Last peak > 200 RFU
Ladder size (bp)75, 200, 300, 400, 500, 700, 1000, 1500, 2000, 3000, 4000, 5000, 7000, 10000, 20000
Quantification using: Lower Marker          Final concentration (ng/uL): 0.5000          Dilution factor: 12.0
```

Sample: SampD10
Well location: D10
Created: Monday, September 20, 2021 6:00:03 PM

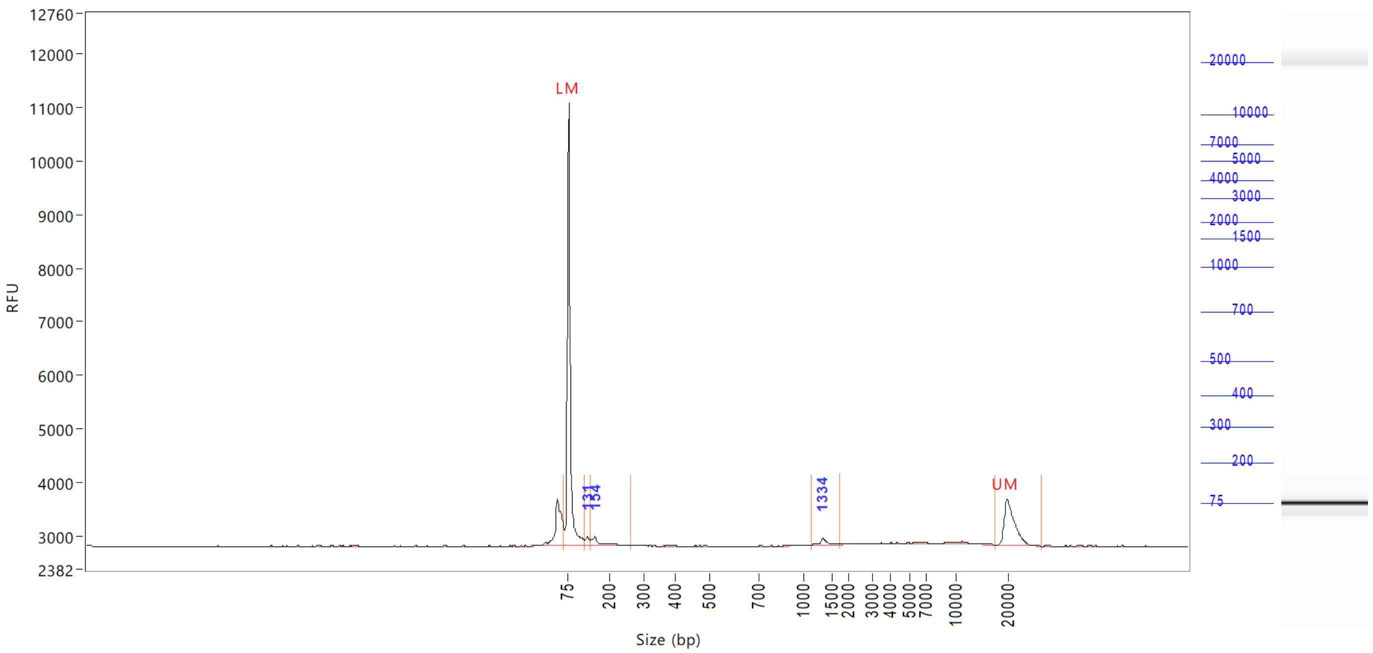

Peak	Size (bp)	Concentration (ng/uL)	From (bp)	To (bp)	Average size (bp)	CV%	RFU	Corrected peak area
1	75 (LM)	0.5000	62	122	76	11.43	8279	58.224
2	131	0.1714	122	141	131	4.33	162	1.663
3	154	0.2952	141	259	167	14.82	162	2.865
4	1334	0.1439	1134	1712	1349	7.57	132	1.396
5	20000 (UM)	0.0952	17683	26770	20600	5.53	853	11.087

TIC:	0.6104	ng/uL
TIM:	5.2309	nmole/L
Total concentration:	0.8840	ng/uL

Sample peak width (sec): 5   Sample min peak height: 50   Sample baseline V to V?: Y   Sample baseline V to V points: 3
Sample filter: Binomial   Number of points for filter: 3 Sample start region (min): 0 Sample end region (min): 30
Marker peak width (sec): 5   Marker min peak height: 200   Marker baseline V to V?: Y   Marker baseline V to V points: 3
Lower marker selection: First peak > 500 RFU   Upper marker selection: Last peak > 200 RFU
Ladder size (bp)75, 200, 300, 400, 500, 700, 1000, 1500, 2000, 3000, 4000, 5000, 7000, 10000, 20000
Quantification using: Lower Marker   Final concentration (ng/uL): 0.5000   Dilution factor: 12.0

Sample: SampE10
Well location: E10
Created: Monday, September 20, 2021 6:00:03 PM

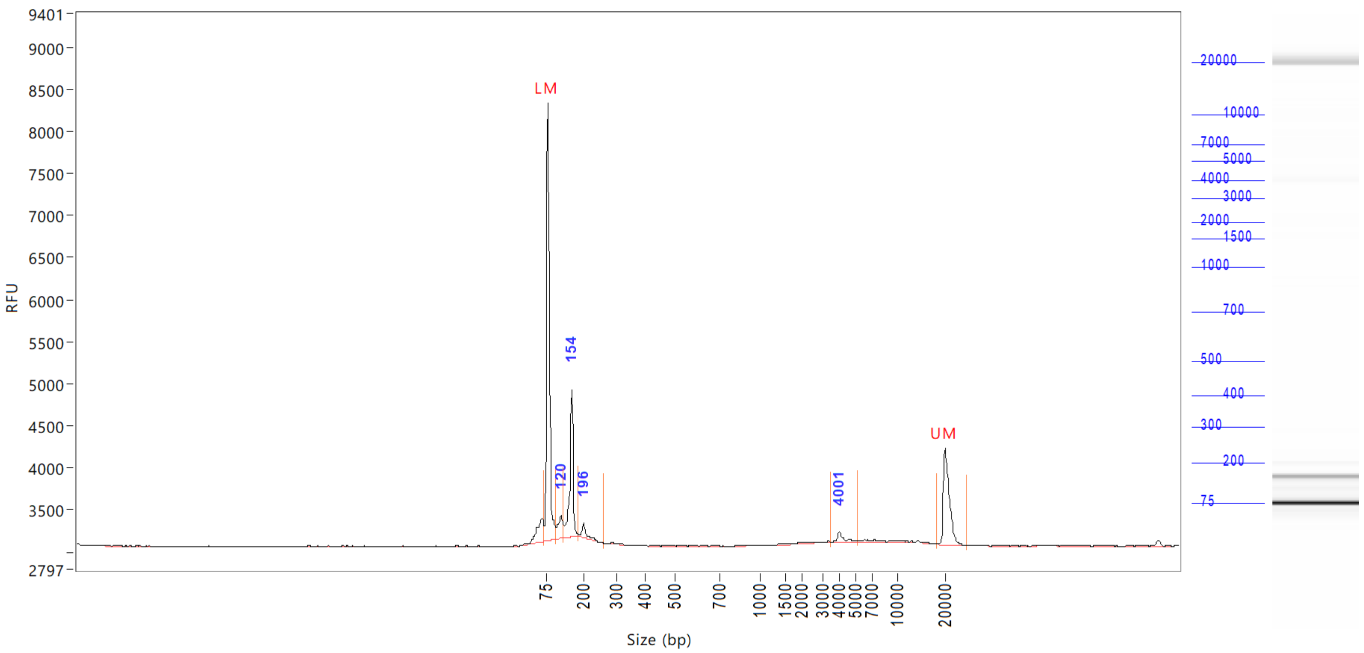

Peak	Size (bp)	Concentration (ng/uL)	From (bp)	To (bp)	Average size (bp)	CV%	RFU	Corrected peak area
1	75 (LM)	0.5000	64	101	75	7.96	5209	35.477
2	120	0.5912	101	129	115	6.99	265	3.496
3	154	2.3384	129	179	151	5.07	1754	13.827
4	196	0.3014	179	259	200	7.15	177	1.782
5	4001	0.1921	3536	5223	4169	7.61	118	1.136
6	20000 (UM)	0.1303	18171	24715	20398	3.56	1151	9.244

TIC:	3.4231	ng/uL
TIM:	36.4509	nmole/L
Total concentration:	3.8901	ng/uL

```
Sample peak width (sec): 5    Sample min peak height: 50    Sample baseline V to V?: Y    Sample baseline V to V points: 3
Sample filter: Binomial       Number of points for filter: 3  Sample start region (min): 0  Sample end region (min): 30
Marker peak width (sec): 5    Marker min peak height: 200   Marker baseline V to V?: Y    Marker baseline V to V points: 3
Lower marker selection: First peak > 500 RFU                Upper marker selection: Last peak > 200 RFU
Ladder size (bp)75, 200, 300, 400, 500, 700, 1000, 1500, 2000, 3000, 4000, 5000, 7000, 10000, 20000
Quantification using: Lower Marker          Final concentration (ng/uL): 0.5000          Dilution factor: 12.0
```

Sample: SampF10
Well location: F10
Created: Monday, September 20, 2021 6:00:03 PM

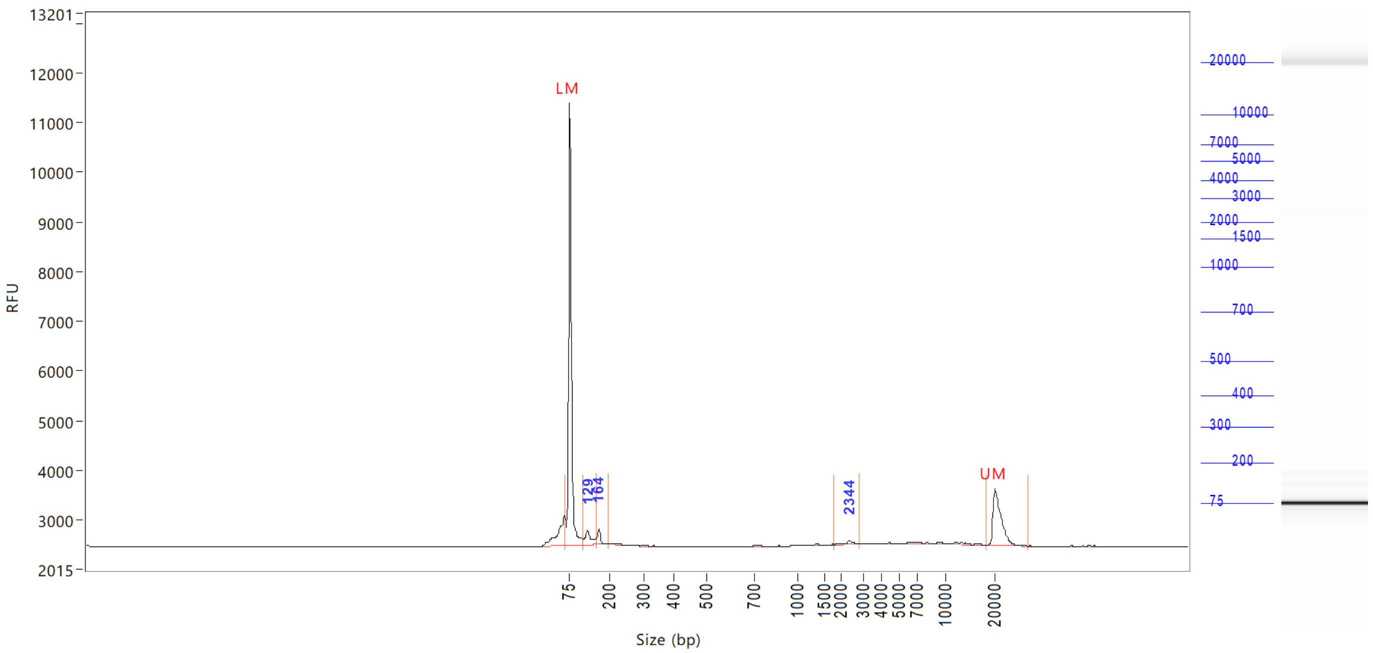

Peak	Size (bp)	Concentration (ng/uL)	From (bp)	To (bp)	Average size (bp)	CV%	RFU	Corrected peak area
1	75 (LM)	0.5000	64	116	75	9.82	8917	58.986
2	129	0.4224	116	156	133	8.32	297	4.152
3	164	0.2054	156	194	163	3.00	297	2.020
4	2344	0.0739	1750	2829	2374	8.78	76	0.726
5	20000 (UM)	0.1000	18171	26529	20595	4.74	1135	11.793

TIC:	0.7016	ng/uL
TIM:	7.3408	nmole/L
Total concentration:	1.0739	ng/uL

```
Sample peak width (sec): 5     Sample min peak height: 50     Sample baseline V to V?: Y     Sample baseline V to V points: 3
Sample filter: Binomial        Number of points for filter: 3  Sample start region (min): 0  Sample end region (min): 30
Marker peak width (sec): 5     Marker min peak height: 200    Marker baseline V to V?: Y     Marker baseline V to V points: 3
Lower marker selection: First peak > 500 RFU                  Upper marker selection: Last peak > 200 RFU
Ladder size (bp)75, 200, 300, 400, 500, 700, 1000, 1500, 2000, 3000, 4000, 5000, 7000, 10000, 20000
Quantification using: Lower Marker          Final concentration (ng/uL): 0.5000          Dilution factor: 12.0
```

Sample: SampG10
Well location: G10
Created: Monday, September 20, 2021 6:00:03 PM

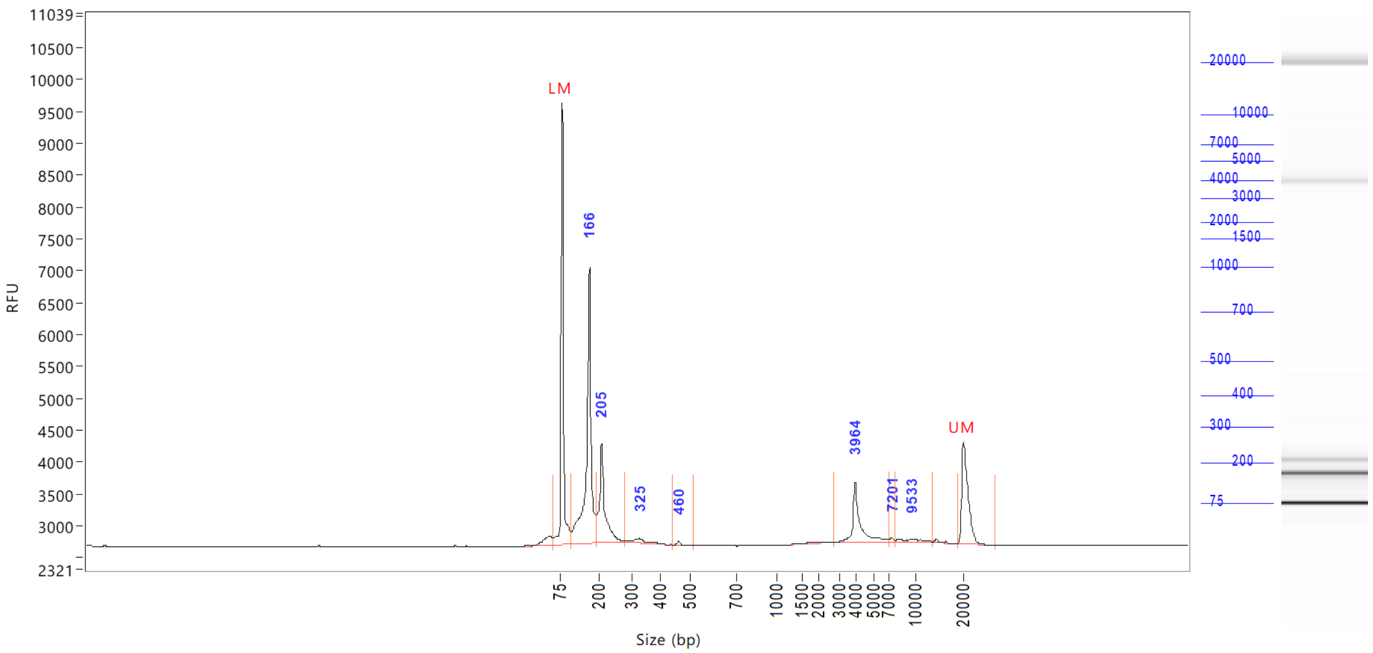

Peak	Size (bp)	Concentration (ng/uL)	From (bp)	To (bp)	Average size (bp)	CV%	RFU	Corrected peak area
1	75 (LM)	0.5000	47	105	75	10.21	6937	43.733
2	166	6.4477	105	189	156	11.14	4324	46.997
3	205	2.6367	189	278	210	7.35	1548	19.218
4	325	0.2571	278	441	331	9.35	68	1.874
5	460	0.0508	441	511	462	2.43	57	0.370
6	3964	1.4592	2772	7000	4258	17.09	951	10.636
7	7201	0.0602	7000	7667	7265	2.91	64	0.439
8	9533	0.2404	7667	13537	9842	15.38	54	1.752
9	20000 (UM)	0.1544	18781	26649	20458	3.78	1593	13.507

TIC:	11.1520	ng/uL
TIM:	90.6554	nmole/L
Total concentration:	11.2460	ng/uL

```
Sample peak width (sec): 5    Sample min peak height: 50    Sample baseline V to V?: Y    Sample baseline V to V points: 3
Sample filter: Binomial       Number of points for filter: 3  Sample start region (min): 0  Sample end region (min): 30
Marker peak width (sec): 5    Marker min peak height: 200   Marker baseline V to V?: Y    Marker baseline V to V points: 3
Lower marker selection: First peak > 500 RFU                Upper marker selection: Last peak > 200 RFU
Ladder size (bp)75, 200, 300, 400, 500, 700, 1000, 1500, 2000, 3000, 4000, 5000, 7000, 10000, 20000
Quantification using: Lower Marker          Final concentration (ng/uL): 0.5000          Dilution factor: 12.0
```

Sample: SampH10
Well location: H10
Created: Monday, September 20, 2021 6:00:03 PM

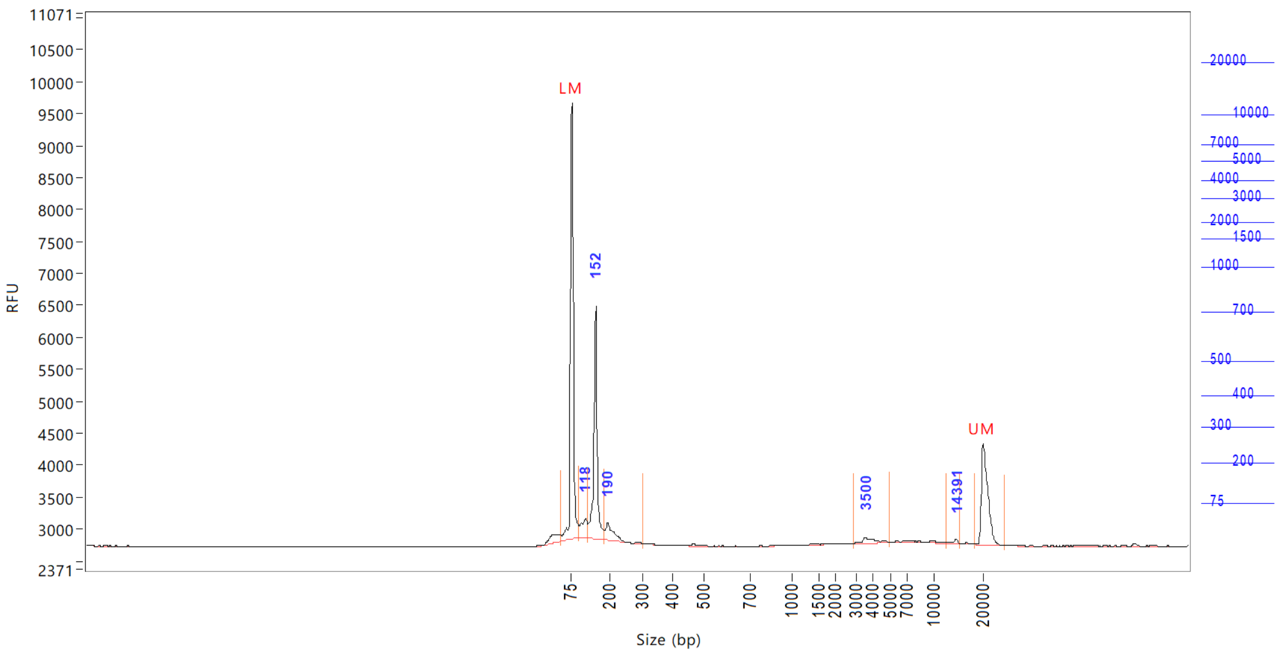

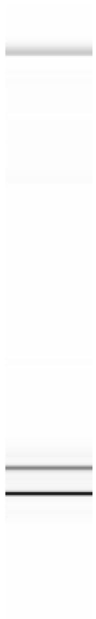

Peak	Size (bp)	Concentration (ng/uL)	From (bp)	To (bp)	Average size (bp)	CV%	RFU	Corrected peak area
1	75 (LM)	0.5000	41	97	73	9.20	6832	45.339
2	118	0.5624	97	126	112	7.58	298	4.250
3	152	3.7409	126	179	149	5.84	3632	28.268
4	190	0.6299	179	300	201	9.38	265	4.760
5	3500	0.2176	2857	4966	3883	12.02	94	1.645
6	14391	0.0574	12562	15366	13902	4.44	65	0.434
7	20000 (UM)	0.1453	18293	24352	20385	3.55	1582	13.174

TIC:	5.2083	ng/uL
TIM:	54.7633	nmole/L
Total concentration:	5.5211	ng/uL

Sample peak width (sec): 5 Sample min peak height: 50 Sample baseline V to V?: Y Sample baseline V to V points: 3
Sample filter: Binomial Number of points for filter: 3 Sample start region (min): 0 Sample end region (min): 30
Marker peak width (sec): 5 Marker min peak height: 200 Marker baseline V to V?: Y Marker baseline V to V points: 3
Lower marker selection: First peak > 500 RFU Upper marker selection: Last peak > 200 RFU
Ladder size (bp)75, 200, 300, 400, 500, 700, 1000, 1500, 2000, 3000, 4000, 5000, 7000, 10000, 20000
Quantification using: Lower Marker Final concentration (ng/uL): 0.5000 Dilution factor: 12.0

Sample: SampA11
Well location: A11
Created: Monday, September 20, 2021 6:00:03 PM

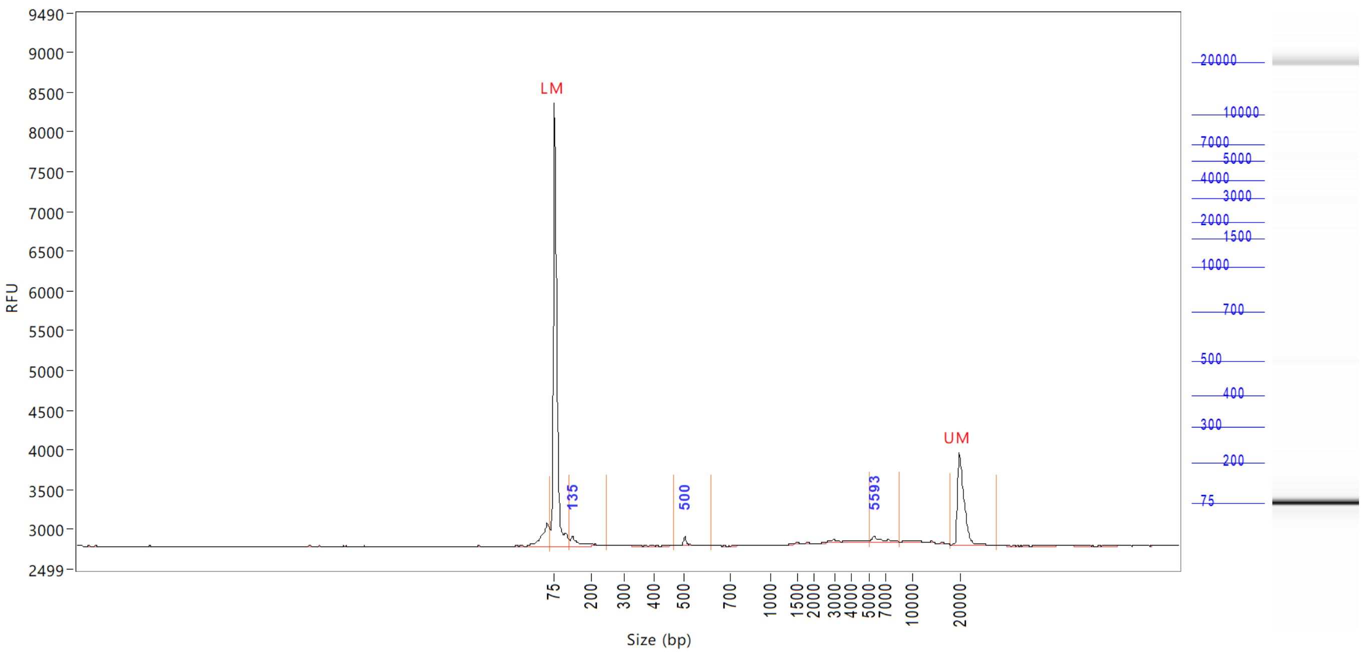

Peak	Size (bp)	Concentration (ng/uL)	From (bp)	To (bp)	Average size (bp)	CV%	RFU	Corrected peak area
1	75 (LM)	0.5000	64	124	77	11.83	5584	48.563
2	135	0.3195	124	242	158	18.98	126	2.586
3	500	0.1201	466	616	509	4.84	120	0.972
4	5593	0.1138	5075	8734	6452	14.69	67	0.921
5	20000 (UM)	0.1023	18171	27738	20442	4.88	1156	9.940

TIC:	0.5534	ng/uL
TIM:	3.7405	nmole/L
Total concentration:	0.8512	ng/uL

Sample peak width (sec): 5 Sample min peak height: 50 Sample baseline V to V?: Y Sample baseline V to V points: 3
Sample filter: Binomial Number of points for filter: 3 Sample start region (min): 0 Sample end region (min): 30
Marker peak width (sec): 5 Marker min peak height: 200 Marker baseline V to V?: Y Marker baseline V to V points: 3
Lower marker selection: First peak > 500 RFU Upper marker selection: Last peak > 200 RFU
Ladder size (bp)75, 200, 300, 400, 500, 700, 1000, 1500, 2000, 3000, 4000, 5000, 7000, 10000, 20000
Quantification using: Lower Marker Final concentration (ng/uL): 0.5000 Dilution factor: 12.0

Sample: SampB11
Well location: B11
Created: Monday, September 20, 2021 6:00:03 PM

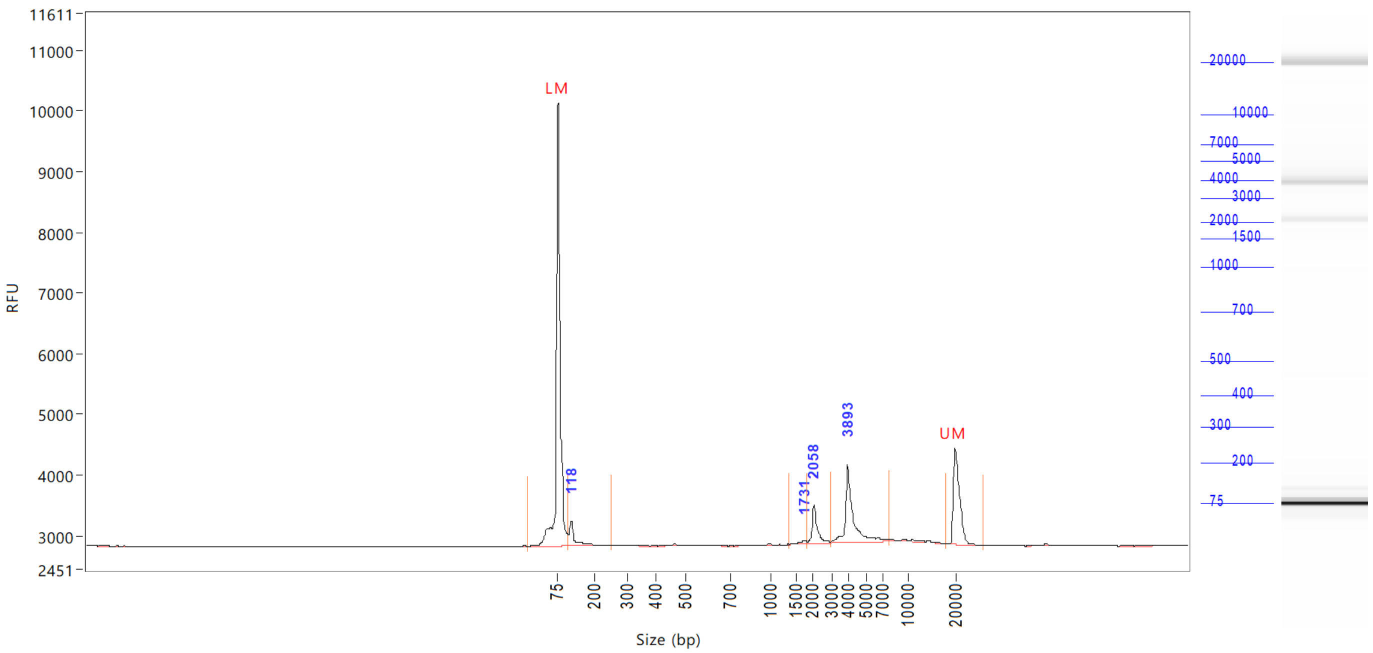

Peak	Size (bp)	Concentration (ng/uL)	From (bp)	To (bp)	Average size (bp)	CV%	RFU	Corrected peak area
1	75 (LM)	0.5000	0	108	71	20.42	7314	64.071
2	118	0.4882	108	251	134	21.09	416	5.214
3	1731	0.0566	1356	1846	1675	6.68	59	0.605
4	2058	0.4902	1846	2886	2144	8.97	634	5.235
5	3893	1.3953	2886	7867	4222	18.89	1295	14.899
6	20000 (UM)	0.1041	18171	25803	20364	3.83	1597	13.338

TIC:	2.4303	ng/uL
TIM:	6.9632	nmole/L
Total concentration:	2.6543	ng/uL

Sample peak width (sec): 5   Sample min peak height: 50   Sample baseline V to V?: Y   Sample baseline V to V points: 3
Sample filter: Binomial   Number of points for filter: 3 Sample start region (min): 0 Sample end region (min): 30
Marker peak width (sec): 5   Marker min peak height: 200   Marker baseline V to V?: Y   Marker baseline V to V points: 3
Lower marker selection: First peak > 500 RFU   Upper marker selection: Last peak > 200 RFU
Ladder size (bp)75, 200, 300, 400, 500, 700, 1000, 1500, 2000, 3000, 4000, 5000, 7000, 10000, 20000
Quantification using: Lower Marker   Final concentration (ng/uL): 0.5000   Dilution factor: 12.0

Sample: SampC11
Well location: C11
Created: Monday, September 20, 2021 6:00:03 PM

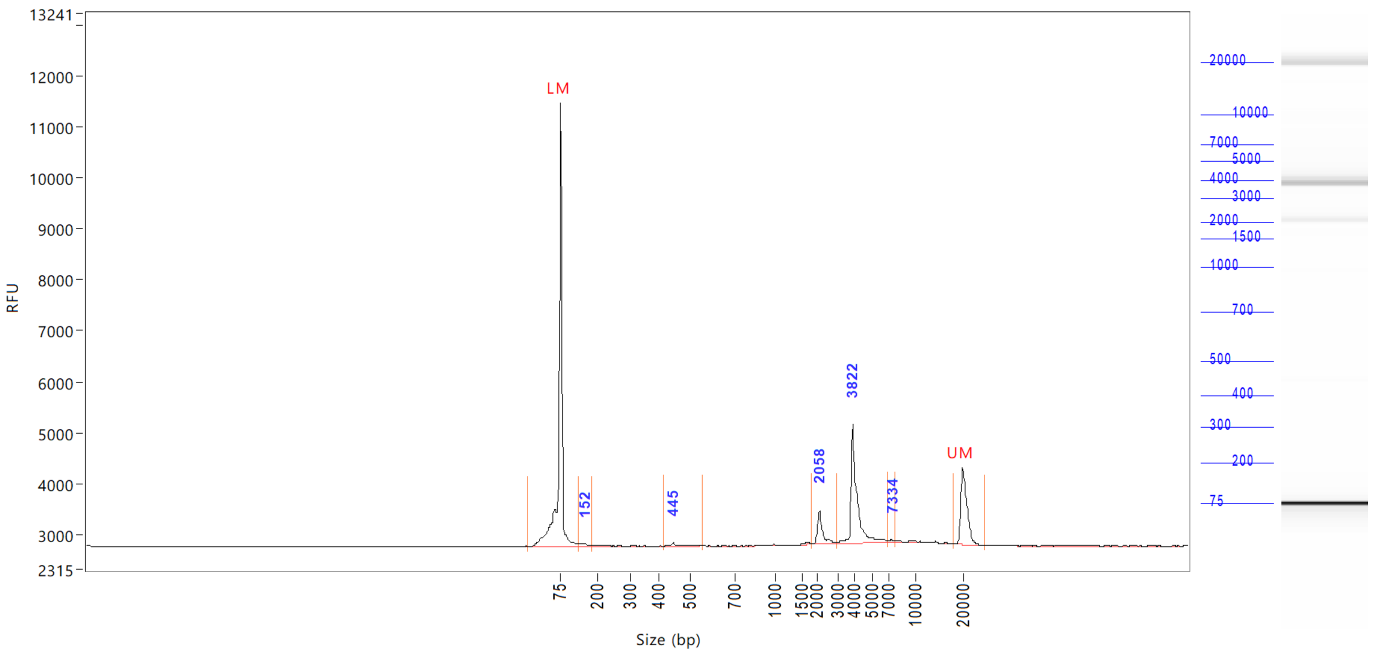

Peak	Size (bp)	Concentration (ng/uL)	From (bp)	To (bp)	Average size (bp)	CV%	RFU	Corrected peak area
1	75 (LM)	0.5000	0	137	66	29.40	8729	70.500
2	152	0.0747	137	179	152	6.79	58	0.878
3	445	0.0610	414	555	457	5.54	63	0.717
4	2058	0.4474	1789	2943	2147	9.94	661	5.257
5	3822	1.7911	2943	6852	4066	12.99	2326	21.045
6	7334	0.0377	6852	7734	7236	3.43	54	0.443
7	20000 (UM)	0.0875	18049	24594	20341	3.49	1504	12.344

TIC:	2.4119	ng/uL
TIM:	2.1043	nmole/L
Total concentration:	2.6191	ng/uL

```
Sample peak width (sec): 5    Sample min peak height: 50    Sample baseline V to V?: Y    Sample baseline V to V points: 3
Sample filter: Binomial       Number of points for filter: 3  Sample start region (min): 0  Sample end region (min): 30
Marker peak width (sec): 5    Marker min peak height: 200   Marker baseline V to V?: Y    Marker baseline V to V points: 3
Lower marker selection: First peak > 500 RFU                Upper marker selection: Last peak > 200 RFU
Ladder size (bp)75, 200, 300, 400, 500, 700, 1000, 1500, 2000, 3000, 4000, 5000, 7000, 10000, 20000
Quantification using: Lower Marker          Final concentration (ng/uL): 0.5000          Dilution factor: 12.0
```

Sample: SampD11
Well location: D11
Created: Monday, September 20, 2021 6:00:03 PM

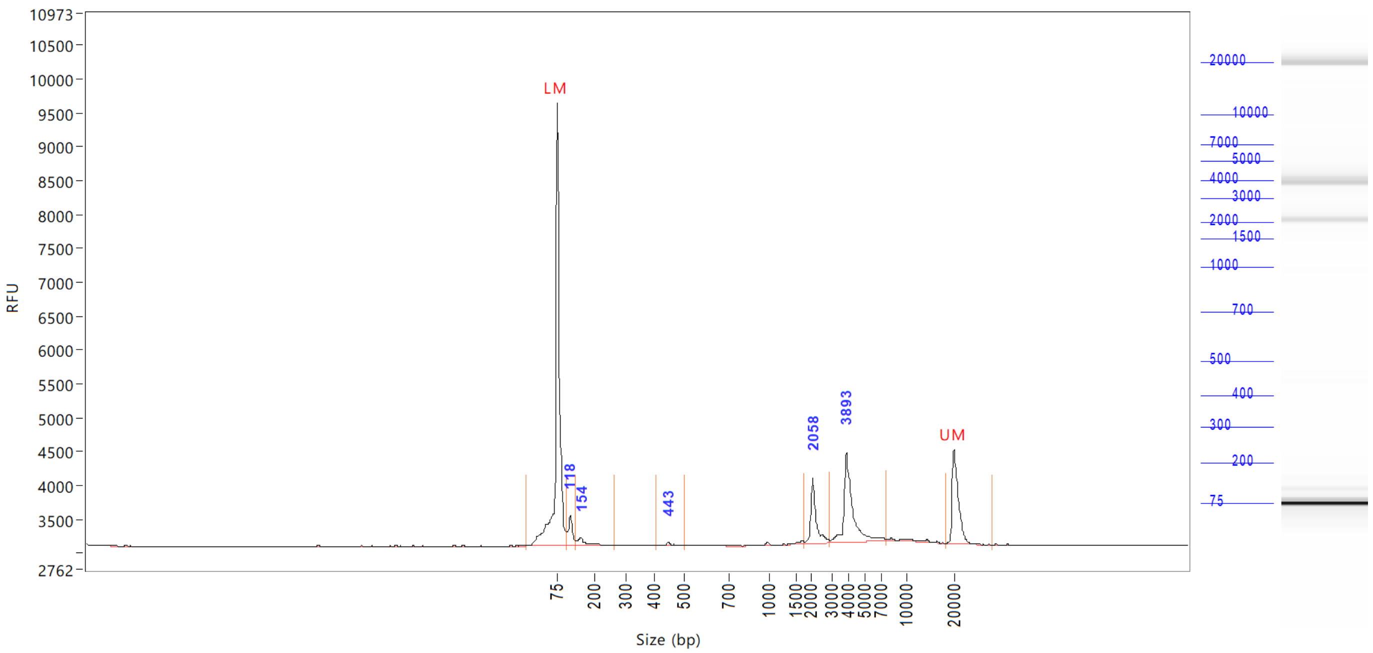

Peak	Size (bp)	Concentration (ng/uL)	From (bp)	To (bp)	Average size (bp)	CV%	RFU	Corrected peak area
1	75 (LM)	0.5000	0	107	68	28.49	6552	61.701
2	118	0.4134	107	137	117	6.35	443	4.251
3	154	0.2478	137	262	170	17.47	123	2.548
4	443	0.0703	403	500	451	4.34	58	0.723
5	2058	0.7977	1789	2914	2151	10.23	979	8.203
6	3893	1.6662	2914	7601	4172	16.75	1332	17.134
7	20000 (UM)	0.0942	18171	28100	20394	4.28	1383	11.621

TIC:	3.1954	ng/uL
TIM:	9.7250	nmole/L
Total concentration:	3.4638	ng/uL

Sample peak width (sec): 5 Sample min peak height: 50 Sample baseline V to V?: Y Sample baseline V to V points: 3
Sample filter: Binomial Number of points for filter: 3 Sample start region (min): 0 Sample end region (min): 30
Marker peak width (sec): 5 Marker min peak height: 200 Marker baseline V to V?: Y Marker baseline V to V points: 3
Lower marker selection: First peak > 500 RFU Upper marker selection: Last peak > 200 RFU
Ladder size (bp)75, 200, 300, 400, 500, 700, 1000, 1500, 2000, 3000, 4000, 5000, 7000, 10000, 20000
Quantification using: Lower Marker Final concentration (ng/uL): 0.5000 Dilution factor: 12.0

Sample: SampE11
Well location: E11
Created: Monday, September 20, 2021 6:00:03 PM

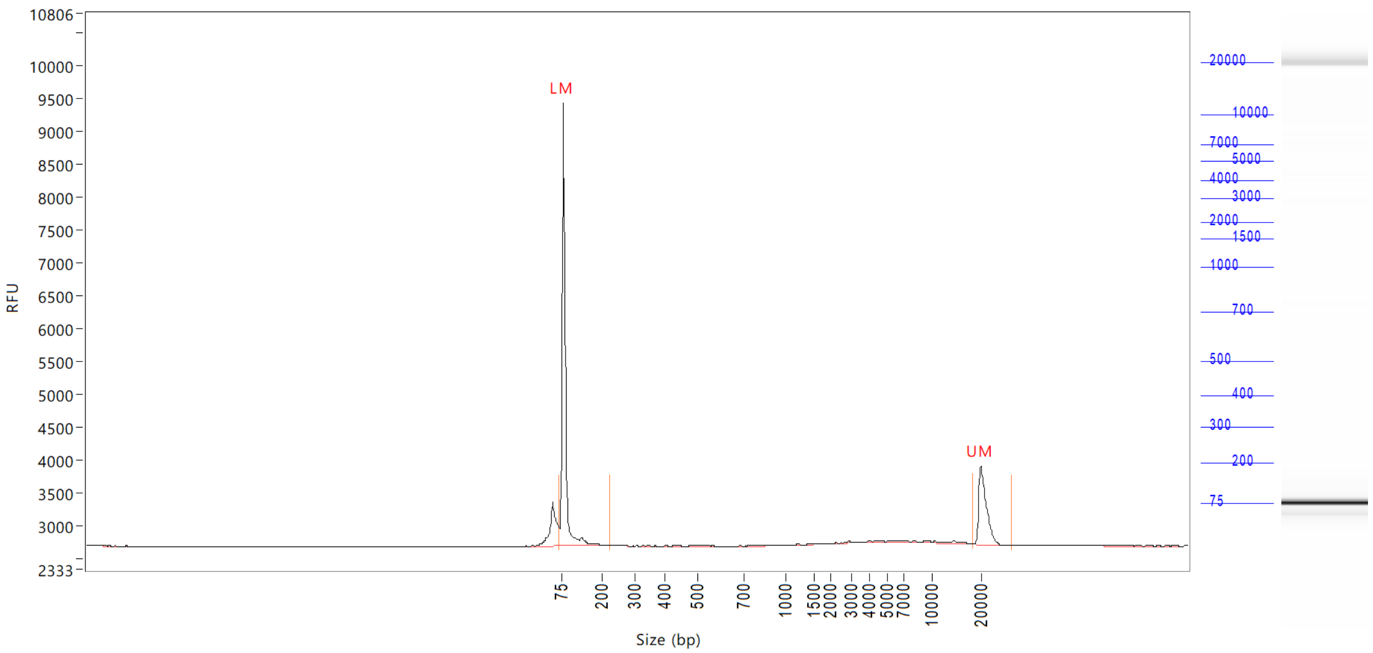

Peak	Size (bp)	Concentration (ng/uL)	From (bp)	To (bp)	Average size (bp)	CV%	RFU	Corrected peak area
1	75 (LM)	0.5000	64	223	79	20.73	6762	54.040
2	20000 (UM)	0.1106	18415	26287	20484	4.52	1202	11.951

TIC:	0.0000	ng/uL
TIM:	0.0000	nmole/L
Total concentration:	0.4364	ng/uL

Sample peak width (sec): 5   Sample min peak height: 50   Sample baseline V to V?: Y   Sample baseline V to V points: 3
Sample filter: Binomial   Number of points for filter: 3 Sample start region (min): 0 Sample end region (min): 30
Marker peak width (sec): 5   Marker min peak height: 200   Marker baseline V to V?: Y   Marker baseline V to V points: 3
Lower marker selection: First peak > 500 RFU   Upper marker selection: Last peak > 200 RFU
Ladder size (bp)75, 200, 300, 400, 500, 700, 1000, 1500, 2000, 3000, 4000, 5000, 7000, 10000, 20000
Quantification using: Lower Marker   Final concentration (ng/uL): 0.5000   Dilution factor: 12.0

Sample: SampF11
Well location: F11
Created: Monday, September 20, 2021 6:00:03 PM

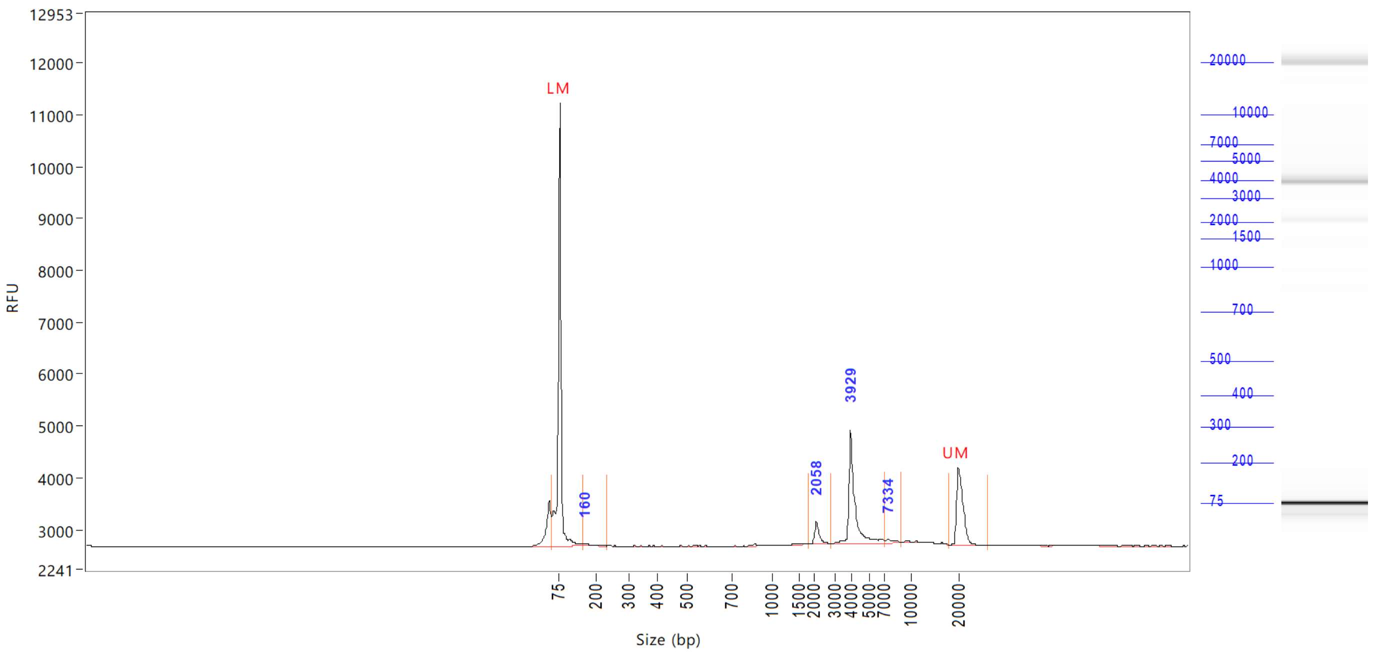

Peak	Size (bp)	Concentration (ng/uL)	From (bp)	To (bp)	Average size (bp)	CV%	RFU	Corrected peak area
1	75 (LM)	0.5000	48	152	74	16.48	8554	60.634
2	160	0.0807	152	233	173	11.19	51	0.816
3	2058	0.3024	1808	2800	2108	6.98	433	3.055
4	3929	1.9249	2800	7001	4163	14.21	2177	19.452
5	7334	0.0838	7001	8867	7738	6.91	77	0.847
6	20000 (UM)	0.1038	18049	26408	20423	3.83	1497	12.582

TIC:	2.3917	ng/uL
TIM:	1.7821	nmole/L
Total concentration:	2.6192	ng/uL

```
Sample peak width (sec): 5    Sample min peak height: 50    Sample baseline V to V?: Y    Sample baseline V to V points: 3
Sample filter: Binomial       Number of points for filter: 3  Sample start region (min): 0  Sample end region (min): 30
Marker peak width (sec): 5    Marker min peak height: 200   Marker baseline V to V?: Y    Marker baseline V to V points: 3
Lower marker selection: First peak > 500 RFU                Upper marker selection: Last peak > 200 RFU
Ladder size (bp)75, 200, 300, 400, 500, 700, 1000, 1500, 2000, 3000, 4000, 5000, 7000, 10000, 20000
Quantification using: Lower Marker          Final concentration (ng/uL): 0.5000          Dilution factor: 12.0
```

Sample: SampG11
Well location: G11
Created: Monday, September 20, 2021 6:00:03 PM

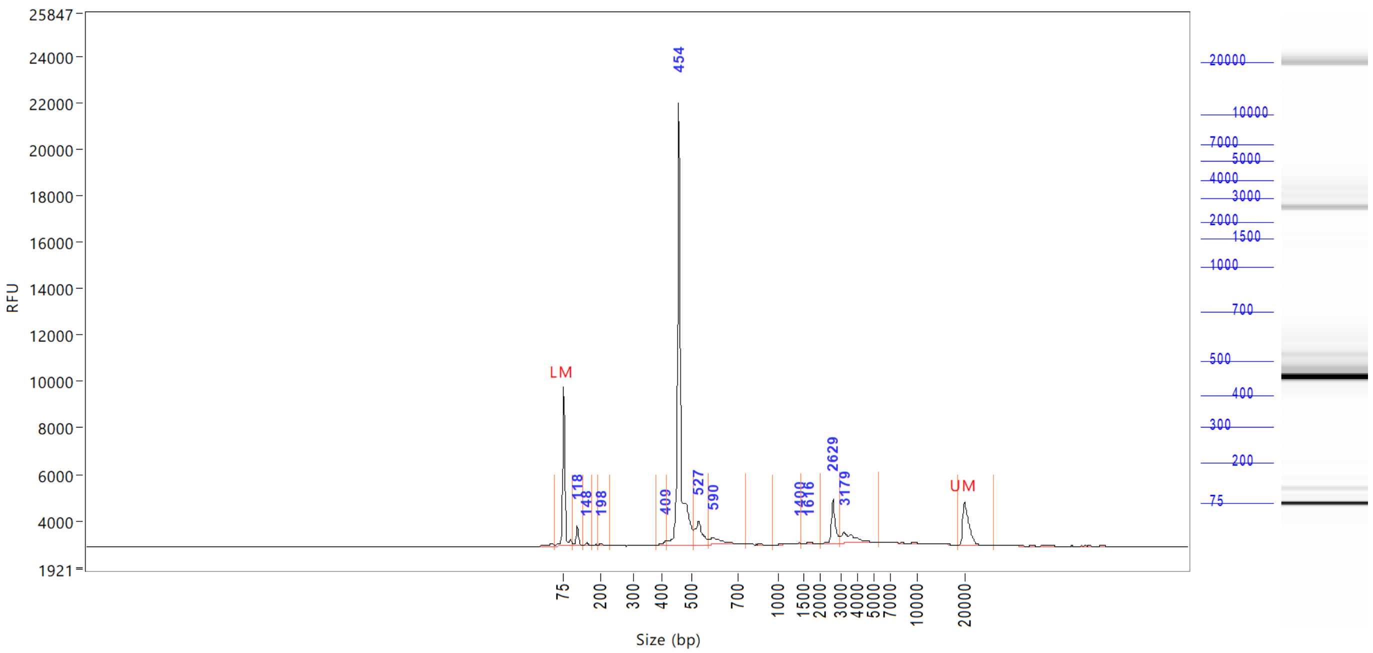

Peak	Size (bp)	Concentration (ng/uL)	From (bp)	To (bp)	Average size (bp)	CV%	RFU	Corrected peak area
1	75 (LM)	0.5000	47	107	74	8.95	6855	40.149
2	118	0.7485	107	141	118	4.53	826	5.009
3	148	0.1168	141	168	149	3.16	122	0.782
4	198	0.0950	189	224	196	2.28	98	0.636
5	409	0.2950	378	414	403	2.02	206	1.974
6	454	18.6598	414	501	457	2.97	19066	124.861
7	527	2.1289	501	567	527	3.24	1001	14.245
8	590	1.0498	567	753	610	5.50	316	7.024
9	1400	0.1163	957	1445	1276	9.76	53	0.778
10	1616	0.1196	1445	2001	1645	8.24	53	0.800
11	2629	1.7161	2001	2943	2627	4.91	1934	11.483
12	3179	1.3603	2943	5445	3556	12.80	452	9.102
13	20000 (UM)	0.1891	18537	26166	20409	3.89	1827	15.187

TIC:	26.4061	ng/uL
TIM:	92.3494	nmole/L
Total concentration:	26.5646	ng/uL

```
Sample peak width (sec): 5     Sample min peak height: 50     Sample baseline V to V?: Y     Sample baseline V to V points: 3
Sample filter: Binomial        Number of points for filter: 3  Sample start region (min): 0  Sample end region (min): 30
Marker peak width (sec): 5     Marker min peak height: 200    Marker baseline V to V?: Y     Marker baseline V to V points: 3
Lower marker selection: First peak > 500 RFU                  Upper marker selection: Last peak > 200 RFU
Ladder size (bp)75, 200, 300, 400, 500, 700, 1000, 1500, 2000, 3000, 4000, 5000, 7000, 10000, 20000
Quantification using: Lower Marker          Final concentration (ng/uL): 0.5000          Dilution factor: 12.0
```

Sample: SampH11
Well location: H11
Created: Monday, September 20, 2021 6:00:03 PM

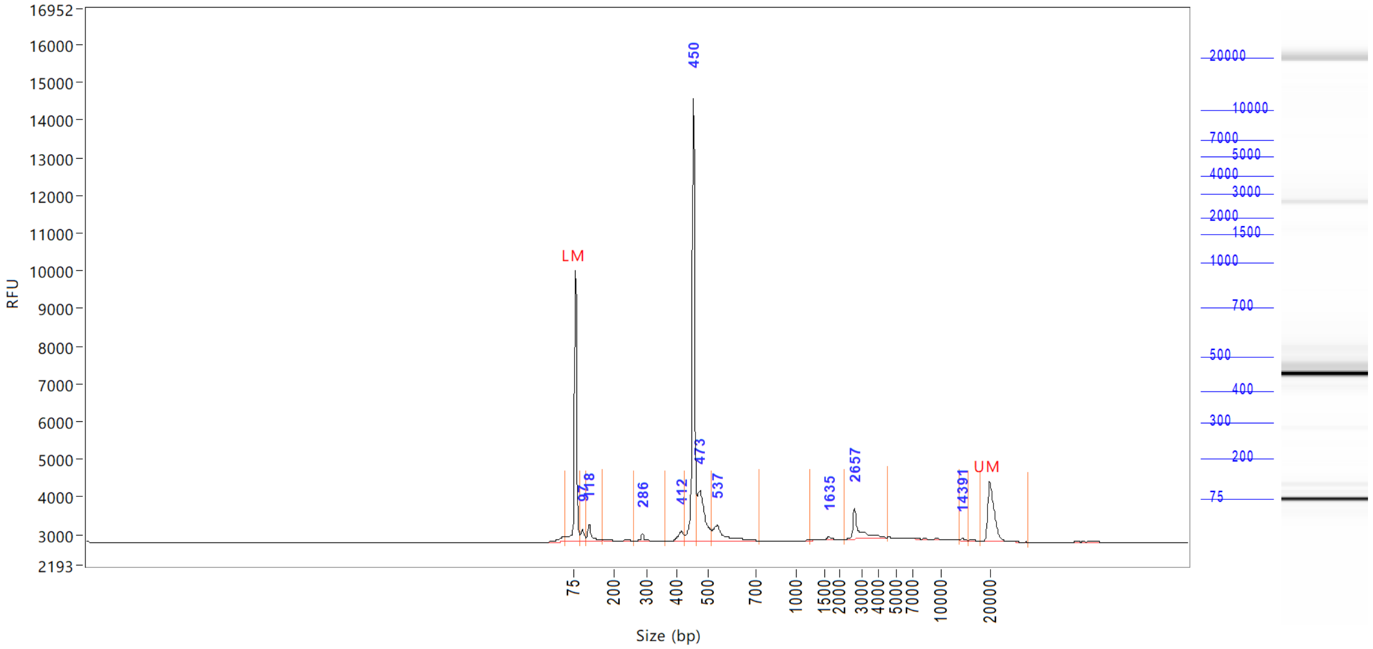

Peak	Size (bp)	Concentration (ng/uL)	From (bp)	To (bp)	Average size (bp)	CV%	RFU	Corrected peak area
1	75 (LM)	0.5000	47	89	73	7.32	7204	42.066
2	97	0.3678	89	108	97	5.46	334	2.579
3	118	0.5540	108	160	120	7.31	434	3.884
4	286	0.2316	259	360	289	5.36	199	1.624
5	412	0.4704	360	424	407	2.56	274	3.298
6	450	8.9080	424	462	448	1.24	11768	62.454
7	473	2.7499	462	511	478	2.63	1333	19.280
8	537	1.2155	511	727	556	7.46	415	8.522
9	1635	0.1371	1256	2144	1598	9.91	86	0.961
10	2657	1.0539	2144	4518	2931	14.29	799	7.389
11	14391	0.0372	13659	15610	14361	2.90	50	0.261
12	20000 (UM)	0.1564	18171	27859	20390	4.03	1581	13.157

TIC:	15.7255	ng/uL
TIM:	63.5486	nmole/L
Total concentration:	15.8141	ng/uL

Sample peak width (sec): 5   Sample min peak height: 50   Sample baseline V to V?: Y   Sample baseline V to V points: 3
Sample filter: Binomial   Number of points for filter: 3   Sample start region (min): 0   Sample end region (min): 30
Marker peak width (sec): 5   Marker min peak height: 200   Marker baseline V to V?: Y   Marker baseline V to V points: 3
Lower marker selection: First peak > 500 RFU   Upper marker selection: Last peak > 200 RFU
Ladder size (bp)75, 200, 300, 400, 500, 700, 1000, 1500, 2000, 3000, 4000, 5000, 7000, 10000, 20000
Quantification using: Lower Marker   Final concentration (ng/uL): 0.5000   Dilution factor: 12.0

Sample: SampA12
Well location: A12
Created: Monday, September 20, 2021 6:00:03 PM

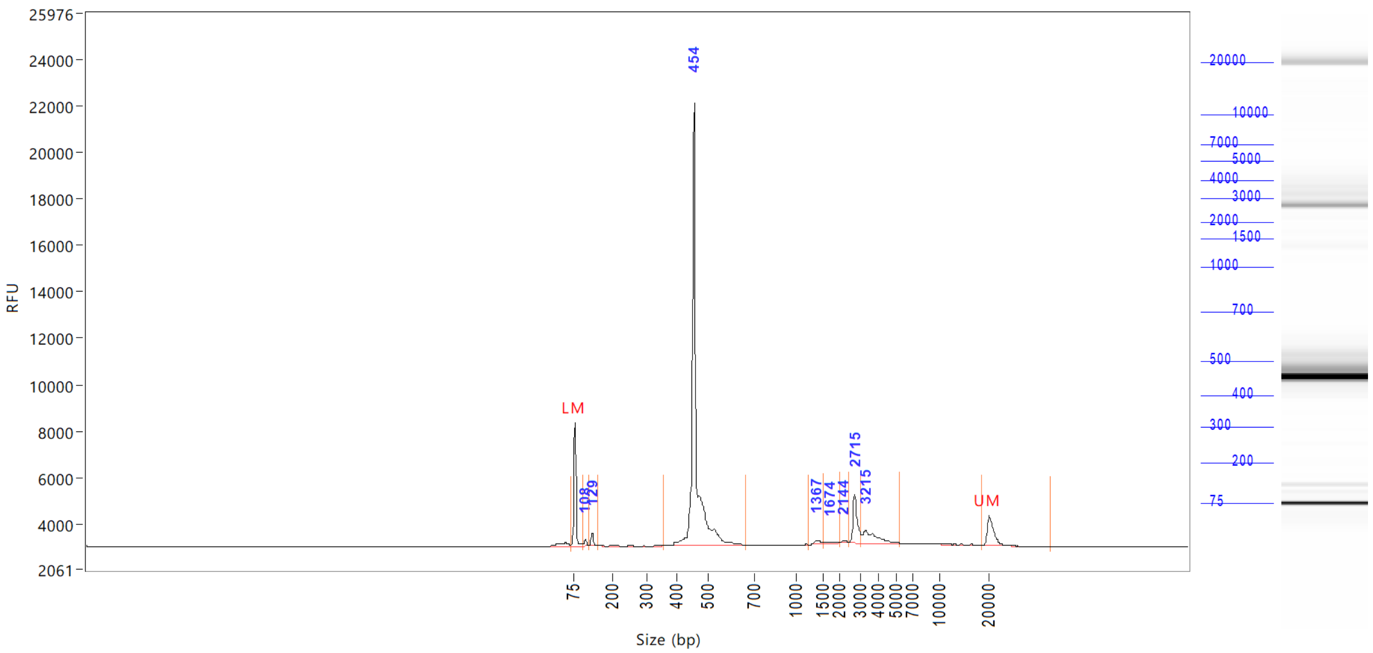

Peak	Size (bp)	Concentration (ng/uL)	From (bp)	To (bp)	Average size (bp)	CV%	RFU	Corrected peak area
1	75 (LM)	0.5000	64	99	74	6.31	5298	29.112
2	108	0.3501	99	120	107	4.66	264	1.699
3	129	0.6938	120	148	129	3.07	564	3.366
4	454	31.1515	360	659	463	6.29	19072	151.145
5	1367	0.3005	1223	1501	1377	3.50	172	1.458
6	1674	0.1251	1501	2000	1712	6.97	61	0.607
7	2144	0.1537	2000	2429	2209	5.56	84	0.746
8	2715	2.9178	2429	3001	2728	4.33	2098	14.157
9	3215	2.1199	3001	5445	3617	12.29	516	10.286
10	20000 (UM)	0.1852	18537	32574	20530	5.29	1271	10.785

TIC:	37.8122	ng/uL
TIM:	128.2376	nmole/L
Total concentration:	38.2663	ng/uL

Sample peak width (sec): 5 Sample min peak height: 50 Sample baseline V to V?: Y Sample baseline V to V points: 3
Sample filter: Binomial Number of points for filter: 3 Sample start region (min): 0 Sample end region (min): 30
Marker peak width (sec): 5 Marker min peak height: 200 Marker baseline V to V?: Y Marker baseline V to V points: 3
Lower marker selection: First peak > 500 RFU Upper marker selection: Last peak > 200 RFU
Ladder size (bp)75, 200, 300, 400, 500, 700, 1000, 1500, 2000, 3000, 4000, 5000, 7000, 10000, 20000
Quantification using: Lower Marker Final concentration (ng/uL): 0.5000 Dilution factor: 12.0

Sample: SampB12
Well location: B12
Created: Monday, September 20, 2021 6:00:03 PM

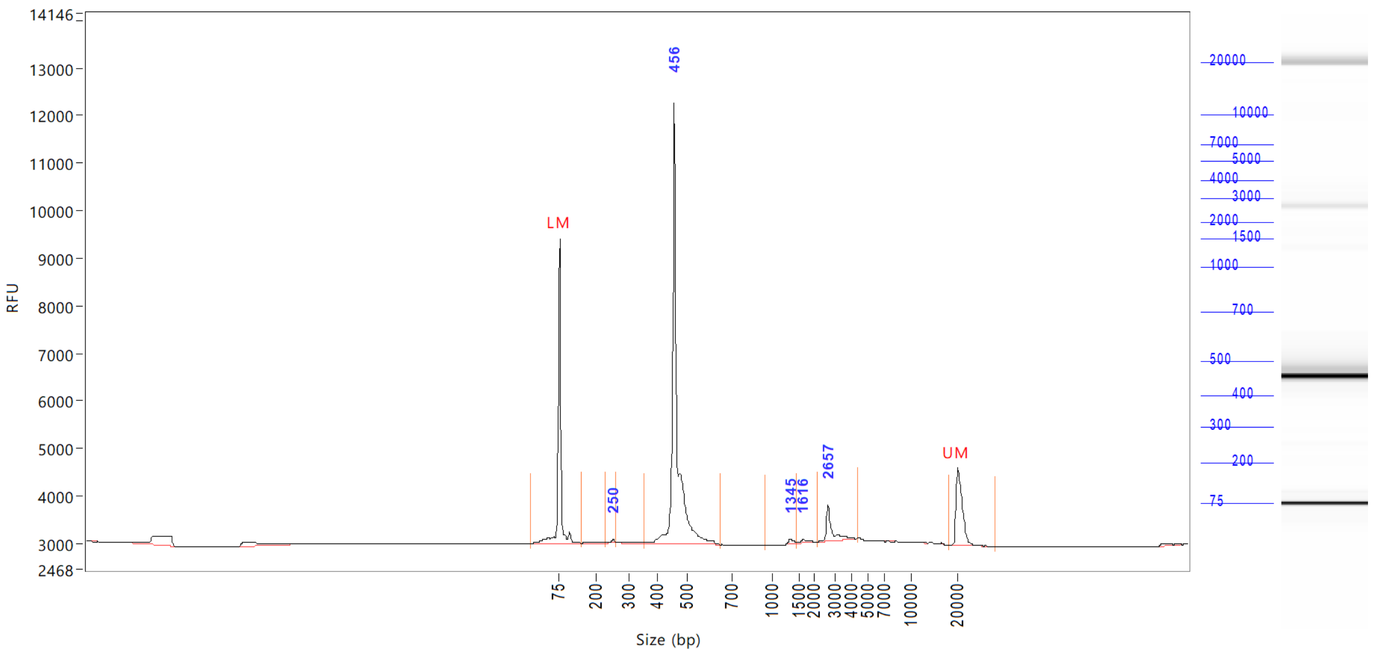

Peak	Size<br>(bp)	Concentration<br>(ng/uL)	From<br>(bp)	To<br>(bp)	Average size<br>(bp)	CV%	RFU	Corrected peak area
1	75 (LM)	0.5000	0	148	71	23.03	6405	42.264
2	250	0.0885	226	262	245	3.10	77	0.623
3	456	11.9938	356	644	465	6.58	9277	84.483
4	1345	0.1362	944	1467	1324	7.21	102	0.959
5	1616	0.1315	1467	2115	1699	8.15	68	0.926
6	2657	0.9316	2115	4345	2867	12.97	748	6.562
7	20000 (UM)	0.1589	18049	27859	20418	3.88	1633	13.432

TIC:	13.2816	ng/uL
TIM:	43.8668	nmole/L
Total concentration:	13.5171	ng/uL

Sample peak width (sec): 5 Sample min peak height: 50 Sample baseline V to V?: Y Sample baseline V to V points: 3
Sample filter: Binomial Number of points for filter: 3 Sample start region (min): 0 Sample end region (min): 30
Marker peak width (sec): 5 Marker min peak height: 200 Marker baseline V to V?: Y Marker baseline V to V points: 3
Lower marker selection: First peak > 500 RFU Upper marker selection: Last peak > 200 RFU
Ladder size (bp)75, 200, 300, 400, 500, 700, 1000, 1500, 2000, 3000, 4000, 5000, 7000, 10000, 20000
Quantification using: Lower Marker Final concentration (ng/uL): 0.5000 Dilution factor: 12.0

Sample: SampC12
Well location: C12
Created: Monday, September 20, 2021 6:00:03 PM

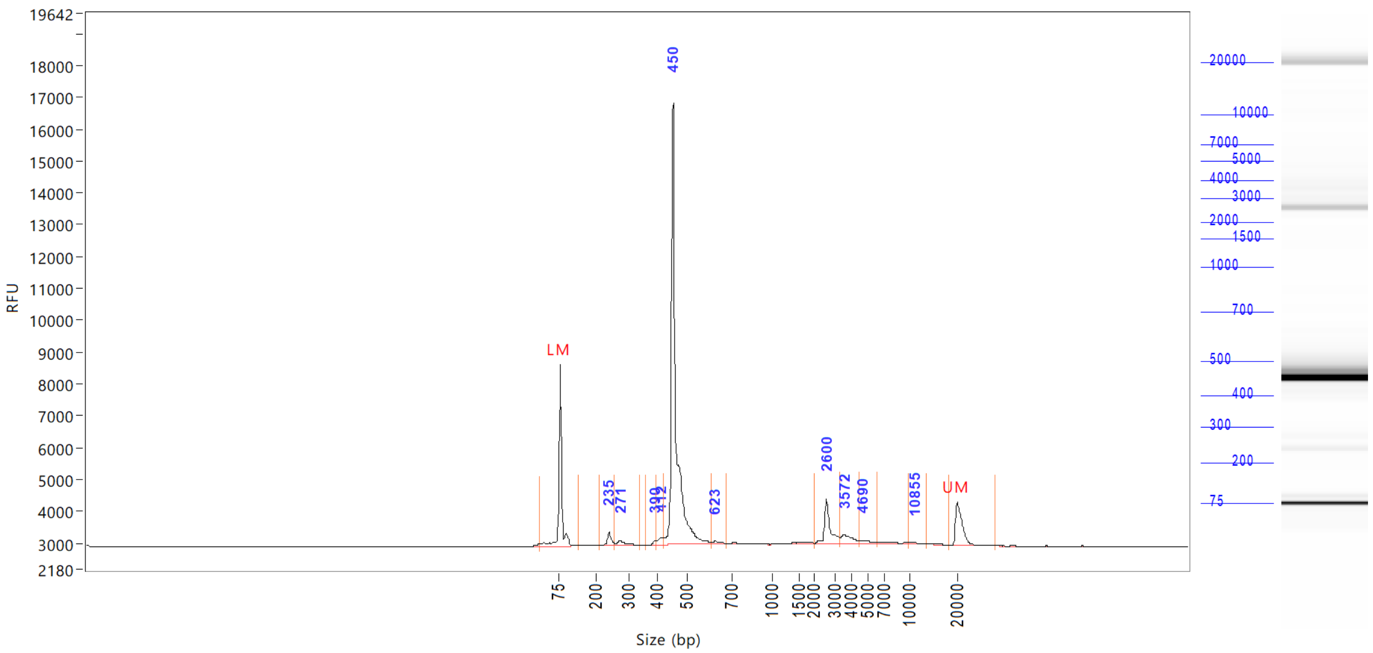

Peak	Size (bp)	Concentration (ng/uL)	From (bp)	To (bp)	Average size (bp)	CV%	RFU	Corrected peak area
1	75 (LM)	0.5000	7	135	71	21.43	5708	40.554
2	235	0.5617	208	255	235	3.59	410	3.797
3	271	0.3827	255	337	277	5.89	164	2.587
4	390	0.1477	360	396	387	1.61	142	0.998
5	412	0.3693	396	420	409	1.72	221	2.496
6	450	18.7478	420	603	460	5.12	13889	126.717
7	623	0.1376	603	672	625	2.38	65	0.930
8	2600	1.8098	2001	3322	2682	8.80	1401	12.232
9	3572	0.6664	3322	4518	3810	8.37	270	4.504
10	4690	0.2075	4518	6112	5027	8.74	94	1.403
11	10855	0.0878	9867	13415	11250	9.28	60	0.593
12	20000 (UM)	0.1470	18171	28100	20559	4.44	1355	11.921

TIC:	23.1182	ng/uL
TIM:	77.2199	nmole/L
Total concentration:	23.4876	ng/uL

```
Sample peak width (sec): 5    Sample min peak height: 50    Sample baseline V to V?: Y    Sample baseline V to V points: 3
Sample filter: Binomial       Number of points for filter: 3  Sample start region (min): 0  Sample end region (min): 30
Marker peak width (sec): 5    Marker min peak height: 200   Marker baseline V to V?: Y    Marker baseline V to V points: 3
Lower marker selection: First peak > 500 RFU                Upper marker selection: Last peak > 200 RFU
Ladder size (bp)75, 200, 300, 400, 500, 700, 1000, 1500, 2000, 3000, 4000, 5000, 7000, 10000, 20000
Quantification using: Lower Marker          Final concentration (ng/uL): 0.5000          Dilution factor: 12.0
```

Sample: SampD12
Well location: D12
Created: Monday, September 20, 2021 6:00:03 PM

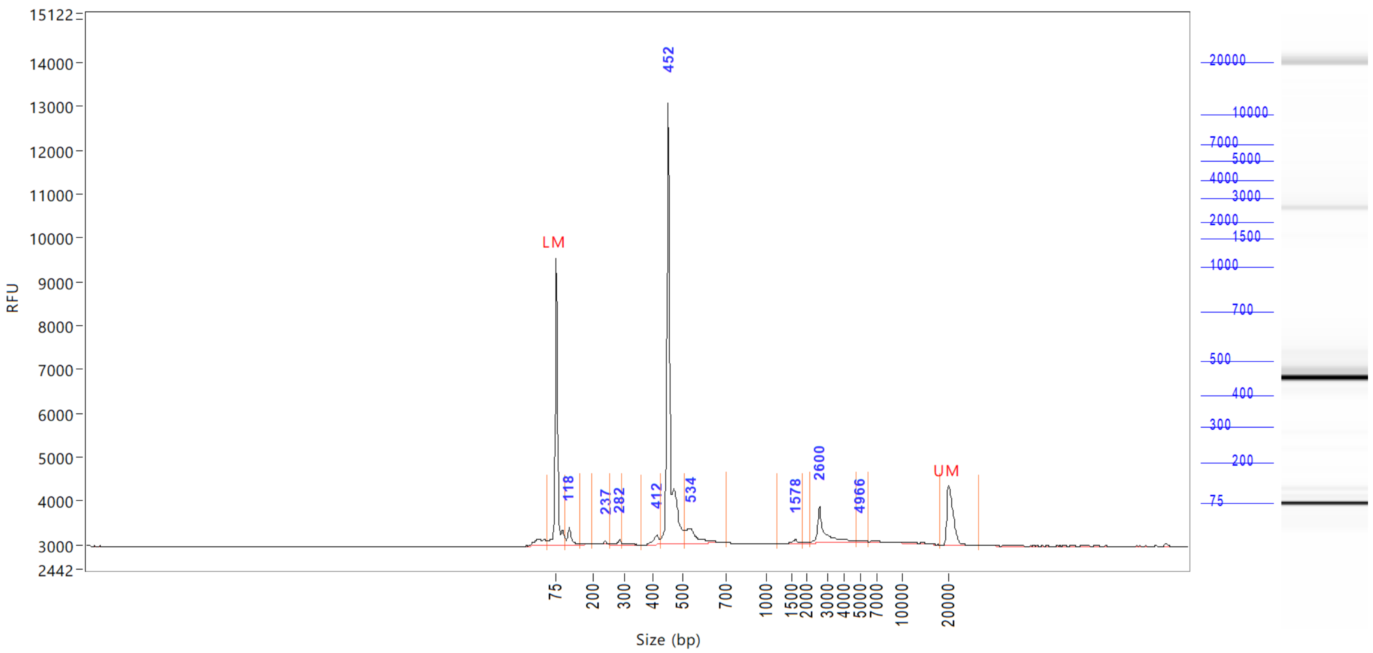

Peak	Size (bp)	Concentration (ng/uL)	From (bp)	To (bp)	Average size (bp)	CV%	RFU	Corrected peak area
1	75 (LM)	0.5000	47	107	74	10.65	6535	41.581
2	118	0.5310	107	156	120	7.94	406	3.680
3	237	0.0892	198	251	234	3.43	76	0.618
4	282	0.1364	251	293	278	3.07	116	0.945
5	412	0.3815	360	424	407	2.64	211	2.644
6	452	10.3533	424	509	455	3.08	10081	71.750
7	534	1.1370	509	697	552	6.85	344	7.880
8	1578	0.1348	1223	1827	1565	8.07	85	0.934
9	2600	1.3425	2144	4724	2967	19.15	825	9.304
10	4966	0.0884	4724	6038	5197	6.96	50	0.613
11	20000 (UM)	0.1358	18171	26649	20454	3.92	1358	11.293

TIC:	14.1941	ng/uL
TIM:	51.9905	nmole/L
Total concentration:	14.5208	ng/uL

```
Sample peak width (sec): 5    Sample min peak height: 50    Sample baseline V to V?: Y    Sample baseline V to V points: 3
Sample filter: Binomial       Number of points for filter: 3  Sample start region (min): 0  Sample end region (min): 30
Marker peak width (sec): 5    Marker min peak height: 200   Marker baseline V to V?: Y    Marker baseline V to V points: 3
Lower marker selection: First peak > 500 RFU                Upper marker selection: Last peak > 200 RFU
Ladder size (bp)75, 200, 300, 400, 500, 700, 1000, 1500, 2000, 3000, 4000, 5000, 7000, 10000, 20000
Quantification using: Lower Marker          Final concentration (ng/uL): 0.5000          Dilution factor: 12.0
```

Sample: SampE12
Well location: E12
Created: Monday, September 20, 2021 6:00:03 PM

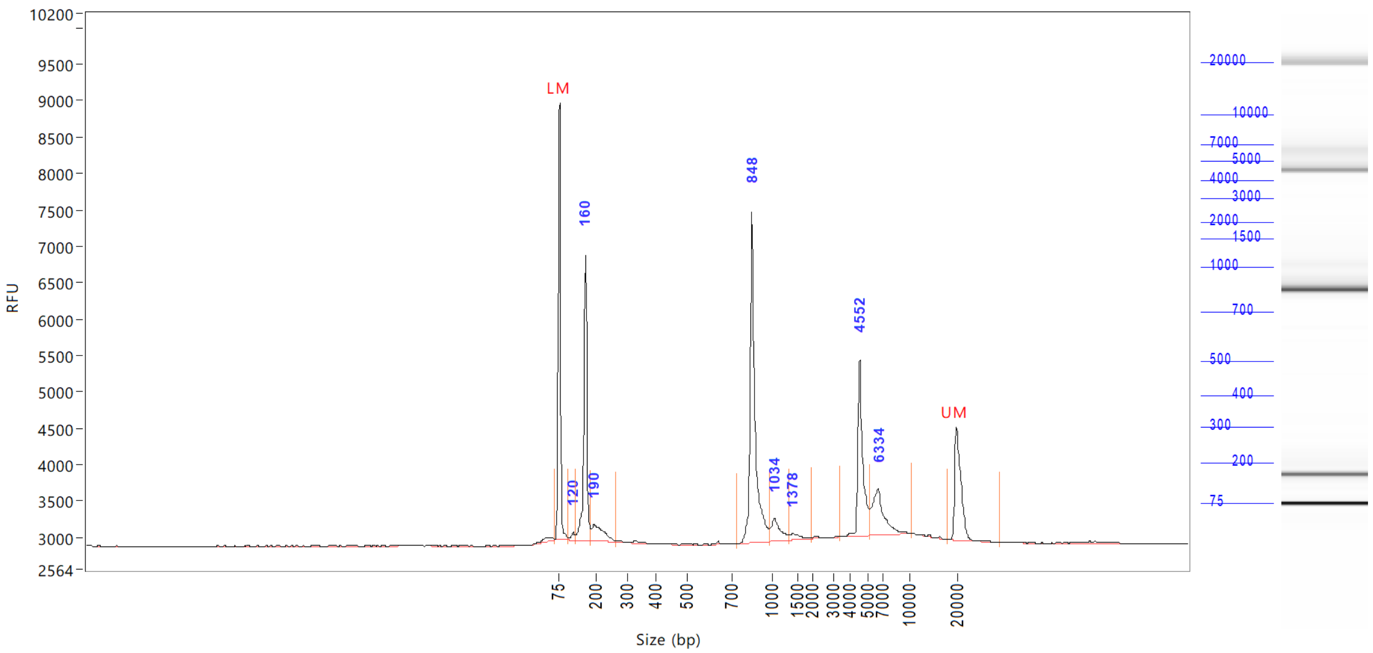

Peak	Size (bp)	Concentration (ng/uL)	From (bp)	To (bp)	Average size (bp)	CV%	RFU	Corrected peak area
1	75 (LM)	0.5000	60	105	73	6.14	6012	33.915
2	120	0.1702	105	128	118	5.19	107	0.962
3	160	4.8860	128	181	159	5.02	3914	27.618
4	190	1.0366	181	260	207	8.71	224	5.859
5	848	6.8694	731	978	859	4.06	4539	38.829
6	1034	0.8860	978	1323	1089	8.67	307	5.008
7	1378	0.2631	1323	1942	1486	9.89	83	1.487
8	4552	3.1895	3322	5223	4612	4.95	2412	18.029
9	6334	1.9534	5223	10367	6486	13.93	631	11.041
10	20000 (UM)	0.1916	18171	29189	20433	4.56	1558	12.996

TIC:	19.2543	ng/uL
TIM:	77.5404	nmole/L
Total concentration:	19.5047	ng/uL

```
Sample peak width (sec): 5    Sample min peak height: 50    Sample baseline V to V?: Y    Sample baseline V to V points: 3
Sample filter: Binomial       Number of points for filter: 3  Sample start region (min): 0  Sample end region (min): 30
Marker peak width (sec): 5    Marker min peak height: 200   Marker baseline V to V?: Y    Marker baseline V to V points: 3
Lower marker selection: First peak > 500 RFU                Upper marker selection: Last peak > 200 RFU
Ladder size (bp)75, 200, 300, 400, 500, 700, 1000, 1500, 2000, 3000, 4000, 5000, 7000, 10000, 20000
Quantification using: Lower Marker         Final concentration (ng/uL): 0.5000         Dilution factor: 12.0
```

Sample: SampF12
Well location: F12
Created: Monday, September 20, 2021 6:00:03 PM

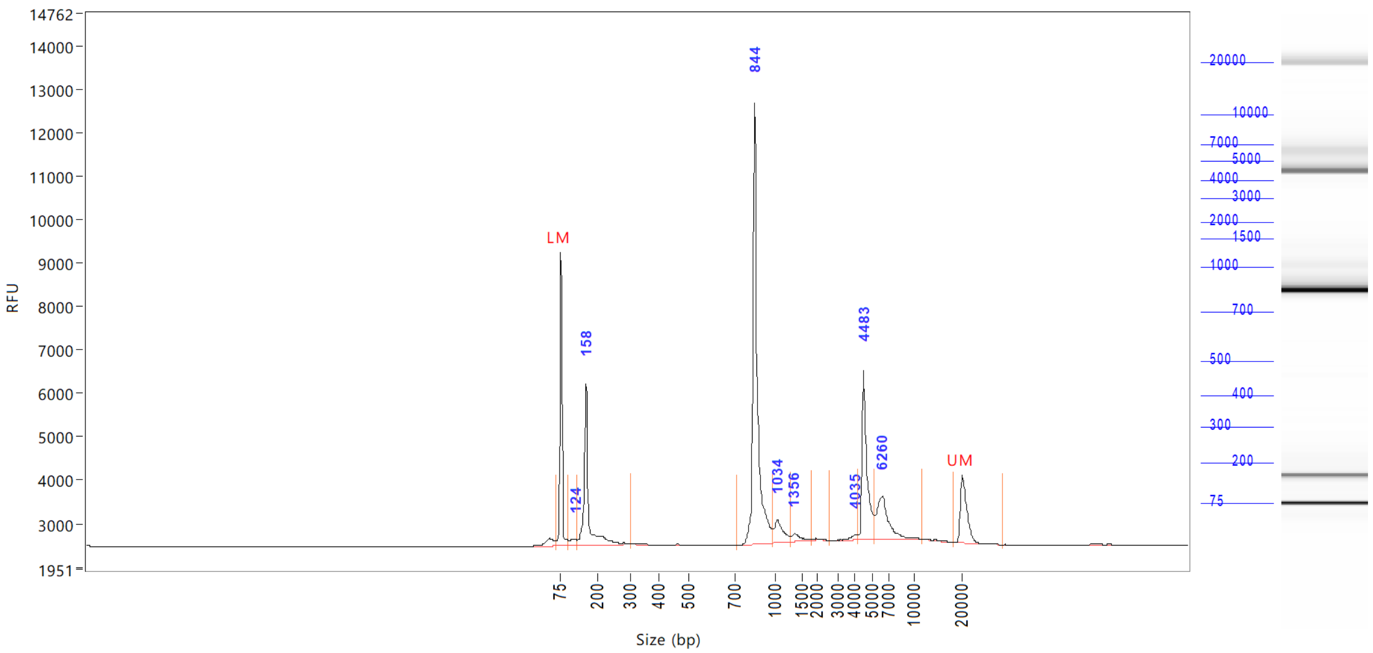

Peak	Size (bp)	Concentration (ng/uL)	From (bp)	To (bp)	Average size (bp)	CV%	RFU	Corrected peak area
1	75 (LM)	0.5000	64	101	74	6.68	6771	38.234
2	124	0.3880	101	129	115	7.48	140	2.472
3	158	5.6703	129	303	168	16.08	3728	36.133
4	844	12.1697	714	983	855	4.16	10189	77.550
5	1034	1.2649	983	1300	1083	8.11	521	8.060
6	1356	0.3871	1300	1827	1428	7.81	173	2.467
7	4035	0.2081	2600	4208	3908	6.20	114	1.326
8	4483	4.6582	4208	5297	4601	4.80	3922	29.684
9	6260	2.7480	5297	11708	6551	14.88	999	17.512
10	20000 (UM)	0.1747	18293	28584	20463	4.03	1549	13.358

TIC:	27.4943	ng/uL
TIM:	89.2634	nmole/L
Total concentration:	27.6293	ng/uL

```
Sample peak width (sec): 5     Sample min peak height: 50     Sample baseline V to V?: Y     Sample baseline V to V points: 3
Sample filter: Binomial        Number of points for filter: 3  Sample start region (min): 0  Sample end region (min): 30
Marker peak width (sec): 5     Marker min peak height: 200    Marker baseline V to V?: Y     Marker baseline V to V points: 3
Lower marker selection: First peak > 500 RFU                  Upper marker selection: Last peak > 200 RFU
Ladder size (bp)75, 200, 300, 400, 500, 700, 1000, 1500, 2000, 3000, 4000, 5000, 7000, 10000, 20000
Quantification using: Lower Marker          Final concentration (ng/uL): 0.5000          Dilution factor: 12.0
```

Sample: SampG12
Well location: G12
Created: Monday, September 20, 2021 6:00:03 PM

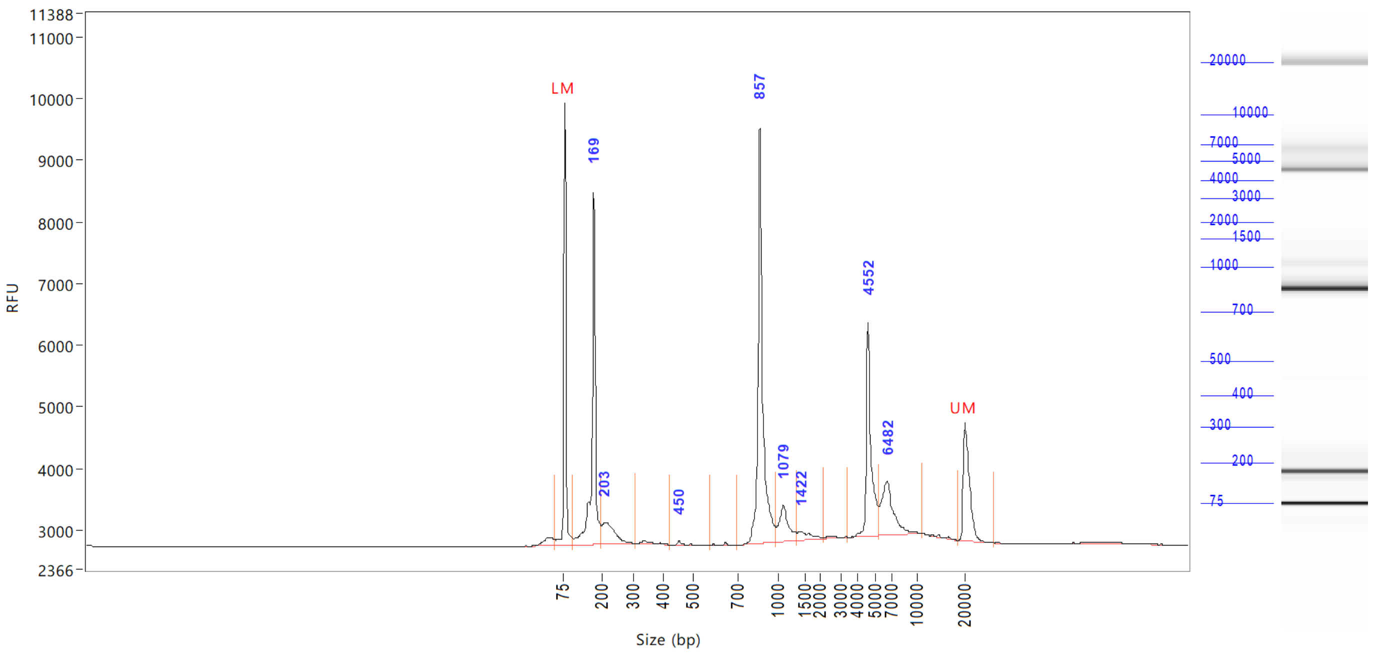

Peak	Size (bp)	Concentration (ng/uL)	From (bp)	To (bp)	Average size (bp)	CV%	RFU	Corrected peak area
1	75 (LM)	0.5000	45	101	73	8.17	7190	41.506
2	169	6.9046	101	194	164	9.47	5702	47.764
3	203	1.4858	194	303	221	9.37	348	10.278
4	450	0.0820	420	572	464	5.91	62	0.567
5	857	7.9892	690	978	862	3.92	6723	55.267
6	1079	1.3332	978	1334	1098	8.46	591	9.223
7	1422	0.4343	1334	2115	1544	11.97	142	3.004
8	4552	3.6293	3393	5371	4652	5.21	3463	25.106
9	6482	2.2068	5371	10733	6629	13.17	875	15.266
10	20000 (UM)	0.1921	18415	26166	20379	3.84	1910	15.951

TIC:	24.0652	ng/uL
TIM:	100.0988	nmole/L
Total concentration:	24.2370	ng/uL

```
Sample peak width (sec): 5    Sample min peak height: 50    Sample baseline V to V?: Y    Sample baseline V to V points: 3
Sample filter: Binomial       Number of points for filter: 3  Sample start region (min): 0  Sample end region (min): 30
Marker peak width (sec): 5    Marker min peak height: 200   Marker baseline V to V?: Y    Marker baseline V to V points: 3
Lower marker selection: First peak > 500 RFU                Upper marker selection: Last peak > 200 RFU
Ladder size (bp)75, 200, 300, 400, 500, 700, 1000, 1500, 2000, 3000, 4000, 5000, 7000, 10000, 20000
Quantification using: Lower Marker          Final concentration (ng/uL): 0.5000          Dilution factor: 12.0
```

Sample: SampH12
Well location: H12
Created: Monday, September 20, 2021 6:00:03 PM

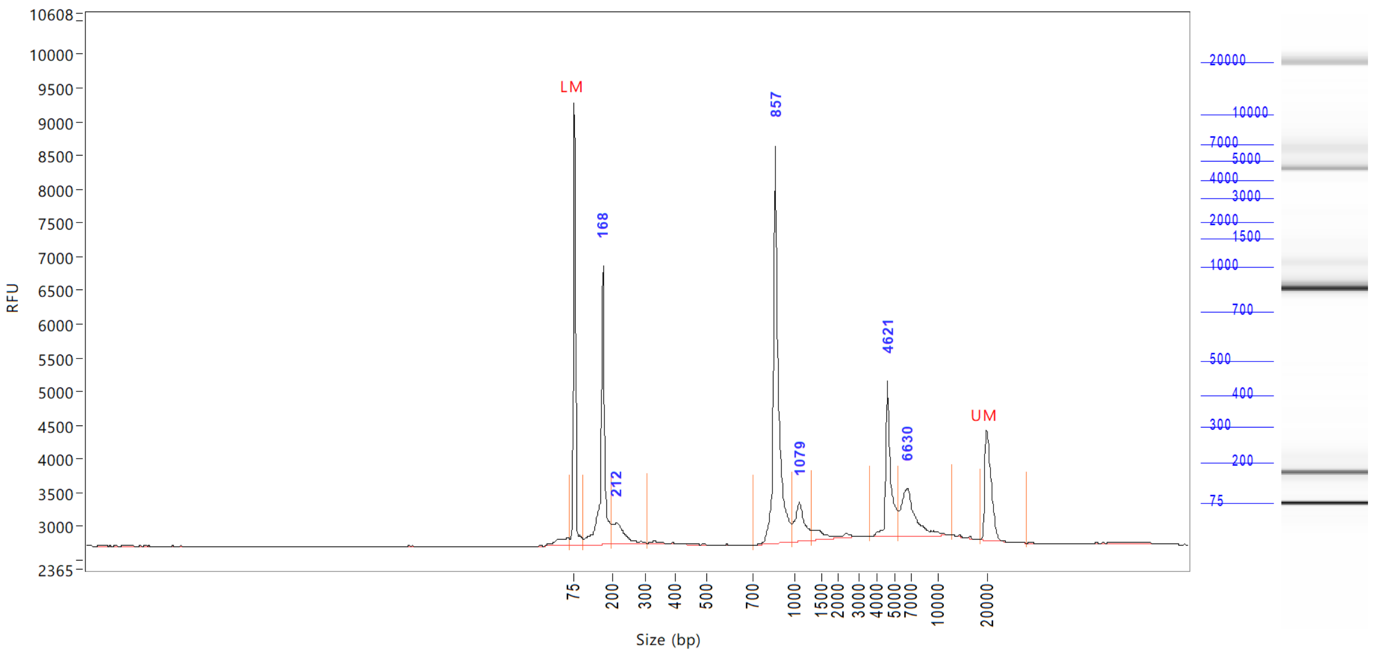

Peak	Size (bp)	Concentration (ng/uL)	From (bp)	To (bp)	Average size (bp)	CV%	RFU	Corrected peak area
1	75 (LM)	0.5000	64	103	74	6.77	6570	36.136
2	168	6.1848	103	196	162	9.82	4125	37.249
3	212	1.3713	196	303	222	9.58	308	8.259
4	857	8.5651	700	978	866	3.96	5882	51.585
5	1079	1.5362	978	1311	1095	8.06	579	9.252
6	4621	2.8679	3643	5519	4713	6.00	2301	17.272
7	6630	2.3405	5519	12928	7021	16.75	712	14.096
8	20000 (UM)	0.1841	18781	28221	20409	3.82	1638	13.306

TIC:	22.8658	ng/uL
TIM:	93.0494	nmole/L
Total concentration:	23.5814	ng/uL

```
Sample peak width (sec): 5    Sample min peak height: 200   Sample baseline V to V?: Y    Sample baseline V to V points: 3
Sample filter: Binomial       Number of points for filter: 3  Sample start region (min): 0  Sample end region (min): 30
Marker peak width (sec): 5    Marker min peak height: 200   Marker baseline V to V?: Y    Marker baseline V to V points: 3
Lower marker selection: First peak > 500 RFU                Upper marker selection: Last peak > 200 RFU
Ladder size (bp)75, 200, 300, 400, 500, 700, 1000, 1500, 2000, 3000, 4000, 5000, 7000, 10000, 20000
Quantification using: Lower Marker          Final concentration (ng/uL): 0.5000          Dilution factor: 12.0
```

Sample: DNA size ladder
Well location: A7
Created: Monday, September 20, 2021 6:00:03 PM
Import from: C:\Users\admin\Desktop\210810_alberto_ladder.SCAL
Fit type: Point to point

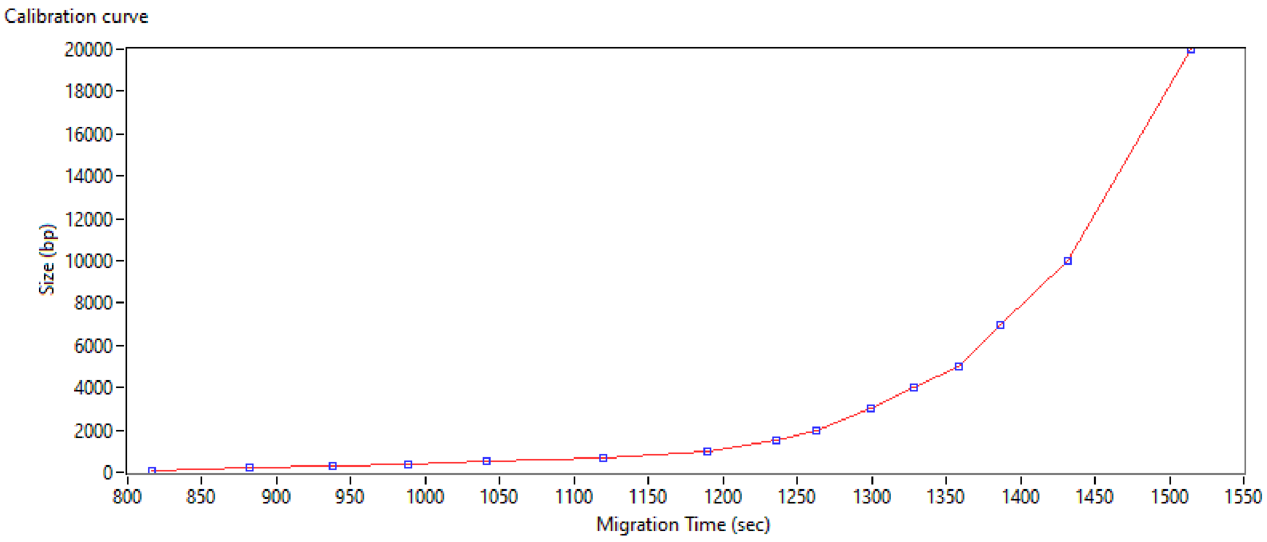

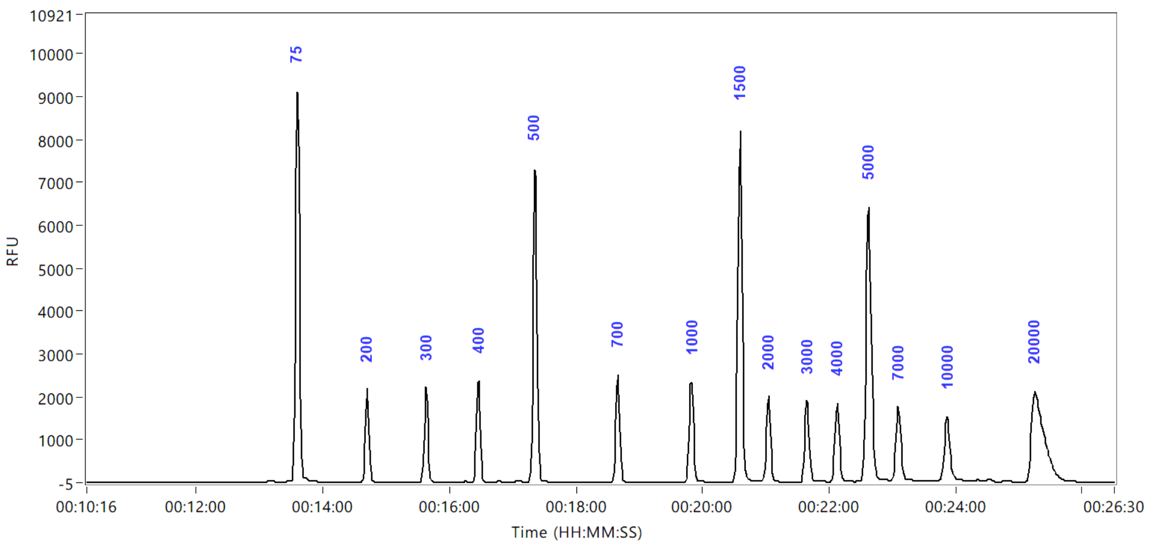

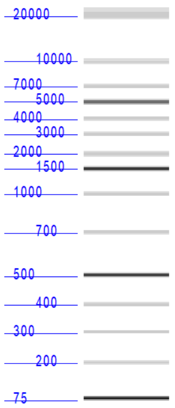

Supplement: Supplementary file 2 — sb3c00292_si_002.zip [file sb3c00292_si_002.zip › dnada_supplementary_material_pks_library_build/ZAG_trials/ZAG_Trial_6/17-39-27_plate1/plate1-t2.pdf]
