# Supplementary material for: Automated Platform for the Plasmid Construction Process
Source: ACS Synth Biol. 2023 Nov 10;12(12):3506–13. doi: 10.1021/acssynbio.3c00292 (PMC10729297; doi:10.1021/acssynbio.3c00292)

Instrument controller software run summary:

Filename and data path: C:\AATI\Data\2021 09 20\19-50-07\2021 09 20 19H 50M..raw
Created: Monday, September 20, 2021 8:00:01 PM
Number of capillaries: 96
Array serial number: 111920-09SFS
Effect length: 33 cm
Array usage count: 226
Instrument type: ZAG DNA Analyzer
Instrument controller software version: 1.0.1.9
Device serial number: Z7597

Method Information

Method name: ZAG130GP33 - DNA 75-20000bp - Gel Prime Only.mthds
Gel prime: Yes
Full conditioning: No
Gel prime to buffer: No
Gel selection: Gel 1
Perform prerun: 8.0 kV, 30 sec.
Rinse: No
Marker 1: Row: A, 5.0 kV, 5 sec.
Rinse: No
Sample injection: 5.0 kV, 5 sec.
Separation: 8.0 kV, 30.0 min.
Tray name: Sample Tray-4

Analysis mode: DNA

Notes

Gel Image

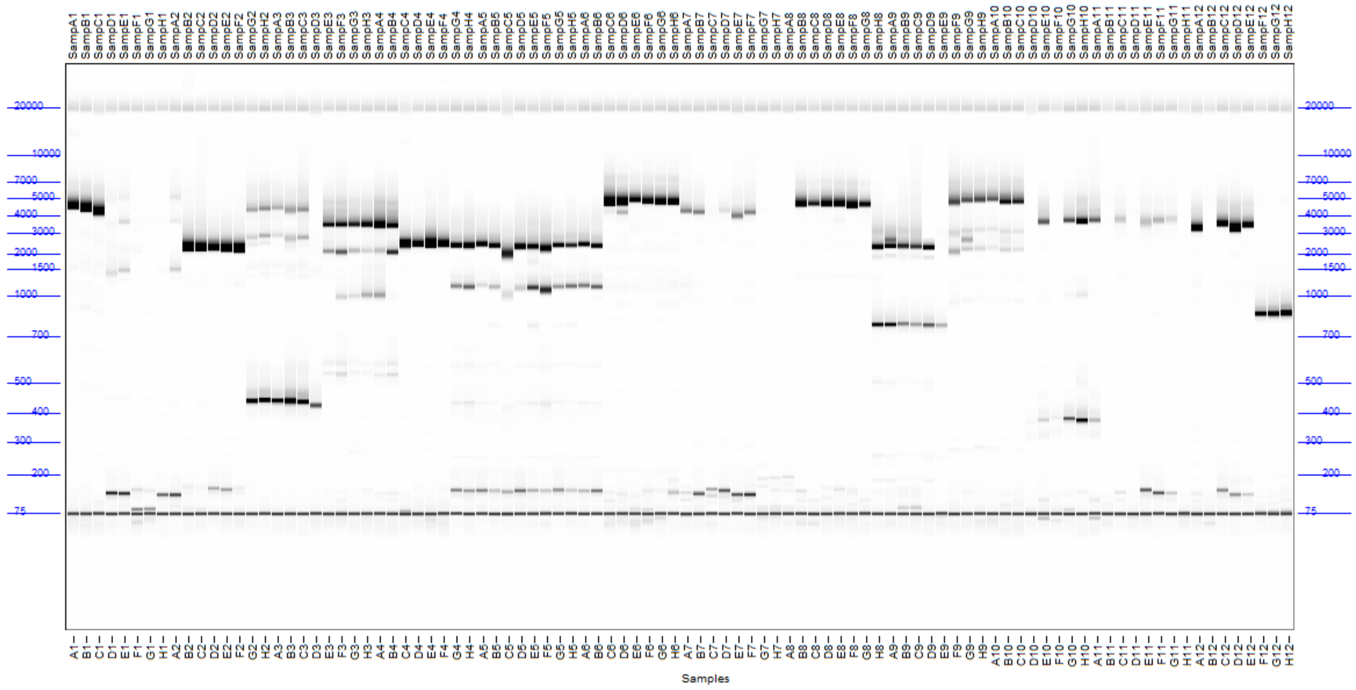
20000
10000
7000
5000
4000
3000
2000
1500
1000
700
500
400
300
200
75
Samples

Filename and data path: C:\AATI\Data\2021 09 20\19-50-07\2021 09 20 19H 50M..raw

A1: SampA1

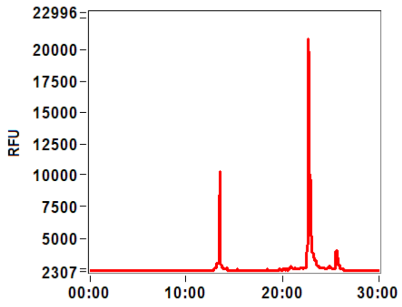

B1: SampB1

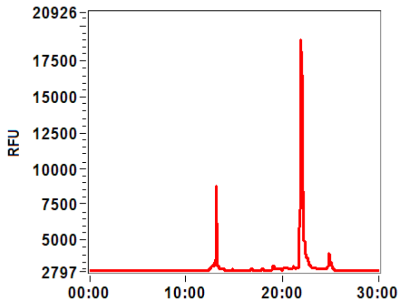

C1: SampC1

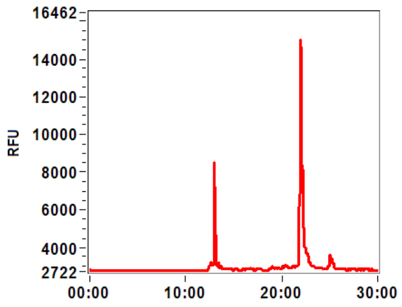

D1: SampD1

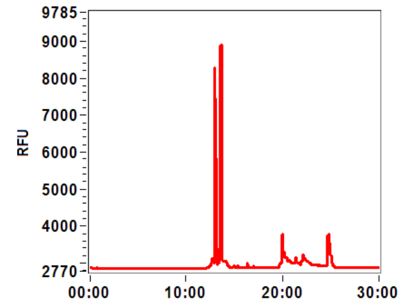

E1: SampE1

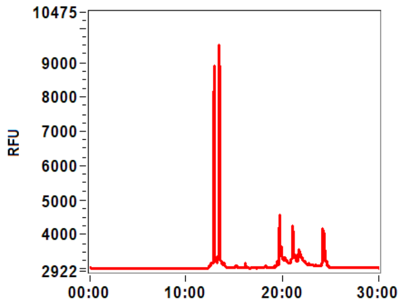

F1: SampF1

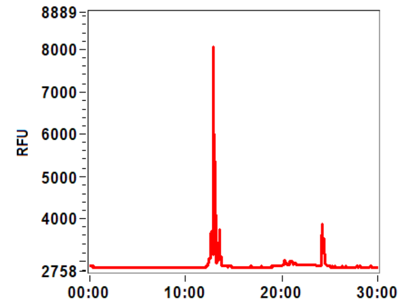

G1: SampG1

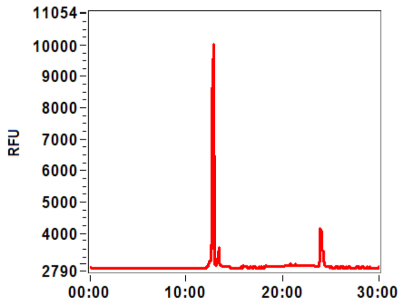

H1: SampH1

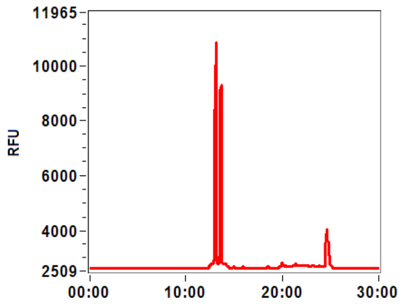

A2: SampA2

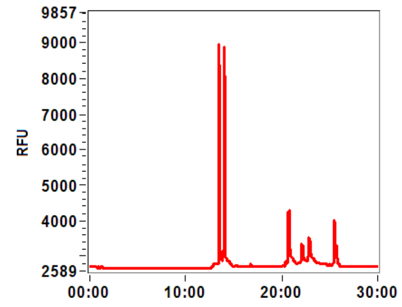

B2: SampB2

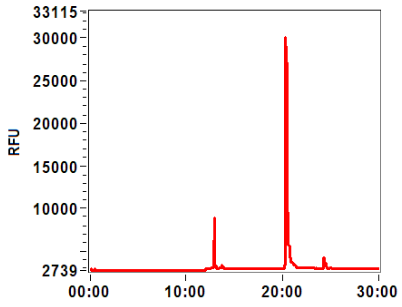

C2: SampC2

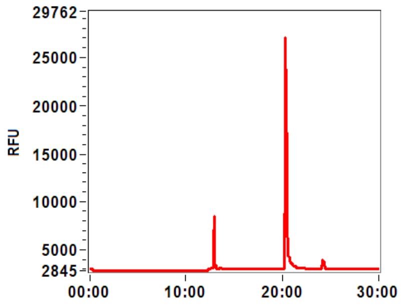

D2: SampD2

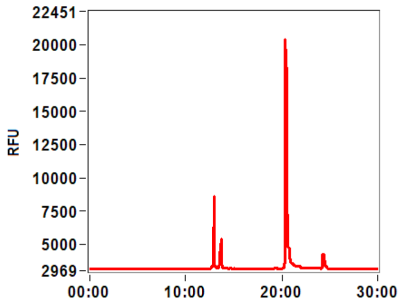

Filename and data path: C:\AATI\Data\2021 09 20\19-50-07\2021 09 20 19H 50M..raw

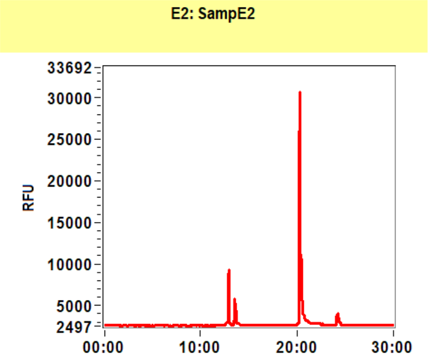

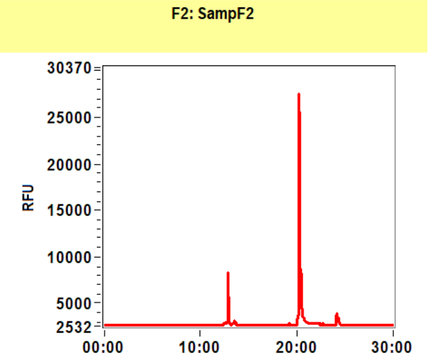

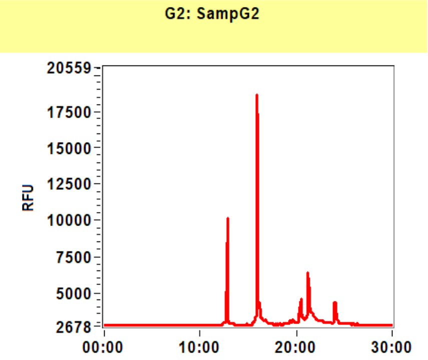

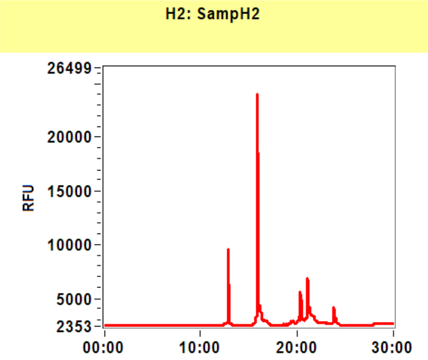

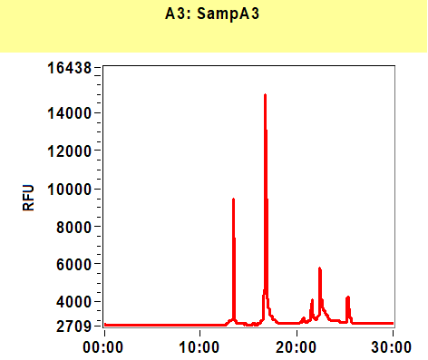

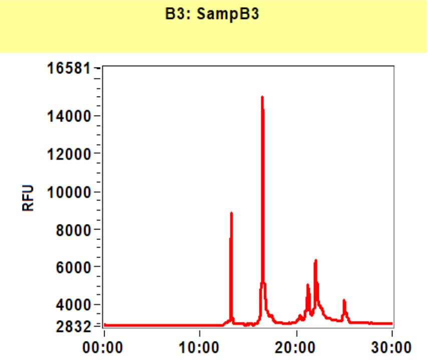

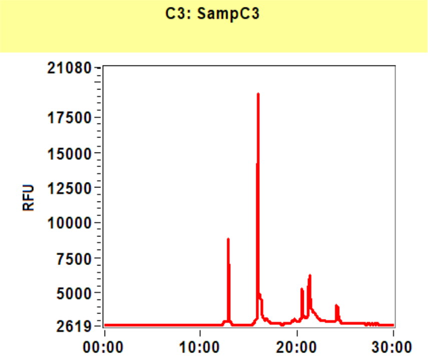

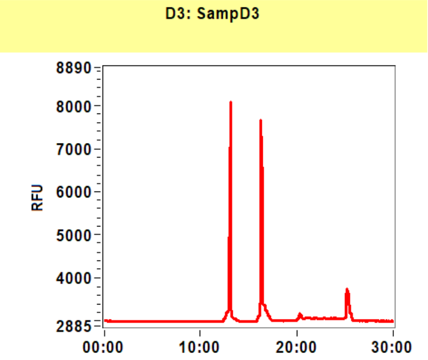

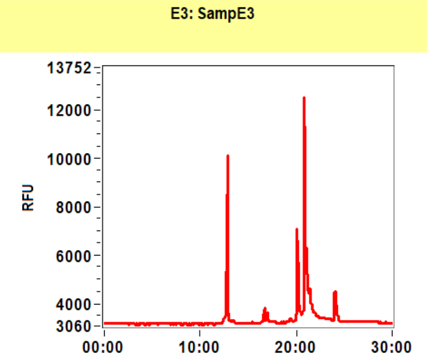

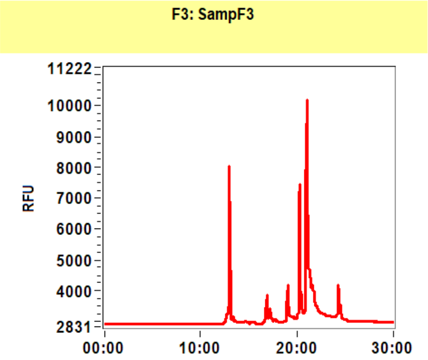

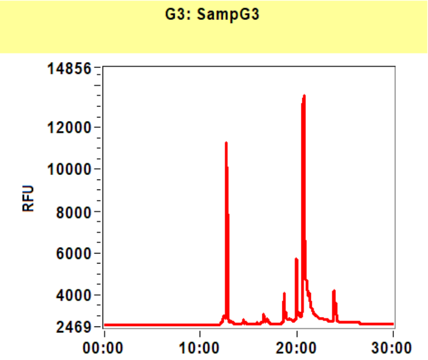

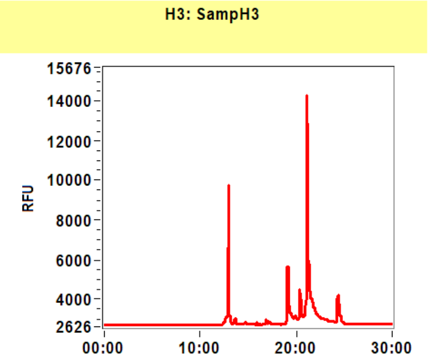

Filename and data path: C:\AATI\Data\2021 09 20\19-50-07\2021 09 20 19H 50M..raw

A4: SampA4

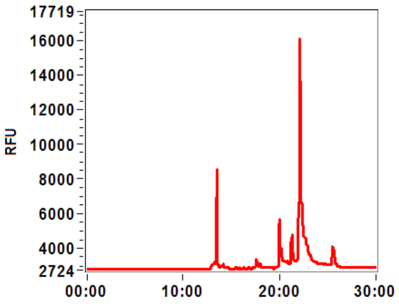

B4: SampB4

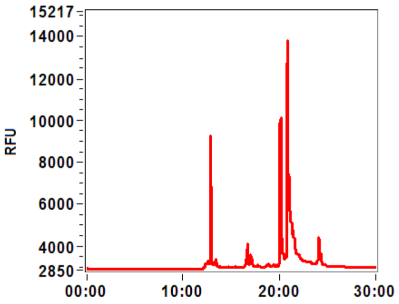

C4: SampC4

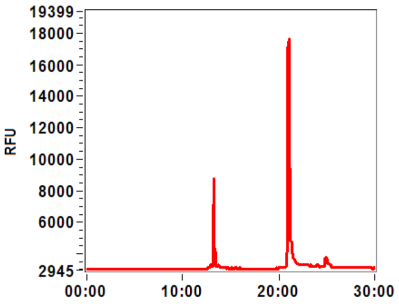

D4: SampD4

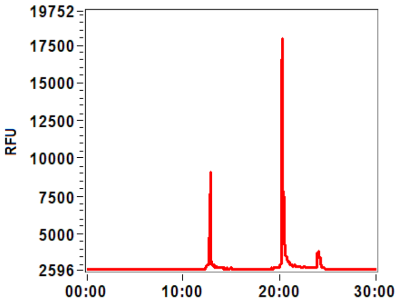

E4: SampE4

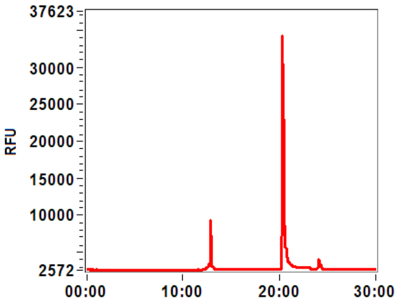

F4: SampF4

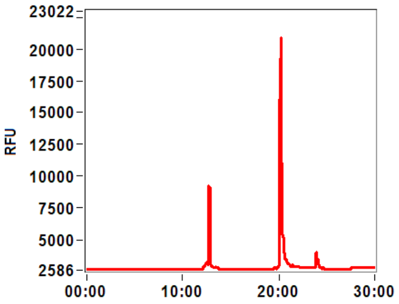

G4: SampG4

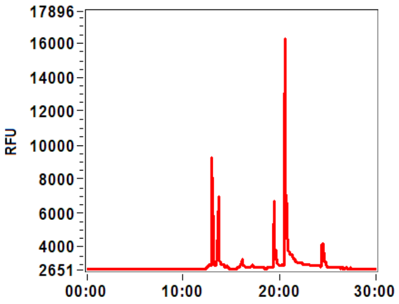

H4: SampH4

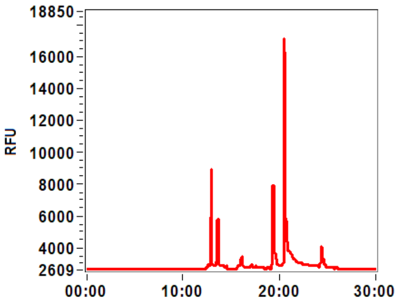

A5: SampA5

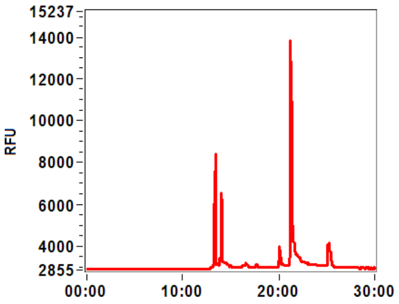

B5: SampB5

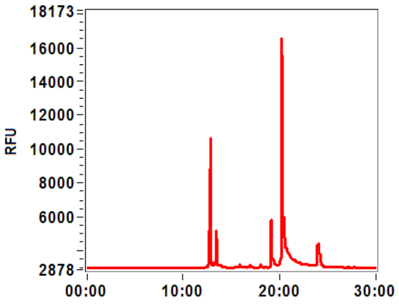

C5: SampC5

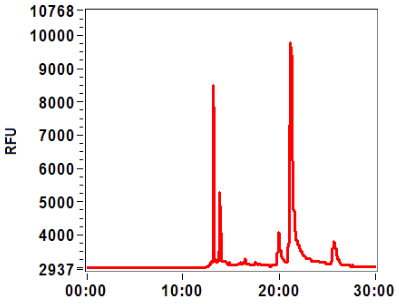

D5: SampD5

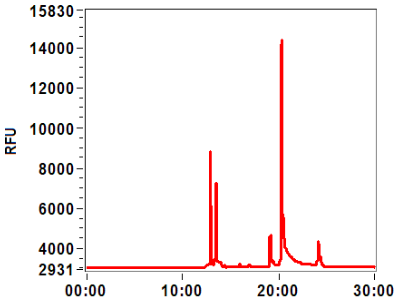

Filename and data path: C:\AATI\Data\2021 09 20\19-50-07\2021 09 20 19H 50M..raw

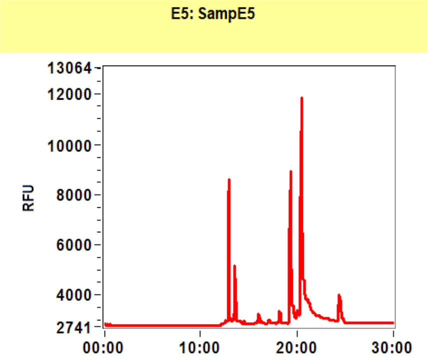

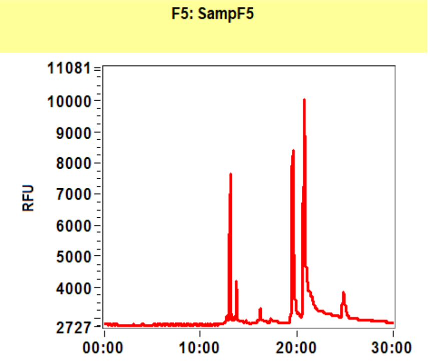

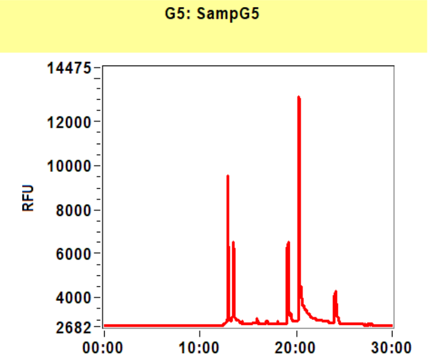

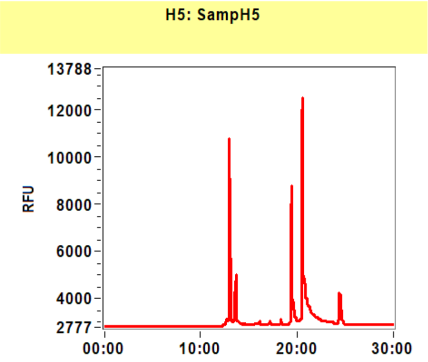

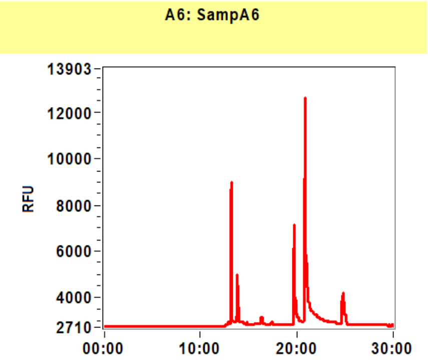

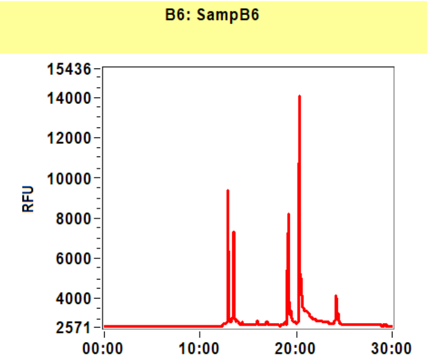

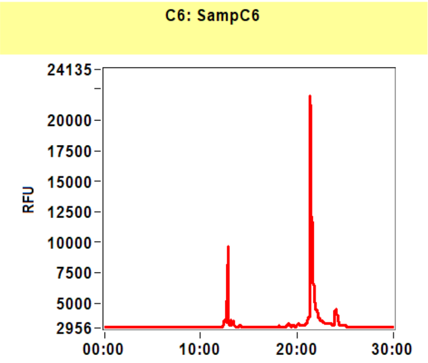

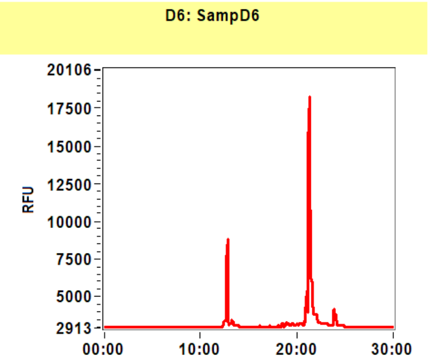

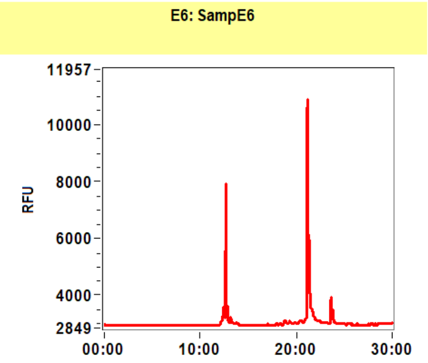

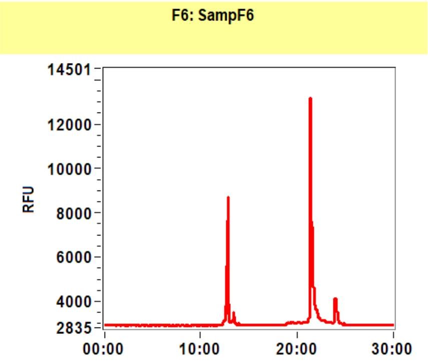

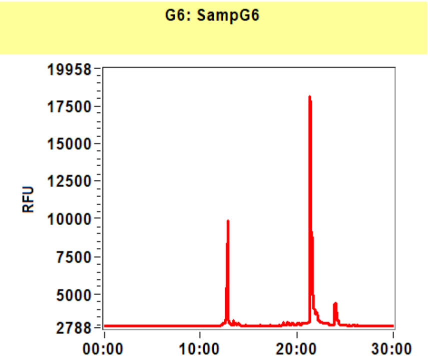

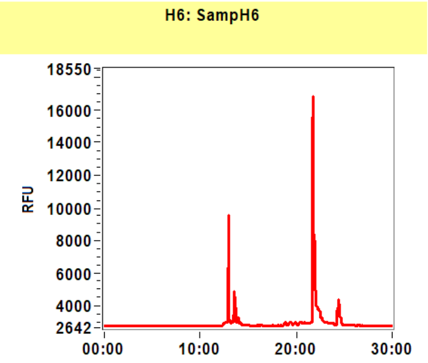

Filename and data path: C:\AATI\Data\2021 09 20\19-50-07\2021 09 20 19H 50M..raw

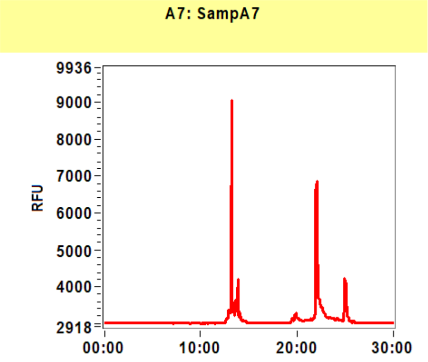

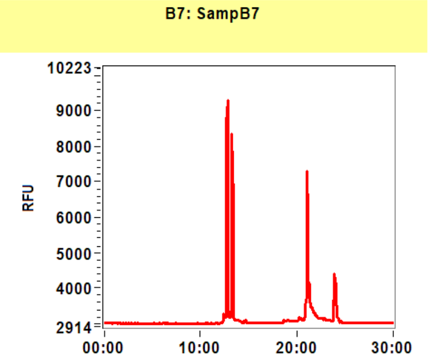

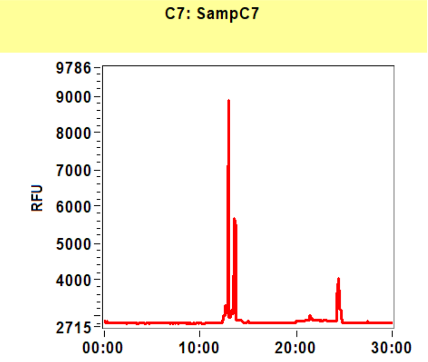

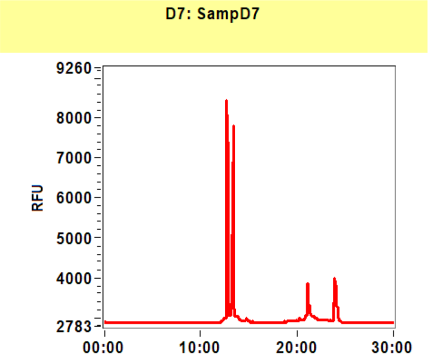

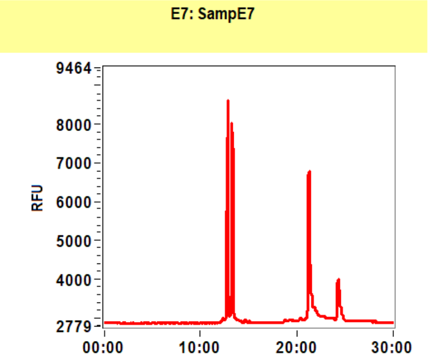

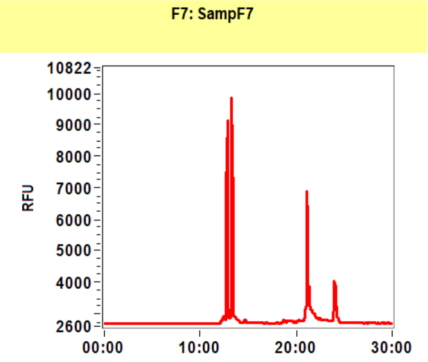

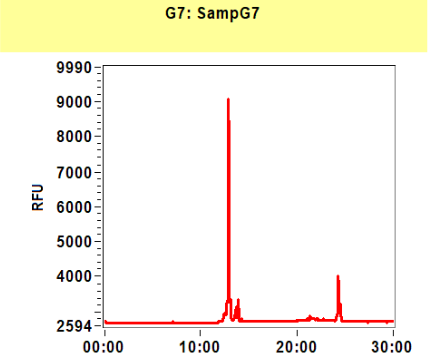

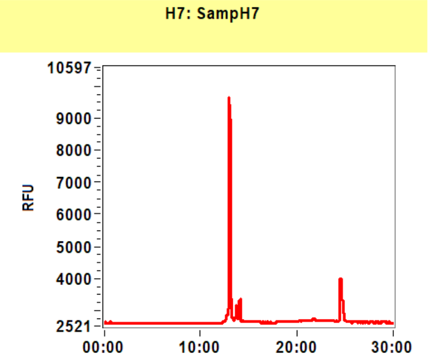

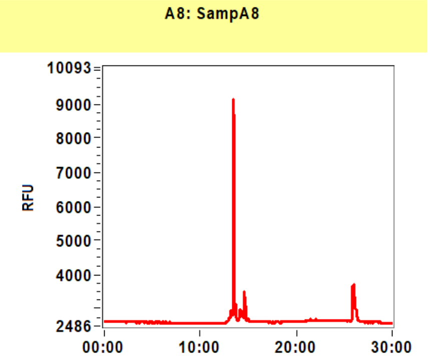

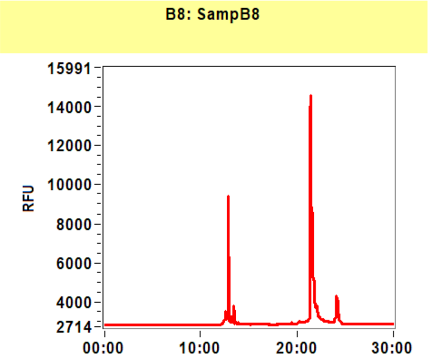

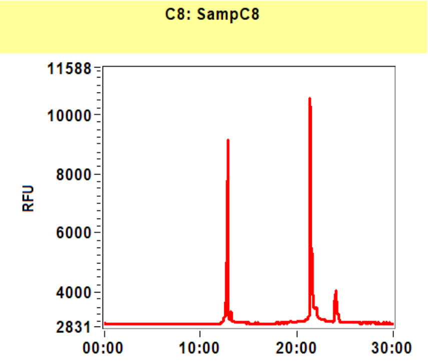

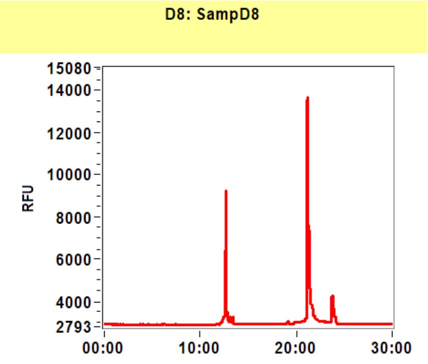

Filename and data path: C:\AATI\Data\2021 09 20\19-50-07\2021 09 20 19H 50M..raw

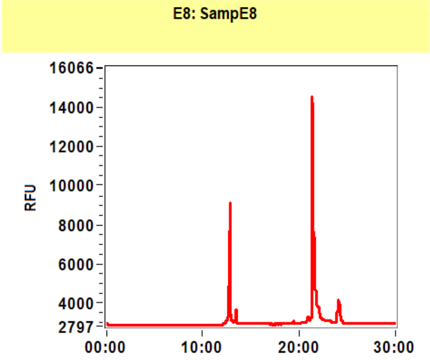

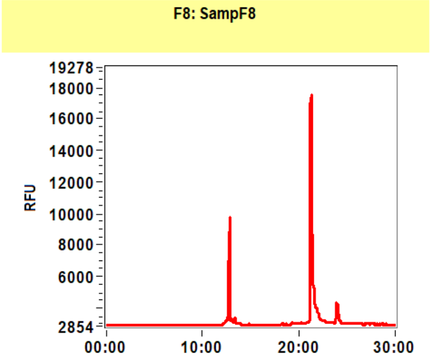

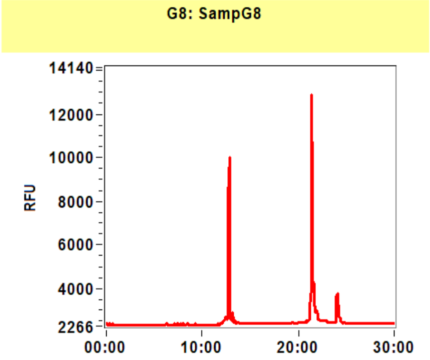

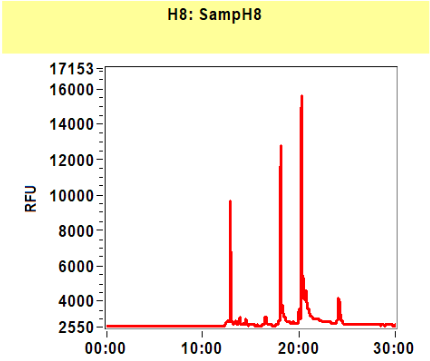

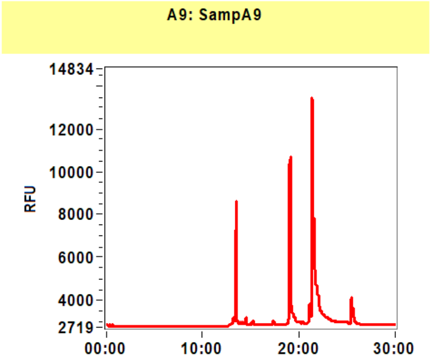

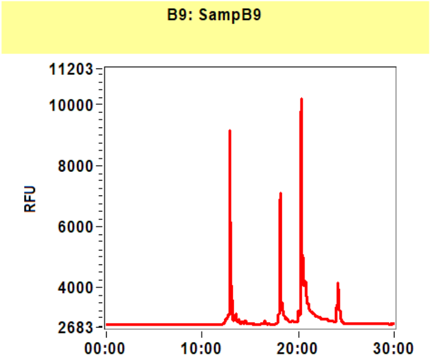

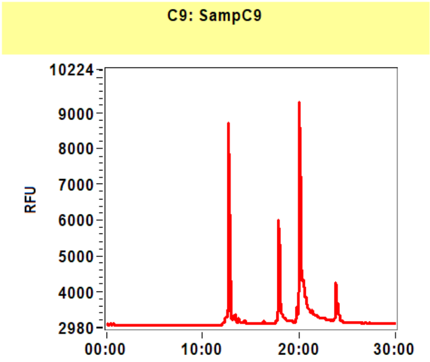

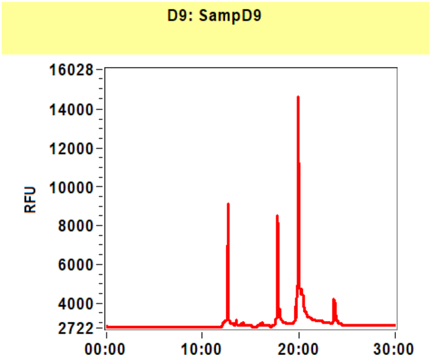

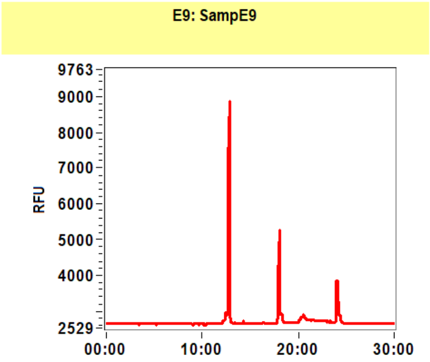

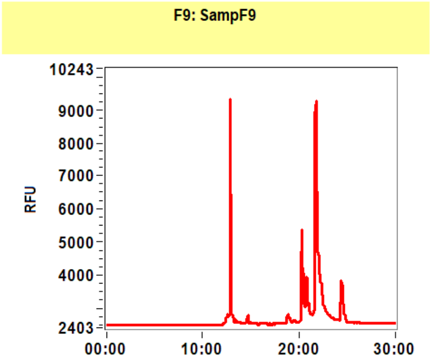

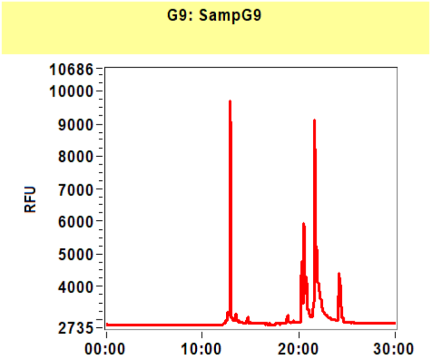

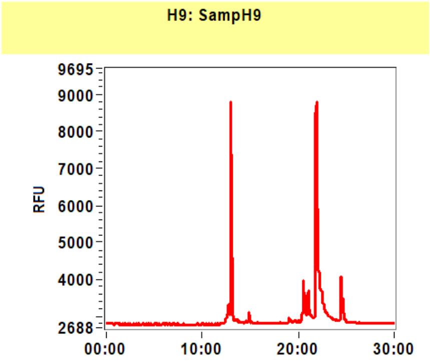

Filename and data path: C:\AATI\Data\2021 09 20\19-50-07\2021 09 20 19H 50M..raw

A10: SampA10

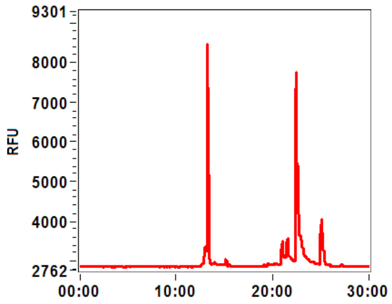

B10: SampB10

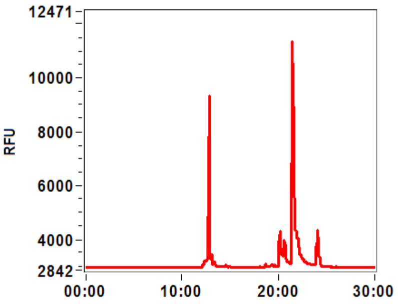

C10: SampC10

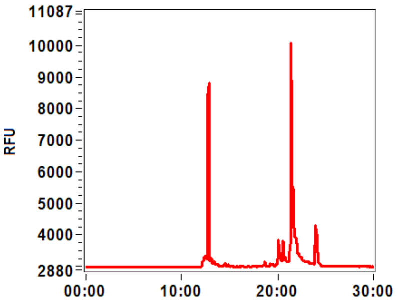

D10: SampD10

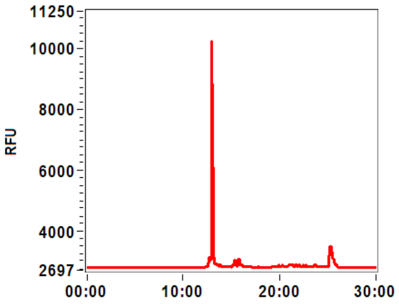

E10: SampE10

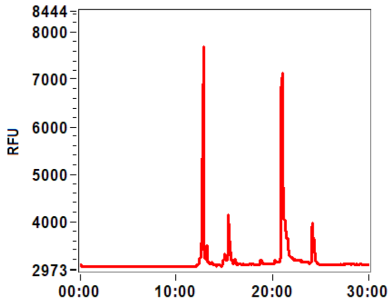

F10: SampF10

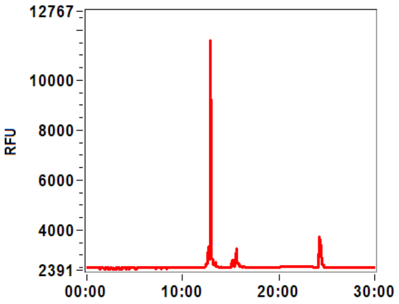

G10: SampG10

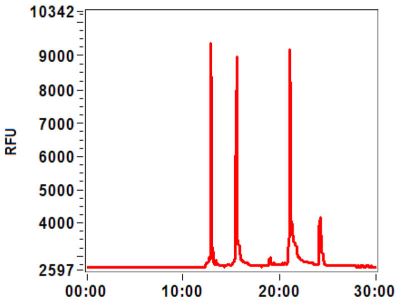

H10: SampH10

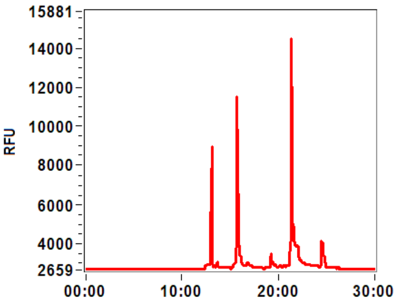

A11: SampA11

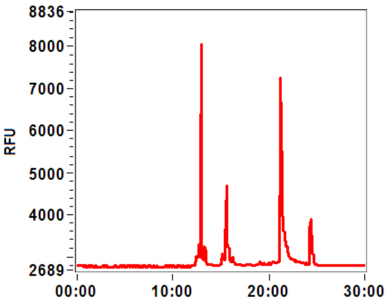

B11: SampB11

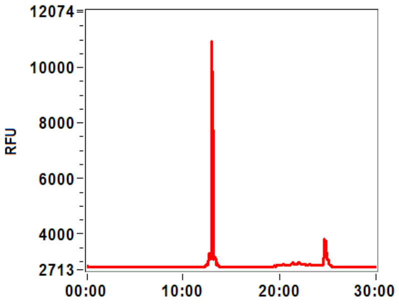

C11: SampC11

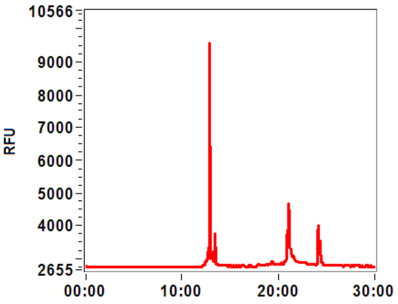

D11: SampD11

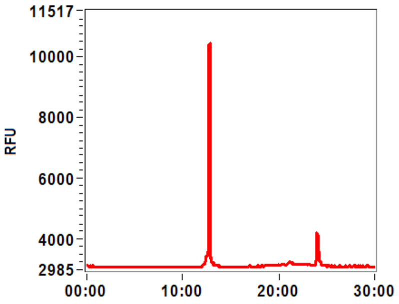

Filename and data path: C:\AATI\Data\2021 09 20\19-50-07\2021 09 20 19H 50M..raw

E11: SampE11

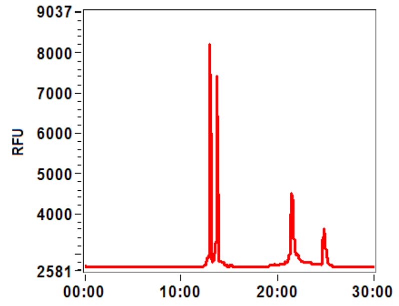

F11: SampF11

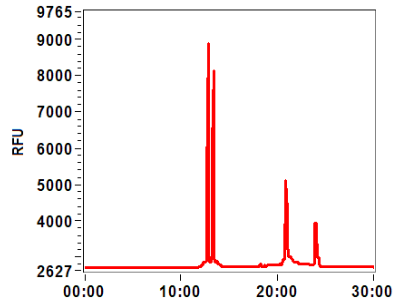

G11: SampG11

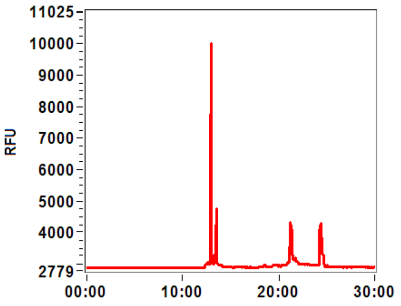

H11: SampH11

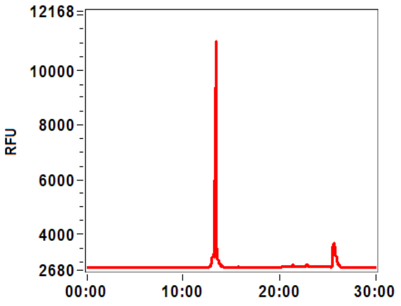

A12: SampA12

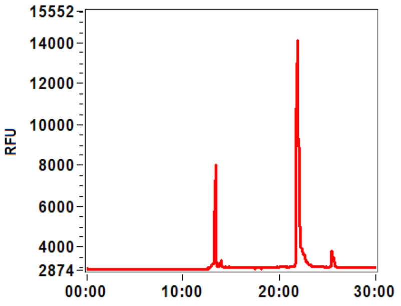

B12: SampB12

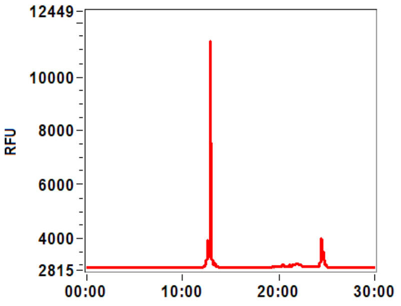

C12: SampC12

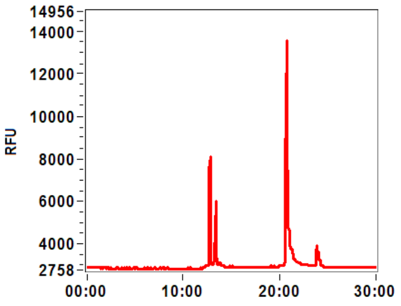

D12: SampD12

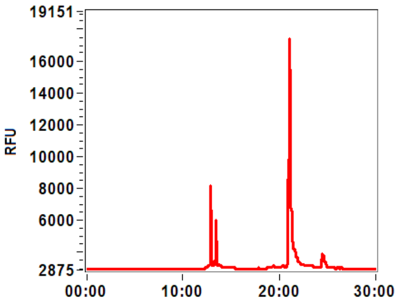

E12: SampE12

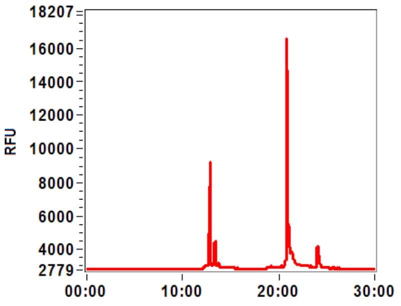

F12: SampF12

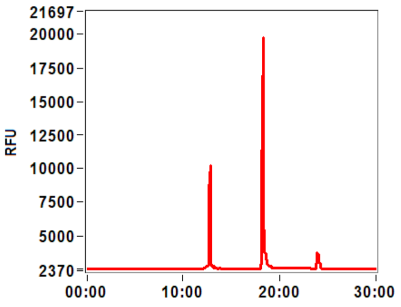

G12: SampG12

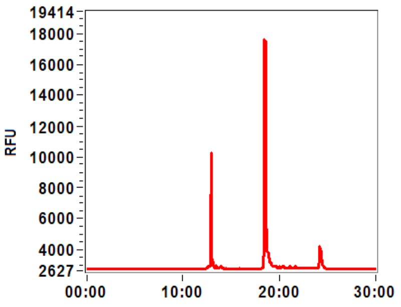

H12: SampH12

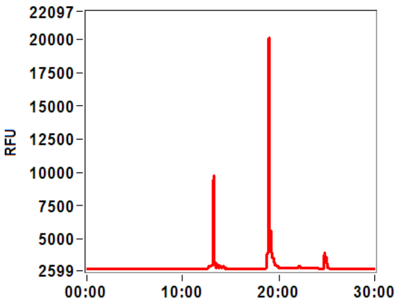

Sample: SampA1
Well location: A1
Created: Monday, September 20, 2021 8:00:01 PM

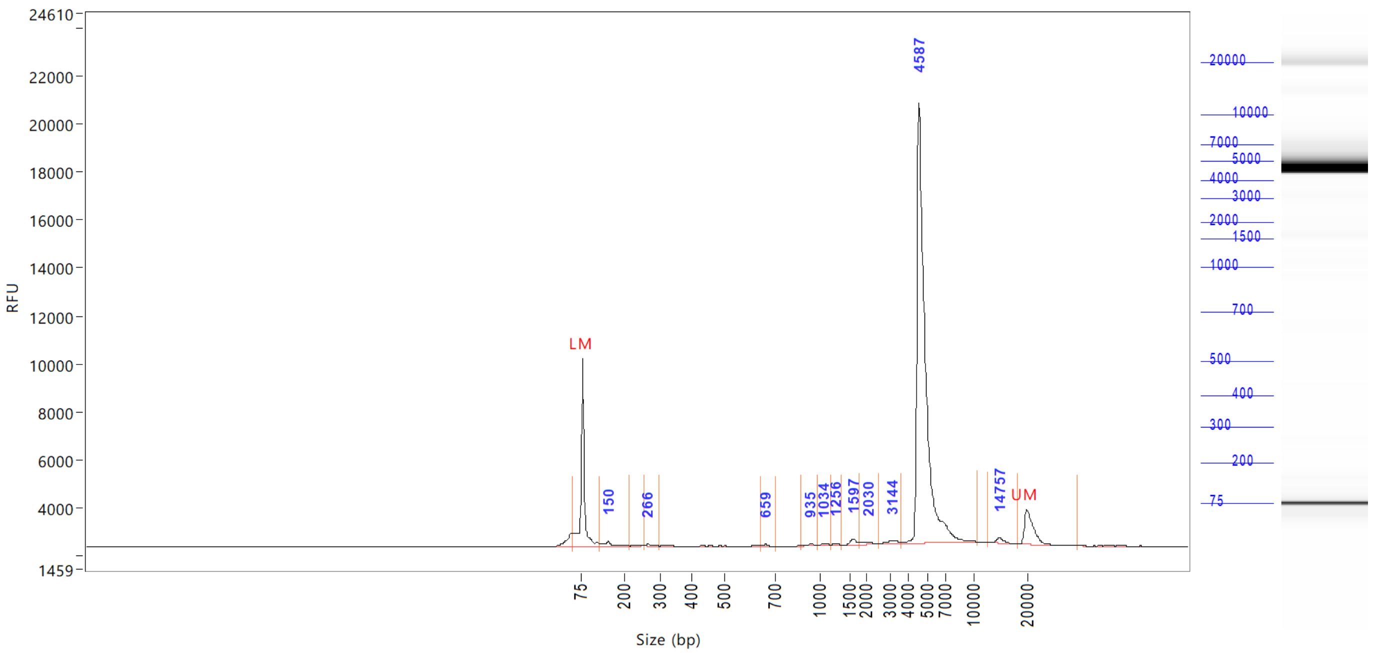

Peak	Size (bp)	Concentration (ng/uL)	From (bp)	To (bp)	Average size (bp)	CV%	RFU	Corrected peak area
1	75 (LM)	0.5000	47	126	74	15.92	7833	59.697
2	150	0.3706	126	215	150	11.63	206	3.687
3	266	0.0629	255	298	266	2.80	72	0.626
4	659	0.0577	641	700	657	1.24	88	0.574
5	935	0.0852	874	983	930	2.20	92	0.848
6	1034	0.0893	983	1178	1064	4.97	78	0.889
7	1256	0.0751	1178	1367	1249	3.00	92	0.747
8	1597	0.2388	1367	1770	1590	5.39	234	2.376
9	2030	0.1768	1770	2486	2010	8.80	97	1.759
10	3144	0.2483	2486	3608	3082	8.57	147	2.470
11	4587	21.0156	3608	10489	4909	14.42	18364	209.095
12	14757	0.2103	12562	18415	14848	5.51	205	2.093
13	20000 (UM)	0.1308	18415	29551	20628	5.23	1433	15.616

TIC:	22.6307	ng/uL
TIM:	12.5776	nmole/L
Total concentration:	22.8045	ng/uL

```
Sample peak width (sec): 5     Sample min peak height: 50     Sample baseline V to V?: Y     Sample baseline V to V points: 3
Sample filter: Binomial        Number of points for filter: 3  Sample start region (min): 0  Sample end region (min): 30
Marker peak width (sec): 5     Marker min peak height: 200    Marker baseline V to V?: Y     Marker baseline V to V points: 3
Lower marker selection: First peak > 500 RFU                  Upper marker selection: Last peak > 200 RFU
Ladder size (bp)75, 200, 300, 400, 500, 700, 1000, 1500, 2000, 3000, 4000, 5000, 7000, 10000, 20000
Quantification using: Lower Marker          Final concentration (ng/uL): 0.5000          Dilution factor: 12.0
```

Sample: SampB1
Well location: B1
Created: Monday, September 20, 2021 8:00:01 PM

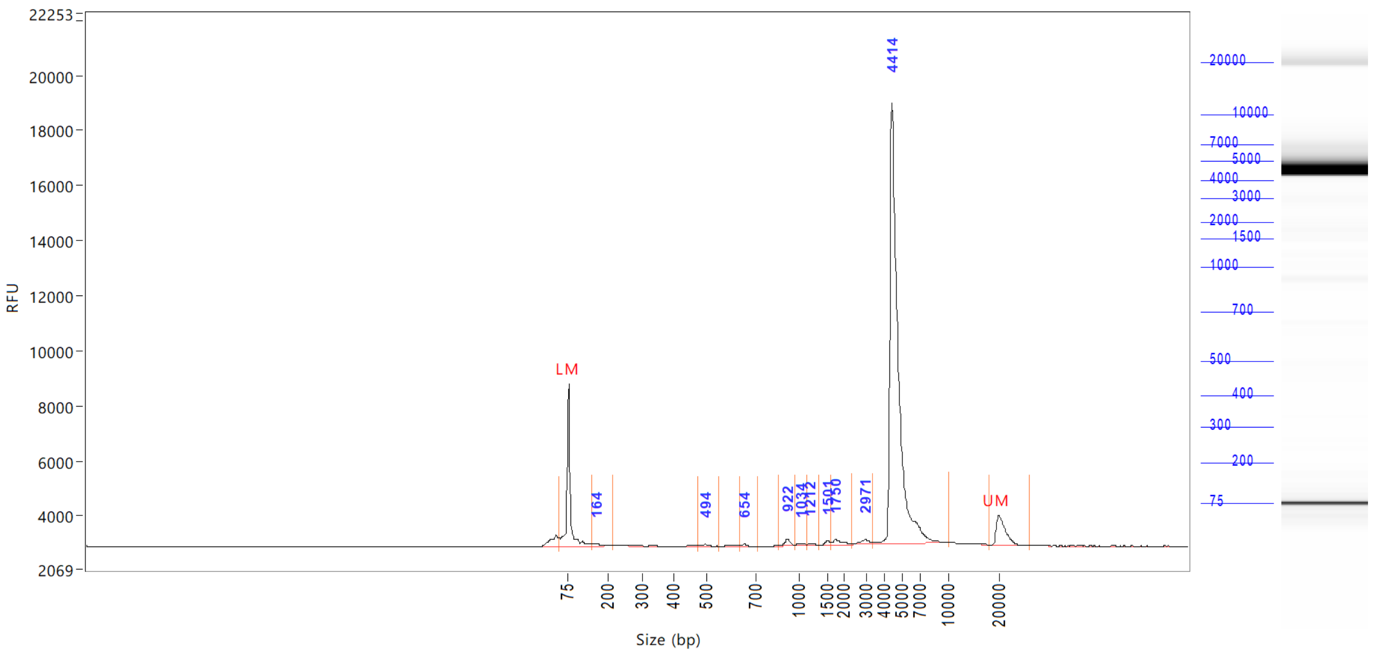

Peak	Size (bp)	Concentration (ng/uL)	From (bp)	To (bp)	Average size (bp)	CV%	RFU	Corrected peak area
1	75 (LM)	0.5000	48	147	76	19.53	5906	46.729
2	164	0.2173	147	210	165	8.34	94	1.692
3	494	0.0937	475	549	496	2.81	73	0.730
4	654	0.0841	636	714	653	1.74	90	0.655
5	922	0.3124	861	974	919	2.35	266	2.433
6	1034	0.1265	974	1134	1032	4.31	76	0.985
7	1212	0.0979	1134	1356	1211	3.66	82	0.763
8	1501	0.1655	1356	1635	1517	4.03	136	1.289
9	1750	0.3667	1635	2344	1878	9.43	183	2.856
10	2971	0.3274	2344	3393	2896	8.55	165	2.550
11	4414	24.0494	3393	10001	4751	14.25	16030	187.302
12	20000 (UM)	0.1313	18049	26045	20618	5.00	1077	12.275

TIC:	25.8409	ng/uL
TIM:	12.6022	nmole/L
Total concentration:	26.1115	ng/uL

```
Sample peak width (sec): 5     Sample min peak height: 50    Sample baseline V to V?: Y    Sample baseline V to V points: 3
Sample filter: Binomial        Number of points for filter: 3  Sample start region (min): 0  Sample end region (min): 30
Marker peak width (sec): 5     Marker min peak height: 200   Marker baseline V to V?: Y    Marker baseline V to V points: 3
Lower marker selection: First peak > 500 RFU                 Upper marker selection: Last peak > 200 RFU
Ladder size (bp)75, 200, 300, 400, 500, 700, 1000, 1500, 2000, 3000, 4000, 5000, 7000, 10000, 20000
Quantification using: Lower Marker          Final concentration (ng/uL): 0.5000          Dilution factor: 12.0
```

Sample: SampC1
Well location: C1
Created: Monday, September 20, 2021 8:00:01 PM

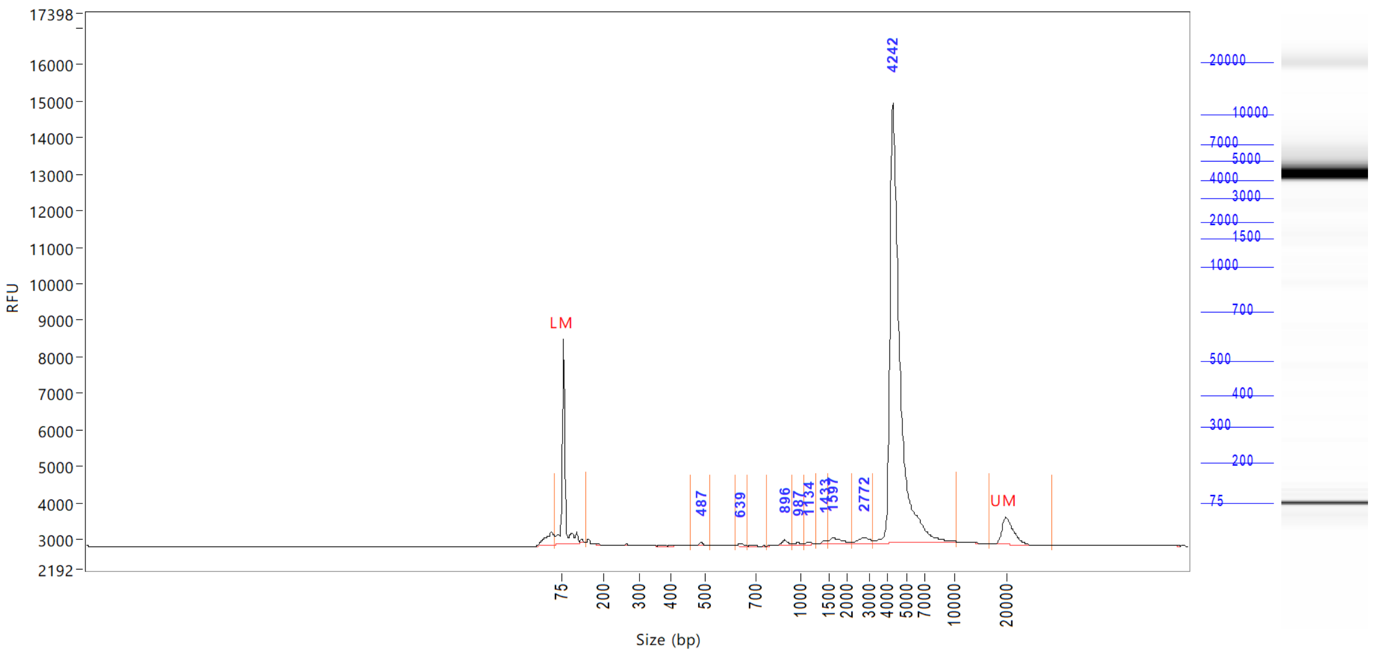

Peak	Size<br>(bp)	Concentration<br>(ng/uL)	From<br>(bp)	To<br>(bp)	Average size<br>(bp)	CV%	RFU	Corrected peak area
1	75 (LM)	0.5000	50	143	77	16.82	5612	42.512
2	487	0.1351	454	519	481	2.27	97	0.957
3	639	0.0825	621	669	638	1.35	71	0.584
4	896	0.2366	770	944	885	3.35	148	1.677
5	987	0.0993	944	1045	984	2.44	57	0.704
6	1134	0.0957	1045	1278	1131	4.26	64	0.678
7	1433	0.1487	1278	1489	1417	3.25	110	1.053
8	1597	0.4451	1489	2258	1739	11.01	183	3.154
9	2772	0.3978	2258	3179	2739	8.39	155	2.818
10	4242	24.1485	3179	10611	4569	15.93	12056	171.100
11	20000 (UM)	0.1266	16708	28826	20481	6.13	737	10.766

TIC:	25.7892	ng/uL
TIM:	10.9543	nmole/L
Total concentration:	26.0778	ng/uL

```
Sample peak width (sec): 5    Sample min peak height: 50    Sample baseline V to V?: Y    Sample baseline V to V points: 3
Sample filter: Binomial       Number of points for filter: 3  Sample start region (min): 0  Sample end region (min): 30
Marker peak width (sec): 5    Marker min peak height: 200   Marker baseline V to V?: Y    Marker baseline V to V points: 3
Lower marker selection: First peak > 500 RFU                Upper marker selection: Last peak > 200 RFU
Ladder size (bp)75, 200, 300, 400, 500, 700, 1000, 1500, 2000, 3000, 4000, 5000, 7000, 10000, 20000
Quantification using: Lower Marker          Final concentration (ng/uL): 0.5000          Dilution factor: 12.0
```

Sample: SampD1
Well location: D1
Created: Monday, September 20, 2021 8:00:01 PM

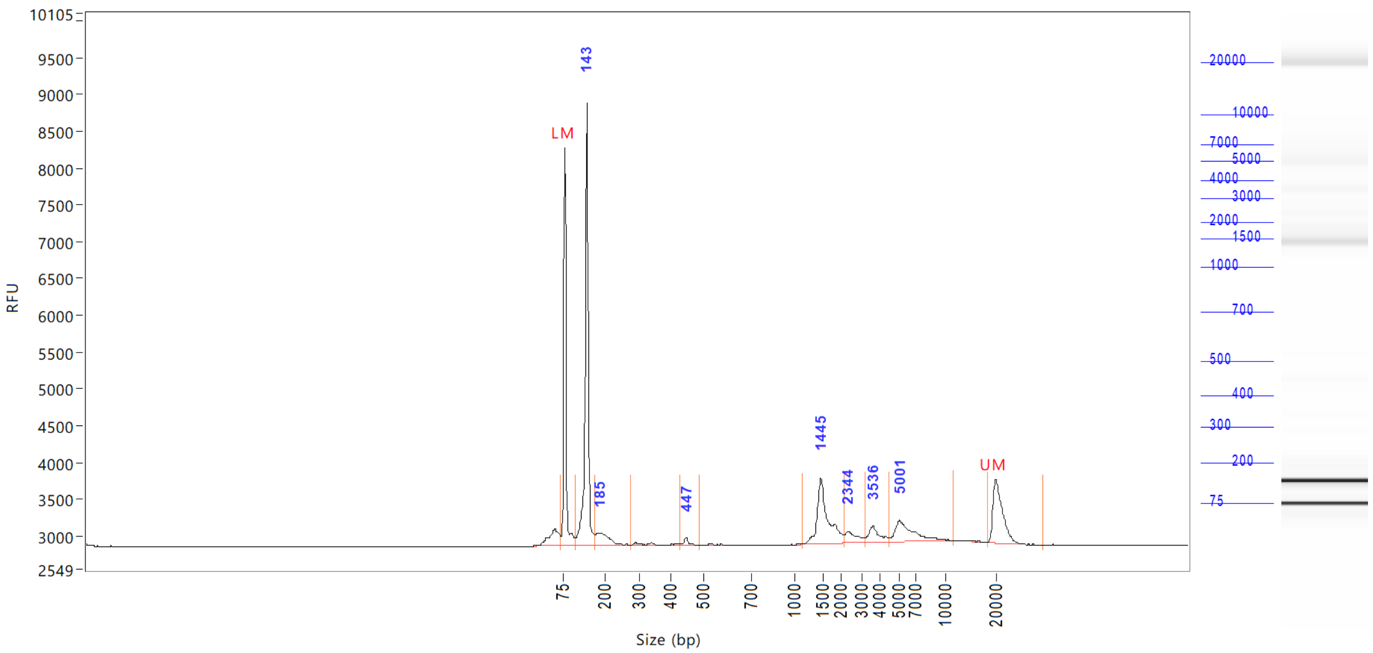

Peak	Size (bp)	Concentration (ng/uL)	From (bp)	To (bp)	Average size (bp)	CV%	RFU	Corrected peak area
1	75 (LM)	0.5000	62	107	74	8.88	5418	33.353
2	143	8.0508	107	168	139	6.01	6024	44.752
3	185	0.9020	168	273	193	9.47	168	5.014
4	447	0.1644	428	487	447	2.16	103	0.914
5	1445	2.6890	1123	2144	1546	13.16	902	14.947
6	2344	0.5166	2144	3108	2548	10.82	157	2.872
7	3536	0.6089	3108	4414	3667	9.07	217	3.385
8	5001	1.2976	4414	11464	6010	21.81	286	7.213
9	20000 (UM)	0.1593	18415	29189	20621	5.52	867	10.626

TIC:	14.2292	ng/uL
TIM:	107.2926	nmole/L
Total concentration:	14.5321	ng/uL

Sample peak width (sec): 5   Sample min peak height: 50   Sample baseline V to V?: Y   Sample baseline V to V points: 3
Sample filter: Binomial   Number of points for filter: 3 Sample start region (min): 0 Sample end region (min): 30
Marker peak width (sec): 5   Marker min peak height: 200   Marker baseline V to V?: Y   Marker baseline V to V points: 3
Lower marker selection: First peak > 500 RFU   Upper marker selection: Last peak > 200 RFU
Ladder size (bp)75, 200, 300, 400, 500, 700, 1000, 1500, 2000, 3000, 4000, 5000, 7000, 10000, 20000
Quantification using: Lower Marker   Final concentration (ng/uL): 0.5000   Dilution factor: 12.0

Sample: SampE1
Well location: E1
Created: Monday, September 20, 2021 8:00:01 PM

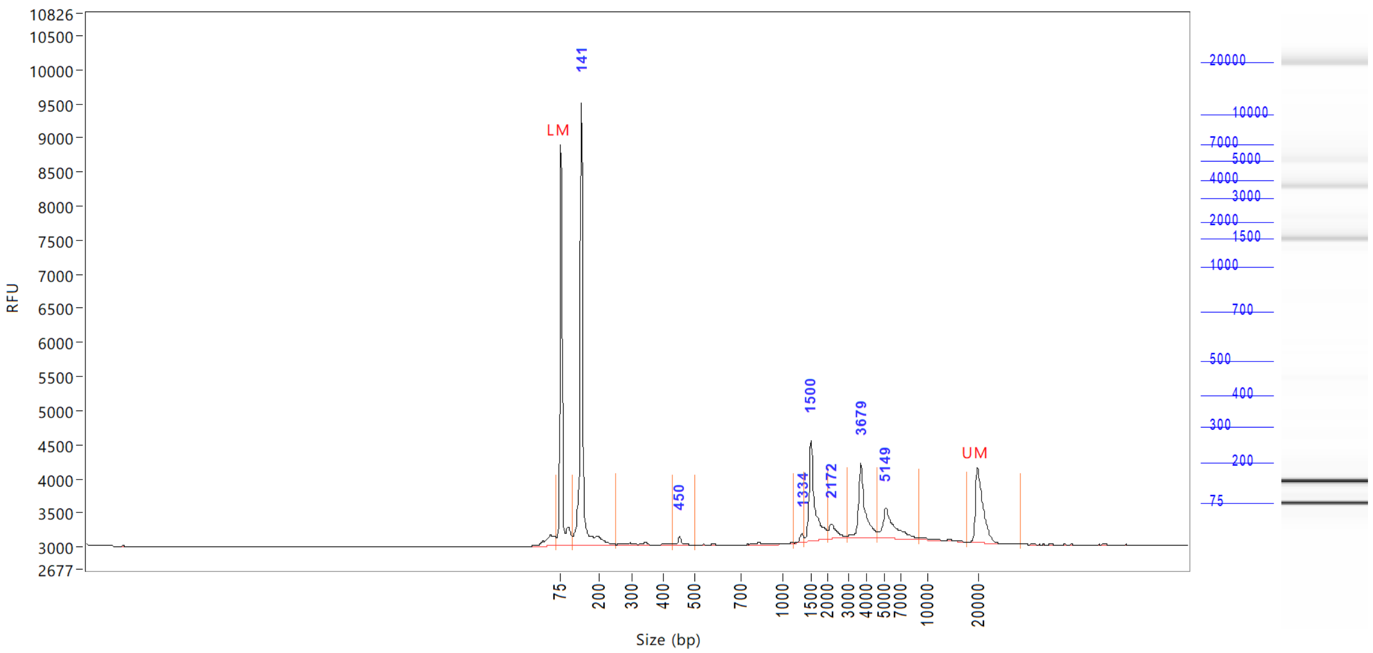

Peak	Size (bp)	Concentration (ng/uL)	From (bp)	To (bp)	Average size (bp)	CV%	RFU	Corrected peak area
1	75 (LM)	0.5000	64	112	76	10.88	5877	36.554
2	141	7.9367	112	246	142	11.38	6495	48.354
3	450	0.1962	431	500	454	3.01	125	1.195
4	1334	0.1368	1190	1389	1321	3.03	124	0.833
5	1500	2.2982	1389	2030	1578	9.39	1476	14.002
6	2172	0.4263	2030	2914	2310	9.24	213	2.597
7	3679	1.7739	2914	4656	3799	8.08	1104	10.807
8	5149	1.2525	4656	8934	5934	17.18	444	7.631
9	20000 (UM)	0.1499	17927	28826	20563	4.82	1100	10.957

TIC:	14.0206	ng/uL
TIM:	96.5499	nmole/L
Total concentration:	14.6116	ng/uL

```
Sample peak width (sec): 5     Sample min peak height: 50     Sample baseline V to V?: Y     Sample baseline V to V points: 3
Sample filter: Binomial        Number of points for filter: 3  Sample start region (min): 0  Sample end region (min): 30
Marker peak width (sec): 5     Marker min peak height: 200    Marker baseline V to V?: Y     Marker baseline V to V points: 3
Lower marker selection: First peak > 500 RFU                  Upper marker selection: Last peak > 200 RFU
Ladder size (bp)75, 200, 300, 400, 500, 700, 1000, 1500, 2000, 3000, 4000, 5000, 7000, 10000, 20000
Quantification using: Lower Marker          Final concentration (ng/uL): 0.5000          Dilution factor: 12.0
```

Sample: SampF1
Well location: F1
Created: Monday, September 20, 2021 8:00:01 PM

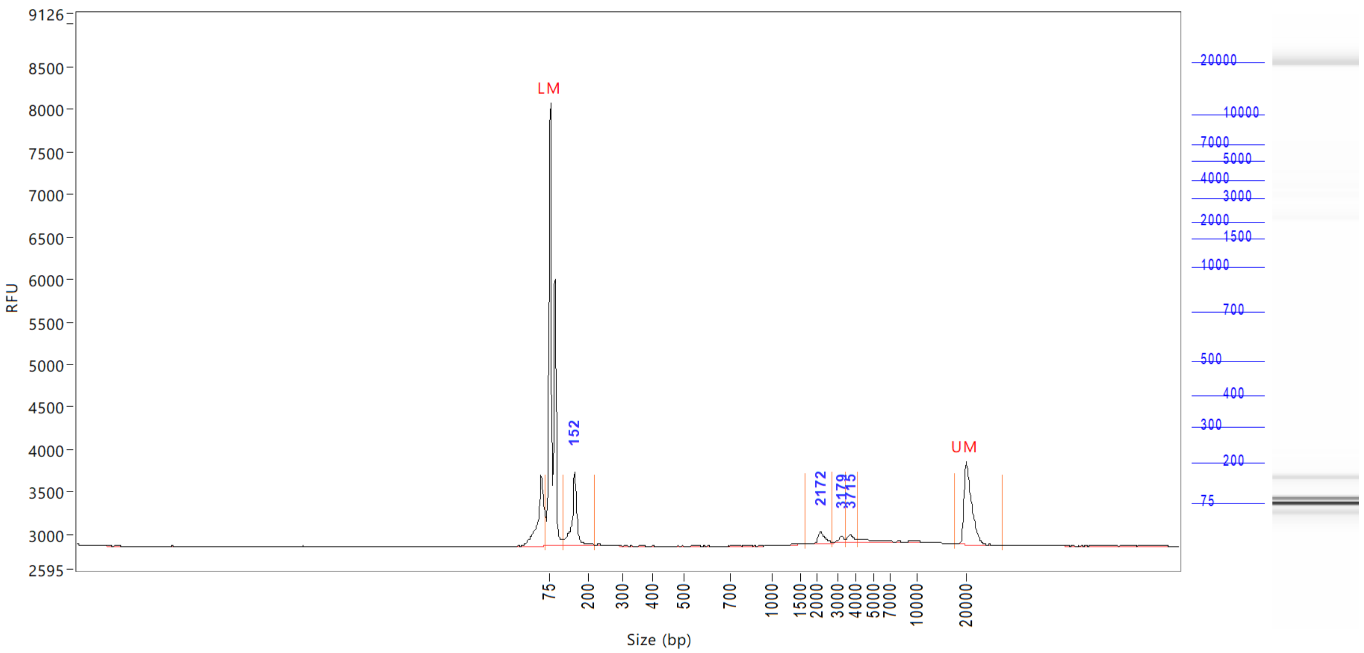

Peak	Size (bp)	Concentration (ng/uL)	From (bp)	To (bp)	Average size (bp)	CV%	RFU	Corrected peak area
1	75 (LM)	0.5000	64	116	79	11.26	5213	50.106
2	152	1.2039	116	215	150	10.42	864	10.054
3	2172	0.1786	1674	2772	2240	8.32	137	1.492
4	3179	0.0829	2772	3429	3145	4.95	84	0.693
5	3715	0.1060	3429	4104	3744	4.64	91	0.886
6	20000 (UM)	0.0974	17561	27254	20500	4.90	976	9.758

TIC:	1.5715	ng/uL
TIM:	13.4124	nmole/L
Total concentration:	1.9095	ng/uL

```
Sample peak width (sec): 5    Sample min peak height: 50    Sample baseline V to V?: Y    Sample baseline V to V points: 3
Sample filter: Binomial       Number of points for filter: 3  Sample start region (min): 0  Sample end region (min): 30
Marker peak width (sec): 5    Marker min peak height: 200   Marker baseline V to V?: Y    Marker baseline V to V points: 3
Lower marker selection: First peak > 500 RFU                Upper marker selection: Last peak > 200 RFU
Ladder size (bp)75, 200, 300, 400, 500, 700, 1000, 1500, 2000, 3000, 4000, 5000, 7000, 10000, 20000
Quantification using: Lower Marker          Final concentration (ng/uL): 0.5000          Dilution factor: 12.0
```

Sample: SampG1
Well location: G1
Created: Monday, September 20, 2021 8:00:01 PM

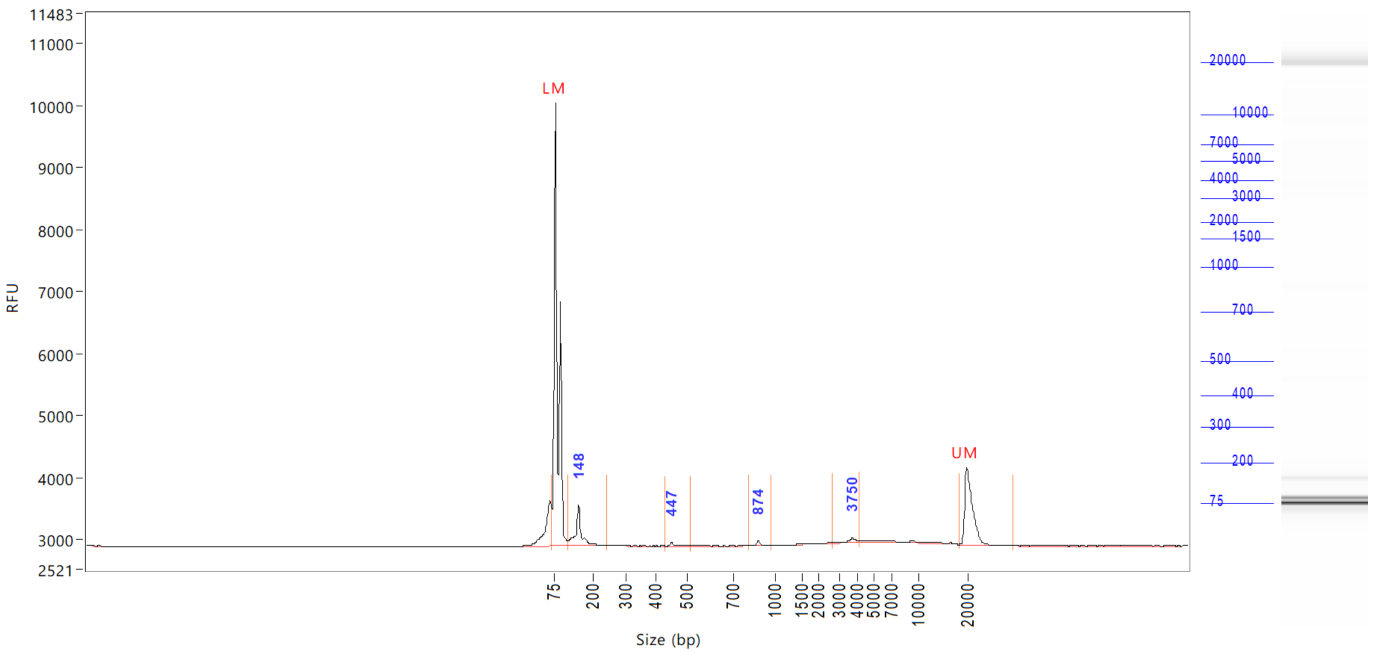

Peak	Size (bp)	Concentration (ng/uL)	From (bp)	To (bp)	Average size (bp)	CV%	RFU	Corrected peak area
1	75 (LM)	0.5000	66	116	79	10.97	7153	68.063
2	148	0.7474	116	241	150	12.23	659	8.478
3	447	0.0648	430	511	459	4.37	67	0.735
4	874	0.0509	805	974	880	3.51	70	0.577
5	3750	0.0911	2657	4104	3530	10.82	74	1.033
6	20000 (UM)	0.0938	18293	29430	20559	5.07	1245	12.764

TIC:	0.9541	ng/uL
TIM:	8.5586	nmole/L
Total concentration:	1.3047	ng/uL

Sample peak width (sec): 5 Sample min peak height: 50 Sample baseline V to V?: Y Sample baseline V to V points: 3
Sample filter: Binomial Number of points for filter: 3 Sample start region (min): 0 Sample end region (min): 30
Marker peak width (sec): 5 Marker min peak height: 200 Marker baseline V to V?: Y Marker baseline V to V points: 3
Lower marker selection: First peak > 500 RFU Upper marker selection: Last peak > 200 RFU
Ladder size (bp)75, 200, 300, 400, 500, 700, 1000, 1500, 2000, 3000, 4000, 5000, 7000, 10000, 20000
Quantification using: Lower Marker Final concentration (ng/uL): 0.5000 Dilution factor: 12.0

Sample: SampH1
Well location: H1
Created: Monday, September 20, 2021 8:00:01 PM

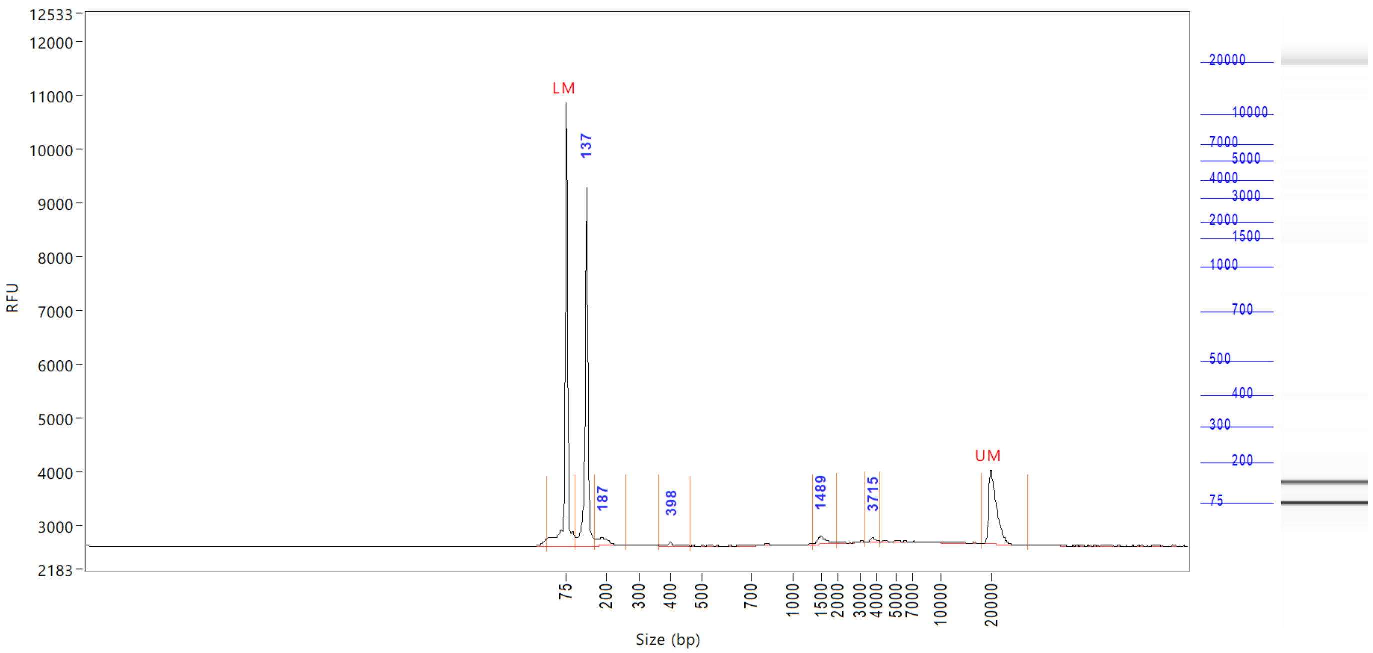

Peak	Size (bp)	Concentration (ng/uL)	From (bp)	To (bp)	Average size (bp)	CV%	RFU	Corrected peak area
1	75 (LM)	0.5000	14	103	70	18.24	8268	56.390
2	137	4.9582	103	164	134	6.00	6662	46.599
3	187	0.4379	164	260	189	8.99	150	4.116
4	398	0.0827	362	462	405	5.24	69	0.777
5	1489	0.2304	1356	2000	1571	9.35	156	2.165
6	3715	0.0840	3286	4139	3719	5.15	82	0.789
7	20000 (UM)	0.1247	18293	27617	20581	4.37	1385	14.066

TIC:	5.7932	ng/uL
TIM:	65.2240	nmole/L
Total concentration:	6.1629	ng/uL

Sample peak width (sec): 5 Sample min peak height: 50 Sample baseline V to V?: Y Sample baseline V to V points: 3
Sample filter: Binomial Number of points for filter: 3 Sample start region (min): 0 Sample end region (min): 30
Marker peak width (sec): 5 Marker min peak height: 200 Marker baseline V to V?: Y Marker baseline V to V points: 3
Lower marker selection: First peak > 500 RFU Upper marker selection: Last peak > 200 RFU
Ladder size (bp)75, 200, 300, 400, 500, 700, 1000, 1500, 2000, 3000, 4000, 5000, 7000, 10000, 20000
Quantification using: Lower Marker Final concentration (ng/uL): 0.5000 Dilution factor: 12.0

Sample: SampA2
Well location: A2
Created: Monday, September 20, 2021 8:00:01 PM

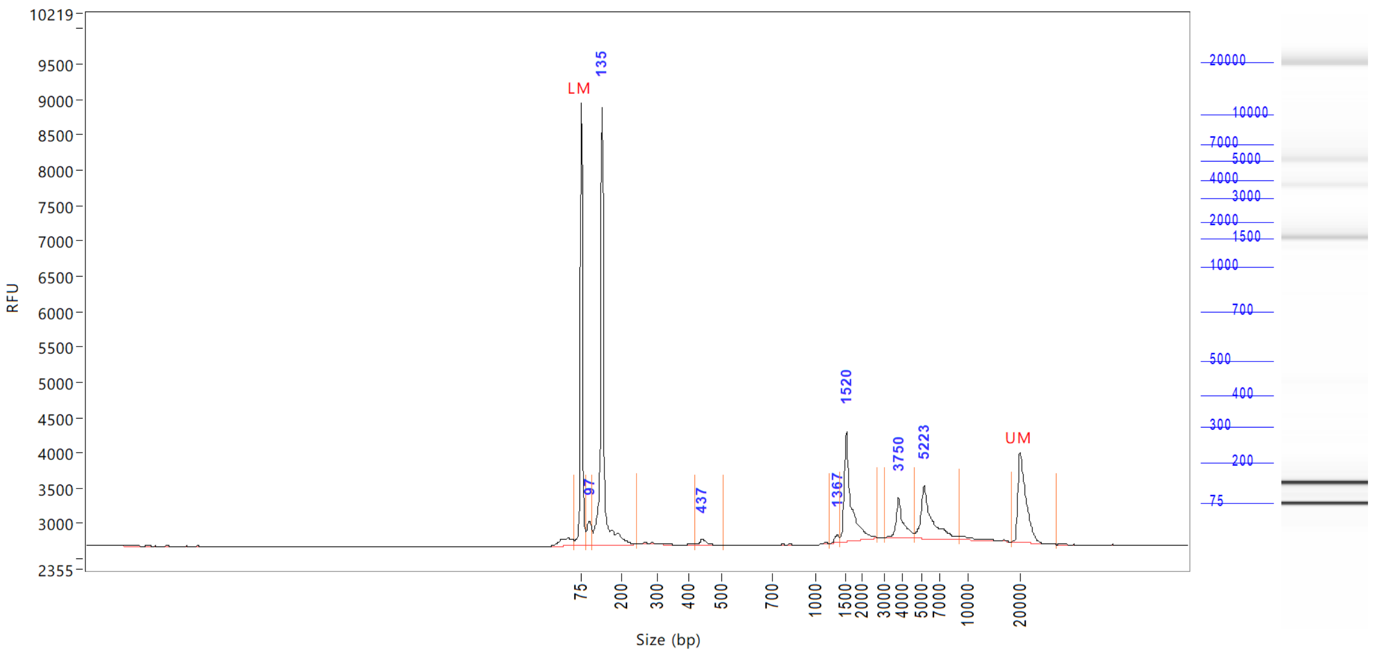

Peak	Size (bp)	Concentration (ng/uL)	From (bp)	To (bp)	Average size (bp)	CV%	RFU	Corrected peak area
1	75 (LM)	0.5000	54	87	73	5.68	6272	35.338
2	97	0.7262	87	110	98	6.77	346	4.277
3	135	8.3769	110	242	138	11.59	6196	49.336
4	437	0.1953	416	501	442	3.29	86	1.150
5	1367	0.1288	1223	1400	1344	2.48	118	0.759
6	1520	2.7133	1400	2686	1638	12.63	1554	15.980
7	3750	0.9909	3001	4621	3873	7.64	564	5.836
8	5223	1.9036	4621	9067	5911	17.50	749	11.212
9	20000 (UM)	0.1826	18171	27012	20512	4.51	1278	12.906

TIC:	15.0350	ng/uL
TIM:	116.4797	nmole/L
Total concentration:	15.4857	ng/uL

```
Sample peak width (sec): 5    Sample min peak height: 50    Sample baseline V to V?: Y    Sample baseline V to V points: 3
Sample filter: Binomial       Number of points for filter: 3  Sample start region (min): 0  Sample end region (min): 30
Marker peak width (sec): 5    Marker min peak height: 200   Marker baseline V to V?: Y    Marker baseline V to V points: 3
Lower marker selection: First peak > 500 RFU                Upper marker selection: Last peak > 200 RFU
Ladder size (bp)75, 200, 300, 400, 500, 700, 1000, 1500, 2000, 3000, 4000, 5000, 7000, 10000, 20000
Quantification using: Lower Marker          Final concentration (ng/uL): 0.5000          Dilution factor: 12.0
```

Sample: SampB2
Well location: B2
Created: Monday, September 20, 2021 8:00:01 PM

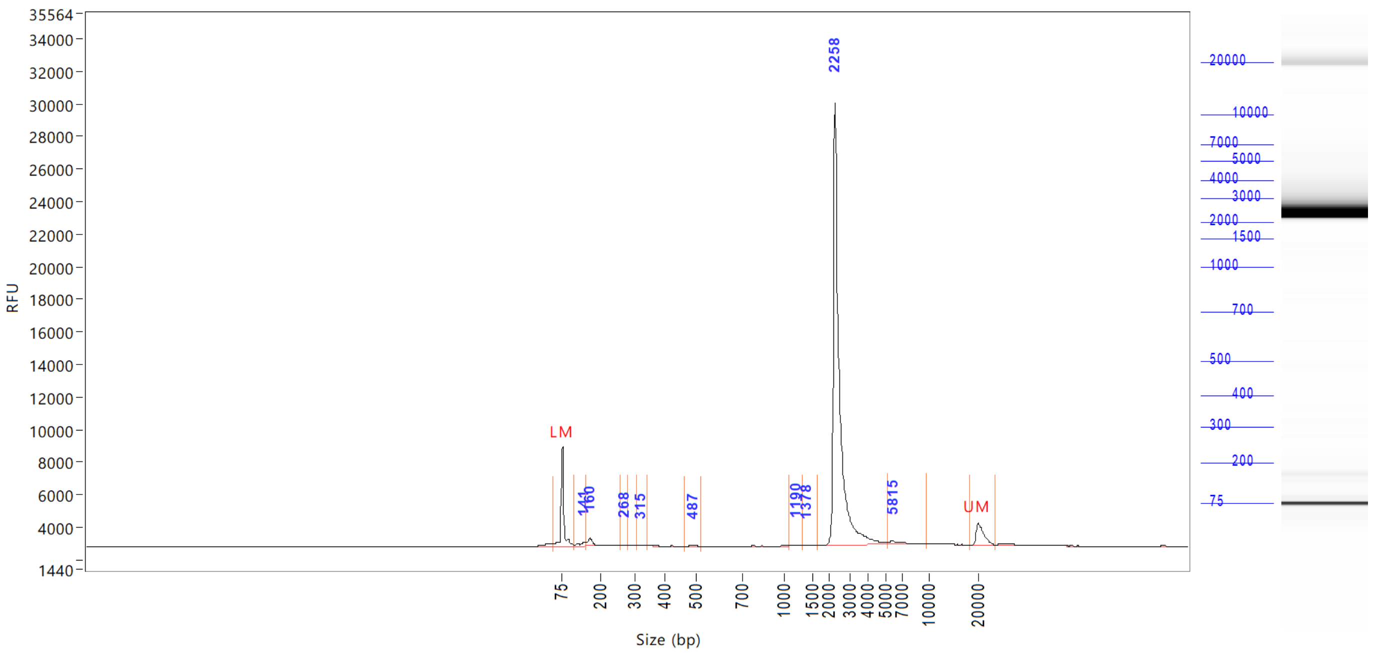

Peak	Size (bp)	Concentration (ng/uL)	From (bp)	To (bp)	Average size (bp)	CV%	RFU	Corrected peak area
1	75 (LM)	0.5000	47	112	74	11.91	6097	41.900
2	141	0.5369	112	150	135	8.27	256	3.749
3	160	0.9855	150	259	172	12.04	489	6.882
4	268	0.0475	259	278	267	1.12	69	0.332
5	315	0.0517	303	343	314	1.91	64	0.361
6	487	0.0759	464	516	487	1.83	52	0.530
7	1190	0.1220	1079	1311	1206	4.56	79	0.852
8	1378	0.0728	1311	1674	1391	4.60	61	0.509
9	2258	36.6571	1674	5297	2476	17.47	27195	255.987
10	5815	0.4351	5297	9800	6521	14.63	160	3.039
11	20000 (UM)	0.1561	18415	23506	20436	4.10	1344	13.083

TIC:	38.9846	ng/uL
TIM:	41.5085	nmole/L
Total concentration:	39.2130	ng/uL

```
Sample peak width (sec): 5     Sample min peak height: 50    Sample baseline V to V?: Y    Sample baseline V to V points: 3
Sample filter: Binomial        Number of points for filter: 3  Sample start region (min): 0  Sample end region (min): 30
Marker peak width (sec): 5     Marker min peak height: 200   Marker baseline V to V?: Y    Marker baseline V to V points: 3
Lower marker selection: First peak > 500 RFU                 Upper marker selection: Last peak > 200 RFU
Ladder size (bp)75, 200, 300, 400, 500, 700, 1000, 1500, 2000, 3000, 4000, 5000, 7000, 10000, 20000
Quantification using: Lower Marker          Final concentration (ng/uL): 0.5000         Dilution factor: 12.0
```

Sample: SampC2
Well location: C2
Created: Monday, September 20, 2021 8:00:01 PM

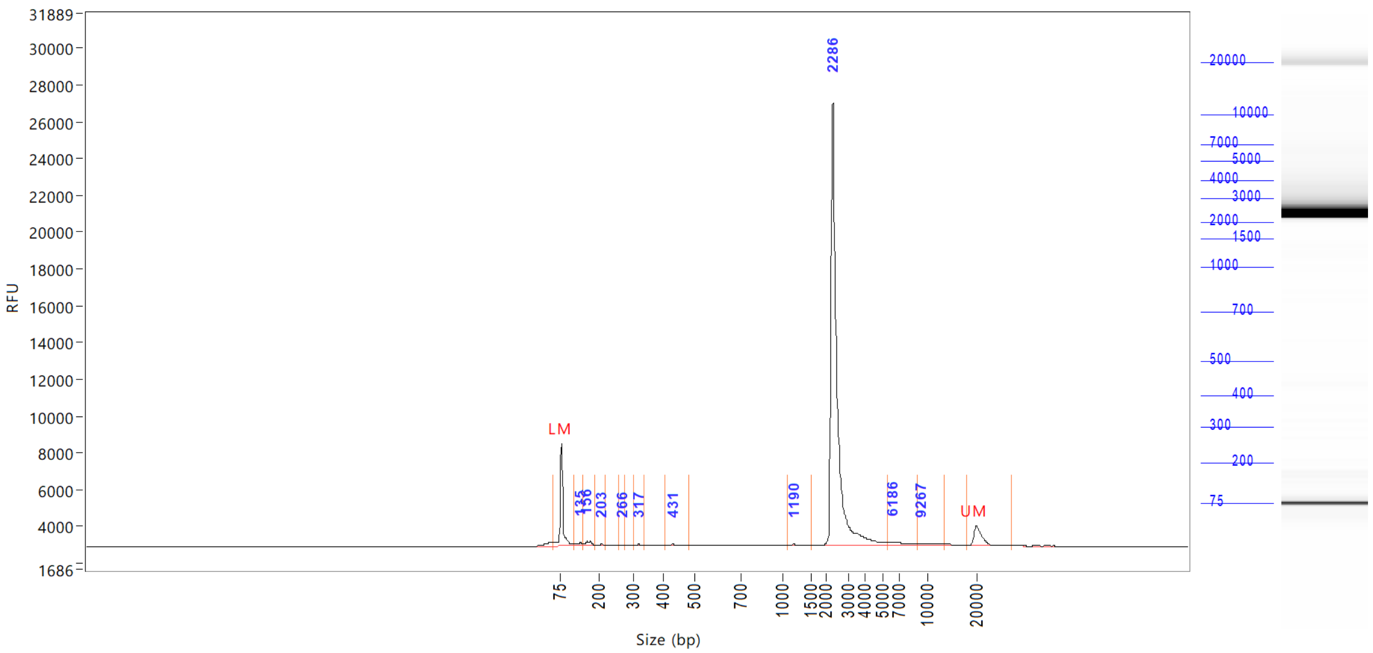

Peak	Size (bp)	Concentration (ng/uL)	From (bp)	To (bp)	Average size (bp)	CV%	RFU	Corrected peak area
1	75 (LM)	0.5000	50	116	74	11.42	5572	38.210
2	135	0.2999	116	145	131	6.21	159	1.910
3	156	0.4430	145	183	159	4.87	218	2.821
4	203	0.0788	183	214	198	3.00	63	0.502
5	266	0.0409	257	275	264	1.12	54	0.260
6	317	0.0677	300	337	316	2.35	65	0.431
7	431	0.1624	407	485	434	3.61	98	1.034
8	1190	0.1840	1079	1539	1268	7.94	73	1.172
9	2286	34.9063	1539	5519	2491	19.53	24082	222.293
10	6186	0.6552	5519	9000	6891	13.68	182	4.173
11	9267	0.2652	9000	13537	10894	12.51	61	1.689
12	20000 (UM)	0.1381	18171	27133	20486	4.38	1084	10.550

TIC:	37.1033	ng/uL
TIM:	33.7216	nmole/L
Total concentration:	37.4532	ng/uL

```
Sample peak width (sec): 5     Sample min peak height: 50    Sample baseline V to V?: Y    Sample baseline V to V points: 3
Sample filter: Binomial        Number of points for filter: 3  Sample start region (min): 0  Sample end region (min): 30
Marker peak width (sec): 5     Marker min peak height: 200   Marker baseline V to V?: Y    Marker baseline V to V points: 3
Lower marker selection: First peak > 500 RFU                 Upper marker selection: Last peak > 200 RFU
Ladder size (bp)75, 200, 300, 400, 500, 700, 1000, 1500, 2000, 3000, 4000, 5000, 7000, 10000, 20000
Quantification using: Lower Marker          Final concentration (ng/uL): 0.5000          Dilution factor: 12.0
```

Sample: SampD2
Well location: D2
Created: Monday, September 20, 2021 8:00:01 PM

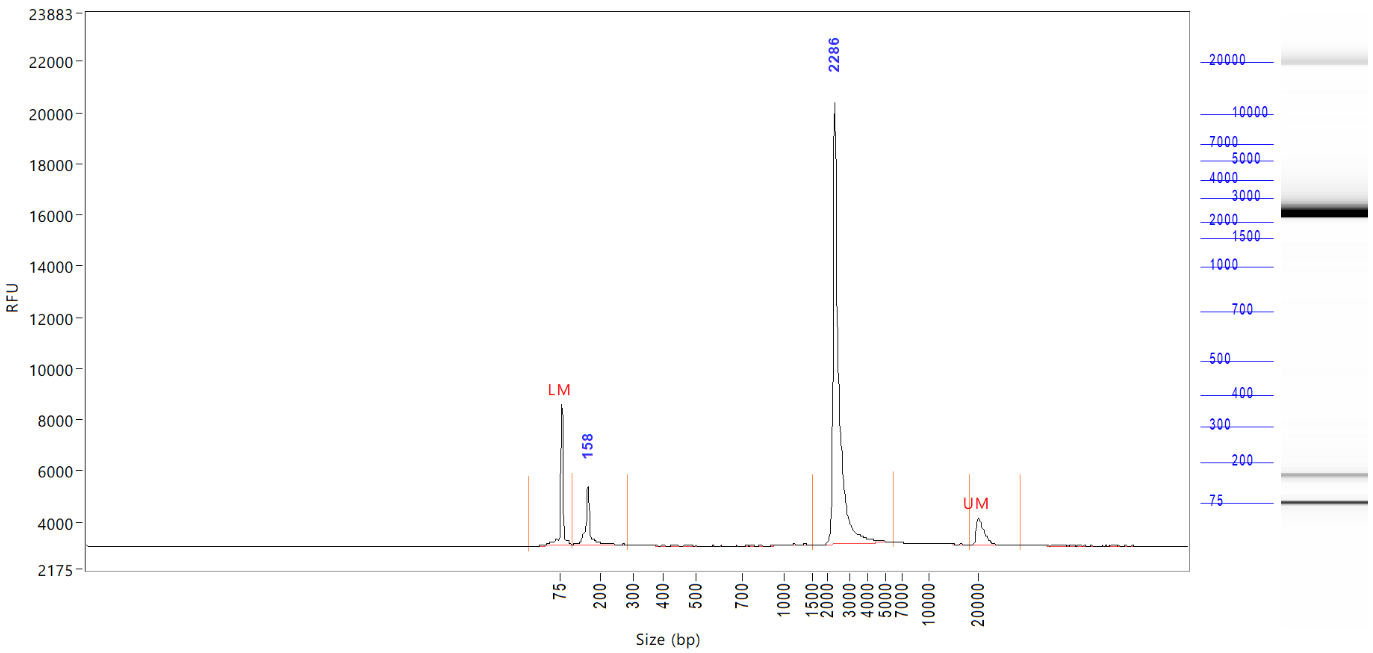

Peak	Size (bp)	Concentration (ng/uL)	From (bp)	To (bp)	Average size (bp)	CV%	RFU	Corrected peak area
1	75 (LM)	0.5000	0	108	70	17.33	5496	35.819
2	158	3.8427	108	282	161	14.18	2286	22.940
3	2286	25.9274	1501	6038	2466	15.90	17269	154.781
4	20000 (UM)	0.1513	18171	28584	20596	4.96	1071	10.835

TIC:	29.7701	ng/uL
TIM:	56.5396	nmole/L
Total concentration:	30.3204	ng/uL

```
Sample peak width (sec): 5    Sample min peak height: 50    Sample baseline V to V?: Y    Sample baseline V to V points: 3
Sample filter: Binomial       Number of points for filter: 3  Sample start region (min): 0  Sample end region (min): 30
Marker peak width (sec): 5    Marker min peak height: 200   Marker baseline V to V?: Y    Marker baseline V to V points: 3
Lower marker selection: First peak > 500 RFU                Upper marker selection: Last peak > 200 RFU
Ladder size (bp)75, 200, 300, 400, 500, 700, 1000, 1500, 2000, 3000, 4000, 5000, 7000, 10000, 20000
Quantification using: Lower Marker          Final concentration (ng/uL): 0.5000          Dilution factor: 12.0
```

Sample: SampE2
Well location: E2
Created: Monday, September 20, 2021 8:00:01 PM

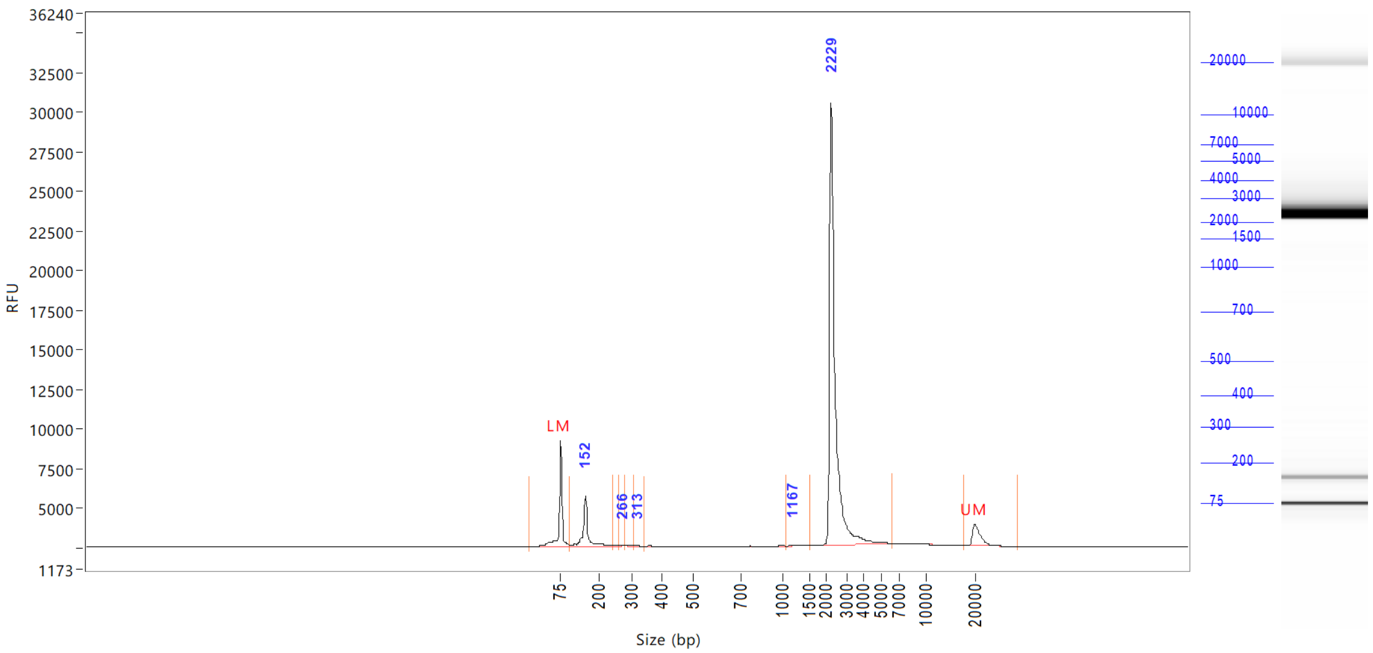

Peak	Size (bp)	Concentration (ng/uL)	From (bp)	To (bp)	Average size (bp)	CV%	RFU	Corrected peak area
1	75 (LM)	0.5000	0	107	68	22.99	6697	49.097
2	152	3.9788	107	239	156	13.73	3194	32.558
3	266	0.0394	259	275	264	1.13	62	0.323
4	313	0.0439	303	337	312	1.66	62	0.360
5	1167	0.1704	1068	1500	1249	7.15	84	1.394
6	2229	29.0173	1500	6334	2417	16.70	27946	237.443
7	20000 (UM)	0.1395	17561	28584	20488	4.71	1349	13.703

TIC:	33.2498	ng/uL
TIM:	62.3857	nmole/L
Total concentration:	33.5572	ng/uL

```
Sample peak width (sec): 5    Sample min peak height: 50    Sample baseline V to V?: Y    Sample baseline V to V points: 3
Sample filter: Binomial       Number of points for filter: 3  Sample start region (min): 0  Sample end region (min): 30
Marker peak width (sec): 5    Marker min peak height: 200   Marker baseline V to V?: Y    Marker baseline V to V points: 3
Lower marker selection: First peak > 500 RFU                Upper marker selection: Last peak > 200 RFU
Ladder size (bp)75, 200, 300, 400, 500, 700, 1000, 1500, 2000, 3000, 4000, 5000, 7000, 10000, 20000
Quantification using: Lower Marker          Final concentration (ng/uL): 0.5000          Dilution factor: 12.0
```

Sample: SampF2
Well location: F2
Created: Monday, September 20, 2021 8:00:01 PM

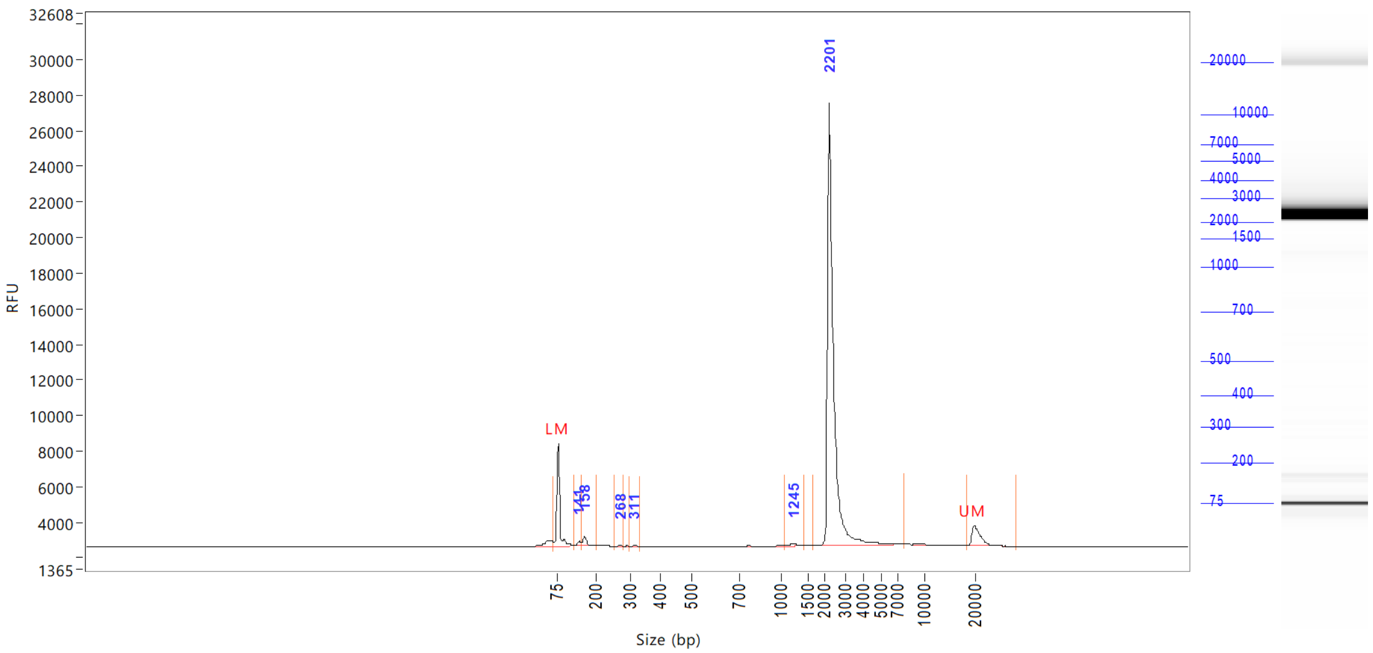

Peak	Size (bp)	Concentration (ng/uL)	From (bp)	To (bp)	Average size (bp)	CV%	RFU	Corrected peak area
1	75 (LM)	0.5000	62	126	75	12.16	5732	37.442
2	141	0.3929	126	148	140	4.25	268	2.452
3	158	0.7336	148	198	159	5.40	494	4.578
4	268	0.0446	253	280	266	1.24	58	0.278
5	311	0.0585	298	333	311	1.65	65	0.365
6	1245	0.3146	1068	1433	1241	6.97	136	1.963
7	2201	36.4403	1635	7734	2376	17.96	24913	227.401
8	20000 (UM)	0.1599	18415	28584	20593	4.93	1116	11.971

TIC:	37.9846	ng/uL
TIM:	38.4455	nmole/L
Total concentration:	38.4273	ng/uL

```
Sample peak width (sec): 5    Sample min peak height: 50    Sample baseline V to V?: Y    Sample baseline V to V points: 3
Sample filter: Binomial       Number of points for filter: 3  Sample start region (min): 0  Sample end region (min): 30
Marker peak width (sec): 5    Marker min peak height: 200   Marker baseline V to V?: Y    Marker baseline V to V points: 3
Lower marker selection: First peak > 500 RFU                Upper marker selection: Last peak > 200 RFU
Ladder size (bp)75, 200, 300, 400, 500, 700, 1000, 1500, 2000, 3000, 4000, 5000, 7000, 10000, 20000
Quantification using: Lower Marker          Final concentration (ng/uL): 0.5000          Dilution factor: 12.0
```

Sample: SampG2
Well location: G2
Created: Monday, September 20, 2021 8:00:01 PM

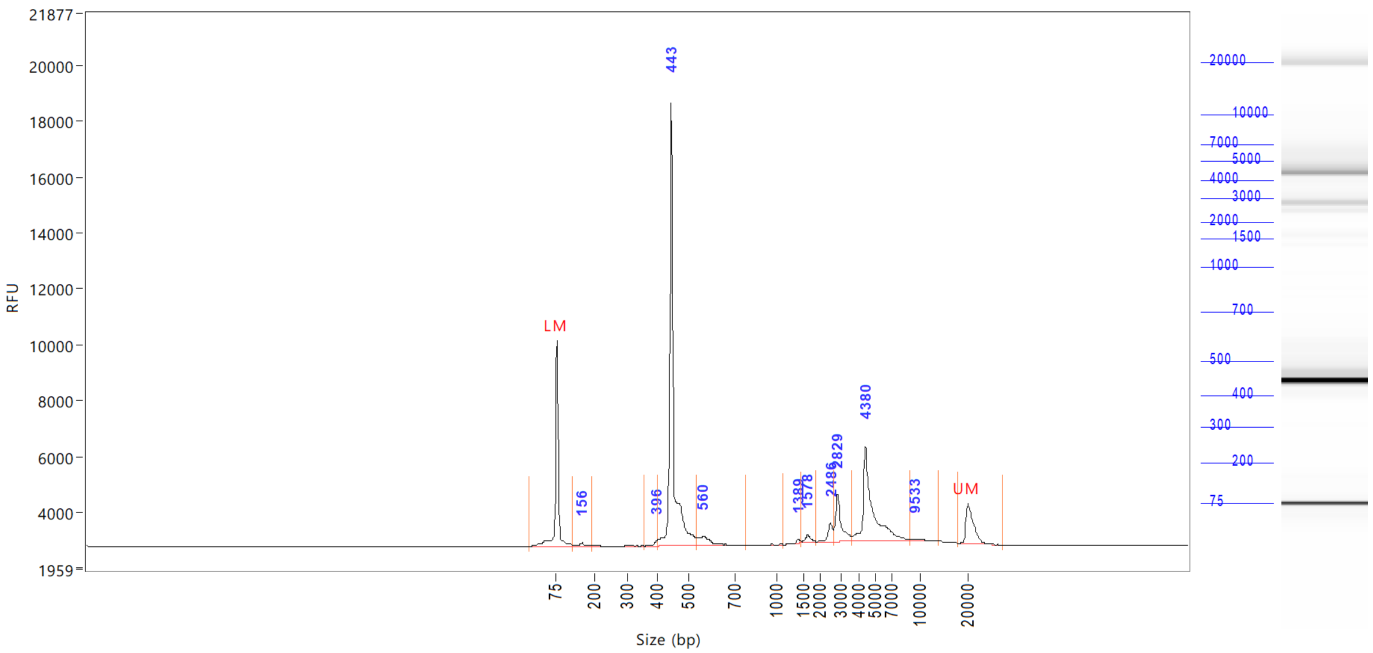

Peak	Size (bp)	Concentration (ng/uL)	From (bp)	To (bp)	Average size (bp)	CV%	RFU	Corrected peak area
1	75 (LM)	0.5000	0	128	70	24.33	7385	52.401
2	156	0.2562	128	187	151	9.37	130	2.237
3	396	0.1422	354	398	390	1.94	203	1.242
4	443	14.2079	398	532	447	4.38	15899	124.084
5	560	0.9691	532	766	579	7.61	323	8.463
6	1389	0.0993	1123	1456	1382	3.27	142	0.867
7	1578	0.2513	1456	1885	1595	5.06	252	2.195
8	2486	0.5449	1885	2629	2444	5.46	644	4.759
9	2829	1.7593	2629	3608	2926	7.84	1684	15.364
10	4380	5.6226	3608	9000	4966	20.14	3416	49.104
11	9533	0.1656	9000	13903	10125	9.72	85	1.446
12	20000 (UM)	0.1353	17805	27012	20504	4.58	1413	14.178

TIC:	24.0183	ng/uL
TIM:	62.0671	nmole/L
Total concentration:	24.0657	ng/uL

Sample peak width (sec): 5   Sample min peak height: 50   Sample baseline V to V?: Y   Sample baseline V to V points: 3
Sample filter: Binomial   Number of points for filter: 3 Sample start region (min): 0 Sample end region (min): 30
Marker peak width (sec): 5   Marker min peak height: 200   Marker baseline V to V?: Y   Marker baseline V to V points: 3
Lower marker selection: First peak > 500 RFU   Upper marker selection: Last peak > 200 RFU
Ladder size (bp)75, 200, 300, 400, 500, 700, 1000, 1500, 2000, 3000, 4000, 5000, 7000, 10000, 20000
Quantification using: Lower Marker   Final concentration (ng/uL): 0.5000   Dilution factor: 12.0

Sample: SampH2
Well location: H2
Created: Monday, September 20, 2021 8:00:01 PM

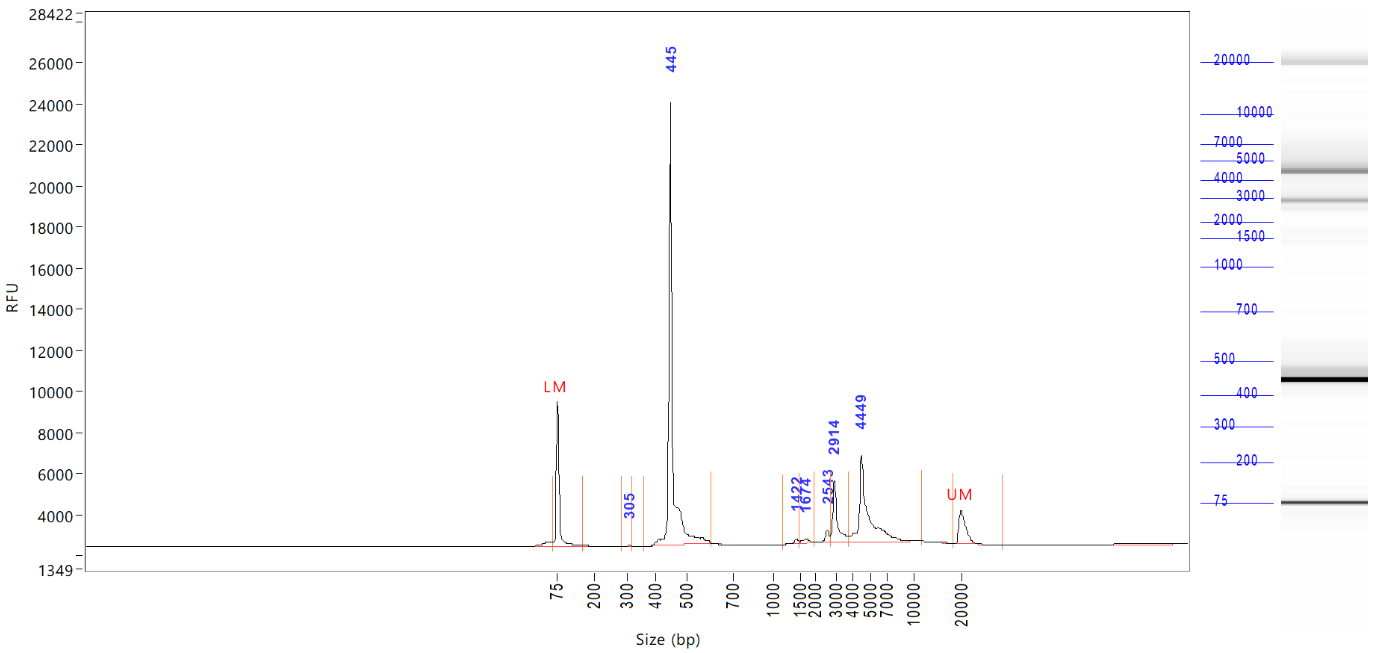

Peak	Size (bp)	Concentration (ng/uL)	From (bp)	To (bp)	Average size (bp)	CV%	RFU	Corrected peak area
1	75 (LM)	0.5000	64	162	78	17.10	7080	46.812
2	305	0.0404	280	315	300	2.08	54	0.315
3	445	20.6582	354	603	452	5.99	21571	161.175
4	1422	0.1816	1178	1478	1413	2.83	248	1.417
5	1674	0.2832	1478	1962	1644	6.20	205	2.209
6	2543	0.4924	1962	2686	2506	5.07	598	3.842
7	2914	2.8494	2686	3715	3000	7.77	2980	22.231
8	4449	7.2305	3715	11586	5069	21.81	4203	56.412
9	20000 (UM)	0.1585	18293	28705	20431	4.37	1595	14.839

TIC:	31.7357	ng/uL
TIM:	80.1542	nmole/L
Total concentration:	31.9452	ng/uL

Sample peak width (sec): 5 Sample min peak height: 50 Sample baseline V to V?: Y Sample baseline V to V points: 3
Sample filter: Binomial Number of points for filter: 3 Sample start region (min): 0 Sample end region (min): 30
Marker peak width (sec): 5 Marker min peak height: 200 Marker baseline V to V?: Y Marker baseline V to V points: 3
Lower marker selection: First peak > 500 RFU Upper marker selection: Last peak > 200 RFU
Ladder size (bp)75, 200, 300, 400, 500, 700, 1000, 1500, 2000, 3000, 4000, 5000, 7000, 10000, 20000
Quantification using: Lower Marker Final concentration (ng/uL): 0.5000 Dilution factor: 12.0

Sample: SampA3
Well location: A3
Created: Monday, September 20, 2021 8:00:01 PM

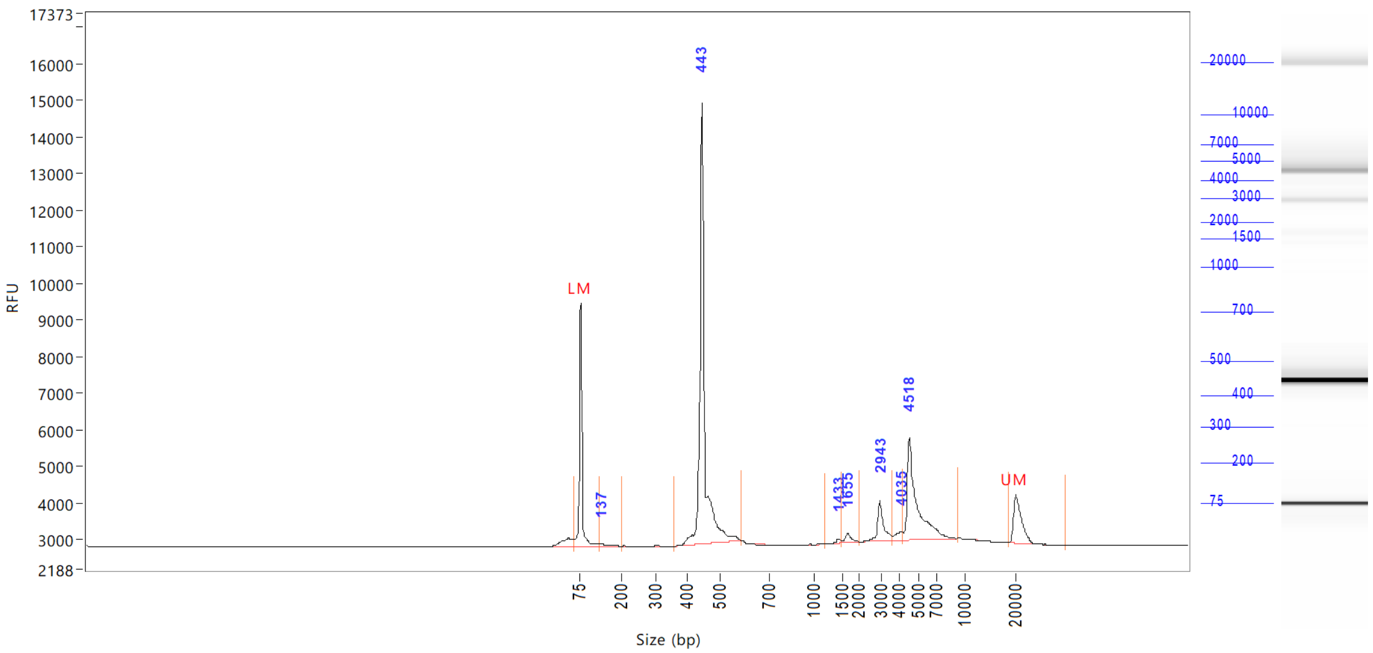

Peak	Size (bp)	Concentration (ng/uL)	From (bp)	To (bp)	Average size (bp)	CV%	RFU	Corrected peak area
1	75 (LM)	0.5000	54	129	74	12.77	6636	42.312
2	137	0.1961	129	200	152	12.26	73	1.383
3	443	14.7387	354	585	448	5.28	12064	103.937
4	1433	0.1176	1201	1500	1424	3.12	124	0.829
5	1655	0.3159	1500	2000	1662	5.54	241	2.228
6	2943	1.3828	2000	3608	2978	7.78	1114	9.752
7	4035	0.3510	3608	4173	3925	4.10	222	2.475
8	4518	5.2272	4173	9334	5065	16.92	2780	36.862
9	20000 (UM)	0.1536	18537	29793	20519	4.74	1330	13.000

TIC:	22.3293	ng/uL
TIM:	59.3118	nmole/L
Total concentration:	22.4437	ng/uL

Sample peak width (sec): 5 Sample min peak height: 50 Sample baseline V to V?: Y Sample baseline V to V points: 3
Sample filter: Binomial Number of points for filter: 3 Sample start region (min): 0 Sample end region (min): 30
Marker peak width (sec): 5 Marker min peak height: 200 Marker baseline V to V?: Y Marker baseline V to V points: 3
Lower marker selection: First peak > 500 RFU Upper marker selection: Last peak > 200 RFU
Ladder size (bp)75, 200, 300, 400, 500, 700, 1000, 1500, 2000, 3000, 4000, 5000, 7000, 10000, 20000
Quantification using: Lower Marker Final concentration (ng/uL): 0.5000 Dilution factor: 12.0

Sample: SampB3
Well location: B3
Created: Monday, September 20, 2021 8:00:01 PM

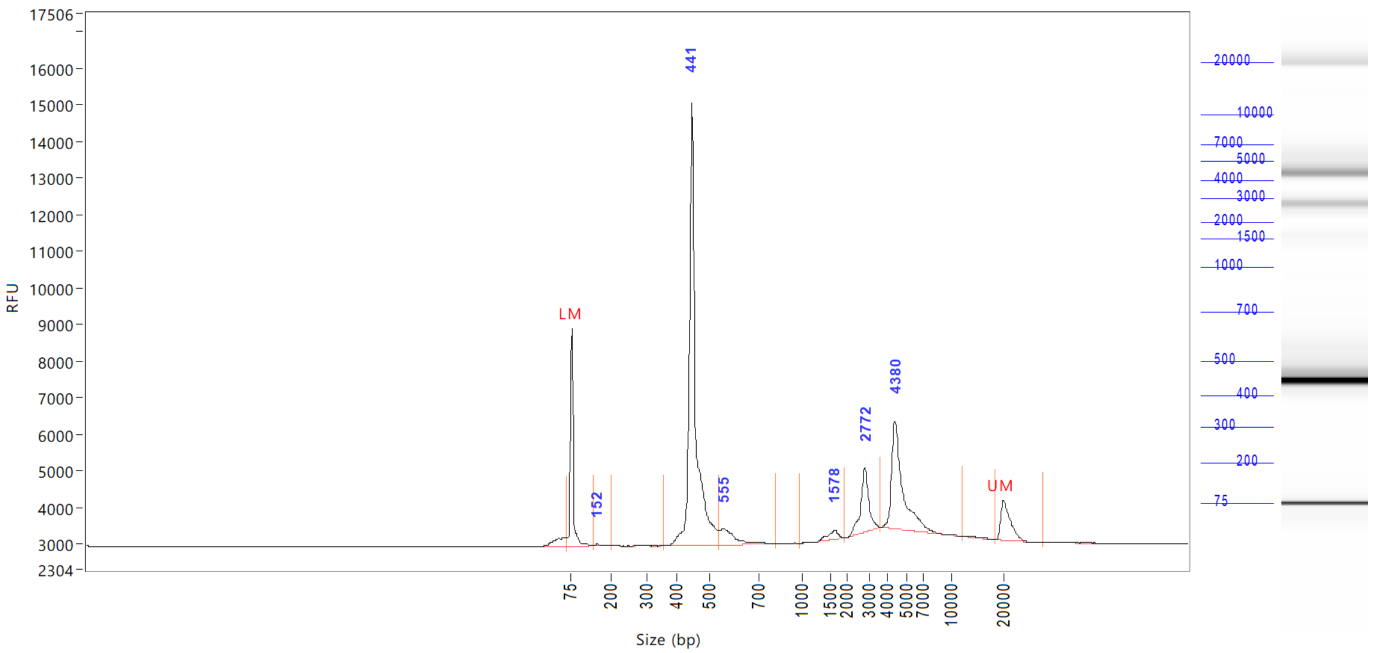

Peak	Size (bp)	Concentration (ng/uL)	From (bp)	To (bp)	Average size (bp)	CV%	RFU	Corrected peak area
1	75 (LM)	0.5000	62	143	75	12.87	5955	39.147
2	152	0.0962	143	196	156	6.86	53	0.627
3	441	22.8832	358	532	448	5.02	12120	149.302
4	555	1.9618	532	814	586	8.66	422	12.800
5	1578	0.6233	983	1904	1504	9.14	259	4.067
6	2772	3.3078	1904	3608	2729	8.48	1755	21.582
7	4380	7.2364	3608	11952	4814	17.58	2957	47.214
8	20000 (UM)	0.1690	18537	27738	20722	5.30	1101	13.234

TIC:	36.1086	ng/uL
TIM:	95.7196	nmole/L
Total concentration:	36.2190	ng/uL

Sample peak width (sec): 5   Sample min peak height: 50   Sample baseline V to V?: Y   Sample baseline V to V points: 3
Sample filter: Binomial   Number of points for filter: 3   Sample start region (min): 0   Sample end region (min): 30
Marker peak width (sec): 5   Marker min peak height: 200   Marker baseline V to V?: Y   Marker baseline V to V points: 3
Lower marker selection: First peak > 500 RFU   Upper marker selection: Last peak > 200 RFU
Ladder size (bp)75, 200, 300, 400, 500, 700, 1000, 1500, 2000, 3000, 4000, 5000, 7000, 10000, 20000
Quantification using: Lower Marker   Final concentration (ng/uL): 0.5000   Dilution factor: 12.0

Sample: SampC3
Well location: C3
Created: Monday, September 20, 2021 8:00:01 PM

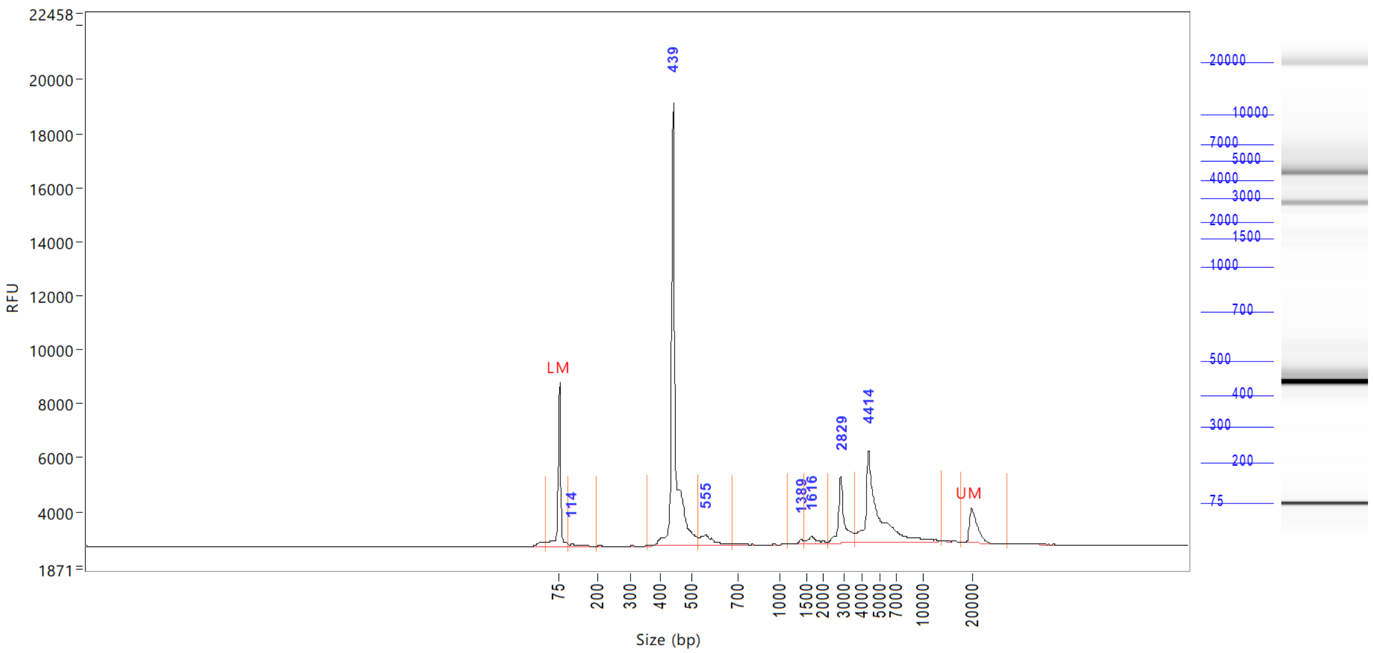

Peak	Size (bp)	Concentration (ng/uL)	From (bp)	To (bp)	Average size (bp)	CV%	RFU	Corrected peak area
1	75 (LM)	0.5000	30	107	71	15.03	6083	40.738
2	114	0.2587	107	192	134	16.67	76	1.757
3	439	20.3383	356	527	445	4.69	16429	138.090
4	555	1.4986	527	672	572	6.09	396	10.175
5	1389	0.2095	1145	1456	1371	4.29	183	1.422
6	1616	0.6085	1456	2201	1706	11.11	255	4.132
7	2829	3.4311	2201	3608	2892	9.42	2458	23.296
8	4414	8.8471	3608	13659	5302	30.27	3385	60.069
9	20000 (UM)	0.1576	17683	27254	20502	4.57	1276	12.844

TIC:	35.1918	ng/uL
TIM:	88.2239	nmole/L
Total concentration:	35.4735	ng/uL

```
Sample peak width (sec): 5    Sample min peak height: 50    Sample baseline V to V?: Y    Sample baseline V to V points: 3
Sample filter: Binomial       Number of points for filter: 3  Sample start region (min): 0  Sample end region (min): 30
Marker peak width (sec): 5    Marker min peak height: 200   Marker baseline V to V?: Y    Marker baseline V to V points: 3
Lower marker selection: First peak > 500 RFU                Upper marker selection: Last peak > 200 RFU
Ladder size (bp)75, 200, 300, 400, 500, 700, 1000, 1500, 2000, 3000, 4000, 5000, 7000, 10000, 20000
Quantification using: Lower Marker          Final concentration (ng/uL): 0.5000          Dilution factor: 12.0
```

Sample: SampD3
Well location: D3
Created: Monday, September 20, 2021 8:00:01 PM

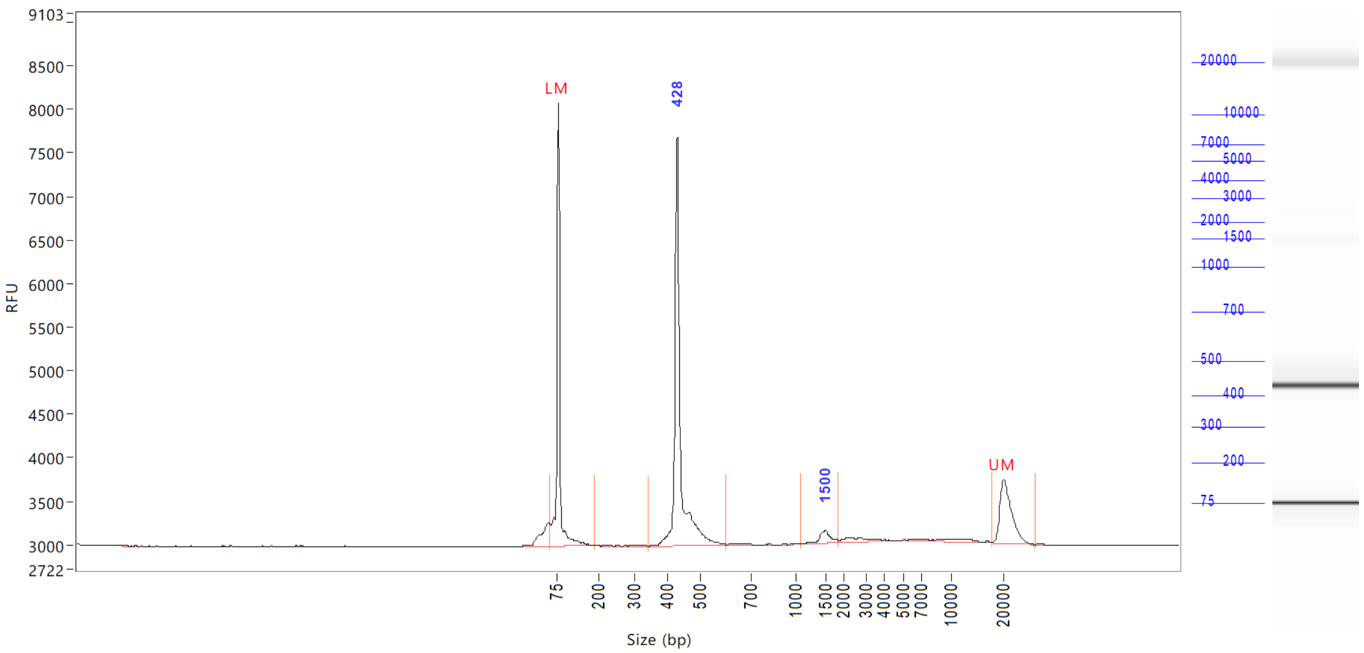

Peak	Size (bp)	Concentration (ng/uL)	From (bp)	To (bp)	Average size (bp)	CV%	RFU	Corrected peak area
1	75 (LM)	0.5000	52	185	76	18.68	5095	37.334
2	428	8.0434	339	598	435	6.38	4697	50.049
3	1500	0.3935	1068	1866	1488	9.85	152	2.448
4	20000 (UM)	0.1439	17927	25924	20570	5.93	736	10.744

TIC:	8.4369	ng/uL
TIM:	30.8594	nmole/L
Total concentration:	9.2418	ng/uL

```
Sample peak width (sec): 5    Sample min peak height: 50    Sample baseline V to V?: Y    Sample baseline V to V points: 3
Sample filter: Binomial       Number of points for filter: 3  Sample start region (min): 0  Sample end region (min): 30
Marker peak width (sec): 5    Marker min peak height: 200   Marker baseline V to V?: Y    Marker baseline V to V points: 3
Lower marker selection: First peak > 500 RFU                Upper marker selection: Last peak > 200 RFU
Ladder size (bp)75, 200, 300, 400, 500, 700, 1000, 1500, 2000, 3000, 4000, 5000, 7000, 10000, 20000
Quantification using: Lower Marker          Final concentration (ng/uL): 0.5000          Dilution factor: 12.0
```

Sample: SampE3
Well location: E3
Created: Monday, September 20, 2021 8:00:01 PM

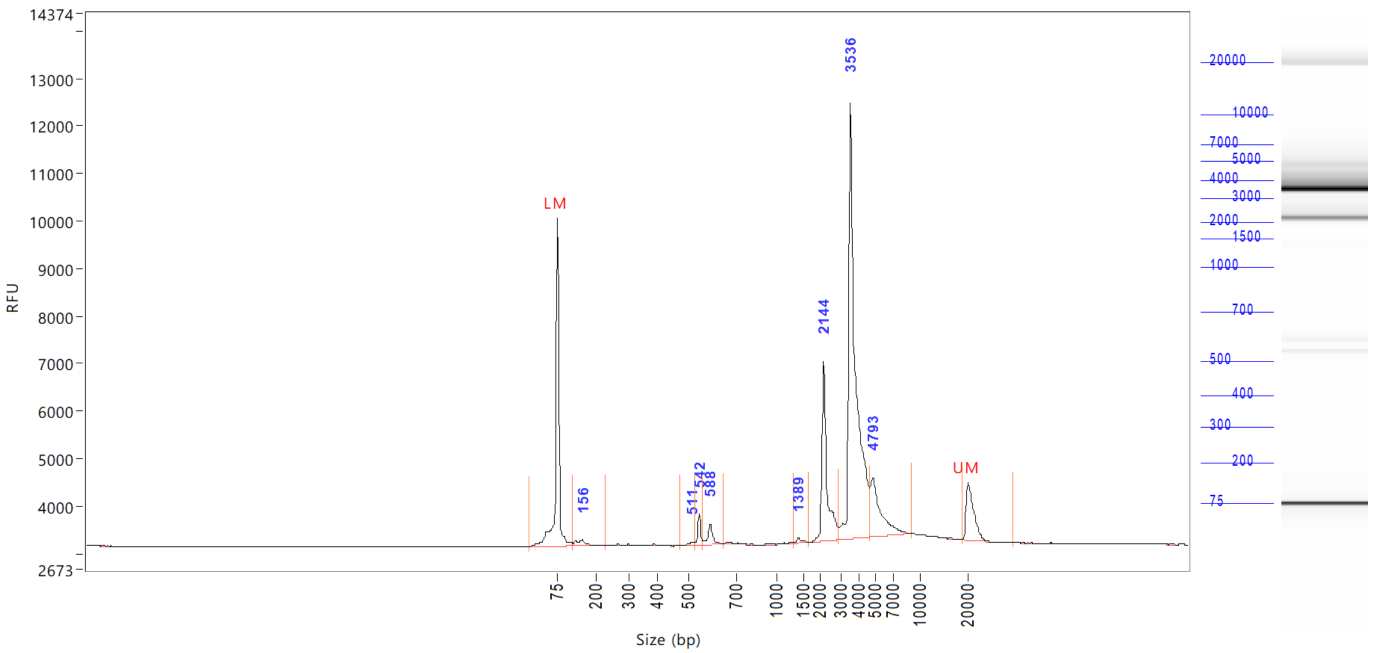

Peak	Size (bp)	Concentration (ng/uL)	From (bp)	To (bp)	Average size (bp)	CV%	RFU	Corrected peak area
1	75 (LM)	0.5000	0	124	69	23.93	6918	53.656
2	156	0.3443	124	226	153	14.45	126	3.079
3	511	0.1004	468	521	505	2.17	78	0.898
4	542	0.4141	521	557	538	1.37	642	3.703
5	588	0.4718	557	646	587	2.75	436	4.219
6	1389	0.0957	1300	1635	1405	4.18	97	0.856
7	2144	3.3825	1635	2886	2233	9.90	3786	30.248
8	3536	11.1740	2886	4621	3739	9.39	9189	99.926
9	4793	2.1781	4621	9067	5397	15.91	1248	19.478
10	20000 (UM)	0.1154	18781	29309	20570	4.76	1221	12.379

TIC:	18.1609	ng/uL
TIM:	14.8048	nmole/L
Total concentration:	18.3272	ng/uL

```
Sample peak width (sec): 5    Sample min peak height: 50    Sample baseline V to V?: Y    Sample baseline V to V points: 3
Sample filter: Binomial       Number of points for filter: 3  Sample start region (min): 0  Sample end region (min): 30
Marker peak width (sec): 5    Marker min peak height: 200   Marker baseline V to V?: Y    Marker baseline V to V points: 3
Lower marker selection: First peak > 500 RFU                Upper marker selection: Last peak > 200 RFU
Ladder size (bp)75, 200, 300, 400, 500, 700, 1000, 1500, 2000, 3000, 4000, 5000, 7000, 10000, 20000
Quantification using: Lower Marker          Final concentration (ng/uL): 0.5000          Dilution factor: 12.0
```

Sample: SampF3
Well location: F3
Created: Monday, September 20, 2021 8:00:01 PM

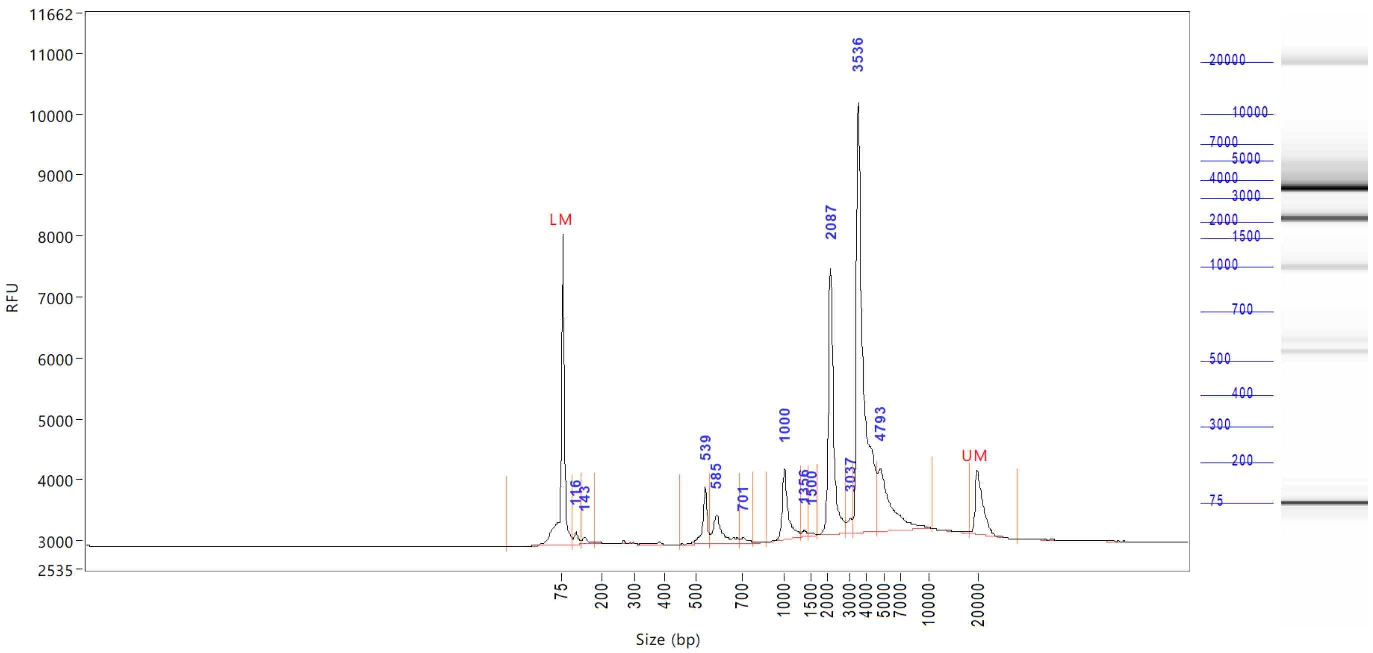

Peak	Size (bp)	Concentration (ng/uL)	From (bp)	To (bp)	Average size (bp)	CV%	RFU	Corrected peak area
1	75 (LM)	0.5000	0	107	68	25.73	5098	43.377
2	116	0.3014	107	133	117	5.98	201	2.179
3	143	0.2032	133	179	149	8.27	110	1.469
4	539	1.1464	447	560	531	3.07	939	8.288
5	585	1.1637	560	687	604	5.44	470	8.413
6	701	0.1480	687	775	713	3.24	90	1.070
7	1000	1.6305	866	1300	1035	7.31	1158	11.788
8	1356	0.1138	1300	1433	1358	2.75	98	0.822
9	1500	0.0667	1433	1693	1496	3.10	56	0.482
10	2087	4.8654	1693	2857	2151	8.55	4368	35.174
11	3037	0.2818	2857	3179	3016	3.15	241	2.038
12	3536	10.2898	3179	4621	3759	9.11	7071	74.390
13	4793	2.7547	4621	10855	5700	20.93	1036	19.915
14	20000 (UM)	0.1251	18415	28100	20629	5.16	1049	10.856

TIC:	22.9654	ng/uL
TIM:	25.5209	nmole/L
Total concentration:	23.0730	ng/uL

Sample peak width (sec): 5   Sample min peak height: 50   Sample baseline V to V?: Y   Sample baseline V to V points: 3
Sample filter: Binomial   Number of points for filter: 3   Sample start region (min): 0   Sample end region (min): 30
Marker peak width (sec): 5   Marker min peak height: 200   Marker baseline V to V?: Y   Marker baseline V to V points: 3
Lower marker selection: First peak > 500 RFU   Upper marker selection: Last peak > 200 RFU
Ladder size (bp)75, 200, 300, 400, 500, 700, 1000, 1500, 2000, 3000, 4000, 5000, 7000, 10000, 20000
Quantification using: Lower Marker   Final concentration (ng/uL): 0.5000   Dilution factor: 12.0

Sample: SampG3
Well location: G3
Created: Monday, September 20, 2021 8:00:01 PM

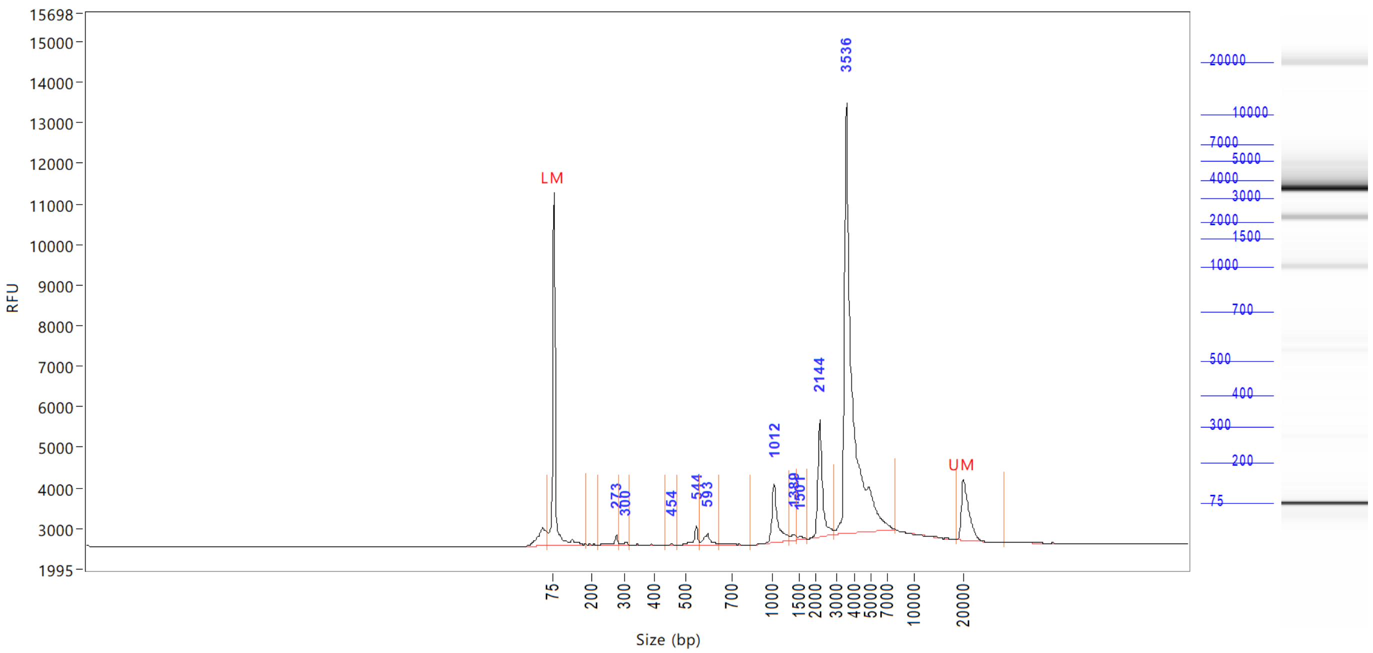

Peak	Size (bp)	Concentration (ng/uL)	From (bp)	To (bp)	Average size (bp)	CV%	RFU	Corrected peak area
1	75 (LM)	0.5000	56	177	76	17.45	8687	57.772
2	273	0.2153	215	282	262	5.52	244	2.073
3	300	0.0764	282	315	296	2.41	81	0.735
4	454	0.0446	433	471	453	1.58	62	0.429
5	544	0.4147	471	562	534	3.60	468	3.993
6	593	0.4118	562	644	593	3.18	290	3.965
7	1012	1.5001	835	1311	1051	7.65	1440	14.444
8	1389	0.1285	1311	1456	1378	2.91	149	1.237
9	1501	0.0789	1456	1750	1519	3.43	89	0.759
10	2144	2.2045	1750	2886	2204	7.60	2902	21.227
11	3536	13.0183	2886	7801	3930	16.42	10629	125.349
12	20000 (UM)	0.1309	18415	28584	20551	4.45	1495	15.123

TIC:	18.0930	ng/uL
TIM:	14.0467	nmole/L
Total concentration:	18.2719	ng/uL

Sample peak width (sec): 5 Sample min peak height: 50 Sample baseline V to V?: Y Sample baseline V to V points: 3
Sample filter: Binomial Number of points for filter: 3 Sample start region (min): 0 Sample end region (min): 30
Marker peak width (sec): 5 Marker min peak height: 200 Marker baseline V to V?: Y Marker baseline V to V points: 3
Lower marker selection: First peak > 500 RFU Upper marker selection: Last peak > 200 RFU
Ladder size (bp)75, 200, 300, 400, 500, 700, 1000, 1500, 2000, 3000, 4000, 5000, 7000, 10000, 20000
Quantification using: Lower Marker Final concentration (ng/uL): 0.5000 Dilution factor: 12.0

Sample: SampH3
Well location: H3
Created: Monday, September 20, 2021 8:00:01 PM

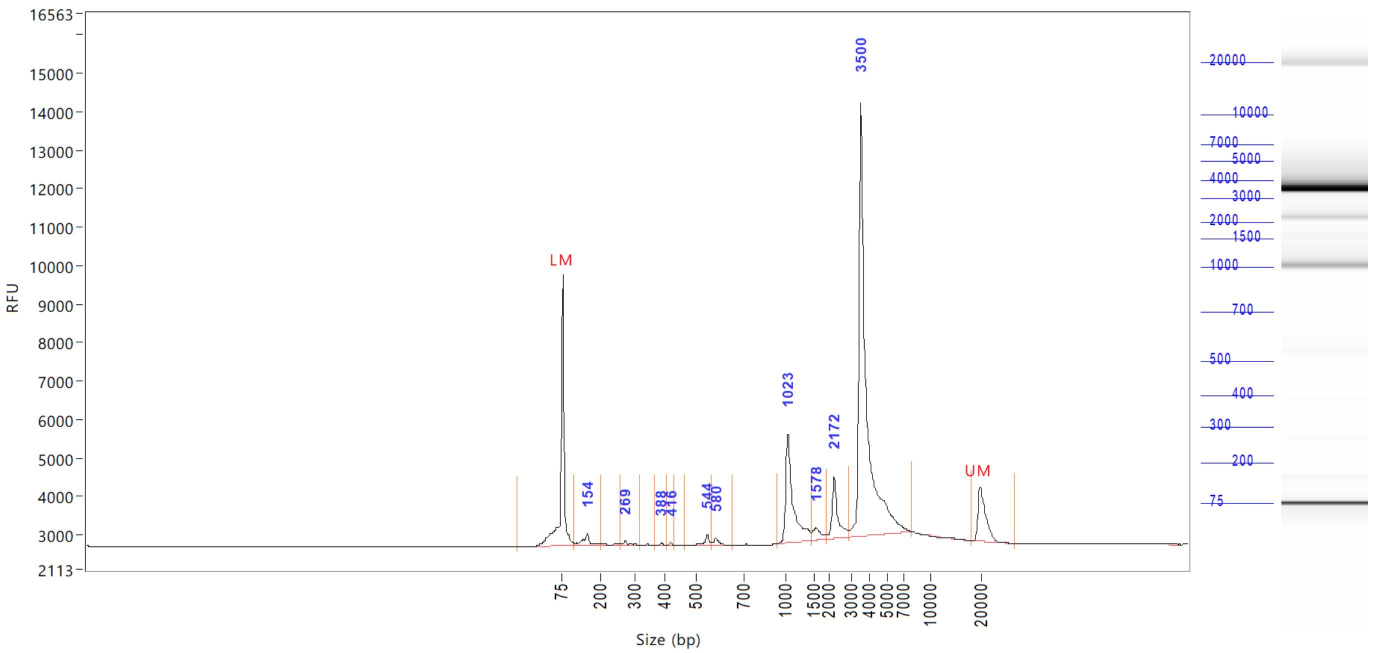

Peak	Size (bp)	Concentration (ng/uL)	From (bp)	To (bp)	Average size (bp)	CV%	RFU	Corrected peak area
1	75 (LM)	0.5000	0	110	67	26.07	7052	56.537
2	154	0.4854	110	196	145	11.61	315	4.574
3	269	0.0989	255	317	275	4.77	97	0.932
4	388	0.0470	368	403	383	1.65	63	0.443
5	416	0.0424	403	428	414	0.99	75	0.400
6	544	0.2825	464	565	533	3.63	267	2.662
7	580	0.2007	565	654	587	3.19	188	1.891
8	1023	3.6814	939	1467	1099	11.00	2825	34.689
9	1578	0.4587	1467	1904	1622	7.65	316	4.322
10	2172	1.5626	1904	2914	2270	10.09	1628	14.724
11	3500	13.2209	2914	7867	3876	16.42	11281	124.578
12	20000 (UM)	0.1257	18293	26891	20450	4.51	1420	14.210

TIC:	20.0806	ng/uL
TIM:	20.6263	nmole/L
Total concentration:	20.1958	ng/uL

Sample peak width (sec): 5   Sample min peak height: 50   Sample baseline V to V?: Y   Sample baseline V to V points: 3
Sample filter: Binomial   Number of points for filter: 3 Sample start region (min): 0 Sample end region (min): 30
Marker peak width (sec): 5   Marker min peak height: 200   Marker baseline V to V?: Y   Marker baseline V to V points: 3
Lower marker selection: First peak > 500 RFU   Upper marker selection: Last peak > 200 RFU
Ladder size (bp)75, 200, 300, 400, 500, 700, 1000, 1500, 2000, 3000, 4000, 5000, 7000, 10000, 20000
Quantification using: Lower Marker   Final concentration (ng/uL): 0.5000   Dilution factor: 12.0

Sample: SampA4
Well location: A4
Created: Monday, September 20, 2021 8:00:01 PM

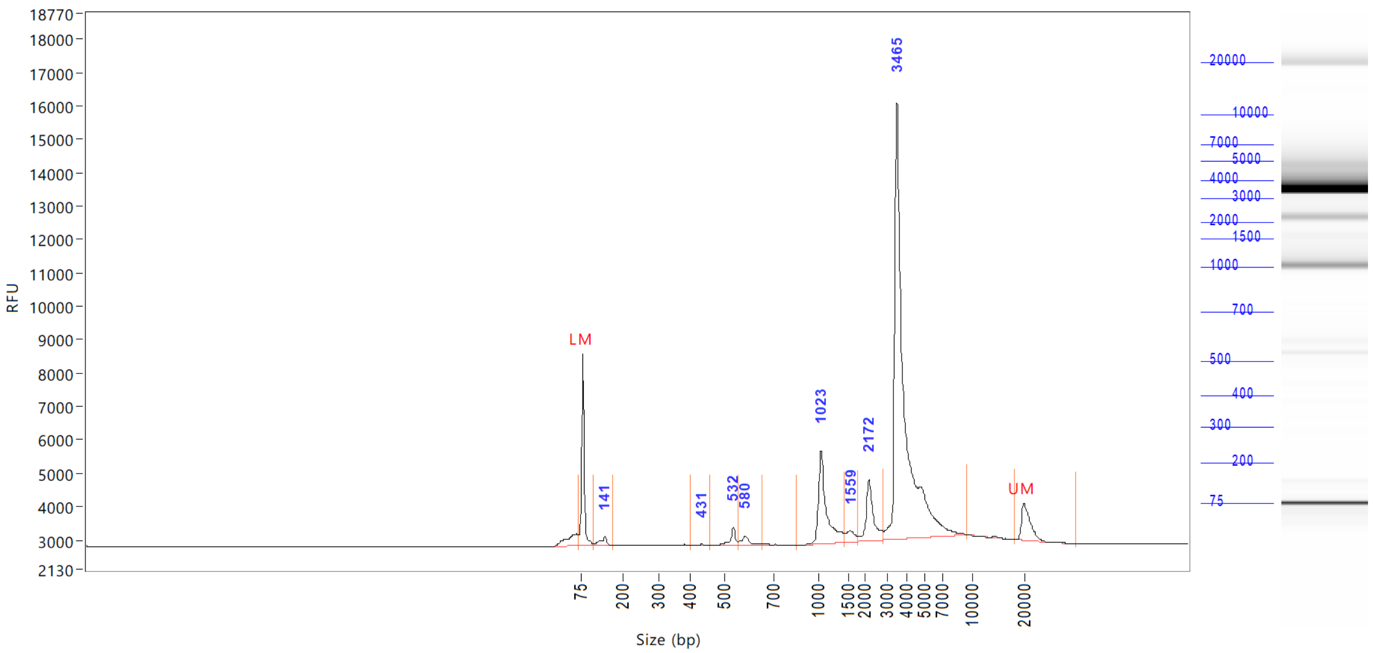

Peak	Size (bp)	Concentration (ng/uL)	From (bp)	To (bp)	Average size (bp)	CV%	RFU	Corrected peak area
1	75 (LM)	0.5000	64	107	74	7.52	5721	35.019
2	141	0.5850	107	166	131	8.49	264	3.414
3	431	0.0600	400	458	430	1.88	53	0.350
4	532	0.8823	458	552	524	3.33	551	5.149
5	580	0.7310	552	649	585	3.74	295	4.267
6	1023	6.1248	848	1456	1098	11.10	2774	35.747
7	1559	0.7735	1456	1789	1582	6.05	337	4.515
8	2172	3.4107	1789	2829	2231	10.00	1852	19.907
9	3465	30.0919	2829	9600	3975	21.43	13080	175.630
10	20000 (UM)	0.1692	18293	29793	20580	4.88	1122	11.850

TIC:	42.6594	ng/uL
TIM:	37.3601	nmole/L
Total concentration:	42.9383	ng/uL

Sample peak width (sec): 5 Sample min peak height: 50 Sample baseline V to V?: Y Sample baseline V to V points: 3
Sample filter: Binomial Number of points for filter: 3 Sample start region (min): 0 Sample end region (min): 30
Marker peak width (sec): 5 Marker min peak height: 200 Marker baseline V to V?: Y Marker baseline V to V points: 3
Lower marker selection: First peak > 500 RFU Upper marker selection: Last peak > 200 RFU
Ladder size (bp)75, 200, 300, 400, 500, 700, 1000, 1500, 2000, 3000, 4000, 5000, 7000, 10000, 20000
Quantification using: Lower Marker Final concentration (ng/uL): 0.5000 Dilution factor: 12.0

Sample: SampB4
Well location: B4
Created: Monday, September 20, 2021 8:00:01 PM

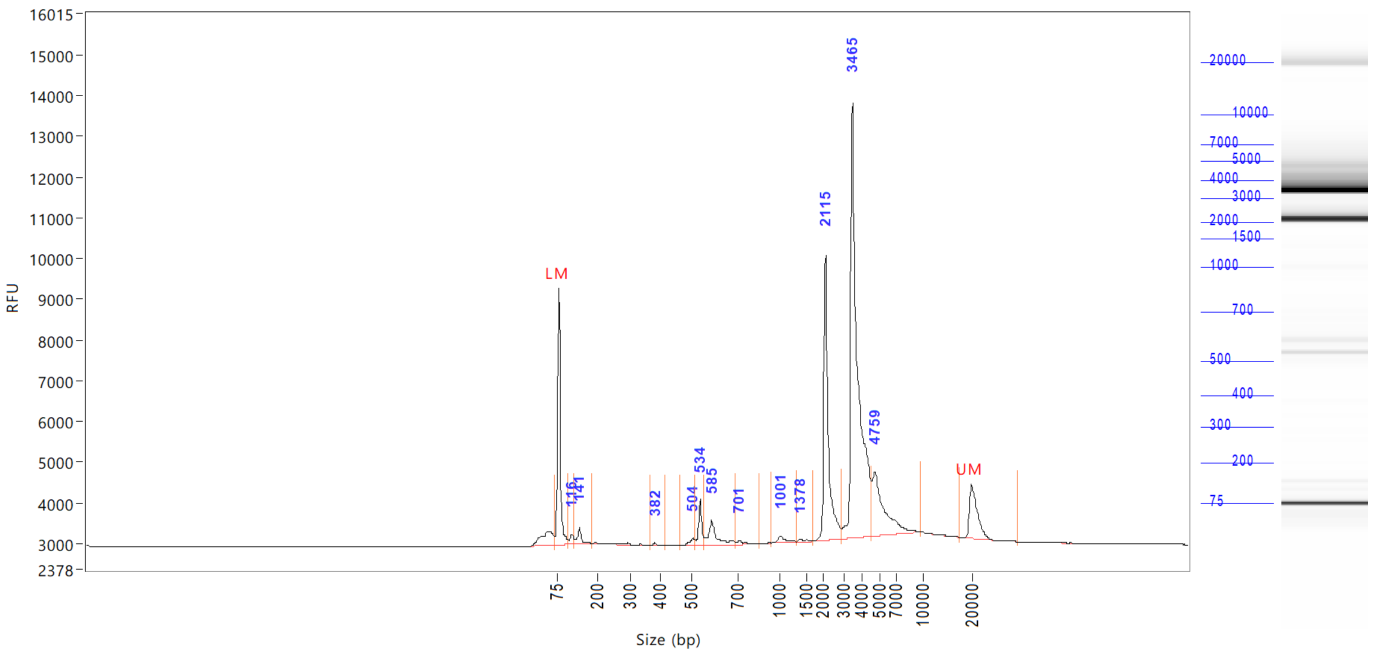

Peak	Size (bp)	Concentration (ng/uL)	From (bp)	To (bp)	Average size (bp)	CV%	RFU	Corrected peak area
1	75 (LM)	0.5000	62	107	74	8.66	6307	40.894
2	116	0.3286	107	126	116	4.55	248	2.240
3	141	0.5420	126	183	140	6.61	400	3.694
4	382	0.0663	366	416	380	2.38	66	0.452
5	504	0.2900	464	516	499	2.16	169	1.977
6	534	1.0173	516	552	533	1.42	1120	6.934
7	585	1.3913	552	690	600	5.71	602	9.483
8	701	0.1982	690	848	728	4.94	109	1.351
9	1001	0.2736	935	1300	1041	7.38	171	1.865
10	1378	0.0982	1300	1693	1425	5.31	63	0.670
11	2115	7.4744	1693	2886	2157	8.83	7011	50.943
12	3465	17.3667	2886	4587	3703	9.70	10690	118.365
13	4759	4.0169	4587	9733	5516	18.49	1579	27.378
14	20000 (UM)	0.1683	17439	29551	20539	5.16	1320	13.765

TIC:	33.0636	ng/uL
TIM:	34.8335	nmole/L
Total concentration:	33.0506	ng/uL

```
Sample peak width (sec): 5    Sample min peak height: 50    Sample baseline V to V?: Y    Sample baseline V to V points: 3
Sample filter: Binomial       Number of points for filter: 3  Sample start region (min): 0  Sample end region (min): 30
Marker peak width (sec): 5    Marker min peak height: 200   Marker baseline V to V?: Y    Marker baseline V to V points: 3
Lower marker selection: First peak > 500 RFU                Upper marker selection: Last peak > 200 RFU
Ladder size (bp)75, 200, 300, 400, 500, 700, 1000, 1500, 2000, 3000, 4000, 5000, 7000, 10000, 20000
Quantification using: Lower Marker          Final concentration (ng/uL): 0.5000          Dilution factor: 12.0
```

Sample: SampC4
Well location: C4
Created: Monday, September 20, 2021 8:00:01 PM

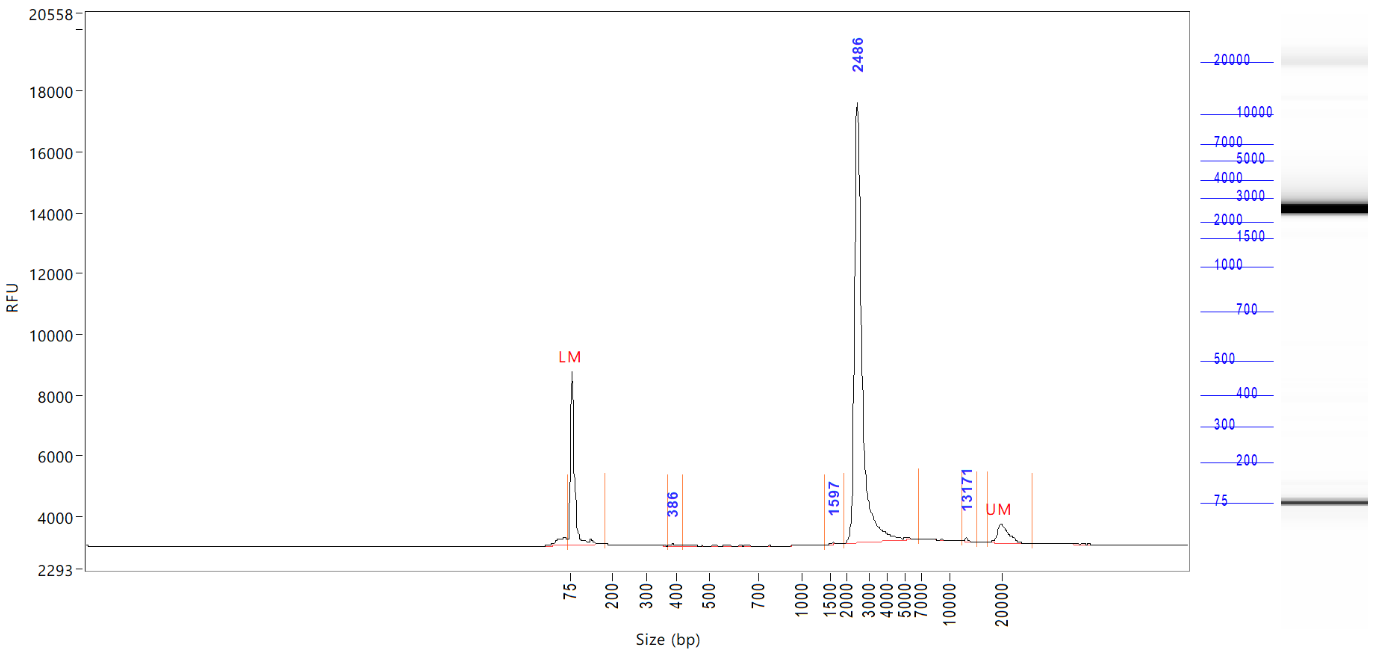

Peak	Size (bp)	Concentration (ng/uL)	From (bp)	To (bp)	Average size (bp)	CV%	RFU	Corrected peak area
1	75 (LM)	0.5000	62	177	78	18.68	5696	51.834
2	386	0.0770	368	414	389	2.72	72	0.665
3	1597	0.0553	1411	1923	1612	6.19	53	0.478
4	2486	18.9976	1923	6704	2630	15.21	14483	164.120
5	13171	0.0596	12318	15366	13090	2.92	112	0.515
6	20000 (UM)	0.0851	17439	26287	20576	5.99	614	8.821

TIC:	19.1894	ng/uL
TIM:	12.2806	nmole/L
Total concentration:	19.4759	ng/uL

Sample peak width (sec): 5 Sample min peak height: 50 Sample baseline V to V?: Y Sample baseline V to V points: 3
Sample filter: Binomial Number of points for filter: 3 Sample start region (min): 0 Sample end region (min): 30
Marker peak width (sec): 5 Marker min peak height: 200 Marker baseline V to V?: Y Marker baseline V to V points: 3
Lower marker selection: First peak > 500 RFU Upper marker selection: Last peak > 200 RFU
Ladder size (bp)75, 200, 300, 400, 500, 700, 1000, 1500, 2000, 3000, 4000, 5000, 7000, 10000, 20000
Quantification using: Lower Marker Final concentration (ng/uL): 0.5000 Dilution factor: 12.0

Sample: SampD4
Well location: D4
Created: Monday, September 20, 2021 8:00:01 PM

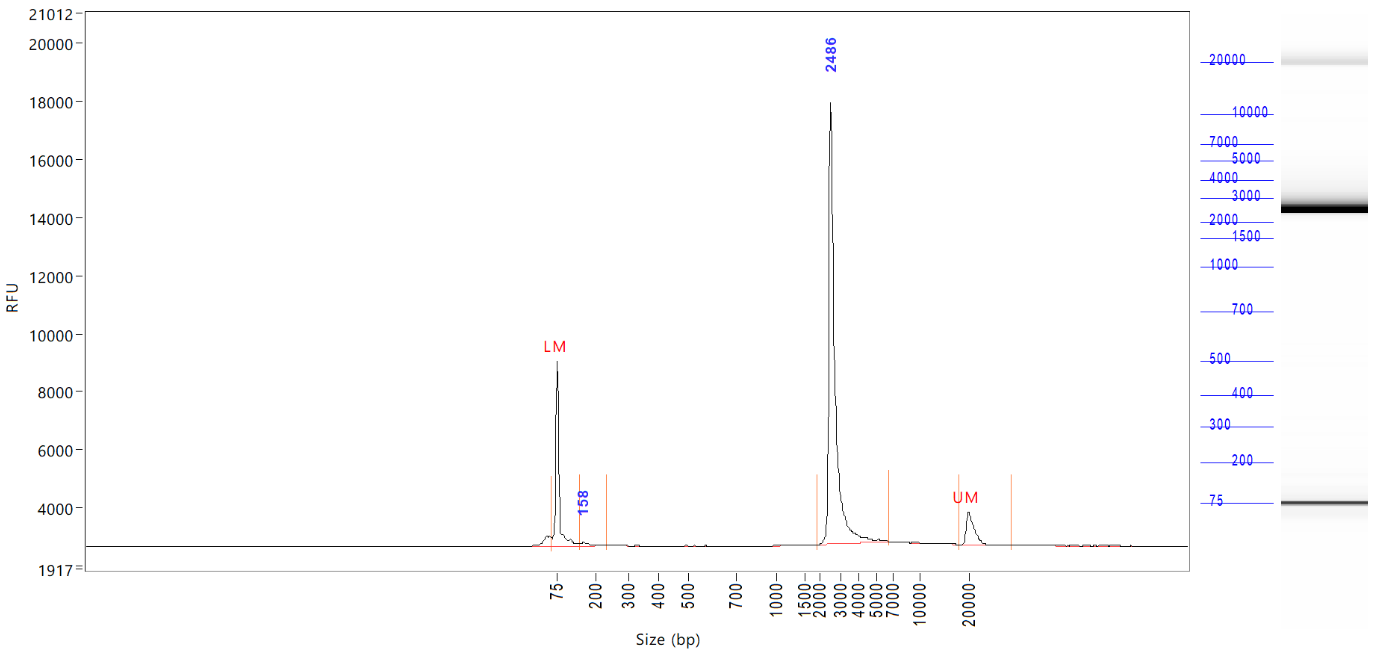

Peak	Size (bp)	Concentration (ng/uL)	From (bp)	To (bp)	Average size (bp)	CV%	RFU	Corrected peak area
1	75 (LM)	0.5000	60	147	78	18.68	6370	48.778
2	158	0.3120	147	232	174	12.97	135	2.537
3	2486	17.3447	1904	6556	2665	16.10	15187	141.008
4	20000 (UM)	0.1176	18049	28826	20560	4.88	1131	11.470

TIC:	17.6567	ng/uL
TIM:	13.6618	nmole/L
Total concentration:	17.9882	ng/uL

```
Sample peak width (sec): 5    Sample min peak height: 50    Sample baseline V to V?: Y    Sample baseline V to V points: 3
Sample filter: Binomial       Number of points for filter: 3  Sample start region (min): 0  Sample end region (min): 30
Marker peak width (sec): 5    Marker min peak height: 200   Marker baseline V to V?: Y    Marker baseline V to V points: 3
Lower marker selection: First peak > 500 RFU                Upper marker selection: Last peak > 200 RFU
Ladder size (bp)75, 200, 300, 400, 500, 700, 1000, 1500, 2000, 3000, 4000, 5000, 7000, 10000, 20000
Quantification using: Lower Marker          Final concentration (ng/uL): 0.5000          Dilution factor: 12.0
```

Sample: SampE4
Well location: E4
Created: Monday, September 20, 2021 8:00:01 PM

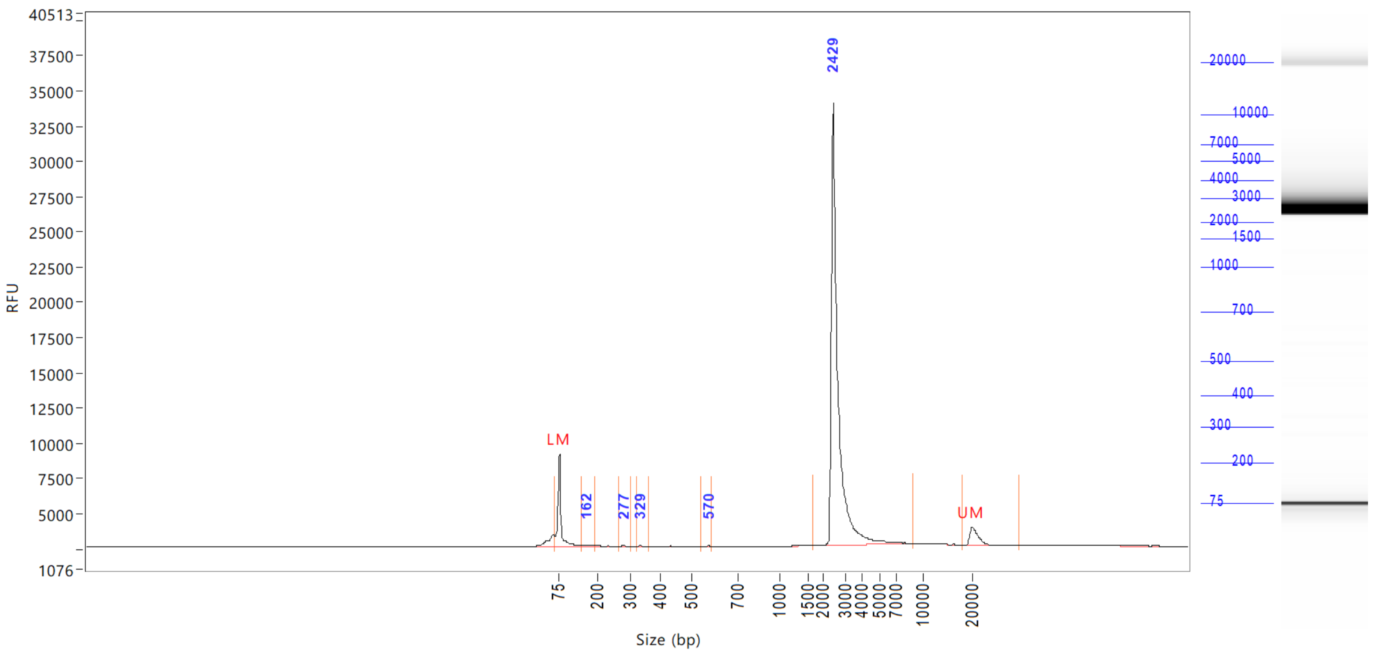

Peak	Size (bp)	Concentration (ng/uL)	From (bp)	To (bp)	Average size (bp)	CV%	RFU	Corrected peak area
1	75 (LM)	0.5000	62	147	77	17.81	6601	50.311
2	162	0.1368	147	189	163	6.77	70	1.147
3	277	0.0583	262	298	275	2.17	75	0.488
4	329	0.0515	319	358	329	1.54	83	0.432
5	570	0.0521	539	583	563	1.73	71	0.437
6	2429	37.2808	1674	8934	2699	21.53	31434	312.606
7	20000 (UM)	0.1339	17927	29551	20522	5.11	1288	13.474

TIC:	37.5795	ng/uL
TIM:	24.8766	nmole/L
Total concentration:	37.9137	ng/uL

Sample peak width (sec): 5   Sample min peak height: 50   Sample baseline V to V?: Y   Sample baseline V to V points: 3
Sample filter: Binomial   Number of points for filter: 3 Sample start region (min): 0 Sample end region (min): 30
Marker peak width (sec): 5   Marker min peak height: 200   Marker baseline V to V?: Y   Marker baseline V to V points: 3
Lower marker selection: First peak > 500 RFU   Upper marker selection: Last peak > 200 RFU
Ladder size (bp)75, 200, 300, 400, 500, 700, 1000, 1500, 2000, 3000, 4000, 5000, 7000, 10000, 20000
Quantification using: Lower Marker   Final concentration (ng/uL): 0.5000   Dilution factor: 12.0

Sample: SampF4
Well location: F4
Created: Monday, September 20, 2021 8:00:01 PM

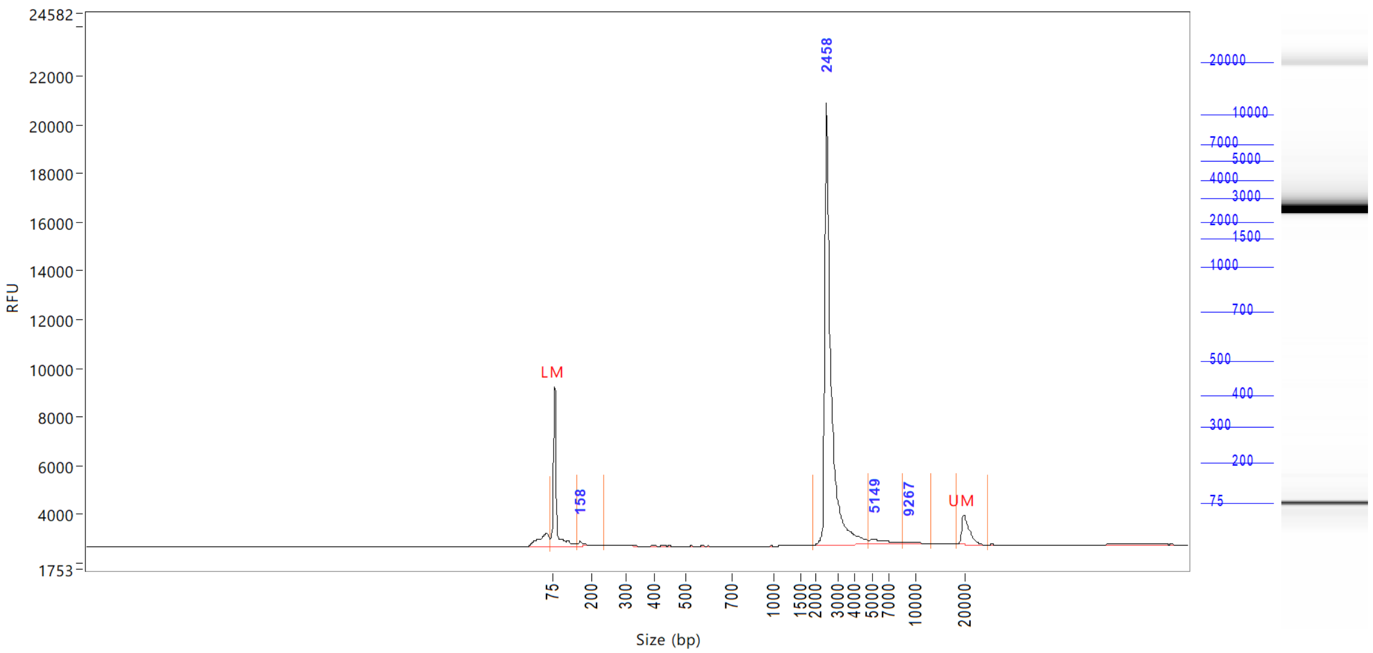

Peak	Size (bp)	Concentration (ng/uL)	From (bp)	To (bp)	Average size (bp)	CV%	RFU	Corrected peak area
1	75 (LM)	0.5000	62	148	79	19.61	6539	50.438
2	158	0.3793	148	235	175	13.68	200	3.189
3	2458	21.5107	1904	4828	2672	15.59	18183	180.827
4	5149	0.5926	4828	8600	6138	17.38	203	4.982
5	9267	0.1147	8600	13293	9717	9.12	50	0.965
6	20000 (UM)	0.1204	18293	25078	20522	4.42	1204	12.146

TIC:	22.5974	ng/uL
TIM:	16.9941	nmole/L
Total concentration:	22.9101	ng/uL

```
Sample peak width (sec): 5    Sample min peak height: 50    Sample baseline V to V?: Y    Sample baseline V to V points: 3
Sample filter: Binomial       Number of points for filter: 3  Sample start region (min): 0  Sample end region (min): 30
Marker peak width (sec): 5    Marker min peak height: 200   Marker baseline V to V?: Y    Marker baseline V to V points: 3
Lower marker selection: First peak > 500 RFU                Upper marker selection: Last peak > 200 RFU
Ladder size (bp)75, 200, 300, 400, 500, 700, 1000, 1500, 2000, 3000, 4000, 5000, 7000, 10000, 20000
Quantification using: Lower Marker          Final concentration (ng/uL): 0.5000          Dilution factor: 12.0
```

Sample: SampG4
Well location: G4
Created: Monday, September 20, 2021 8:00:01 PM

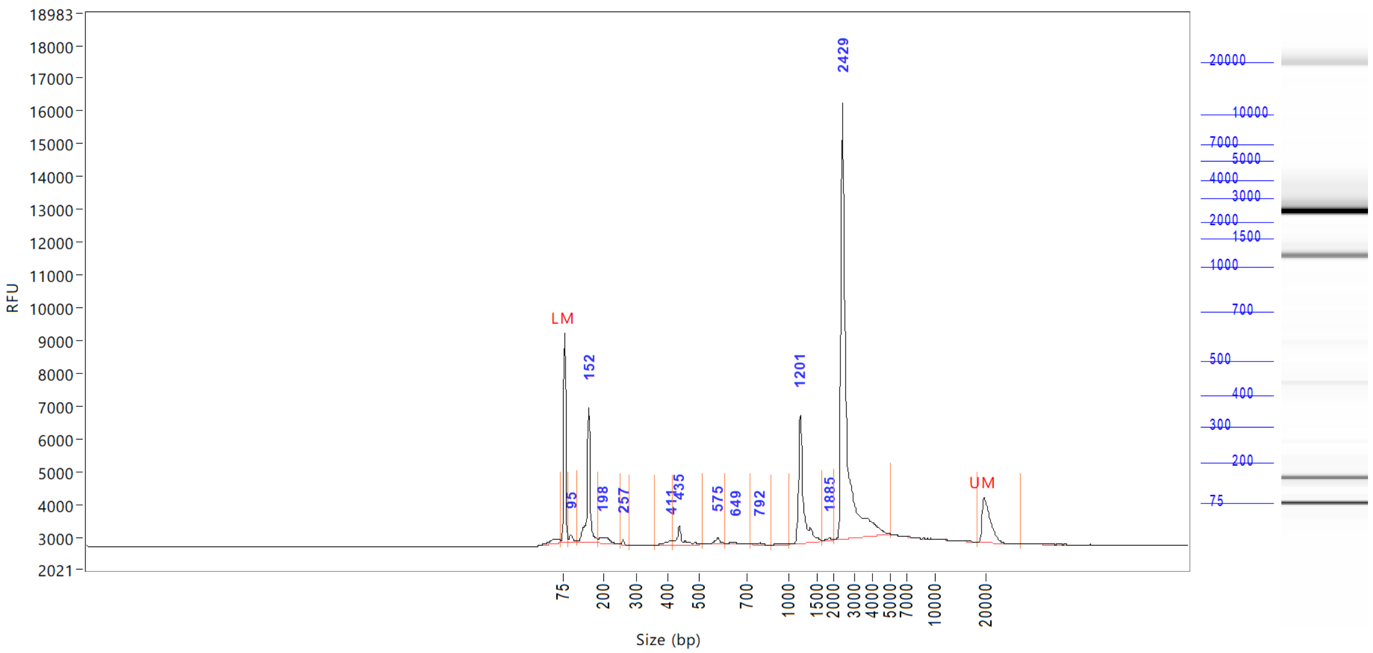

Peak	Size (bp)	Concentration (ng/uL)	From (bp)	To (bp)	Average size (bp)	CV%	RFU	Corrected peak area
1	75 (LM)	0.5000	66	87	74	4.16	6385	34.851
2	95	0.2961	87	116	95	6.92	206	1.720
3	152	5.4042	116	181	149	6.39	4100	31.390
4	198	0.7019	181	248	201	6.43	166	4.077
5	257	0.1196	248	277	255	1.24	154	0.695
6	411	0.3430	358	416	398	3.28	122	1.992
7	435	0.9773	416	514	442	4.19	560	5.677
8	575	0.3832	514	608	570	3.12	212	2.226
9	649	0.2470	608	723	651	4.17	70	1.435
10	792	0.1050	723	870	777	3.58	50	0.610
11	1201	5.2638	1001	1655	1228	6.92	3908	30.575
12	1885	0.1072	1655	2001	1824	4.64	66	0.623
13	2429	19.1748	2001	5001	2655	17.69	13330	111.375
14	20000 (UM)	0.2052	18659	27254	20501	4.58	1382	14.301

TIC:	33.1232	ng/uL
TIM:	97.2908	nmole/L
Total concentration:	33.3263	ng/uL

```
Sample peak width (sec): 5    Sample min peak height: 50    Sample baseline V to V?: Y    Sample baseline V to V points: 3
Sample filter: Binomial       Number of points for filter: 3  Sample start region (min): 0  Sample end region (min): 30
Marker peak width (sec): 5    Marker min peak height: 200   Marker baseline V to V?: Y    Marker baseline V to V points: 3
Lower marker selection: First peak > 500 RFU                Upper marker selection: Last peak > 200 RFU
Ladder size (bp)75, 200, 300, 400, 500, 700, 1000, 1500, 2000, 3000, 4000, 5000, 7000, 10000, 20000
Quantification using: Lower Marker          Final concentration (ng/uL): 0.5000          Dilution factor: 12.0
```

Sample: SampH4
Well location: H4
Created: Monday, September 20, 2021 8:00:01 PM

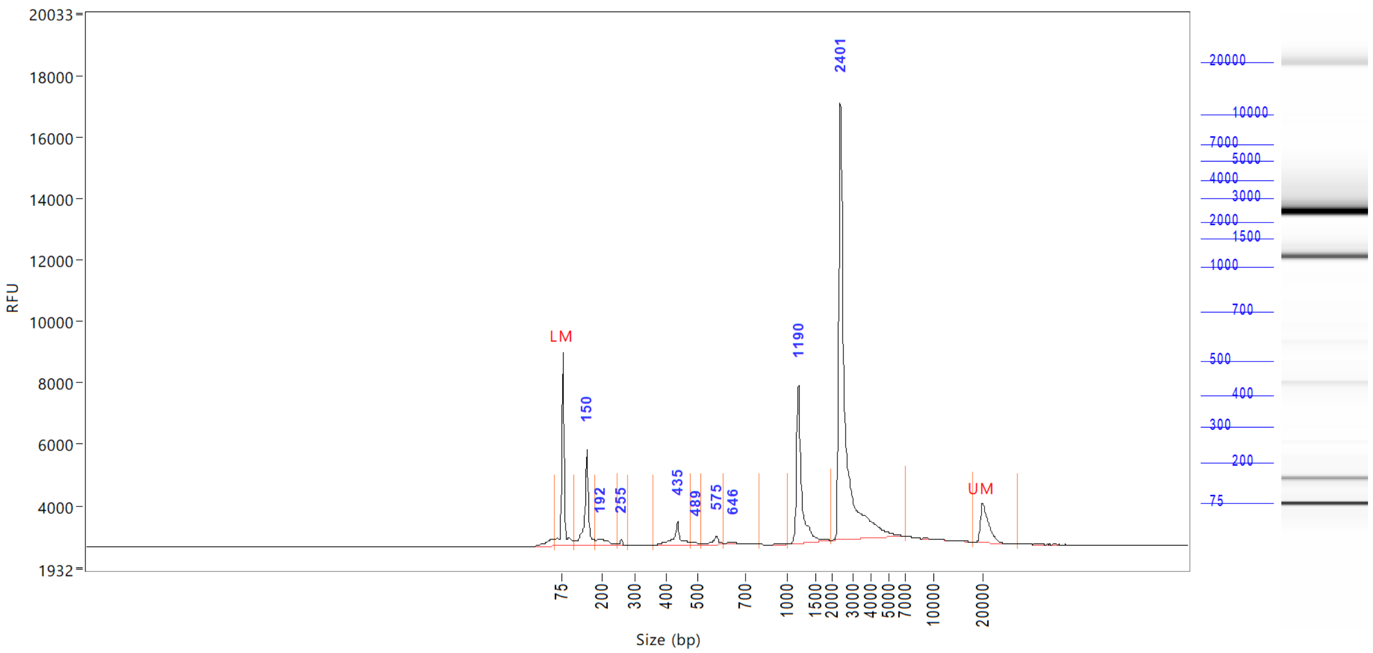

Peak	Size (bp)	Concentration (ng/uL)	From (bp)	To (bp)	Average size (bp)	CV%	RFU	Corrected peak area
1	75 (LM)	0.5000	48	108	73	11.67	6278	41.683
2	150	4.0413	108	177	145	8.16	3115	28.076
3	192	0.6712	177	246	199	7.37	175	4.663
4	255	0.1273	246	275	254	1.27	181	0.885
5	435	1.3704	358	477	428	5.15	761	9.521
6	489	0.1184	477	509	488	1.50	74	0.823
7	575	0.4248	509	608	568	3.46	249	2.951
8	646	0.2739	608	792	656	5.41	92	1.902
9	1190	6.2043	1000	2000	1223	8.65	5145	43.102
10	2401	20.0946	2000	7000	2714	21.91	14245	139.599
11	20000 (UM)	0.1606	18049	26891	20516	4.61	1287	13.385

TIC:	33.3263	ng/uL
TIM:	80.2987	nmole/L
Total concentration:	33.4752	ng/uL

```
Sample peak width (sec): 5    Sample min peak height: 50    Sample baseline V to V?: Y    Sample baseline V to V points: 3
Sample filter: Binomial       Number of points for filter: 3  Sample start region (min): 0  Sample end region (min): 30
Marker peak width (sec): 5    Marker min peak height: 200   Marker baseline V to V?: Y    Marker baseline V to V points: 3
Lower marker selection: First peak > 500 RFU                Upper marker selection: Last peak > 200 RFU
Ladder size (bp)75, 200, 300, 400, 500, 700, 1000, 1500, 2000, 3000, 4000, 5000, 7000, 10000, 20000
Quantification using: Lower Marker          Final concentration (ng/uL): 0.5000          Dilution factor: 12.0
```

Sample: SampA5
Well location: A5
Created: Monday, September 20, 2021 8:00:01 PM

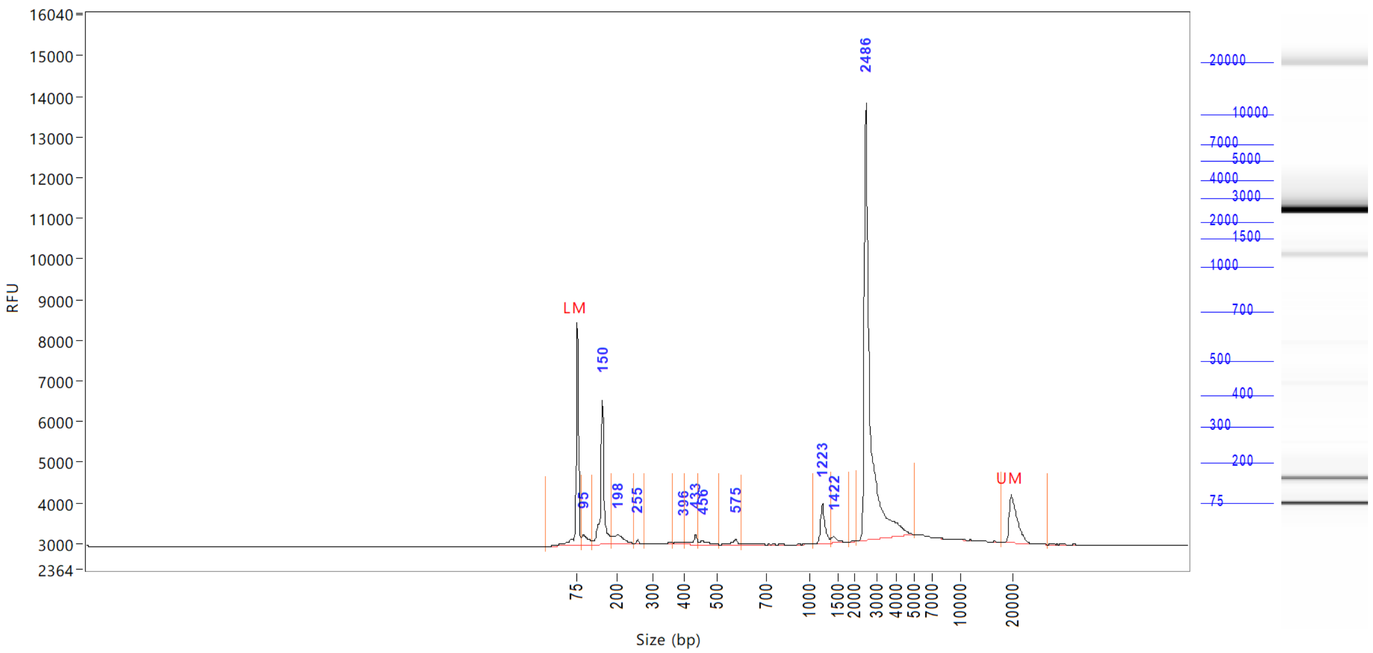

Peak	Size (bp)	Concentration (ng/uL)	From (bp)	To (bp)	Average size (bp)	CV%	RFU	Corrected peak area
1	75 (LM)	0.5000	0	87	71	16.02	5497	33.003
2	95	0.6961	87	122	101	9.89	249	3.829
3	150	5.1584	122	179	149	6.29	3562	28.374
4	198	0.9496	179	248	200	6.79	208	5.223
5	255	0.0743	248	277	253	1.09	92	0.409
6	396	0.1856	366	403	386	2.87	58	1.021
7	433	0.3830	403	445	427	2.54	228	2.107
8	456	0.2457	445	509	463	2.82	82	1.352
9	575	0.2736	509	600	565	3.28	142	1.505
10	1223	1.2869	1068	1367	1227	4.09	979	7.079
11	1422	0.2911	1367	1808	1453	5.23	153	1.601
12	2486	17.3392	2058	5001	2711	16.38	10776	95.374
13	20000 (UM)	0.1777	17927	27012	20505	4.46	1182	11.727

TIC:	26.8835	ng/uL
TIM:	93.0270	nmole/L
Total concentration:	27.3249	ng/uL

```
Sample peak width (sec): 5    Sample min peak height: 50    Sample baseline V to V?: Y    Sample baseline V to V points: 3
Sample filter: Binomial       Number of points for filter: 3  Sample start region (min): 0  Sample end region (min): 30
Marker peak width (sec): 5    Marker min peak height: 200   Marker baseline V to V?: Y    Marker baseline V to V points: 3
Lower marker selection: First peak > 500 RFU                Upper marker selection: Last peak > 200 RFU
Ladder size (bp)75, 200, 300, 400, 500, 700, 1000, 1500, 2000, 3000, 4000, 5000, 7000, 10000, 20000
Quantification using: Lower Marker          Final concentration (ng/uL): 0.5000          Dilution factor: 12.0
```

Sample: SampB5
Well location: B5
Created: Monday, September 20, 2021 8:00:01 PM

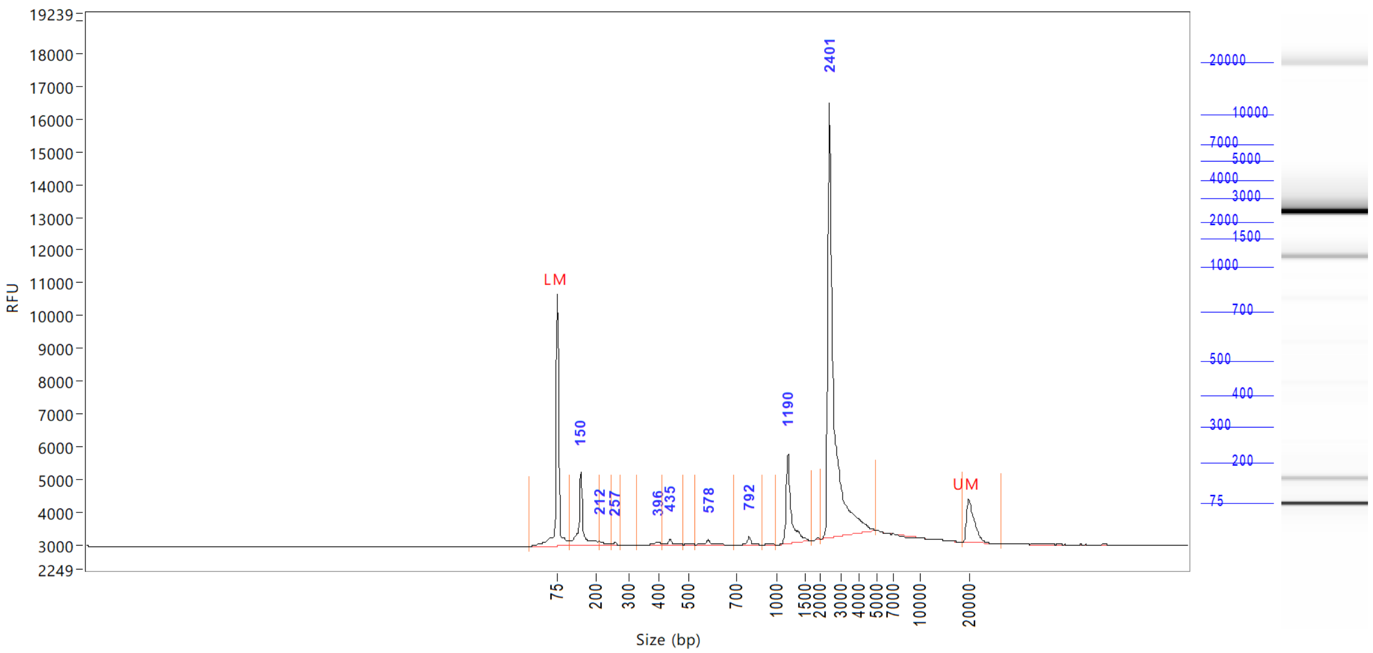

Peak	Size (bp)	Concentration (ng/uL)	From (bp)	To (bp)	Average size (bp)	CV%	RFU	Corrected peak area
1	75 (LM)	0.5000	0	114	70	21.60	7676	54.910
2	150	2.5489	114	212	151	12.47	2232	23.327
3	212	0.1408	212	248	224	4.42	109	1.288
4	257	0.0575	248	273	255	1.64	87	0.526
5	396	0.1857	323	411	384	5.06	84	1.700
6	435	0.2417	411	479	436	3.67	199	2.212
7	578	0.2434	524	692	581	5.48	172	2.228
8	792	0.1976	692	887	788	3.41	246	1.809
9	1190	2.5517	987	1731	1221	7.30	2754	23.352
10	2401	13.7316	2000	5000	2667	17.39	13309	125.667
11	20000 (UM)	0.1215	18781	26649	20508	4.45	1336	13.348

TIC:	19.8990	ng/uL
TIM:	43.8734	nmole/L
Total concentration:	20.0326	ng/uL

```
Sample peak width (sec): 5    Sample min peak height: 50    Sample baseline V to V?: Y    Sample baseline V to V points: 3
Sample filter: Binomial       Number of points for filter: 3  Sample start region (min): 0  Sample end region (min): 30
Marker peak width (sec): 5    Marker min peak height: 200   Marker baseline V to V?: Y    Marker baseline V to V points: 3
Lower marker selection: First peak > 500 RFU                Upper marker selection: Last peak > 200 RFU
Ladder size (bp)75, 200, 300, 400, 500, 700, 1000, 1500, 2000, 3000, 4000, 5000, 7000, 10000, 20000
Quantification using: Lower Marker          Final concentration (ng/uL): 0.5000          Dilution factor: 12.0
```

Sample: SampC5
Well location: C5
Created: Monday, September 20, 2021 8:00:01 PM

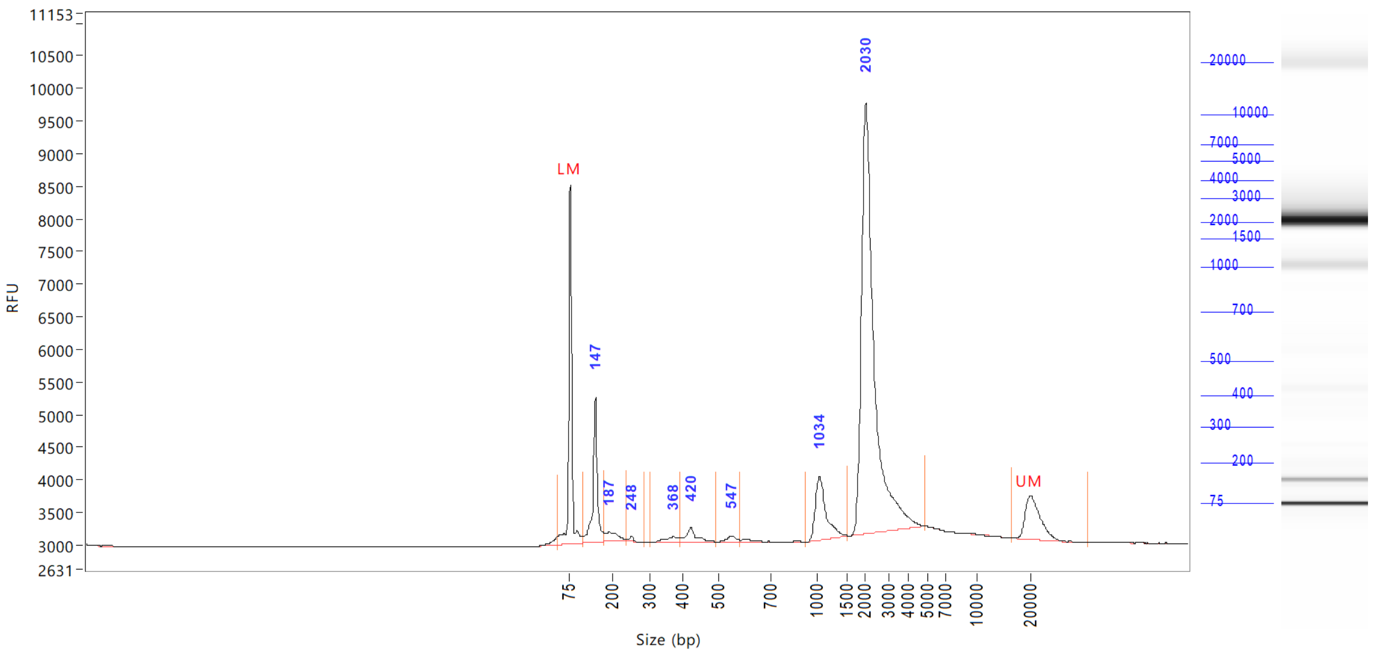

Peak	Size (bp)	Concentration (ng/uL)	From (bp)	To (bp)	Average size (bp)	CV%	RFU	Corrected peak area
1	75 (LM)	0.5000	39	110	72	12.62	5510	39.674
2	147	3.4106	110	173	143	7.42	2224	22.552
3	187	0.5914	173	235	195	7.52	136	3.910
4	248	0.0956	235	282	246	2.53	78	0.632
5	368	0.3658	301	390	358	5.84	86	2.419
6	420	0.6659	390	492	424	4.82	228	4.403
7	547	0.2519	492	583	545	3.53	100	1.666
8	1034	2.4706	926	1520	1083	9.42	980	16.337
9	2030	18.8112	1520	4862	2261	20.38	6615	124.387
10	20000 (UM)	0.1571	16464	30519	20636	7.38	672	12.467

TIC:	26.6631	ng/uL
TIM:	67.2974	nmole/L
Total concentration:	26.8816	ng/uL

```
Sample peak width (sec): 5    Sample min peak height: 50    Sample baseline V to V?: Y    Sample baseline V to V points: 3
Sample filter: Binomial       Number of points for filter: 3  Sample start region (min): 0  Sample end region (min): 30
Marker peak width (sec): 5    Marker min peak height: 200   Marker baseline V to V?: Y    Marker baseline V to V points: 3
Lower marker selection: First peak > 500 RFU                Upper marker selection: Last peak > 200 RFU
Ladder size (bp)75, 200, 300, 400, 500, 700, 1000, 1500, 2000, 3000, 4000, 5000, 7000, 10000, 20000
Quantification using: Lower Marker          Final concentration (ng/uL): 0.5000          Dilution factor: 12.0
```

Sample: SampD5
Well location: D5
Created: Monday, September 20, 2021 8:00:01 PM

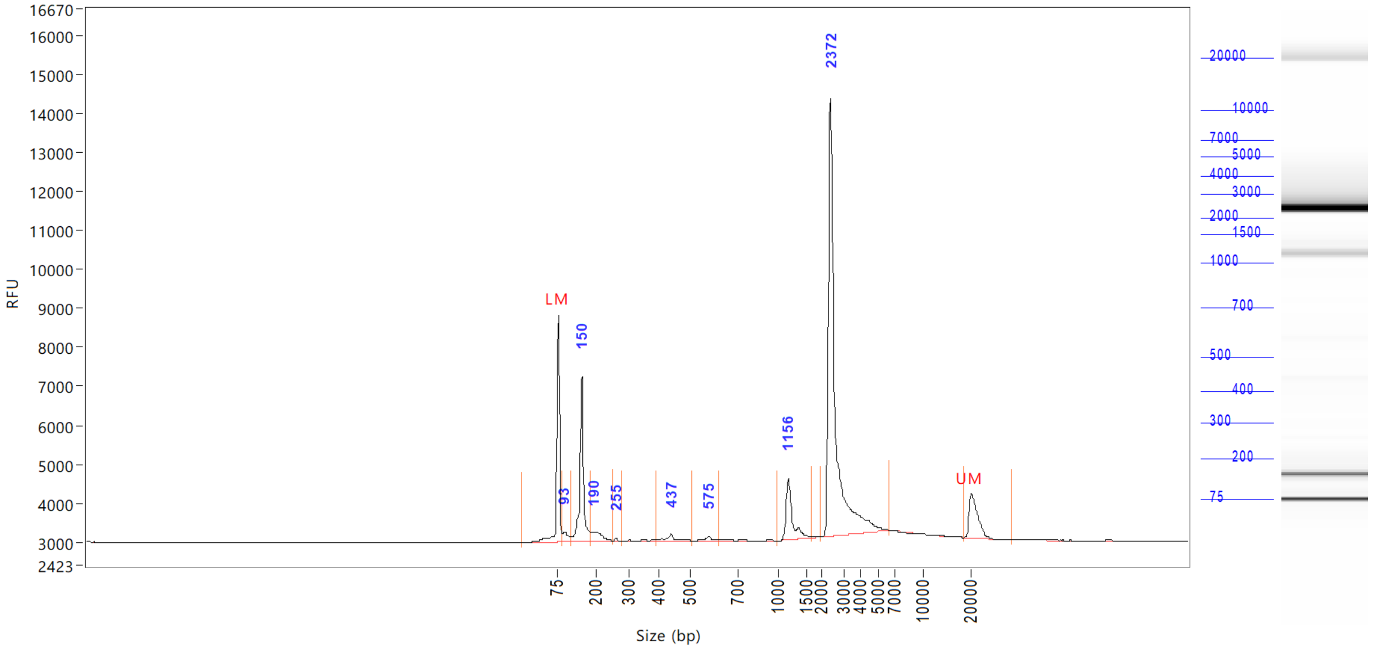

Peak	Size (bp)	Concentration (ng/uL)	From (bp)	To (bp)	Average size (bp)	CV%	RFU	Corrected peak area
1	75 (LM)	0.5000	0	86	68	21.75	5786	37.500
2	93	0.6158	86	118	99	9.60	262	3.849
3	150	5.8864	118	181	148	6.94	4202	36.790
4	190	0.8721	181	246	200	6.66	235	5.451
5	255	0.0749	246	275	254	1.54	86	0.468
6	437	0.4693	390	504	432	5.52	171	2.933
7	575	0.2461	504	618	570	3.65	122	1.538
8	1156	2.6243	991	1655	1201	8.24	1586	16.402
9	2372	18.7754	1942	6334	2662	21.67	11229	117.346
10	20000 (UM)	0.1685	18415	28342	20638	4.69	1159	12.640

TIC:	29.5643	ng/uL
TIM:	100.9397	nmole/L
Total concentration:	29.9266	ng/uL

```
Sample peak width (sec): 5    Sample min peak height: 50    Sample baseline V to V?: Y    Sample baseline V to V points: 3
Sample filter: Binomial       Number of points for filter: 3  Sample start region (min): 0  Sample end region (min): 30
Marker peak width (sec): 5    Marker min peak height: 200   Marker baseline V to V?: Y    Marker baseline V to V points: 3
Lower marker selection: First peak > 500 RFU                Upper marker selection: Last peak > 200 RFU
Ladder size (bp)75, 200, 300, 400, 500, 700, 1000, 1500, 2000, 3000, 4000, 5000, 7000, 10000, 20000
Quantification using: Lower Marker          Final concentration (ng/uL): 0.5000          Dilution factor: 12.0
```

Sample: SampE5
Well location: E5
Created: Monday, September 20, 2021 8:00:01 PM

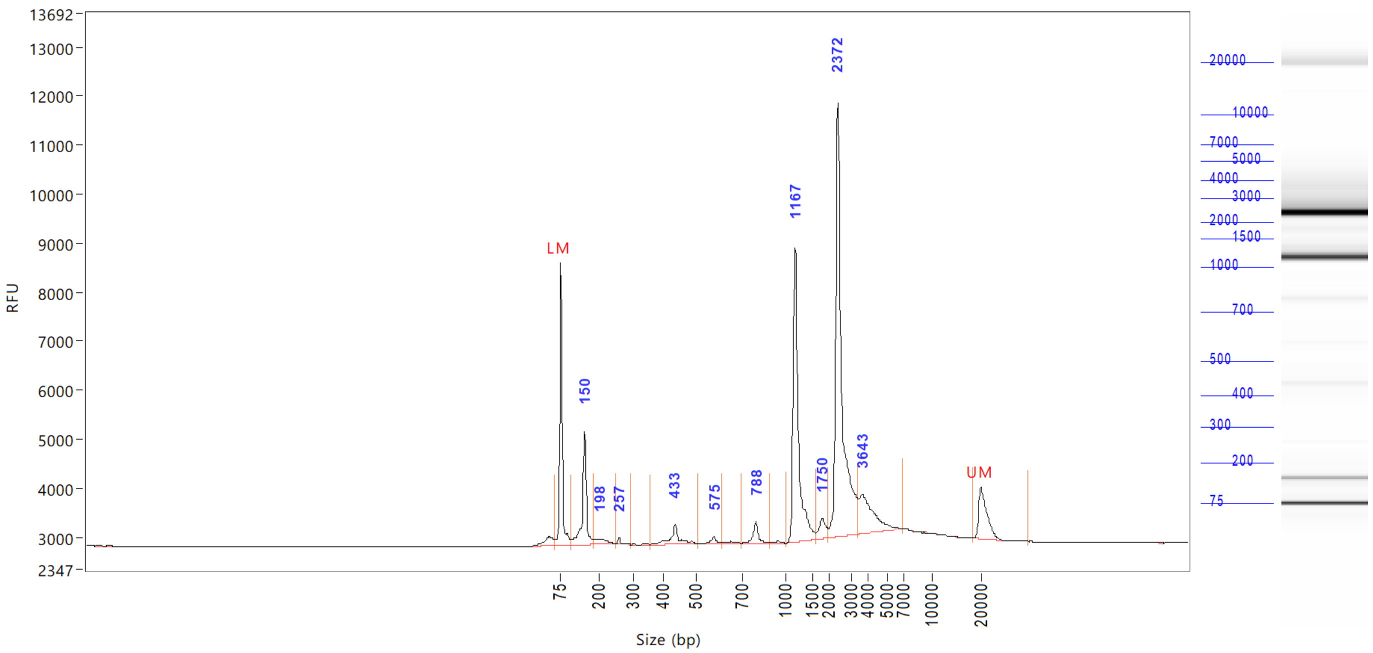

Peak	Size (bp)	Concentration (ng/uL)	From (bp)	To (bp)	Average size (bp)	CV%	RFU	Corrected peak area
1	75 (LM)	0.5000	56	108	75	9.61	5754	36.688
2	150	3.3461	108	179	146	8.42	2316	20.461
3	198	0.4766	179	246	201	7.13	112	2.914
4	257	0.1124	246	291	257	3.30	122	0.687
5	433	1.0634	354	506	430	6.48	408	6.502
6	575	0.2643	506	611	568	3.43	154	1.616
7	788	0.6499	692	883	782	3.65	453	3.974
8	1167	8.4266	1000	1559	1201	7.16	5998	51.526
9	1750	0.7850	1559	1981	1771	6.13	416	4.800
10	2372	14.4114	1981	3429	2515	11.80	8866	88.122
11	3643	2.8391	3429	6926	4059	13.20	793	17.360
12	20000 (UM)	0.1556	18415	29430	20573	5.04	1055	11.420

TIC:	32.3748	ng/uL
TIM:	71.3487	nmole/L
Total concentration:	32.4723	ng/uL

```
Sample peak width (sec): 5     Sample min peak height: 50    Sample baseline V to V?: Y    Sample baseline V to V points: 3
Sample filter: Binomial        Number of points for filter: 3  Sample start region (min): 0  Sample end region (min): 30
Marker peak width (sec): 5     Marker min peak height: 200   Marker baseline V to V?: Y    Marker baseline V to V points: 3
Lower marker selection: First peak > 500 RFU                 Upper marker selection: Last peak > 200 RFU
Ladder size (bp)75, 200, 300, 400, 500, 700, 1000, 1500, 2000, 3000, 4000, 5000, 7000, 10000, 20000
Quantification using: Lower Marker          Final concentration (ng/uL): 0.5000          Dilution factor: 12.0
```

Sample: SampF5
Well location: F5
Created: Monday, September 20, 2021 8:00:01 PM

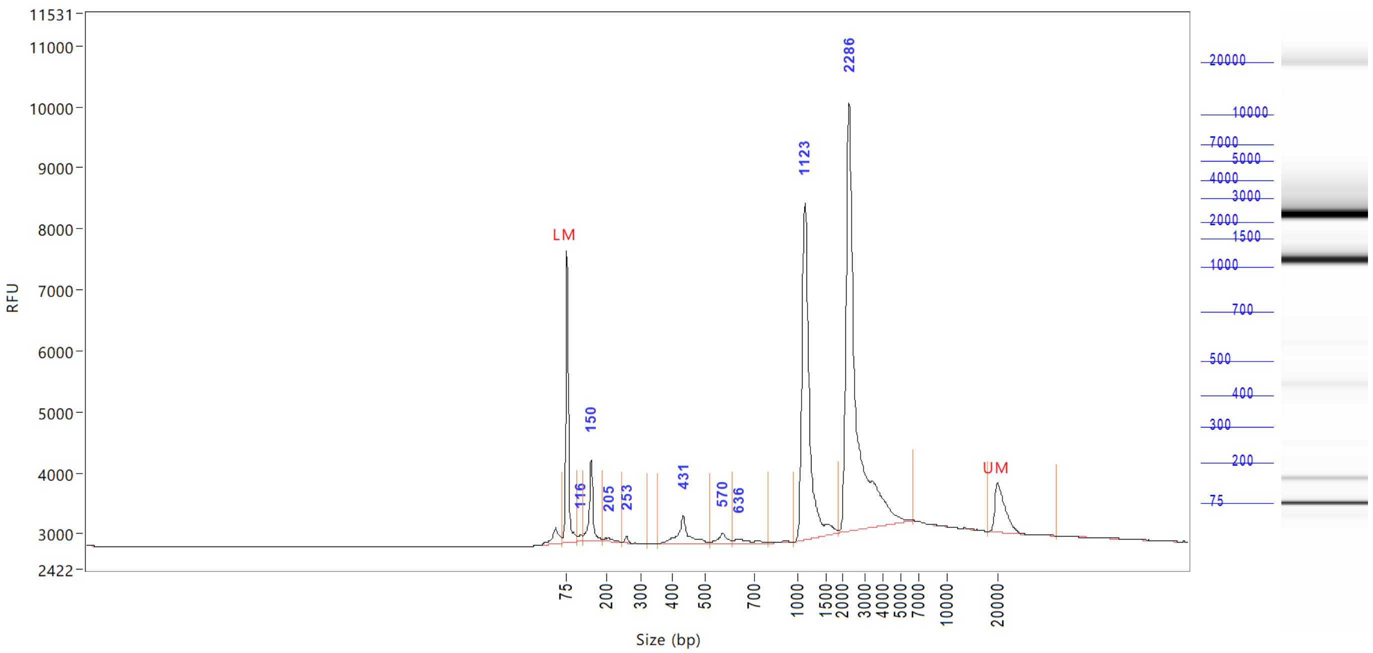

Peak	Size (bp)	Concentration (ng/uL)	From (bp)	To (bp)	Average size (bp)	CV%	RFU	Corrected peak area
1	75 (LM)	0.5000	62	108	75	8.63	4777	32.042
2	116	0.1807	108	124	116	4.08	103	0.965
3	150	2.2894	124	189	148	6.86	1325	12.226
4	205	0.1901	189	241	205	5.71	51	1.015
5	253	0.1601	241	319	260	6.65	107	0.855
6	431	1.7519	349	516	431	6.99	455	9.356
7	570	0.5038	516	611	567	3.81	161	2.691
8	636	0.3732	611	801	670	7.21	67	1.993
9	1123	12.2595	970	1885	1162	10.31	5508	65.470
10	2286	19.7991	1885	6482	2630	23.04	7028	105.734
11	20000 (UM)	0.1651	18171	31607	20805	6.71	815	10.580

TIC:	37.5080	ng/uL
TIM:	69.3462	nmole/L
Total concentration:	37.6558	ng/uL

Sample peak width (sec): 5 Sample min peak height: 50 Sample baseline V to V?: Y Sample baseline V to V points: 3
Sample filter: Binomial Number of points for filter: 3 Sample start region (min): 0 Sample end region (min): 30
Marker peak width (sec): 5 Marker min peak height: 200 Marker baseline V to V?: Y Marker baseline V to V points: 3
Lower marker selection: First peak > 500 RFU Upper marker selection: Last peak > 200 RFU
Ladder size (bp)75, 200, 300, 400, 500, 700, 1000, 1500, 2000, 3000, 4000, 5000, 7000, 10000, 20000
Quantification using: Lower Marker Final concentration (ng/uL): 0.5000 Dilution factor: 12.0

Sample: SampG5
Well location: G5
Created: Monday, September 20, 2021 8:00:01 PM

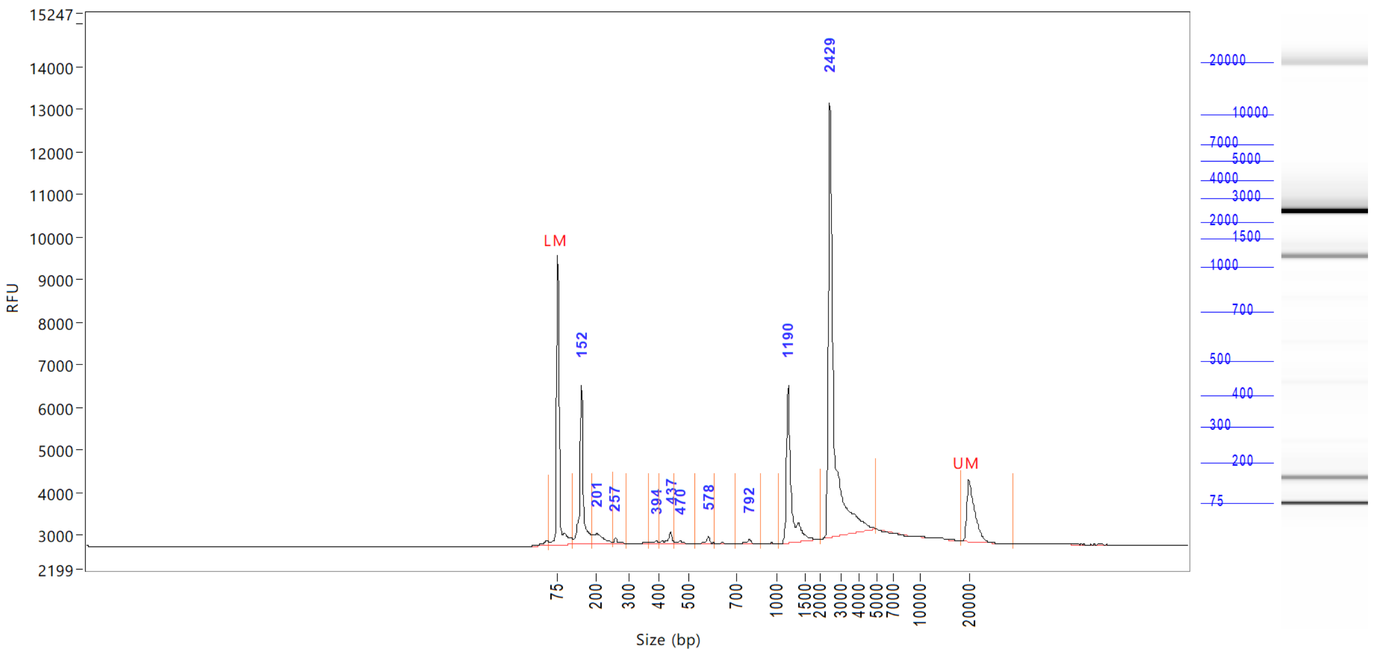

Peak	Size (bp)	Concentration (ng/uL)	From (bp)	To (bp)	Average size (bp)	CV%	RFU	Corrected peak area
1	75 (LM)	0.5000	47	124	76	14.05	6789	43.825
2	152	4.2636	124	187	151	7.06	3705	31.142
3	201	0.7107	187	250	208	7.25	219	5.191
4	257	0.1207	250	293	259	2.99	134	0.881
5	394	0.1195	368	401	386	2.55	60	0.873
6	437	0.3883	401	452	430	2.87	299	2.836
7	470	0.1441	452	519	470	2.53	69	1.053
8	578	0.2141	519	608	572	2.85	178	1.564
9	792	0.1674	700	879	780	4.09	128	1.223
10	1190	4.0229	1012	2000	1234	8.88	3704	29.383
11	2429	11.9086	2000	5000	2687	17.62	10215	86.982
12	20000 (UM)	0.1715	18293	29189	20648	5.12	1447	15.035

TIC:	22.0597	ng/uL
TIM:	68.9213	nmole/L
Total concentration:	22.2689	ng/uL

Sample peak width (sec): 5 Sample min peak height: 50 Sample baseline V to V?: Y Sample baseline V to V points: 3
Sample filter: Binomial Number of points for filter: 3 Sample start region (min): 0 Sample end region (min): 30
Marker peak width (sec): 5 Marker min peak height: 200 Marker baseline V to V?: Y Marker baseline V to V points: 3
Lower marker selection: First peak > 500 RFU Upper marker selection: Last peak > 200 RFU
Ladder size (bp)75, 200, 300, 400, 500, 700, 1000, 1500, 2000, 3000, 4000, 5000, 7000, 10000, 20000
Quantification using: Lower Marker Final concentration (ng/uL): 0.5000 Dilution factor: 12.0

Sample: SampH5
Well location: H5
Created: Monday, September 20, 2021 8:00:01 PM

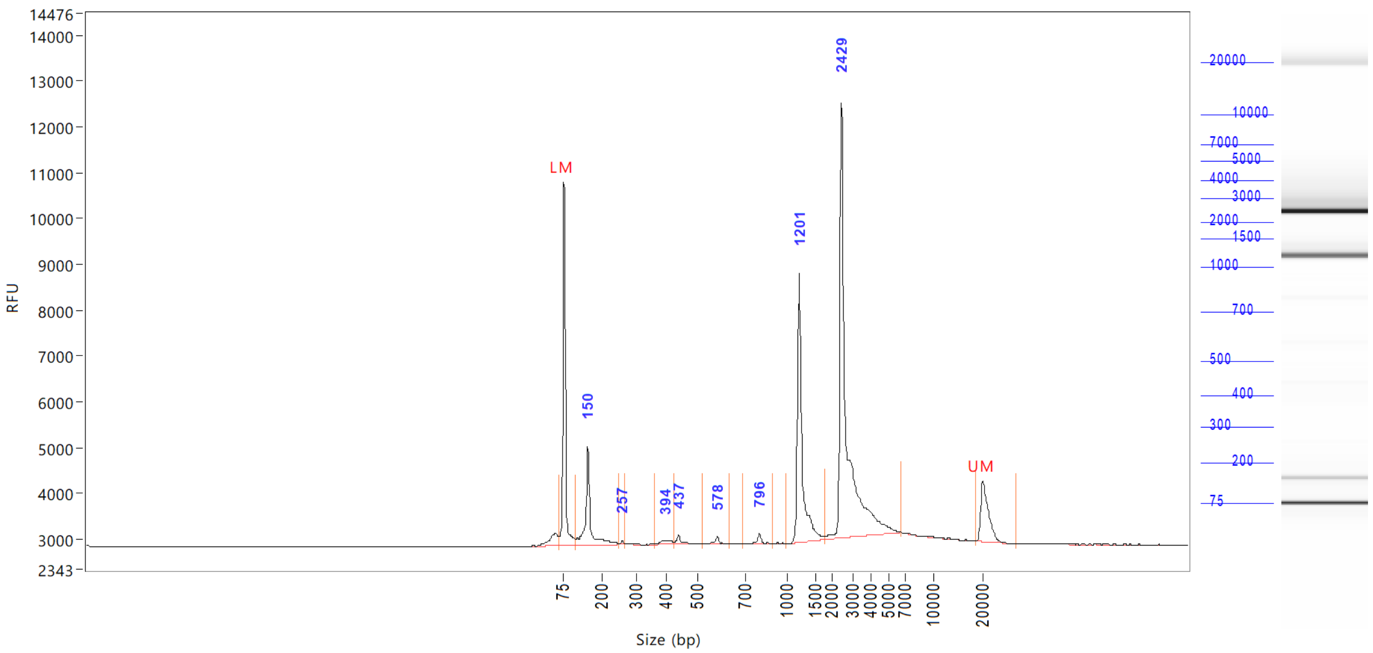

Peak	Size (bp)	Concentration (ng/uL)	From (bp)	To (bp)	Average size (bp)	CV%	RFU	Corrected peak area
1	75 (LM)	0.5000	60	110	75	9.13	7943	50.675
2	150	2.7790	110	248	155	15.63	2163	23.471
3	257	0.0592	248	266	255	1.25	94	0.500
4	394	0.2307	360	422	395	3.91	90	1.948
5	437	0.2537	422	516	445	4.43	214	2.142
6	578	0.1847	516	631	576	3.62	172	1.560
7	796	0.2272	685	892	790	3.83	244	1.919
8	1201	5.3566	991	1789	1236	8.37	5872	45.241
9	2429	11.3616	1789	6482	2809	22.49	9509	95.959
10	20000 (UM)	0.1293	18659	26770	20529	4.50	1307	13.107

TIC:	20.4527	ng/uL
TIM:	46.5418	nmole/L
Total concentration:	20.6007	ng/uL

```
Sample peak width (sec): 5    Sample min peak height: 50    Sample baseline V to V?: Y    Sample baseline V to V points: 3
Sample filter: Binomial       Number of points for filter: 3  Sample start region (min): 0  Sample end region (min): 30
Marker peak width (sec): 5    Marker min peak height: 200   Marker baseline V to V?: Y    Marker baseline V to V points: 3
Lower marker selection: First peak > 500 RFU                Upper marker selection: Last peak > 200 RFU
Ladder size (bp)75, 200, 300, 400, 500, 700, 1000, 1500, 2000, 3000, 4000, 5000, 7000, 10000, 20000
Quantification using: Lower Marker          Final concentration (ng/uL): 0.5000          Dilution factor: 12.0
```

Sample: SampA6
Well location: A6
Created: Monday, September 20, 2021 8:00:01 PM

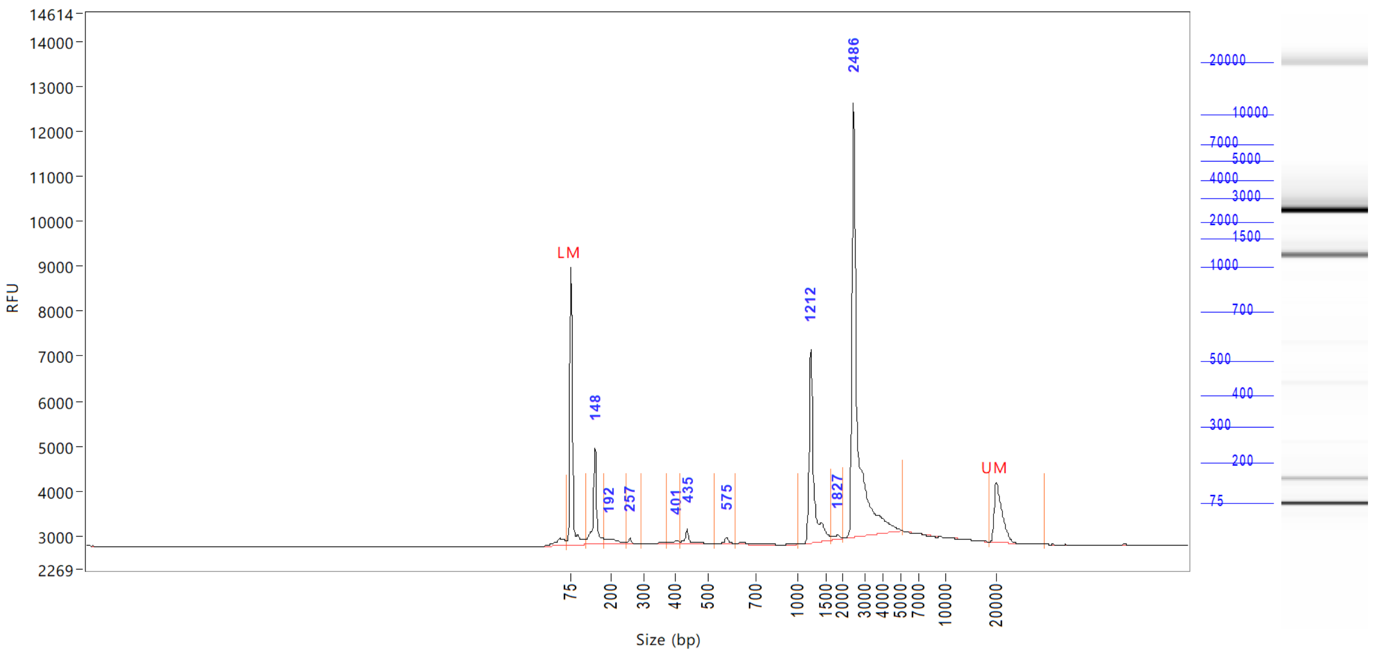

Peak	Size (bp)	Concentration (ng/uL)	From (bp)	To (bp)	Average size (bp)	CV%	RFU	Corrected peak area
1	75 (LM)	0.5000	62	122	76	12.58	6163	38.014
2	148	2.7628	122	179	147	6.68	2136	17.504
3	192	0.4724	179	248	205	8.82	100	2.993
4	257	0.1164	248	289	257	3.01	119	0.737
5	401	0.1824	370	412	393	3.17	58	1.156
6	435	0.5150	412	527	441	4.37	322	3.263
7	575	0.2211	527	613	573	2.99	158	1.401
8	1212	5.1120	996	1635	1242	7.13	4295	32.388
9	1827	0.1604	1635	2001	1789	5.37	88	1.016
10	2486	13.1442	2001	5075	2736	17.58	9659	83.277
11	20000 (UM)	0.1749	18659	29672	20548	4.69	1335	13.295

TIC:	22.6868	ng/uL
TIM:	53.5731	nmole/L
Total concentration:	22.9810	ng/uL

Sample peak width (sec): 5   Sample min peak height: 50   Sample baseline V to V?: Y   Sample baseline V to V points: 3
Sample filter: Binomial   Number of points for filter: 3 Sample start region (min): 0 Sample end region (min): 30
Marker peak width (sec): 5   Marker min peak height: 200   Marker baseline V to V?: Y   Marker baseline V to V points: 3
Lower marker selection: First peak > 500 RFU   Upper marker selection: Last peak > 200 RFU
Ladder size (bp)75, 200, 300, 400, 500, 700, 1000, 1500, 2000, 3000, 4000, 5000, 7000, 10000, 20000
Quantification using: Lower Marker   Final concentration (ng/uL): 0.5000   Dilution factor: 12.0

Sample: SampB6
Well location: B6
Created: Monday, September 20, 2021 8:00:01 PM

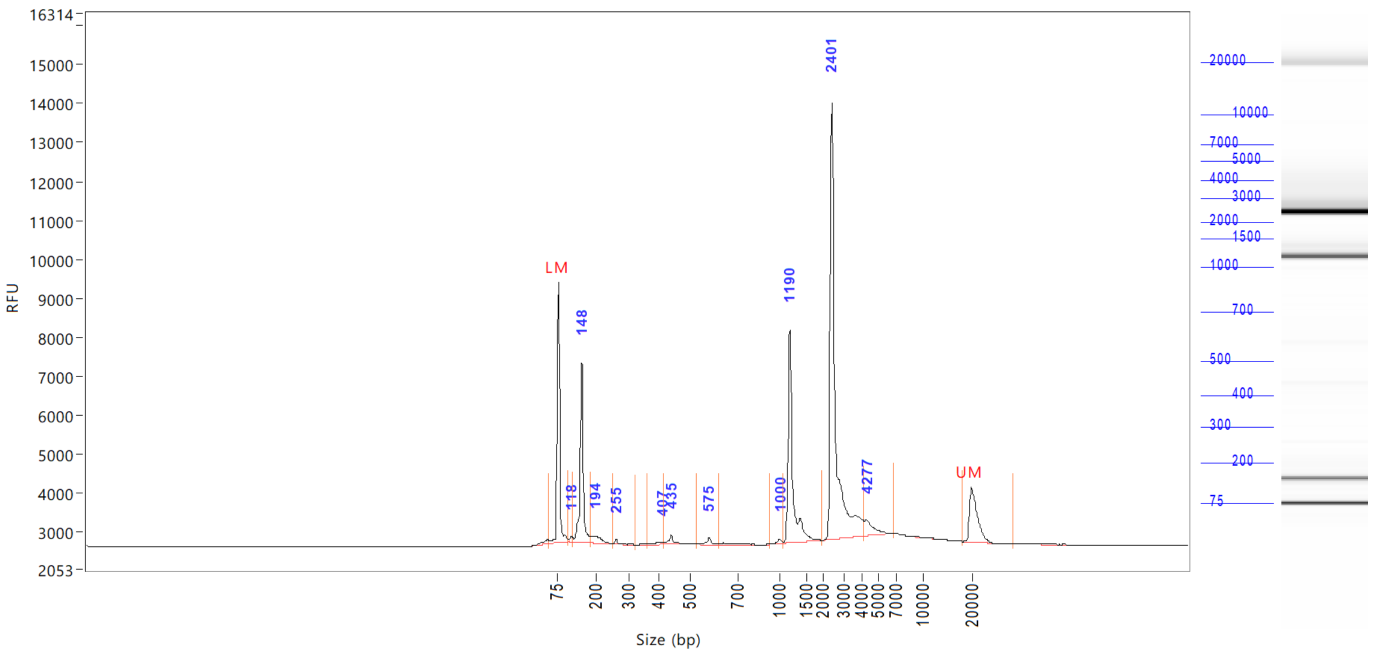

Peak	Size (bp)	Concentration (ng/uL)	From (bp)	To (bp)	Average size (bp)	CV%	RFU	Corrected peak area
1	75 (LM)	0.5000	48	108	74	8.54	6666	39.659
2	118	0.1574	108	124	116	3.78	133	1.040
3	148	5.2930	124	179	148	5.63	4587	34.986
4	194	0.6583	179	246	199	7.00	191	4.351
5	255	0.1307	246	315	263	6.38	134	0.864
6	407	0.1597	358	414	393	3.65	56	1.056
7	435	0.4138	414	524	441	4.69	244	2.735
8	575	0.2580	524	618	573	3.18	180	1.705
9	1000	0.1271	935	1045	991	1.97	124	0.840
10	1190	6.2019	1045	1981	1226	8.60	5450	40.993
11	2401	13.7762	1981	4139	2634	16.53	11214	91.058
12	4277	0.8452	4139	6778	4571	9.05	400	5.586
13	20000 (UM)	0.1817	18049	28342	20523	4.67	1406	14.411

TIC:	28.0213	ng/uL
TIM:	87.6670	nmole/L
Total concentration:	28.1947	ng/uL

```
Sample peak width (sec): 5     Sample min peak height: 50     Sample baseline V to V?: Y     Sample baseline V to V points: 3
Sample filter: Binomial        Number of points for filter: 3  Sample start region (min): 0  Sample end region (min): 30
Marker peak width (sec): 5     Marker min peak height: 200    Marker baseline V to V?: Y     Marker baseline V to V points: 3
Lower marker selection: First peak > 500 RFU                  Upper marker selection: Last peak > 200 RFU
Ladder size (bp)75, 200, 300, 400, 500, 700, 1000, 1500, 2000, 3000, 4000, 5000, 7000, 10000, 20000
Quantification using: Lower Marker          Final concentration (ng/uL): 0.5000          Dilution factor: 12.0
```

Sample: SampC6
Well location: C6
Created: Monday, September 20, 2021 8:00:01 PM

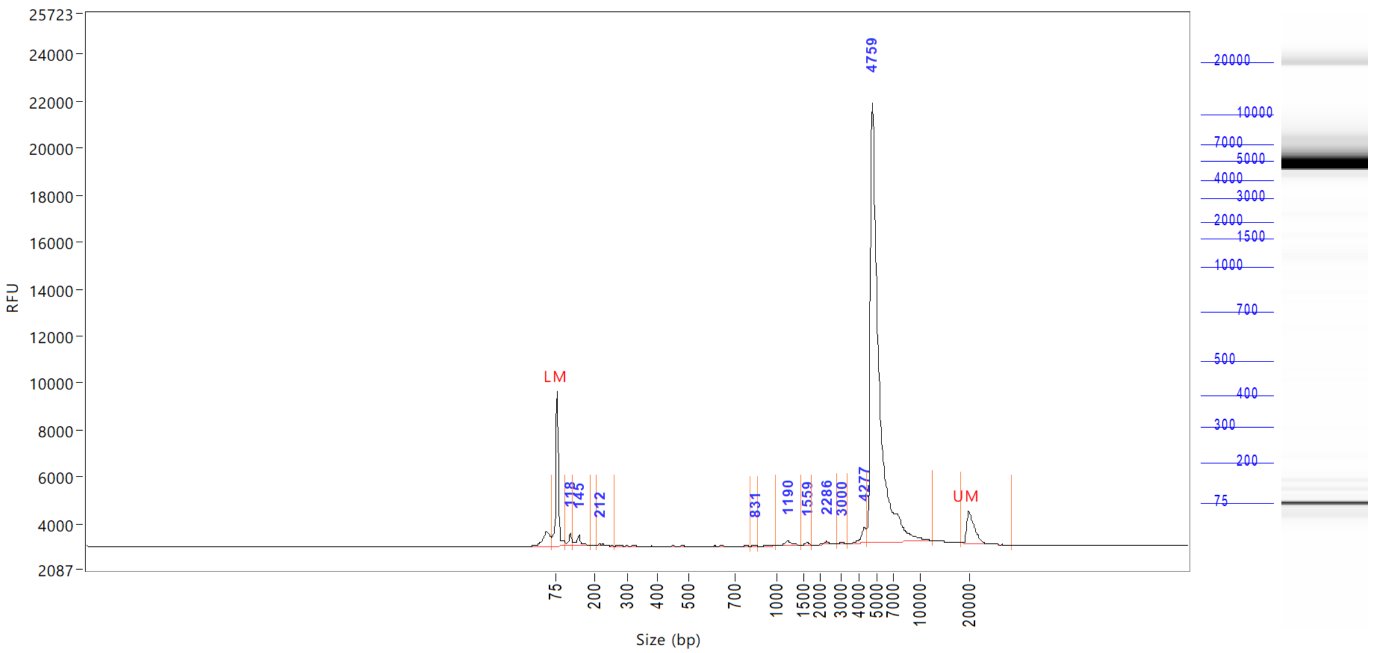

Peak	Size<br>(bp)	Concentration<br>(ng/uL)	From<br>(bp)	To<br>(bp)	Average size<br>(bp)	CV%	RFU	Corrected peak area
1	75 (LM)	0.5000	58	105	74	9.15	6555	42.231
2	118	0.5761	105	128	116	4.76	499	4.055
3	145	0.5929	128	183	144	6.91	432	4.173
4	212	0.1134	203	257	214	3.67	87	0.798
5	831	0.0503	805	861	828	1.24	69	0.354
6	1190	0.3428	996	1445	1197	6.77	181	2.413
7	1559	0.0926	1445	1731	1553	3.31	96	0.652
8	2286	0.1783	1731	2800	2330	8.83	122	1.255
9	3000	0.0585	2800	3358	2975	3.49	51	0.411
10	4277	0.7425	3358	4449	4213	4.29	662	5.226
11	4759	32.8184	4449	12684	5322	18.41	18726	230.990
12	20000 (UM)	0.1609	18293	28947	20559	4.69	1345	13.590

TIC:	35.5659	ng/uL
TIM:	27.0666	nmole/L
Total concentration:	35.8296	ng/uL

```
Sample peak width (sec): 5    Sample min peak height: 50    Sample baseline V to V?: Y    Sample baseline V to V points: 3
Sample filter: Binomial       Number of points for filter: 3  Sample start region (min): 0  Sample end region (min): 30
Marker peak width (sec): 5    Marker min peak height: 200   Marker baseline V to V?: Y    Marker baseline V to V points: 3
Lower marker selection: First peak > 500 RFU                Upper marker selection: Last peak > 200 RFU
Ladder size (bp)75, 200, 300, 400, 500, 700, 1000, 1500, 2000, 3000, 4000, 5000, 7000, 10000, 20000
Quantification using: Lower Marker          Final concentration (ng/uL): 0.5000          Dilution factor: 12.0
```

Sample: SampD6
Well location: D6
Created: Monday, September 20, 2021 8:00:01 PM

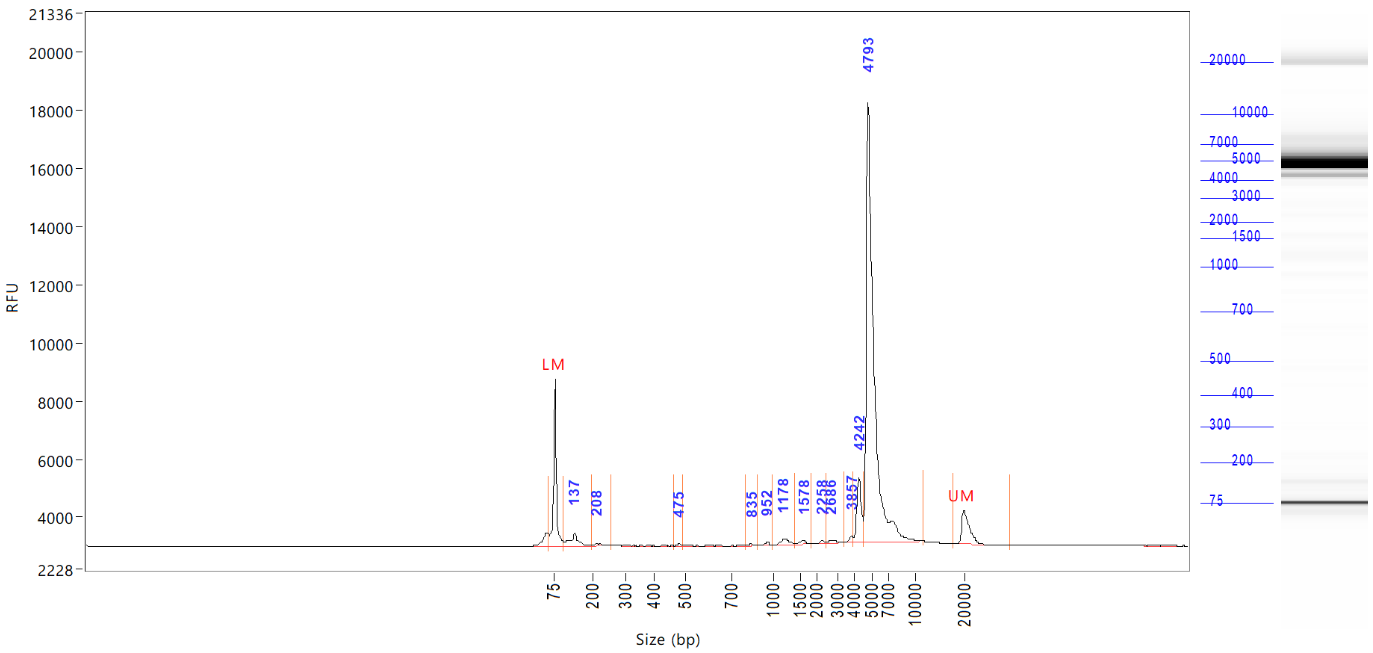

Peak	Size (bp)	Concentration (ng/uL)	From (bp)	To (bp)	Average size (bp)	CV%	RFU	Corrected peak area
1	75 (LM)	0.5000	58	105	74	9.49	5760	39.758
2	137	1.3073	105	196	137	14.44	461	8.663
3	208	0.1829	196	253	212	4.29	102	1.212
4	475	0.0591	460	489	474	1.14	69	0.392
5	835	0.0604	792	883	827	1.68	69	0.400
6	952	0.1291	883	987	942	1.67	125	0.856
7	1178	0.4424	987	1400	1202	6.68	190	2.931
8	1578	0.1884	1400	1846	1531	4.70	137	1.249
9	2258	0.1192	1846	2458	2214	5.49	111	0.790
10	2686	0.1836	2458	3429	2768	6.74	99	1.217
11	3857	0.1863	3429	3964	3791	2.99	206	1.234
12	4242	2.2351	3964	4483	4236	2.87	2228	14.810
13	4793	25.2962	4483	11586	5221	16.10	15129	167.619
14	20000 (UM)	0.1501	17683	29672	20622	4.98	1154	11.938

TIC:	30.3901	ng/uL
TIM:	27.5823	nmole/L
Total concentration:	30.5580	ng/uL

Sample peak width (sec): 5 Sample min peak height: 50 Sample baseline V to V?: Y Sample baseline V to V points: 3
Sample filter: Binomial Number of points for filter: 3 Sample start region (min): 0 Sample end region (min): 30
Marker peak width (sec): 5 Marker min peak height: 200 Marker baseline V to V?: Y Marker baseline V to V points: 3
Lower marker selection: First peak > 500 RFU Upper marker selection: Last peak > 200 RFU
Ladder size (bp)75, 200, 300, 400, 500, 700, 1000, 1500, 2000, 3000, 4000, 5000, 7000, 10000, 20000
Quantification using: Lower Marker Final concentration (ng/uL): 0.5000 Dilution factor: 12.0

Sample: SampE6
Well location: E6
Created: Monday, September 20, 2021 8:00:01 PM

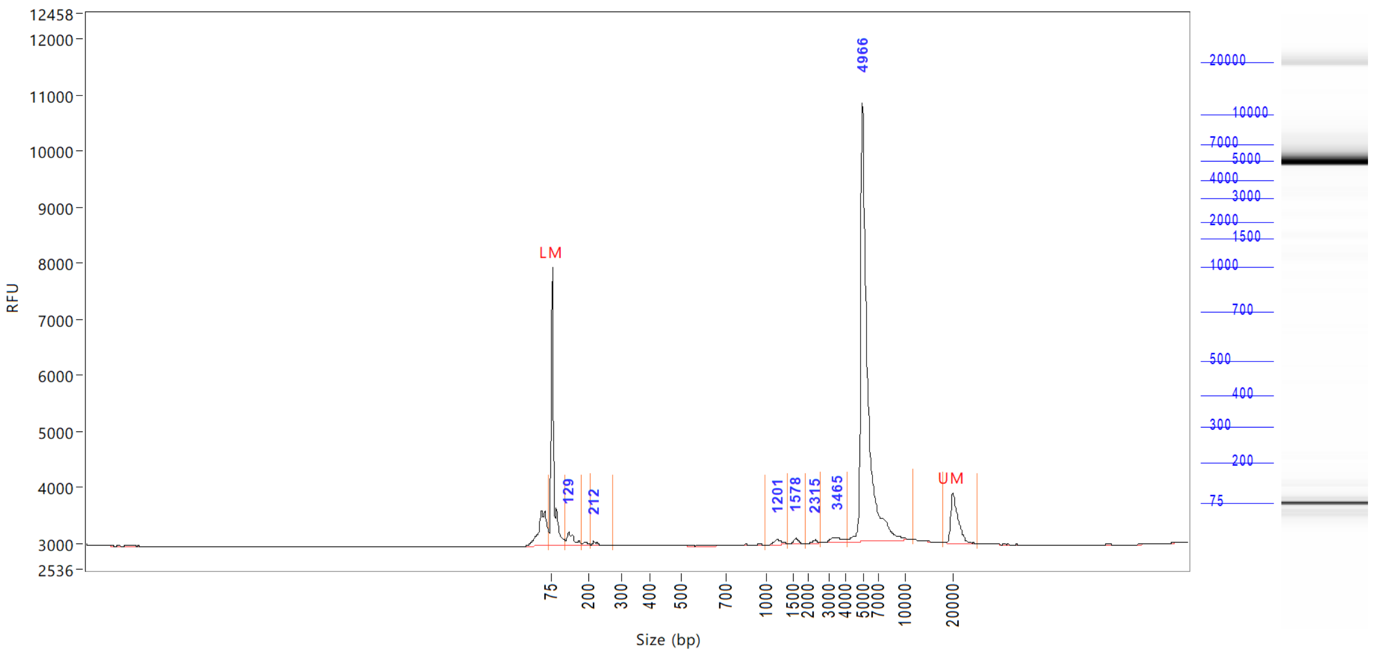

Peak	Size (bp)	Concentration (ng/uL)	From (bp)	To (bp)	Average size (bp)	CV%	RFU	Corrected peak area
1	75 (LM)	0.5000	66	118	77	11.92	4957	33.789
2	129	0.6636	118	173	138	9.71	251	3.737
3	212	0.0960	201	271	217	6.06	59	0.541
4	1201	0.2445	991	1411	1207	6.55	102	1.377
5	1578	0.1388	1411	1923	1575	4.69	99	0.781
6	2315	0.0750	1923	2629	2289	5.61	59	0.422
7	3465	0.3276	2629	4070	3422	9.96	94	1.845
8	4966	14.3610	4070	11708	5450	16.02	7840	80.874
9	20000 (UM)	0.1301	17805	25078	20487	4.43	898	8.791

TIC:	15.9065	ng/uL
TIM:	13.6574	nmole/L
Total concentration:	16.2929	ng/uL

```
Sample peak width (sec): 5    Sample min peak height: 50    Sample baseline V to V?: Y    Sample baseline V to V points: 3
Sample filter: Binomial       Number of points for filter: 3  Sample start region (min): 0  Sample end region (min): 30
Marker peak width (sec): 5    Marker min peak height: 200   Marker baseline V to V?: Y    Marker baseline V to V points: 3
Lower marker selection: First peak > 500 RFU                Upper marker selection: Last peak > 200 RFU
Ladder size (bp)75, 200, 300, 400, 500, 700, 1000, 1500, 2000, 3000, 4000, 5000, 7000, 10000, 20000
Quantification using: Lower Marker          Final concentration (ng/uL): 0.5000          Dilution factor: 12.0
```

Sample: SampF6
Well location: F6
Created: Monday, September 20, 2021 8:00:01 PM

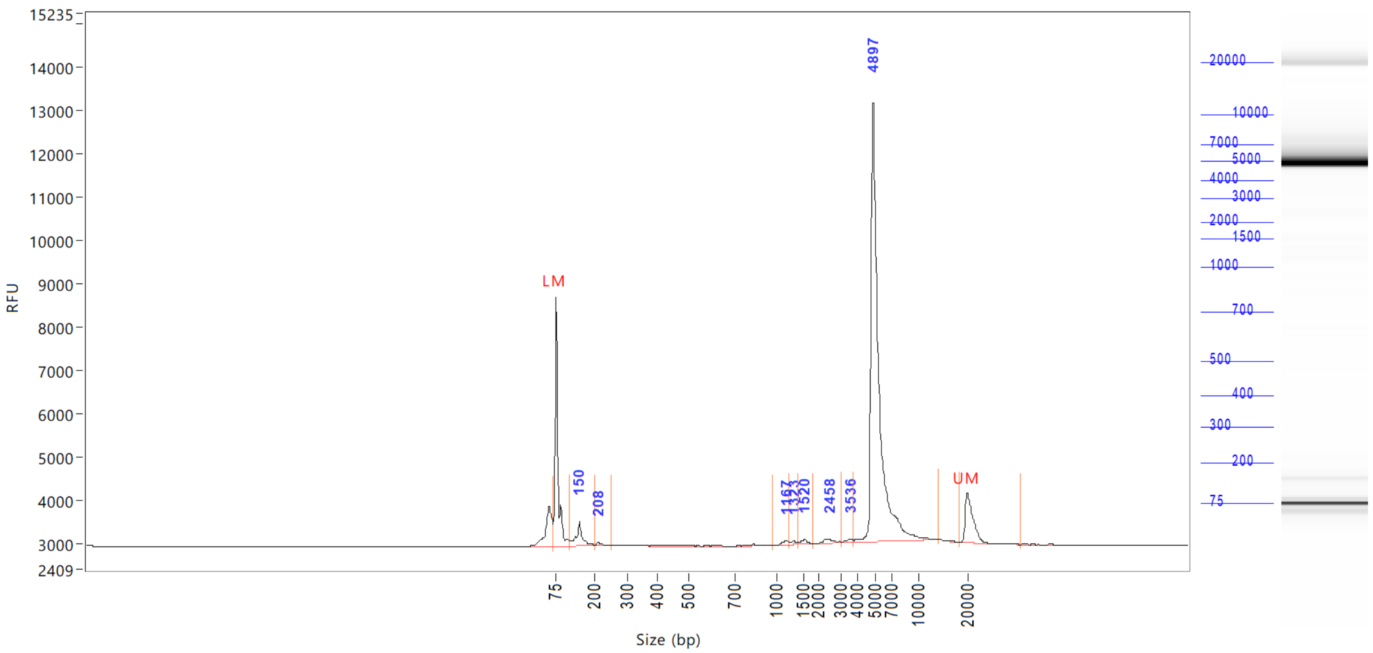

Peak	Size (bp)	Concentration (ng/uL)	From (bp)	To (bp)	Average size (bp)	CV%	RFU	Corrected peak area
1	75 (LM)	0.5000	66	118	76	12.33	5742	41.601
2	150	1.1419	118	200	147	10.84	573	7.918
3	208	0.1369	200	251	213	4.72	83	0.949
4	1167	0.1618	974	1245	1143	5.07	105	1.122
5	1323	0.0903	1245	1400	1315	3.22	77	0.626
6	1520	0.1465	1400	1789	1510	4.58	97	1.016
7	2458	0.2171	1789	3072	2447	11.17	88	1.506
8	3536	0.1106	3072	3786	3472	5.51	66	0.767
9	4897	16.4425	3786	13903	5400	18.81	10139	114.005
10	20000 (UM)	0.1420	18537	30760	20571	5.57	1144	11.812

TIC:	18.4476	ng/uL
TIM:	19.5407	nmole/L
Total concentration:	18.7307	ng/uL

```
Sample peak width (sec): 5    Sample min peak height: 50    Sample baseline V to V?: Y    Sample baseline V to V points: 3
Sample filter: Binomial       Number of points for filter: 3  Sample start region (min): 0  Sample end region (min): 30
Marker peak width (sec): 5    Marker min peak height: 200   Marker baseline V to V?: Y    Marker baseline V to V points: 3
Lower marker selection: First peak > 500 RFU                Upper marker selection: Last peak > 200 RFU
Ladder size (bp)75, 200, 300, 400, 500, 700, 1000, 1500, 2000, 3000, 4000, 5000, 7000, 10000, 20000
Quantification using: Lower Marker          Final concentration (ng/uL): 0.5000          Dilution factor: 12.0
```

Sample: SampG6
Well location: G6
Created: Monday, September 20, 2021 8:00:01 PM

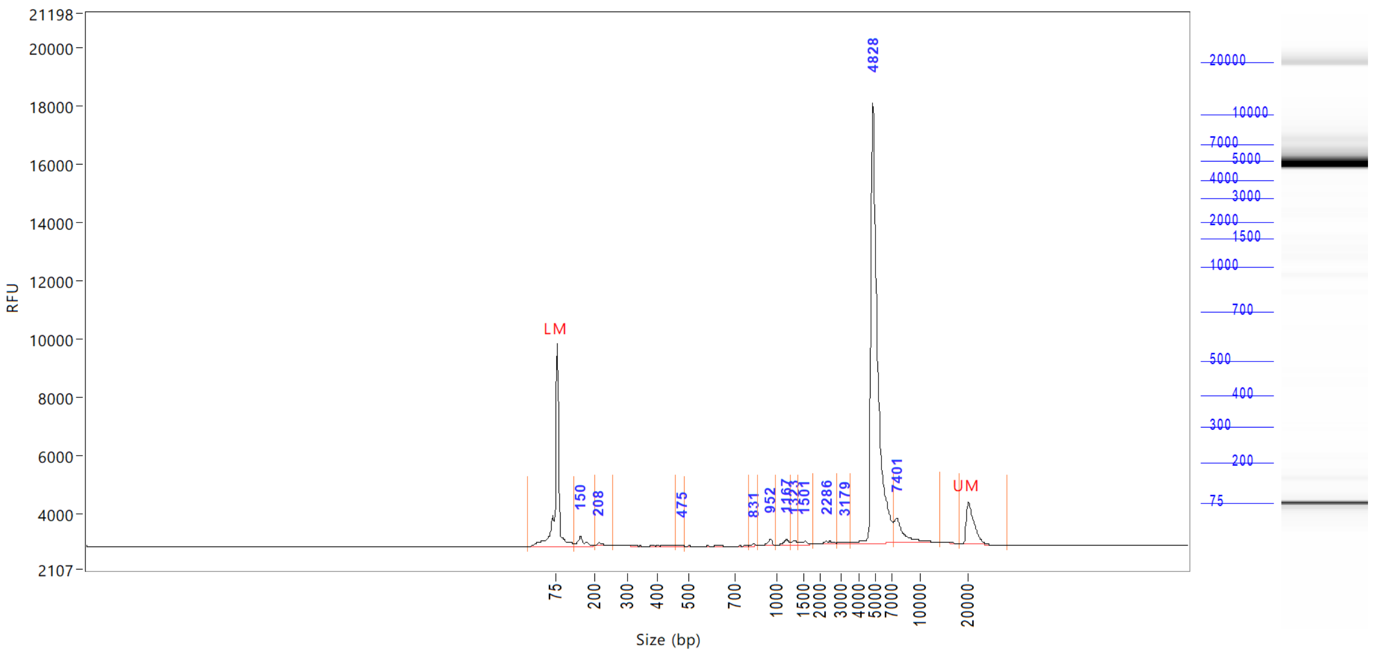

Peak	Size (bp)	Concentration (ng/uL)	From (bp)	To (bp)	Average size (bp)	CV%	RFU	Corrected peak area
1	75 (LM)	0.5000	0	131	69	28.07	6969	60.923
2	150	0.5143	131	196	155	9.63	347	5.222
3	208	0.1418	196	253	213	5.00	120	1.440
4	475	0.0364	458	487	472	1.24	54	0.369
5	831	0.0413	792	857	824	1.28	82	0.420
6	952	0.1690	857	996	942	1.94	228	1.716
7	1167	0.2017	996	1256	1153	5.05	196	2.049
8	1323	0.1272	1256	1400	1322	2.96	161	1.291
9	1501	0.1205	1400	1789	1497	4.53	110	1.224
10	2286	0.1454	1789	2772	2350	8.73	101	1.476
11	3179	0.0925	2772	3500	3125	6.60	63	0.939
12	4828	14.5981	3500	7068	5093	10.34	15147	148.227
13	7401	1.1715	7068	14147	8132	13.66	831	11.896
14	20000 (UM)	0.1206	18171	28100	20600	4.47	1448	14.696

TIC:	17.3596	ng/uL
TIM:	12.7376	nmole/L
Total concentration:	17.4750	ng/uL

```
Sample peak width (sec): 5     Sample min peak height: 50     Sample baseline V to V?: Y     Sample baseline V to V points: 3
Sample filter: Binomial        Number of points for filter: 3  Sample start region (min): 0  Sample end region (min): 30
Marker peak width (sec): 5     Marker min peak height: 200    Marker baseline V to V?: Y     Marker baseline V to V points: 3
Lower marker selection: First peak > 500 RFU                  Upper marker selection: Last peak > 200 RFU
Ladder size (bp)75, 200, 300, 400, 500, 700, 1000, 1500, 2000, 3000, 4000, 5000, 7000, 10000, 20000
Quantification using: Lower Marker          Final concentration (ng/uL): 0.5000          Dilution factor: 12.0
```

Sample: SampH6
Well location: H6
Created: Monday, September 20, 2021 8:00:01 PM

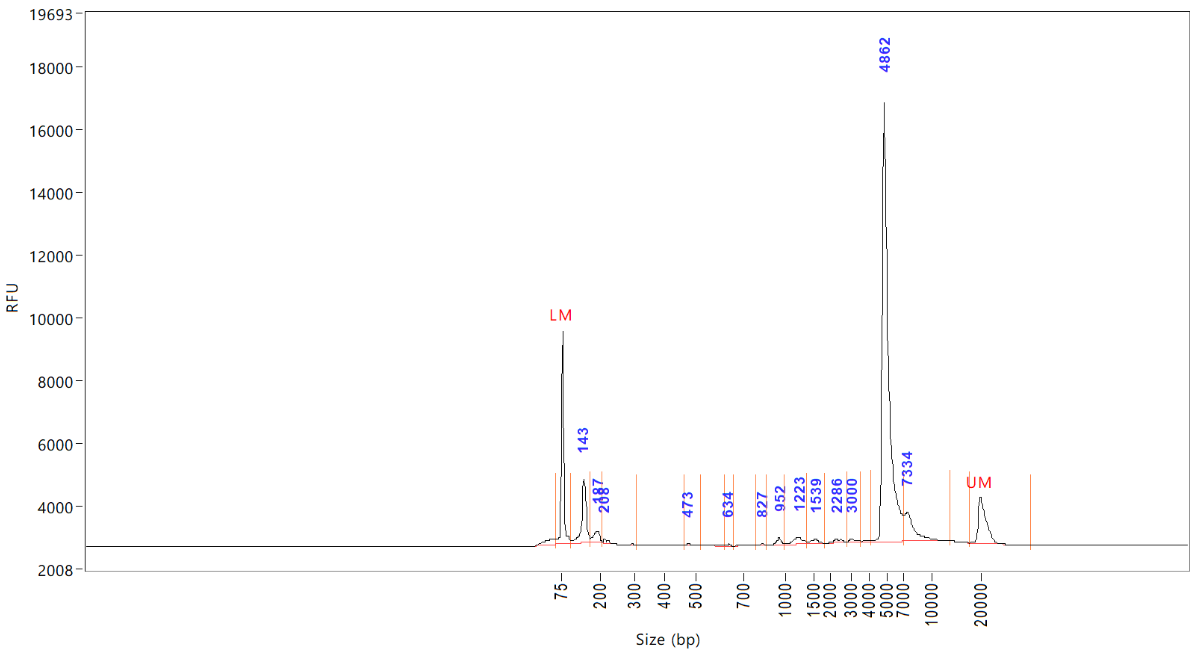

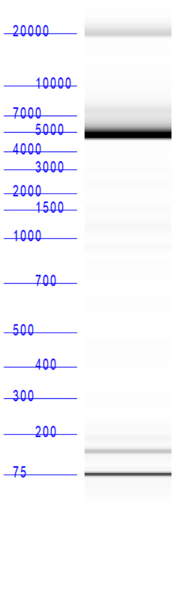

Peak	Size (bp)	Concentration (ng/uL)	From (bp)	To (bp)	Average size (bp)	CV%	RFU	Corrected peak area
1	75 (LM)	0.5000	56	103	74	7.69	6762	41.422
2	143	3.0525	103	164	140	7.62	2001	21.073
3	187	0.7503	164	200	181	4.55	366	5.180
4	208	0.2313	200	303	222	11.97	135	1.597
5	473	0.0725	462	514	475	2.07	72	0.501
6	634	0.0430	618	657	631	0.96	52	0.297
7	827	0.0635	788	853	820	1.41	83	0.438
8	952	0.2680	853	991	942	2.29	227	1.850
9	1223	0.5358	991	1389	1199	7.58	222	3.699
10	1539	0.2358	1389	1846	1516	5.27	137	1.628
11	2286	0.1977	1846	2772	2307	7.83	116	1.365
12	3000	0.1196	2772	3572	3081	5.61	89	0.826
13	4862	18.4550	4139	7000	5084	9.78	13980	127.407
14	7334	2.0351	7000	13903	7970	13.05	935	14.049
15	20000 (UM)	0.1824	17683	30156	20473	4.90	1480	15.108

TIC:	26.0601	ng/uL
TIM:	52.9099	nmole/L
Total concentration:	26.1653	ng/uL

Sample peak width (sec): 5   Sample min peak height: 50   Sample baseline V to V?: Y   Sample baseline V to V points: 3
Sample filter: Binomial   Number of points for filter: 3 Sample start region (min): 0 Sample end region (min): 30
Marker peak width (sec): 5   Marker min peak height: 200   Marker baseline V to V?: Y   Marker baseline V to V points: 3
Lower marker selection: First peak > 500 RFU   Upper marker selection: Last peak > 200 RFU
Ladder size (bp)75, 200, 300, 400, 500, 700, 1000, 1500, 2000, 3000, 4000, 5000, 7000, 10000, 20000
Quantification using: Lower Marker   Final concentration (ng/uL): 0.5000   Dilution factor: 12.0

Sample: SampA7
Well location: A7
Created: Monday, September 20, 2021 8:00:01 PM

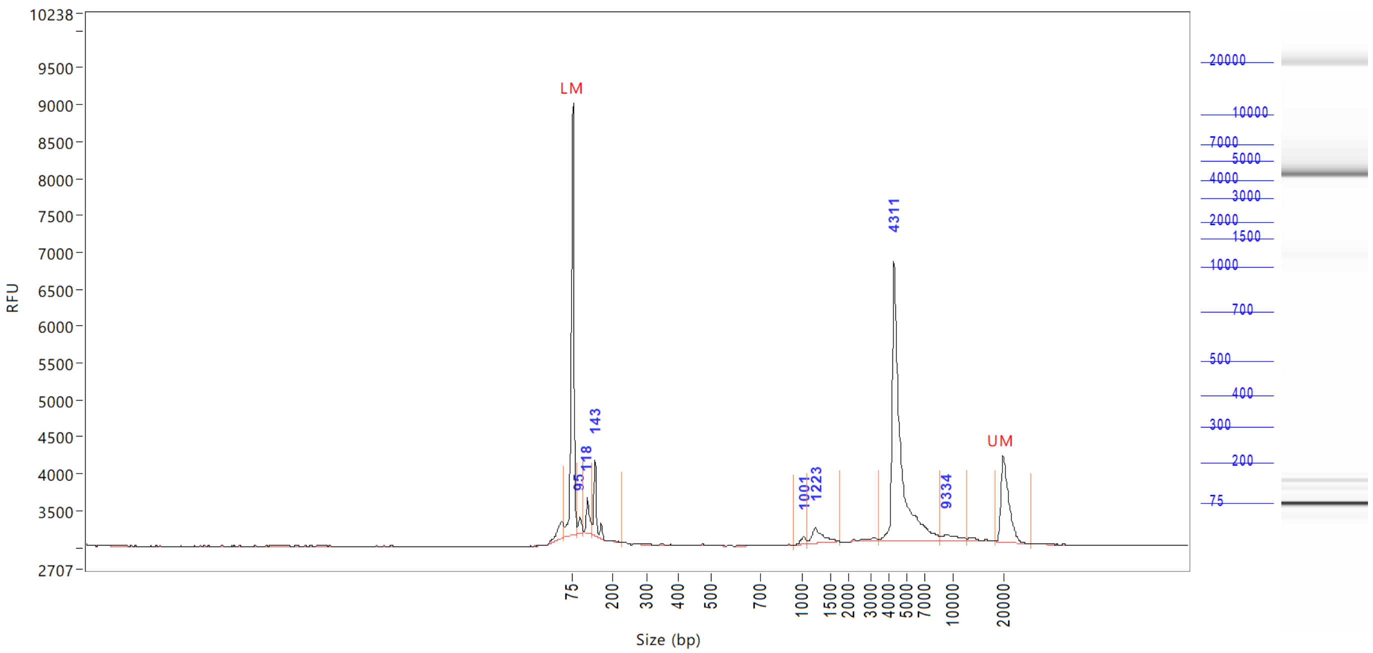

Peak	Size (bp)	Concentration (ng/uL)	From (bp)	To (bp)	Average size (bp)	CV%	RFU	Corrected peak area
1	75 (LM)	0.5000	48	87	71	7.21	5861	37.645
2	95	0.2856	87	108	95	4.62	222	1.792
3	118	0.5962	108	133	120	4.99	476	3.741
4	143	1.1306	133	226	146	8.15	1015	7.093
5	1001	0.1349	931	1068	1000	2.60	104	0.847
6	1223	0.5589	1068	1750	1276	9.67	218	3.507
7	4311	6.9151	3429	8800	4790	18.80	3779	43.386
8	9334	0.2759	8800	12928	10253	11.14	73	1.731
9	20000 (UM)	0.1535	18537	25440	20515	4.38	1168	11.560

TIC:	9.8973	ng/uL
TIM:	29.1885	nmole/L
Total concentration:	10.2861	ng/uL

```
Sample peak width (sec): 5     Sample min peak height: 50     Sample baseline V to V?: Y     Sample baseline V to V points: 3
Sample filter: Binomial        Number of points for filter: 3  Sample start region (min): 0  Sample end region (min): 30
Marker peak width (sec): 5     Marker min peak height: 200    Marker baseline V to V?: Y     Marker baseline V to V points: 3
Lower marker selection: First peak > 500 RFU                  Upper marker selection: Last peak > 200 RFU
Ladder size (bp)75, 200, 300, 400, 500, 700, 1000, 1500, 2000, 3000, 4000, 5000, 7000, 10000, 20000
Quantification using: Lower Marker          Final concentration (ng/uL): 0.5000          Dilution factor: 12.0
```

Sample: SampB7
Well location: B7
Created: Monday, September 20, 2021 8:00:01 PM

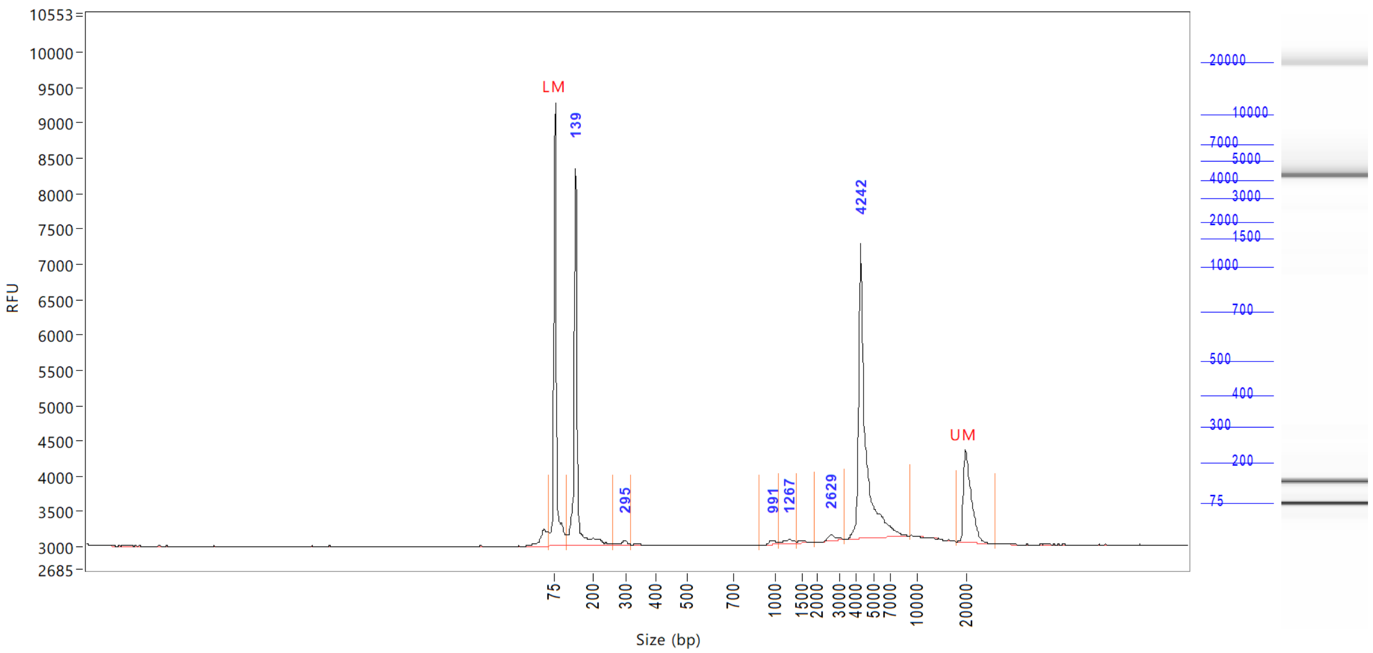

Peak	Size (bp)	Concentration (ng/uL)	From (bp)	To (bp)	Average size (bp)	CV%	RFU	Corrected peak area
1	75 (LM)	0.5000	58	112	75	11.60	6280	42.066
2	139	5.9309	112	259	143	13.84	5345	41.581
3	295	0.1313	259	315	286	4.12	68	0.921
4	991	0.1051	883	1068	985	2.95	61	0.737
5	1267	0.1358	1068	1400	1234	6.96	61	0.952
6	2629	0.1559	1904	3322	2665	8.17	87	1.093
7	4242	6.1402	3322	9200	4619	16.44	4180	43.049
8	20000 (UM)	0.1634	18171	26166	20521	4.67	1319	13.750

TIC:	12.5991	ng/uL
TIM:	71.5550	nmole/L
Total concentration:	12.8264	ng/uL

Sample peak width (sec): 5   Sample min peak height: 50   Sample baseline V to V?: Y   Sample baseline V to V points: 3
Sample filter: Binomial   Number of points for filter: 3   Sample start region (min): 0   Sample end region (min): 30
Marker peak width (sec): 5   Marker min peak height: 200   Marker baseline V to V?: Y   Marker baseline V to V points: 3
Lower marker selection: First peak > 500 RFU   Upper marker selection: Last peak > 200 RFU
Ladder size (bp)75, 200, 300, 400, 500, 700, 1000, 1500, 2000, 3000, 4000, 5000, 7000, 10000, 20000
Quantification using: Lower Marker   Final concentration (ng/uL): 0.5000   Dilution factor: 12.0

Sample: SampC7
Well location: C7
Created: Monday, September 20, 2021 8:00:01 PM

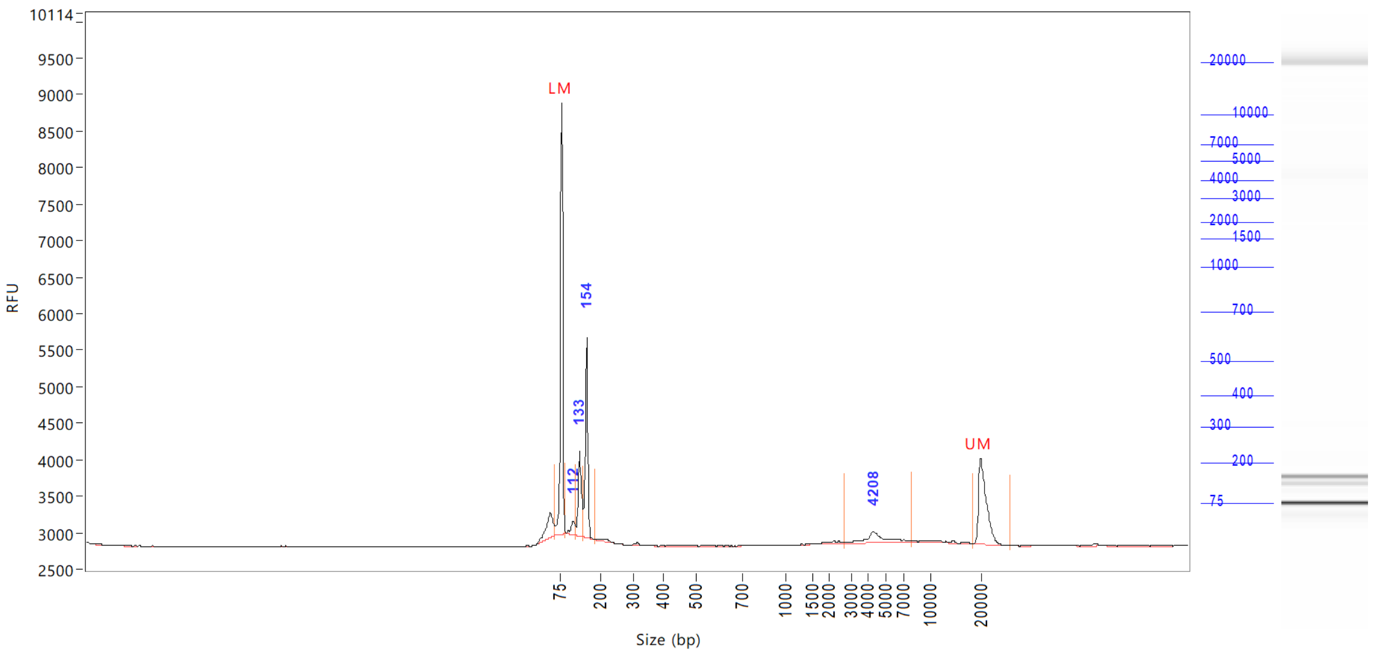

Peak	Size (bp)	Concentration (ng/uL)	From (bp)	To (bp)	Average size (bp)	CV%	RFU	Corrected peak area
1	75 (LM)	0.5000	56	89	73	5.22	5918	35.627
2	112	0.3538	89	120	108	6.74	196	2.101
3	133	1.5864	120	145	133	4.30	1159	9.420
4	154	2.6882	145	181	153	2.46	2746	15.962
5	4208	0.6906	2686	7867	4613	24.08	155	4.101
6	20000 (UM)	0.1687	18415	26045	20571	4.42	1179	12.021

TIC:	5.3190	ng/uL
TIM:	54.1039	nmole/L
Total concentration:	5.8919	ng/uL

```
Sample peak width (sec): 5     Sample min peak height: 50     Sample baseline V to V?: Y     Sample baseline V to V points: 3
Sample filter: Binomial        Number of points for filter: 3  Sample start region (min): 0  Sample end region (min): 30
Marker peak width (sec): 5     Marker min peak height: 200    Marker baseline V to V?: Y     Marker baseline V to V points: 3
Lower marker selection: First peak > 500 RFU                  Upper marker selection: Last peak > 200 RFU
Ladder size (bp)75, 200, 300, 400, 500, 700, 1000, 1500, 2000, 3000, 4000, 5000, 7000, 10000, 20000
Quantification using: Lower Marker          Final concentration (ng/uL): 0.5000          Dilution factor: 12.0
```

Sample: SampD7
Well location: D7
Created: Monday, September 20, 2021 8:00:01 PM

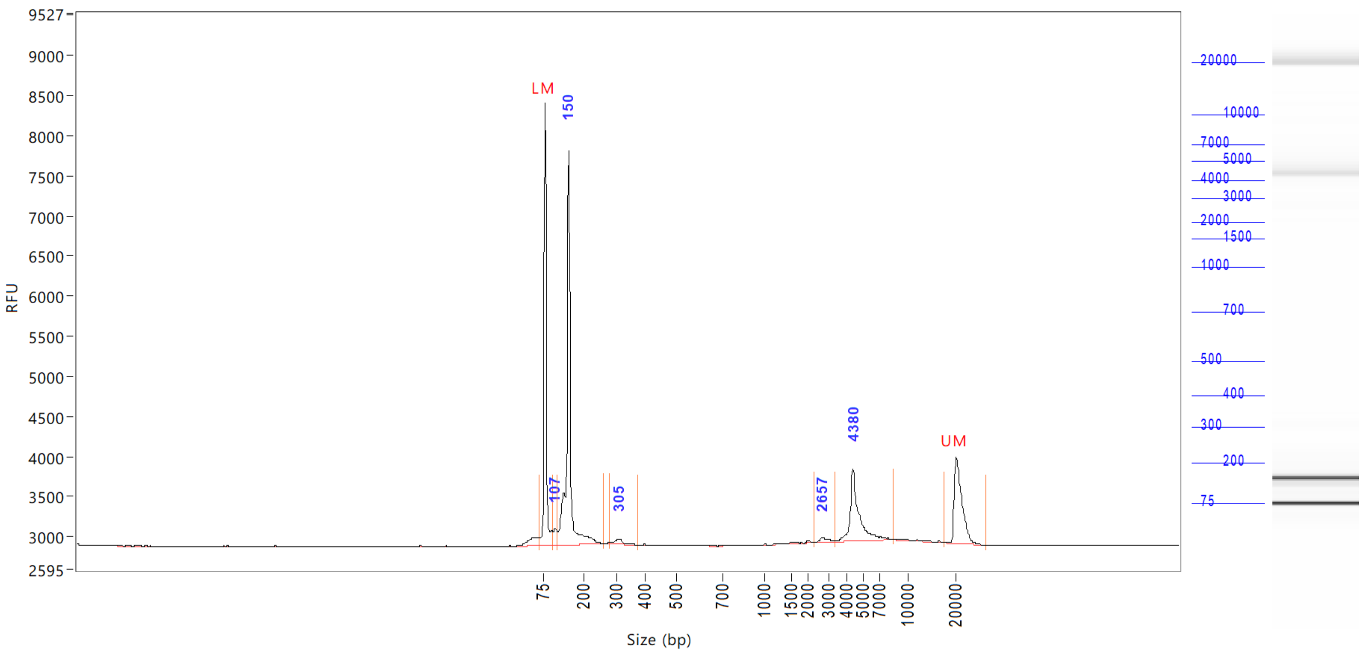

Peak	Size (bp)	Concentration (ng/uL)	From (bp)	To (bp)	Average size (bp)	CV%	RFU	Corrected peak area
1	75 (LM)	0.5000	58	101	74	8.30	5527	33.271
2	107	0.3772	101	116	108	4.49	206	2.092
3	150	7.6195	116	260	151	12.77	4920	42.252
4	305	0.2150	278	374	308	5.76	71	1.192
5	2657	0.1472	2315	3393	2808	8.60	55	0.816
6	4380	2.1135	3393	8401	4673	15.27	903	11.720
7	20000 (UM)	0.1672	17439	26287	20615	4.65	1087	11.125

TIC:	10.4723	ng/uL
TIM:	90.6484	nmole/L
Total concentration:	10.7743	ng/uL

```
Sample peak width (sec): 5    Sample min peak height: 50    Sample baseline V to V?: Y    Sample baseline V to V points: 3
Sample filter: Binomial       Number of points for filter: 3  Sample start region (min): 0  Sample end region (min): 30
Marker peak width (sec): 5    Marker min peak height: 200   Marker baseline V to V?: Y    Marker baseline V to V points: 3
Lower marker selection: First peak > 500 RFU                Upper marker selection: Last peak > 200 RFU
Ladder size (bp)75, 200, 300, 400, 500, 700, 1000, 1500, 2000, 3000, 4000, 5000, 7000, 10000, 20000
Quantification using: Lower Marker          Final concentration (ng/uL): 0.5000          Dilution factor: 12.0
```

Sample: SampE7
Well location: E7
Created: Monday, September 20, 2021 8:00:01 PM

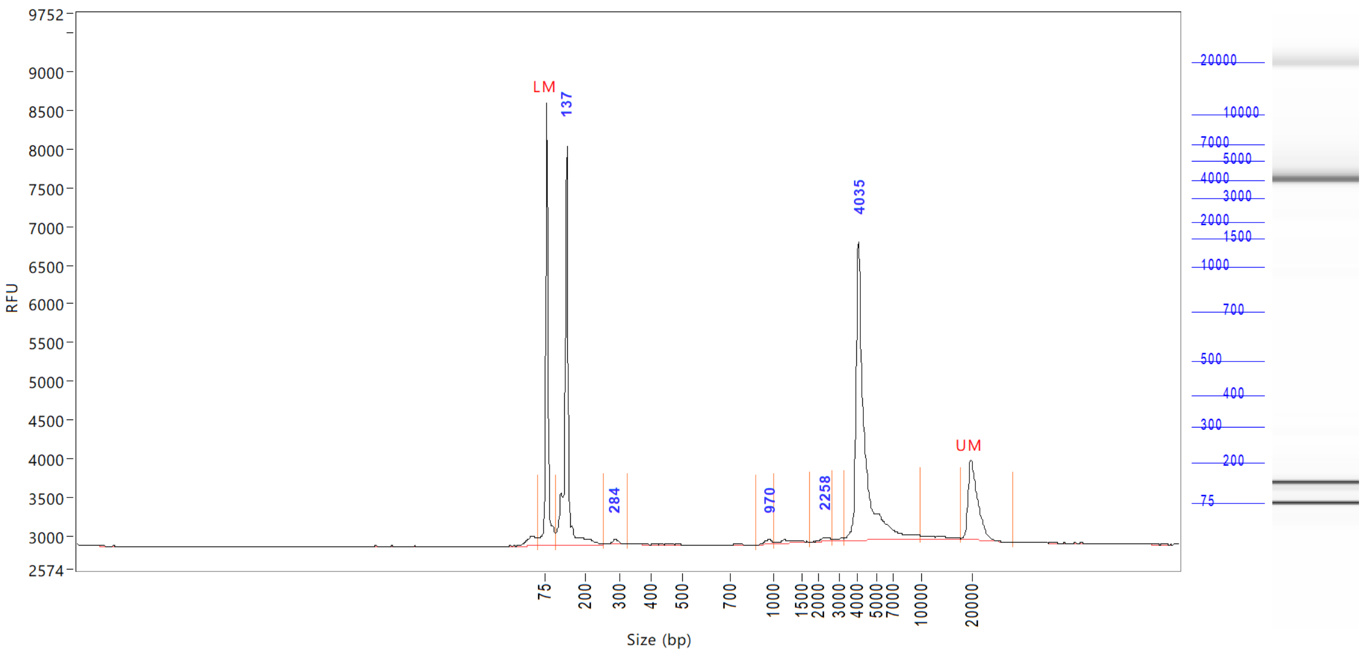

Peak	Size (bp)	Concentration (ng/uL)	From (bp)	To (bp)	Average size (bp)	CV%	RFU	Corrected peak area
1	75 (LM)	0.5000	50	103	75	9.18	5725	37.213
2	137	7.0183	103	251	137	13.97	5146	43.529
3	284	0.1631	251	325	284	4.59	67	1.011
4	970	0.1196	874	1012	958	2.57	62	0.742
5	2258	0.1253	1731	2657	2299	8.41	56	0.777
6	4035	7.8575	3215	10000	4484	22.21	3846	48.734
7	20000 (UM)	0.1740	17927	28221	20521	5.44	1027	12.951

TIC:	15.2838	ng/uL
TIM:	88.3057	nmole/L
Total concentration:	15.8157	ng/uL

```
Sample peak width (sec): 5     Sample min peak height: 50    Sample baseline V to V?: Y    Sample baseline V to V points: 3
Sample filter: Binomial        Number of points for filter: 3  Sample start region (min): 0  Sample end region (min): 30
Marker peak width (sec): 5     Marker min peak height: 200   Marker baseline V to V?: Y    Marker baseline V to V points: 3
Lower marker selection: First peak > 500 RFU                 Upper marker selection: Last peak > 200 RFU
Ladder size (bp)75, 200, 300, 400, 500, 700, 1000, 1500, 2000, 3000, 4000, 5000, 7000, 10000, 20000
Quantification using: Lower Marker          Final concentration (ng/uL): 0.5000          Dilution factor: 12.0
```

Sample: SampF7
Well location: F7
Created: Monday, September 20, 2021 8:00:01 PM

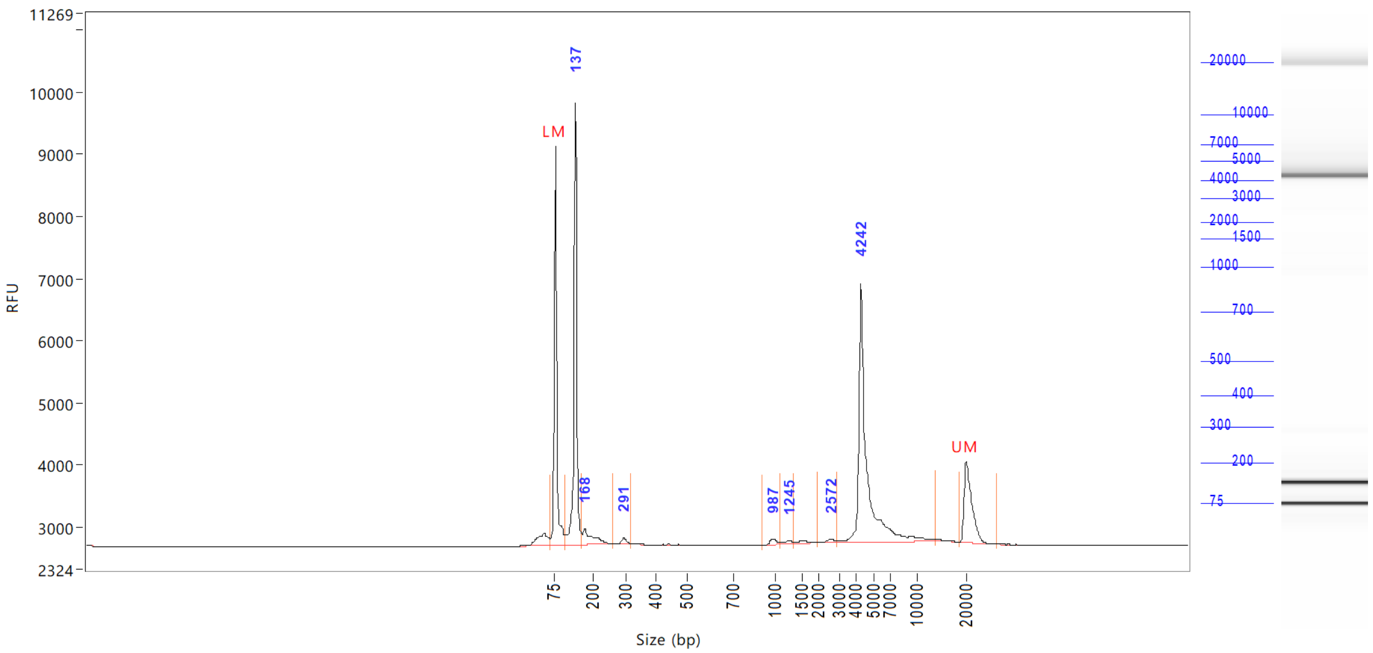

Peak	Size (bp)	Concentration (ng/uL)	From (bp)	To (bp)	Average size (bp)	CV%	RFU	Corrected peak area
1	75 (LM)	0.5000	58	107	74	9.87	6425	42.202
2	137	7.0523	107	160	135	5.34	7122	49.604
3	168	0.7921	160	257	188	11.12	252	5.572
4	291	0.1467	257	313	287	2.85	96	1.032
5	987	0.1493	905	1068	985	2.97	85	1.050
6	1245	0.1057	1068	1345	1210	6.29	52	0.743
7	2572	0.1005	1962	2886	2547	7.69	53	0.707
8	4242	6.8156	2886	13781	4899	29.94	4157	47.939
9	20000 (UM)	0.1582	18415	26287	20519	4.52	1304	13.354

TIC:	15.1623	ng/uL
TIM:	96.3563	nmole/L
Total concentration:	15.4304	ng/uL

```
Sample peak width (sec): 5    Sample min peak height: 50    Sample baseline V to V?: Y    Sample baseline V to V points: 3
Sample filter: Binomial       Number of points for filter: 3  Sample start region (min): 0  Sample end region (min): 30
Marker peak width (sec): 5    Marker min peak height: 200   Marker baseline V to V?: Y    Marker baseline V to V points: 3
Lower marker selection: First peak > 500 RFU                Upper marker selection: Last peak > 200 RFU
Ladder size (bp)75, 200, 300, 400, 500, 700, 1000, 1500, 2000, 3000, 4000, 5000, 7000, 10000, 20000
Quantification using: Lower Marker          Final concentration (ng/uL): 0.5000          Dilution factor: 12.0
```

Sample: SampG7
Well location: G7
Created: Monday, September 20, 2021 8:00:01 PM

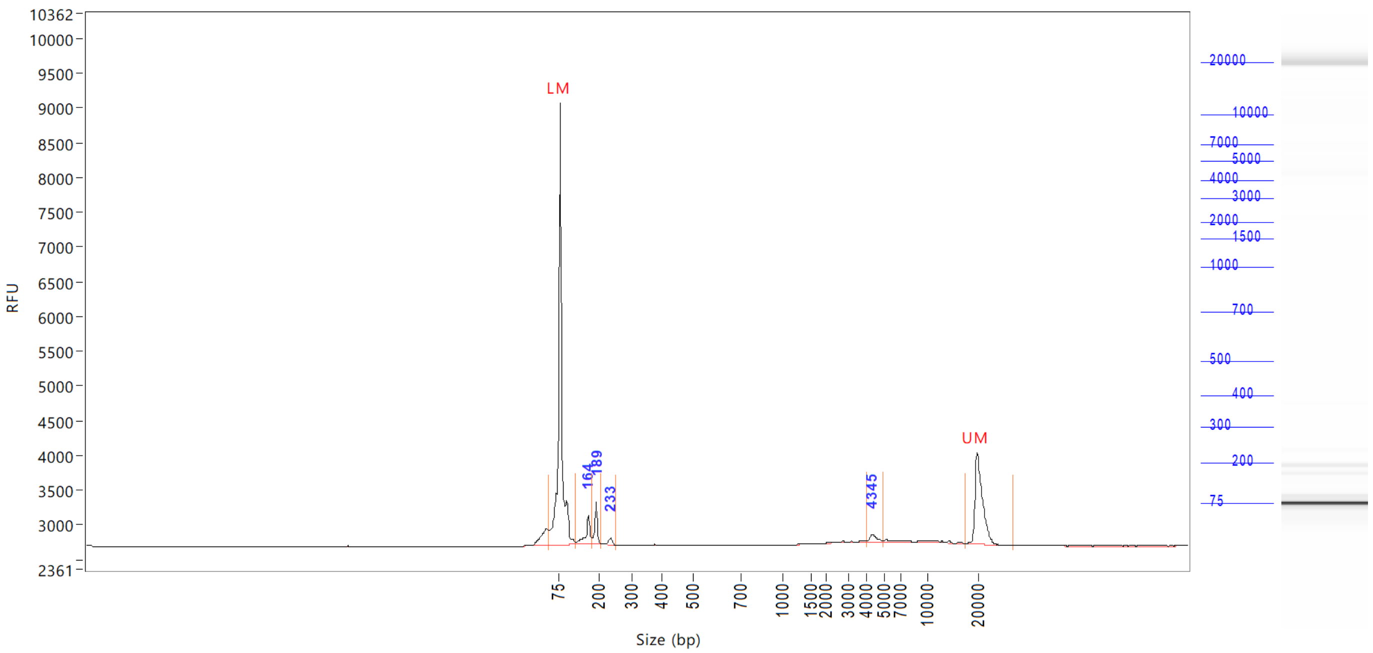

Peak	Size (bp)	Concentration (ng/uL)	From (bp)	To (bp)	Average size (bp)	CV%	RFU	Corrected peak area
1	75 (LM)	0.5000	41	126	74	15.50	6370	52.325
2	164	0.4657	126	177	157	7.68	409	4.062
3	189	0.4027	177	205	187	2.35	604	3.512
4	233	0.1035	205	248	229	3.27	94	0.903
5	4345	0.1189	4070	4966	4455	4.87	98	1.037
6	20000 (UM)	0.1232	17683	27133	20435	4.47	1308	12.888

TIC:	1.0908	ng/uL
TIM:	9.2034	nmole/L
Total concentration:	1.4585	ng/uL

```
Sample peak width (sec): 5    Sample min peak height: 50    Sample baseline V to V?: Y    Sample baseline V to V points: 3
Sample filter: Binomial       Number of points for filter: 3  Sample start region (min): 0  Sample end region (min): 30
Marker peak width (sec): 5    Marker min peak height: 200   Marker baseline V to V?: Y    Marker baseline V to V points: 3
Lower marker selection: First peak > 500 RFU                Upper marker selection: Last peak > 200 RFU
Ladder size (bp)75, 200, 300, 400, 500, 700, 1000, 1500, 2000, 3000, 4000, 5000, 7000, 10000, 20000
Quantification using: Lower Marker          Final concentration (ng/uL): 0.5000          Dilution factor: 12.0
```

Sample: SampH7
Well location: H7
Created: Monday, September 20, 2021 8:00:01 PM

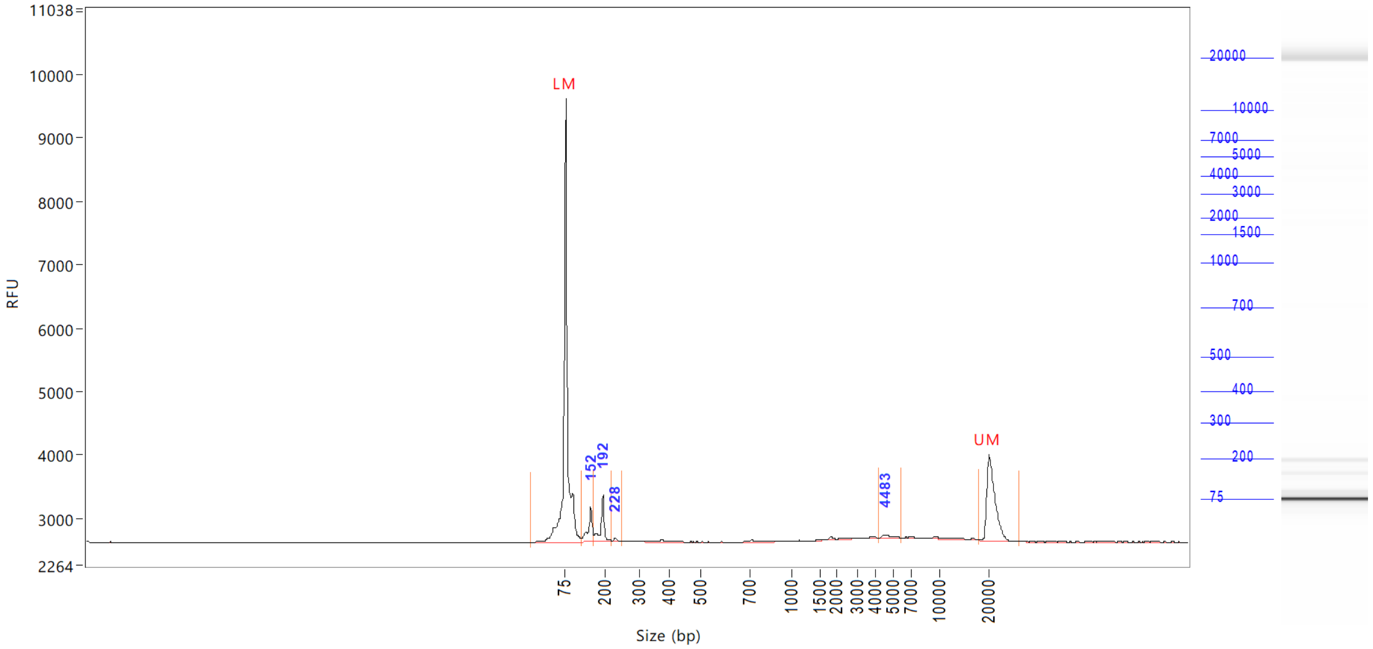

Peak	Size (bp)	Concentration (ng/uL)	From (bp)	To (bp)	Average size (bp)	CV%	RFU	Corrected peak area
1	75 (LM)	0.5000	0	124	73	21.79	7007	60.405
2	152	0.5230	124	164	147	6.45	557	5.265
3	192	0.5808	164	219	187	5.49	739	5.847
4	228	0.0455	219	250	228	2.61	50	0.459
5	4483	0.0820	4173	5889	4727	8.20	62	0.825
6	20000 (UM)	0.1171	17927	26166	20543	4.58	1352	14.151

TIC:	1.2313	ng/uL
TIM:	11.3106	nmole/L
Total concentration:	1.6198	ng/uL

```
Sample peak width (sec): 5    Sample min peak height: 50    Sample baseline V to V?: Y    Sample baseline V to V points: 3
Sample filter: Binomial       Number of points for filter: 3  Sample start region (min): 0  Sample end region (min): 30
Marker peak width (sec): 5    Marker min peak height: 200   Marker baseline V to V?: Y    Marker baseline V to V points: 3
Lower marker selection: First peak > 500 RFU                Upper marker selection: Last peak > 200 RFU
Ladder size (bp)75, 200, 300, 400, 500, 700, 1000, 1500, 2000, 3000, 4000, 5000, 7000, 10000, 20000
Quantification using: Lower Marker          Final concentration (ng/uL): 0.5000          Dilution factor: 12.0
```

Sample: SampA8
Well location: A8
Created: Monday, September 20, 2021 8:00:01 PM

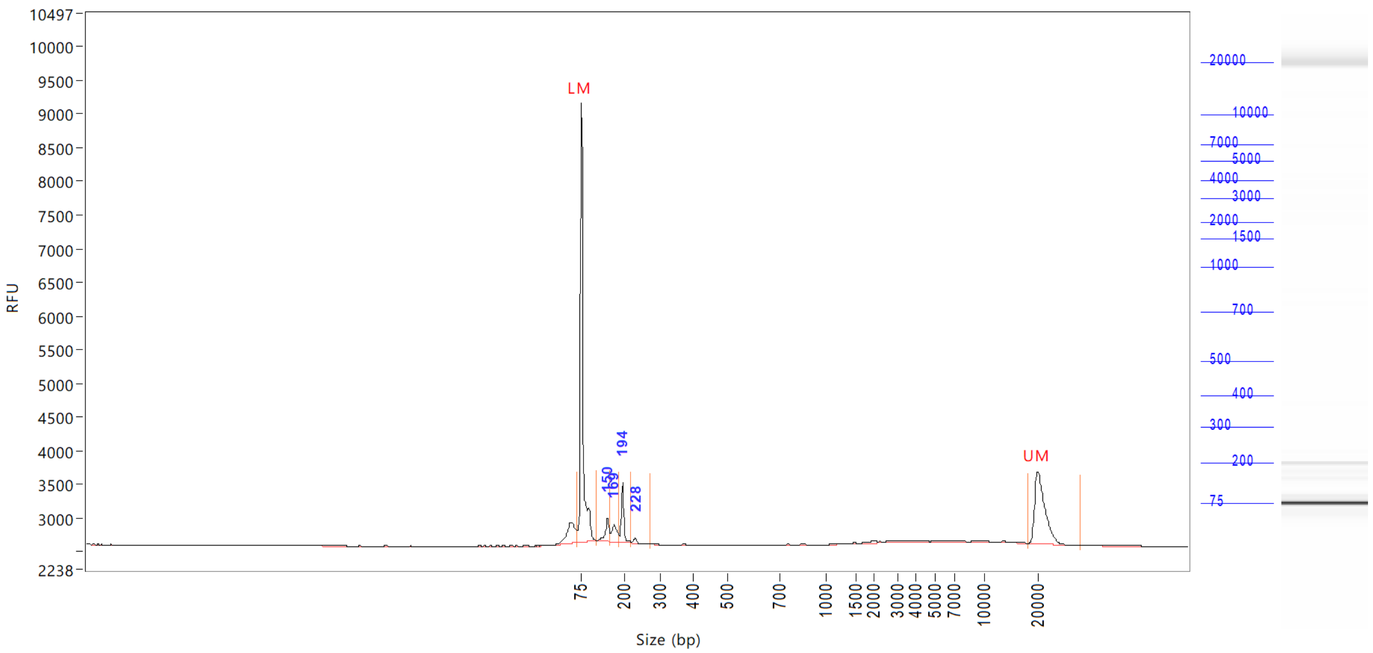

Peak	Size (bp)	Concentration (ng/uL)	From (bp)	To (bp)	Average size (bp)	CV%	RFU	Corrected peak area
1	75 (LM)	0.5000	62	118	76	9.68	6534	42.608
2	150	0.4190	118	158	146	5.32	339	2.975
3	169	0.4499	158	185	170	4.35	259	3.195
4	194	0.7323	185	219	194	2.68	884	5.200
5	228	0.0714	219	271	228	3.81	73	0.507
6	20000 (UM)	0.1568	18171	27979	20540	5.21	1072	13.365

TIC:	1.6725	ng/uL
TIM:	15.7875	nmole/L
Total concentration:	2.2625	ng/uL

Sample peak width (sec): 5   Sample min peak height: 50   Sample baseline V to V?: Y   Sample baseline V to V points: 3
Sample filter: Binomial   Number of points for filter: 3 Sample start region (min): 0 Sample end region (min): 30
Marker peak width (sec): 5   Marker min peak height: 200   Marker baseline V to V?: Y   Marker baseline V to V points: 3
Lower marker selection: First peak > 500 RFU   Upper marker selection: Last peak > 200 RFU
Ladder size (bp)75, 200, 300, 400, 500, 700, 1000, 1500, 2000, 3000, 4000, 5000, 7000, 10000, 20000
Quantification using: Lower Marker   Final concentration (ng/uL): 0.5000   Dilution factor: 12.0

Sample: SampB8
Well location: B8
Created: Monday, September 20, 2021 8:00:01 PM

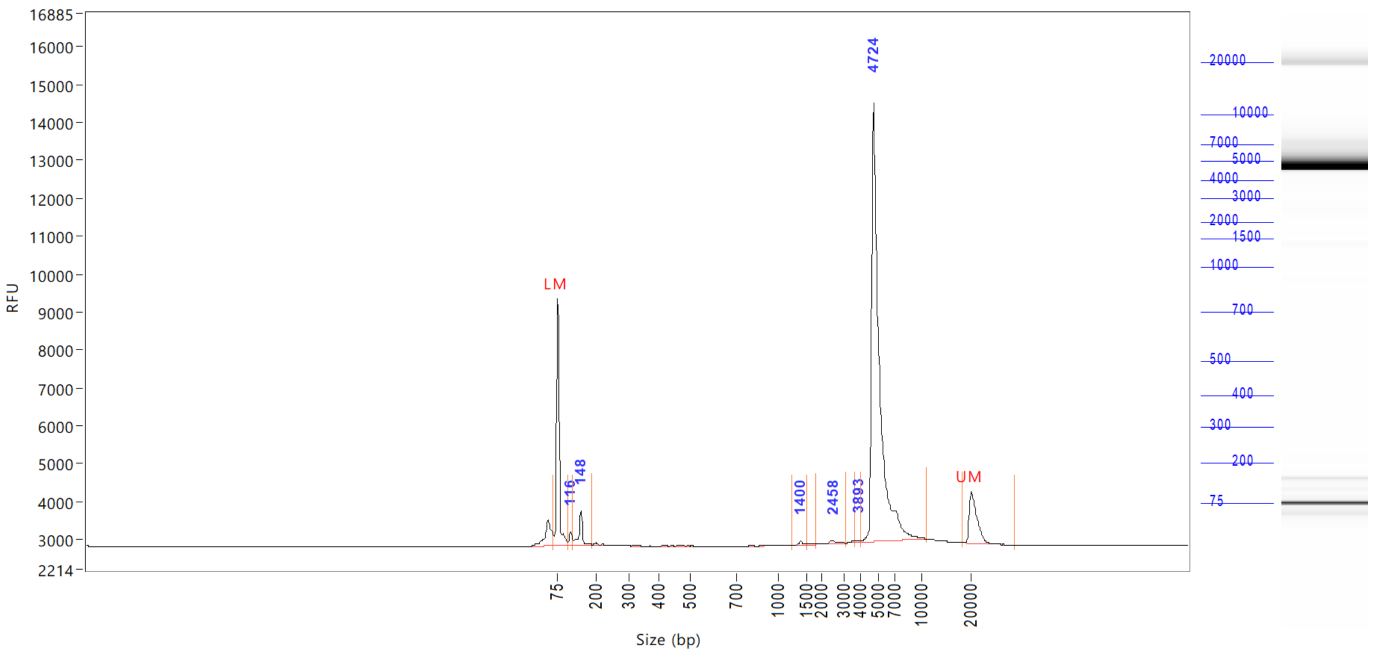

Peak	Size (bp)	Concentration (ng/uL)	From (bp)	To (bp)	Average size (bp)	CV%	RFU	Corrected peak area
1	75 (LM)	0.5000	64	108	75	8.73	6531	40.357
2	116	0.3746	108	126	116	3.95	348	2.520
3	148	1.0817	126	187	145	5.98	891	7.276
4	1400	0.0983	1245	1501	1392	2.90	100	0.661
5	2458	0.1557	1808	3108	2477	9.45	83	1.047
6	3893	0.0653	3679	4035	3874	2.72	61	0.439
7	4724	19.2788	4035	10855	5137	15.86	11596	129.673
8	20000 (UM)	0.1702	18293	28705	20576	4.68	1361	13.740

TIC:	21.0544	ng/uL
TIM:	23.9904	nmole/L
Total concentration:	21.3768	ng/uL

Sample peak width (sec): 5 Sample min peak height: 50 Sample baseline V to V?: Y Sample baseline V to V points: 3
Sample filter: Binomial Number of points for filter: 3 Sample start region (min): 0 Sample end region (min): 30
Marker peak width (sec): 5 Marker min peak height: 200 Marker baseline V to V?: Y Marker baseline V to V points: 3
Lower marker selection: First peak > 500 RFU Upper marker selection: Last peak > 200 RFU
Ladder size (bp)75, 200, 300, 400, 500, 700, 1000, 1500, 2000, 3000, 4000, 5000, 7000, 10000, 20000
Quantification using: Lower Marker Final concentration (ng/uL): 0.5000 Dilution factor: 12.0

Sample: SampC8
Well location: C8
Created: Monday, September 20, 2021 8:00:01 PM

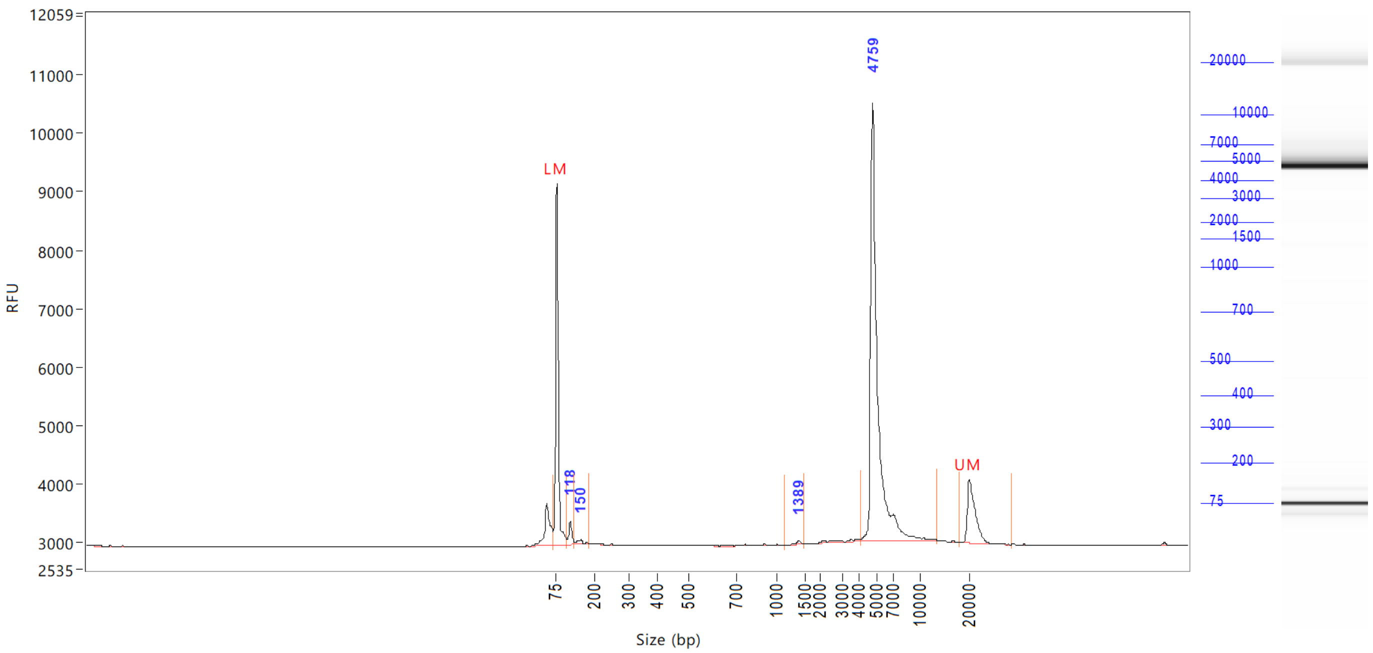

Peak	Size (bp)	Concentration (ng/uL)	From (bp)	To (bp)	Average size (bp)	CV%	RFU	Corrected peak area
1	75 (LM)	0.5000	64	108	74	8.99	6203	40.409
2	118	0.4385	108	131	117	4.36	396	2.953
3	150	0.1673	131	179	147	7.18	74	1.127
4	1389	0.0614	1134	1478	1367	3.74	64	0.413
5	4759	11.1560	4035	13293	5201	18.27	7521	75.134
6	20000 (UM)	0.1385	17927	28826	20604	4.87	1085	11.195

TIC:	11.8232	ng/uL
TIM:	11.6324	nmole/L
Total concentration:	12.3395	ng/uL

Sample peak width (sec): 5 Sample min peak height: 50 Sample baseline V to V?: Y Sample baseline V to V points: 3
Sample filter: Binomial Number of points for filter: 3 Sample start region (min): 0 Sample end region (min): 30
Marker peak width (sec): 5 Marker min peak height: 200 Marker baseline V to V?: Y Marker baseline V to V points: 3
Lower marker selection: First peak > 500 RFU Upper marker selection: Last peak > 200 RFU
Ladder size (bp)75, 200, 300, 400, 500, 700, 1000, 1500, 2000, 3000, 4000, 5000, 7000, 10000, 20000
Quantification using: Lower Marker Final concentration (ng/uL): 0.5000 Dilution factor: 12.0

Sample: SampD8
Well location: D8
Created: Monday, September 20, 2021 8:00:01 PM

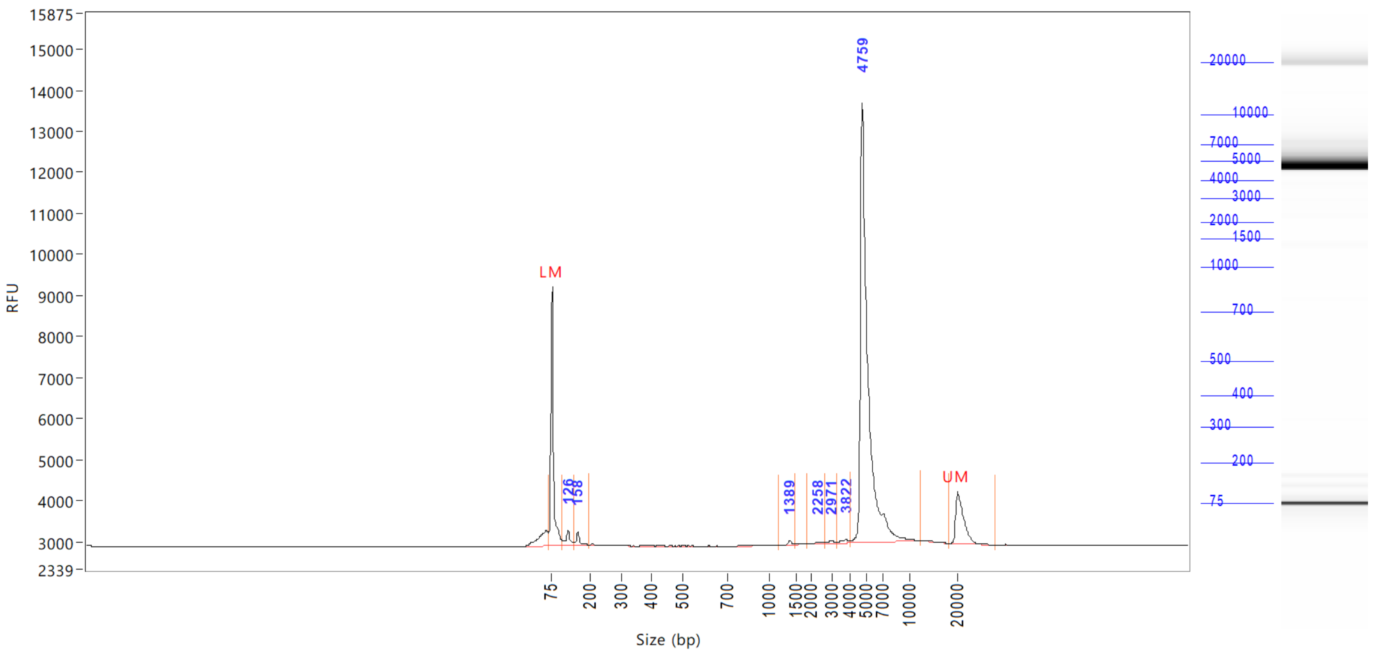

Peak	Size (bp)	Concentration (ng/uL)	From (bp)	To (bp)	Average size (bp)	CV%	RFU	Corrected peak area
1	75 (LM)	0.5000	64	110	75	10.28	6296	42.318
2	126	0.5148	110	148	127	7.18	353	3.631
3	158	0.3179	148	198	158	4.69	307	2.242
4	1389	0.0849	1167	1489	1377	2.76	95	0.599
5	2258	0.0965	1866	2629	2307	7.55	56	0.680
6	2971	0.0937	2629	3251	2930	5.43	53	0.661
7	3822	0.1370	3251	4001	3680	5.67	91	0.966
8	4759	16.9816	4001	12318	5158	16.72	10721	119.770
9	20000 (UM)	0.1566	18049	27859	20641	4.99	1261	13.255

TIC:	18.2264	ng/uL
TIM:	15.6712	nmole/L
Total concentration:	18.4861	ng/uL

Sample peak width (sec): 5   Sample min peak height: 50   Sample baseline V to V?: Y   Sample baseline V to V points: 3
Sample filter: Binomial   Number of points for filter: 3 Sample start region (min): 0 Sample end region (min): 30
Marker peak width (sec): 5   Marker min peak height: 200   Marker baseline V to V?: Y   Marker baseline V to V points: 3
Lower marker selection: First peak > 500 RFU   Upper marker selection: Last peak > 200 RFU
Ladder size (bp)75, 200, 300, 400, 500, 700, 1000, 1500, 2000, 3000, 4000, 5000, 7000, 10000, 20000
Quantification using: Lower Marker   Final concentration (ng/uL): 0.5000   Dilution factor: 12.0

Sample: SampE8
Well location: E8
Created: Monday, September 20, 2021 8:00:01 PM

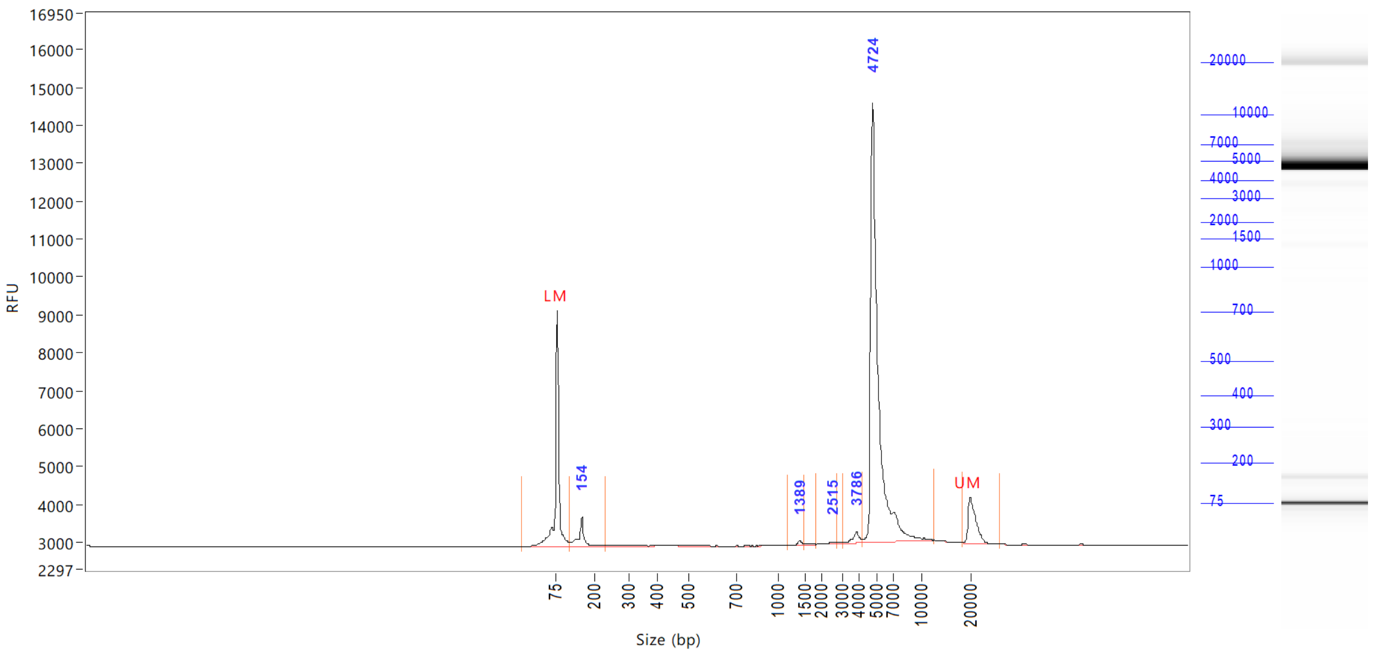

Peak	Size (bp)	Concentration (ng/uL)	From (bp)	To (bp)	Average size (bp)	CV%	RFU	Corrected peak area
1	75 (LM)	0.5000	0	118	70	24.77	6213	50.921
2	154	1.1623	118	232	155	14.82	767	9.864
3	1389	0.0925	1178	1489	1384	3.01	120	0.785
4	2515	0.0940	1827	2772	2411	9.76	61	0.798
5	3786	0.3552	3037	4139	3723	6.50	300	3.014
6	4724	14.8419	4139	12562	5180	17.73	11603	125.961
7	20000 (UM)	0.1210	18537	26045	20585	4.39	1202	12.327

TIC:	16.5458	ng/uL
TIM:	17.3731	nmole/L
Total concentration:	16.8952	ng/uL

```
Sample peak width (sec): 5    Sample min peak height: 50    Sample baseline V to V?: Y    Sample baseline V to V points: 3
Sample filter: Binomial       Number of points for filter: 3  Sample start region (min): 0  Sample end region (min): 30
Marker peak width (sec): 5    Marker min peak height: 200   Marker baseline V to V?: Y    Marker baseline V to V points: 3
Lower marker selection: First peak > 500 RFU                Upper marker selection: Last peak > 200 RFU
Ladder size (bp)75, 200, 300, 400, 500, 700, 1000, 1500, 2000, 3000, 4000, 5000, 7000, 10000, 20000
Quantification using: Lower Marker          Final concentration (ng/uL): 0.5000          Dilution factor: 12.0
```

Sample: SampF8
Well location: F8
Created: Monday, September 20, 2021 8:00:01 PM

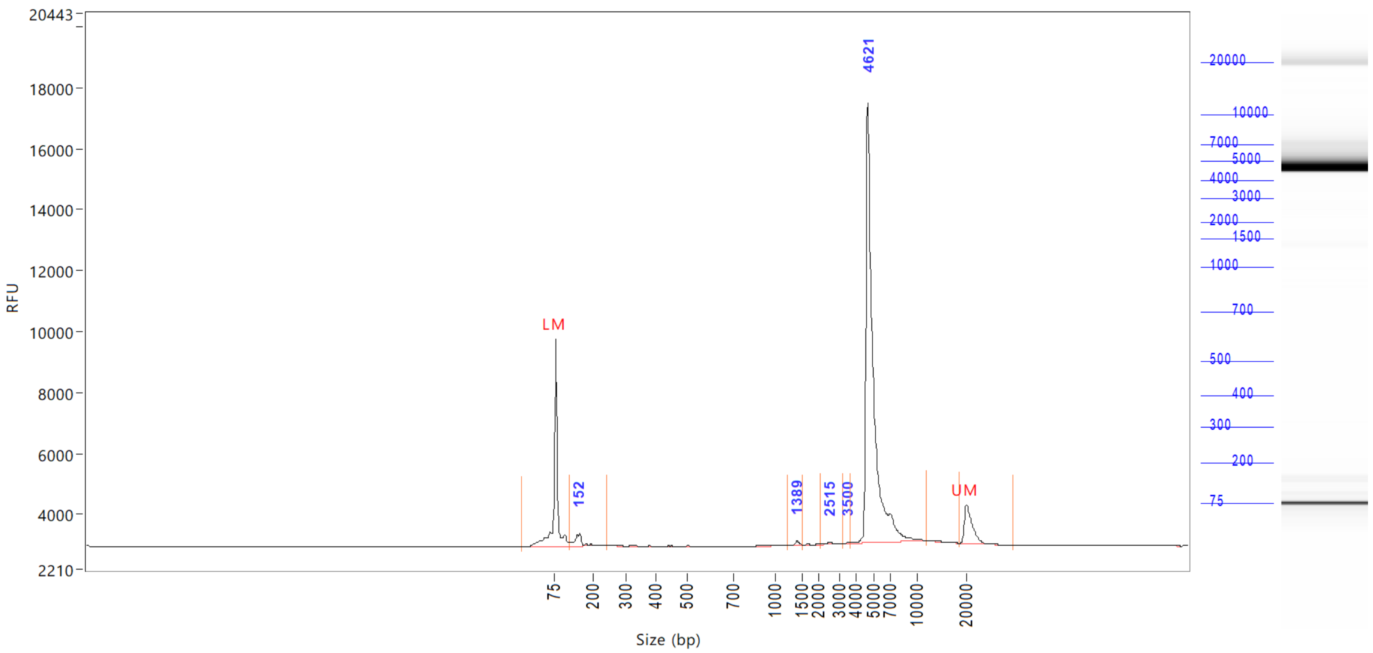

Peak	Size (bp)	Concentration (ng/uL)	From (bp)	To (bp)	Average size (bp)	CV%	RFU	Corrected peak area
1	75 (LM)	0.5000	0	122	69	28.91	6815	56.707
2	152	0.7603	122	239	150	12.40	409	7.186
3	1389	0.1039	1223	1500	1390	3.25	145	0.982
4	2515	0.0831	2058	3215	2538	8.89	60	0.785
5	3500	0.0310	3215	3679	3492	2.94	51	0.293
6	4621	16.3985	3679	11708	5051	16.89	14451	154.985
7	20000 (UM)	0.1172	18659	29551	20628	5.01	1274	13.293

TIC:	17.3767	ng/uL
TIM:	13.8664	nmole/L
Total concentration:	17.6356	ng/uL

Sample peak width (sec): 5 Sample min peak height: 50 Sample baseline V to V?: Y Sample baseline V to V points: 3
Sample filter: Binomial Number of points for filter: 3 Sample start region (min): 0 Sample end region (min): 30
Marker peak width (sec): 5 Marker min peak height: 200 Marker baseline V to V?: Y Marker baseline V to V points: 3
Lower marker selection: First peak > 500 RFU Upper marker selection: Last peak > 200 RFU
Ladder size (bp)75, 200, 300, 400, 500, 700, 1000, 1500, 2000, 3000, 4000, 5000, 7000, 10000, 20000
Quantification using: Lower Marker Final concentration (ng/uL): 0.5000 Dilution factor: 12.0

Sample: SampG8
Well location: G8
Created: Monday, September 20, 2021 8:00:01 PM

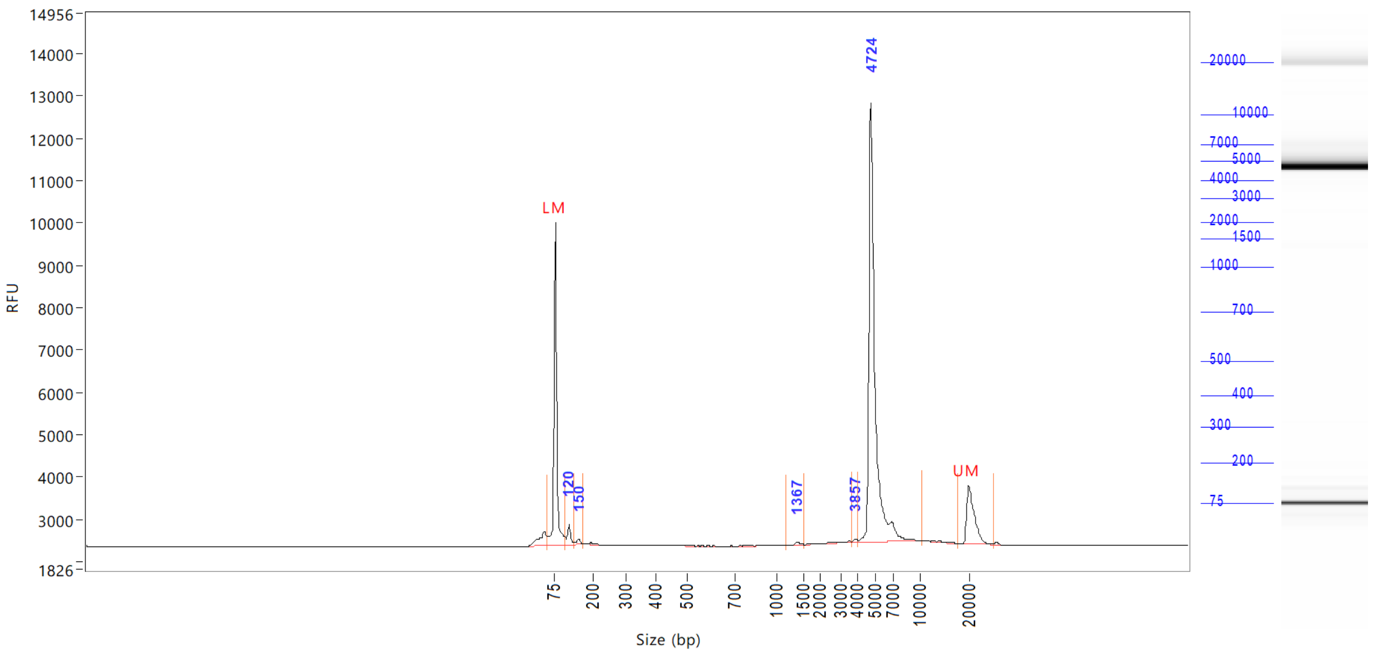

Peak	Size (bp)	Concentration (ng/uL)	From (bp)	To (bp)	Average size (bp)	CV%	RFU	Corrected peak area
1	75 (LM)	0.5000	52	110	75	10.61	7612	53.601
2	120	0.4271	110	135	119	4.59	495	3.815
3	150	0.1396	135	164	146	3.68	133	1.247
4	1367	0.0640	1178	1520	1371	3.54	79	0.571
5	3857	0.0608	3643	4001	3844	2.66	87	0.543
6	4724	10.1390	4001	10367	4968	13.39	10386	90.577
7	20000 (UM)	0.1296	17683	24957	20530	4.52	1392	13.897

TIC:	10.8305	ng/uL
TIM:	10.9298	nmole/L
Total concentration:	11.1614	ng/uL

```
Sample peak width (sec): 5    Sample min peak height: 50    Sample baseline V to V?: Y    Sample baseline V to V points: 3
Sample filter: Binomial       Number of points for filter: 3  Sample start region (min): 0  Sample end region (min): 30
Marker peak width (sec): 5    Marker min peak height: 200   Marker baseline V to V?: Y    Marker baseline V to V points: 3
Lower marker selection: First peak > 500 RFU                Upper marker selection: Last peak > 200 RFU
Ladder size (bp)75, 200, 300, 400, 500, 700, 1000, 1500, 2000, 3000, 4000, 5000, 7000, 10000, 20000
Quantification using: Lower Marker          Final concentration (ng/uL): 0.5000          Dilution factor: 12.0
```

Sample: SampH8
Well location: H8
Created: Monday, September 20, 2021 8:00:01 PM

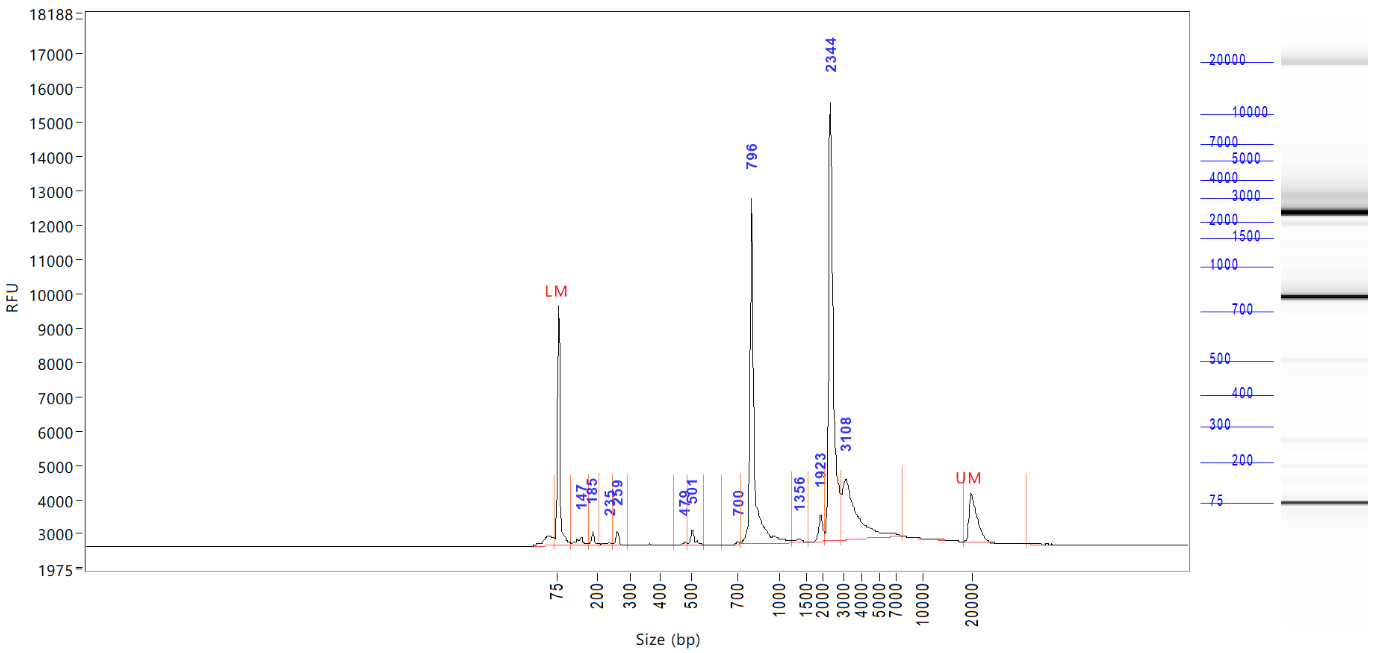

Peak	Size (bp)	Concentration (ng/uL)	From (bp)	To (bp)	Average size (bp)	CV%	RFU	Corrected peak area
1	75 (LM)	0.5000	64	116	75	9.59	6995	42.890
2	147	0.4931	116	171	140	9.20	232	3.525
3	185	0.3278	171	206	183	2.37	392	2.343
4	235	0.0862	206	244	232	3.38	68	0.616
5	259	0.3868	244	293	258	2.24	365	2.765
6	479	0.1440	445	489	474	2.01	112	1.029
7	501	0.5476	489	552	507	2.64	445	3.915
8	700	0.0815	629	718	697	1.76	74	0.583
9	796	10.2525	718	1234	814	7.29	10083	73.289
10	1356	0.1886	1234	1559	1342	4.41	120	1.348
11	1923	0.7573	1559	2115	1932	4.20	784	5.413
12	2344	12.9111	2115	2857	2404	6.47	12799	92.293
13	3108	5.6027	2857	7801	3642	22.74	1767	40.050
14	20000 (UM)	0.1734	18293	31244	20570	5.26	1422	14.877

TIC:	31.7792	ng/uL
TIM:	47.2591	nmole/L
Total concentration:	31.8315	ng/uL

```
Sample peak width (sec): 5    Sample min peak height: 50    Sample baseline V to V?: Y    Sample baseline V to V points: 3
Sample filter: Binomial       Number of points for filter: 3  Sample start region (min): 0  Sample end region (min): 30
Marker peak width (sec): 5    Marker min peak height: 200   Marker baseline V to V?: Y    Marker baseline V to V points: 3
Lower marker selection: First peak > 500 RFU                Upper marker selection: Last peak > 200 RFU
Ladder size (bp)75, 200, 300, 400, 500, 700, 1000, 1500, 2000, 3000, 4000, 5000, 7000, 10000, 20000
Quantification using: Lower Marker         Final concentration (ng/uL): 0.5000         Dilution factor: 12.0
```

Sample: SampA9
Well location: A9
Created: Monday, September 20, 2021 8:00:01 PM

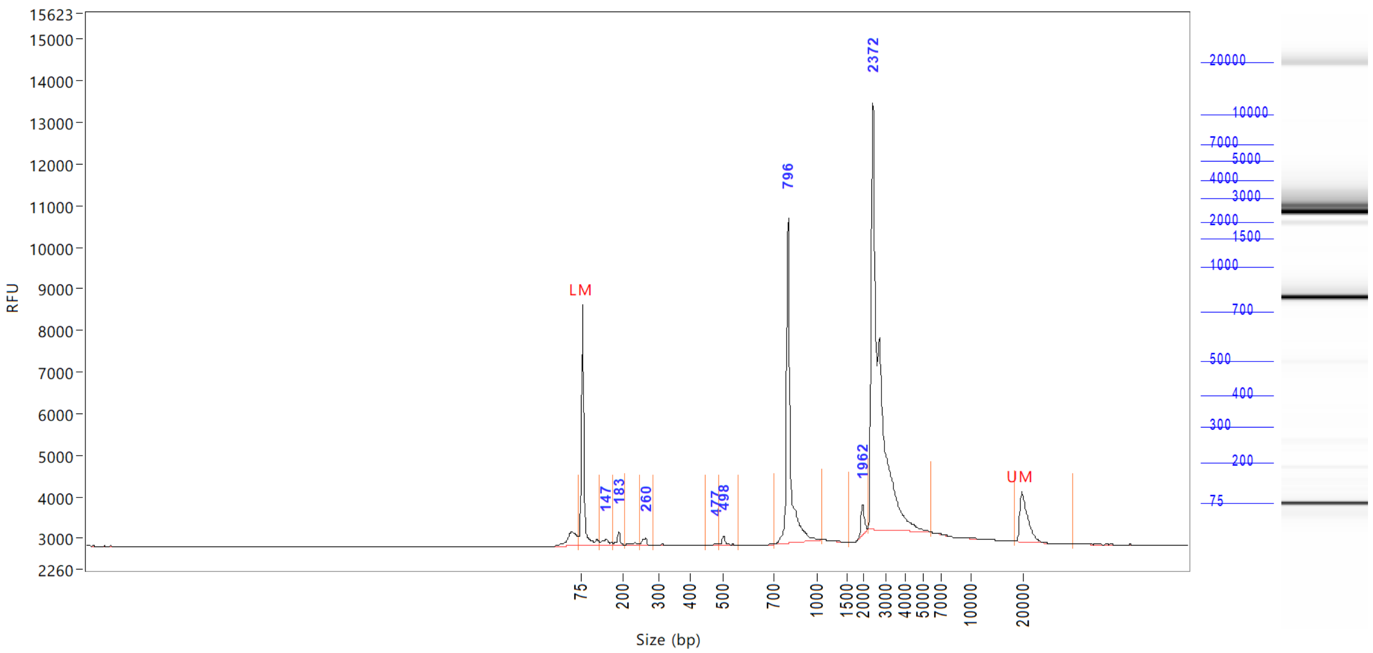

Peak	Size (bp)	Concentration (ng/uL)	From (bp)	To (bp)	Average size (bp)	CV%	RFU	Corrected peak area
1	75 (LM)	0.5000	62	126	75	13.01	5796	35.586
2	147	0.3961	126	168	143	7.60	153	2.349
3	183	0.3453	168	201	180	2.68	324	2.048
4	260	0.2587	246	282	255	2.02	174	1.534
5	477	0.1364	443	487	471	2.29	67	0.809
6	498	0.3354	487	560	504	3.03	229	1.989
7	796	8.9297	705	1068	805	4.62	7808	52.961
8	1962	0.6390	1559	2172	1955	2.79	727	3.790
9	2372	20.2972	2172	5815	2723	17.31	10272	120.381
10	20000 (UM)	0.1683	18537	29672	20577	5.20	1203	11.976

TIC:	31.3377	ng/uL
TIM:	42.0110	nmole/L
Total concentration:	31.7541	ng/uL

Sample peak width (sec): 5 Sample min peak height: 50 Sample baseline V to V?: Y Sample baseline V to V points: 3
Sample filter: Binomial Number of points for filter: 3 Sample start region (min): 0 Sample end region (min): 30
Marker peak width (sec): 5 Marker min peak height: 200 Marker baseline V to V?: Y Marker baseline V to V points: 3
Lower marker selection: First peak > 500 RFU Upper marker selection: Last peak > 200 RFU
Ladder size (bp)75, 200, 300, 400, 500, 700, 1000, 1500, 2000, 3000, 4000, 5000, 7000, 10000, 20000
Quantification using: Lower Marker Final concentration (ng/uL): 0.5000 Dilution factor: 12.0

Sample: SampB9
Well location: B9
Created: Monday, September 20, 2021 8:00:01 PM

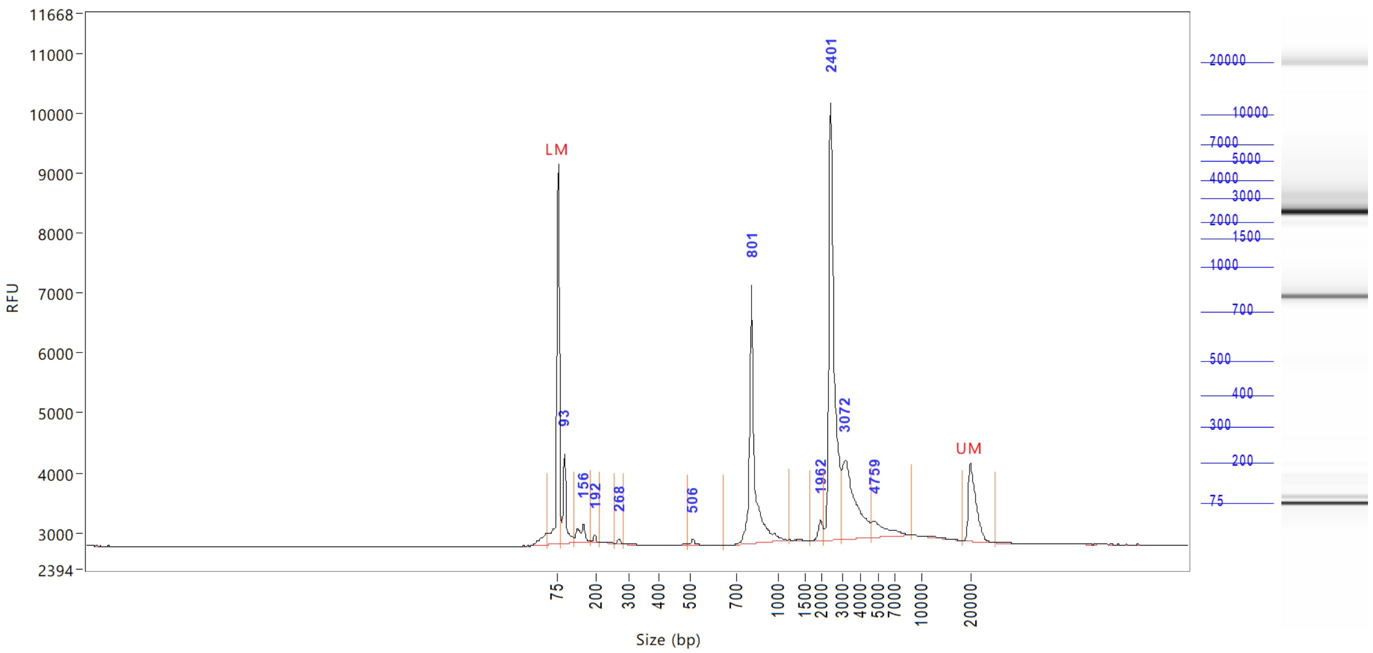

Peak	Size (bp)	Concentration (ng/uL)	From (bp)	To (bp)	Average size (bp)	CV%	RFU	Corrected peak area
1	75 (LM)	0.5000	41	84	71	9.70	6323	39.125
2	93	1.8151	84	126	95	9.43	1490	11.836
3	156	0.7142	126	181	145	7.53	292	4.657
4	192	0.0972	181	205	190	1.76	117	0.634
5	268	0.0884	253	282	264	1.52	86	0.576
6	506	0.1689	492	639	522	5.82	111	1.101
7	801	5.8595	639	1190	816	6.32	4298	38.209
8	1962	0.4188	1597	2087	1944	4.16	345	2.731
9	2401	9.4011	2087	2914	2463	7.07	7306	61.304
10	3072	4.1511	2914	4587	3470	13.07	1320	27.069
11	4759	0.9387	4587	8867	5620	17.18	277	6.121
12	20000 (UM)	0.1665	18171	25199	20482	4.32	1298	13.027

TIC:	23.6529	ng/uL
TIM:	62.0905	nmole/L
Total concentration:	23.7070	ng/uL

Sample peak width (sec): 5 Sample min peak height: 50 Sample baseline V to V?: Y Sample baseline V to V points: 3
Sample filter: Binomial Number of points for filter: 3 Sample start region (min): 0 Sample end region (min): 30
Marker peak width (sec): 5 Marker min peak height: 200 Marker baseline V to V?: Y Marker baseline V to V points: 3
Lower marker selection: First peak > 500 RFU Upper marker selection: Last peak > 200 RFU
Ladder size (bp)75, 200, 300, 400, 500, 700, 1000, 1500, 2000, 3000, 4000, 5000, 7000, 10000, 20000
Quantification using: Lower Marker Final concentration (ng/uL): 0.5000 Dilution factor: 12.0

Sample: SampC9
Well location: C9
Created: Monday, September 20, 2021 8:00:01 PM

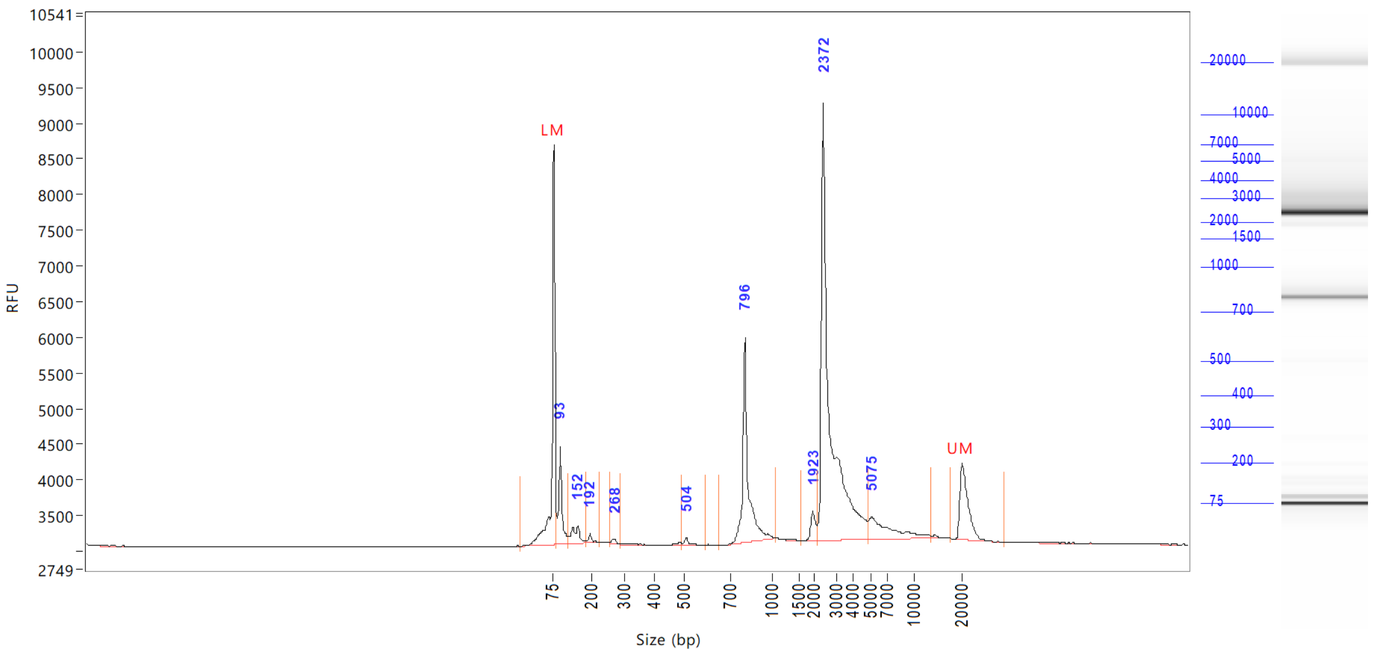

Peak	Size (bp)	Concentration (ng/uL)	From (bp)	To (bp)	Average size (bp)	CV%	RFU	Corrected peak area
1	75 (LM)	0.5000	0	84	66	24.06	5600	41.228
2	93	1.6568	84	124	95	8.95	1371	11.384
3	152	0.6363	124	183	144	9.31	249	4.372
4	192	0.1283	183	221	192	3.30	123	0.881
5	268	0.0805	251	284	263	2.00	64	0.553
6	504	0.1652	490	585	514	4.19	107	1.135
7	796	3.9476	646	1068	811	5.88	2864	27.125
8	1923	0.4606	1539	2087	1934	4.11	416	3.165
9	2372	11.8767	2087	4862	2832	22.44	6143	81.609
10	5075	1.4689	4862	13659	7203	28.94	309	10.093
11	20000 (UM)	0.1401	17683	28705	20640	5.27	1078	11.552

TIC:	20.4209	ng/uL
TIM:	53.6692	nmole/L
Total concentration:	20.5858	ng/uL

```
Sample peak width (sec): 5    Sample min peak height: 50    Sample baseline V to V?: Y    Sample baseline V to V points: 3
Sample filter: Binomial       Number of points for filter: 3  Sample start region (min): 0  Sample end region (min): 30
Marker peak width (sec): 5    Marker min peak height: 200   Marker baseline V to V?: Y    Marker baseline V to V points: 3
Lower marker selection: First peak > 500 RFU                Upper marker selection: Last peak > 200 RFU
Ladder size (bp)75, 200, 300, 400, 500, 700, 1000, 1500, 2000, 3000, 4000, 5000, 7000, 10000, 20000
Quantification using: Lower Marker          Final concentration (ng/uL): 0.5000          Dilution factor: 12.0
```

Sample: SampD9
Well location: D9
Created: Monday, September 20, 2021 8:00:01 PM

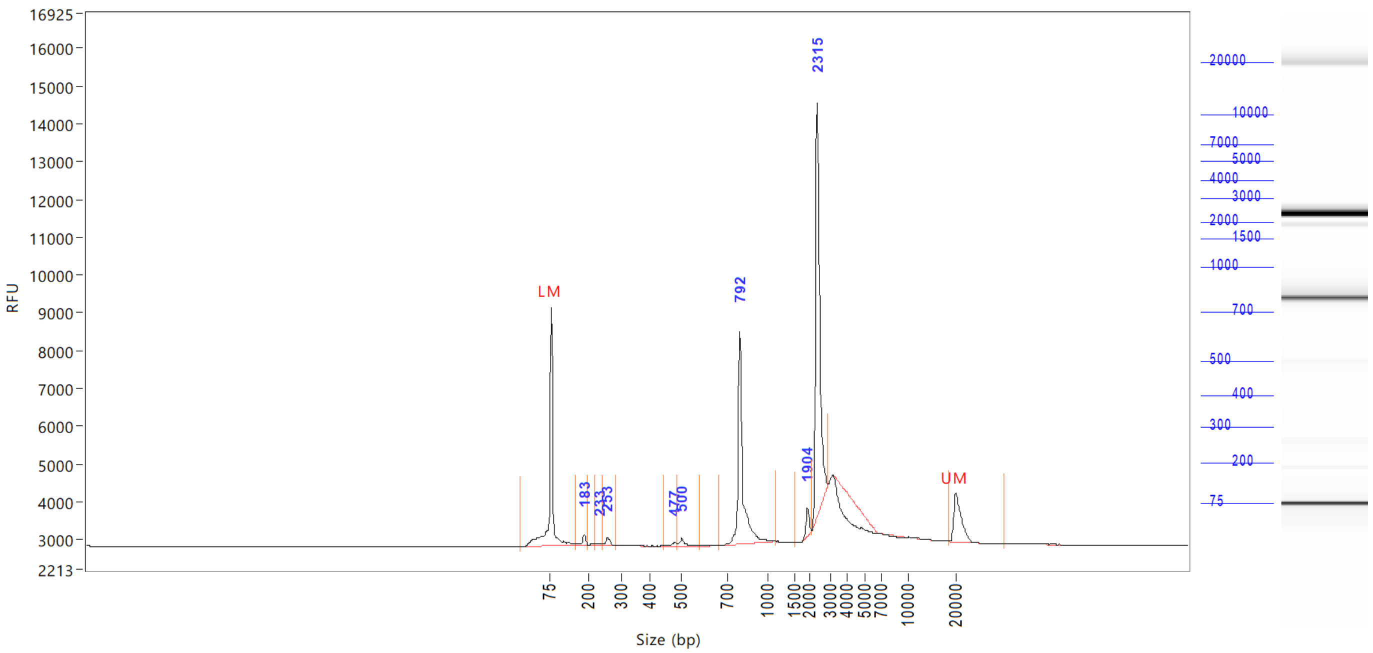

Peak	Size (bp)	Concentration (ng/uL)	From (bp)	To (bp)	Average size (bp)	CV%	RFU	Corrected peak area
1	75 (LM)	0.5000	0	156	68	33.11	6315	52.271
2	183	0.2425	156	194	177	4.38	288	2.113
3	233	0.0654	215	242	230	3.22	53	0.569
4	253	0.2269	242	282	255	2.35	199	1.976
5	477	0.1139	445	487	472	2.07	100	0.992
6	500	0.2482	487	580	509	4.13	211	2.163
7	792	5.2183	664	1134	805	5.84	5642	45.461
8	1904	0.4607	1520	2058	1893	2.66	746	4.013
9	2315	7.9852	2058	2857	2344	4.91	10985	69.566
10	20000 (UM)	0.1232	18415	29914	20537	4.84	1295	12.884

TIC:	14.5610	ng/uL
TIM:	22.0597	nmole/L
Total concentration:	14.7455	ng/uL

```
Sample peak width (sec): 5     Sample min peak height: 50     Sample baseline V to V?: Y     Sample baseline V to V points: 3
Sample filter: Binomial        Number of points for filter: 3  Sample start region (min): 0  Sample end region (min): 30
Marker peak width (sec): 5     Marker min peak height: 200    Marker baseline V to V?: Y     Marker baseline V to V points: 3
Lower marker selection: First peak > 500 RFU                  Upper marker selection: Last peak > 200 RFU
Ladder size (bp)75, 200, 300, 400, 500, 700, 1000, 1500, 2000, 3000, 4000, 5000, 7000, 10000, 20000
Quantification using: Lower Marker          Final concentration (ng/uL): 0.5000          Dilution factor: 12.0
```

Sample: SampE9
Well location: E9
Created: Monday, September 20, 2021 8:00:01 PM

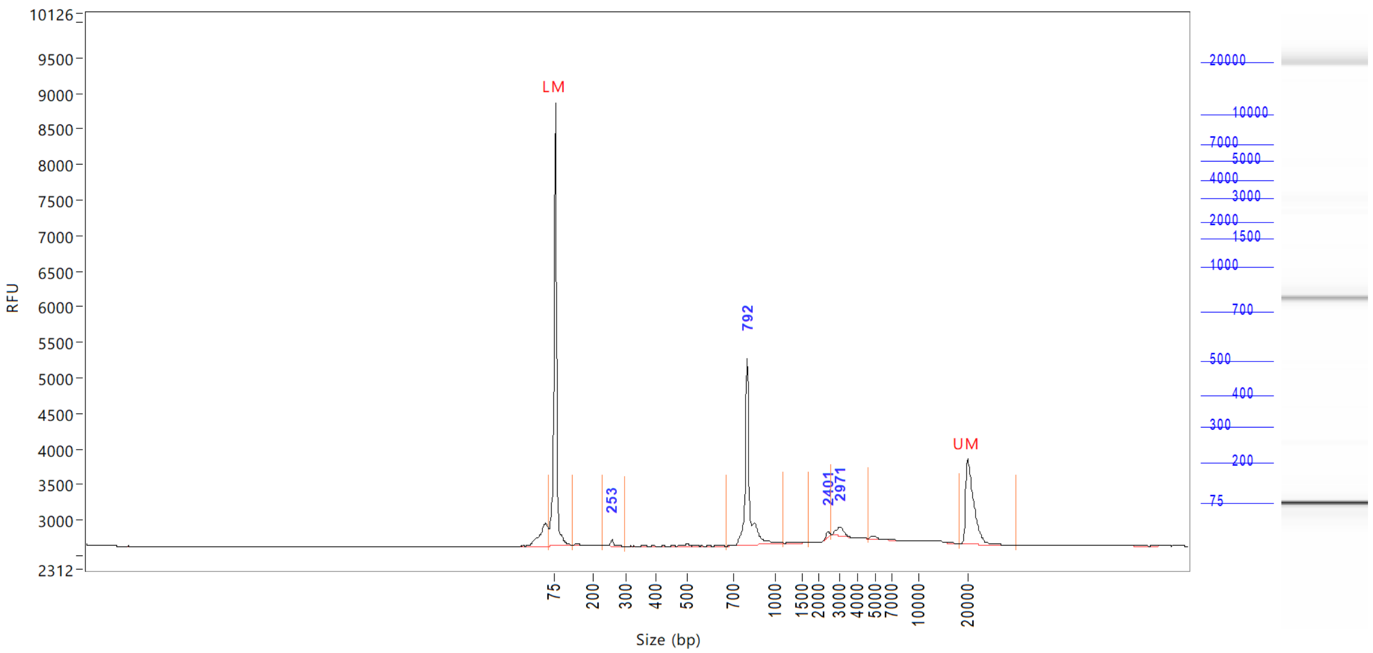

Peak	Size (bp)	Concentration (ng/uL)	From (bp)	To (bp)	Average size (bp)	CV%	RFU	Corrected peak area
1	75 (LM)	0.5000	56	129	74	10.00	6238	43.291
2	253	0.0911	228	293	253	3.98	88	0.657
3	792	2.8260	664	1145	798	5.51	2615	20.390
4	2401	0.0643	1674	2600	2354	5.76	84	0.464
5	2971	0.2227	2600	4621	3050	10.67	121	1.607
6	20000 (UM)	0.1474	18171	29914	20702	5.46	1198	12.765

TIC:	3.2040	ng/uL
TIM:	6.5857	nmole/L
Total concentration:	3.5982	ng/uL

```
Sample peak width (sec): 5    Sample min peak height: 50    Sample baseline V to V?: Y    Sample baseline V to V points: 3
Sample filter: Binomial       Number of points for filter: 3  Sample start region (min): 0  Sample end region (min): 30
Marker peak width (sec): 5    Marker min peak height: 200   Marker baseline V to V?: Y    Marker baseline V to V points: 3
Lower marker selection: First peak > 500 RFU                Upper marker selection: Last peak > 200 RFU
Ladder size (bp)75, 200, 300, 400, 500, 700, 1000, 1500, 2000, 3000, 4000, 5000, 7000, 10000, 20000
Quantification using: Lower Marker          Final concentration (ng/uL): 0.5000          Dilution factor: 12.0
```

Sample: SampF9
Well location: F9
Created: Monday, September 20, 2021 8:00:01 PM

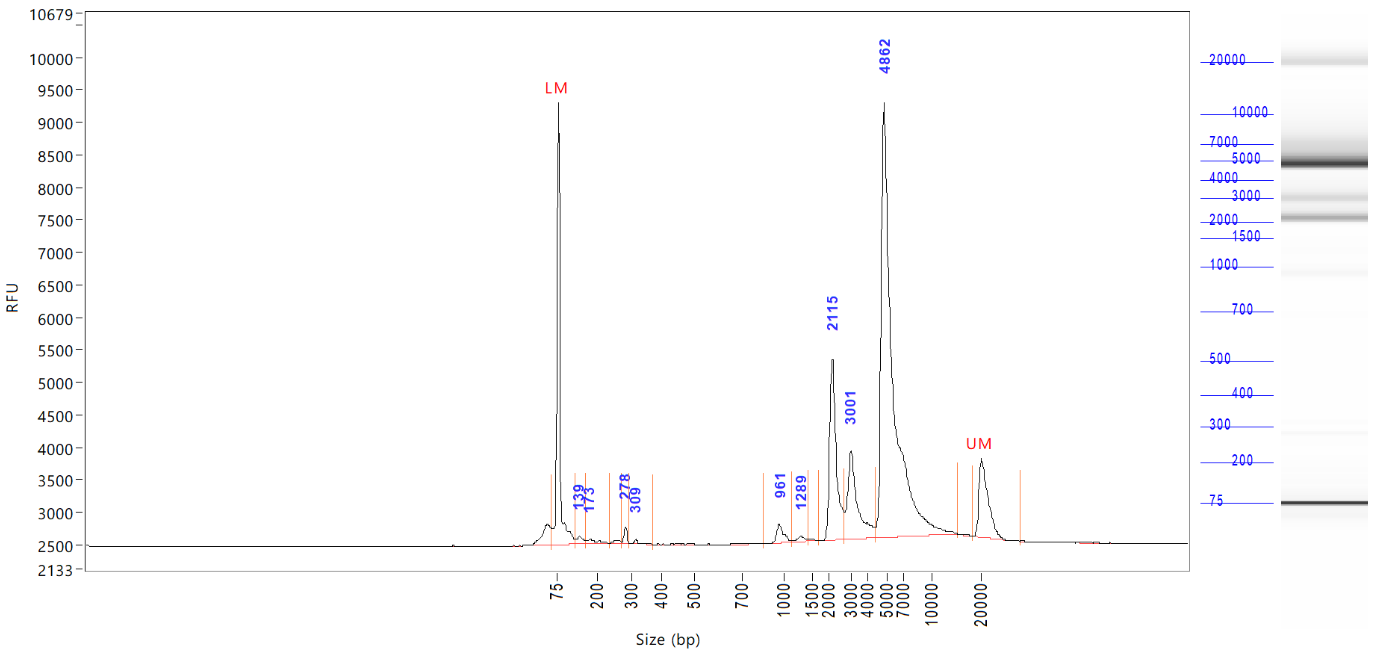

Peak	Size (bp)	Concentration (ng/uL)	From (bp)	To (bp)	Average size (bp)	CV%	RFU	Corrected peak area
1	75 (LM)	0.5000	56	129	75	14.42	6804	48.273
2	139	0.2164	129	164	144	6.57	123	1.741
3	173	0.1795	164	232	186	8.66	66	1.444
4	278	0.1996	268	293	277	1.43	264	1.606
5	309	0.0716	293	368	317	5.29	66	0.576
6	961	0.4112	848	1112	970	3.54	286	3.308
7	1289	0.1253	1112	1422	1273	5.82	78	1.008
8	2115	3.6775	1674	2715	2195	8.67	2781	29.587
9	3001	2.5524	2715	4380	3227	12.61	1349	20.535
10	4862	13.9842	4380	15244	5749	24.70	6674	112.510
11	20000 (UM)	0.1497	18415	27738	20630	5.05	1210	14.449

TIC:	21.4178	ng/uL
TIM:	14.5375	nmole/L
Total concentration:	21.6191	ng/uL

```
Sample peak width (sec): 5    Sample min peak height: 50    Sample baseline V to V?: Y    Sample baseline V to V points: 3
Sample filter: Binomial       Number of points for filter: 3  Sample start region (min): 0  Sample end region (min): 30
Marker peak width (sec): 5    Marker min peak height: 200   Marker baseline V to V?: Y    Marker baseline V to V points: 3
Lower marker selection: First peak > 500 RFU                Upper marker selection: Last peak > 200 RFU
Ladder size (bp)75, 200, 300, 400, 500, 700, 1000, 1500, 2000, 3000, 4000, 5000, 7000, 10000, 20000
Quantification using: Lower Marker          Final concentration (ng/uL): 0.5000          Dilution factor: 12.0
```

Sample: SampG9
Well location: G9
Created: Monday, September 20, 2021 8:00:01 PM

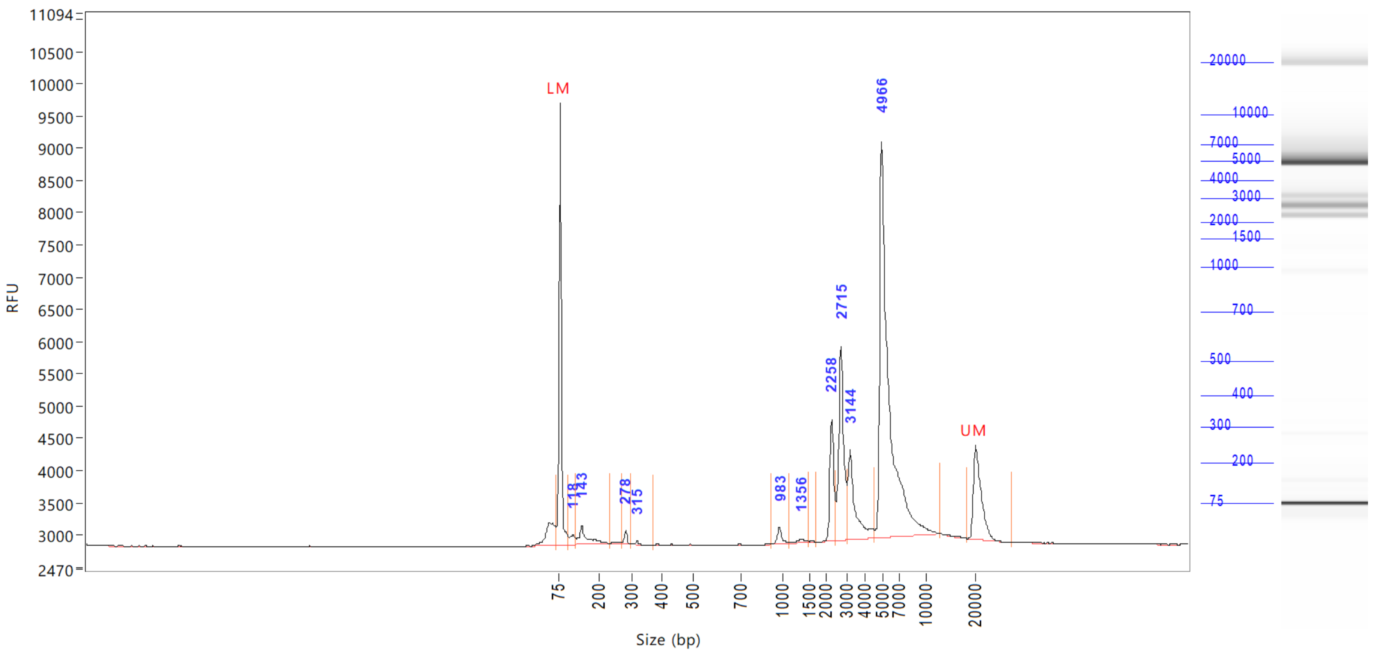

Peak	Size (bp)	Concentration (ng/uL)	From (bp)	To (bp)	Average size (bp)	CV%	RFU	Corrected peak area
1	75 (LM)	0.5000	64	103	74	7.54	6870	40.866
2	118	0.2771	103	126	114	5.87	157	1.888
3	143	0.7574	126	230	157	14.51	298	5.159
4	278	0.1664	268	295	277	1.32	203	1.133
5	315	0.0483	295	374	316	4.45	50	0.329
6	983	0.2893	918	1134	985	3.48	258	1.971
7	1356	0.1135	1134	1489	1323	5.97	63	0.773
8	2258	1.7647	1674	2458	2255	4.43	1879	12.020
9	2715	3.5270	2458	3000	2713	4.80	2996	24.023
10	3144	2.3291	3000	4552	3413	12.05	1388	15.864
11	4966	11.8056	4552	12806	5756	20.39	6145	80.408
12	20000 (UM)	0.1792	18293	27254	20548	4.53	1452	14.650

TIC:	21.0785	ng/uL
TIM:	21.7149	nmole/L
Total concentration:	21.1238	ng/uL

```
Sample peak width (sec): 5     Sample min peak height: 50     Sample baseline V to V?: Y     Sample baseline V to V points: 3
Sample filter: Binomial        Number of points for filter: 3  Sample start region (min): 0  Sample end region (min): 30
Marker peak width (sec): 5     Marker min peak height: 200    Marker baseline V to V?: Y     Marker baseline V to V points: 3
Lower marker selection: First peak > 500 RFU                  Upper marker selection: Last peak > 200 RFU
Ladder size (bp)75, 200, 300, 400, 500, 700, 1000, 1500, 2000, 3000, 4000, 5000, 7000, 10000, 20000
Quantification using: Lower Marker          Final concentration (ng/uL): 0.5000          Dilution factor: 12.0
```

Sample: SampH9
Well location: H9
Created: Monday, September 20, 2021 8:00:01 PM

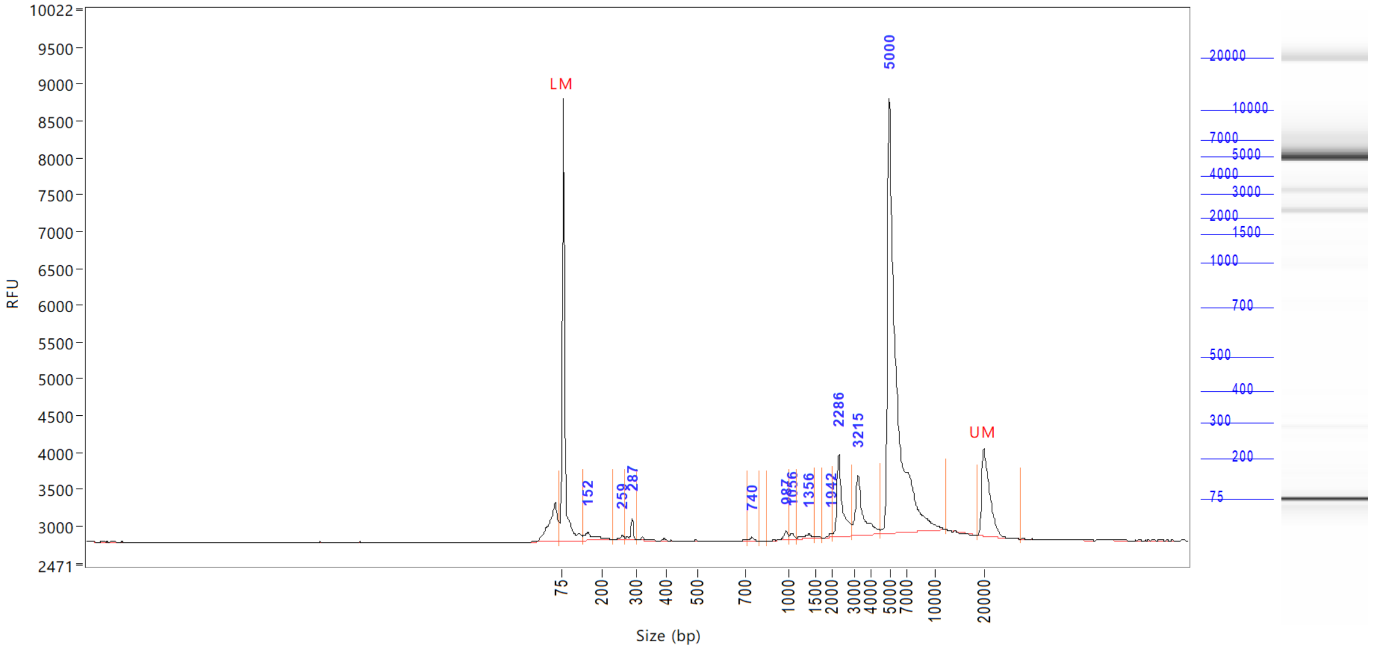

Peak	Size (bp)	Concentration (ng/uL)	From (bp)	To (bp)	Average size (bp)	CV%	RFU	Corrected peak area
1	75 (LM)	0.5000	66	137	77	14.87	6018	38.865
2	152	0.3905	137	230	166	13.17	114	2.530
3	259	0.0801	230	266	255	2.94	58	0.519
4	287	0.2914	266	303	284	2.30	300	1.888
5	740	0.0616	710	788	736	2.25	51	0.399
6	987	0.1379	848	1001	974	2.37	124	0.893
7	1056	0.1007	1001	1145	1058	3.59	87	0.652
8	1356	0.1362	1145	1489	1314	6.60	58	0.882
9	1942	0.0486	1693	2001	1933	3.01	54	0.315
10	2286	1.5163	2001	2914	2371	8.81	1123	9.822
11	3215	1.4208	2914	4483	3405	10.82	817	9.203
12	5000	11.5984	4483	12440	5744	19.71	5896	75.129
13	20000 (UM)	0.1567	18781	27254	20530	4.72	1192	12.178

TIC:	15.7825	ng/uL
TIM:	11.8751	nmole/L
Total concentration:	15.9961	ng/uL

Sample peak width (sec): 5   Sample min peak height: 50   Sample baseline V to V?: Y   Sample baseline V to V points: 3
Sample filter: Binomial   Number of points for filter: 3 Sample start region (min): 0 Sample end region (min): 30
Marker peak width (sec): 5   Marker min peak height: 200   Marker baseline V to V?: Y   Marker baseline V to V points: 3
Lower marker selection: First peak > 500 RFU   Upper marker selection: Last peak > 200 RFU
Ladder size (bp)75, 200, 300, 400, 500, 700, 1000, 1500, 2000, 3000, 4000, 5000, 7000, 10000, 20000
Quantification using: Lower Marker   Final concentration (ng/uL): 0.5000   Dilution factor: 12.0

Sample: SampA10
Well location: A10
Created: Monday, September 20, 2021 8:00:01 PM

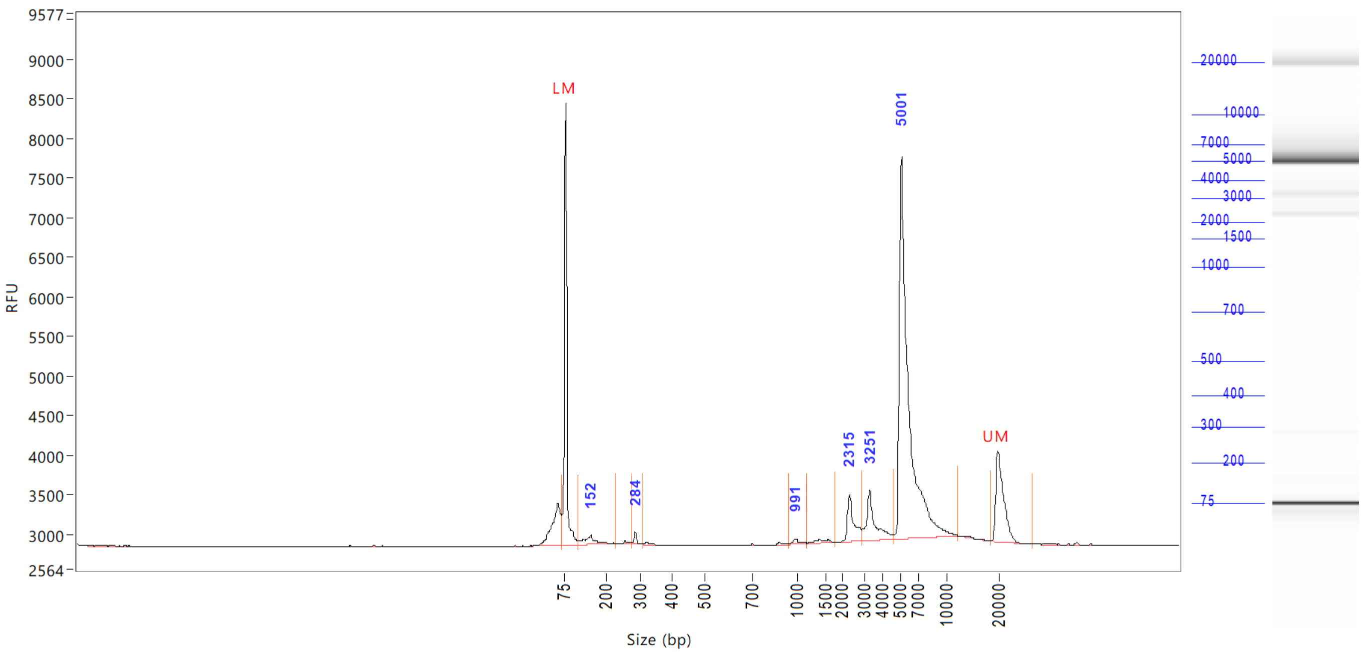

Peak	Size (bp)	Concentration (ng/uL)	From (bp)	To (bp)	Average size (bp)	CV%	RFU	Corrected peak area
1	75 (LM)	0.5000	64	114	74	9.16	5587	34.042
2	152	0.4880	114	226	150	15.92	112	2.769
3	284	0.1484	275	305	282	1.39	159	0.842
4	991	0.1266	944	1178	1011	5.25	71	0.718
5	2315	0.9934	1770	2886	2429	8.58	598	5.636
6	3251	1.3734	2886	4587	3458	11.97	631	7.792
7	5001	10.6852	4587	12196	5797	18.94	4828	60.624
8	20000 (UM)	0.1692	18415	26770	20481	4.58	1149	11.521

TIC:	13.8150	ng/uL
TIM:	10.7803	nmole/L
Total concentration:	14.2953	ng/uL

Sample peak width (sec): 5   Sample min peak height: 50   Sample baseline V to V?: Y   Sample baseline V to V points: 3
Sample filter: Binomial   Number of points for filter: 3 Sample start region (min): 0 Sample end region (min): 30
Marker peak width (sec): 5   Marker min peak height: 200   Marker baseline V to V?: Y   Marker baseline V to V points: 3
Lower marker selection: First peak > 500 RFU   Upper marker selection: Last peak > 200 RFU
Ladder size (bp)75, 200, 300, 400, 500, 700, 1000, 1500, 2000, 3000, 4000, 5000, 7000, 10000, 20000
Quantification using: Lower Marker   Final concentration (ng/uL): 0.5000   Dilution factor: 12.0

Sample: SampB10
Well location: B10
Created: Monday, September 20, 2021 8:00:01 PM

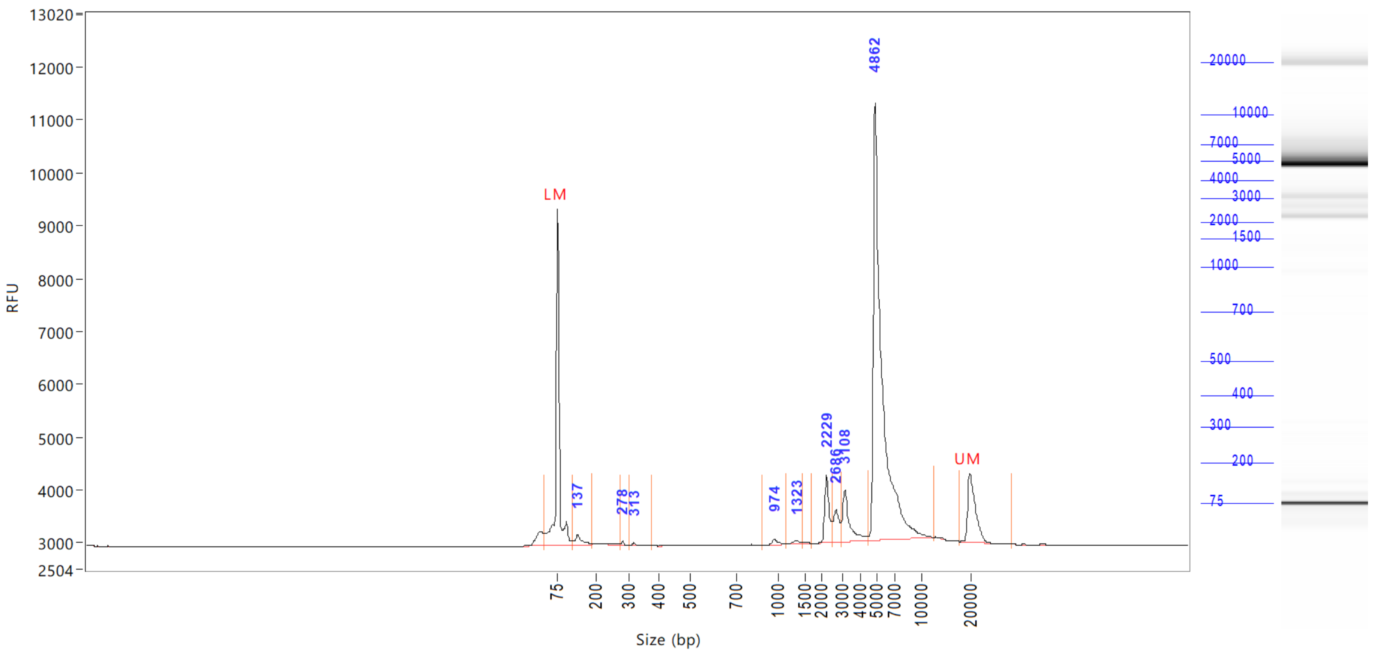

Peak	Size (bp)	Concentration (ng/uL)	From (bp)	To (bp)	Average size (bp)	CV%	RFU	Corrected peak area
1	75 (LM)	0.5000	33	122	73	18.60	6355	48.475
2	137	0.3926	122	185	144	10.05	199	3.172
3	278	0.0448	271	300	277	1.37	69	0.362
4	313	0.0541	300	378	325	6.34	56	0.437
5	974	0.1303	883	1134	979	3.65	117	1.053
6	1323	0.0947	1134	1456	1308	5.32	69	0.765
7	2229	1.1431	1693	2515	2246	5.62	1290	9.235
8	2686	0.7194	2515	2914	2703	4.27	613	5.812
9	3108	1.3096	2914	4449	3319	11.36	977	10.581
10	4862	12.8684	4449	12684	5556	19.42	8286	103.966
11	20000 (UM)	0.1350	17805	28342	20508	4.65	1302	13.091

TIC:	16.7570	ng/uL
TIM:	11.0974	nmole/L
Total concentration:	16.9271	ng/uL

Sample peak width (sec): 5 Sample min peak height: 50 Sample baseline V to V?: Y Sample baseline V to V points: 3
Sample filter: Binomial Number of points for filter: 3 Sample start region (min): 0 Sample end region (min): 30
Marker peak width (sec): 5 Marker min peak height: 200 Marker baseline V to V?: Y Marker baseline V to V points: 3
Lower marker selection: First peak > 500 RFU Upper marker selection: Last peak > 200 RFU
Ladder size (bp)75, 200, 300, 400, 500, 700, 1000, 1500, 2000, 3000, 4000, 5000, 7000, 10000, 20000
Quantification using: Lower Marker Final concentration (ng/uL): 0.5000 Dilution factor: 12.0

Sample: SampC10
Well location: C10
Created: Monday, September 20, 2021 8:00:01 PM

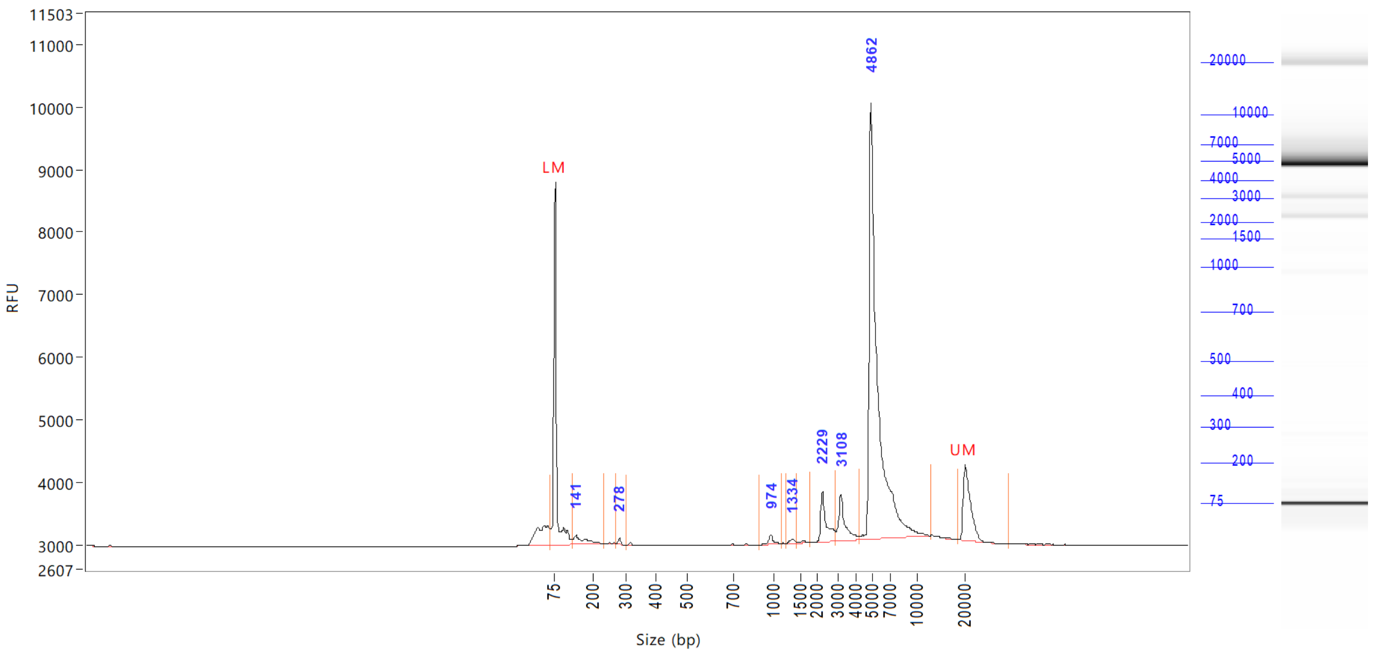

Peak	Size (bp)	Concentration (ng/uL)	From (bp)	To (bp)	Average size (bp)	CV%	RFU	Corrected peak area
1	75 (LM)	0.5000	62	131	77	16.18	5802	39.123
2	141	0.5219	131	233	161	14.20	144	3.403
3	278	0.0752	268	301	276	1.56	93	0.490
4	974	0.1922	883	1123	980	3.80	153	1.253
5	1334	0.0883	1201	1411	1314	3.58	67	0.576
6	2229	1.1616	1750	2886	2360	9.77	820	7.574
7	3108	1.1289	2886	4242	3288	9.99	748	7.361
8	4862	12.8977	4242	12928	5565	19.64	6992	84.099
9	20000 (UM)	0.1509	18659	29068	20548	4.61	1206	11.807

TIC:	16.0658	ng/uL
TIM:	11.4004	nmole/L
Total concentration:	16.3795	ng/uL

Sample peak width (sec): 5 Sample min peak height: 50 Sample baseline V to V?: Y Sample baseline V to V points: 3
Sample filter: Binomial Number of points for filter: 3 Sample start region (min): 0 Sample end region (min): 30
Marker peak width (sec): 5 Marker min peak height: 200 Marker baseline V to V?: Y Marker baseline V to V points: 3
Lower marker selection: First peak > 500 RFU Upper marker selection: Last peak > 200 RFU
Ladder size (bp)75, 200, 300, 400, 500, 700, 1000, 1500, 2000, 3000, 4000, 5000, 7000, 10000, 20000
Quantification using: Lower Marker Final concentration (ng/uL): 0.5000 Dilution factor: 12.0

Sample: SampD10
Well location: D10
Created: Monday, September 20, 2021 8:00:01 PM

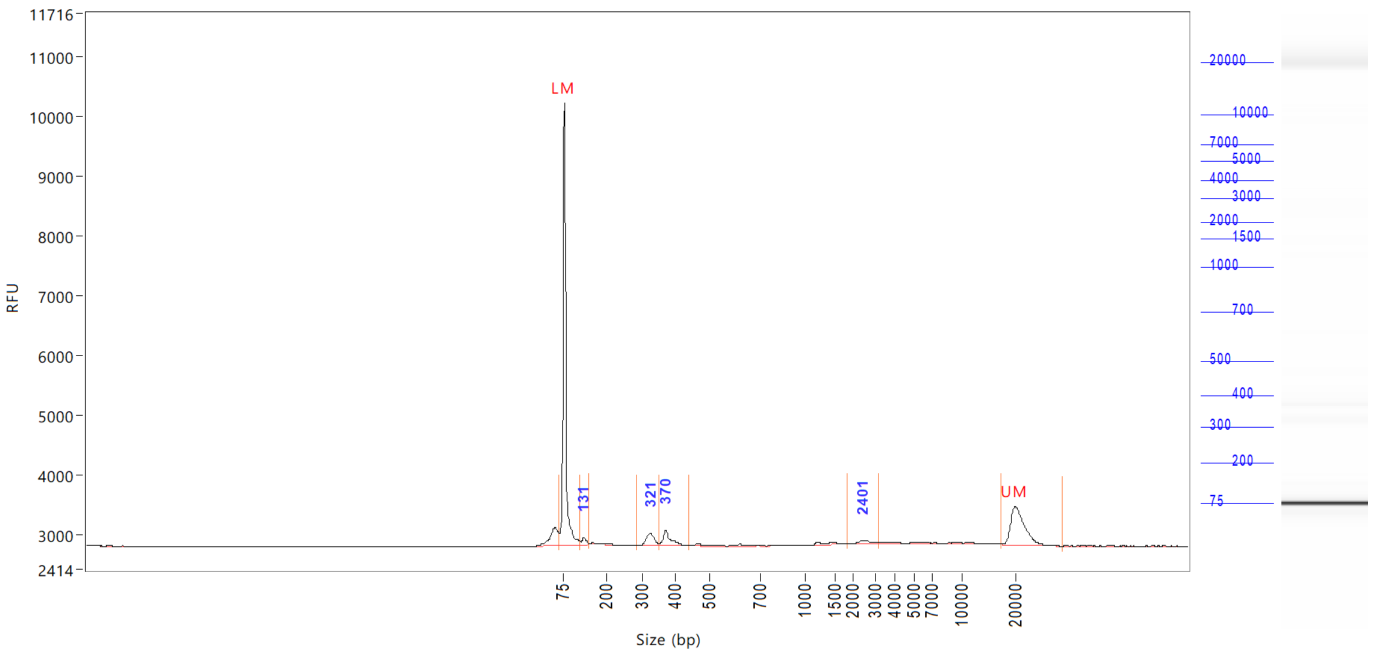

Peak	Size (bp)	Concentration (ng/uL)	From (bp)	To (bp)	Average size (bp)	CV%	RFU	Corrected peak area
1	75 (LM)	0.5000	58	124	75	11.02	7415	52.285
2	131	0.1339	124	150	132	4.67	117	1.166
3	321	0.4126	286	349	320	3.91	210	3.595
4	370	0.4901	349	441	379	5.07	263	4.271
5	2401	0.1237	1866	3108	2491	11.60	57	1.078
6	20000 (UM)	0.1046	17317	28947	20666	6.82	641	10.939

TIC:	1.1603	ng/uL
TIM:	5.9966	nmole/L
Total concentration:	1.7214	ng/uL

Sample peak width (sec): 5   Sample min peak height: 50   Sample baseline V to V?: Y   Sample baseline V to V points: 3
Sample filter: Binomial   Number of points for filter: 3   Sample start region (min): 0   Sample end region (min): 30
Marker peak width (sec): 5   Marker min peak height: 200   Marker baseline V to V?: Y   Marker baseline V to V points: 3
Lower marker selection: First peak > 500 RFU   Upper marker selection: Last peak > 200 RFU
Ladder size (bp)75, 200, 300, 400, 500, 700, 1000, 1500, 2000, 3000, 4000, 5000, 7000, 10000, 20000
Quantification using: Lower Marker   Final concentration (ng/uL): 0.5000   Dilution factor: 12.0

Sample: SampE10
Well location: E10
Created: Monday, September 20, 2021 8:00:01 PM

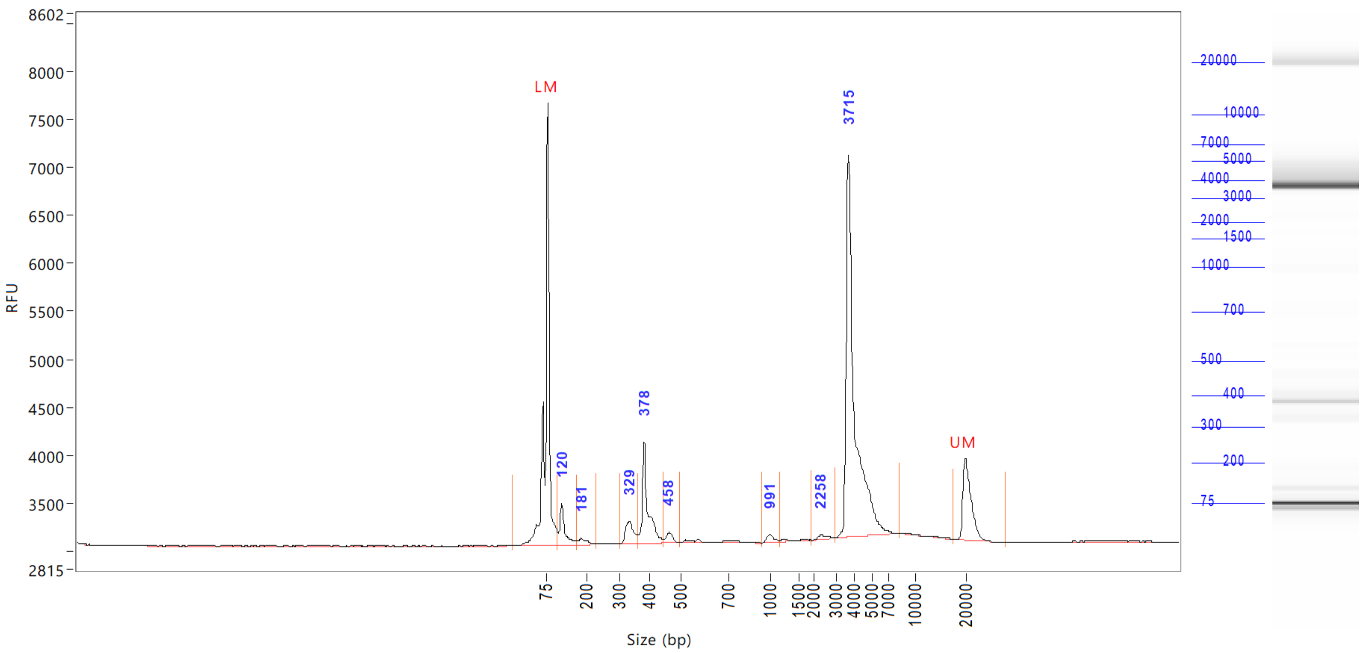

Peak	Size (bp)	Concentration (ng/uL)	From (bp)	To (bp)	Average size (bp)	CV%	RFU	Corrected peak area
1	75 (LM)	0.5000	0	107	67	23.63	4612	44.649
2	120	0.7053	107	169	126	12.31	426	5.249
3	181	0.1576	169	228	188	6.77	66	1.172
4	329	0.5394	301	358	330	3.98	234	4.014
5	378	1.6419	358	443	387	4.41	1050	12.218
6	458	0.1840	443	492	459	1.90	107	1.369
7	991	0.1363	935	1145	1004	4.52	80	1.014
8	2258	0.0941	1923	2971	2371	9.47	50	0.700
9	3715	6.6284	2971	8201	4030	14.09	3980	49.325
10	20000 (UM)	0.1015	17683	28342	20554	4.82	855	9.063

TIC:	10.0870	ng/uL
TIM:	23.9002	nmole/L
Total concentration:	10.4080	ng/uL

Sample peak width (sec): 5 Sample min peak height: 50 Sample baseline V to V?: Y Sample baseline V to V points: 3
Sample filter: Binomial Number of points for filter: 3 Sample start region (min): 0 Sample end region (min): 30
Marker peak width (sec): 5 Marker min peak height: 200 Marker baseline V to V?: Y Marker baseline V to V points: 3
Lower marker selection: First peak > 500 RFU Upper marker selection: Last peak > 200 RFU
Ladder size (bp)75, 200, 300, 400, 500, 700, 1000, 1500, 2000, 3000, 4000, 5000, 7000, 10000, 20000
Quantification using: Lower Marker Final concentration (ng/uL): 0.5000 Dilution factor: 12.0

Sample: SampF10
Well location: F10
Created: Monday, September 20, 2021 8:00:01 PM

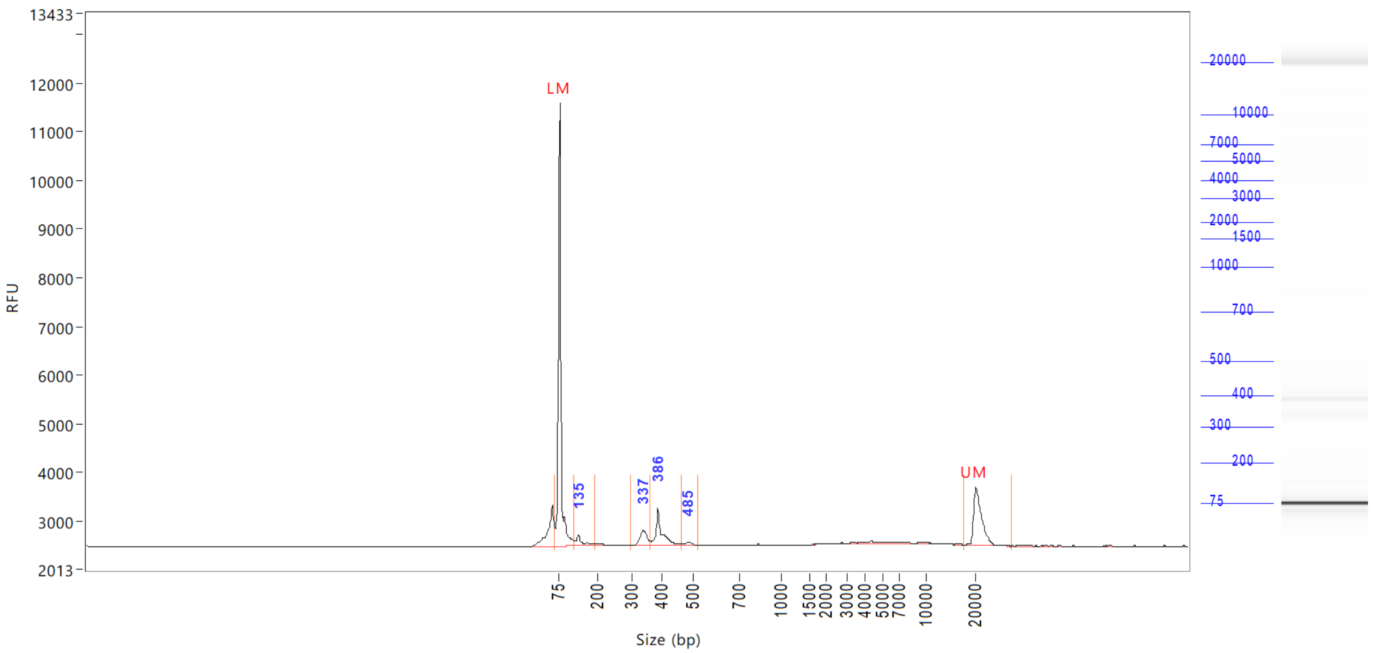

Peak	Size (bp)	Concentration (ng/uL)	From (bp)	To (bp)	Average size (bp)	CV%	RFU	Corrected peak area
1	75 (LM)	0.5000	62	124	76	12.02	9118	58.560
2	135	0.3297	124	189	145	11.86	213	3.218
3	337	0.5017	296	362	336	3.56	304	4.897
4	386	0.9459	362	462	396	5.07	770	9.232
5	485	0.1199	462	519	483	2.56	82	1.171
6	20000 (UM)	0.1047	17561	27254	20619	4.79	1196	12.266

TIC:	1.8973	ng/uL
TIM:	10.5324	nmole/L
Total concentration:	2.3843	ng/uL

Sample peak width (sec): 5 Sample min peak height: 50 Sample baseline V to V?: Y Sample baseline V to V points: 3
Sample filter: Binomial Number of points for filter: 3 Sample start region (min): 0 Sample end region (min): 30
Marker peak width (sec): 5 Marker min peak height: 200 Marker baseline V to V?: Y Marker baseline V to V points: 3
Lower marker selection: First peak > 500 RFU Upper marker selection: Last peak > 200 RFU
Ladder size (bp)75, 200, 300, 400, 500, 700, 1000, 1500, 2000, 3000, 4000, 5000, 7000, 10000, 20000
Quantification using: Lower Marker Final concentration (ng/uL): 0.5000 Dilution factor: 12.0

Sample: SampG10
Well location: G10
Created: Monday, September 20, 2021 8:00:01 PM

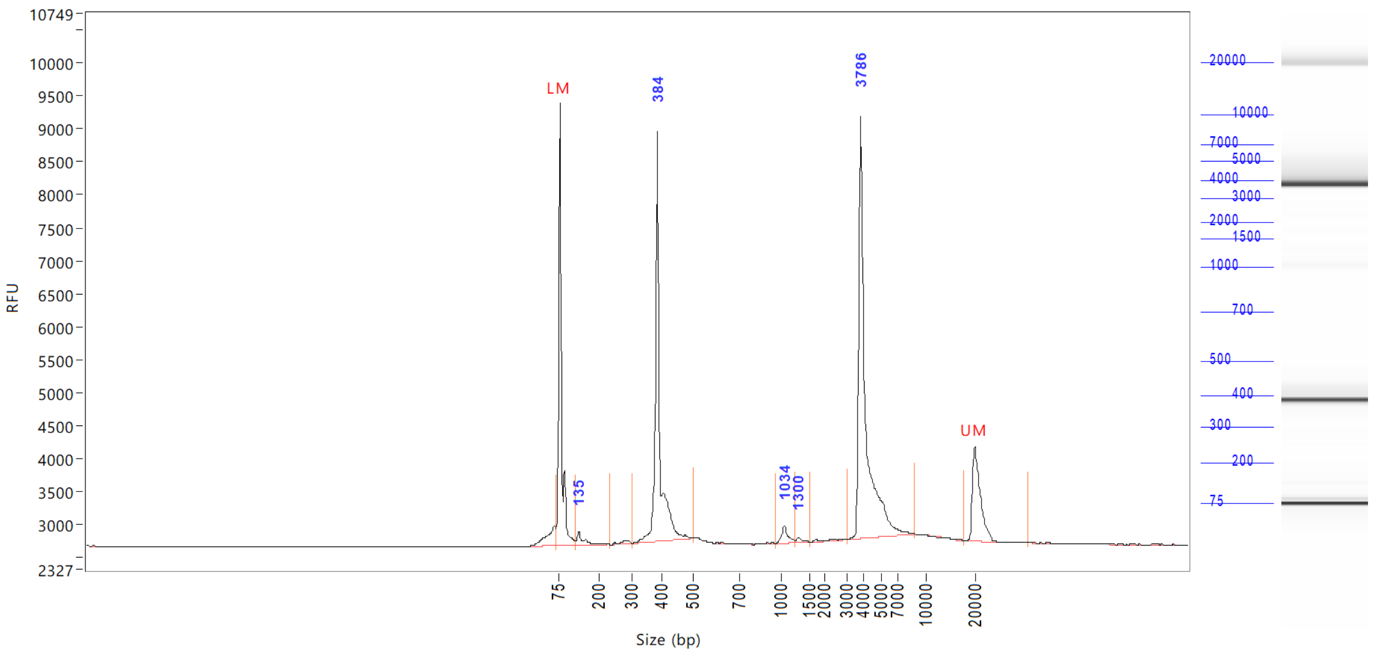

Peak	Size (bp)	Concentration (ng/uL)	From (bp)	To (bp)	Average size (bp)	CV%	RFU	Corrected peak area
1	75 (LM)	0.5000	64	124	76	11.92	6718	48.075
2	135	0.3909	124	232	150	14.95	204	3.132
3	384	6.7407	300	501	388	5.54	6215	54.010
4	1034	0.3466	957	1234	1054	6.24	269	2.777
5	1300	0.0955	1234	1501	1323	4.98	81	0.765
6	3786	8.1341	3037	8800	4145	15.89	6388	65.175
7	20000 (UM)	0.1540	17561	30760	20548	5.39	1424	14.811

TIC:	15.7078	ng/uL
TIM:	36.7569	nmole/L
Total concentration:	15.9819	ng/uL

Sample peak width (sec): 5   Sample min peak height: 50   Sample baseline V to V?: Y   Sample baseline V to V points: 3
Sample filter: Binomial   Number of points for filter: 3   Sample start region (min): 0   Sample end region (min): 30
Marker peak width (sec): 5   Marker min peak height: 200   Marker baseline V to V?: Y   Marker baseline V to V points: 3
Lower marker selection: First peak > 500 RFU   Upper marker selection: Last peak > 200 RFU
Ladder size (bp)75, 200, 300, 400, 500, 700, 1000, 1500, 2000, 3000, 4000, 5000, 7000, 10000, 20000
Quantification using: Lower Marker   Final concentration (ng/uL): 0.5000   Dilution factor: 12.0

Sample: SampH10
Well location: H10
Created: Monday, September 20, 2021 8:00:01 PM

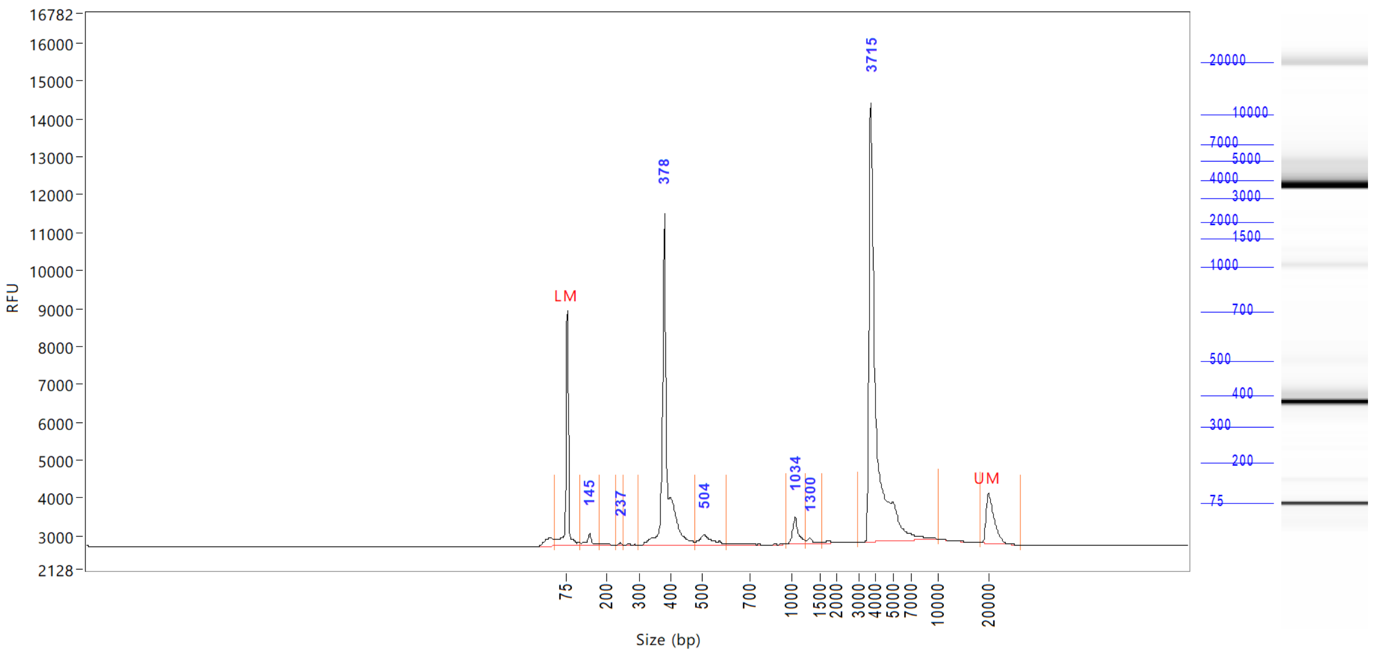

Peak	Size (bp)	Concentration (ng/uL)	From (bp)	To (bp)	Average size (bp)	CV%	RFU	Corrected peak area
1	75 (LM)	0.5000	37	116	72	13.67	6206	40.493
2	145	0.5136	116	177	141	8.48	328	3.466
3	237	0.0481	224	248	236	1.41	62	0.324
4	378	12.1347	293	473	382	5.82	8737	81.896
5	504	0.9937	473	600	519	6.00	274	6.706
6	1034	0.9845	961	1234	1056	5.94	711	6.645
7	1300	0.2072	1234	1539	1318	4.98	150	1.398
8	3715	17.3597	3001	10000	4115	19.16	11585	117.159
9	20000 (UM)	0.1656	18415	26529	20520	4.40	1320	13.409

TIC:	32.2415	ng/uL
TIM:	70.4727	nmole/L
Total concentration:	32.7700	ng/uL

```
Sample peak width (sec): 5    Sample min peak height: 50    Sample baseline V to V?: Y    Sample baseline V to V points: 3
Sample filter: Binomial       Number of points for filter: 3  Sample start region (min): 0  Sample end region (min): 30
Marker peak width (sec): 5    Marker min peak height: 200    Marker baseline V to V?: Y    Marker baseline V to V points: 3
Lower marker selection: First peak > 500 RFU                  Upper marker selection: Last peak > 200 RFU
Ladder size (bp)75, 200, 300, 400, 500, 700, 1000, 1500, 2000, 3000, 4000, 5000, 7000, 10000, 20000
Quantification using: Lower Marker          Final concentration (ng/uL): 0.5000          Dilution factor: 12.0
```

Sample: SampA11
Well location: A11
Created: Monday, September 20, 2021 8:00:01 PM

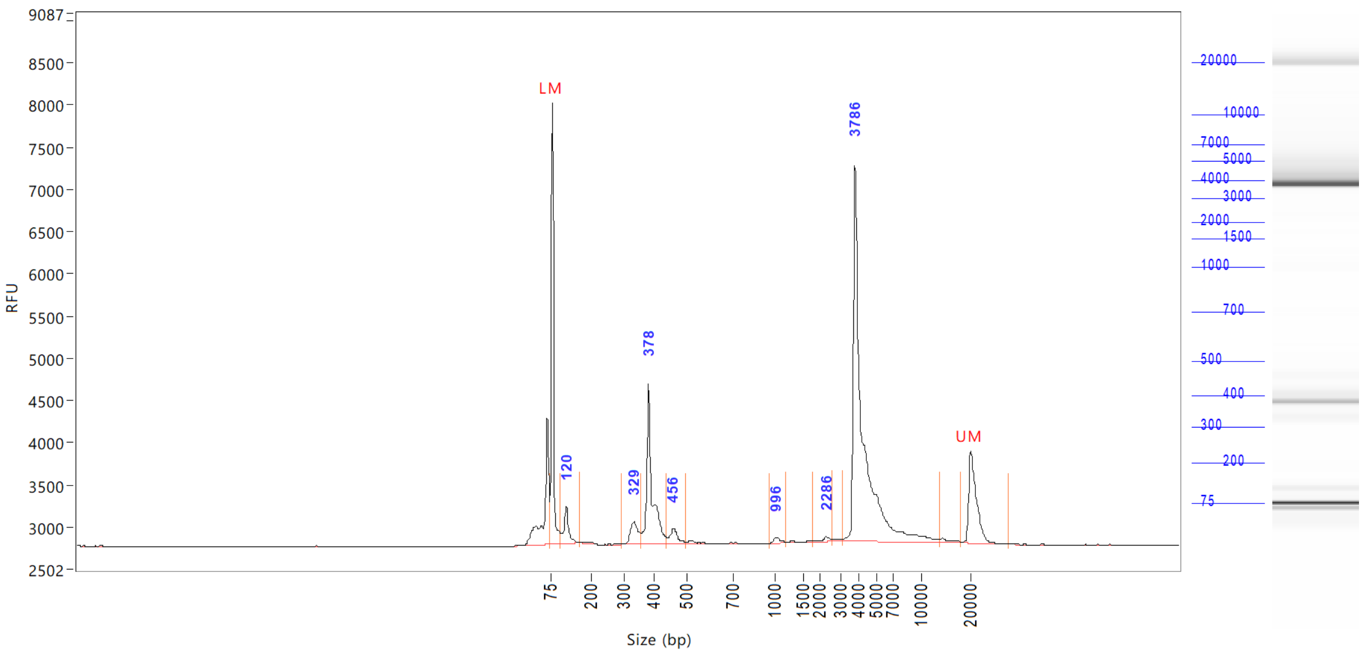

Peak	Size (bp)	Concentration (ng/uL)	From (bp)	To (bp)	Average size (bp)	CV%	RFU	Corrected peak area
1	75 (LM)	0.5000	67	103	75	7.46	5235	32.007
2	120	0.8977	103	164	119	8.14	441	4.789
3	329	0.8265	289	352	329	3.69	271	4.409
4	378	3.8782	352	437	385	4.38	1895	20.688
5	456	0.5069	437	498	458	2.77	176	2.704
6	996	0.1482	952	1178	1027	5.07	65	0.790
7	2286	0.1182	1770	2543	2231	8.48	56	0.631
8	3786	11.1049	3037	13781	4563	32.56	4442	59.240
9	20000 (UM)	0.1722	18171	27617	20579	4.89	1088	11.025

TIC:	17.4807	ng/uL
TIM:	39.2514	nmole/L
Total concentration:	17.9586	ng/uL

```
Sample peak width (sec): 5    Sample min peak height: 50    Sample baseline V to V?: Y    Sample baseline V to V points: 3
Sample filter: Binomial       Number of points for filter: 3  Sample start region (min): 0  Sample end region (min): 30
Marker peak width (sec): 5    Marker min peak height: 200   Marker baseline V to V?: Y    Marker baseline V to V points: 3
Lower marker selection: First peak > 500 RFU                Upper marker selection: Last peak > 200 RFU
Ladder size (bp)75, 200, 300, 400, 500, 700, 1000, 1500, 2000, 3000, 4000, 5000, 7000, 10000, 20000
Quantification using: Lower Marker          Final concentration (ng/uL): 0.5000          Dilution factor: 12.0
```

Sample: SampB11
Well location: B11
Created: Monday, September 20, 2021 8:00:01 PM

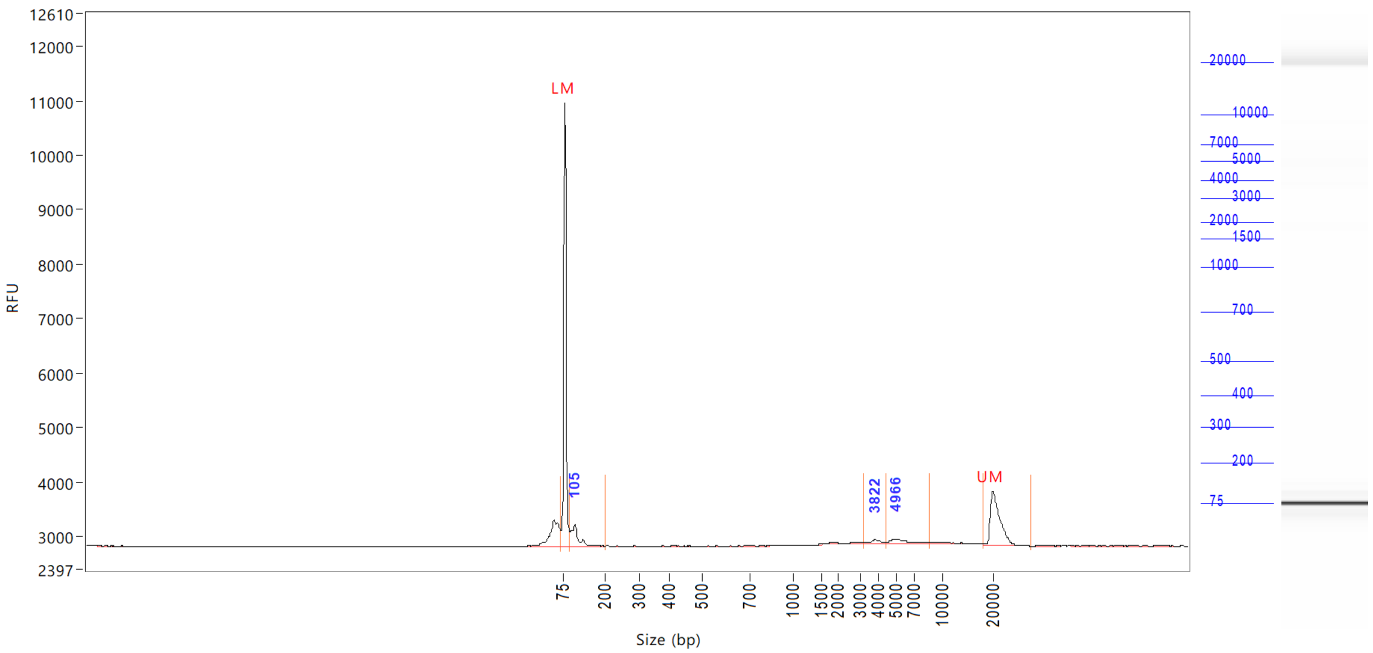

Peak	Size (bp)	Concentration (ng/uL)	From (bp)	To (bp)	Average size (bp)	CV%	RFU	Corrected peak area
1	75 (LM)	0.5000	62	89	73	5.69	8159	50.182
2	105	0.8088	89	200	108	15.47	389	6.765
3	3822	0.1136	3179	4449	3875	7.58	66	0.950
4	4966	0.2266	4449	8667	5674	19.02	84	1.896
5	20000 (UM)	0.1195	18293	27738	20709	5.29	987	11.996

TIC:	1.1491	ng/uL
TIM:	12.4142	nmole/L
Total concentration:	1.5001	ng/uL

```
Sample peak width (sec): 5    Sample min peak height: 50    Sample baseline V to V?: Y    Sample baseline V to V points: 3
Sample filter: Binomial       Number of points for filter: 3  Sample start region (min): 0  Sample end region (min): 30
Marker peak width (sec): 5    Marker min peak height: 200   Marker baseline V to V?: Y    Marker baseline V to V points: 3
Lower marker selection: First peak > 500 RFU                Upper marker selection: Last peak > 200 RFU
Ladder size (bp)75, 200, 300, 400, 500, 700, 1000, 1500, 2000, 3000, 4000, 5000, 7000, 10000, 20000
Quantification using: Lower Marker          Final concentration (ng/uL): 0.5000          Dilution factor: 12.0
```

Sample: SampC11
Well location: C11
Created: Monday, September 20, 2021 8:00:01 PM

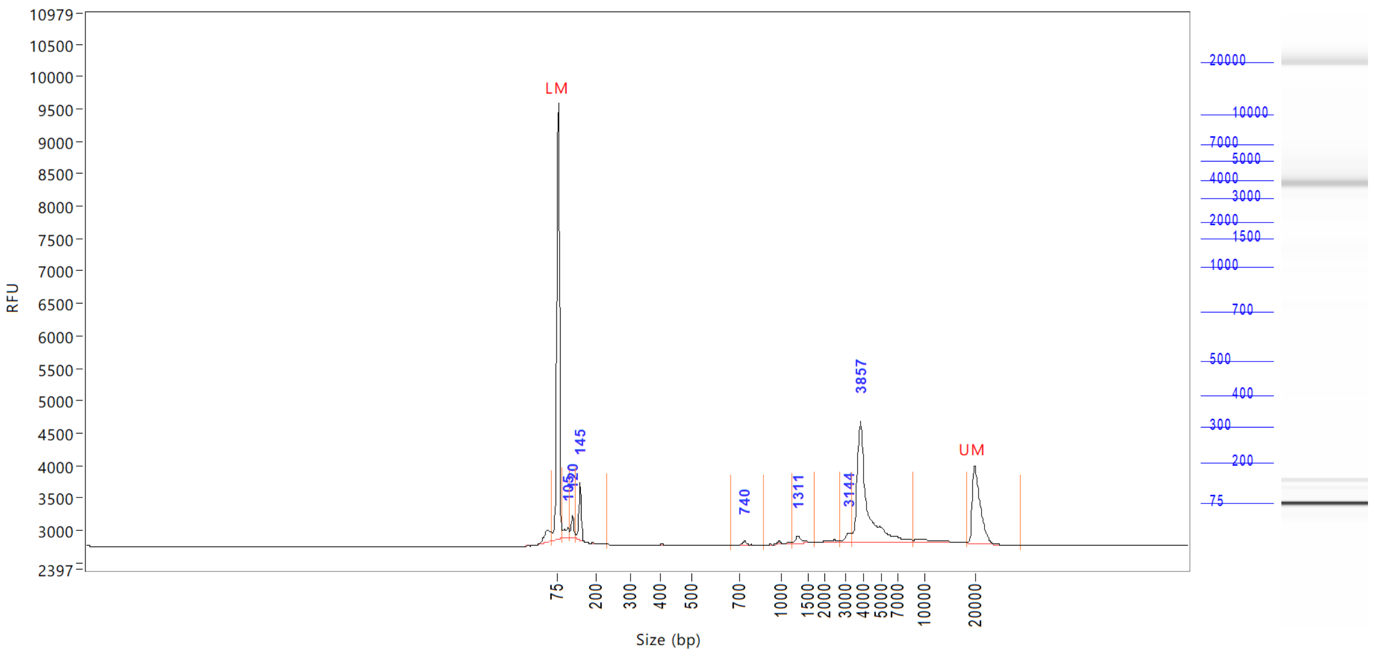

Peak	Size (bp)	Concentration (ng/uL)	From (bp)	To (bp)	Average size (bp)	CV%	RFU	Corrected peak area
1	75 (LM)	0.5000	58	87	72	5.47	6744	41.535
2	105	0.2818	87	112	100	7.49	150	1.950
3	120	0.3175	112	129	119	3.47	334	2.198
4	145	0.8074	129	228	144	8.46	890	5.589
5	740	0.1058	667	879	740	4.44	78	0.732
6	1311	0.1818	1201	1712	1339	6.49	118	1.259
7	3144	0.1980	2715	3322	3093	4.72	142	1.371
8	3857	3.9815	3322	8667	4281	21.26	1862	27.562
9	20000 (UM)	0.1496	18537	29430	20606	4.78	1205	12.423

TIC:	5.8738	ng/uL
TIM:	20.3198	nmole/L
Total concentration:	6.3318	ng/uL

```
Sample peak width (sec): 5     Sample min peak height: 50    Sample baseline V to V?: Y    Sample baseline V to V points: 3
Sample filter: Binomial        Number of points for filter: 3  Sample start region (min): 0  Sample end region (min): 30
Marker peak width (sec): 5     Marker min peak height: 200   Marker baseline V to V?: Y    Marker baseline V to V points: 3
Lower marker selection: First peak > 500 RFU                 Upper marker selection: Last peak > 200 RFU
Ladder size (bp)75, 200, 300, 400, 500, 700, 1000, 1500, 2000, 3000, 4000, 5000, 7000, 10000, 20000
Quantification using: Lower Marker          Final concentration (ng/uL): 0.5000          Dilution factor: 12.0
```

Sample: SampD11
Well location: D11
Created: Monday, September 20, 2021 8:00:01 PM

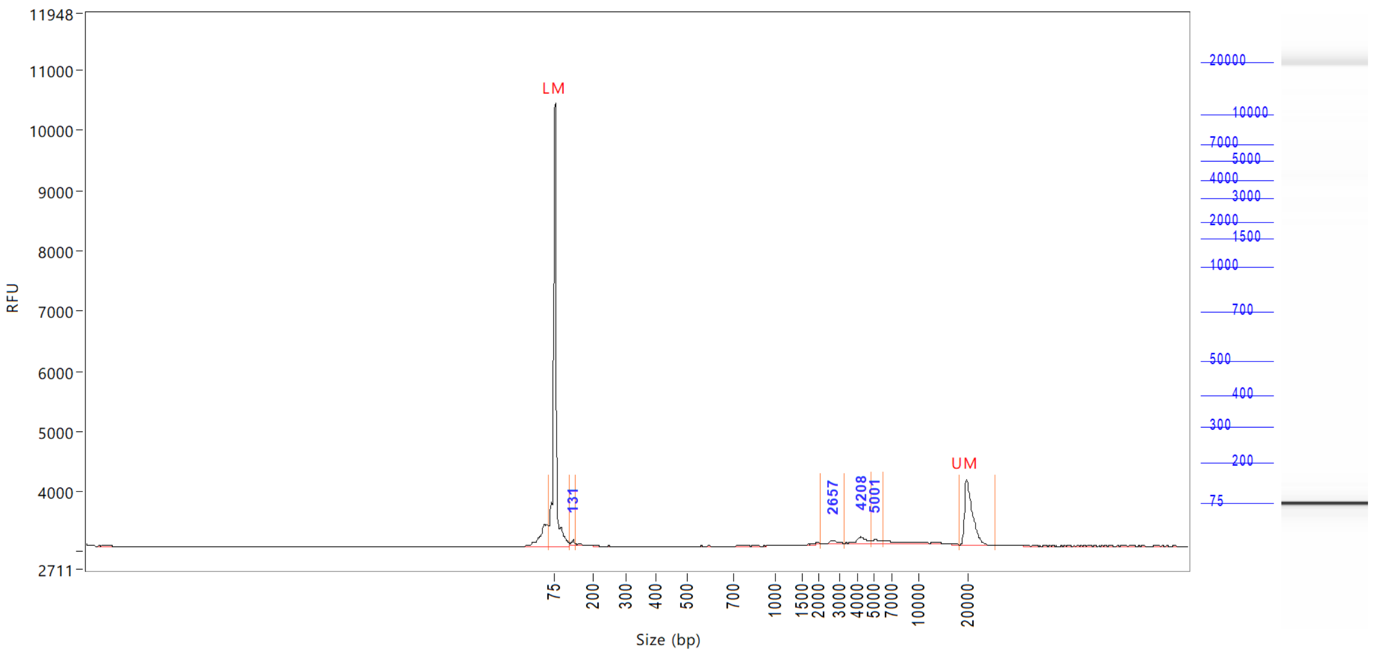

Peak	Size (bp)	Concentration (ng/uL)	From (bp)	To (bp)	Average size (bp)	CV%	RFU	Corrected peak area
1	75 (LM)	0.5000	54	122	73	12.12	7376	52.091
2	131	0.1000	122	143	130	4.05	104	0.868
3	2657	0.0875	2115	3215	2662	8.66	54	0.760
4	4208	0.1865	3215	4828	4225	7.67	104	1.619
5	5001	0.0766	4828	6112	5297	7.36	57	0.665
6	20000 (UM)	0.1090	18537	25803	20528	4.70	1093	11.355

TIC:	0.4505	ng/uL
TIM:	1.4139	nmole/L
Total concentration:	0.8485	ng/uL

Sample peak width (sec): 5 Sample min peak height: 50 Sample baseline V to V?: Y Sample baseline V to V points: 3
Sample filter: Binomial Number of points for filter: 3 Sample start region (min): 0 Sample end region (min): 30
Marker peak width (sec): 5 Marker min peak height: 200 Marker baseline V to V?: Y Marker baseline V to V points: 3
Lower marker selection: First peak > 500 RFU Upper marker selection: Last peak > 200 RFU
Ladder size (bp)75, 200, 300, 400, 500, 700, 1000, 1500, 2000, 3000, 4000, 5000, 7000, 10000, 20000
Quantification using: Lower Marker Final concentration (ng/uL): 0.5000 Dilution factor: 12.0

Sample: SampE11
Well location: E11
Created: Monday, September 20, 2021 8:00:01 PM

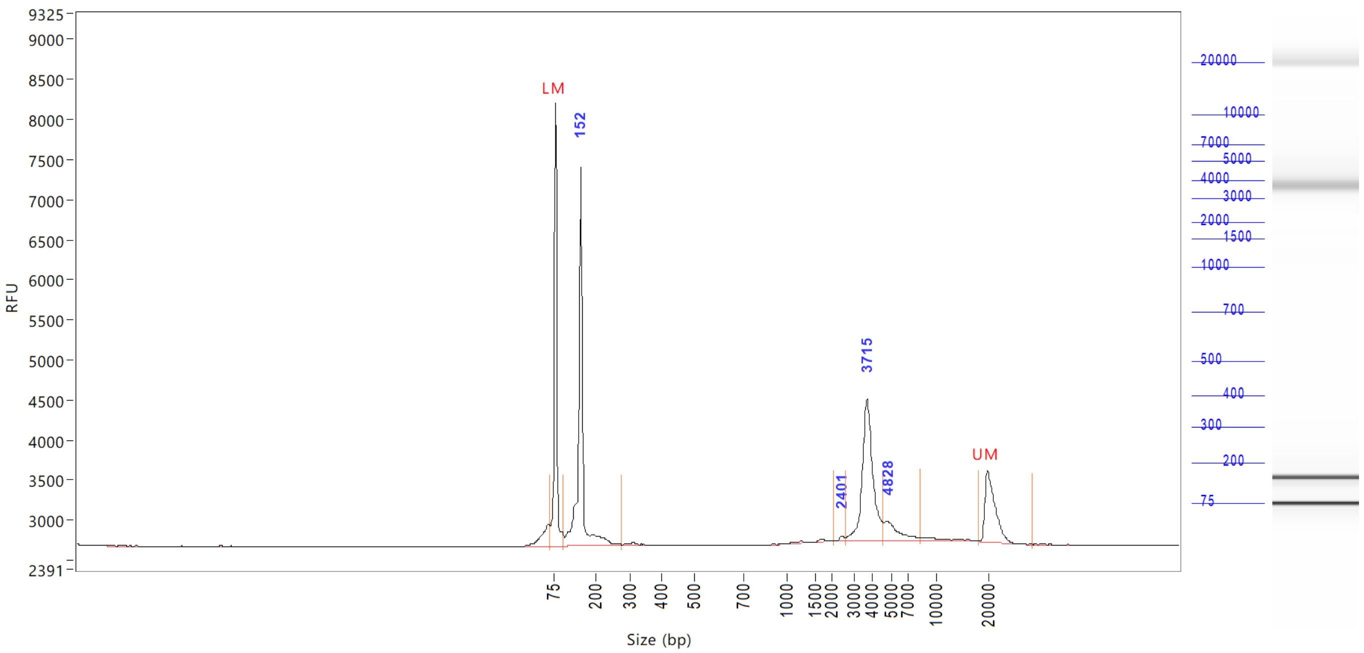

Peak	Size<br>(bp)	Concentration<br>(ng/uL)	From<br>(bp)	To<br>(bp)	Average size<br>(bp)	CV%	RFU	Corrected peak area
1	75 (LM)	0.5000	58	101	73	8.19	5537	37.024
2	152	7.3430	101	275	152	15.09	4725	45.311
3	2401	0.0665	2058	2572	2402	4.06	62	0.410
4	3715	4.5132	2572	4621	3723	9.64	1759	27.850
5	4828	0.8071	4621	8334	5575	17.44	236	4.980
6	20000 (UM)	0.1549	18293	28826	20699	5.63	886	11.469

TIC:	12.7299	ng/uL
TIM:	81.6786	nmole/L
Total concentration:	13.2937	ng/uL

```
Sample peak width (sec): 5    Sample min peak height: 50    Sample baseline V to V?: Y    Sample baseline V to V points: 3
Sample filter: Binomial       Number of points for filter: 3  Sample start region (min): 0  Sample end region (min): 30
Marker peak width (sec): 5    Marker min peak height: 200   Marker baseline V to V?: Y    Marker baseline V to V points: 3
Lower marker selection: First peak > 500 RFU                Upper marker selection: Last peak > 200 RFU
Ladder size (bp)75, 200, 300, 400, 500, 700, 1000, 1500, 2000, 3000, 4000, 5000, 7000, 10000, 20000
Quantification using: Lower Marker          Final concentration (ng/uL): 0.5000          Dilution factor: 12.0
```

Sample: SampF11
Well location: F11
Created: Monday, September 20, 2021 8:00:01 PM

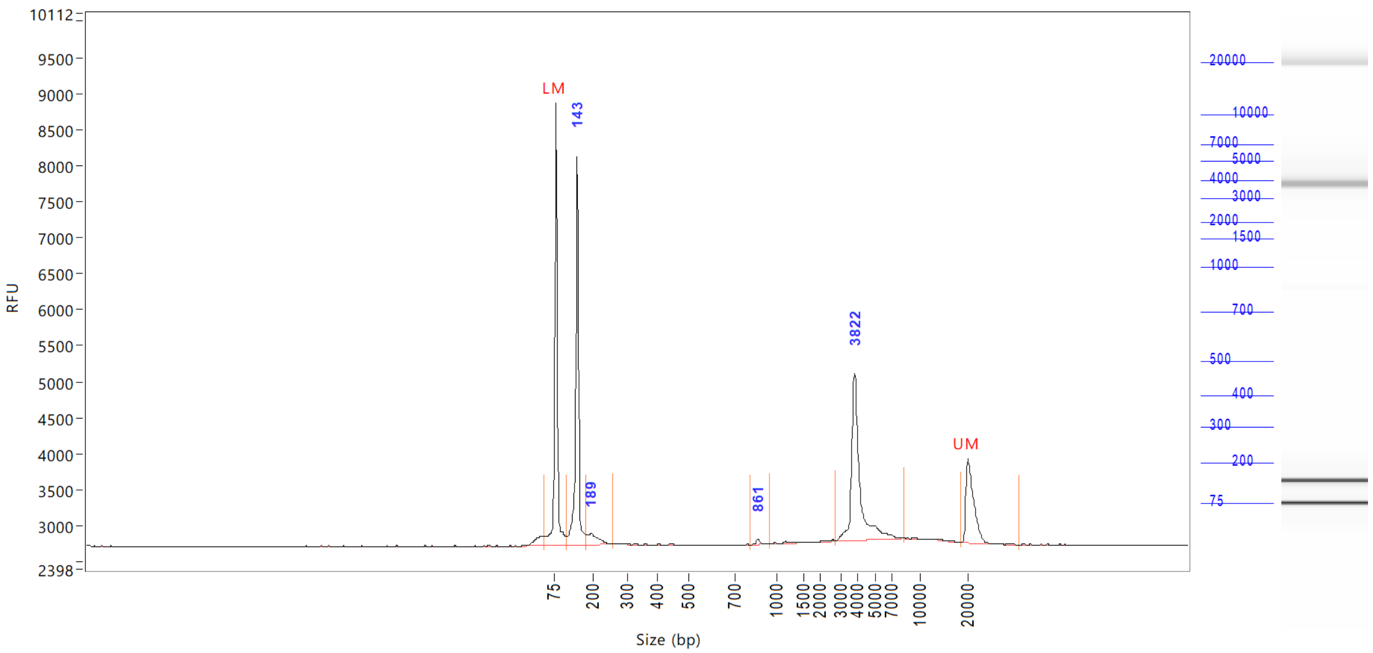

Peak	Size (bp)	Concentration (ng/uL)	From (bp)	To (bp)	Average size (bp)	CV%	RFU	Corrected peak area
1	75 (LM)	0.5000	39	110	74	12.28	6150	41.731
2	143	5.6517	110	173	140	6.29	5380	39.308
3	189	0.5824	173	259	196	8.40	161	4.051
4	861	0.0849	809	952	869	2.90	82	0.590
5	3822	4.2354	2772	8334	4037	16.87	2317	29.458
6	20000 (UM)	0.1438	18537	30519	20568	5.37	1175	12.003

TIC:	10.5544	ng/uL
TIM:	73.1130	nmole/L
Total concentration:	10.8413	ng/uL

Sample peak width (sec): 5   Sample min peak height: 50   Sample baseline V to V?: Y   Sample baseline V to V points: 3
Sample filter: Binomial   Number of points for filter: 3   Sample start region (min): 0   Sample end region (min): 30
Marker peak width (sec): 5   Marker min peak height: 200   Marker baseline V to V?: Y   Marker baseline V to V points: 3
Lower marker selection: First peak > 500 RFU   Upper marker selection: Last peak > 200 RFU
Ladder size (bp)75, 200, 300, 400, 500, 700, 1000, 1500, 2000, 3000, 4000, 5000, 7000, 10000, 20000
Quantification using: Lower Marker   Final concentration (ng/uL): 0.5000   Dilution factor: 12.0

Sample: SampG11
Well location: G11
Created: Monday, September 20, 2021 8:00:01 PM

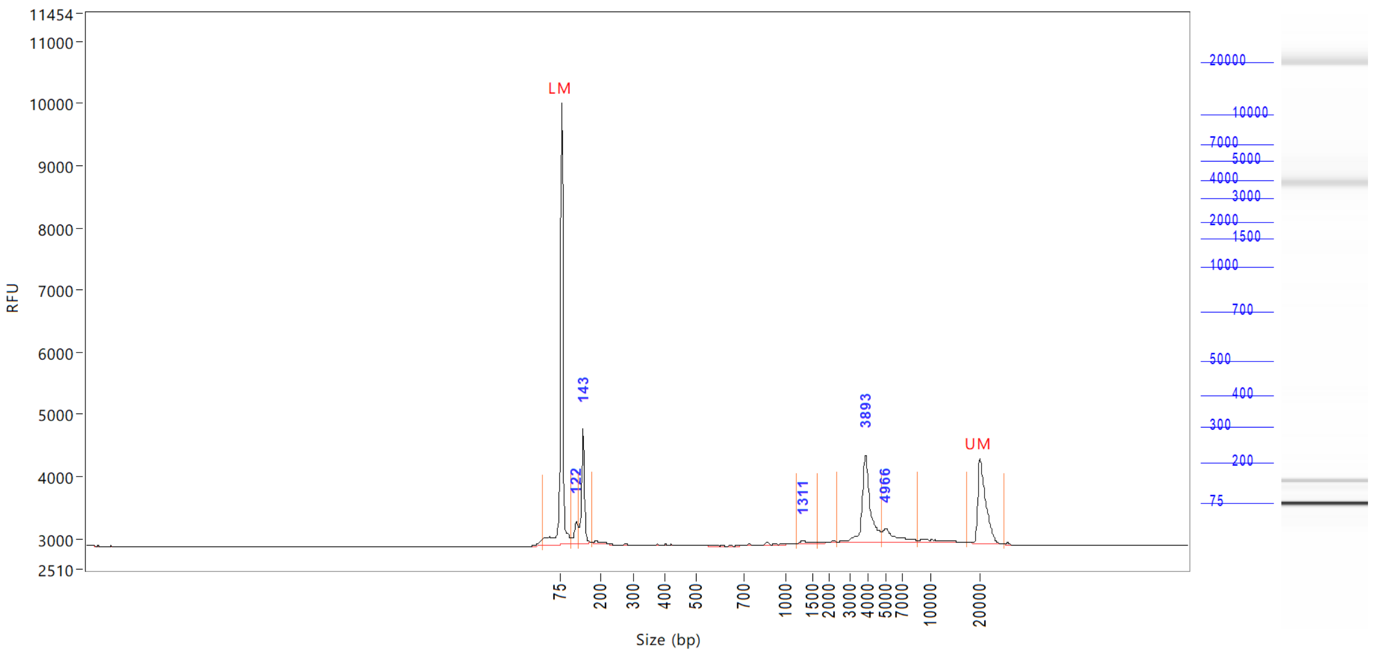

Peak	Size (bp)	Concentration (ng/uL)	From (bp)	To (bp)	Average size (bp)	CV%	RFU	Corrected peak area
1	75 (LM)	0.5000	14	105	70	17.08	7114	49.626
2	122	0.4447	105	129	119	5.51	362	3.678
3	143	1.5231	129	169	140	3.57	1831	12.598
4	1311	0.0834	1212	1655	1355	7.01	50	0.690
5	3893	2.1405	2344	4724	3870	9.49	1398	17.704
6	4966	0.5148	4724	8600	5891	18.67	209	4.258
7	20000 (UM)	0.1412	17561	25078	20573	4.50	1357	14.013

TIC:	4.7065	ng/uL
TIM:	25.1734	nmole/L
Total concentration:	5.2485	ng/uL

Sample peak width (sec): 5   Sample min peak height: 50   Sample baseline V to V?: Y   Sample baseline V to V points: 3
Sample filter: Binomial   Number of points for filter: 3   Sample start region (min): 0   Sample end region (min): 30
Marker peak width (sec): 5   Marker min peak height: 200   Marker baseline V to V?: Y   Marker baseline V to V points: 3
Lower marker selection: First peak > 500 RFU   Upper marker selection: Last peak > 200 RFU
Ladder size (bp)75, 200, 300, 400, 500, 700, 1000, 1500, 2000, 3000, 4000, 5000, 7000, 10000, 20000
Quantification using: Lower Marker   Final concentration (ng/uL): 0.5000   Dilution factor: 12.0

Sample: SampH11
Well location: H11
Created: Monday, September 20, 2021 8:00:01 PM

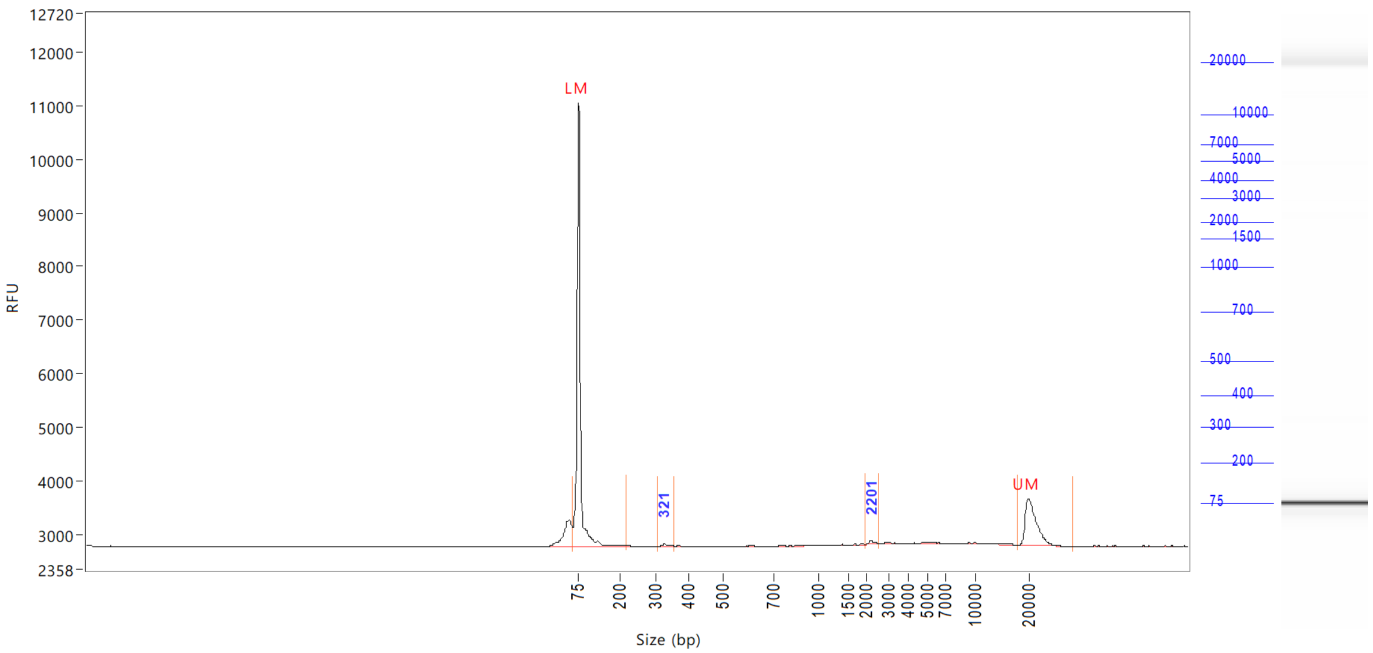

Peak	Size (bp)	Concentration (ng/uL)	From (bp)	To (bp)	Average size (bp)	CV%	RFU	Corrected peak area
1	75 (LM)	0.5000	56	215	76	18.73	8278	65.737
2	321	0.0460	301	356	324	3.52	54	0.504
3	2201	0.0445	1981	2515	2227	5.55	62	0.487
4	20000 (UM)	0.0910	18049	28221	20743	5.80	880	11.969

TIC:	0.0905	ng/uL
TIM:	0.2665	nmole/L
Total concentration:	0.3668	ng/uL

Sample peak width (sec): 5   Sample min peak height: 50   Sample baseline V to V?: Y   Sample baseline V to V points: 3
Sample filter: Binomial   Number of points for filter: 3 Sample start region (min): 0 Sample end region (min): 30
Marker peak width (sec): 5   Marker min peak height: 200   Marker baseline V to V?: Y   Marker baseline V to V points: 3
Lower marker selection: First peak > 500 RFU   Upper marker selection: Last peak > 200 RFU
Ladder size (bp)75, 200, 300, 400, 500, 700, 1000, 1500, 2000, 3000, 4000, 5000, 7000, 10000, 20000
Quantification using: Lower Marker   Final concentration (ng/uL): 0.5000   Dilution factor: 12.0

Sample: SampA12
Well location: A12
Created: Monday, September 20, 2021 8:00:01 PM

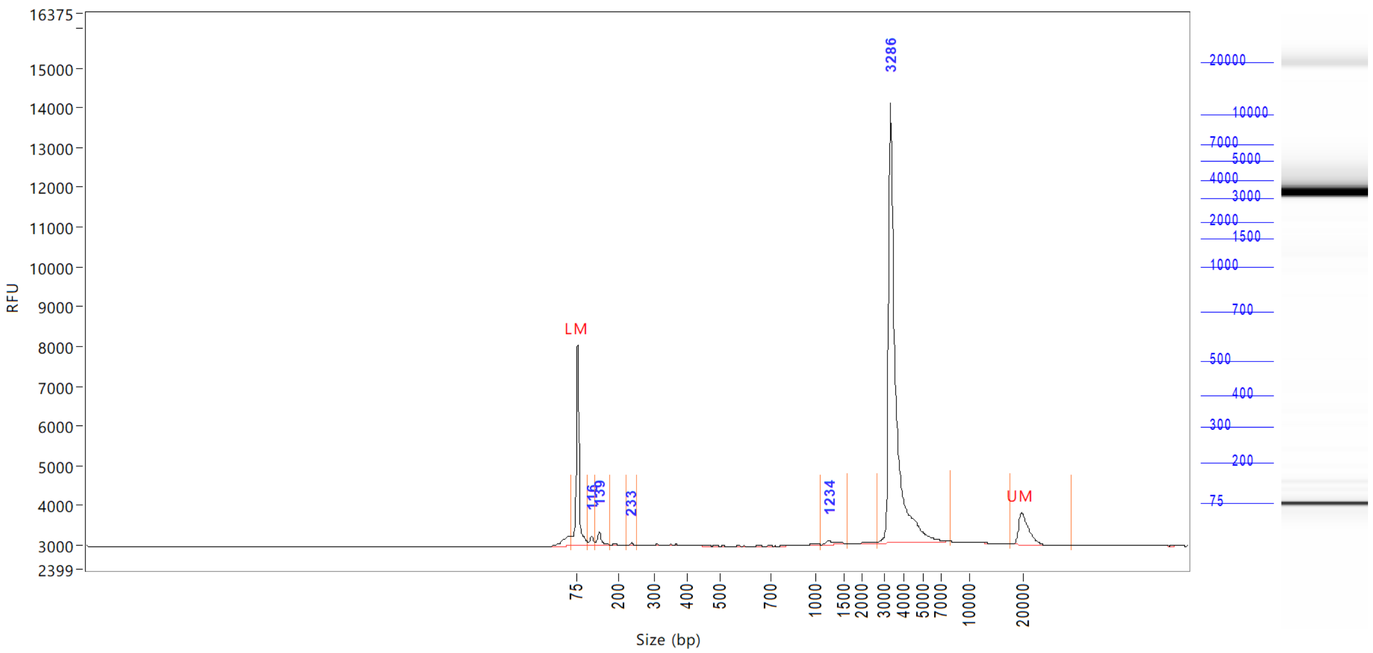

Peak	Size (bp)	Concentration (ng/uL)	From (bp)	To (bp)	Average size (bp)	CV%	RFU	Corrected peak area
1	75 (LM)	0.5000	56	105	73	9.36	5035	35.730
2	116	0.3435	105	126	114	4.87	223	2.046
3	139	0.5250	126	173	139	5.72	335	3.127
4	233	0.0636	219	250	232	1.92	61	0.379
5	1234	0.3038	1090	1597	1297	8.33	94	1.809
6	3286	19.5034	2657	8001	3540	14.84	11080	116.143
7	20000 (UM)	0.1395	17805	29309	20511	5.63	801	9.969

TIC:	20.7393	ng/uL
TIM:	21.0633	nmole/L
Total concentration:	21.3738	ng/uL

Sample peak width (sec): 5 Sample min peak height: 50 Sample baseline V to V?: Y Sample baseline V to V points: 3
Sample filter: Binomial Number of points for filter: 3 Sample start region (min): 0 Sample end region (min): 30
Marker peak width (sec): 5 Marker min peak height: 200 Marker baseline V to V?: Y Marker baseline V to V points: 3
Lower marker selection: First peak > 500 RFU Upper marker selection: Last peak > 200 RFU
Ladder size (bp)75, 200, 300, 400, 500, 700, 1000, 1500, 2000, 3000, 4000, 5000, 7000, 10000, 20000
Quantification using: Lower Marker Final concentration (ng/uL): 0.5000 Dilution factor: 12.0

Sample: SampB12
Well location: B12
Created: Monday, September 20, 2021 8:00:01 PM

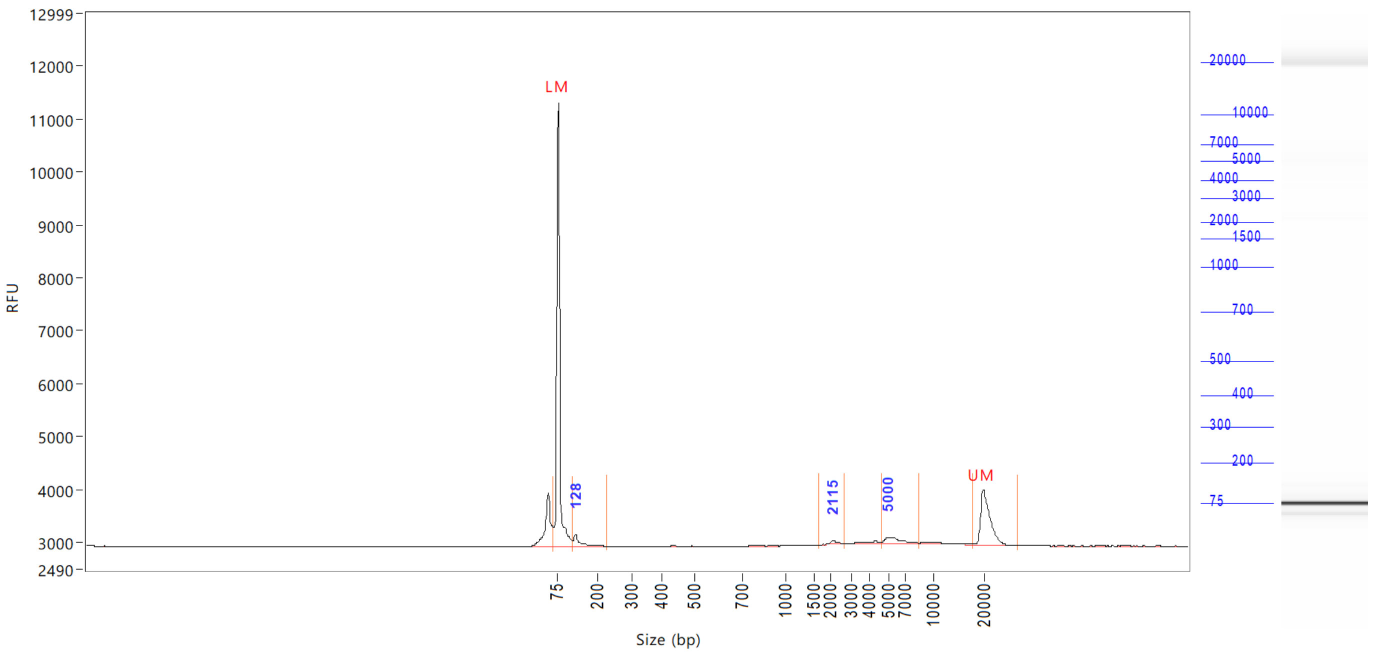

Peak	Size (bp)	Concentration (ng/uL)	From (bp)	To (bp)	Average size (bp)	CV%	RFU	Corrected peak area
1	75 (LM)	0.5000	62	120	75	11.82	8395	57.386
2	128	0.3211	120	224	141	15.38	222	3.071
3	2115	0.0719	1635	2657	2158	7.79	67	0.687
4	5000	0.2567	4621	8467	5696	15.21	112	2.455
5	20000 (UM)	0.1084	17927	26770	20584	5.26	1048	12.441

TIC:	0.6497	ng/uL
TIM:	3.8712	nmole/L
Total concentration:	0.9849	ng/uL

Sample peak width (sec): 5   Sample min peak height: 50   Sample baseline V to V?: Y   Sample baseline V to V points: 3
Sample filter: Binomial   Number of points for filter: 3   Sample start region (min): 0   Sample end region (min): 30
Marker peak width (sec): 5   Marker min peak height: 200   Marker baseline V to V?: Y   Marker baseline V to V points: 3
Lower marker selection: First peak > 500 RFU   Upper marker selection: Last peak > 200 RFU
Ladder size (bp)75, 200, 300, 400, 500, 700, 1000, 1500, 2000, 3000, 4000, 5000, 7000, 10000, 20000
Quantification using: Lower Marker   Final concentration (ng/uL): 0.5000   Dilution factor: 12.0

Sample: SampC12
Well location: C12
Created: Monday, September 20, 2021 8:00:01 PM

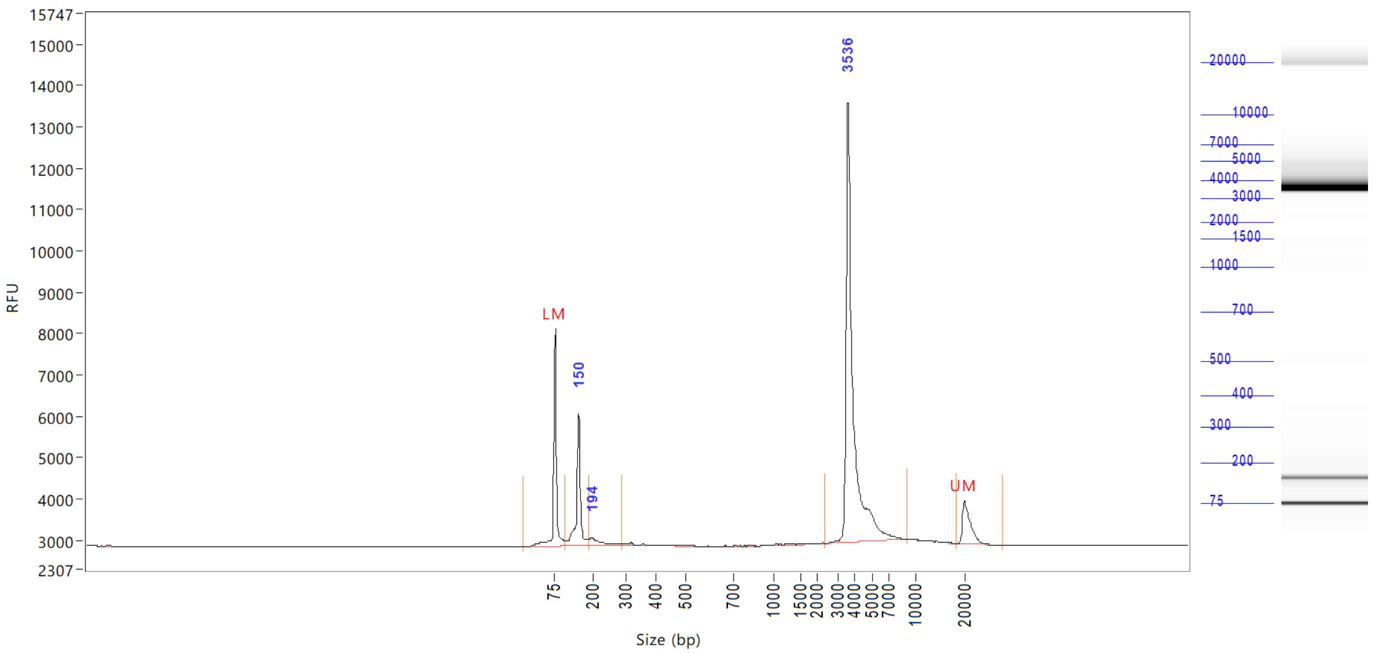

Peak	Size (bp)	Concentration (ng/uL)	From (bp)	To (bp)	Average size (bp)	CV%	RFU	Corrected peak area
1	75 (LM)	0.5000	0	110	71	21.65	5246	37.145
2	150	4.7253	110	183	147	8.29	3175	29.253
3	194	0.8171	183	286	214	12.06	197	5.059
4	3536	17.9885	2401	9000	3879	16.61	10636	111.362
5	20000 (UM)	0.1457	18415	27979	20633	5.15	1030	10.822

TIC:	23.5309	ng/uL
TIM:	66.7422	nmole/L
Total concentration:	23.9772	ng/uL

```
Sample peak width (sec): 5    Sample min peak height: 50    Sample baseline V to V?: Y    Sample baseline V to V points: 3
Sample filter: Binomial       Number of points for filter: 3  Sample start region (min): 0  Sample end region (min): 30
Marker peak width (sec): 5    Marker min peak height: 200   Marker baseline V to V?: Y    Marker baseline V to V points: 3
Lower marker selection: First peak > 500 RFU                Upper marker selection: Last peak > 200 RFU
Ladder size (bp)75, 200, 300, 400, 500, 700, 1000, 1500, 2000, 3000, 4000, 5000, 7000, 10000, 20000
Quantification using: Lower Marker          Final concentration (ng/uL): 0.5000          Dilution factor: 12.0
```

Sample: SampD12
Well location: D12
Created: Monday, September 20, 2021 8:00:01 PM

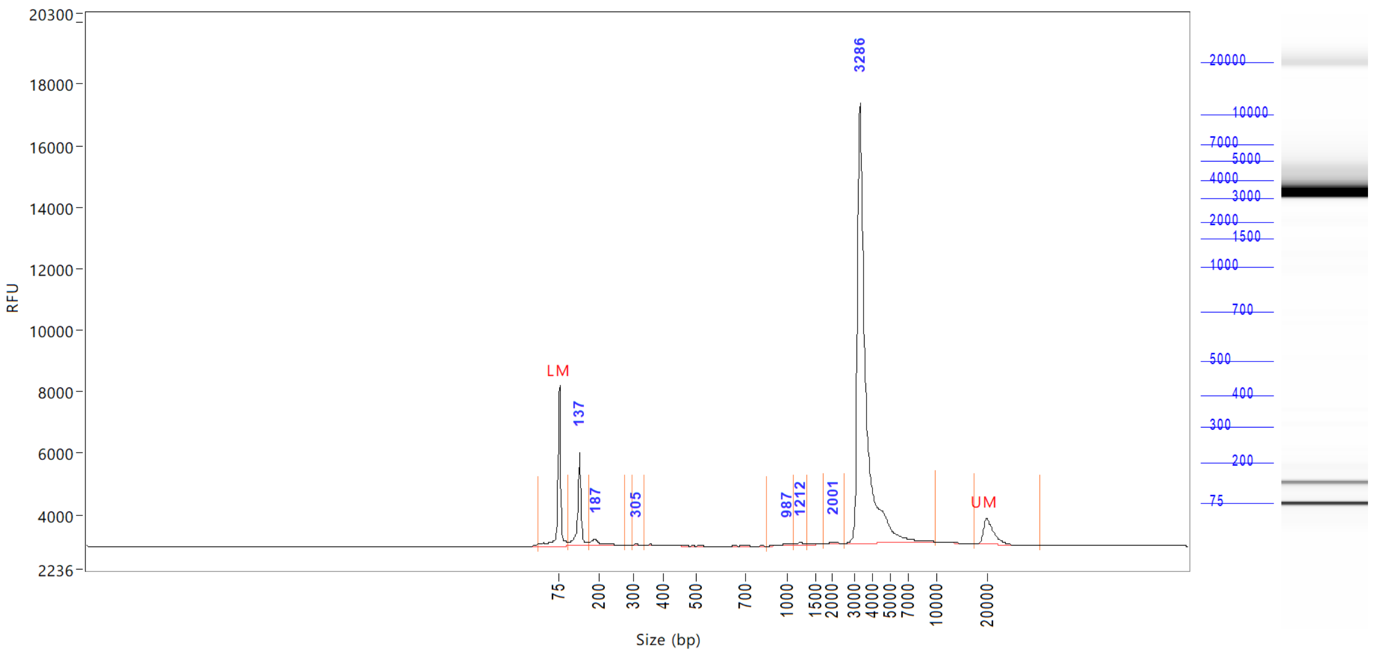

Peak	Size (bp)	Concentration (ng/uL)	From (bp)	To (bp)	Average size (bp)	CV%	RFU	Corrected peak area
1	75 (LM)	0.5000	12	101	70	18.84	5218	37.576
2	137	4.0325	101	168	134	7.93	3006	25.254
3	187	0.7153	168	275	194	11.49	191	4.480
4	305	0.0568	295	333	304	1.69	62	0.356
5	987	0.1493	844	1090	985	4.99	62	0.935
6	1212	0.1562	1090	1345	1202	5.13	78	0.978
7	2001	0.1390	1750	2515	2059	6.82	77	0.870
8	3286	28.4703	2515	9933	3602	18.81	14338	178.299
9	20000 (UM)	0.1434	17561	30519	20643	6.10	827	10.777

TIC:	33.7193	ng/uL
TIM:	69.4048	nmole/L
Total concentration:	34.0086	ng/uL

```
Sample peak width (sec): 5     Sample min peak height: 50     Sample baseline V to V?: Y     Sample baseline V to V points: 3
Sample filter: Binomial        Number of points for filter: 3  Sample start region (min): 0  Sample end region (min): 30
Marker peak width (sec): 5     Marker min peak height: 200    Marker baseline V to V?: Y     Marker baseline V to V points: 3
Lower marker selection: First peak > 500 RFU                  Upper marker selection: Last peak > 200 RFU
Ladder size (bp)75, 200, 300, 400, 500, 700, 1000, 1500, 2000, 3000, 4000, 5000, 7000, 10000, 20000
Quantification using: Lower Marker          Final concentration (ng/uL): 0.5000          Dilution factor: 12.0
```

Sample: SampE12
Well location: E12
Created: Monday, September 20, 2021 8:00:01 PM

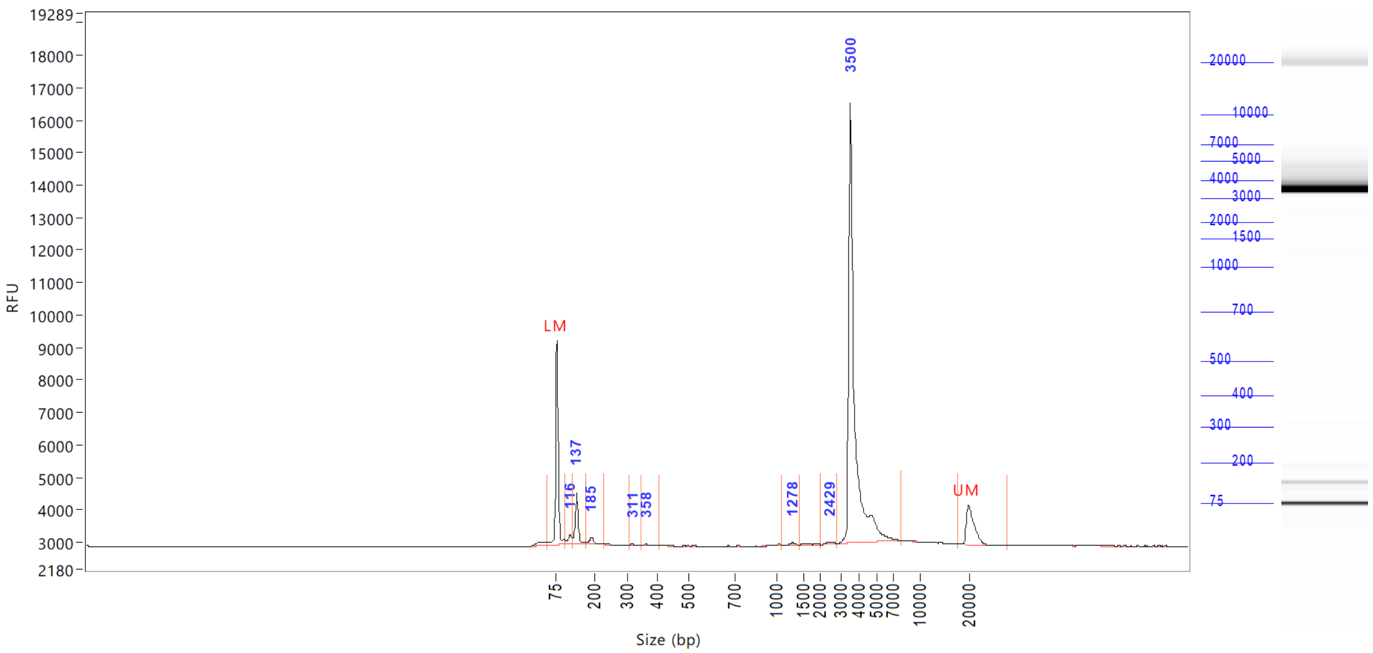

Peak	Size (bp)	Concentration (ng/uL)	From (bp)	To (bp)	Average size (bp)	CV%	RFU	Corrected peak area
1	75 (LM)	0.5000	45	105	73	9.13	6274	38.944
2	116	0.3850	105	124	115	4.67	266	2.499
3	137	1.7466	124	171	137	5.57	1578	11.337
4	185	0.3197	171	224	185	4.24	199	2.075
5	311	0.0506	301	343	311	2.52	57	0.329
6	358	0.0619	343	403	361	3.06	53	0.402
7	1278	0.1321	1079	1400	1254	5.72	73	0.858
8	2429	0.1015	2030	2800	2401	6.17	63	0.659
9	3500	18.6703	2800	7867	3741	14.00	13563	121.183
10	20000 (UM)	0.1591	17805	27979	20466	4.45	1228	12.395

TIC:	21.4677	ng/uL
TIM:	38.2987	nmole/L
Total concentration:	21.7956	ng/uL

Sample peak width (sec): 5 Sample min peak height: 50 Sample baseline V to V?: Y Sample baseline V to V points: 3
Sample filter: Binomial Number of points for filter: 3 Sample start region (min): 0 Sample end region (min): 30
Marker peak width (sec): 5 Marker min peak height: 200 Marker baseline V to V?: Y Marker baseline V to V points: 3
Lower marker selection: First peak > 500 RFU Upper marker selection: Last peak > 200 RFU
Ladder size (bp)75, 200, 300, 400, 500, 700, 1000, 1500, 2000, 3000, 4000, 5000, 7000, 10000, 20000
Quantification using: Lower Marker Final concentration (ng/uL): 0.5000 Dilution factor: 12.0

Sample: SampF12
Well location: F12
Created: Monday, September 20, 2021 8:00:01 PM

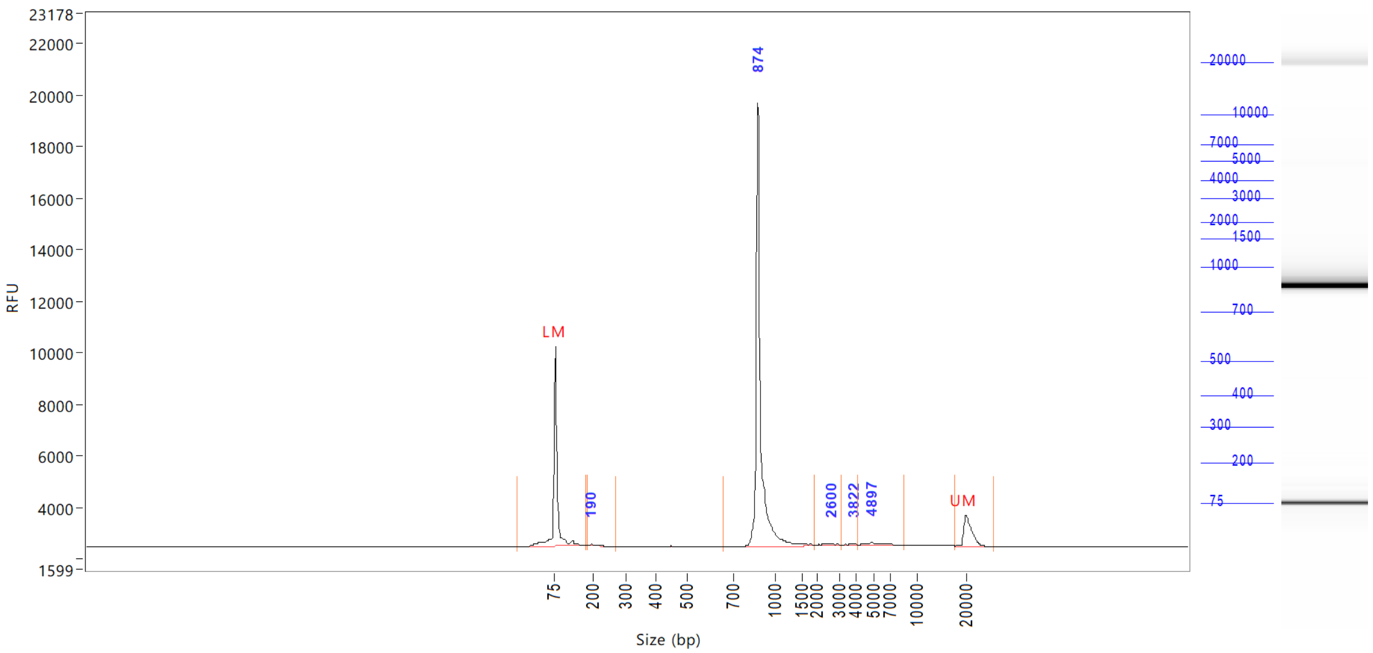

Peak	Size (bp)	Concentration (ng/uL)	From (bp)	To (bp)	Average size (bp)	CV%	RFU	Corrected peak area
1	75 (LM)	0.5000	0	173	73	27.65	7740	64.796
2	190	0.0549	181	269	206	9.20	57	0.593
3	874	12.4265	654	1904	905	11.64	17243	134.199
4	2600	0.1727	1904	3072	2443	14.22	57	1.866
5	3822	0.0992	3072	4139	3623	8.56	53	1.072
6	4897	0.2169	4139	8600	5442	19.15	106	2.342
7	20000 (UM)	0.0954	17805	25803	20570	4.49	1214	12.360

TIC:	12.9703	ng/uL
TIM:	23.2653	nmole/L
Total concentration:	13.0857	ng/uL

Sample peak width (sec): 5 Sample min peak height: 50 Sample baseline V to V?: Y Sample baseline V to V points: 3
Sample filter: Binomial Number of points for filter: 3 Sample start region (min): 0 Sample end region (min): 30
Marker peak width (sec): 5 Marker min peak height: 200 Marker baseline V to V?: Y Marker baseline V to V points: 3
Lower marker selection: First peak > 500 RFU Upper marker selection: Last peak > 200 RFU
Ladder size (bp)75, 200, 300, 400, 500, 700, 1000, 1500, 2000, 3000, 4000, 5000, 7000, 10000, 20000
Quantification using: Lower Marker Final concentration (ng/uL): 0.5000 Dilution factor: 12.0

Sample: SampG12
Well location: G12
Created: Monday, September 20, 2021 8:00:01 PM

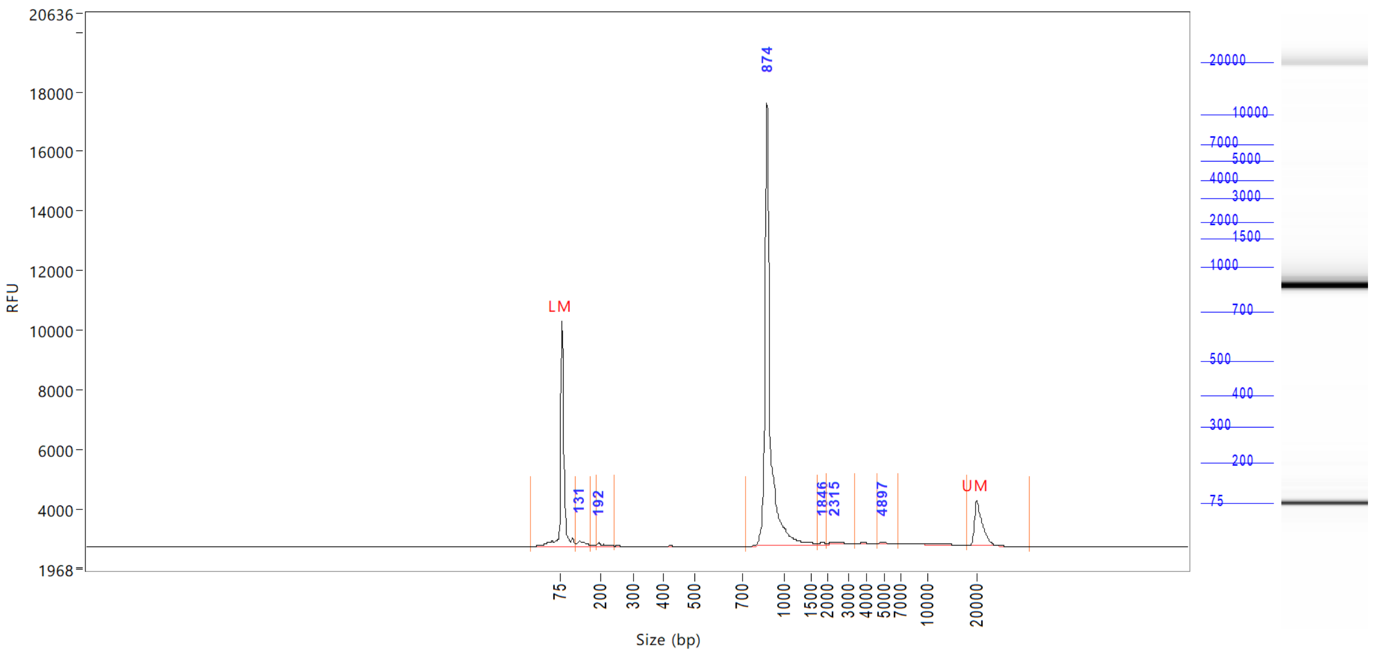

Peak	Size (bp)	Concentration (ng/uL)	From (bp)	To (bp)	Average size (bp)	CV%	RFU	Corrected peak area
1	75 (LM)	0.5000	0	122	73	20.22	7563	63.408
2	131	0.3020	122	166	139	8.30	207	3.192
3	192	0.1295	185	242	201	6.42	117	1.369
4	874	12.3183	723	1674	903	9.67	14897	130.180
5	1846	0.0664	1674	1962	1817	4.57	65	0.702
6	2315	0.1464	1962	3322	2447	13.63	64	1.547
7	4897	0.0758	4656	6704	5229	10.30	70	0.801
8	20000 (UM)	0.1215	17805	30881	20564	5.30	1502	15.409

TIC:	13.0384	ng/uL
TIM:	27.2647	nmole/L
Total concentration:	13.3544	ng/uL

```
Sample peak width (sec): 5    Sample min peak height: 50    Sample baseline V to V?: Y    Sample baseline V to V points: 3
Sample filter: Binomial       Number of points for filter: 3  Sample start region (min): 0  Sample end region (min): 30
Marker peak width (sec): 5    Marker min peak height: 200   Marker baseline V to V?: Y    Marker baseline V to V points: 3
Lower marker selection: First peak > 500 RFU                Upper marker selection: Last peak > 200 RFU
Ladder size (bp)75, 200, 300, 400, 500, 700, 1000, 1500, 2000, 3000, 4000, 5000, 7000, 10000, 20000
Quantification using: Lower Marker          Final concentration (ng/uL): 0.5000          Dilution factor: 12.0
```

Sample: SampH12
Well location: H12
Created: Monday, September 20, 2021 8:00:01 PM

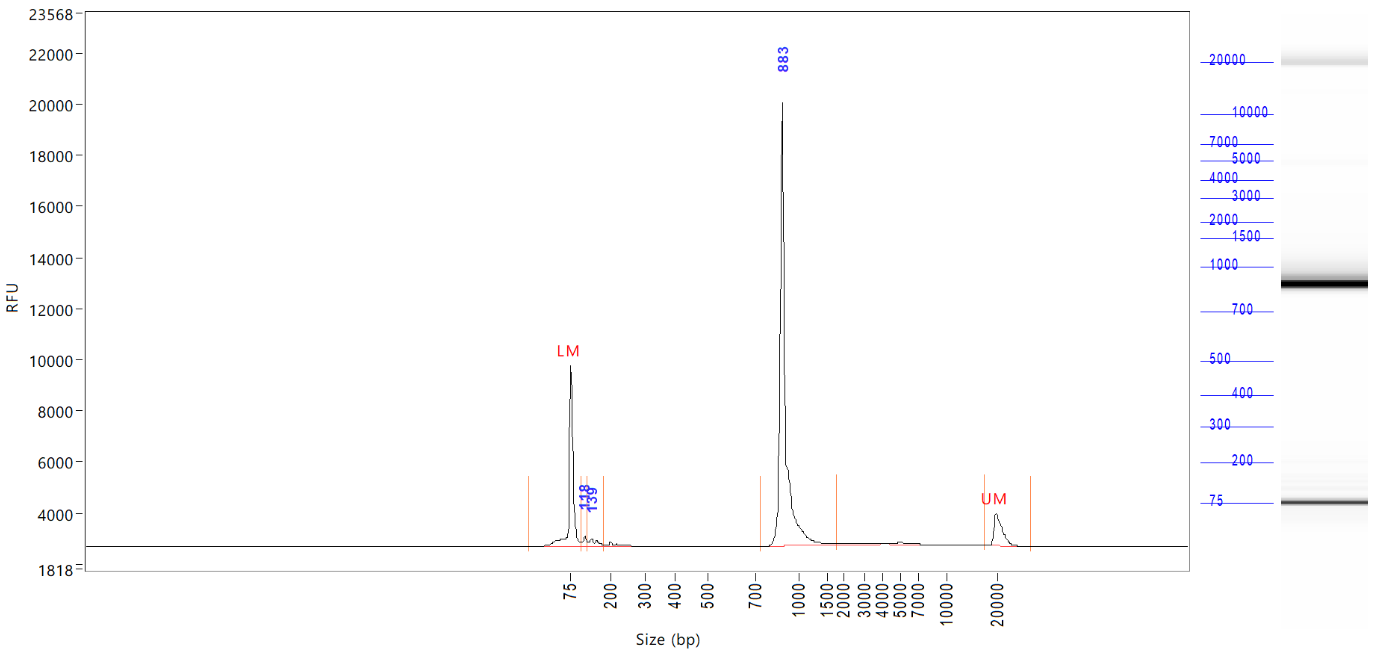

Peak	Size (bp)	Concentration (ng/uL)	From (bp)	To (bp)	Average size (bp)	CV%	RFU	Corrected peak area
1	75 (LM)	0.5000	0	108	70	24.41	7077	64.972
2	118	0.2729	108	128	117	4.43	370	2.955
3	139	0.4405	128	177	147	8.89	299	4.770
4	883	14.1467	731	1808	909	9.89	17365	153.190
5	20000 (UM)	0.0998	17561	26770	20431	4.39	1266	12.972

TIC:	14.8601	ng/uL
TIM:	34.3712	nmole/L
Total concentration:	15.5338	ng/uL

Sample peak width (sec): 5   Sample min peak height: 200   Sample baseline V to V?: Y   Sample baseline V to V points: 3
Sample filter: Binomial   Number of points for filter: 3   Sample start region (min): 0   Sample end region (min): 30
Marker peak width (sec): 5   Marker min peak height: 200   Marker baseline V to V?: Y   Marker baseline V to V points: 3
Lower marker selection: First peak > 500 RFU   Upper marker selection: Last peak > 200 RFU
Ladder size (bp)75, 200, 300, 400, 500, 700, 1000, 1500, 2000, 3000, 4000, 5000, 7000, 10000, 20000
Quantification using: Lower Marker   Final concentration (ng/uL): 0.5000   Dilution factor: 12.0

Sample: DNA size ladder
Well location: A7
Created: Monday, September 20, 2021 8:00:01 PM
Import from: C:\Users\admin\Desktop\210810_alberto_ladder.SCAL
Fit type: Point to point

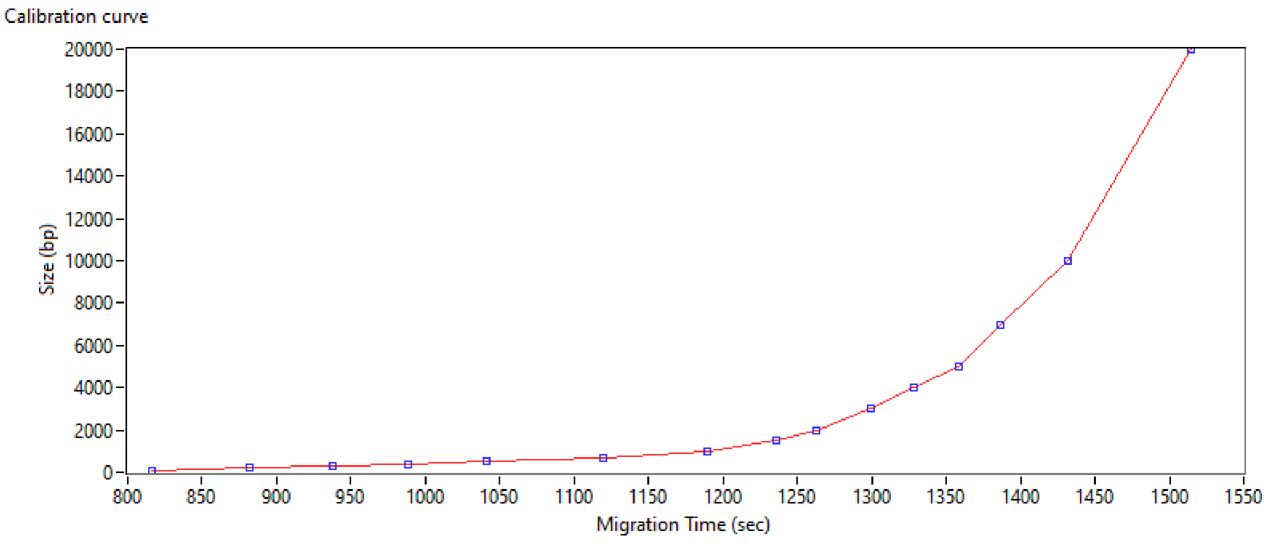

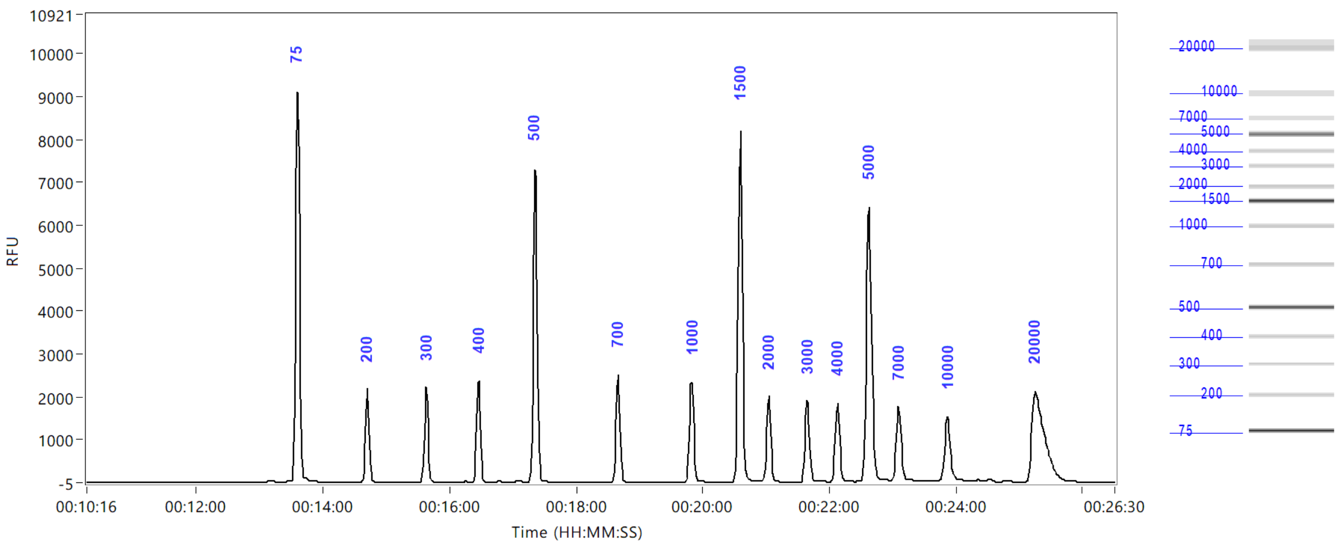

Supplement: Supplementary file 2 — sb3c00292_si_002.zip [file sb3c00292_si_002.zip › dnada_supplementary_material_pks_library_build/ZAG_trials/ZAG_Trial_6/19-50-07_plate4/plate4-t2.pdf]
