# Supplementary material for: Automated Platform for the Plasmid Construction Process
Source: ACS Synth Biol. 2023 Nov 10;12(12):3506–13. doi: 10.1021/acssynbio.3c00292 (PMC10729297; doi:10.1021/acssynbio.3c00292)

Instrument controller software run summary:

Filename and data path: C:\AATI\Data\2021 10 21\18-38-27\2021 10 21 18H 38M..raw
Created: Thursday, October 21, 2021 6:48:20 PM
Number of capillaries: 96
Array serial number: 111920-09SFS
Effect length: 33 cm
Array usage count: 267
Instrument type: ZAG DNA Analyzer
Instrument controller software version: 1.0.1.9
Device serial number: Z7597

Method Information

Method name: ZAG130GP33 - DNA 75-20000bp - Gel Prime Only.mthds
Gel prime: Yes
Full conditioning: No
Gel prime to buffer: No
Gel selection: Gel 1
Perform prerun: 8.0 kV, 30 sec.
Rinse: No
Marker 1: Row: A, 5.0 kV, 5 sec.
Rinse: No
Sample injection: 5.0 kV, 5 sec.
Separation: 8.0 kV, 30.0 min.
Tray name: Sample Tray-3

Analysis mode: DNA

Notes

Gel Image

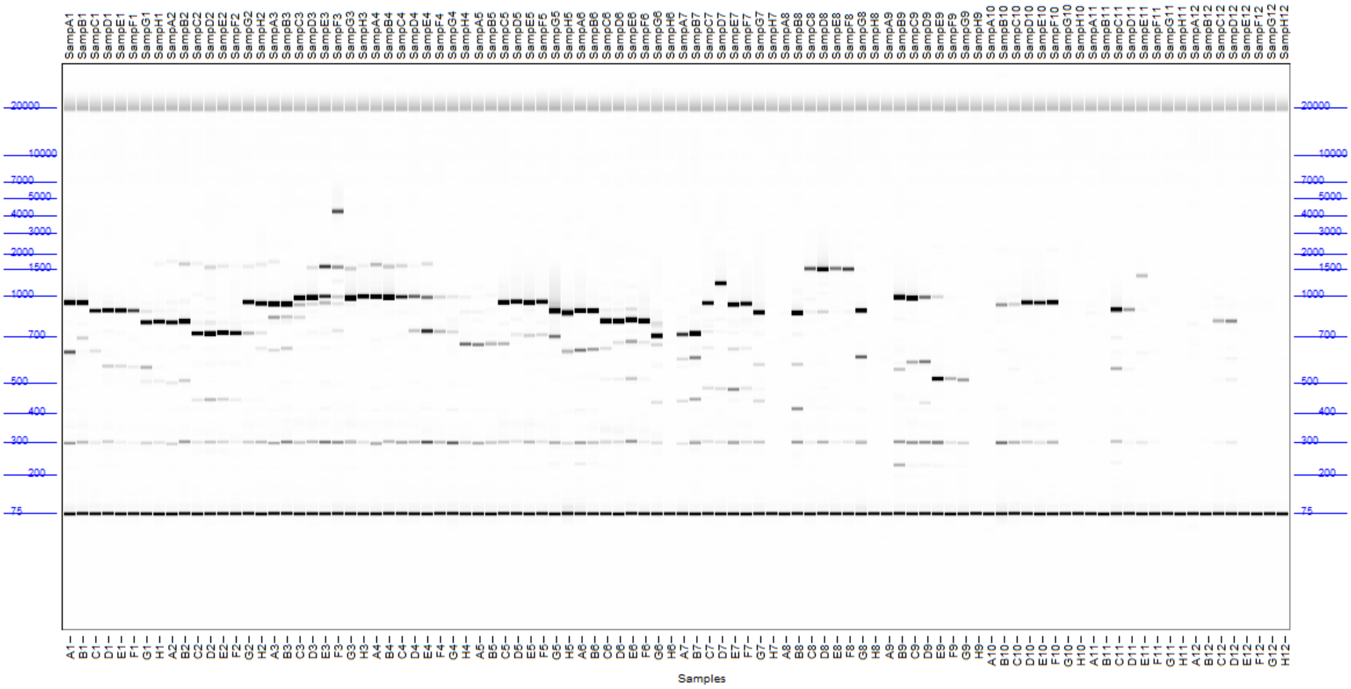

Filename and data path: C:\AATI\Data\2021 10 21\18-38-27\2021 10 21 18H 38M..raw

A1: SampA1

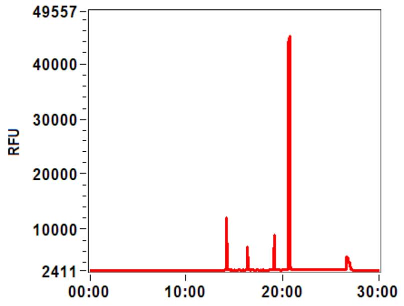

B1: SampB1

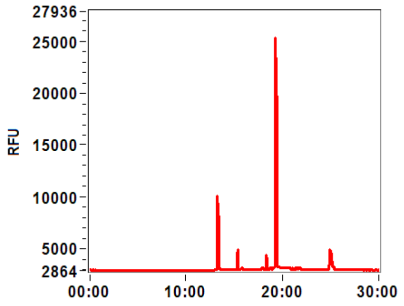

C1: SampC1

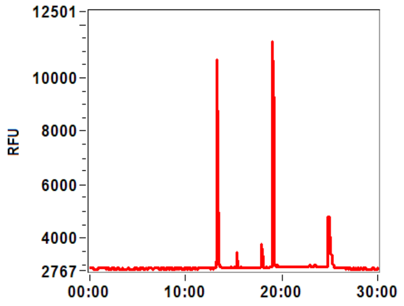

D1: SampD1

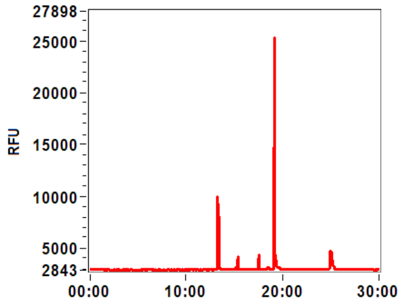

E1: SampE1

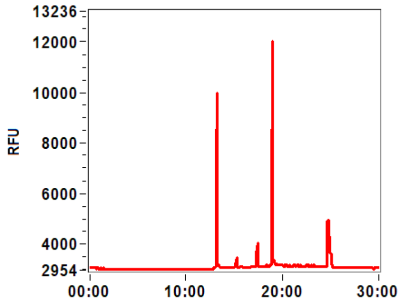

F1: SampF1

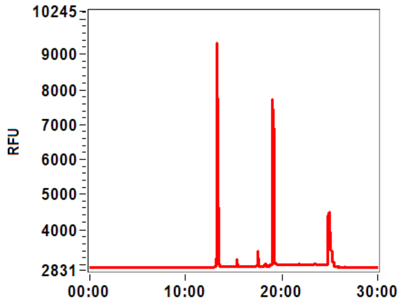

G1: SampG1

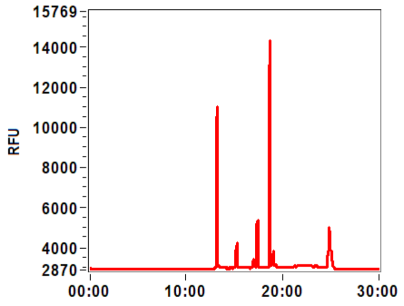

H1: SampH1

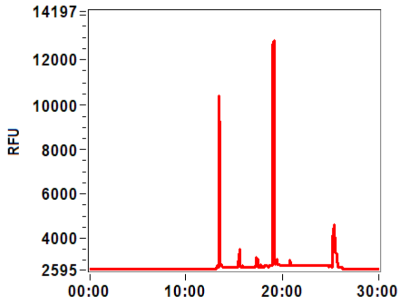

A2: SampA2

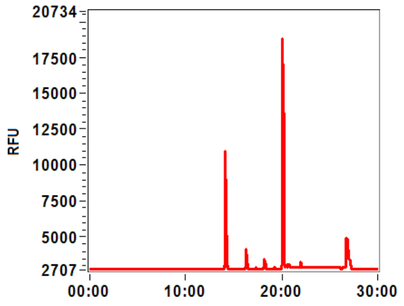

B2: SampB2

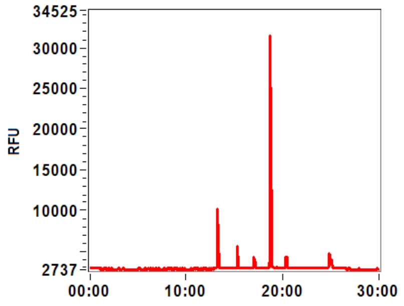

C2: SampC2

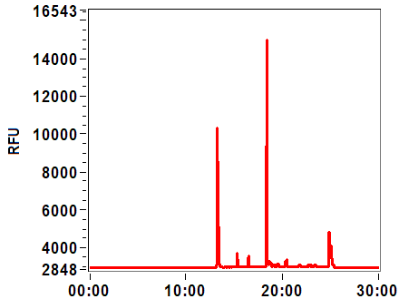

D2: SampD2

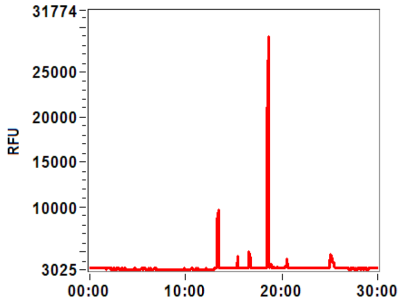

Filename and data path: C:\AATI\Data\2021 10 21\18-38-27\2021 10 21 18H 38M..raw

E2: SampE2

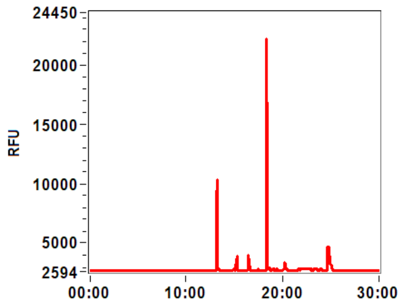

F2: SampF2

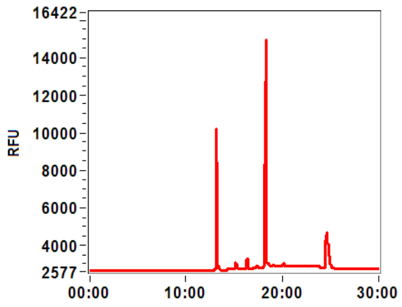

G2: SampG2

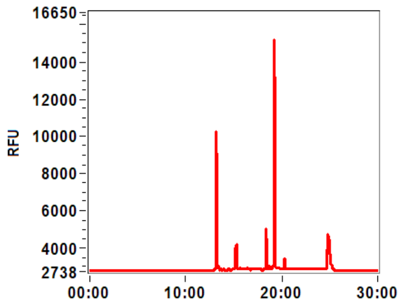

H2: SampH2

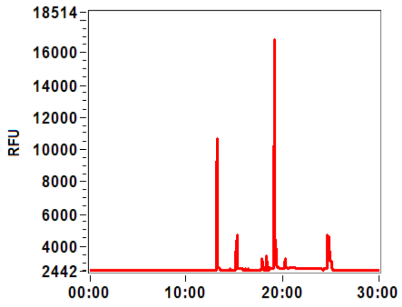

A3: SampA3

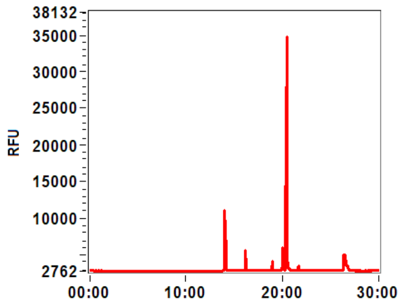

B3: SampB3

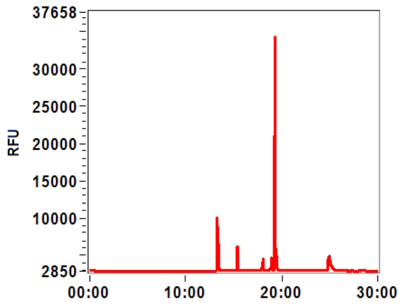

C3: SampC3

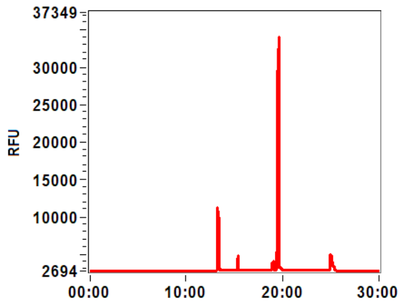

D3: SampD3

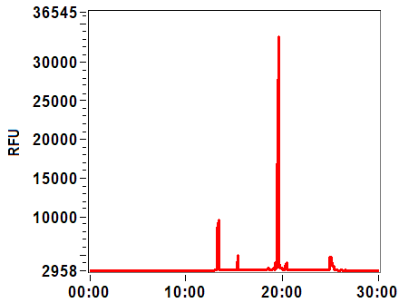

E3: SampE3

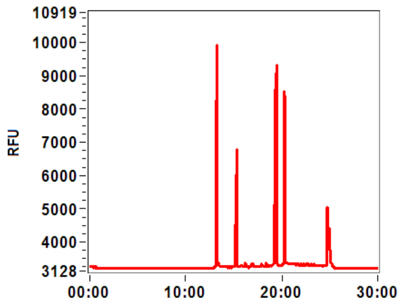

F3: SampF3

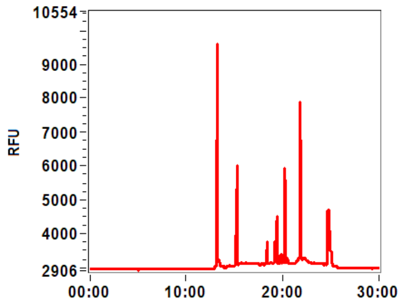

G3: SampG3

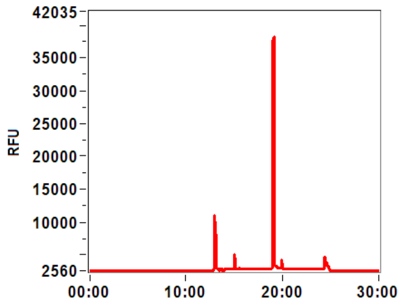

H3: SampH3

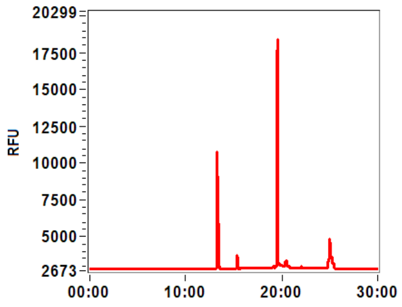

Filename and data path: C:\AATI\Data\2021 10 21\18-38-27\2021 10 21 18H 38M..raw

A4: SampA4

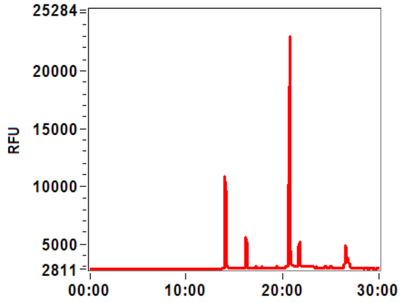

B4: SampB4

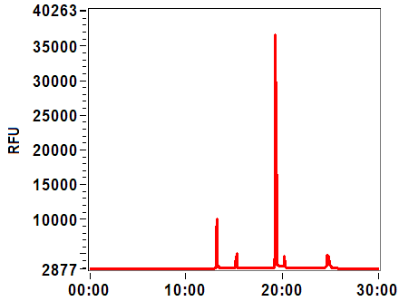

C4: SampC4

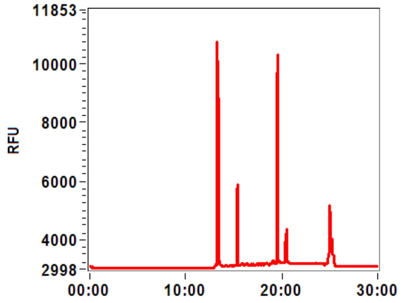

D4: SampD4

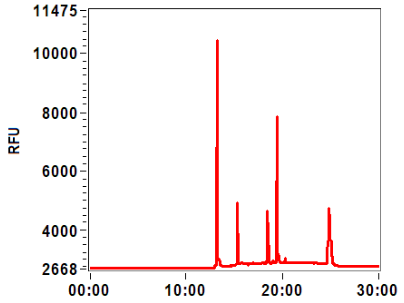

E4: SampE4

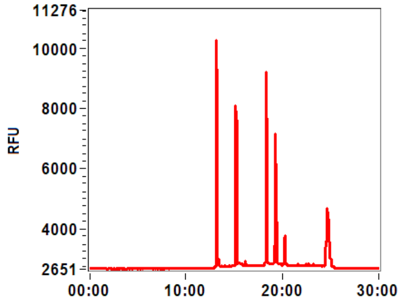

F4: SampF4

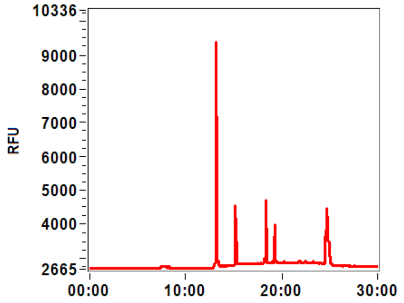

G4: SampG4

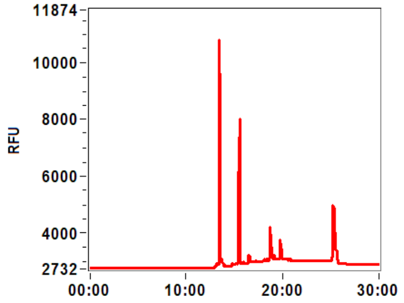

H4: SampH4

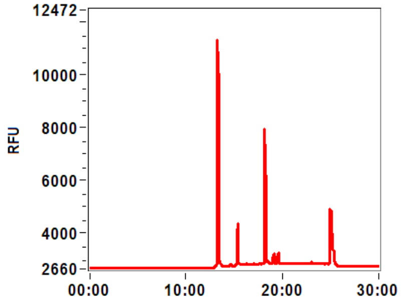

A5: SampA5

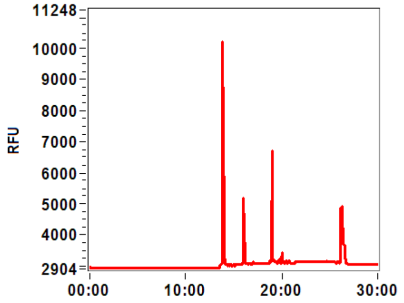

B5: SampB5

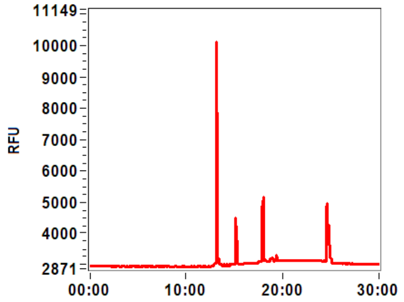

C5: SampC5

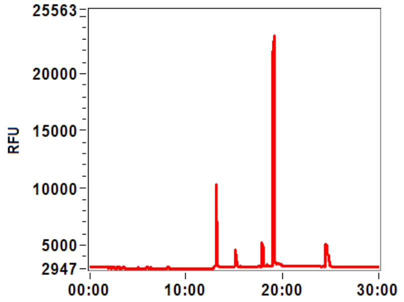

D5: SampD5

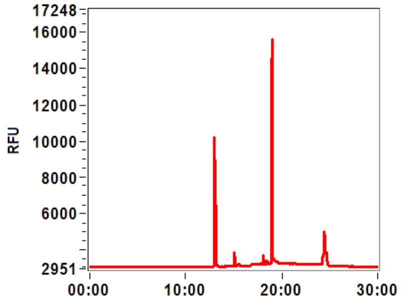

Filename and data path: C:\AATI\Data\2021 10 21\18-38-27\2021 10 21 18H 38M..raw

E5: SampE5

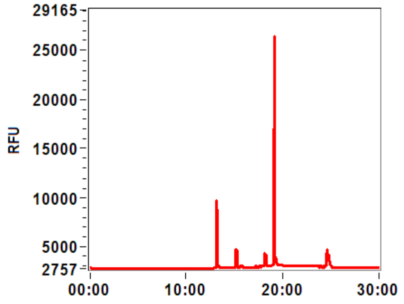

F5: SampF5

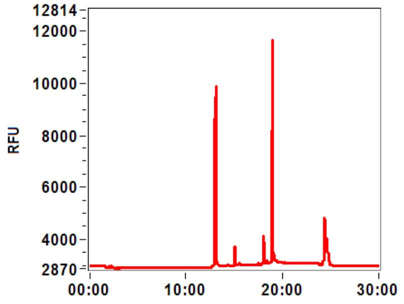

G5: SampG5

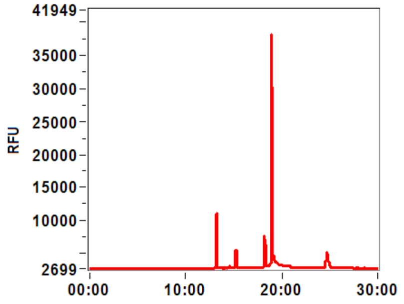

H5: SampH5

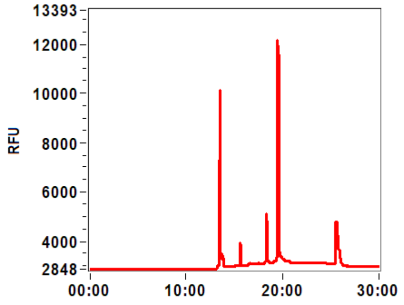

A6: SampA6

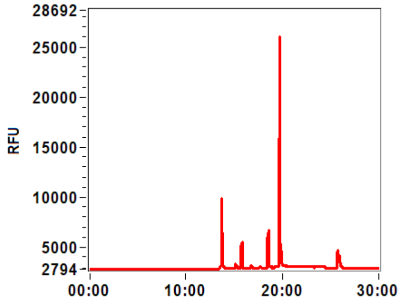

B6: SampB6

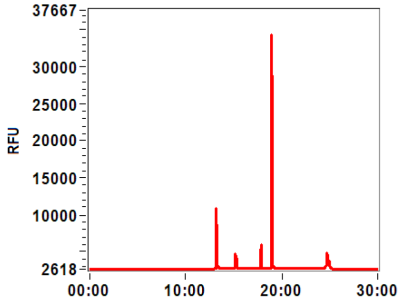

C6: SampC6

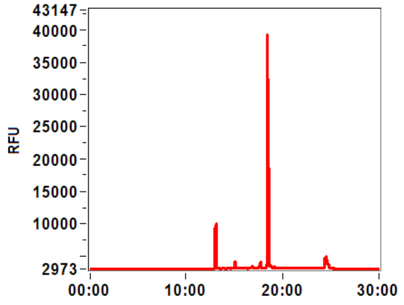

D6: SampD6

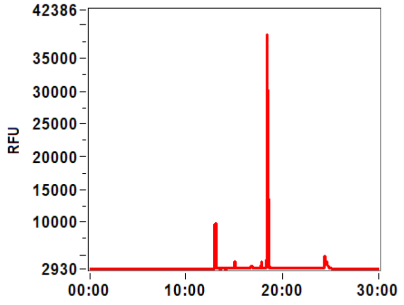

E6: SampE6

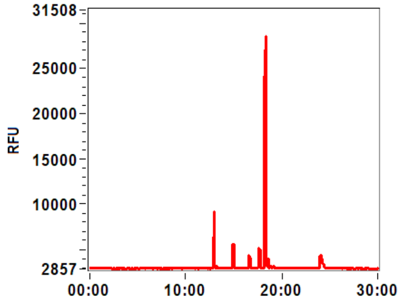

F6: SampF6

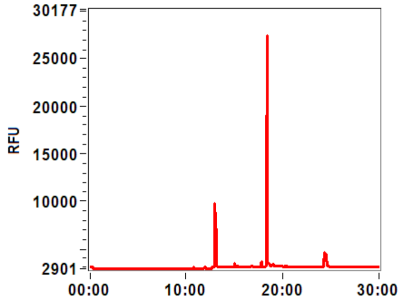

G6: SampG6

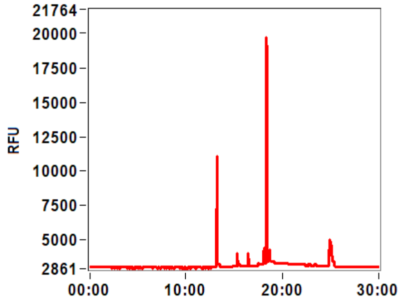

H6: SampH6

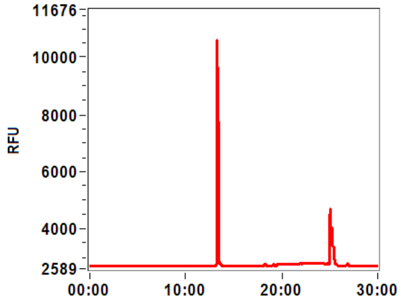

Filename and data path: C:\AATI\Data\2021 10 21\18-38-27\2021 10 21 18H 38M..raw

A7: SampA7

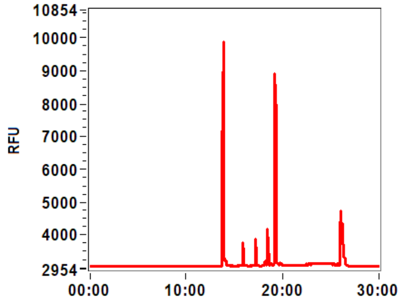

B7: SampB7

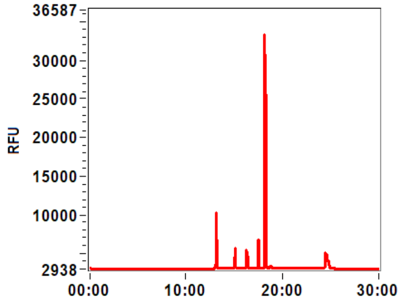

C7: SampC7

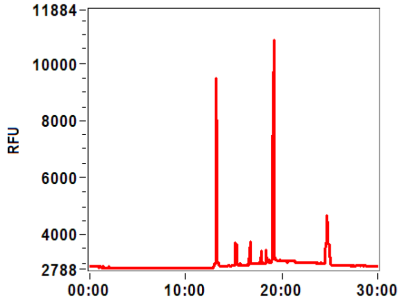

D7: SampD7

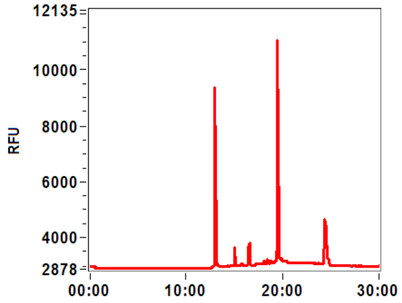

E7: SampE7

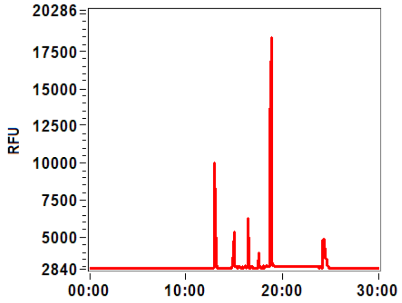

F7: SampF7

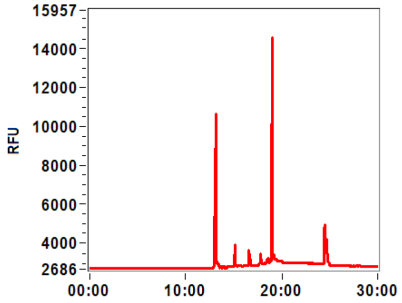

G7: SampG7

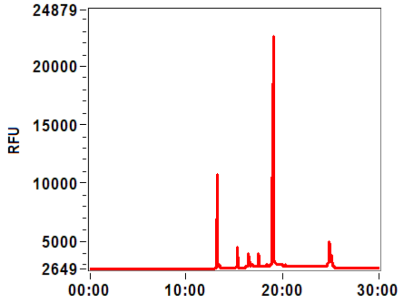

H7: SampH7

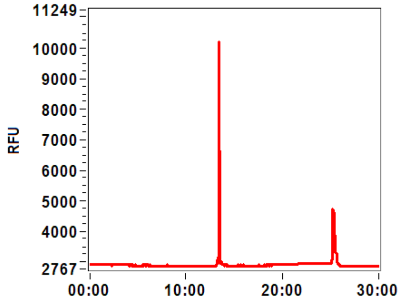

A8: SampA8

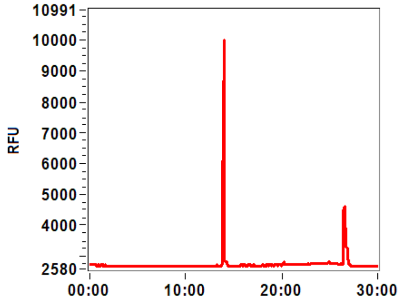

B8: SampB8

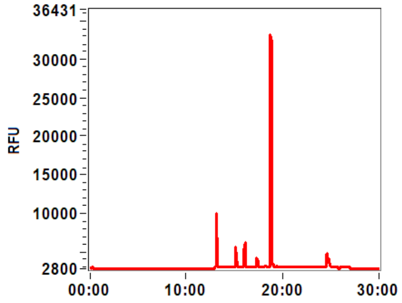

C8: SampC8

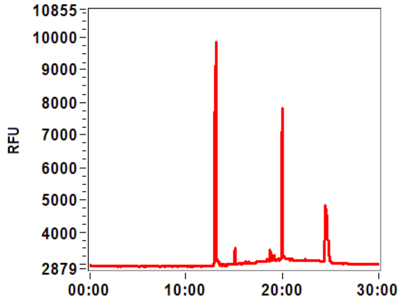

D8: SampD8

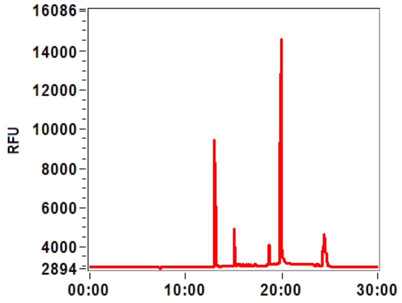

Filename and data path: C:\AATI\Data\2021 10 21\18-38-27\2021 10 21 18H 38M..raw

E8: SampE8

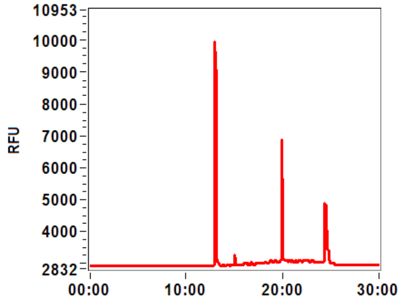

F8: SampF8

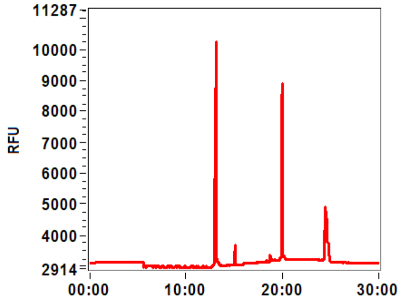

G8: SampG8

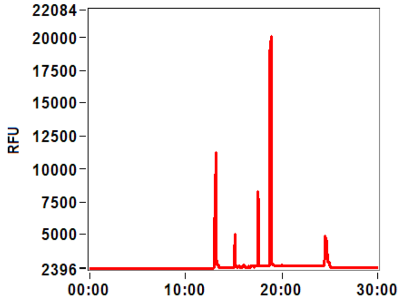

H8: SampH8

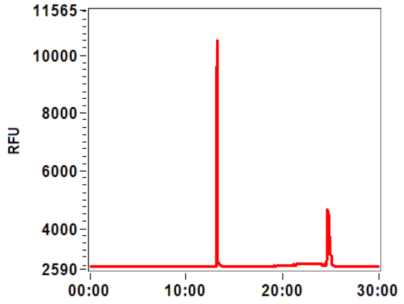

A9: SampA9

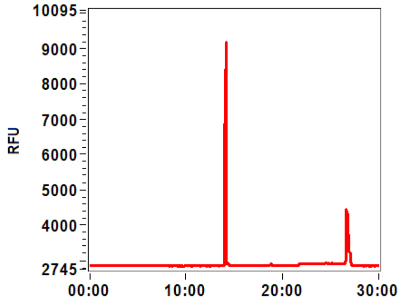

B9: SampB9

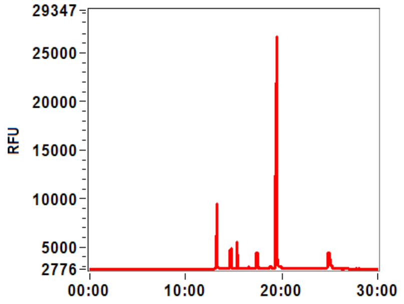

C9: SampC9

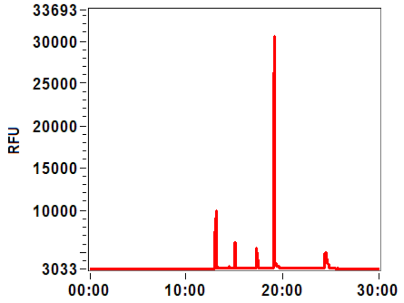

D9: SampD9

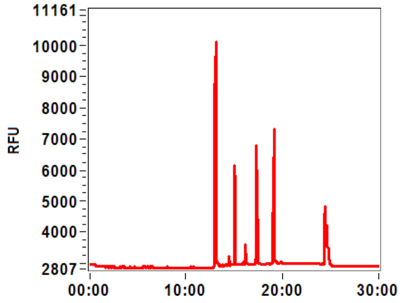

E9: SampE9

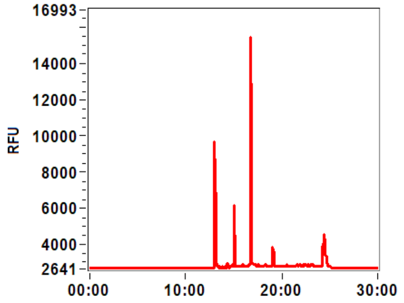

F9: SampF9

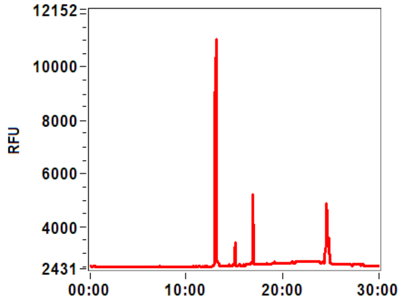

G9: SampG9

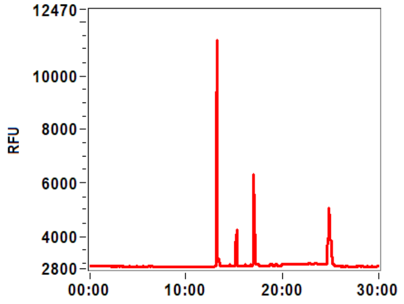

H9: SampH9

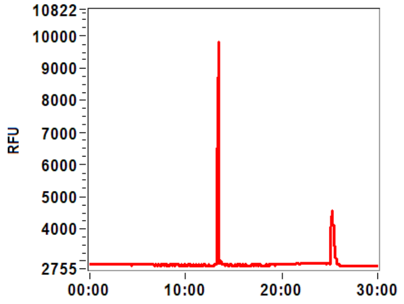

Filename and data path: C:\AATI\Data\2021 10 21\18-38-27\2021 10 21 18H 38M..raw

A10: SampA10

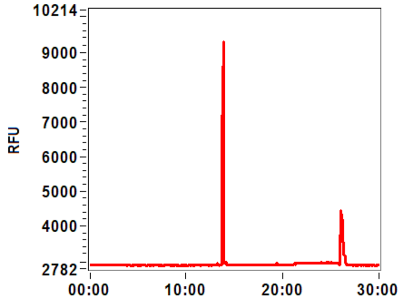

B10: SampB10

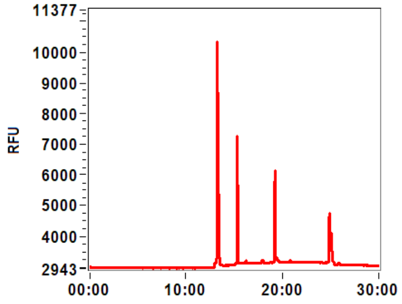

C10: SampC10

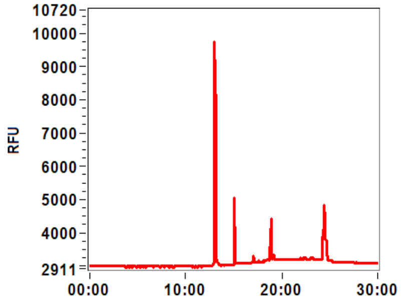

D10: SampD10

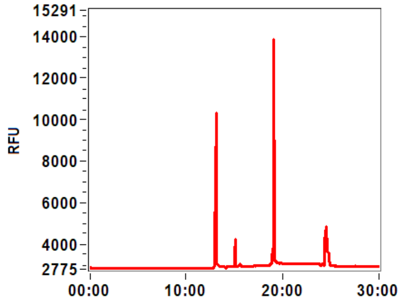

E10: SampE10

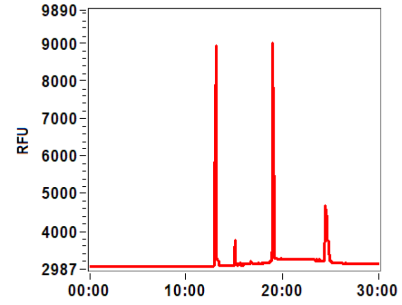

F10: SampF10

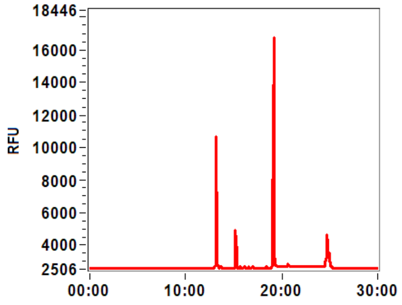

G10: SampG10

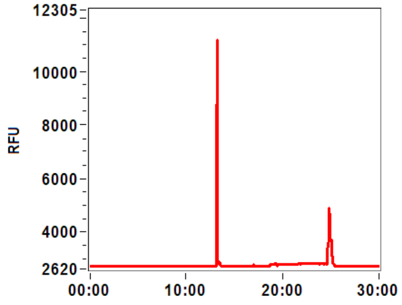

H10: SampH10

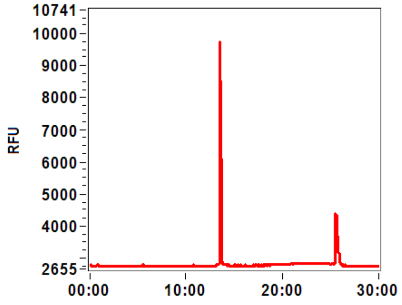

A11: SampA11

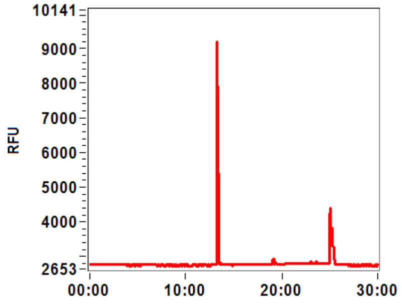

B11: SampB11

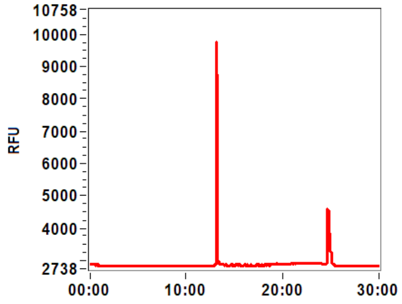

C11: SampC11

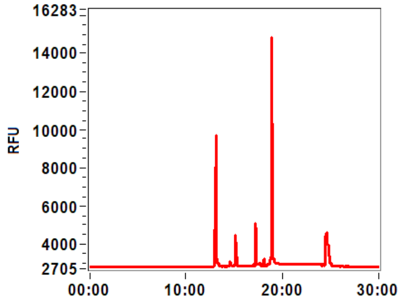

D11: SampD11

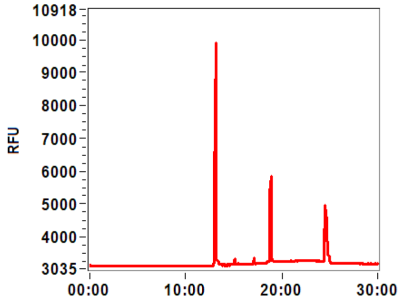

Filename and data path: C:\AATI\Data\2021 10 21\18-38-27\2021 10 21 18H 38M..raw

E11: SampE11

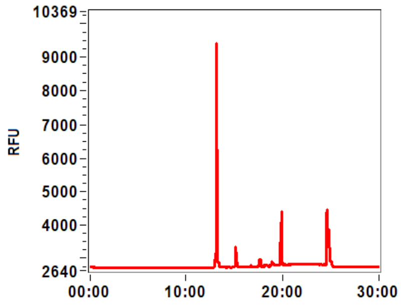

F11: SampF11

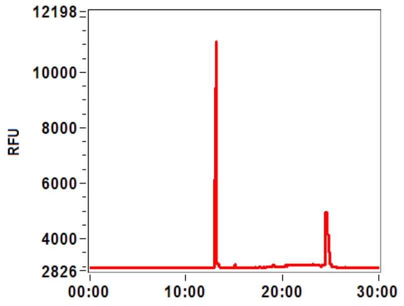

G11: SampG11

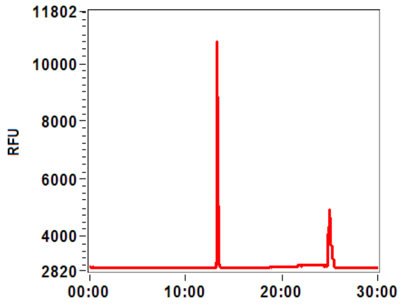

H11: SampH11

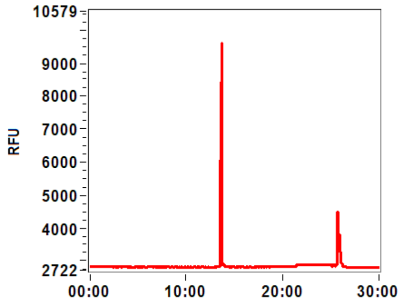

A12: SampA12

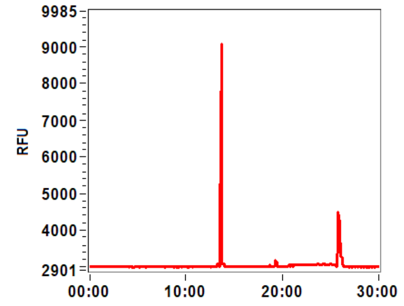

B12: SampB12

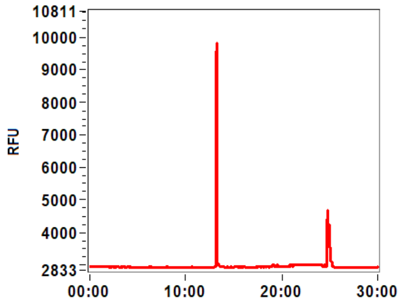

C12: SampC12

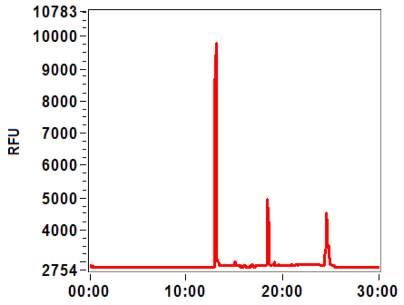

D12: SampD12

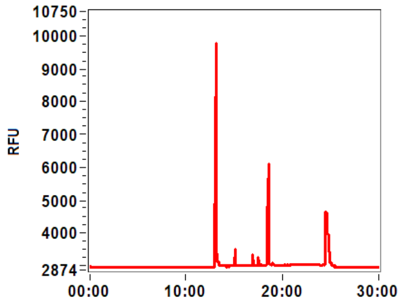

E12: SampE12

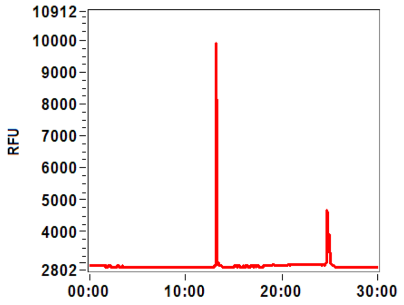

F12: SampF12

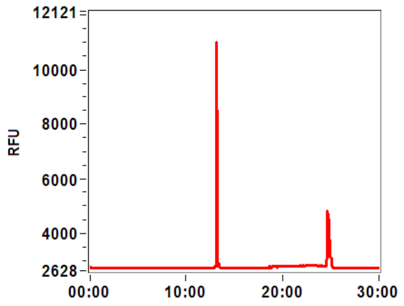

G12: SampG12

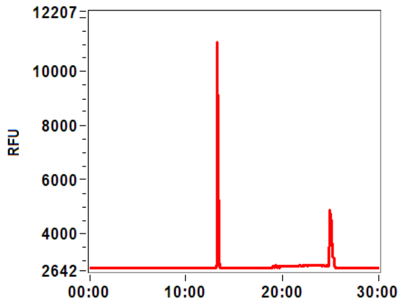

H12: SampH12

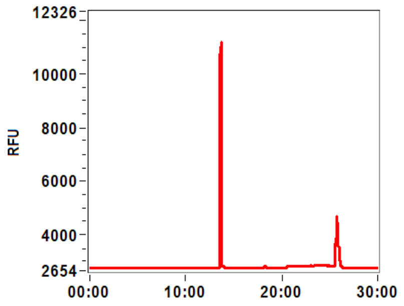

Sample: SampA1
Well location: A1
Created: Thursday, October 21, 2021 6:48:20 PM

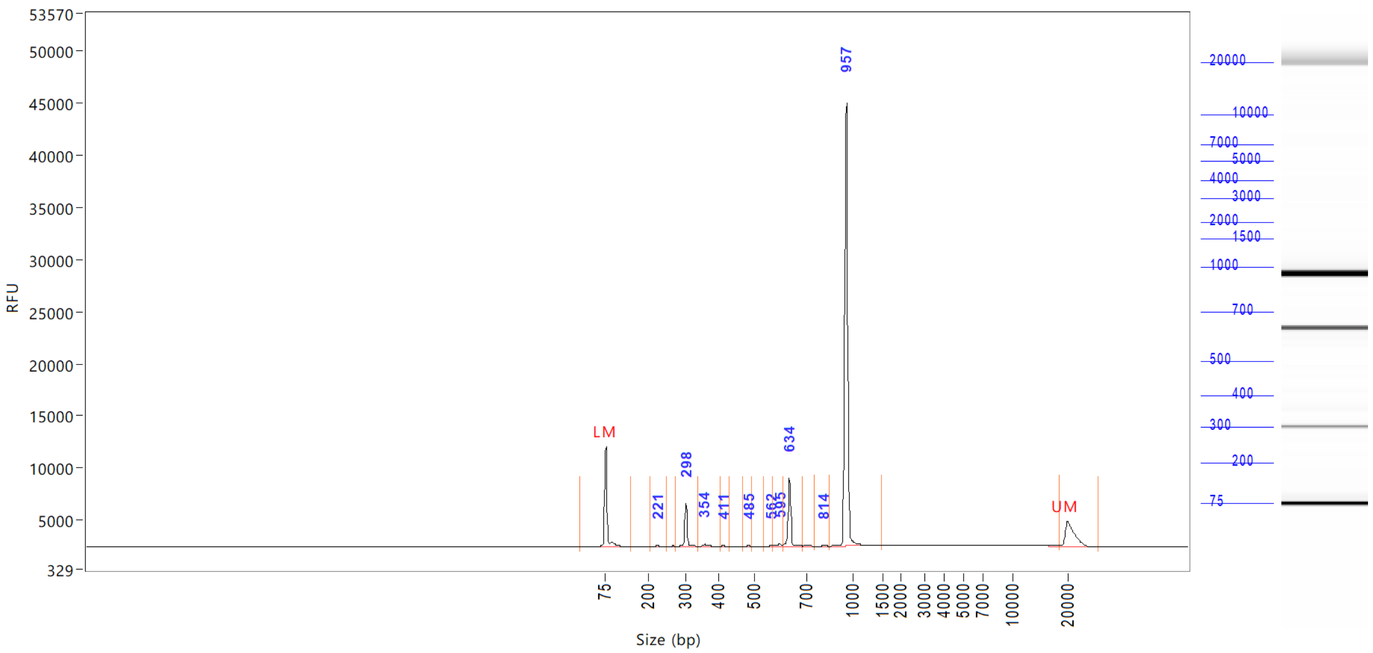

Peak	Size	Concentration	Relative concentration	Molarity	From	To	CV%	RFU
	(bp)	(ng/uL)	Percent	(nmole/L)	(bp)	(bp)		
1	75 (LM)	0.5000		10.9378	1	148	12.58	9543
2	221	0.0587	0.2	0.4370	201	248	4.15	64
3	298	2.0842	7.9	11.5043	273	333	1.68	4055
4	354	0.1979	0.8	0.9069	333	401	3.59	196
5	411	0.0528	0.2	0.2122	401	428	0.89	107
6	485	0.0351	0.1	0.1205	466	494	1.14	58
7	562	0.0578	0.2	0.1698	534	572	1.36	96
8	595	0.1882	0.7	0.5232	572	608	1.56	193
9	634	3.2217	12.3	8.4023	608	687	1.13	6479
10	814	0.0373	0.1	0.0761	753	844	1.88	59
11	957	20.3429	77.4	35.0237	844	1478	2.98	42504
12	20000 (UM)	0.2254		0.0180	18537	25561	4.54	2355

TIC:	26.2765	ng/uL
TIM:	57.3761	nmole/L
Total concentration:	26.7833	ng/uL

```
Sample peak width (sec): 5    Sample min peak height: 50    Sample baseline V to V?: Y    Sample baseline V to V points: 3
Sample filter: Binomial       Number of points for filter: 3  Sample start region (min): 0  Sample end region (min): 30
Marker peak width (sec): 5    Marker min peak height: 200   Marker baseline V to V?: Y    Marker baseline V to V points: 3
Lower marker selection: First peak > 500 RFU                Upper marker selection: Last peak > 200 RFU
Ladder size (bp)75, 200, 300, 400, 500, 700, 1000, 1500, 2000, 3000, 4000, 5000, 7000, 10000, 20000
Quantification using: Lower Marker          Final concentration (ng/uL): 0.5000          Dilution factor: 12.0
```

Sample: SampB1
Well location: B1
Created: Thursday, October 21, 2021 6:48:20 PM

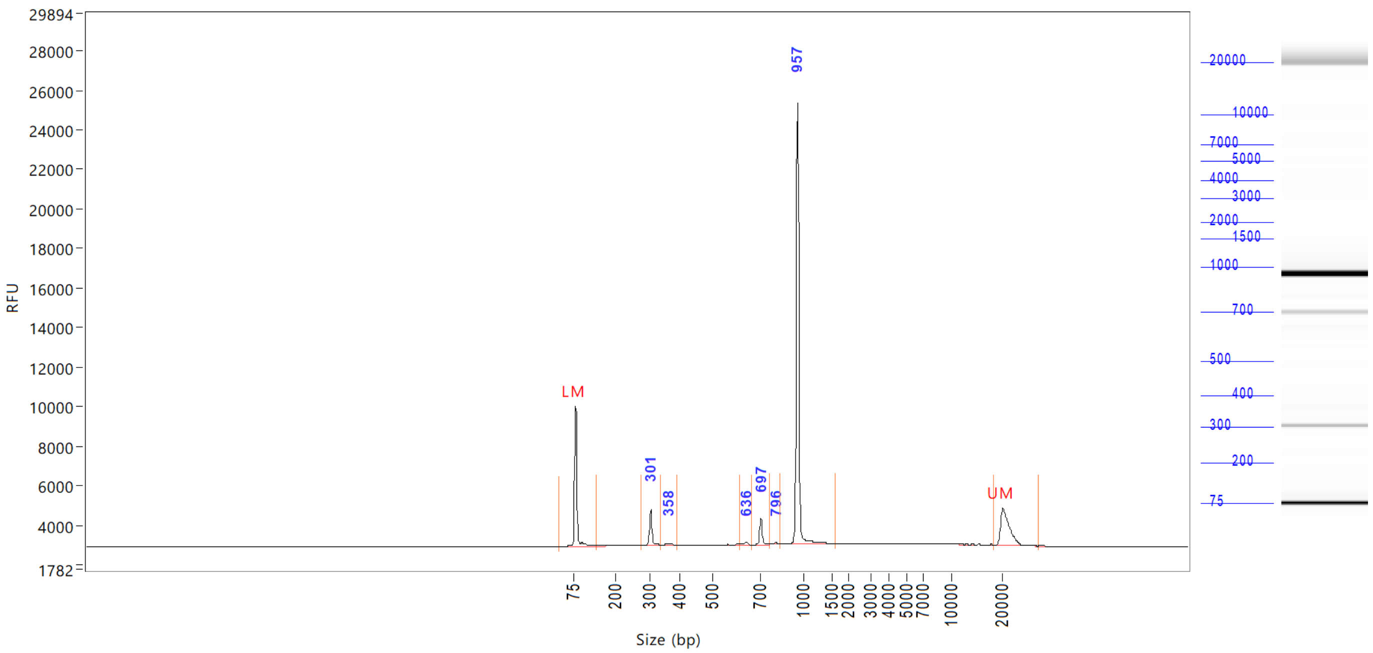

Peak	Size	Concentration	Relative concentration	Molarity	From	To	CV%	RFU
	(bp)	(ng/uL)	Percent	(nmole/L)	(bp)	(bp)		
1	75 (LM)	0.5000		10.9378	26	141	12.10	7110
2	301	1.3398	8.0	7.3221	275	337	2.15	1855
3	358	0.1841	1.1	0.8415	337	388	3.02	114
4	636	0.1488	0.9	0.3886	611	664	1.47	152
5	697	0.8642	5.2	2.0435	664	762	1.06	1302
6	796	0.0790	0.5	0.1638	762	827	1.28	113
7	957	14.0873	84.3	24.1526	827	1597	4.30	22342
8	20000 (UM)	0.2393		0.0190	18293	27012	4.58	1873

TIC:	16.7032	ng/uL
TIM:	34.9120	nmole/L
Total concentration:	17.1804	ng/uL

```
Sample peak width (sec): 5    Sample min peak height: 50    Sample baseline V to V?: Y    Sample baseline V to V points: 3
Sample filter: Binomial       Number of points for filter: 3  Sample start region (min): 0  Sample end region (min): 30
Marker peak width (sec): 5    Marker min peak height: 200   Marker baseline V to V?: Y    Marker baseline V to V points: 3
Lower marker selection: First peak > 500 RFU                Upper marker selection: Last peak > 200 RFU
Ladder size (bp)75, 200, 300, 400, 500, 700, 1000, 1500, 2000, 3000, 4000, 5000, 7000, 10000, 20000
Quantification using: Lower Marker          Final concentration (ng/uL): 0.5000          Dilution factor: 12.0
```

Sample: SampC1
Well location: C1
Created: Thursday, October 21, 2021 6:48:20 PM

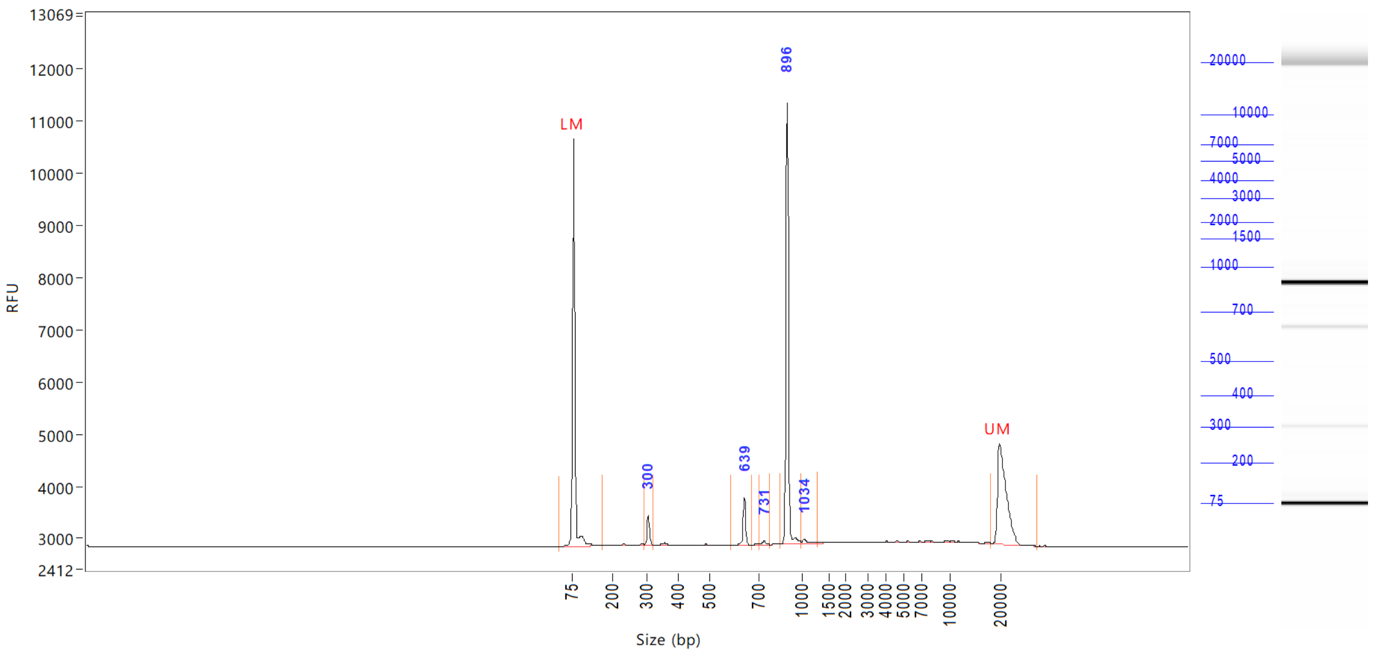

Peak	Size	Concentration	Relative concentration	Molarity	From	To	CV%	RFU
	(bp)	(ng/uL)	Percent	(nmole/L)	(bp)	(bp)		
1	75 (LM)	0.5000		10.9378	31	164	13.66	7817
2	300	0.3661	6.1	2.0211	287	319	1.26	561
3	639	0.5607	9.4	1.4508	583	674	1.31	902
4	731	0.0463	0.8	0.1043	705	779	1.58	63
5	896	4.9099	82.2	9.0293	848	996	1.59	8471
6	1034	0.0920	1.5	0.1417	996	1289	6.86	79
7	20000 (UM)	0.2307		0.0184	18293	27375	4.68	1919

TIC:	5.9751	ng/uL
TIM:	12.7472	nmole/L
Total concentration:	6.5277	ng/uL

```
Sample peak width (sec): 5    Sample min peak height: 50    Sample baseline V to V?: Y    Sample baseline V to V points: 3
Sample filter: Binomial       Number of points for filter: 3  Sample start region (min): 0  Sample end region (min): 30
Marker peak width (sec): 5    Marker min peak height: 200   Marker baseline V to V?: Y    Marker baseline V to V points: 3
Lower marker selection: First peak > 500 RFU                Upper marker selection: Last peak > 200 RFU
Ladder size (bp)75, 200, 300, 400, 500, 700, 1000, 1500, 2000, 3000, 4000, 5000, 7000, 10000, 20000
Quantification using: Lower Marker          Final concentration (ng/uL): 0.5000          Dilution factor: 12.0
```

Sample: SampD1
Well location: D1
Created: Thursday, October 21, 2021 6:48:20 PM

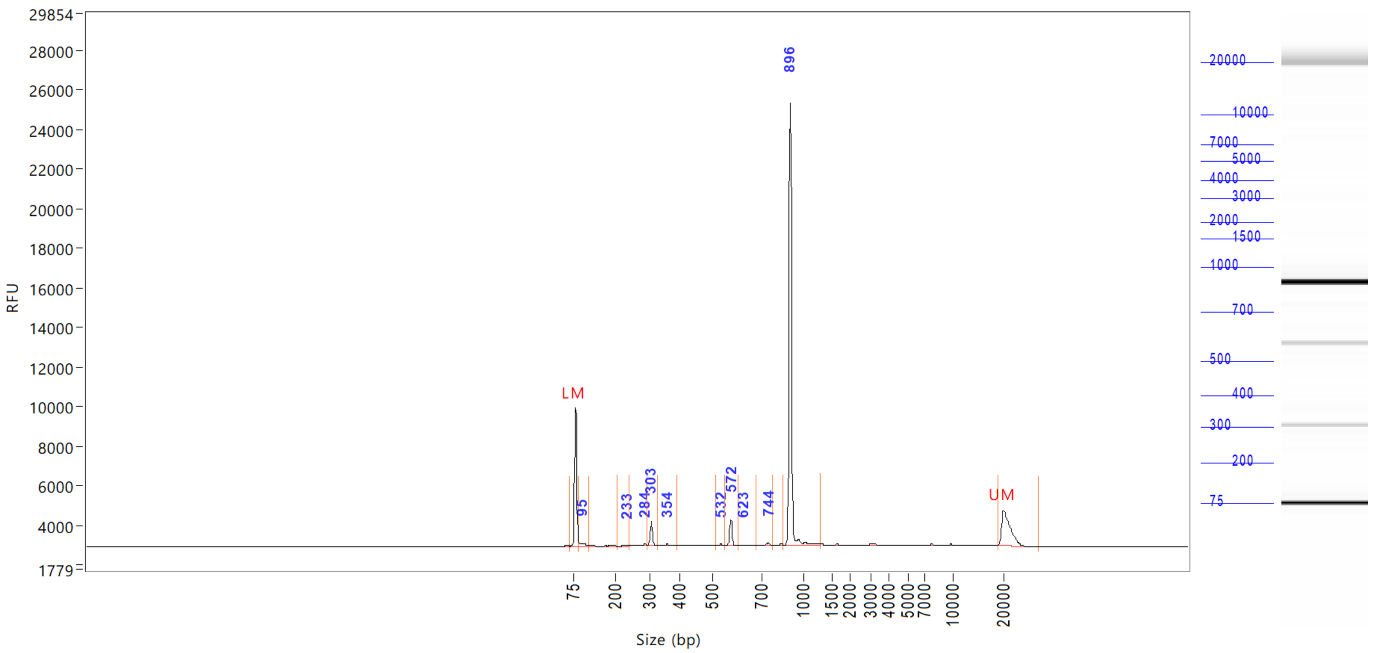

Peak	Size	Concentration	Relative concentration	Molarity	From	To	CV%	RFU
	(bp)	(ng/uL)	Percent	(nmole/L)	(bp)	(bp)		
1	75 (LM)	0.5000		11.0851	60	87	4.42	7012
2	95	0.3144	1.7	5.2673	87	120	7.78	164
3	233	0.0728	0.4	0.5250	205	242	2.66	60
4	284	0.1666	0.9	0.9855	242	293	4.34	113
5	303	0.9643	5.2	5.2699	293	327	1.23	1273
6	354	0.1529	0.8	0.7104	327	388	3.09	71
7	532	0.0880	0.5	0.2729	511	552	1.69	72
8	572	0.9798	5.3	2.8286	552	603	0.96	1347
9	623	0.0956	0.5	0.2484	603	680	2.34	61
10	744	0.1400	0.8	0.3140	680	783	2.49	129
11	896	15.4200	83.8	28.2308	857	1289	2.29	22367
12	20000 (UM)	0.2549		0.0203	18903	26770	4.61	1787

TIC:	18.3943	ng/uL
TIM:	44.6528	nmole/L
Total concentration:	18.9941	ng/uL

Sample peak width (sec): 5   Sample min peak height: 50   Sample baseline V to V?: Y   Sample baseline V to V points: 3
Sample filter: Binomial   Number of points for filter: 3   Sample start region (min): 0   Sample end region (min): 30
Marker peak width (sec): 5   Marker min peak height: 200   Marker baseline V to V?: Y   Marker baseline V to V points: 3
Lower marker selection: First peak > 500 RFU   Upper marker selection: Last peak > 200 RFU
Ladder size (bp)75, 200, 300, 400, 500, 700, 1000, 1500, 2000, 3000, 4000, 5000, 7000, 10000, 20000
Quantification using: Lower Marker   Final concentration (ng/uL): 0.5000   Dilution factor: 12.0

Sample: SampE1
Well location: E1
Created: Thursday, October 21, 2021 6:48:20 PM

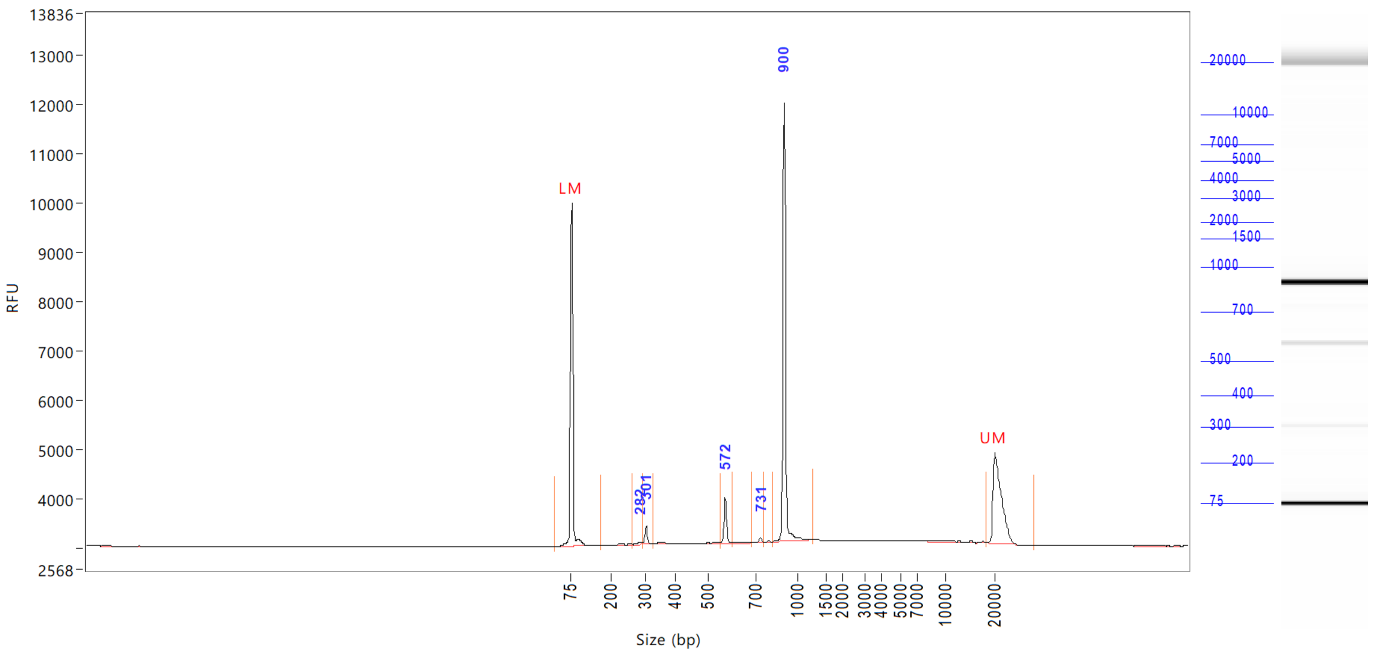

Peak	Size	Concentration	Relative concentration	Molarity	From	To	CV%	RFU
	(bp)	(ng/uL)	Percent	(nmole/L)	(bp)	(bp)		
1	75 (LM)	0.5000		11.0851	24	168	12.53	6940
2	282	0.0786	1.1	0.4618	262	291	2.48	57
3	301	0.2849	4.0	1.5621	291	323	1.29	372
4	572	0.6355	9.0	1.8347	549	600	1.06	940
5	731	0.0763	1.1	0.1737	682	753	1.80	93
6	900	6.0190	84.8	11.0195	814	1278	2.55	8900
7	20000 (UM)	0.2421		0.0193	18171	27738	4.60	1836

TIC:	7.0943	ng/uL
TIM:	15.0517	nmole/L
Total concentration:	7.6942	ng/uL

Sample peak width (sec): 5 Sample min peak height: 50 Sample baseline V to V?: Y Sample baseline V to V points: 3
Sample filter: Binomial Number of points for filter: 3 Sample start region (min): 0 Sample end region (min): 30
Marker peak width (sec): 5 Marker min peak height: 200 Marker baseline V to V?: Y Marker baseline V to V points: 3
Lower marker selection: First peak > 500 RFU Upper marker selection: Last peak > 200 RFU
Ladder size (bp)75, 200, 300, 400, 500, 700, 1000, 1500, 2000, 3000, 4000, 5000, 7000, 10000, 20000
Quantification using: Lower Marker Final concentration (ng/uL): 0.5000 Dilution factor: 12.0

Sample: SampF1
Well location: F1
Created: Thursday, October 21, 2021 6:48:20 PM

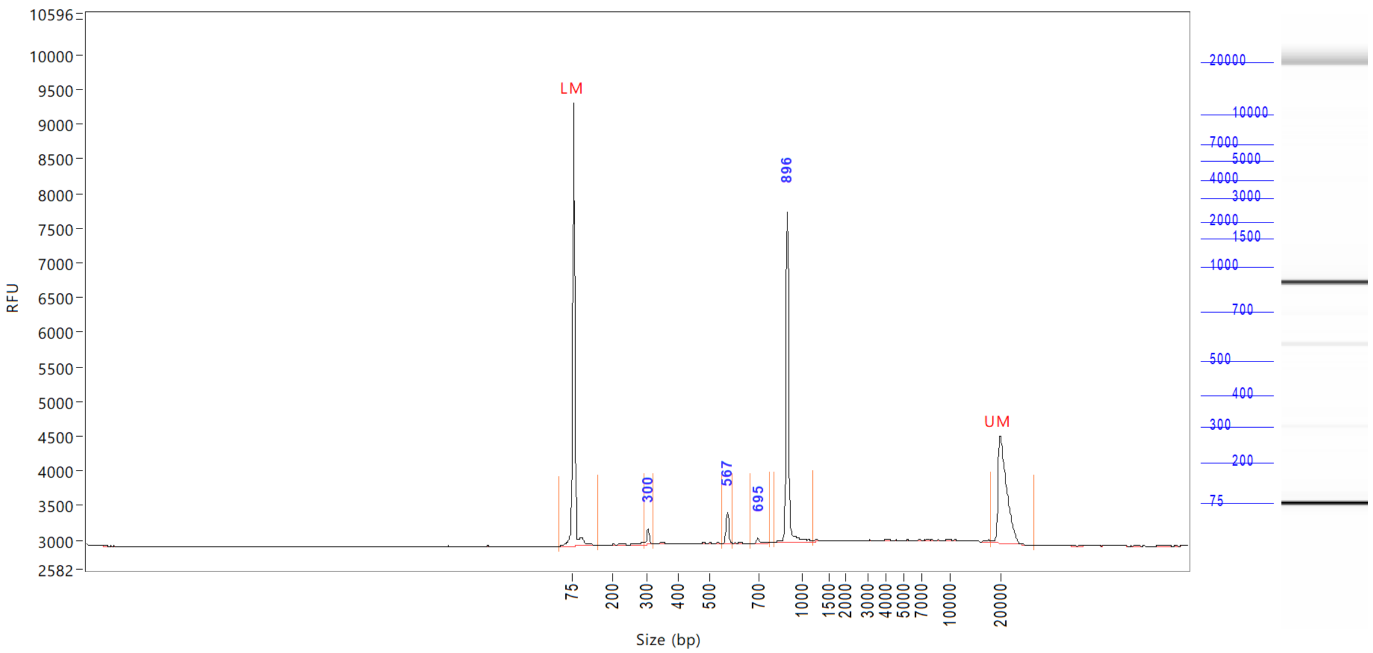

Peak	Size	Concentration	Relative concentration	Molarity	From	To	CV%	RFU
	(bp)	(ng/uL)	Percent	(nmole/L)	(bp)	(bp)		
1	75 (LM)	0.5000		11.0851	31	150	11.69	6399
2	300	0.1934	4.4	1.0674	289	317	1.29	228
3	567	0.3543	8.1	1.0301	549	590	0.98	455
4	695	0.0865	2.0	0.2029	667	770	2.69	78
5	896	3.7600	85.6	6.8761	805	1201	3.56	4755
6	20000 (UM)	0.2247		0.0179	18293	26770	4.64	1546

TIC:	4.3942	ng/uL
TIM:	9.1765	nmole/L
Total concentration:	4.9958	ng/uL

```
Sample peak width (sec): 5     Sample min peak height: 50    Sample baseline V to V?: Y    Sample baseline V to V points: 3
Sample filter: Binomial        Number of points for filter: 3  Sample start region (min): 0  Sample end region (min): 30
Marker peak width (sec): 5     Marker min peak height: 200   Marker baseline V to V?: Y    Marker baseline V to V points: 3
Lower marker selection: First peak > 500 RFU                 Upper marker selection: Last peak > 200 RFU
Ladder size (bp)75, 200, 300, 400, 500, 700, 1000, 1500, 2000, 3000, 4000, 5000, 7000, 10000, 20000
Quantification using: Lower Marker          Final concentration (ng/uL): 0.5000          Dilution factor: 12.0
```

Sample: SampG1
Well location: G1
Created: Thursday, October 21, 2021 6:48:20 PM

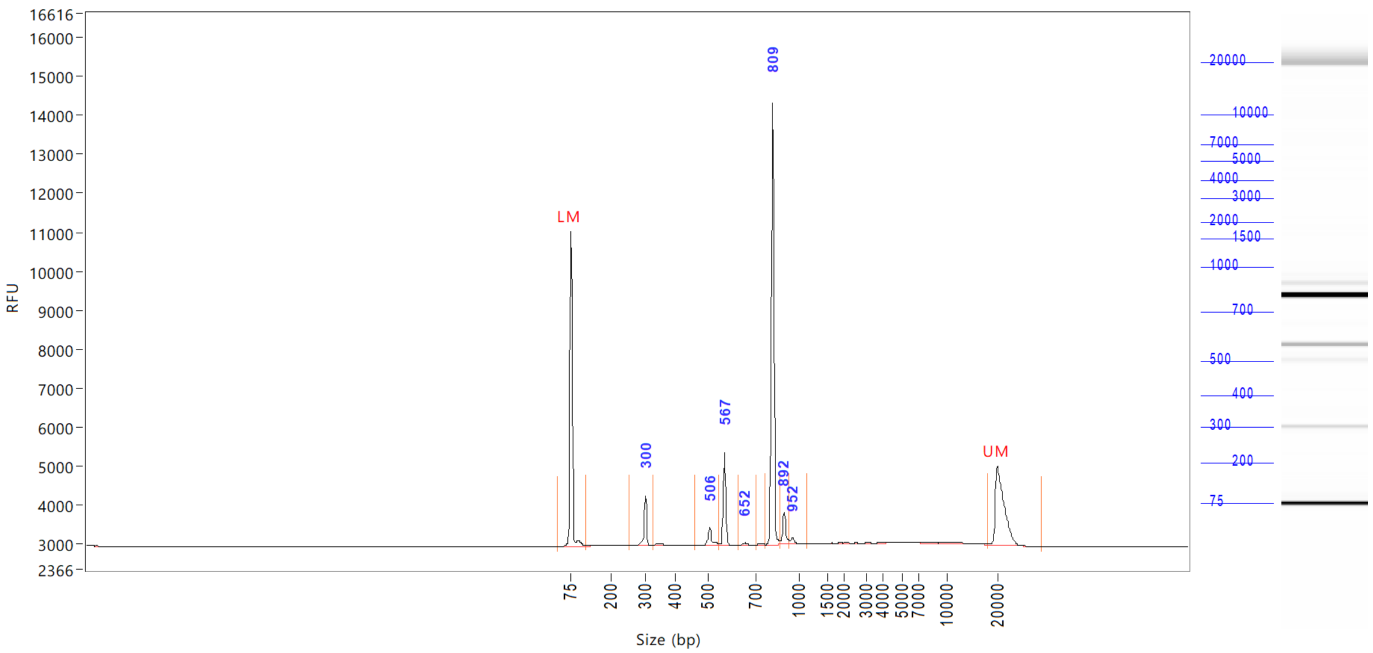

Peak	Size	Concentration	Relative concentration	Molarity	From	To	CV%	RFU
	(bp)	(ng/uL)	Percent	(nmole/L)	(bp)	(bp)		
1	75 (LM)	0.5000		11.0851	33	124	8.51	8074
2	300	0.9287	8.6	5.1608	255	323	2.01	1287
3	506	0.4558	4.2	1.4794	460	547	2.58	464
4	567	1.5058	13.9	4.3858	547	629	1.11	2391
5	652	0.0568	0.5	0.1433	629	697	1.65	53
6	809	7.0730	65.4	14.4250	762	861	1.25	11340
7	892	0.6325	5.8	1.1671	861	935	1.73	825
8	952	0.1701	1.6	0.2904	935	1123	3.29	172
9	20000 (UM)	0.2470		0.0197	18171	28826	5.01	2006

TIC:	10.8227	ng/uL
TIM:	27.0518	nmole/L
Total concentration:	11.4464	ng/uL

```
Sample peak width (sec): 5    Sample min peak height: 50    Sample baseline V to V?: Y    Sample baseline V to V points: 3
Sample filter: Binomial       Number of points for filter: 3  Sample start region (min): 0  Sample end region (min): 30
Marker peak width (sec): 5    Marker min peak height: 200   Marker baseline V to V?: Y    Marker baseline V to V points: 3
Lower marker selection: First peak > 500 RFU                Upper marker selection: Last peak > 200 RFU
Ladder size (bp)75, 200, 300, 400, 500, 700, 1000, 1500, 2000, 3000, 4000, 5000, 7000, 10000, 20000
Quantification using: Lower Marker          Final concentration (ng/uL): 0.5000          Dilution factor: 12.0
```

Sample: SampH1
Well location: H1
Created: Thursday, October 21, 2021 6:48:20 PM

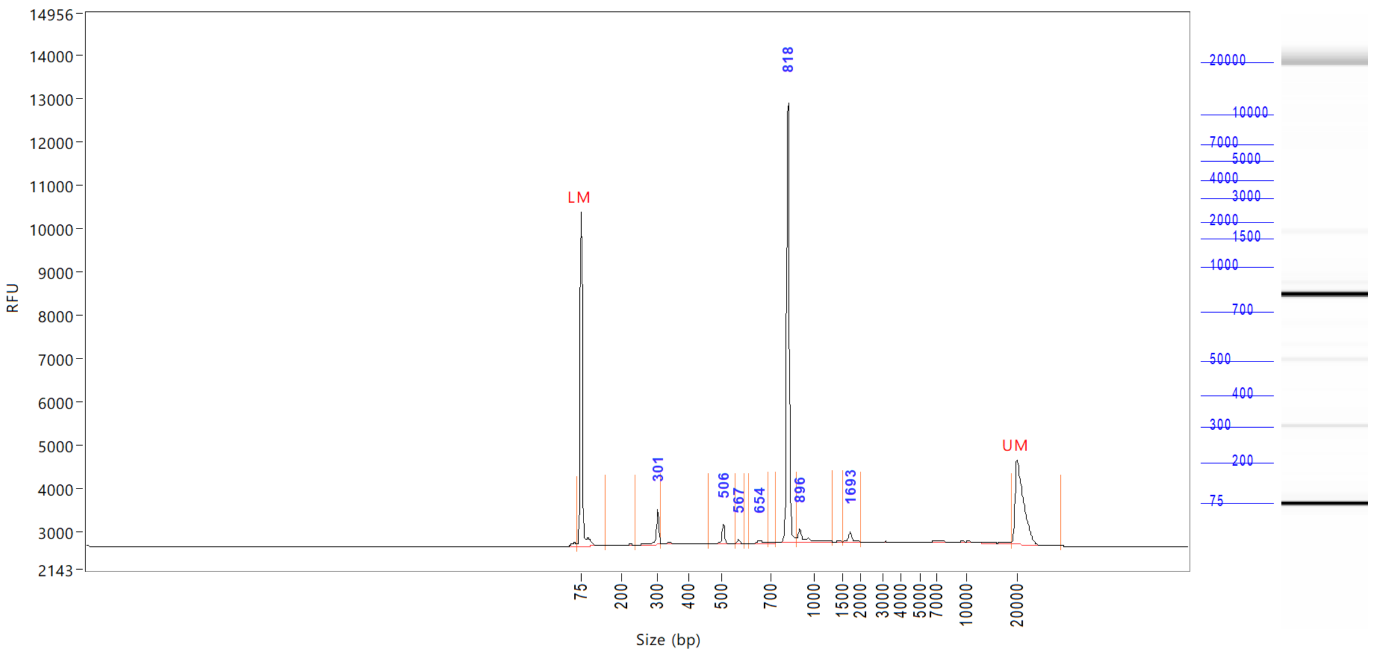

Peak	Size	Concentration	Relative concentration	Molarity	From	To	CV%	RFU
	(bp)	(ng/uL)	Percent	(nmole/L)	(bp)	(bp)		
1	75 (LM)	0.5000		11.0851	62	148	10.65	7712
2	301	0.6021	7.6	3.3461	237	313	3.29	825
3	506	0.3145	4.0	1.0269	458	552	2.03	446
4	567	0.0631	0.8	0.1837	552	593	1.01	97
5	654	0.0899	1.1	0.2262	613	690	1.96	74
6	818	6.3039	79.2	12.7774	736	874	1.43	10153
7	896	0.4209	5.3	0.7393	874	1311	7.56	310
8	1693	0.1638	2.1	0.1595	1489	2058	4.57	241
9	20000 (UM)	0.2296		0.0183	18781	28463	4.75	1953

TIC:	7.9582	ng/uL
TIM:	18.4591	nmole/L
Total concentration:	8.4965	ng/uL

Sample peak width (sec): 5 Sample min peak height: 50 Sample baseline V to V?: Y Sample baseline V to V points: 3
Sample filter: Binomial Number of points for filter: 3 Sample start region (min): 0 Sample end region (min): 30
Marker peak width (sec): 5 Marker min peak height: 200 Marker baseline V to V?: Y Marker baseline V to V points: 3
Lower marker selection: First peak > 500 RFU Upper marker selection: Last peak > 200 RFU
Ladder size (bp)75, 200, 300, 400, 500, 700, 1000, 1500, 2000, 3000, 4000, 5000, 7000, 10000, 20000
Quantification using: Lower Marker Final concentration (ng/uL): 0.5000 Dilution factor: 12.0

Sample: SampA2
Well location: A2
Created: Thursday, October 21, 2021 6:48:20 PM

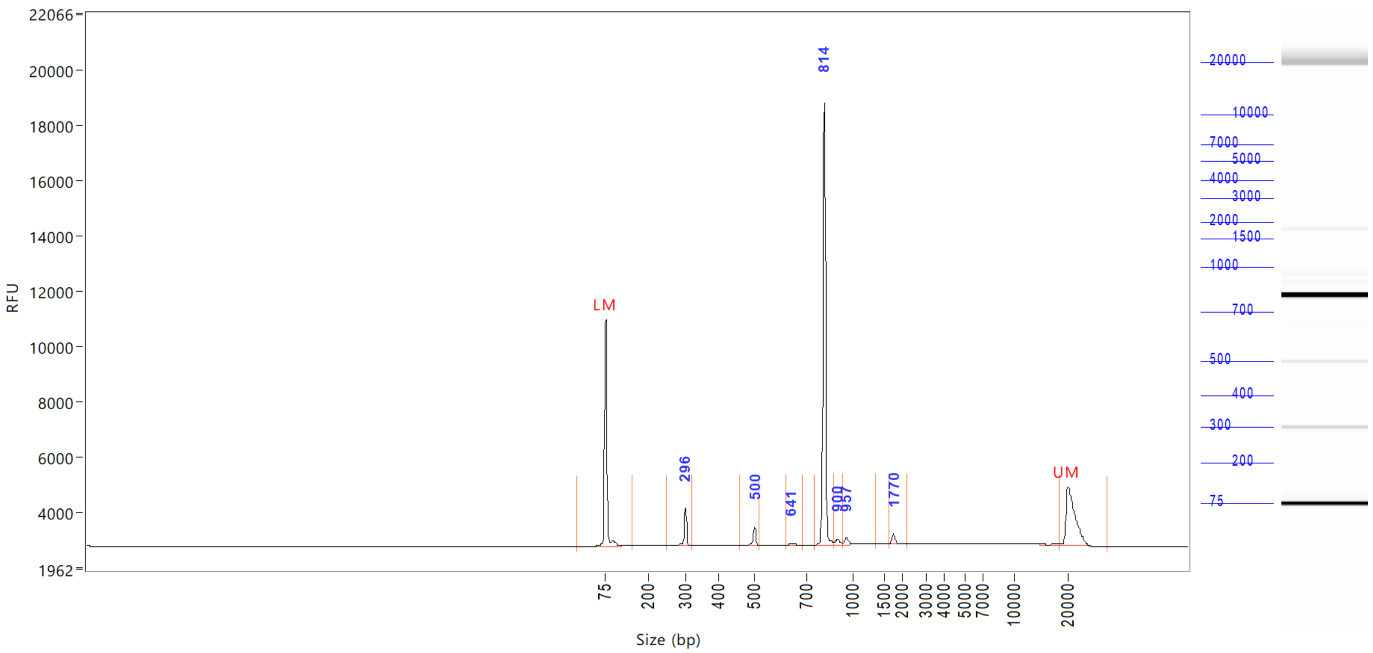

Peak	Size	Concentration	Relative concentration	Molarity	From	To	CV%	RFU
	(bp)	(ng/uL)	Percent	(nmole/L)	(bp)	(bp)		
1	75 (LM)	0.5000		11.2364	0	152	10.25	8207
2	296	0.8853	8.1	4.9534	248	317	2.52	1370
3	500	0.4080	3.7	1.3509	458	514	1.27	677
4	641	0.0883	0.8	0.2264	621	682	1.67	77
5	814	8.8355	81.0	18.0195	749	874	1.32	16006
6	900	0.1929	1.8	0.3535	874	931	1.59	217
7	957	0.2546	2.3	0.4236	931	1367	7.94	238
8	1770	0.2477	2.3	0.2291	1635	2229	4.99	376
9	20000 (UM)	0.2453		0.0195	18537	27133	4.51	2129

TIC:	10.9123	ng/uL
TIM:	25.5563	nmole/L
Total concentration:	11.4623	ng/uL

Sample peak width (sec): 5   Sample min peak height: 50   Sample baseline V to V?: Y   Sample baseline V to V points: 3
Sample filter: Binomial   Number of points for filter: 3 Sample start region (min): 0 Sample end region (min): 30
Marker peak width (sec): 5   Marker min peak height: 200   Marker baseline V to V?: Y   Marker baseline V to V points: 3
Lower marker selection: First peak > 500 RFU   Upper marker selection: Last peak > 200 RFU
Ladder size (bp)75, 200, 300, 400, 500, 700, 1000, 1500, 2000, 3000, 4000, 5000, 7000, 10000, 20000
Quantification using: Lower Marker   Final concentration (ng/uL): 0.5000   Dilution factor: 12.0

Sample: SampB2
Well location: B2
Created: Thursday, October 21, 2021 6:48:20 PM

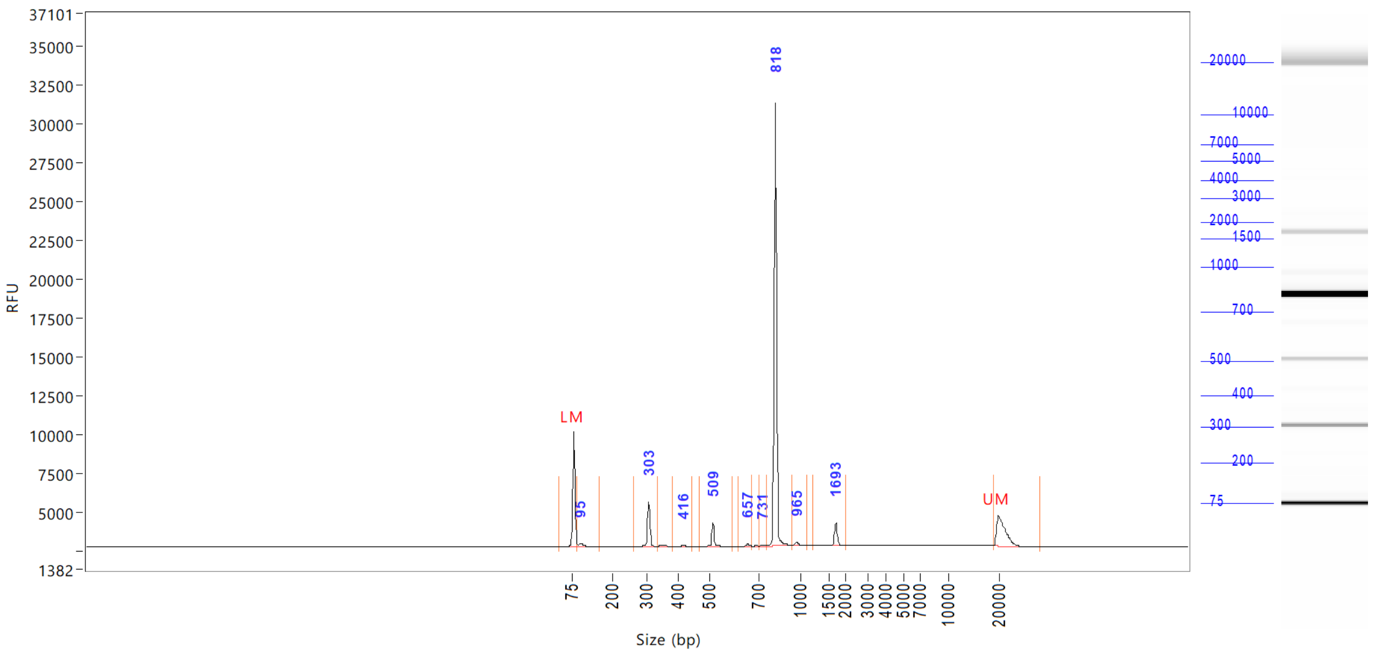

Peak	Size	Concentration	Relative concentration	Molarity	From	To	CV%	RFU
	(bp)	(ng/uL)	Percent	(nmole/L)	(bp)	(bp)		
1	75 (LM)	0.5000		11.0851	30	87	4.92	7437
2	95	0.4404	1.9	6.7597	87	158	16.79	181
3	303	2.1090	9.0	11.5257	259	331	1.75	2862
4	416	0.0902	0.4	0.3593	384	443	2.59	77
5	509	1.0458	4.4	3.3944	466	590	1.94	1465
6	657	0.1239	0.5	0.3117	618	677	1.20	131
7	731	0.0610	0.3	0.1374	701	753	1.74	59
8	818	18.3171	77.8	36.9900	753	935	1.57	28514
9	965	0.2542	1.1	0.4326	935	1112	2.11	237
10	1693	1.0976	4.7	1.0819	1223	2001	5.65	1447
11	20000 (UM)	0.2598		0.0207	18903	28100	5.00	1925

TIC:	23.5392	ng/uL
TIM:	60.9928	nmole/L
Total concentration:	24.1955	ng/uL

```
Sample peak width (sec): 5    Sample min peak height: 50    Sample baseline V to V?: Y    Sample baseline V to V points: 3
Sample filter: Binomial       Number of points for filter: 3  Sample start region (min): 0  Sample end region (min): 30
Marker peak width (sec): 5    Marker min peak height: 200   Marker baseline V to V?: Y    Marker baseline V to V points: 3
Lower marker selection: First peak > 500 RFU                Upper marker selection: Last peak > 200 RFU
Ladder size (bp)75, 200, 300, 400, 500, 700, 1000, 1500, 2000, 3000, 4000, 5000, 7000, 10000, 20000
Quantification using: Lower Marker          Final concentration (ng/uL): 0.5000          Dilution factor: 12.0
```

Sample: SampC2
Well location: C2
Created: Thursday, October 21, 2021 6:48:20 PM

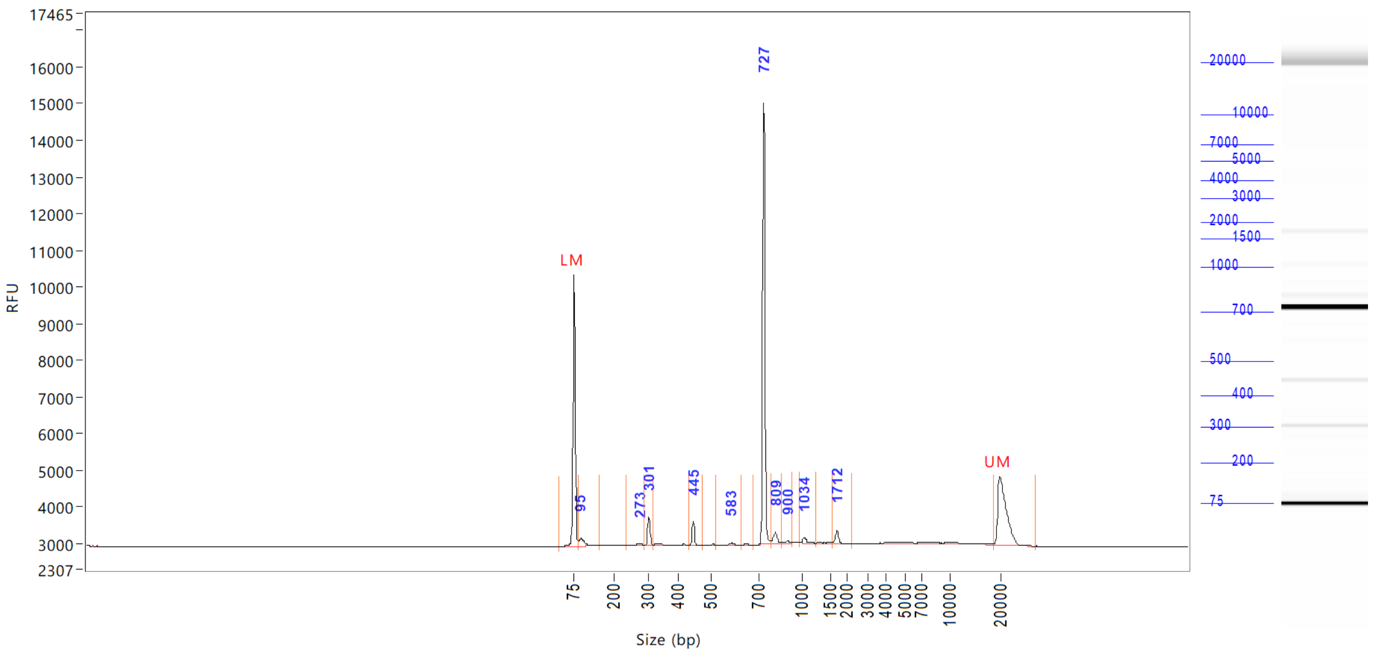

Peak	Size	Concentration	Relative concentration	Molarity	From	To	CV%	RFU
	(bp)	(ng/uL)	Percent	(nmole/L)	(bp)	(bp)		
1	75 (LM)	0.5000		11.3919	30	87	5.48	7393
2	95	0.4355	4.5	7.0113	87	152	13.36	208
3	273	0.0910	0.9	0.5544	235	289	3.99	54
4	301	0.5351	5.5	2.9338	289	315	1.13	769
5	445	0.4231	4.3	1.5752	433	473	0.79	646
6	583	0.0819	0.8	0.2324	521	626	3.01	59
7	727	7.4003	76.1	16.8222	674	779	1.26	12056
8	809	0.3259	3.3	0.6662	779	853	1.77	309
9	900	0.0645	0.7	0.1192	853	935	1.86	63
10	1034	0.1192	1.2	0.1891	991	1245	3.77	156
11	1712	0.2521	2.6	0.2451	1578	2229	3.61	370
12	20000 (UM)	0.2436		0.0195	18781	26891	4.52	1867

TIC:	9.7286	ng/uL
TIM:	30.3489	nmole/L
Total concentration:	10.3449	ng/uL

Sample peak width (sec): 5 Sample min peak height: 50 Sample baseline V to V?: Y Sample baseline V to V points: 3
Sample filter: Binomial Number of points for filter: 3 Sample start region (min): 0 Sample end region (min): 30
Marker peak width (sec): 5 Marker min peak height: 200 Marker baseline V to V?: Y Marker baseline V to V points: 3
Lower marker selection: First peak > 500 RFU Upper marker selection: Last peak > 200 RFU
Ladder size (bp)75, 200, 300, 400, 500, 700, 1000, 1500, 2000, 3000, 4000, 5000, 7000, 10000, 20000
Quantification using: Lower Marker Final concentration (ng/uL): 0.5000 Dilution factor: 12.0

Sample: SampD2
Well location: D2
Created: Thursday, October 21, 2021 6:48:20 PM

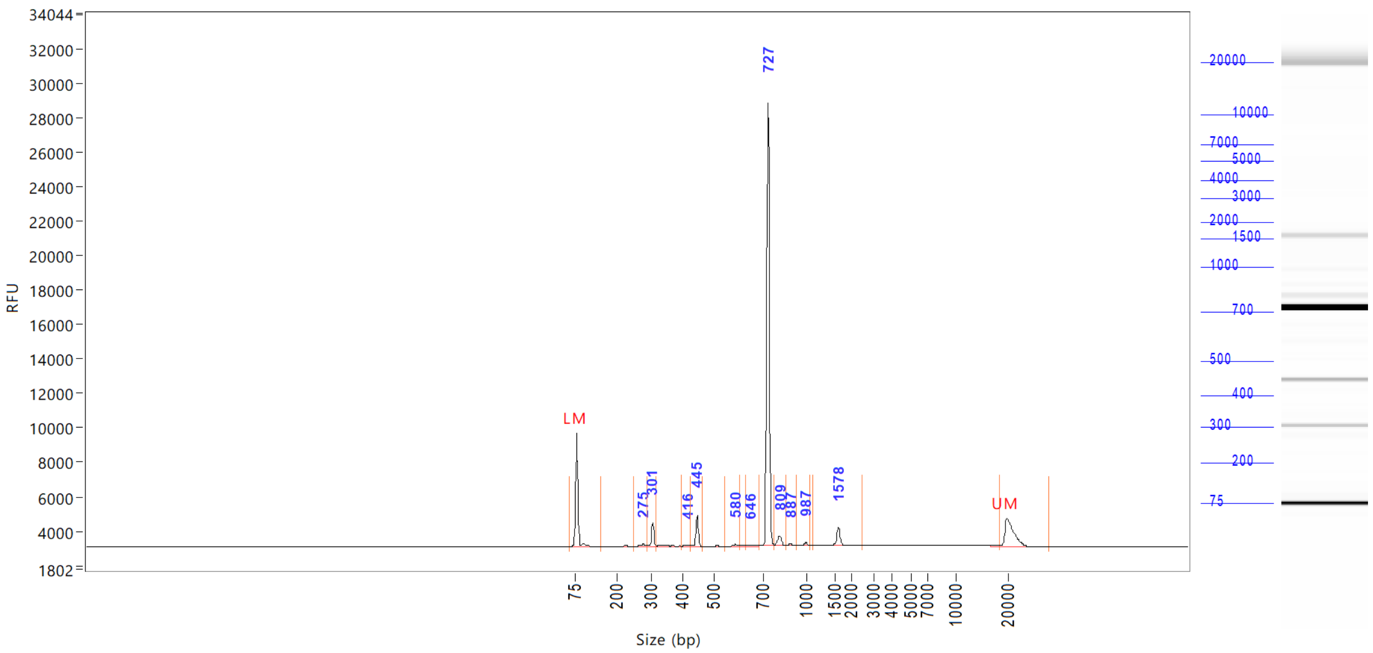

Peak	Size	Concentration	Relative concentration	Molarity	From	To	CV%	RFU
	(bp)	(ng/uL)	Percent	(nmole/L)	(bp)	(bp)		
1	75 (LM)	0.5000		10.9378	54	152	11.25	6558
2	275	0.1724	0.7	1.0423	246	287	2.64	109
3	301	1.0899	4.7	5.9759	287	315	1.09	1395
4	416	0.0686	0.3	0.2733	398	424	1.62	51
5	445	1.3768	6.0	5.1136	424	464	0.91	1821
6	580	0.1211	0.5	0.3441	542	603	1.52	100
7	646	0.0987	0.4	0.2517	623	685	1.58	72
8	727	18.1575	78.6	41.3320	685	775	1.22	25734
9	809	0.5845	2.5	1.1980	775	853	1.56	549
10	887	0.1091	0.5	0.2035	853	922	1.37	129
11	987	0.1710	0.7	0.2871	922	1045	2.17	173
12	1578	1.1574	5.0	1.2444	1112	2458	9.60	1074
13	20000 (UM)	0.2353		0.0188	18537	28100	4.77	1614

TIC:	23.1067	ng/uL
TIM:	57.2659	nmole/L
Total concentration:	23.8897	ng/uL

```
Sample peak width (sec): 5    Sample min peak height: 50    Sample baseline V to V?: Y    Sample baseline V to V points: 3
Sample filter: Binomial       Number of points for filter: 3  Sample start region (min): 0  Sample end region (min): 30
Marker peak width (sec): 5    Marker min peak height: 200   Marker baseline V to V?: Y    Marker baseline V to V points: 3
Lower marker selection: First peak > 500 RFU                Upper marker selection: Last peak > 200 RFU
Ladder size (bp)75, 200, 300, 400, 500, 700, 1000, 1500, 2000, 3000, 4000, 5000, 7000, 10000, 20000
Quantification using: Lower Marker          Final concentration (ng/uL): 0.5000          Dilution factor: 12.0
```

Sample: SampE2
Well location: E2
Created: Thursday, October 21, 2021 6:48:20 PM

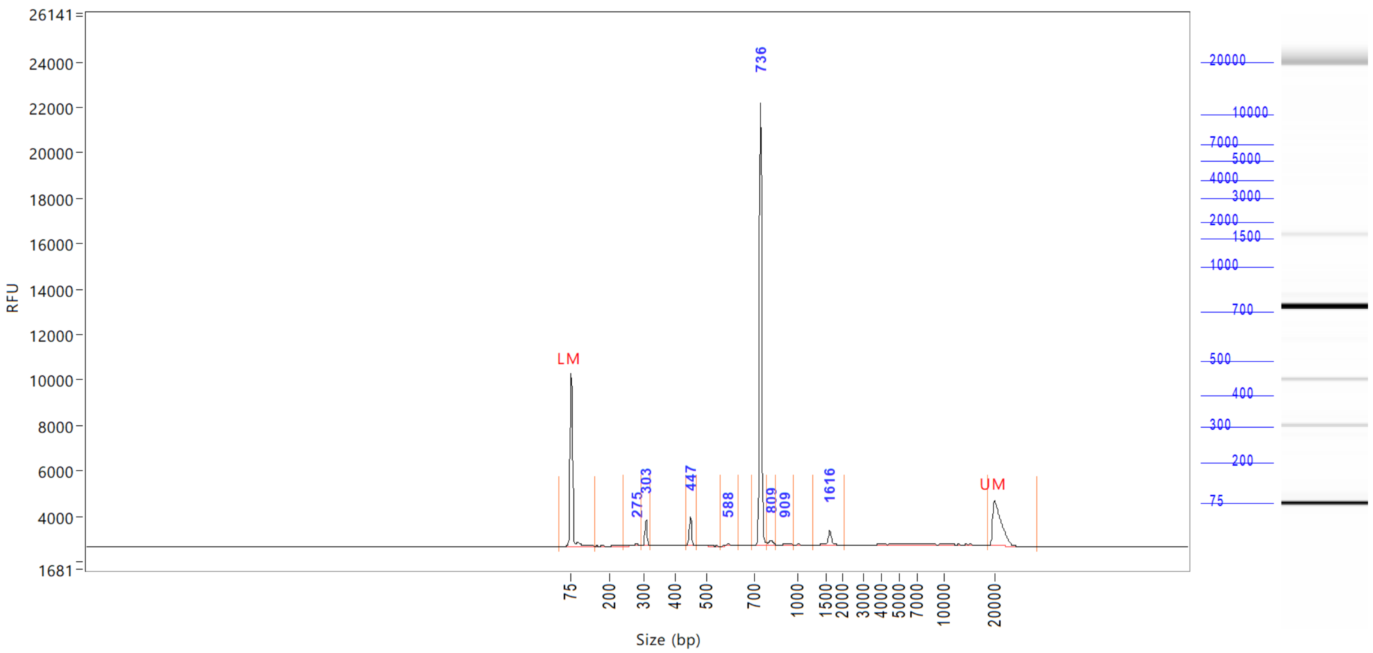

Peak	Size	Concentration	Relative concentration	Molarity	From	To	CV%	RFU
	(bp)	(ng/uL)	Percent	(nmole/L)	(bp)	(bp)		
1	75 (LM)	0.5000		10.9378	39	148	11.27	7625
2	275	0.1219	0.9	0.7347	239	291	3.16	83
3	303	0.7541	5.4	4.1211	291	315	1.03	1158
4	447	0.7513	5.4	2.7779	435	466	0.77	1247
5	588	0.1014	0.7	0.2833	552	631	2.32	86
6	736	11.3439	81.6	25.5748	687	783	1.21	19511
7	809	0.2465	1.8	0.5034	783	844	1.70	232
8	909	0.1126	0.8	0.2039	844	978	3.78	57
9	1616	0.4734	3.4	0.4888	1289	2144	5.08	663
10	20000 (UM)	0.2448		0.0195	18537	28584	4.61	1986

TIC:	13.9052	ng/uL
TIM:	34.6879	nmole/L
Total concentration:	14.6023	ng/uL

Sample peak width (sec): 5 Sample min peak height: 50 Sample baseline V to V?: Y Sample baseline V to V points: 3
Sample filter: Binomial Number of points for filter: 3 Sample start region (min): 0 Sample end region (min): 30
Marker peak width (sec): 5 Marker min peak height: 200 Marker baseline V to V?: Y Marker baseline V to V points: 3
Lower marker selection: First peak > 500 RFU Upper marker selection: Last peak > 200 RFU
Ladder size (bp)75, 200, 300, 400, 500, 700, 1000, 1500, 2000, 3000, 4000, 5000, 7000, 10000, 20000
Quantification using: Lower Marker Final concentration (ng/uL): 0.5000 Dilution factor: 12.0

Sample: SampF2
Well location: F2
Created: Thursday, October 21, 2021 6:48:20 PM

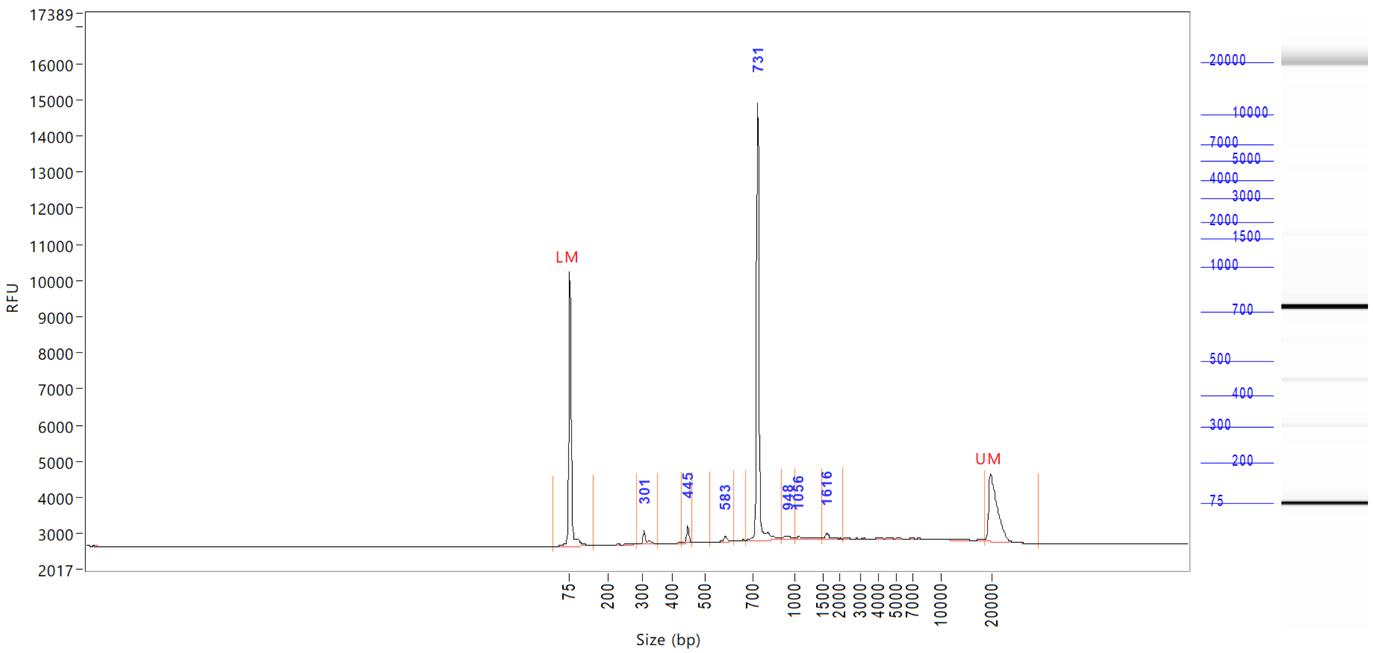

Peak	Size	Concentration	Relative concentration	Molarity	From	To	CV%	RFU
	(bp)	(ng/uL)	Percent	(nmole/L)	(bp)	(bp)		
1	75 (LM)	0.5000		10.9378	26	150	12.82	7581
2	301	0.3640	4.1	1.9503	284	349	3.60	383
3	445	0.3028	3.4	1.1274	428	458	0.86	493
4	583	0.1351	1.5	0.3826	521	621	2.03	131
5	731	7.5207	84.5	16.8859	669	909	3.61	12118
6	948	0.1925	2.2	0.3320	909	1001	2.88	92
7	1056	0.2385	2.7	0.3223	1001	1500	11.94	67
8	1616	0.1517	1.7	0.1524	1500	2144	6.45	172
9	20000 (UM)	0.2296		0.0183	18903	29551	5.08	1870

TIC:	8.9052	ng/uL
TIM:	21.1529	nmole/L
Total concentration:	9.4789	ng/uL

```
Sample peak width (sec): 5    Sample min peak height: 50    Sample baseline V to V?: Y    Sample baseline V to V points: 3
Sample filter: Binomial       Number of points for filter: 3  Sample start region (min): 0  Sample end region (min): 30
Marker peak width (sec): 5    Marker min peak height: 200   Marker baseline V to V?: Y    Marker baseline V to V points: 3
Lower marker selection: First peak > 500 RFU                Upper marker selection: Last peak > 200 RFU
Ladder size (bp)75, 200, 300, 400, 500, 700, 1000, 1500, 2000, 3000, 4000, 5000, 7000, 10000, 20000
Quantification using: Lower Marker          Final concentration (ng/uL): 0.5000          Dilution factor: 12.0
```

Sample: SampG2
Well location: G2
Created: Thursday, October 21, 2021 6:48:20 PM

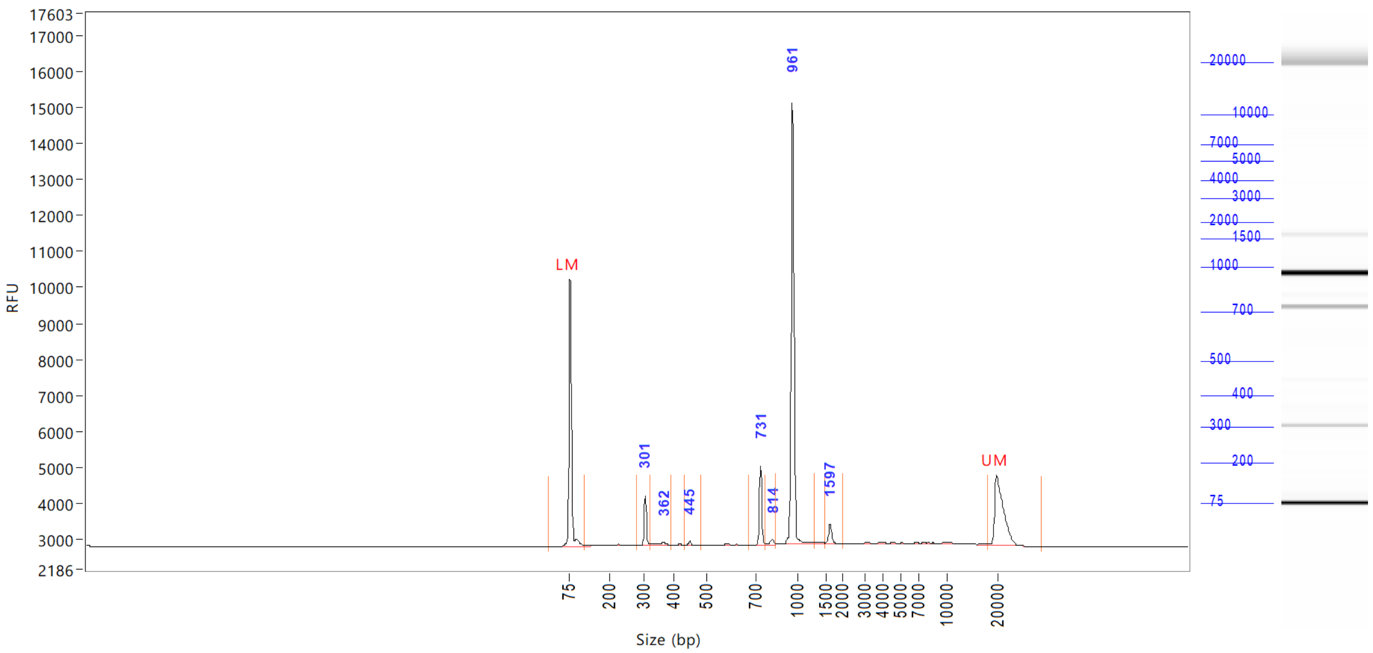

Peak	Size	Concentration	Relative concentration	Molarity	From	To	CV%	RFU
	(bp)	(ng/uL)	Percent	(nmole/L)	(bp)	(bp)		
1	75 (LM)	0.5000		10.9378	12	122	9.37	7423
2	301	0.9217	8.7	5.0541	280	317	1.38	1380
3	362	0.1977	1.9	0.9320	317	386	4.87	90
4	445	0.0763	0.7	0.2820	431	483	1.87	105
5	731	1.2895	12.1	2.9231	672	762	1.20	2191
6	814	0.1321	1.2	0.2704	762	840	1.78	160
7	961	7.6322	71.8	13.0717	840	1289	3.00	12259
8	1597	0.3811	3.6	0.3926	1478	2001	4.24	551
9	20000 (UM)	0.2397		0.0191	18293	28705	4.85	1931

TIC:	10.6305	ng/uL
TIM:	22.9258	nmole/L
Total concentration:	11.2205	ng/uL

```
Sample peak width (sec): 5    Sample min peak height: 50    Sample baseline V to V?: Y    Sample baseline V to V points: 3
Sample filter: Binomial       Number of points for filter: 3  Sample start region (min): 0  Sample end region (min): 30
Marker peak width (sec): 5    Marker min peak height: 200   Marker baseline V to V?: Y    Marker baseline V to V points: 3
Lower marker selection: First peak > 500 RFU                Upper marker selection: Last peak > 200 RFU
Ladder size (bp)75, 200, 300, 400, 500, 700, 1000, 1500, 2000, 3000, 4000, 5000, 7000, 10000, 20000
Quantification using: Lower Marker          Final concentration (ng/uL): 0.5000          Dilution factor: 12.0
```

Sample: SampH2
Well location: H2
Created: Thursday, October 21, 2021 6:48:20 PM

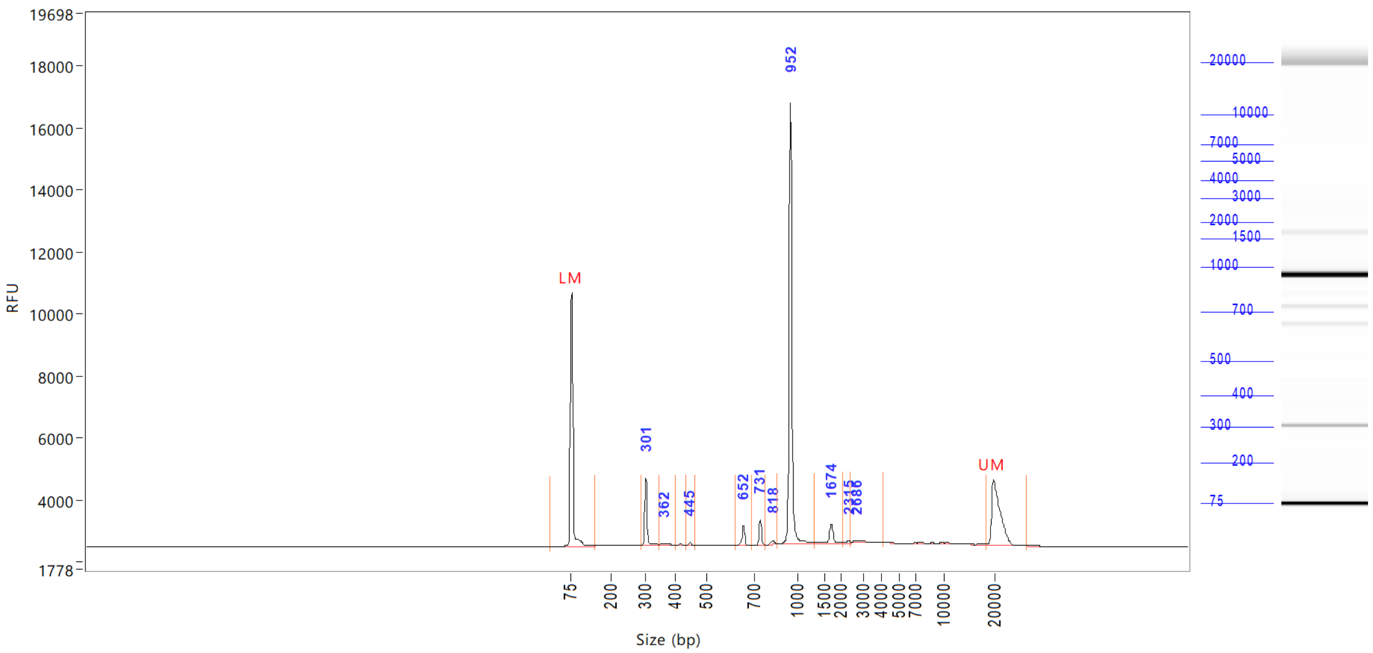

Peak	Size	Concentration	Relative concentration	Molarity	From	To	CV%	RFU
	(bp)	(ng/uL)	Percent	(nmole/L)	(bp)	(bp)		
1	75 (LM)	0.5000		10.9378	11	150	12.90	8160
2	301	1.3297	11.6	7.2429	287	345	2.44	2160
3	362	0.1134	1.0	0.5096	345	400	3.42	56
4	445	0.0467	0.4	0.1736	433	464	1.00	79
5	652	0.3713	3.2	0.9445	616	685	0.97	633
6	731	0.4277	3.7	0.9669	685	770	1.16	797
7	818	0.1029	0.9	0.2073	770	853	1.55	140
8	952	8.4024	73.0	14.5421	853	1311	2.92	14243
9	1674	0.4761	4.1	0.4724	1311	2058	7.35	622
10	2315	0.0595	0.5	0.0433	2058	2429	4.68	60
11	2686	0.1781	1.5	0.0990	2429	4104	12.51	62
12	20000 (UM)	0.2317		0.0185	18537	26649	4.62	2106

TIC:	11.5079	ng/uL
TIM:	25.2015	nmole/L
Total concentration:	11.9095	ng/uL

Sample peak width (sec): 5 Sample min peak height: 50 Sample baseline V to V?: Y Sample baseline V to V points: 3
Sample filter: Binomial Number of points for filter: 3 Sample start region (min): 0 Sample end region (min): 30
Marker peak width (sec): 5 Marker min peak height: 200 Marker baseline V to V?: Y Marker baseline V to V points: 3
Lower marker selection: First peak > 500 RFU Upper marker selection: Last peak > 200 RFU
Ladder size (bp)75, 200, 300, 400, 500, 700, 1000, 1500, 2000, 3000, 4000, 5000, 7000, 10000, 20000
Quantification using: Lower Marker Final concentration (ng/uL): 0.5000 Dilution factor: 12.0

Sample: SampA3
Well location: A3
Created: Thursday, October 21, 2021 6:48:20 PM

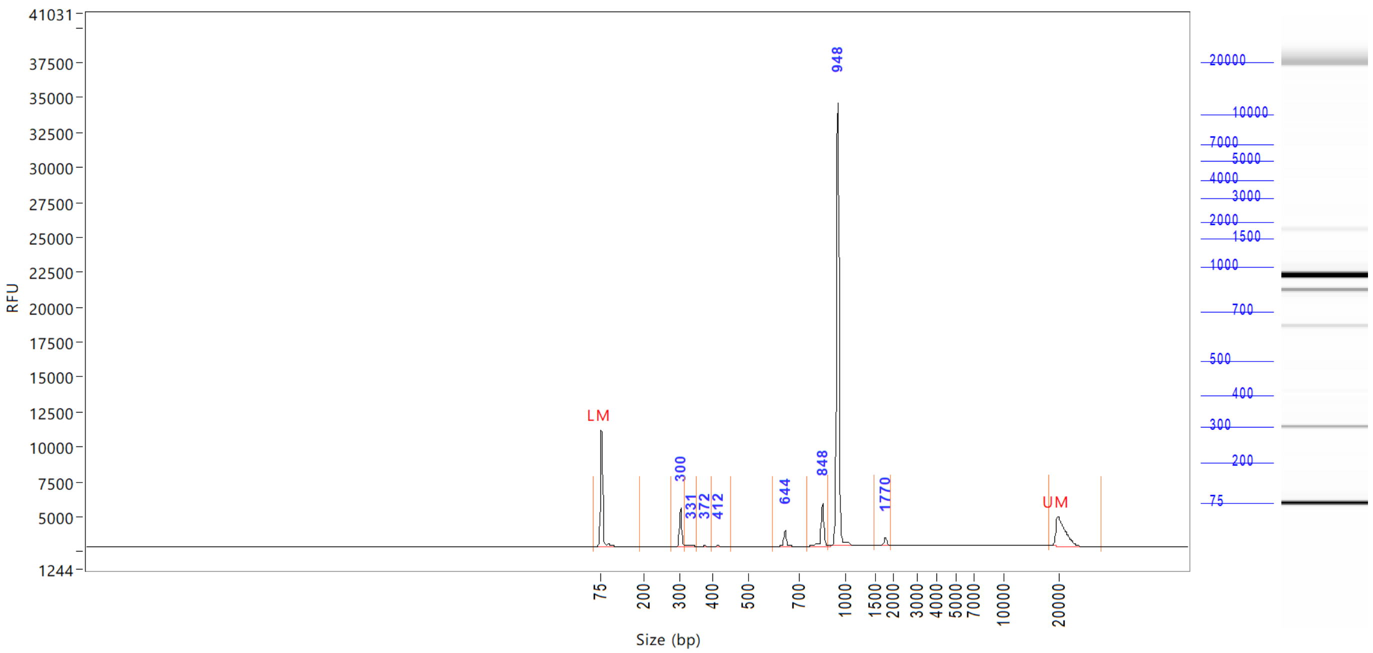

Peak	Size	Concentration	Relative concentration	Molarity	From	To	CV%	RFU
	(bp)	(ng/uL)	Percent	(nmole/L)	(bp)	(bp)		
1	75 (LM)	0.5000		10.7944	54	187	14.39	8330
2	300	1.5706	7.2	8.6985	273	311	1.15	2754
3	331	0.1150	0.5	0.5786	311	347	3.00	56
4	372	0.0819	0.4	0.3653	347	396	3.17	53
5	412	0.0737	0.3	0.2928	396	450	2.42	94
6	644	0.7011	3.2	1.8000	595	744	1.83	1170
7	848	1.8343	8.4	3.5940	744	883	1.82	3088
8	948	17.0332	78.4	29.6668	883	1467	2.20	31758
9	1770	0.3247	1.5	0.3066	1467	1923	3.18	549
10	20000 (UM)	0.2442		0.0195	18415	27979	4.84	2136

TIC:	21.7345	ng/uL
TIM:	45.3027	nmole/L
Total concentration:	22.1771	ng/uL

```
Sample peak width (sec): 5    Sample min peak height: 50    Sample baseline V to V?: Y    Sample baseline V to V points: 3
Sample filter: Binomial       Number of points for filter: 3  Sample start region (min): 0  Sample end region (min): 30
Marker peak width (sec): 5    Marker min peak height: 200   Marker baseline V to V?: Y    Marker baseline V to V points: 3
Lower marker selection: First peak > 500 RFU                Upper marker selection: Last peak > 200 RFU
Ladder size (bp)75, 200, 300, 400, 500, 700, 1000, 1500, 2000, 3000, 4000, 5000, 7000, 10000, 20000
Quantification using: Lower Marker          Final concentration (ng/uL): 0.5000          Dilution factor: 12.0
```

Sample: SampB3
Well location: B3
Created: Thursday, October 21, 2021 6:48:20 PM

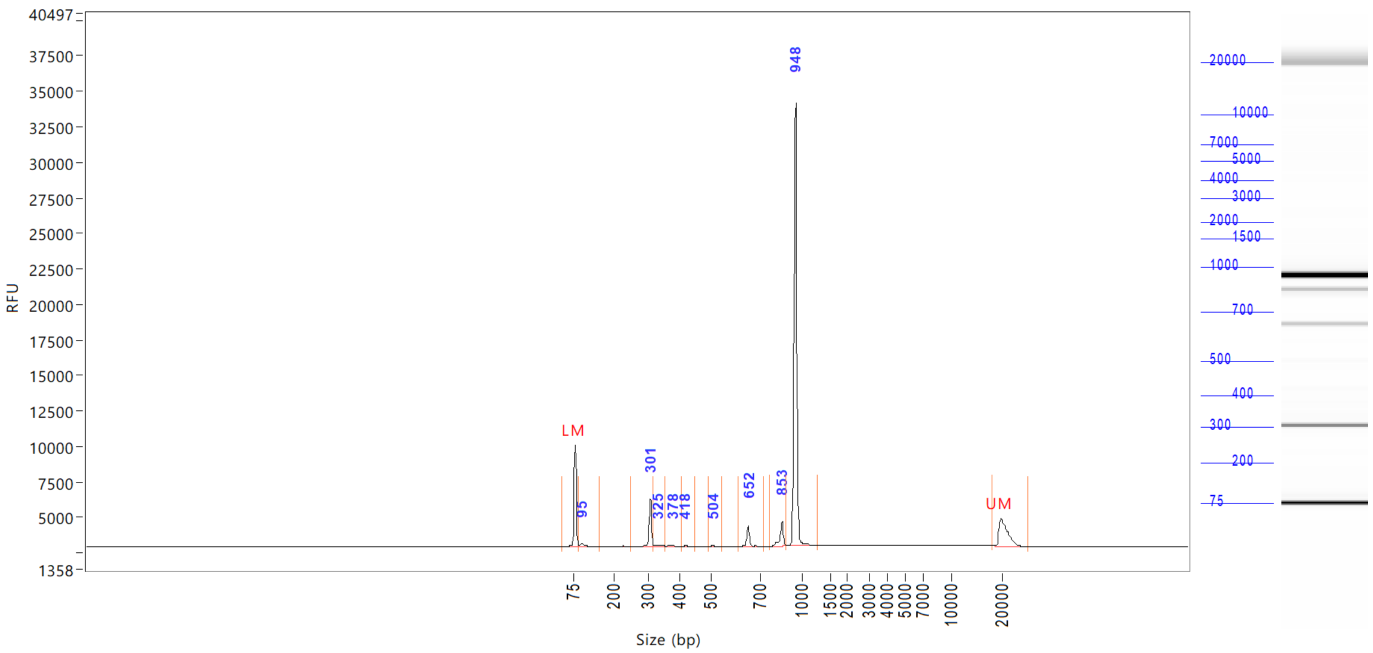

Peak	Size	Concentration	Relative concentration	Molarity	From	To	CV%	RFU
	(bp)	(ng/uL)	Percent	(nmole/L)	(bp)	(bp)		
1	75 (LM)	0.5000		11.0851	39	87	5.07	7172
2	95	0.4431	1.7	6.9299	87	150	14.74	195
3	301	2.4837	9.4	13.6182	248	315	1.75	3330
4	325	0.2142	0.8	1.0645	315	354	3.20	85
5	378	0.1693	0.6	0.7409	354	409	3.81	73
6	418	0.0827	0.3	0.3256	409	449	1.66	95
7	504	0.0907	0.3	0.2960	492	544	1.79	107
8	652	1.0370	3.9	2.6336	611	723	1.34	1478
9	853	1.3824	5.3	2.7053	770	883	2.05	1706
10	948	20.3990	77.6	35.5289	883	1256	2.05	31235
11	20000 (UM)	0.2555		0.0204	18171	25199	4.53	1913

TIC:	26.3019	ng/uL
TIM:	63.8430	nmole/L
Total concentration:	26.9750	ng/uL

Sample peak width (sec): 5   Sample min peak height: 50   Sample baseline V to V?: Y   Sample baseline V to V points: 3
Sample filter: Binomial   Number of points for filter: 3   Sample start region (min): 0   Sample end region (min): 30
Marker peak width (sec): 5   Marker min peak height: 200   Marker baseline V to V?: Y   Marker baseline V to V points: 3
Lower marker selection: First peak > 500 RFU   Upper marker selection: Last peak > 200 RFU
Ladder size (bp)75, 200, 300, 400, 500, 700, 1000, 1500, 2000, 3000, 4000, 5000, 7000, 10000, 20000
Quantification using: Lower Marker   Final concentration (ng/uL): 0.5000   Dilution factor: 12.0

Sample: SampC3
Well location: C3
Created: Thursday, October 21, 2021 6:48:20 PM

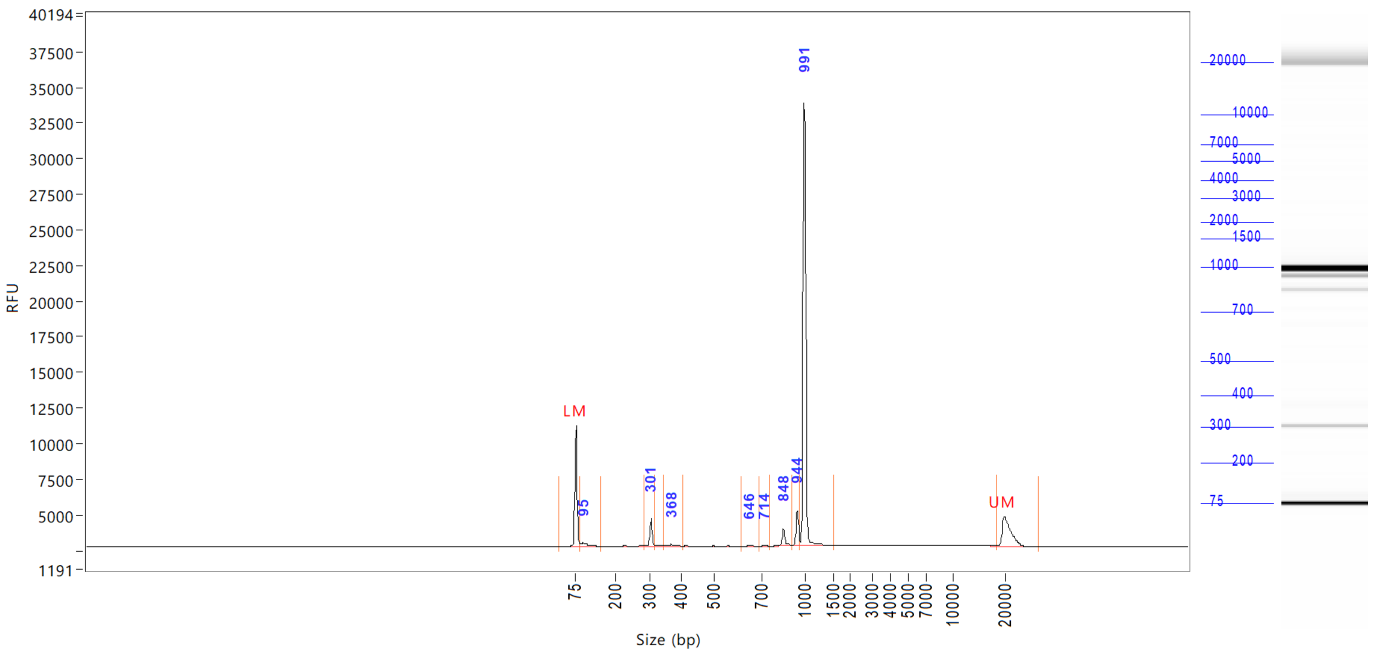

Peak	Size	Concentration	Relative concentration	Molarity	From	To	CV%	RFU
	(bp)	(ng/uL)	Percent	(nmole/L)	(bp)	(bp)		
1	75 (LM)	0.5000		11.2364	24	87	5.88	8443
2	95	0.5323	2.4	8.4049	87	152	14.26	260
3	301	1.2181	5.6	6.7013	282	315	1.17	1903
4	368	0.2134	1.0	0.9594	345	405	2.91	118
5	646	0.0775	0.4	0.1972	616	690	1.92	83
6	714	0.0443	0.2	0.1023	690	749	1.41	58
7	848	0.8483	3.9	1.6465	749	905	2.06	1233
8	944	1.4427	6.6	2.5289	905	965	1.12	2487
9	991	17.5423	80.0	29.0478	965	1520	3.72	31114
10	20000 (UM)	0.2479		0.0198	18415	26649	4.70	2097

TIC:	21.9191	ng/uL
TIM:	49.5883	nmole/L
Total concentration:	22.5334	ng/uL

```
Sample peak width (sec): 5    Sample min peak height: 50    Sample baseline V to V?: Y    Sample baseline V to V points: 3
Sample filter: Binomial       Number of points for filter: 3  Sample start region (min): 0  Sample end region (min): 30
Marker peak width (sec): 5    Marker min peak height: 200   Marker baseline V to V?: Y    Marker baseline V to V points: 3
Lower marker selection: First peak > 500 RFU                Upper marker selection: Last peak > 200 RFU
Ladder size (bp)75, 200, 300, 400, 500, 700, 1000, 1500, 2000, 3000, 4000, 5000, 7000, 10000, 20000
Quantification using: Lower Marker          Final concentration (ng/uL): 0.5000          Dilution factor: 12.0
```

Sample: SampD3
Well location: D3
Created: Thursday, October 21, 2021 6:48:20 PM

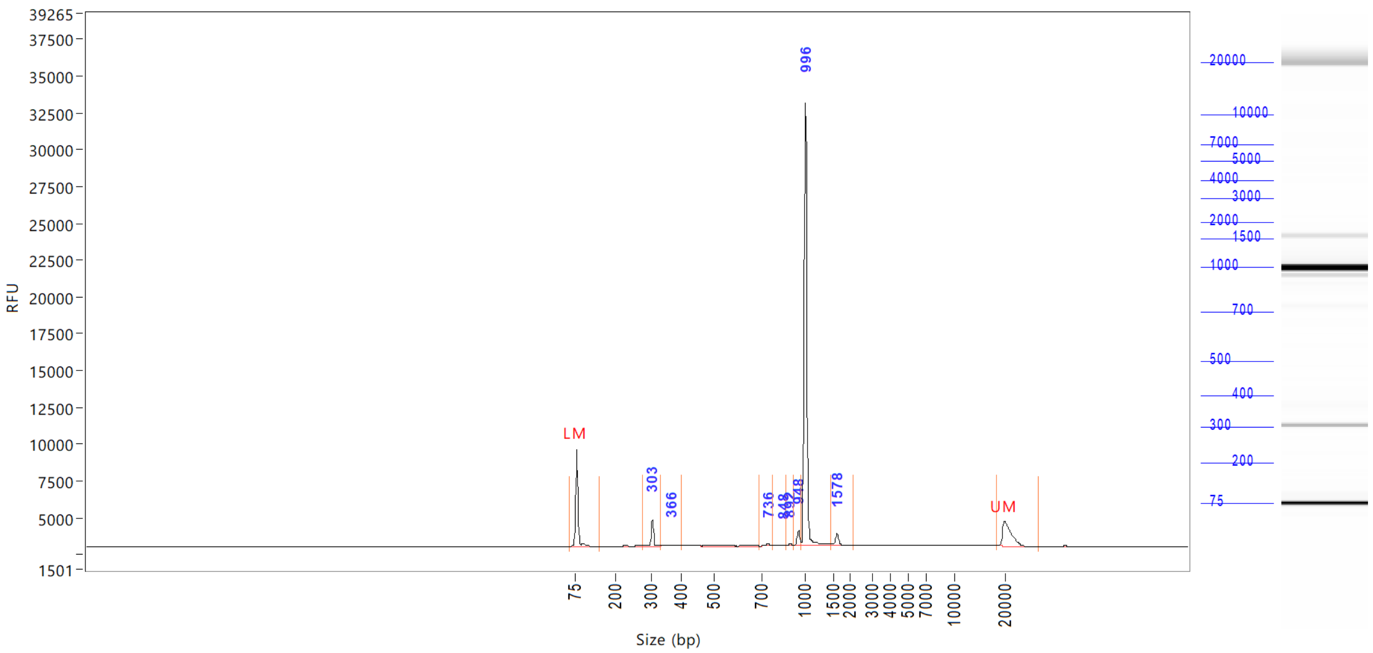

Peak	Size	Concentration	Relative concentration	Molarity	From	To	CV%	RFU
	(bp)	(ng/uL)	Percent	(nmole/L)	(bp)	(bp)		
1	75 (LM)	0.5000		10.9378	54	148	11.56	6588
2	303	1.4139	5.9	7.7267	275	333	1.63	1821
3	366	0.2272	0.9	1.0243	333	400	3.61	96
4	736	0.1714	0.7	0.3881	690	770	1.93	166
5	848	0.0603	0.3	0.1190	770	870	2.36	51
6	892	0.0912	0.4	0.1693	870	913	1.07	122
7	948	0.7481	3.1	1.3002	913	970	1.30	998
8	996	20.5620	85.8	33.9115	970	1433	3.81	30100
9	1578	0.6789	2.8	0.7118	1433	2087	5.71	821
10	20000 (UM)	0.2427		0.0194	18293	26529	4.68	1677

TIC:	23.9531	ng/uL
TIM:	45.3508	nmole/L
Total concentration:	24.5554	ng/uL

Sample peak width (sec): 5 Sample min peak height: 50 Sample baseline V to V?: Y Sample baseline V to V points: 3
Sample filter: Binomial Number of points for filter: 3 Sample start region (min): 0 Sample end region (min): 30
Marker peak width (sec): 5 Marker min peak height: 200 Marker baseline V to V?: Y Marker baseline V to V points: 3
Lower marker selection: First peak > 500 RFU Upper marker selection: Last peak > 200 RFU
Ladder size (bp)75, 200, 300, 400, 500, 700, 1000, 1500, 2000, 3000, 4000, 5000, 7000, 10000, 20000
Quantification using: Lower Marker Final concentration (ng/uL): 0.5000 Dilution factor: 12.0

Sample: SampE3
Well location: E3
Created: Thursday, October 21, 2021 6:48:20 PM

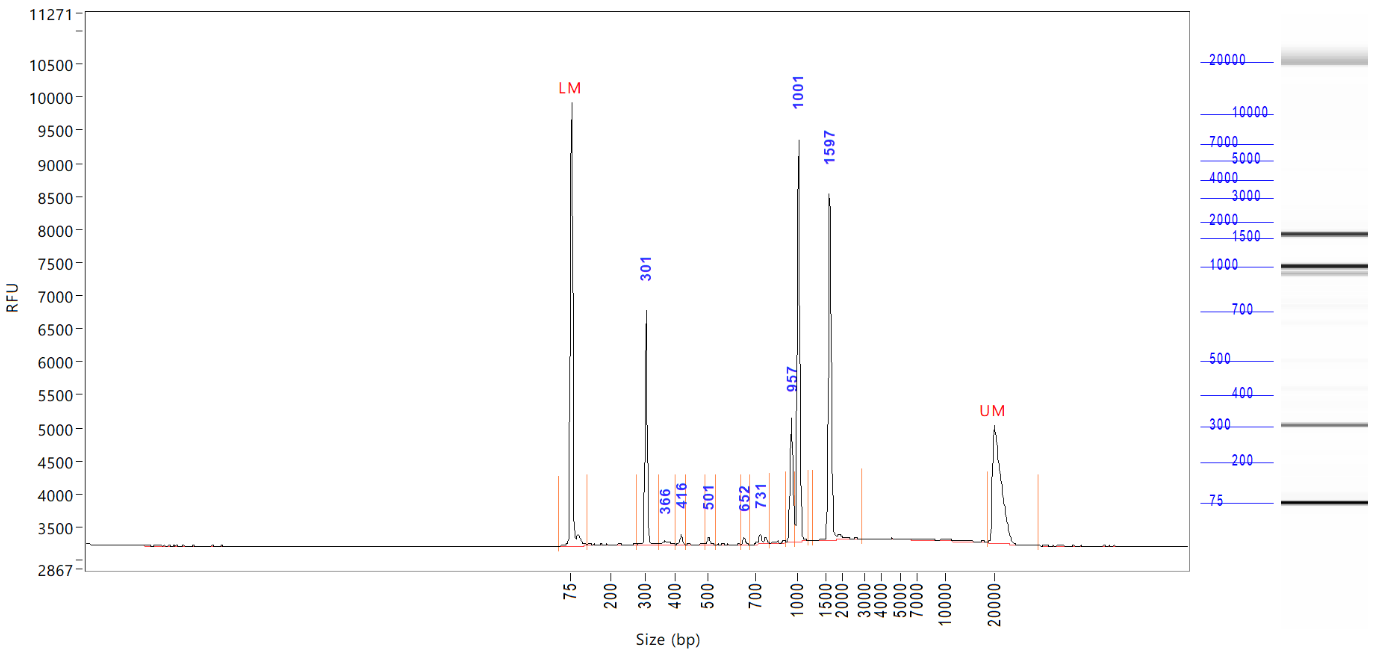

Peak	Size	Concentration	Relative concentration	Molarity	From	To	CV%	RFU
	(bp)	(ng/uL)	Percent	(nmole/L)	(bp)	(bp)		
1	75 (LM)	0.5000		11.0851	37	124	9.38	6713
2	301	2.6645	21.1	14.5614	277	345	1.67	3547
3	366	0.1248	1.0	0.5581	345	400	3.20	51
4	416	0.1265	1.0	0.5026	400	431	1.19	146
5	501	0.0905	0.7	0.2972	492	532	1.20	118
6	652	0.0816	0.6	0.2064	639	677	0.90	102
7	731	0.2027	1.6	0.4532	677	801	3.36	147
8	957	1.4982	11.9	2.5847	918	983	1.28	1865
9	1001	4.0830	32.3	6.6936	983	1178	1.56	6075
10	1597	3.7646	29.8	3.8634	1267	2914	7.08	5242
11	20000 (UM)	0.2543		0.0203	18415	28584	4.67	1769

TIC:	12.6364	ng/uL
TIM:	29.7206	nmole/L
Total concentration:	13.1968	ng/uL

```
Sample peak width (sec): 5    Sample min peak height: 50    Sample baseline V to V?: Y    Sample baseline V to V points: 3
Sample filter: Binomial       Number of points for filter: 3  Sample start region (min): 0  Sample end region (min): 30
Marker peak width (sec): 5    Marker min peak height: 200   Marker baseline V to V?: Y    Marker baseline V to V points: 3
Lower marker selection: First peak > 500 RFU                Upper marker selection: Last peak > 200 RFU
Ladder size (bp)75, 200, 300, 400, 500, 700, 1000, 1500, 2000, 3000, 4000, 5000, 7000, 10000, 20000
Quantification using: Lower Marker          Final concentration (ng/uL): 0.5000          Dilution factor: 12.0
```

Sample: SampF3
Well location: F3
Created: Thursday, October 21, 2021 6:48:20 PM

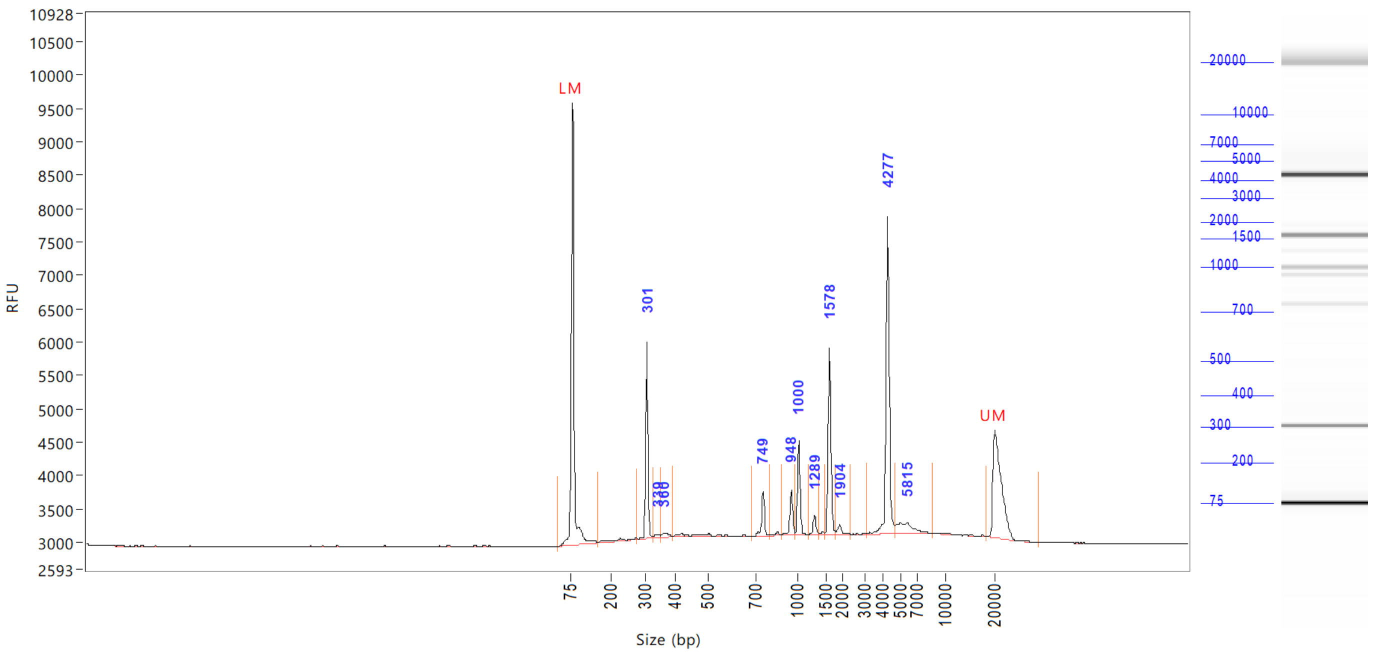

Peak	Size	Concentration	Relative concentration	Molarity	From	To	CV%	RFU
	(bp)	(ng/uL)	Percent	(nmole/L)	(bp)	(bp)		
1	75 (LM)	0.5000		10.7944	30	156	15.36	6640
2	301	1.9762	19.8	10.8359	275	323	1.33	2953
3	339	0.0763	0.8	0.3748	323	347	2.05	51
4	360	0.1219	1.2	0.5508	347	388	2.86	61
5	749	0.4870	4.9	1.0816	685	801	2.19	668
6	948	0.4675	4.7	0.8160	879	974	1.49	680
7	1000	0.8745	8.8	1.4408	974	1167	1.45	1404
8	1289	0.1883	1.9	0.2417	1167	1378	2.37	278
9	1578	1.7896	18.0	1.8728	1478	1789	2.96	2805
10	1904	0.1290	1.3	0.1118	1789	2372	4.40	150
11	4277	3.3226	33.3	1.2873	3108	4690	3.64	4755
12	5815	0.5315	5.3	0.1535	4690	8600	14.87	157
13	20000 (UM)	0.2040		0.0163	18293	28584	4.66	1622

TIC:	9.9645	ng/uL
TIM:	18.7673	nmole/L
Total concentration:	10.4391	ng/uL

```
Sample peak width (sec): 5     Sample min peak height: 50     Sample baseline V to V?: Y     Sample baseline V to V points: 3
Sample filter: Binomial        Number of points for filter: 3  Sample start region (min): 0  Sample end region (min): 30
Marker peak width (sec): 5     Marker min peak height: 200    Marker baseline V to V?: Y     Marker baseline V to V points: 3
Lower marker selection: First peak > 500 RFU                  Upper marker selection: Last peak > 200 RFU
Ladder size (bp)75, 200, 300, 400, 500, 700, 1000, 1500, 2000, 3000, 4000, 5000, 7000, 10000, 20000
Quantification using: Lower Marker          Final concentration (ng/uL): 0.5000          Dilution factor: 12.0
```

Sample: SampG3
Well location: G3
Created: Thursday, October 21, 2021 6:48:20 PM

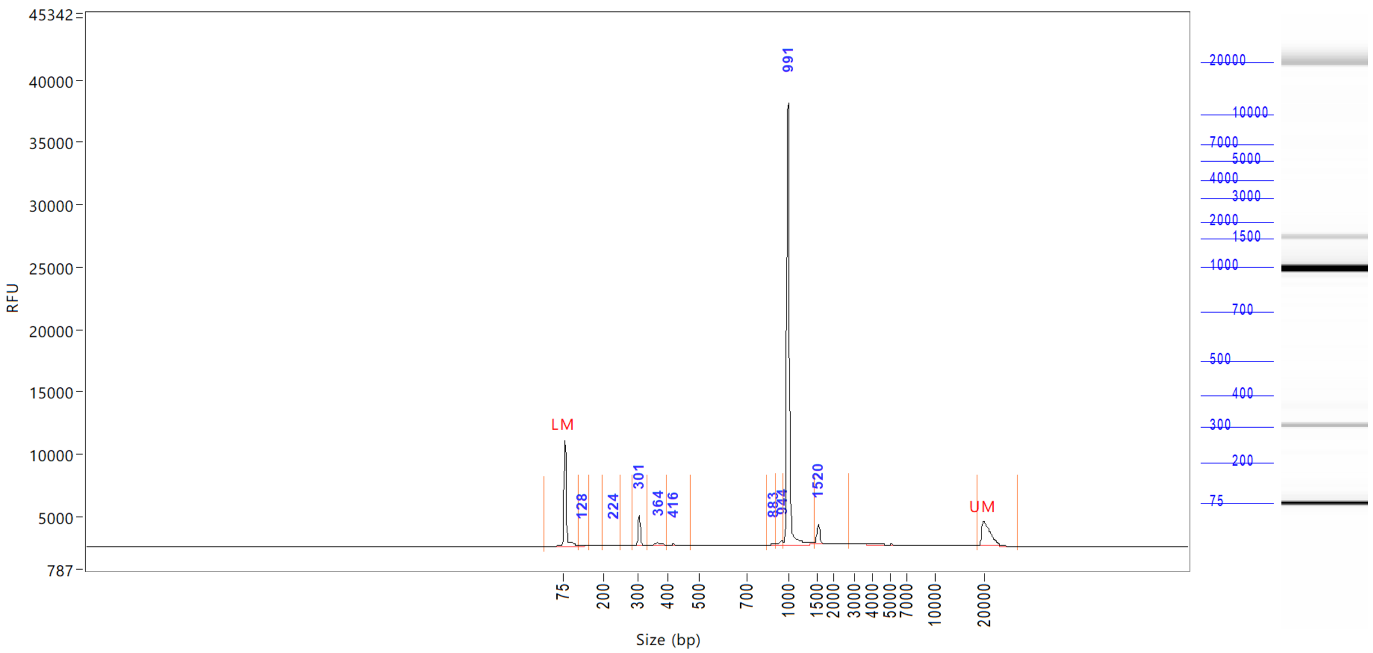

Peak	Size	Concentration	Relative concentration	Molarity	From	To	CV%	RFU
	(bp)	(ng/uL)	Percent	(nmole/L)	(bp)	(bp)		
1	75 (LM)	0.5000		10.9378	11	116	10.14	8419
2	128	0.0869	0.4	1.0895	116	154	7.64	54
3	224	0.0671	0.3	0.4969	192	250	5.20	64
4	301	1.3582	6.0	7.4227	284	333	1.70	2359
5	364	0.2887	1.3	1.3083	333	394	3.45	171
6	416	0.0960	0.4	0.3762	394	471	3.95	53
7	883	0.0545	0.2	0.1018	835	905	1.52	88
8	944	0.2310	1.0	0.4040	905	961	1.46	334
9	991	19.3080	85.8	31.8433	961	1456	5.11	35460
10	1520	1.0165	4.5	1.0630	1456	2772	9.80	1564
11	20000 (UM)	0.2140		0.0171	18659	26770	4.63	2015

TIC:	22.5068	ng/uL
TIM:	44.1058	nmole/L
Total concentration:	22.9183	ng/uL

```
Sample peak width (sec): 5    Sample min peak height: 50    Sample baseline V to V?: Y    Sample baseline V to V points: 3
Sample filter: Binomial       Number of points for filter: 3  Sample start region (min): 0  Sample end region (min): 30
Marker peak width (sec): 5    Marker min peak height: 200    Marker baseline V to V?: Y    Marker baseline V to V points: 3
Lower marker selection: First peak > 500 RFU                  Upper marker selection: Last peak > 200 RFU
Ladder size (bp)75, 200, 300, 400, 500, 700, 1000, 1500, 2000, 3000, 4000, 5000, 7000, 10000, 20000
Quantification using: Lower Marker          Final concentration (ng/uL): 0.5000          Dilution factor: 12.0
```

Sample: SampH3
Well location: H3
Created: Thursday, October 21, 2021 6:48:20 PM

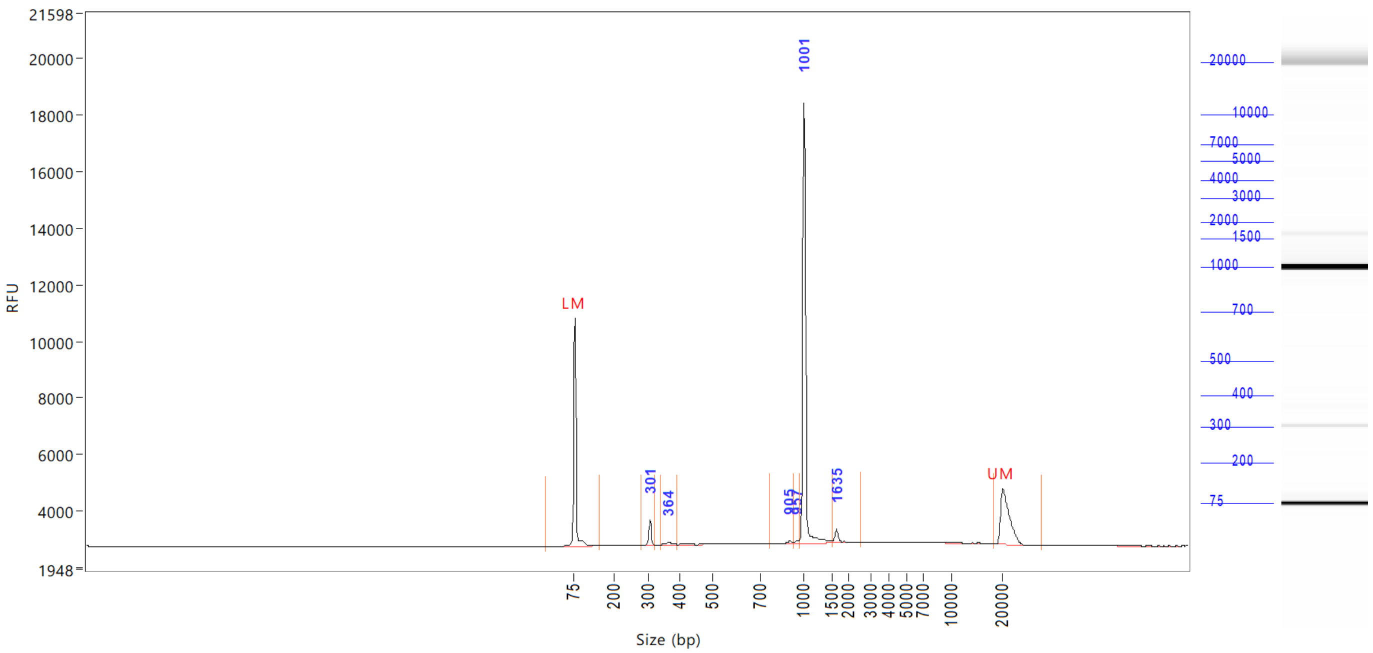

Peak	Size	Concentration	Relative concentration	Molarity	From	To	CV%	RFU
	(bp)	(ng/uL)	Percent	(nmole/L)	(bp)	(bp)		
1	75 (LM)	0.5000		11.0851	0	150	11.63	8057
2	301	0.5449	5.3	2.9879	278	317	1.27	890
3	364	0.1541	1.5	0.7024	335	392	3.27	81
4	905	0.1011	1.0	0.1873	757	926	3.57	85
5	957	0.0914	0.9	0.1583	926	970	1.30	108
6	1001	9.0653	87.6	14.6572	970	1501	6.89	15594
7	1635	0.3947	3.8	0.3861	1501	2600	10.11	474
8	20000 (UM)	0.2305		0.0183	18293	27738	4.71	1989

TIC:	10.3516	ng/uL
TIM:	19.0793	nmole/L
Total concentration:	10.7707	ng/uL

Sample peak width (sec): 5 Sample min peak height: 50 Sample baseline V to V?: Y Sample baseline V to V points: 3
Sample filter: Binomial Number of points for filter: 3 Sample start region (min): 0 Sample end region (min): 30
Marker peak width (sec): 5 Marker min peak height: 200 Marker baseline V to V?: Y Marker baseline V to V points: 3
Lower marker selection: First peak > 500 RFU Upper marker selection: Last peak > 200 RFU
Ladder size (bp)75, 200, 300, 400, 500, 700, 1000, 1500, 2000, 3000, 4000, 5000, 7000, 10000, 20000
Quantification using: Lower Marker Final concentration (ng/uL): 0.5000 Dilution factor: 12.0

Sample: SampA4
Well location: A4
Created: Thursday, October 21, 2021 6:48:20 PM

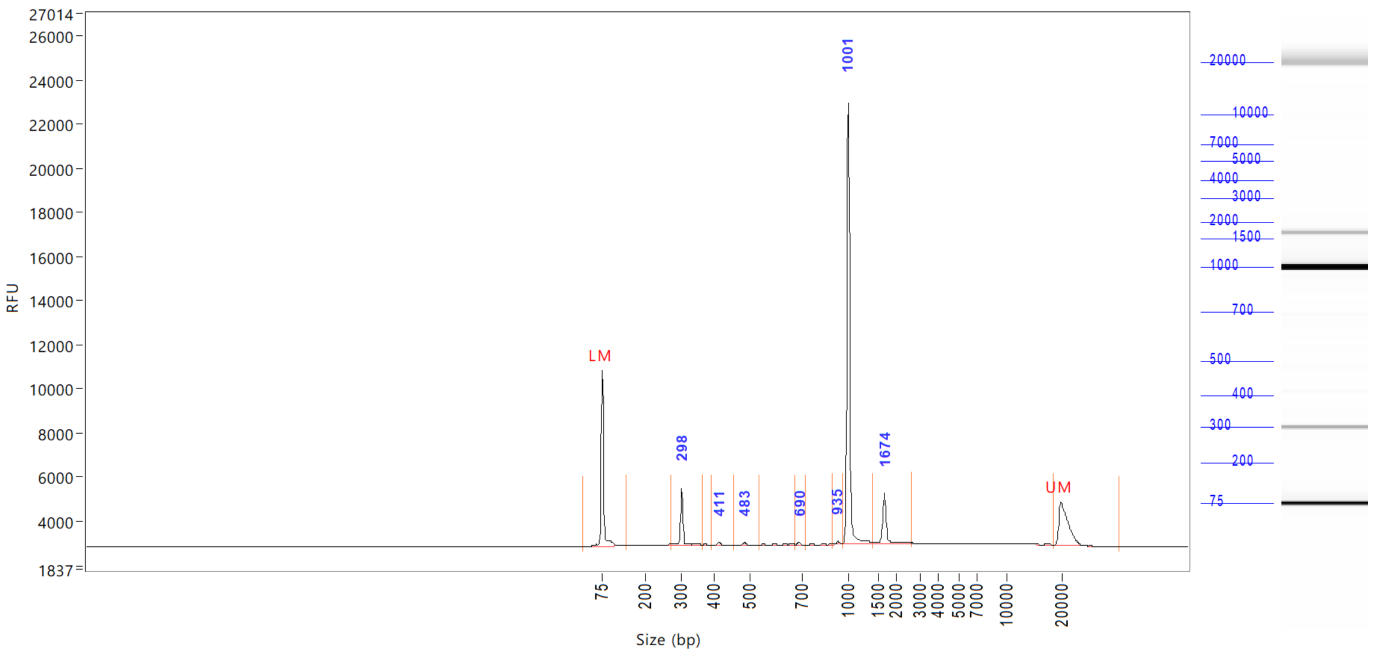

Peak	Size	Concentration	Relative concentration	Molarity	From	To	CV%	RFU
	(bp)	(ng/uL)	Percent	(nmole/L)	(bp)	(bp)		
1	75 (LM)	0.5000		10.9378	20	145	12.81	8000
2	298	1.6129	10.6	8.9330	269	362	3.01	2581
3	411	0.0950	0.6	0.3814	388	454	2.38	125
4	483	0.0890	0.6	0.3008	454	537	2.98	93
5	690	0.0658	0.4	0.1576	677	718	0.73	122
6	935	0.1144	0.8	0.2016	900	965	1.57	138
7	1001	11.6223	76.6	18.9214	965	1422	5.20	20020
8	1674	1.5750	10.4	1.5572	1422	2686	7.30	2265
9	20000 (UM)	0.2237		0.0178	18659	30760	5.21	1937

TIC:	15.1745	ng/uL
TIM:	30.4531	nmole/L
Total concentration:	15.5735	ng/uL

Sample peak width (sec): 5 Sample min peak height: 50 Sample baseline V to V?: Y Sample baseline V to V points: 3
Sample filter: Binomial Number of points for filter: 3 Sample start region (min): 0 Sample end region (min): 30
Marker peak width (sec): 5 Marker min peak height: 200 Marker baseline V to V?: Y Marker baseline V to V points: 3
Lower marker selection: First peak > 500 RFU Upper marker selection: Last peak > 200 RFU
Ladder size (bp)75, 200, 300, 400, 500, 700, 1000, 1500, 2000, 3000, 4000, 5000, 7000, 10000, 20000
Quantification using: Lower Marker Final concentration (ng/uL): 0.5000 Dilution factor: 12.0

Sample: SampB4
Well location: B4
Created: Thursday, October 21, 2021 6:48:20 PM

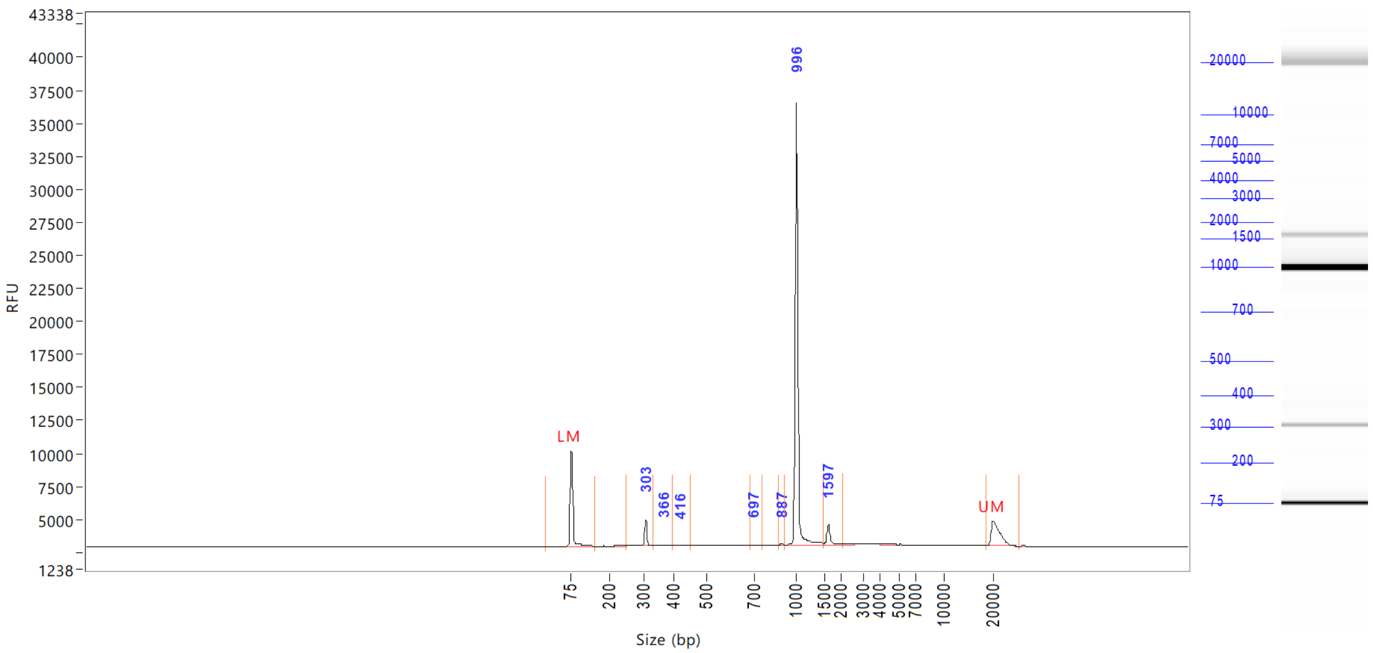

Peak	Size	Concentration	Relative concentration	Molarity	From	To	CV%	RFU
	(bp)	(ng/uL)	Percent	(nmole/L)	(bp)	(bp)		
1	75 (LM)	0.5000		10.7944	0	150	13.08	7184
2	303	1.3651	5.8	7.4352	246	329	2.31	2007
3	366	0.2117	0.9	0.9569	329	394	4.00	97
4	416	0.1077	0.5	0.4248	394	452	3.10	57
5	697	0.0526	0.2	0.1237	685	757	1.58	66
6	887	0.0721	0.3	0.1336	866	918	1.46	95
7	996	20.5147	87.5	33.5645	918	1489	5.74	33528
8	1597	1.1299	4.8	1.1531	1489	2058	6.11	1556
9	20000 (UM)	0.2284		0.0182	18537	25320	4.45	1866

TIC:	23.4538	ng/uL
TIM:	43.7919	nmole/L
Total concentration:	24.0178	ng/uL

```
Sample peak width (sec): 5     Sample min peak height: 50     Sample baseline V to V?: Y     Sample baseline V to V points: 3
Sample filter: Binomial        Number of points for filter: 3  Sample start region (min): 0  Sample end region (min): 30
Marker peak width (sec): 5     Marker min peak height: 200    Marker baseline V to V?: Y     Marker baseline V to V points: 3
Lower marker selection: First peak > 500 RFU                  Upper marker selection: Last peak > 200 RFU
Ladder size (bp)75, 200, 300, 400, 500, 700, 1000, 1500, 2000, 3000, 4000, 5000, 7000, 10000, 20000
Quantification using: Lower Marker          Final concentration (ng/uL): 0.5000          Dilution factor: 12.0
```

Sample: SampC4
Well location: C4
Created: Thursday, October 21, 2021 6:48:20 PM

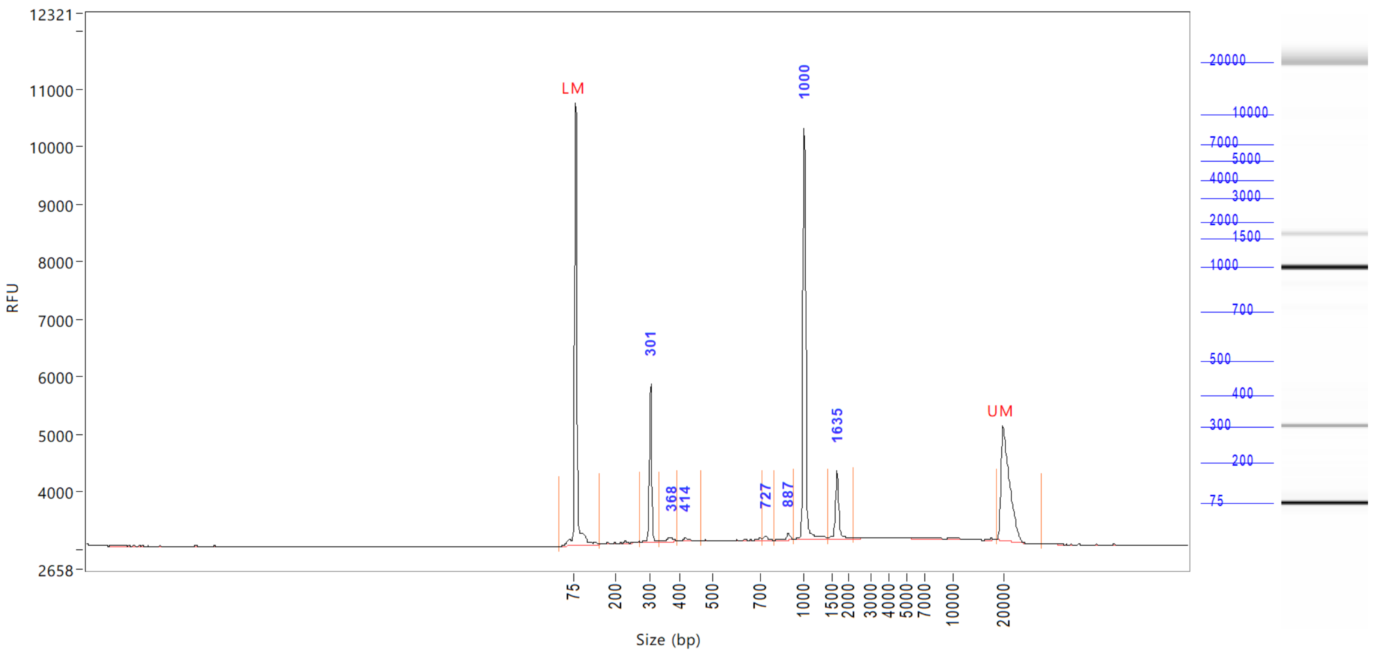

Peak	Size	Concentration	Relative concentration	Molarity	From	To	CV%	RFU
	(bp)	(ng/uL)	Percent	(nmole/L)	(bp)	(bp)		
1	75 (LM)	0.5000		10.9378	24	150	13.52	7708
2	301	1.6919	23.9	9.3081	273	329	1.62	2752
3	368	0.1430	2.0	0.6536	329	390	4.00	64
4	414	0.1020	1.4	0.4016	390	464	3.78	64
5	727	0.0693	1.0	0.1574	701	788	2.06	82
6	887	0.1164	1.6	0.2180	788	931	3.08	125
7	1000	4.1345	58.4	6.7444	931	1433	5.38	7144
8	1635	0.8230	11.6	0.8322	1433	2172	5.71	1200
9	20000 (UM)	0.2257		0.0180	18781	27617	4.65	2003

TIC:	7.0803	ng/uL
TIM:	18.3153	nmole/L
Total concentration:	7.4608	ng/uL

```
Sample peak width (sec): 5     Sample min peak height: 50     Sample baseline V to V?: Y     Sample baseline V to V points: 3
Sample filter: Binomial        Number of points for filter: 3  Sample start region (min): 0  Sample end region (min): 30
Marker peak width (sec): 5     Marker min peak height: 200    Marker baseline V to V?: Y     Marker baseline V to V points: 3
Lower marker selection: First peak > 500 RFU                  Upper marker selection: Last peak > 200 RFU
Ladder size (bp)75, 200, 300, 400, 500, 700, 1000, 1500, 2000, 3000, 4000, 5000, 7000, 10000, 20000
Quantification using: Lower Marker          Final concentration (ng/uL): 0.5000          Dilution factor: 12.0
```

Sample: SampD4
Well location: D4
Created: Thursday, October 21, 2021 6:48:20 PM

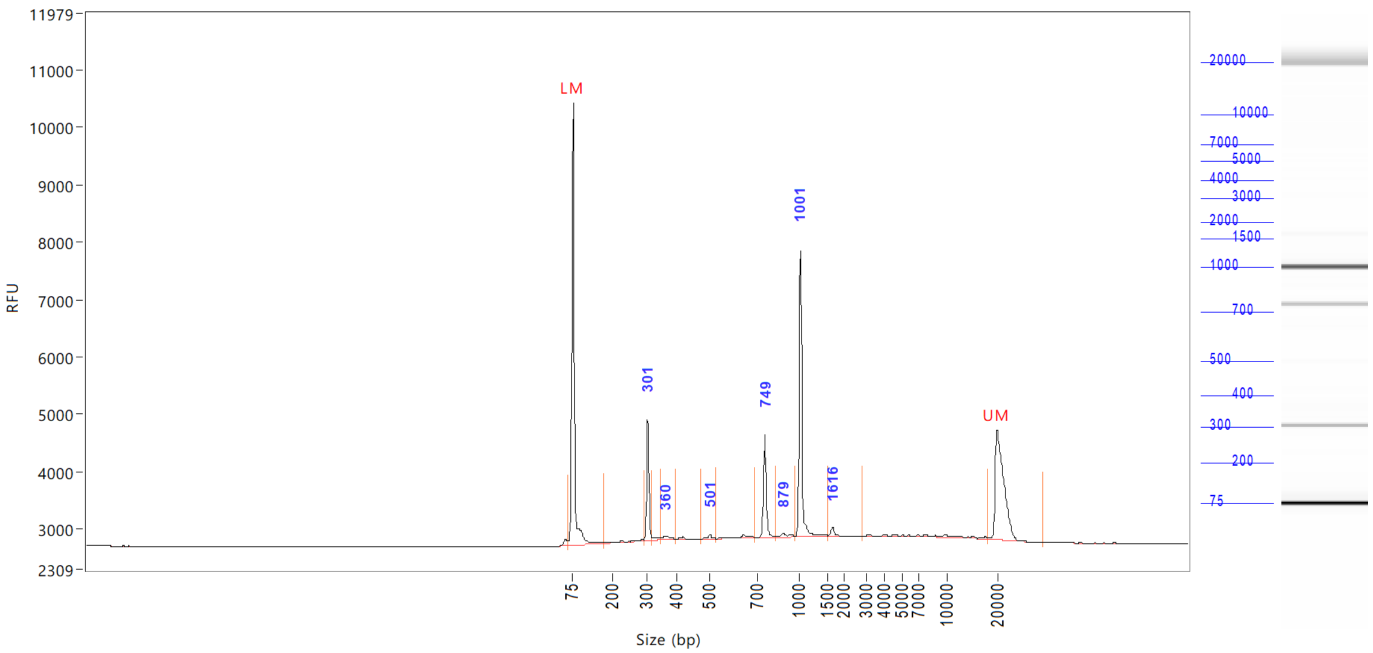

Peak	Size	Concentration	Relative concentration	Molarity	From	To	CV%	RFU
	(bp)	(ng/uL)	Percent	(nmole/L)	(bp)	(bp)		
1	75 (LM)	0.5000		10.7944	60	173	15.43	7706
2	301	1.2933	22.0	7.0678	291	317	1.11	2106
3	360	0.1250	2.1	0.5650	345	394	3.26	67
4	501	0.0600	1.0	0.1981	473	529	1.59	77
5	749	1.1033	18.8	2.4374	687	827	1.77	1794
6	879	0.1168	2.0	0.2136	827	970	4.05	66
7	1001	3.0255	51.5	4.8917	970	1500	6.37	4979
8	1616	0.1557	2.6	0.1492	1500	2800	14.29	168
9	20000 (UM)	0.2226		0.0178	18171	29189	4.70	1906

TIC:	5.8796	ng/uL
TIM:	15.5228	nmole/L
Total concentration:	6.5384	ng/uL

```
Sample peak width (sec): 5    Sample min peak height: 50    Sample baseline V to V?: Y    Sample baseline V to V points: 3
Sample filter: Binomial       Number of points for filter: 3  Sample start region (min): 0  Sample end region (min): 30
Marker peak width (sec): 5    Marker min peak height: 200   Marker baseline V to V?: Y    Marker baseline V to V points: 3
Lower marker selection: First peak > 500 RFU                Upper marker selection: Last peak > 200 RFU
Ladder size (bp)75, 200, 300, 400, 500, 700, 1000, 1500, 2000, 3000, 4000, 5000, 7000, 10000, 20000
Quantification using: Lower Marker          Final concentration (ng/uL): 0.5000          Dilution factor: 12.0
```

Sample: SampE4
Well location: E4
Created: Thursday, October 21, 2021 6:48:20 PM

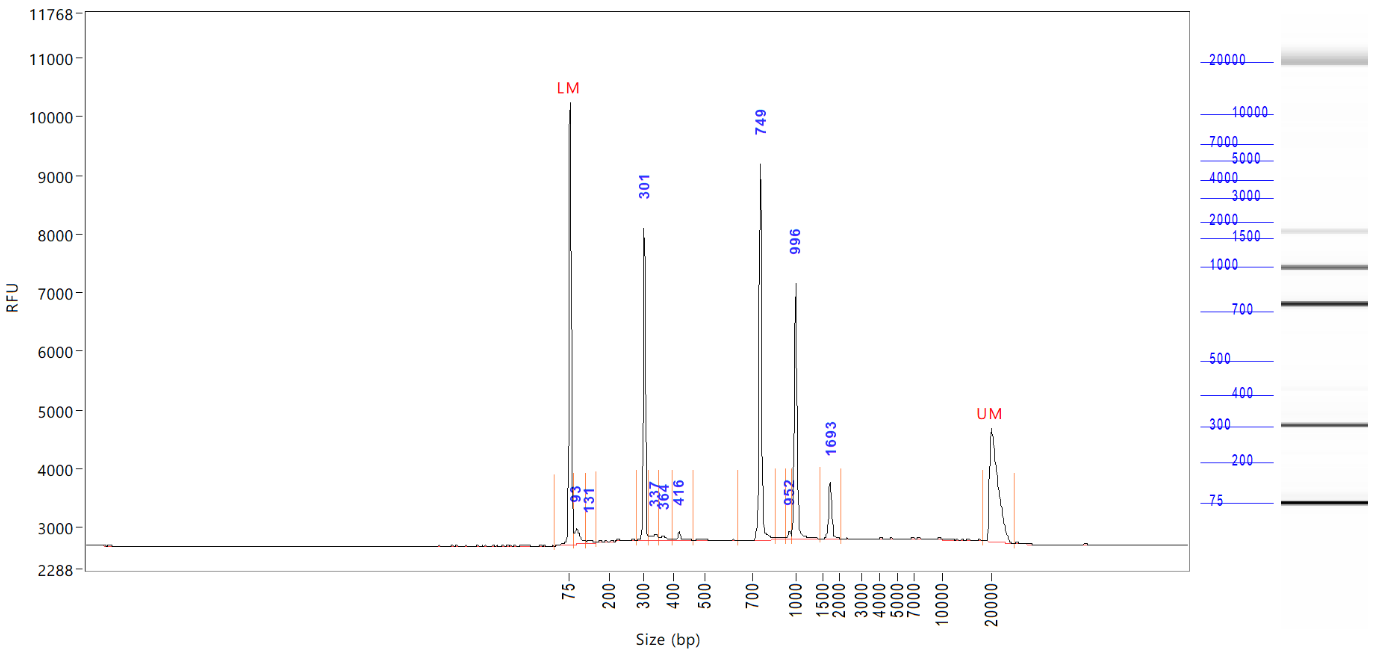

Peak	Size	Concentration	Relative concentration	Molarity	From	To	CV%	RFU
	(bp)	(ng/uL)	Percent	(nmole/L)	(bp)	(bp)		
1	75 (LM)	0.5000		11.3919	26	86	5.59	7551
2	93	0.5005	4.0	8.4723	86	124	9.67	264
3	131	0.1136	0.9	1.3526	124	160	7.44	59
4	301	3.5756	28.3	19.6053	278	315	1.08	5332
5	337	0.2213	1.8	1.0932	315	352	2.99	110
6	364	0.1343	1.1	0.6038	352	396	2.90	75
7	416	0.1401	1.1	0.5500	396	460	2.73	145
8	749	4.2561	33.7	9.4275	639	857	2.08	6403
9	952	0.0954	0.8	0.1649	931	970	1.14	124
10	996	2.9275	23.2	4.8137	970	1456	3.78	4368
11	1693	0.6600	5.2	0.6470	1456	2058	3.67	953
12	20000 (UM)	0.2502		0.0200	18293	24473	4.40	1924

TIC:	12.6243	ng/uL
TIM:	46.7302	nmole/L
Total concentration:	13.0997	ng/uL

```
Sample peak width (sec): 5    Sample min peak height: 50    Sample baseline V to V?: Y    Sample baseline V to V points: 3
Sample filter: Binomial       Number of points for filter: 3  Sample start region (min): 0  Sample end region (min): 30
Marker peak width (sec): 5    Marker min peak height: 200   Marker baseline V to V?: Y    Marker baseline V to V points: 3
Lower marker selection: First peak > 500 RFU                Upper marker selection: Last peak > 200 RFU
Ladder size (bp)75, 200, 300, 400, 500, 700, 1000, 1500, 2000, 3000, 4000, 5000, 7000, 10000, 20000
Quantification using: Lower Marker          Final concentration (ng/uL): 0.5000          Dilution factor: 12.0
```

Sample: SampF4
Well location: F4
Created: Thursday, October 21, 2021 6:48:20 PM

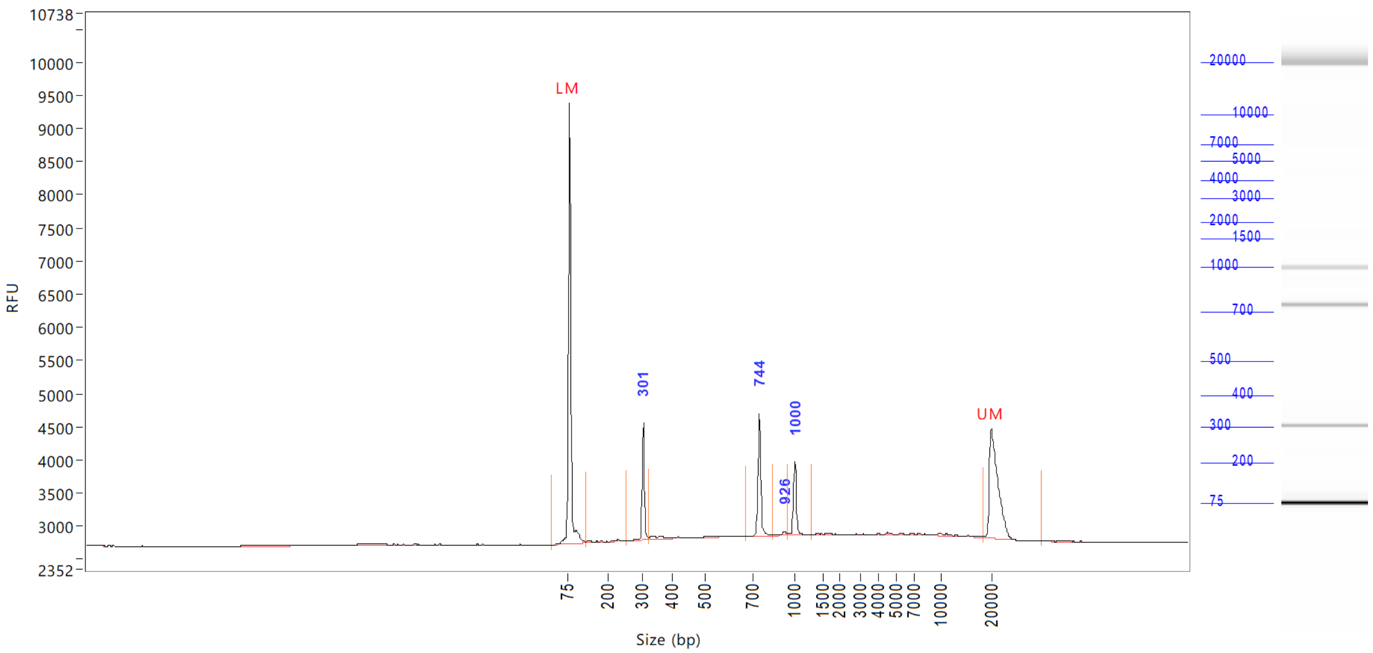

Peak	Size	Concentration	Relative concentration	Molarity	From	To	CV%	RFU
	(bp)	(ng/uL)	Percent	(nmole/L)	(bp)	(bp)		
1	75 (LM)	0.5000		10.9378	24	126	10.48	6662
2	301	1.2588	36.0	6.9250	251	319	1.98	1758
3	744	1.3641	39.1	3.0297	672	835	2.06	1850
4	926	0.0762	2.2	0.1382	835	948	3.11	62
5	1000	0.7935	22.7	1.3034	948	1300	3.35	1107
6	20000 (UM)	0.2234		0.0178	18293	30035	5.00	1650

TIC:	3.4926	ng/uL
TIM:	11.3964	nmole/L
Total concentration:	4.2320	ng/uL

```
Sample peak width (sec): 5    Sample min peak height: 50    Sample baseline V to V?: Y    Sample baseline V to V points: 3
Sample filter: Binomial       Number of points for filter: 3  Sample start region (min): 0  Sample end region (min): 30
Marker peak width (sec): 5    Marker min peak height: 200   Marker baseline V to V?: Y    Marker baseline V to V points: 3
Lower marker selection: First peak > 500 RFU                Upper marker selection: Last peak > 200 RFU
Ladder size (bp)75, 200, 300, 400, 500, 700, 1000, 1500, 2000, 3000, 4000, 5000, 7000, 10000, 20000
Quantification using: Lower Marker          Final concentration (ng/uL): 0.5000          Dilution factor: 12.0
```

Sample: SampG4
Well location: G4
Created: Thursday, October 21, 2021 6:48:20 PM

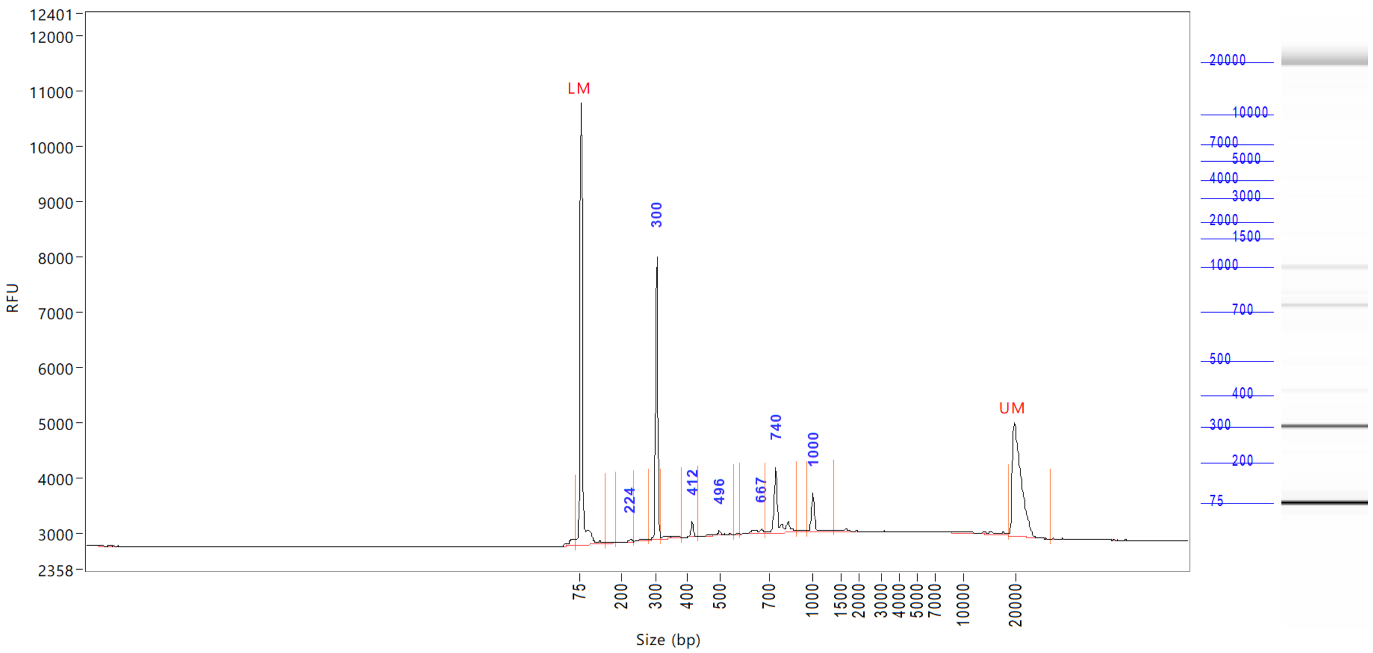

Peak	Size	Concentration	Relative concentration	Molarity	From	To	CV%	RFU
	(bp)	(ng/uL)	Percent	(nmole/L)	(bp)	(bp)		
1	75 (LM)	0.5000		10.7944	60	148	12.30	8000
2	224	0.0465	1.0	0.3508	185	233	4.46	53
3	300	2.8978	60.3	15.9955	277	315	1.14	5115
4	412	0.1545	3.2	0.6201	382	433	1.03	270
5	496	0.0907	1.9	0.3009	433	552	4.23	76
6	667	0.1472	3.1	0.3755	580	685	3.36	70
7	740	1.0242	21.3	2.2237	685	883	5.44	1189
8	1000	0.4487	9.3	0.7277	965	1356	5.70	699
9	20000 (UM)	0.2242		0.0179	18903	27012	4.69	2035

TIC:	4.8096	ng/uL
TIM:	20.5941	nmole/L
Total concentration:	5.4268	ng/uL

Sample peak width (sec): 5 Sample min peak height: 50 Sample baseline V to V?: Y Sample baseline V to V points: 3
Sample filter: Binomial Number of points for filter: 3 Sample start region (min): 0 Sample end region (min): 30
Marker peak width (sec): 5 Marker min peak height: 200 Marker baseline V to V?: Y Marker baseline V to V points: 3
Lower marker selection: First peak > 500 RFU Upper marker selection: Last peak > 200 RFU
Ladder size (bp)75, 200, 300, 400, 500, 700, 1000, 1500, 2000, 3000, 4000, 5000, 7000, 10000, 20000
Quantification using: Lower Marker Final concentration (ng/uL): 0.5000 Dilution factor: 12.0

Sample: SampH4
Well location: H4
Created: Thursday, October 21, 2021 6:48:20 PM

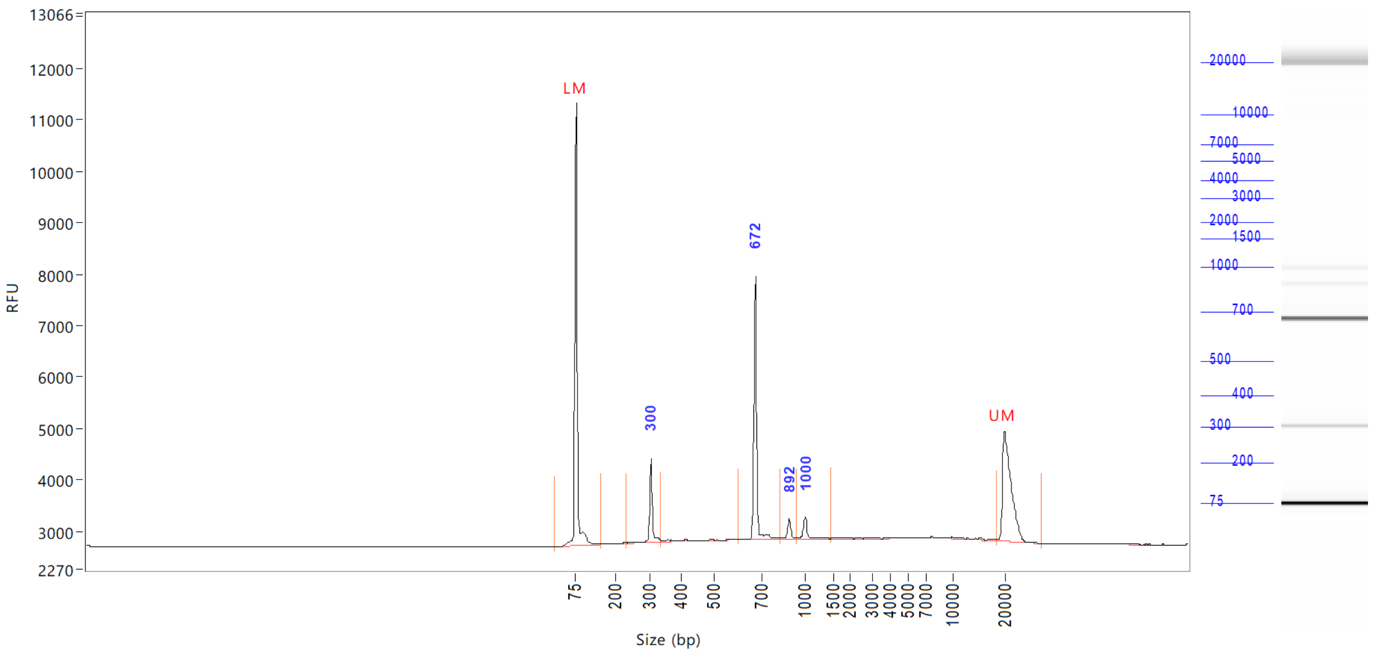

Peak	Size	Concentration	Relative concentration	Molarity	From	To	CV%	RFU
	(bp)	(ng/uL)	Percent	(nmole/L)	(bp)	(bp)		
1	75 (LM)	0.5000		10.9378	11	152	13.27	8613
2	300	1.0683	23.8	5.8577	232	335	3.28	1626
3	672	2.8203	62.7	6.8864	606	822	2.92	5101
4	892	0.2860	6.4	0.5330	822	939	2.31	403
5	1000	0.3227	7.2	0.5233	939	1445	7.11	427
6	20000 (UM)	0.2276		0.0181	18415	27496	4.76	2120

TIC:	4.4973	ng/uL
TIM:	13.8004	nmole/L
Total concentration:	4.9867	ng/uL

```
Sample peak width (sec): 5    Sample min peak height: 50    Sample baseline V to V?: Y    Sample baseline V to V points: 3
Sample filter: Binomial       Number of points for filter: 3  Sample start region (min): 0  Sample end region (min): 30
Marker peak width (sec): 5    Marker min peak height: 200   Marker baseline V to V?: Y    Marker baseline V to V points: 3
Lower marker selection: First peak > 500 RFU                Upper marker selection: Last peak > 200 RFU
Ladder size (bp)75, 200, 300, 400, 500, 700, 1000, 1500, 2000, 3000, 4000, 5000, 7000, 10000, 20000
Quantification using: Lower Marker          Final concentration (ng/uL): 0.5000          Dilution factor: 12.0
```

Sample: SampA5
Well location: A5
Created: Thursday, October 21, 2021 6:48:20 PM

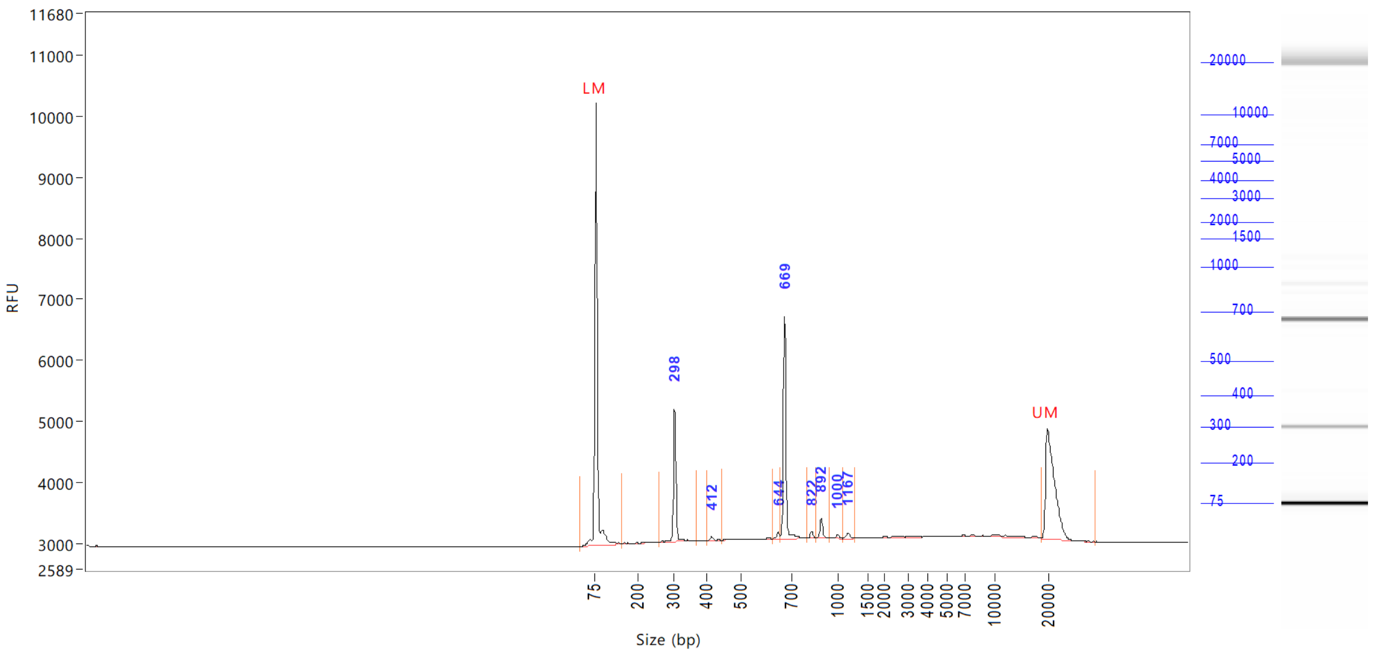

Peak	Size	Concentration	Relative concentration	Molarity	From	To	CV%	RFU
	(bp)	(ng/uL)	Percent	(nmole/L)	(bp)	(bp)		
1	75 (LM)	0.5000		11.0851	30	150	12.95	7250
2	298	1.5294	34.8	8.4420	257	368	2.67	2161
3	412	0.0472	1.1	0.1886	400	443	1.61	65
4	644	0.0780	1.8	0.2004	621	652	1.03	109
5	669	2.3212	52.8	5.7102	652	801	2.00	3628
6	822	0.0744	1.7	0.1490	801	853	1.23	109
7	892	0.2194	5.0	0.4076	853	948	1.32	328
8	1000	0.0485	1.1	0.0801	948	1079	1.87	61
9	1167	0.0809	1.8	0.1152	1079	1278	2.77	98
10	20000 (UM)	0.2242		0.0179	18903	28947	4.82	1810

TIC:	4.3992	ng/uL
TIM:	15.2931	nmole/L
Total concentration:	4.8228	ng/uL

```
Sample peak width (sec): 5    Sample min peak height: 50    Sample baseline V to V?: Y    Sample baseline V to V points: 3
Sample filter: Binomial       Number of points for filter: 3  Sample start region (min): 0  Sample end region (min): 30
Marker peak width (sec): 5    Marker min peak height: 200   Marker baseline V to V?: Y    Marker baseline V to V points: 3
Lower marker selection: First peak > 500 RFU                Upper marker selection: Last peak > 200 RFU
Ladder size (bp)75, 200, 300, 400, 500, 700, 1000, 1500, 2000, 3000, 4000, 5000, 7000, 10000, 20000
Quantification using: Lower Marker          Final concentration (ng/uL): 0.5000          Dilution factor: 12.0
```

Sample: SampB5
Well location: B5
Created: Thursday, October 21, 2021 6:48:20 PM

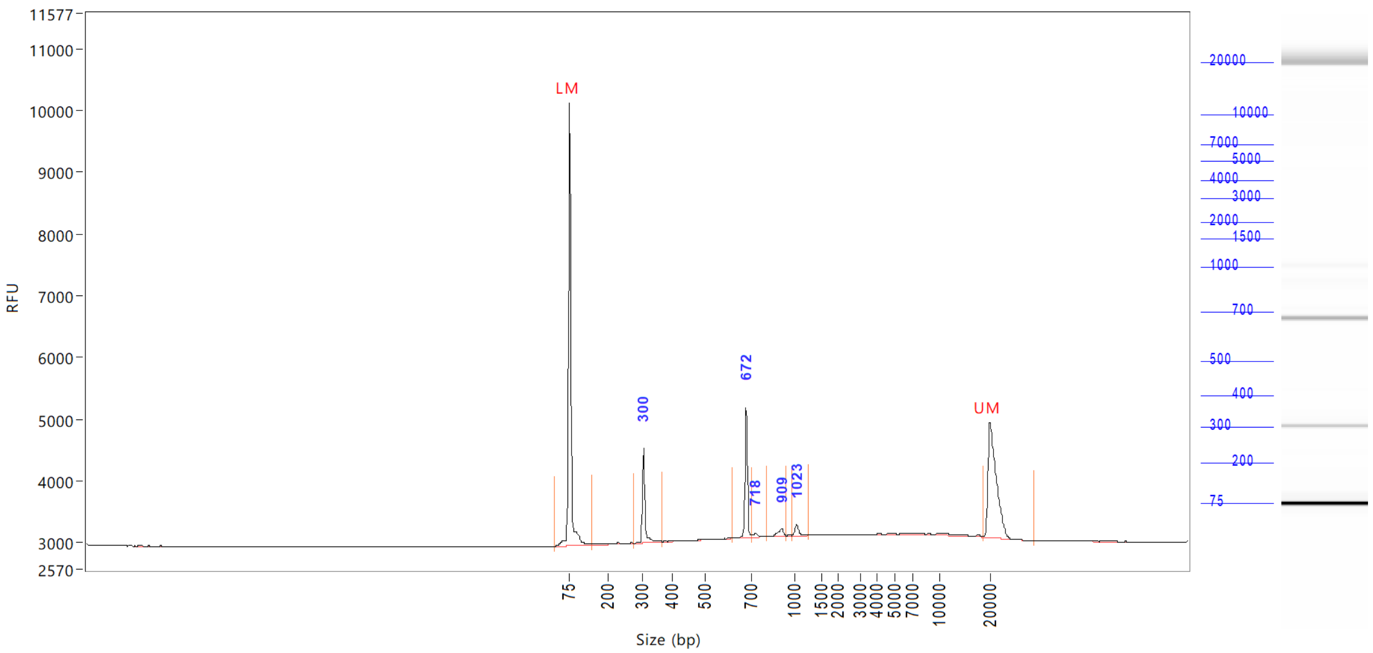

Peak	Size	Concentration	Relative concentration	Molarity	From	To	CV%	RFU
	(bp)	(ng/uL)	Percent	(nmole/L)	(bp)	(bp)		
1	75 (LM)	0.5000		11.0851	28	148	13.82	7188
2	300	1.1467	41.0	6.2458	273	364	3.33	1528
3	672	1.2634	45.2	3.0986	616	697	1.05	2109
4	718	0.0845	3.0	0.1924	697	805	3.06	74
5	909	0.1427	5.1	0.2636	805	944	2.59	121
6	1023	0.1585	5.7	0.2521	983	1245	4.12	186
7	20000 (UM)	0.2227		0.0177	18903	28947	4.56	1859

TIC:	2.7958	ng/uL
TIM:	10.0526	nmole/L
Total concentration:	3.2995	ng/uL

Sample peak width (sec): 5   Sample min peak height: 50   Sample baseline V to V?: Y   Sample baseline V to V points: 3
Sample filter: Binomial   Number of points for filter: 3   Sample start region (min): 0   Sample end region (min): 30
Marker peak width (sec): 5   Marker min peak height: 200   Marker baseline V to V?: Y   Marker baseline V to V points: 3
Lower marker selection: First peak > 500 RFU   Upper marker selection: Last peak > 200 RFU
Ladder size (bp)75, 200, 300, 400, 500, 700, 1000, 1500, 2000, 3000, 4000, 5000, 7000, 10000, 20000
Quantification using: Lower Marker   Final concentration (ng/uL): 0.5000   Dilution factor: 12.0

Sample: SampC5
Well location: C5
Created: Thursday, October 21, 2021 6:48:20 PM

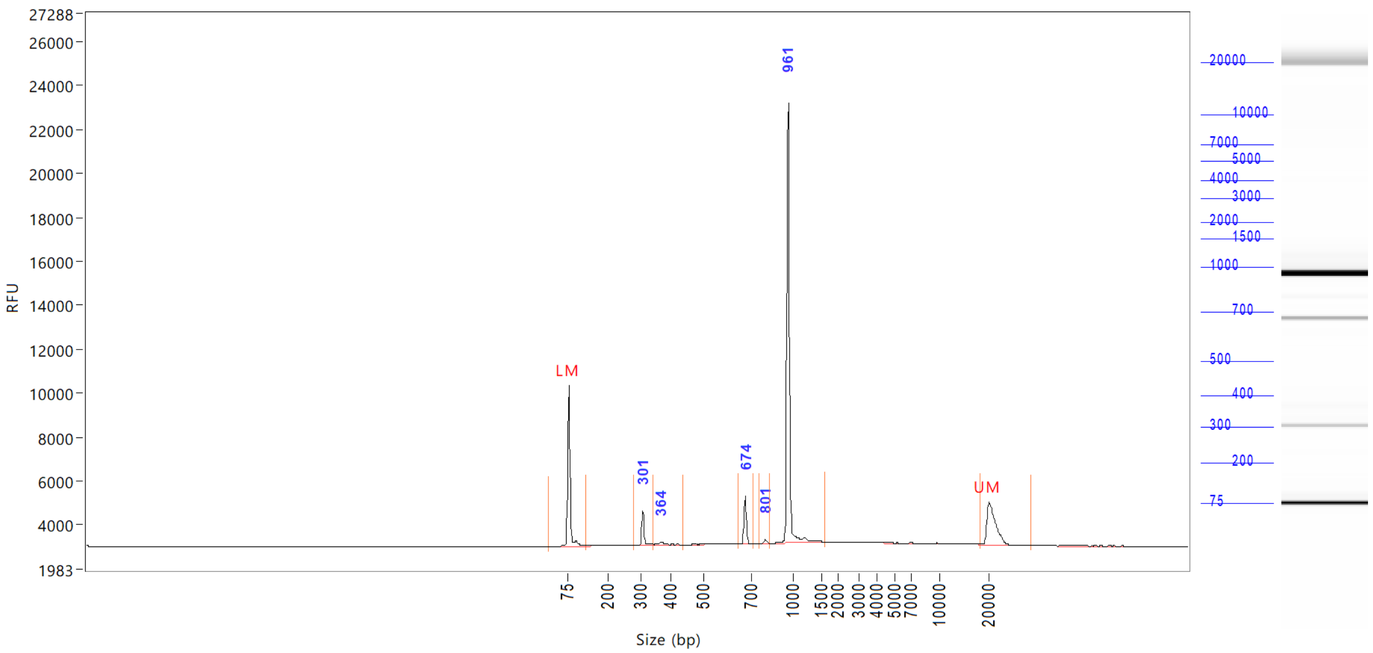

Peak	Size	Concentration	Relative concentration	Molarity	From	To	CV%	RFU
	(bp)	(ng/uL)	Percent	(nmole/L)	(bp)	(bp)		
1	75 (LM)	0.5000		10.9378	14	128	9.88	7321
2	301	1.1207	7.4	6.1041	275	339	2.67	1535
3	364	0.2601	1.7	1.1597	339	433	5.33	138
4	674	1.3103	8.6	3.2136	644	710	0.85	2179
5	801	0.1199	0.8	0.2482	757	835	1.32	175
6	961	12.4291	81.6	21.1554	835	1597	5.42	20061
7	20000 (UM)	0.2404		0.0192	18415	28463	4.68	1975

TIC:	15.2400	ng/uL
TIM:	31.8810	nmole/L
Total concentration:	15.7478	ng/uL

Sample peak width (sec): 5 Sample min peak height: 50 Sample baseline V to V?: Y Sample baseline V to V points: 3
Sample filter: Binomial Number of points for filter: 3 Sample start region (min): 0 Sample end region (min): 30
Marker peak width (sec): 5 Marker min peak height: 200 Marker baseline V to V?: Y Marker baseline V to V points: 3
Lower marker selection: First peak > 500 RFU Upper marker selection: Last peak > 200 RFU
Ladder size (bp)75, 200, 300, 400, 500, 700, 1000, 1500, 2000, 3000, 4000, 5000, 7000, 10000, 20000
Quantification using: Lower Marker Final concentration (ng/uL): 0.5000 Dilution factor: 12.0

Sample: SampD5
Well location: D5
Created: Thursday, October 21, 2021 6:48:20 PM

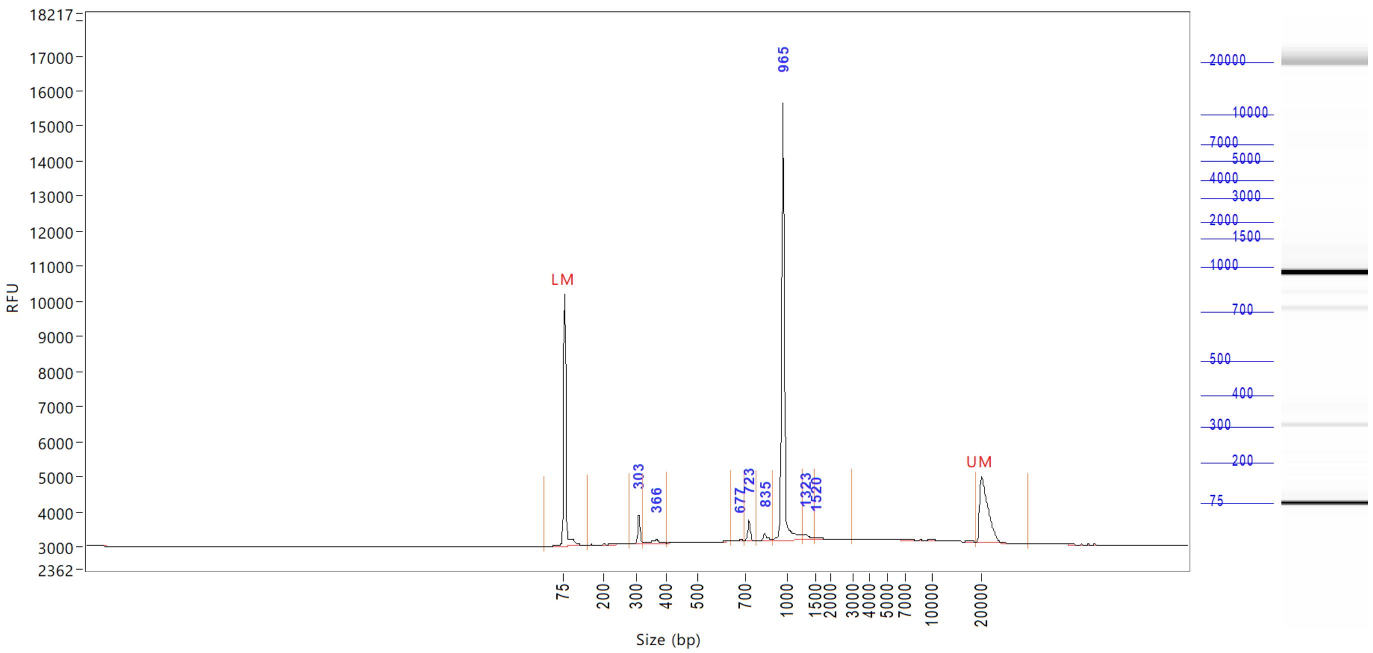

Peak	Size	Concentration	Relative concentration	Molarity	From	To	CV%	RFU
	(bp)	(ng/uL)	Percent	(nmole/L)	(bp)	(bp)		
1	75 (LM)	0.5000		10.9378	12	150	12.50	7203
2	303	0.5806	5.7	3.1518	280	319	1.48	809
3	366	0.2468	2.4	1.1470	319	398	4.64	105
4	677	0.0412	0.4	0.1004	639	692	1.17	61
5	723	0.3598	3.6	0.8270	692	775	1.40	577
6	835	0.1812	1.8	0.3554	775	887	2.26	204
7	965	8.2891	81.9	14.0219	887	1278	4.79	12491
8	1323	0.2418	2.4	0.2925	1278	1489	4.54	159
9	1520	0.1821	1.8	0.1624	1489	3000	18.49	66
10	20000 (UM)	0.2293		0.0183	18903	29430	4.82	1890

TIC:	10.1226	ng/uL
TIM:	20.0583	nmole/L
Total concentration:	10.4885	ng/uL

```
Sample peak width (sec): 5     Sample min peak height: 50     Sample baseline V to V?: Y     Sample baseline V to V points: 3
Sample filter: Binomial        Number of points for filter: 3  Sample start region (min): 0  Sample end region (min): 30
Marker peak width (sec): 5     Marker min peak height: 200    Marker baseline V to V?: Y     Marker baseline V to V points: 3
Lower marker selection: First peak > 500 RFU                  Upper marker selection: Last peak > 200 RFU
Ladder size (bp)75, 200, 300, 400, 500, 700, 1000, 1500, 2000, 3000, 4000, 5000, 7000, 10000, 20000
Quantification using: Lower Marker          Final concentration (ng/uL): 0.5000          Dilution factor: 12.0
```

Sample: SampE5
Well location: E5
Created: Thursday, October 21, 2021 6:48:20 PM

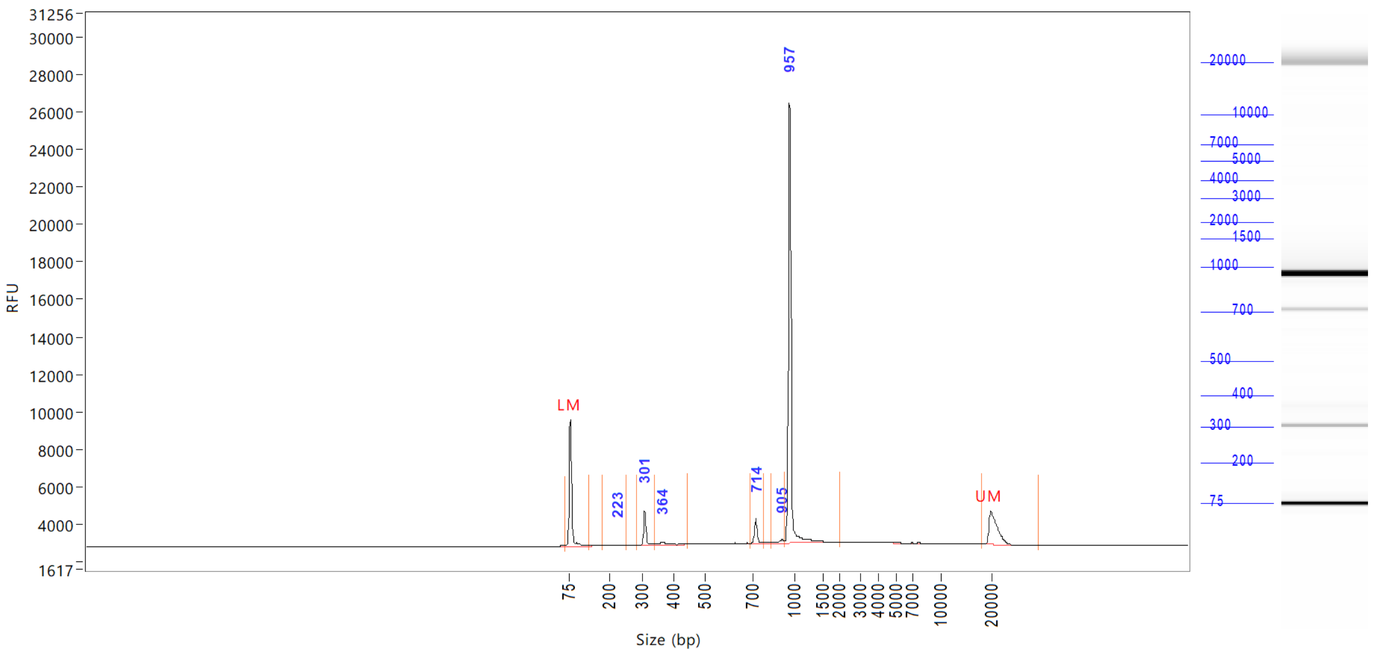

Peak	Size	Concentration	Relative concentration	Molarity	From	To	CV%	RFU
	(bp)	(ng/uL)	Percent	(nmole/L)	(bp)	(bp)		
1	75 (LM)	0.5000		11.0851	60	137	9.95	6782
2	223	0.0746	0.4	0.5630	175	248	6.34	51
3	301	1.4726	7.3	8.0210	280	337	2.34	1849
4	364	0.3776	1.9	1.6974	337	443	5.28	177
5	714	0.9870	4.9	2.2814	687	779	1.88	1319
6	905	0.2485	1.2	0.4580	831	922	2.37	203
7	957	16.8977	84.2	28.6134	922	2030	8.39	23503
8	20000 (UM)	0.2361		0.0188	18293	29430	4.86	1755

TIC:	20.0580	ng/uL
TIM:	41.6342	nmole/L
Total concentration:	20.5555	ng/uL

```
Sample peak width (sec): 5    Sample min peak height: 50    Sample baseline V to V?: Y    Sample baseline V to V points: 3
Sample filter: Binomial       Number of points for filter: 3  Sample start region (min): 0  Sample end region (min): 30
Marker peak width (sec): 5    Marker min peak height: 200   Marker baseline V to V?: Y    Marker baseline V to V points: 3
Lower marker selection: First peak > 500 RFU                Upper marker selection: Last peak > 200 RFU
Ladder size (bp)75, 200, 300, 400, 500, 700, 1000, 1500, 2000, 3000, 4000, 5000, 7000, 10000, 20000
Quantification using: Lower Marker          Final concentration (ng/uL): 0.5000         Dilution factor: 12.0
```

Sample: SampF5
Well location: F5
Created: Thursday, October 21, 2021 6:48:20 PM

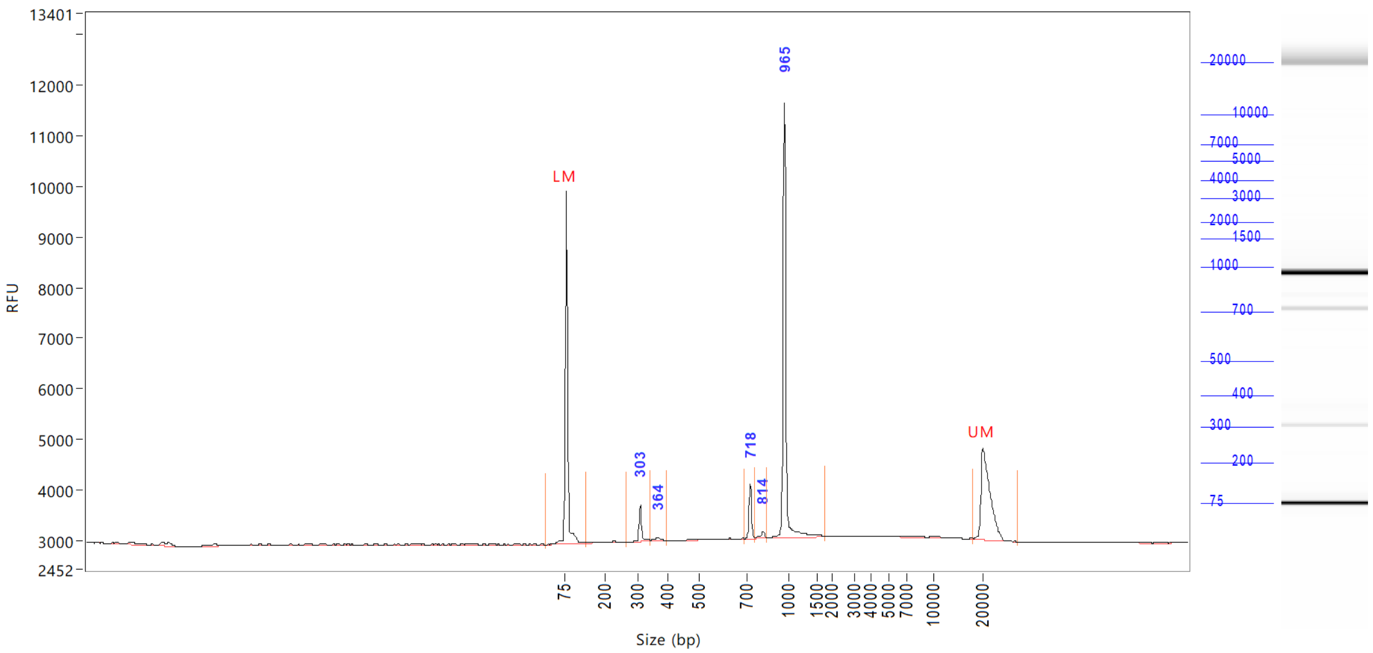

Peak	Size	Concentration	Relative concentration	Molarity	From	To	CV%	RFU
	(bp)	(ng/uL)	Percent	(nmole/L)	(bp)	(bp)		
1	75 (LM)	0.5000		10.9378	9	139	11.34	6945
2	303	0.5924	7.6	3.2054	264	339	3.05	735
3	364	0.1467	1.9	0.6702	339	392	3.20	76
4	718	0.7051	9.1	1.6230	690	753	1.31	1069
5	814	0.1374	1.8	0.2823	753	844	2.45	139
6	965	6.1721	79.6	10.3662	844	1770	8.74	8578
7	20000 (UM)	0.2513		0.0199	18171	26891	5.09	1804

TIC:	7.7536	ng/uL
TIM:	16.1470	nmole/L
Total concentration:	8.1915	ng/uL

```
Sample peak width (sec): 5     Sample min peak height: 50    Sample baseline V to V?: Y    Sample baseline V to V points: 3
Sample filter: Binomial        Number of points for filter: 3  Sample start region (min): 0  Sample end region (min): 30
Marker peak width (sec): 5     Marker min peak height: 200   Marker baseline V to V?: Y    Marker baseline V to V points: 3
Lower marker selection: First peak > 500 RFU                 Upper marker selection: Last peak > 200 RFU
Ladder size (bp)75, 200, 300, 400, 500, 700, 1000, 1500, 2000, 3000, 4000, 5000, 7000, 10000, 20000
Quantification using: Lower Marker          Final concentration (ng/uL): 0.5000          Dilution factor: 12.0
```

Sample: SampG5
Well location: G5
Created: Thursday, October 21, 2021 6:48:20 PM

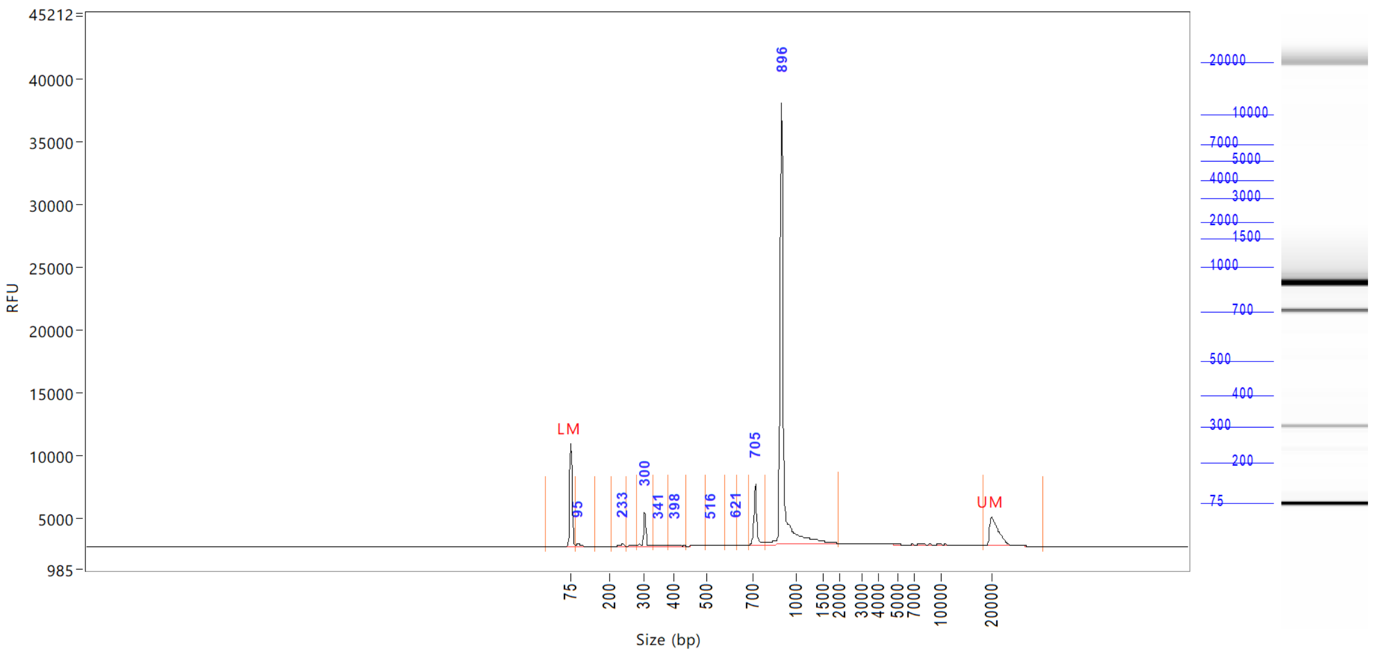

Peak	Size	Concentration	Relative concentration	Molarity	From	To	CV%	RFU
	(bp)	(ng/uL)	Percent	(nmole/L)	(bp)	(bp)		
1	75 (LM)	0.5000		11.3919	0	87	5.62	8236
2	95	0.4099	1.3	6.5354	87	150	14.10	204
3	233	0.1842	0.6	1.3170	203	248	3.13	209
4	300	1.7994	5.7	9.8995	277	331	2.20	2649
5	341	0.1602	0.5	0.7507	331	378	3.48	67
6	398	0.0981	0.3	0.4036	378	437	3.07	66
7	516	0.0801	0.3	0.2520	496	578	2.88	71
8	621	0.0583	0.2	0.1562	578	631	1.80	64
9	705	3.1645	10.0	7.3559	680	779	2.38	4796
10	896	25.6357	81.2	45.5656	779	1962	11.85	35179
11	20000 (UM)	0.2816		0.0223	18293	30277	5.22	2276

TIC:	31.5904	ng/uL
TIM:	72.2360	nmole/L
Total concentration:	32.0108	ng/uL

```
Sample peak width (sec): 5    Sample min peak height: 50    Sample baseline V to V?: Y    Sample baseline V to V points: 3
Sample filter: Binomial       Number of points for filter: 3  Sample start region (min): 0  Sample end region (min): 30
Marker peak width (sec): 5    Marker min peak height: 200   Marker baseline V to V?: Y    Marker baseline V to V points: 3
Lower marker selection: First peak > 500 RFU                Upper marker selection: Last peak > 200 RFU
Ladder size (bp)75, 200, 300, 400, 500, 700, 1000, 1500, 2000, 3000, 4000, 5000, 7000, 10000, 20000
Quantification using: Lower Marker          Final concentration (ng/uL): 0.5000          Dilution factor: 12.0
```

Sample: SampH5
Well location: H5
Created: Thursday, October 21, 2021 6:48:20 PM

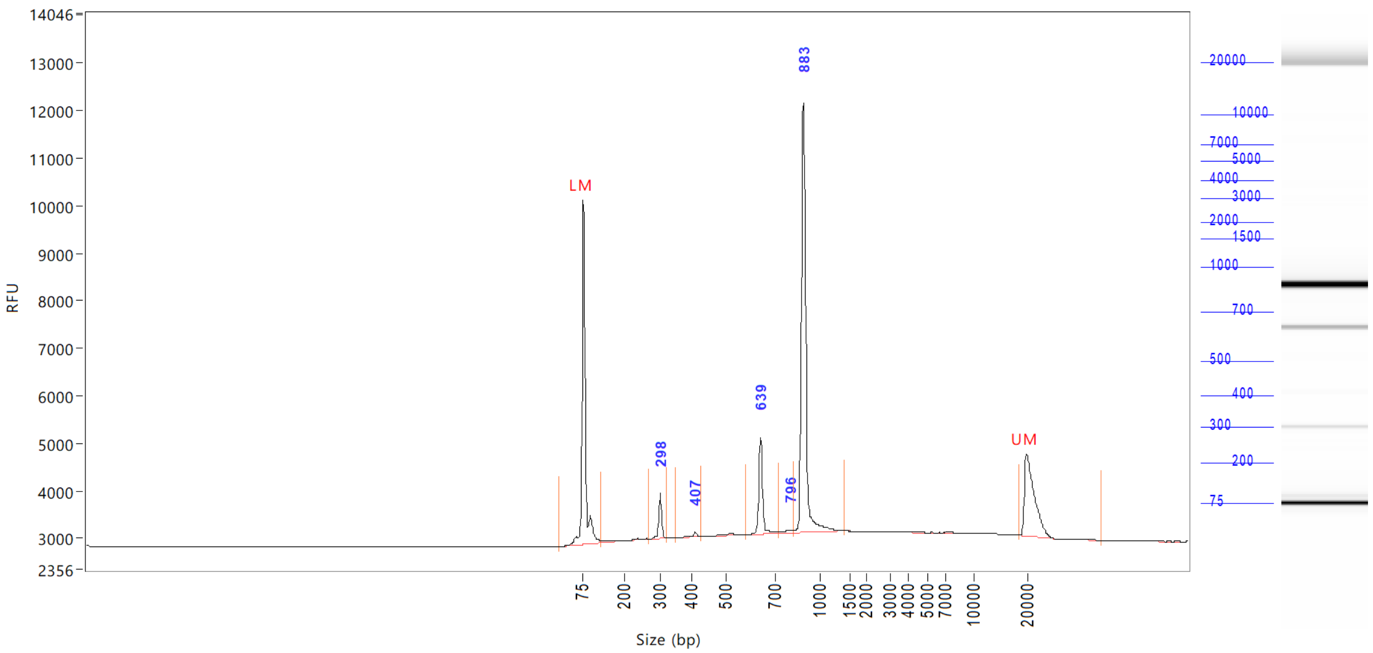

Peak	Size	Concentration	Relative concentration	Molarity	From	To	CV%	RFU
	(bp)	(ng/uL)	Percent	(nmole/L)	(bp)	(bp)		
1	75 (LM)	0.5000		10.9378	3	128	13.75	7248
2	298	0.6225	6.6	3.4593	266	317	1.94	942
3	407	0.0808	0.9	0.3322	349	424	3.99	91
4	639	1.5372	16.2	3.9651	578	718	2.30	2028
5	796	0.1110	1.2	0.2364	718	814	3.50	69
6	883	7.1367	75.2	13.2425	814	1411	4.71	9047
7	20000 (UM)	0.1930		0.0153	18537	34146	6.48	1734

TIC:	9.4882	ng/uL
TIM:	21.2355	nmole/L
Total concentration:	9.9111	ng/uL

Sample peak width (sec): 5   Sample min peak height: 50   Sample baseline V to V?: Y   Sample baseline V to V points: 3
Sample filter: Binomial   Number of points for filter: 3 Sample start region (min): 0 Sample end region (min): 30
Marker peak width (sec): 5   Marker min peak height: 200   Marker baseline V to V?: Y   Marker baseline V to V points: 3
Lower marker selection: First peak > 500 RFU   Upper marker selection: Last peak > 200 RFU
Ladder size (bp)75, 200, 300, 400, 500, 700, 1000, 1500, 2000, 3000, 4000, 5000, 7000, 10000, 20000
Quantification using: Lower Marker   Final concentration (ng/uL): 0.5000   Dilution factor: 12.0

Sample: SampA6
Well location: A6
Created: Thursday, October 21, 2021 6:48:20 PM

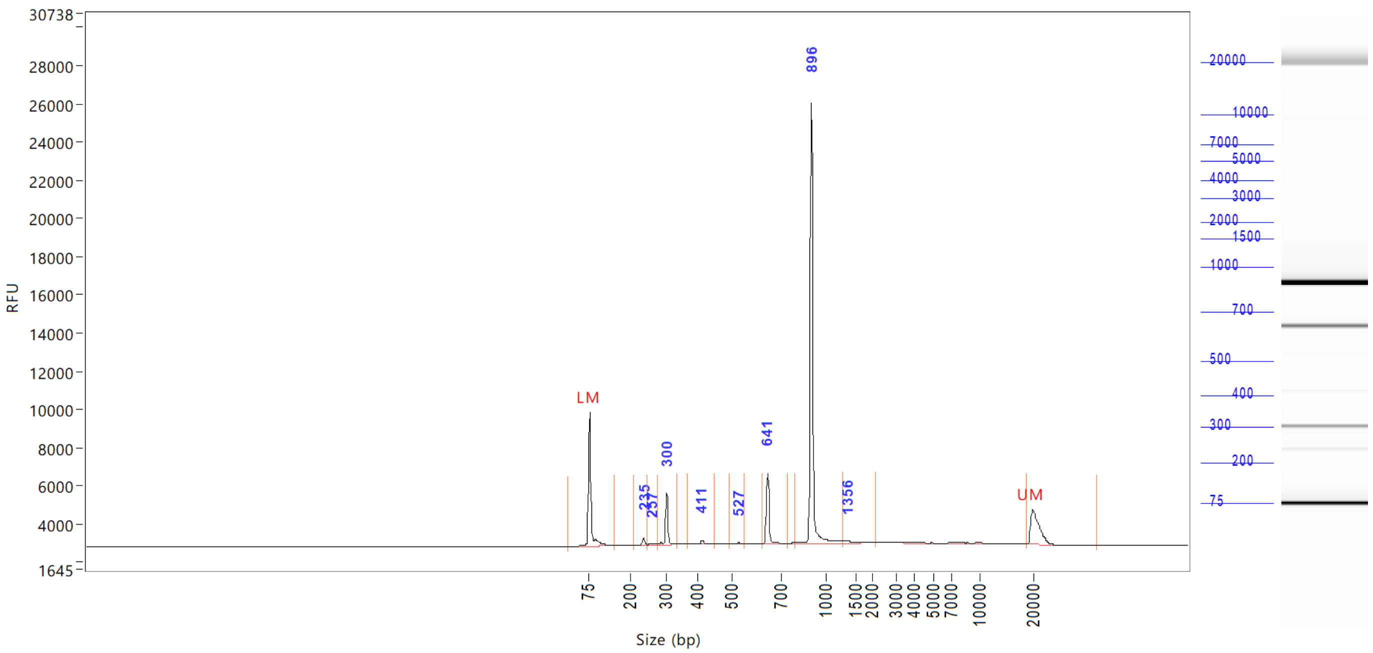

Peak	Size	Concentration	Relative concentration	Molarity	From	To	CV%	RFU
	(bp)	(ng/uL)	Percent	(nmole/L)	(bp)	(bp)		
1	75 (LM)	0.5000		10.7944	12	152	15.34	6977
2	235	0.2879	1.5	2.0406	206	246	2.26	405
3	257	0.0870	0.5	0.5522	246	275	2.94	53
4	300	1.7953	9.4	9.9097	275	331	1.89	2666
5	411	0.1971	1.0	0.7968	368	450	2.68	214
6	527	0.0847	0.4	0.2695	494	549	1.92	60
7	641	2.2702	11.9	5.8193	623	736	1.50	3671
8	896	14.1542	74.0	25.7418	788	1289	5.38	23084
9	1356	0.2610	1.4	0.2930	1289	2115	12.47	121
10	20000 (UM)	0.2152		0.0171	18903	32211	4.95	1838

TIC:	19.1372	ng/uL
TIM:	45.4228	nmole/L
Total concentration:	19.6893	ng/uL

```
Sample peak width (sec): 5    Sample min peak height: 50    Sample baseline V to V?: Y    Sample baseline V to V points: 3
Sample filter: Binomial       Number of points for filter: 3  Sample start region (min): 0  Sample end region (min): 30
Marker peak width (sec): 5    Marker min peak height: 200   Marker baseline V to V?: Y    Marker baseline V to V points: 3
Lower marker selection: First peak > 500 RFU                Upper marker selection: Last peak > 200 RFU
Ladder size (bp)75, 200, 300, 400, 500, 700, 1000, 1500, 2000, 3000, 4000, 5000, 7000, 10000, 20000
Quantification using: Lower Marker          Final concentration (ng/uL): 0.5000          Dilution factor: 12.0
```

Sample: SampB6
Well location: B6
Created: Thursday, October 21, 2021 6:48:20 PM

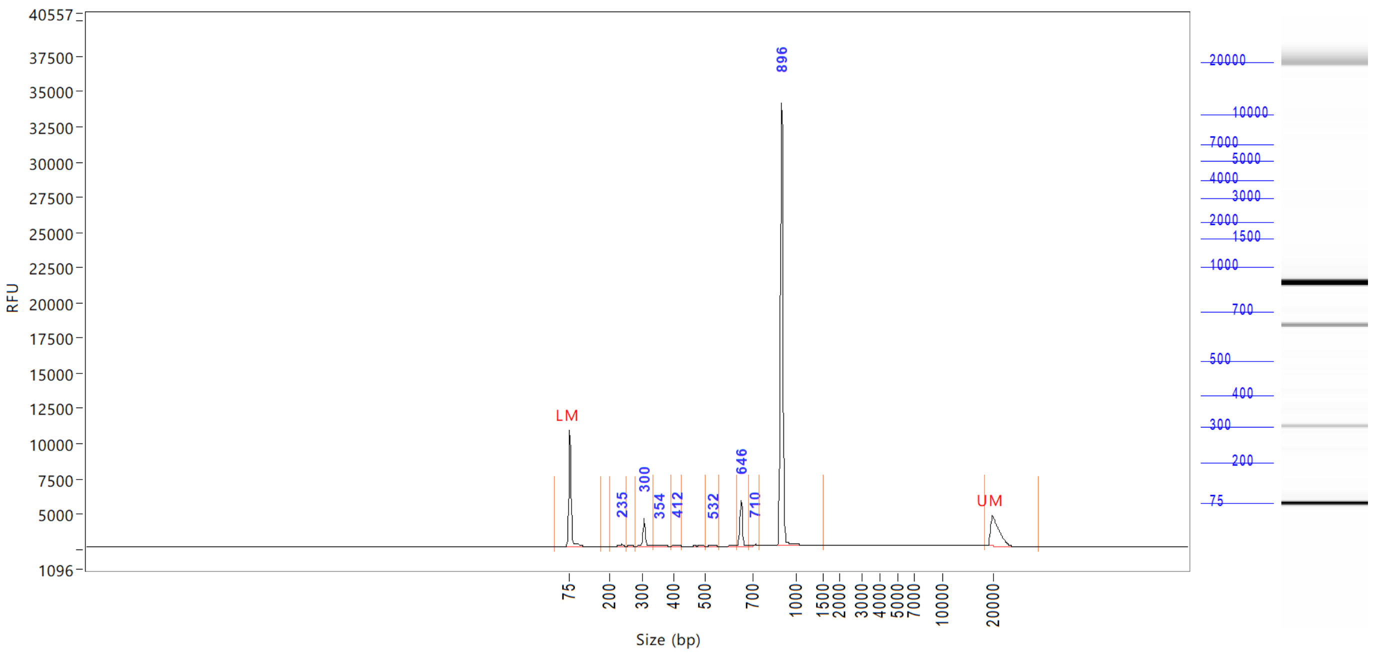

Peak	Size	Concentration	Relative concentration	Molarity	From	To	CV%	RFU
	(bp)	(ng/uL)	Percent	(nmole/L)	(bp)	(bp)		
1	75 (LM)	0.5000		10.9378	31	175	12.63	8230
2	235	0.1224	0.6	0.8716	203	248	3.75	125
3	300	1.2998	6.3	7.1507	277	331	2.03	1942
4	354	0.1733	0.8	0.8056	331	390	3.10	92
5	412	0.0805	0.4	0.3254	390	426	1.63	102
6	532	0.0710	0.3	0.2227	498	555	1.90	66
7	646	1.7406	8.4	4.4480	629	682	0.84	3191
8	710	0.0791	0.4	0.1849	682	740	1.38	110
9	896	17.2167	82.8	31.5905	740	1501	3.43	31490
10	20000 (UM)	0.2494		0.0199	18537	29309	4.76	2183

TIC:	20.7835	ng/uL
TIM:	45.5994	nmole/L
Total concentration:	21.1903	ng/uL

```
Sample peak width (sec): 5    Sample min peak height: 50    Sample baseline V to V?: Y    Sample baseline V to V points: 3
Sample filter: Binomial       Number of points for filter: 3  Sample start region (min): 0  Sample end region (min): 30
Marker peak width (sec): 5    Marker min peak height: 200   Marker baseline V to V?: Y    Marker baseline V to V points: 3
Lower marker selection: First peak > 500 RFU                Upper marker selection: Last peak > 200 RFU
Ladder size (bp)75, 200, 300, 400, 500, 700, 1000, 1500, 2000, 3000, 4000, 5000, 7000, 10000, 20000
Quantification using: Lower Marker          Final concentration (ng/uL): 0.5000          Dilution factor: 12.0
```

Sample: SampC6
Well location: C6
Created: Thursday, October 21, 2021 6:48:20 PM

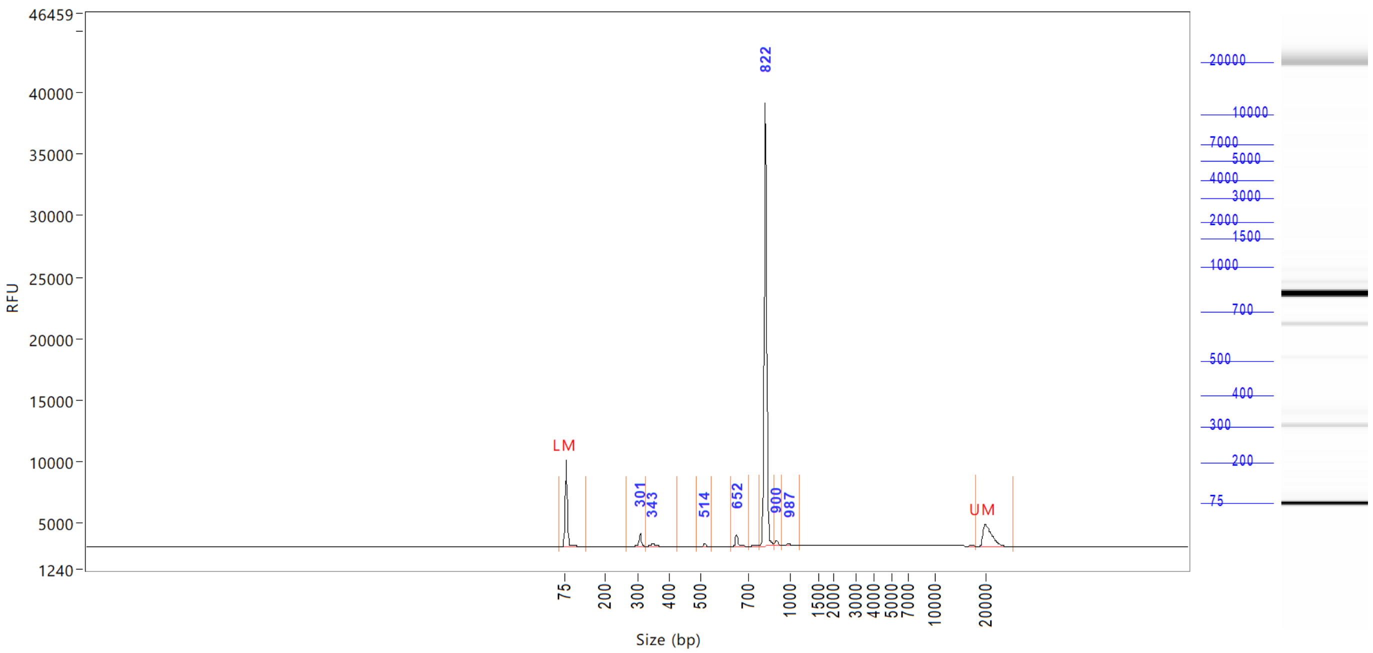

Peak	Size	Concentration	Relative concentration	Molarity	From	To	CV%	RFU
	(bp)	(ng/uL)	Percent	(nmole/L)	(bp)	(bp)		
1	75 (LM)	0.5000		11.0851	54	141	9.49	7033
2	301	0.8747	3.3	4.7963	260	323	2.36	1048
3	343	0.4370	1.6	2.0599	323	424	4.94	194
4	514	0.1987	0.7	0.6398	487	542	1.38	263
5	652	0.6662	2.5	1.6868	623	701	1.25	934
6	822	23.7506	89.3	47.7869	770	883	1.26	36100
7	900	0.4245	1.6	0.7754	883	939	1.51	466
8	987	0.2454	0.9	0.4096	939	1178	4.20	170
9	20000 (UM)	0.2498		0.0199	18171	25682	4.70	1793

TIC:	26.5972	ng/uL
TIM:	58.1547	nmole/L
Total concentration:	27.1712	ng/uL

```
Sample peak width (sec): 5    Sample min peak height: 50    Sample baseline V to V?: Y    Sample baseline V to V points: 3
Sample filter: Binomial       Number of points for filter: 3  Sample start region (min): 0  Sample end region (min): 30
Marker peak width (sec): 5    Marker min peak height: 200   Marker baseline V to V?: Y    Marker baseline V to V points: 3
Lower marker selection: First peak > 500 RFU                Upper marker selection: Last peak > 200 RFU
Ladder size (bp)75, 200, 300, 400, 500, 700, 1000, 1500, 2000, 3000, 4000, 5000, 7000, 10000, 20000
Quantification using: Lower Marker          Final concentration (ng/uL): 0.5000          Dilution factor: 12.0
```

Sample: SampD6
Well location: D6
Created: Thursday, October 21, 2021 6:48:20 PM

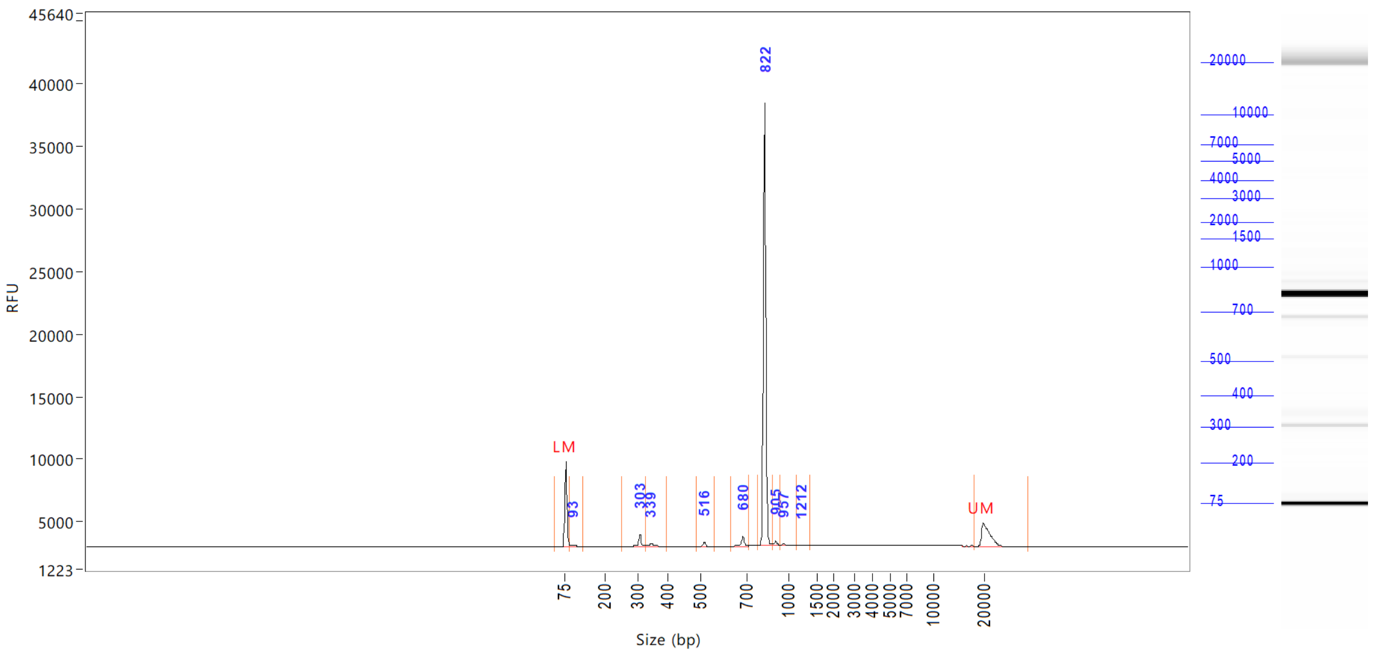

Peak	Size	Concentration	Relative concentration	Molarity	From	To	CV%	RFU
	(bp)	(ng/uL)	Percent	(nmole/L)	(bp)	(bp)		
1	75 (LM)	0.5000		11.3919	39	86	4.86	6807
2	93	0.3371	1.2	5.7060	86	128	9.74	166
3	303	0.8894	3.2	4.8928	248	321	3.20	937
4	339	0.3960	1.4	1.8992	321	394	3.88	185
5	516	0.2781	1.0	0.8919	487	560	1.62	362
6	680	0.6511	2.4	1.5922	631	701	1.35	816
7	822	24.3189	88.4	49.0501	775	879	1.14	35444
8	905	0.3053	1.1	0.5583	879	931	1.40	340
9	957	0.2313	0.8	0.3889	931	1112	4.32	132
10	1212	0.1068	0.4	0.1439	1112	1389	4.79	65
11	20000 (UM)	0.2544		0.0202	18293	29068	4.82	1820

TIC:	27.5139	ng/uL
TIM:	65.1235	nmole/L
Total concentration:	28.1117	ng/uL

Sample peak width (sec): 5   Sample min peak height: 50   Sample baseline V to V?: Y   Sample baseline V to V points: 3
Sample filter: Binomial   Number of points for filter: 3 Sample start region (min): 0 Sample end region (min): 30
Marker peak width (sec): 5   Marker min peak height: 200   Marker baseline V to V?: Y   Marker baseline V to V points: 3
Lower marker selection: First peak > 500 RFU   Upper marker selection: Last peak > 200 RFU
Ladder size (bp)75, 200, 300, 400, 500, 700, 1000, 1500, 2000, 3000, 4000, 5000, 7000, 10000, 20000
Quantification using: Lower Marker   Final concentration (ng/uL): 0.5000   Dilution factor: 12.0

Sample: SampE6
Well location: E6
Created: Thursday, October 21, 2021 6:48:20 PM

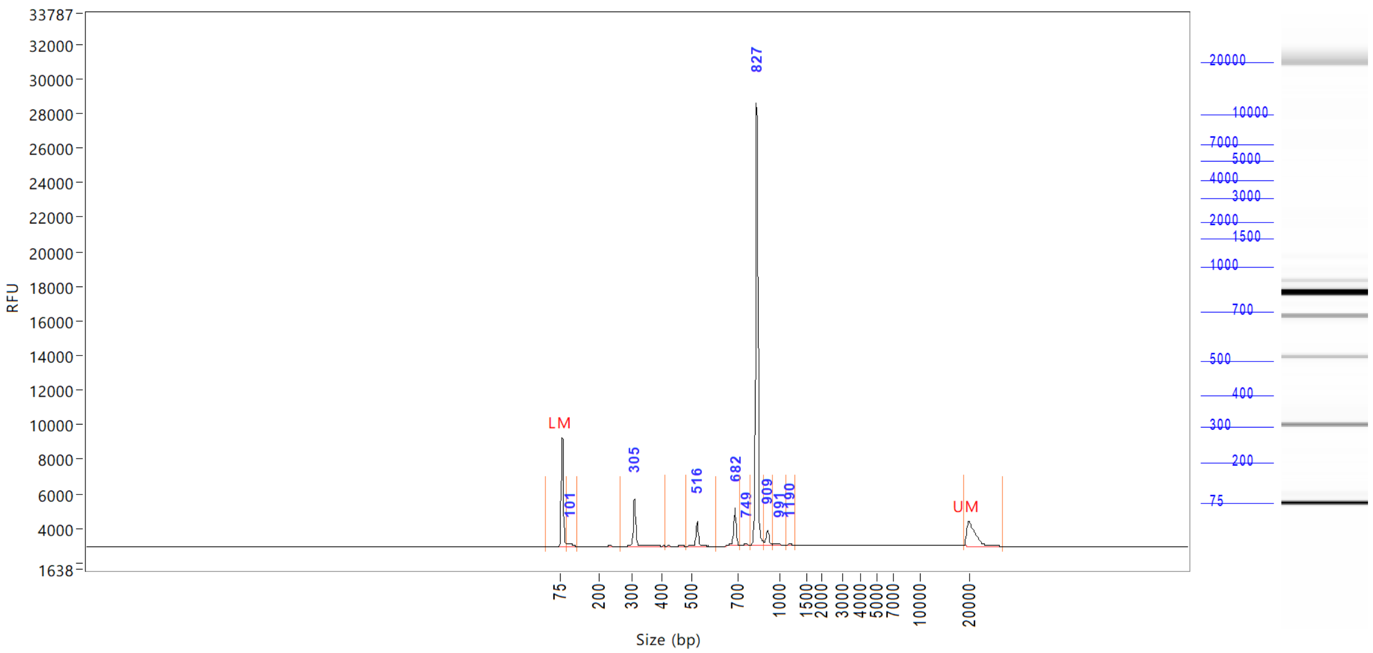

Peak	Size	Concentration	Relative concentration	Molarity	From	To	CV%	RFU
	(bp)	(ng/uL)	Percent	(nmole/L)	(bp)	(bp)		
1	75 (LM)	0.5000		11.0851	24	89	5.15	6272
2	101	0.3838	1.5	6.3023	89	124	7.54	190
3	305	2.6708	10.2	14.2641	264	411	5.68	2706
4	516	1.2322	4.7	3.9296	479	600	2.13	1463
5	682	1.7295	6.6	4.1920	600	710	1.17	2165
6	749	0.0961	0.4	0.2117	710	783	2.09	74
7	827	18.9996	72.9	37.9034	783	887	1.28	25624
8	909	0.7234	2.8	1.3126	887	948	1.25	836
9	991	0.1570	0.6	0.2623	948	1123	3.19	93
10	1190	0.0871	0.3	0.1220	1123	1278	2.00	109
11	20000 (UM)	0.2262		0.0179	18903	27133	5.69	1435

TIC:	26.0795	ng/uL
TIM:	68.5000	nmole/L
Total concentration:	26.6281	ng/uL

Sample peak width (sec): 5 Sample min peak height: 50 Sample baseline V to V?: Y Sample baseline V to V points: 3
Sample filter: Binomial Number of points for filter: 3 Sample start region (min): 0 Sample end region (min): 30
Marker peak width (sec): 5 Marker min peak height: 200 Marker baseline V to V?: Y Marker baseline V to V points: 3
Lower marker selection: First peak > 500 RFU Upper marker selection: Last peak > 200 RFU
Ladder size (bp)75, 200, 300, 400, 500, 700, 1000, 1500, 2000, 3000, 4000, 5000, 7000, 10000, 20000
Quantification using: Lower Marker Final concentration (ng/uL): 0.5000 Dilution factor: 12.0

Sample: SampF6
Well location: F6
Created: Thursday, October 21, 2021 6:48:20 PM

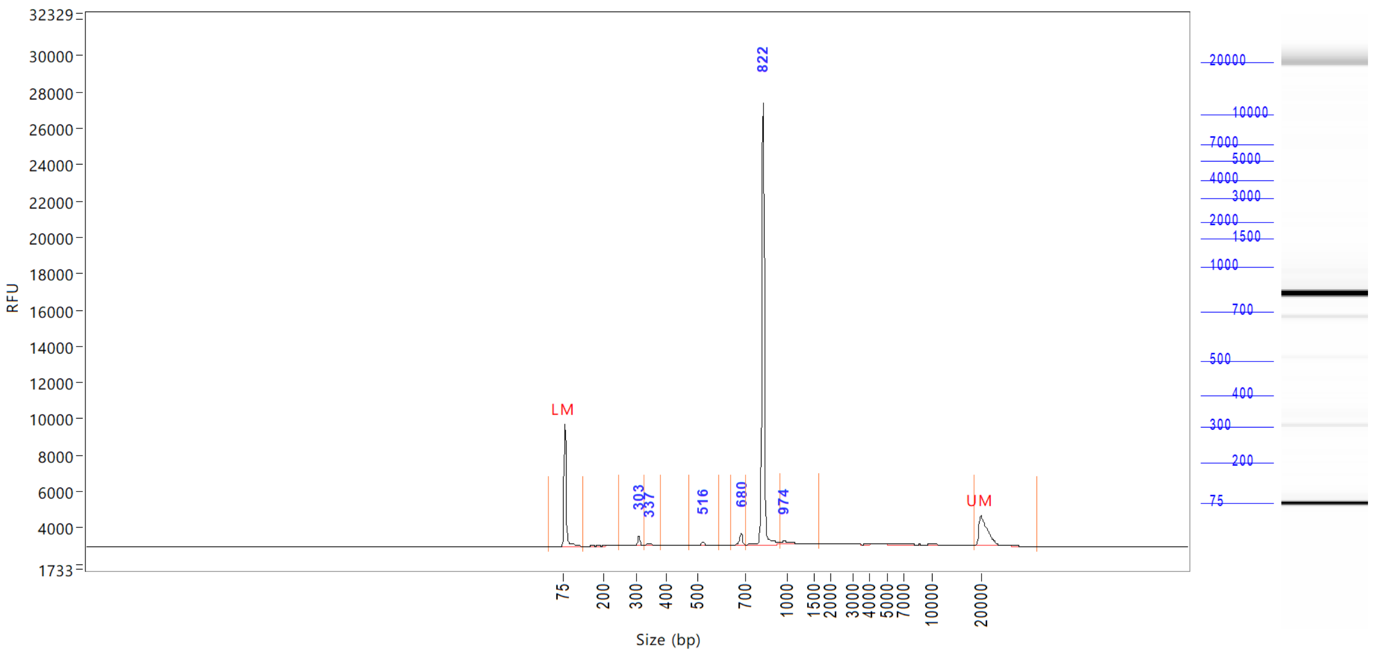

Peak	Size	Concentration	Relative concentration	Molarity	From	To	CV%	RFU
	(bp)	(ng/uL)	Percent	(nmole/L)	(bp)	(bp)		
1	75 (LM)	0.5000		11.0851	24	131	9.55	6710
2	303	0.5141	2.7	2.8285	242	321	4.02	500
3	337	0.2625	1.4	1.2699	321	380	3.50	117
4	516	0.1998	1.0	0.6410	471	585	2.82	215
5	680	0.5099	2.6	1.2431	639	700	1.20	638
6	822	17.2908	89.6	34.7048	700	948	2.25	24356
7	974	0.5110	2.6	0.7935	948	1616	11.47	194
8	20000 (UM)	0.2336		0.0185	18781	31486	5.75	1633

TIC:	19.2881	ng/uL
TIM:	41.4809	nmole/L
Total concentration:	19.7280	ng/uL

Sample peak width (sec): 5 Sample min peak height: 50 Sample baseline V to V?: Y Sample baseline V to V points: 3
Sample filter: Binomial Number of points for filter: 3 Sample start region (min): 0 Sample end region (min): 30
Marker peak width (sec): 5 Marker min peak height: 200 Marker baseline V to V?: Y Marker baseline V to V points: 3
Lower marker selection: First peak > 500 RFU Upper marker selection: Last peak > 200 RFU
Ladder size (bp)75, 200, 300, 400, 500, 700, 1000, 1500, 2000, 3000, 4000, 5000, 7000, 10000, 20000
Quantification using: Lower Marker Final concentration (ng/uL): 0.5000 Dilution factor: 12.0

Sample: SampG6
Well location: G6
Created: Thursday, October 21, 2021 6:48:20 PM

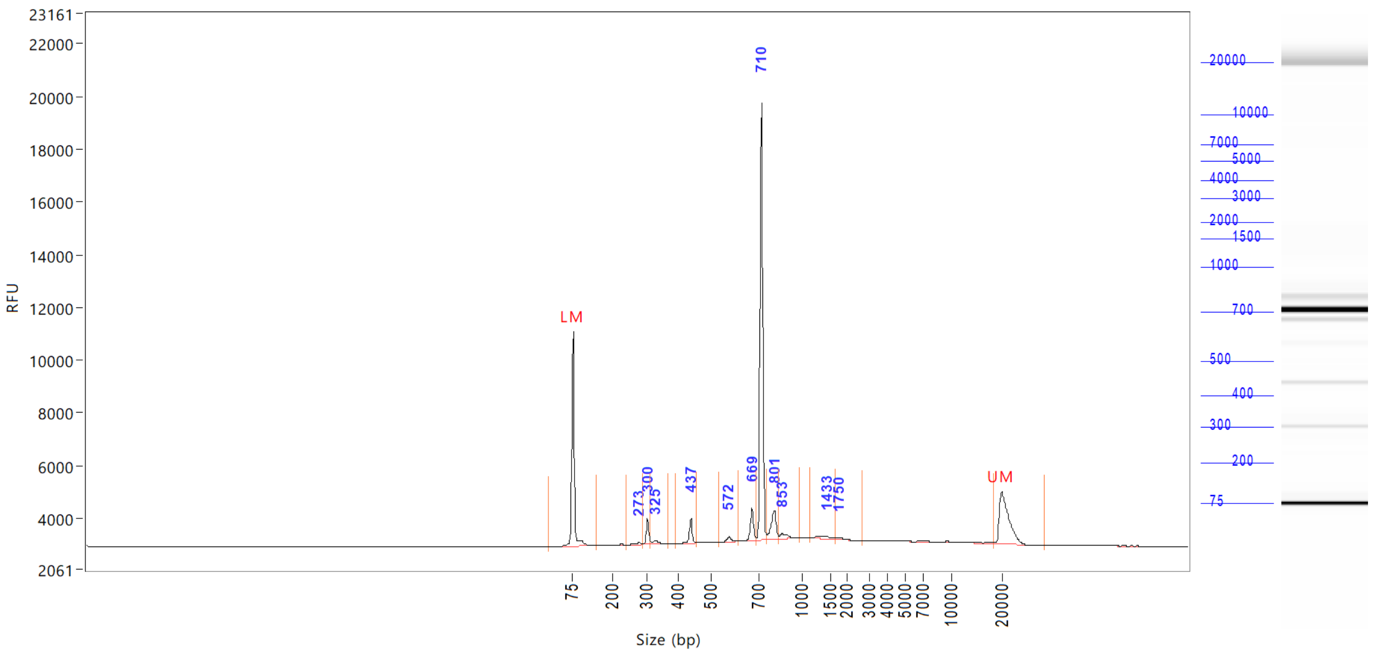

Peak	Size	Concentration	Relative concentration	Molarity	From	To	CV%	RFU
	(bp)	(ng/uL)	Percent	(nmole/L)	(bp)	(bp)		
1	75 (LM)	0.5000		11.0851	3	148	11.75	8146
2	273	0.1248	0.9	0.7659	237	287	4.07	66
3	300	0.6312	4.3	3.4844	287	309	1.25	987
4	325	0.2224	1.5	1.1222	309	366	3.73	108
5	437	0.6016	4.1	2.2808	394	454	1.44	916
6	572	0.1953	1.3	0.5609	532	613	1.88	195
7	669	0.8426	5.8	2.0852	613	687	1.33	1247
8	710	10.0470	69.1	23.3544	687	753	1.25	16618
9	801	1.1951	8.2	2.4835	753	835	2.23	1100
10	853	0.3104	2.1	0.5845	835	987	3.29	194
11	1433	0.2482	1.7	0.2973	1145	1635	9.11	75
12	1750	0.1110	0.8	0.1003	1635	2715	10.01	73
13	20000 (UM)	0.2385		0.0190	18415	28584	5.06	1969

TIC:	14.5296	ng/uL
TIM:	37.1194	nmole/L
Total concentration:	14.9287	ng/uL

```
Sample peak width (sec): 5    Sample min peak height: 50    Sample baseline V to V?: Y    Sample baseline V to V points: 3
Sample filter: Binomial       Number of points for filter: 3  Sample start region (min): 0  Sample end region (min): 30
Marker peak width (sec): 5    Marker min peak height: 200   Marker baseline V to V?: Y    Marker baseline V to V points: 3
Lower marker selection: First peak > 500 RFU                Upper marker selection: Last peak > 200 RFU
Ladder size (bp)75, 200, 300, 400, 500, 700, 1000, 1500, 2000, 3000, 4000, 5000, 7000, 10000, 20000
Quantification using: Lower Marker          Final concentration (ng/uL): 0.5000          Dilution factor: 12.0
```

Sample: SampH6
Well location: H6
Created: Thursday, October 21, 2021 6:48:20 PM

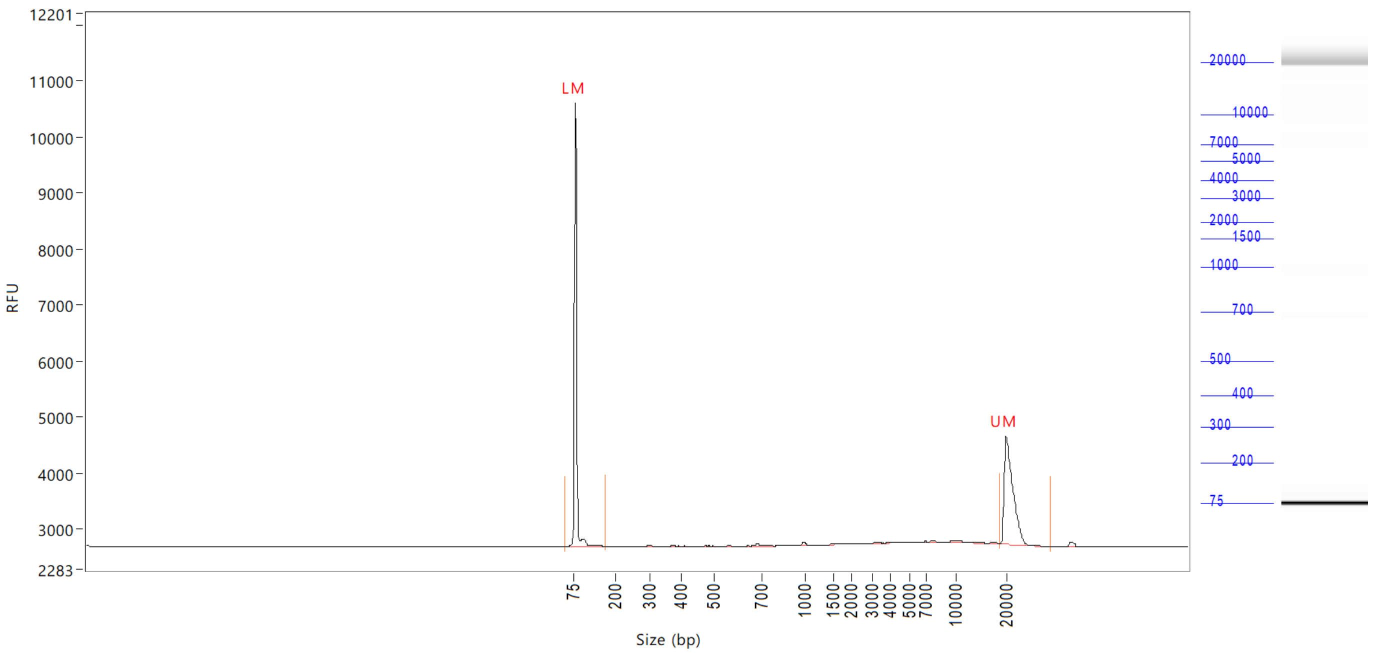

Peak	Size	Concentration	Relative concentration	Molarity	From	To	CV%	RFU
	(bp)	(ng/uL)	Percent	(nmole/L)	(bp)	(bp)		
1	75 (LM)	0.5000		11.0851	45	171	9.65	7926
2	20000 (UM)	0.2438		0.0194	18781	28584	4.99	1940

TIC:	0.0000	ng/uL
TIM:	0.0000	nmole/L
Total concentration:	0.5570	ng/uL

Sample peak width (sec): 5 Sample min peak height: 50 Sample baseline V to V?: Y Sample baseline V to V points: 3
Sample filter: Binomial Number of points for filter: 3 Sample start region (min): 0 Sample end region (min): 30
Marker peak width (sec): 5 Marker min peak height: 200 Marker baseline V to V?: Y Marker baseline V to V points: 3
Lower marker selection: First peak > 500 RFU Upper marker selection: Last peak > 200 RFU
Ladder size (bp)75, 200, 300, 400, 500, 700, 1000, 1500, 2000, 3000, 4000, 5000, 7000, 10000, 20000
Quantification using: Lower Marker Final concentration (ng/uL): 0.5000 Dilution factor: 12.0

Sample: SampA7
Well location: A7
Created: Thursday, October 21, 2021 6:48:20 PM

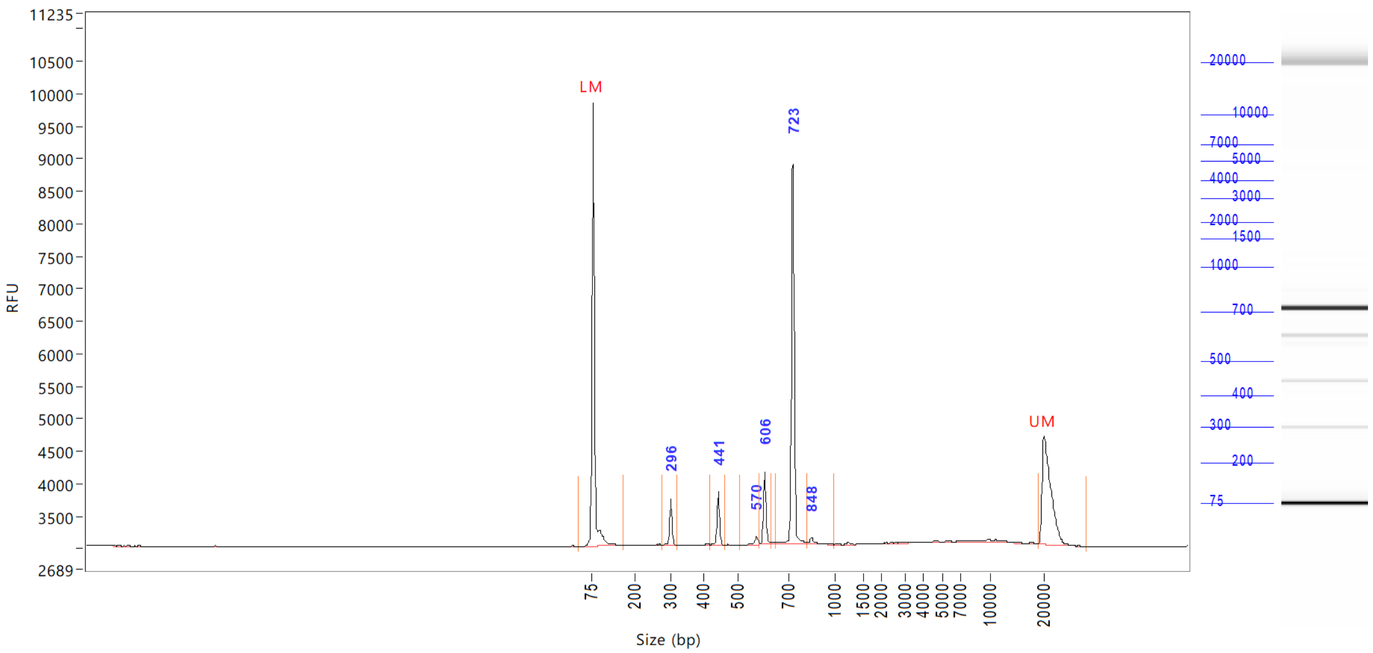

Peak	Size	Concentration	Relative concentration	Molarity	From	To	CV%	RFU
	(bp)	(ng/uL)	Percent	(nmole/L)	(bp)	(bp)		
1	75 (LM)	0.5000		10.9378	33	162	11.75	6825
2	296	0.5318	8.5	2.9650	273	315	1.63	721
3	441	0.5823	9.4	2.1824	418	464	1.05	815
4	570	0.1168	1.9	0.3403	506	585	2.01	127
5	606	0.7720	12.4	2.1104	585	631	1.02	1120
6	723	4.1003	65.9	9.4116	649	822	2.01	5847
7	848	0.1199	1.9	0.2292	822	1000	3.72	103
8	20000 (UM)	0.2234		0.0178	18903	27979	4.84	1673

TIC:	6.2231	ng/uL
TIM:	17.2389	nmole/L
Total concentration:	6.7882	ng/uL

Sample peak width (sec): 5 Sample min peak height: 50 Sample baseline V to V?: Y Sample baseline V to V points: 3
Sample filter: Binomial Number of points for filter: 3 Sample start region (min): 0 Sample end region (min): 30
Marker peak width (sec): 5 Marker min peak height: 200 Marker baseline V to V?: Y Marker baseline V to V points: 3
Lower marker selection: First peak > 500 RFU Upper marker selection: Last peak > 200 RFU
Ladder size (bp)75, 200, 300, 400, 500, 700, 1000, 1500, 2000, 3000, 4000, 5000, 7000, 10000, 20000
Quantification using: Lower Marker Final concentration (ng/uL): 0.5000 Dilution factor: 12.0

Sample: SampB7
Well location: B7
Created: Thursday, October 21, 2021 6:48:20 PM

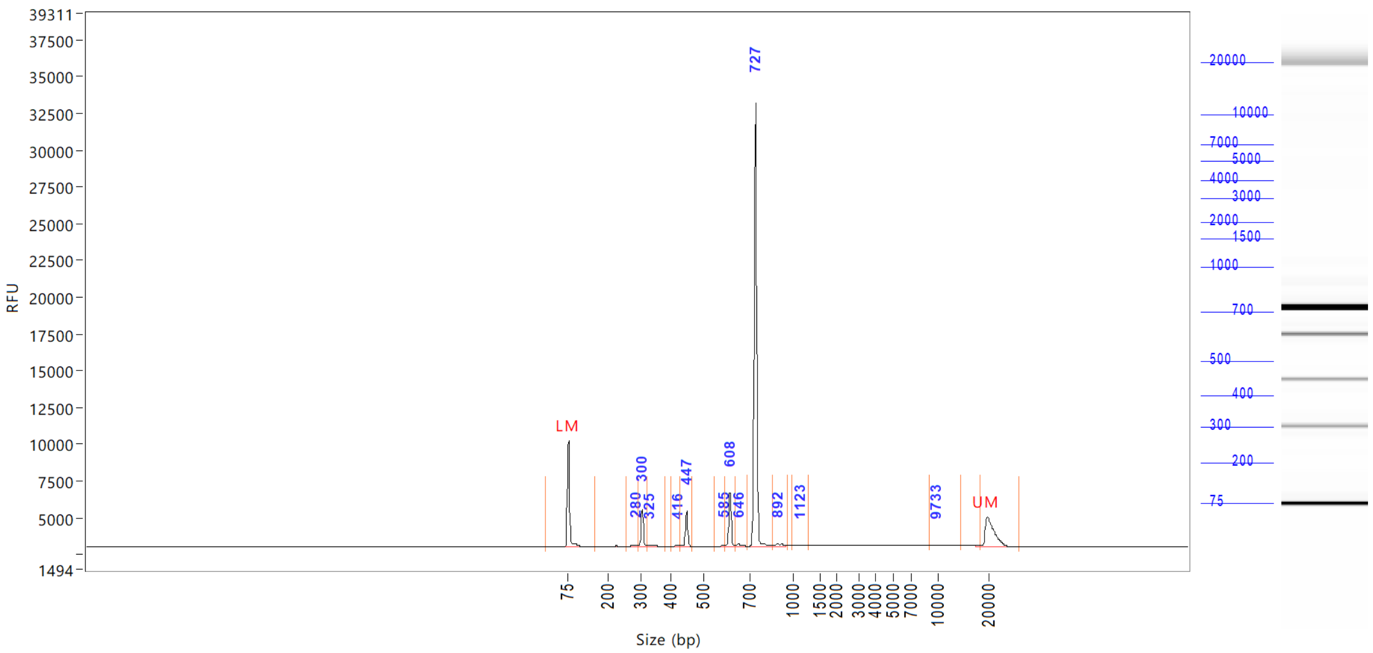

Peak	Size	Concentration	Relative concentration	Molarity	From	To	CV%	RFU
	(bp)	(ng/uL)	Percent	(nmole/L)	(bp)	(bp)		
1	75 (LM)	0.5000		11.0851	3	160	13.53	7243
2	280	0.1637	0.6	0.9721	253	289	2.66	112
3	300	1.7565	6.9	9.6635	289	317	1.32	2548
4	325	0.1390	0.5	0.6828	317	380	4.18	60
5	416	0.0531	0.2	0.2112	396	426	1.70	52
6	447	1.5455	6.0	5.7147	426	466	0.88	2420
7	585	0.1164	0.5	0.3297	549	593	1.39	120
8	608	2.3708	9.3	6.4276	593	634	0.90	3613
9	646	0.1824	0.7	0.4618	634	687	1.62	143
10	727	18.7337	73.2	42.5262	687	861	1.68	30199
11	892	0.2251	0.9	0.4116	861	961	2.27	166
12	1123	0.0837	0.3	0.1263	996	1289	4.93	55
13	9733	0.2185	0.9	0.0314	9000	14635	15.07	55
14	20000 (UM)	0.2531		0.0202	18537	26287	4.72	1951

TIC:	25.5886	ng/uL
TIM:	67.5588	nmole/L
Total concentration:	25.9968	ng/uL

Sample peak width (sec): 5 Sample min peak height: 50 Sample baseline V to V?: Y Sample baseline V to V points: 3
Sample filter: Binomial Number of points for filter: 3 Sample start region (min): 0 Sample end region (min): 30
Marker peak width (sec): 5 Marker min peak height: 200 Marker baseline V to V?: Y Marker baseline V to V points: 3
Lower marker selection: First peak > 500 RFU Upper marker selection: Last peak > 200 RFU
Ladder size (bp)75, 200, 300, 400, 500, 700, 1000, 1500, 2000, 3000, 4000, 5000, 7000, 10000, 20000
Quantification using: Lower Marker Final concentration (ng/uL): 0.5000 Dilution factor: 12.0

Sample: SampC7
Well location: C7
Created: Thursday, October 21, 2021 6:48:20 PM

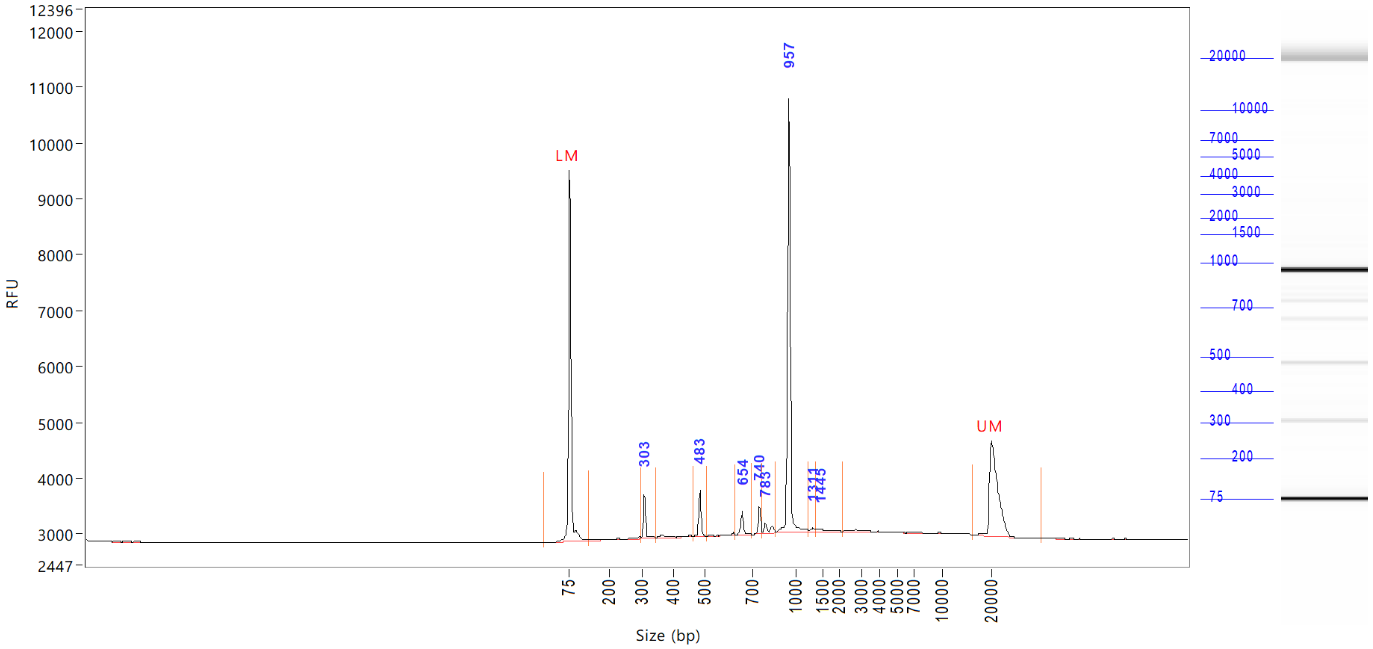

Peak	Size (bp)	Concentration (ng/uL)	Relative concentration Percent	Molarity (nmole/L)	From (bp)	To (bp)	CV%	RFU
1	75 (LM)	0.5000		11.0851	0	135	10.98	6645
2	303	0.6524	8.1	3.5184	295	341	2.67	796
3	483	0.5910	7.4	2.0218	462	504	0.95	822
4	654	0.3177	4.0	0.8018	629	695	1.24	426
5	740	0.3191	4.0	0.7117	695	762	1.17	486
6	783	0.2515	3.1	0.5162	762	857	3.21	184
7	957	5.6616	70.7	9.7678	857	1234	3.21	7773
8	1311	0.0698	0.9	0.0887	1234	1356	2.64	67
9	1445	0.1457	1.8	0.1509	1356	2115	12.61	53
10	20000 (UM)	0.2364		0.0189	16098	30035	5.62	1717

TIC:	8.0089	ng/uL
TIM:	17.5773	nmole/L
Total concentration:	8.6431	ng/uL

Sample peak width (sec): 5   Sample min peak height: 50   Sample baseline V to V?: Y   Sample baseline V to V points: 3
Sample filter: Binomial   Number of points for filter: 3   Sample start region (min): 0   Sample end region (min): 30
Marker peak width (sec): 5   Marker min peak height: 200   Marker baseline V to V?: Y   Marker baseline V to V points: 3
Lower marker selection: First peak > 500 RFU   Upper marker selection: Last peak > 200 RFU
Ladder size (bp)75, 200, 300, 400, 500, 700, 1000, 1500, 2000, 3000, 4000, 5000, 7000, 10000, 20000
Quantification using: Lower Marker   Final concentration (ng/uL): 0.5000   Dilution factor: 12.0

Sample: SampD7
Well location: D7
Created: Thursday, October 21, 2021 6:48:20 PM

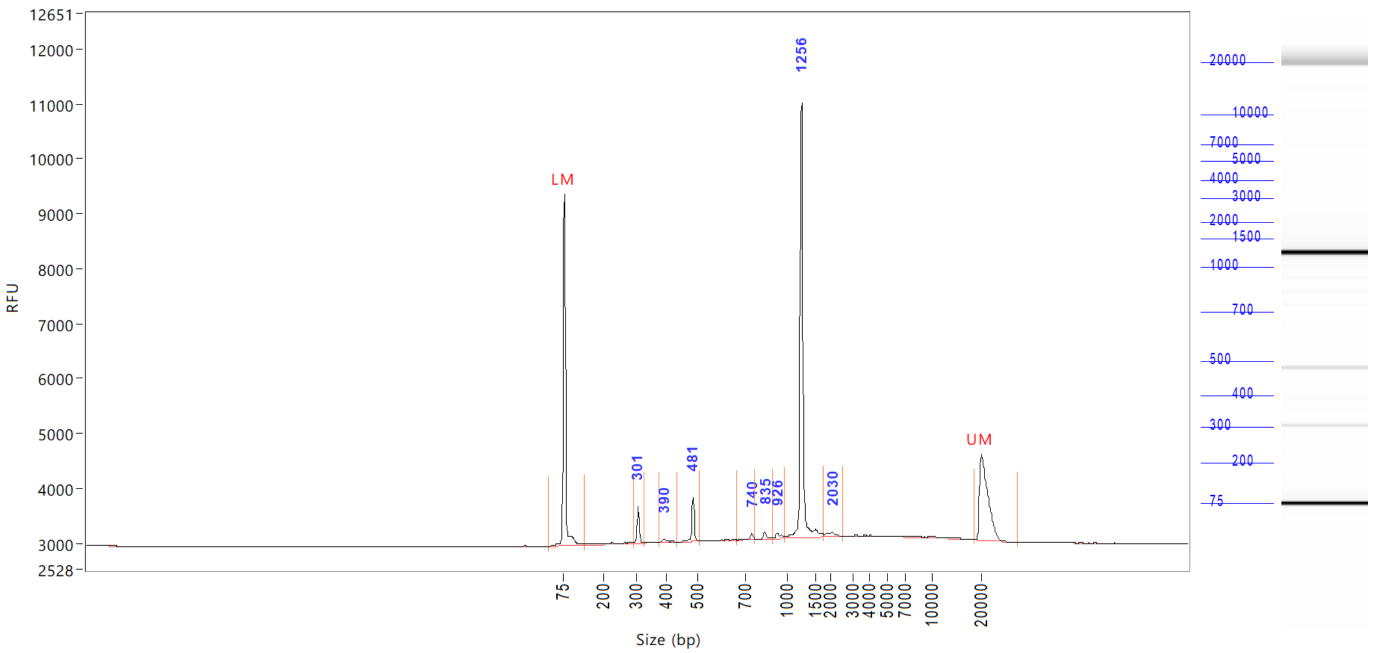

Peak	Size	Concentration	Relative concentration	Molarity	From	To	CV%	RFU
	(bp)	(ng/uL)	Percent	(nmole/L)	(bp)	(bp)		
1	75 (LM)	0.5000		11.0851	26	139	11.63	6393
2	301	0.5300	6.6	2.8966	291	325	1.53	669
3	390	0.1089	1.3	0.4547	374	431	3.06	59
4	481	0.6384	7.9	2.1931	431	504	1.50	801
5	740	0.0969	1.2	0.2184	664	766	2.71	123
6	835	0.1307	1.6	0.2583	766	887	2.11	144
7	926	0.1430	1.8	0.2515	887	978	2.29	108
8	1256	6.2610	77.6	8.2183	978	1789	6.43	7931
9	2030	0.1601	2.0	0.1319	1789	2515	7.17	78
10	20000 (UM)	0.2233		0.0178	18537	27133	4.71	1581

TIC:	8.0691	ng/uL
TIM:	14.6228	nmole/L
Total concentration:	8.6350	ng/uL

```
Sample peak width (sec): 5    Sample min peak height: 50    Sample baseline V to V?: Y    Sample baseline V to V points: 3
Sample filter: Binomial       Number of points for filter: 3  Sample start region (min): 0  Sample end region (min): 30
Marker peak width (sec): 5    Marker min peak height: 200   Marker baseline V to V?: Y    Marker baseline V to V points: 3
Lower marker selection: First peak > 500 RFU                Upper marker selection: Last peak > 200 RFU
Ladder size (bp)75, 200, 300, 400, 500, 700, 1000, 1500, 2000, 3000, 4000, 5000, 7000, 10000, 20000
Quantification using: Lower Marker          Final concentration (ng/uL): 0.5000          Dilution factor: 12.0
```

Sample: SampE7
Well location: E7
Created: Thursday, October 21, 2021 6:48:20 PM

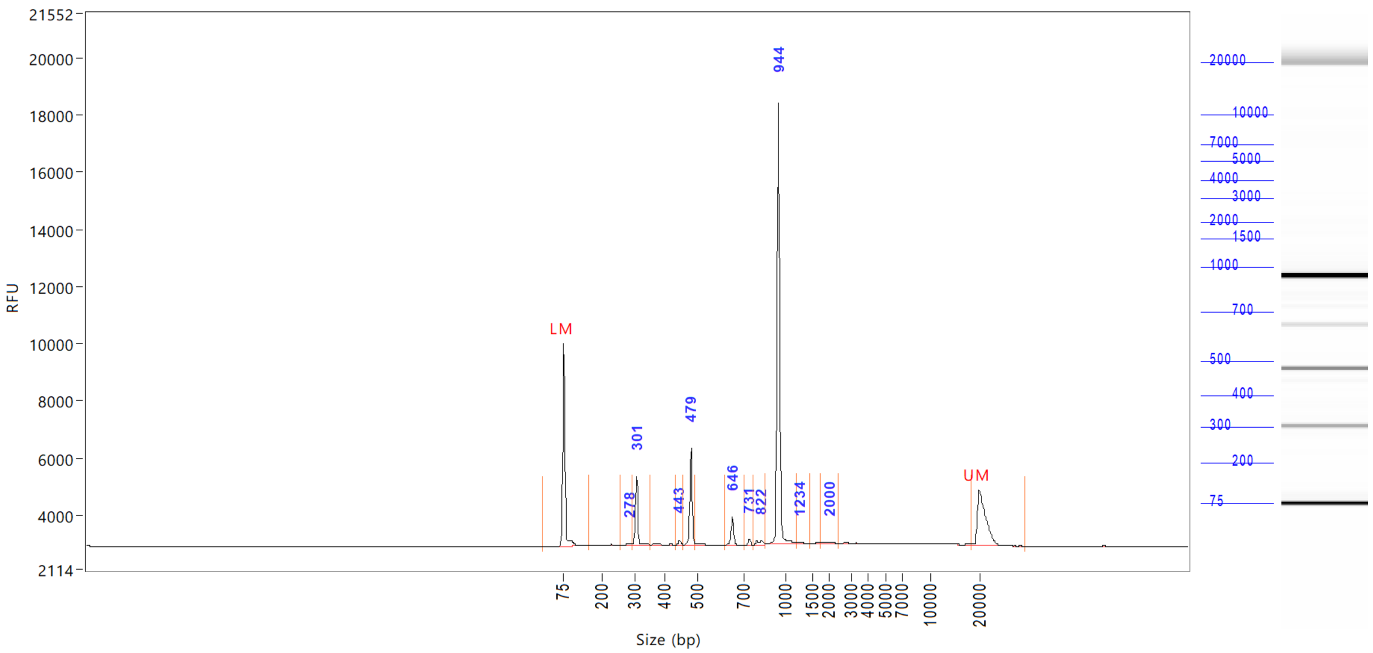

Peak	Size	Concentration	Relative concentration	Molarity	From	To	CV%	RFU
	(bp)	(ng/uL)	Percent	(nmole/L)	(bp)	(bp)		
1	75 (LM)	0.5000		11.0851	11	154	10.38	7078
2	278	0.1303	0.8	0.7736	253	289	2.92	72
3	301	1.7826	11.4	9.7744	289	347	2.47	2394
4	443	0.1325	0.8	0.4943	431	456	1.08	164
5	479	2.2429	14.3	7.7372	456	494	0.84	3376
6	646	0.6924	4.4	1.7667	618	701	1.10	981
7	731	0.1177	0.8	0.2656	701	762	1.11	186
8	822	0.2117	1.4	0.4318	762	853	2.67	147
9	944	10.1545	64.9	17.7424	853	1178	2.25	15454
10	1234	0.0814	0.5	0.1058	1178	1456	5.42	60
11	2000	0.0930	0.6	0.0782	1731	2401	7.10	56
12	20000 (UM)	0.2536		0.0202	18415	29430	5.02	1915

TIC:	15.6390	ng/uL
TIM:	39.1700	nmole/L
Total concentration:	16.2037	ng/uL

```
Sample peak width (sec): 5    Sample min peak height: 50    Sample baseline V to V?: Y    Sample baseline V to V points: 3
Sample filter: Binomial       Number of points for filter: 3  Sample start region (min): 0  Sample end region (min): 30
Marker peak width (sec): 5    Marker min peak height: 200   Marker baseline V to V?: Y    Marker baseline V to V points: 3
Lower marker selection: First peak > 500 RFU                Upper marker selection: Last peak > 200 RFU
Ladder size (bp)75, 200, 300, 400, 500, 700, 1000, 1500, 2000, 3000, 4000, 5000, 7000, 10000, 20000
Quantification using: Lower Marker          Final concentration (ng/uL): 0.5000          Dilution factor: 12.0
```

Sample: SampF7
Well location: F7
Created: Thursday, October 21, 2021 6:48:20 PM

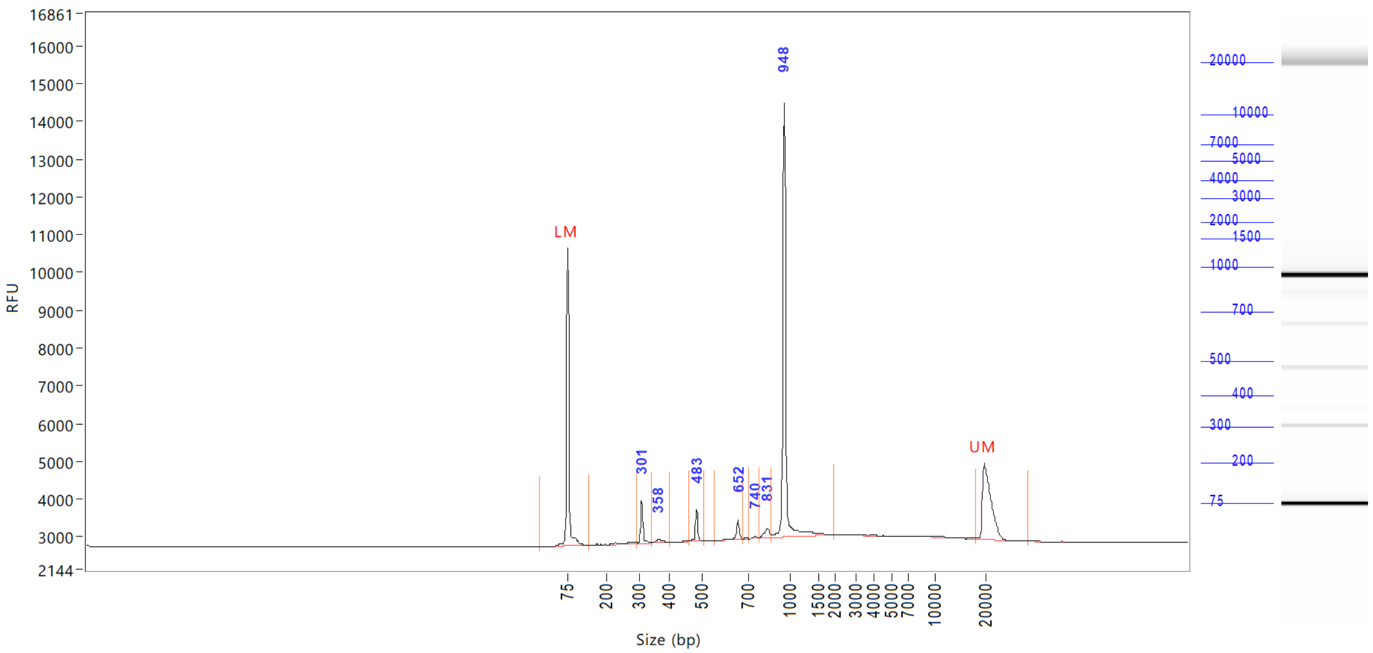

Peak	Size	Concentration	Relative concentration	Molarity	From	To	CV%	RFU
	(bp)	(ng/uL)	Percent	(nmole/L)	(bp)	(bp)		
1	75 (LM)	0.5000		11.2364	0	145	11.87	7890
2	301	0.7212	7.7	3.9284	291	339	2.24	1121
3	358	0.1394	1.5	0.6372	339	398	2.91	87
4	483	0.4876	5.2	1.6716	458	506	1.10	827
5	652	0.3156	3.4	0.8027	555	672	1.64	526
6	740	0.0426	0.5	0.0951	701	766	1.61	67
7	831	0.2820	3.0	0.5661	766	861	2.61	254
8	948	7.3816	78.8	12.5124	861	2001	10.27	11511
9	20000 (UM)	0.2277		0.0182	18171	28705	4.88	2028

TIC:	9.3701	ng/uL
TIM:	20.2133	nmole/L
Total concentration:	9.7559	ng/uL

```
Sample peak width (sec): 5    Sample min peak height: 50    Sample baseline V to V?: Y    Sample baseline V to V points: 3
Sample filter: Binomial       Number of points for filter: 3  Sample start region (min): 0  Sample end region (min): 30
Marker peak width (sec): 5    Marker min peak height: 200   Marker baseline V to V?: Y    Marker baseline V to V points: 3
Lower marker selection: First peak > 500 RFU                Upper marker selection: Last peak > 200 RFU
Ladder size (bp)75, 200, 300, 400, 500, 700, 1000, 1500, 2000, 3000, 4000, 5000, 7000, 10000, 20000
Quantification using: Lower Marker          Final concentration (ng/uL): 0.5000          Dilution factor: 12.0
```

Sample: SampG7
Well location: G7
Created: Thursday, October 21, 2021 6:48:20 PM

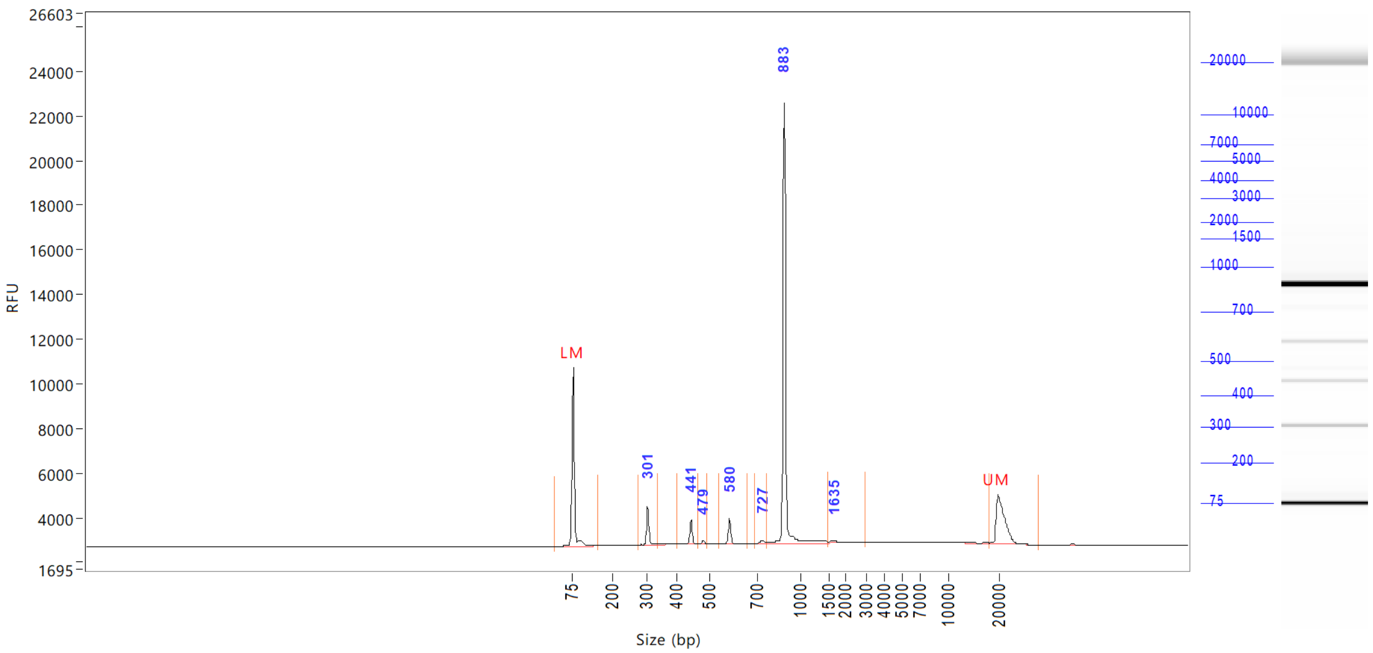

Peak	Size	Concentration	Relative concentration	Molarity	From	To	CV%	RFU
	(bp)	(ng/uL)	Percent	(nmole/L)	(bp)	(bp)		
1	75 (LM)	0.5000		10.7944	22	152	13.90	8022
2	301	1.0633	7.3	5.8300	277	337	1.77	1736
3	441	0.6802	4.7	2.5554	398	466	1.63	1115
4	479	0.1006	0.7	0.3463	466	490	0.99	154
5	580	0.6888	4.7	1.9612	539	659	1.97	1146
6	727	0.1380	0.9	0.3137	687	762	2.09	155
7	883	11.6768	79.9	21.4493	762	1500	8.13	19765
8	1635	0.2658	1.8	0.2254	1500	2943	19.09	82
9	20000 (UM)	0.2387		0.0191	18293	27979	4.42	2190

TIC:	14.6135	ng/uL
TIM:	32.6813	nmole/L
Total concentration:	14.9782	ng/uL

```
Sample peak width (sec): 5    Sample min peak height: 50    Sample baseline V to V?: Y    Sample baseline V to V points: 3
Sample filter: Binomial       Number of points for filter: 3  Sample start region (min): 0  Sample end region (min): 30
Marker peak width (sec): 5    Marker min peak height: 200   Marker baseline V to V?: Y    Marker baseline V to V points: 3
Lower marker selection: First peak > 500 RFU                Upper marker selection: Last peak > 200 RFU
Ladder size (bp)75, 200, 300, 400, 500, 700, 1000, 1500, 2000, 3000, 4000, 5000, 7000, 10000, 20000
Quantification using: Lower Marker          Final concentration (ng/uL): 0.5000          Dilution factor: 12.0
```

Sample: SampH7
Well location: H7
Created: Thursday, October 21, 2021 6:48:20 PM

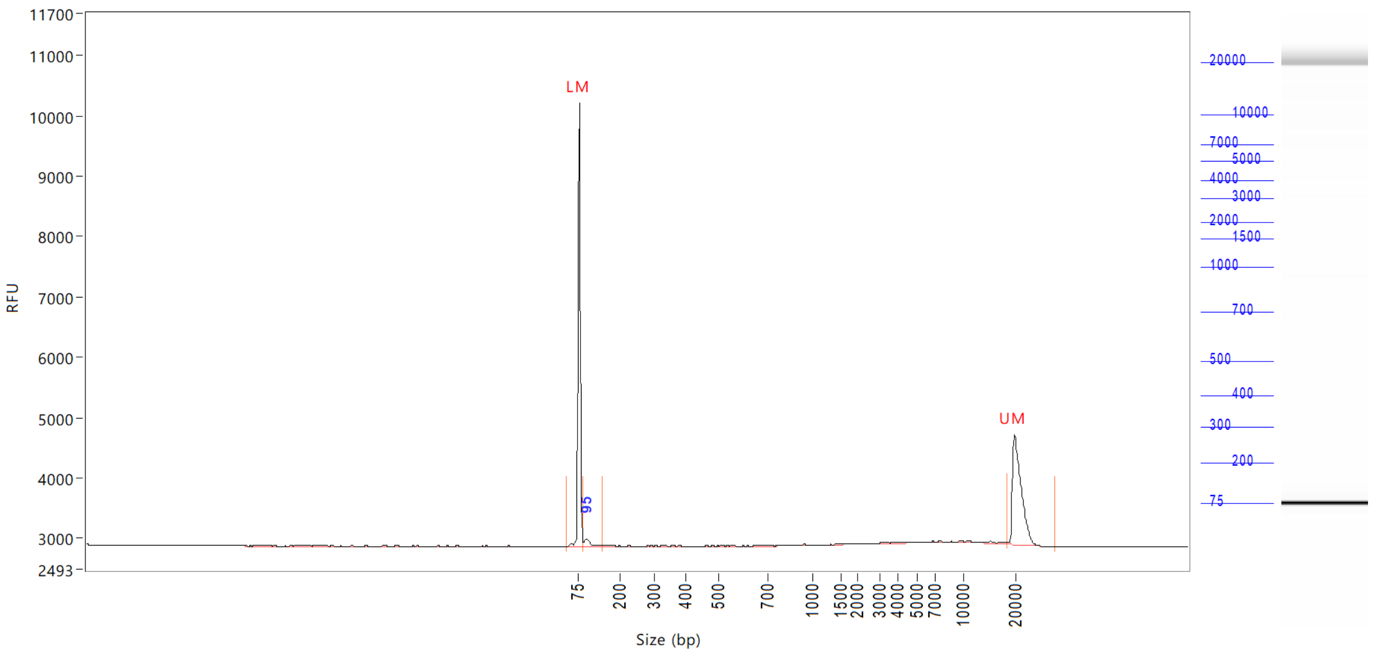

Peak	Size	Concentration	Relative concentration	Molarity	From	To	CV%	RFU
	(bp)	(ng/uL)	Percent	(nmole/L)	(bp)	(bp)		
1	75 (LM)	0.5000		11.2364	39	87	5.37	7356
2	95	0.2671	100.0	4.3004	87	145	12.33	125
3	20000 (UM)	0.2455		0.0196	18415	27859	4.64	1833

TIC:	0.2671	ng/uL
TIM:	4.3004	nmole/L
Total concentration:	0.9384	ng/uL

Sample peak width (sec): 5 Sample min peak height: 50 Sample baseline V to V?: Y Sample baseline V to V points: 3
Sample filter: Binomial Number of points for filter: 3 Sample start region (min): 0 Sample end region (min): 30
Marker peak width (sec): 5 Marker min peak height: 200 Marker baseline V to V?: Y Marker baseline V to V points: 3
Lower marker selection: First peak > 500 RFU Upper marker selection: Last peak > 200 RFU
Ladder size (bp)75, 200, 300, 400, 500, 700, 1000, 1500, 2000, 3000, 4000, 5000, 7000, 10000, 20000
Quantification using: Lower Marker Final concentration (ng/uL): 0.5000 Dilution factor: 12.0

Sample: SampA8
Well location: A8
Created: Thursday, October 21, 2021 6:48:20 PM

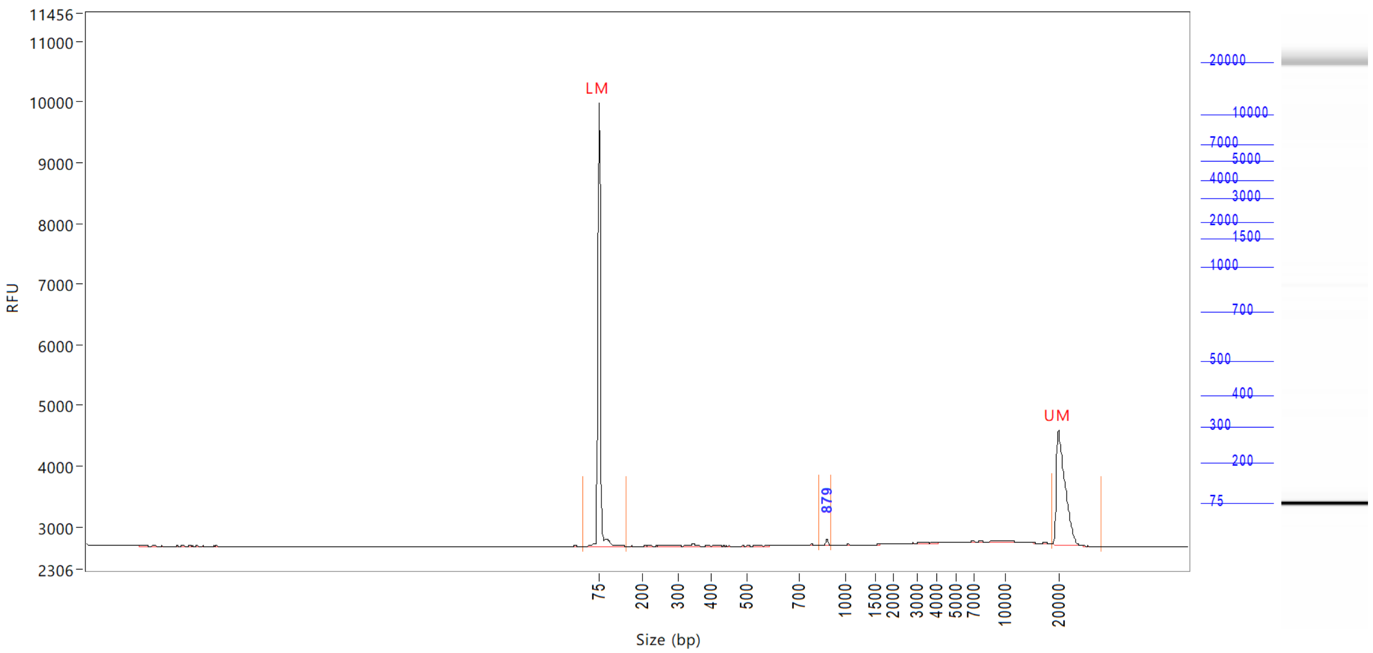

Peak	Size	Concentration	Relative concentration	Molarity	From	To	CV%	RFU
	(bp)	(ng/uL)	Percent	(nmole/L)	(bp)	(bp)		
1	75 (LM)	0.5000		11.2364	31	152	9.69	7313
2	879	0.0692	100.0	0.1304	831	905	1.10	108
3	20000 (UM)	0.2403		0.0193	18903	27859	4.38	1877

TIC:	0.0692	ng/uL
TIM:	0.1304	nmole/L
Total concentration:	0.7616	ng/uL

Sample peak width (sec): 5 Sample min peak height: 50 Sample baseline V to V?: Y Sample baseline V to V points: 3
Sample filter: Binomial Number of points for filter: 3 Sample start region (min): 0 Sample end region (min): 30
Marker peak width (sec): 5 Marker min peak height: 200 Marker baseline V to V?: Y Marker baseline V to V points: 3
Lower marker selection: First peak > 500 RFU Upper marker selection: Last peak > 200 RFU
Ladder size (bp)75, 200, 300, 400, 500, 700, 1000, 1500, 2000, 3000, 4000, 5000, 7000, 10000, 20000
Quantification using: Lower Marker Final concentration (ng/uL): 0.5000 Dilution factor: 12.0

Sample: SampB8
Well location: B8
Created: Thursday, October 21, 2021 6:48:20 PM

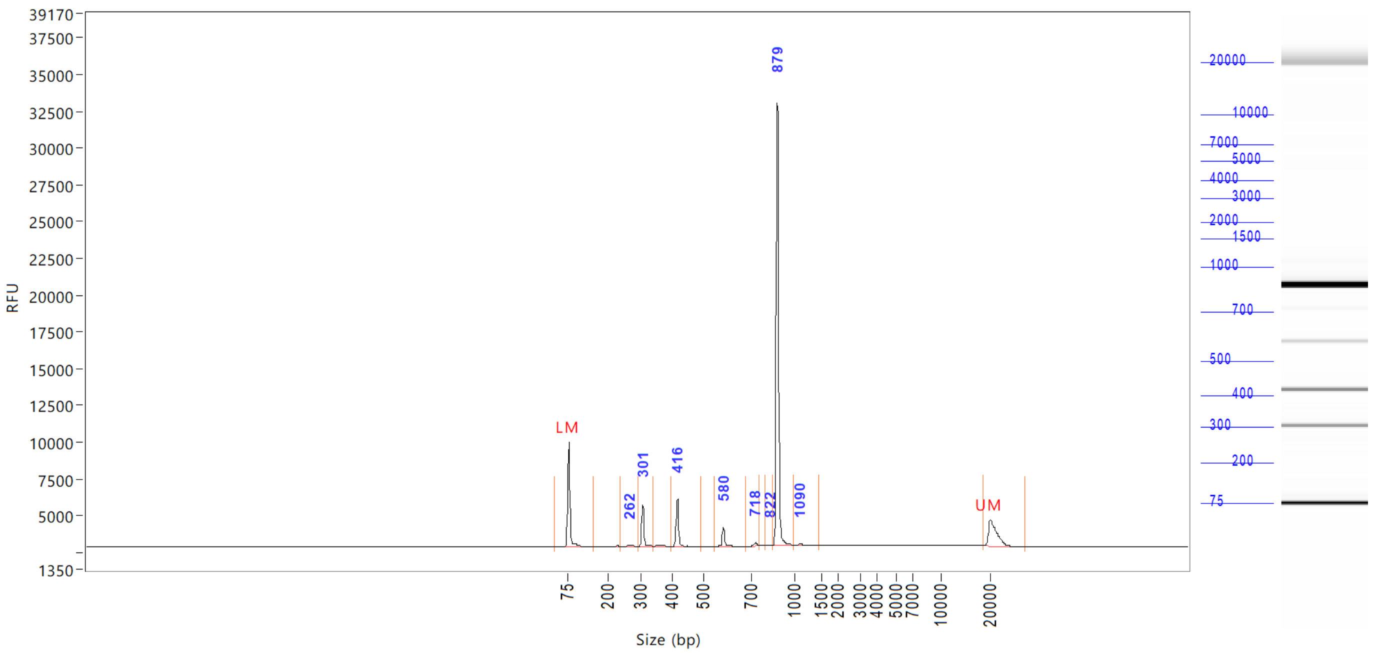

Peak	Size (bp)	Concentration (ng/uL)	Relative concentration Percent	Molarity (nmole/L)	From (bp)	To (bp)	CV%	RFU
1	75 (LM)	0.5000		10.7944	33	154	12.41	7066
2	262	0.1332	0.5	0.8331	237	289	4.31	61
3	301	2.0238	8.2	11.0597	289	337	1.70	2846
4	416	2.1851	8.9	8.6841	396	490	1.76	3225
5	580	0.8604	3.5	2.4455	544	674	1.63	1308
6	718	0.1675	0.7	0.3844	674	757	1.61	207
7	822	0.0739	0.3	0.1478	792	848	1.75	75
8	879	18.9360	77.0	35.4969	848	996	1.45	30177
9	1090	0.2173	0.9	0.3219	996	1456	8.54	134
10	20000 (UM)	0.2290		0.0182	18659	27133	4.62	1790

TIC:	24.5972	ng/uL
TIM:	59.3736	nmole/L
Total concentration:	25.1050	ng/uL

Sample peak width (sec): 5   Sample min peak height: 50   Sample baseline V to V?: Y   Sample baseline V to V points: 3
Sample filter: Binomial   Number of points for filter: 3 Sample start region (min): 0 Sample end region (min): 30
Marker peak width (sec): 5   Marker min peak height: 200   Marker baseline V to V?: Y   Marker baseline V to V points: 3
Lower marker selection: First peak > 500 RFU   Upper marker selection: Last peak > 200 RFU
Ladder size (bp)75, 200, 300, 400, 500, 700, 1000, 1500, 2000, 3000, 4000, 5000, 7000, 10000, 20000
Quantification using: Lower Marker   Final concentration (ng/uL): 0.5000   Dilution factor: 12.0

Sample: SampC8
Well location: C8
Created: Thursday, October 21, 2021 6:48:20 PM

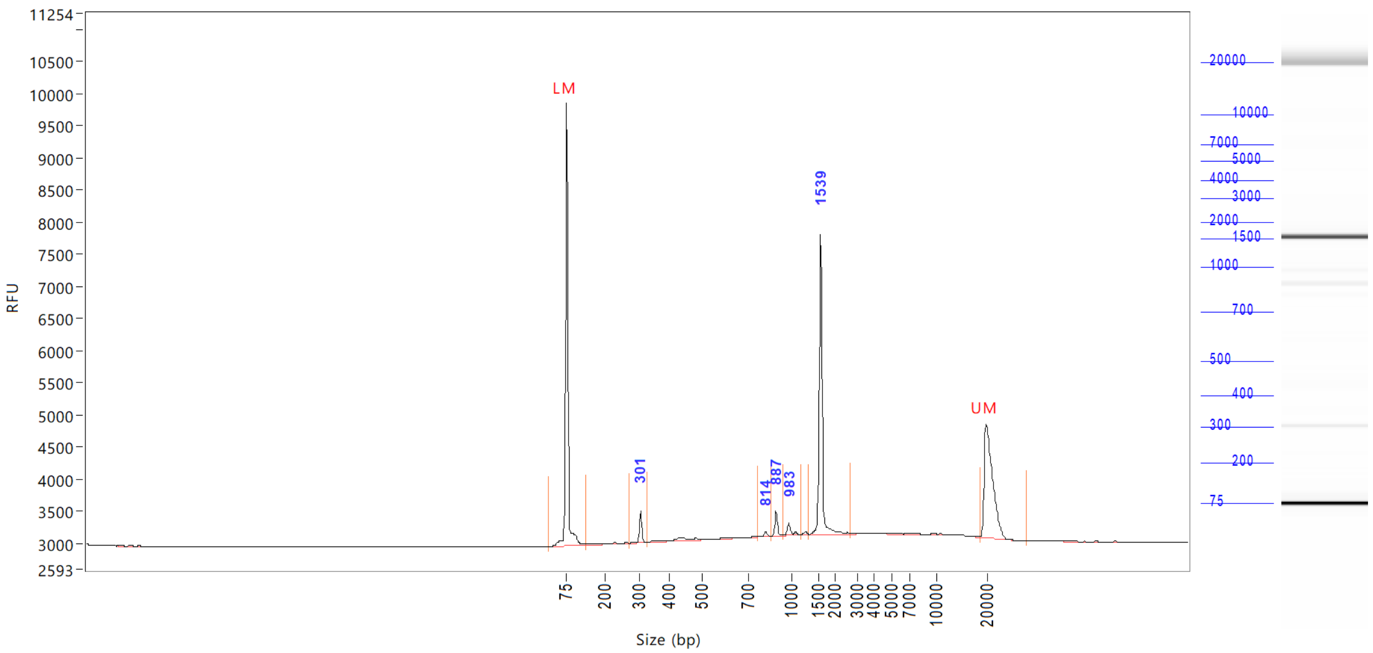

Peak	Size	Concentration	Relative concentration	Molarity	From	To	CV%	RFU
	(bp)	(ng/uL)	Percent	(nmole/L)	(bp)	(bp)		
1	75 (LM)	0.5000		11.0851	18	137	12.46	6913
2	301	0.3873	9.3	2.1308	268	327	2.18	507
3	814	0.0592	1.4	0.1204	762	857	1.80	70
4	887	0.2506	6.0	0.4645	857	939	1.41	386
5	983	0.1824	4.4	0.3008	939	1167	4.42	188
6	1539	3.2954	78.9	3.4862	1323	2743	9.25	4683
7	20000 (UM)	0.2258		0.0180	18781	28100	4.72	1773

TIC:	4.1749	ng/uL
TIM:	6.5027	nmole/L
Total concentration:	4.8898	ng/uL

Sample peak width (sec): 5   Sample min peak height: 50   Sample baseline V to V?: Y   Sample baseline V to V points: 3
Sample filter: Binomial   Number of points for filter: 3   Sample start region (min): 0   Sample end region (min): 30
Marker peak width (sec): 5   Marker min peak height: 200   Marker baseline V to V?: Y   Marker baseline V to V points: 3
Lower marker selection: First peak > 500 RFU   Upper marker selection: Last peak > 200 RFU
Ladder size (bp)75, 200, 300, 400, 500, 700, 1000, 1500, 2000, 3000, 4000, 5000, 7000, 10000, 20000
Quantification using: Lower Marker   Final concentration (ng/uL): 0.5000   Dilution factor: 12.0

Sample: SampD8
Well location: D8
Created: Thursday, October 21, 2021 6:48:20 PM

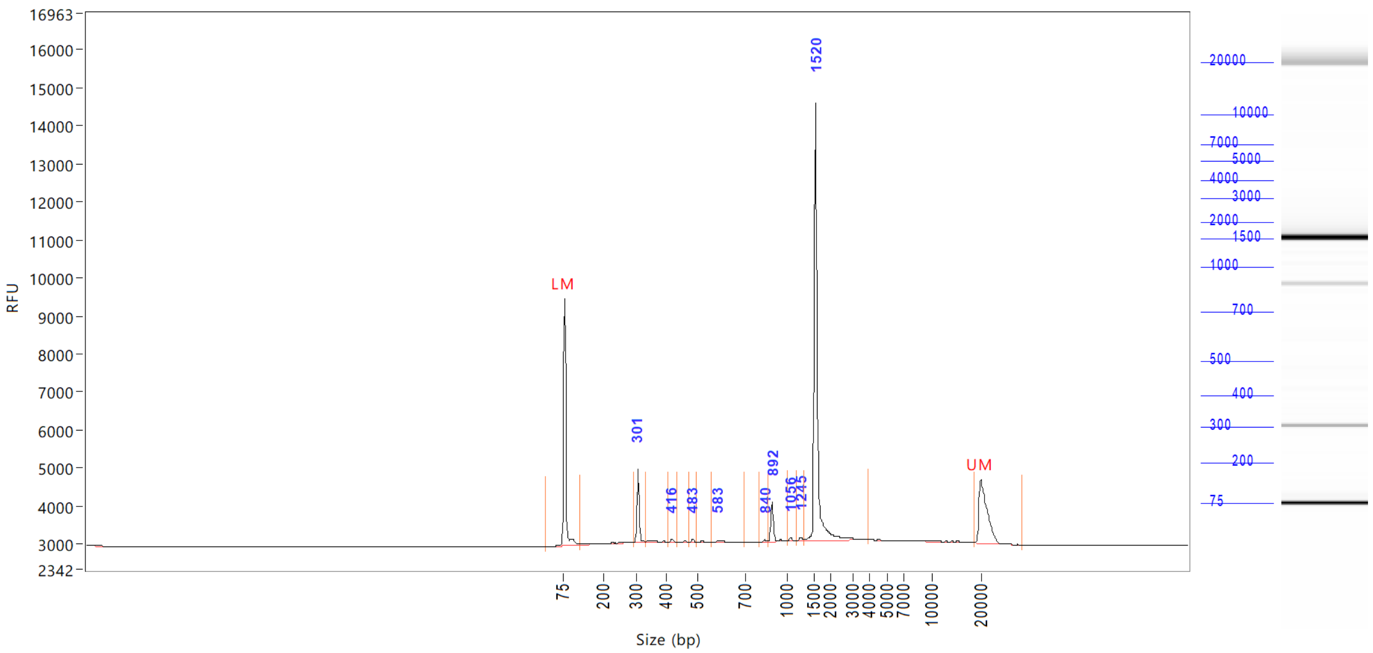

Peak	Size	Concentration	Relative concentration	Molarity	From	To	CV%	RFU
	(bp)	(ng/uL)	Percent	(nmole/L)	(bp)	(bp)		
1	75 (LM)	0.5000		10.9378	16	126	9.77	6489
2	301	1.4446	12.1	7.8945	291	329	1.57	1932
3	416	0.0649	0.5	0.2560	403	433	1.35	75
4	483	0.0670	0.6	0.2292	471	494	0.99	87
5	583	0.1063	0.9	0.2885	555	697	4.75	53
6	840	0.0452	0.4	0.0889	796	861	1.37	57
7	892	0.7922	6.6	1.4618	861	1001	2.54	1049
8	1056	0.0667	0.6	0.1046	1001	1156	2.24	95
9	1245	0.0793	0.7	0.1056	1156	1300	2.35	98
10	1520	9.2674	77.7	9.6796	1300	3893	14.30	11529
11	20000 (UM)	0.2302		0.0184	18781	28463	4.66	1666

TIC:	11.9336	ng/uL
TIM:	20.1087	nmole/L
Total concentration:	12.5937	ng/uL

Sample peak width (sec): 5 Sample min peak height: 50 Sample baseline V to V?: Y Sample baseline V to V points: 3
Sample filter: Binomial Number of points for filter: 3 Sample start region (min): 0 Sample end region (min): 30
Marker peak width (sec): 5 Marker min peak height: 200 Marker baseline V to V?: Y Marker baseline V to V points: 3
Lower marker selection: First peak > 500 RFU Upper marker selection: Last peak > 200 RFU
Ladder size (bp)75, 200, 300, 400, 500, 700, 1000, 1500, 2000, 3000, 4000, 5000, 7000, 10000, 20000
Quantification using: Lower Marker Final concentration (ng/uL): 0.5000 Dilution factor: 12.0

Sample: SampE8
Well location: E8
Created: Thursday, October 21, 2021 6:48:20 PM

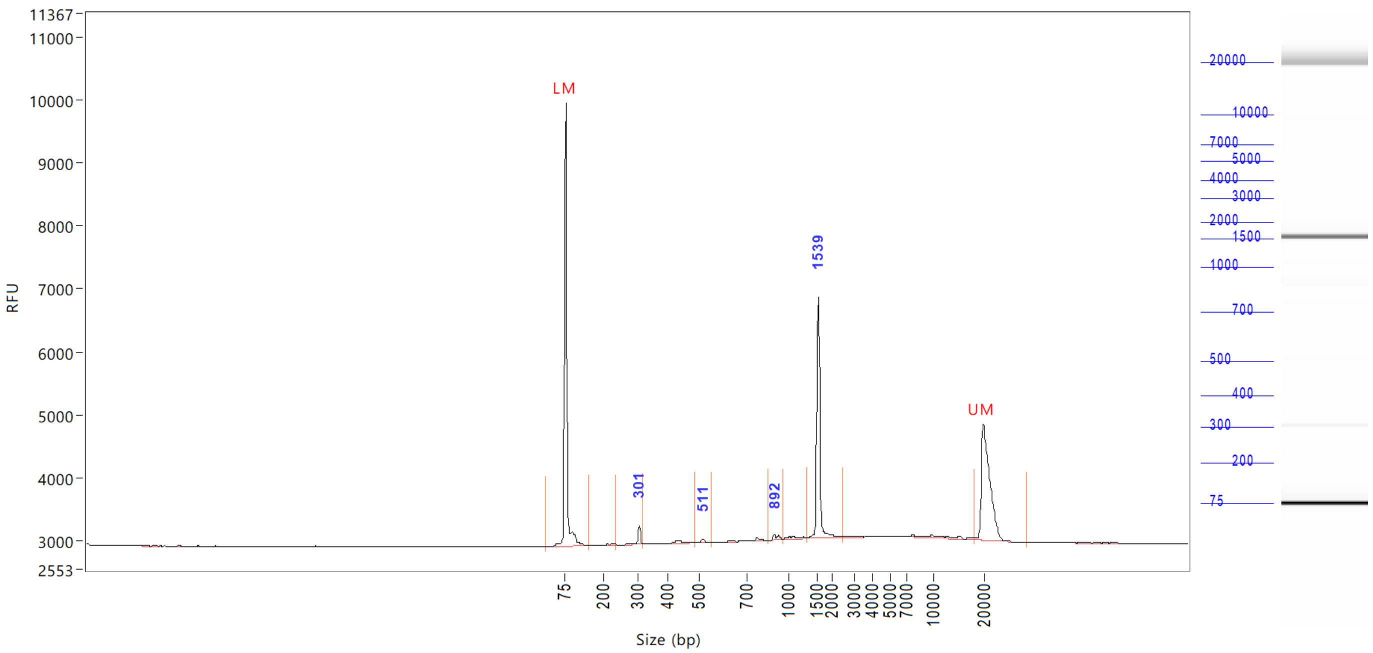

Peak	Size	Concentration	Relative concentration	Molarity	From	To	CV%	RFU
	(bp)	(ng/uL)	Percent	(nmole/L)	(bp)	(bp)		
1	75 (LM)	0.5000		10.9378	12	150	12.86	7040
2	301	0.2346	7.8	1.2950	235	313	2.94	301
3	511	0.0580	1.9	0.1874	483	549	2.19	62
4	892	0.1079	3.6	0.1961	853	957	2.24	87
5	1539	2.5943	86.6	2.7480	1323	2486	7.71	3808
6	20000 (UM)	0.2374		0.0189	18293	28705	4.84	1848

TIC:	2.9948	ng/uL
TIM:	4.4265	nmole/L
Total concentration:	3.7746	ng/uL

```
Sample peak width (sec): 5    Sample min peak height: 50    Sample baseline V to V?: Y    Sample baseline V to V points: 3
Sample filter: Binomial       Number of points for filter: 3  Sample start region (min): 0  Sample end region (min): 30
Marker peak width (sec): 5    Marker min peak height: 200   Marker baseline V to V?: Y    Marker baseline V to V points: 3
Lower marker selection: First peak > 500 RFU                Upper marker selection: Last peak > 200 RFU
Ladder size (bp)75, 200, 300, 400, 500, 700, 1000, 1500, 2000, 3000, 4000, 5000, 7000, 10000, 20000
Quantification using: Lower Marker          Final concentration (ng/uL): 0.5000          Dilution factor: 12.0
```

Sample: SampF8
Well location: F8
Created: Thursday, October 21, 2021 6:48:20 PM

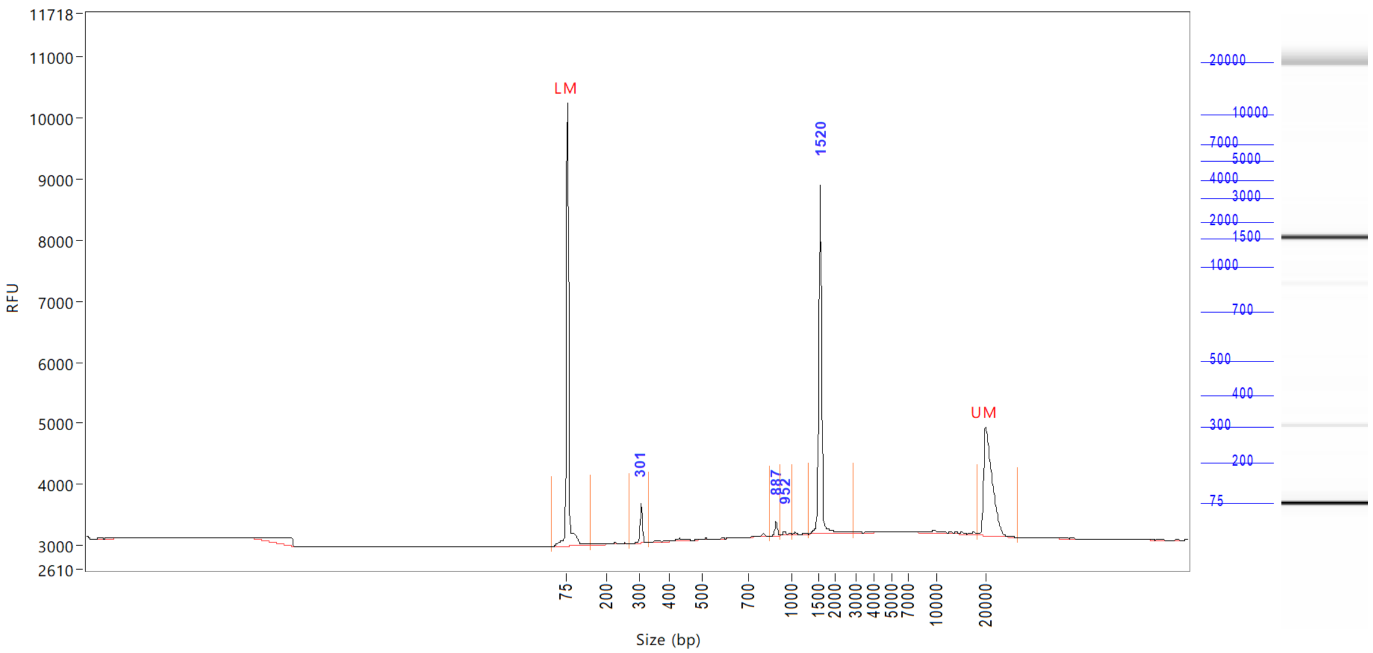

Peak	Size	Concentration	Relative concentration	Molarity	From	To	CV%	RFU
	(bp)	(ng/uL)	Percent	(nmole/L)	(bp)	(bp)		
1	75 (LM)	0.5000		10.9378	24	150	13.45	7273
2	301	0.4545	10.4	2.4837	269	329	1.71	649
3	887	0.1353	3.1	0.2514	844	918	1.12	227
4	952	0.0731	1.7	0.1252	918	1012	2.35	67
5	1520	3.6880	84.8	3.9497	1311	2857	8.60	5715
6	20000 (UM)	0.2194		0.0175	18293	26287	4.70	1790

TIC:	4.3509	ng/uL
TIM:	6.8101	nmole/L
Total concentration:	5.0233	ng/uL

```
Sample peak width (sec): 5    Sample min peak height: 50    Sample baseline V to V?: Y    Sample baseline V to V points: 3
Sample filter: Binomial       Number of points for filter: 3  Sample start region (min): 0  Sample end region (min): 30
Marker peak width (sec): 5    Marker min peak height: 200   Marker baseline V to V?: Y    Marker baseline V to V points: 3
Lower marker selection: First peak > 500 RFU                Upper marker selection: Last peak > 200 RFU
Ladder size (bp)75, 200, 300, 400, 500, 700, 1000, 1500, 2000, 3000, 4000, 5000, 7000, 10000, 20000
Quantification using: Lower Marker          Final concentration (ng/uL): 0.5000          Dilution factor: 12.0
```

Sample: SampG8
Well location: G8
Created: Thursday, October 21, 2021 6:48:20 PM

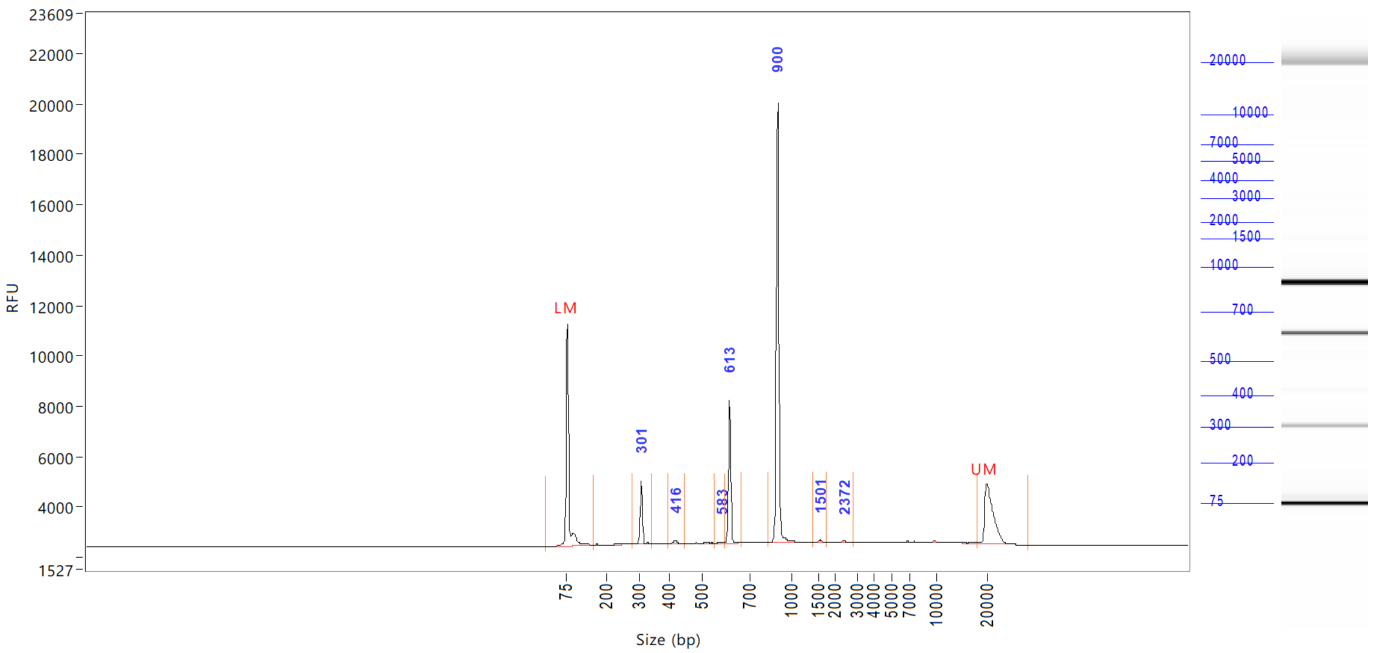

Peak	Size	Concentration	Relative concentration	Molarity	From	To	CV%	RFU
	(bp)	(ng/uL)	Percent	(nmole/L)	(bp)	(bp)		
1	75 (LM)	0.5000		10.7944	7	160	16.09	8846
2	301	1.3449	10.8	7.3742	277	337	1.88	2534
3	416	0.1166	0.9	0.4666	390	447	1.94	143
4	583	0.0595	0.5	0.1703	552	593	1.61	69
5	613	2.6055	20.9	7.0175	593	664	1.03	5690
6	900	8.2552	66.1	15.1303	835	1389	2.99	17493
7	1501	0.0501	0.4	0.0550	1389	1712	2.82	92
8	2372	0.0563	0.5	0.0400	1712	2857	11.42	50
9	20000 (UM)	0.2242		0.0178	18171	28221	4.63	2389

TIC:	12.4880	ng/uL
TIM:	30.2540	nmole/L
Total concentration:	12.9776	ng/uL

```
Sample peak width (sec): 5    Sample min peak height: 50    Sample baseline V to V?: Y    Sample baseline V to V points: 3
Sample filter: Binomial       Number of points for filter: 3  Sample start region (min): 0  Sample end region (min): 30
Marker peak width (sec): 5    Marker min peak height: 200   Marker baseline V to V?: Y    Marker baseline V to V points: 3
Lower marker selection: First peak > 500 RFU                Upper marker selection: Last peak > 200 RFU
Ladder size (bp)75, 200, 300, 400, 500, 700, 1000, 1500, 2000, 3000, 4000, 5000, 7000, 10000, 20000
Quantification using: Lower Marker          Final concentration (ng/uL): 0.5000          Dilution factor: 12.0
```

Sample: SampH8
Well location: H8
Created: Thursday, October 21, 2021 6:48:20 PM

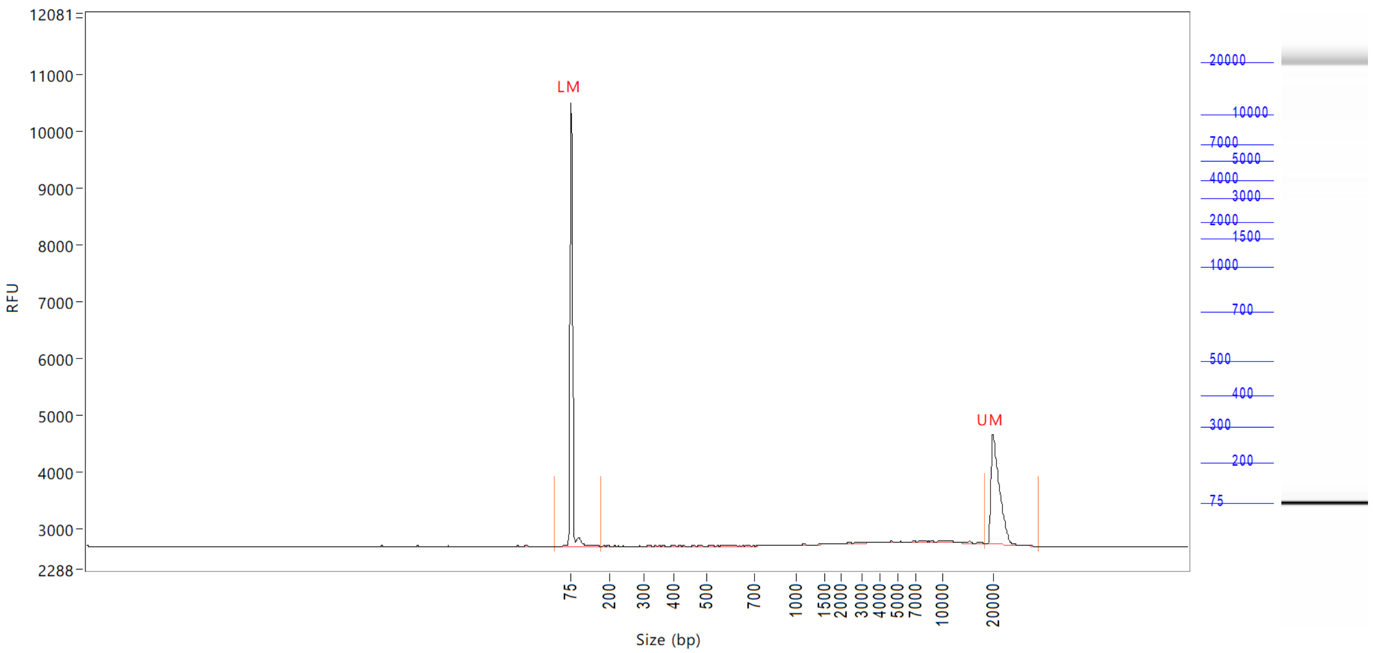

Peak	Size	Concentration	Relative concentration	Molarity	From	To	CV%	RFU
	(bp)	(ng/uL)	Percent	(nmole/L)	(bp)	(bp)		
1	75 (LM)	0.5000		10.9378	24	171	11.52	7823
2	20000 (UM)	0.2379		0.0189	18415	29309	4.79	1929

TIC:	0.0000	ng/uL
TIM:	0.0000	nmole/L
Total concentration:	0.4720	ng/uL

Sample peak width (sec): 5 Sample min peak height: 50 Sample baseline V to V?: Y Sample baseline V to V points: 3
Sample filter: Binomial Number of points for filter: 3 Sample start region (min): 0 Sample end region (min): 30
Marker peak width (sec): 5 Marker min peak height: 200 Marker baseline V to V?: Y Marker baseline V to V points: 3
Lower marker selection: First peak > 500 RFU Upper marker selection: Last peak > 200 RFU
Ladder size (bp)75, 200, 300, 400, 500, 700, 1000, 1500, 2000, 3000, 4000, 5000, 7000, 10000, 20000
Quantification using: Lower Marker Final concentration (ng/uL): 0.5000 Dilution factor: 12.0

Sample: SampA9
Well location: A9
Created: Thursday, October 21, 2021 6:48:20 PM

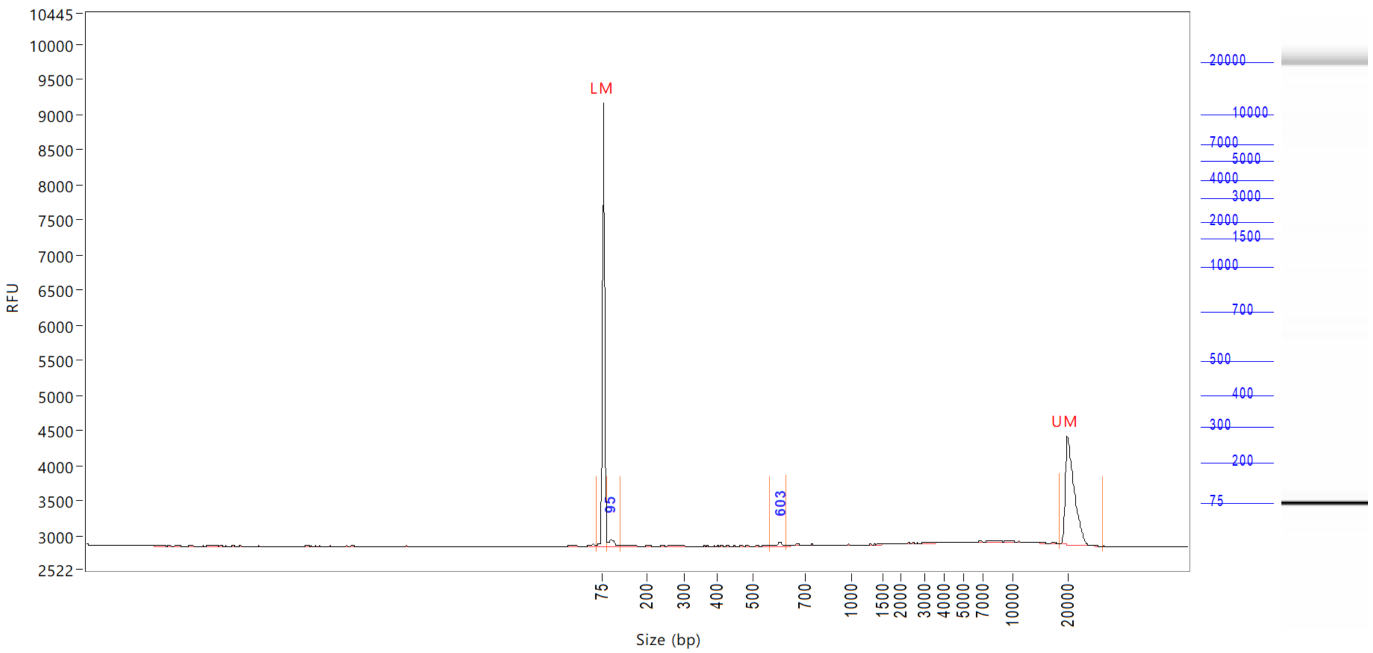

Peak	Size	Concentration	Relative concentration	Molarity	From	To	CV%	RFU
	(bp)	(ng/uL)	Percent	(nmole/L)	(bp)	(bp)		
1	75 (LM)	0.5000		11.2364	54	86	4.45	6330
2	95	0.2372	72.5	3.9750	86	126	8.91	93
3	603	0.0899	27.5	0.2471	565	631	2.38	60
4	20000 (UM)	0.2478		0.0198	18659	26408	4.47	1554

TIC:	0.3272	ng/uL
TIM:	4.2221	nmole/L
Total concentration:	1.0800	ng/uL

```
Sample peak width (sec): 5    Sample min peak height: 50    Sample baseline V to V?: Y    Sample baseline V to V points: 3
Sample filter: Binomial       Number of points for filter: 3  Sample start region (min): 0  Sample end region (min): 30
Marker peak width (sec): 5    Marker min peak height: 200   Marker baseline V to V?: Y    Marker baseline V to V points: 3
Lower marker selection: First peak > 500 RFU                Upper marker selection: Last peak > 200 RFU
Ladder size (bp)75, 200, 300, 400, 500, 700, 1000, 1500, 2000, 3000, 4000, 5000, 7000, 10000, 20000
Quantification using: Lower Marker          Final concentration (ng/uL): 0.5000          Dilution factor: 12.0
```

Sample: SampB9
Well location: B9
Created: Thursday, October 21, 2021 6:48:20 PM

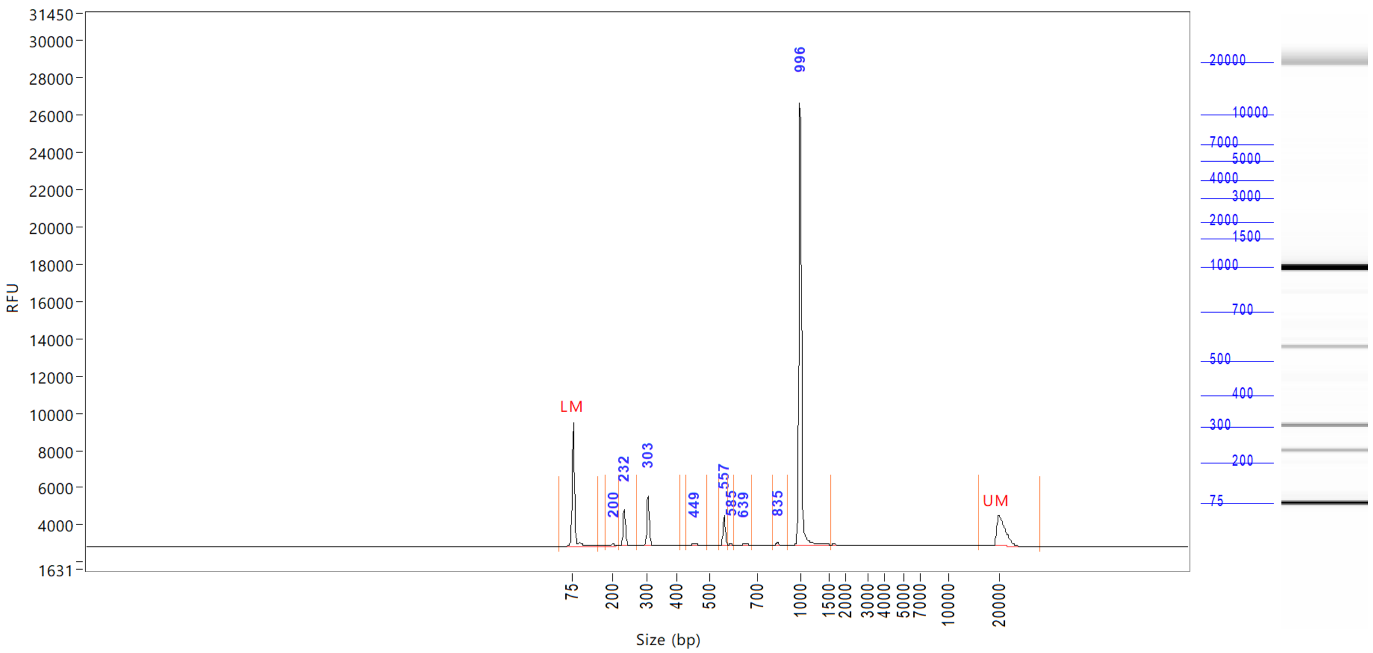

Peak	Size	Concentration	Relative concentration	Molarity	From	To	CV%	RFU
	(bp)	(ng/uL)	Percent	(nmole/L)	(bp)	(bp)		
1	75 (LM)	0.5000		10.7944	33	152	13.26	6613
2	200	0.1484	0.7	1.2387	179	215	3.77	106
3	232	1.5457	7.2	11.0519	215	271	2.34	1989
4	303	2.1175	9.9	11.4203	271	409	5.73	2702
5	449	0.2196	1.0	0.7995	426	490	2.60	114
6	557	1.0481	4.9	3.1020	537	575	0.88	1590
7	585	0.1048	0.5	0.2952	575	600	0.95	146
8	639	0.1883	0.9	0.4842	600	677	2.73	72
9	835	0.1651	0.8	0.3204	801	909	3.08	156
10	996	15.8052	74.1	25.9366	909	1539	4.97	23778
11	20000 (UM)	0.2165		0.0173	15976	28221	4.74	1664

TIC:	21.3427	ng/uL
TIM:	54.6488	nmole/L
Total concentration:	21.7578	ng/uL

```
Sample peak width (sec): 5    Sample min peak height: 50    Sample baseline V to V?: Y    Sample baseline V to V points: 3
Sample filter: Binomial       Number of points for filter: 3  Sample start region (min): 0  Sample end region (min): 30
Marker peak width (sec): 5    Marker min peak height: 200   Marker baseline V to V?: Y    Marker baseline V to V points: 3
Lower marker selection: First peak > 500 RFU                Upper marker selection: Last peak > 200 RFU
Ladder size (bp)75, 200, 300, 400, 500, 700, 1000, 1500, 2000, 3000, 4000, 5000, 7000, 10000, 20000
Quantification using: Lower Marker          Final concentration (ng/uL): 0.5000          Dilution factor: 12.0
```

Sample: SampC9
Well location: C9
Created: Thursday, October 21, 2021 6:48:20 PM

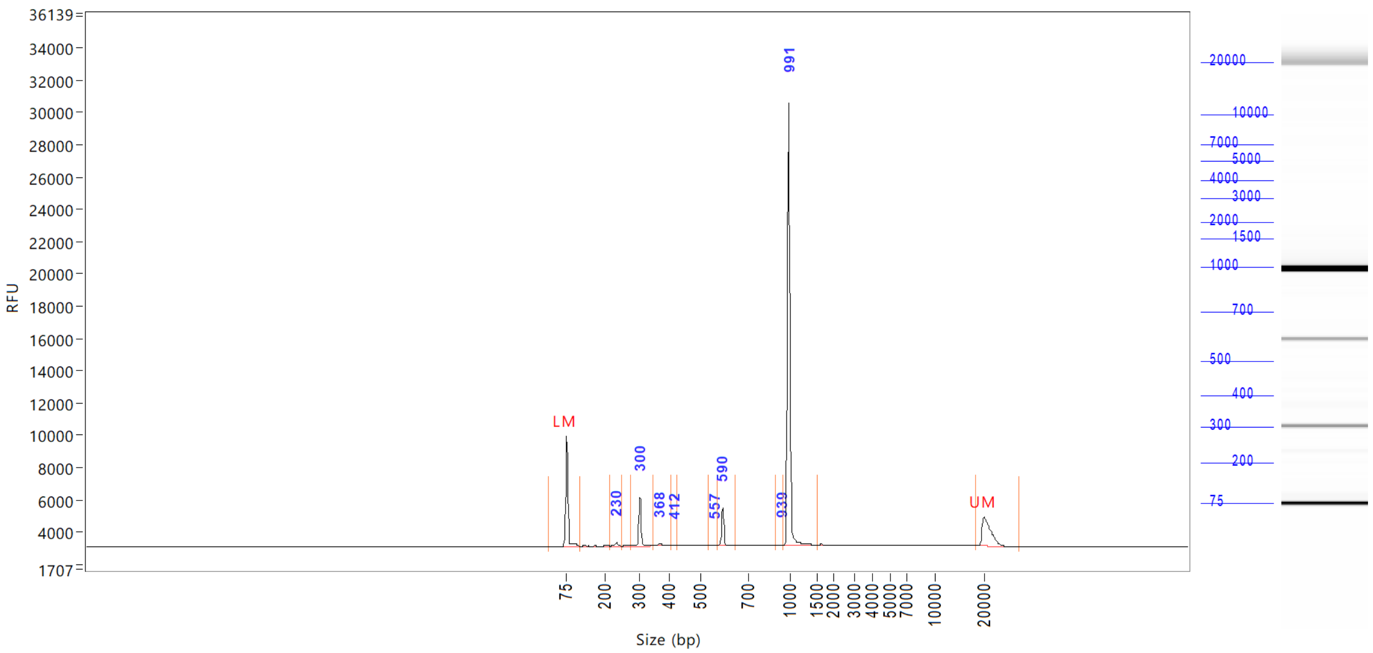

Peak	Size	Concentration	Relative concentration	Molarity	From	To	CV%	RFU
	(bp)	(ng/uL)	Percent	(nmole/L)	(bp)	(bp)		
1	75 (LM)	0.5000		11.0851	20	118	9.35	6858
2	230	0.1806	0.8	1.3025	212	246	1.93	199
3	300	2.3302	10.2	12.8197	275	347	1.93	3000
4	368	0.2180	1.0	0.9747	347	403	2.74	113
5	412	0.0450	0.2	0.1801	403	422	0.85	62
6	557	0.0427	0.2	0.1264	529	567	1.26	53
7	590	1.6229	7.1	4.5419	567	644	1.35	2322
8	939	0.0583	0.3	0.1027	905	957	1.51	51
9	991	18.4381	80.4	30.5617	957	1501	4.33	27452
10	20000 (UM)	0.2506		0.0200	18415	27133	4.72	1820

TIC:	22.9358	ng/uL
TIM:	50.6096	nmole/L
Total concentration:	23.6823	ng/uL

```
Sample peak width (sec): 5    Sample min peak height: 50    Sample baseline V to V?: Y    Sample baseline V to V points: 3
Sample filter: Binomial       Number of points for filter: 3  Sample start region (min): 0  Sample end region (min): 30
Marker peak width (sec): 5    Marker min peak height: 200   Marker baseline V to V?: Y    Marker baseline V to V points: 3
Lower marker selection: First peak > 500 RFU                Upper marker selection: Last peak > 200 RFU
Ladder size (bp)75, 200, 300, 400, 500, 700, 1000, 1500, 2000, 3000, 4000, 5000, 7000, 10000, 20000
Quantification using: Lower Marker          Final concentration (ng/uL): 0.5000          Dilution factor: 12.0
```

Sample: SampD9
Well location: D9
Created: Thursday, October 21, 2021 6:48:20 PM

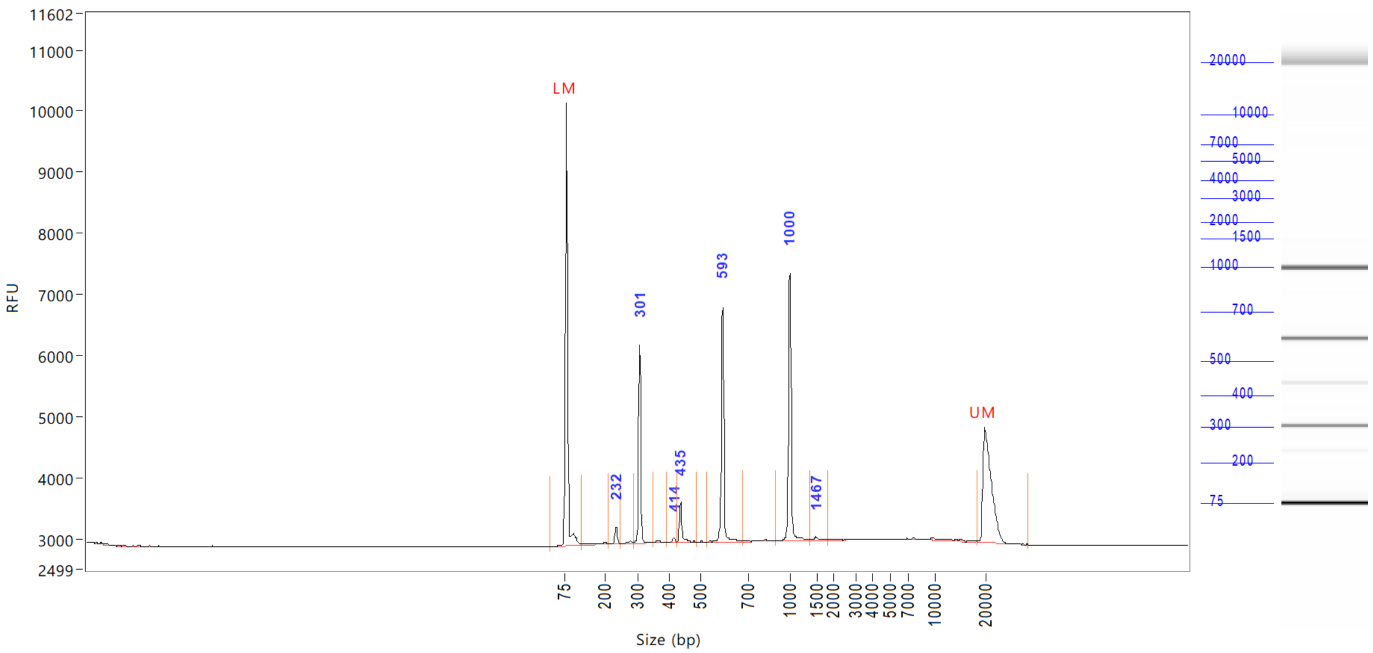

Peak	Size	Concentration	Relative concentration	Molarity	From	To	CV%	RFU
	(bp)	(ng/uL)	Percent	(nmole/L)	(bp)	(bp)		
1	75 (LM)	0.5000		10.9378	24	126	9.50	7266
2	232	0.2200	2.5	1.5797	210	246	1.73	278
3	301	2.2530	26.0	12.3535	282	345	1.56	3252
4	414	0.0576	0.7	0.2300	392	424	1.18	81
5	435	0.4974	5.7	1.8771	424	489	2.14	659
6	593	2.6404	30.5	7.3522	527	680	1.92	3846
7	1000	2.9263	33.8	4.8020	900	1378	4.42	4370
8	1467	0.0661	0.8	0.0728	1378	1808	6.25	57
9	20000 (UM)	0.2393		0.0190	18415	28705	4.58	1870

TIC:	8.6608	ng/uL
TIM:	28.2674	nmole/L
Total concentration:	9.2243	ng/uL

Sample peak width (sec): 5   Sample min peak height: 50   Sample baseline V to V?: Y   Sample baseline V to V points: 3
Sample filter: Binomial   Number of points for filter: 3   Sample start region (min): 0   Sample end region (min): 30
Marker peak width (sec): 5   Marker min peak height: 200   Marker baseline V to V?: Y   Marker baseline V to V points: 3
Lower marker selection: First peak > 500 RFU   Upper marker selection: Last peak > 200 RFU
Ladder size (bp)75, 200, 300, 400, 500, 700, 1000, 1500, 2000, 3000, 4000, 5000, 7000, 10000, 20000
Quantification using: Lower Marker   Final concentration (ng/uL): 0.5000   Dilution factor: 12.0

Sample: SampE9
Well location: E9
Created: Thursday, October 21, 2021 6:48:20 PM

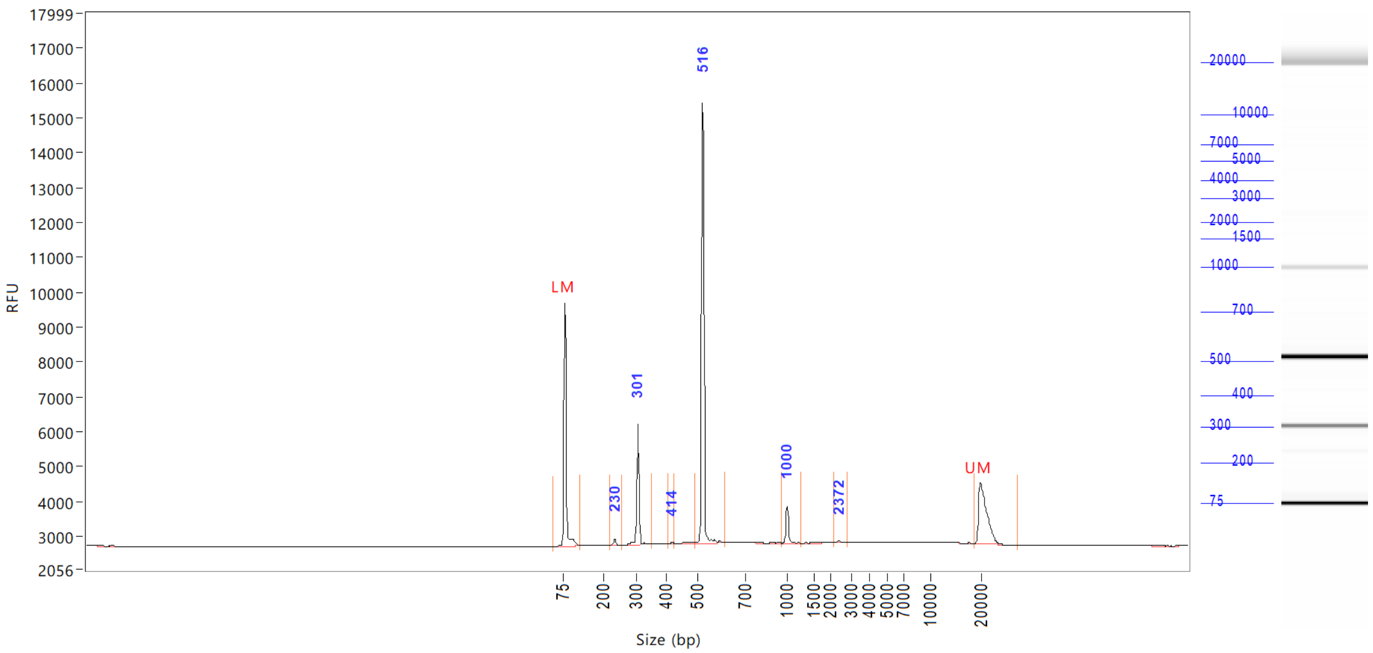

Peak	Size	Concentration	Relative concentration	Molarity	From	To	CV%	RFU
	(bp)	(ng/uL)	Percent	(nmole/L)	(bp)	(bp)		
1	75 (LM)	0.5000		10.9378	37	124	10.10	6970
2	230	0.1453	1.2	1.0478	215	253	2.04	180
3	301	2.5026	21.2	13.7677	253	349	2.18	3438
4	414	0.0342	0.3	0.1367	405	424	0.75	52
5	516	8.3803	70.9	26.7248	489	613	2.00	12648
6	1000	0.6958	5.9	1.1441	961	1245	2.16	1069
7	2372	0.0593	0.5	0.0409	2144	2829	6.01	64
8	20000 (UM)	0.2298		0.0183	18903	27496	4.76	1751

TIC:	11.8175	ng/uL
TIM:	42.8620	nmole/L
Total concentration:	12.4584	ng/uL

```
Sample peak width (sec): 5    Sample min peak height: 50    Sample baseline V to V?: Y    Sample baseline V to V points: 3
Sample filter: Binomial       Number of points for filter: 3  Sample start region (min): 0  Sample end region (min): 30
Marker peak width (sec): 5    Marker min peak height: 200   Marker baseline V to V?: Y    Marker baseline V to V points: 3
Lower marker selection: First peak > 500 RFU                Upper marker selection: Last peak > 200 RFU
Ladder size (bp)75, 200, 300, 400, 500, 700, 1000, 1500, 2000, 3000, 4000, 5000, 7000, 10000, 20000
Quantification using: Lower Marker          Final concentration (ng/uL): 0.5000         Dilution factor: 12.0
```

Sample: SampF9
Well location: F9
Created: Thursday, October 21, 2021 6:48:20 PM

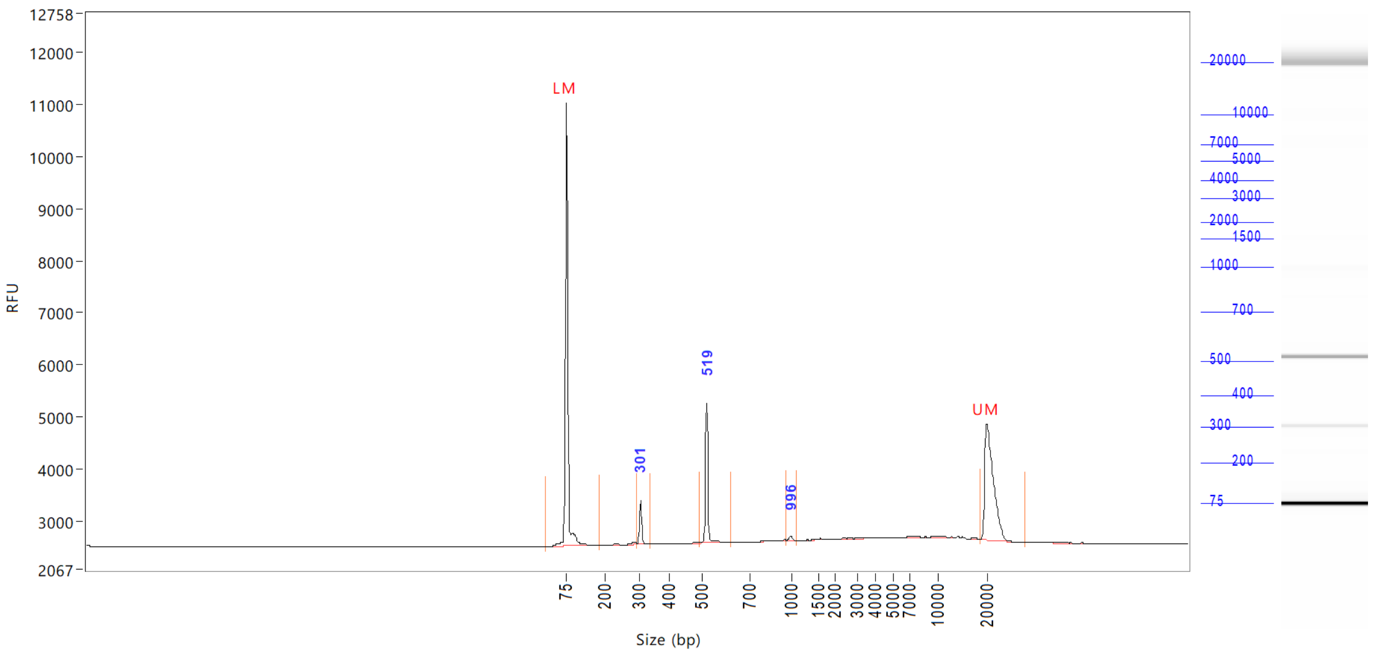

Peak	Size	Concentration	Relative concentration	Molarity	From	To	CV%	RFU
	(bp)	(ng/uL)	Percent	(nmole/L)	(bp)	(bp)		
1	75 (LM)	0.5000		11.0851	11	181	14.17	8534
2	301	0.4820	23.9	2.6430	291	333	1.49	851
3	519	1.4736	73.0	4.6902	492	621	2.45	2689
4	996	0.0624	3.1	0.1038	961	1079	1.52	88
5	20000 (UM)	0.2333		0.0186	18781	27738	4.64	2238

TIC:	2.0180	ng/uL
TIM:	7.4369	nmole/L
Total concentration:	2.4377	ng/uL

```
Sample peak width (sec): 5    Sample min peak height: 50    Sample baseline V to V?: Y    Sample baseline V to V points: 3
Sample filter: Binomial       Number of points for filter: 3  Sample start region (min): 0  Sample end region (min): 30
Marker peak width (sec): 5    Marker min peak height: 200   Marker baseline V to V?: Y    Marker baseline V to V points: 3
Lower marker selection: First peak > 500 RFU                Upper marker selection: Last peak > 200 RFU
Ladder size (bp)75, 200, 300, 400, 500, 700, 1000, 1500, 2000, 3000, 4000, 5000, 7000, 10000, 20000
Quantification using: Lower Marker          Final concentration (ng/uL): 0.5000          Dilution factor: 12.0
```

Sample: SampG9
Well location: G9
Created: Thursday, October 21, 2021 6:48:20 PM

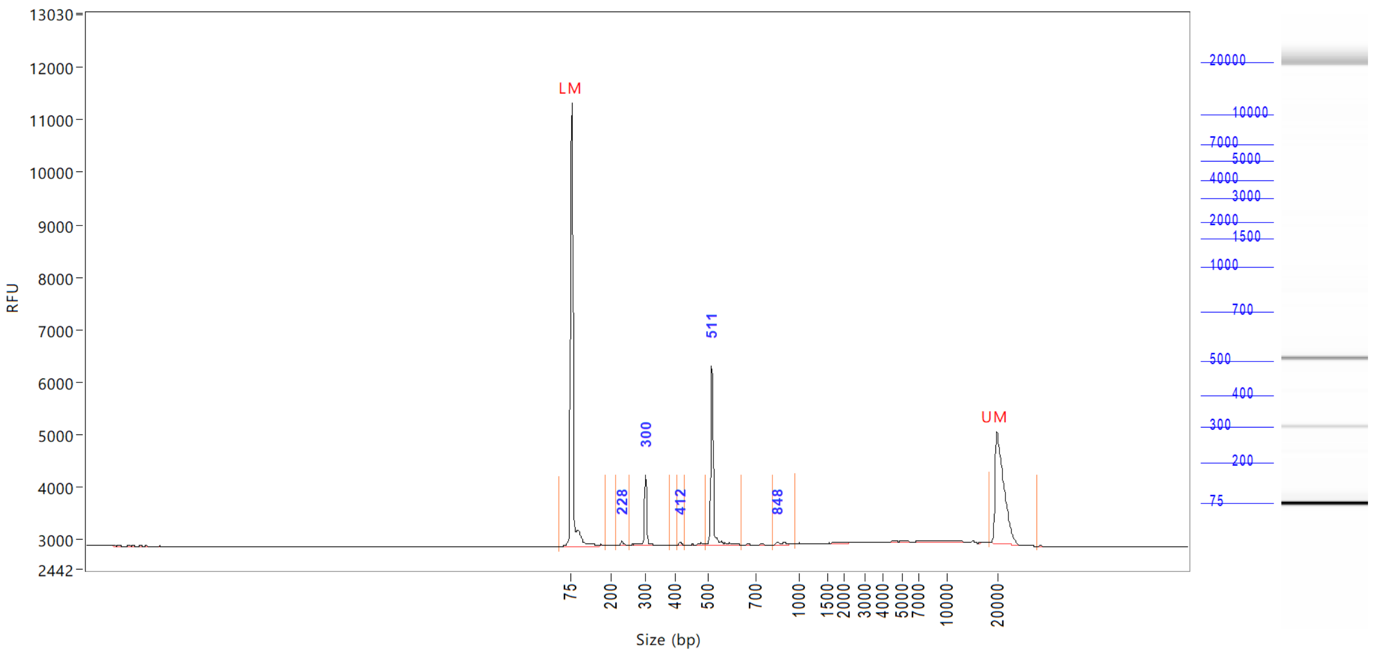

Peak	Size	Concentration	Relative concentration	Molarity	From	To	CV%	RFU
	(bp)	(ng/uL)	Percent	(nmole/L)	(bp)	(bp)		
1	75 (LM)	0.5000		10.7944	37	183	17.88	8461
2	228	0.0900	3.0	0.6438	212	253	4.19	88
3	300	0.8398	27.6	4.6355	253	380	3.81	1348
4	412	0.0341	1.1	0.1367	403	426	0.83	63
5	511	1.9966	65.7	6.3919	489	639	3.09	3410
6	848	0.0805	2.6	0.1511	814	974	3.99	58
7	20000 (UM)	0.2275		0.0181	18415	27979	4.63	2117

TIC:	3.0410	ng/uL
TIM:	11.9590	nmole/L
Total concentration:	3.4407	ng/uL

```
Sample peak width (sec): 5     Sample min peak height: 50     Sample baseline V to V?: Y     Sample baseline V to V points: 3
Sample filter: Binomial        Number of points for filter: 3  Sample start region (min): 0  Sample end region (min): 30
Marker peak width (sec): 5     Marker min peak height: 200    Marker baseline V to V?: Y     Marker baseline V to V points: 3
Lower marker selection: First peak > 500 RFU                  Upper marker selection: Last peak > 200 RFU
Ladder size (bp)75, 200, 300, 400, 500, 700, 1000, 1500, 2000, 3000, 4000, 5000, 7000, 10000, 20000
Quantification using: Lower Marker          Final concentration (ng/uL): 0.5000          Dilution factor: 12.0
```

Sample: SampH9
Well location: H9
Created: Thursday, October 21, 2021 6:48:20 PM

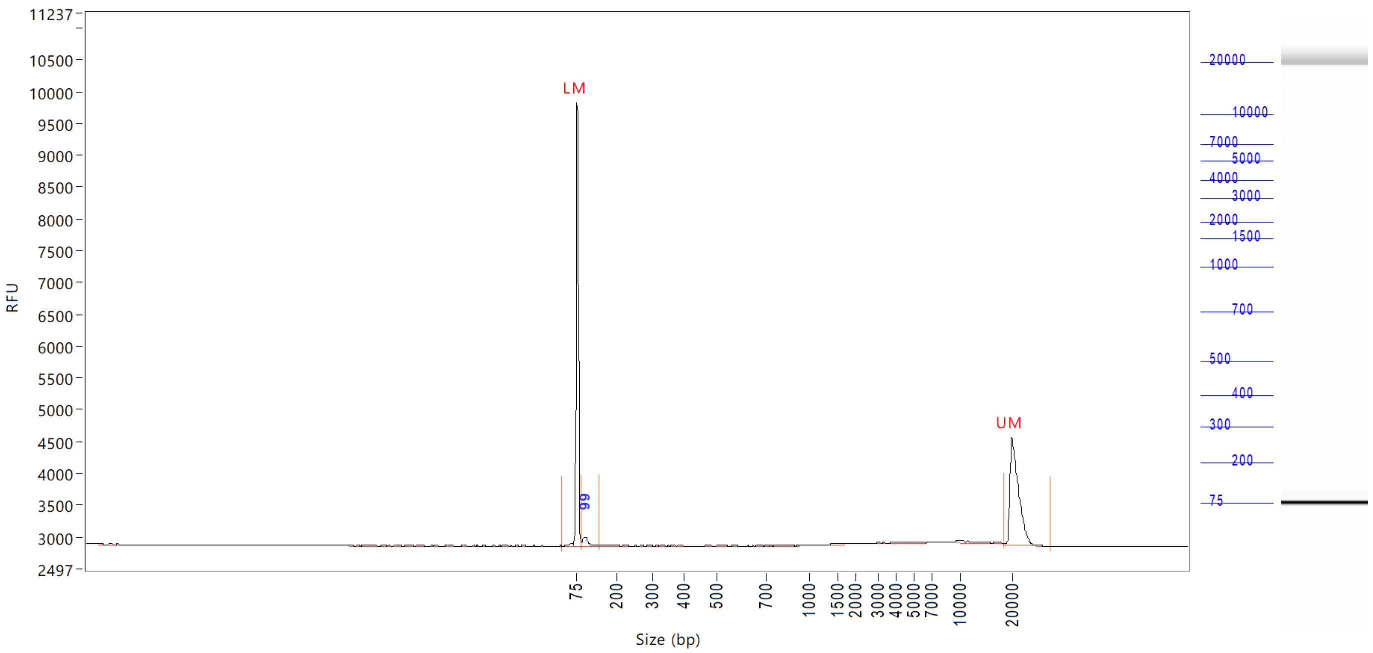

Peak	Size	Concentration	Relative concentration	Molarity	From	To	CV%	RFU
	(bp)	(ng/uL)	Percent	(nmole/L)	(bp)	(bp)		
1	75 (LM)	0.5000		11.0851	31	87	5.12	6980
2	99	0.2742	100.0	4.4579	87	141	10.80	140
3	20000 (UM)	0.2323		0.0185	18293	27496	4.81	1690

TIC:	0.2742	ng/uL
TIM:	4.4579	nmole/L
Total concentration:	1.0175	ng/uL

```
Sample peak width (sec): 5    Sample min peak height: 50    Sample baseline V to V?: Y    Sample baseline V to V points: 3
Sample filter: Binomial       Number of points for filter: 3  Sample start region (min): 0  Sample end region (min): 30
Marker peak width (sec): 5    Marker min peak height: 200   Marker baseline V to V?: Y    Marker baseline V to V points: 3
Lower marker selection: First peak > 500 RFU                Upper marker selection: Last peak > 200 RFU
Ladder size (bp)75, 200, 300, 400, 500, 700, 1000, 1500, 2000, 3000, 4000, 5000, 7000, 10000, 20000
Quantification using: Lower Marker          Final concentration (ng/uL): 0.5000          Dilution factor: 12.0
```

Sample: SampA10
Well location: A10
Created: Thursday, October 21, 2021 6:48:20 PM

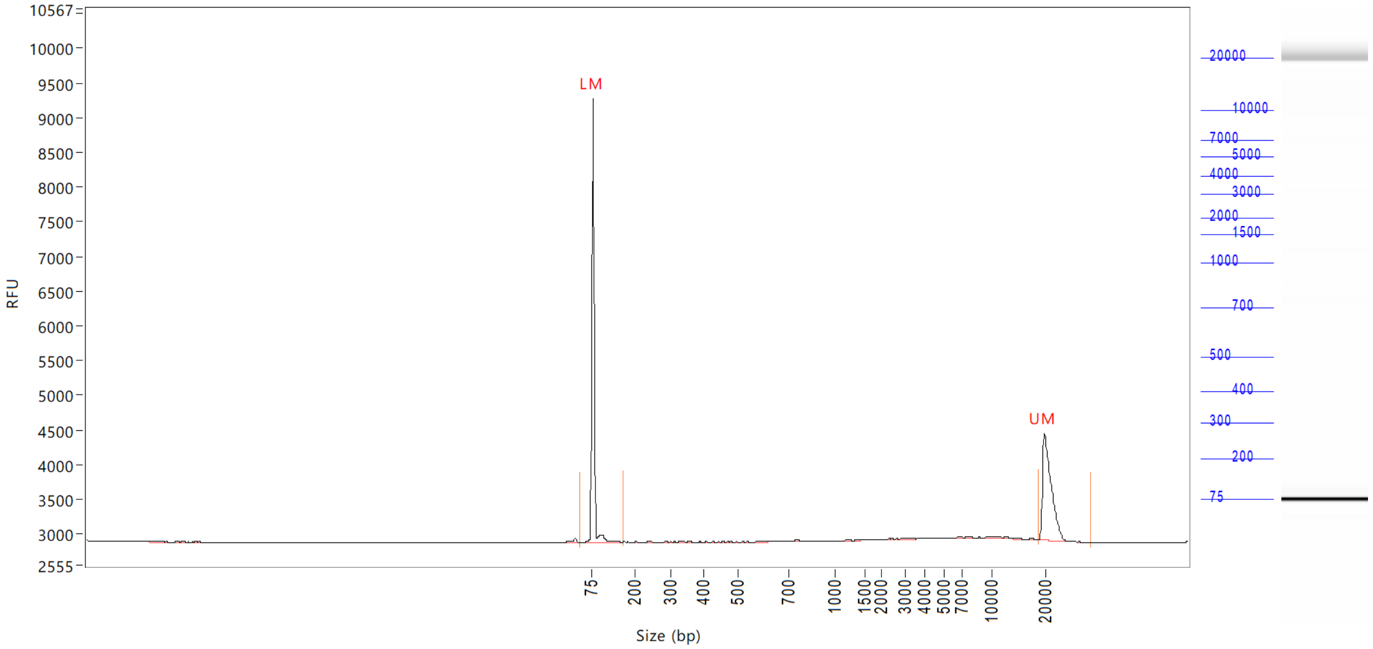

Peak	Size	Concentration	Relative concentration	Molarity	From	To	CV%	RFU
	(bp)	(ng/uL)	Percent	(nmole/L)	(bp)	(bp)		
1	75 (LM)	0.5000		11.0851	37	162	9.68	6402
2	20000 (UM)	0.2306		0.0184	18781	28584	4.67	1539

TIC:	0.0000	ng/uL
TIM:	0.0000	nmole/L
Total concentration:	0.6823	ng/uL

Sample peak width (sec): 5 Sample min peak height: 50 Sample baseline V to V?: Y Sample baseline V to V points: 3
Sample filter: Binomial Number of points for filter: 3 Sample start region (min): 0 Sample end region (min): 30
Marker peak width (sec): 5 Marker min peak height: 200 Marker baseline V to V?: Y Marker baseline V to V points: 3
Lower marker selection: First peak > 500 RFU Upper marker selection: Last peak > 200 RFU
Ladder size (bp)75, 200, 300, 400, 500, 700, 1000, 1500, 2000, 3000, 4000, 5000, 7000, 10000, 20000
Quantification using: Lower Marker Final concentration (ng/uL): 0.5000 Dilution factor: 12.0

Sample: SampB10
Well location: B10
Created: Thursday, October 21, 2021 6:48:20 PM

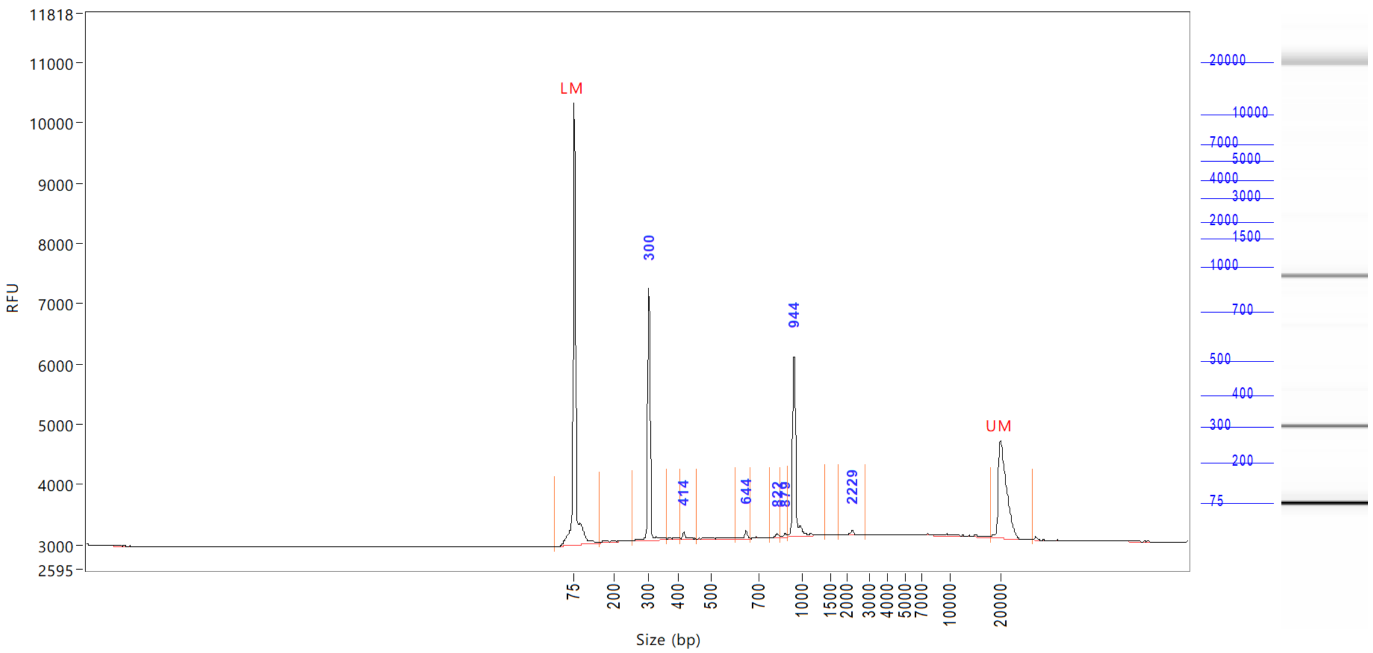

Peak	Size	Concentration	Relative concentration	Molarity	From	To	CV%	RFU
	(bp)	(ng/uL)	Percent	(nmole/L)	(bp)	(bp)		
1	75 (LM)	0.5000		10.9378	14	152	16.08	7348
2	300	2.5450	53.2	14.0013	253	360	2.93	4180
3	414	0.1013	2.1	0.3988	403	456	2.60	127
4	644	0.0823	1.7	0.2123	603	664	1.42	127
5	822	0.0461	1.0	0.0927	775	844	1.60	61
6	879	0.0480	1.0	0.0905	844	896	1.58	61
7	944	1.8894	39.5	3.2804	896	1400	4.75	2993
8	2229	0.0701	1.5	0.0534	1750	2857	7.82	78
9	20000 (UM)	0.1709		0.0137	18049	26287	4.37	1625

TIC:	4.7823	ng/uL
TIM:	18.1293	nmole/L
Total concentration:	5.2405	ng/uL

Sample peak width (sec): 5   Sample min peak height: 50   Sample baseline V to V?: Y   Sample baseline V to V points: 3
Sample filter: Binomial   Number of points for filter: 3   Sample start region (min): 0   Sample end region (min): 30
Marker peak width (sec): 5   Marker min peak height: 200   Marker baseline V to V?: Y   Marker baseline V to V points: 3
Lower marker selection: First peak > 500 RFU   Upper marker selection: Last peak > 200 RFU
Ladder size (bp)75, 200, 300, 400, 500, 700, 1000, 1500, 2000, 3000, 4000, 5000, 7000, 10000, 20000
Quantification using: Lower Marker   Final concentration (ng/uL): 0.5000   Dilution factor: 12.0

Sample: SampC10
Well location: C10
Created: Thursday, October 21, 2021 6:48:20 PM

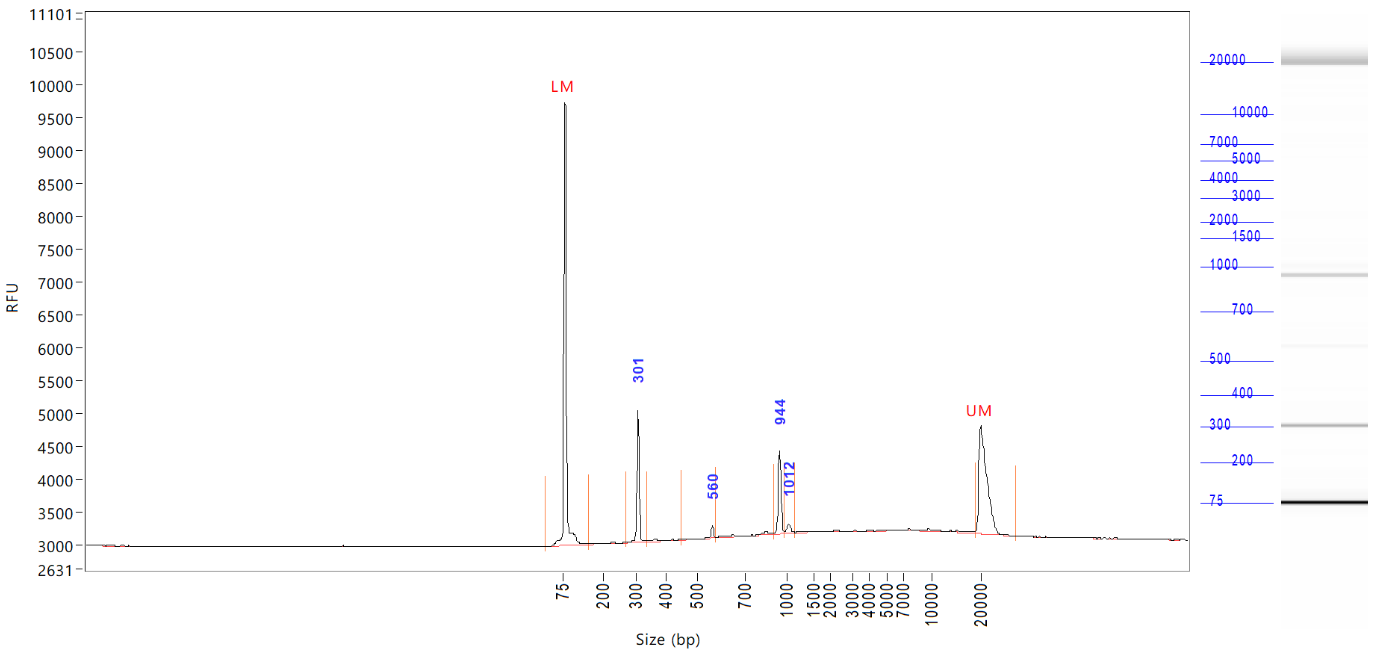

Peak	Size	Concentration	Relative concentration	Molarity	From	To	CV%	RFU
	(bp)	(ng/uL)	Percent	(nmole/L)	(bp)	(bp)		
1	75 (LM)	0.5000		10.7944	14	150	13.46	6757
2	301	1.3934	55.7	7.6657	266	331	1.73	2010
3	560	0.1671	6.7	0.5122	443	572	6.61	189
4	944	0.8130	32.5	1.4220	905	983	1.10	1254
5	1012	0.1293	5.2	0.2089	983	1145	3.11	147
6	20000 (UM)	0.2004		0.0160	19024	27133	4.56	1653

TIC:	2.5028	ng/uL
TIM:	9.8087	nmole/L
Total concentration:	3.2197	ng/uL

Sample peak width (sec): 5 Sample min peak height: 50 Sample baseline V to V?: Y Sample baseline V to V points: 3
Sample filter: Binomial Number of points for filter: 3 Sample start region (min): 0 Sample end region (min): 30
Marker peak width (sec): 5 Marker min peak height: 200 Marker baseline V to V?: Y Marker baseline V to V points: 3
Lower marker selection: First peak > 500 RFU Upper marker selection: Last peak > 200 RFU
Ladder size (bp)75, 200, 300, 400, 500, 700, 1000, 1500, 2000, 3000, 4000, 5000, 7000, 10000, 20000
Quantification using: Lower Marker Final concentration (ng/uL): 0.5000 Dilution factor: 12.0

Sample: SampD10
Well location: D10
Created: Thursday, October 21, 2021 6:48:20 PM

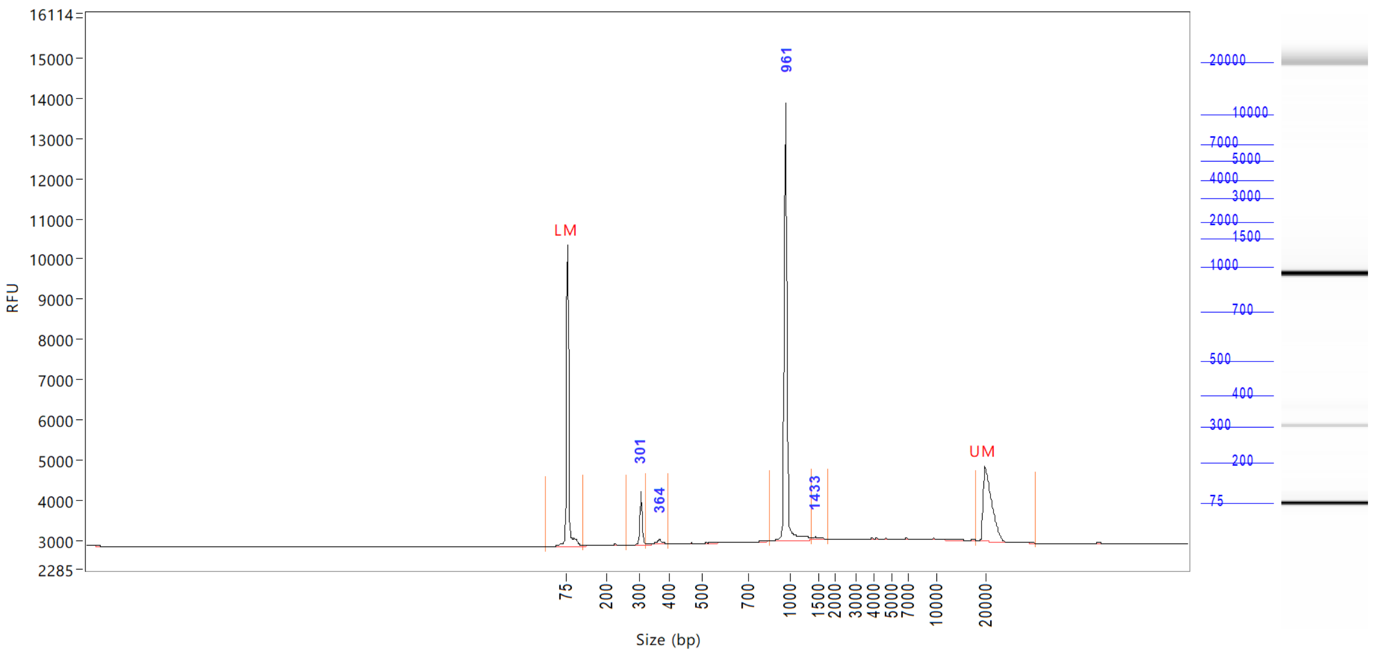

Peak	Size	Concentration	Relative concentration	Molarity	From	To	CV%	RFU
	(bp)	(ng/uL)	Percent	(nmole/L)	(bp)	(bp)		
1	75 (LM)	0.5000		10.9378	7	128	10.88	7495
2	301	0.9006	10.5	4.9383	259	319	1.57	1339
3	364	0.1946	2.3	0.8945	319	392	3.94	114
4	961	7.3783	85.8	12.5715	844	1378	5.61	10912
5	1433	0.1295	1.5	0.1418	1378	1789	7.08	70
6	20000 (UM)	0.2282		0.0182	18171	30277	5.08	1860

TIC:	8.6031	ng/uL
TIM:	18.5461	nmole/L
Total concentration:	9.2054	ng/uL

Sample peak width (sec): 5 Sample min peak height: 50 Sample baseline V to V?: Y Sample baseline V to V points: 3
Sample filter: Binomial Number of points for filter: 3 Sample start region (min): 0 Sample end region (min): 30
Marker peak width (sec): 5 Marker min peak height: 200 Marker baseline V to V?: Y Marker baseline V to V points: 3
Lower marker selection: First peak > 500 RFU Upper marker selection: Last peak > 200 RFU
Ladder size (bp)75, 200, 300, 400, 500, 700, 1000, 1500, 2000, 3000, 4000, 5000, 7000, 10000, 20000
Quantification using: Lower Marker Final concentration (ng/uL): 0.5000 Dilution factor: 12.0

Sample: SampE10
Well location: E10
Created: Thursday, October 21, 2021 6:48:20 PM

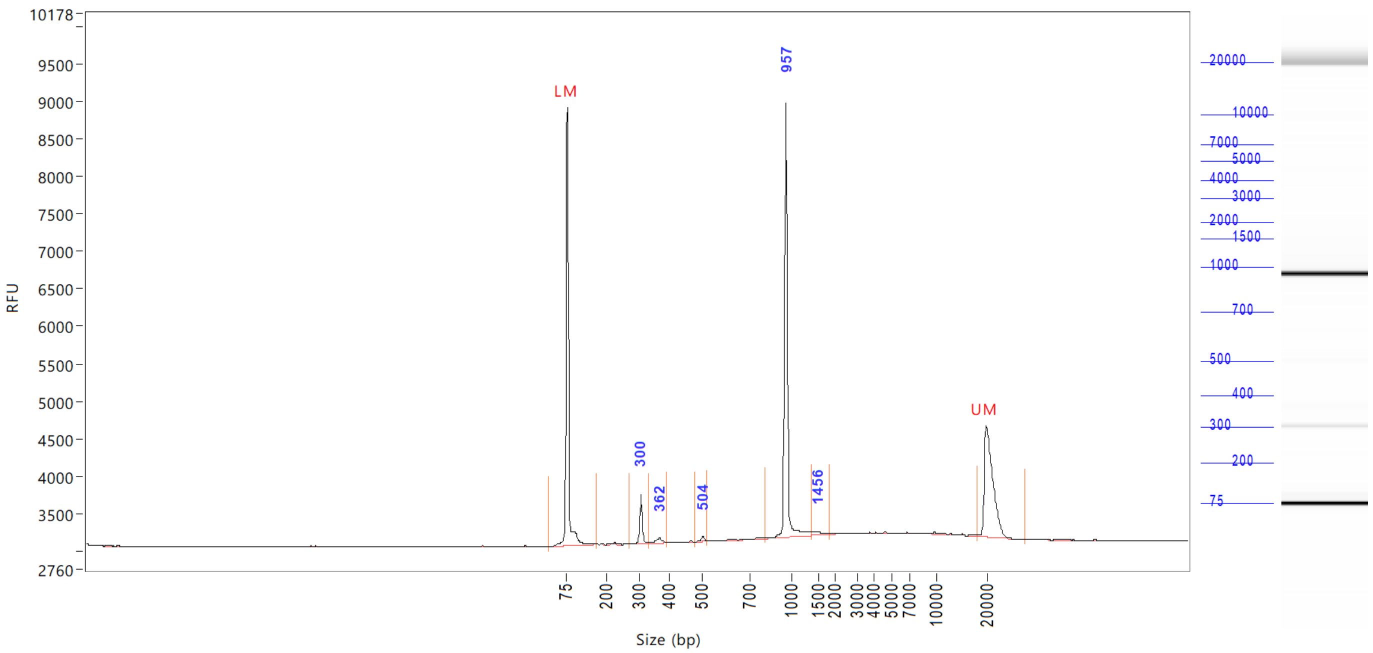

Peak	Size	Concentration	Relative concentration	Molarity	From	To	CV%	RFU
	(bp)	(ng/uL)	Percent	(nmole/L)	(bp)	(bp)		
1	75 (LM)	0.5000		11.0851	16	166	13.08	5843
2	300	0.5955	10.2	3.2759	268	327	2.12	666
3	362	0.1494	2.6	0.6903	327	386	3.45	64
4	504	0.0621	1.1	0.2045	475	519	1.48	63
5	957	4.9136	83.9	8.3720	814	1367	6.07	5806
6	1456	0.1354	2.3	0.1476	1367	1827	8.05	55
7	20000 (UM)	0.2214		0.0177	18171	27738	4.90	1488

TIC:	5.8559	ng/uL
TIM:	12.6903	nmole/L
Total concentration:	6.2857	ng/uL

Sample peak width (sec): 5 Sample min peak height: 50 Sample baseline V to V?: Y Sample baseline V to V points: 3
Sample filter: Binomial Number of points for filter: 3 Sample start region (min): 0 Sample end region (min): 30
Marker peak width (sec): 5 Marker min peak height: 200 Marker baseline V to V?: Y Marker baseline V to V points: 3
Lower marker selection: First peak > 500 RFU Upper marker selection: Last peak > 200 RFU
Ladder size (bp)75, 200, 300, 400, 500, 700, 1000, 1500, 2000, 3000, 4000, 5000, 7000, 10000, 20000
Quantification using: Lower Marker Final concentration (ng/uL): 0.5000 Dilution factor: 12.0

Sample: SampF10
Well location: F10
Created: Thursday, October 21, 2021 6:48:20 PM

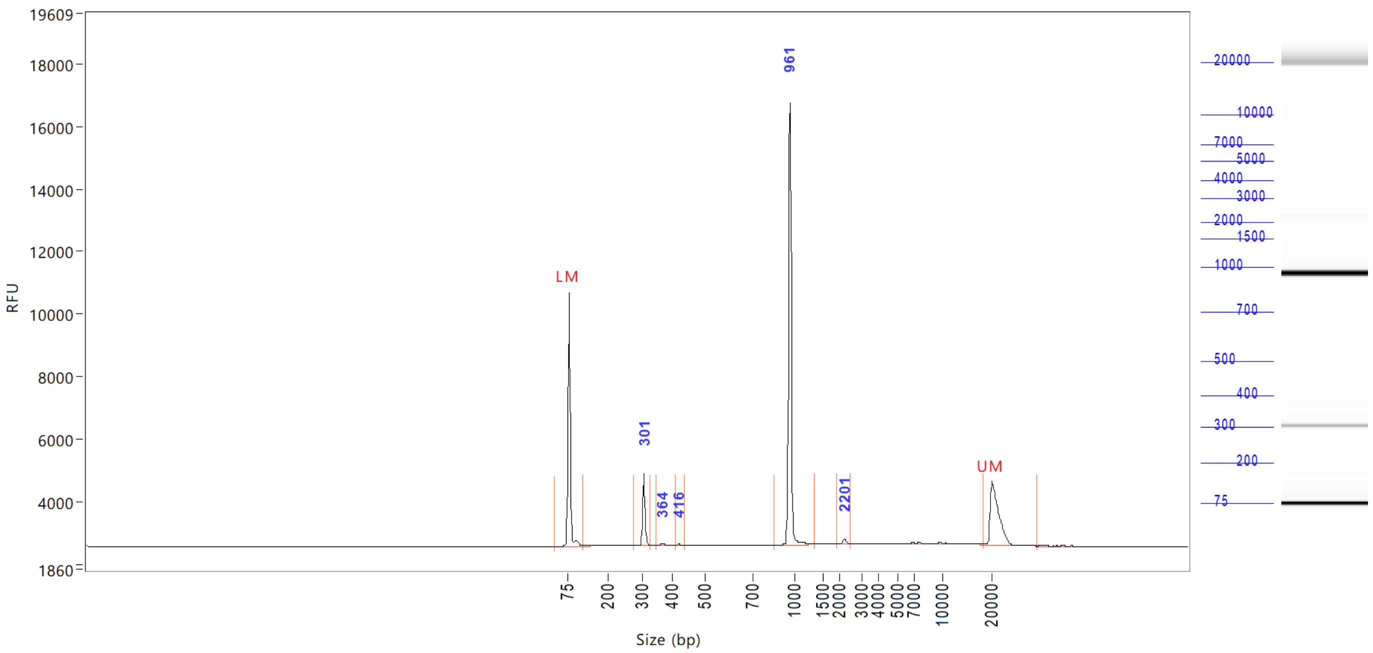

Peak	Size	Concentration	Relative concentration	Molarity	From	To	CV%	RFU
	(bp)	(ng/uL)	Percent	(nmole/L)	(bp)	(bp)		
1	75 (LM)	0.5000		11.0851	33	120	8.68	8078
2	301	1.5132	14.6	8.2968	271	323	1.46	2308
3	364	0.0868	0.8	0.3881	345	407	3.69	70
4	416	0.0429	0.4	0.1697	407	437	1.30	63
5	961	8.6035	83.1	14.7507	853	1345	3.23	14136
6	2201	0.1079	1.0	0.0814	1923	2515	4.66	160
7	20000 (UM)	0.2374		0.0189	18293	29068	4.76	2030

TIC:	10.3543	ng/uL
TIM:	23.6867	nmole/L
Total concentration:	10.9670	ng/uL

```
Sample peak width (sec): 5    Sample min peak height: 50    Sample baseline V to V?: Y    Sample baseline V to V points: 3
Sample filter: Binomial       Number of points for filter: 3  Sample start region (min): 0  Sample end region (min): 30
Marker peak width (sec): 5    Marker min peak height: 200   Marker baseline V to V?: Y    Marker baseline V to V points: 3
Lower marker selection: First peak > 500 RFU                Upper marker selection: Last peak > 200 RFU
Ladder size (bp)75, 200, 300, 400, 500, 700, 1000, 1500, 2000, 3000, 4000, 5000, 7000, 10000, 20000
Quantification using: Lower Marker          Final concentration (ng/uL): 0.5000          Dilution factor: 12.0
```

Sample: SampG10
Well location: G10
Created: Thursday, October 21, 2021 6:48:20 PM

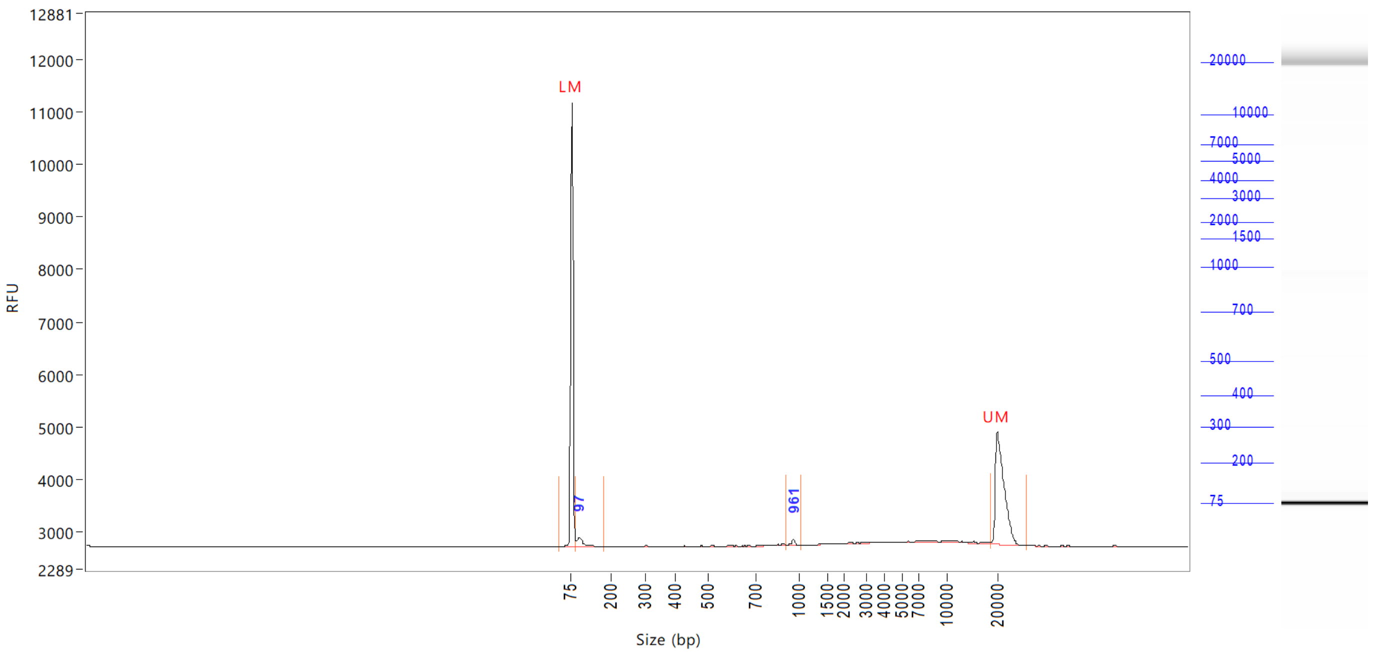

Peak	Size	Concentration	Relative concentration	Molarity	From	To	CV%	RFU
	(bp)	(ng/uL)	Percent	(nmole/L)	(bp)	(bp)		
1	75 (LM)	0.5000		11.2364	39	87	4.72	8471
2	97	0.3802	80.6	5.8362	87	177	17.54	173
3	961	0.0915	19.4	0.1582	909	1023	1.75	109
4	20000 (UM)	0.2598		0.0207	18537	25682	4.61	2159

TIC:	0.4717	ng/uL
TIM:	5.9944	nmole/L
Total concentration:	1.1136	ng/uL

Sample peak width (sec): 5 Sample min peak height: 50 Sample baseline V to V?: Y Sample baseline V to V points: 3
Sample filter: Binomial Number of points for filter: 3 Sample start region (min): 0 Sample end region (min): 30
Marker peak width (sec): 5 Marker min peak height: 200 Marker baseline V to V?: Y Marker baseline V to V points: 3
Lower marker selection: First peak > 500 RFU Upper marker selection: Last peak > 200 RFU
Ladder size (bp)75, 200, 300, 400, 500, 700, 1000, 1500, 2000, 3000, 4000, 5000, 7000, 10000, 20000
Quantification using: Lower Marker Final concentration (ng/uL): 0.5000 Dilution factor: 12.0

Sample: SampH10
Well location: H10
Created: Thursday, October 21, 2021 6:48:20 PM

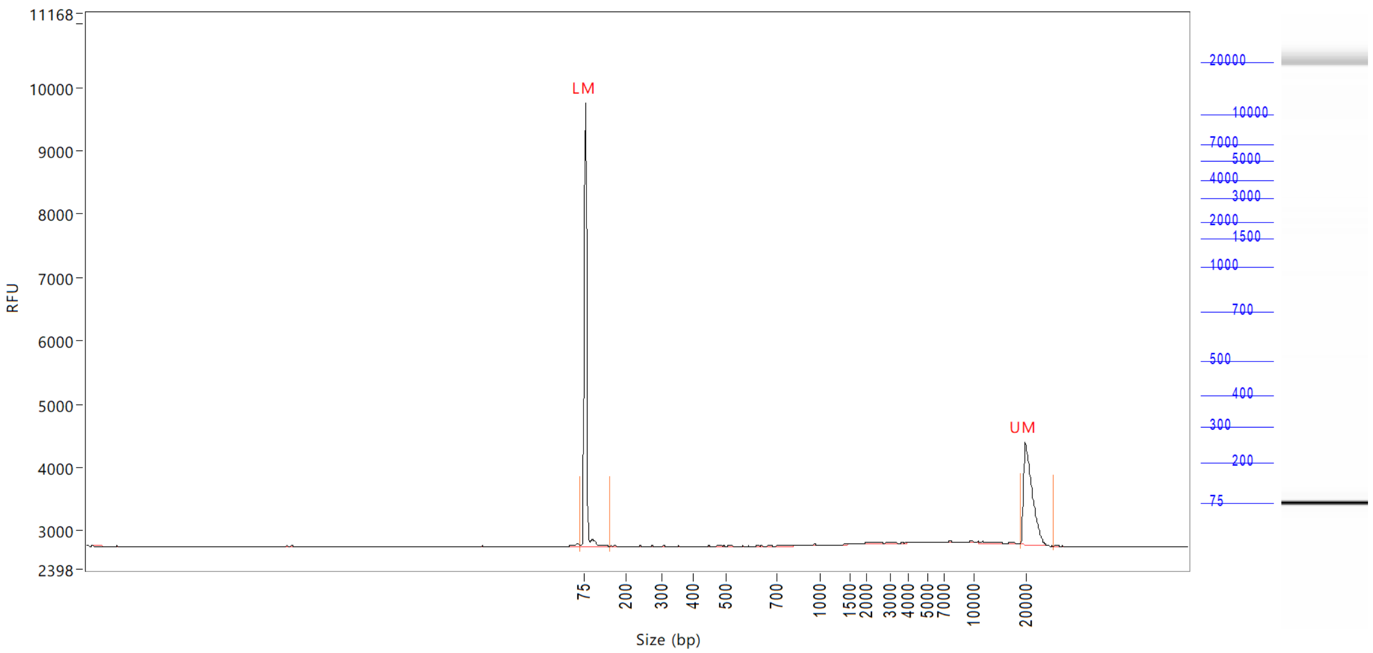

Peak	Size	Concentration	Relative concentration	Molarity	From	To	CV%	RFU
	(bp)	(ng/uL)	Percent	(nmole/L)	(bp)	(bp)		
1	75 (LM)	0.5000		10.9378	60	148	8.77	7008
2	20000 (UM)	0.2170		0.0173	18903	25320	4.40	1606

TIC:	0.0000	ng/uL
TIM:	0.0000	nmole/L
Total concentration:	0.6297	ng/uL

Sample peak width (sec): 5 Sample min peak height: 50 Sample baseline V to V?: Y Sample baseline V to V points: 3
Sample filter: Binomial Number of points for filter: 3 Sample start region (min): 0 Sample end region (min): 30
Marker peak width (sec): 5 Marker min peak height: 200 Marker baseline V to V?: Y Marker baseline V to V points: 3
Lower marker selection: First peak > 500 RFU Upper marker selection: Last peak > 200 RFU
Ladder size (bp)75, 200, 300, 400, 500, 700, 1000, 1500, 2000, 3000, 4000, 5000, 7000, 10000, 20000
Quantification using: Lower Marker Final concentration (ng/uL): 0.5000 Dilution factor: 12.0

Sample: SampA11
Well location: A11
Created: Thursday, October 21, 2021 6:48:20 PM

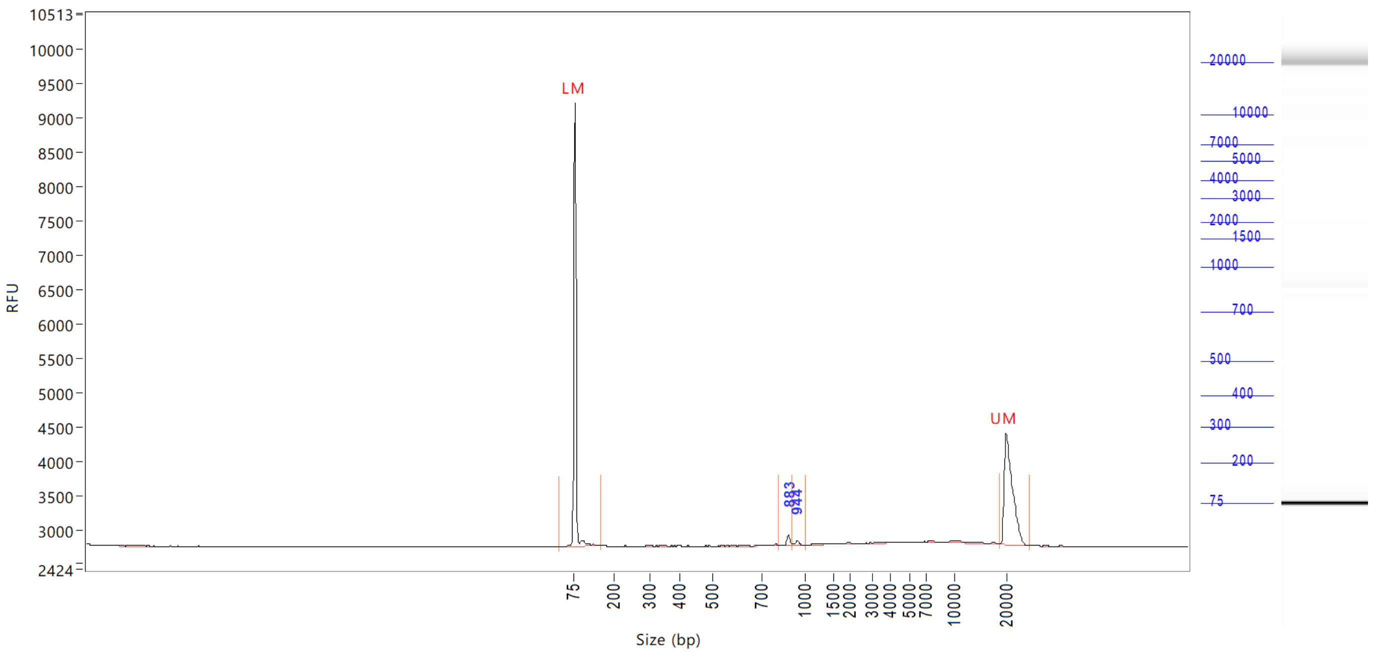

Peak	Size	Concentration	Relative concentration	Molarity	From	To	CV%	RFU
	(bp)	(ng/uL)	Percent	(nmole/L)	(bp)	(bp)		
1	75 (LM)	0.5000		11.0851	30	156	9.81	6461
2	883	0.1402	66.2	0.2636	814	909	1.75	163
3	944	0.0715	33.8	0.1252	909	1000	1.71	61
4	20000 (UM)	0.2388		0.0191	18659	24594	4.34	1627

TIC:	0.2117	ng/uL
TIM:	0.3889	nmole/L
Total concentration:	0.7642	ng/uL

Sample peak width (sec): 5 Sample min peak height: 50 Sample baseline V to V?: Y Sample baseline V to V points: 3
Sample filter: Binomial Number of points for filter: 3 Sample start region (min): 0 Sample end region (min): 30
Marker peak width (sec): 5 Marker min peak height: 200 Marker baseline V to V?: Y Marker baseline V to V points: 3
Lower marker selection: First peak > 500 RFU Upper marker selection: Last peak > 200 RFU
Ladder size (bp)75, 200, 300, 400, 500, 700, 1000, 1500, 2000, 3000, 4000, 5000, 7000, 10000, 20000
Quantification using: Lower Marker Final concentration (ng/uL): 0.5000 Dilution factor: 12.0

Sample: SampB11
Well location: B11
Created: Thursday, October 21, 2021 6:48:20 PM

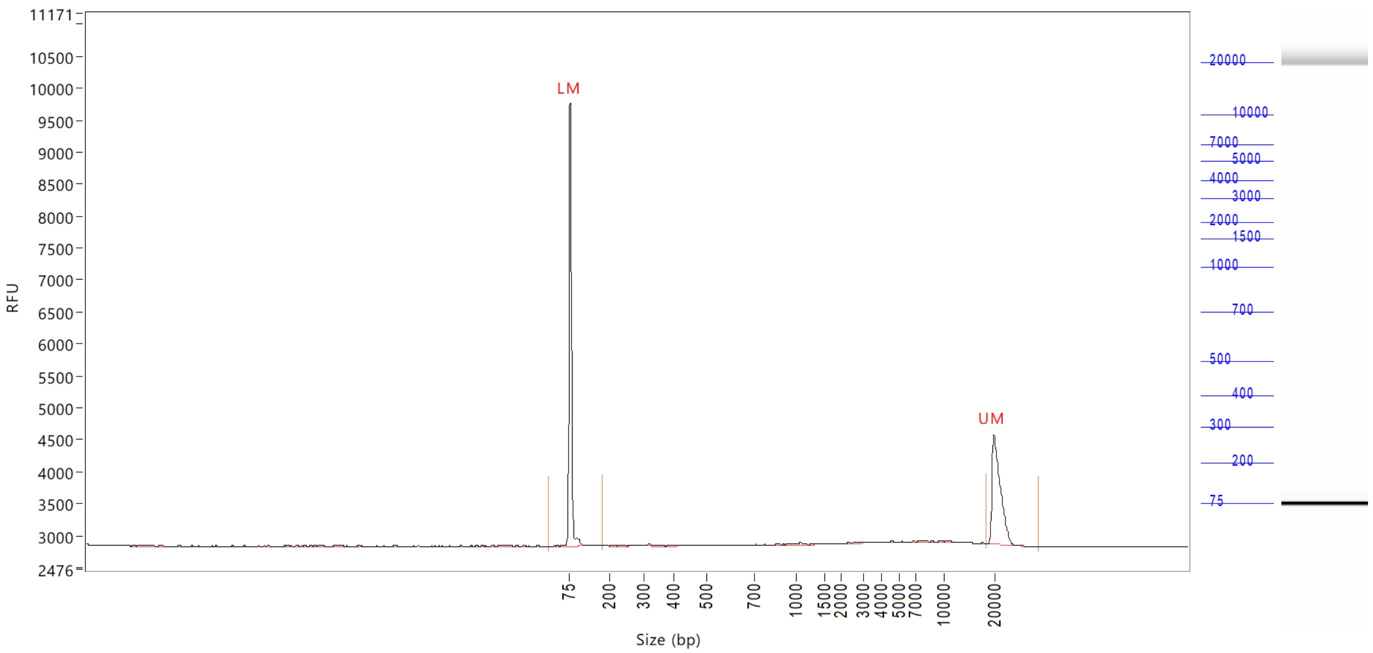

Peak	Size	Concentration	Relative concentration	Molarity	From	To	CV%	RFU
	(bp)	(ng/uL)	Percent	(nmole/L)	(bp)	(bp)		
1	75 (LM)	0.5000		11.2364	11	177	10.11	6943
2	20000 (UM)	0.2266		0.0181	18537	28947	4.66	1717

TIC:	0.0000	ng/uL
TIM:	0.0000	nmole/L
Total concentration:	0.6192	ng/uL

Sample peak width (sec): 5 Sample min peak height: 50 Sample baseline V to V?: Y Sample baseline V to V points: 3
Sample filter: Binomial Number of points for filter: 3 Sample start region (min): 0 Sample end region (min): 30
Marker peak width (sec): 5 Marker min peak height: 200 Marker baseline V to V?: Y Marker baseline V to V points: 3
Lower marker selection: First peak > 500 RFU Upper marker selection: Last peak > 200 RFU
Ladder size (bp)75, 200, 300, 400, 500, 700, 1000, 1500, 2000, 3000, 4000, 5000, 7000, 10000, 20000
Quantification using: Lower Marker Final concentration (ng/uL): 0.5000 Dilution factor: 12.0

Sample: SampC11
Well location: C11
Created: Thursday, October 21, 2021 6:48:20 PM

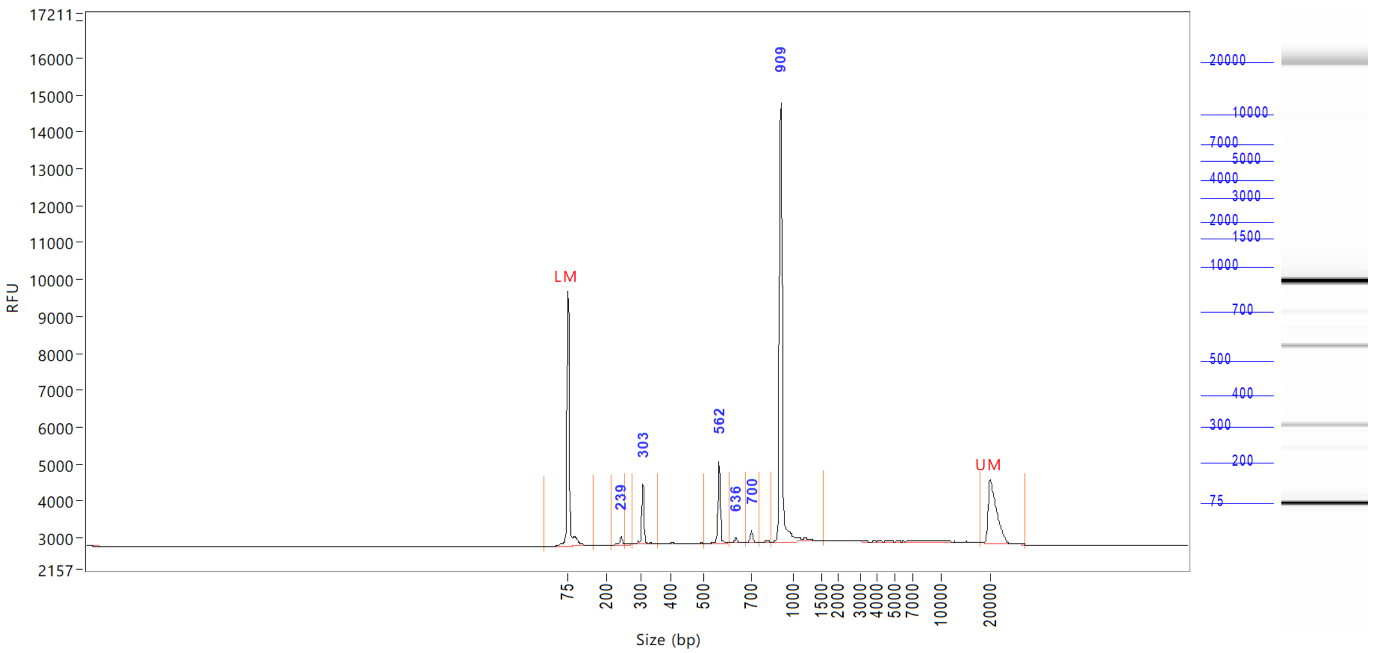

Peak	Size	Concentration	Relative concentration	Molarity	From	To	CV%	RFU
	(bp)	(ng/uL)	Percent	(nmole/L)	(bp)	(bp)		
1	75 (LM)	0.5000		10.9378	0	156	13.85	6917
2	239	0.1697	1.5	1.1822	212	251	2.31	221
3	303	1.2233	10.9	6.6414	273	352	2.76	1616
4	562	1.4476	12.9	4.2538	501	608	1.60	2210
5	636	0.1130	1.0	0.2948	608	677	1.81	122
6	700	0.2139	1.9	0.5043	677	757	1.06	336
7	909	8.0578	71.8	14.4943	835	1520	6.03	11907
8	20000 (UM)	0.2191		0.0175	18171	27133	4.66	1744

TIC:	11.2253	ng/uL
TIM:	27.3709	nmole/L
Total concentration:	11.7457	ng/uL

```
Sample peak width (sec): 5    Sample min peak height: 50    Sample baseline V to V?: Y    Sample baseline V to V points: 3
Sample filter: Binomial       Number of points for filter: 3  Sample start region (min): 0  Sample end region (min): 30
Marker peak width (sec): 5    Marker min peak height: 200   Marker baseline V to V?: Y    Marker baseline V to V points: 3
Lower marker selection: First peak > 500 RFU                Upper marker selection: Last peak > 200 RFU
Ladder size (bp)75, 200, 300, 400, 500, 700, 1000, 1500, 2000, 3000, 4000, 5000, 7000, 10000, 20000
Quantification using: Lower Marker          Final concentration (ng/uL): 0.5000          Dilution factor: 12.0
```

Sample: SampD11
Well location: D11
Created: Thursday, October 21, 2021 6:48:20 PM

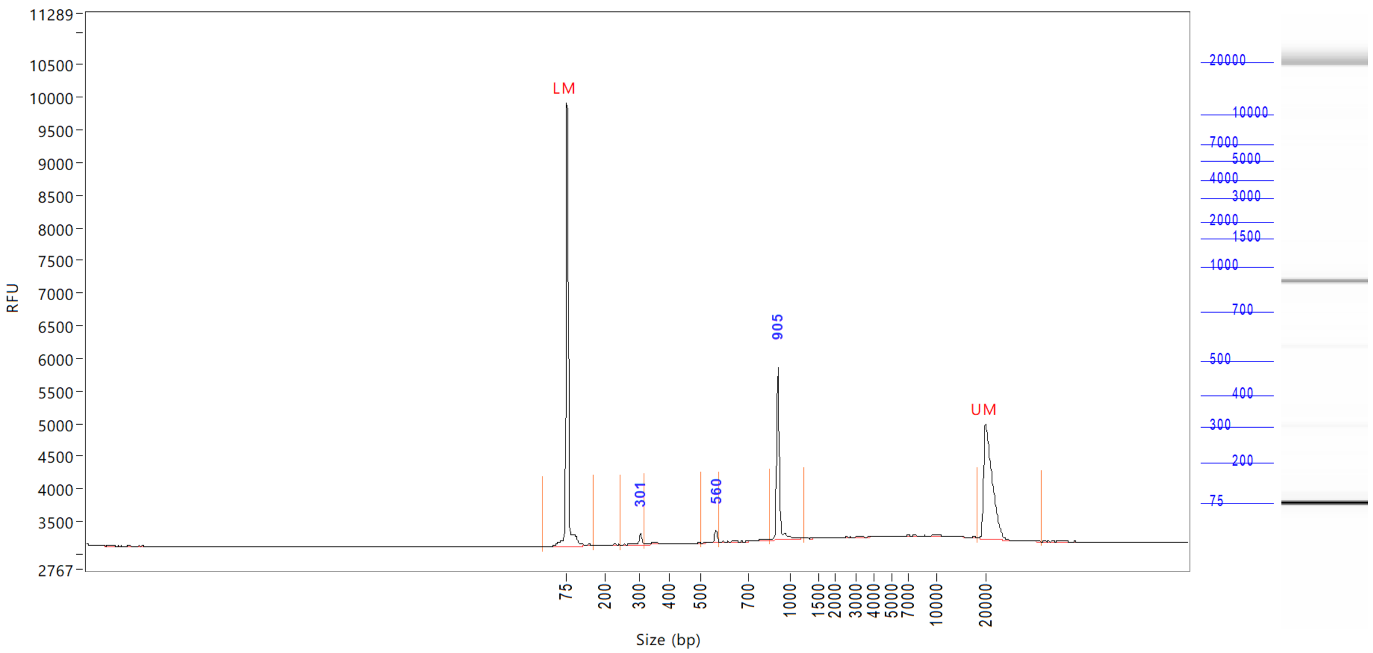

Peak	Size	Concentration	Relative concentration	Molarity	From	To	CV%	RFU
	(bp)	(ng/uL)	Percent	(nmole/L)	(bp)	(bp)		
1	75 (LM)	0.5000		10.9378	1	162	14.76	6808
2	301	0.1839	8.6	1.0325	244	315	4.73	178
3	560	0.1364	6.4	0.4051	501	575	2.14	189
4	905	1.8061	84.9	3.2739	853	1245	2.91	2634
5	20000 (UM)	0.2257		0.0180	18293	31123	5.42	1755

TIC:	2.1264	ng/uL
TIM:	4.7115	nmole/L
Total concentration:	2.6931	ng/uL

```
Sample peak width (sec): 5    Sample min peak height: 50    Sample baseline V to V?: Y    Sample baseline V to V points: 3
Sample filter: Binomial       Number of points for filter: 3  Sample start region (min): 0  Sample end region (min): 30
Marker peak width (sec): 5    Marker min peak height: 200   Marker baseline V to V?: Y    Marker baseline V to V points: 3
Lower marker selection: First peak > 500 RFU                Upper marker selection: Last peak > 200 RFU
Ladder size (bp)75, 200, 300, 400, 500, 700, 1000, 1500, 2000, 3000, 4000, 5000, 7000, 10000, 20000
Quantification using: Lower Marker          Final concentration (ng/uL): 0.5000          Dilution factor: 12.0
```

Sample: SampE11
Well location: E11
Created: Thursday, October 21, 2021 6:48:20 PM

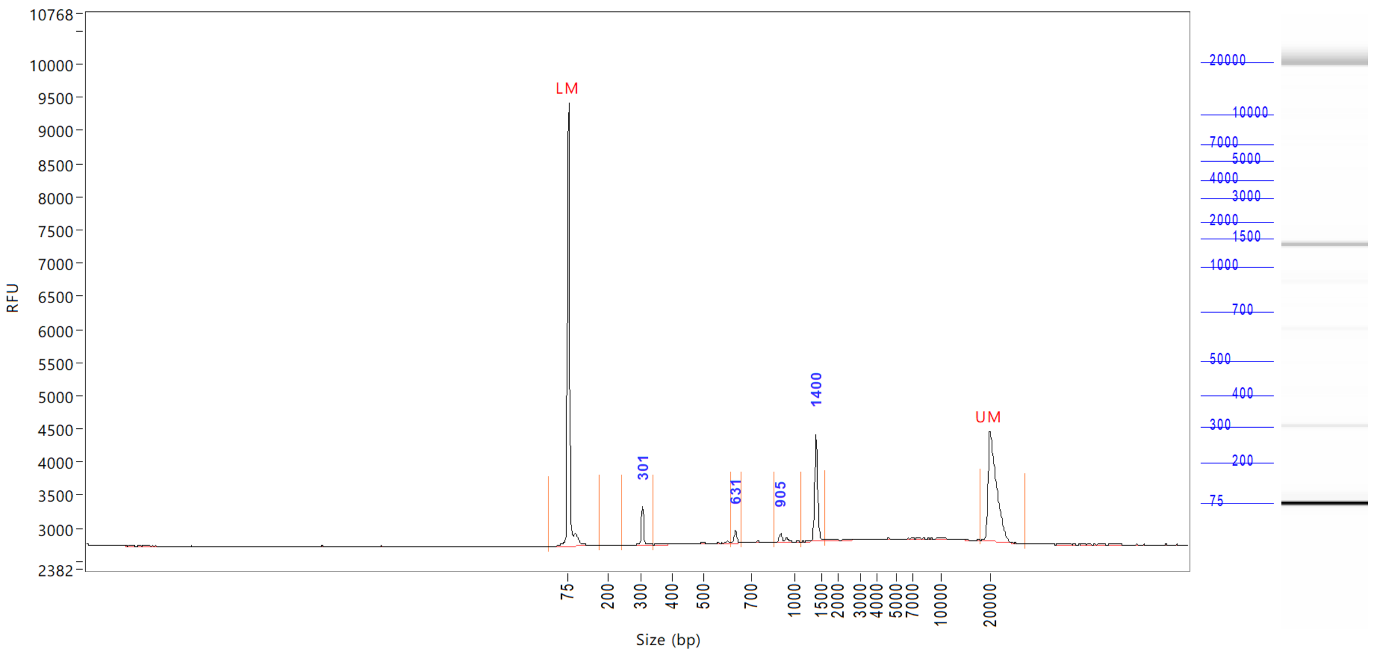

Peak	Size	Concentration	Relative concentration	Molarity	From	To	CV%	RFU
	(bp)	(ng/uL)	Percent	(nmole/L)	(bp)	(bp)		
1	75 (LM)	0.5000		11.0851	14	173	12.26	6698
2	301	0.4883	24.9	2.6866	241	335	3.27	575
3	631	0.1450	7.4	0.3800	613	654	0.89	193
4	905	0.1730	8.8	0.3085	857	1123	4.01	127
5	1400	1.1538	58.9	1.3693	1123	1597	2.63	1607
6	20000 (UM)	0.2235		0.0179	18171	27133	4.59	1652

TIC:	1.9602	ng/uL
TIM:	4.7444	nmole/L
Total concentration:	2.4073	ng/uL

Sample peak width (sec): 5 Sample min peak height: 50 Sample baseline V to V?: Y Sample baseline V to V points: 3
Sample filter: Binomial Number of points for filter: 3 Sample start region (min): 0 Sample end region (min): 30
Marker peak width (sec): 5 Marker min peak height: 200 Marker baseline V to V?: Y Marker baseline V to V points: 3
Lower marker selection: First peak > 500 RFU Upper marker selection: Last peak > 200 RFU
Ladder size (bp)75, 200, 300, 400, 500, 700, 1000, 1500, 2000, 3000, 4000, 5000, 7000, 10000, 20000
Quantification using: Lower Marker Final concentration (ng/uL): 0.5000 Dilution factor: 12.0

Sample: SampF11
Well location: F11
Created: Thursday, October 21, 2021 6:48:20 PM

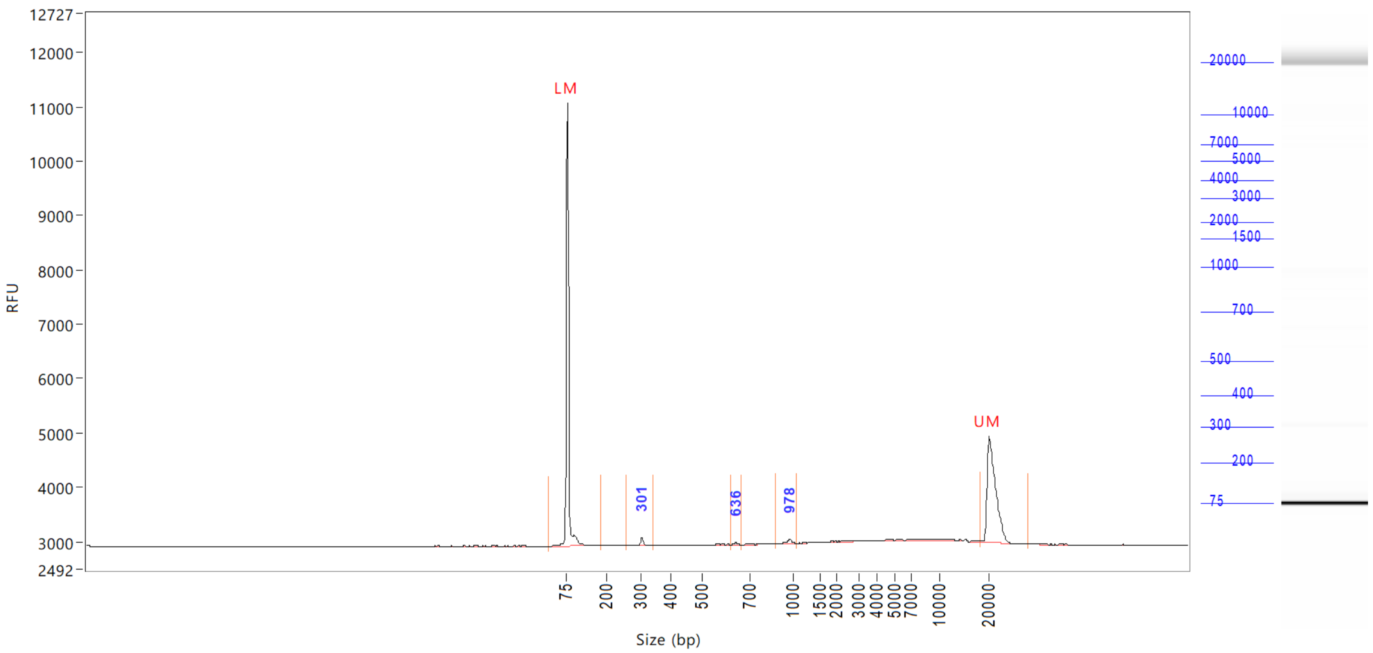

Peak	Size	Concentration	Relative concentration	Molarity	From	To	CV%	RFU
	(bp)	(ng/uL)	Percent	(nmole/L)	(bp)	(bp)		
1	75 (LM)	0.5000		10.9378	18	183	12.93	8172
2	301	0.1216	49.7	0.6643	257	341	3.87	142
3	636	0.0346	14.2	0.0899	618	659	1.03	53
4	978	0.0886	36.2	0.1520	879	1045	3.06	72
5	20000 (UM)	0.2226		0.0177	18415	27859	4.60	1970

TIC:	0.2448	ng/uL
TIM:	0.9063	nmole/L
Total concentration:	0.6585	ng/uL

Sample peak width (sec): 5 Sample min peak height: 50 Sample baseline V to V?: Y Sample baseline V to V points: 3
Sample filter: Binomial Number of points for filter: 3 Sample start region (min): 0 Sample end region (min): 30
Marker peak width (sec): 5 Marker min peak height: 200 Marker baseline V to V?: Y Marker baseline V to V points: 3
Lower marker selection: First peak > 500 RFU Upper marker selection: Last peak > 200 RFU
Ladder size (bp)75, 200, 300, 400, 500, 700, 1000, 1500, 2000, 3000, 4000, 5000, 7000, 10000, 20000
Quantification using: Lower Marker Final concentration (ng/uL): 0.5000 Dilution factor: 12.0

Sample: SampG11
Well location: G11
Created: Thursday, October 21, 2021 6:48:20 PM

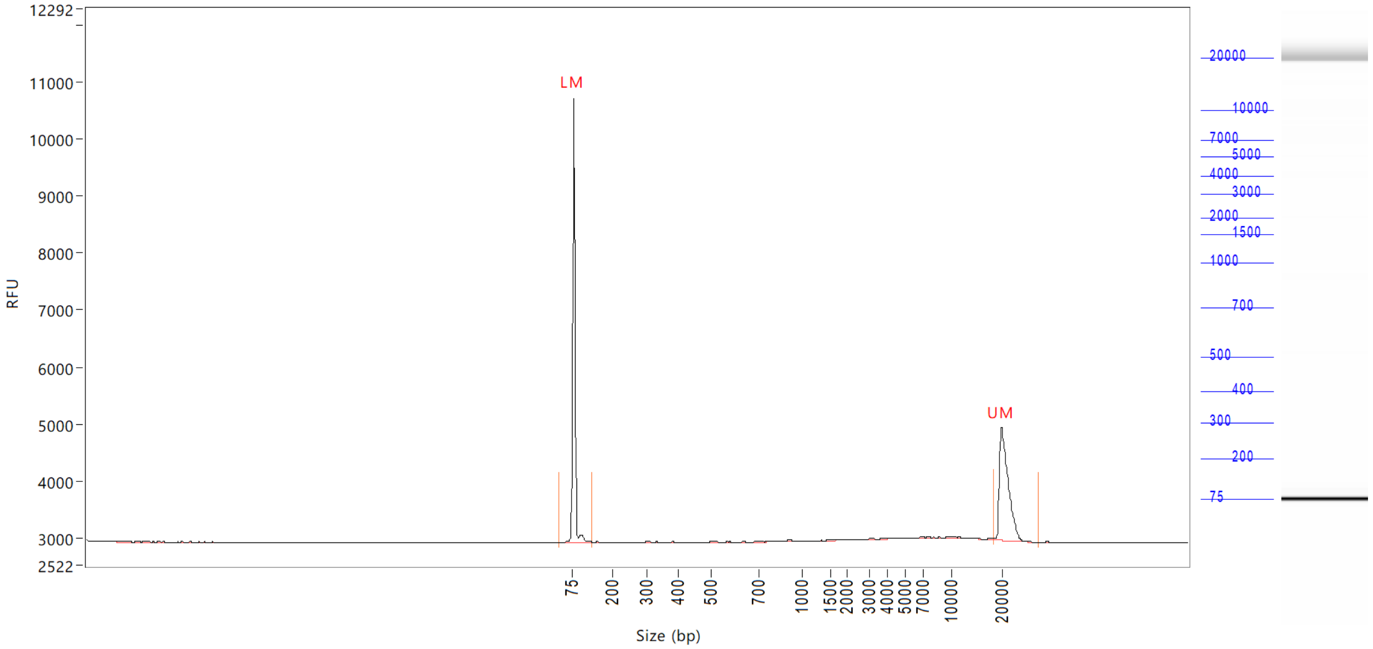

Peak	Size	Concentration	Relative concentration	Molarity	From	To	CV%	RFU
	(bp)	(ng/uL)	Percent	(nmole/L)	(bp)	(bp)		
1	75 (LM)	0.5000		11.0851	31	135	8.74	7811
2	20000 (UM)	0.2474		0.0198	18415	27133	4.71	1989

TIC:	0.0000	ng/uL
TIM:	0.0000	nmole/L
Total concentration:	0.6194	ng/uL

Sample peak width (sec): 5 Sample min peak height: 50 Sample baseline V to V?: Y Sample baseline V to V points: 3
Sample filter: Binomial Number of points for filter: 3 Sample start region (min): 0 Sample end region (min): 30
Marker peak width (sec): 5 Marker min peak height: 200 Marker baseline V to V?: Y Marker baseline V to V points: 3
Lower marker selection: First peak > 500 RFU Upper marker selection: Last peak > 200 RFU
Ladder size (bp)75, 200, 300, 400, 500, 700, 1000, 1500, 2000, 3000, 4000, 5000, 7000, 10000, 20000
Quantification using: Lower Marker Final concentration (ng/uL): 0.5000 Dilution factor: 12.0

Sample: SampH11
Well location: H11
Created: Thursday, October 21, 2021 6:48:20 PM

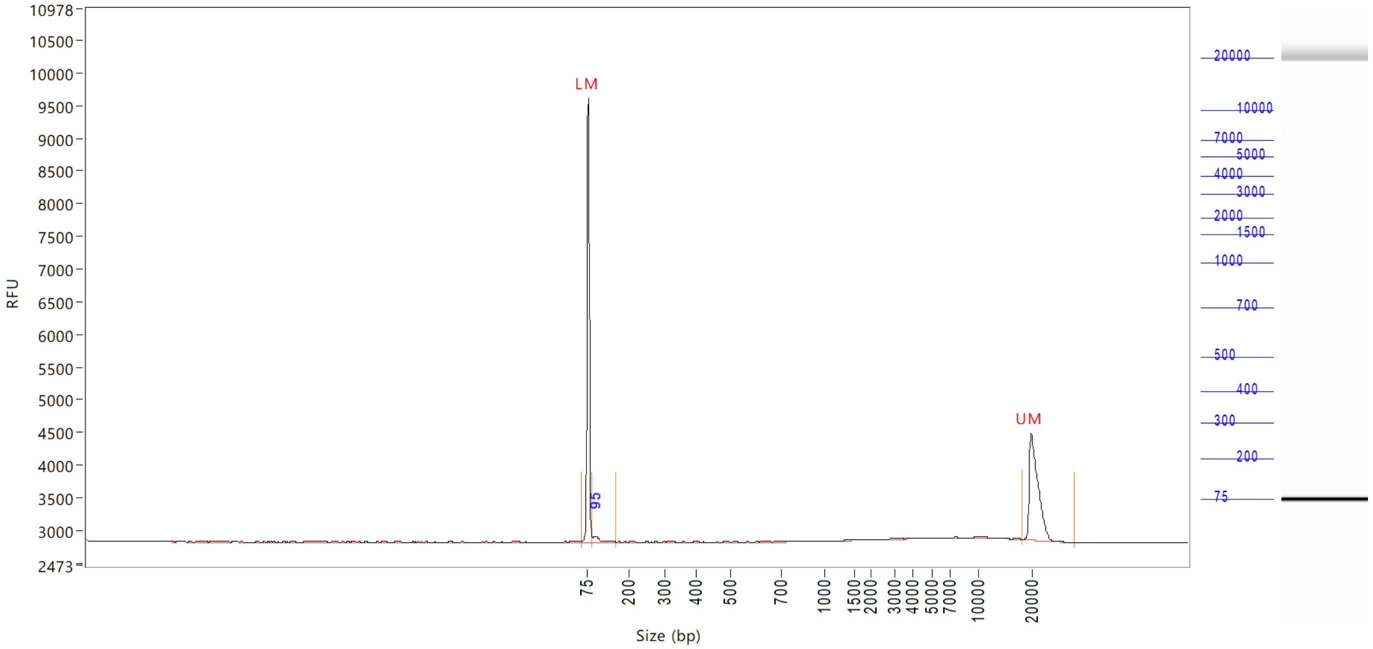

Peak	Size	Concentration	Relative concentration	Molarity	From	To	CV%	RFU
	(bp)	(ng/uL)	Percent	(nmole/L)	(bp)	(bp)		
1	75 (LM)	0.5000		11.3919	54	87	4.70	6793
2	95	0.2228	100.0	3.5872	87	160	14.59	93
3	20000 (UM)	0.2362		0.0188	18293	28342	4.61	1638

TIC:	0.2228	ng/uL
TIM:	3.5872	nmole/L
Total concentration:	0.7347	ng/uL

Sample peak width (sec): 5 Sample min peak height: 50 Sample baseline V to V?: Y Sample baseline V to V points: 3
Sample filter: Binomial Number of points for filter: 3 Sample start region (min): 0 Sample end region (min): 30
Marker peak width (sec): 5 Marker min peak height: 200 Marker baseline V to V?: Y Marker baseline V to V points: 3
Lower marker selection: First peak > 500 RFU Upper marker selection: Last peak > 200 RFU
Ladder size (bp)75, 200, 300, 400, 500, 700, 1000, 1500, 2000, 3000, 4000, 5000, 7000, 10000, 20000
Quantification using: Lower Marker Final concentration (ng/uL): 0.5000 Dilution factor: 12.0

Sample: SampA12
Well location: A12
Created: Thursday, October 21, 2021 6:48:20 PM

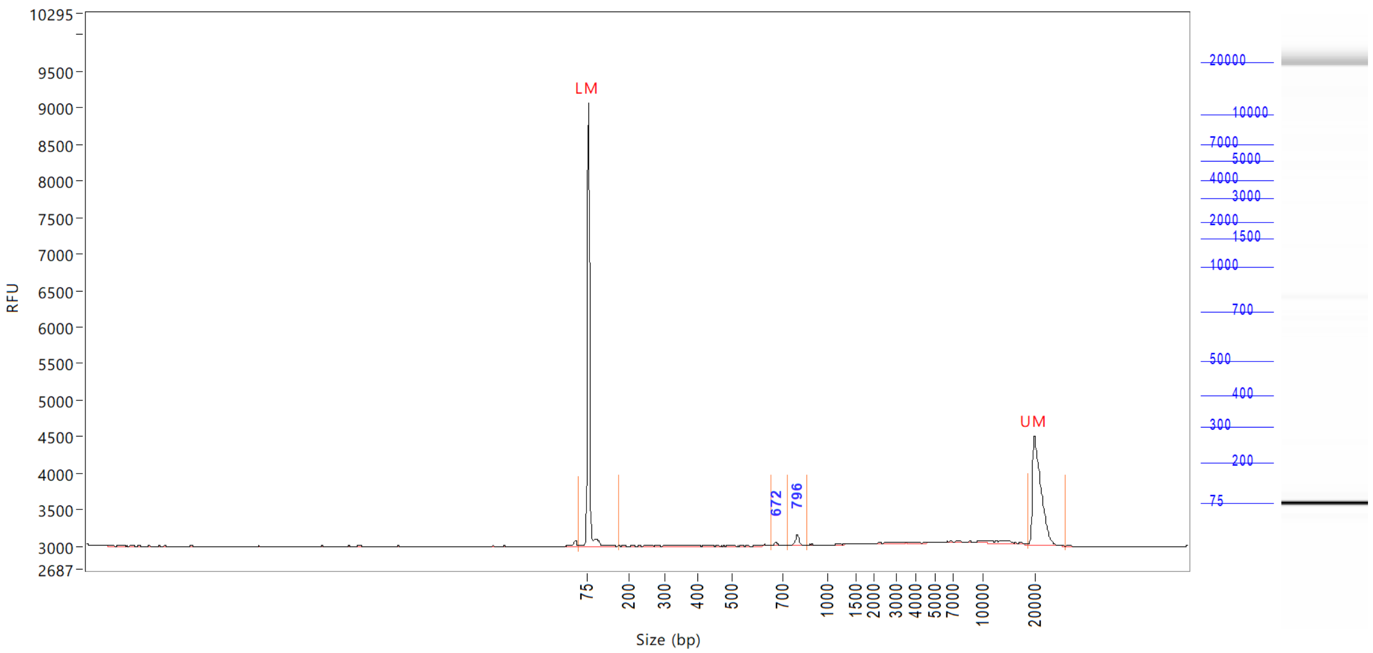

Peak	Size	Concentration	Relative concentration	Molarity	From	To	CV%	RFU
	(bp)	(ng/uL)	Percent	(nmole/L)	(bp)	(bp)		
1	75 (LM)	0.5000		10.9378	48	166	10.01	6077
2	672	0.0701	30.0	0.1702	657	731	2.06	53
3	796	0.1639	70.0	0.3405	731	861	2.11	145
4	20000 (UM)	0.2311		0.0185	18659	25682	4.53	1485

TIC:	0.2340	ng/uL
TIM:	0.5107	nmole/L
Total concentration:	0.9667	ng/uL

Sample peak width (sec): 5   Sample min peak height: 50   Sample baseline V to V?: Y   Sample baseline V to V points: 3
Sample filter: Binomial   Number of points for filter: 3   Sample start region (min): 0   Sample end region (min): 30
Marker peak width (sec): 5   Marker min peak height: 200   Marker baseline V to V?: Y   Marker baseline V to V points: 3
Lower marker selection: First peak > 500 RFU   Upper marker selection: Last peak > 200 RFU
Ladder size (bp)75, 200, 300, 400, 500, 700, 1000, 1500, 2000, 3000, 4000, 5000, 7000, 10000, 20000
Quantification using: Lower Marker   Final concentration (ng/uL): 0.5000   Dilution factor: 12.0

Sample: SampB12
Well location: B12
Created: Thursday, October 21, 2021 6:48:20 PM

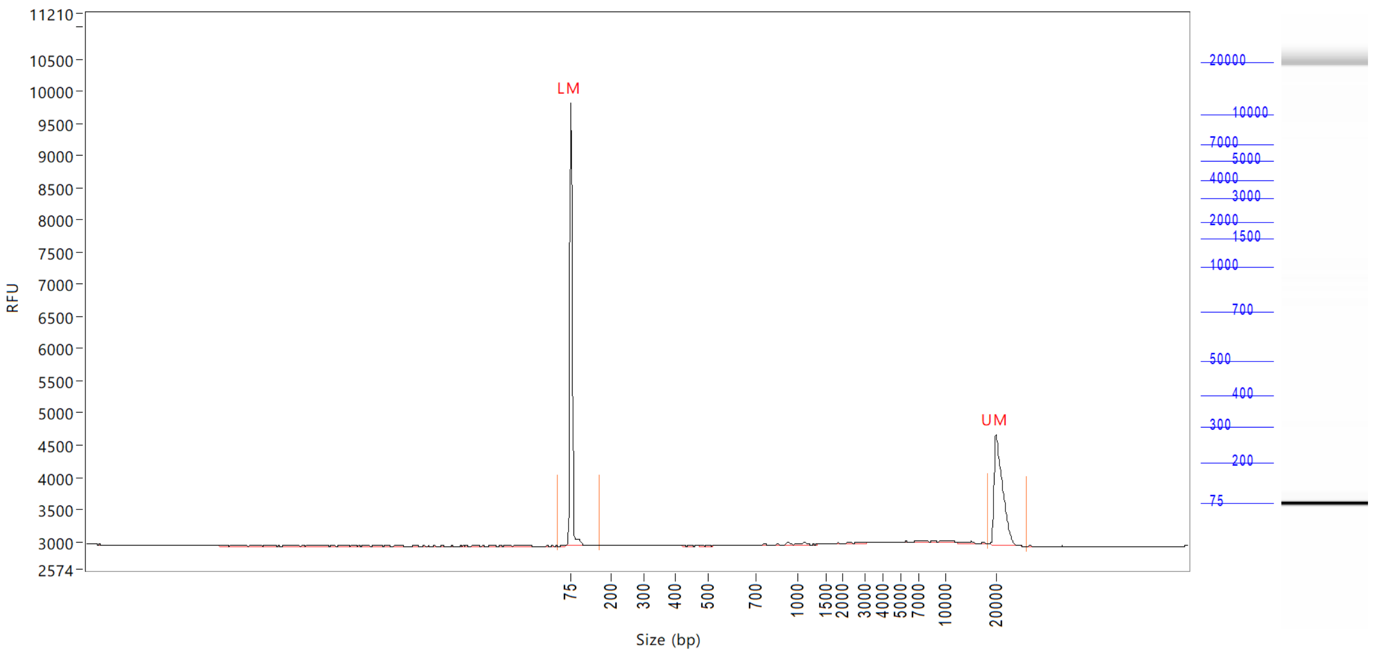

Peak	Size	Concentration	Relative concentration	Molarity	From	To	CV%	RFU
	(bp)	(ng/uL)	Percent	(nmole/L)	(bp)	(bp)		
1	75 (LM)	0.5000		11.0851	35	164	9.10	6894
2	20000 (UM)	0.2369		0.0190	18293	26045	4.46	1703

TIC:	0.0000	ng/uL
TIM:	0.0000	nmole/L
Total concentration:	0.6755	ng/uL

Sample peak width (sec): 5 Sample min peak height: 50 Sample baseline V to V?: Y Sample baseline V to V points: 3
Sample filter: Binomial Number of points for filter: 3 Sample start region (min): 0 Sample end region (min): 30
Marker peak width (sec): 5 Marker min peak height: 200 Marker baseline V to V?: Y Marker baseline V to V points: 3
Lower marker selection: First peak > 500 RFU Upper marker selection: Last peak > 200 RFU
Ladder size (bp)75, 200, 300, 400, 500, 700, 1000, 1500, 2000, 3000, 4000, 5000, 7000, 10000, 20000
Quantification using: Lower Marker Final concentration (ng/uL): 0.5000 Dilution factor: 12.0

Sample: SampC12
Well location: C12
Created: Thursday, October 21, 2021 6:48:20 PM

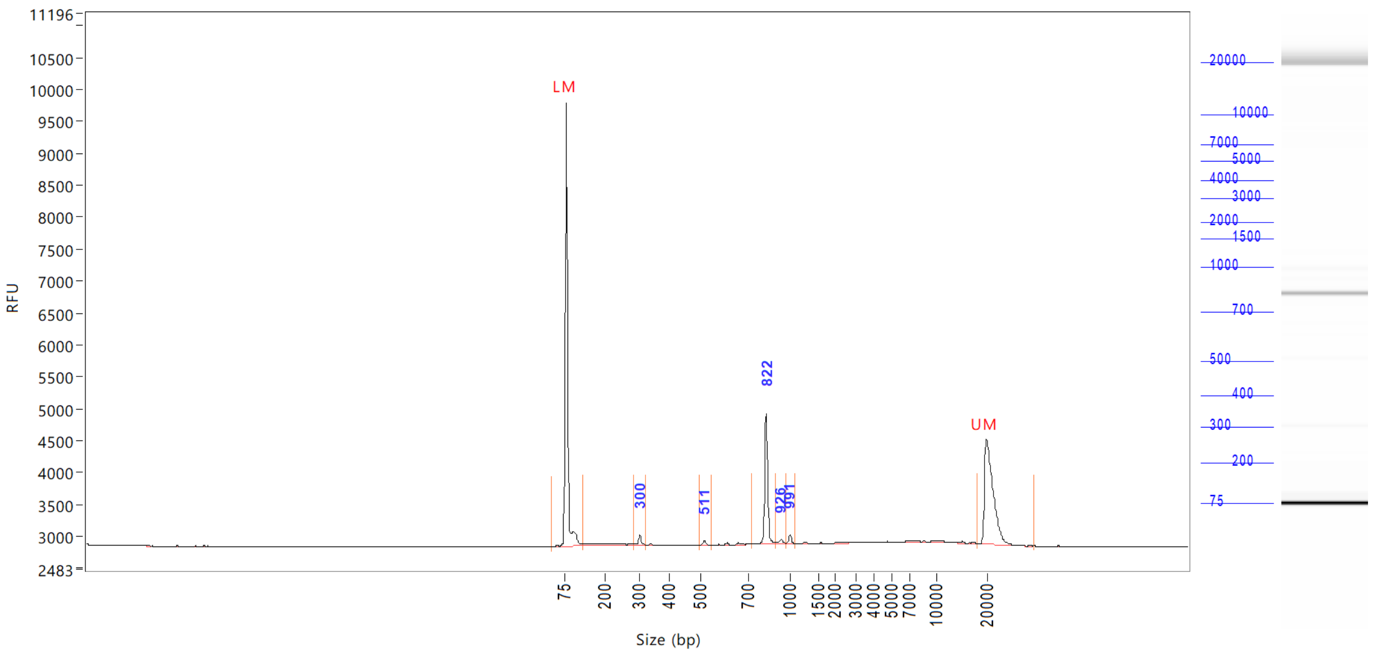

Peak	Size	Concentration	Relative concentration	Molarity	From	To	CV%	RFU
	(bp)	(ng/uL)	Percent	(nmole/L)	(bp)	(bp)		
1	75 (LM)	0.5000		10.9378	33	126	10.57	6960
2	300	0.1289	7.1	0.7092	282	321	1.88	154
3	511	0.0604	3.3	0.1949	494	539	1.48	72
4	822	1.4554	79.7	2.9283	714	896	2.02	2049
5	926	0.0737	4.0	0.1312	896	961	1.64	69
6	991	0.1080	5.9	0.1800	961	1079	1.28	141
7	20000 (UM)	0.2160		0.0172	18171	29430	5.38	1654

TIC:	1.8264	ng/uL
TIM:	4.1436	nmole/L
Total concentration:	2.4574	ng/uL

Sample peak width (sec): 5   Sample min peak height: 50   Sample baseline V to V?: Y   Sample baseline V to V points: 3
Sample filter: Binomial   Number of points for filter: 3   Sample start region (min): 0   Sample end region (min): 30
Marker peak width (sec): 5   Marker min peak height: 200   Marker baseline V to V?: Y   Marker baseline V to V points: 3
Lower marker selection: First peak > 500 RFU   Upper marker selection: Last peak > 200 RFU
Ladder size (bp)75, 200, 300, 400, 500, 700, 1000, 1500, 2000, 3000, 4000, 5000, 7000, 10000, 20000
Quantification using: Lower Marker   Final concentration (ng/uL): 0.5000   Dilution factor: 12.0

Sample: SampD12
Well location: D12
Created: Thursday, October 21, 2021 6:48:20 PM

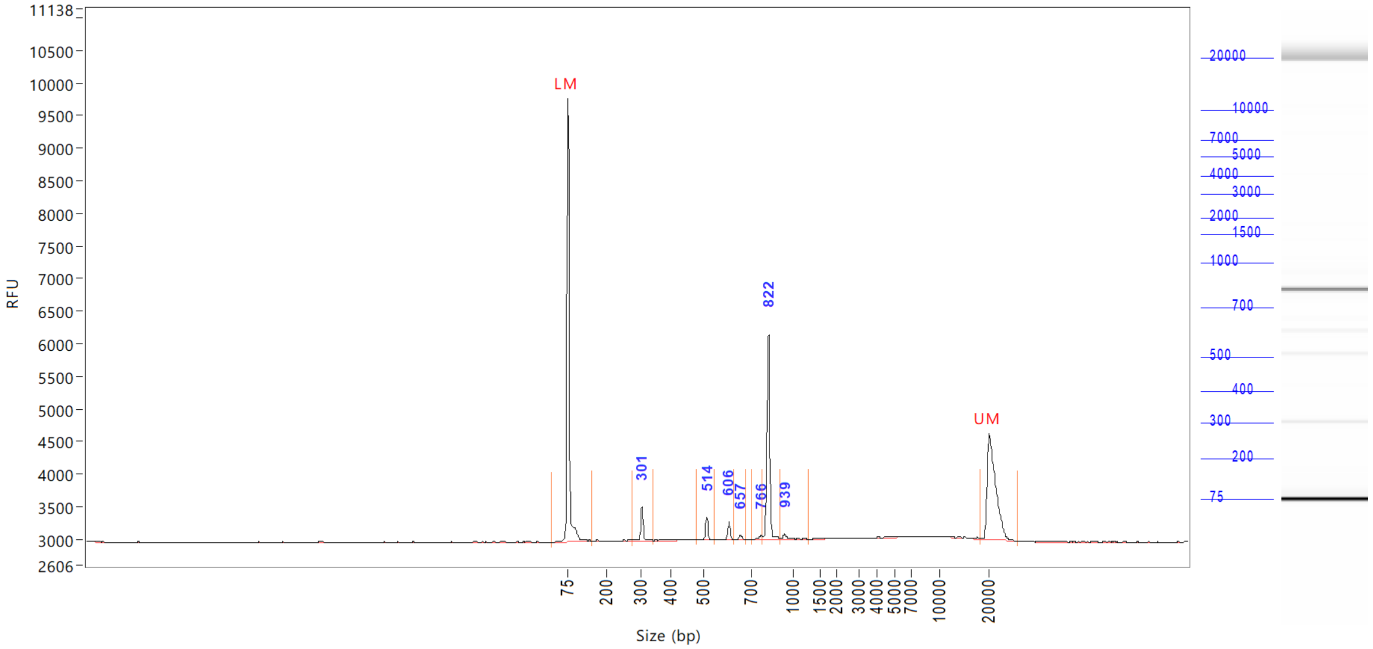

Peak	Size	Concentration	Relative concentration	Molarity	From	To	CV%	RFU
	(bp)	(ng/uL)	Percent	(nmole/L)	(bp)	(bp)		
1	75 (LM)	0.5000		10.9378	24	152	12.02	6811
2	301	0.4127	12.2	2.2705	275	339	2.16	513
3	514	0.2699	8.0	0.8725	477	544	1.47	357
4	606	0.2084	6.2	0.5715	544	626	1.70	271
5	657	0.0504	1.5	0.1269	626	680	1.23	66
6	766	0.0740	2.2	0.1608	705	783	2.45	59
7	822	2.2212	65.9	4.4691	783	913	1.74	3131
8	939	0.1332	4.0	0.2235	913	1278	7.80	80
9	20000 (UM)	0.2206		0.0176	18415	25803	4.54	1638

TIC:	3.3698	ng/uL
TIM:	8.6949	nmole/L
Total concentration:	3.7240	ng/uL

Sample peak width (sec): 5   Sample min peak height: 50   Sample baseline V to V?: Y   Sample baseline V to V points: 3
Sample filter: Binomial   Number of points for filter: 3 Sample start region (min): 0 Sample end region (min): 30
Marker peak width (sec): 5   Marker min peak height: 200   Marker baseline V to V?: Y   Marker baseline V to V points: 3
Lower marker selection: First peak > 500 RFU   Upper marker selection: Last peak > 200 RFU
Ladder size (bp)75, 200, 300, 400, 500, 700, 1000, 1500, 2000, 3000, 4000, 5000, 7000, 10000, 20000
Quantification using: Lower Marker   Final concentration (ng/uL): 0.5000   Dilution factor: 12.0

Sample: SampE12
Well location: E12
Created: Thursday, October 21, 2021 6:48:20 PM

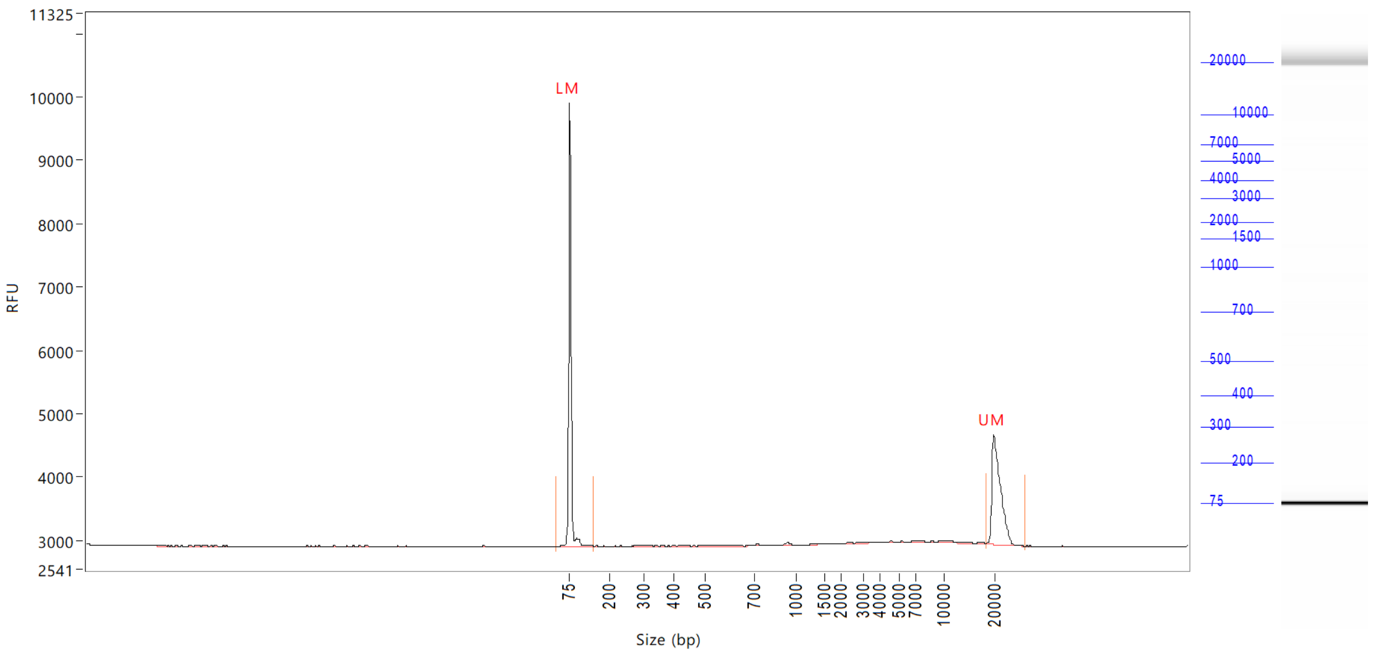

Peak	Size	Concentration	Relative concentration	Molarity	From	To	CV%	RFU
	(bp)	(ng/uL)	Percent	(nmole/L)	(bp)	(bp)		
1	75 (LM)	0.5000		11.0851	35	150	10.24	7021
2	20000 (UM)	0.2398		0.0191	18415	26166	4.59	1730

TIC:	0.0000	ng/uL
TIM:	0.0000	nmole/L
Total concentration:	0.6680	ng/uL

```
Sample peak width (sec): 5    Sample min peak height: 50    Sample baseline V to V?: Y    Sample baseline V to V points: 3
Sample filter: Binomial       Number of points for filter: 3  Sample start region (min): 0  Sample end region (min): 30
Marker peak width (sec): 5    Marker min peak height: 200   Marker baseline V to V?: Y    Marker baseline V to V points: 3
Lower marker selection: First peak > 500 RFU                Upper marker selection: Last peak > 200 RFU
Ladder size (bp)75, 200, 300, 400, 500, 700, 1000, 1500, 2000, 3000, 4000, 5000, 7000, 10000, 20000
Quantification using: Lower Marker          Final concentration (ng/uL): 0.5000          Dilution factor: 12.0
```

Sample: SampF12
Well location: F12
Created: Thursday, October 21, 2021 6:48:20 PM

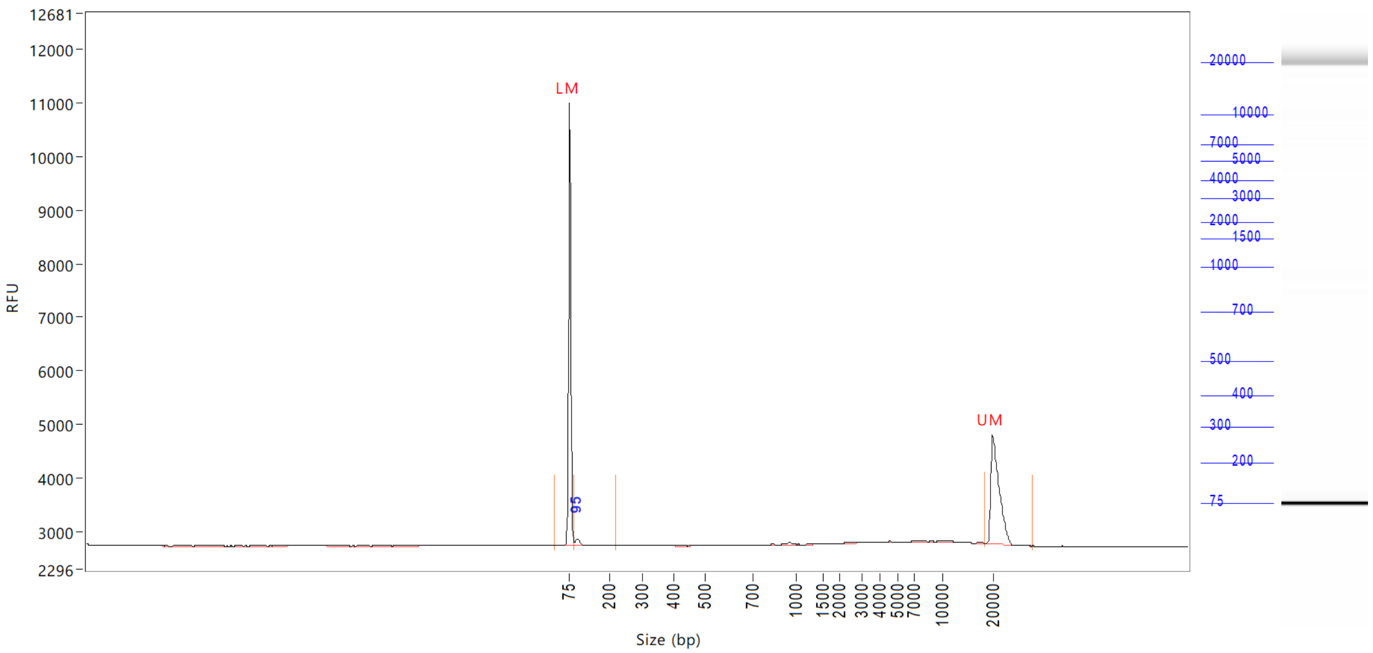

Peak	Size	Concentration	Relative concentration	Molarity	From	To	CV%	RFU
	(bp)	(ng/uL)	Percent	(nmole/L)	(bp)	(bp)		
1	75 (LM)	0.5000		11.3919	30	87	4.87	8289
2	95	0.2737	100.0	4.0139	87	221	26.76	130
3	20000 (UM)	0.2414		0.0193	18537	28100	4.42	2027

TIC:	0.2737	ng/uL
TIM:	4.0139	nmole/L
Total concentration:	0.8291	ng/uL

```
Sample peak width (sec): 5    Sample min peak height: 50    Sample baseline V to V?: Y    Sample baseline V to V points: 3
Sample filter: Binomial       Number of points for filter: 3  Sample start region (min): 0  Sample end region (min): 30
Marker peak width (sec): 5    Marker min peak height: 200   Marker baseline V to V?: Y    Marker baseline V to V points: 3
Lower marker selection: First peak > 500 RFU                Upper marker selection: Last peak > 200 RFU
Ladder size (bp)75, 200, 300, 400, 500, 700, 1000, 1500, 2000, 3000, 4000, 5000, 7000, 10000, 20000
Quantification using: Lower Marker          Final concentration (ng/uL): 0.5000          Dilution factor: 12.0
```

Sample: SampG12
Well location: G12
Created: Thursday, October 21, 2021 6:48:20 PM

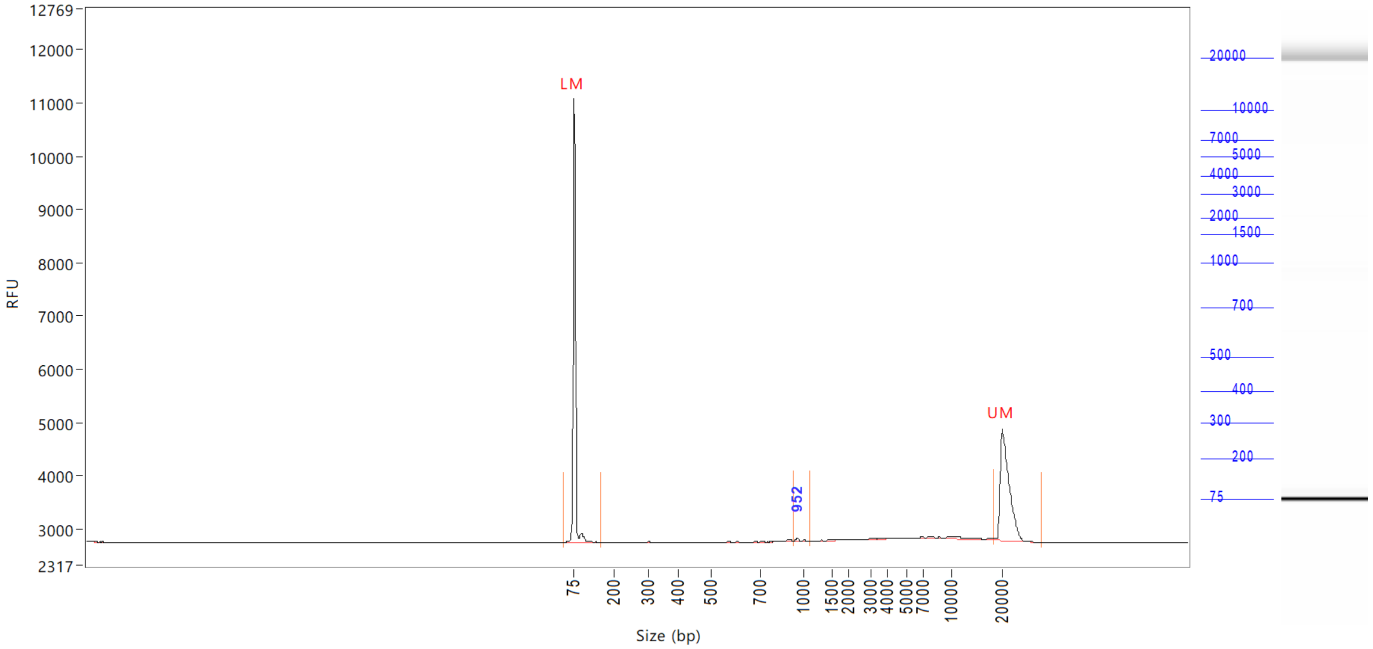

Peak	Size	Concentration	Relative concentration	Molarity	From	To	CV%	RFU
	(bp)	(ng/uL)	Percent	(nmole/L)	(bp)	(bp)		
1	75 (LM)	0.5000		11.0851	45	160	9.01	8350
2	952	0.0610	100.0	0.1042	926	1123	2.99	67
3	20000 (UM)	0.2500		0.0199	18293	27617	4.73	2098

TIC:	0.0610	ng/uL
TIM:	0.1042	nmole/L
Total concentration:	0.6877	ng/uL

Sample peak width (sec): 5 Sample min peak height: 50 Sample baseline V to V?: Y Sample baseline V to V points: 3
Sample filter: Binomial Number of points for filter: 3 Sample start region (min): 0 Sample end region (min): 30
Marker peak width (sec): 5 Marker min peak height: 200 Marker baseline V to V?: Y Marker baseline V to V points: 3
Lower marker selection: First peak > 500 RFU Upper marker selection: Last peak > 200 RFU
Ladder size (bp)75, 200, 300, 400, 500, 700, 1000, 1500, 2000, 3000, 4000, 5000, 7000, 10000, 20000
Quantification using: Lower Marker Final concentration (ng/uL): 0.5000 Dilution factor: 12.0

Sample: SampH12
Well location: H12
Created: Thursday, October 21, 2021 6:48:20 PM

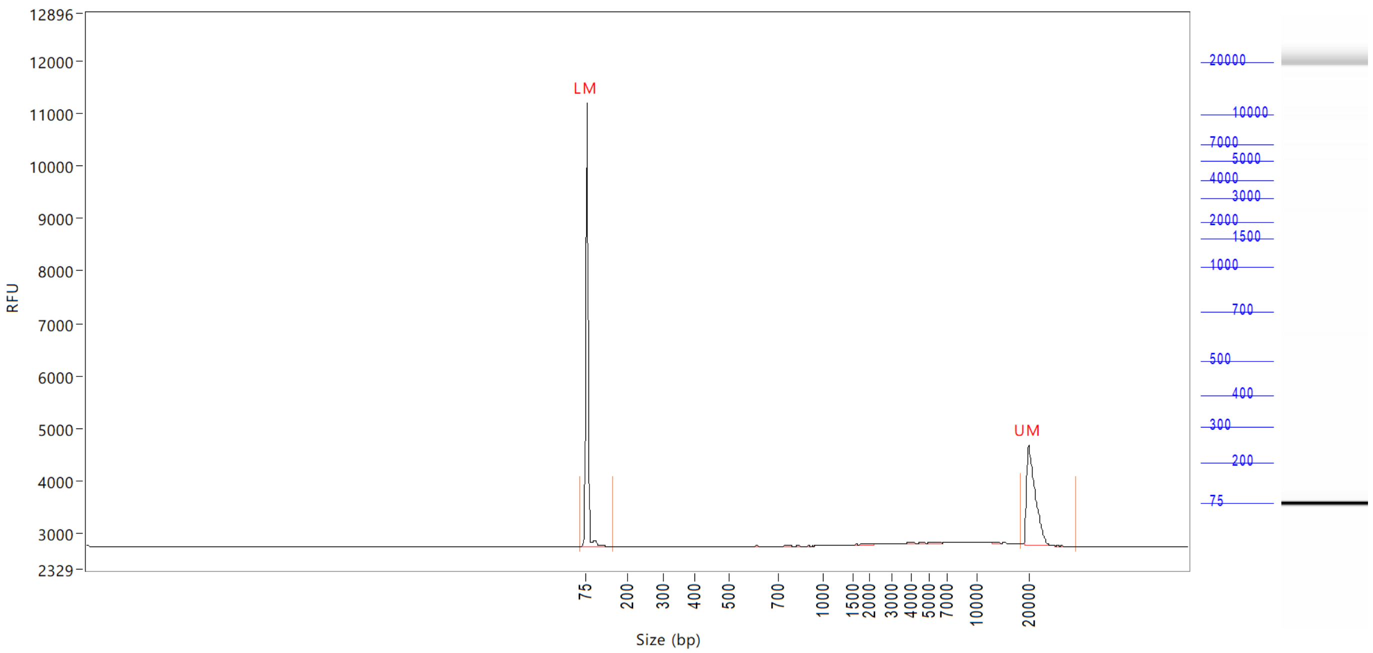

Peak	Size	Concentration	Relative concentration	Molarity	From	To	CV%	RFU
	(bp)	(ng/uL)	Percent	(nmole/L)	(bp)	(bp)		
1	75 (LM)	0.5000		11.2364	54	154	8.01	8448
2	20000 (UM)	0.2129		0.0170	18415	29189	4.80	1883

TIC:	0.0000	ng/uL
TIM:	0.0000	nmole/L
Total concentration:	0.4365	ng/uL

Sample peak width (sec): 5 Sample min peak height: 200 Sample baseline V to V?: Y Sample baseline V to V points: 3
Sample filter: Binomial Number of points for filter: 3 Sample start region (min): 0 Sample end region (min): 30
Marker peak width (sec): 5 Marker min peak height: 200 Marker baseline V to V?: Y Marker baseline V to V points: 3
Lower marker selection: First peak > 500 RFU Upper marker selection: Last peak > 200 RFU
Ladder size (bp)75, 200, 300, 400, 500, 700, 1000, 1500, 2000, 3000, 4000, 5000, 7000, 10000, 20000
Quantification using: Lower Marker Final concentration (ng/uL): 0.5000 Dilution factor: 12.0

Sample: DNA size ladder
Well location: A7
Created: Thursday, October 21, 2021 6:48:20 PM
Import from: C:\Users\admin\Desktop\210810_alberto_ladder.SCAL
Fit type: Point to point

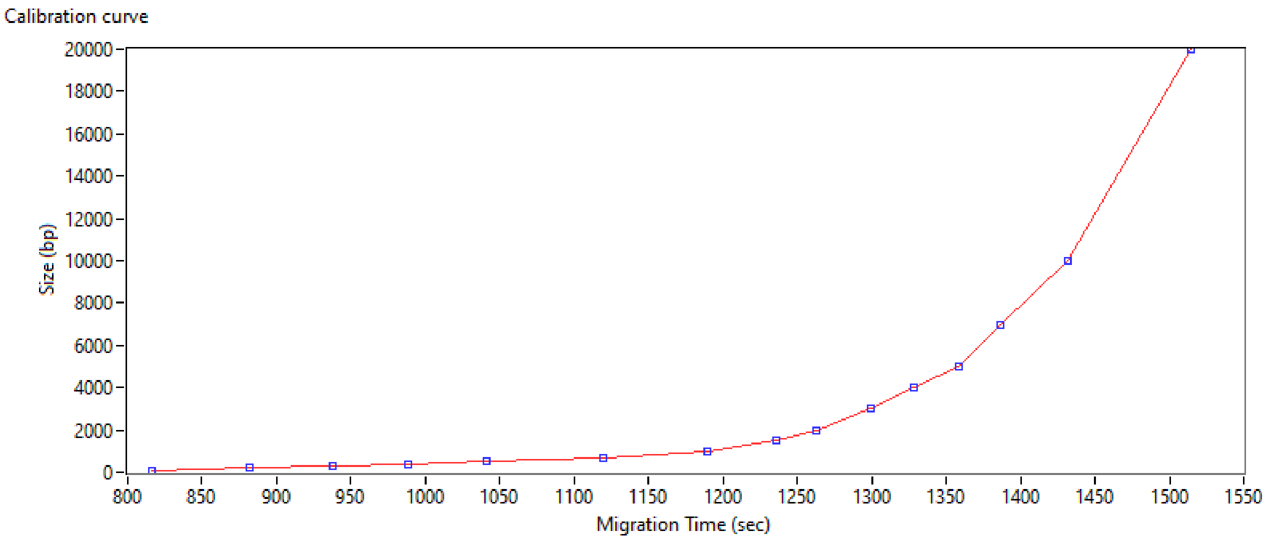

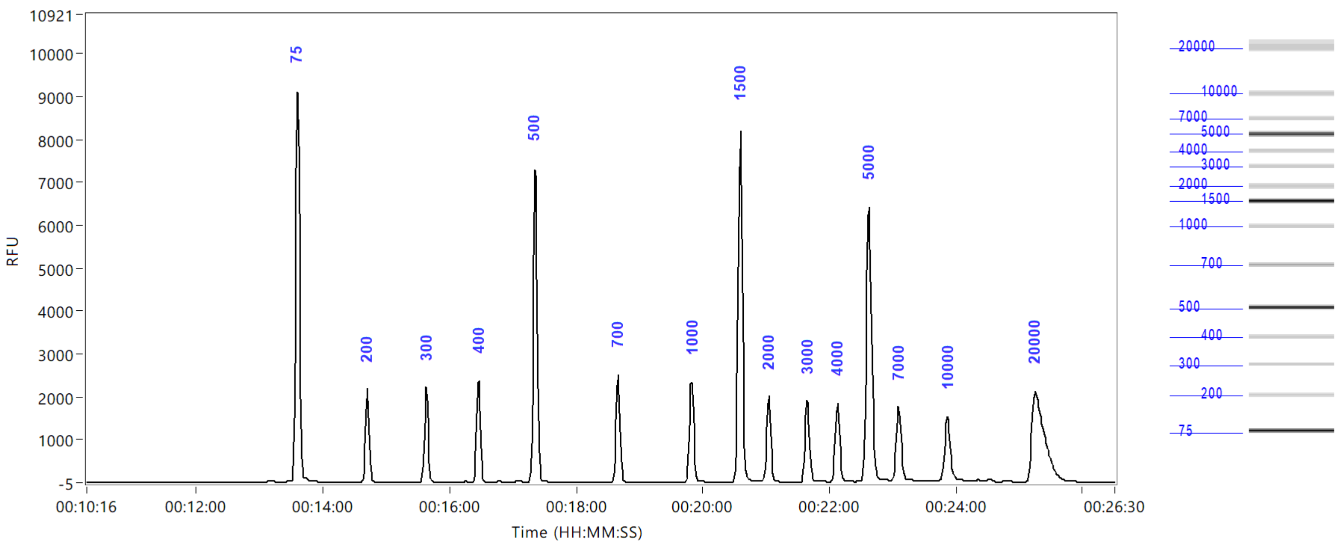

Supplement: Supplementary file 2 — sb3c00292_si_002.zip [file sb3c00292_si_002.zip › dnada_supplementary_material_pks_library_build/colony_pcr/ZAG/18-38-27_plate7/colonypcrplate_7.pdf]
