# Supplementary material for: Automated Platform for the Plasmid Construction Process
Source: ACS Synth Biol. 2023 Nov 10;12(12):3506–13. doi: 10.1021/acssynbio.3c00292 (PMC10729297; doi:10.1021/acssynbio.3c00292)

Instrument controller software run summary:

Filename and data path: C:\AATI\Data\2021 10 20\16-28-48\2021 10 20 16H 28M..raw
Created: Wednesday, October 20, 2021 4:38:54 PM
Number of capillaries: 96
Array serial number: 111920-09SFS
Effect length: 33 cm
Array usage count: 261
Instrument type: ZAG DNA Analyzer
Instrument controller software version: 1.0.1.9
Device serial number: Z7597

Method Information

Method name: ZAG130GP33 - DNA 75-20000bp - Gel Prime Only.mthds
Gel prime: Yes
Full conditioning: No
Gel prime to buffer: No
Gel selection: Gel 1
Perform prerun: 8.0 kV, 30 sec.
Rinse: No
Marker 1: Row: A, 5.0 kV, 5 sec.
Rinse: No
Sample injection: 5.0 kV, 5 sec.
Separation: 8.0 kV, 30.0 min.
Tray name: Sample Tray-1

Analysis mode: DNA

Notes

Gel Image

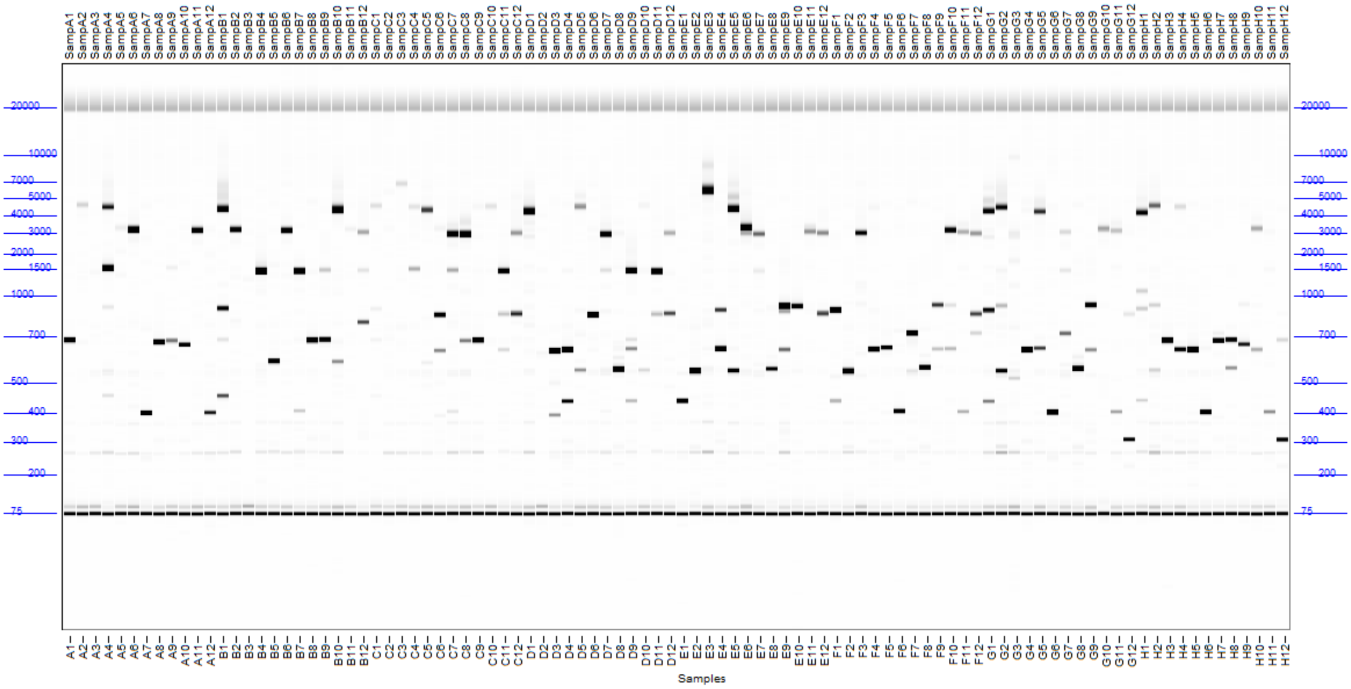
20000
10000
7000
5000
4000
3000
2000
1500
1000
700
500
400
300
200
75
Samples

Filename and data path: C:\AATI\Data\2021 10 20\16-28-48\2021 10 20 16H 28M..raw

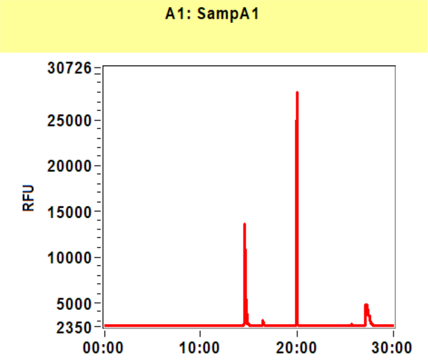

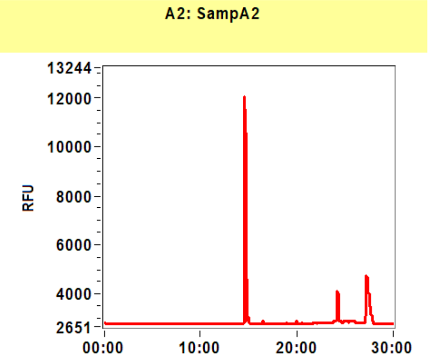

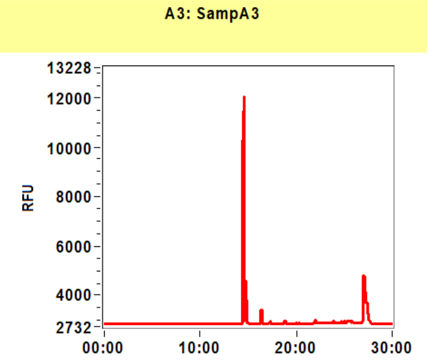

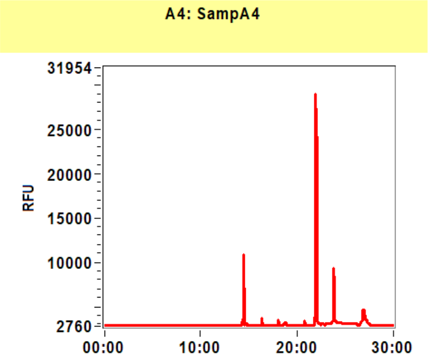

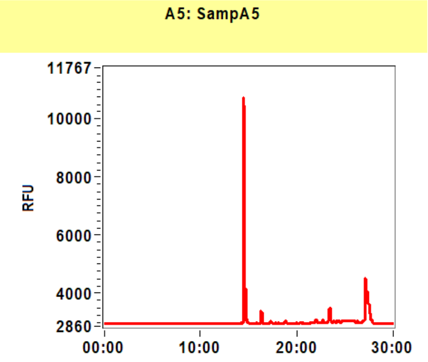

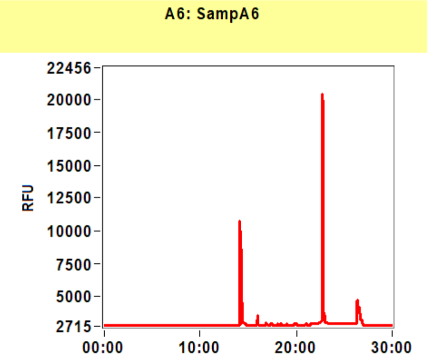

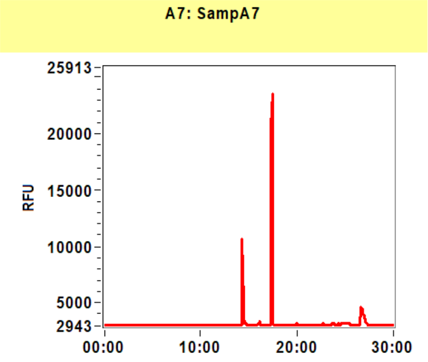

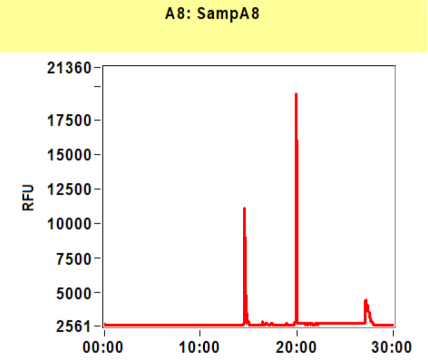

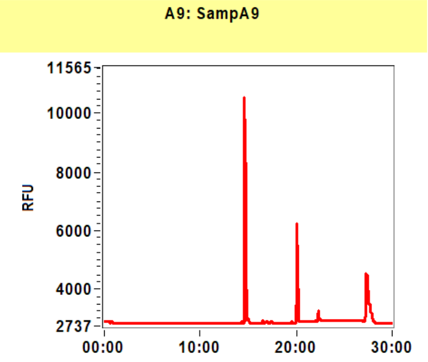

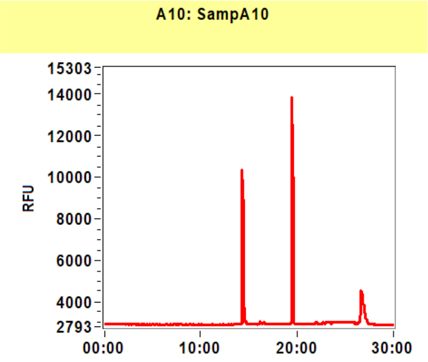

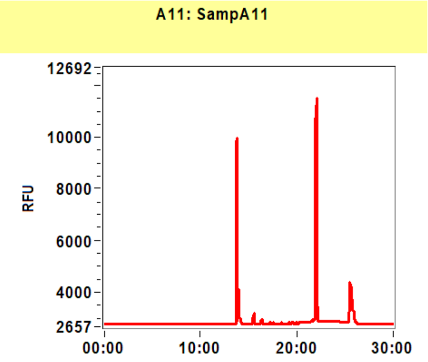

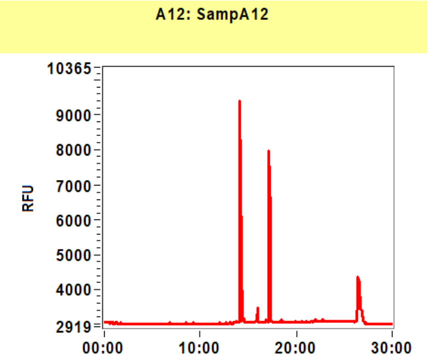

Filename and data path: C:\AATI\Data\2021 10 20\16-28-48\2021 10 20 16H 28M..raw

B1: SampB1

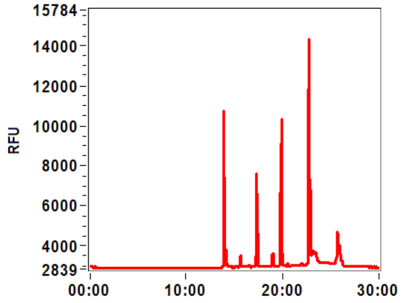

B2: SampB2

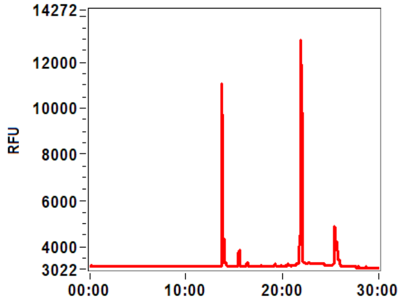

B3: SampB3

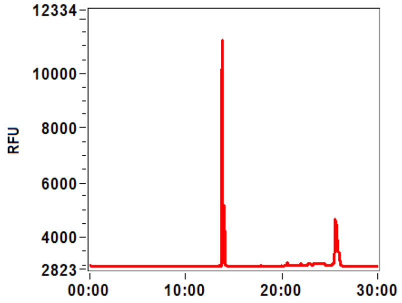

B4: SampB4

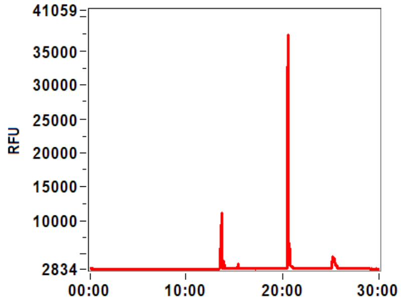

B5: SampB5

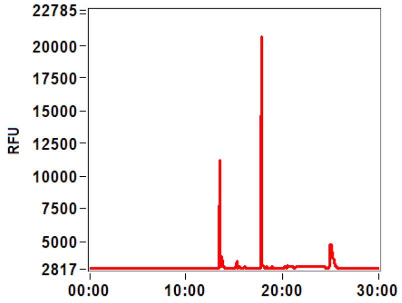

B6: SampB6

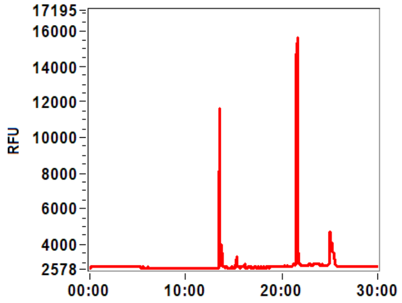

B7: SampB7

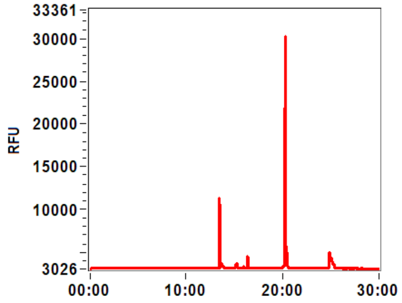

B8: SampB8

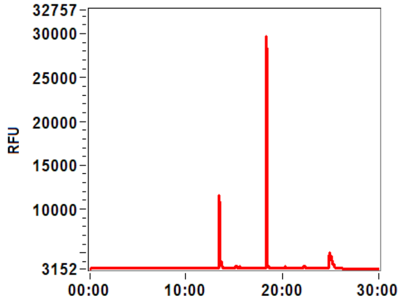

B9: SampB9

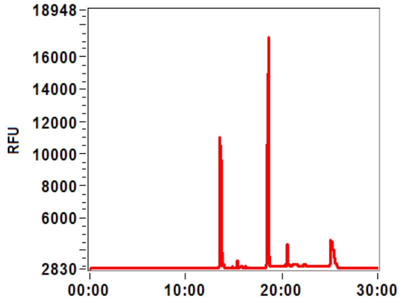

B10: SampB10

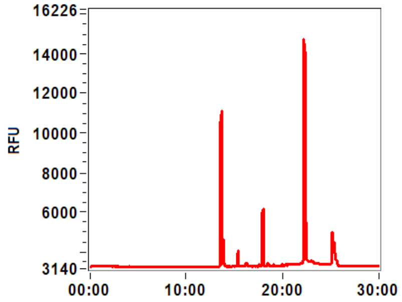

B11: SampB11

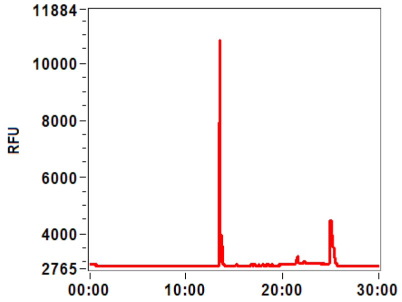

B12: SampB12

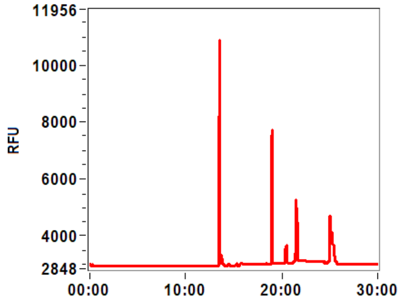

Filename and data path: C:\AATI\Data\2021 10 20\16-28-48\2021 10 20 16H 28M..raw

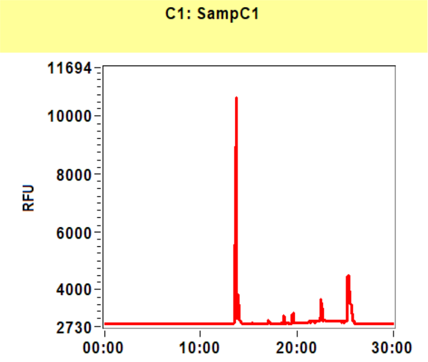

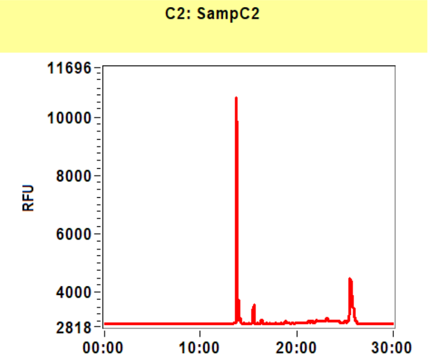

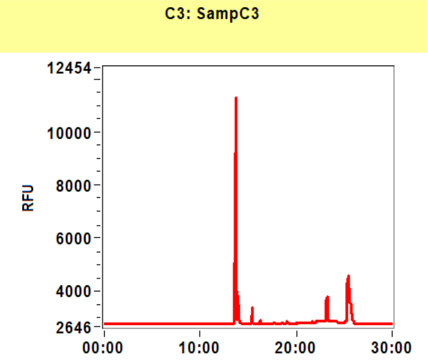

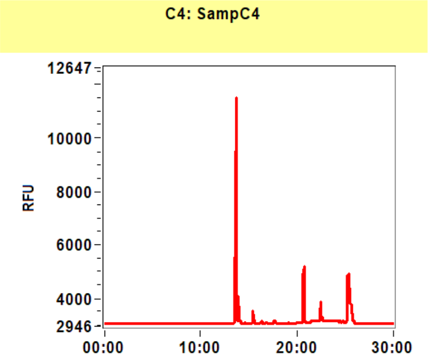

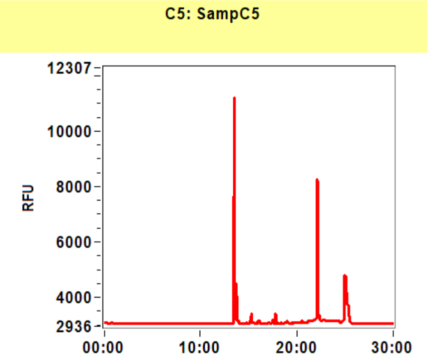

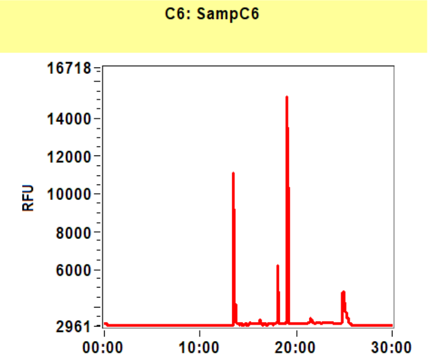

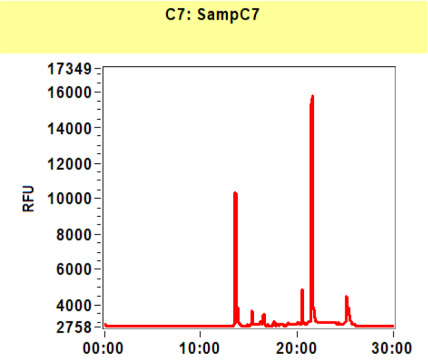

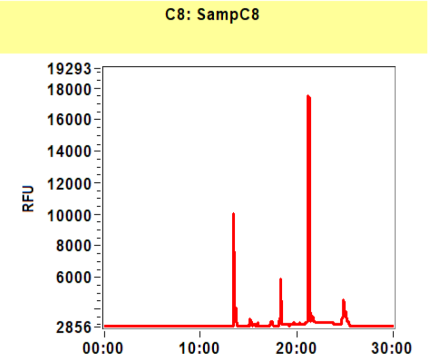

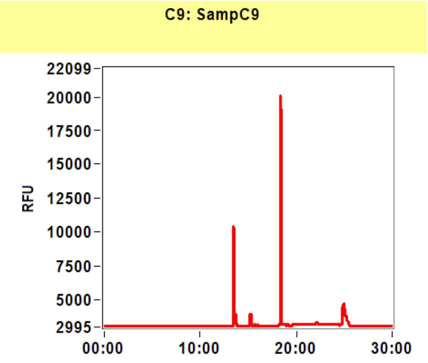

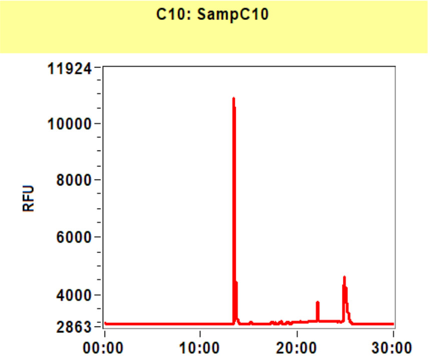

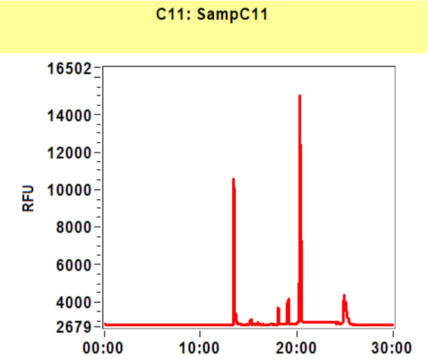

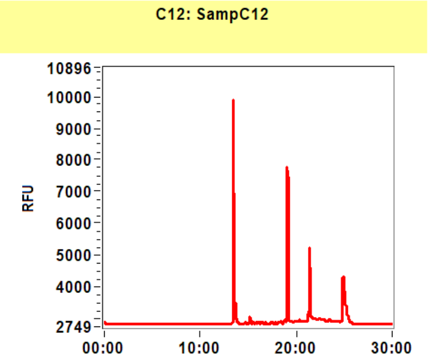

Filename and data path: C:\AATI\Data\2021 10 20\16-28-48\2021 10 20 16H 28M..raw

D1: SampD1

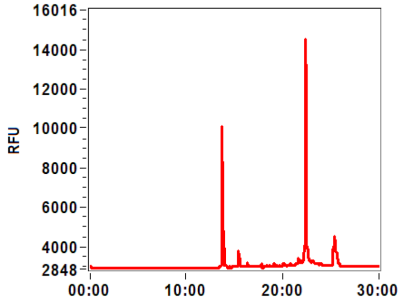

D2: SampD2

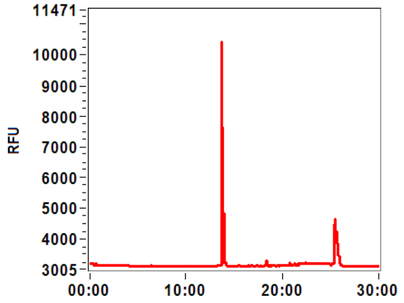

D3: SampD3

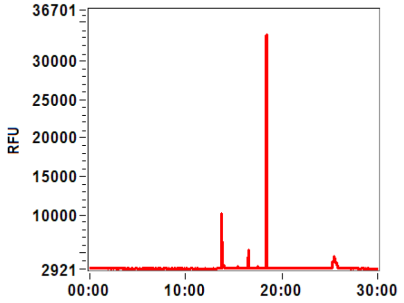

D4: SampD4

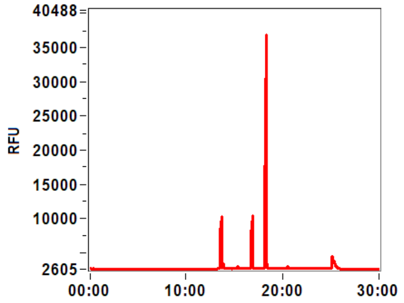

D5: SampD5

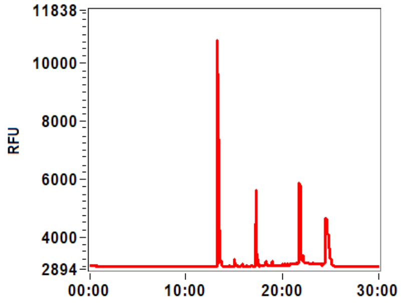

D6: SampD6

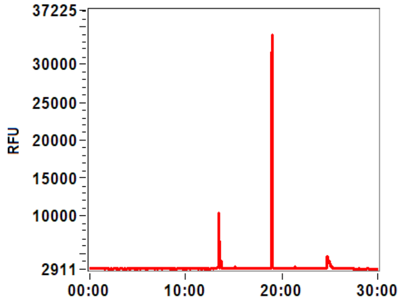

D7: SampD7

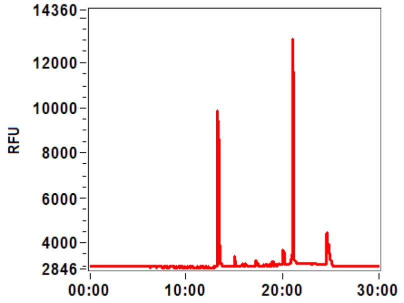

D8: SampD8

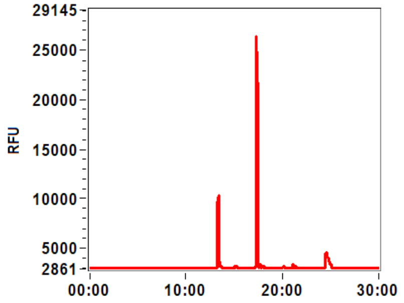

D9: SampD9

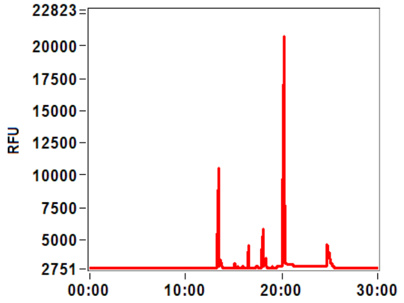

D10: SampD10

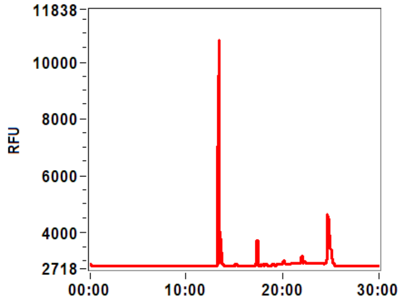

D11: SampD11

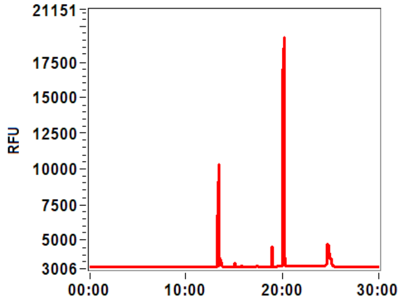

D12: SampD12

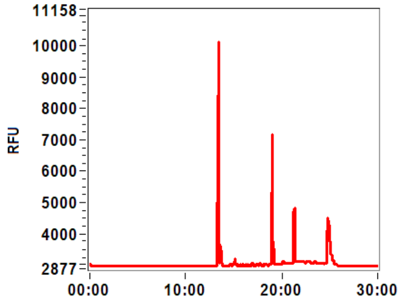

Filename and data path: C:\AATI\Data\2021 10 20\16-28-48\2021 10 20 16H 28M..raw

E1: SampE1

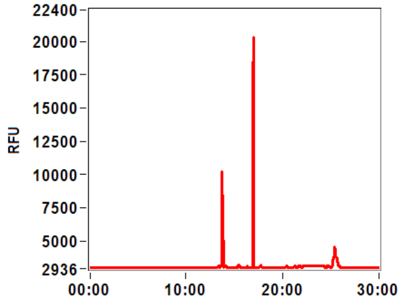

E2: SampE2

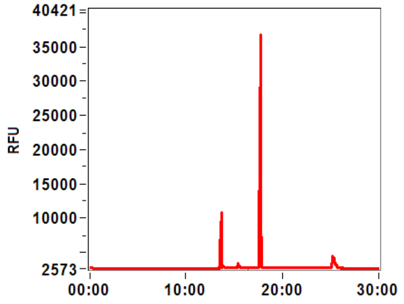

E3: SampE3

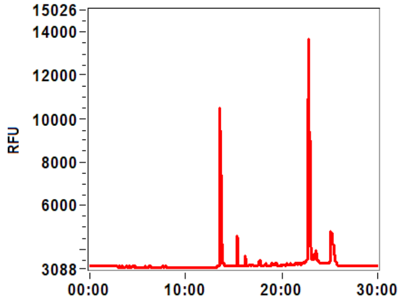

E4: SampE4

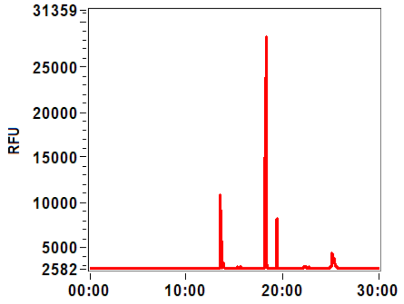

E5: SampE5

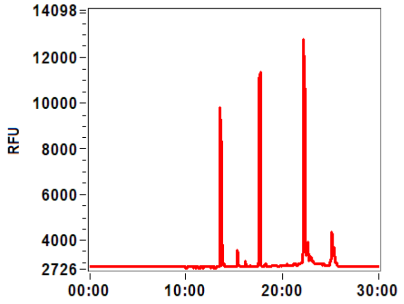

E6: SampE6

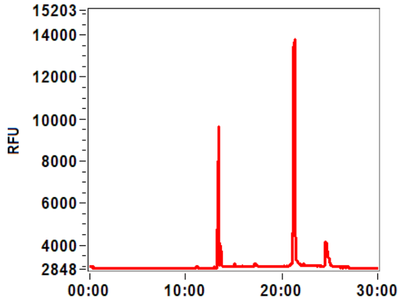

E7: SampE7

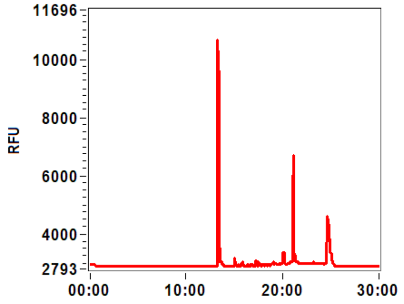

E8: SampE8

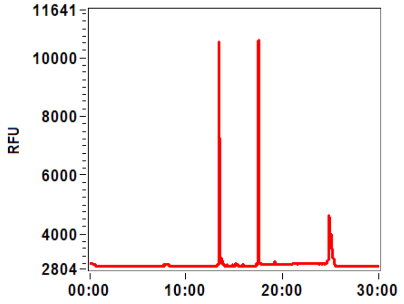

E9: SampE9

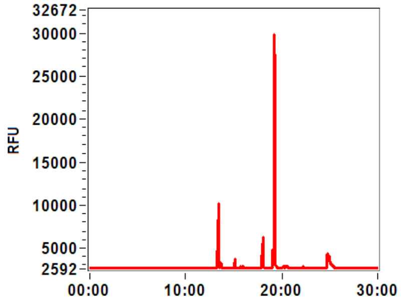

E10: SampE10

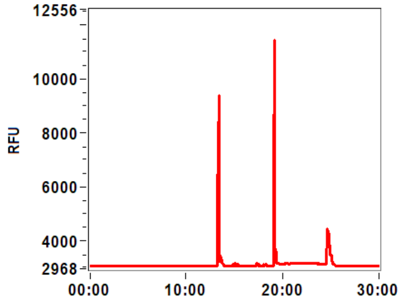

E11: SampE11

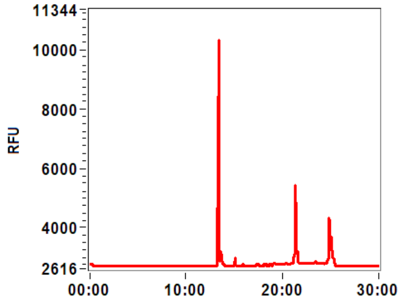

E12: SampE12

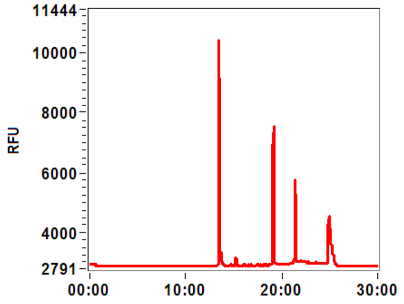

Filename and data path: C:\AATI\Data\2021 10 20\16-28-48\2021 10 20 16H 28M..raw

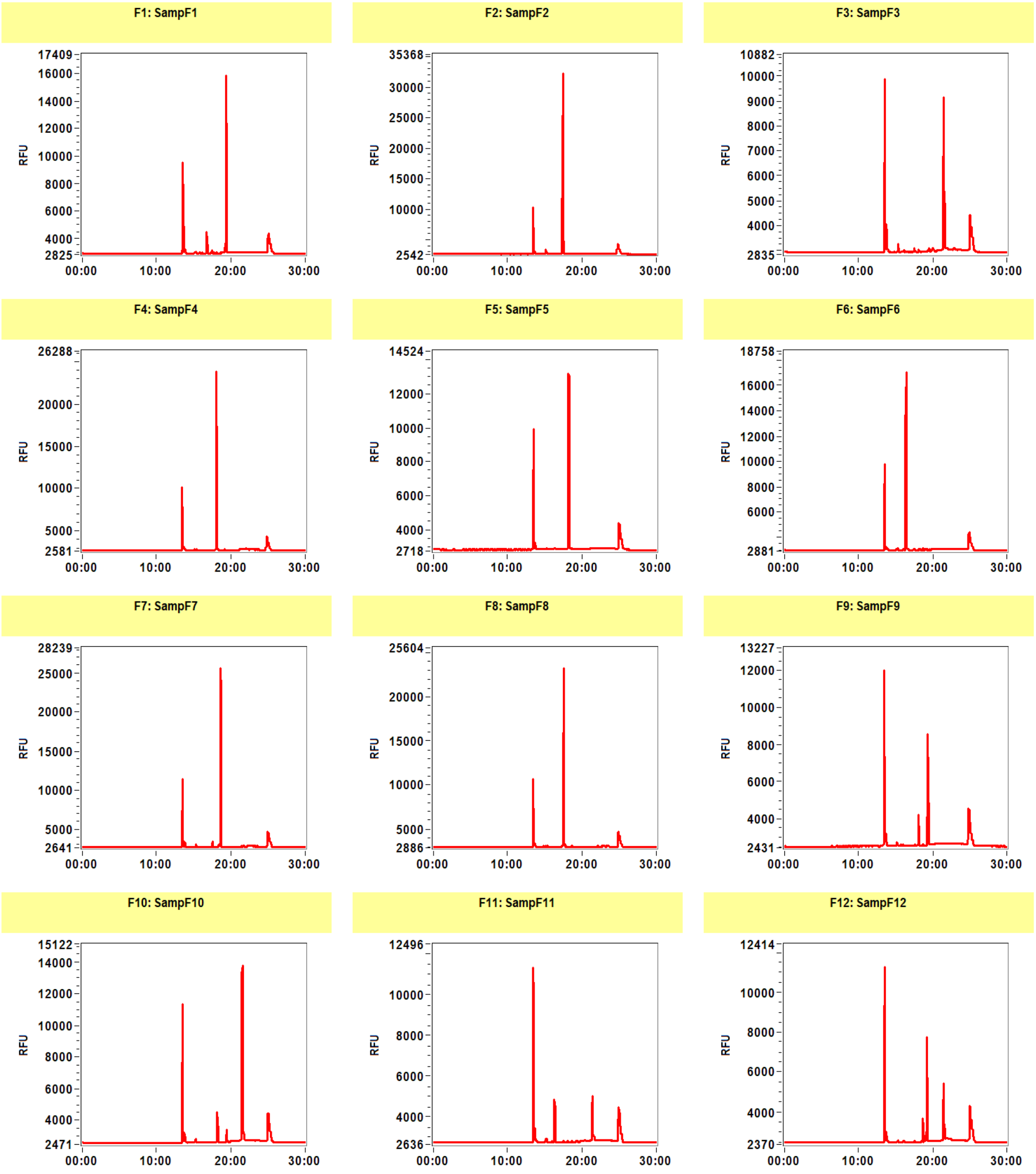

Filename and data path: C:\AATI\Data\2021 10 20\16-28-48\2021 10 20 16H 28M..raw

G1: SampG1

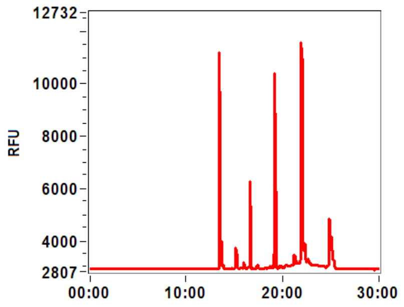

G2: SampG2

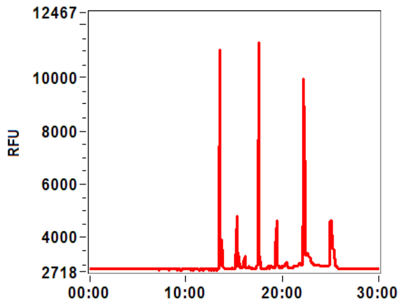

G3: SampG3

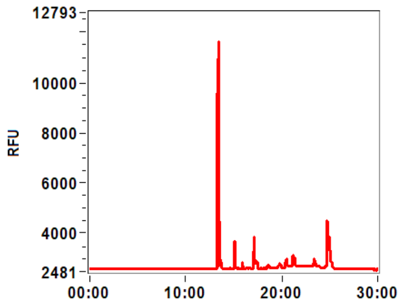

G4: SampG4

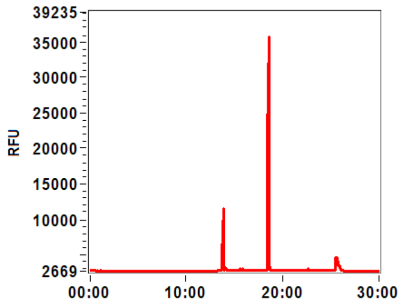

G5: SampG5

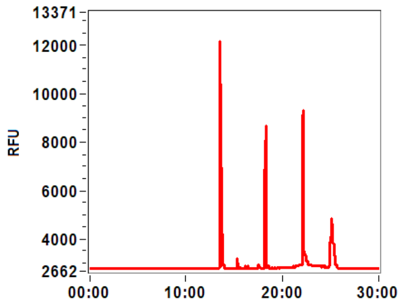

G6: SampG6

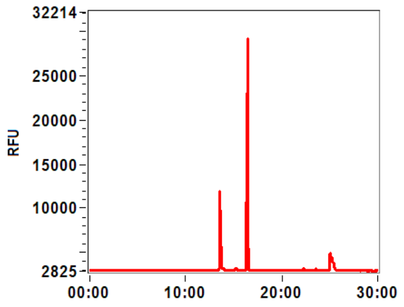

G7: SampG7

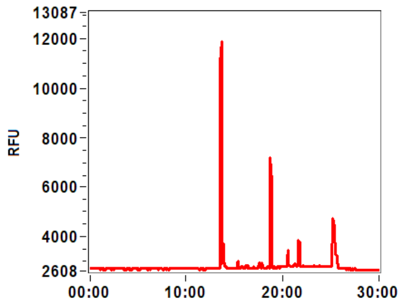

G8: SampG8

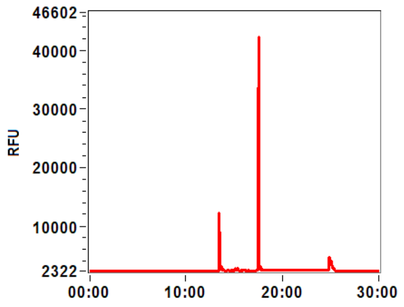

G9: SampG9

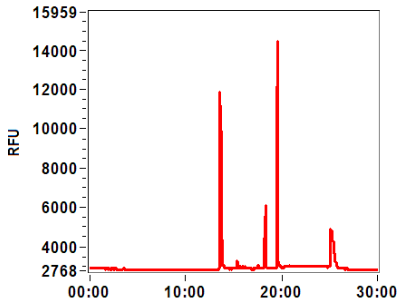

G10: SampG10

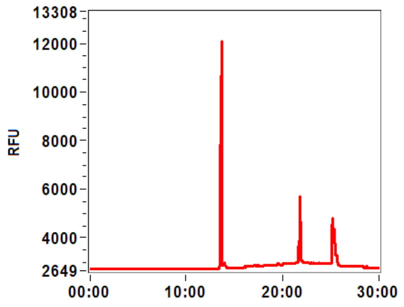

G11: SampG11

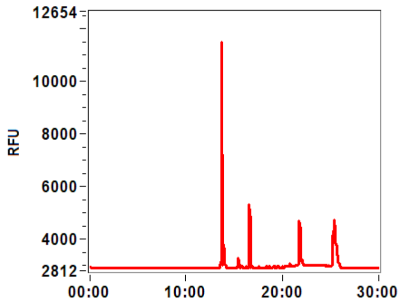

G12: SampG12

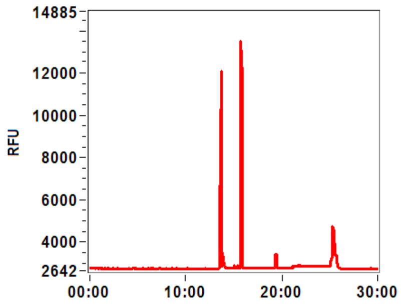

Filename and data path: C:\AATI\Data\2021 10 20\16-28-48\2021 10 20 16H 28M..raw

H1: SampH1

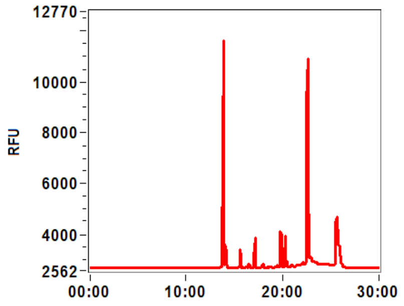

H2: SampH2

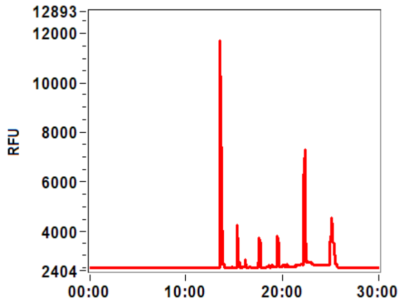

H3: SampH3

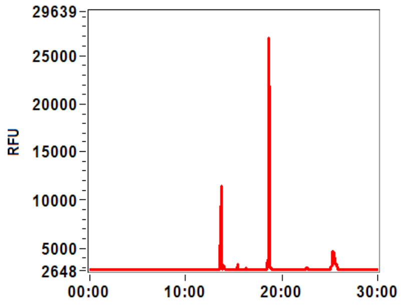

H4: SampH4

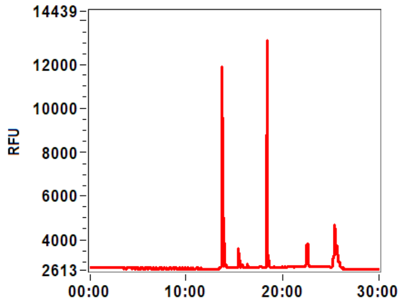

H5: SampH5

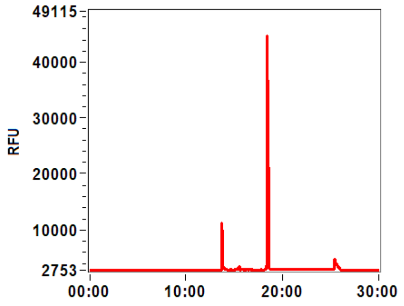

H6: SampH6

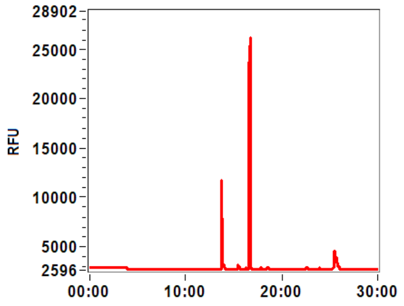

H7: SampH7

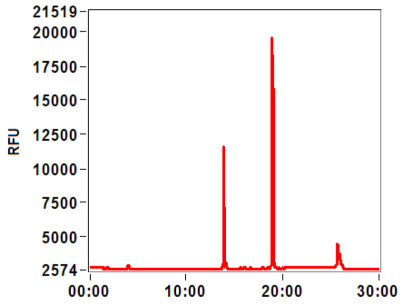

H8: SampH8

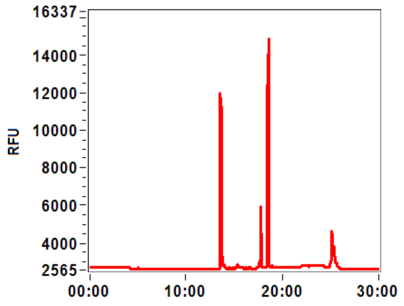

H9: SampH9

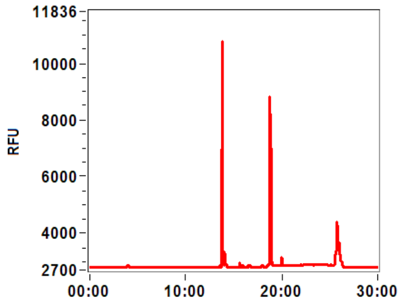

H10: SampH10

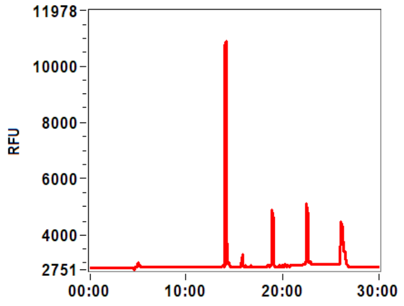

H11: SampH11

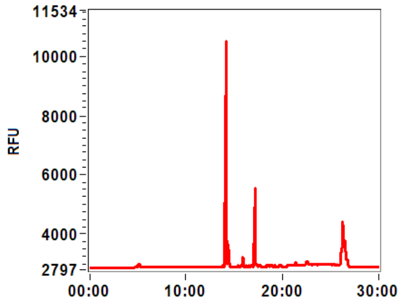

H12: SampH12

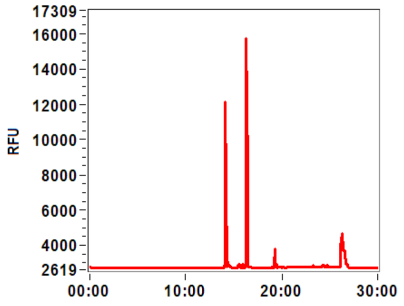

Sample: SampA1
Well location: A1
Created: Wednesday, October 20, 2021 4:38:54 PM

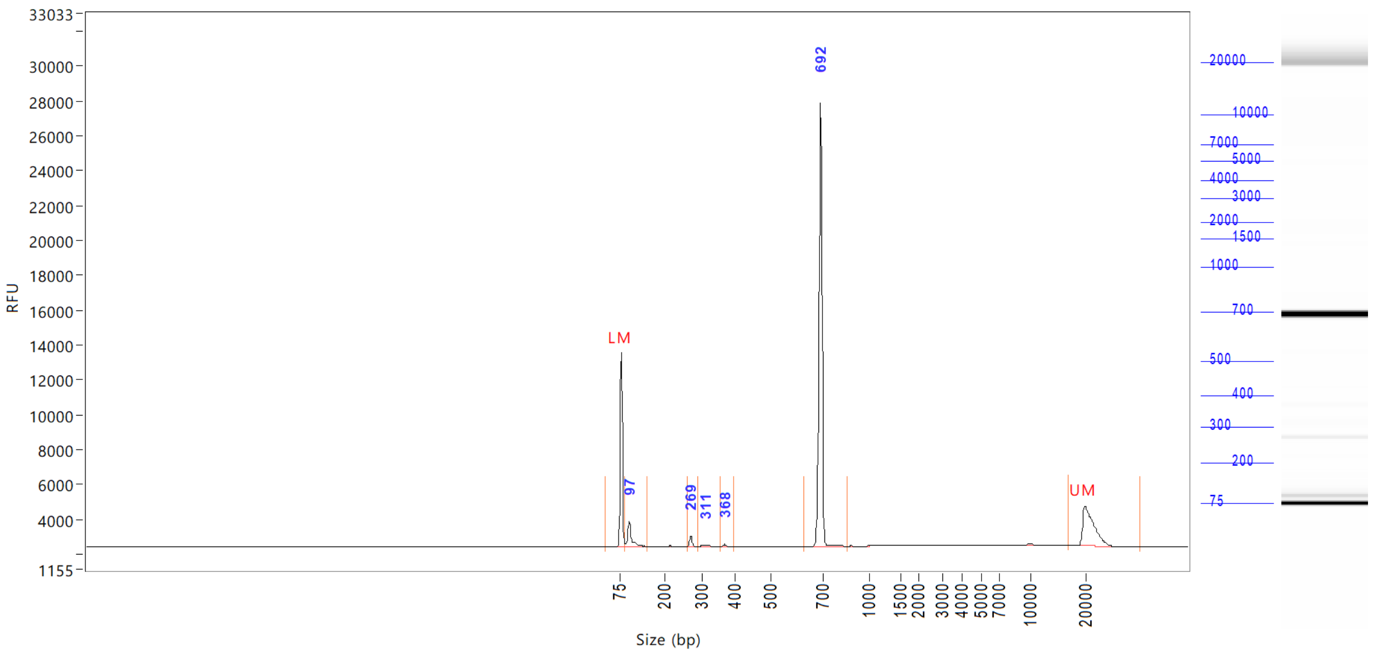

Peak	Size (bp)	Concentration (ng/uL)	From (bp)	To (bp)	Average size (bp)	CV%	RFU	Corrected peak area
1	75 (LM)	0.5000	33	86	73	4.44	11169	56.094
2	97	1.2602	86	148	99	10.44	1415	11.782
3	269	0.2976	260	289	267	1.33	584	2.783
4	311	0.1192	289	354	311	3.84	87	1.115
5	368	0.0698	354	396	366	1.64	118	0.653
6	692	13.6796	626	853	688	1.38	25468	127.890
7	20000 (UM)	0.2650	17073	29914	20868	5.70	2283	29.727

TIC:	15.4265	ng/uL
TIM:	56.4026	nmole/L
Total concentration:	15.9910	ng/uL

Sample peak width (sec): 5   Sample min peak height: 50   Sample baseline V to V?: Y   Sample baseline V to V points: 3
Sample filter: Binomial   Number of points for filter: 3 Sample start region (min): 0 Sample end region (min): 30
Marker peak width (sec): 5   Marker min peak height: 200   Marker baseline V to V?: Y   Marker baseline V to V points: 3
Lower marker selection: First peak > 500 RFU   Upper marker selection: Last peak > 200 RFU
Ladder size (bp)75, 200, 300, 400, 500, 700, 1000, 1500, 2000, 3000, 4000, 5000, 7000, 10000, 20000
Quantification using: Lower Marker   Final concentration (ng/uL): 0.5000   Dilution factor: 12.0

Sample: SampA2
Well location: A2
Created: Wednesday, October 20, 2021 4:38:54 PM

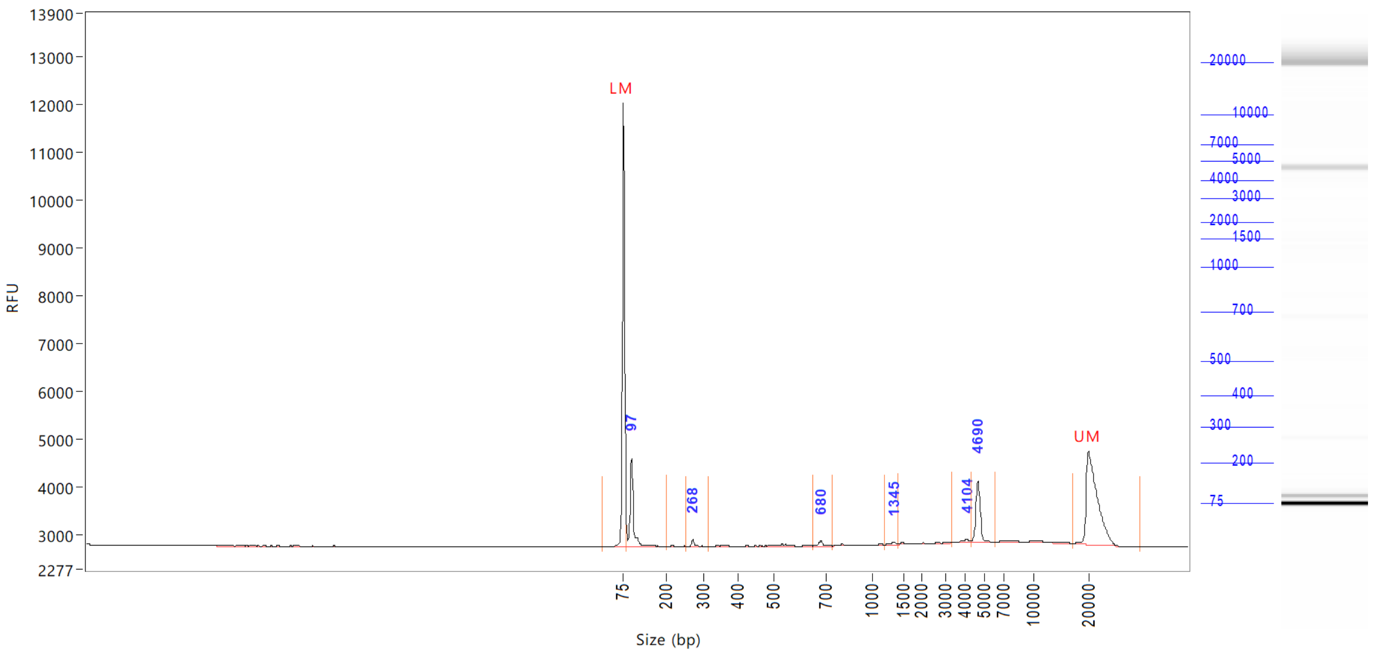

Peak	Size (bp)	Concentration (ng/uL)	From (bp)	To (bp)	Average size (bp)	CV%	RFU	Corrected peak area
1	75 (LM)	0.5000	14	86	73	5.38	9288	47.062
2	97	1.6882	86	196	98	11.47	1846	13.242
3	268	0.1017	251	311	268	3.19	141	0.798
4	680	0.0899	649	740	678	1.54	117	0.705
5	1345	0.0538	1201	1411	1305	3.60	55	0.422
6	4104	0.0972	3322	4311	3967	5.51	75	0.763
7	4690	0.9824	4311	6186	4667	3.51	1298	7.706
8	20000 (UM)	0.2678	16952	29430	20820	5.80	1969	25.204

TIC:	3.0132	ng/uL
TIM:	29.5832	nmole/L
Total concentration:	3.5926	ng/uL

```
Sample peak width (sec): 5    Sample min peak height: 50    Sample baseline V to V?: Y    Sample baseline V to V points: 3
Sample filter: Binomial       Number of points for filter: 3  Sample start region (min): 0  Sample end region (min): 30
Marker peak width (sec): 5    Marker min peak height: 200   Marker baseline V to V?: Y    Marker baseline V to V points: 3
Lower marker selection: First peak > 500 RFU                Upper marker selection: Last peak > 200 RFU
Ladder size (bp)75, 200, 300, 400, 500, 700, 1000, 1500, 2000, 3000, 4000, 5000, 7000, 10000, 20000
Quantification using: Lower Marker          Final concentration (ng/uL): 0.5000          Dilution factor: 12.0
```

Sample: SampA3
Well location: A3
Created: Wednesday, October 20, 2021 4:38:54 PM

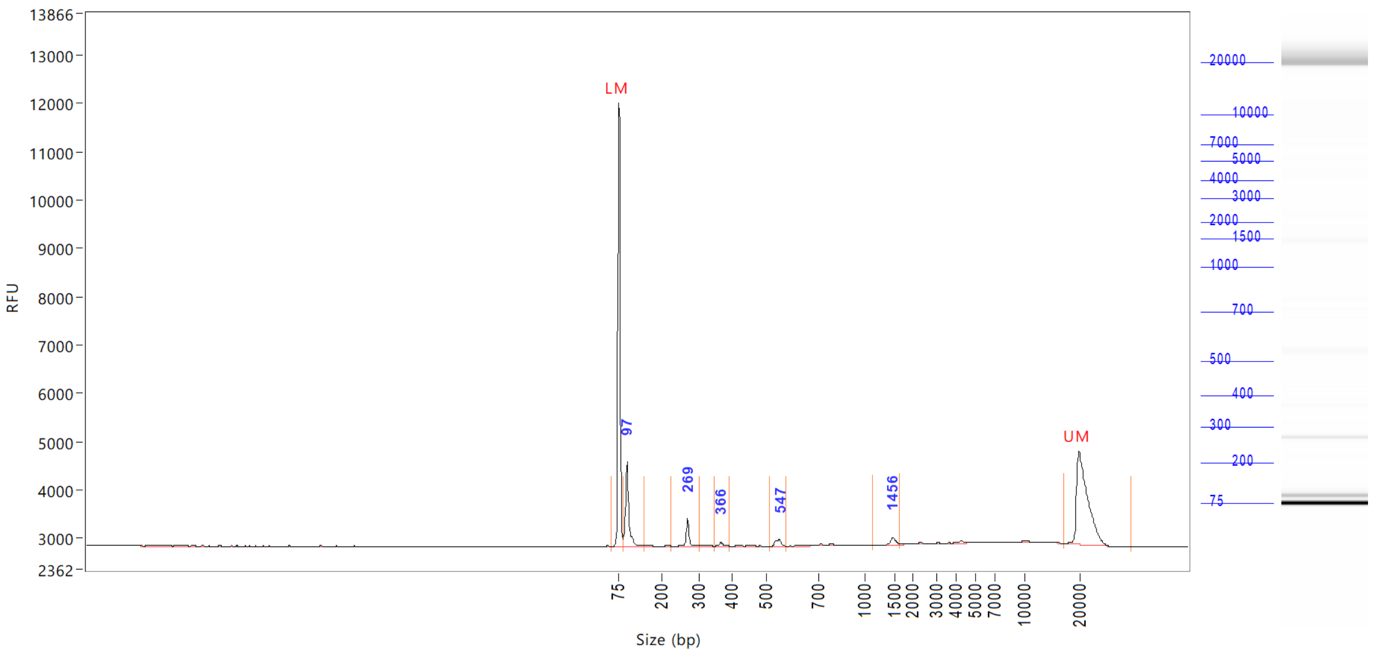

Peak	Size (bp)	Concentration (ng/uL)	From (bp)	To (bp)	Average size (bp)	CV%	RFU	Corrected peak area
1	75 (LM)	0.5000	56	87	74	4.55	9192	47.326
2	97	1.6001	87	147	98	8.14	1750	12.621
3	269	0.4004	226	301	267	2.78	579	3.158
4	366	0.0780	345	394	366	2.16	94	0.615
5	547	0.1770	509	575	538	1.87	148	1.396
6	1456	0.1533	1134	1635	1439	5.73	156	1.209
7	20000 (UM)	0.2629	17317	29551	20884	5.80	1919	24.885

TIC:	2.4088	ng/uL
TIM:	30.3441	nmole/L
Total concentration:	3.0128	ng/uL

Sample peak width (sec): 5 Sample min peak height: 50 Sample baseline V to V?: Y Sample baseline V to V points: 3
Sample filter: Binomial Number of points for filter: 3 Sample start region (min): 0 Sample end region (min): 30
Marker peak width (sec): 5 Marker min peak height: 200 Marker baseline V to V?: Y Marker baseline V to V points: 3
Lower marker selection: First peak > 500 RFU Upper marker selection: Last peak > 200 RFU
Ladder size (bp)75, 200, 300, 400, 500, 700, 1000, 1500, 2000, 3000, 4000, 5000, 7000, 10000, 20000
Quantification using: Lower Marker Final concentration (ng/uL): 0.5000 Dilution factor: 12.0

Sample: SampA4
Well location: A4
Created: Wednesday, October 20, 2021 4:38:54 PM

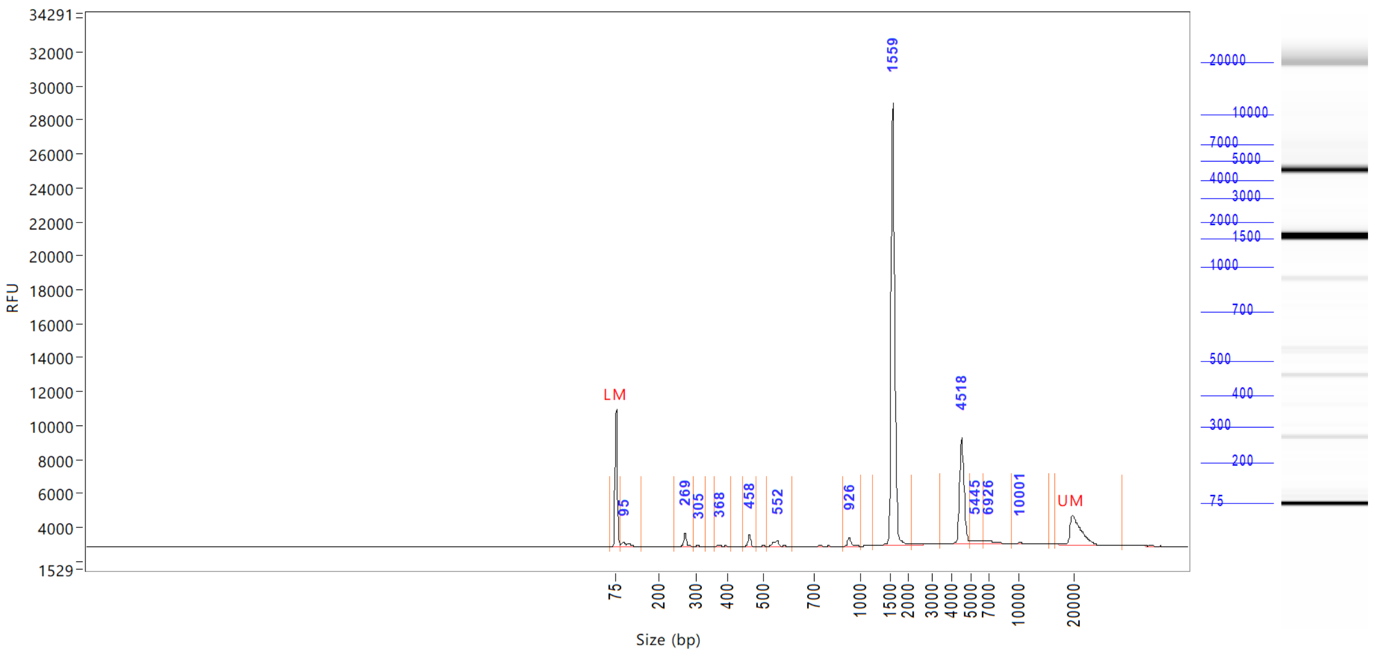

Peak	Size (bp)	Concentration (ng/uL)	From (bp)	To (bp)	Average size (bp)	CV%	RFU	Corrected peak area
1	75 (LM)	0.5000	56	87	73	4.78	8096	42.321
2	95	0.5803	87	148	103	11.91	273	4.093
3	269	0.6489	239	295	268	2.52	814	4.577
4	305	0.0600	295	327	305	2.27	67	0.423
5	368	0.1113	356	405	373	2.80	114	0.785
6	458	0.5033	441	481	457	0.89	717	3.550
7	552	0.5054	514	611	547	2.89	351	3.565
8	926	0.3983	887	1000	922	1.36	536	2.809
9	1559	19.6955	1201	2144	1546	3.96	26110	138.923
10	4518	5.4668	3429	5001	4530	3.72	6285	38.560
11	5445	0.3728	5001	6334	5636	7.12	210	2.630
12	6926	0.5223	6334	9267	7373	10.46	217	3.684
13	10001	0.2196	9267	15488	10992	12.40	72	1.549
14	20000 (UM)	0.2629	16708	29068	20782	5.78	1747	22.254

TIC:	29.0846	ng/uL
TIM:	41.3091	nmole/L
Total concentration:	29.4718	ng/uL

Sample peak width (sec): 5 Sample min peak height: 50 Sample baseline V to V?: Y Sample baseline V to V points: 3
Sample filter: Binomial Number of points for filter: 3 Sample start region (min): 0 Sample end region (min): 30
Marker peak width (sec): 5 Marker min peak height: 200 Marker baseline V to V?: Y Marker baseline V to V points: 3
Lower marker selection: First peak > 500 RFU Upper marker selection: Last peak > 200 RFU
Ladder size (bp)75, 200, 300, 400, 500, 700, 1000, 1500, 2000, 3000, 4000, 5000, 7000, 10000, 20000
Quantification using: Lower Marker Final concentration (ng/uL): 0.5000 Dilution factor: 12.0

Sample: SampA5
Well location: A5
Created: Wednesday, October 20, 2021 4:38:54 PM

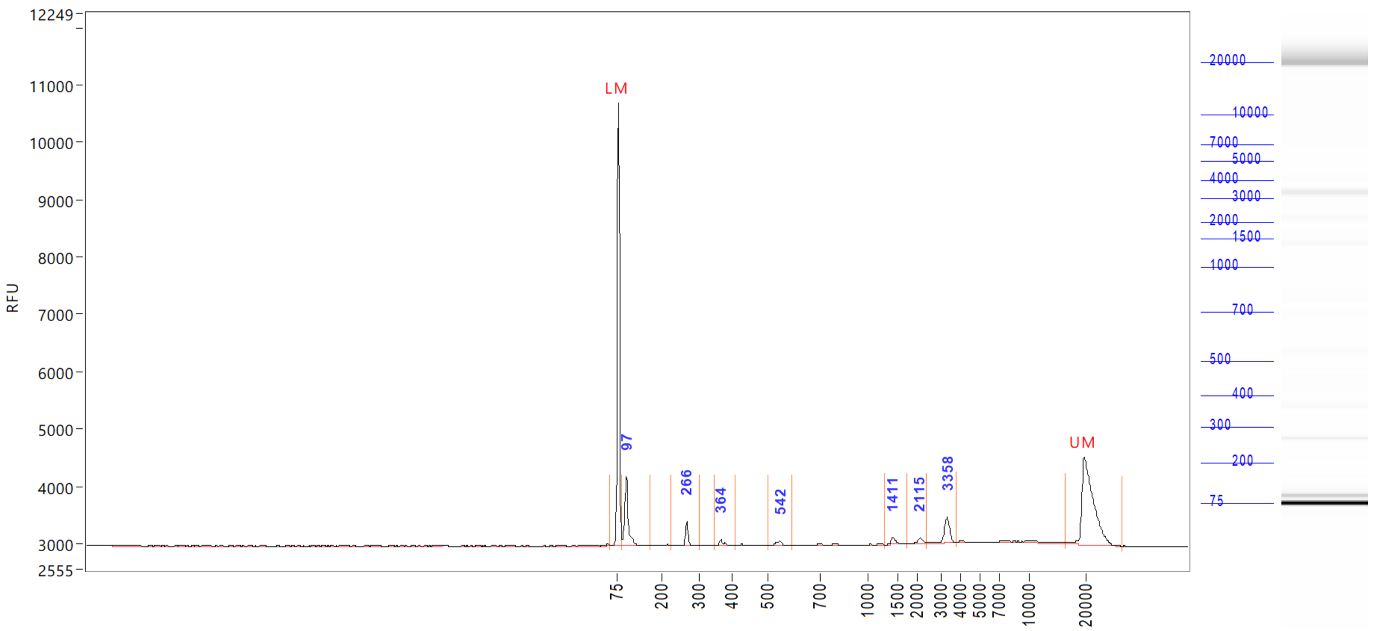

Peak	Size (bp)	Concentration (ng/uL)	From (bp)	To (bp)	Average size (bp)	CV%	RFU	Corrected peak area
1	75 (LM)	0.5000	52	86	73	4.50	7738	39.933
2	97	1.3929	86	169	99	11.18	1198	9.270
3	266	0.3702	223	300	264	3.23	431	2.464
4	364	0.1283	343	405	365	2.68	100	0.854
5	542	0.1116	501	590	537	2.27	79	0.743
6	1411	0.1380	1289	1750	1444	6.03	118	0.919
7	2115	0.1187	1750	2429	2104	6.40	98	0.790
8	3358	0.4992	2429	3786	3261	6.19	450	3.322
9	20000 (UM)	0.2591	16586	26770	20816	6.11	1531	20.695

TIC:	2.7589	ng/uL
TIM:	26.8321	nmole/L
Total concentration:	3.3886	ng/uL

Sample peak width (sec): 5   Sample min peak height: 50   Sample baseline V to V?: Y   Sample baseline V to V points: 3
Sample filter: Binomial   Number of points for filter: 3 Sample start region (min): 0 Sample end region (min): 30
Marker peak width (sec): 5   Marker min peak height: 200   Marker baseline V to V?: Y   Marker baseline V to V points: 3
Lower marker selection: First peak > 500 RFU   Upper marker selection: Last peak > 200 RFU
Ladder size (bp)75, 200, 300, 400, 500, 700, 1000, 1500, 2000, 3000, 4000, 5000, 7000, 10000, 20000
Quantification using: Lower Marker   Final concentration (ng/uL): 0.5000   Dilution factor: 12.0

Sample: SampA6
Well location: A6
Created: Wednesday, October 20, 2021 4:38:54 PM

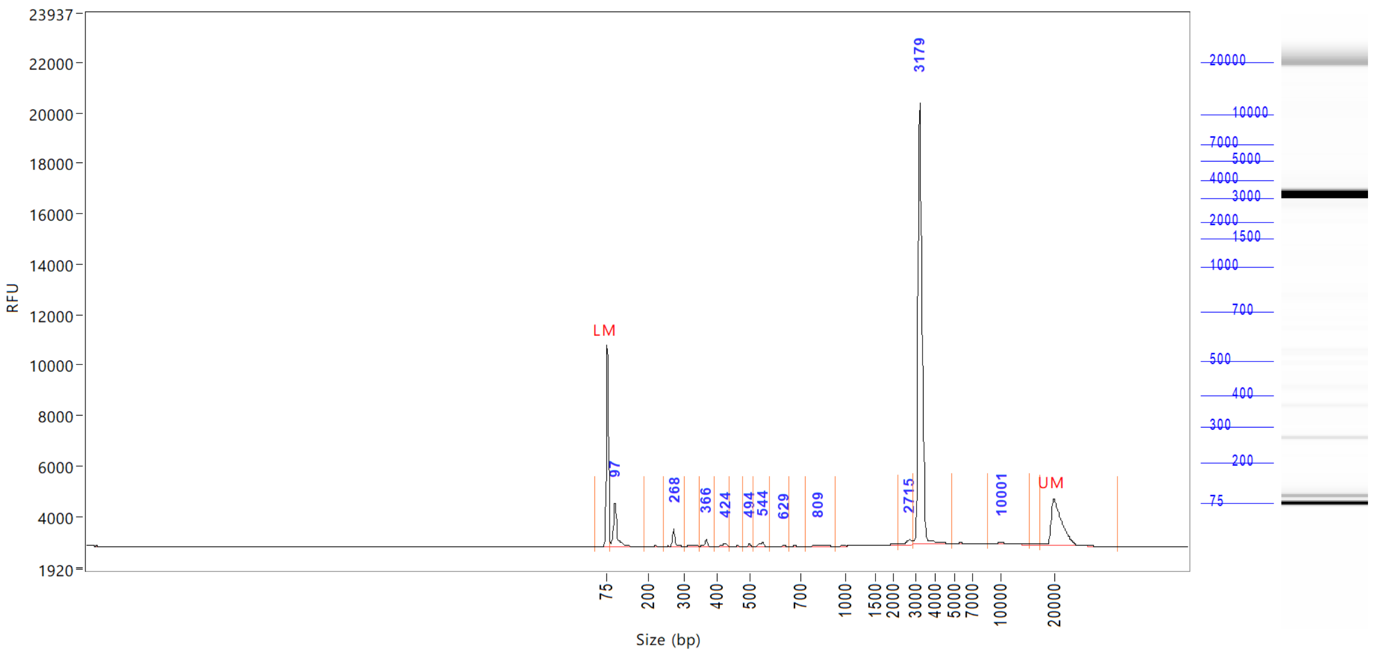

Peak	Size (bp)	Concentration (ng/uL)	From (bp)	To (bp)	Average size (bp)	CV%	RFU	Corrected peak area
1	75 (LM)	0.5000	41	86	74	4.31	7969	40.636
2	97	2.1664	86	183	100	11.95	1723	14.673
3	268	0.5306	241	298	267	2.44	709	3.594
4	366	0.1987	347	394	363	1.57	257	1.346
5	424	0.1418	394	437	419	1.69	125	0.960
6	494	0.0599	477	511	493	0.99	85	0.405
7	544	0.1880	511	575	537	1.77	152	1.273
8	629	0.0538	575	649	623	2.06	62	0.364
9	809	0.1570	723	926	812	4.46	68	1.063
10	2715	0.2525	2229	2886	2658	5.85	185	1.710
11	3179	14.7308	2886	4897	3195	5.20	17513	99.768
12	10001	0.2453	8667	15488	11669	16.87	58	1.661
13	20000 (UM)	0.2871	17317	32090	20748	6.00	1858	23.331

TIC:	18.7247	ng/uL
TIM:	49.3179	nmole/L
Total concentration:	19.1854	ng/uL

```
Sample peak width (sec): 5    Sample min peak height: 50    Sample baseline V to V?: Y    Sample baseline V to V points: 3
Sample filter: Binomial       Number of points for filter: 3  Sample start region (min): 0  Sample end region (min): 30
Marker peak width (sec): 5    Marker min peak height: 200   Marker baseline V to V?: Y    Marker baseline V to V points: 3
Lower marker selection: First peak > 500 RFU                Upper marker selection: Last peak > 200 RFU
Ladder size (bp)75, 200, 300, 400, 500, 700, 1000, 1500, 2000, 3000, 4000, 5000, 7000, 10000, 20000
Quantification using: Lower Marker          Final concentration (ng/uL): 0.5000          Dilution factor: 12.0
```

Sample: SampA7
Well location: A7
Created: Wednesday, October 20, 2021 4:38:54 PM

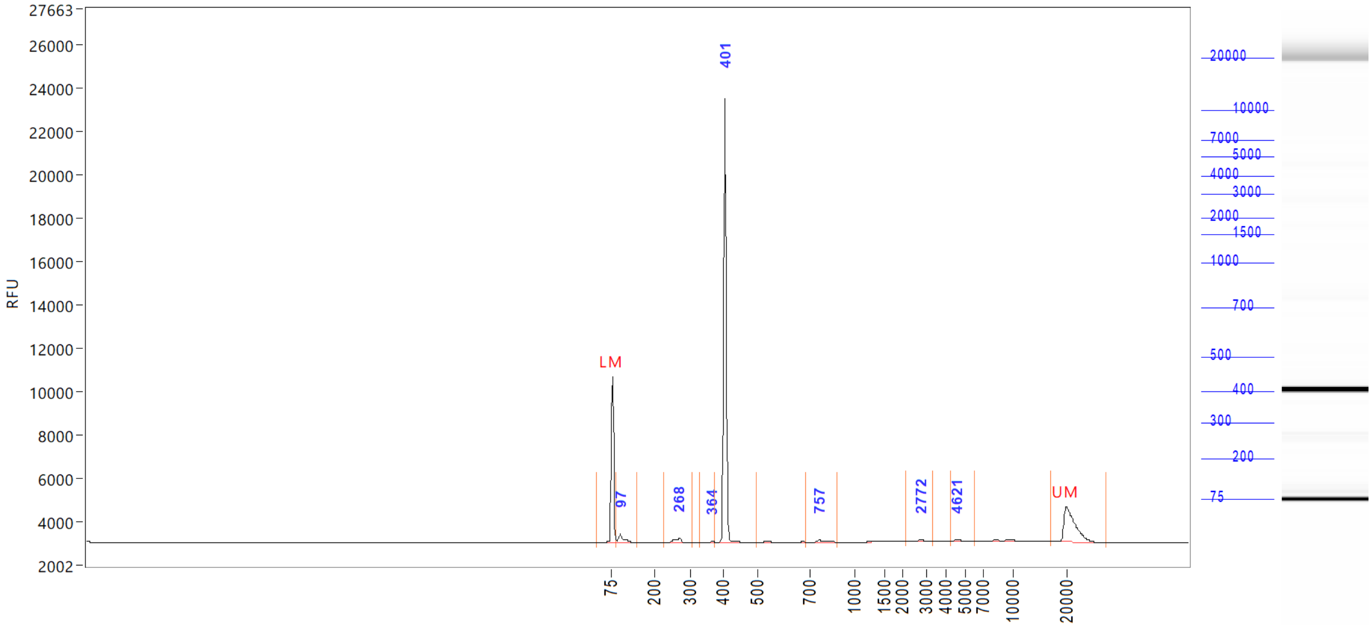

Peak	Size (bp)	Concentration (ng/uL)	From (bp)	To (bp)	Average size (bp)	CV%	RFU	Corrected peak area
1	75 (LM)	0.5000	30	87	73	4.77	7649	39.115
2	97	0.7196	87	150	102	11.50	387	4.691
3	268	0.4126	224	301	258	4.35	219	2.690
4	364	0.0558	325	374	357	2.99	61	0.363
5	401	14.9674	374	496	401	1.34	20509	97.576
6	757	0.1814	687	887	780	4.60	89	1.182
7	2772	0.1208	2144	3322	2761	7.78	83	0.787
8	4621	0.1038	4277	6112	4692	6.14	79	0.676
9	20000 (UM)	0.2699	17073	27254	20842	5.89	1623	21.111

TIC:	16.5613	ng/uL
TIM:	76.3751	nmole/L
Total concentration:	17.1213	ng/uL

Sample peak width (sec): 5 Sample min peak height: 50 Sample baseline V to V?: Y Sample baseline V to V points: 3
Sample filter: Binomial Number of points for filter: 3 Sample start region (min): 0 Sample end region (min): 30
Marker peak width (sec): 5 Marker min peak height: 200 Marker baseline V to V?: Y Marker baseline V to V points: 3
Lower marker selection: First peak > 500 RFU Upper marker selection: Last peak > 200 RFU
Ladder size (bp)75, 200, 300, 400, 500, 700, 1000, 1500, 2000, 3000, 4000, 5000, 7000, 10000, 20000
Quantification using: Lower Marker Final concentration (ng/uL): 0.5000 Dilution factor: 12.0

Sample: SampA8
Well location: A8
Created: Wednesday, October 20, 2021 4:38:54 PM

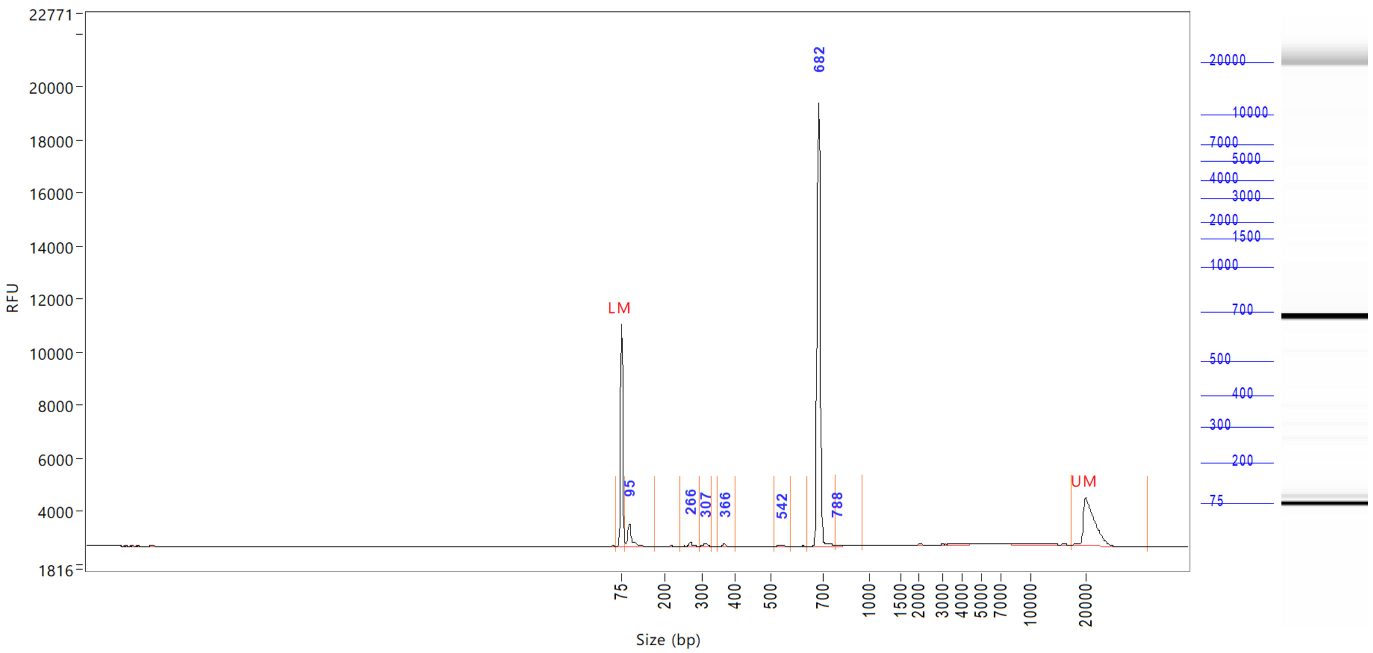

Peak	Size (bp)	Concentration (ng/uL)	From (bp)	To (bp)	Average size (bp)	CV%	RFU	Corrected peak area
1	75 (LM)	0.5000	58	86	73	4.59	8401	42.637
2	95	1.0751	86	171	98	10.65	857	7.640
3	266	0.1758	241	293	266	3.55	179	1.249
4	307	0.1421	293	327	306	2.05	112	1.010
5	366	0.0942	345	398	363	1.79	102	0.669
6	542	0.0878	514	575	535	2.07	72	0.624
7	682	11.6171	634	775	679	1.27	16739	82.553
8	788	0.1591	775	952	843	5.97	53	1.131
9	20000 (UM)	0.2755	17439	31244	20872	5.96	1807	23.489

TIC:	13.3513	ng/uL
TIM:	49.0288	nmole/L
Total concentration:	13.9927	ng/uL

Sample peak width (sec): 5 Sample min peak height: 50 Sample baseline V to V?: Y Sample baseline V to V points: 3
Sample filter: Binomial Number of points for filter: 3 Sample start region (min): 0 Sample end region (min): 30
Marker peak width (sec): 5 Marker min peak height: 200 Marker baseline V to V?: Y Marker baseline V to V points: 3
Lower marker selection: First peak > 500 RFU Upper marker selection: Last peak > 200 RFU
Ladder size (bp)75, 200, 300, 400, 500, 700, 1000, 1500, 2000, 3000, 4000, 5000, 7000, 10000, 20000
Quantification using: Lower Marker Final concentration (ng/uL): 0.5000 Dilution factor: 12.0

Sample: SampA9
Well location: A9
Created: Wednesday, October 20, 2021 4:38:54 PM

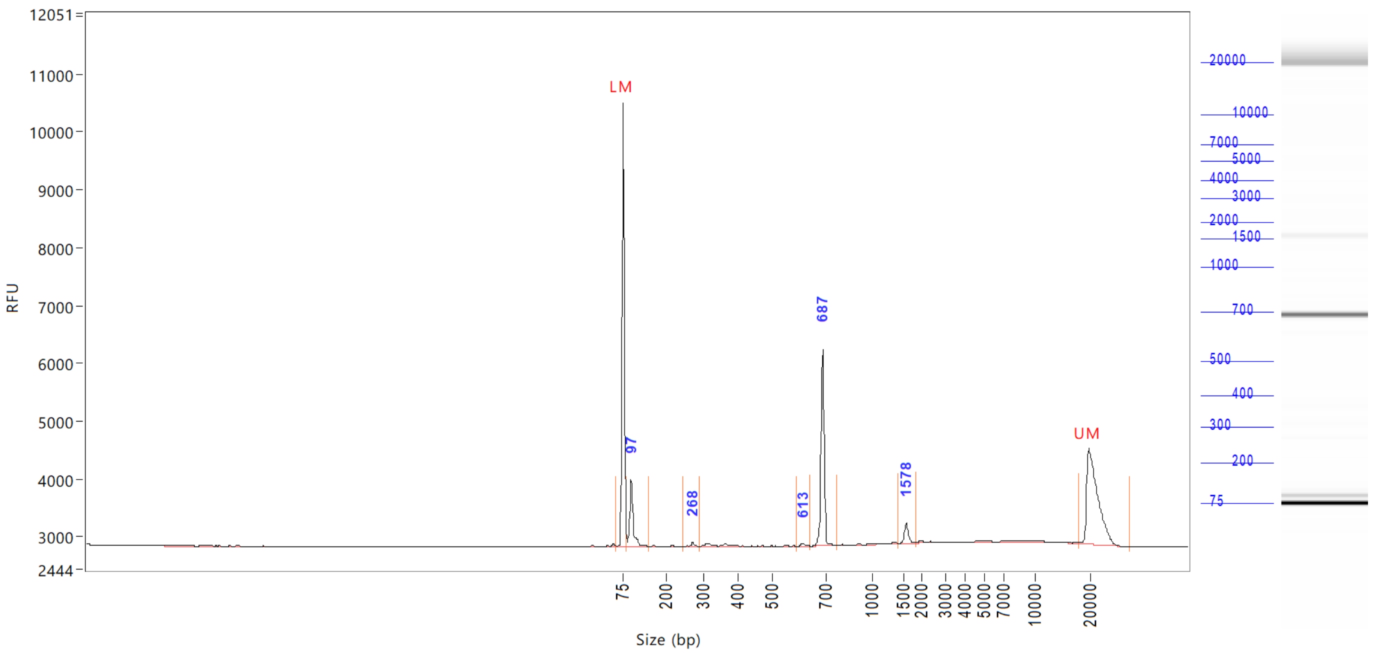

Peak	Size (bp)	Concentration (ng/uL)	From (bp)	To (bp)	Average size (bp)	CV%	RFU	Corrected peak area
1	75 (LM)	0.5000	58	86	73	4.28	7677	38.861
2	97	1.3255	86	148	98	8.35	1149	8.585
3	268	0.0655	244	289	265	2.92	68	0.424
4	613	0.0479	590	644	611	1.38	50	0.310
5	687	2.7455	644	775	685	1.29	3398	17.782
6	1578	0.3111	1433	1846	1565	4.02	344	2.015
7	20000 (UM)	0.2719	18049	27375	20810	5.58	1661	21.131

TIC:	4.4955	ng/uL
TIM:	29.6680	nmole/L
Total concentration:	5.2559	ng/uL

```
Sample peak width (sec): 5    Sample min peak height: 50    Sample baseline V to V?: Y    Sample baseline V to V points: 3
Sample filter: Binomial       Number of points for filter: 3  Sample start region (min): 0  Sample end region (min): 30
Marker peak width (sec): 5    Marker min peak height: 200   Marker baseline V to V?: Y    Marker baseline V to V points: 3
Lower marker selection: First peak > 500 RFU                Upper marker selection: Last peak > 200 RFU
Ladder size (bp)75, 200, 300, 400, 500, 700, 1000, 1500, 2000, 3000, 4000, 5000, 7000, 10000, 20000
Quantification using: Lower Marker          Final concentration (ng/uL): 0.5000          Dilution factor: 12.0
```

Sample: SampA10
Well location: A10
Created: Wednesday, October 20, 2021 4:38:54 PM

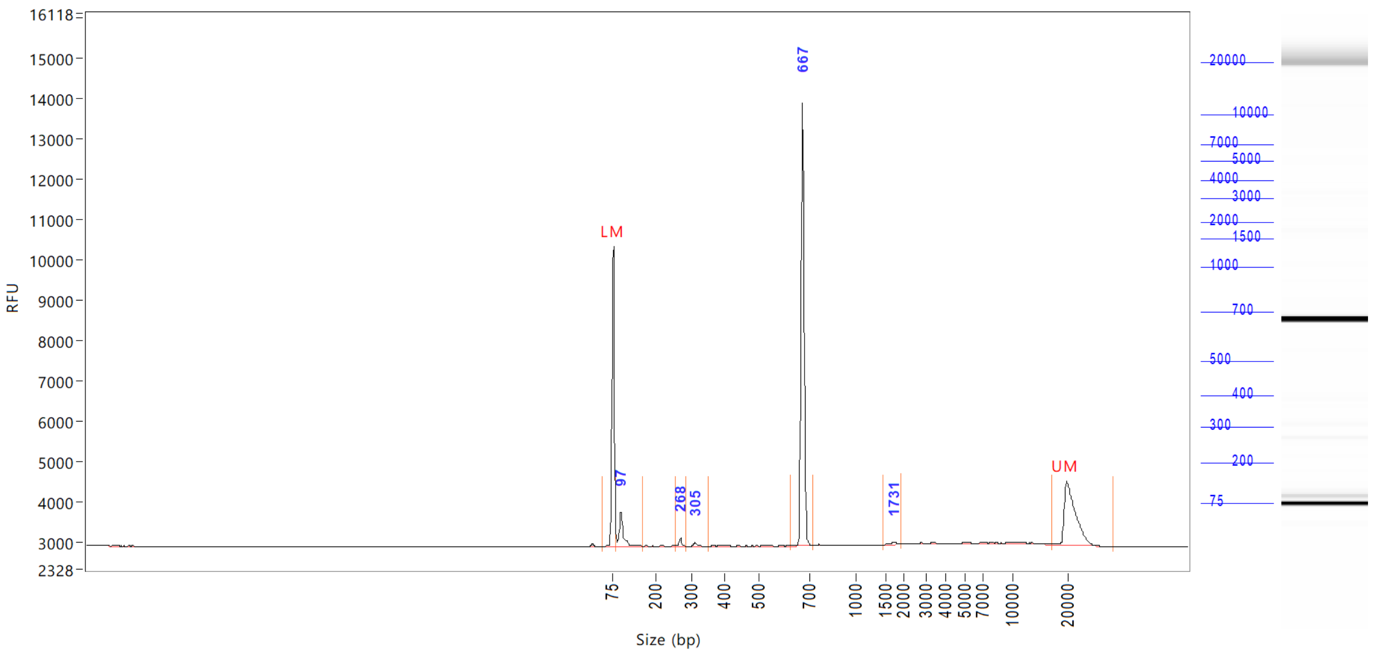

Peak	Size (bp)	Concentration (ng/uL)	From (bp)	To (bp)	Average size (bp)	CV%	RFU	Corrected peak area
1	75 (LM)	0.5000	45	86	72	4.69	7428	38.410
2	97	1.1581	86	162	99	11.12	824	7.414
3	268	0.1571	255	284	266	1.60	198	1.006
4	305	0.1188	284	352	307	3.07	93	0.761
5	667	8.6475	626	714	667	0.90	11008	55.358
6	1731	0.0849	1467	1942	1684	5.86	64	0.543
7	20000 (UM)	0.2730	17195	28584	20863	5.95	1583	20.969

TIC:	10.1664	ng/uL
TIM:	42.2358	nmole/L
Total concentration:	10.9674	ng/uL

```
Sample peak width (sec): 5    Sample min peak height: 50    Sample baseline V to V?: Y    Sample baseline V to V points: 3
Sample filter: Binomial       Number of points for filter: 3  Sample start region (min): 0  Sample end region (min): 30
Marker peak width (sec): 5    Marker min peak height: 200   Marker baseline V to V?: Y    Marker baseline V to V points: 3
Lower marker selection: First peak > 500 RFU                Upper marker selection: Last peak > 200 RFU
Ladder size (bp)75, 200, 300, 400, 500, 700, 1000, 1500, 2000, 3000, 4000, 5000, 7000, 10000, 20000
Quantification using: Lower Marker         Final concentration (ng/uL): 0.5000         Dilution factor: 12.0
```

Sample: SampA11
Well location: A11
Created: Wednesday, October 20, 2021 4:38:54 PM

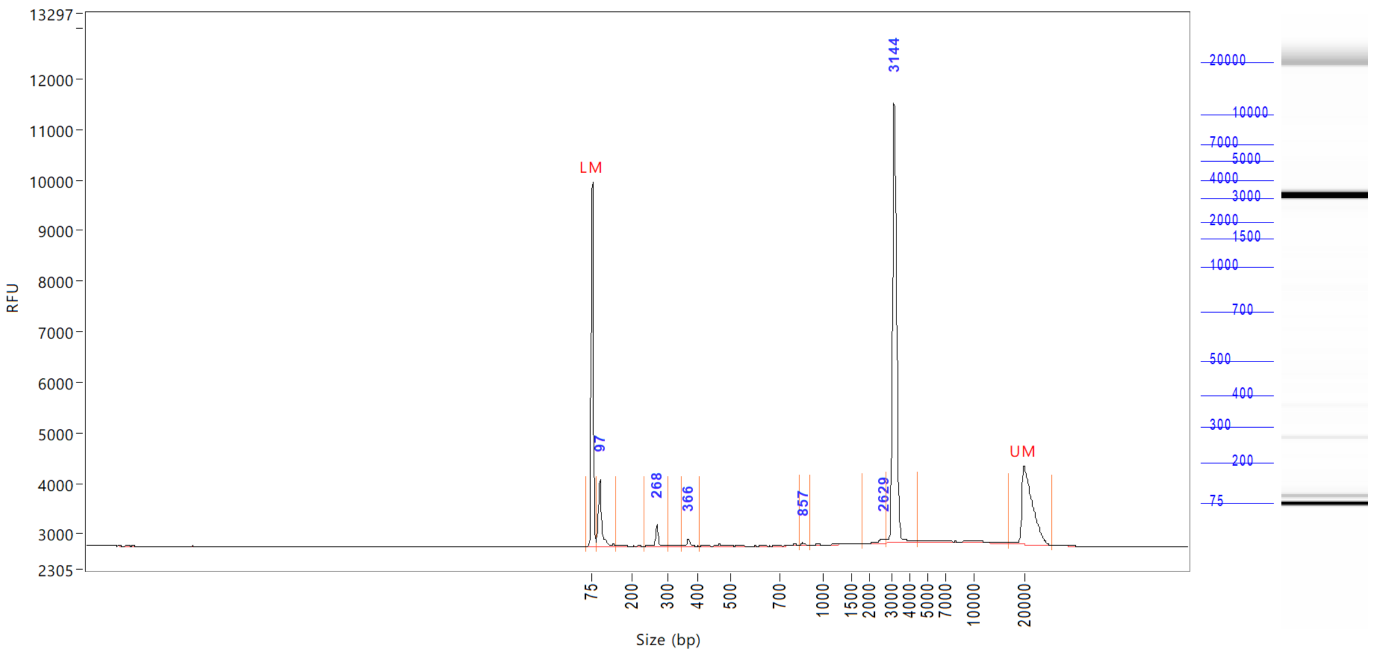

Peak	Size (bp)	Concentration (ng/uL)	From (bp)	To (bp)	Average size (bp)	CV%	RFU	Corrected peak area
1	75 (LM)	0.5000	56	86	72	4.45	7201	38.231
2	97	1.6056	86	147	98	10.18	1327	10.231
3	268	0.3857	230	303	267	3.26	433	2.458
4	366	0.1454	345	403	365	2.52	149	0.926
5	857	0.0591	831	905	857	1.62	54	0.377
6	2629	0.1526	1827	2800	2513	8.71	89	0.973
7	3144	7.8368	2800	4449	3158	4.48	8712	49.935
8	20000 (UM)	0.2656	16952	25561	20793	5.71	1559	20.306

TIC:	10.1853	ng/uL
TIM:	34.2322	nmole/L
Total concentration:	10.9461	ng/uL

Sample peak width (sec): 5 Sample min peak height: 50 Sample baseline V to V?: Y Sample baseline V to V points: 3
Sample filter: Binomial Number of points for filter: 3 Sample start region (min): 0 Sample end region (min): 30
Marker peak width (sec): 5 Marker min peak height: 200 Marker baseline V to V?: Y Marker baseline V to V points: 3
Lower marker selection: First peak > 500 RFU Upper marker selection: Last peak > 200 RFU
Ladder size (bp)75, 200, 300, 400, 500, 700, 1000, 1500, 2000, 3000, 4000, 5000, 7000, 10000, 20000
Quantification using: Lower Marker Final concentration (ng/uL): 0.5000 Dilution factor: 12.0

Sample: SampA12
Well location: A12
Created: Wednesday, October 20, 2021 4:38:54 PM

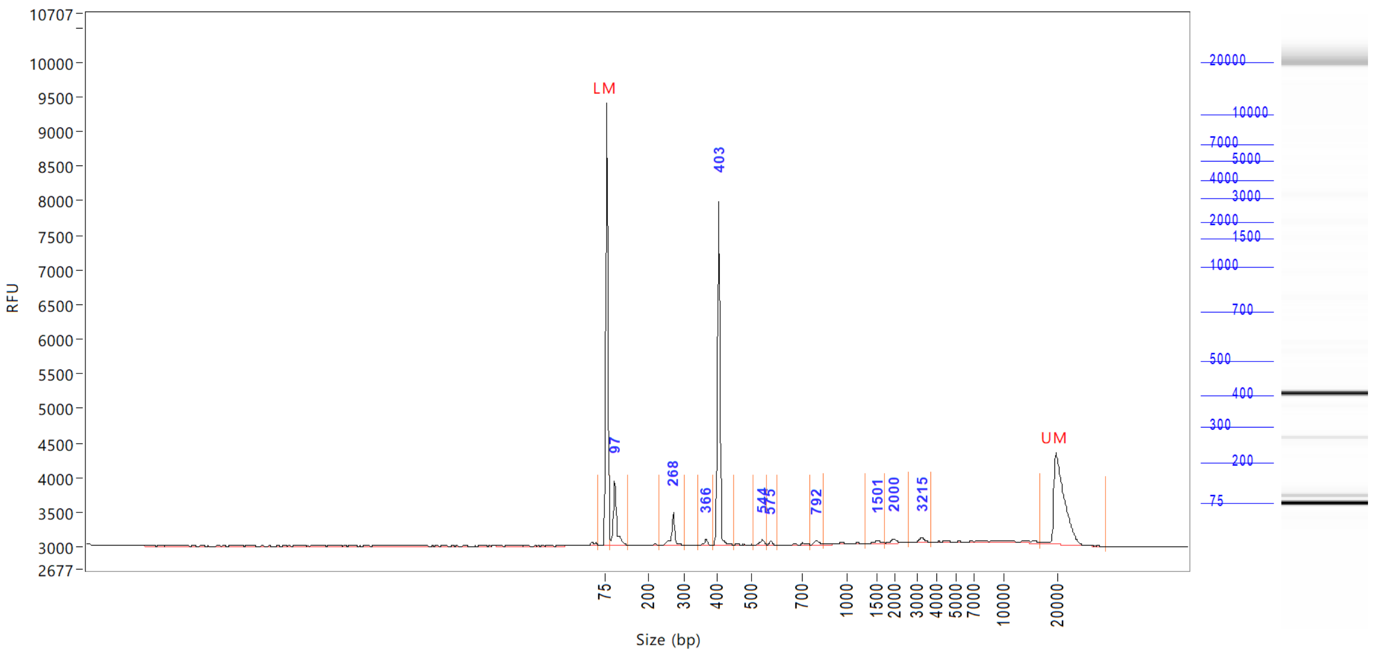

Peak	Size (bp)	Concentration (ng/uL)	From (bp)	To (bp)	Average size (bp)	CV%	RFU	Corrected peak area
1	75 (LM)	0.5000	52	87	74	4.40	6404	33.248
2	97	1.2918	87	139	99	8.39	937	7.158
3	268	0.5638	228	301	263	3.40	471	3.124
4	366	0.0992	343	386	363	2.11	86	0.550
5	403	4.3278	386	449	402	1.20	4964	23.981
6	544	0.1102	506	560	536	2.03	78	0.611
7	575	0.0574	560	603	574	1.21	55	0.318
8	792	0.0934	753	848	795	2.41	63	0.518
9	1501	0.0836	1300	1750	1508	5.46	51	0.463
10	2000	0.1035	1750	2600	2017	7.49	66	0.574
11	3215	0.1147	2600	3750	3186	6.24	73	0.636
12	20000 (UM)	0.2499	16952	29189	20781	5.78	1324	16.617

TIC:	6.8454	ng/uL
TIM:	44.0458	nmole/L
Total concentration:	7.4129	ng/uL

```
Sample peak width (sec): 5    Sample min peak height: 50    Sample baseline V to V?: Y    Sample baseline V to V points: 3
Sample filter: Binomial       Number of points for filter: 3  Sample start region (min): 0  Sample end region (min): 30
Marker peak width (sec): 5    Marker min peak height: 200   Marker baseline V to V?: Y    Marker baseline V to V points: 3
Lower marker selection: First peak > 500 RFU                Upper marker selection: Last peak > 200 RFU
Ladder size (bp)75, 200, 300, 400, 500, 700, 1000, 1500, 2000, 3000, 4000, 5000, 7000, 10000, 20000
Quantification using: Lower Marker          Final concentration (ng/uL): 0.5000          Dilution factor: 12.0
```

Sample: SampB1
Well location: B1
Created: Wednesday, October 20, 2021 4:38:54 PM

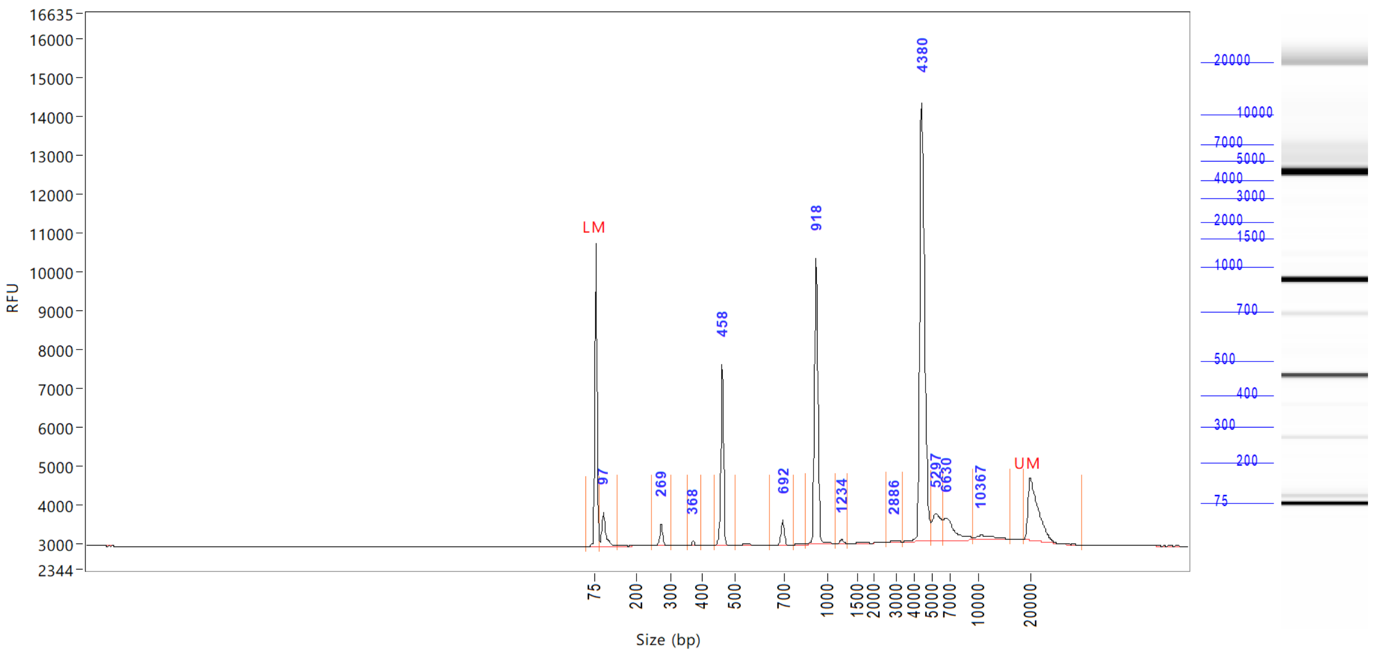

Peak	Size (bp)	Concentration (ng/uL)	From (bp)	To (bp)	Average size (bp)	CV%	RFU	Corrected peak area
1	75 (LM)	0.5000	43	86	72	4.90	7810	41.009
2	97	1.1592	86	141	99	9.44	891	7.923
3	269	0.5227	242	296	267	2.52	574	3.573
4	368	0.1010	354	396	366	1.44	124	0.690
5	458	3.4976	435	498	456	1.06	4654	23.906
6	692	0.5239	636	753	689	1.40	643	3.581
7	918	5.9216	844	1112	913	1.71	7340	40.474
8	1234	0.0887	1112	1323	1218	2.78	99	0.606
9	2886	0.0767	2572	3358	2927	4.38	52	0.524
10	4380	11.0808	3358	4897	4420	3.26	11289	75.736
11	5297	1.3209	4897	6186	5459	7.32	689	9.028
12	6630	1.3117	6186	9467	7165	11.40	572	8.965
13	10367	0.3823	9467	16098	11531	13.08	112	2.613
14	20000 (UM)	0.2465	18781	30156	20937	5.81	1616	20.221

TIC:	25.9873	ng/uL
TIM:	52.4934	nmole/L
Total concentration:	26.3429	ng/uL

Sample peak width (sec): 5 Sample min peak height: 50 Sample baseline V to V?: Y Sample baseline V to V points: 3
Sample filter: Binomial Number of points for filter: 3 Sample start region (min): 0 Sample end region (min): 30
Marker peak width (sec): 5 Marker min peak height: 200 Marker baseline V to V?: Y Marker baseline V to V points: 3
Lower marker selection: First peak > 500 RFU Upper marker selection: Last peak > 200 RFU
Ladder size (bp)75, 200, 300, 400, 500, 700, 1000, 1500, 2000, 3000, 4000, 5000, 7000, 10000, 20000
Quantification using: Lower Marker Final concentration (ng/uL): 0.5000 Dilution factor: 12.0

Sample: SampB2
Well location: B2
Created: Wednesday, October 20, 2021 4:38:54 PM

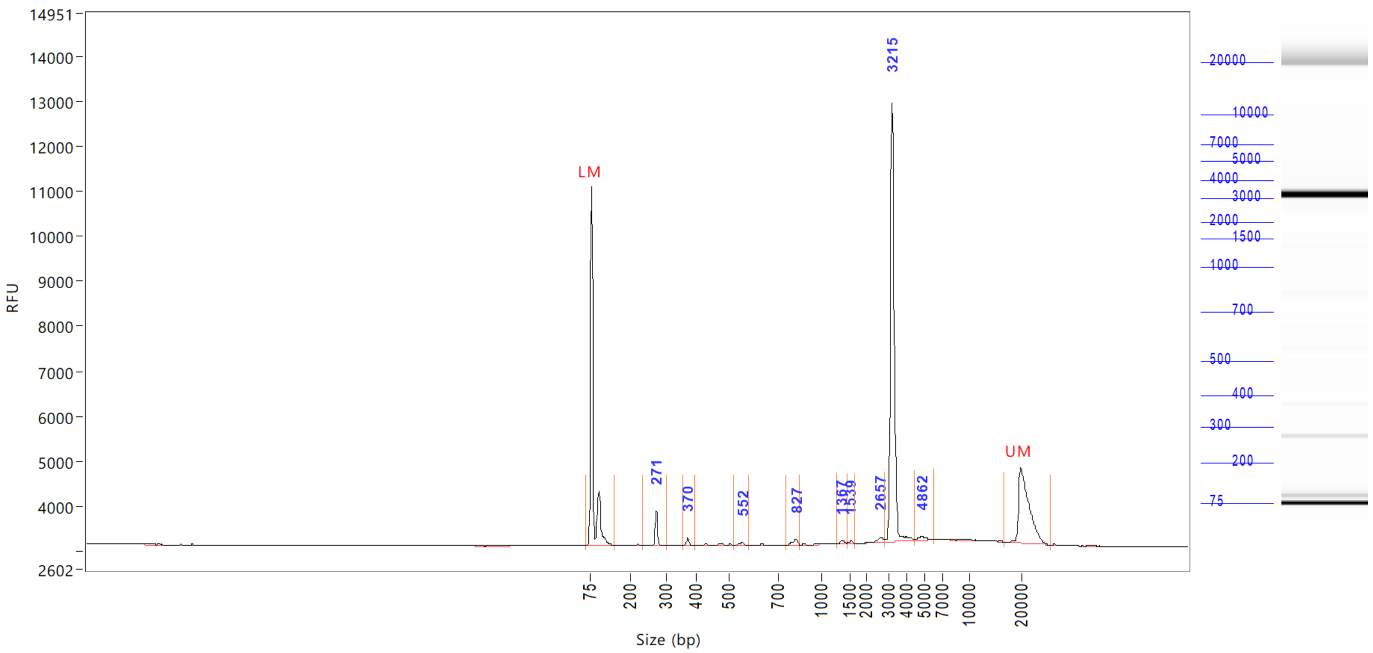

Peak	Size<br>(bp)	Concentration<br>(ng/uL)	From<br>(bp)	To<br>(bp)	Average size<br>(bp)	CV%	RFU	Corrected peak area
1	75 (LM)	0.5000	60	147	79	14.87	7974	53.221
2	271	0.4493	232	301	270	2.44	755	3.985
3	370	0.0982	356	392	367	1.21	173	0.871
4	552	0.0762	516	580	543	1.91	71	0.676
5	827	0.1301	757	857	810	2.47	142	1.154
6	1367	0.0562	1278	1456	1367	2.68	80	0.499
7	1539	0.0360	1456	1674	1520	2.64	51	0.319
8	2657	0.1259	1674	2886	2594	8.27	111	1.116
9	3215	6.2053	2886	4449	3214	5.14	9762	55.042
10	4862	0.1146	4449	6112	4934	7.09	84	1.017
11	20000 (UM)	0.2010	16830	25924	20862	5.59	1676	21.398

TIC:	7.2919	ng/uL
TIM:	7.0761	nmole/L
Total concentration:	7.7399	ng/uL

```
Sample peak width (sec): 5     Sample min peak height: 50     Sample baseline V to V?: Y     Sample baseline V to V points: 3
Sample filter: Binomial        Number of points for filter: 3  Sample start region (min): 0  Sample end region (min): 30
Marker peak width (sec): 5     Marker min peak height: 200    Marker baseline V to V?: Y     Marker baseline V to V points: 3
Lower marker selection: First peak > 500 RFU                  Upper marker selection: Last peak > 200 RFU
Ladder size (bp)75, 200, 300, 400, 500, 700, 1000, 1500, 2000, 3000, 4000, 5000, 7000, 10000, 20000
Quantification using: Lower Marker          Final concentration (ng/uL): 0.5000          Dilution factor: 12.0
```

Sample: SampB3
Well location: B3
Created: Wednesday, October 20, 2021 4:38:54 PM

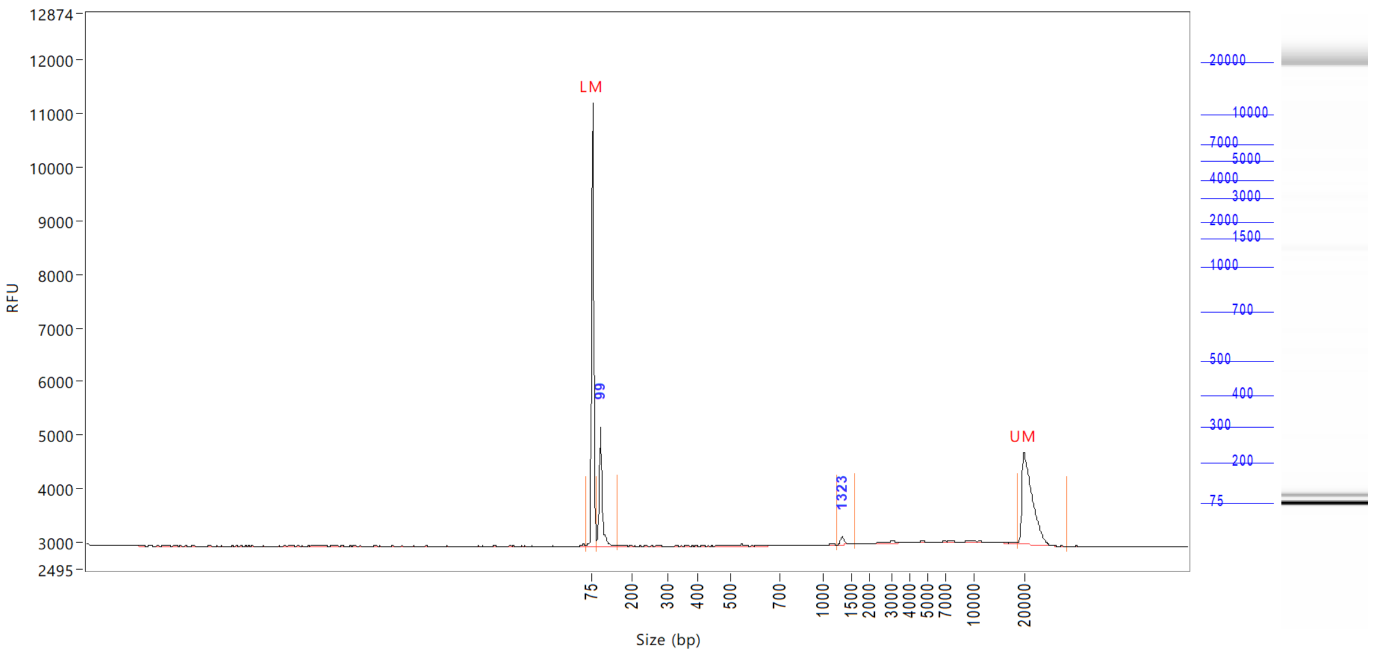

Peak	Size (bp)	Concentration (ng/uL)	From (bp)	To (bp)	Average size (bp)	CV%	RFU	Corrected peak area
1	75 (LM)	0.5000	54	87	74	4.51	8290	43.859
2	99	2.0875	87	152	100	8.59	2232	15.259
3	1323	0.1232	1245	1578	1330	3.67	144	0.901
4	20000 (UM)	0.2482	18659	28221	20842	5.63	1713	21.771

TIC:	2.2108	ng/uL
TIM:	34.4318	nmole/L
Total concentration:	2.8595	ng/uL

Sample peak width (sec): 5 Sample min peak height: 50 Sample baseline V to V?: Y Sample baseline V to V points: 3
Sample filter: Binomial Number of points for filter: 3 Sample start region (min): 0 Sample end region (min): 30
Marker peak width (sec): 5 Marker min peak height: 200 Marker baseline V to V?: Y Marker baseline V to V points: 3
Lower marker selection: First peak > 500 RFU Upper marker selection: Last peak > 200 RFU
Ladder size (bp)75, 200, 300, 400, 500, 700, 1000, 1500, 2000, 3000, 4000, 5000, 7000, 10000, 20000
Quantification using: Lower Marker Final concentration (ng/uL): 0.5000 Dilution factor: 12.0

Sample: SampB4
Well location: B4
Created: Wednesday, October 20, 2021 4:38:54 PM

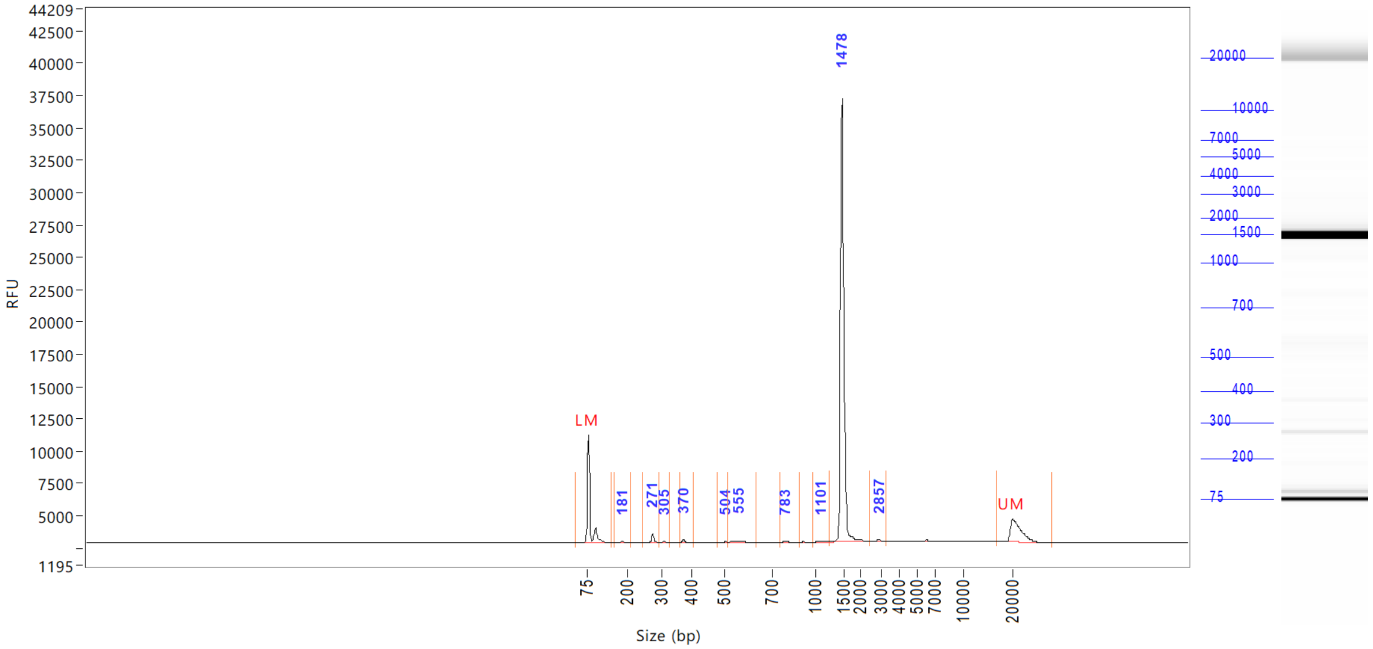

Peak	Size (bp)	Concentration (ng/uL)	From (bp)	To (bp)	Average size (bp)	CV%	RFU	Corrected peak area
1	75 (LM)	0.5000	37	147	78	14.90	8300	53.568
2	181	0.1000	158	210	179	5.63	91	0.893
3	271	0.3969	244	293	270	2.46	622	3.544
4	305	0.0766	293	323	306	2.32	96	0.684
5	370	0.1273	358	403	371	2.37	189	1.136
6	504	0.0640	477	514	497	1.53	76	0.571
7	555	0.2726	514	631	553	3.62	151	2.433
8	783	0.0829	753	887	791	3.10	78	0.740
9	1101	0.1393	987	1245	1119	5.96	108	1.244
10	1478	20.7272	1245	2429	1481	5.01	34328	185.052
11	2857	0.1284	2429	3286	2784	7.92	68	1.146
12	20000 (UM)	0.2138	16708	27617	20796	5.68	1748	22.907

TIC:	22.1152	ng/uL
TIM:	28.8267	nmole/L
Total concentration:	22.6276	ng/uL

Sample peak width (sec): 5 Sample min peak height: 50 Sample baseline V to V?: Y Sample baseline V to V points: 3
Sample filter: Binomial Number of points for filter: 3 Sample start region (min): 0 Sample end region (min): 30
Marker peak width (sec): 5 Marker min peak height: 200 Marker baseline V to V?: Y Marker baseline V to V points: 3
Lower marker selection: First peak > 500 RFU Upper marker selection: Last peak > 200 RFU
Ladder size (bp)75, 200, 300, 400, 500, 700, 1000, 1500, 2000, 3000, 4000, 5000, 7000, 10000, 20000
Quantification using: Lower Marker Final concentration (ng/uL): 0.5000 Dilution factor: 12.0

Sample: SampB5
Well location: B5
Created: Wednesday, October 20, 2021 4:38:54 PM

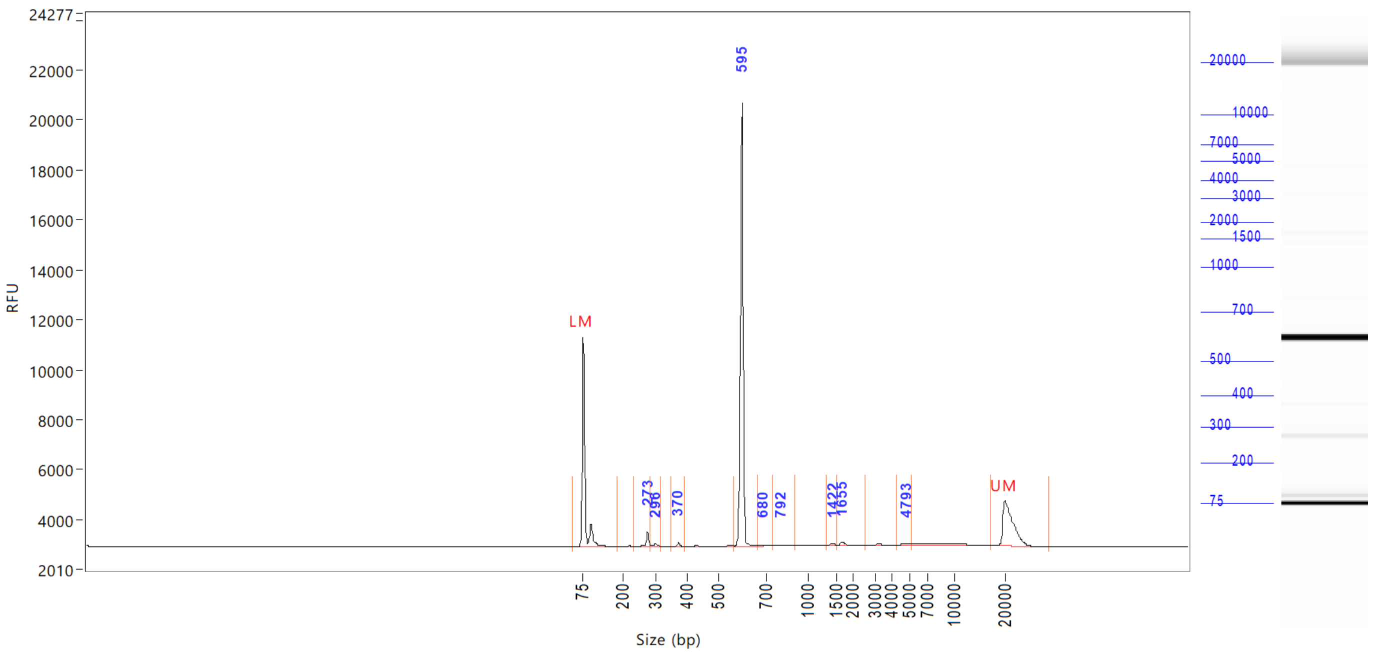

Peak	Size (bp)	Concentration (ng/uL)	From (bp)	To (bp)	Average size (bp)	CV%	RFU	Corrected peak area
1	75 (LM)	0.5000	41	183	78	16.49	8393	52.442
2	273	0.3508	230	284	270	2.33	584	3.066
3	296	0.1028	284	313	295	2.18	89	0.899
4	370	0.0926	345	392	367	1.61	149	0.810
5	595	9.7773	565	662	593	1.18	17783	85.457
6	680	0.0799	662	740	687	2.60	59	0.698
7	792	0.0732	740	900	805	4.05	50	0.640
8	1422	0.0558	1323	1520	1423	2.97	64	0.488
9	1655	0.1337	1520	2600	1745	11.28	144	1.168
10	4793	0.0475	4242	5075	4704	3.88	51	0.416
11	20000 (UM)	0.2241	17073	28947	20891	5.84	1830	23.505

TIC:	10.7136	ng/uL
TIM:	30.8066	nmole/L
Total concentration:	11.1465	ng/uL

Sample peak width (sec): 5   Sample min peak height: 50   Sample baseline V to V?: Y   Sample baseline V to V points: 3
Sample filter: Binomial   Number of points for filter: 3 Sample start region (min): 0 Sample end region (min): 30
Marker peak width (sec): 5   Marker min peak height: 200   Marker baseline V to V?: Y   Marker baseline V to V points: 3
Lower marker selection: First peak > 500 RFU   Upper marker selection: Last peak > 200 RFU
Ladder size (bp)75, 200, 300, 400, 500, 700, 1000, 1500, 2000, 3000, 4000, 5000, 7000, 10000, 20000
Quantification using: Lower Marker   Final concentration (ng/uL): 0.5000   Dilution factor: 12.0

Sample: SampB6
Well location: B6
Created: Wednesday, October 20, 2021 4:38:54 PM

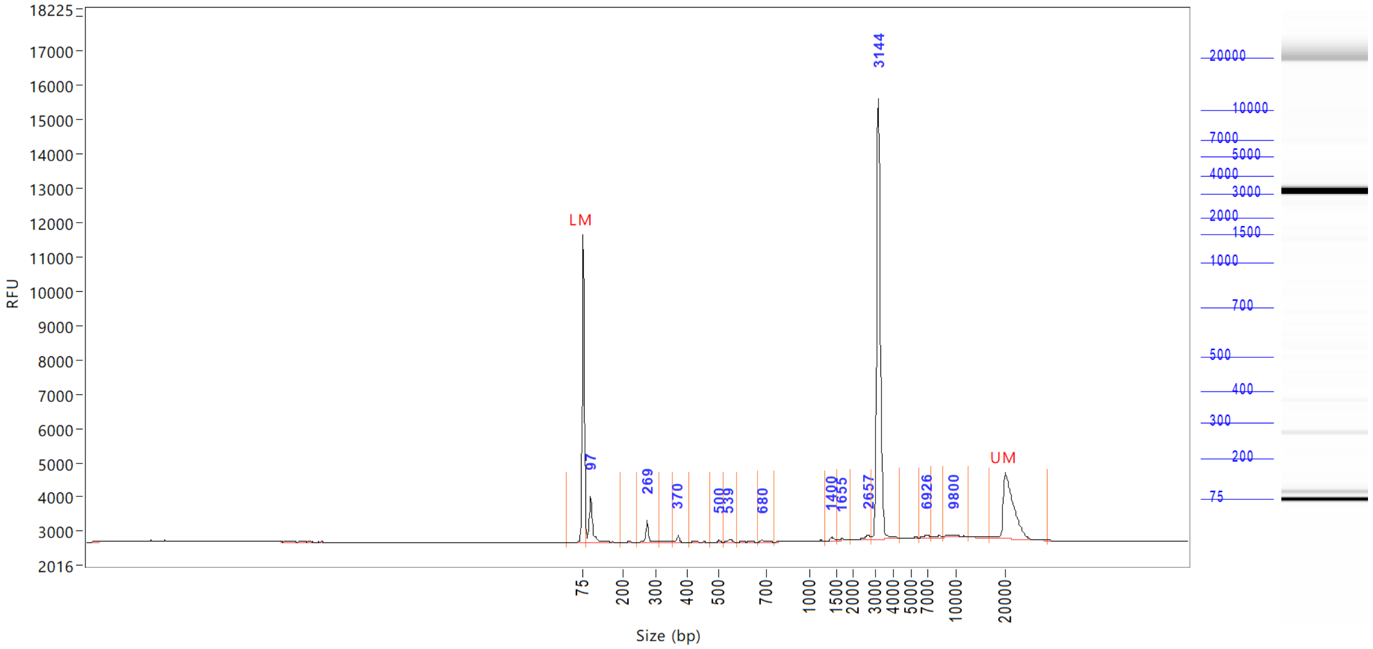

Peak	Size (bp)	Concentration (ng/uL)	From (bp)	To (bp)	Average size (bp)	CV%	RFU	Corrected peak area
1	75 (LM)	0.5000	26	86	73	5.29	8956	46.680
2	97	1.4170	86	190	99	13.07	1324	11.024
3	269	0.4491	242	307	270	3.01	620	3.494
4	370	0.1501	354	405	368	2.09	198	1.168
5	500	0.0424	473	516	497	1.54	58	0.330
6	539	0.0834	516	575	541	1.81	63	0.649
7	680	0.0535	662	749	682	2.05	64	0.416
8	1400	0.0888	1289	1501	1403	2.94	102	0.691
9	1655	0.0540	1501	1942	1654	5.09	53	0.420
10	2657	0.1482	1942	2829	2584	6.81	115	1.153
11	3144	9.2964	2829	4380	3153	4.44	12858	72.327
12	6926	0.0678	5963	7334	6816	3.82	82	0.527
13	9800	0.1778	8600	12562	10065	10.15	70	1.383
14	20000 (UM)	0.2709	16708	28342	20889	6.42	1924	25.290

TIC:	12.0285	ng/uL
TIM:	32.5842	nmole/L
Total concentration:	12.5022	ng/uL

Sample peak width (sec): 5   Sample min peak height: 50   Sample baseline V to V?: Y   Sample baseline V to V points: 3
Sample filter: Binomial   Number of points for filter: 3 Sample start region (min): 0 Sample end region (min): 30
Marker peak width (sec): 5   Marker min peak height: 200   Marker baseline V to V?: Y   Marker baseline V to V points: 3
Lower marker selection: First peak > 500 RFU   Upper marker selection: Last peak > 200 RFU
Ladder size (bp)75, 200, 300, 400, 500, 700, 1000, 1500, 2000, 3000, 4000, 5000, 7000, 10000, 20000
Quantification using: Lower Marker   Final concentration (ng/uL): 0.5000   Dilution factor: 12.0

Sample: SampB7
Well location: B7
Created: Wednesday, October 20, 2021 4:38:54 PM

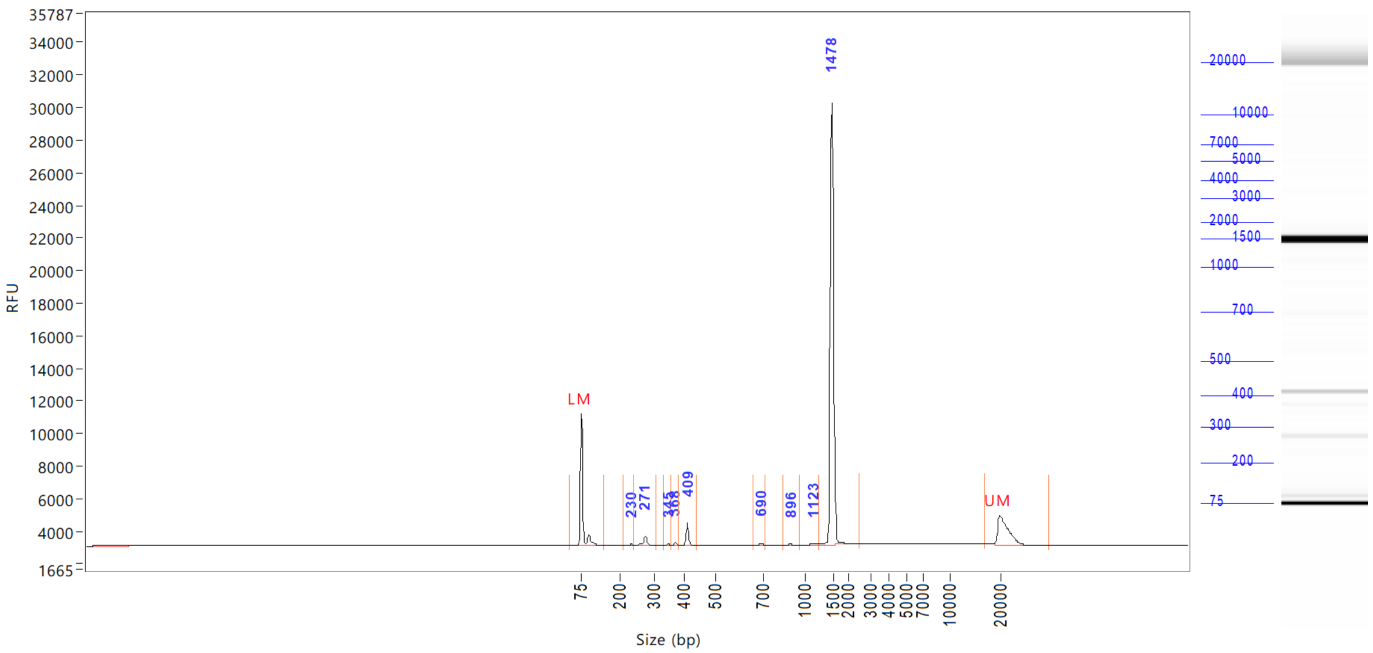

Peak	Size (bp)	Concentration (ng/uL)	From (bp)	To (bp)	Average size (bp)	CV%	RFU	Corrected peak area
1	75 (LM)	0.5000	41	148	77	13.64	8119	48.320
2	230	0.0617	208	241	225	2.99	52	0.497
3	271	0.4979	241	305	270	3.18	542	4.009
4	345	0.0480	331	356	343	1.23	77	0.386
5	368	0.1085	356	384	367	1.24	170	0.874
6	409	0.8174	384	439	407	1.25	1338	6.583
7	690	0.1003	659	714	688	1.29	127	0.808
8	896	0.0495	835	965	891	2.72	52	0.398
9	1123	0.0945	965	1245	1114	6.45	72	0.761
10	1478	17.3900	1245	2458	1476	3.30	27152	140.048
11	20000 (UM)	0.2453	16830	29793	21036	7.24	1761	23.710

TIC:	19.1676	ng/uL
TIM:	27.3689	nmole/L
Total concentration:	19.7389	ng/uL

Sample peak width (sec): 5 Sample min peak height: 50 Sample baseline V to V?: Y Sample baseline V to V points: 3
Sample filter: Binomial Number of points for filter: 3 Sample start region (min): 0 Sample end region (min): 30
Marker peak width (sec): 5 Marker min peak height: 200 Marker baseline V to V?: Y Marker baseline V to V points: 3
Lower marker selection: First peak > 500 RFU Upper marker selection: Last peak > 200 RFU
Ladder size (bp)75, 200, 300, 400, 500, 700, 1000, 1500, 2000, 3000, 4000, 5000, 7000, 10000, 20000
Quantification using: Lower Marker Final concentration (ng/uL): 0.5000 Dilution factor: 12.0

Sample: SampB8
Well location: B8
Created: Wednesday, October 20, 2021 4:38:54 PM

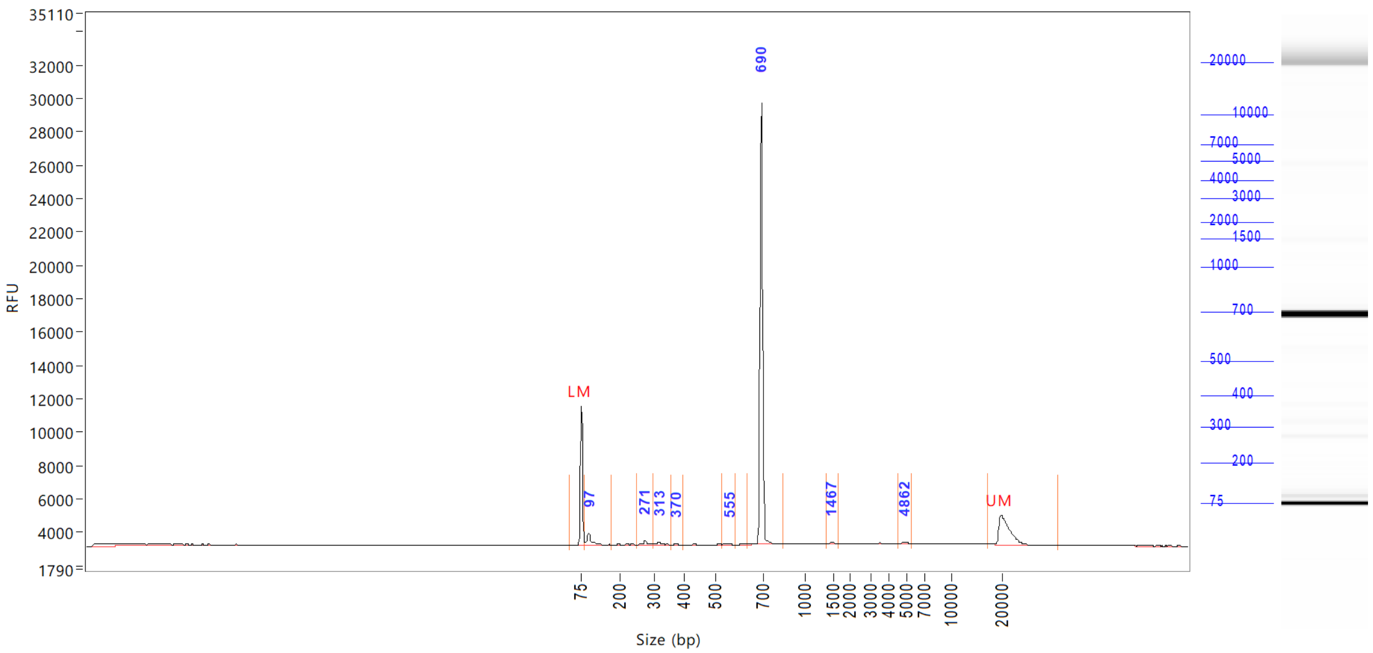

Peak	Size (bp)	Concentration (ng/uL)	From (bp)	To (bp)	Average size (bp)	CV%	RFU	Corrected peak area
1	75 (LM)	0.5000	39	86	73	4.44	8337	43.312
2	97	1.0195	86	169	101	13.54	702	7.359
3	271	0.2299	248	298	271	3.13	257	1.659
4	313	0.1878	298	354	315	3.49	137	1.355
5	370	0.0711	354	394	369	1.77	84	0.513
6	555	0.0886	521	580	544	1.98	70	0.639
7	690	17.5899	629	840	688	1.46	26505	126.977
8	1467	0.1126	1367	1655	1482	3.81	119	0.813
9	4862	0.0940	4552	5519	4849	2.67	104	0.679
10	20000 (UM)	0.2867	17195	31365	21135	8.37	1781	24.838

TIC:	19.3934	ng/uL
TIM:	61.7696	nmole/L
Total concentration:	20.0896	ng/uL

Sample peak width (sec): 5   Sample min peak height: 50   Sample baseline V to V?: Y   Sample baseline V to V points: 3
Sample filter: Binomial   Number of points for filter: 3   Sample start region (min): 0   Sample end region (min): 30
Marker peak width (sec): 5   Marker min peak height: 200   Marker baseline V to V?: Y   Marker baseline V to V points: 3
Lower marker selection: First peak > 500 RFU   Upper marker selection: Last peak > 200 RFU
Ladder size (bp)75, 200, 300, 400, 500, 700, 1000, 1500, 2000, 3000, 4000, 5000, 7000, 10000, 20000
Quantification using: Lower Marker   Final concentration (ng/uL): 0.5000   Dilution factor: 12.0

Sample: SampB9
Well location: B9
Created: Wednesday, October 20, 2021 4:38:54 PM

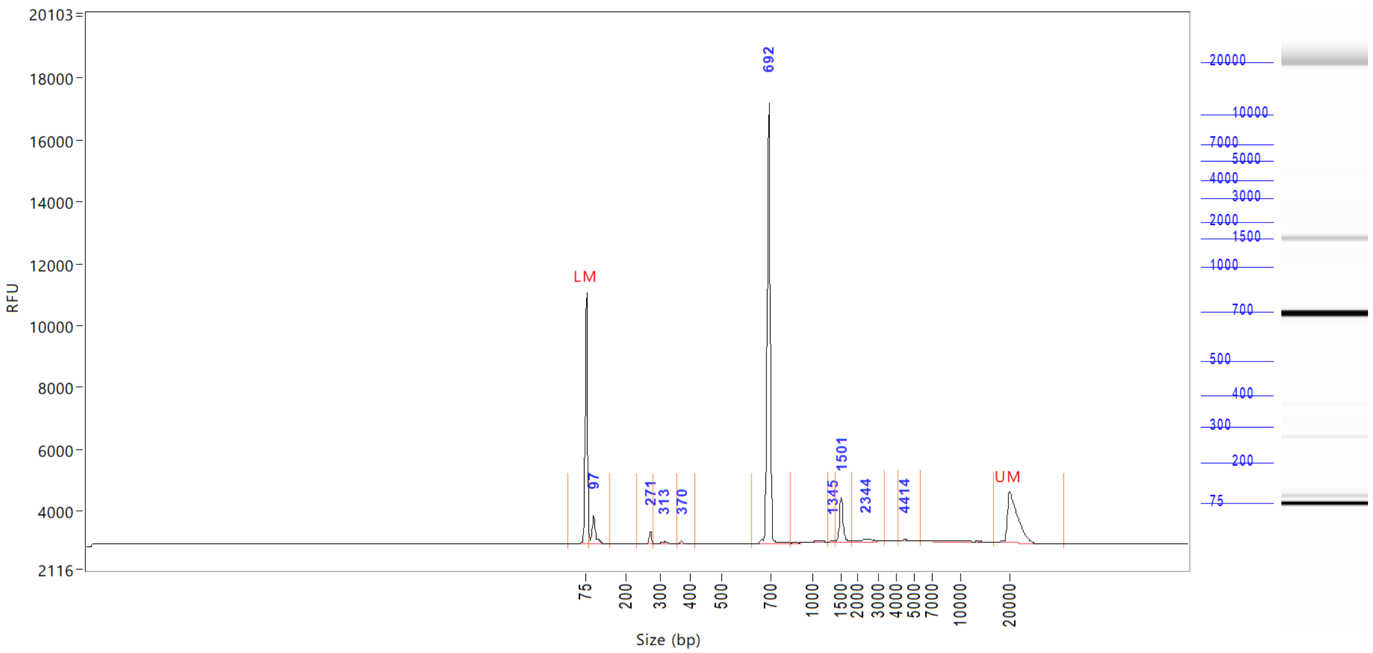

Peak	Size (bp)	Concentration (ng/uL)	From (bp)	To (bp)	Average size (bp)	CV%	RFU	Corrected peak area
1	75 (LM)	0.5000	20	86	72	5.03	8131	43.160
2	97	1.0961	86	147	98	9.62	929	7.885
3	271	0.2984	230	282	267	2.71	382	2.147
4	313	0.1706	282	354	312	4.54	81	1.228
5	370	0.1169	354	416	378	4.06	97	0.841
6	692	10.0045	623	844	690	1.51	14276	71.966
7	1345	0.0621	1278	1411	1345	2.71	63	0.447
8	1501	1.1445	1411	1846	1507	3.63	1423	8.233
9	2344	0.2459	1846	3358	2469	14.35	65	1.769
10	4414	0.1057	4070	5667	4572	5.78	65	0.760
11	20000 (UM)	0.2469	16952	30760	20827	5.90	1665	21.313

TIC:	13.2447	ng/uL
TIM:	47.0018	nmole/L
Total concentration:	13.7203	ng/uL

```
Sample peak width (sec): 5    Sample min peak height: 50    Sample baseline V to V?: Y    Sample baseline V to V points: 3
Sample filter: Binomial       Number of points for filter: 3  Sample start region (min): 0  Sample end region (min): 30
Marker peak width (sec): 5    Marker min peak height: 200   Marker baseline V to V?: Y    Marker baseline V to V points: 3
Lower marker selection: First peak > 500 RFU                Upper marker selection: Last peak > 200 RFU
Ladder size (bp)75, 200, 300, 400, 500, 700, 1000, 1500, 2000, 3000, 4000, 5000, 7000, 10000, 20000
Quantification using: Lower Marker          Final concentration (ng/uL): 0.5000          Dilution factor: 12.0
```

Sample: SampB10
Well location: B10
Created: Wednesday, October 20, 2021 4:38:54 PM

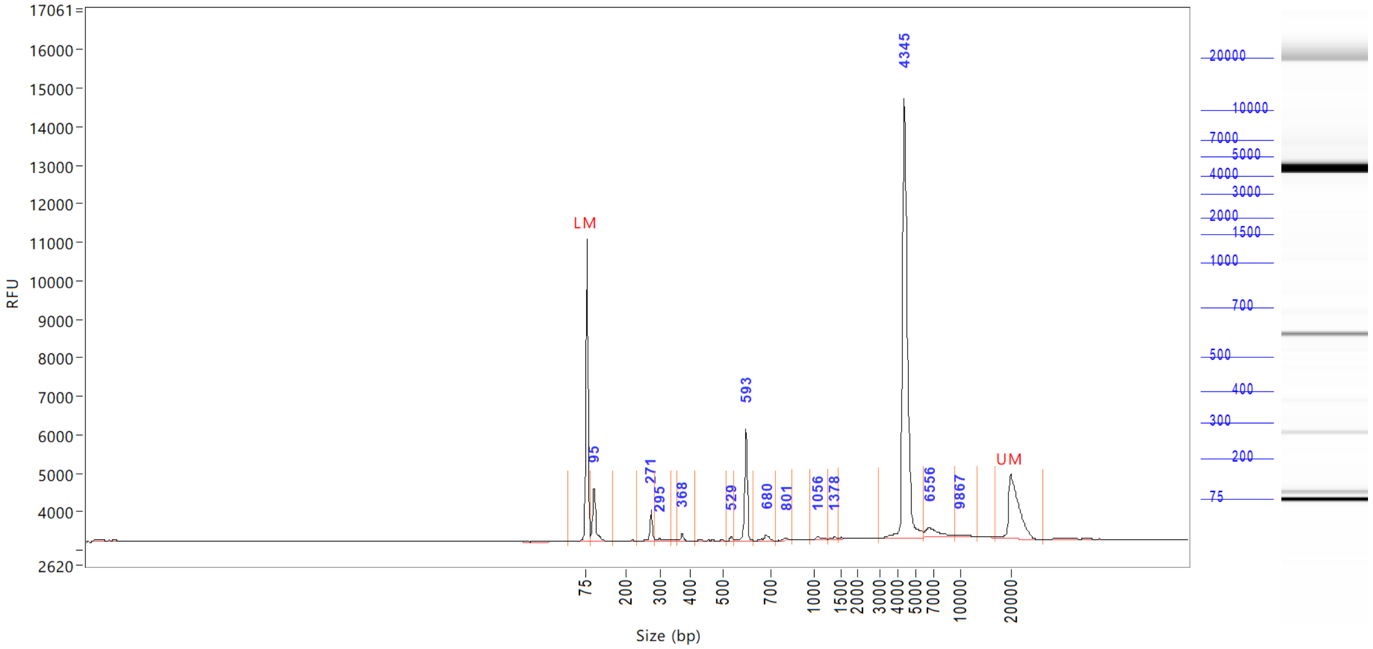

Peak	Size (bp)	Concentration (ng/uL)	From (bp)	To (bp)	Average size (bp)	CV%	RFU	Corrected peak area
1	75 (LM)	0.5000	20	86	72	5.24	7856	41.729
2	95	1.6099	86	158	98	10.36	1370	11.197
3	271	0.6160	232	282	268	2.27	807	4.284
4	295	0.1535	282	331	302	4.39	60	1.067
5	368	0.1654	352	412	369	2.76	180	1.150
6	529	0.0900	514	544	530	1.36	102	0.626
7	593	2.1387	544	626	589	1.42	2893	14.874
8	680	0.2055	626	736	673	2.43	139	1.429
9	801	0.0864	736	844	798	2.40	73	0.601
10	1056	0.1216	978	1245	1095	5.76	72	0.846
11	1378	0.0725	1245	1445	1347	3.69	56	0.504
12	4345	11.7827	2971	5963	4391	5.36	11419	81.946
13	6556	0.6821	5963	9400	7207	12.14	250	4.744
14	9867	0.1181	9400	13293	10561	9.40	57	0.821
15	20000 (UM)	0.2565	16830	26408	20806	5.71	1667	21.405

TIC:	17.8424	ng/uL
TIM:	44.1272	nmole/L
Total concentration:	18.2468	ng/uL

```
Sample peak width (sec): 5     Sample min peak height: 50    Sample baseline V to V?: Y    Sample baseline V to V points: 3
Sample filter: Binomial        Number of points for filter: 3  Sample start region (min): 0  Sample end region (min): 30
Marker peak width (sec): 5     Marker min peak height: 200   Marker baseline V to V?: Y    Marker baseline V to V points: 3
Lower marker selection: First peak > 500 RFU                 Upper marker selection: Last peak > 200 RFU
Ladder size (bp)75, 200, 300, 400, 500, 700, 1000, 1500, 2000, 3000, 4000, 5000, 7000, 10000, 20000
Quantification using: Lower Marker          Final concentration (ng/uL): 0.5000          Dilution factor: 12.0
```

Sample: SampB11
Well location: B11
Created: Wednesday, October 20, 2021 4:38:54 PM

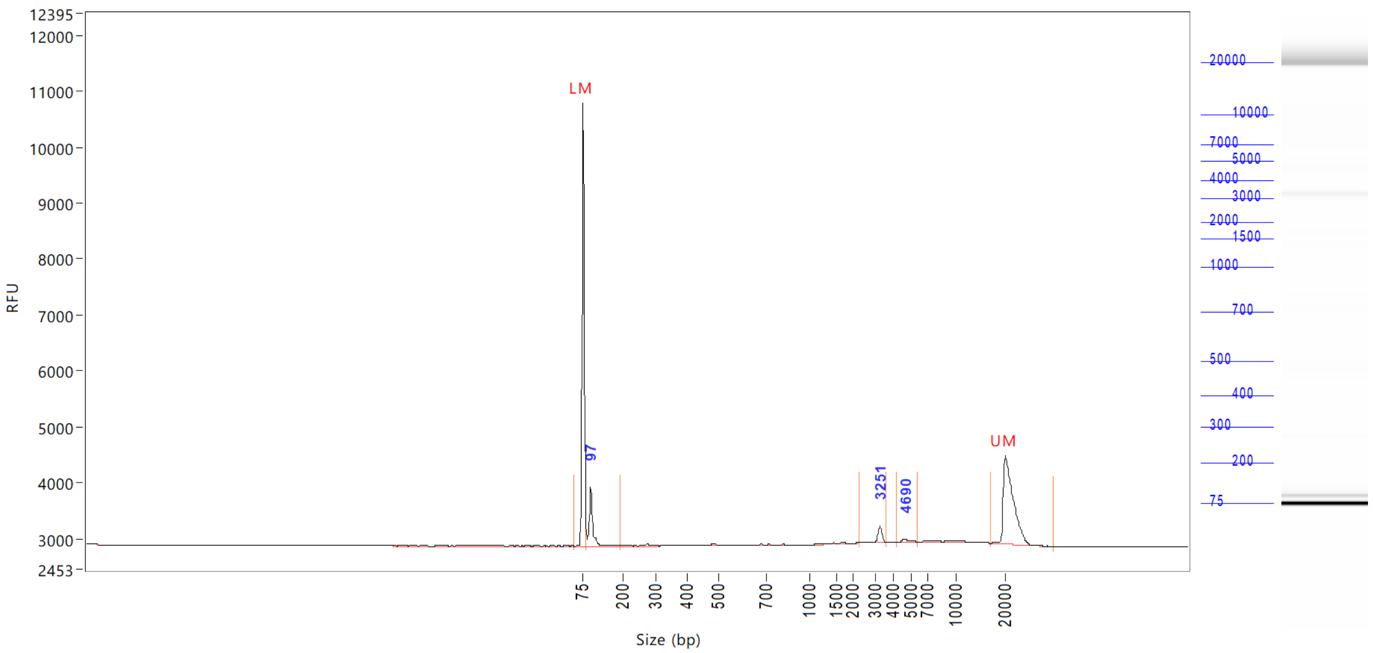

Peak	Size (bp)	Concentration (ng/uL)	From (bp)	To (bp)	Average size (bp)	CV%	RFU	Corrected peak area
1	75 (LM)	0.5000	48	86	73	4.48	7938	42.026
2	97	1.1911	86	194	100	13.55	1047	8.342
3	3251	0.2621	2286	3643	3194	5.00	294	1.836
4	4690	0.0926	4208	5741	4788	6.30	61	0.649
5	20000 (UM)	0.2389	16952	29551	20835	5.70	1583	20.079

TIC:	1.5457	ng/uL
TIM:	19.7251	nmole/L
Total concentration:	2.1471	ng/uL

Sample peak width (sec): 5 Sample min peak height: 50 Sample baseline V to V?: Y Sample baseline V to V points: 3
Sample filter: Binomial Number of points for filter: 3 Sample start region (min): 0 Sample end region (min): 30
Marker peak width (sec): 5 Marker min peak height: 200 Marker baseline V to V?: Y Marker baseline V to V points: 3
Lower marker selection: First peak > 500 RFU Upper marker selection: Last peak > 200 RFU
Ladder size (bp)75, 200, 300, 400, 500, 700, 1000, 1500, 2000, 3000, 4000, 5000, 7000, 10000, 20000
Quantification using: Lower Marker Final concentration (ng/uL): 0.5000 Dilution factor: 12.0

Sample: SampB12
Well location: B12
Created: Wednesday, October 20, 2021 4:38:54 PM

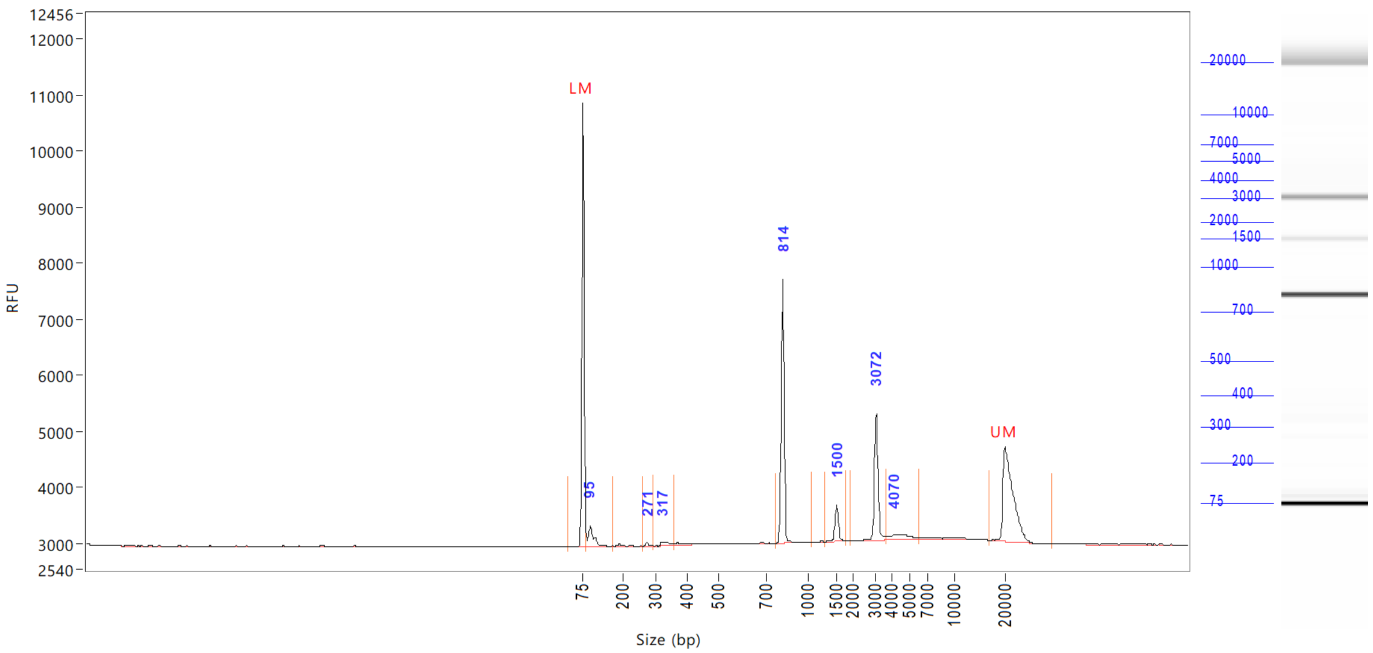

Peak	Size (bp)	Concentration (ng/uL)	From (bp)	To (bp)	Average size (bp)	CV%	RFU	Corrected peak area
1	75 (LM)	0.5000	31	86	72	4.87	7931	43.165
2	95	0.6641	86	171	102	14.71	385	4.778
3	271	0.0566	259	293	270	2.14	73	0.407
4	317	0.1887	293	358	328	4.22	70	1.358
5	814	3.2727	762	1045	811	2.07	4696	23.544
6	1500	0.5199	1300	1789	1492	3.32	658	3.740
7	3072	1.8126	1942	3679	3054	5.13	2249	13.040
8	4070	0.3348	3679	5963	4432	11.02	84	2.409
9	20000 (UM)	0.2525	16952	29551	20901	5.77	1682	21.800

TIC:	6.8494	ng/uL
TIM:	20.3002	nmole/L
Total concentration:	7.2432	ng/uL

Sample peak width (sec): 5 Sample min peak height: 50 Sample baseline V to V?: Y Sample baseline V to V points: 3
Sample filter: Binomial Number of points for filter: 3 Sample start region (min): 0 Sample end region (min): 30
Marker peak width (sec): 5 Marker min peak height: 200 Marker baseline V to V?: Y Marker baseline V to V points: 3
Lower marker selection: First peak > 500 RFU Upper marker selection: Last peak > 200 RFU
Ladder size (bp)75, 200, 300, 400, 500, 700, 1000, 1500, 2000, 3000, 4000, 5000, 7000, 10000, 20000
Quantification using: Lower Marker Final concentration (ng/uL): 0.5000 Dilution factor: 12.0

Sample: SampC1
Well location: C1
Created: Wednesday, October 20, 2021 4:38:54 PM

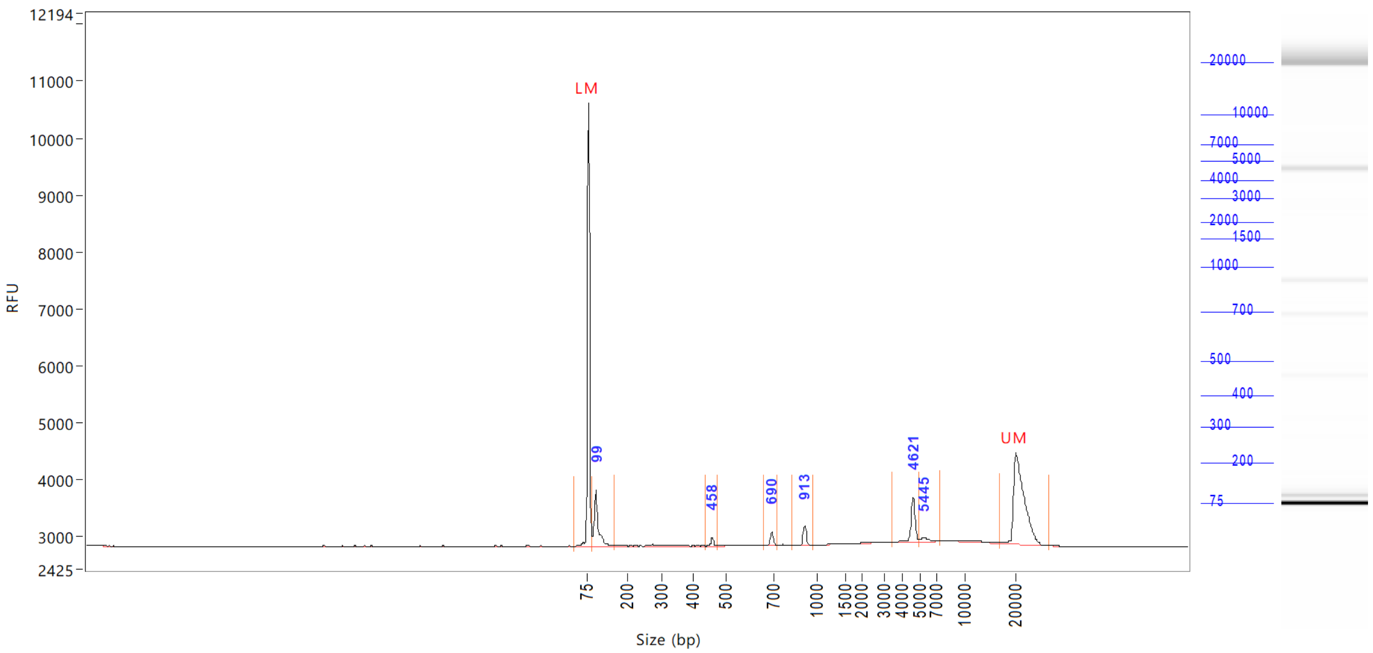

Peak	Size (bp)	Concentration (ng/uL)	From (bp)	To (bp)	Average size (bp)	CV%	RFU	Corrected peak area
1	75 (LM)	0.5000	31	87	74	5.34	7803	41.997
2	99	1.2914	87	156	101	10.96	996	9.039
3	458	0.1175	439	473	456	1.21	155	0.823
4	690	0.1942	659	718	687	1.00	256	1.359
5	913	0.2534	822	974	909	1.41	344	1.774
6	4621	0.6775	3465	4966	4564	4.21	786	4.742
7	5445	0.1296	4966	7334	5818	11.49	65	0.907
8	20000 (UM)	0.2540	16830	26529	20853	5.81	1622	21.334

TIC:	2.6636	ng/uL
TIM:	22.6260	nmole/L
Total concentration:	3.1470	ng/uL

```
Sample peak width (sec): 5    Sample min peak height: 50    Sample baseline V to V?: Y    Sample baseline V to V points: 3
Sample filter: Binomial       Number of points for filter: 3  Sample start region (min): 0  Sample end region (min): 30
Marker peak width (sec): 5    Marker min peak height: 200   Marker baseline V to V?: Y    Marker baseline V to V points: 3
Lower marker selection: First peak > 500 RFU                Upper marker selection: Last peak > 200 RFU
Ladder size (bp)75, 200, 300, 400, 500, 700, 1000, 1500, 2000, 3000, 4000, 5000, 7000, 10000, 20000
Quantification using: Lower Marker          Final concentration (ng/uL): 0.5000          Dilution factor: 12.0
```

Sample: SampC2
Well location: C2
Created: Wednesday, October 20, 2021 4:38:54 PM

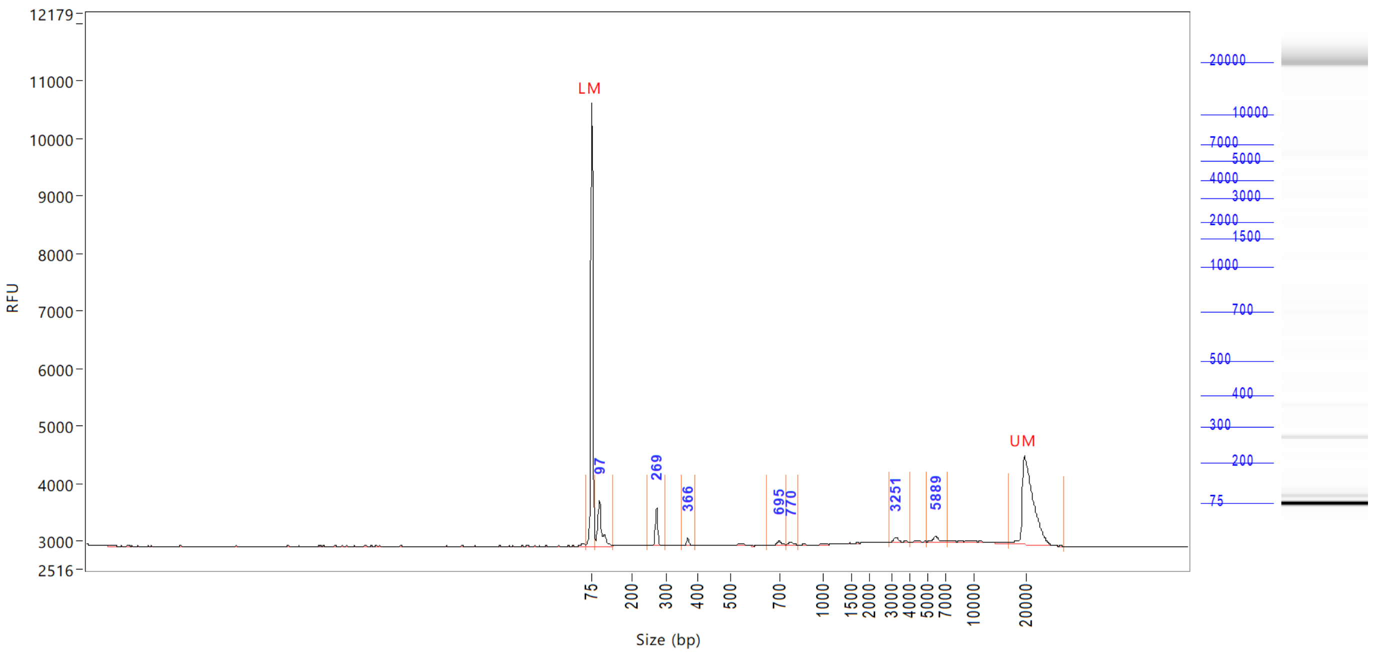

Peak	Size (bp)	Concentration (ng/uL)	From (bp)	To (bp)	Average size (bp)	CV%	RFU	Corrected peak area
1	75 (LM)	0.5000	56	86	73	4.67	7717	40.782
2	97	1.0604	86	141	100	10.02	788	7.208
3	269	0.5201	244	293	267	1.87	670	3.535
4	366	0.1077	345	388	363	1.62	130	0.732
5	695	0.1233	644	736	692	2.66	87	0.838
6	770	0.0755	736	822	771	2.53	52	0.513
7	3251	0.1012	2914	4035	3300	6.78	82	0.688
8	5889	0.1069	4966	7268	5923	7.91	92	0.727
9	20000 (UM)	0.2536	16830	27496	20803	6.08	1549	20.682

TIC:	2.0951	ng/uL
TIM:	21.6394	nmole/L
Total concentration:	2.7290	ng/uL

Sample peak width (sec): 5 Sample min peak height: 50 Sample baseline V to V?: Y Sample baseline V to V points: 3
Sample filter: Binomial Number of points for filter: 3 Sample start region (min): 0 Sample end region (min): 30
Marker peak width (sec): 5 Marker min peak height: 200 Marker baseline V to V?: Y Marker baseline V to V points: 3
Lower marker selection: First peak > 500 RFU Upper marker selection: Last peak > 200 RFU
Ladder size (bp)75, 200, 300, 400, 500, 700, 1000, 1500, 2000, 3000, 4000, 5000, 7000, 10000, 20000
Quantification using: Lower Marker Final concentration (ng/uL): 0.5000 Dilution factor: 12.0

Sample: SampC3
Well location: C3
Created: Wednesday, October 20, 2021 4:38:54 PM

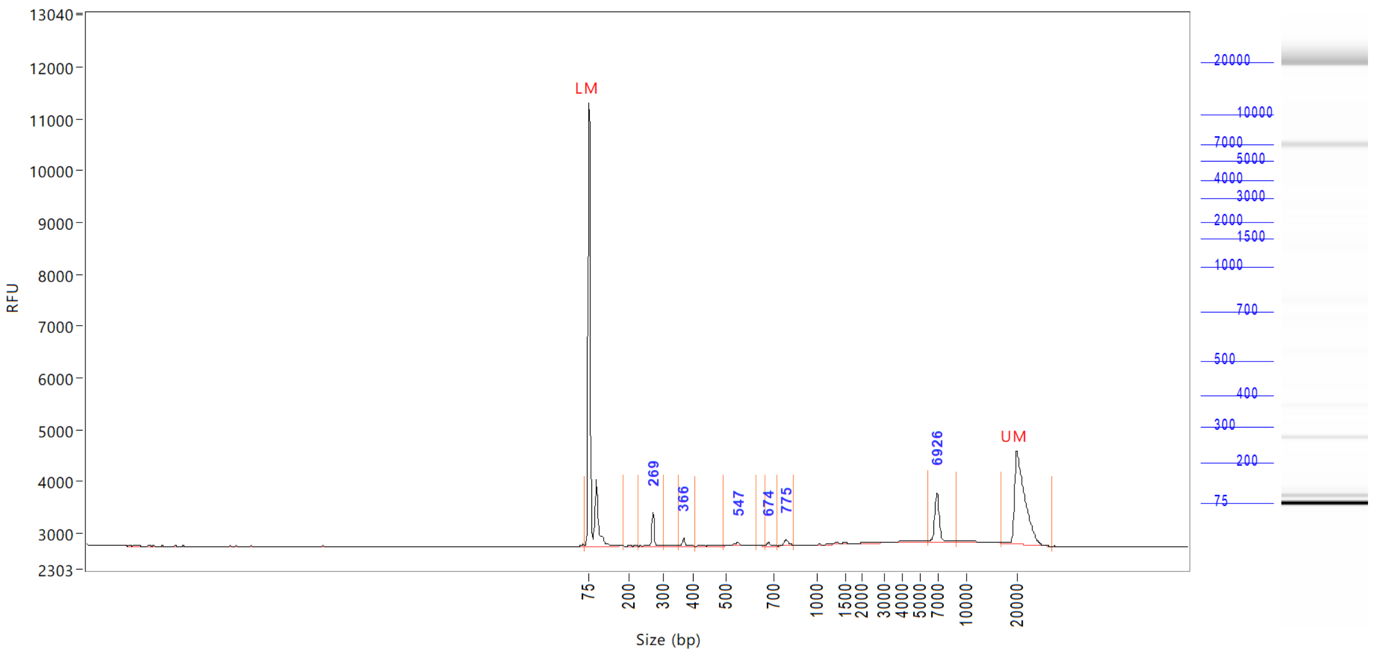

Peak	Size (bp)	Concentration (ng/uL)	From (bp)	To (bp)	Average size (bp)	CV%	RFU	Corrected peak area
1	75 (LM)	0.5000	64	183	79	16.12	8579	56.102
2	269	0.3645	226	303	267	2.68	642	3.408
3	366	0.0898	352	403	366	2.06	148	0.840
4	547	0.1011	492	626	547	4.98	70	0.946
5	674	0.0496	659	718	674	1.48	66	0.463
6	775	0.1046	718	831	776	2.43	114	0.978
7	6926	0.6403	5815	8934	6917	5.87	947	5.987
8	20000 (UM)	0.2048	16952	27012	20894	5.59	1795	22.981

TIC:	1.3499	ng/uL
TIM:	3.4487	nmole/L
Total concentration:	1.8026	ng/uL

Sample peak width (sec): 5 Sample min peak height: 50 Sample baseline V to V?: Y Sample baseline V to V points: 3
Sample filter: Binomial Number of points for filter: 3 Sample start region (min): 0 Sample end region (min): 30
Marker peak width (sec): 5 Marker min peak height: 200 Marker baseline V to V?: Y Marker baseline V to V points: 3
Lower marker selection: First peak > 500 RFU Upper marker selection: Last peak > 200 RFU
Ladder size (bp)75, 200, 300, 400, 500, 700, 1000, 1500, 2000, 3000, 4000, 5000, 7000, 10000, 20000
Quantification using: Lower Marker Final concentration (ng/uL): 0.5000 Dilution factor: 12.0

Sample: SampC4
Well location: C4
Created: Wednesday, October 20, 2021 4:38:54 PM

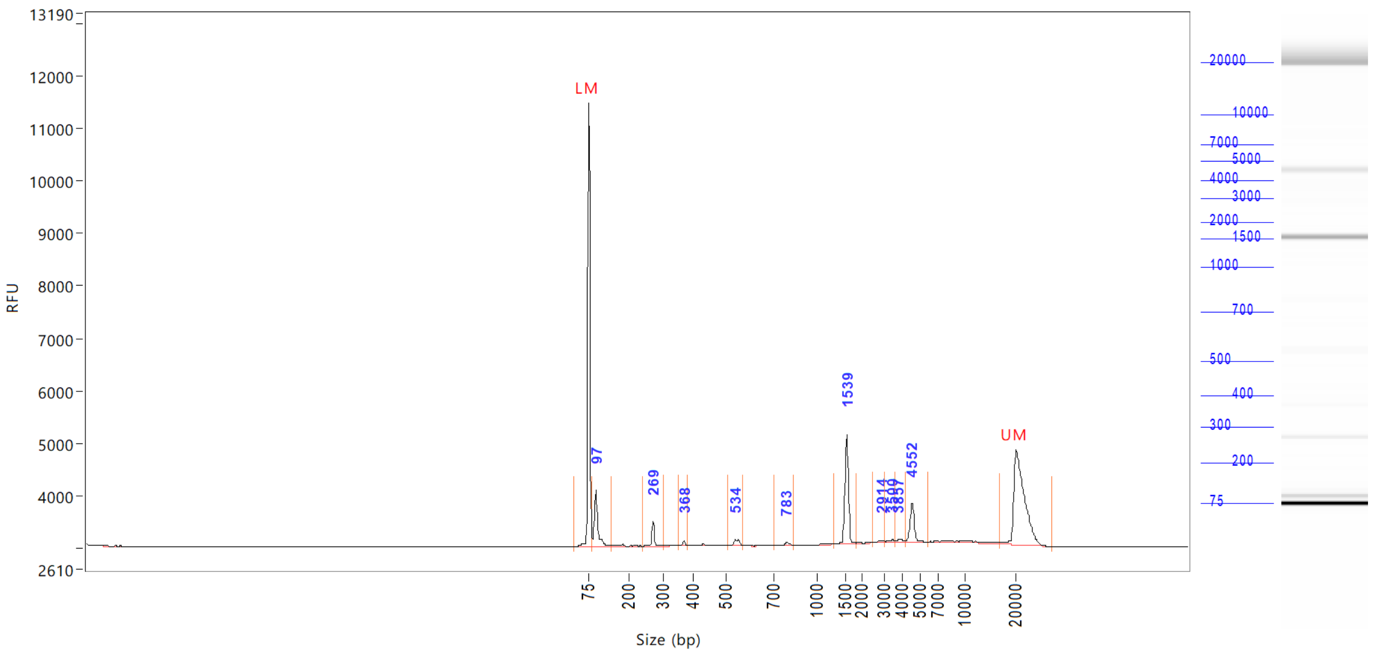

Peak	Size (bp)	Concentration (ng/uL)	From (bp)	To (bp)	Average size (bp)	CV%	RFU	Corrected peak area
1	75 (LM)	0.5000	30	86	73	5.45	8455	44.337
2	97	1.1841	86	145	98	9.23	1067	8.750
3	269	0.3372	239	300	267	2.44	465	2.492
4	368	0.0740	349	378	365	1.26	110	0.547
5	534	0.1598	506	565	539	1.90	120	1.181
6	783	0.0678	697	831	779	2.53	58	0.501
7	1539	1.5282	1289	1827	1522	3.19	2084	11.293
8	2914	0.0578	2486	3072	2845	4.69	51	0.427
9	3500	0.0714	3072	3679	3394	5.18	52	0.528
10	3857	0.0690	3679	4173	3905	3.50	56	0.510
11	4552	0.6044	4173	5963	4544	3.26	739	4.466
12	20000 (UM)	0.2690	16830	27254	20882	5.84	1822	23.854

TIC:	4.1536	ng/uL
TIM:	24.8502	nmole/L
Total concentration:	4.6765	ng/uL

```
Sample peak width (sec): 5     Sample min peak height: 50     Sample baseline V to V?: Y     Sample baseline V to V points: 3
Sample filter: Binomial        Number of points for filter: 3  Sample start region (min): 0  Sample end region (min): 30
Marker peak width (sec): 5     Marker min peak height: 200    Marker baseline V to V?: Y     Marker baseline V to V points: 3
Lower marker selection: First peak > 500 RFU                  Upper marker selection: Last peak > 200 RFU
Ladder size (bp)75, 200, 300, 400, 500, 700, 1000, 1500, 2000, 3000, 4000, 5000, 7000, 10000, 20000
Quantification using: Lower Marker          Final concentration (ng/uL): 0.5000          Dilution factor: 12.0
```

Sample: SampC5
Well location: C5
Created: Wednesday, October 20, 2021 4:38:54 PM

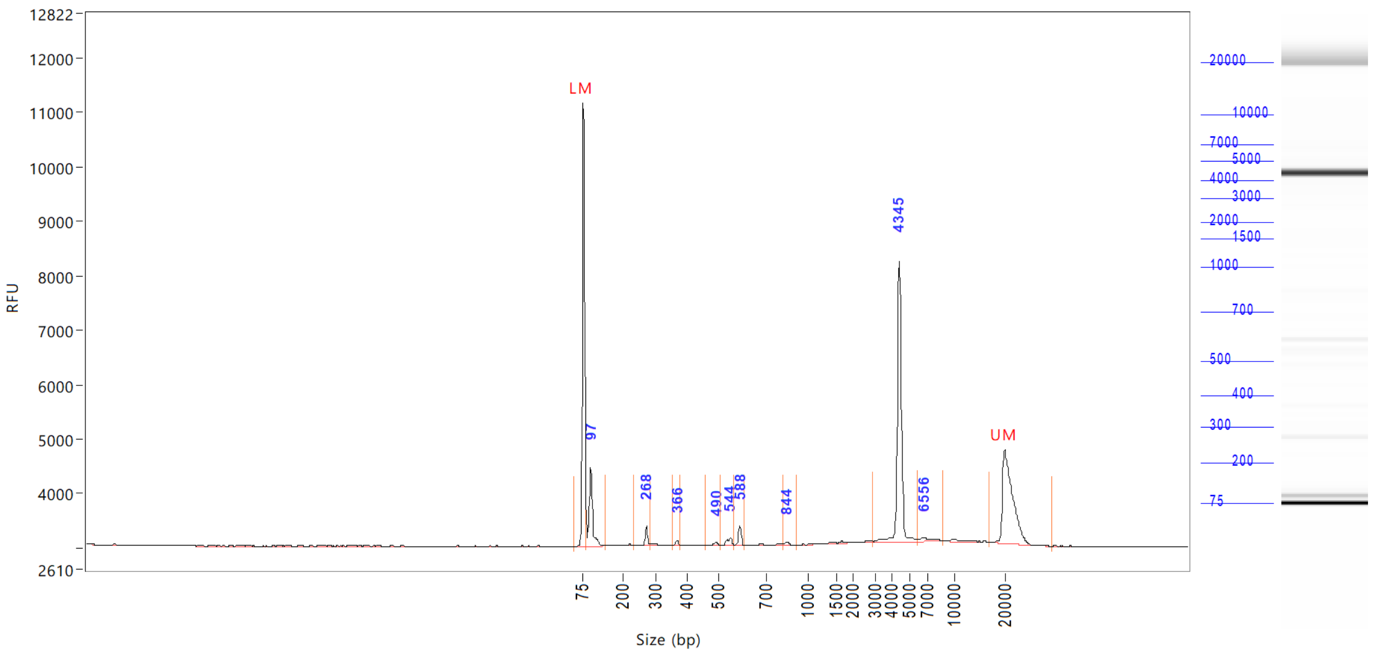

Peak	Size (bp)	Concentration (ng/uL)	From (bp)	To (bp)	Average size (bp)	CV%	RFU	Corrected peak area
1	75 (LM)	0.5000	47	86	74	4.31	8156	43.431
2	97	1.6204	86	147	97	8.44	1447	11.729
3	268	0.2711	230	280	266	2.37	360	1.962
4	366	0.0752	352	378	363	1.20	102	0.544
5	490	0.0684	460	506	486	1.78	66	0.495
6	544	0.1839	506	565	538	2.02	145	1.331
7	588	0.2480	565	606	585	1.01	360	1.795
8	844	0.0654	814	918	842	2.15	66	0.473
9	4345	4.3237	2943	5889	4347	5.43	5157	31.297
10	6556	0.1643	5889	8667	7065	10.40	67	1.189
11	20000 (UM)	0.2589	16830	29430	20823	5.91	1712	22.492

TIC:	7.0203	ng/uL
TIM:	32.7407	nmole/L
Total concentration:	7.5714	ng/uL

Sample peak width (sec): 5 Sample min peak height: 50 Sample baseline V to V?: Y Sample baseline V to V points: 3
Sample filter: Binomial Number of points for filter: 3 Sample start region (min): 0 Sample end region (min): 30
Marker peak width (sec): 5 Marker min peak height: 200 Marker baseline V to V?: Y Marker baseline V to V points: 3
Lower marker selection: First peak > 500 RFU Upper marker selection: Last peak > 200 RFU
Ladder size (bp)75, 200, 300, 400, 500, 700, 1000, 1500, 2000, 3000, 4000, 5000, 7000, 10000, 20000
Quantification using: Lower Marker Final concentration (ng/uL): 0.5000 Dilution factor: 12.0

Sample: SampC6
Well location: C6
Created: Wednesday, October 20, 2021 4:38:54 PM

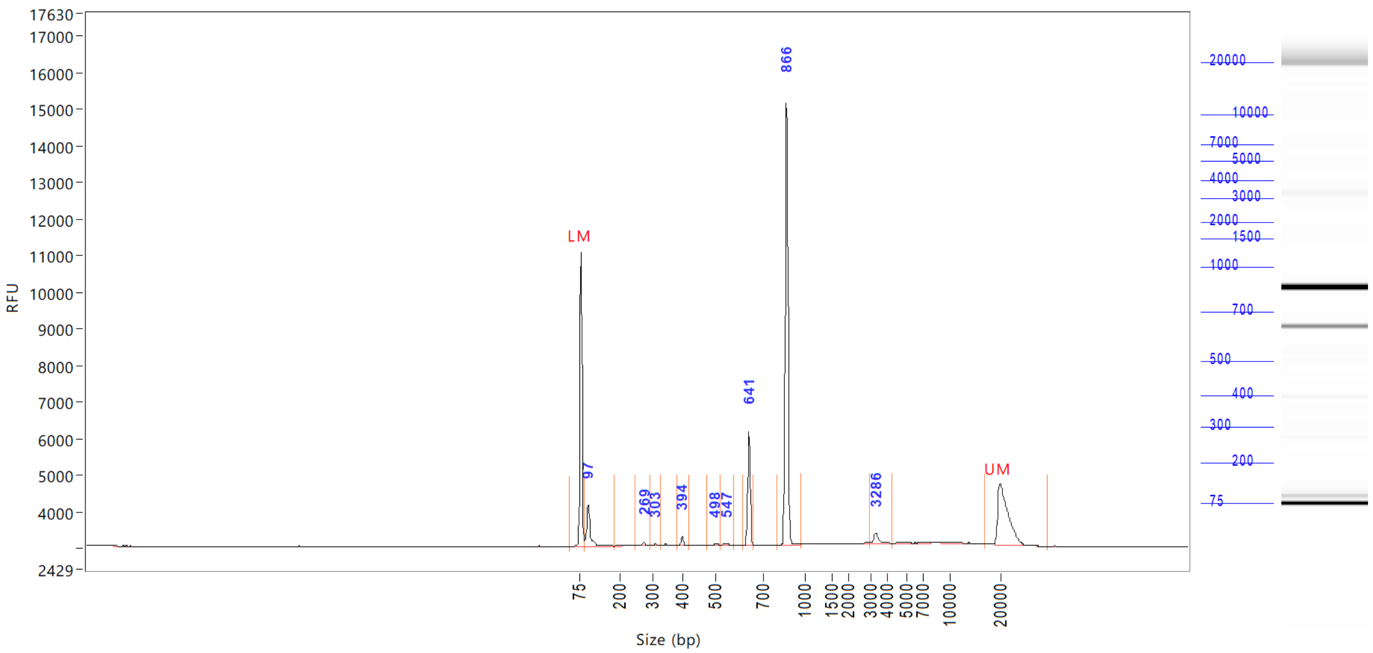

Peak	Size (bp)	Concentration (ng/uL)	From (bp)	To (bp)	Average size (bp)	CV%	RFU	Corrected peak area
1	75 (LM)	0.5000	41	86	73	4.44	8048	42.023
2	97	1.3660	86	181	99	11.97	1118	9.568
3	269	0.1375	246	291	268	3.42	117	0.963
4	303	0.0683	291	321	304	2.35	55	0.478
5	394	0.1756	380	418	392	1.29	243	1.230
6	498	0.0799	473	521	500	1.82	67	0.559
7	547	0.0833	521	578	540	2.01	54	0.583
8	641	2.0908	611	662	638	0.83	3110	14.644
9	866	8.4053	805	970	863	1.20	12103	58.870
10	3286	0.3062	3000	4173	3309	5.81	280	2.145
11	20000 (UM)	0.2594	16830	29672	20883	5.84	1678	21.801

TIC:	12.7129	ng/uL
TIM:	46.7001	nmole/L
Total concentration:	13.3185	ng/uL

```
Sample peak width (sec): 5     Sample min peak height: 50     Sample baseline V to V?: Y     Sample baseline V to V points: 3
Sample filter: Binomial        Number of points for filter: 3  Sample start region (min): 0  Sample end region (min): 30
Marker peak width (sec): 5     Marker min peak height: 200    Marker baseline V to V?: Y     Marker baseline V to V points: 3
Lower marker selection: First peak > 500 RFU                  Upper marker selection: Last peak > 200 RFU
Ladder size (bp)75, 200, 300, 400, 500, 700, 1000, 1500, 2000, 3000, 4000, 5000, 7000, 10000, 20000
Quantification using: Lower Marker          Final concentration (ng/uL): 0.5000          Dilution factor: 12.0
```

Sample: SampC7
Well location: C7
Created: Wednesday, October 20, 2021 4:38:54 PM

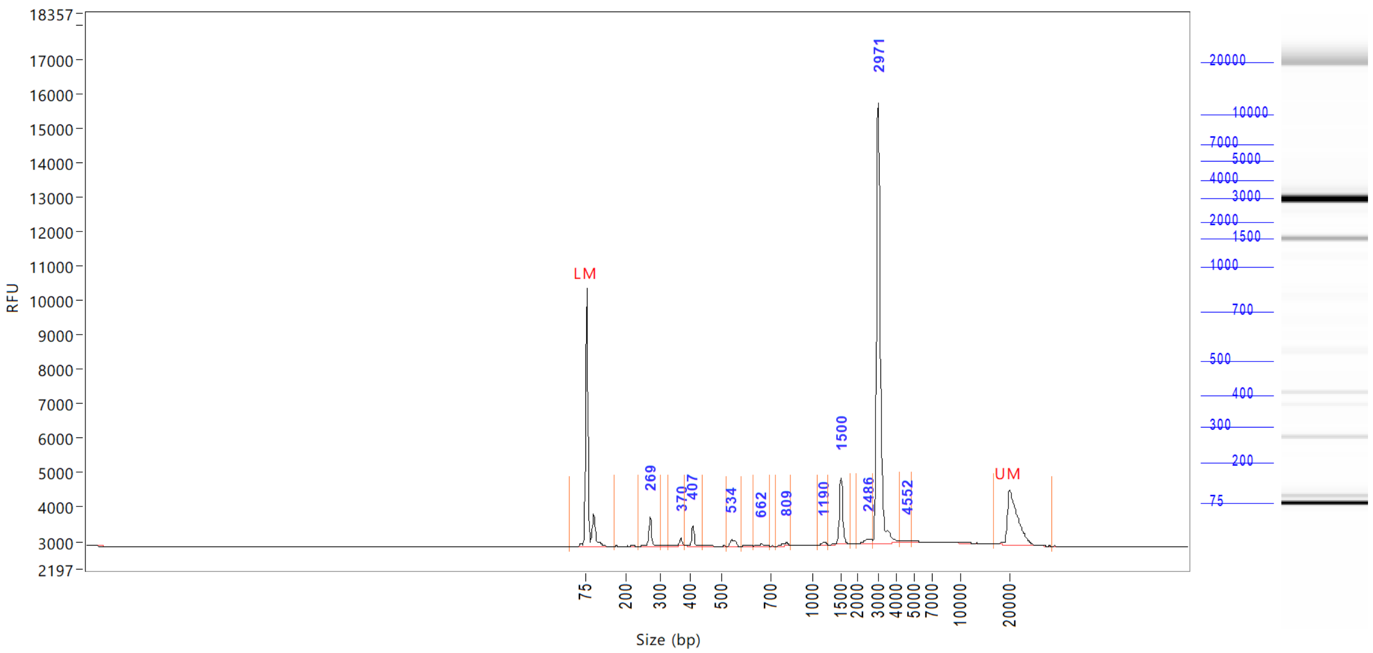

Peak	Size (bp)	Concentration (ng/uL)	From (bp)	To (bp)	Average size (bp)	CV%	RFU	Corrected peak area
1	75 (LM)	0.5000	24	164	78	13.86	7511	49.420
2	269	0.5711	237	303	269	2.60	847	4.704
3	370	0.1788	325	380	362	3.17	232	1.473
4	407	0.3961	380	439	405	2.09	568	3.263
5	534	0.2199	516	578	539	1.81	194	1.811
6	662	0.0801	629	697	662	2.08	57	0.659
7	809	0.1194	740	844	798	2.71	96	0.984
8	1190	0.0669	1090	1278	1179	2.85	81	0.551
9	1500	1.2722	1278	1789	1495	2.59	1947	10.479
10	2486	0.1765	1981	2715	2477	5.98	138	1.454
11	2971	9.2298	2715	4173	2988	5.38	12822	76.023
12	4552	0.0838	4173	4828	4490	4.23	54	0.690
13	20000 (UM)	0.2085	16952	28463	20796	5.87	1583	20.609

TIC:	12.3947	ng/uL
TIM:	13.7579	nmole/L
Total concentration:	12.7720	ng/uL

```
Sample peak width (sec): 5     Sample min peak height: 50     Sample baseline V to V?: Y     Sample baseline V to V points: 3
Sample filter: Binomial        Number of points for filter: 3  Sample start region (min): 0  Sample end region (min): 30
Marker peak width (sec): 5     Marker min peak height: 200    Marker baseline V to V?: Y     Marker baseline V to V points: 3
Lower marker selection: First peak > 500 RFU                  Upper marker selection: Last peak > 200 RFU
Ladder size (bp)75, 200, 300, 400, 500, 700, 1000, 1500, 2000, 3000, 4000, 5000, 7000, 10000, 20000
Quantification using: Lower Marker          Final concentration (ng/uL): 0.5000          Dilution factor: 12.0
```

Sample: SampC8
Well location: C8
Created: Wednesday, October 20, 2021 4:38:54 PM

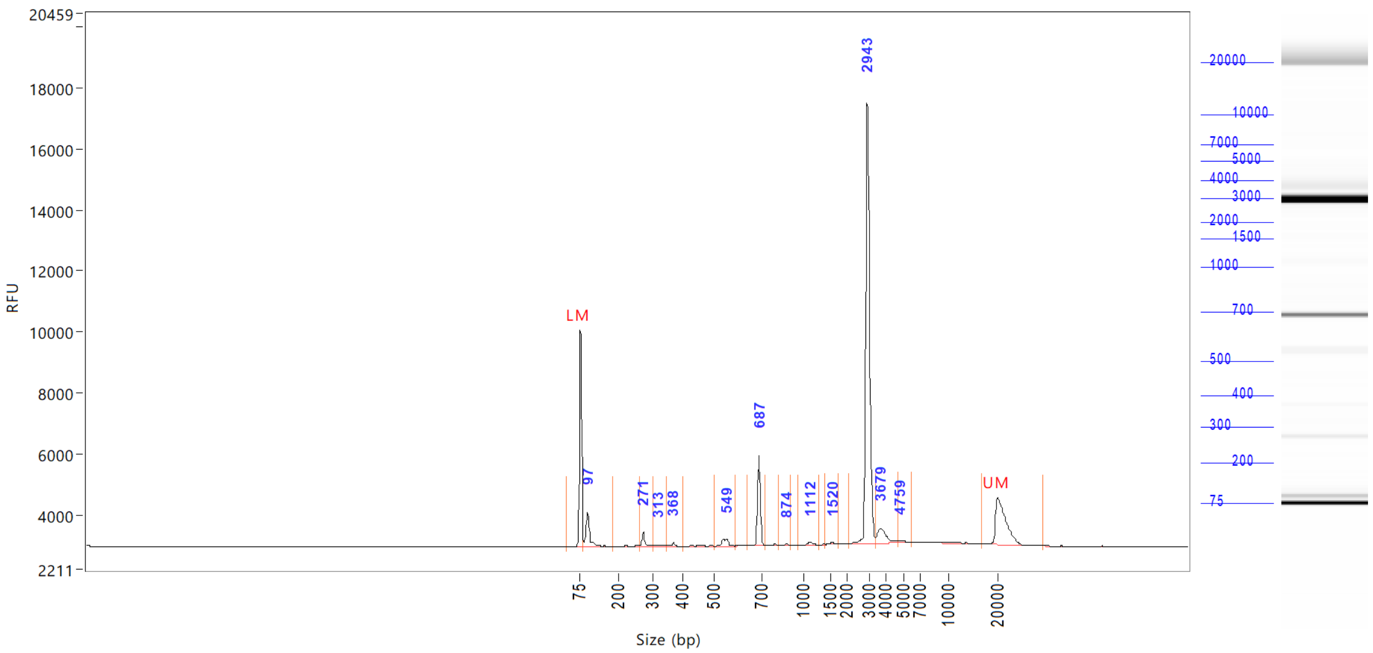

Peak	Size (bp)	Concentration (ng/uL)	From (bp)	To (bp)	Average size (bp)	CV%	RFU	Corrected peak area
1	75 (LM)	0.5000	33	86	74	4.33	7098	38.314
2	97	1.5782	86	181	99	13.01	1137	10.078
3	271	0.4122	259	301	272	2.91	450	2.632
4	313	0.1081	301	347	320	3.85	55	0.690
5	368	0.1221	347	401	366	2.22	121	0.780
6	549	0.3842	500	585	540	2.02	238	2.453
7	687	2.3337	639	727	685	0.98	2963	14.902
8	874	0.0724	818	913	862	2.42	53	0.462
9	1112	0.1339	961	1289	1103	5.23	88	0.855
10	1520	0.0572	1400	1731	1523	3.28	56	0.365
11	2943	13.4119	2087	3393	2945	3.57	14484	85.644
12	3679	0.9824	3393	4656	3807	7.49	486	6.273
13	4759	0.1007	4656	5963	4931	4.60	57	0.643
14	20000 (UM)	0.2651	16830	29189	20933	5.80	1560	20.317

TIC:	19.6967	ng/uL
TIM:	44.9062	nmole/L
Total concentration:	20.1678	ng/uL

Sample peak width (sec): 5   Sample min peak height: 50   Sample baseline V to V?: Y   Sample baseline V to V points: 3
Sample filter: Binomial   Number of points for filter: 3 Sample start region (min): 0 Sample end region (min): 30
Marker peak width (sec): 5   Marker min peak height: 200   Marker baseline V to V?: Y   Marker baseline V to V points: 3
Lower marker selection: First peak > 500 RFU   Upper marker selection: Last peak > 200 RFU
Ladder size (bp)75, 200, 300, 400, 500, 700, 1000, 1500, 2000, 3000, 4000, 5000, 7000, 10000, 20000
Quantification using: Lower Marker   Final concentration (ng/uL): 0.5000   Dilution factor: 12.0

Sample: SampC9
Well location: C9
Created: Wednesday, October 20, 2021 4:38:54 PM

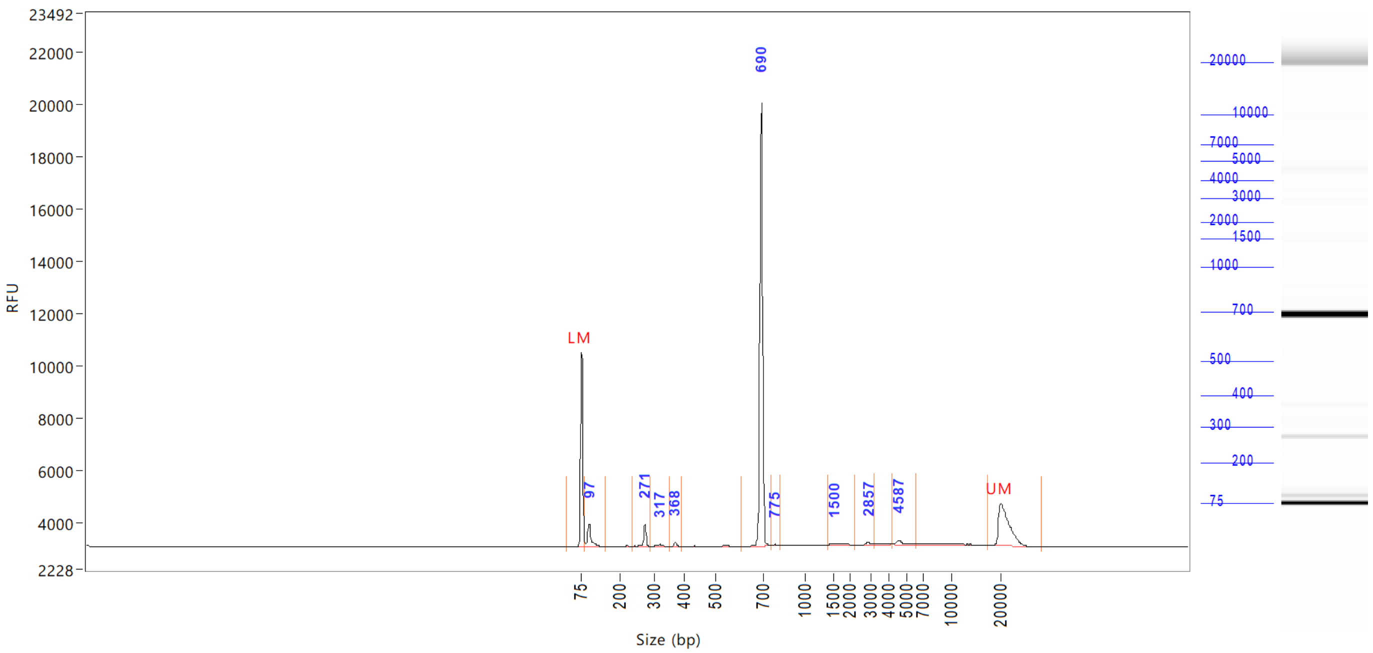

Peak	Size (bp)	Concentration (ng/uL)	From (bp)	To (bp)	Average size (bp)	CV%	RFU	Corrected peak area
1	75 (LM)	0.5000	31	86	74	4.29	7414	39.750
2	97	1.1360	86	150	100	10.46	865	7.526
3	271	0.6537	233	287	268	2.41	824	4.331
4	317	0.1503	287	349	314	3.90	74	0.996
5	368	0.1089	349	392	366	1.34	139	0.721
6	690	12.7301	603	749	687	0.97	16978	84.337
7	775	0.0814	749	822	778	2.42	55	0.539
8	1500	0.1363	1400	2229	1609	10.67	63	0.903
9	2857	0.0814	2229	3215	2827	5.37	77	0.539
10	4587	0.2438	4139	6186	4737	8.40	170	1.615
11	20000 (UM)	0.2722	17317	28342	20942	5.88	1622	21.642

TIC:	15.3218	ng/uL
TIM:	54.8824	nmole/L
Total concentration:	15.9068	ng/uL

Sample peak width (sec): 5 Sample min peak height: 50 Sample baseline V to V?: Y Sample baseline V to V points: 3
Sample filter: Binomial Number of points for filter: 3 Sample start region (min): 0 Sample end region (min): 30
Marker peak width (sec): 5 Marker min peak height: 200 Marker baseline V to V?: Y Marker baseline V to V points: 3
Lower marker selection: First peak > 500 RFU Upper marker selection: Last peak > 200 RFU
Ladder size (bp)75, 200, 300, 400, 500, 700, 1000, 1500, 2000, 3000, 4000, 5000, 7000, 10000, 20000
Quantification using: Lower Marker Final concentration (ng/uL): 0.5000 Dilution factor: 12.0

Sample: SampC10
Well location: C10
Created: Wednesday, October 20, 2021 4:38:54 PM

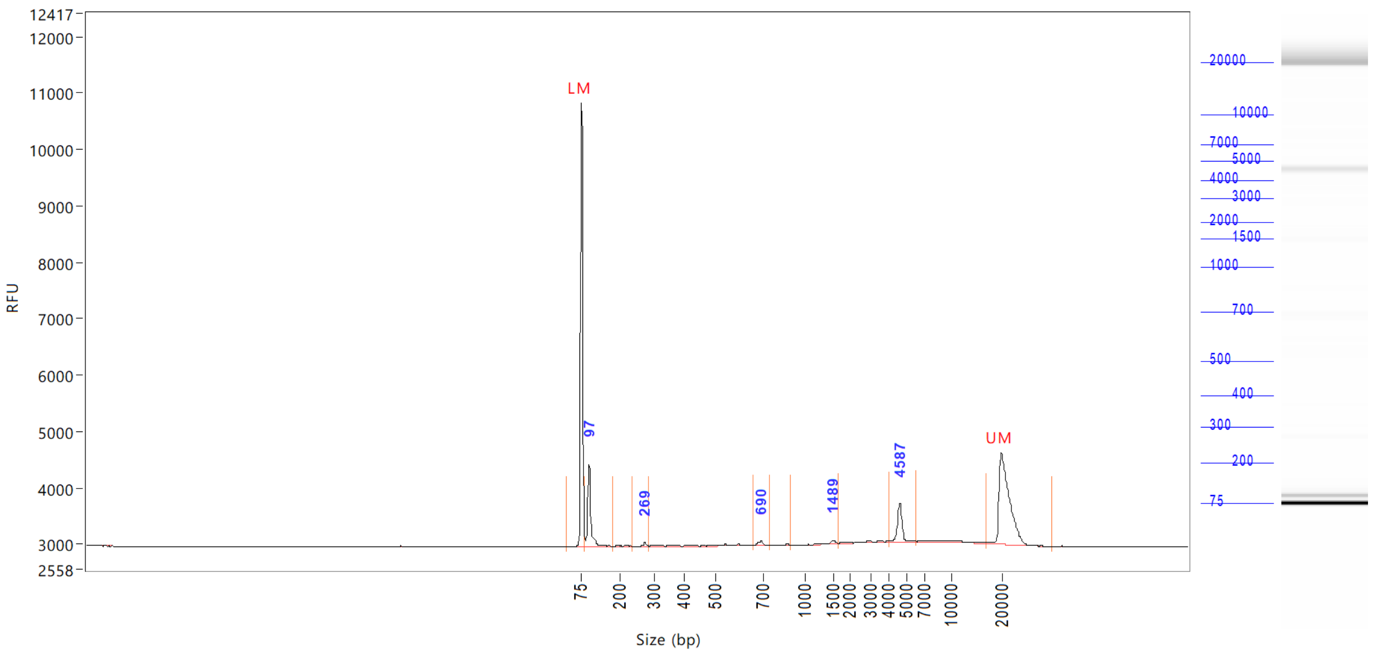

Peak	Size (bp)	Concentration (ng/uL)	From (bp)	To (bp)	Average size (bp)	CV%	RFU	Corrected peak area
1	75 (LM)	0.5000	30	86	74	4.34	7880	41.566
2	97	1.5882	86	177	99	11.02	1453	11.002
3	269	0.0621	235	282	267	3.00	67	0.430
4	690	0.1113	657	740	682	1.62	88	0.771
5	1489	0.1128	896	1635	1343	14.53	66	0.781
6	4587	0.6663	4001	6112	4574	4.01	684	4.616
7	20000 (UM)	0.2544	17073	30277	20912	6.05	1626	21.148

TIC:	2.5407	ng/uL
TIM:	27.3714	nmole/L
Total concentration:	3.1154	ng/uL

Sample peak width (sec): 5   Sample min peak height: 50   Sample baseline V to V?: Y   Sample baseline V to V points: 3
Sample filter: Binomial   Number of points for filter: 3 Sample start region (min): 0 Sample end region (min): 30
Marker peak width (sec): 5   Marker min peak height: 200   Marker baseline V to V?: Y   Marker baseline V to V points: 3
Lower marker selection: First peak > 500 RFU   Upper marker selection: Last peak > 200 RFU
Ladder size (bp)75, 200, 300, 400, 500, 700, 1000, 1500, 2000, 3000, 4000, 5000, 7000, 10000, 20000
Quantification using: Lower Marker   Final concentration (ng/uL): 0.5000   Dilution factor: 12.0

Sample: SampC11
Well location: C11
Created: Wednesday, October 20, 2021 4:38:54 PM

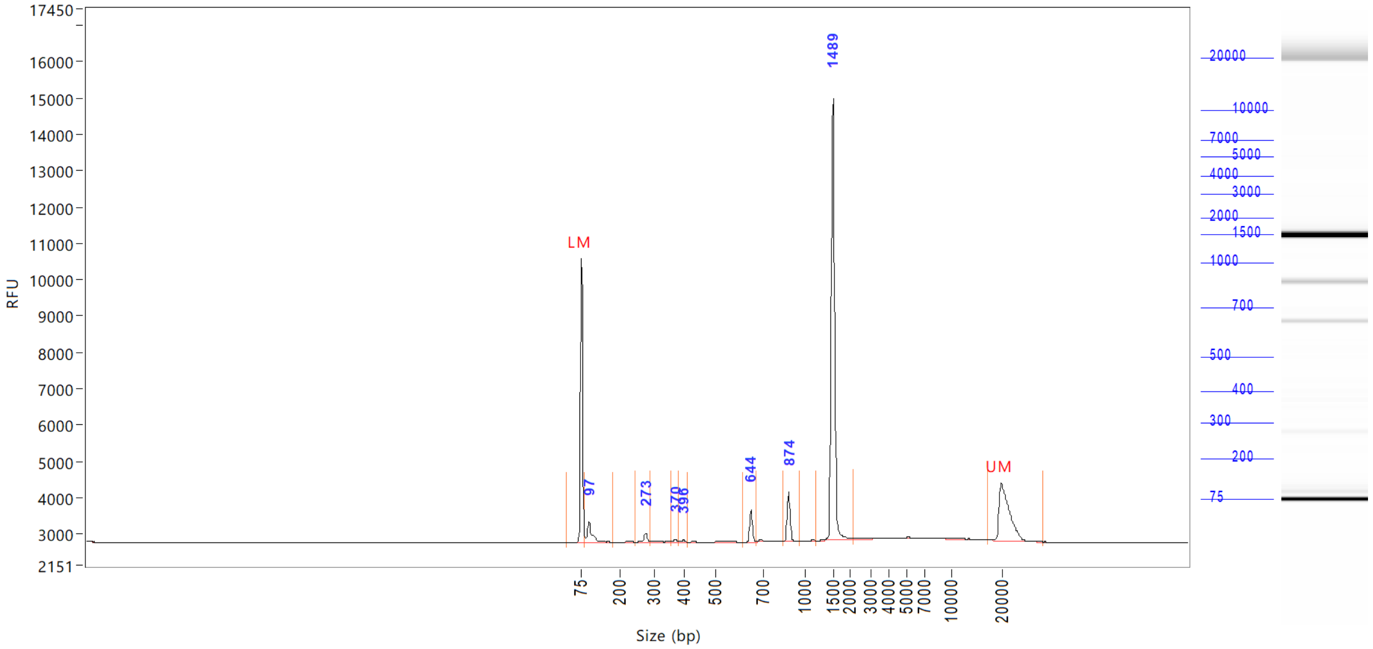

Peak	Size (bp)	Concentration (ng/uL)	From (bp)	To (bp)	Average size (bp)	CV%	RFU	Corrected peak area
1	75 (LM)	0.5000	30	86	73	4.42	7797	40.850
2	97	0.9359	86	177	103	13.94	552	6.372
3	273	0.2397	244	289	270	2.07	247	1.632
4	370	0.0601	356	380	367	1.17	81	0.409
5	396	0.0481	380	411	393	1.42	52	0.328
6	644	0.6514	608	664	642	0.88	906	4.435
7	874	1.0400	831	961	871	1.49	1354	7.080
8	1489	9.4309	1178	2115	1486	4.01	12165	64.208
9	20000 (UM)	0.2521	17195	28463	20892	5.76	1576	20.594

TIC:	12.4060	ng/uL
TIM:	30.9347	nmole/L
Total concentration:	13.1173	ng/uL

Sample peak width (sec): 5 Sample min peak height: 50 Sample baseline V to V?: Y Sample baseline V to V points: 3
Sample filter: Binomial Number of points for filter: 3 Sample start region (min): 0 Sample end region (min): 30
Marker peak width (sec): 5 Marker min peak height: 200 Marker baseline V to V?: Y Marker baseline V to V points: 3
Lower marker selection: First peak > 500 RFU Upper marker selection: Last peak > 200 RFU
Ladder size (bp)75, 200, 300, 400, 500, 700, 1000, 1500, 2000, 3000, 4000, 5000, 7000, 10000, 20000
Quantification using: Lower Marker Final concentration (ng/uL): 0.5000 Dilution factor: 12.0

Sample: SampC12
Well location: C12
Created: Wednesday, October 20, 2021 4:38:54 PM

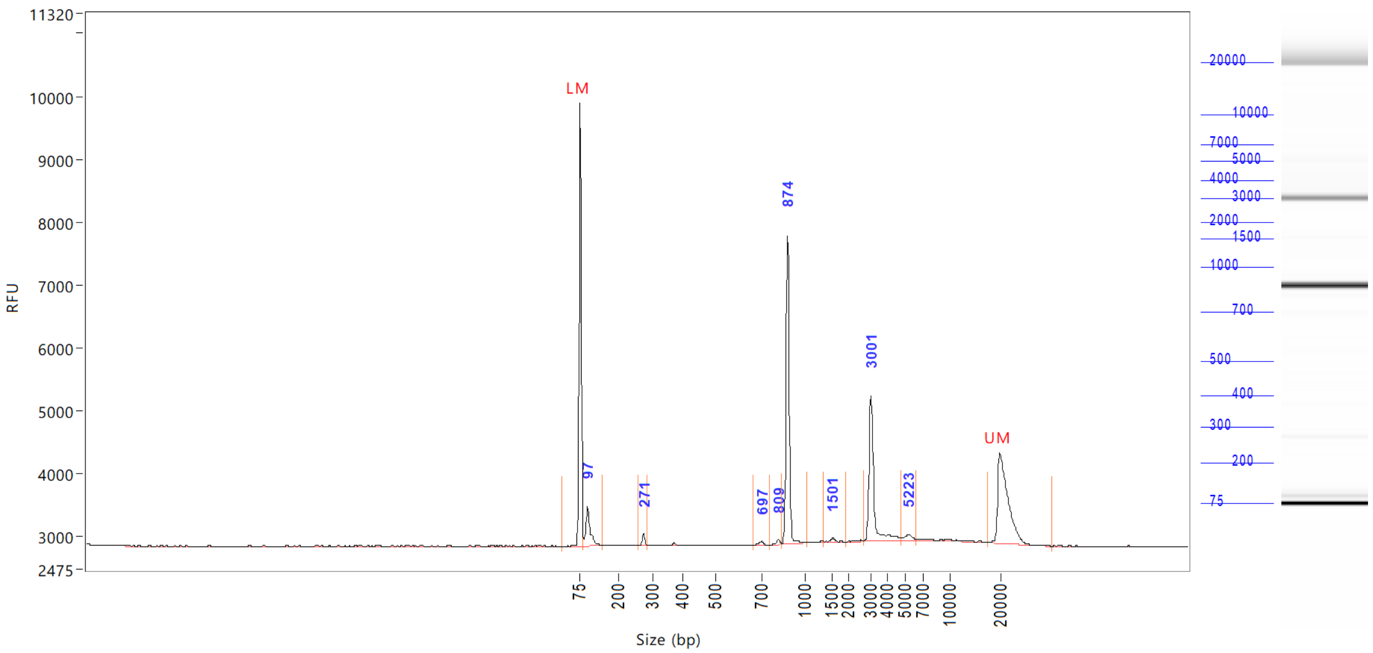

Peak	Size (bp)	Concentration (ng/uL)	From (bp)	To (bp)	Average size (bp)	CV%	RFU	Corrected peak area
1	75 (LM)	0.5000	20	86	73	5.07	7064	37.950
2	97	1.0143	86	147	99	10.41	638	6.416
3	271	0.1669	255	282	269	1.49	197	1.056
4	697	0.0908	662	744	689	1.74	64	0.574
5	809	0.0760	744	831	804	1.86	83	0.481
6	874	4.2086	831	1023	873	1.81	4900	26.619
7	1501	0.1055	1356	1904	1529	7.26	68	0.668
8	3001	2.6089	2686	4724	3173	12.51	2312	16.501
9	5223	0.2050	4724	6260	5264	7.53	107	1.296
10	20000 (UM)	0.2459	17561	30639	20963	6.39	1433	18.667

TIC:	8.4761	ng/uL
TIM:	27.6829	nmole/L
Total concentration:	8.9932	ng/uL

Sample peak width (sec): 5   Sample min peak height: 50   Sample baseline V to V?: Y   Sample baseline V to V points: 3
Sample filter: Binomial   Number of points for filter: 3 Sample start region (min): 0 Sample end region (min): 30
Marker peak width (sec): 5   Marker min peak height: 200   Marker baseline V to V?: Y   Marker baseline V to V points: 3
Lower marker selection: First peak > 500 RFU   Upper marker selection: Last peak > 200 RFU
Ladder size (bp)75, 200, 300, 400, 500, 700, 1000, 1500, 2000, 3000, 4000, 5000, 7000, 10000, 20000
Quantification using: Lower Marker   Final concentration (ng/uL): 0.5000   Dilution factor: 12.0

Sample: SampD1
Well location: D1
Created: Wednesday, October 20, 2021 4:38:54 PM

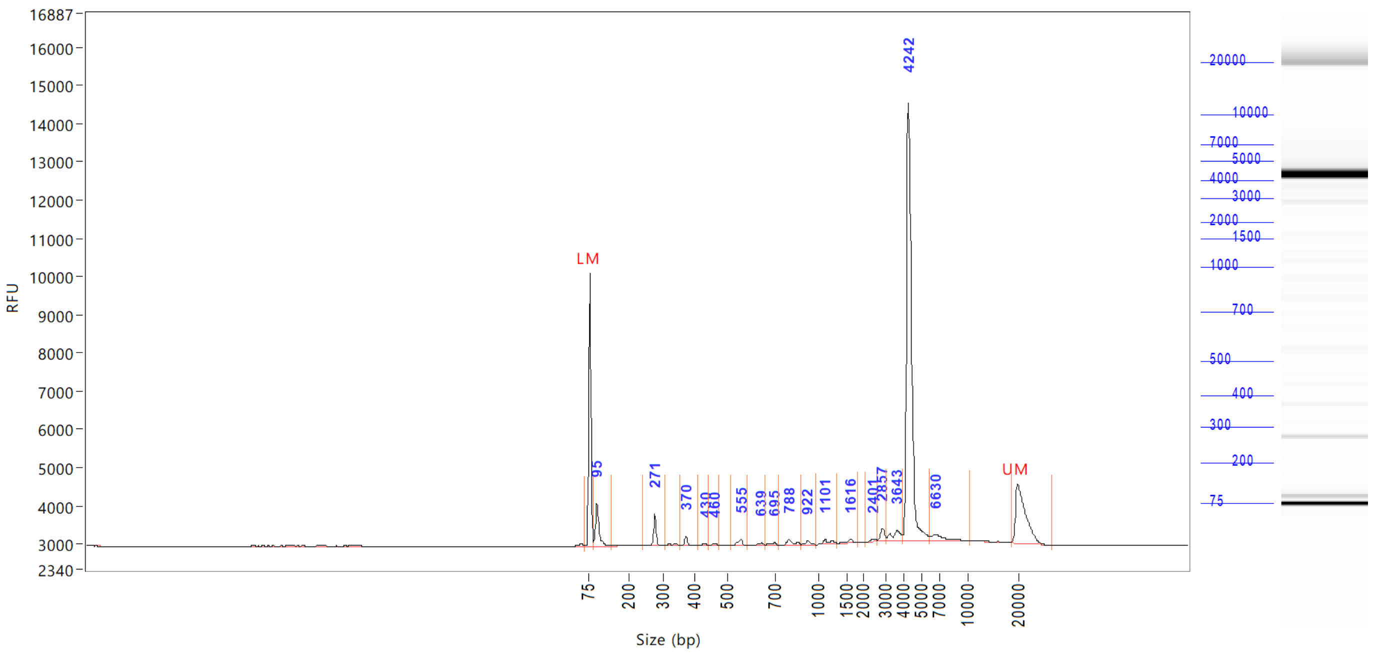

Peak	Size (bp)	Concentration (ng/uL)	From (bp)	To (bp)	Average size (bp)	CV%	RFU	Corrected peak area
1	75 (LM)	0.5000	60	86	72	4.49	7158	38.006
2	95	1.4134	86	143	97	8.51	1104	8.953
3	271	0.7145	235	303	269	2.76	829	4.526
4	370	0.2285	352	409	369	2.25	254	1.447
5	430	0.0693	409	439	426	1.41	58	0.439
6	460	0.0780	439	475	456	1.72	54	0.494
7	555	0.2165	514	583	545	1.76	158	1.371
8	639	0.1193	583	654	629	2.26	75	0.755
9	695	0.1033	654	714	682	1.98	70	0.655
10	788	0.2645	714	874	800	4.02	161	1.676
11	922	0.1122	874	983	919	1.89	97	0.711
12	1101	0.1985	983	1334	1143	6.80	116	1.258
13	1616	0.1384	1334	1808	1547	5.27	103	0.877
14	2401	0.0748	2087	2629	2404	5.01	56	0.474
15	2857	0.3042	2629	3037	2848	3.19	327	1.927
16	3643	0.4933	3037	3964	3534	7.42	254	3.125
17	4242	12.4506	3964	5889	4309	5.04	11460	78.867
18	6630	0.3909	5889	10367	6989	12.00	142	2.476
19	20000 (UM)	0.2556	18781	26770	20849	5.45	1542	19.430

TIC:	17.3703	ng/uL
TIM:	37.5601	nmole/L
Total concentration:	17.6025	ng/uL

```
Sample peak width (sec): 5     Sample min peak height: 50     Sample baseline V to V?: Y     Sample baseline V to V points: 3
Sample filter: Binomial        Number of points for filter: 3  Sample start region (min): 0  Sample end region (min): 30
Marker peak width (sec): 5     Marker min peak height: 200    Marker baseline V to V?: Y     Marker baseline V to V points: 3
Lower marker selection: First peak > 500 RFU                  Upper marker selection: Last peak > 200 RFU
Ladder size (bp)75, 200, 300, 400, 500, 700, 1000, 1500, 2000, 3000, 4000, 5000, 7000, 10000, 20000
Quantification using: Lower Marker          Final concentration (ng/uL): 0.5000          Dilution factor: 12.0
```

Sample: SampD2
Well location: D2
Created: Wednesday, October 20, 2021 4:38:54 PM

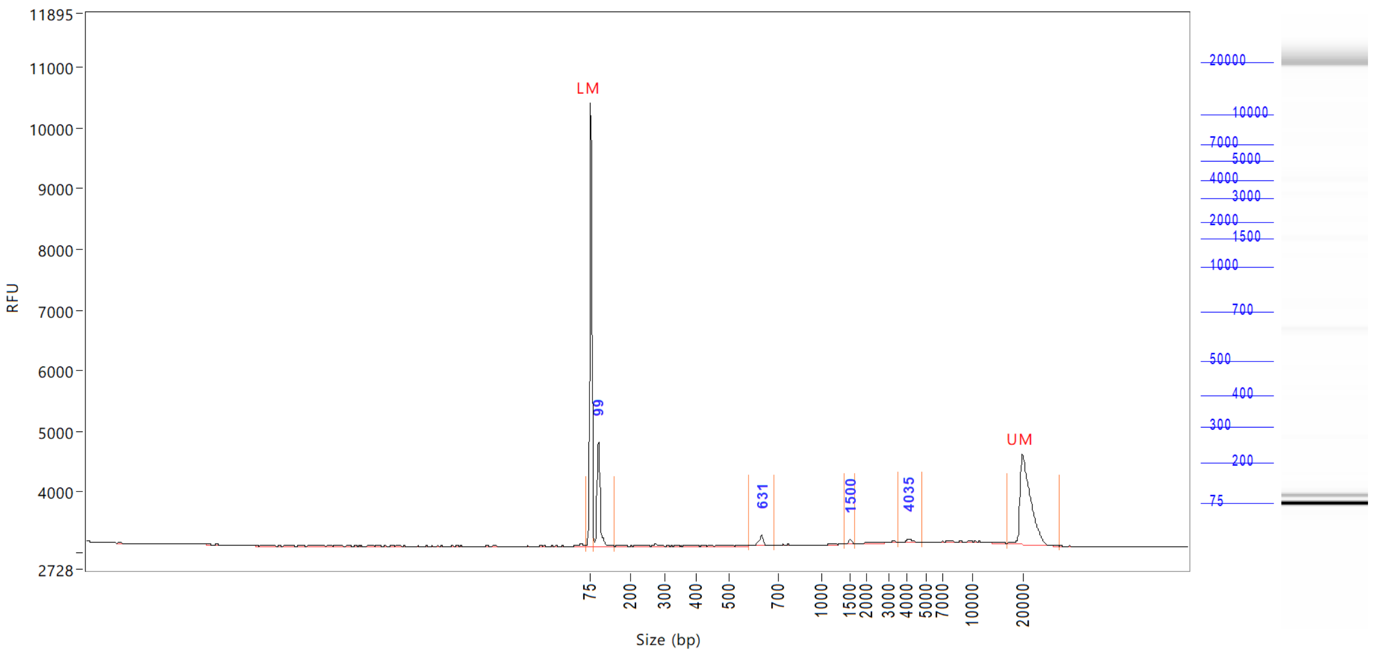

Peak	Size (bp)	Concentration (ng/uL)	From (bp)	To (bp)	Average size (bp)	CV%	RFU	Corrected peak area
1	75 (LM)	0.5000	60	86	73	4.20	7324	37.874
2	99	1.8510	86	148	98	7.41	1711	11.684
3	631	0.2359	580	682	625	2.18	182	1.489
4	1500	0.0653	1400	1655	1496	2.41	78	0.412
5	4035	0.0938	3500	4862	4156	6.31	62	0.592
6	20000 (UM)	0.2557	16830	27133	20769	5.75	1502	19.371

TIC:	2.2460	ng/uL
TIM:	31.7438	nmole/L
Total concentration:	2.8845	ng/uL

```
Sample peak width (sec): 5    Sample min peak height: 50    Sample baseline V to V?: Y    Sample baseline V to V points: 3
Sample filter: Binomial       Number of points for filter: 3  Sample start region (min): 0  Sample end region (min): 30
Marker peak width (sec): 5    Marker min peak height: 200   Marker baseline V to V?: Y    Marker baseline V to V points: 3
Lower marker selection: First peak > 500 RFU                Upper marker selection: Last peak > 200 RFU
Ladder size (bp)75, 200, 300, 400, 500, 700, 1000, 1500, 2000, 3000, 4000, 5000, 7000, 10000, 20000
Quantification using: Lower Marker         Final concentration (ng/uL): 0.5000         Dilution factor: 12.0
```

Sample: SampD3
Well location: D3
Created: Wednesday, October 20, 2021 4:38:54 PM

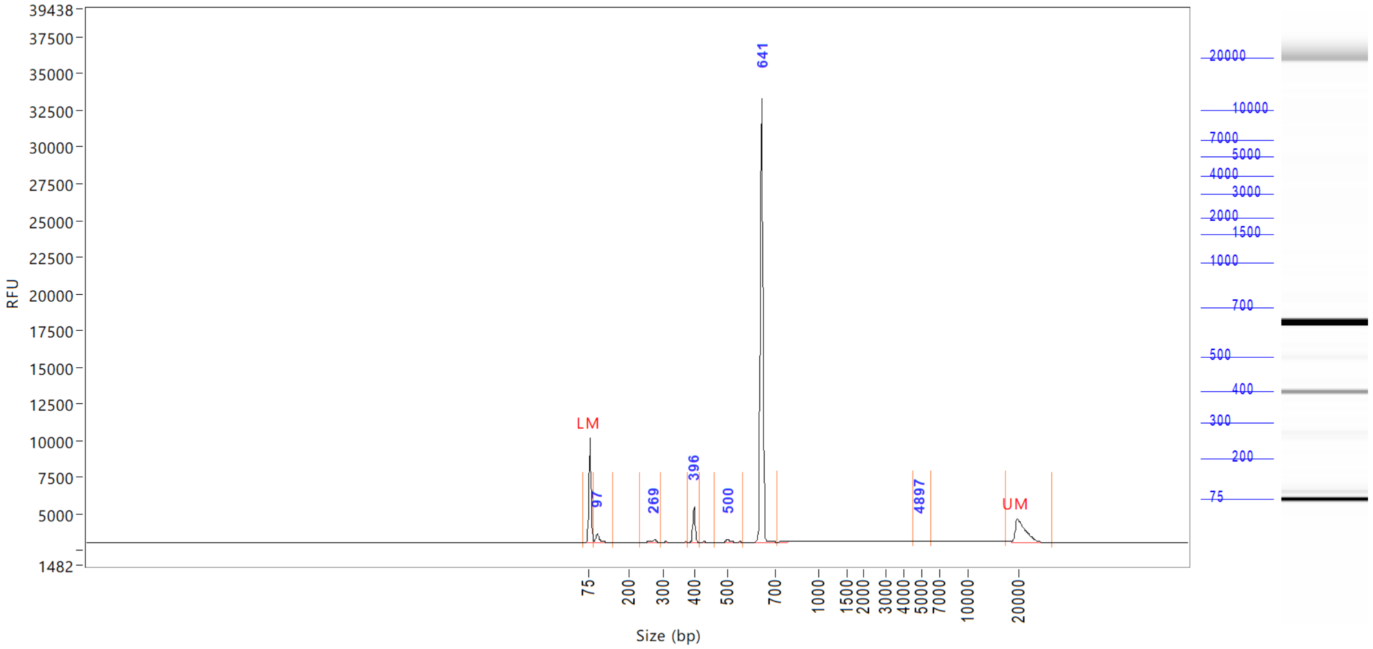

Peak	Size (bp)	Concentration (ng/uL)	From (bp)	To (bp)	Average size (bp)	CV%	RFU	Corrected peak area
1	75 (LM)	0.5000	56	86	73	4.52	7158	37.633
2	97	0.8823	86	145	99	10.18	586	5.534
3	269	0.3479	228	291	262	3.64	205	2.182
4	396	1.8138	376	412	394	1.00	2436	11.376
5	500	0.3240	458	562	506	3.53	204	2.032
6	641	23.3900	562	710	638	0.94	30319	146.705
7	4897	0.0840	4552	6112	4888	4.48	50	0.527
8	20000 (UM)	0.2621	17683	26770	20778	5.49	1561	19.728

TIC:	26.8419	ng/uL
TIM:	85.8079	nmole/L
Total concentration:	27.6264	ng/uL

```
Sample peak width (sec): 5    Sample min peak height: 50    Sample baseline V to V?: Y    Sample baseline V to V points: 3
Sample filter: Binomial       Number of points for filter: 3  Sample start region (min): 0  Sample end region (min): 30
Marker peak width (sec): 5    Marker min peak height: 200   Marker baseline V to V?: Y    Marker baseline V to V points: 3
Lower marker selection: First peak > 500 RFU                Upper marker selection: Last peak > 200 RFU
Ladder size (bp)75, 200, 300, 400, 500, 700, 1000, 1500, 2000, 3000, 4000, 5000, 7000, 10000, 20000
Quantification using: Lower Marker          Final concentration (ng/uL): 0.5000          Dilution factor: 12.0
```

Sample: SampD4
Well location: D4
Created: Wednesday, October 20, 2021 4:38:54 PM

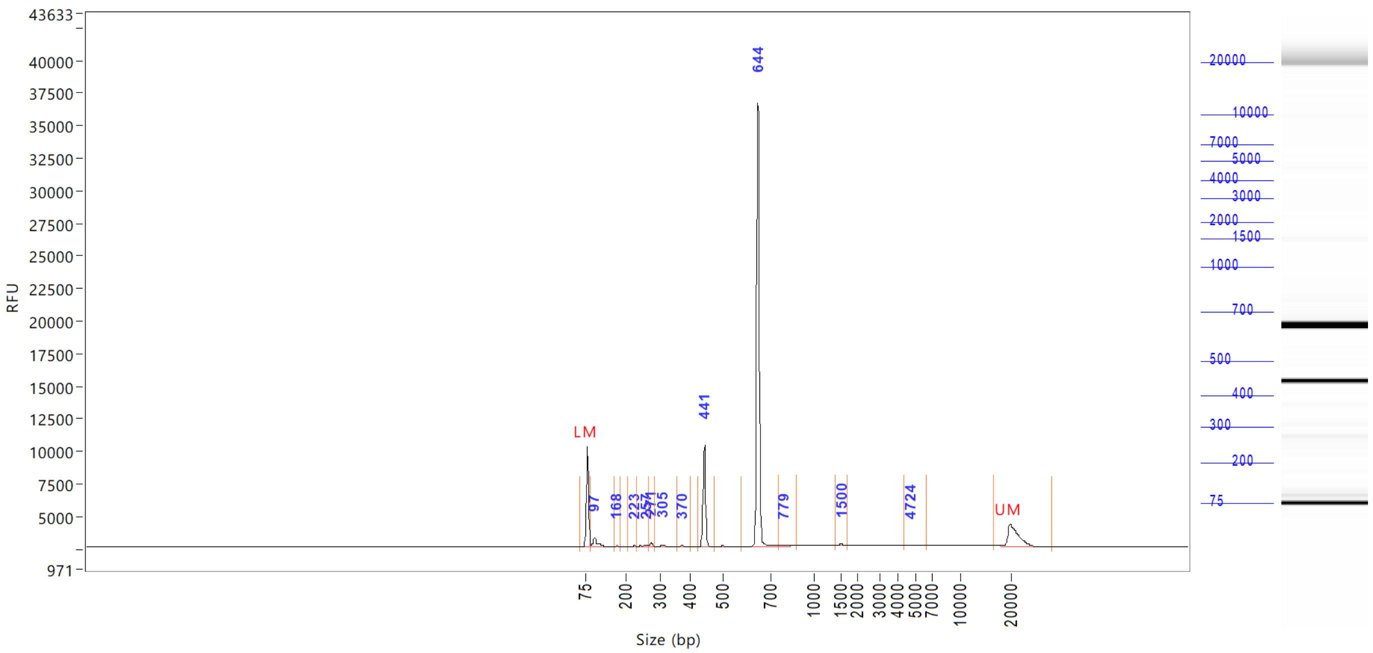

Peak	Size (bp)	Concentration (ng/uL)	From (bp)	To (bp)	Average size (bp)	CV%	RFU	Corrected peak area
1	75 (LM)	0.5000	56	87	74	4.40	7668	41.334
2	97	1.0081	87	160	102	11.94	653	6.945
3	168	0.0488	160	179	167	2.33	68	0.336
4	223	0.0569	203	232	219	2.56	51	0.392
5	257	0.2165	232	264	251	3.67	130	1.491
6	271	0.2517	264	282	269	1.26	332	1.734
7	305	0.1859	282	354	304	3.21	151	1.281
8	370	0.0757	354	400	370	2.28	90	0.522
9	441	5.2529	422	473	439	0.88	7746	36.187
10	644	23.9006	578	749	643	1.19	34074	164.650
11	779	0.1636	749	883	795	4.13	99	1.127
12	1500	0.1229	1400	1655	1487	2.21	157	0.847
13	4724	0.0917	4345	6260	4772	6.77	65	0.632
14	20000 (UM)	0.2618	16830	28342	20818	5.78	1686	21.640

TIC:	31.3753	ng/uL
TIM:	102.8037	nmole/L
Total concentration:	31.8887	ng/uL

```
Sample peak width (sec): 5    Sample min peak height: 50    Sample baseline V to V?: Y    Sample baseline V to V points: 3
Sample filter: Binomial       Number of points for filter: 3  Sample start region (min): 0  Sample end region (min): 30
Marker peak width (sec): 5    Marker min peak height: 200   Marker baseline V to V?: Y    Marker baseline V to V points: 3
Lower marker selection: First peak > 500 RFU                Upper marker selection: Last peak > 200 RFU
Ladder size (bp)75, 200, 300, 400, 500, 700, 1000, 1500, 2000, 3000, 4000, 5000, 7000, 10000, 20000
Quantification using: Lower Marker          Final concentration (ng/uL): 0.5000          Dilution factor: 12.0
```

Sample: SampD5
Well location: D5
Created: Wednesday, October 20, 2021 4:38:54 PM

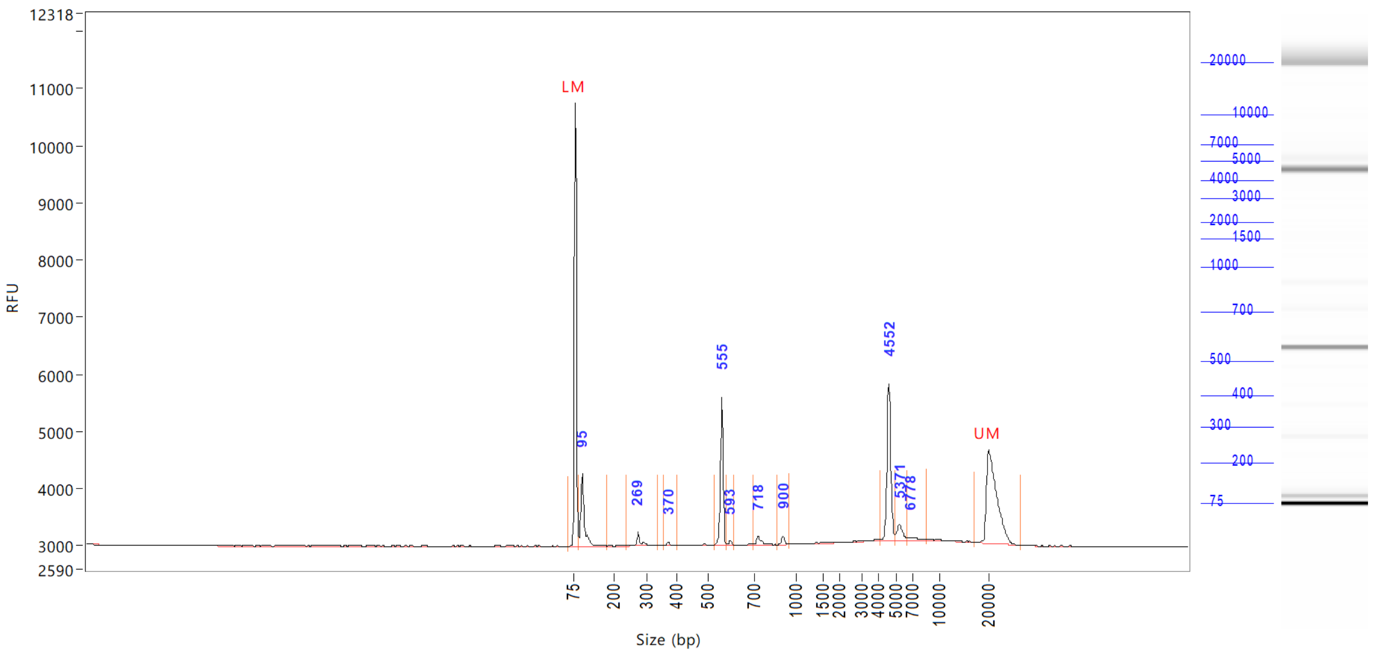

Peak	Size (bp)	Concentration (ng/uL)	From (bp)	To (bp)	Average size (bp)	CV%	RFU	Corrected peak area
1	75 (LM)	0.5000	56	86	73	4.43	7768	41.168
2	95	1.5712	86	177	99	11.99	1262	10.780
3	269	0.2915	239	335	276	5.75	236	2.000
4	370	0.0635	358	403	371	2.52	71	0.436
5	555	1.9504	524	578	552	1.13	2602	13.382
6	593	0.0650	578	611	591	1.00	85	0.446
7	718	0.2045	695	857	734	4.49	155	1.403
8	900	0.1259	857	948	897	1.45	158	0.864
9	4552	2.3148	4070	4966	4557	2.74	2753	15.883
10	5371	0.3472	4966	6260	5403	5.95	281	2.382
11	6778	0.1108	6260	8600	7021	7.19	58	0.760
12	20000 (UM)	0.2620	16952	26529	20823	5.59	1643	21.573

TIC:	7.0448	ng/uL
TIM:	35.7314	nmole/L
Total concentration:	7.4272	ng/uL

```
Sample peak width (sec): 5    Sample min peak height: 50    Sample baseline V to V?: Y    Sample baseline V to V points: 3
Sample filter: Binomial       Number of points for filter: 3  Sample start region (min): 0  Sample end region (min): 30
Marker peak width (sec): 5    Marker min peak height: 200   Marker baseline V to V?: Y    Marker baseline V to V points: 3
Lower marker selection: First peak > 500 RFU                Upper marker selection: Last peak > 200 RFU
Ladder size (bp)75, 200, 300, 400, 500, 700, 1000, 1500, 2000, 3000, 4000, 5000, 7000, 10000, 20000
Quantification using: Lower Marker          Final concentration (ng/uL): 0.5000          Dilution factor: 12.0
```

Sample: SampD6
Well location: D6
Created: Wednesday, October 20, 2021 4:38:54 PM

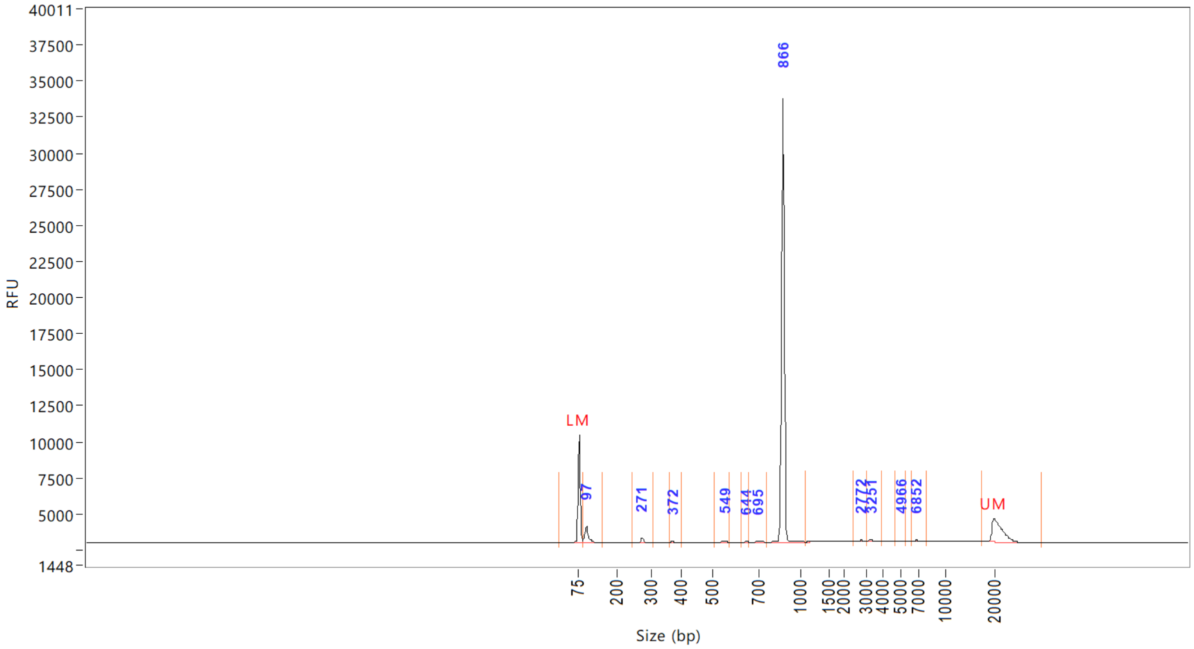

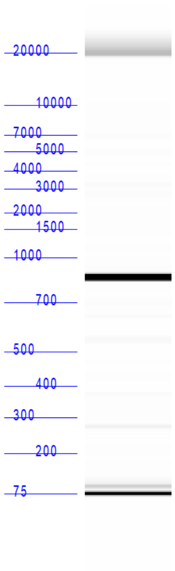

Peak	Size (bp)	Concentration (ng/uL)	From (bp)	To (bp)	Average size (bp)	CV%	RFU	Corrected peak area
1	75 (LM)	0.5000	14	86	73	5.05	7452	39.875
2	97	1.4696	86	148	99	10.04	1111	9.766
3	271	0.3212	244	309	272	4.09	312	2.135
4	372	0.0876	360	403	370	1.88	104	0.582
5	549	0.1827	506	572	544	1.66	137	1.214
6	644	0.0691	621	657	641	0.91	95	0.459
7	695	0.1212	657	753	696	2.77	69	0.806
8	866	21.9650	753	1079	863	1.32	30798	145.975
9	2772	0.0959	2372	3001	2740	5.62	74	0.637
10	3251	0.1140	3001	3964	3282	6.29	97	0.758
11	4966	0.0422	4690	5445	4943	2.20	53	0.281
12	6852	0.0723	6186	7867	6939	5.46	60	0.481
13	20000 (UM)	0.2635	17317	29430	20837	5.76	1595	21.012

TIC:	24.5409	ng/uL
TIM:	69.7600	nmole/L
Total concentration:	25.0675	ng/uL

```
Sample peak width (sec): 5     Sample min peak height: 50     Sample baseline V to V?: Y     Sample baseline V to V points: 3
Sample filter: Binomial        Number of points for filter: 3  Sample start region (min): 0  Sample end region (min): 30
Marker peak width (sec): 5     Marker min peak height: 200    Marker baseline V to V?: Y     Marker baseline V to V points: 3
Lower marker selection: First peak > 500 RFU                  Upper marker selection: Last peak > 200 RFU
Ladder size (bp)75, 200, 300, 400, 500, 700, 1000, 1500, 2000, 3000, 4000, 5000, 7000, 10000, 20000
Quantification using: Lower Marker          Final concentration (ng/uL): 0.5000          Dilution factor: 12.0
```

Sample: SampD7
Well location: D7
Created: Wednesday, October 20, 2021 4:38:54 PM

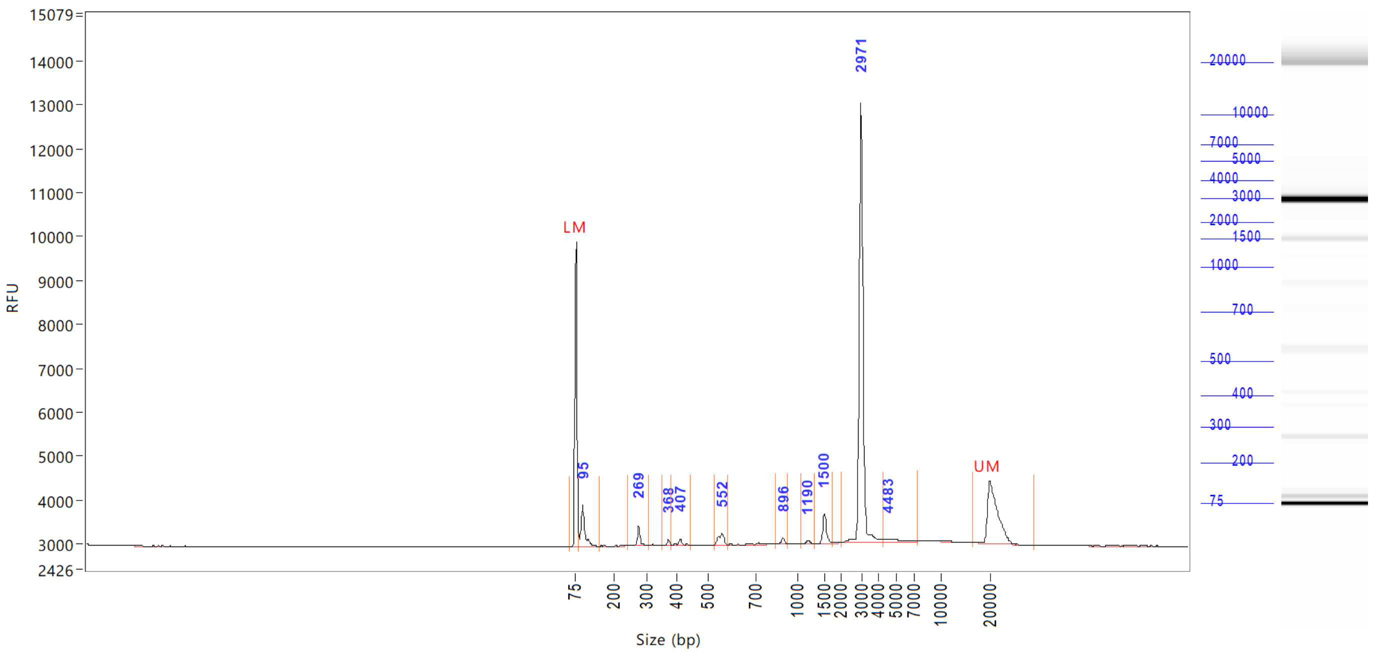

Peak	Size (bp)	Concentration (ng/uL)	From (bp)	To (bp)	Average size (bp)	CV%	RFU	Corrected peak area
1	75 (LM)	0.5000	58	86	73	4.50	6919	36.739
2	95	1.3446	86	148	98	10.08	946	8.234
3	269	0.4413	241	303	269	2.74	462	2.702
4	368	0.1115	347	380	366	1.58	121	0.683
5	407	0.1815	380	439	407	2.77	152	1.111
6	552	0.4857	521	578	547	1.98	275	2.974
7	896	0.1459	844	935	890	1.49	156	0.893
8	1190	0.0758	1079	1323	1180	2.92	72	0.464
9	1500	0.6476	1323	1750	1500	3.21	675	3.965
10	2971	9.6465	2058	4208	2966	5.99	10022	59.067
11	4483	0.2579	4208	7468	5246	17.71	60	1.579
12	20000 (UM)	0.2541	16708	29068	20898	5.89	1434	18.674

TIC:	13.3381	ng/uL
TIM:	34.4449	nmole/L
Total concentration:	13.7410	ng/uL

```
Sample peak width (sec): 5    Sample min peak height: 50    Sample baseline V to V?: Y    Sample baseline V to V points: 3
Sample filter: Binomial       Number of points for filter: 3  Sample start region (min): 0  Sample end region (min): 30
Marker peak width (sec): 5    Marker min peak height: 200   Marker baseline V to V?: Y    Marker baseline V to V points: 3
Lower marker selection: First peak > 500 RFU                Upper marker selection: Last peak > 200 RFU
Ladder size (bp)75, 200, 300, 400, 500, 700, 1000, 1500, 2000, 3000, 4000, 5000, 7000, 10000, 20000
Quantification using: Lower Marker          Final concentration (ng/uL): 0.5000          Dilution factor: 12.0
```

Sample: SampD8
Well location: D8
Created: Wednesday, October 20, 2021 4:38:54 PM

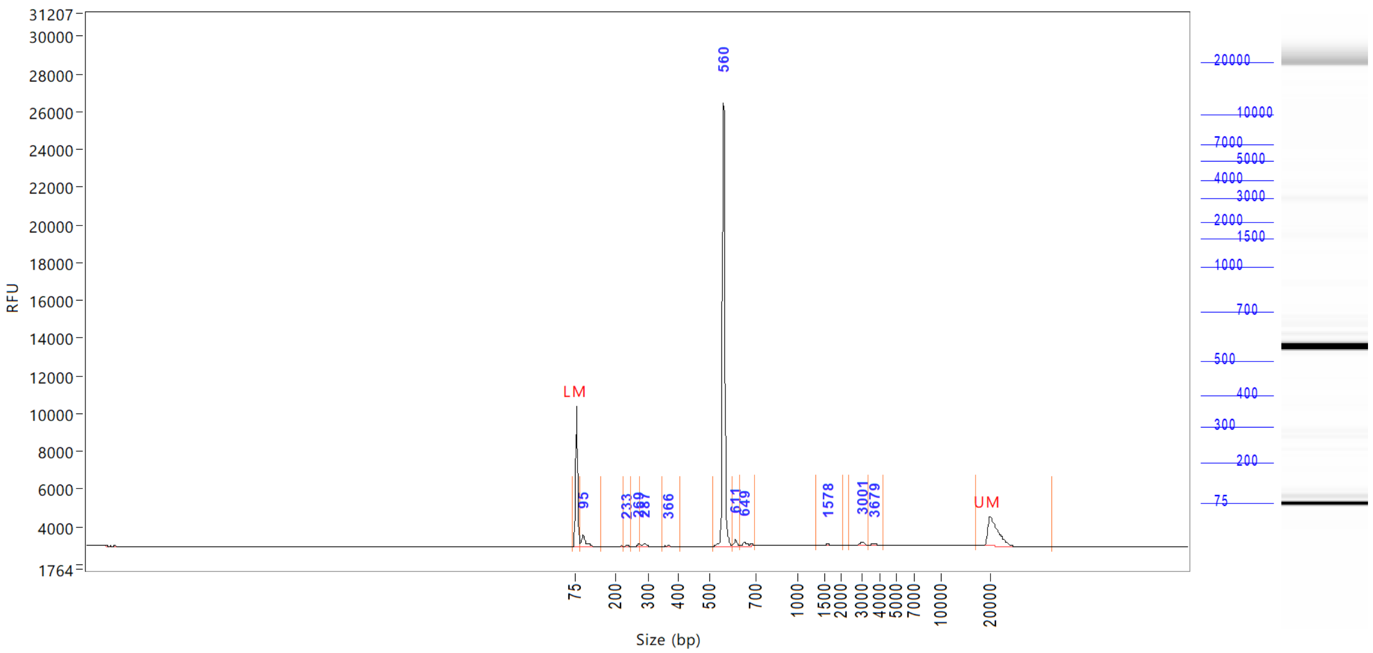

Peak	Size (bp)	Concentration (ng/uL)	From (bp)	To (bp)	Average size (bp)	CV%	RFU	Corrected peak area
1	75 (LM)	0.5000	64	86	73	4.32	7402	38.773
2	95	0.8992	86	154	98	9.85	604	5.811
3	233	0.0728	224	246	233	1.54	90	0.471
4	269	0.1508	246	277	268	2.10	172	0.974
5	287	0.2363	277	345	291	4.80	153	1.527
6	366	0.0871	345	405	367	3.72	55	0.563
7	560	17.9260	514	595	557	1.19	23522	115.840
8	611	0.2953	595	629	608	1.11	337	1.908
9	649	0.3533	629	695	657	2.31	176	2.283
10	1578	0.1797	1345	2058	1687	10.44	98	1.161
11	3001	0.2605	2372	3322	2909	6.50	203	1.684
12	3679	0.0973	3322	4208	3679	4.88	63	0.629
13	20000 (UM)	0.2605	17195	32816	20987	6.35	1562	20.202

TIC:	20.5583	ng/uL
TIM:	73.2430	nmole/L
Total concentration:	21.0439	ng/uL

Sample peak width (sec): 5 Sample min peak height: 50 Sample baseline V to V?: Y Sample baseline V to V points: 3
Sample filter: Binomial Number of points for filter: 3 Sample start region (min): 0 Sample end region (min): 30
Marker peak width (sec): 5 Marker min peak height: 200 Marker baseline V to V?: Y Marker baseline V to V points: 3
Lower marker selection: First peak > 500 RFU Upper marker selection: Last peak > 200 RFU
Ladder size (bp)75, 200, 300, 400, 500, 700, 1000, 1500, 2000, 3000, 4000, 5000, 7000, 10000, 20000
Quantification using: Lower Marker Final concentration (ng/uL): 0.5000 Dilution factor: 12.0

Sample: SampD9
Well location: D9
Created: Wednesday, October 20, 2021 4:38:54 PM

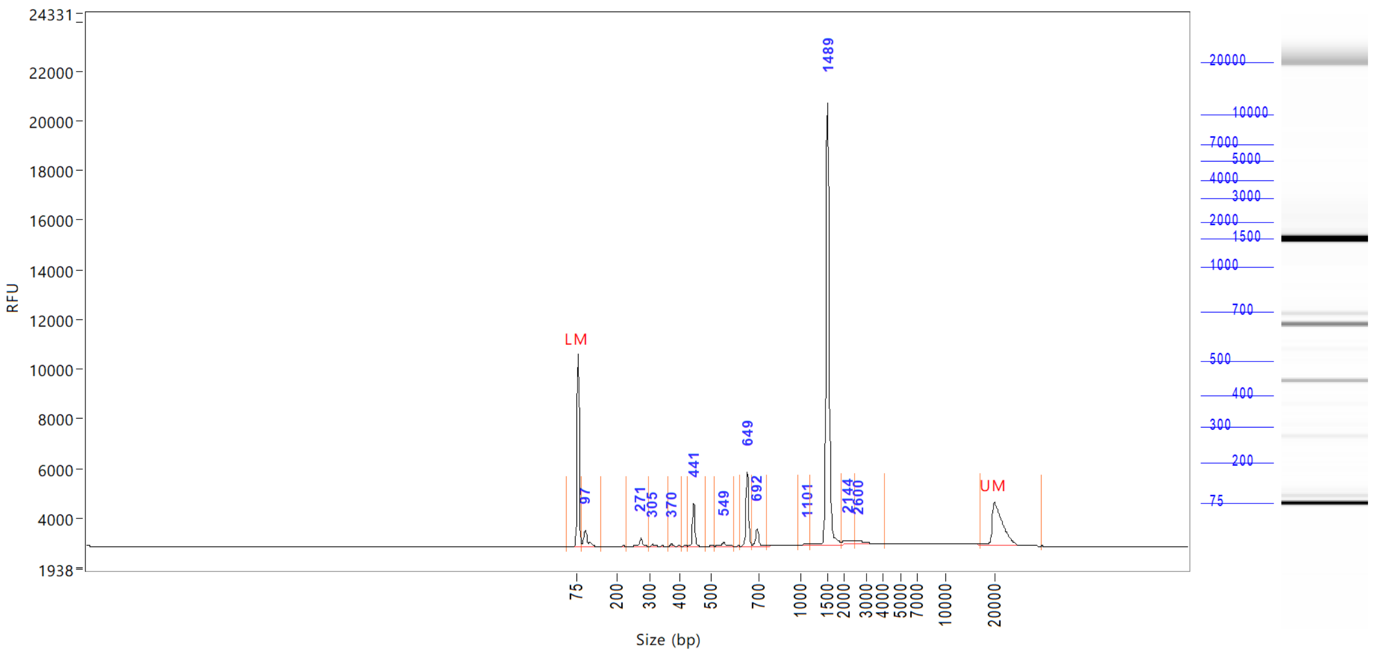

Peak	Size (bp)	Concentration (ng/uL)	From (bp)	To (bp)	Average size (bp)	CV%	RFU	Corrected peak area
1	75 (LM)	0.5000	41	86	74	4.22	7781	41.621
2	97	0.9181	86	148	101	10.70	664	6.369
3	271	0.3578	230	296	268	3.84	364	2.482
4	305	0.1004	296	360	312	4.45	83	0.697
5	370	0.1030	360	403	374	2.85	102	0.714
6	441	1.2086	424	483	440	1.10	1702	8.384
7	549	0.1791	511	595	548	2.94	147	1.242
8	649	2.1014	621	669	647	0.91	2971	14.577
9	692	0.6138	669	753	691	1.65	715	4.258
10	1101	0.0782	983	1167	1088	4.12	60	0.542
11	1489	13.6068	1167	1885	1488	3.57	17831	94.388
12	2144	0.4051	1885	2515	2166	8.46	177	2.810
13	2600	0.2869	2515	4104	2906	11.76	117	1.991
14	20000 (UM)	0.2732	16952	29430	20859	5.82	1736	22.744

TIC:	19.9593	ng/uL
TIM:	45.6111	nmole/L
Total concentration:	20.3735	ng/uL

```
Sample peak width (sec): 5     Sample min peak height: 50    Sample baseline V to V?: Y    Sample baseline V to V points: 3
Sample filter: Binomial        Number of points for filter: 3  Sample start region (min): 0  Sample end region (min): 30
Marker peak width (sec): 5     Marker min peak height: 200   Marker baseline V to V?: Y    Marker baseline V to V points: 3
Lower marker selection: First peak > 500 RFU                 Upper marker selection: Last peak > 200 RFU
Ladder size (bp)75, 200, 300, 400, 500, 700, 1000, 1500, 2000, 3000, 4000, 5000, 7000, 10000, 20000
Quantification using: Lower Marker          Final concentration (ng/uL): 0.5000          Dilution factor: 12.0
```

Sample: SampD10
Well location: D10
Created: Wednesday, October 20, 2021 4:38:54 PM

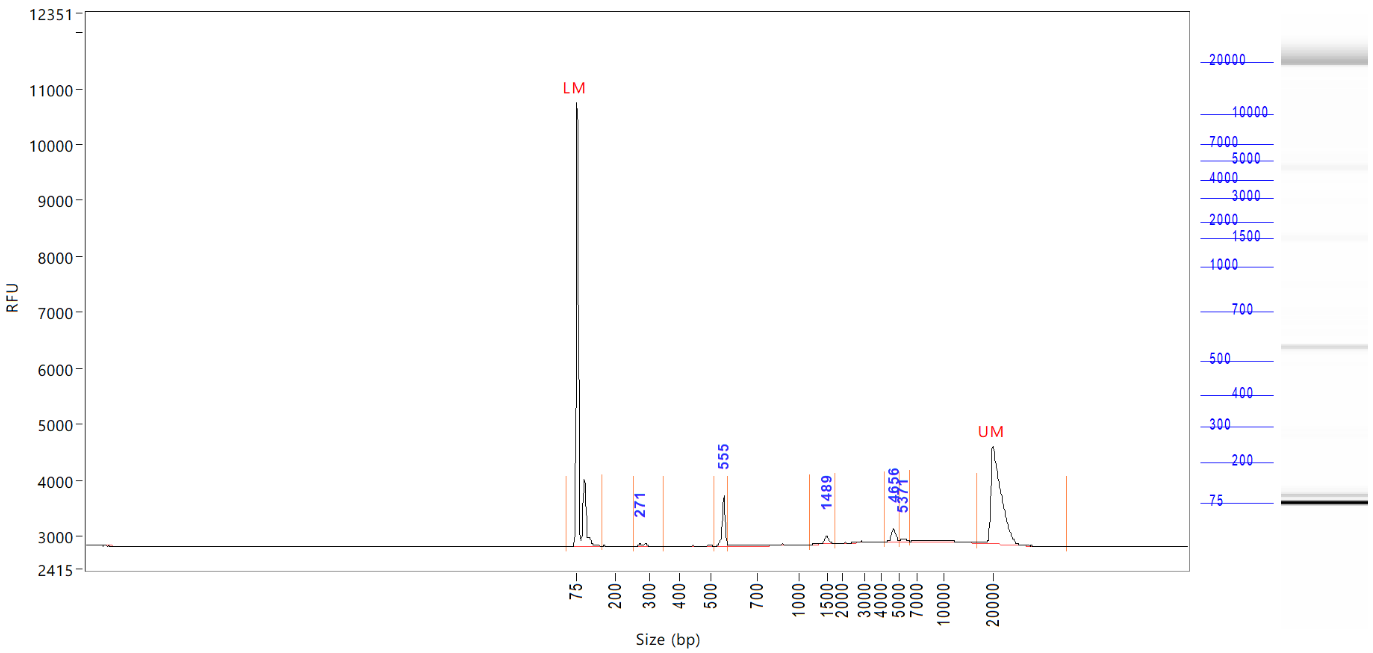

Peak	Size (bp)	Concentration (ng/uL)	From (bp)	To (bp)	Average size (bp)	CV%	RFU	Corrected peak area
1	75 (LM)	0.5000	43	156	78	13.48	7943	51.417
2	271	0.0901	251	349	286	6.89	52	0.772
3	555	0.5748	511	575	551	1.39	891	4.926
4	1489	0.1252	1190	1750	1481	5.46	139	1.073
5	4656	0.1790	4104	5000	4627	3.19	234	1.534
6	5371	0.0492	5000	6260	5393	5.09	52	0.421
7	20000 (UM)	0.2228	16830	34871	20950	6.60	1742	22.908

TIC:	1.0183	ng/uL
TIM:	2.4529	nmole/L
Total concentration:	1.4393	ng/uL

Sample peak width (sec): 5   Sample min peak height: 50   Sample baseline V to V?: Y   Sample baseline V to V points: 3
Sample filter: Binomial   Number of points for filter: 3 Sample start region (min): 0 Sample end region (min): 30
Marker peak width (sec): 5   Marker min peak height: 200   Marker baseline V to V?: Y   Marker baseline V to V points: 3
Lower marker selection: First peak > 500 RFU   Upper marker selection: Last peak > 200 RFU
Ladder size (bp)75, 200, 300, 400, 500, 700, 1000, 1500, 2000, 3000, 4000, 5000, 7000, 10000, 20000
Quantification using: Lower Marker   Final concentration (ng/uL): 0.5000   Dilution factor: 12.0

Sample: SampD11
Well location: D11
Created: Wednesday, October 20, 2021 4:38:54 PM

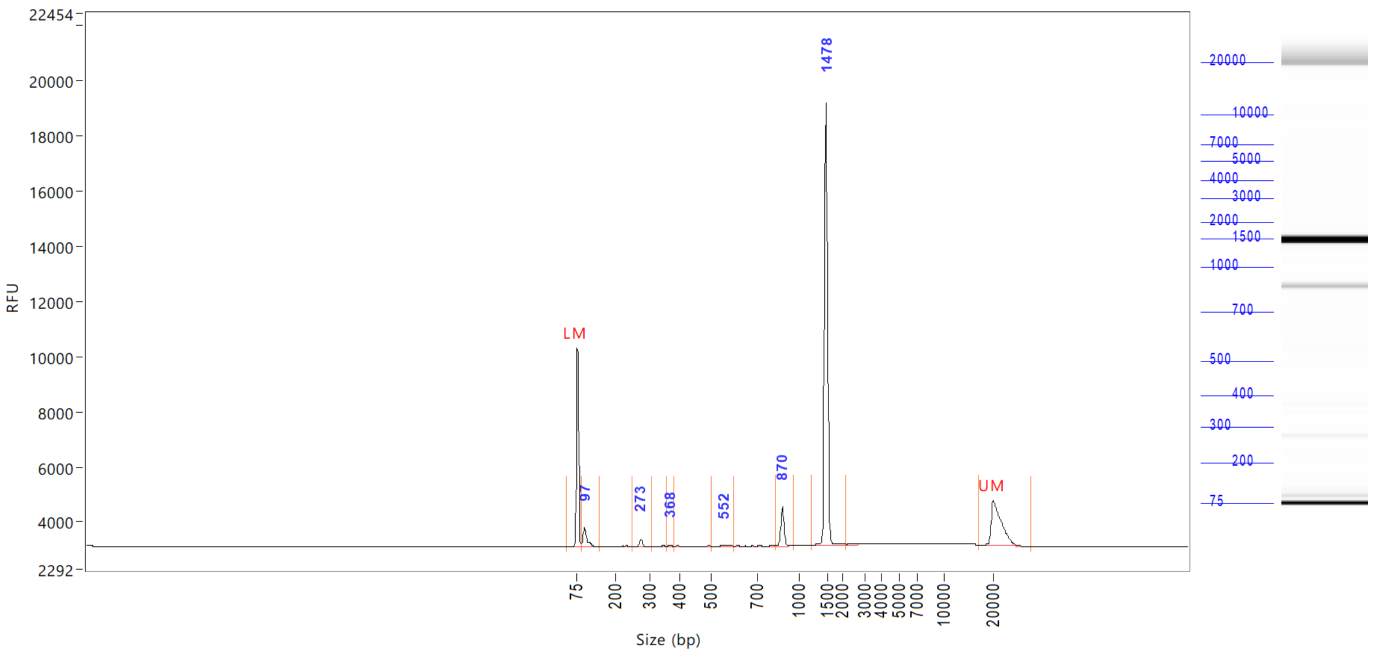

Peak	Size (bp)	Concentration (ng/uL)	From (bp)	To (bp)	Average size (bp)	CV%	RFU	Corrected peak area
1	75 (LM)	0.5000	43	87	74	4.32	7185	39.014
2	97	0.9758	87	147	101	10.52	687	6.345
3	273	0.2913	250	307	272	2.58	285	1.894
4	368	0.0597	356	380	366	1.20	70	0.388
5	552	0.1129	504	598	557	3.61	51	0.734
6	870	1.1654	831	961	870	1.51	1418	7.578
7	1478	12.8318	1212	2115	1469	2.93	16067	83.437
8	20000 (UM)	0.2675	16952	27617	20945	5.71	1602	20.870

TIC:	15.4369	ng/uL
TIM:	34.8122	nmole/L
Total concentration:	16.2254	ng/uL

Sample peak width (sec): 5 Sample min peak height: 50 Sample baseline V to V?: Y Sample baseline V to V points: 3
Sample filter: Binomial Number of points for filter: 3 Sample start region (min): 0 Sample end region (min): 30
Marker peak width (sec): 5 Marker min peak height: 200 Marker baseline V to V?: Y Marker baseline V to V points: 3
Lower marker selection: First peak > 500 RFU Upper marker selection: Last peak > 200 RFU
Ladder size (bp)75, 200, 300, 400, 500, 700, 1000, 1500, 2000, 3000, 4000, 5000, 7000, 10000, 20000
Quantification using: Lower Marker Final concentration (ng/uL): 0.5000 Dilution factor: 12.0

Sample: SampD12
Well location: D12
Created: Wednesday, October 20, 2021 4:38:54 PM

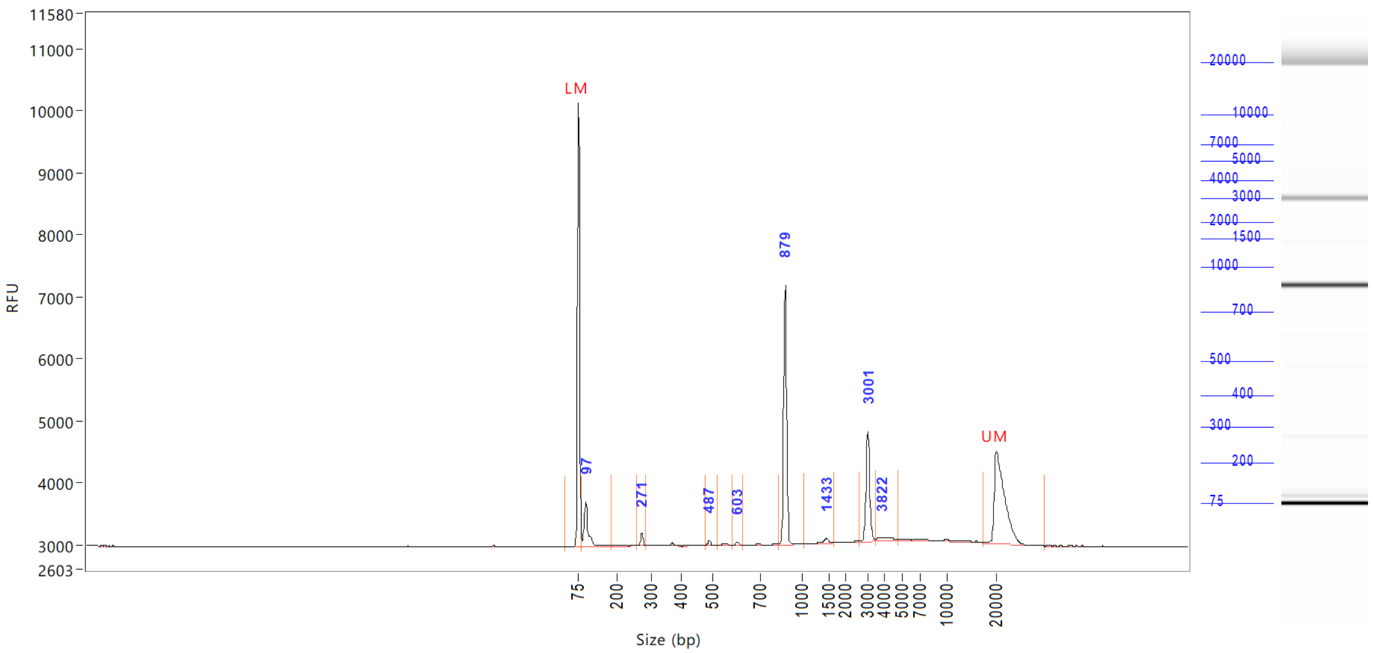

Peak	Size (bp)	Concentration (ng/uL)	From (bp)	To (bp)	Average size (bp)	CV%	RFU	Corrected peak area
1	75 (LM)	0.5000	37	86	73	4.29	7172	37.547
2	97	1.0611	86	179	101	13.15	721	6.640
3	271	0.1750	257	284	269	1.61	207	1.095
4	487	0.0850	477	516	487	1.05	95	0.532
5	603	0.0690	583	623	601	1.20	67	0.432
6	879	3.5012	835	1045	875	1.62	4184	21.910
7	1433	0.1182	1045	1655	1405	7.10	82	0.740
8	3001	1.6280	2629	3465	3009	3.27	1772	10.188
9	3822	0.1998	3465	4724	4044	8.42	52	1.250
10	20000 (UM)	0.2640	17195	29793	20959	6.10	1503	19.827

TIC:	6.8374	ng/uL
TIM:	26.4945	nmole/L
Total concentration:	7.5107	ng/uL

```
Sample peak width (sec): 5    Sample min peak height: 50    Sample baseline V to V?: Y    Sample baseline V to V points: 3
Sample filter: Binomial       Number of points for filter: 3  Sample start region (min): 0  Sample end region (min): 30
Marker peak width (sec): 5    Marker min peak height: 200   Marker baseline V to V?: Y    Marker baseline V to V points: 3
Lower marker selection: First peak > 500 RFU                Upper marker selection: Last peak > 200 RFU
Ladder size (bp)75, 200, 300, 400, 500, 700, 1000, 1500, 2000, 3000, 4000, 5000, 7000, 10000, 20000
Quantification using: Lower Marker          Final concentration (ng/uL): 0.5000          Dilution factor: 12.0
```

Sample: SampE1
Well location: E1
Created: Wednesday, October 20, 2021 4:38:54 PM

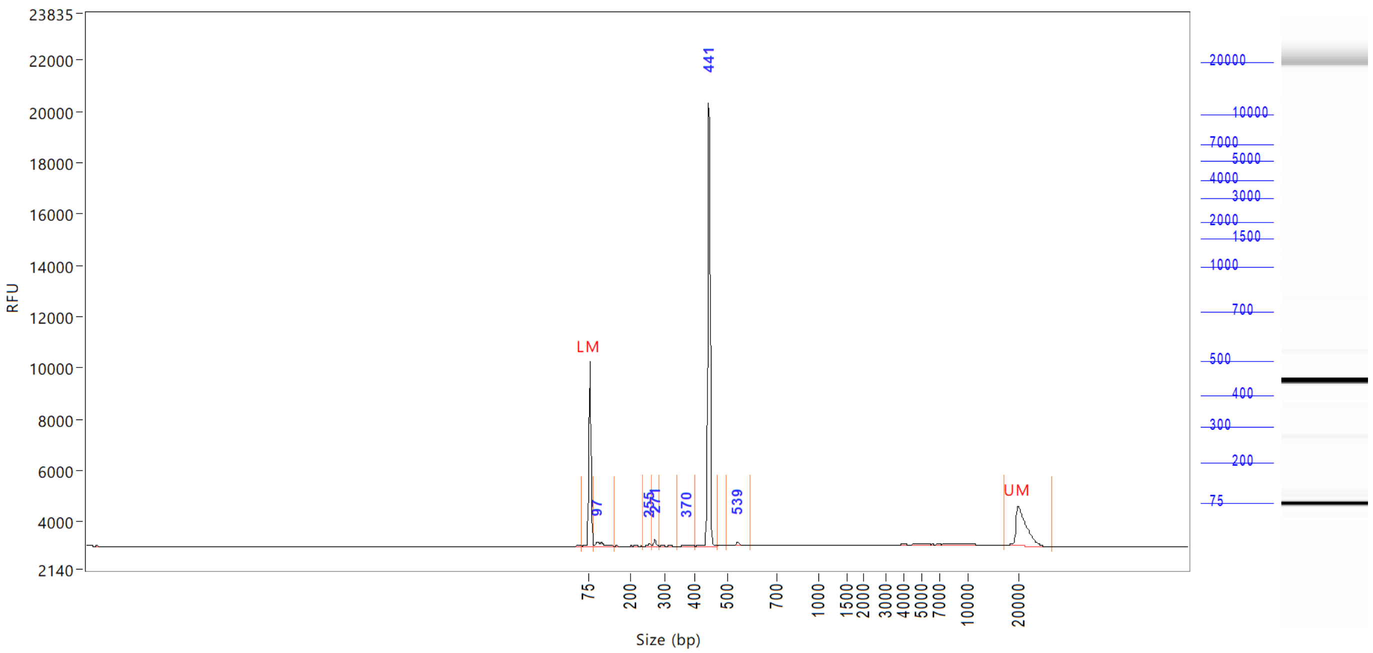

Peak	Size (bp)	Concentration (ng/uL)	From (bp)	To (bp)	Average size (bp)	CV%	RFU	Corrected peak area
1	75 (LM)	0.5000	50	87	72	4.70	7215	38.215
2	97	0.4205	87	150	105	12.04	176	2.678
3	255	0.0957	237	262	254	2.11	73	0.609
4	271	0.2076	262	286	269	1.41	251	1.322
5	370	0.0677	343	400	370	2.68	60	0.431
6	441	12.8308	400	468	440	0.86	17320	81.721
7	539	0.1727	498	593	540	2.28	171	1.100
8	20000 (UM)	0.2624	17317	26649	20793	5.70	1566	20.056

TIC:	13.7949	ng/uL
TIM:	57.2737	nmole/L
Total concentration:	14.5604	ng/uL

```
Sample peak width (sec): 5    Sample min peak height: 50    Sample baseline V to V?: Y    Sample baseline V to V points: 3
Sample filter: Binomial       Number of points for filter: 3  Sample start region (min): 0  Sample end region (min): 30
Marker peak width (sec): 5    Marker min peak height: 200   Marker baseline V to V?: Y    Marker baseline V to V points: 3
Lower marker selection: First peak > 500 RFU                Upper marker selection: Last peak > 200 RFU
Ladder size (bp)75, 200, 300, 400, 500, 700, 1000, 1500, 2000, 3000, 4000, 5000, 7000, 10000, 20000
Quantification using: Lower Marker          Final concentration (ng/uL): 0.5000          Dilution factor: 12.0
```

Sample: SampE2
Well location: E2
Created: Wednesday, October 20, 2021 4:38:54 PM

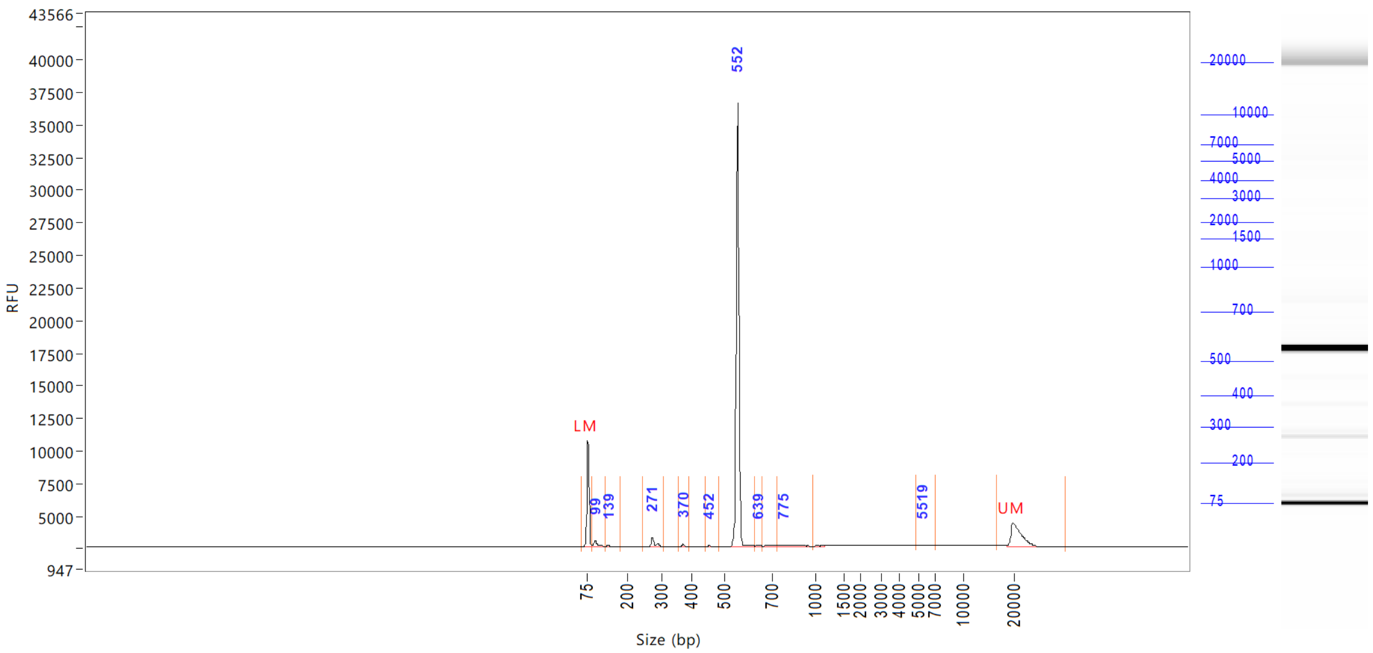

Peak	Size (bp)	Concentration (ng/uL)	From (bp)	To (bp)	Average size (bp)	CV%	RFU	Corrected peak area
1	75 (LM)	0.5000	58	87	74	4.23	8109	43.227
2	99	0.6243	87	129	101	9.00	448	4.498
3	139	0.1117	129	177	143	7.68	78	0.804
4	271	0.7068	242	307	273	3.58	712	5.092
5	370	0.1191	356	392	368	1.29	166	0.858
6	452	0.0695	439	483	452	1.43	80	0.501
7	552	23.5793	483	623	550	1.26	34061	169.879
8	639	0.0762	623	659	639	1.54	62	0.549
9	775	0.2768	727	987	809	6.66	118	1.995
10	5519	0.1602	4897	7068	5870	11.04	71	1.154
11	20000 (UM)	0.2571	16830	30398	20807	5.70	1772	22.227

TIC:	25.7240	ng/uL
TIM:	87.8310	nmole/L
Total concentration:	26.3998	ng/uL

```
Sample peak width (sec): 5     Sample min peak height: 50     Sample baseline V to V?: Y     Sample baseline V to V points: 3
Sample filter: Binomial        Number of points for filter: 3  Sample start region (min): 0  Sample end region (min): 30
Marker peak width (sec): 5     Marker min peak height: 200    Marker baseline V to V?: Y     Marker baseline V to V points: 3
Lower marker selection: First peak > 500 RFU                  Upper marker selection: Last peak > 200 RFU
Ladder size (bp)75, 200, 300, 400, 500, 700, 1000, 1500, 2000, 3000, 4000, 5000, 7000, 10000, 20000
Quantification using: Lower Marker          Final concentration (ng/uL): 0.5000          Dilution factor: 12.0
```

Sample: SampE3
Well location: E3
Created: Wednesday, October 20, 2021 4:38:54 PM

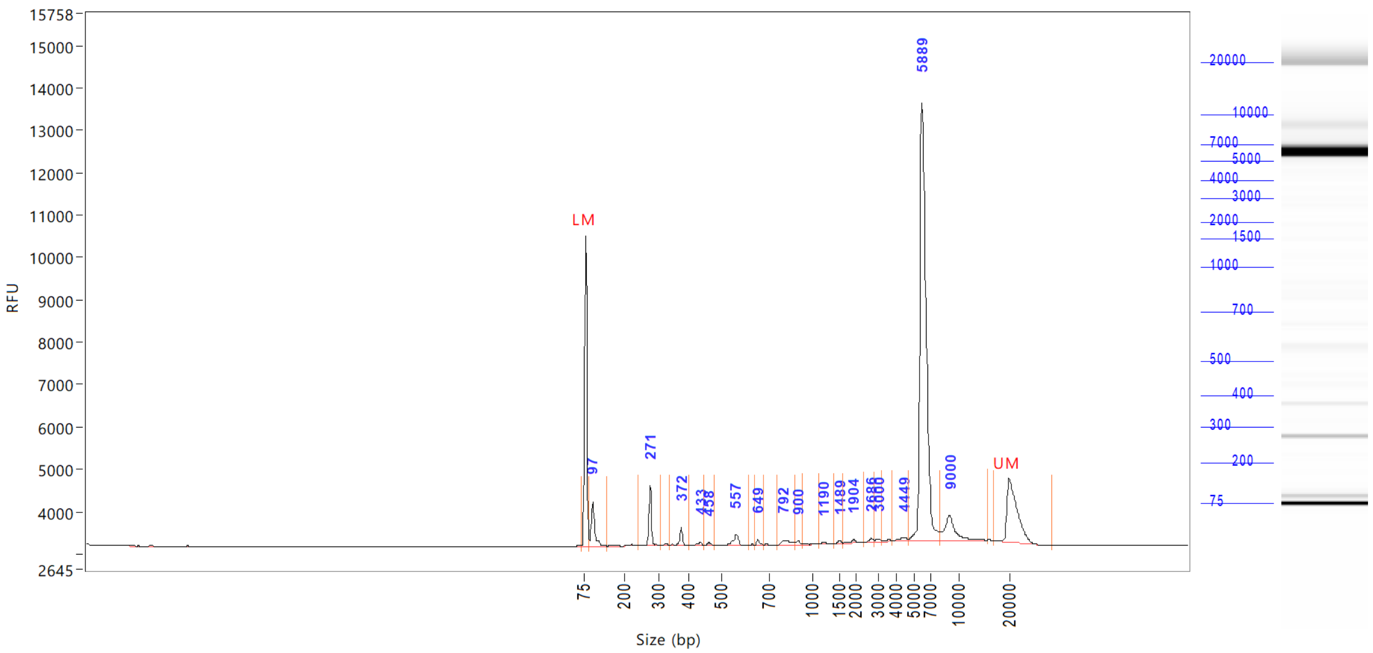

Peak	Size (bp)	Concentration (ng/uL)	From (bp)	To (bp)	Average size (bp)	CV%	RFU	Corrected peak area
1	75 (LM)	0.5000	62	86	73	4.44	7329	38.492
2	97	1.3729	86	141	97	8.37	1055	8.808
3	271	1.1515	237	305	270	2.34	1407	7.387
4	372	0.4090	333	398	366	2.66	445	2.624
5	433	0.1168	398	443	427	1.66	93	0.750
6	458	0.0897	443	475	457	1.31	79	0.575
7	557	0.4443	475	611	548	3.41	257	2.850
8	649	0.1166	636	674	650	1.34	132	0.748
9	792	0.2592	753	874	813	3.67	117	1.663
10	900	0.0944	874	931	895	1.34	101	0.605
11	1190	0.0648	1112	1400	1208	4.70	51	0.416
12	1489	0.0536	1400	1578	1478	2.03	69	0.344
13	1904	0.0944	1578	2344	1890	8.17	76	0.606
14	2686	0.0876	2344	2829	2655	3.95	88	0.562
15	3000	0.0873	2829	3215	2977	3.06	77	0.560
16	4449	0.1592	3822	4656	4306	4.79	84	1.021
17	5889	13.7084	4656	8001	6076	7.40	10350	87.945
18	9000	1.2829	8001	15732	9262	11.61	590	8.230
19	20000 (UM)	0.2501	16952	28584	20831	5.58	1512	19.258

TIC:	19.5925	ng/uL
TIM:	39.5296	nmole/L
Total concentration:	19.8537	ng/uL

```
Sample peak width (sec): 5    Sample min peak height: 50    Sample baseline V to V?: Y    Sample baseline V to V points: 3
Sample filter: Binomial       Number of points for filter: 3  Sample start region (min): 0  Sample end region (min): 30
Marker peak width (sec): 5    Marker min peak height: 200   Marker baseline V to V?: Y    Marker baseline V to V points: 3
Lower marker selection: First peak > 500 RFU                Upper marker selection: Last peak > 200 RFU
Ladder size (bp)75, 200, 300, 400, 500, 700, 1000, 1500, 2000, 3000, 4000, 5000, 7000, 10000, 20000
Quantification using: Lower Marker          Final concentration (ng/uL): 0.5000          Dilution factor: 12.0
```

Sample: SampE4
Well location: E4
Created: Wednesday, October 20, 2021 4:38:54 PM

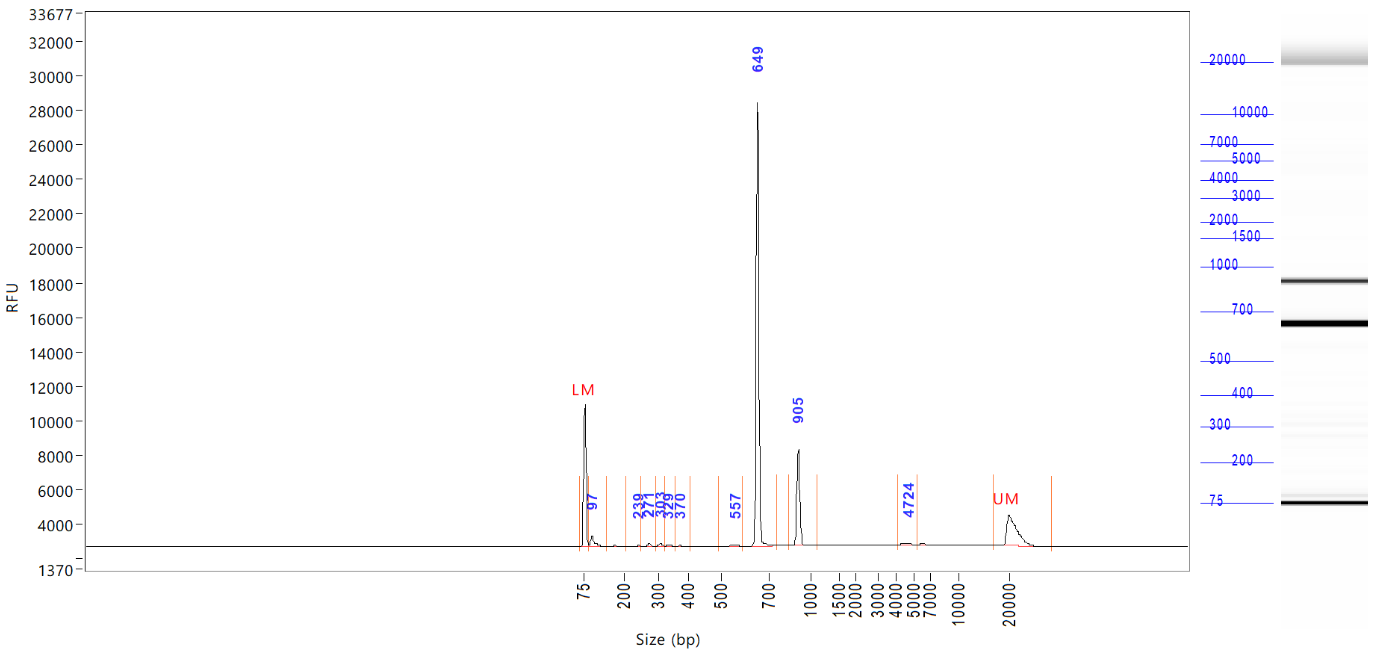

Peak	Size (bp)	Concentration (ng/uL)	From (bp)	To (bp)	Average size (bp)	CV%	RFU	Corrected peak area
1	75 (LM)	0.5000	62	86	73	4.26	8251	42.867
2	97	0.7988	86	145	99	9.93	606	5.707
3	239	0.0763	205	248	230	4.84	52	0.545
4	271	0.1506	248	291	268	3.03	152	1.076
5	303	0.1595	291	319	303	1.91	156	1.140
6	329	0.0974	319	356	331	2.17	68	0.696
7	370	0.0403	356	407	368	1.83	54	0.288
8	557	0.1224	492	588	548	3.31	69	0.874
9	649	17.2631	588	753	648	1.18	25804	123.336
10	905	3.9722	835	1101	901	1.61	5567	28.380
11	4724	0.1094	4139	5445	4623	5.50	65	0.782
12	20000 (UM)	0.2652	16952	28584	20850	5.68	1766	22.734

TIC:	22.7901	ng/uL
TIM:	67.7547	nmole/L
Total concentration:	23.2308	ng/uL

Sample peak width (sec): 5 Sample min peak height: 50 Sample baseline V to V?: Y Sample baseline V to V points: 3
Sample filter: Binomial Number of points for filter: 3 Sample start region (min): 0 Sample end region (min): 30
Marker peak width (sec): 5 Marker min peak height: 200 Marker baseline V to V?: Y Marker baseline V to V points: 3
Lower marker selection: First peak > 500 RFU Upper marker selection: Last peak > 200 RFU
Ladder size (bp)75, 200, 300, 400, 500, 700, 1000, 1500, 2000, 3000, 4000, 5000, 7000, 10000, 20000
Quantification using: Lower Marker Final concentration (ng/uL): 0.5000 Dilution factor: 12.0

Sample: SampE5
Well location: E5
Created: Wednesday, October 20, 2021 4:38:54 PM

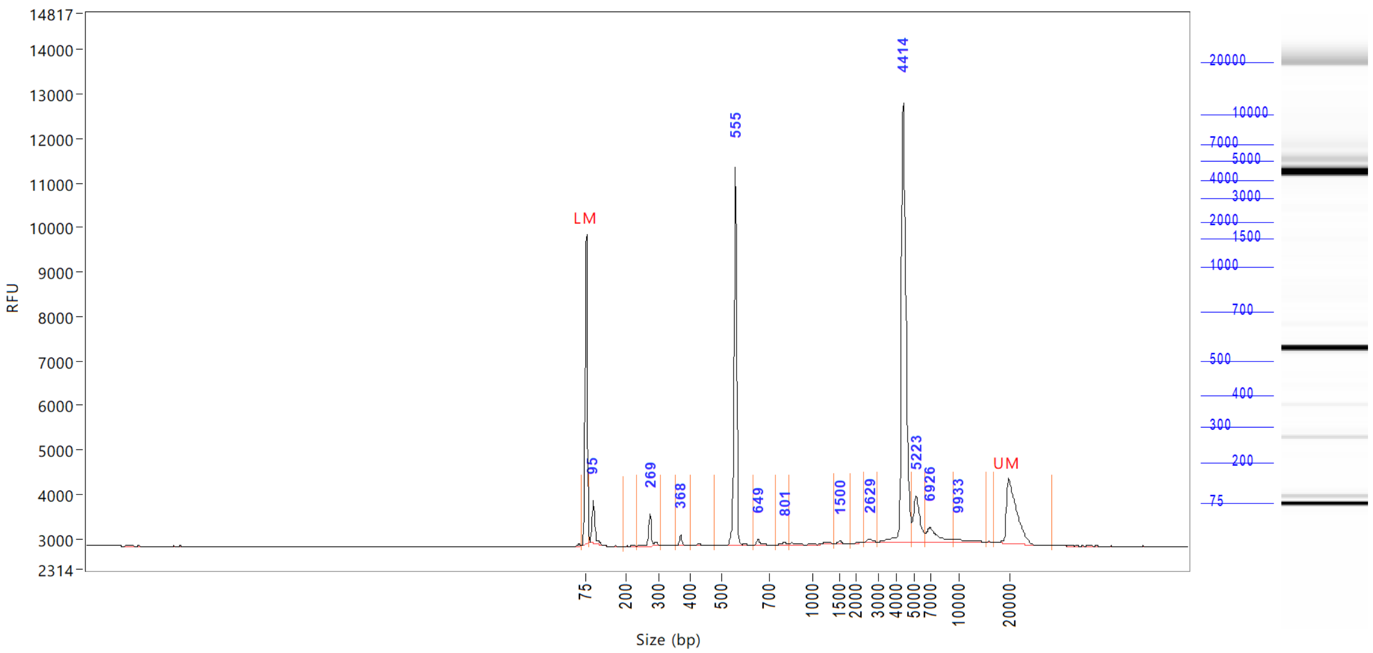

Peak	Size (bp)	Concentration (ng/uL)	From (bp)	To (bp)	Average size (bp)	CV%	RFU	Corrected peak area
1	75 (LM)	0.5000	62	86	72	4.13	6952	36.486
2	95	1.0969	86	192	97	11.36	938	6.670
3	269	0.7337	230	305	270	3.33	727	4.462
4	368	0.2166	352	400	366	1.34	258	1.317
5	555	7.0504	477	631	551	1.47	8504	42.873
6	649	0.1854	631	744	659	3.20	135	1.127
7	801	0.1027	744	831	793	2.55	63	0.624
8	1500	0.0830	1400	1827	1525	5.83	67	0.505
9	2629	0.1076	2315	2943	2601	4.99	74	0.654
10	4414	11.0894	2943	4897	4415	4.06	9890	67.434
11	5223	1.5629	4897	6260	5332	6.52	1033	9.504
12	6926	0.8288	6260	9400	7357	10.94	322	5.040
13	9933	0.1831	9400	15488	10975	13.33	63	1.113
14	20000 (UM)	0.2559	17195	28826	20861	5.64	1478	18.674

TIC:	23.2404	ng/uL
TIM:	50.7312	nmole/L
Total concentration:	23.6023	ng/uL

Sample peak width (sec): 5 Sample min peak height: 50 Sample baseline V to V?: Y Sample baseline V to V points: 3
Sample filter: Binomial Number of points for filter: 3 Sample start region (min): 0 Sample end region (min): 30
Marker peak width (sec): 5 Marker min peak height: 200 Marker baseline V to V?: Y Marker baseline V to V points: 3
Lower marker selection: First peak > 500 RFU Upper marker selection: Last peak > 200 RFU
Ladder size (bp)75, 200, 300, 400, 500, 700, 1000, 1500, 2000, 3000, 4000, 5000, 7000, 10000, 20000
Quantification using: Lower Marker Final concentration (ng/uL): 0.5000 Dilution factor: 12.0

Sample: SampE6
Well location: E6
Created: Wednesday, October 20, 2021 4:38:54 PM

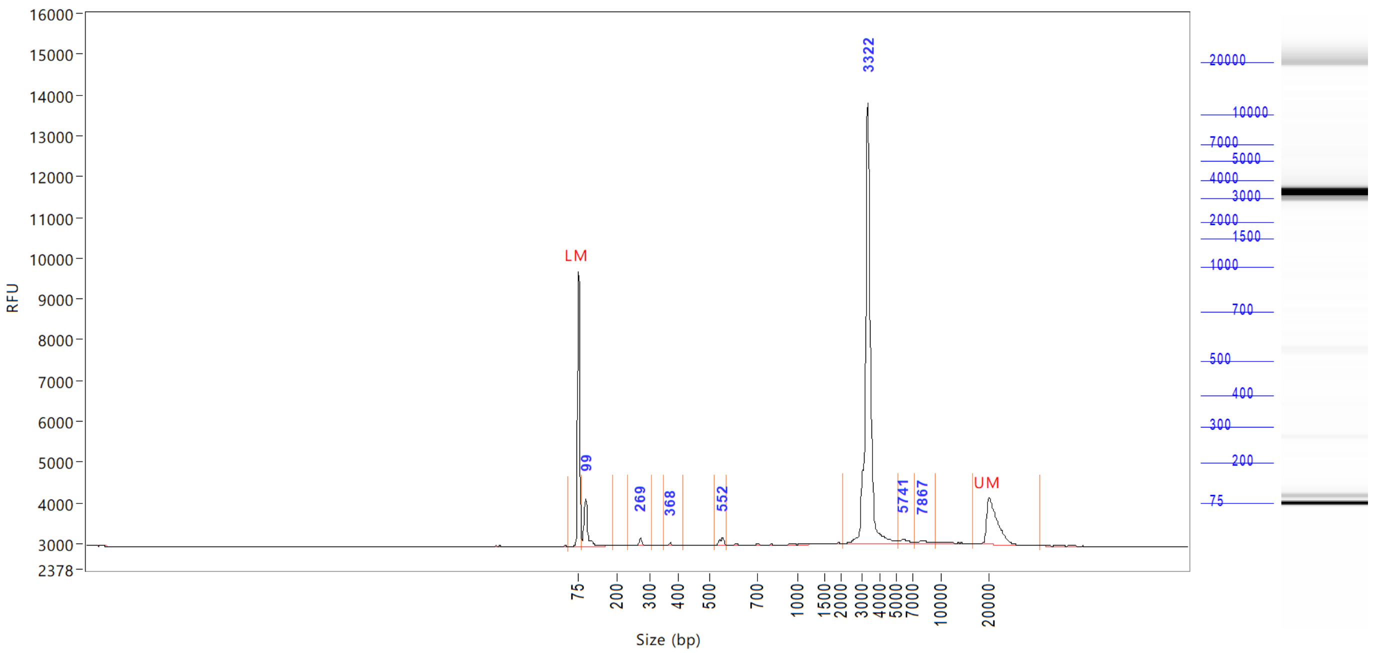

Peak	Size (bp)	Concentration (ng/uL)	From (bp)	To (bp)	Average size (bp)	CV%	RFU	Corrected peak area
1	75 (LM)	0.5000	45	86	74	4.32	6716	36.239
2	99	1.6477	86	189	99	11.53	1174	9.952
3	269	0.2472	233	307	272	4.62	191	1.493
4	368	0.0975	347	411	370	3.46	72	0.589
5	552	0.2878	514	570	543	1.87	185	1.738
6	3322	13.8675	2058	5075	3320	8.53	10814	83.757
7	5741	0.2301	5075	7134	5990	9.56	102	1.390
8	7867	0.1721	7134	9533	8230	8.11	52	1.039
9	20000 (UM)	0.2162	16830	30398	20965	6.43	1163	15.667

TIC:	16.5499	ng/uL
TIM:	37.1034	nmole/L
Total concentration:	17.1646	ng/uL

Sample peak width (sec): 5   Sample min peak height: 50   Sample baseline V to V?: Y   Sample baseline V to V points: 3
Sample filter: Binomial   Number of points for filter: 3 Sample start region (min): 0 Sample end region (min): 30
Marker peak width (sec): 5   Marker min peak height: 200   Marker baseline V to V?: Y   Marker baseline V to V points: 3
Lower marker selection: First peak > 500 RFU   Upper marker selection: Last peak > 200 RFU
Ladder size (bp)75, 200, 300, 400, 500, 700, 1000, 1500, 2000, 3000, 4000, 5000, 7000, 10000, 20000
Quantification using: Lower Marker   Final concentration (ng/uL): 0.5000   Dilution factor: 12.0

Sample: SampE7
Well location: E7
Created: Wednesday, October 20, 2021 4:38:54 PM

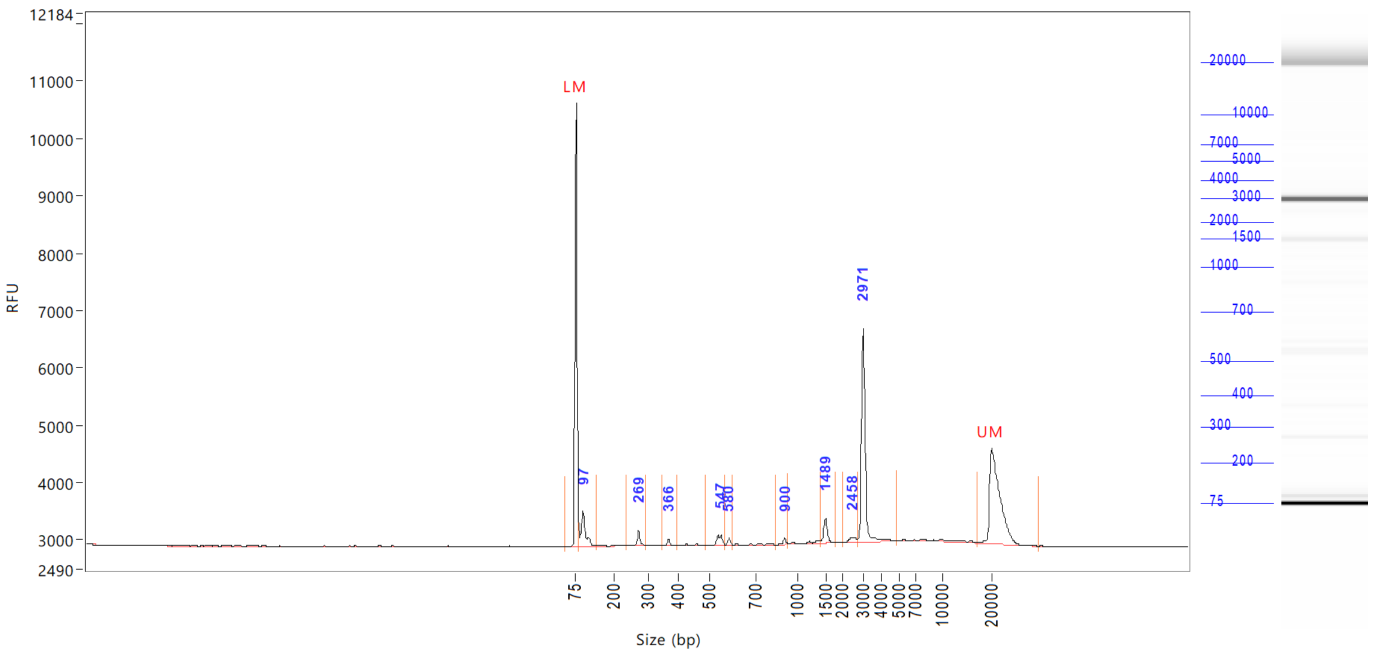

Peak	Size (bp)	Concentration (ng/uL)	From (bp)	To (bp)	Average size (bp)	CV%	RFU	Corrected peak area
1	75 (LM)	0.5000	45	86	73	4.38	7745	40.489
2	97	0.9153	86	141	99	10.23	611	6.176
3	269	0.2517	235	293	268	2.94	280	1.699
4	366	0.1118	347	400	367	2.56	123	0.754
5	547	0.2981	489	565	536	2.33	194	2.012
6	580	0.1102	565	598	578	1.19	126	0.744
7	900	0.0899	840	926	892	1.64	110	0.607
8	1489	0.3988	1389	1808	1487	3.28	437	2.691
9	2458	0.1477	2030	2715	2451	6.28	92	0.997
10	2971	3.2379	2715	4897	3015	8.00	3711	21.850
11	20000 (UM)	0.2754	16952	29430	20869	6.24	1660	22.304

TIC:	5.5614	ng/uL
TIM:	20.9303	nmole/L
Total concentration:	6.2227	ng/uL

```
Sample peak width (sec): 5     Sample min peak height: 50    Sample baseline V to V?: Y    Sample baseline V to V points: 3
Sample filter: Binomial        Number of points for filter: 3  Sample start region (min): 0  Sample end region (min): 30
Marker peak width (sec): 5     Marker min peak height: 200   Marker baseline V to V?: Y    Marker baseline V to V points: 3
Lower marker selection: First peak > 500 RFU                 Upper marker selection: Last peak > 200 RFU
Ladder size (bp)75, 200, 300, 400, 500, 700, 1000, 1500, 2000, 3000, 4000, 5000, 7000, 10000, 20000
Quantification using: Lower Marker          Final concentration (ng/uL): 0.5000          Dilution factor: 12.0
```

Sample: SampE8
Well location: E8
Created: Wednesday, October 20, 2021 4:38:54 PM

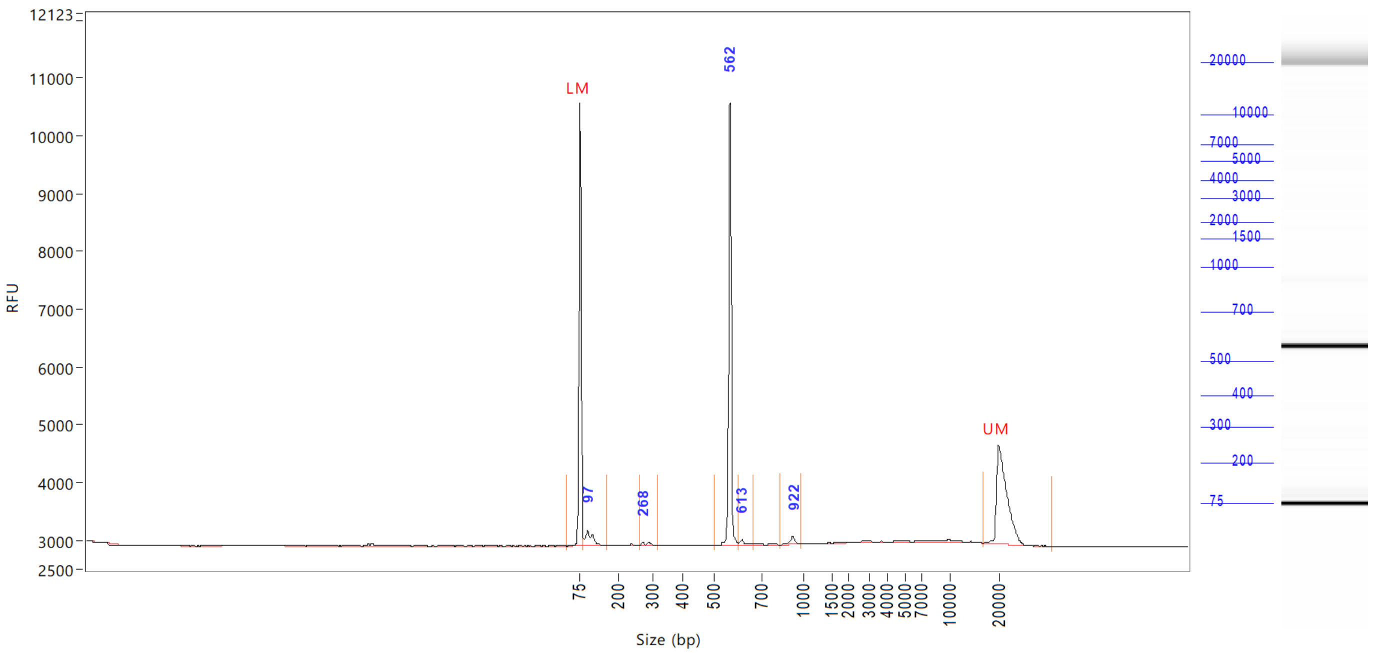

Peak	Size (bp)	Concentration (ng/uL)	From (bp)	To (bp)	Average size (bp)	CV%	RFU	Corrected peak area
1	75 (LM)	0.5000	33	86	72	4.62	7668	41.023
2	97	0.6480	86	160	105	13.38	280	4.431
3	268	0.1309	259	317	279	4.12	65	0.895
4	562	5.5928	500	600	559	1.27	7671	38.240
5	613	0.1260	600	664	622	2.73	99	0.862
6	922	0.1558	831	978	910	2.54	130	1.065
7	20000 (UM)	0.2710	16830	30760	20910	6.19	1701	22.231

TIC:	6.6536	ng/uL
TIM:	27.9870	nmole/L
Total concentration:	7.2707	ng/uL

Sample peak width (sec): 5 Sample min peak height: 50 Sample baseline V to V?: Y Sample baseline V to V points: 3
Sample filter: Binomial Number of points for filter: 3 Sample start region (min): 0 Sample end region (min): 30
Marker peak width (sec): 5 Marker min peak height: 200 Marker baseline V to V?: Y Marker baseline V to V points: 3
Lower marker selection: First peak > 500 RFU Upper marker selection: Last peak > 200 RFU
Ladder size (bp)75, 200, 300, 400, 500, 700, 1000, 1500, 2000, 3000, 4000, 5000, 7000, 10000, 20000
Quantification using: Lower Marker Final concentration (ng/uL): 0.5000 Dilution factor: 12.0

Sample: SampE9
Well location: E9
Created: Wednesday, October 20, 2021 4:38:54 PM

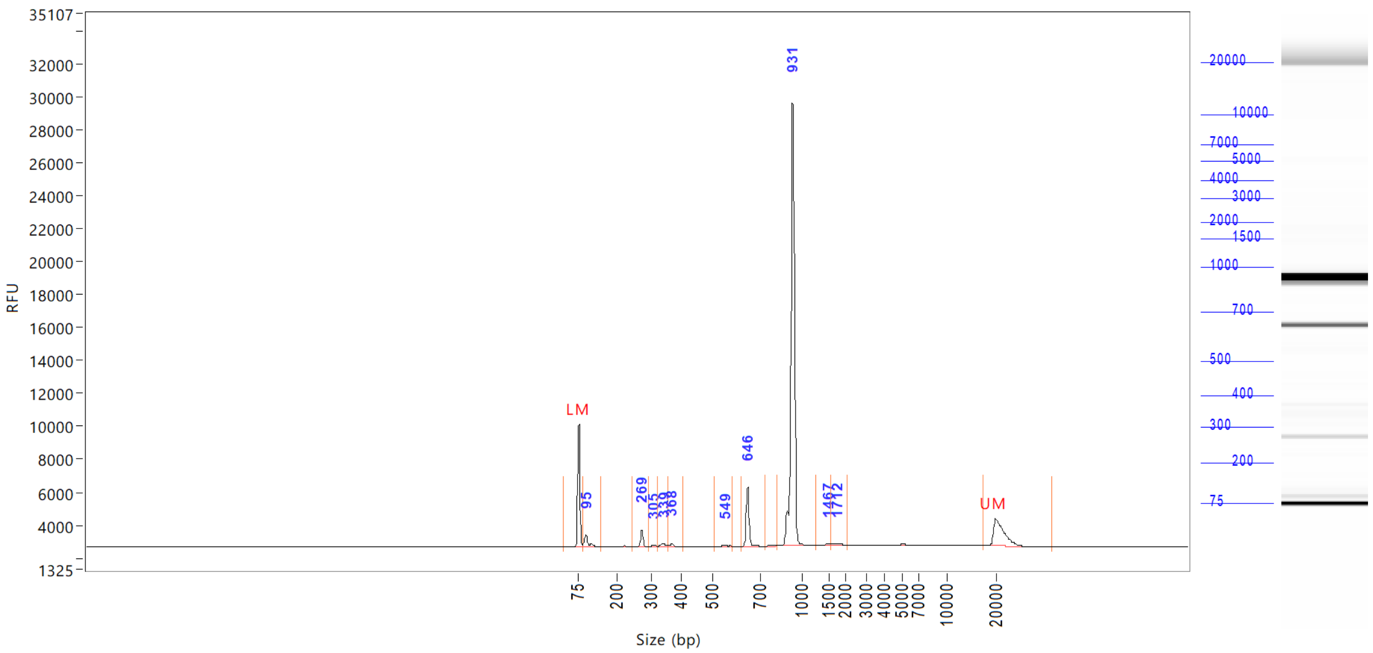

Peak	Size (bp)	Concentration (ng/uL)	From (bp)	To (bp)	Average size (bp)	CV%	RFU	Corrected peak area
1	75 (LM)	0.5000	26	86	72	4.80	7447	40.801
2	95	0.9627	86	147	99	10.95	692	6.546
3	269	0.8040	242	293	269	1.91	1017	5.468
4	305	0.0949	293	319	305	2.20	69	0.646
5	339	0.2717	319	358	338	2.78	141	1.847
6	368	0.1956	358	407	368	1.95	212	1.330
7	549	0.1236	504	583	548	2.84	75	0.840
8	646	2.7506	621	731	644	1.19	3564	18.705
9	931	23.1781	827	1245	927	2.01	26955	157.614
10	1467	0.1087	1245	1539	1428	5.07	61	0.739
11	1712	0.1699	1539	2058	1744	7.08	78	1.156
12	20000 (UM)	0.2701	17317	31486	20933	6.28	1644	22.041

TIC:	28.6599	ng/uL
TIM:	72.4313	nmole/L
Total concentration:	29.2635	ng/uL

```
Sample peak width (sec): 5     Sample min peak height: 50     Sample baseline V to V?: Y     Sample baseline V to V points: 3
Sample filter: Binomial        Number of points for filter: 3  Sample start region (min): 0  Sample end region (min): 30
Marker peak width (sec): 5     Marker min peak height: 200    Marker baseline V to V?: Y     Marker baseline V to V points: 3
Lower marker selection: First peak > 500 RFU                  Upper marker selection: Last peak > 200 RFU
Ladder size (bp)75, 200, 300, 400, 500, 700, 1000, 1500, 2000, 3000, 4000, 5000, 7000, 10000, 20000
Quantification using: Lower Marker          Final concentration (ng/uL): 0.5000          Dilution factor: 12.0
```

Sample: SampE10
Well location: E10
Created: Wednesday, October 20, 2021 4:38:54 PM

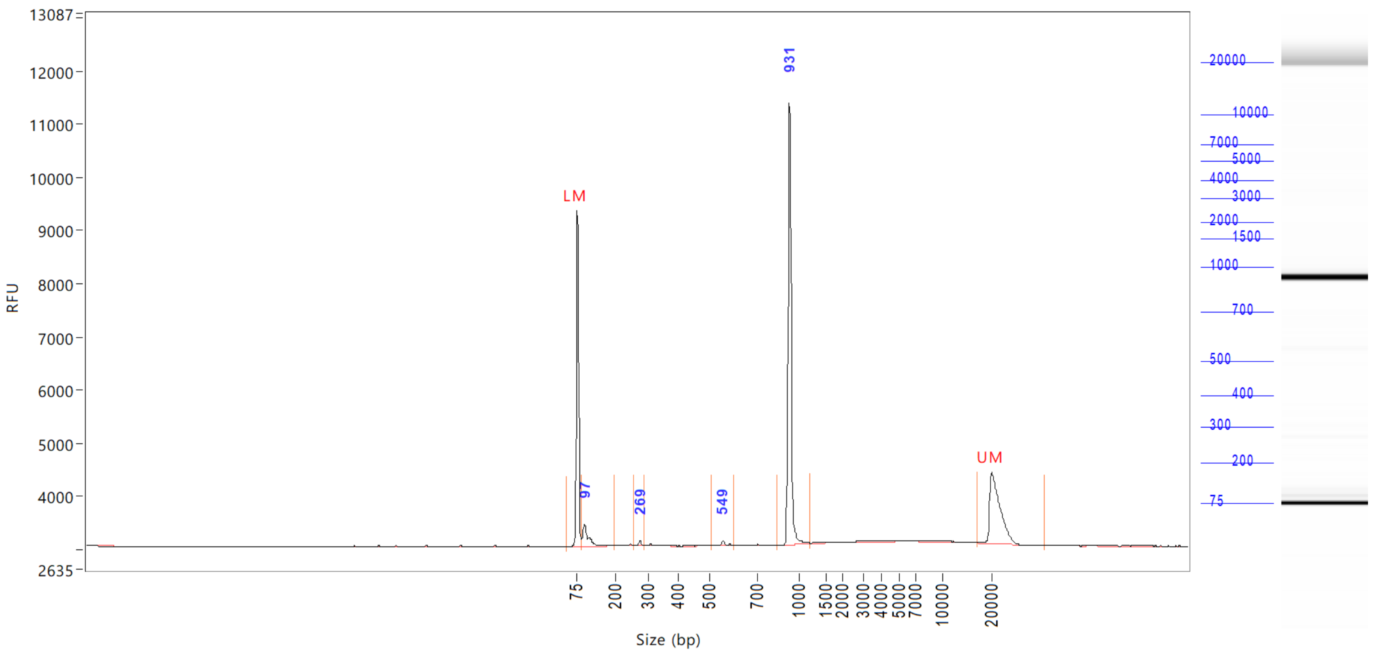

Peak	Size (bp)	Concentration (ng/uL)	From (bp)	To (bp)	Average size (bp)	CV%	RFU	Corrected peak area
1	75 (LM)	0.5000	41	87	74	4.56	6316	34.374
2	97	0.9207	87	194	106	15.97	411	5.274
3	269	0.0975	251	282	268	2.01	95	0.558
4	549	0.1551	501	598	554	3.32	99	0.889
5	931	7.8146	840	1212	932	2.48	8321	44.769
6	20000 (UM)	0.2648	16830	30881	20898	6.30	1357	18.204

TIC:	8.9878	ng/uL
TIM:	29.1238	nmole/L
Total concentration:	9.7946	ng/uL

```
Sample peak width (sec): 5    Sample min peak height: 50    Sample baseline V to V?: Y    Sample baseline V to V points: 3
Sample filter: Binomial       Number of points for filter: 3  Sample start region (min): 0  Sample end region (min): 30
Marker peak width (sec): 5    Marker min peak height: 200   Marker baseline V to V?: Y    Marker baseline V to V points: 3
Lower marker selection: First peak > 500 RFU                Upper marker selection: Last peak > 200 RFU
Ladder size (bp)75, 200, 300, 400, 500, 700, 1000, 1500, 2000, 3000, 4000, 5000, 7000, 10000, 20000
Quantification using: Lower Marker          Final concentration (ng/uL): 0.5000          Dilution factor: 12.0
```

Sample: SampE11
Well location: E11
Created: Wednesday, October 20, 2021 4:38:54 PM

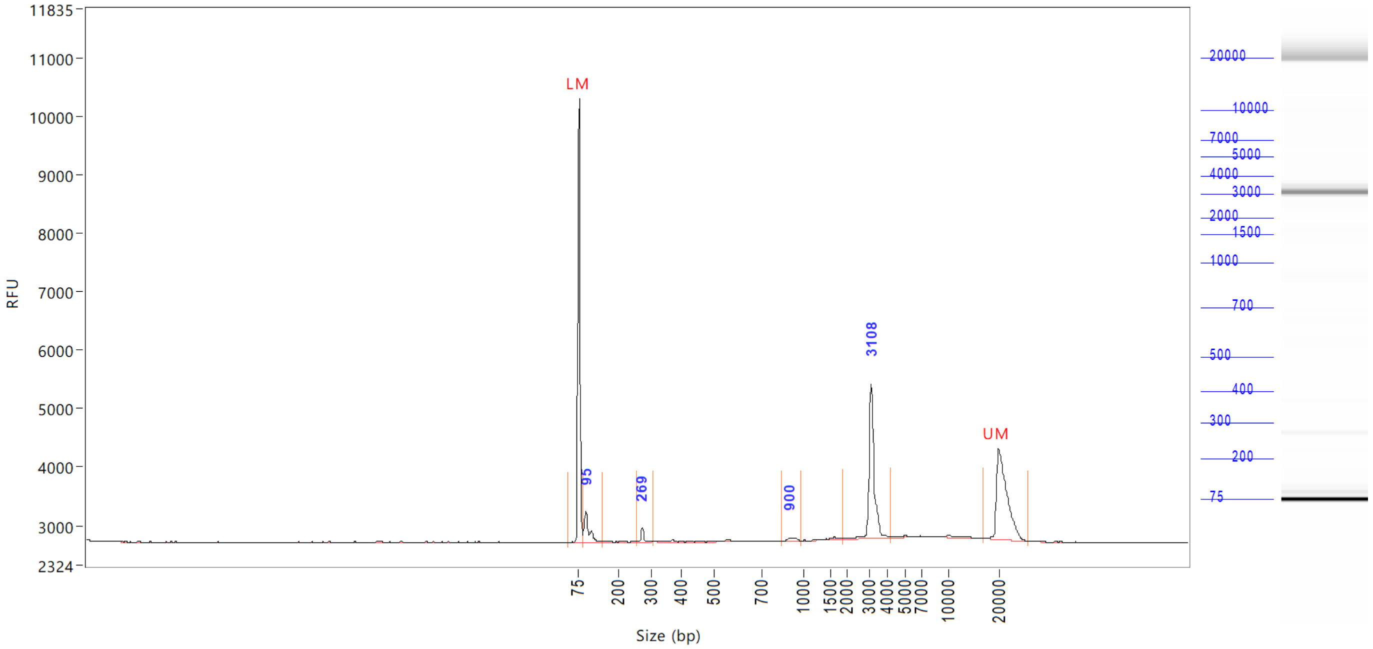

Peak	Size (bp)	Concentration (ng/uL)	From (bp)	To (bp)	Average size (bp)	CV%	RFU	Corrected peak area
1	75 (LM)	0.5000	45	86	73	4.46	7598	40.307
2	95	0.8762	86	148	101	12.30	526	5.886
3	269	0.1968	255	303	269	2.33	250	1.322
4	900	0.1167	840	983	913	2.81	62	0.784
5	3108	2.8791	1866	4242	3122	7.58	2642	19.341
6	20000 (UM)	0.2537	16952	25803	20888	5.75	1567	20.449

TIC:	4.0688	ng/uL
TIM:	17.1779	nmole/L
Total concentration:	4.7266	ng/uL

Sample peak width (sec): 5 Sample min peak height: 50 Sample baseline V to V?: Y Sample baseline V to V points: 3
Sample filter: Binomial Number of points for filter: 3 Sample start region (min): 0 Sample end region (min): 30
Marker peak width (sec): 5 Marker min peak height: 200 Marker baseline V to V?: Y Marker baseline V to V points: 3
Lower marker selection: First peak > 500 RFU Upper marker selection: Last peak > 200 RFU
Ladder size (bp)75, 200, 300, 400, 500, 700, 1000, 1500, 2000, 3000, 4000, 5000, 7000, 10000, 20000
Quantification using: Lower Marker Final concentration (ng/uL): 0.5000 Dilution factor: 12.0

Sample: SampE12
Well location: E12
Created: Wednesday, October 20, 2021 4:38:54 PM

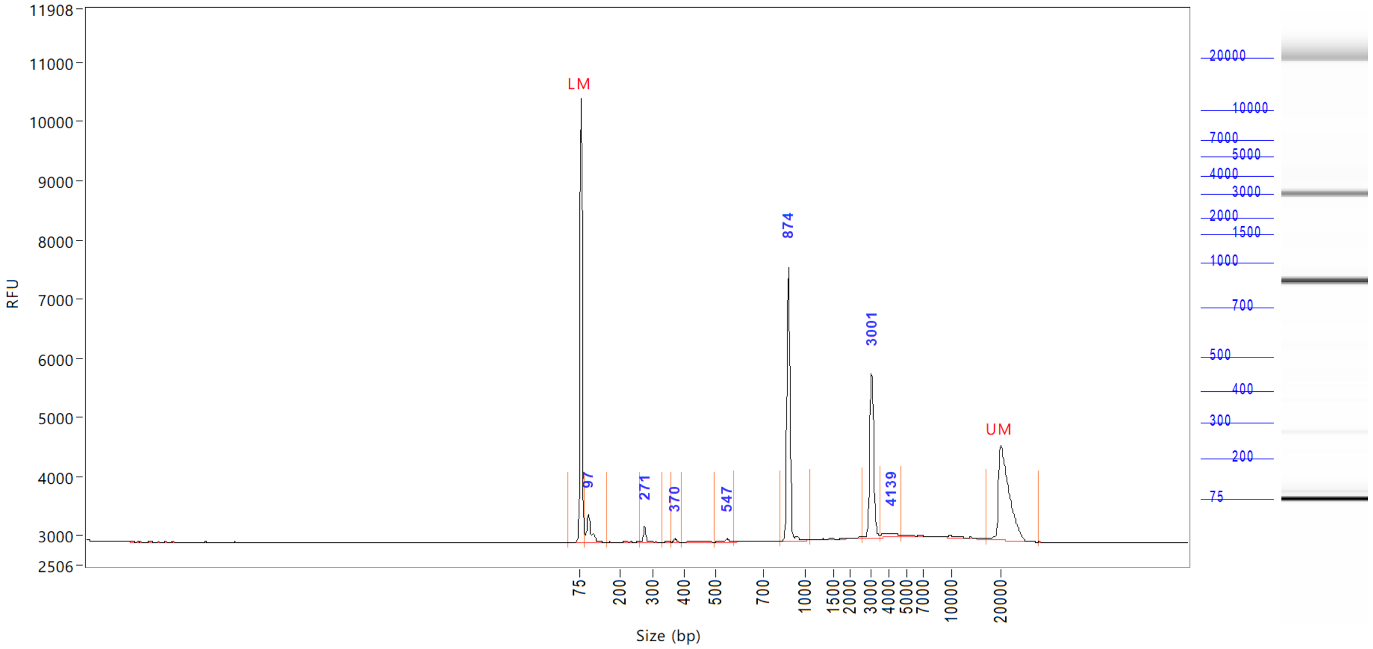

Peak	Size (bp)	Concentration (ng/uL)	From (bp)	To (bp)	Average size (bp)	CV%	RFU	Corrected peak area
1	75 (LM)	0.5000	39	87	74	4.52	7517	41.040
2	97	0.7288	87	160	102	11.74	469	4.985
3	271	0.2255	259	329	272	3.25	273	1.542
4	370	0.0431	358	392	369	1.47	56	0.295
5	547	0.0705	494	578	542	2.94	53	0.482
6	874	3.6780	822	1090	876	1.77	4619	25.157
7	3001	2.3483	2629	3500	3015	3.28	2778	16.062
8	4139	0.2153	3500	4690	4058	7.85	67	1.473
9	20000 (UM)	0.2605	17073	27496	20941	5.92	1605	21.386

TIC:	7.3095	ng/uL
TIM:	21.7835	nmole/L
Total concentration:	7.8456	ng/uL

```
Sample peak width (sec): 5    Sample min peak height: 50    Sample baseline V to V?: Y    Sample baseline V to V points: 3
Sample filter: Binomial       Number of points for filter: 3  Sample start region (min): 0  Sample end region (min): 30
Marker peak width (sec): 5    Marker min peak height: 200   Marker baseline V to V?: Y    Marker baseline V to V points: 3
Lower marker selection: First peak > 500 RFU                Upper marker selection: Last peak > 200 RFU
Ladder size (bp)75, 200, 300, 400, 500, 700, 1000, 1500, 2000, 3000, 4000, 5000, 7000, 10000, 20000
Quantification using: Lower Marker          Final concentration (ng/uL): 0.5000         Dilution factor: 12.0
```

Sample: SampF1
Well location: F1
Created: Wednesday, October 20, 2021 4:38:54 PM

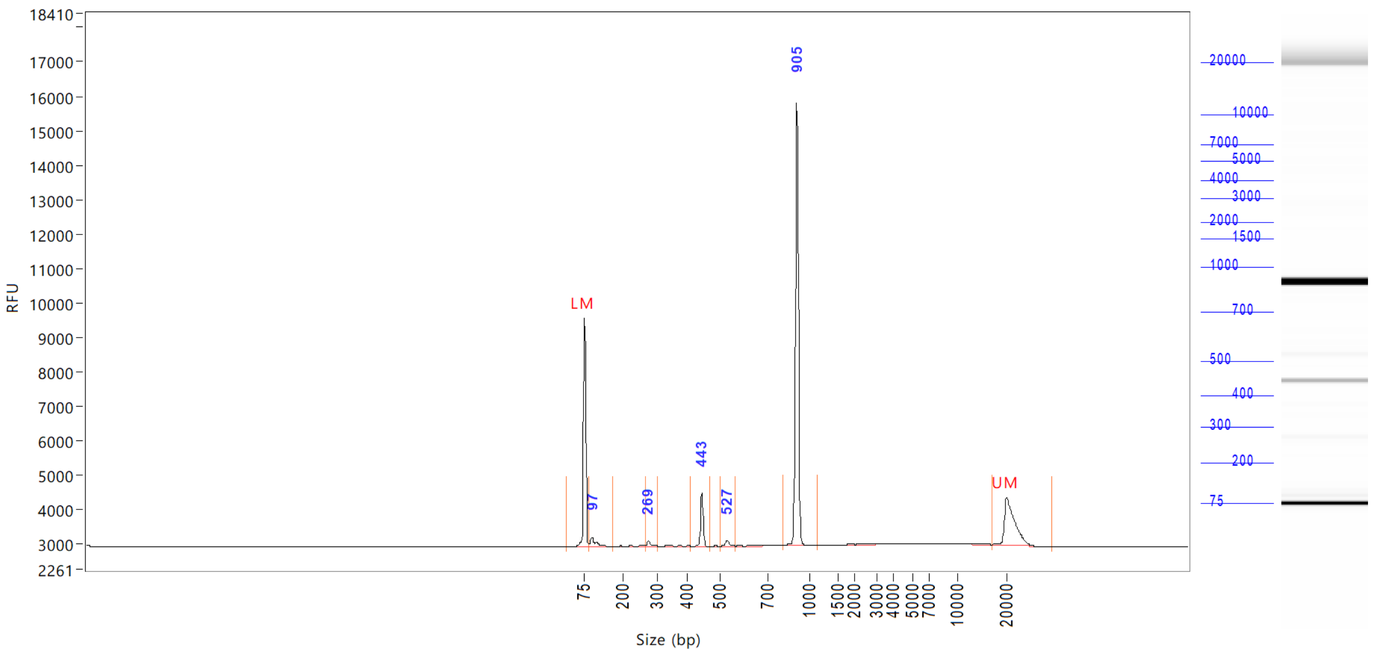

Peak	Size (bp)	Concentration (ng/uL)	From (bp)	To (bp)	Average size (bp)	CV%	RFU	Corrected peak area
1	75 (LM)	0.5000	20	87	73	5.62	6627	35.316
2	97	0.5351	87	168	103	12.68	271	3.149
3	269	0.1656	262	301	271	2.68	159	0.974
4	443	1.2348	409	470	440	1.00	1532	7.268
5	527	0.1966	500	562	527	1.89	174	1.157
6	905	10.9648	801	1112	900	1.41	12880	64.538
7	20000 (UM)	0.2585	17073	28947	20830	5.98	1404	18.261

TIC:	13.0968	ng/uL
TIM:	34.8191	nmole/L
Total concentration:	13.8153	ng/uL

```
Sample peak width (sec): 5     Sample min peak height: 50     Sample baseline V to V?: Y     Sample baseline V to V points: 3
Sample filter: Binomial        Number of points for filter: 3  Sample start region (min): 0  Sample end region (min): 30
Marker peak width (sec): 5     Marker min peak height: 200    Marker baseline V to V?: Y     Marker baseline V to V points: 3
Lower marker selection: First peak > 500 RFU                  Upper marker selection: Last peak > 200 RFU
Ladder size (bp)75, 200, 300, 400, 500, 700, 1000, 1500, 2000, 3000, 4000, 5000, 7000, 10000, 20000
Quantification using: Lower Marker          Final concentration (ng/uL): 0.5000          Dilution factor: 12.0
```

Sample: SampF2
Well location: F2
Created: Wednesday, October 20, 2021 4:38:54 PM

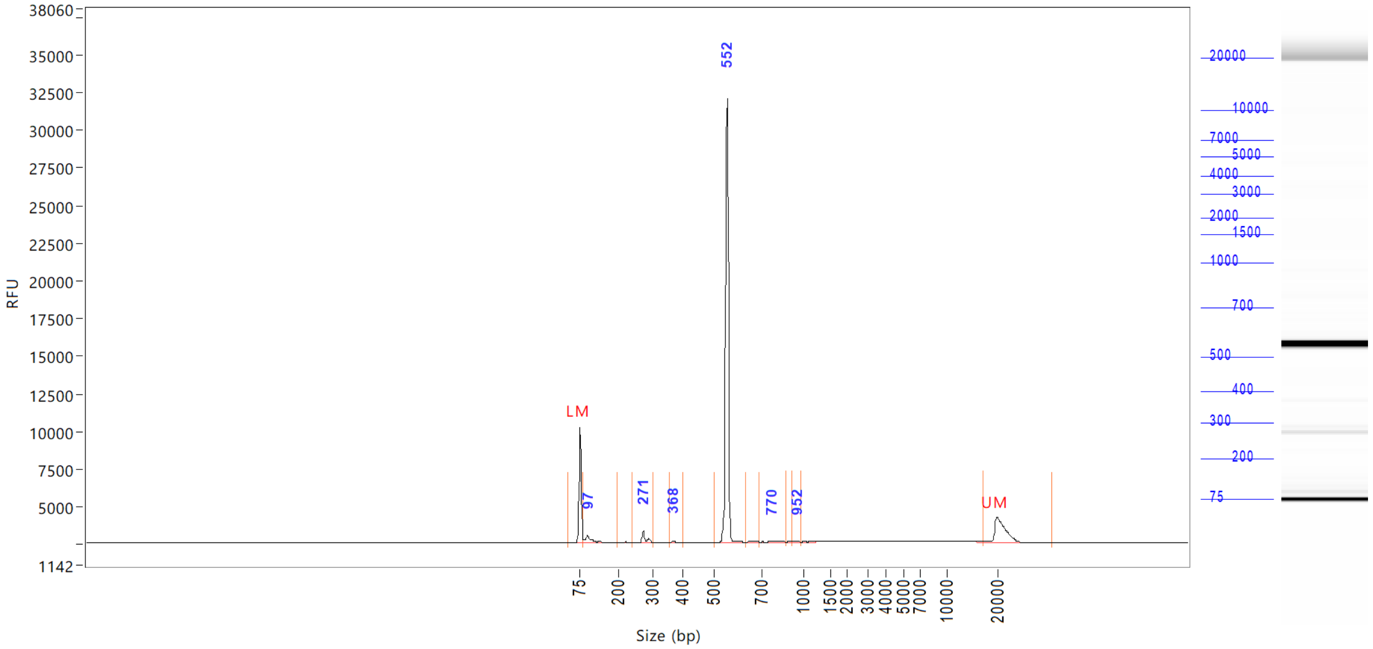

Peak	Size (bp)	Concentration (ng/uL)	From (bp)	To (bp)	Average size (bp)	CV%	RFU	Corrected peak area
1	75 (LM)	0.5000	41	86	73	4.49	7630	40.533
2	97	0.9923	86	196	107	18.34	515	6.704
3	271	0.7924	239	303	273	3.57	745	5.353
4	368	0.1094	356	400	367	1.36	136	0.739
5	552	21.5812	501	634	549	1.29	29498	145.791
6	770	0.1648	695	879	782	4.96	85	1.114
7	952	0.0570	918	987	947	1.38	61	0.385
8	20000 (UM)	0.2703	17073	31002	20938	5.96	1683	21.908

TIC:	23.6972	ng/uL
TIM:	85.6298	nmole/L
Total concentration:	24.5523	ng/uL

Sample peak width (sec): 5   Sample min peak height: 50   Sample baseline V to V?: Y   Sample baseline V to V points: 3
Sample filter: Binomial   Number of points for filter: 3 Sample start region (min): 0 Sample end region (min): 30
Marker peak width (sec): 5   Marker min peak height: 200   Marker baseline V to V?: Y   Marker baseline V to V points: 3
Lower marker selection: First peak > 500 RFU   Upper marker selection: Last peak > 200 RFU
Ladder size (bp)75, 200, 300, 400, 500, 700, 1000, 1500, 2000, 3000, 4000, 5000, 7000, 10000, 20000
Quantification using: Lower Marker   Final concentration (ng/uL): 0.5000   Dilution factor: 12.0

Sample: SampF3
Well location: F3
Created: Wednesday, October 20, 2021 4:38:54 PM

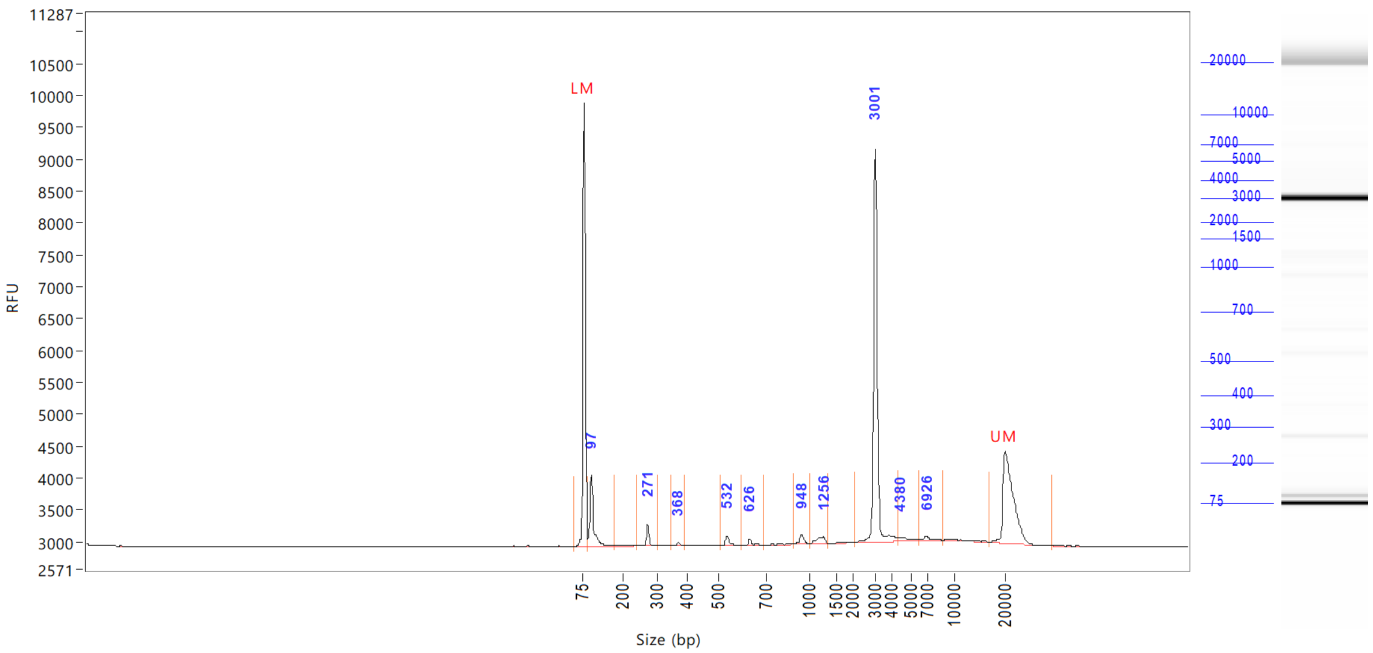

Peak	Size (bp)	Concentration (ng/uL)	From (bp)	To (bp)	Average size (bp)	CV%	RFU	Corrected peak area
1	75 (LM)	0.5000	45	86	73	4.77	6965	37.436
2	97	1.5565	86	173	101	13.14	1116	9.712
3	271	0.2991	237	300	270	2.45	345	1.866
4	368	0.0625	347	392	366	2.15	57	0.390
5	532	0.1872	504	590	534	2.34	156	1.168
6	626	0.1426	590	687	634	2.53	112	0.889
7	948	0.1844	896	1012	944	2.05	147	1.151
8	1256	0.2417	1012	1334	1176	5.93	111	1.508
9	3001	5.6685	2087	4311	3043	7.19	6153	35.368
10	4380	0.1630	4311	6112	4826	9.32	62	1.017
11	6926	0.1142	6112	8667	7070	8.08	72	0.713
12	20000 (UM)	0.2561	16952	29551	20901	5.97	1455	19.175

TIC:	8.6198	ng/uL
TIM:	32.1660	nmole/L
Total concentration:	8.9778	ng/uL

```
Sample peak width (sec): 5    Sample min peak height: 50    Sample baseline V to V?: Y    Sample baseline V to V points: 3
Sample filter: Binomial       Number of points for filter: 3  Sample start region (min): 0  Sample end region (min): 30
Marker peak width (sec): 5    Marker min peak height: 200   Marker baseline V to V?: Y    Marker baseline V to V points: 3
Lower marker selection: First peak > 500 RFU                Upper marker selection: Last peak > 200 RFU
Ladder size (bp)75, 200, 300, 400, 500, 700, 1000, 1500, 2000, 3000, 4000, 5000, 7000, 10000, 20000
Quantification using: Lower Marker          Final concentration (ng/uL): 0.5000          Dilution factor: 12.0
```

Sample: SampF4
Well location: F4
Created: Wednesday, October 20, 2021 4:38:54 PM

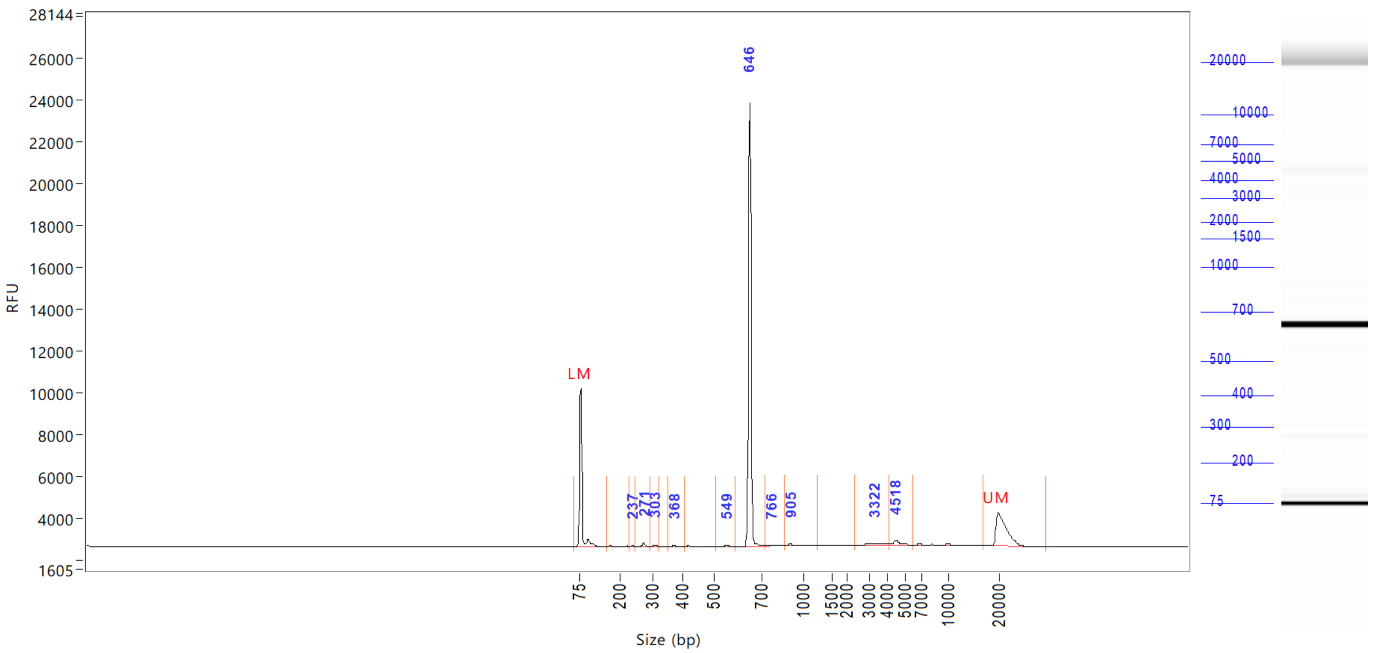

Peak	Size (bp)	Concentration (ng/uL)	From (bp)	To (bp)	Average size (bp)	CV%	RFU	Corrected peak area
1	75 (LM)	0.5000	56	158	75	12.87	7529	44.200
2	237	0.0378	228	246	236	1.70	51	0.278
3	271	0.1428	246	291	267	2.46	174	1.052
4	303	0.0842	291	319	303	1.52	93	0.621
5	368	0.0454	349	403	367	2.00	61	0.335
6	549	0.0654	506	583	546	2.42	53	0.482
7	646	13.7329	583	718	645	1.02	21197	101.165
8	766	0.1360	718	861	788	5.05	51	1.002
9	905	0.1788	861	1234	940	7.89	109	1.317
10	3322	0.1807	2286	4139	3277	13.70	62	1.331
11	4518	0.2466	4139	5963	4597	6.20	205	1.817
12	20000 (UM)	0.2372	17073	29672	20906	5.88	1602	20.968

TIC:	14.8507	ng/uL
TIM:	37.8164	nmole/L
Total concentration:	15.2349	ng/uL

```
Sample peak width (sec): 5    Sample min peak height: 50    Sample baseline V to V?: Y    Sample baseline V to V points: 3
Sample filter: Binomial       Number of points for filter: 3  Sample start region (min): 0  Sample end region (min): 30
Marker peak width (sec): 5    Marker min peak height: 200   Marker baseline V to V?: Y    Marker baseline V to V points: 3
Lower marker selection: First peak > 500 RFU                Upper marker selection: Last peak > 200 RFU
Ladder size (bp)75, 200, 300, 400, 500, 700, 1000, 1500, 2000, 3000, 4000, 5000, 7000, 10000, 20000
Quantification using: Lower Marker          Final concentration (ng/uL): 0.5000          Dilution factor: 12.0
```

Sample: SampF5
Well location: F5
Created: Wednesday, October 20, 2021 4:38:54 PM

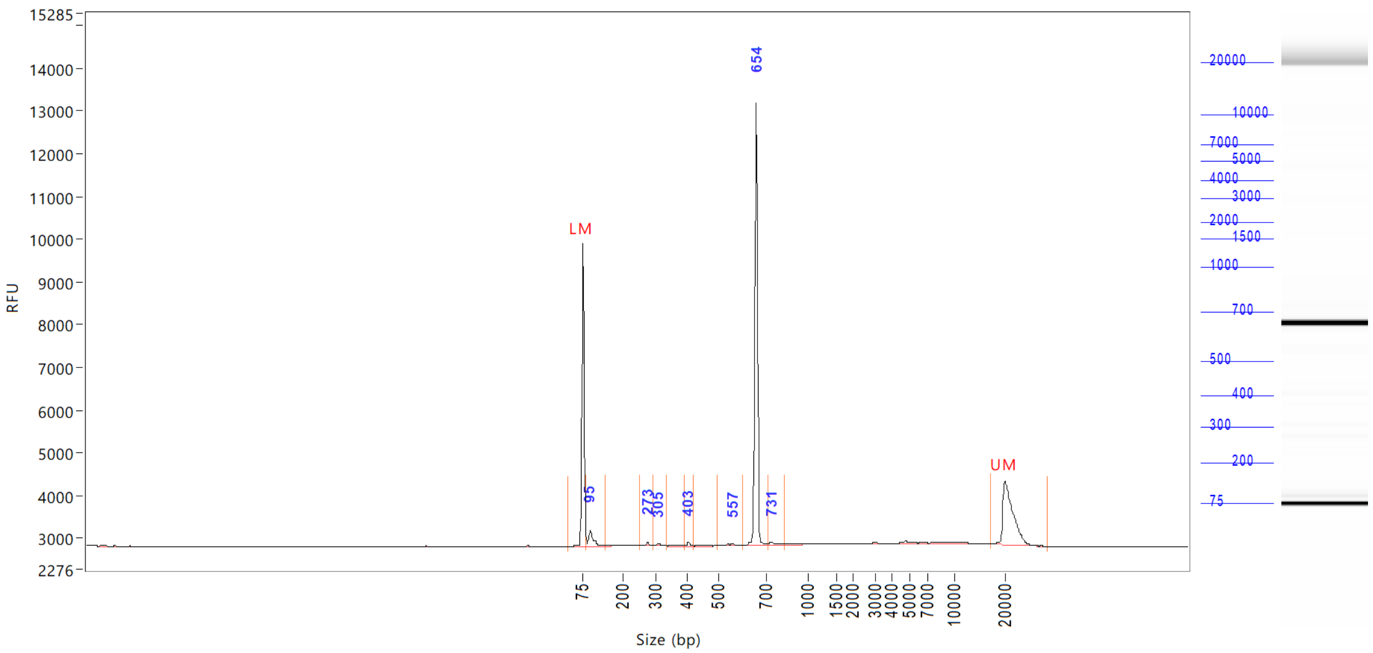

Peak	Size (bp)	Concentration (ng/uL)	From (bp)	To (bp)	Average size (bp)	CV%	RFU	Corrected peak area
1	75 (LM)	0.5000	31	86	72	4.92	7072	38.581
2	95	0.6310	86	148	100	10.66	384	4.058
3	273	0.1070	248	293	270	2.95	104	0.688
4	305	0.0677	293	333	304	2.05	57	0.435
5	403	0.0713	390	422	402	1.07	88	0.459
6	557	0.1080	494	600	545	3.43	50	0.694
7	654	7.9859	600	705	653	1.11	10378	51.350
8	731	0.1634	705	831	756	4.79	78	1.051
9	20000 (UM)	0.2512	17317	28463	20885	5.77	1493	19.384

TIC:	9.1344	ng/uL
TIM:	32.4804	nmole/L
Total concentration:	9.6487	ng/uL

Sample peak width (sec): 5 Sample min peak height: 50 Sample baseline V to V?: Y Sample baseline V to V points: 3
Sample filter: Binomial Number of points for filter: 3 Sample start region (min): 0 Sample end region (min): 30
Marker peak width (sec): 5 Marker min peak height: 200 Marker baseline V to V?: Y Marker baseline V to V points: 3
Lower marker selection: First peak > 500 RFU Upper marker selection: Last peak > 200 RFU
Ladder size (bp)75, 200, 300, 400, 500, 700, 1000, 1500, 2000, 3000, 4000, 5000, 7000, 10000, 20000
Quantification using: Lower Marker Final concentration (ng/uL): 0.5000 Dilution factor: 12.0

Sample: SampF6
Well location: F6
Created: Wednesday, October 20, 2021 4:38:54 PM

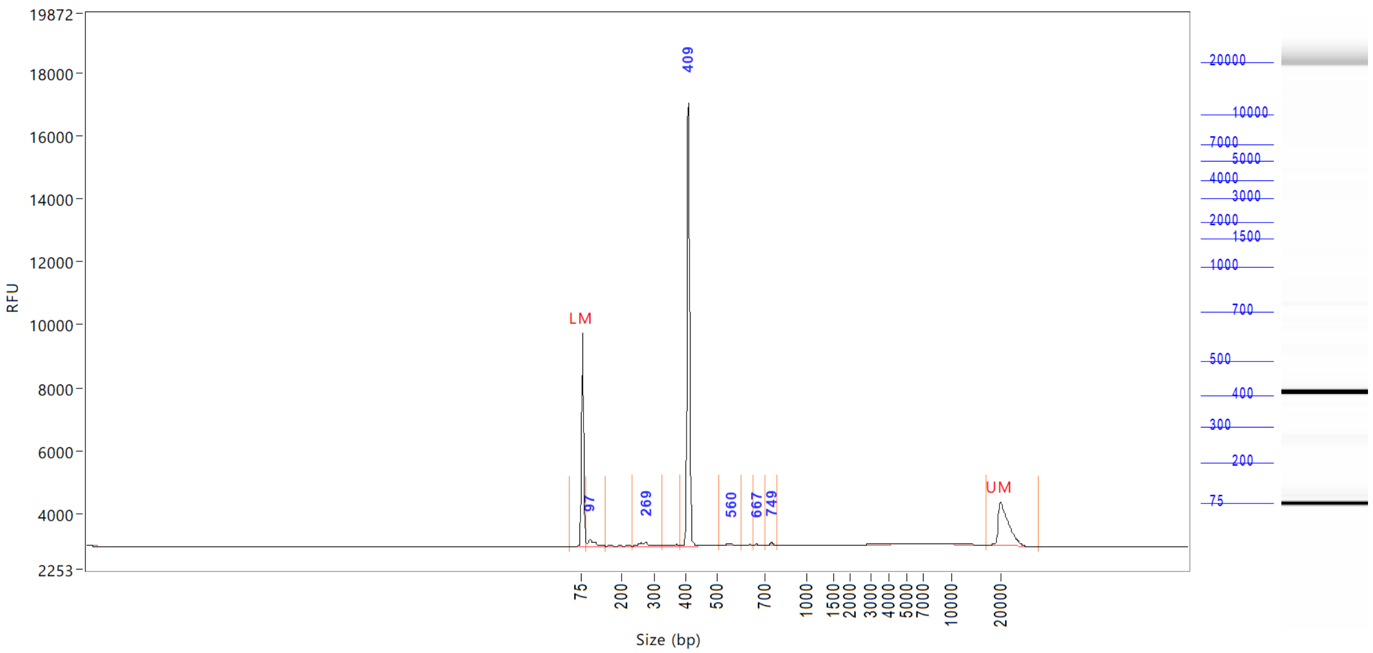

Peak	Size (bp)	Concentration (ng/uL)	From (bp)	To (bp)	Average size (bp)	CV%	RFU	Corrected peak area
1	75 (LM)	0.5000	37	87	73	4.71	6757	35.769
2	97	0.5209	87	148	103	11.49	244	3.105
3	269	0.3466	232	321	264	5.90	136	2.066
4	409	11.4018	382	504	406	1.34	14066	67.972
5	560	0.1433	504	603	547	2.91	71	0.854
6	667	0.0649	654	701	667	1.45	57	0.387
7	749	0.1196	701	788	745	1.87	125	0.713
8	20000 (UM)	0.2461	17073	27738	20855	5.76	1369	17.607

TIC:	12.5970	ng/uL
TIM:	57.5254	nmole/L
Total concentration:	13.3299	ng/uL

Sample peak width (sec): 5   Sample min peak height: 50   Sample baseline V to V?: Y   Sample baseline V to V points: 3
Sample filter: Binomial   Number of points for filter: 3 Sample start region (min): 0 Sample end region (min): 30
Marker peak width (sec): 5   Marker min peak height: 200   Marker baseline V to V?: Y   Marker baseline V to V points: 3
Lower marker selection: First peak > 500 RFU   Upper marker selection: Last peak > 200 RFU
Ladder size (bp)75, 200, 300, 400, 500, 700, 1000, 1500, 2000, 3000, 4000, 5000, 7000, 10000, 20000
Quantification using: Lower Marker   Final concentration (ng/uL): 0.5000   Dilution factor: 12.0

Sample: SampF7
Well location: F7
Created: Wednesday, October 20, 2021 4:38:54 PM

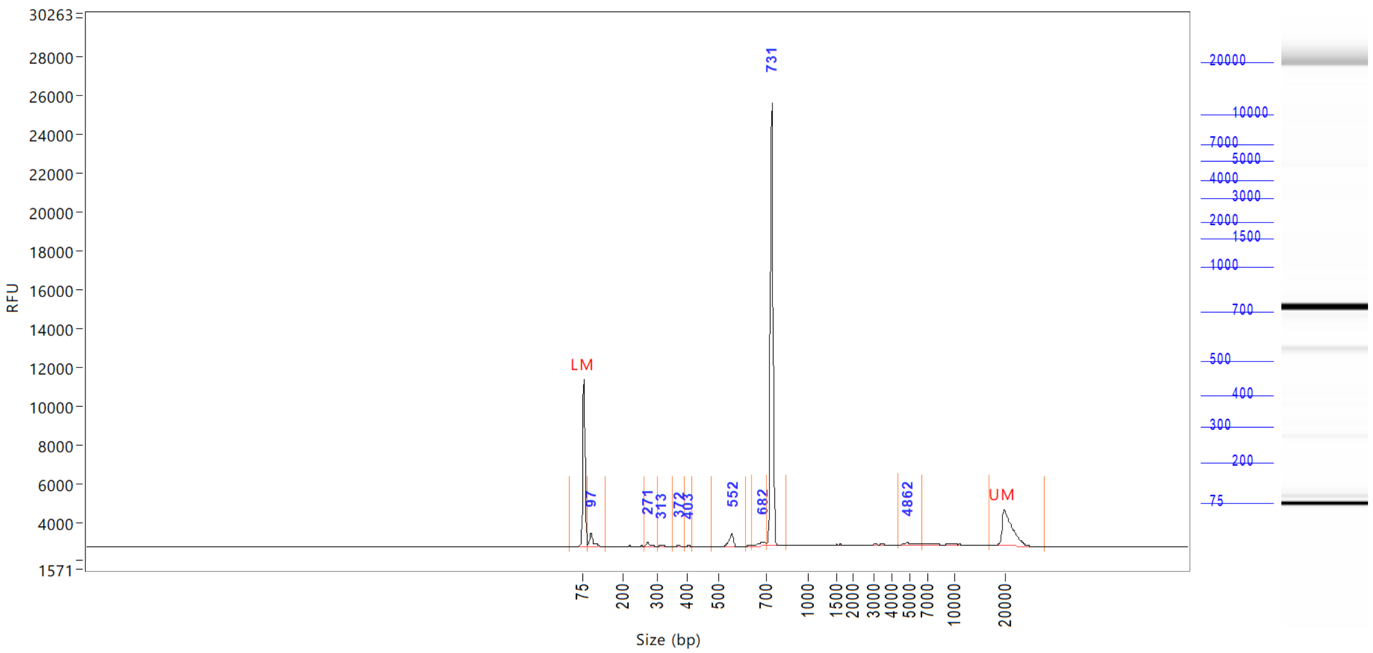

Peak	Size (bp)	Concentration (ng/uL)	From (bp)	To (bp)	Average size (bp)	CV%	RFU	Corrected peak area
1	75 (LM)	0.5000	33	86	73	4.68	8654	45.384
2	97	0.8478	86	145	99	9.52	669	6.413
3	271	0.2162	262	300	273	2.70	247	1.635
4	313	0.0817	300	352	317	3.72	60	0.618
5	372	0.0748	352	392	369	1.62	95	0.566
6	403	0.0439	392	416	402	1.14	58	0.332
7	552	0.6765	477	611	544	2.13	687	5.117
8	682	0.3659	641	700	678	2.08	227	2.768
9	731	14.5684	700	835	727	1.42	22899	110.196
10	4862	0.1380	4311	6408	5080	9.38	102	1.044
11	20000 (UM)	0.2633	16952	27979	20874	5.86	1850	23.897

TIC:	17.0132	ng/uL
TIM:	52.2601	nmole/L
Total concentration:	17.5790	ng/uL

```
Sample peak width (sec): 5    Sample min peak height: 50    Sample baseline V to V?: Y    Sample baseline V to V points: 3
Sample filter: Binomial       Number of points for filter: 3  Sample start region (min): 0  Sample end region (min): 30
Marker peak width (sec): 5    Marker min peak height: 200   Marker baseline V to V?: Y    Marker baseline V to V points: 3
Lower marker selection: First peak > 500 RFU                Upper marker selection: Last peak > 200 RFU
Ladder size (bp)75, 200, 300, 400, 500, 700, 1000, 1500, 2000, 3000, 4000, 5000, 7000, 10000, 20000
Quantification using: Lower Marker          Final concentration (ng/uL): 0.5000         Dilution factor: 12.0
```

Sample: SampF8
Well location: F8
Created: Wednesday, October 20, 2021 4:38:54 PM

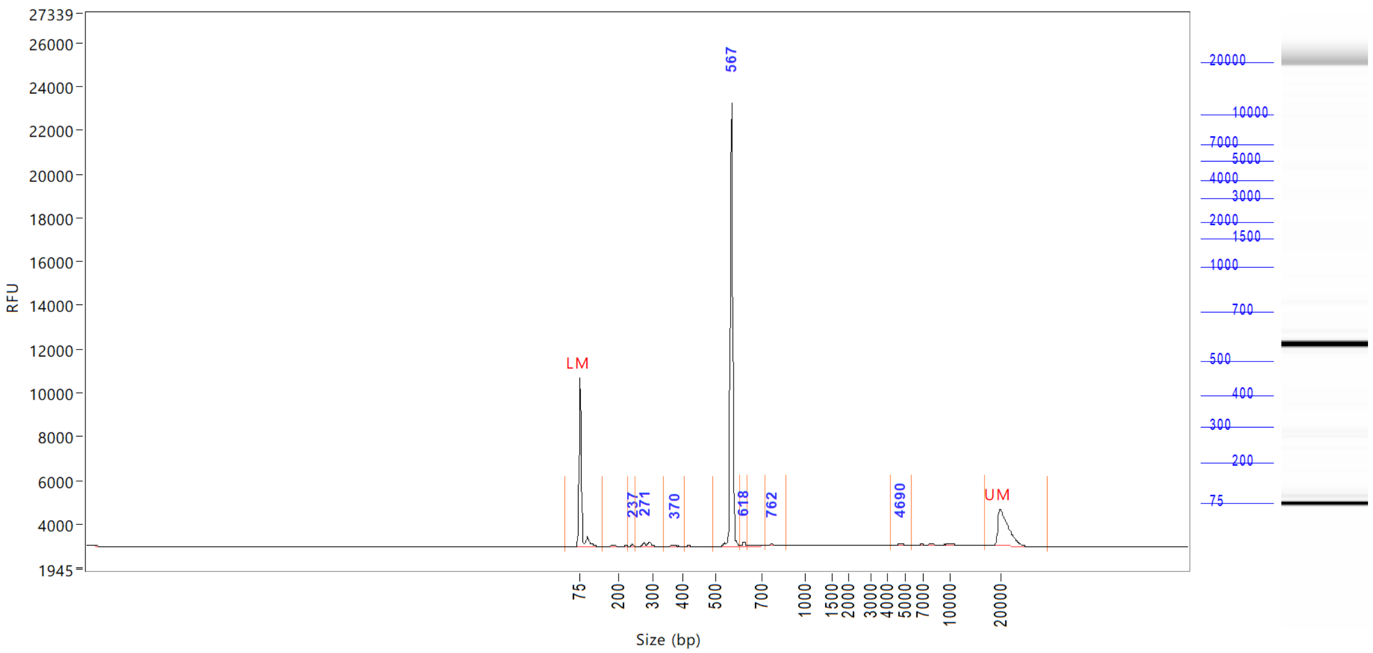

Peak	Size (bp)	Concentration (ng/uL)	From (bp)	To (bp)	Average size (bp)	CV%	RFU	Corrected peak area
1	75 (LM)	0.5000	31	147	75	12.42	7711	45.990
2	237	0.0581	224	248	235	1.64	86	0.445
3	271	0.3274	248	337	280	4.45	174	2.510
4	370	0.0757	337	403	368	3.79	51	0.580
5	567	12.7107	494	606	565	1.17	20284	97.428
6	618	0.1313	606	636	618	1.04	182	1.007
7	762	0.0943	714	866	767	2.98	102	0.723
8	4690	0.0626	4173	5593	4714	4.60	60	0.480
9	20000 (UM)	0.2389	16952	29551	20916	5.83	1694	21.976

TIC:	13.4600	ng/uL
TIM:	40.2626	nmole/L
Total concentration:	14.0121	ng/uL

Sample peak width (sec): 5   Sample min peak height: 50   Sample baseline V to V?: Y   Sample baseline V to V points: 3
Sample filter: Binomial   Number of points for filter: 3 Sample start region (min): 0 Sample end region (min): 30
Marker peak width (sec): 5   Marker min peak height: 200   Marker baseline V to V?: Y   Marker baseline V to V points: 3
Lower marker selection: First peak > 500 RFU   Upper marker selection: Last peak > 200 RFU
Ladder size (bp)75, 200, 300, 400, 500, 700, 1000, 1500, 2000, 3000, 4000, 5000, 7000, 10000, 20000
Quantification using: Lower Marker   Final concentration (ng/uL): 0.5000   Dilution factor: 12.0

Sample: SampF9
Well location: F9
Created: Wednesday, October 20, 2021 4:38:54 PM

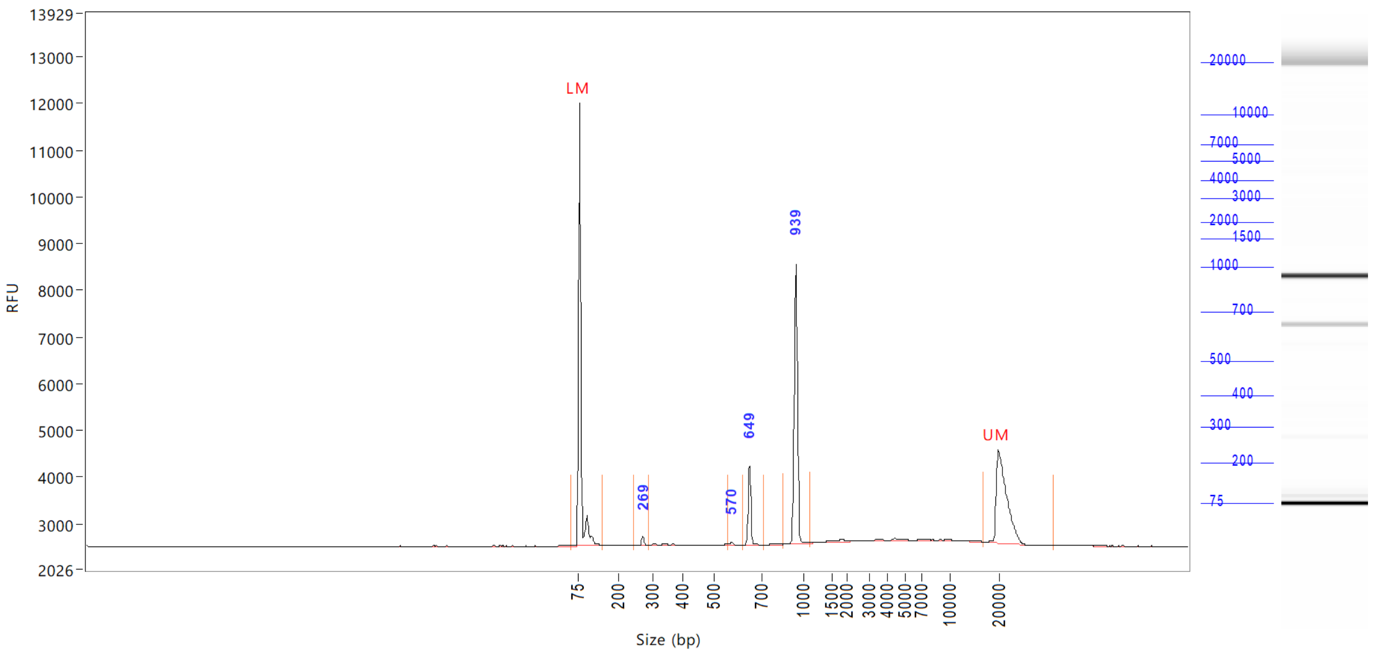

Peak	Size (bp)	Concentration (ng/uL)	From (bp)	To (bp)	Average size (bp)	CV%	RFU	Corrected peak area
1	75 (LM)	0.5000	48	148	76	13.72	9502	56.562
2	269	0.1167	242	289	268	2.25	198	1.100
3	570	0.0504	557	618	570	1.48	78	0.476
4	649	0.8988	618	714	646	1.03	1701	8.473
5	939	3.4179	848	1112	938	1.43	5992	32.221
6	20000 (UM)	0.2320	16830	31002	20850	5.92	2000	26.240

TIC:	4.4839	ng/uL
TIM:	9.1490	nmole/L
Total concentration:	5.1449	ng/uL

Sample peak width (sec): 5 Sample min peak height: 50 Sample baseline V to V?: Y Sample baseline V to V points: 3
Sample filter: Binomial Number of points for filter: 3 Sample start region (min): 0 Sample end region (min): 30
Marker peak width (sec): 5 Marker min peak height: 200 Marker baseline V to V?: Y Marker baseline V to V points: 3
Lower marker selection: First peak > 500 RFU Upper marker selection: Last peak > 200 RFU
Ladder size (bp)75, 200, 300, 400, 500, 700, 1000, 1500, 2000, 3000, 4000, 5000, 7000, 10000, 20000
Quantification using: Lower Marker Final concentration (ng/uL): 0.5000 Dilution factor: 12.0

Sample: SampF10
Well location: F10
Created: Wednesday, October 20, 2021 4:38:54 PM

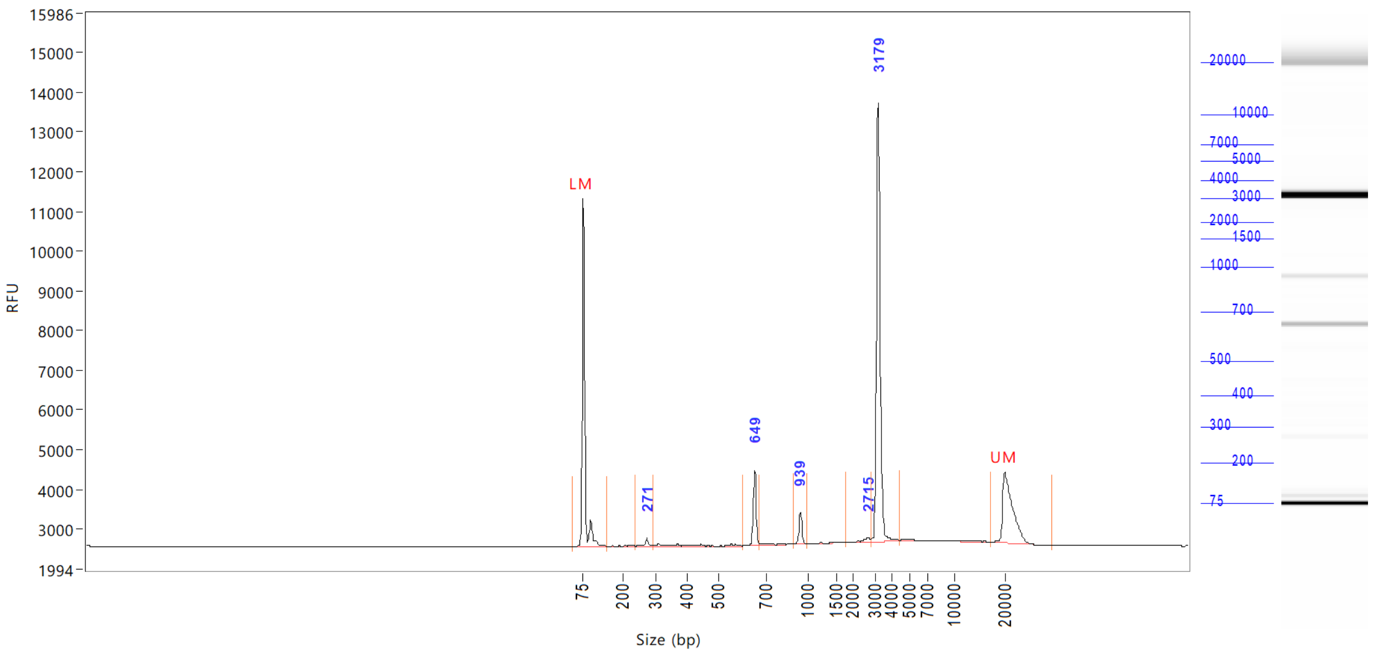

Peak	Size (bp)	Concentration (ng/uL)	From (bp)	To (bp)	Average size (bp)	CV%	RFU	Corrected peak area
1	75 (LM)	0.5000	45	150	76	12.40	8768	51.897
2	271	0.1321	237	289	268	2.44	211	1.142
3	649	1.0738	598	669	647	0.96	1890	9.288
4	939	0.4654	887	987	935	1.21	789	4.025
5	2715	0.1159	1789	2829	2560	8.48	93	1.003
6	3179	7.1299	2829	4449	3178	4.70	11061	61.670
7	20000 (UM)	0.2200	17195	29430	20822	5.68	1798	22.830

TIC:	8.9171	ng/uL
TIM:	8.1289	nmole/L
Total concentration:	9.5061	ng/uL

Sample peak width (sec): 5 Sample min peak height: 50 Sample baseline V to V?: Y Sample baseline V to V points: 3
Sample filter: Binomial Number of points for filter: 3 Sample start region (min): 0 Sample end region (min): 30
Marker peak width (sec): 5 Marker min peak height: 200 Marker baseline V to V?: Y Marker baseline V to V points: 3
Lower marker selection: First peak > 500 RFU Upper marker selection: Last peak > 200 RFU
Ladder size (bp)75, 200, 300, 400, 500, 700, 1000, 1500, 2000, 3000, 4000, 5000, 7000, 10000, 20000
Quantification using: Lower Marker Final concentration (ng/uL): 0.5000 Dilution factor: 12.0

Sample: SampF11
Well location: F11
Created: Wednesday, October 20, 2021 4:38:54 PM

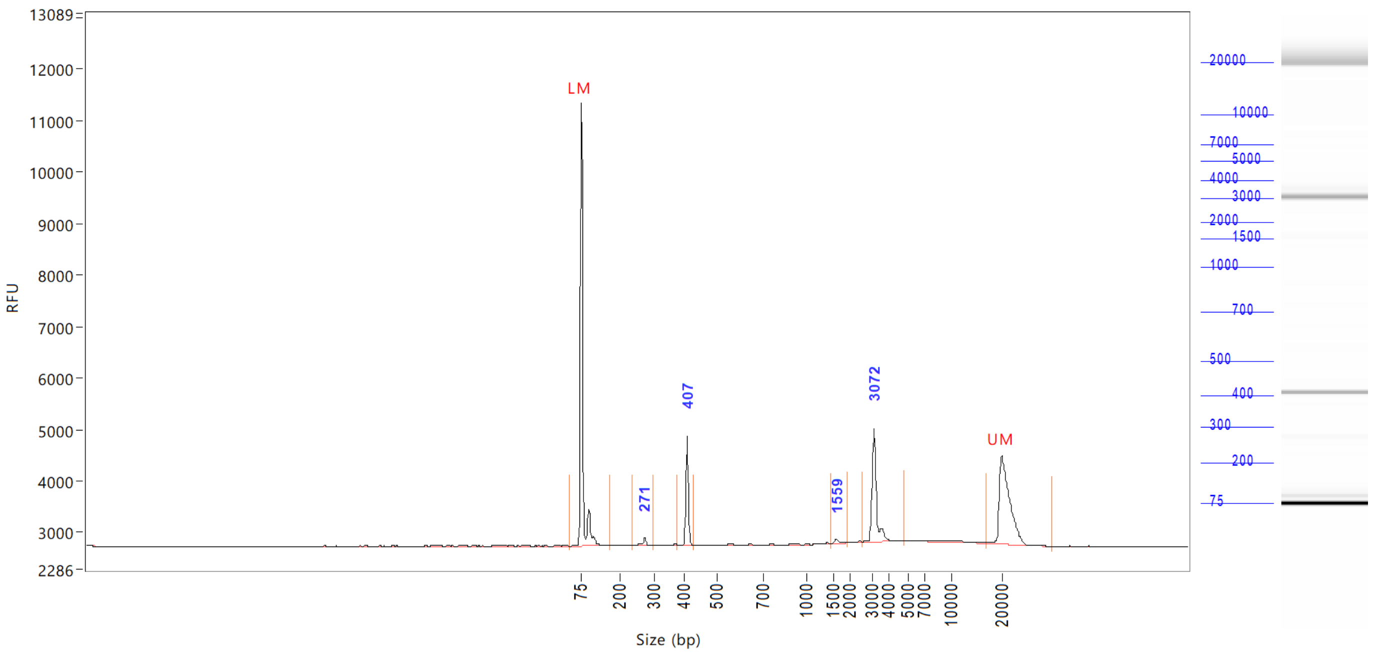

Peak	Size (bp)	Concentration (ng/uL)	From (bp)	To (bp)	Average size (bp)	CV%	RFU	Corrected peak area
1	75 (LM)	0.5000	41	164	76	13.42	8624	51.890
2	271	0.1519	233	296	265	4.22	148	1.314
3	407	1.1943	378	428	405	1.08	2133	10.329
4	1559	0.0655	1456	1904	1563	5.00	84	0.567
5	3072	1.6567	2572	4828	3142	7.65	2210	14.328
6	20000 (UM)	0.2151	16830	30035	20821	5.98	1719	22.319

TIC:	3.0685	ng/uL
TIM:	6.7318	nmole/L
Total concentration:	3.5597	ng/uL

Sample peak width (sec): 5 Sample min peak height: 50 Sample baseline V to V?: Y Sample baseline V to V points: 3
Sample filter: Binomial Number of points for filter: 3 Sample start region (min): 0 Sample end region (min): 30
Marker peak width (sec): 5 Marker min peak height: 200 Marker baseline V to V?: Y Marker baseline V to V points: 3
Lower marker selection: First peak > 500 RFU Upper marker selection: Last peak > 200 RFU
Ladder size (bp)75, 200, 300, 400, 500, 700, 1000, 1500, 2000, 3000, 4000, 5000, 7000, 10000, 20000
Quantification using: Lower Marker Final concentration (ng/uL): 0.5000 Dilution factor: 12.0

Sample: SampF12
Well location: F12
Created: Wednesday, October 20, 2021 4:38:54 PM

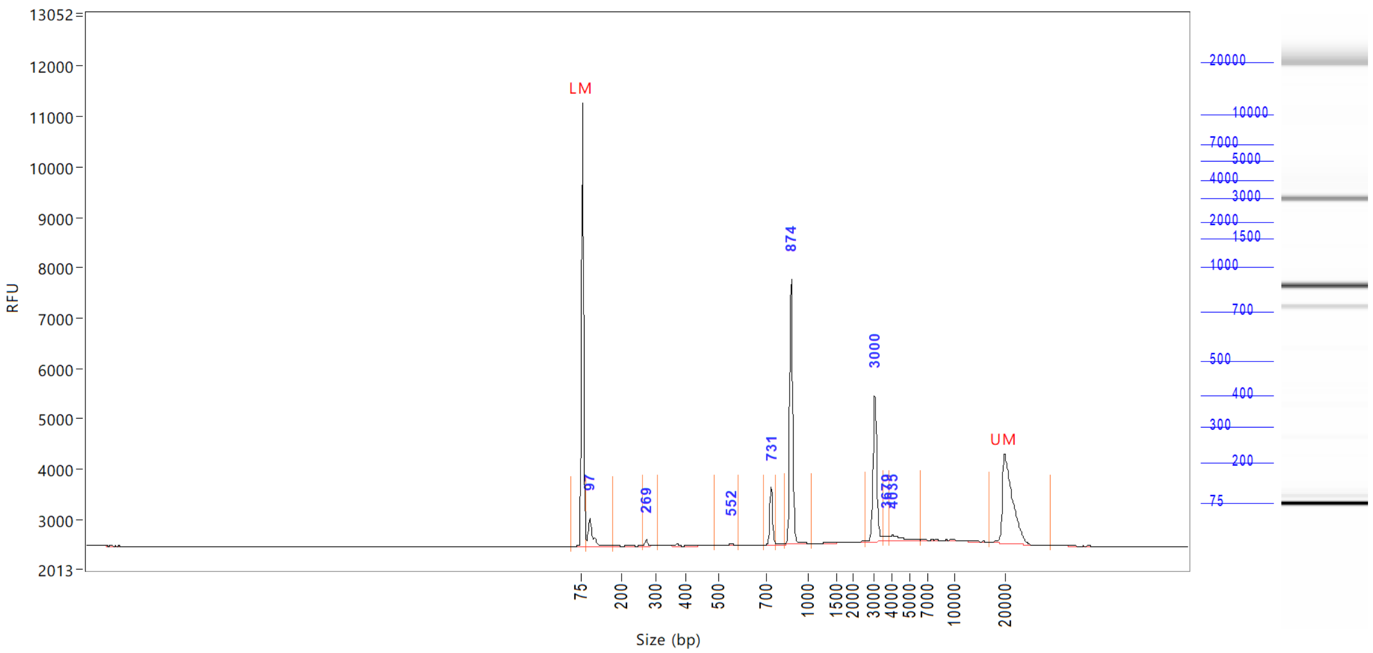

Peak	Size (bp)	Concentration (ng/uL)	From (bp)	To (bp)	Average size (bp)	CV%	RFU	Corrected peak area
1	75 (LM)	0.5000	39	86	73	4.54	8821	46.456
2	97	0.7585	86	173	101	13.47	570	5.873
3	269	0.0934	259	303	270	2.86	122	0.723
4	552	0.0475	489	580	545	2.57	51	0.368
5	731	0.7435	690	770	727	1.39	1145	5.756
6	874	3.5752	831	1056	873	1.81	5258	27.682
7	3000	2.1551	2600	3465	2997	3.44	2883	16.687
8	3679	0.1195	3465	3893	3678	3.55	109	0.926
9	4035	0.2591	3893	6186	4419	10.36	110	2.006
10	20000 (UM)	0.2461	16830	29068	20782	5.79	1770	22.862

TIC:	7.7518	ng/uL
TIM:	22.8012	nmole/L
Total concentration:	8.0947	ng/uL

```
Sample peak width (sec): 5    Sample min peak height: 50    Sample baseline V to V?: Y    Sample baseline V to V points: 3
Sample filter: Binomial       Number of points for filter: 3  Sample start region (min): 0  Sample end region (min): 30
Marker peak width (sec): 5    Marker min peak height: 200   Marker baseline V to V?: Y    Marker baseline V to V points: 3
Lower marker selection: First peak > 500 RFU                Upper marker selection: Last peak > 200 RFU
Ladder size (bp)75, 200, 300, 400, 500, 700, 1000, 1500, 2000, 3000, 4000, 5000, 7000, 10000, 20000
Quantification using: Lower Marker          Final concentration (ng/uL): 0.5000          Dilution factor: 12.0
```

Sample: SampG1
Well location: G1
Created: Wednesday, October 20, 2021 4:38:54 PM

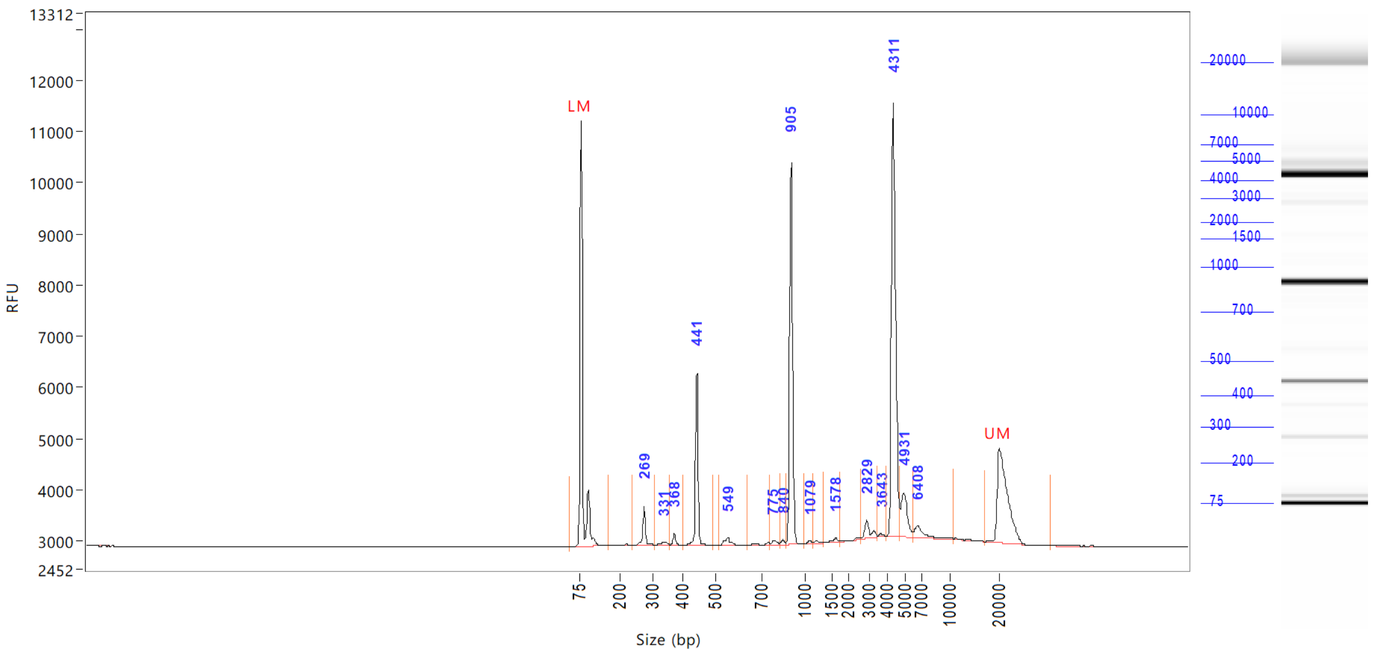

Peak	Size (bp)	Concentration (ng/uL)	From (bp)	To (bp)	Average size (bp)	CV%	RFU	Corrected peak area
1	75 (LM)	0.5000	43	162	78	13.40	8314	53.473
2	269	0.5358	237	305	269	3.23	777	4.775
3	331	0.1080	305	352	330	3.33	66	0.962
4	368	0.1659	352	400	367	2.28	247	1.479
5	441	1.9487	400	494	438	1.46	3355	17.367
6	549	0.1905	514	636	551	3.62	151	1.698
7	775	0.0863	753	818	782	2.24	80	0.770
8	840	0.0606	818	861	838	1.37	91	0.540
9	905	4.2553	861	991	898	1.28	7472	37.924
10	1079	0.0462	991	1134	1071	3.25	65	0.412
11	1578	0.0635	1356	1731	1545	4.61	75	0.565
12	2829	0.3185	2600	3429	2915	6.27	363	2.838
13	3643	0.0409	3429	3964	3631	3.47	58	0.364
14	4311	5.7832	3964	4690	4303	2.84	8485	51.541
15	4931	0.8836	4690	5815	4971	4.72	866	7.874
16	6408	0.3726	5815	10733	7102	16.55	233	3.320
17	20000 (UM)	0.2235	16952	30277	20905	5.80	1839	23.898

TIC:	14.8594	ng/uL
TIM:	23.4758	nmole/L
Total concentration:	15.0157	ng/uL

```
Sample peak width (sec): 5     Sample min peak height: 50    Sample baseline V to V?: Y    Sample baseline V to V points: 3
Sample filter: Binomial        Number of points for filter: 3  Sample start region (min): 0  Sample end region (min): 30
Marker peak width (sec): 5     Marker min peak height: 200   Marker baseline V to V?: Y    Marker baseline V to V points: 3
Lower marker selection: First peak > 500 RFU                 Upper marker selection: Last peak > 200 RFU
Ladder size (bp)75, 200, 300, 400, 500, 700, 1000, 1500, 2000, 3000, 4000, 5000, 7000, 10000, 20000
Quantification using: Lower Marker          Final concentration (ng/uL): 0.5000          Dilution factor: 12.0
```

Sample: SampG2
Well location: G2
Created: Wednesday, October 20, 2021 4:38:54 PM

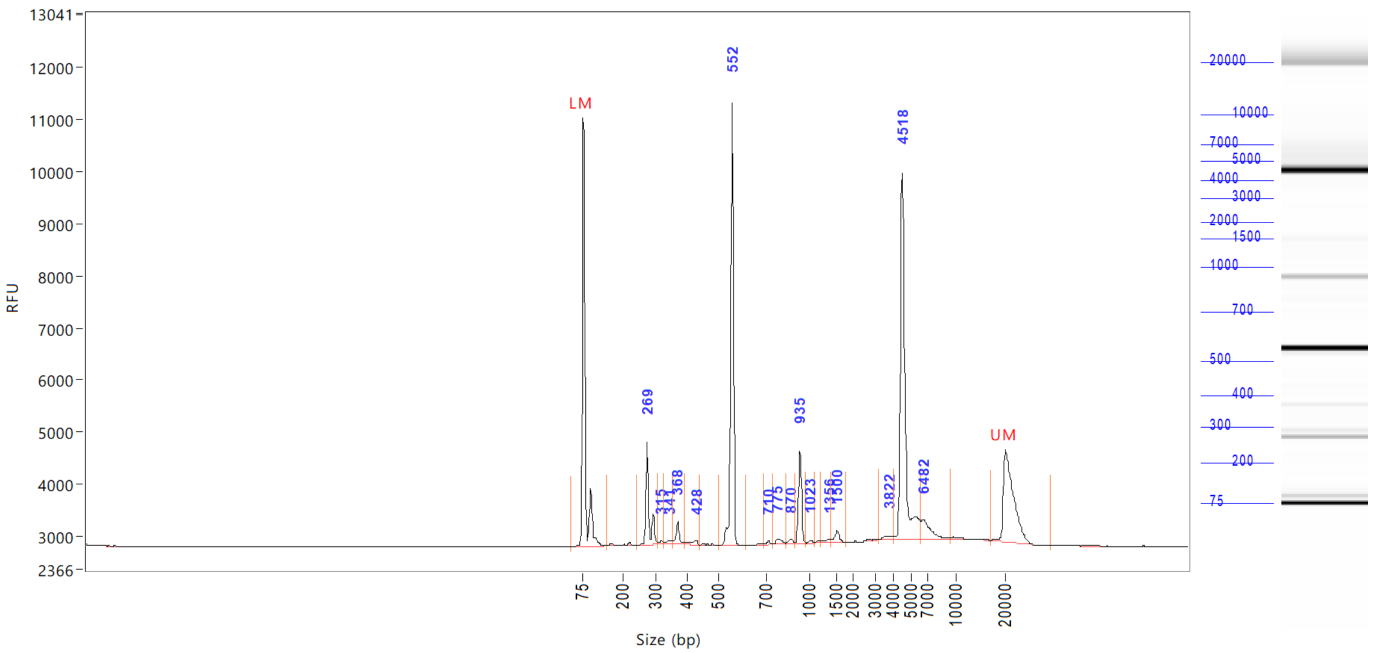

Peak	Size (bp)	Concentration (ng/uL)	From (bp)	To (bp)	Average size (bp)	CV%	RFU	Corrected peak area
1	75 (LM)	0.5000	37	150	78	13.82	8238	53.558
2	269	1.4832	241	303	273	3.54	1982	13.239
3	315	0.0558	303	323	313	1.91	53	0.498
4	341	0.0877	323	352	338	2.43	64	0.783
5	368	0.2958	352	392	367	2.01	422	2.640
6	428	0.1356	392	437	414	2.68	86	1.211
7	552	5.0285	501	608	549	1.39	8511	44.886
8	710	0.0415	690	740	706	1.19	66	0.371
9	775	0.1141	740	831	782	2.49	105	1.019
10	870	0.0799	831	900	865	1.98	92	0.713
11	935	1.0686	900	978	932	1.15	1785	9.539
12	1023	0.0384	978	1090	1016	2.25	58	0.342
13	1356	0.0833	1212	1400	1317	4.04	74	0.743
14	1500	0.1899	1400	1789	1493	3.73	228	1.695
15	3822	0.1182	3144	4035	3701	5.84	81	1.055
16	4518	5.5219	4035	6186	4641	7.79	7034	49.290
17	6482	0.6021	6186	9400	7102	10.91	379	5.375
18	20000 (UM)	0.2145	17195	29068	20853	5.79	1773	22.974

TIC:	14.9447	ng/uL
TIM:	31.4967	nmole/L
Total concentration:	15.1871	ng/uL

```
Sample peak width (sec): 5     Sample min peak height: 50     Sample baseline V to V?: Y     Sample baseline V to V points: 3
Sample filter: Binomial        Number of points for filter: 3  Sample start region (min): 0  Sample end region (min): 30
Marker peak width (sec): 5     Marker min peak height: 200    Marker baseline V to V?: Y     Marker baseline V to V points: 3
Lower marker selection: First peak > 500 RFU                  Upper marker selection: Last peak > 200 RFU
Ladder size (bp)75, 200, 300, 400, 500, 700, 1000, 1500, 2000, 3000, 4000, 5000, 7000, 10000, 20000
Quantification using: Lower Marker          Final concentration (ng/uL): 0.5000          Dilution factor: 12.0
```

Sample: SampG3
Well location: G3
Created: Wednesday, October 20, 2021 4:38:54 PM

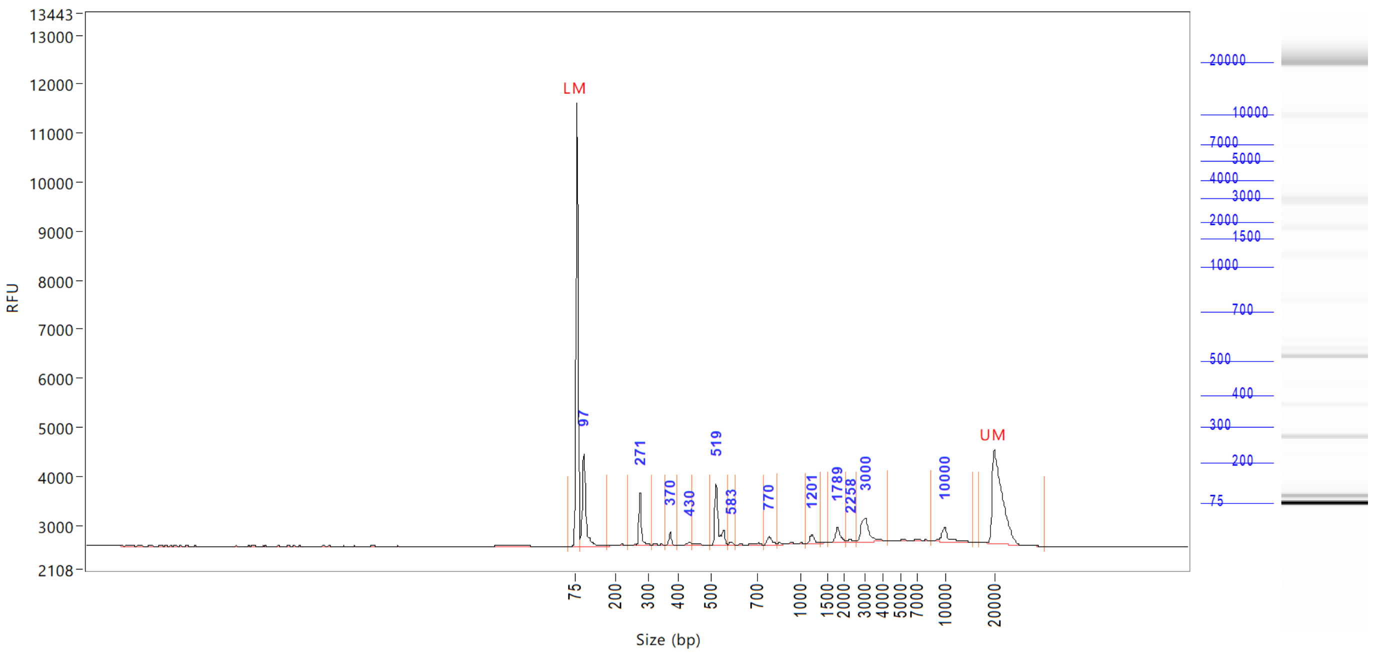

Peak	Size (bp)	Concentration (ng/uL)	From (bp)	To (bp)	Average size (bp)	CV%	RFU	Corrected peak area
1	75 (LM)	0.5000	48	86	74	4.05	9054	48.956
2	97	1.8639	86	171	99	11.77	1888	15.208
3	271	0.7707	235	307	270	2.93	1086	6.288
4	370	0.1728	352	396	367	1.39	272	1.410
5	430	0.0714	396	441	427	1.84	70	0.583
6	519	1.0404	498	570	526	2.79	1252	8.489
7	583	0.0660	570	606	582	1.12	75	0.538
8	770	0.1776	736	835	781	2.78	172	1.449
9	1201	0.1641	1101	1367	1203	3.52	179	1.339
10	1789	0.3037	1501	2087	1810	5.35	301	2.478
11	2258	0.0599	2087	2572	2282	5.57	54	0.489
12	3000	0.7342	2572	4242	3029	9.10	486	5.990
13	10000	0.3596	8467	15732	10339	11.91	304	2.934
14	20000 (UM)	0.2521	16708	30035	20861	5.82	1903	24.686

TIC:	5.7842	ng/uL
TIM:	41.4756	nmole/L
Total concentration:	6.2531	ng/uL

```
Sample peak width (sec): 5     Sample min peak height: 50    Sample baseline V to V?: Y    Sample baseline V to V points: 3
Sample filter: Binomial        Number of points for filter: 3  Sample start region (min): 0  Sample end region (min): 30
Marker peak width (sec): 5     Marker min peak height: 200   Marker baseline V to V?: Y    Marker baseline V to V points: 3
Lower marker selection: First peak > 500 RFU                 Upper marker selection: Last peak > 200 RFU
Ladder size (bp)75, 200, 300, 400, 500, 700, 1000, 1500, 2000, 3000, 4000, 5000, 7000, 10000, 20000
Quantification using: Lower Marker          Final concentration (ng/uL): 0.5000          Dilution factor: 12.0
```

Sample: SampG4
Well location: G4
Created: Wednesday, October 20, 2021 4:38:54 PM

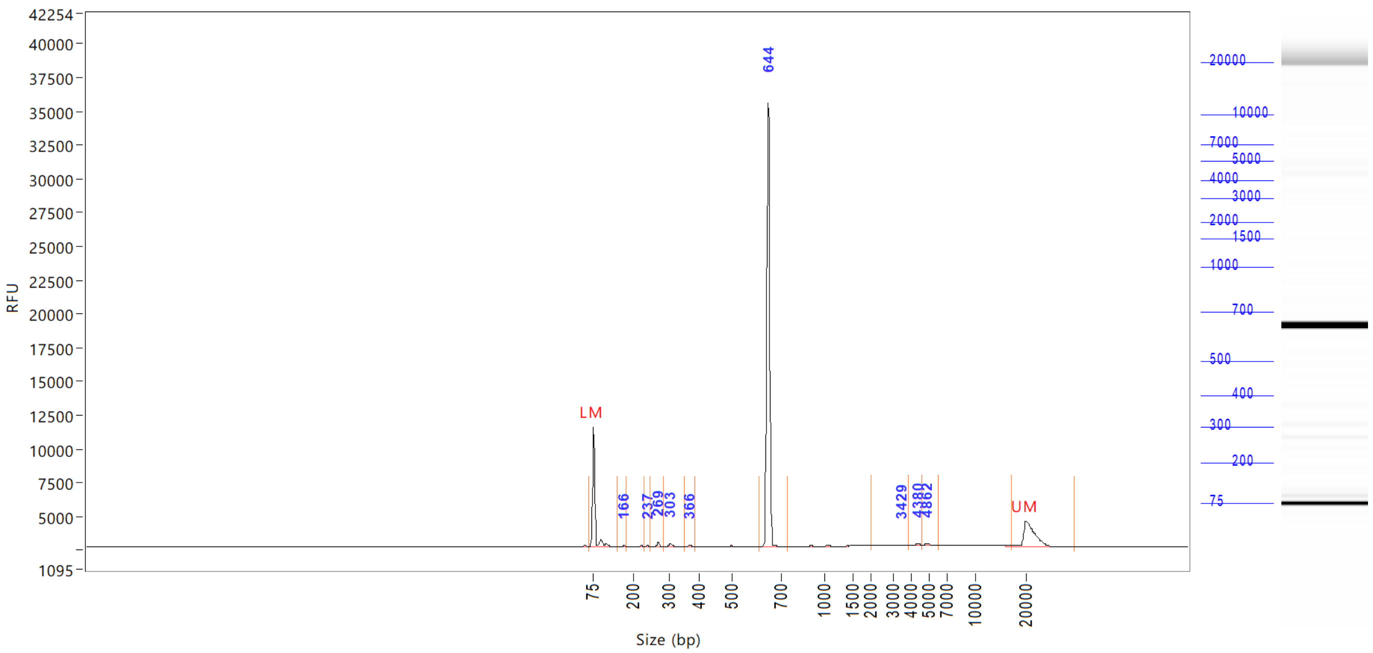

Peak	Size (bp)	Concentration (ng/uL)	From (bp)	To (bp)	Average size (bp)	CV%	RFU	Corrected peak area
1	75 (LM)	0.5000	60	150	76	13.32	8799	51.100
2	166	0.0450	150	175	164	2.69	70	0.384
3	237	0.0468	228	244	235	1.60	74	0.399
4	269	0.2095	244	284	265	2.55	294	1.784
5	303	0.1846	284	349	305	3.18	190	1.572
6	366	0.0771	349	382	364	1.22	125	0.657
7	644	18.6561	611	736	642	0.89	32889	158.888
8	3429	0.1127	2030	3857	3114	13.61	57	0.960
9	4380	0.1335	3857	4621	4332	3.62	161	1.137
10	4862	0.1299	4621	6038	4906	4.20	122	1.106
11	20000 (UM)	0.2386	17195	29309	20858	5.96	1883	24.388

TIC:	19.5953	ng/uL
TIM:	51.4006	nmole/L
Total concentration:	20.1164	ng/uL

```
Sample peak width (sec): 5     Sample min peak height: 50     Sample baseline V to V?: Y     Sample baseline V to V points: 3
Sample filter: Binomial        Number of points for filter: 3  Sample start region (min): 0  Sample end region (min): 30
Marker peak width (sec): 5     Marker min peak height: 200    Marker baseline V to V?: Y     Marker baseline V to V points: 3
Lower marker selection: First peak > 500 RFU                  Upper marker selection: Last peak > 200 RFU
Ladder size (bp)75, 200, 300, 400, 500, 700, 1000, 1500, 2000, 3000, 4000, 5000, 7000, 10000, 20000
Quantification using: Lower Marker          Final concentration (ng/uL): 0.5000          Dilution factor: 12.0
```

Sample: SampG5
Well location: G5
Created: Wednesday, October 20, 2021 4:38:54 PM

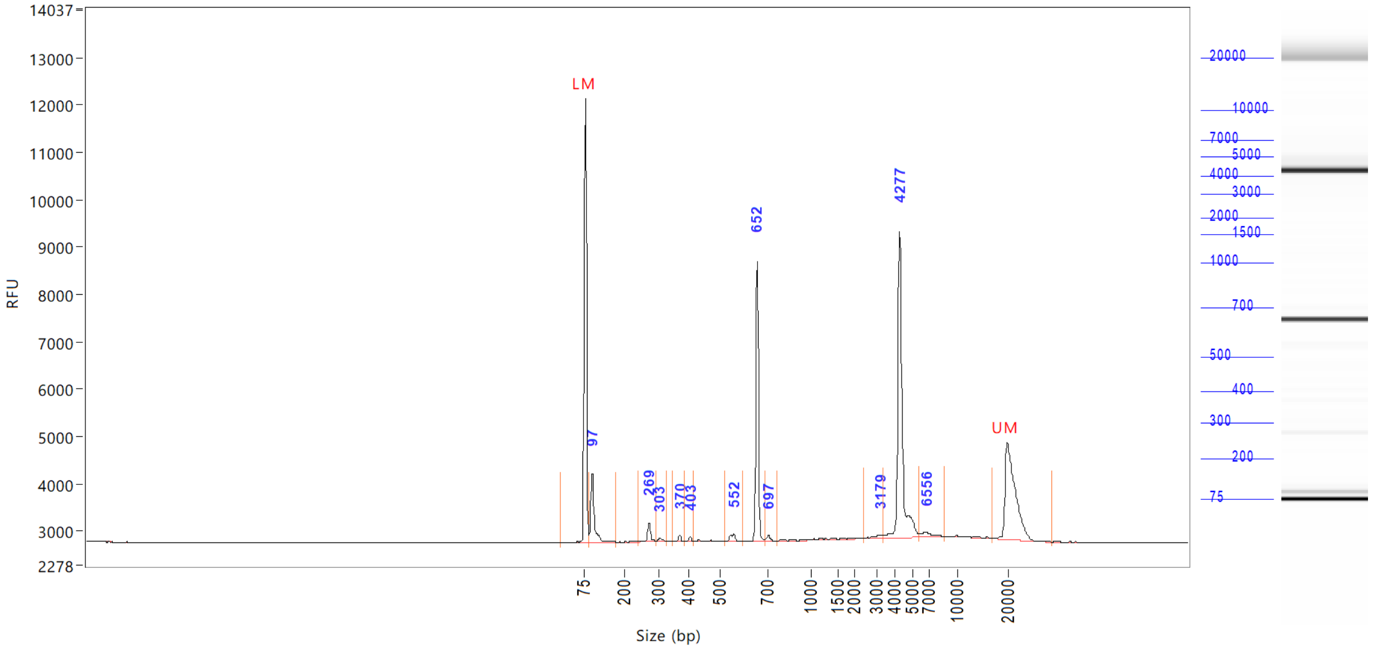

Peak	Size (bp)	Concentration (ng/uL)	From (bp)	To (bp)	Average size (bp)	CV%	RFU	Corrected peak area
1	75 (LM)	0.5000	0	86	73	5.33	9400	49.224
2	97	1.5014	86	175	98	11.74	1456	12.318
3	269	0.2951	241	295	269	2.98	404	2.421
4	303	0.0832	295	327	306	2.51	72	0.683
5	370	0.0908	347	386	365	1.58	130	0.745
6	403	0.0571	386	414	400	0.93	88	0.468
7	552	0.1766	514	593	544	1.85	157	1.449
8	652	3.3548	593	685	649	0.94	5917	27.523
9	697	0.0979	685	757	700	1.78	121	0.803
10	3179	0.1240	2401	3322	2958	8.14	73	1.017
11	4277	5.2125	3322	5815	4325	6.35	6467	42.763
12	6556	0.1915	5815	8667	6789	9.62	95	1.571
13	20000 (UM)	0.2683	16952	28947	20853	5.81	2046	26.411

TIC:	11.1848	ng/uL
TIM:	39.4229	nmole/L
Total concentration:	11.5701	ng/uL

Sample peak width (sec): 5   Sample min peak height: 50   Sample baseline V to V?: Y   Sample baseline V to V points: 3
Sample filter: Binomial   Number of points for filter: 3 Sample start region (min): 0 Sample end region (min): 30
Marker peak width (sec): 5   Marker min peak height: 200   Marker baseline V to V?: Y   Marker baseline V to V points: 3
Lower marker selection: First peak > 500 RFU   Upper marker selection: Last peak > 200 RFU
Ladder size (bp)75, 200, 300, 400, 500, 700, 1000, 1500, 2000, 3000, 4000, 5000, 7000, 10000, 20000
Quantification using: Lower Marker   Final concentration (ng/uL): 0.5000   Dilution factor: 12.0

Sample: SampG6
Well location: G6
Created: Wednesday, October 20, 2021 4:38:54 PM

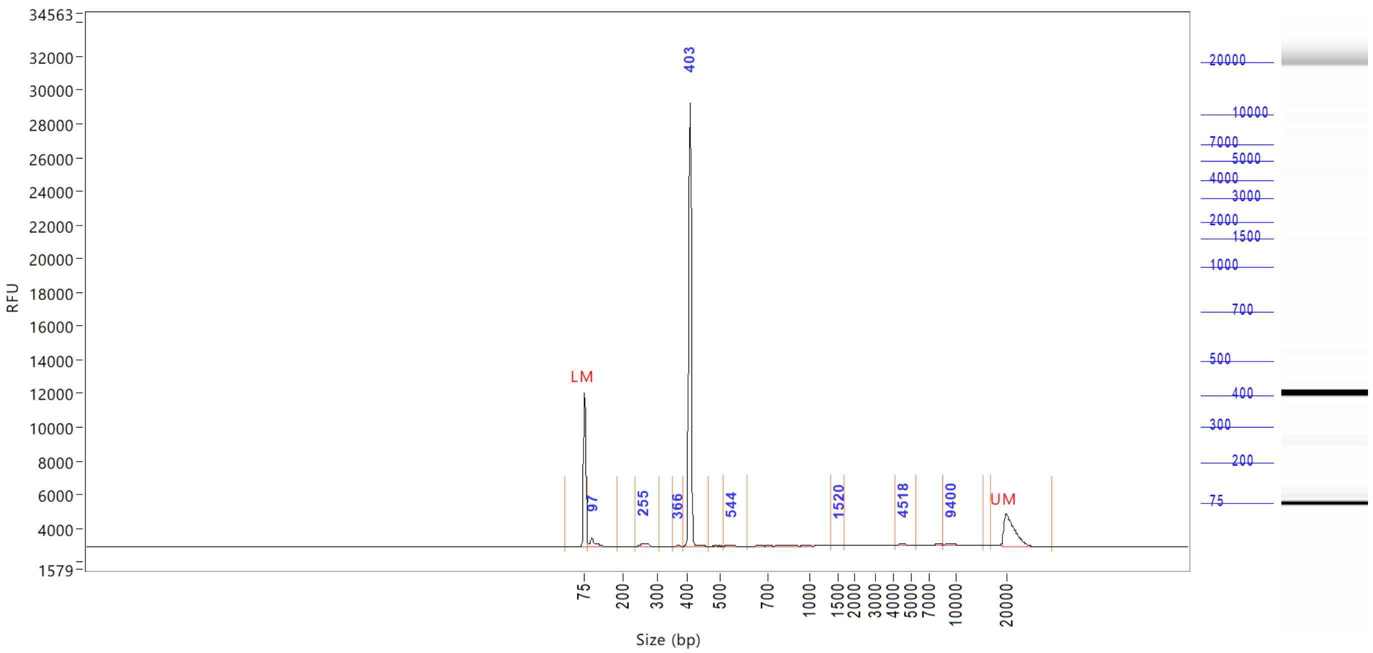

Peak	Size (bp)	Concentration (ng/uL)	From (bp)	To (bp)	Average size (bp)	CV%	RFU	Corrected peak area
1	75 (LM)	0.5000	18	86	73	5.11	9088	47.683
2	97	0.8495	86	181	105	16.83	504	6.751
3	255	0.4067	232	303	258	4.39	196	3.232
4	366	0.0578	347	382	364	1.92	69	0.459
5	403	16.0933	382	464	403	1.10	26350	127.895
6	544	0.1036	514	616	542	2.72	94	0.824
7	1520	0.0531	1389	1674	1500	3.68	56	0.422
8	4518	0.1089	4104	5519	4507	5.02	96	0.865
9	9400	0.2321	8667	15366	11143	17.74	65	1.844
10	20000 (UM)	0.2722	16952	29189	20856	6.07	1954	25.955

TIC:	17.9049	ng/uL
TIM:	82.2911	nmole/L
Total concentration:	18.4549	ng/uL

```
Sample peak width (sec): 5    Sample min peak height: 50    Sample baseline V to V?: Y    Sample baseline V to V points: 3
Sample filter: Binomial       Number of points for filter: 3  Sample start region (min): 0  Sample end region (min): 30
Marker peak width (sec): 5    Marker min peak height: 200   Marker baseline V to V?: Y    Marker baseline V to V points: 3
Lower marker selection: First peak > 500 RFU                Upper marker selection: Last peak > 200 RFU
Ladder size (bp)75, 200, 300, 400, 500, 700, 1000, 1500, 2000, 3000, 4000, 5000, 7000, 10000, 20000
Quantification using: Lower Marker          Final concentration (ng/uL): 0.5000          Dilution factor: 12.0
```

Sample: SampG7
Well location: G7
Created: Wednesday, October 20, 2021 4:38:54 PM

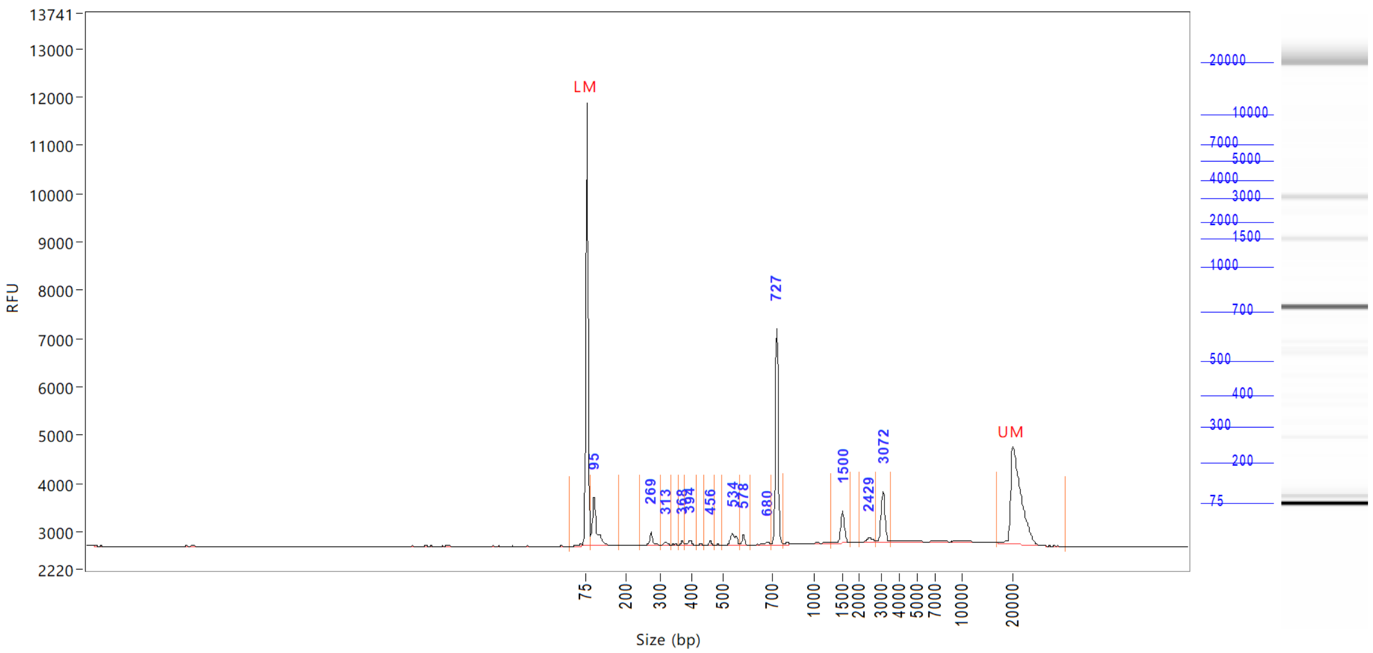

Peak	Size (bp)	Concentration (ng/uL)	From (bp)	To (bp)	Average size (bp)	CV%	RFU	Corrected peak area
1	75 (LM)	0.5000	22	86	73	5.14	9194	48.572
2	95	1.2635	86	175	100	12.38	1008	10.229
3	269	0.2226	235	300	269	3.18	290	1.802
4	313	0.0712	300	331	313	2.17	73	0.576
5	368	0.0563	358	378	367	1.16	90	0.456
6	394	0.0845	378	414	393	1.68	102	0.684
7	456	0.0598	437	471	455	1.13	87	0.484
8	534	0.3412	492	565	536	2.26	253	2.762
9	578	0.1323	565	608	577	1.04	211	1.071
10	680	0.0816	608	697	668	2.79	58	0.661
11	727	2.7610	697	779	725	1.38	4465	22.351
12	1500	0.4932	1278	1731	1482	3.55	672	3.993
13	2429	0.1161	2000	2772	2446	6.27	90	0.940
14	3072	0.7446	2772	3536	3055	3.30	1062	6.027
15	20000 (UM)	0.2649	16830	30519	20889	5.89	2016	25.731

TIC:	6.4278	ng/uL
TIM:	32.2259	nmole/L
Total concentration:	6.8302	ng/uL

```
Sample peak width (sec): 5    Sample min peak height: 50    Sample baseline V to V?: Y    Sample baseline V to V points: 3
Sample filter: Binomial       Number of points for filter: 3  Sample start region (min): 0  Sample end region (min): 30
Marker peak width (sec): 5    Marker min peak height: 200   Marker baseline V to V?: Y    Marker baseline V to V points: 3
Lower marker selection: First peak > 500 RFU                Upper marker selection: Last peak > 200 RFU
Ladder size (bp)75, 200, 300, 400, 500, 700, 1000, 1500, 2000, 3000, 4000, 5000, 7000, 10000, 20000
Quantification using: Lower Marker          Final concentration (ng/uL): 0.5000          Dilution factor: 12.0
```

Sample: SampG8
Well location: G8
Created: Wednesday, October 20, 2021 4:38:54 PM

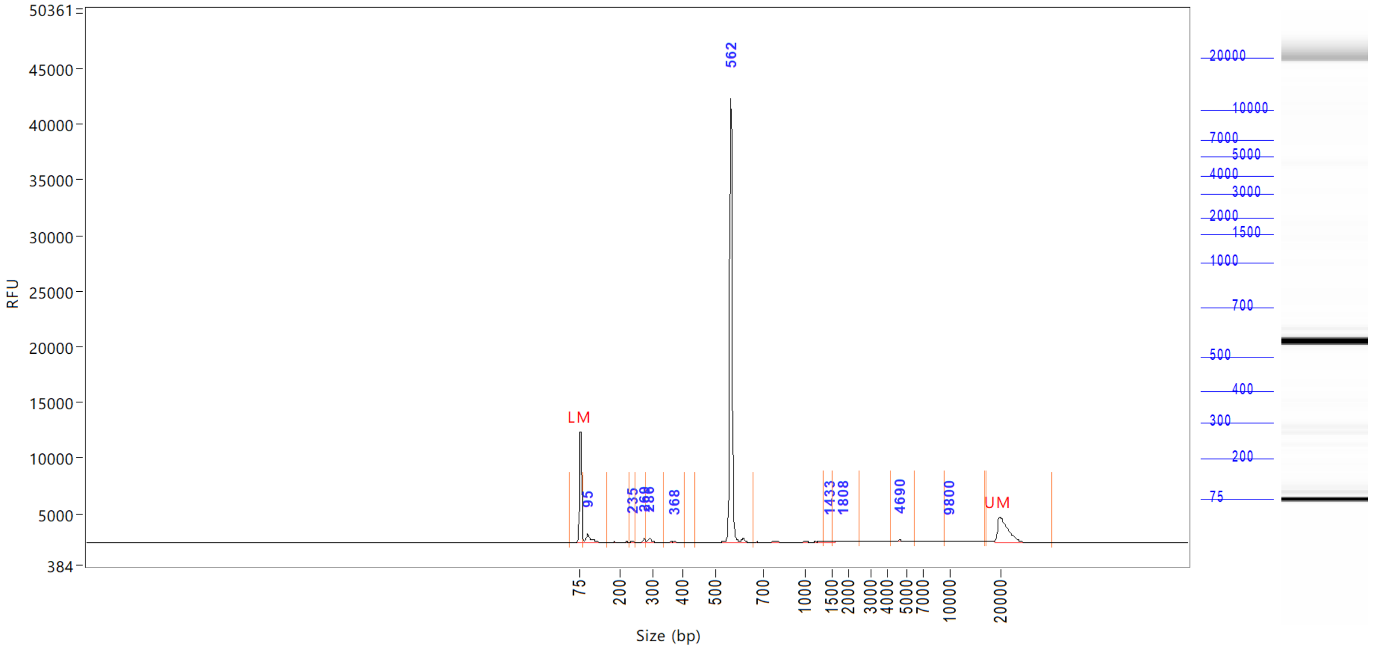

Peak	Size (bp)	Concentration (ng/uL)	From (bp)	To (bp)	Average size (bp)	CV%	RFU	Corrected peak area
1	75 (LM)	0.5000	43	84	72	4.36	9952	53.861
2	95	1.0183	84	158	101	13.28	735	9.141
3	235	0.0908	226	246	234	1.52	165	0.815
4	269	0.2215	246	277	268	2.08	360	1.988
5	286	0.3200	277	331	287	2.97	320	2.873
6	368	0.1318	331	403	364	4.07	104	1.183
7	562	21.6745	437	662	561	1.60	39933	194.568
8	1433	0.0411	1345	1500	1424	2.63	55	0.369
9	1808	0.1023	1500	2486	1804	10.13	54	0.918
10	4690	0.0977	4139	5963	4675	4.44	122	0.877
11	9800	0.1525	9400	17073	11765	16.71	58	1.369
12	20000 (UM)	0.2684	17317	30398	20892	5.81	2249	28.908

TIC:	23.8506	ng/uL
TIM:	84.7585	nmole/L
Total concentration:	24.2854	ng/uL

Sample peak width (sec): 5 Sample min peak height: 50 Sample baseline V to V?: Y Sample baseline V to V points: 3
Sample filter: Binomial Number of points for filter: 3 Sample start region (min): 0 Sample end region (min): 30
Marker peak width (sec): 5 Marker min peak height: 200 Marker baseline V to V?: Y Marker baseline V to V points: 3
Lower marker selection: First peak > 500 RFU Upper marker selection: Last peak > 200 RFU
Ladder size (bp)75, 200, 300, 400, 500, 700, 1000, 1500, 2000, 3000, 4000, 5000, 7000, 10000, 20000
Quantification using: Lower Marker Final concentration (ng/uL): 0.5000 Dilution factor: 12.0

Sample: SampG9
Well location: G9
Created: Wednesday, October 20, 2021 4:38:54 PM

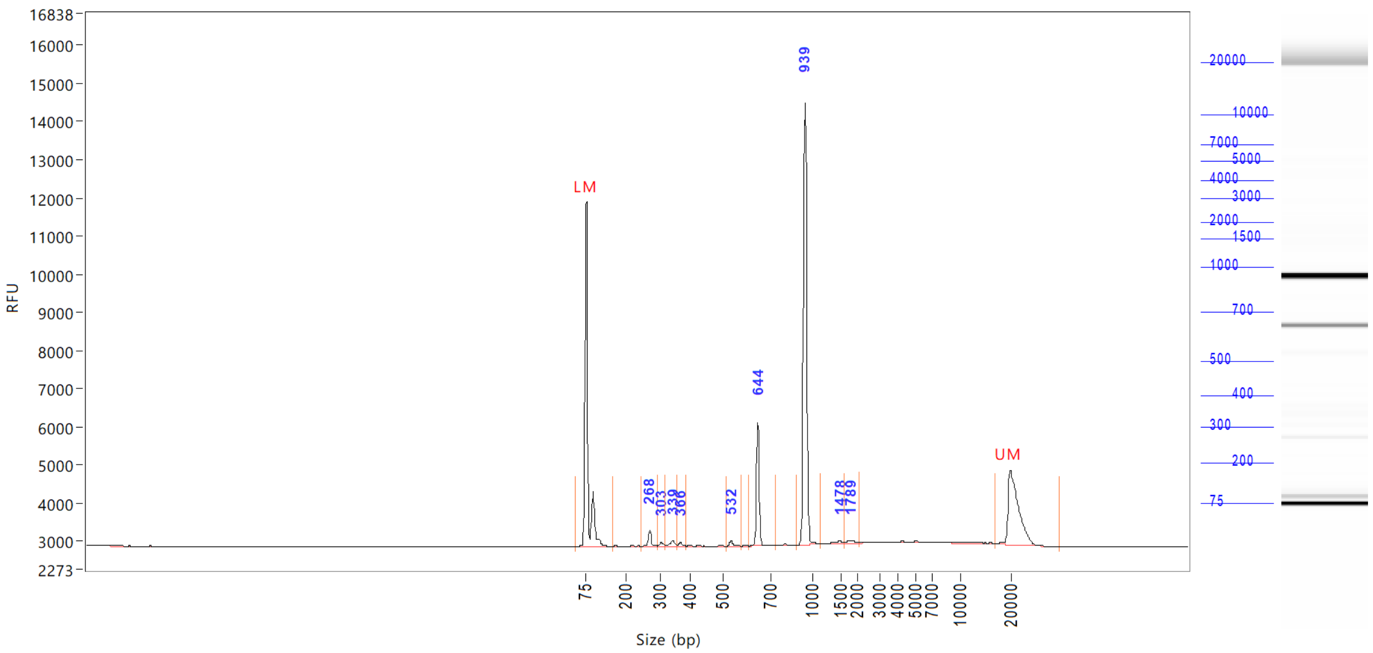

Peak	Size (bp)	Concentration (ng/uL)	From (bp)	To (bp)	Average size (bp)	CV%	RFU	Corrected peak area
1	75 (LM)	0.5000	41	156	77	14.70	9026	61.207
2	268	0.2222	244	291	267	2.02	415	2.267
3	303	0.0671	291	317	303	1.95	90	0.685
4	339	0.1362	317	356	337	2.62	125	1.390
5	366	0.0572	356	386	365	1.68	88	0.583
6	532	0.0919	514	575	533	1.77	141	0.938
7	644	1.5580	611	731	643	1.22	3222	15.893
8	939	5.9314	887	1112	938	1.31	11592	60.508
9	1478	0.0961	1112	1578	1400	7.82	61	0.981
10	1789	0.0930	1578	2058	1782	6.74	66	0.949
11	20000 (UM)	0.2094	16952	29914	20853	5.87	1961	25.631

TIC:	8.2532	ng/uL
TIM:	17.5340	nmole/L
Total concentration:	8.6522	ng/uL

```
Sample peak width (sec): 5    Sample min peak height: 50    Sample baseline V to V?: Y    Sample baseline V to V points: 3
Sample filter: Binomial       Number of points for filter: 3  Sample start region (min): 0  Sample end region (min): 30
Marker peak width (sec): 5    Marker min peak height: 200   Marker baseline V to V?: Y    Marker baseline V to V points: 3
Lower marker selection: First peak > 500 RFU                Upper marker selection: Last peak > 200 RFU
Ladder size (bp)75, 200, 300, 400, 500, 700, 1000, 1500, 2000, 3000, 4000, 5000, 7000, 10000, 20000
Quantification using: Lower Marker          Final concentration (ng/uL): 0.5000          Dilution factor: 12.0
```

Sample: SampG10
Well location: G10
Created: Wednesday, October 20, 2021 4:38:54 PM

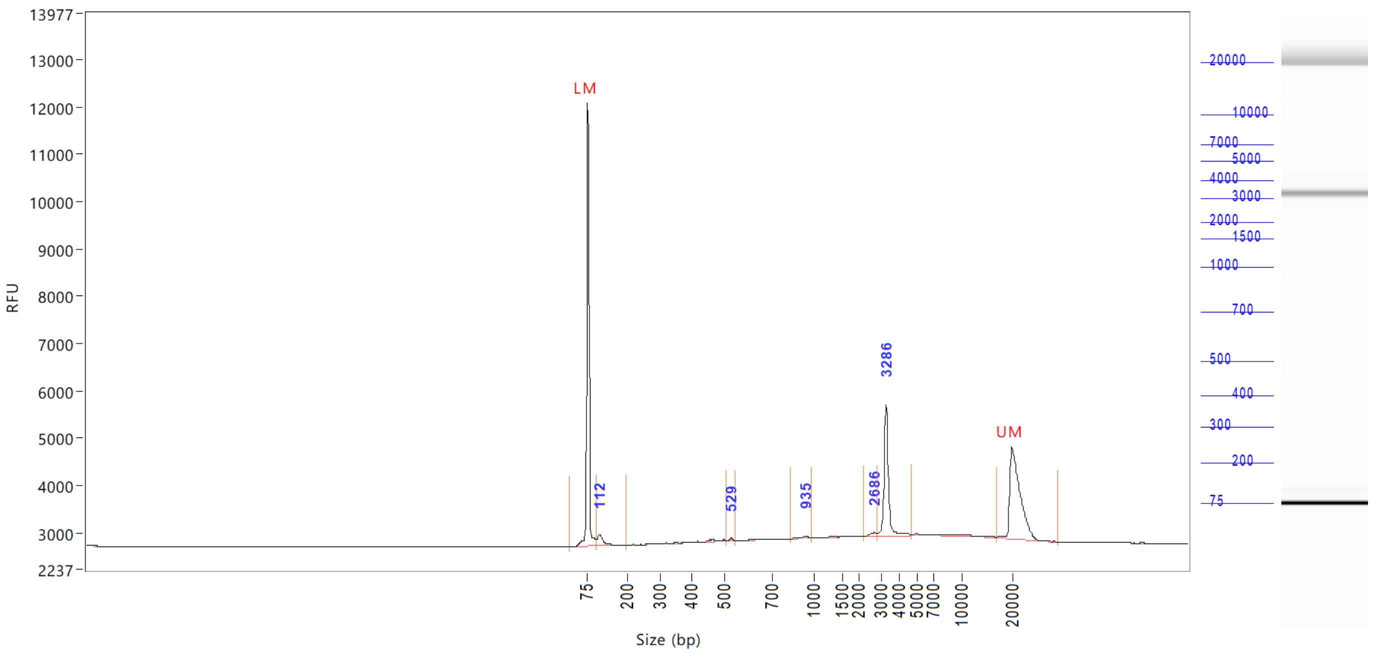

Peak	Size (bp)	Concentration (ng/uL)	From (bp)	To (bp)	Average size (bp)	CV%	RFU	Corrected peak area
1	75 (LM)	0.5000	20	101	74	7.58	9373	52.914
2	112	0.3705	101	194	117	13.36	234	3.267
3	529	0.0392	511	547	525	1.21	58	0.346
4	935	0.0464	831	978	926	2.83	53	0.409
5	2686	0.0852	2172	2800	2590	5.66	77	0.751
6	3286	2.0490	2800	4690	3292	7.70	2768	18.070
7	20000 (UM)	0.2377	16830	29309	20809	5.92	1945	25.160

TIC:	2.5902	ng/uL
TIM:	6.4857	nmole/L
Total concentration:	3.0370	ng/uL

Sample peak width (sec): 5 Sample min peak height: 50 Sample baseline V to V?: Y Sample baseline V to V points: 3
Sample filter: Binomial Number of points for filter: 3 Sample start region (min): 0 Sample end region (min): 30
Marker peak width (sec): 5 Marker min peak height: 200 Marker baseline V to V?: Y Marker baseline V to V points: 3
Lower marker selection: First peak > 500 RFU Upper marker selection: Last peak > 200 RFU
Ladder size (bp)75, 200, 300, 400, 500, 700, 1000, 1500, 2000, 3000, 4000, 5000, 7000, 10000, 20000
Quantification using: Lower Marker Final concentration (ng/uL): 0.5000 Dilution factor: 12.0

Sample: SampG11
Well location: G11
Created: Wednesday, October 20, 2021 4:38:54 PM

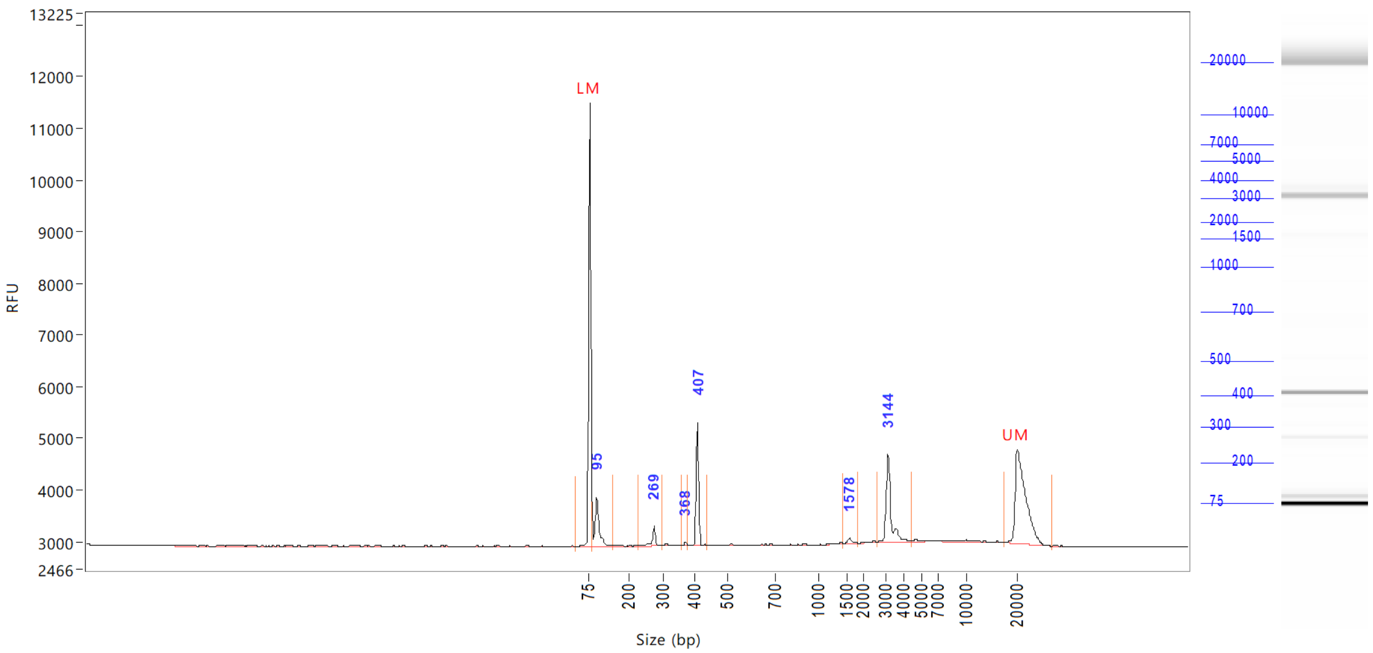

Peak	Size (bp)	Concentration (ng/uL)	From (bp)	To (bp)	Average size (bp)	CV%	RFU	Corrected peak area
1	75 (LM)	0.5000	30	84	72	4.96	8594	46.398
2	95	1.1057	84	148	97	9.86	957	8.550
3	269	0.3235	224	293	264	3.57	385	2.502
4	368	0.0452	356	378	367	1.31	66	0.349
5	407	1.5523	378	435	404	1.20	2395	12.004
6	1578	0.0864	1445	1866	1575	4.48	99	0.668
7	3144	1.5900	2629	4483	3229	8.57	1697	12.295
8	20000 (UM)	0.2599	17317	26770	20942	5.73	1817	24.113

TIC:	4.7031	ng/uL
TIM:	28.1571	nmole/L
Total concentration:	5.2738	ng/uL

Sample peak width (sec): 5 Sample min peak height: 50 Sample baseline V to V?: Y Sample baseline V to V points: 3
Sample filter: Binomial Number of points for filter: 3 Sample start region (min): 0 Sample end region (min): 30
Marker peak width (sec): 5 Marker min peak height: 200 Marker baseline V to V?: Y Marker baseline V to V points: 3
Lower marker selection: First peak > 500 RFU Upper marker selection: Last peak > 200 RFU
Ladder size (bp)75, 200, 300, 400, 500, 700, 1000, 1500, 2000, 3000, 4000, 5000, 7000, 10000, 20000
Quantification using: Lower Marker Final concentration (ng/uL): 0.5000 Dilution factor: 12.0

Sample: SampG12
Well location: G12
Created: Wednesday, October 20, 2021 4:38:54 PM

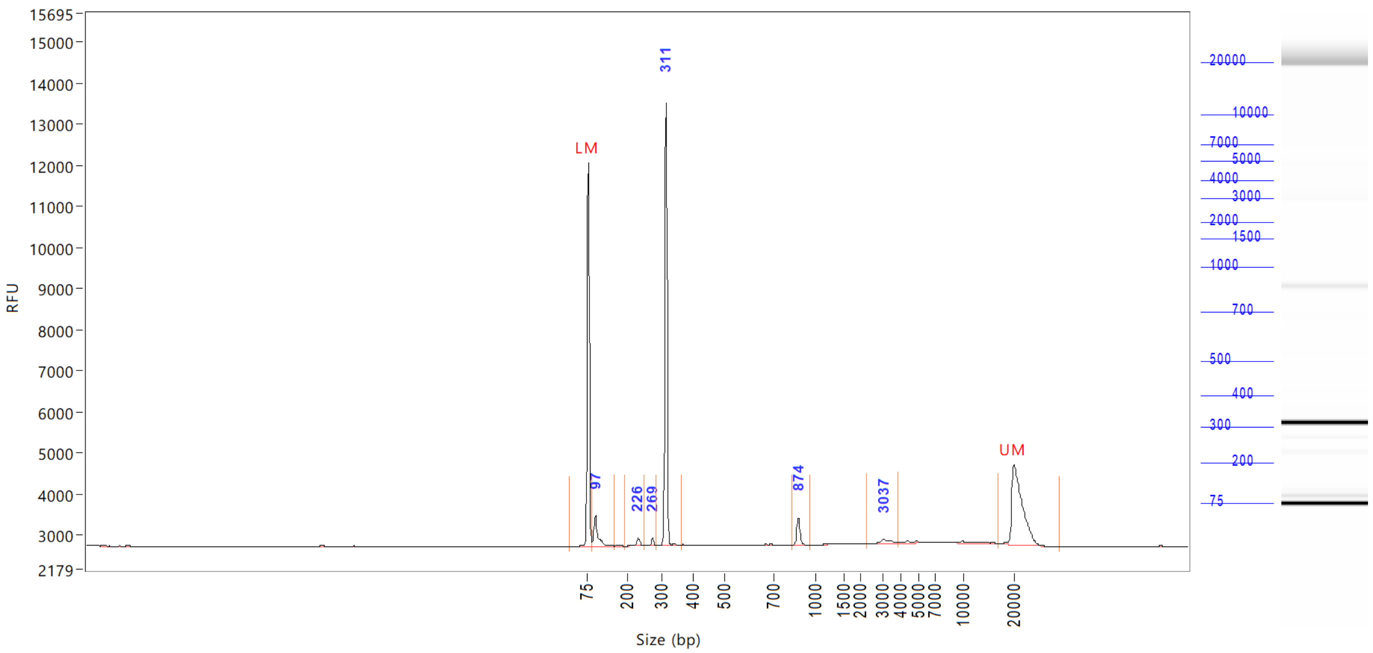

Peak	Size (bp)	Concentration (ng/uL)	From (bp)	To (bp)	Average size (bp)	CV%	RFU	Corrected peak area
1	75 (LM)	0.5000	18	86	73	4.76	9318	48.526
2	97	0.8416	86	156	99	10.43	741	6.806
3	226	0.1707	190	246	225	2.75	184	1.381
4	269	0.1192	246	282	266	1.69	189	0.964
5	311	6.3649	282	362	309	1.61	10785	51.477
6	874	0.4591	827	961	871	1.71	668	3.713
7	3037	0.1614	2286	3893	3182	9.78	98	1.306
8	20000 (UM)	0.2630	16952	29068	20874	5.86	1957	25.526

TIC:	8.1169	ng/uL
TIM:	50.7780	nmole/L
Total concentration:	8.6777	ng/uL

```
Sample peak width (sec): 5    Sample min peak height: 50    Sample baseline V to V?: Y    Sample baseline V to V points: 3
Sample filter: Binomial       Number of points for filter: 3  Sample start region (min): 0  Sample end region (min): 30
Marker peak width (sec): 5    Marker min peak height: 200   Marker baseline V to V?: Y    Marker baseline V to V points: 3
Lower marker selection: First peak > 500 RFU                Upper marker selection: Last peak > 200 RFU
Ladder size (bp)75, 200, 300, 400, 500, 700, 1000, 1500, 2000, 3000, 4000, 5000, 7000, 10000, 20000
Quantification using: Lower Marker          Final concentration (ng/uL): 0.5000          Dilution factor: 12.0
```

Sample: SampH1
Well location: H1
Created: Wednesday, October 20, 2021 4:38:54 PM

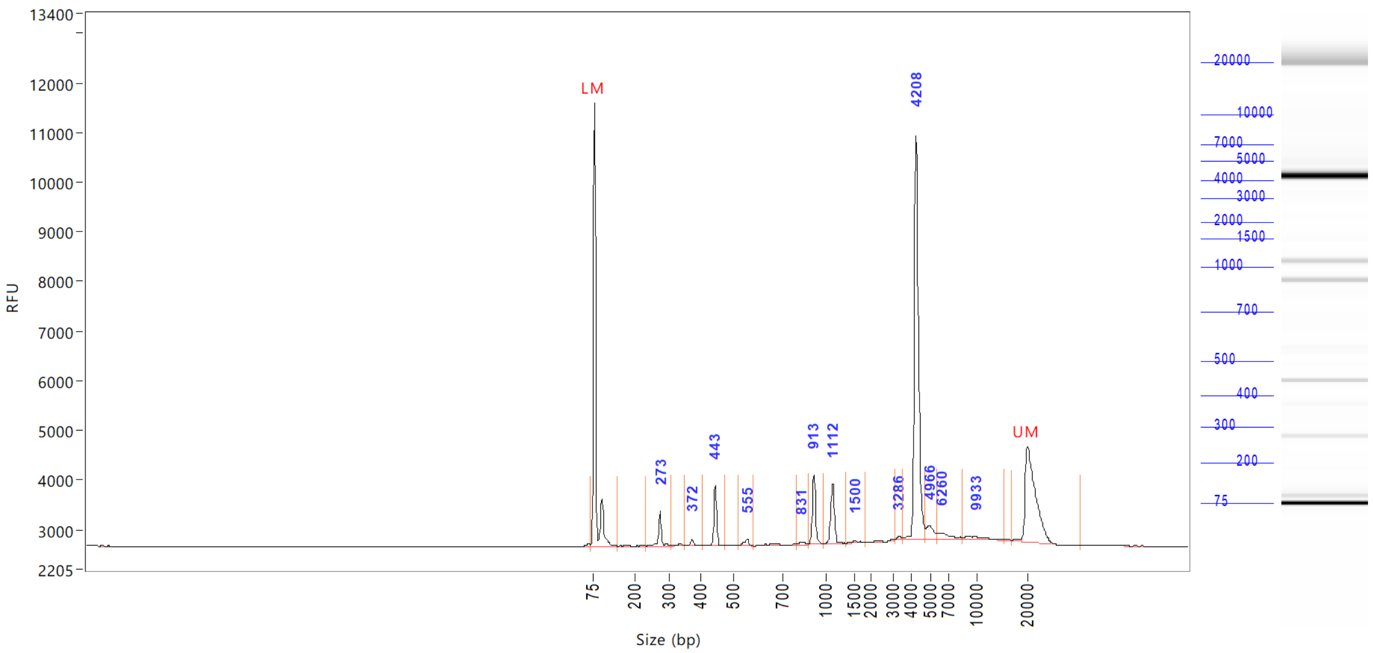

Peak	Size (bp)	Concentration (ng/uL)	From (bp)	To (bp)	Average size (bp)	CV%	RFU	Corrected peak area
1	75 (LM)	0.5000	64	148	77	13.30	8952	54.803
2	273	0.4684	232	301	270	3.30	697	4.278
3	372	0.1074	349	407	370	2.61	143	0.981
4	443	0.6593	407	475	441	1.14	1203	6.022
5	555	0.1319	516	583	545	2.04	140	1.205
6	831	0.0663	801	883	837	2.51	65	0.606
7	913	0.8249	883	983	911	1.36	1394	7.534
8	1112	0.7586	983	1345	1097	3.45	1200	6.929
9	1500	0.0571	1345	1808	1531	5.33	58	0.522
10	3286	0.0437	3037	3536	3249	3.56	67	0.399
11	4208	5.4090	3536	4724	4213	3.21	8119	49.405
12	4966	0.3171	4724	5741	5057	5.41	269	2.897
13	6260	0.2491	5741	8467	6806	11.10	125	2.275
14	9933	0.1843	8467	15366	10519	15.45	59	1.684
15	20000 (UM)	0.2256	16952	30398	20866	6.05	1934	24.731

TIC:	9.2771	ng/uL
TIM:	11.3373	nmole/L
Total concentration:	9.5514	ng/uL

Sample peak width (sec): 5 Sample min peak height: 50 Sample baseline V to V?: Y Sample baseline V to V points: 3
Sample filter: Binomial Number of points for filter: 3 Sample start region (min): 0 Sample end region (min): 30
Marker peak width (sec): 5 Marker min peak height: 200 Marker baseline V to V?: Y Marker baseline V to V points: 3
Lower marker selection: First peak > 500 RFU Upper marker selection: Last peak > 200 RFU
Ladder size (bp)75, 200, 300, 400, 500, 700, 1000, 1500, 2000, 3000, 4000, 5000, 7000, 10000, 20000
Quantification using: Lower Marker Final concentration (ng/uL): 0.5000 Dilution factor: 12.0

Sample: SampH2
Well location: H2
Created: Wednesday, October 20, 2021 4:38:54 PM

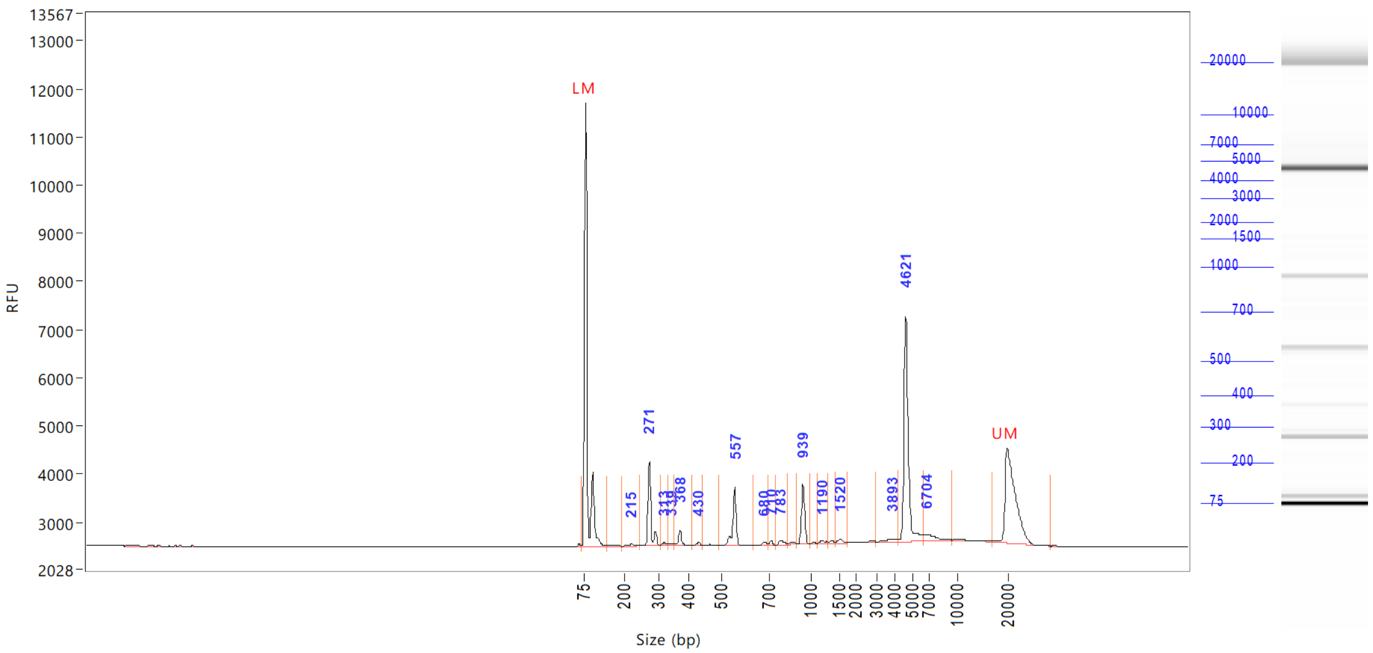

Peak	Size (bp)	Concentration (ng/uL)	From (bp)	To (bp)	Average size (bp)	CV%	RFU	Corrected peak area
1	75 (LM)	0.5000	62	145	77	14.19	9226	60.932
2	215	0.0566	190	241	213	4.86	56	0.575
3	271	1.0463	241	303	271	3.05	1759	10.625
4	313	0.0573	303	329	315	2.26	64	0.582
5	339	0.0461	329	349	339	1.74	56	0.468
6	368	0.2071	349	409	366	2.20	329	2.103
7	430	0.0491	409	441	425	1.27	71	0.498
8	557	0.7863	492	634	549	2.55	1229	7.986
9	680	0.0595	634	695	676	1.86	69	0.604
10	710	0.0516	695	753	707	1.37	90	0.524
11	783	0.0817	753	831	787	2.22	98	0.830
12	939	0.6220	900	987	936	1.12	1235	6.316
13	1190	0.0591	1090	1311	1208	4.62	60	0.600
14	1520	0.0675	1433	1770	1520	3.67	93	0.685
15	3893	0.0992	2971	4139	3681	8.41	63	1.008
16	4621	2.8623	4139	6260	4682	6.11	4680	29.067
17	6704	0.2496	6260	9467	7312	10.98	143	2.535
18	20000 (UM)	0.2095	16952	28705	20846	5.77	1977	25.527

TIC:	6.4014	ng/uL
TIM:	13.5787	nmole/L
Total concentration:	6.6451	ng/uL

```
Sample peak width (sec): 5     Sample min peak height: 50    Sample baseline V to V?: Y    Sample baseline V to V points: 3
Sample filter: Binomial        Number of points for filter: 3  Sample start region (min): 0  Sample end region (min): 30
Marker peak width (sec): 5     Marker min peak height: 200   Marker baseline V to V?: Y    Marker baseline V to V points: 3
Lower marker selection: First peak > 500 RFU                 Upper marker selection: Last peak > 200 RFU
Ladder size (bp)75, 200, 300, 400, 500, 700, 1000, 1500, 2000, 3000, 4000, 5000, 7000, 10000, 20000
Quantification using: Lower Marker          Final concentration (ng/uL): 0.5000          Dilution factor: 12.0
```

Sample: SampH3
Well location: H3
Created: Wednesday, October 20, 2021 4:38:54 PM

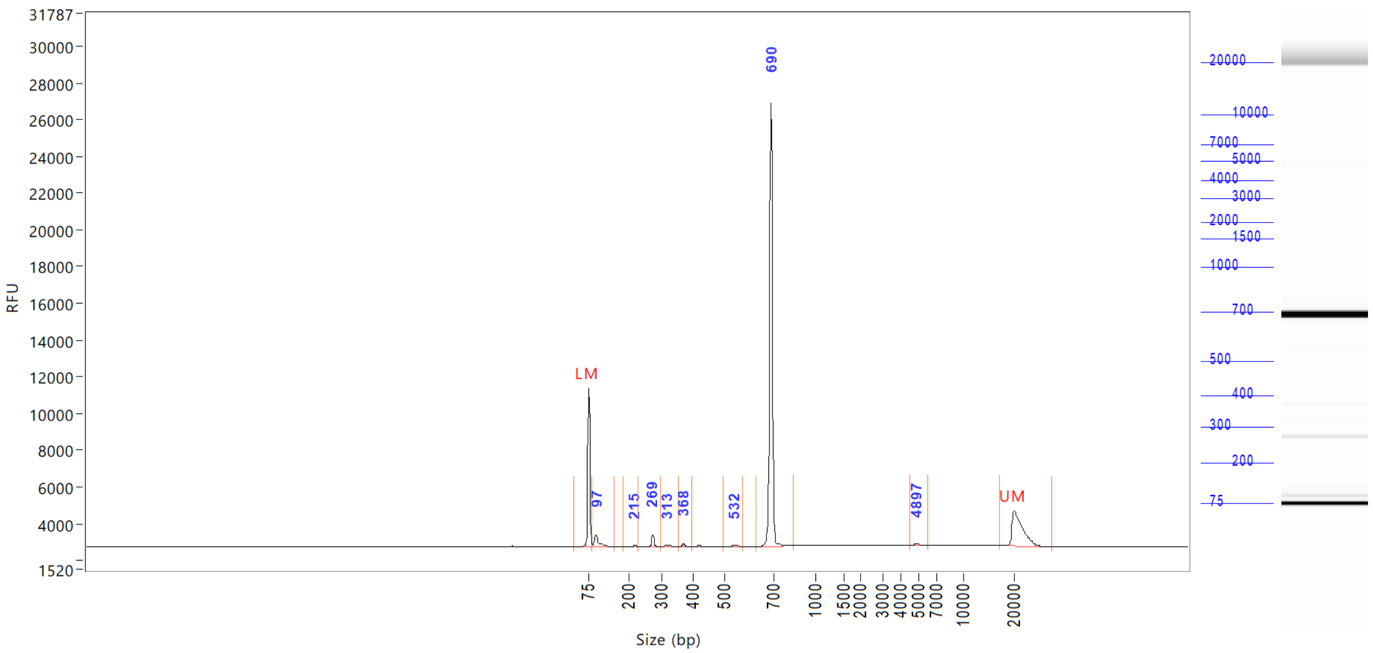

Peak	Size (bp)	Concentration (ng/uL)	From (bp)	To (bp)	Average size (bp)	CV%	RFU	Corrected peak area
1	75 (LM)	0.5000	31	86	73	4.59	8654	45.010
2	97	0.8677	86	156	101	11.85	690	6.510
3	215	0.0482	183	226	210	3.84	57	0.361
4	269	0.5080	226	293	268	2.85	677	3.811
5	313	0.1628	293	354	316	4.07	95	1.221
6	368	0.1382	354	398	367	2.01	188	1.037
7	532	0.0965	494	567	532	2.60	58	0.724
8	690	16.3471	626	840	687	1.66	24175	122.632
9	4897	0.0835	4552	6112	4899	4.32	78	0.626
10	20000 (UM)	0.2788	17195	27496	20909	5.81	1919	25.099

TIC:	18.2519	ng/uL
TIM:	58.5566	nmole/L
Total concentration:	18.8563	ng/uL

Sample peak width (sec): 5   Sample min peak height: 50   Sample baseline V to V?: Y   Sample baseline V to V points: 3
Sample filter: Binomial   Number of points for filter: 3 Sample start region (min): 0 Sample end region (min): 30
Marker peak width (sec): 5   Marker min peak height: 200   Marker baseline V to V?: Y   Marker baseline V to V points: 3
Lower marker selection: First peak > 500 RFU   Upper marker selection: Last peak > 200 RFU
Ladder size (bp)75, 200, 300, 400, 500, 700, 1000, 1500, 2000, 3000, 4000, 5000, 7000, 10000, 20000
Quantification using: Lower Marker   Final concentration (ng/uL): 0.5000   Dilution factor: 12.0

Sample: SampH4
Well location: H4
Created: Wednesday, October 20, 2021 4:38:54 PM

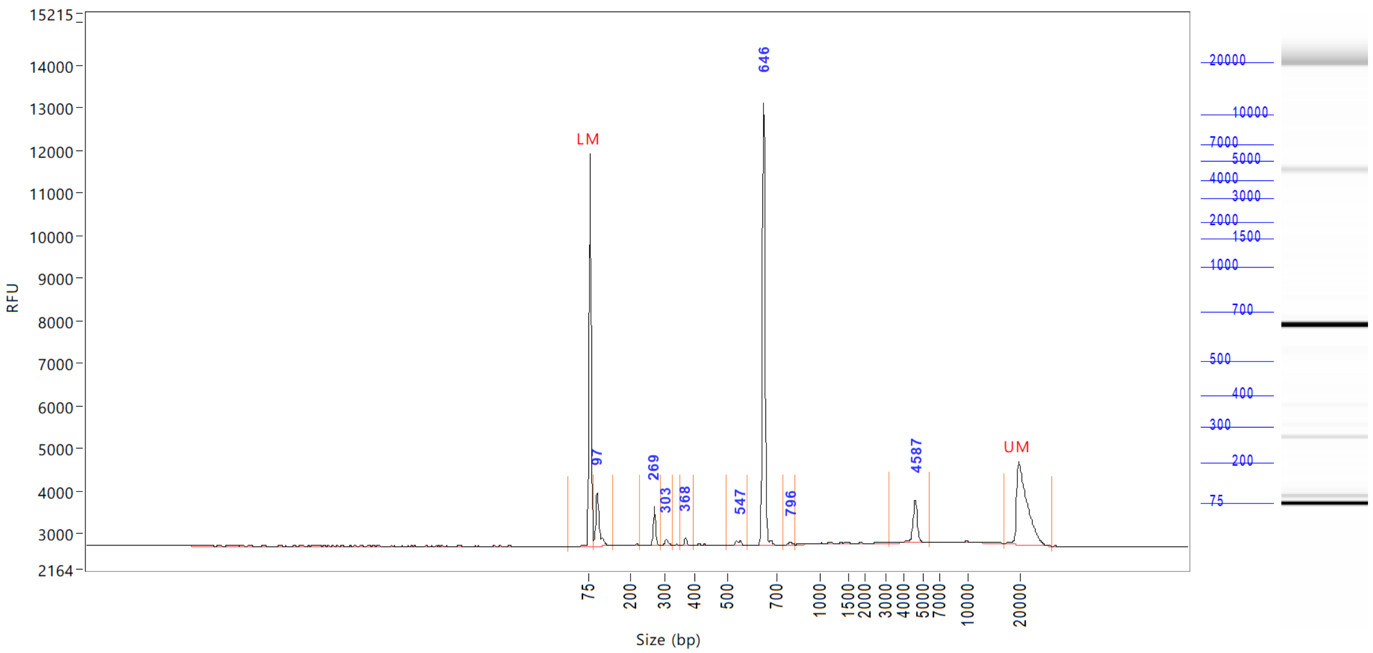

Peak	Size (bp)	Concentration (ng/uL)	From (bp)	To (bp)	Average size (bp)	CV%	RFU	Corrected peak area
1	75 (LM)	0.5000	11	86	73	5.14	9202	47.484
2	97	1.2842	86	147	98	9.31	1251	10.163
3	269	0.6081	228	291	267	2.56	916	4.813
4	303	0.1433	291	325	304	2.19	141	1.134
5	368	0.1329	349	396	366	1.62	190	1.052
6	547	0.1515	496	578	535	2.73	101	1.199
7	646	6.5306	578	749	644	1.10	10402	51.683
8	796	0.0555	749	831	790	1.97	63	0.439
9	4587	0.8571	3108	5667	4492	5.60	1003	6.783
10	20000 (UM)	0.2728	17073	26529	20880	6.01	1960	25.906

TIC:	9.7632	ng/uL
TIM:	44.2199	nmole/L
Total concentration:	10.3469	ng/uL

Sample peak width (sec): 5 Sample min peak height: 50 Sample baseline V to V?: Y Sample baseline V to V points: 3
Sample filter: Binomial Number of points for filter: 3 Sample start region (min): 0 Sample end region (min): 30
Marker peak width (sec): 5 Marker min peak height: 200 Marker baseline V to V?: Y Marker baseline V to V points: 3
Lower marker selection: First peak > 500 RFU Upper marker selection: Last peak > 200 RFU
Ladder size (bp)75, 200, 300, 400, 500, 700, 1000, 1500, 2000, 3000, 4000, 5000, 7000, 10000, 20000
Quantification using: Lower Marker Final concentration (ng/uL): 0.5000 Dilution factor: 12.0

Sample: SampH5
Well location: H5
Created: Wednesday, October 20, 2021 4:38:54 PM

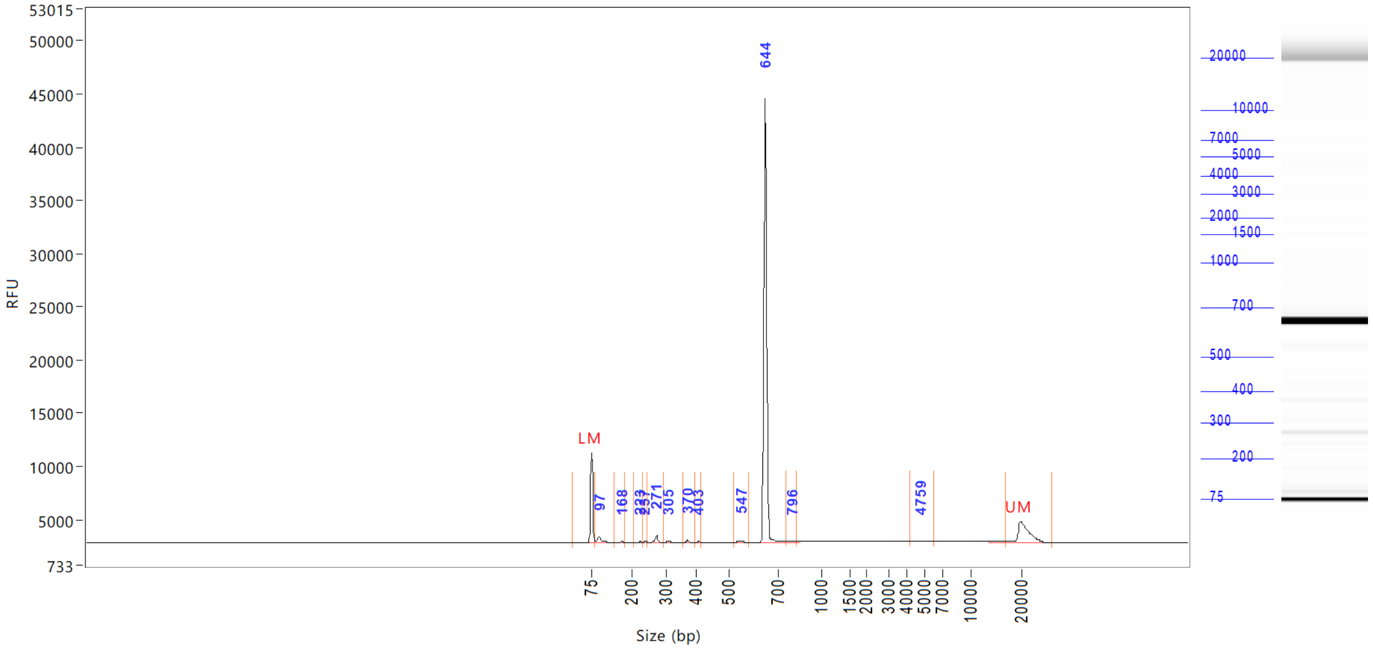

Peak	Size (bp)	Concentration (ng/uL)	From (bp)	To (bp)	Average size (bp)	CV%	RFU	Corrected peak area
1	75 (LM)	0.5000	18	86	72	5.16	8464	45.433
2	97	0.7430	86	147	101	11.40	523	5.626
3	168	0.0845	147	177	165	3.50	113	0.640
4	223	0.0534	203	230	219	2.23	58	0.405
5	237	0.0731	230	246	237	1.61	102	0.554
6	271	0.4917	246	293	268	2.60	619	3.723
7	305	0.1504	293	354	312	4.98	103	1.138
8	370	0.1454	354	392	367	1.42	204	1.101
9	403	0.0667	392	411	401	0.94	95	0.505
10	547	0.1807	514	578	539	1.73	169	1.368
11	644	27.0315	578	749	643	1.20	41768	204.686
12	796	0.0871	749	831	787	2.81	52	0.660
13	4759	0.0890	4208	6038	4761	5.83	79	0.674
14	20000 (UM)	0.2787	16952	26166	20814	5.85	1938	25.322

TIC:	29.1964	ng/uL
TIM:	88.5151	nmole/L
Total concentration:	29.7106	ng/uL

```
Sample peak width (sec): 5    Sample min peak height: 50    Sample baseline V to V?: Y    Sample baseline V to V points: 3
Sample filter: Binomial       Number of points for filter: 3  Sample start region (min): 0  Sample end region (min): 30
Marker peak width (sec): 5    Marker min peak height: 200   Marker baseline V to V?: Y    Marker baseline V to V points: 3
Lower marker selection: First peak > 500 RFU                Upper marker selection: Last peak > 200 RFU
Ladder size (bp)75, 200, 300, 400, 500, 700, 1000, 1500, 2000, 3000, 4000, 5000, 7000, 10000, 20000
Quantification using: Lower Marker          Final concentration (ng/uL): 0.5000          Dilution factor: 12.0
```

Sample: SampH6
Well location: H6
Created: Wednesday, October 20, 2021 4:38:54 PM

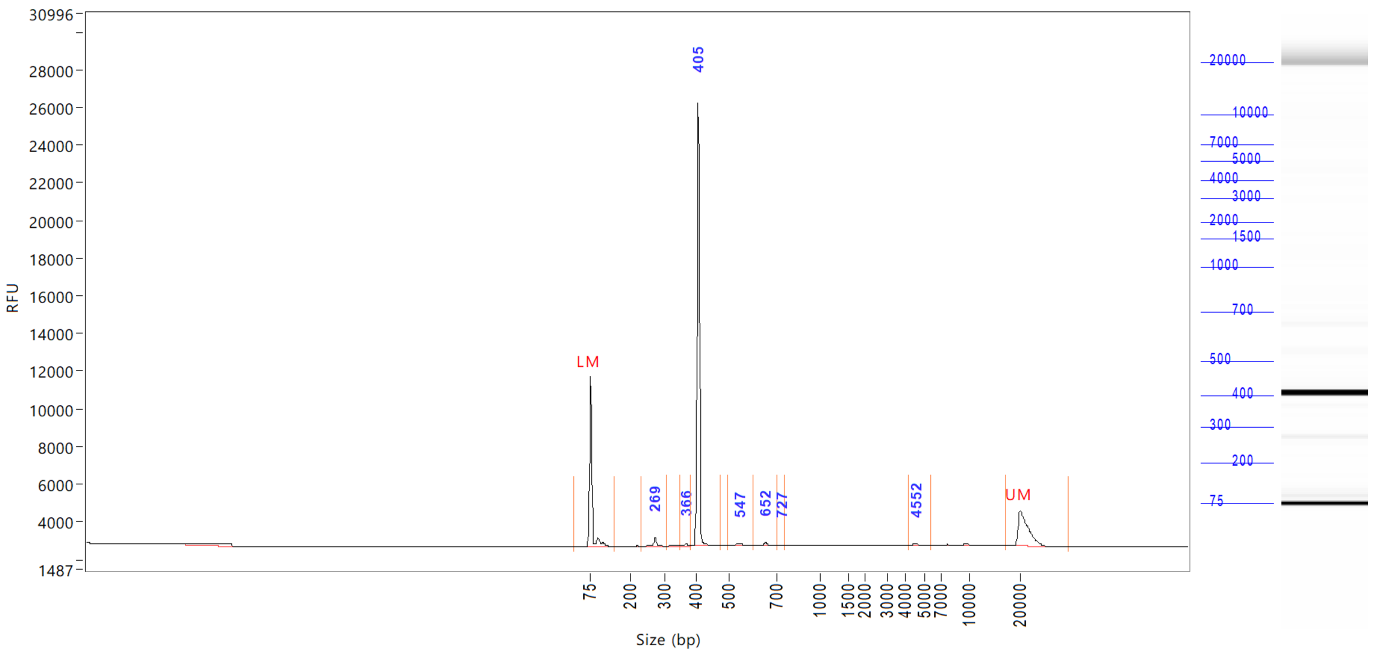

Peak	Size (bp)	Concentration (ng/uL)	From (bp)	To (bp)	Average size (bp)	CV%	RFU	Corrected peak area
1	75 (LM)	0.5000	24	148	76	13.48	9049	52.654
2	269	0.3642	232	303	266	3.49	471	3.196
3	366	0.0887	345	382	363	1.36	152	0.778
4	405	12.7119	382	473	404	1.27	23557	111.555
5	547	0.1630	498	603	539	2.96	123	1.431
6	652	0.1783	603	700	654	2.43	189	1.565
7	727	0.0444	700	753	723	1.65	66	0.390
8	4552	0.0726	4208	5889	4659	6.27	71	0.637
9	20000 (UM)	0.2225	17073	29430	20892	5.69	1851	23.428

TIC:	13.6231	ng/uL
TIM:	55.4968	nmole/L
Total concentration:	14.1425	ng/uL

```
Sample peak width (sec): 5     Sample min peak height: 50    Sample baseline V to V?: Y    Sample baseline V to V points: 3
Sample filter: Binomial        Number of points for filter: 3  Sample start region (min): 0  Sample end region (min): 30
Marker peak width (sec): 5     Marker min peak height: 200   Marker baseline V to V?: Y    Marker baseline V to V points: 3
Lower marker selection: First peak > 500 RFU                 Upper marker selection: Last peak > 200 RFU
Ladder size (bp)75, 200, 300, 400, 500, 700, 1000, 1500, 2000, 3000, 4000, 5000, 7000, 10000, 20000
Quantification using: Lower Marker          Final concentration (ng/uL): 0.5000          Dilution factor: 12.0
```

Sample: SampH7
Well location: H7
Created: Wednesday, October 20, 2021 4:38:54 PM

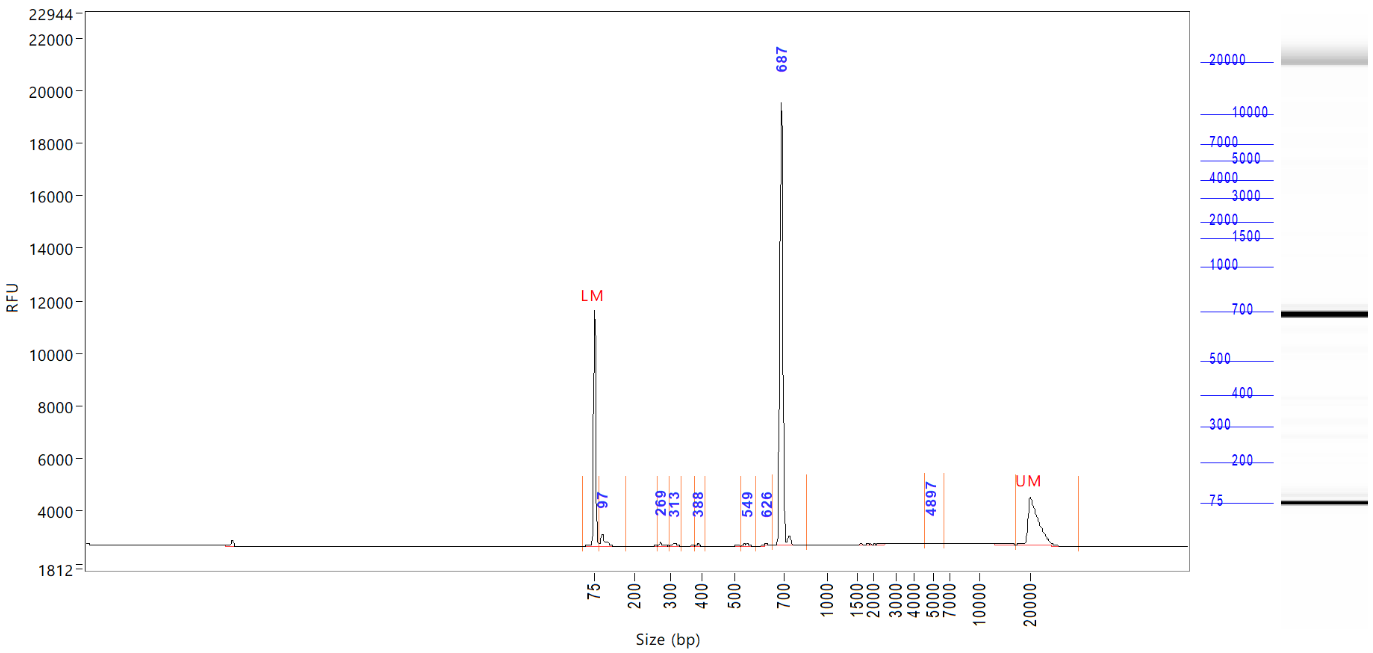

Peak	Size (bp)	Concentration (ng/uL)	From (bp)	To (bp)	Average size (bp)	CV%	RFU	Corrected peak area
1	75 (LM)	0.5000	39	87	73	5.28	8956	46.365
2	97	0.6471	87	169	103	13.13	458	5.001
3	269	0.1076	260	296	271	2.70	143	0.831
4	313	0.1115	296	333	311	2.01	100	0.861
5	388	0.0701	378	411	387	1.32	102	0.542
6	549	0.1142	524	588	543	1.75	95	0.882
7	626	0.0775	588	657	624	1.50	75	0.599
8	687	10.9251	657	853	687	1.37	16854	84.424
9	4897	0.0630	4552	6408	4984	6.70	51	0.487
10	20000 (UM)	0.2536	17073	29430	20844	5.92	1824	23.520

TIC:	12.1160	ng/uL
TIM:	38.6007	nmole/L
Total concentration:	12.5775	ng/uL

```
Sample peak width (sec): 5    Sample min peak height: 50    Sample baseline V to V?: Y    Sample baseline V to V points: 3
Sample filter: Binomial       Number of points for filter: 3  Sample start region (min): 0  Sample end region (min): 30
Marker peak width (sec): 5    Marker min peak height: 200   Marker baseline V to V?: Y    Marker baseline V to V points: 3
Lower marker selection: First peak > 500 RFU                Upper marker selection: Last peak > 200 RFU
Ladder size (bp)75, 200, 300, 400, 500, 700, 1000, 1500, 2000, 3000, 4000, 5000, 7000, 10000, 20000
Quantification using: Lower Marker          Final concentration (ng/uL): 0.5000         Dilution factor: 12.0
```

Sample: SampH8
Well location: H8
Created: Wednesday, October 20, 2021 4:38:54 PM

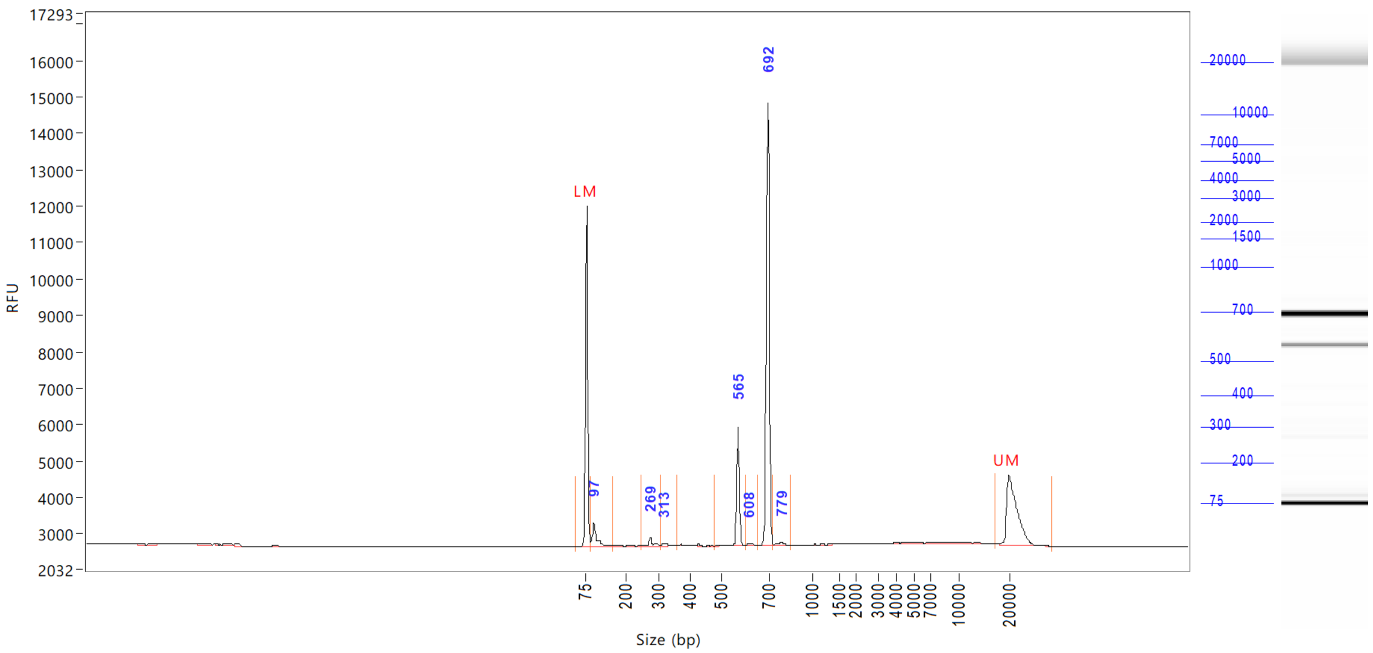

Peak	Size (bp)	Concentration (ng/uL)	From (bp)	To (bp)	Average size (bp)	CV%	RFU	Corrected peak area
1	75 (LM)	0.5000	41	87	73	4.33	9321	48.396
2	97	0.7778	87	158	102	11.77	624	6.274
3	269	0.2375	246	301	275	4.22	224	1.915
4	313	0.1023	301	356	318	3.82	63	0.825
5	565	1.9731	475	595	561	1.55	3247	15.915
6	608	0.0684	595	649	616	1.81	50	0.551
7	692	7.5277	649	723	689	0.86	12169	60.719
8	779	0.1038	723	848	771	3.15	87	0.837
9	20000 (UM)	0.2597	17317	28705	20927	5.83	1936	25.138

TIC:	10.7905	ng/uL
TIM:	38.6448	nmole/L
Total concentration:	11.3532	ng/uL

Sample peak width (sec): 5 Sample min peak height: 50 Sample baseline V to V?: Y Sample baseline V to V points: 3
Sample filter: Binomial Number of points for filter: 3 Sample start region (min): 0 Sample end region (min): 30
Marker peak width (sec): 5 Marker min peak height: 200 Marker baseline V to V?: Y Marker baseline V to V points: 3
Lower marker selection: First peak > 500 RFU Upper marker selection: Last peak > 200 RFU
Ladder size (bp)75, 200, 300, 400, 500, 700, 1000, 1500, 2000, 3000, 4000, 5000, 7000, 10000, 20000
Quantification using: Lower Marker Final concentration (ng/uL): 0.5000 Dilution factor: 12.0

Sample: SampH9
Well location: H9
Created: Wednesday, October 20, 2021 4:38:54 PM

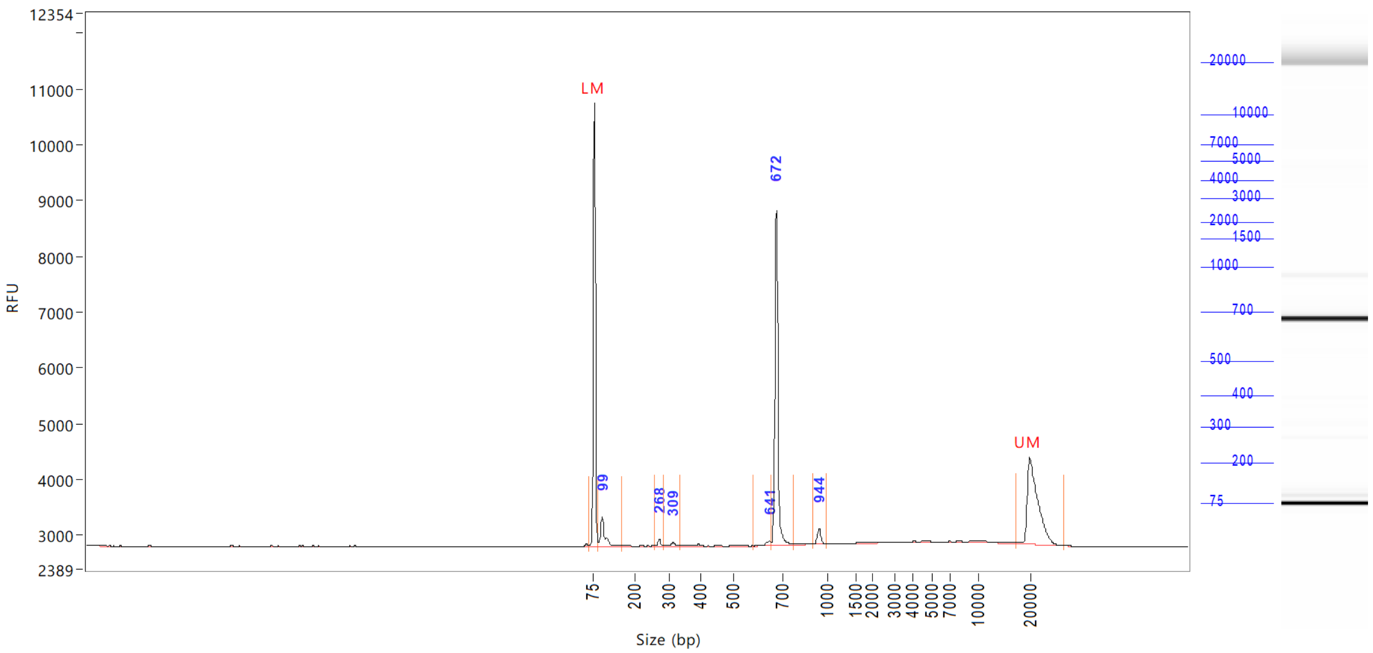

Peak	Size (bp)	Concentration (ng/uL)	From (bp)	To (bp)	Average size (bp)	CV%	RFU	Corrected peak area
1	75 (LM)	0.5000	58	87	73	4.31	7961	40.686
2	99	0.7546	87	158	102	11.34	515	5.117
3	268	0.0938	255	284	266	1.50	124	0.636
4	309	0.0737	284	331	307	2.94	58	0.499
5	641	0.1063	580	652	635	1.84	80	0.721
6	672	4.7830	652	766	670	1.53	5999	32.433
7	944	0.2324	909	1000	942	1.26	280	1.576
8	20000 (UM)	0.2477	17439	26649	20908	5.76	1565	20.154

TIC:	6.0438	ng/uL
TIM:	25.5538	nmole/L
Total concentration:	6.6855	ng/uL

Sample peak width (sec): 5 Sample min peak height: 50 Sample baseline V to V?: Y Sample baseline V to V points: 3
Sample filter: Binomial Number of points for filter: 3 Sample start region (min): 0 Sample end region (min): 30
Marker peak width (sec): 5 Marker min peak height: 200 Marker baseline V to V?: Y Marker baseline V to V points: 3
Lower marker selection: First peak > 500 RFU Upper marker selection: Last peak > 200 RFU
Ladder size (bp)75, 200, 300, 400, 500, 700, 1000, 1500, 2000, 3000, 4000, 5000, 7000, 10000, 20000
Quantification using: Lower Marker Final concentration (ng/uL): 0.5000 Dilution factor: 12.0

Sample: SampH10
Well location: H10
Created: Wednesday, October 20, 2021 4:38:54 PM

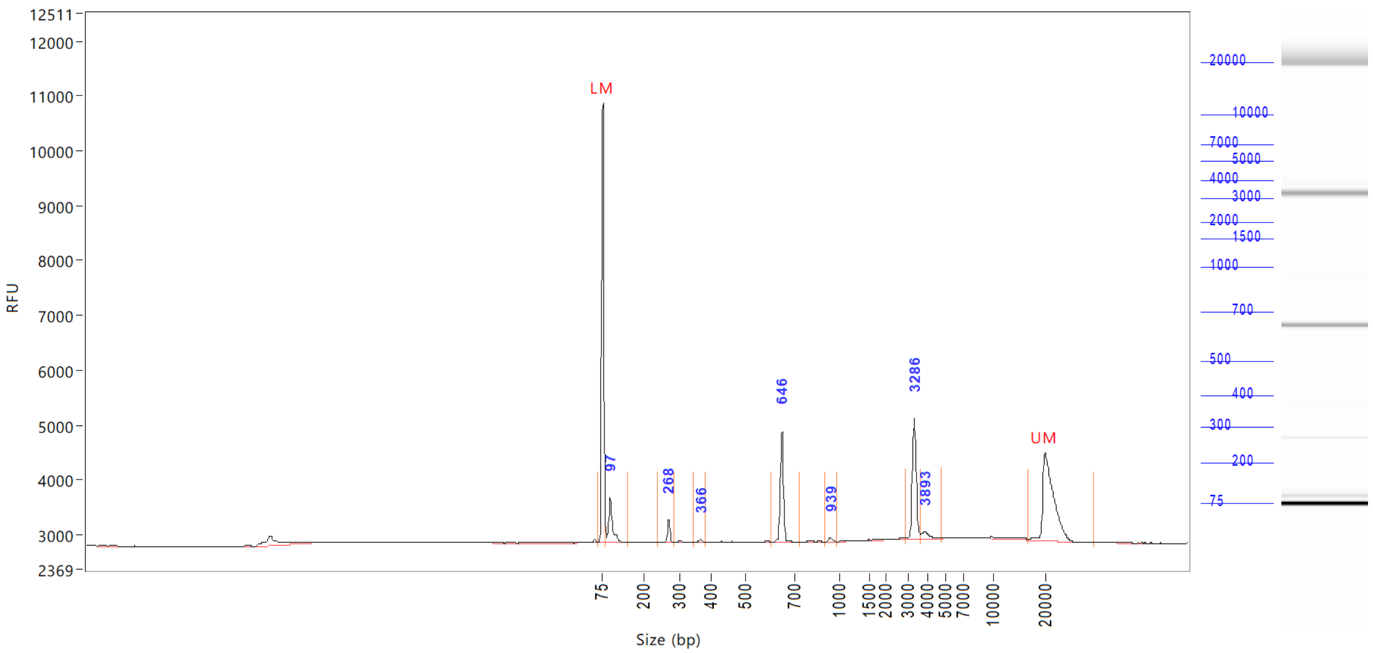

Peak	Size (bp)	Concentration (ng/uL)	From (bp)	To (bp)	Average size (bp)	CV%	RFU	Corrected peak area
1	75 (LM)	0.5000	60	86	72	4.41	8037	42.445
2	97	0.9943	86	150	99	9.47	826	7.034
3	268	0.3057	239	286	266	1.69	421	2.162
4	366	0.0641	343	382	362	1.88	63	0.454
5	646	1.5038	606	723	643	1.31	2020	10.638
6	939	0.0625	900	983	936	1.39	73	0.442
7	3286	1.8005	2857	3679	3260	3.78	2193	12.737
8	3893	0.2070	3679	4793	3993	6.31	134	1.465
9	20000 (UM)	0.2514	16586	29189	20822	6.23	1613	21.345

TIC:	4.9379	ng/uL
TIM:	23.6270	nmole/L
Total concentration:	5.5060	ng/uL

```
Sample peak width (sec): 5    Sample min peak height: 50    Sample baseline V to V?: Y    Sample baseline V to V points: 3
Sample filter: Binomial       Number of points for filter: 3  Sample start region (min): 0  Sample end region (min): 30
Marker peak width (sec): 5    Marker min peak height: 200   Marker baseline V to V?: Y    Marker baseline V to V points: 3
Lower marker selection: First peak > 500 RFU                Upper marker selection: Last peak > 200 RFU
Ladder size (bp)75, 200, 300, 400, 500, 700, 1000, 1500, 2000, 3000, 4000, 5000, 7000, 10000, 20000
Quantification using: Lower Marker          Final concentration (ng/uL): 0.5000          Dilution factor: 12.0
```

Sample: SampH11
Well location: H11
Created: Wednesday, October 20, 2021 4:38:54 PM

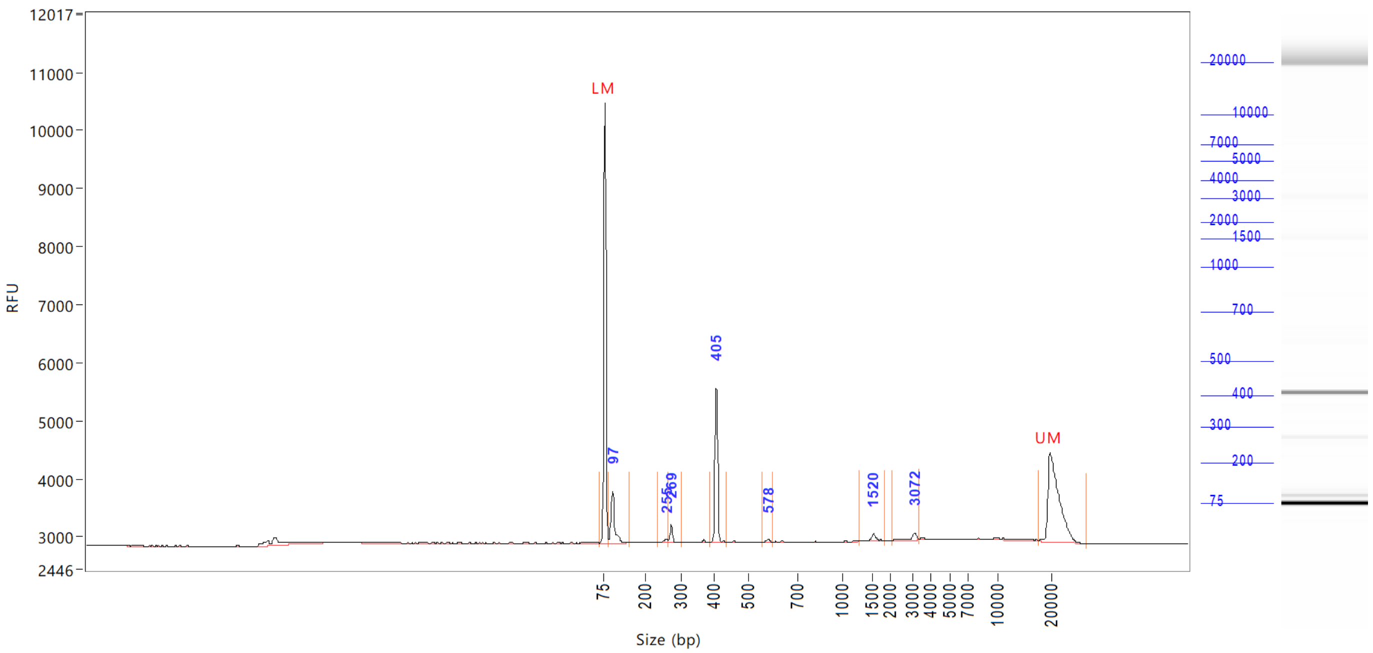

Peak	Size (bp)	Concentration (ng/uL)	From (bp)	To (bp)	Average size (bp)	CV%	RFU	Corrected peak area
1	75 (LM)	0.5000	58	86	73	4.19	7590	38.808
2	97	1.0993	86	148	99	9.09	895	7.110
3	255	0.0822	232	260	251	2.55	54	0.532
4	269	0.2581	260	298	268	1.71	321	1.669
5	405	2.0244	386	437	404	1.03	2664	13.094
6	578	0.0594	560	598	577	1.43	55	0.384
7	1520	0.1436	1267	1846	1524	6.26	123	0.929
8	3072	0.1569	2058	3358	2954	9.05	127	1.015
9	20000 (UM)	0.2613	17805	26770	20923	5.80	1531	20.277

TIC:	3.8239	ng/uL
TIM:	29.0127	nmole/L
Total concentration:	4.4637	ng/uL

```
Sample peak width (sec): 5    Sample min peak height: 50    Sample baseline V to V?: Y    Sample baseline V to V points: 3
Sample filter: Binomial       Number of points for filter: 3  Sample start region (min): 0  Sample end region (min): 30
Marker peak width (sec): 5    Marker min peak height: 200   Marker baseline V to V?: Y    Marker baseline V to V points: 3
Lower marker selection: First peak > 500 RFU                Upper marker selection: Last peak > 200 RFU
Ladder size (bp)75, 200, 300, 400, 500, 700, 1000, 1500, 2000, 3000, 4000, 5000, 7000, 10000, 20000
Quantification using: Lower Marker          Final concentration (ng/uL): 0.5000          Dilution factor: 12.0
```

Sample: SampH12
Well location: H12
Created: Wednesday, October 20, 2021 4:38:54 PM

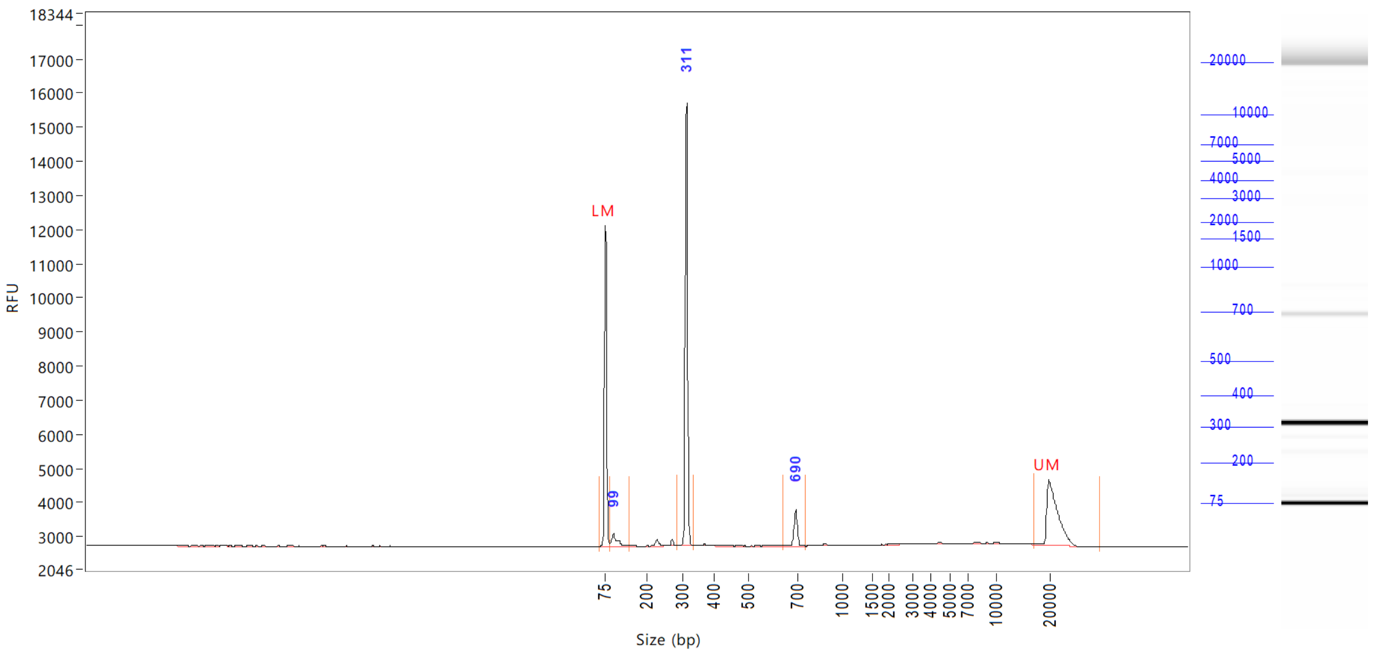

Peak	Size (bp)	Concentration (ng/uL)	From (bp)	To (bp)	Average size (bp)	CV%	RFU	Corrected peak area
1	75 (LM)	0.5000	56	87	74	4.16	9384	48.027
2	99	0.5563	87	147	104	11.45	374	4.453
3	311	7.8014	284	335	308	1.27	13010	62.446
4	690	0.7148	644	749	688	1.13	1093	5.721
5	20000 (UM)	0.2569	17195	29672	20933	5.86	1915	24.674

TIC:	9.0725	ng/uL
TIM:	52.1609	nmole/L
Total concentration:	9.9875	ng/uL

```
Sample peak width (sec): 5    Sample min peak height: 200   Sample baseline V to V?: Y    Sample baseline V to V points: 3
Sample filter: Binomial       Number of points for filter: 3  Sample start region (min): 0  Sample end region (min): 30
Marker peak width (sec): 5    Marker min peak height: 200   Marker baseline V to V?: Y    Marker baseline V to V points: 3
Lower marker selection: First peak > 500 RFU                Upper marker selection: Last peak > 200 RFU
Ladder size (bp)75, 200, 300, 400, 500, 700, 1000, 1500, 2000, 3000, 4000, 5000, 7000, 10000, 20000
Quantification using: Lower Marker          Final concentration (ng/uL): 0.5000         Dilution factor: 12.0
```

Sample: DNA size ladder
Well location: A7
Created: Wednesday, October 20, 2021 4:38:54 PM
Import from: C:\Users\admin\Desktop\210810_alberto_ladder.SCAL
Fit type: Point to point

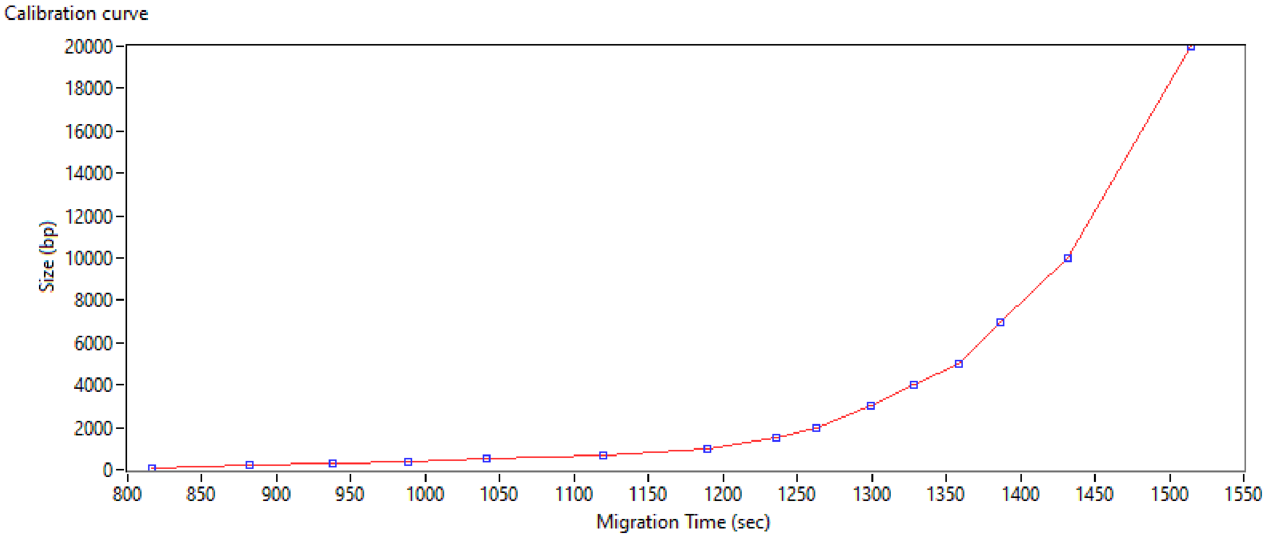

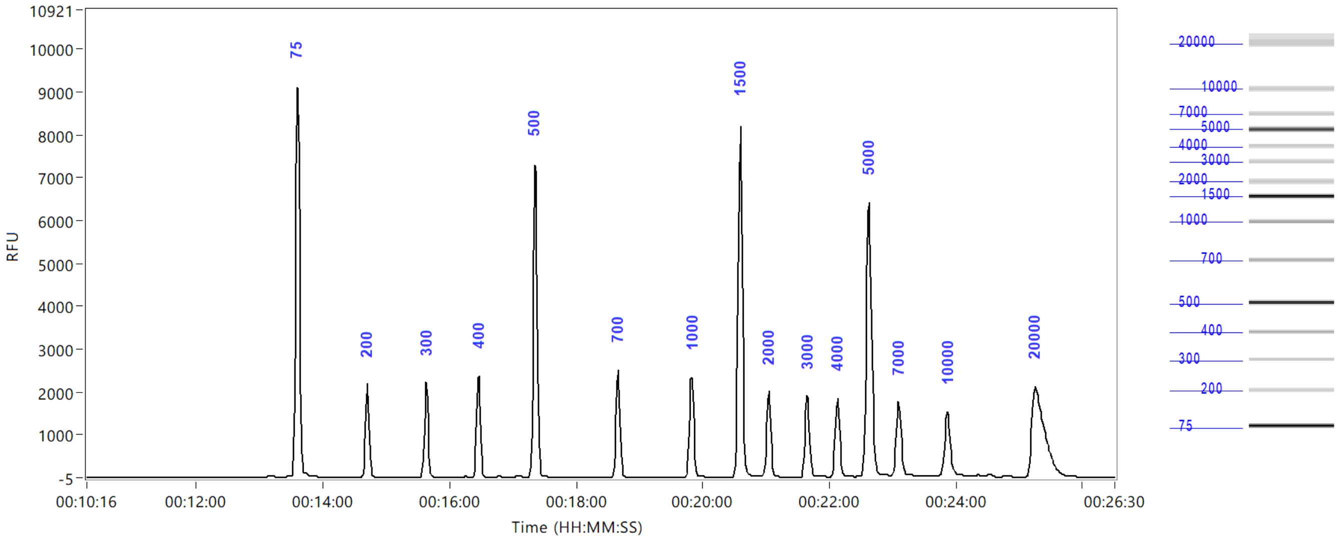

Supplement: Supplementary file 2 — sb3c00292_si_002.zip [file sb3c00292_si_002.zip › dnada_supplementary_material_pks_library_build/colony_pcr/ZAG/16-28-48_plate1/colonypcrplate_1.pdf]
