# Supplementary material for: Automated Platform for the Plasmid Construction Process
Source: ACS Synth Biol. 2023 Nov 10;12(12):3506–13. doi: 10.1021/acssynbio.3c00292 (PMC10729297; doi:10.1021/acssynbio.3c00292)

Instrument controller software run summary:

Filename and data path: C:\AATI\Data\2021 10 20\17-48-56\2021 10 20 17H 48M..raw
Created: Wednesday, October 20, 2021 5:58:44 PM
Number of capillaries: 96
Array serial number: 111920-09SFS
Effect length: 33 cm
Array usage count: 263
Instrument type: ZAG DNA Analyzer
Instrument controller software version: 1.0.1.9
Device serial number: Z7597

Method Information

Method name: ZAG130GP33 - DNA 75-20000bp - Gel Prime Only.mthds
Gel prime: Yes
Full conditioning: No
Gel prime to buffer: No
Gel selection: Gel 1
Perform prerun: 8.0 kV, 30 sec.
Rinse: No
Marker 1: Row: A, 5.0 kV, 5 sec.
Rinse: No
Sample injection: 5.0 kV, 5 sec.
Separation: 8.0 kV, 30.0 min.
Tray name: Sample Tray-3

Analysis mode: DNA

Notes

Gel Image

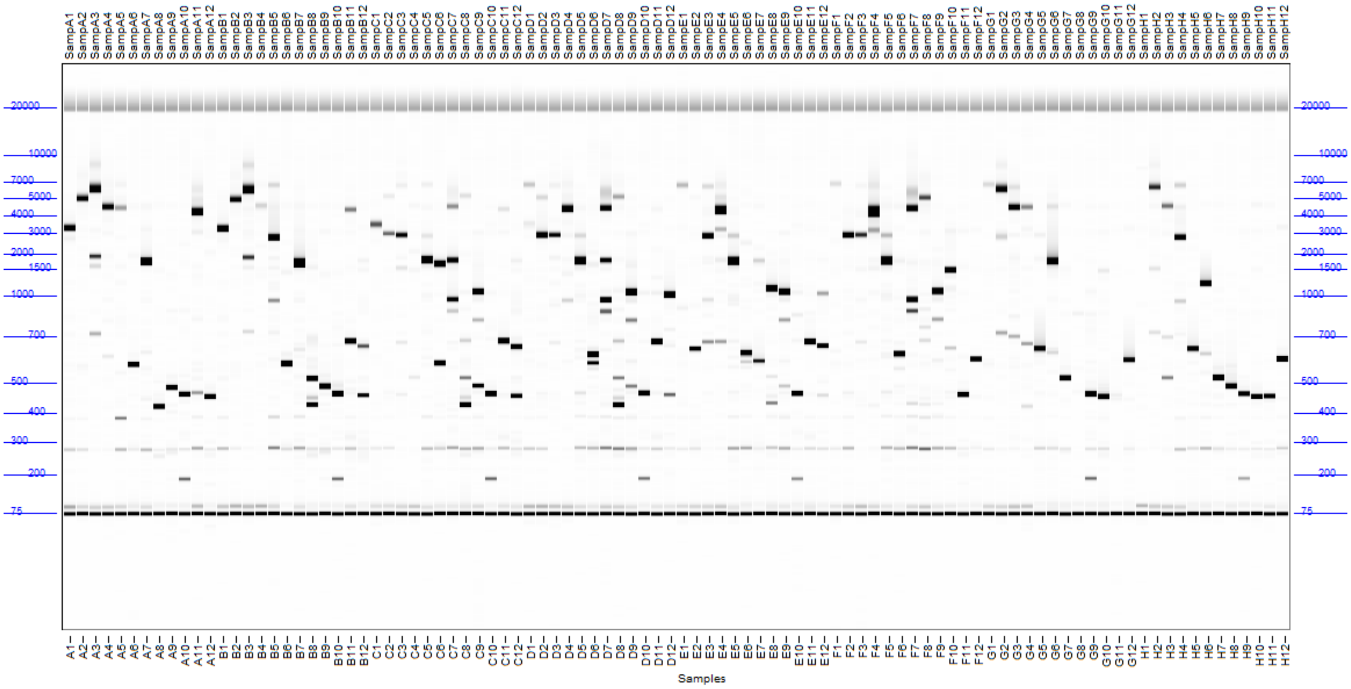

20000
10000
7000
5000
4000
3000
2000
1500
1000
700
500
400
300
200
75
Samples

Filename and data path: C:\AATI\Data\2021 10 20\17-48-56\2021 10 20 17H 48M..raw

A1: SampA1

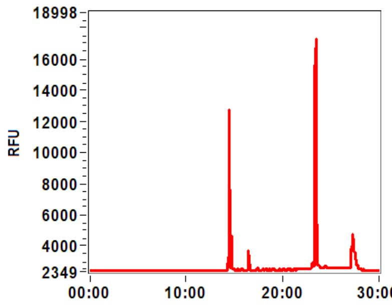

A2: SampA2

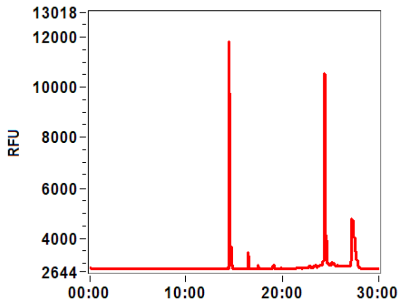

A3: SampA3

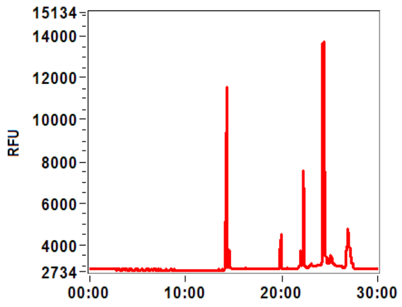

A4: SampA4

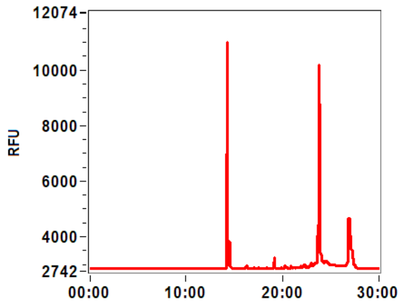

A5: SampA5

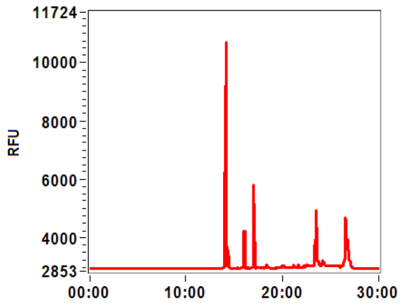

A6: SampA6

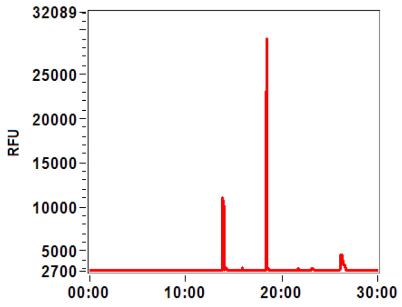

A7: SampA7

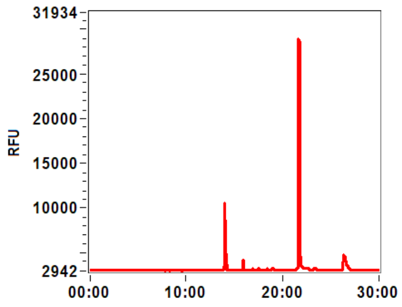

A8: SampA8

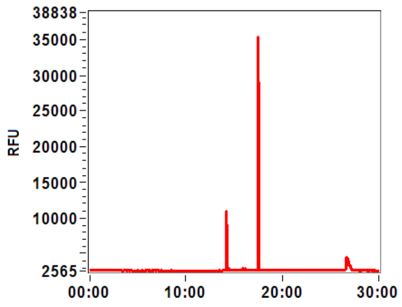

A9: SampA9

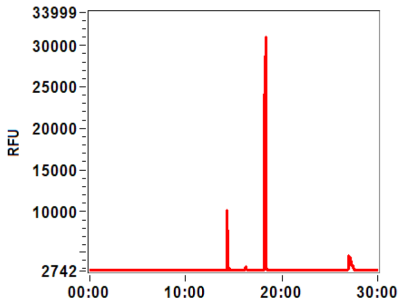

A10: SampA10

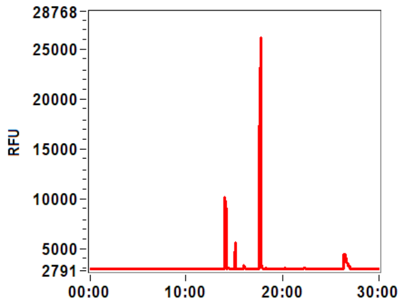

A11: SampA11

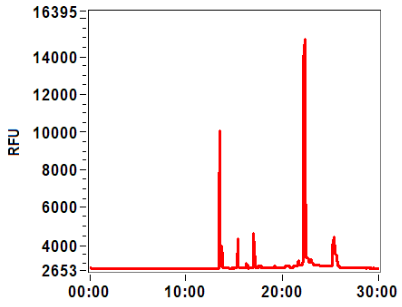

A12: SampA12

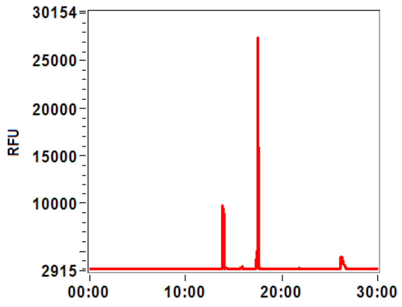

Filename and data path: C:\AATI\Data\2021 10 20\17-48-56\2021 10 20 17H 48M..raw

B1: SampB1

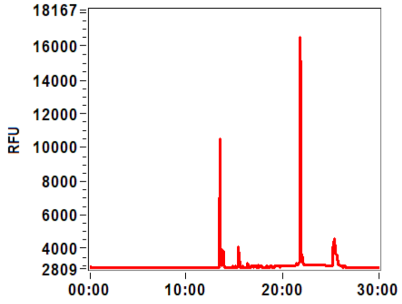

B2: SampB2

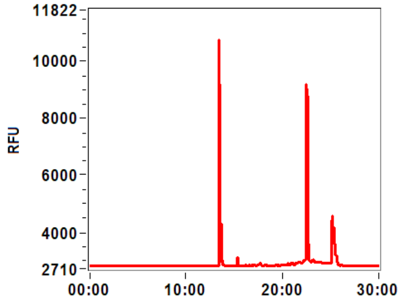

B3: SampB3

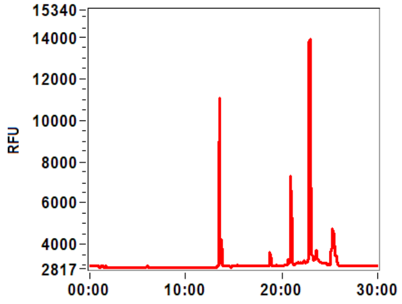

B4: SampB4

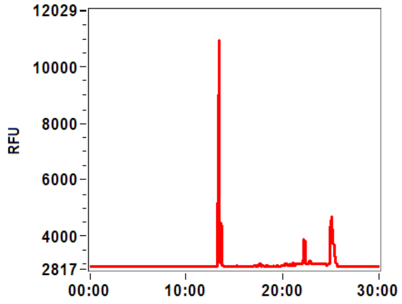

B5: SampB5

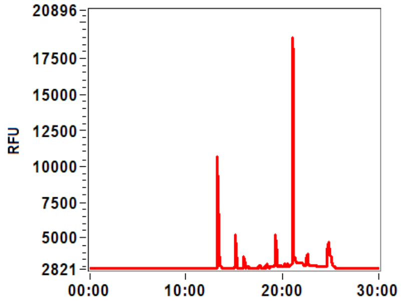

B6: SampB6

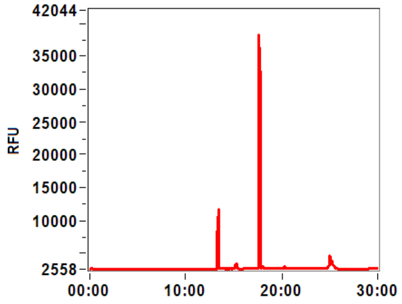

B7: SampB7

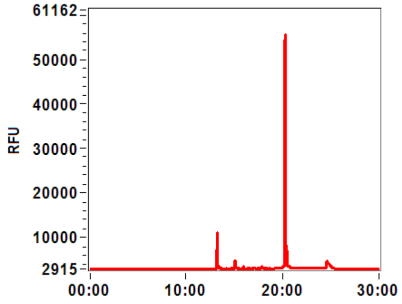

B8: SampB8

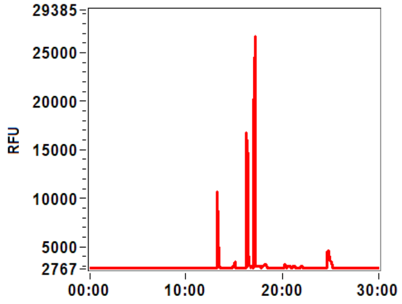

B9: SampB9

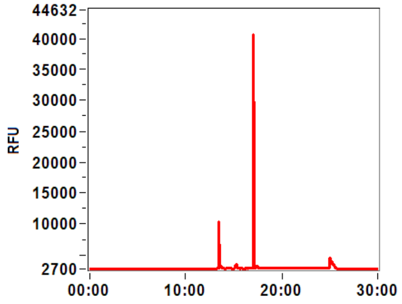

B10: SampB10

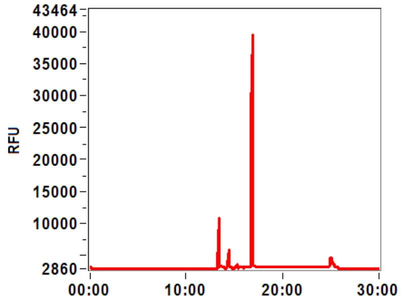

B11: SampB11

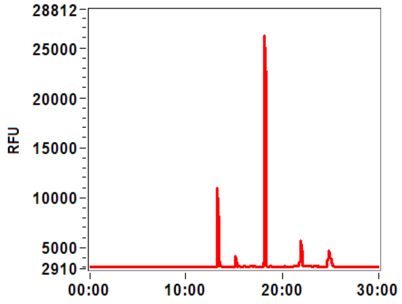

B12: SampB12

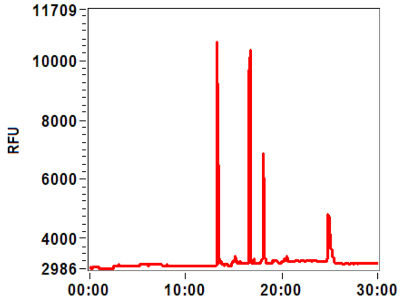

Filename and data path: C:\AATI\Data\2021 10 20\17-48-56\2021 10 20 17H 48M..raw

C1: SampC1

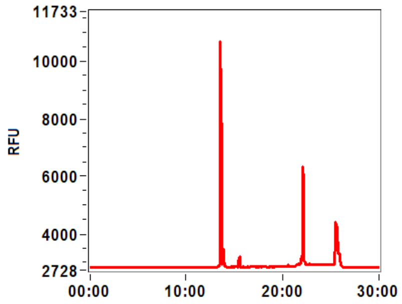

C2: SampC2

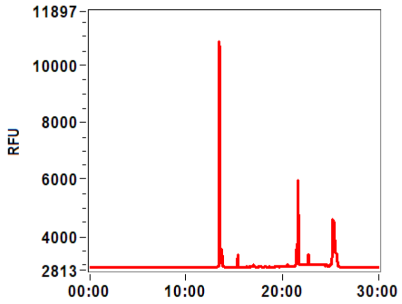

C3: SampC3

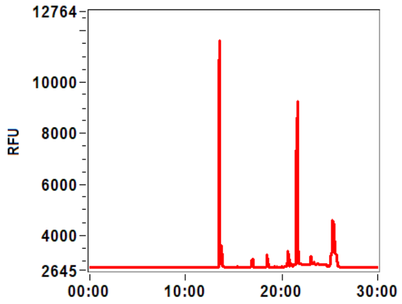

C4: SampC4

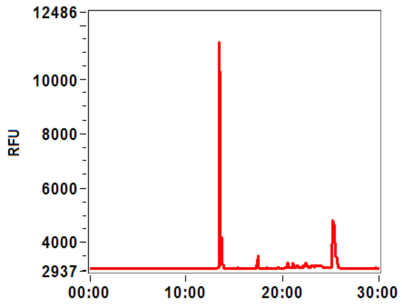

C5: SampC5

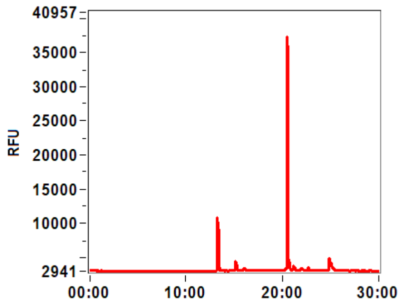

C6: SampC6

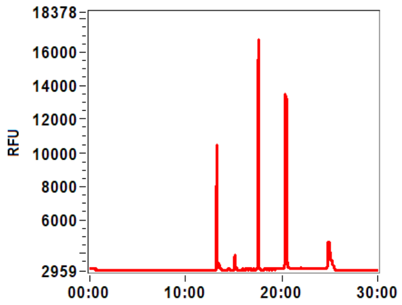

C7: SampC7

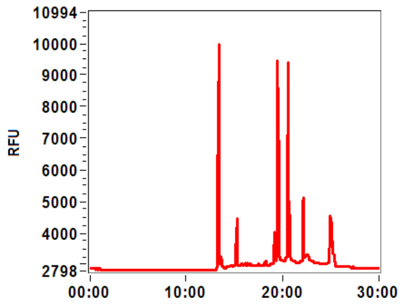

C8: SampC8

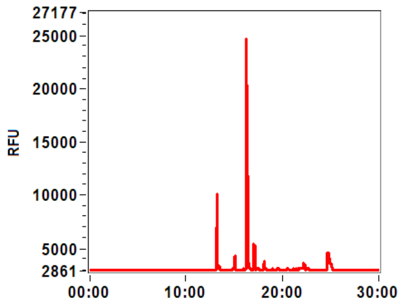

C9: SampC9

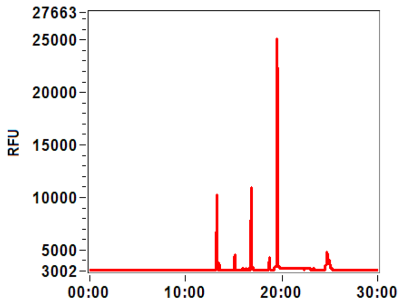

C10: SampC10

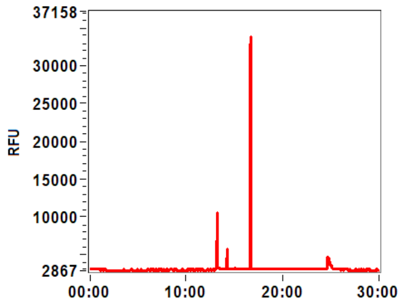

C11: SampC11

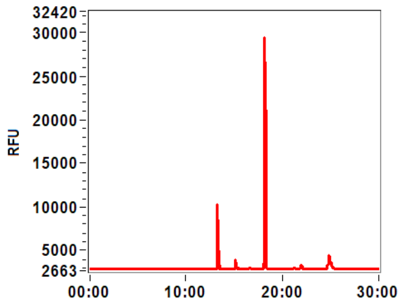

C12: SampC12

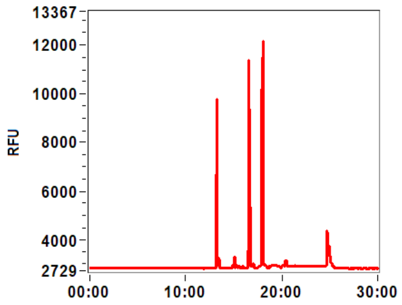

Filename and data path: C:\AATI\Data\2021 10 20\17-48-56\2021 10 20 17H 48M..raw

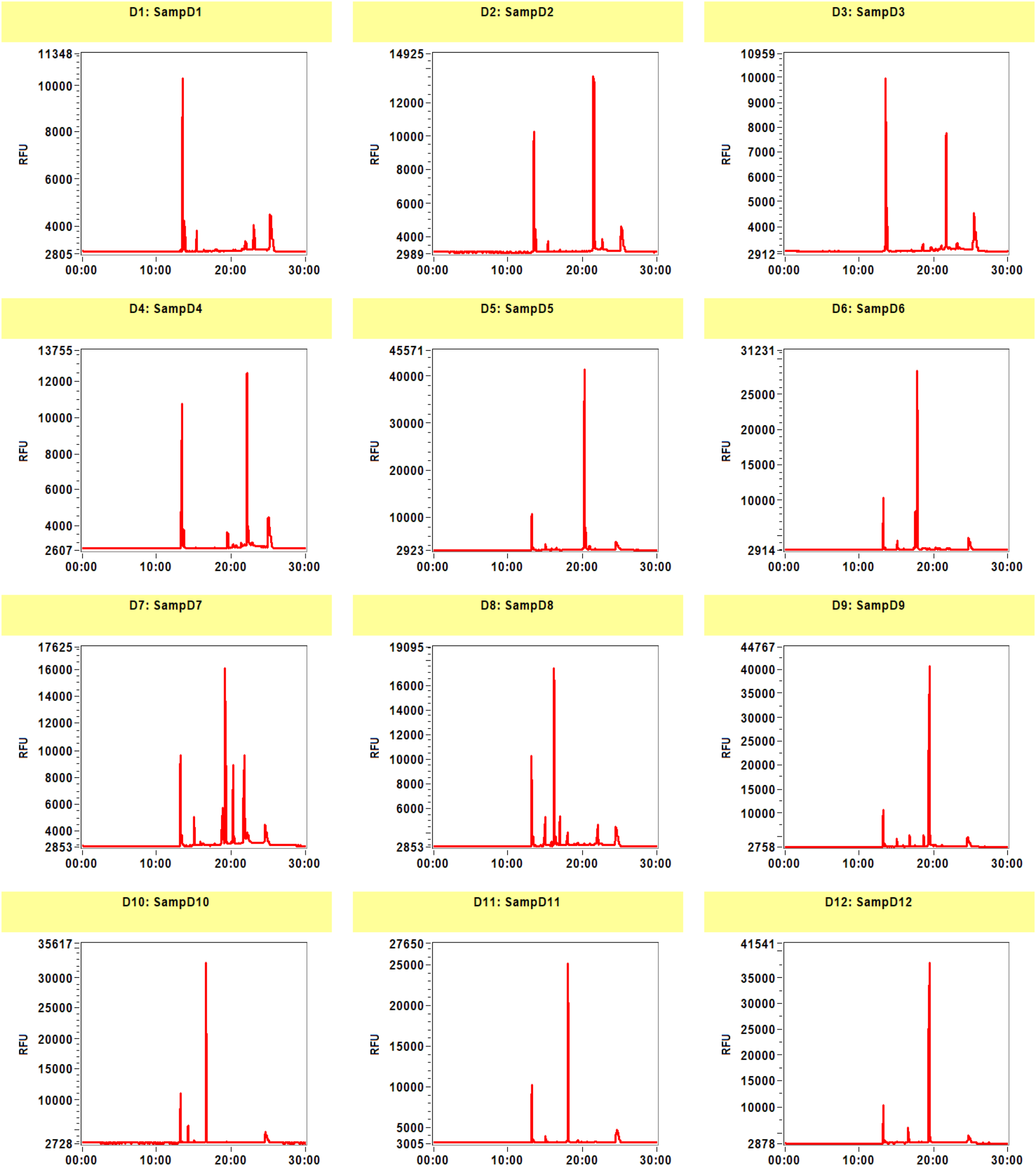

Filename and data path: C:\AATI\Data\2021 10 20\17-48-56\2021 10 20 17H 48M..raw

E1: SampE1

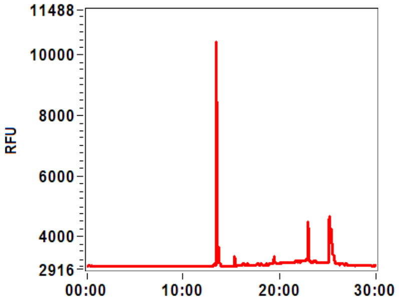

E2: SampE2

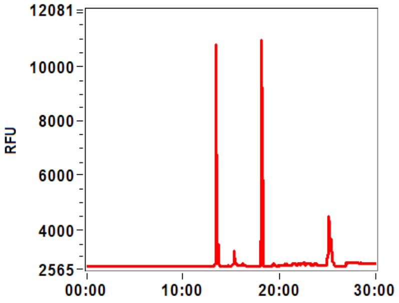

E3: SampE3

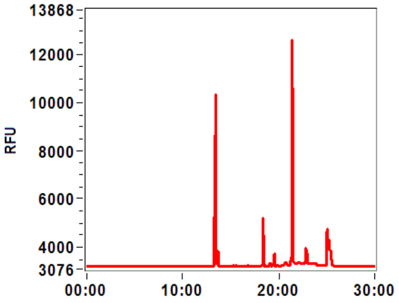

E4: SampE4

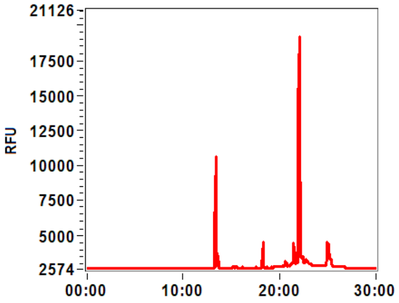

E5: SampE5

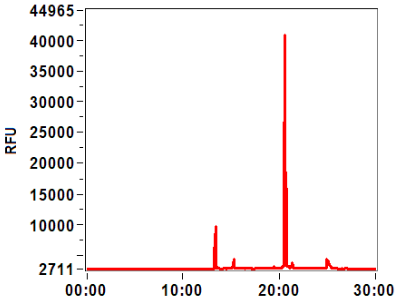

E6: SampE6

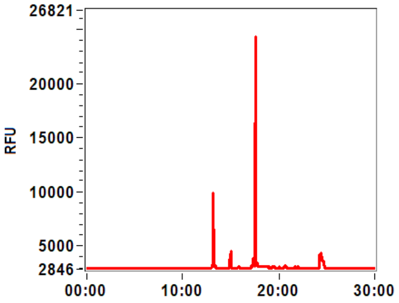

E7: SampE7

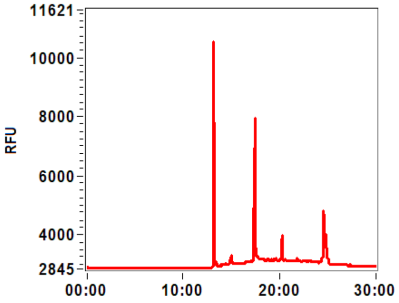

E8: SampE8

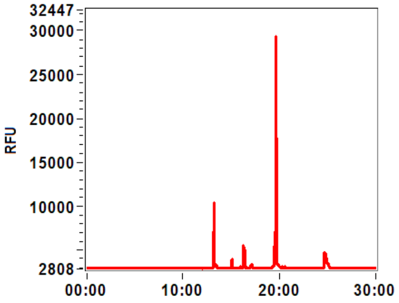

E9: SampE9

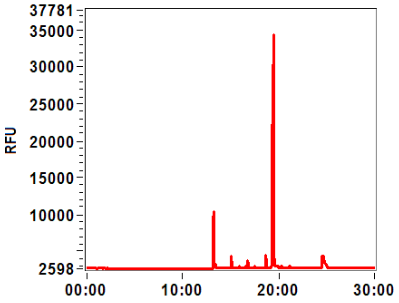

E10: SampE10

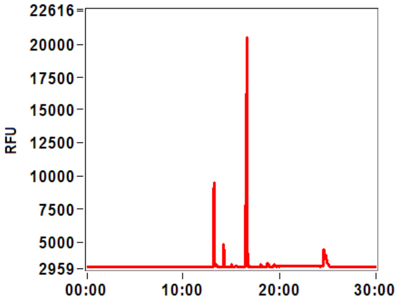

E11: SampE11

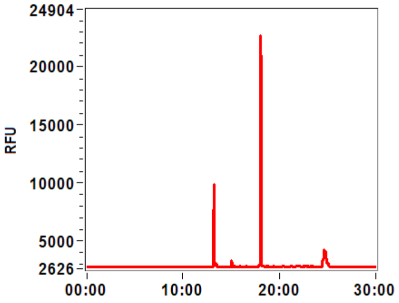

E12: SampE12

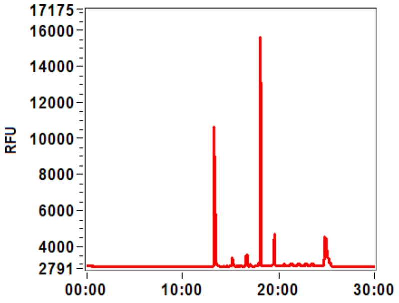

Filename and data path: C:\AATI\Data\2021 10 20\17-48-56\2021 10 20 17H 48M..raw

F1: SampF1

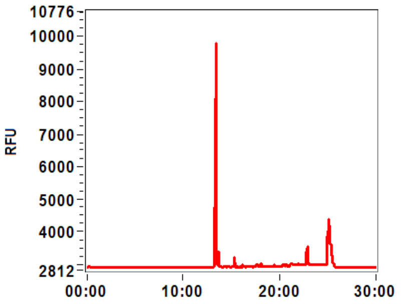

F2: SampF2

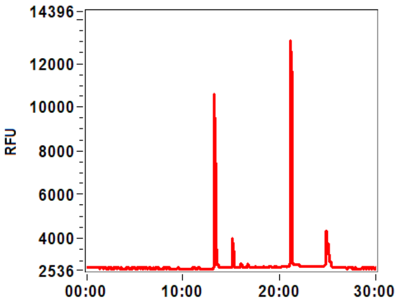

F3: SampF3

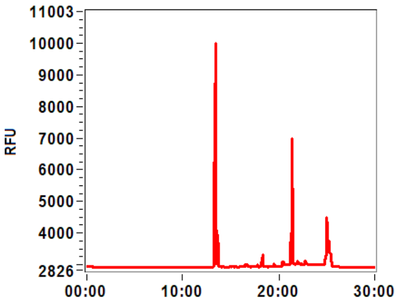

F4: SampF4

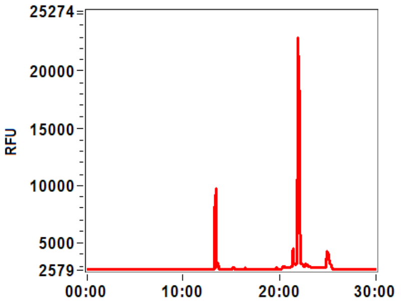

F5: SampF5

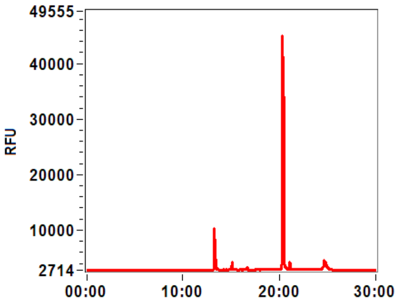

F6: SampF6

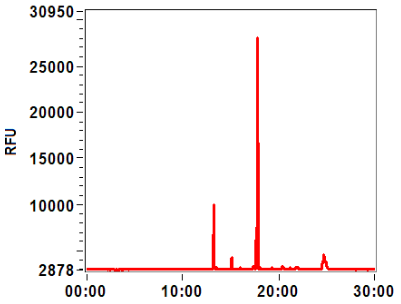

F7: SampF7

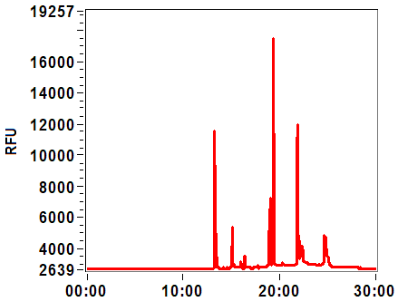

F8: SampF8

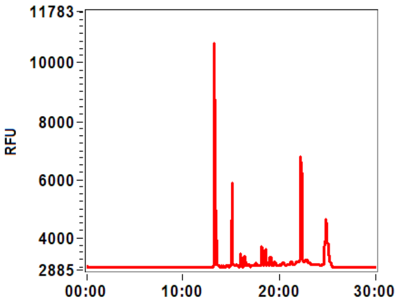

F9: SampF9

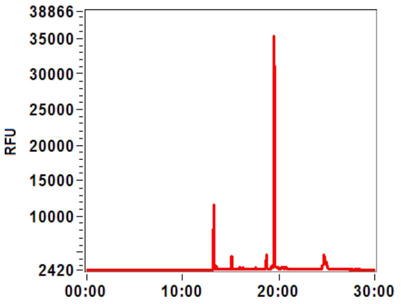

F10: SampF10

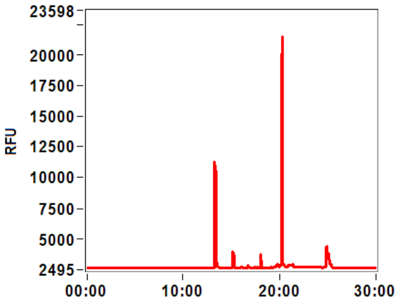

F11: SampF11

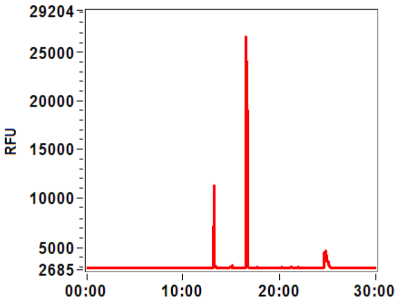

F12: SampF12

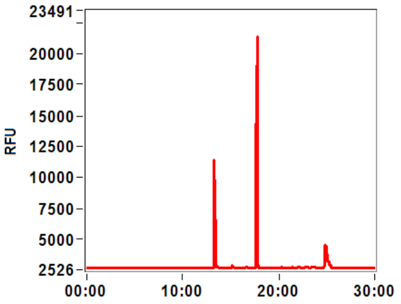

Filename and data path: C:\AATI\Data\2021 10 20\17-48-56\2021 10 20 17H 48M..raw

G1: SampG1

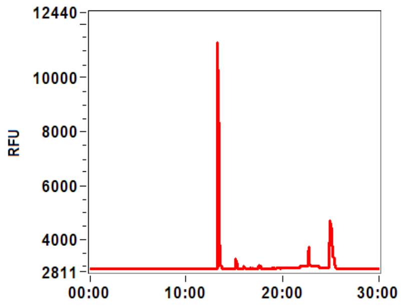

G2: SampG2

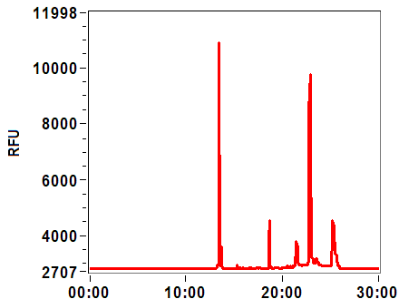

G3: SampG3

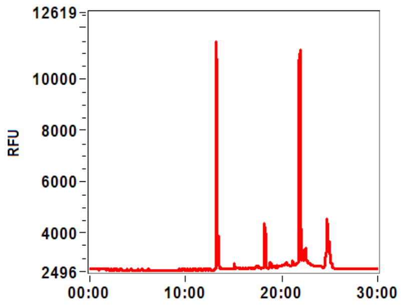

G4: SampG4

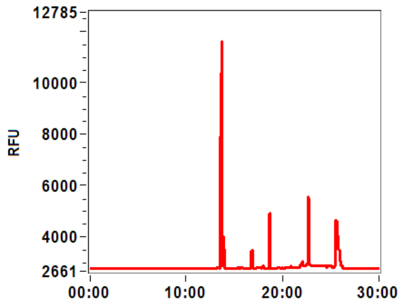

G5: SampG5

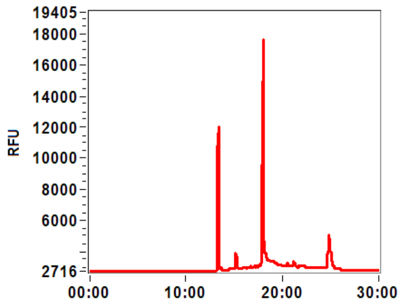

G6: SampG6

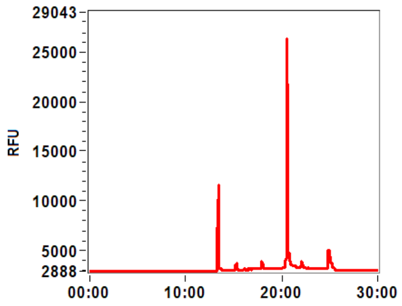

G7: SampG7

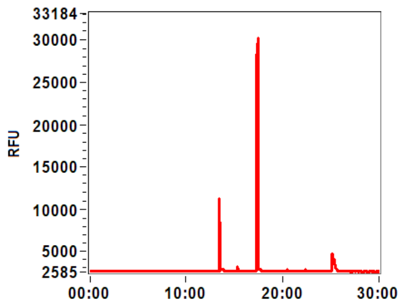

G8: SampG8

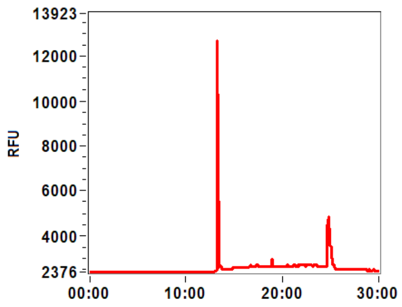

G9: SampG9

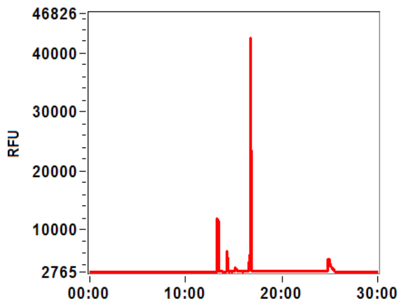

G10: SampG10

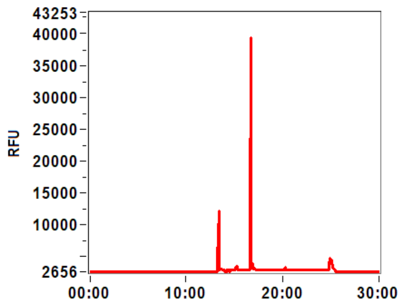

G11: SampG11

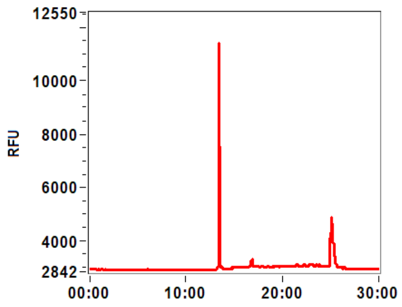

G12: SampG12

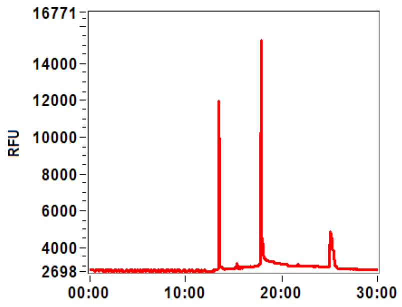

Filename and data path: C:\AATI\Data\2021 10 20\17-48-56\2021 10 20 17H 48M..raw

H1: SampH1

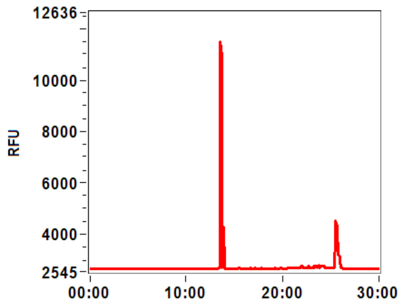

H2: SampH2

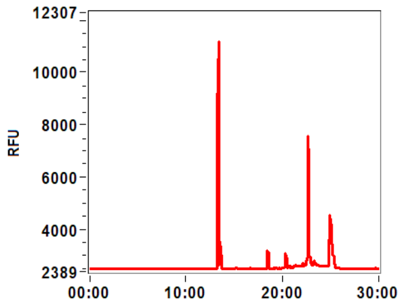

H3: SampH3

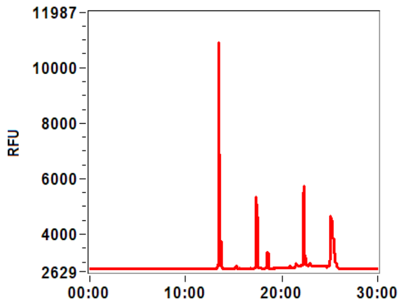

H4: SampH4

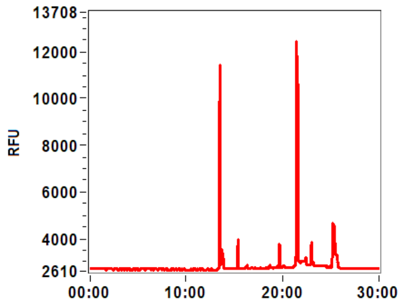

H5: SampH5

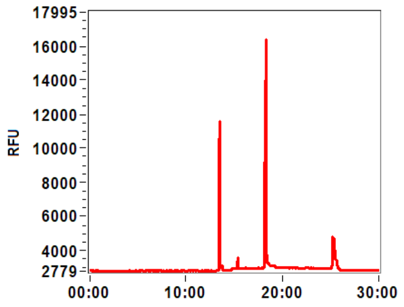

H6: SampH6

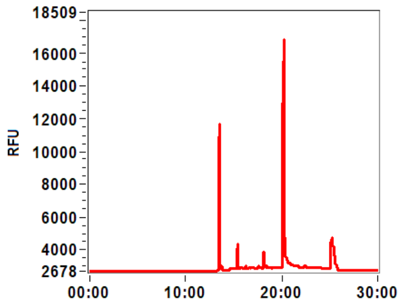

H7: SampH7

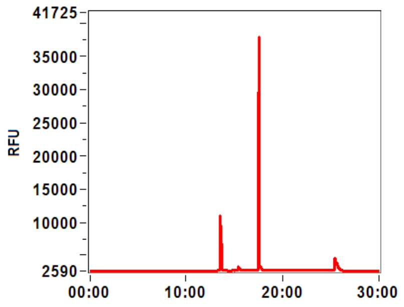

H8: SampH8

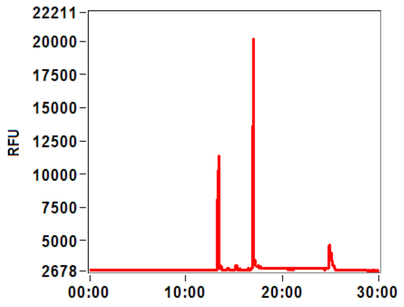

H9: SampH9

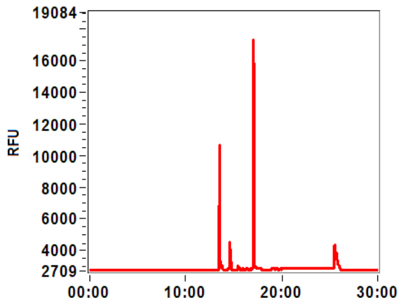

H10: SampH10

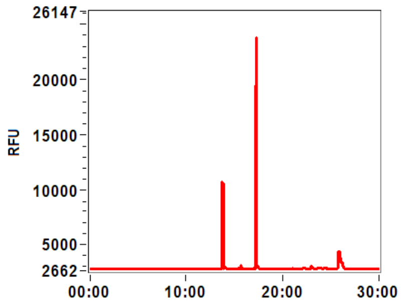

H11: SampH11

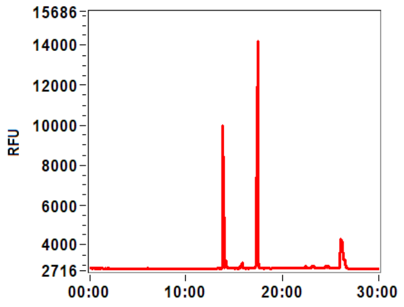

H12: SampH12

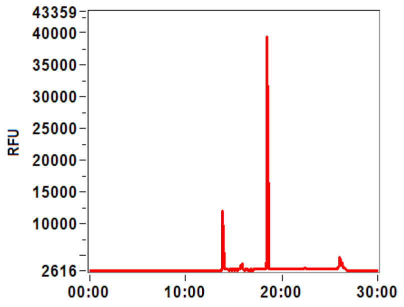

Sample: SampA1
Well location: A1
Created: Wednesday, October 20, 2021 5:58:44 PM

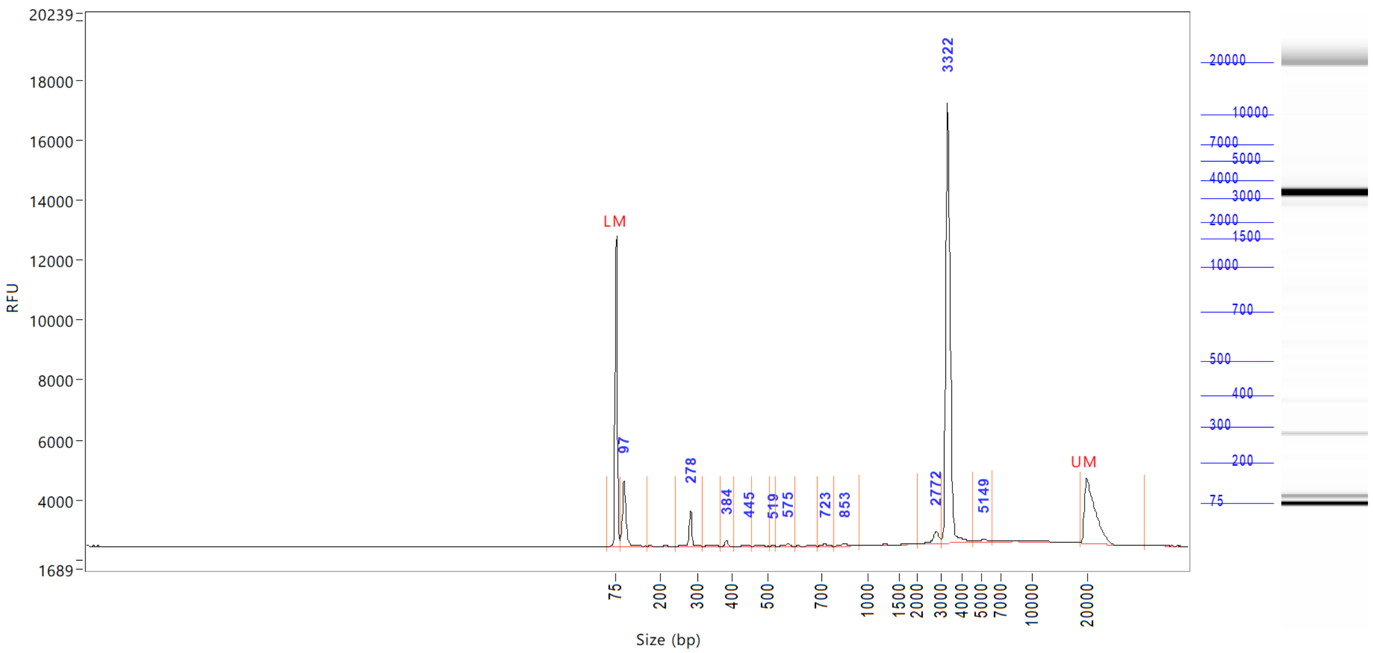

Peak	Size (bp)	Concentration (ng/uL)	From (bp)	To (bp)	Average size (bp)	CV%	RFU	Corrected peak area
1	75 (LM)	0.5000	50	84	72	4.50	10349	53.182
2	97	1.9784	84	162	97	10.61	2207	17.536
3	278	0.6965	239	313	277	2.49	1205	6.173
4	384	0.1200	366	405	381	1.42	198	1.064
5	445	0.0561	405	456	438	2.12	58	0.497
6	519	0.0282	506	532	516	0.86	53	0.250
7	575	0.0955	532	600	567	2.07	95	0.846
8	723	0.0798	687	783	718	2.32	97	0.707
9	853	0.1148	783	944	851	3.65	81	1.018
10	2772	0.3718	2001	3000	2720	6.88	384	3.296
11	3322	9.7117	3000	4621	3310	5.15	14711	86.081
12	5149	0.1242	4621	6186	5165	7.31	85	1.101
13	20000 (UM)	0.2543	18781	30277	20829	5.51	2176	27.044

TIC:	13.3769	ng/uL
TIM:	44.2204	nmole/L
Total concentration:	13.9207	ng/uL

```
Sample peak width (sec): 5    Sample min peak height: 50    Sample baseline V to V?: Y    Sample baseline V to V points: 3
Sample filter: Binomial       Number of points for filter: 3  Sample start region (min): 0  Sample end region (min): 30
Marker peak width (sec): 5    Marker min peak height: 200   Marker baseline V to V?: Y    Marker baseline V to V points: 3
Lower marker selection: First peak > 500 RFU                Upper marker selection: Last peak > 200 RFU
Ladder size (bp)75, 200, 300, 400, 500, 700, 1000, 1500, 2000, 3000, 4000, 5000, 7000, 10000, 20000
Quantification using: Lower Marker          Final concentration (ng/uL): 0.5000          Dilution factor: 12.0
```

Sample: SampA2
Well location: A2
Created: Wednesday, October 20, 2021 5:58:44 PM

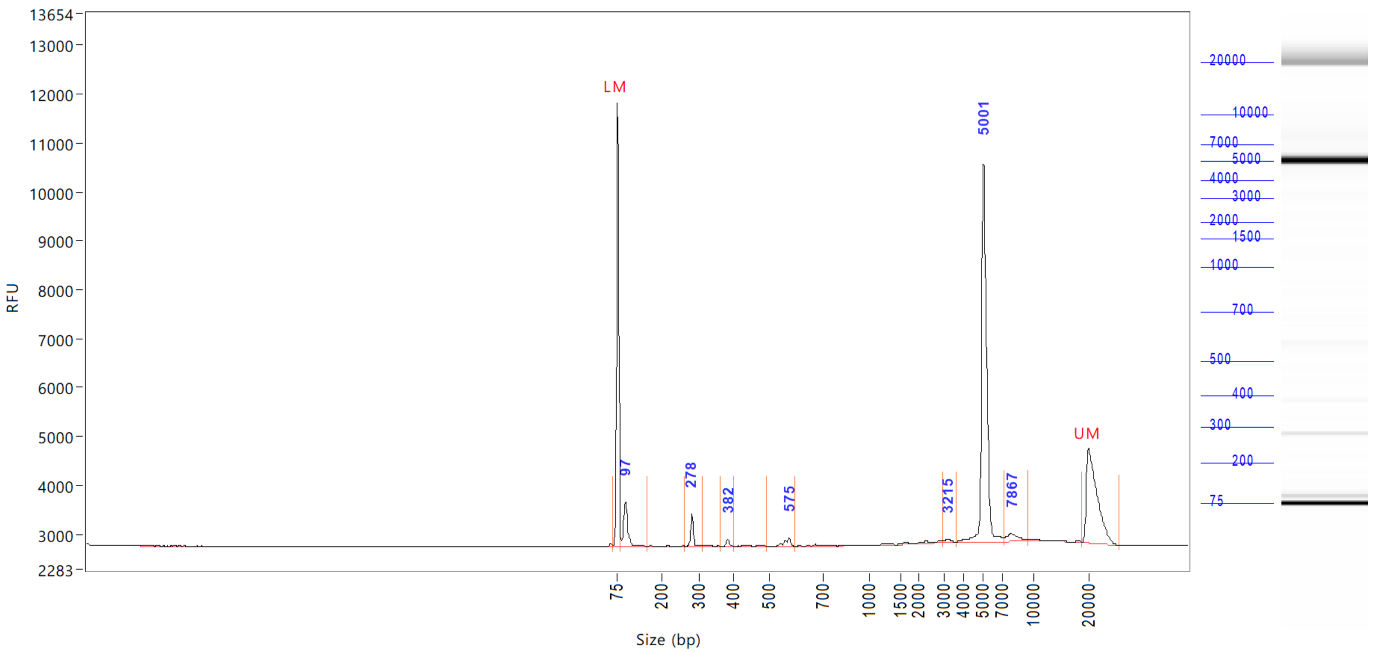

Peak	Size (bp)	Concentration (ng/uL)	From (bp)	To (bp)	Average size (bp)	CV%	RFU	Corrected peak area
1	75 (LM)	0.5000	64	84	73	4.12	9089	45.470
2	97	1.0785	84	158	98	11.33	907	8.173
3	278	0.4494	262	309	278	2.03	661	3.406
4	382	0.1009	362	401	381	1.31	144	0.765
5	575	0.2184	494	595	560	2.87	159	1.655
6	3215	0.0739	2971	3643	3198	4.21	70	0.560
7	5001	6.6682	3643	7201	5107	6.75	7717	50.533
8	7867	0.3277	7201	9600	8133	7.46	158	2.484
9	20000 (UM)	0.2625	18781	25440	20815	5.23	1950	23.876

TIC:	8.9171	ng/uL
TIM:	24.0605	nmole/L
Total concentration:	9.5189	ng/uL

```
Sample peak width (sec): 5    Sample min peak height: 50    Sample baseline V to V?: Y    Sample baseline V to V points: 3
Sample filter: Binomial       Number of points for filter: 3  Sample start region (min): 0  Sample end region (min): 30
Marker peak width (sec): 5    Marker min peak height: 200   Marker baseline V to V?: Y    Marker baseline V to V points: 3
Lower marker selection: First peak > 500 RFU                Upper marker selection: Last peak > 200 RFU
Ladder size (bp)75, 200, 300, 400, 500, 700, 1000, 1500, 2000, 3000, 4000, 5000, 7000, 10000, 20000
Quantification using: Lower Marker          Final concentration (ng/uL): 0.5000          Dilution factor: 12.0
```

Sample: SampA3
Well location: A3
Created: Wednesday, October 20, 2021 5:58:44 PM

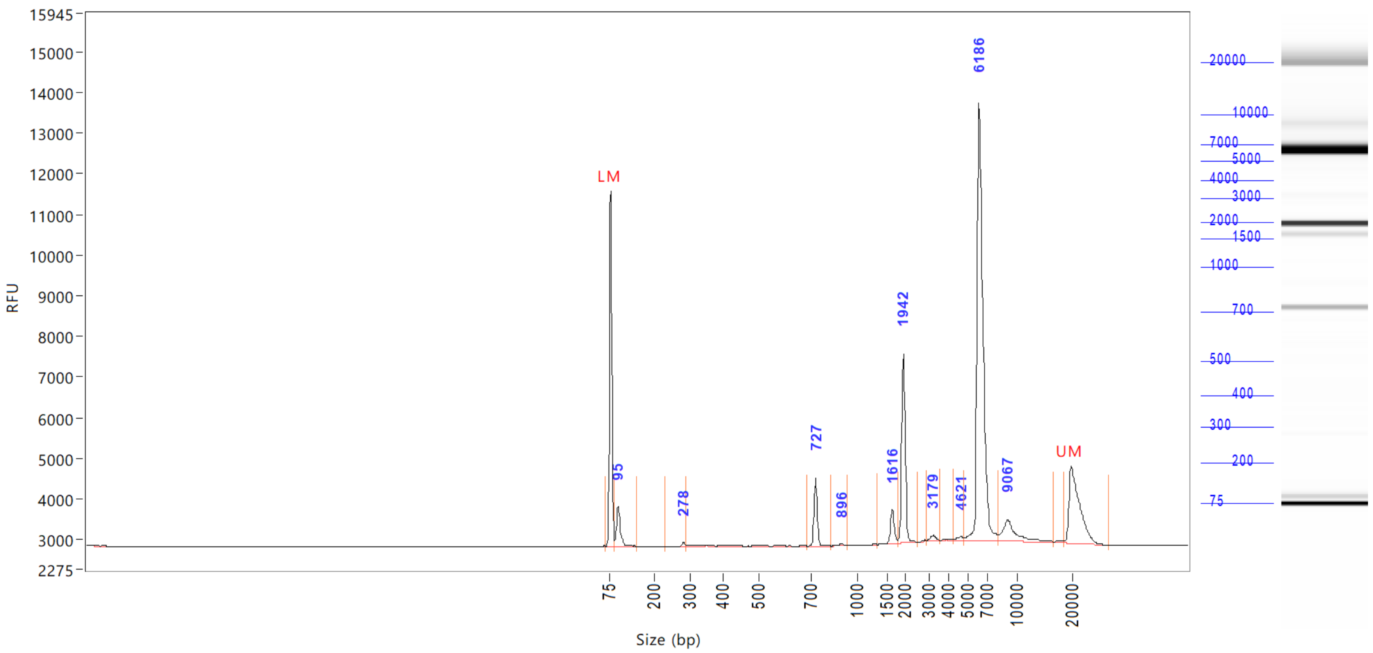

Peak	Size (bp)	Concentration (ng/uL)	From (bp)	To (bp)	Average size (bp)	CV%	RFU	Corrected peak area
1	75 (LM)	0.5000	62	86	73	4.65	8739	45.156
2	95	1.1671	86	152	96	9.19	991	8.784
3	278	0.0794	230	287	271	4.91	92	0.597
4	727	1.1350	685	831	722	1.63	1682	8.542
5	896	0.0518	831	935	888	2.09	52	0.390
6	1616	0.6452	1334	1770	1604	4.06	839	4.856
7	1942	3.3332	1770	2515	1928	3.09	4633	25.086
8	3179	0.1274	2914	3572	3143	3.86	128	0.959
9	4621	0.1049	4242	4828	4570	3.54	83	0.789
10	6186	11.3099	4828	8201	6271	6.67	10778	85.118
11	9067	1.1688	8201	16708	9976	18.02	533	8.796
12	20000 (UM)	0.2633	18659	26770	20784	5.59	1883	23.775

TIC:	19.1228	ng/uL
TIM:	29.9014	nmole/L
Total concentration:	19.5401	ng/uL

```
Sample peak width (sec): 5    Sample min peak height: 50    Sample baseline V to V?: Y    Sample baseline V to V points: 3
Sample filter: Binomial       Number of points for filter: 3  Sample start region (min): 0  Sample end region (min): 30
Marker peak width (sec): 5    Marker min peak height: 200   Marker baseline V to V?: Y    Marker baseline V to V points: 3
Lower marker selection: First peak > 500 RFU                Upper marker selection: Last peak > 200 RFU
Ladder size (bp)75, 200, 300, 400, 500, 700, 1000, 1500, 2000, 3000, 4000, 5000, 7000, 10000, 20000
Quantification using: Lower Marker          Final concentration (ng/uL): 0.5000          Dilution factor: 12.0
```

Sample: SampA4
Well location: A4
Created: Wednesday, October 20, 2021 5:58:44 PM

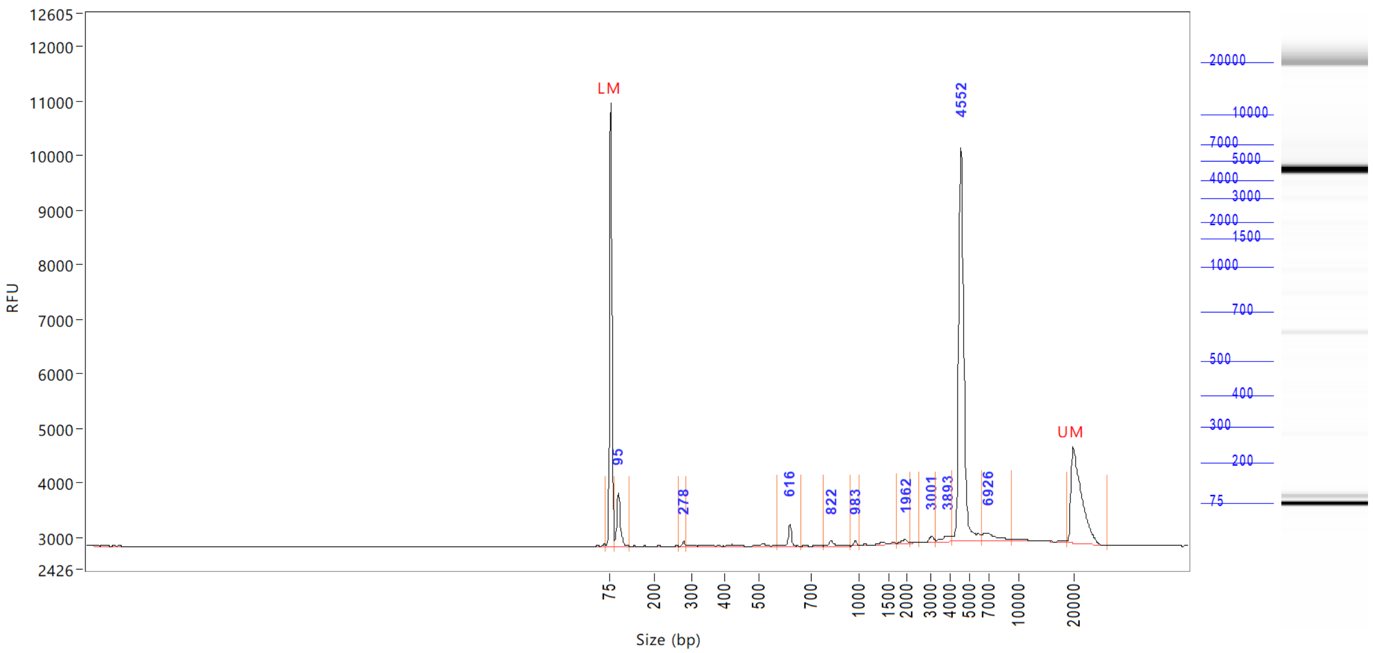

Peak	Size (bp)	Concentration (ng/uL)	From (bp)	To (bp)	Average size (bp)	CV%	RFU	Corrected peak area
1	75 (LM)	0.5000	64	86	73	4.49	8136	41.407
2	95	1.1603	86	131	95	6.37	985	8.007
3	278	0.0703	268	287	277	1.42	98	0.485
4	616	0.3070	567	659	612	1.50	409	2.119
5	822	0.1175	770	948	836	3.94	96	0.811
6	983	0.0639	948	1001	976	1.02	87	0.441
7	1962	0.0976	1712	2144	1901	4.71	75	0.673
8	3001	0.1131	2543	3286	3021	4.14	117	0.781
9	3893	0.1820	3286	4070	3772	5.70	105	1.256
10	4552	7.0877	4070	6334	4585	5.52	7215	48.913
11	6926	0.3566	6334	9334	7387	10.71	143	2.461
12	20000 (UM)	0.2641	18781	26287	20797	5.44	1753	21.870

TIC:	9.5559	ng/uL
TIM:	24.4845	nmole/L
Total concentration:	10.0875	ng/uL

```
Sample peak width (sec): 5     Sample min peak height: 50    Sample baseline V to V?: Y    Sample baseline V to V points: 3
Sample filter: Binomial        Number of points for filter: 3  Sample start region (min): 0  Sample end region (min): 30
Marker peak width (sec): 5     Marker min peak height: 200   Marker baseline V to V?: Y    Marker baseline V to V points: 3
Lower marker selection: First peak > 500 RFU                 Upper marker selection: Last peak > 200 RFU
Ladder size (bp)75, 200, 300, 400, 500, 700, 1000, 1500, 2000, 3000, 4000, 5000, 7000, 10000, 20000
Quantification using: Lower Marker          Final concentration (ng/uL): 0.5000          Dilution factor: 12.0
```

Sample: SampA5
Well location: A5
Created: Wednesday, October 20, 2021 5:58:44 PM

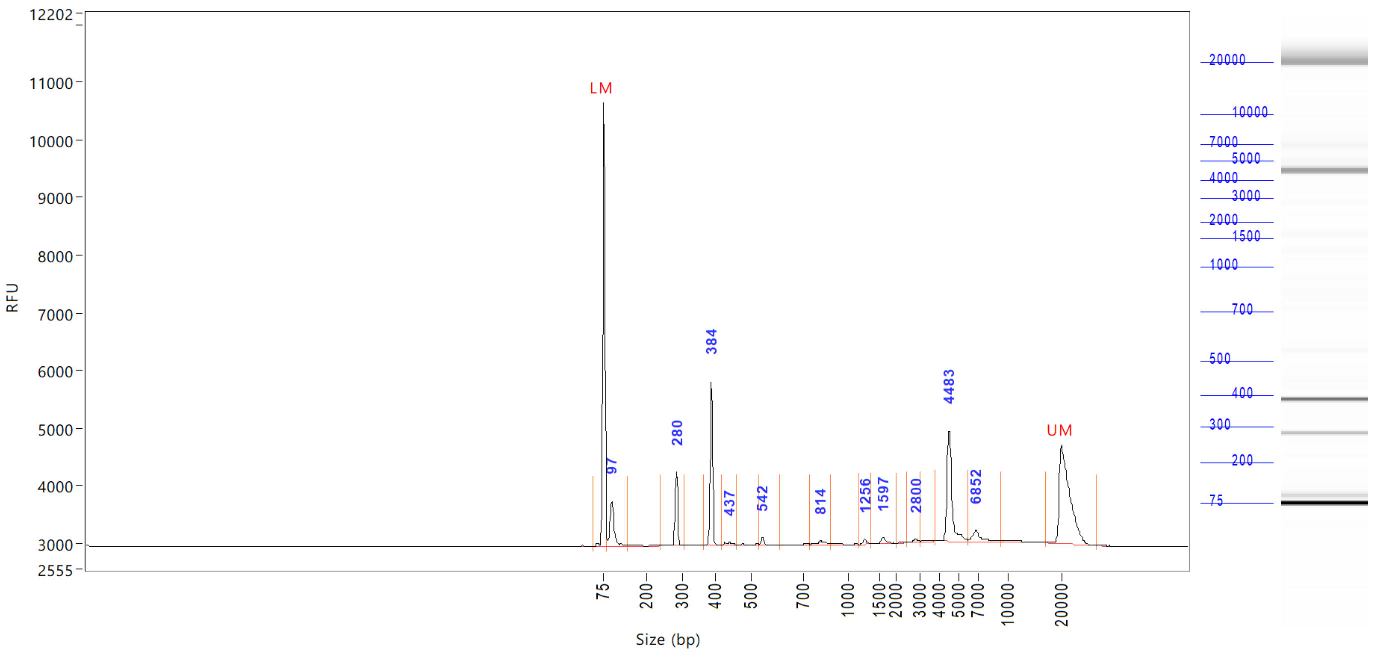

Peak	Size (bp)	Concentration (ng/uL)	From (bp)	To (bp)	Average size (bp)	CV%	RFU	Corrected peak area
1	75 (LM)	0.5000	47	86	73	4.84	7712	39.748
2	97	1.0512	86	145	98	9.54	778	6.964
3	280	0.9725	237	301	277	2.02	1269	6.442
4	384	2.0144	360	412	383	1.05	2830	13.344
5	437	0.1139	412	460	435	2.51	50	0.755
6	542	0.1334	529	608	547	2.72	130	0.884
7	814	0.1570	744	879	812	3.94	68	1.040
8	1256	0.0766	1167	1378	1258	2.67	84	0.507
9	1597	0.1245	1378	2001	1611	6.39	102	0.825
10	2800	0.0601	2429	3001	2763	4.01	60	0.398
11	4483	1.8939	3786	5963	4537	5.24	1916	12.547
12	6852	0.3220	5963	9334	7117	10.15	192	2.133
13	20000 (UM)	0.2677	17073	26408	20814	5.54	1700	21.280

TIC:	6.9195	ng/uL
TIM:	34.2159	nmole/L
Total concentration:	7.3737	ng/uL

Sample peak width (sec): 5 Sample min peak height: 50 Sample baseline V to V?: Y Sample baseline V to V points: 3
Sample filter: Binomial Number of points for filter: 3 Sample start region (min): 0 Sample end region (min): 30
Marker peak width (sec): 5 Marker min peak height: 200 Marker baseline V to V?: Y Marker baseline V to V points: 3
Lower marker selection: First peak > 500 RFU Upper marker selection: Last peak > 200 RFU
Ladder size (bp)75, 200, 300, 400, 500, 700, 1000, 1500, 2000, 3000, 4000, 5000, 7000, 10000, 20000
Quantification using: Lower Marker Final concentration (ng/uL): 0.5000 Dilution factor: 12.0

Sample: SampA6
Well location: A6
Created: Wednesday, October 20, 2021 5:58:44 PM

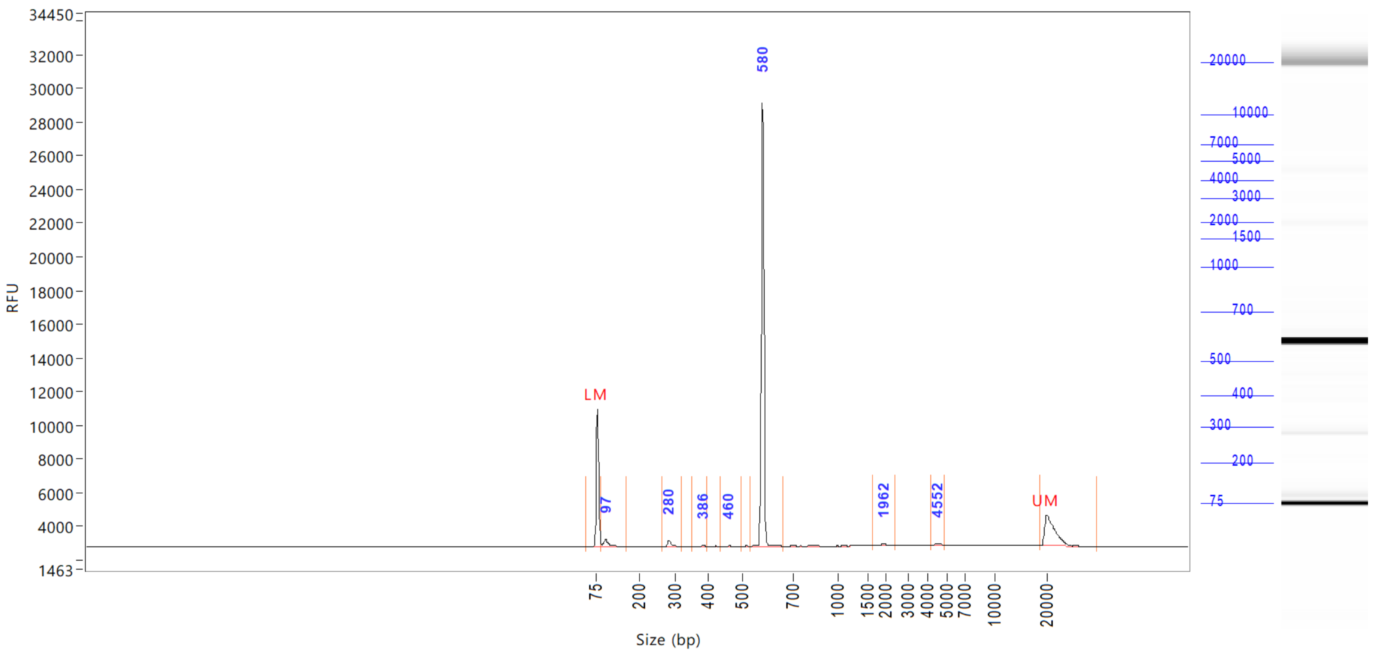

Peak	Size (bp)	Concentration (ng/uL)	From (bp)	To (bp)	Average size (bp)	CV%	RFU	Corrected peak area
1	75 (LM)	0.5000	41	87	73	4.53	8187	42.358
2	97	0.7575	87	164	104	12.80	403	5.348
3	280	0.3984	262	317	283	2.86	368	2.813
4	386	0.0562	352	398	383	1.88	52	0.397
5	460	0.0824	435	496	466	2.95	56	0.582
6	580	17.5983	534	662	578	1.19	26356	124.238
7	1962	0.1605	1655	2429	1942	6.32	126	1.133
8	4552	0.1203	4208	4931	4559	3.27	98	0.849
9	20000 (UM)	0.2618	18781	29309	20775	5.43	1805	22.175

TIC:	19.1736	ng/uL
TIM:	65.0930	nmole/L
Total concentration:	19.7772	ng/uL

```
Sample peak width (sec): 5    Sample min peak height: 50    Sample baseline V to V?: Y    Sample baseline V to V points: 3
Sample filter: Binomial       Number of points for filter: 3  Sample start region (min): 0  Sample end region (min): 30
Marker peak width (sec): 5    Marker min peak height: 200   Marker baseline V to V?: Y    Marker baseline V to V points: 3
Lower marker selection: First peak > 500 RFU                Upper marker selection: Last peak > 200 RFU
Ladder size (bp)75, 200, 300, 400, 500, 700, 1000, 1500, 2000, 3000, 4000, 5000, 7000, 10000, 20000
Quantification using: Lower Marker          Final concentration (ng/uL): 0.5000          Dilution factor: 12.0
```

Sample: SampA7
Well location: A7
Created: Wednesday, October 20, 2021 5:58:44 PM

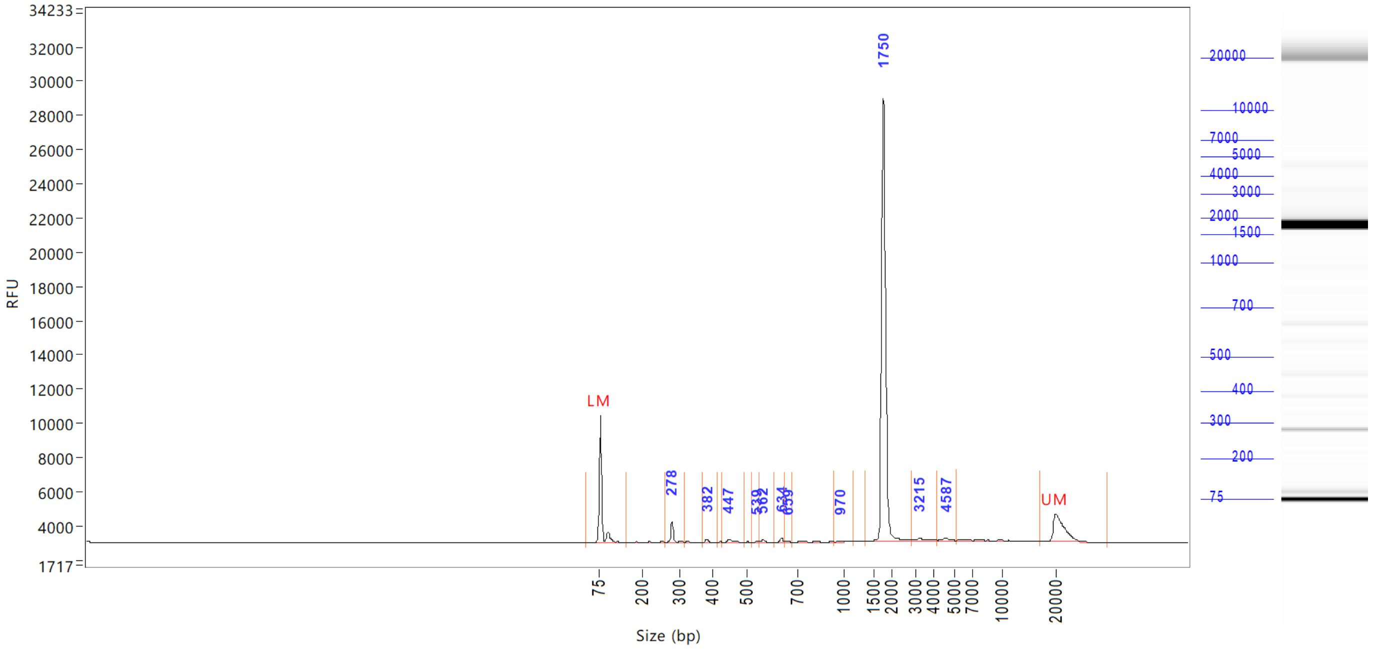

Peak	Size (bp)	Concentration (ng/uL)	From (bp)	To (bp)	Average size (bp)	CV%	RFU	Corrected peak area
1	75 (LM)	0.5000	33	148	76	13.24	7438	44.822
2	278	0.8522	260	315	278	2.72	1195	6.366
3	382	0.1524	372	412	383	1.93	192	1.139
4	447	0.2402	430	492	453	2.76	186	1.795
5	539	0.0497	519	547	536	1.21	62	0.371
6	562	0.1323	547	608	562	1.88	134	0.988
7	634	0.1779	608	649	631	1.06	255	1.329
8	659	0.0465	649	677	658	0.85	60	0.347
9	970	0.1010	939	1145	995	4.31	70	0.754
10	1750	20.6833	1345	2829	1763	5.91	25932	154.512
11	3215	0.2586	2829	4173	3286	9.98	153	1.932
12	4587	0.1629	4173	5223	4562	3.75	136	1.217
13	20000 (UM)	0.2392	17073	29672	20849	6.15	1637	21.439

TIC:	22.8570	ng/uL
TIM:	27.3571	nmole/L
Total concentration:	23.4549	ng/uL

Sample peak width (sec): 5   Sample min peak height: 50   Sample baseline V to V?: Y   Sample baseline V to V points: 3
Sample filter: Binomial   Number of points for filter: 3 Sample start region (min): 0 Sample end region (min): 30
Marker peak width (sec): 5   Marker min peak height: 200   Marker baseline V to V?: Y   Marker baseline V to V points: 3
Lower marker selection: First peak > 500 RFU   Upper marker selection: Last peak > 200 RFU
Ladder size (bp)75, 200, 300, 400, 500, 700, 1000, 1500, 2000, 3000, 4000, 5000, 7000, 10000, 20000
Quantification using: Lower Marker   Final concentration (ng/uL): 0.5000   Dilution factor: 12.0

Sample: SampA8
Well location: A8
Created: Wednesday, October 20, 2021 5:58:44 PM

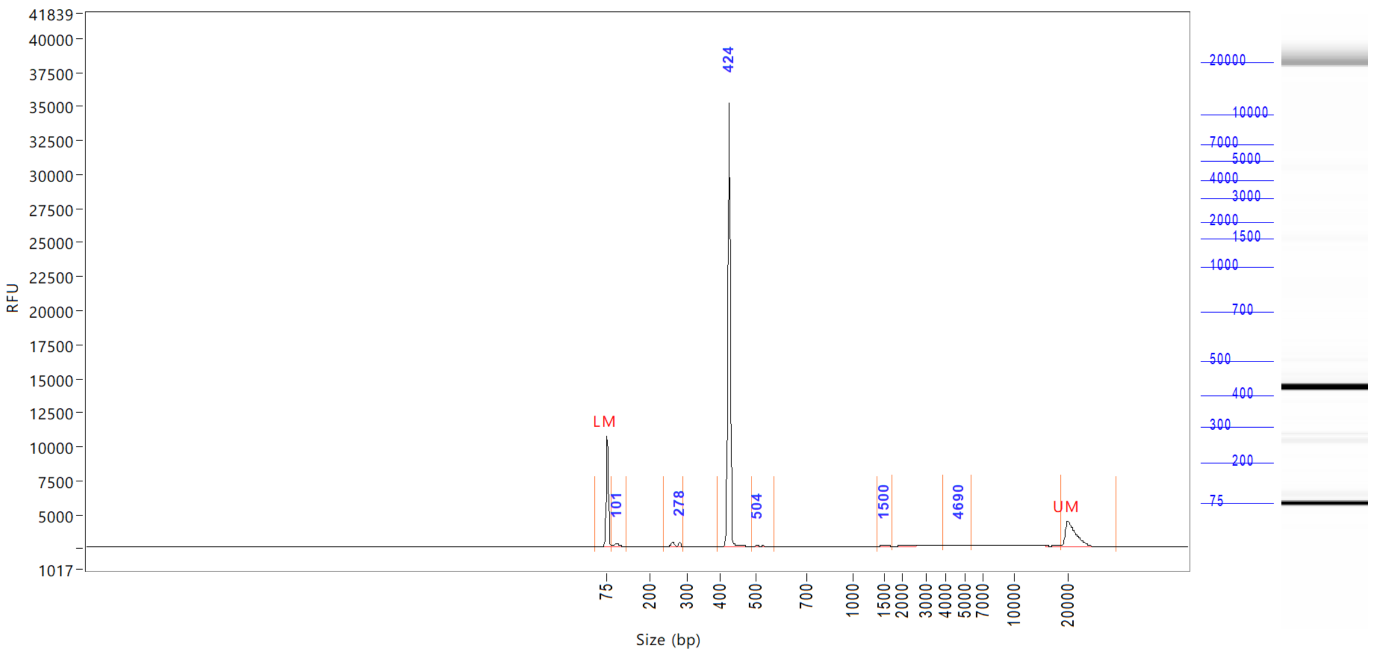

Peak	Size (bp)	Concentration (ng/uL)	From (bp)	To (bp)	Average size (bp)	CV%	RFU	Corrected peak area
1	75 (LM)	0.5000	39	86	73	4.94	8159	41.948
2	101	0.4467	86	133	101	9.49	220	3.123
3	278	0.5426	237	289	264	3.88	301	3.793
4	424	22.0684	390	492	422	1.26	32633	154.286
5	504	0.1631	492	570	517	3.63	118	1.140
6	1500	0.0981	1389	1712	1506	4.14	89	0.686
7	4690	0.0999	3929	5741	4664	6.72	64	0.698
8	20000 (UM)	0.2711	18659	28705	20751	5.49	1833	22.746

TIC:	23.4187	ng/uL
TIM:	97.3471	nmole/L
Total concentration:	24.0060	ng/uL

```
Sample peak width (sec): 5    Sample min peak height: 50    Sample baseline V to V?: Y    Sample baseline V to V points: 3
Sample filter: Binomial       Number of points for filter: 3  Sample start region (min): 0  Sample end region (min): 30
Marker peak width (sec): 5    Marker min peak height: 200   Marker baseline V to V?: Y    Marker baseline V to V points: 3
Lower marker selection: First peak > 500 RFU                Upper marker selection: Last peak > 200 RFU
Ladder size (bp)75, 200, 300, 400, 500, 700, 1000, 1500, 2000, 3000, 4000, 5000, 7000, 10000, 20000
Quantification using: Lower Marker          Final concentration (ng/uL): 0.5000          Dilution factor: 12.0
```

Sample: SampA9
Well location: A9
Created: Wednesday, October 20, 2021 5:58:44 PM

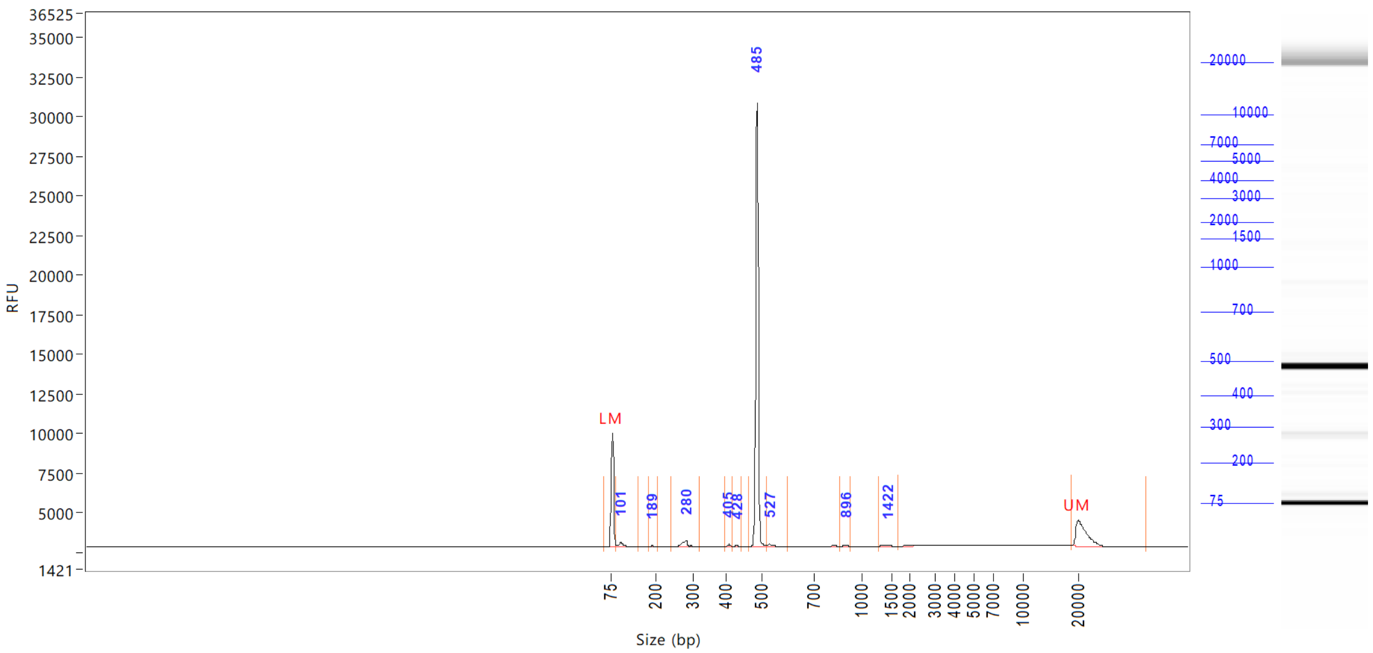

Peak	Size (bp)	Concentration (ng/uL)	From (bp)	To (bp)	Average size (bp)	CV%	RFU	Corrected peak area
1	75 (LM)	0.5000	54	87	74	4.35	7181	38.044
2	101	0.4482	87	150	102	11.05	246	2.842
3	189	0.0587	179	206	189	3.38	60	0.372
4	280	0.6602	239	317	275	3.54	401	4.186
5	405	0.1183	394	418	404	1.15	136	0.750
6	428	0.0788	418	441	426	1.07	88	0.499
7	485	20.7214	464	514	483	0.81	28053	131.386
8	527	0.2113	514	595	533	3.42	134	1.340
9	896	0.1001	857	935	892	1.38	113	0.634
10	1422	0.0849	1267	1674	1390	4.26	50	0.538
11	20000 (UM)	0.2631	18781	32332	20802	5.73	1618	20.022

TIC:	22.4818	ng/uL
TIM:	83.9926	nmole/L
Total concentration:	23.1209	ng/uL

```
Sample peak width (sec): 5    Sample min peak height: 50    Sample baseline V to V?: Y    Sample baseline V to V points: 3
Sample filter: Binomial       Number of points for filter: 3  Sample start region (min): 0  Sample end region (min): 30
Marker peak width (sec): 5    Marker min peak height: 200   Marker baseline V to V?: Y    Marker baseline V to V points: 3
Lower marker selection: First peak > 500 RFU                Upper marker selection: Last peak > 200 RFU
Ladder size (bp)75, 200, 300, 400, 500, 700, 1000, 1500, 2000, 3000, 4000, 5000, 7000, 10000, 20000
Quantification using: Lower Marker          Final concentration (ng/uL): 0.5000          Dilution factor: 12.0
```

Sample: SampA10
Well location: A10
Created: Wednesday, October 20, 2021 5:58:44 PM

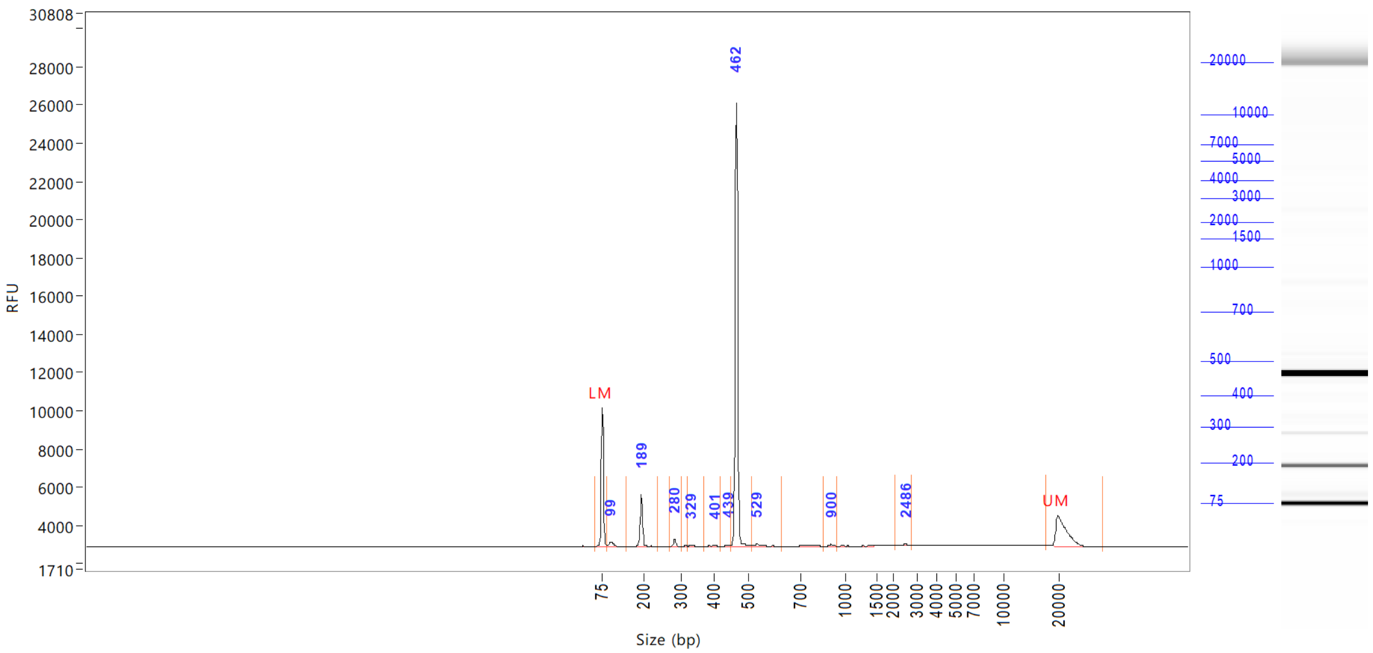

Peak	Size (bp)	Concentration (ng/uL)	From (bp)	To (bp)	Average size (bp)	CV%	RFU	Corrected peak area
1	75 (LM)	0.5000	54	89	73	4.85	7277	38.055
2	99	0.4523	89	145	104	11.23	229	2.869
3	189	2.3383	145	232	186	3.35	2728	14.831
4	280	0.3114	266	301	278	1.29	404	1.975
5	329	0.1185	317	368	331	2.70	76	0.752
6	401	0.0896	368	416	392	2.39	53	0.568
7	439	0.1215	416	449	439	1.34	95	0.770
8	462	17.9430	449	514	462	1.07	23251	113.802
9	529	0.1949	514	626	546	4.94	120	1.236
10	900	0.0911	848	939	896	1.64	94	0.578
11	2486	0.0927	2001	2772	2452	6.41	70	0.588
12	20000 (UM)	0.2662	17805	28342	20819	5.63	1589	20.262

TIC:	21.7533	ng/uL
TIM:	95.7955	nmole/L
Total concentration:	22.5218	ng/uL

```
Sample peak width (sec): 5    Sample min peak height: 50    Sample baseline V to V?: Y    Sample baseline V to V points: 3
Sample filter: Binomial       Number of points for filter: 3  Sample start region (min): 0  Sample end region (min): 30
Marker peak width (sec): 5    Marker min peak height: 200   Marker baseline V to V?: Y    Marker baseline V to V points: 3
Lower marker selection: First peak > 500 RFU                Upper marker selection: Last peak > 200 RFU
Ladder size (bp)75, 200, 300, 400, 500, 700, 1000, 1500, 2000, 3000, 4000, 5000, 7000, 10000, 20000
Quantification using: Lower Marker          Final concentration (ng/uL): 0.5000          Dilution factor: 12.0
```

Sample: SampA11
Well location: A11
Created: Wednesday, October 20, 2021 5:58:44 PM

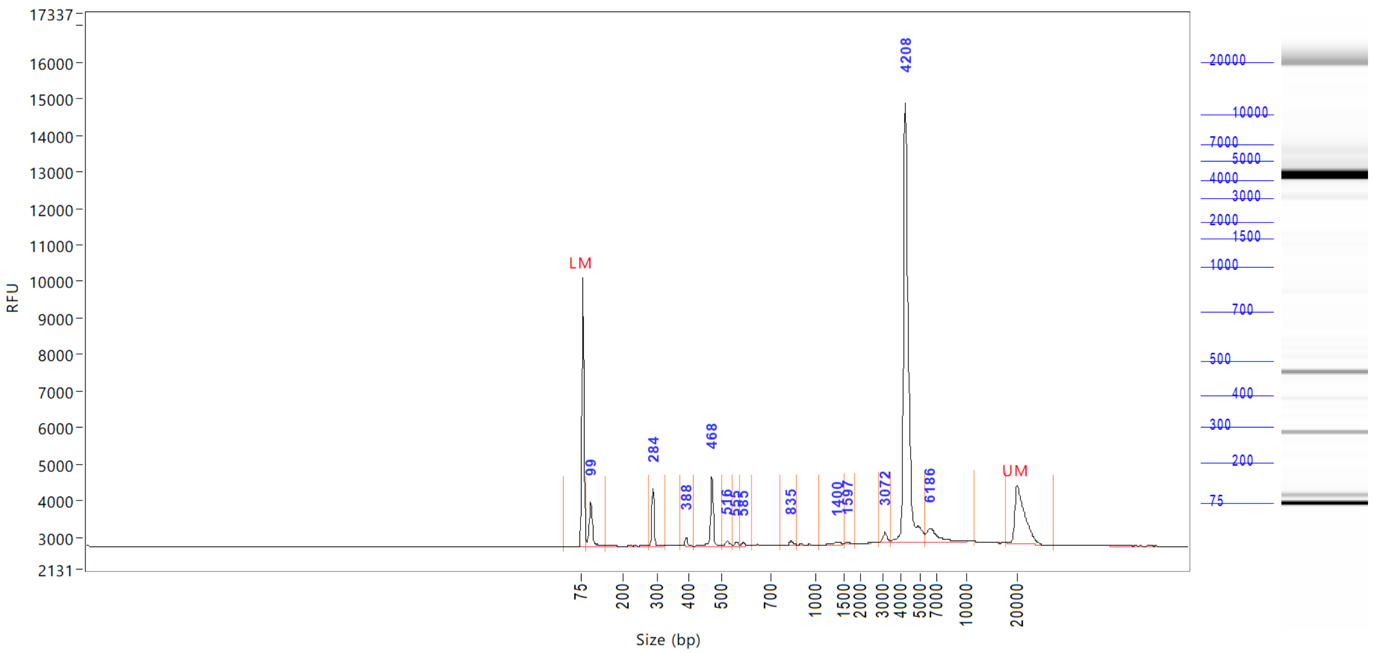

Peak	Size (bp)	Concentration (ng/uL)	From (bp)	To (bp)	Average size (bp)	CV%	RFU	Corrected peak area
1	75 (LM)	0.5000	18	86	73	5.27	7354	39.301
2	99	1.5728	86	147	100	10.12	1215	10.302
3	284	1.2046	271	323	282	1.66	1575	7.890
4	388	0.1929	370	411	387	1.29	243	1.263
5	468	1.5709	411	500	466	1.70	1910	10.290
6	516	0.1537	500	539	518	1.70	139	1.006
7	555	0.1229	539	570	554	1.39	116	0.805
8	585	0.0965	570	618	583	1.37	86	0.632
9	835	0.1397	757	879	835	2.01	126	0.915
10	1400	0.1719	1056	1500	1341	7.05	70	1.126
11	1597	0.0838	1500	1827	1589	3.92	77	0.549
12	3072	0.2894	2829	3393	3084	3.95	281	1.896
13	4208	12.9551	3393	5519	4273	5.36	12042	84.858
14	6186	0.9891	5519	11708	6879	17.06	366	6.479
15	20000 (UM)	0.2511	17805	27133	20779	5.29	1605	19.734

TIC:	19.5432	ng/uL
TIM:	46.3002	nmole/L
Total concentration:	19.9728	ng/uL

Sample peak width (sec): 5   Sample min peak height: 50   Sample baseline V to V?: Y   Sample baseline V to V points: 3
Sample filter: Binomial   Number of points for filter: 3   Sample start region (min): 0   Sample end region (min): 30
Marker peak width (sec): 5   Marker min peak height: 200   Marker baseline V to V?: Y   Marker baseline V to V points: 3
Lower marker selection: First peak > 500 RFU   Upper marker selection: Last peak > 200 RFU
Ladder size (bp)75, 200, 300, 400, 500, 700, 1000, 1500, 2000, 3000, 4000, 5000, 7000, 10000, 20000
Quantification using: Lower Marker   Final concentration (ng/uL): 0.5000   Dilution factor: 12.0

Sample: SampA12
Well location: A12
Created: Wednesday, October 20, 2021 5:58:44 PM

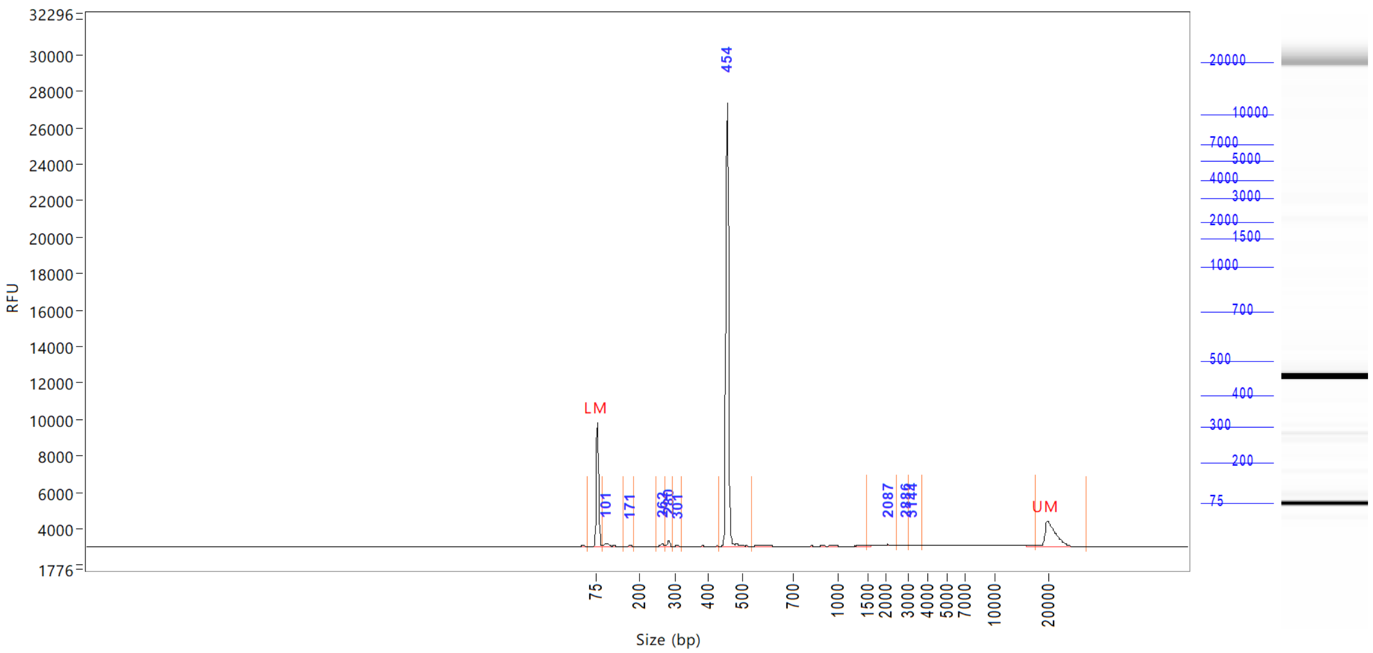

Peak	Size (bp)	Concentration (ng/uL)	From (bp)	To (bp)	Average size (bp)	CV%	RFU	Corrected peak area
1	75 (LM)	0.5000	48	91	73	4.96	6791	35.409
2	101	0.4107	91	152	106	11.99	170	2.424
3	171	0.0927	152	185	168	2.85	91	0.547
4	262	0.2046	244	271	261	2.25	123	1.208
5	280	0.2594	271	293	278	1.29	295	1.531
6	301	0.0791	293	319	301	1.28	77	0.467
7	454	20.1045	433	534	453	1.19	24385	118.646
8	2087	0.1243	1478	2458	2017	8.50	79	0.734
9	2886	0.0660	2458	3000	2852	3.76	60	0.389
10	3144	0.0853	3000	3679	3206	5.57	57	0.503
11	20000 (UM)	0.2447	17683	27254	20749	5.42	1392	17.332

TIC:	21.4266	ng/uL
TIM:	83.7354	nmole/L
Total concentration:	22.2498	ng/uL

```
Sample peak width (sec): 5     Sample min peak height: 50     Sample baseline V to V?: Y     Sample baseline V to V points: 3
Sample filter: Binomial        Number of points for filter: 3  Sample start region (min): 0  Sample end region (min): 30
Marker peak width (sec): 5     Marker min peak height: 200    Marker baseline V to V?: Y     Marker baseline V to V points: 3
Lower marker selection: First peak > 500 RFU                  Upper marker selection: Last peak > 200 RFU
Ladder size (bp)75, 200, 300, 400, 500, 700, 1000, 1500, 2000, 3000, 4000, 5000, 7000, 10000, 20000
Quantification using: Lower Marker          Final concentration (ng/uL): 0.5000          Dilution factor: 12.0
```

Sample: SampB1
Well location: B1
Created: Wednesday, October 20, 2021 5:58:44 PM

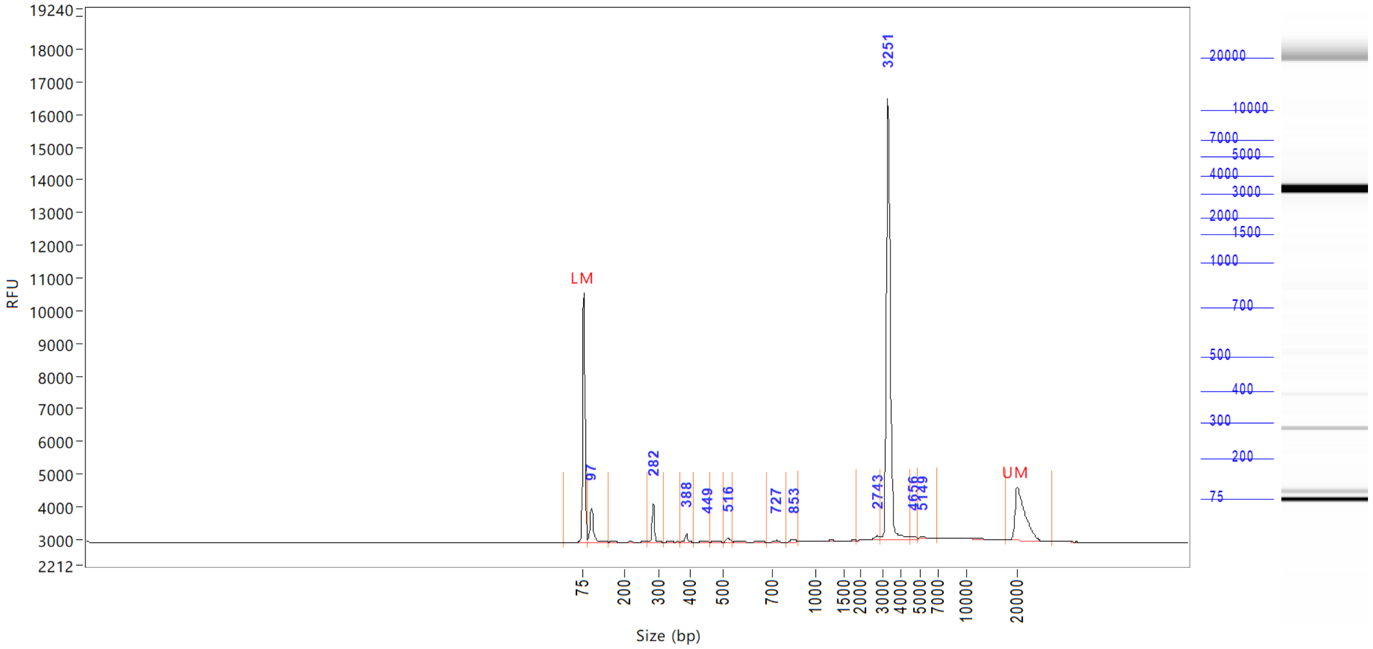

Peak	Size (bp)	Concentration (ng/uL)	From (bp)	To (bp)	Average size (bp)	CV%	RFU	Corrected peak area
1	75 (LM)	0.5000	14	86	72	5.42	7633	40.715
2	97	1.3407	86	150	99	10.02	1059	9.098
3	282	0.8787	264	313	281	1.59	1191	5.962
4	388	0.1991	366	411	385	1.56	241	1.351
5	449	0.0754	411	458	441	1.98	53	0.511
6	516	0.1277	500	539	516	1.55	110	0.867
7	727	0.0593	677	796	721	2.51	56	0.402
8	853	0.1050	796	879	839	2.02	72	0.713
9	2743	0.1974	1866	2886	2605	8.53	126	1.340
10	3251	11.6563	2886	4449	3263	4.97	13526	79.097
11	4656	0.0827	4449	4897	4659	2.86	64	0.561
12	5149	0.1050	4897	7001	5420	8.49	64	0.712
13	20000 (UM)	0.2495	17805	26770	20841	5.38	1642	20.316

TIC:	14.8273	ng/uL
TIM:	35.3279	nmole/L
Total concentration:	15.4099	ng/uL

```
Sample peak width (sec): 5     Sample min peak height: 50     Sample baseline V to V?: Y     Sample baseline V to V points: 3
Sample filter: Binomial        Number of points for filter: 3  Sample start region (min): 0  Sample end region (min): 30
Marker peak width (sec): 5     Marker min peak height: 200    Marker baseline V to V?: Y     Marker baseline V to V points: 3
Lower marker selection: First peak > 500 RFU                  Upper marker selection: Last peak > 200 RFU
Ladder size (bp)75, 200, 300, 400, 500, 700, 1000, 1500, 2000, 3000, 4000, 5000, 7000, 10000, 20000
Quantification using: Lower Marker          Final concentration (ng/uL): 0.5000          Dilution factor: 12.0
```

Sample: SampB2
Well location: B2
Created: Wednesday, October 20, 2021 5:58:44 PM

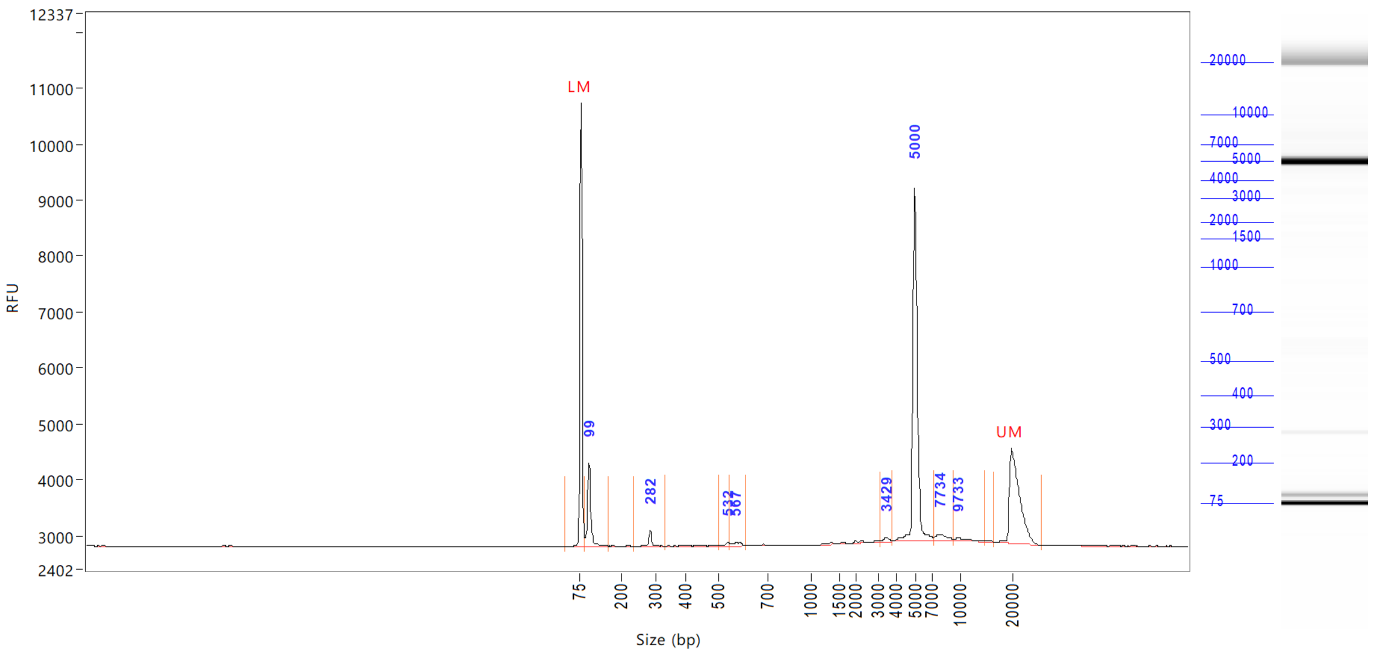

Peak	Size (bp)	Concentration (ng/uL)	From (bp)	To (bp)	Average size (bp)	CV%	RFU	Corrected peak area
1	75 (LM)	0.5000	28	87	74	5.03	7944	43.187
2	99	1.6486	87	160	100	9.71	1483	11.867
3	282	0.2245	233	329	281	3.27	289	1.616
4	532	0.0644	500	542	528	1.79	57	0.463
5	567	0.1448	542	606	566	2.59	68	1.042
6	3429	0.0908	3072	3750	3390	4.84	62	0.654
7	5000	5.8037	3750	7134	5039	6.88	6313	41.775
8	7734	0.2776	7134	9200	8025	6.88	128	1.998
9	9733	0.1671	9200	14757	10886	13.49	58	1.203
10	20000 (UM)	0.2476	16464	25924	20820	5.51	1700	21.386

TIC:	8.4216	ng/uL
TIM:	31.0309	nmole/L
Total concentration:	8.8708	ng/uL

Sample peak width (sec): 5   Sample min peak height: 50   Sample baseline V to V?: Y   Sample baseline V to V points: 3
Sample filter: Binomial   Number of points for filter: 3   Sample start region (min): 0   Sample end region (min): 30
Marker peak width (sec): 5   Marker min peak height: 200   Marker baseline V to V?: Y   Marker baseline V to V points: 3
Lower marker selection: First peak > 500 RFU   Upper marker selection: Last peak > 200 RFU
Ladder size (bp)75, 200, 300, 400, 500, 700, 1000, 1500, 2000, 3000, 4000, 5000, 7000, 10000, 20000
Quantification using: Lower Marker   Final concentration (ng/uL): 0.5000   Dilution factor: 12.0

Sample: SampB3
Well location: B3
Created: Wednesday, October 20, 2021 5:58:44 PM

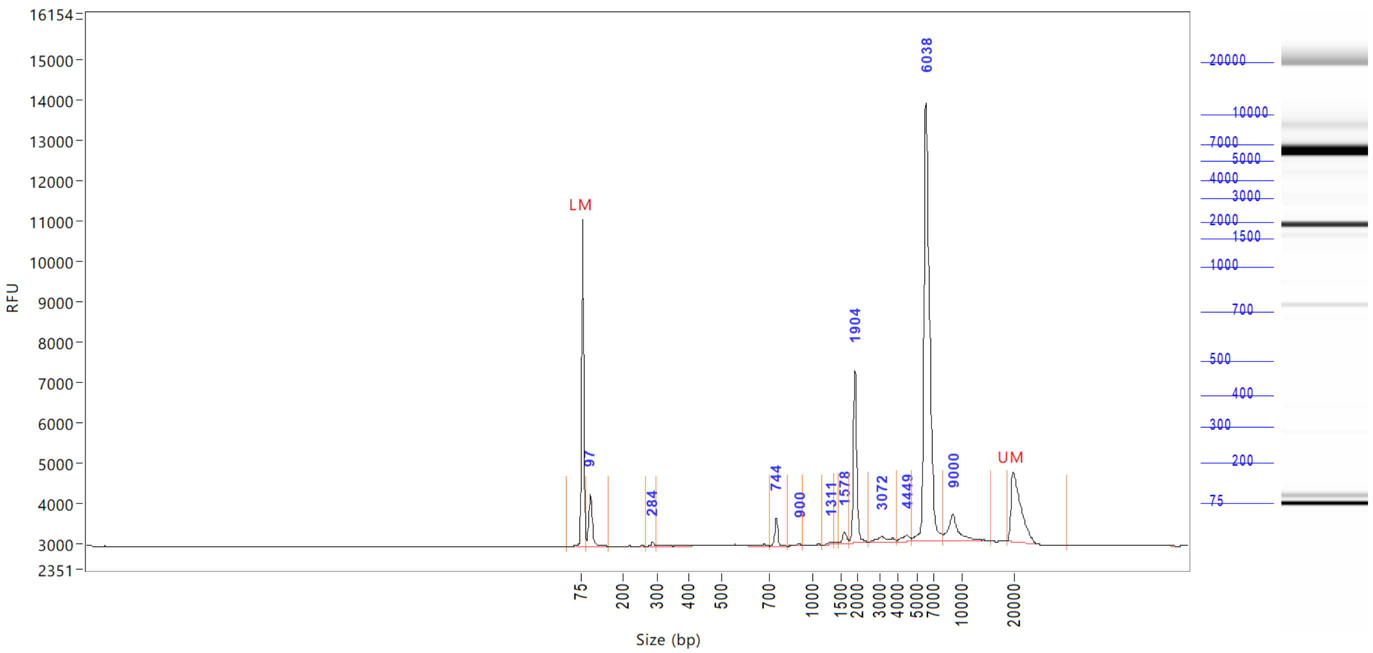

Peak	Size (bp)	Concentration (ng/uL)	From (bp)	To (bp)	Average size (bp)	CV%	RFU	Corrected peak area
1	75 (LM)	0.5000	28	86	73	5.47	8126	43.307
2	97	1.6304	86	156	99	9.69	1304	11.768
3	284	0.0866	264	296	281	1.86	110	0.625
4	744	0.5136	700	822	739	1.79	706	3.707
5	900	0.0496	822	922	889	2.27	56	0.358
6	1311	0.0602	1145	1356	1284	3.47	50	0.434
7	1578	0.2558	1456	1712	1573	3.96	284	1.846
8	1904	3.1925	1712	2458	1899	3.45	4294	23.043
9	3072	0.3282	2458	3893	3177	10.76	125	2.369
10	4449	0.2546	3893	4724	4334	4.98	161	1.837
11	6038	12.1992	4724	8001	6146	6.86	10902	88.053
12	9000	1.2354	8001	15366	9325	11.49	659	8.917
13	20000 (UM)	0.2438	18781	30398	20795	5.42	1735	21.116

TIC:	19.8060	ng/uL
TIM:	35.6497	nmole/L
Total concentration:	20.2342	ng/uL

```
Sample peak width (sec): 5     Sample min peak height: 50    Sample baseline V to V?: Y    Sample baseline V to V points: 3
Sample filter: Binomial        Number of points for filter: 3  Sample start region (min): 0  Sample end region (min): 30
Marker peak width (sec): 5     Marker min peak height: 200   Marker baseline V to V?: Y    Marker baseline V to V points: 3
Lower marker selection: First peak > 500 RFU                 Upper marker selection: Last peak > 200 RFU
Ladder size (bp)75, 200, 300, 400, 500, 700, 1000, 1500, 2000, 3000, 4000, 5000, 7000, 10000, 20000
Quantification using: Lower Marker          Final concentration (ng/uL): 0.5000          Dilution factor: 12.0
```

Sample: SampB4
Well location: B4
Created: Wednesday, October 20, 2021 5:58:44 PM

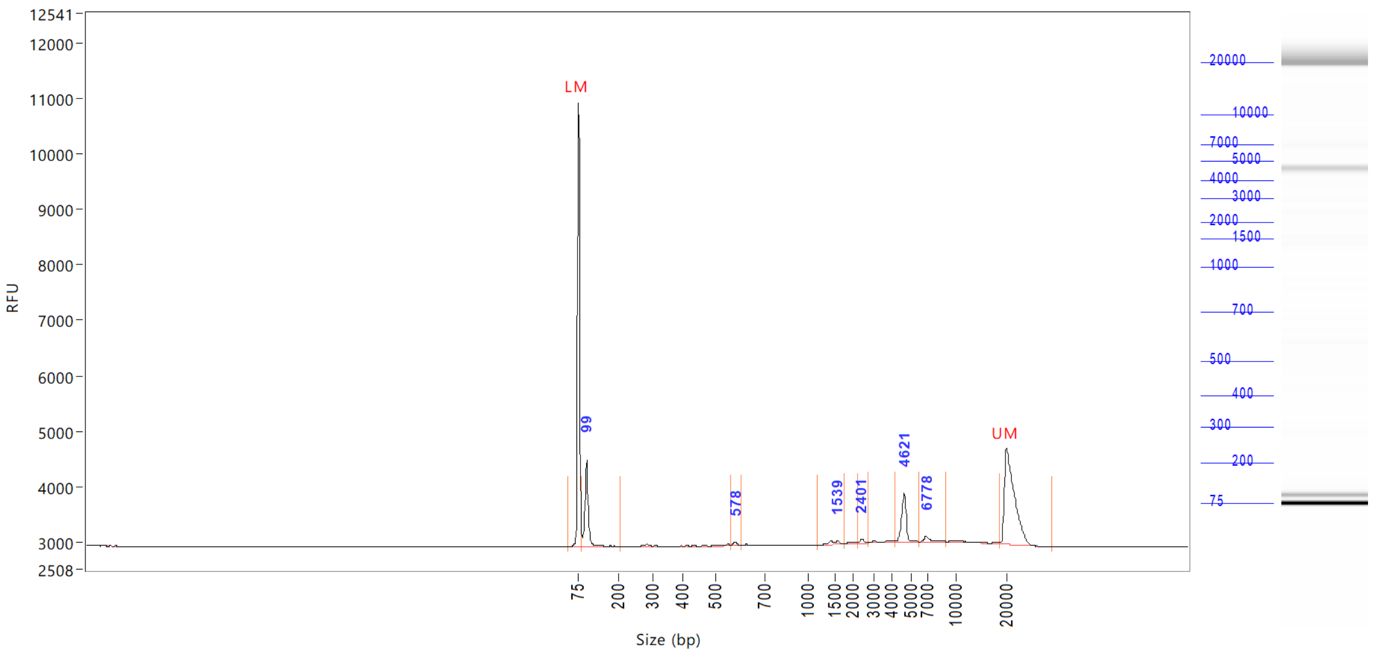

Peak	Size (bp)	Concentration (ng/uL)	From (bp)	To (bp)	Average size (bp)	CV%	RFU	Corrected peak area
1	75 (LM)	0.5000	45	86	73	4.55	8020	42.832
2	99	1.7055	86	203	100	11.46	1570	12.175
3	578	0.0742	560	603	576	1.51	65	0.530
4	1539	0.1360	1167	1731	1459	7.12	68	0.971
5	2401	0.0677	2201	2743	2395	4.19	72	0.484
6	4621	0.8738	4139	6038	4606	4.33	893	6.238
7	6778	0.1467	6038	9000	7143	9.41	100	1.047
8	20000 (UM)	0.2566	18537	28826	20808	5.48	1729	21.980

TIC:	3.0040	ng/uL
TIM:	28.7634	nmole/L
Total concentration:	3.6126	ng/uL

```
Sample peak width (sec): 5    Sample min peak height: 50    Sample baseline V to V?: Y    Sample baseline V to V points: 3
Sample filter: Binomial       Number of points for filter: 3  Sample start region (min): 0  Sample end region (min): 30
Marker peak width (sec): 5    Marker min peak height: 200   Marker baseline V to V?: Y    Marker baseline V to V points: 3
Lower marker selection: First peak > 500 RFU                Upper marker selection: Last peak > 200 RFU
Ladder size (bp)75, 200, 300, 400, 500, 700, 1000, 1500, 2000, 3000, 4000, 5000, 7000, 10000, 20000
Quantification using: Lower Marker          Final concentration (ng/uL): 0.5000          Dilution factor: 12.0
```

Sample: SampB5
Well location: B5
Created: Wednesday, October 20, 2021 5:58:44 PM

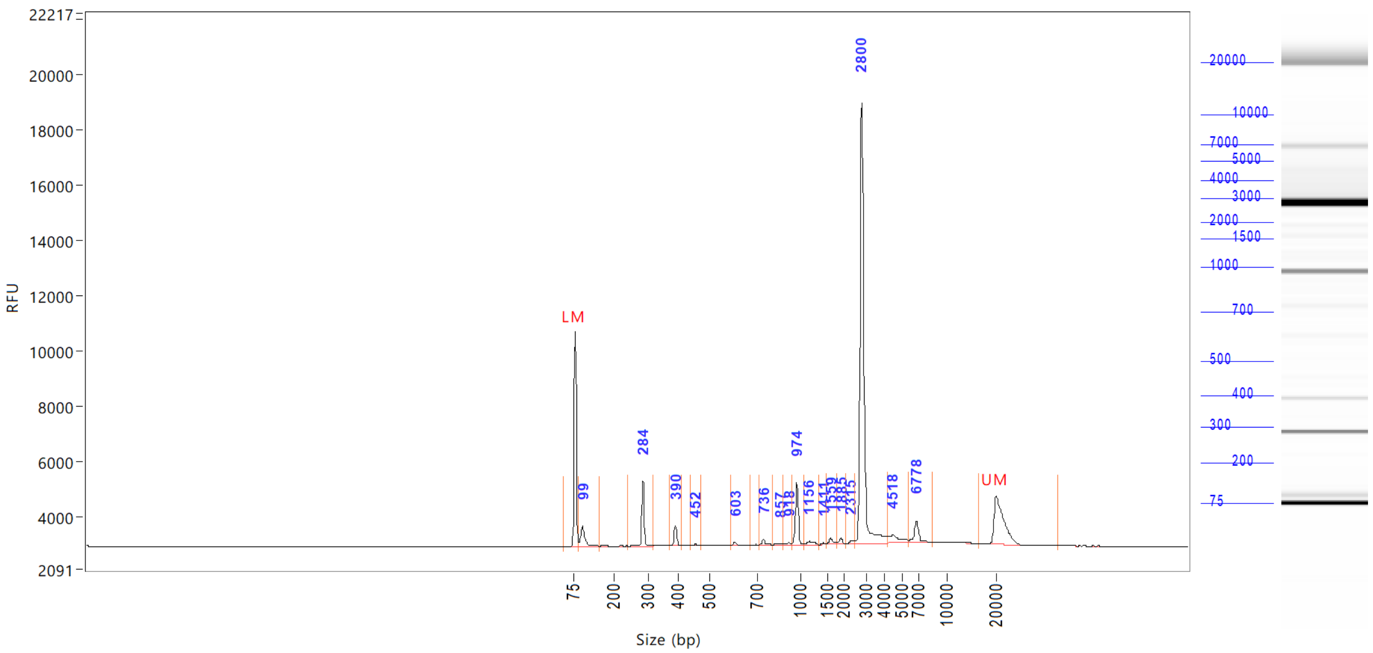

Peak	Size (bp)	Concentration (ng/uL)	From (bp)	To (bp)	Average size (bp)	CV%	RFU	Corrected peak area
1	75 (LM)	0.5000	41	87	74	4.51	7796	43.012
2	99	1.0848	87	154	101	11.94	748	7.777
3	284	1.7083	241	319	283	2.28	2342	12.246
4	390	0.5117	370	412	387	1.18	720	3.668
5	452	0.0686	437	470	450	1.40	71	0.492
6	603	0.1437	588	669	607	2.68	145	1.030
7	736	0.2287	701	801	738	2.72	225	1.639
8	857	0.1006	801	883	842	2.35	66	0.721
9	918	0.0898	883	939	911	1.63	84	0.644
10	974	1.5434	939	1056	970	1.19	2239	11.064
11	1156	0.2124	1056	1345	1183	5.10	121	1.523
12	1411	0.0393	1345	1478	1410	2.23	51	0.281
13	1559	0.1844	1478	1770	1583	4.91	200	1.322
14	1885	0.1474	1770	2030	1866	2.54	197	1.057
15	2315	0.1040	2030	2486	2318	4.49	100	0.746
16	2800	13.4517	2486	4208	2856	8.97	15976	96.430
17	4518	0.6506	4208	5815	4710	8.58	286	4.664
18	6778	0.7801	5815	8600	6742	6.52	797	5.592
19	20000 (UM)	0.2517	16586	32211	20820	6.12	1735	21.651

TIC:	21.0497	ng/uL
TIM:	42.7790	nmole/L
Total concentration:	21.4419	ng/uL

```
Sample peak width (sec): 5    Sample min peak height: 50    Sample baseline V to V?: Y    Sample baseline V to V points: 3
Sample filter: Binomial       Number of points for filter: 3  Sample start region (min): 0  Sample end region (min): 30
Marker peak width (sec): 5    Marker min peak height: 200   Marker baseline V to V?: Y    Marker baseline V to V points: 3
Lower marker selection: First peak > 500 RFU                Upper marker selection: Last peak > 200 RFU
Ladder size (bp)75, 200, 300, 400, 500, 700, 1000, 1500, 2000, 3000, 4000, 5000, 7000, 10000, 20000
Quantification using: Lower Marker          Final concentration (ng/uL): 0.5000          Dilution factor: 12.0
```

Sample: SampB6
Well location: B6
Created: Wednesday, October 20, 2021 5:58:44 PM

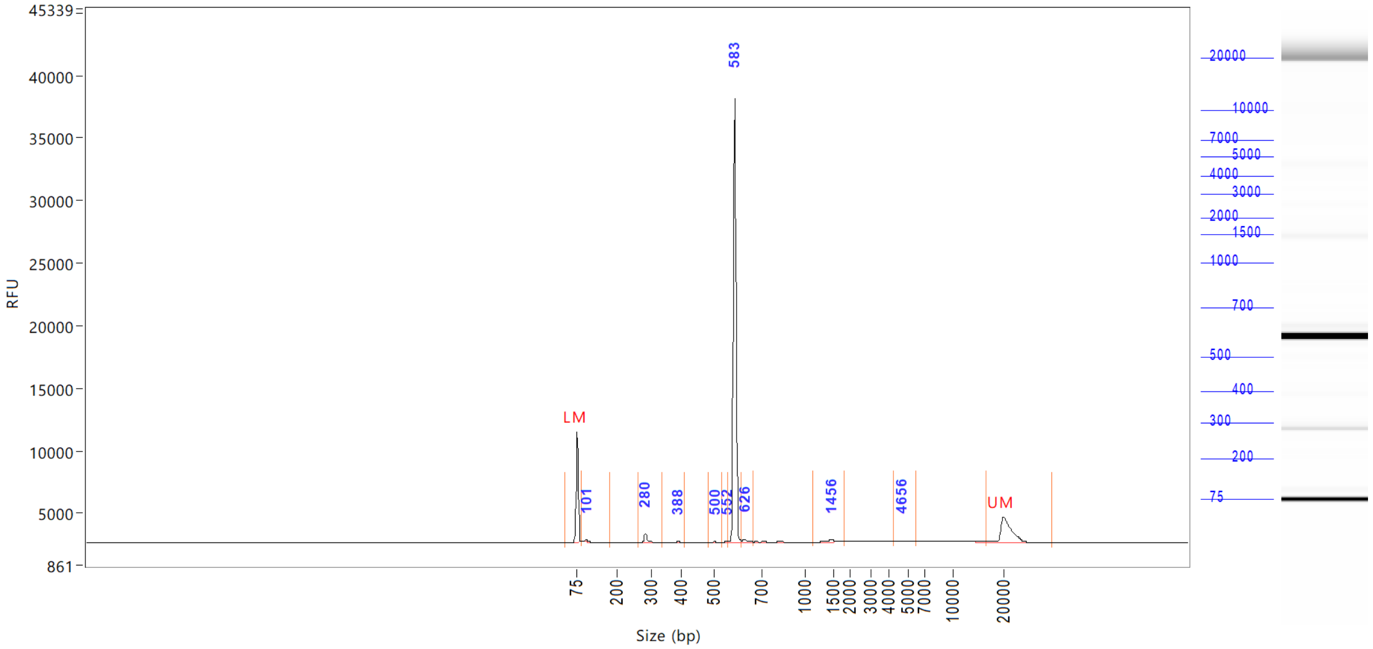

Peak	Size (bp)	Concentration (ng/uL)	From (bp)	To (bp)	Average size (bp)	CV%	RFU	Corrected peak area
1	75 (LM)	0.5000	37	89	73	4.23	8833	46.420
2	101	0.1924	89	175	103	10.18	146	1.489
3	280	0.6780	262	337	283	2.93	735	5.246
4	388	0.0815	337	411	389	2.79	74	0.631
5	500	0.0559	483	532	500	1.65	51	0.433
6	552	0.0687	532	560	550	1.10	98	0.531
7	583	23.7808	560	611	581	0.97	35537	183.985
8	626	0.2684	611	664	625	1.66	256	2.076
9	1456	0.2397	1156	1846	1484	6.91	220	1.855
10	4656	0.1098	4242	5963	4644	5.19	93	0.849
11	20000 (UM)	0.2890	16586	29551	20860	6.00	2053	26.832

TIC:	25.4752	ng/uL
TIM:	76.1110	nmole/L
Total concentration:	25.9843	ng/uL

```
Sample peak width (sec): 5    Sample min peak height: 50    Sample baseline V to V?: Y    Sample baseline V to V points: 3
Sample filter: Binomial       Number of points for filter: 3  Sample start region (min): 0  Sample end region (min): 30
Marker peak width (sec): 5    Marker min peak height: 200   Marker baseline V to V?: Y    Marker baseline V to V points: 3
Lower marker selection: First peak > 500 RFU                Upper marker selection: Last peak > 200 RFU
Ladder size (bp)75, 200, 300, 400, 500, 700, 1000, 1500, 2000, 3000, 4000, 5000, 7000, 10000, 20000
Quantification using: Lower Marker          Final concentration (ng/uL): 0.5000          Dilution factor: 12.0
```

Sample: SampB7
Well location: B7
Created: Wednesday, October 20, 2021 5:58:44 PM

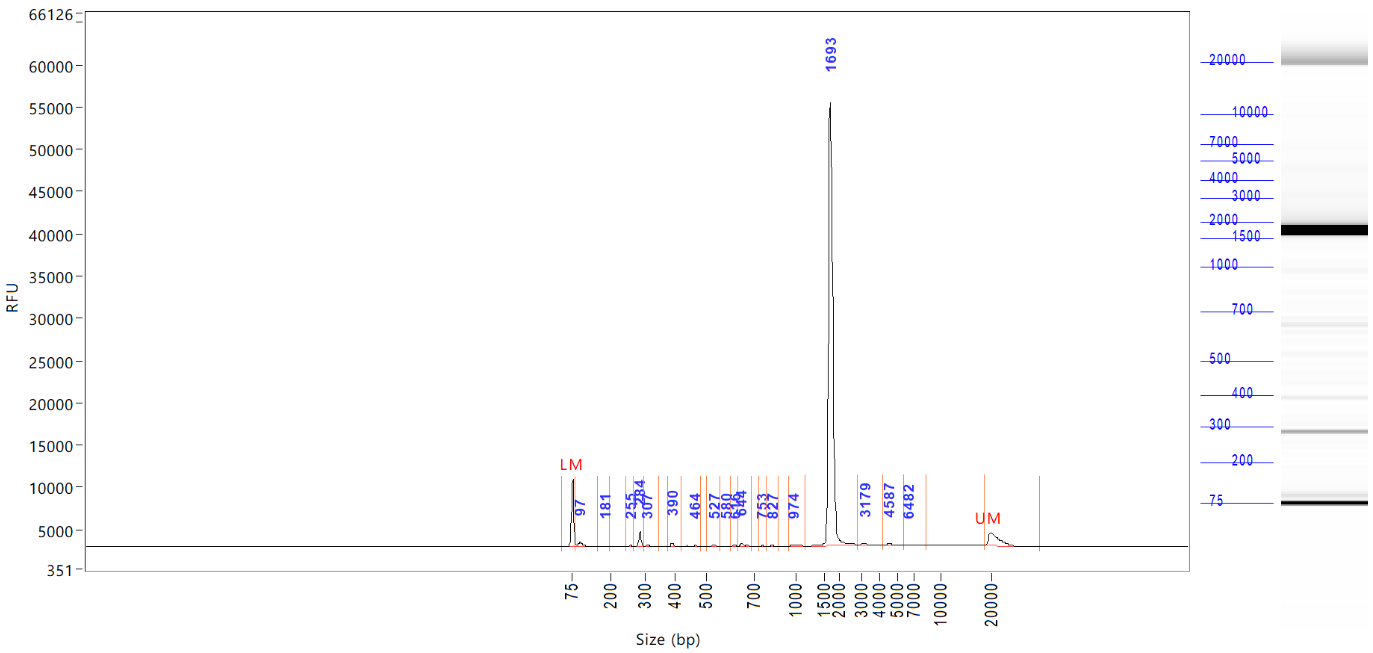

Peak	Size (bp)	Concentration (ng/uL)	From (bp)	To (bp)	Average size (bp)	CV%	RFU	Corrected peak area
1	75 (LM)	0.5000	43	86	73	4.54	8025	43.222
2	97	0.9100	86	154	101	12.95	573	6.556
3	181	0.0715	154	190	174	5.09	63	0.515
4	255	0.0791	244	264	254	1.88	85	0.570
5	284	1.2240	264	295	282	1.50	1750	8.817
6	307	0.1808	295	345	311	3.66	98	1.302
7	390	0.3134	376	418	389	1.44	423	2.257
8	464	0.2047	418	481	450	3.43	78	1.474
9	527	0.1464	500	557	524	1.76	136	1.055
10	580	0.0668	557	598	578	1.57	56	0.481
11	616	0.1089	598	629	613	1.13	129	0.784
12	644	0.3984	629	690	649	1.93	415	2.870
13	753	0.0624	727	783	747	1.49	68	0.449
14	827	0.0700	783	870	827	1.71	71	0.504
15	974	0.1971	944	1156	996	4.15	149	1.420
16	1693	47.6565	1156	2857	1705	6.50	52507	343.302
17	3179	0.4718	2857	4208	3407	11.80	161	3.399
18	4587	0.2799	4208	5889	4734	8.38	141	2.016
19	6482	0.1460	5889	8401	6958	9.99	66	1.052
20	20000 (UM)	0.2329	18781	30035	20948	5.90	1576	20.130

TIC:	52.5876	ng/uL
TIM:	75.0781	nmole/L
Total concentration:	52.8431	ng/uL

Sample peak width (sec): 5   Sample min peak height: 50   Sample baseline V to V?: Y   Sample baseline V to V points: 3
Sample filter: Binomial   Number of points for filter: 3 Sample start region (min): 0 Sample end region (min): 30
Marker peak width (sec): 5   Marker min peak height: 200   Marker baseline V to V?: Y   Marker baseline V to V points: 3
Lower marker selection: First peak > 500 RFU   Upper marker selection: Last peak > 200 RFU
Ladder size (bp)75, 200, 300, 400, 500, 700, 1000, 1500, 2000, 3000, 4000, 5000, 7000, 10000, 20000
Quantification using: Lower Marker   Final concentration (ng/uL): 0.5000   Dilution factor: 12.0

Sample: SampB8
Well location: B8
Created: Wednesday, October 20, 2021 5:58:44 PM

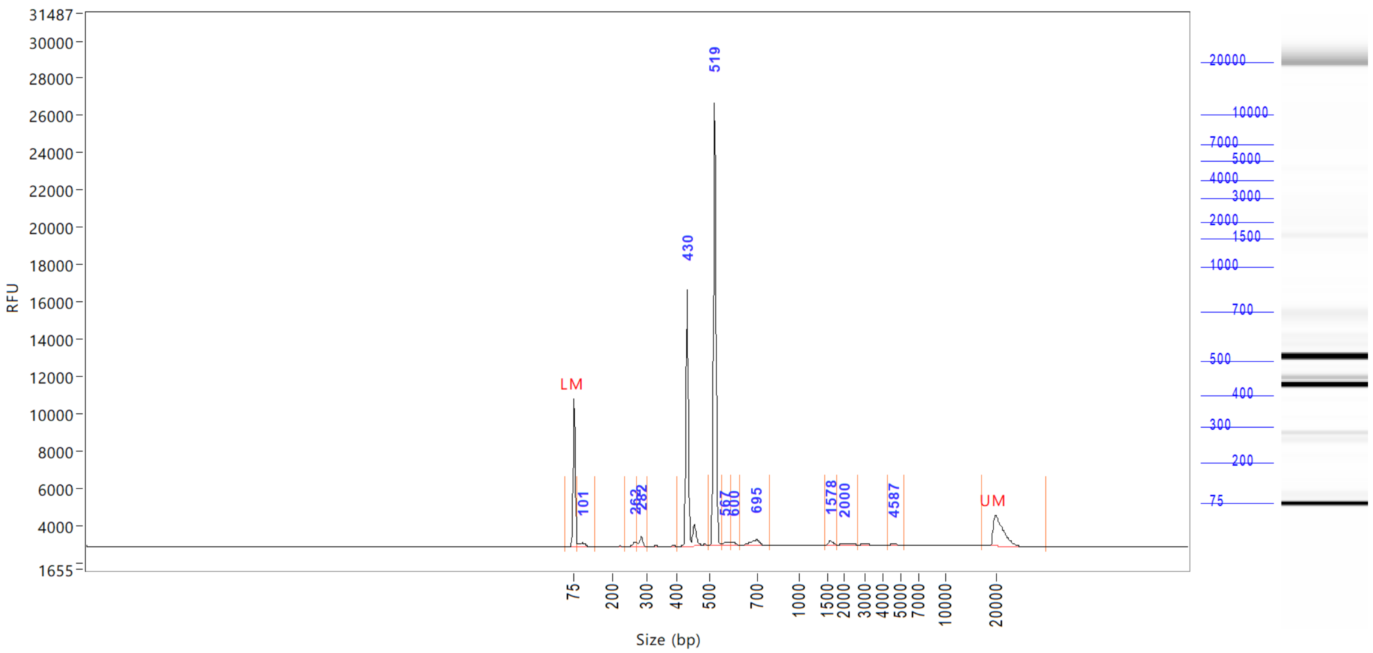

Peak	Size (bp)	Concentration (ng/uL)	From (bp)	To (bp)	Average size (bp)	CV%	RFU	Corrected peak area
1	75 (LM)	0.5000	47	86	73	4.50	7956	42.907
2	101	0.4713	86	139	100	10.52	211	3.370
3	262	0.3279	232	271	261	2.49	259	2.345
4	282	0.5091	271	301	279	1.62	549	3.641
5	430	10.4009	396	496	430	2.28	13758	74.379
6	519	15.9901	496	547	516	1.03	23800	114.349
7	567	0.3207	547	588	568	2.02	204	2.293
8	600	0.2407	588	629	601	1.62	178	1.722
9	695	0.7684	629	792	684	3.37	307	5.495
10	1578	0.2537	1467	1789	1589	4.78	238	1.814
11	2000	0.2498	1789	2686	2148	11.11	90	1.786
12	4587	0.0607	4242	5371	4590	3.61	60	0.434
13	20000 (UM)	0.2445	17317	30035	20758	5.59	1682	20.983

TIC:	29.5933	ng/uL
TIM:	107.5109	nmole/L
Total concentration:	30.0616	ng/uL

```
Sample peak width (sec): 5    Sample min peak height: 50    Sample baseline V to V?: Y    Sample baseline V to V points: 3
Sample filter: Binomial       Number of points for filter: 3  Sample start region (min): 0  Sample end region (min): 30
Marker peak width (sec): 5    Marker min peak height: 200   Marker baseline V to V?: Y    Marker baseline V to V points: 3
Lower marker selection: First peak > 500 RFU                Upper marker selection: Last peak > 200 RFU
Ladder size (bp)75, 200, 300, 400, 500, 700, 1000, 1500, 2000, 3000, 4000, 5000, 7000, 10000, 20000
Quantification using: Lower Marker          Final concentration (ng/uL): 0.5000          Dilution factor: 12.0
```

Sample: SampB9
Well location: B9
Created: Wednesday, October 20, 2021 5:58:44 PM

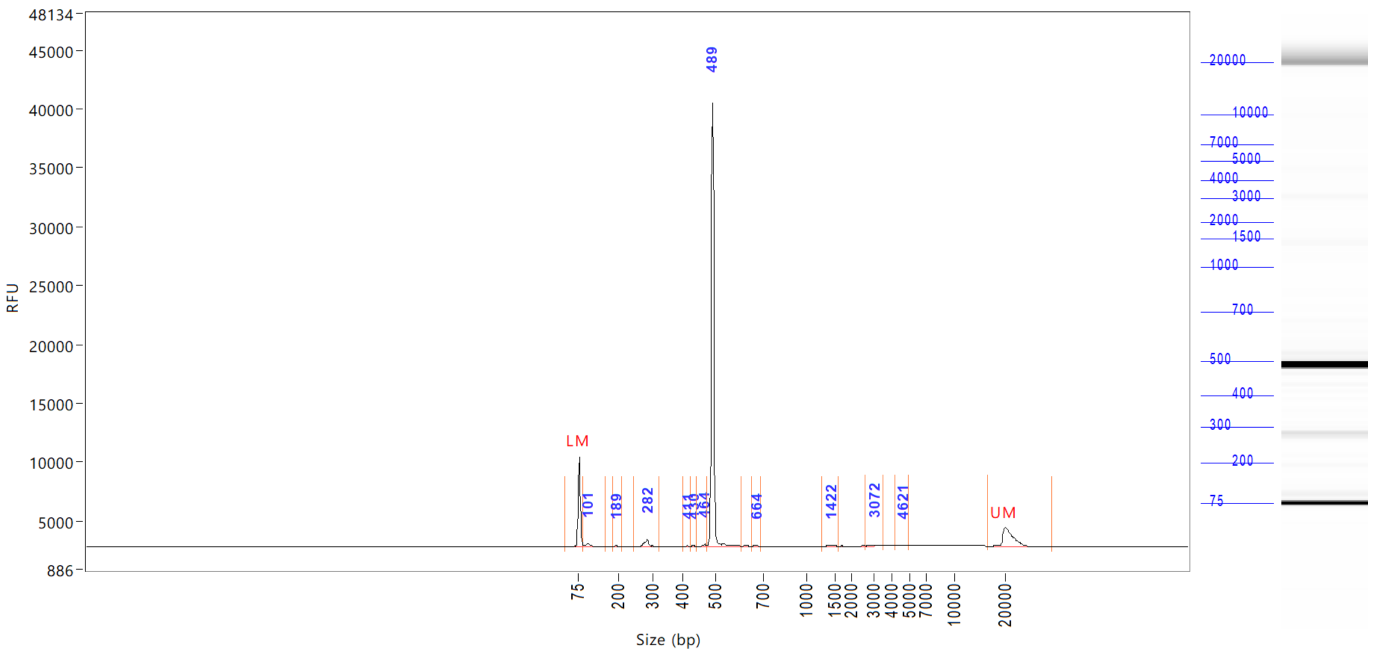

Peak	Size (bp)	Concentration (ng/uL)	From (bp)	To (bp)	Average size (bp)	CV%	RFU	Corrected peak area
1	75 (LM)	0.5000	35	89	74	4.95	7643	41.986
2	101	0.4709	89	160	105	12.38	258	3.295
3	189	0.0943	179	208	189	3.14	106	0.660
4	282	0.8688	244	319	277	3.68	575	6.079
5	411	0.0445	398	420	409	0.91	64	0.311
6	430	0.0768	420	441	429	0.89	116	0.537
7	464	0.1388	441	471	461	1.24	164	0.971
8	489	27.0627	471	603	488	1.70	37751	189.376
9	664	0.0540	649	685	662	1.17	56	0.378
10	1422	0.1318	1278	1597	1424	3.98	108	0.923
11	3072	0.1323	2657	3536	3077	5.07	123	0.926
12	4621	0.0775	4208	5000	4584	3.66	67	0.542
13	20000 (UM)	0.2451	16708	29309	20852	5.61	1650	20.578

TIC:	29.1523	ng/uL
TIM:	105.9503	nmole/L
Total concentration:	29.7068	ng/uL

Sample peak width (sec): 5 Sample min peak height: 50 Sample baseline V to V?: Y Sample baseline V to V points: 3
Sample filter: Binomial Number of points for filter: 3 Sample start region (min): 0 Sample end region (min): 30
Marker peak width (sec): 5 Marker min peak height: 200 Marker baseline V to V?: Y Marker baseline V to V points: 3
Lower marker selection: First peak > 500 RFU Upper marker selection: Last peak > 200 RFU
Ladder size (bp)75, 200, 300, 400, 500, 700, 1000, 1500, 2000, 3000, 4000, 5000, 7000, 10000, 20000
Quantification using: Lower Marker Final concentration (ng/uL): 0.5000 Dilution factor: 12.0

Sample: SampB10
Well location: B10
Created: Wednesday, October 20, 2021 5:58:44 PM

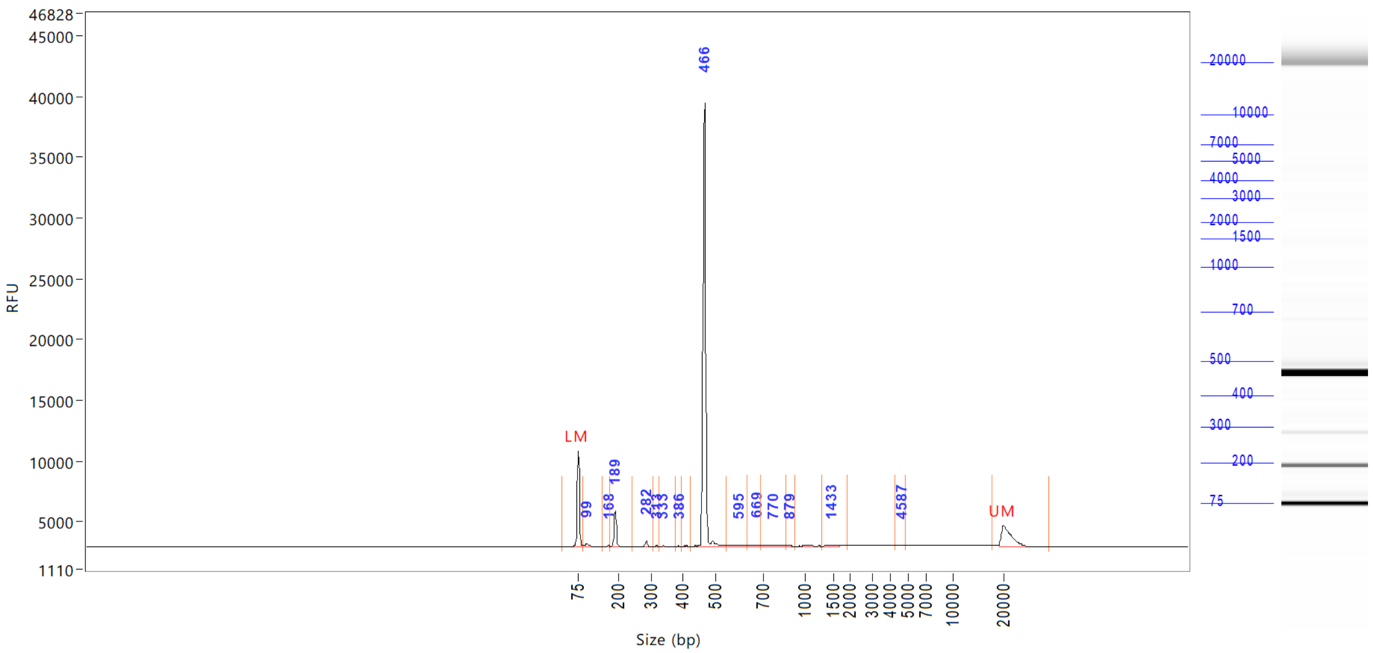

Peak	Size (bp)	Concentration (ng/uL)	From (bp)	To (bp)	Average size (bp)	CV%	RFU	Corrected peak area
1	75 (LM)	0.5000	28	89	72	5.33	7917	43.570
2	99	0.4490	89	150	105	12.33	249	3.260
3	168	0.0899	150	175	166	3.65	81	0.653
4	189	2.1819	175	241	187	2.88	2958	15.844
5	282	0.3539	241	301	278	2.62	466	2.570
6	313	0.0449	301	321	311	1.25	70	0.326
7	333	0.0816	321	376	338	3.31	53	0.592
8	386	0.0344	376	394	384	1.03	55	0.250
9	466	24.9415	426	547	464	1.56	36530	181.115
10	595	0.2088	547	634	585	4.05	62	1.516
11	669	0.1292	634	690	663	1.92	121	0.938
12	770	0.1981	690	857	763	5.49	71	1.439
13	879	0.0416	857	926	877	1.35	56	0.302
14	1433	0.1020	1289	1942	1485	8.15	60	0.741
15	4587	0.0715	4277	4897	4563	3.20	65	0.519
16	20000 (UM)	0.2427	17805	28947	20777	5.47	1692	21.146

TIC:	28.9281	ng/uL
TIM:	119.9713	nmole/L
Total concentration:	29.3802	ng/uL

Sample peak width (sec): 5   Sample min peak height: 50   Sample baseline V to V?: Y   Sample baseline V to V points: 3
Sample filter: Binomial   Number of points for filter: 3 Sample start region (min): 0 Sample end region (min): 30
Marker peak width (sec): 5   Marker min peak height: 200   Marker baseline V to V?: Y   Marker baseline V to V points: 3
Lower marker selection: First peak > 500 RFU   Upper marker selection: Last peak > 200 RFU
Ladder size (bp)75, 200, 300, 400, 500, 700, 1000, 1500, 2000, 3000, 4000, 5000, 7000, 10000, 20000
Quantification using: Lower Marker   Final concentration (ng/uL): 0.5000   Dilution factor: 12.0

Sample: SampB11
Well location: B11
Created: Wednesday, October 20, 2021 5:58:44 PM

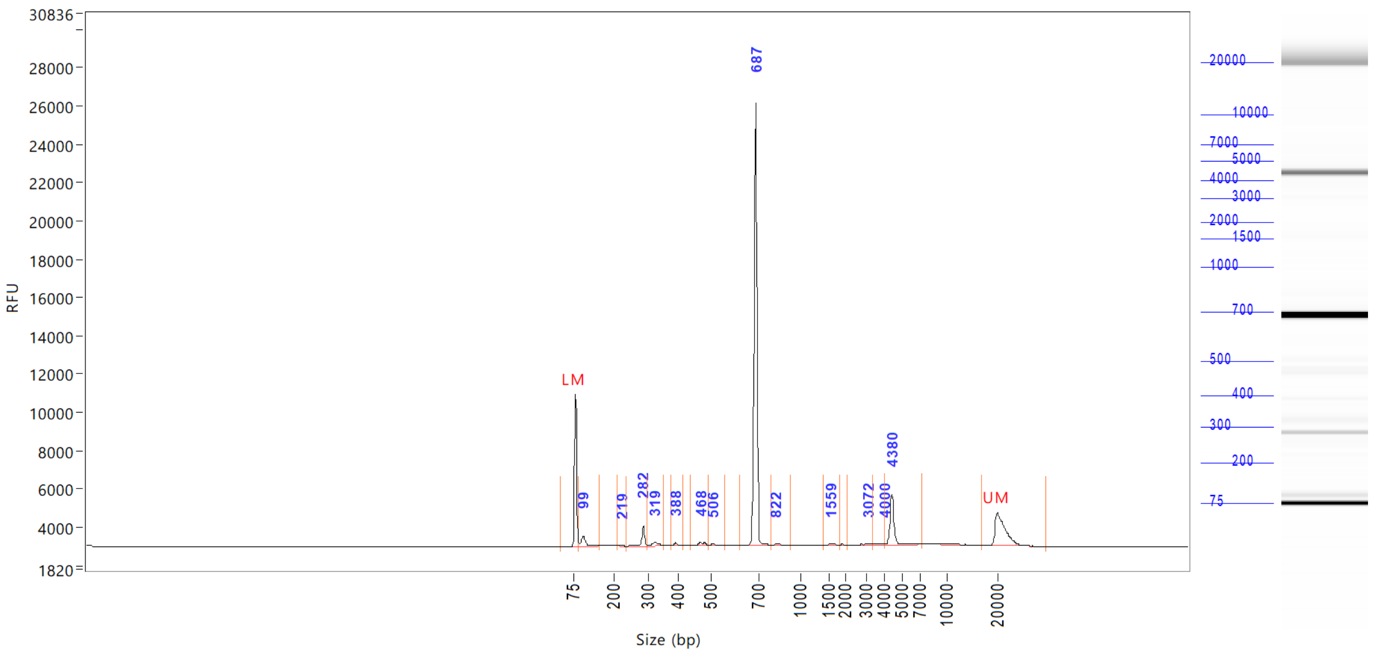

Peak	Size (bp)	Concentration (ng/uL)	From (bp)	To (bp)	Average size (bp)	CV%	RFU	Corrected peak area
1	75 (LM)	0.5000	31	87	73	4.51	7948	42.319
2	99	0.7718	87	152	100	10.12	553	5.444
3	219	0.0561	208	232	217	2.07	54	0.396
4	282	1.0247	232	298	276	4.35	1089	7.227
5	319	0.3736	298	347	319	3.36	207	2.635
6	388	0.1135	376	412	387	1.40	149	0.801
7	468	0.3106	437	492	469	2.14	193	2.191
8	506	0.1181	492	555	507	2.06	106	0.833
9	687	16.5483	623	783	684	1.12	23133	116.718
10	822	0.1081	783	926	833	3.76	60	0.763
11	1559	0.0753	1389	1827	1572	6.00	55	0.531
12	3072	0.0882	2115	3286	2942	7.76	75	0.622
13	4000	0.1200	3286	4035	3721	5.78	62	0.846
14	4380	2.2423	4035	7134	4460	8.90	2632	15.816
15	20000 (UM)	0.2561	16830	29672	20769	5.73	1697	21.679

TIC:	21.9505	ng/uL
TIM:	64.1256	nmole/L
Total concentration:	22.3518	ng/uL

Sample peak width (sec): 5 Sample min peak height: 50 Sample baseline V to V?: Y Sample baseline V to V points: 3
Sample filter: Binomial Number of points for filter: 3 Sample start region (min): 0 Sample end region (min): 30
Marker peak width (sec): 5 Marker min peak height: 200 Marker baseline V to V?: Y Marker baseline V to V points: 3
Lower marker selection: First peak > 500 RFU Upper marker selection: Last peak > 200 RFU
Ladder size (bp)75, 200, 300, 400, 500, 700, 1000, 1500, 2000, 3000, 4000, 5000, 7000, 10000, 20000
Quantification using: Lower Marker Final concentration (ng/uL): 0.5000 Dilution factor: 12.0

Sample: SampB12
Well location: B12
Created: Wednesday, October 20, 2021 5:58:44 PM

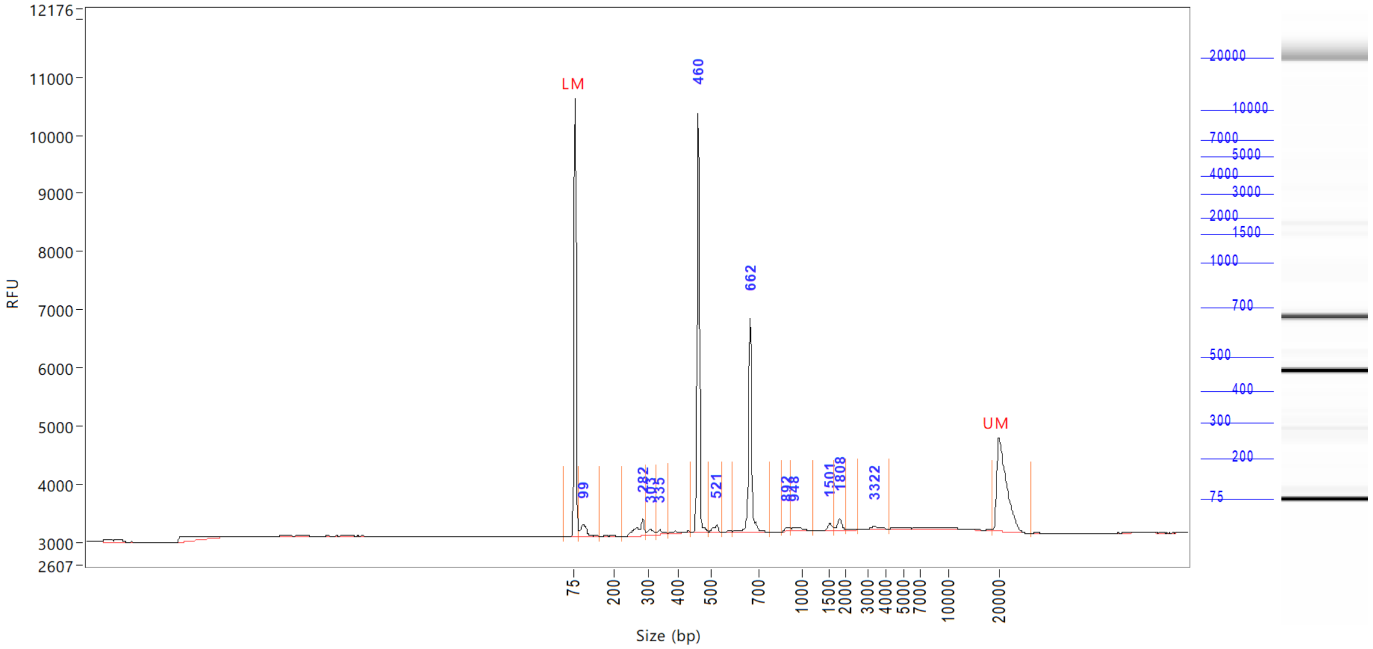

Peak	Size (bp)	Concentration (ng/uL)	From (bp)	To (bp)	Average size (bp)	CV%	RFU	Corrected peak area
1	75 (LM)	0.5000	43	89	74	4.54	7555	40.496
2	99	0.5001	89	154	106	13.57	216	3.376
3	282	0.6383	221	293	267	5.57	288	4.308
4	303	0.1969	293	325	306	2.93	101	1.329
5	335	0.1230	325	362	339	2.82	86	0.830
6	460	5.1277	439	492	458	1.00	7209	34.608
7	521	0.1711	492	542	512	1.92	121	1.155
8	662	3.0387	588	770	660	1.81	3670	20.509
9	892	0.0880	857	926	898	1.90	62	0.594
10	948	0.1635	926	1212	979	4.37	61	1.104
11	1501	0.1407	1212	1635	1502	4.56	132	0.949
12	1808	0.2168	1635	2001	1792	4.24	210	1.463
13	3322	0.1291	2572	4139	3375	10.62	57	0.872
14	20000 (UM)	0.2439	18659	26408	20840	5.20	1603	19.756

TIC:	10.5338	ng/uL
TIM:	40.7373	nmole/L
Total concentration:	11.0731	ng/uL

```
Sample peak width (sec): 5     Sample min peak height: 50    Sample baseline V to V?: Y    Sample baseline V to V points: 3
Sample filter: Binomial        Number of points for filter: 3  Sample start region (min): 0  Sample end region (min): 30
Marker peak width (sec): 5     Marker min peak height: 200   Marker baseline V to V?: Y    Marker baseline V to V points: 3
Lower marker selection: First peak > 500 RFU                 Upper marker selection: Last peak > 200 RFU
Ladder size (bp)75, 200, 300, 400, 500, 700, 1000, 1500, 2000, 3000, 4000, 5000, 7000, 10000, 20000
Quantification using: Lower Marker          Final concentration (ng/uL): 0.5000          Dilution factor: 12.0
```

Sample: SampC1
Well location: C1
Created: Wednesday, October 20, 2021 5:58:44 PM

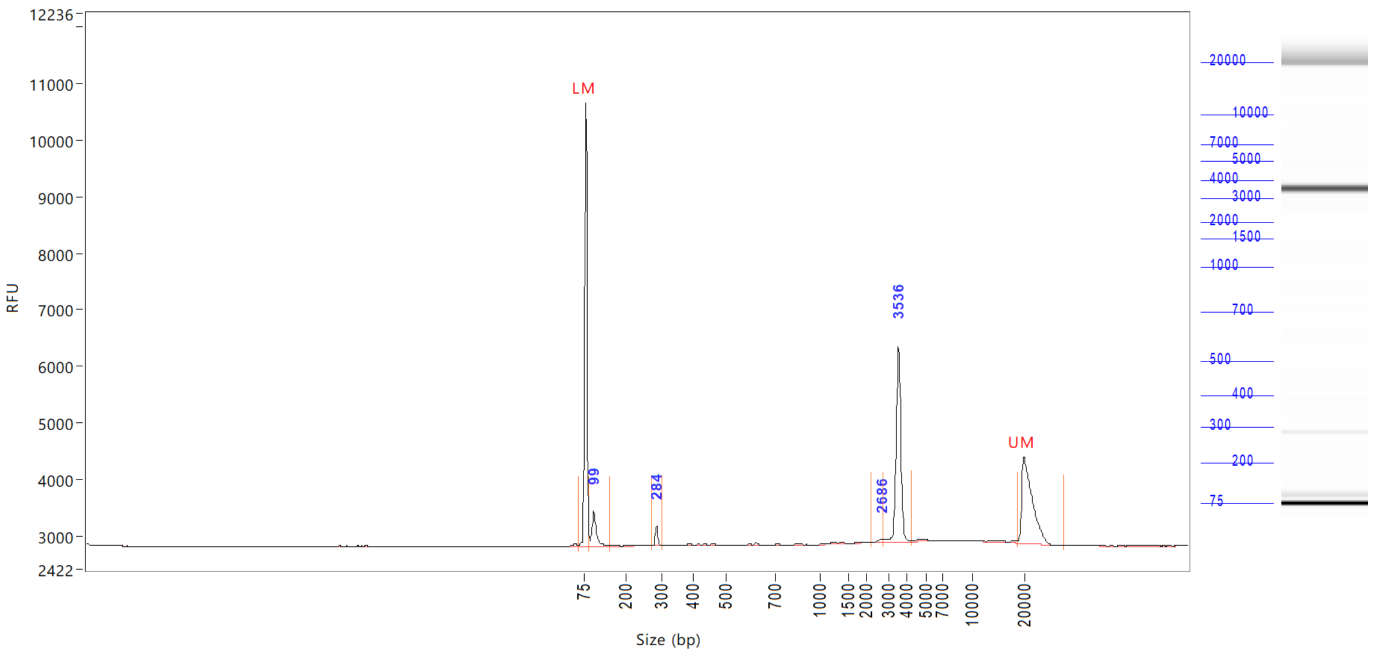

Peak	Size (bp)	Concentration (ng/uL)	From (bp)	To (bp)	Average size (bp)	CV%	RFU	Corrected peak area
1	75 (LM)	0.5000	52	87	73	4.92	7841	42.385
2	99	0.9074	87	148	101	10.34	615	6.410
3	284	0.2657	271	300	282	1.28	356	1.877
4	2686	0.0634	2201	2772	2593	4.79	52	0.448
5	3536	3.3210	2772	4208	3506	4.51	3455	23.460
6	20000 (UM)	0.2340	18659	27738	20911	5.59	1523	19.835

TIC:	4.5575	ng/uL
TIM:	17.9028	nmole/L
Total concentration:	5.4023	ng/uL

```
Sample peak width (sec): 5    Sample min peak height: 50    Sample baseline V to V?: Y    Sample baseline V to V points: 3
Sample filter: Binomial       Number of points for filter: 3  Sample start region (min): 0  Sample end region (min): 30
Marker peak width (sec): 5    Marker min peak height: 200   Marker baseline V to V?: Y    Marker baseline V to V points: 3
Lower marker selection: First peak > 500 RFU                Upper marker selection: Last peak > 200 RFU
Ladder size (bp)75, 200, 300, 400, 500, 700, 1000, 1500, 2000, 3000, 4000, 5000, 7000, 10000, 20000
Quantification using: Lower Marker          Final concentration (ng/uL): 0.5000          Dilution factor: 12.0
```

Sample: SampC2
Well location: C2
Created: Wednesday, October 20, 2021 5:58:44 PM

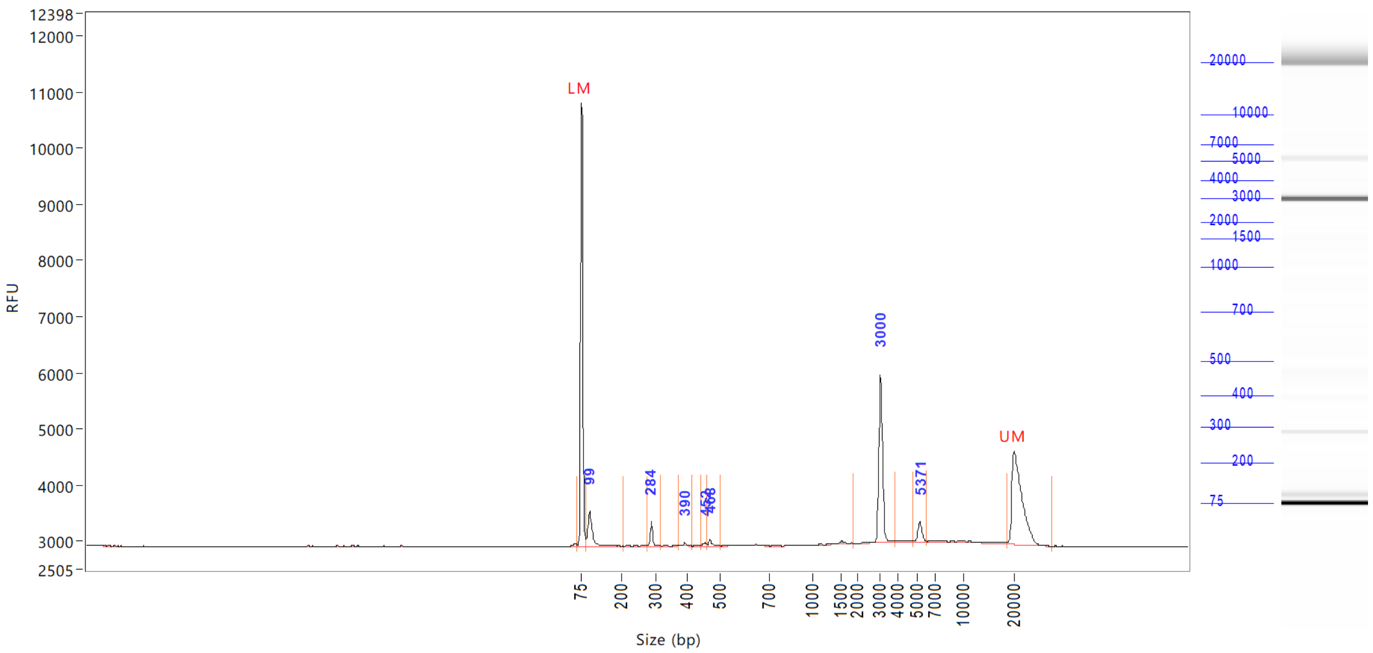

Peak	Size (bp)	Concentration (ng/uL)	From (bp)	To (bp)	Average size (bp)	CV%	RFU	Corrected peak area
1	75 (LM)	0.5000	62	87	74	4.33	7906	42.958
2	99	0.9594	87	205	105	17.38	635	6.869
3	284	0.3085	273	315	283	1.79	445	2.209
4	390	0.0465	372	412	388	1.49	54	0.333
5	452	0.0577	439	460	450	1.21	59	0.413
6	468	0.1014	460	500	469	1.48	111	0.726
7	3000	2.2711	1866	3786	2987	4.41	2981	16.260
8	5371	0.2784	4793	6186	5274	4.04	358	1.993
9	20000 (UM)	0.2397	18659	27496	20848	5.38	1663	20.593

TIC:	4.0229	ng/uL
TIM:	18.9009	nmole/L
Total concentration:	4.6901	ng/uL

Sample peak width (sec): 5 Sample min peak height: 50 Sample baseline V to V?: Y Sample baseline V to V points: 3
Sample filter: Binomial Number of points for filter: 3 Sample start region (min): 0 Sample end region (min): 30
Marker peak width (sec): 5 Marker min peak height: 200 Marker baseline V to V?: Y Marker baseline V to V points: 3
Lower marker selection: First peak > 500 RFU Upper marker selection: Last peak > 200 RFU
Ladder size (bp)75, 200, 300, 400, 500, 700, 1000, 1500, 2000, 3000, 4000, 5000, 7000, 10000, 20000
Quantification using: Lower Marker Final concentration (ng/uL): 0.5000 Dilution factor: 12.0

Sample: SampC3
Well location: C3
Created: Wednesday, October 20, 2021 5:58:44 PM

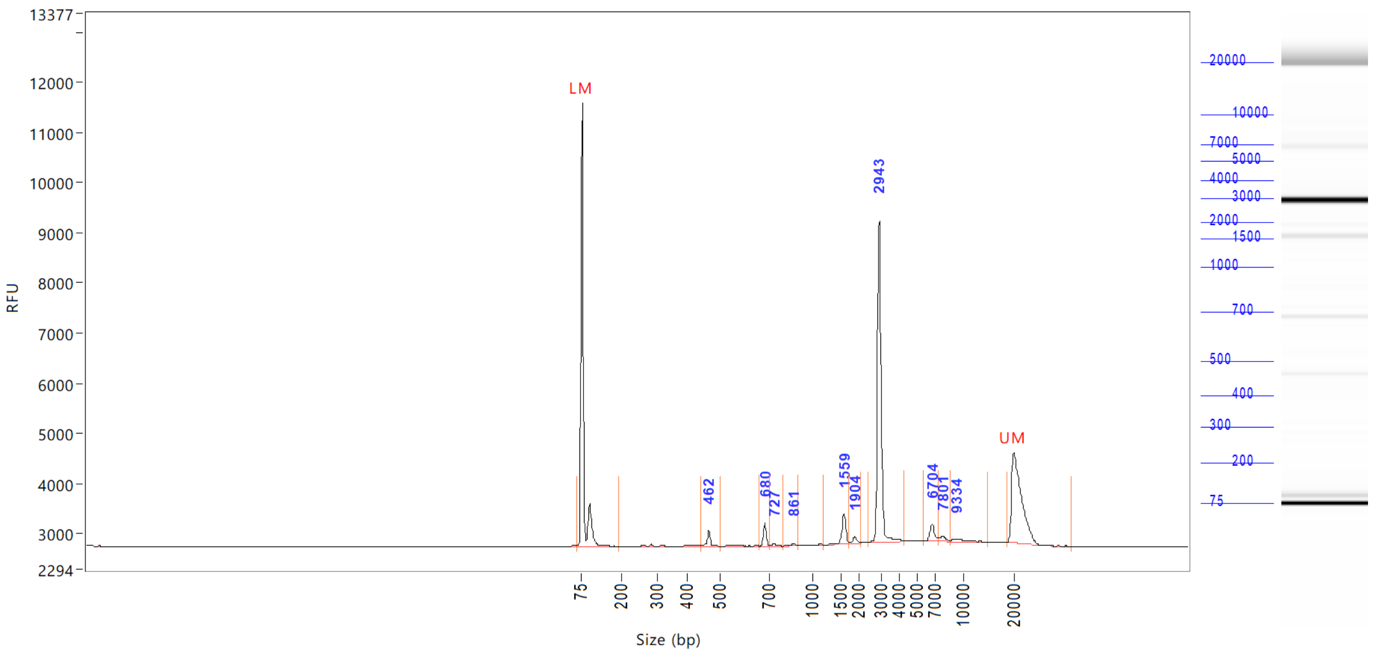

Peak	Size (bp)	Concentration (ng/uL)	From (bp)	To (bp)	Average size (bp)	CV%	RFU	Corrected peak area
1	75 (LM)	0.5000	62	189	77	14.92	8861	54.861
2	462	0.2196	437	500	460	1.81	313	2.008
3	680	0.2497	659	701	677	0.87	465	2.283
4	727	0.0392	701	788	727	1.99	61	0.359
5	861	0.0409	788	896	853	2.48	57	0.374
6	1559	0.3950	1190	1731	1536	4.46	596	3.612
7	1904	0.0971	1731	2087	1886	3.78	131	0.888
8	2943	3.9094	2429	4277	2934	5.34	6391	35.745
9	6704	0.2510	5741	7334	6685	4.34	343	2.295
10	7801	0.1061	7334	8600	7848	4.18	98	0.970
11	9334	0.1348	8600	14879	10488	15.59	58	1.232
12	20000 (UM)	0.2029	18659	31244	20828	5.76	1786	22.260

TIC:	5.4428	ng/uL
TIM:	4.3671	nmole/L
Total concentration:	5.7206	ng/uL

```
Sample peak width (sec): 5     Sample min peak height: 50     Sample baseline V to V?: Y     Sample baseline V to V points: 3
Sample filter: Binomial        Number of points for filter: 3  Sample start region (min): 0  Sample end region (min): 30
Marker peak width (sec): 5     Marker min peak height: 200    Marker baseline V to V?: Y     Marker baseline V to V points: 3
Lower marker selection: First peak > 500 RFU                  Upper marker selection: Last peak > 200 RFU
Ladder size (bp)75, 200, 300, 400, 500, 700, 1000, 1500, 2000, 3000, 4000, 5000, 7000, 10000, 20000
Quantification using: Lower Marker          Final concentration (ng/uL): 0.5000          Dilution factor: 12.0
```

Sample: SampC4
Well location: C4
Created: Wednesday, October 20, 2021 5:58:44 PM

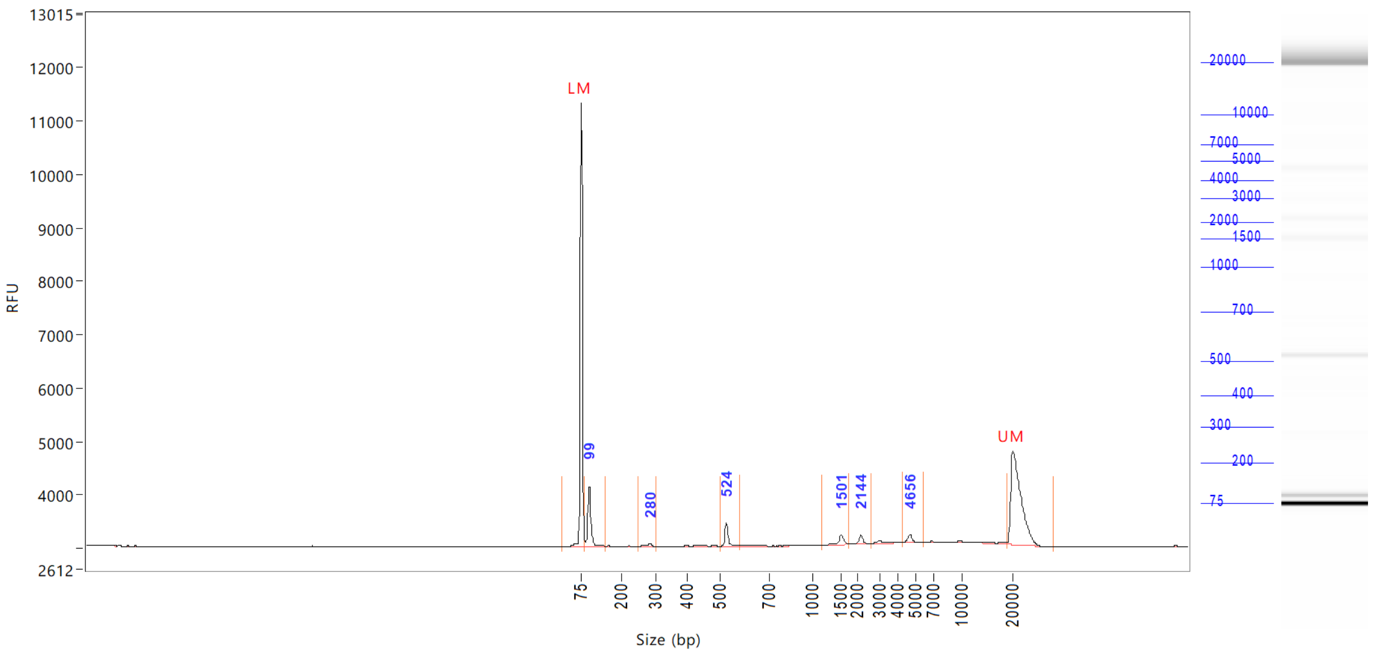

Peak	Size (bp)	Concentration (ng/uL)	From (bp)	To (bp)	Average size (bp)	CV%	RFU	Corrected peak area
1	75 (LM)	0.5000	16	86	72	5.97	8316	44.962
2	99	1.1502	86	150	98	8.36	1124	8.619
3	280	0.0718	248	298	273	3.55	52	0.538
4	524	0.3153	500	580	524	2.10	436	2.362
5	1501	0.2025	1167	1731	1484	5.62	176	1.518
6	2144	0.1518	1731	2600	2101	6.61	160	1.137
7	4656	0.1374	4242	5815	4633	3.74	154	1.030
8	20000 (UM)	0.2472	18781	27979	20895	5.46	1761	22.233

TIC:	2.0290	ng/uL
TIM:	21.0864	nmole/L
Total concentration:	2.4859	ng/uL

```
Sample peak width (sec): 5     Sample min peak height: 50     Sample baseline V to V?: Y     Sample baseline V to V points: 3
Sample filter: Binomial        Number of points for filter: 3  Sample start region (min): 0  Sample end region (min): 30
Marker peak width (sec): 5     Marker min peak height: 200    Marker baseline V to V?: Y     Marker baseline V to V points: 3
Lower marker selection: First peak > 500 RFU                  Upper marker selection: Last peak > 200 RFU
Ladder size (bp)75, 200, 300, 400, 500, 700, 1000, 1500, 2000, 3000, 4000, 5000, 7000, 10000, 20000
Quantification using: Lower Marker          Final concentration (ng/uL): 0.5000          Dilution factor: 12.0
```

Sample: SampC5
Well location: C5
Created: Wednesday, October 20, 2021 5:58:44 PM

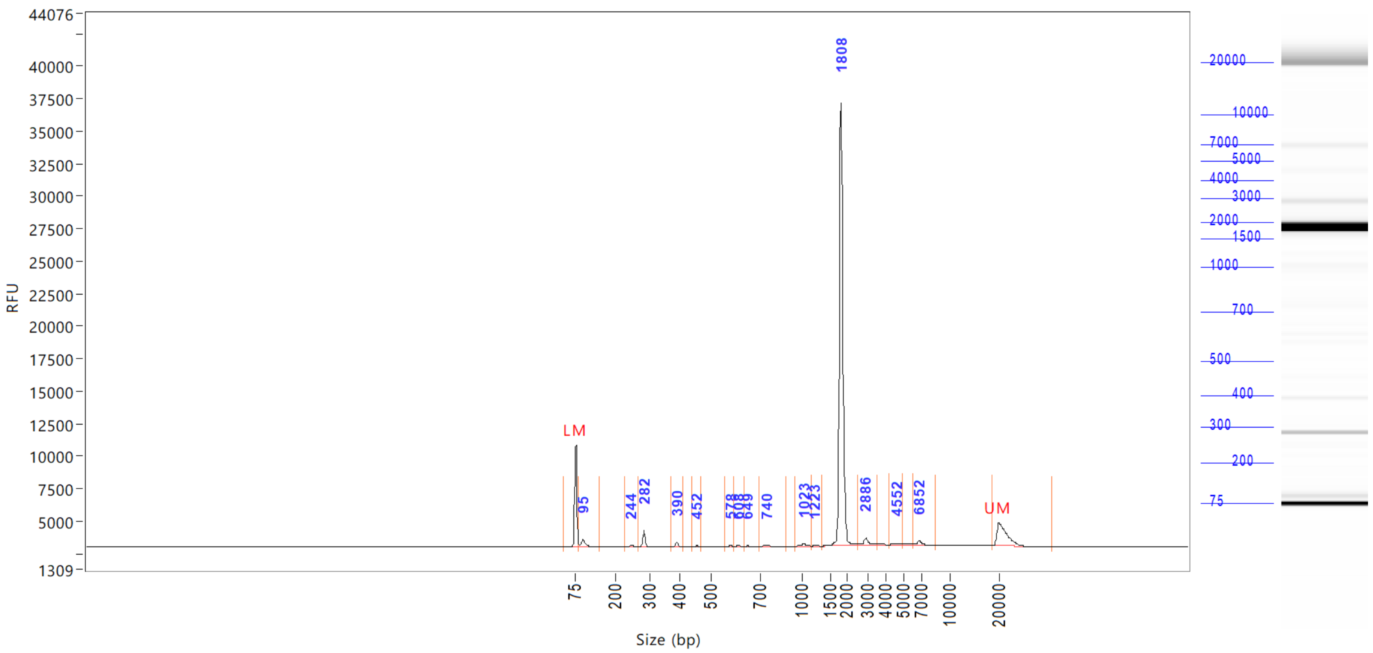

Peak	Size (bp)	Concentration (ng/uL)	From (bp)	To (bp)	Average size (bp)	CV%	RFU	Corrected peak area
1	75 (LM)	0.5000	39	86	72	4.67	7772	42.848
2	95	0.8245	86	150	99	11.46	553	5.888
3	244	0.1179	228	266	248	3.56	69	0.842
4	282	0.9335	266	370	283	3.69	1257	6.667
5	390	0.1996	374	409	387	0.98	300	1.425
6	452	0.0505	437	468	451	1.32	51	0.361
7	578	0.0655	549	593	574	1.31	76	0.468
8	608	0.1009	593	636	610	1.41	120	0.721
9	649	0.0545	636	695	656	2.17	54	0.389
10	740	0.1615	695	887	761	5.46	85	1.153
11	1023	0.2084	948	1156	1013	3.80	148	1.488
12	1223	0.0429	1156	1345	1221	2.35	53	0.306
13	1808	26.7439	1345	2515	1805	4.03	34116	190.986
14	2886	0.5796	2515	3465	2886	6.16	527	4.139
15	4552	0.1265	4104	4862	4508	3.27	124	0.904
16	6852	0.2752	5963	8467	6810	5.30	297	1.965
17	20000 (UM)	0.2530	18659	30519	20817	5.54	1743	21.682

TIC:	30.4849	ng/uL
TIM:	47.0921	nmole/L
Total concentration:	30.8896	ng/uL

```
Sample peak width (sec): 5    Sample min peak height: 50    Sample baseline V to V?: Y    Sample baseline V to V points: 3
Sample filter: Binomial       Number of points for filter: 3  Sample start region (min): 0  Sample end region (min): 30
Marker peak width (sec): 5    Marker min peak height: 200   Marker baseline V to V?: Y    Marker baseline V to V points: 3
Lower marker selection: First peak > 500 RFU                Upper marker selection: Last peak > 200 RFU
Ladder size (bp)75, 200, 300, 400, 500, 700, 1000, 1500, 2000, 3000, 4000, 5000, 7000, 10000, 20000
Quantification using: Lower Marker          Final concentration (ng/uL): 0.5000          Dilution factor: 12.0
```

Sample: SampC6
Well location: C6
Created: Wednesday, October 20, 2021 5:58:44 PM

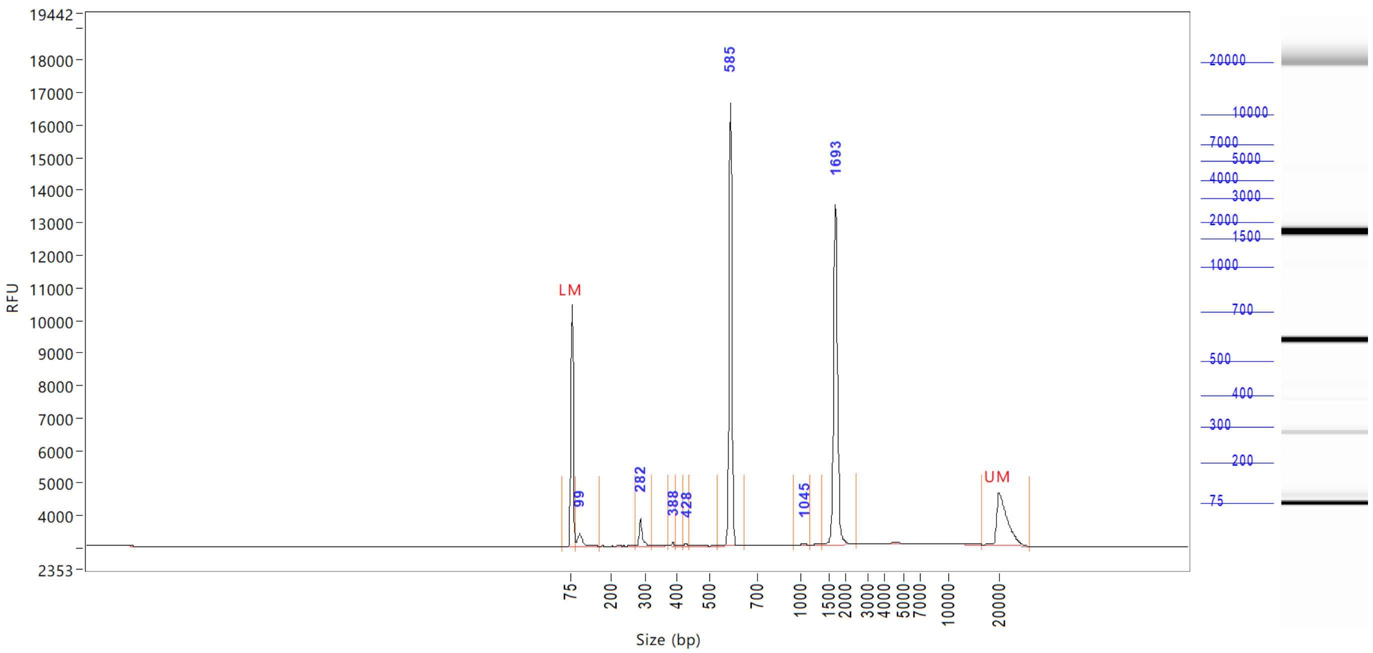

Peak	Size (bp)	Concentration (ng/uL)	From (bp)	To (bp)	Average size (bp)	CV%	RFU	Corrected peak area
1	75 (LM)	0.5000	45	87	74	4.38	7422	41.260
2	99	0.7173	87	164	103	13.06	392	4.933
3	282	0.8170	269	317	284	2.39	861	5.618
4	388	0.0753	374	398	386	1.22	98	0.518
5	428	0.0657	418	439	427	1.17	69	0.452
6	585	10.7026	532	646	584	1.08	13641	73.598
7	1045	0.0612	957	1167	1037	3.69	57	0.421
8	1693	9.7054	1389	2515	1686	4.82	10443	66.741
9	20000 (UM)	0.2632	16586	26045	20792	5.92	1614	21.718

TIC:	22.1444	ng/uL
TIM:	56.4735	nmole/L
Total concentration:	22.9232	ng/uL

Sample peak width (sec): 5   Sample min peak height: 50   Sample baseline V to V?: Y   Sample baseline V to V points: 3
Sample filter: Binomial   Number of points for filter: 3   Sample start region (min): 0   Sample end region (min): 30
Marker peak width (sec): 5   Marker min peak height: 200   Marker baseline V to V?: Y   Marker baseline V to V points: 3
Lower marker selection: First peak > 500 RFU   Upper marker selection: Last peak > 200 RFU
Ladder size (bp)75, 200, 300, 400, 500, 700, 1000, 1500, 2000, 3000, 4000, 5000, 7000, 10000, 20000
Quantification using: Lower Marker   Final concentration (ng/uL): 0.5000   Dilution factor: 12.0

Sample: SampC7
Well location: C7
Created: Wednesday, October 20, 2021 5:58:44 PM

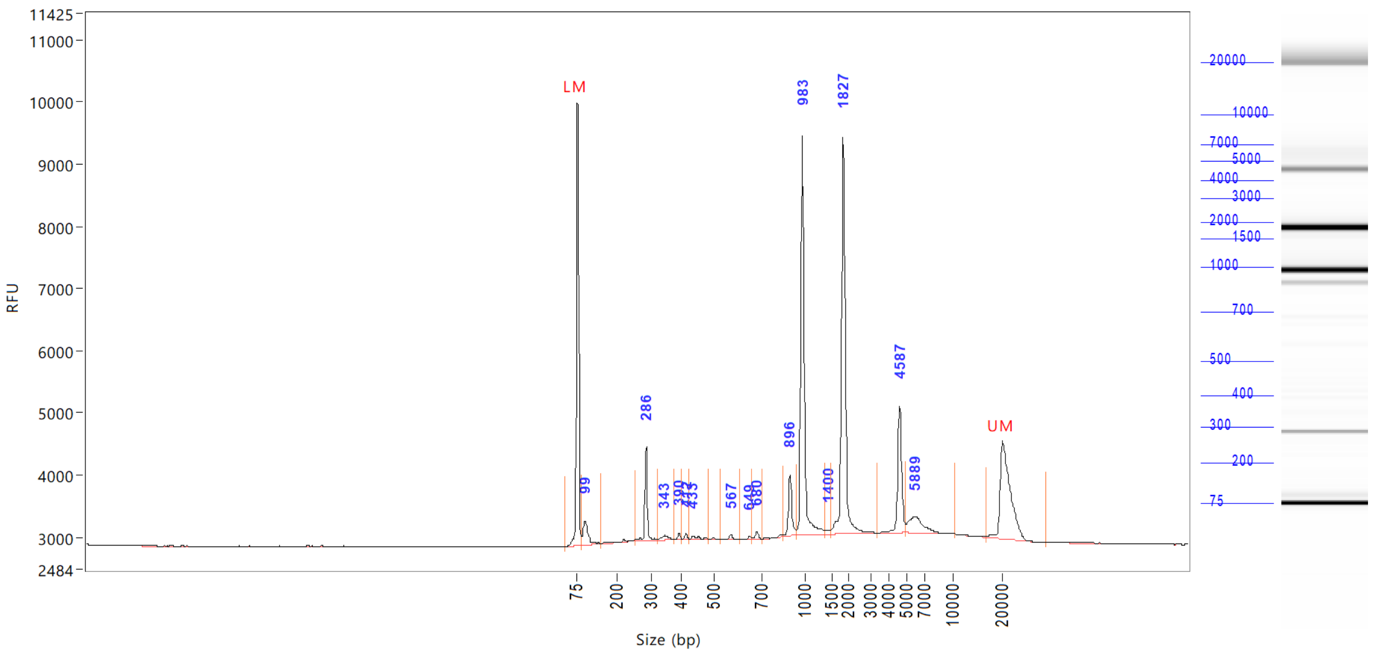

Peak	Size (bp)	Concentration (ng/uL)	From (bp)	To (bp)	Average size (bp)	CV%	RFU	Corrected peak area
1	75 (LM)	0.5000	37	87	74	5.57	7133	40.512
2	99	0.6938	87	148	101	10.65	389	4.684
3	286	1.2017	255	319	283	2.53	1505	8.114
4	343	0.1592	319	376	344	3.48	73	1.075
5	390	0.0800	376	400	388	1.00	112	0.540
6	412	0.0801	400	424	411	1.11	96	0.541
7	433	0.1377	424	483	449	3.69	63	0.930
8	567	0.0859	524	611	565	2.25	78	0.580
9	649	0.0417	611	662	647	1.09	51	0.282
10	680	0.0982	662	700	676	0.90	122	0.663
11	896	0.8556	848	939	894	1.66	987	5.777
12	983	5.4876	939	1367	995	5.96	6424	37.053
13	1400	0.0777	1367	1456	1411	2.02	72	0.525
14	1827	5.7616	1456	3322	1821	8.30	6372	38.903
15	4587	1.8167	3322	4931	4546	3.88	2030	12.267
16	5889	0.8253	4931	10611	6165	14.87	258	5.572
17	20000 (UM)	0.2499	16708	28584	20761	5.79	1591	20.249

TIC:	17.4029	ng/uL
TIM:	37.6157	nmole/L
Total concentration:	17.5891	ng/uL

```
Sample peak width (sec): 5     Sample min peak height: 50     Sample baseline V to V?: Y     Sample baseline V to V points: 3
Sample filter: Binomial        Number of points for filter: 3  Sample start region (min): 0  Sample end region (min): 30
Marker peak width (sec): 5     Marker min peak height: 200    Marker baseline V to V?: Y     Marker baseline V to V points: 3
Lower marker selection: First peak > 500 RFU                  Upper marker selection: Last peak > 200 RFU
Ladder size (bp)75, 200, 300, 400, 500, 700, 1000, 1500, 2000, 3000, 4000, 5000, 7000, 10000, 20000
Quantification using: Lower Marker          Final concentration (ng/uL): 0.5000          Dilution factor: 12.0
```

Sample: SampC8
Well location: C8
Created: Wednesday, October 20, 2021 5:58:44 PM

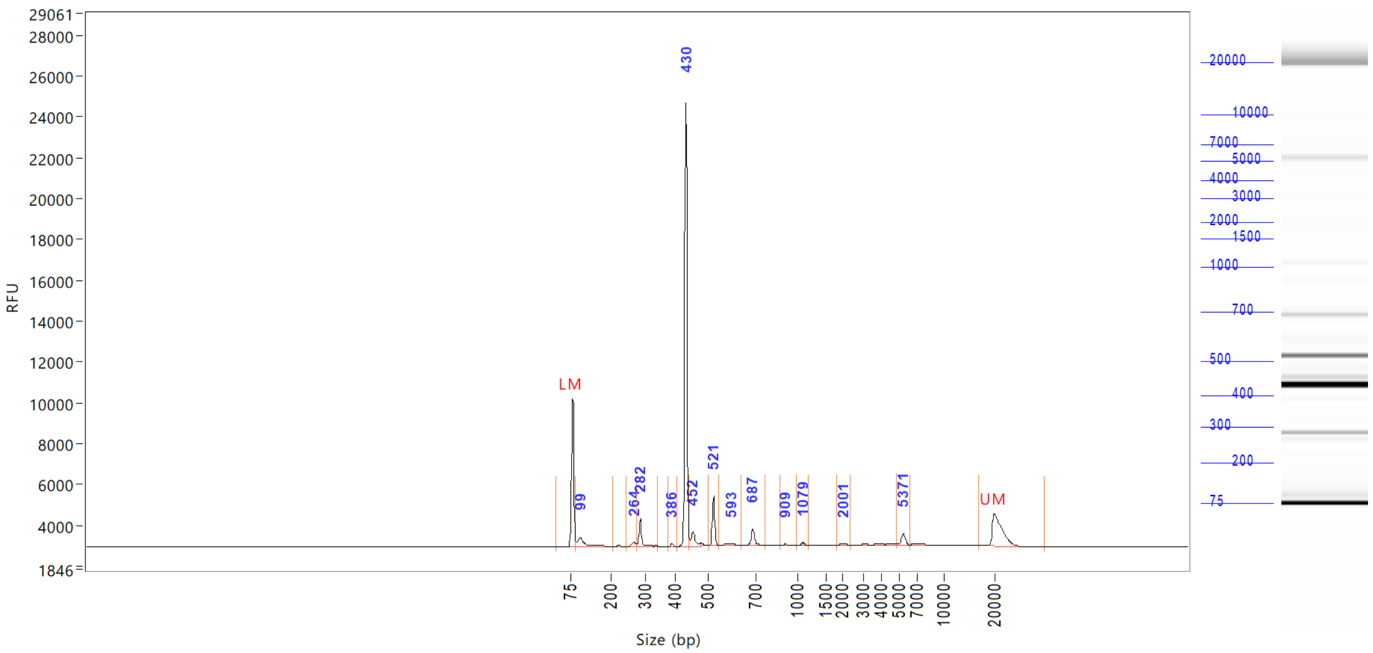

Peak	Size (bp)	Concentration (ng/uL)	From (bp)	To (bp)	Average size (bp)	CV%	RFU	Corrected peak area
1	75 (LM)	0.5000	28	87	74	4.88	7210	40.011
2	99	1.1993	87	201	113	21.07	462	7.997
3	264	0.2986	241	273	262	2.43	221	1.991
4	282	1.0838	273	341	283	3.45	1333	7.227
5	386	0.0787	372	401	384	1.03	107	0.525
6	430	15.5043	401	443	427	0.87	21733	103.390
7	452	0.9974	443	501	458	2.80	695	6.651
8	521	1.7358	501	542	519	1.01	2434	11.575
9	593	0.3104	542	639	585	3.40	117	2.070
10	687	0.7880	639	766	687	1.84	828	5.255
11	909	0.0868	874	996	914	2.34	91	0.579
12	1079	0.1262	996	1190	1069	2.80	147	0.842
13	2001	0.0871	1866	2401	2027	5.13	65	0.581
14	5371	0.4757	4862	6186	5327	4.20	550	3.172
15	20000 (UM)	0.2502	16952	30035	20804	5.62	1601	20.018

TIC:	22.7723	ng/uL
TIM:	98.1035	nmole/L
Total concentration:	23.2231	ng/uL

```
Sample peak width (sec): 5     Sample min peak height: 50     Sample baseline V to V?: Y     Sample baseline V to V points: 3
Sample filter: Binomial        Number of points for filter: 3  Sample start region (min): 0  Sample end region (min): 30
Marker peak width (sec): 5     Marker min peak height: 200    Marker baseline V to V?: Y     Marker baseline V to V points: 3
Lower marker selection: First peak > 500 RFU                  Upper marker selection: Last peak > 200 RFU
Ladder size (bp)75, 200, 300, 400, 500, 700, 1000, 1500, 2000, 3000, 4000, 5000, 7000, 10000, 20000
Quantification using: Lower Marker          Final concentration (ng/uL): 0.5000          Dilution factor: 12.0
```

Sample: SampC9
Well location: C9
Created: Wednesday, October 20, 2021 5:58:44 PM

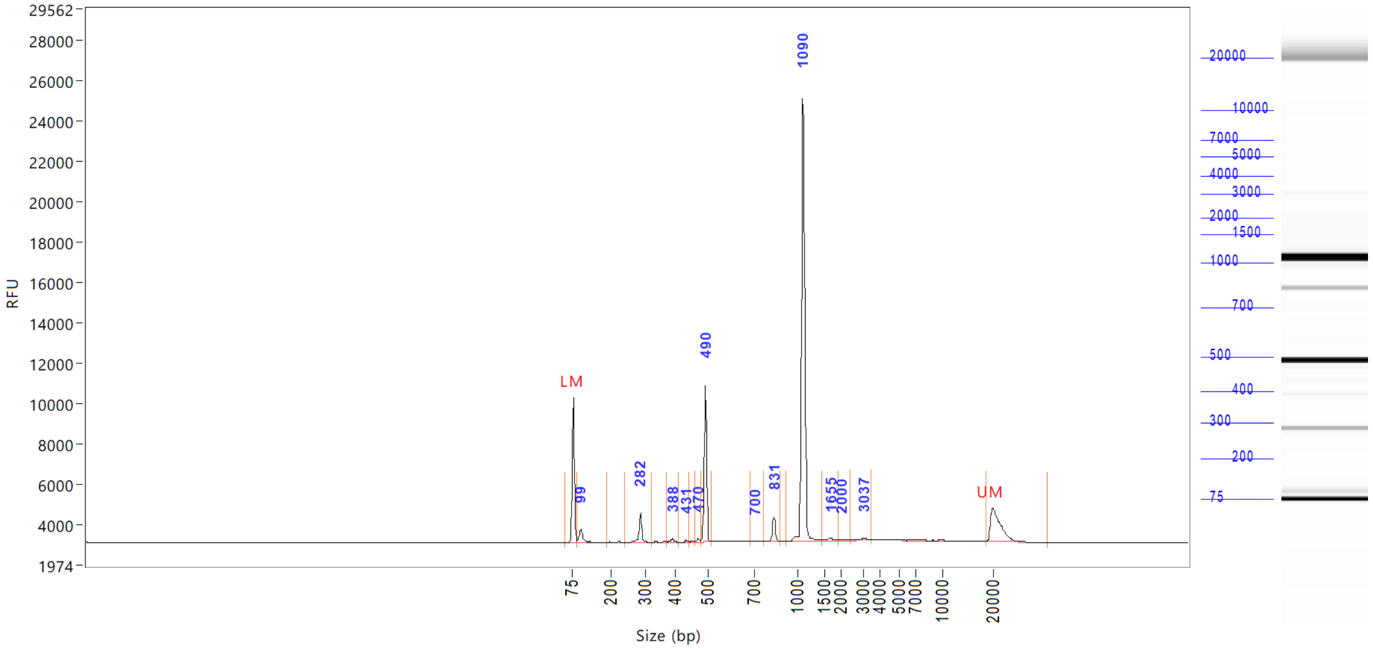

Peak	Size (bp)	Concentration (ng/uL)	From (bp)	To (bp)	Average size (bp)	CV%	RFU	Corrected peak area
1	75 (LM)	0.5000	50	87	74	4.21	7201	39.407
2	99	0.9212	87	183	101	12.42	638	6.050
3	282	1.3599	239	317	280	2.60	1479	8.932
4	388	0.1883	370	407	386	1.67	183	1.237
5	431	0.0801	407	443	429	1.22	97	0.526
6	470	0.1397	460	477	469	0.95	167	0.918
7	490	5.7149	477	514	489	0.75	7738	37.535
8	700	0.0698	682	762	707	2.04	53	0.459
9	831	0.9644	762	874	825	1.23	1230	6.334
10	1090	17.3594	926	1456	1086	4.04	21981	114.014
11	1655	0.2760	1456	1904	1645	7.99	118	1.813
12	2000	0.1329	1904	2458	2091	7.25	72	0.873
13	3037	0.1578	2458	3465	2918	6.51	123	1.037
14	20000 (UM)	0.2693	18659	31002	20887	5.97	1658	21.221

TIC:	27.3646	ng/uL
TIM:	72.6642	nmole/L
Total concentration:	27.9051	ng/uL

```
Sample peak width (sec): 5     Sample min peak height: 50     Sample baseline V to V?: Y     Sample baseline V to V points: 3
Sample filter: Binomial        Number of points for filter: 3  Sample start region (min): 0  Sample end region (min): 30
Marker peak width (sec): 5     Marker min peak height: 200    Marker baseline V to V?: Y     Marker baseline V to V points: 3
Lower marker selection: First peak > 500 RFU                  Upper marker selection: Last peak > 200 RFU
Ladder size (bp)75, 200, 300, 400, 500, 700, 1000, 1500, 2000, 3000, 4000, 5000, 7000, 10000, 20000
Quantification using: Lower Marker          Final concentration (ng/uL): 0.5000          Dilution factor: 12.0
```

Sample: SampC10
Well location: C10
Created: Wednesday, October 20, 2021 5:58:44 PM

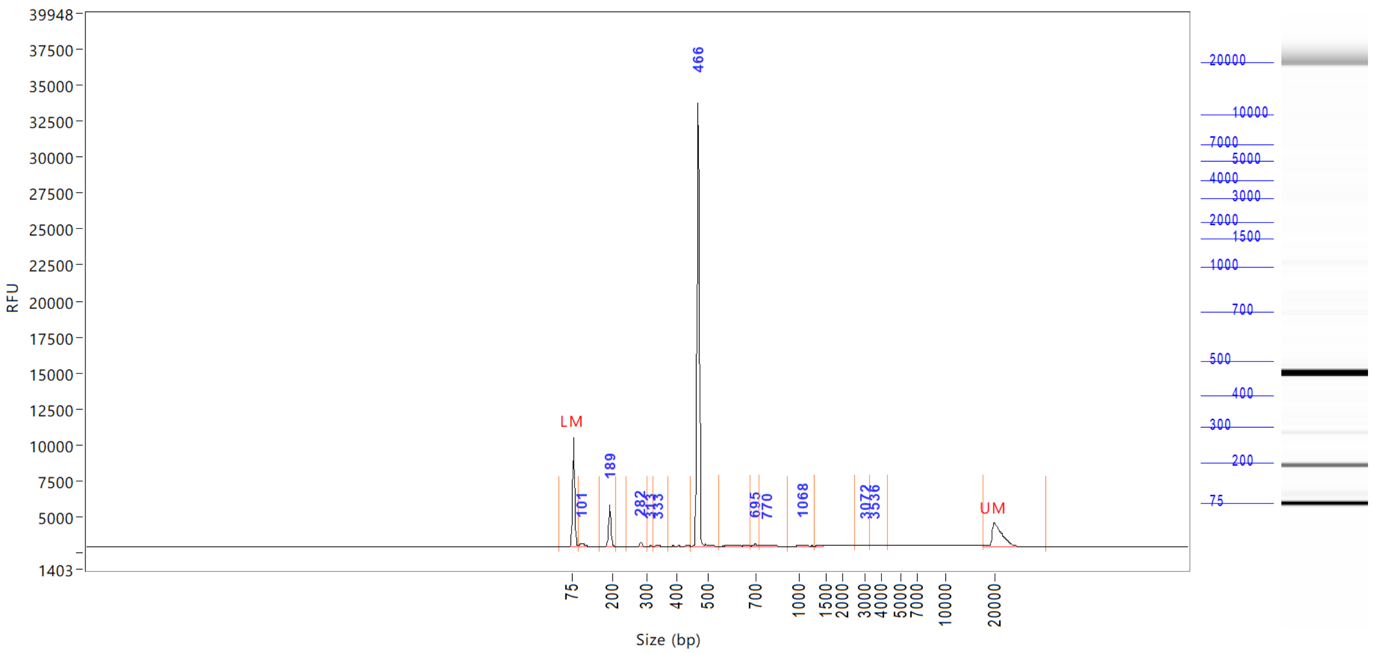

Peak	Size (bp)	Concentration (ng/uL)	From (bp)	To (bp)	Average size (bp)	CV%	RFU	Corrected peak area
1	75 (LM)	0.5000	31	89	73	5.06	7580	40.854
2	101	0.4692	89	158	107	14.11	219	3.195
3	189	2.3322	158	210	187	2.50	2901	15.880
4	282	0.2671	237	303	278	3.16	302	1.818
5	313	0.0426	303	319	311	1.24	58	0.290
6	333	0.0955	319	368	334	2.65	60	0.650
7	466	21.7219	445	539	464	1.05	30791	147.906
8	695	0.1064	677	718	694	1.12	126	0.724
9	770	0.1546	718	922	785	5.05	58	1.053
10	1068	0.1357	922	1278	1053	5.41	109	0.924
11	3072	0.0756	2572	3322	3048	4.73	66	0.515
12	3536	0.0881	3322	4277	3678	6.63	52	0.600
13	20000 (UM)	0.2449	17683	30277	20812	5.68	1613	20.008

TIC:	25.4890	ng/uL
TIM:	107.8811	nmole/L
Total concentration:	26.0821	ng/uL

```
Sample peak width (sec): 5     Sample min peak height: 50     Sample baseline V to V?: Y     Sample baseline V to V points: 3
Sample filter: Binomial        Number of points for filter: 3  Sample start region (min): 0  Sample end region (min): 30
Marker peak width (sec): 5     Marker min peak height: 200    Marker baseline V to V?: Y     Marker baseline V to V points: 3
Lower marker selection: First peak > 500 RFU                  Upper marker selection: Last peak > 200 RFU
Ladder size (bp)75, 200, 300, 400, 500, 700, 1000, 1500, 2000, 3000, 4000, 5000, 7000, 10000, 20000
Quantification using: Lower Marker          Final concentration (ng/uL): 0.5000          Dilution factor: 12.0
```

Sample: SampC11
Well location: C11
Created: Wednesday, October 20, 2021 5:58:44 PM

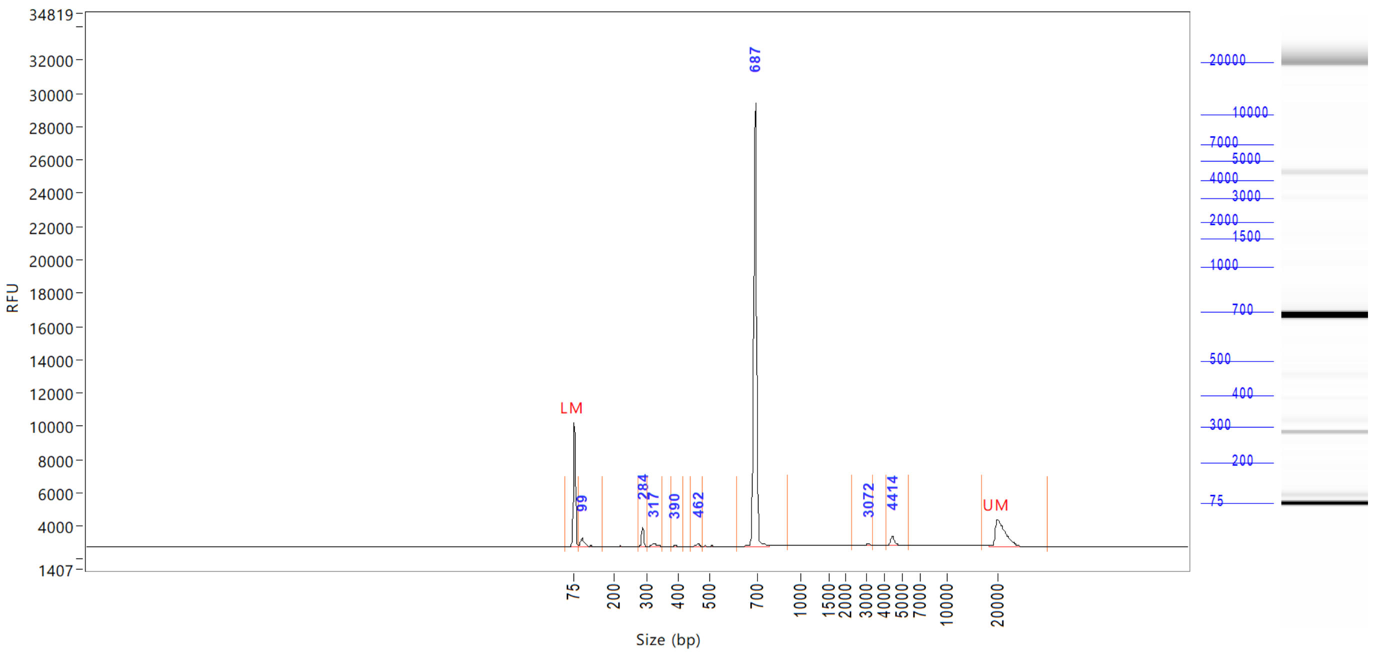

Peak	Size (bp)	Concentration (ng/uL)	From (bp)	To (bp)	Average size (bp)	CV%	RFU	Corrected peak area
1	75 (LM)	0.5000	47	87	73	4.39	7489	40.087
2	99	0.8354	87	166	103	13.00	514	5.581
3	284	0.8688	271	298	282	1.24	1188	5.804
4	317	0.2914	298	347	318	2.97	177	1.947
5	390	0.0715	376	414	389	1.39	97	0.478
6	462	0.1799	441	475	458	1.41	151	1.202
7	687	19.9110	611	913	686	1.95	26682	133.028
8	3072	0.1074	2315	3322	2998	5.49	109	0.717
9	4414	0.4897	4104	5815	4427	3.61	539	3.272
10	20000 (UM)	0.2512	16952	29914	20814	5.56	1622	20.140

TIC:	22.7550	ng/uL
TIM:	68.8503	nmole/L
Total concentration:	23.4027	ng/uL

```
Sample peak width (sec): 5    Sample min peak height: 50    Sample baseline V to V?: Y    Sample baseline V to V points: 3
Sample filter: Binomial       Number of points for filter: 3  Sample start region (min): 0  Sample end region (min): 30
Marker peak width (sec): 5    Marker min peak height: 200   Marker baseline V to V?: Y    Marker baseline V to V points: 3
Lower marker selection: First peak > 500 RFU                Upper marker selection: Last peak > 200 RFU
Ladder size (bp)75, 200, 300, 400, 500, 700, 1000, 1500, 2000, 3000, 4000, 5000, 7000, 10000, 20000
Quantification using: Lower Marker          Final concentration (ng/uL): 0.5000          Dilution factor: 12.0
```

Sample: SampC12
Well location: C12
Created: Wednesday, October 20, 2021 5:58:44 PM

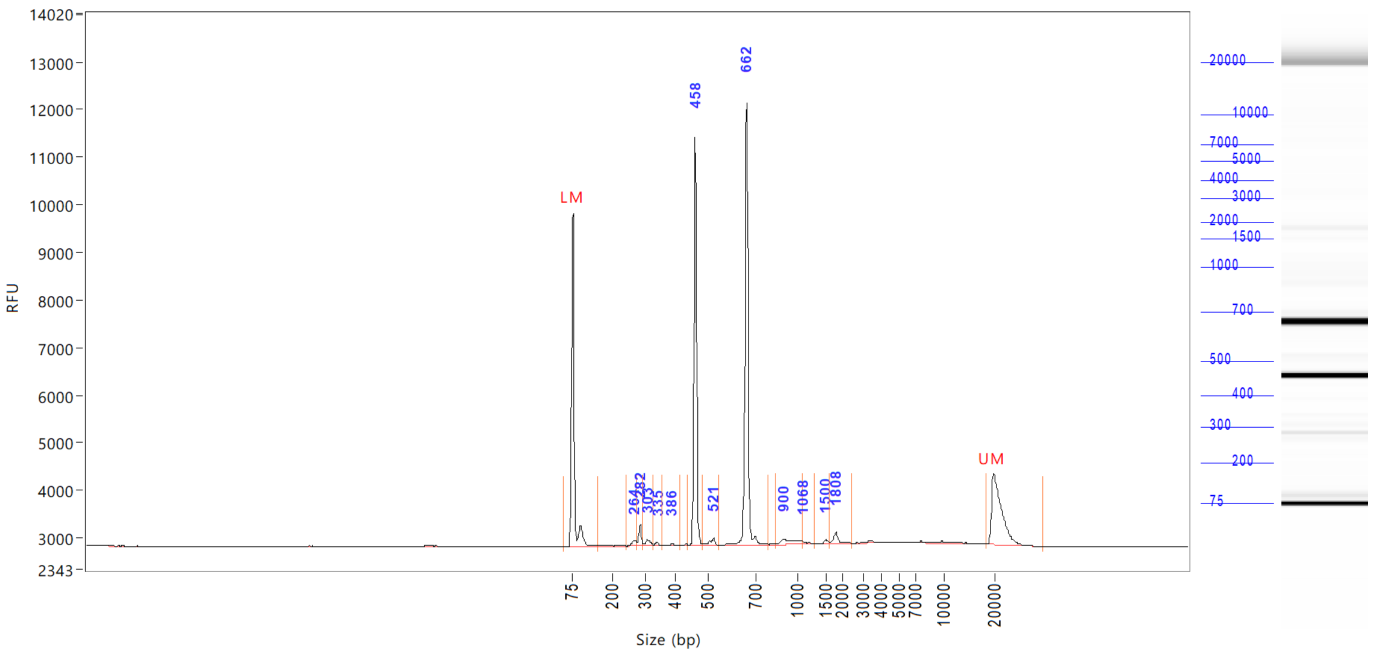

Peak	Size (bp)	Concentration (ng/uL)	From (bp)	To (bp)	Average size (bp)	CV%	RFU	Corrected peak area
1	75 (LM)	0.5000	47	156	76	13.59	6981	44.332
2	264	0.1641	241	273	263	2.59	119	1.212
3	282	0.3483	273	291	280	1.35	460	2.574
4	303	0.1785	291	323	304	2.47	123	1.319
5	335	0.0686	323	352	332	1.38	81	0.507
6	386	0.0513	352	412	387	2.21	56	0.379
7	458	5.8798	437	481	456	0.95	8564	43.444
8	521	0.2256	481	547	508	2.62	151	1.667
9	662	7.0949	547	788	658	1.96	9302	52.421
10	900	0.3738	835	1068	939	5.77	121	2.762
11	1068	0.0690	1068	1289	1139	4.82	63	0.510
12	1500	0.0864	1289	1616	1500	3.90	88	0.638
13	1808	0.2576	1616	2458	1824	7.47	234	1.903
14	20000 (UM)	0.2136	18537	29914	20893	6.11	1495	18.936

TIC:	14.7979	ng/uL
TIM:	45.3704	nmole/L
Total concentration:	15.2672	ng/uL

```
Sample peak width (sec): 5     Sample min peak height: 50     Sample baseline V to V?: Y     Sample baseline V to V points: 3
Sample filter: Binomial        Number of points for filter: 3  Sample start region (min): 0  Sample end region (min): 30
Marker peak width (sec): 5     Marker min peak height: 200    Marker baseline V to V?: Y     Marker baseline V to V points: 3
Lower marker selection: First peak > 500 RFU                  Upper marker selection: Last peak > 200 RFU
Ladder size (bp)75, 200, 300, 400, 500, 700, 1000, 1500, 2000, 3000, 4000, 5000, 7000, 10000, 20000
Quantification using: Lower Marker          Final concentration (ng/uL): 0.5000          Dilution factor: 12.0
```

Sample: SampD1
Well location: D1
Created: Wednesday, October 20, 2021 5:58:44 PM

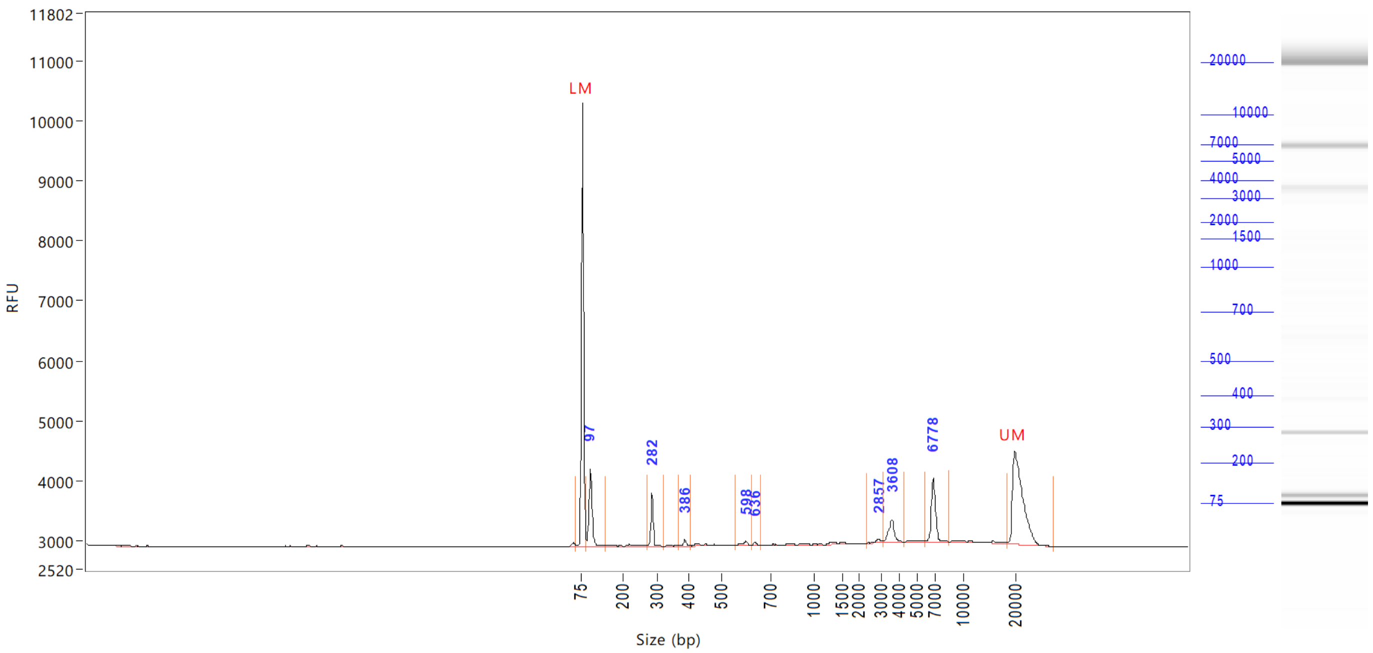

Peak	Size (bp)	Concentration (ng/uL)	From (bp)	To (bp)	Average size (bp)	CV%	RFU	Corrected peak area
1	75 (LM)	0.5000	56	86	73	4.40	7419	39.023
2	97	1.5326	86	147	98	7.75	1303	9.968
3	282	0.6748	269	321	281	1.61	895	4.389
4	386	0.0943	368	407	385	1.56	103	0.613
5	598	0.1336	555	621	587	2.54	74	0.869
6	636	0.0583	621	659	634	1.05	65	0.379
7	2857	0.0691	2315	3072	2842	5.14	56	0.450
8	3608	0.4982	3072	4277	3526	5.23	385	3.240
9	6778	0.9470	5741	8467	6743	4.01	1055	6.159
10	20000 (UM)	0.2500	18659	27496	20862	5.32	1556	19.514

TIC:	4.0079	ng/uL
TIM:	31.0617	nmole/L
Total concentration:	4.7989	ng/uL

Sample peak width (sec): 5   Sample min peak height: 50   Sample baseline V to V?: Y   Sample baseline V to V points: 3
Sample filter: Binomial   Number of points for filter: 3 Sample start region (min): 0 Sample end region (min): 30
Marker peak width (sec): 5   Marker min peak height: 200   Marker baseline V to V?: Y   Marker baseline V to V points: 3
Lower marker selection: First peak > 500 RFU   Upper marker selection: Last peak > 200 RFU
Ladder size (bp)75, 200, 300, 400, 500, 700, 1000, 1500, 2000, 3000, 4000, 5000, 7000, 10000, 20000
Quantification using: Lower Marker   Final concentration (ng/uL): 0.5000   Dilution factor: 12.0

Sample: SampD2
Well location: D2
Created: Wednesday, October 20, 2021 5:58:44 PM

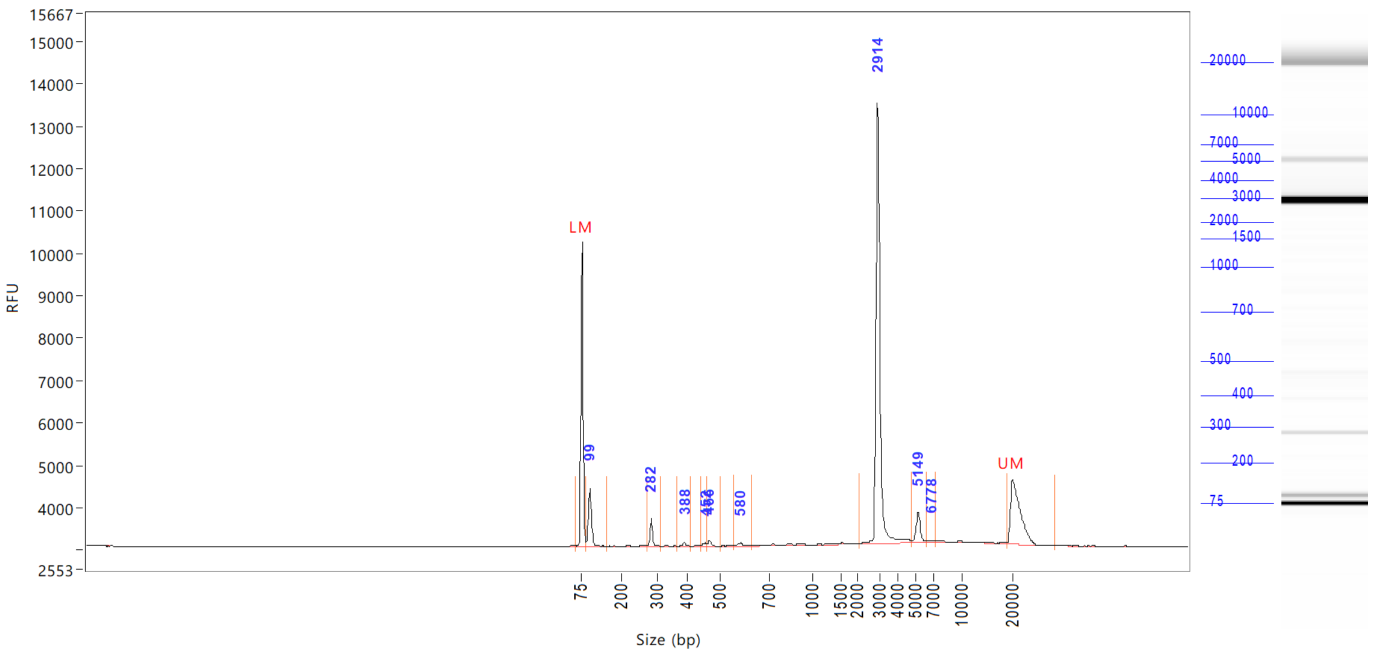

Peak	Size (bp)	Concentration (ng/uL)	From (bp)	To (bp)	Average size (bp)	CV%	RFU	Corrected peak area
1	75 (LM)	0.5000	58	86	73	4.41	7191	37.978
2	99	1.6858	86	152	98	7.85	1378	10.671
3	282	0.5177	269	313	281	1.61	662	3.277
4	388	0.1061	364	411	386	1.79	109	0.672
5	452	0.0898	439	458	449	1.21	77	0.568
6	466	0.1371	458	498	466	1.12	141	0.868
7	580	0.1285	555	626	581	2.67	77	0.814
8	2914	9.9073	2058	4759	2961	8.87	10410	62.710
9	5149	0.7100	4759	6260	5191	5.32	705	4.494
10	6778	0.0737	6260	7201	6721	4.13	52	0.467
11	20000 (UM)	0.2453	18781	28342	20801	5.35	1511	18.636

TIC:	13.3560	ng/uL
TIM:	38.6566	nmole/L
Total concentration:	14.1376	ng/uL

```
Sample peak width (sec): 5     Sample min peak height: 50    Sample baseline V to V?: Y    Sample baseline V to V points: 3
Sample filter: Binomial        Number of points for filter: 3  Sample start region (min): 0  Sample end region (min): 30
Marker peak width (sec): 5     Marker min peak height: 200   Marker baseline V to V?: Y    Marker baseline V to V points: 3
Lower marker selection: First peak > 500 RFU                 Upper marker selection: Last peak > 200 RFU
Ladder size (bp)75, 200, 300, 400, 500, 700, 1000, 1500, 2000, 3000, 4000, 5000, 7000, 10000, 20000
Quantification using: Lower Marker          Final concentration (ng/uL): 0.5000         Dilution factor: 12.0
```

Sample: SampD3
Well location: D3
Created: Wednesday, October 20, 2021 5:58:44 PM

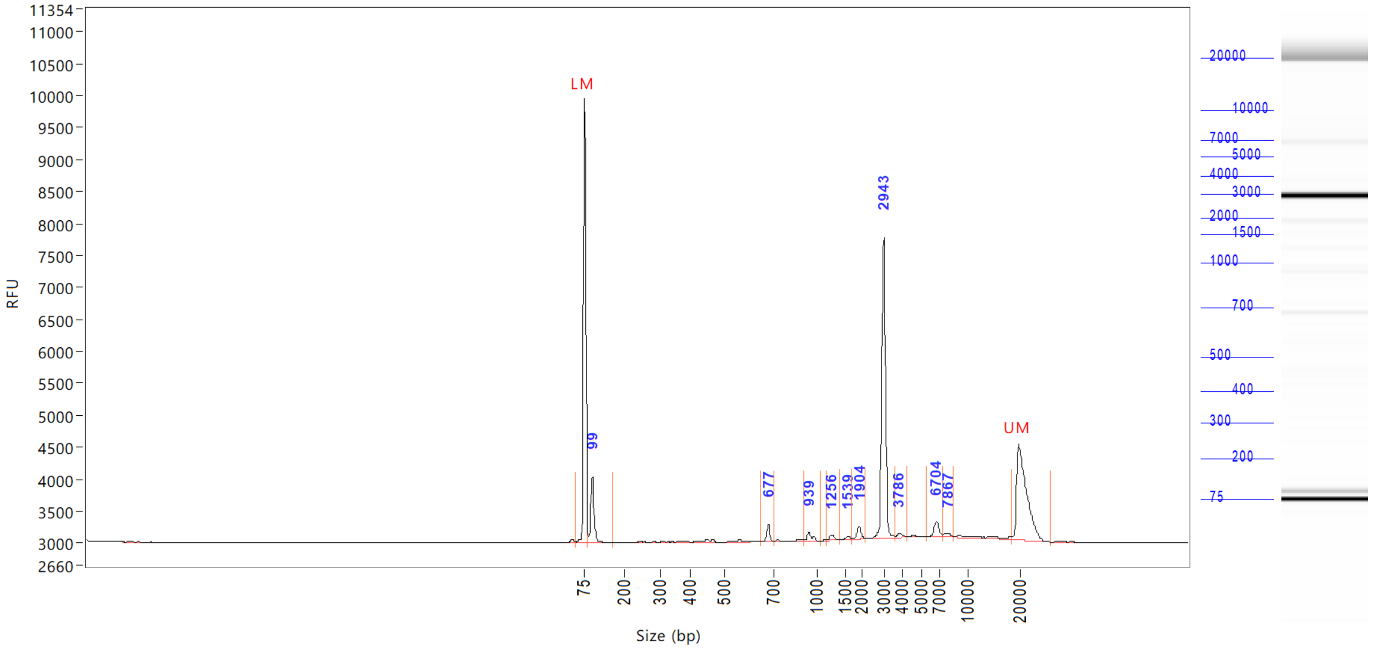

Peak	Size (bp)	Concentration (ng/uL)	From (bp)	To (bp)	Average size (bp)	CV%	RFU	Corrected peak area
1	75 (LM)	0.5000	50	86	73	4.77	6953	36.740
2	99	1.3460	86	162	98	8.97	1037	8.242
3	677	0.2231	649	701	675	0.90	275	1.366
4	939	0.1832	913	1068	949	2.19	143	1.122
5	1256	0.0950	1167	1400	1252	3.05	83	0.582
6	1539	0.0563	1400	1674	1525	3.50	55	0.345
7	1904	0.2008	1674	2144	1882	3.69	201	1.229
8	2943	4.2090	2144	3572	2915	3.53	4688	25.774
9	3786	0.0923	3572	4242	3840	4.08	64	0.565
10	6704	0.2758	5519	7401	6690	4.78	239	1.689
11	7867	0.0900	7401	8534	7876	3.84	57	0.551
12	20000 (UM)	0.2534	18659	26166	20794	5.34	1492	18.623

TIC:	6.7714	ng/uL
TIM:	26.2783	nmole/L
Total concentration:	7.5412	ng/uL

```
Sample peak width (sec): 5     Sample min peak height: 50     Sample baseline V to V?: Y     Sample baseline V to V points: 3
Sample filter: Binomial        Number of points for filter: 3  Sample start region (min): 0  Sample end region (min): 30
Marker peak width (sec): 5     Marker min peak height: 200    Marker baseline V to V?: Y     Marker baseline V to V points: 3
Lower marker selection: First peak > 500 RFU                  Upper marker selection: Last peak > 200 RFU
Ladder size (bp)75, 200, 300, 400, 500, 700, 1000, 1500, 2000, 3000, 4000, 5000, 7000, 10000, 20000
Quantification using: Lower Marker          Final concentration (ng/uL): 0.5000          Dilution factor: 12.0
```

Sample: SampD4
Well location: D4
Created: Wednesday, October 20, 2021 5:58:44 PM

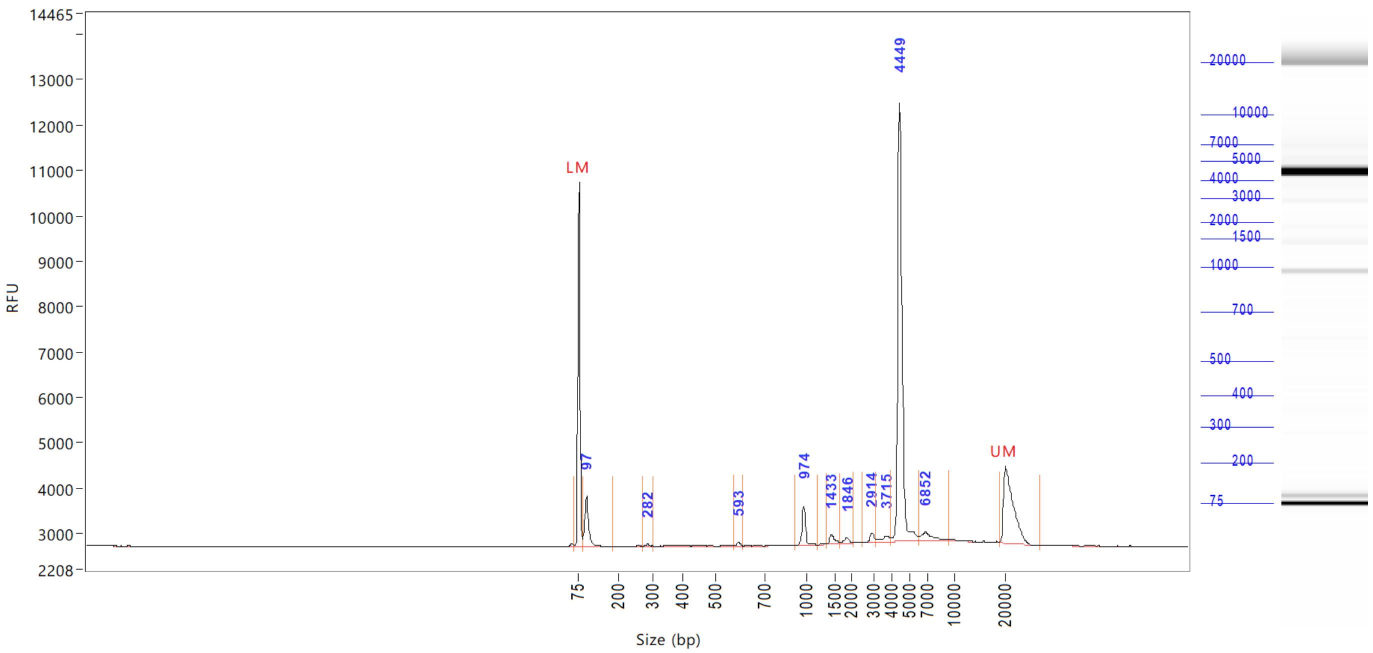

Peak	Size (bp)	Concentration (ng/uL)	From (bp)	To (bp)	Average size (bp)	CV%	RFU	Corrected peak area
1	75 (LM)	0.5000	62	86	73	4.39	8048	42.367
2	97	1.4639	86	181	99	12.12	1117	10.337
3	282	0.0479	269	301	281	2.01	58	0.338
4	593	0.0818	575	611	590	1.12	96	0.577
5	974	0.6818	918	1190	975	2.62	870	4.814
6	1433	0.2371	1345	1635	1452	4.21	195	1.674
7	1846	0.1498	1635	2030	1809	4.66	128	1.058
8	2914	0.1770	2486	3108	2903	3.43	197	1.250
9	3715	0.2225	3108	3929	3568	6.47	132	1.571
10	4449	8.8168	3929	6112	4455	4.94	9683	62.257
11	6852	0.4354	6112	9334	7251	10.78	185	3.074
12	20000 (UM)	0.2471	18781	26891	20854	5.28	1694	20.941

TIC:	12.3140	ng/uL
TIM:	29.9051	nmole/L
Total concentration:	12.7620	ng/uL

```
Sample peak width (sec): 5    Sample min peak height: 50    Sample baseline V to V?: Y    Sample baseline V to V points: 3
Sample filter: Binomial       Number of points for filter: 3  Sample start region (min): 0  Sample end region (min): 30
Marker peak width (sec): 5    Marker min peak height: 200   Marker baseline V to V?: Y    Marker baseline V to V points: 3
Lower marker selection: First peak > 500 RFU                Upper marker selection: Last peak > 200 RFU
Ladder size (bp)75, 200, 300, 400, 500, 700, 1000, 1500, 2000, 3000, 4000, 5000, 7000, 10000, 20000
Quantification using: Lower Marker          Final concentration (ng/uL): 0.5000          Dilution factor: 12.0
```

Sample: SampD5
Well location: D5
Created: Wednesday, October 20, 2021 5:58:44 PM

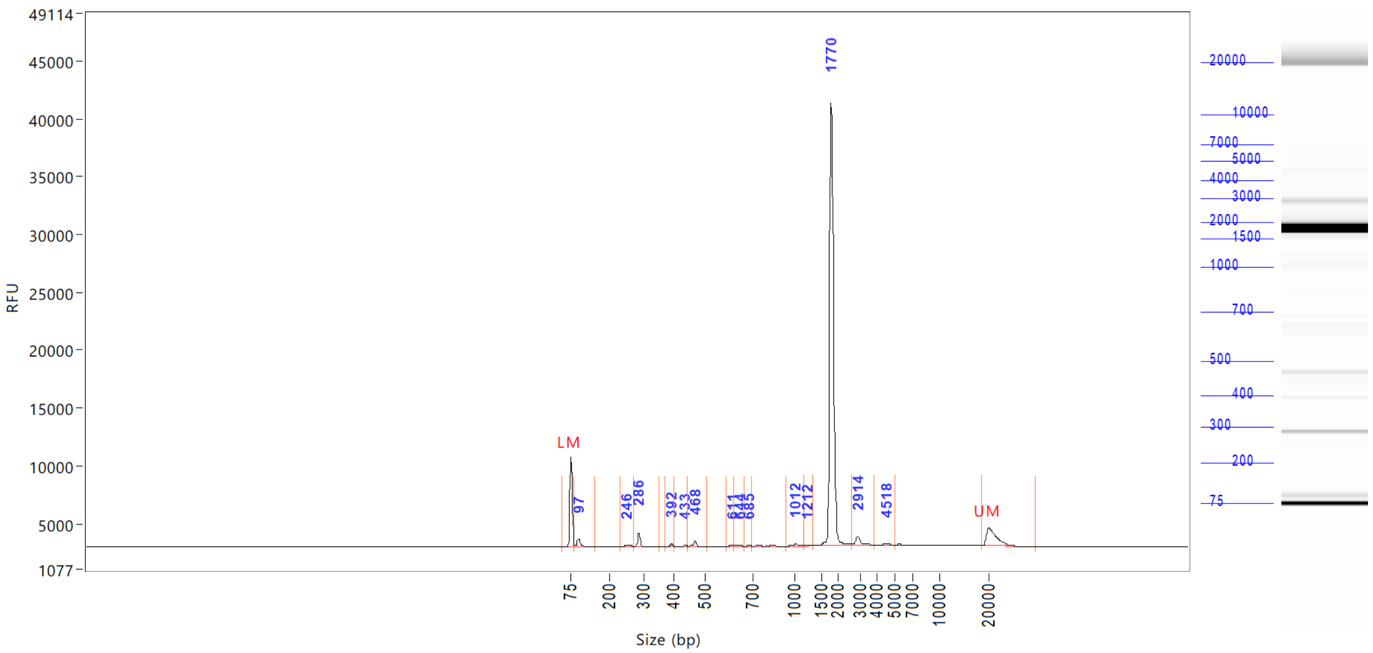

Peak	Size (bp)	Concentration (ng/uL)	From (bp)	To (bp)	Average size (bp)	CV%	RFU	Corrected peak area
1	75 (LM)	0.5000	50	86	73	4.36	7725	41.378
2	97	0.9486	86	152	100	11.82	648	6.542
3	246	0.1684	230	271	253	3.93	68	1.161
4	286	0.9942	271	352	286	3.61	1208	6.856
5	392	0.1840	372	401	389	1.12	248	1.269
6	433	0.1308	401	443	428	2.48	100	0.902
7	468	0.4841	443	504	465	1.89	512	3.338
8	611	0.1132	590	621	608	1.06	142	0.780
9	644	0.1521	621	664	639	1.40	134	1.049
10	685	0.0842	664	700	682	0.97	110	0.581
11	1012	0.2752	935	1156	1022	4.85	159	1.898
12	1212	0.0704	1156	1323	1208	2.57	75	0.485
13	1770	32.9160	1323	2629	1777	4.86	38330	226.999
14	2914	0.9209	2629	3893	2988	8.87	771	6.351
15	4518	0.2445	3893	5001	4422	5.99	148	1.686
16	20000 (UM)	0.2370	18659	29430	20833	5.80	1564	19.612

TIC:	37.6867	ng/uL
TIM:	57.9138	nmole/L
Total concentration:	38.0550	ng/uL

Sample peak width (sec): 5 Sample min peak height: 50 Sample baseline V to V?: Y Sample baseline V to V points: 3
Sample filter: Binomial Number of points for filter: 3 Sample start region (min): 0 Sample end region (min): 30
Marker peak width (sec): 5 Marker min peak height: 200 Marker baseline V to V?: Y Marker baseline V to V points: 3
Lower marker selection: First peak > 500 RFU Upper marker selection: Last peak > 200 RFU
Ladder size (bp)75, 200, 300, 400, 500, 700, 1000, 1500, 2000, 3000, 4000, 5000, 7000, 10000, 20000
Quantification using: Lower Marker Final concentration (ng/uL): 0.5000 Dilution factor: 12.0

Sample: SampD6
Well location: D6
Created: Wednesday, October 20, 2021 5:58:44 PM

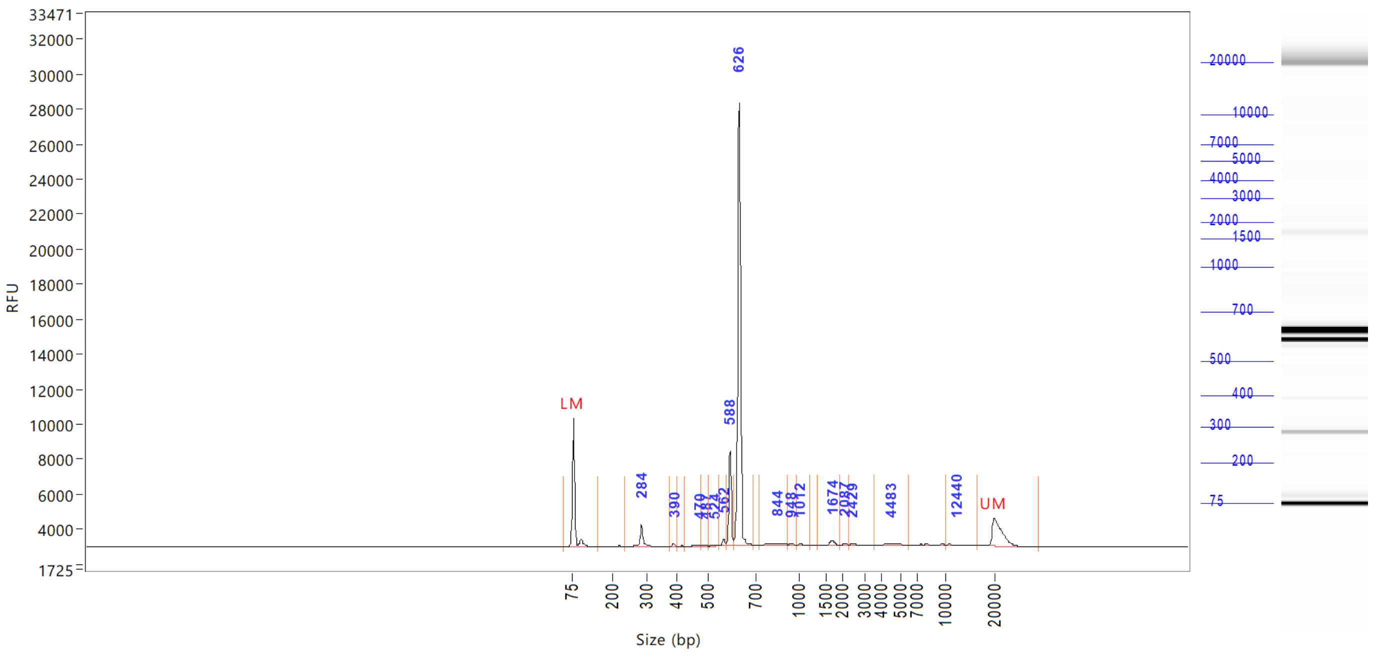

Peak	Size (bp)	Concentration (ng/uL)	From (bp)	To (bp)	Average size (bp)	CV%	RFU	Corrected peak area
1	75 (LM)	0.5000	45	154	76	13.51	7326	44.317
2	284	1.1796	235	376	284	4.99	1287	8.713
3	390	0.1087	376	400	387	0.93	171	0.803
4	470	0.0810	424	477	458	2.82	58	0.598
5	487	0.0515	477	500	485	0.83	76	0.380
6	524	0.0511	500	539	522	1.21	74	0.377
7	562	0.2831	539	572	561	1.25	378	2.091
8	588	3.5087	572	603	585	0.96	5377	25.916
9	626	17.0138	603	690	623	1.08	25308	125.667
10	844	0.3394	723	926	835	5.71	110	2.507
11	948	0.0886	926	983	947	1.39	100	0.654
12	1012	0.0792	983	1190	1034	3.81	63	0.585
13	1674	0.3257	1334	1923	1641	6.03	279	2.406
14	2087	0.1048	1923	2258	2071	3.91	119	0.774
15	2429	0.1303	2258	3536	2676	11.78	63	0.962
16	4483	0.1584	3536	6038	4500	8.89	63	1.170
17	12440	0.1568	10245	16708	12695	13.36	51	1.158
18	20000 (UM)	0.2241	16708	28947	20749	5.74	1613	19.867

TIC:	23.6606	ng/uL
TIM:	65.0812	nmole/L
Total concentration:	23.9338	ng/uL

```
Sample peak width (sec): 5     Sample min peak height: 50    Sample baseline V to V?: Y    Sample baseline V to V points: 3
Sample filter: Binomial        Number of points for filter: 3  Sample start region (min): 0  Sample end region (min): 30
Marker peak width (sec): 5     Marker min peak height: 200   Marker baseline V to V?: Y    Marker baseline V to V points: 3
Lower marker selection: First peak > 500 RFU                 Upper marker selection: Last peak > 200 RFU
Ladder size (bp)75, 200, 300, 400, 500, 700, 1000, 1500, 2000, 3000, 4000, 5000, 7000, 10000, 20000
Quantification using: Lower Marker          Final concentration (ng/uL): 0.5000          Dilution factor: 12.0
```

Sample: SampD7
Well location: D7
Created: Wednesday, October 20, 2021 5:58:44 PM

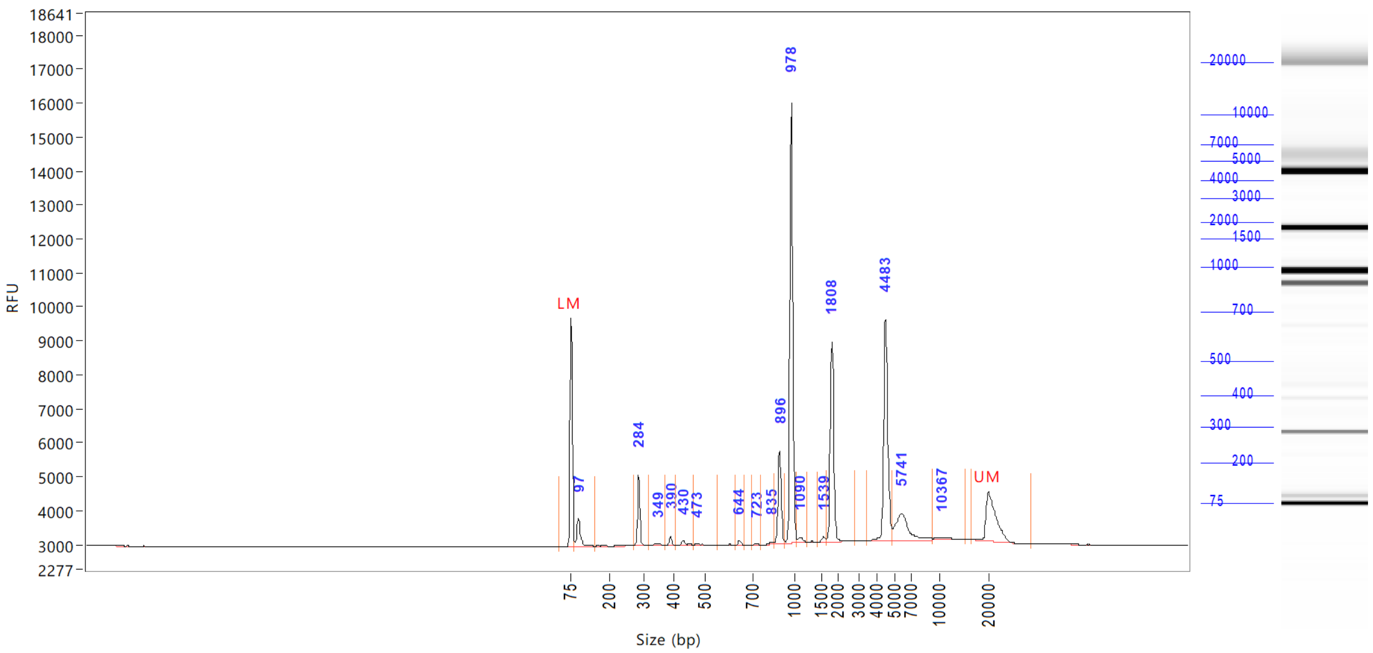

Peak	Size (bp)	Concentration (ng/uL)	From (bp)	To (bp)	Average size (bp)	CV%	RFU	Corrected peak area
1	75 (LM)	0.5000	41	86	73	4.39	6731	35.991
2	97	1.2629	86	150	99	10.49	812	7.575
3	284	1.7798	271	317	283	1.68	2091	10.676
4	349	0.1494	317	374	344	3.59	55	0.896
5	390	0.2402	374	407	387	1.05	292	1.441
6	430	0.2363	407	462	434	2.64	135	1.417
7	473	0.1495	462	549	487	3.75	55	0.897
8	644	0.1348	626	664	643	0.93	151	0.808
9	723	0.0553	697	762	719	1.73	50	0.332
10	835	0.1139	762	857	832	1.92	80	0.683
11	896	2.3977	857	935	890	1.35	2748	14.382
12	978	10.8177	935	1045	976	1.10	12977	64.890
13	1090	0.1952	1045	1245	1099	3.62	160	1.171
14	1539	0.1501	1411	1635	1539	3.30	144	0.900
15	1808	5.2962	1635	2772	1804	4.09	5883	31.769
16	4483	6.7574	3393	4897	4467	3.32	6521	40.534
17	5741	2.4305	4897	9334	6043	15.36	807	14.579
18	10367	0.2396	9334	15366	10978	11.77	67	1.437
19	20000 (UM)	0.2555	16464	28584	20781	5.73	1446	18.395

TIC:	32.4064	ng/uL
TIM:	66.2754	nmole/L
Total concentration:	32.6496	ng/uL

```
Sample peak width (sec): 5    Sample min peak height: 50    Sample baseline V to V?: Y    Sample baseline V to V points: 3
Sample filter: Binomial       Number of points for filter: 3  Sample start region (min): 0  Sample end region (min): 30
Marker peak width (sec): 5    Marker min peak height: 200   Marker baseline V to V?: Y    Marker baseline V to V points: 3
Lower marker selection: First peak > 500 RFU                Upper marker selection: Last peak > 200 RFU
Ladder size (bp)75, 200, 300, 400, 500, 700, 1000, 1500, 2000, 3000, 4000, 5000, 7000, 10000, 20000
Quantification using: Lower Marker          Final concentration (ng/uL): 0.5000          Dilution factor: 12.0
```

Sample: SampD8
Well location: D8
Created: Wednesday, October 20, 2021 5:58:44 PM

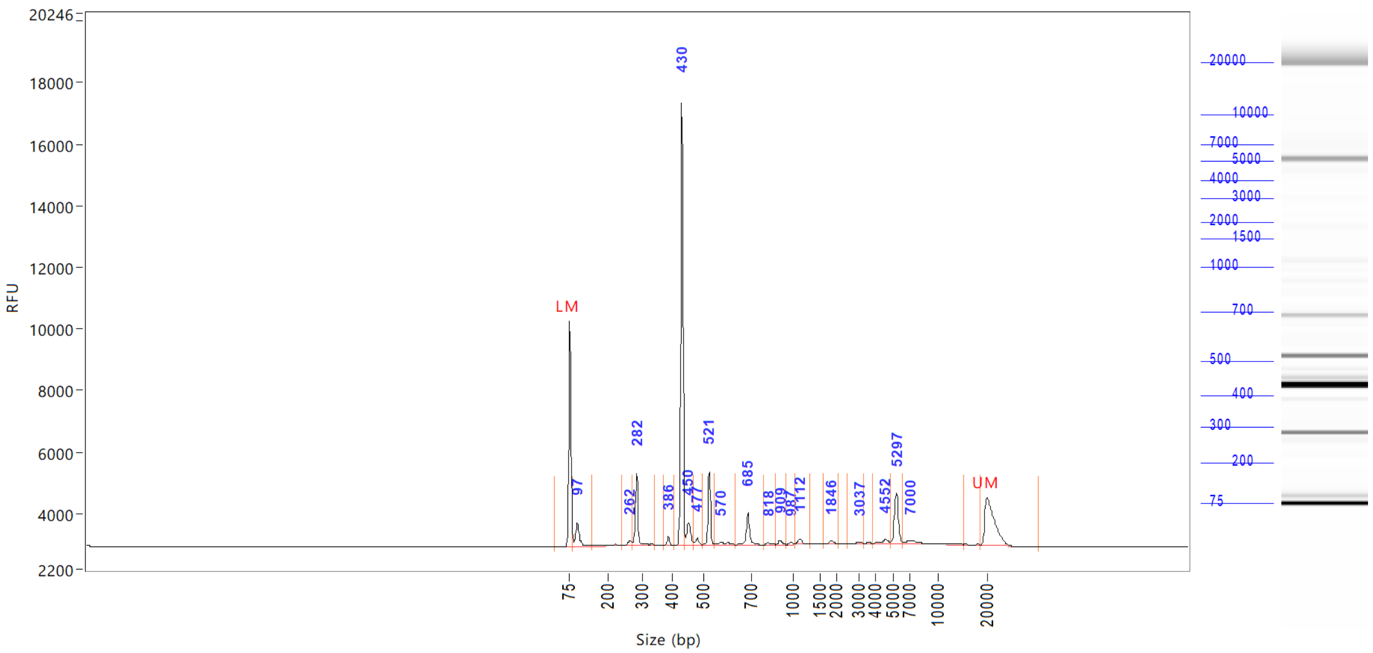

Peak	Size (bp)	Concentration (ng/uL)	From (bp)	To (bp)	Average size (bp)	CV%	RFU	Corrected peak area
1	75 (LM)	0.5000	30	86	73	4.44	7314	38.931
2	97	1.1715	86	148	99	11.09	786	7.602
3	262	0.2397	237	271	260	2.65	170	1.556
4	282	1.9950	271	339	283	3.65	2359	12.945
5	386	0.2208	372	403	384	1.08	289	1.433
6	430	10.4196	403	441	427	0.95	14382	67.608
7	450	0.8433	441	468	450	1.32	745	5.471
8	477	0.2183	468	494	476	1.04	217	1.416
9	521	1.7134	494	544	518	1.00	2359	11.117
10	570	0.1710	544	636	582	3.23	60	1.109
11	685	0.9918	636	788	684	2.13	1040	6.435
12	818	0.0938	788	870	825	2.13	59	0.609
13	909	0.1259	870	952	905	1.47	144	0.817
14	987	0.0684	952	1023	986	1.39	74	0.444
15	1112	0.2144	1023	1300	1117	4.65	187	1.391
16	1846	0.1141	1578	2087	1821	4.29	95	0.740
17	3037	0.0604	2515	3322	2960	5.42	51	0.392
18	4552	0.1770	3857	4828	4481	4.69	119	1.149
19	5297	1.4274	4828	6186	5244	4.35	1647	9.262
20	7000	0.4011	6186	15122	8454	24.92	97	2.602
21	20000 (UM)	0.2461	18781	30398	20832	5.88	1541	19.164

TIC:	20.6668	ng/uL
TIM:	87.3644	nmole/L
Total concentration:	20.8747	ng/uL

```
Sample peak width (sec): 5    Sample min peak height: 50    Sample baseline V to V?: Y    Sample baseline V to V points: 3
Sample filter: Binomial       Number of points for filter: 3  Sample start region (min): 0  Sample end region (min): 30
Marker peak width (sec): 5    Marker min peak height: 200   Marker baseline V to V?: Y    Marker baseline V to V points: 3
Lower marker selection: First peak > 500 RFU                Upper marker selection: Last peak > 200 RFU
Ladder size (bp)75, 200, 300, 400, 500, 700, 1000, 1500, 2000, 3000, 4000, 5000, 7000, 10000, 20000
Quantification using: Lower Marker          Final concentration (ng/uL): 0.5000          Dilution factor: 12.0
```

Sample: SampD9
Well location: D9
Created: Wednesday, October 20, 2021 5:58:44 PM

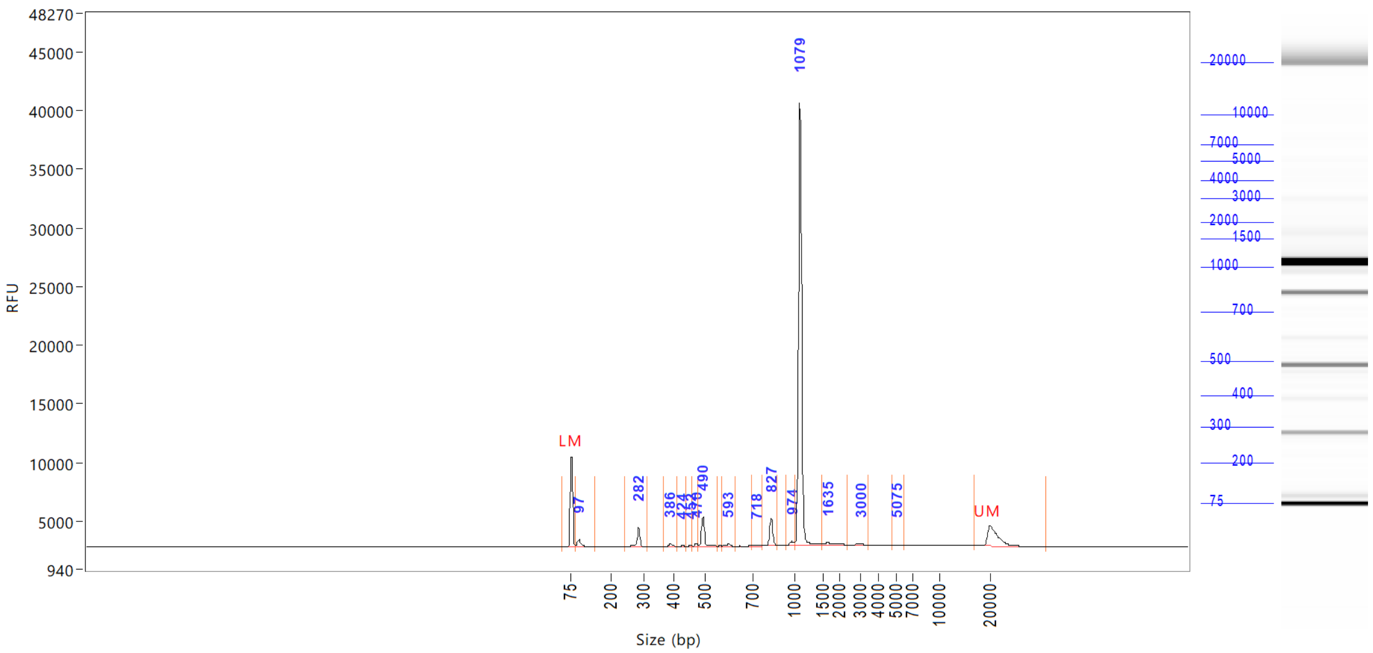

Peak	Size (bp)	Concentration (ng/uL)	From (bp)	To (bp)	Average size (bp)	CV%	RFU	Corrected peak area
1	75 (LM)	0.5000	45	86	72	4.48	7674	42.731
2	97	0.8181	86	148	99	10.26	577	5.826
3	282	1.3641	241	311	279	2.68	1644	9.715
4	386	0.2441	364	409	386	1.79	230	1.739
5	424	0.0567	409	435	423	1.15	64	0.404
6	452	0.0603	435	458	448	1.21	70	0.429
7	470	0.1816	458	477	468	1.04	214	1.294
8	490	1.9010	477	547	489	1.71	2449	13.539
9	593	0.2252	567	626	590	1.53	238	1.604
10	718	0.0558	697	762	715	1.76	55	0.397
11	827	1.8080	762	870	823	1.38	2369	12.876
12	974	0.3726	931	1000	976	1.60	352	2.654
13	1079	28.3135	1000	1500	1078	4.09	37749	201.646
14	1635	0.5220	1500	2429	1791	12.96	230	3.718
15	3000	0.2133	2429	3393	2856	6.98	144	1.519
16	5075	0.0576	4759	5963	5121	4.63	51	0.410
17	20000 (UM)	0.2658	16830	31486	20932	6.44	1755	22.718

TIC:	36.1938	ng/uL
TIM:	78.9820	nmole/L
Total concentration:	36.6640	ng/uL

```
Sample peak width (sec): 5    Sample min peak height: 50    Sample baseline V to V?: Y    Sample baseline V to V points: 3
Sample filter: Binomial       Number of points for filter: 3  Sample start region (min): 0  Sample end region (min): 30
Marker peak width (sec): 5    Marker min peak height: 200   Marker baseline V to V?: Y    Marker baseline V to V points: 3
Lower marker selection: First peak > 500 RFU                Upper marker selection: Last peak > 200 RFU
Ladder size (bp)75, 200, 300, 400, 500, 700, 1000, 1500, 2000, 3000, 4000, 5000, 7000, 10000, 20000
Quantification using: Lower Marker          Final concentration (ng/uL): 0.5000          Dilution factor: 12.0
```

Sample: SampD10
Well location: D10
Created: Wednesday, October 20, 2021 5:58:44 PM

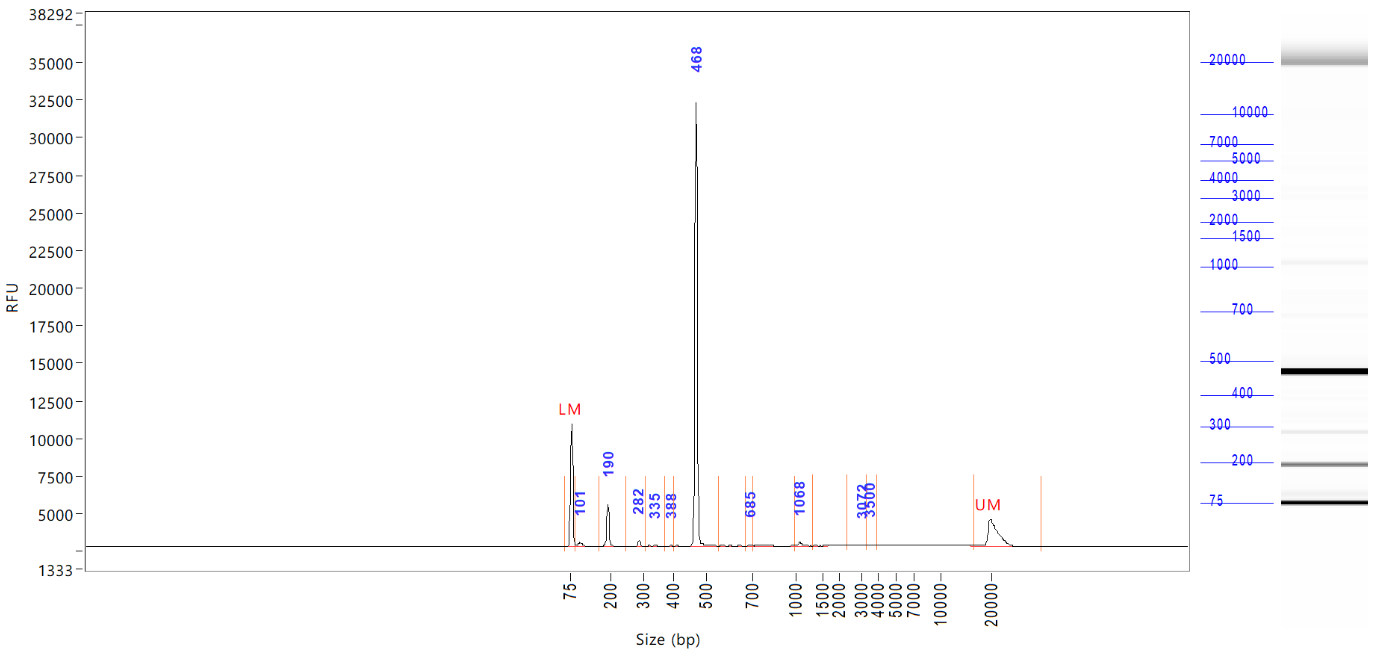

Peak	Size (bp)	Concentration (ng/uL)	From (bp)	To (bp)	Average size (bp)	CV%	RFU	Corrected peak area
1	75 (LM)	0.5000	54	89	74	4.55	8113	43.980
2	101	0.4991	89	166	107	15.39	265	3.658
3	190	2.1521	166	244	189	3.31	2824	15.775
4	282	0.3159	244	303	279	2.48	418	2.315
5	335	0.1327	303	368	329	3.82	69	0.972
6	388	0.0431	368	396	385	1.31	62	0.316
7	468	19.6867	396	552	466	1.50	29535	144.304
8	685	0.0761	667	700	682	0.96	103	0.558
9	1068	0.2022	996	1300	1069	4.14	228	1.482
10	3072	0.0553	2372	3286	3028	5.33	57	0.405
11	3500	0.0569	3286	3893	3495	3.61	64	0.417
12	20000 (UM)	0.2580	16586	30156	20779	5.98	1765	22.696

TIC:	23.2202	ng/uL
TIM:	99.1572	nmole/L
Total concentration:	23.8331	ng/uL

```
Sample peak width (sec): 5    Sample min peak height: 50    Sample baseline V to V?: Y    Sample baseline V to V points: 3
Sample filter: Binomial       Number of points for filter: 3  Sample start region (min): 0  Sample end region (min): 30
Marker peak width (sec): 5    Marker min peak height: 200   Marker baseline V to V?: Y    Marker baseline V to V points: 3
Lower marker selection: First peak > 500 RFU                Upper marker selection: Last peak > 200 RFU
Ladder size (bp)75, 200, 300, 400, 500, 700, 1000, 1500, 2000, 3000, 4000, 5000, 7000, 10000, 20000
Quantification using: Lower Marker          Final concentration (ng/uL): 0.5000          Dilution factor: 12.0
```

Sample: SampD11
Well location: D11
Created: Wednesday, October 20, 2021 5:58:44 PM

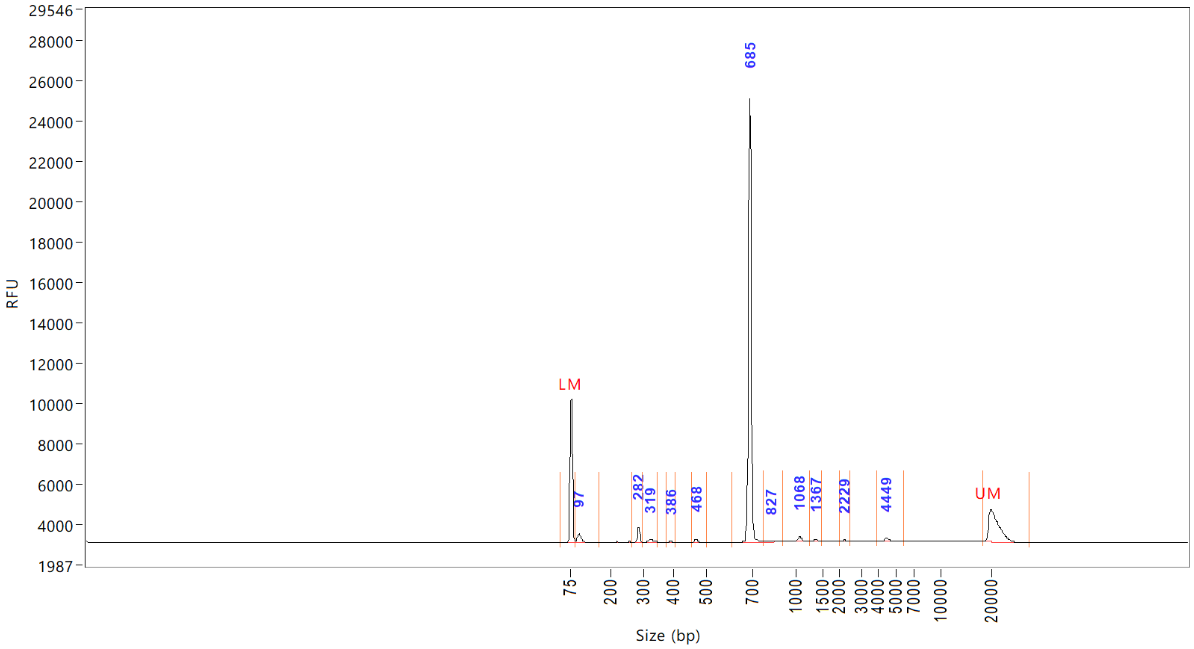

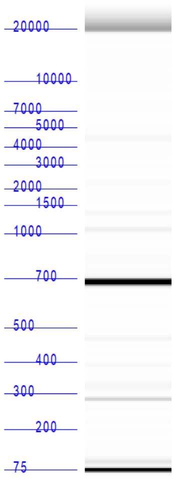

Peak	Size (bp)	Concentration (ng/uL)	From (bp)	To (bp)	Average size (bp)	CV%	RFU	Corrected peak area
1	75 (LM)	0.5000	41	86	72	4.45	7099	39.339
2	97	0.7320	86	166	101	13.66	459	4.799
3	282	0.6056	264	295	280	1.41	769	3.971
4	319	0.2736	295	345	319	3.20	151	1.794
5	386	0.0654	374	401	385	1.17	86	0.429
6	468	0.1666	456	501	469	1.82	168	1.093
7	685	16.9272	611	770	682	1.10	22011	110.982
8	827	0.1345	770	909	822	3.76	59	0.882
9	1068	0.2712	909	1267	1066	5.37	259	1.778
10	1367	0.1096	1267	1467	1352	2.44	124	0.719
11	2229	0.0567	2001	2515	2186	4.27	53	0.371
12	4449	0.1801	3893	5889	4468	5.82	141	1.181
13	20000 (UM)	0.2606	18659	27859	20901	5.44	1621	20.504

TIC:	19.5225	ng/uL
TIM:	59.5114	nmole/L
Total concentration:	20.0557	ng/uL

Sample peak width (sec): 5 Sample min peak height: 50 Sample baseline V to V?: Y Sample baseline V to V points: 3
Sample filter: Binomial Number of points for filter: 3 Sample start region (min): 0 Sample end region (min): 30
Marker peak width (sec): 5 Marker min peak height: 200 Marker baseline V to V?: Y Marker baseline V to V points: 3
Lower marker selection: First peak > 500 RFU Upper marker selection: Last peak > 200 RFU
Ladder size (bp)75, 200, 300, 400, 500, 700, 1000, 1500, 2000, 3000, 4000, 5000, 7000, 10000, 20000
Quantification using: Lower Marker Final concentration (ng/uL): 0.5000 Dilution factor: 12.0

Sample: SampD12
Well location: D12
Created: Wednesday, October 20, 2021 5:58:44 PM

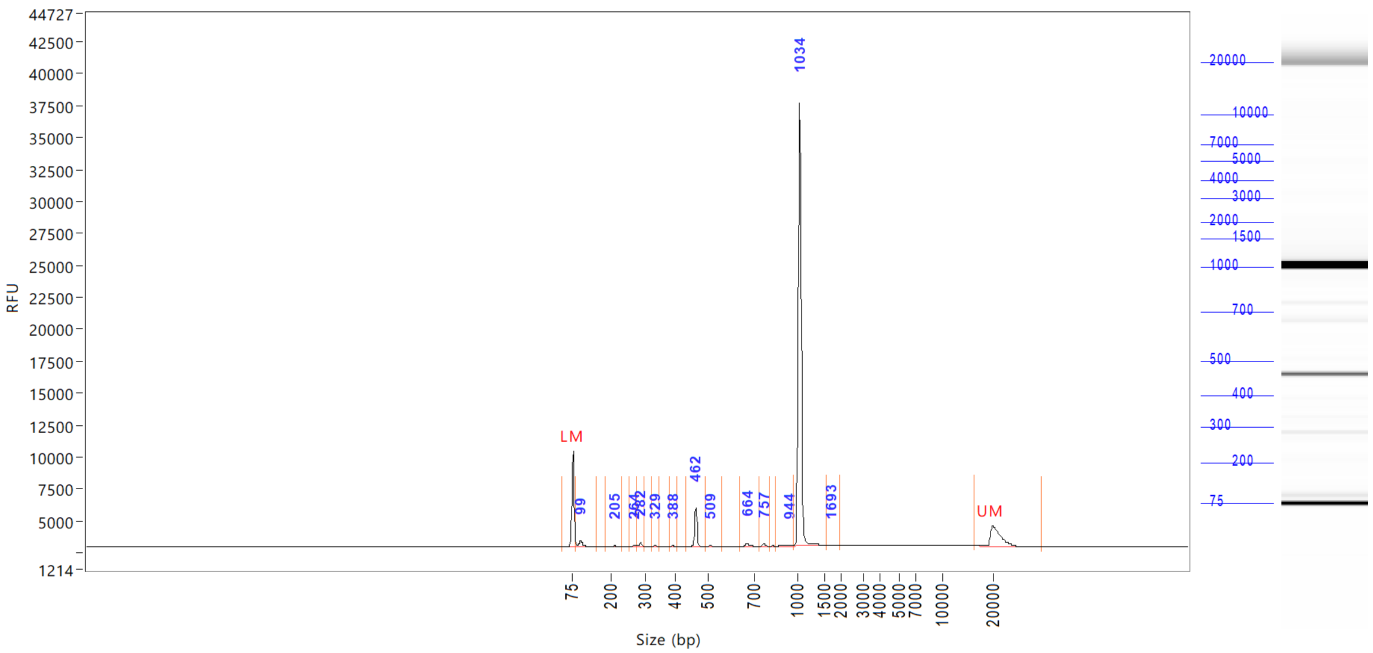

Peak	Size (bp)	Concentration (ng/uL)	From (bp)	To (bp)	Average size (bp)	CV%	RFU	Corrected peak area
1	75 (LM)	0.5000	45	86	73	4.41	7453	39.636
2	99	0.7377	86	148	100	10.74	458	4.873
3	205	0.1291	177	230	203	5.57	57	0.853
4	264	0.0870	251	273	264	2.05	65	0.575
5	282	0.2917	273	293	281	1.21	363	1.927
6	329	0.0729	319	345	327	1.47	90	0.482
7	388	0.0702	378	403	387	1.20	71	0.464
8	462	2.2645	433	494	460	0.94	3028	14.959
9	509	0.0693	494	557	513	2.83	54	0.458
10	664	0.2938	634	727	667	1.91	253	1.941
11	757	0.1694	727	801	755	1.39	222	1.119
12	944	0.1776	848	970	923	3.18	80	1.173
13	1034	27.4539	970	1539	1029	4.42	34734	181.361
14	1693	0.1341	1539	1942	1723	6.74	58	0.886
15	20000 (UM)	0.2588	16342	29914	20824	5.77	1608	20.514

TIC:	31.9512	ng/uL
TIM:	69.8500	nmole/L
Total concentration:	32.4655	ng/uL

```
Sample peak width (sec): 5     Sample min peak height: 50     Sample baseline V to V?: Y     Sample baseline V to V points: 3
Sample filter: Binomial        Number of points for filter: 3  Sample start region (min): 0  Sample end region (min): 30
Marker peak width (sec): 5     Marker min peak height: 200    Marker baseline V to V?: Y     Marker baseline V to V points: 3
Lower marker selection: First peak > 500 RFU                  Upper marker selection: Last peak > 200 RFU
Ladder size (bp)75, 200, 300, 400, 500, 700, 1000, 1500, 2000, 3000, 4000, 5000, 7000, 10000, 20000
Quantification using: Lower Marker          Final concentration (ng/uL): 0.5000          Dilution factor: 12.0
```

Sample: SampE1
Well location: E1
Created: Wednesday, October 20, 2021 5:58:44 PM

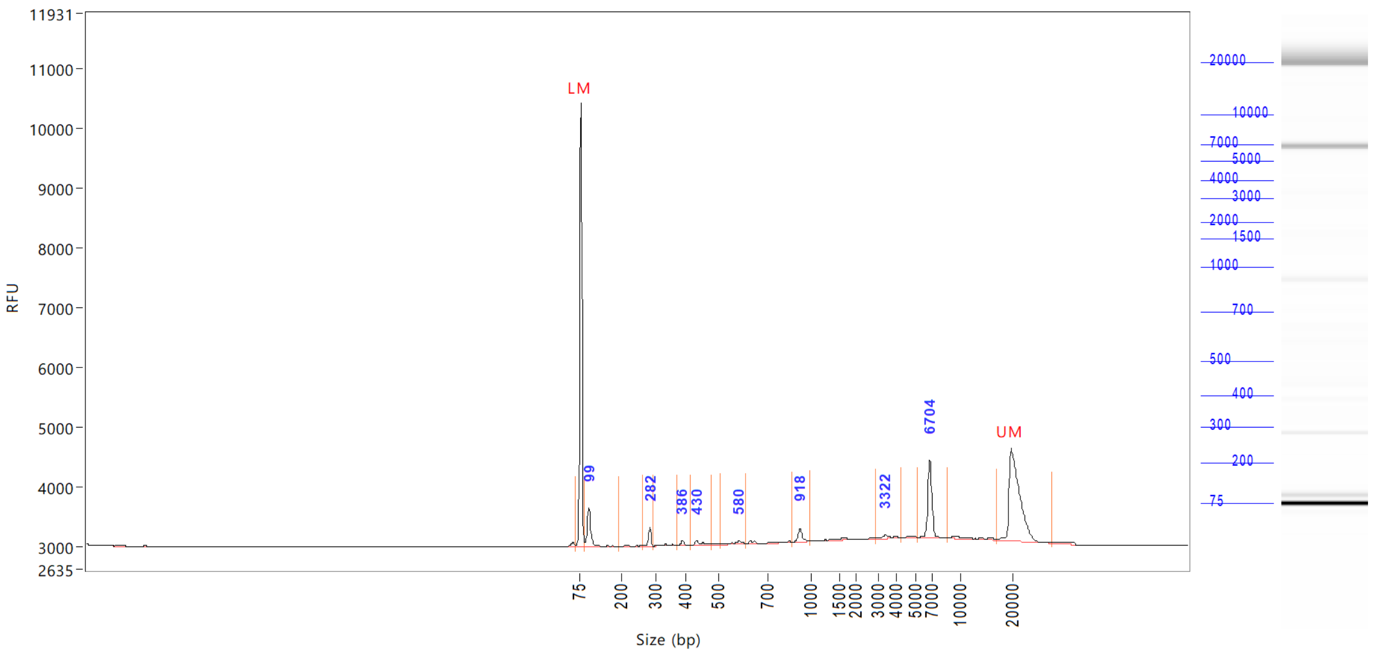

Peak	Size (bp)	Concentration (ng/uL)	From (bp)	To (bp)	Average size (bp)	CV%	RFU	Corrected peak area
1	75 (LM)	0.5000	58	86	73	4.35	7432	39.330
2	99	0.8638	86	192	100	11.65	650	5.662
3	282	0.2296	262	293	279	1.24	302	1.505
4	386	0.0782	368	414	385	1.92	79	0.512
5	430	0.1339	414	473	441	3.27	65	0.878
6	580	0.1354	501	608	563	4.59	53	0.887
7	918	0.2430	870	991	919	2.06	230	1.593
8	3322	0.1225	2914	4208	3475	9.42	58	0.803
9	6704	1.1951	5297	8600	6690	4.99	1299	7.834
10	20000 (UM)	0.2584	17073	27979	20830	6.15	1547	20.329

TIC:	3.0014	ng/uL
TIM:	17.5550	nmole/L
Total concentration:	3.6830	ng/uL

```
Sample peak width (sec): 5     Sample min peak height: 50     Sample baseline V to V?: Y     Sample baseline V to V points: 3
Sample filter: Binomial        Number of points for filter: 3  Sample start region (min): 0  Sample end region (min): 30
Marker peak width (sec): 5     Marker min peak height: 200    Marker baseline V to V?: Y     Marker baseline V to V points: 3
Lower marker selection: First peak > 500 RFU                  Upper marker selection: Last peak > 200 RFU
Ladder size (bp)75, 200, 300, 400, 500, 700, 1000, 1500, 2000, 3000, 4000, 5000, 7000, 10000, 20000
Quantification using: Lower Marker          Final concentration (ng/uL): 0.5000          Dilution factor: 12.0
```

Sample: SampE2
Well location: E2
Created: Wednesday, October 20, 2021 5:58:44 PM

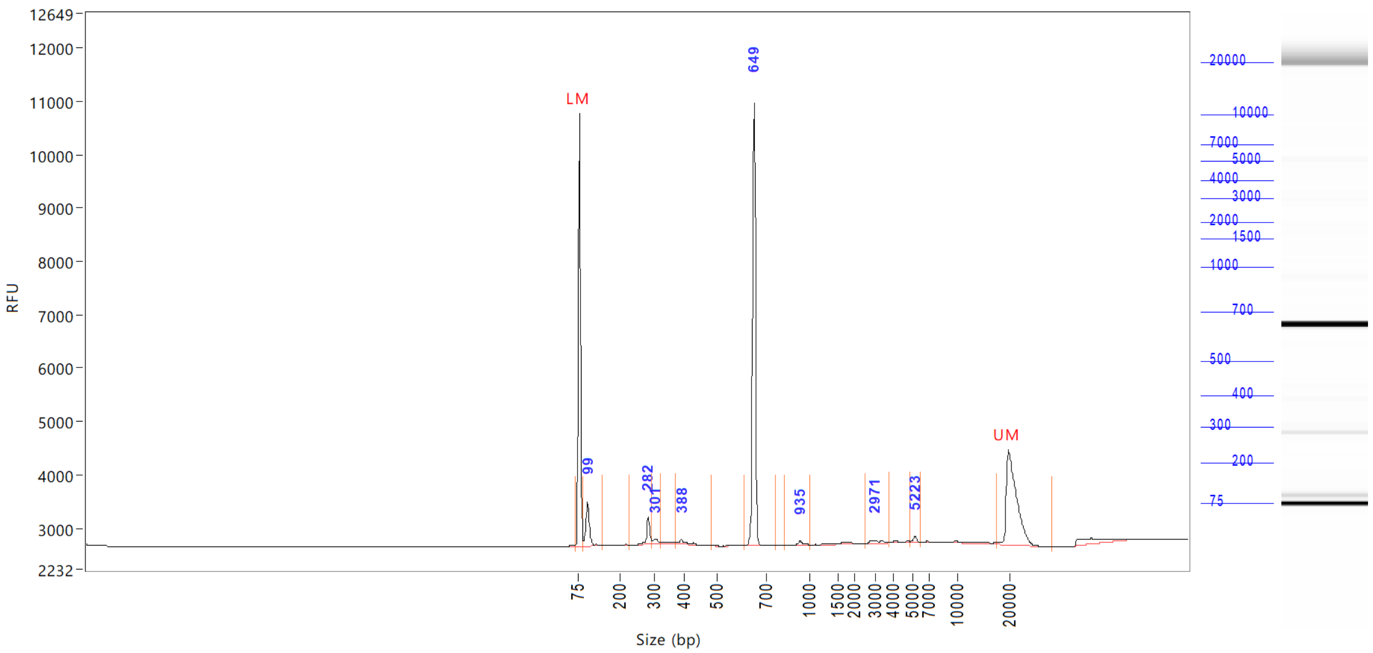

Peak	Size (bp)	Concentration (ng/uL)	From (bp)	To (bp)	Average size (bp)	CV%	RFU	Corrected peak area
1	75 (LM)	0.5000	62	86	72	4.39	8123	44.022
2	99	0.9903	86	148	99	9.35	837	7.266
3	282	0.4248	230	293	279	2.70	513	3.117
4	301	0.1354	293	323	303	2.41	85	0.993
5	388	0.1674	372	481	401	5.10	89	1.228
6	649	5.5844	608	766	647	1.16	8313	40.973
7	935	0.1036	827	1012	927	4.05	77	0.760
8	2971	0.1161	2543	3715	3028	8.19	57	0.852
9	5223	0.0890	4862	5889	5166	3.64	112	0.653
10	20000 (UM)	0.2603	17561	28463	20841	5.74	1792	22.915

TIC:	7.6111	ng/uL
TIM:	34.8325	nmole/L
Total concentration:	8.1693	ng/uL

Sample peak width (sec): 5   Sample min peak height: 50   Sample baseline V to V?: Y   Sample baseline V to V points: 3
Sample filter: Binomial   Number of points for filter: 3   Sample start region (min): 0   Sample end region (min): 30
Marker peak width (sec): 5   Marker min peak height: 200   Marker baseline V to V?: Y   Marker baseline V to V points: 3
Lower marker selection: First peak > 500 RFU   Upper marker selection: Last peak > 200 RFU
Ladder size (bp)75, 200, 300, 400, 500, 700, 1000, 1500, 2000, 3000, 4000, 5000, 7000, 10000, 20000
Quantification using: Lower Marker   Final concentration (ng/uL): 0.5000   Dilution factor: 12.0

Sample: SampE3
Well location: E3
Created: Wednesday, October 20, 2021 5:58:44 PM

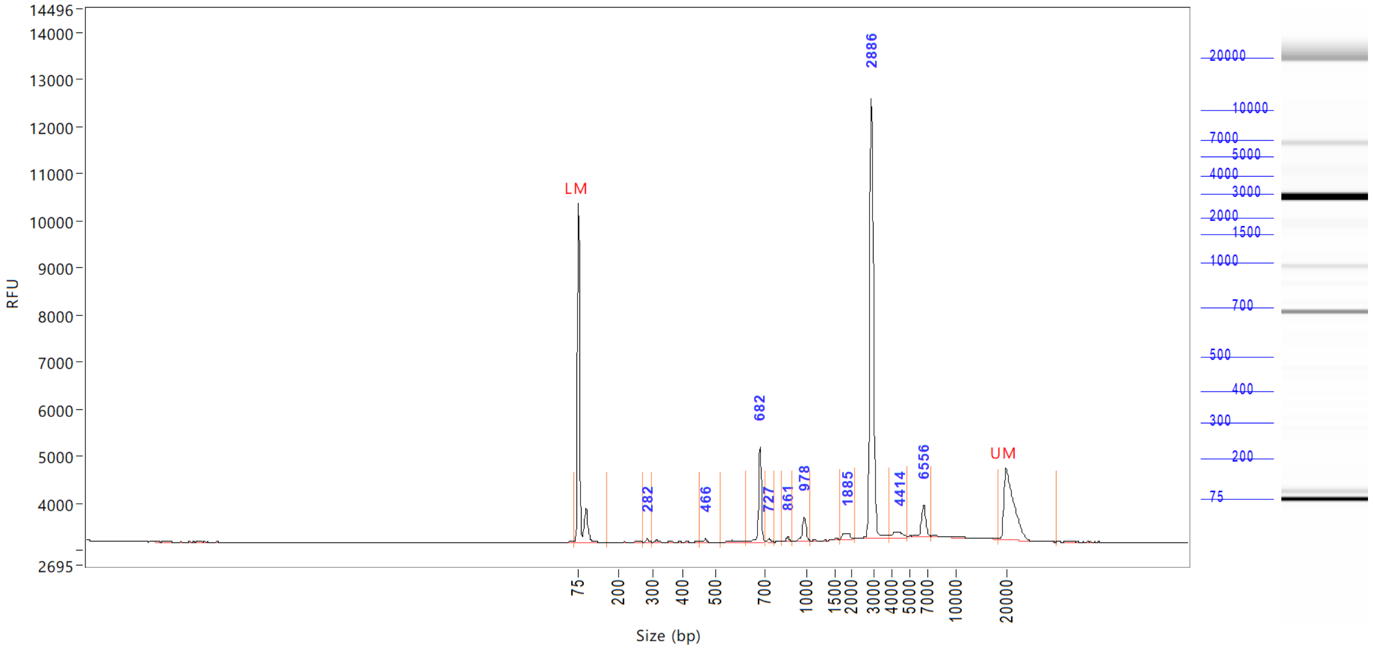

Peak	Size (bp)	Concentration (ng/uL)	From (bp)	To (bp)	Average size (bp)	CV%	RFU	Corrected peak area
1	75 (LM)	0.5000	64	164	77	13.56	7199	44.626
2	282	0.0601	271	298	282	1.77	76	0.447
3	466	0.0693	449	519	465	2.44	76	0.516
4	682	1.3416	623	701	678	1.21	2003	9.978
5	727	0.0530	701	770	724	1.49	72	0.394
6	861	0.0768	822	896	860	1.46	101	0.572
7	978	0.4301	896	1056	971	1.98	509	3.199
8	1885	0.1978	1616	2115	1810	5.36	132	1.471
9	2886	6.8474	2115	3857	2879	4.16	9346	50.928
10	4414	0.2332	3857	4862	4297	5.61	113	1.734
11	6556	0.5849	4862	7401	6424	6.93	668	4.351
12	20000 (UM)	0.2118	18537	29914	20826	5.49	1520	18.899

TIC:	9.8943	ng/uL
TIM:	9.1833	nmole/L
Total concentration:	10.3329	ng/uL

```
Sample peak width (sec): 5     Sample min peak height: 50     Sample baseline V to V?: Y     Sample baseline V to V points: 3
Sample filter: Binomial        Number of points for filter: 3  Sample start region (min): 0  Sample end region (min): 30
Marker peak width (sec): 5     Marker min peak height: 200    Marker baseline V to V?: Y     Marker baseline V to V points: 3
Lower marker selection: First peak > 500 RFU                  Upper marker selection: Last peak > 200 RFU
Ladder size (bp)75, 200, 300, 400, 500, 700, 1000, 1500, 2000, 3000, 4000, 5000, 7000, 10000, 20000
Quantification using: Lower Marker          Final concentration (ng/uL): 0.5000          Dilution factor: 12.0
```

Sample: SampE4
Well location: E4
Created: Wednesday, October 20, 2021 5:58:44 PM

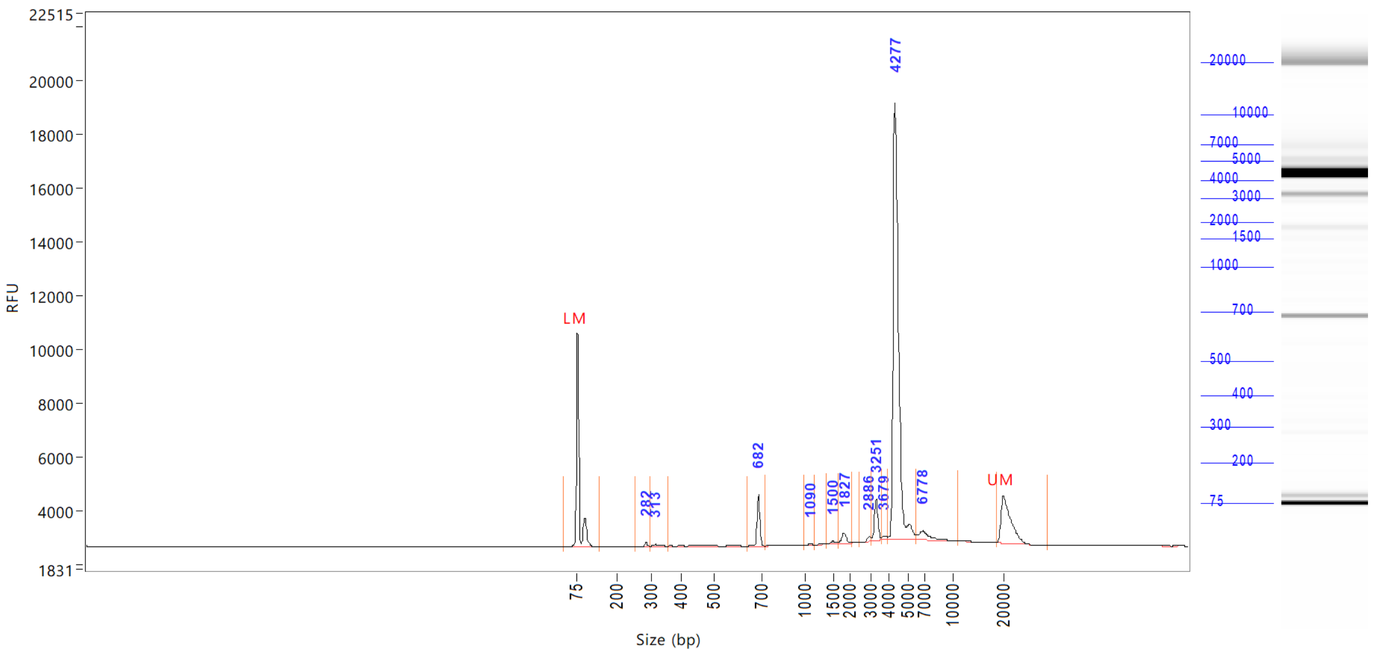

Peak	Size (bp)	Concentration (ng/uL)	From (bp)	To (bp)	Average size (bp)	CV%	RFU	Corrected peak area
1	75 (LM)	0.5000	35	147	78	13.01	7952	52.604
2	282	0.0930	251	300	280	3.27	126	0.815
3	313	0.0854	300	354	318	3.82	68	0.749
4	682	1.0765	636	714	680	0.97	1905	9.438
5	1090	0.0489	996	1178	1074	3.39	65	0.429
6	1500	0.1052	1378	1655	1513	4.35	97	0.922
7	1827	0.3079	1655	2115	1814	4.08	374	2.700
8	2886	0.1130	2458	3000	2865	3.05	187	0.990
9	3251	0.9472	3000	3536	3213	3.21	1534	8.305
10	3679	0.0984	3536	3893	3702	2.84	130	0.862
11	4277	13.9761	3893	5963	4368	5.44	16263	122.535
12	6778	0.5866	5963	10977	7134	12.02	331	5.143
13	20000 (UM)	0.2078	18659	28705	20827	5.33	1778	21.863

TIC:	17.4382	ng/uL
TIM:	10.0590	nmole/L
Total concentration:	17.7758	ng/uL

```
Sample peak width (sec): 5    Sample min peak height: 50    Sample baseline V to V?: Y    Sample baseline V to V points: 3
Sample filter: Binomial       Number of points for filter: 3  Sample start region (min): 0  Sample end region (min): 30
Marker peak width (sec): 5    Marker min peak height: 200   Marker baseline V to V?: Y    Marker baseline V to V points: 3
Lower marker selection: First peak > 500 RFU                Upper marker selection: Last peak > 200 RFU
Ladder size (bp)75, 200, 300, 400, 500, 700, 1000, 1500, 2000, 3000, 4000, 5000, 7000, 10000, 20000
Quantification using: Lower Marker          Final concentration (ng/uL): 0.5000          Dilution factor: 12.0
```

Sample: SampE5
Well location: E5
Created: Wednesday, October 20, 2021 5:58:44 PM

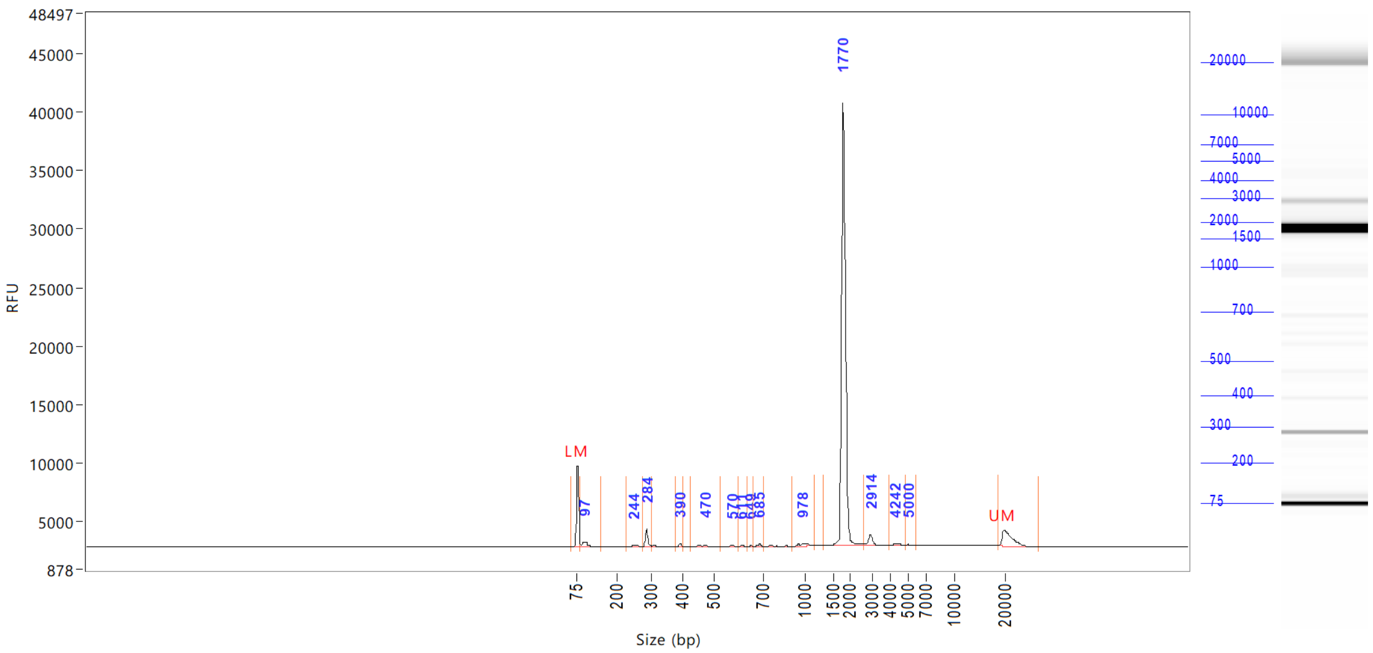

Peak	Size (bp)	Concentration (ng/uL)	From (bp)	To (bp)	Average size (bp)	CV%	RFU	Corrected peak area
1	75 (LM)	0.5000	54	84	72	4.59	6881	37.919
2	97	0.8549	84	147	98	11.32	426	5.403
3	244	0.1730	224	271	250	4.15	74	1.093
4	284	1.2109	271	298	282	1.34	1522	7.653
5	390	0.1634	376	400	387	0.94	219	1.033
6	470	0.2459	426	524	462	3.31	161	1.554
7	570	0.1398	524	598	565	1.94	134	0.884
8	611	0.1013	598	636	610	1.10	127	0.640
9	649	0.0422	636	662	649	0.85	59	0.267
10	685	0.1587	662	701	681	1.00	189	1.003
11	978	0.4703	900	1156	983	4.09	195	2.972
12	1770	35.7276	1300	2600	1769	4.85	37998	225.795
13	2914	1.0589	2600	3929	2945	8.35	887	6.692
14	4242	0.1995	3929	4828	4334	5.22	94	1.261
15	5000	0.0654	4828	5889	5074	4.63	60	0.413
16	20000 (UM)	0.2227	18781	26891	20779	5.21	1407	16.888

TIC:	40.6118	ng/uL
TIM:	59.9879	nmole/L
Total concentration:	41.2989	ng/uL

```
Sample peak width (sec): 5    Sample min peak height: 50    Sample baseline V to V?: Y    Sample baseline V to V points: 3
Sample filter: Binomial       Number of points for filter: 3  Sample start region (min): 0  Sample end region (min): 30
Marker peak width (sec): 5    Marker min peak height: 200   Marker baseline V to V?: Y    Marker baseline V to V points: 3
Lower marker selection: First peak > 500 RFU                Upper marker selection: Last peak > 200 RFU
Ladder size (bp)75, 200, 300, 400, 500, 700, 1000, 1500, 2000, 3000, 4000, 5000, 7000, 10000, 20000
Quantification using: Lower Marker          Final concentration (ng/uL): 0.5000          Dilution factor: 12.0
```

Sample: SampE6
Well location: E6
Created: Wednesday, October 20, 2021 5:58:44 PM

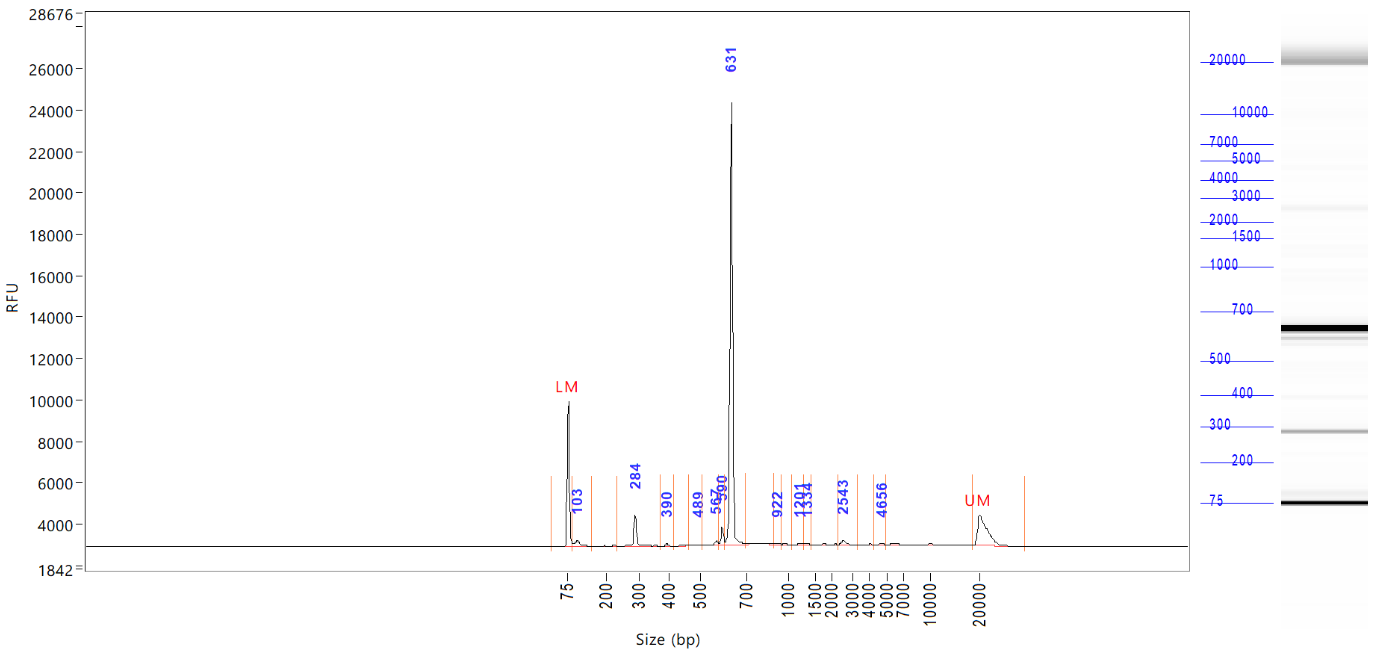

Peak	Size (bp)	Concentration (ng/uL)	From (bp)	To (bp)	Average size (bp)	CV%	RFU	Corrected peak area
1	75 (LM)	0.5000	24	87	73	5.36	6991	37.978
2	103	0.6249	87	154	104	12.30	263	3.956
3	284	1.3667	232	372	285	4.43	1503	8.651
4	390	0.0817	372	414	390	1.39	105	0.517
5	489	0.1020	464	509	482	1.86	60	0.645
6	567	0.1746	509	578	564	1.73	193	1.105
7	590	0.6587	578	603	590	0.92	834	4.169
8	631	16.8290	603	697	629	1.19	21376	106.522
9	922	0.0482	896	948	918	1.34	50	0.305
10	1201	0.0587	1056	1267	1196	3.54	56	0.372
11	1334	0.0546	1267	1422	1321	2.37	58	0.345
12	2543	0.2133	2315	3322	2568	5.67	197	1.350
13	4656	0.0504	4277	4931	4622	2.51	57	0.319
14	20000 (UM)	0.2403	18659	29309	20938	5.95	1453	18.254

TIC:	20.2628	ng/uL
TIM:	65.2157	nmole/L
Total concentration:	20.8241	ng/uL

Sample peak width (sec): 5 Sample min peak height: 50 Sample baseline V to V?: Y Sample baseline V to V points: 3
Sample filter: Binomial Number of points for filter: 3 Sample start region (min): 0 Sample end region (min): 30
Marker peak width (sec): 5 Marker min peak height: 200 Marker baseline V to V?: Y Marker baseline V to V points: 3
Lower marker selection: First peak > 500 RFU Upper marker selection: Last peak > 200 RFU
Ladder size (bp)75, 200, 300, 400, 500, 700, 1000, 1500, 2000, 3000, 4000, 5000, 7000, 10000, 20000
Quantification using: Lower Marker Final concentration (ng/uL): 0.5000 Dilution factor: 12.0

Sample: SampE7
Well location: E7
Created: Wednesday, October 20, 2021 5:58:44 PM

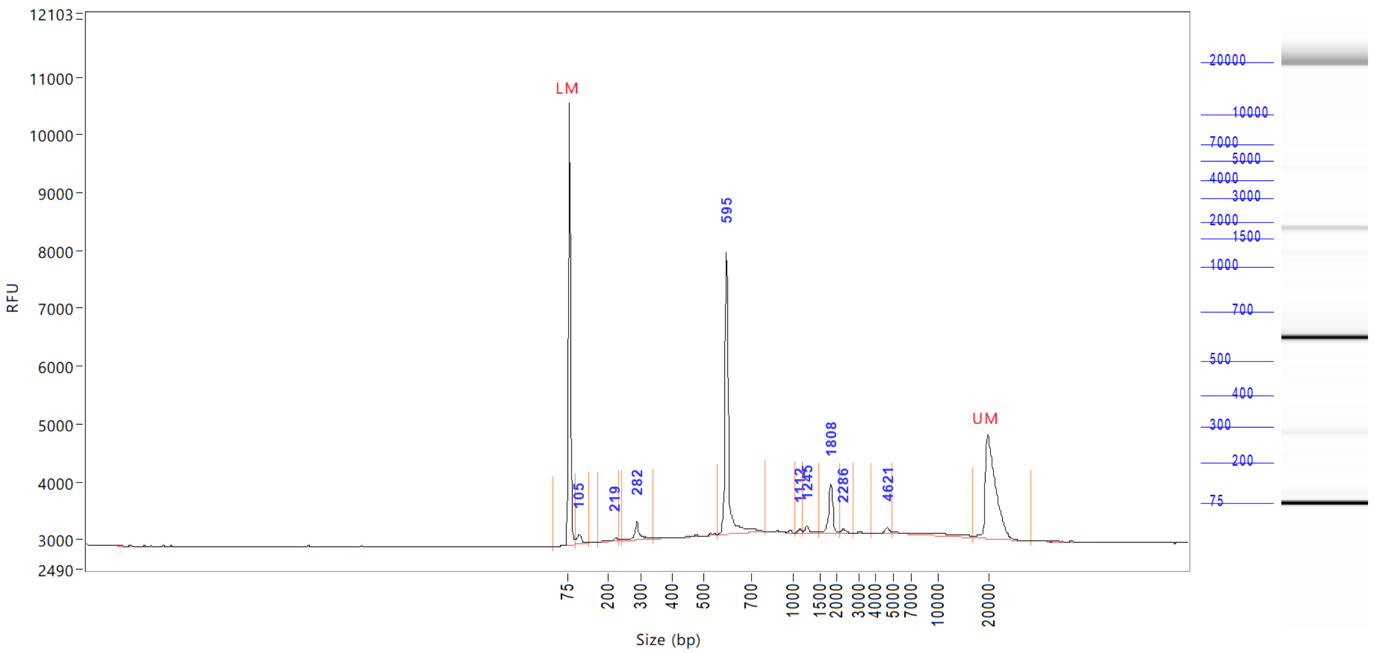

Peak	Size (bp)	Concentration (ng/uL)	From (bp)	To (bp)	Average size (bp)	CV%	RFU	Corrected peak area
1	75 (LM)	0.5000	26	93	74	5.04	7658	42.112
2	105	0.2832	93	137	105	7.66	164	1.988
3	219	0.1071	166	230	208	7.69	53	0.752
4	282	0.5082	241	337	285	6.55	309	3.567
5	595	4.3445	555	801	602	4.46	4882	30.492
6	1112	0.0505	1023	1167	1105	2.73	63	0.354
7	1245	0.1169	1167	1467	1257	5.27	121	0.820
8	1808	0.7627	1467	2115	1775	4.65	859	5.353
9	2286	0.0685	2115	2772	2293	4.35	69	0.481
10	4621	0.1138	3786	4931	4542	5.01	107	0.799
11	20000 (UM)	0.2822	16830	28826	20835	6.17	1808	23.764

TIC:	6.3554	ng/uL
TIM:	21.1118	nmole/L
Total concentration:	6.9777	ng/uL

```
Sample peak width (sec): 5    Sample min peak height: 50    Sample baseline V to V?: Y    Sample baseline V to V points: 3
Sample filter: Binomial       Number of points for filter: 3  Sample start region (min): 0  Sample end region (min): 30
Marker peak width (sec): 5    Marker min peak height: 200   Marker baseline V to V?: Y    Marker baseline V to V points: 3
Lower marker selection: First peak > 500 RFU                Upper marker selection: Last peak > 200 RFU
Ladder size (bp)75, 200, 300, 400, 500, 700, 1000, 1500, 2000, 3000, 4000, 5000, 7000, 10000, 20000
Quantification using: Lower Marker          Final concentration (ng/uL): 0.5000          Dilution factor: 12.0
```

Sample: SampE8
Well location: E8
Created: Wednesday, October 20, 2021 5:58:44 PM

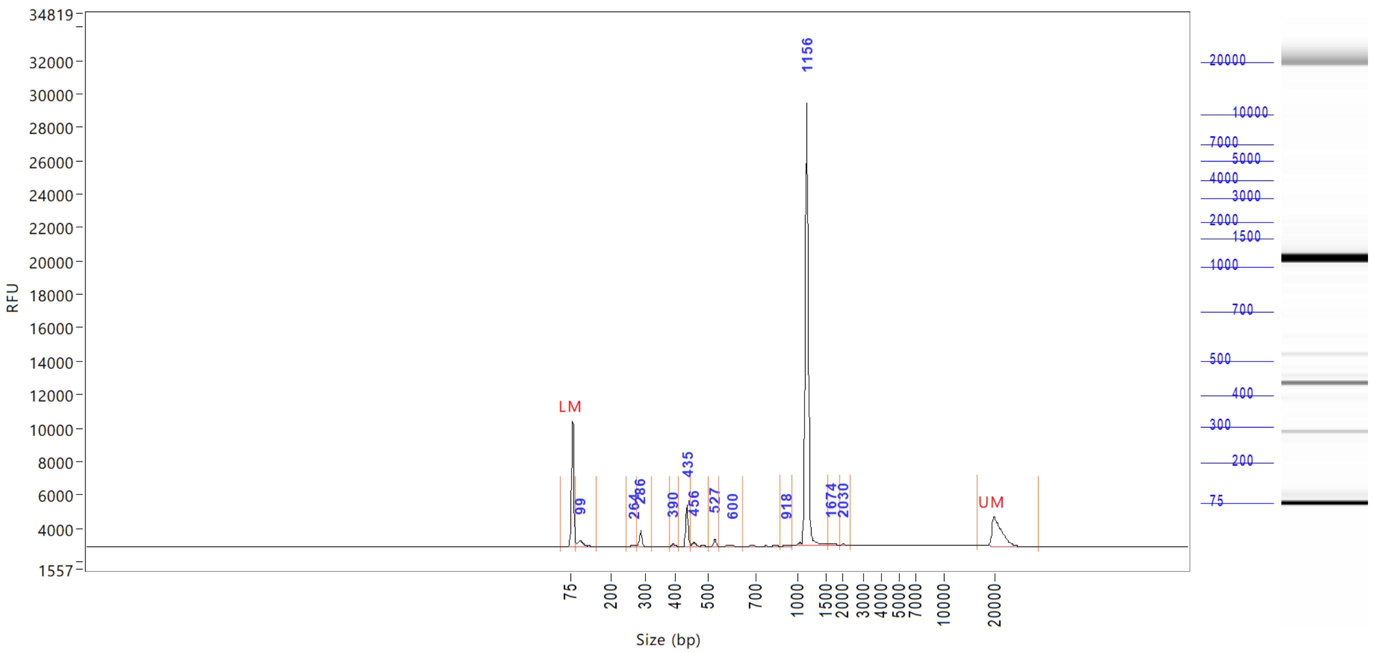

Peak	Size (bp)	Concentration (ng/uL)	From (bp)	To (bp)	Average size (bp)	CV%	RFU	Corrected peak area
1	75 (LM)	0.5000	41	86	74	4.39	7498	41.367
2	99	0.7954	86	154	102	12.82	351	5.484
3	264	0.1365	241	275	263	2.70	87	0.941
4	286	0.7512	275	319	284	1.83	986	5.179
5	390	0.1651	378	411	389	1.39	182	1.138
6	435	1.7552	411	447	433	0.87	2528	12.101
7	456	0.3534	447	501	462	2.68	260	2.436
8	527	0.3044	501	547	524	0.98	444	2.099
9	600	0.0965	547	644	590	2.61	51	0.665
10	918	0.0708	874	961	921	1.91	66	0.488
11	1156	19.6447	961	1578	1150	4.03	26542	135.440
12	1674	0.1198	1578	1923	1720	5.49	72	0.826
13	2030	0.0615	1923	2401	2049	4.37	58	0.424
14	20000 (UM)	0.2622	16708	29068	20821	5.68	1728	21.696

TIC:	24.2544	ng/uL
TIM:	56.2691	nmole/L
Total concentration:	24.7605	ng/uL

Sample peak width (sec): 5 Sample min peak height: 50 Sample baseline V to V?: Y Sample baseline V to V points: 3
Sample filter: Binomial Number of points for filter: 3 Sample start region (min): 0 Sample end region (min): 30
Marker peak width (sec): 5 Marker min peak height: 200 Marker baseline V to V?: Y Marker baseline V to V points: 3
Lower marker selection: First peak > 500 RFU Upper marker selection: Last peak > 200 RFU
Ladder size (bp)75, 200, 300, 400, 500, 700, 1000, 1500, 2000, 3000, 4000, 5000, 7000, 10000, 20000
Quantification using: Lower Marker Final concentration (ng/uL): 0.5000 Dilution factor: 12.0

Sample: SampE9
Well location: E9
Created: Wednesday, October 20, 2021 5:58:44 PM

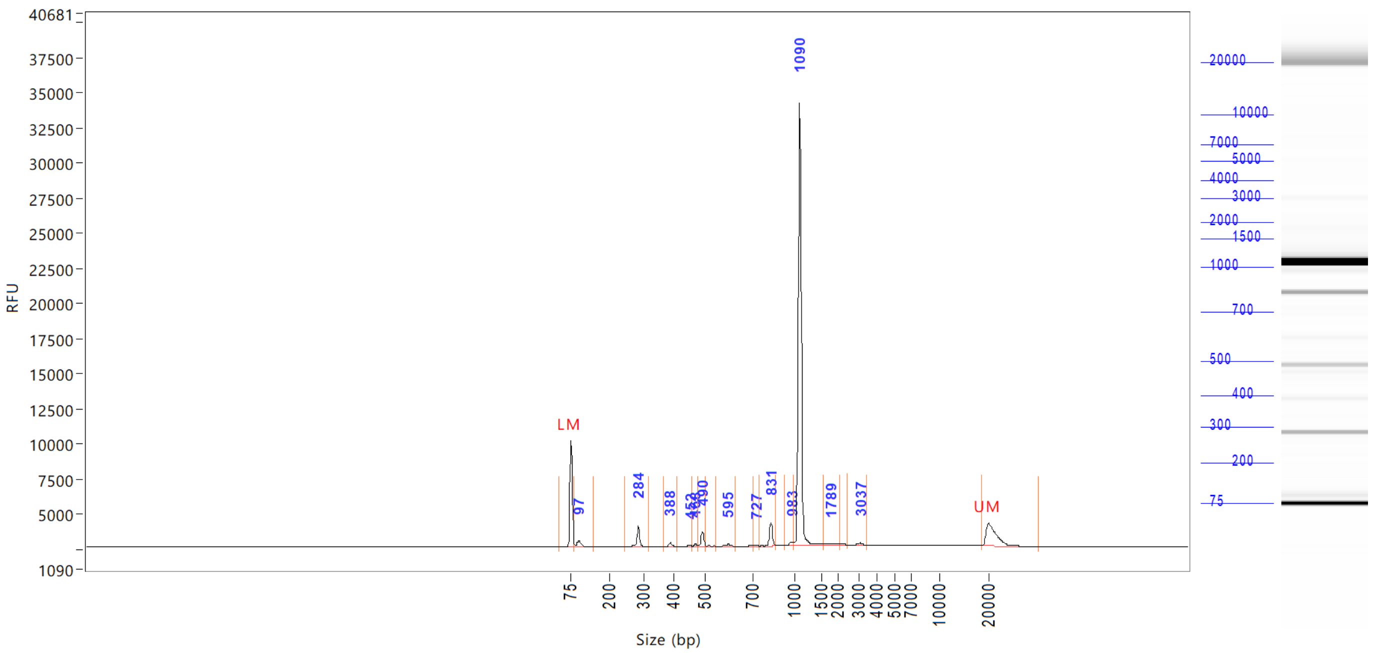

Peak	Size<br>(bp)	Concentration<br>(ng/uL)	From<br>(bp)	To<br>(bp)	Average size<br>(bp)	CV%	RFU	Corrected peak area
1	75 (LM)	0.5000	41	86	73	4.32	7588	40.403
2	97	0.7000	86	148	101	11.43	395	4.714
3	284	1.3069	242	317	281	2.83	1520	8.801
4	388	0.2533	368	409	386	1.60	258	1.706
5	452	0.0995	409	460	445	2.30	67	0.670
6	468	0.1509	460	477	468	0.98	185	1.016
7	490	0.7913	477	504	488	0.84	1064	5.329
8	595	0.1933	547	629	590	2.41	167	1.302
9	727	0.0506	701	749	720	1.60	56	0.340
10	831	1.3158	749	870	825	1.43	1686	8.861
11	983	0.2909	931	1000	979	1.36	283	1.959
12	1090	24.5134	1000	1539	1083	4.13	31577	165.070
13	1789	0.1855	1539	2058	1763	7.31	104	1.249
14	3037	0.1309	2458	3393	2961	4.68	135	0.881
15	20000 (UM)	0.2577	18659	30156	20906	6.22	1615	20.827

TIC:	29.9824	ng/uL
TIM:	64.9490	nmole/L
Total concentration:	30.5810	ng/uL

```
Sample peak width (sec): 5     Sample min peak height: 50    Sample baseline V to V?: Y    Sample baseline V to V points: 3
Sample filter: Binomial        Number of points for filter: 3  Sample start region (min): 0  Sample end region (min): 30
Marker peak width (sec): 5     Marker min peak height: 200   Marker baseline V to V?: Y    Marker baseline V to V points: 3
Lower marker selection: First peak > 500 RFU                 Upper marker selection: Last peak > 200 RFU
Ladder size (bp)75, 200, 300, 400, 500, 700, 1000, 1500, 2000, 3000, 4000, 5000, 7000, 10000, 20000
Quantification using: Lower Marker          Final concentration (ng/uL): 0.5000          Dilution factor: 12.0
```

Sample: SampE10
Well location: E10
Created: Wednesday, October 20, 2021 5:58:44 PM

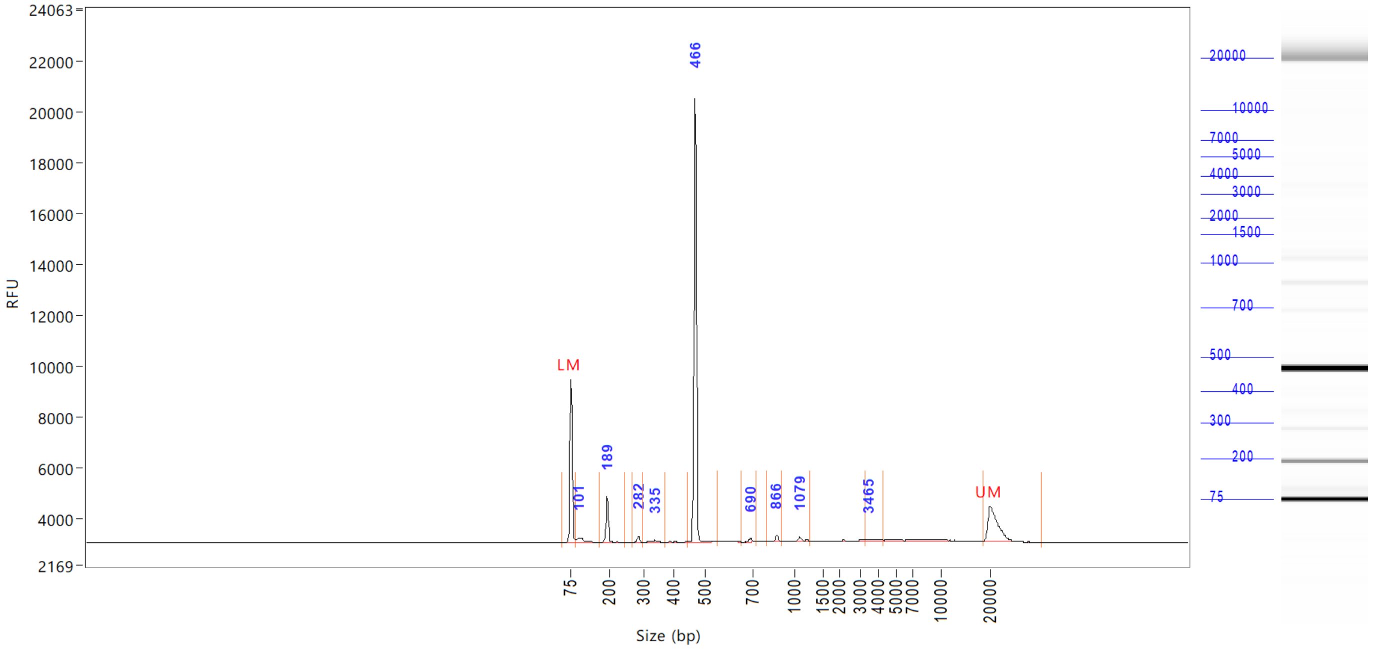

Peak	Size (bp)	Concentration (ng/uL)	From (bp)	To (bp)	Average size (bp)	CV%	RFU	Corrected peak area
1	75 (LM)	0.5000	50	89	73	4.96	6401	35.114
2	101	0.6051	89	168	110	16.33	200	3.541
3	189	1.7448	168	244	188	3.68	1810	10.212
4	282	0.2442	264	295	279	1.62	254	1.429
5	335	0.1889	295	372	330	4.04	81	1.106
6	466	14.4735	445	547	464	0.99	17486	84.705
7	690	0.1435	652	714	687	1.19	142	0.840
8	866	0.2580	796	905	858	2.01	258	1.510
9	1079	0.2455	905	1278	1100	5.80	179	1.437
10	3465	0.0965	3251	4277	3620	7.54	58	0.565
11	20000 (UM)	0.2474	18537	30277	20822	5.77	1375	17.377

TIC:	18.0000	ng/uL
TIM:	79.2510	nmole/L
Total concentration:	18.5869	ng/uL

Sample peak width (sec): 5   Sample min peak height: 50   Sample baseline V to V?: Y   Sample baseline V to V points: 3
Sample filter: Binomial   Number of points for filter: 3 Sample start region (min): 0 Sample end region (min): 30
Marker peak width (sec): 5   Marker min peak height: 200   Marker baseline V to V?: Y   Marker baseline V to V points: 3
Lower marker selection: First peak > 500 RFU   Upper marker selection: Last peak > 200 RFU
Ladder size (bp)75, 200, 300, 400, 500, 700, 1000, 1500, 2000, 3000, 4000, 5000, 7000, 10000, 20000
Quantification using: Lower Marker   Final concentration (ng/uL): 0.5000   Dilution factor: 12.0

Sample: SampE11
Well location: E11
Created: Wednesday, October 20, 2021 5:58:44 PM

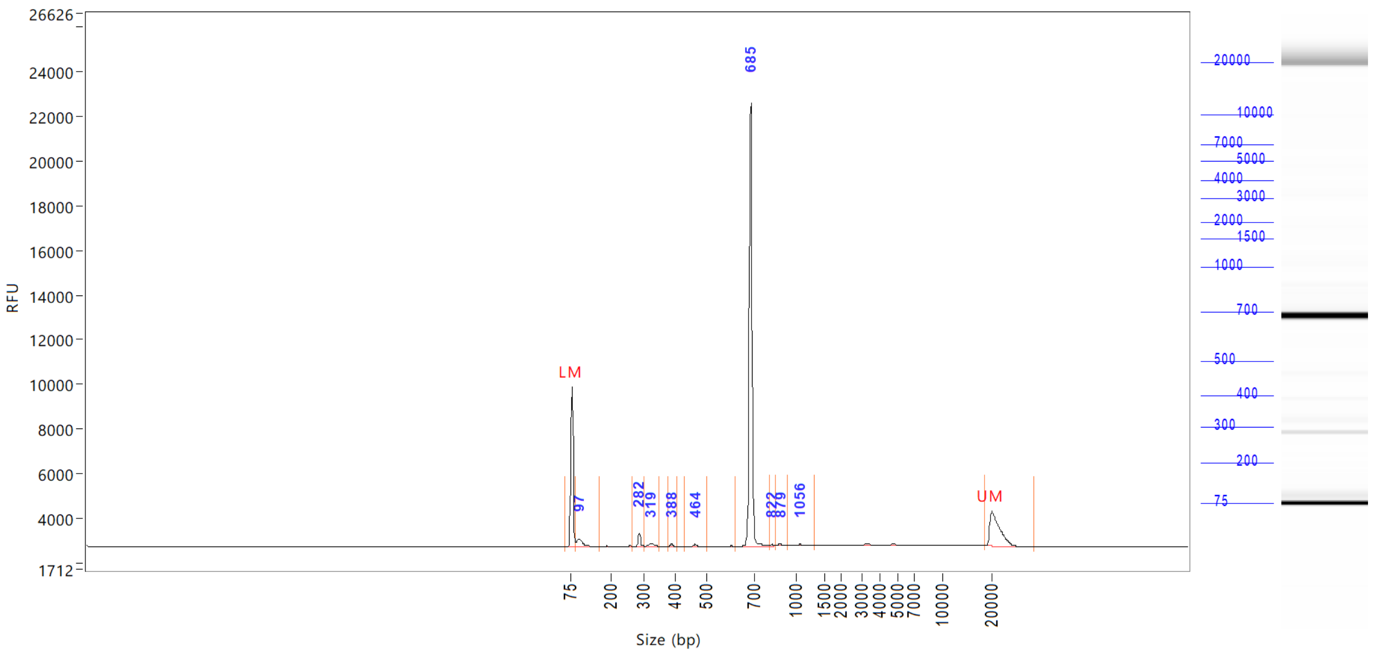

Peak	Size (bp)	Concentration (ng/uL)	From (bp)	To (bp)	Average size (bp)	CV%	RFU	Corrected peak area
1	75 (LM)	0.5000	54	86	74	4.32	7167	38.913
2	97	0.8743	86	166	102	14.14	364	5.670
3	282	0.4937	264	300	281	1.65	598	3.202
4	319	0.2336	300	349	320	3.03	128	1.515
5	388	0.0834	376	405	386	1.03	109	0.541
6	464	0.1288	428	501	462	2.86	91	0.835
7	685	15.8239	621	805	682	1.59	19900	102.625
8	822	0.0943	805	857	828	1.84	71	0.612
9	879	0.0852	857	939	878	1.64	86	0.553
10	1056	0.1226	939	1334	1089	7.87	59	0.795
11	20000 (UM)	0.2528	18659	28463	20822	5.62	1543	19.676

TIC:	17.9397	ng/uL
TIM:	57.6975	nmole/L
Total concentration:	18.6160	ng/uL

Sample peak width (sec): 5 Sample min peak height: 50 Sample baseline V to V?: Y Sample baseline V to V points: 3
Sample filter: Binomial Number of points for filter: 3 Sample start region (min): 0 Sample end region (min): 30
Marker peak width (sec): 5 Marker min peak height: 200 Marker baseline V to V?: Y Marker baseline V to V points: 3
Lower marker selection: First peak > 500 RFU Upper marker selection: Last peak > 200 RFU
Ladder size (bp)75, 200, 300, 400, 500, 700, 1000, 1500, 2000, 3000, 4000, 5000, 7000, 10000, 20000
Quantification using: Lower Marker Final concentration (ng/uL): 0.5000 Dilution factor: 12.0

Sample: SampE12
Well location: E12
Created: Wednesday, October 20, 2021 5:58:44 PM

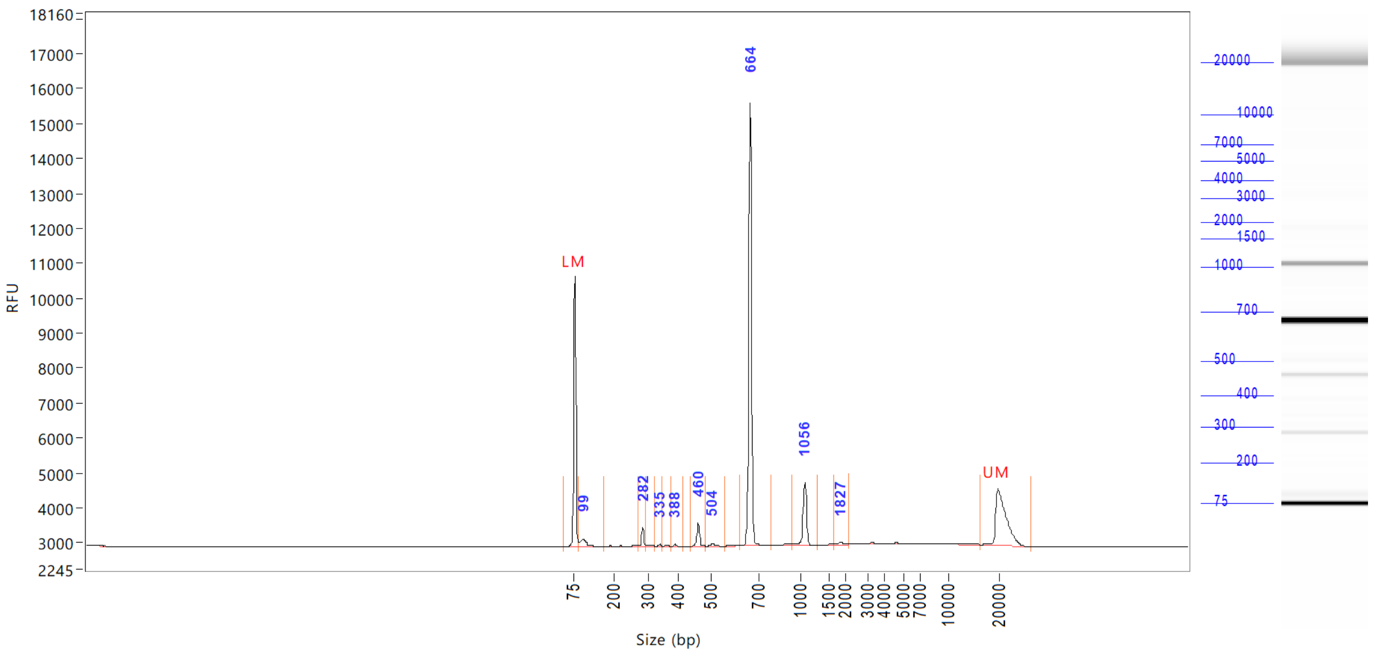

Peak	Size (bp)	Concentration (ng/uL)	From (bp)	To (bp)	Average size (bp)	CV%	RFU	Corrected peak area
1	75 (LM)	0.5000	43	89	73	4.95	7734	41.818
2	99	0.4629	89	166	105	14.26	229	3.226
3	282	0.3856	269	293	280	1.29	533	2.688
4	335	0.0443	321	345	333	1.31	58	0.309
5	388	0.0521	374	414	388	1.77	60	0.363
6	460	0.5218	439	485	458	1.15	680	3.637
7	504	0.1212	485	555	507	2.14	89	0.844
8	664	9.2898	623	783	662	1.21	12699	64.747
9	1056	1.4673	935	1300	1044	3.34	1786	10.227
10	1827	0.0888	1655	2172	1821	5.29	74	0.619
11	20000 (UM)	0.2562	16586	26529	20784	5.74	1639	21.428

TIC:	12.4338	ng/uL
TIM:	37.7003	nmole/L
Total concentration:	13.1506	ng/uL

Sample peak width (sec): 5   Sample min peak height: 50   Sample baseline V to V?: Y   Sample baseline V to V points: 3
Sample filter: Binomial   Number of points for filter: 3 Sample start region (min): 0 Sample end region (min): 30
Marker peak width (sec): 5   Marker min peak height: 200   Marker baseline V to V?: Y   Marker baseline V to V points: 3
Lower marker selection: First peak > 500 RFU   Upper marker selection: Last peak > 200 RFU
Ladder size (bp)75, 200, 300, 400, 500, 700, 1000, 1500, 2000, 3000, 4000, 5000, 7000, 10000, 20000
Quantification using: Lower Marker   Final concentration (ng/uL): 0.5000   Dilution factor: 12.0

Sample: SampF1
Well location: F1
Created: Wednesday, October 20, 2021 5:58:44 PM

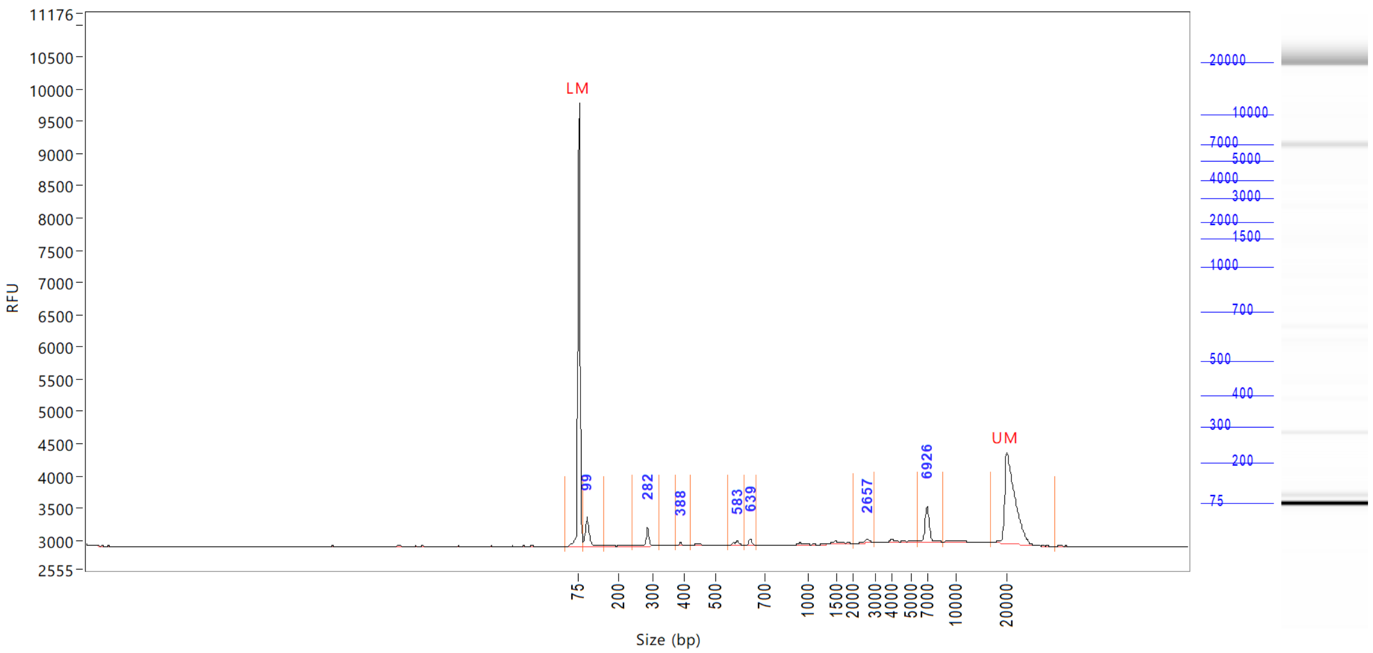

Peak	Size (bp)	Concentration (ng/uL)	From (bp)	To (bp)	Average size (bp)	CV%	RFU	Corrected peak area
1	75 (LM)	0.5000	31	87	73	5.97	6890	37.307
2	99	0.6646	87	152	100	10.42	448	4.133
3	282	0.2833	239	317	279	3.31	299	1.761
4	388	0.0583	372	422	388	2.23	60	0.363
5	583	0.1176	547	616	577	2.21	66	0.731
6	639	0.1011	616	664	638	1.04	109	0.628
7	2657	0.0786	2030	2971	2619	6.89	58	0.489
8	6926	0.5736	5741	8534	6881	5.17	564	3.566
9	20000 (UM)	0.2451	16830	29430	20899	5.76	1420	18.285

TIC:	1.8771	ng/uL
TIM:	13.6141	nmole/L
Total concentration:	2.5330	ng/uL

Sample peak width (sec): 5 Sample min peak height: 50 Sample baseline V to V?: Y Sample baseline V to V points: 3
Sample filter: Binomial Number of points for filter: 3 Sample start region (min): 0 Sample end region (min): 30
Marker peak width (sec): 5 Marker min peak height: 200 Marker baseline V to V?: Y Marker baseline V to V points: 3
Lower marker selection: First peak > 500 RFU Upper marker selection: Last peak > 200 RFU
Ladder size (bp)75, 200, 300, 400, 500, 700, 1000, 1500, 2000, 3000, 4000, 5000, 7000, 10000, 20000
Quantification using: Lower Marker Final concentration (ng/uL): 0.5000 Dilution factor: 12.0

Sample: SampF2
Well location: F2
Created: Wednesday, October 20, 2021 5:58:44 PM

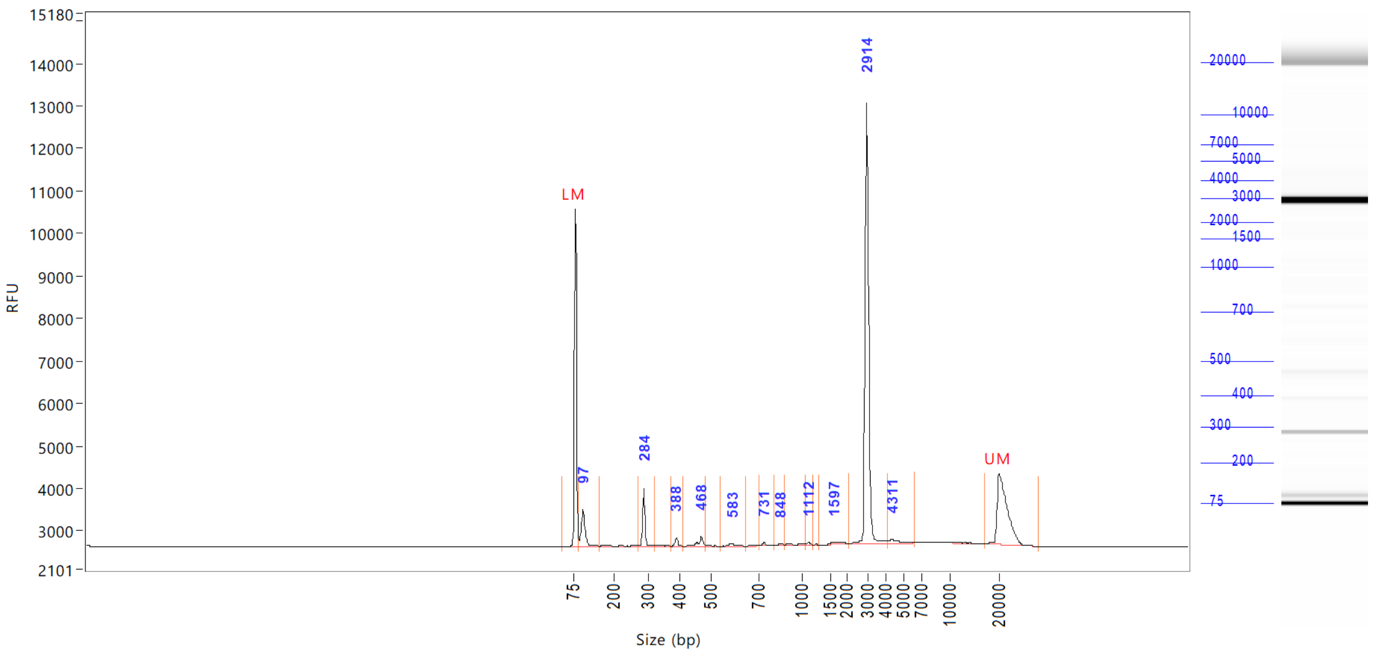

Peak	Size (bp)	Concentration (ng/uL)	From (bp)	To (bp)	Average size (bp)	CV%	RFU	Corrected peak area
1	75 (LM)	0.5000	33	86	73	4.61	7939	42.154
2	97	1.1907	86	150	99	10.63	870	8.366
3	284	0.9858	269	317	282	1.81	1366	6.926
4	388	0.1463	370	412	387	1.50	187	1.028
5	468	0.2698	412	483	459	2.66	236	1.895
6	583	0.1177	537	641	582	3.28	64	0.827
7	731	0.0730	700	805	730	2.31	81	0.513
8	848	0.0604	805	874	845	1.84	51	0.424
9	1112	0.0515	1045	1178	1095	2.45	65	0.362
10	1597	0.1359	1289	2087	1696	8.88	57	0.955
11	2914	8.2465	2087	4070	2922	5.10	10388	57.937
12	4311	0.1936	4070	6260	4648	11.23	84	1.360
13	20000 (UM)	0.2501	17195	27979	20823	5.43	1656	21.086

TIC:	11.4713	ng/uL
TIM:	32.6284	nmole/L
Total concentration:	12.0302	ng/uL

```
Sample peak width (sec): 5     Sample min peak height: 50    Sample baseline V to V?: Y    Sample baseline V to V points: 3
Sample filter: Binomial        Number of points for filter: 3  Sample start region (min): 0  Sample end region (min): 30
Marker peak width (sec): 5     Marker min peak height: 200   Marker baseline V to V?: Y    Marker baseline V to V points: 3
Lower marker selection: First peak > 500 RFU                 Upper marker selection: Last peak > 200 RFU
Ladder size (bp)75, 200, 300, 400, 500, 700, 1000, 1500, 2000, 3000, 4000, 5000, 7000, 10000, 20000
Quantification using: Lower Marker          Final concentration (ng/uL): 0.5000          Dilution factor: 12.0
```

Sample: SampF3
Well location: F3
Created: Wednesday, October 20, 2021 5:58:44 PM

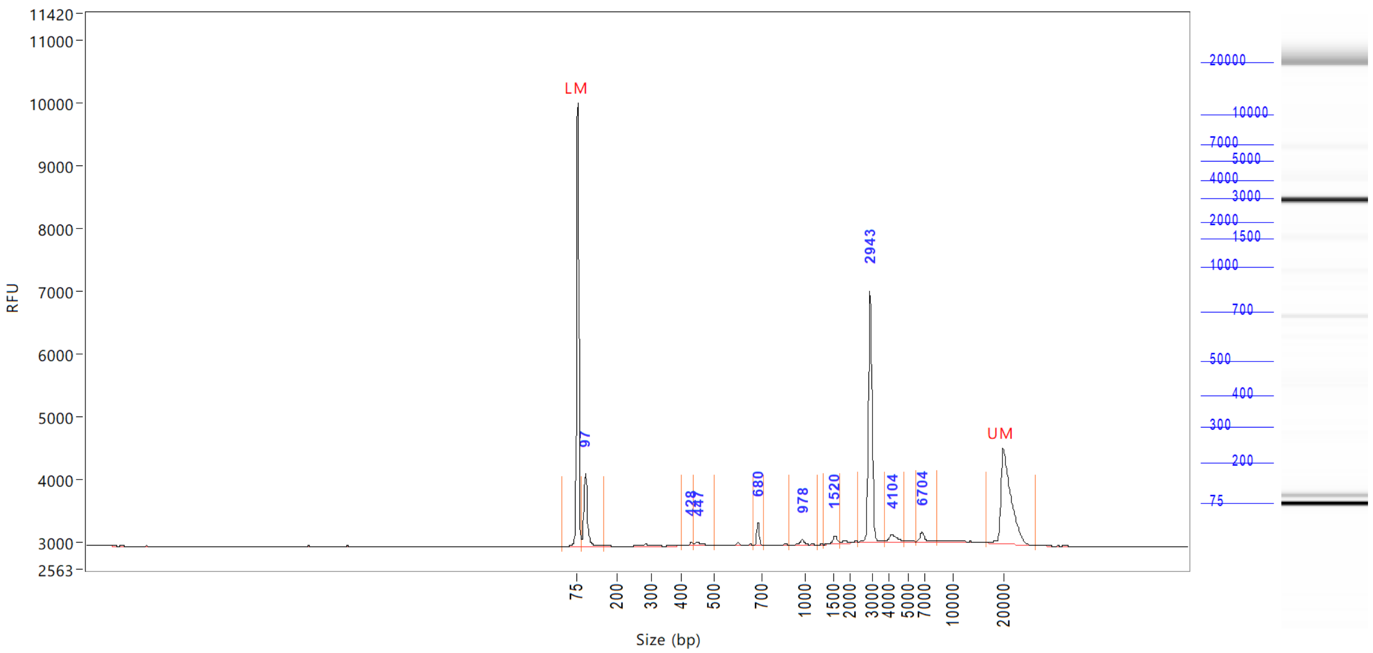

Peak	Size (bp)	Concentration (ng/uL)	From (bp)	To (bp)	Average size (bp)	CV%	RFU	Corrected peak area
1	75 (LM)	0.5000	30	86	72	5.21	7080	38.846
2	97	1.4764	86	156	99	9.47	1152	9.559
3	428	0.0806	400	435	422	2.13	66	0.522
4	447	0.1309	435	501	453	2.77	55	0.848
5	680	0.2808	662	705	679	0.88	363	1.818
6	978	0.1463	887	1212	983	5.18	101	0.947
7	1520	0.1490	1323	1693	1524	3.83	132	0.965
8	2943	3.3576	2401	3750	2923	3.62	3994	21.738
9	4104	0.2059	3750	4793	4186	5.06	117	1.333
10	6704	0.1716	5889	8334	6821	7.07	149	1.111
11	20000 (UM)	0.2474	16708	26529	20815	5.61	1524	19.218

TIC:	5.9991	ng/uL
TIM:	28.3777	nmole/L
Total concentration:	6.5018	ng/uL

```
Sample peak width (sec): 5    Sample min peak height: 50    Sample baseline V to V?: Y    Sample baseline V to V points: 3
Sample filter: Binomial       Number of points for filter: 3  Sample start region (min): 0  Sample end region (min): 30
Marker peak width (sec): 5    Marker min peak height: 200   Marker baseline V to V?: Y    Marker baseline V to V points: 3
Lower marker selection: First peak > 500 RFU                Upper marker selection: Last peak > 200 RFU
Ladder size (bp)75, 200, 300, 400, 500, 700, 1000, 1500, 2000, 3000, 4000, 5000, 7000, 10000, 20000
Quantification using: Lower Marker          Final concentration (ng/uL): 0.5000          Dilution factor: 12.0
```

Sample: SampF4
Well location: F4
Created: Wednesday, October 20, 2021 5:58:44 PM

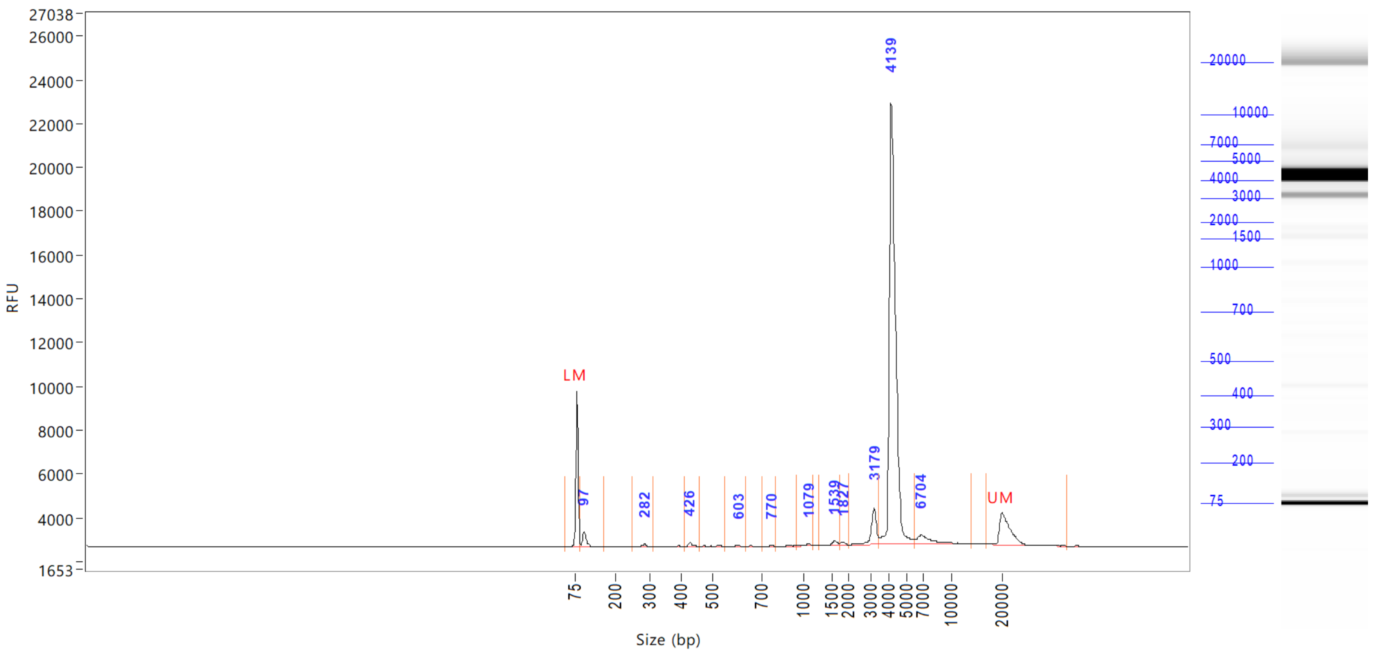

Peak	Size (bp)	Concentration (ng/uL)	From (bp)	To (bp)	Average size (bp)	CV%	RFU	Corrected peak area
1	75 (LM)	0.5000	39	87	74	4.72	7091	39.618
2	97	0.9720	87	160	100	11.23	663	6.418
3	282	0.1137	246	311	278	3.91	90	0.751
4	426	0.1756	409	456	428	2.13	166	1.159
5	603	0.0787	544	636	594	2.77	59	0.520
6	770	0.0545	710	801	759	2.25	54	0.360
7	1079	0.1087	952	1167	1051	3.93	95	0.718
8	1539	0.2477	1267	1712	1557	4.92	206	1.636
9	1827	0.1348	1712	2001	1809	3.36	126	0.890
10	3179	1.9549	2001	3500	3130	6.34	1699	12.908
11	4139	27.3834	3500	5889	4234	5.54	20191	180.815
12	6704	1.3307	5889	14025	7552	18.36	421	8.787
13	20000 (UM)	0.2463	16830	33058	20868	6.30	1492	19.520

TIC:	32.5548	ng/uL
TIM:	30.1657	nmole/L
Total concentration:	32.8208	ng/uL

Sample peak width (sec): 5 Sample min peak height: 50 Sample baseline V to V?: Y Sample baseline V to V points: 3
Sample filter: Binomial Number of points for filter: 3 Sample start region (min): 0 Sample end region (min): 30
Marker peak width (sec): 5 Marker min peak height: 200 Marker baseline V to V?: Y Marker baseline V to V points: 3
Lower marker selection: First peak > 500 RFU Upper marker selection: Last peak > 200 RFU
Ladder size (bp)75, 200, 300, 400, 500, 700, 1000, 1500, 2000, 3000, 4000, 5000, 7000, 10000, 20000
Quantification using: Lower Marker Final concentration (ng/uL): 0.5000 Dilution factor: 12.0

Sample: SampF5
Well location: F5
Created: Wednesday, October 20, 2021 5:58:44 PM

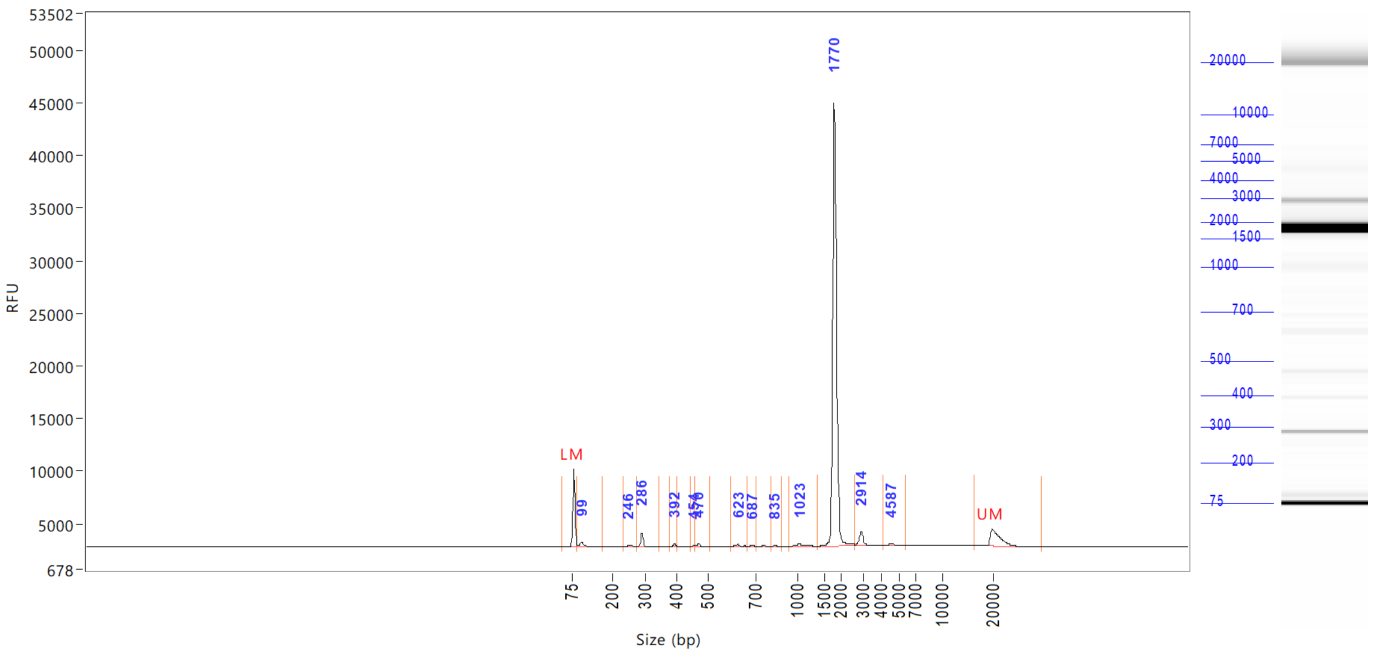

Peak	Size (bp)	Concentration (ng/uL)	From (bp)	To (bp)	Average size (bp)	CV%	RFU	Corrected peak area
1	75 (LM)	0.5000	43	87	74	4.33	7324	39.673
2	99	0.7071	87	168	102	12.87	425	4.676
3	246	0.1717	230	273	253	3.96	76	1.135
4	286	1.1073	273	343	286	3.47	1315	7.322
5	392	0.1711	378	403	390	0.95	238	1.132
6	454	0.0851	443	460	452	1.08	79	0.563
7	470	0.2588	460	506	470	1.50	295	1.711
8	623	0.2959	593	664	621	2.22	197	1.956
9	687	0.1016	664	701	682	1.06	113	0.672
10	835	0.0737	814	887	843	2.12	50	0.487
11	1023	0.4254	944	1356	1052	8.01	195	2.813
12	1770	39.0309	1356	2629	1777	4.99	42174	258.079
13	2914	1.4488	2629	4104	2988	9.36	1340	9.580
14	4587	0.3132	4104	5889	4689	7.08	152	2.071
15	20000 (UM)	0.2539	16342	29793	20786	6.05	1581	20.144

TIC:	44.1905	ng/uL
TIM:	59.7101	nmole/L
Total concentration:	44.5843	ng/uL

Sample peak width (sec): 5 Sample min peak height: 50 Sample baseline V to V?: Y Sample baseline V to V points: 3
Sample filter: Binomial Number of points for filter: 3 Sample start region (min): 0 Sample end region (min): 30
Marker peak width (sec): 5 Marker min peak height: 200 Marker baseline V to V?: Y Marker baseline V to V points: 3
Lower marker selection: First peak > 500 RFU Upper marker selection: Last peak > 200 RFU
Ladder size (bp)75, 200, 300, 400, 500, 700, 1000, 1500, 2000, 3000, 4000, 5000, 7000, 10000, 20000
Quantification using: Lower Marker Final concentration (ng/uL): 0.5000 Dilution factor: 12.0

Sample: SampF6
Well location: F6
Created: Wednesday, October 20, 2021 5:58:44 PM

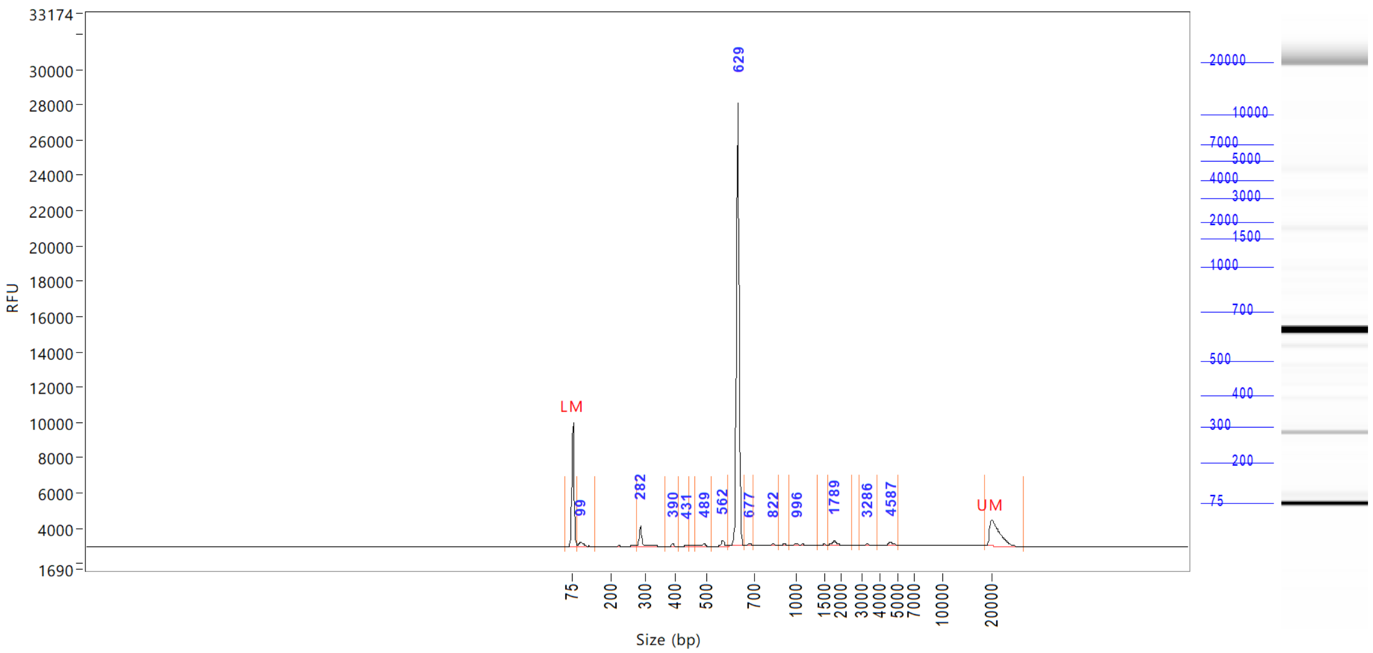

Peak	Size (bp)	Concentration (ng/uL)	From (bp)	To (bp)	Average size (bp)	CV%	RFU	Corrected peak area
1	75 (LM)	0.5000	52	87	73	4.58	7014	37.876
2	99	0.5121	87	147	103	12.08	235	3.233
3	282	1.1569	271	364	287	5.16	1186	7.303
4	390	0.1294	364	411	387	1.70	152	0.817
5	431	0.0656	411	443	431	1.43	63	0.414
6	489	0.1861	458	519	479	2.23	120	1.175
7	562	0.2796	519	585	560	1.48	309	1.765
8	629	19.6178	585	654	625	0.92	25124	123.842
9	677	0.1472	654	695	671	1.28	124	0.929
10	822	0.1104	695	870	813	4.85	51	0.697
11	996	0.1438	944	1378	1036	7.29	85	0.908
12	1789	0.2956	1578	2515	1806	6.84	225	1.866
13	3286	0.0762	2857	3893	3230	5.98	57	0.481
14	4587	0.1948	3893	5001	4594	4.20	148	1.230
15	20000 (UM)	0.2503	18781	26408	20918	5.48	1509	18.958

TIC:	22.9156	ng/uL
TIM:	69.9037	nmole/L
Total concentration:	23.4028	ng/uL

```
Sample peak width (sec): 5    Sample min peak height: 50    Sample baseline V to V?: Y    Sample baseline V to V points: 3
Sample filter: Binomial       Number of points for filter: 3  Sample start region (min): 0  Sample end region (min): 30
Marker peak width (sec): 5    Marker min peak height: 200   Marker baseline V to V?: Y    Marker baseline V to V points: 3
Lower marker selection: First peak > 500 RFU                Upper marker selection: Last peak > 200 RFU
Ladder size (bp)75, 200, 300, 400, 500, 700, 1000, 1500, 2000, 3000, 4000, 5000, 7000, 10000, 20000
Quantification using: Lower Marker          Final concentration (ng/uL): 0.5000          Dilution factor: 12.0
```

Sample: SampF7
Well location: F7
Created: Wednesday, October 20, 2021 5:58:44 PM

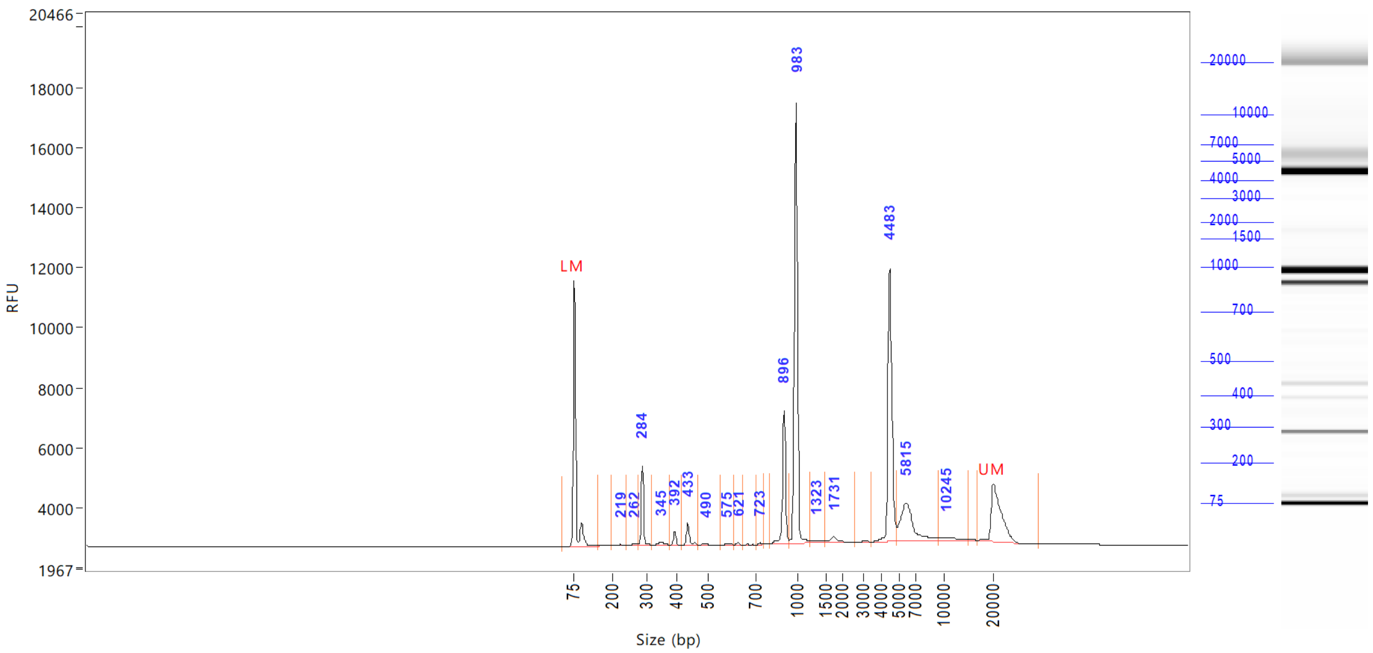

Peak	Size (bp)	Concentration (ng/uL)	From (bp)	To (bp)	Average size (bp)	CV%	RFU	Corrected peak area
1	75 (LM)	0.5000	39	152	77	12.83	8845	54.157
2	219	0.0424	194	239	219	3.39	51	0.383
3	262	0.0916	239	273	260	3.06	66	0.827
4	284	1.5346	273	317	284	1.75	2650	13.851
5	345	0.1718	317	376	345	3.80	84	1.550
6	392	0.2547	376	416	389	1.18	451	2.299
7	433	0.5104	416	466	435	2.01	740	4.607
8	490	0.1076	466	547	491	3.36	60	0.971
9	575	0.0817	547	606	579	2.19	62	0.738
10	621	0.0649	606	646	620	1.11	102	0.585
11	723	0.0325	697	753	719	1.47	56	0.293
12	896	2.6250	801	935	890	1.81	4413	23.694
13	983	8.4427	935	1212	980	2.21	14675	76.206
14	1323	0.1327	1212	1478	1337	5.63	80	1.197
15	1731	0.2498	1478	2543	1730	9.23	196	2.254
16	4483	6.1436	3393	4897	4456	3.26	9072	55.453
17	5815	2.5755	4897	9467	6126	16.49	1281	23.247
18	10245	0.2652	9467	14879	11220	12.48	106	2.394
19	20000 (UM)	0.2302	16586	29189	20834	5.78	1932	24.932

TIC:	23.3268	ng/uL
TIM:	36.8884	nmole/L
Total concentration:	23.3848	ng/uL

```
Sample peak width (sec): 5    Sample min peak height: 50    Sample baseline V to V?: Y    Sample baseline V to V points: 3
Sample filter: Binomial       Number of points for filter: 3  Sample start region (min): 0  Sample end region (min): 30
Marker peak width (sec): 5    Marker min peak height: 200   Marker baseline V to V?: Y    Marker baseline V to V points: 3
Lower marker selection: First peak > 500 RFU                Upper marker selection: Last peak > 200 RFU
Ladder size (bp)75, 200, 300, 400, 500, 700, 1000, 1500, 2000, 3000, 4000, 5000, 7000, 10000, 20000
Quantification using: Lower Marker          Final concentration (ng/uL): 0.5000          Dilution factor: 12.0
```

Sample: SampF8
Well location: F8
Created: Wednesday, October 20, 2021 5:58:44 PM

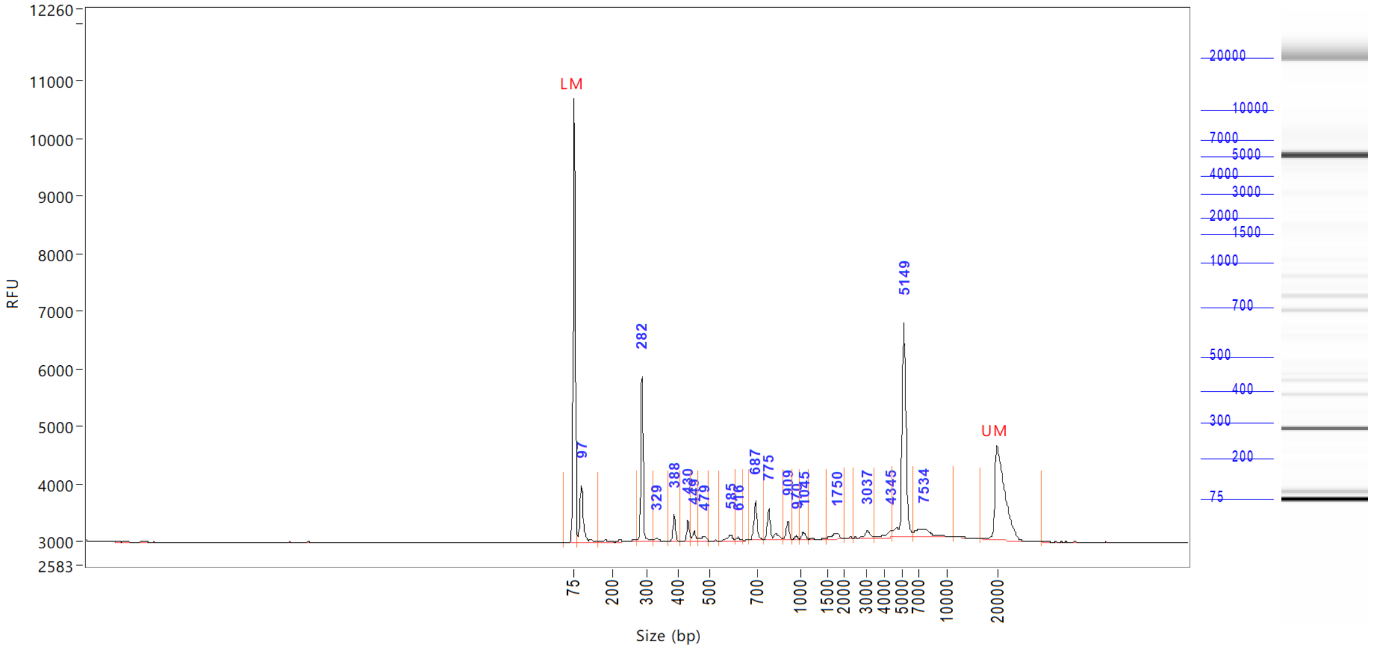

Peak	Size (bp)	Concentration (ng/uL)	From (bp)	To (bp)	Average size (bp)	CV%	RFU	Corrected peak area
1	75 (LM)	0.5000	45	86	73	4.42	7735	41.084
2	97	1.2692	86	148	98	10.38	986	8.691
3	282	2.1675	269	315	281	2.02	2871	14.842
4	329	0.1162	315	368	333	3.81	72	0.796
5	388	0.3462	368	407	385	1.15	470	2.371
6	430	0.2782	407	439	428	1.09	368	1.905
7	449	0.1649	439	460	448	1.17	171	1.129
8	479	0.1435	460	498	475	1.85	80	0.982
9	585	0.1363	539	603	577	2.08	95	0.933
10	616	0.0463	603	639	614	1.00	54	0.317
11	687	0.5484	659	744	687	1.43	681	3.755
12	775	0.5372	744	874	787	3.68	558	3.678
13	909	0.2405	874	939	904	1.21	305	1.647
14	970	0.0452	939	996	965	1.15	62	0.310
15	1045	0.1072	996	1145	1042	3.16	126	0.734
16	1750	0.1894	1467	1981	1666	6.76	102	1.297
17	3037	0.1429	2372	3465	3017	6.55	125	0.978
18	4345	0.1674	3465	4449	4135	6.17	111	1.146
19	5149	3.2919	4449	6186	5137	4.81	3725	22.541
20	7534	0.4398	6186	11098	7462	11.33	134	3.011
21	20000 (UM)	0.2513	16708	29068	20850	5.63	1634	20.645

TIC:	10.3781	ng/uL
TIM:	43.2964	nmole/L
Total concentration:	10.6524	ng/uL

```
Sample peak width (sec): 5    Sample min peak height: 50    Sample baseline V to V?: Y    Sample baseline V to V points: 3
Sample filter: Binomial       Number of points for filter: 3  Sample start region (min): 0  Sample end region (min): 30
Marker peak width (sec): 5    Marker min peak height: 200   Marker baseline V to V?: Y    Marker baseline V to V points: 3
Lower marker selection: First peak > 500 RFU                Upper marker selection: Last peak > 200 RFU
Ladder size (bp)75, 200, 300, 400, 500, 700, 1000, 1500, 2000, 3000, 4000, 5000, 7000, 10000, 20000
Quantification using: Lower Marker          Final concentration (ng/uL): 0.5000          Dilution factor: 12.0
```

Sample: SampF9
Well location: F9
Created: Wednesday, October 20, 2021 5:58:44 PM

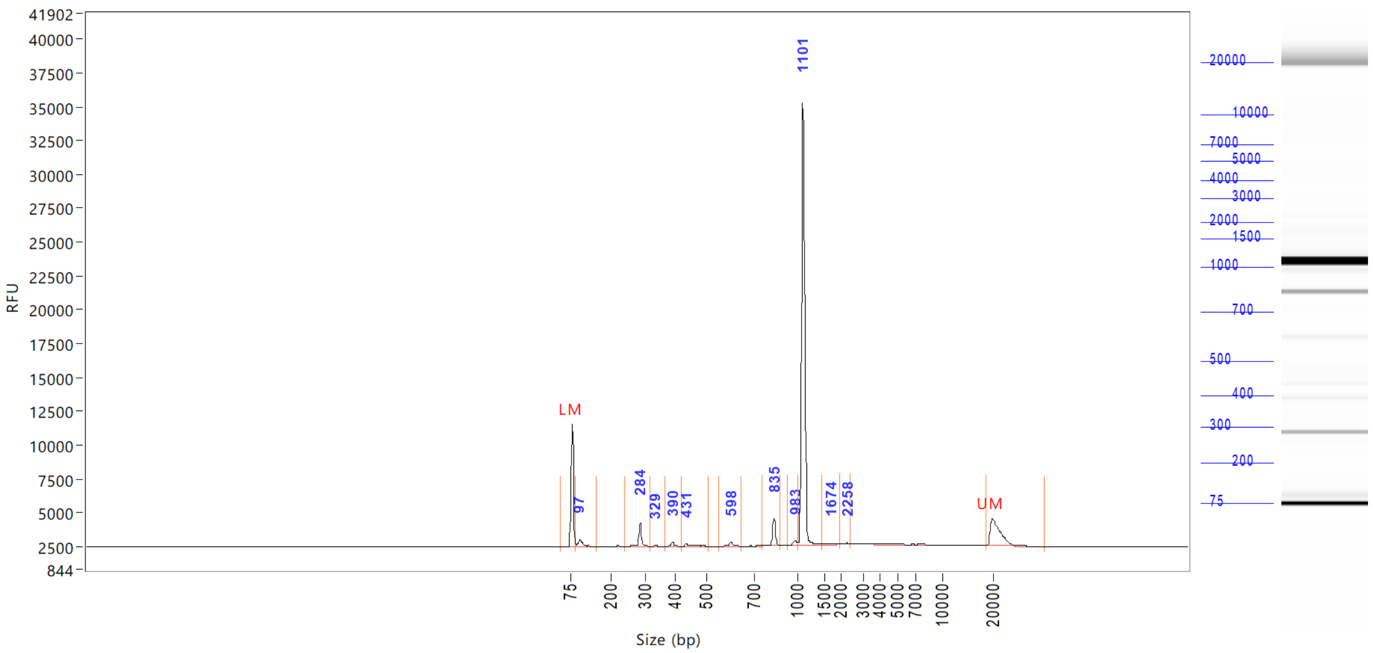

Peak	Size (bp)	Concentration (ng/uL)	From (bp)	To (bp)	Average size (bp)	CV%	RFU	Corrected peak area
1	75 (LM)	0.5000	39	86	73	4.36	9102	48.705
2	97	0.7292	86	150	100	11.26	502	5.919
3	284	1.2325	241	317	281	2.87	1769	10.004
4	329	0.0736	317	368	337	4.06	52	0.597
5	390	0.2633	368	420	389	1.80	316	2.137
6	431	0.2786	420	501	449	4.48	205	2.262
7	598	0.2834	544	641	594	2.45	315	2.301
8	835	1.2967	753	874	830	1.36	2048	10.526
9	983	0.2868	935	1001	981	1.38	322	2.328
10	1101	20.4123	1001	1445	1096	3.36	32731	165.696
11	1674	0.2293	1445	1962	1677	8.21	118	1.862
12	2258	0.0974	1962	2458	2179	5.26	104	0.790
13	20000 (UM)	0.2612	18659	30639	20833	6.00	2024	25.443

TIC:	25.1831	ng/uL
TIM:	56.4731	nmole/L
Total concentration:	25.6835	ng/uL

Sample peak width (sec): 5 Sample min peak height: 50 Sample baseline V to V?: Y Sample baseline V to V points: 3
Sample filter: Binomial Number of points for filter: 3 Sample start region (min): 0 Sample end region (min): 30
Marker peak width (sec): 5 Marker min peak height: 200 Marker baseline V to V?: Y Marker baseline V to V points: 3
Lower marker selection: First peak > 500 RFU Upper marker selection: Last peak > 200 RFU
Ladder size (bp)75, 200, 300, 400, 500, 700, 1000, 1500, 2000, 3000, 4000, 5000, 7000, 10000, 20000
Quantification using: Lower Marker Final concentration (ng/uL): 0.5000 Dilution factor: 12.0

Sample: SampF10
Well location: F10
Created: Wednesday, October 20, 2021 5:58:44 PM

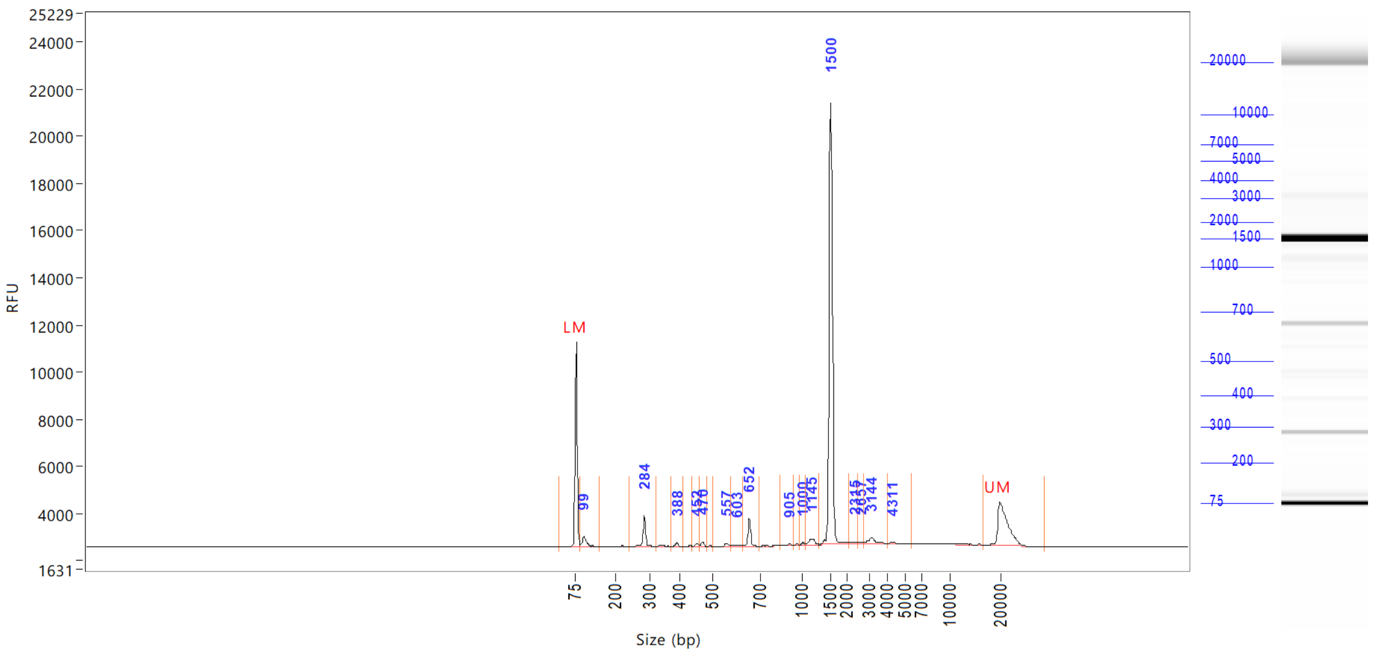

Peak	Size (bp)	Concentration (ng/uL)	From (bp)	To (bp)	Average size (bp)	CV%	RFU	Corrected peak area
1	75 (LM)	0.5000	24	87	73	4.62	8666	46.249
2	99	0.5871	87	148	101	10.53	421	4.526
3	284	0.9221	242	321	281	2.68	1323	7.108
4	388	0.1037	368	411	385	1.44	149	0.799
5	452	0.0983	439	460	451	1.03	135	0.758
6	470	0.1378	460	483	468	0.87	202	1.062
7	557	0.1119	504	575	552	2.47	136	0.863
8	603	0.0987	575	629	600	2.09	73	0.761
9	652	0.7234	629	697	650	0.99	1162	5.576
10	905	0.0571	835	931	896	2.02	83	0.440
11	1000	0.0681	978	1045	1004	1.82	90	0.525
12	1145	0.3134	1045	1278	1139	3.88	258	2.416
13	1500	12.4999	1278	2058	1499	3.56	18768	96.351
14	2315	0.1274	2058	2486	2284	5.48	92	0.982
15	2657	0.0878	2486	2743	2616	3.09	91	0.677
16	3144	0.3644	2743	4035	3150	8.49	254	2.809
17	4311	0.0614	4035	5815	4407	6.40	54	0.473
18	20000 (UM)	0.2463	16830	29068	20835	5.50	1818	22.781

TIC:	16.3626	ng/uL
TIM:	33.4220	nmole/L
Total concentration:	16.6827	ng/uL

```
Sample peak width (sec): 5    Sample min peak height: 50    Sample baseline V to V?: Y    Sample baseline V to V points: 3
Sample filter: Binomial       Number of points for filter: 3  Sample start region (min): 0  Sample end region (min): 30
Marker peak width (sec): 5    Marker min peak height: 200   Marker baseline V to V?: Y    Marker baseline V to V points: 3
Lower marker selection: First peak > 500 RFU                Upper marker selection: Last peak > 200 RFU
Ladder size (bp)75, 200, 300, 400, 500, 700, 1000, 1500, 2000, 3000, 4000, 5000, 7000, 10000, 20000
Quantification using: Lower Marker          Final concentration (ng/uL): 0.5000          Dilution factor: 12.0
```

Sample: SampF11
Well location: F11
Created: Wednesday, October 20, 2021 5:58:44 PM

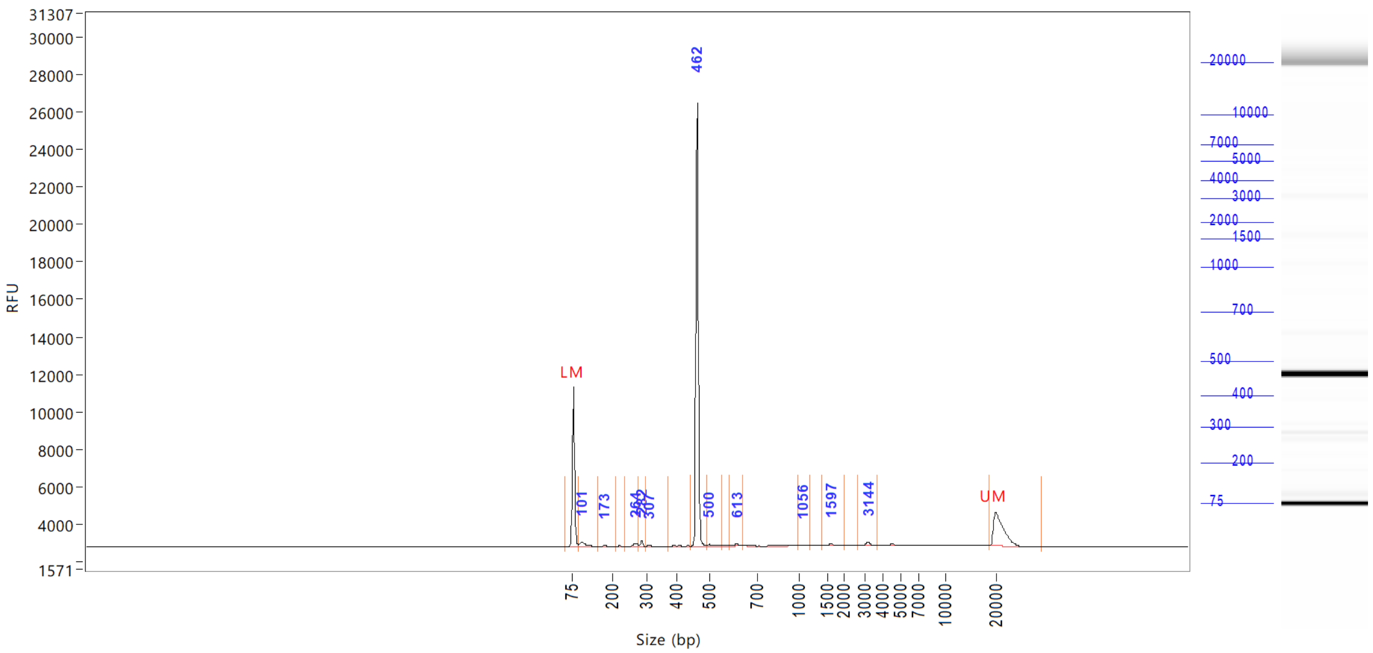

Peak	Size (bp)	Concentration (ng/uL)	From (bp)	To (bp)	Average size (bp)	CV%	RFU	Corrected peak area
1	75 (LM)	0.5000	48	89	73	4.73	8537	46.704
2	101	0.4759	89	154	106	13.47	260	3.704
3	173	0.0976	154	208	175	7.13	85	0.760
4	264	0.1940	235	275	263	2.67	145	1.510
5	282	0.2150	275	298	281	1.47	318	1.674
6	307	0.0749	298	368	312	4.54	78	0.583
7	462	14.5925	439	492	460	0.91	23747	113.588
8	500	0.1340	492	549	512	3.39	88	1.043
9	613	0.0945	585	644	609	1.64	99	0.735
10	1056	0.0591	991	1190	1051	3.67	59	0.460
11	1597	0.1037	1411	2058	1625	7.52	88	0.808
12	3144	0.1279	2686	3679	3141	5.40	133	0.995
13	20000 (UM)	0.2449	18659	29068	20820	5.44	1820	22.877

TIC:	16.1690	ng/uL
TIM:	64.3048	nmole/L
Total concentration:	16.6462	ng/uL

```
Sample peak width (sec): 5    Sample min peak height: 50    Sample baseline V to V?: Y    Sample baseline V to V points: 3
Sample filter: Binomial       Number of points for filter: 3  Sample start region (min): 0  Sample end region (min): 30
Marker peak width (sec): 5    Marker min peak height: 200   Marker baseline V to V?: Y    Marker baseline V to V points: 3
Lower marker selection: First peak > 500 RFU                Upper marker selection: Last peak > 200 RFU
Ladder size (bp)75, 200, 300, 400, 500, 700, 1000, 1500, 2000, 3000, 4000, 5000, 7000, 10000, 20000
Quantification using: Lower Marker          Final concentration (ng/uL): 0.5000          Dilution factor: 12.0
```

Sample: SampF12
Well location: F12
Created: Wednesday, October 20, 2021 5:58:44 PM

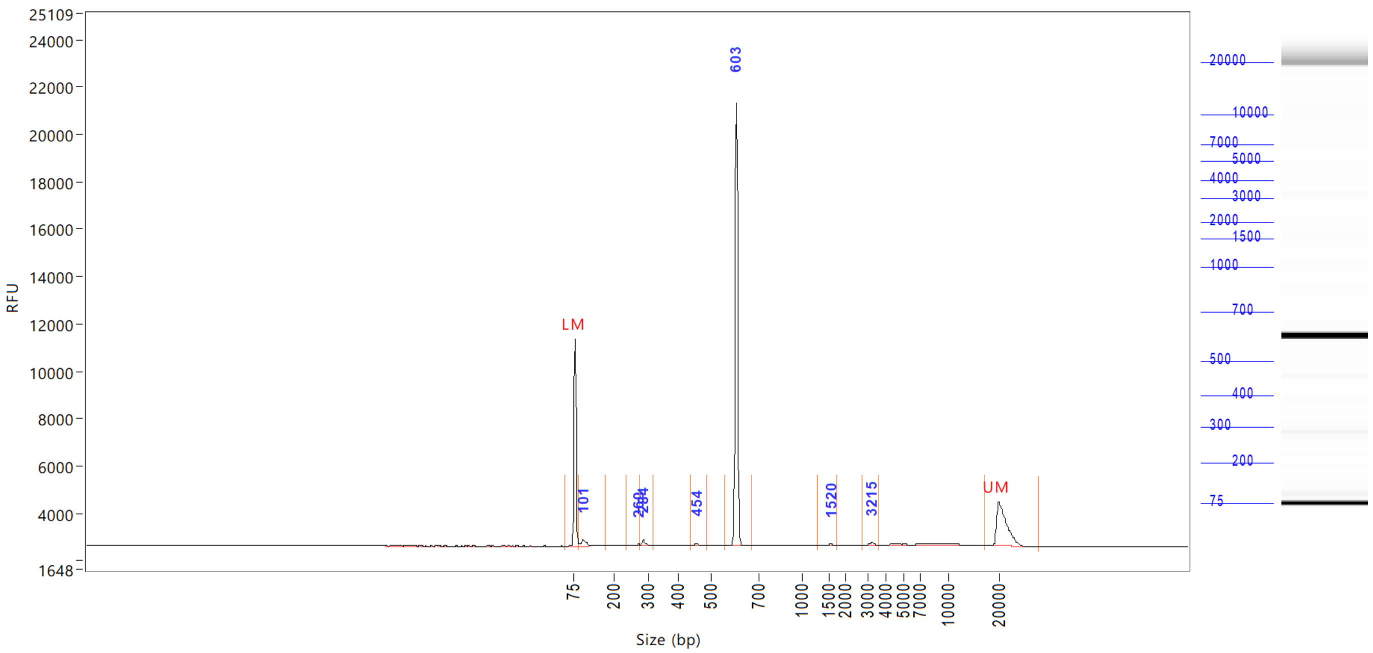

Peak	Size (bp)	Concentration (ng/uL)	From (bp)	To (bp)	Average size (bp)	CV%	RFU	Corrected peak area
1	75 (LM)	0.5000	48	87	73	4.26	8708	46.589
2	101	0.4820	87	169	107	15.05	268	3.742
3	269	0.0700	237	275	264	2.85	65	0.544
4	284	0.2494	275	317	286	2.54	263	1.936
5	454	0.1144	439	487	455	1.77	110	0.888
6	603	11.7794	555	674	602	1.02	18726	91.464
7	1520	0.0654	1289	1750	1541	6.16	50	0.508
8	3215	0.0751	2743	3572	3162	4.48	87	0.583
9	20000 (UM)	0.2484	17195	27979	20828	5.46	1848	23.141

TIC:	12.8357	ng/uL
TIM:	41.9915	nmole/L
Total concentration:	13.4194	ng/uL

Sample peak width (sec): 5 Sample min peak height: 50 Sample baseline V to V?: Y Sample baseline V to V points: 3
Sample filter: Binomial Number of points for filter: 3 Sample start region (min): 0 Sample end region (min): 30
Marker peak width (sec): 5 Marker min peak height: 200 Marker baseline V to V?: Y Marker baseline V to V points: 3
Lower marker selection: First peak > 500 RFU Upper marker selection: Last peak > 200 RFU
Ladder size (bp)75, 200, 300, 400, 500, 700, 1000, 1500, 2000, 3000, 4000, 5000, 7000, 10000, 20000
Quantification using: Lower Marker Final concentration (ng/uL): 0.5000 Dilution factor: 12.0

Sample: SampG1
Well location: G1
Created: Wednesday, October 20, 2021 5:58:44 PM

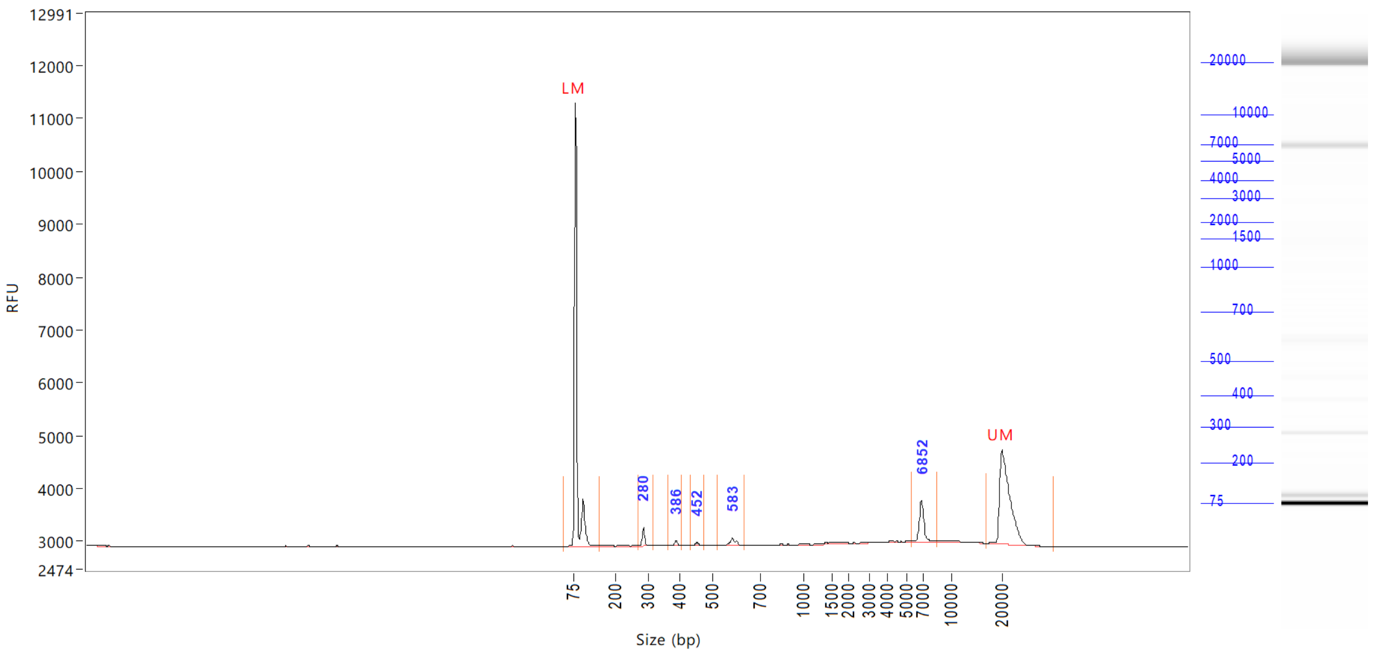

Peak	Size (bp)	Concentration (ng/uL)	From (bp)	To (bp)	Average size (bp)	CV%	RFU	Corrected peak area
1	75 (LM)	0.5000	41	150	77	13.53	8405	52.366
2	280	0.2313	266	309	280	2.10	360	2.019
3	386	0.0586	360	407	382	1.77	86	0.511
4	452	0.0590	433	473	452	1.73	69	0.515
5	583	0.1922	521	631	580	3.02	143	1.677
6	6852	0.5634	5667	8534	6818	5.14	772	4.917
7	20000 (UM)	0.2184	16830	30156	20843	5.66	1781	22.869

TIC:	1.1046	ng/uL
TIM:	2.5075	nmole/L
Total concentration:	1.6619	ng/uL

Sample peak width (sec): 5   Sample min peak height: 50   Sample baseline V to V?: Y   Sample baseline V to V points: 3
Sample filter: Binomial   Number of points for filter: 3 Sample start region (min): 0 Sample end region (min): 30
Marker peak width (sec): 5   Marker min peak height: 200   Marker baseline V to V?: Y   Marker baseline V to V points: 3
Lower marker selection: First peak > 500 RFU   Upper marker selection: Last peak > 200 RFU
Ladder size (bp)75, 200, 300, 400, 500, 700, 1000, 1500, 2000, 3000, 4000, 5000, 7000, 10000, 20000
Quantification using: Lower Marker   Final concentration (ng/uL): 0.5000   Dilution factor: 12.0

Sample: SampG2
Well location: G2
Created: Wednesday, October 20, 2021 5:58:44 PM

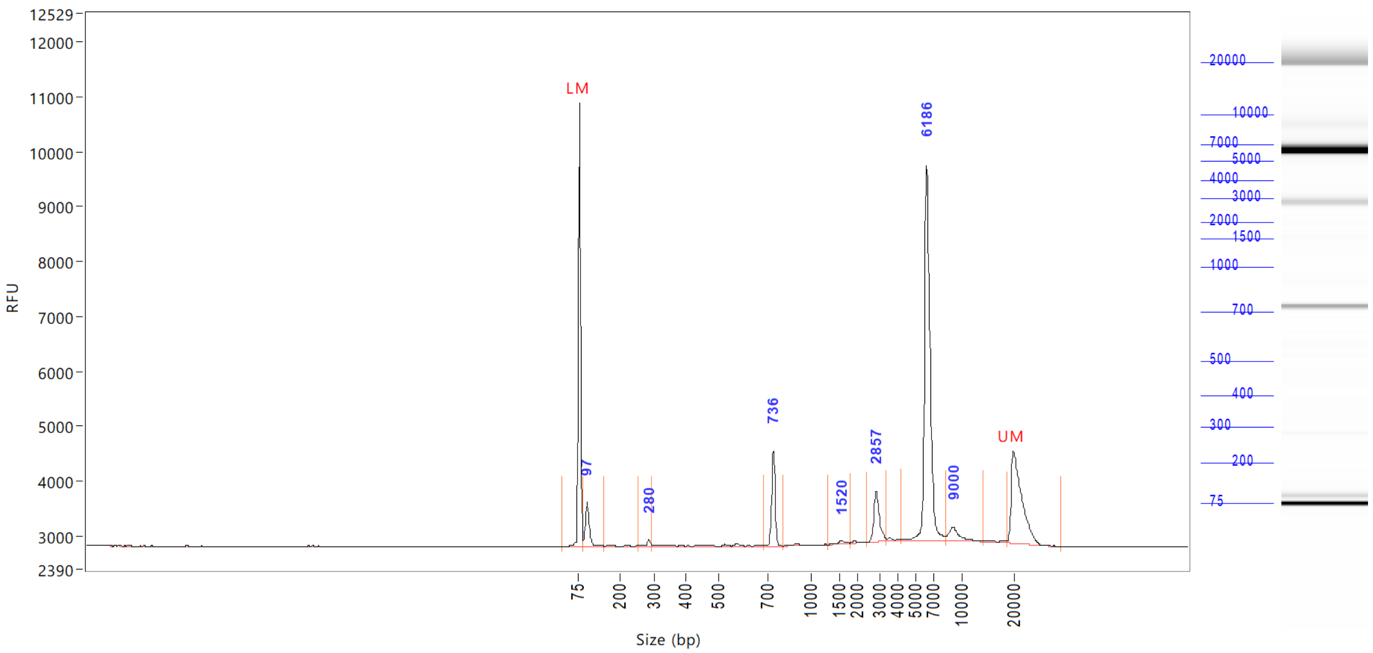

Peak	Size (bp)	Concentration (ng/uL)	From (bp)	To (bp)	Average size (bp)	CV%	RFU	Corrected peak area
1	75 (LM)	0.5000	24	86	72	5.76	8106	44.548
2	97	0.9998	86	148	98	9.67	809	7.423
3	280	0.1099	251	291	276	2.63	129	0.816
4	736	1.4260	685	796	728	1.56	1741	10.587
5	1520	0.0862	1278	1789	1505	7.10	55	0.640
6	2857	1.0325	2401	3393	2865	5.04	928	7.666
7	6186	7.2502	4173	8267	6261	7.01	6832	53.830
8	9000	0.4996	8267	14269	9424	10.71	262	3.709
9	20000 (UM)	0.2468	18659	29189	20946	5.82	1675	21.985

TIC:	11.4042	ng/uL
TIM:	23.3118	nmole/L
Total concentration:	11.9972	ng/uL

```
Sample peak width (sec): 5     Sample min peak height: 50    Sample baseline V to V?: Y    Sample baseline V to V points: 3
Sample filter: Binomial        Number of points for filter: 3  Sample start region (min): 0  Sample end region (min): 30
Marker peak width (sec): 5     Marker min peak height: 200   Marker baseline V to V?: Y    Marker baseline V to V points: 3
Lower marker selection: First peak > 500 RFU                 Upper marker selection: Last peak > 200 RFU
Ladder size (bp)75, 200, 300, 400, 500, 700, 1000, 1500, 2000, 3000, 4000, 5000, 7000, 10000, 20000
Quantification using: Lower Marker          Final concentration (ng/uL): 0.5000          Dilution factor: 12.0
```

Sample: SampG3
Well location: G3
Created: Wednesday, October 20, 2021 5:58:44 PM

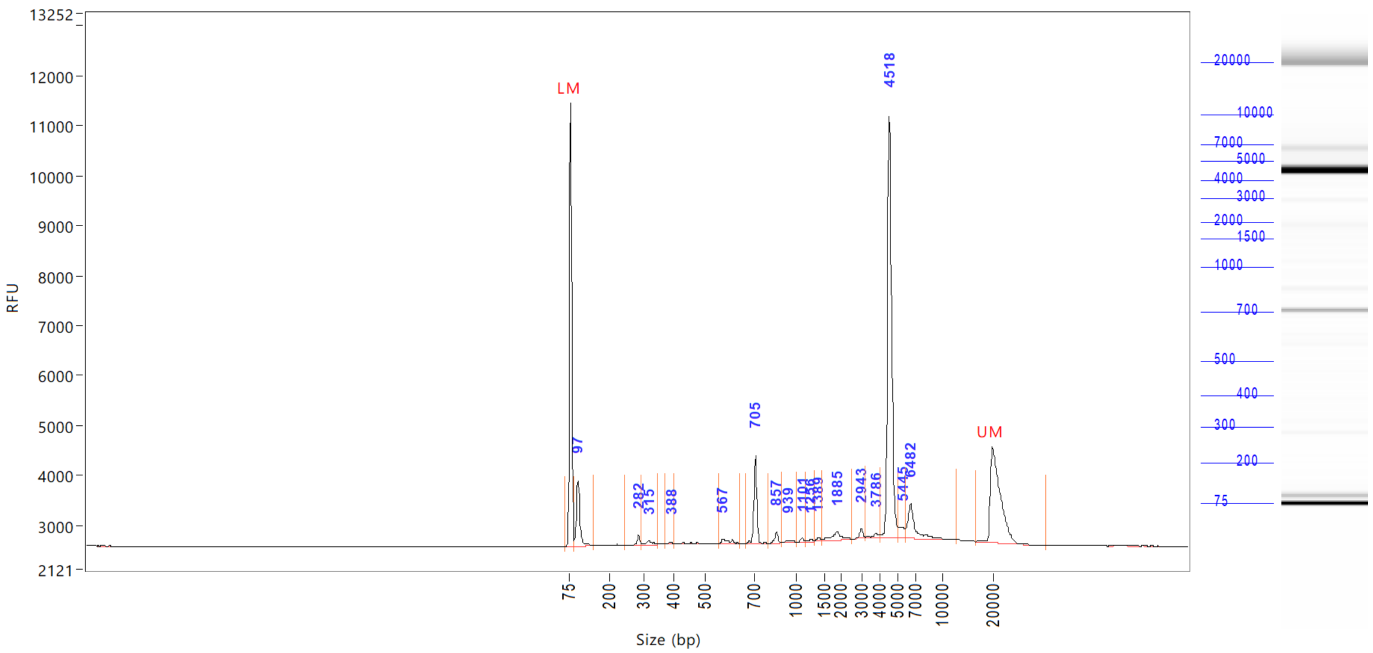

Peak	Size (bp)	Concentration (ng/uL)	From (bp)	To (bp)	Average size (bp)	CV%	RFU	Corrected peak area
1	75 (LM)	0.5000	58	86	73	4.20	8894	47.054
2	97	1.4580	86	150	98	9.08	1320	11.434
3	282	0.1433	242	293	278	3.14	189	1.124
4	315	0.1386	293	347	315	4.00	69	1.087
5	388	0.0462	372	398	384	1.43	53	0.362
6	567	0.2042	549	644	587	3.67	104	1.602
7	705	1.1724	667	801	705	1.72	1765	9.194
8	857	0.1726	801	896	849	1.59	227	1.354
9	939	0.0847	896	1000	948	2.34	52	0.664
10	1101	0.0653	1000	1167	1089	2.98	92	0.512
11	1256	0.0468	1167	1323	1246	3.10	51	0.367
12	1389	0.0615	1323	1456	1390	2.55	69	0.483
13	1885	0.3532	1456	2543	1842	11.64	178	2.770
14	2943	0.1193	2543	3144	2905	2.66	186	0.935
15	3786	0.0756	3144	4001	3732	5.32	72	0.593
16	4518	6.7220	4001	5001	4523	2.89	8428	52.716
17	5445	0.2579	5001	5889	5441	5.05	215	2.022
18	6482	0.9058	5889	12806	6951	14.71	705	7.104
19	20000 (UM)	0.2617	16586	30760	20795	5.73	1912	24.631

TIC:	12.0273	ng/uL
TIM:	33.3778	nmole/L
Total concentration:	12.2116	ng/uL

```
Sample peak width (sec): 5    Sample min peak height: 50    Sample baseline V to V?: Y    Sample baseline V to V points: 3
Sample filter: Binomial       Number of points for filter: 3  Sample start region (min): 0  Sample end region (min): 30
Marker peak width (sec): 5    Marker min peak height: 200   Marker baseline V to V?: Y    Marker baseline V to V points: 3
Lower marker selection: First peak > 500 RFU                Upper marker selection: Last peak > 200 RFU
Ladder size (bp)75, 200, 300, 400, 500, 700, 1000, 1500, 2000, 3000, 4000, 5000, 7000, 10000, 20000
Quantification using: Lower Marker          Final concentration (ng/uL): 0.5000          Dilution factor: 12.0
```

Sample: SampG4
Well location: G4
Created: Wednesday, October 20, 2021 5:58:44 PM

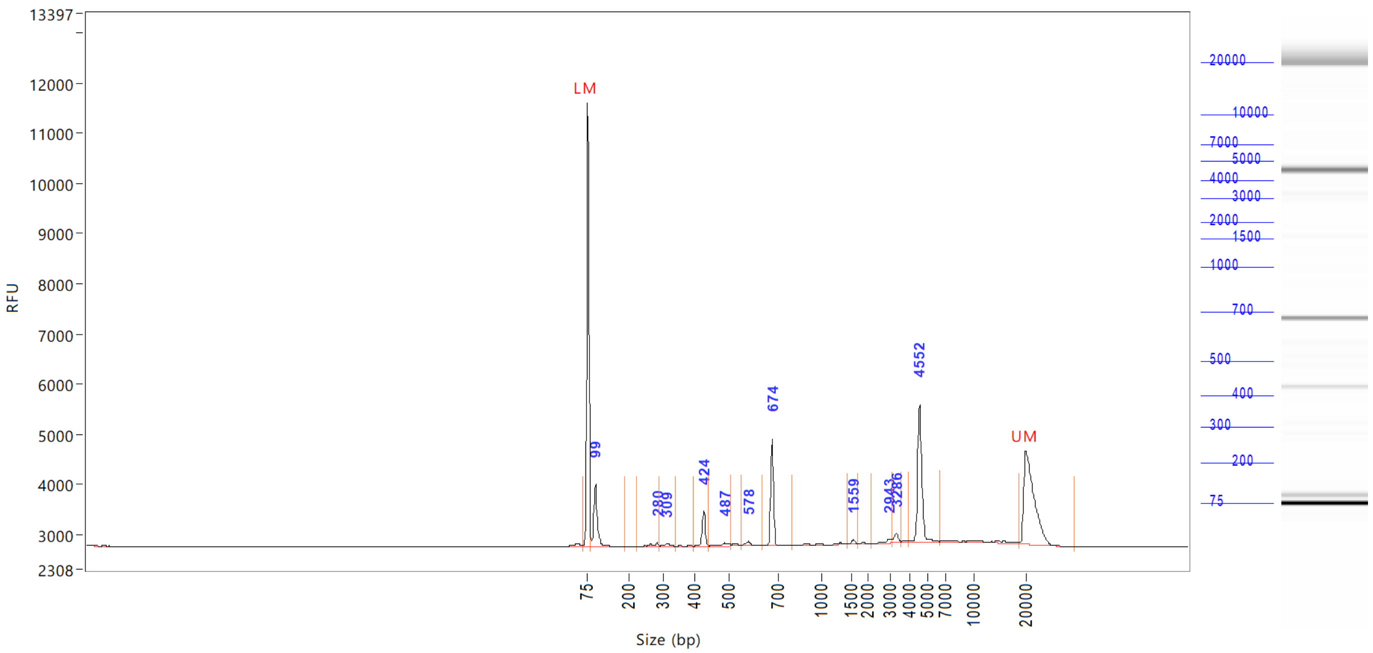

Peak	Size (bp)	Concentration (ng/uL)	From (bp)	To (bp)	Average size (bp)	CV%	RFU	Corrected peak area
1	75 (LM)	0.5000	62	86	73	4.17	8865	46.149
2	99	1.3407	86	189	99	10.25	1235	10.312
3	280	0.1058	221	287	267	5.05	81	0.814
4	309	0.0791	287	335	308	3.04	62	0.609
5	424	0.5177	394	441	421	1.25	710	3.982
6	487	0.1021	441	506	484	2.95	61	0.785
7	578	0.1087	549	634	574	2.36	86	0.836
8	674	1.4008	634	796	672	1.30	2132	10.774
9	1559	0.0619	1445	1712	1543	3.19	87	0.476
10	2943	0.0848	2115	3037	2844	6.88	85	0.652
11	3286	0.1557	3037	3572	3247	3.70	186	1.197
12	4552	2.1186	4001	6334	4544	3.83	2752	16.296
13	20000 (UM)	0.2565	18537	29309	20792	5.50	1863	23.671

TIC:	6.0759	ng/uL
TIM:	30.3860	nmole/L
Total concentration:	6.5665	ng/uL

```
Sample peak width (sec): 5     Sample min peak height: 50     Sample baseline V to V?: Y     Sample baseline V to V points: 3
Sample filter: Binomial        Number of points for filter: 3  Sample start region (min): 0  Sample end region (min): 30
Marker peak width (sec): 5     Marker min peak height: 200    Marker baseline V to V?: Y     Marker baseline V to V points: 3
Lower marker selection: First peak > 500 RFU                  Upper marker selection: Last peak > 200 RFU
Ladder size (bp)75, 200, 300, 400, 500, 700, 1000, 1500, 2000, 3000, 4000, 5000, 7000, 10000, 20000
Quantification using: Lower Marker          Final concentration (ng/uL): 0.5000          Dilution factor: 12.0
```

Sample: SampG5
Well location: G5
Created: Wednesday, October 20, 2021 5:58:44 PM

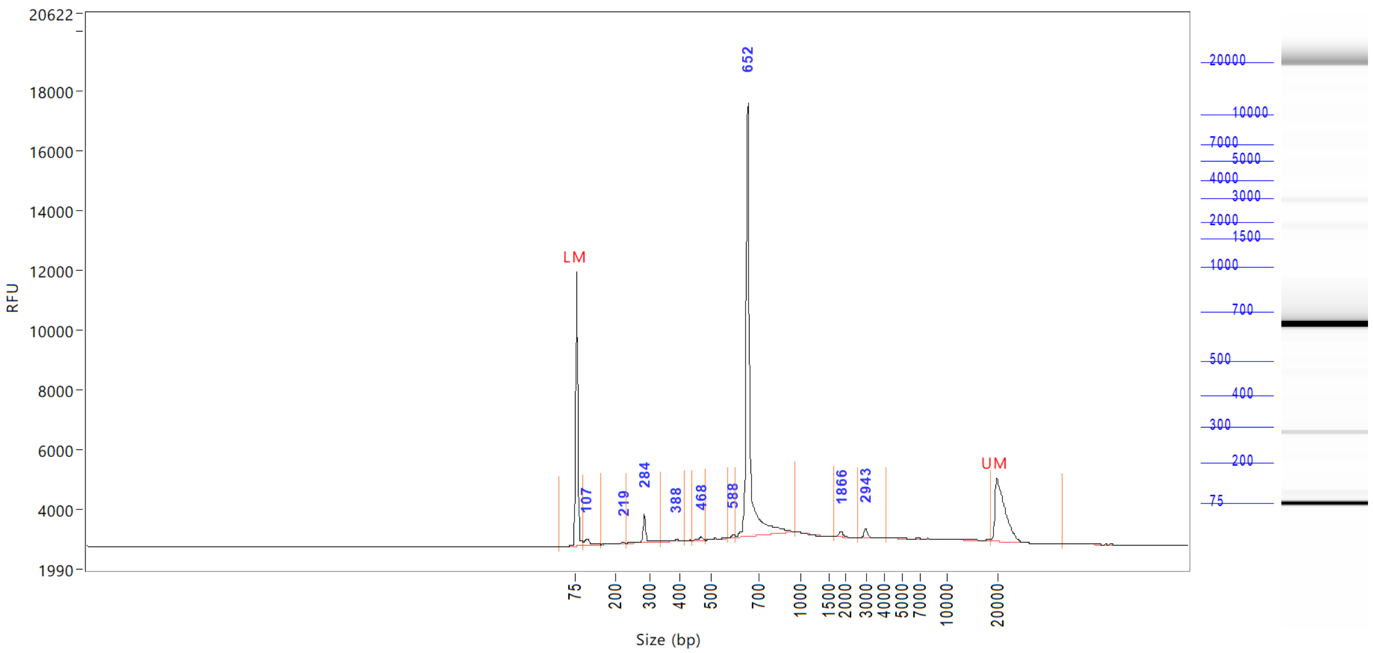

Peak	Size (bp)	Concentration (ng/uL)	From (bp)	To (bp)	Average size (bp)	CV%	RFU	Corrected peak area
1	75 (LM)	0.5000	24	95	73	5.83	9201	50.093
2	107	0.3284	95	152	108	10.42	220	2.741
3	219	0.0966	152	230	198	11.44	59	0.807
4	284	0.7942	230	337	283	5.36	968	6.630
5	388	0.1136	337	416	378	5.24	52	0.948
6	468	0.1140	437	483	460	2.04	112	0.952
7	588	0.0861	565	603	588	1.37	105	0.719
8	652	12.0510	603	957	664	6.59	14549	100.612
9	1866	0.1600	1655	2600	1857	5.65	188	1.335
10	2943	0.2253	2600	4070	2965	6.55	296	1.881
11	20000 (UM)	0.2670	18903	33179	20942	5.86	2101	26.748

TIC:	13.9691	ng/uL
TIM:	41.6899	nmole/L
Total concentration:	14.2759	ng/uL

```
Sample peak width (sec): 5    Sample min peak height: 50    Sample baseline V to V?: Y    Sample baseline V to V points: 3
Sample filter: Binomial       Number of points for filter: 3  Sample start region (min): 0  Sample end region (min): 30
Marker peak width (sec): 5    Marker min peak height: 200   Marker baseline V to V?: Y    Marker baseline V to V points: 3
Lower marker selection: First peak > 500 RFU                Upper marker selection: Last peak > 200 RFU
Ladder size (bp)75, 200, 300, 400, 500, 700, 1000, 1500, 2000, 3000, 4000, 5000, 7000, 10000, 20000
Quantification using: Lower Marker          Final concentration (ng/uL): 0.5000          Dilution factor: 12.0
```

Sample: SampG6
Well location: G6
Created: Wednesday, October 20, 2021 5:58:44 PM

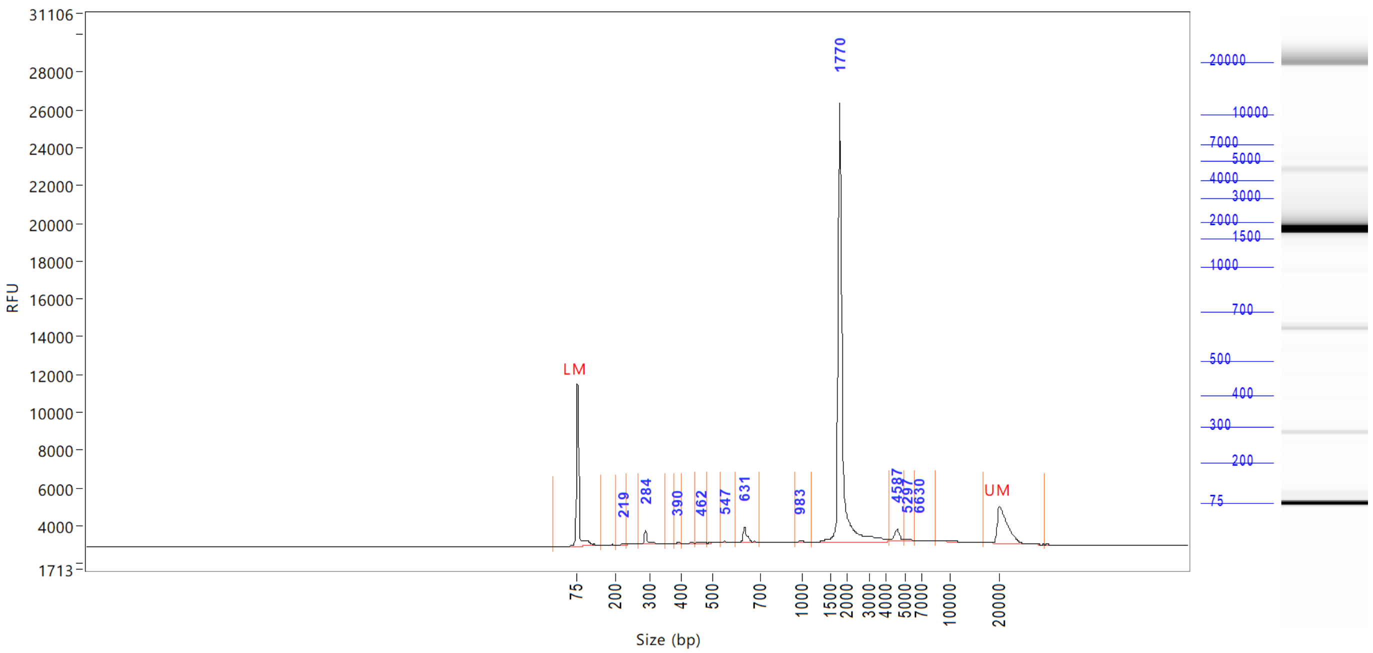

Peak	Size (bp)	Concentration (ng/uL)	From (bp)	To (bp)	Average size (bp)	CV%	RFU	Corrected peak area
1	75 (LM)	0.5000	0	150	77	13.32	8575	53.609
2	219	0.0412	196	226	214	3.32	50	0.368
3	284	0.5462	264	349	288	4.78	686	4.881
4	390	0.0355	376	400	389	1.13	62	0.318
5	462	0.0615	443	483	461	1.54	69	0.549
6	547	0.0862	532	595	554	2.70	97	0.770
7	631	0.7315	595	697	636	2.35	823	6.535
8	983	0.0917	948	1145	996	3.58	85	0.820
9	1770	17.8196	1145	4104	1869	18.94	23245	159.216
10	4587	0.6058	4104	4966	4530	4.19	640	5.413
11	5297	0.1527	4966	6186	5409	6.49	131	1.364
12	6630	0.0995	6186	8534	7013	7.73	59	0.889
13	20000 (UM)	0.2334	16830	29068	20859	5.67	1931	25.020

TIC:	20.2714	ng/uL
TIM:	22.0913	nmole/L
Total concentration:	20.5204	ng/uL

```
Sample peak width (sec): 5    Sample min peak height: 50    Sample baseline V to V?: Y    Sample baseline V to V points: 3
Sample filter: Binomial       Number of points for filter: 3  Sample start region (min): 0  Sample end region (min): 30
Marker peak width (sec): 5    Marker min peak height: 200   Marker baseline V to V?: Y    Marker baseline V to V points: 3
Lower marker selection: First peak > 500 RFU                Upper marker selection: Last peak > 200 RFU
Ladder size (bp)75, 200, 300, 400, 500, 700, 1000, 1500, 2000, 3000, 4000, 5000, 7000, 10000, 20000
Quantification using: Lower Marker          Final concentration (ng/uL): 0.5000          Dilution factor: 12.0
```

Sample: SampG7
Well location: G7
Created: Wednesday, October 20, 2021 5:58:44 PM

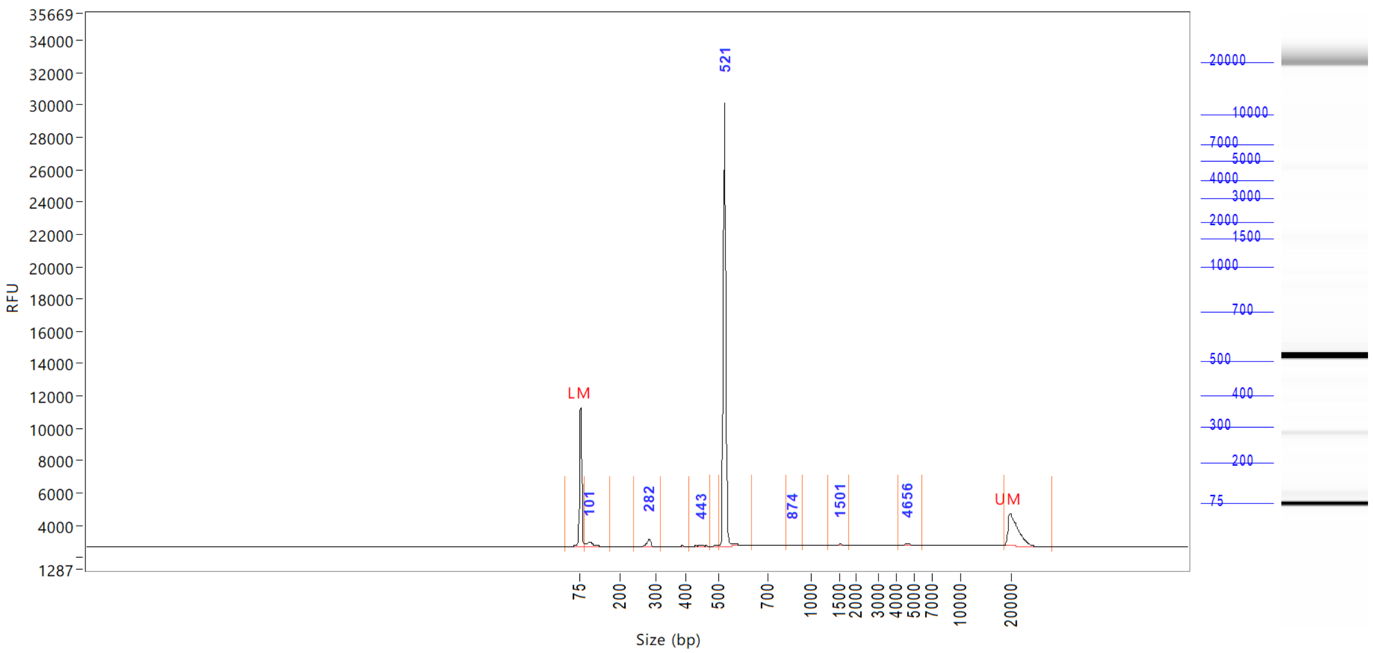

Peak	Size (bp)	Concentration (ng/uL)	From (bp)	To (bp)	Average size (bp)	CV%	RFU	Corrected peak area
1	75 (LM)	0.5000	26	86	72	5.55	8620	47.555
2	101	0.6217	86	168	105	15.09	273	4.927
3	282	0.4876	235	313	276	3.25	481	3.864
4	443	0.0869	407	473	440	2.46	73	0.689
5	521	16.8084	498	639	520	1.93	27451	133.222
6	874	0.0634	835	944	876	2.53	62	0.502
7	1501	0.1362	1289	1808	1501	6.58	96	1.079
8	4656	0.1098	4173	5963	4705	5.97	116	0.870
9	20000 (UM)	0.2617	18659	28221	20803	5.34	2013	24.891

TIC:	18.3139	ng/uL
TIM:	66.4514	nmole/L
Total concentration:	18.7984	ng/uL

```
Sample peak width (sec): 5    Sample min peak height: 50    Sample baseline V to V?: Y    Sample baseline V to V points: 3
Sample filter: Binomial       Number of points for filter: 3  Sample start region (min): 0  Sample end region (min): 30
Marker peak width (sec): 5    Marker min peak height: 200   Marker baseline V to V?: Y    Marker baseline V to V points: 3
Lower marker selection: First peak > 500 RFU                Upper marker selection: Last peak > 200 RFU
Ladder size (bp)75, 200, 300, 400, 500, 700, 1000, 1500, 2000, 3000, 4000, 5000, 7000, 10000, 20000
Quantification using: Lower Marker          Final concentration (ng/uL): 0.5000          Dilution factor: 12.0
```

Sample: SampG8
Well location: G8
Created: Wednesday, October 20, 2021 5:58:44 PM

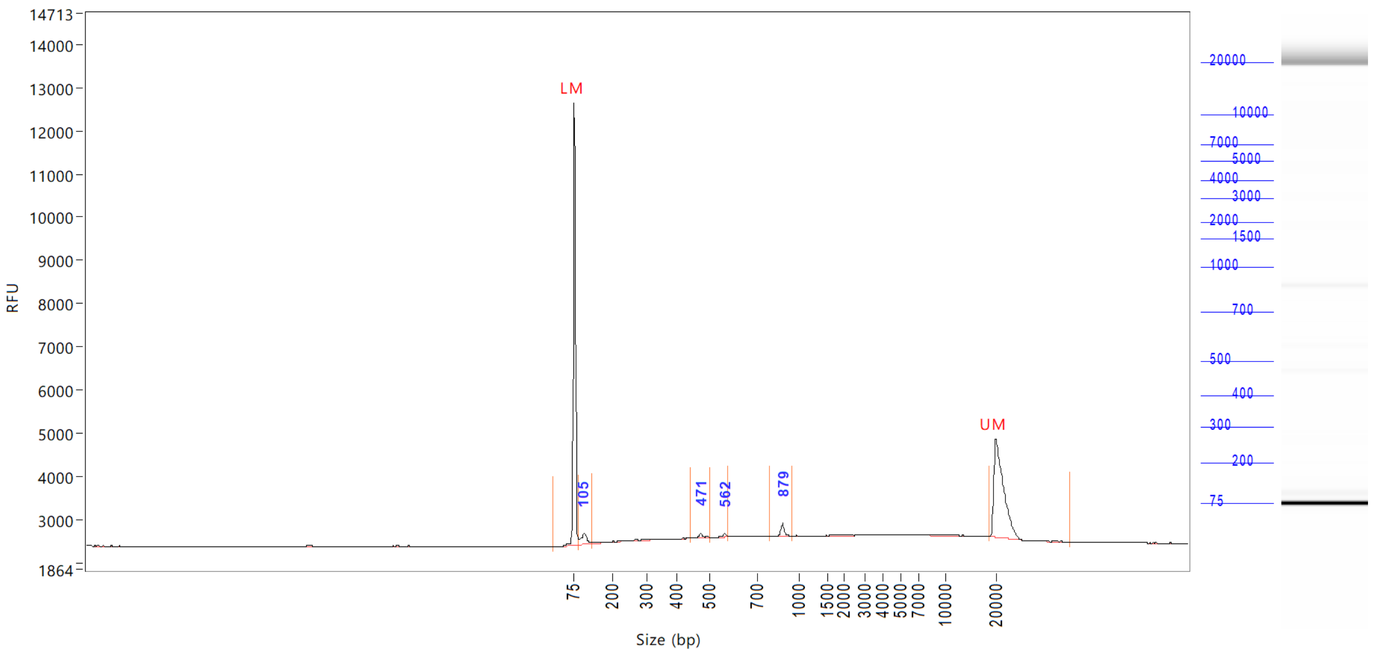

Peak	Size (bp)	Concentration (ng/uL)	From (bp)	To (bp)	Average size (bp)	CV%	RFU	Corrected peak area
1	75 (LM)	0.5000	12	89	73	5.30	10241	54.889
2	105	0.3894	89	131	104	8.84	249	3.563
3	471	0.1022	441	501	474	2.11	121	0.935
4	562	0.0605	501	578	555	2.83	83	0.553
5	879	0.1874	792	944	872	2.12	289	1.714
6	20000 (UM)	0.2568	18659	34871	20930	6.28	2268	28.190

TIC:	0.7395	ng/uL
TIM:	7.0375	nmole/L
Total concentration:	1.2049	ng/uL

Sample peak width (sec): 5   Sample min peak height: 50   Sample baseline V to V?: Y   Sample baseline V to V points: 3
Sample filter: Binomial   Number of points for filter: 3 Sample start region (min): 0 Sample end region (min): 30
Marker peak width (sec): 5   Marker min peak height: 200   Marker baseline V to V?: Y   Marker baseline V to V points: 3
Lower marker selection: First peak > 500 RFU   Upper marker selection: Last peak > 200 RFU
Ladder size (bp)75, 200, 300, 400, 500, 700, 1000, 1500, 2000, 3000, 4000, 5000, 7000, 10000, 20000
Quantification using: Lower Marker   Final concentration (ng/uL): 0.5000   Dilution factor: 12.0

Sample: SampG9
Well location: G9
Created: Wednesday, October 20, 2021 5:58:44 PM

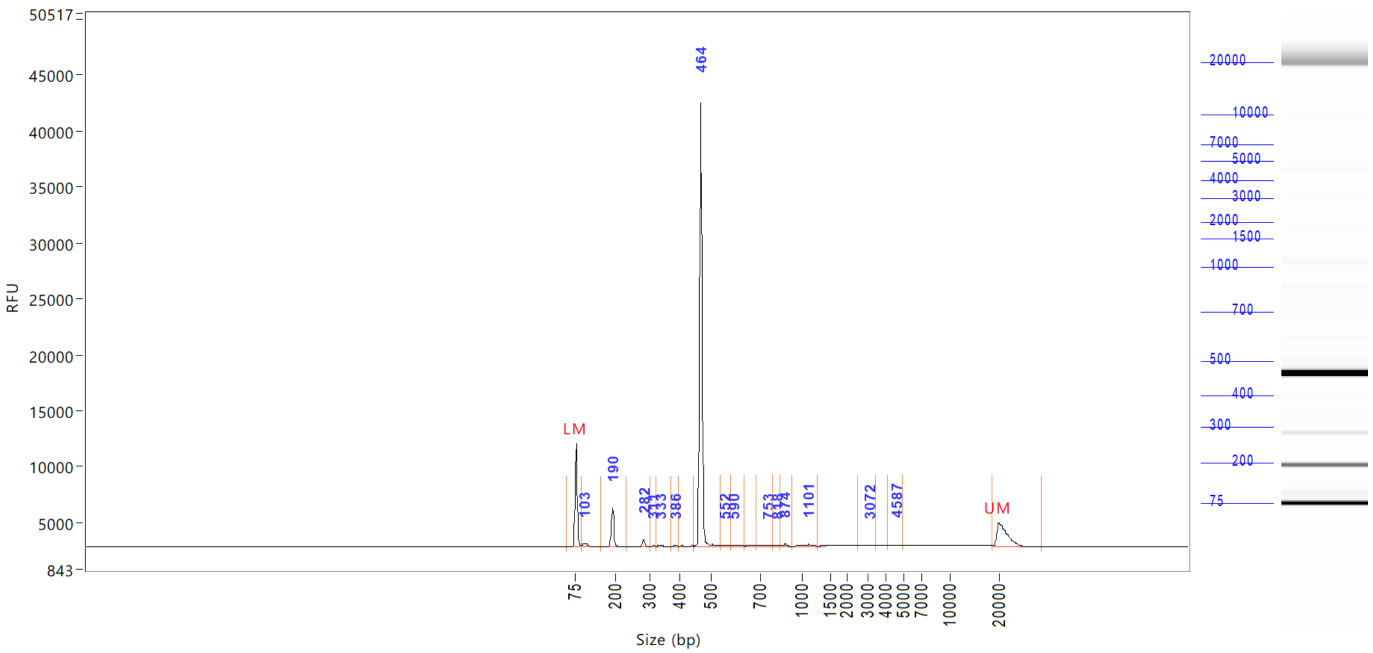

Peak	Size (bp)	Concentration (ng/uL)	From (bp)	To (bp)	Average size (bp)	CV%	RFU	Corrected peak area
1	75 (LM)	0.5000	47	91	74	4.92	9211	49.927
2	103	0.4537	91	154	107	12.19	249	3.775
3	190	2.2436	154	232	188	3.04	3411	18.669
4	282	0.4270	232	301	278	2.79	612	3.553
5	311	0.0531	301	319	310	1.41	82	0.442
6	333	0.0998	319	372	335	3.08	70	0.830
7	386	0.0437	372	396	384	1.06	79	0.363
8	464	23.2907	443	532	463	1.19	39674	193.804
9	552	0.0832	532	578	552	2.22	66	0.692
10	590	0.0826	578	636	592	2.02	107	0.687
11	753	0.1772	685	792	736	4.09	94	1.475
12	818	0.0717	792	844	815	1.73	76	0.596
13	874	0.1208	844	926	869	1.51	167	1.005
14	1101	0.1729	926	1278	1063	6.14	147	1.439
15	3072	0.0606	2515	3429	2993	5.98	57	0.504
16	4587	0.0735	4035	4862	4519	3.57	83	0.611
17	20000 (UM)	0.2639	18659	28463	20825	5.47	2058	26.352

TIC:	27.4541	ng/uL
TIM:	114.4176	nmole/L
Total concentration:	27.8858	ng/uL

Sample peak width (sec): 5 Sample min peak height: 50 Sample baseline V to V?: Y Sample baseline V to V points: 3
Sample filter: Binomial Number of points for filter: 3 Sample start region (min): 0 Sample end region (min): 30
Marker peak width (sec): 5 Marker min peak height: 200 Marker baseline V to V?: Y Marker baseline V to V points: 3
Lower marker selection: First peak > 500 RFU Upper marker selection: Last peak > 200 RFU
Ladder size (bp)75, 200, 300, 400, 500, 700, 1000, 1500, 2000, 3000, 4000, 5000, 7000, 10000, 20000
Quantification using: Lower Marker Final concentration (ng/uL): 0.5000 Dilution factor: 12.0

Sample: SampG10
Well location: G10
Created: Wednesday, October 20, 2021 5:58:44 PM

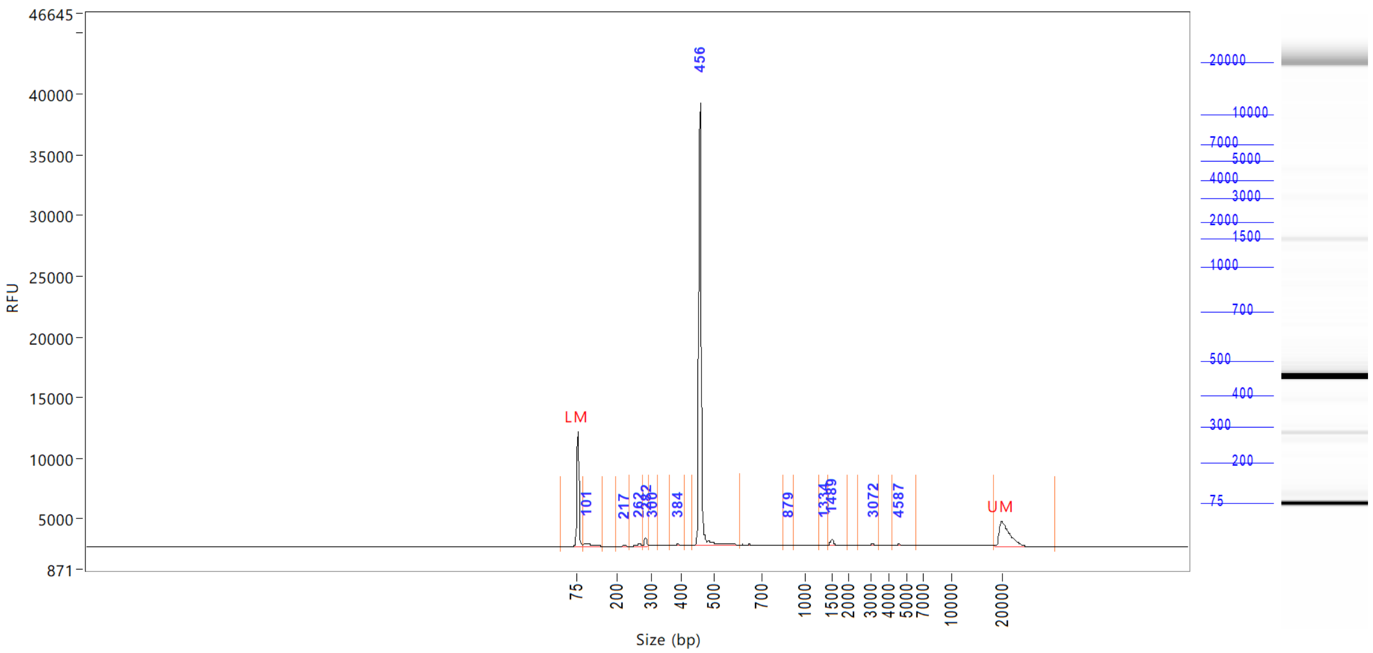

Peak	Size (bp)	Concentration (ng/uL)	From (bp)	To (bp)	Average size (bp)	CV%	RFU	Corrected peak area
1	75 (LM)	0.5000	22	89	73	4.99	9443	50.729
2	101	0.6182	89	150	108	13.27	294	5.227
3	217	0.0458	198	237	217	2.75	53	0.387
4	262	0.2172	237	273	262	2.55	177	1.837
5	282	0.4533	273	293	280	1.35	730	3.833
6	300	0.0853	293	319	301	1.99	109	0.721
7	384	0.1352	360	411	386	2.61	125	1.143
8	456	21.8261	433	608	456	2.41	36488	184.537
9	879	0.0522	853	918	878	1.57	61	0.441
10	1334	0.0552	1245	1411	1338	2.78	79	0.467
11	1489	0.3452	1411	1981	1502	4.94	476	2.919
12	3072	0.0987	2429	3429	2998	5.86	104	0.834
13	4587	0.0788	4173	6038	4602	5.34	86	0.667
14	20000 (UM)	0.2588	18293	30639	20798	5.64	2048	26.258

TIC:	24.0113	ng/uL
TIM:	94.2005	nmole/L
Total concentration:	24.4717	ng/uL

```
Sample peak width (sec): 5     Sample min peak height: 50     Sample baseline V to V?: Y     Sample baseline V to V points: 3
Sample filter: Binomial        Number of points for filter: 3  Sample start region (min): 0  Sample end region (min): 30
Marker peak width (sec): 5     Marker min peak height: 200    Marker baseline V to V?: Y     Marker baseline V to V points: 3
Lower marker selection: First peak > 500 RFU                  Upper marker selection: Last peak > 200 RFU
Ladder size (bp)75, 200, 300, 400, 500, 700, 1000, 1500, 2000, 3000, 4000, 5000, 7000, 10000, 20000
Quantification using: Lower Marker          Final concentration (ng/uL): 0.5000          Dilution factor: 12.0
```

Sample: SampG11
Well location: G11
Created: Wednesday, October 20, 2021 5:58:44 PM

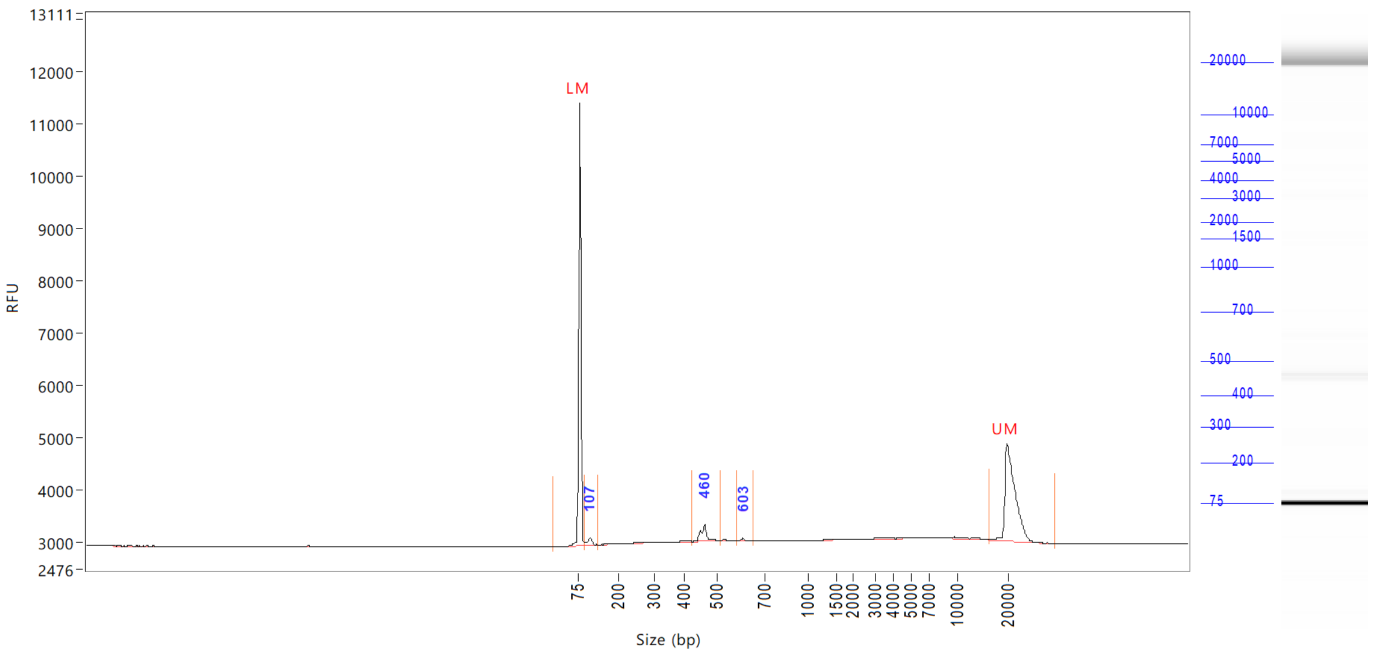

Peak	Size (bp)	Concentration (ng/uL)	From (bp)	To (bp)	Average size (bp)	CV%	RFU	Corrected peak area
1	75 (LM)	0.5000	0	91	73	6.08	8486	46.399
2	107	0.2062	91	133	104	7.28	135	1.595
3	460	0.4754	422	511	459	3.37	320	3.676
4	603	0.0601	580	649	606	2.54	53	0.465
5	20000 (UM)	0.2593	16464	29430	20780	5.86	1863	24.065

TIC:	0.7417	ng/uL
TIM:	5.1235	nmole/L
Total concentration:	1.3546	ng/uL

```
Sample peak width (sec): 5    Sample min peak height: 50    Sample baseline V to V?: Y    Sample baseline V to V points: 3
Sample filter: Binomial       Number of points for filter: 3  Sample start region (min): 0  Sample end region (min): 30
Marker peak width (sec): 5    Marker min peak height: 200   Marker baseline V to V?: Y    Marker baseline V to V points: 3
Lower marker selection: First peak > 500 RFU                Upper marker selection: Last peak > 200 RFU
Ladder size (bp)75, 200, 300, 400, 500, 700, 1000, 1500, 2000, 3000, 4000, 5000, 7000, 10000, 20000
Quantification using: Lower Marker          Final concentration (ng/uL): 0.5000          Dilution factor: 12.0
```

Sample: SampG12
Well location: G12
Created: Wednesday, October 20, 2021 5:58:44 PM

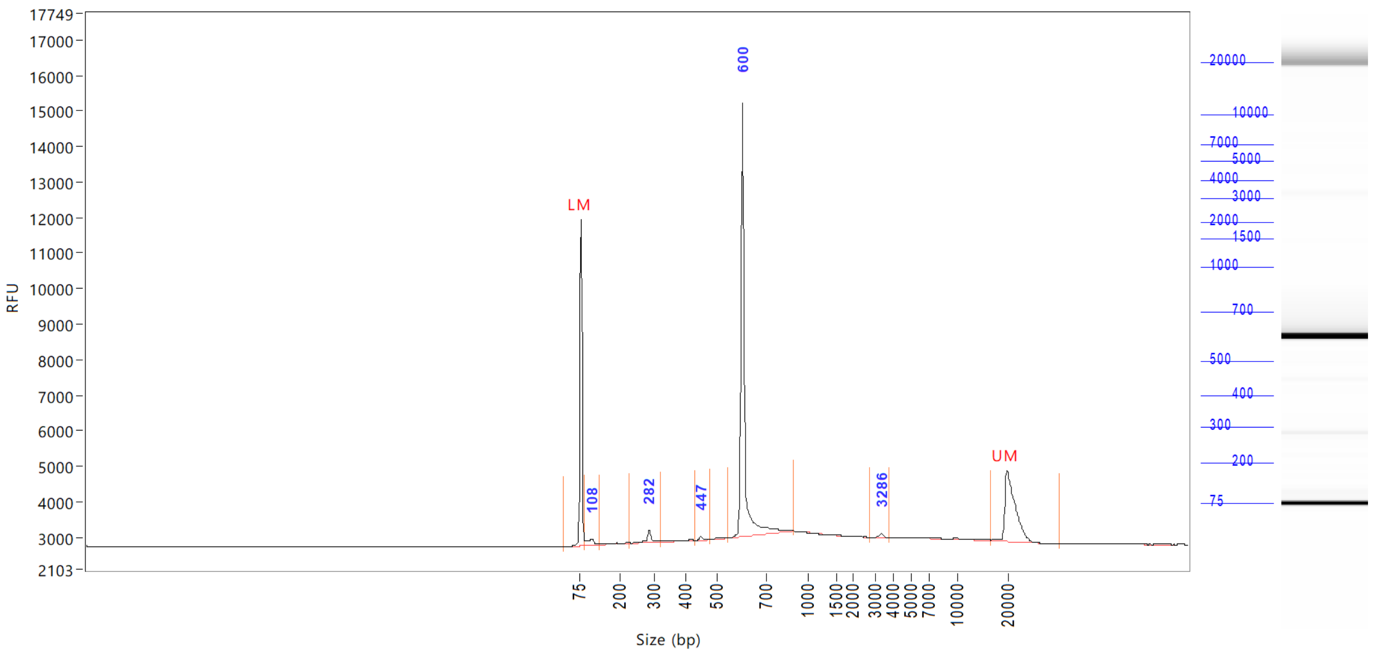

Peak	Size (bp)	Concentration (ng/uL)	From (bp)	To (bp)	Average size (bp)	CV%	RFU	Corrected peak area
1	75 (LM)	0.5000	24	87	73	5.00	9183	49.461
2	108	0.3803	87	133	104	10.03	176	3.135
3	282	0.3875	226	319	278	5.59	329	3.194
4	447	0.0921	431	477	447	1.54	118	0.759
5	600	9.5586	544	892	611	6.07	12228	78.796
6	3286	0.0942	2743	3715	3235	4.44	109	0.777
7	20000 (UM)	0.2653	16708	30398	20806	6.10	1991	26.240

TIC:	10.5127	ng/uL
TIM:	34.4296	nmole/L
Total concentration:	10.9983	ng/uL

Sample peak width (sec): 5   Sample min peak height: 50   Sample baseline V to V?: Y   Sample baseline V to V points: 3
Sample filter: Binomial   Number of points for filter: 3 Sample start region (min): 0 Sample end region (min): 30
Marker peak width (sec): 5   Marker min peak height: 200   Marker baseline V to V?: Y   Marker baseline V to V points: 3
Lower marker selection: First peak > 500 RFU   Upper marker selection: Last peak > 200 RFU
Ladder size (bp)75, 200, 300, 400, 500, 700, 1000, 1500, 2000, 3000, 4000, 5000, 7000, 10000, 20000
Quantification using: Lower Marker   Final concentration (ng/uL): 0.5000   Dilution factor: 12.0

Sample: SampH1
Well location: H1
Created: Wednesday, October 20, 2021 5:58:44 PM

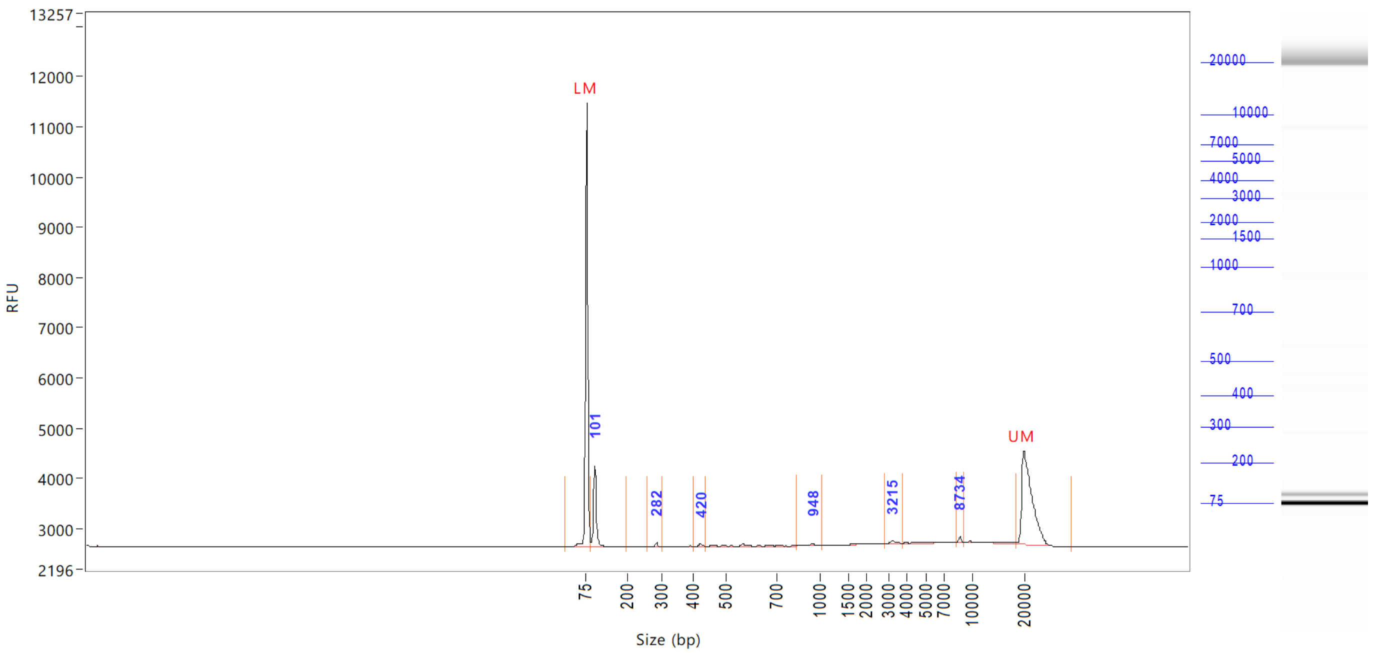

Peak	Size (bp)	Concentration (ng/uL)	From (bp)	To (bp)	Average size (bp)	CV%	RFU	Corrected peak area
1	75 (LM)	0.5000	14	87	73	6.41	8845	47.798
2	101	1.3953	87	198	101	10.15	1604	11.116
3	282	0.0525	257	298	278	2.39	72	0.418
4	420	0.0483	400	437	418	1.53	52	0.385
5	948	0.0511	840	1012	932	3.23	55	0.407
6	3215	0.0578	2857	3715	3214	5.62	50	0.461
7	8734	0.0490	8401	9067	8663	1.52	121	0.390
8	20000 (UM)	0.2447	18537	29189	20862	5.33	1855	23.389

TIC:	1.6540	ng/uL
TIM:	23.3158	nmole/L
Total concentration:	2.1814	ng/uL

```
Sample peak width (sec): 5    Sample min peak height: 50    Sample baseline V to V?: Y    Sample baseline V to V points: 3
Sample filter: Binomial       Number of points for filter: 3  Sample start region (min): 0  Sample end region (min): 30
Marker peak width (sec): 5    Marker min peak height: 200   Marker baseline V to V?: Y    Marker baseline V to V points: 3
Lower marker selection: First peak > 500 RFU                Upper marker selection: Last peak > 200 RFU
Ladder size (bp)75, 200, 300, 400, 500, 700, 1000, 1500, 2000, 3000, 4000, 5000, 7000, 10000, 20000
Quantification using: Lower Marker          Final concentration (ng/uL): 0.5000          Dilution factor: 12.0
```

Sample: SampH2
Well location: H2
Created: Wednesday, October 20, 2021 5:58:44 PM

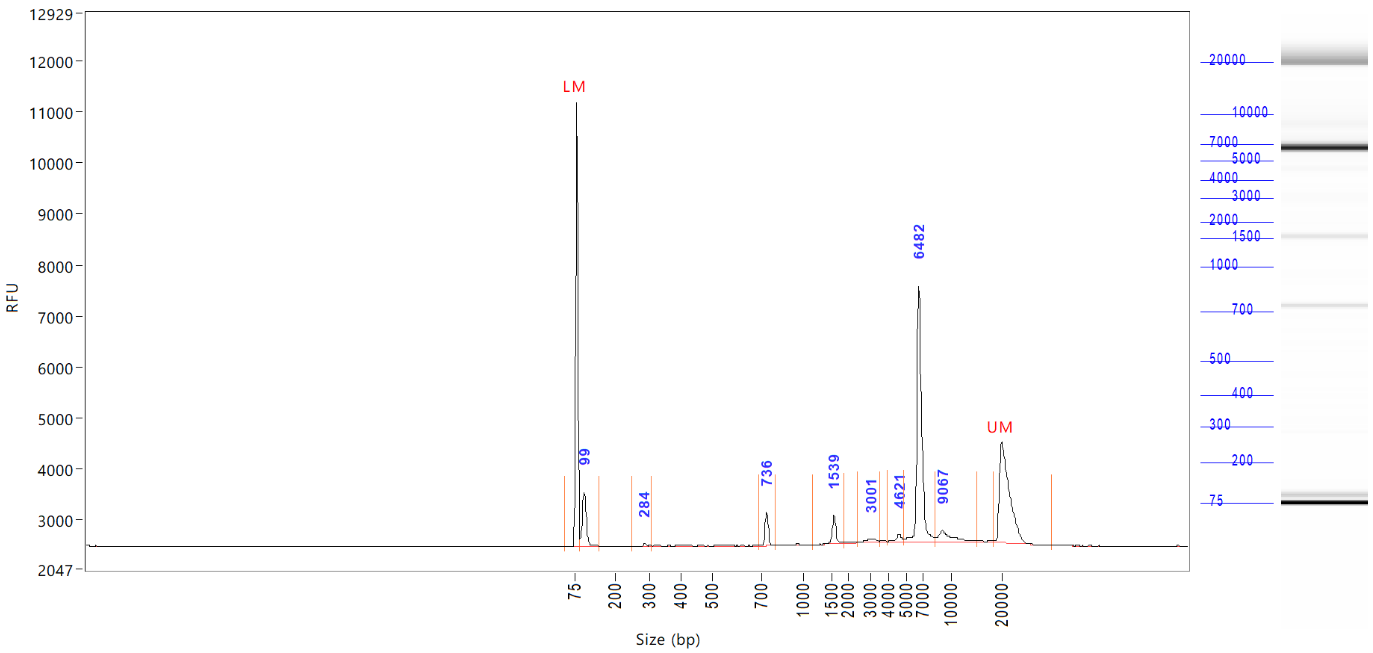

Peak	Size (bp)	Concentration (ng/uL)	From (bp)	To (bp)	Average size (bp)	CV%	RFU	Corrected peak area
1	75 (LM)	0.5000	39	86	74	4.27	8704	47.625
2	99	1.2701	86	148	98	8.73	1067	10.082
3	284	0.0611	246	305	281	4.33	57	0.485
4	736	0.4336	695	805	733	1.61	659	3.442
5	1539	0.4331	1167	1866	1540	5.27	555	3.438
6	3001	0.1243	2429	3536	3005	7.30	70	0.986
7	4621	0.1260	4000	4862	4556	3.73	136	1.000
8	6482	4.2025	4862	8267	6511	6.17	4983	33.358
9	9067	0.4781	8267	15122	10014	15.94	213	3.795
10	20000 (UM)	0.2606	18537	29793	20873	5.70	1977	24.820

TIC:	7.1288	ng/uL
TIM:	24.3300	nmole/L
Total concentration:	7.5025	ng/uL

Sample peak width (sec): 5 Sample min peak height: 50 Sample baseline V to V?: Y Sample baseline V to V points: 3
Sample filter: Binomial Number of points for filter: 3 Sample start region (min): 0 Sample end region (min): 30
Marker peak width (sec): 5 Marker min peak height: 200 Marker baseline V to V?: Y Marker baseline V to V points: 3
Lower marker selection: First peak > 500 RFU Upper marker selection: Last peak > 200 RFU
Ladder size (bp)75, 200, 300, 400, 500, 700, 1000, 1500, 2000, 3000, 4000, 5000, 7000, 10000, 20000
Quantification using: Lower Marker Final concentration (ng/uL): 0.5000 Dilution factor: 12.0

Sample: SampH3
Well location: H3
Created: Wednesday, October 20, 2021 5:58:44 PM

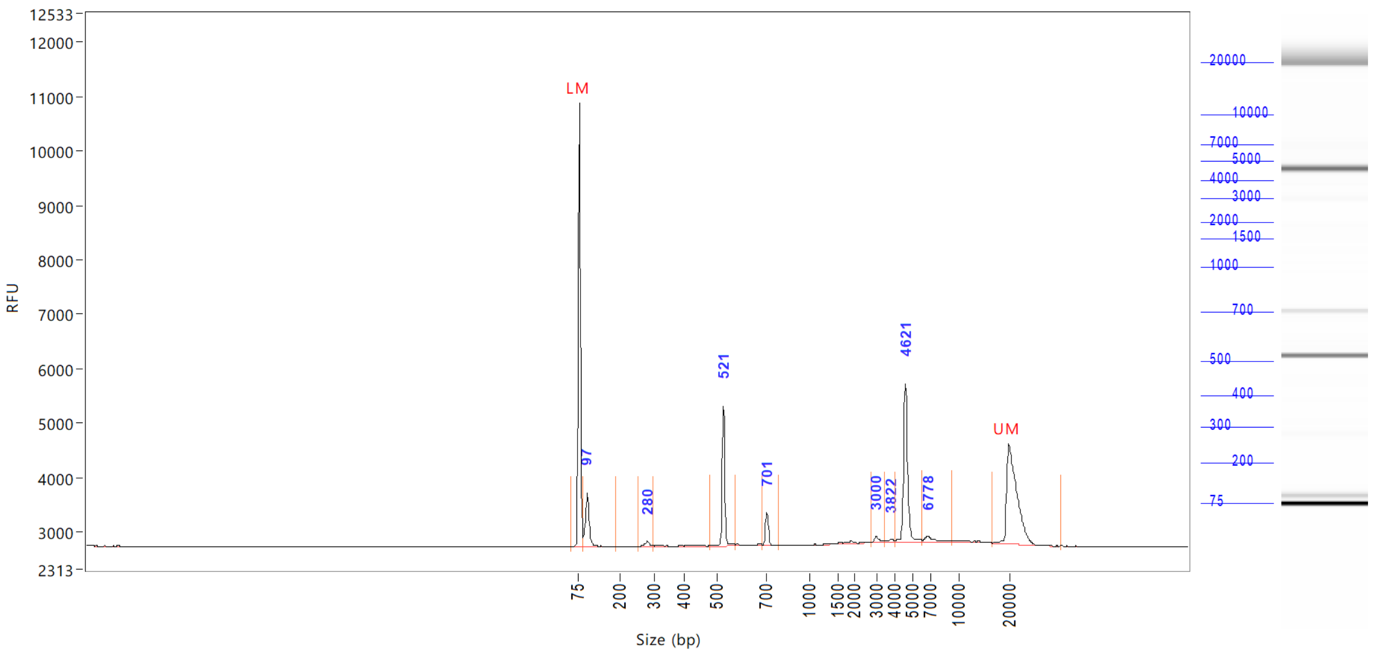

Peak	Size (bp)	Concentration (ng/uL)	From (bp)	To (bp)	Average size (bp)	CV%	RFU	Corrected peak area
1	75 (LM)	0.5000	50	86	72	4.67	8172	44.737
2	97	1.1706	86	189	98	9.86	993	8.728
3	280	0.1198	255	298	277	2.96	91	0.893
4	521	1.7249	477	572	520	1.70	2569	12.861
5	701	0.4185	685	788	701	1.36	612	3.120
6	3000	0.1185	2743	3500	3058	5.53	106	0.884
7	3822	0.0635	3500	4001	3789	3.81	53	0.473
8	4621	2.3203	4001	6112	4609	4.36	2914	17.300
9	6778	0.1813	6112	9334	7261	11.17	94	1.352
10	20000 (UM)	0.2647	16708	30156	20782	5.82	1838	23.688

TIC:	6.1173	ng/uL
TIM:	27.7264	nmole/L
Total concentration:	6.6022	ng/uL

```
Sample peak width (sec): 5     Sample min peak height: 50     Sample baseline V to V?: Y     Sample baseline V to V points: 3
Sample filter: Binomial        Number of points for filter: 3  Sample start region (min): 0  Sample end region (min): 30
Marker peak width (sec): 5     Marker min peak height: 200    Marker baseline V to V?: Y     Marker baseline V to V points: 3
Lower marker selection: First peak > 500 RFU                  Upper marker selection: Last peak > 200 RFU
Ladder size (bp)75, 200, 300, 400, 500, 700, 1000, 1500, 2000, 3000, 4000, 5000, 7000, 10000, 20000
Quantification using: Lower Marker          Final concentration (ng/uL): 0.5000          Dilution factor: 12.0
```

Sample: SampH4
Well location: H4
Created: Wednesday, October 20, 2021 5:58:44 PM

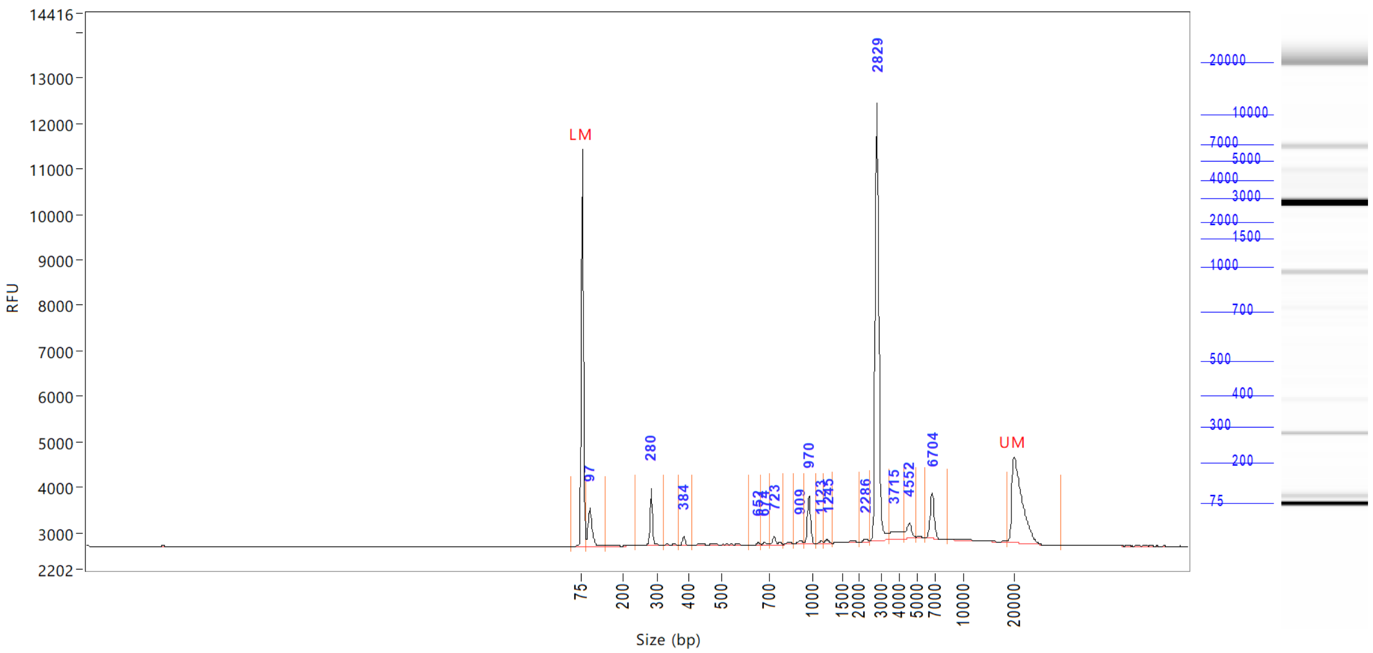

Peak	Size (bp)	Concentration (ng/uL)	From (bp)	To (bp)	Average size (bp)	CV%	RFU	Corrected peak area
1	75 (LM)	0.5000	39	84	72	4.67	8729	46.969
2	97	1.0940	84	148	97	10.20	842	8.564
3	280	0.8726	235	319	279	3.02	1263	6.831
4	384	0.1566	370	409	383	1.32	212	1.226
5	652	0.0380	611	664	649	1.26	56	0.298
6	674	0.0465	664	695	676	1.08	62	0.364
7	723	0.1609	695	788	728	2.80	170	1.260
8	909	0.0726	861	931	901	1.81	69	0.568
9	970	0.7189	931	1045	965	1.18	1055	5.627
10	1123	0.0358	1045	1167	1121	2.55	50	0.280
11	1245	0.0668	1167	1334	1225	2.66	75	0.523
12	2286	0.0609	2001	2515	2292	4.96	64	0.477
13	2829	6.8781	2515	3429	2817	3.70	9637	53.842
14	3715	0.3897	3429	4277	3851	6.34	180	3.051
15	4552	0.3355	4277	4931	4515	3.05	333	2.626
16	6704	0.7163	5815	8334	6670	3.53	985	5.607
17	20000 (UM)	0.2535	18537	29068	20882	5.58	1897	23.817

TIC:	11.6433	ng/uL
TIM:	30.9394	nmole/L
Total concentration:	12.2260	ng/uL

Sample peak width (sec): 5 Sample min peak height: 50 Sample baseline V to V?: Y Sample baseline V to V points: 3
Sample filter: Binomial Number of points for filter: 3 Sample start region (min): 0 Sample end region (min): 30
Marker peak width (sec): 5 Marker min peak height: 200 Marker baseline V to V?: Y Marker baseline V to V points: 3
Lower marker selection: First peak > 500 RFU Upper marker selection: Last peak > 200 RFU
Ladder size (bp)75, 200, 300, 400, 500, 700, 1000, 1500, 2000, 3000, 4000, 5000, 7000, 10000, 20000
Quantification using: Lower Marker Final concentration (ng/uL): 0.5000 Dilution factor: 12.0

Sample: SampH5
Well location: H5
Created: Wednesday, October 20, 2021 5:58:44 PM

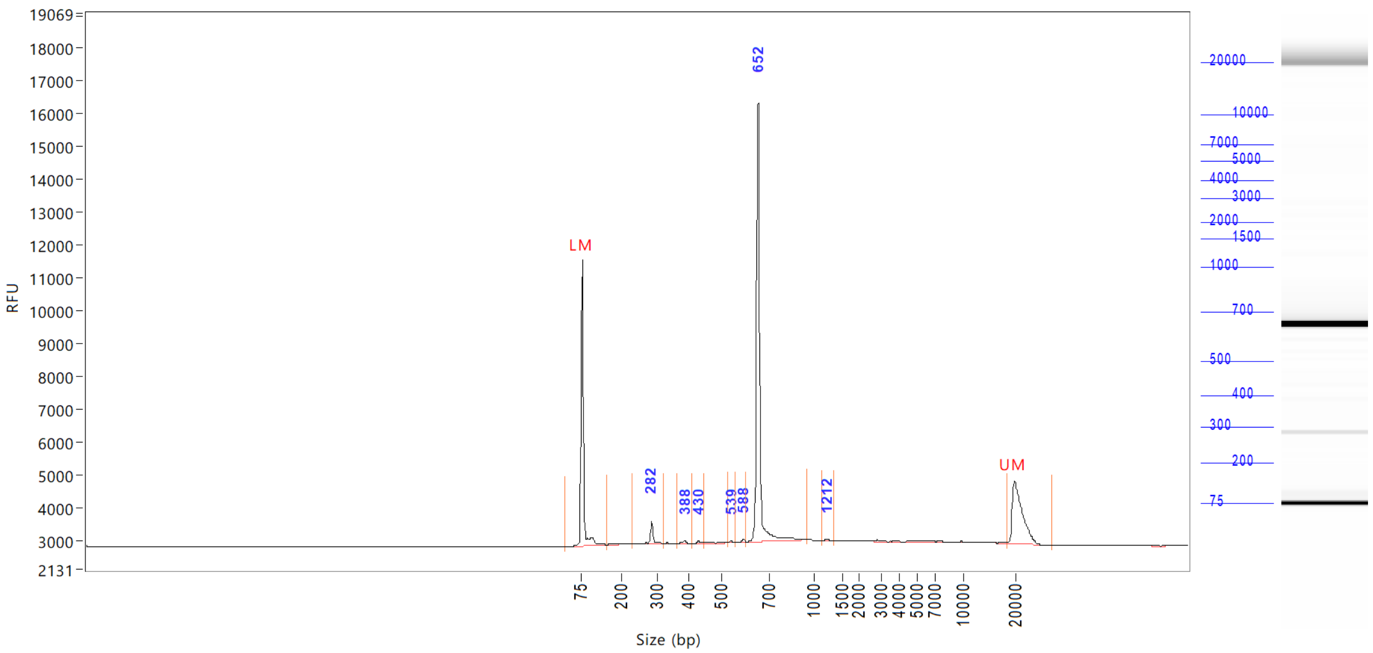

Peak	Size (bp)	Concentration (ng/uL)	From (bp)	To (bp)	Average size (bp)	CV%	RFU	Corrected peak area
1	75 (LM)	0.5000	22	152	76	12.84	8714	51.622
2	282	0.5174	228	321	280	4.41	704	4.452
3	388	0.0806	364	411	384	2.22	87	0.693
4	430	0.0497	411	447	430	1.62	58	0.427
5	539	0.0367	524	555	536	1.02	57	0.316
6	588	0.0684	555	603	585	1.27	101	0.588
7	652	9.1703	603	961	655	4.91	13391	78.898
8	1212	0.0411	1156	1367	1225	2.90	55	0.354
9	20000 (UM)	0.2285	18659	27133	20806	5.36	1904	23.586

TIC:	9.9642	ng/uL
TIM:	26.9757	nmole/L
Total concentration:	10.4631	ng/uL

```
Sample peak width (sec): 5    Sample min peak height: 50    Sample baseline V to V?: Y    Sample baseline V to V points: 3
Sample filter: Binomial       Number of points for filter: 3  Sample start region (min): 0  Sample end region (min): 30
Marker peak width (sec): 5    Marker min peak height: 200   Marker baseline V to V?: Y    Marker baseline V to V points: 3
Lower marker selection: First peak > 500 RFU                Upper marker selection: Last peak > 200 RFU
Ladder size (bp)75, 200, 300, 400, 500, 700, 1000, 1500, 2000, 3000, 4000, 5000, 7000, 10000, 20000
Quantification using: Lower Marker          Final concentration (ng/uL): 0.5000          Dilution factor: 12.0
```

Sample: SampH6
Well location: H6
Created: Wednesday, October 20, 2021 5:58:44 PM

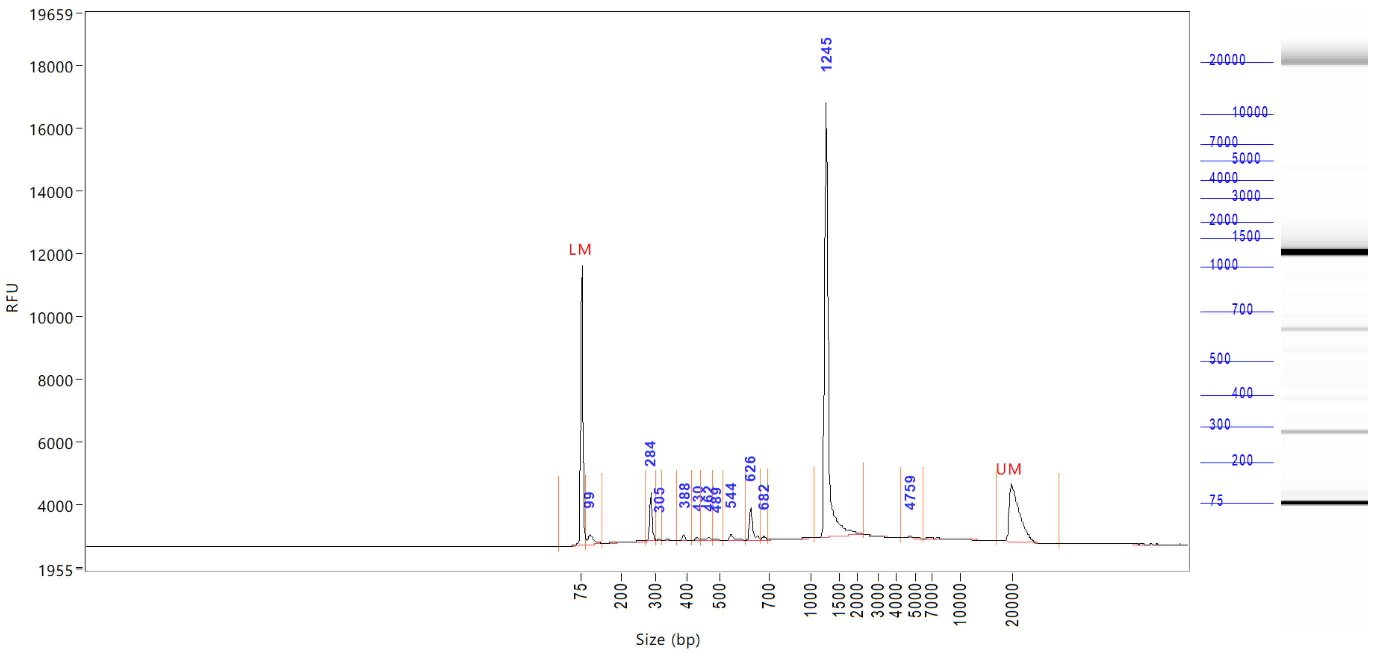

Peak	Size (bp)	Concentration (ng/uL)	From (bp)	To (bp)	Average size (bp)	CV%	RFU	Corrected peak area
1	75 (LM)	0.5000	7	89	73	5.60	8907	47.937
2	99	0.5494	89	139	104	10.57	318	4.389
3	284	1.0220	269	298	281	1.37	1540	8.166
4	305	0.0559	298	315	304	1.42	67	0.446
5	388	0.1245	366	414	386	1.59	173	0.994
6	430	0.0707	414	439	428	1.17	95	0.565
7	462	0.1269	439	479	460	2.15	91	1.014
8	489	0.0510	479	511	488	1.17	51	0.407
9	544	0.1961	511	603	549	2.79	184	1.567
10	626	0.7723	603	669	629	1.79	1017	6.170
11	682	0.0875	669	700	681	1.09	104	0.699
12	1245	10.8332	1034	2315	1277	8.95	13856	86.552
13	4759	0.0656	4242	6038	4751	5.44	65	0.524
14	20000 (UM)	0.2424	16952	29551	20848	5.74	1842	23.239

TIC:	13.9549	ng/uL
TIM:	33.1938	nmole/L
Total concentration:	14.2935	ng/uL

```
Sample peak width (sec): 5    Sample min peak height: 50    Sample baseline V to V?: Y    Sample baseline V to V points: 3
Sample filter: Binomial       Number of points for filter: 3  Sample start region (min): 0  Sample end region (min): 30
Marker peak width (sec): 5    Marker min peak height: 200   Marker baseline V to V?: Y    Marker baseline V to V points: 3
Lower marker selection: First peak > 500 RFU                Upper marker selection: Last peak > 200 RFU
Ladder size (bp)75, 200, 300, 400, 500, 700, 1000, 1500, 2000, 3000, 4000, 5000, 7000, 10000, 20000
Quantification using: Lower Marker          Final concentration (ng/uL): 0.5000          Dilution factor: 12.0
```

Sample: SampH7
Well location: H7
Created: Wednesday, October 20, 2021 5:58:44 PM

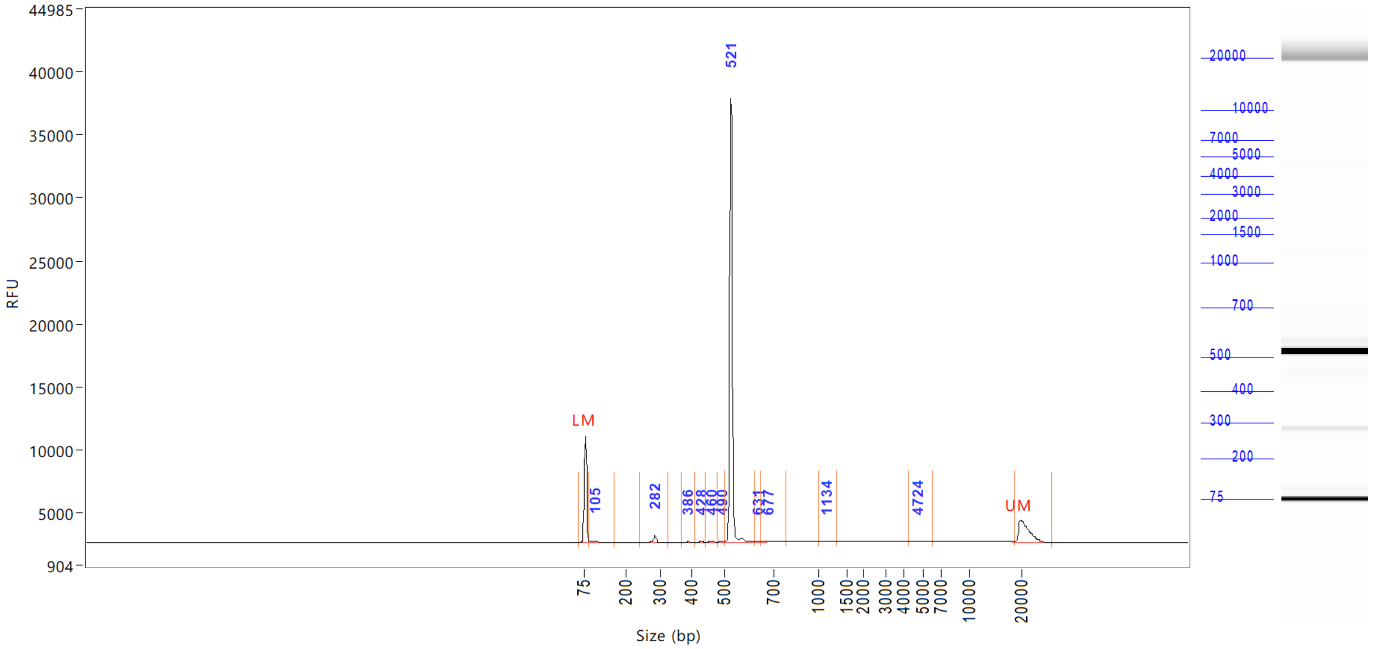

Peak	Size (bp)	Concentration (ng/uL)	From (bp)	To (bp)	Average size (bp)	CV%	RFU	Corrected peak area
1	75 (LM)	0.5000	58	89	74	4.51	8387	45.483
2	105	0.3733	89	166	107	13.22	174	2.830
3	282	0.4979	237	321	277	3.23	550	3.774
4	386	0.0467	368	409	385	1.23	72	0.354
5	428	0.0707	409	439	425	0.84	106	0.536
6	460	0.1351	439	475	456	1.32	117	1.024
7	490	0.0490	475	498	488	1.06	56	0.372
8	521	23.0011	498	618	521	1.80	35203	174.360
9	631	0.0686	618	644	631	1.23	60	0.520
10	677	0.1922	644	783	683	3.83	56	1.457
11	1134	0.0764	1001	1311	1155	5.79	67	0.579
12	4724	0.0869	4208	6186	4762	6.88	64	0.659
13	20000 (UM)	0.2448	18781	26045	20858	5.27	1778	22.266

TIC:	24.5980	ng/uL
TIM:	83.2408	nmole/L
Total concentration:	25.0034	ng/uL

Sample peak width (sec): 5 Sample min peak height: 50 Sample baseline V to V?: Y Sample baseline V to V points: 3
Sample filter: Binomial Number of points for filter: 3 Sample start region (min): 0 Sample end region (min): 30
Marker peak width (sec): 5 Marker min peak height: 200 Marker baseline V to V?: Y Marker baseline V to V points: 3
Lower marker selection: First peak > 500 RFU Upper marker selection: Last peak > 200 RFU
Ladder size (bp)75, 200, 300, 400, 500, 700, 1000, 1500, 2000, 3000, 4000, 5000, 7000, 10000, 20000
Quantification using: Lower Marker Final concentration (ng/uL): 0.5000 Dilution factor: 12.0

Sample: SampH8
Well location: H8
Created: Wednesday, October 20, 2021 5:58:44 PM

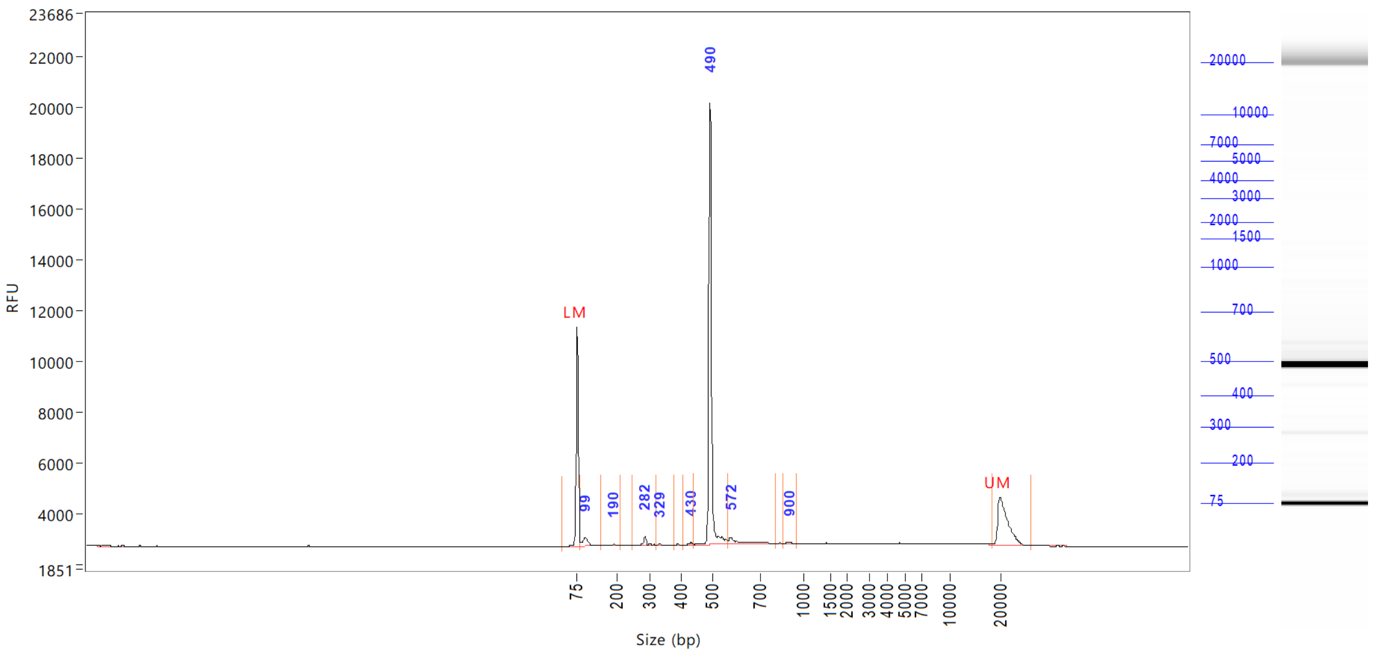

Peak	Size (bp)	Concentration (ng/uL)	From (bp)	To (bp)	Average size (bp)	CV%	RFU	Corrected peak area
1	75 (LM)	0.5000	28	86	73	4.46	8610	45.331
2	99	0.6241	86	148	103	12.31	340	4.715
3	190	0.0920	148	208	181	8.31	66	0.695
4	282	0.3110	244	319	280	3.77	340	2.350
5	329	0.0598	319	378	337	4.53	57	0.452
6	430	0.0738	407	439	427	1.10	108	0.558
7	490	12.2439	439	560	490	2.20	17373	92.505
8	572	0.7843	560	801	627	9.61	277	5.925
9	900	0.0804	857	948	891	1.99	72	0.607
10	20000 (UM)	0.2540	18415	26408	20842	5.38	1855	23.024

TIC:	14.2692	ng/uL
TIM:	56.5126	nmole/L
Total concentration:	14.7626	ng/uL

```
Sample peak width (sec): 5    Sample min peak height: 50    Sample baseline V to V?: Y    Sample baseline V to V points: 3
Sample filter: Binomial       Number of points for filter: 3  Sample start region (min): 0  Sample end region (min): 30
Marker peak width (sec): 5    Marker min peak height: 200   Marker baseline V to V?: Y    Marker baseline V to V points: 3
Lower marker selection: First peak > 500 RFU                Upper marker selection: Last peak > 200 RFU
Ladder size (bp)75, 200, 300, 400, 500, 700, 1000, 1500, 2000, 3000, 4000, 5000, 7000, 10000, 20000
Quantification using: Lower Marker          Final concentration (ng/uL): 0.5000          Dilution factor: 12.0
```

Sample: SampH9
Well location: H9
Created: Wednesday, October 20, 2021 5:58:44 PM

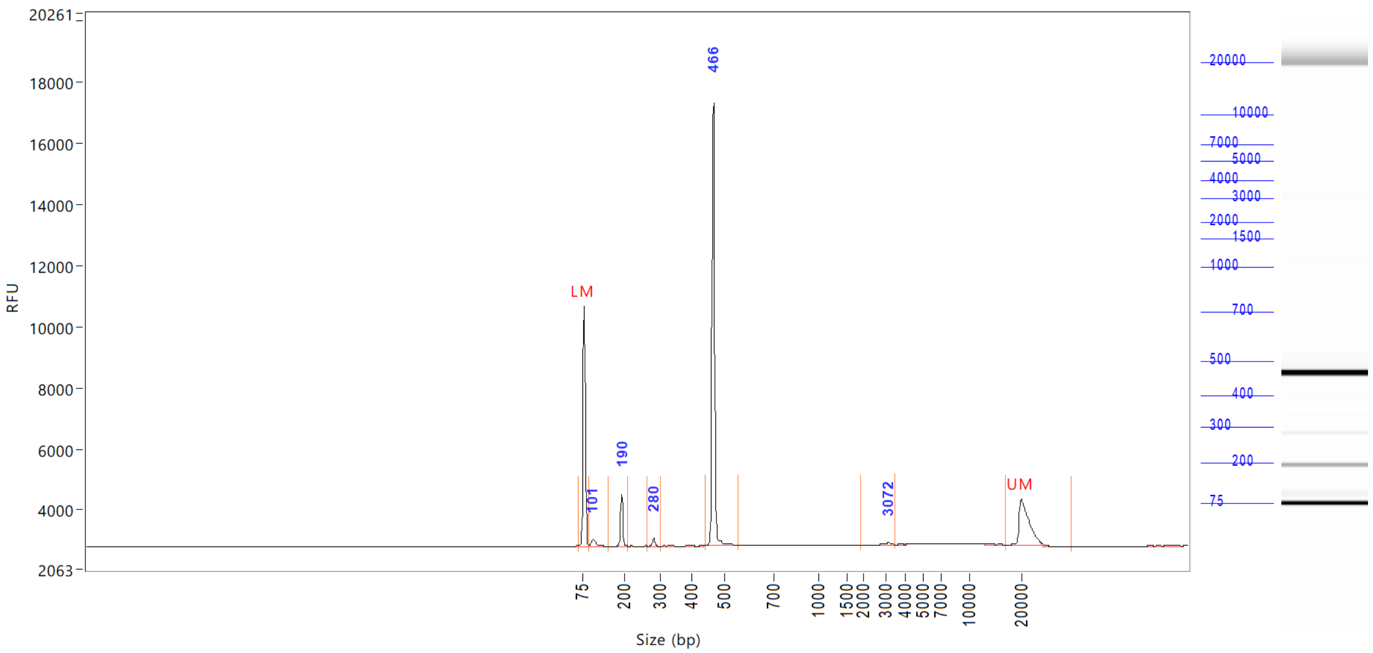

Peak	Size (bp)	Concentration (ng/uL)	From (bp)	To (bp)	Average size (bp)	CV%	RFU	Corrected peak area
1	75 (LM)	0.5000	60	89	74	4.34	7853	41.769
2	101	0.4262	89	148	104	9.50	243	2.967
3	190	1.2994	148	208	188	2.41	1709	9.046
4	280	0.2090	266	303	279	1.51	277	1.455
5	466	10.6578	443	555	464	1.56	14521	74.194
6	3072	0.1039	1923	3536	2877	12.63	65	0.723
7	20000 (UM)	0.2283	17073	29672	20822	5.73	1518	19.068

TIC:	12.6963	ng/uL
TIM:	57.1800	nmole/L
Total concentration:	13.3194	ng/uL

```
Sample peak width (sec): 5    Sample min peak height: 50    Sample baseline V to V?: Y    Sample baseline V to V points: 3
Sample filter: Binomial       Number of points for filter: 3  Sample start region (min): 0  Sample end region (min): 30
Marker peak width (sec): 5    Marker min peak height: 200   Marker baseline V to V?: Y    Marker baseline V to V points: 3
Lower marker selection: First peak > 500 RFU                Upper marker selection: Last peak > 200 RFU
Ladder size (bp)75, 200, 300, 400, 500, 700, 1000, 1500, 2000, 3000, 4000, 5000, 7000, 10000, 20000
Quantification using: Lower Marker          Final concentration (ng/uL): 0.5000          Dilution factor: 12.0
```

Sample: SampH10
Well location: H10
Created: Wednesday, October 20, 2021 5:58:44 PM

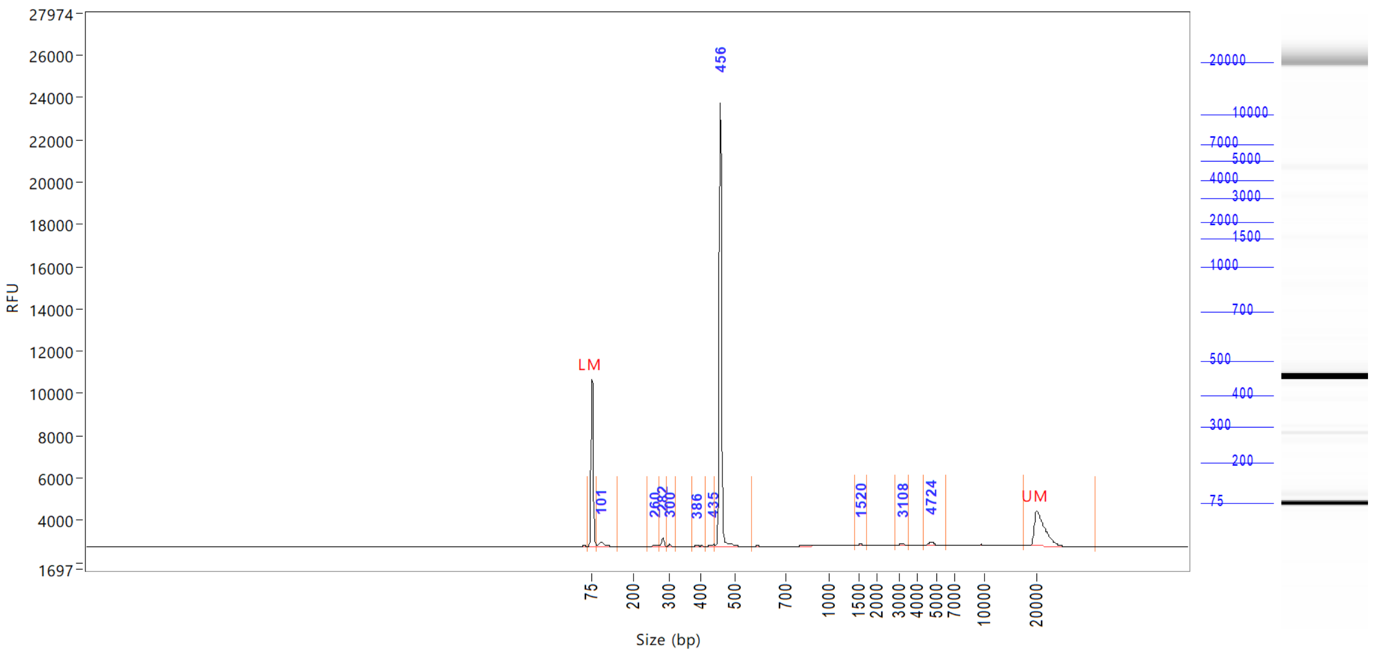

Peak	Size (bp)	Concentration (ng/uL)	From (bp)	To (bp)	Average size (bp)	CV%	RFU	Corrected peak area
1	75 (LM)	0.5000	62	91	74	4.48	7917	42.298
2	101	0.4279	91	150	106	11.57	237	3.016
3	260	0.1297	237	273	259	2.84	90	0.915
4	282	0.2721	273	291	280	1.07	408	1.918
5	300	0.0673	291	321	299	1.13	99	0.474
6	386	0.0828	372	414	390	2.32	71	0.584
7	435	0.1168	414	441	431	1.46	94	0.823
8	456	13.8447	441	567	455	1.55	21000	97.600
9	1520	0.0599	1433	1731	1510	2.70	77	0.423
10	3108	0.0734	2829	3500	3086	3.75	72	0.518
11	4724	0.1649	4277	6038	4704	4.47	166	1.162
12	20000 (UM)	0.2475	17561	31244	20805	5.88	1660	20.941

TIC:	15.2394	ng/uL
TIM:	60.4454	nmole/L
Total concentration:	15.8148	ng/uL

Sample peak width (sec): 5   Sample min peak height: 50   Sample baseline V to V?: Y   Sample baseline V to V points: 3
Sample filter: Binomial   Number of points for filter: 3   Sample start region (min): 0   Sample end region (min): 30
Marker peak width (sec): 5   Marker min peak height: 200   Marker baseline V to V?: Y   Marker baseline V to V points: 3
Lower marker selection: First peak > 500 RFU   Upper marker selection: Last peak > 200 RFU
Ladder size (bp)75, 200, 300, 400, 500, 700, 1000, 1500, 2000, 3000, 4000, 5000, 7000, 10000, 20000
Quantification using: Lower Marker   Final concentration (ng/uL): 0.5000   Dilution factor: 12.0

Sample: SampH11
Well location: H11
Created: Wednesday, October 20, 2021 5:58:44 PM

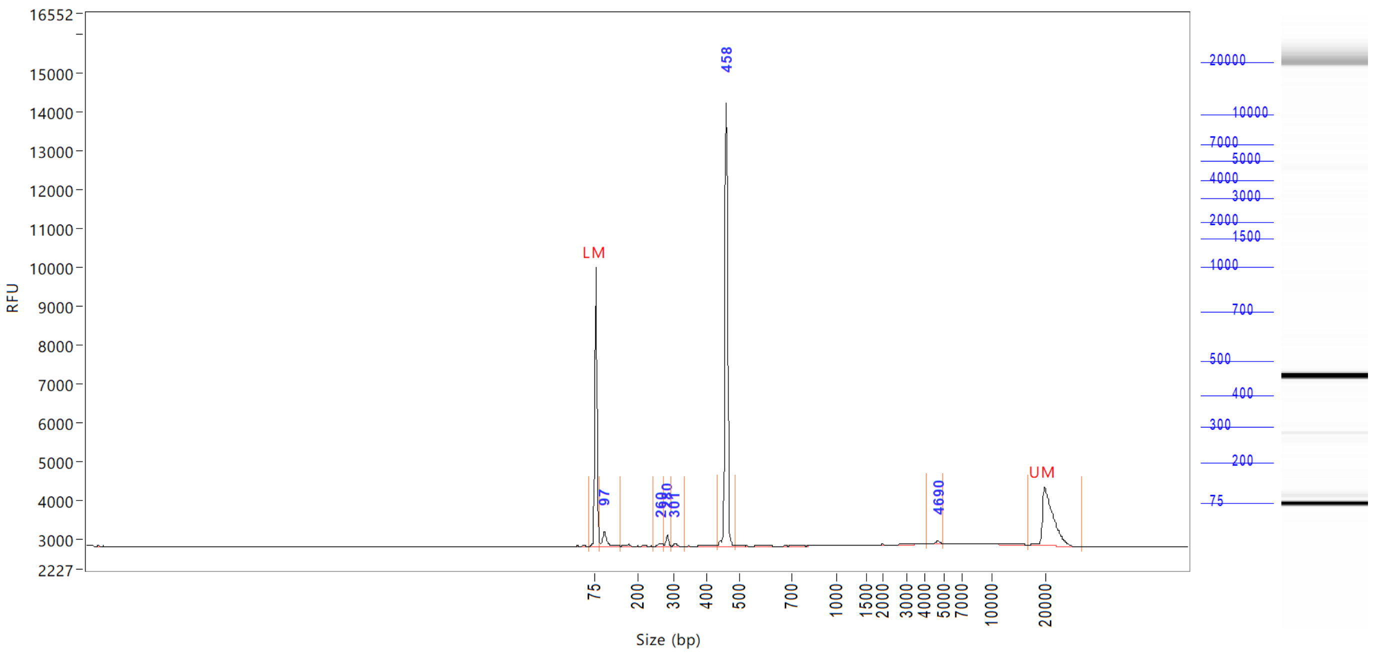

Peak	Size (bp)	Concentration (ng/uL)	From (bp)	To (bp)	Average size (bp)	CV%	RFU	Corrected peak area
1	75 (LM)	0.5000	54	86	72	4.54	7198	37.858
2	97	0.6155	86	147	100	11.05	373	3.883
3	260	0.1171	241	273	260	2.67	73	0.739
4	280	0.2281	273	291	279	1.01	294	1.439
5	301	0.0622	291	329	302	1.82	63	0.392
6	458	9.0113	431	485	455	0.98	11441	56.858
7	4690	0.0935	4104	5000	4614	3.53	78	0.590
8	20000 (UM)	0.2573	16830	27012	20839	5.81	1500	19.485

TIC:	10.1276	ng/uL
TIM:	45.1516	nmole/L
Total concentration:	10.8722	ng/uL

```
Sample peak width (sec): 5    Sample min peak height: 50    Sample baseline V to V?: Y    Sample baseline V to V points: 3
Sample filter: Binomial       Number of points for filter: 3  Sample start region (min): 0  Sample end region (min): 30
Marker peak width (sec): 5    Marker min peak height: 200   Marker baseline V to V?: Y    Marker baseline V to V points: 3
Lower marker selection: First peak > 500 RFU                Upper marker selection: Last peak > 200 RFU
Ladder size (bp)75, 200, 300, 400, 500, 700, 1000, 1500, 2000, 3000, 4000, 5000, 7000, 10000, 20000
Quantification using: Lower Marker          Final concentration (ng/uL): 0.5000          Dilution factor: 12.0
```

Sample: SampH12
Well location: H12
Created: Wednesday, October 20, 2021 5:58:44 PM

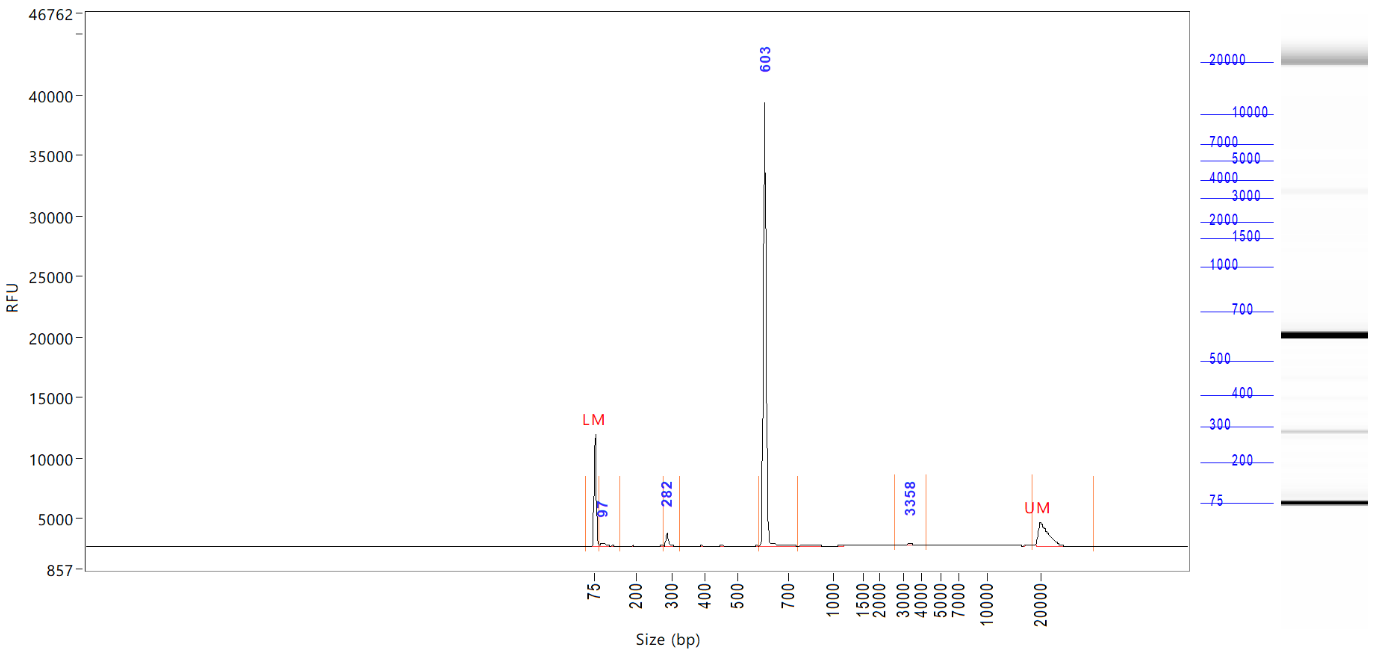

Peak	Size (bp)	Concentration (ng/uL)	From (bp)	To (bp)	Average size (bp)	CV%	RFU	Corrected peak area
1	75 (LM)	0.5000	50	86	73	4.34	9286	47.783
2	97	0.5628	86	148	103	13.50	276	4.482
3	282	0.7772	275	321	284	2.16	1088	6.189
4	603	22.1797	580	757	602	1.56	36674	176.635
5	3358	0.2093	2629	4208	3361	7.41	225	1.666
6	20000 (UM)	0.2513	18537	30277	20878	5.59	1927	24.015

TIC:	23.7289	ng/uL
TIM:	74.2084	nmole/L
Total concentration:	24.6194	ng/uL

```
Sample peak width (sec): 5    Sample min peak height: 200   Sample baseline V to V?: Y    Sample baseline V to V points: 3
Sample filter: Binomial       Number of points for filter: 3  Sample start region (min): 0  Sample end region (min): 30
Marker peak width (sec): 5    Marker min peak height: 200   Marker baseline V to V?: Y    Marker baseline V to V points: 3
Lower marker selection: First peak > 500 RFU                Upper marker selection: Last peak > 200 RFU
Ladder size (bp)75, 200, 300, 400, 500, 700, 1000, 1500, 2000, 3000, 4000, 5000, 7000, 10000, 20000
Quantification using: Lower Marker          Final concentration (ng/uL): 0.5000          Dilution factor: 12.0
```

Sample: DNA size ladder
Well location: A7
Created: Wednesday, October 20, 2021 5:58:44 PM
Import from: C:\Users\admin\Desktop\210810_alberto_ladder.SCAL
Fit type: Point to point

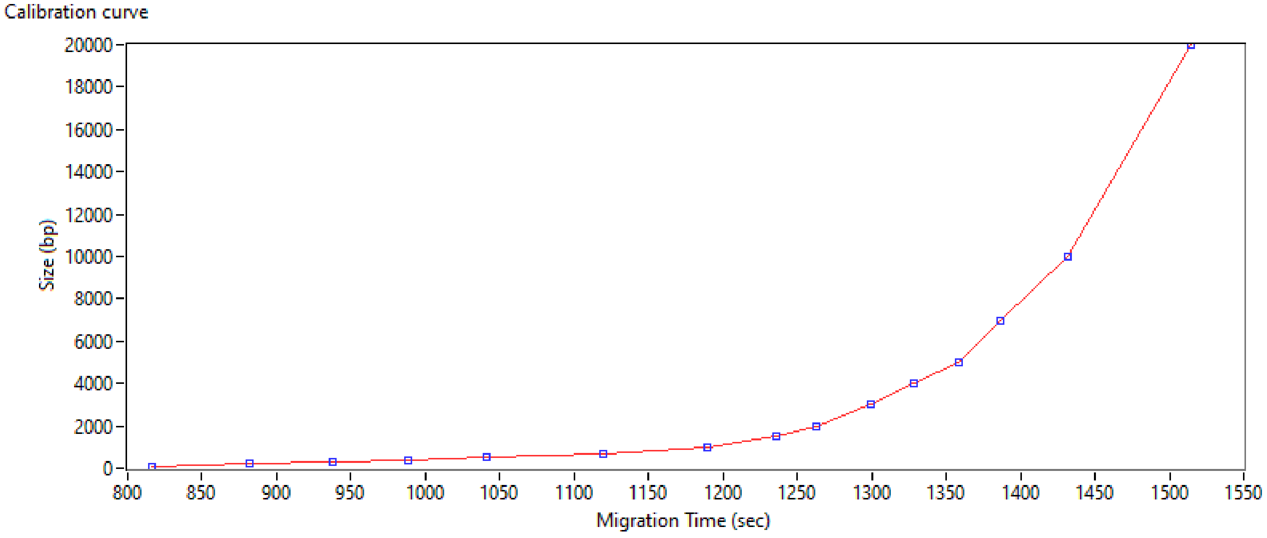

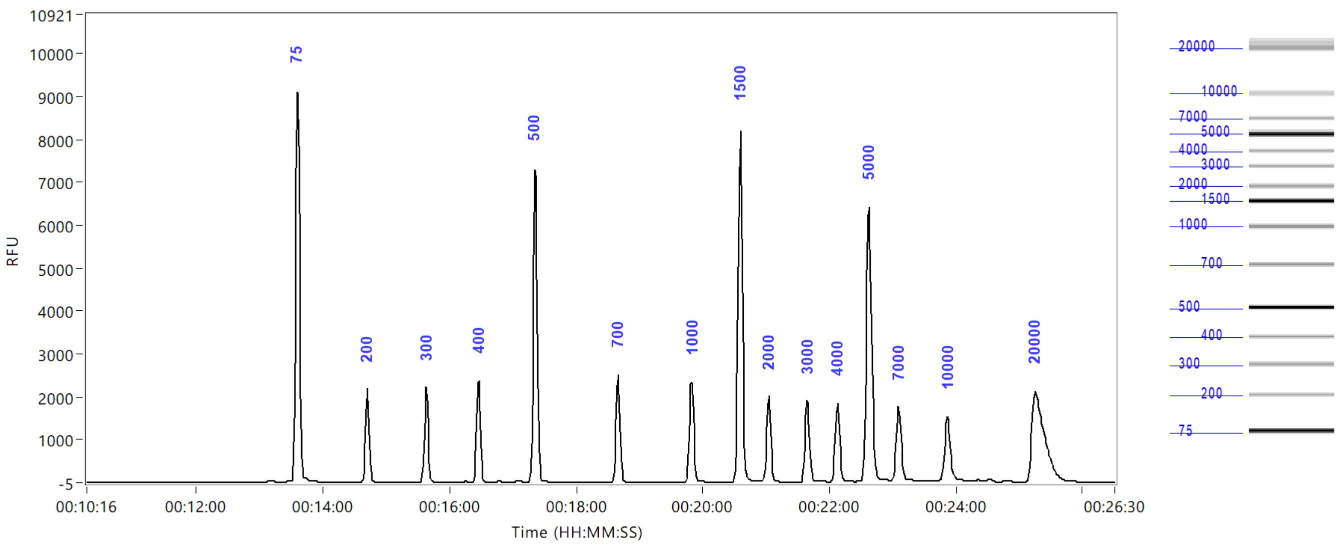

Supplement: Supplementary file 2 — sb3c00292_si_002.zip [file sb3c00292_si_002.zip › dnada_supplementary_material_pks_library_build/colony_pcr/ZAG/17-48-56_plate3/colonypcrplate_3.pdf]
