# Supplementary material for: Automated Platform for the Plasmid Construction Process
Source: ACS Synth Biol. 2023 Nov 10;12(12):3506–13. doi: 10.1021/acssynbio.3c00292 (PMC10729297; doi:10.1021/acssynbio.3c00292)

Instrument controller software run summary:

Filename and data path: C:\AATI\Data\2021 10 20\17-09-04\2021 10 20 17H 09M..raw
Created: Wednesday, October 20, 2021 5:18:46 PM
Number of capillaries: 96
Array serial number: 111920-09SFS
Effect length: 33 cm
Array usage count: 262
Instrument type: ZAG DNA Analyzer
Instrument controller software version: 1.0.1.9
Device serial number: Z7597

Method Information
Method name: ZAG130GP33 - DNA 75-20000bp - Gel Prime Only.mthds
Gel prime: Yes
Full conditioning: No
Gel prime to buffer: No
Gel selection: Gel 1
Perform prerun: 8.0 kV, 30 sec.
Rinse: No
Marker 1: Row: A, 5.0 kV, 5 sec.
Rinse: No
Sample injection: 5.0 kV, 5 sec.
Separation: 8.0 kV, 30.0 min.
Tray name: Sample Tray-2

Analysis mode: DNA

Notes

Gel Image

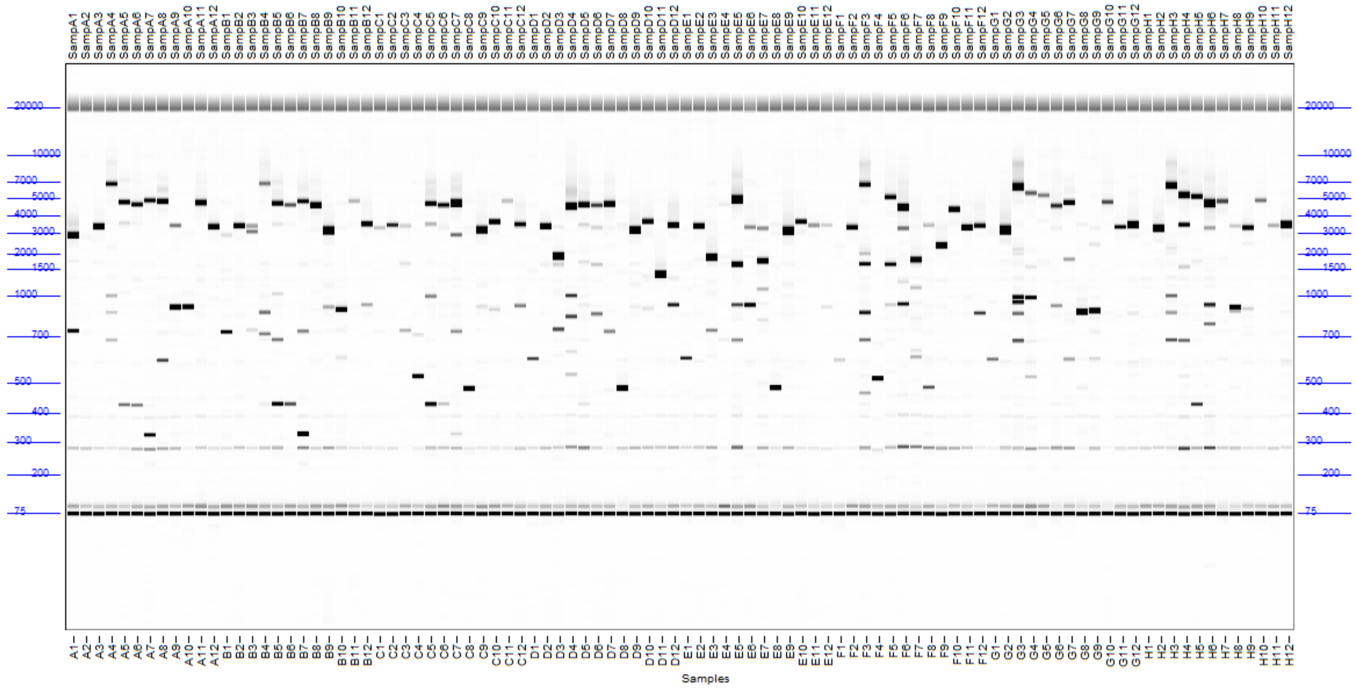

Filename and data path: C:\AATI\Data\2021 10 20\17-09-04\2021 10 20 17H 09M..raw

A1: SampA1

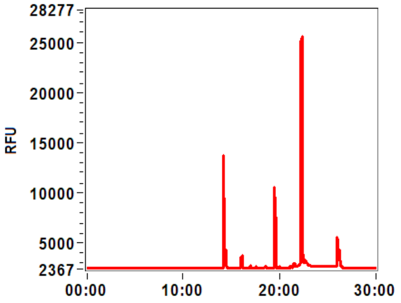

A2: SampA2

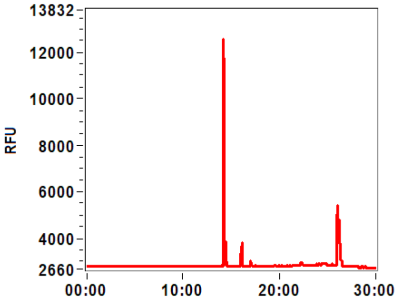

A3: SampA3

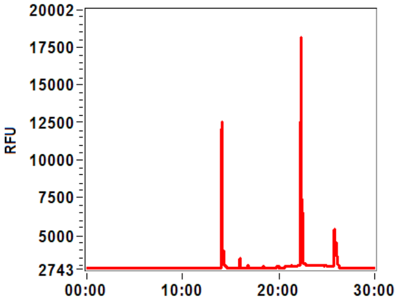

A4: SampA4

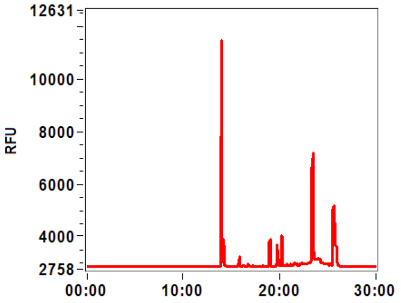

A5: SampA5

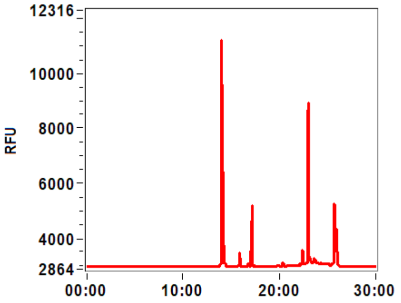

A6: SampA6

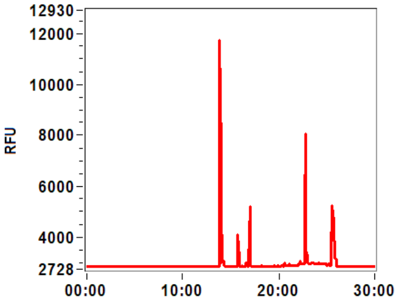

A7: SampA7

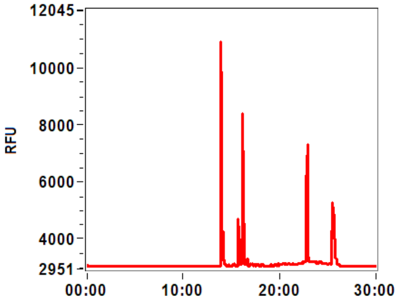

A8: SampA8

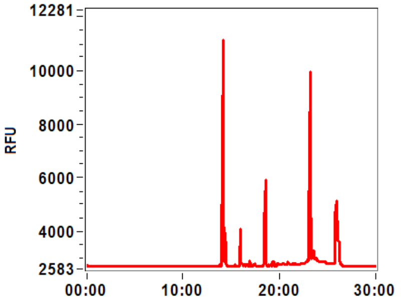

A9: SampA9

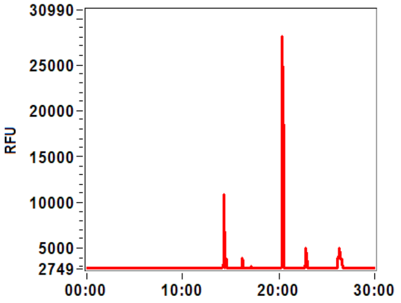

A10: SampA10

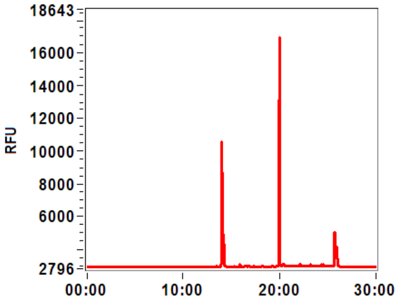

A11: SampA11

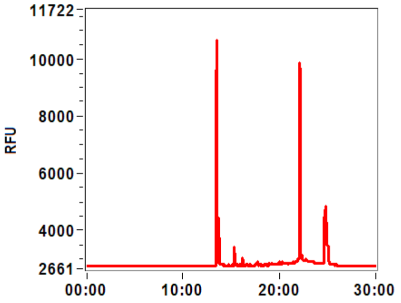

A12: SampA12

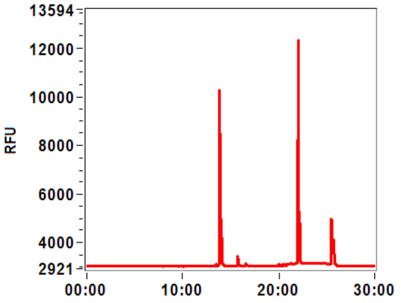

Filename and data path: C:\AATI\Data\2021 10 20\17-09-04\2021 10 20 17H 09M..raw

B1: SampB1

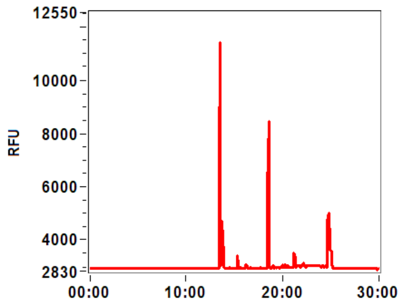

B2: SampB2

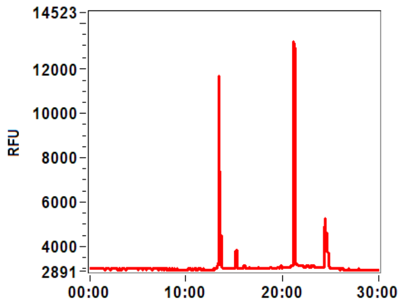

B3: SampB3

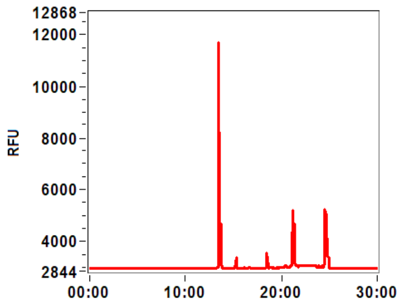

B4: SampB4

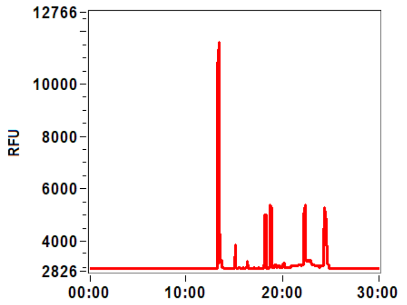

B5: SampB5

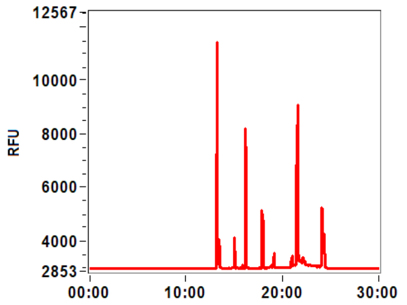

B6: SampB6

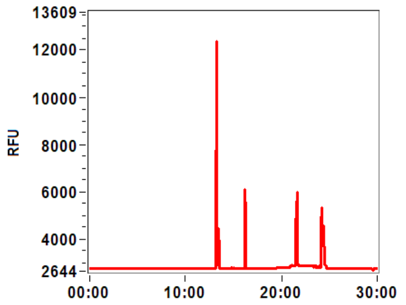

B7: SampB7

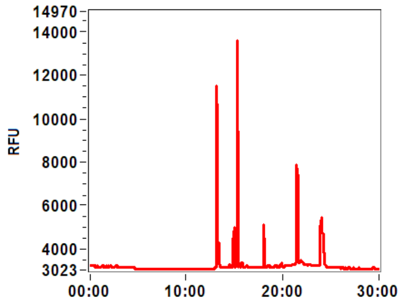

B8: SampB8

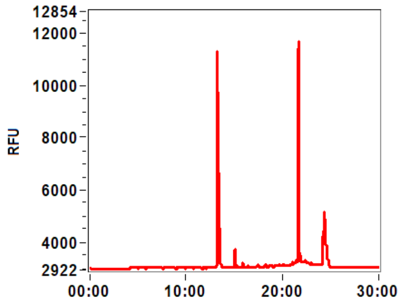

B9: SampB9

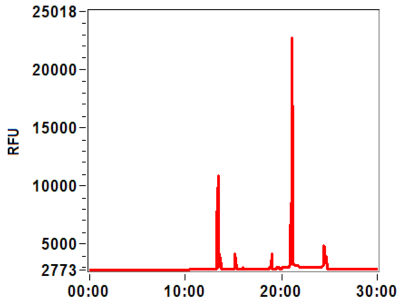

B10: SampB10

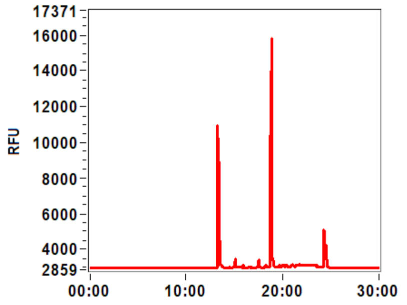

B11: SampB11

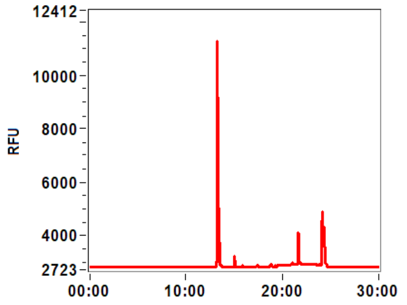

B12: SampB12

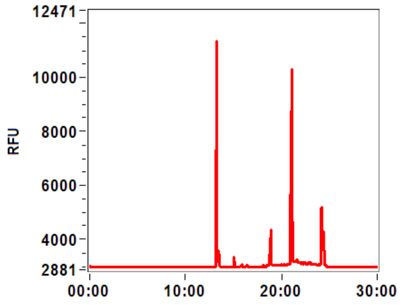

Filename and data path: C:\AATI\Data\2021 10 20\17-09-04\2021 10 20 17H 09M..raw

C1: SampC1

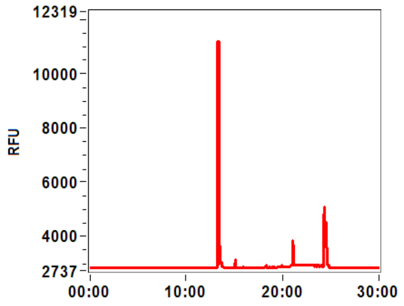

C2: SampC2

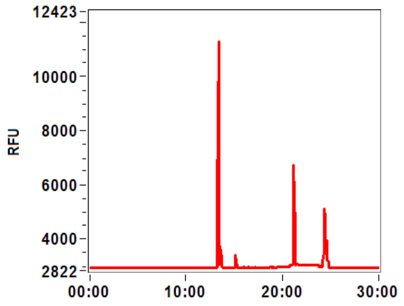

C3: SampC3

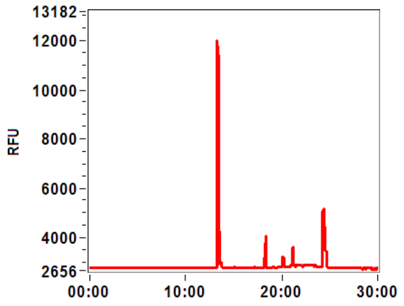

C4: SampC4

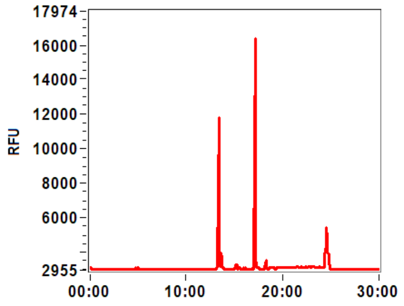

C5: SampC5

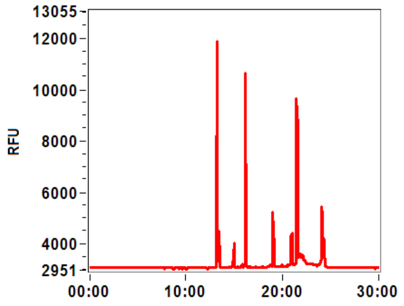

C6: SampC6

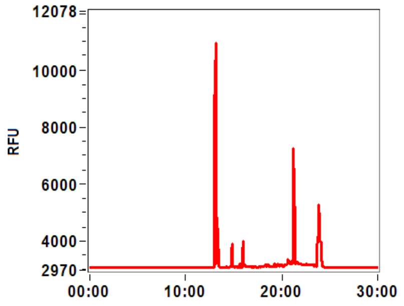

C7: SampC7

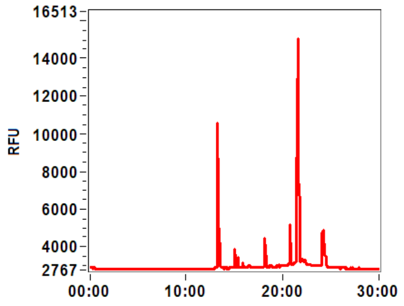

C8: SampC8

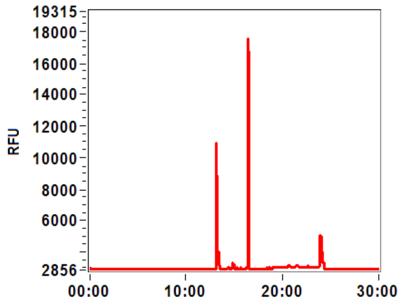

C9: SampC9

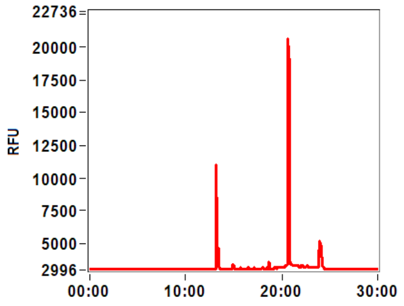

C10: SampC10

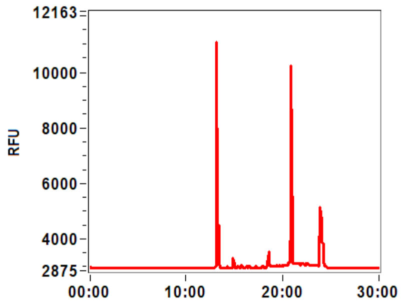

C11: SampC11

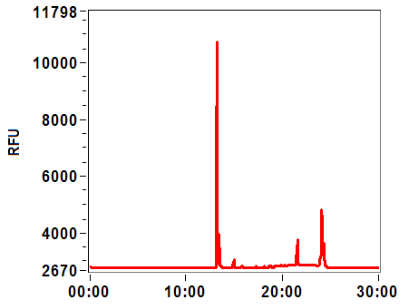

C12: SampC12

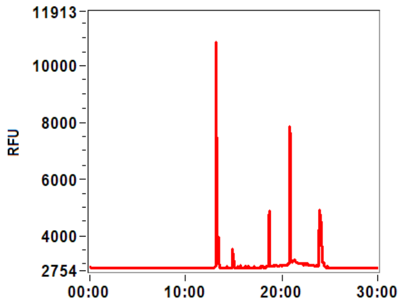

Filename and data path: C:\AATI\Data\2021 10 20\17-09-04\2021 10 20 17H 09M..raw

D1: SampD1

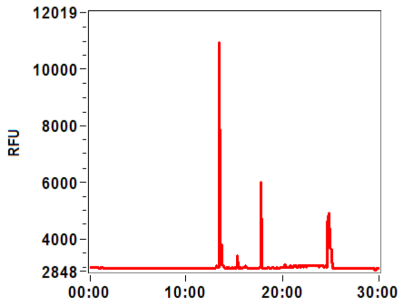

D2: SampD2

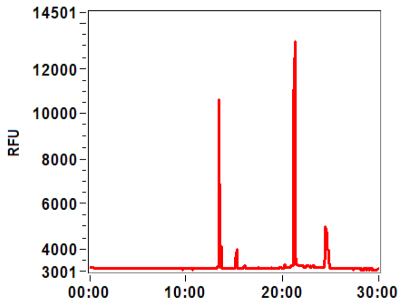

D3: SampD3

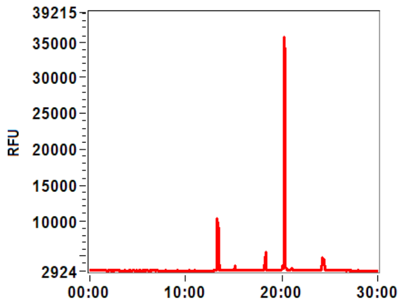

D4: SampD4

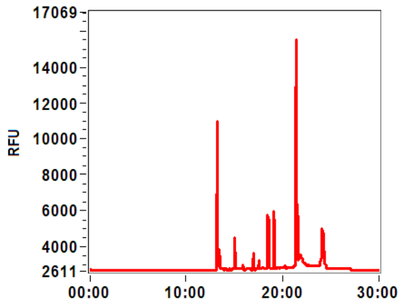

D5: SampD5

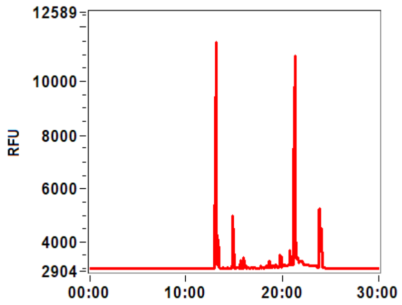

D6: SampD6

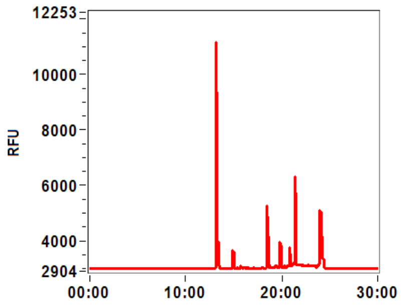

D7: SampD7

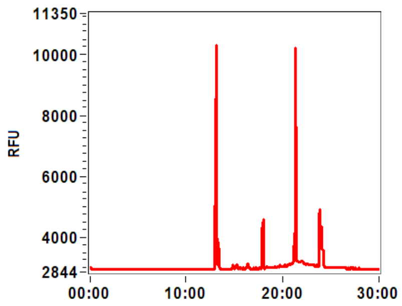

D8: SampD8

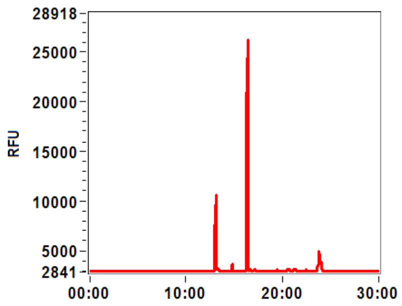

D9: SampD9

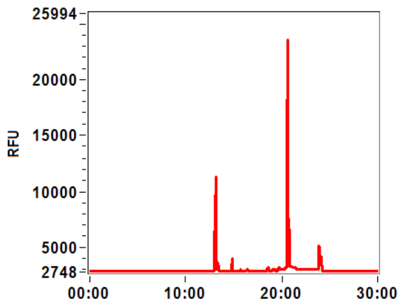

D10: SampD10

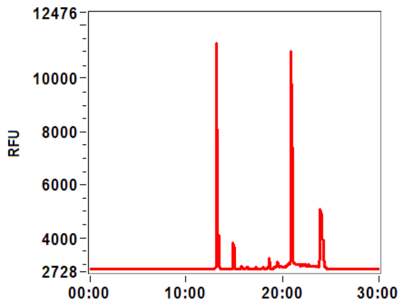

D11: SampD11

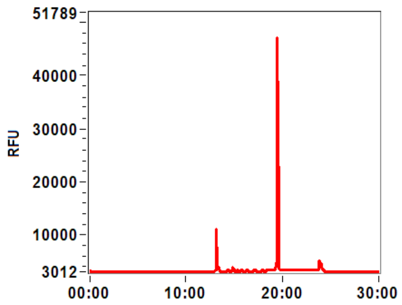

D12: SampD12

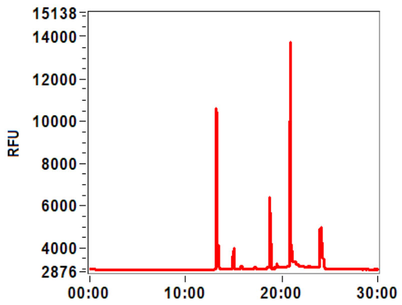

Filename and data path: C:\AATI\Data\2021 10 20\17-09-04\2021 10 20 17H 09M..raw

E1: SampE1

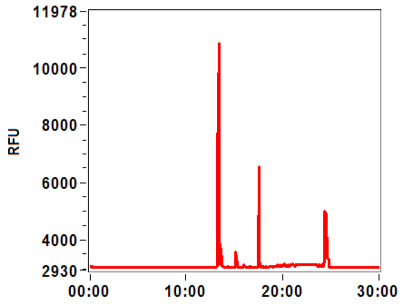

E2: SampE2

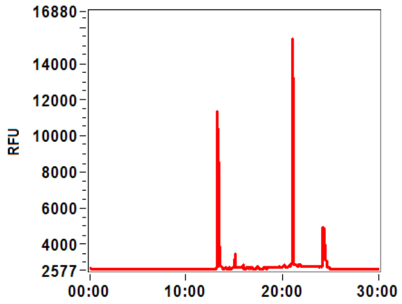

E3: SampE3

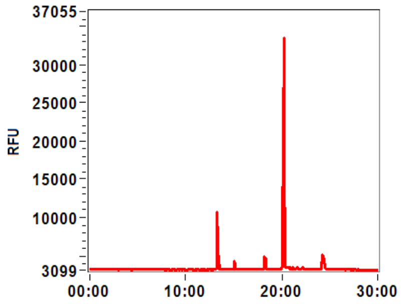

E4: SampE4

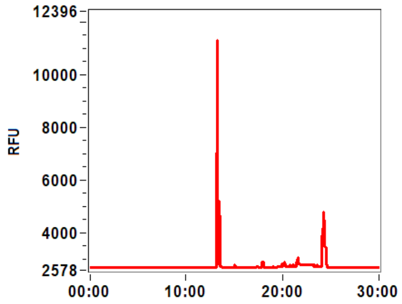

E5: SampE5

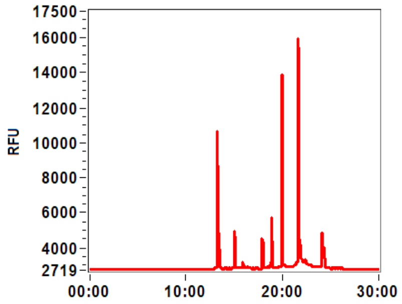

E6: SampE6

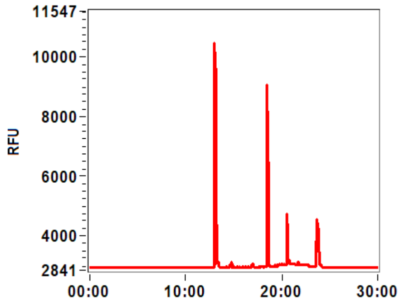

E7: SampE7

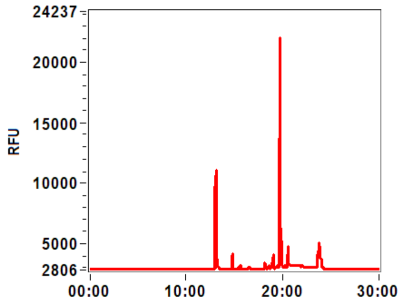

E8: SampE8

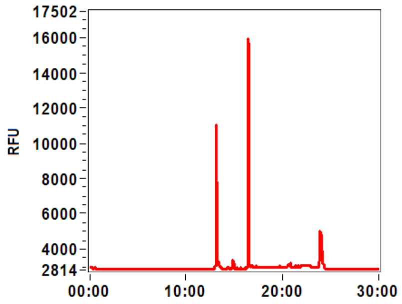

E9: SampE9

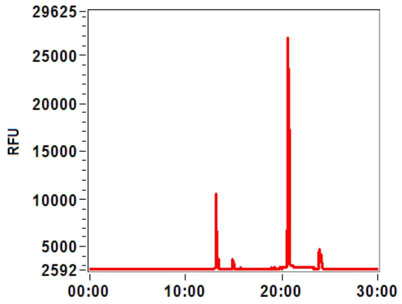

E10: SampE10

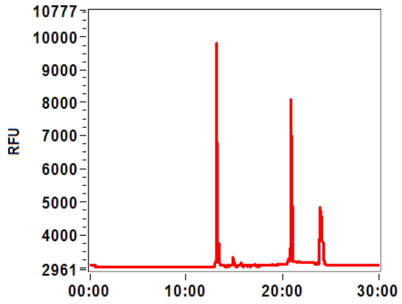

E11: SampE11

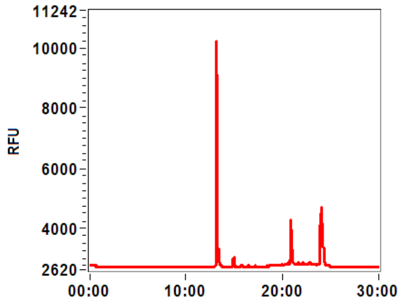

E12: SampE12

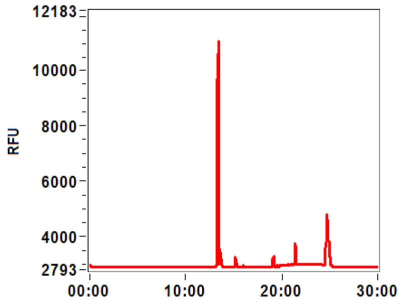

Filename and data path: C:\AATI\Data\2021 10 20\17-09-04\2021 10 20 17H 09M..raw

F1: SampF1

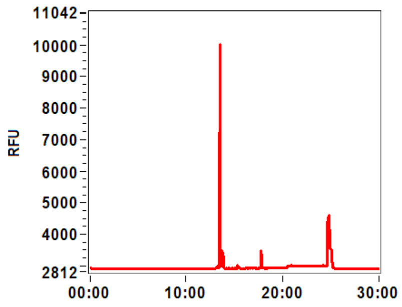

F2: SampF2

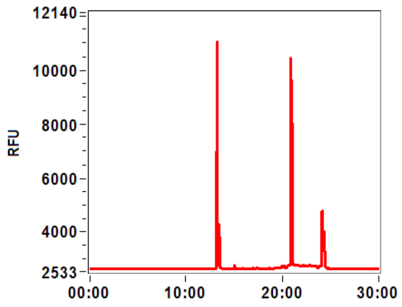

F3: SampF3

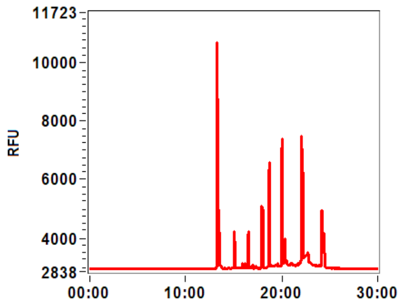

F4: SampF4

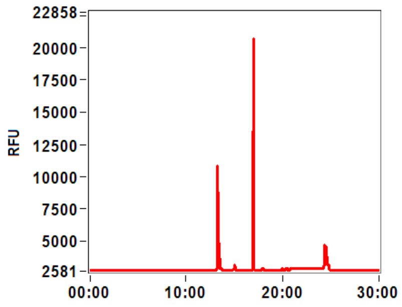

F5: SampF5

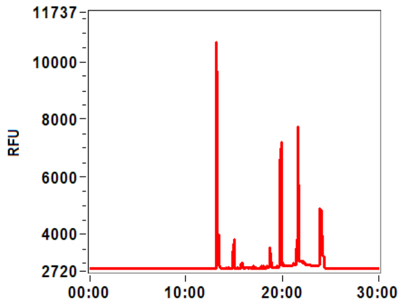

F6: SampF6

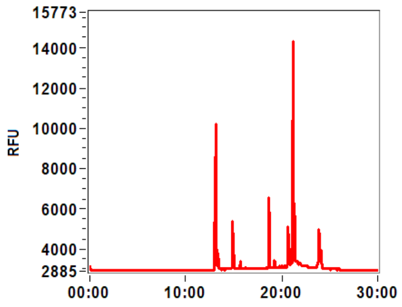

F7: SampF7

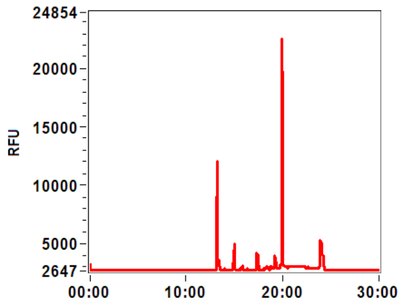

F8: SampF8

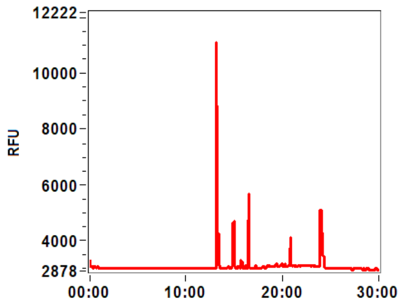

F9: SampF9

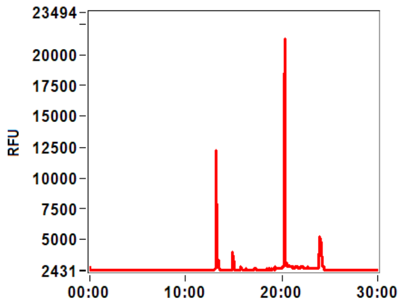

F10: SampF10

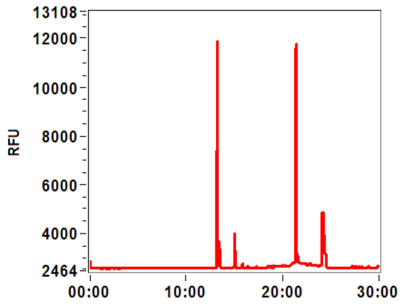

F11: SampF11

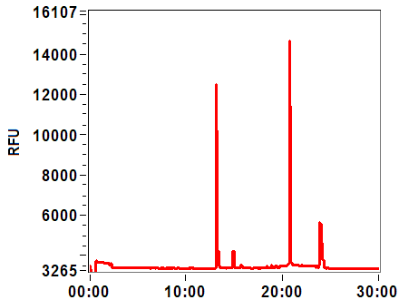

F12: SampF12

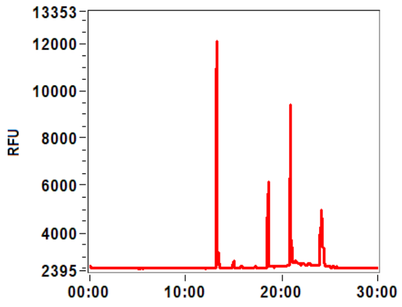

Filename and data path: C:\AATI\Data\2021 10 20\17-09-04\2021 10 20 17H 09M..raw

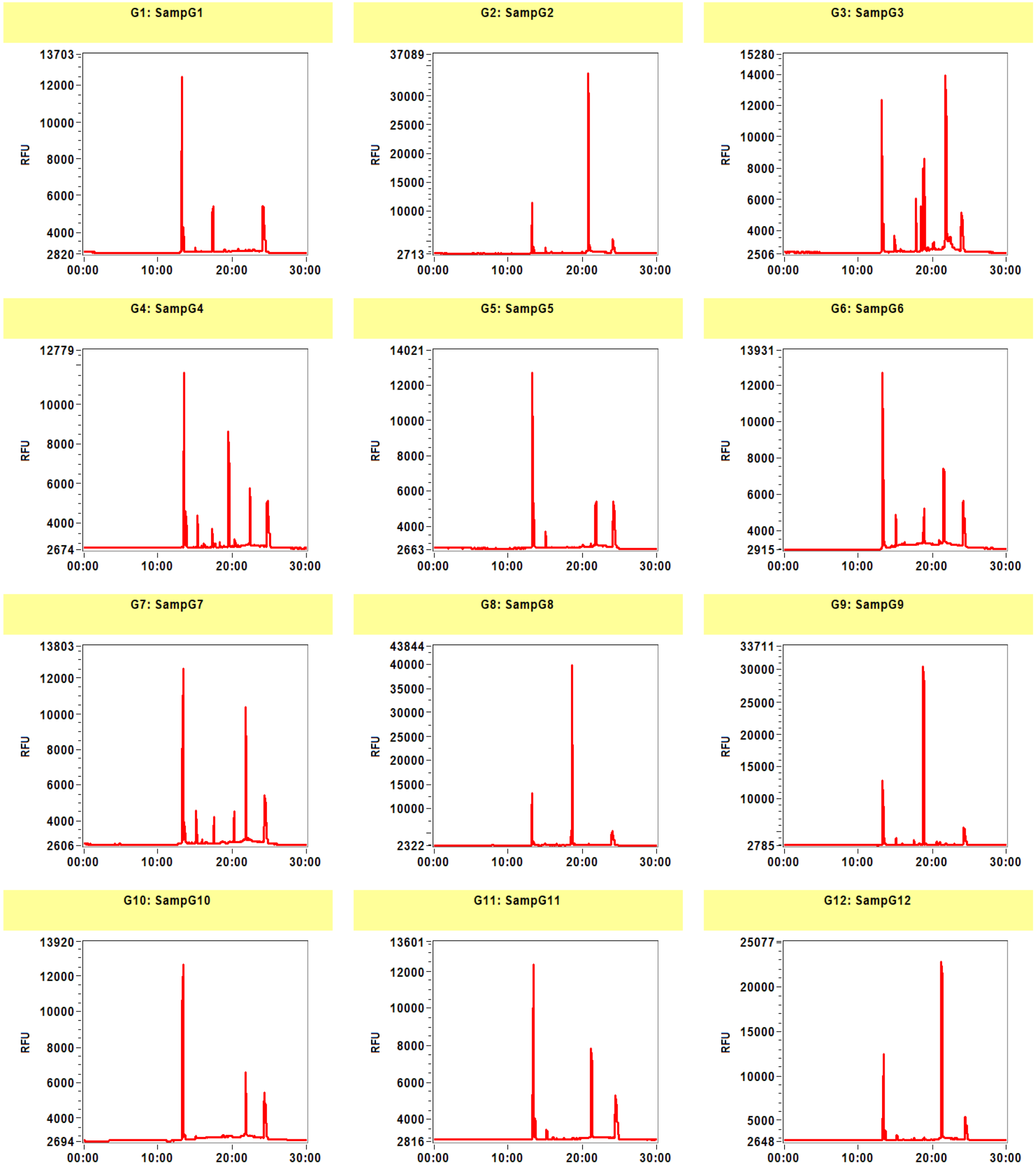

Filename and data path: C:\AATI\Data\2021 10 20\17-09-04\2021 10 20 17H 09M..raw

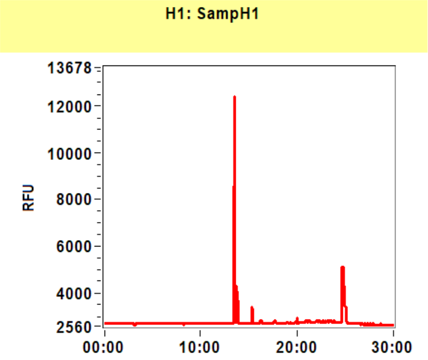

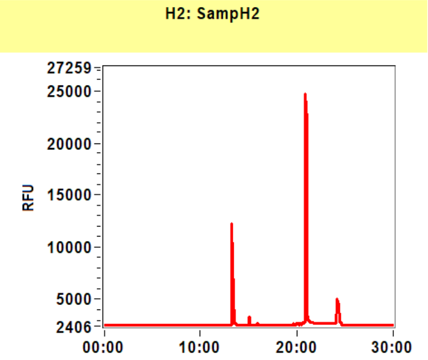

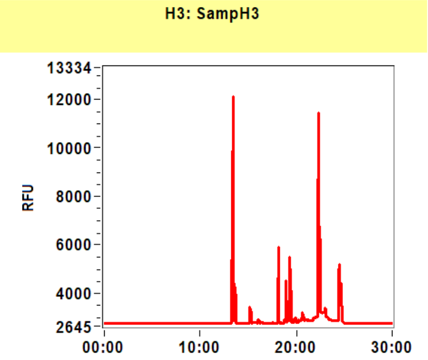

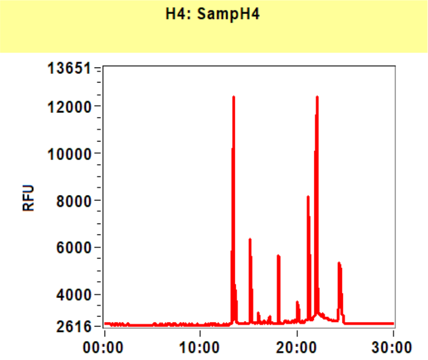

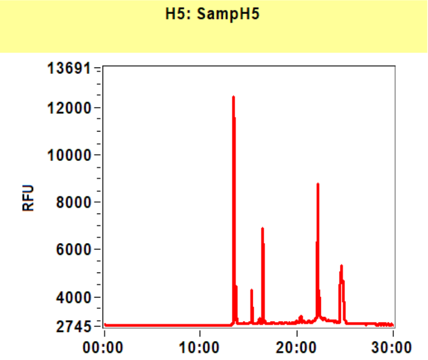

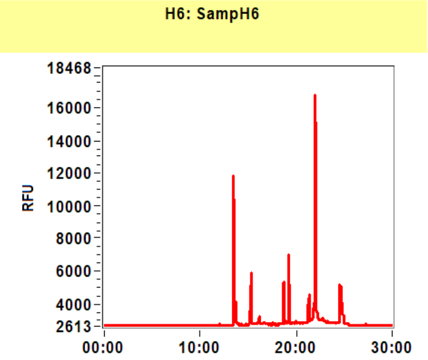

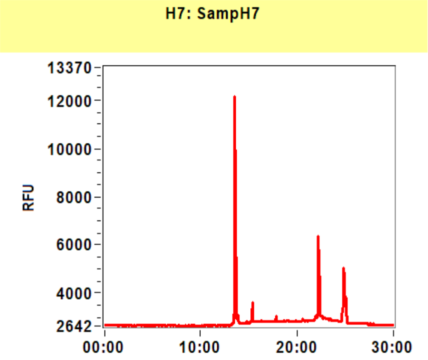

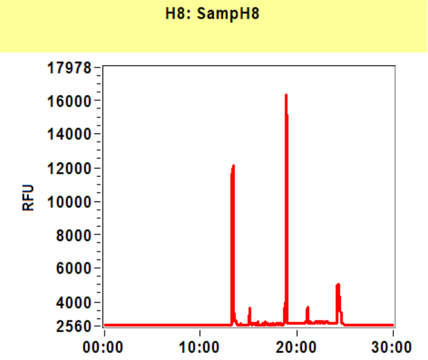

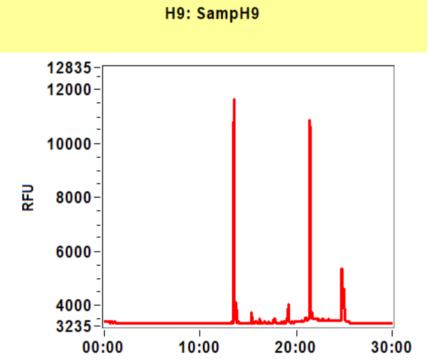

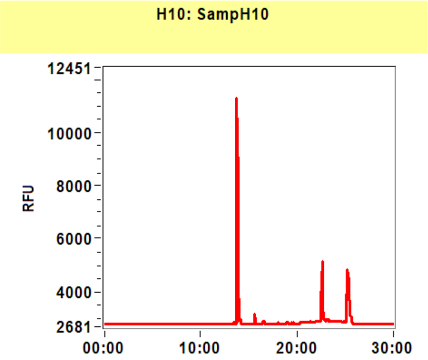

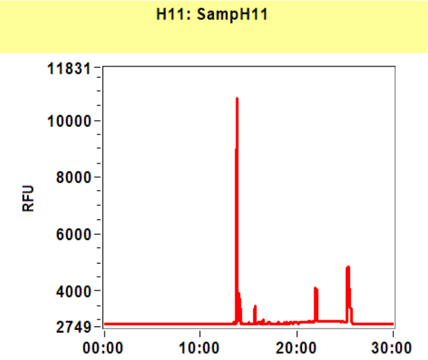

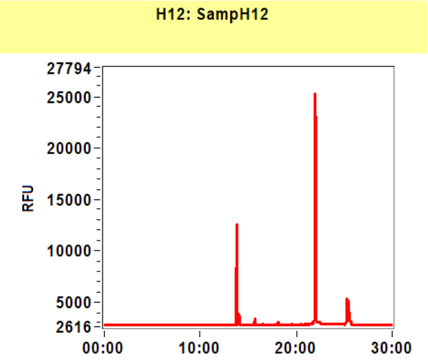

Sample: SampA1
Well location: A1
Created: Wednesday, October 20, 2021 5:18:46 PM

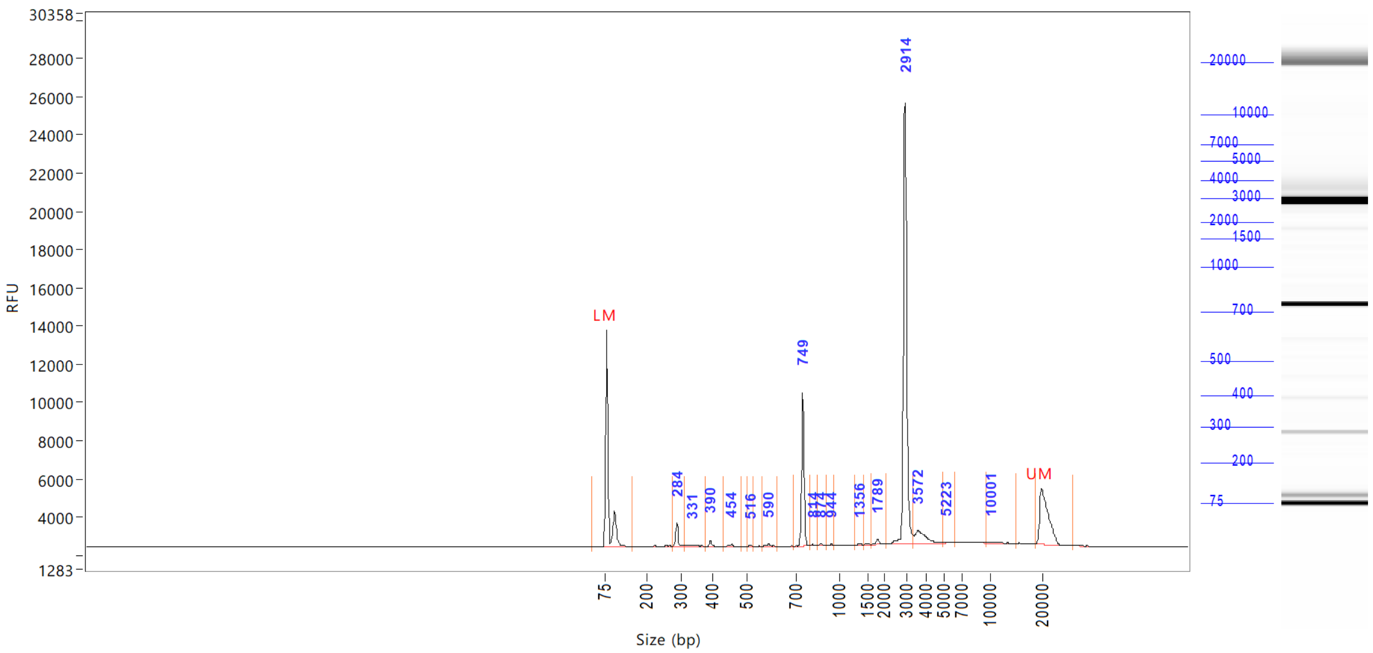

Peak	Size (bp)	Concentration (ng/uL)	From (bp)	To (bp)	Average size (bp)	CV%	RFU	Corrected peak area
1	75 (LM)	0.5000	30	152	79	14.82	11320	73.138
2	284	0.4736	271	309	283	1.87	1229	5.773
3	331	0.0879	309	372	336	5.00	54	1.071
4	390	0.1414	372	430	389	2.03	328	1.723
5	454	0.0675	430	485	453	2.35	106	0.823
6	516	0.0230	501	529	513	1.09	59	0.280
7	590	0.0830	562	626	587	1.85	156	1.012
8	749	2.5827	695	792	744	1.10	8032	31.482
9	814	0.0493	792	844	819	1.85	75	0.601
10	874	0.0492	844	909	864	1.53	77	0.600
11	944	0.0247	909	965	937	1.19	57	0.301
12	1356	0.0344	1256	1422	1339	2.15	84	0.419
13	1789	0.1358	1578	2030	1782	4.44	294	1.655
14	2914	9.3588	2030	3358	2892	3.70	23098	114.080
15	3572	0.8457	3358	4931	3811	9.08	672	10.308
16	5223	0.0461	4931	6186	5323	5.82	54	0.562
17	10001	0.0689	9667	15122	11145	11.02	55	0.840
18	20000 (UM)	0.2117	18659	26166	20634	4.29	2911	30.960

TIC:	14.0719	ng/uL
TIM:	16.1665	nmole/L
Total concentration:	14.2670	ng/uL

```
Sample peak width (sec): 5    Sample min peak height: 50    Sample baseline V to V?: Y    Sample baseline V to V points: 3
Sample filter: Binomial       Number of points for filter: 3  Sample start region (min): 0  Sample end region (min): 30
Marker peak width (sec): 5    Marker min peak height: 200   Marker baseline V to V?: Y    Marker baseline V to V points: 3
Lower marker selection: First peak > 500 RFU                Upper marker selection: Last peak > 200 RFU
Ladder size (bp)75, 200, 300, 400, 500, 700, 1000, 1500, 2000, 3000, 4000, 5000, 7000, 10000, 20000
Quantification using: Lower Marker          Final concentration (ng/uL): 0.5000          Dilution factor: 12.0
```

Sample: SampA2
Well location: A2
Created: Wednesday, October 20, 2021 5:18:46 PM

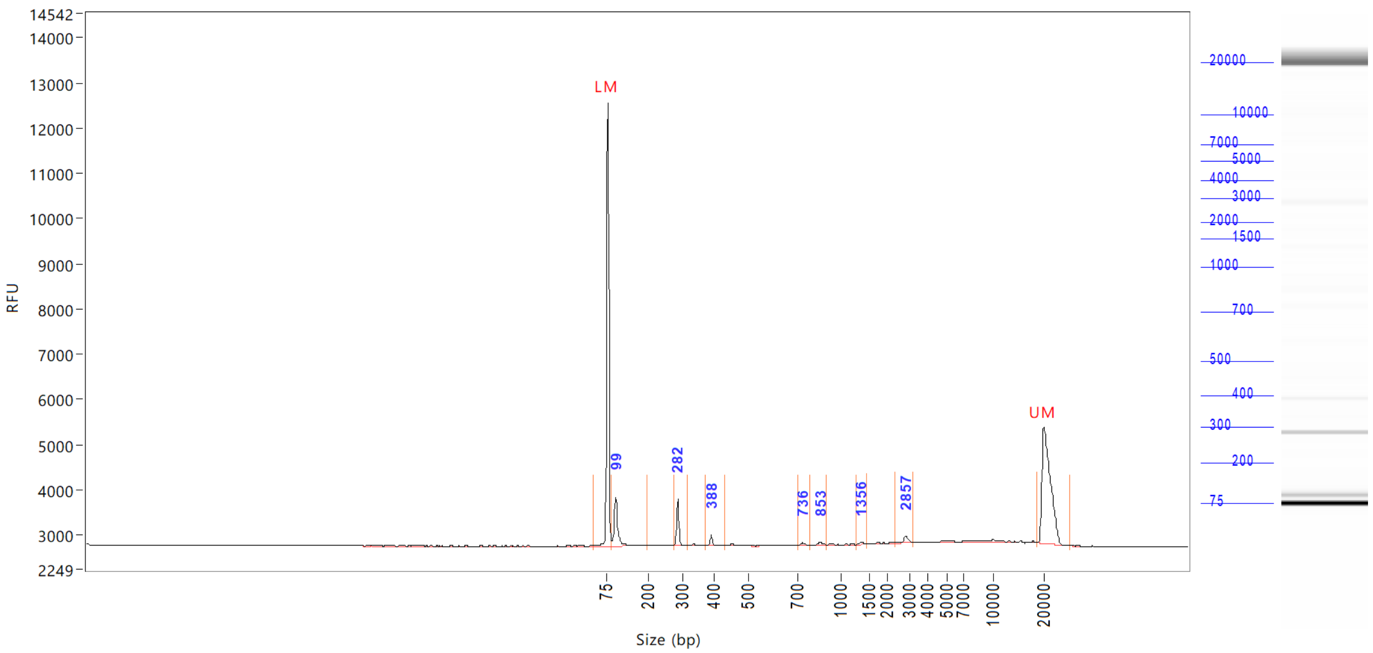

Peak	Size (bp)	Concentration (ng/uL)	From (bp)	To (bp)	Average size (bp)	CV%	RFU	Corrected peak area
1	75 (LM)	0.5000	33	86	73	5.20	9815	49.850
2	99	1.1001	86	196	102	15.24	1084	9.140
3	282	0.5789	271	309	281	1.58	1029	4.810
4	388	0.1513	370	428	388	2.14	236	1.257
5	736	0.0463	701	788	732	1.91	57	0.385
6	853	0.0615	788	900	852	2.17	64	0.511
7	1356	0.0386	1256	1445	1343	2.68	54	0.321
8	2857	0.1223	2372	3179	2833	4.39	151	1.016
9	20000 (UM)	0.2684	18537	24957	20567	4.13	2581	26.760

TIC:	2.0991	ng/uL
TIM:	22.0829	nmole/L
Total concentration:	2.7016	ng/uL

Sample peak width (sec): 5 Sample min peak height: 50 Sample baseline V to V?: Y Sample baseline V to V points: 3
Sample filter: Binomial Number of points for filter: 3 Sample start region (min): 0 Sample end region (min): 30
Marker peak width (sec): 5 Marker min peak height: 200 Marker baseline V to V?: Y Marker baseline V to V points: 3
Lower marker selection: First peak > 500 RFU Upper marker selection: Last peak > 200 RFU
Ladder size (bp)75, 200, 300, 400, 500, 700, 1000, 1500, 2000, 3000, 4000, 5000, 7000, 10000, 20000
Quantification using: Lower Marker Final concentration (ng/uL): 0.5000 Dilution factor: 12.0

Sample: SampA3
Well location: A3
Created: Wednesday, October 20, 2021 5:18:46 PM

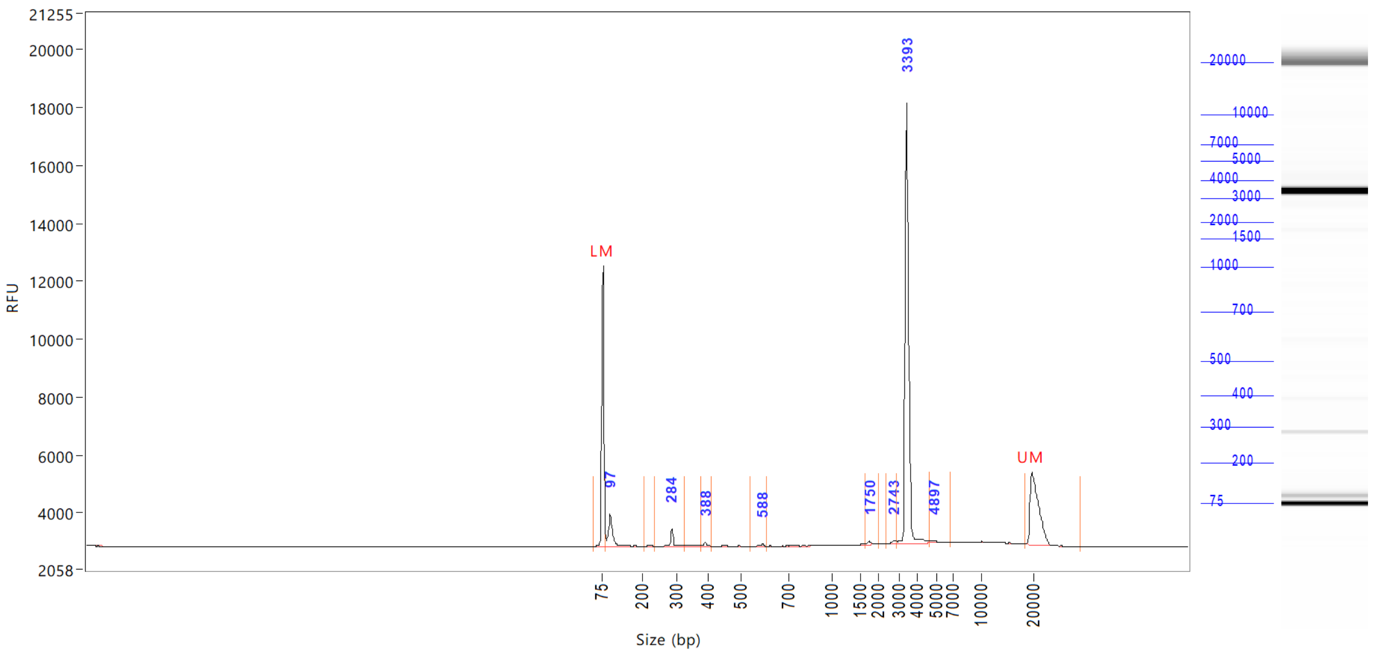

Peak	Size (bp)	Concentration (ng/uL)	From (bp)	To (bp)	Average size (bp)	CV%	RFU	Corrected peak area
1	75 (LM)	0.5000	47	86	73	4.72	9688	49.883
2	97	1.2062	86	201	100	13.70	1107	10.028
3	284	0.3851	235	321	283	3.74	589	3.201
4	388	0.0846	376	411	387	1.40	138	0.703
5	588	0.0677	539	608	579	1.86	81	0.563
6	1750	0.0890	1597	2001	1759	4.34	116	0.740
7	2743	0.0841	2372	2886	2730	3.64	101	0.699
8	3393	9.2502	2886	4621	3386	4.83	15241	76.905
9	4897	0.1309	4621	6778	5217	10.69	84	1.089
10	20000 (UM)	0.2672	18537	29430	20573	4.58	2506	26.660

TIC:	11.2978	ng/uL
TIM:	27.2697	nmole/L
Total concentration:	11.7194	ng/uL

Sample peak width (sec): 5 Sample min peak height: 50 Sample baseline V to V?: Y Sample baseline V to V points: 3
Sample filter: Binomial Number of points for filter: 3 Sample start region (min): 0 Sample end region (min): 30
Marker peak width (sec): 5 Marker min peak height: 200 Marker baseline V to V?: Y Marker baseline V to V points: 3
Lower marker selection: First peak > 500 RFU Upper marker selection: Last peak > 200 RFU
Ladder size (bp)75, 200, 300, 400, 500, 700, 1000, 1500, 2000, 3000, 4000, 5000, 7000, 10000, 20000
Quantification using: Lower Marker Final concentration (ng/uL): 0.5000 Dilution factor: 12.0

Sample: SampA4
Well location: A4
Created: Wednesday, October 20, 2021 5:18:46 PM

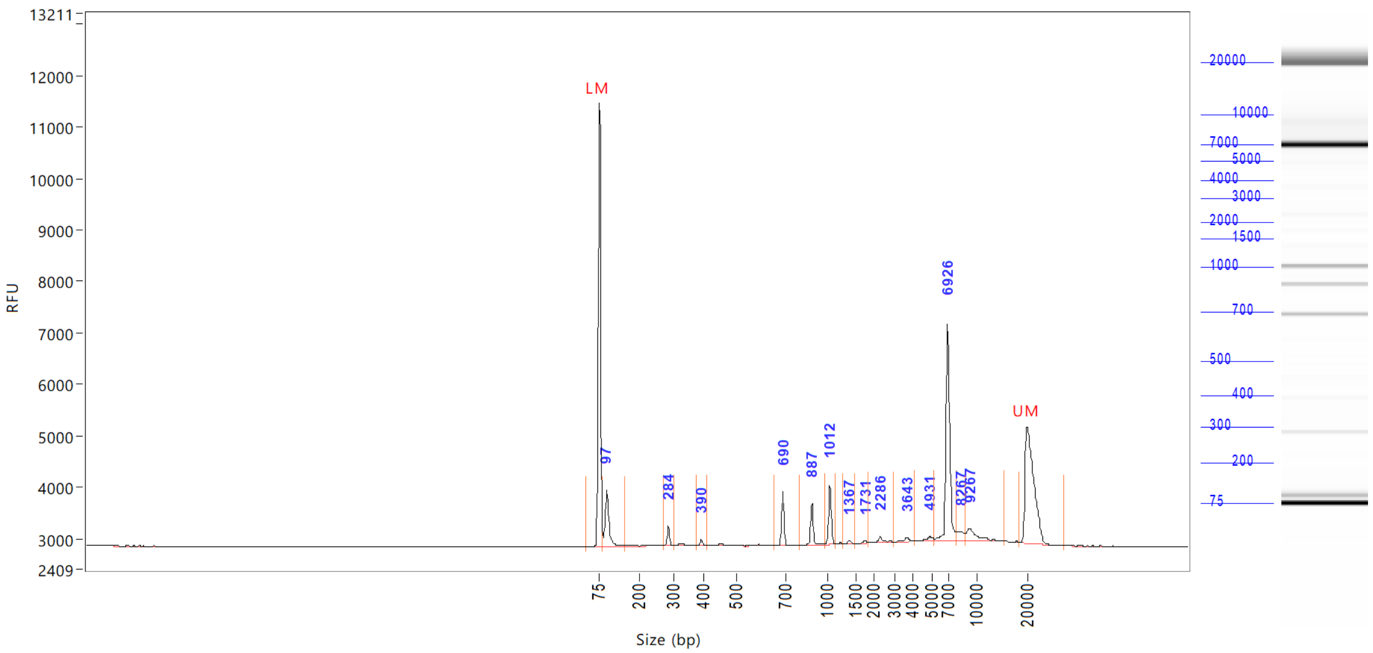

Peak	Size (bp)	Concentration (ng/uL)	From (bp)	To (bp)	Average size (bp)	CV%	RFU	Corrected peak area
1	75 (LM)	0.5000	35	87	74	4.79	8633	45.156
2	97	1.2438	87	154	99	9.15	1090	9.361
3	284	0.2433	269	301	282	1.51	388	1.831
4	390	0.0806	374	407	387	1.30	120	0.607
5	690	0.5874	654	796	689	1.56	1058	4.421
6	887	0.4937	796	978	884	2.37	811	3.716
7	1012	0.6378	978	1123	1010	1.78	1159	4.800
8	1367	0.0501	1256	1456	1351	2.50	62	0.377
9	1731	0.0382	1456	1846	1701	3.87	53	0.287
10	2286	0.1143	1846	2914	2324	9.19	115	0.860
11	3643	0.0750	2914	4035	3541	6.18	74	0.565
12	4931	0.1188	4035	5297	4820	5.32	94	0.894
13	6926	2.9360	5297	7801	6846	4.37	4231	22.096
14	8267	0.2319	7801	8734	8254	3.44	174	1.746
15	9267	0.4538	8734	15244	9903	11.85	237	3.415
16	20000 (UM)	0.2679	18415	27133	20564	4.39	2265	24.196

TIC:	7.3047	ng/uL
TIM:	26.8349	nmole/L
Total concentration:	7.5720	ng/uL

```
Sample peak width (sec): 5     Sample min peak height: 50    Sample baseline V to V?: Y    Sample baseline V to V points: 3
Sample filter: Binomial        Number of points for filter: 3  Sample start region (min): 0  Sample end region (min): 30
Marker peak width (sec): 5     Marker min peak height: 200   Marker baseline V to V?: Y    Marker baseline V to V points: 3
Lower marker selection: First peak > 500 RFU                 Upper marker selection: Last peak > 200 RFU
Ladder size (bp)75, 200, 300, 400, 500, 700, 1000, 1500, 2000, 3000, 4000, 5000, 7000, 10000, 20000
Quantification using: Lower Marker          Final concentration (ng/uL): 0.5000          Dilution factor: 12.0
```

Sample: SampA5
Well location: A5
Created: Wednesday, October 20, 2021 5:18:46 PM

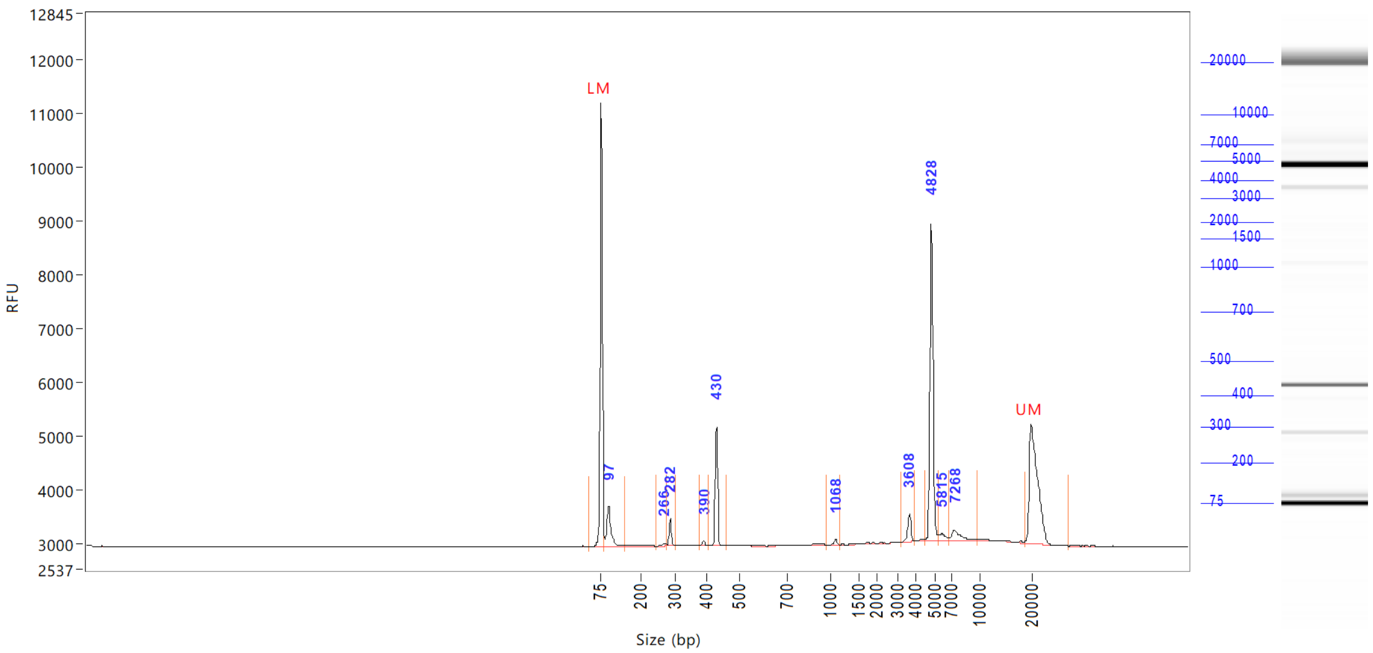

Peak	Size (bp)	Concentration (ng/uL)	From (bp)	To (bp)	Average size (bp)	CV%	RFU	Corrected peak area
1	75 (LM)	0.5000	41	86	73	4.71	8237	42.352
2	97	1.0079	86	148	99	9.81	745	7.115
3	266	0.0604	242	273	263	2.40	52	0.426
4	282	0.3313	273	300	281	1.22	505	2.338
5	390	0.0598	378	403	388	1.36	83	0.422
6	430	1.3644	403	460	427	1.09	2196	9.630
7	1068	0.0788	965	1145	1053	3.13	109	0.556
8	3608	0.3179	3108	3893	3558	2.90	501	2.244
9	4828	3.7745	4449	5371	4812	2.08	5888	26.643
10	5815	0.1745	5371	6704	5918	6.27	125	1.232
11	7268	0.3290	6704	9733	7634	8.76	201	2.322
12	20000 (UM)	0.2702	18659	27496	20595	4.28	2207	22.887

TIC:	7.4985	ng/uL
TIM:	26.2267	nmole/L
Total concentration:	8.0375	ng/uL

```
Sample peak width (sec): 5    Sample min peak height: 50    Sample baseline V to V?: Y    Sample baseline V to V points: 3
Sample filter: Binomial       Number of points for filter: 3  Sample start region (min): 0  Sample end region (min): 30
Marker peak width (sec): 5    Marker min peak height: 200   Marker baseline V to V?: Y    Marker baseline V to V points: 3
Lower marker selection: First peak > 500 RFU                Upper marker selection: Last peak > 200 RFU
Ladder size (bp)75, 200, 300, 400, 500, 700, 1000, 1500, 2000, 3000, 4000, 5000, 7000, 10000, 20000
Quantification using: Lower Marker          Final concentration (ng/uL): 0.5000          Dilution factor: 12.0
```

Sample: SampA6
Well location: A6
Created: Wednesday, October 20, 2021 5:18:46 PM

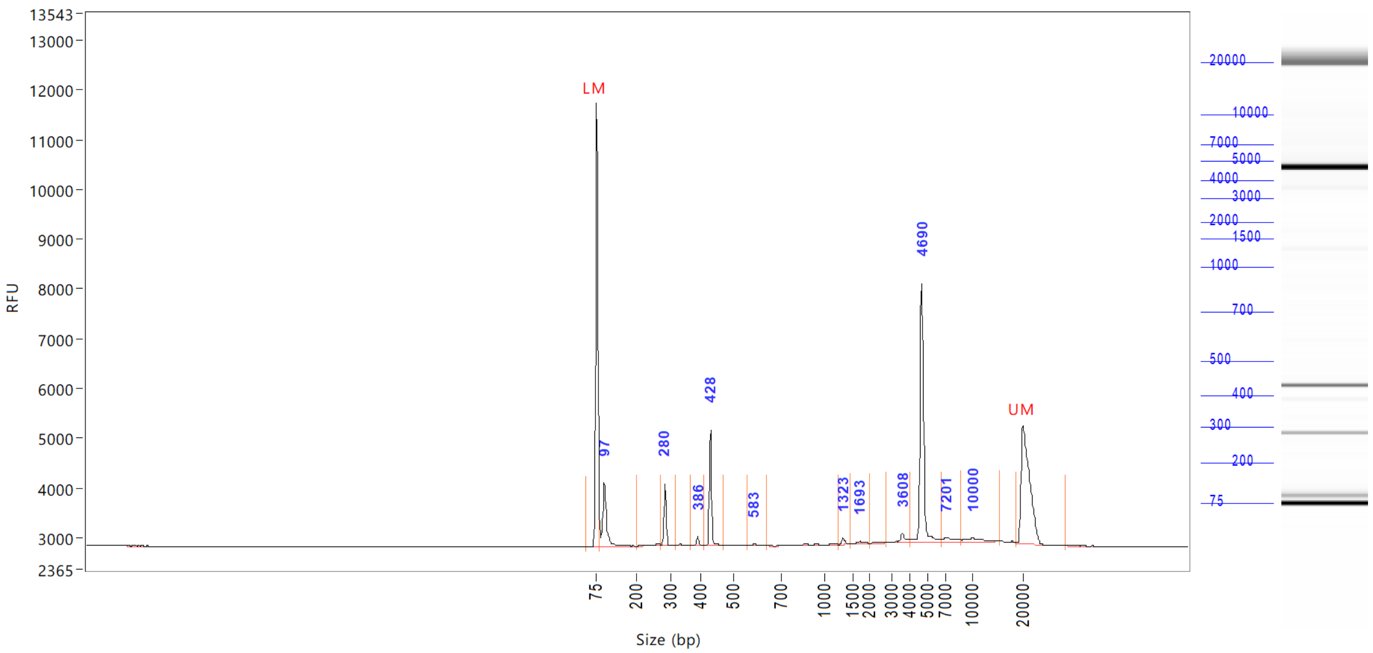

Peak	Size (bp)	Concentration (ng/uL)	From (bp)	To (bp)	Average size (bp)	CV%	RFU	Corrected peak area
1	75 (LM)	0.5000	45	86	73	4.36	8934	45.640
2	97	1.5179	86	198	101	14.01	1285	11.546
3	280	0.7804	269	313	280	1.55	1249	5.937
4	386	0.1093	364	407	383	1.40	176	0.831
5	428	1.3363	407	468	426	1.25	2331	10.165
6	583	0.0498	555	636	587	2.87	53	0.379
7	1323	0.0916	1245	1467	1320	2.75	135	0.696
8	1693	0.0660	1467	2001	1709	6.90	52	0.502
9	3608	0.2027	2743	4035	3600	7.33	180	1.542
10	4690	3.3389	4035	6630	4718	5.71	5189	25.398
11	7201	0.1922	6630	8800	7577	7.85	92	1.462
12	10000	0.1839	8800	15366	10728	14.27	71	1.399
13	20000 (UM)	0.2746	18537	28463	20607	4.44	2360	25.063

TIC:	7.8690	ng/uL
TIM:	36.5398	nmole/L
Total concentration:	8.2141	ng/uL

```
Sample peak width (sec): 5    Sample min peak height: 50    Sample baseline V to V?: Y    Sample baseline V to V points: 3
Sample filter: Binomial       Number of points for filter: 3  Sample start region (min): 0  Sample end region (min): 30
Marker peak width (sec): 5    Marker min peak height: 200   Marker baseline V to V?: Y    Marker baseline V to V points: 3
Lower marker selection: First peak > 500 RFU                Upper marker selection: Last peak > 200 RFU
Ladder size (bp)75, 200, 300, 400, 500, 700, 1000, 1500, 2000, 3000, 4000, 5000, 7000, 10000, 20000
Quantification using: Lower Marker          Final concentration (ng/uL): 0.5000          Dilution factor: 12.0
```

Sample: SampA7
Well location: A7
Created: Wednesday, October 20, 2021 5:18:46 PM

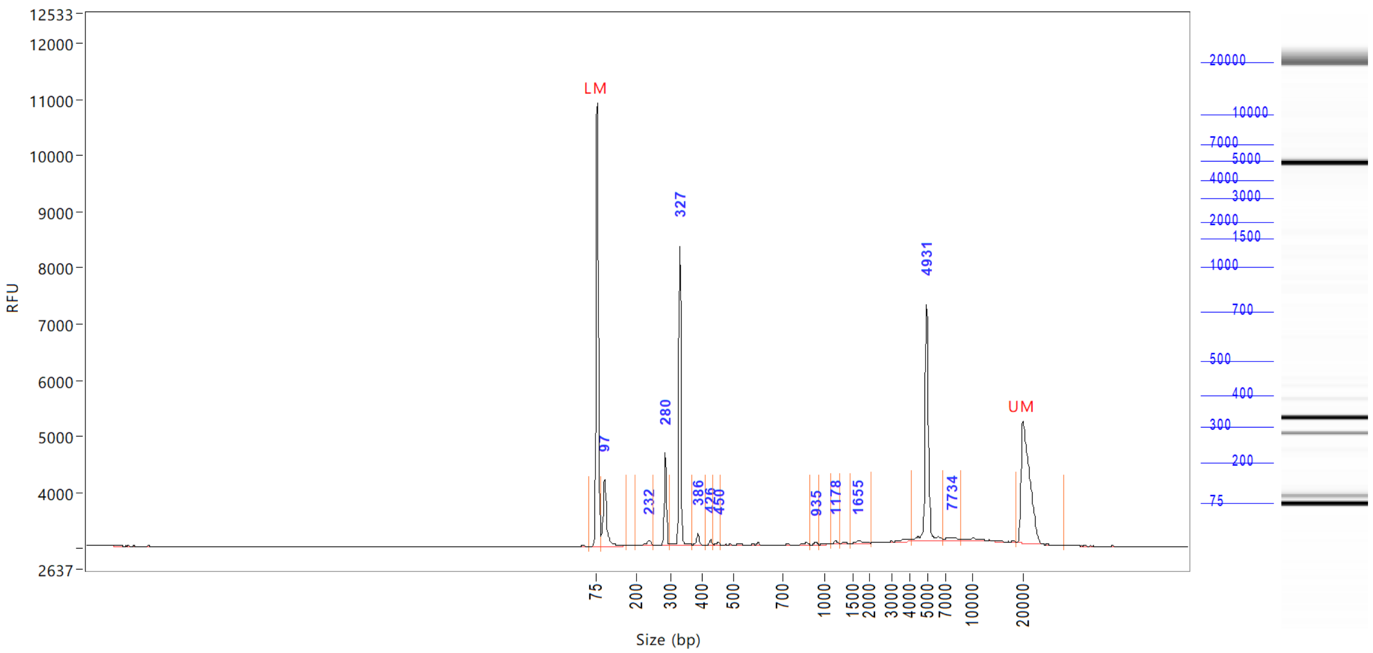

Peak	Size (bp)	Concentration (ng/uL)	From (bp)	To (bp)	Average size (bp)	CV%	RFU	Corrected peak area
1	75 (LM)	0.5000	48	86	72	4.64	7906	41.663
2	97	1.4589	86	164	98	11.28	1198	10.130
3	232	0.1398	192	246	228	3.82	97	0.971
4	280	1.1096	246	295	278	1.77	1657	7.705
5	327	3.5205	295	370	325	1.75	5323	24.445
6	386	0.1778	370	411	385	1.49	228	1.234
7	426	0.0672	411	435	425	0.93	104	0.466
8	450	0.0540	435	458	446	1.07	64	0.375
9	935	0.0405	900	957	928	1.14	52	0.281
10	1178	0.0549	1101	1267	1175	3.11	61	0.381
11	1655	0.0790	1456	2030	1686	7.19	55	0.549
12	4931	2.9951	4070	6852	4936	5.36	4208	20.797
13	7734	0.1128	6852	8800	7692	6.43	56	0.783
14	20000 (UM)	0.2755	18537	28100	20598	4.35	2180	22.955

TIC:	9.8098	ng/uL
TIM:	52.3042	nmole/L
Total concentration:	10.4003	ng/uL

```
Sample peak width (sec): 5    Sample min peak height: 50    Sample baseline V to V?: Y    Sample baseline V to V points: 3
Sample filter: Binomial       Number of points for filter: 3  Sample start region (min): 0  Sample end region (min): 30
Marker peak width (sec): 5    Marker min peak height: 200   Marker baseline V to V?: Y    Marker baseline V to V points: 3
Lower marker selection: First peak > 500 RFU                Upper marker selection: Last peak > 200 RFU
Ladder size (bp)75, 200, 300, 400, 500, 700, 1000, 1500, 2000, 3000, 4000, 5000, 7000, 10000, 20000
Quantification using: Lower Marker          Final concentration (ng/uL): 0.5000          Dilution factor: 12.0
```

Sample: SampA8
Well location: A8
Created: Wednesday, October 20, 2021 5:18:46 PM

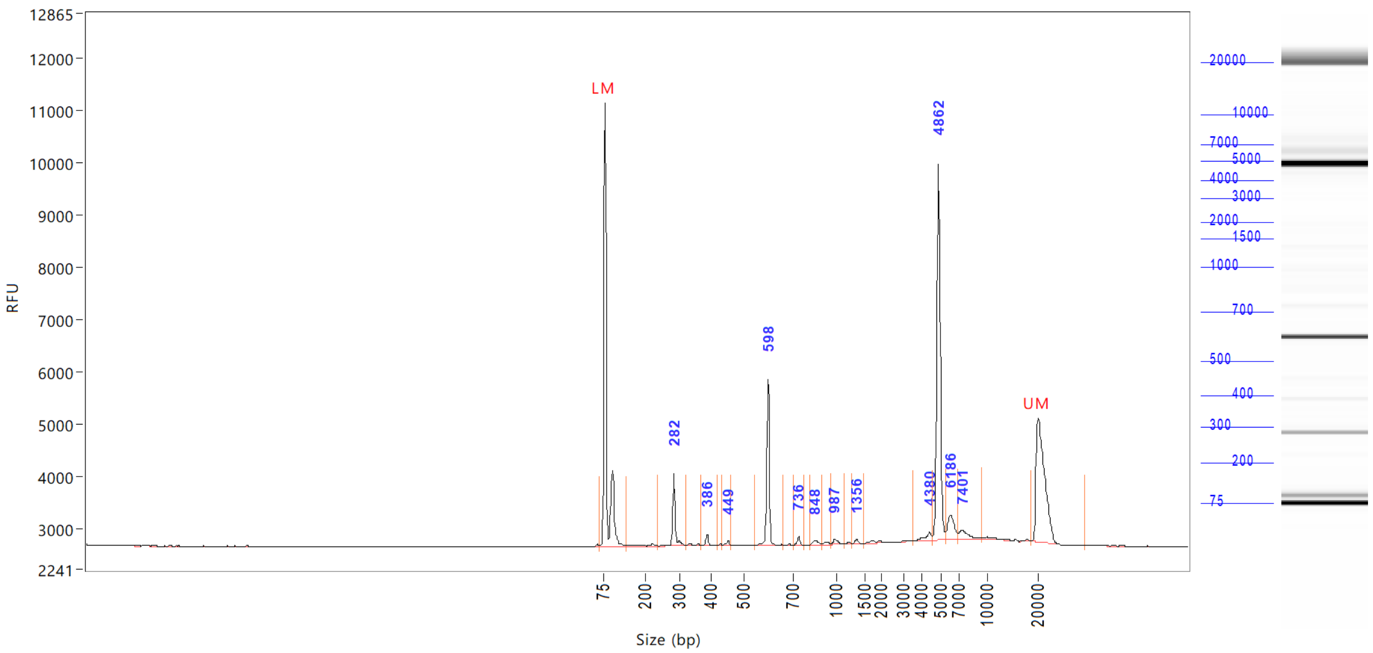

Peak	Size (bp)	Concentration (ng/uL)	From (bp)	To (bp)	Average size (bp)	CV%	RFU	Corrected peak area
1	75 (LM)	0.5000	58	145	79	14.08	8492	55.253
2	282	0.7918	237	317	281	2.88	1396	7.292
3	386	0.1236	368	420	385	1.94	216	1.138
4	449	0.0600	433	460	445	1.13	87	0.552
5	598	1.4895	544	662	595	1.49	3188	13.716
6	736	0.0843	701	775	730	1.48	158	0.777
7	848	0.0856	814	900	855	2.42	72	0.788
8	987	0.0786	961	1145	996	2.71	94	0.724
9	1356	0.0520	1278	1489	1349	2.59	93	0.479
10	4380	0.1448	3465	4518	4194	5.56	155	1.333
11	4862	3.7899	4518	5519	4863	2.36	7174	34.900
12	6186	0.4743	5519	7001	6189	6.04	467	4.368
13	7401	0.2169	7001	9533	7710	7.47	162	1.997
14	20000 (UM)	0.2210	18537	29189	20649	4.27	2369	24.417

TIC:	7.3912	ng/uL
TIM:	11.5649	nmole/L
Total concentration:	7.8107	ng/uL

Sample peak width (sec): 5   Sample min peak height: 50   Sample baseline V to V?: Y   Sample baseline V to V points: 3
Sample filter: Binomial   Number of points for filter: 3   Sample start region (min): 0   Sample end region (min): 30
Marker peak width (sec): 5   Marker min peak height: 200   Marker baseline V to V?: Y   Marker baseline V to V points: 3
Lower marker selection: First peak > 500 RFU   Upper marker selection: Last peak > 200 RFU
Ladder size (bp)75, 200, 300, 400, 500, 700, 1000, 1500, 2000, 3000, 4000, 5000, 7000, 10000, 20000
Quantification using: Lower Marker   Final concentration (ng/uL): 0.5000   Dilution factor: 12.0

Sample: SampA9
Well location: A9
Created: Wednesday, October 20, 2021 5:18:46 PM

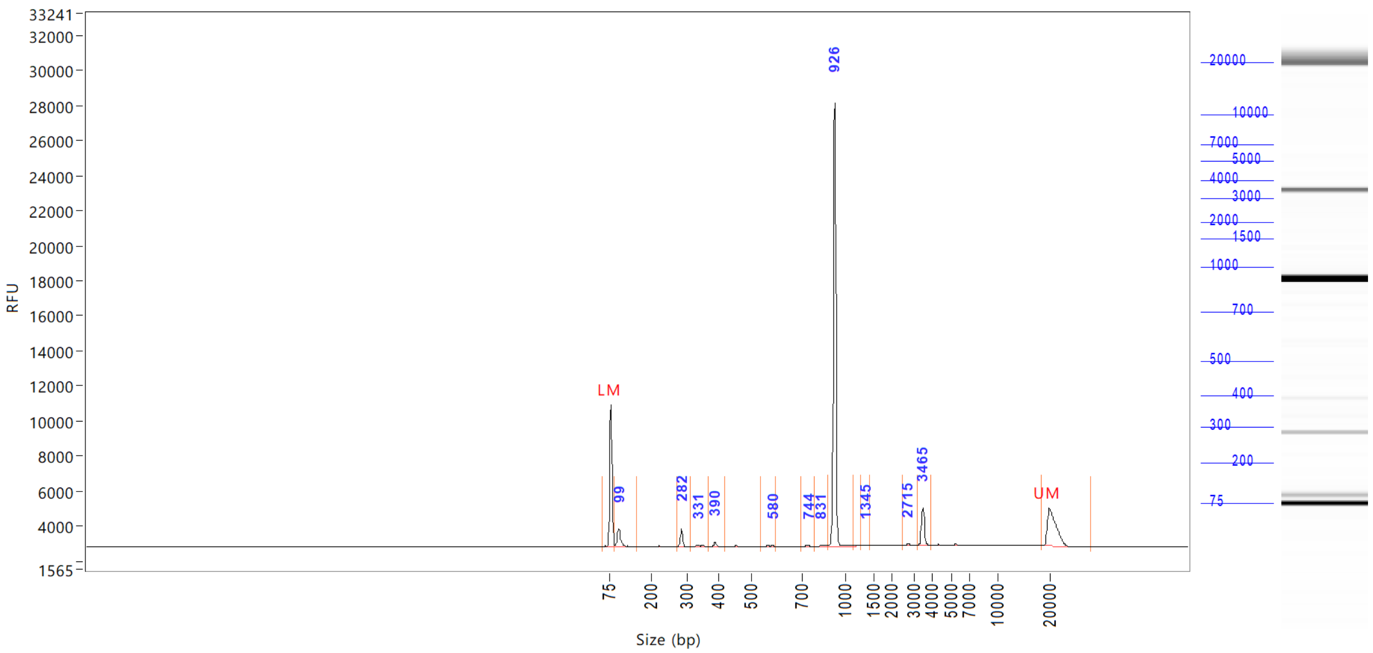

Peak	Size (bp)	Concentration (ng/uL)	From (bp)	To (bp)	Average size (bp)	CV%	RFU	Corrected peak area
1	75 (LM)	0.5000	50	86	73	4.63	8082	40.747
2	99	1.2127	86	152	99	8.51	1021	8.236
3	282	0.7187	269	313	282	1.78	1035	4.881
4	331	0.1270	313	368	339	3.86	86	0.863
5	390	0.1750	368	418	387	1.69	227	1.188
6	580	0.0874	537	598	570	2.00	69	0.594
7	744	0.0524	700	788	736	1.97	64	0.356
8	831	0.0971	788	883	846	2.52	69	0.660
9	926	15.0429	883	1123	921	1.24	25297	102.160
10	1345	0.0429	1256	1422	1331	2.15	51	0.291
11	2715	0.0987	2515	3144	2804	6.16	53	0.670
12	3465	1.2711	3144	3929	3438	2.45	2138	8.632
13	20000 (UM)	0.2734	18537	27859	20587	4.29	2145	22.280

TIC:	18.9260	ng/uL
TIM:	53.8278	nmole/L
Total concentration:	19.5816	ng/uL

```
Sample peak width (sec): 5    Sample min peak height: 50    Sample baseline V to V?: Y    Sample baseline V to V points: 3
Sample filter: Binomial       Number of points for filter: 3  Sample start region (min): 0  Sample end region (min): 30
Marker peak width (sec): 5    Marker min peak height: 200   Marker baseline V to V?: Y    Marker baseline V to V points: 3
Lower marker selection: First peak > 500 RFU                Upper marker selection: Last peak > 200 RFU
Ladder size (bp)75, 200, 300, 400, 500, 700, 1000, 1500, 2000, 3000, 4000, 5000, 7000, 10000, 20000
Quantification using: Lower Marker          Final concentration (ng/uL): 0.5000          Dilution factor: 12.0
```

Sample: SampA10
Well location: A10
Created: Wednesday, October 20, 2021 5:18:46 PM

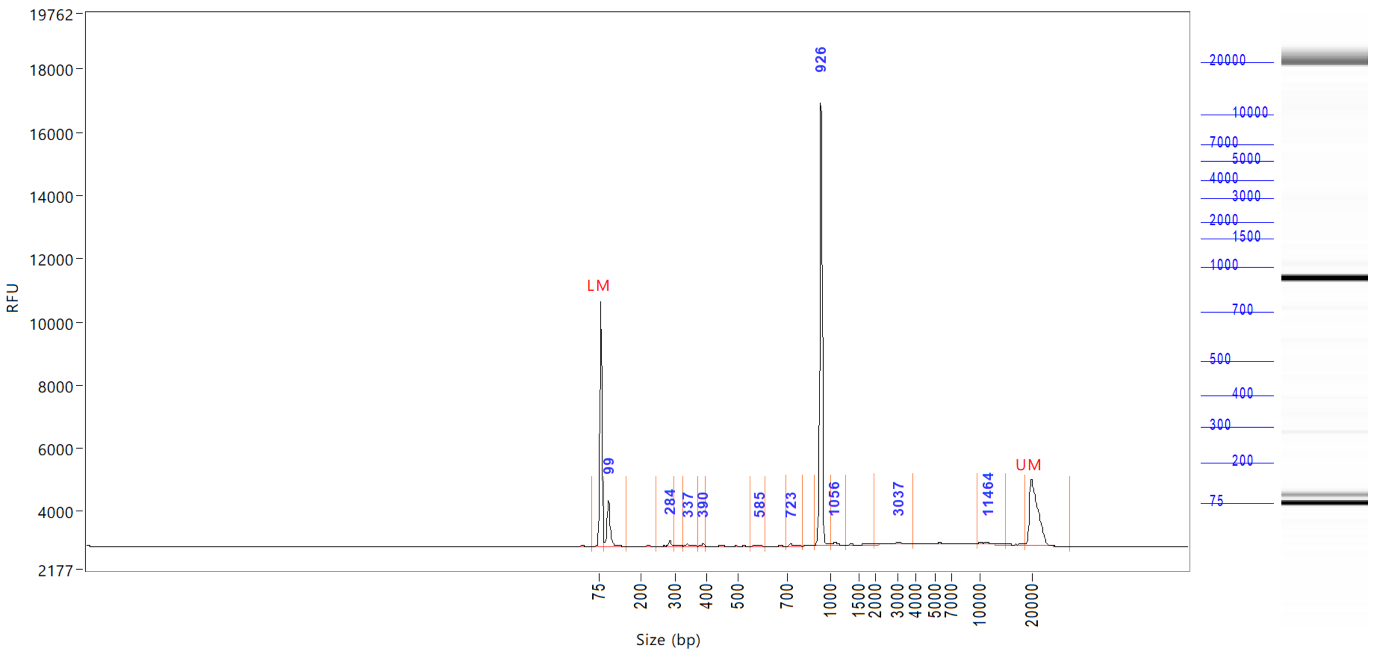

Peak	Size (bp)	Concentration (ng/uL)	From (bp)	To (bp)	Average size (bp)	CV%	RFU	Corrected peak area
1	75 (LM)	0.5000	50	87	74	4.46	7735	40.642
2	99	1.5758	87	154	99	7.34	1438	10.674
3	284	0.1460	244	295	279	3.18	172	0.989
4	337	0.1046	323	372	343	3.33	66	0.708
5	390	0.0529	372	400	386	1.39	71	0.359
6	585	0.0728	544	606	574	2.11	54	0.493
7	723	0.0781	692	801	725	2.55	86	0.529
8	926	8.4732	887	1000	924	1.03	14027	57.395
9	1056	0.1178	1000	1267	1069	5.03	108	0.798
10	3037	0.1637	1981	3857	2806	14.58	59	1.109
11	11464	0.1926	9667	15000	11968	12.98	54	1.304
12	20000 (UM)	0.2681	18781	27738	20654	4.30	2076	21.793

TIC:	10.9775	ng/uL
TIM:	43.5075	nmole/L
Total concentration:	11.4993	ng/uL

```
Sample peak width (sec): 5    Sample min peak height: 50    Sample baseline V to V?: Y    Sample baseline V to V points: 3
Sample filter: Binomial       Number of points for filter: 3  Sample start region (min): 0  Sample end region (min): 30
Marker peak width (sec): 5    Marker min peak height: 200   Marker baseline V to V?: Y    Marker baseline V to V points: 3
Lower marker selection: First peak > 500 RFU                Upper marker selection: Last peak > 200 RFU
Ladder size (bp)75, 200, 300, 400, 500, 700, 1000, 1500, 2000, 3000, 4000, 5000, 7000, 10000, 20000
Quantification using: Lower Marker          Final concentration (ng/uL): 0.5000          Dilution factor: 12.0
```

Sample: SampA11
Well location: A11
Created: Wednesday, October 20, 2021 5:18:46 PM

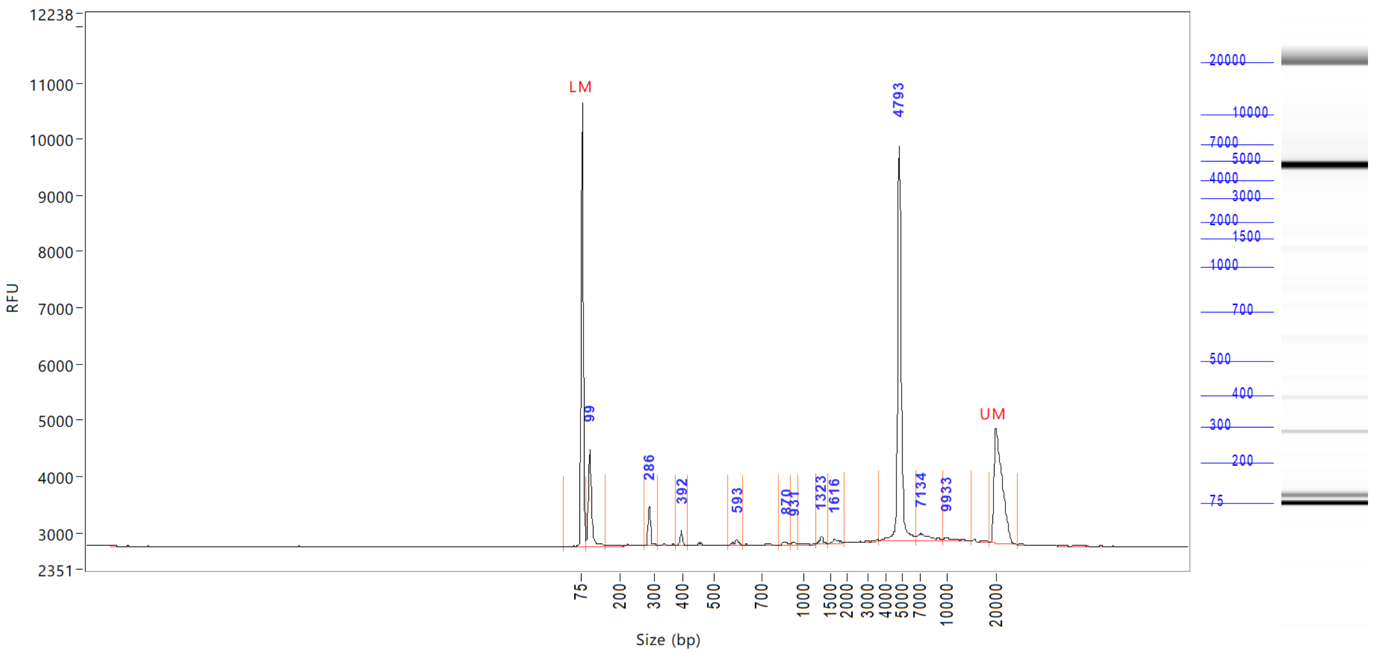

Peak	Size (bp)	Concentration (ng/uL)	From (bp)	To (bp)	Average size (bp)	CV%	RFU	Corrected peak area
1	75 (LM)	0.5000	18	86	74	4.74	7900	41.933
2	99	1.9425	86	152	99	9.52	1718	13.576
3	286	0.4904	273	311	284	1.62	690	3.427
4	392	0.1925	374	412	389	1.34	264	1.345
5	593	0.1523	552	618	585	2.21	116	1.065
6	870	0.0856	822	909	863	2.28	65	0.598
7	931	0.0488	909	961	929	1.35	52	0.341
8	1323	0.1255	1223	1456	1317	3.52	130	0.877
9	1616	0.1053	1456	1923	1659	7.16	72	0.736
10	4793	5.5503	3679	6630	4809	5.81	7026	38.790
11	7134	0.3464	6630	9533	7758	10.13	131	2.421
12	9933	0.1315	9533	15000	11104	12.32	60	0.919
13	20000 (UM)	0.2536	18781	24352	20662	4.21	2045	21.266

TIC:	9.1710	ng/uL
TIM:	38.8053	nmole/L
Total concentration:	9.6954	ng/uL

```
Sample peak width (sec): 5    Sample min peak height: 50    Sample baseline V to V?: Y    Sample baseline V to V points: 3
Sample filter: Binomial       Number of points for filter: 3  Sample start region (min): 0  Sample end region (min): 30
Marker peak width (sec): 5    Marker min peak height: 200   Marker baseline V to V?: Y    Marker baseline V to V points: 3
Lower marker selection: First peak > 500 RFU                Upper marker selection: Last peak > 200 RFU
Ladder size (bp)75, 200, 300, 400, 500, 700, 1000, 1500, 2000, 3000, 4000, 5000, 7000, 10000, 20000
Quantification using: Lower Marker          Final concentration (ng/uL): 0.5000          Dilution factor: 12.0
```

Sample: SampA12
Well location: A12
Created: Wednesday, October 20, 2021 5:18:46 PM

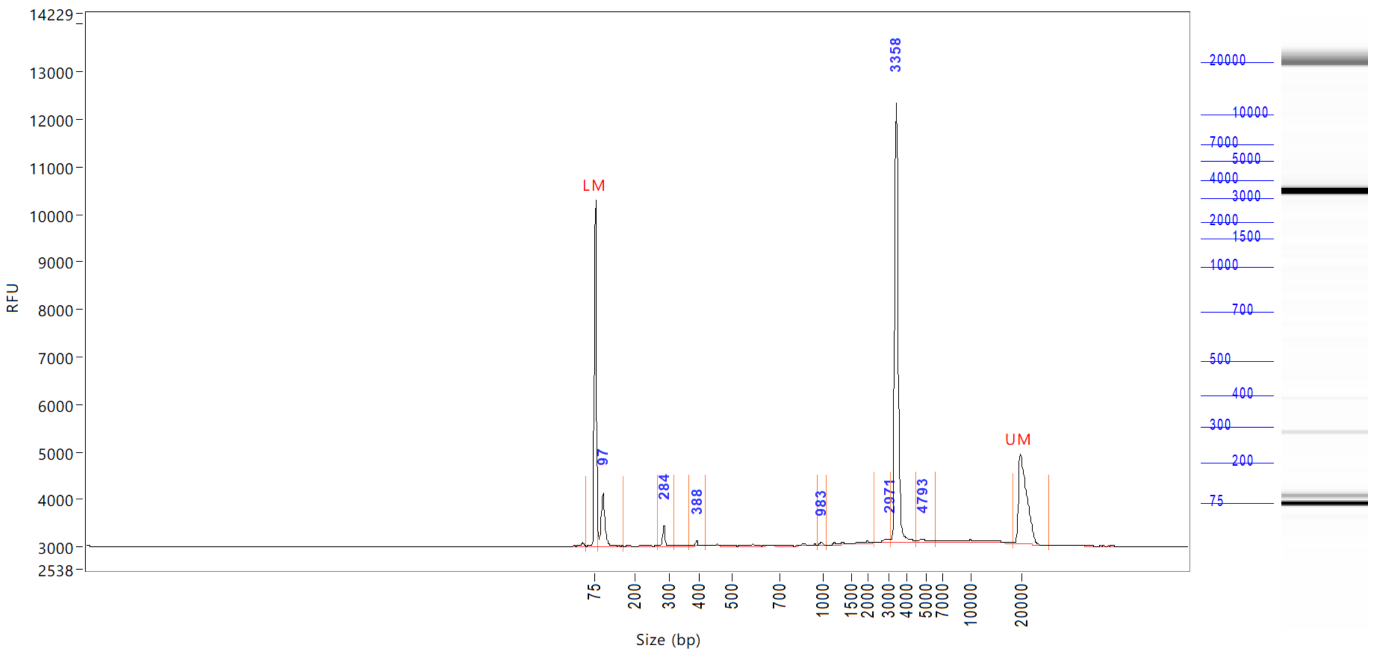

Peak	Size (bp)	Concentration (ng/uL)	From (bp)	To (bp)	Average size (bp)	CV%	RFU	Corrected peak area
1	75 (LM)	0.5000	47	84	73	4.58	7269	37.526
2	97	1.5737	84	164	97	9.04	1137	9.842
3	284	0.3527	268	313	283	2.24	432	2.206
4	388	0.0974	366	418	385	1.82	113	0.609
5	983	0.0592	957	1056	983	1.36	62	0.370
6	2971	0.1375	2344	3037	2807	5.97	75	0.860
7	3358	7.3346	3037	4449	3378	4.28	9260	45.873
8	4793	0.1392	4449	6260	5035	9.06	52	0.870
9	20000 (UM)	0.2610	18415	25561	20521	4.15	1891	19.585

TIC:	9.6941	ng/uL
TIM:	32.9040	nmole/L
Total concentration:	10.6344	ng/uL

```
Sample peak width (sec): 5    Sample min peak height: 50    Sample baseline V to V?: Y    Sample baseline V to V points: 3
Sample filter: Binomial       Number of points for filter: 3  Sample start region (min): 0  Sample end region (min): 30
Marker peak width (sec): 5    Marker min peak height: 200   Marker baseline V to V?: Y    Marker baseline V to V points: 3
Lower marker selection: First peak > 500 RFU                Upper marker selection: Last peak > 200 RFU
Ladder size (bp)75, 200, 300, 400, 500, 700, 1000, 1500, 2000, 3000, 4000, 5000, 7000, 10000, 20000
Quantification using: Lower Marker          Final concentration (ng/uL): 0.5000          Dilution factor: 12.0
```

Sample: SampB1
Well location: B1
Created: Wednesday, October 20, 2021 5:18:46 PM

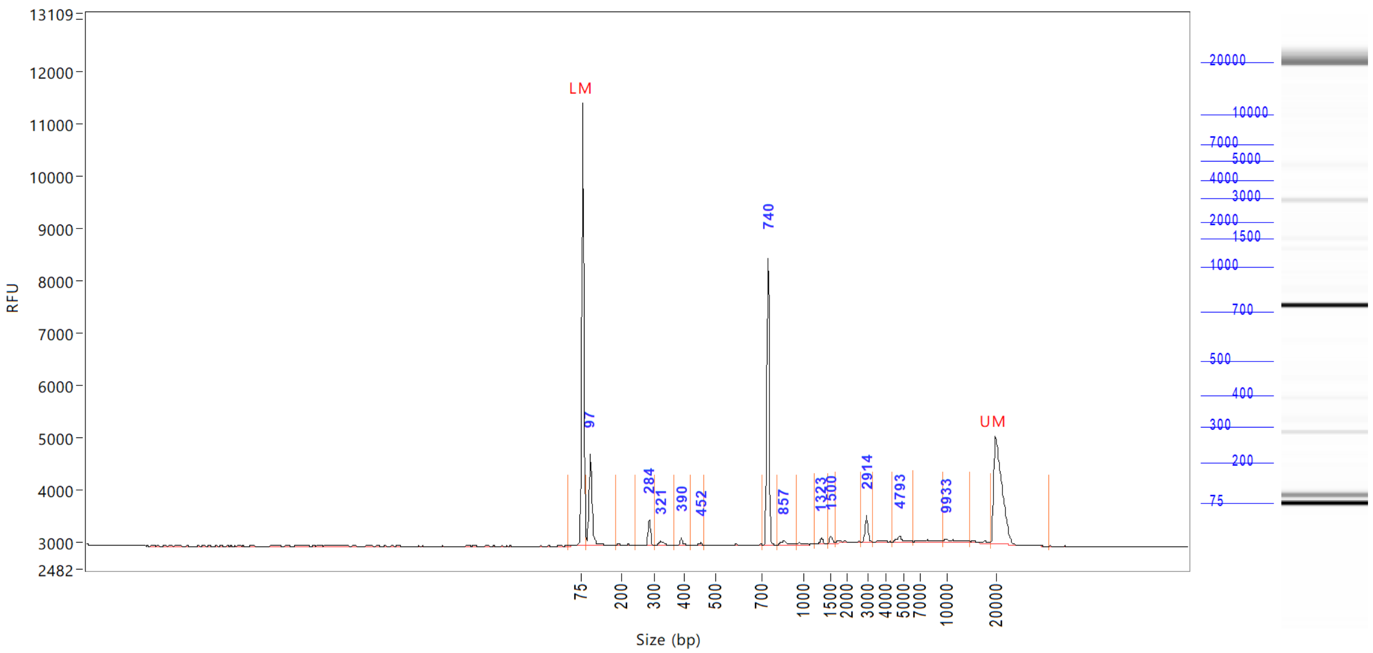

Peak	Size (bp)	Concentration (ng/uL)	From (bp)	To (bp)	Average size (bp)	CV%	RFU	Corrected peak area
1	75 (LM)	0.5000	28	86	73	5.12	8482	44.438
2	97	1.8478	86	181	100	12.02	1749	13.685
3	284	0.3526	242	300	280	2.77	495	2.611
4	321	0.1534	300	368	326	4.81	79	1.136
5	390	0.1295	368	418	389	1.97	155	0.959
6	452	0.0540	418	464	446	2.07	54	0.400
7	740	2.9480	701	809	736	1.16	5485	21.833
8	857	0.1209	809	948	865	4.07	69	0.896
9	1323	0.0736	1212	1433	1311	2.63	109	0.545
10	1500	0.0899	1433	1655	1511	3.28	138	0.666
11	2914	0.2941	2657	3286	2904	2.56	504	2.178
12	4793	0.1140	4345	6186	4817	5.42	114	0.845
13	9933	0.1345	9533	14879	11706	13.97	51	0.996
14	20000 (UM)	0.2442	18903	30760	20643	4.75	2059	21.704

TIC:	6.3122	ng/uL
TIM:	41.1714	nmole/L
Total concentration:	6.7836	ng/uL

```
Sample peak width (sec): 5     Sample min peak height: 50     Sample baseline V to V?: Y     Sample baseline V to V points: 3
Sample filter: Binomial        Number of points for filter: 3  Sample start region (min): 0  Sample end region (min): 30
Marker peak width (sec): 5     Marker min peak height: 200    Marker baseline V to V?: Y     Marker baseline V to V points: 3
Lower marker selection: First peak > 500 RFU                  Upper marker selection: Last peak > 200 RFU
Ladder size (bp)75, 200, 300, 400, 500, 700, 1000, 1500, 2000, 3000, 4000, 5000, 7000, 10000, 20000
Quantification using: Lower Marker          Final concentration (ng/uL): 0.5000          Dilution factor: 12.0
```

Sample: SampB2
Well location: B2
Created: Wednesday, October 20, 2021 5:18:46 PM

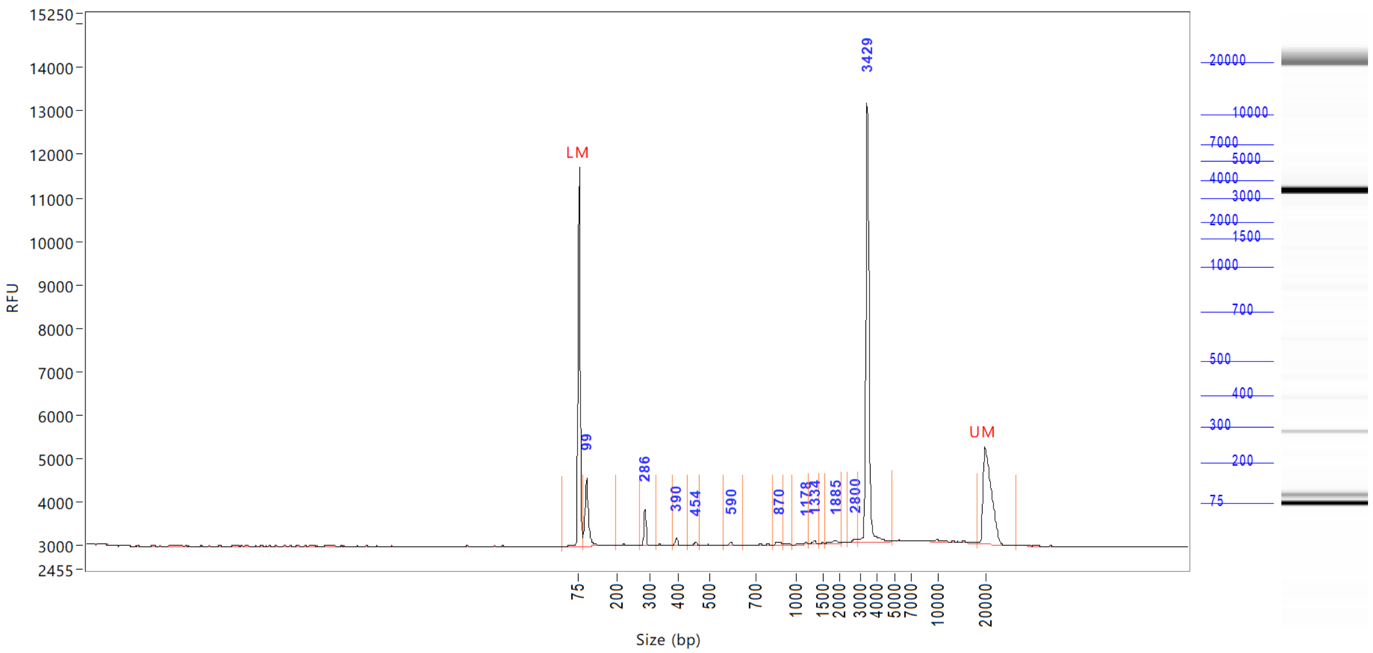

Peak	Size (bp)	Concentration (ng/uL)	From (bp)	To (bp)	Average size (bp)	CV%	RFU	Corrected peak area
1	75 (LM)	0.5000	24	86	73	5.02	8704	46.143
2	99	1.7577	86	198	101	14.09	1572	13.518
3	286	0.5362	271	323	284	1.74	848	4.124
4	390	0.1232	380	428	392	1.93	166	0.948
5	454	0.0689	428	468	451	1.46	78	0.530
6	590	0.0974	557	646	591	2.99	92	0.749
7	870	0.0995	822	909	865	2.11	80	0.766
8	1178	0.0613	965	1234	1117	5.95	57	0.471
9	1334	0.0649	1234	1422	1317	2.66	81	0.499
10	1885	0.0969	1539	2144	1786	7.03	58	0.745
11	2800	0.0960	2401	2943	2763	4.49	87	0.738
12	3429	6.4678	2943	4897	3440	4.89	10128	49.741
13	20000 (UM)	0.2593	18415	26408	20580	4.29	2230	23.934

TIC:	9.4698	ng/uL
TIM:	36.3256	nmole/L
Total concentration:	10.0098	ng/uL

```
Sample peak width (sec): 5    Sample min peak height: 50    Sample baseline V to V?: Y    Sample baseline V to V points: 3
Sample filter: Binomial       Number of points for filter: 3  Sample start region (min): 0  Sample end region (min): 30
Marker peak width (sec): 5    Marker min peak height: 200   Marker baseline V to V?: Y    Marker baseline V to V points: 3
Lower marker selection: First peak > 500 RFU                Upper marker selection: Last peak > 200 RFU
Ladder size (bp)75, 200, 300, 400, 500, 700, 1000, 1500, 2000, 3000, 4000, 5000, 7000, 10000, 20000
Quantification using: Lower Marker          Final concentration (ng/uL): 0.5000          Dilution factor: 12.0
```

Sample: SampB3
Well location: B3
Created: Wednesday, October 20, 2021 5:18:46 PM

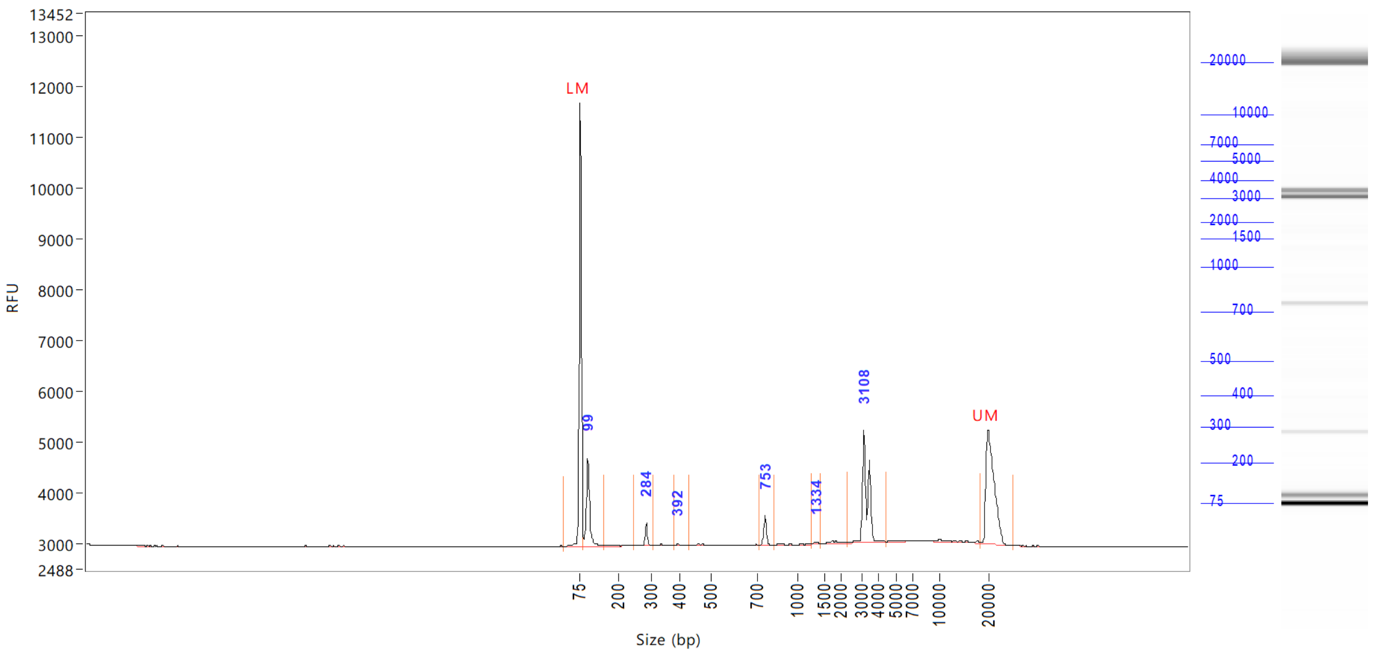

Peak	Size (bp)	Concentration (ng/uL)	From (bp)	To (bp)	Average size (bp)	CV%	RFU	Corrected peak area
1	75 (LM)	0.5000	24	86	73	5.53	8757	46.401
2	99	1.7761	86	154	99	9.37	1727	13.735
3	284	0.2925	244	305	282	2.24	455	2.262
4	392	0.0576	378	428	395	2.45	53	0.445
5	753	0.3651	714	827	750	1.56	599	2.824
6	1334	0.0433	1245	1433	1320	2.82	52	0.335
7	3108	2.2828	2286	4414	3227	7.55	2210	17.654
8	20000 (UM)	0.2545	18537	25199	20642	4.17	2244	23.616

TIC:	4.8174	ng/uL
TIM:	33.4247	nmole/L
Total concentration:	5.5132	ng/uL

```
Sample peak width (sec): 5    Sample min peak height: 50    Sample baseline V to V?: Y    Sample baseline V to V points: 3
Sample filter: Binomial       Number of points for filter: 3  Sample start region (min): 0  Sample end region (min): 30
Marker peak width (sec): 5    Marker min peak height: 200   Marker baseline V to V?: Y    Marker baseline V to V points: 3
Lower marker selection: First peak > 500 RFU                Upper marker selection: Last peak > 200 RFU
Ladder size (bp)75, 200, 300, 400, 500, 700, 1000, 1500, 2000, 3000, 4000, 5000, 7000, 10000, 20000
Quantification using: Lower Marker          Final concentration (ng/uL): 0.5000          Dilution factor: 12.0
```

Sample: SampB4
Well location: B4
Created: Wednesday, October 20, 2021 5:18:46 PM

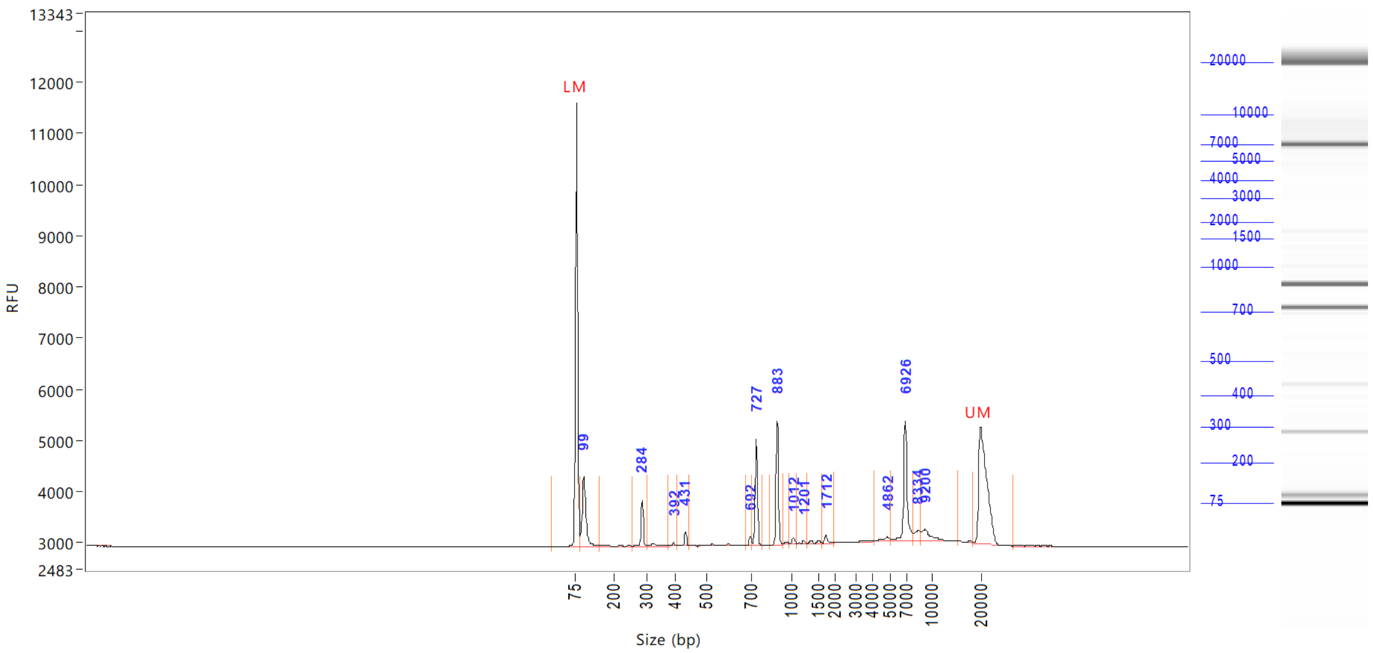

Peak	Size (bp)	Concentration (ng/uL)	From (bp)	To (bp)	Average size (bp)	CV%	RFU	Corrected peak area
1	75 (LM)	0.5000	0	86	73	6.59	8682	46.604
2	99	1.5566	86	148	99	9.41	1360	12.090
3	284	0.5624	253	301	282	1.77	898	4.368
4	392	0.0376	376	403	390	1.00	66	0.292
5	431	0.1754	403	443	428	1.26	274	1.363
6	692	0.0917	674	701	691	0.82	166	0.713
7	727	1.1418	701	770	722	1.25	2071	8.868
8	883	1.3083	827	926	882	1.08	2426	10.162
9	1012	0.0714	983	1068	1006	1.50	127	0.555
10	1201	0.0404	1068	1256	1158	3.97	52	0.314
11	1712	0.0957	1597	1962	1703	3.60	155	0.743
12	4862	0.1133	4070	5075	4694	4.85	88	0.880
13	6926	1.6042	5075	7734	6855	5.39	2347	12.460
14	8334	0.2430	7734	8667	8218	3.39	205	1.887
15	9200	0.4299	8667	15244	9872	12.97	233	3.339
16	20000 (UM)	0.2613	18537	26770	20656	4.28	2284	24.355

TIC:	7.4717	ng/uL
TIM:	36.0061	nmole/L
Total concentration:	7.9479	ng/uL

```
Sample peak width (sec): 5    Sample min peak height: 50    Sample baseline V to V?: Y    Sample baseline V to V points: 3
Sample filter: Binomial       Number of points for filter: 3  Sample start region (min): 0  Sample end region (min): 30
Marker peak width (sec): 5    Marker min peak height: 200   Marker baseline V to V?: Y    Marker baseline V to V points: 3
Lower marker selection: First peak > 500 RFU                Upper marker selection: Last peak > 200 RFU
Ladder size (bp)75, 200, 300, 400, 500, 700, 1000, 1500, 2000, 3000, 4000, 5000, 7000, 10000, 20000
Quantification using: Lower Marker          Final concentration (ng/uL): 0.5000          Dilution factor: 12.0
```

Sample: SampB5
Well location: B5
Created: Wednesday, October 20, 2021 5:18:46 PM

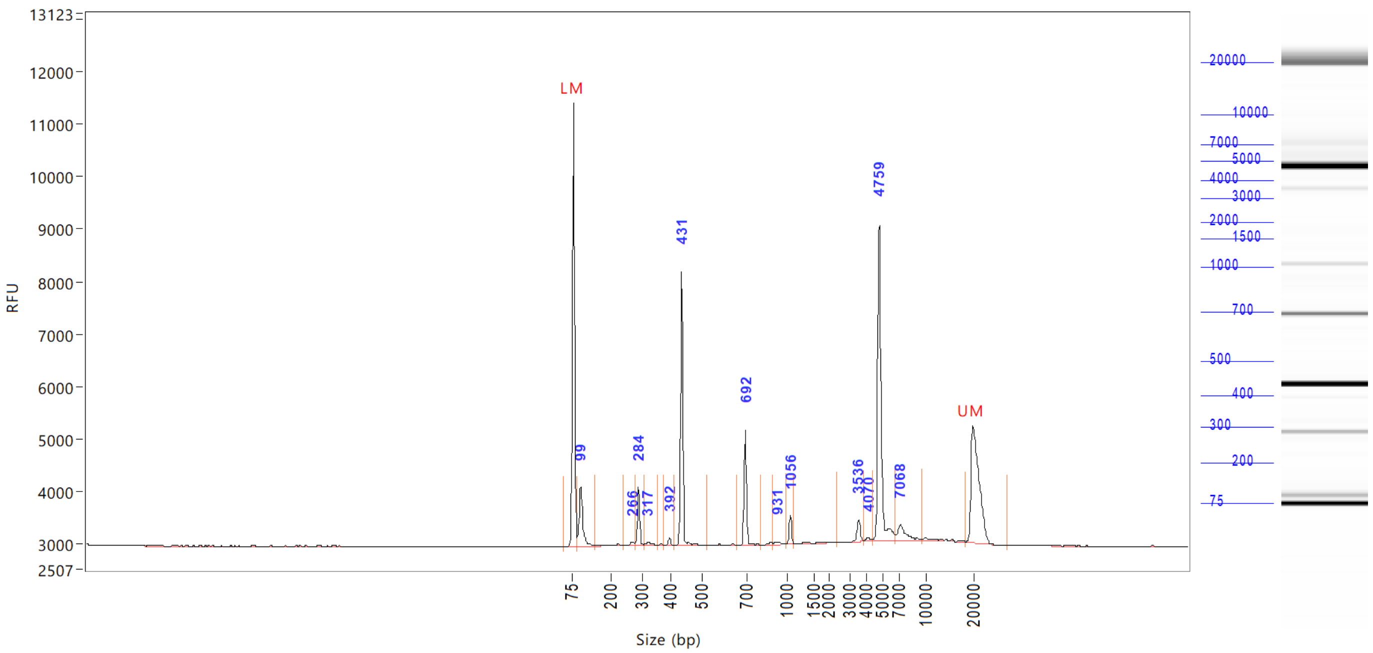

Peak	Size (bp)	Concentration (ng/uL)	From (bp)	To (bp)	Average size (bp)	CV%	RFU	Corrected peak area
1	75 (LM)	0.5000	45	86	73	4.66	8483	45.730
2	99	1.3348	86	145	99	9.63	1124	10.174
3	266	0.0769	237	275	264	2.95	63	0.586
4	284	0.6974	275	301	283	1.34	1115	5.315
5	317	0.0815	301	352	318	3.45	61	0.621
6	392	0.0838	374	411	390	1.20	131	0.639
7	431	3.2087	411	519	431	1.56	5219	24.455
8	692	1.3010	657	801	690	1.61	2191	9.916
9	931	0.0844	892	987	927	2.39	59	0.643
10	1056	0.3087	987	1123	1042	2.11	537	2.353
11	3536	0.2739	2372	3857	3480	5.07	412	2.088
12	4070	0.0682	3857	4345	4107	3.26	61	0.520
13	4759	4.2134	4345	6556	4823	6.55	6031	32.113
14	7068	0.4493	6556	9600	7446	9.16	296	3.424
15	20000 (UM)	0.2571	18415	27133	20598	4.29	2223	23.517

TIC:	12.1820	ng/uL
TIM:	45.1319	nmole/L
Total concentration:	12.5514	ng/uL

```
Sample peak width (sec): 5     Sample min peak height: 50    Sample baseline V to V?: Y    Sample baseline V to V points: 3
Sample filter: Binomial        Number of points for filter: 3  Sample start region (min): 0  Sample end region (min): 30
Marker peak width (sec): 5     Marker min peak height: 200   Marker baseline V to V?: Y    Marker baseline V to V points: 3
Lower marker selection: First peak > 500 RFU                 Upper marker selection: Last peak > 200 RFU
Ladder size (bp)75, 200, 300, 400, 500, 700, 1000, 1500, 2000, 3000, 4000, 5000, 7000, 10000, 20000
Quantification using: Lower Marker          Final concentration (ng/uL): 0.5000          Dilution factor: 12.0
```

Sample: SampB6
Well location: B6
Created: Wednesday, October 20, 2021 5:18:46 PM

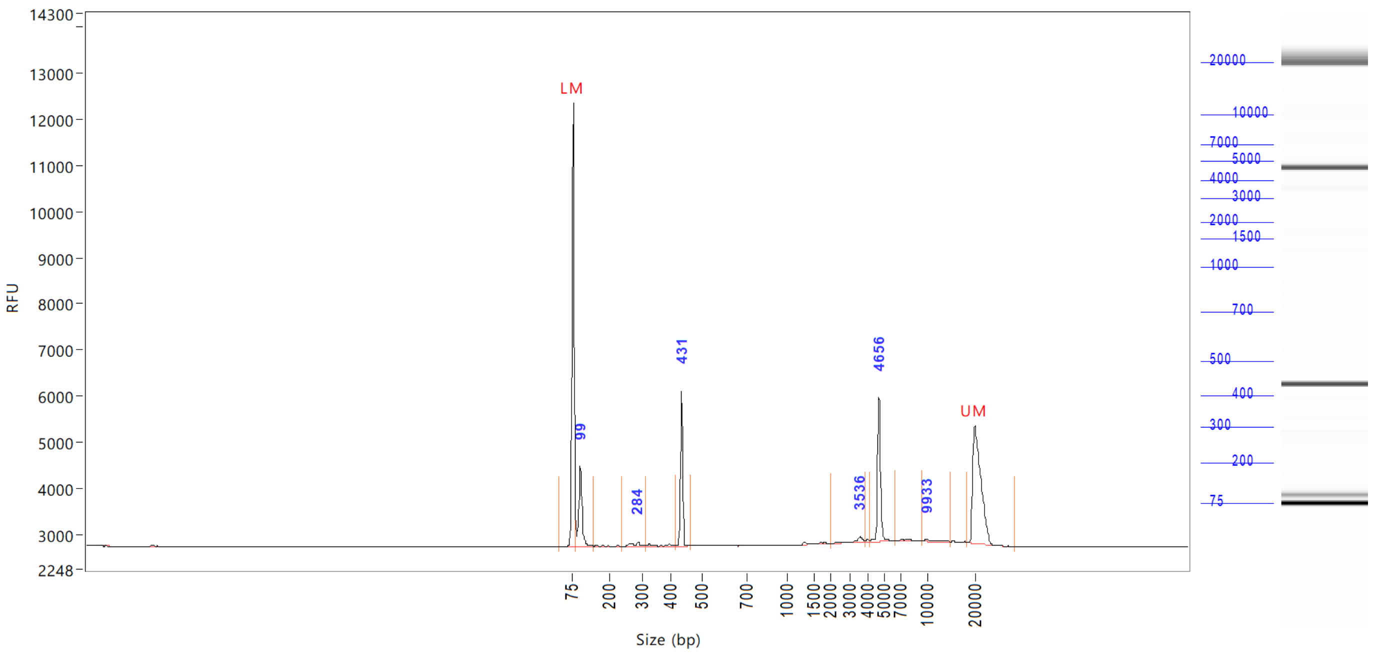

Peak	Size (bp)	Concentration (ng/uL)	From (bp)	To (bp)	Average size (bp)	CV%	RFU	Corrected peak area
1	75 (LM)	0.5000	31	86	73	4.76	9633	51.128
2	99	1.6152	86	145	98	7.34	1741	13.764
3	284	0.1586	235	309	271	5.15	102	1.351
4	431	1.8046	412	462	430	0.97	3344	15.378
5	3536	0.1291	2058	3822	3353	11.45	132	1.100
6	4656	1.6951	4139	6334	4652	3.05	3112	14.444
7	9933	0.0920	9533	15000	11217	12.41	51	0.784
8	20000 (UM)	0.2650	18415	28342	20581	4.26	2558	27.099

TIC:	5.4946	ng/uL
TIM:	35.6081	nmole/L
Total concentration:	5.9624	ng/uL

Sample peak width (sec): 5   Sample min peak height: 50   Sample baseline V to V?: Y   Sample baseline V to V points: 3
Sample filter: Binomial   Number of points for filter: 3   Sample start region (min): 0   Sample end region (min): 30
Marker peak width (sec): 5   Marker min peak height: 200   Marker baseline V to V?: Y   Marker baseline V to V points: 3
Lower marker selection: First peak > 500 RFU   Upper marker selection: Last peak > 200 RFU
Ladder size (bp)75, 200, 300, 400, 500, 700, 1000, 1500, 2000, 3000, 4000, 5000, 7000, 10000, 20000
Quantification using: Lower Marker   Final concentration (ng/uL): 0.5000   Dilution factor: 12.0

Sample: SampB7
Well location: B7
Created: Wednesday, October 20, 2021 5:18:46 PM

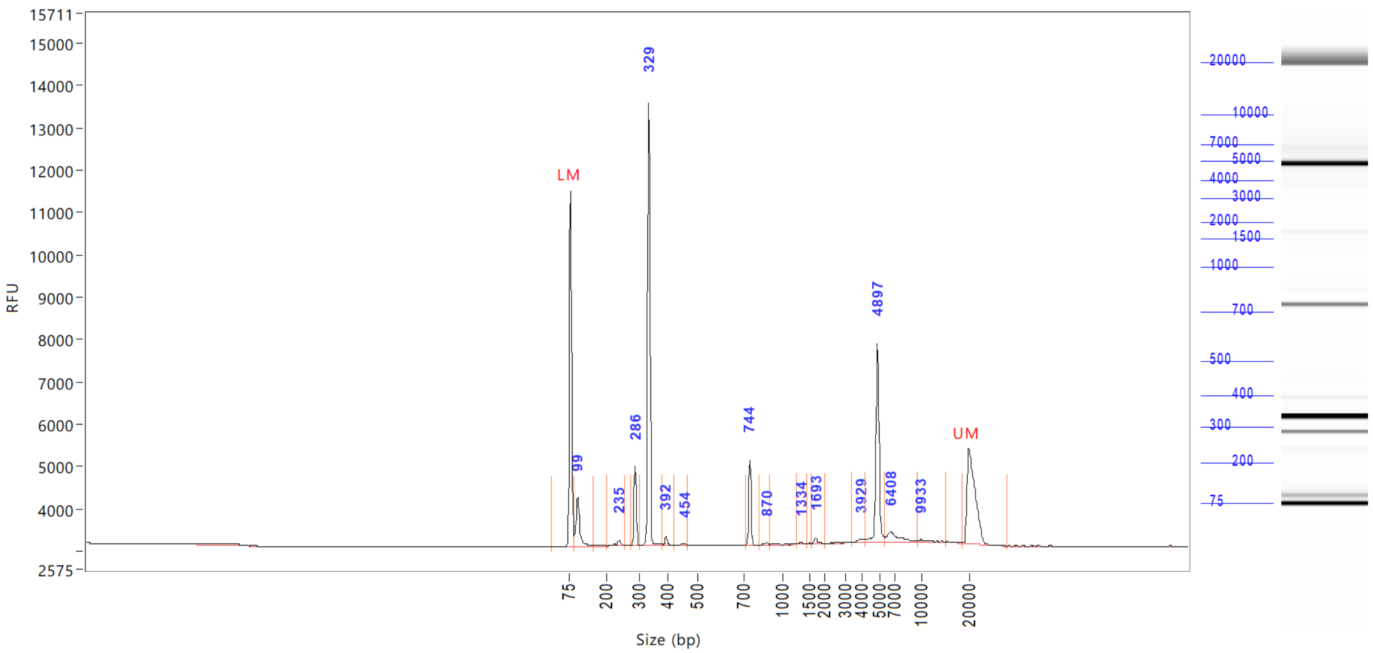

Peak	Size (bp)	Concentration (ng/uL)	From (bp)	To (bp)	Average size (bp)	CV%	RFU	Corrected peak area
1	75 (LM)	0.5000	14	86	73	5.06	8383	44.700
2	99	1.4425	86	152	99	9.98	1160	10.747
3	235	0.1571	198	253	231	3.43	131	1.170
4	286	1.2058	271	301	283	1.17	1859	8.983
5	329	6.5908	301	380	329	1.65	10473	49.101
6	392	0.1572	380	422	390	1.32	220	1.171
7	454	0.0673	422	464	446	1.85	52	0.501
8	744	1.1917	710	818	741	1.36	2021	8.878
9	870	0.0629	818	896	858	1.98	52	0.469
10	1334	0.0484	1234	1456	1342	3.94	55	0.361
11	1693	0.1154	1539	2001	1680	4.58	161	0.860
12	3929	0.1312	3393	4208	3895	5.15	82	0.977
13	4897	3.1862	4208	5741	4871	3.47	4693	23.737
14	6408	0.5733	5741	9533	7054	13.52	236	4.271
15	9933	0.1255	9533	15000	11152	12.26	52	0.935
16	20000 (UM)	0.2687	18659	28100	20686	4.53	2249	24.022

TIC:	15.0552	ng/uL
TIM:	70.1434	nmole/L
Total concentration:	15.3735	ng/uL

```
Sample peak width (sec): 5    Sample min peak height: 50    Sample baseline V to V?: Y    Sample baseline V to V points: 3
Sample filter: Binomial       Number of points for filter: 3  Sample start region (min): 0  Sample end region (min): 30
Marker peak width (sec): 5    Marker min peak height: 200   Marker baseline V to V?: Y    Marker baseline V to V points: 3
Lower marker selection: First peak > 500 RFU                Upper marker selection: Last peak > 200 RFU
Ladder size (bp)75, 200, 300, 400, 500, 700, 1000, 1500, 2000, 3000, 4000, 5000, 7000, 10000, 20000
Quantification using: Lower Marker          Final concentration (ng/uL): 0.5000          Dilution factor: 12.0
```

Sample: SampB8
Well location: B8
Created: Wednesday, October 20, 2021 5:18:46 PM

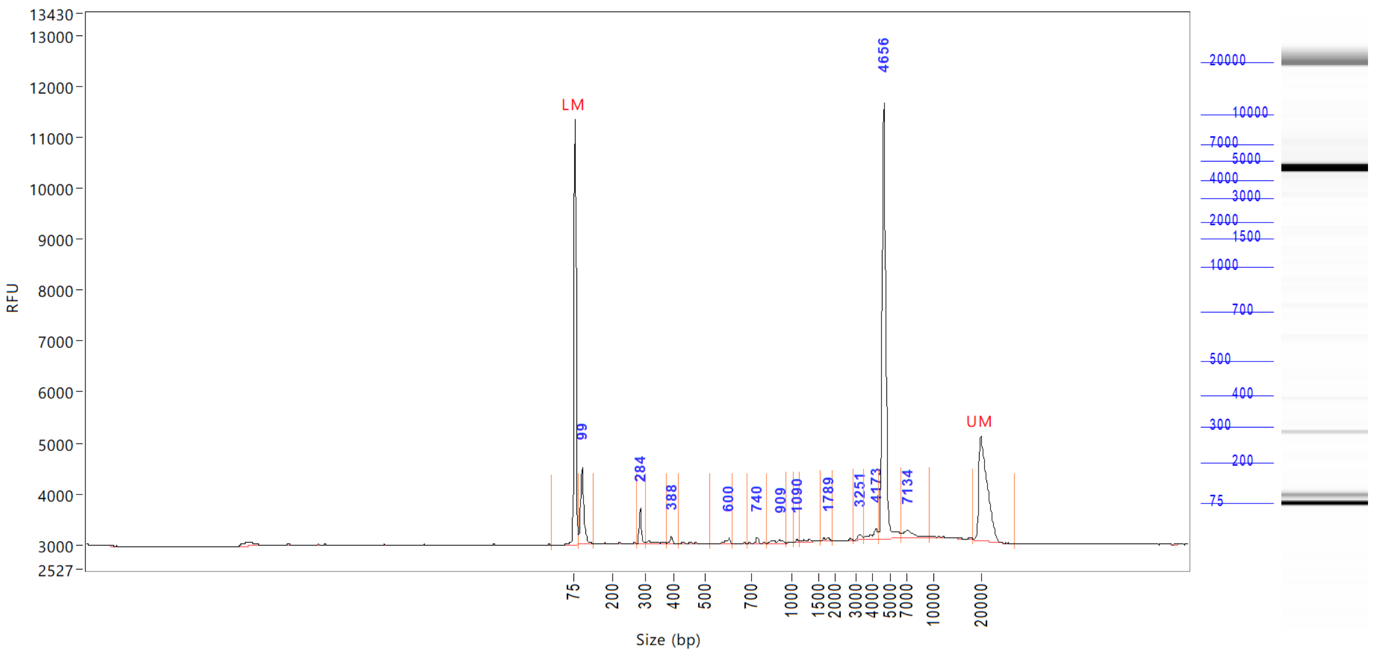

Peak	Size (bp)	Concentration (ng/uL)	From (bp)	To (bp)	Average size (bp)	CV%	RFU	Corrected peak area
1	75 (LM)	0.5000	1	86	73	5.44	8325	44.494
2	99	1.6531	86	139	98	7.47	1513	12.259
3	284	0.4335	271	300	282	1.44	691	3.215
4	388	0.0990	372	412	386	1.52	140	0.734
5	600	0.1090	519	618	586	2.92	104	0.808
6	740	0.0891	682	814	737	3.24	120	0.661
7	909	0.1272	814	957	882	3.77	63	0.943
8	1090	0.0330	1023	1123	1074	2.40	50	0.244
9	1789	0.0776	1539	1923	1706	5.03	67	0.575
10	3251	0.0960	2914	3465	3223	3.83	115	0.712
11	4173	0.2521	3465	4311	3984	6.02	194	1.869
12	4656	6.2583	4311	6408	4666	4.91	8564	46.410
13	7134	0.3201	6408	9600	7460	10.20	160	2.373
14	20000 (UM)	0.2552	18415	27133	20626	4.59	2042	22.712

TIC:	9.5479	ng/uL
TIM:	33.9480	nmole/L
Total concentration:	10.0374	ng/uL

Sample peak width (sec): 5 Sample min peak height: 50 Sample baseline V to V?: Y Sample baseline V to V points: 3
Sample filter: Binomial Number of points for filter: 3 Sample start region (min): 0 Sample end region (min): 30
Marker peak width (sec): 5 Marker min peak height: 200 Marker baseline V to V?: Y Marker baseline V to V points: 3
Lower marker selection: First peak > 500 RFU Upper marker selection: Last peak > 200 RFU
Ladder size (bp)75, 200, 300, 400, 500, 700, 1000, 1500, 2000, 3000, 4000, 5000, 7000, 10000, 20000
Quantification using: Lower Marker Final concentration (ng/uL): 0.5000 Dilution factor: 12.0

Sample: SampB9
Well location: B9
Created: Wednesday, October 20, 2021 5:18:46 PM

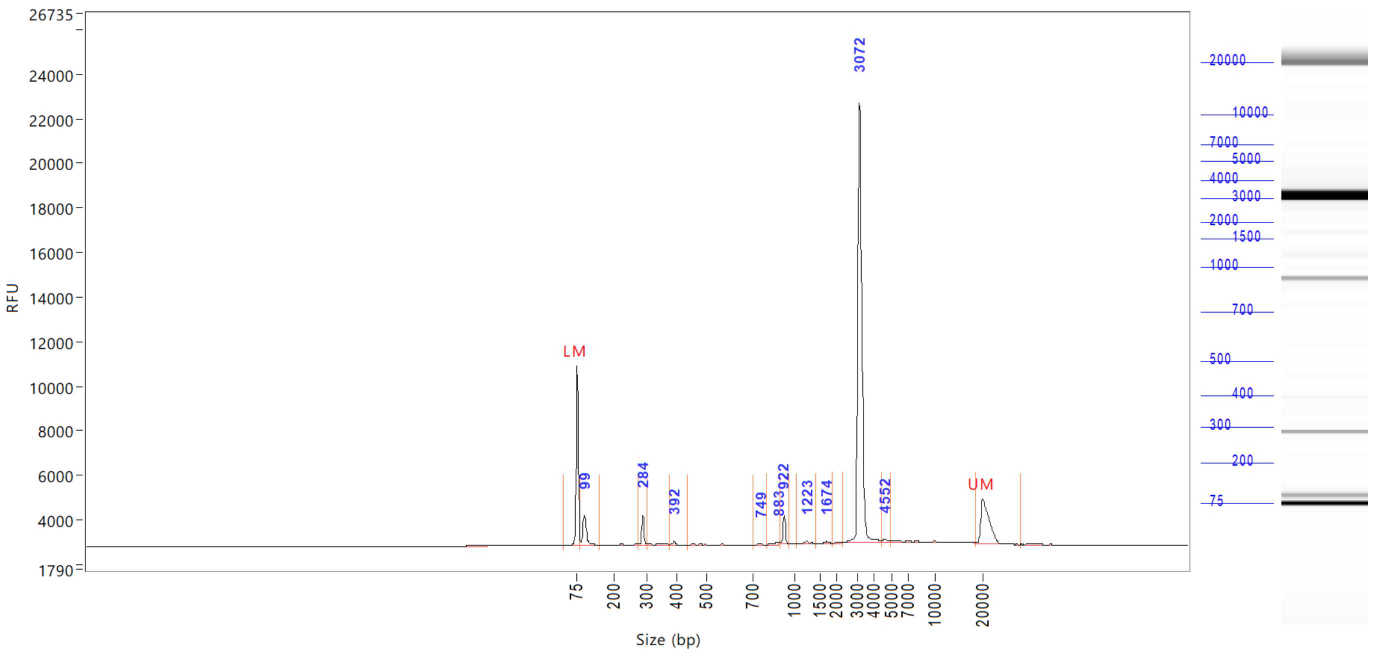

Peak	Size (bp)	Concentration (ng/uL)	From (bp)	To (bp)	Average size (bp)	CV%	RFU	Corrected peak area
1	75 (LM)	0.5000	31	86	73	5.21	8031	42.587
2	99	1.5498	86	150	98	8.87	1344	11.000
3	284	0.8893	271	296	283	1.15	1349	6.312
4	392	0.1349	374	437	391	2.66	176	0.958
5	749	0.0523	701	792	741	1.92	65	0.371
6	883	0.1322	792	896	862	2.86	89	0.938
7	922	0.7951	896	970	921	1.10	1295	5.644
8	1223	0.1669	1034	1400	1227	4.66	139	1.185
9	1674	0.0886	1400	1885	1624	5.62	89	0.629
10	3072	16.8495	2286	4380	3116	5.58	19764	119.594
11	4552	0.1452	4380	4966	4625	3.48	112	1.030
12	20000 (UM)	0.2514	18415	27979	20628	4.42	2000	21.411

TIC:	20.8038	ng/uL
TIM:	42.7605	nmole/L
Total concentration:	21.5345	ng/uL

```
Sample peak width (sec): 5    Sample min peak height: 50    Sample baseline V to V?: Y    Sample baseline V to V points: 3
Sample filter: Binomial       Number of points for filter: 3  Sample start region (min): 0  Sample end region (min): 30
Marker peak width (sec): 5    Marker min peak height: 200   Marker baseline V to V?: Y    Marker baseline V to V points: 3
Lower marker selection: First peak > 500 RFU                Upper marker selection: Last peak > 200 RFU
Ladder size (bp)75, 200, 300, 400, 500, 700, 1000, 1500, 2000, 3000, 4000, 5000, 7000, 10000, 20000
Quantification using: Lower Marker          Final concentration (ng/uL): 0.5000          Dilution factor: 12.0
```

Sample: SampB10
Well location: B10
Created: Wednesday, October 20, 2021 5:18:46 PM

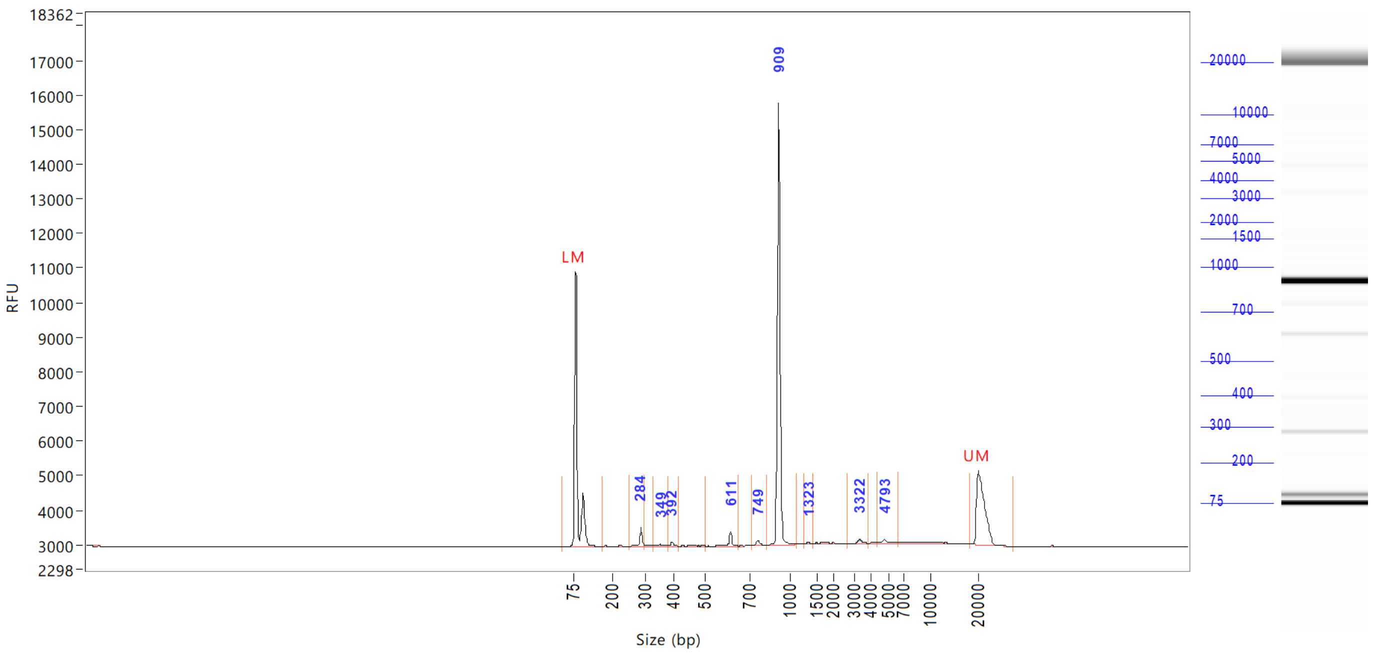

Peak	Size (bp)	Concentration (ng/uL)	From (bp)	To (bp)	Average size (bp)	CV%	RFU	Corrected peak area
1	75 (LM)	0.5000	33	166	80	15.31	7947	56.295
2	284	0.2887	250	296	283	2.00	533	2.709
3	349	0.0902	325	378	347	3.58	51	0.847
4	392	0.0709	378	412	391	1.41	124	0.666
5	611	0.2367	504	646	603	2.80	405	2.220
6	749	0.1147	701	814	750	2.13	154	1.077
7	909	6.2499	814	1090	904	1.55	12798	58.640
8	1323	0.0339	1234	1422	1309	2.87	50	0.318
9	3322	0.0742	2686	3893	3330	4.91	101	0.696
10	4793	0.0844	4380	6260	4948	7.95	108	0.792
11	20000 (UM)	0.1995	18293	27133	20573	4.24	2127	22.461

TIC:	7.2438	ng/uL
TIM:	14.7887	nmole/L
Total concentration:	7.5956	ng/uL

```
Sample peak width (sec): 5     Sample min peak height: 50     Sample baseline V to V?: Y     Sample baseline V to V points: 3
Sample filter: Binomial        Number of points for filter: 3  Sample start region (min): 0  Sample end region (min): 30
Marker peak width (sec): 5     Marker min peak height: 200    Marker baseline V to V?: Y     Marker baseline V to V points: 3
Lower marker selection: First peak > 500 RFU                  Upper marker selection: Last peak > 200 RFU
Ladder size (bp)75, 200, 300, 400, 500, 700, 1000, 1500, 2000, 3000, 4000, 5000, 7000, 10000, 20000
Quantification using: Lower Marker          Final concentration (ng/uL): 0.5000          Dilution factor: 12.0
```

Sample: SampB11
Well location: B11
Created: Wednesday, October 20, 2021 5:18:46 PM

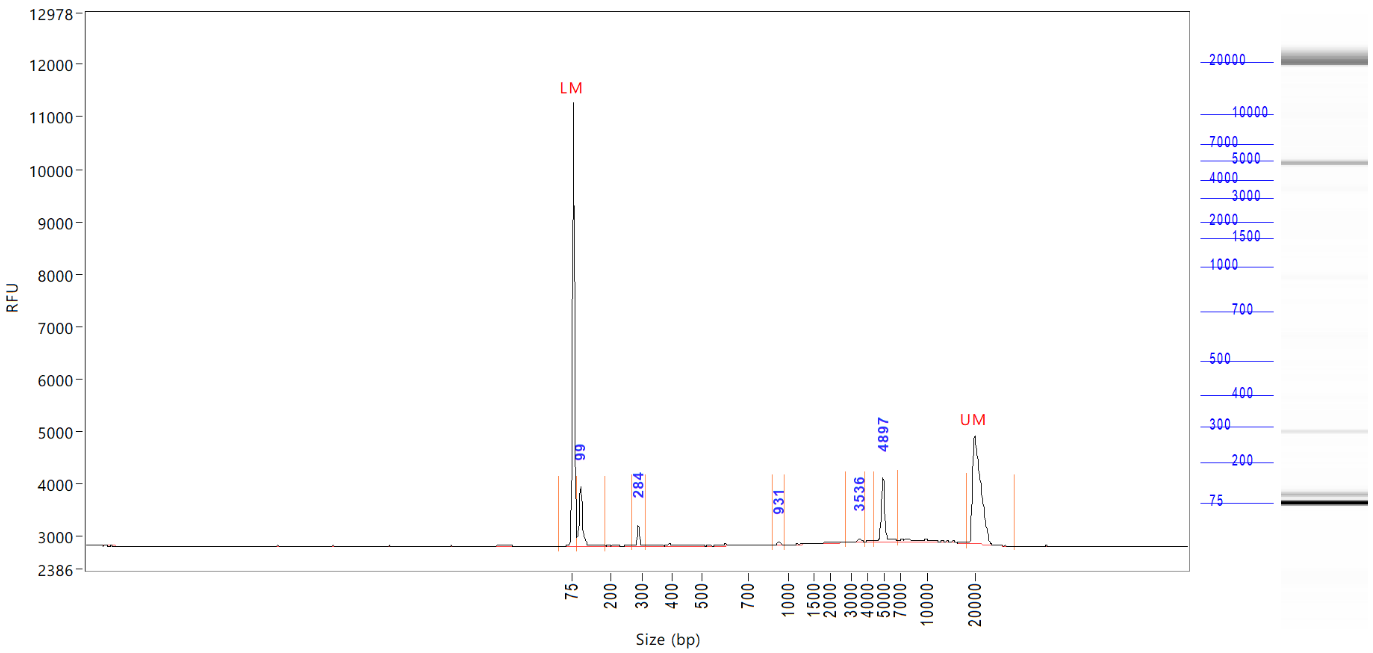

Peak	Size (bp)	Concentration (ng/uL)	From (bp)	To (bp)	Average size (bp)	CV%	RFU	Corrected peak area
1	75 (LM)	0.5000	28	86	73	4.86	8463	45.069
2	99	1.2782	86	181	101	13.67	1120	9.601
3	284	0.2785	266	307	283	1.97	401	2.092
4	931	0.0537	887	978	927	1.59	72	0.404
5	3536	0.0635	2772	3822	3442	6.09	70	0.477
6	4897	0.8154	4414	6704	4966	6.57	1215	6.125
7	20000 (UM)	0.2431	18415	28342	20619	4.35	2043	21.912

TIC:	2.4893	ng/uL
TIM:	22.7964	nmole/L
Total concentration:	3.1276	ng/uL

Sample peak width (sec): 5   Sample min peak height: 50   Sample baseline V to V?: Y   Sample baseline V to V points: 3
Sample filter: Binomial   Number of points for filter: 3 Sample start region (min): 0 Sample end region (min): 30
Marker peak width (sec): 5   Marker min peak height: 200   Marker baseline V to V?: Y   Marker baseline V to V points: 3
Lower marker selection: First peak > 500 RFU   Upper marker selection: Last peak > 200 RFU
Ladder size (bp)75, 200, 300, 400, 500, 700, 1000, 1500, 2000, 3000, 4000, 5000, 7000, 10000, 20000
Quantification using: Lower Marker   Final concentration (ng/uL): 0.5000   Dilution factor: 12.0

Sample: SampB12
Well location: B12
Created: Wednesday, October 20, 2021 5:18:46 PM

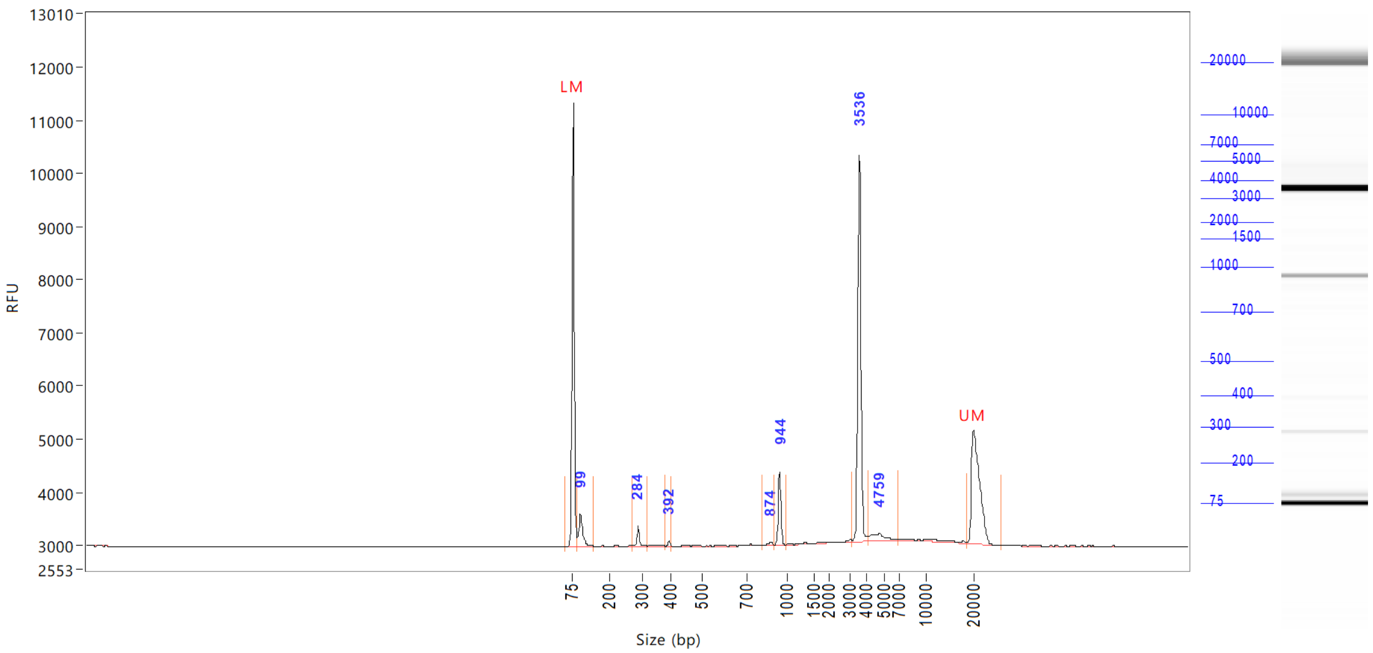

Peak	Size (bp)	Concentration (ng/uL)	From (bp)	To (bp)	Average size (bp)	CV%	RFU	Corrected peak area
1	75 (LM)	0.5000	48	87	73	4.55	8360	44.796
2	99	0.7805	87	145	100	8.31	608	5.827
3	284	0.2551	269	315	284	1.97	372	1.905
4	392	0.0547	378	401	389	1.03	89	0.409
5	874	0.0629	809	905	864	1.93	62	0.469
6	944	0.7933	905	987	938	1.00	1375	5.923
7	3536	4.5496	3144	4070	3540	3.11	7257	33.967
8	4759	0.3800	4070	6852	4768	12.02	140	2.837
9	20000 (UM)	0.2546	18415	25803	20576	4.31	2137	22.809

TIC:	6.8760	ng/uL
TIM:	18.2834	nmole/L
Total concentration:	7.4679	ng/uL

Sample peak width (sec): 5 Sample min peak height: 50 Sample baseline V to V?: Y Sample baseline V to V points: 3
Sample filter: Binomial Number of points for filter: 3 Sample start region (min): 0 Sample end region (min): 30
Marker peak width (sec): 5 Marker min peak height: 200 Marker baseline V to V?: Y Marker baseline V to V points: 3
Lower marker selection: First peak > 500 RFU Upper marker selection: Last peak > 200 RFU
Ladder size (bp)75, 200, 300, 400, 500, 700, 1000, 1500, 2000, 3000, 4000, 5000, 7000, 10000, 20000
Quantification using: Lower Marker Final concentration (ng/uL): 0.5000 Dilution factor: 12.0

Sample: SampC1
Well location: C1
Created: Wednesday, October 20, 2021 5:18:46 PM

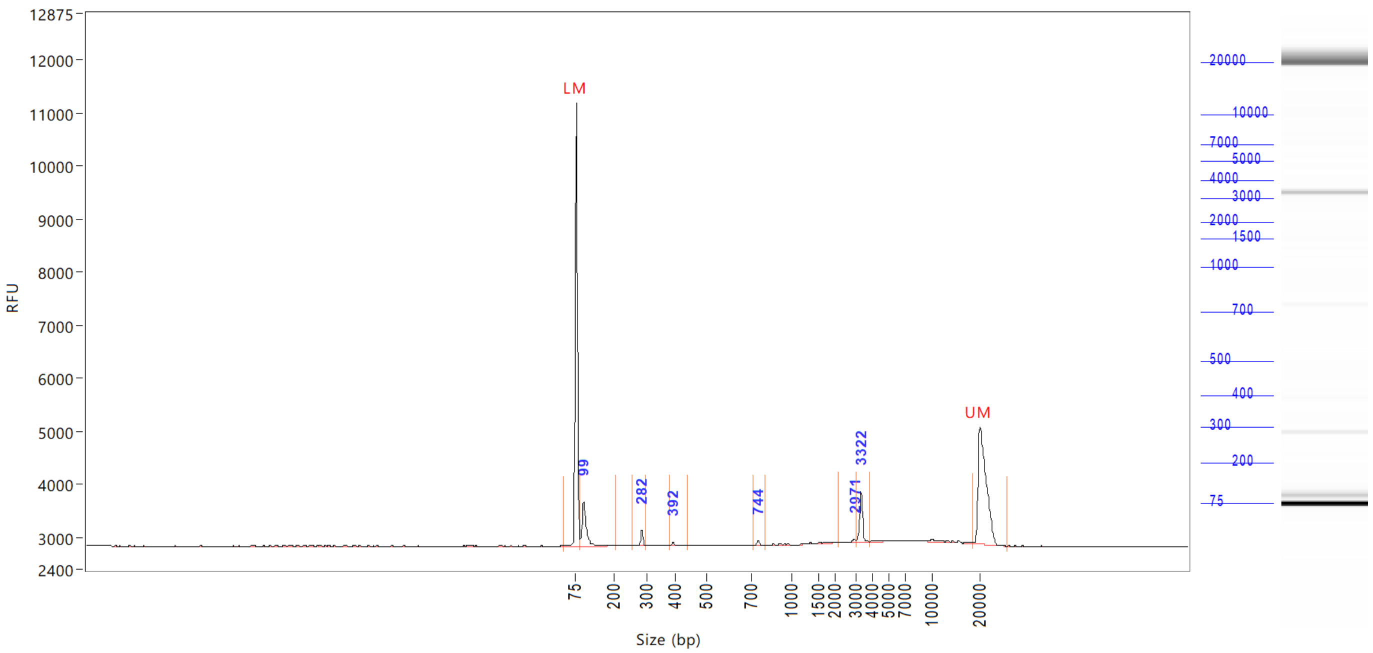

Peak	Size (bp)	Concentration (ng/uL)	From (bp)	To (bp)	Average size (bp)	CV%	RFU	Corrected peak area
1	75 (LM)	0.5000	33	86	72	5.27	8366	46.671
2	99	1.0661	86	205	103	16.86	827	8.293
3	282	0.1914	255	298	281	1.80	289	1.489
4	392	0.0493	378	437	393	2.73	60	0.383
5	744	0.0574	710	801	742	1.59	99	0.446
6	2971	0.0476	2201	3072	2844	7.08	54	0.370
7	3322	0.5500	3072	3857	3303	3.16	942	4.278
8	20000 (UM)	0.2520	18537	25682	20655	4.26	2209	23.525

TIC:	1.9618	ng/uL
TIM:	18.7537	nmole/L
Total concentration:	2.4195	ng/uL

Sample peak width (sec): 5   Sample min peak height: 50   Sample baseline V to V?: Y   Sample baseline V to V points: 3
Sample filter: Binomial   Number of points for filter: 3   Sample start region (min): 0   Sample end region (min): 30
Marker peak width (sec): 5   Marker min peak height: 200   Marker baseline V to V?: Y   Marker baseline V to V points: 3
Lower marker selection: First peak > 500 RFU   Upper marker selection: Last peak > 200 RFU
Ladder size (bp)75, 200, 300, 400, 500, 700, 1000, 1500, 2000, 3000, 4000, 5000, 7000, 10000, 20000
Quantification using: Lower Marker   Final concentration (ng/uL): 0.5000   Dilution factor: 12.0

Sample: SampC2
Well location: C2
Created: Wednesday, October 20, 2021 5:18:46 PM

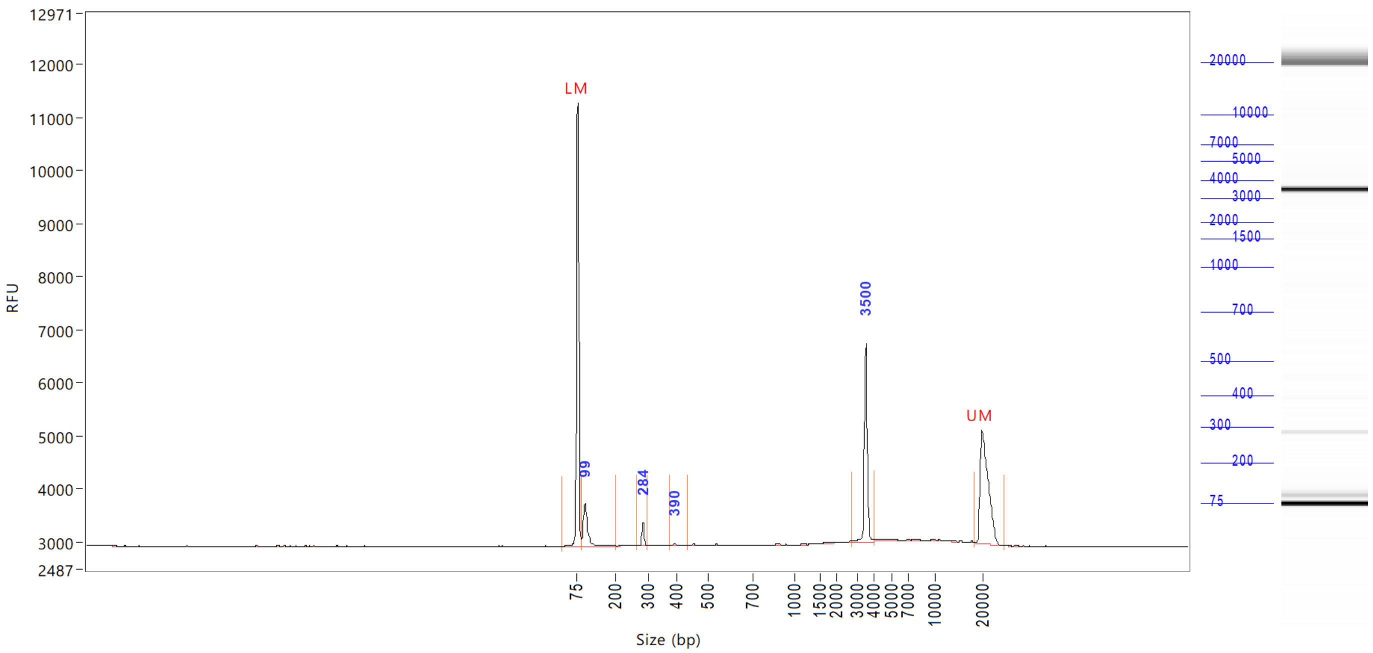

Peak	Size (bp)	Concentration (ng/uL)	From (bp)	To (bp)	Average size (bp)	CV%	RFU	Corrected peak area
1	75 (LM)	0.5000	28	86	72	5.52	8374	45.872
2	99	1.0448	86	203	102	15.45	804	7.988
3	284	0.2885	264	298	281	1.44	442	2.206
4	390	0.0444	374	431	393	2.77	53	0.339
5	3500	2.2230	2743	4001	3461	3.76	3733	16.996
6	20000 (UM)	0.2464	18415	24594	20557	4.16	2145	22.608

TIC:	3.6006	ng/uL
TIM:	19.7527	nmole/L
Total concentration:	4.1909	ng/uL

Sample peak width (sec): 5   Sample min peak height: 50   Sample baseline V to V?: Y   Sample baseline V to V points: 3
Sample filter: Binomial   Number of points for filter: 3 Sample start region (min): 0 Sample end region (min): 30
Marker peak width (sec): 5   Marker min peak height: 200   Marker baseline V to V?: Y   Marker baseline V to V points: 3
Lower marker selection: First peak > 500 RFU   Upper marker selection: Last peak > 200 RFU
Ladder size (bp)75, 200, 300, 400, 500, 700, 1000, 1500, 2000, 3000, 4000, 5000, 7000, 10000, 20000
Quantification using: Lower Marker   Final concentration (ng/uL): 0.5000   Dilution factor: 12.0

Sample: SampC3
Well location: C3
Created: Wednesday, October 20, 2021 5:18:46 PM

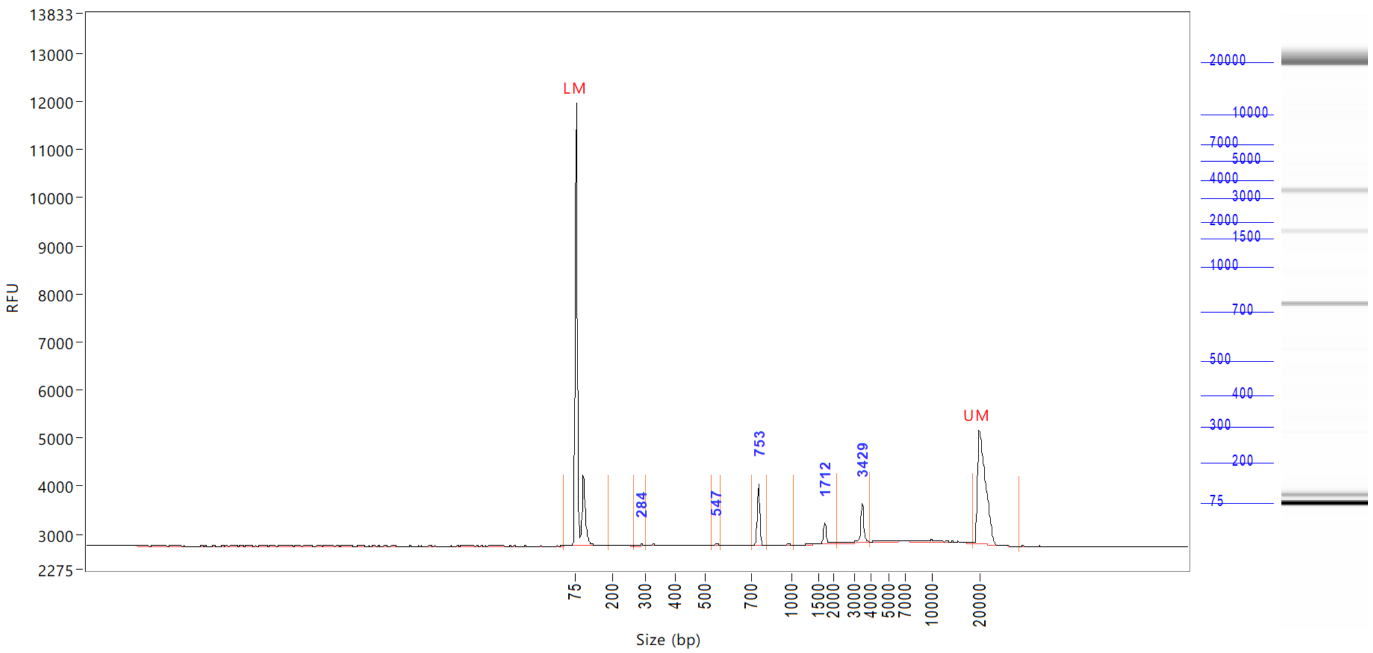

Peak	Size (bp)	Concentration (ng/uL)	From (bp)	To (bp)	Average size (bp)	CV%	RFU	Corrected peak area
1	75 (LM)	0.5000	35	183	79	15.34	9225	61.215
2	284	0.0344	260	298	282	2.63	51	0.351
3	547	0.0256	529	562	545	1.02	54	0.261
4	753	0.5866	700	818	746	1.41	1292	5.985
5	1712	0.2545	1023	2115	1662	9.49	446	2.597
6	3429	0.4499	2115	3929	3304	9.44	811	4.590
7	20000 (UM)	0.2037	18537	28463	20646	4.19	2366	24.942

TIC:	1.3510	ng/uL
TIM:	2.0485	nmole/L
Total concentration:	1.7331	ng/uL

Sample peak width (sec): 5 Sample min peak height: 50 Sample baseline V to V?: Y Sample baseline V to V points: 3
Sample filter: Binomial Number of points for filter: 3 Sample start region (min): 0 Sample end region (min): 30
Marker peak width (sec): 5 Marker min peak height: 200 Marker baseline V to V?: Y Marker baseline V to V points: 3
Lower marker selection: First peak > 500 RFU Upper marker selection: Last peak > 200 RFU
Ladder size (bp)75, 200, 300, 400, 500, 700, 1000, 1500, 2000, 3000, 4000, 5000, 7000, 10000, 20000
Quantification using: Lower Marker Final concentration (ng/uL): 0.5000 Dilution factor: 12.0

Sample: SampC4
Well location: C4
Created: Wednesday, October 20, 2021 5:18:46 PM

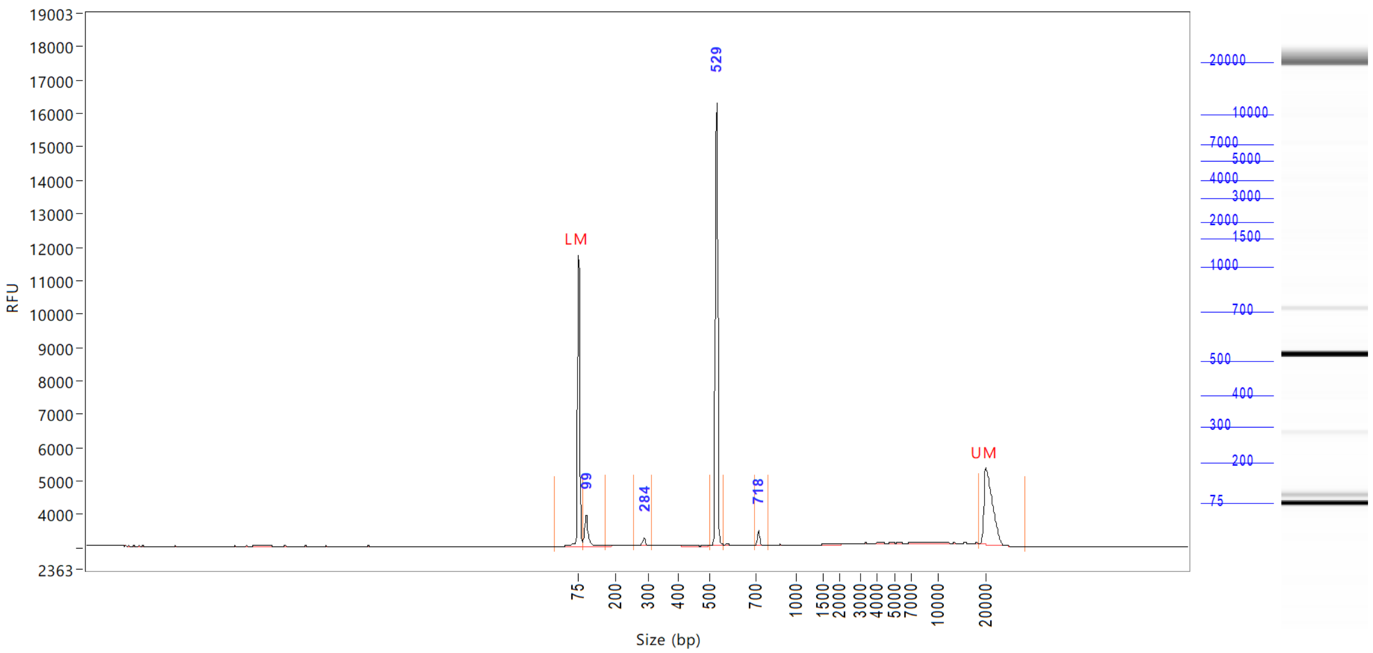

Peak	Size (bp)	Concentration (ng/uL)	From (bp)	To (bp)	Average size (bp)	CV%	RFU	Corrected peak area
1	75 (LM)	0.5000	1	87	73	6.62	8712	47.256
2	99	0.9841	87	162	100	9.71	929	7.751
3	284	0.2219	250	309	279	2.77	249	1.748
4	529	7.1019	504	562	526	0.94	13282	55.934
5	718	0.2573	697	792	716	1.66	469	2.026
6	20000 (UM)	0.2623	18537	27979	20668	4.37	2304	24.789

TIC:	8.5652	ng/uL
TIM:	40.2775	nmole/L
Total concentration:	9.3137	ng/uL

```
Sample peak width (sec): 5    Sample min peak height: 50    Sample baseline V to V?: Y    Sample baseline V to V points: 3
Sample filter: Binomial       Number of points for filter: 3  Sample start region (min): 0  Sample end region (min): 30
Marker peak width (sec): 5    Marker min peak height: 200   Marker baseline V to V?: Y    Marker baseline V to V points: 3
Lower marker selection: First peak > 500 RFU                Upper marker selection: Last peak > 200 RFU
Ladder size (bp)75, 200, 300, 400, 500, 700, 1000, 1500, 2000, 3000, 4000, 5000, 7000, 10000, 20000
Quantification using: Lower Marker          Final concentration (ng/uL): 0.5000          Dilution factor: 12.0
```

Sample: SampC5
Well location: C5
Created: Wednesday, October 20, 2021 5:18:46 PM

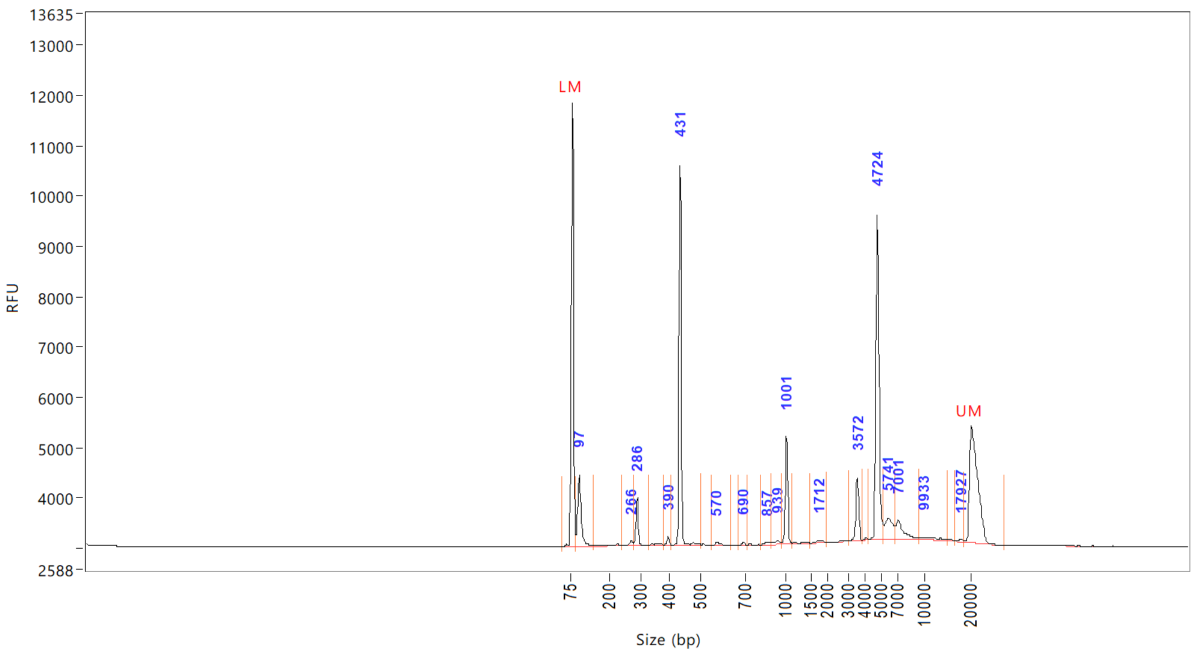

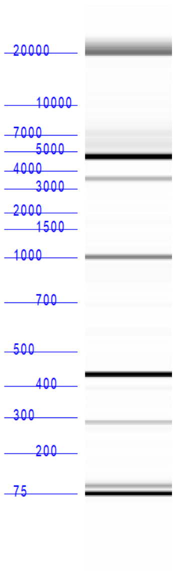

Peak	Size<br>(bp)	Concentration<br>(ng/uL)	From<br>(bp)	To<br>(bp)	Average size<br>(bp)	CV%	RFU	Corrected peak area
1	75 (LM)	0.5000	41	86	73	4.86	8828	47.051
2	97	1.5673	86	147	98	8.56	1424	12.291
3	266	0.0943	237	275	265	2.68	85	0.740
4	286	0.5953	275	323	283	1.83	949	4.668
5	390	0.0972	378	405	389	1.05	160	0.762
6	431	4.3155	405	504	430	1.46	7527	33.842
7	570	0.0763	547	636	578	2.71	72	0.598
8	690	0.0455	672	710	686	0.82	73	0.356
9	857	0.0803	814	896	860	2.41	60	0.630
10	939	0.0990	896	970	931	2.10	84	0.776
11	1001	1.1925	970	1123	1005	1.79	2134	9.352
12	1712	0.0619	1467	1962	1715	6.29	53	0.485
13	3572	0.6810	3072	3857	3515	2.64	1249	5.341
14	4724	4.0755	4242	5223	4735	2.39	6451	31.960
15	5741	0.6281	5223	6630	5891	6.80	424	4.925
16	7001	0.5812	6630	9533	7450	9.27	376	4.558
17	9933	0.1424	9533	15000	11669	13.46	50	1.117
18	17927	0.0428	16464	18537	17674	2.92	50	0.336
19	20000 (UM)	0.2617	18537	26891	20672	4.26	2319	24.628

TIC:	14.3763	ng/uL
TIM:	51.9621	nmole/L
Total concentration:	14.6407	ng/uL

```
Sample peak width (sec): 5    Sample min peak height: 50    Sample baseline V to V?: Y    Sample baseline V to V points: 3
Sample filter: Binomial       Number of points for filter: 3  Sample start region (min): 0  Sample end region (min): 30
Marker peak width (sec): 5    Marker min peak height: 200   Marker baseline V to V?: Y    Marker baseline V to V points: 3
Lower marker selection: First peak > 500 RFU                Upper marker selection: Last peak > 200 RFU
Ladder size (bp)75, 200, 300, 400, 500, 700, 1000, 1500, 2000, 3000, 4000, 5000, 7000, 10000, 20000
Quantification using: Lower Marker          Final concentration (ng/uL): 0.5000          Dilution factor: 12.0
```

Sample: SampC6
Well location: C6
Created: Wednesday, October 20, 2021 5:18:46 PM

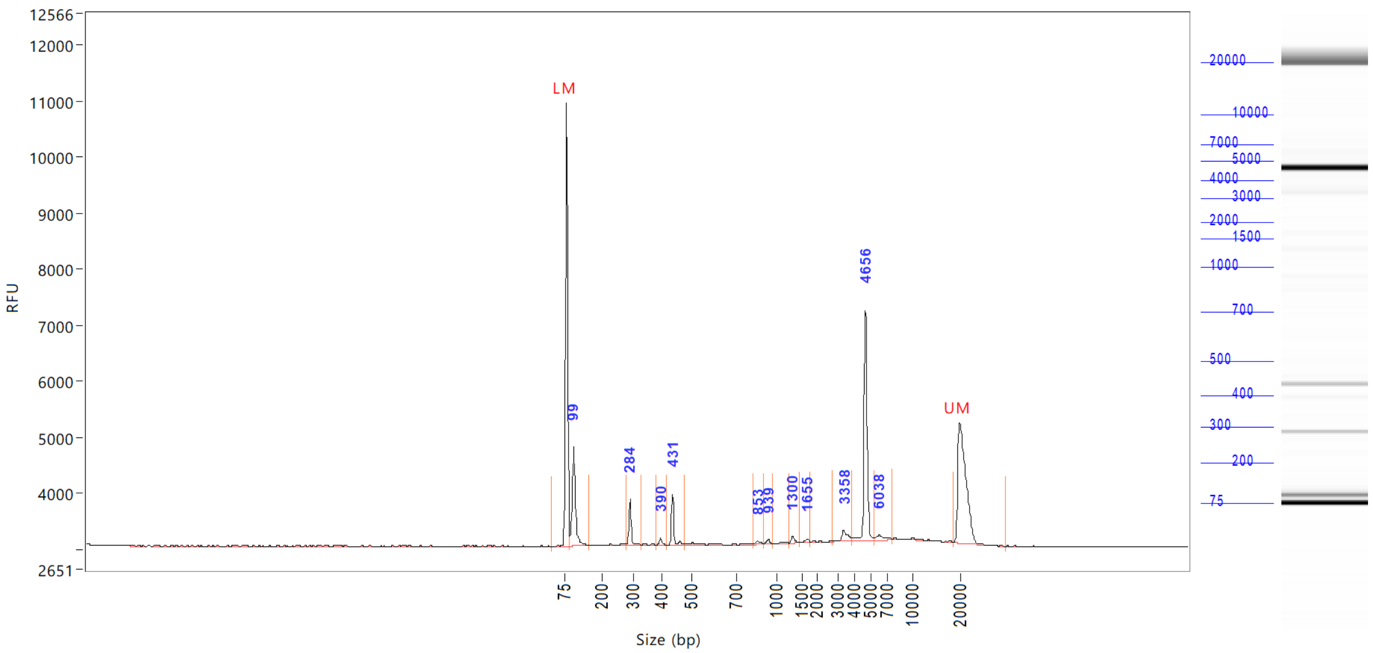

Peak	Size (bp)	Concentration (ng/uL)	From (bp)	To (bp)	Average size (bp)	CV%	RFU	Corrected peak area
1	75 (LM)	0.5000	28	87	74	4.63	7917	43.864
2	99	1.9290	87	150	100	8.68	1764	14.102
3	284	0.5727	275	323	284	2.05	813	4.187
4	390	0.0869	376	411	390	1.24	125	0.635
5	431	0.6186	411	471	432	1.95	910	4.523
6	853	0.0734	822	905	860	2.39	58	0.536
7	939	0.0710	905	978	931	1.36	87	0.519
8	1300	0.0951	1234	1445	1314	3.12	112	0.696
9	1655	0.0638	1445	1770	1607	4.66	69	0.466
10	3358	0.2389	2715	3893	3404	6.60	201	1.747
11	4656	2.7318	3893	5297	4625	3.03	4092	19.971
12	6038	0.1652	5297	7534	6241	9.79	90	1.208
13	20000 (UM)	0.2645	18659	30035	20713	4.84	2152	23.200

TIC:	6.6465	ng/uL
TIM:	39.2974	nmole/L
Total concentration:	7.1540	ng/uL

Sample peak width (sec): 5   Sample min peak height: 50   Sample baseline V to V?: Y   Sample baseline V to V points: 3
Sample filter: Binomial   Number of points for filter: 3   Sample start region (min): 0   Sample end region (min): 30
Marker peak width (sec): 5   Marker min peak height: 200   Marker baseline V to V?: Y   Marker baseline V to V points: 3
Lower marker selection: First peak > 500 RFU   Upper marker selection: Last peak > 200 RFU
Ladder size (bp)75, 200, 300, 400, 500, 700, 1000, 1500, 2000, 3000, 4000, 5000, 7000, 10000, 20000
Quantification using: Lower Marker   Final concentration (ng/uL): 0.5000   Dilution factor: 12.0

Sample: SampC7
Well location: C7
Created: Wednesday, October 20, 2021 5:18:46 PM

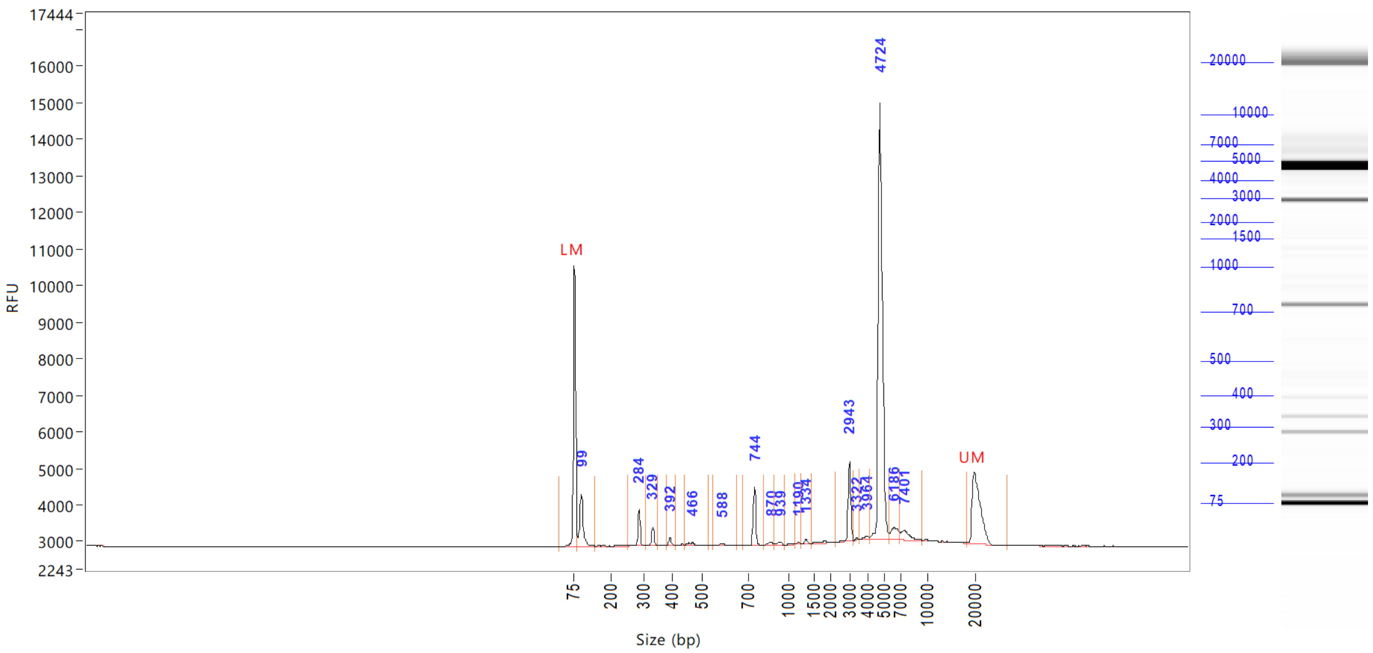

Peak	Size (bp)	Concentration (ng/uL)	From (bp)	To (bp)	Average size (bp)	CV%	RFU	Corrected peak area
1	75 (LM)	0.5000	26	86	73	5.96	7666	42.354
2	99	1.7703	86	145	98	8.71	1435	12.497
3	284	0.7226	250	307	281	2.43	992	5.101
4	329	0.3919	307	347	328	1.64	522	2.766
5	392	0.1717	378	411	390	1.31	221	1.212
6	466	0.1771	439	524	467	4.09	77	1.250
7	588	0.0974	544	649	583	3.46	55	0.688
8	744	1.0349	677	818	741	1.90	1602	7.305
9	870	0.1181	818	900	859	2.21	97	0.834
10	939	0.0914	900	974	929	1.55	83	0.645
11	1190	0.0396	1123	1256	1183	2.19	59	0.280
12	1334	0.0848	1256	1445	1325	2.23	117	0.598
13	2943	1.3029	2229	3144	2910	2.45	2187	9.197
14	3322	0.0309	3144	3536	3316	2.17	56	0.218
15	3964	0.0986	3536	4104	3904	3.49	88	0.696
16	4724	10.4694	4104	5519	4738	3.02	11964	73.904
17	6186	0.5343	5519	7000	6251	6.53	332	3.772
18	7401	0.4418	7000	9533	7606	6.60	255	3.119
19	20000 (UM)	0.2462	18415	26891	20581	4.32	1963	20.854

TIC:	17.5778	ng/uL
TIM:	44.9958	nmole/L
Total concentration:	17.9733	ng/uL

```
Sample peak width (sec): 5    Sample min peak height: 50    Sample baseline V to V?: Y    Sample baseline V to V points: 3
Sample filter: Binomial       Number of points for filter: 3  Sample start region (min): 0  Sample end region (min): 30
Marker peak width (sec): 5    Marker min peak height: 200   Marker baseline V to V?: Y    Marker baseline V to V points: 3
Lower marker selection: First peak > 500 RFU                Upper marker selection: Last peak > 200 RFU
Ladder size (bp)75, 200, 300, 400, 500, 700, 1000, 1500, 2000, 3000, 4000, 5000, 7000, 10000, 20000
Quantification using: Lower Marker          Final concentration (ng/uL): 0.5000          Dilution factor: 12.0
```

Sample: SampC8
Well location: C8
Created: Wednesday, October 20, 2021 5:18:46 PM

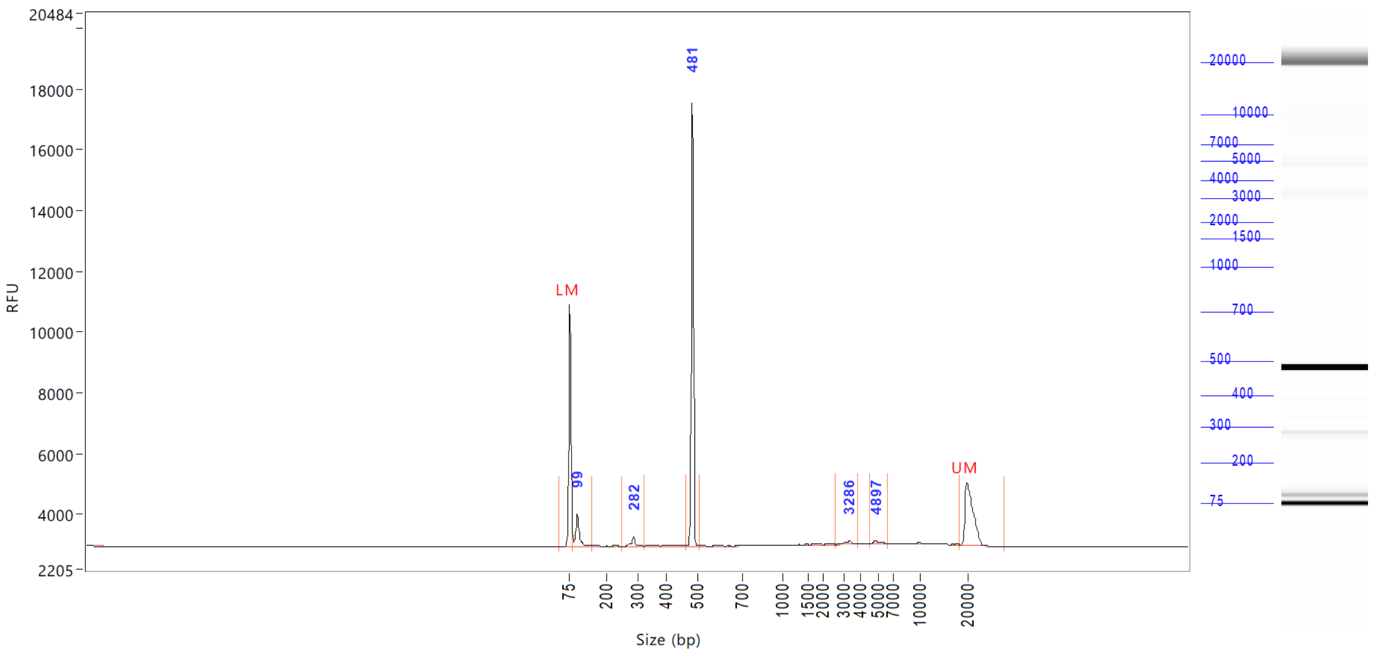

Peak	Size (bp)	Concentration (ng/uL)	From (bp)	To (bp)	Average size (bp)	CV%	RFU	Corrected peak area
1	75 (LM)	0.5000	41	86	73	4.46	7967	42.188
2	99	1.2931	86	150	100	9.75	1075	9.092
3	282	0.3573	248	315	278	3.25	340	2.513
4	481	9.1194	462	509	480	0.79	14596	64.121
5	3286	0.1680	2600	3857	3211	6.74	111	1.181
6	4897	0.1545	4552	6260	5052	6.64	106	1.086
7	20000 (UM)	0.2613	18537	27859	20625	4.39	2082	22.047

TIC:	11.0922	ng/uL
TIM:	54.7458	nmole/L
Total concentration:	11.8200	ng/uL

Sample peak width (sec): 5 Sample min peak height: 50 Sample baseline V to V?: Y Sample baseline V to V points: 3
Sample filter: Binomial Number of points for filter: 3 Sample start region (min): 0 Sample end region (min): 30
Marker peak width (sec): 5 Marker min peak height: 200 Marker baseline V to V?: Y Marker baseline V to V points: 3
Lower marker selection: First peak > 500 RFU Upper marker selection: Last peak > 200 RFU
Ladder size (bp)75, 200, 300, 400, 500, 700, 1000, 1500, 2000, 3000, 4000, 5000, 7000, 10000, 20000
Quantification using: Lower Marker Final concentration (ng/uL): 0.5000 Dilution factor: 12.0

Sample: SampC9
Well location: C9
Created: Wednesday, October 20, 2021 5:18:46 PM

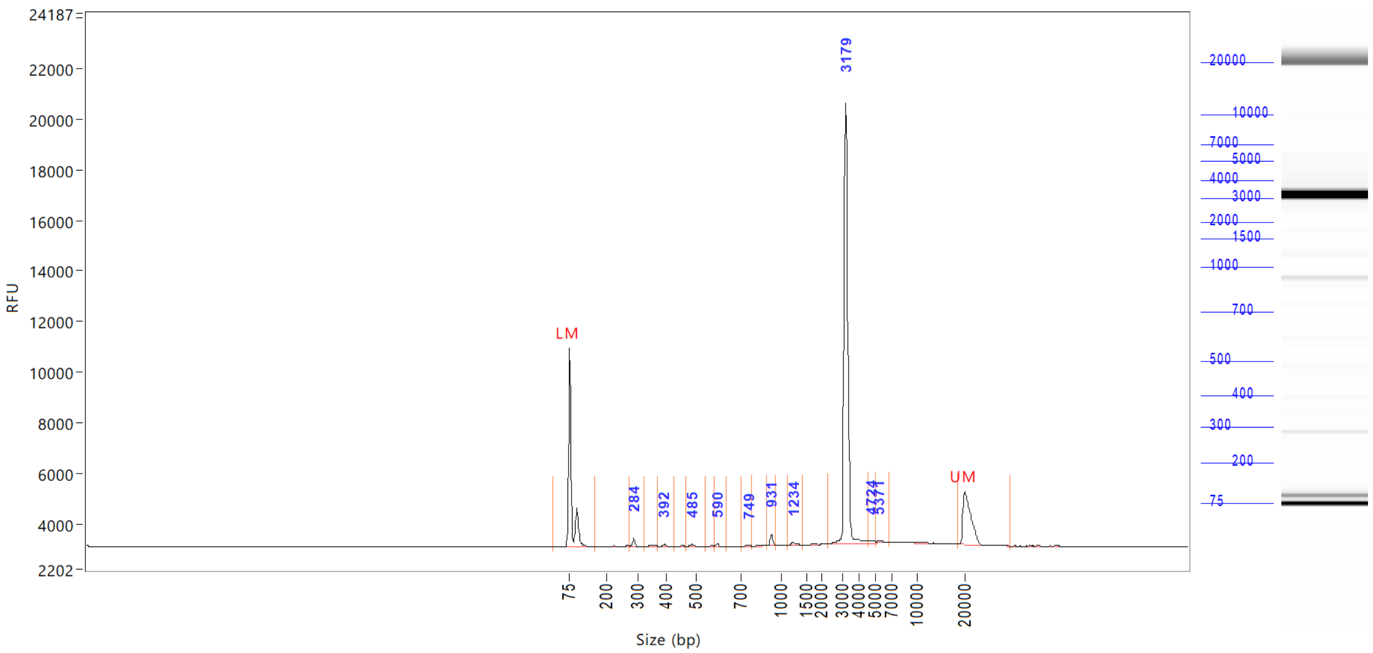

Peak	Size (bp)	Concentration (ng/uL)	From (bp)	To (bp)	Average size (bp)	CV%	RFU	Corrected peak area
1	75 (LM)	0.5000	22	162	79	15.76	7889	54.982
2	284	0.2030	273	319	285	2.93	327	1.860
3	392	0.0612	370	422	388	2.12	88	0.561
4	485	0.0714	464	539	487	3.03	76	0.654
5	590	0.0589	578	631	591	1.61	92	0.539
6	749	0.0364	697	779	741	2.05	55	0.334
7	931	0.2433	892	965	923	1.19	468	2.230
8	1234	0.1001	1123	1411	1237	4.39	111	0.917
9	3179	10.8930	2286	4518	3178	5.90	17464	99.820
10	4724	0.0923	4518	5000	4742	3.01	86	0.846
11	5371	0.0895	5000	6630	5537	7.38	78	0.820
12	20000 (UM)	0.2035	18537	29793	20698	4.52	2083	22.376

TIC:	11.8490	ng/uL
TIM:	8.1852	nmole/L
Total concentration:	12.2536	ng/uL

```
Sample peak width (sec): 5    Sample min peak height: 50    Sample baseline V to V?: Y    Sample baseline V to V points: 3
Sample filter: Binomial       Number of points for filter: 3  Sample start region (min): 0  Sample end region (min): 30
Marker peak width (sec): 5    Marker min peak height: 200   Marker baseline V to V?: Y    Marker baseline V to V points: 3
Lower marker selection: First peak > 500 RFU                Upper marker selection: Last peak > 200 RFU
Ladder size (bp)75, 200, 300, 400, 500, 700, 1000, 1500, 2000, 3000, 4000, 5000, 7000, 10000, 20000
Quantification using: Lower Marker          Final concentration (ng/uL): 0.5000          Dilution factor: 12.0
```

Sample: SampC10
Well location: C10
Created: Wednesday, October 20, 2021 5:18:46 PM

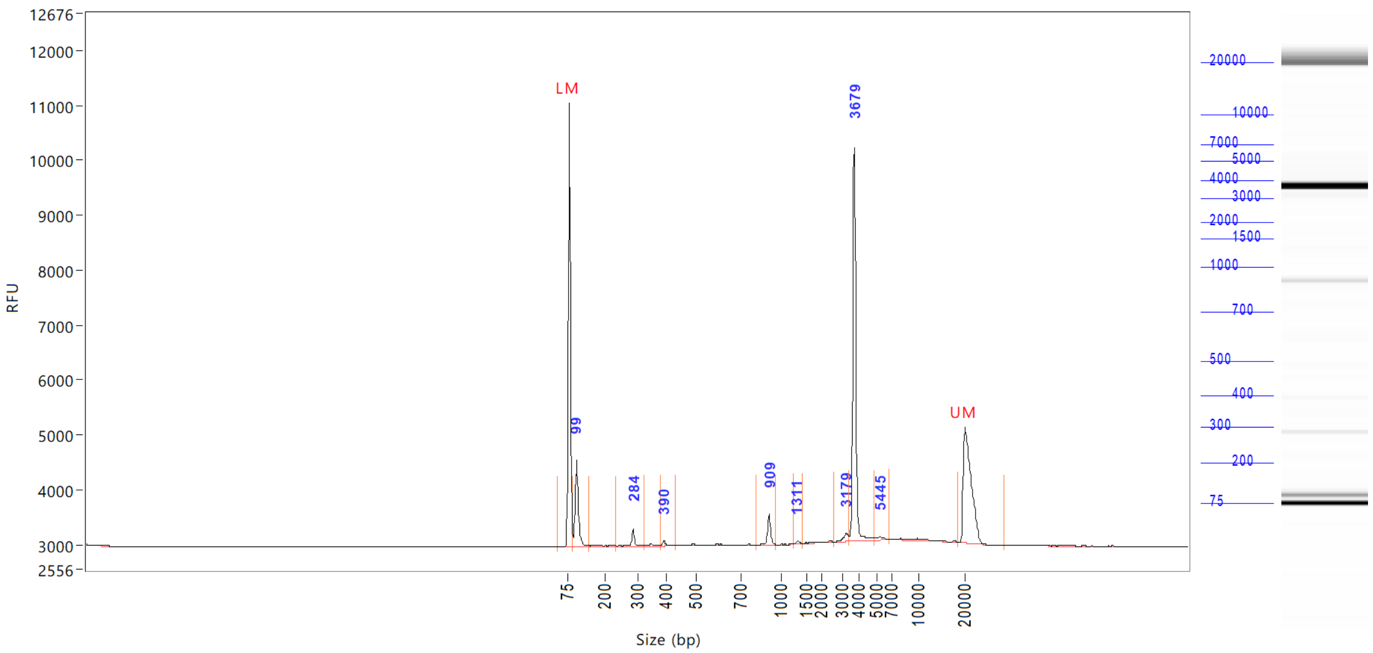

Peak	Size (bp)	Concentration (ng/uL)	From (bp)	To (bp)	Average size (bp)	CV%	RFU	Corrected peak area
1	75 (LM)	0.5000	35	86	73	4.43	8088	43.244
2	99	1.7138	86	145	99	7.78	1565	12.352
3	284	0.2725	232	323	282	3.90	327	1.964
4	390	0.0844	380	430	391	1.83	102	0.608
5	909	0.4304	809	961	902	2.04	573	3.102
6	1311	0.0471	1223	1422	1311	3.17	51	0.340
7	3179	0.1663	2600	3393	3126	5.53	139	1.199
8	3679	4.7760	3393	4897	3692	4.53	7159	34.423
9	5445	0.0917	4897	6556	5382	6.94	60	0.661
10	20000 (UM)	0.2596	18415	28463	20639	4.56	2100	22.452

TIC:	7.5822	ng/uL
TIM:	33.4599	nmole/L
Total concentration:	8.1401	ng/uL

Sample peak width (sec): 5   Sample min peak height: 50   Sample baseline V to V?: Y   Sample baseline V to V points: 3
Sample filter: Binomial   Number of points for filter: 3 Sample start region (min): 0 Sample end region (min): 30
Marker peak width (sec): 5   Marker min peak height: 200   Marker baseline V to V?: Y   Marker baseline V to V points: 3
Lower marker selection: First peak > 500 RFU   Upper marker selection: Last peak > 200 RFU
Ladder size (bp)75, 200, 300, 400, 500, 700, 1000, 1500, 2000, 3000, 4000, 5000, 7000, 10000, 20000
Quantification using: Lower Marker   Final concentration (ng/uL): 0.5000   Dilution factor: 12.0

Sample: SampC11
Well location: C11
Created: Wednesday, October 20, 2021 5:18:46 PM

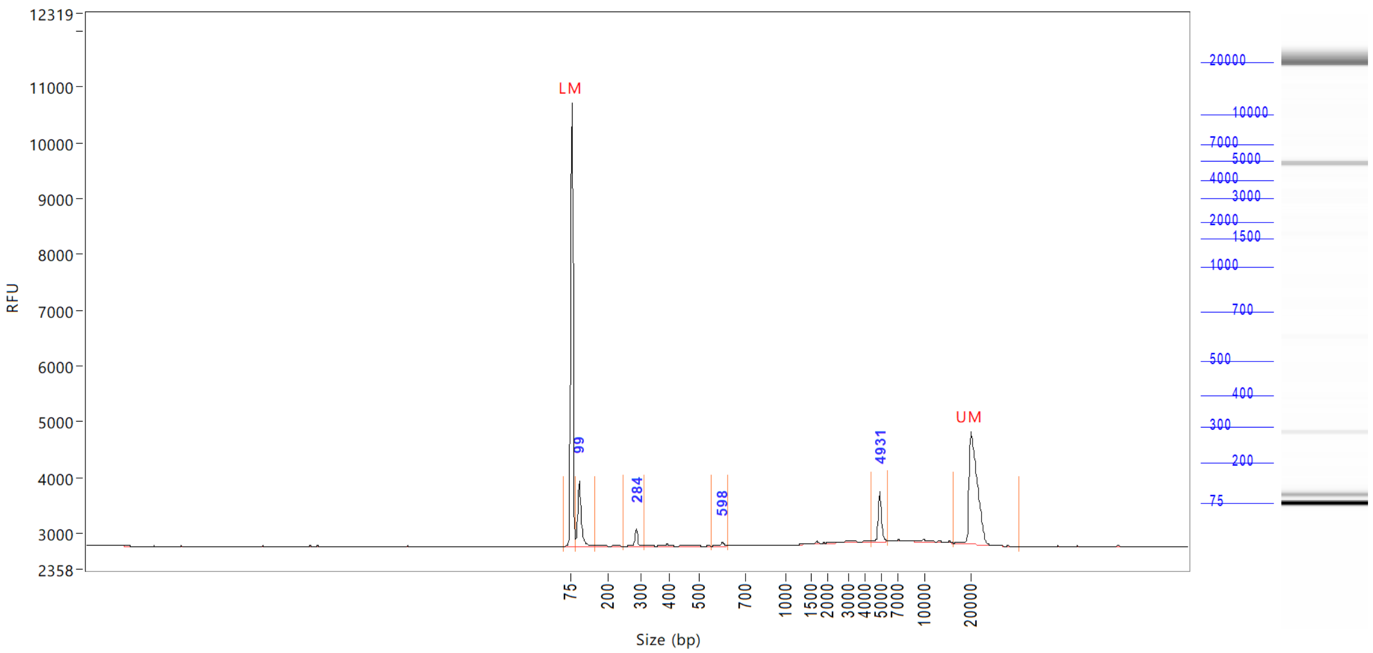

Peak	Size (bp)	Concentration (ng/uL)	From (bp)	To (bp)	Average size (bp)	CV%	RFU	Corrected peak area
1	75 (LM)	0.5000	48	86	73	4.45	7958	42.784
2	99	1.4245	86	154	100	9.43	1181	10.158
3	284	0.2404	242	311	282	2.65	305	1.714
4	598	0.0606	549	623	589	2.20	62	0.432
5	4931	0.6167	4414	5667	4882	2.81	910	4.398
6	20000 (UM)	0.2524	16342	30035	20574	4.87	2010	21.595

TIC:	2.3422	ng/uL
TIM:	25.1713	nmole/L
Total concentration:	3.0022	ng/uL

Sample peak width (sec): 5   Sample min peak height: 50   Sample baseline V to V?: Y   Sample baseline V to V points: 3
Sample filter: Binomial   Number of points for filter: 3 Sample start region (min): 0 Sample end region (min): 30
Marker peak width (sec): 5   Marker min peak height: 200   Marker baseline V to V?: Y   Marker baseline V to V points: 3
Lower marker selection: First peak > 500 RFU   Upper marker selection: Last peak > 200 RFU
Ladder size (bp)75, 200, 300, 400, 500, 700, 1000, 1500, 2000, 3000, 4000, 5000, 7000, 10000, 20000
Quantification using: Lower Marker   Final concentration (ng/uL): 0.5000   Dilution factor: 12.0

Sample: SampC12
Well location: C12
Created: Wednesday, October 20, 2021 5:18:46 PM

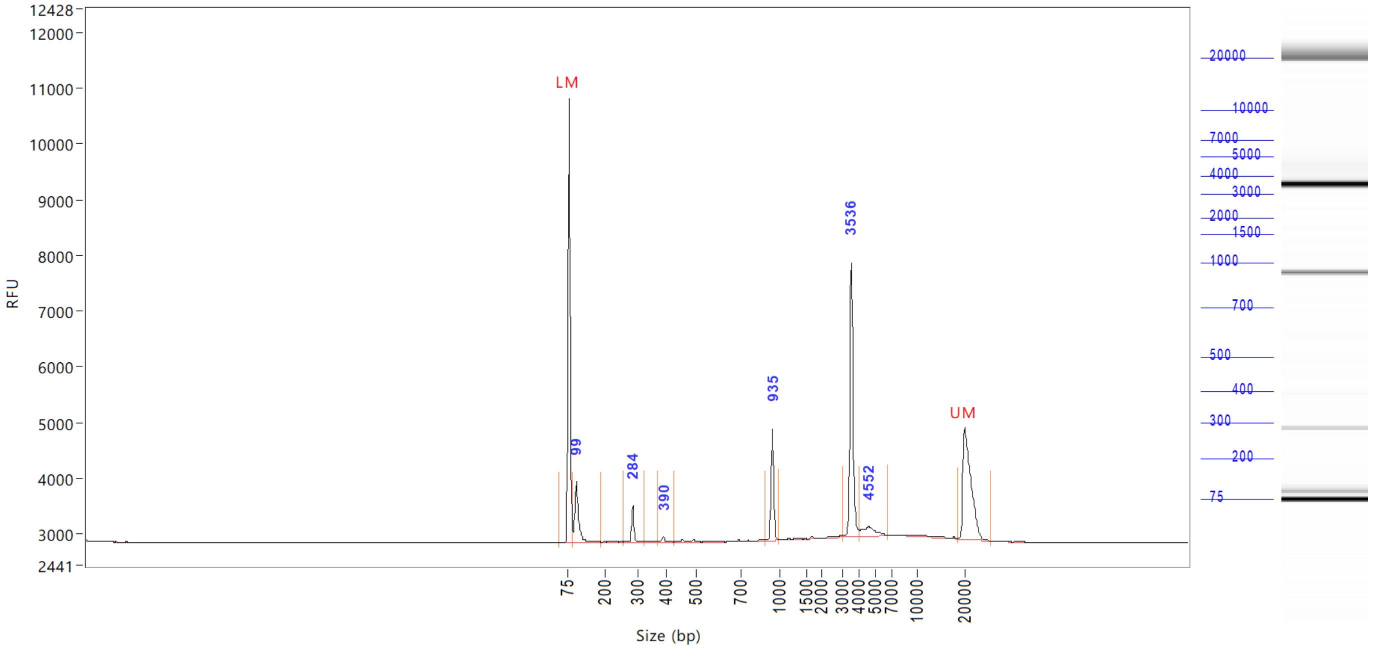

Peak	Size (bp)	Concentration (ng/uL)	From (bp)	To (bp)	Average size (bp)	CV%	RFU	Corrected peak area
1	75 (LM)	0.5000	43	86	73	4.58	7981	43.387
2	99	1.3800	86	181	101	12.52	1084	9.979
3	284	0.4928	251	323	282	2.84	659	3.564
4	390	0.0816	370	424	387	2.01	93	0.590
5	935	1.2741	879	987	932	1.21	2001	9.213
6	3536	3.4094	3037	4035	3524	3.65	4905	24.654
7	4552	0.4701	4035	6482	4673	10.34	168	3.399
8	20000 (UM)	0.2487	18537	25682	20710	4.54	1999	21.583

TIC:	7.1080	ng/uL
TIM:	29.6666	nmole/L
Total concentration:	7.7550	ng/uL

```
Sample peak width (sec): 5    Sample min peak height: 50    Sample baseline V to V?: Y    Sample baseline V to V points: 3
Sample filter: Binomial       Number of points for filter: 3  Sample start region (min): 0  Sample end region (min): 30
Marker peak width (sec): 5    Marker min peak height: 200   Marker baseline V to V?: Y    Marker baseline V to V points: 3
Lower marker selection: First peak > 500 RFU                Upper marker selection: Last peak > 200 RFU
Ladder size (bp)75, 200, 300, 400, 500, 700, 1000, 1500, 2000, 3000, 4000, 5000, 7000, 10000, 20000
Quantification using: Lower Marker          Final concentration (ng/uL): 0.5000          Dilution factor: 12.0
```

Sample: SampD1
Well location: D1
Created: Wednesday, October 20, 2021 5:18:46 PM

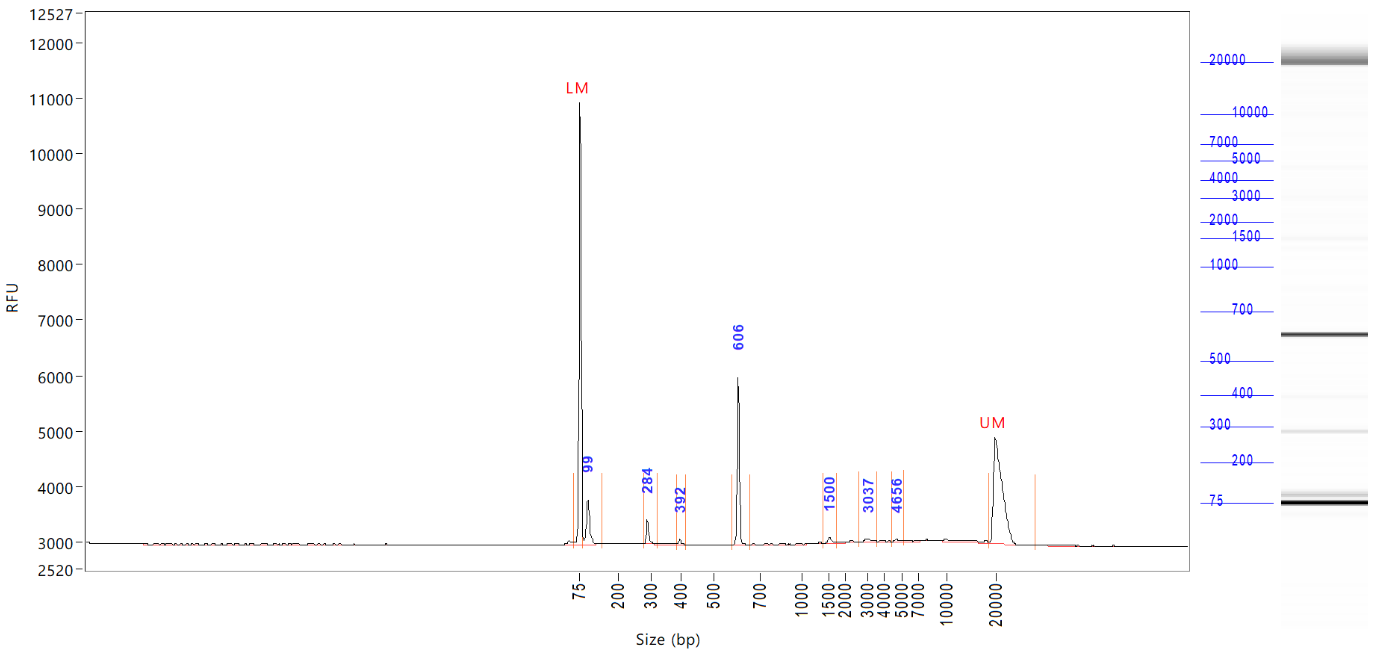

Peak	Size (bp)	Concentration (ng/uL)	From (bp)	To (bp)	Average size (bp)	CV%	RFU	Corrected peak area
1	75 (LM)	0.5000	56	86	73	4.26	7964	41.614
2	99	0.9288	86	147	99	7.41	801	6.442
3	284	0.3351	273	317	284	2.03	446	2.324
4	392	0.0769	382	412	391	1.32	94	0.534
5	606	1.7419	578	657	603	0.88	3018	12.081
6	1500	0.0973	1400	1693	1504	3.55	110	0.675
7	3037	0.0999	2629	3572	3076	6.38	63	0.693
8	4656	0.0406	4414	5149	4680	2.89	54	0.281
9	20000 (UM)	0.2591	18659	27979	20727	4.87	1910	21.564

TIC:	3.3206	ng/uL
TIM:	22.5981	nmole/L
Total concentration:	4.0244	ng/uL

Sample peak width (sec): 5 Sample min peak height: 50 Sample baseline V to V?: Y Sample baseline V to V points: 3
Sample filter: Binomial Number of points for filter: 3 Sample start region (min): 0 Sample end region (min): 30
Marker peak width (sec): 5 Marker min peak height: 200 Marker baseline V to V?: Y Marker baseline V to V points: 3
Lower marker selection: First peak > 500 RFU Upper marker selection: Last peak > 200 RFU
Ladder size (bp)75, 200, 300, 400, 500, 700, 1000, 1500, 2000, 3000, 4000, 5000, 7000, 10000, 20000
Quantification using: Lower Marker Final concentration (ng/uL): 0.5000 Dilution factor: 12.0

Sample: SampD2
Well location: D2
Created: Wednesday, October 20, 2021 5:18:46 PM

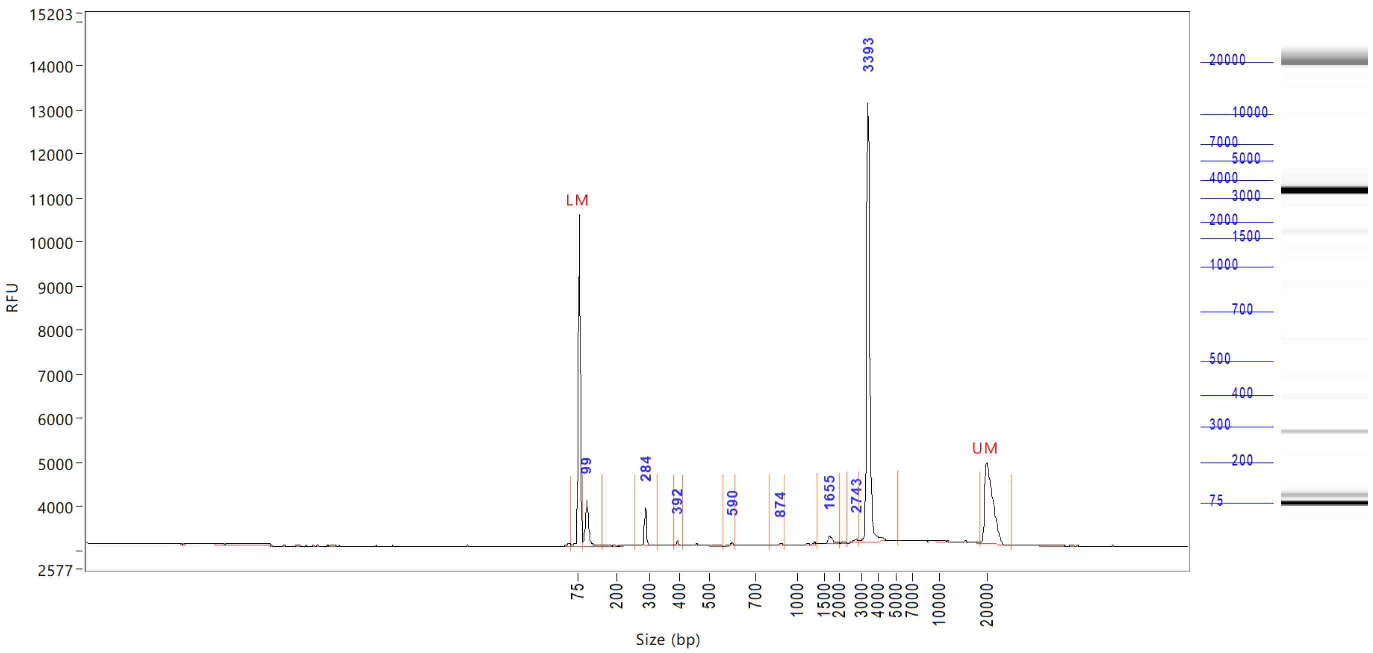

Peak	Size (bp)	Concentration (ng/uL)	From (bp)	To (bp)	Average size (bp)	CV%	RFU	Corrected peak area
1	75 (LM)	0.5000	48	86	73	4.97	7515	40.013
2	99	1.2241	86	150	98	8.36	1030	8.163
3	284	0.6286	255	323	283	2.09	856	4.192
4	392	0.0753	382	411	390	1.01	110	0.502
5	590	0.0686	557	608	586	1.52	73	0.458
6	874	0.0603	792	909	862	3.16	50	0.402
7	1655	0.1873	1378	2000	1682	5.95	183	1.249
8	2743	0.0704	2344	2914	2737	3.93	67	0.469
9	3393	7.3440	2914	5371	3402	4.74	9992	48.975
10	20000 (UM)	0.2387	18537	25199	20564	4.15	1830	19.105

TIC:	9.6585	ng/uL
TIM:	28.5681	nmole/L
Total concentration:	10.1231	ng/uL

```
Sample peak width (sec): 5    Sample min peak height: 50    Sample baseline V to V?: Y    Sample baseline V to V points: 3
Sample filter: Binomial       Number of points for filter: 3  Sample start region (min): 0  Sample end region (min): 30
Marker peak width (sec): 5    Marker min peak height: 200   Marker baseline V to V?: Y    Marker baseline V to V points: 3
Lower marker selection: First peak > 500 RFU                Upper marker selection: Last peak > 200 RFU
Ladder size (bp)75, 200, 300, 400, 500, 700, 1000, 1500, 2000, 3000, 4000, 5000, 7000, 10000, 20000
Quantification using: Lower Marker          Final concentration (ng/uL): 0.5000          Dilution factor: 12.0
```

Sample: SampD3
Well location: D3
Created: Wednesday, October 20, 2021 5:18:46 PM

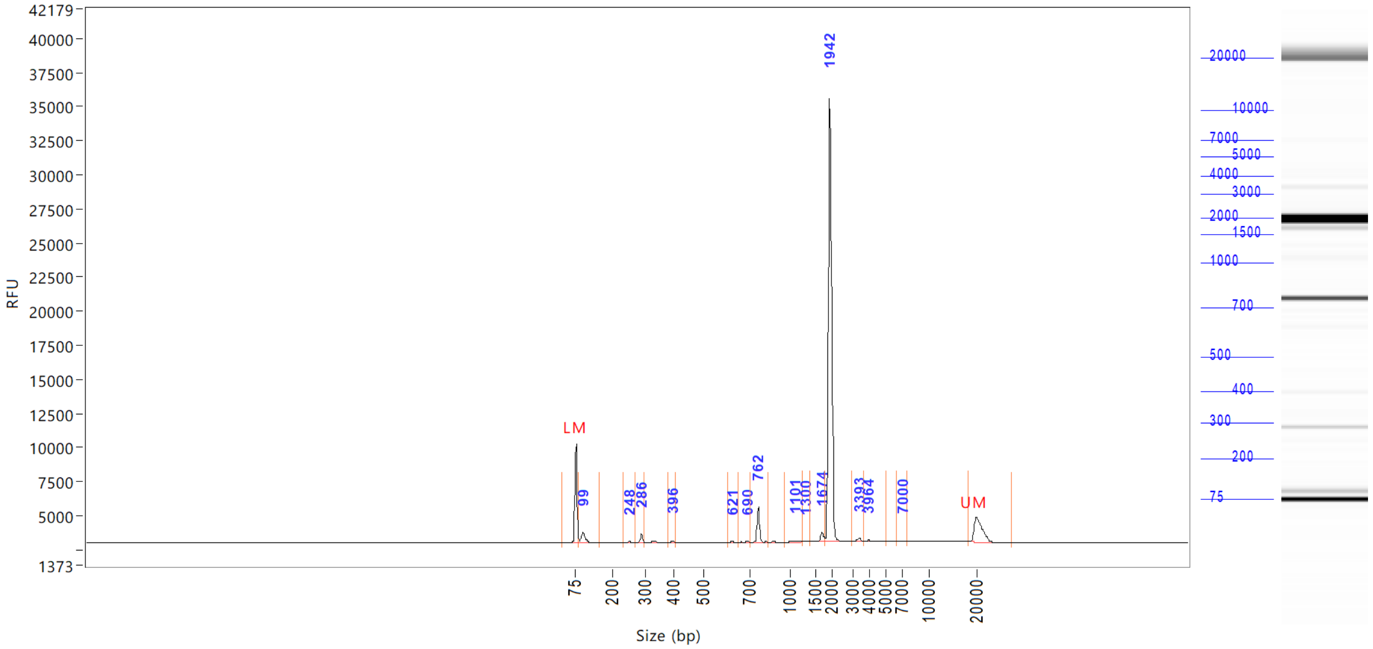

Peak	Size (bp)	Concentration (ng/uL)	From (bp)	To (bp)	Average size (bp)	CV%	RFU	Corrected peak area
1	75 (LM)	0.5000	33	86	72	6.04	7248	39.271
2	99	1.1123	86	154	99	10.31	793	7.280
3	248	0.0878	232	269	251	3.29	53	0.575
4	286	0.4795	269	298	284	1.46	645	3.138
5	396	0.1007	378	405	393	1.00	135	0.659
6	621	0.0806	606	649	622	1.42	92	0.527
7	690	0.0767	649	705	677	2.16	68	0.502
8	762	1.7501	705	840	756	1.74	2568	11.455
9	1101	0.1662	957	1223	1079	5.37	115	1.088
10	1300	0.0616	1223	1389	1295	2.50	65	0.403
11	1674	0.5471	1389	1770	1660	3.93	667	3.581
12	1942	25.4702	1770	3000	1926	3.78	32550	166.706
13	3393	0.1812	3000	3679	3314	3.71	232	1.186
14	3964	0.1368	3679	5000	4241	7.36	67	0.895
15	7000	0.0443	6334	7468	6926	3.49	53	0.290
16	20000 (UM)	0.2468	18415	27617	20576	4.48	1854	19.387

TIC:	30.2949	ng/uL
TIM:	49.2294	nmole/L
Total concentration:	30.8574	ng/uL

Sample peak width (sec): 5   Sample min peak height: 50   Sample baseline V to V?: Y   Sample baseline V to V points: 3
Sample filter: Binomial   Number of points for filter: 3 Sample start region (min): 0 Sample end region (min): 30
Marker peak width (sec): 5   Marker min peak height: 200   Marker baseline V to V?: Y   Marker baseline V to V points: 3
Lower marker selection: First peak > 500 RFU   Upper marker selection: Last peak > 200 RFU
Ladder size (bp)75, 200, 300, 400, 500, 700, 1000, 1500, 2000, 3000, 4000, 5000, 7000, 10000, 20000
Quantification using: Lower Marker   Final concentration (ng/uL): 0.5000   Dilution factor: 12.0

Sample: SampD4
Well location: D4
Created: Wednesday, October 20, 2021 5:18:46 PM

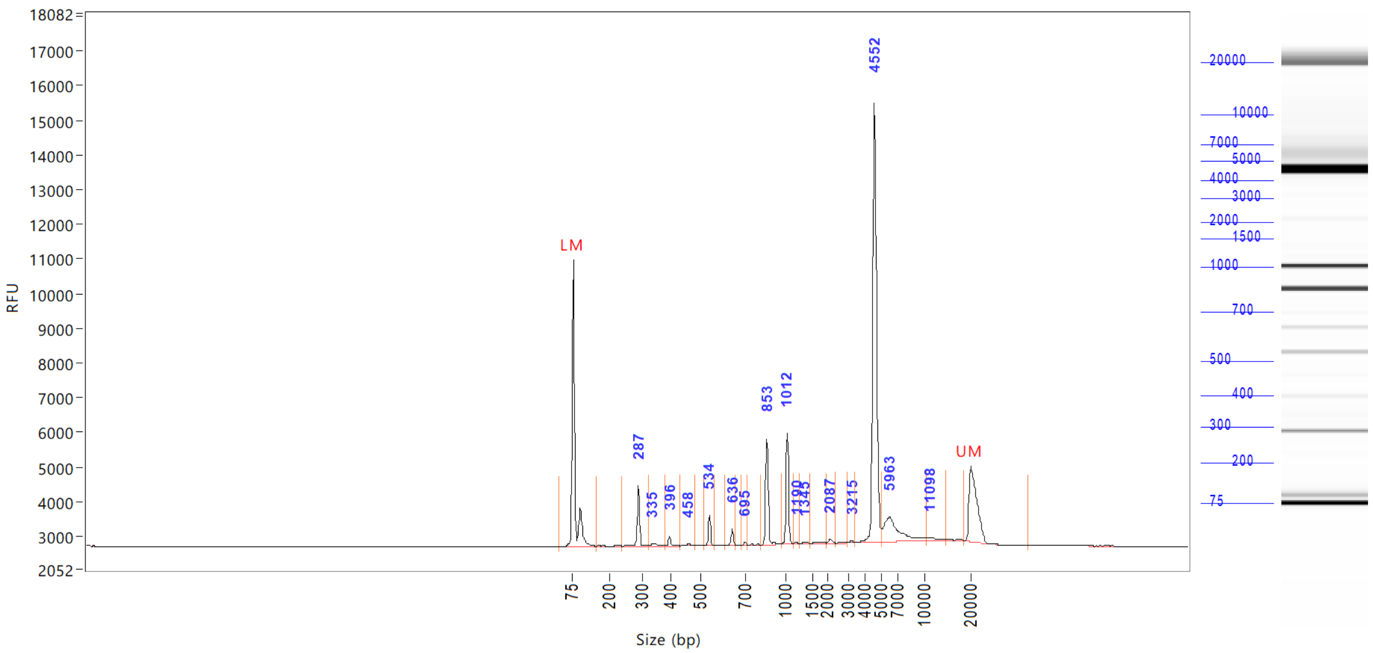

Peak	Size (bp)	Concentration (ng/uL)	From (bp)	To (bp)	Average size (bp)	CV%	RFU	Corrected peak area
1	75 (LM)	0.5000	30	152	79	15.42	8287	56.046
2	287	0.9833	237	321	285	3.06	1731	9.185
3	335	0.1365	321	382	345	4.59	82	1.275
4	396	0.1410	382	430	394	1.41	263	1.317
5	458	0.0518	430	479	456	2.08	67	0.484
6	534	0.4000	511	557	534	0.96	860	3.737
7	636	0.2297	606	657	634	1.07	473	2.145
8	695	0.0471	680	710	693	0.75	101	0.440
9	853	1.4441	809	965	854	1.96	3035	13.489
10	1012	1.5046	965	1134	1012	1.89	3190	14.054
11	1190	0.0421	1134	1245	1179	2.36	69	0.393
12	1345	0.0533	1245	1467	1348	3.67	53	0.498
13	2087	0.0783	1962	2401	2096	3.74	126	0.731
14	3215	0.0364	2943	3429	3169	3.52	64	0.340
15	4552	7.9615	3429	5000	4548	3.05	12684	74.369
16	5963	1.6798	5000	10489	6663	19.85	702	15.691
17	11098	0.1563	10489	14757	12157	9.60	87	1.460
18	20000 (UM)	0.2015	18537	31969	20591	4.95	2172	22.586

TIC:	14.9458	ng/uL
TIM:	17.7953	nmole/L
Total concentration:	15.1577	ng/uL

```
Sample peak width (sec): 5     Sample min peak height: 50     Sample baseline V to V?: Y     Sample baseline V to V points: 3
Sample filter: Binomial        Number of points for filter: 3  Sample start region (min): 0  Sample end region (min): 30
Marker peak width (sec): 5     Marker min peak height: 200    Marker baseline V to V?: Y     Marker baseline V to V points: 3
Lower marker selection: First peak > 500 RFU                  Upper marker selection: Last peak > 200 RFU
Ladder size (bp)75, 200, 300, 400, 500, 700, 1000, 1500, 2000, 3000, 4000, 5000, 7000, 10000, 20000
Quantification using: Lower Marker          Final concentration (ng/uL): 0.5000          Dilution factor: 12.0
```

Sample: SampD5
Well location: D5
Created: Wednesday, October 20, 2021 5:18:46 PM

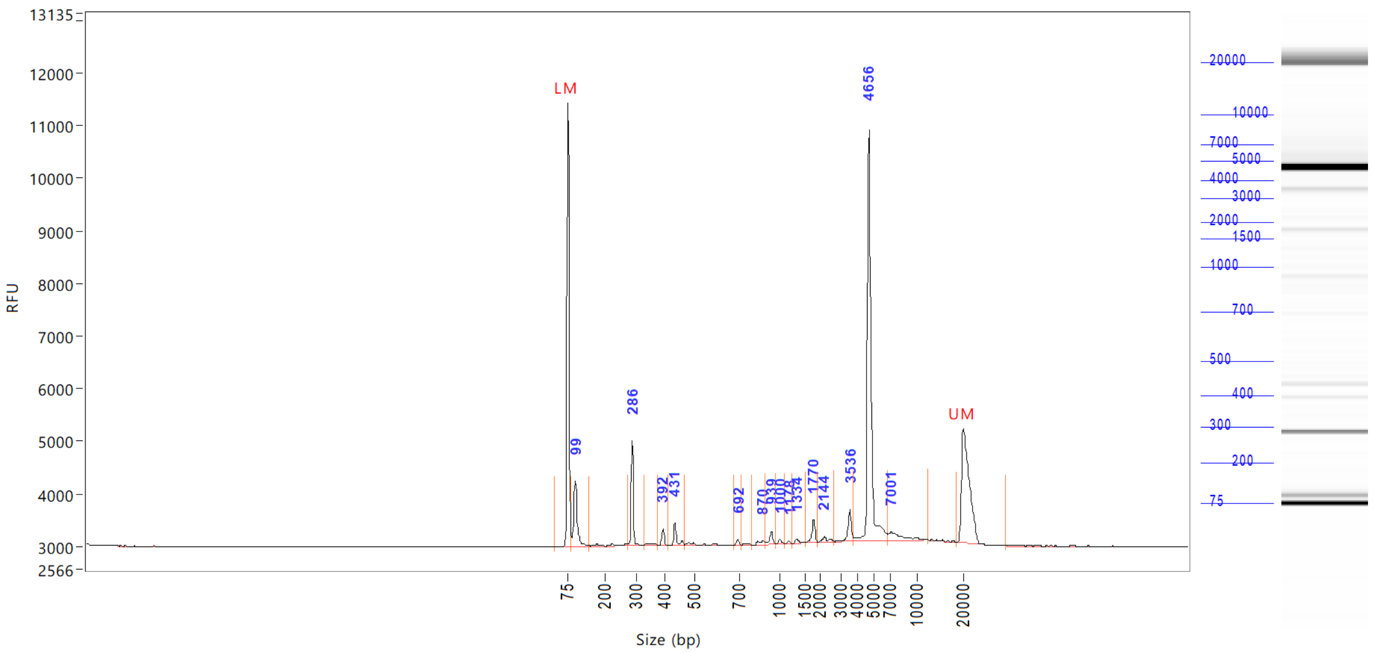

Peak	Size (bp)	Concentration (ng/uL)	From (bp)	To (bp)	Average size (bp)	CV%	RFU	Corrected peak area
1	75 (LM)	0.5000	31	86	73	4.55	8449	45.165
2	99	1.4449	86	148	99	9.00	1236	10.876
3	286	1.2738	273	323	283	1.77	1987	9.589
4	392	0.1850	376	412	390	1.08	300	1.393
5	431	0.3016	412	466	434	2.30	410	2.271
6	692	0.0717	672	705	689	0.84	119	0.539
7	870	0.1109	796	887	851	2.52	80	0.835
8	939	0.1984	887	970	929	1.79	252	1.493
9	1000	0.0637	970	1101	1006	2.38	83	0.479
10	1178	0.0319	1101	1245	1160	2.18	53	0.240
11	1334	0.0792	1245	1501	1351	4.51	85	0.596
12	1770	0.2952	1501	1923	1723	3.76	425	2.222
13	2144	0.1164	1923	2686	2210	7.93	104	0.876
14	3536	0.4193	2686	3750	3454	4.14	609	3.156
15	4656	5.9306	3750	6704	4732	7.37	7817	44.642
16	7001	0.4299	6704	12440	8138	15.19	155	3.236
17	20000 (UM)	0.2521	18659	29068	20622	4.41	2171	22.776

TIC:	10.9525	ng/uL
TIM:	36.9947	nmole/L
Total concentration:	11.3451	ng/uL

Sample peak width (sec): 5 Sample min peak height: 50 Sample baseline V to V?: Y Sample baseline V to V points: 3
Sample filter: Binomial Number of points for filter: 3 Sample start region (min): 0 Sample end region (min): 30
Marker peak width (sec): 5 Marker min peak height: 200 Marker baseline V to V?: Y Marker baseline V to V points: 3
Lower marker selection: First peak > 500 RFU Upper marker selection: Last peak > 200 RFU
Ladder size (bp)75, 200, 300, 400, 500, 700, 1000, 1500, 2000, 3000, 4000, 5000, 7000, 10000, 20000
Quantification using: Lower Marker Final concentration (ng/uL): 0.5000 Dilution factor: 12.0

Sample: SampD6
Well location: D6
Created: Wednesday, October 20, 2021 5:18:46 PM

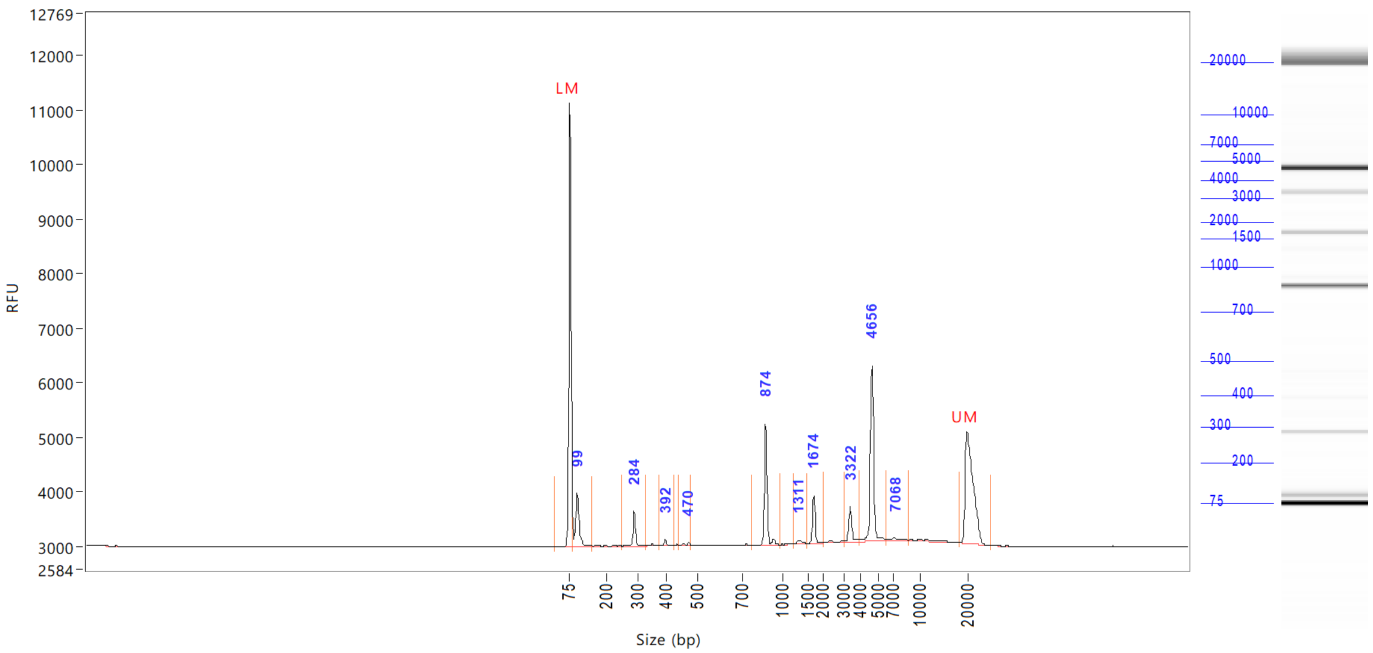

Peak	Size (bp)	Concentration (ng/uL)	From (bp)	To (bp)	Average size (bp)	CV%	RFU	Corrected peak area
1	75 (LM)	0.5000	26	86	73	4.76	8141	44.004
2	99	1.1546	86	152	99	8.87	973	8.468
3	284	0.4630	248	323	283	2.61	637	3.396
4	392	0.1005	370	420	391	2.33	112	0.737
5	470	0.0752	439	479	461	2.24	57	0.552
6	874	1.3932	766	978	873	2.05	2227	10.218
7	1311	0.0795	1212	1467	1327	3.78	72	0.583
8	1674	0.5367	1467	2000	1654	3.34	878	3.936
9	3322	0.4419	3037	3893	3345	4.44	653	3.241
10	4656	2.1630	3893	6038	4639	4.45	3199	15.863
11	7068	0.1133	6038	8667	7194	9.70	51	0.831
12	20000 (UM)	0.2462	18415	24957	20628	4.19	2057	21.672

TIC:	6.5208	ng/uL
TIM:	26.8026	nmole/L
Total concentration:	6.9634	ng/uL

```
Sample peak width (sec): 5    Sample min peak height: 50    Sample baseline V to V?: Y    Sample baseline V to V points: 3
Sample filter: Binomial       Number of points for filter: 3  Sample start region (min): 0  Sample end region (min): 30
Marker peak width (sec): 5    Marker min peak height: 200    Marker baseline V to V?: Y    Marker baseline V to V points: 3
Lower marker selection: First peak > 500 RFU                Upper marker selection: Last peak > 200 RFU
Ladder size (bp)75, 200, 300, 400, 500, 700, 1000, 1500, 2000, 3000, 4000, 5000, 7000, 10000, 20000
Quantification using: Lower Marker          Final concentration (ng/uL): 0.5000          Dilution factor: 12.0
```

Sample: SampD7
Well location: D7
Created: Wednesday, October 20, 2021 5:18:46 PM

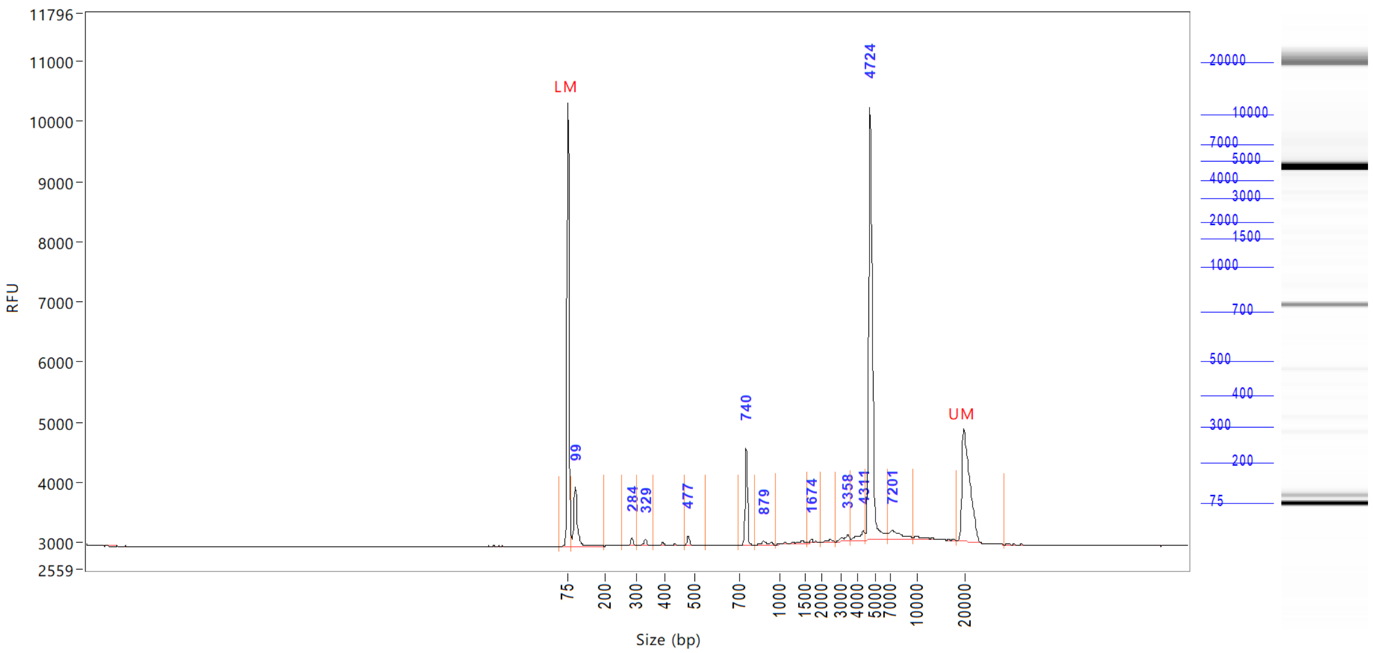

Peak	Size<br>(bp)	Concentration<br>(ng/uL)	From<br>(bp)	To<br>(bp)	Average size<br>(bp)	CV%	RFU	Corrected peak area
1	75 (LM)	0.5000	47	86	73	4.35	7378	39.399
2	99	1.3204	86	198	100	12.63	985	8.670
3	284	0.1179	251	301	281	2.73	124	0.774
4	329	0.1216	301	356	326	2.98	100	0.798
5	477	0.1466	462	544	478	2.59	165	0.963
6	740	1.0856	692	809	739	1.36	1617	7.129
7	879	0.1037	809	965	886	3.60	59	0.681
8	1674	0.0619	1501	2000	1699	5.66	57	0.406
9	3358	0.1424	2715	3536	3170	6.16	107	0.935
10	4311	0.2362	3536	4414	4079	5.90	159	1.551
11	4724	6.0232	4414	6556	4754	5.39	7187	39.552
12	7201	0.4005	6556	9533	7666	10.14	146	2.630
13	20000 (UM)	0.2497	18537	28584	20603	4.57	1864	19.677

TIC:	9.7599	ng/uL
TIM:	28.5015	nmole/L
Total concentration:	10.1926	ng/uL

Sample peak width (sec): 5 Sample min peak height: 50 Sample baseline V to V?: Y Sample baseline V to V points: 3
Sample filter: Binomial Number of points for filter: 3 Sample start region (min): 0 Sample end region (min): 30
Marker peak width (sec): 5 Marker min peak height: 200 Marker baseline V to V?: Y Marker baseline V to V points: 3
Lower marker selection: First peak > 500 RFU Upper marker selection: Last peak > 200 RFU
Ladder size (bp)75, 200, 300, 400, 500, 700, 1000, 1500, 2000, 3000, 4000, 5000, 7000, 10000, 20000
Quantification using: Lower Marker Final concentration (ng/uL): 0.5000 Dilution factor: 12.0

Sample: SampD8
Well location: D8
Created: Wednesday, October 20, 2021 5:18:46 PM

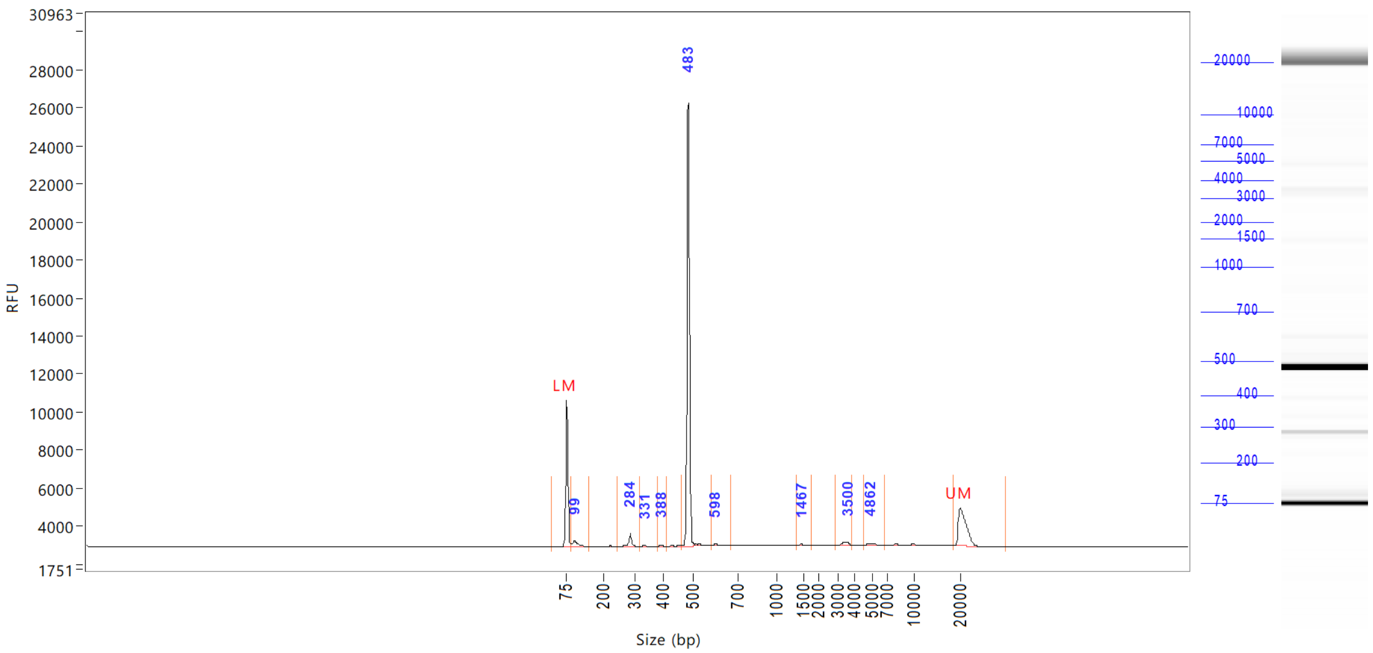

Peak	Size (bp)	Concentration (ng/uL)	From (bp)	To (bp)	Average size (bp)	CV%	RFU	Corrected peak area
1	75 (LM)	0.5000	24	89	73	5.04	7680	41.306
2	99	0.5971	89	150	105	11.63	301	4.111
3	284	0.6331	244	313	279	2.80	712	4.359
4	331	0.0715	313	378	336	4.16	61	0.492
5	388	0.0622	378	409	389	1.40	83	0.428
6	483	15.8563	460	585	482	1.55	23323	109.161
7	598	0.1219	585	669	605	2.85	121	0.839
8	1467	0.0698	1367	1770	1484	4.68	74	0.481
9	3500	0.2224	2857	3822	3358	5.03	177	1.531
10	4862	0.1259	4518	6704	5098	9.61	101	0.867
11	20000 (UM)	0.2512	18537	29914	20636	4.55	1989	20.749

TIC:	17.7602	ng/uL
TIM:	68.3743	nmole/L
Total concentration:	18.3271	ng/uL

Sample peak width (sec): 5   Sample min peak height: 50   Sample baseline V to V?: Y   Sample baseline V to V points: 3
Sample filter: Binomial   Number of points for filter: 3 Sample start region (min): 0 Sample end region (min): 30
Marker peak width (sec): 5   Marker min peak height: 200   Marker baseline V to V?: Y   Marker baseline V to V points: 3
Lower marker selection: First peak > 500 RFU   Upper marker selection: Last peak > 200 RFU
Ladder size (bp)75, 200, 300, 400, 500, 700, 1000, 1500, 2000, 3000, 4000, 5000, 7000, 10000, 20000
Quantification using: Lower Marker   Final concentration (ng/uL): 0.5000   Dilution factor: 12.0

Sample: SampD9
Well location: D9
Created: Wednesday, October 20, 2021 5:18:46 PM

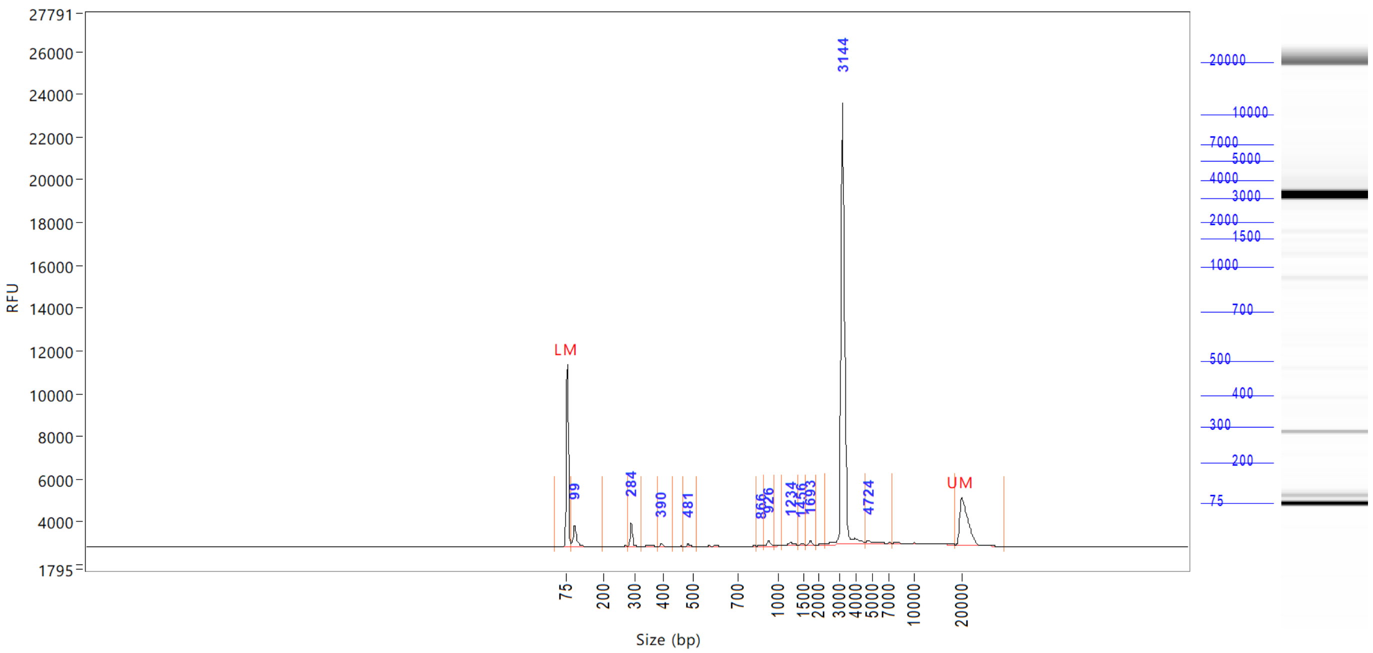

Peak	Size (bp)	Concentration (ng/uL)	From (bp)	To (bp)	Average size (bp)	CV%	RFU	Corrected peak area
1	75 (LM)	0.5000	33	87	73	4.79	8502	45.594
2	99	1.1431	87	192	101	12.66	954	8.687
3	284	0.7414	273	319	284	2.07	1101	5.634
4	390	0.1050	378	428	391	1.89	134	0.798
5	481	0.0941	462	511	480	1.69	110	0.715
6	866	0.0516	831	887	864	1.59	59	0.392
7	926	0.2100	887	970	923	1.48	284	1.596
8	1234	0.1618	1056	1389	1242	4.68	143	1.229
9	1456	0.0606	1389	1539	1448	1.94	84	0.460
10	1693	0.1187	1539	1904	1686	3.12	184	0.902
11	3144	15.5000	2286	4518	3168	6.16	20679	117.785
12	4724	0.3279	4518	7334	5352	14.84	129	2.492
13	20000 (UM)	0.2593	18659	29189	20699	4.54	2224	23.644

TIC:	18.5141	ng/uL
TIM:	32.6717	nmole/L
Total concentration:	19.0179	ng/uL

Sample peak width (sec): 5 Sample min peak height: 50 Sample baseline V to V?: Y Sample baseline V to V points: 3
Sample filter: Binomial Number of points for filter: 3 Sample start region (min): 0 Sample end region (min): 30
Marker peak width (sec): 5 Marker min peak height: 200 Marker baseline V to V?: Y Marker baseline V to V points: 3
Lower marker selection: First peak > 500 RFU Upper marker selection: Last peak > 200 RFU
Ladder size (bp)75, 200, 300, 400, 500, 700, 1000, 1500, 2000, 3000, 4000, 5000, 7000, 10000, 20000
Quantification using: Lower Marker Final concentration (ng/uL): 0.5000 Dilution factor: 12.0

Sample: SampD10
Well location: D10
Created: Wednesday, October 20, 2021 5:18:46 PM

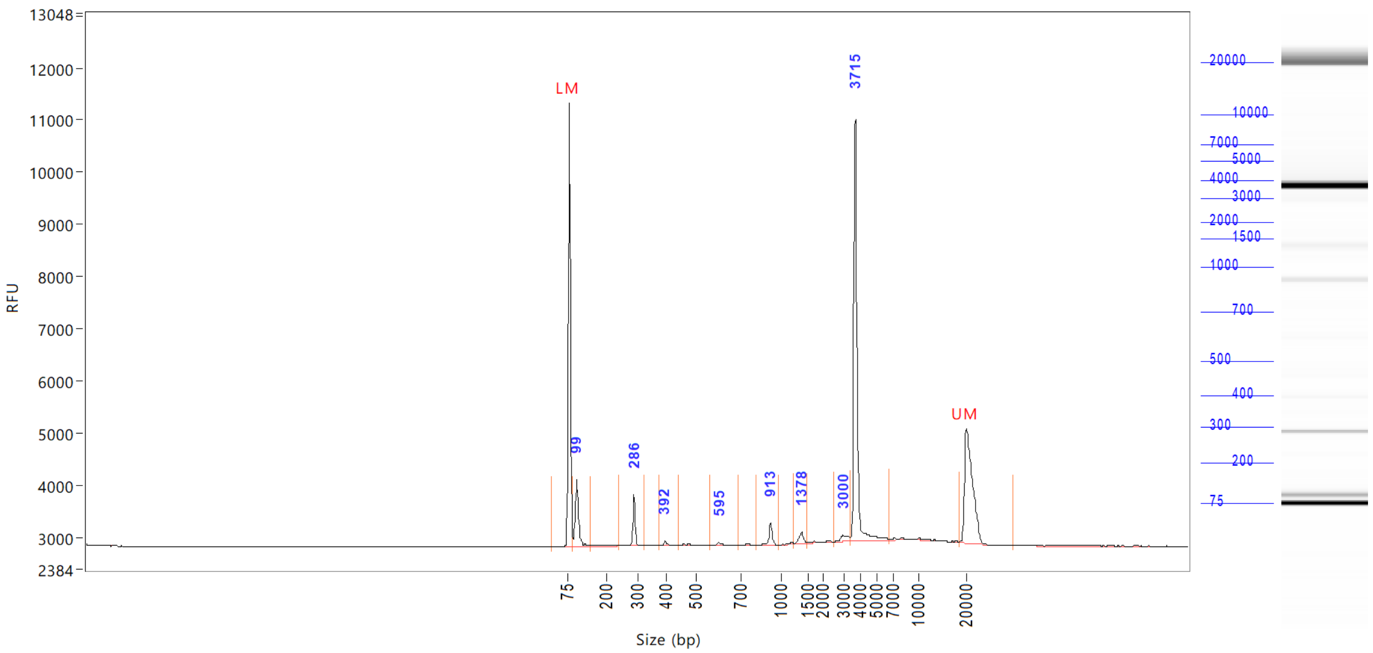

Peak	Size (bp)	Concentration (ng/uL)	From (bp)	To (bp)	Average size (bp)	CV%	RFU	Corrected peak area
1	75 (LM)	0.5000	20	86	73	4.42	8522	45.522
2	99	1.3683	86	148	99	8.37	1269	10.381
3	286	0.6592	237	319	284	2.50	983	5.002
4	392	0.1036	372	439	393	2.57	107	0.786
5	595	0.0964	560	682	602	4.27	63	0.731
6	913	0.3165	809	978	908	2.21	414	2.401
7	1378	0.2196	1234	1478	1359	3.43	218	1.666
8	3000	0.1783	2486	3358	3034	5.73	118	1.353
9	3715	5.5403	3358	6556	3765	8.80	8104	42.035
10	20000 (UM)	0.2572	18415	29914	20645	4.53	2195	23.415

TIC:	8.4824	ng/uL
TIM:	30.5699	nmole/L
Total concentration:	8.9876	ng/uL

Sample peak width (sec): 5   Sample min peak height: 50   Sample baseline V to V?: Y   Sample baseline V to V points: 3
Sample filter: Binomial   Number of points for filter: 3   Sample start region (min): 0   Sample end region (min): 30
Marker peak width (sec): 5   Marker min peak height: 200   Marker baseline V to V?: Y   Marker baseline V to V points: 3
Lower marker selection: First peak > 500 RFU   Upper marker selection: Last peak > 200 RFU
Ladder size (bp)75, 200, 300, 400, 500, 700, 1000, 1500, 2000, 3000, 4000, 5000, 7000, 10000, 20000
Quantification using: Lower Marker   Final concentration (ng/uL): 0.5000   Dilution factor: 12.0

Sample: SampD11
Well location: D11
Created: Wednesday, October 20, 2021 5:18:46 PM

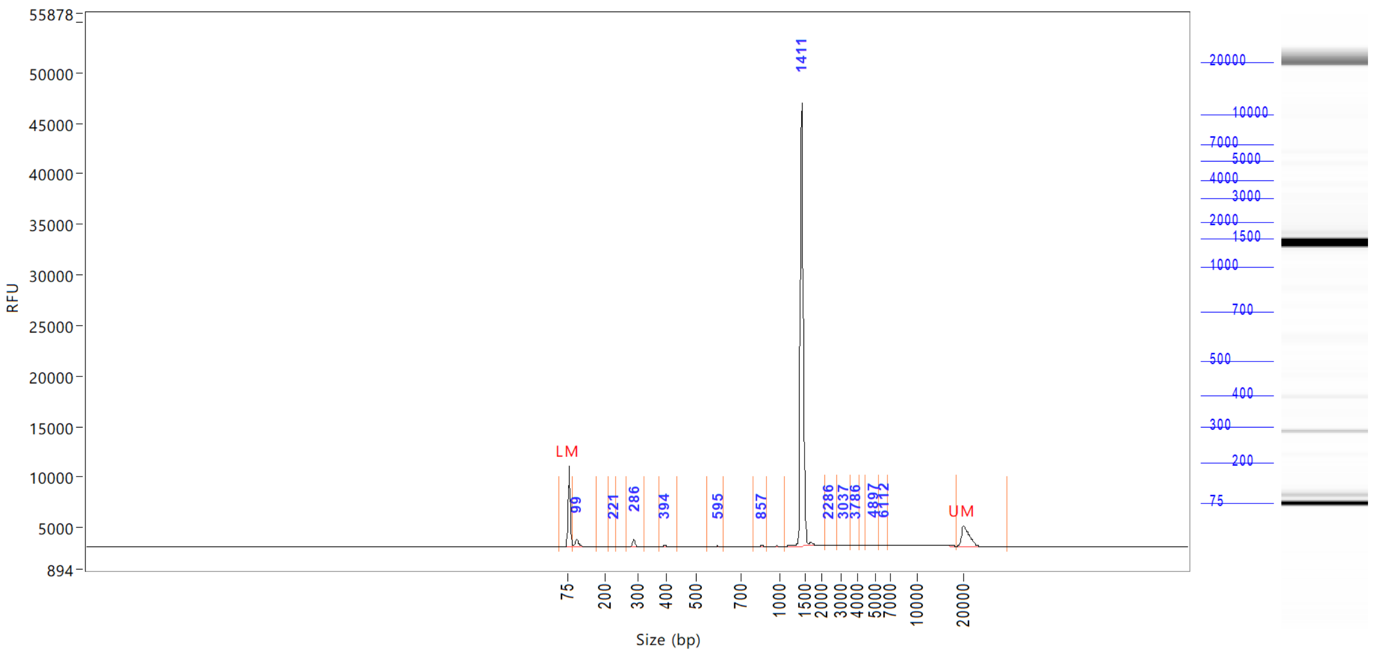

Peak	Size (bp)	Concentration (ng/uL)	From (bp)	To (bp)	Average size (bp)	CV%	RFU	Corrected peak area
1	75 (LM)	0.5000	43	87	73	4.64	7942	42.344
2	99	0.9941	87	166	102	11.48	747	7.016
3	221	0.0507	205	233	219	2.14	60	0.358
4	286	0.6154	264	319	285	2.28	802	4.343
5	394	0.1547	378	437	394	2.13	195	1.092
6	595	0.1329	547	626	587	2.39	77	0.938
7	857	0.0721	796	905	855	2.28	58	0.509
8	1411	31.1784	1090	2172	1414	3.92	43897	220.039
9	2286	0.1455	2172	2743	2432	6.80	74	1.027
10	3037	0.1024	2743	3500	3029	5.63	68	0.723
11	3786	0.0552	3500	4035	3752	3.00	62	0.390
12	4897	0.0588	4380	5371	4834	2.91	71	0.415
13	6112	0.0475	5371	6556	6018	3.53	72	0.335
14	20000 (UM)	0.2448	18537	29309	20623	4.34	1992	20.730

TIC:	33.6077	ng/uL
TIM:	57.6016	nmole/L
Total concentration:	34.1066	ng/uL

Sample peak width (sec): 5 Sample min peak height: 50 Sample baseline V to V?: Y Sample baseline V to V points: 3
Sample filter: Binomial Number of points for filter: 3 Sample start region (min): 0 Sample end region (min): 30
Marker peak width (sec): 5 Marker min peak height: 200 Marker baseline V to V?: Y Marker baseline V to V points: 3
Lower marker selection: First peak > 500 RFU Upper marker selection: Last peak > 200 RFU
Ladder size (bp)75, 200, 300, 400, 500, 700, 1000, 1500, 2000, 3000, 4000, 5000, 7000, 10000, 20000
Quantification using: Lower Marker Final concentration (ng/uL): 0.5000 Dilution factor: 12.0

Sample: SampD12
Well location: D12
Created: Wednesday, October 20, 2021 5:18:46 PM

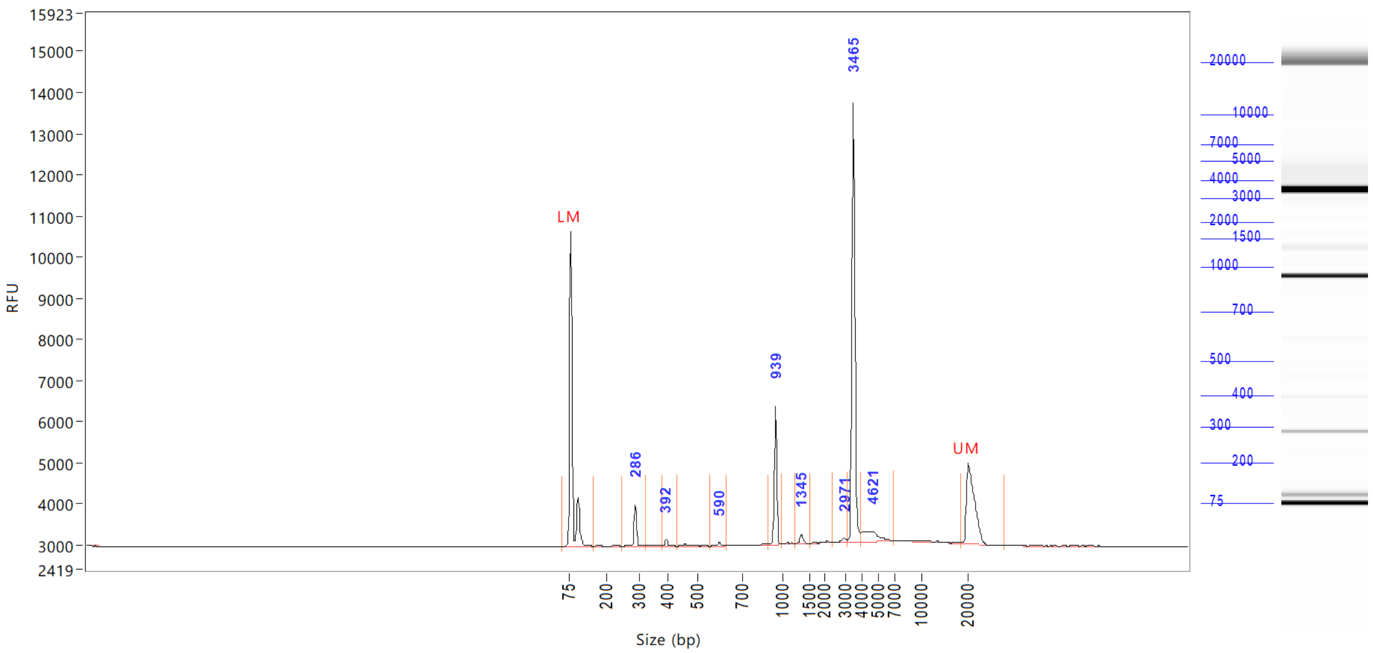

Peak	Size (bp)	Concentration (ng/uL)	From (bp)	To (bp)	Average size (bp)	CV%	RFU	Corrected peak area
1	75 (LM)	0.5000	50	154	79	14.42	7664	51.325
2	286	0.6080	242	321	284	2.71	1016	5.201
3	392	0.1091	380	428	392	1.80	171	0.933
4	590	0.0648	552	626	587	1.93	82	0.554
5	939	1.7034	887	991	938	1.13	3375	14.571
6	1345	0.1682	1223	1539	1340	3.33	230	1.438
7	2971	0.0825	2372	3072	2887	4.12	98	0.706
8	3465	6.0746	3072	3929	3463	3.08	10685	51.963
9	4621	0.7328	3929	6852	4589	11.90	255	6.269
10	20000 (UM)	0.2021	18537	27496	20676	4.39	1958	20.750

TIC:	9.5435	ng/uL
TIM:	10.5543	nmole/L
Total concentration:	10.0949	ng/uL

Sample peak width (sec): 5   Sample min peak height: 50   Sample baseline V to V?: Y   Sample baseline V to V points: 3
Sample filter: Binomial   Number of points for filter: 3 Sample start region (min): 0 Sample end region (min): 30
Marker peak width (sec): 5   Marker min peak height: 200   Marker baseline V to V?: Y   Marker baseline V to V points: 3
Lower marker selection: First peak > 500 RFU   Upper marker selection: Last peak > 200 RFU
Ladder size (bp)75, 200, 300, 400, 500, 700, 1000, 1500, 2000, 3000, 4000, 5000, 7000, 10000, 20000
Quantification using: Lower Marker   Final concentration (ng/uL): 0.5000   Dilution factor: 12.0

Sample: SampE1
Well location: E1
Created: Wednesday, October 20, 2021 5:18:46 PM

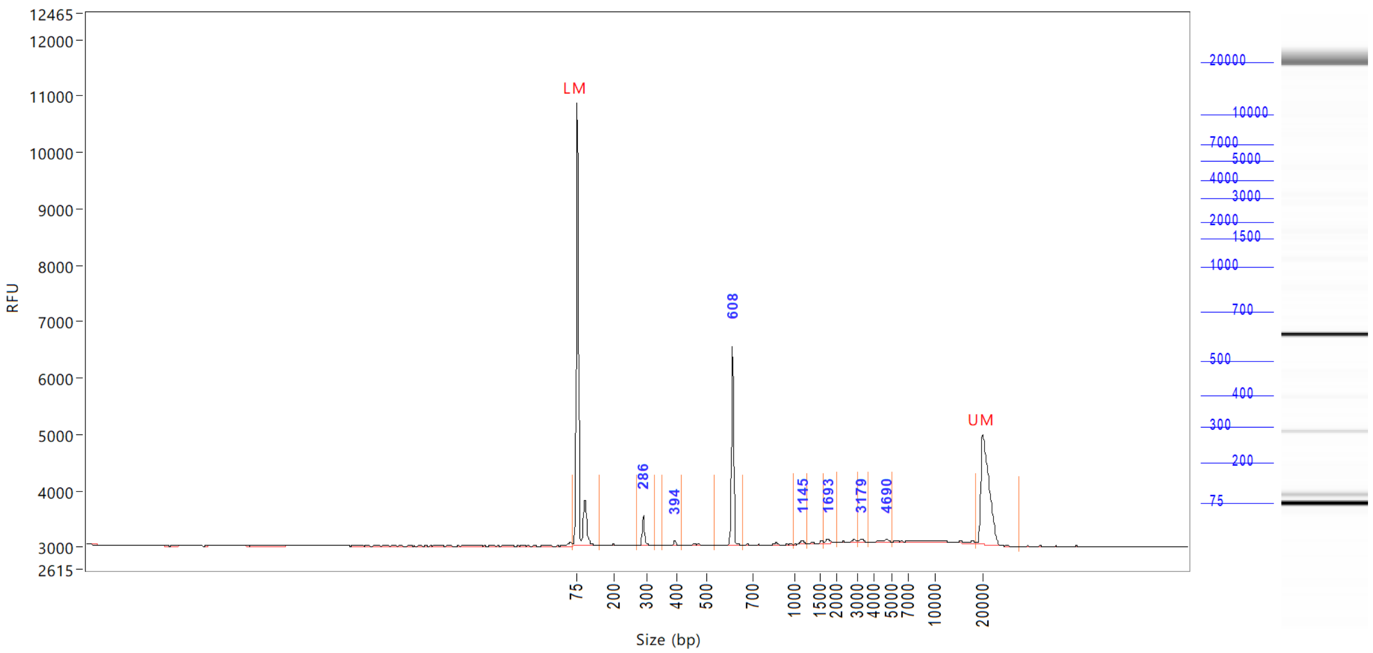

Peak	Size (bp)	Concentration (ng/uL)	From (bp)	To (bp)	Average size (bp)	CV%	RFU	Corrected peak area
1	75 (LM)	0.5000	60	147	77	13.26	7863	47.896
2	286	0.3479	266	323	285	2.17	532	2.777
3	394	0.0778	352	412	386	3.04	94	0.621
4	608	1.8752	527	652	605	1.09	3517	14.969
5	1145	0.0558	996	1234	1132	2.93	74	0.445
6	1693	0.0737	1578	2001	1732	5.67	72	0.588
7	3179	0.0493	3000	3679	3202	4.46	61	0.393
8	4690	0.0556	3679	5001	4504	6.69	50	0.444
9	20000 (UM)	0.2156	18537	27617	20598	4.28	1947	20.649

TIC:	2.5352	ng/uL
TIM:	7.6370	nmole/L
Total concentration:	3.1607	ng/uL

```
Sample peak width (sec): 5    Sample min peak height: 50    Sample baseline V to V?: Y    Sample baseline V to V points: 3
Sample filter: Binomial       Number of points for filter: 3  Sample start region (min): 0  Sample end region (min): 30
Marker peak width (sec): 5    Marker min peak height: 200   Marker baseline V to V?: Y    Marker baseline V to V points: 3
Lower marker selection: First peak > 500 RFU                Upper marker selection: Last peak > 200 RFU
Ladder size (bp)75, 200, 300, 400, 500, 700, 1000, 1500, 2000, 3000, 4000, 5000, 7000, 10000, 20000
Quantification using: Lower Marker          Final concentration (ng/uL): 0.5000          Dilution factor: 12.0
```

Sample: SampE2
Well location: E2
Created: Wednesday, October 20, 2021 5:18:46 PM

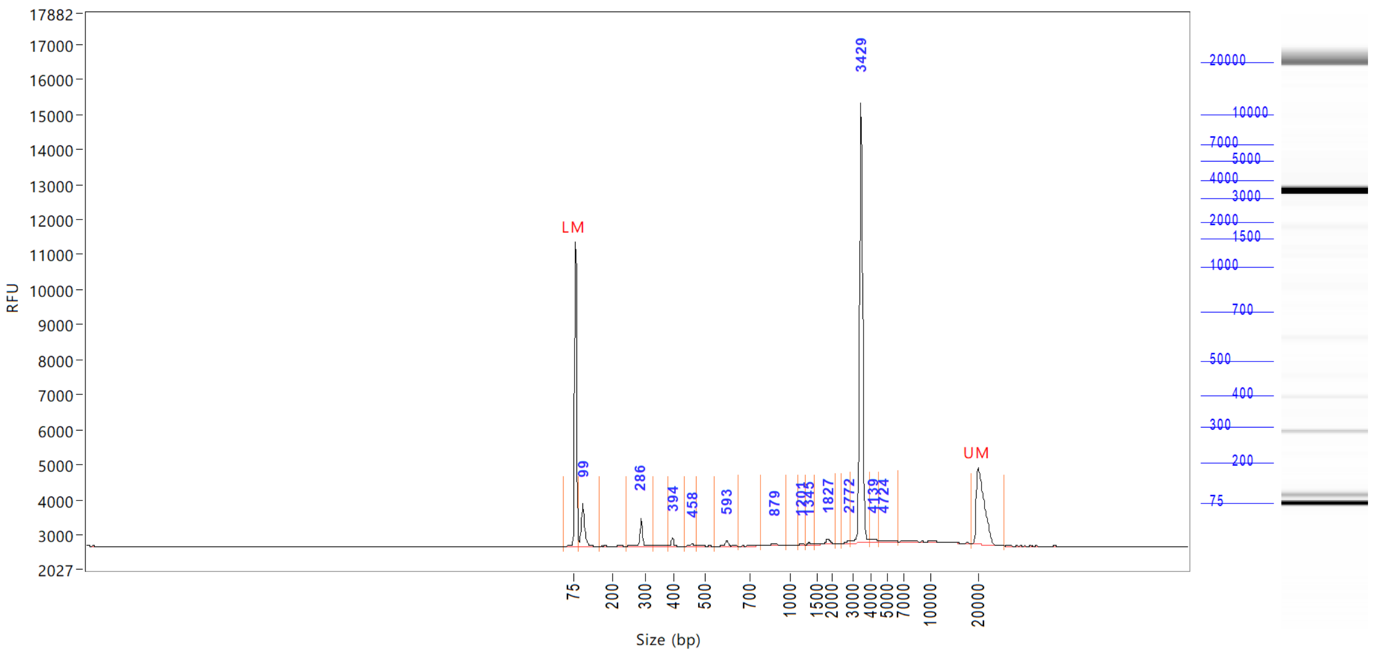

Peak	Size (bp)	Concentration (ng/uL)	From (bp)	To (bp)	Average size (bp)	CV%	RFU	Corrected peak area
1	75 (LM)	0.5000	37	86	73	4.97	8686	46.457
2	99	1.2931	86	154	99	8.55	1211	10.012
3	286	0.5996	239	325	284	3.70	810	4.643
4	394	0.1517	378	433	392	1.53	236	1.175
5	458	0.0653	433	471	453	1.62	68	0.505
6	593	0.1407	542	652	592	2.45	143	1.089
7	879	0.1238	779	965	879	3.93	75	0.959
8	1201	0.0429	1134	1267	1198	2.53	61	0.332
9	1345	0.0504	1267	1445	1340	2.95	66	0.390
10	1827	0.1703	1445	2087	1776	7.01	148	1.319
11	2772	0.0756	2401	2886	2736	3.85	88	0.585
12	3429	7.9092	2886	3964	3401	3.12	12569	61.240
13	4139	0.1194	3964	4483	4185	3.65	85	0.924
14	4724	0.1241	4483	6408	5006	8.45	57	0.961
15	20000 (UM)	0.2475	18537	25682	20636	4.11	2208	22.999

TIC:	10.8660	ng/uL
TIM:	30.6568	nmole/L
Total concentration:	11.2356	ng/uL

```
Sample peak width (sec): 5    Sample min peak height: 50    Sample baseline V to V?: Y    Sample baseline V to V points: 3
Sample filter: Binomial       Number of points for filter: 3  Sample start region (min): 0  Sample end region (min): 30
Marker peak width (sec): 5    Marker min peak height: 200   Marker baseline V to V?: Y    Marker baseline V to V points: 3
Lower marker selection: First peak > 500 RFU                Upper marker selection: Last peak > 200 RFU
Ladder size (bp)75, 200, 300, 400, 500, 700, 1000, 1500, 2000, 3000, 4000, 5000, 7000, 10000, 20000
Quantification using: Lower Marker          Final concentration (ng/uL): 0.5000          Dilution factor: 12.0
```

Sample: SampE3
Well location: E3
Created: Wednesday, October 20, 2021 5:18:46 PM

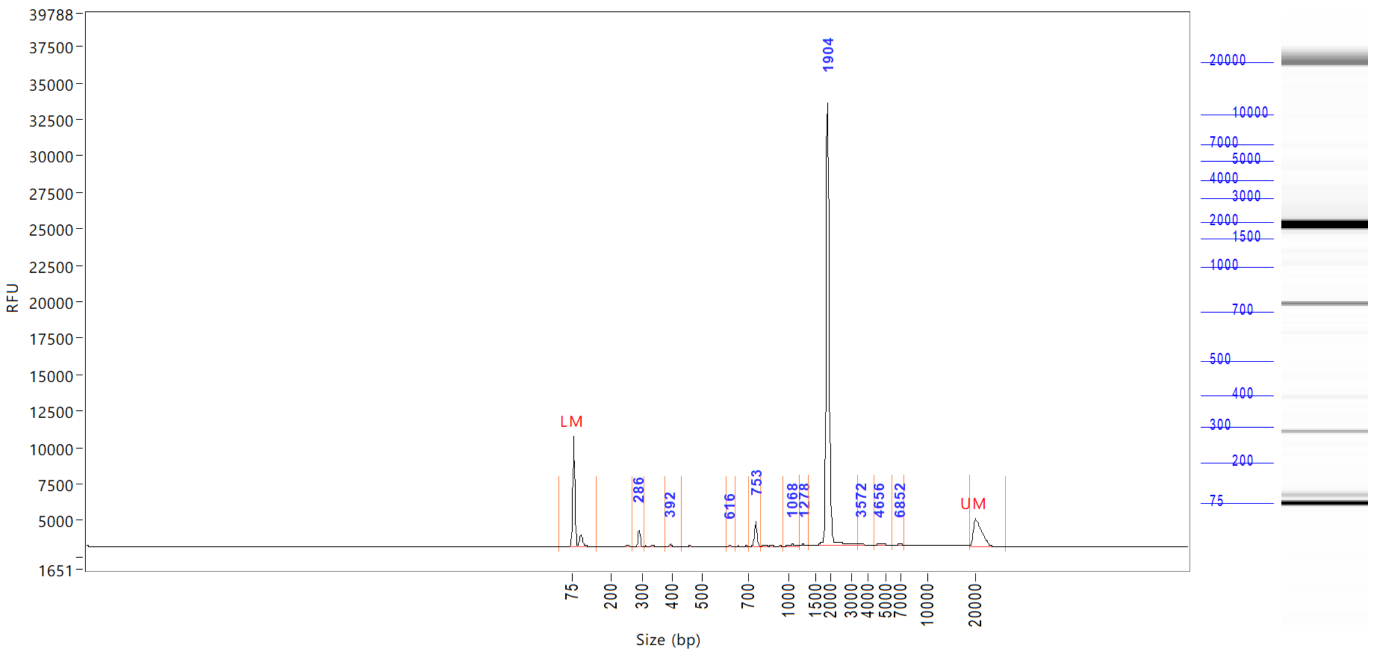

Peak	Size (bp)	Concentration (ng/uL)	From (bp)	To (bp)	Average size (bp)	CV%	RFU	Corrected peak area
1	75 (LM)	0.5000	26	148	77	14.22	7578	47.865
2	286	0.6871	264	300	284	1.39	1150	5.482
3	392	0.1044	378	430	393	1.88	155	0.833
4	616	0.0679	603	646	618	1.44	100	0.541
5	753	0.9584	701	792	748	1.30	1738	7.646
6	1068	0.1422	961	1212	1070	5.41	121	1.134
7	1278	0.0834	1212	1378	1271	2.52	104	0.665
8	1904	19.7196	1378	3393	1904	7.38	30425	157.313
9	3572	0.1402	3393	4311	3734	6.76	103	1.118
10	4656	0.1306	4311	5889	4807	6.69	87	1.042
11	6852	0.0639	5889	7334	6732	4.42	93	0.510
12	20000 (UM)	0.2090	18659	26408	20598	4.33	1860	20.008

TIC:	22.0976	ng/uL
TIM:	24.2040	nmole/L
Total concentration:	22.7312	ng/uL

```
Sample peak width (sec): 5    Sample min peak height: 50    Sample baseline V to V?: Y    Sample baseline V to V points: 3
Sample filter: Binomial       Number of points for filter: 3  Sample start region (min): 0  Sample end region (min): 30
Marker peak width (sec): 5    Marker min peak height: 200   Marker baseline V to V?: Y    Marker baseline V to V points: 3
Lower marker selection: First peak > 500 RFU                Upper marker selection: Last peak > 200 RFU
Ladder size (bp)75, 200, 300, 400, 500, 700, 1000, 1500, 2000, 3000, 4000, 5000, 7000, 10000, 20000
Quantification using: Lower Marker          Final concentration (ng/uL): 0.5000          Dilution factor: 12.0
```

Sample: SampE4
Well location: E4
Created: Wednesday, October 20, 2021 5:18:46 PM

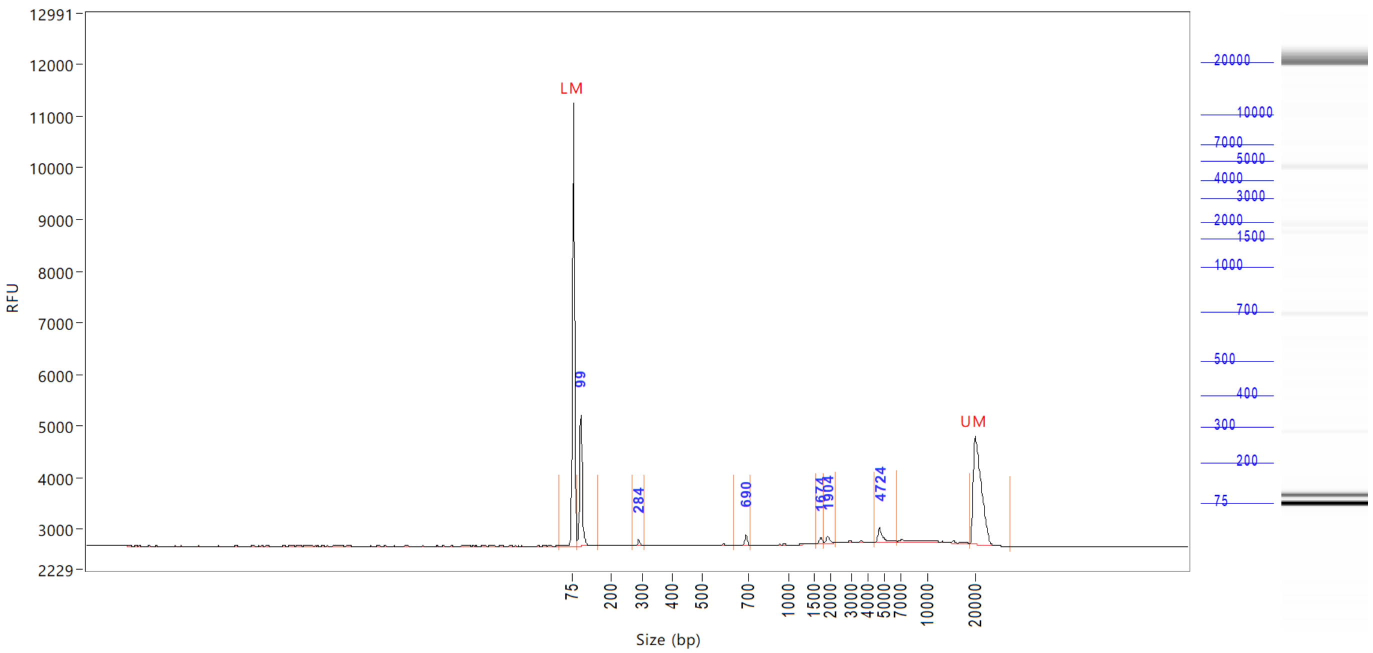

Peak	Size (bp)	Concentration (ng/uL)	From (bp)	To (bp)	Average size (bp)	CV%	RFU	Corrected peak area
1	75 (LM)	0.5000	26	86	72	5.30	8593	45.877
2	99	2.1722	86	158	98	6.76	2530	16.609
3	284	0.0919	264	303	283	2.09	124	0.703
4	690	0.1343	639	714	688	1.21	225	1.027
5	1674	0.0698	1539	1789	1667	3.00	109	0.534
6	1904	0.1105	1789	2258	1922	3.75	139	0.845
7	4724	0.2190	4414	6556	4799	6.03	275	1.675
8	20000 (UM)	0.2404	18781	27496	20665	4.18	2084	22.060

TIC:	2.7977	ng/uL
TIM:	37.4891	nmole/L
Total concentration:	3.3482	ng/uL

Sample peak width (sec): 5 Sample min peak height: 50 Sample baseline V to V?: Y Sample baseline V to V points: 3
Sample filter: Binomial Number of points for filter: 3 Sample start region (min): 0 Sample end region (min): 30
Marker peak width (sec): 5 Marker min peak height: 200 Marker baseline V to V?: Y Marker baseline V to V points: 3
Lower marker selection: First peak > 500 RFU Upper marker selection: Last peak > 200 RFU
Ladder size (bp)75, 200, 300, 400, 500, 700, 1000, 1500, 2000, 3000, 4000, 5000, 7000, 10000, 20000
Quantification using: Lower Marker Final concentration (ng/uL): 0.5000 Dilution factor: 12.0

Sample: SampE5
Well location: E5
Created: Wednesday, October 20, 2021 5:18:46 PM

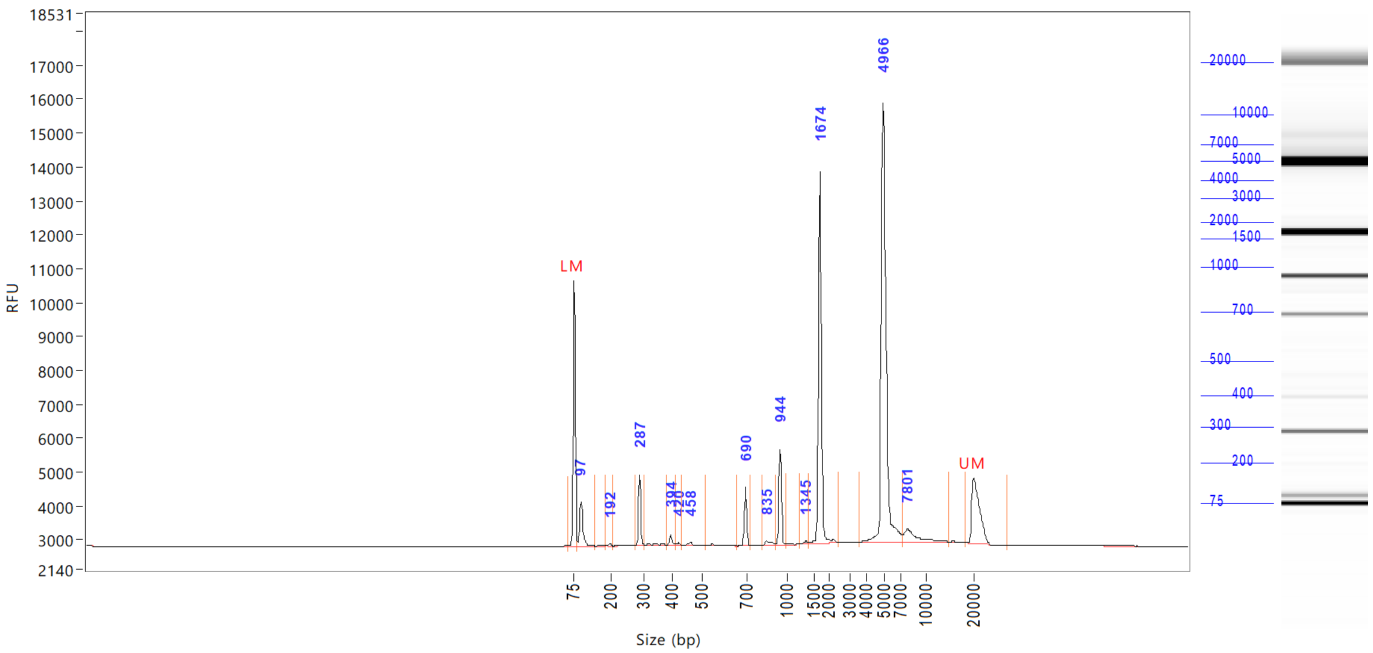

Peak	Size (bp)	Concentration (ng/uL)	From (bp)	To (bp)	Average size (bp)	CV%	RFU	Corrected peak area
1	75 (LM)	0.5000	54	86	73	4.62	7839	41.535
2	97	1.5592	86	145	97	7.78	1304	10.793
3	192	0.0551	183	201	191	1.56	78	0.381
4	287	1.4707	273	301	284	1.31	2089	10.181
5	394	0.2554	380	412	394	1.64	324	1.768
6	420	0.0615	412	428	419	0.92	81	0.426
7	458	0.1644	428	509	459	3.73	94	1.138
8	690	1.0446	654	714	687	0.75	1717	7.232
9	835	0.1827	805	909	856	2.92	122	1.265
10	944	1.7542	909	987	938	1.03	2827	12.143
11	1345	0.0878	1223	1400	1319	3.48	84	0.608
12	1674	7.4920	1400	2401	1665	4.76	10979	51.864
13	4966	13.3014	3536	7201	5044	8.03	12974	92.079
14	7801	0.9939	7201	14635	8661	15.51	376	6.880
15	20000 (UM)	0.2378	18293	27012	20580	4.13	1931	19.752

TIC:	28.4227	ng/uL
TIM:	55.2603	nmole/L
Total concentration:	28.9267	ng/uL

Sample peak width (sec): 5 Sample min peak height: 50 Sample baseline V to V?: Y Sample baseline V to V points: 3
Sample filter: Binomial Number of points for filter: 3 Sample start region (min): 0 Sample end region (min): 30
Marker peak width (sec): 5 Marker min peak height: 200 Marker baseline V to V?: Y Marker baseline V to V points: 3
Lower marker selection: First peak > 500 RFU Upper marker selection: Last peak > 200 RFU
Ladder size (bp)75, 200, 300, 400, 500, 700, 1000, 1500, 2000, 3000, 4000, 5000, 7000, 10000, 20000
Quantification using: Lower Marker Final concentration (ng/uL): 0.5000 Dilution factor: 12.0

Sample: SampE6
Well location: E6
Created: Wednesday, October 20, 2021 5:18:46 PM

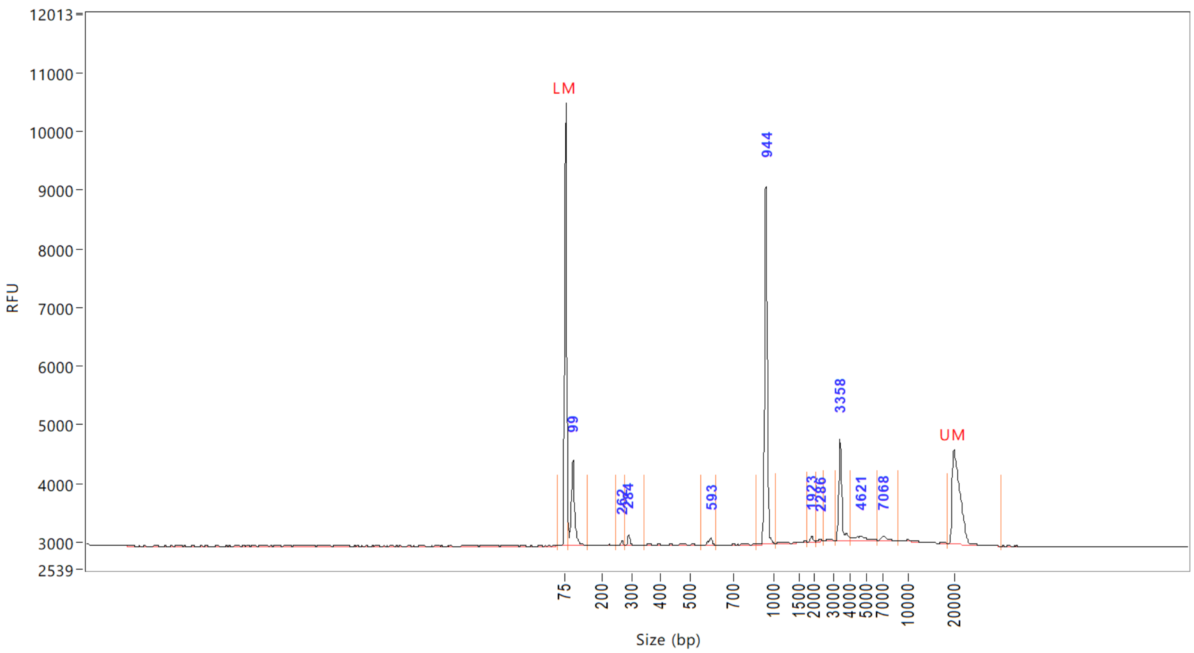

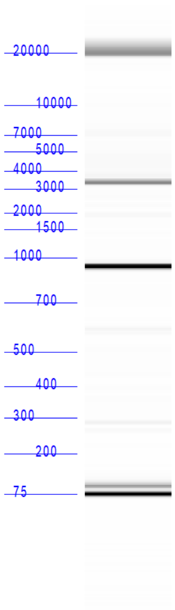

Peak	Size (bp)	Concentration (ng/uL)	From (bp)	To (bp)	Average size (bp)	CV%	RFU	Corrected peak area
1	75 (LM)	0.5000	48	87	73	4.68	7562	40.030
2	99	1.7378	87	152	100	8.55	1463	11.594
3	262	0.0616	244	273	261	1.75	80	0.411
4	284	0.1764	273	337	289	4.62	182	1.177
5	593	0.1528	544	618	585	1.84	141	1.019
6	944	4.1656	861	1034	938	1.23	6095	27.792
7	1923	0.0684	1789	2058	1903	2.57	93	0.456
8	2286	0.0365	2058	2486	2260	3.75	50	0.244
9	3358	1.2948	3037	4035	3387	5.07	1731	8.638
10	4621	0.2617	4035	6334	4728	11.33	77	1.746
11	7068	0.1381	6334	8800	7260	7.00	89	0.921
12	20000 (UM)	0.2120	18537	30277	20603	4.80	1609	16.975

TIC:	8.0936	ng/uL
TIM:	38.5056	nmole/L
Total concentration:	8.6042	ng/uL

```
Sample peak width (sec): 5     Sample min peak height: 50     Sample baseline V to V?: Y     Sample baseline V to V points: 3
Sample filter: Binomial        Number of points for filter: 3  Sample start region (min): 0  Sample end region (min): 30
Marker peak width (sec): 5     Marker min peak height: 200    Marker baseline V to V?: Y     Marker baseline V to V points: 3
Lower marker selection: First peak > 500 RFU                  Upper marker selection: Last peak > 200 RFU
Ladder size (bp)75, 200, 300, 400, 500, 700, 1000, 1500, 2000, 3000, 4000, 5000, 7000, 10000, 20000
Quantification using: Lower Marker          Final concentration (ng/uL): 0.5000          Dilution factor: 12.0
```

Sample: SampE7
Well location: E7
Created: Wednesday, October 20, 2021 5:18:46 PM

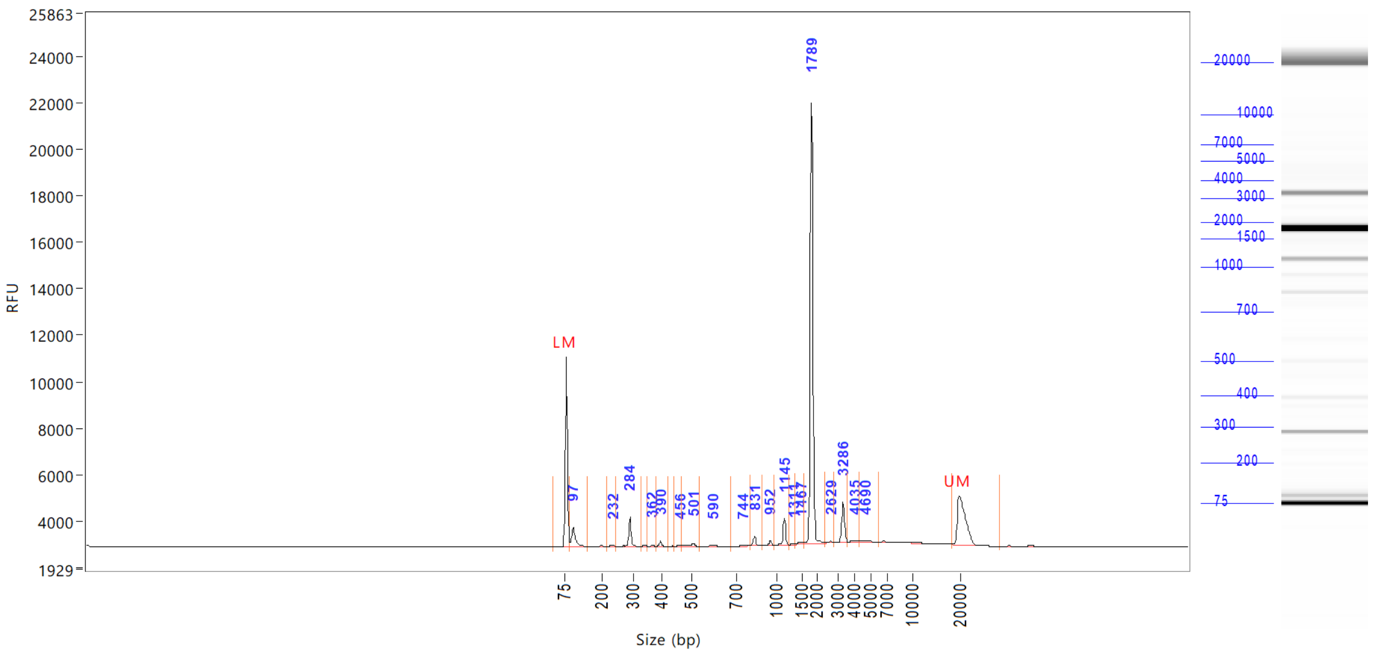

Peak	Size (bp)	Concentration (ng/uL)	From (bp)	To (bp)	Average size (bp)	CV%	RFU	Corrected peak area
1	75 (LM)	0.5000	31	87	74	4.74	8147	43.399
2	97	1.1022	87	148	100	9.87	846	7.972
3	232	0.0567	214	242	227	2.43	56	0.410
4	284	0.8812	242	323	283	2.49	1245	6.374
5	362	0.0602	347	376	361	1.48	77	0.436
6	390	0.1569	376	420	389	1.16	241	1.135
7	456	0.0508	441	466	454	1.34	55	0.367
8	501	0.1682	466	532	494	2.37	145	1.217
9	590	0.0964	532	674	598	4.33	68	0.697
10	744	0.0810	674	801	752	3.31	55	0.586
11	831	0.2594	801	887	830	1.79	392	1.876
12	952	0.1443	887	983	939	1.79	194	1.043
13	1145	0.7132	983	1245	1127	2.80	1144	5.159
14	1311	0.0388	1245	1367	1311	2.37	50	0.281
15	1467	0.1163	1367	1520	1450	2.82	103	0.841
16	1789	11.9214	1520	2344	1776	3.04	19010	86.229
17	2629	0.0515	2344	2800	2578	3.85	62	0.373
18	3286	1.0687	2800	3608	3259	3.02	1727	7.730
19	4035	0.1279	3608	4277	3977	4.69	76	0.925
20	4690	0.1911	4277	6038	4749	8.02	86	1.382
21	20000 (UM)	0.2614	18415	28705	20579	4.43	2136	22.693

TIC:	17.2862	ng/uL
TIM:	39.4874	nmole/L
Total concentration:	17.5369	ng/uL

```
Sample peak width (sec): 5    Sample min peak height: 50    Sample baseline V to V?: Y    Sample baseline V to V points: 3
Sample filter: Binomial       Number of points for filter: 3  Sample start region (min): 0  Sample end region (min): 30
Marker peak width (sec): 5    Marker min peak height: 200   Marker baseline V to V?: Y    Marker baseline V to V points: 3
Lower marker selection: First peak > 500 RFU                Upper marker selection: Last peak > 200 RFU
Ladder size (bp)75, 200, 300, 400, 500, 700, 1000, 1500, 2000, 3000, 4000, 5000, 7000, 10000, 20000
Quantification using: Lower Marker          Final concentration (ng/uL): 0.5000          Dilution factor: 12.0
```

Sample: SampE8
Well location: E8
Created: Wednesday, October 20, 2021 5:18:46 PM

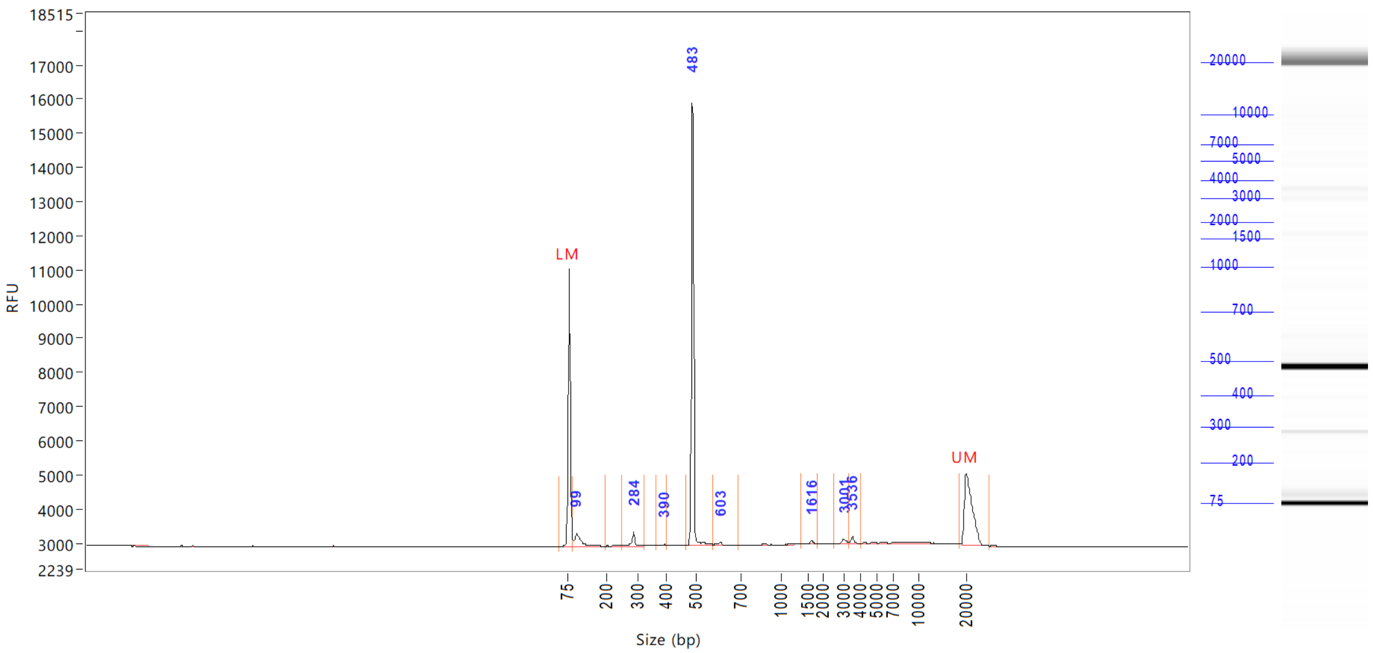

Peak	Size (bp)	Concentration (ng/uL)	From (bp)	To (bp)	Average size (bp)	CV%	RFU	Corrected peak area
1	75 (LM)	0.5000	41	86	73	4.63	8142	43.501
2	99	0.7886	86	196	106	16.33	381	5.717
3	284	0.3435	248	319	281	3.02	411	2.491
4	390	0.0404	362	400	386	1.69	55	0.293
5	483	8.3531	466	570	484	1.67	12985	60.562
6	603	0.1184	570	682	604	3.95	83	0.858
7	1616	0.0740	1378	1808	1587	4.44	91	0.537
8	3001	0.1126	2543	3286	3023	3.86	120	0.816
9	3536	0.1386	3286	4001	3508	3.53	191	1.005
10	20000 (UM)	0.2534	18415	24957	20572	4.17	2091	22.049

TIC:	9.9692	ng/uL
TIM:	43.3254	nmole/L
Total concentration:	10.4218	ng/uL

```
Sample peak width (sec): 5    Sample min peak height: 50    Sample baseline V to V?: Y    Sample baseline V to V points: 3
Sample filter: Binomial       Number of points for filter: 3  Sample start region (min): 0  Sample end region (min): 30
Marker peak width (sec): 5    Marker min peak height: 200   Marker baseline V to V?: Y    Marker baseline V to V points: 3
Lower marker selection: First peak > 500 RFU                Upper marker selection: Last peak > 200 RFU
Ladder size (bp)75, 200, 300, 400, 500, 700, 1000, 1500, 2000, 3000, 4000, 5000, 7000, 10000, 20000
Quantification using: Lower Marker          Final concentration (ng/uL): 0.5000          Dilution factor: 12.0
```

Sample: SampE9
Well location: E9
Created: Wednesday, October 20, 2021 5:18:46 PM

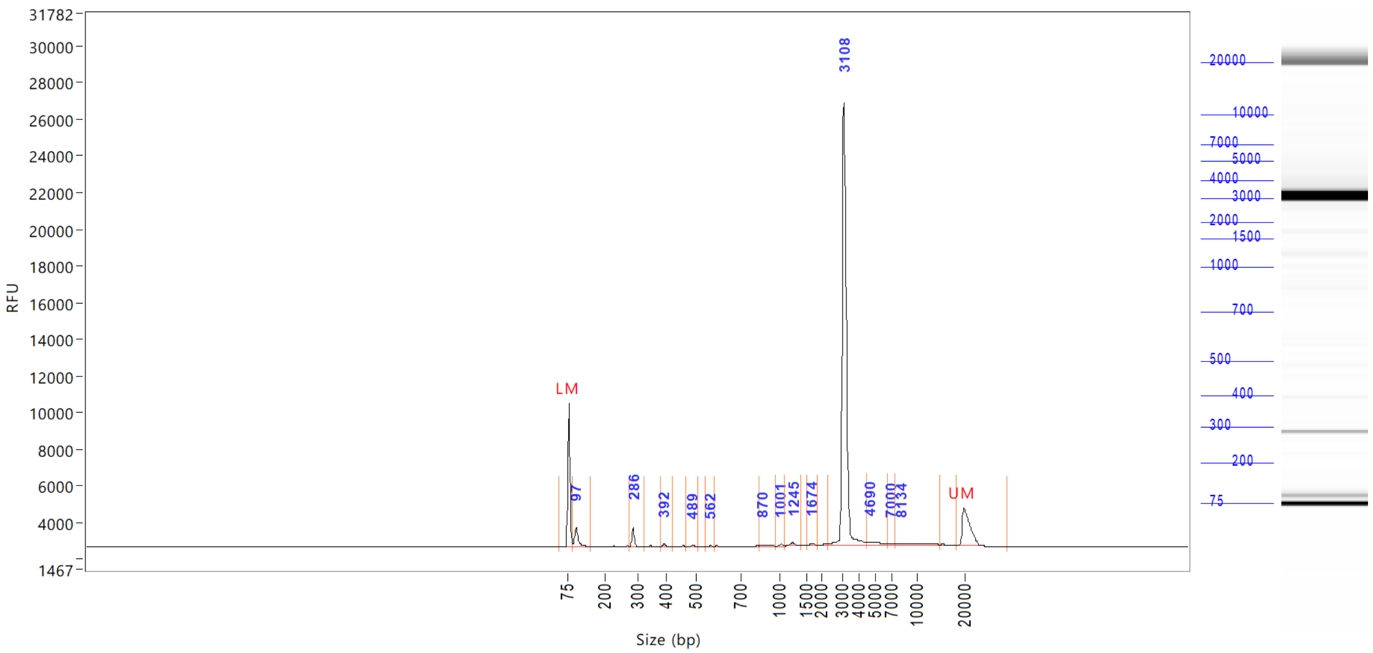

Peak	Size (bp)	Concentration (ng/uL)	From (bp)	To (bp)	Average size (bp)	CV%	RFU	Corrected peak area
1	75 (LM)	0.5000	43	84	72	4.51	7806	42.613
2	97	1.3245	84	147	98	9.35	1051	9.407
3	286	0.7780	273	319	284	2.06	1083	5.526
4	392	0.1165	378	420	390	1.51	154	0.827
5	489	0.0753	466	511	485	1.66	71	0.535
6	562	0.0497	544	580	565	1.37	59	0.353
7	870	0.1527	835	961	887	3.50	89	1.085
8	1001	0.0675	961	1090	1003	1.55	96	0.480
9	1245	0.1939	1090	1400	1232	4.21	174	1.377
10	1674	0.0976	1539	1885	1681	3.79	119	0.693
11	3108	20.9115	2286	4483	3119	6.14	24132	148.516
12	4690	0.3401	4483	6556	5148	11.05	134	2.415
13	7000	0.0651	6556	7401	6976	3.54	53	0.462
14	8134	0.2713	7401	14757	9892	19.12	56	1.927
15	20000 (UM)	0.2480	18415	29430	20693	4.68	1999	21.132

TIC:	24.4438	ng/uL
TIM:	39.5452	nmole/L
Total concentration:	24.8672	ng/uL

```
Sample peak width (sec): 5     Sample min peak height: 50    Sample baseline V to V?: Y    Sample baseline V to V points: 3
Sample filter: Binomial        Number of points for filter: 3  Sample start region (min): 0  Sample end region (min): 30
Marker peak width (sec): 5     Marker min peak height: 200   Marker baseline V to V?: Y    Marker baseline V to V points: 3
Lower marker selection: First peak > 500 RFU                 Upper marker selection: Last peak > 200 RFU
Ladder size (bp)75, 200, 300, 400, 500, 700, 1000, 1500, 2000, 3000, 4000, 5000, 7000, 10000, 20000
Quantification using: Lower Marker          Final concentration (ng/uL): 0.5000          Dilution factor: 12.0
```

Sample: SampE10
Well location: E10
Created: Wednesday, October 20, 2021 5:18:46 PM

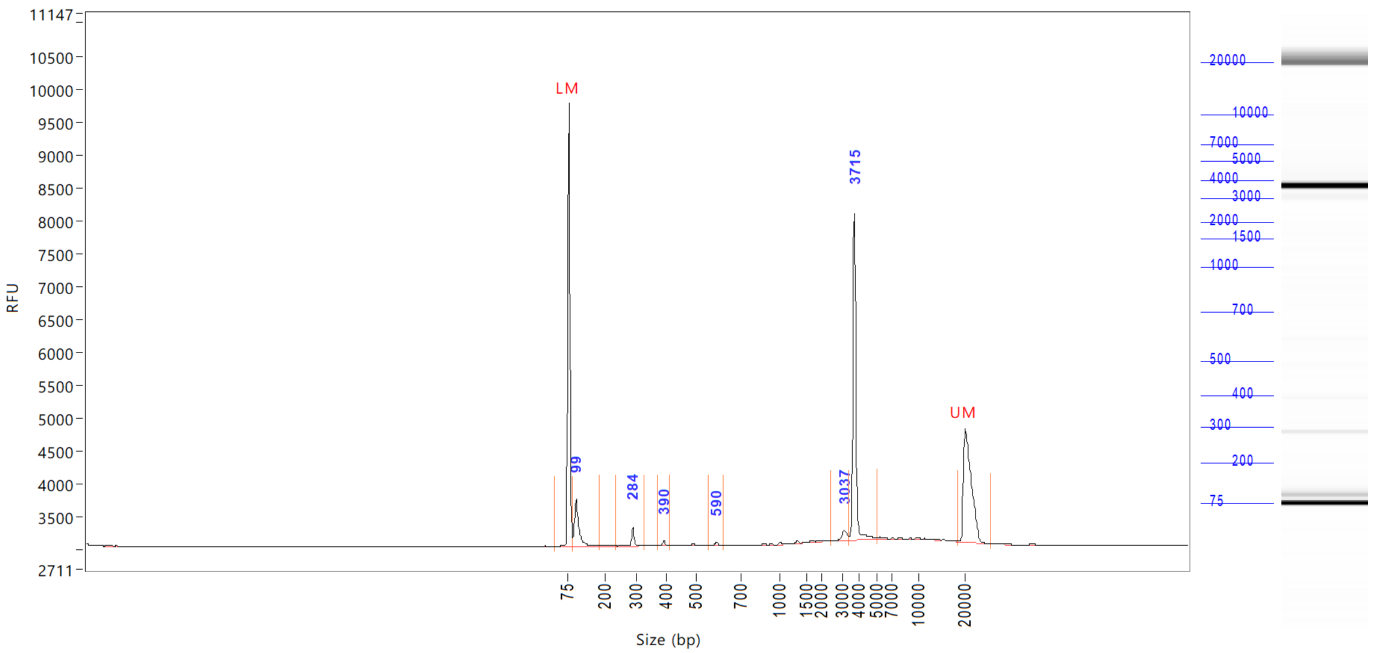

Peak	Size (bp)	Concentration (ng/uL)	From (bp)	To (bp)	Average size (bp)	CV%	RFU	Corrected peak area
1	75 (LM)	0.5000	28	86	74	4.63	6741	37.156
2	99	1.2495	86	181	104	14.67	717	7.738
3	284	0.2794	233	325	282	4.13	291	1.730
4	390	0.0802	368	412	388	1.68	86	0.496
5	590	0.0751	552	621	582	2.24	58	0.465
6	3037	0.1952	2458	3393	3085	4.82	147	1.209
7	3715	3.8143	3393	5149	3722	5.46	4956	23.621
8	20000 (UM)	0.2450	18415	25561	20637	4.27	1729	18.206

TIC:	5.6936	ng/uL
TIM:	23.7041	nmole/L
Total concentration:	6.2244	ng/uL

Sample peak width (sec): 5   Sample min peak height: 50   Sample baseline V to V?: Y   Sample baseline V to V points: 3
Sample filter: Binomial   Number of points for filter: 3 Sample start region (min): 0 Sample end region (min): 30
Marker peak width (sec): 5   Marker min peak height: 200   Marker baseline V to V?: Y   Marker baseline V to V points: 3
Lower marker selection: First peak > 500 RFU   Upper marker selection: Last peak > 200 RFU
Ladder size (bp)75, 200, 300, 400, 500, 700, 1000, 1500, 2000, 3000, 4000, 5000, 7000, 10000, 20000
Quantification using: Lower Marker   Final concentration (ng/uL): 0.5000   Dilution factor: 12.0

Sample: SampE11
Well location: E11
Created: Wednesday, October 20, 2021 5:18:46 PM

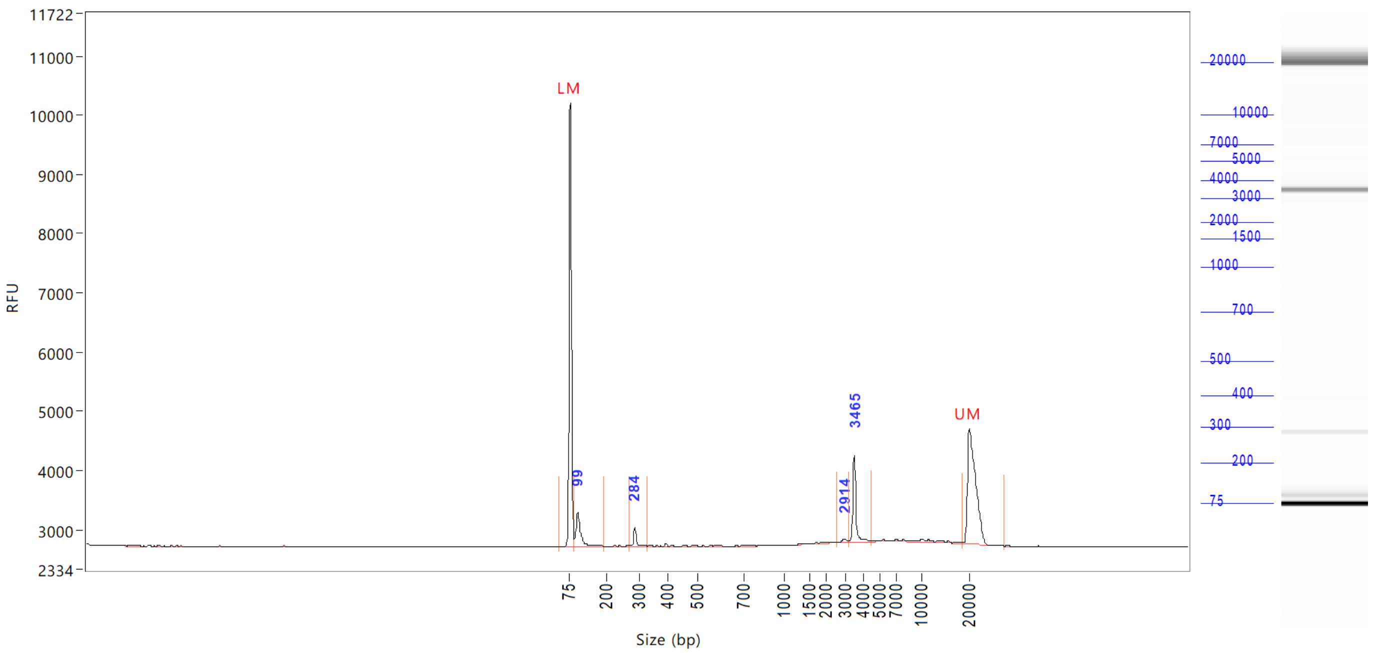

Peak	Size (bp)	Concentration (ng/uL)	From (bp)	To (bp)	Average size (bp)	CV%	RFU	Corrected peak area
1	75 (LM)	0.5000	39	86	72	4.75	7504	41.350
2	99	0.9414	86	187	103	15.21	574	6.488
3	284	0.2450	266	325	283	2.74	316	1.688
4	2914	0.0647	2515	3179	2913	4.64	53	0.446
5	3465	1.0367	3179	4449	3497	5.15	1470	7.144
6	20000 (UM)	0.2524	18415	27496	20657	4.58	1946	20.872

TIC:	2.2877	ng/uL
TIM:	16.9576	nmole/L
Total concentration:	2.8714	ng/uL

```
Sample peak width (sec): 5    Sample min peak height: 50    Sample baseline V to V?: Y    Sample baseline V to V points: 3
Sample filter: Binomial       Number of points for filter: 3  Sample start region (min): 0  Sample end region (min): 30
Marker peak width (sec): 5    Marker min peak height: 200   Marker baseline V to V?: Y    Marker baseline V to V points: 3
Lower marker selection: First peak > 500 RFU                Upper marker selection: Last peak > 200 RFU
Ladder size (bp)75, 200, 300, 400, 500, 700, 1000, 1500, 2000, 3000, 4000, 5000, 7000, 10000, 20000
Quantification using: Lower Marker          Final concentration (ng/uL): 0.5000          Dilution factor: 12.0
```

Sample: SampE12
Well location: E12
Created: Wednesday, October 20, 2021 5:18:46 PM

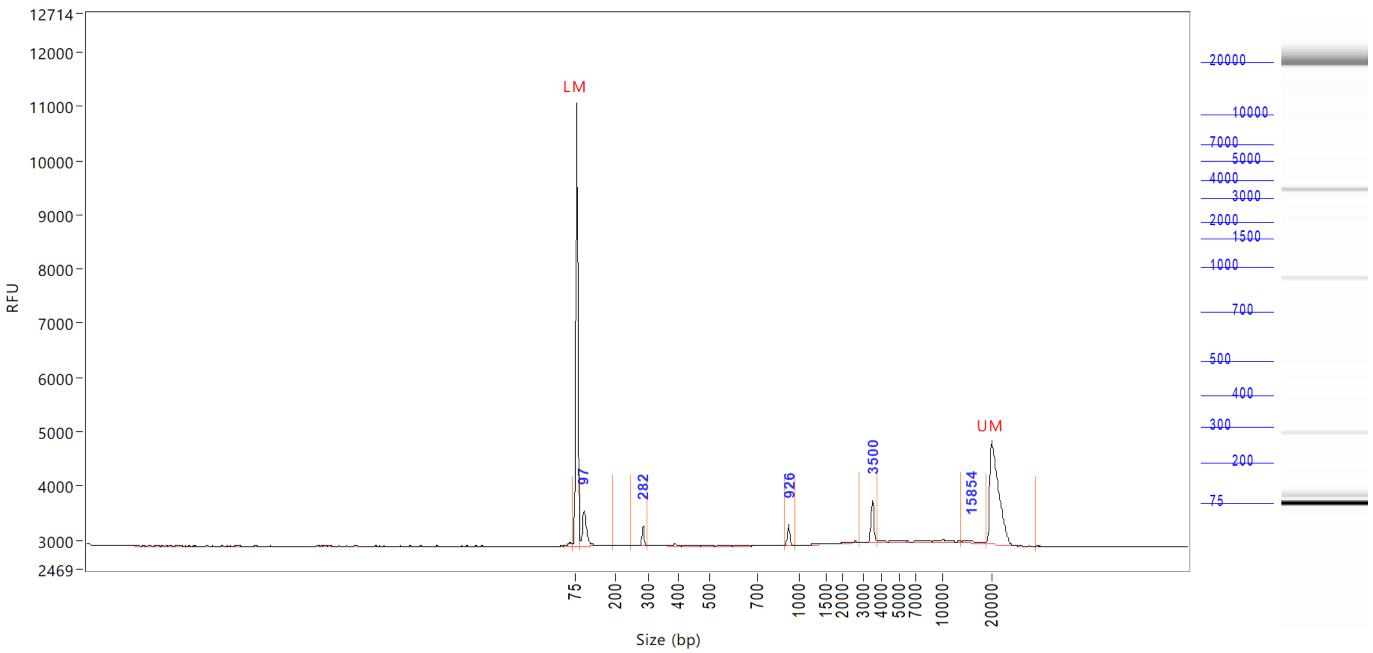

Peak	Size (bp)	Concentration (ng/uL)	From (bp)	To (bp)	Average size (bp)	CV%	RFU	Corrected peak area
1	75 (LM)	0.5000	62	86	73	4.13	8182	42.894
2	97	0.8840	86	190	101	12.88	643	6.320
3	282	0.2424	248	293	278	2.02	359	1.733
4	926	0.2182	896	974	922	0.99	385	1.560
5	3500	0.4629	2800	3786	3445	3.59	762	3.309
6	15854	0.1069	13781	18781	16122	8.80	51	0.765
7	20000 (UM)	0.2538	18781	28705	20684	4.95	1895	21.774

TIC:	1.9145	ng/uL
TIM:	16.4293	nmole/L
Total concentration:	2.4420	ng/uL

Sample peak width (sec): 5   Sample min peak height: 50   Sample baseline V to V?: Y   Sample baseline V to V points: 3
Sample filter: Binomial   Number of points for filter: 3   Sample start region (min): 0   Sample end region (min): 30
Marker peak width (sec): 5   Marker min peak height: 200   Marker baseline V to V?: Y   Marker baseline V to V points: 3
Lower marker selection: First peak > 500 RFU   Upper marker selection: Last peak > 200 RFU
Ladder size (bp)75, 200, 300, 400, 500, 700, 1000, 1500, 2000, 3000, 4000, 5000, 7000, 10000, 20000
Quantification using: Lower Marker   Final concentration (ng/uL): 0.5000   Dilution factor: 12.0

Sample: SampF1
Well location: F1
Created: Wednesday, October 20, 2021 5:18:46 PM

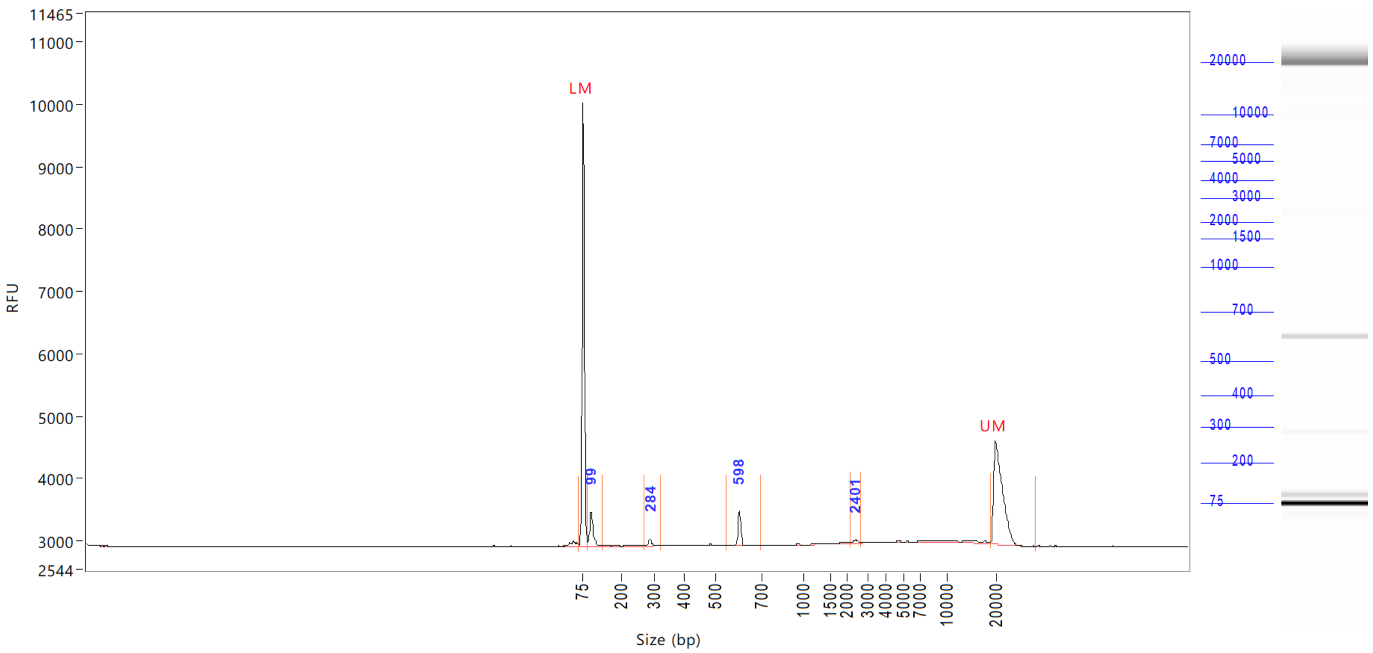

Peak	Size (bp)	Concentration (ng/uL)	From (bp)	To (bp)	Average size (bp)	CV%	RFU	Corrected peak area
1	75 (LM)	0.5000	62	87	73	4.32	7125	37.210
2	99	0.7280	87	137	99	7.14	550	4.515
3	284	0.1291	269	319	284	2.65	119	0.801
4	598	0.4636	547	695	598	2.01	557	2.875
5	2401	0.0527	2144	2629	2378	4.28	51	0.327
6	20000 (UM)	0.2527	18903	28100	20747	5.00	1653	18.807

TIC:	1.3735	ng/uL
TIM:	14.1352	nmole/L
Total concentration:	2.0676	ng/uL

Sample peak width (sec): 5 Sample min peak height: 50 Sample baseline V to V?: Y Sample baseline V to V points: 3
Sample filter: Binomial Number of points for filter: 3 Sample start region (min): 0 Sample end region (min): 30
Marker peak width (sec): 5 Marker min peak height: 200 Marker baseline V to V?: Y Marker baseline V to V points: 3
Lower marker selection: First peak > 500 RFU Upper marker selection: Last peak > 200 RFU
Ladder size (bp)75, 200, 300, 400, 500, 700, 1000, 1500, 2000, 3000, 4000, 5000, 7000, 10000, 20000
Quantification using: Lower Marker Final concentration (ng/uL): 0.5000 Dilution factor: 12.0

Sample: SampF2
Well location: F2
Created: Wednesday, October 20, 2021 5:18:46 PM

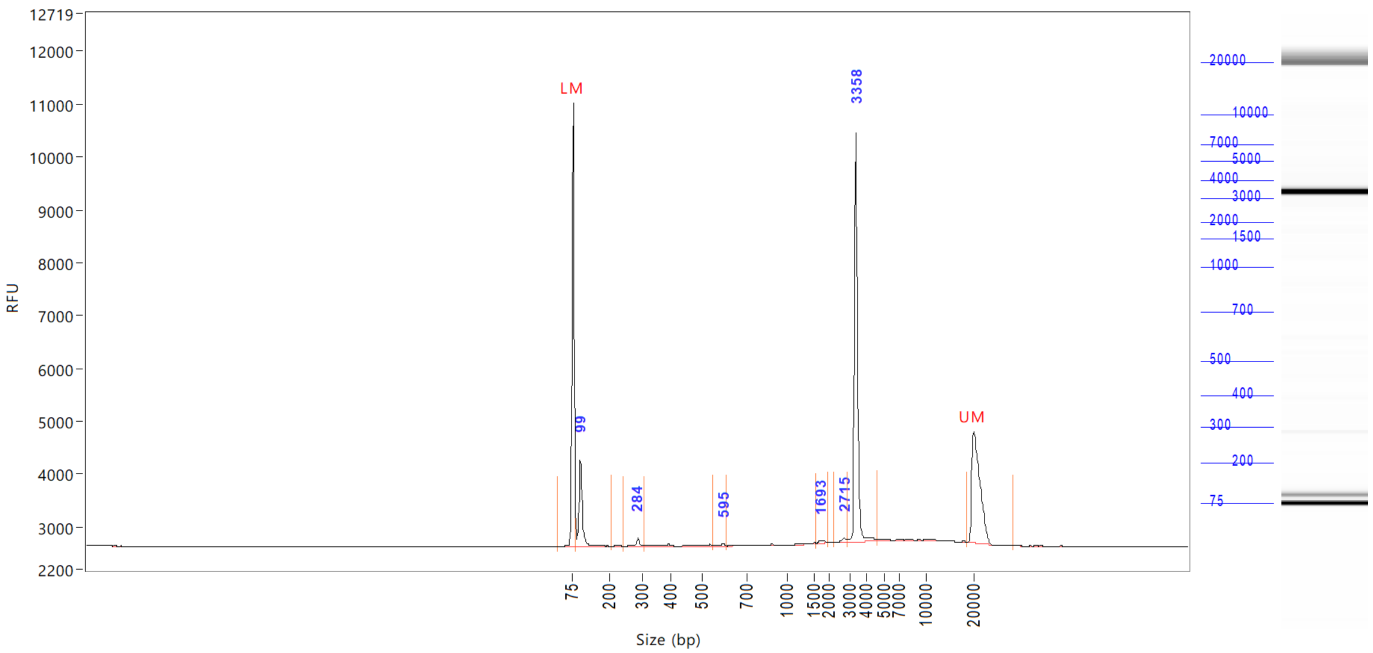

Peak	Size (bp)	Concentration (ng/uL)	From (bp)	To (bp)	Average size (bp)	CV%	RFU	Corrected peak area
1	75 (LM)	0.5000	24	86	73	4.89	8404	44.729
2	99	1.7468	86	203	100	10.77	1641	13.022
3	284	0.1354	242	303	280	3.38	167	1.009
4	595	0.0540	549	611	584	2.24	52	0.403
5	1693	0.0603	1578	1981	1742	5.06	52	0.450
6	2715	0.0783	2258	2857	2678	4.62	72	0.583
7	3358	5.0318	2857	4621	3353	5.38	7731	37.512
8	20000 (UM)	0.2508	18415	28221	20643	4.30	2115	22.436

TIC:	7.1067	ng/uL
TIM:	32.2078	nmole/L
Total concentration:	7.6966	ng/uL

```
Sample peak width (sec): 5    Sample min peak height: 50    Sample baseline V to V?: Y    Sample baseline V to V points: 3
Sample filter: Binomial       Number of points for filter: 3  Sample start region (min): 0  Sample end region (min): 30
Marker peak width (sec): 5    Marker min peak height: 200   Marker baseline V to V?: Y    Marker baseline V to V points: 3
Lower marker selection: First peak > 500 RFU                Upper marker selection: Last peak > 200 RFU
Ladder size (bp)75, 200, 300, 400, 500, 700, 1000, 1500, 2000, 3000, 4000, 5000, 7000, 10000, 20000
Quantification using: Lower Marker          Final concentration (ng/uL): 0.5000          Dilution factor: 12.0
```

Sample: SampF3
Well location: F3
Created: Wednesday, October 20, 2021 5:18:46 PM

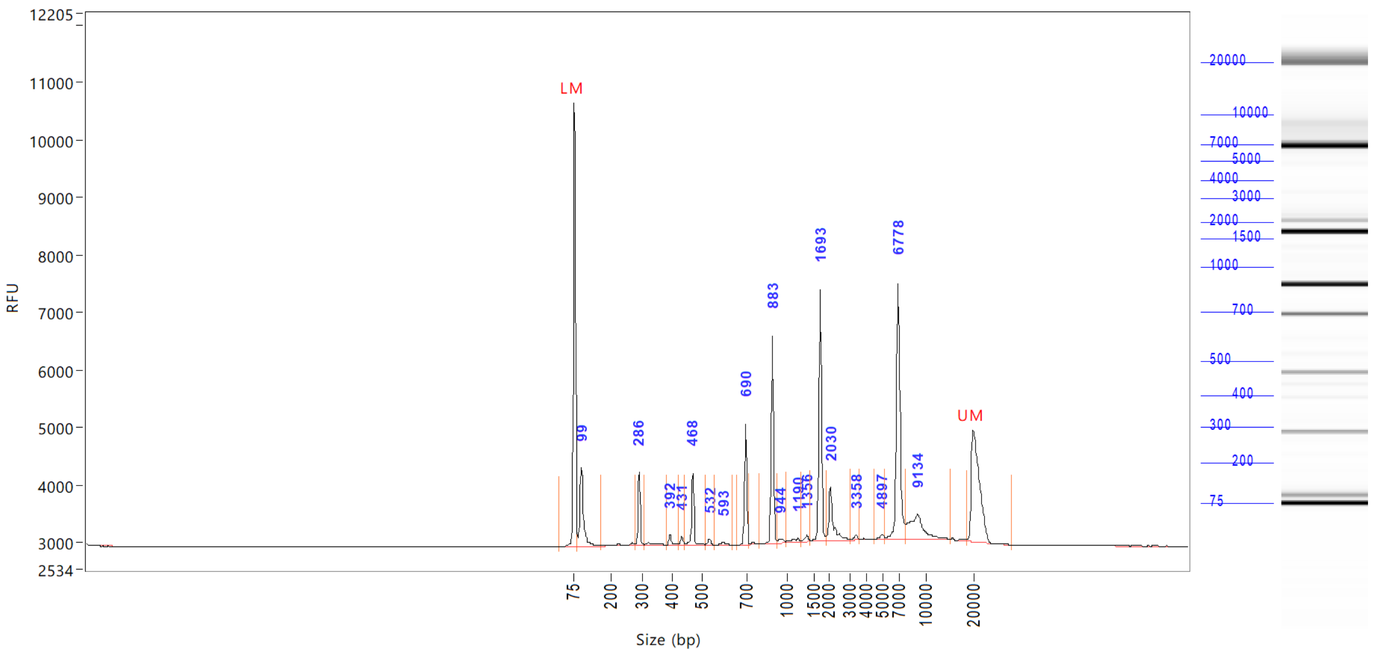

Peak	Size (bp)	Concentration (ng/uL)	From (bp)	To (bp)	Average size (bp)	CV%	RFU	Corrected peak area
1	75 (LM)	0.5000	26	86	73	5.05	7725	41.588
2	99	1.7582	86	166	101	11.65	1363	12.187
3	286	0.8638	273	300	283	1.34	1263	5.987
4	392	0.1517	380	420	395	2.45	174	1.052
5	431	0.1160	420	441	429	1.07	159	0.804
6	468	0.8837	441	509	466	1.66	1257	6.125
7	532	0.1054	509	552	526	1.35	108	0.731
8	593	0.0807	552	631	589	2.30	61	0.560
9	690	1.2956	654	714	688	0.77	2104	8.980
10	883	2.2059	792	922	881	1.23	3600	15.290
11	944	0.0970	922	987	950	1.84	74	0.672
12	1190	0.1237	987	1267	1112	6.89	63	0.858
13	1356	0.0894	1267	1445	1344	2.44	110	0.620
14	1693	2.7298	1445	1904	1679	2.98	4371	18.921
15	2030	0.9077	1904	3001	2152	10.91	929	6.292
16	3358	0.0793	3001	3608	3287	4.16	87	0.550
17	4897	0.0820	4414	5223	4871	3.31	83	0.569
18	6778	3.5859	5223	7667	6781	4.56	4457	24.855
19	9134	1.2593	7667	15244	9138	13.23	437	8.729
20	20000 (UM)	0.2490	18537	28221	20670	4.46	1936	20.708

TIC:	16.4154	ng/uL
TIM:	50.5765	nmole/L
Total concentration:	16.7033	ng/uL

Sample peak width (sec): 5 Sample min peak height: 50 Sample baseline V to V?: Y Sample baseline V to V points: 3
Sample filter: Binomial Number of points for filter: 3 Sample start region (min): 0 Sample end region (min): 30
Marker peak width (sec): 5 Marker min peak height: 200 Marker baseline V to V?: Y Marker baseline V to V points: 3
Lower marker selection: First peak > 500 RFU Upper marker selection: Last peak > 200 RFU
Ladder size (bp)75, 200, 300, 400, 500, 700, 1000, 1500, 2000, 3000, 4000, 5000, 7000, 10000, 20000
Quantification using: Lower Marker Final concentration (ng/uL): 0.5000 Dilution factor: 12.0

Sample: SampF4
Well location: F4
Created: Wednesday, October 20, 2021 5:18:46 PM

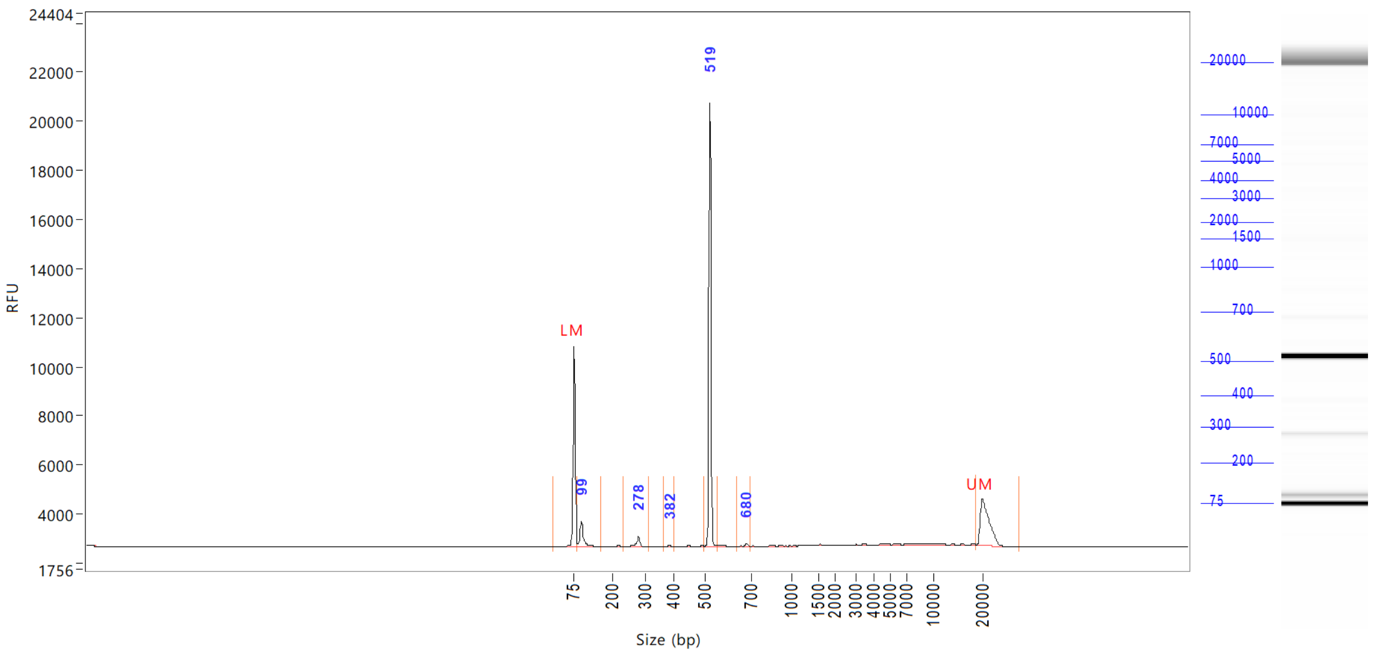

Peak	Size (bp)	Concentration (ng/uL)	From (bp)	To (bp)	Average size (bp)	CV%	RFU	Corrected peak area
1	75 (LM)	0.5000	7	86	72	5.77	8136	43.903
2	99	1.1751	86	162	100	10.93	1025	8.598
3	278	0.3812	235	311	274	2.99	410	2.789
4	382	0.0350	364	400	381	1.37	50	0.256
5	519	10.1781	496	552	516	0.89	18092	74.475
6	680	0.0733	639	697	673	1.29	109	0.536
7	20000 (UM)	0.2513	18781	27617	20721	4.73	1931	22.064

TIC:	11.8427	ng/uL
TIM:	54.3734	nmole/L
Total concentration:	12.5776	ng/uL

Sample peak width (sec): 5   Sample min peak height: 50   Sample baseline V to V?: Y   Sample baseline V to V points: 3
Sample filter: Binomial   Number of points for filter: 3   Sample start region (min): 0   Sample end region (min): 30
Marker peak width (sec): 5   Marker min peak height: 200   Marker baseline V to V?: Y   Marker baseline V to V points: 3
Lower marker selection: First peak > 500 RFU   Upper marker selection: Last peak > 200 RFU
Ladder size (bp)75, 200, 300, 400, 500, 700, 1000, 1500, 2000, 3000, 4000, 5000, 7000, 10000, 20000
Quantification using: Lower Marker   Final concentration (ng/uL): 0.5000   Dilution factor: 12.0

Sample: SampF5
Well location: F5
Created: Wednesday, October 20, 2021 5:18:46 PM

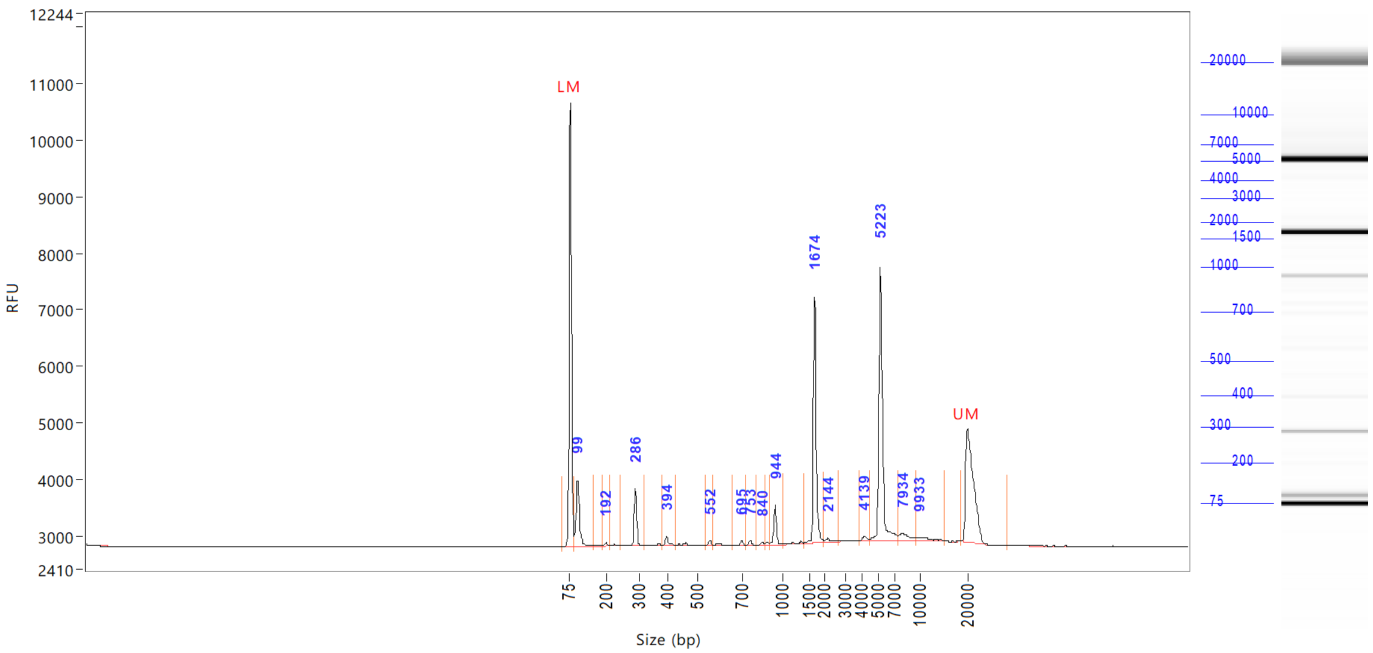

Peak	Size (bp)	Concentration (ng/uL)	From (bp)	To (bp)	Average size (bp)	CV%	RFU	Corrected peak area
1	75 (LM)	0.5000	48	86	73	4.60	7858	42.536
2	99	1.4016	86	152	98	8.43	1163	9.937
3	192	0.0457	183	206	192	2.12	59	0.324
4	286	0.7233	241	317	284	2.08	1011	5.127
5	394	0.1425	380	426	396	2.28	154	1.010
6	552	0.0653	529	567	548	1.34	83	0.463
7	695	0.0583	654	714	690	1.08	88	0.414
8	753	0.0620	714	792	747	1.63	83	0.439
9	840	0.0473	792	861	839	1.65	55	0.335
10	944	0.4663	896	1000	937	1.41	707	3.306
11	1674	2.8339	1389	2000	1669	3.75	4342	20.090
12	2144	0.0748	2000	2715	2176	6.94	65	0.530
13	4139	0.0828	3893	4483	4191	3.35	80	0.587
14	5223	3.6540	4483	7334	5303	8.35	4837	25.904
15	7934	0.2764	7334	9533	8240	7.34	129	1.960
16	9933	0.1431	9533	15000	11106	12.58	59	1.015
17	20000 (UM)	0.2480	18537	28584	20615	4.45	2006	21.099

TIC:	10.0773	ng/uL
TIM:	34.1356	nmole/L
Total concentration:	10.4392	ng/uL

Sample peak width (sec): 5   Sample min peak height: 50   Sample baseline V to V?: Y   Sample baseline V to V points: 3
Sample filter: Binomial   Number of points for filter: 3 Sample start region (min): 0 Sample end region (min): 30
Marker peak width (sec): 5   Marker min peak height: 200   Marker baseline V to V?: Y   Marker baseline V to V points: 3
Lower marker selection: First peak > 500 RFU   Upper marker selection: Last peak > 200 RFU
Ladder size (bp)75, 200, 300, 400, 500, 700, 1000, 1500, 2000, 3000, 4000, 5000, 7000, 10000, 20000
Quantification using: Lower Marker   Final concentration (ng/uL): 0.5000   Dilution factor: 12.0

Sample: SampF6
Well location: F6
Created: Wednesday, October 20, 2021 5:18:46 PM

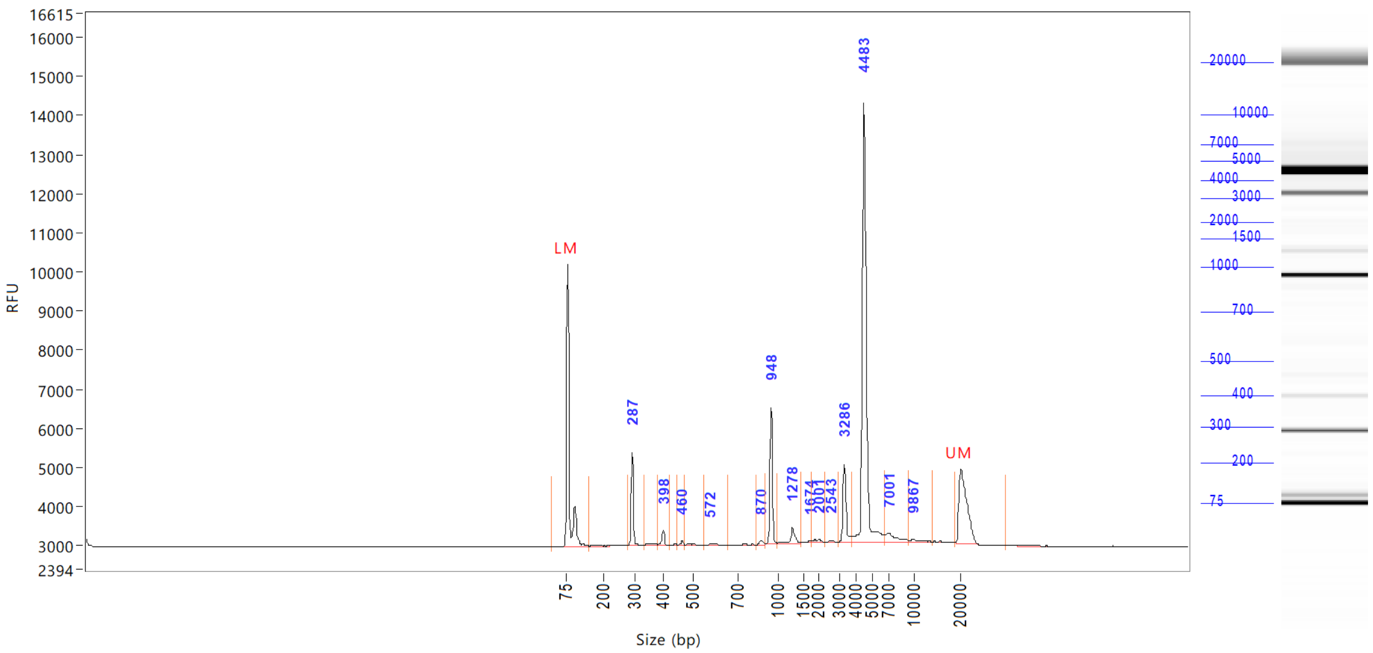

Peak	Size (bp)	Concentration (ng/uL)	From (bp)	To (bp)	Average size (bp)	CV%	RFU	Corrected peak area
1	75 (LM)	0.5000	26	150	79	14.51	7220	49.212
2	287	1.3872	275	327	287	1.54	2374	11.377
3	398	0.2455	376	418	394	1.40	385	2.014
4	460	0.0883	447	470	458	1.16	114	0.724
5	572	0.1056	547	654	591	3.66	52	0.866
6	870	0.0950	831	905	868	1.85	92	0.779
7	948	1.8545	905	996	945	1.05	3497	15.211
8	1278	0.3654	996	1456	1255	6.89	407	2.997
9	1674	0.0493	1456	1770	1656	4.57	61	0.404
10	2001	0.1039	1770	2286	1935	5.05	85	0.852
11	2543	0.0472	2286	3000	2609	5.48	57	0.387
12	3286	1.1534	3000	3822	3304	4.45	1987	9.460
13	4483	8.5866	3822	6482	4535	6.62	11244	70.427
14	7001	0.4267	6482	9467	7505	10.28	221	3.499
15	9867	0.0814	9467	14025	10451	8.66	56	0.668
16	20000 (UM)	0.2057	18659	29672	20673	4.78	1904	20.248

TIC:	14.5900	ng/uL
TIM:	17.4415	nmole/L
Total concentration:	14.9458	ng/uL

```
Sample peak width (sec): 5     Sample min peak height: 50    Sample baseline V to V?: Y    Sample baseline V to V points: 3
Sample filter: Binomial        Number of points for filter: 3  Sample start region (min): 0  Sample end region (min): 30
Marker peak width (sec): 5     Marker min peak height: 200   Marker baseline V to V?: Y    Marker baseline V to V points: 3
Lower marker selection: First peak > 500 RFU                Upper marker selection: Last peak > 200 RFU
Ladder size (bp)75, 200, 300, 400, 500, 700, 1000, 1500, 2000, 3000, 4000, 5000, 7000, 10000, 20000
Quantification using: Lower Marker          Final concentration (ng/uL): 0.5000          Dilution factor: 12.0
```

Sample: SampF7
Well location: F7
Created: Wednesday, October 20, 2021 5:18:46 PM

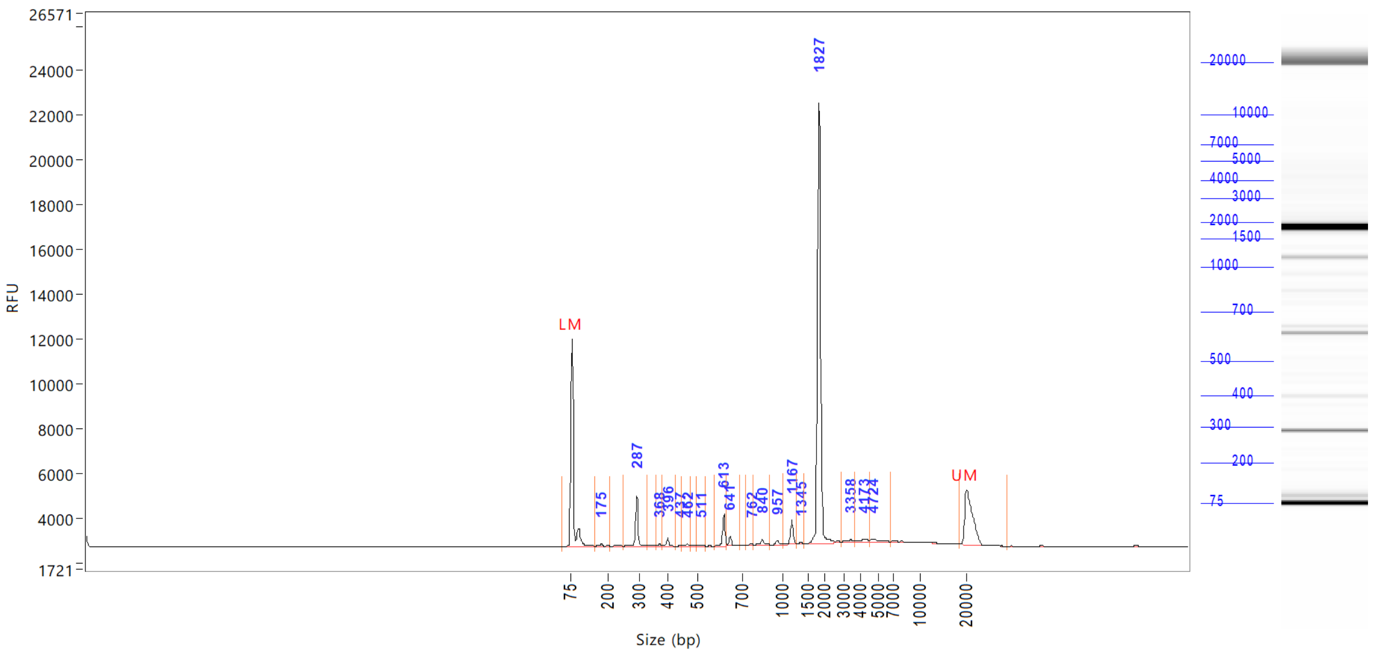

Peak	Size (bp)	Concentration (ng/uL)	From (bp)	To (bp)	Average size (bp)	CV%	RFU	Corrected peak area
1	75 (LM)	0.5000	43	154	78	13.91	9254	58.817
2	175	0.0888	154	205	177	6.58	98	0.870
3	287	1.1666	244	325	287	2.29	2257	11.436
4	368	0.0475	358	378	366	1.21	88	0.466
5	396	0.1820	378	424	394	1.39	356	1.784
6	437	0.0369	424	445	436	1.00	71	0.362
7	462	0.0630	445	471	458	1.39	95	0.618
8	511	0.0562	492	532	507	1.65	69	0.551
9	613	0.6945	570	626	609	1.27	1440	6.808
10	641	0.2131	626	687	640	1.29	422	2.089
11	762	0.0604	723	779	752	1.63	95	0.592
12	840	0.2044	779	905	836	3.30	249	2.004
13	957	0.1198	905	1000	952	1.81	185	1.174
14	1167	0.5221	1000	1267	1154	3.10	1075	5.119
15	1345	0.0464	1267	1411	1339	2.11	93	0.454
16	1827	9.8906	1411	2857	1827	5.93	19723	96.956
17	3358	0.1409	2857	3643	3252	6.85	115	1.381
18	4173	0.1906	3643	4518	4105	5.83	134	1.868
19	4724	0.1754	4518	6630	5062	10.03	111	1.720
20	20000 (UM)	0.2178	18537	28705	20671	4.33	2448	25.623

TIC:	13.8991	ng/uL
TIM:	22.1163	nmole/L
Total concentration:	14.1647	ng/uL

```
Sample peak width (sec): 5    Sample min peak height: 50    Sample baseline V to V?: Y    Sample baseline V to V points: 3
Sample filter: Binomial       Number of points for filter: 3  Sample start region (min): 0  Sample end region (min): 30
Marker peak width (sec): 5    Marker min peak height: 200   Marker baseline V to V?: Y    Marker baseline V to V points: 3
Lower marker selection: First peak > 500 RFU                Upper marker selection: Last peak > 200 RFU
Ladder size (bp)75, 200, 300, 400, 500, 700, 1000, 1500, 2000, 3000, 4000, 5000, 7000, 10000, 20000
Quantification using: Lower Marker          Final concentration (ng/uL): 0.5000          Dilution factor: 12.0
```

Sample: SampF8
Well location: F8
Created: Wednesday, October 20, 2021 5:18:46 PM

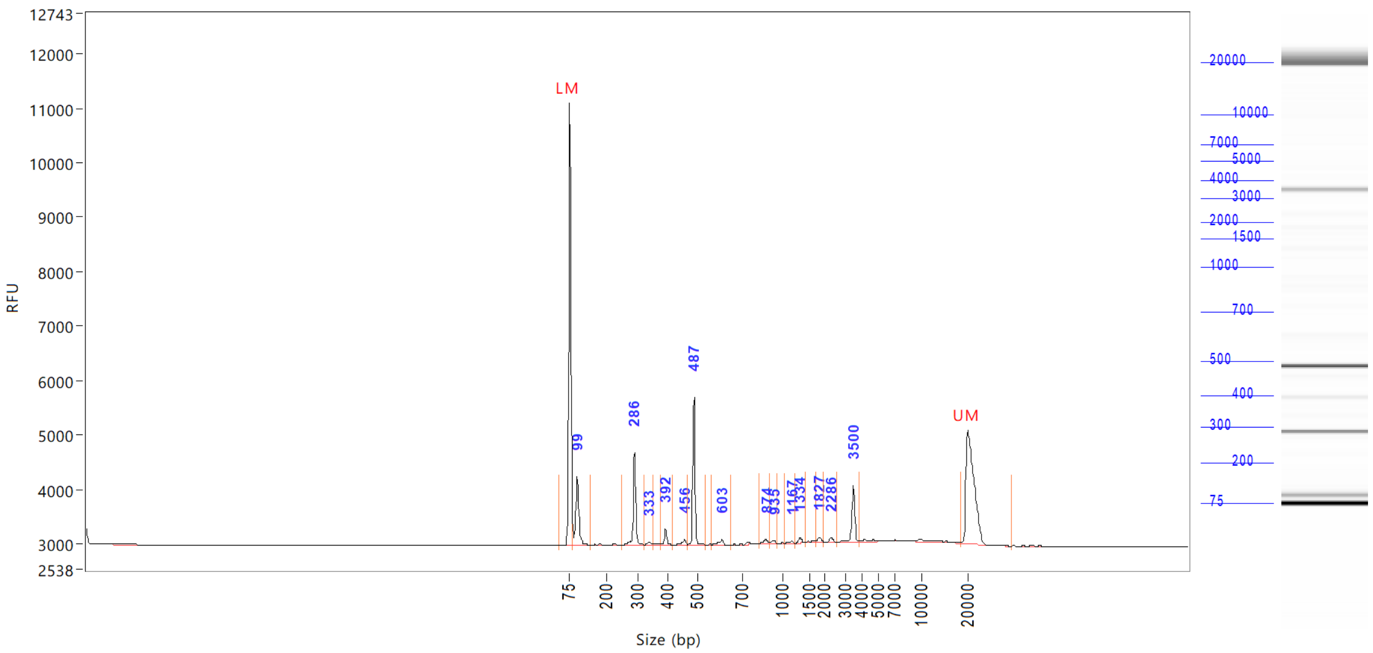

Peak	Size (bp)	Concentration (ng/uL)	From (bp)	To (bp)	Average size (bp)	CV%	RFU	Corrected peak area
1	75 (LM)	0.5000	41	86	73	4.45	8141	43.210
2	99	1.3849	86	145	99	8.15	1257	9.973
3	286	1.2432	246	319	283	2.39	1688	8.953
4	333	0.0671	319	352	334	2.46	56	0.484
5	392	0.2116	374	416	390	1.48	287	1.524
6	456	0.1076	416	464	448	2.00	105	0.775
7	487	1.6973	464	534	485	1.06	2701	12.223
8	603	0.1207	562	646	595	2.34	96	0.869
9	874	0.0954	818	900	863	2.27	81	0.687
10	935	0.0606	900	957	924	1.48	57	0.437
11	1167	0.0359	1023	1223	1130	3.93	50	0.259
12	1334	0.0784	1223	1422	1315	2.65	97	0.564
13	1827	0.0827	1712	1981	1812	3.08	97	0.595
14	2286	0.0635	1981	2572	2263	4.80	75	0.457
15	3500	0.6731	2572	3893	3436	4.30	1031	4.847
16	20000 (UM)	0.2579	18415	29309	20561	4.47	2095	22.289

TIC:	5.9220	ng/uL
TIM:	38.7905	nmole/L
Total concentration:	6.4960	ng/uL

Sample peak width (sec): 5 Sample min peak height: 50 Sample baseline V to V?: Y Sample baseline V to V points: 3
Sample filter: Binomial Number of points for filter: 3 Sample start region (min): 0 Sample end region (min): 30
Marker peak width (sec): 5 Marker min peak height: 200 Marker baseline V to V?: Y Marker baseline V to V points: 3
Lower marker selection: First peak > 500 RFU Upper marker selection: Last peak > 200 RFU
Ladder size (bp)75, 200, 300, 400, 500, 700, 1000, 1500, 2000, 3000, 4000, 5000, 7000, 10000, 20000
Quantification using: Lower Marker Final concentration (ng/uL): 0.5000 Dilution factor: 12.0

Sample: SampF9
Well location: F9
Created: Wednesday, October 20, 2021 5:18:46 PM

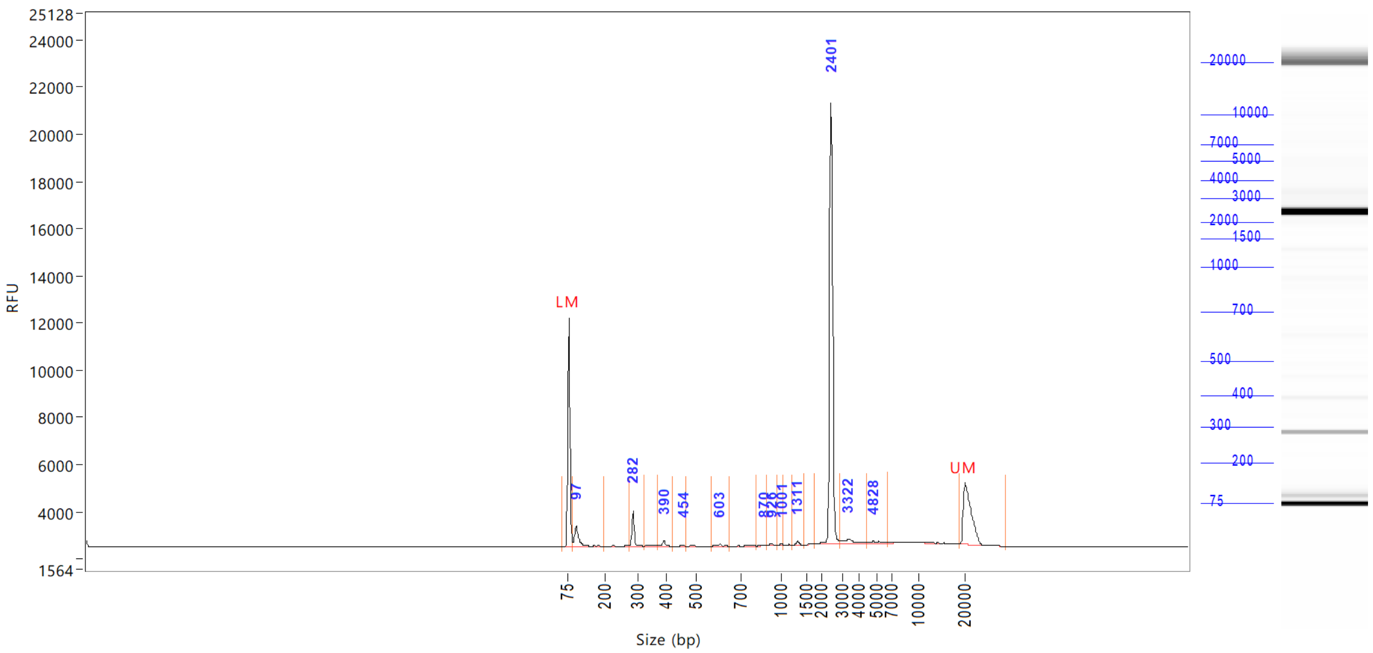

Peak	Size (bp)	Concentration (ng/uL)	From (bp)	To (bp)	Average size (bp)	CV%	RFU	Corrected peak area
1	75 (LM)	0.5000	54	84	72	4.33	9654	53.350
2	97	1.0495	84	194	102	15.78	869	9.332
3	282	0.8500	271	319	282	1.51	1518	7.557
4	390	0.1466	368	422	388	1.73	238	1.303
5	454	0.0480	422	464	449	1.60	71	0.427
6	603	0.0936	565	649	603	2.86	87	0.832
7	870	0.0507	818	896	860	2.13	59	0.451
8	926	0.0747	896	974	924	1.69	102	0.664
9	1001	0.0259	974	1045	1000	1.36	52	0.230
10	1311	0.0853	1212	1445	1305	2.73	145	0.759
11	2401	10.0210	1750	2857	2390	3.82	18729	89.103
12	3322	0.4425	2857	4414	3433	12.30	202	3.935
13	4828	0.1554	4414	6408	5052	9.57	78	1.382
14	20000 (UM)	0.2592	18659	28584	20630	4.34	2618	27.653

TIC:	13.0431	ng/uL
TIM:	30.4525	nmole/L
Total concentration:	13.4223	ng/uL

Sample peak width (sec): 5   Sample min peak height: 50   Sample baseline V to V?: Y   Sample baseline V to V points: 3
Sample filter: Binomial   Number of points for filter: 3 Sample start region (min): 0 Sample end region (min): 30
Marker peak width (sec): 5   Marker min peak height: 200   Marker baseline V to V?: Y   Marker baseline V to V points: 3
Lower marker selection: First peak > 500 RFU   Upper marker selection: Last peak > 200 RFU
Ladder size (bp)75, 200, 300, 400, 500, 700, 1000, 1500, 2000, 3000, 4000, 5000, 7000, 10000, 20000
Quantification using: Lower Marker   Final concentration (ng/uL): 0.5000   Dilution factor: 12.0

Sample: SampF10
Well location: F10
Created: Wednesday, October 20, 2021 5:18:46 PM

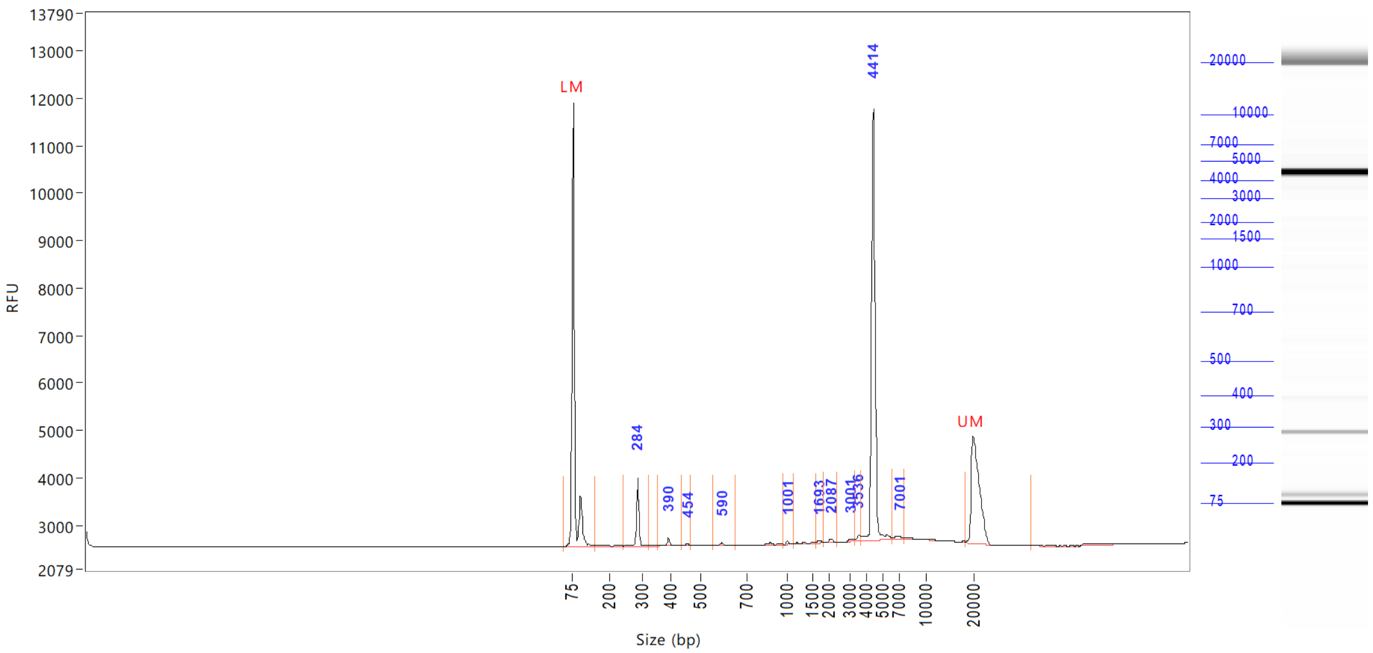

Peak	Size (bp)	Concentration (ng/uL)	From (bp)	To (bp)	Average size (bp)	CV%	RFU	Corrected peak area
1	75 (LM)	0.5000	47	147	78	14.03	9360	59.103
2	284	0.7432	241	321	282	2.59	1444	7.321
3	390	0.1271	349	431	385	3.81	164	1.252
4	454	0.0377	431	462	448	1.41	55	0.371
5	590	0.0606	549	646	589	2.99	63	0.597
6	1001	0.0427	978	1123	1021	3.47	74	0.421
7	1693	0.0369	1559	1827	1675	3.95	53	0.364
8	2087	0.0480	1827	2429	2049	4.88	73	0.472
9	3001	0.0434	2429	3358	3087	4.75	51	0.428
10	3536	0.0634	3358	3679	3535	2.51	118	0.624
11	4414	4.8445	3679	6038	4401	4.33	9094	47.721
12	7001	0.0785	6038	7534	6770	5.75	63	0.773
13	20000 (UM)	0.2016	18415	32332	20660	4.87	2257	23.827

TIC:	6.1261	ng/uL
TIM:	7.2134	nmole/L
Total concentration:	6.4831	ng/uL

Sample peak width (sec): 5 Sample min peak height: 50 Sample baseline V to V?: Y Sample baseline V to V points: 3
Sample filter: Binomial Number of points for filter: 3 Sample start region (min): 0 Sample end region (min): 30
Marker peak width (sec): 5 Marker min peak height: 200 Marker baseline V to V?: Y Marker baseline V to V points: 3
Lower marker selection: First peak > 500 RFU Upper marker selection: Last peak > 200 RFU
Ladder size (bp)75, 200, 300, 400, 500, 700, 1000, 1500, 2000, 3000, 4000, 5000, 7000, 10000, 20000
Quantification using: Lower Marker Final concentration (ng/uL): 0.5000 Dilution factor: 12.0

Sample: SampF11
Well location: F11
Created: Wednesday, October 20, 2021 5:18:46 PM

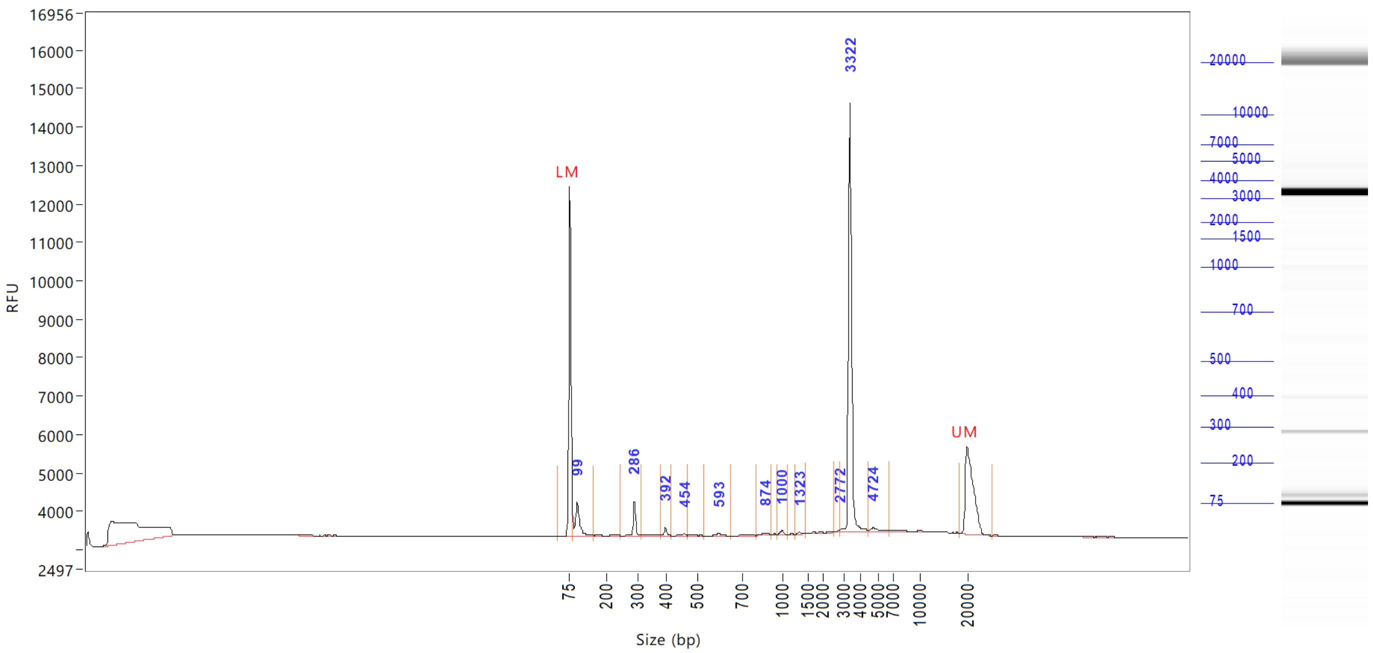

Peak	Size (bp)	Concentration (ng/uL)	From (bp)	To (bp)	Average size (bp)	CV%	RFU	Corrected peak area
1	75 (LM)	0.5000	37	86	73	4.56	9077	48.280
2	99	1.0739	86	156	101	11.08	871	8.641
3	286	0.5788	242	309	283	2.18	886	4.657
4	392	0.1232	380	414	390	1.22	199	0.991
5	454	0.0481	414	466	450	1.98	53	0.387
6	593	0.0828	529	646	588	2.68	70	0.667
7	874	0.0881	796	913	870	2.48	71	0.709
8	1000	0.0926	961	1090	992	1.60	118	0.745
9	1323	0.0406	1234	1445	1322	2.96	54	0.326
10	2772	0.0399	2515	2800	2709	2.66	68	0.321
11	3322	6.9888	2800	4483	3327	4.88	11183	56.237
12	4724	0.1826	4483	6334	5039	9.23	103	1.470
13	20000 (UM)	0.2497	18537	25320	20674	4.24	2265	24.107

TIC:	9.3394	ng/uL
TIM:	25.6639	nmole/L
Total concentration:	9.8403	ng/uL

```
Sample peak width (sec): 5     Sample min peak height: 50     Sample baseline V to V?: Y     Sample baseline V to V points: 3
Sample filter: Binomial        Number of points for filter: 3  Sample start region (min): 0  Sample end region (min): 30
Marker peak width (sec): 5     Marker min peak height: 200    Marker baseline V to V?: Y     Marker baseline V to V points: 3
Lower marker selection: First peak > 500 RFU                  Upper marker selection: Last peak > 200 RFU
Ladder size (bp)75, 200, 300, 400, 500, 700, 1000, 1500, 2000, 3000, 4000, 5000, 7000, 10000, 20000
Quantification using: Lower Marker          Final concentration (ng/uL): 0.5000          Dilution factor: 12.0
```

Sample: SampF12
Well location: F12
Created: Wednesday, October 20, 2021 5:18:46 PM

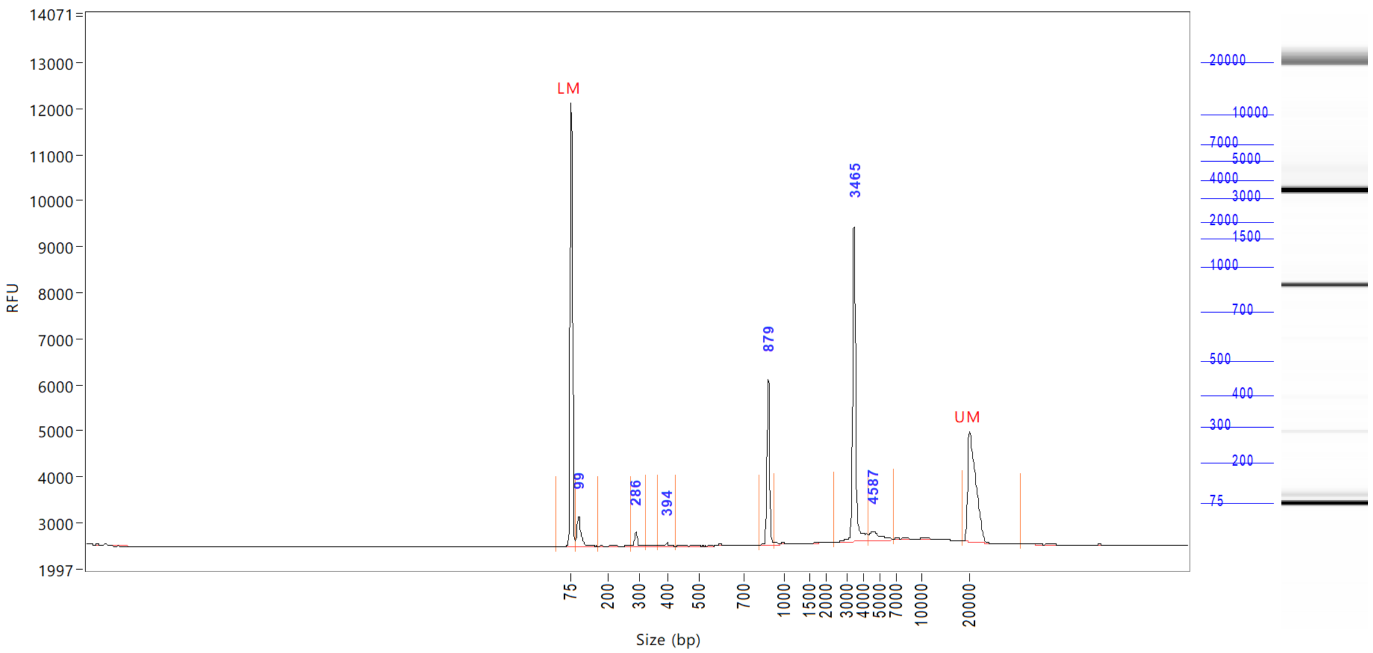

Peak	Size (bp)	Concentration (ng/uL)	From (bp)	To (bp)	Average size (bp)	CV%	RFU	Corrected peak area
1	75 (LM)	0.5000	26	89	74	4.92	9651	51.897
2	99	0.7586	89	166	102	10.89	655	6.561
3	286	0.1932	269	317	285	2.24	319	1.671
4	394	0.0583	364	422	388	2.52	68	0.504
5	879	1.8140	818	922	876	1.12	3599	15.690
6	3465	3.9436	2429	4311	3461	5.45	6834	34.110
7	4587	0.3860	4311	6704	4907	11.34	208	3.338
8	20000 (UM)	0.2461	18537	30881	20701	4.62	2402	25.548

TIC:	7.1536	ng/uL
TIM:	18.9884	nmole/L
Total concentration:	7.5947	ng/uL

Sample peak width (sec): 5 Sample min peak height: 50 Sample baseline V to V?: Y Sample baseline V to V points: 3
Sample filter: Binomial Number of points for filter: 3 Sample start region (min): 0 Sample end region (min): 30
Marker peak width (sec): 5 Marker min peak height: 200 Marker baseline V to V?: Y Marker baseline V to V points: 3
Lower marker selection: First peak > 500 RFU Upper marker selection: Last peak > 200 RFU
Ladder size (bp)75, 200, 300, 400, 500, 700, 1000, 1500, 2000, 3000, 4000, 5000, 7000, 10000, 20000
Quantification using: Lower Marker Final concentration (ng/uL): 0.5000 Dilution factor: 12.0

Sample: SampG1
Well location: G1
Created: Wednesday, October 20, 2021 5:18:46 PM

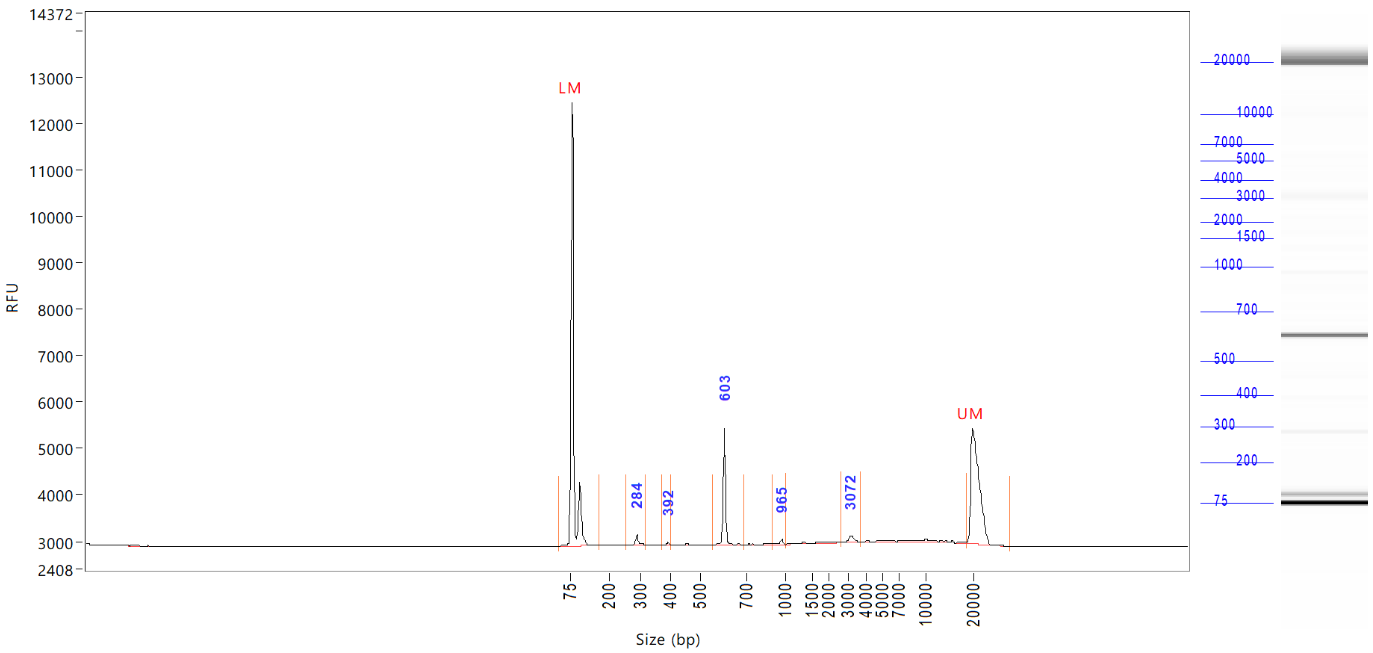

Peak	Size (bp)	Concentration (ng/uL)	From (bp)	To (bp)	Average size (bp)	CV%	RFU	Corrected peak area
1	75 (LM)	0.5000	33	164	78	15.42	9554	62.168
2	284	0.1333	253	311	282	3.14	223	1.381
3	392	0.0270	372	400	387	1.39	52	0.279
4	603	1.0914	549	690	602	1.96	2512	11.308
5	965	0.0590	900	991	950	1.99	107	0.611
6	3072	0.1201	2657	3715	3108	5.39	142	1.245
7	20000 (UM)	0.2172	18537	27859	20654	4.42	2487	27.001

TIC:	1.4308	ng/uL
TIM:	4.0416	nmole/L
Total concentration:	1.9072	ng/uL

Sample peak width (sec): 5 Sample min peak height: 50 Sample baseline V to V?: Y Sample baseline V to V points: 3
Sample filter: Binomial Number of points for filter: 3 Sample start region (min): 0 Sample end region (min): 30
Marker peak width (sec): 5 Marker min peak height: 200 Marker baseline V to V?: Y Marker baseline V to V points: 3
Lower marker selection: First peak > 500 RFU Upper marker selection: Last peak > 200 RFU
Ladder size (bp)75, 200, 300, 400, 500, 700, 1000, 1500, 2000, 3000, 4000, 5000, 7000, 10000, 20000
Quantification using: Lower Marker Final concentration (ng/uL): 0.5000 Dilution factor: 12.0

Sample: SampG2
Well location: G2
Created: Wednesday, October 20, 2021 5:18:46 PM

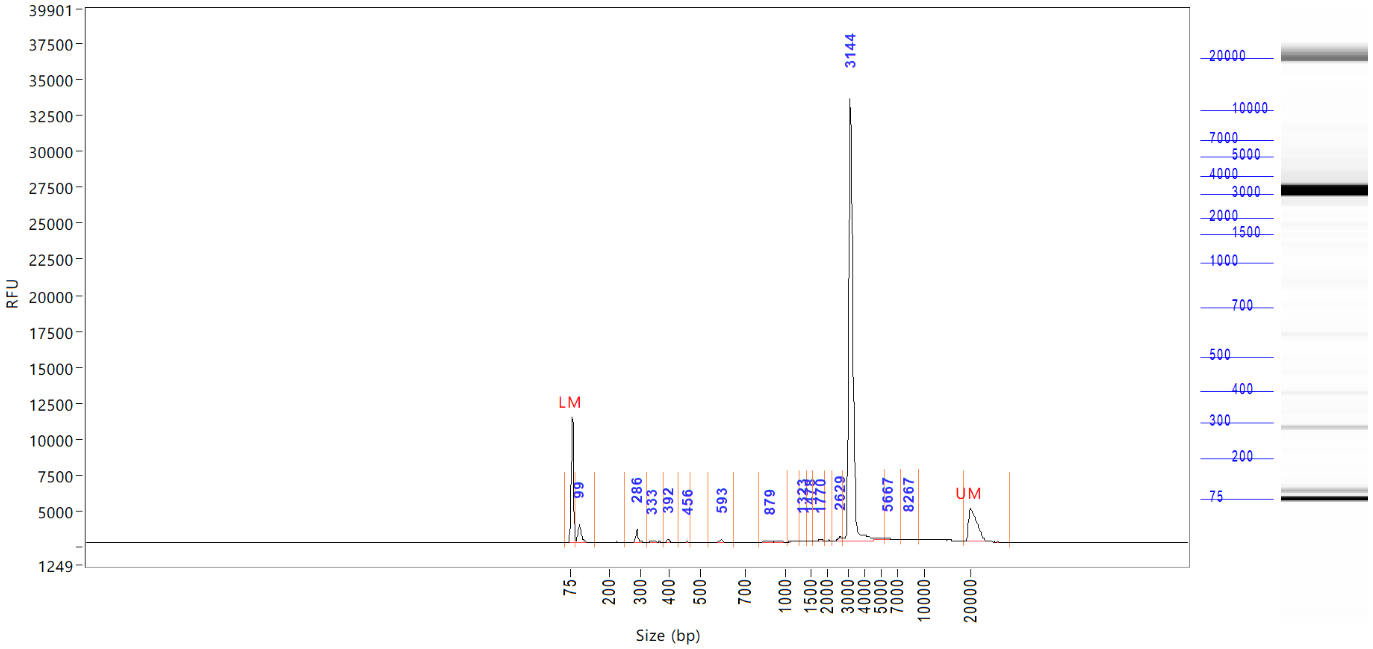

Peak	Size (bp)	Concentration (ng/uL)	From (bp)	To (bp)	Average size (bp)	CV%	RFU	Corrected peak area
1	75 (LM)	0.5000	50	86	74	4.25	8732	47.279
2	99	1.3437	86	150	99	8.41	1245	10.588
3	286	0.6554	246	321	284	3.38	906	5.165
4	333	0.1516	321	380	347	4.56	61	1.195
5	392	0.1722	380	428	392	1.90	235	1.357
6	456	0.0512	428	466	450	1.48	58	0.404
7	593	0.1818	534	649	589	2.73	198	1.432
8	879	0.1716	805	1023	885	4.88	88	1.352
9	1323	0.0542	1256	1400	1310	1.92	86	0.427
10	1478	0.0398	1400	1539	1477	1.77	66	0.314
11	1770	0.1096	1539	1942	1730	4.53	130	0.864
12	2629	0.2129	2229	2772	2615	3.89	247	1.678
13	3144	25.3906	2772	5297	3188	7.97	30771	200.073
14	5667	0.2183	5297	7401	6164	9.96	121	1.720
15	8267	0.0842	7401	9467	8081	5.27	68	0.663
16	20000 (UM)	0.2585	18293	28221	20548	4.37	2298	24.447

TIC:	28.8372	ng/uL
TIM:	42.0758	nmole/L
Total concentration:	29.0806	ng/uL

```
Sample peak width (sec): 5     Sample min peak height: 50     Sample baseline V to V?: Y     Sample baseline V to V points: 3
Sample filter: Binomial        Number of points for filter: 3  Sample start region (min): 0  Sample end region (min): 30
Marker peak width (sec): 5     Marker min peak height: 200    Marker baseline V to V?: Y     Marker baseline V to V points: 3
Lower marker selection: First peak > 500 RFU                  Upper marker selection: Last peak > 200 RFU
Ladder size (bp)75, 200, 300, 400, 500, 700, 1000, 1500, 2000, 3000, 4000, 5000, 7000, 10000, 20000
Quantification using: Lower Marker          Final concentration (ng/uL): 0.5000          Dilution factor: 12.0
```

Sample: SampG3
Well location: G3
Created: Wednesday, October 20, 2021 5:18:46 PM

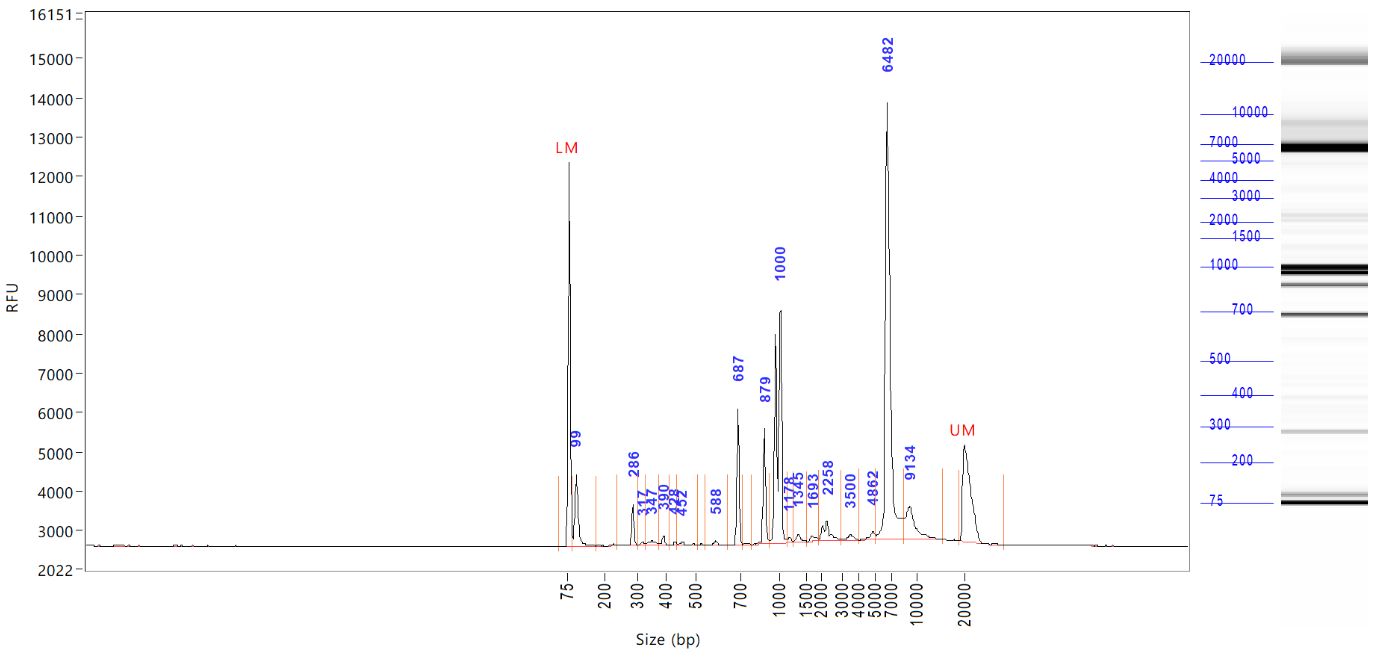

Peak	Size (bp)	Concentration (ng/uL)	From (bp)	To (bp)	Average size (bp)	CV%	RFU	Corrected peak area
1	75 (LM)	0.5000	41	86	74	4.31	9741	52.236
2	99	1.7623	86	166	100	10.12	1820	15.343
3	286	0.6328	237	300	281	2.09	1048	5.509
4	317	0.0830	300	325	314	2.18	80	0.723
5	347	0.2136	325	376	348	3.68	102	1.859
6	390	0.1502	376	409	388	1.34	239	1.308
7	428	0.0550	409	439	427	1.23	97	0.479
8	452	0.1221	439	506	464	4.02	90	1.063
9	588	0.1090	542	641	585	2.70	114	0.949
10	687	1.6646	641	705	684	0.79	3459	14.492
11	879	1.5128	783	922	874	1.51	2918	13.170
12	1000	5.5555	922	1123	979	2.51	5923	48.367
13	1178	0.0827	1123	1234	1166	2.44	124	0.720
14	1345	0.1871	1234	1501	1339	3.92	208	1.629
15	1693	0.1508	1501	1923	1717	5.76	149	1.313
16	2258	0.6006	1923	2971	2217	9.62	504	5.229
17	3500	0.1429	2971	4001	3457	5.88	127	1.244
18	4862	0.1802	4001	5001	4747	4.32	209	1.569
19	6482	9.3784	5001	8467	6576	7.74	11121	81.649
20	9134	1.3036	8467	15244	9520	11.18	827	11.349
21	20000 (UM)	0.2534	18781	28463	20708	4.53	2472	26.472

TIC:	23.8874	ng/uL
TIM:	55.5116	nmole/L
Total concentration:	23.9231	ng/uL

Sample peak width (sec): 5 Sample min peak height: 50 Sample baseline V to V?: Y Sample baseline V to V points: 3
Sample filter: Binomial Number of points for filter: 3 Sample start region (min): 0 Sample end region (min): 30
Marker peak width (sec): 5 Marker min peak height: 200 Marker baseline V to V?: Y Marker baseline V to V points: 3
Lower marker selection: First peak > 500 RFU Upper marker selection: Last peak > 200 RFU
Ladder size (bp)75, 200, 300, 400, 500, 700, 1000, 1500, 2000, 3000, 4000, 5000, 7000, 10000, 20000
Quantification using: Lower Marker Final concentration (ng/uL): 0.5000 Dilution factor: 12.0

Sample: SampG4
Well location: G4
Created: Wednesday, October 20, 2021 5:18:46 PM

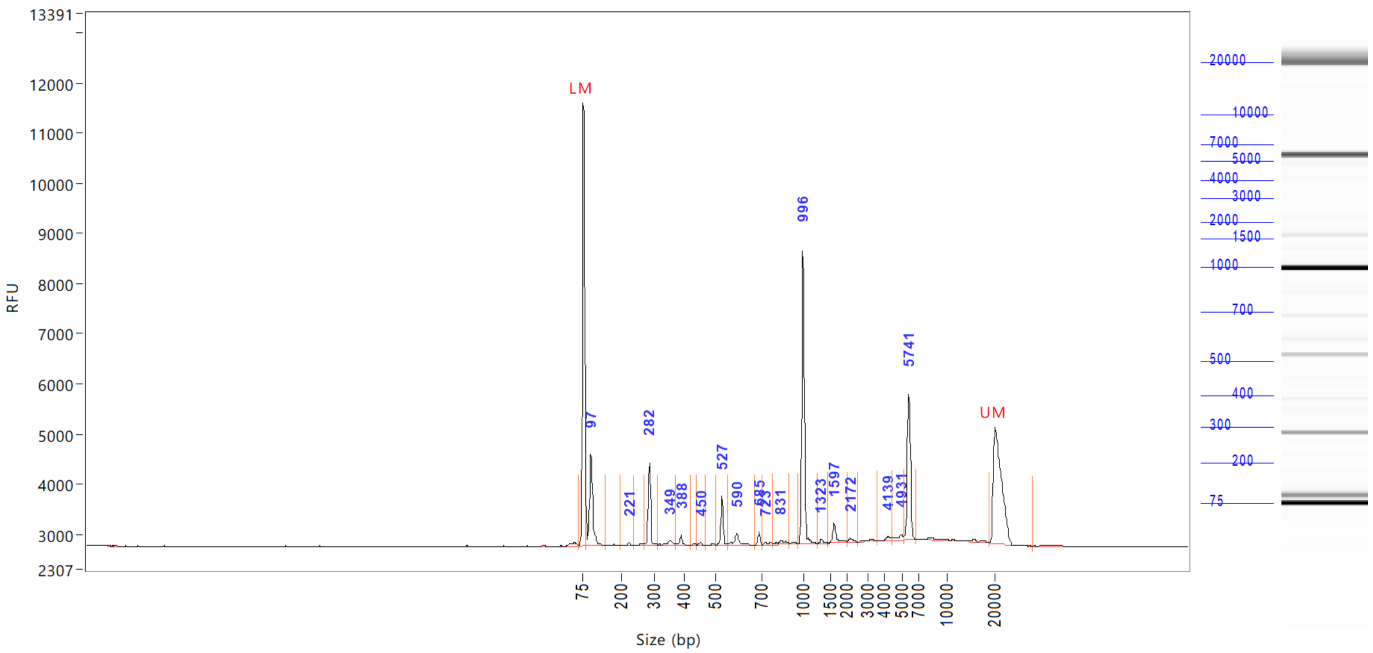

Peak	Size (bp)	Concentration (ng/uL)	From (bp)	To (bp)	Average size (bp)	CV%	RFU	Corrected peak area
1	75 (LM)	0.5000	62	86	74	4.08	8849	47.228
2	97	1.8321	86	145	99	8.09	1824	14.421
3	221	0.0518	194	237	218	2.97	55	0.408
4	282	1.0026	266	307	281	1.64	1640	7.892
5	349	0.1498	307	372	344	4.26	85	1.179
6	388	0.1346	372	418	386	1.66	198	1.059
7	450	0.0394	439	466	448	1.05	56	0.310
8	527	0.5523	500	549	523	1.17	966	4.347
9	590	0.2690	549	667	590	3.48	231	2.117
10	685	0.1373	667	700	682	0.77	254	1.081
11	723	0.0639	700	779	733	2.95	50	0.503
12	831	0.1203	779	900	840	3.49	83	0.947
13	996	3.0785	961	1256	996	2.19	5843	24.232
14	1323	0.0606	1256	1445	1325	2.69	80	0.477
15	1597	0.2855	1445	2001	1619	6.68	398	2.247
16	2172	0.0657	2001	2515	2167	4.33	77	0.517
17	4139	0.0831	3536	4449	4145	4.57	74	0.654
18	4931	0.1242	4449	5075	4799	3.60	119	0.978
19	5741	1.6179	5075	6630	5666	3.57	2900	12.735
20	20000 (UM)	0.2600	18903	27617	20648	4.26	2324	24.562

TIC:	9.6686	ng/uL
TIM:	47.3289	nmole/L
Total concentration:	10.0910	ng/uL

```
Sample peak width (sec): 5    Sample min peak height: 50    Sample baseline V to V?: Y    Sample baseline V to V points: 3
Sample filter: Binomial       Number of points for filter: 3  Sample start region (min): 0  Sample end region (min): 30
Marker peak width (sec): 5    Marker min peak height: 200   Marker baseline V to V?: Y    Marker baseline V to V points: 3
Lower marker selection: First peak > 500 RFU                Upper marker selection: Last peak > 200 RFU
Ladder size (bp)75, 200, 300, 400, 500, 700, 1000, 1500, 2000, 3000, 4000, 5000, 7000, 10000, 20000
Quantification using: Lower Marker          Final concentration (ng/uL): 0.5000          Dilution factor: 12.0
```

Sample: SampG5
Well location: G5
Created: Wednesday, October 20, 2021 5:18:46 PM

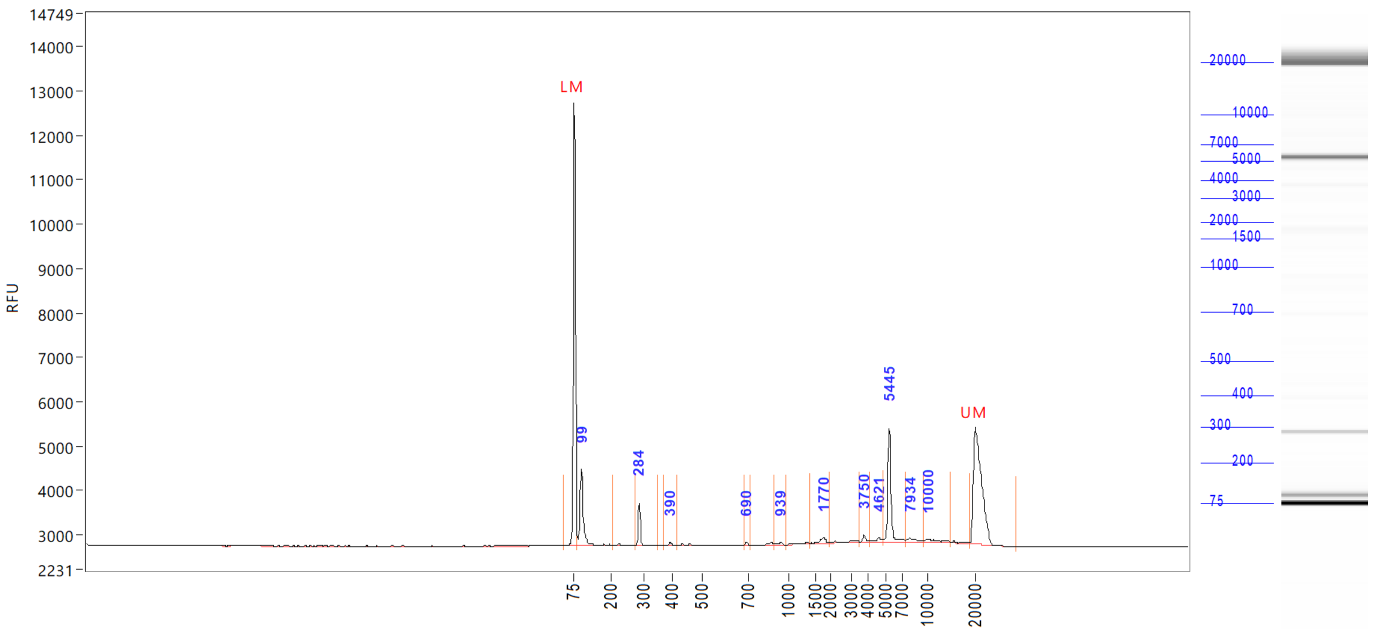

Peak	Size (bp)	Concentration (ng/uL)	From (bp)	To (bp)	Average size (bp)	CV%	RFU	Corrected peak area
1	75 (LM)	0.5000	43	86	73	4.46	9987	53.075
2	99	1.6125	86	203	102	15.17	1719	14.264
3	284	0.5239	273	349	284	2.96	952	4.634
4	390	0.0546	372	414	390	1.67	84	0.483
5	690	0.0352	680	714	688	0.72	78	0.311
6	939	0.0440	896	987	929	1.78	53	0.390
7	1770	0.1444	1389	1942	1686	7.05	135	1.277
8	3750	0.0803	3500	4070	3737	2.74	153	0.710
9	4621	0.0771	4070	4862	4568	4.19	86	0.682
10	5445	1.4113	4862	7468	5479	7.36	2558	12.484
11	7934	0.1235	7468	9533	8347	6.89	74	1.093
12	10000	0.1384	9533	14757	11507	13.22	68	1.224
13	20000 (UM)	0.2650	18659	28584	20599	4.36	2618	28.135

TIC:	4.2451	ng/uL
TIM:	30.0592	nmole/L
Total concentration:	4.6194	ng/uL

Sample peak width (sec): 5   Sample min peak height: 50   Sample baseline V to V?: Y   Sample baseline V to V points: 3
Sample filter: Binomial   Number of points for filter: 3 Sample start region (min): 0 Sample end region (min): 30
Marker peak width (sec): 5   Marker min peak height: 200   Marker baseline V to V?: Y   Marker baseline V to V points: 3
Lower marker selection: First peak > 500 RFU   Upper marker selection: Last peak > 200 RFU
Ladder size (bp)75, 200, 300, 400, 500, 700, 1000, 1500, 2000, 3000, 4000, 5000, 7000, 10000, 20000
Quantification using: Lower Marker   Final concentration (ng/uL): 0.5000   Dilution factor: 12.0

Sample: SampG6
Well location: G6
Created: Wednesday, October 20, 2021 5:18:46 PM

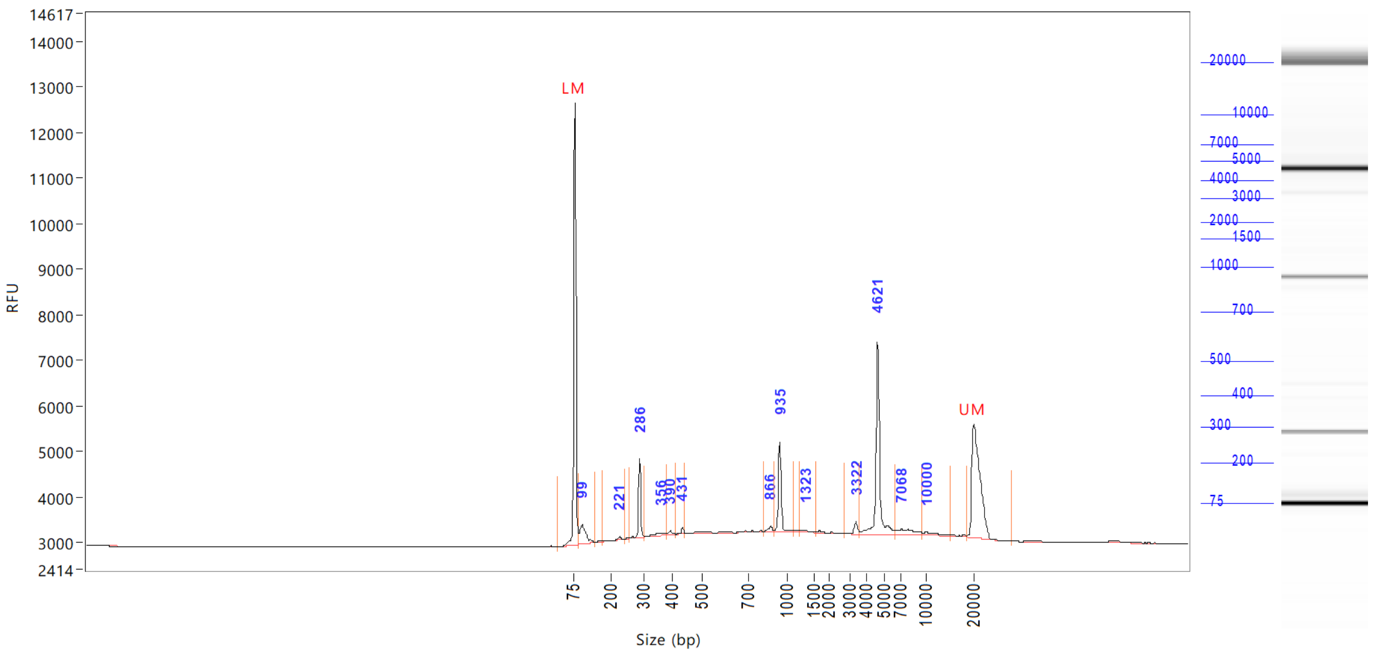

Peak	Size (bp)	Concentration (ng/uL)	From (bp)	To (bp)	Average size (bp)	CV%	RFU	Corrected peak area
1	75 (LM)	0.5000	18	89	73	6.77	9717	53.647
2	99	0.6637	89	143	104	10.64	423	5.934
3	221	0.0725	166	239	206	10.65	60	0.648
4	286	0.9487	257	300	282	1.73	1725	8.483
5	356	0.1704	300	380	342	6.25	65	1.523
6	390	0.0662	380	411	390	1.60	92	0.592
7	431	0.0689	411	441	429	1.19	133	0.616
8	866	0.0886	814	896	860	2.01	110	0.792
9	935	0.9977	896	1090	934	2.11	1961	8.920
10	1323	0.0798	1223	1520	1352	6.32	50	0.714
11	3322	0.1681	2715	3572	3274	4.72	267	1.503
12	4621	2.7537	3572	6482	4646	7.95	4239	24.622
13	7068	0.3334	6482	9600	7840	10.80	117	2.981
14	10000	0.1557	9600	15000	11399	12.98	76	1.392
15	20000 (UM)	0.2484	18537	27979	20666	4.43	2496	26.648

TIC:	6.5674	ng/uL
TIM:	21.1334	nmole/L
Total concentration:	6.9157	ng/uL

```
Sample peak width (sec): 5    Sample min peak height: 50    Sample baseline V to V?: Y    Sample baseline V to V points: 3
Sample filter: Binomial       Number of points for filter: 3  Sample start region (min): 0  Sample end region (min): 30
Marker peak width (sec): 5    Marker min peak height: 200   Marker baseline V to V?: Y    Marker baseline V to V points: 3
Lower marker selection: First peak > 500 RFU                Upper marker selection: Last peak > 200 RFU
Ladder size (bp)75, 200, 300, 400, 500, 700, 1000, 1500, 2000, 3000, 4000, 5000, 7000, 10000, 20000
Quantification using: Lower Marker          Final concentration (ng/uL): 0.5000          Dilution factor: 12.0
```

Sample: SampG7
Well location: G7
Created: Wednesday, October 20, 2021 5:18:46 PM

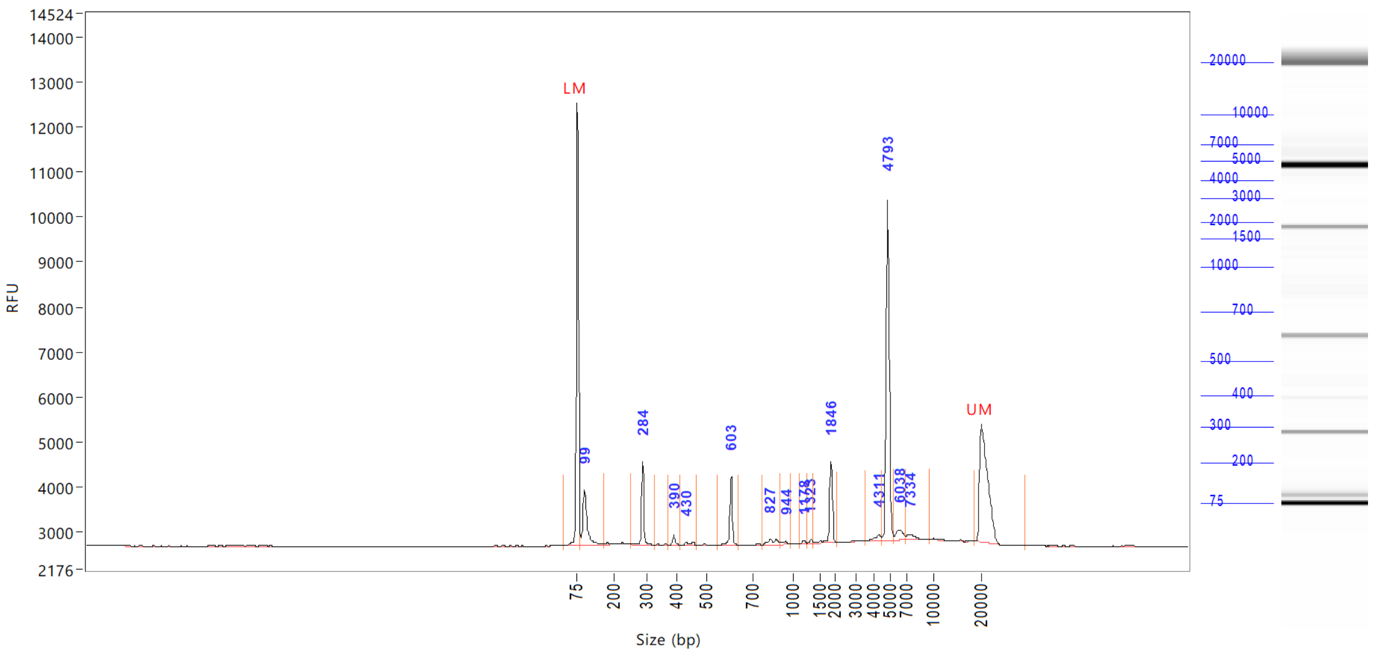

Peak	Size (bp)	Concentration (ng/uL)	From (bp)	To (bp)	Average size (bp)	CV%	RFU	Corrected peak area
1	75 (LM)	0.5000	33	86	73	5.79	9842	52.994
2	99	1.3624	86	166	102	13.26	1213	12.033
3	284	1.0359	250	323	282	2.21	1841	9.150
4	390	0.1310	372	412	389	1.41	229	1.157
5	430	0.0932	412	466	439	2.64	73	0.823
6	603	0.8232	544	639	599	1.51	1526	7.271
7	827	0.2118	762	900	838	3.89	132	1.870
8	944	0.0699	900	983	929	1.83	69	0.618
9	1178	0.0419	1112	1256	1179	2.69	71	0.370
10	1323	0.0507	1256	1378	1317	2.18	85	0.448
11	1846	0.9209	1378	2001	1800	5.36	1785	8.134
12	4311	0.1155	3500	4483	4205	4.49	139	1.020
13	4793	4.1175	4483	5371	4792	2.14	7562	36.367
14	6038	0.2872	5371	6926	6077	6.69	229	2.537
15	7334	0.1686	6926	9467	7512	6.31	126	1.489
16	20000 (UM)	0.2607	18537	28947	20594	4.30	2623	27.630

TIC:	9.4296	ng/uL
TIM:	34.2195	nmole/L
Total concentration:	9.7244	ng/uL

Sample peak width (sec): 5 Sample min peak height: 50 Sample baseline V to V?: Y Sample baseline V to V points: 3
Sample filter: Binomial Number of points for filter: 3 Sample start region (min): 0 Sample end region (min): 30
Marker peak width (sec): 5 Marker min peak height: 200 Marker baseline V to V?: Y Marker baseline V to V points: 3
Lower marker selection: First peak > 500 RFU Upper marker selection: Last peak > 200 RFU
Ladder size (bp)75, 200, 300, 400, 500, 700, 1000, 1500, 2000, 3000, 4000, 5000, 7000, 10000, 20000
Quantification using: Lower Marker Final concentration (ng/uL): 0.5000 Dilution factor: 12.0

Sample: SampG8
Well location: G8
Created: Wednesday, October 20, 2021 5:18:46 PM

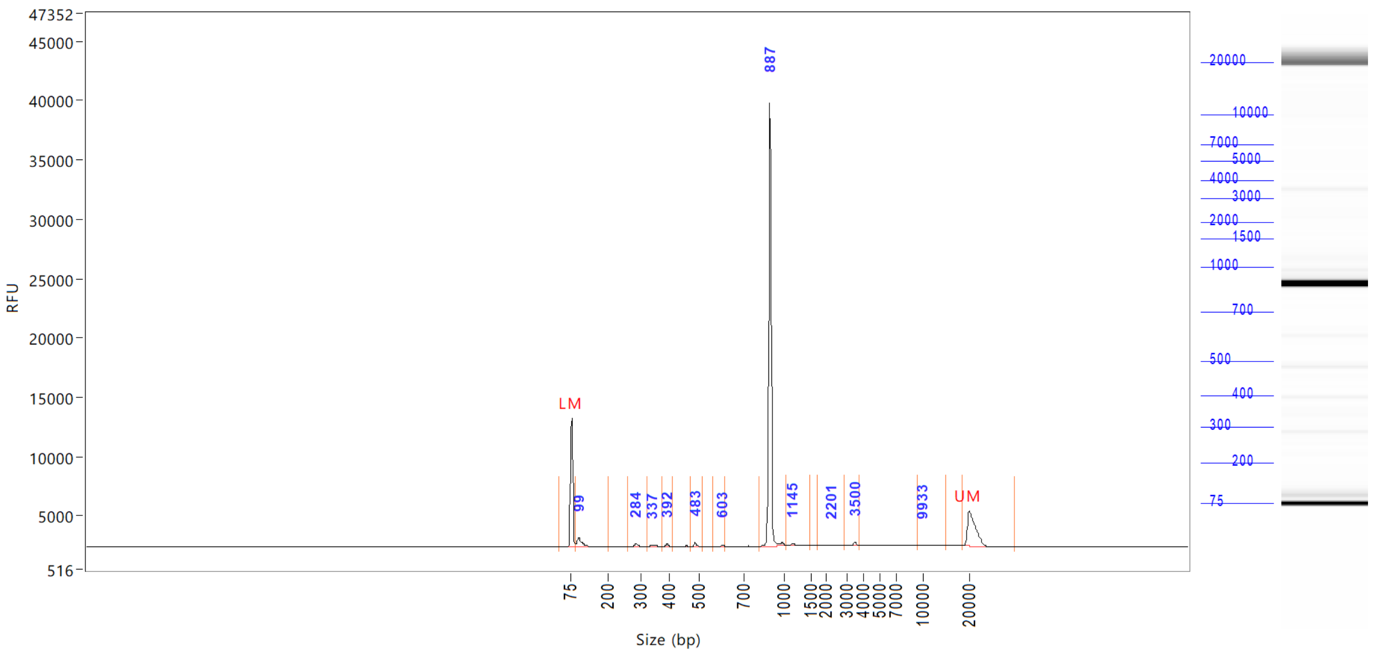

Peak	Size (bp)	Concentration (ng/uL)	From (bp)	To (bp)	Average size (bp)	CV%	RFU	Corrected peak area
1	75 (LM)	0.5000	33	86	73	4.41	10876	57.824
2	99	0.9568	86	200	106	17.76	718	9.221
3	284	0.2057	260	321	285	4.23	256	1.983
4	337	0.1175	321	376	342	3.24	84	1.133
5	392	0.1127	376	412	390	1.10	221	1.086
6	483	0.1735	473	511	484	1.10	314	1.672
7	603	0.0868	560	616	597	1.72	161	0.837
8	887	17.0966	809	1045	887	1.63	37398	164.766
9	1145	0.1821	1045	1500	1185	7.95	134	1.755
10	2201	0.1010	1693	2943	2342	13.43	57	0.973
11	3500	0.1486	2943	3822	3417	5.05	255	1.432
12	9933	0.1227	9467	15000	11937	13.98	57	1.182
13	20000 (UM)	0.2692	18537	29551	20646	4.56	2903	31.133

TIC:	19.3040	ng/uL
TIM:	50.0179	nmole/L
Total concentration:	19.6298	ng/uL

Sample peak width (sec): 5 Sample min peak height: 50 Sample baseline V to V?: Y Sample baseline V to V points: 3
Sample filter: Binomial Number of points for filter: 3 Sample start region (min): 0 Sample end region (min): 30
Marker peak width (sec): 5 Marker min peak height: 200 Marker baseline V to V?: Y Marker baseline V to V points: 3
Lower marker selection: First peak > 500 RFU Upper marker selection: Last peak > 200 RFU
Ladder size (bp)75, 200, 300, 400, 500, 700, 1000, 1500, 2000, 3000, 4000, 5000, 7000, 10000, 20000
Quantification using: Lower Marker Final concentration (ng/uL): 0.5000 Dilution factor: 12.0

Sample: SampG9
Well location: G9
Created: Wednesday, October 20, 2021 5:18:46 PM

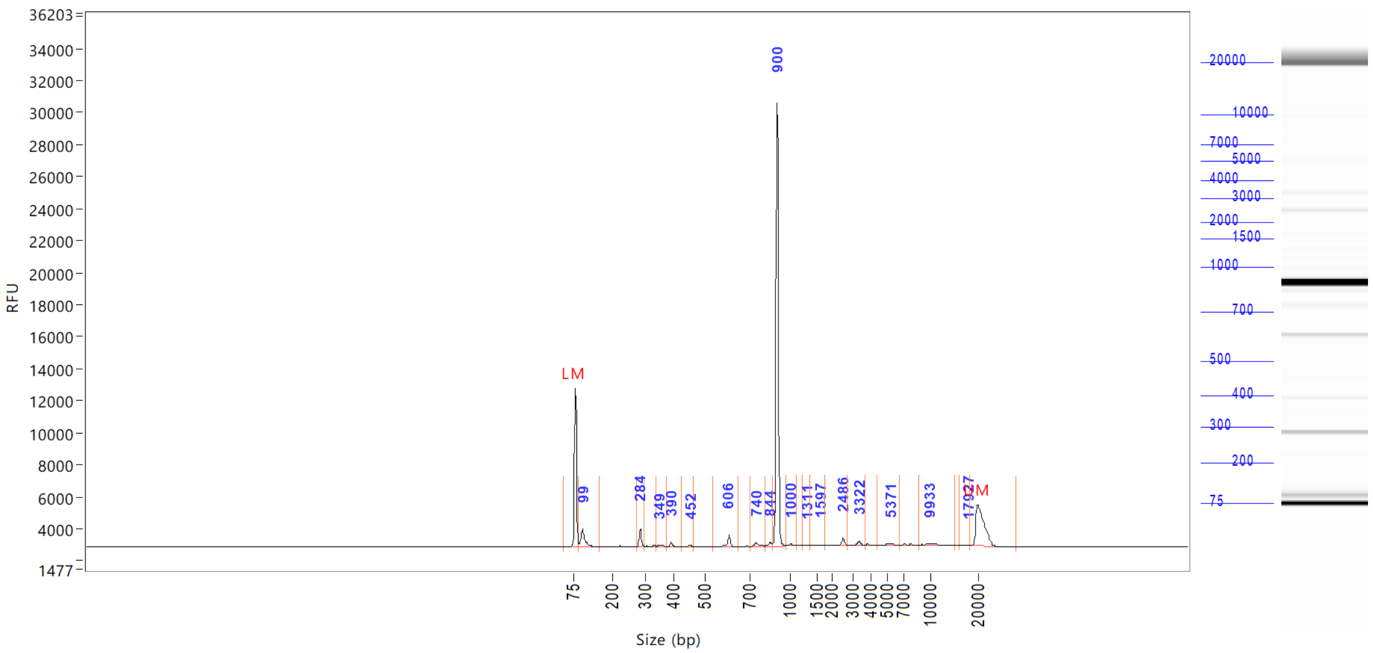

Peak	Size (bp)	Concentration (ng/uL)	From (bp)	To (bp)	Average size (bp)	CV%	RFU	Corrected peak area
1	75 (LM)	0.5000	39	86	73	4.77	9929	52.430
2	99	1.1750	86	152	100	10.63	1111	10.268
3	284	0.6047	271	298	281	1.39	1129	5.284
4	349	0.0579	337	372	351	2.30	54	0.506
5	390	0.1453	372	422	387	1.32	269	1.270
6	452	0.0579	422	462	446	1.86	63	0.506
7	606	0.4254	537	649	600	2.01	711	3.717
8	740	0.1706	701	805	740	2.53	205	1.491
9	844	0.1444	805	861	841	1.49	223	1.262
10	900	13.2375	861	974	895	1.10	27727	115.674
11	1000	0.0705	974	1090	998	1.69	111	0.616
12	1311	0.0376	1223	1378	1297	2.36	60	0.328
13	1597	0.0490	1378	1731	1551	5.36	52	0.428
14	2486	0.2578	1731	2715	2376	8.19	434	2.253
15	3322	0.1523	2715	3643	3243	5.18	234	1.331
16	5371	0.0907	4414	6482	5173	8.19	80	0.793
17	9933	0.1896	8734	15122	11379	16.17	69	1.657
18	17927	0.0468	16220	18415	17427	3.42	52	0.409
19	20000 (UM)	0.2665	18415	27979	20568	4.30	2601	27.950

TIC:	16.9132	ng/uL
TIM:	50.6420	nmole/L
Total concentration:	17.2644	ng/uL

```
Sample peak width (sec): 5     Sample min peak height: 50    Sample baseline V to V?: Y    Sample baseline V to V points: 3
Sample filter: Binomial        Number of points for filter: 3  Sample start region (min): 0  Sample end region (min): 30
Marker peak width (sec): 5     Marker min peak height: 200   Marker baseline V to V?: Y    Marker baseline V to V points: 3
Lower marker selection: First peak > 500 RFU                 Upper marker selection: Last peak > 200 RFU
Ladder size (bp)75, 200, 300, 400, 500, 700, 1000, 1500, 2000, 3000, 4000, 5000, 7000, 10000, 20000
Quantification using: Lower Marker          Final concentration (ng/uL): 0.5000          Dilution factor: 12.0
```

Sample: SampG10
Well location: G10
Created: Wednesday, October 20, 2021 5:18:46 PM

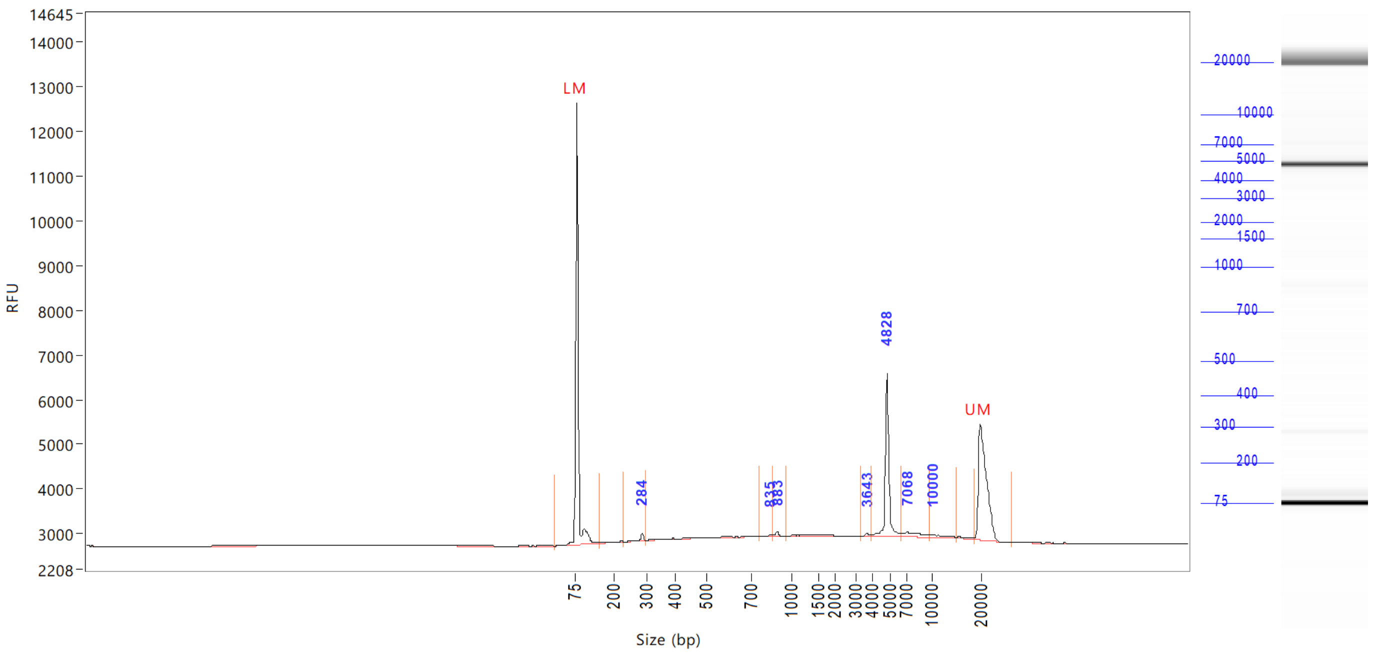

Peak	Size (bp)	Concentration (ng/uL)	From (bp)	To (bp)	Average size (bp)	CV%	RFU	Corrected peak area
1	75 (LM)	0.5000	3	150	76	13.51	9901	59.883
2	284	0.1139	230	298	278	4.62	180	1.137
3	835	0.0365	757	853	824	2.33	54	0.364
4	883	0.0657	853	952	882	1.93	101	0.656
5	3643	0.0485	3358	3929	3681	3.65	65	0.484
6	4828	1.9677	3929	6408	4848	5.97	3658	19.638
7	7068	0.2441	6408	9600	7815	11.03	111	2.436
8	10000	0.1198	9600	15000	11587	14.08	68	1.196
9	20000 (UM)	0.2289	18659	26408	20590	4.24	2598	27.414

TIC:	2.5962	ng/uL
TIM:	1.6280	nmole/L
Total concentration:	2.9735	ng/uL

Sample peak width (sec): 5   Sample min peak height: 50   Sample baseline V to V?: Y   Sample baseline V to V points: 3
Sample filter: Binomial   Number of points for filter: 3 Sample start region (min): 0 Sample end region (min): 30
Marker peak width (sec): 5   Marker min peak height: 200   Marker baseline V to V?: Y   Marker baseline V to V points: 3
Lower marker selection: First peak > 500 RFU   Upper marker selection: Last peak > 200 RFU
Ladder size (bp)75, 200, 300, 400, 500, 700, 1000, 1500, 2000, 3000, 4000, 5000, 7000, 10000, 20000
Quantification using: Lower Marker   Final concentration (ng/uL): 0.5000   Dilution factor: 12.0

Sample: SampG11
Well location: G11
Created: Wednesday, October 20, 2021 5:18:46 PM

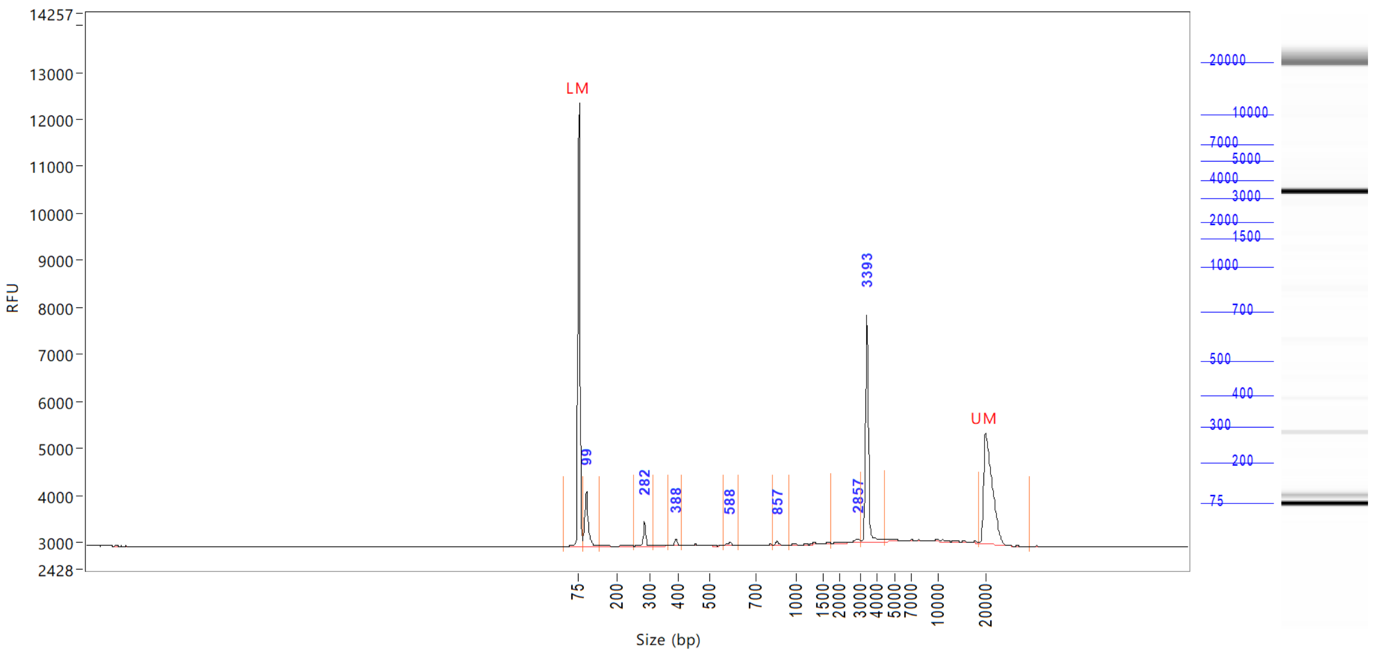

Peak	Size (bp)	Concentration (ng/uL)	From (bp)	To (bp)	Average size (bp)	CV%	RFU	Corrected peak area
1	75 (LM)	0.5000	28	86	73	5.09	9453	49.997
2	99	1.1503	86	145	98	8.22	1169	9.585
3	282	0.3309	251	309	281	2.42	518	2.757
4	388	0.0914	366	409	387	1.44	151	0.761
5	588	0.0800	557	623	582	1.88	93	0.667
6	857	0.0672	822	952	861	2.90	81	0.560
7	2857	0.1227	1750	3037	2585	14.12	73	1.022
8	3393	2.6717	3037	4449	3387	5.06	4835	22.263
9	20000 (UM)	0.2586	18415	29068	20640	4.65	2357	25.857

TIC:	4.5141	ng/uL
TIM:	23.3296	nmole/L
Total concentration:	5.1025	ng/uL

Sample peak width (sec): 5 Sample min peak height: 50 Sample baseline V to V?: Y Sample baseline V to V points: 3
Sample filter: Binomial Number of points for filter: 3 Sample start region (min): 0 Sample end region (min): 30
Marker peak width (sec): 5 Marker min peak height: 200 Marker baseline V to V?: Y Marker baseline V to V points: 3
Lower marker selection: First peak > 500 RFU Upper marker selection: Last peak > 200 RFU
Ladder size (bp)75, 200, 300, 400, 500, 700, 1000, 1500, 2000, 3000, 4000, 5000, 7000, 10000, 20000
Quantification using: Lower Marker Final concentration (ng/uL): 0.5000 Dilution factor: 12.0

Sample: SampG12
Well location: G12
Created: Wednesday, October 20, 2021 5:18:46 PM

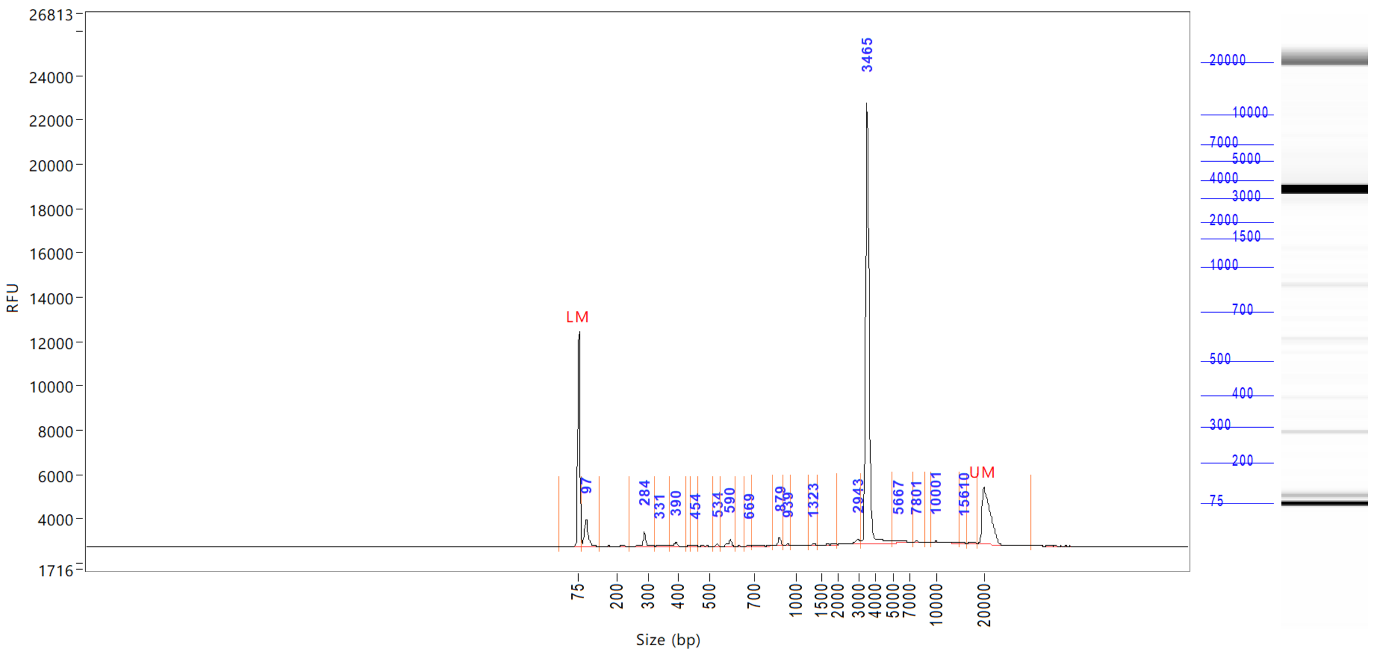

Peak	Size (bp)	Concentration (ng/uL)	From (bp)	To (bp)	Average size (bp)	CV%	RFU	Corrected peak area
1	75 (LM)	0.5000	12	84	72	4.84	9728	51.847
2	97	1.2450	84	145	98	8.31	1217	10.758
3	284	0.4293	241	317	282	3.44	664	3.709
4	331	0.0892	317	370	340	4.06	58	0.771
5	390	0.1270	370	424	388	1.91	197	1.097
6	454	0.0451	441	464	450	1.10	62	0.390
7	534	0.0452	514	549	531	0.88	94	0.390
8	590	0.2180	549	613	583	1.60	291	1.884
9	669	0.0305	652	685	669	0.83	62	0.263
10	879	0.2184	831	909	873	1.47	385	1.887
11	939	0.0514	909	961	933	1.13	84	0.444
12	1323	0.0462	1245	1400	1321	1.97	86	0.399
13	2943	0.2150	2000	3108	2835	7.53	196	1.858
14	3465	12.5453	3108	4966	3494	5.49	19934	108.407
15	5667	0.2156	4966	7401	5874	11.26	110	1.863
16	7801	0.0595	7401	8800	7931	4.38	76	0.514
17	10001	0.1485	9467	15000	11851	13.97	57	1.283
18	15610	0.0313	15000	16342	15500	2.08	55	0.271
19	20000 (UM)	0.2621	18537	29914	20638	4.39	2551	27.182

TIC:	15.7605	ng/uL
TIM:	32.0219	nmole/L
Total concentration:	16.0167	ng/uL

```
Sample peak width (sec): 5    Sample min peak height: 50    Sample baseline V to V?: Y    Sample baseline V to V points: 3
Sample filter: Binomial       Number of points for filter: 3  Sample start region (min): 0  Sample end region (min): 30
Marker peak width (sec): 5    Marker min peak height: 200   Marker baseline V to V?: Y    Marker baseline V to V points: 3
Lower marker selection: First peak > 500 RFU                Upper marker selection: Last peak > 200 RFU
Ladder size (bp)75, 200, 300, 400, 500, 700, 1000, 1500, 2000, 3000, 4000, 5000, 7000, 10000, 20000
Quantification using: Lower Marker          Final concentration (ng/uL): 0.5000          Dilution factor: 12.0
```

Sample: SampH1
Well location: H1
Created: Wednesday, October 20, 2021 5:18:46 PM

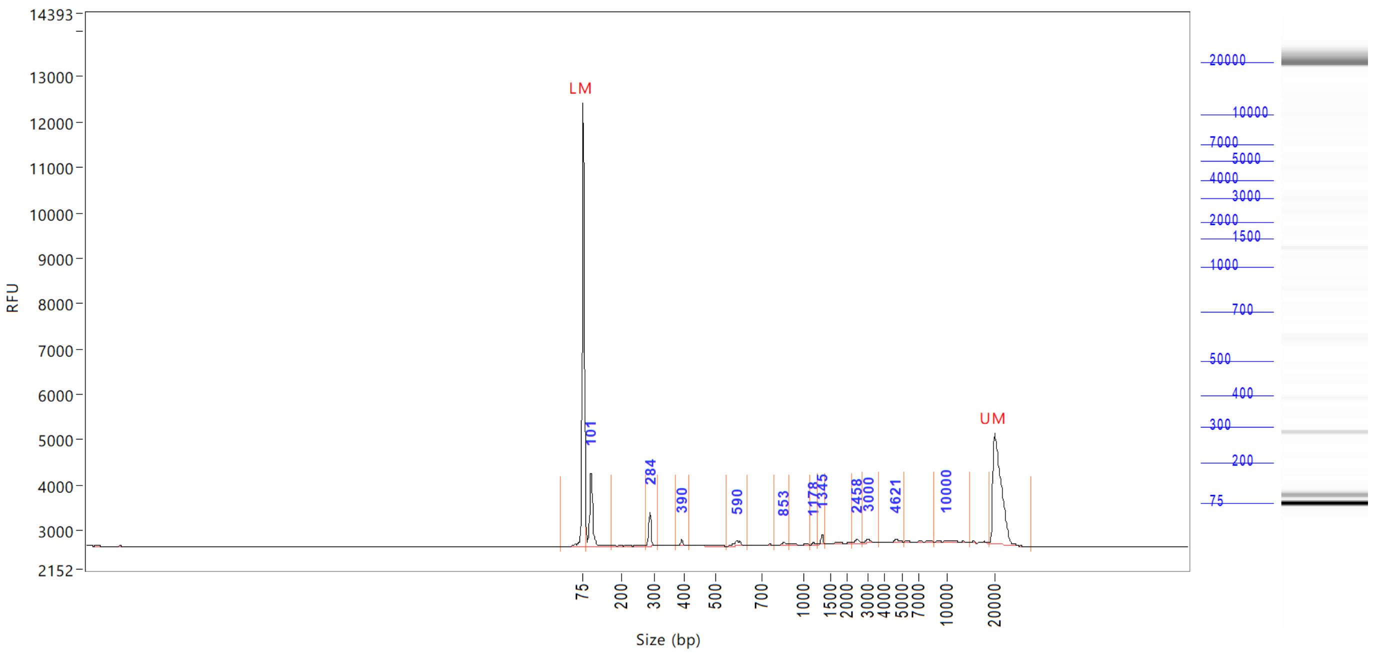

Peak	Size (bp)	Concentration (ng/uL)	From (bp)	To (bp)	Average size (bp)	CV%	RFU	Corrected peak area
1	75 (LM)	0.5000	5	86	72	6.74	9780	51.504
2	101	1.4037	86	166	99	8.64	1611	12.050
3	284	0.4201	273	311	282	1.64	747	3.606
4	390	0.0900	370	416	388	1.43	158	0.772
5	590	0.1554	547	634	588	2.32	123	1.334
6	853	0.0700	783	900	852	2.78	71	0.601
7	1178	0.0330	1112	1256	1177	3.02	53	0.283
8	1345	0.0906	1256	1400	1324	1.69	208	0.777
9	2458	0.0583	2258	2743	2464	3.95	80	0.501
10	3000	0.0821	2743	3643	3034	5.22	93	0.705
11	4621	0.0776	3643	5075	4545	6.69	62	0.666
12	10000	0.1357	8667	14879	11105	15.65	53	1.165
13	20000 (UM)	0.2515	18903	27375	20607	4.47	2424	25.903

TIC:	2.6164	ng/uL
TIM:	26.9751	nmole/L
Total concentration:	3.0401	ng/uL

Sample peak width (sec): 5   Sample min peak height: 50   Sample baseline V to V?: Y   Sample baseline V to V points: 3
Sample filter: Binomial   Number of points for filter: 3   Sample start region (min): 0   Sample end region (min): 30
Marker peak width (sec): 5   Marker min peak height: 200   Marker baseline V to V?: Y   Marker baseline V to V points: 3
Lower marker selection: First peak > 500 RFU   Upper marker selection: Last peak > 200 RFU
Ladder size (bp)75, 200, 300, 400, 500, 700, 1000, 1500, 2000, 3000, 4000, 5000, 7000, 10000, 20000
Quantification using: Lower Marker   Final concentration (ng/uL): 0.5000   Dilution factor: 12.0

Sample: SampH2
Well location: H2
Created: Wednesday, October 20, 2021 5:18:46 PM

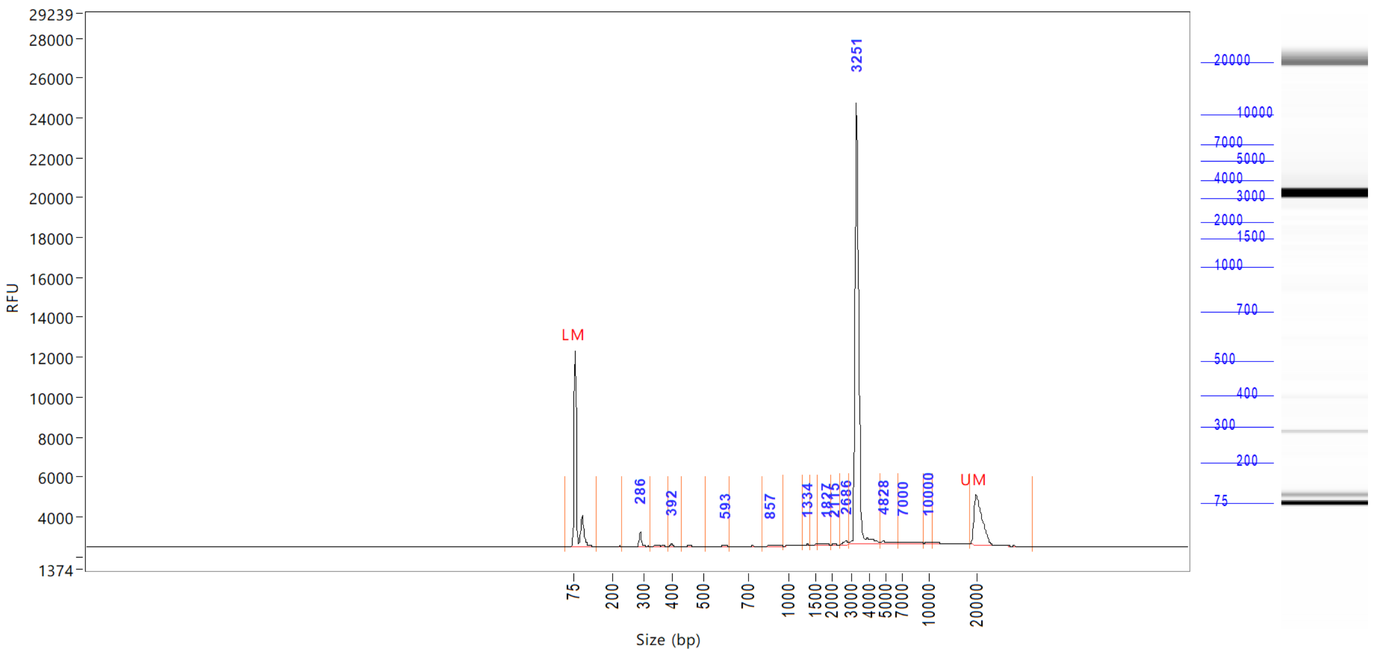

Peak	Size (bp)	Concentration (ng/uL)	From (bp)	To (bp)	Average size (bp)	CV%	RFU	Corrected peak area
1	75 (LM)	0.5000	47	145	79	14.19	9801	64.988
2	286	0.3947	228	321	283	4.26	768	4.275
3	392	0.0790	380	426	392	1.63	166	0.856
4	593	0.0482	506	613	581	3.29	62	0.522
5	857	0.0973	796	957	878	3.67	71	1.054
6	1334	0.0328	1245	1400	1315	2.29	64	0.355
7	1827	0.0722	1559	1962	1729	5.59	75	0.782
8	2115	0.0247	1962	2372	2099	3.22	60	0.267
9	2686	0.1081	2372	2829	2672	3.67	161	1.171
10	3251	11.3758	2829	4656	3273	6.03	22162	123.215
11	4828	0.1867	4656	6630	5316	10.67	120	2.022
12	7000	0.1130	6630	9467	7741	9.72	60	1.224
13	10000	0.0342	9467	10611	9992	2.95	51	0.371
14	20000 (UM)	0.2052	18659	31849	20600	4.94	2508	26.674

TIC:	12.5666	ng/uL
TIM:	8.9495	nmole/L
Total concentration:	12.9212	ng/uL

```
Sample peak width (sec): 5    Sample min peak height: 50    Sample baseline V to V?: Y    Sample baseline V to V points: 3
Sample filter: Binomial       Number of points for filter: 3  Sample start region (min): 0  Sample end region (min): 30
Marker peak width (sec): 5    Marker min peak height: 200   Marker baseline V to V?: Y    Marker baseline V to V points: 3
Lower marker selection: First peak > 500 RFU                Upper marker selection: Last peak > 200 RFU
Ladder size (bp)75, 200, 300, 400, 500, 700, 1000, 1500, 2000, 3000, 4000, 5000, 7000, 10000, 20000
Quantification using: Lower Marker          Final concentration (ng/uL): 0.5000          Dilution factor: 12.0
```

Sample: SampH3
Well location: H3
Created: Wednesday, October 20, 2021 5:18:46 PM

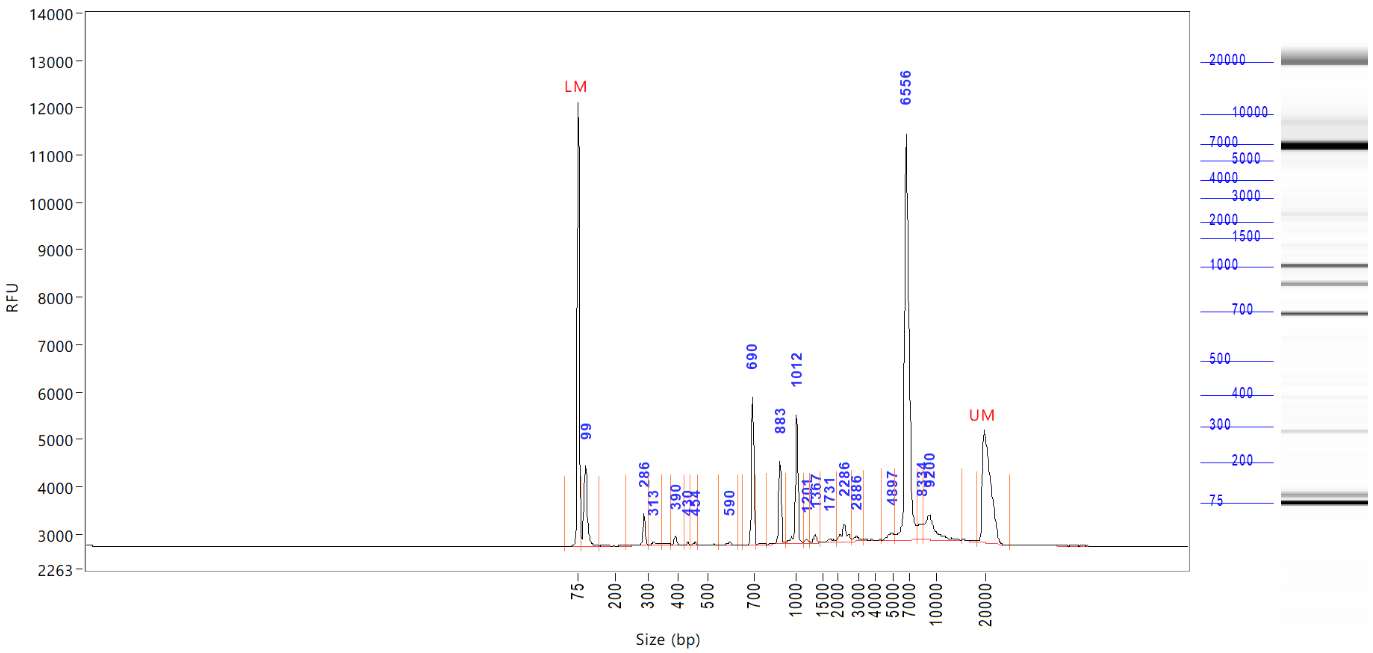

Peak	Size (bp)	Concentration (ng/uL)	From (bp)	To (bp)	Average size (bp)	CV%	RFU	Corrected peak area
1	75 (LM)	0.5000	31	86	73	4.84	9383	49.319
2	99	1.6676	86	147	98	8.16	1691	13.708
3	286	0.4410	230	300	282	2.76	677	3.625
4	313	0.1238	300	343	320	3.69	70	1.018
5	390	0.1157	378	418	389	1.41	187	0.951
6	430	0.0343	418	439	428	0.87	65	0.282
7	454	0.0433	439	464	451	1.03	60	0.356
8	590	0.0650	542	631	582	2.16	72	0.534
9	690	1.5587	649	710	688	0.67	3130	12.812
10	883	0.9009	788	926	880	1.37	1743	7.405
11	1012	1.4942	926	1134	1006	2.36	2707	12.282
12	1201	0.0615	1134	1256	1191	2.60	90	0.506
13	1367	0.1190	1256	1467	1349	2.66	171	0.978
14	1731	0.0955	1467	1942	1750	6.20	80	0.785
15	2286	0.3826	1942	2657	2261	7.72	380	3.145
16	2886	0.1180	2657	3251	2891	5.23	98	0.970
17	4897	0.1646	4345	5075	4814	3.44	155	1.353
18	6556	6.6352	5075	7801	6634	5.24	8551	54.540
19	8334	0.3445	7801	8600	8202	3.00	341	2.832
20	9200	0.8608	8600	15244	9645	10.97	533	7.076
21	20000 (UM)	0.2563	18537	25320	20588	4.19	2394	25.278

TIC:	15.2262	ng/uL
TIM:	42.5560	nmole/L
Total concentration:	15.4210	ng/uL

```
Sample peak width (sec): 5    Sample min peak height: 50    Sample baseline V to V?: Y    Sample baseline V to V points: 3
Sample filter: Binomial       Number of points for filter: 3  Sample start region (min): 0  Sample end region (min): 30
Marker peak width (sec): 5    Marker min peak height: 200   Marker baseline V to V?: Y    Marker baseline V to V points: 3
Lower marker selection: First peak > 500 RFU                Upper marker selection: Last peak > 200 RFU
Ladder size (bp)75, 200, 300, 400, 500, 700, 1000, 1500, 2000, 3000, 4000, 5000, 7000, 10000, 20000
Quantification using: Lower Marker          Final concentration (ng/uL): 0.5000          Dilution factor: 12.0
```

Sample: SampH4
Well location: H4
Created: Wednesday, October 20, 2021 5:18:46 PM

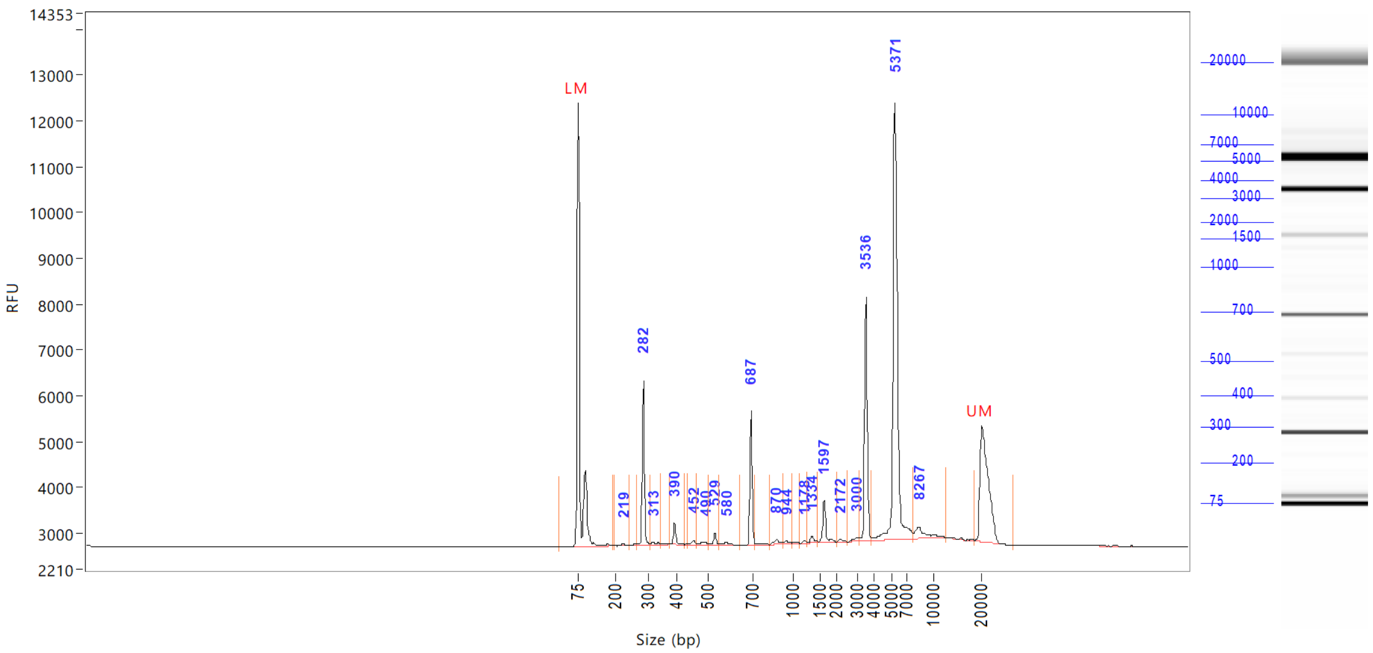

Peak	Size (bp)	Concentration (ng/uL)	From (bp)	To (bp)	Average size (bp)	CV%	RFU	Corrected peak area
1	75 (LM)	0.5000	12	189	78	17.61	9691	66.962
2	219	0.0347	194	239	218	3.07	53	0.387
3	282	1.5099	266	303	281	1.46	3604	16.851
4	313	0.0772	303	343	320	3.47	69	0.861
5	390	0.1970	372	422	388	1.26	464	2.199
6	452	0.0539	435	462	449	1.18	98	0.602
7	490	0.0582	462	501	482	1.95	58	0.650
8	529	0.1228	501	544	525	1.42	269	1.370
9	580	0.0816	544	641	579	3.43	61	0.911
10	687	1.0931	641	710	686	0.75	2918	12.199
11	870	0.0986	814	922	868	3.03	100	1.100
12	944	0.0404	922	983	944	1.67	77	0.451
13	1178	0.0263	1112	1256	1182	2.67	58	0.294
14	1334	0.0880	1256	1456	1342	3.35	155	0.982
15	1597	0.4144	1456	2001	1602	5.43	912	4.625
16	2172	0.0338	2001	2543	2185	4.56	57	0.377
17	3000	0.0599	2543	3108	2912	4.30	81	0.669
18	3536	2.0189	3108	3893	3489	2.73	5308	22.532
19	5371	5.3756	3893	7601	5456	8.39	9554	59.993
20	8267	0.3308	7601	12318	8736	10.63	256	3.692
21	20000 (UM)	0.2042	18537	26529	20688	4.30	2535	27.342

TIC:	11.7151	ng/uL
TIM:	17.4916	nmole/L
Total concentration:	11.8178	ng/uL

```
Sample peak width (sec): 5    Sample min peak height: 50    Sample baseline V to V?: Y    Sample baseline V to V points: 3
Sample filter: Binomial       Number of points for filter: 3  Sample start region (min): 0  Sample end region (min): 30
Marker peak width (sec): 5    Marker min peak height: 200   Marker baseline V to V?: Y    Marker baseline V to V points: 3
Lower marker selection: First peak > 500 RFU                Upper marker selection: Last peak > 200 RFU
Ladder size (bp)75, 200, 300, 400, 500, 700, 1000, 1500, 2000, 3000, 4000, 5000, 7000, 10000, 20000
Quantification using: Lower Marker          Final concentration (ng/uL): 0.5000          Dilution factor: 12.0
```

Sample: SampH5
Well location: H5
Created: Wednesday, October 20, 2021 5:18:46 PM

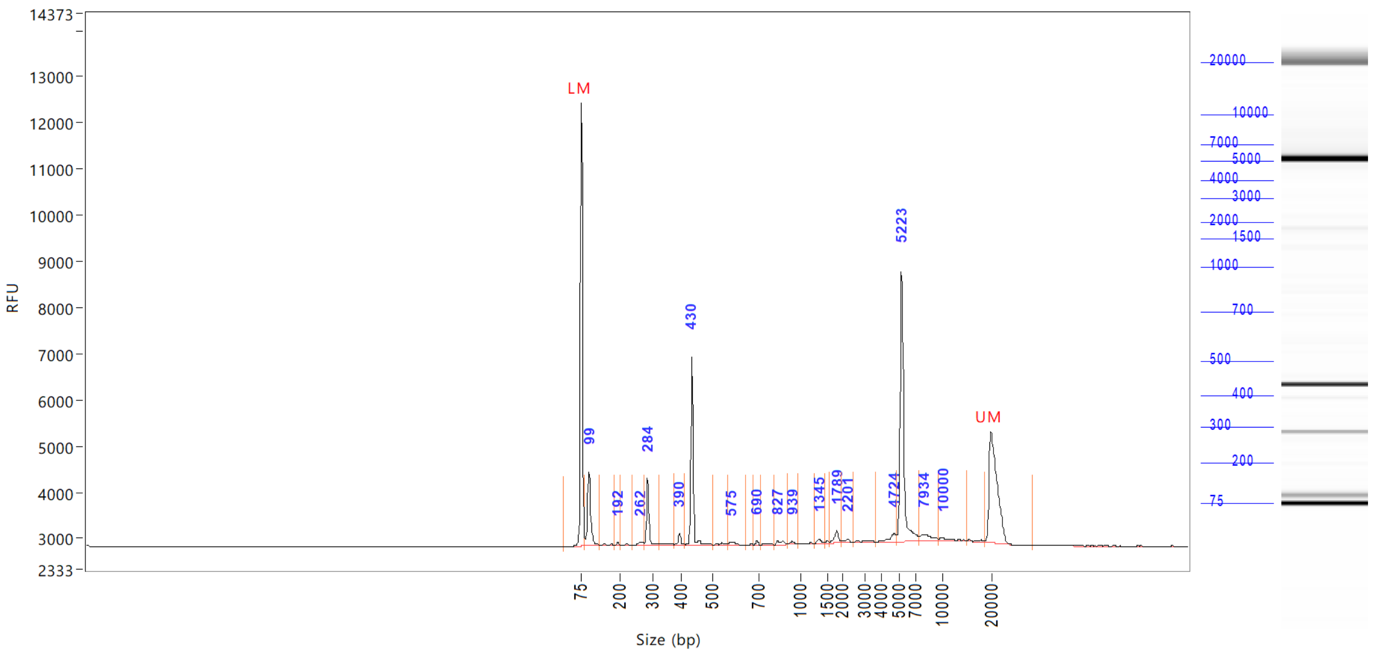

Peak	Size (bp)	Concentration (ng/uL)	From (bp)	To (bp)	Average size (bp)	CV%	RFU	Corrected peak area
1	75 (LM)	0.5000	20	86	73	4.78	9612	50.245
2	99	1.4908	86	135	98	7.08	1603	12.485
3	192	0.0440	181	201	191	1.60	77	0.369
4	262	0.0993	239	275	262	3.18	67	0.832
5	284	0.8429	275	323	283	2.02	1442	7.058
6	390	0.1333	374	411	389	1.27	236	1.116
7	430	2.2518	411	501	430	1.93	4051	18.857
8	575	0.0976	562	644	586	2.60	68	0.817
9	690	0.0443	677	705	687	0.70	84	0.371
10	827	0.1105	805	905	850	2.86	85	0.926
11	939	0.0613	905	987	941	2.17	62	0.513
12	1345	0.0878	1256	1456	1346	3.34	108	0.735
13	1789	0.2115	1539	1942	1750	4.80	271	1.771
14	2201	0.0685	1942	2486	2124	5.13	79	0.574
15	4724	0.2066	3679	4897	4578	5.71	171	1.730
16	5223	3.7493	4897	7401	5368	8.11	5845	31.397
17	7934	0.2945	7401	9600	8297	7.25	154	2.466
18	10000	0.1501	9600	15000	11222	12.94	78	1.257
19	20000 (UM)	0.2513	18781	28705	20647	4.34	2398	25.249

TIC:	9.9443	ng/uL
TIM:	42.4266	nmole/L
Total concentration:	10.3059	ng/uL

```
Sample peak width (sec): 5    Sample min peak height: 50    Sample baseline V to V?: Y    Sample baseline V to V points: 3
Sample filter: Binomial       Number of points for filter: 3  Sample start region (min): 0  Sample end region (min): 30
Marker peak width (sec): 5    Marker min peak height: 200   Marker baseline V to V?: Y    Marker baseline V to V points: 3
Lower marker selection: First peak > 500 RFU                Upper marker selection: Last peak > 200 RFU
Ladder size (bp)75, 200, 300, 400, 500, 700, 1000, 1500, 2000, 3000, 4000, 5000, 7000, 10000, 20000
Quantification using: Lower Marker          Final concentration (ng/uL): 0.5000          Dilution factor: 12.0
```

Sample: SampH6
Well location: H6
Created: Wednesday, October 20, 2021 5:18:46 PM

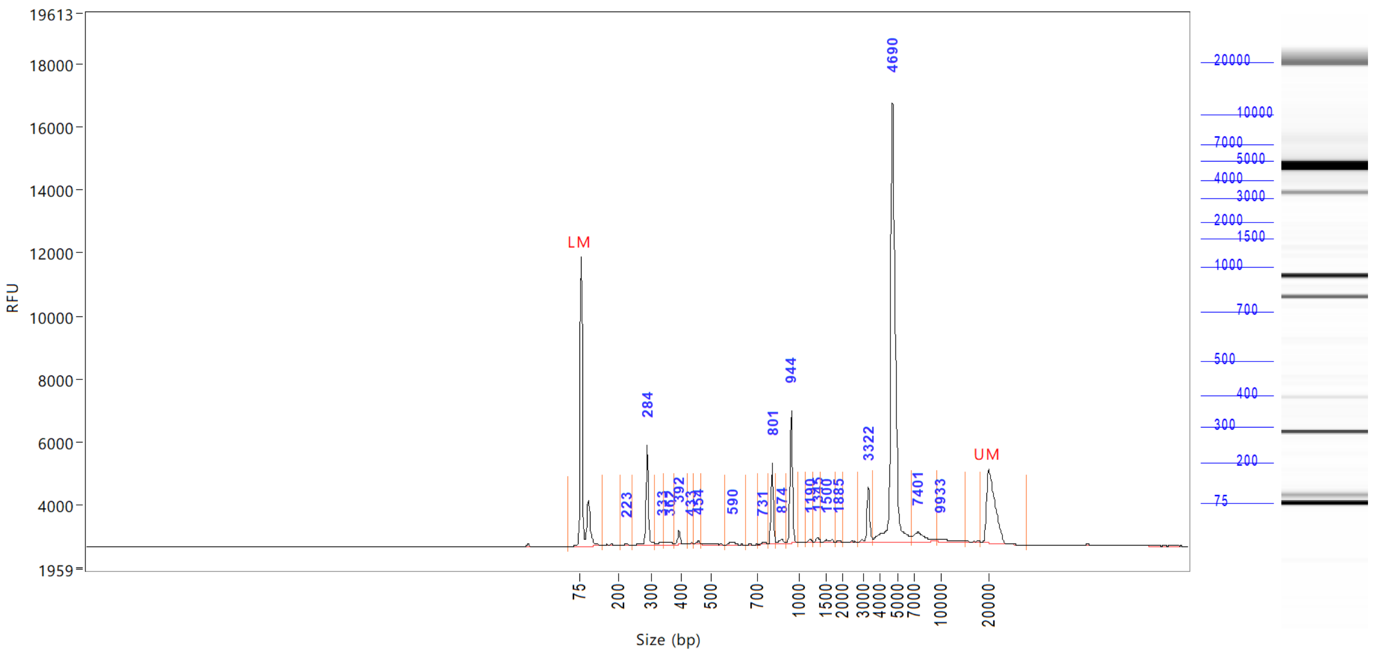

Peak	Size (bp)	Concentration (ng/uL)	From (bp)	To (bp)	Average size (bp)	CV%	RFU	Corrected peak area
1	75 (LM)	0.5000	33	147	79	14.83	9200	62.986
2	223	0.0471	203	241	220	3.14	59	0.494
3	284	1.5473	241	311	283	2.31	3192	16.243
4	333	0.1000	311	343	329	2.84	84	1.049
5	362	0.1083	343	376	358	2.48	88	1.137
6	392	0.2328	376	422	390	1.53	470	2.444
7	433	0.0294	422	439	432	0.87	67	0.309
8	454	0.0653	439	464	450	1.16	106	0.685
9	590	0.0998	555	649	587	2.88	105	1.047
10	731	0.0452	697	770	735	1.97	70	0.475
11	801	0.9962	770	827	795	1.00	2584	10.458
12	874	0.0943	827	900	860	1.92	134	0.990
13	944	1.6793	900	991	939	1.07	4193	17.628
14	1190	0.0470	1123	1256	1185	2.13	99	0.493
15	1345	0.0794	1256	1411	1331	2.39	159	0.833
16	1500	0.0817	1411	1789	1565	6.07	86	0.858
17	1885	0.0343	1789	2058	1884	2.76	63	0.360
18	3322	0.7209	2743	3572	3279	3.44	1721	7.567
19	4690	9.1195	3572	6778	4744	6.47	13956	95.735
20	7401	0.4631	6778	9600	7793	9.30	295	4.862
21	9933	0.1429	9600	15000	11401	13.61	83	1.500
22	20000 (UM)	0.1993	18293	27738	20564	4.37	2336	25.106

TIC:	15.7338	ng/uL
TIM:	21.1672	nmole/L

```
Sample peak width (sec): 5    Sample min peak height: 50    Sample baseline V to V?: Y    Sample baseline V to V points: 3
Sample filter: Binomial       Number of points for filter: 3  Sample start region (min): 0  Sample end region (min): 30
Marker peak width (sec): 5    Marker min peak height: 200   Marker baseline V to V?: Y    Marker baseline V to V points: 3
Lower marker selection: First peak > 500 RFU                Upper marker selection: Last peak > 200 RFU
Ladder size (bp)75, 200, 300, 400, 500, 700, 1000, 1500, 2000, 3000, 4000, 5000, 7000, 10000, 20000
Quantification using: Lower Marker          Final concentration (ng/uL): 0.5000          Dilution factor: 12.0
```

Sample: SampH6
Well location: H6
Created: Wednesday, October 20, 2021 5:18:46 PM

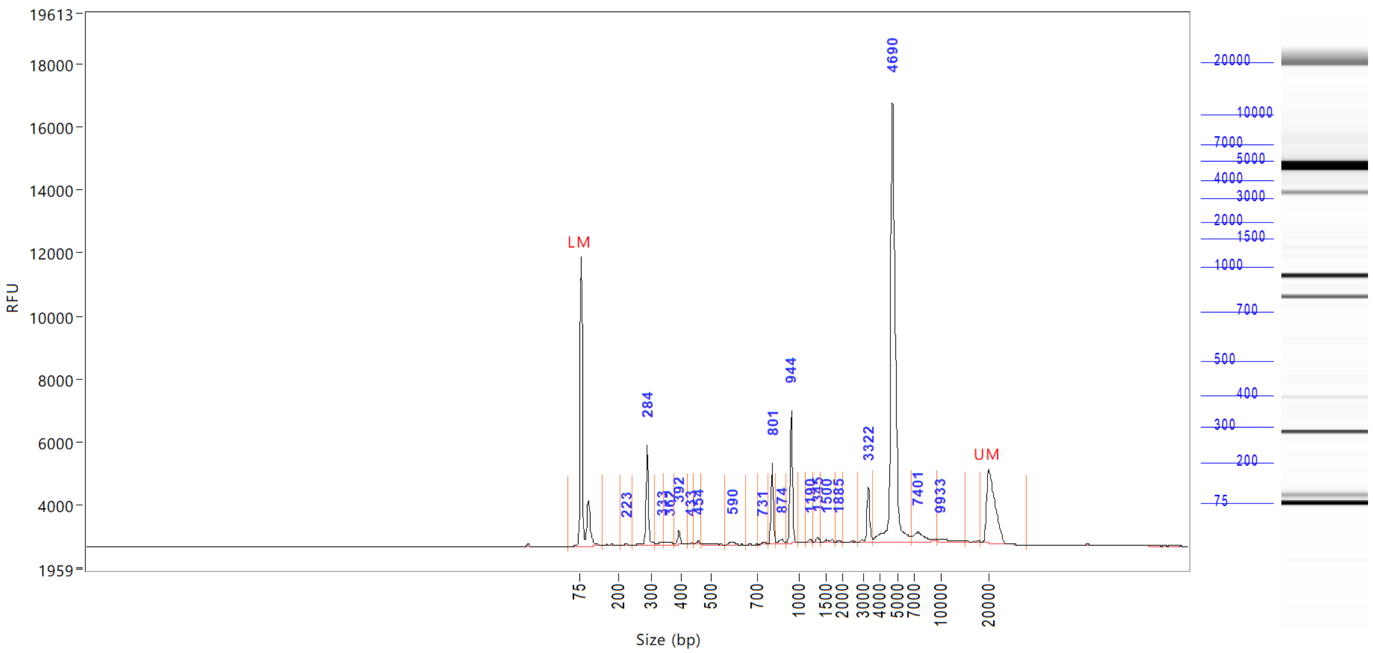

Peak	Size (bp)	Concentration (ng/uL)	From (bp)	To (bp)	Average size (bp)	CV%	RFU	Corrected peak area
	Total concentration:		15.9027	ng/uL				

```
Sample peak width (sec): 5    Sample min peak height: 50    Sample baseline V to V?: Y    Sample baseline V to V points: 3
Sample filter: Binomial       Number of points for filter: 3  Sample start region (min): 0  Sample end region (min): 30
Marker peak width (sec): 5    Marker min peak height: 200   Marker baseline V to V?: Y    Marker baseline V to V points: 3
Lower marker selection: First peak > 500 RFU                Upper marker selection: Last peak > 200 RFU
Ladder size (bp)75, 200, 300, 400, 500, 700, 1000, 1500, 2000, 3000, 4000, 5000, 7000, 10000, 20000
Quantification using: Lower Marker          Final concentration (ng/uL): 0.5000          Dilution factor: 12.0
```

Sample: SampH7
Well location: H7
Created: Wednesday, October 20, 2021 5:18:46 PM

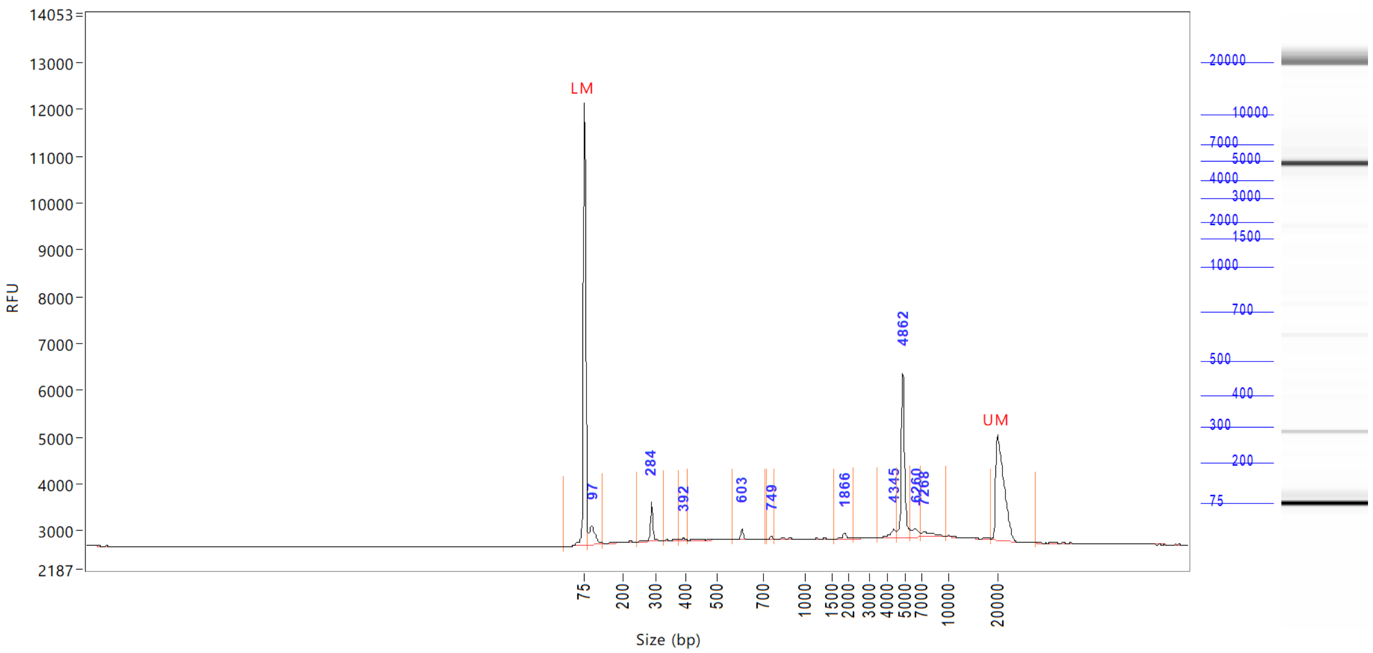

Peak	Size (bp)	Concentration (ng/uL)	From (bp)	To (bp)	Average size (bp)	CV%	RFU	Corrected peak area
1	75 (LM)	0.5000	9	86	73	6.43	9456	51.179
2	97	0.6812	86	135	100	10.20	406	5.811
3	284	0.5498	239	323	283	3.41	841	4.690
4	392	0.0382	374	407	389	1.67	53	0.326
5	603	0.1619	565	701	606	3.38	239	1.381
6	749	0.0399	714	775	744	1.20	77	0.341
7	1866	0.0929	1559	2229	1830	5.88	123	0.793
8	4345	0.1780	3429	4518	4238	5.09	173	1.518
9	4862	2.0787	4518	5667	4887	3.25	3496	17.731
10	6260	0.2090	5667	6926	6217	5.73	171	1.783
11	7268	0.2126	6926	9667	7812	8.16	100	1.813
12	20000 (UM)	0.2298	18781	27738	20664	4.34	2240	23.526

TIC:	4.2422	ng/uL
TIM:	16.0245	nmole/L
Total concentration:	4.6221	ng/uL

Sample peak width (sec): 5 Sample min peak height: 50 Sample baseline V to V?: Y Sample baseline V to V points: 3
Sample filter: Binomial Number of points for filter: 3 Sample start region (min): 0 Sample end region (min): 30
Marker peak width (sec): 5 Marker min peak height: 200 Marker baseline V to V?: Y Marker baseline V to V points: 3
Lower marker selection: First peak > 500 RFU Upper marker selection: Last peak > 200 RFU
Ladder size (bp)75, 200, 300, 400, 500, 700, 1000, 1500, 2000, 3000, 4000, 5000, 7000, 10000, 20000
Quantification using: Lower Marker Final concentration (ng/uL): 0.5000 Dilution factor: 12.0

Sample: SampH8
Well location: H8
Created: Wednesday, October 20, 2021 5:18:46 PM

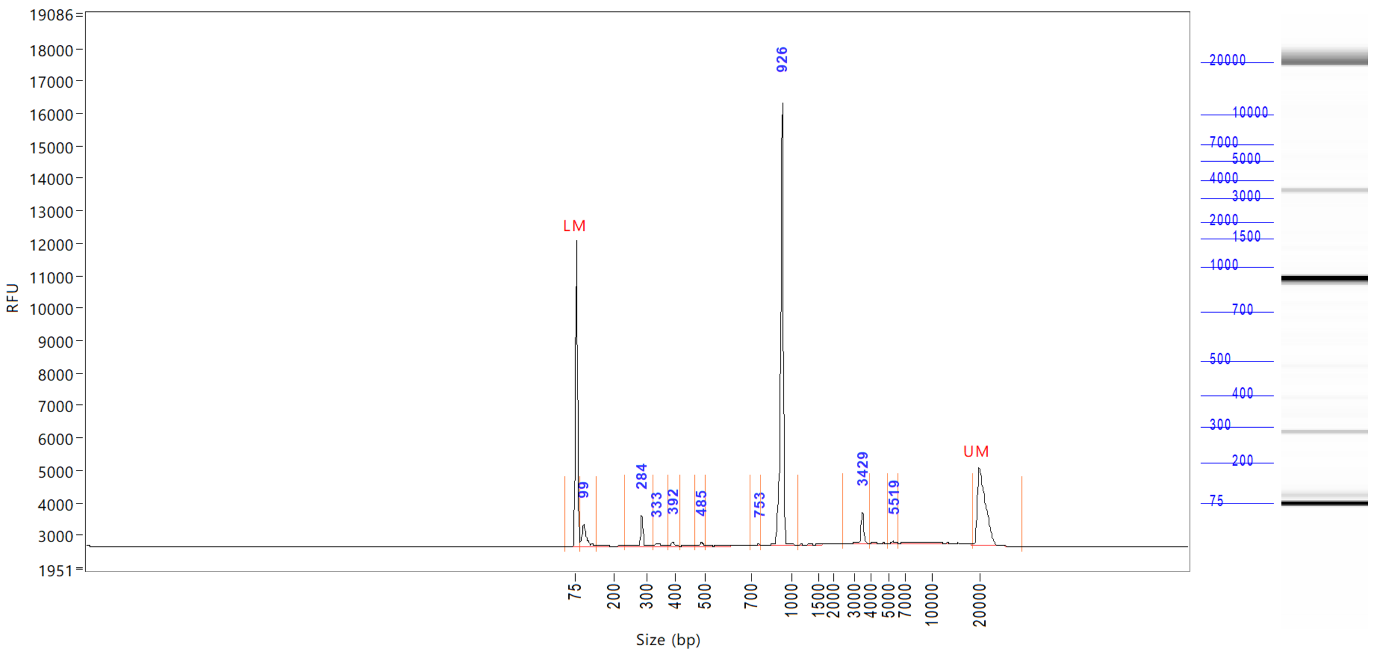

Peak	Size (bp)	Concentration (ng/uL)	From (bp)	To (bp)	Average size (bp)	CV%	RFU	Corrected peak area
1	75 (LM)	0.5000	41	86	73	4.40	9443	49.946
2	99	0.8259	86	143	100	10.30	660	6.875
3	284	0.6124	232	321	282	3.30	943	5.098
4	333	0.1235	321	372	342	3.60	78	1.028
5	392	0.0926	372	414	389	1.58	135	0.771
6	485	0.0878	466	500	483	1.26	127	0.731
7	753	0.0387	697	770	738	2.01	51	0.322
8	926	7.4996	770	1123	918	1.65	13655	62.429
9	3429	0.5579	2401	3893	3333	7.10	950	4.644
10	5519	0.0466	4931	6260	5483	5.31	59	0.388
11	20000 (UM)	0.2545	18537	28947	20658	4.45	2373	25.426

TIC:	9.8851	ng/uL
TIM:	32.2412	nmole/L
Total concentration:	10.4427	ng/uL

```
Sample peak width (sec): 5     Sample min peak height: 50     Sample baseline V to V?: Y     Sample baseline V to V points: 3
Sample filter: Binomial        Number of points for filter: 3  Sample start region (min): 0  Sample end region (min): 30
Marker peak width (sec): 5     Marker min peak height: 200    Marker baseline V to V?: Y     Marker baseline V to V points: 3
Lower marker selection: First peak > 500 RFU                  Upper marker selection: Last peak > 200 RFU
Ladder size (bp)75, 200, 300, 400, 500, 700, 1000, 1500, 2000, 3000, 4000, 5000, 7000, 10000, 20000
Quantification using: Lower Marker          Final concentration (ng/uL): 0.5000          Dilution factor: 12.0
```

Sample: SampH9
Well location: H9
Created: Wednesday, October 20, 2021 5:18:46 PM

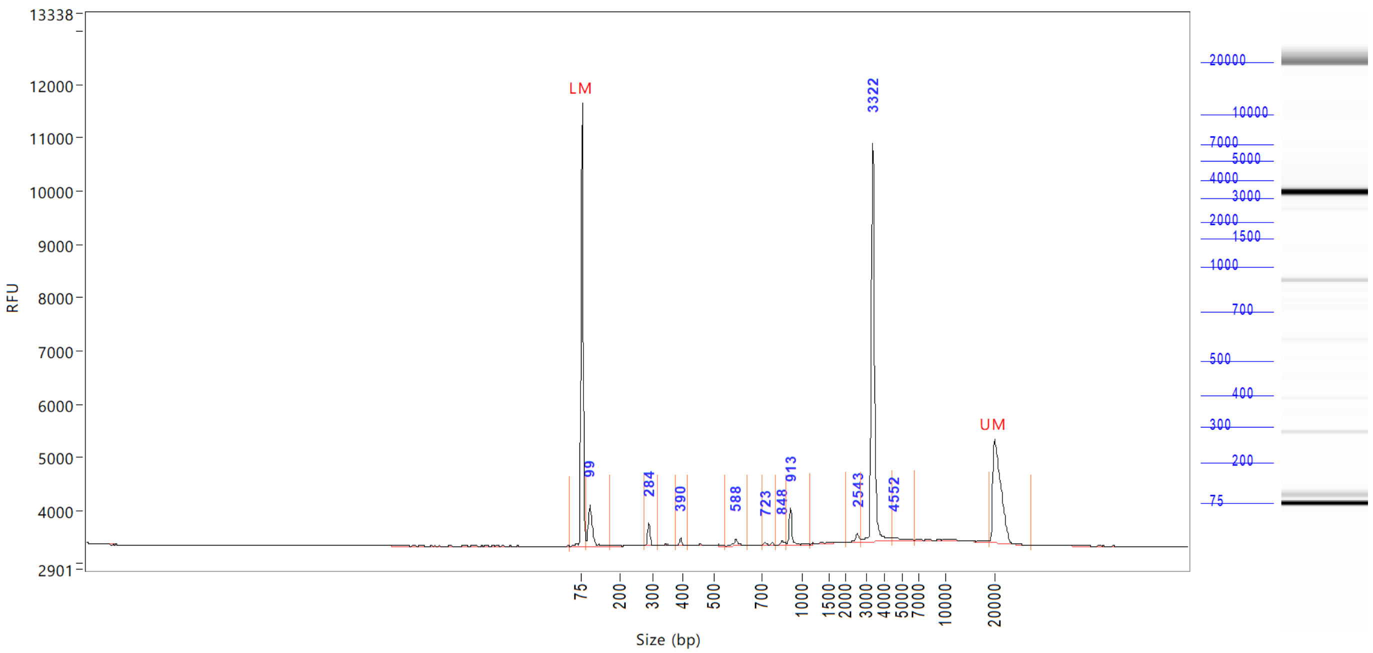

Peak	Size (bp)	Concentration (ng/uL)	From (bp)	To (bp)	Average size (bp)	CV%	RFU	Corrected peak area
1	75 (LM)	0.5000	35	86	73	4.85	8335	43.561
2	99	1.0415	86	164	101	10.56	782	7.561
3	284	0.3066	271	311	284	2.16	430	2.226
4	390	0.0944	376	411	389	1.14	140	0.685
5	588	0.1540	547	639	588	2.49	137	1.118
6	723	0.0879	701	801	745	3.53	63	0.638
7	848	0.0855	801	879	849	1.97	89	0.621
8	913	0.4696	879	1134	915	3.12	694	3.409
9	2543	0.1236	2000	2743	2507	5.38	155	0.897
10	3322	4.9462	2743	4414	3332	5.33	7485	35.910
11	4552	0.1524	4414	6482	4969	10.15	71	1.106
12	20000 (UM)	0.2323	19024	27617	20691	4.59	1957	20.237

TIC:	7.4615	ng/uL
TIM:	23.3190	nmole/L
Total concentration:	8.0555	ng/uL

```
Sample peak width (sec): 5    Sample min peak height: 50    Sample baseline V to V?: Y    Sample baseline V to V points: 3
Sample filter: Binomial       Number of points for filter: 3  Sample start region (min): 0  Sample end region (min): 30
Marker peak width (sec): 5    Marker min peak height: 200   Marker baseline V to V?: Y    Marker baseline V to V points: 3
Lower marker selection: First peak > 500 RFU                Upper marker selection: Last peak > 200 RFU
Ladder size (bp)75, 200, 300, 400, 500, 700, 1000, 1500, 2000, 3000, 4000, 5000, 7000, 10000, 20000
Quantification using: Lower Marker          Final concentration (ng/uL): 0.5000          Dilution factor: 12.0
```

Sample: SampH10
Well location: H10
Created: Wednesday, October 20, 2021 5:18:46 PM

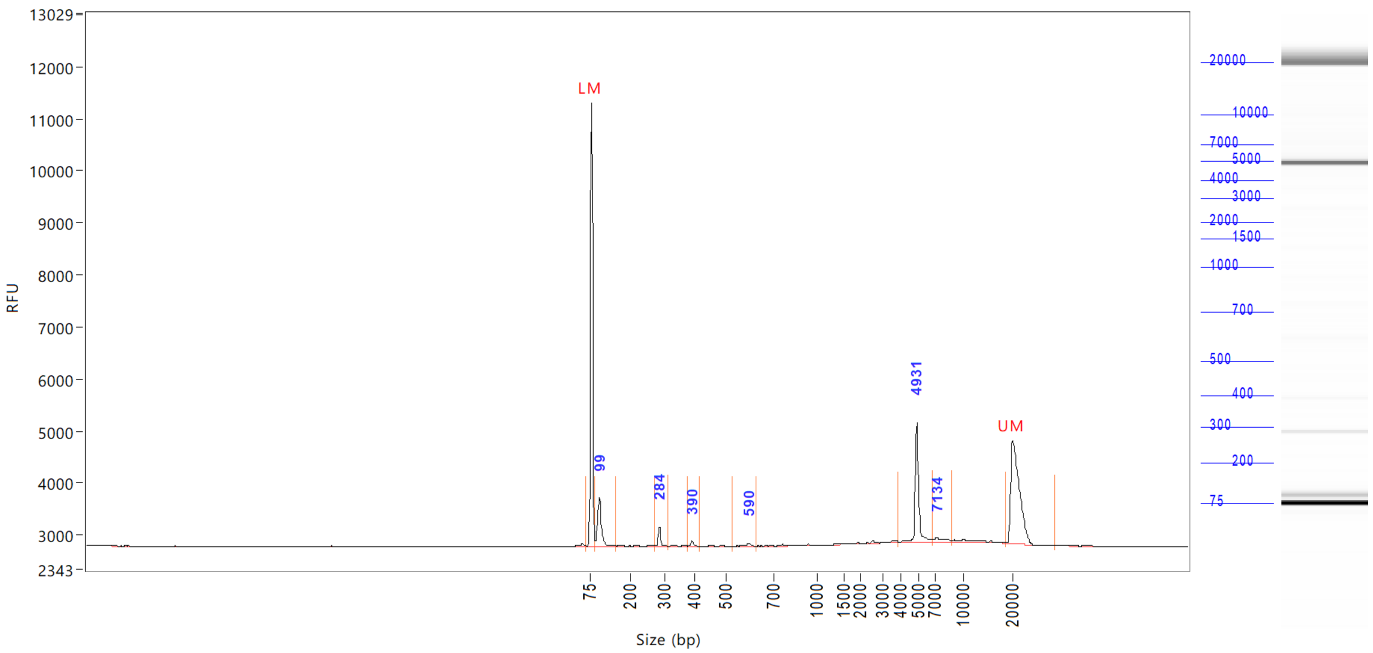

Peak	Size (bp)	Concentration (ng/uL)	From (bp)	To (bp)	Average size (bp)	CV%	RFU	Corrected peak area
1	75 (LM)	0.5000	62	87	74	4.20	8537	45.090
2	99	1.0576	87	154	100	8.50	925	7.948
3	284	0.2462	273	309	283	1.41	377	1.850
4	390	0.0804	376	416	388	1.43	115	0.604
5	590	0.0893	519	621	580	3.46	63	0.671
6	4931	1.5376	3893	6704	4969	6.54	2287	11.555
7	7134	0.1370	6704	8800	7590	7.47	69	1.029
8	20000 (UM)	0.2380	18659	28463	20633	4.48	1991	21.461

TIC:	3.1481	ng/uL
TIM:	19.9314	nmole/L
Total concentration:	3.7086	ng/uL

```
Sample peak width (sec): 5    Sample min peak height: 50    Sample baseline V to V?: Y    Sample baseline V to V points: 3
Sample filter: Binomial       Number of points for filter: 3  Sample start region (min): 0  Sample end region (min): 30
Marker peak width (sec): 5    Marker min peak height: 200   Marker baseline V to V?: Y    Marker baseline V to V points: 3
Lower marker selection: First peak > 500 RFU                Upper marker selection: Last peak > 200 RFU
Ladder size (bp)75, 200, 300, 400, 500, 700, 1000, 1500, 2000, 3000, 4000, 5000, 7000, 10000, 20000
Quantification using: Lower Marker          Final concentration (ng/uL): 0.5000          Dilution factor: 12.0
```

Sample: SampH11
Well location: H11
Created: Wednesday, October 20, 2021 5:18:46 PM

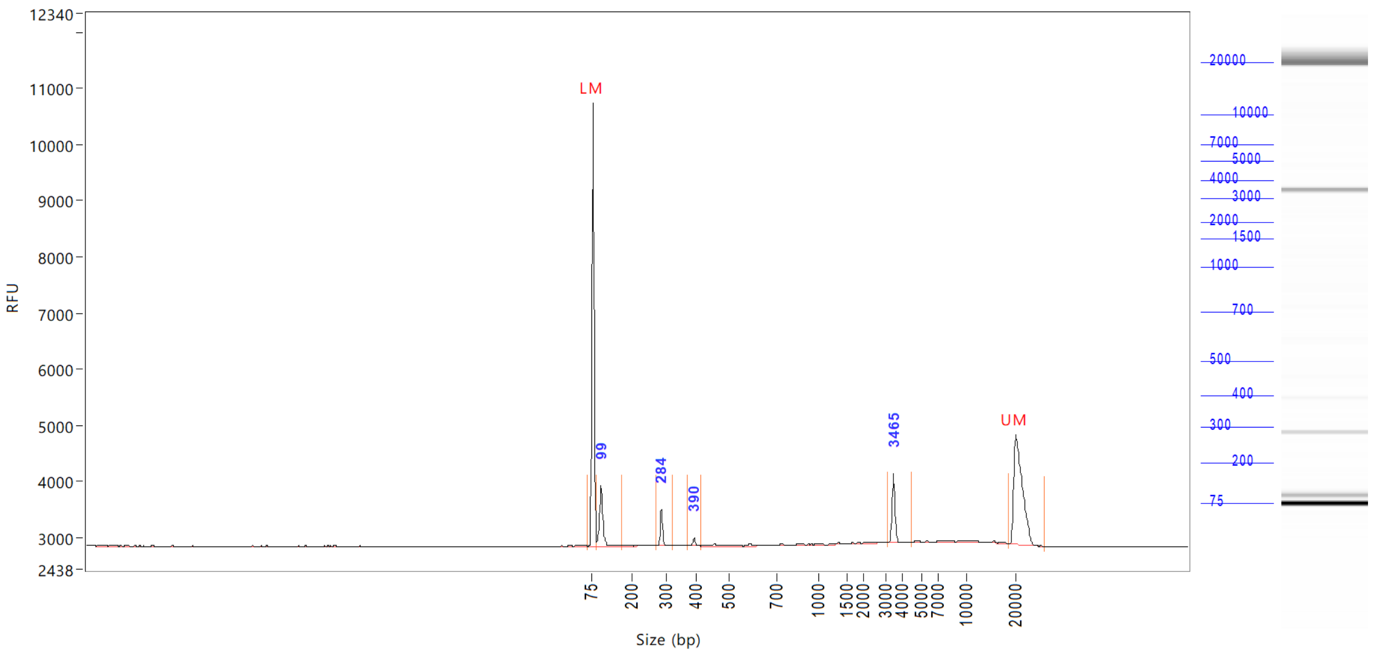

Peak	Size (bp)	Concentration (ng/uL)	From (bp)	To (bp)	Average size (bp)	CV%	RFU	Corrected peak area
1	75 (LM)	0.5000	58	86	73	4.25	7907	40.628
2	99	1.2394	86	166	99	9.37	1067	8.393
3	284	0.4543	269	319	282	1.83	640	3.076
4	390	0.1001	370	411	388	1.32	140	0.678
5	3465	0.7759	3108	4449	3454	3.89	1218	5.254
6	20000 (UM)	0.2546	18659	25803	20587	4.19	1951	20.684

TIC:	2.5697	ng/uL
TIM:	24.0017	nmole/L
Total concentration:	3.3622	ng/uL

```
Sample peak width (sec): 5    Sample min peak height: 50    Sample baseline V to V?: Y    Sample baseline V to V points: 3
Sample filter: Binomial       Number of points for filter: 3  Sample start region (min): 0  Sample end region (min): 30
Marker peak width (sec): 5    Marker min peak height: 200   Marker baseline V to V?: Y    Marker baseline V to V points: 3
Lower marker selection: First peak > 500 RFU                Upper marker selection: Last peak > 200 RFU
Ladder size (bp)75, 200, 300, 400, 500, 700, 1000, 1500, 2000, 3000, 4000, 5000, 7000, 10000, 20000
Quantification using: Lower Marker          Final concentration (ng/uL): 0.5000          Dilution factor: 12.0
```

Sample: SampH12
Well location: H12
Created: Wednesday, October 20, 2021 5:18:46 PM

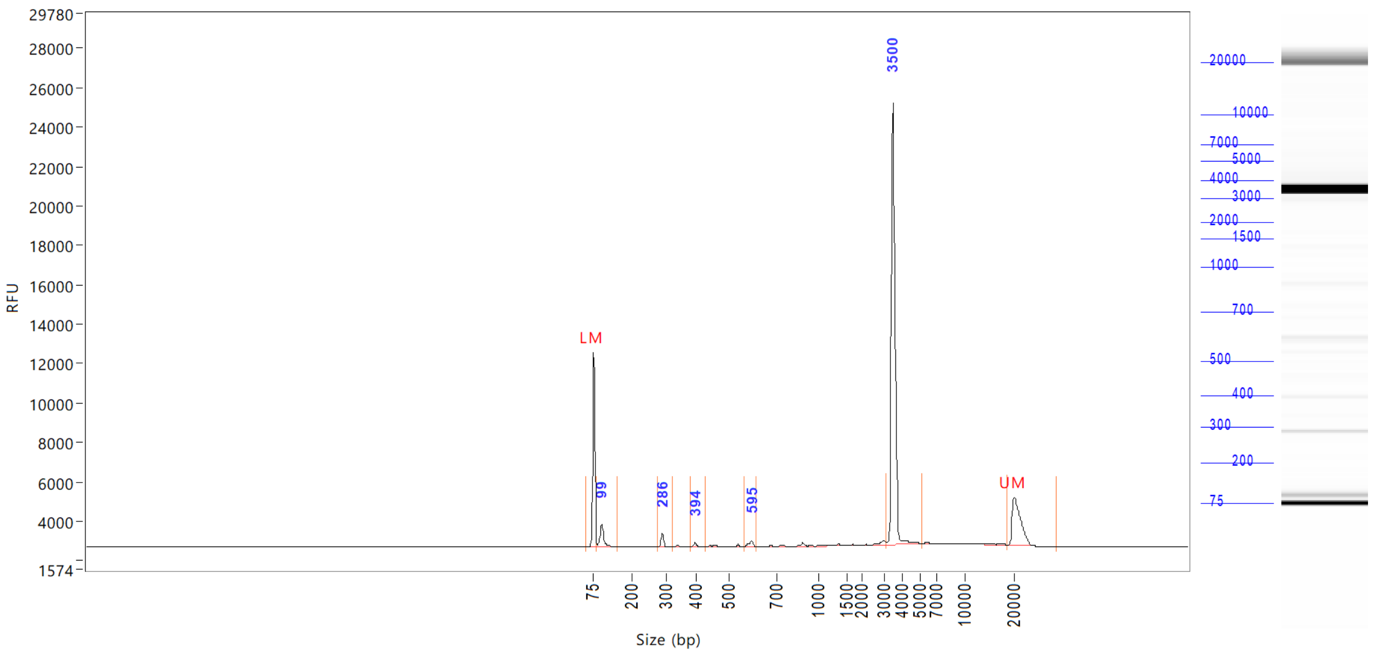

Peak	Size (bp)	Concentration (ng/uL)	From (bp)	To (bp)	Average size (bp)	CV%	RFU	Corrected peak area
1	75 (LM)	0.5000	52	86	73	4.12	9865	50.525
2	99	1.1647	86	152	99	9.40	1140	9.808
3	286	0.3918	273	317	286	2.21	657	3.299
4	394	0.1353	378	424	392	1.38	227	1.140
5	595	0.2674	562	616	587	1.61	328	2.252
6	3500	14.4074	3108	5223	3510	5.08	22444	121.323
7	20000 (UM)	0.2513	18659	28584	20638	4.30	2466	25.391

TIC:	16.3666	ng/uL
TIM:	29.6464	nmole/L
Total concentration:	17.6689	ng/uL

Sample peak width (sec): 5 Sample min peak height: 200 Sample baseline V to V?: Y Sample baseline V to V points: 3
Sample filter: Binomial Number of points for filter: 3 Sample start region (min): 0 Sample end region (min): 30
Marker peak width (sec): 5 Marker min peak height: 200 Marker baseline V to V?: Y Marker baseline V to V points: 3
Lower marker selection: First peak > 500 RFU Upper marker selection: Last peak > 200 RFU
Ladder size (bp)75, 200, 300, 400, 500, 700, 1000, 1500, 2000, 3000, 4000, 5000, 7000, 10000, 20000
Quantification using: Lower Marker Final concentration (ng/uL): 0.5000 Dilution factor: 12.0

Sample: DNA size ladder
Well location: A7
Created: Wednesday, October 20, 2021 5:18:46 PM
Import from: C:\Users\admin\Desktop\210810_alberto_ladder.SCAL
Fit type: Point to point

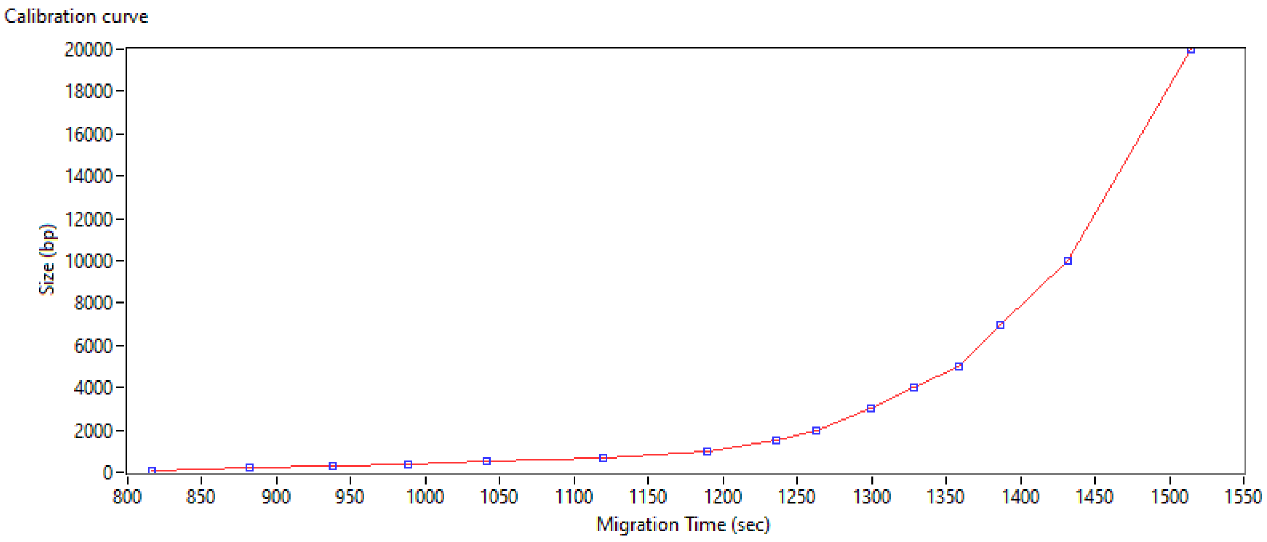

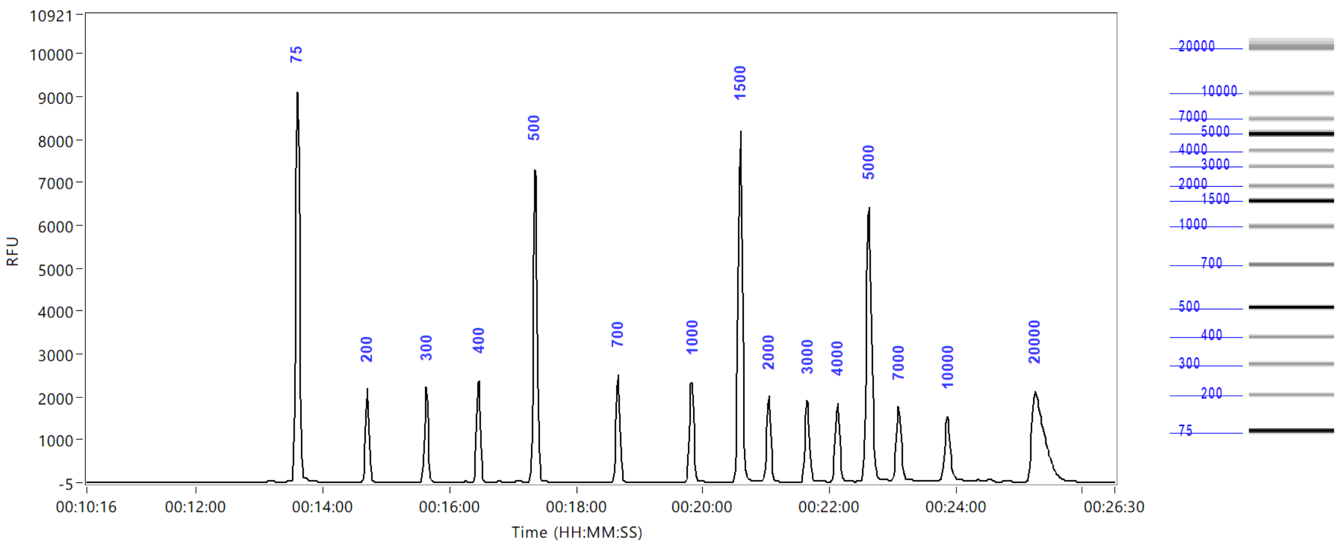

Supplement: Supplementary file 2 — sb3c00292_si_002.zip [file sb3c00292_si_002.zip › dnada_supplementary_material_pks_library_build/colony_pcr/ZAG/17-09-04_plate2/colonypcrplate_2.pdf]
