# Supplementary material for: Automated Platform for the Plasmid Construction Process
Source: ACS Synth Biol. 2023 Nov 10;12(12):3506–13. doi: 10.1021/acssynbio.3c00292 (PMC10729297; doi:10.1021/acssynbio.3c00292)

Instrument controller software run summary:

Filename and data path: C:\AATI\Data\2021 10 21\17-18-29\2021 10 21 17H 18M..raw
Created: Thursday, October 21, 2021 5:28:30 PM
Number of capillaries: 96
Array serial number: 111920-09SFS
Effect length: 33 cm
Array usage count: 265
Instrument type: ZAG DNA Analyzer
Instrument controller software version: 1.0.1.9
Device serial number: Z7597

Method Information

Method name: ZAG130GP33 - DNA 75-20000bp - Gel Prime Only.mthds
Gel prime: Yes
Full conditioning: No
Gel prime to buffer: No
Gel selection: Gel 1
Perform prerun: 8.0 kV, 30 sec.
Rinse: No
Marker 1: Row: A, 5.0 kV, 5 sec.
Rinse: No
Sample injection: 5.0 kV, 5 sec.
Separation: 8.0 kV, 30.0 min.
Tray name: Sample Tray-1

Analysis mode: DNA

Notes

Gel Image

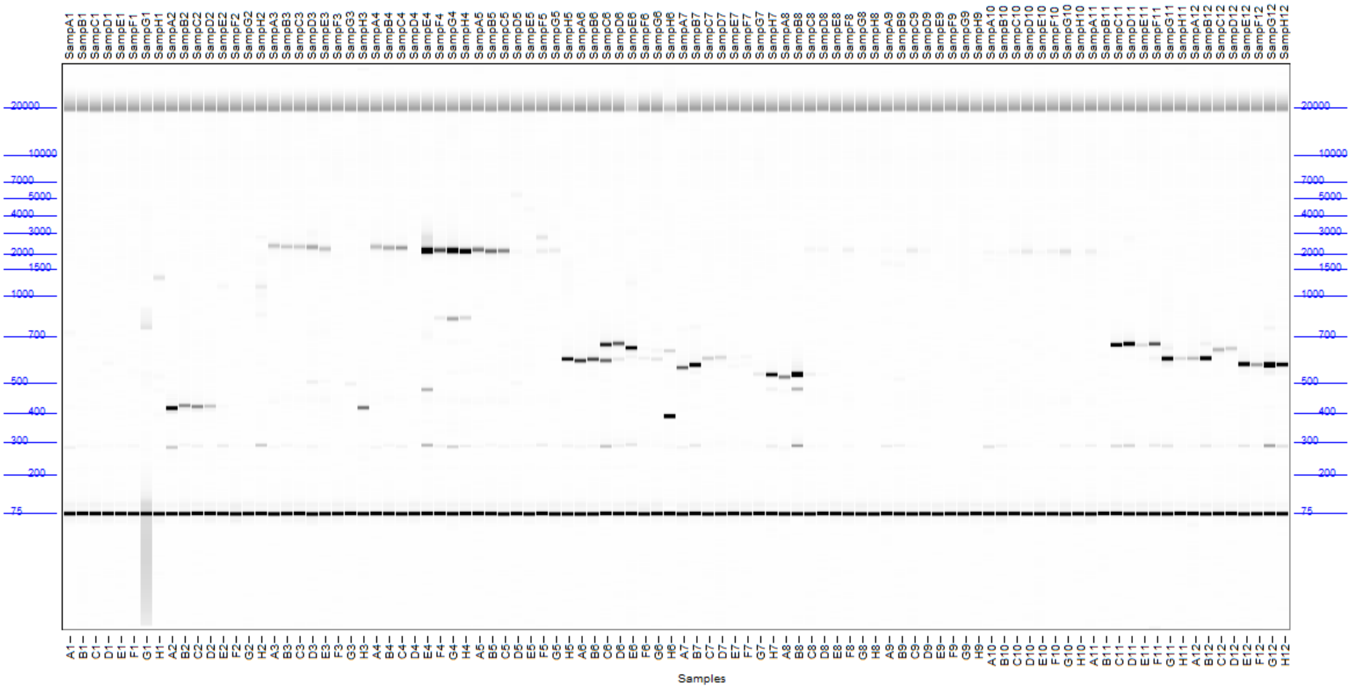
SampA1 SampB1 SampC1 SampD1 SampE1 SampF1 SampG1 SampH1 SampA2 SampB2 SampC2 SampD2 SampE2 SampF2 SampG2 SampH2 SampA3 SampB3 SampC3 SampD3 SampE3 SampF3 SampG3 SampH3 SampA4 SampB4 SampC4 SampD4 SampE4 SampF4 SampG4 SampH4 SampA5 SampB5 SampC5 SampD5 SampE5 SampF5 SampG5 SampH5 SampA6 SampB6 SampC6 SampD6 SampE6 SampF6 SampG6 SampH6 SampA7 SampB7 SampC7 SampD7 SampE7 SampF7 SampG7 SampH7 SampA8 SampB8 SampC8 SampD8 SampE8 SampF8 SampG8 SampH8 SampA9 SampB9 SampC9 SampD9 SampE9 SampF9 SampG9 SampH9 SampA10 SampB10 SampC10 SampD10 SampE10 SampF10 SampG10 SampH10 SampA11 SampB11 SampC11 SampD11 SampE11 SampF11 SampG11 SampH11 SampA12 SampB12 SampC12 SampD12 SampE12 SampF12 SampG12 SampH12
20000
10000
7000
5000
4000
3000
2000
1500
1000
700
500
400
300
200
75
A1 B1 C1 D1 E1 F1 G1 H1 A2 B2 C2 D2 E2 F2 G2 H2 A3 B3 C3 D3 E3 F3 G3 H3 A4 B4 C4 D4 E4 F4 G4 H4 A5 B5 C5 D5 E5 F5 G5 H5 A6 B6 C6 D6 E6 F6 G6 H6 A7 B7 C7 D7 E7 F7 G7 H7 A8 B8 C8 D8 E8 F8 G8 H8 A9 B9 C9 D9 E9 F9 G9 H9 A10 B10 C10 D10 E10 F10 G10 H10 A11 B11 C11 D11 E11 F11 G11 H11 A12 B12 C12 D12 E12 F12 G12 H12
Samples

Filename and data path: C:\AATI\Data\2021 10 21\17-18-29\2021 10 21 17H 18M..raw

A1: SampA1

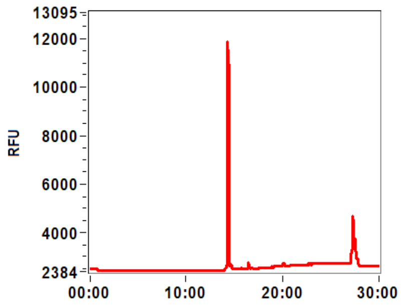

B1: SampB1

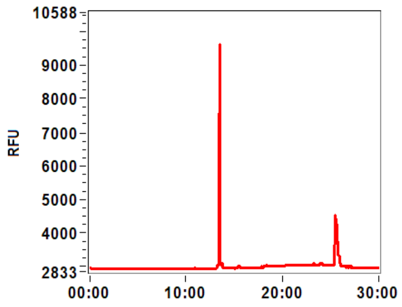

C1: SampC1

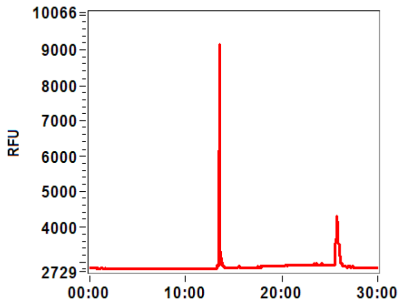

D1: SampD1

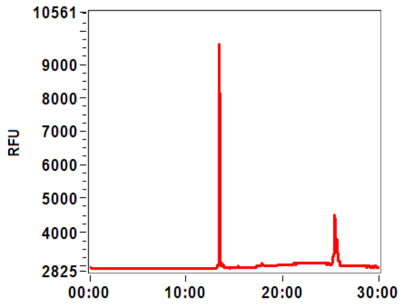

E1: SampE1

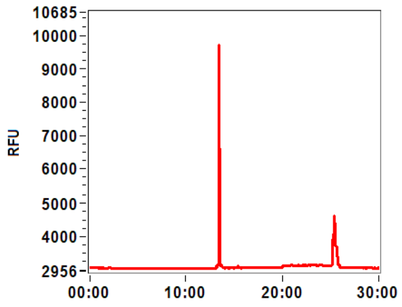

F1: SampF1

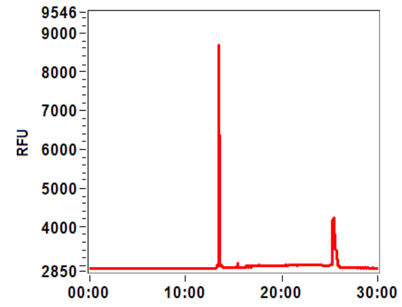

G1: SampG1

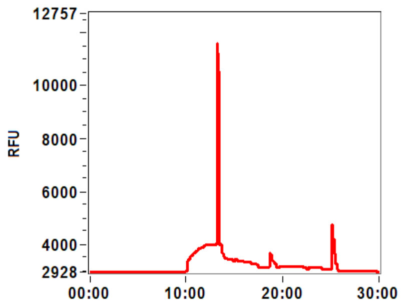

H1: SampH1

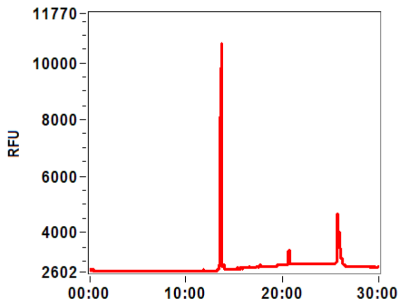

A2: SampA2

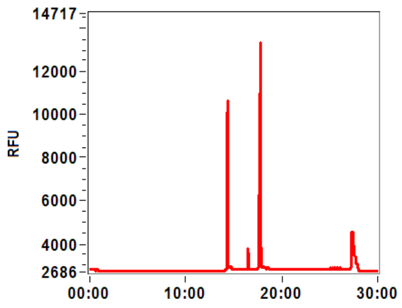

B2: SampB2

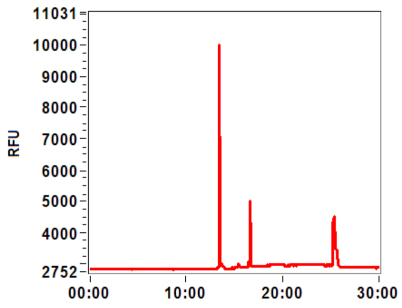

C2: SampC2

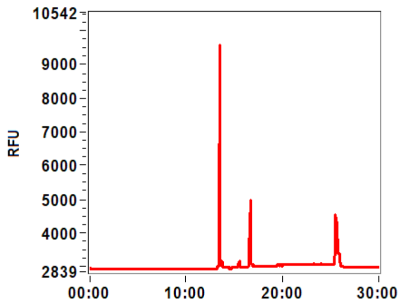

D2: SampD2

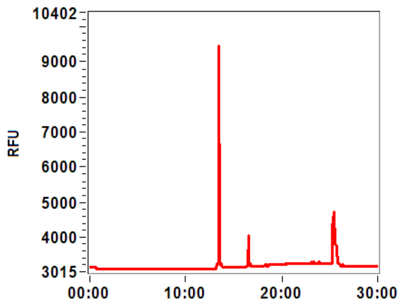

Filename and data path: C:\AATI\Data\2021 10 21\17-18-29\2021 10 21 17H 18M..raw

E2: SampE2

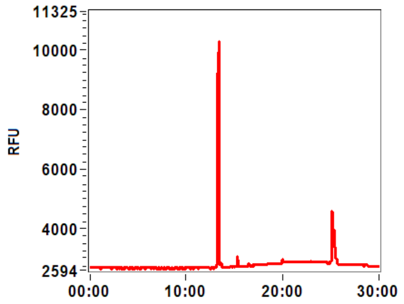

F2: SampF2

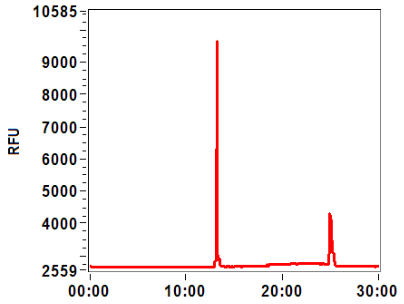

G2: SampG2

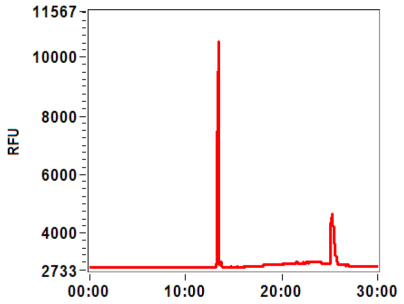

H2: SampH2

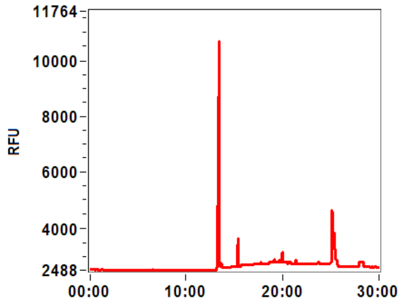

A3: SampA3

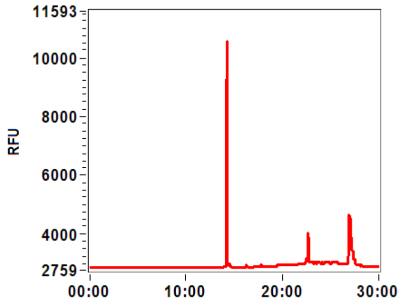

B3: SampB3

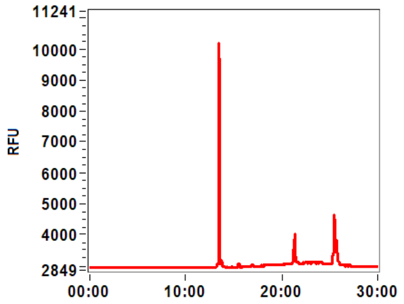

C3: SampC3

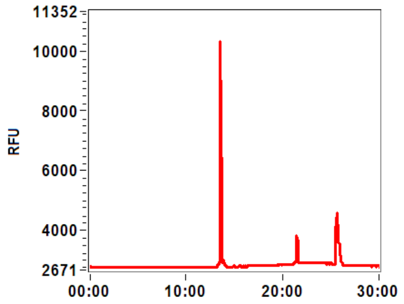

D3: SampD3

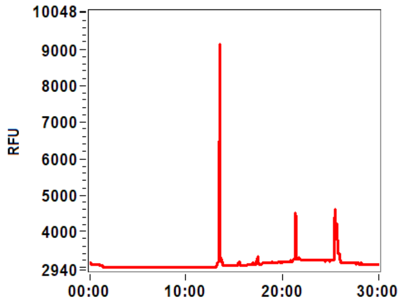

E3: SampE3

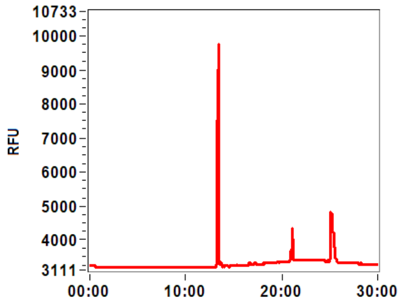

F3: SampF3

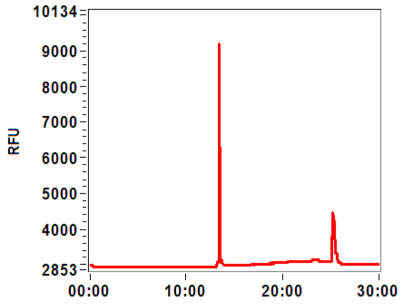

G3: SampG3

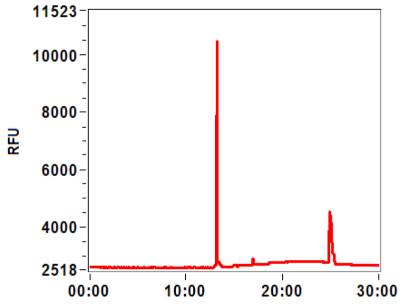

H3: SampH3

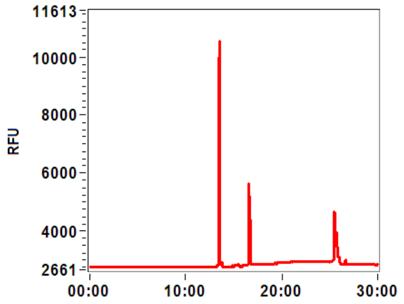

Filename and data path: C:\AATI\Data\2021 10 21\17-18-29\2021 10 21 17H 18M..raw

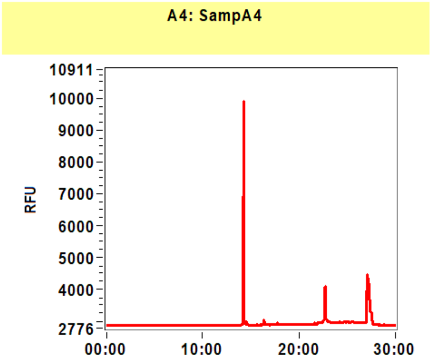

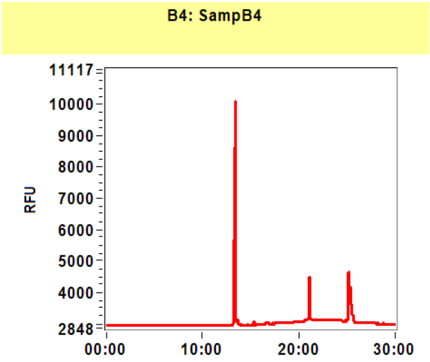

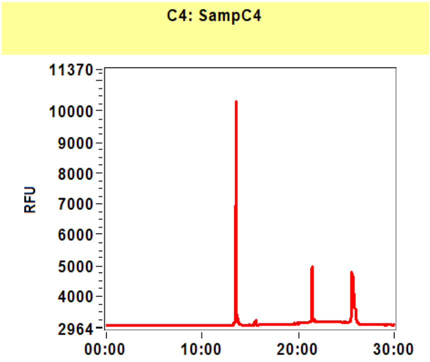

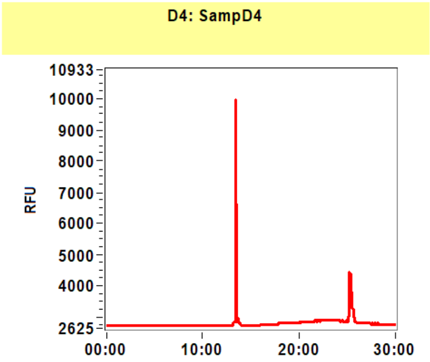

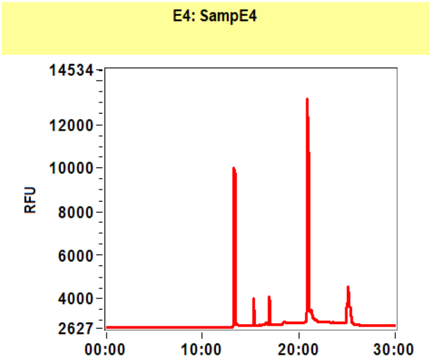

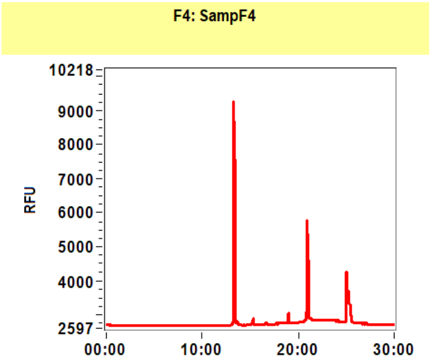

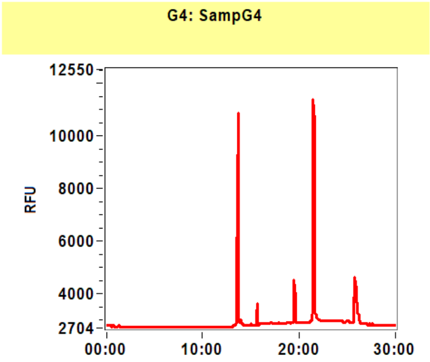

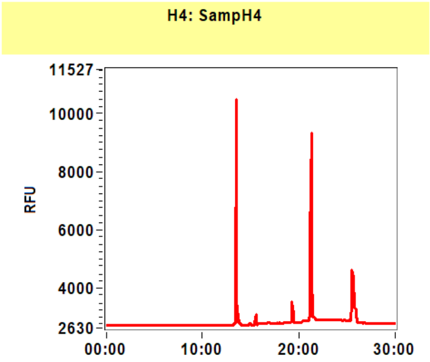

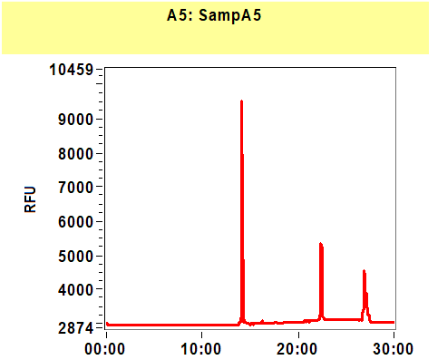

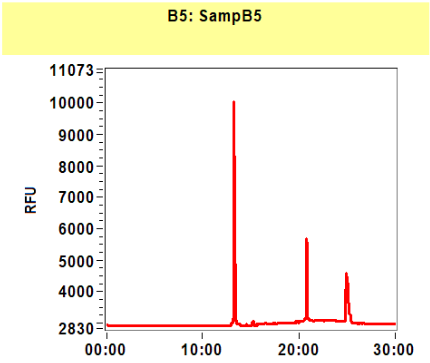

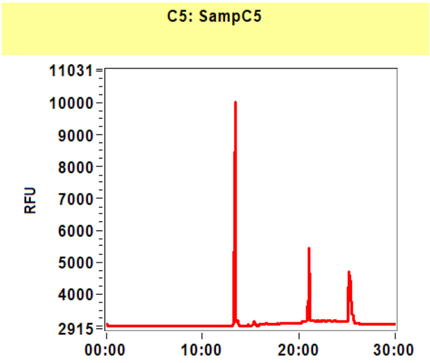

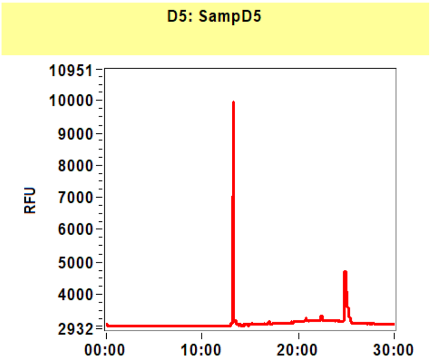

Filename and data path: C:\AATI\Data\2021 10 21\17-18-29\2021 10 21 17H 18M..raw

E5: SampE5

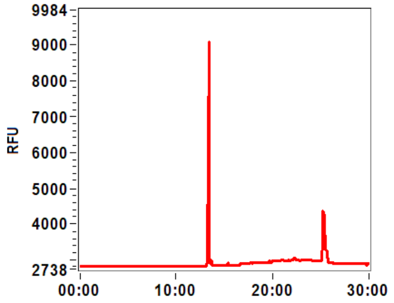

F5: SampF5

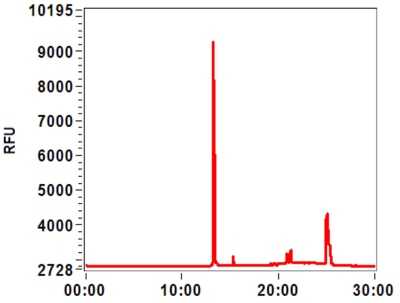

G5: SampG5

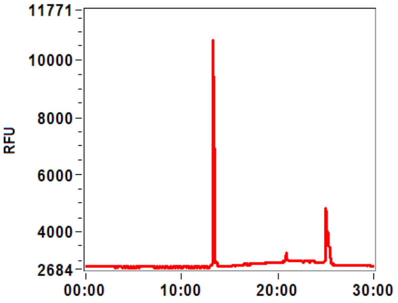

H5: SampH5

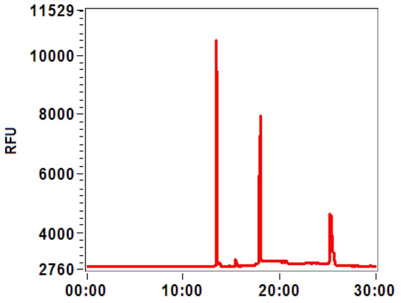

A6: SampA6

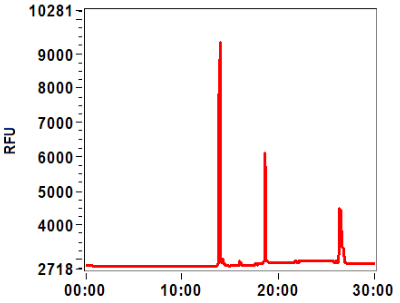

B6: SampB6

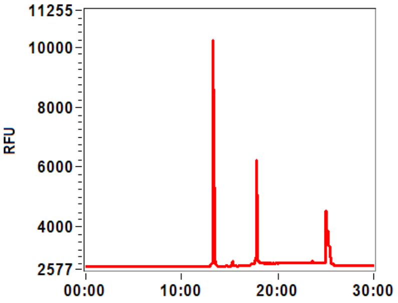

C6: SampC6

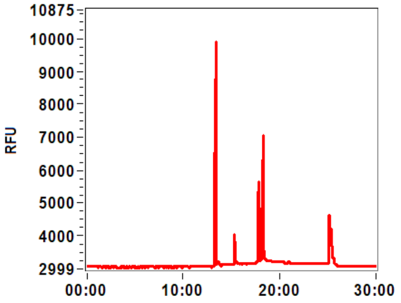

D6: SampD6

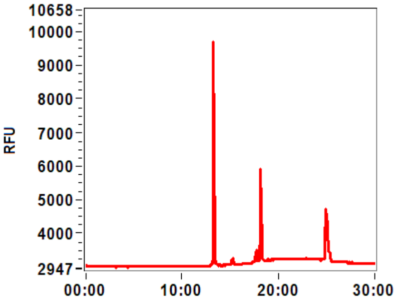

E6: SampE6

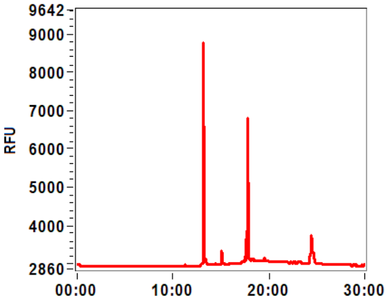

F6: SampF6

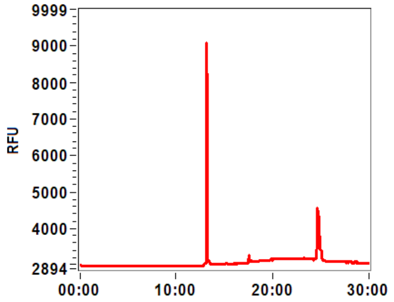

G6: SampG6

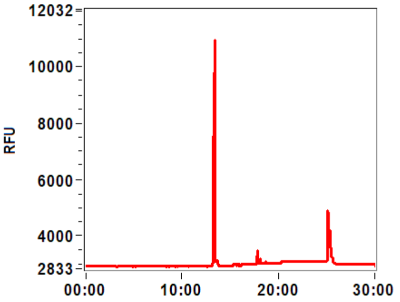

H6: SampH6

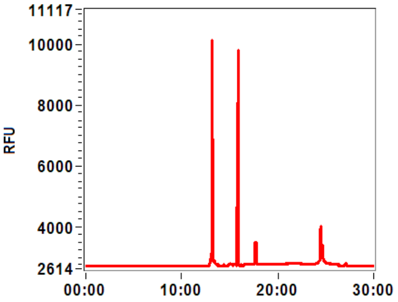

Filename and data path: C:\AATI\Data\2021 10 21\17-18-29\2021 10 21 17H 18M..raw

A7: SampA7

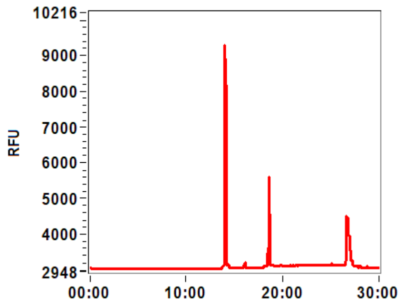

B7: SampB7

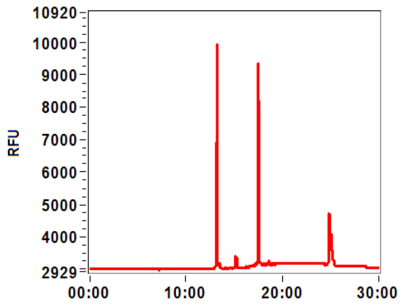

C7: SampC7

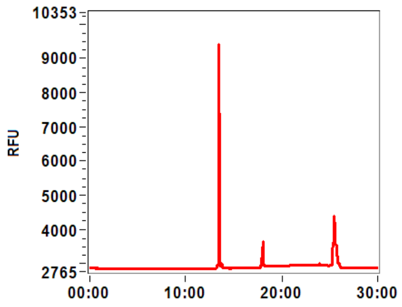

D7: SampD7

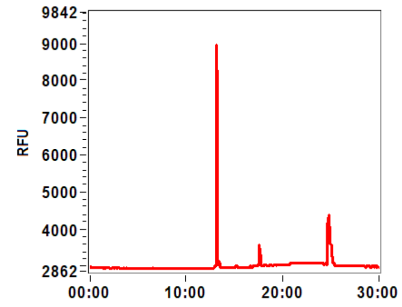

E7: SampE7

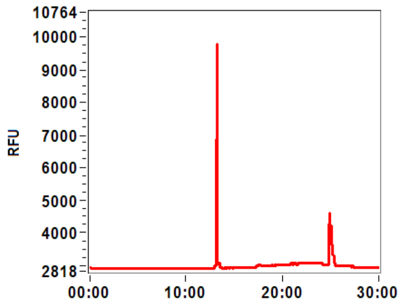

F7: SampF7

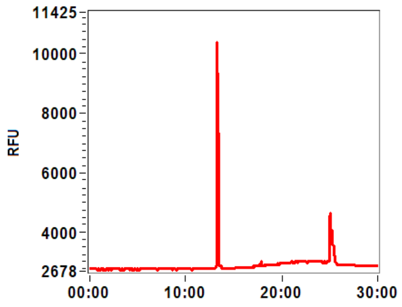

G7: SampG7

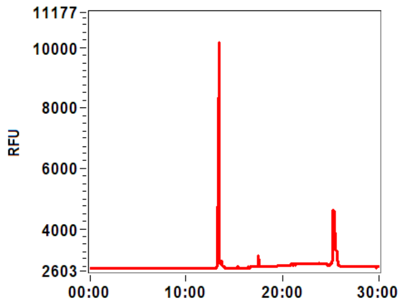

H7: SampH7

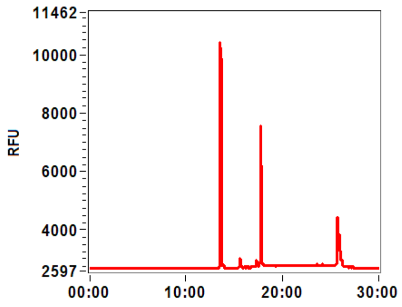

A8: SampA8

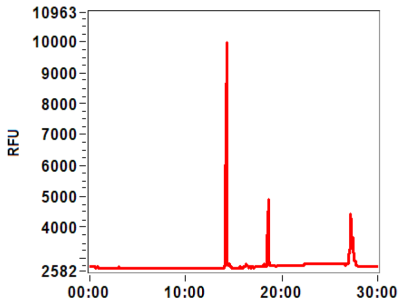

B8: SampB8

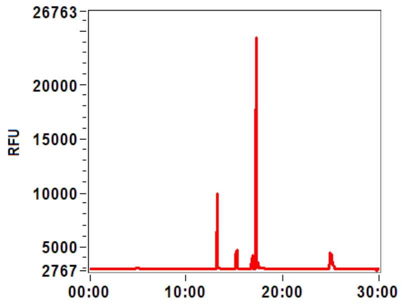

C8: SampC8

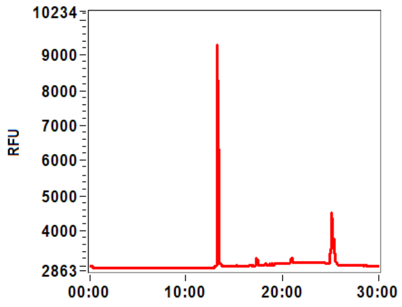

D8: SampD8

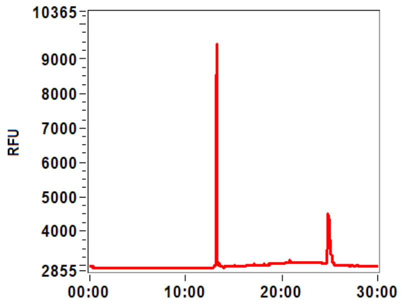

Filename and data path: C:\AATI\Data\2021 10 21\17-18-29\2021 10 21 17H 18M..raw

E8: SampE8

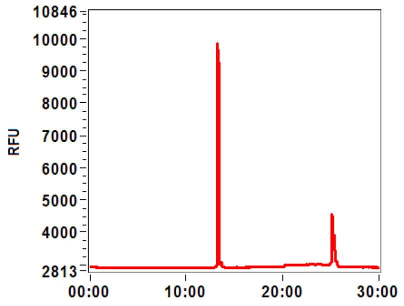

F8: SampF8

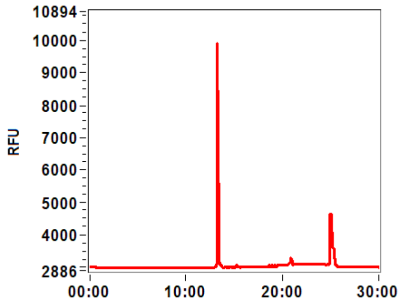

G8: SampG8

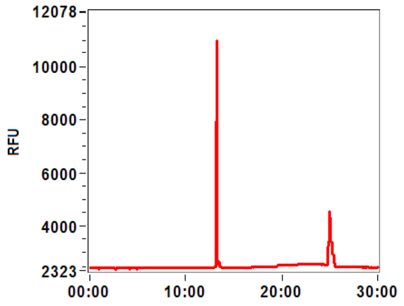

H8: SampH8

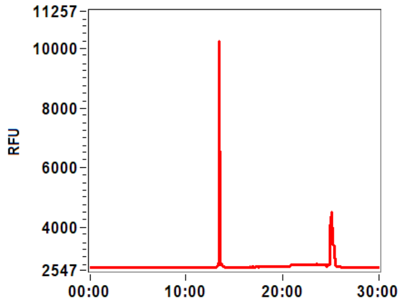

A9: SampA9

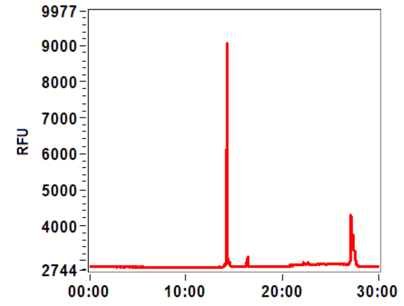

B9: SampB9

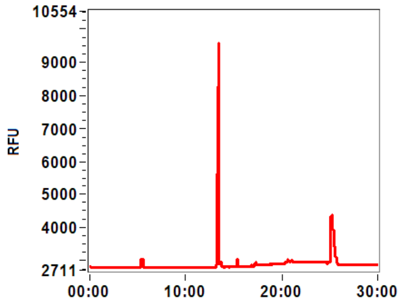

C9: SampC9

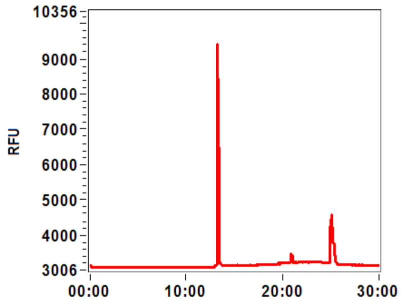

D9: SampD9

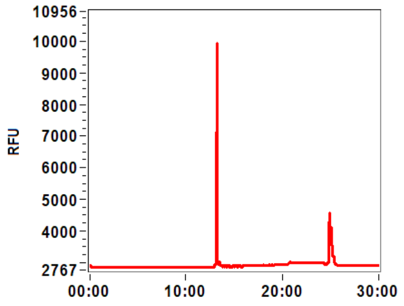

E9: SampE9

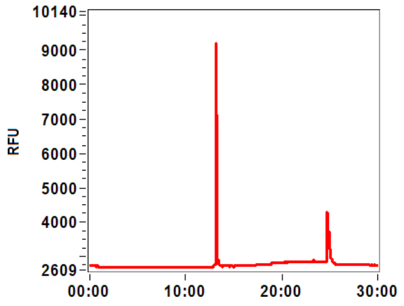

F9: SampF9

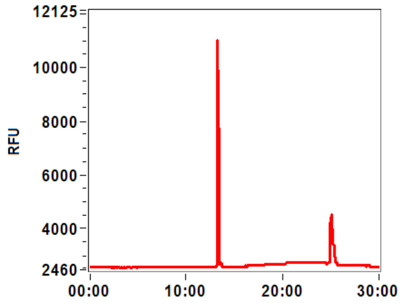

G9: SampG9

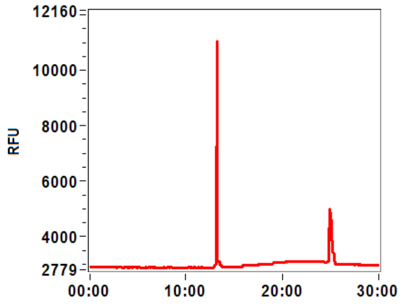

H9: SampH9

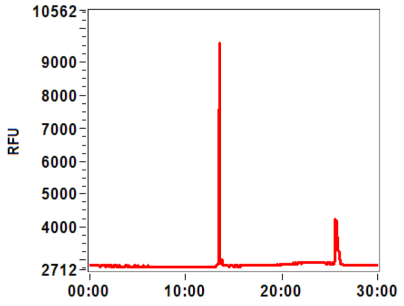

Filename and data path: C:\AATI\Data\2021 10 21\17-18-29\2021 10 21 17H 18M..raw

A10: SampA10

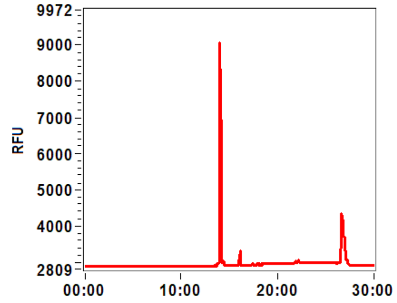

B10: SampB10

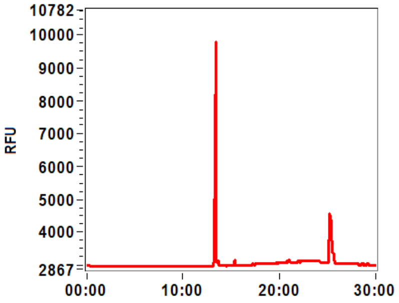

C10: SampC10

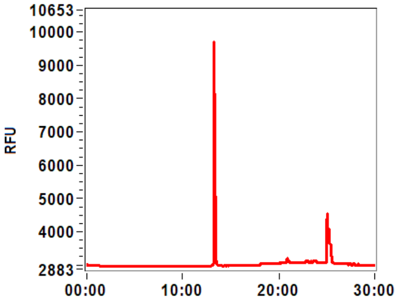

D10: SampD10

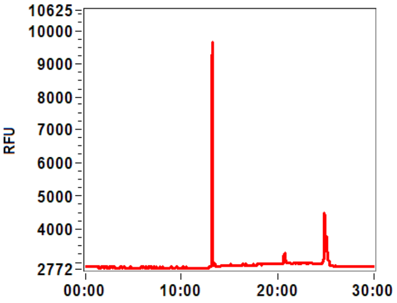

E10: SampE10

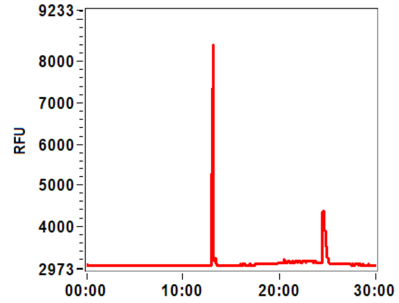

F10: SampF10

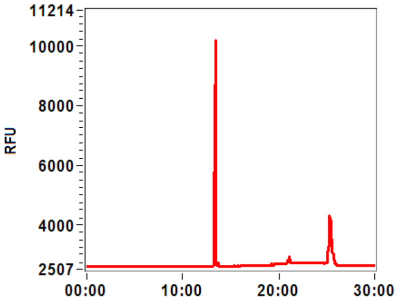

G10: SampG10

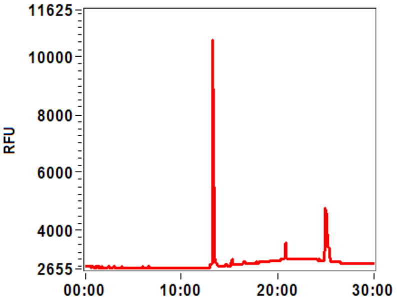

H10: SampH10

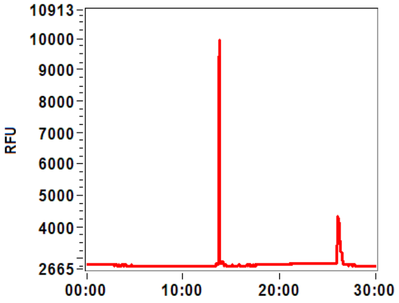

A11: SampA11

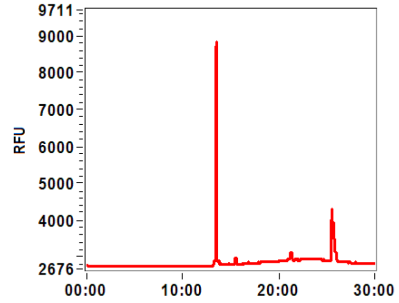

B11: SampB11

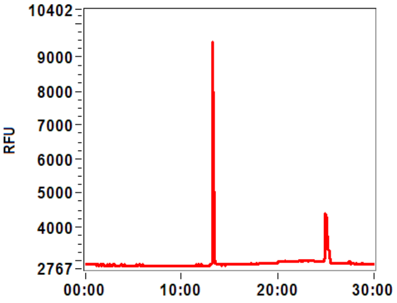

C11: SampC11

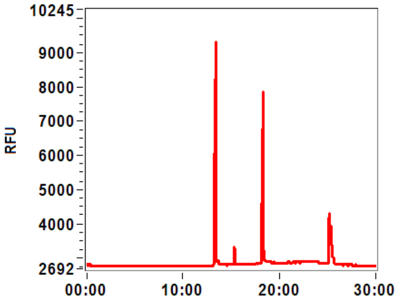

D11: SampD11

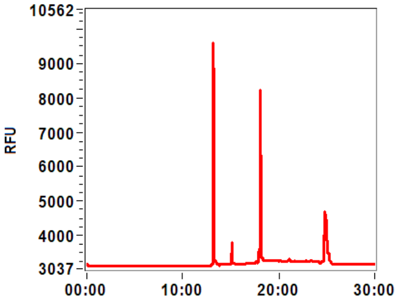

Filename and data path: C:\AATI\Data\2021 10 21\17-18-29\2021 10 21 17H 18M..raw

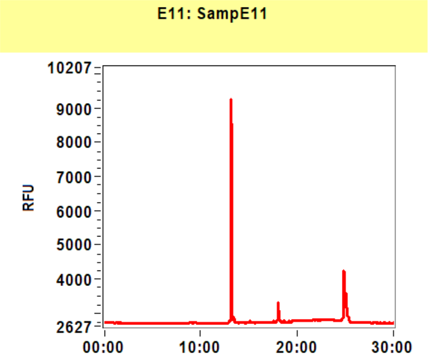

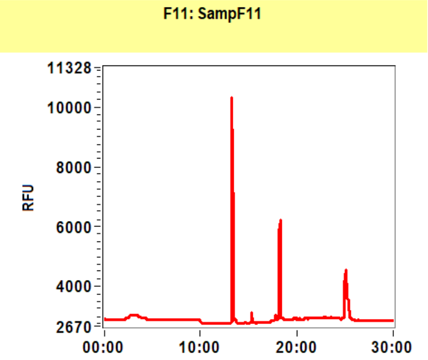

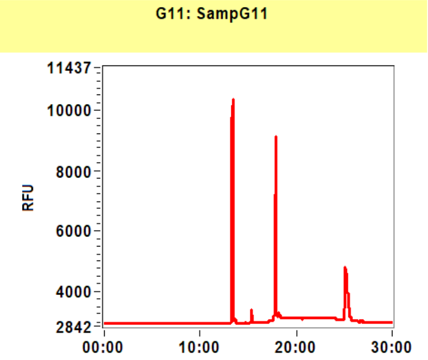

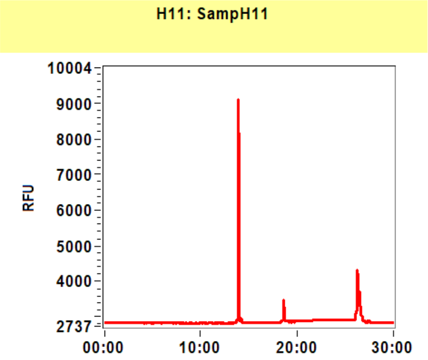

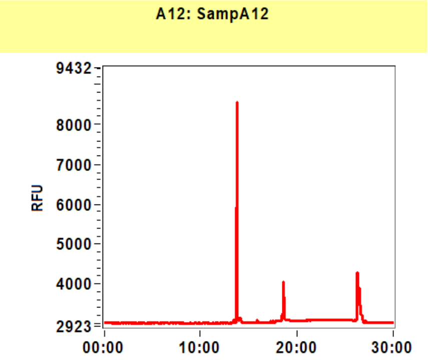

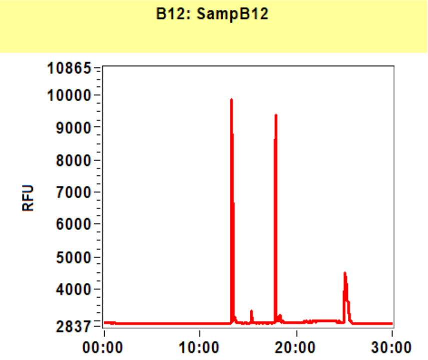

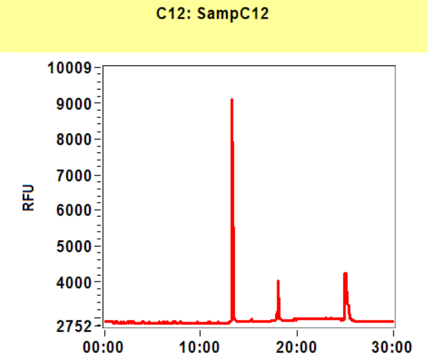

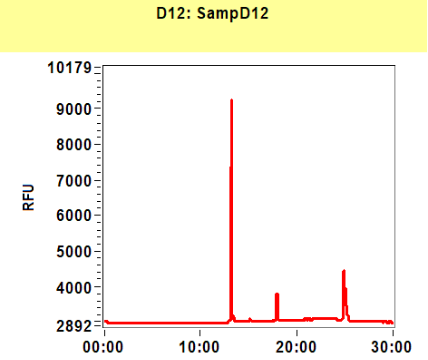

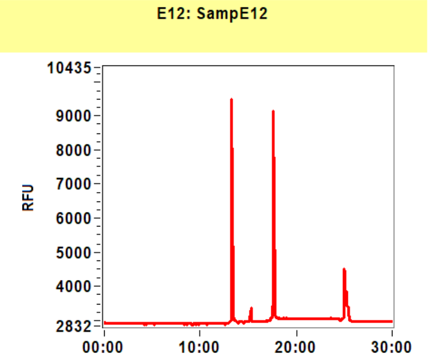

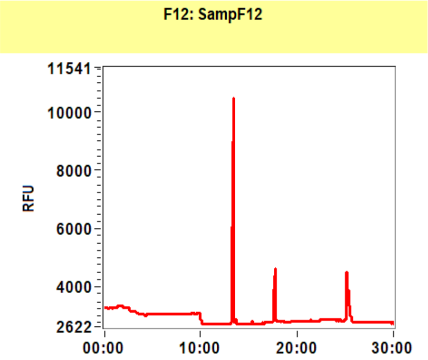

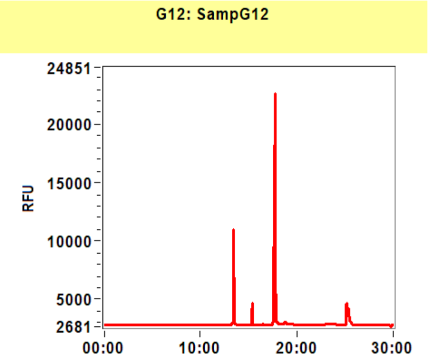

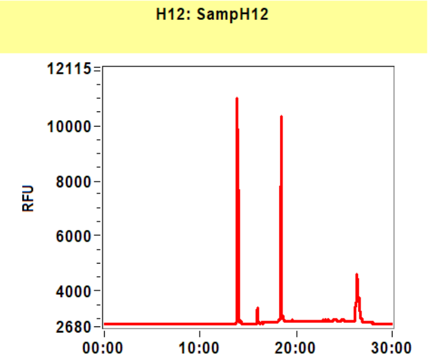

Sample: SampA1
Well location: A1
Created: Thursday, October 21, 2021 5:28:30 PM

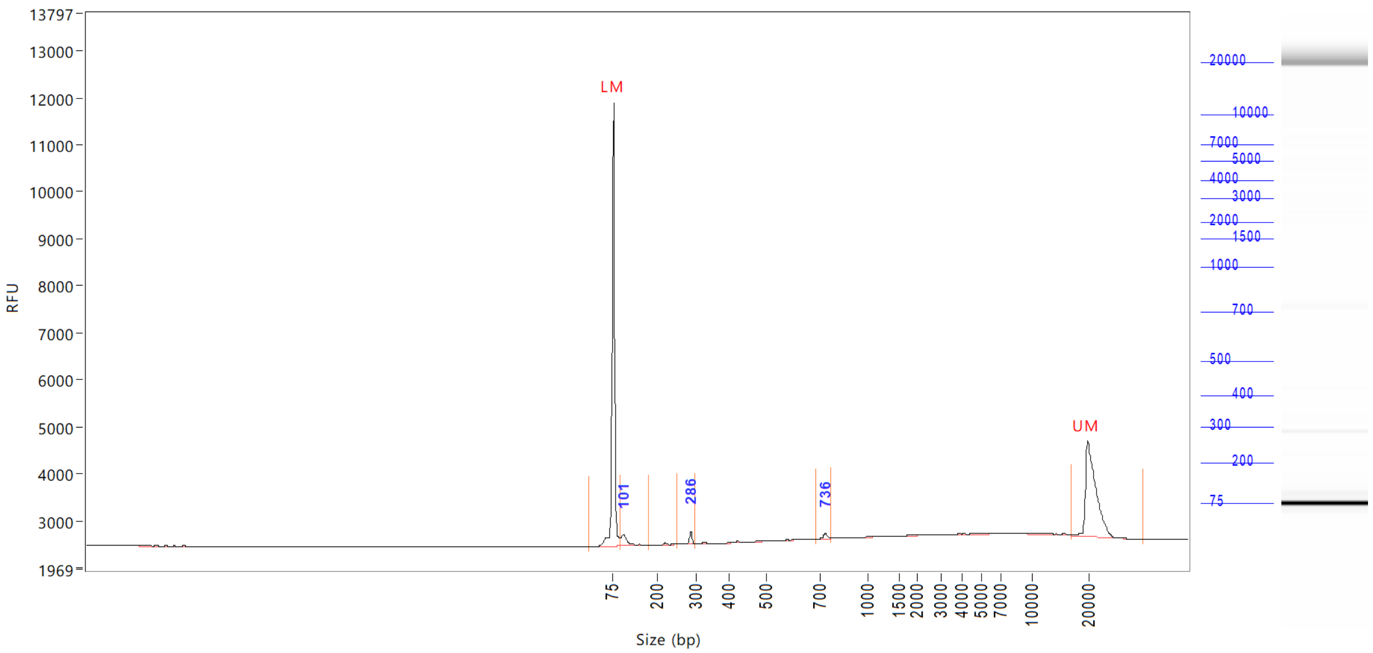

Peak	Size	Concentration	Relative concentration	Molarity	From	To	CV%	RFU
	(bp)	(ng/uL)	Percent	(nmole/L)	(bp)	(bp)		
1	75 (LM)	0.5000		11.3919	7	93	8.48	9445
2	101	0.4091	62.8	6.1080	93	173	15.16	242
3	286	0.1558	23.9	0.9053	251	296	1.75	278
4	736	0.0867	13.3	0.1961	687	770	1.93	122
5	20000 (UM)	0.2294		0.0182	16952	29914	5.63	2031

TIC:	0.6516	ng/uL
TIM:	7.2094	nmole/L
Total concentration:	1.1832	ng/uL

```
Sample peak width (sec): 5    Sample min peak height: 50    Sample baseline V to V?: Y    Sample baseline V to V points: 3
Sample filter: Binomial       Number of points for filter: 3  Sample start region (min): 0  Sample end region (min): 30
Marker peak width (sec): 5    Marker min peak height: 200   Marker baseline V to V?: Y    Marker baseline V to V points: 3
Lower marker selection: First peak > 500 RFU                Upper marker selection: Last peak > 200 RFU
Ladder size (bp)75, 200, 300, 400, 500, 700, 1000, 1500, 2000, 3000, 4000, 5000, 7000, 10000, 20000
Quantification using: Lower Marker          Final concentration (ng/uL): 0.5000          Dilution factor: 12.0
```

Sample: SampB1
Well location: B1
Created: Thursday, October 21, 2021 5:28:30 PM

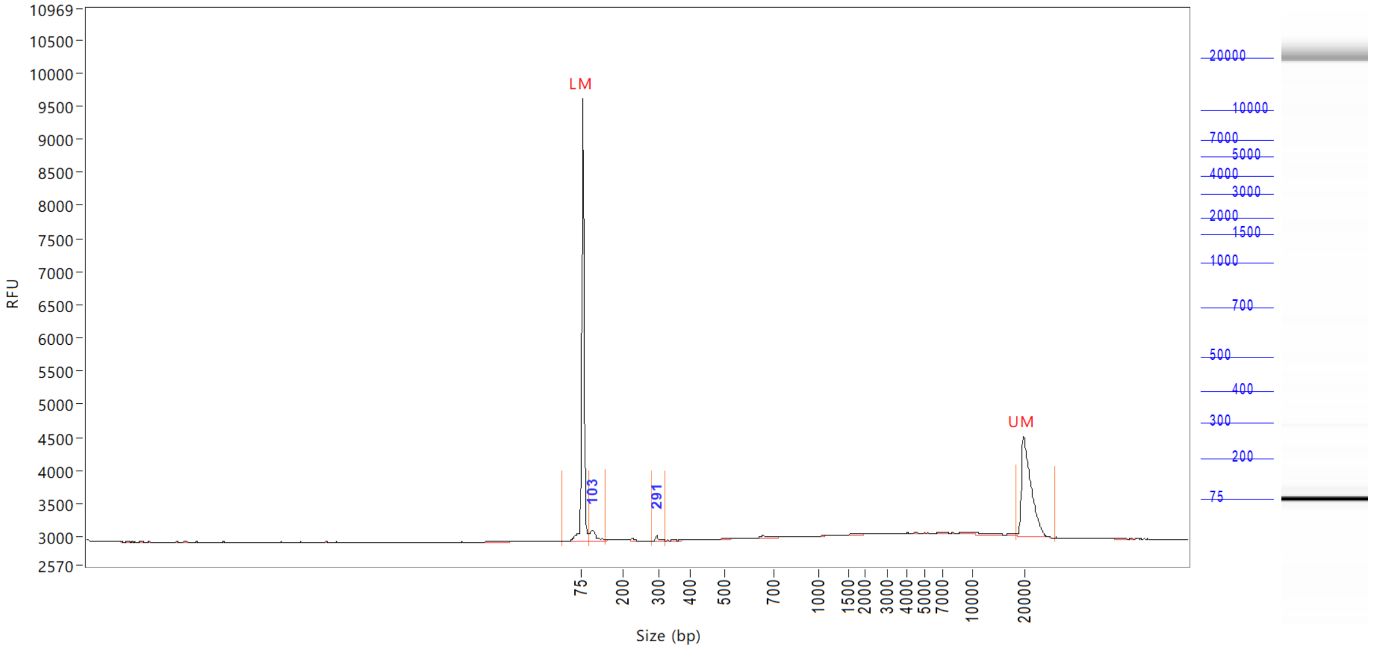

Peak	Size	Concentration	Relative concentration	Molarity	From	To	CV%	RFU
	(bp)	(ng/uL)	Percent	(nmole/L)	(bp)	(bp)		
1	75 (LM)	0.5000		11.2364	16	93	7.57	6690
2	103	0.3661	82.1	5.6196	93	145	10.98	158
3	291	0.0800	17.9	0.4504	280	319	2.39	82
4	20000 (UM)	0.2308		0.0184	18537	25803	4.93	1515

TIC:	0.4461	ng/uL
TIM:	6.0700	nmole/L
Total concentration:	1.1897	ng/uL

Sample peak width (sec): 5 Sample min peak height: 50 Sample baseline V to V?: Y Sample baseline V to V points: 3
Sample filter: Binomial Number of points for filter: 3 Sample start region (min): 0 Sample end region (min): 30
Marker peak width (sec): 5 Marker min peak height: 200 Marker baseline V to V?: Y Marker baseline V to V points: 3
Lower marker selection: First peak > 500 RFU Upper marker selection: Last peak > 200 RFU
Ladder size (bp)75, 200, 300, 400, 500, 700, 1000, 1500, 2000, 3000, 4000, 5000, 7000, 10000, 20000
Quantification using: Lower Marker Final concentration (ng/uL): 0.5000 Dilution factor: 12.0

Sample: SampC1
Well location: C1
Created: Thursday, October 21, 2021 5:28:30 PM

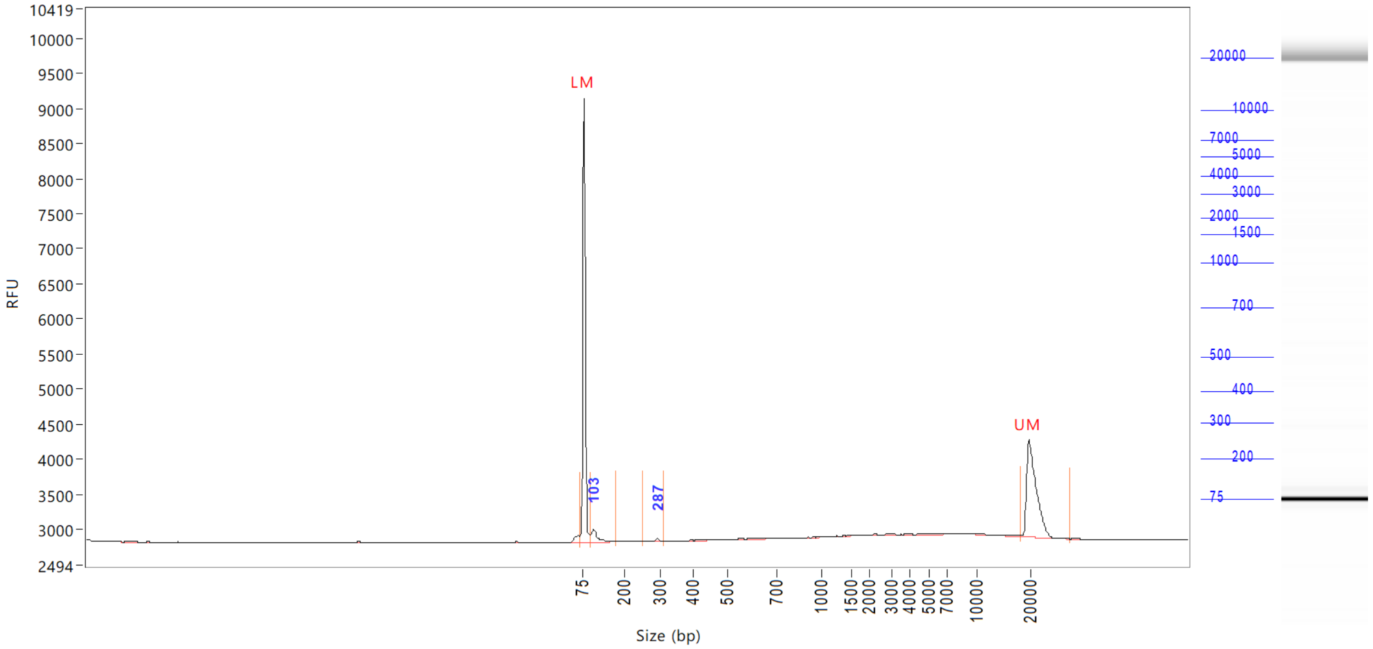

Peak	Size	Concentration	Relative concentration	Molarity	From	To	CV%	RFU
	(bp)	(ng/uL)	Percent	(nmole/L)	(bp)	(bp)		
1	75 (LM)	0.5000		11.0851	64	95	5.52	6333
2	103	0.4669	86.4	6.8480	95	173	15.46	179
3	287	0.0738	13.6	0.4288	248	309	3.87	55
4	20000 (UM)	0.2351		0.0188	18537	27738	5.07	1390

TIC:	0.5407	ng/uL
TIM:	7.2769	nmole/L
Total concentration:	1.1658	ng/uL

Sample peak width (sec): 5   Sample min peak height: 50   Sample baseline V to V?: Y   Sample baseline V to V points: 3
Sample filter: Binomial   Number of points for filter: 3   Sample start region (min): 0   Sample end region (min): 30
Marker peak width (sec): 5   Marker min peak height: 200   Marker baseline V to V?: Y   Marker baseline V to V points: 3
Lower marker selection: First peak > 500 RFU   Upper marker selection: Last peak > 200 RFU
Ladder size (bp)75, 200, 300, 400, 500, 700, 1000, 1500, 2000, 3000, 4000, 5000, 7000, 10000, 20000
Quantification using: Lower Marker   Final concentration (ng/uL): 0.5000   Dilution factor: 12.0

Sample: SampD1
Well location: D1
Created: Thursday, October 21, 2021 5:28:30 PM

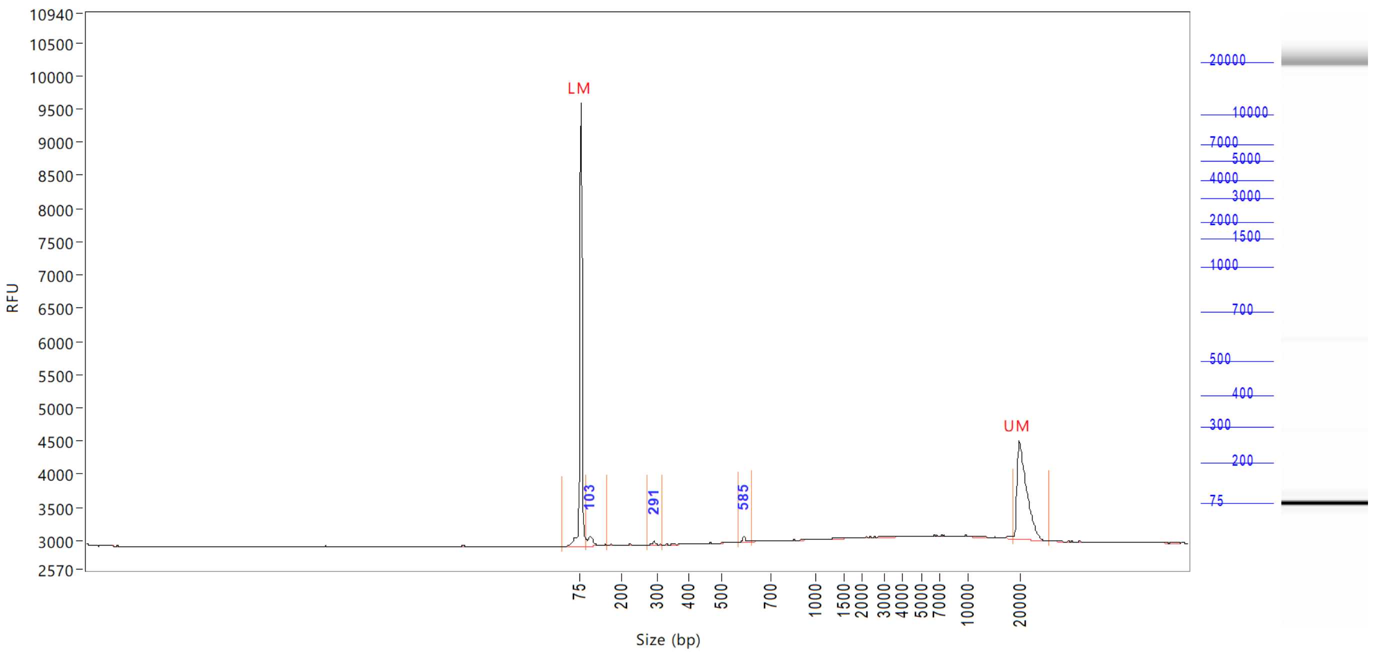

Peak	Size	Concentration	Relative concentration	Molarity	From	To	CV%	RFU
	(bp)	(ng/uL)	Percent	(nmole/L)	(bp)	(bp)		
1	75 (LM)	0.5000		11.2364	22	93	7.89	6686
2	103	0.3356	70.0	5.0574	93	156	13.92	152
3	291	0.0651	13.6	0.3708	269	315	2.89	54
4	585	0.0785	16.4	0.2200	567	618	1.70	83
5	20000 (UM)	0.2332		0.0186	18659	25803	5.01	1490

TIC:	0.4793	ng/uL
TIM:	5.6482	nmole/L
Total concentration:	1.0454	ng/uL

```
Sample peak width (sec): 5    Sample min peak height: 50    Sample baseline V to V?: Y    Sample baseline V to V points: 3
Sample filter: Binomial       Number of points for filter: 3  Sample start region (min): 0  Sample end region (min): 30
Marker peak width (sec): 5    Marker min peak height: 200   Marker baseline V to V?: Y    Marker baseline V to V points: 3
Lower marker selection: First peak > 500 RFU                Upper marker selection: Last peak > 200 RFU
Ladder size (bp)75, 200, 300, 400, 500, 700, 1000, 1500, 2000, 3000, 4000, 5000, 7000, 10000, 20000
Quantification using: Lower Marker          Final concentration (ng/uL): 0.5000          Dilution factor: 12.0
```

Sample: SampE1
Well location: E1
Created: Thursday, October 21, 2021 5:28:30 PM

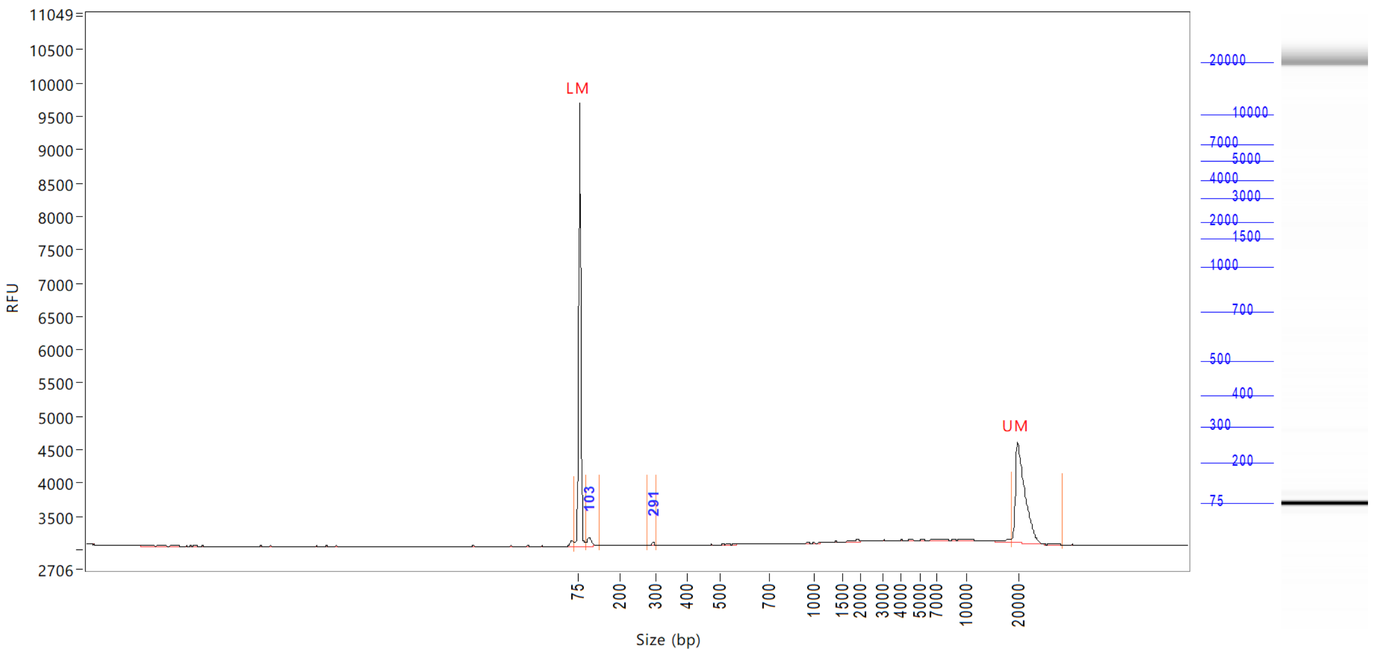

Peak	Size	Concentration	Relative concentration	Molarity	From	To	CV%	RFU
	(bp)	(ng/uL)	Percent	(nmole/L)	(bp)	(bp)		
1	75 (LM)	0.5000		11.2364	58	93	4.99	6664
2	103	0.2511	82.2	3.9273	93	135	8.17	125
3	291	0.0545	17.8	0.3115	273	300	1.74	54
4	20000 (UM)	0.2483		0.0197	18781	28584	5.30	1501

TIC:	0.3056	ng/uL
TIM:	4.2388	nmole/L
Total concentration:	1.0216	ng/uL

```
Sample peak width (sec): 5    Sample min peak height: 50    Sample baseline V to V?: Y    Sample baseline V to V points: 3
Sample filter: Binomial       Number of points for filter: 3  Sample start region (min): 0  Sample end region (min): 30
Marker peak width (sec): 5    Marker min peak height: 200   Marker baseline V to V?: Y    Marker baseline V to V points: 3
Lower marker selection: First peak > 500 RFU                Upper marker selection: Last peak > 200 RFU
Ladder size (bp)75, 200, 300, 400, 500, 700, 1000, 1500, 2000, 3000, 4000, 5000, 7000, 10000, 20000
Quantification using: Lower Marker          Final concentration (ng/uL): 0.5000          Dilution factor: 12.0
```

Sample: SampF1
Well location: F1
Created: Thursday, October 21, 2021 5:28:30 PM

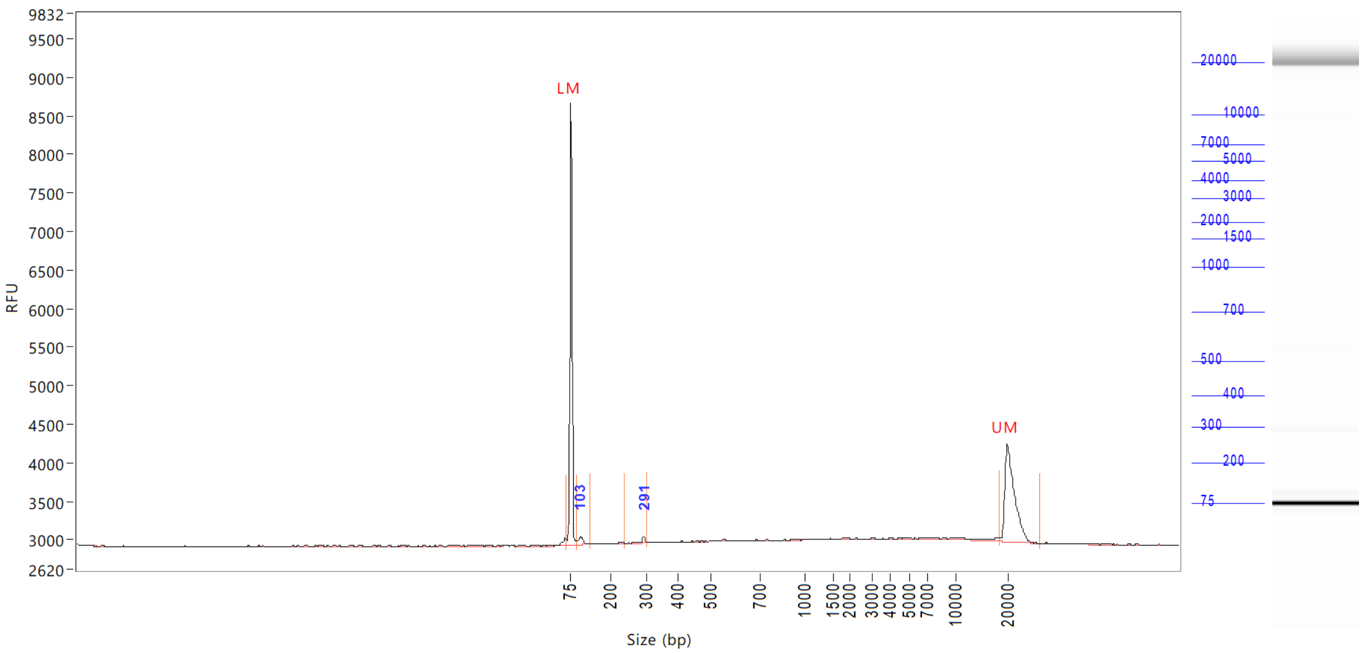

Peak	Size	Concentration	Relative concentration	Molarity	From	To	CV%	RFU
	(bp)	(ng/uL)	Percent	(nmole/L)	(bp)	(bp)		
1	75 (LM)	0.5000		11.2364	64	93	4.65	5746
2	103	0.2319	65.8	3.6276	93	135	8.03	101
3	291	0.1208	34.2	0.7019	237	301	4.34	90
4	20000 (UM)	0.2493		0.0198	18537	26408	5.16	1264

TIC:	0.3527	ng/uL
TIM:	4.3294	nmole/L
Total concentration:	1.1174	ng/uL

```
Sample peak width (sec): 5    Sample min peak height: 50    Sample baseline V to V?: Y    Sample baseline V to V points: 3
Sample filter: Binomial       Number of points for filter: 3  Sample start region (min): 0  Sample end region (min): 30
Marker peak width (sec): 5    Marker min peak height: 200   Marker baseline V to V?: Y    Marker baseline V to V points: 3
Lower marker selection: First peak > 500 RFU                Upper marker selection: Last peak > 200 RFU
Ladder size (bp)75, 200, 300, 400, 500, 700, 1000, 1500, 2000, 3000, 4000, 5000, 7000, 10000, 20000
Quantification using: Lower Marker          Final concentration (ng/uL): 0.5000          Dilution factor: 12.0
```

Sample: SampG1
Well location: G1
Created: Thursday, October 21, 2021 5:28:30 PM

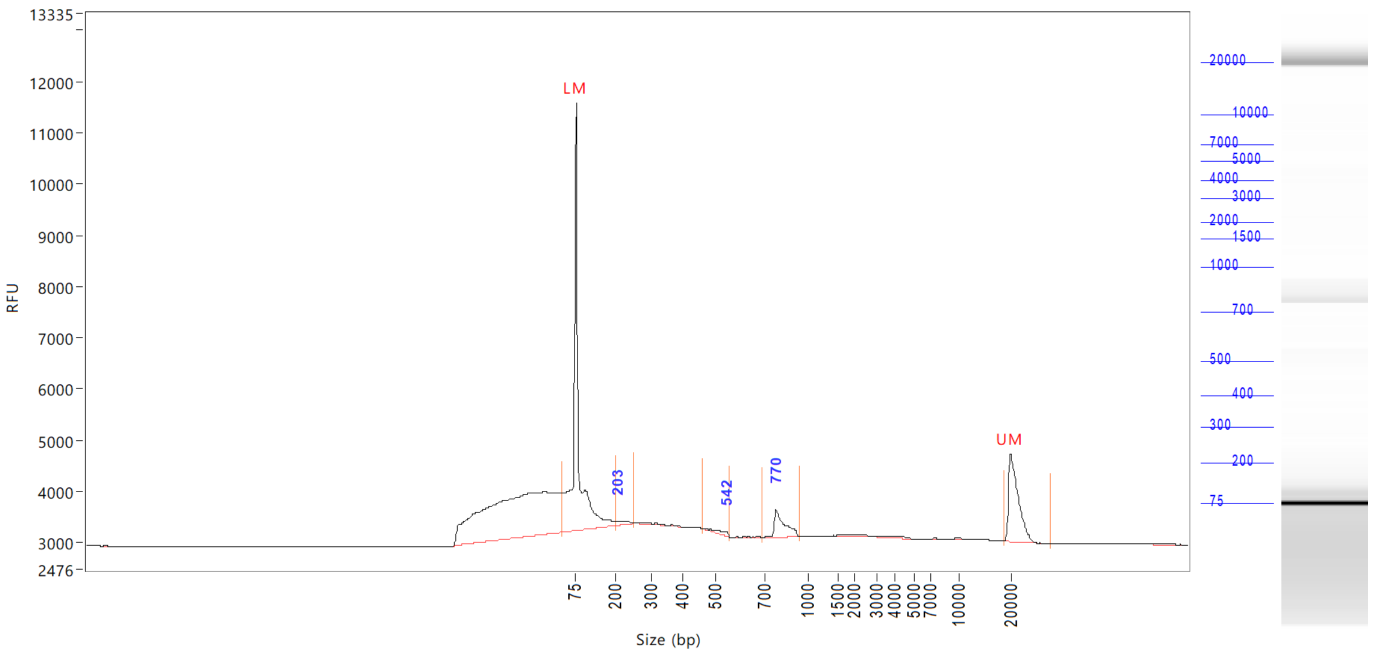

Peak	Size	Concentration	Relative concentration	Molarity	From	To	CV%	RFU
	(bp)	(ng/uL)	Percent	(nmole/L)	(bp)	(bp)		
1	75 (LM)	0.5000		10.1302	35	198	34.75	8366
2	203	0.1128	13.2	0.8584	198	250	5.73	74
3	542	0.1267	14.8	0.4072	460	555	4.37	78
4	770	0.6167	72.0	1.2485	687	935	5.72	543
5	20000 (UM)	0.1137		0.0090	18537	27617	5.13	1719

TIC:	0.8562	ng/uL
TIM:	2.5141	nmole/L
Total concentration:	1.2279	ng/uL

Sample peak width (sec): 5   Sample min peak height: 50   Sample baseline V to V?: Y   Sample baseline V to V points: 3
Sample filter: Binomial   Number of points for filter: 3 Sample start region (min): 0 Sample end region (min): 30
Marker peak width (sec): 5   Marker min peak height: 200   Marker baseline V to V?: Y   Marker baseline V to V points: 3
Lower marker selection: First peak > 500 RFU   Upper marker selection: Last peak > 200 RFU
Ladder size (bp)75, 200, 300, 400, 500, 700, 1000, 1500, 2000, 3000, 4000, 5000, 7000, 10000, 20000
Quantification using: Lower Marker   Final concentration (ng/uL): 0.5000   Dilution factor: 12.0

Sample: SampH1
Well location: H1
Created: Thursday, October 21, 2021 5:28:30 PM

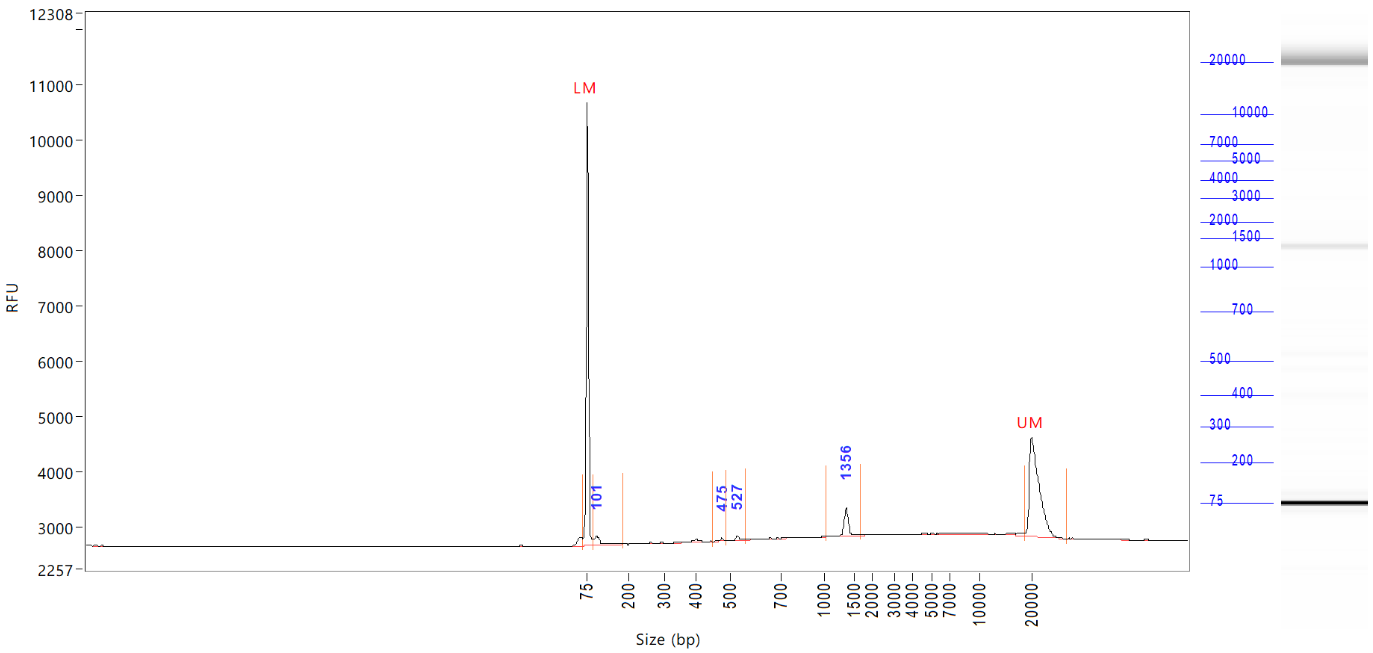

Peak	Size	Concentration	Relative concentration	Molarity	From	To	CV%	RFU
	(bp)	(ng/uL)	Percent	(nmole/L)	(bp)	(bp)		
1	75 (LM)	0.5000		11.2364	62	93	5.23	8021
2	101	0.3258	34.0	4.8643	93	183	16.46	166
3	475	0.0467	4.9	0.1624	449	489	1.47	55
4	527	0.0956	10.0	0.2996	489	560	2.71	80
5	1356	0.4910	51.2	0.5969	1001	1655	5.28	518
6	20000 (UM)	0.2488		0.0197	18659	26770	5.22	1797

TIC:	0.9590	ng/uL
TIM:	5.9231	nmole/L
Total concentration:	1.5758	ng/uL

Sample peak width (sec): 5 Sample min peak height: 50 Sample baseline V to V?: Y Sample baseline V to V points: 3
Sample filter: Binomial Number of points for filter: 3 Sample start region (min): 0 Sample end region (min): 30
Marker peak width (sec): 5 Marker min peak height: 200 Marker baseline V to V?: Y Marker baseline V to V points: 3
Lower marker selection: First peak > 500 RFU Upper marker selection: Last peak > 200 RFU
Ladder size (bp)75, 200, 300, 400, 500, 700, 1000, 1500, 2000, 3000, 4000, 5000, 7000, 10000, 20000
Quantification using: Lower Marker Final concentration (ng/uL): 0.5000 Dilution factor: 12.0

Sample: SampA2
Well location: A2
Created: Thursday, October 21, 2021 5:28:30 PM

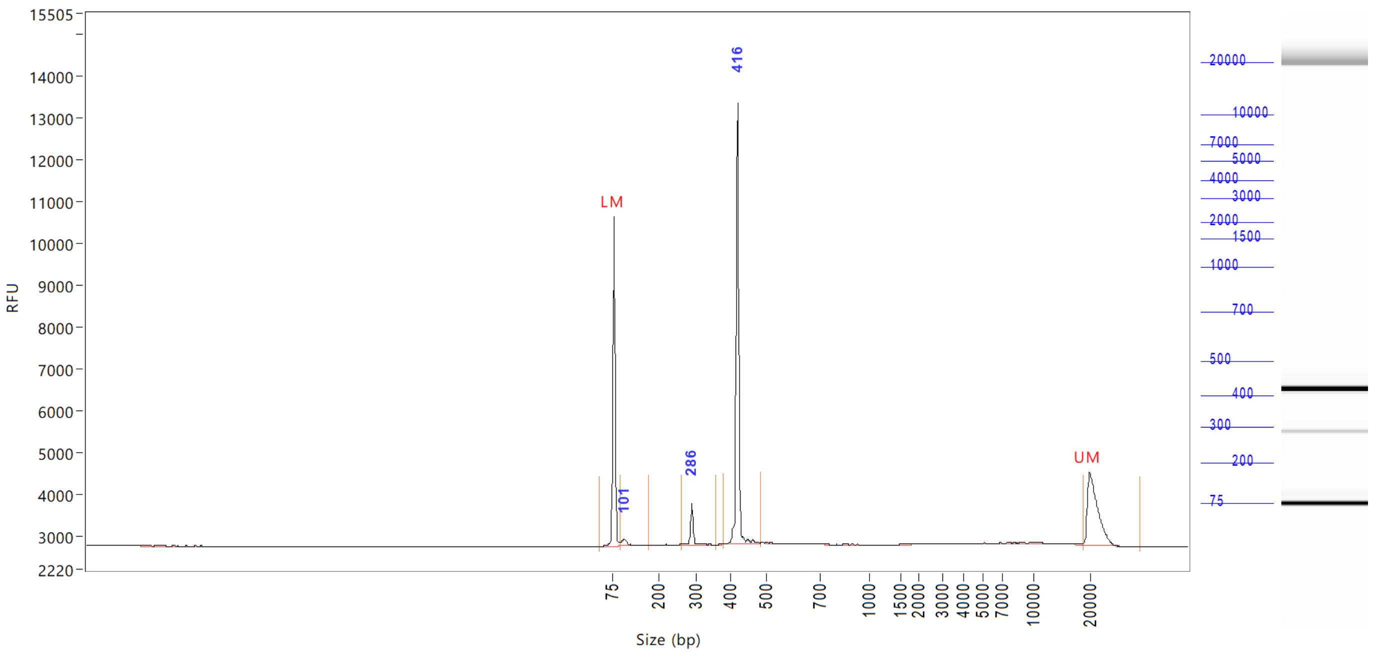

Peak	Size	Concentration	Relative concentration	Molarity	From	To	CV%	RFU
	(bp)	(ng/uL)	Percent	(nmole/L)	(bp)	(bp)		
1	75 (LM)	0.5000		11.2364	35	93	5.57	7878
2	101	0.3171	3.6	4.7775	93	171	14.71	154
3	286	0.8599	9.7	4.9454	260	358	3.98	1005
4	416	7.6830	86.7	30.3873	378	483	1.81	10555
5	20000 (UM)	0.2501		0.0199	18781	28947	5.15	1757

TIC:	8.8599	ng/uL
TIM:	40.1101	nmole/L
Total concentration:	9.3681	ng/uL

```
Sample peak width (sec): 5    Sample min peak height: 50    Sample baseline V to V?: Y    Sample baseline V to V points: 3
Sample filter: Binomial       Number of points for filter: 3  Sample start region (min): 0  Sample end region (min): 30
Marker peak width (sec): 5    Marker min peak height: 200    Marker baseline V to V?: Y    Marker baseline V to V points: 3
Lower marker selection: First peak > 500 RFU                 Upper marker selection: Last peak > 200 RFU
Ladder size (bp)75, 200, 300, 400, 500, 700, 1000, 1500, 2000, 3000, 4000, 5000, 7000, 10000, 20000
Quantification using: Lower Marker          Final concentration (ng/uL): 0.5000          Dilution factor: 12.0
```

Sample: SampB2
Well location: B2
Created: Thursday, October 21, 2021 5:28:30 PM

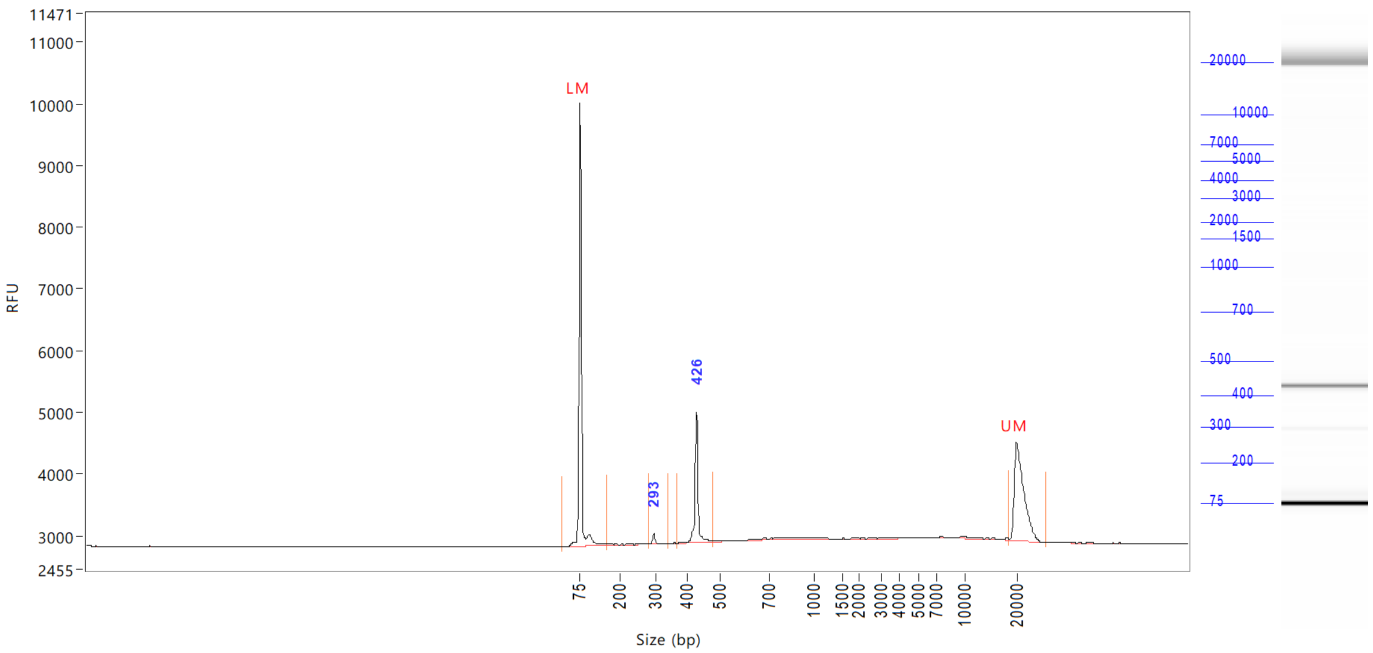

Peak	Size	Concentration	Relative concentration	Molarity	From	To	CV%	RFU
	(bp)	(ng/uL)	Percent	(nmole/L)	(bp)	(bp)		
1	75 (LM)	0.5000		10.9378	24	158	14.42	7200
2	293	0.1303	6.8	0.7316	280	337	2.62	163
3	426	1.7914	93.2	6.9354	366	479	2.60	2107
4	20000 (UM)	0.2225		0.0177	18537	25803	5.03	1607

TIC:	1.9218	ng/uL
TIM:	7.6671	nmole/L
Total concentration:	2.7016	ng/uL

```
Sample peak width (sec): 5    Sample min peak height: 50    Sample baseline V to V?: Y    Sample baseline V to V points: 3
Sample filter: Binomial       Number of points for filter: 3  Sample start region (min): 0  Sample end region (min): 30
Marker peak width (sec): 5    Marker min peak height: 200   Marker baseline V to V?: Y    Marker baseline V to V points: 3
Lower marker selection: First peak > 500 RFU                Upper marker selection: Last peak > 200 RFU
Ladder size (bp)75, 200, 300, 400, 500, 700, 1000, 1500, 2000, 3000, 4000, 5000, 7000, 10000, 20000
Quantification using: Lower Marker          Final concentration (ng/uL): 0.5000          Dilution factor: 12.0
```

Sample: SampC2
Well location: C2
Created: Thursday, October 21, 2021 5:28:30 PM

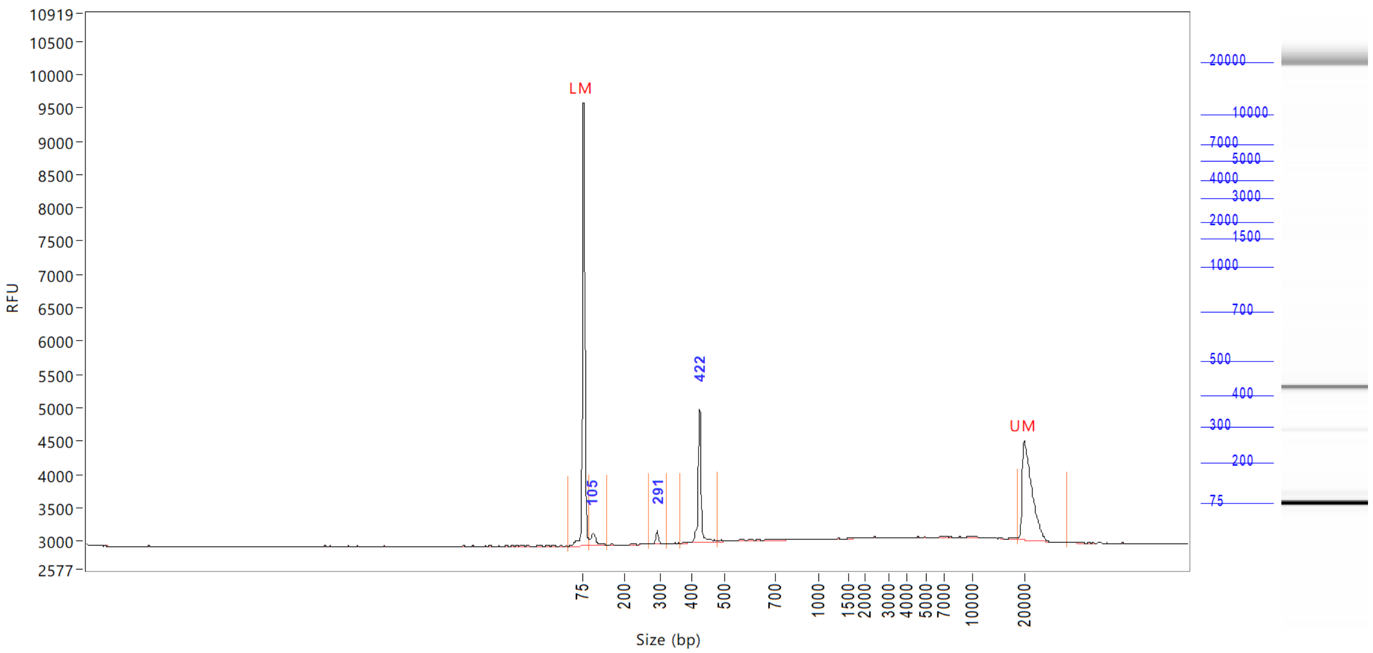

Peak	Size	Concentration	Relative concentration	Molarity	From	To	CV%	RFU
	(bp)	(ng/uL)	Percent	(nmole/L)	(bp)	(bp)		
1	75 (LM)	0.5000		11.0851	31	95	7.27	6659
2	105	0.3731	15.3	5.6741	95	147	10.48	196
3	291	0.1730	7.1	0.9848	268	321	1.97	200
4	422	1.8866	77.5	7.3731	364	475	2.51	2006
5	20000 (UM)	0.2291		0.0182	18781	28100	4.98	1501

TIC:	2.4327	ng/uL
TIM:	14.0320	nmole/L
Total concentration:	3.0281	ng/uL

```
Sample peak width (sec): 5    Sample min peak height: 50    Sample baseline V to V?: Y    Sample baseline V to V points: 3
Sample filter: Binomial       Number of points for filter: 3  Sample start region (min): 0  Sample end region (min): 30
Marker peak width (sec): 5    Marker min peak height: 200   Marker baseline V to V?: Y    Marker baseline V to V points: 3
Lower marker selection: First peak > 500 RFU                Upper marker selection: Last peak > 200 RFU
Ladder size (bp)75, 200, 300, 400, 500, 700, 1000, 1500, 2000, 3000, 4000, 5000, 7000, 10000, 20000
Quantification using: Lower Marker          Final concentration (ng/uL): 0.5000          Dilution factor: 12.0
```

Sample: SampD2
Well location: D2
Created: Thursday, October 21, 2021 5:28:30 PM

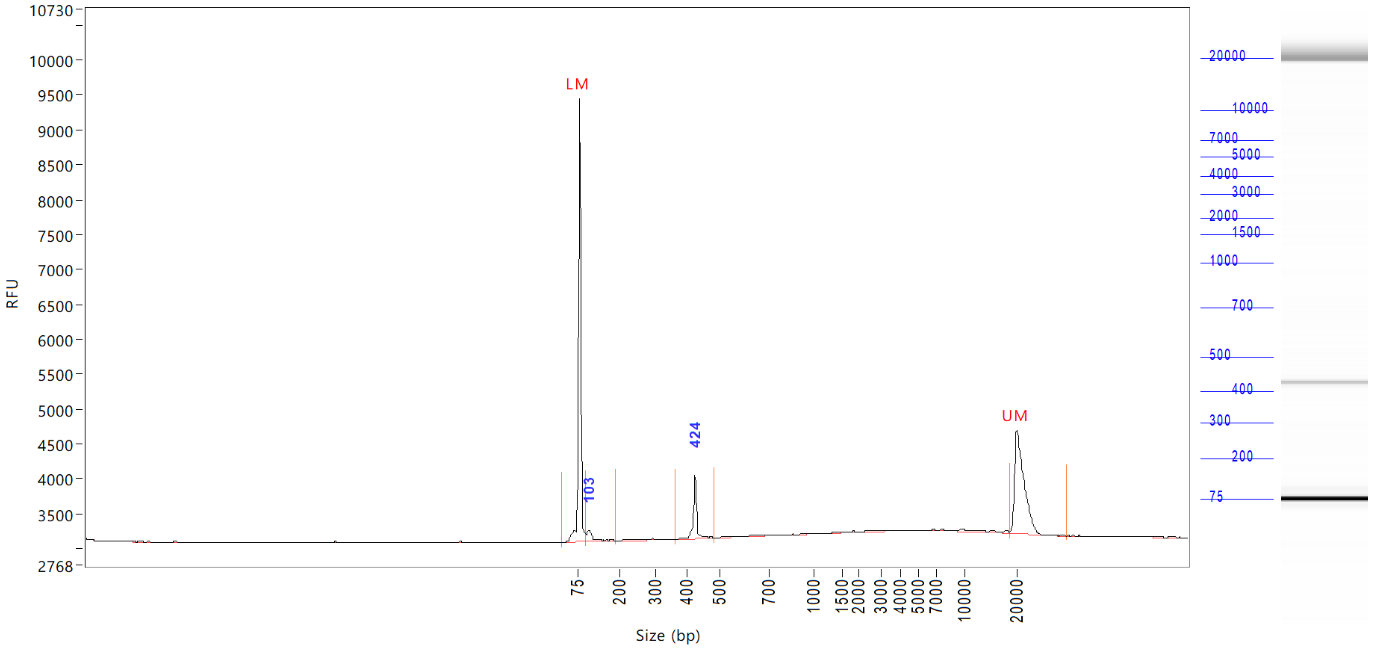

Peak	Size	Concentration	Relative concentration	Molarity	From	To	CV%	RFU
	(bp)	(ng/uL)	Percent	(nmole/L)	(bp)	(bp)		
1	75 (LM)	0.5000		11.2364	26	95	8.47	6354
2	103	0.3290	25.6	4.8688	95	189	15.96	160
3	424	0.9561	74.4	3.7279	364	481	3.11	914
4	20000 (UM)	0.2303		0.0183	18537	29551	5.35	1487

TIC:	1.2852	ng/uL
TIM:	8.5968	nmole/L
Total concentration:	1.9918	ng/uL

Sample peak width (sec): 5   Sample min peak height: 50   Sample baseline V to V?: Y   Sample baseline V to V points: 3
Sample filter: Binomial   Number of points for filter: 3 Sample start region (min): 0 Sample end region (min): 30
Marker peak width (sec): 5   Marker min peak height: 200   Marker baseline V to V?: Y   Marker baseline V to V points: 3
Lower marker selection: First peak > 500 RFU   Upper marker selection: Last peak > 200 RFU
Ladder size (bp)75, 200, 300, 400, 500, 700, 1000, 1500, 2000, 3000, 4000, 5000, 7000, 10000, 20000
Quantification using: Lower Marker   Final concentration (ng/uL): 0.5000   Dilution factor: 12.0

Sample: SampE2
Well location: E2
Created: Thursday, October 21, 2021 5:28:30 PM

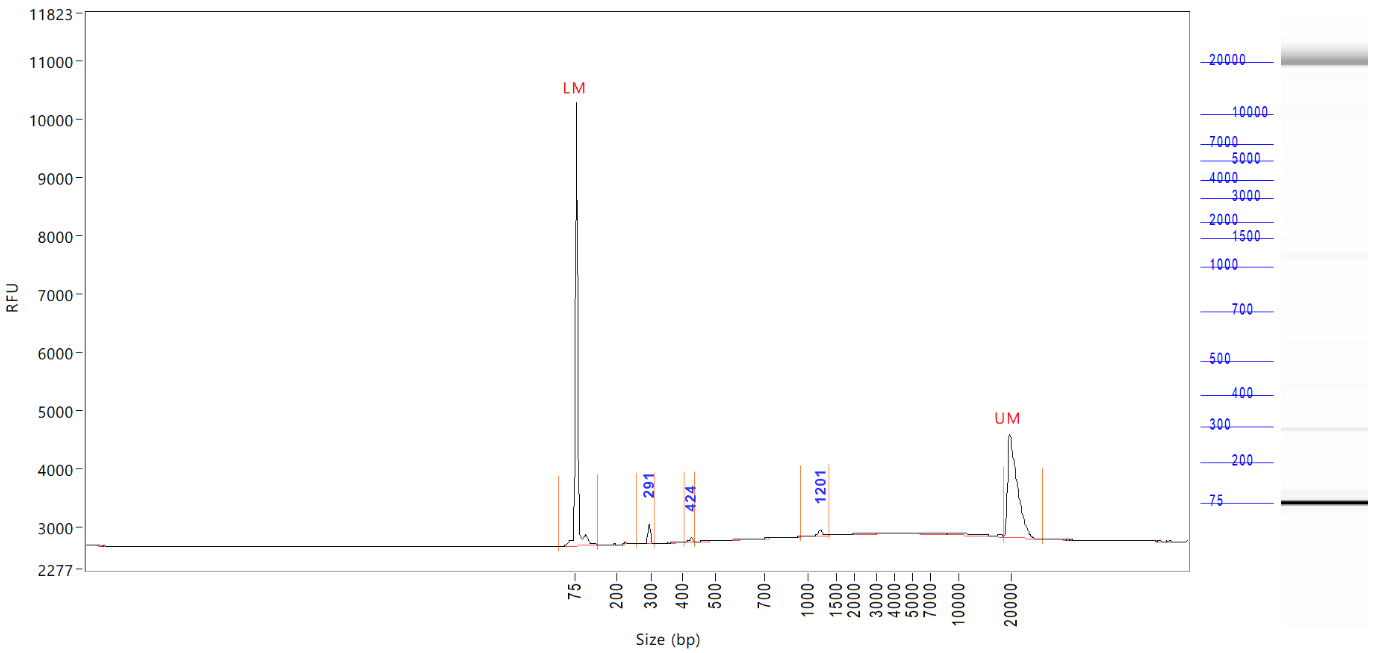

Peak	Size	Concentration	Relative concentration	Molarity	From	To	CV%	RFU
	(bp)	(ng/uL)	Percent	(nmole/L)	(bp)	(bp)		
1	75 (LM)	0.5000		11.0851	22	141	11.90	7621
2	291	0.2424	58.6	1.3843	257	307	2.25	341
3	424	0.0626	15.1	0.2452	405	433	1.42	78
4	1201	0.1088	26.3	0.1538	952	1356	6.64	104
5	20000 (UM)	0.2381		0.0189	18659	26287	5.13	1780

TIC:	0.4137	ng/uL
TIM:	1.7833	nmole/L
Total concentration:	1.0191	ng/uL

```
Sample peak width (sec): 5    Sample min peak height: 50    Sample baseline V to V?: Y    Sample baseline V to V points: 3
Sample filter: Binomial       Number of points for filter: 3  Sample start region (min): 0  Sample end region (min): 30
Marker peak width (sec): 5    Marker min peak height: 200   Marker baseline V to V?: Y    Marker baseline V to V points: 3
Lower marker selection: First peak > 500 RFU                Upper marker selection: Last peak > 200 RFU
Ladder size (bp)75, 200, 300, 400, 500, 700, 1000, 1500, 2000, 3000, 4000, 5000, 7000, 10000, 20000
Quantification using: Lower Marker          Final concentration (ng/uL): 0.5000          Dilution factor: 12.0
```

Sample: SampF2
Well location: F2
Created: Thursday, October 21, 2021 5:28:30 PM

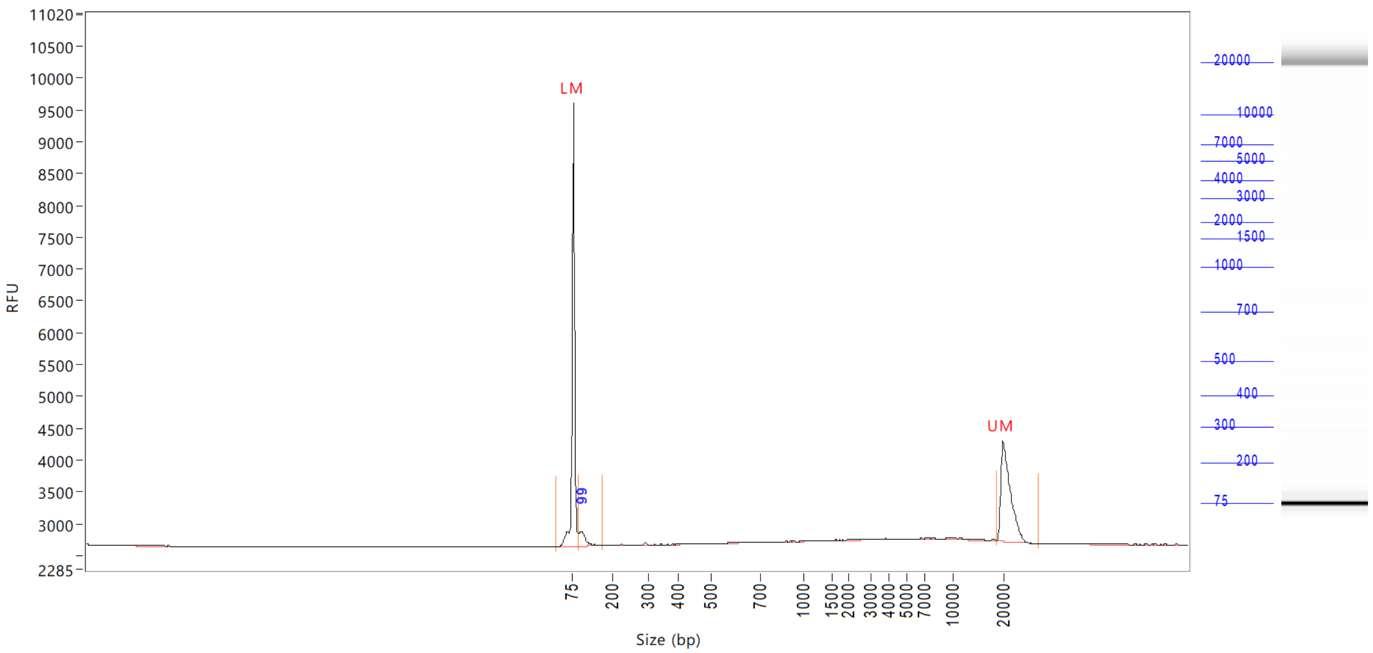

Peak	Size	Concentration	Relative concentration	Molarity	From	To	CV%	RFU
	(bp)	(ng/uL)	Percent	(nmole/L)	(bp)	(bp)		
1	75 (LM)	0.5000		11.5518	22	89	9.50	6978
2	99	0.5616	100.0	8.7012	89	168	15.60	235
3	20000 (UM)	0.2249		0.0178	18659	27133	5.23	1578

TIC:	0.5616	ng/uL
TIM:	8.7012	nmole/L
Total concentration:	1.1736	ng/uL

Sample peak width (sec): 5   Sample min peak height: 50   Sample baseline V to V?: Y   Sample baseline V to V points: 3
Sample filter: Binomial   Number of points for filter: 3 Sample start region (min): 0 Sample end region (min): 30
Marker peak width (sec): 5   Marker min peak height: 200   Marker baseline V to V?: Y   Marker baseline V to V points: 3
Lower marker selection: First peak > 500 RFU   Upper marker selection: Last peak > 200 RFU
Ladder size (bp)75, 200, 300, 400, 500, 700, 1000, 1500, 2000, 3000, 4000, 5000, 7000, 10000, 20000
Quantification using: Lower Marker   Final concentration (ng/uL): 0.5000   Dilution factor: 12.0

Sample: SampG2
Well location: G2
Created: Thursday, October 21, 2021 5:28:30 PM

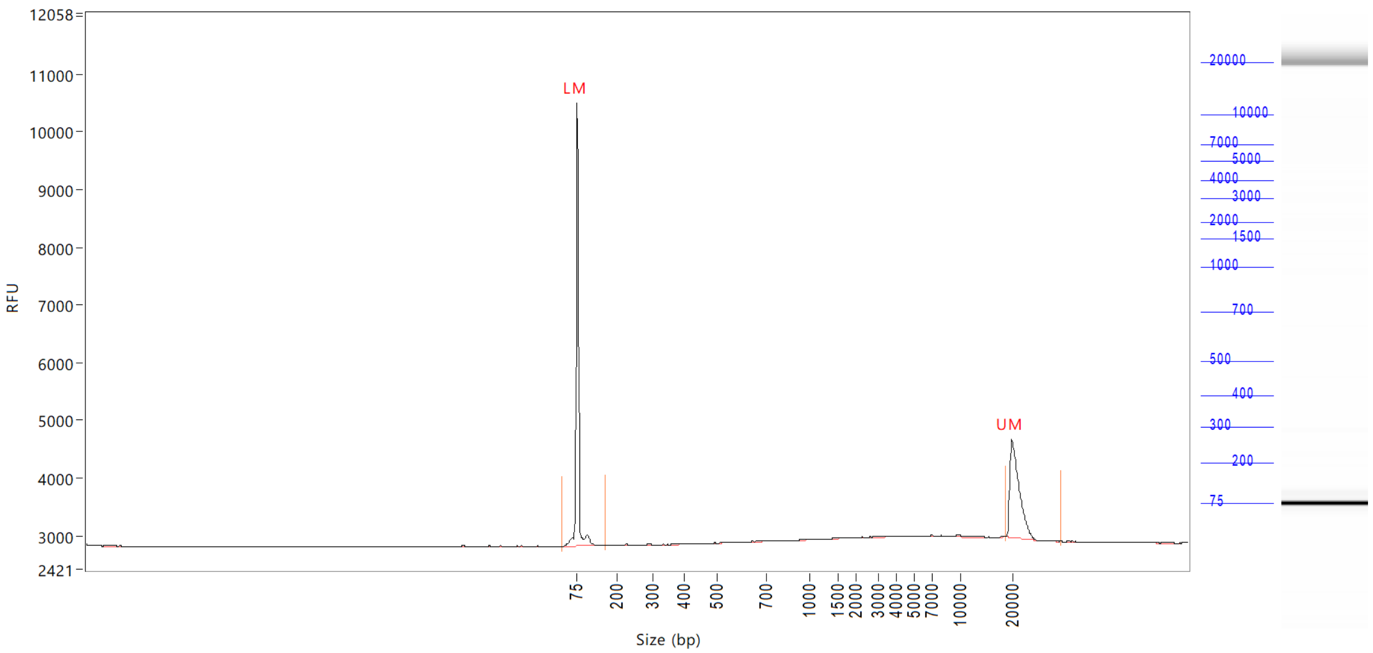

Peak	Size	Concentration	Relative concentration	Molarity	From	To	CV%	RFU
	(bp)	(ng/uL)	Percent	(nmole/L)	(bp)	(bp)		
1	75 (LM)	0.5000		11.0851	31	164	12.37	7693
2	20000 (UM)	0.2169		0.0172	18659	29551	5.21	1693

TIC:	0.0000	ng/uL
TIM:	0.0000	nmole/L
Total concentration:	0.5680	ng/uL

Sample peak width (sec): 5 Sample min peak height: 50 Sample baseline V to V?: Y Sample baseline V to V points: 3
Sample filter: Binomial Number of points for filter: 3 Sample start region (min): 0 Sample end region (min): 30
Marker peak width (sec): 5 Marker min peak height: 200 Marker baseline V to V?: Y Marker baseline V to V points: 3
Lower marker selection: First peak > 500 RFU Upper marker selection: Last peak > 200 RFU
Ladder size (bp)75, 200, 300, 400, 500, 700, 1000, 1500, 2000, 3000, 4000, 5000, 7000, 10000, 20000
Quantification using: Lower Marker Final concentration (ng/uL): 0.5000 Dilution factor: 12.0

Sample: SampH2
Well location: H2
Created: Thursday, October 21, 2021 5:28:30 PM

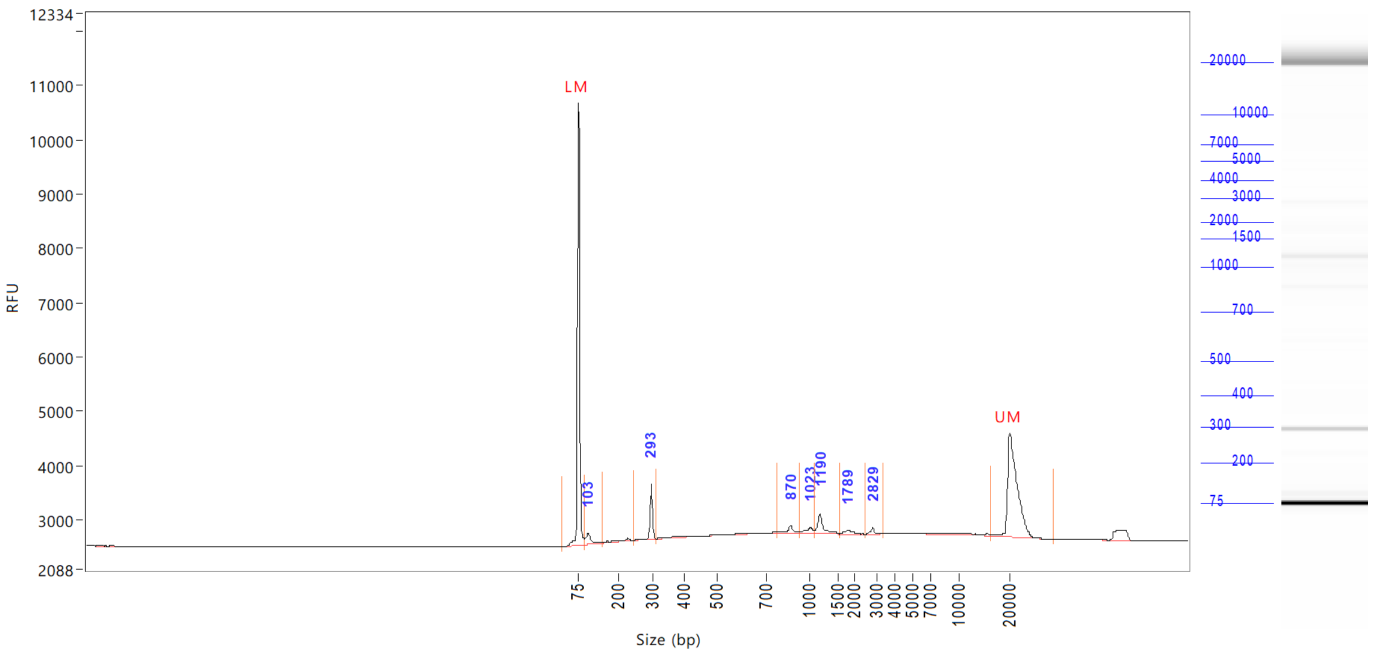

Peak	Size	Concentration	Relative concentration	Molarity	From	To	CV%	RFU
	(bp)	(ng/uL)	Percent	(nmole/L)	(bp)	(bp)		
1	75 (LM)	0.5000		11.2364	28	93	6.64	8167
2	103	0.3750	18.1	5.5989	93	152	13.33	210
3	293	0.6632	32.0	3.7615	241	307	2.21	1008
4	870	0.1945	9.4	0.3704	775	935	4.13	144
5	1023	0.1869	9.0	0.3052	935	1101	4.39	105
6	1190	0.4422	21.3	0.5966	1101	1539	7.30	380
7	1789	0.1219	5.9	0.1093	1539	2486	8.10	63
8	2829	0.0889	4.3	0.0518	2486	3358	5.17	113
9	20000 (UM)	0.2520		0.0200	16342	28463	5.50	1925

TIC:	2.0724	ng/uL
TIM:	10.7938	nmole/L
Total concentration:	2.6568	ng/uL

```
Sample peak width (sec): 5    Sample min peak height: 50    Sample baseline V to V?: Y    Sample baseline V to V points: 3
Sample filter: Binomial       Number of points for filter: 3  Sample start region (min): 0  Sample end region (min): 30
Marker peak width (sec): 5    Marker min peak height: 200   Marker baseline V to V?: Y    Marker baseline V to V points: 3
Lower marker selection: First peak > 500 RFU                Upper marker selection: Last peak > 200 RFU
Ladder size (bp)75, 200, 300, 400, 500, 700, 1000, 1500, 2000, 3000, 4000, 5000, 7000, 10000, 20000
Quantification using: Lower Marker          Final concentration (ng/uL): 0.5000          Dilution factor: 12.0
```

Sample: SampA3
Well location: A3
Created: Thursday, October 21, 2021 5:28:30 PM

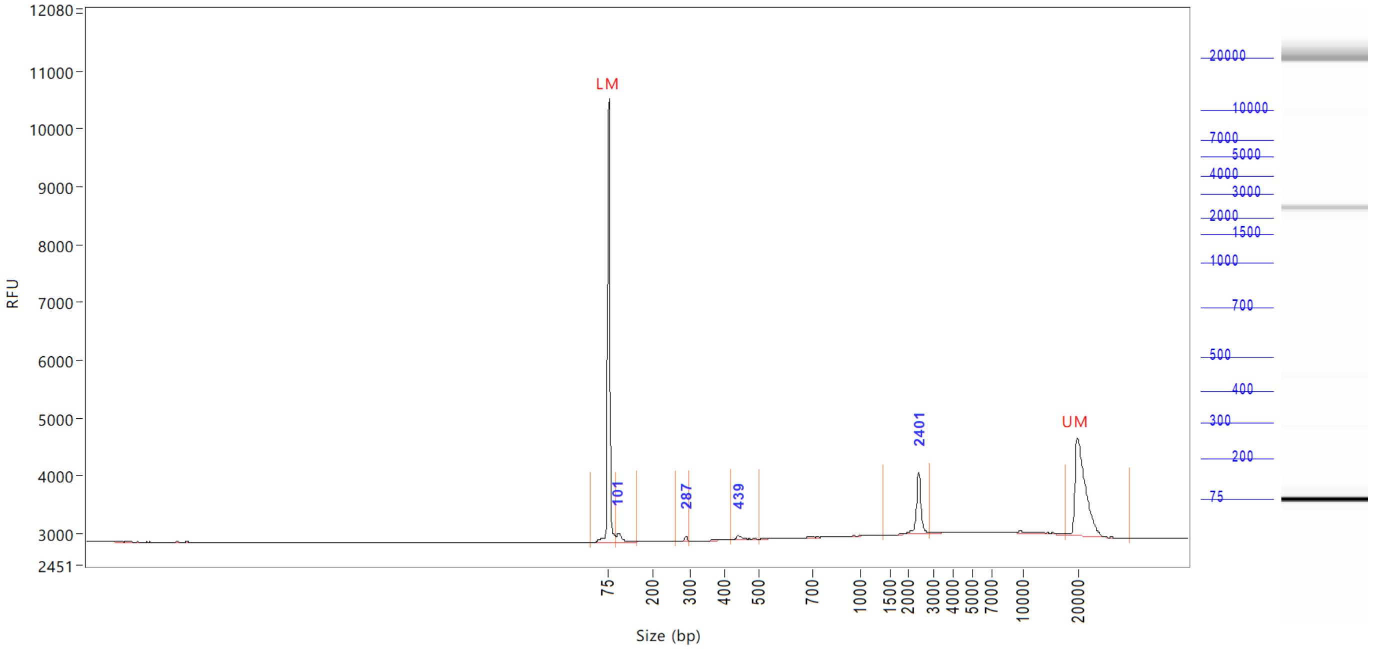

Peak	Size	Concentration	Relative concentration	Molarity	From	To	CV%	RFU
	(bp)	(ng/uL)	Percent	(nmole/L)	(bp)	(bp)		
1	75 (LM)	0.5000		11.3919	24	93	7.05	7695
2	101	0.2983	20.0	4.5779	93	154	12.43	159
3	287	0.0669	4.5	0.3875	259	296	1.98	88
4	439	0.1263	8.5	0.4648	418	498	3.57	72
5	2401	1.0002	67.1	0.7022	1378	2829	7.45	1061
6	20000 (UM)	0.2414		0.0192	17927	29430	5.56	1701

TIC:	1.4917	ng/uL
TIM:	6.1324	nmole/L
Total concentration:	1.9704	ng/uL

```
Sample peak width (sec): 5    Sample min peak height: 50    Sample baseline V to V?: Y    Sample baseline V to V points: 3
Sample filter: Binomial       Number of points for filter: 3  Sample start region (min): 0  Sample end region (min): 30
Marker peak width (sec): 5    Marker min peak height: 200   Marker baseline V to V?: Y    Marker baseline V to V points: 3
Lower marker selection: First peak > 500 RFU                Upper marker selection: Last peak > 200 RFU
Ladder size (bp)75, 200, 300, 400, 500, 700, 1000, 1500, 2000, 3000, 4000, 5000, 7000, 10000, 20000
Quantification using: Lower Marker          Final concentration (ng/uL): 0.5000          Dilution factor: 12.0
```

Sample: SampB3
Well location: B3
Created: Thursday, October 21, 2021 5:28:30 PM

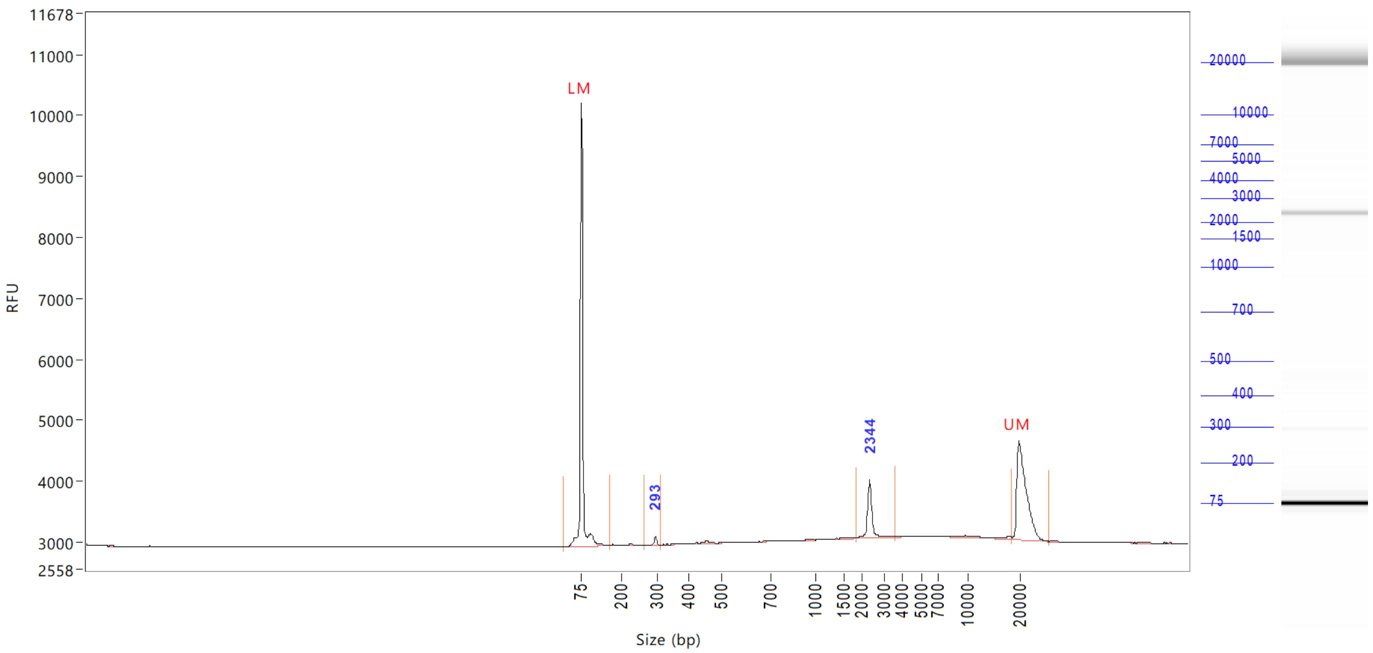

Peak	Size	Concentration	Relative concentration	Molarity	From	To	CV%	RFU
	(bp)	(ng/uL)	Percent	(nmole/L)	(bp)	(bp)		
1	75 (LM)	0.5000		10.9378	24	160	15.29	7286
2	293	0.1007	10.4	0.5714	264	311	2.28	131
3	2344	0.8680	89.6	0.6022	1846	3643	9.23	959
4	20000 (UM)	0.2137		0.0170	18659	25924	5.03	1622

TIC:	0.9688	ng/uL
TIM:	1.1735	nmole/L
Total concentration:	1.5815	ng/uL

```
Sample peak width (sec): 5    Sample min peak height: 50    Sample baseline V to V?: Y    Sample baseline V to V points: 3
Sample filter: Binomial       Number of points for filter: 3  Sample start region (min): 0  Sample end region (min): 30
Marker peak width (sec): 5    Marker min peak height: 200   Marker baseline V to V?: Y    Marker baseline V to V points: 3
Lower marker selection: First peak > 500 RFU                Upper marker selection: Last peak > 200 RFU
Ladder size (bp)75, 200, 300, 400, 500, 700, 1000, 1500, 2000, 3000, 4000, 5000, 7000, 10000, 20000
Quantification using: Lower Marker          Final concentration (ng/uL): 0.5000          Dilution factor: 12.0
```

Sample: SampC3
Well location: C3
Created: Thursday, October 21, 2021 5:28:30 PM

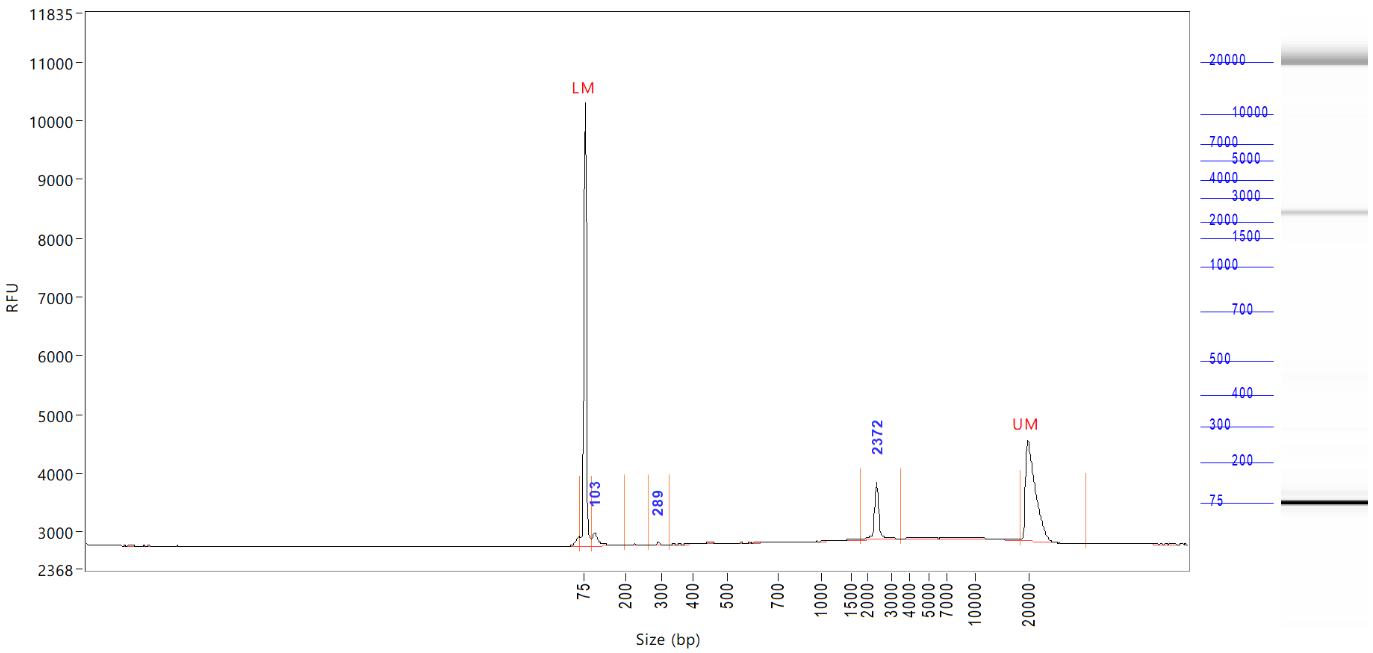

Peak	Size	Concentration	Relative concentration	Molarity	From	To	CV%	RFU
	(bp)	(ng/uL)	Percent	(nmole/L)	(bp)	(bp)		
1	75 (LM)	0.5000		11.0851	62	95	5.52	7563
2	103	0.4699	31.6	6.7710	95	194	17.93	220
3	289	0.0600	4.0	0.3426	260	321	3.35	57
4	2372	0.9588	64.4	0.6694	1770	3536	9.34	978
5	20000 (UM)	0.2421		0.0192	18659	31244	5.72	1711

TIC:	1.4887	ng/uL
TIM:	7.7830	nmole/L
Total concentration:	2.0704	ng/uL

```
Sample peak width (sec): 5    Sample min peak height: 50    Sample baseline V to V?: Y    Sample baseline V to V points: 3
Sample filter: Binomial       Number of points for filter: 3  Sample start region (min): 0  Sample end region (min): 30
Marker peak width (sec): 5    Marker min peak height: 200   Marker baseline V to V?: Y    Marker baseline V to V points: 3
Lower marker selection: First peak > 500 RFU                Upper marker selection: Last peak > 200 RFU
Ladder size (bp)75, 200, 300, 400, 500, 700, 1000, 1500, 2000, 3000, 4000, 5000, 7000, 10000, 20000
Quantification using: Lower Marker          Final concentration (ng/uL): 0.5000          Dilution factor: 12.0
```

Sample: SampD3
Well location: D3
Created: Thursday, October 21, 2021 5:28:30 PM

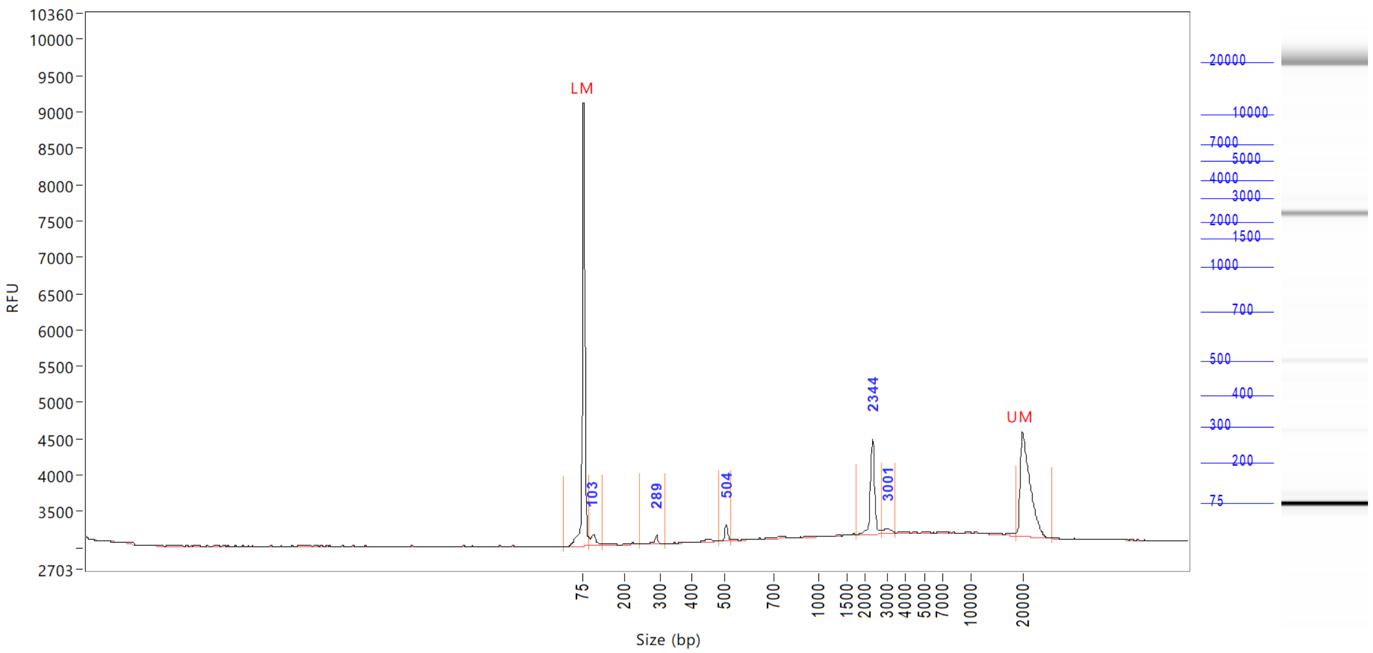

Peak	Size	Concentration	Relative concentration	Molarity	From	To	CV%	RFU
	(bp)	(ng/uL)	Percent	(nmole/L)	(bp)	(bp)		
1	75 (LM)	0.5000		11.5518	14	93	9.69	6113
2	103	0.3175	15.1	5.0129	93	131	8.15	158
3	289	0.1505	7.1	0.8745	244	317	3.63	120
4	504	0.1930	9.2	0.6327	483	524	1.17	219
5	2344	1.3110	62.2	0.9346	1750	2772	5.60	1305
6	3001	0.1354	6.4	0.0739	2772	3465	5.27	74
7	20000 (UM)	0.2344		0.0186	18659	25682	5.11	1451

TIC:	2.1073	ng/uL
TIM:	7.5287	nmole/L
Total concentration:	2.7887	ng/uL

```
Sample peak width (sec): 5     Sample min peak height: 50     Sample baseline V to V?: Y     Sample baseline V to V points: 3
Sample filter: Binomial        Number of points for filter: 3  Sample start region (min): 0  Sample end region (min): 30
Marker peak width (sec): 5     Marker min peak height: 200    Marker baseline V to V?: Y     Marker baseline V to V points: 3
Lower marker selection: First peak > 500 RFU                  Upper marker selection: Last peak > 200 RFU
Ladder size (bp)75, 200, 300, 400, 500, 700, 1000, 1500, 2000, 3000, 4000, 5000, 7000, 10000, 20000
Quantification using: Lower Marker          Final concentration (ng/uL): 0.5000          Dilution factor: 12.0
```

Sample: SampE3
Well location: E3
Created: Thursday, October 21, 2021 5:28:30 PM

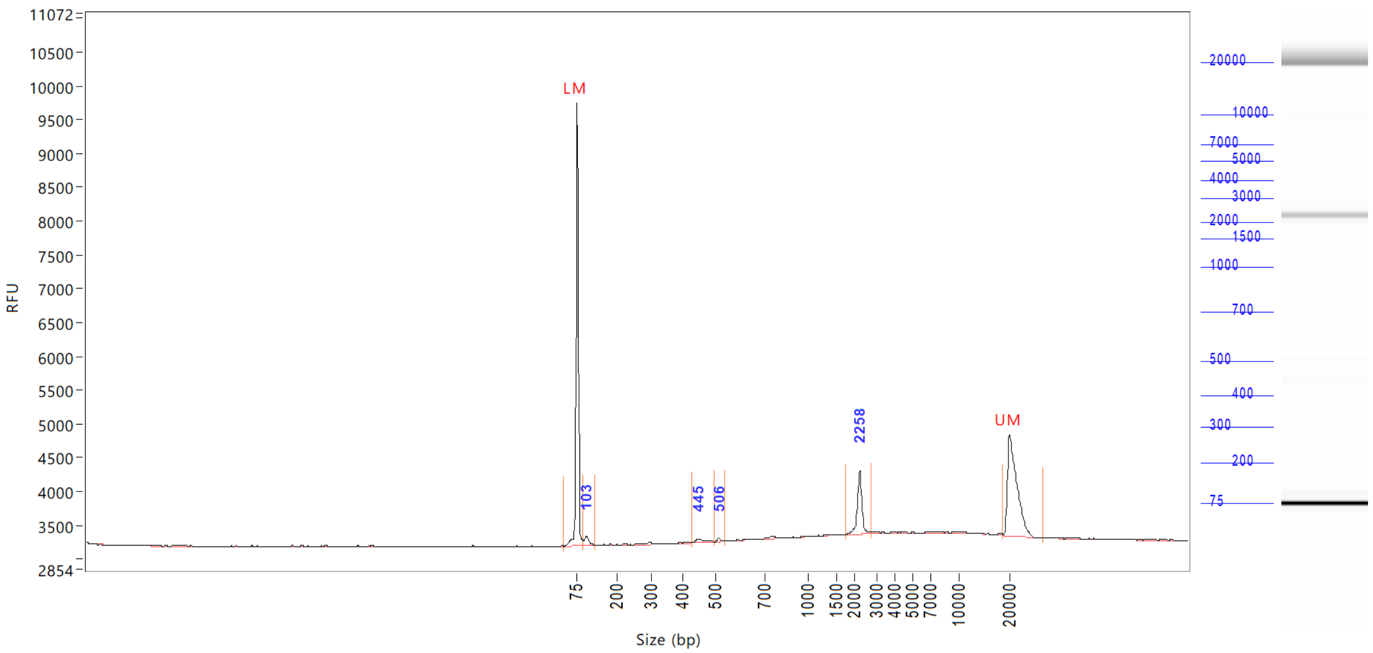

Peak	Size	Concentration	Relative concentration	Molarity	From	To	CV%	RFU
	(bp)	(ng/uL)	Percent	(nmole/L)	(bp)	(bp)		
1	75 (LM)	0.5000		11.3919	33	93	7.10	6558
2	103	0.2493	16.8	3.9370	93	131	7.33	133
3	445	0.1384	9.3	0.5061	426	496	3.04	59
4	506	0.0492	3.3	0.1601	496	532	1.08	62
5	2258	1.0512	70.6	0.7777	1750	2800	6.53	946
6	20000 (UM)	0.2398		0.0190	18659	26408	5.07	1489

TIC:	1.4881	ng/uL
TIM:	5.3809	nmole/L
Total concentration:	2.1835	ng/uL

Sample peak width (sec): 5 Sample min peak height: 50 Sample baseline V to V?: Y Sample baseline V to V points: 3
Sample filter: Binomial Number of points for filter: 3 Sample start region (min): 0 Sample end region (min): 30
Marker peak width (sec): 5 Marker min peak height: 200 Marker baseline V to V?: Y Marker baseline V to V points: 3
Lower marker selection: First peak > 500 RFU Upper marker selection: Last peak > 200 RFU
Ladder size (bp)75, 200, 300, 400, 500, 700, 1000, 1500, 2000, 3000, 4000, 5000, 7000, 10000, 20000
Quantification using: Lower Marker Final concentration (ng/uL): 0.5000 Dilution factor: 12.0

Sample: SampF3
Well location: F3
Created: Thursday, October 21, 2021 5:28:30 PM

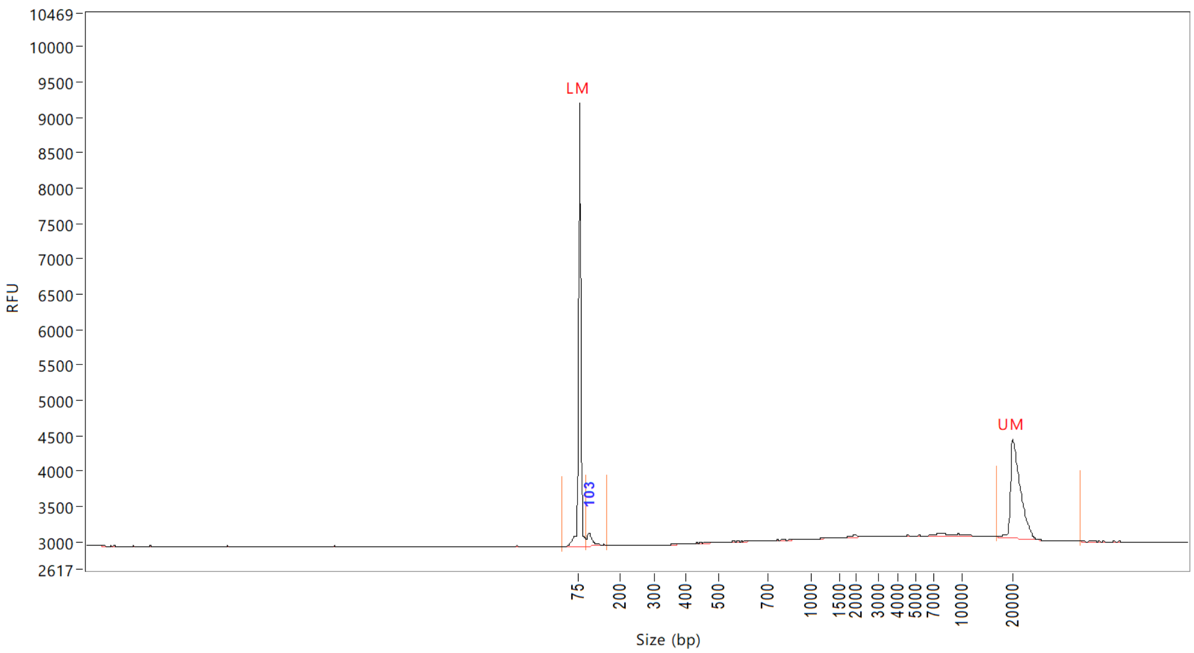

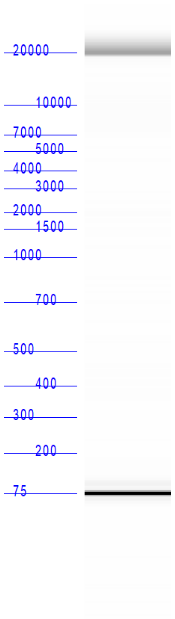

Peak	Size	Concentration	Relative concentration	Molarity	From	To	CV%	RFU
	(bp)	(ng/uL)	Percent	(nmole/L)	(bp)	(bp)		
1	75 (LM)	0.5000		11.2364	22	93	8.05	6271
2	103	0.4357	100.0	6.5652	93	158	13.10	185
3	20000 (UM)	0.2390		0.0189	16952	33299	7.00	1390

TIC:	0.4357	ng/uL
TIM:	6.5652	nmole/L
Total concentration:	1.1206	ng/uL

Sample peak width (sec): 5 Sample min peak height: 50 Sample baseline V to V?: Y Sample baseline V to V points: 3
Sample filter: Binomial Number of points for filter: 3 Sample start region (min): 0 Sample end region (min): 30
Marker peak width (sec): 5 Marker min peak height: 200 Marker baseline V to V?: Y Marker baseline V to V points: 3
Lower marker selection: First peak > 500 RFU Upper marker selection: Last peak > 200 RFU
Ladder size (bp)75, 200, 300, 400, 500, 700, 1000, 1500, 2000, 3000, 4000, 5000, 7000, 10000, 20000
Quantification using: Lower Marker Final concentration (ng/uL): 0.5000 Dilution factor: 12.0

Sample: SampG3
Well location: G3
Created: Thursday, October 21, 2021 5:28:30 PM

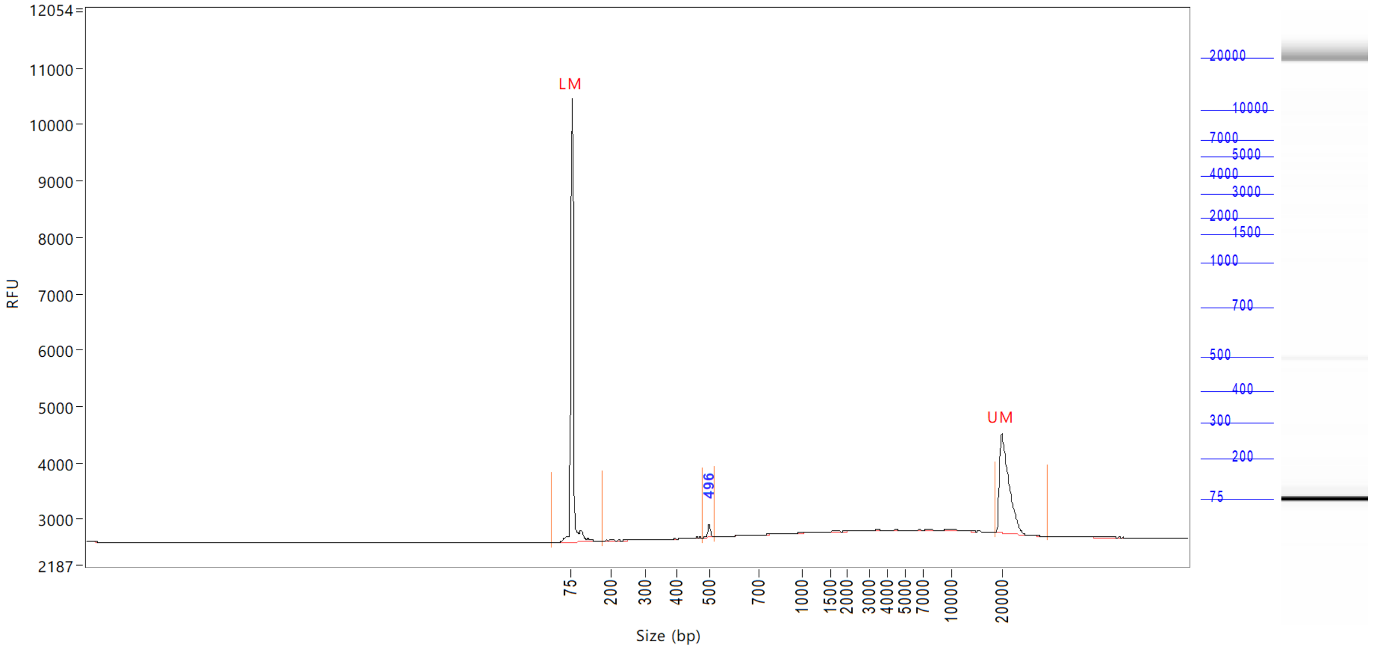

Peak	Size	Concentration	Relative concentration	Molarity	From	To	CV%	RFU
	(bp)	(ng/uL)	Percent	(nmole/L)	(bp)	(bp)		
1	75 (LM)	0.5000		10.7944	12	173	14.45	7881
2	496	0.1440	100.0	0.4786	477	516	0.95	224
3	20000 (UM)	0.2127		0.0169	18659	29068	5.19	1768

TIC:	0.1440	ng/uL
TIM:	0.4786	nmole/L
Total concentration:	0.5866	ng/uL

Sample peak width (sec): 5 Sample min peak height: 50 Sample baseline V to V?: Y Sample baseline V to V points: 3
Sample filter: Binomial Number of points for filter: 3 Sample start region (min): 0 Sample end region (min): 30
Marker peak width (sec): 5 Marker min peak height: 200 Marker baseline V to V?: Y Marker baseline V to V points: 3
Lower marker selection: First peak > 500 RFU Upper marker selection: Last peak > 200 RFU
Ladder size (bp)75, 200, 300, 400, 500, 700, 1000, 1500, 2000, 3000, 4000, 5000, 7000, 10000, 20000
Quantification using: Lower Marker Final concentration (ng/uL): 0.5000 Dilution factor: 12.0

Sample: SampH3
Well location: H3
Created: Thursday, October 21, 2021 5:28:30 PM

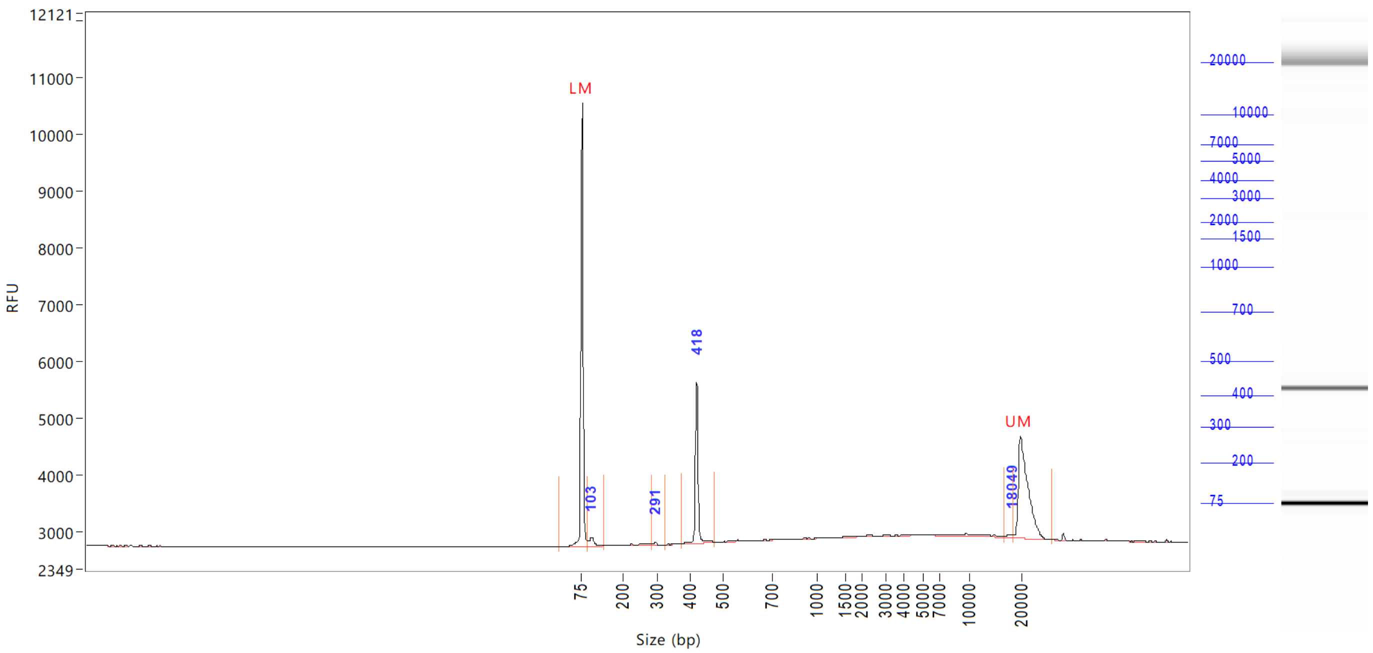

Peak	Size	Concentration	Relative concentration	Molarity	From	To	CV%	RFU
	(bp)	(ng/uL)	Percent	(nmole/L)	(bp)	(bp)		
1	75 (LM)	0.5000		11.2364	5	93	7.38	7808
2	103	0.2720	10.6	4.2140	93	143	10.21	150
3	291	0.0517	2.0	0.2921	282	321	2.47	59
4	418	2.1755	84.9	8.5633	374	473	2.11	2821
5	18049	0.0622	2.4	0.0058	16708	18537	2.92	52
6	20000 (UM)	0.2508		0.0199	18537	26045	5.11	1789

TIC:	2.5614	ng/uL
TIM:	13.0753	nmole/L
Total concentration:	3.3387	ng/uL

```
Sample peak width (sec): 5    Sample min peak height: 50    Sample baseline V to V?: Y    Sample baseline V to V points: 3
Sample filter: Binomial       Number of points for filter: 3  Sample start region (min): 0  Sample end region (min): 30
Marker peak width (sec): 5    Marker min peak height: 200   Marker baseline V to V?: Y    Marker baseline V to V points: 3
Lower marker selection: First peak > 500 RFU                Upper marker selection: Last peak > 200 RFU
Ladder size (bp)75, 200, 300, 400, 500, 700, 1000, 1500, 2000, 3000, 4000, 5000, 7000, 10000, 20000
Quantification using: Lower Marker          Final concentration (ng/uL): 0.5000          Dilution factor: 12.0
```

Sample: SampA4
Well location: A4
Created: Thursday, October 21, 2021 5:28:30 PM

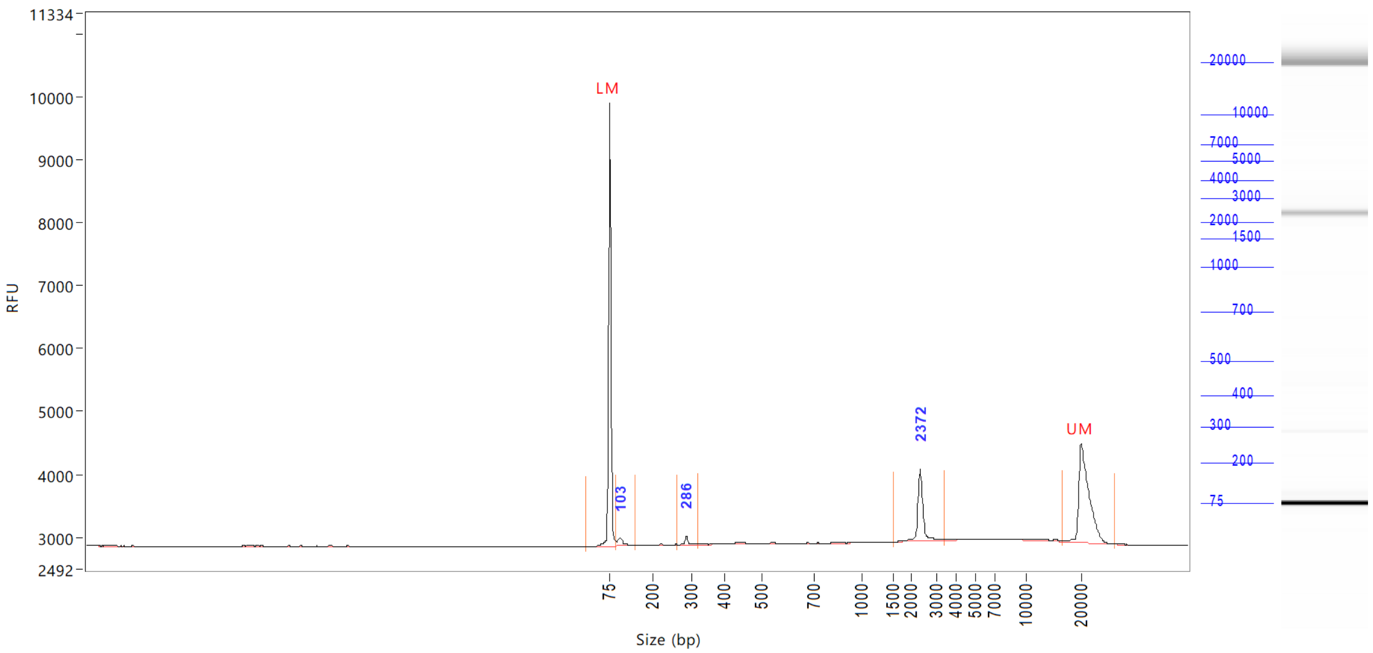

Peak	Size	Concentration	Relative concentration	Molarity	From	To	CV%	RFU
	(bp)	(ng/uL)	Percent	(nmole/L)	(bp)	(bp)		
1	75 (LM)	0.5000		11.2364	11	93	6.44	7061
2	103	0.2628	15.4	4.0339	93	147	10.66	127
3	286	0.1446	8.5	0.8317	264	317	3.12	145
4	2372	1.2994	76.1	0.9052	1501	3429	8.98	1148
5	20000 (UM)	0.2462		0.0196	16464	26045	5.34	1569

TIC:	1.7068	ng/uL
TIM:	5.7708	nmole/L
Total concentration:	2.2502	ng/uL

Sample peak width (sec): 5   Sample min peak height: 50   Sample baseline V to V?: Y   Sample baseline V to V points: 3
Sample filter: Binomial   Number of points for filter: 3   Sample start region (min): 0   Sample end region (min): 30
Marker peak width (sec): 5   Marker min peak height: 200   Marker baseline V to V?: Y   Marker baseline V to V points: 3
Lower marker selection: First peak > 500 RFU   Upper marker selection: Last peak > 200 RFU
Ladder size (bp)75, 200, 300, 400, 500, 700, 1000, 1500, 2000, 3000, 4000, 5000, 7000, 10000, 20000
Quantification using: Lower Marker   Final concentration (ng/uL): 0.5000   Dilution factor: 12.0

Sample: SampB4
Well location: B4
Created: Thursday, October 21, 2021 5:28:30 PM

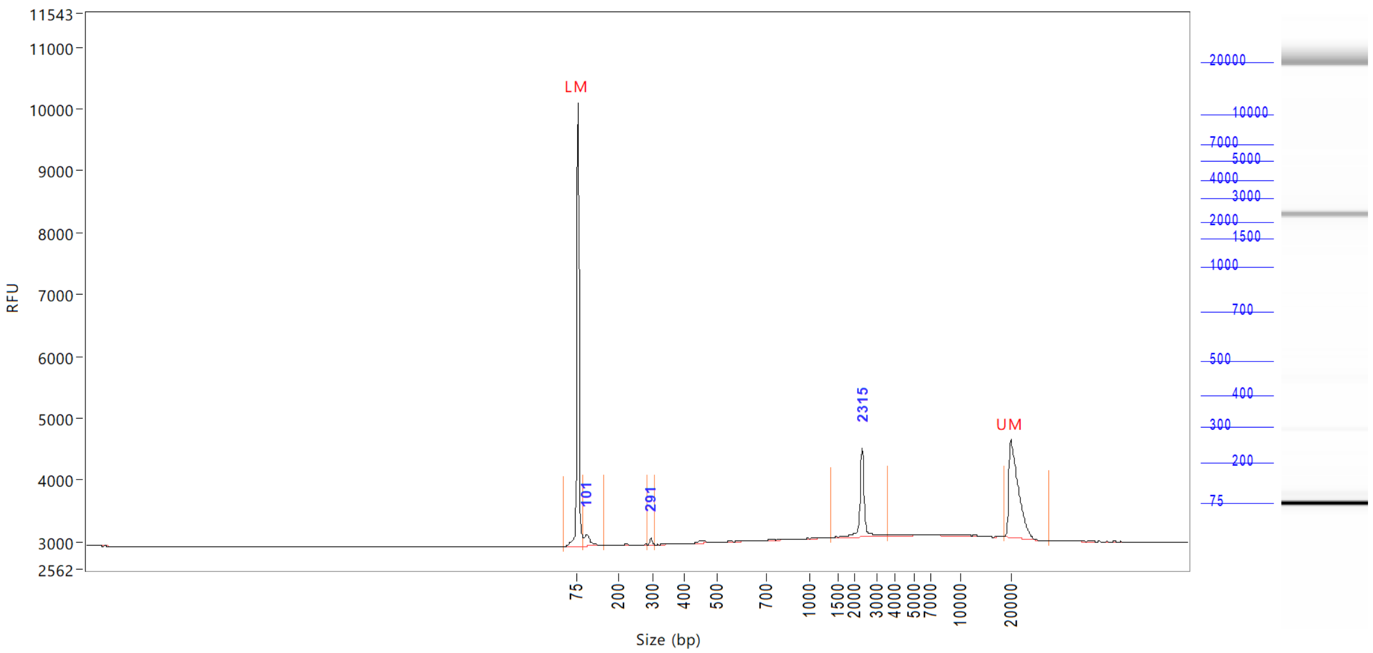

Peak	Size	Concentration	Relative concentration	Molarity	From	To	CV%	RFU
	(bp)	(ng/uL)	Percent	(nmole/L)	(bp)	(bp)		
1	75 (LM)	0.5000		11.2364	31	93	7.16	7173
2	101	0.4123	20.8	6.2702	93	156	12.68	193
3	291	0.0927	4.7	0.5242	284	305	1.06	122
4	2315	1.4741	74.5	1.0587	1367	3608	11.71	1423
5	20000 (UM)	0.2298		0.0182	18659	27496	5.17	1576

TIC:	1.9792	ng/uL
TIM:	7.8531	nmole/L
Total concentration:	2.5229	ng/uL

```
Sample peak width (sec): 5    Sample min peak height: 50    Sample baseline V to V?: Y    Sample baseline V to V points: 3
Sample filter: Binomial       Number of points for filter: 3  Sample start region (min): 0  Sample end region (min): 30
Marker peak width (sec): 5    Marker min peak height: 200   Marker baseline V to V?: Y    Marker baseline V to V points: 3
Lower marker selection: First peak > 500 RFU                Upper marker selection: Last peak > 200 RFU
Ladder size (bp)75, 200, 300, 400, 500, 700, 1000, 1500, 2000, 3000, 4000, 5000, 7000, 10000, 20000
Quantification using: Lower Marker          Final concentration (ng/uL): 0.5000          Dilution factor: 12.0
```

Sample: SampC4
Well location: C4
Created: Thursday, October 21, 2021 5:28:30 PM

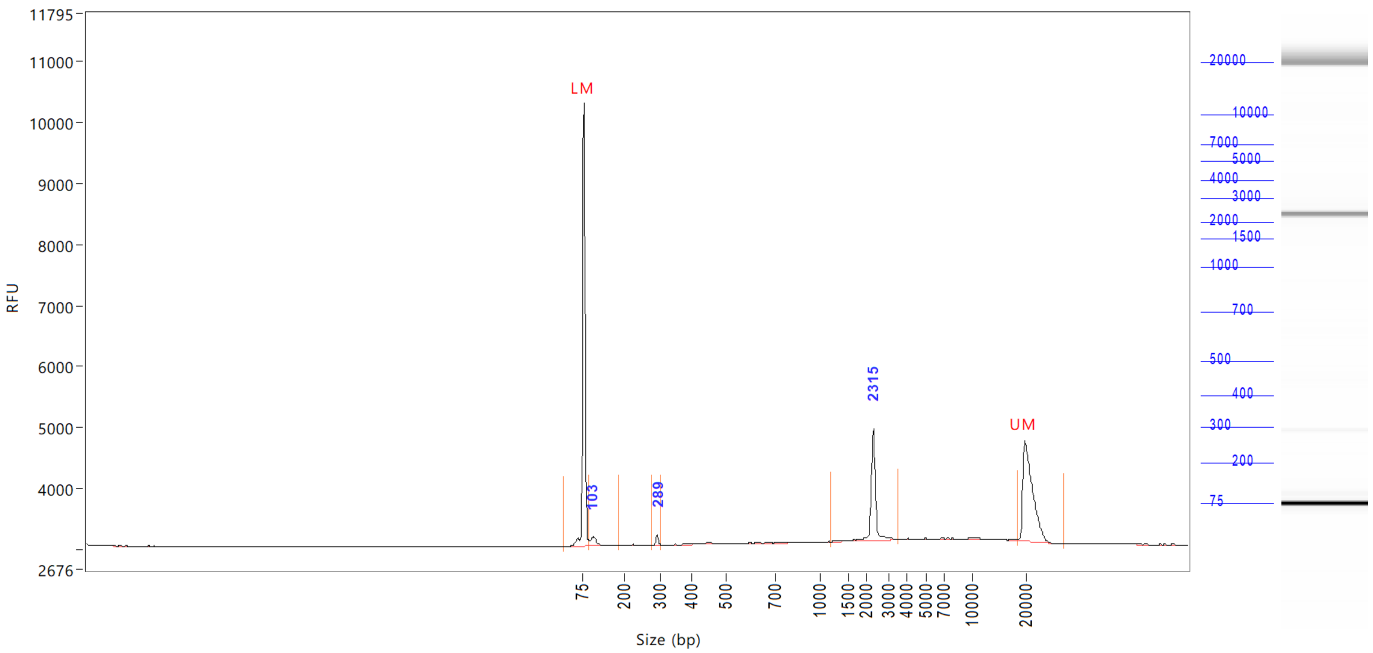

Peak	Size	Concentration	Relative concentration	Molarity	From	To	CV%	RFU
	(bp)	(ng/uL)	Percent	(nmole/L)	(bp)	(bp)		
1	75 (LM)	0.5000		11.3919	14	93	7.54	7281
2	103	0.2935	13.2	4.4641	93	179	14.06	148
3	289	0.1327	5.9	0.7605	277	301	1.17	180
4	2315	1.8043	80.9	1.3010	1178	3500	11.28	1835
5	20000 (UM)	0.2431		0.0193	18659	27496	5.04	1656

TIC:	2.2305	ng/uL
TIM:	6.5256	nmole/L
Total concentration:	2.6922	ng/uL

Sample peak width (sec): 5 Sample min peak height: 50 Sample baseline V to V?: Y Sample baseline V to V points: 3
Sample filter: Binomial Number of points for filter: 3 Sample start region (min): 0 Sample end region (min): 30
Marker peak width (sec): 5 Marker min peak height: 200 Marker baseline V to V?: Y Marker baseline V to V points: 3
Lower marker selection: First peak > 500 RFU Upper marker selection: Last peak > 200 RFU
Ladder size (bp)75, 200, 300, 400, 500, 700, 1000, 1500, 2000, 3000, 4000, 5000, 7000, 10000, 20000
Quantification using: Lower Marker Final concentration (ng/uL): 0.5000 Dilution factor: 12.0

Sample: SampD4
Well location: D4
Created: Thursday, October 21, 2021 5:28:30 PM

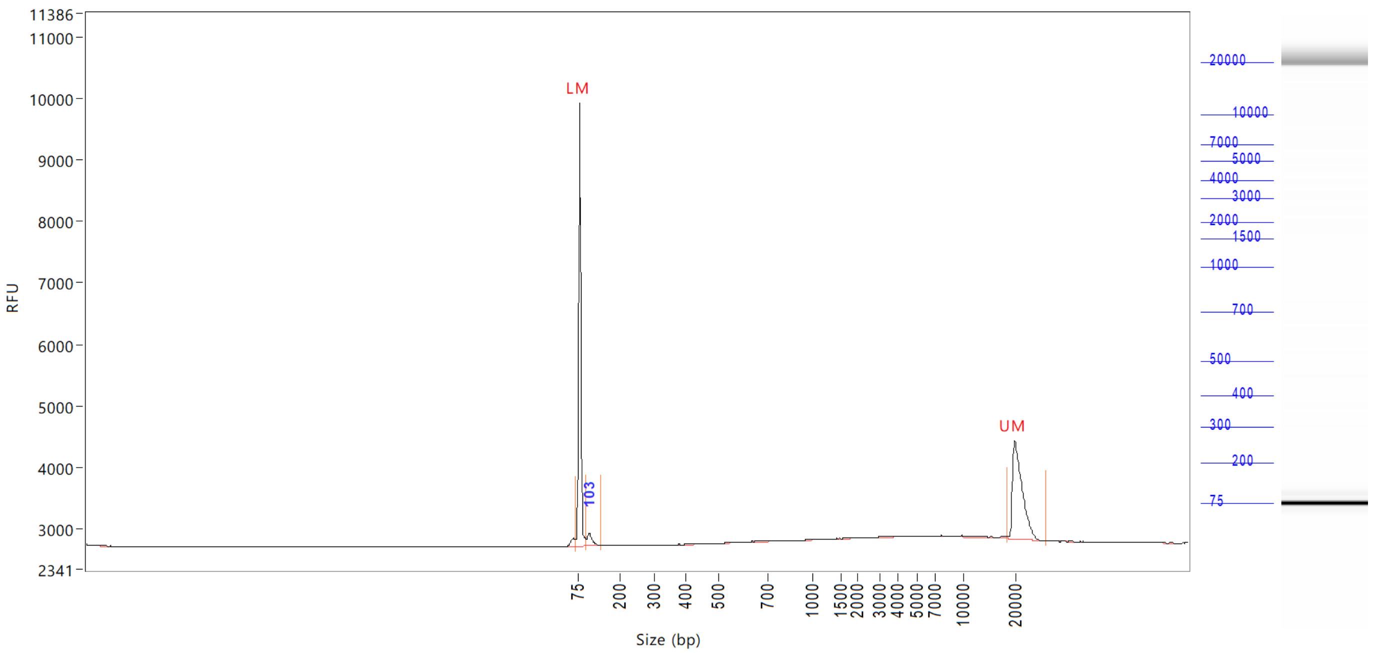

Peak	Size	Concentration	Relative concentration	Molarity	From	To	CV%	RFU
	(bp)	(ng/uL)	Percent	(nmole/L)	(bp)	(bp)		
1	75 (LM)	0.5000		11.0851	62	95	5.58	7223
2	103	0.3579	100.0	5.5454	95	139	7.95	199
3	20000 (UM)	0.2411		0.0191	18659	26166	4.99	1603

TIC:	0.3579	ng/uL
TIM:	5.5454	nmole/L
Total concentration:	0.9672	ng/uL

```
Sample peak width (sec): 5    Sample min peak height: 50    Sample baseline V to V?: Y    Sample baseline V to V points: 3
Sample filter: Binomial       Number of points for filter: 3  Sample start region (min): 0  Sample end region (min): 30
Marker peak width (sec): 5    Marker min peak height: 200   Marker baseline V to V?: Y    Marker baseline V to V points: 3
Lower marker selection: First peak > 500 RFU                Upper marker selection: Last peak > 200 RFU
Ladder size (bp)75, 200, 300, 400, 500, 700, 1000, 1500, 2000, 3000, 4000, 5000, 7000, 10000, 20000
Quantification using: Lower Marker          Final concentration (ng/uL): 0.5000          Dilution factor: 12.0
```

Sample: SampE4
Well location: E4
Created: Thursday, October 21, 2021 5:28:30 PM

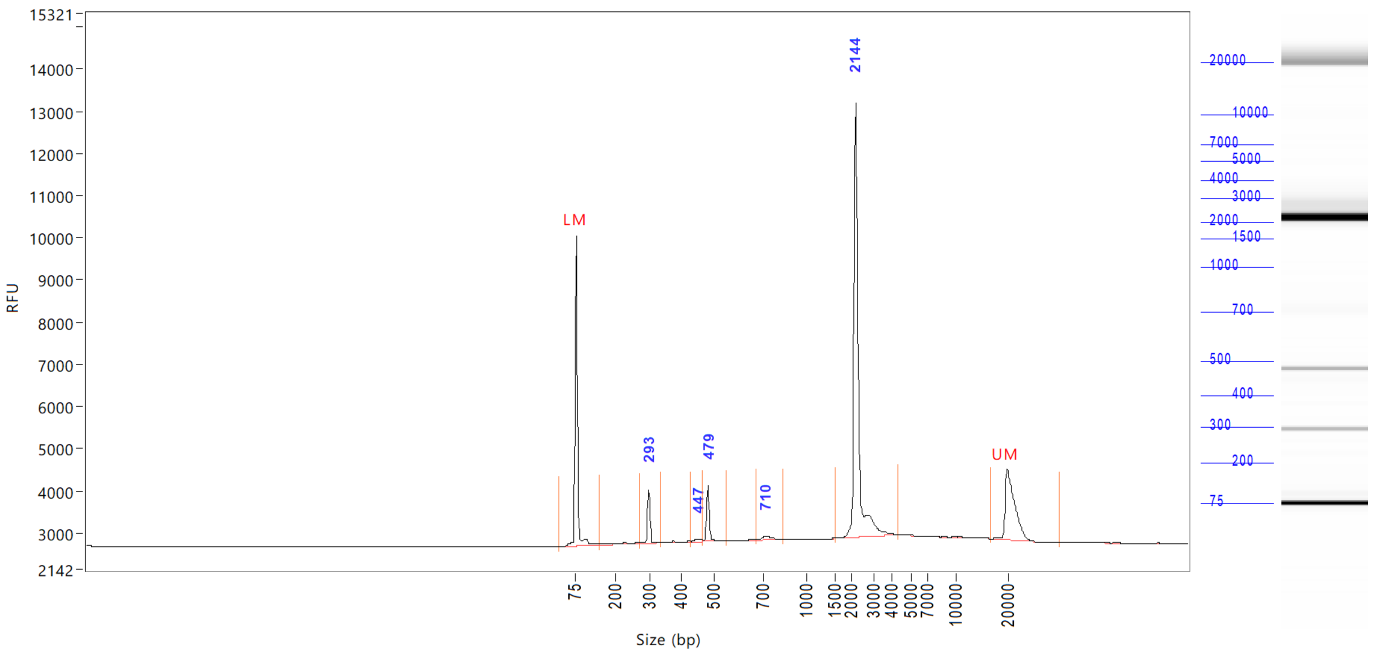

Peak	Size	Concentration	Relative concentration	Molarity	From	To	CV%	RFU
	(bp)	(ng/uL)	Percent	(nmole/L)	(bp)	(bp)		
1	75 (LM)	0.5000		10.9378	22	150	12.72	7343
2	293	0.8953	7.5	5.0605	268	329	2.01	1267
3	447	0.1213	1.0	0.4466	426	464	2.06	67
4	479	0.9743	8.2	3.3538	464	547	1.66	1321
5	710	0.2202	1.9	0.4971	667	831	5.20	89
6	2144	9.6630	81.4	7.1045	1500	4380	13.07	10314
7	20000 (UM)	0.2288		0.0181	16830	30156	5.40	1673

TIC:	11.8740	ng/uL
TIM:	16.4625	nmole/L
Total concentration:	12.2532	ng/uL

Sample peak width (sec): 5 Sample min peak height: 50 Sample baseline V to V?: Y Sample baseline V to V points: 3
Sample filter: Binomial Number of points for filter: 3 Sample start region (min): 0 Sample end region (min): 30
Marker peak width (sec): 5 Marker min peak height: 200 Marker baseline V to V?: Y Marker baseline V to V points: 3
Lower marker selection: First peak > 500 RFU Upper marker selection: Last peak > 200 RFU
Ladder size (bp)75, 200, 300, 400, 500, 700, 1000, 1500, 2000, 3000, 4000, 5000, 7000, 10000, 20000
Quantification using: Lower Marker Final concentration (ng/uL): 0.5000 Dilution factor: 12.0

Sample: SampF4
Well location: F4
Created: Thursday, October 21, 2021 5:28:30 PM

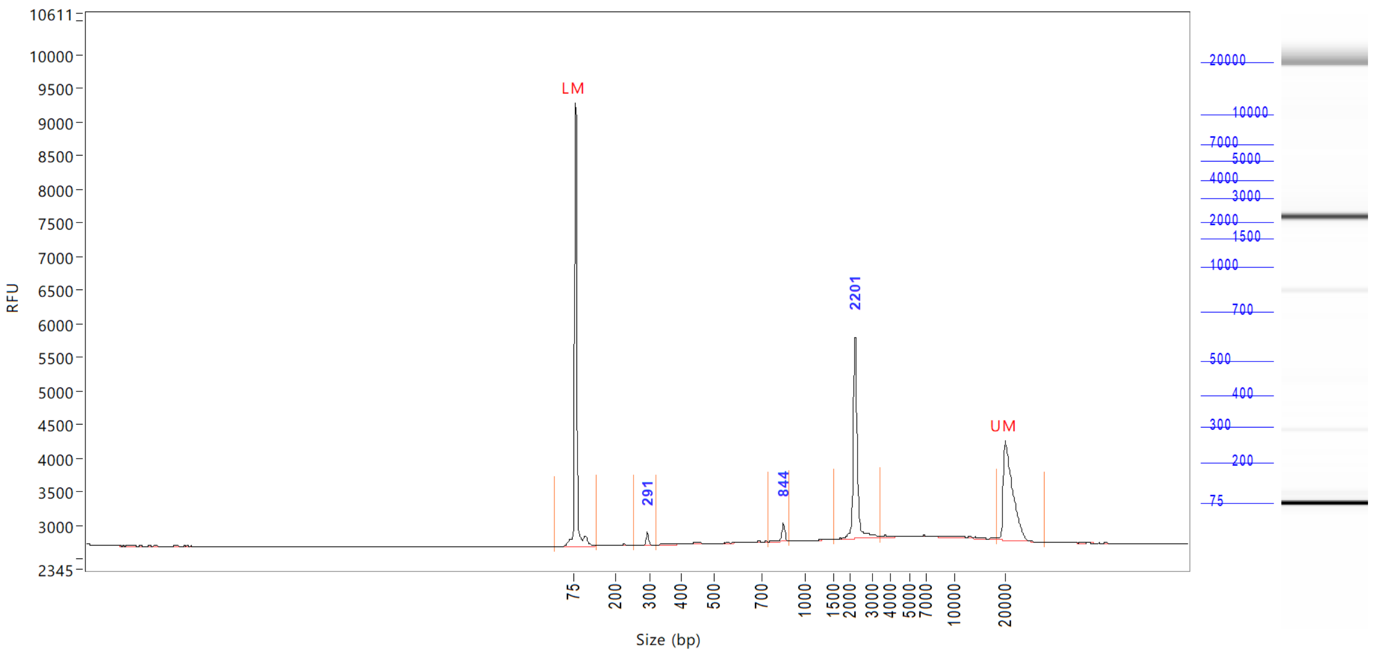

Peak	Size	Concentration	Relative concentration	Molarity	From	To	CV%	RFU
	(bp)	(ng/uL)	Percent	(nmole/L)	(bp)	(bp)		
1	75 (LM)	0.5000		10.9378	14	143	12.13	6604
2	291	0.1590	4.9	0.9050	253	319	2.10	191
3	844	0.2232	6.9	0.4394	736	892	2.37	268
4	2201	2.8573	88.2	2.1370	1520	3393	9.06	2983
5	20000 (UM)	0.2219		0.0176	18293	27859	5.22	1478

TIC:	3.2394	ng/uL
TIM:	3.4813	nmole/L
Total concentration:	3.8872	ng/uL

```
Sample peak width (sec): 5     Sample min peak height: 50     Sample baseline V to V?: Y     Sample baseline V to V points: 3
Sample filter: Binomial        Number of points for filter: 3  Sample start region (min): 0  Sample end region (min): 30
Marker peak width (sec): 5     Marker min peak height: 200    Marker baseline V to V?: Y     Marker baseline V to V points: 3
Lower marker selection: First peak > 500 RFU                  Upper marker selection: Last peak > 200 RFU
Ladder size (bp)75, 200, 300, 400, 500, 700, 1000, 1500, 2000, 3000, 4000, 5000, 7000, 10000, 20000
Quantification using: Lower Marker          Final concentration (ng/uL): 0.5000          Dilution factor: 12.0
```

Sample: SampG4
Well location: G4
Created: Thursday, October 21, 2021 5:28:30 PM

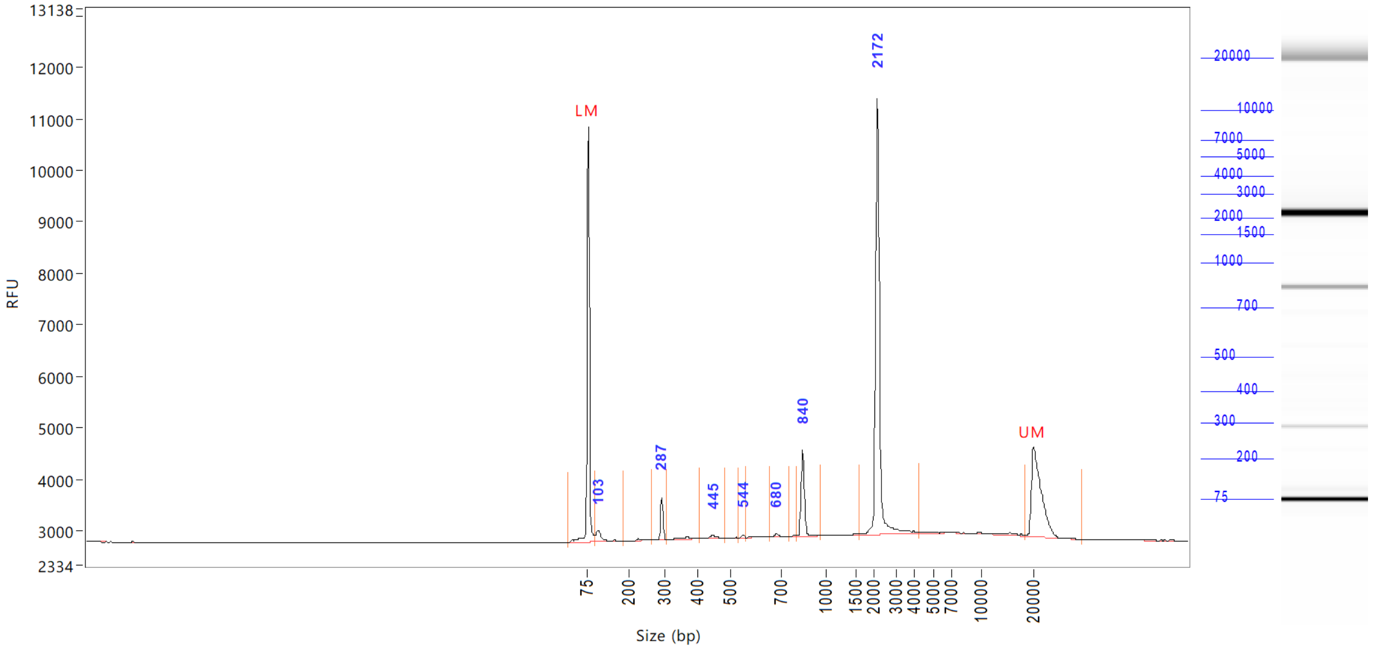

Peak	Size	Concentration	Relative concentration	Molarity	From	To	CV%	RFU
	(bp)	(ng/uL)	Percent	(nmole/L)	(bp)	(bp)		
1	75 (LM)	0.5000		11.3919	14	95	9.89	8060
2	103	0.3884	4.2	5.7480	95	181	15.39	211
3	287	0.5252	5.7	3.0208	260	301	1.23	820
4	445	0.1094	1.2	0.4074	405	483	3.43	51
5	544	0.0384	0.4	0.1166	527	560	0.97	60
6	680	0.0594	0.6	0.1437	649	744	1.93	58
7	840	1.2020	13.1	2.3665	801	957	1.88	1669
8	2172	6.8852	74.8	5.1472	1597	4242	12.09	8481
9	20000 (UM)	0.2321		0.0184	18415	29189	5.30	1751

TIC:	9.2081	ng/uL
TIM:	16.9503	nmole/L
Total concentration:	9.7239	ng/uL

Sample peak width (sec): 5 Sample min peak height: 50 Sample baseline V to V?: Y Sample baseline V to V points: 3
Sample filter: Binomial Number of points for filter: 3 Sample start region (min): 0 Sample end region (min): 30
Marker peak width (sec): 5 Marker min peak height: 200 Marker baseline V to V?: Y Marker baseline V to V points: 3
Lower marker selection: First peak > 500 RFU Upper marker selection: Last peak > 200 RFU
Ladder size (bp)75, 200, 300, 400, 500, 700, 1000, 1500, 2000, 3000, 4000, 5000, 7000, 10000, 20000
Quantification using: Lower Marker Final concentration (ng/uL): 0.5000 Dilution factor: 12.0

Sample: SampH4
Well location: H4
Created: Thursday, October 21, 2021 5:28:30 PM

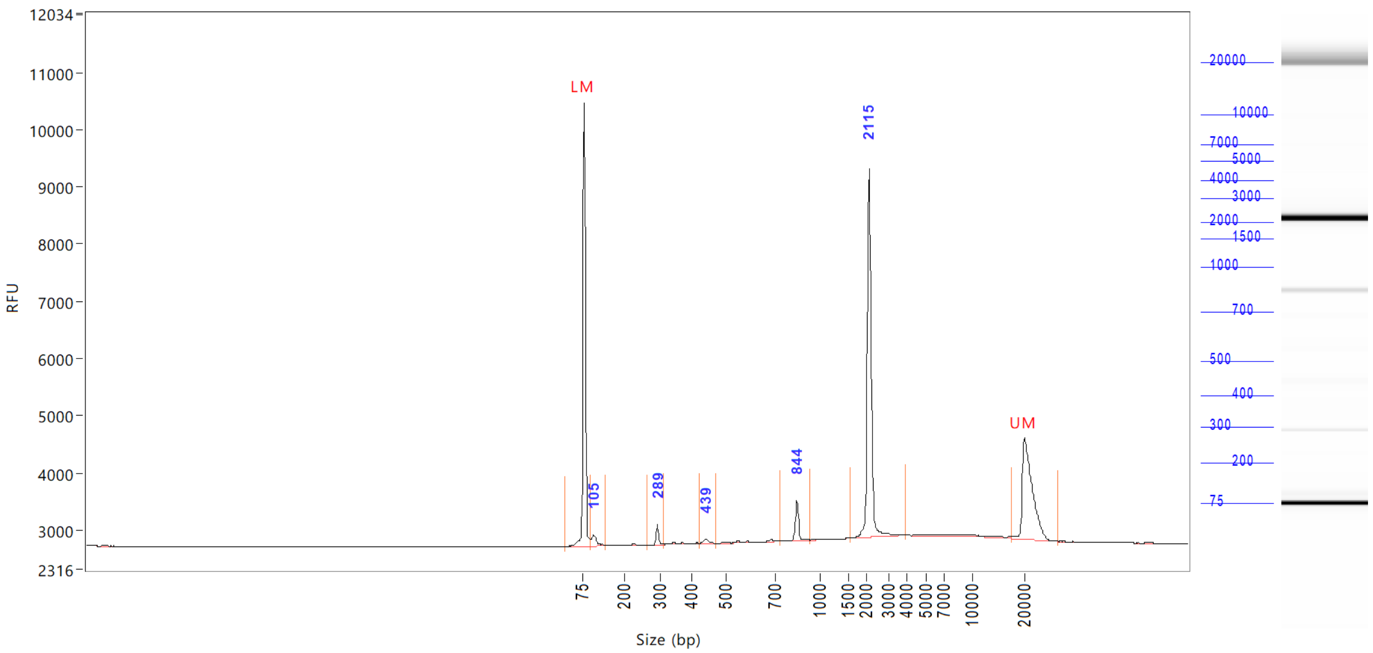

Peak	Size	Concentration	Relative concentration	Molarity	From	To	CV%	RFU
	(bp)	(ng/uL)	Percent	(nmole/L)	(bp)	(bp)		
1	75 (LM)	0.5000		11.2364	22	95	7.50	7763
2	105	0.3192	4.9	4.8995	95	139	9.17	185
3	289	0.2350	3.6	1.3424	262	311	1.50	347
4	439	0.1138	1.8	0.4255	420	471	2.02	78
5	844	0.5252	8.1	1.0278	736	935	2.01	707
6	2115	5.2973	81.6	4.0864	1520	3929	7.59	6450
7	20000 (UM)	0.2479		0.0197	17561	26529	5.35	1780

TIC:	6.4906	ng/uL
TIM:	11.7816	nmole/L
Total concentration:	7.1109	ng/uL

```
Sample peak width (sec): 5     Sample min peak height: 50     Sample baseline V to V?: Y     Sample baseline V to V points: 3
Sample filter: Binomial        Number of points for filter: 3  Sample start region (min): 0  Sample end region (min): 30
Marker peak width (sec): 5     Marker min peak height: 200    Marker baseline V to V?: Y     Marker baseline V to V points: 3
Lower marker selection: First peak > 500 RFU                  Upper marker selection: Last peak > 200 RFU
Ladder size (bp)75, 200, 300, 400, 500, 700, 1000, 1500, 2000, 3000, 4000, 5000, 7000, 10000, 20000
Quantification using: Lower Marker          Final concentration (ng/uL): 0.5000          Dilution factor: 12.0
```

Sample: SampA5
Well location: A5
Created: Thursday, October 21, 2021 5:28:30 PM

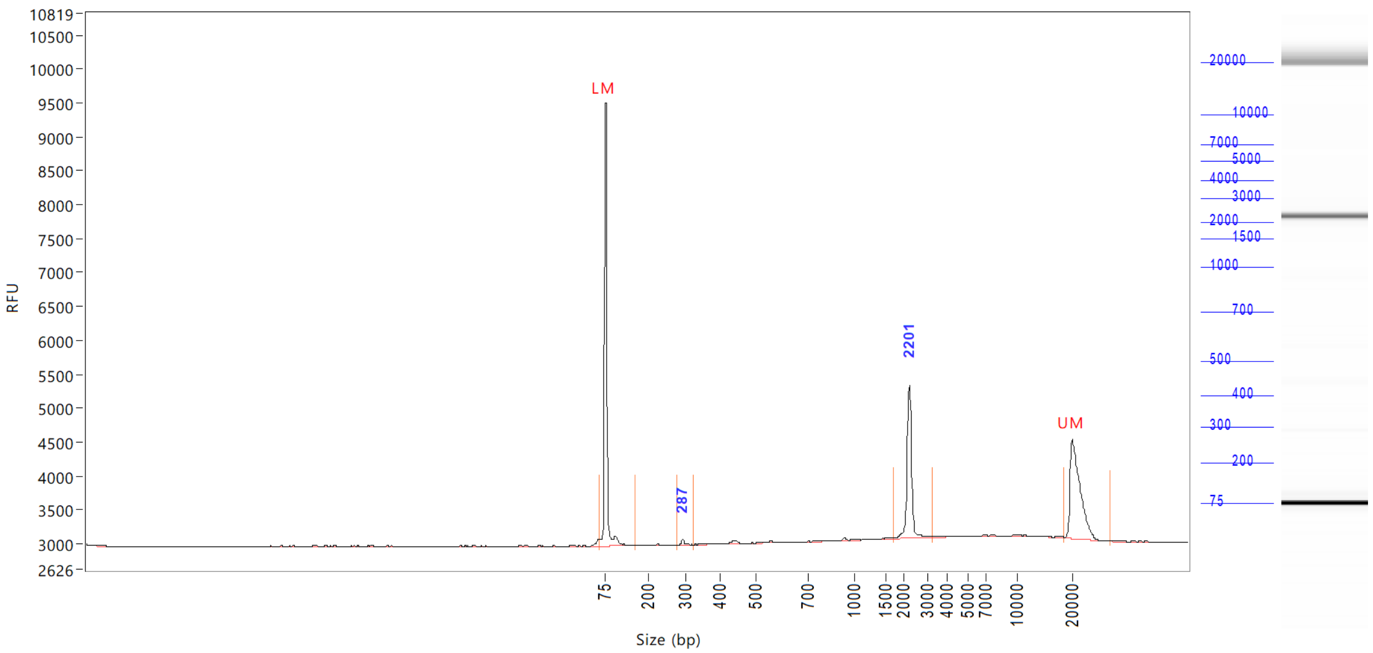

Peak	Size	Concentration	Relative concentration	Molarity	From	To	CV%	RFU
	(bp)	(ng/uL)	Percent	(nmole/L)	(bp)	(bp)		
1	75 (LM)	0.5000		10.7944	62	162	11.45	6542
2	287	0.0765	3.3	0.4351	275	319	2.50	82
3	2201	2.2527	96.7	1.6856	1712	3179	7.23	2251
4	20000 (UM)	0.2265		0.0180	18537	26891	5.09	1471

TIC:	2.3292	ng/uL
TIM:	2.1207	nmole/L
Total concentration:	2.9765	ng/uL

```
Sample peak width (sec): 5    Sample min peak height: 50    Sample baseline V to V?: Y    Sample baseline V to V points: 3
Sample filter: Binomial       Number of points for filter: 3  Sample start region (min): 0  Sample end region (min): 30
Marker peak width (sec): 5    Marker min peak height: 200   Marker baseline V to V?: Y    Marker baseline V to V points: 3
Lower marker selection: First peak > 500 RFU                Upper marker selection: Last peak > 200 RFU
Ladder size (bp)75, 200, 300, 400, 500, 700, 1000, 1500, 2000, 3000, 4000, 5000, 7000, 10000, 20000
Quantification using: Lower Marker          Final concentration (ng/uL): 0.5000          Dilution factor: 12.0
```

Sample: SampB5
Well location: B5
Created: Thursday, October 21, 2021 5:28:30 PM

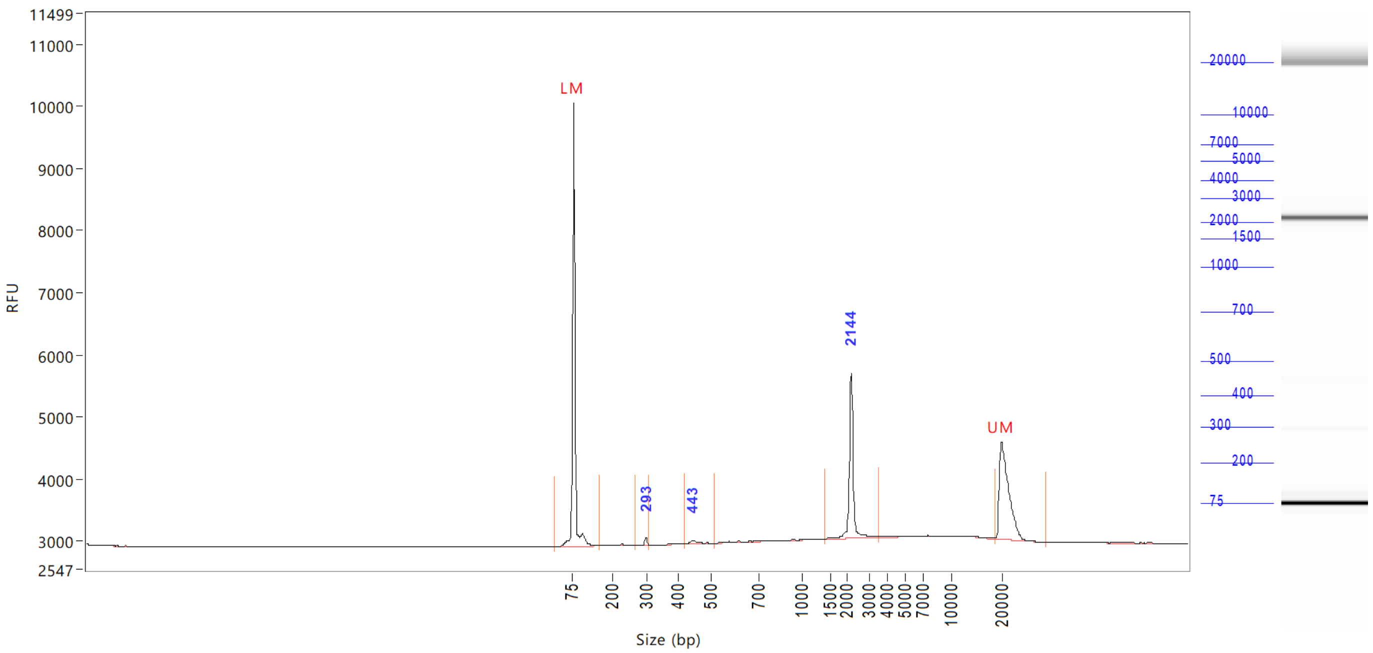

Peak	Size	Concentration	Relative concentration	Molarity	From	To	CV%	RFU
	(bp)	(ng/uL)	Percent	(nmole/L)	(bp)	(bp)		
1	75 (LM)	0.5000		10.9378	20	156	13.90	7154
2	293	0.0906	3.6	0.5136	264	303	2.08	121
3	443	0.1244	5.0	0.4548	420	511	3.92	60
4	2144	2.2698	91.3	1.7411	1389	3465	10.04	2653
5	20000 (UM)	0.2146		0.0171	18781	28705	5.27	1577

TIC:	2.4848	ng/uL
TIM:	2.7096	nmole/L
Total concentration:	2.9222	ng/uL

Sample peak width (sec): 5   Sample min peak height: 50   Sample baseline V to V?: Y   Sample baseline V to V points: 3
Sample filter: Binomial   Number of points for filter: 3   Sample start region (min): 0   Sample end region (min): 30
Marker peak width (sec): 5   Marker min peak height: 200   Marker baseline V to V?: Y   Marker baseline V to V points: 3
Lower marker selection: First peak > 500 RFU   Upper marker selection: Last peak > 200 RFU
Ladder size (bp)75, 200, 300, 400, 500, 700, 1000, 1500, 2000, 3000, 4000, 5000, 7000, 10000, 20000
Quantification using: Lower Marker   Final concentration (ng/uL): 0.5000   Dilution factor: 12.0

Sample: SampC5
Well location: C5
Created: Thursday, October 21, 2021 5:28:30 PM

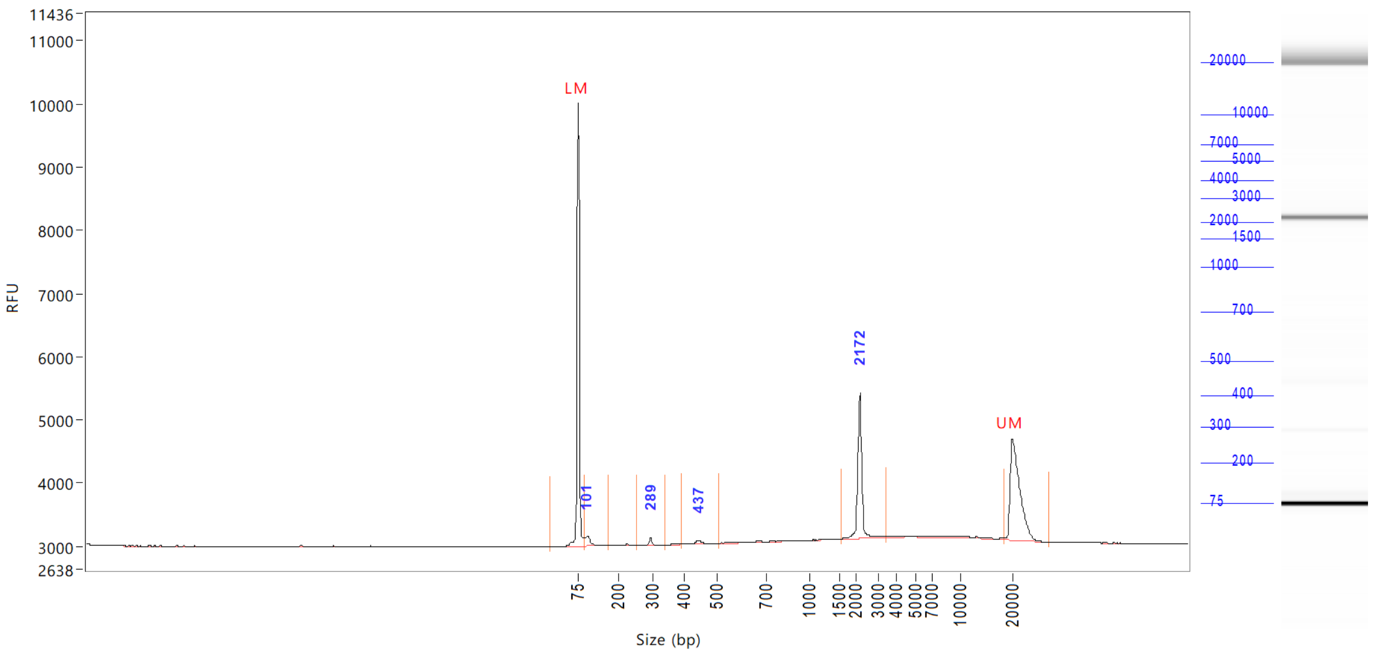

Peak	Size	Concentration	Relative concentration	Molarity	From	To	CV%	RFU
	(bp)	(ng/uL)	Percent	(nmole/L)	(bp)	(bp)		
1	75 (LM)	0.5000		11.3919	0	95	8.05	7027
2	101	0.3009	11.4	4.4934	95	166	14.33	153
3	289	0.1118	4.2	0.6387	250	335	3.41	127
4	437	0.1455	5.5	0.5428	392	501	4.44	62
5	2172	2.0880	78.9	1.5906	1559	3465	9.23	2301
6	20000 (UM)	0.2371		0.0188	18415	27254	5.23	1601

TIC:	2.6462	ng/uL
TIM:	7.2654	nmole/L
Total concentration:	3.1680	ng/uL

```
Sample peak width (sec): 5    Sample min peak height: 50    Sample baseline V to V?: Y    Sample baseline V to V points: 3
Sample filter: Binomial       Number of points for filter: 3  Sample start region (min): 0  Sample end region (min): 30
Marker peak width (sec): 5    Marker min peak height: 200   Marker baseline V to V?: Y    Marker baseline V to V points: 3
Lower marker selection: First peak > 500 RFU                Upper marker selection: Last peak > 200 RFU
Ladder size (bp)75, 200, 300, 400, 500, 700, 1000, 1500, 2000, 3000, 4000, 5000, 7000, 10000, 20000
Quantification using: Lower Marker          Final concentration (ng/uL): 0.5000          Dilution factor: 12.0
```

Sample: SampD5
Well location: D5
Created: Thursday, October 21, 2021 5:28:30 PM

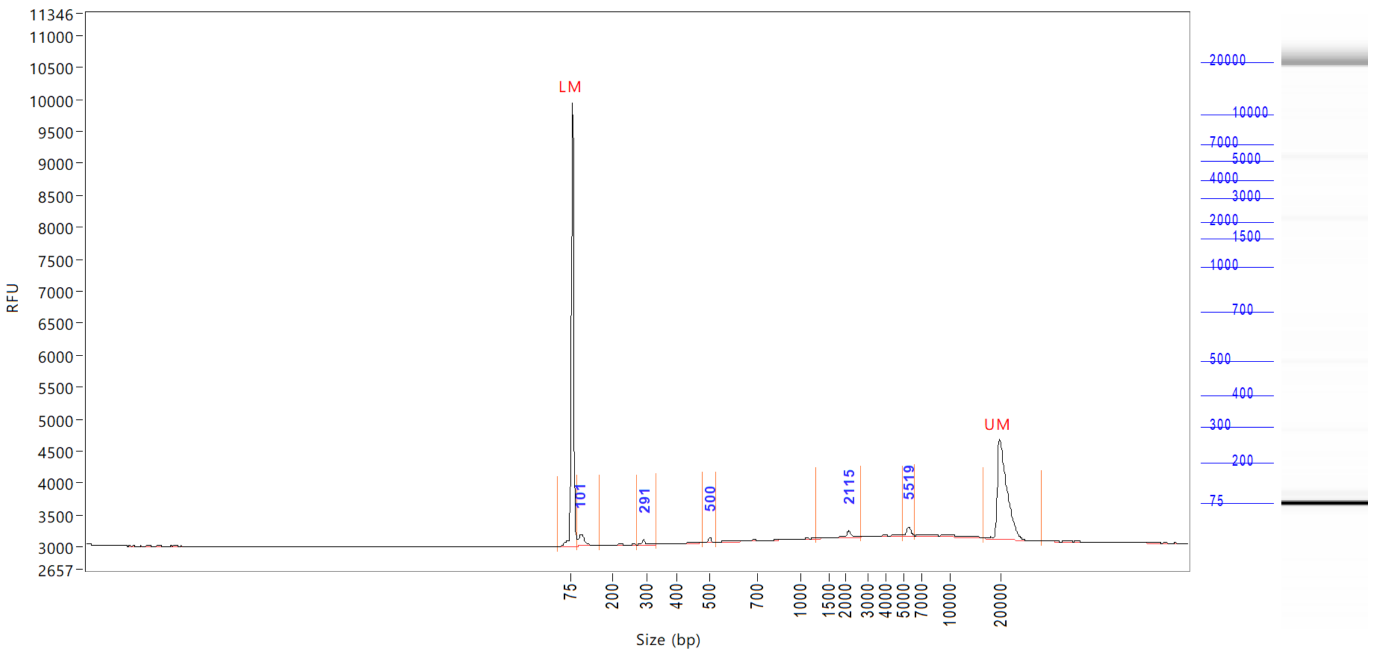

Peak	Size	Concentration	Relative concentration	Molarity	From	To	CV%	RFU
	(bp)	(ng/uL)	Percent	(nmole/L)	(bp)	(bp)		
1	75 (LM)	0.5000		11.2364	30	91	6.79	6940
2	101	0.4142	45.3	6.2990	91	162	14.01	177
3	291	0.0950	10.4	0.5370	269	329	3.35	76
4	500	0.0865	9.5	0.2868	477	527	1.38	84
5	2115	0.1872	20.5	0.1581	1256	2657	17.36	102
6	5519	0.1320	14.4	0.0399	4966	6260	4.33	143
7	20000 (UM)	0.2437		0.0194	16586	28100	5.57	1562

TIC:	0.9149	ng/uL
TIM:	7.3207	nmole/L
Total concentration:	1.5393	ng/uL

```
Sample peak width (sec): 5    Sample min peak height: 50    Sample baseline V to V?: Y    Sample baseline V to V points: 3
Sample filter: Binomial       Number of points for filter: 3  Sample start region (min): 0  Sample end region (min): 30
Marker peak width (sec): 5    Marker min peak height: 200   Marker baseline V to V?: Y    Marker baseline V to V points: 3
Lower marker selection: First peak > 500 RFU                Upper marker selection: Last peak > 200 RFU
Ladder size (bp)75, 200, 300, 400, 500, 700, 1000, 1500, 2000, 3000, 4000, 5000, 7000, 10000, 20000
Quantification using: Lower Marker          Final concentration (ng/uL): 0.5000          Dilution factor: 12.0
```

Sample: SampE5
Well location: E5
Created: Thursday, October 21, 2021 5:28:30 PM

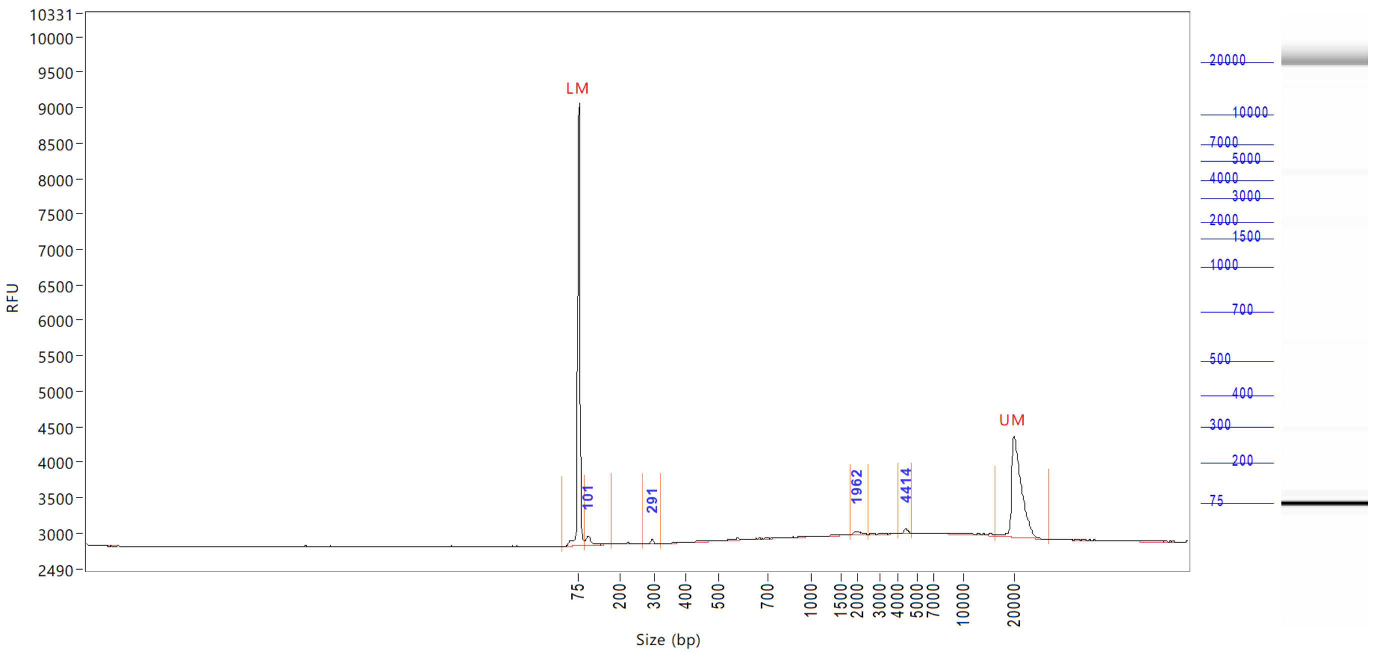

Peak	Size	Concentration	Relative concentration	Molarity	From	To	CV%	RFU
	(bp)	(ng/uL)	Percent	(nmole/L)	(bp)	(bp)		
1	75 (LM)	0.5000		11.3919	26	91	7.60	6253
2	101	0.2776	51.5	4.3016	91	173	13.39	129
3	291	0.0731	13.6	0.4149	264	319	2.66	71
4	1962	0.0976	18.1	0.0796	1789	2486	7.03	52
5	4414	0.0911	16.9	0.0342	4001	4759	3.45	76
6	20000 (UM)	0.2416		0.0192	16464	26891	5.41	1432

TIC:	0.5394	ng/uL
TIM:	4.8303	nmole/L
Total concentration:	1.1390	ng/uL

```
Sample peak width (sec): 5    Sample min peak height: 50    Sample baseline V to V?: Y    Sample baseline V to V points: 3
Sample filter: Binomial       Number of points for filter: 3  Sample start region (min): 0  Sample end region (min): 30
Marker peak width (sec): 5    Marker min peak height: 200   Marker baseline V to V?: Y    Marker baseline V to V points: 3
Lower marker selection: First peak > 500 RFU                Upper marker selection: Last peak > 200 RFU
Ladder size (bp)75, 200, 300, 400, 500, 700, 1000, 1500, 2000, 3000, 4000, 5000, 7000, 10000, 20000
Quantification using: Lower Marker          Final concentration (ng/uL): 0.5000          Dilution factor: 12.0
```

Sample: SampF5
Well location: F5
Created: Thursday, October 21, 2021 5:28:30 PM

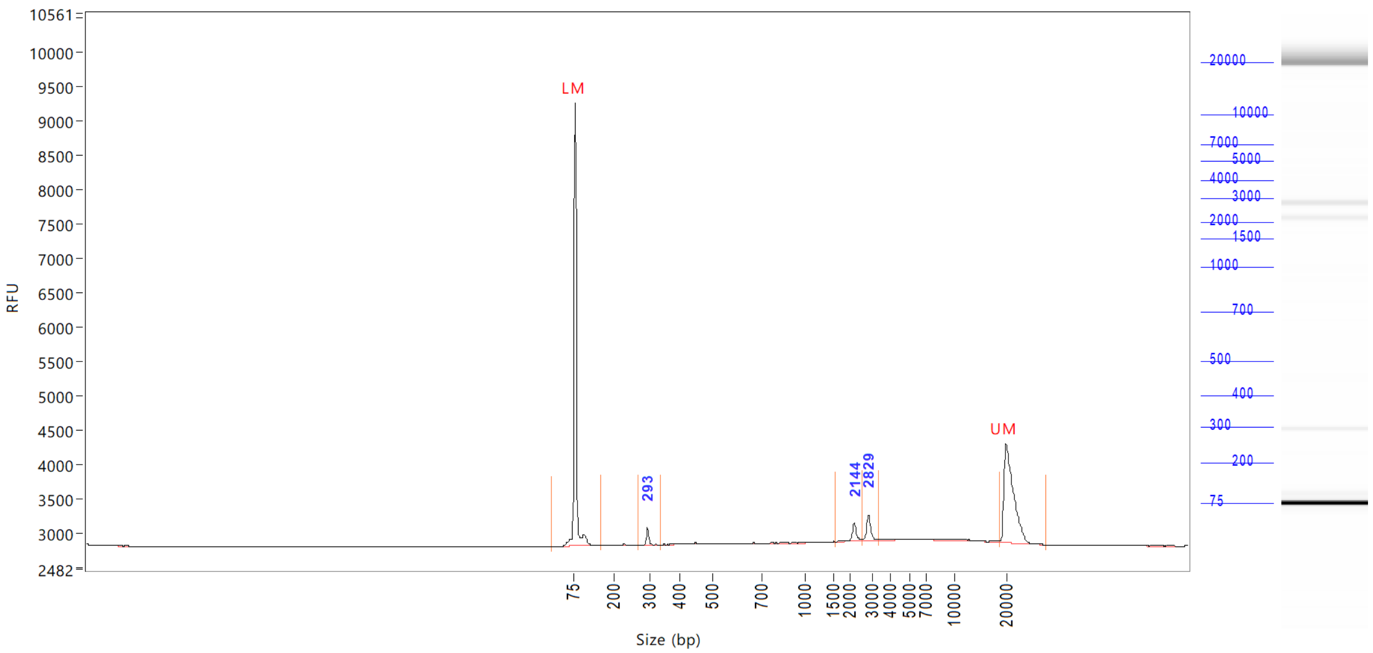

Peak	Size	Concentration	Relative concentration	Molarity	From	To	CV%	RFU
	(bp)	(ng/uL)	Percent	(nmole/L)	(bp)	(bp)		
1	75 (LM)	0.5000		10.9378	7	158	10.87	6447
2	293	0.1983	23.5	1.1168	268	333	2.19	247
3	2144	0.2734	32.5	0.2124	1520	2515	6.99	263
4	2829	0.3708	44.0	0.2172	2515	3322	4.38	376
5	20000 (UM)	0.2226		0.0176	18659	27859	5.22	1448

TIC:	0.8425	ng/uL
TIM:	1.5464	nmole/L
Total concentration:	1.2492	ng/uL

```
Sample peak width (sec): 5    Sample min peak height: 50    Sample baseline V to V?: Y    Sample baseline V to V points: 3
Sample filter: Binomial       Number of points for filter: 3  Sample start region (min): 0  Sample end region (min): 30
Marker peak width (sec): 5    Marker min peak height: 200   Marker baseline V to V?: Y    Marker baseline V to V points: 3
Lower marker selection: First peak > 500 RFU                Upper marker selection: Last peak > 200 RFU
Ladder size (bp)75, 200, 300, 400, 500, 700, 1000, 1500, 2000, 3000, 4000, 5000, 7000, 10000, 20000
Quantification using: Lower Marker          Final concentration (ng/uL): 0.5000          Dilution factor: 12.0
```

Sample: SampG5
Well location: G5
Created: Thursday, October 21, 2021 5:28:30 PM

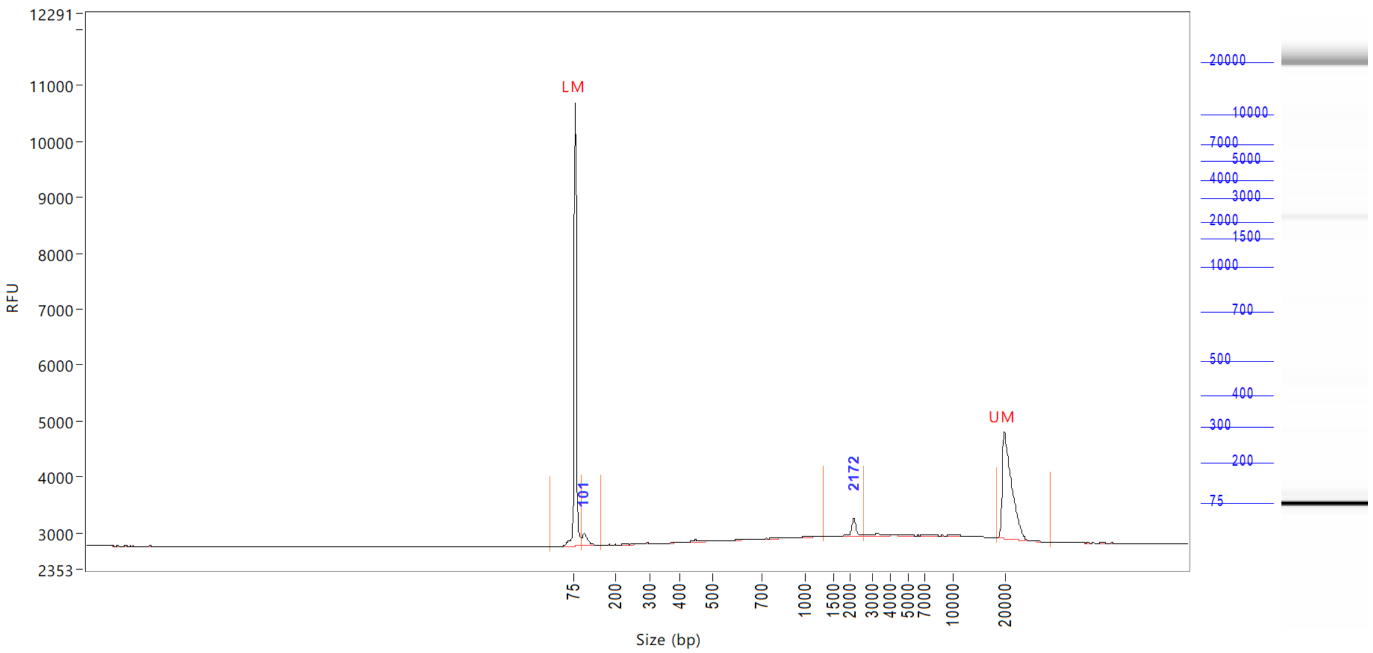

Peak	Size	Concentration	Relative concentration	Molarity	From	To	CV%	RFU
	(bp)	(ng/uL)	Percent	(nmole/L)	(bp)	(bp)		
1	75 (LM)	0.5000		11.3919	0	93	7.73	7934
2	101	0.3735	53.7	5.7870	93	154	10.55	216
3	2172	0.3222	46.3	0.2519	1345	2629	10.59	330
4	20000 (UM)	0.2442		0.0193	18659	29189	5.23	1900

TIC:	0.6957	ng/uL
TIM:	6.0390	nmole/L
Total concentration:	1.2597	ng/uL

Sample peak width (sec): 5   Sample min peak height: 50   Sample baseline V to V?: Y   Sample baseline V to V points: 3
Sample filter: Binomial   Number of points for filter: 3 Sample start region (min): 0 Sample end region (min): 30
Marker peak width (sec): 5   Marker min peak height: 200   Marker baseline V to V?: Y   Marker baseline V to V points: 3
Lower marker selection: First peak > 500 RFU   Upper marker selection: Last peak > 200 RFU
Ladder size (bp)75, 200, 300, 400, 500, 700, 1000, 1500, 2000, 3000, 4000, 5000, 7000, 10000, 20000
Quantification using: Lower Marker   Final concentration (ng/uL): 0.5000   Dilution factor: 12.0

Sample: SampH5
Well location: H5
Created: Thursday, October 21, 2021 5:28:30 PM

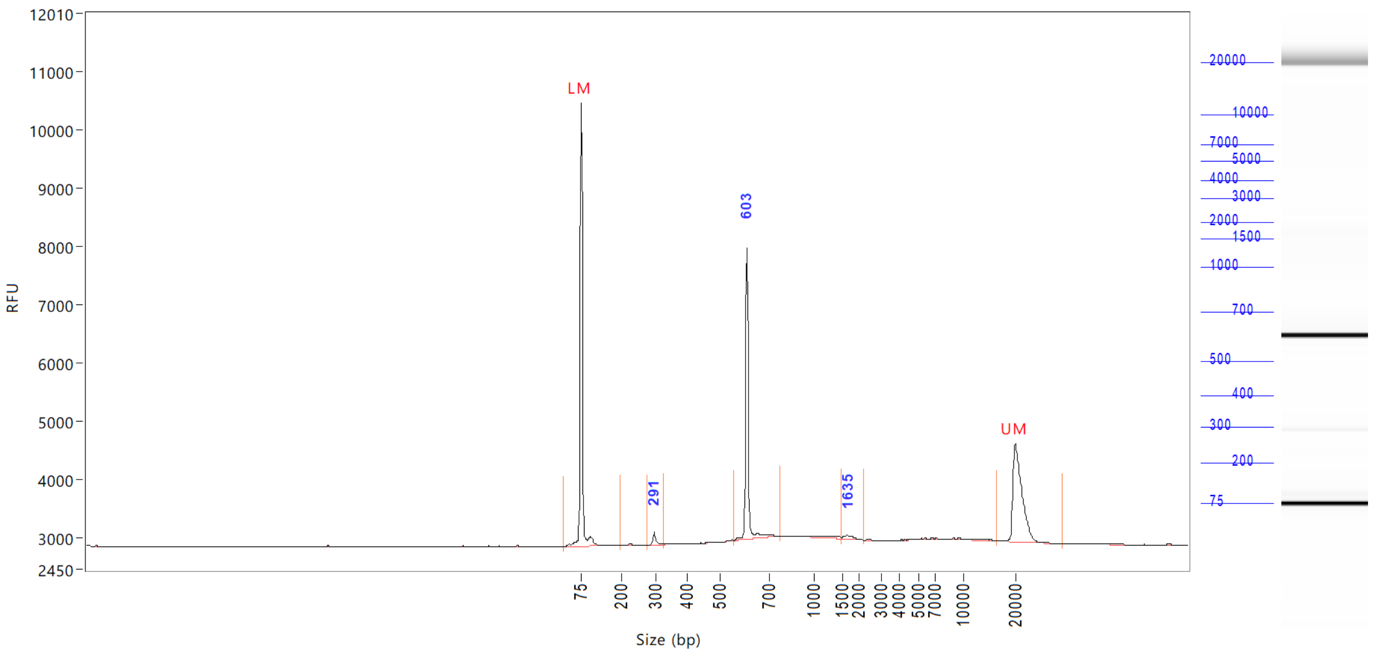

Peak	Size	Concentration	Relative concentration	Molarity	From	To	CV%	RFU
	(bp)	(ng/uL)	Percent	(nmole/L)	(bp)	(bp)		
1	75 (LM)	0.5000		11.0851	20	196	15.24	7635
2	291	0.1856	5.2	1.0455	271	323	2.57	215
3	603	3.2756	91.4	8.9098	552	762	2.80	5004
4	1635	0.1219	3.4	0.1191	1489	2201	7.62	65
5	20000 (UM)	0.2109		0.0168	16220	29189	5.38	1699

TIC:	3.5831	ng/uL
TIM:	10.0745	nmole/L
Total concentration:	4.0972	ng/uL

Sample peak width (sec): 5 Sample min peak height: 50 Sample baseline V to V?: Y Sample baseline V to V points: 3
Sample filter: Binomial Number of points for filter: 3 Sample start region (min): 0 Sample end region (min): 30
Marker peak width (sec): 5 Marker min peak height: 200 Marker baseline V to V?: Y Marker baseline V to V points: 3
Lower marker selection: First peak > 500 RFU Upper marker selection: Last peak > 200 RFU
Ladder size (bp)75, 200, 300, 400, 500, 700, 1000, 1500, 2000, 3000, 4000, 5000, 7000, 10000, 20000
Quantification using: Lower Marker Final concentration (ng/uL): 0.5000 Dilution factor: 12.0

Sample: SampA6
Well location: A6
Created: Thursday, October 21, 2021 5:28:30 PM

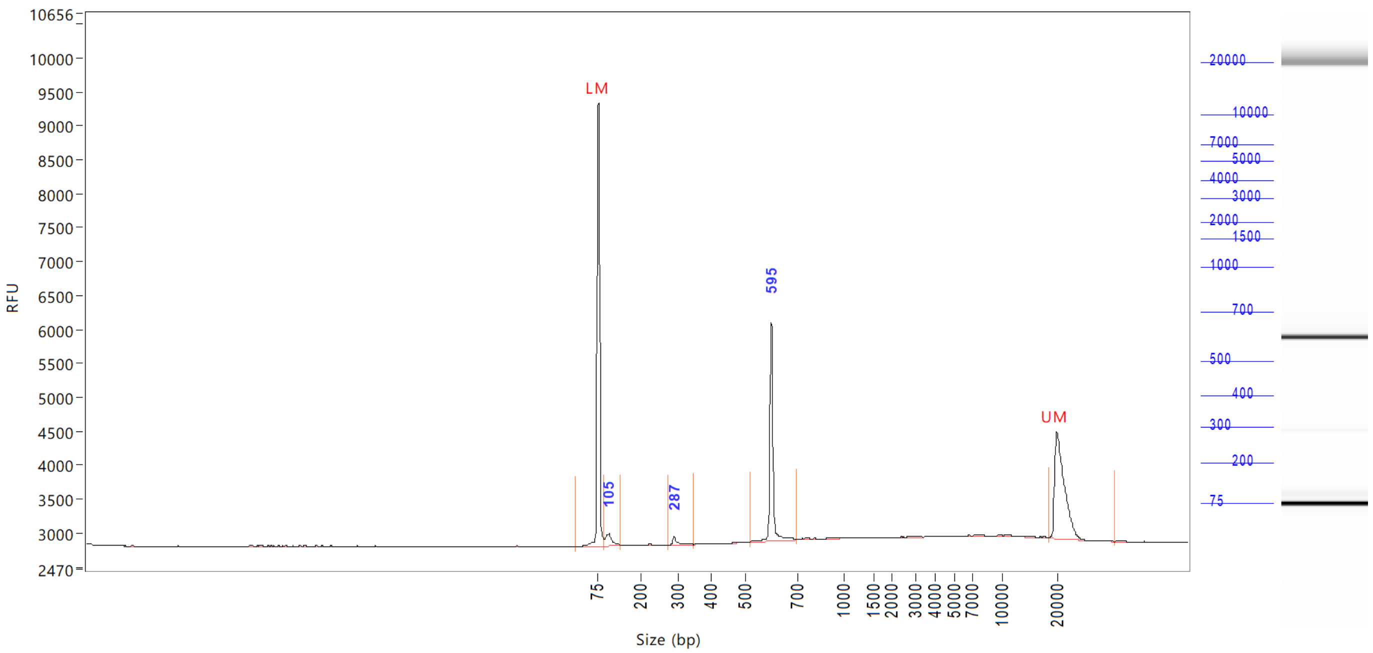

Peak	Size	Concentration	Relative concentration	Molarity	From	To	CV%	RFU
	(bp)	(ng/uL)	Percent	(nmole/L)	(bp)	(bp)		
1	75 (LM)	0.5000		11.3919	9	89	7.65	6538
2	105	0.4437	12.3	7.0066	89	139	10.07	176
3	287	0.1996	5.5	1.1128	273	345	4.88	129
4	595	2.9607	82.2	8.1748	514	692	2.74	3229
5	20000 (UM)	0.2525		0.0201	18537	30639	5.47	1584

TIC:	3.6039	ng/uL
TIM:	16.2942	nmole/L
Total concentration:	4.1254	ng/uL

```
Sample peak width (sec): 5    Sample min peak height: 50    Sample baseline V to V?: Y    Sample baseline V to V points: 3
Sample filter: Binomial       Number of points for filter: 3  Sample start region (min): 0  Sample end region (min): 30
Marker peak width (sec): 5    Marker min peak height: 200   Marker baseline V to V?: Y    Marker baseline V to V points: 3
Lower marker selection: First peak > 500 RFU                Upper marker selection: Last peak > 200 RFU
Ladder size (bp)75, 200, 300, 400, 500, 700, 1000, 1500, 2000, 3000, 4000, 5000, 7000, 10000, 20000
Quantification using: Lower Marker          Final concentration (ng/uL): 0.5000          Dilution factor: 12.0
```

Sample: SampB6
Well location: B6
Created: Thursday, October 21, 2021 5:28:30 PM

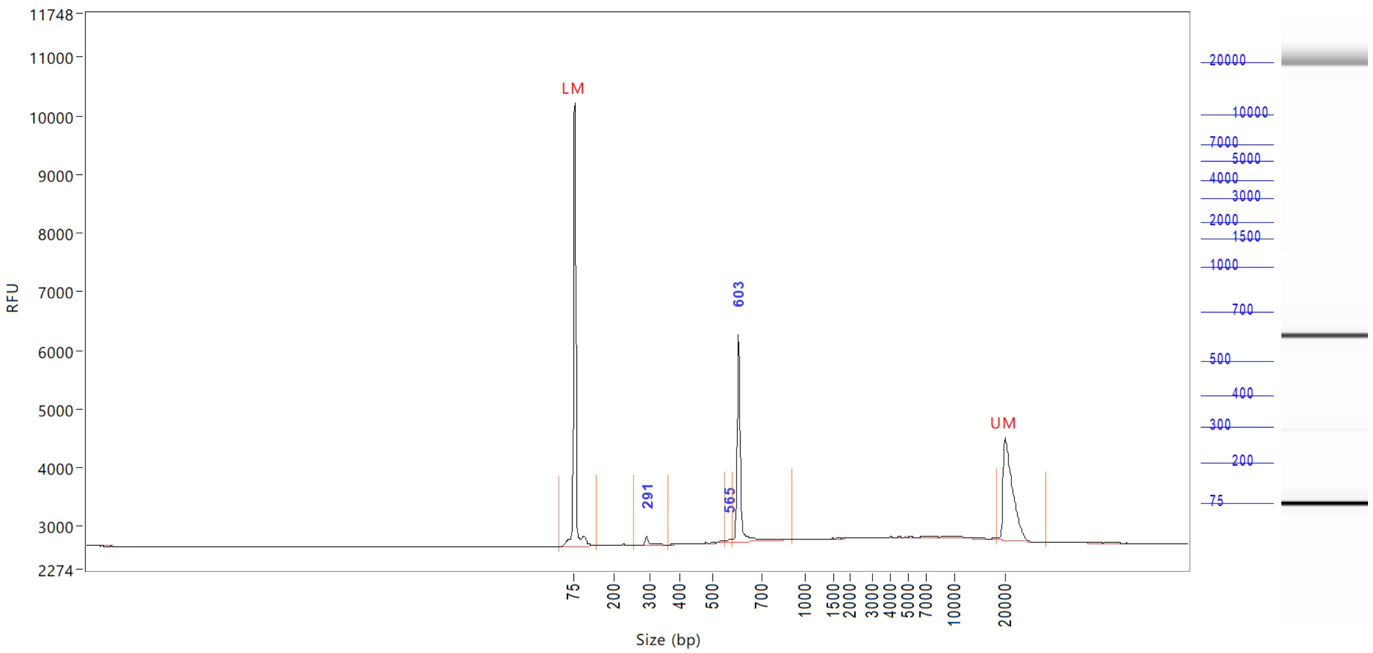

Peak	Size	Concentration	Relative concentration	Molarity	From	To	CV%	RFU
	(bp)	(ng/uL)	Percent	(nmole/L)	(bp)	(bp)		
1	75 (LM)	0.5000		11.2364	26	143	12.41	7568
2	291	0.1877	6.3	1.0464	251	358	5.28	159
3	565	0.0552	1.9	0.1615	549	575	1.29	57
4	603	2.7285	91.8	7.3370	575	905	6.54	3523
5	20000 (UM)	0.2273		0.0181	18415	27979	5.23	1750

TIC:	2.9714	ng/uL
TIM:	8.5449	nmole/L
Total concentration:	3.4336	ng/uL

Sample peak width (sec): 5 Sample min peak height: 50 Sample baseline V to V?: Y Sample baseline V to V points: 3
Sample filter: Binomial Number of points for filter: 3 Sample start region (min): 0 Sample end region (min): 30
Marker peak width (sec): 5 Marker min peak height: 200 Marker baseline V to V?: Y Marker baseline V to V points: 3
Lower marker selection: First peak > 500 RFU Upper marker selection: Last peak > 200 RFU
Ladder size (bp)75, 200, 300, 400, 500, 700, 1000, 1500, 2000, 3000, 4000, 5000, 7000, 10000, 20000
Quantification using: Lower Marker Final concentration (ng/uL): 0.5000 Dilution factor: 12.0

Sample: SampC6
Well location: C6
Created: Thursday, October 21, 2021 5:28:30 PM

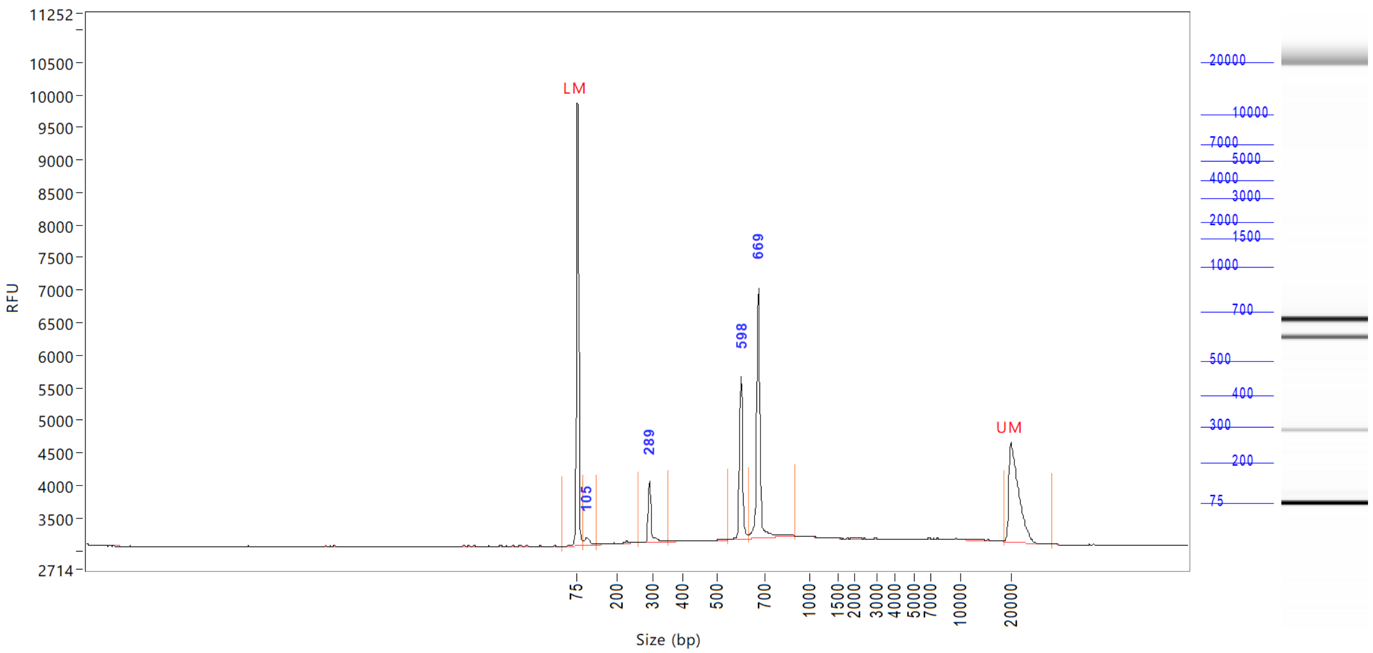

Peak	Size	Concentration	Relative concentration	Molarity	From	To	CV%	RFU
	(bp)	(ng/uL)	Percent	(nmole/L)	(bp)	(bp)		
1	75 (LM)	0.5000		11.0851	31	93	5.15	6814
2	105	0.2478	3.5	3.8389	93	135	9.26	118
3	289	0.9104	12.9	5.1286	260	349	4.23	937
4	598	2.1640	30.7	5.9852	547	629	1.38	2498
5	669	3.7228	52.8	9.1306	629	900	3.77	3845
6	20000 (UM)	0.2420		0.0191	18659	27979	5.25	1528

TIC:	7.0450	ng/uL
TIM:	24.0833	nmole/L
Total concentration:	7.5620	ng/uL

```
Sample peak width (sec): 5    Sample min peak height: 50    Sample baseline V to V?: Y    Sample baseline V to V points: 3
Sample filter: Binomial       Number of points for filter: 3  Sample start region (min): 0  Sample end region (min): 30
Marker peak width (sec): 5    Marker min peak height: 200   Marker baseline V to V?: Y    Marker baseline V to V points: 3
Lower marker selection: First peak > 500 RFU                Upper marker selection: Last peak > 200 RFU
Ladder size (bp)75, 200, 300, 400, 500, 700, 1000, 1500, 2000, 3000, 4000, 5000, 7000, 10000, 20000
Quantification using: Lower Marker          Final concentration (ng/uL): 0.5000          Dilution factor: 12.0
```

Sample: SampD6
Well location: D6
Created: Thursday, October 21, 2021 5:28:30 PM

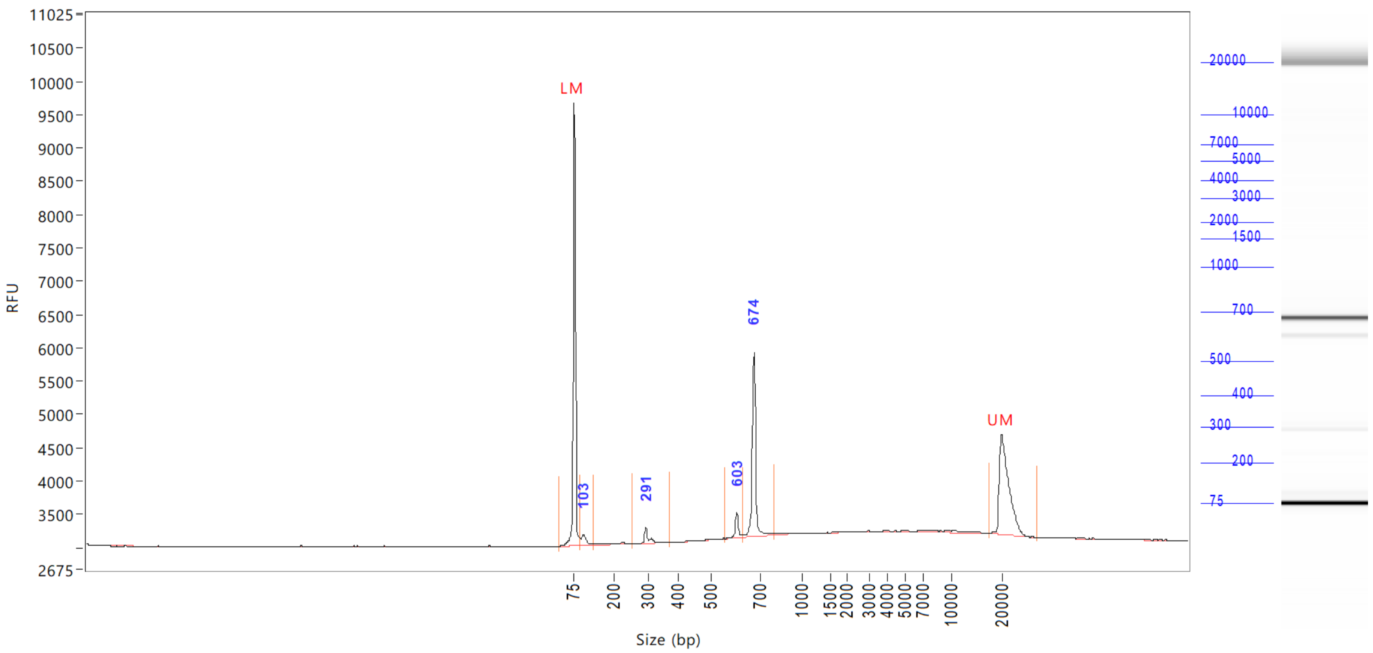

Peak	Size	Concentration	Relative concentration	Molarity	From	To	CV%	RFU
	(bp)	(ng/uL)	Percent	(nmole/L)	(bp)	(bp)		
1	75 (LM)	0.5000		11.2364	28	95	7.20	6663
2	103	0.3013	8.5	4.6690	95	135	8.67	162
3	291	0.3231	9.1	1.7834	253	368	5.05	232
4	603	0.3537	9.9	0.9701	555	626	1.71	375
5	674	2.5836	72.5	6.3086	626	801	2.71	2760
6	20000 (UM)	0.2378		0.0189	17439	27012	5.23	1505

TIC:	3.5618	ng/uL
TIM:	13.7310	nmole/L
Total concentration:	4.2068	ng/uL

Sample peak width (sec): 5 Sample min peak height: 50 Sample baseline V to V?: Y Sample baseline V to V points: 3
Sample filter: Binomial Number of points for filter: 3 Sample start region (min): 0 Sample end region (min): 30
Marker peak width (sec): 5 Marker min peak height: 200 Marker baseline V to V?: Y Marker baseline V to V points: 3
Lower marker selection: First peak > 500 RFU Upper marker selection: Last peak > 200 RFU
Ladder size (bp)75, 200, 300, 400, 500, 700, 1000, 1500, 2000, 3000, 4000, 5000, 7000, 10000, 20000
Quantification using: Lower Marker Final concentration (ng/uL): 0.5000 Dilution factor: 12.0

Sample: SampE6
Well location: E6
Created: Thursday, October 21, 2021 5:28:30 PM

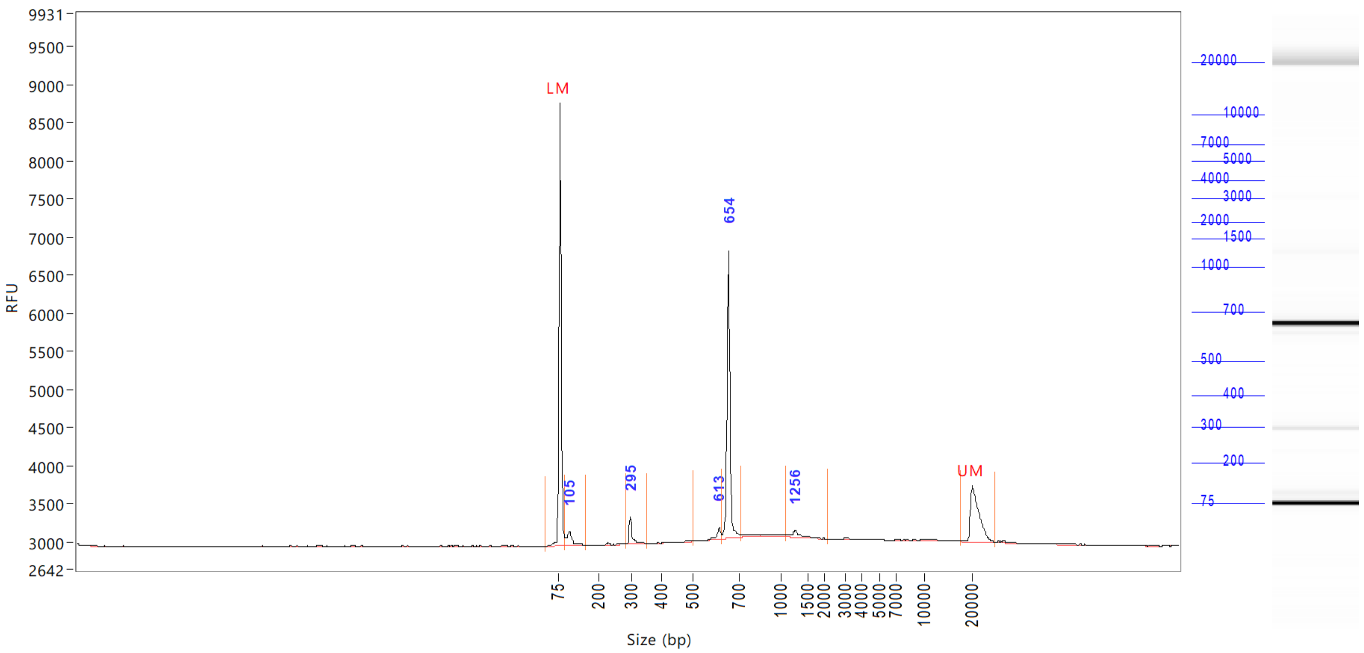

Peak	Size	Concentration	Relative concentration	Molarity	From	To	CV%	RFU
	(bp)	(ng/uL)	Percent	(nmole/L)	(bp)	(bp)		
1	75 (LM)	0.5000		11.2364	28	91	6.06	5818
2	105	0.4575	8.8	7.0216	91	158	12.18	181
3	295	0.4393	8.5	2.4246	284	349	3.62	358
4	613	0.2187	4.2	0.5979	501	626	3.60	153
5	654	3.8294	74.0	9.6657	626	714	1.52	3762
6	1256	0.2329	4.5	0.2872	1101	2201	12.93	87
7	20000 (UM)	0.1277		0.0101	17683	24715	4.96	733

TIC:	5.1777	ng/uL
TIM:	19.9970	nmole/L
Total concentration:	5.6873	ng/uL

```
Sample peak width (sec): 5    Sample min peak height: 50    Sample baseline V to V?: Y    Sample baseline V to V points: 3
Sample filter: Binomial       Number of points for filter: 3  Sample start region (min): 0  Sample end region (min): 30
Marker peak width (sec): 5    Marker min peak height: 200   Marker baseline V to V?: Y    Marker baseline V to V points: 3
Lower marker selection: First peak > 500 RFU                Upper marker selection: Last peak > 200 RFU
Ladder size (bp)75, 200, 300, 400, 500, 700, 1000, 1500, 2000, 3000, 4000, 5000, 7000, 10000, 20000
Quantification using: Lower Marker          Final concentration (ng/uL): 0.5000          Dilution factor: 12.0
```

Sample: SampF6
Well location: F6
Created: Thursday, October 21, 2021 5:28:30 PM

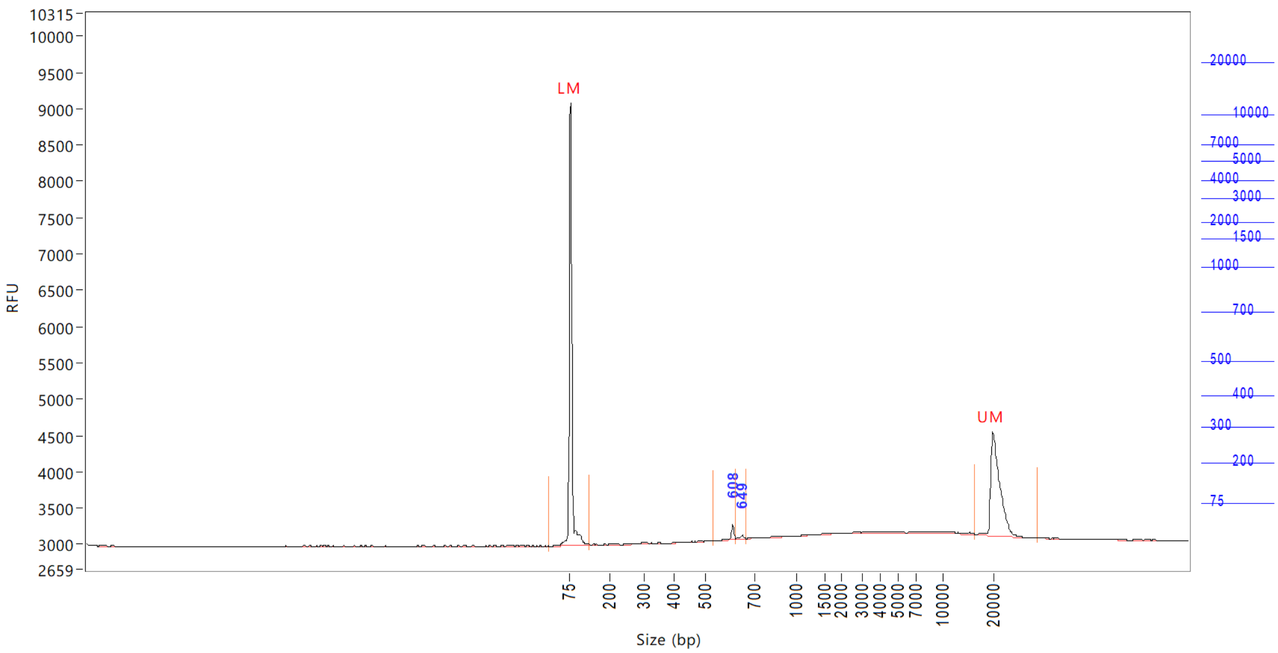

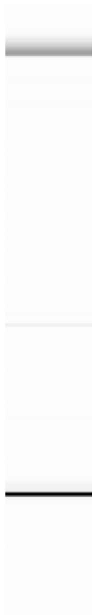

Peak	Size	Concentration	Relative concentration	Molarity	From	To	CV%	RFU
	(bp)	(ng/uL)	Percent	(nmole/L)	(bp)	(bp)		
1	75 (LM)	0.5000		11.0851	11	135	12.02	6109
2	608	0.1787	74.8	0.4884	529	623	2.06	212
3	649	0.0603	25.2	0.1540	623	669	1.49	58
4	20000 (UM)	0.2259		0.0180	16464	28826	5.50	1438

TIC:	0.2390	ng/uL
TIM:	0.6424	nmole/L
Total concentration:	0.9315	ng/uL

Sample peak width (sec): 5 Sample min peak height: 50 Sample baseline V to V?: Y Sample baseline V to V points: 3
Sample filter: Binomial Number of points for filter: 3 Sample start region (min): 0 Sample end region (min): 30
Marker peak width (sec): 5 Marker min peak height: 200 Marker baseline V to V?: Y Marker baseline V to V points: 3
Lower marker selection: First peak > 500 RFU Upper marker selection: Last peak > 200 RFU
Ladder size (bp)75, 200, 300, 400, 500, 700, 1000, 1500, 2000, 3000, 4000, 5000, 7000, 10000, 20000
Quantification using: Lower Marker Final concentration (ng/uL): 0.5000 Dilution factor: 12.0

Sample: SampG6
Well location: G6
Created: Thursday, October 21, 2021 5:28:30 PM

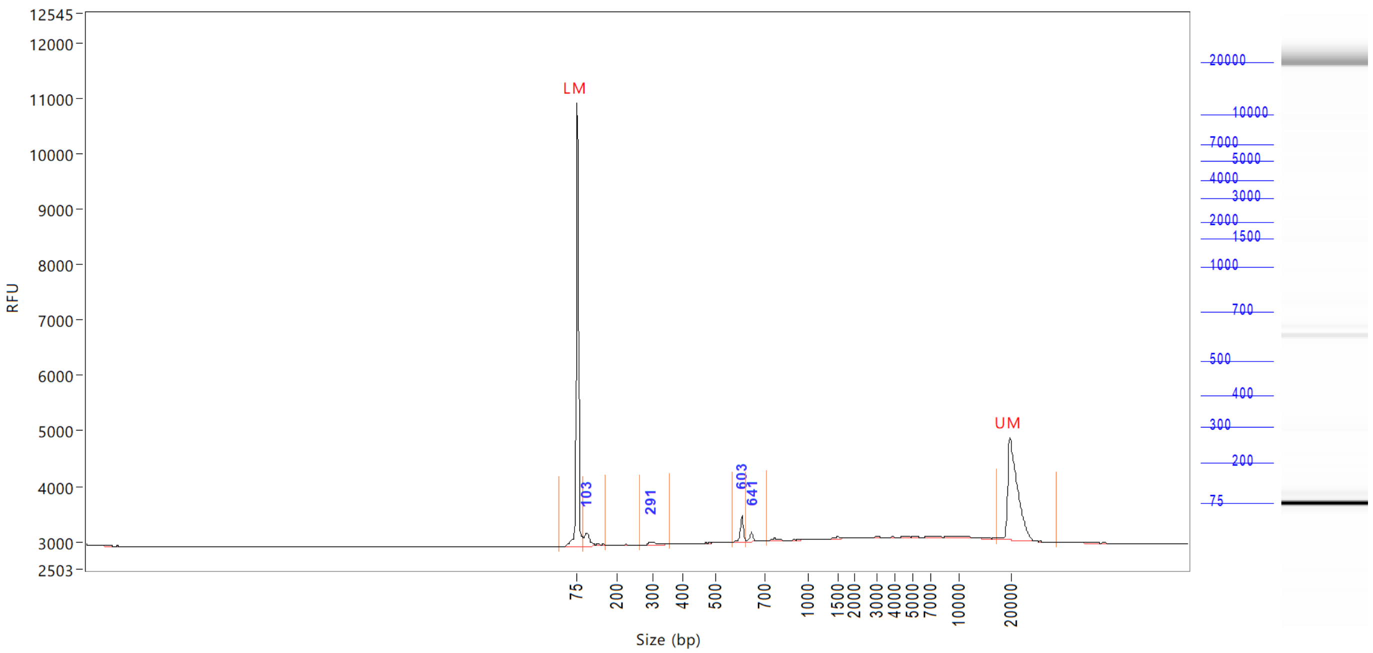

Peak	Size	Concentration	Relative concentration	Molarity	From	To	CV%	RFU
	(bp)	(ng/uL)	Percent	(nmole/L)	(bp)	(bp)		
1	75 (LM)	0.5000		11.2364	22	93	7.46	8023
2	103	0.4694	42.9	7.0082	93	164	14.62	233
3	291	0.1283	11.7	0.6921	264	356	5.68	56
4	603	0.3381	30.9	0.9273	562	618	1.32	478
5	641	0.1573	14.4	0.4025	618	705	2.42	164
6	20000 (UM)	0.2476		0.0197	17195	28947	5.45	1845

TIC:	1.0930	ng/uL
TIM:	9.0301	nmole/L
Total concentration:	1.6228	ng/uL

Sample peak width (sec): 5 Sample min peak height: 50 Sample baseline V to V?: Y Sample baseline V to V points: 3
Sample filter: Binomial Number of points for filter: 3 Sample start region (min): 0 Sample end region (min): 30
Marker peak width (sec): 5 Marker min peak height: 200 Marker baseline V to V?: Y Marker baseline V to V points: 3
Lower marker selection: First peak > 500 RFU Upper marker selection: Last peak > 200 RFU
Ladder size (bp)75, 200, 300, 400, 500, 700, 1000, 1500, 2000, 3000, 4000, 5000, 7000, 10000, 20000
Quantification using: Lower Marker Final concentration (ng/uL): 0.5000 Dilution factor: 12.0

Sample: SampH6
Well location: H6
Created: Thursday, October 21, 2021 5:28:30 PM

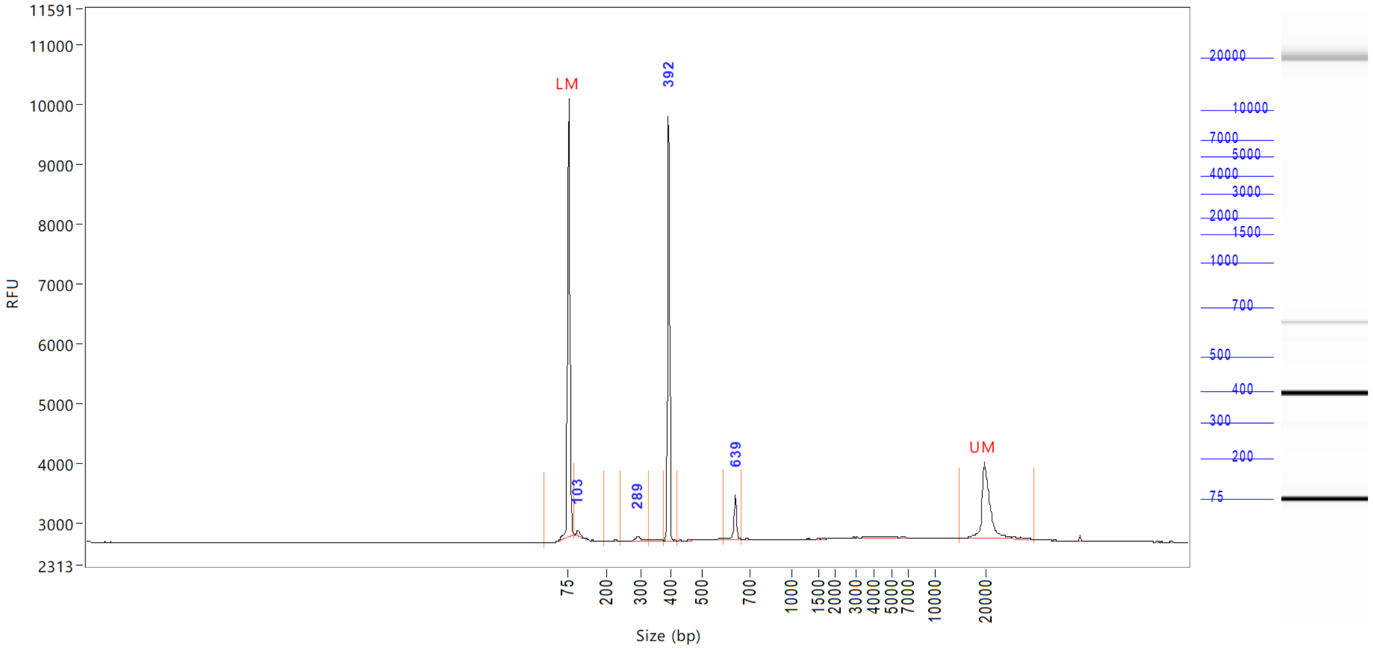

Peak	Size	Concentration	Relative concentration	Molarity	From	To	CV%	RFU
	(bp)	(ng/uL)	Percent	(nmole/L)	(bp)	(bp)		
1	75 (LM)	0.5000		11.2364	0	93	6.04	7323
2	103	0.1391	2.7	2.0766	93	189	15.07	91
3	289	0.1447	2.8	0.8265	235	325	5.57	81
4	392	4.3229	84.4	18.2367	376	420	0.98	7072
5	639	0.5162	10.1	1.3379	590	667	1.51	755
6	20000 (UM)	0.1557		0.0125	14757	29914	8.24	1268

TIC:	5.1229	ng/uL
TIM:	22.4778	nmole/L
Total concentration:	5.5239	ng/uL

Sample peak width (sec): 5 Sample min peak height: 50 Sample baseline V to V?: Y Sample baseline V to V points: 3
Sample filter: Binomial Number of points for filter: 3 Sample start region (min): 0 Sample end region (min): 30
Marker peak width (sec): 5 Marker min peak height: 200 Marker baseline V to V?: Y Marker baseline V to V points: 3
Lower marker selection: First peak > 500 RFU Upper marker selection: Last peak > 200 RFU
Ladder size (bp)75, 200, 300, 400, 500, 700, 1000, 1500, 2000, 3000, 4000, 5000, 7000, 10000, 20000
Quantification using: Lower Marker Final concentration (ng/uL): 0.5000 Dilution factor: 12.0

Sample: SampA7
Well location: A7
Created: Thursday, October 21, 2021 5:28:30 PM

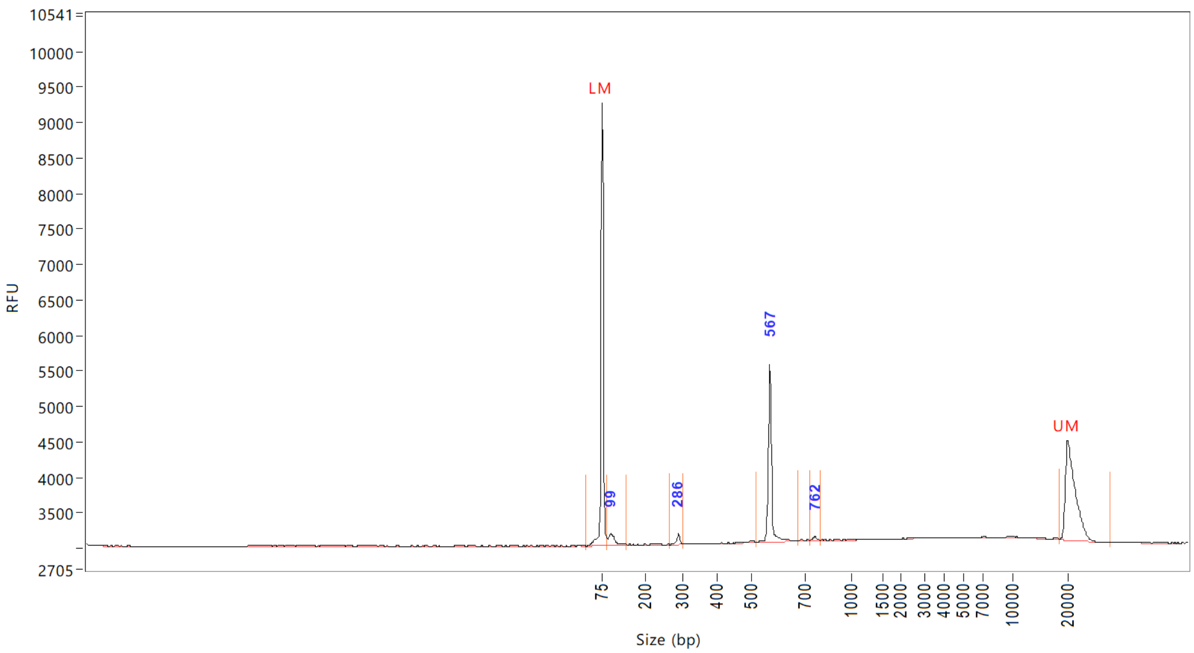

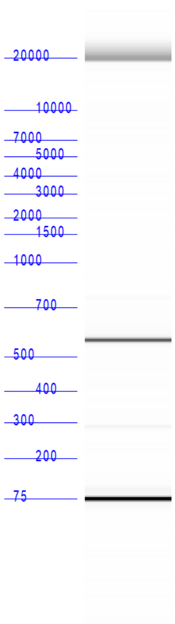

Peak	Size	Concentration	Relative concentration	Molarity	From	To	CV%	RFU
	(bp)	(ng/uL)	Percent	(nmole/L)	(bp)	(bp)		
1	75 (LM)	0.5000		11.3919	30	89	7.21	6251
2	99	0.3952	12.8	6.3017	89	147	11.56	162
3	286	0.1682	5.4	0.9778	264	300	1.97	155
4	567	2.4776	79.9	7.1909	514	677	2.99	2493
5	762	0.0585	1.9	0.1273	727	796	1.68	51
6	20000 (UM)	0.2363		0.0188	18415	27496	5.17	1400

TIC:	3.0997	ng/uL
TIM:	14.5977	nmole/L
Total concentration:	3.6119	ng/uL

```
Sample peak width (sec): 5    Sample min peak height: 50    Sample baseline V to V?: Y    Sample baseline V to V points: 3
Sample filter: Binomial       Number of points for filter: 3  Sample start region (min): 0  Sample end region (min): 30
Marker peak width (sec): 5    Marker min peak height: 200   Marker baseline V to V?: Y    Marker baseline V to V points: 3
Lower marker selection: First peak > 500 RFU                Upper marker selection: Last peak > 200 RFU
Ladder size (bp)75, 200, 300, 400, 500, 700, 1000, 1500, 2000, 3000, 4000, 5000, 7000, 10000, 20000
Quantification using: Lower Marker          Final concentration (ng/uL): 0.5000          Dilution factor: 12.0
```

Sample: SampB7
Well location: B7
Created: Thursday, October 21, 2021 5:28:30 PM

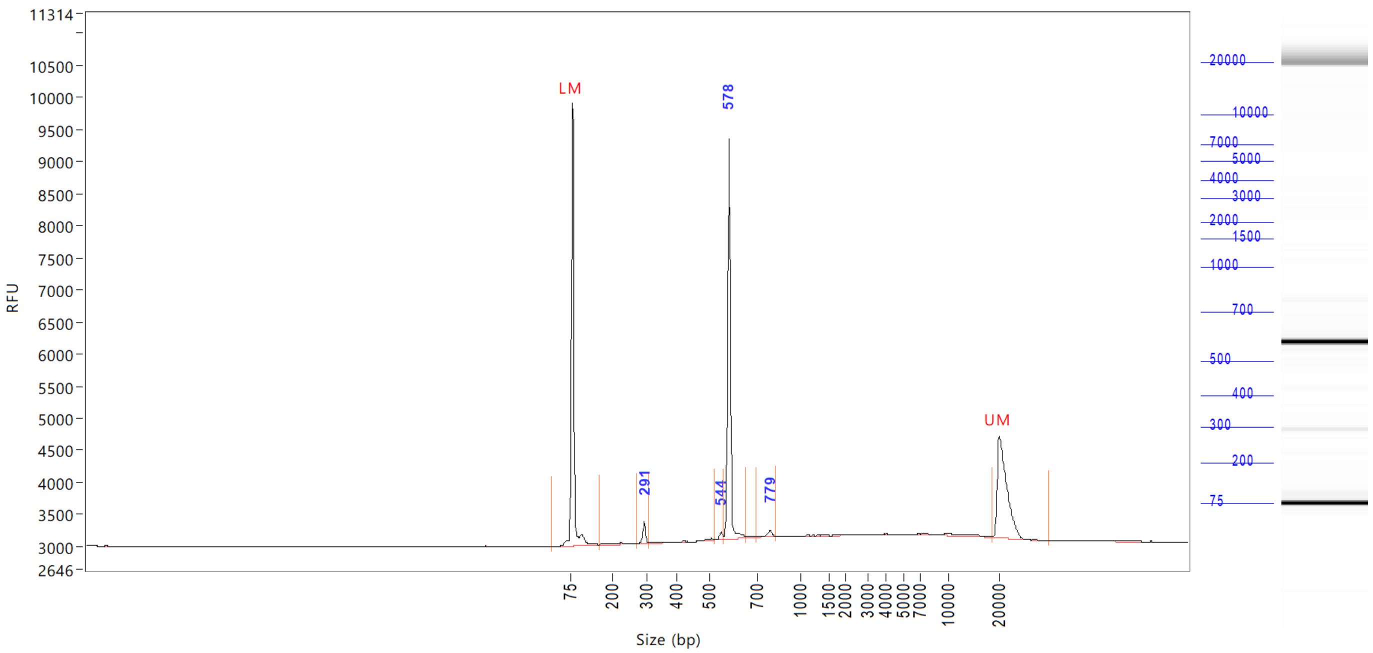

Peak	Size	Concentration	Relative concentration	Molarity	From	To	CV%	RFU
	(bp)	(ng/uL)	Percent	(nmole/L)	(bp)	(bp)		
1	75 (LM)	0.5000		10.9378	14	158	13.62	6921
2	291	0.2868	5.6	1.6267	271	305	1.66	338
3	544	0.1017	2.0	0.3078	519	557	1.32	118
4	578	4.5768	90.0	13.0532	557	649	1.77	6237
5	779	0.1226	2.4	0.2661	692	822	3.97	103
6	20000 (UM)	0.2241		0.0177	18537	29914	5.46	1583

TIC:	5.0879	ng/uL
TIM:	15.2538	nmole/L
Total concentration:	5.7770	ng/uL

```
Sample peak width (sec): 5    Sample min peak height: 50    Sample baseline V to V?: Y    Sample baseline V to V points: 3
Sample filter: Binomial       Number of points for filter: 3  Sample start region (min): 0  Sample end region (min): 30
Marker peak width (sec): 5    Marker min peak height: 200   Marker baseline V to V?: Y    Marker baseline V to V points: 3
Lower marker selection: First peak > 500 RFU                Upper marker selection: Last peak > 200 RFU
Ladder size (bp)75, 200, 300, 400, 500, 700, 1000, 1500, 2000, 3000, 4000, 5000, 7000, 10000, 20000
Quantification using: Lower Marker          Final concentration (ng/uL): 0.5000          Dilution factor: 12.0
```

Sample: SampC7
Well location: C7
Created: Thursday, October 21, 2021 5:28:30 PM

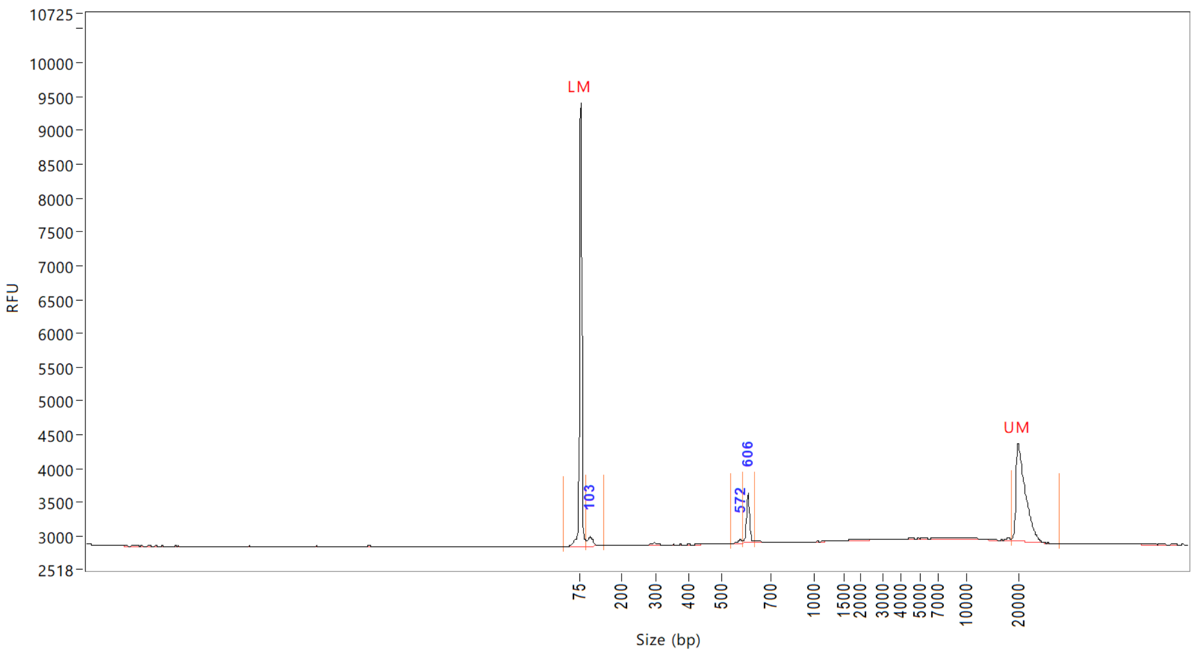

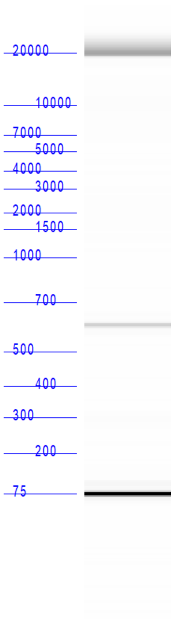

Peak	Size	Concentration	Relative concentration	Molarity	From	To	CV%	RFU
	(bp)	(ng/uL)	Percent	(nmole/L)	(bp)	(bp)		
1	75 (LM)	0.5000		11.3919	24	93	7.02	6556
2	103	0.2925	28.3	4.4902	93	147	11.33	135
3	572	0.0576	5.6	0.1665	537	583	1.74	51
4	606	0.6825	66.1	1.8595	583	634	1.27	736
5	20000 (UM)	0.2314		0.0184	18659	27979	5.27	1447

TIC:	1.0326	ng/uL
TIM:	6.5162	nmole/L
Total concentration:	1.7814	ng/uL

```
Sample peak width (sec): 5    Sample min peak height: 50    Sample baseline V to V?: Y    Sample baseline V to V points: 3
Sample filter: Binomial       Number of points for filter: 3  Sample start region (min): 0  Sample end region (min): 30
Marker peak width (sec): 5    Marker min peak height: 200   Marker baseline V to V?: Y    Marker baseline V to V points: 3
Lower marker selection: First peak > 500 RFU                Upper marker selection: Last peak > 200 RFU
Ladder size (bp)75, 200, 300, 400, 500, 700, 1000, 1500, 2000, 3000, 4000, 5000, 7000, 10000, 20000
Quantification using: Lower Marker          Final concentration (ng/uL): 0.5000          Dilution factor: 12.0
```

Sample: SampD7
Well location: D7
Created: Thursday, October 21, 2021 5:28:30 PM

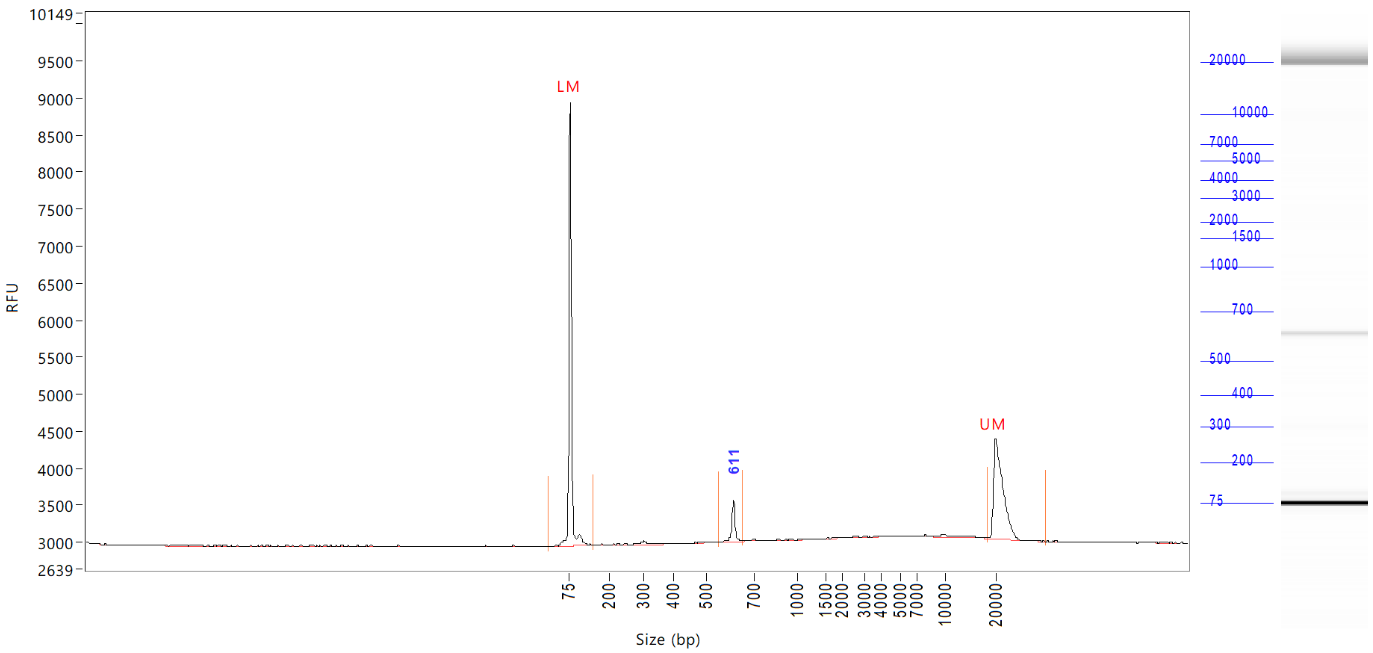

Peak	Size	Concentration	Relative concentration	Molarity	From	To	CV%	RFU
	(bp)	(ng/uL)	Percent	(nmole/L)	(bp)	(bp)		
1	75 (LM)	0.5000		11.0851	11	147	13.20	5997
2	611	0.5699	100.0	1.5452	547	649	1.97	551
3	20000 (UM)	0.2217		0.0175	18537	29914	5.25	1344

TIC:	0.5699	ng/uL
TIM:	1.5452	nmole/L
Total concentration:	1.3255	ng/uL

```
Sample peak width (sec): 5    Sample min peak height: 50    Sample baseline V to V?: Y    Sample baseline V to V points: 3
Sample filter: Binomial       Number of points for filter: 3  Sample start region (min): 0  Sample end region (min): 30
Marker peak width (sec): 5    Marker min peak height: 200   Marker baseline V to V?: Y    Marker baseline V to V points: 3
Lower marker selection: First peak > 500 RFU                Upper marker selection: Last peak > 200 RFU
Ladder size (bp)75, 200, 300, 400, 500, 700, 1000, 1500, 2000, 3000, 4000, 5000, 7000, 10000, 20000
Quantification using: Lower Marker          Final concentration (ng/uL): 0.5000          Dilution factor: 12.0
```

Sample: SampE7
Well location: E7
Created: Thursday, October 21, 2021 5:28:30 PM

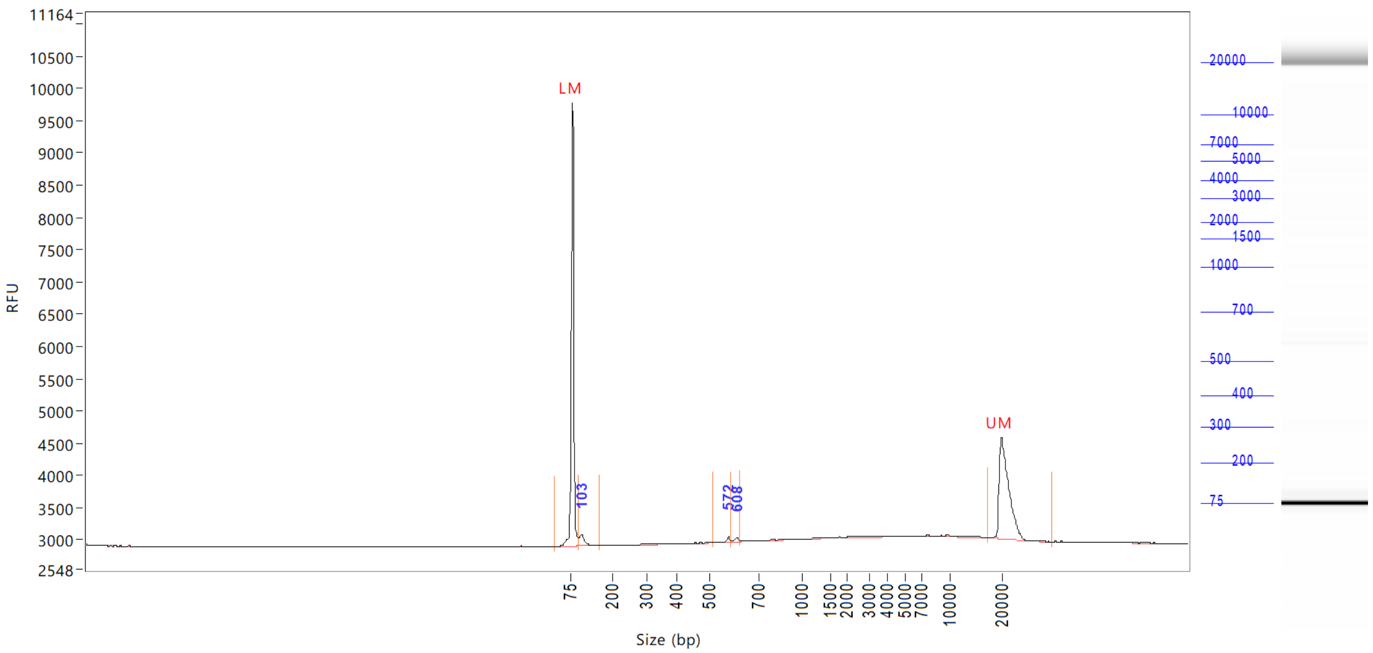

Peak	Size	Concentration	Relative concentration	Molarity	From	To	CV%	RFU
	(bp)	(ng/uL)	Percent	(nmole/L)	(bp)	(bp)		
1	75 (LM)	0.5000		11.2364	24	95	7.43	6880
2	103	0.3275	70.7	5.0272	95	158	10.96	176
3	572	0.0706	15.2	0.2053	509	585	2.14	79
4	608	0.0651	14.1	0.1776	585	623	1.34	58
5	20000 (UM)	0.2400		0.0190	17317	30156	5.51	1570

TIC:	0.4632	ng/uL
TIM:	5.4101	nmole/L
Total concentration:	1.0945	ng/uL

Sample peak width (sec): 5 Sample min peak height: 50 Sample baseline V to V?: Y Sample baseline V to V points: 3
Sample filter: Binomial Number of points for filter: 3 Sample start region (min): 0 Sample end region (min): 30
Marker peak width (sec): 5 Marker min peak height: 200 Marker baseline V to V?: Y Marker baseline V to V points: 3
Lower marker selection: First peak > 500 RFU Upper marker selection: Last peak > 200 RFU
Ladder size (bp)75, 200, 300, 400, 500, 700, 1000, 1500, 2000, 3000, 4000, 5000, 7000, 10000, 20000
Quantification using: Lower Marker Final concentration (ng/uL): 0.5000 Dilution factor: 12.0

Sample: SampF7
Well location: F7
Created: Thursday, October 21, 2021 5:28:30 PM

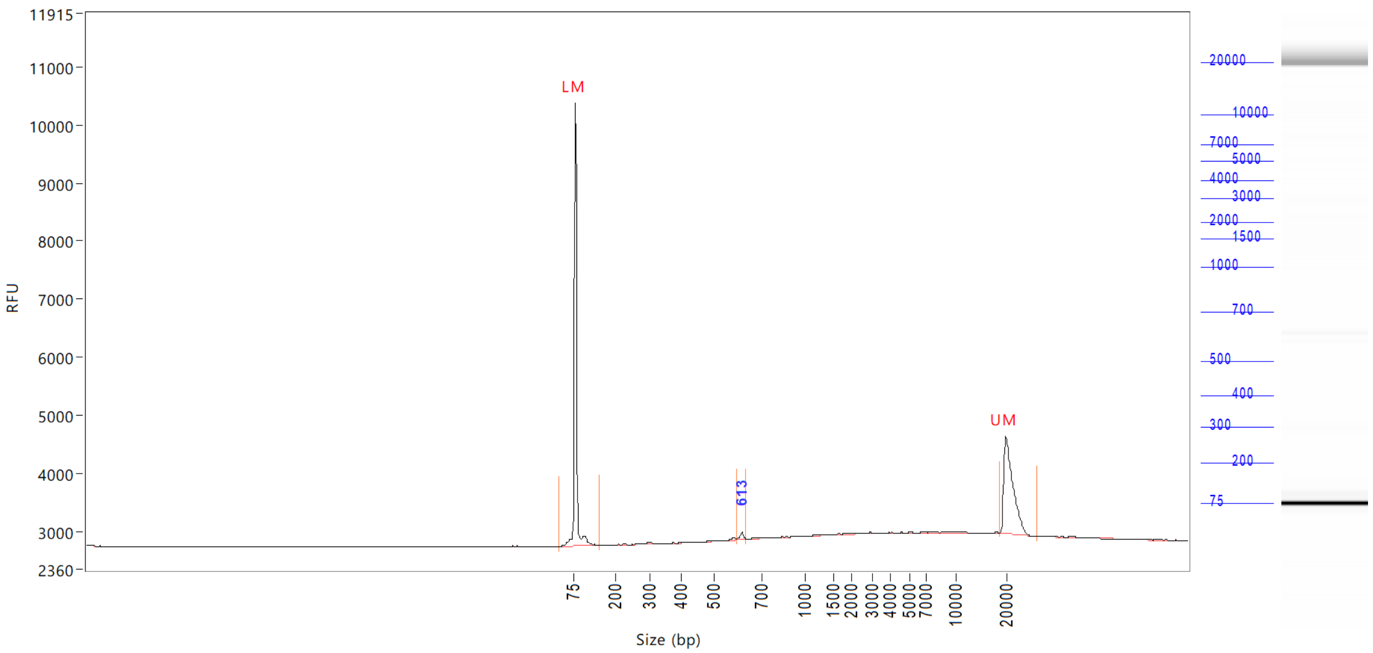

Peak	Size	Concentration	Relative concentration	Molarity	From	To	CV%	RFU
	(bp)	(ng/uL)	Percent	(nmole/L)	(bp)	(bp)		
1	75 (LM)	0.5000		11.0851	26	152	12.24	7627
2	613	0.1045	100.0	0.2819	593	634	1.26	127
3	20000 (UM)	0.2181		0.0173	18781	26166	5.02	1668

TIC:	0.1045	ng/uL
TIM:	0.2819	nmole/L
Total concentration:	0.6484	ng/uL

```
Sample peak width (sec): 5     Sample min peak height: 50     Sample baseline V to V?: Y     Sample baseline V to V points: 3
Sample filter: Binomial        Number of points for filter: 3  Sample start region (min): 0  Sample end region (min): 30
Marker peak width (sec): 5     Marker min peak height: 200    Marker baseline V to V?: Y     Marker baseline V to V points: 3
Lower marker selection: First peak > 500 RFU                  Upper marker selection: Last peak > 200 RFU
Ladder size (bp)75, 200, 300, 400, 500, 700, 1000, 1500, 2000, 3000, 4000, 5000, 7000, 10000, 20000
Quantification using: Lower Marker          Final concentration (ng/uL): 0.5000          Dilution factor: 12.0
```

Sample: SampG7
Well location: G7
Created: Thursday, October 21, 2021 5:28:30 PM

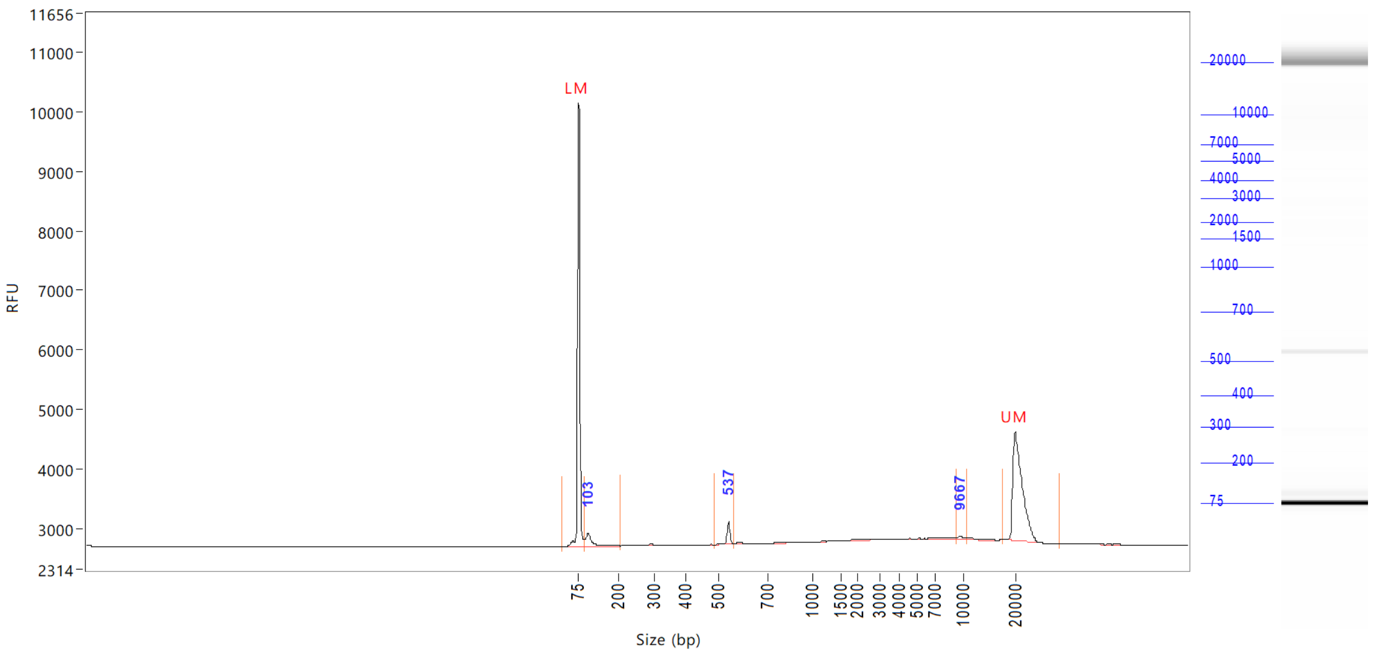

Peak	Size	Concentration	Relative concentration	Molarity	From	To	CV%	RFU
	(bp)	(ng/uL)	Percent	(nmole/L)	(bp)	(bp)		
1	75 (LM)	0.5000		11.0851	30	93	6.47	7466
2	103	0.4673	56.3	6.7934	93	203	18.62	214
3	537	0.2917	35.1	0.8973	485	560	1.50	391
4	9667	0.0712	8.6	0.0120	9200	10733	4.16	54
5	20000 (UM)	0.2508		0.0200	17561	28463	5.03	1822

TIC:	0.8303	ng/uL
TIM:	7.7027	nmole/L
Total concentration:	1.4016	ng/uL

```
Sample peak width (sec): 5    Sample min peak height: 50    Sample baseline V to V?: Y    Sample baseline V to V points: 3
Sample filter: Binomial       Number of points for filter: 3  Sample start region (min): 0  Sample end region (min): 30
Marker peak width (sec): 5    Marker min peak height: 200   Marker baseline V to V?: Y    Marker baseline V to V points: 3
Lower marker selection: First peak > 500 RFU                Upper marker selection: Last peak > 200 RFU
Ladder size (bp)75, 200, 300, 400, 500, 700, 1000, 1500, 2000, 3000, 4000, 5000, 7000, 10000, 20000
Quantification using: Lower Marker          Final concentration (ng/uL): 0.5000          Dilution factor: 12.0
```

Sample: SampH7
Well location: H7
Created: Thursday, October 21, 2021 5:28:30 PM

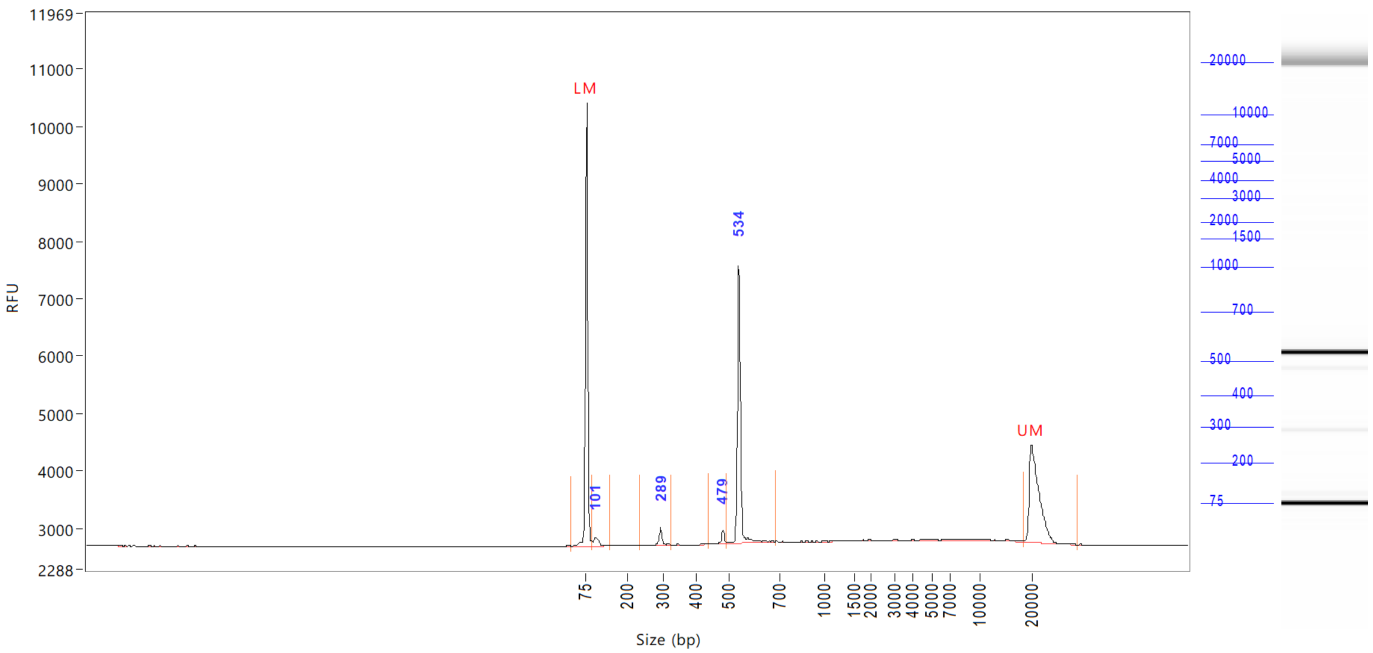

Peak	Size	Concentration	Relative concentration	Molarity	From	To	CV%	RFU
	(bp)	(ng/uL)	Percent	(nmole/L)	(bp)	(bp)		
1	75 (LM)	0.5000		11.2364	31	93	6.25	7733
2	101	0.2844	6.3	4.4068	93	145	10.60	155
3	289	0.3029	6.7	1.7298	233	321	3.69	304
4	479	0.1909	4.3	0.6587	439	492	1.42	233
5	534	3.7120	82.7	11.4175	492	685	2.80	4825
6	20000 (UM)	0.2435		0.0194	18415	28705	5.32	1695

TIC:	4.4903	ng/uL
TIM:	18.2129	nmole/L
Total concentration:	4.9983	ng/uL

```
Sample peak width (sec): 5    Sample min peak height: 50    Sample baseline V to V?: Y    Sample baseline V to V points: 3
Sample filter: Binomial       Number of points for filter: 3  Sample start region (min): 0  Sample end region (min): 30
Marker peak width (sec): 5    Marker min peak height: 200   Marker baseline V to V?: Y    Marker baseline V to V points: 3
Lower marker selection: First peak > 500 RFU                Upper marker selection: Last peak > 200 RFU
Ladder size (bp)75, 200, 300, 400, 500, 700, 1000, 1500, 2000, 3000, 4000, 5000, 7000, 10000, 20000
Quantification using: Lower Marker          Final concentration (ng/uL): 0.5000          Dilution factor: 12.0
```

Sample: SampA8
Well location: A8
Created: Thursday, October 21, 2021 5:28:30 PM

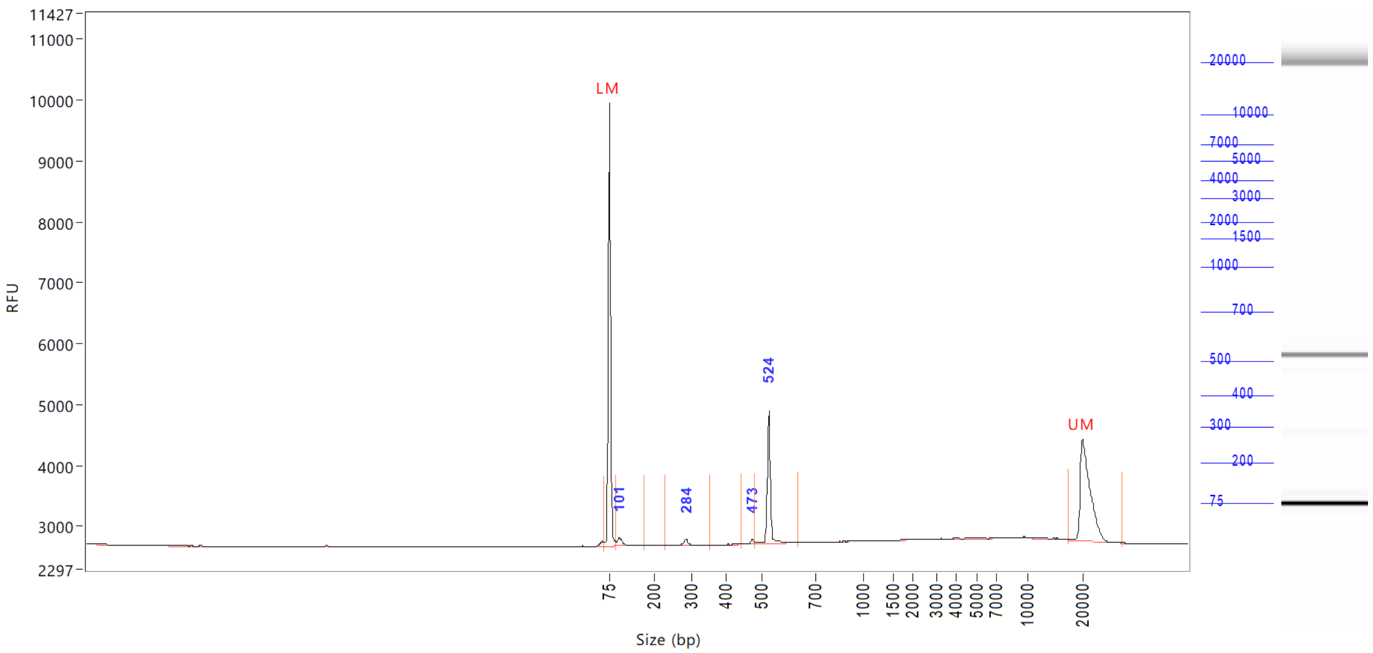

Peak	Size	Concentration	Relative concentration	Molarity	From	To	CV%	RFU
	(bp)	(ng/uL)	Percent	(nmole/L)	(bp)	(bp)		
1	75 (LM)	0.5000		11.2364	62	93	4.99	7291
2	101	0.2609	11.1	4.0044	93	173	12.82	130
3	284	0.1836	7.8	1.0823	228	352	6.18	106
4	473	0.0763	3.2	0.2682	441	481	1.87	75
5	524	1.8306	77.9	5.7378	481	636	3.03	2172
6	20000 (UM)	0.2555		0.0204	17317	27012	5.09	1677

TIC:	2.3513	ng/uL
TIM:	11.0926	nmole/L
Total concentration:	2.7242	ng/uL

```
Sample peak width (sec): 5    Sample min peak height: 50    Sample baseline V to V?: Y    Sample baseline V to V points: 3
Sample filter: Binomial       Number of points for filter: 3  Sample start region (min): 0  Sample end region (min): 30
Marker peak width (sec): 5    Marker min peak height: 200   Marker baseline V to V?: Y    Marker baseline V to V points: 3
Lower marker selection: First peak > 500 RFU                Upper marker selection: Last peak > 200 RFU
Ladder size (bp)75, 200, 300, 400, 500, 700, 1000, 1500, 2000, 3000, 4000, 5000, 7000, 10000, 20000
Quantification using: Lower Marker          Final concentration (ng/uL): 0.5000          Dilution factor: 12.0
```

Sample: SampB8
Well location: B8
Created: Thursday, October 21, 2021 5:28:30 PM

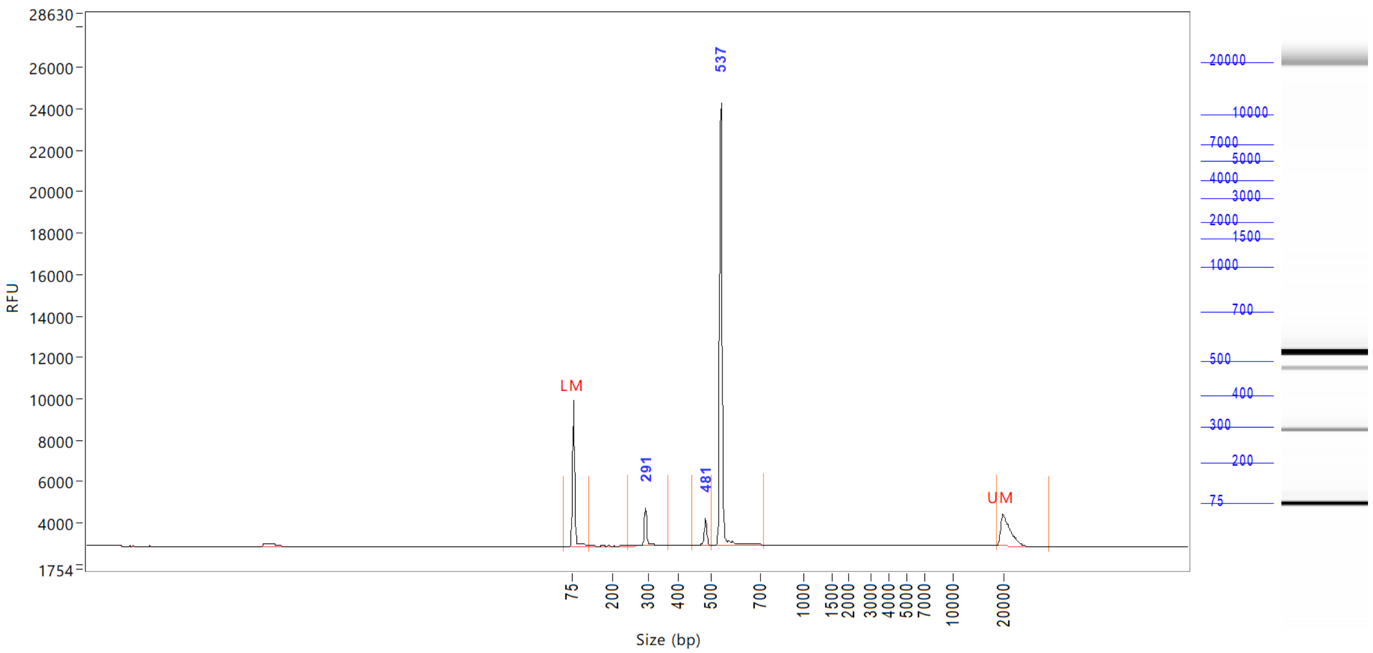

Peak	Size	Concentration	Relative concentration	Molarity	From	To	CV%	RFU
	(bp)	(ng/uL)	Percent	(nmole/L)	(bp)	(bp)		
1	75 (LM)	0.5000		11.0851	45	122	9.55	7109
2	291	1.6592	8.9	9.3465	242	362	3.64	1837
3	481	0.9887	5.3	3.4034	441	501	1.16	1279
4	537	15.9750	85.8	49.1362	501	727	2.42	21411
5	20000 (UM)	0.2269		0.0180	18659	29068	5.22	1542

TIC:	18.6229	ng/uL
TIM:	61.8862	nmole/L
Total concentration:	19.1650	ng/uL

Sample peak width (sec): 5   Sample min peak height: 50   Sample baseline V to V?: Y   Sample baseline V to V points: 3
Sample filter: Binomial   Number of points for filter: 3 Sample start region (min): 0 Sample end region (min): 30
Marker peak width (sec): 5   Marker min peak height: 200   Marker baseline V to V?: Y   Marker baseline V to V points: 3
Lower marker selection: First peak > 500 RFU   Upper marker selection: Last peak > 200 RFU
Ladder size (bp)75, 200, 300, 400, 500, 700, 1000, 1500, 2000, 3000, 4000, 5000, 7000, 10000, 20000
Quantification using: Lower Marker   Final concentration (ng/uL): 0.5000   Dilution factor: 12.0

Sample: SampC8
Well location: C8
Created: Thursday, October 21, 2021 5:28:30 PM

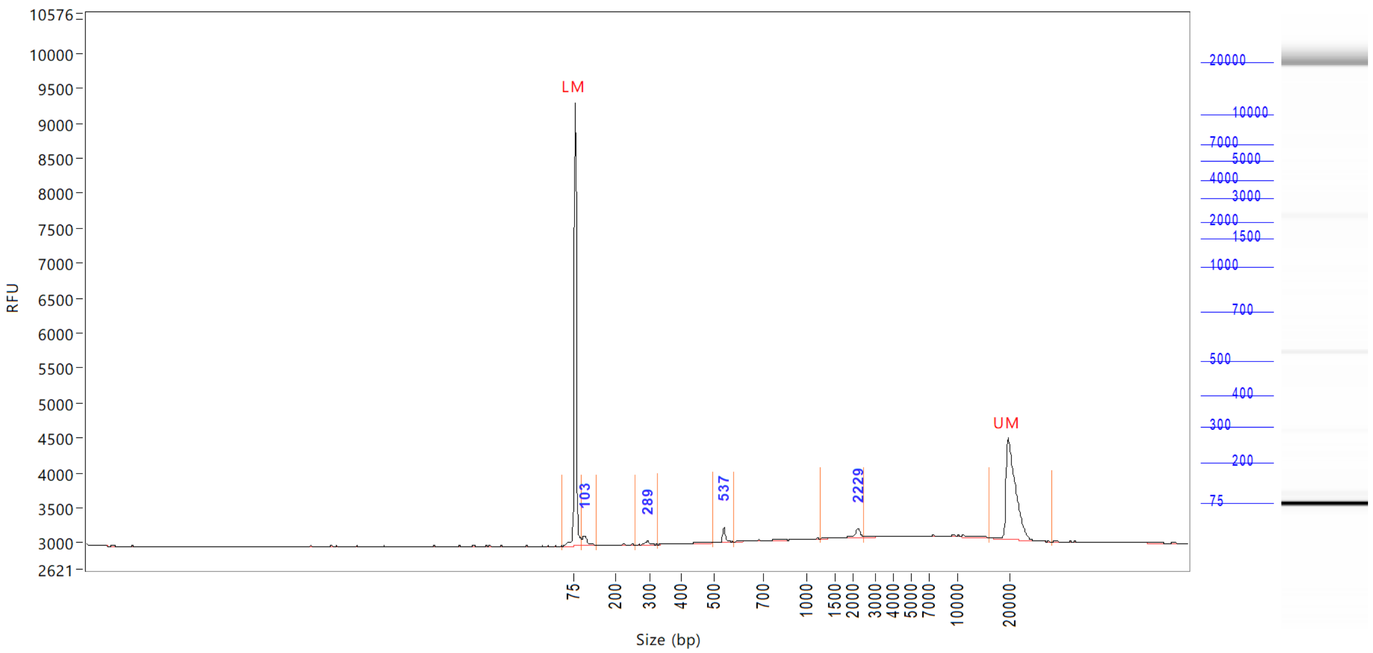

Peak	Size	Concentration	Relative concentration	Molarity	From	To	CV%	RFU
	(bp)	(ng/uL)	Percent	(nmole/L)	(bp)	(bp)		
1	75 (LM)	0.5000		11.3919	35	95	6.92	6351
2	103	0.2859	35.3	4.3887	95	141	10.19	146
3	289	0.0930	11.5	0.5349	255	321	3.43	62
4	537	0.2031	25.1	0.6237	496	580	1.88	221
5	2229	0.2272	28.1	0.1859	1223	2515	16.01	128
6	20000 (UM)	0.2417		0.0192	16220	28463	5.51	1459

TIC:	0.8093	ng/uL
TIM:	5.7332	nmole/L
Total concentration:	1.3749	ng/uL

Sample peak width (sec): 5 Sample min peak height: 50 Sample baseline V to V?: Y Sample baseline V to V points: 3
Sample filter: Binomial Number of points for filter: 3 Sample start region (min): 0 Sample end region (min): 30
Marker peak width (sec): 5 Marker min peak height: 200 Marker baseline V to V?: Y Marker baseline V to V points: 3
Lower marker selection: First peak > 500 RFU Upper marker selection: Last peak > 200 RFU
Ladder size (bp)75, 200, 300, 400, 500, 700, 1000, 1500, 2000, 3000, 4000, 5000, 7000, 10000, 20000
Quantification using: Lower Marker Final concentration (ng/uL): 0.5000 Dilution factor: 12.0

Sample: SampD8
Well location: D8
Created: Thursday, October 21, 2021 5:28:30 PM

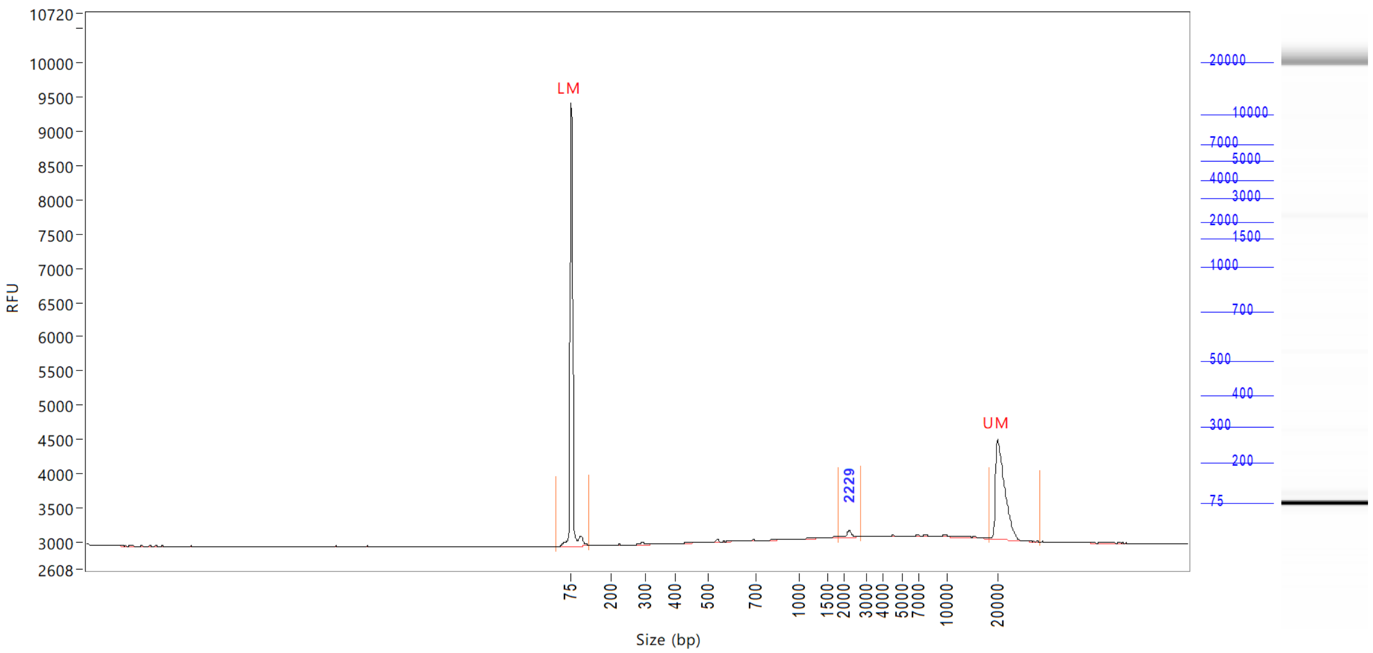

Peak	Size	Concentration	Relative concentration	Molarity	From	To	CV%	RFU
	(bp)	(ng/uL)	Percent	(nmole/L)	(bp)	(bp)		
1	75 (LM)	0.5000		10.9378	30	133	11.01	6479
2	2229	0.1421	100.0	0.1052	1827	2743	8.28	108
3	20000 (UM)	0.2203		0.0175	18293	28463	4.91	1468

TIC:	0.1421	ng/uL
TIM:	0.1052	nmole/L
Total concentration:	0.8269	ng/uL

Sample peak width (sec): 5   Sample min peak height: 50   Sample baseline V to V?: Y   Sample baseline V to V points: 3
Sample filter: Binomial   Number of points for filter: 3 Sample start region (min): 0 Sample end region (min): 30
Marker peak width (sec): 5   Marker min peak height: 200   Marker baseline V to V?: Y   Marker baseline V to V points: 3
Lower marker selection: First peak > 500 RFU   Upper marker selection: Last peak > 200 RFU
Ladder size (bp)75, 200, 300, 400, 500, 700, 1000, 1500, 2000, 3000, 4000, 5000, 7000, 10000, 20000
Quantification using: Lower Marker   Final concentration (ng/uL): 0.5000   Dilution factor: 12.0

Sample: SampE8
Well location: E8
Created: Thursday, October 21, 2021 5:28:30 PM

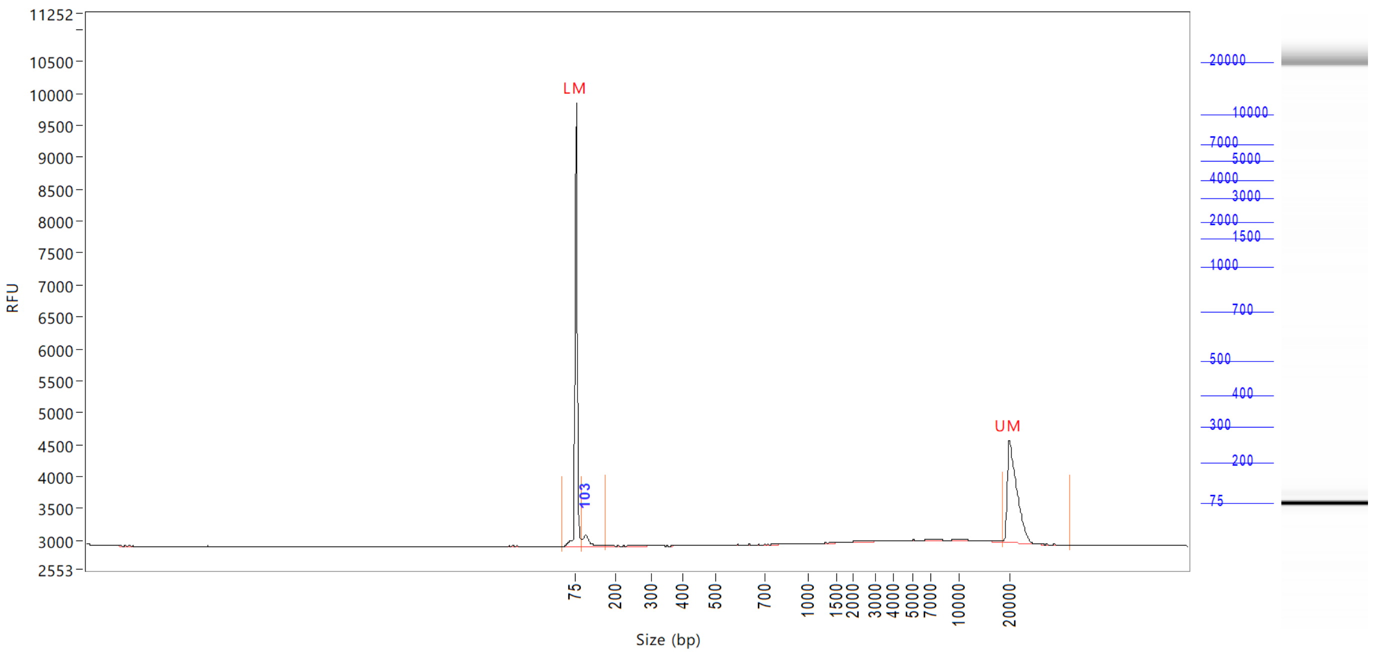

Peak	Size	Concentration	Relative concentration	Molarity	From	To	CV%	RFU
	(bp)	(ng/uL)	Percent	(nmole/L)	(bp)	(bp)		
1	75 (LM)	0.5000		11.2364	35	93	6.87	6951
2	103	0.3954	100.0	5.9580	93	164	12.65	178
3	20000 (UM)	0.2487		0.0197	18659	31849	5.95	1605

TIC:	0.3954	ng/uL
TIM:	5.9580	nmole/L
Total concentration:	0.9131	ng/uL

Sample peak width (sec): 5 Sample min peak height: 50 Sample baseline V to V?: Y Sample baseline V to V points: 3
Sample filter: Binomial Number of points for filter: 3 Sample start region (min): 0 Sample end region (min): 30
Marker peak width (sec): 5 Marker min peak height: 200 Marker baseline V to V?: Y Marker baseline V to V points: 3
Lower marker selection: First peak > 500 RFU Upper marker selection: Last peak > 200 RFU
Ladder size (bp)75, 200, 300, 400, 500, 700, 1000, 1500, 2000, 3000, 4000, 5000, 7000, 10000, 20000
Quantification using: Lower Marker Final concentration (ng/uL): 0.5000 Dilution factor: 12.0

Sample: SampF8
Well location: F8
Created: Thursday, October 21, 2021 5:28:30 PM

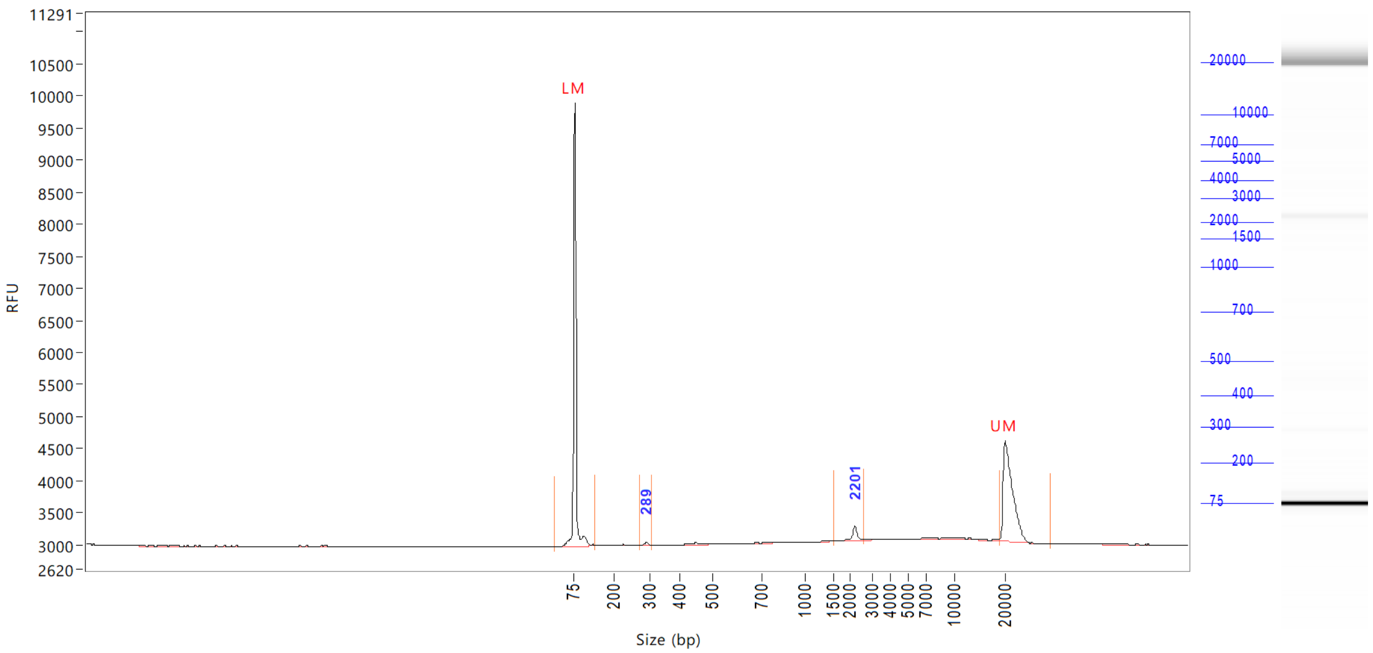

Peak	Size	Concentration	Relative concentration	Molarity	From	To	CV%	RFU
	(bp)	(ng/uL)	Percent	(nmole/L)	(bp)	(bp)		
1	75 (LM)	0.5000		11.0851	14	139	12.08	6926
2	289	0.0660	20.7	0.3781	271	305	2.17	60
3	2201	0.2529	79.3	0.1949	1520	2600	8.20	220
4	20000 (UM)	0.2258		0.0179	18781	28705	5.26	1576

TIC:	0.3189	ng/uL
TIM:	0.5730	nmole/L
Total concentration:	0.9186	ng/uL

```
Sample peak width (sec): 5    Sample min peak height: 50    Sample baseline V to V?: Y    Sample baseline V to V points: 3
Sample filter: Binomial       Number of points for filter: 3  Sample start region (min): 0  Sample end region (min): 30
Marker peak width (sec): 5    Marker min peak height: 200   Marker baseline V to V?: Y    Marker baseline V to V points: 3
Lower marker selection: First peak > 500 RFU                Upper marker selection: Last peak > 200 RFU
Ladder size (bp)75, 200, 300, 400, 500, 700, 1000, 1500, 2000, 3000, 4000, 5000, 7000, 10000, 20000
Quantification using: Lower Marker          Final concentration (ng/uL): 0.5000          Dilution factor: 12.0
```

Sample: SampG8
Well location: G8
Created: Thursday, October 21, 2021 5:28:30 PM

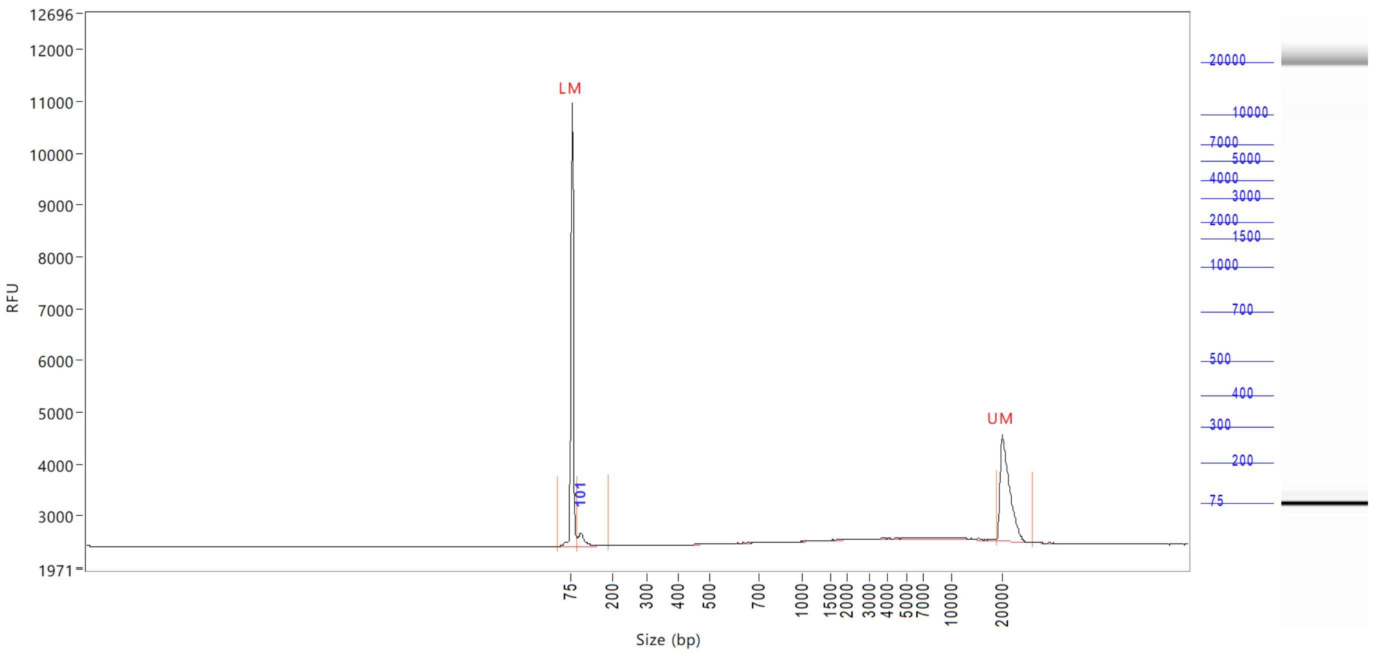

Peak	Size	Concentration	Relative concentration	Molarity	From	To	CV%	RFU
	(bp)	(ng/uL)	Percent	(nmole/L)	(bp)	(bp)		
1	75 (LM)	0.5000		11.3919	31	91	6.85	8567
2	101	0.4838	100.0	7.2906	91	187	16.95	254
3	20000 (UM)	0.2497		0.0198	18781	25924	5.03	2051

TIC:	0.4838	ng/uL
TIM:	7.2906	nmole/L
Total concentration:	0.9974	ng/uL

```
Sample peak width (sec): 5    Sample min peak height: 50    Sample baseline V to V?: Y    Sample baseline V to V points: 3
Sample filter: Binomial       Number of points for filter: 3  Sample start region (min): 0  Sample end region (min): 30
Marker peak width (sec): 5    Marker min peak height: 200   Marker baseline V to V?: Y    Marker baseline V to V points: 3
Lower marker selection: First peak > 500 RFU                Upper marker selection: Last peak > 200 RFU
Ladder size (bp)75, 200, 300, 400, 500, 700, 1000, 1500, 2000, 3000, 4000, 5000, 7000, 10000, 20000
Quantification using: Lower Marker          Final concentration (ng/uL): 0.5000          Dilution factor: 12.0
```

Sample: SampH8
Well location: H8
Created: Thursday, October 21, 2021 5:28:30 PM

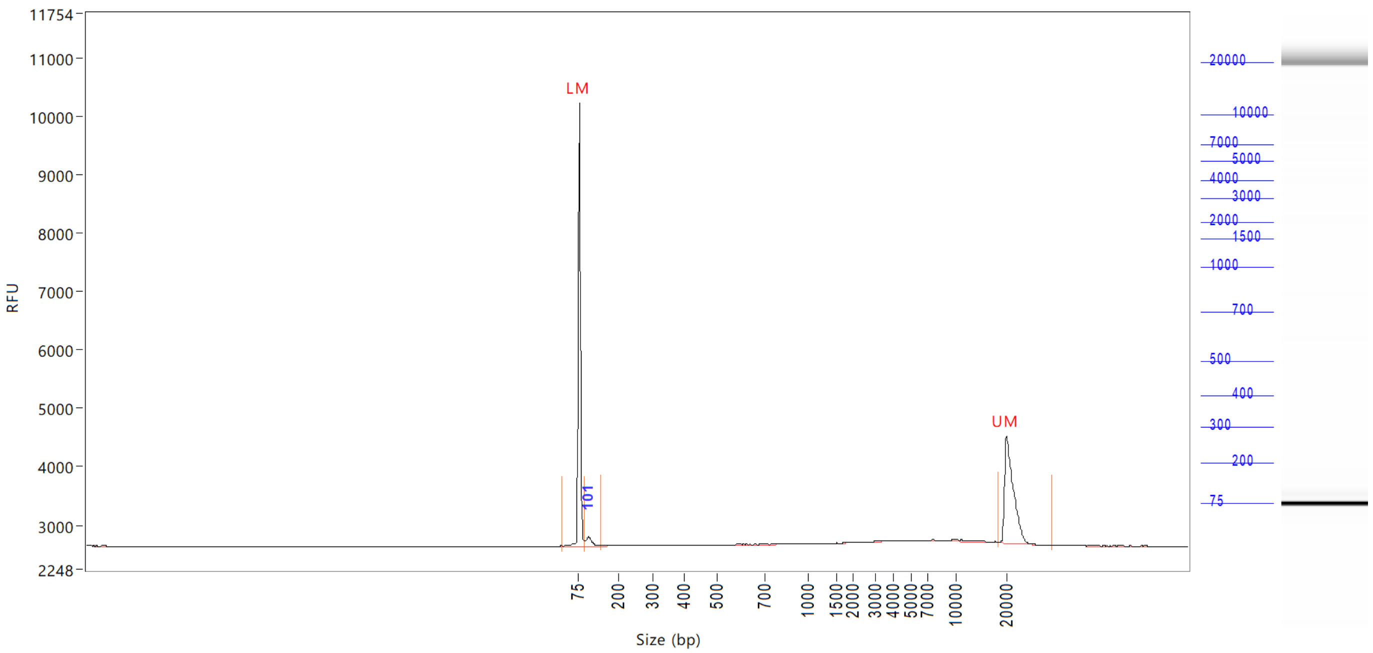

Peak	Size	Concentration	Relative concentration	Molarity	From	To	CV%	RFU
	(bp)	(ng/uL)	Percent	(nmole/L)	(bp)	(bp)		
1	75 (LM)	0.5000		11.3919	24	91	6.90	7595
2	101	0.3169	100.0	4.9573	91	143	10.81	162
3	20000 (UM)	0.2416		0.0192	18415	29068	4.98	1820

TIC:	0.3169	ng/uL
TIM:	4.9573	nmole/L
Total concentration:	0.8592	ng/uL

Sample peak width (sec): 5 Sample min peak height: 50 Sample baseline V to V?: Y Sample baseline V to V points: 3
Sample filter: Binomial Number of points for filter: 3 Sample start region (min): 0 Sample end region (min): 30
Marker peak width (sec): 5 Marker min peak height: 200 Marker baseline V to V?: Y Marker baseline V to V points: 3
Lower marker selection: First peak > 500 RFU Upper marker selection: Last peak > 200 RFU
Ladder size (bp)75, 200, 300, 400, 500, 700, 1000, 1500, 2000, 3000, 4000, 5000, 7000, 10000, 20000
Quantification using: Lower Marker Final concentration (ng/uL): 0.5000 Dilution factor: 12.0

Sample: SampA9
Well location: A9
Created: Thursday, October 21, 2021 5:28:30 PM

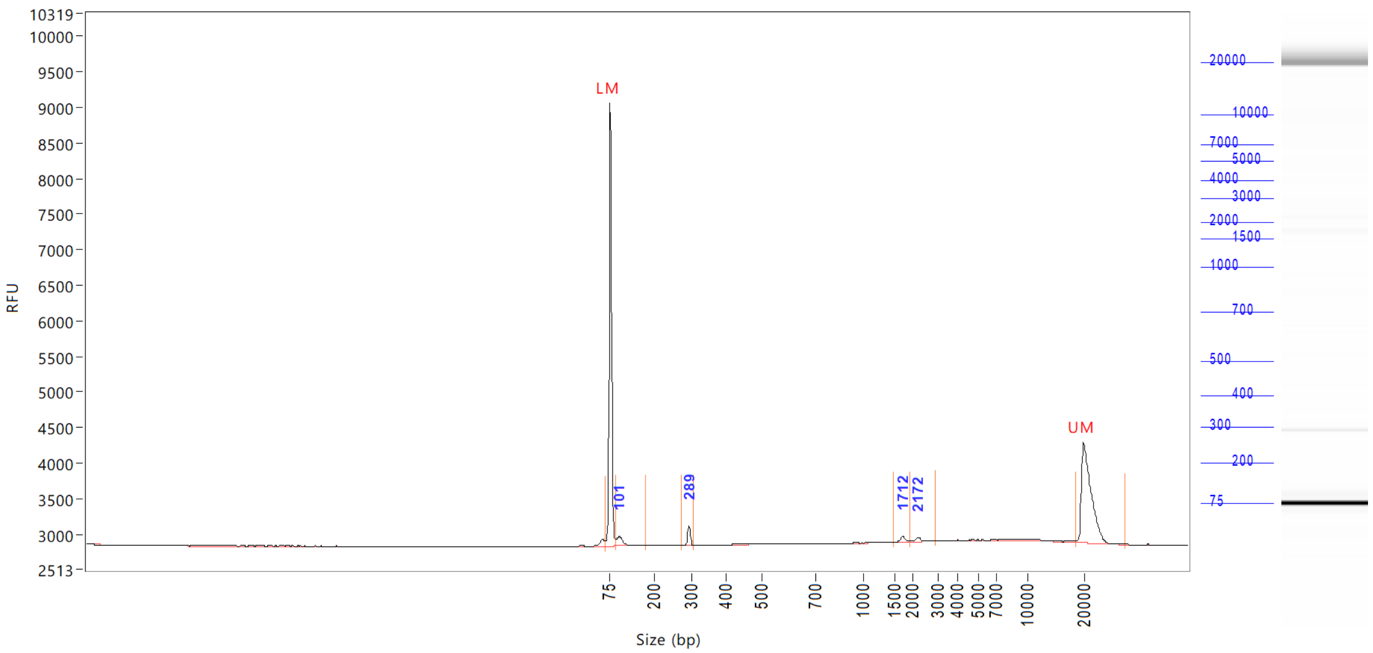

Peak	Size	Concentration	Relative concentration	Molarity	From	To	CV%	RFU
	(bp)	(ng/uL)	Percent	(nmole/L)	(bp)	(bp)		
1	75 (LM)	0.5000		11.0851	64	91	4.82	6233
2	101	0.3369	42.2	5.2696	91	173	13.25	131
3	289	0.2484	31.1	1.4185	271	301	1.34	274
4	1712	0.1103	13.8	0.1068	1478	1923	5.38	83
5	2172	0.1037	13.0	0.0760	1923	2914	10.40	57
6	20000 (UM)	0.2429		0.0194	18659	27375	5.05	1413

TIC:	0.7993	ng/uL
TIM:	6.8709	nmole/L
Total concentration:	1.4574	ng/uL

```
Sample peak width (sec): 5    Sample min peak height: 50    Sample baseline V to V?: Y    Sample baseline V to V points: 3
Sample filter: Binomial       Number of points for filter: 3  Sample start region (min): 0  Sample end region (min): 30
Marker peak width (sec): 5    Marker min peak height: 200   Marker baseline V to V?: Y    Marker baseline V to V points: 3
Lower marker selection: First peak > 500 RFU                Upper marker selection: Last peak > 200 RFU
Ladder size (bp)75, 200, 300, 400, 500, 700, 1000, 1500, 2000, 3000, 4000, 5000, 7000, 10000, 20000
Quantification using: Lower Marker          Final concentration (ng/uL): 0.5000          Dilution factor: 12.0
```

Sample: SampB9
Well location: B9
Created: Thursday, October 21, 2021 5:28:30 PM

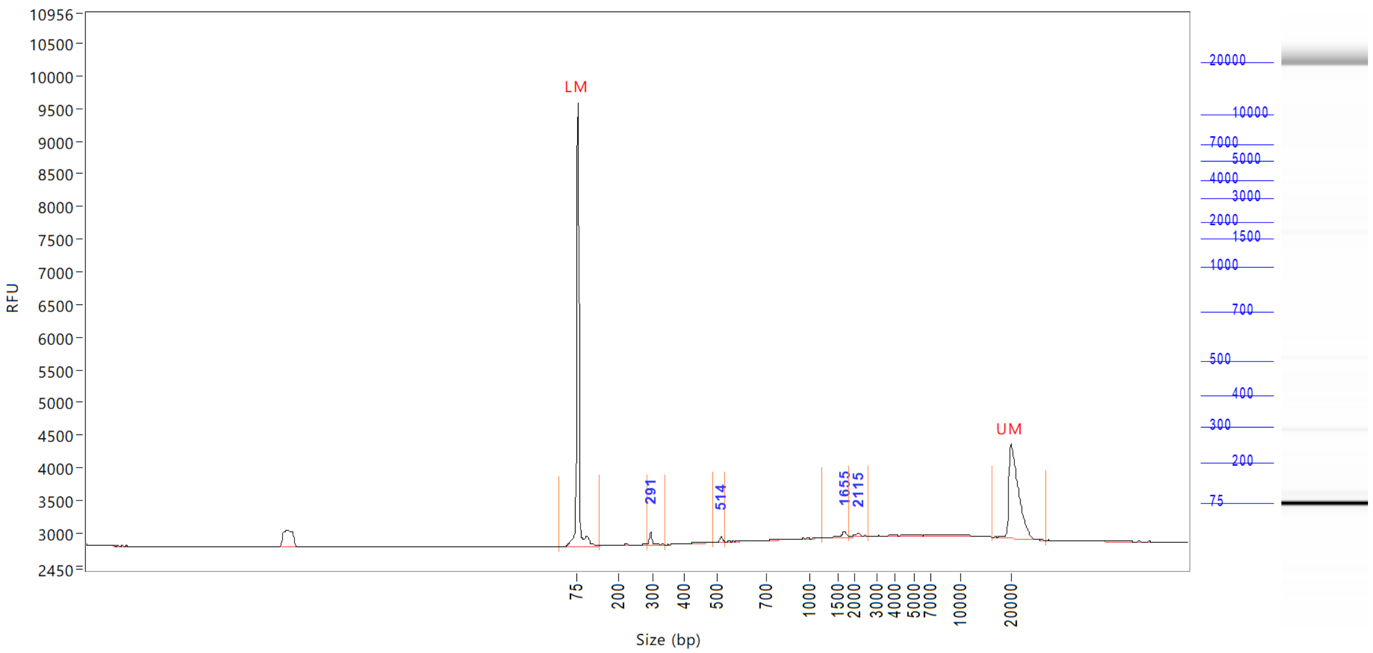

Peak	Size	Concentration	Relative concentration	Molarity	From	To	CV%	RFU
	(bp)	(ng/uL)	Percent	(nmole/L)	(bp)	(bp)		
1	75 (LM)	0.5000		11.0851	16	145	12.91	6798
2	291	0.1695	36.8	0.9546	284	339	2.85	208
3	514	0.0743	16.1	0.2387	489	534	1.30	91
4	1655	0.1241	26.9	0.1282	1212	1827	8.30	91
5	2115	0.0931	20.2	0.0721	1827	2629	9.29	51
6	20000 (UM)	0.2140		0.0170	16220	26770	5.25	1459

TIC:	0.4609	ng/uL
TIM:	1.3936	nmole/L
Total concentration:	0.9455	ng/uL

Sample peak width (sec): 5 Sample min peak height: 50 Sample baseline V to V?: Y Sample baseline V to V points: 3
Sample filter: Binomial Number of points for filter: 3 Sample start region (min): 0 Sample end region (min): 30
Marker peak width (sec): 5 Marker min peak height: 200 Marker baseline V to V?: Y Marker baseline V to V points: 3
Lower marker selection: First peak > 500 RFU Upper marker selection: Last peak > 200 RFU
Ladder size (bp)75, 200, 300, 400, 500, 700, 1000, 1500, 2000, 3000, 4000, 5000, 7000, 10000, 20000
Quantification using: Lower Marker Final concentration (ng/uL): 0.5000 Dilution factor: 12.0

Sample: SampC9
Well location: C9
Created: Thursday, October 21, 2021 5:28:30 PM

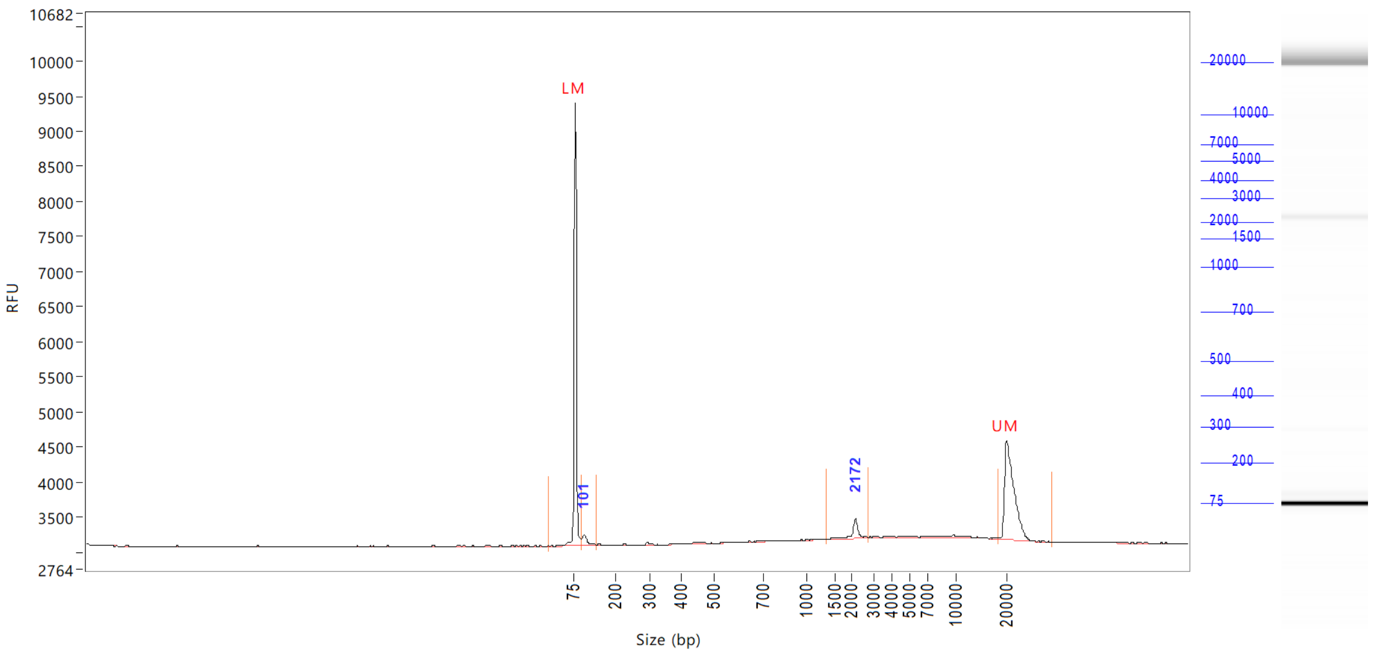

Peak	Size	Concentration	Relative concentration	Molarity	From	To	CV%	RFU
	(bp)	(ng/uL)	Percent	(nmole/L)	(bp)	(bp)		
1	75 (LM)	0.5000		11.3919	0	93	7.57	6317
2	101	0.3167	43.5	4.9076	93	139	10.21	148
3	2172	0.4120	56.5	0.3239	1367	2772	12.35	280
4	20000 (UM)	0.2358		0.0187	18293	28826	5.44	1409

TIC:	0.7288	ng/uL
TIM:	5.2315	nmole/L
Total concentration:	1.3800	ng/uL

```
Sample peak width (sec): 5    Sample min peak height: 50    Sample baseline V to V?: Y    Sample baseline V to V points: 3
Sample filter: Binomial       Number of points for filter: 3  Sample start region (min): 0  Sample end region (min): 30
Marker peak width (sec): 5    Marker min peak height: 200   Marker baseline V to V?: Y    Marker baseline V to V points: 3
Lower marker selection: First peak > 500 RFU                Upper marker selection: Last peak > 200 RFU
Ladder size (bp)75, 200, 300, 400, 500, 700, 1000, 1500, 2000, 3000, 4000, 5000, 7000, 10000, 20000
Quantification using: Lower Marker          Final concentration (ng/uL): 0.5000          Dilution factor: 12.0
```

Sample: SampD9
Well location: D9
Created: Thursday, October 21, 2021 5:28:30 PM

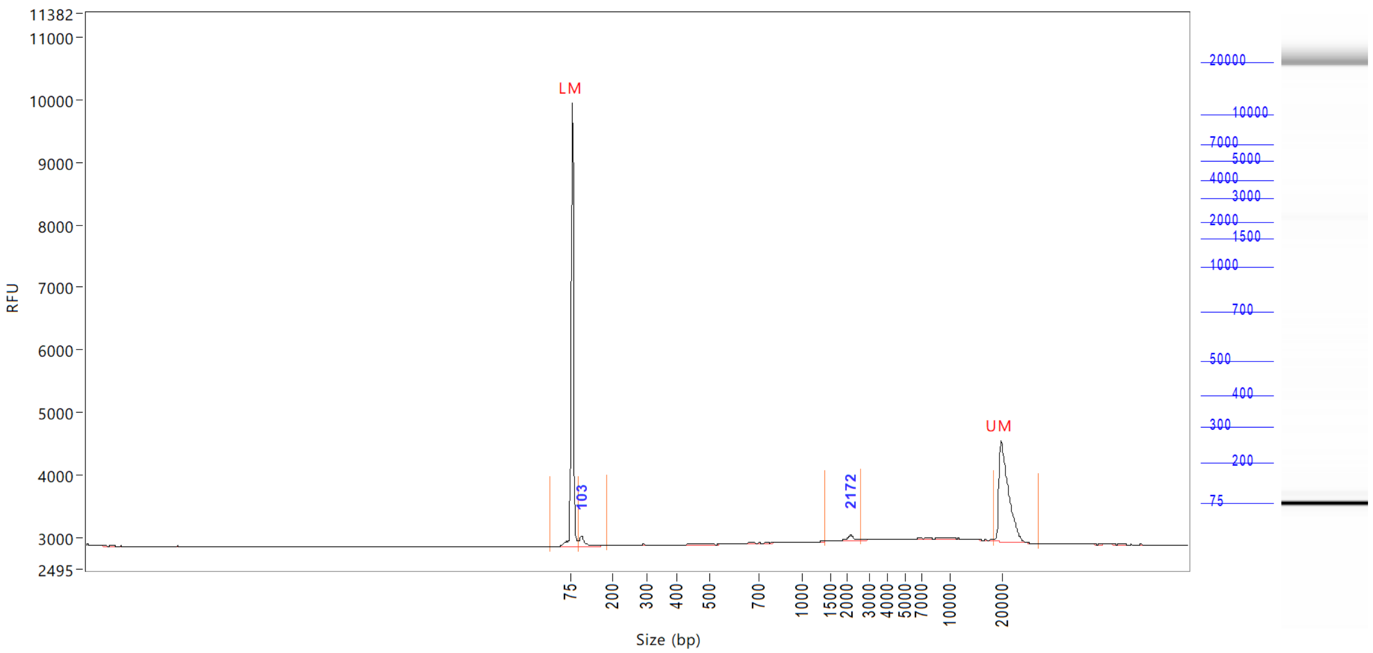

Peak	Size	Concentration	Relative concentration	Molarity	From	To	CV%	RFU
	(bp)	(ng/uL)	Percent	(nmole/L)	(bp)	(bp)		
1	75 (LM)	0.5000		11.2364	7	93	7.49	7100
2	103	0.3411	67.3	5.0028	93	185	18.00	157
3	2172	0.1656	32.7	0.1319	1422	2657	13.49	92
4	20000 (UM)	0.2438		0.0193	18415	27375	5.19	1607

TIC:	0.5067	ng/uL
TIM:	5.1347	nmole/L
Total concentration:	1.0981	ng/uL

Sample peak width (sec): 5 Sample min peak height: 50 Sample baseline V to V?: Y Sample baseline V to V points: 3
Sample filter: Binomial Number of points for filter: 3 Sample start region (min): 0 Sample end region (min): 30
Marker peak width (sec): 5 Marker min peak height: 200 Marker baseline V to V?: Y Marker baseline V to V points: 3
Lower marker selection: First peak > 500 RFU Upper marker selection: Last peak > 200 RFU
Ladder size (bp)75, 200, 300, 400, 500, 700, 1000, 1500, 2000, 3000, 4000, 5000, 7000, 10000, 20000
Quantification using: Lower Marker Final concentration (ng/uL): 0.5000 Dilution factor: 12.0

Sample: SampE9
Well location: E9
Created: Thursday, October 21, 2021 5:28:30 PM

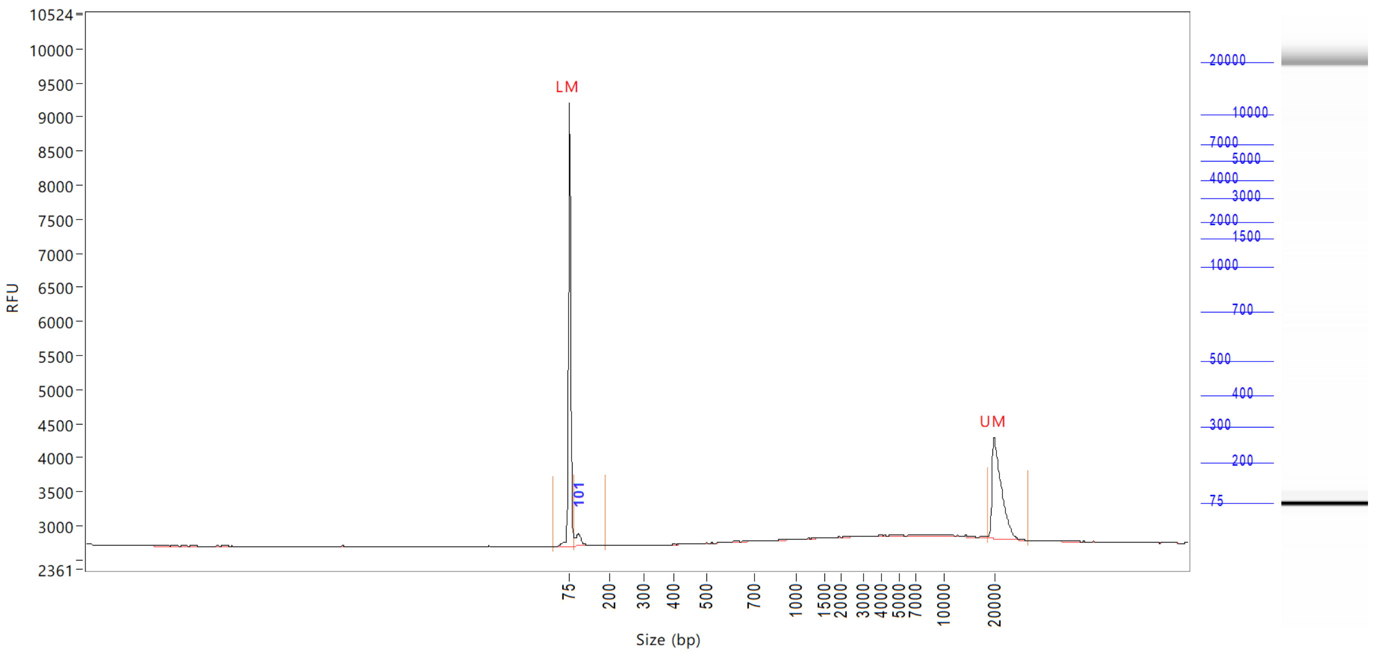

Peak	Size	Concentration	Relative concentration	Molarity	From	To	CV%	RFU
	(bp)	(ng/uL)	Percent	(nmole/L)	(bp)	(bp)		
1	75 (LM)	0.5000		11.3919	26	89	6.50	6519
2	101	0.4127	100.0	6.5169	89	187	14.60	176
3	20000 (UM)	0.2395		0.0190	18781	26649	5.30	1488

TIC:	0.4127	ng/uL
TIM:	6.5169	nmole/L
Total concentration:	1.0588	ng/uL

Sample peak width (sec): 5   Sample min peak height: 50   Sample baseline V to V?: Y   Sample baseline V to V points: 3
Sample filter: Binomial   Number of points for filter: 3   Sample start region (min): 0   Sample end region (min): 30
Marker peak width (sec): 5   Marker min peak height: 200   Marker baseline V to V?: Y   Marker baseline V to V points: 3
Lower marker selection: First peak > 500 RFU   Upper marker selection: Last peak > 200 RFU
Ladder size (bp)75, 200, 300, 400, 500, 700, 1000, 1500, 2000, 3000, 4000, 5000, 7000, 10000, 20000
Quantification using: Lower Marker   Final concentration (ng/uL): 0.5000   Dilution factor: 12.0

Sample: SampF9
Well location: F9
Created: Thursday, October 21, 2021 5:28:30 PM

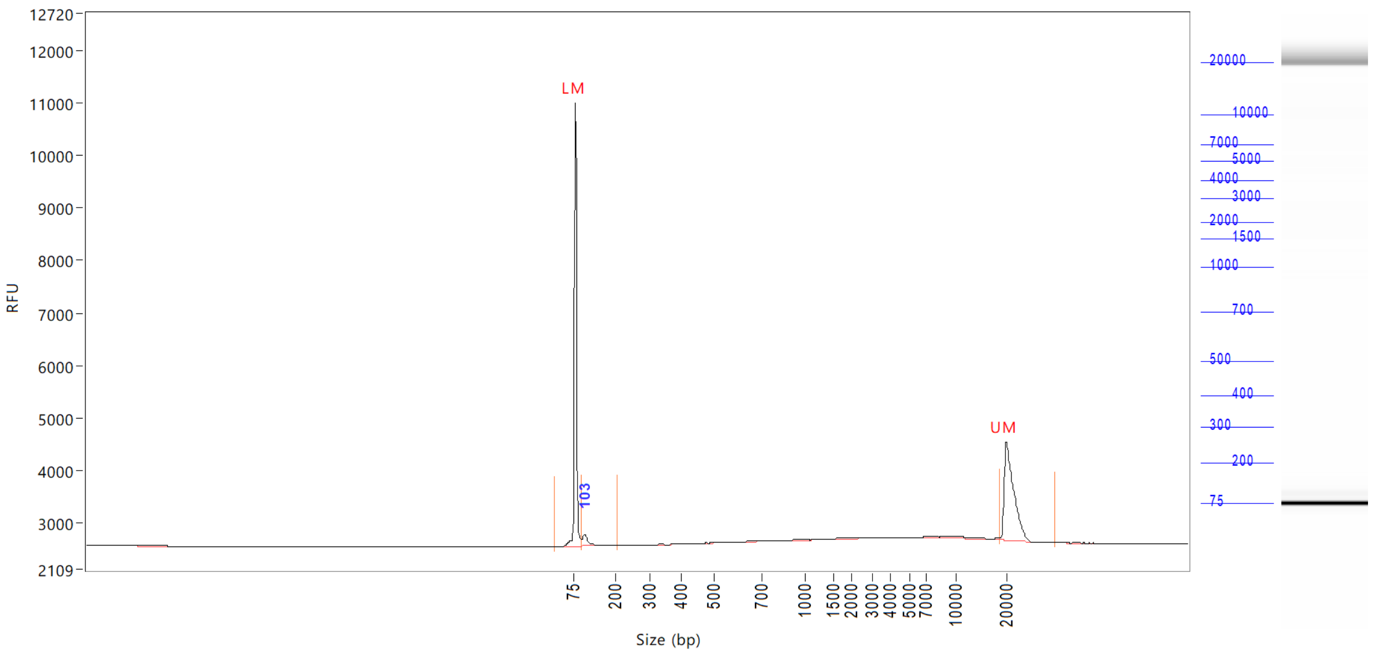

Peak	Size	Concentration	Relative concentration	Molarity	From	To	CV%	RFU
	(bp)	(ng/uL)	Percent	(nmole/L)	(bp)	(bp)		
1	75 (LM)	0.5000		11.2364	14	93	6.81	8474
2	103	0.3981	100.0	5.8381	93	206	18.82	215
3	20000 (UM)	0.2403		0.0190	18659	29672	5.24	1874

TIC:	0.3981	ng/uL
TIM:	5.8381	nmole/L
Total concentration:	1.0414	ng/uL

Sample peak width (sec): 5   Sample min peak height: 50   Sample baseline V to V?: Y   Sample baseline V to V points: 3
Sample filter: Binomial   Number of points for filter: 3   Sample start region (min): 0   Sample end region (min): 30
Marker peak width (sec): 5   Marker min peak height: 200   Marker baseline V to V?: Y   Marker baseline V to V points: 3
Lower marker selection: First peak > 500 RFU   Upper marker selection: Last peak > 200 RFU
Ladder size (bp)75, 200, 300, 400, 500, 700, 1000, 1500, 2000, 3000, 4000, 5000, 7000, 10000, 20000
Quantification using: Lower Marker   Final concentration (ng/uL): 0.5000   Dilution factor: 12.0

Sample: SampG9
Well location: G9
Created: Thursday, October 21, 2021 5:28:30 PM

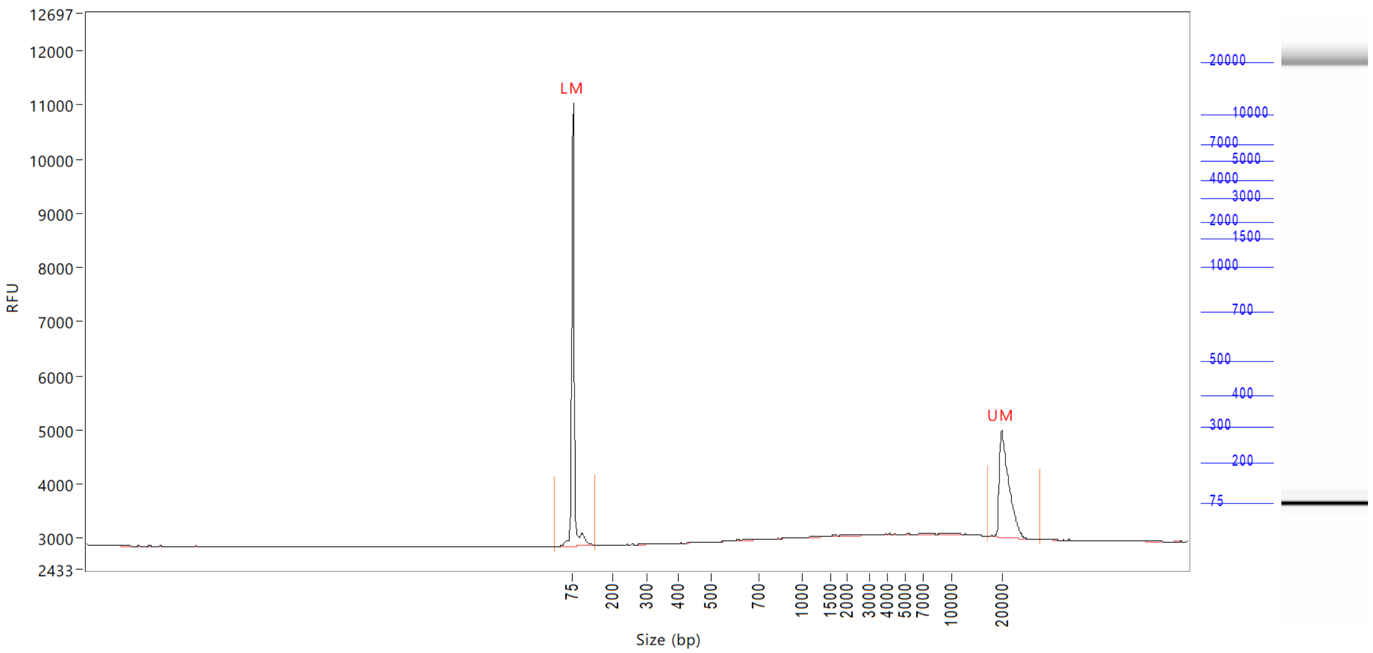

Peak	Size	Concentration	Relative concentration	Molarity	From	To	CV%	RFU
	(bp)	(ng/uL)	Percent	(nmole/L)	(bp)	(bp)		
1	75 (LM)	0.5000		11.0851	18	145	13.30	8196
2	20000 (UM)	0.2380		0.0189	17317	27617	5.33	1965

TIC:	0.0000	ng/uL
TIM:	0.0000	nmole/L
Total concentration:	0.5766	ng/uL

Sample peak width (sec): 5 Sample min peak height: 50 Sample baseline V to V?: Y Sample baseline V to V points: 3
Sample filter: Binomial Number of points for filter: 3 Sample start region (min): 0 Sample end region (min): 30
Marker peak width (sec): 5 Marker min peak height: 200 Marker baseline V to V?: Y Marker baseline V to V points: 3
Lower marker selection: First peak > 500 RFU Upper marker selection: Last peak > 200 RFU
Ladder size (bp)75, 200, 300, 400, 500, 700, 1000, 1500, 2000, 3000, 4000, 5000, 7000, 10000, 20000
Quantification using: Lower Marker Final concentration (ng/uL): 0.5000 Dilution factor: 12.0

Sample: SampH9
Well location: H9
Created: Thursday, October 21, 2021 5:28:30 PM

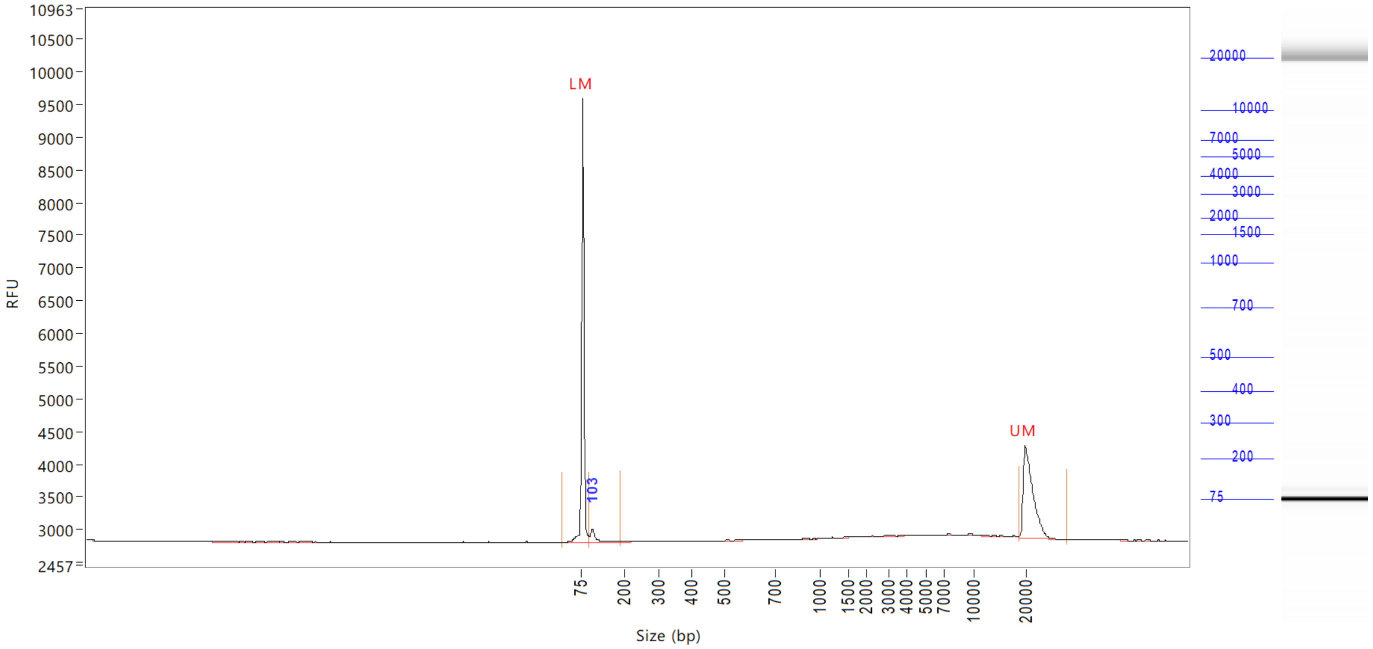

Peak	Size	Concentration	Relative concentration	Molarity	From	To	CV%	RFU
	(bp)	(ng/uL)	Percent	(nmole/L)	(bp)	(bp)		
1	75 (LM)	0.5000		11.2364	16	95	7.98	6797
2	103	0.4088	100.0	5.8394	95	189	18.52	198
3	20000 (UM)	0.2156		0.0171	18659	27859	5.29	1405

TIC:	0.4088	ng/uL
TIM:	5.8394	nmole/L
Total concentration:	0.9670	ng/uL

```
Sample peak width (sec): 5    Sample min peak height: 50    Sample baseline V to V?: Y    Sample baseline V to V points: 3
Sample filter: Binomial       Number of points for filter: 3  Sample start region (min): 0  Sample end region (min): 30
Marker peak width (sec): 5    Marker min peak height: 200   Marker baseline V to V?: Y    Marker baseline V to V points: 3
Lower marker selection: First peak > 500 RFU                Upper marker selection: Last peak > 200 RFU
Ladder size (bp)75, 200, 300, 400, 500, 700, 1000, 1500, 2000, 3000, 4000, 5000, 7000, 10000, 20000
Quantification using: Lower Marker          Final concentration (ng/uL): 0.5000          Dilution factor: 12.0
```

Sample: SampA10
Well location: A10
Created: Thursday, October 21, 2021 5:28:30 PM

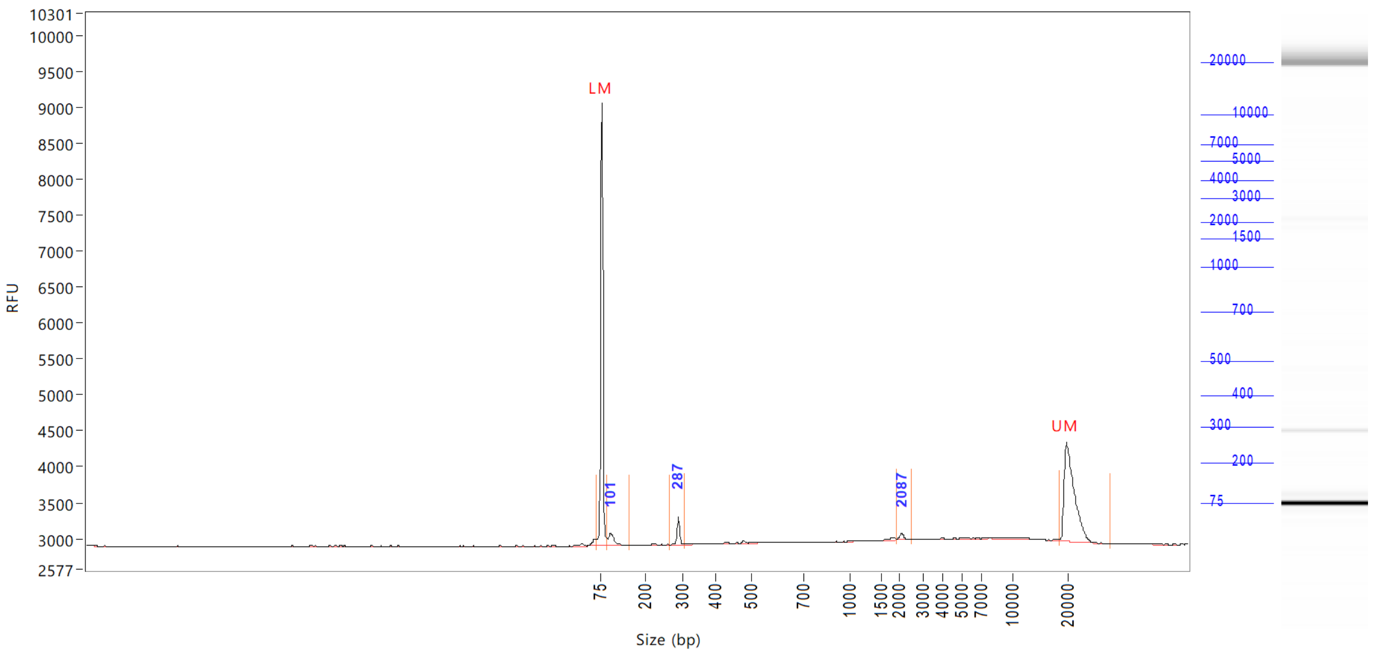

Peak	Size	Concentration	Relative concentration	Molarity	From	To	CV%	RFU
	(bp)	(ng/uL)	Percent	(nmole/L)	(bp)	(bp)		
1	75 (LM)	0.5000		11.2364	62	91	5.05	6162
2	101	0.3777	44.2	6.0214	91	152	9.87	166
3	287	0.3706	43.3	2.1313	266	303	1.48	397
4	2087	0.1068	12.5	0.0839	1942	2486	5.17	88
5	20000 (UM)	0.2496		0.0198	18659	27738	5.29	1378

TIC:	0.8550	ng/uL
TIM:	8.2366	nmole/L
Total concentration:	1.4640	ng/uL

```
Sample peak width (sec): 5    Sample min peak height: 50    Sample baseline V to V?: Y    Sample baseline V to V points: 3
Sample filter: Binomial       Number of points for filter: 3  Sample start region (min): 0  Sample end region (min): 30
Marker peak width (sec): 5    Marker min peak height: 200   Marker baseline V to V?: Y    Marker baseline V to V points: 3
Lower marker selection: First peak > 500 RFU                Upper marker selection: Last peak > 200 RFU
Ladder size (bp)75, 200, 300, 400, 500, 700, 1000, 1500, 2000, 3000, 4000, 5000, 7000, 10000, 20000
Quantification using: Lower Marker          Final concentration (ng/uL): 0.5000          Dilution factor: 12.0
```

Sample: SampB10
Well location: B10
Created: Thursday, October 21, 2021 5:28:30 PM

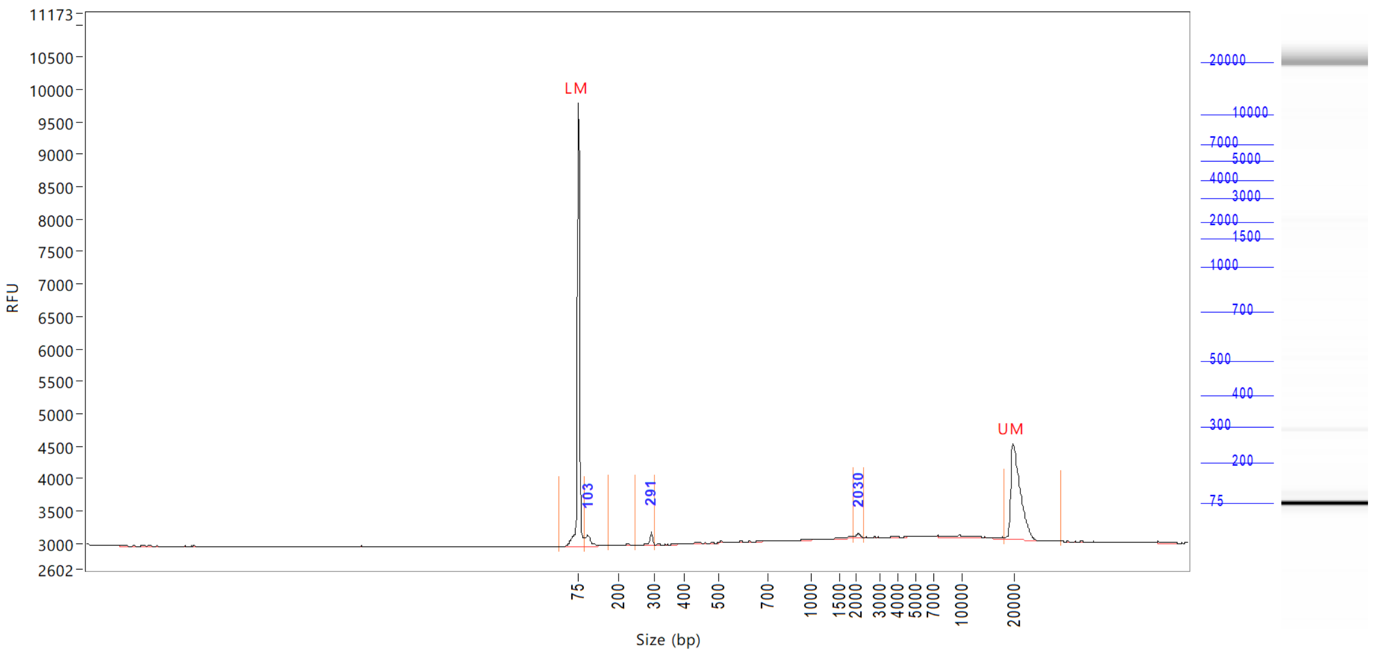

Peak	Size	Concentration	Relative concentration	Molarity	From	To	CV%	RFU
	(bp)	(ng/uL)	Percent	(nmole/L)	(bp)	(bp)		
1	75 (LM)	0.5000		11.3919	16	93	8.72	6849
2	103	0.3907	60.2	5.7821	93	166	15.79	167
3	291	0.1893	29.1	1.0886	244	303	3.58	206
4	2030	0.0695	10.7	0.0563	1885	2286	4.23	66
5	20000 (UM)	0.2202		0.0176	18171	29189	5.13	1483

TIC:	0.6495	ng/uL
TIM:	6.9269	nmole/L
Total concentration:	1.3035	ng/uL

```
Sample peak width (sec): 5    Sample min peak height: 50    Sample baseline V to V?: Y    Sample baseline V to V points: 3
Sample filter: Binomial       Number of points for filter: 3  Sample start region (min): 0  Sample end region (min): 30
Marker peak width (sec): 5    Marker min peak height: 200   Marker baseline V to V?: Y    Marker baseline V to V points: 3
Lower marker selection: First peak > 500 RFU                Upper marker selection: Last peak > 200 RFU
Ladder size (bp)75, 200, 300, 400, 500, 700, 1000, 1500, 2000, 3000, 4000, 5000, 7000, 10000, 20000
Quantification using: Lower Marker          Final concentration (ng/uL): 0.5000          Dilution factor: 12.0
```

Sample: SampC10
Well location: C10
Created: Thursday, October 21, 2021 5:28:30 PM

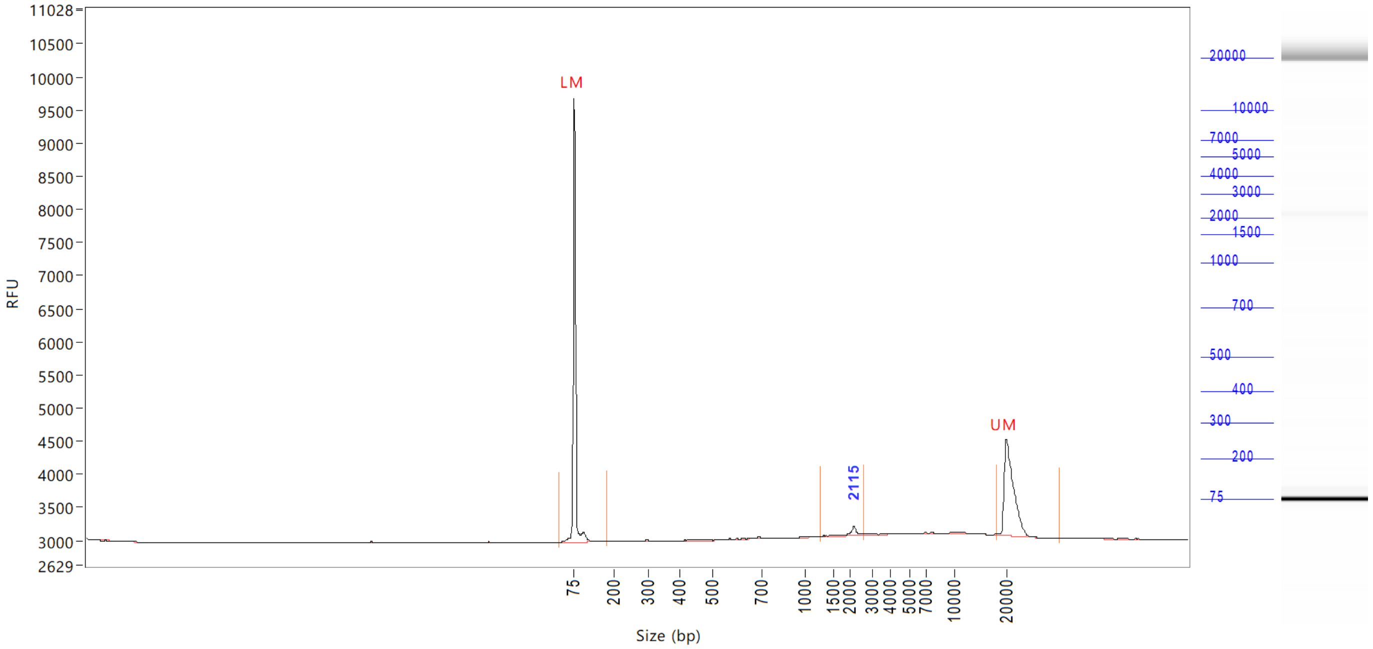

Peak	Size	Concentration	Relative concentration	Molarity	From	To	CV%	RFU
	(bp)	(ng/uL)	Percent	(nmole/L)	(bp)	(bp)		
1	75 (LM)	0.5000		10.9378	28	177	12.81	6707
2	2115	0.2555	100.0	0.2144	1256	2572	15.74	142
3	20000 (UM)	0.2196		0.0174	18293	30398	5.57	1456

TIC:	0.2555	ng/uL
TIM:	0.2144	nmole/L
Total concentration:	0.8517	ng/uL

```
Sample peak width (sec): 5    Sample min peak height: 50    Sample baseline V to V?: Y    Sample baseline V to V points: 3
Sample filter: Binomial       Number of points for filter: 3  Sample start region (min): 0  Sample end region (min): 30
Marker peak width (sec): 5    Marker min peak height: 200   Marker baseline V to V?: Y    Marker baseline V to V points: 3
Lower marker selection: First peak > 500 RFU                Upper marker selection: Last peak > 200 RFU
Ladder size (bp)75, 200, 300, 400, 500, 700, 1000, 1500, 2000, 3000, 4000, 5000, 7000, 10000, 20000
Quantification using: Lower Marker          Final concentration (ng/uL): 0.5000          Dilution factor: 12.0
```

Sample: SampD10
Well location: D10
Created: Thursday, October 21, 2021 5:28:30 PM

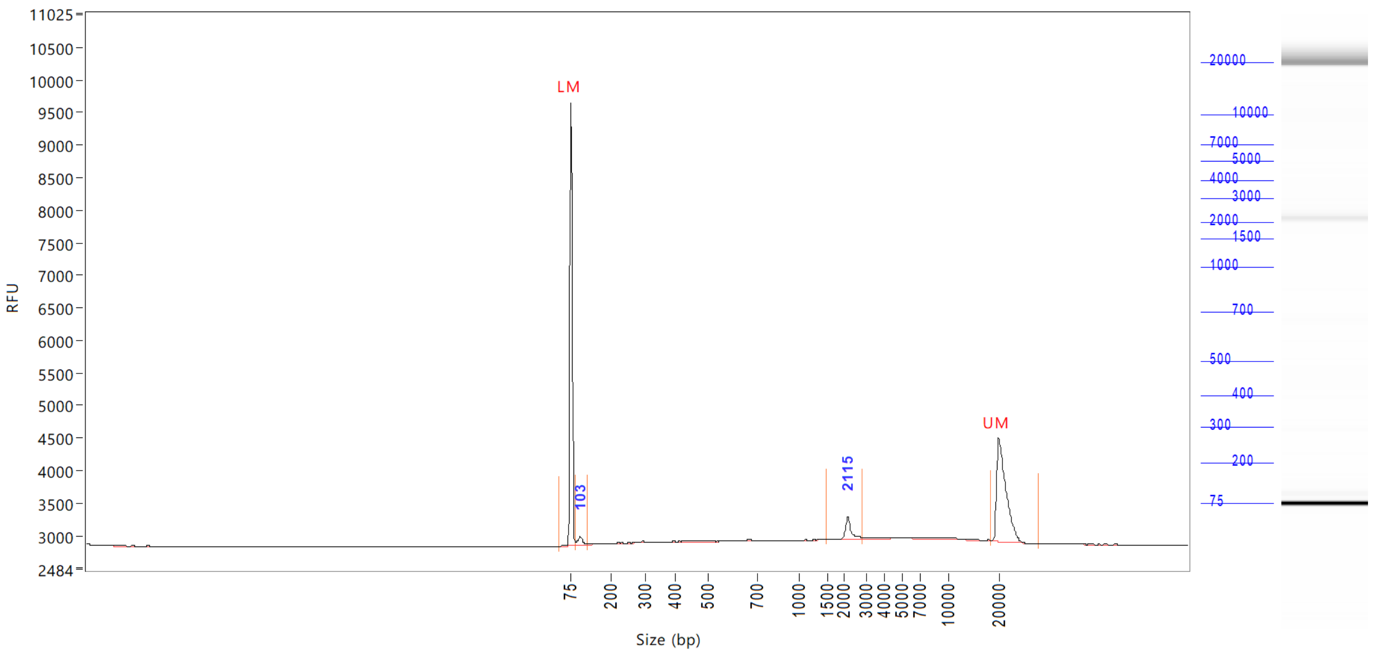

Peak	Size	Concentration	Relative concentration	Molarity	From	To	CV%	RFU
	(bp)	(ng/uL)	Percent	(nmole/L)	(bp)	(bp)		
1	75 (LM)	0.5000		11.2364	37	89	4.77	6816
2	103	0.2666	34.2	4.2929	89	126	7.95	132
3	2115	0.5136	65.8	0.3904	1478	2829	10.05	351
4	20000 (UM)	0.2568		0.0204	18537	27859	5.07	1589

TIC:	0.7803	ng/uL
TIM:	4.6833	nmole/L
Total concentration:	1.5619	ng/uL

```
Sample peak width (sec): 5    Sample min peak height: 50    Sample baseline V to V?: Y    Sample baseline V to V points: 3
Sample filter: Binomial       Number of points for filter: 3  Sample start region (min): 0  Sample end region (min): 30
Marker peak width (sec): 5    Marker min peak height: 200   Marker baseline V to V?: Y    Marker baseline V to V points: 3
Lower marker selection: First peak > 500 RFU                Upper marker selection: Last peak > 200 RFU
Ladder size (bp)75, 200, 300, 400, 500, 700, 1000, 1500, 2000, 3000, 4000, 5000, 7000, 10000, 20000
Quantification using: Lower Marker          Final concentration (ng/uL): 0.5000          Dilution factor: 12.0
```

Sample: SampE10
Well location: E10
Created: Thursday, October 21, 2021 5:28:30 PM

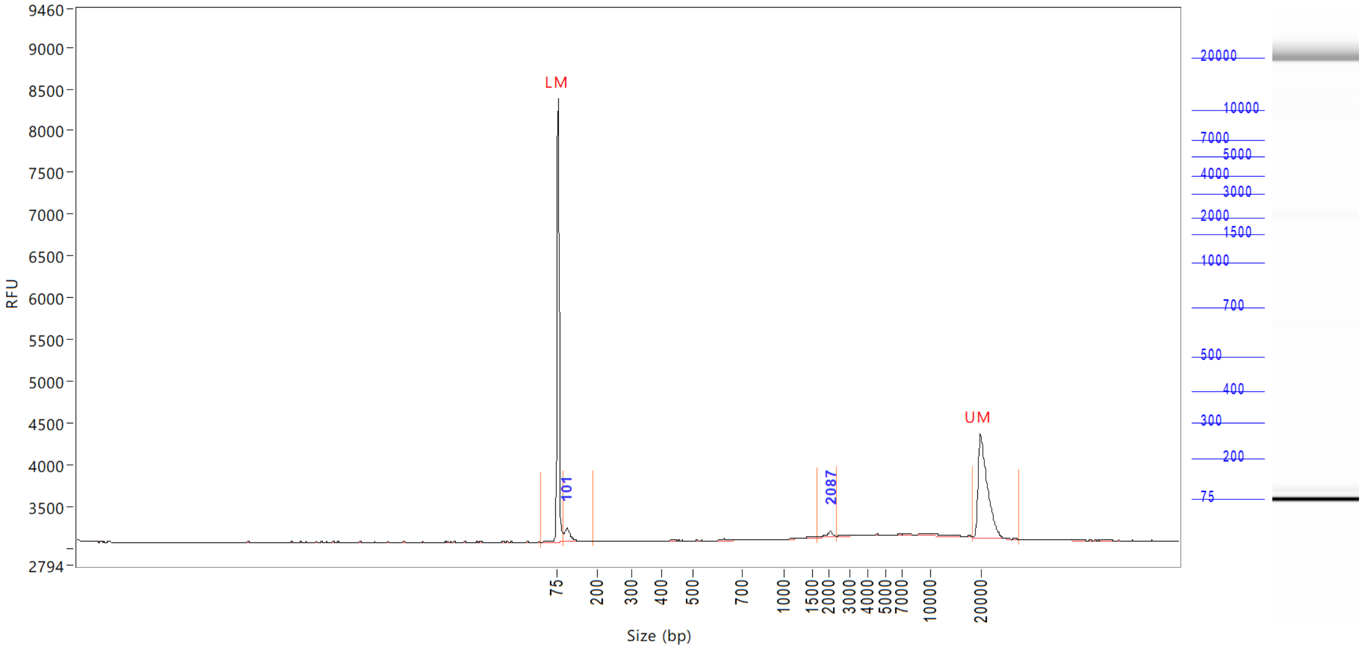

Peak	Size	Concentration	Relative concentration	Molarity	From	To	CV%	RFU
	(bp)	(ng/uL)	Percent	(nmole/L)	(bp)	(bp)		
1	75 (LM)	0.5000		11.2364	22	91	6.20	5322
2	101	0.5145	78.9	7.8235	91	185	14.14	171
3	2087	0.1377	21.1	0.1132	1655	2372	7.54	74
4	20000 (UM)	0.2547		0.0202	18415	27738	5.44	1254

TIC:	0.6522	ng/uL
TIM:	7.9368	nmole/L
Total concentration:	1.4548	ng/uL

```
Sample peak width (sec): 5    Sample min peak height: 50    Sample baseline V to V?: Y    Sample baseline V to V points: 3
Sample filter: Binomial       Number of points for filter: 3  Sample start region (min): 0  Sample end region (min): 30
Marker peak width (sec): 5    Marker min peak height: 200   Marker baseline V to V?: Y    Marker baseline V to V points: 3
Lower marker selection: First peak > 500 RFU                Upper marker selection: Last peak > 200 RFU
Ladder size (bp)75, 200, 300, 400, 500, 700, 1000, 1500, 2000, 3000, 4000, 5000, 7000, 10000, 20000
Quantification using: Lower Marker          Final concentration (ng/uL): 0.5000          Dilution factor: 12.0
```

Sample: SampF10
Well location: F10
Created: Thursday, October 21, 2021 5:28:30 PM

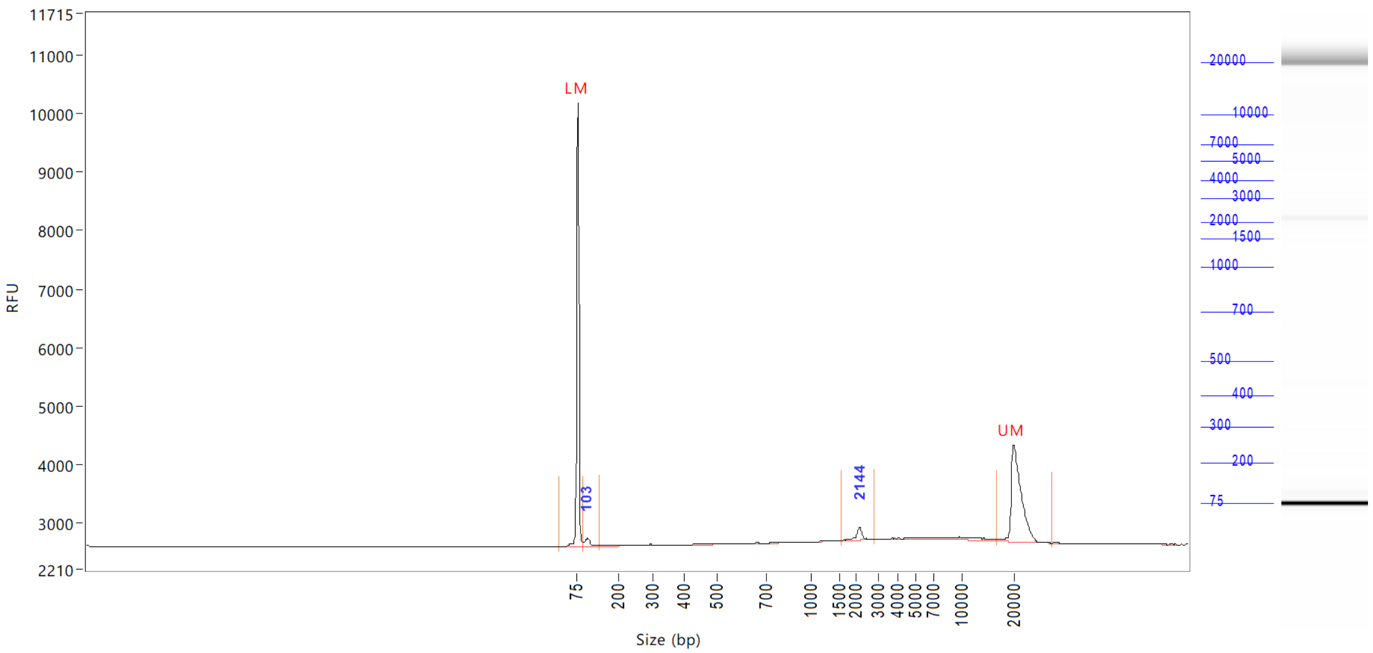

Peak	Size	Concentration	Relative concentration	Molarity	From	To	CV%	RFU
	(bp)	(ng/uL)	Percent	(nmole/L)	(bp)	(bp)		
1	75 (LM)	0.5000		11.2364	18	93	6.32	7595
2	103	0.2401	45.4	3.7197	93	141	9.37	137
3	2144	0.2890	54.6	0.2274	1559	2772	9.62	226
4	20000 (UM)	0.2471		0.0196	16830	27375	5.57	1654

TIC:	0.5291	ng/uL
TIM:	3.9472	nmole/L
Total concentration:	1.2273	ng/uL

Sample peak width (sec): 5 Sample min peak height: 50 Sample baseline V to V?: Y Sample baseline V to V points: 3
Sample filter: Binomial Number of points for filter: 3 Sample start region (min): 0 Sample end region (min): 30
Marker peak width (sec): 5 Marker min peak height: 200 Marker baseline V to V?: Y Marker baseline V to V points: 3
Lower marker selection: First peak > 500 RFU Upper marker selection: Last peak > 200 RFU
Ladder size (bp)75, 200, 300, 400, 500, 700, 1000, 1500, 2000, 3000, 4000, 5000, 7000, 10000, 20000
Quantification using: Lower Marker Final concentration (ng/uL): 0.5000 Dilution factor: 12.0

Sample: SampG10
Well location: G10
Created: Thursday, October 21, 2021 5:28:30 PM

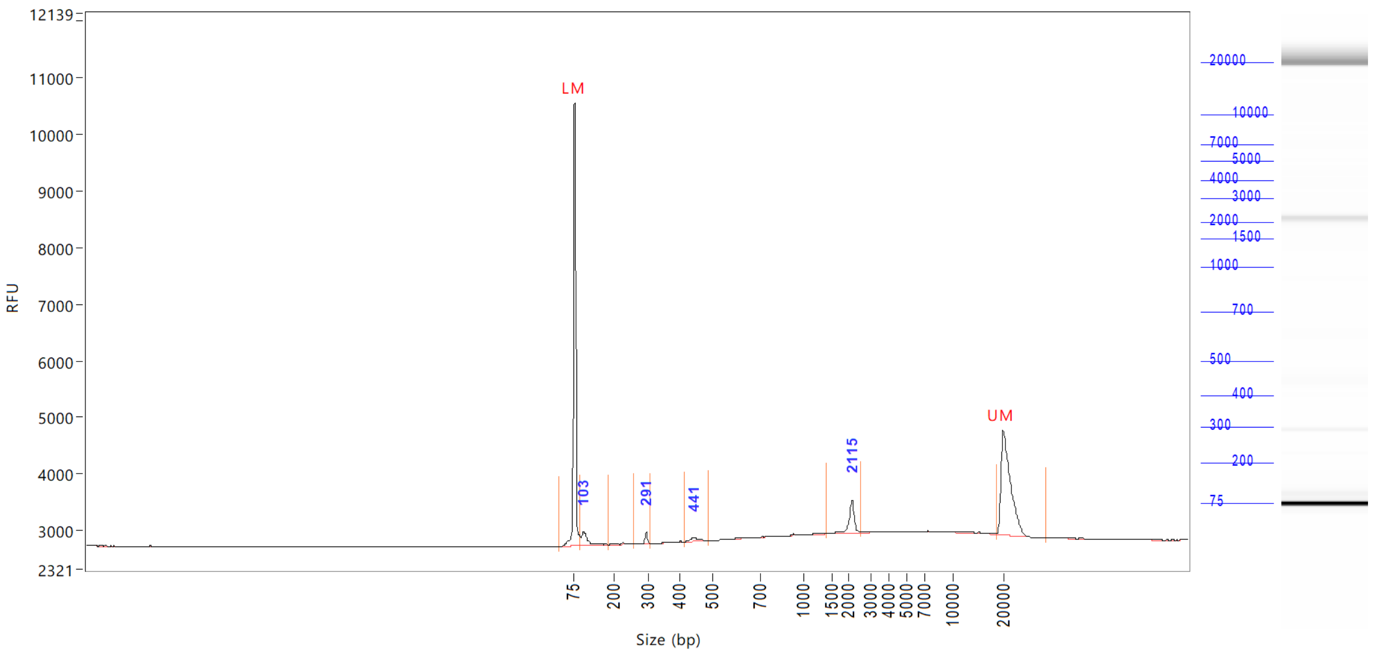

Peak	Size	Concentration	Relative concentration	Molarity	From	To	CV%	RFU
	(bp)	(ng/uL)	Percent	(nmole/L)	(bp)	(bp)		
1	75 (LM)	0.5000		11.3919	28	93	7.33	7836
2	103	0.4641	33.3	6.9302	93	179	15.87	242
3	291	0.1486	10.7	0.8487	255	301	2.10	213
4	441	0.1501	10.8	0.5550	412	490	3.37	67
5	2115	0.6292	45.2	0.5055	1389	2543	8.83	587
6	20000 (UM)	0.2408		0.0191	18659	28584	5.21	1860

TIC:	1.3920	ng/uL
TIM:	8.8394	nmole/L
Total concentration:	1.8500	ng/uL

Sample peak width (sec): 5   Sample min peak height: 50   Sample baseline V to V?: Y   Sample baseline V to V points: 3
Sample filter: Binomial   Number of points for filter: 3 Sample start region (min): 0 Sample end region (min): 30
Marker peak width (sec): 5   Marker min peak height: 200   Marker baseline V to V?: Y   Marker baseline V to V points: 3
Lower marker selection: First peak > 500 RFU   Upper marker selection: Last peak > 200 RFU
Ladder size (bp)75, 200, 300, 400, 500, 700, 1000, 1500, 2000, 3000, 4000, 5000, 7000, 10000, 20000
Quantification using: Lower Marker   Final concentration (ng/uL): 0.5000   Dilution factor: 12.0

Sample: SampH10
Well location: H10
Created: Thursday, October 21, 2021 5:28:30 PM

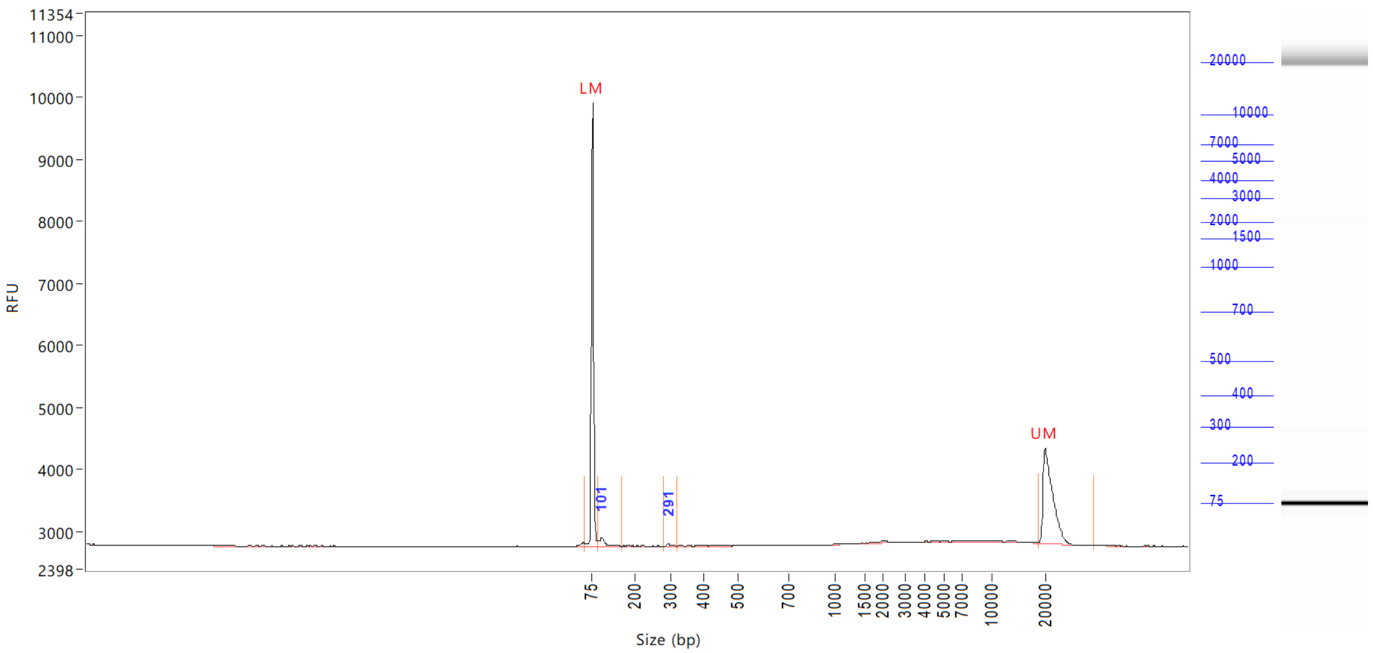

Peak	Size	Concentration	Relative concentration	Molarity	From	To	CV%	RFU
	(bp)	(ng/uL)	Percent	(nmole/L)	(bp)	(bp)		
1	75 (LM)	0.5000		11.2364	54	91	5.09	7156
2	101	0.2776	85.1	4.3005	91	160	12.34	132
3	291	0.0488	14.9	0.2747	280	315	2.57	50
4	20000 (UM)	0.2384		0.0189	18781	29189	5.25	1536

TIC:	0.3263	ng/uL
TIM:	4.5752	nmole/L
Total concentration:	0.8795	ng/uL

```
Sample peak width (sec): 5    Sample min peak height: 50    Sample baseline V to V?: Y    Sample baseline V to V points: 3
Sample filter: Binomial       Number of points for filter: 3  Sample start region (min): 0  Sample end region (min): 30
Marker peak width (sec): 5    Marker min peak height: 200    Marker baseline V to V?: Y    Marker baseline V to V points: 3
Lower marker selection: First peak > 500 RFU                 Upper marker selection: Last peak > 200 RFU
Ladder size (bp)75, 200, 300, 400, 500, 700, 1000, 1500, 2000, 3000, 4000, 5000, 7000, 10000, 20000
Quantification using: Lower Marker          Final concentration (ng/uL): 0.5000          Dilution factor: 12.0
```

Sample: SampA11
Well location: A11
Created: Thursday, October 21, 2021 5:28:30 PM

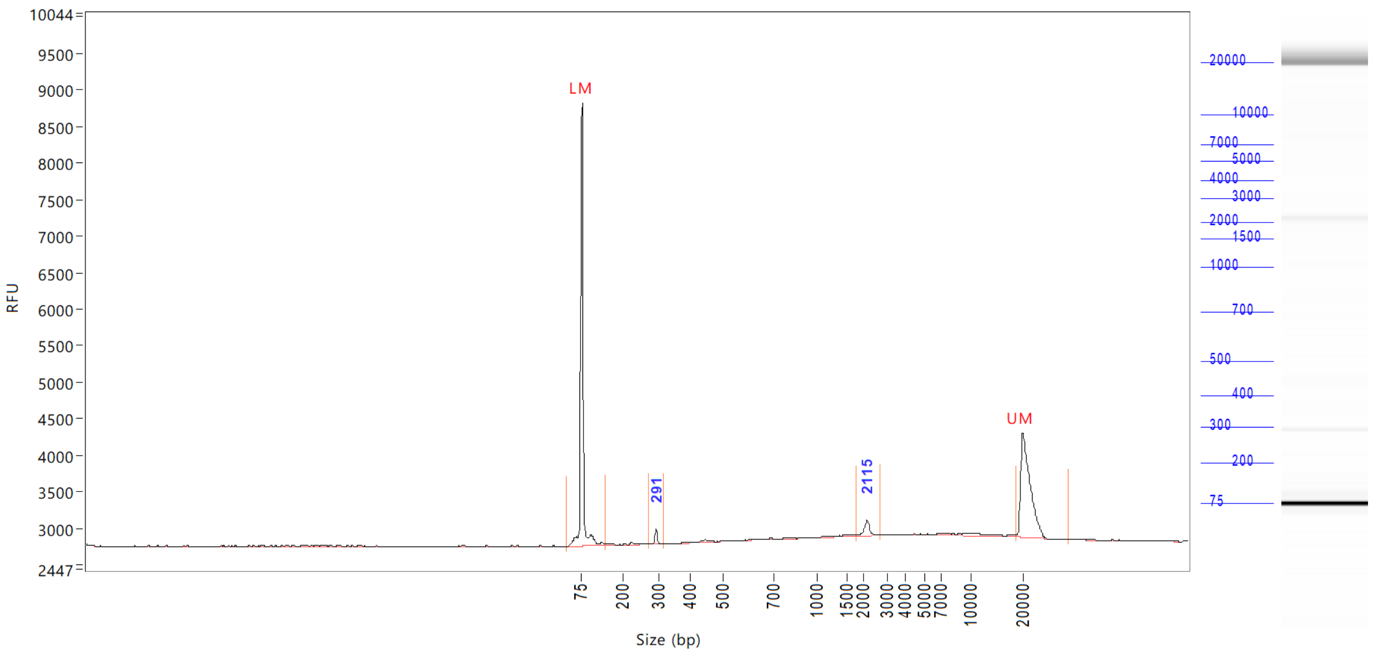

Peak	Size	Concentration	Relative concentration	Molarity	From	To	CV%	RFU
	(bp)	(ng/uL)	Percent	(nmole/L)	(bp)	(bp)		
1	75 (LM)	0.5000		11.0851	31	145	14.44	6066
2	291	0.1710	36.8	0.9698	273	317	1.57	201
3	2115	0.2931	63.2	0.2292	1770	2743	7.82	208
4	20000 (UM)	0.2214		0.0175	18659	28826	5.07	1418

TIC:	0.4641	ng/uL
TIM:	1.1990	nmole/L
Total concentration:	1.3300	ng/uL

Sample peak width (sec): 5   Sample min peak height: 50   Sample baseline V to V?: Y   Sample baseline V to V points: 3
Sample filter: Binomial   Number of points for filter: 3 Sample start region (min): 0 Sample end region (min): 30
Marker peak width (sec): 5   Marker min peak height: 200   Marker baseline V to V?: Y   Marker baseline V to V points: 3
Lower marker selection: First peak > 500 RFU   Upper marker selection: Last peak > 200 RFU
Ladder size (bp)75, 200, 300, 400, 500, 700, 1000, 1500, 2000, 3000, 4000, 5000, 7000, 10000, 20000
Quantification using: Lower Marker   Final concentration (ng/uL): 0.5000   Dilution factor: 12.0

Sample: SampB11
Well location: B11
Created: Thursday, October 21, 2021 5:28:30 PM

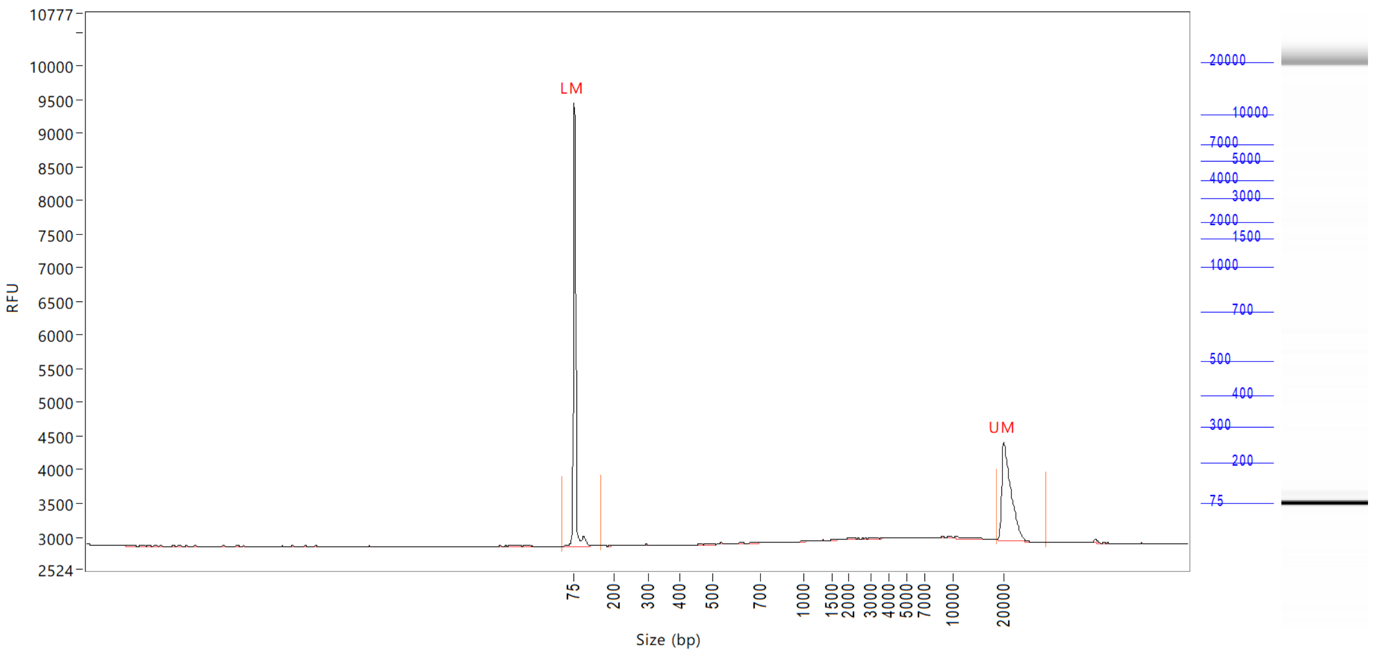

Peak	Size	Concentration	Relative concentration	Molarity	From	To	CV%	RFU
	(bp)	(ng/uL)	Percent	(nmole/L)	(bp)	(bp)		
1	75 (LM)	0.5000		10.9378	39	160	10.12	6594
2	20000 (UM)	0.2221		0.0176	18781	28463	5.07	1451

TIC:	0.0000	ng/uL
TIM:	0.0000	nmole/L
Total concentration:	0.6782	ng/uL

Sample peak width (sec): 5   Sample min peak height: 50   Sample baseline V to V?: Y   Sample baseline V to V points: 3
Sample filter: Binomial   Number of points for filter: 3   Sample start region (min): 0   Sample end region (min): 30
Marker peak width (sec): 5   Marker min peak height: 200   Marker baseline V to V?: Y   Marker baseline V to V points: 3
Lower marker selection: First peak > 500 RFU   Upper marker selection: Last peak > 200 RFU
Ladder size (bp)75, 200, 300, 400, 500, 700, 1000, 1500, 2000, 3000, 4000, 5000, 7000, 10000, 20000
Quantification using: Lower Marker   Final concentration (ng/uL): 0.5000   Dilution factor: 12.0

Sample: SampC11
Well location: C11
Created: Thursday, October 21, 2021 5:28:30 PM

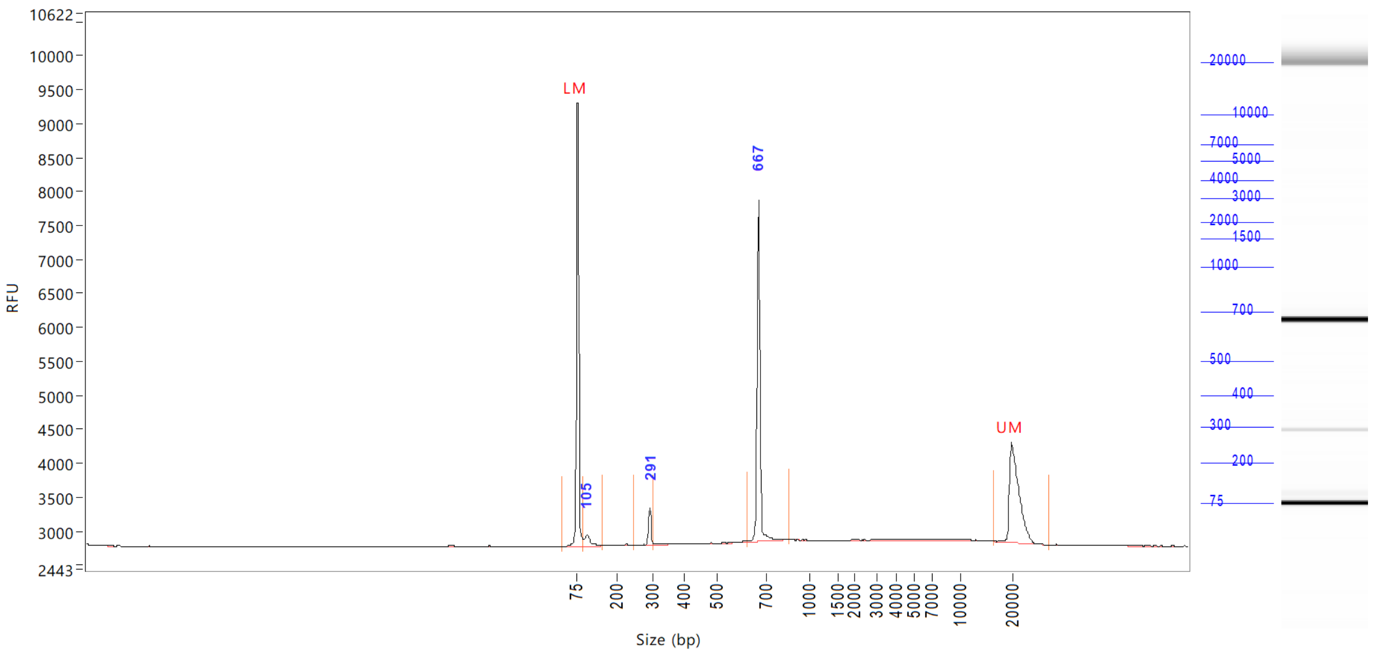

Peak	Size	Concentration	Relative concentration	Molarity	From	To	CV%	RFU
	(bp)	(ng/uL)	Percent	(nmole/L)	(bp)	(bp)		
1	75 (LM)	0.5000		11.0851	31	95	6.23	6537
2	105	0.3831	7.5	5.7731	95	152	11.47	172
3	291	0.4640	9.1	2.6501	248	303	2.08	552
4	667	4.2692	83.4	10.5178	623	857	2.19	5026
5	20000 (UM)	0.2321		0.0184	16342	27375	5.15	1467

TIC:	5.1163	ng/uL
TIM:	18.9410	nmole/L
Total concentration:	5.5898	ng/uL

Sample peak width (sec): 5   Sample min peak height: 50   Sample baseline V to V?: Y   Sample baseline V to V points: 3
Sample filter: Binomial   Number of points for filter: 3 Sample start region (min): 0 Sample end region (min): 30
Marker peak width (sec): 5   Marker min peak height: 200   Marker baseline V to V?: Y   Marker baseline V to V points: 3
Lower marker selection: First peak > 500 RFU   Upper marker selection: Last peak > 200 RFU
Ladder size (bp)75, 200, 300, 400, 500, 700, 1000, 1500, 2000, 3000, 4000, 5000, 7000, 10000, 20000
Quantification using: Lower Marker   Final concentration (ng/uL): 0.5000   Dilution factor: 12.0

Sample: SampD11
Well location: D11
Created: Thursday, October 21, 2021 5:28:30 PM

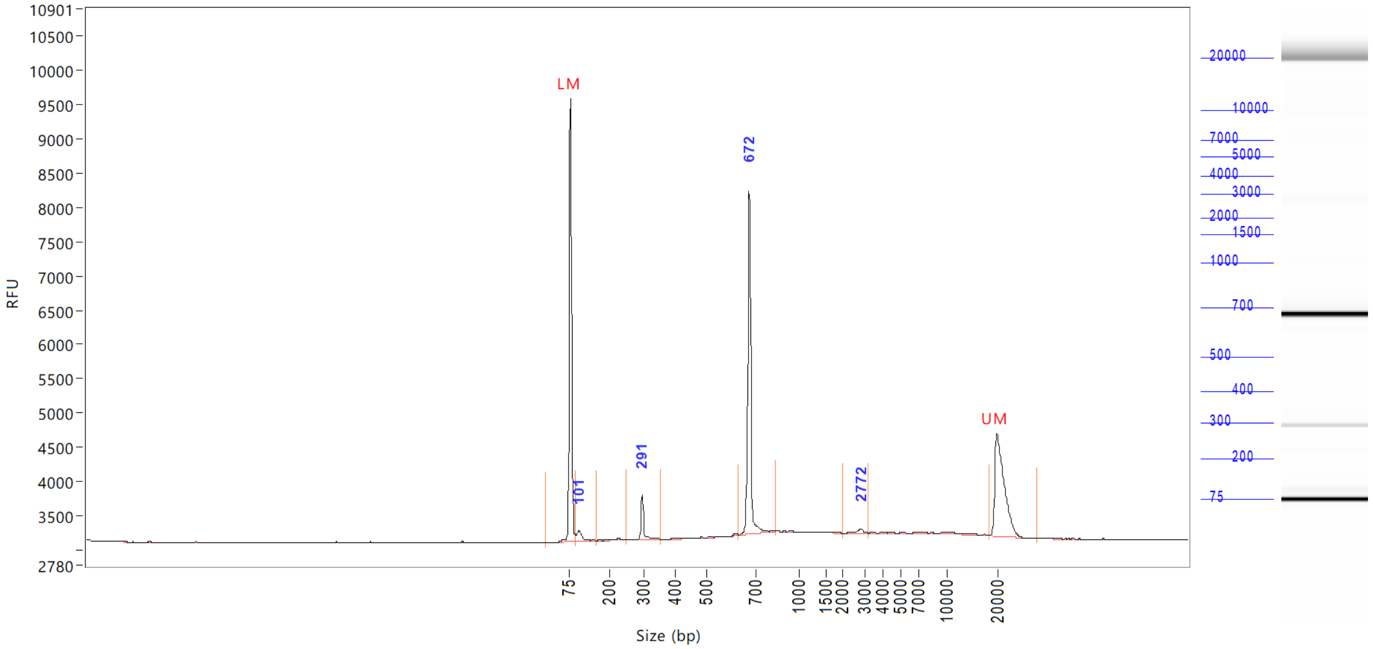

Peak	Size	Concentration	Relative concentration	Molarity	From	To	CV%	RFU
	(bp)	(ng/uL)	Percent	(nmole/L)	(bp)	(bp)		
1	75 (LM)	0.5000		11.2364	1	91	6.38	6480
2	101	0.3602	6.2	5.6341	91	154	12.22	162
3	291	0.6555	11.3	3.6803	248	354	3.96	653
4	672	4.6527	80.5	11.3945	631	840	2.10	4995
5	2772	0.1080	1.9	0.0664	1981	3179	8.14	68
6	20000 (UM)	0.2472		0.0196	18537	27859	5.17	1502

TIC:	5.7765	ng/uL
TIM:	20.7753	nmole/L
Total concentration:	6.2485	ng/uL

Sample peak width (sec): 5   Sample min peak height: 50   Sample baseline V to V?: Y   Sample baseline V to V points: 3
Sample filter: Binomial   Number of points for filter: 3 Sample start region (min): 0 Sample end region (min): 30
Marker peak width (sec): 5   Marker min peak height: 200   Marker baseline V to V?: Y   Marker baseline V to V points: 3
Lower marker selection: First peak > 500 RFU   Upper marker selection: Last peak > 200 RFU
Ladder size (bp)75, 200, 300, 400, 500, 700, 1000, 1500, 2000, 3000, 4000, 5000, 7000, 10000, 20000
Quantification using: Lower Marker   Final concentration (ng/uL): 0.5000   Dilution factor: 12.0

Sample: SampE11
Well location: E11
Created: Thursday, October 21, 2021 5:28:30 PM

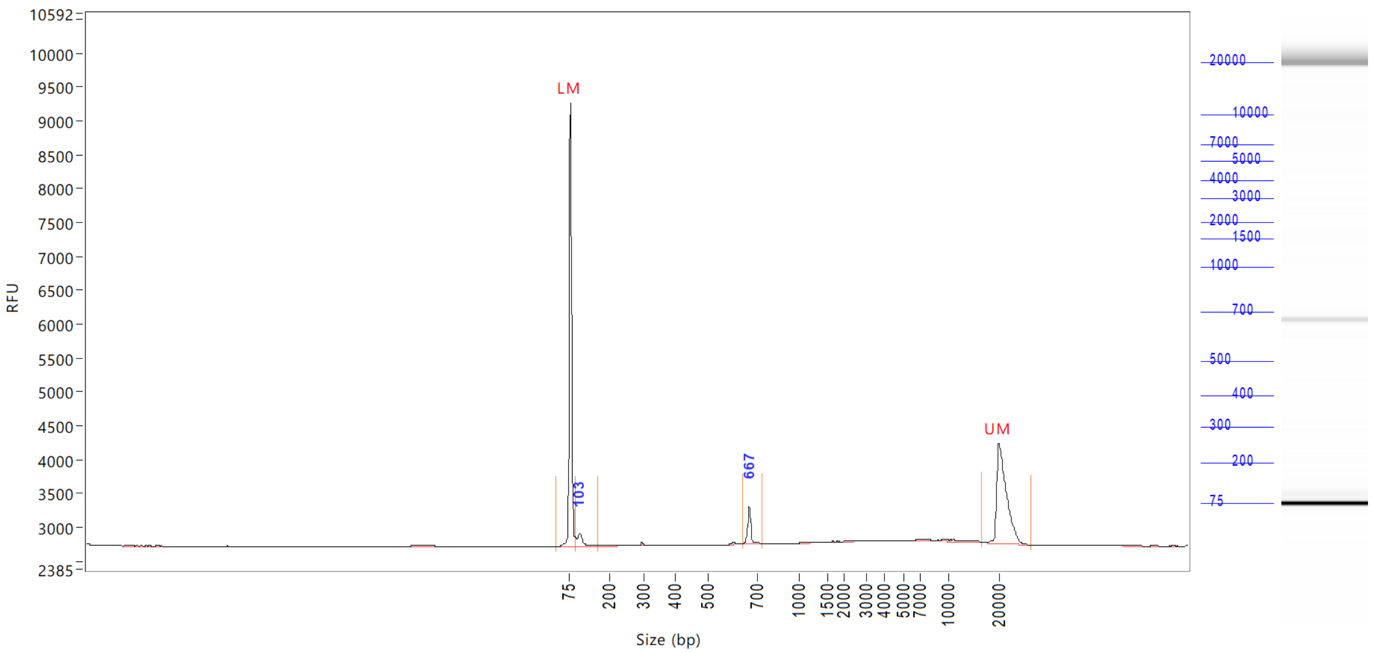

Peak	Size	Concentration	Relative concentration	Molarity	From	To	CV%	RFU
	(bp)	(ng/uL)	Percent	(nmole/L)	(bp)	(bp)		
1	75 (LM)	0.5000		11.2364	31	93	6.48	6559
2	103	0.4074	41.9	6.2529	93	160	12.07	192
3	667	0.5644	58.1	1.3925	639	740	1.82	566
4	20000 (UM)	0.2478		0.0197	16586	26166	5.50	1475

TIC:	0.9717	ng/uL
TIM:	7.6453	nmole/L
Total concentration:	1.6596	ng/uL

```
Sample peak width (sec): 5    Sample min peak height: 50    Sample baseline V to V?: Y    Sample baseline V to V points: 3
Sample filter: Binomial       Number of points for filter: 3  Sample start region (min): 0  Sample end region (min): 30
Marker peak width (sec): 5    Marker min peak height: 200   Marker baseline V to V?: Y    Marker baseline V to V points: 3
Lower marker selection: First peak > 500 RFU                Upper marker selection: Last peak > 200 RFU
Ladder size (bp)75, 200, 300, 400, 500, 700, 1000, 1500, 2000, 3000, 4000, 5000, 7000, 10000, 20000
Quantification using: Lower Marker          Final concentration (ng/uL): 0.5000          Dilution factor: 12.0
```

Sample: SampF11
Well location: F11
Created: Thursday, October 21, 2021 5:28:30 PM

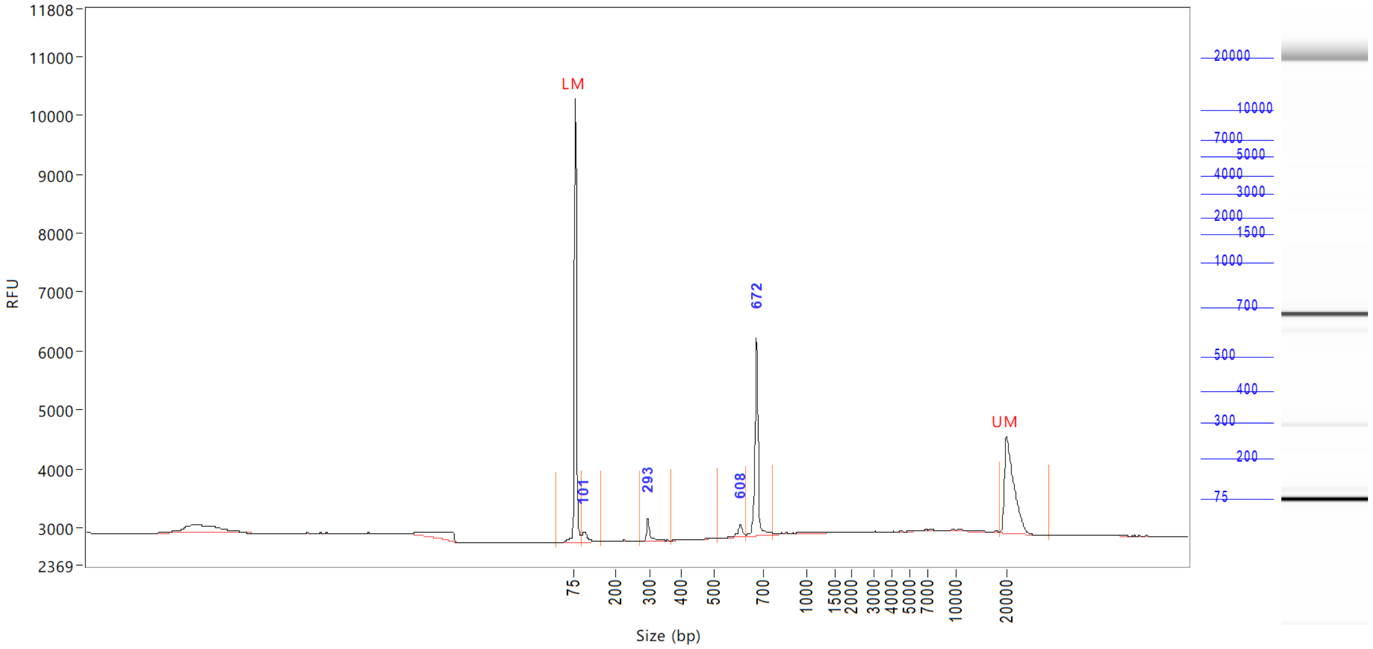

Peak	Size	Concentration	Relative concentration	Molarity	From	To	CV%	RFU
	(bp)	(ng/uL)	Percent	(nmole/L)	(bp)	(bp)		
1	75 (LM)	0.5000		11.2364	18	93	6.62	7541
2	101	0.3421	9.1	5.2505	93	156	11.60	179
3	293	0.3775	10.0	2.0978	269	366	4.49	375
4	608	0.2622	7.0	0.7203	511	631	3.39	201
5	672	2.7792	73.9	6.8063	631	770	2.10	3371
6	20000 (UM)	0.2326		0.0185	18537	28221	5.25	1639

TIC:	3.7610	ng/uL
TIM:	14.8749	nmole/L
Total concentration:	4.3475	ng/uL

Sample peak width (sec): 5   Sample min peak height: 50   Sample baseline V to V?: Y   Sample baseline V to V points: 3
Sample filter: Binomial   Number of points for filter: 3 Sample start region (min): 0 Sample end region (min): 30
Marker peak width (sec): 5   Marker min peak height: 200   Marker baseline V to V?: Y   Marker baseline V to V points: 3
Lower marker selection: First peak > 500 RFU   Upper marker selection: Last peak > 200 RFU
Ladder size (bp)75, 200, 300, 400, 500, 700, 1000, 1500, 2000, 3000, 4000, 5000, 7000, 10000, 20000
Quantification using: Lower Marker   Final concentration (ng/uL): 0.5000   Dilution factor: 12.0

Sample: SampG11
Well location: G11
Created: Thursday, October 21, 2021 5:28:30 PM

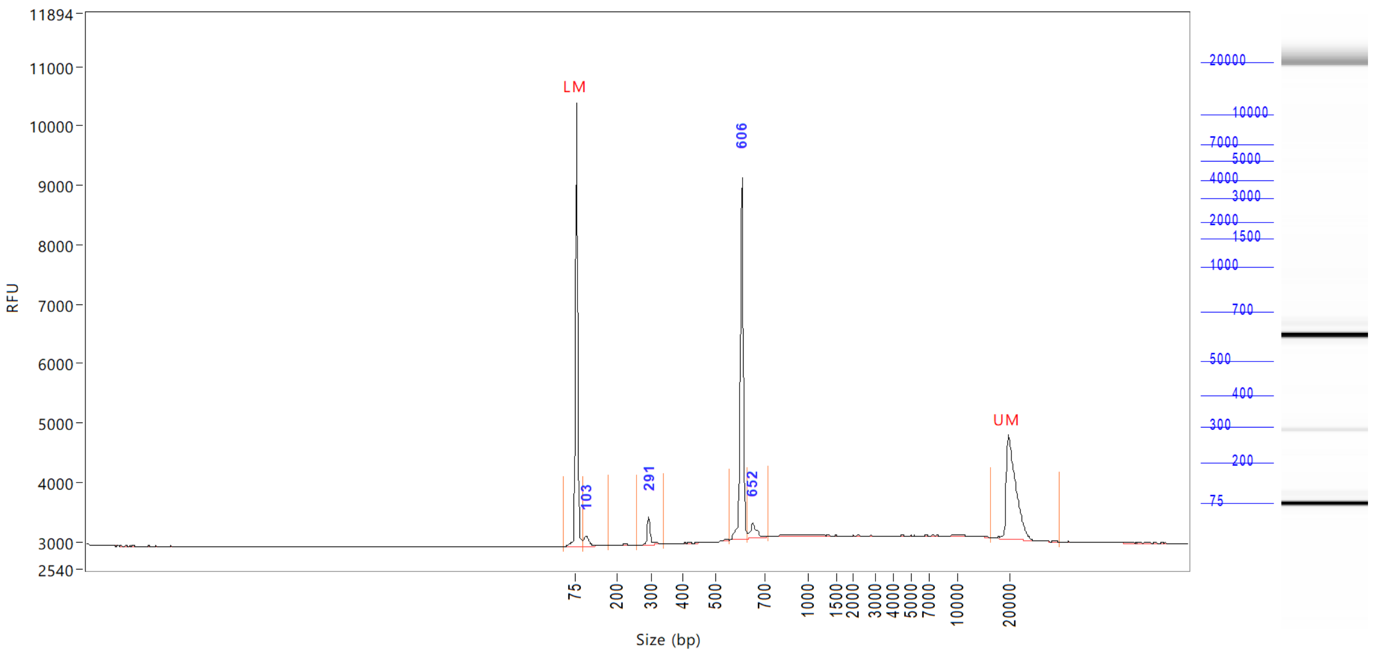

Peak	Size	Concentration	Relative concentration	Molarity	From	To	CV%	RFU
	(bp)	(ng/uL)	Percent	(nmole/L)	(bp)	(bp)		
1	75 (LM)	0.5000		11.2364	35	93	6.37	7475
2	103	0.3515	6.0	5.2488	93	171	15.29	169
3	291	0.4408	7.5	2.4834	255	341	3.66	463
4	606	4.6803	79.6	12.7943	557	631	1.37	6084
5	652	0.4038	6.9	1.0101	631	727	2.84	247
6	20000 (UM)	0.2554		0.0203	16464	29914	5.70	1761

TIC:	5.8765	ng/uL
TIM:	21.5366	nmole/L
Total concentration:	6.4661	ng/uL

Sample peak width (sec): 5 Sample min peak height: 50 Sample baseline V to V?: Y Sample baseline V to V points: 3
Sample filter: Binomial Number of points for filter: 3 Sample start region (min): 0 Sample end region (min): 30
Marker peak width (sec): 5 Marker min peak height: 200 Marker baseline V to V?: Y Marker baseline V to V points: 3
Lower marker selection: First peak > 500 RFU Upper marker selection: Last peak > 200 RFU
Ladder size (bp)75, 200, 300, 400, 500, 700, 1000, 1500, 2000, 3000, 4000, 5000, 7000, 10000, 20000
Quantification using: Lower Marker Final concentration (ng/uL): 0.5000 Dilution factor: 12.0

Sample: SampH11
Well location: H11
Created: Thursday, October 21, 2021 5:28:30 PM

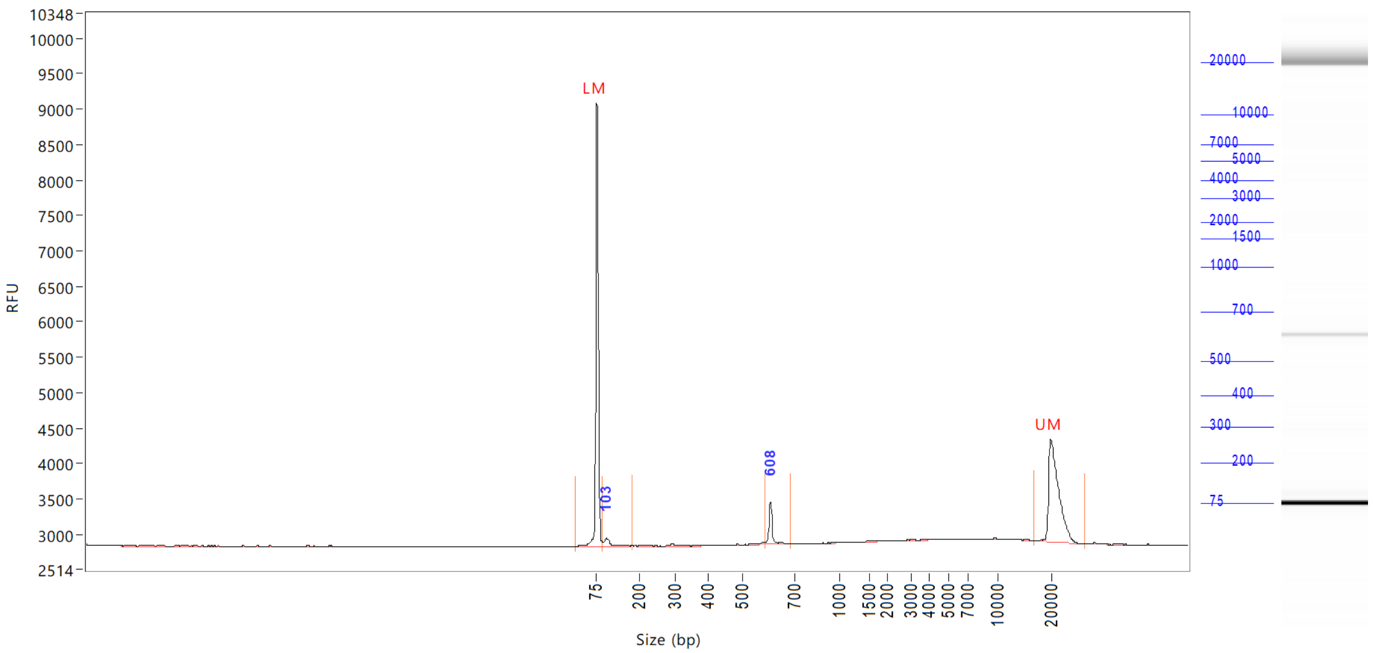

Peak	Size	Concentration	Relative concentration	Molarity	From	To	CV%	RFU
	(bp)	(ng/uL)	Percent	(nmole/L)	(bp)	(bp)		
1	75 (LM)	0.5000		11.2364	16	93	8.06	6260
2	103	0.2576	30.2	3.9177	93	181	13.35	114
3	608	0.5943	69.8	1.6058	585	687	2.58	601
4	20000 (UM)	0.2436		0.0193	16952	26408	5.24	1439

TIC:	0.8519	ng/uL
TIM:	5.5235	nmole/L
Total concentration:	1.3900	ng/uL

```
Sample peak width (sec): 5    Sample min peak height: 50    Sample baseline V to V?: Y    Sample baseline V to V points: 3
Sample filter: Binomial       Number of points for filter: 3  Sample start region (min): 0  Sample end region (min): 30
Marker peak width (sec): 5    Marker min peak height: 200   Marker baseline V to V?: Y    Marker baseline V to V points: 3
Lower marker selection: First peak > 500 RFU                Upper marker selection: Last peak > 200 RFU
Ladder size (bp)75, 200, 300, 400, 500, 700, 1000, 1500, 2000, 3000, 4000, 5000, 7000, 10000, 20000
Quantification using: Lower Marker          Final concentration (ng/uL): 0.5000          Dilution factor: 12.0
```

Sample: SampA12
Well location: A12
Created: Thursday, October 21, 2021 5:28:30 PM

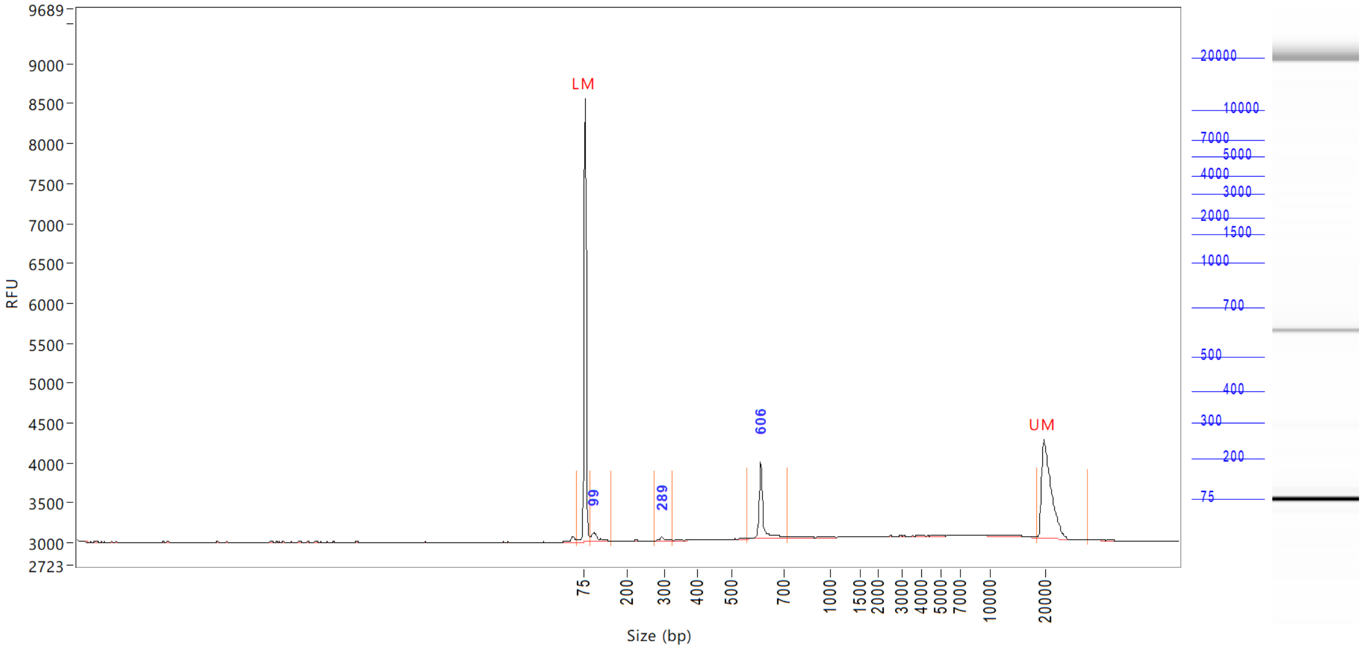

Peak	Size	Concentration	Relative concentration	Molarity	From	To	CV%	RFU
	(bp)	(ng/uL)	Percent	(nmole/L)	(bp)	(bp)		
1	75 (LM)	0.5000		11.2364	50	89	4.95	5561
2	99	0.3521	19.5	5.4557	89	148	12.42	114
3	289	0.0960	5.3	0.5407	273	323	3.40	56
4	606	1.3613	75.2	3.6487	555	718	4.03	968
5	20000 (UM)	0.2395		0.0191	18781	28100	5.16	1229

TIC:	1.8094	ng/uL
TIM:	9.6451	nmole/L
Total concentration:	2.4394	ng/uL

```
Sample peak width (sec): 5    Sample min peak height: 50    Sample baseline V to V?: Y    Sample baseline V to V points: 3
Sample filter: Binomial       Number of points for filter: 3  Sample start region (min): 0  Sample end region (min): 30
Marker peak width (sec): 5    Marker min peak height: 200   Marker baseline V to V?: Y    Marker baseline V to V points: 3
Lower marker selection: First peak > 500 RFU                Upper marker selection: Last peak > 200 RFU
Ladder size (bp)75, 200, 300, 400, 500, 700, 1000, 1500, 2000, 3000, 4000, 5000, 7000, 10000, 20000
Quantification using: Lower Marker          Final concentration (ng/uL): 0.5000          Dilution factor: 12.0
```

Sample: SampB12
Well location: B12
Created: Thursday, October 21, 2021 5:28:30 PM

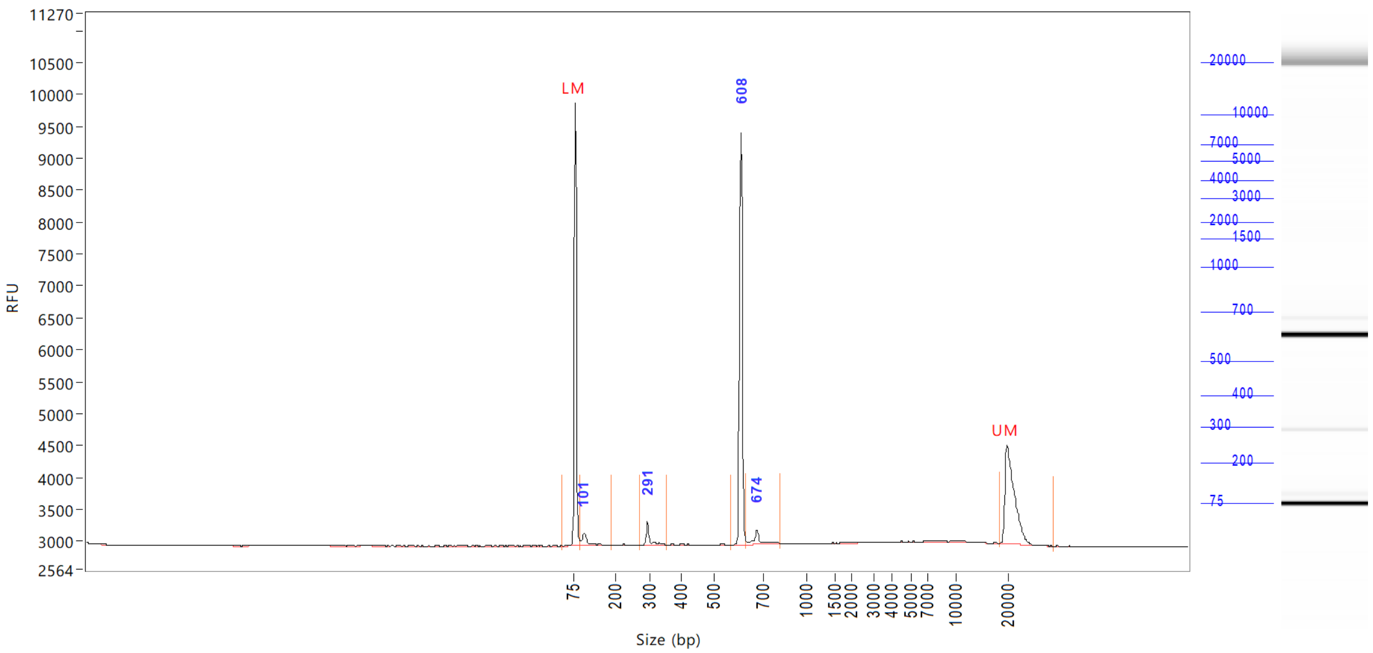

Peak	Size	Concentration	Relative concentration	Molarity	From	To	CV%	RFU
	(bp)	(ng/uL)	Percent	(nmole/L)	(bp)	(bp)		
1	75 (LM)	0.5000		11.2364	37	91	4.94	6947
2	101	0.3871	6.2	5.7804	91	187	17.60	189
3	291	0.4351	6.9	2.4015	269	354	5.57	370
4	608	5.0442	80.2	13.7206	565	631	0.93	6446
5	674	0.4259	6.8	1.0202	631	822	6.15	229
6	20000 (UM)	0.2428		0.0193	18415	28947	5.29	1544

TIC:	6.2922	ng/uL
TIM:	22.9227	nmole/L
Total concentration:	6.8031	ng/uL

```
Sample peak width (sec): 5    Sample min peak height: 50    Sample baseline V to V?: Y    Sample baseline V to V points: 3
Sample filter: Binomial       Number of points for filter: 3  Sample start region (min): 0  Sample end region (min): 30
Marker peak width (sec): 5    Marker min peak height: 200   Marker baseline V to V?: Y    Marker baseline V to V points: 3
Lower marker selection: First peak > 500 RFU                Upper marker selection: Last peak > 200 RFU
Ladder size (bp)75, 200, 300, 400, 500, 700, 1000, 1500, 2000, 3000, 4000, 5000, 7000, 10000, 20000
Quantification using: Lower Marker          Final concentration (ng/uL): 0.5000          Dilution factor: 12.0
```

Sample: SampC12
Well location: C12
Created: Thursday, October 21, 2021 5:28:30 PM

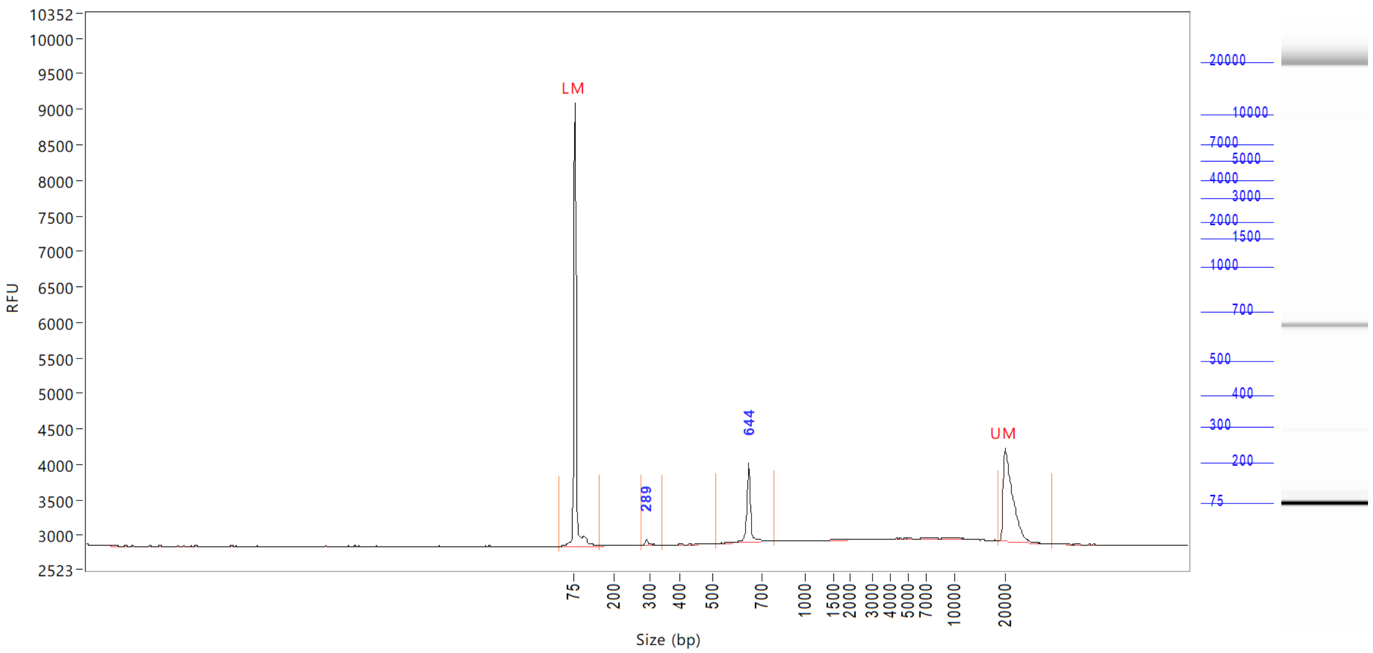

Peak	Size	Concentration	Relative concentration	Molarity	From	To	CV%	RFU
	(bp)	(ng/uL)	Percent	(nmole/L)	(bp)	(bp)		
1	75 (LM)	0.5000		10.9378	26	152	13.15	6257
2	289	0.1149	8.4	0.6427	277	341	3.91	92
3	644	1.2452	91.6	3.1969	509	788	3.49	1118
4	20000 (UM)	0.2111		0.0167	18537	29189	6.03	1313

TIC:	1.3601	ng/uL
TIM:	3.8395	nmole/L
Total concentration:	1.8640	ng/uL

```
Sample peak width (sec): 5    Sample min peak height: 50    Sample baseline V to V?: Y    Sample baseline V to V points: 3
Sample filter: Binomial       Number of points for filter: 3  Sample start region (min): 0  Sample end region (min): 30
Marker peak width (sec): 5    Marker min peak height: 200   Marker baseline V to V?: Y    Marker baseline V to V points: 3
Lower marker selection: First peak > 500 RFU                Upper marker selection: Last peak > 200 RFU
Ladder size (bp)75, 200, 300, 400, 500, 700, 1000, 1500, 2000, 3000, 4000, 5000, 7000, 10000, 20000
Quantification using: Lower Marker          Final concentration (ng/uL): 0.5000          Dilution factor: 12.0
```

Sample: SampD12
Well location: D12
Created: Thursday, October 21, 2021 5:28:30 PM

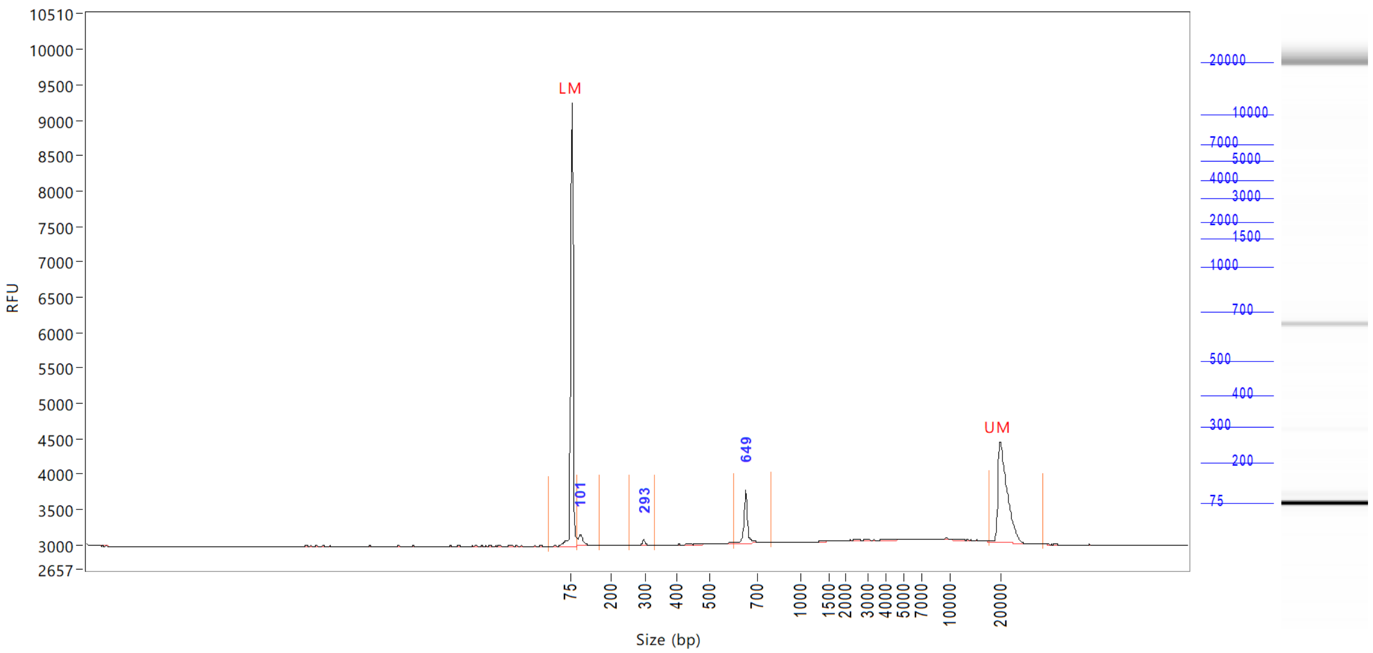

Peak	Size	Concentration	Relative concentration	Molarity	From	To	CV%	RFU
	(bp)	(ng/uL)	Percent	(nmole/L)	(bp)	(bp)		
1	75 (LM)	0.5000		11.2364	7	93	7.91	6271
2	101	0.3582	27.7	5.4467	93	164	14.37	167
3	293	0.1073	8.3	0.6023	251	329	4.11	80
4	649	0.8287	64.0	2.0949	603	796	3.23	761
5	20000 (UM)	0.2322		0.0184	17805	28584	5.15	1416

TIC:	1.2941	ng/uL
TIM:	8.1439	nmole/L
Total concentration:	1.8104	ng/uL

Sample peak width (sec): 5 Sample min peak height: 50 Sample baseline V to V?: Y Sample baseline V to V points: 3
Sample filter: Binomial Number of points for filter: 3 Sample start region (min): 0 Sample end region (min): 30
Marker peak width (sec): 5 Marker min peak height: 200 Marker baseline V to V?: Y Marker baseline V to V points: 3
Lower marker selection: First peak > 500 RFU Upper marker selection: Last peak > 200 RFU
Ladder size (bp)75, 200, 300, 400, 500, 700, 1000, 1500, 2000, 3000, 4000, 5000, 7000, 10000, 20000
Quantification using: Lower Marker Final concentration (ng/uL): 0.5000 Dilution factor: 12.0

Sample: SampE12
Well location: E12
Created: Thursday, October 21, 2021 5:28:30 PM

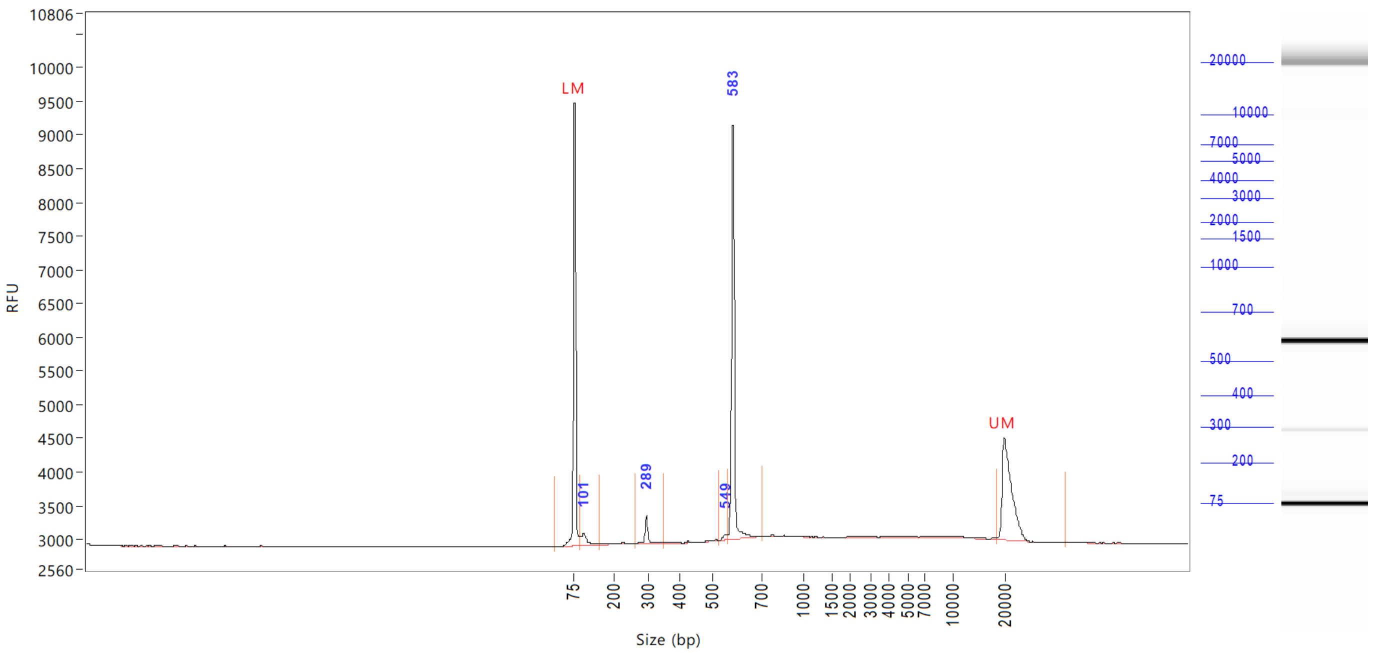

Peak	Size	Concentration	Relative concentration	Molarity	From	To	CV%	RFU
	(bp)	(ng/uL)	Percent	(nmole/L)	(bp)	(bp)		
1	75 (LM)	0.5000		11.3919	16	93	7.04	6581
2	101	0.3962	6.4	5.9696	93	154	13.51	175
3	289	0.4193	6.8	2.3539	260	345	4.96	412
4	549	0.0996	1.6	0.2997	524	560	1.45	80
5	583	5.2750	85.2	14.9152	560	710	2.29	6140
6	20000 (UM)	0.2378		0.0188	18537	31969	5.78	1512

TIC:	6.1901	ng/uL
TIM:	23.5384	nmole/L
Total concentration:	6.9100	ng/uL

Sample peak width (sec): 5 Sample min peak height: 50 Sample baseline V to V?: Y Sample baseline V to V points: 3
Sample filter: Binomial Number of points for filter: 3 Sample start region (min): 0 Sample end region (min): 30
Marker peak width (sec): 5 Marker min peak height: 200 Marker baseline V to V?: Y Marker baseline V to V points: 3
Lower marker selection: First peak > 500 RFU Upper marker selection: Last peak > 200 RFU
Ladder size (bp)75, 200, 300, 400, 500, 700, 1000, 1500, 2000, 3000, 4000, 5000, 7000, 10000, 20000
Quantification using: Lower Marker Final concentration (ng/uL): 0.5000 Dilution factor: 12.0

Sample: SampF12
Well location: F12
Created: Thursday, October 21, 2021 5:28:30 PM

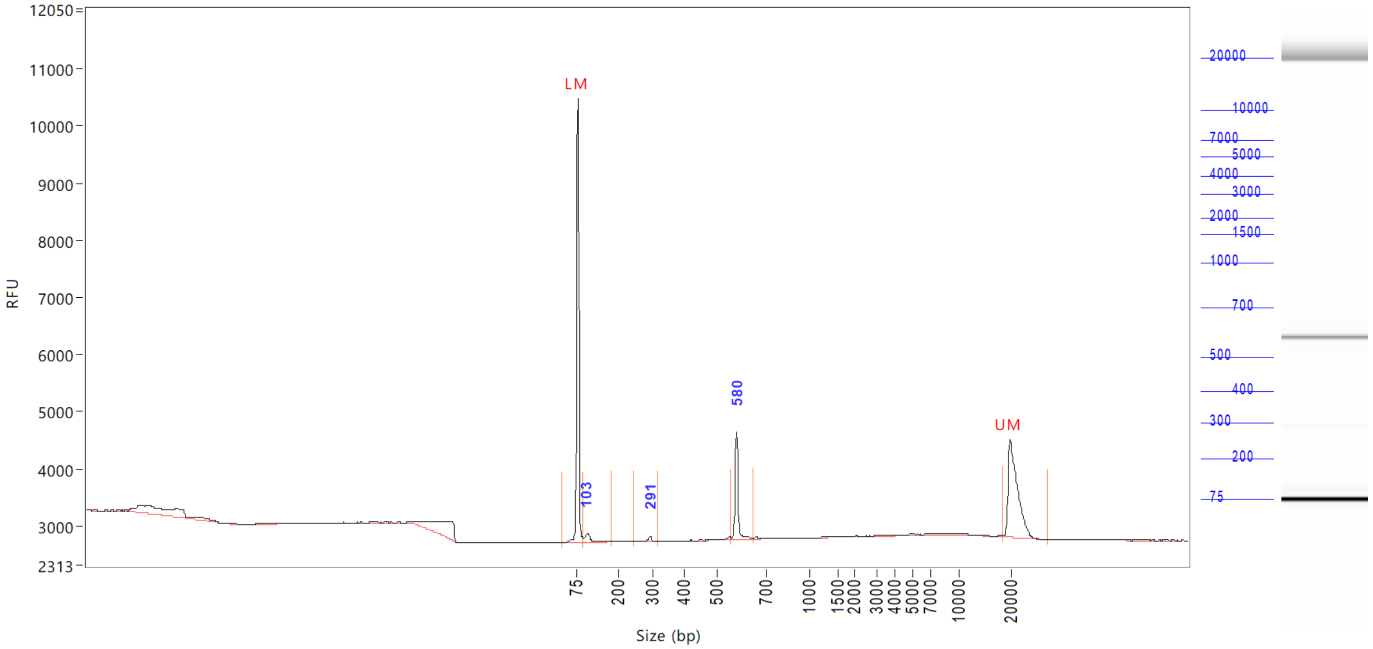

Peak	Size	Concentration	Relative concentration	Molarity	From	To	CV%	RFU
	(bp)	(ng/uL)	Percent	(nmole/L)	(bp)	(bp)		
1	75 (LM)	0.5000		11.2364	30	93	6.10	7783
2	103	0.3048	16.0	4.5110	93	177	16.26	155
3	291	0.1005	5.3	0.5777	242	313	3.70	96
4	580	1.4974	78.7	4.2487	557	652	2.33	1872
5	20000 (UM)	0.2350		0.0186	18537	27133	5.12	1701

TIC:	1.9027	ng/uL
TIM:	9.3374	nmole/L
Total concentration:	2.4754	ng/uL

```
Sample peak width (sec): 5    Sample min peak height: 50    Sample baseline V to V?: Y    Sample baseline V to V points: 3
Sample filter: Binomial       Number of points for filter: 3  Sample start region (min): 0  Sample end region (min): 30
Marker peak width (sec): 5    Marker min peak height: 200   Marker baseline V to V?: Y    Marker baseline V to V points: 3
Lower marker selection: First peak > 500 RFU                Upper marker selection: Last peak > 200 RFU
Ladder size (bp)75, 200, 300, 400, 500, 700, 1000, 1500, 2000, 3000, 4000, 5000, 7000, 10000, 20000
Quantification using: Lower Marker          Final concentration (ng/uL): 0.5000          Dilution factor: 12.0
```

Sample: SampG12
Well location: G12
Created: Thursday, October 21, 2021 5:28:30 PM

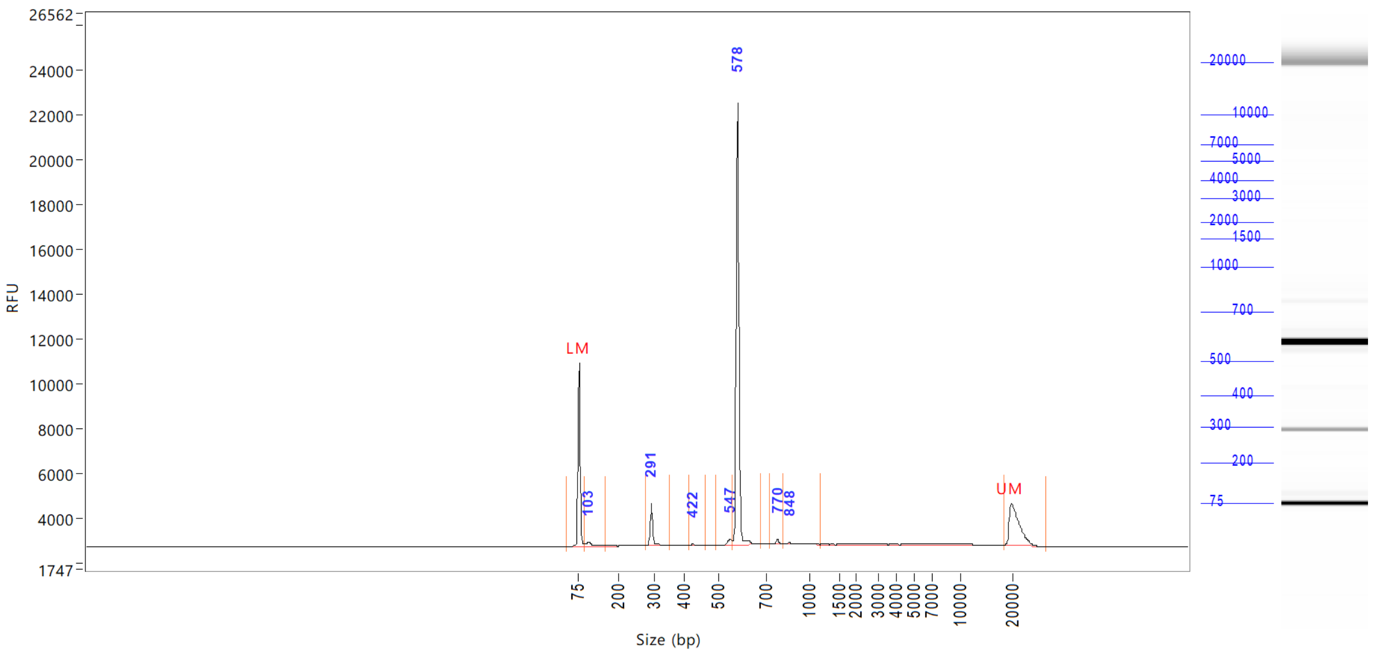

Peak	Size	Concentration	Relative concentration	Molarity	From	To	CV%	RFU
	(bp)	(ng/uL)	Percent	(nmole/L)	(bp)	(bp)		
1	75 (LM)	0.5000		11.2364	37	93	5.52	8171
2	103	0.3725	2.3	5.5617	93	156	13.51	204
3	291	1.3374	8.3	7.5595	275	347	2.68	1880
4	422	0.0504	0.3	0.1972	411	460	1.78	66
5	547	0.2860	1.8	0.8667	494	557	1.67	255
6	578	13.6831	85.4	39.0245	557	674	1.67	19757
7	770	0.1526	1.0	0.3288	714	805	1.86	193
8	848	0.1349	0.8	0.2538	805	1167	5.78	53
9	20000 (UM)	0.2648		0.0210	18537	26649	5.32	1908

TIC:	16.0169	ng/uL
TIM:	53.7921	nmole/L
Total concentration:	16.4987	ng/uL

Sample peak width (sec): 5 Sample min peak height: 50 Sample baseline V to V?: Y Sample baseline V to V points: 3
Sample filter: Binomial Number of points for filter: 3 Sample start region (min): 0 Sample end region (min): 30
Marker peak width (sec): 5 Marker min peak height: 200 Marker baseline V to V?: Y Marker baseline V to V points: 3
Lower marker selection: First peak > 500 RFU Upper marker selection: Last peak > 200 RFU
Ladder size (bp)75, 200, 300, 400, 500, 700, 1000, 1500, 2000, 3000, 4000, 5000, 7000, 10000, 20000
Quantification using: Lower Marker Final concentration (ng/uL): 0.5000 Dilution factor: 12.0

Sample: SampH12
Well location: H12
Created: Thursday, October 21, 2021 5:28:30 PM

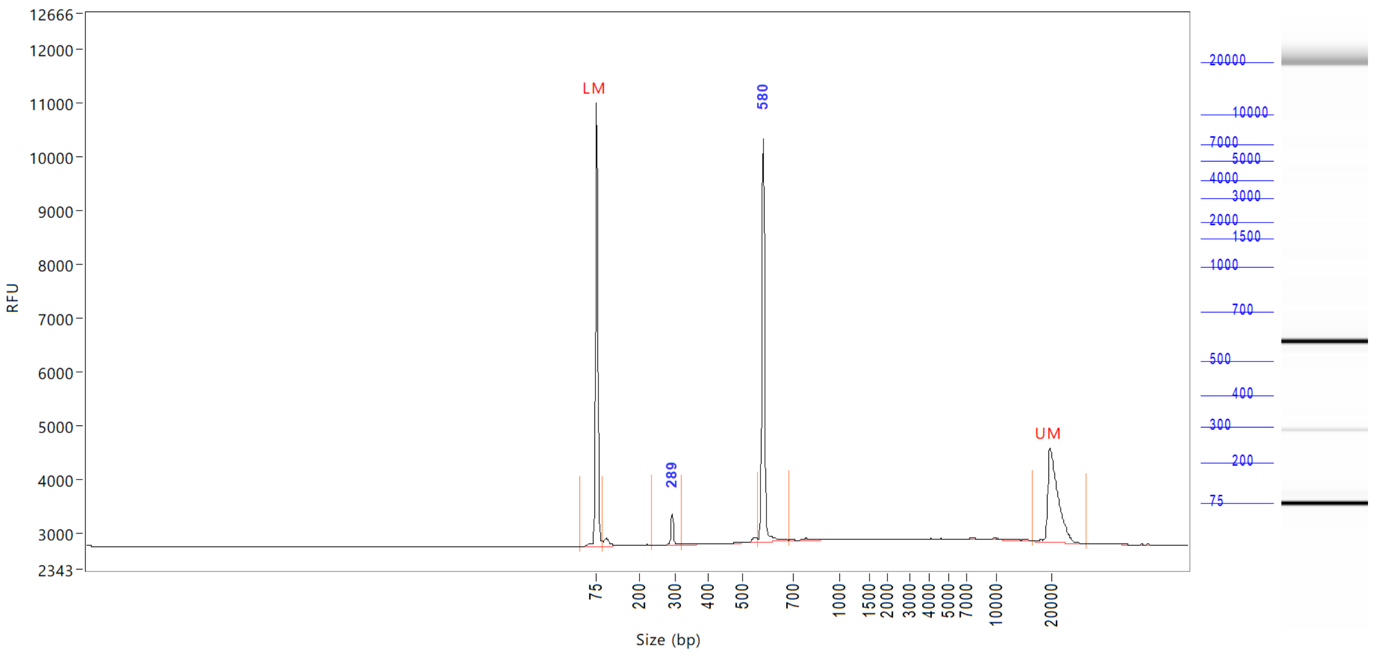

Peak	Size	Concentration	Relative concentration	Molarity	From	To	CV%	RFU
	(bp)	(ng/uL)	Percent	(nmole/L)	(bp)	(bp)		
1	75 (LM)	0.5000		11.2364	28	91	6.27	8247
2	289	0.4427	7.8	2.5372	233	319	3.07	582
3	580	5.2458	92.2	14.9096	557	680	2.09	7490
4	20000 (UM)	0.2409		0.0191	16830	26770	5.60	1759

TIC:	5.6885	ng/uL
TIM:	17.4468	nmole/L
Total concentration:	6.6493	ng/uL

Sample peak width (sec): 5 Sample min peak height: 200 Sample baseline V to V?: Y Sample baseline V to V points: 3
Sample filter: Binomial Number of points for filter: 3 Sample start region (min): 0 Sample end region (min): 30
Marker peak width (sec): 5 Marker min peak height: 200 Marker baseline V to V?: Y Marker baseline V to V points: 3
Lower marker selection: First peak > 500 RFU Upper marker selection: Last peak > 200 RFU
Ladder size (bp)75, 200, 300, 400, 500, 700, 1000, 1500, 2000, 3000, 4000, 5000, 7000, 10000, 20000
Quantification using: Lower Marker Final concentration (ng/uL): 0.5000 Dilution factor: 12.0

Sample: DNA size ladder
Well location: A7
Created: Thursday, October 21, 2021 5:28:30 PM
Import from: C:\Users\admin\Desktop\210810_alberto_ladder.SCAL
Fit type: Point to point

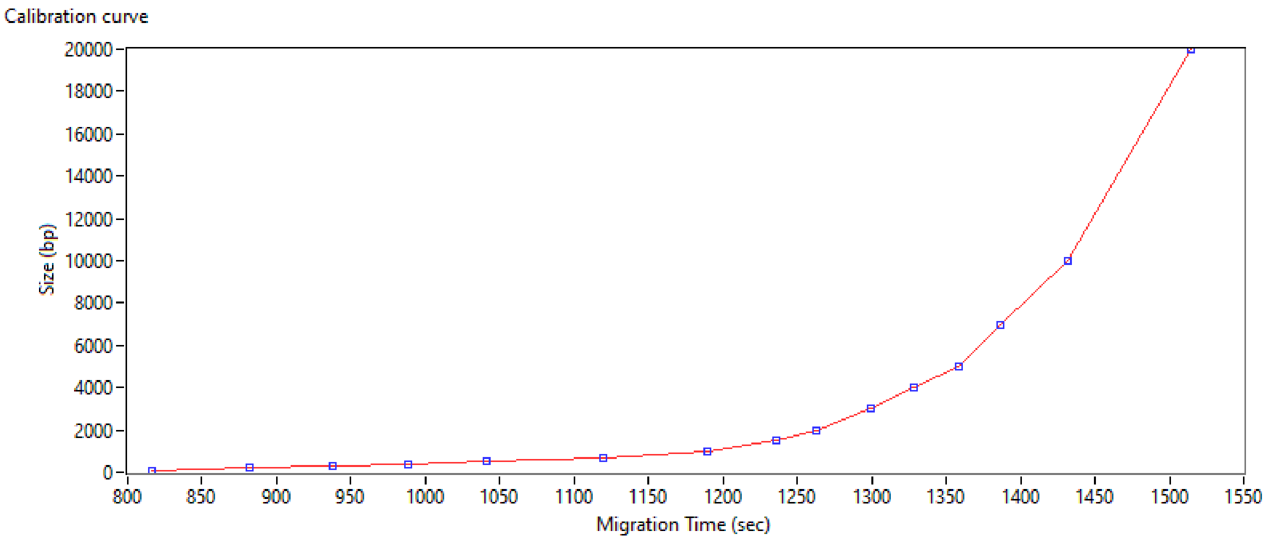

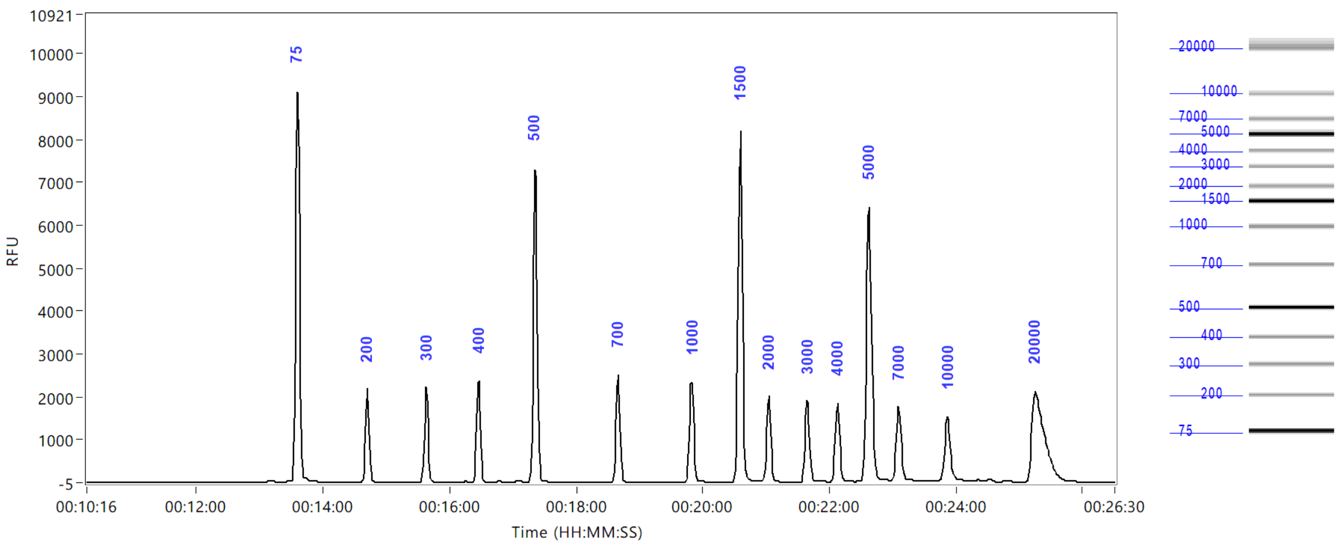

Supplement: Supplementary file 2 — sb3c00292_si_002.zip [file sb3c00292_si_002.zip › dnada_supplementary_material_pks_library_build/colony_pcr/ZAG/17-18-29_plate5/colonypcrplate_5.pdf]
