# Supplementary material for: Automated Platform for the Plasmid Construction Process
Source: ACS Synth Biol. 2023 Nov 10;12(12):3506–13. doi: 10.1021/acssynbio.3c00292 (PMC10729297; doi:10.1021/acssynbio.3c00292)

Instrument controller software run summary:

Filename and data path: C:\AATI\Data\2021 10 21\19-18-30\2021 10 21 19H 18M..raw
Created: Thursday, October 21, 2021 7:28:10 PM
Number of capillaries: 96
Array serial number: 111920-09SFS
Effect length: 33 cm
Array usage count: 268
Instrument type: ZAG DNA Analyzer
Instrument controller software version: 1.0.1.9
Device serial number: Z7597

Method Information

Method name: ZAG130GP33 - DNA 75-20000bp - Gel Prime Only.mthds
Gel prime: Yes
Full conditioning: No
Gel prime to buffer: No
Gel selection: Gel 1
Perform prerun: 8.0 kV, 30 sec.
Rinse: No
Marker 1: Row: A, 5.0 kV, 5 sec.
Rinse: No
Sample injection: 5.0 kV, 5 sec.
Separation: 8.0 kV, 30.0 min.
Tray name: Sample Tray-4

Analysis mode: DNA

Notes

Gel Image

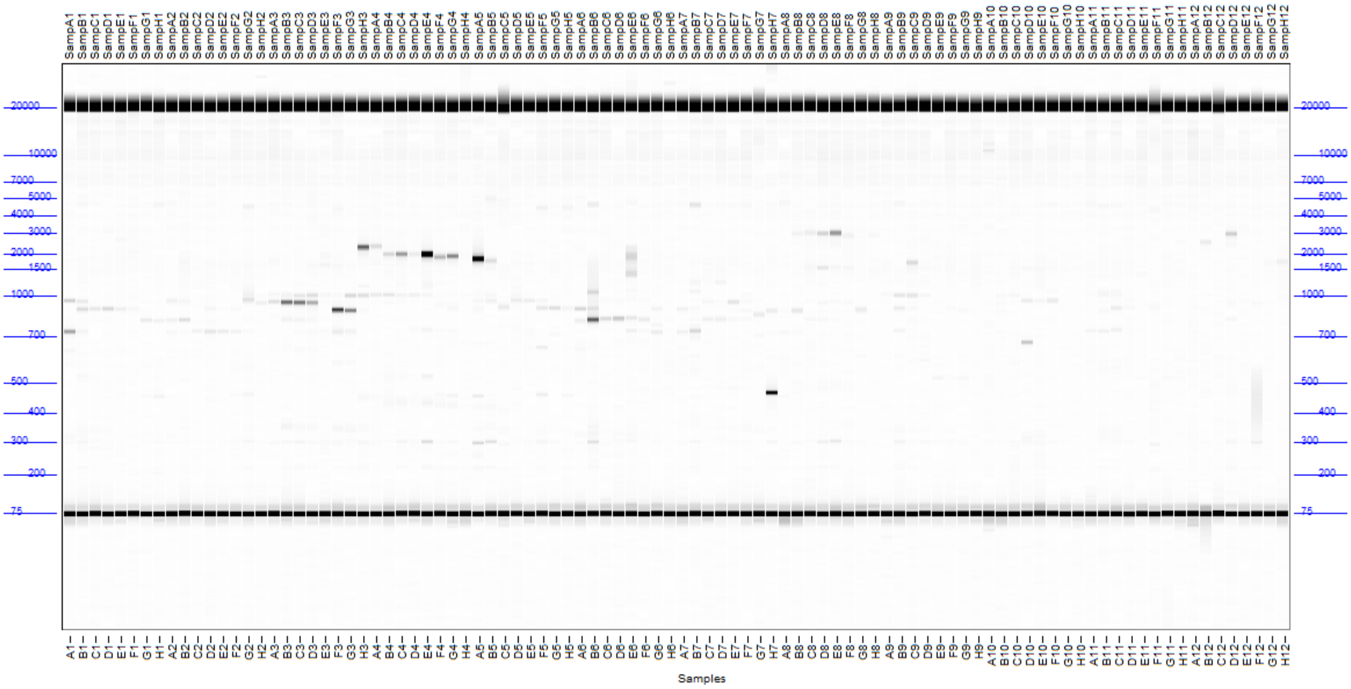

Filename and data path: C:\AATI\Data\2021 10 21\19-18-30\2021 10 21 19H 18M..raw

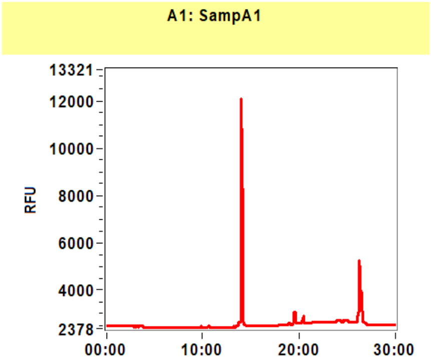

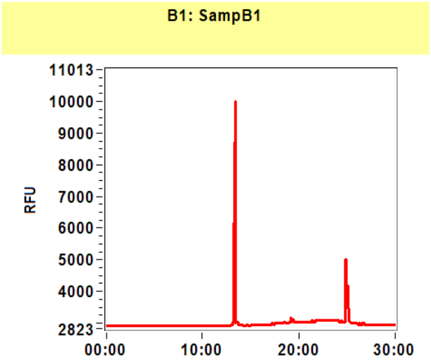

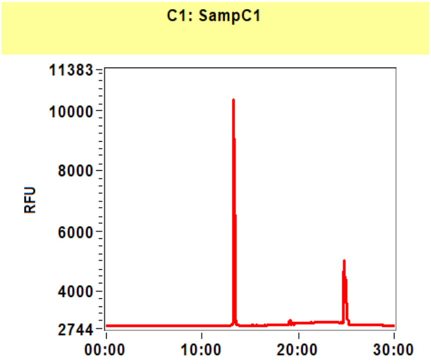

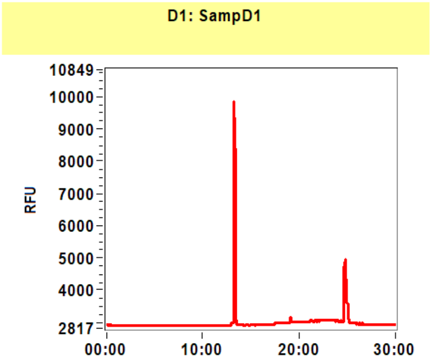

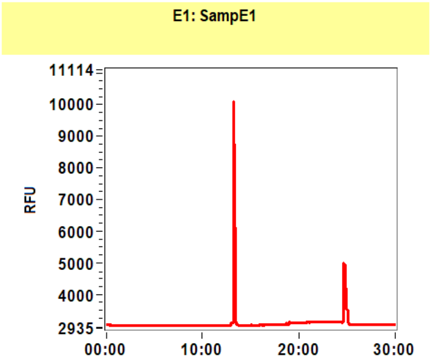

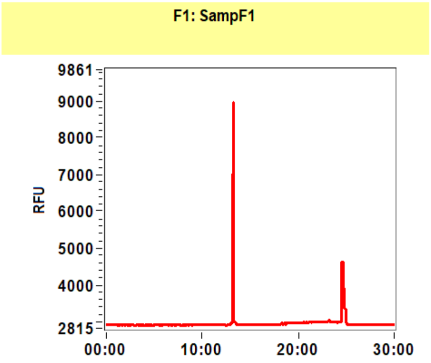

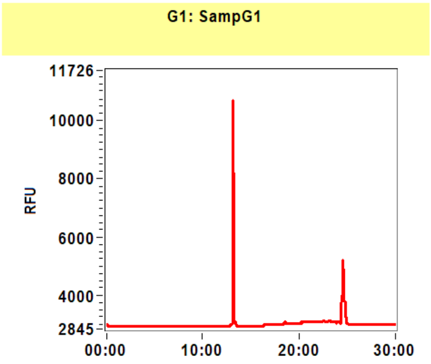

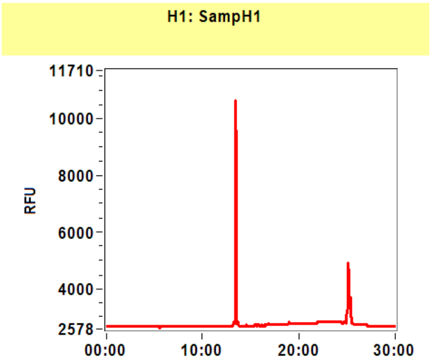

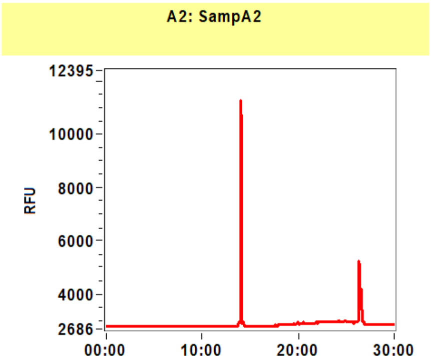

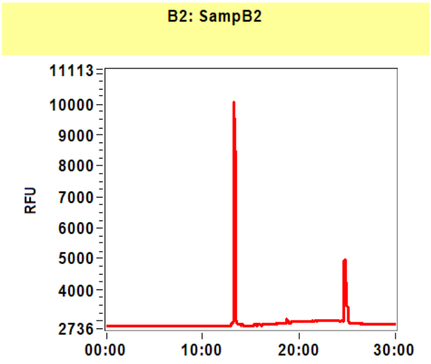

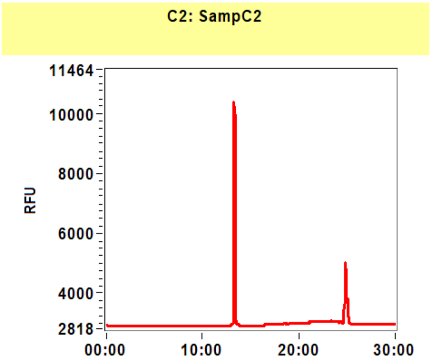

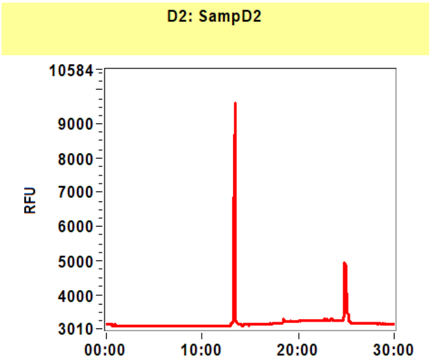

Filename and data path: C:\AATI\Data\2021 10 21\19-18-30\2021 10 21 19H 18M..raw

E2: SampE2

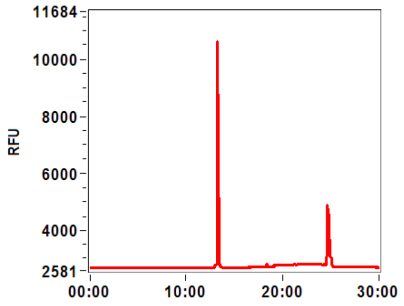

F2: SampF2

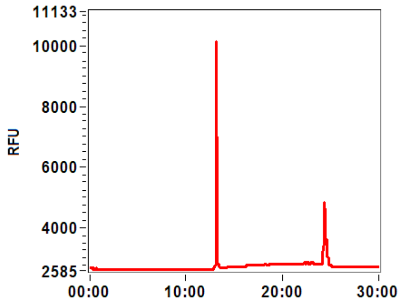

G2: SampG2

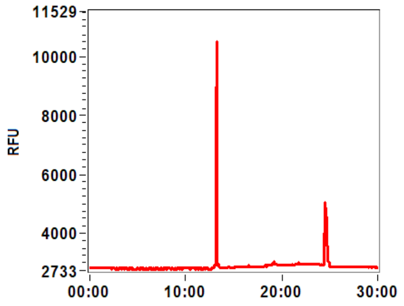

H2: SampH2

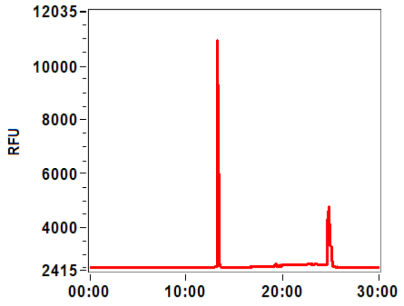

A3: SampA3

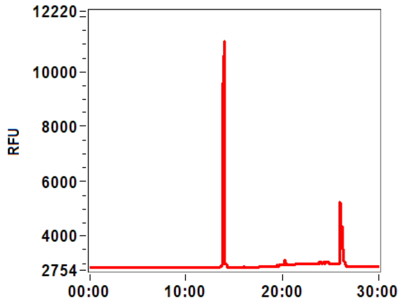

B3: SampB3

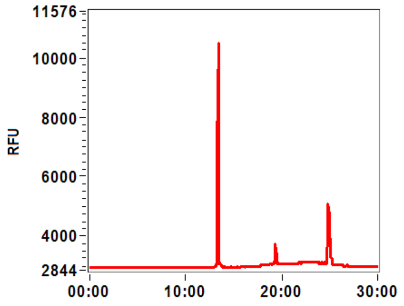

C3: SampC3

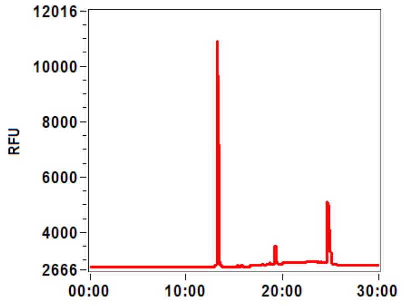

D3: SampD3

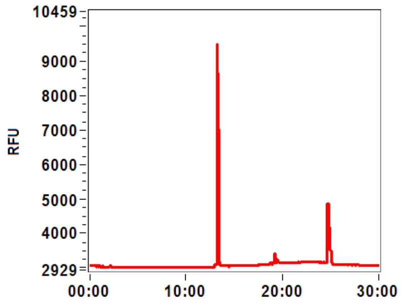

E3: SampE3

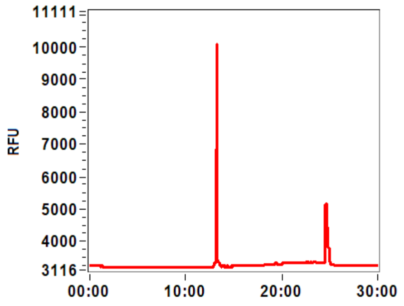

F3: SampF3

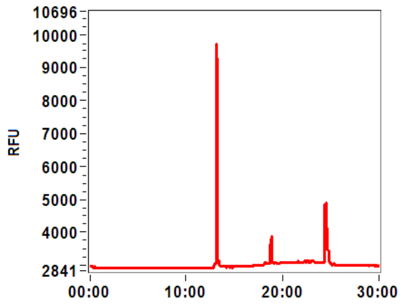

G3: SampG3

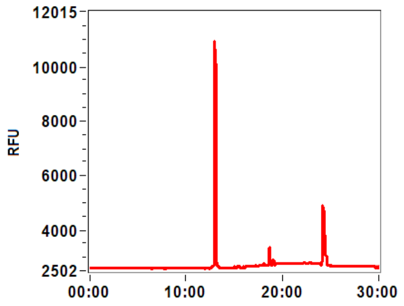

H3: SampH3

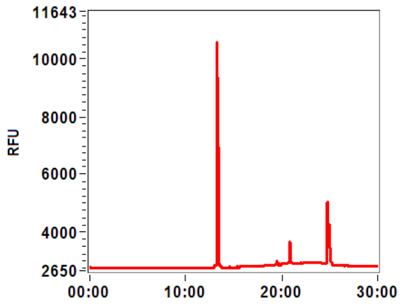

Filename and data path: C:\AATI\Data\2021 10 21\19-18-30\2021 10 21 19H 18M..raw

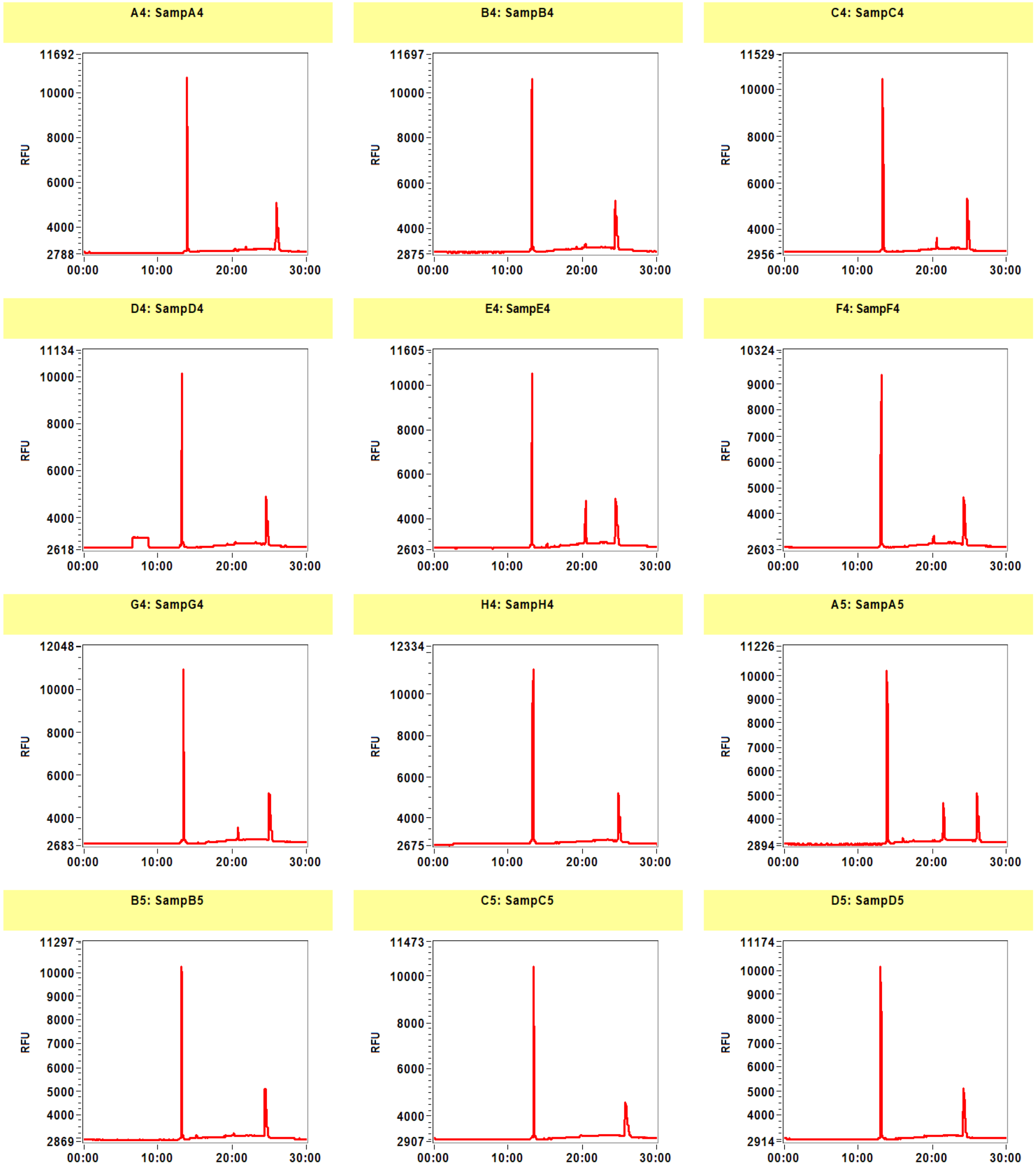

Filename and data path: C:\AATI\Data\2021 10 21\19-18-30\2021 10 21 19H 18M..raw

E5: SampE5

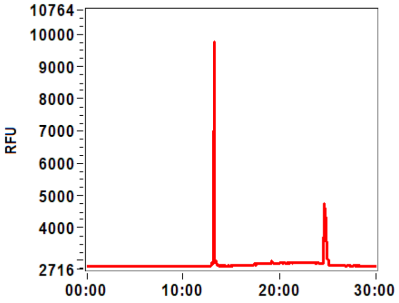

F5: SampF5

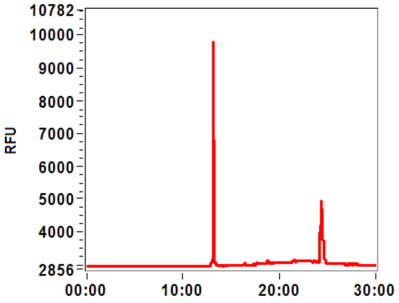

G5: SampG5

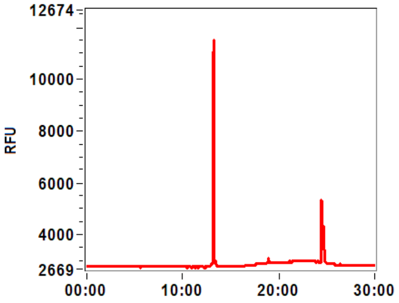

H5: SampH5

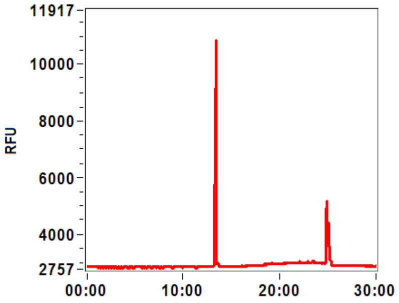

A6: SampA6

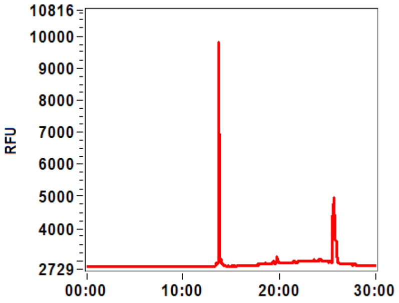

B6: SampB6

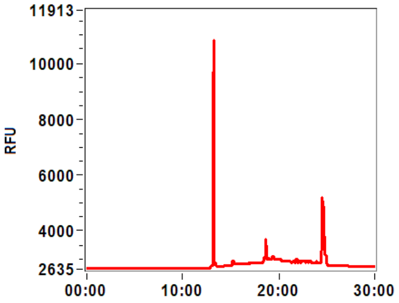

C6: SampC6

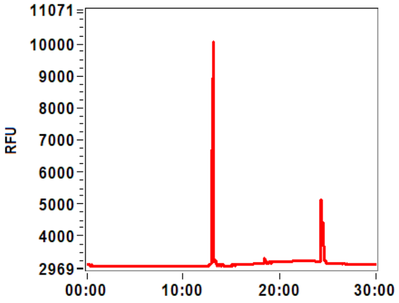

D6: SampD6

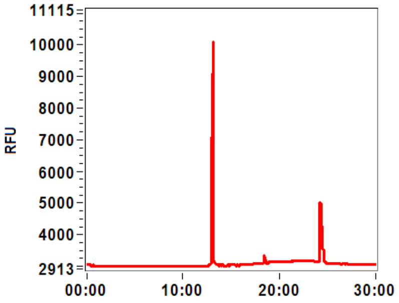

E6: SampE6

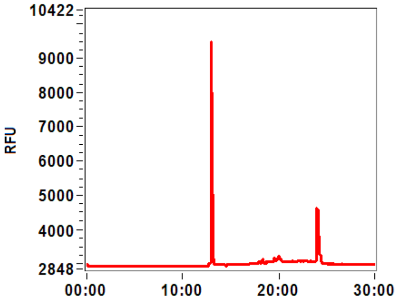

F6: SampF6

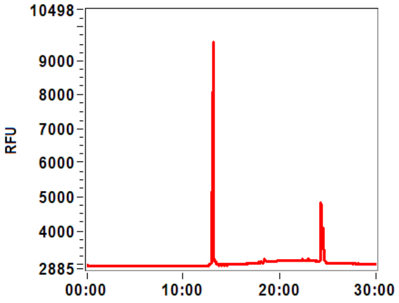

G6: SampG6

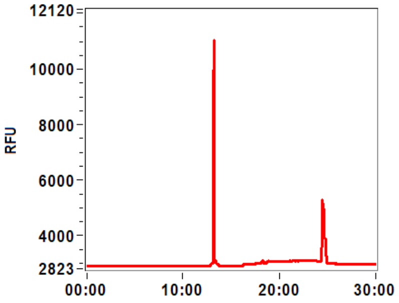

H6: SampH6

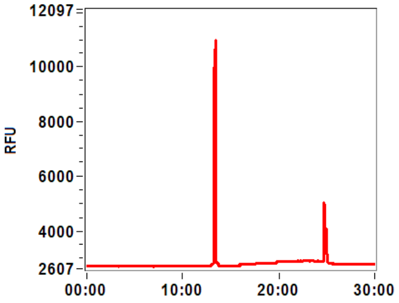

Filename and data path: C:\AATI\Data\2021 10 21\19-18-30\2021 10 21 19H 18M..raw

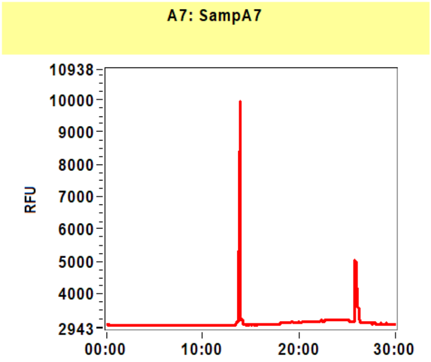

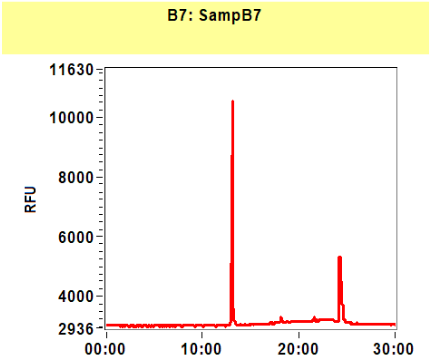

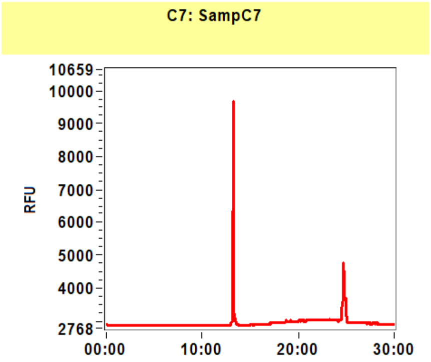

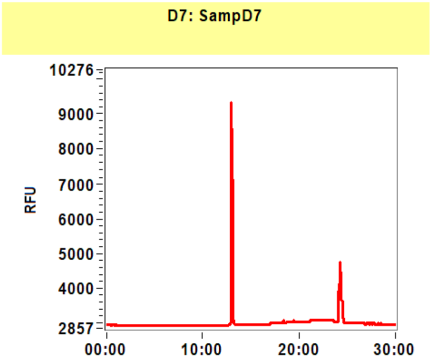

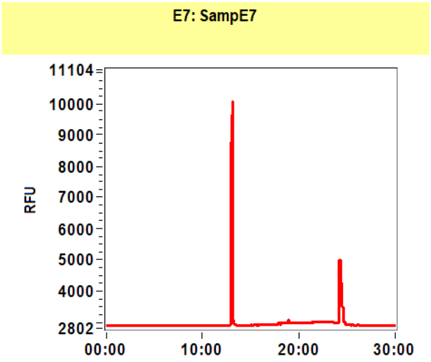

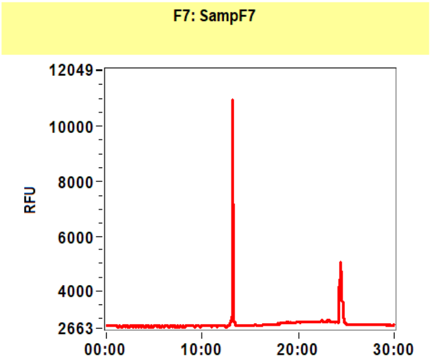

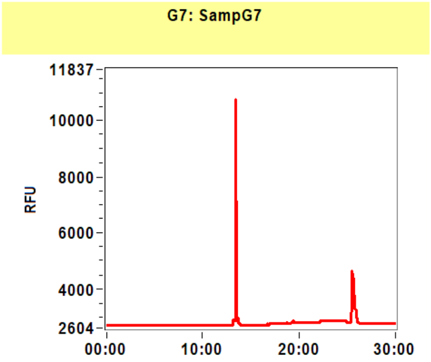

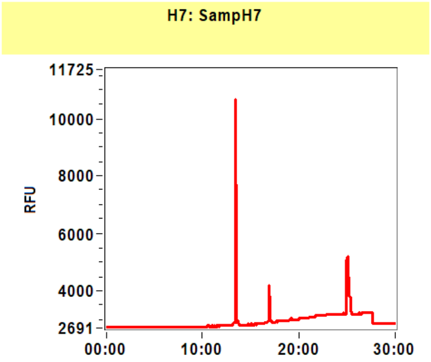

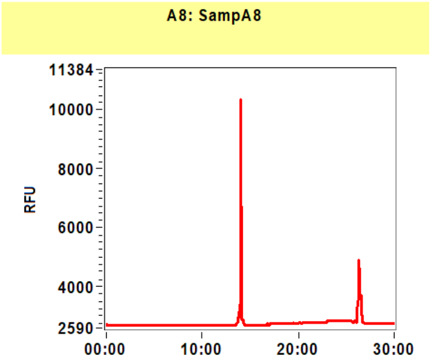

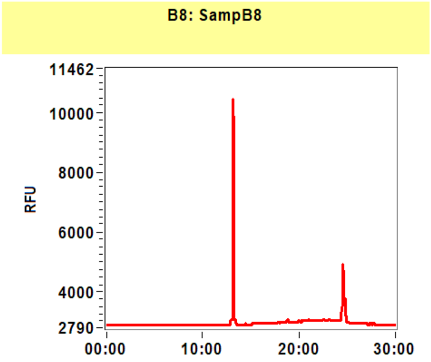

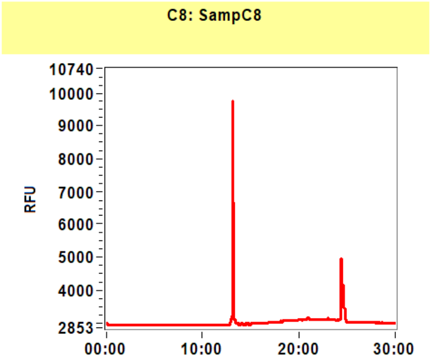

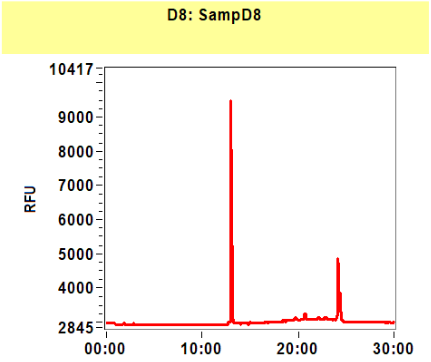

Filename and data path: C:\AATI\Data\2021 10 21\19-18-30\2021 10 21 19H 18M..raw

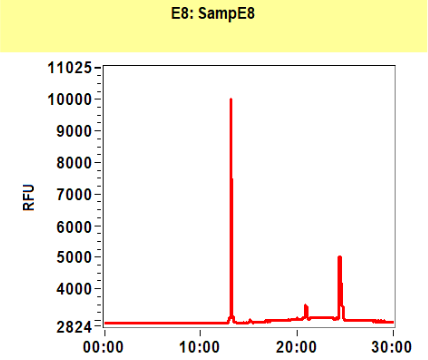

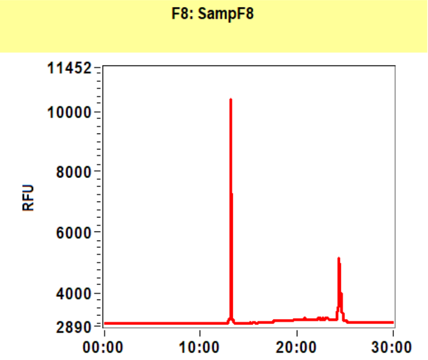

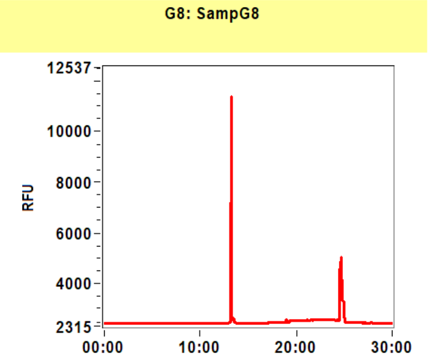

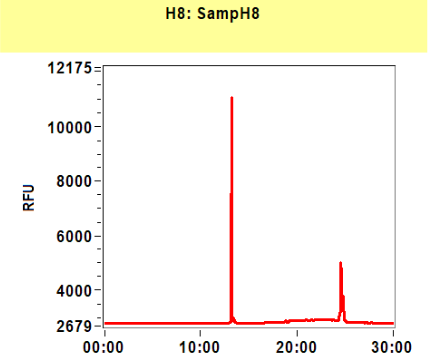

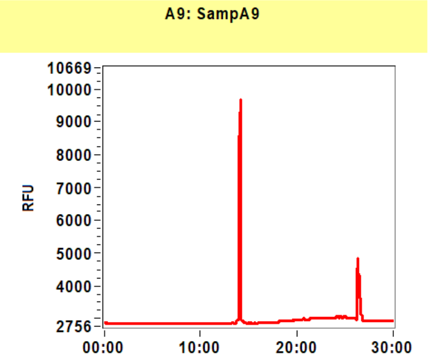

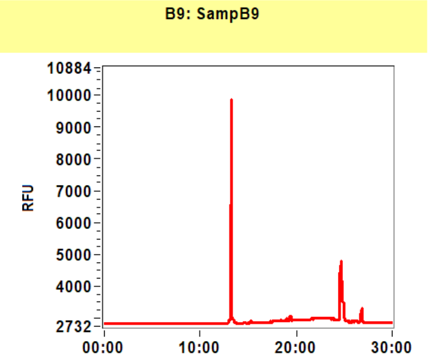

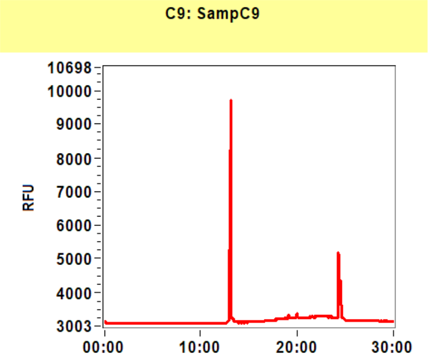

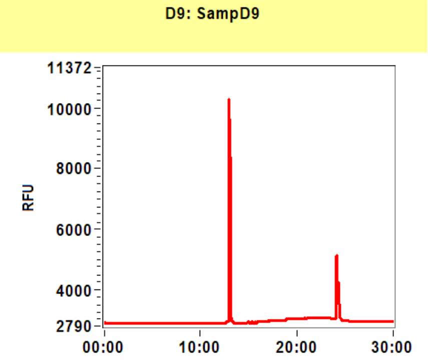

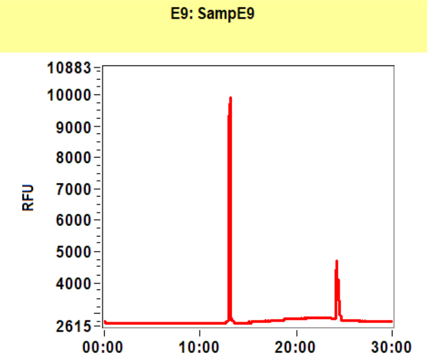

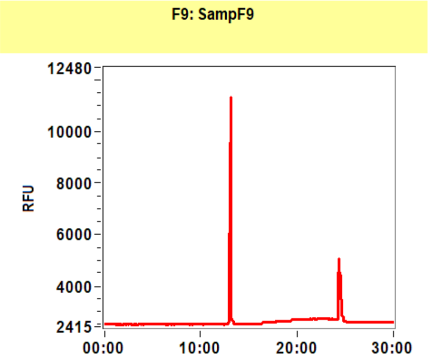

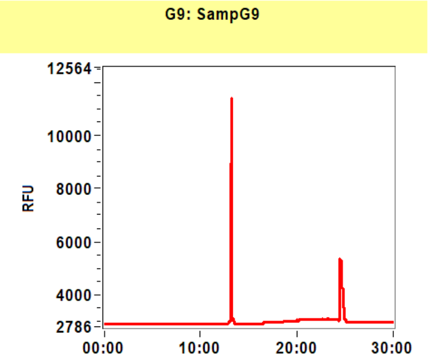

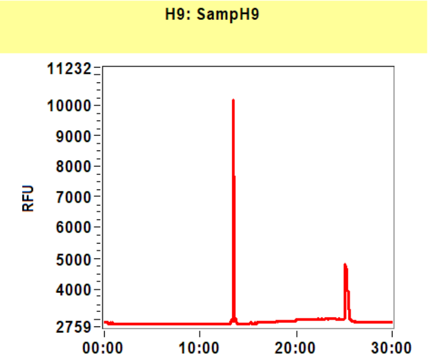

Filename and data path: C:\AATI\Data\2021 10 21\19-18-30\2021 10 21 19H 18M..raw

A10: SampA10

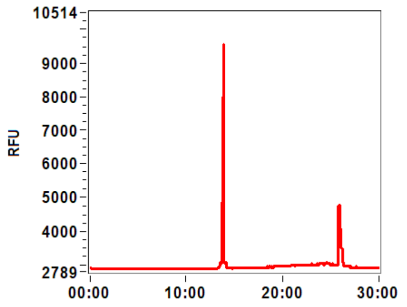

B10: SampB10

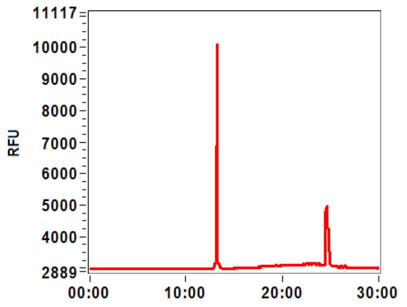

C10: SampC10

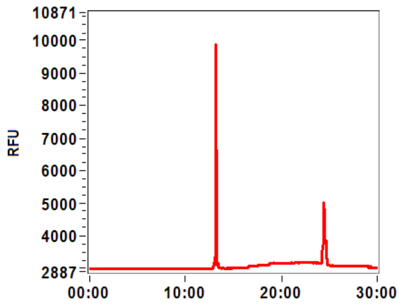

D10: SampD10

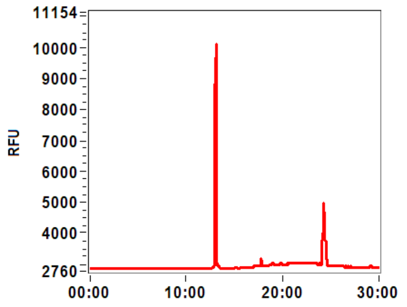

E10: SampE10

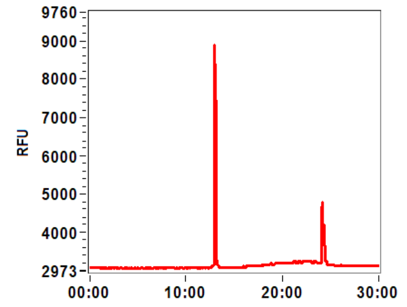

F10: SampF10

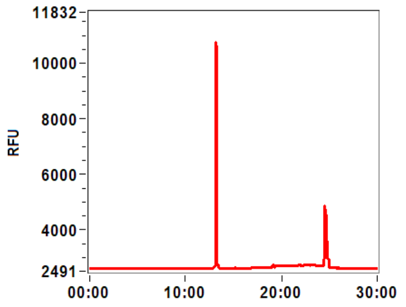

G10: SampG10

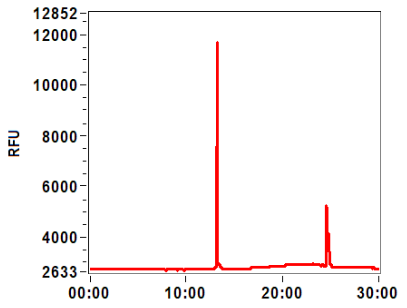

H10: SampH10

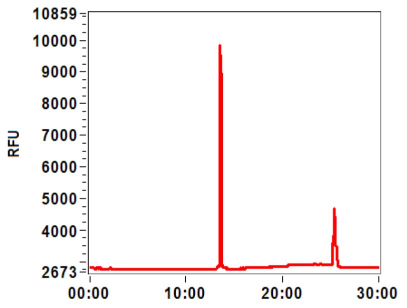

A11: SampA11

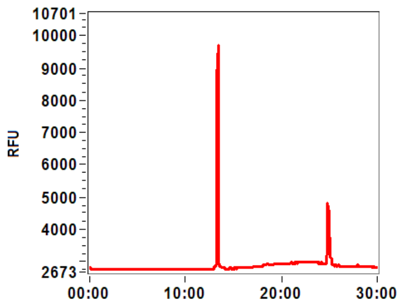

B11: SampB11

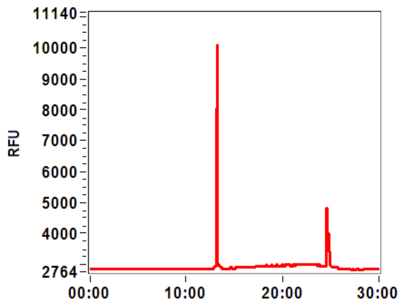

C11: SampC11

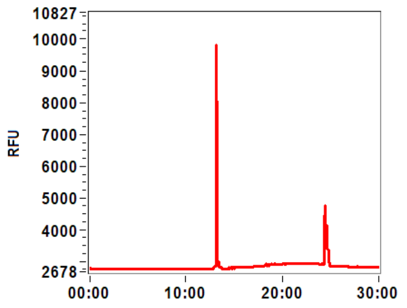

D11: SampD11

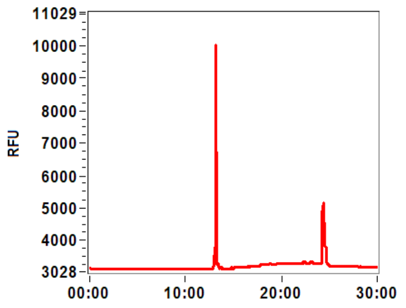

Filename and data path: C:\AATI\Data\2021 10 21\19-18-30\2021 10 21 19H 18M..raw

E11: SampE11

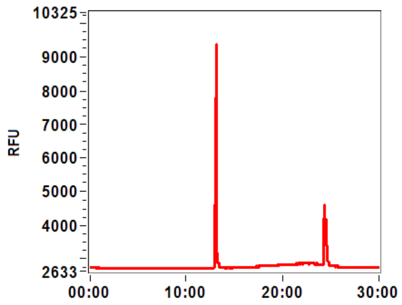

F11: SampF11

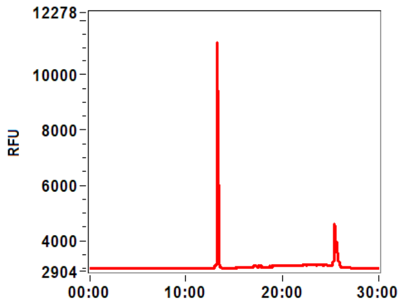

G11: SampG11

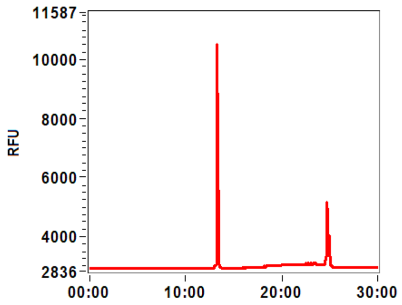

H11: SampH11

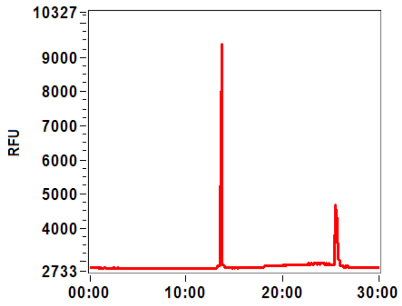

A12: SampA12

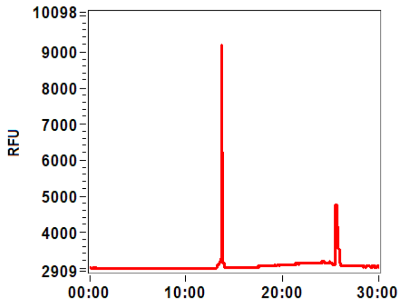

B12: SampB12

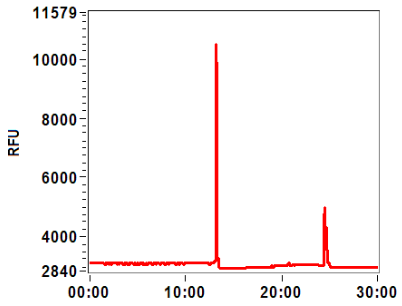

C12: SampC12

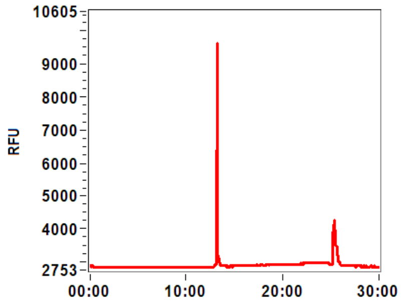

D12: SampD12

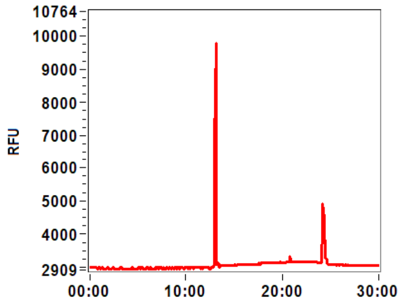

E12: SampE12

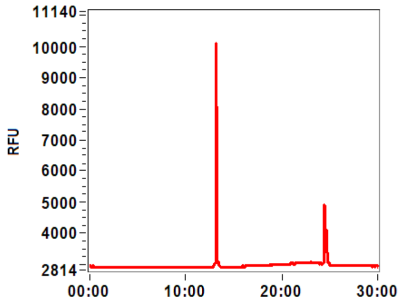

F12: SampF12

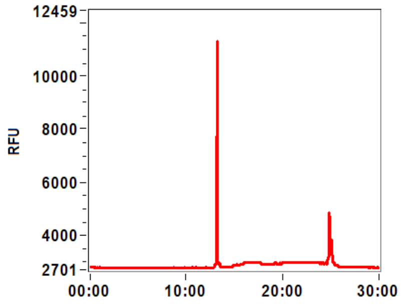

G12: SampG12

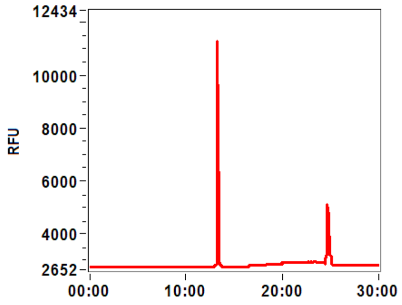

H12: SampH12

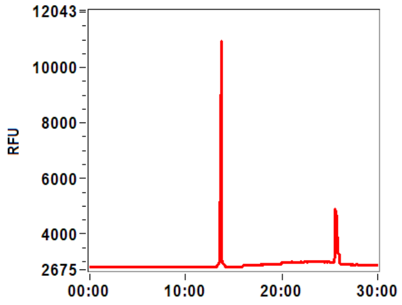

Sample: SampA1
Well location: A1
Created: Thursday, October 21, 2021 7:28:10 PM

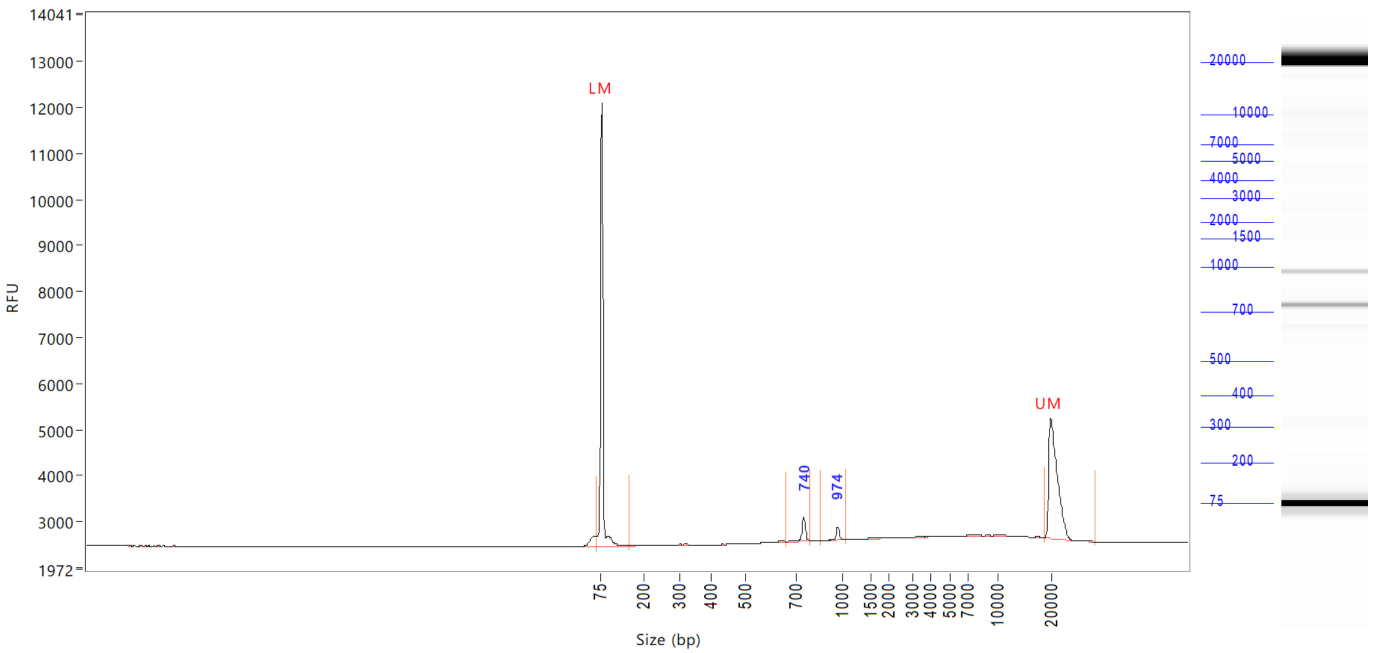

Peak	Size	Concentration	Relative concentration	Molarity	From	To	CV%	RFU
	(bp)	(ng/uL)	Percent	(nmole/L)	(bp)	(bp)		
1	75 (LM)	0.5000		11.0851	58	156	11.15	9641
2	740	0.3290	66.5	0.7367	662	788	2.50	517
3	974	0.1661	33.5	0.2826	861	1068	2.12	291
4	20000 (UM)	0.2392		0.0191	18903	28463	4.35	2609

TIC:	0.4951	ng/uL
TIM:	1.0193	nmole/L
Total concentration:	0.9617	ng/uL

```
Sample peak width (sec): 5    Sample min peak height: 50    Sample baseline V to V?: Y    Sample baseline V to V points: 3
Sample filter: Binomial       Number of points for filter: 3  Sample start region (min): 0  Sample end region (min): 30
Marker peak width (sec): 5    Marker min peak height: 200   Marker baseline V to V?: Y    Marker baseline V to V points: 3
Lower marker selection: First peak > 500 RFU                Upper marker selection: Last peak > 200 RFU
Ladder size (bp)75, 200, 300, 400, 500, 700, 1000, 1500, 2000, 3000, 4000, 5000, 7000, 10000, 20000
Quantification using: Lower Marker          Final concentration (ng/uL): 0.5000          Dilution factor: 12.0
```

Sample: SampB1
Well location: B1
Created: Thursday, October 21, 2021 7:28:10 PM

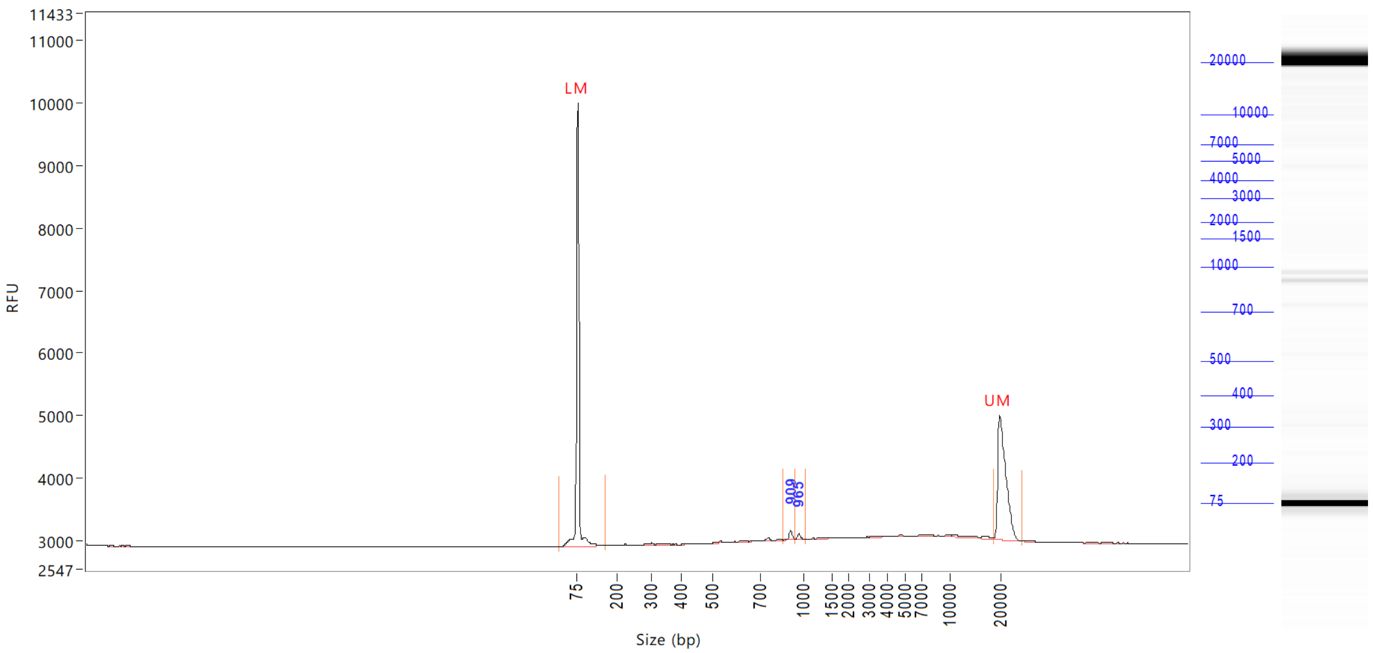

Peak	Size	Concentration	Relative concentration	Molarity	From	To	CV%	RFU
	(bp)	(ng/uL)	Percent	(nmole/L)	(bp)	(bp)		
1	75 (LM)	0.5000		11.2364	20	164	15.18	7099
2	909	0.1164	63.2	0.2120	853	939	1.63	150
3	965	0.0678	36.8	0.1158	939	1034	1.30	86
4	20000 (UM)	0.2360		0.0189	18659	24473	4.13	1981

TIC:	0.1843	ng/uL
TIM:	0.3278	nmole/L
Total concentration:	0.9579	ng/uL

```
Sample peak width (sec): 5    Sample min peak height: 50    Sample baseline V to V?: Y    Sample baseline V to V points: 3
Sample filter: Binomial       Number of points for filter: 3  Sample start region (min): 0  Sample end region (min): 30
Marker peak width (sec): 5    Marker min peak height: 200   Marker baseline V to V?: Y    Marker baseline V to V points: 3
Lower marker selection: First peak > 500 RFU                Upper marker selection: Last peak > 200 RFU
Ladder size (bp)75, 200, 300, 400, 500, 700, 1000, 1500, 2000, 3000, 4000, 5000, 7000, 10000, 20000
Quantification using: Lower Marker          Final concentration (ng/uL): 0.5000          Dilution factor: 12.0
```

Sample: SampC1
Well location: C1
Created: Thursday, October 21, 2021 7:28:10 PM

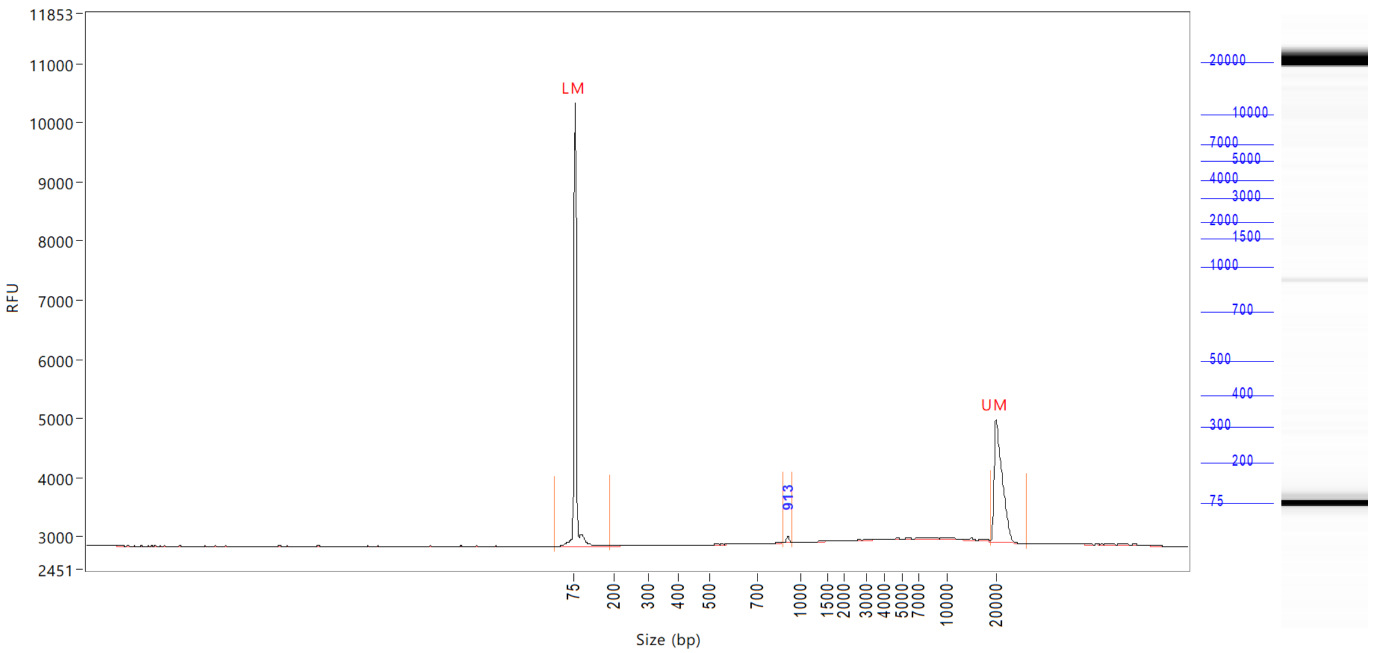

Peak	Size	Concentration	Relative concentration	Molarity	From	To	CV%	RFU
	(bp)	(ng/uL)	Percent	(nmole/L)	(bp)	(bp)		
1	75 (LM)	0.5000		10.9378	16	185	15.13	7512
2	913	0.0722	100.0	0.1307	874	944	1.30	106
3	20000 (UM)	0.2303		0.0185	18903	26166	4.16	2076

TIC:	0.0722	ng/uL
TIM:	0.1307	nmole/L
Total concentration:	0.5942	ng/uL

```
Sample peak width (sec): 5    Sample min peak height: 50    Sample baseline V to V?: Y    Sample baseline V to V points: 3
Sample filter: Binomial       Number of points for filter: 3  Sample start region (min): 0  Sample end region (min): 30
Marker peak width (sec): 5    Marker min peak height: 200   Marker baseline V to V?: Y    Marker baseline V to V points: 3
Lower marker selection: First peak > 500 RFU                Upper marker selection: Last peak > 200 RFU
Ladder size (bp)75, 200, 300, 400, 500, 700, 1000, 1500, 2000, 3000, 4000, 5000, 7000, 10000, 20000
Quantification using: Lower Marker          Final concentration (ng/uL): 0.5000          Dilution factor: 12.0
```

Sample: SampD1
Well location: D1
Created: Thursday, October 21, 2021 7:28:10 PM

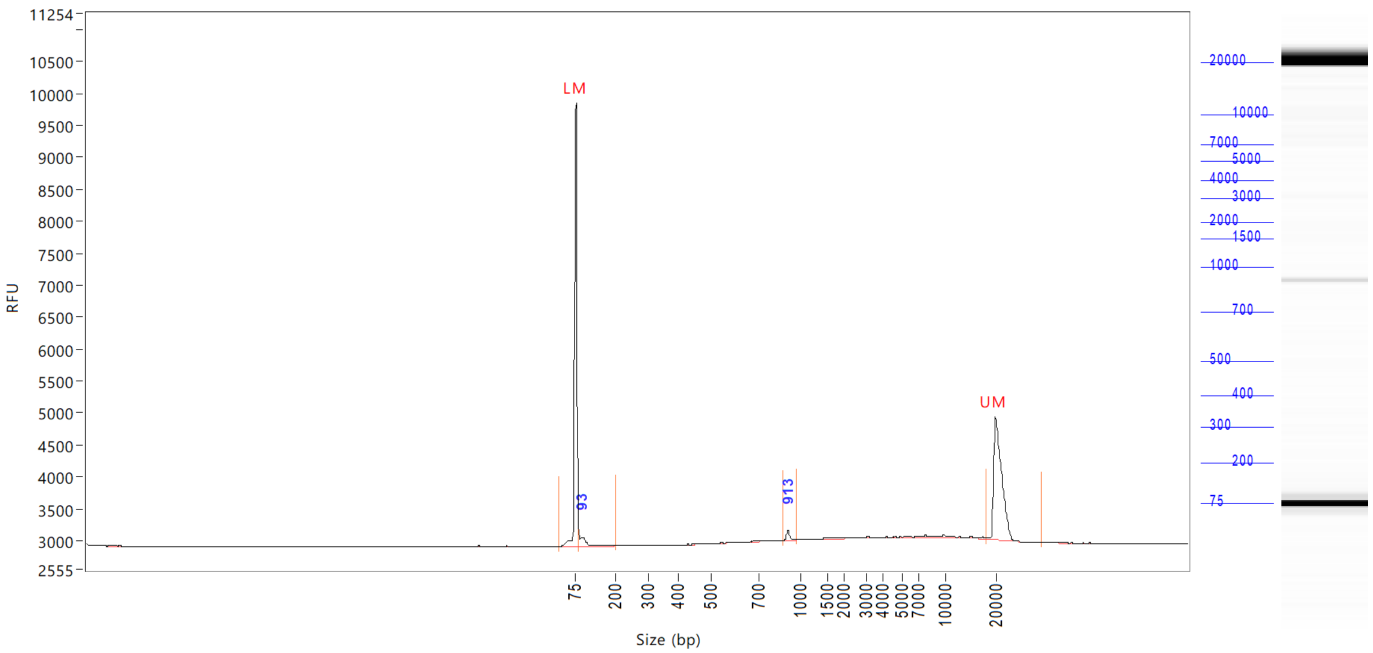

Peak	Size	Concentration	Relative concentration	Molarity	From	To	CV%	RFU
	(bp)	(ng/uL)	Percent	(nmole/L)	(bp)	(bp)		
1	75 (LM)	0.5000		11.5518	22	86	7.95	6951
2	93	0.3603	73.6	5.4285	86	200	23.61	145
3	913	0.1295	26.4	0.2342	870	970	1.66	160
4	20000 (UM)	0.2455		0.0197	18049	29189	4.45	1933

TIC:	0.4897	ng/uL
TIM:	5.6626	nmole/L
Total concentration:	1.0674	ng/uL

```
Sample peak width (sec): 5    Sample min peak height: 50    Sample baseline V to V?: Y    Sample baseline V to V points: 3
Sample filter: Binomial       Number of points for filter: 3  Sample start region (min): 0  Sample end region (min): 30
Marker peak width (sec): 5    Marker min peak height: 200   Marker baseline V to V?: Y    Marker baseline V to V points: 3
Lower marker selection: First peak > 500 RFU                Upper marker selection: Last peak > 200 RFU
Ladder size (bp)75, 200, 300, 400, 500, 700, 1000, 1500, 2000, 3000, 4000, 5000, 7000, 10000, 20000
Quantification using: Lower Marker          Final concentration (ng/uL): 0.5000          Dilution factor: 12.0
```

Sample: SampE1
Well location: E1
Created: Thursday, October 21, 2021 7:28:10 PM

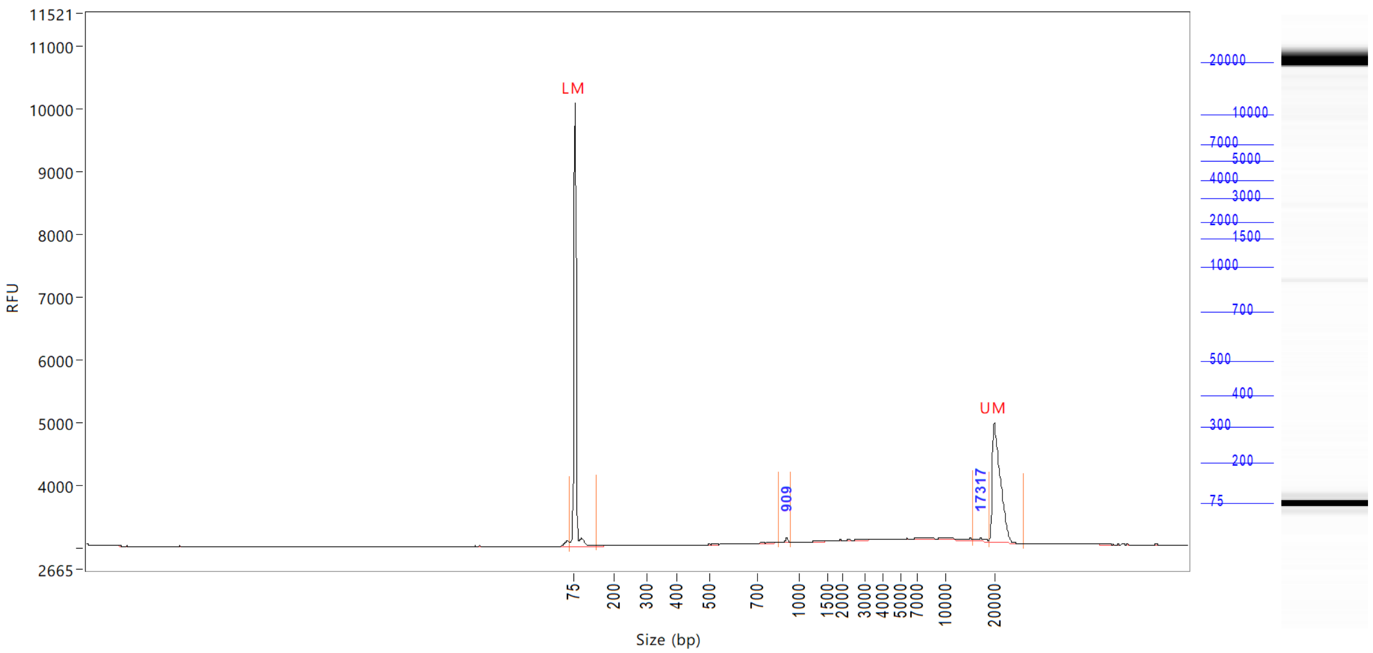

Peak	Size	Concentration	Relative concentration	Molarity	From	To	CV%	RFU
	(bp)	(ng/uL)	Percent	(nmole/L)	(bp)	(bp)		
1	75 (LM)	0.5000		11.0851	62	143	8.57	7075
2	909	0.0552	39.4	0.1007	848	939	1.70	68
3	17317	0.0848	60.6	0.0081	15732	18903	5.09	50
4	20000 (UM)	0.2480		0.0199	18903	25803	4.28	1907

TIC:	0.1400	ng/uL
TIM:	0.1088	nmole/L
Total concentration:	0.7084	ng/uL

Sample peak width (sec): 5 Sample min peak height: 50 Sample baseline V to V?: Y Sample baseline V to V points: 3
Sample filter: Binomial Number of points for filter: 3 Sample start region (min): 0 Sample end region (min): 30
Marker peak width (sec): 5 Marker min peak height: 200 Marker baseline V to V?: Y Marker baseline V to V points: 3
Lower marker selection: First peak > 500 RFU Upper marker selection: Last peak > 200 RFU
Ladder size (bp)75, 200, 300, 400, 500, 700, 1000, 1500, 2000, 3000, 4000, 5000, 7000, 10000, 20000
Quantification using: Lower Marker Final concentration (ng/uL): 0.5000 Dilution factor: 12.0

Sample: SampF1
Well location: F1
Created: Thursday, October 21, 2021 7:28:10 PM

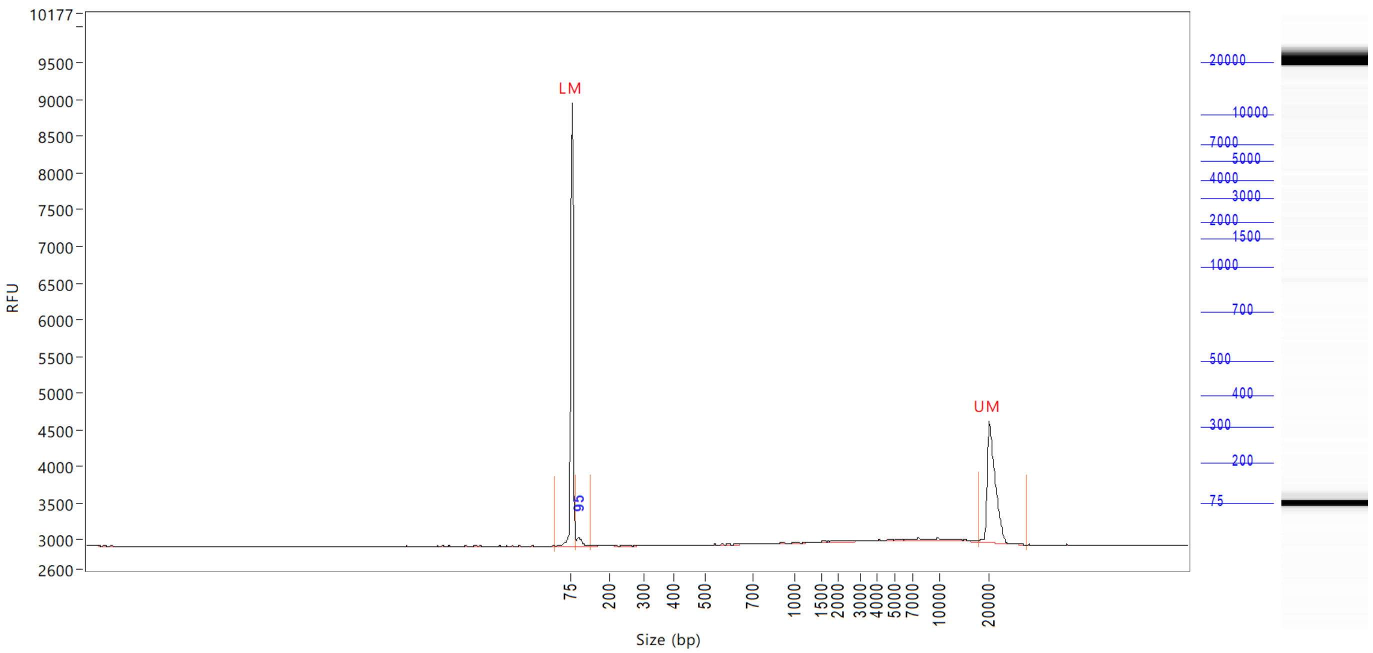

Peak	Size	Concentration	Relative concentration	Molarity	From	To	CV%	RFU
	(bp)	(ng/uL)	Percent	(nmole/L)	(bp)	(bp)		
1	75 (LM)	0.5000		11.2364	24	87	6.61	6051
2	95	0.2572	100.0	4.2237	87	137	10.98	108
3	20000 (UM)	0.2418		0.0193	18049	27617	4.45	1651

TIC:	0.2572	ng/uL
TIM:	4.2237	nmole/L
Total concentration:	0.9156	ng/uL

Sample peak width (sec): 5 Sample min peak height: 50 Sample baseline V to V?: Y Sample baseline V to V points: 3
Sample filter: Binomial Number of points for filter: 3 Sample start region (min): 0 Sample end region (min): 30
Marker peak width (sec): 5 Marker min peak height: 200 Marker baseline V to V?: Y Marker baseline V to V points: 3
Lower marker selection: First peak > 500 RFU Upper marker selection: Last peak > 200 RFU
Ladder size (bp)75, 200, 300, 400, 500, 700, 1000, 1500, 2000, 3000, 4000, 5000, 7000, 10000, 20000
Quantification using: Lower Marker Final concentration (ng/uL): 0.5000 Dilution factor: 12.0

Sample: SampG1
Well location: G1
Created: Thursday, October 21, 2021 7:28:10 PM

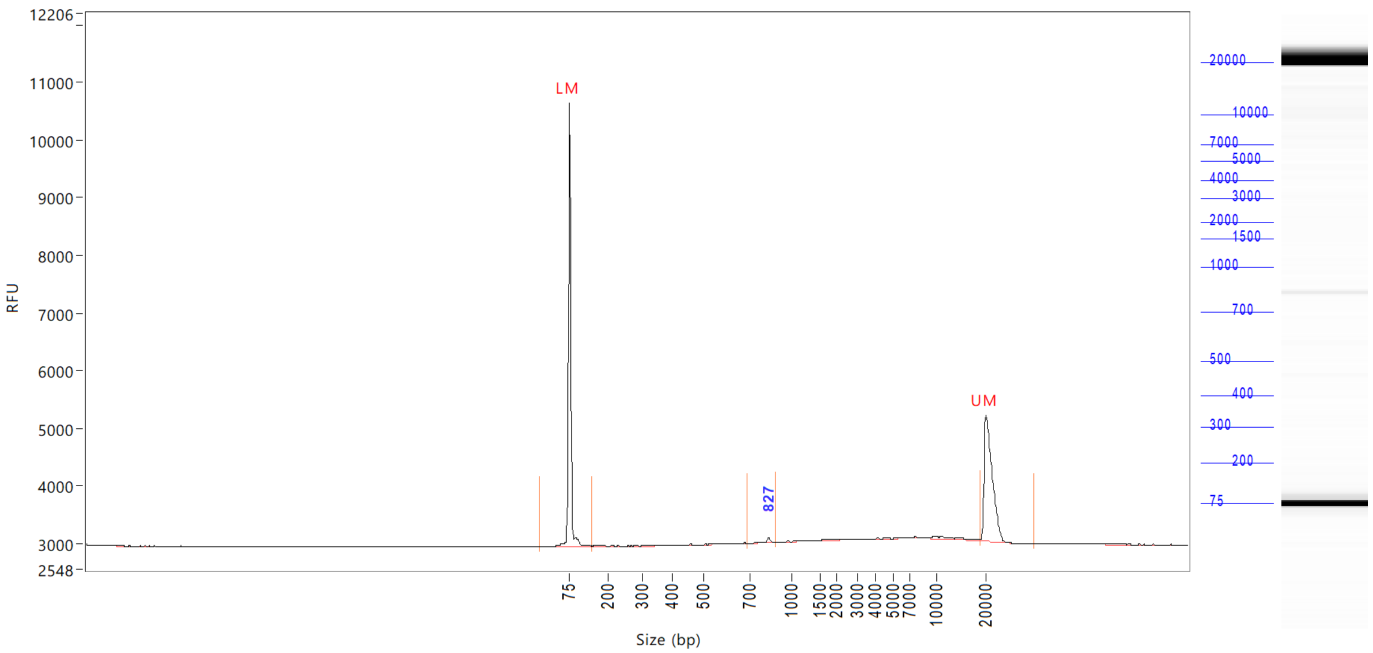

Peak	Size	Concentration	Relative concentration	Molarity	From	To	CV%	RFU
	(bp)	(ng/uL)	Percent	(nmole/L)	(bp)	(bp)		
1	75 (LM)	0.5000		11.2364	0	147	11.90	7721
2	827	0.0764	100.0	0.1546	687	874	3.87	91
3	20000 (UM)	0.2455		0.0195	18903	29793	4.56	2190

TIC:	0.0764	ng/uL
TIM:	0.1546	nmole/L
Total concentration:	0.6228	ng/uL

```
Sample peak width (sec): 5    Sample min peak height: 50    Sample baseline V to V?: Y    Sample baseline V to V points: 3
Sample filter: Binomial       Number of points for filter: 3  Sample start region (min): 0  Sample end region (min): 30
Marker peak width (sec): 5    Marker min peak height: 200   Marker baseline V to V?: Y    Marker baseline V to V points: 3
Lower marker selection: First peak > 500 RFU                Upper marker selection: Last peak > 200 RFU
Ladder size (bp)75, 200, 300, 400, 500, 700, 1000, 1500, 2000, 3000, 4000, 5000, 7000, 10000, 20000
Quantification using: Lower Marker          Final concentration (ng/uL): 0.5000          Dilution factor: 12.0
```

Sample: SampH1
Well location: H1
Created: Thursday, October 21, 2021 7:28:10 PM

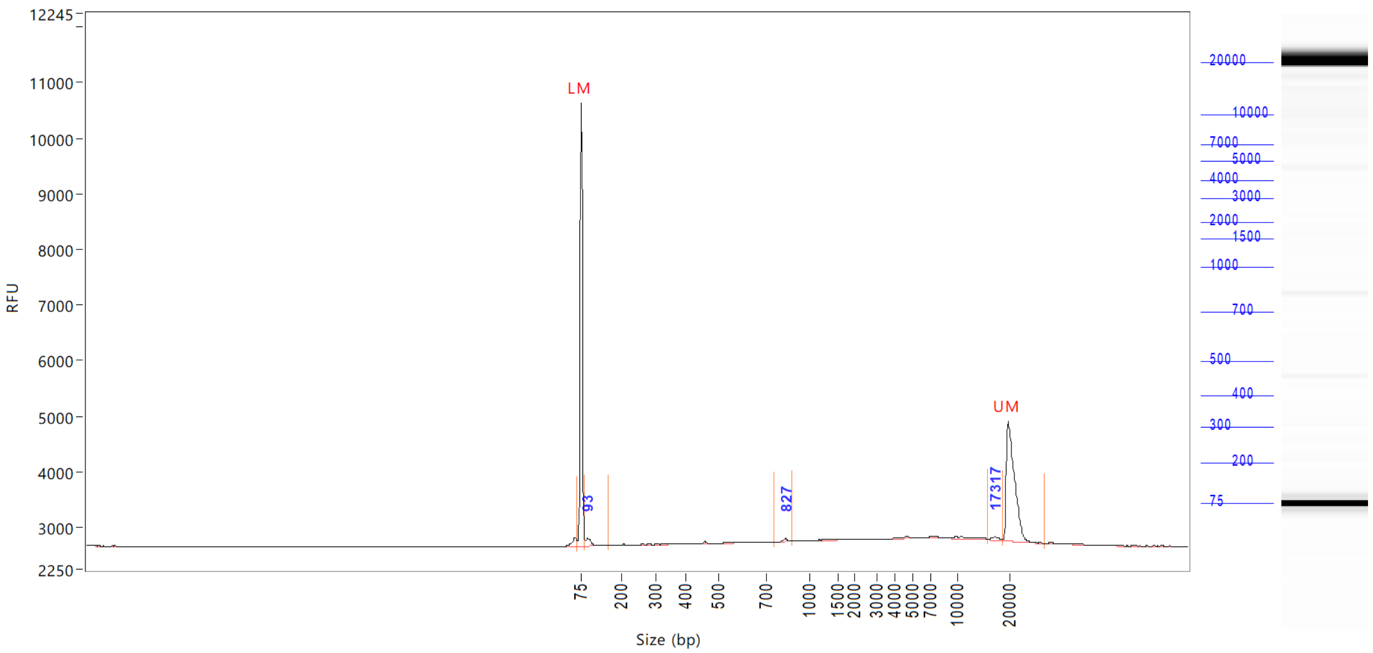

Peak	Size	Concentration	Relative concentration	Molarity	From	To	CV%	RFU
	(bp)	(ng/uL)	Percent	(nmole/L)	(bp)	(bp)		
1	75 (LM)	0.5000		11.3919	62	86	4.47	7982
2	93	0.2771	66.8	4.4612	86	158	16.22	138
3	827	0.0487	11.7	0.0982	749	870	2.08	58
4	17317	0.0891	21.5	0.0084	15854	18903	4.51	65
5	20000 (UM)	0.2548		0.0204	18903	27254	4.43	2164

TIC:	0.4150	ng/uL
TIM:	4.5678	nmole/L
Total concentration:	1.0437	ng/uL

```
Sample peak width (sec): 5    Sample min peak height: 50    Sample baseline V to V?: Y    Sample baseline V to V points: 3
Sample filter: Binomial       Number of points for filter: 3  Sample start region (min): 0  Sample end region (min): 30
Marker peak width (sec): 5    Marker min peak height: 200   Marker baseline V to V?: Y    Marker baseline V to V points: 3
Lower marker selection: First peak > 500 RFU                Upper marker selection: Last peak > 200 RFU
Ladder size (bp)75, 200, 300, 400, 500, 700, 1000, 1500, 2000, 3000, 4000, 5000, 7000, 10000, 20000
Quantification using: Lower Marker          Final concentration (ng/uL): 0.5000          Dilution factor: 12.0
```

Sample: SampA2
Well location: A2
Created: Thursday, October 21, 2021 7:28:10 PM

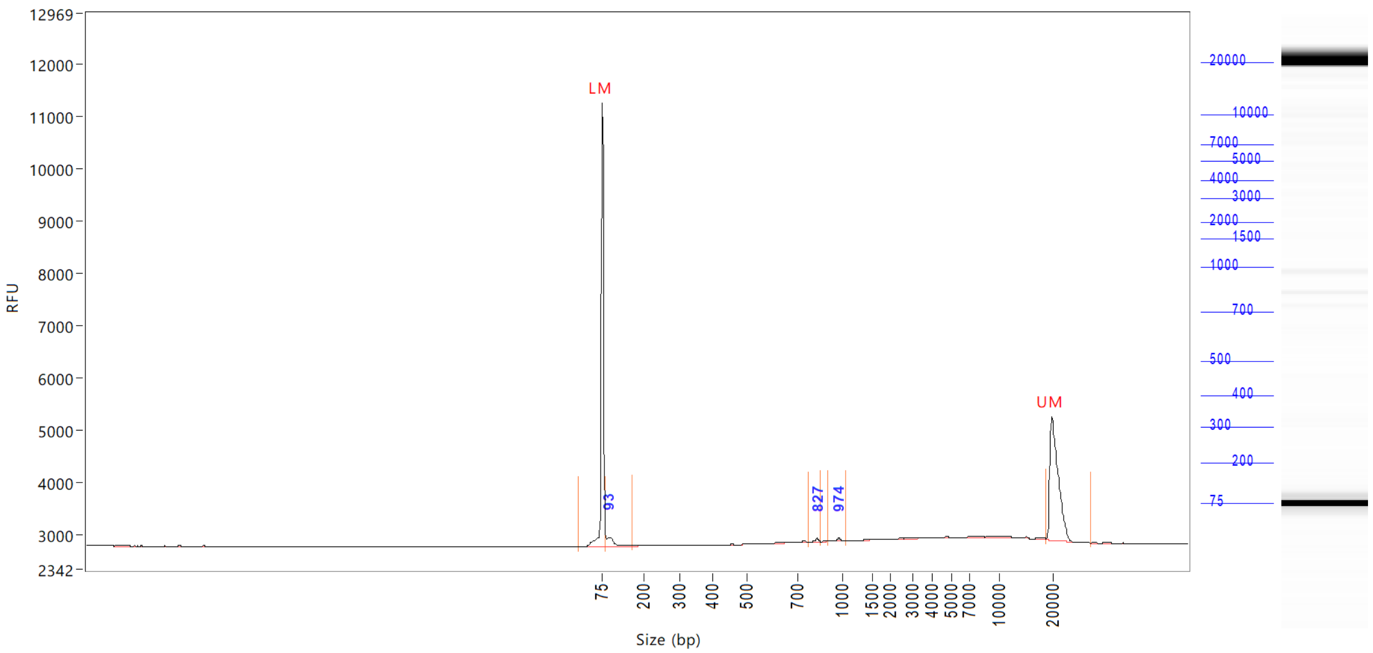

Peak	Size	Concentration	Relative concentration	Molarity	From	To	CV%	RFU
	(bp)	(ng/uL)	Percent	(nmole/L)	(bp)	(bp)		
1	75 (LM)	0.5000		11.3919	5	87	9.25	8490
2	93	0.3302	77.3	5.3166	87	166	14.92	174
3	827	0.0478	11.2	0.0961	766	853	1.92	67
4	974	0.0492	11.5	0.0836	896	1045	1.95	60
5	20000 (UM)	0.2523		0.0202	18903	27375	4.31	2356

TIC:	0.4272	ng/uL
TIM:	5.4964	nmole/L
Total concentration:	0.9246	ng/uL

Sample peak width (sec): 5 Sample min peak height: 50 Sample baseline V to V?: Y Sample baseline V to V points: 3
Sample filter: Binomial Number of points for filter: 3 Sample start region (min): 0 Sample end region (min): 30
Marker peak width (sec): 5 Marker min peak height: 200 Marker baseline V to V?: Y Marker baseline V to V points: 3
Lower marker selection: First peak > 500 RFU Upper marker selection: Last peak > 200 RFU
Ladder size (bp)75, 200, 300, 400, 500, 700, 1000, 1500, 2000, 3000, 4000, 5000, 7000, 10000, 20000
Quantification using: Lower Marker Final concentration (ng/uL): 0.5000 Dilution factor: 12.0

Sample: SampB2
Well location: B2
Created: Thursday, October 21, 2021 7:28:10 PM

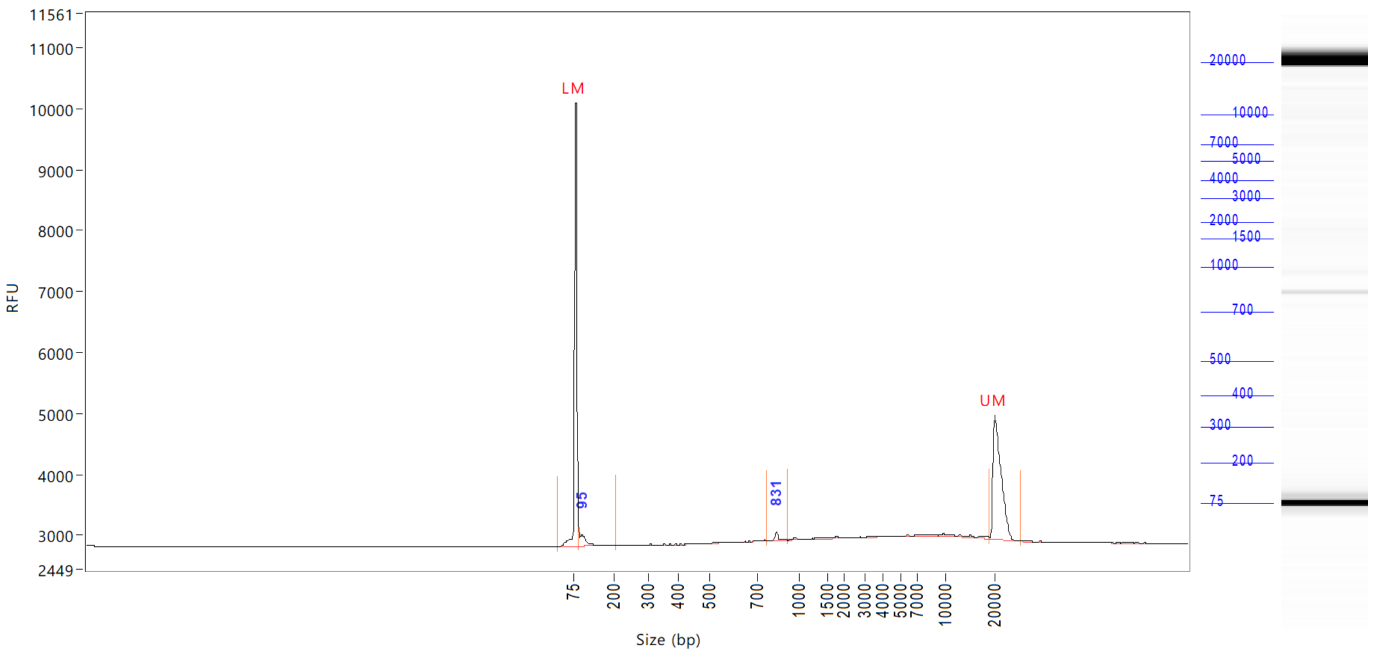

Peak	Size	Concentration	Relative concentration	Molarity	From	To	CV%	RFU
	(bp)	(ng/uL)	Percent	(nmole/L)	(bp)	(bp)		
1	75 (LM)	0.5000		11.2364	18	87	8.21	7277
2	95	0.4408	79.9	6.6428	87	201	21.74	183
3	831	0.1108	20.1	0.2203	762	918	2.62	137
4	20000 (UM)	0.2454		0.0197	18903	25199	4.20	2041

TIC:	0.5517	ng/uL
TIM:	6.8631	nmole/L
Total concentration:	1.2536	ng/uL

Sample peak width (sec): 5   Sample min peak height: 50   Sample baseline V to V?: Y   Sample baseline V to V points: 3
Sample filter: Binomial   Number of points for filter: 3 Sample start region (min): 0 Sample end region (min): 30
Marker peak width (sec): 5   Marker min peak height: 200   Marker baseline V to V?: Y   Marker baseline V to V points: 3
Lower marker selection: First peak > 500 RFU   Upper marker selection: Last peak > 200 RFU
Ladder size (bp)75, 200, 300, 400, 500, 700, 1000, 1500, 2000, 3000, 4000, 5000, 7000, 10000, 20000
Quantification using: Lower Marker   Final concentration (ng/uL): 0.5000   Dilution factor: 12.0

Sample: SampC2
Well location: C2
Created: Thursday, October 21, 2021 7:28:10 PM

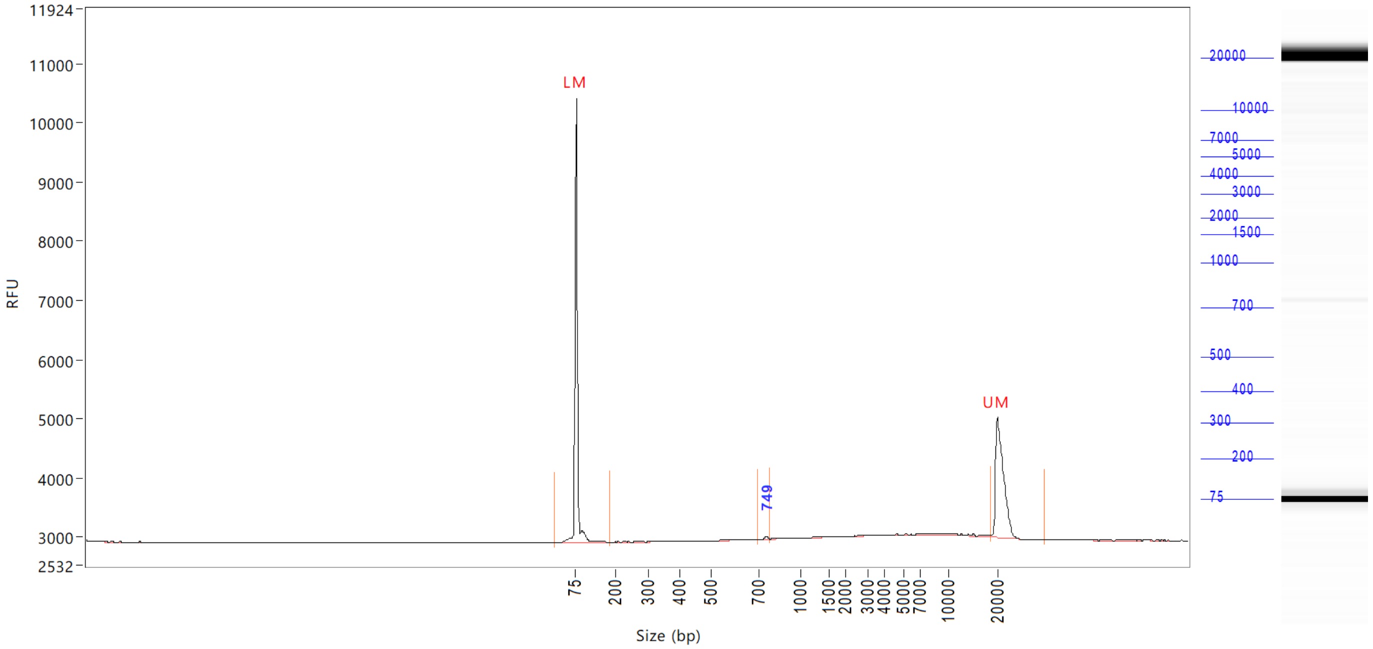

Peak	Size	Concentration	Relative concentration	Molarity	From	To	CV%	RFU
	(bp)	(ng/uL)	Percent	(nmole/L)	(bp)	(bp)		
1	75 (LM)	0.5000		11.0851	12	181	12.10	7506
2	749	0.0369	100.0	0.0820	692	775	2.01	51
3	20000 (UM)	0.2305		0.0184	18781	29430	4.44	2025

TIC:	0.0369	ng/uL
TIM:	0.0820	nmole/L
Total concentration:	0.5245	ng/uL

Sample peak width (sec): 5   Sample min peak height: 50   Sample baseline V to V?: Y   Sample baseline V to V points: 3
Sample filter: Binomial   Number of points for filter: 3   Sample start region (min): 0   Sample end region (min): 30
Marker peak width (sec): 5   Marker min peak height: 200   Marker baseline V to V?: Y   Marker baseline V to V points: 3
Lower marker selection: First peak > 500 RFU   Upper marker selection: Last peak > 200 RFU
Ladder size (bp)75, 200, 300, 400, 500, 700, 1000, 1500, 2000, 3000, 4000, 5000, 7000, 10000, 20000
Quantification using: Lower Marker   Final concentration (ng/uL): 0.5000   Dilution factor: 12.0

Sample: SampD2
Well location: D2
Created: Thursday, October 21, 2021 7:28:10 PM

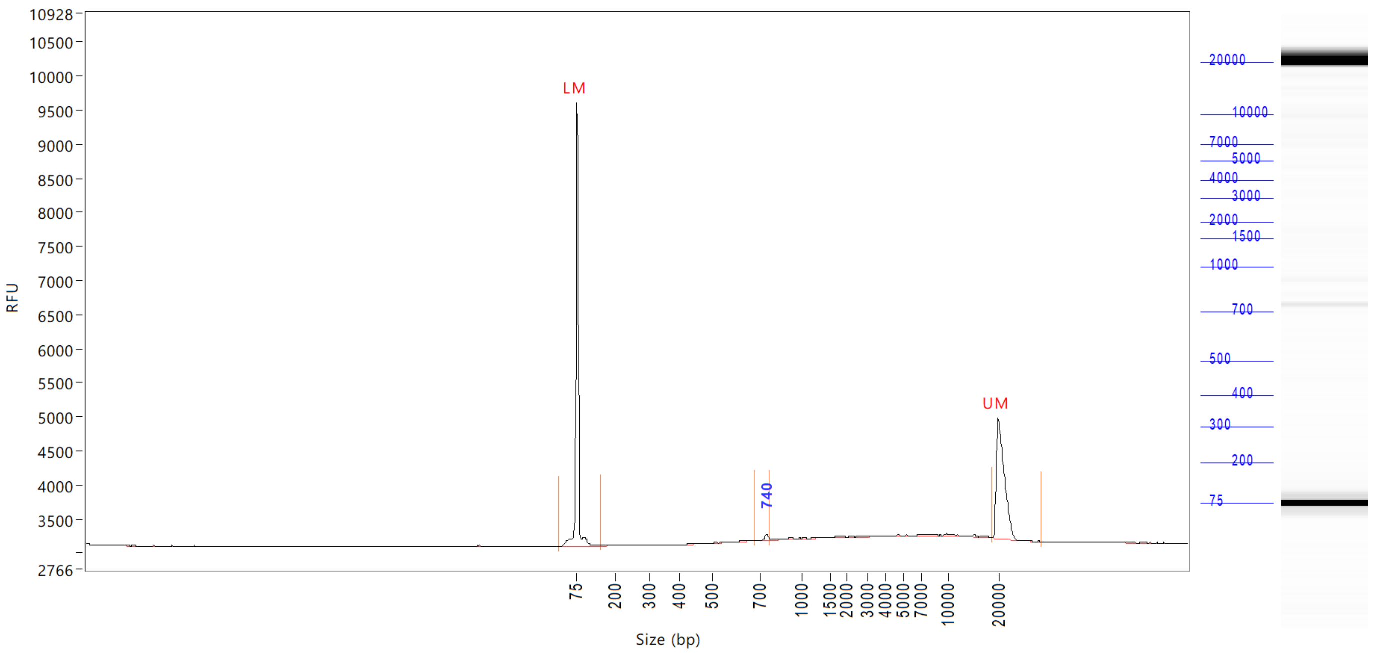

Peak	Size	Concentration	Relative concentration	Molarity	From	To	CV%	RFU
	(bp)	(ng/uL)	Percent	(nmole/L)	(bp)	(bp)		
1	75 (LM)	0.5000		11.0851	22	152	13.28	6521
2	740	0.0842	100.0	0.1890	674	770	2.50	90
3	20000 (UM)	0.2259		0.0181	18781	28705	4.29	1762

TIC:	0.0842	ng/uL
TIM:	0.1890	nmole/L
Total concentration:	0.7104	ng/uL

```
Sample peak width (sec): 5    Sample min peak height: 50    Sample baseline V to V?: Y    Sample baseline V to V points: 3
Sample filter: Binomial       Number of points for filter: 3  Sample start region (min): 0  Sample end region (min): 30
Marker peak width (sec): 5    Marker min peak height: 200   Marker baseline V to V?: Y    Marker baseline V to V points: 3
Lower marker selection: First peak > 500 RFU                Upper marker selection: Last peak > 200 RFU
Ladder size (bp)75, 200, 300, 400, 500, 700, 1000, 1500, 2000, 3000, 4000, 5000, 7000, 10000, 20000
Quantification using: Lower Marker          Final concentration (ng/uL): 0.5000          Dilution factor: 12.0
```

Sample: SampE2
Well location: E2
Created: Thursday, October 21, 2021 7:28:10 PM

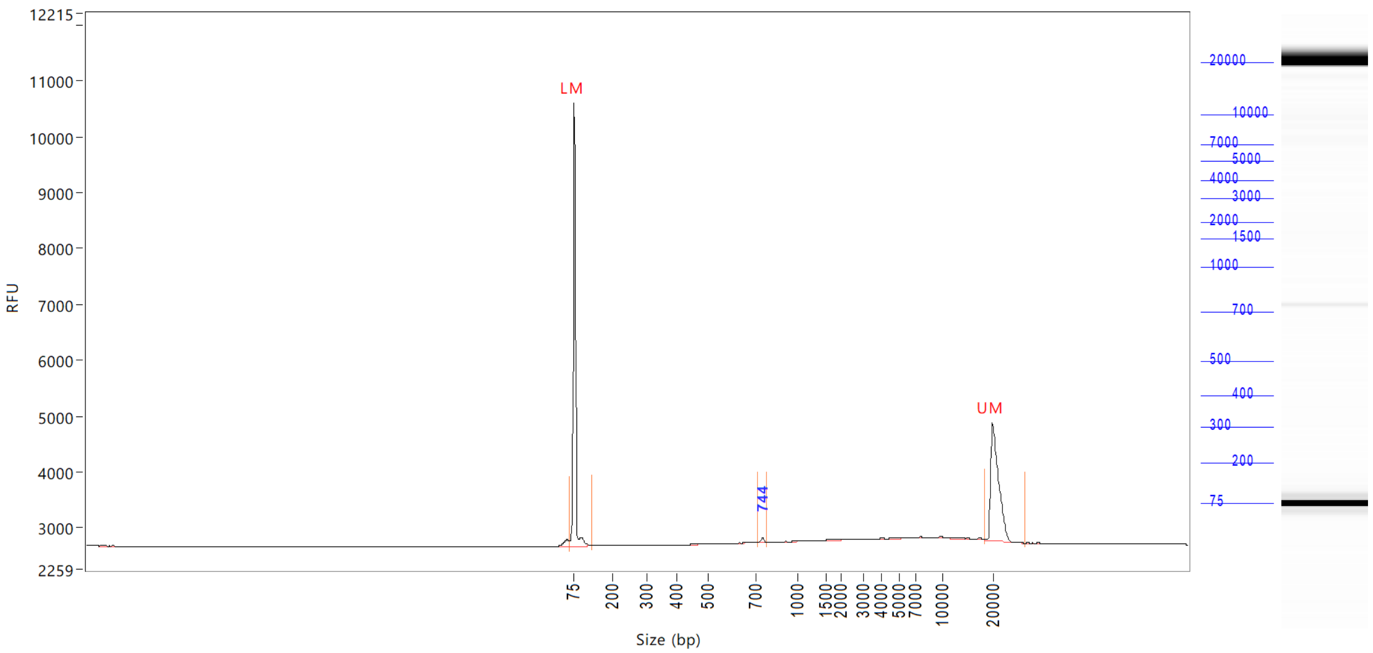

Peak	Size	Concentration	Relative concentration	Molarity	From	To	CV%	RFU
	(bp)	(ng/uL)	Percent	(nmole/L)	(bp)	(bp)		
1	75 (LM)	0.5000		11.0851	62	133	9.08	7952
2	744	0.0615	100.0	0.1367	714	779	1.49	89
3	20000 (UM)	0.2372		0.0190	18537	26649	4.22	2099

TIC:	0.0615	ng/uL
TIM:	0.1367	nmole/L
Total concentration:	0.5014	ng/uL

```
Sample peak width (sec): 5    Sample min peak height: 50    Sample baseline V to V?: Y    Sample baseline V to V points: 3
Sample filter: Binomial       Number of points for filter: 3  Sample start region (min): 0  Sample end region (min): 30
Marker peak width (sec): 5    Marker min peak height: 200   Marker baseline V to V?: Y    Marker baseline V to V points: 3
Lower marker selection: First peak > 500 RFU                Upper marker selection: Last peak > 200 RFU
Ladder size (bp)75, 200, 300, 400, 500, 700, 1000, 1500, 2000, 3000, 4000, 5000, 7000, 10000, 20000
Quantification using: Lower Marker          Final concentration (ng/uL): 0.5000          Dilution factor: 12.0
```

Sample: SampF2
Well location: F2
Created: Thursday, October 21, 2021 7:28:10 PM

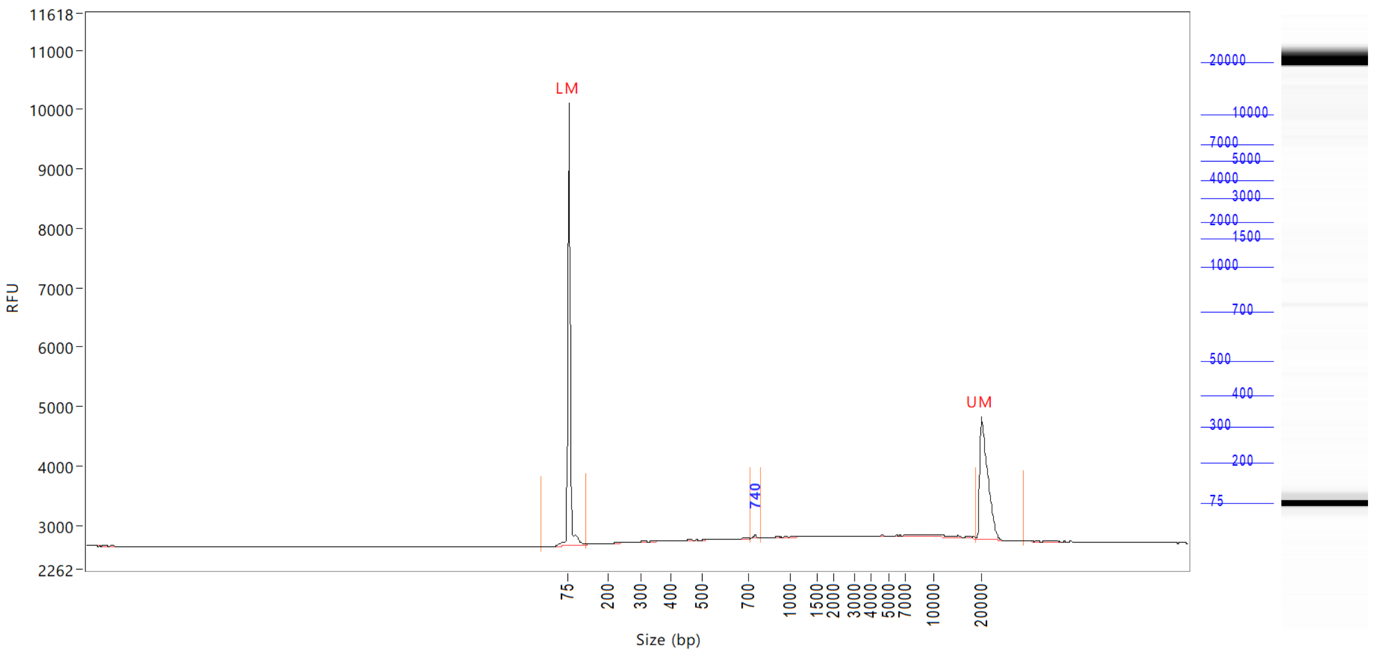

Peak	Size	Concentration	Relative concentration	Molarity	From	To	CV%	RFU
	(bp)	(ng/uL)	Percent	(nmole/L)	(bp)	(bp)		
1	75 (LM)	0.5000		11.2364	0	129	10.94	7462
2	740	0.0389	100.0	0.0864	710	792	1.89	51
3	20000 (UM)	0.2416		0.0193	18903	28705	4.49	2060

TIC:	0.0389	ng/uL
TIM:	0.0864	nmole/L
Total concentration:	0.6757	ng/uL

Sample peak width (sec): 5   Sample min peak height: 50   Sample baseline V to V?: Y   Sample baseline V to V points: 3
Sample filter: Binomial   Number of points for filter: 3 Sample start region (min): 0 Sample end region (min): 30
Marker peak width (sec): 5   Marker min peak height: 200   Marker baseline V to V?: Y   Marker baseline V to V points: 3
Lower marker selection: First peak > 500 RFU   Upper marker selection: Last peak > 200 RFU
Ladder size (bp)75, 200, 300, 400, 500, 700, 1000, 1500, 2000, 3000, 4000, 5000, 7000, 10000, 20000
Quantification using: Lower Marker   Final concentration (ng/uL): 0.5000   Dilution factor: 12.0

Sample: SampG2
Well location: G2
Created: Thursday, October 21, 2021 7:28:10 PM

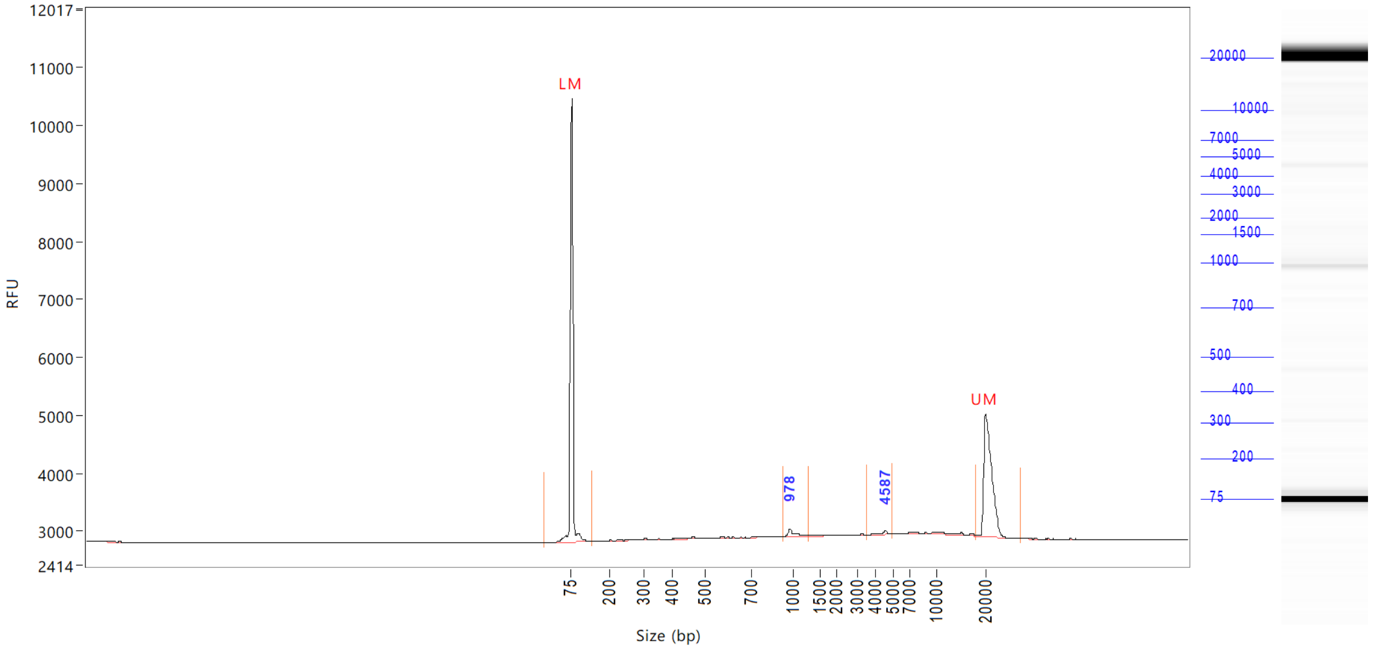

Peak	Size	Concentration	Relative concentration	Molarity	From	To	CV%	RFU
	(bp)	(ng/uL)	Percent	(nmole/L)	(bp)	(bp)		
1	75 (LM)	0.5000		11.2364	0	141	12.25	7669
2	978	0.2087	74.2	0.3345	922	1278	7.96	141
3	4587	0.0727	25.8	0.0273	3536	4966	6.73	61
4	20000 (UM)	0.2369		0.0190	17927	27133	4.26	2127

TIC:	0.2814	ng/uL
TIM:	0.3618	nmole/L
Total concentration:	0.9515	ng/uL

Sample peak width (sec): 5 Sample min peak height: 50 Sample baseline V to V?: Y Sample baseline V to V points: 3
Sample filter: Binomial Number of points for filter: 3 Sample start region (min): 0 Sample end region (min): 30
Marker peak width (sec): 5 Marker min peak height: 200 Marker baseline V to V?: Y Marker baseline V to V points: 3
Lower marker selection: First peak > 500 RFU Upper marker selection: Last peak > 200 RFU
Ladder size (bp)75, 200, 300, 400, 500, 700, 1000, 1500, 2000, 3000, 4000, 5000, 7000, 10000, 20000
Quantification using: Lower Marker Final concentration (ng/uL): 0.5000 Dilution factor: 12.0

Sample: SampH2
Well location: H2
Created: Thursday, October 21, 2021 7:28:10 PM

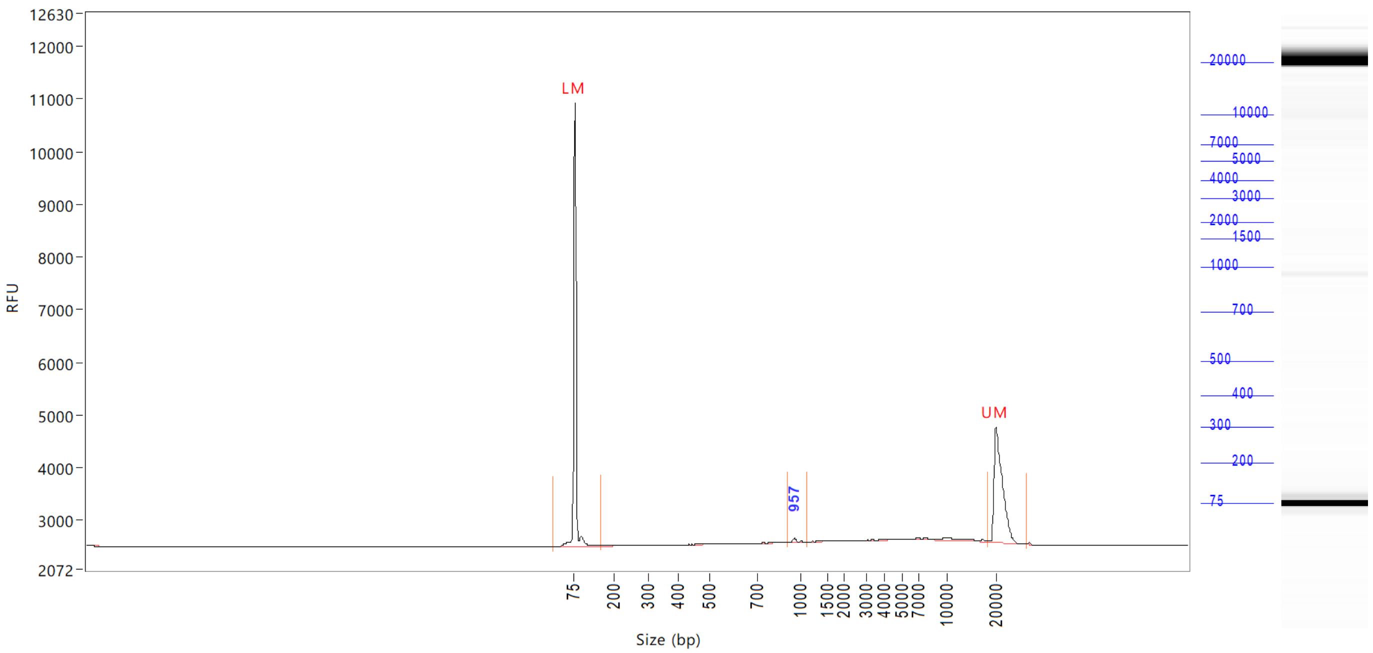

Peak	Size	Concentration	Relative concentration	Molarity	From	To	CV%	RFU
	(bp)	(ng/uL)	Percent	(nmole/L)	(bp)	(bp)		
1	75 (LM)	0.5000		11.0851	11	158	12.11	8438
2	957	0.0612	100.0	0.1048	913	1112	2.60	73
3	20000 (UM)	0.2368		0.0189	18415	26166	4.51	2183

TIC:	0.0612	ng/uL
TIM:	0.1048	nmole/L
Total concentration:	0.5941	ng/uL

Sample peak width (sec): 5   Sample min peak height: 50   Sample baseline V to V?: Y   Sample baseline V to V points: 3
Sample filter: Binomial   Number of points for filter: 3 Sample start region (min): 0 Sample end region (min): 30
Marker peak width (sec): 5   Marker min peak height: 200   Marker baseline V to V?: Y   Marker baseline V to V points: 3
Lower marker selection: First peak > 500 RFU   Upper marker selection: Last peak > 200 RFU
Ladder size (bp)75, 200, 300, 400, 500, 700, 1000, 1500, 2000, 3000, 4000, 5000, 7000, 10000, 20000
Quantification using: Lower Marker   Final concentration (ng/uL): 0.5000   Dilution factor: 12.0

Sample: SampA3
Well location: A3
Created: Thursday, October 21, 2021 7:28:10 PM

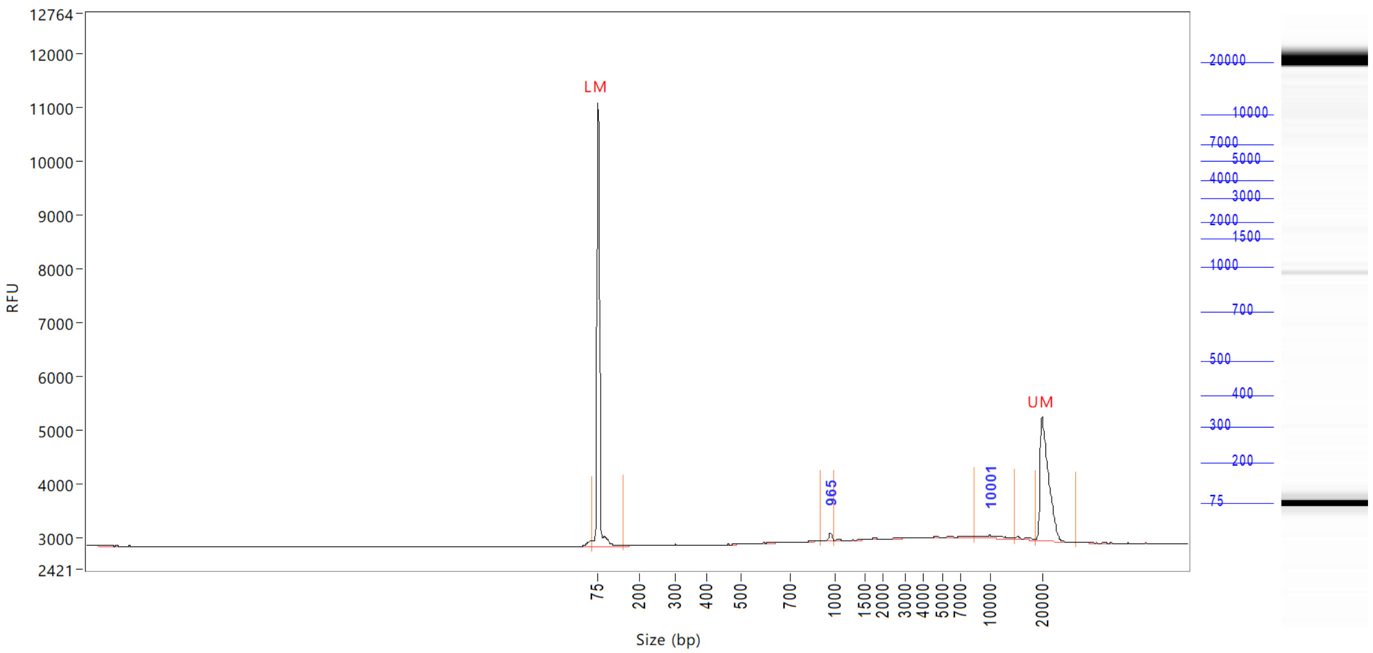

Peak	Size	Concentration	Relative concentration	Molarity	From	To	CV%	RFU
	(bp)	(ng/uL)	Percent	(nmole/L)	(bp)	(bp)		
1	75 (LM)	0.5000		10.9378	60	150	9.50	8263
2	965	0.1005	33.6	0.1725	896	991	1.37	155
3	10001	0.1990	66.4	0.0292	8467	14757	15.93	52
4	20000 (UM)	0.2555		0.0205	18781	26408	4.37	2299

TIC:	0.2995	ng/uL
TIM:	0.2017	nmole/L
Total concentration:	0.8355	ng/uL

Sample peak width (sec): 5 Sample min peak height: 50 Sample baseline V to V?: Y Sample baseline V to V points: 3
Sample filter: Binomial Number of points for filter: 3 Sample start region (min): 0 Sample end region (min): 30
Marker peak width (sec): 5 Marker min peak height: 200 Marker baseline V to V?: Y Marker baseline V to V points: 3
Lower marker selection: First peak > 500 RFU Upper marker selection: Last peak > 200 RFU
Ladder size (bp)75, 200, 300, 400, 500, 700, 1000, 1500, 2000, 3000, 4000, 5000, 7000, 10000, 20000
Quantification using: Lower Marker Final concentration (ng/uL): 0.5000 Dilution factor: 12.0

Sample: SampB3
Well location: B3
Created: Thursday, October 21, 2021 7:28:10 PM

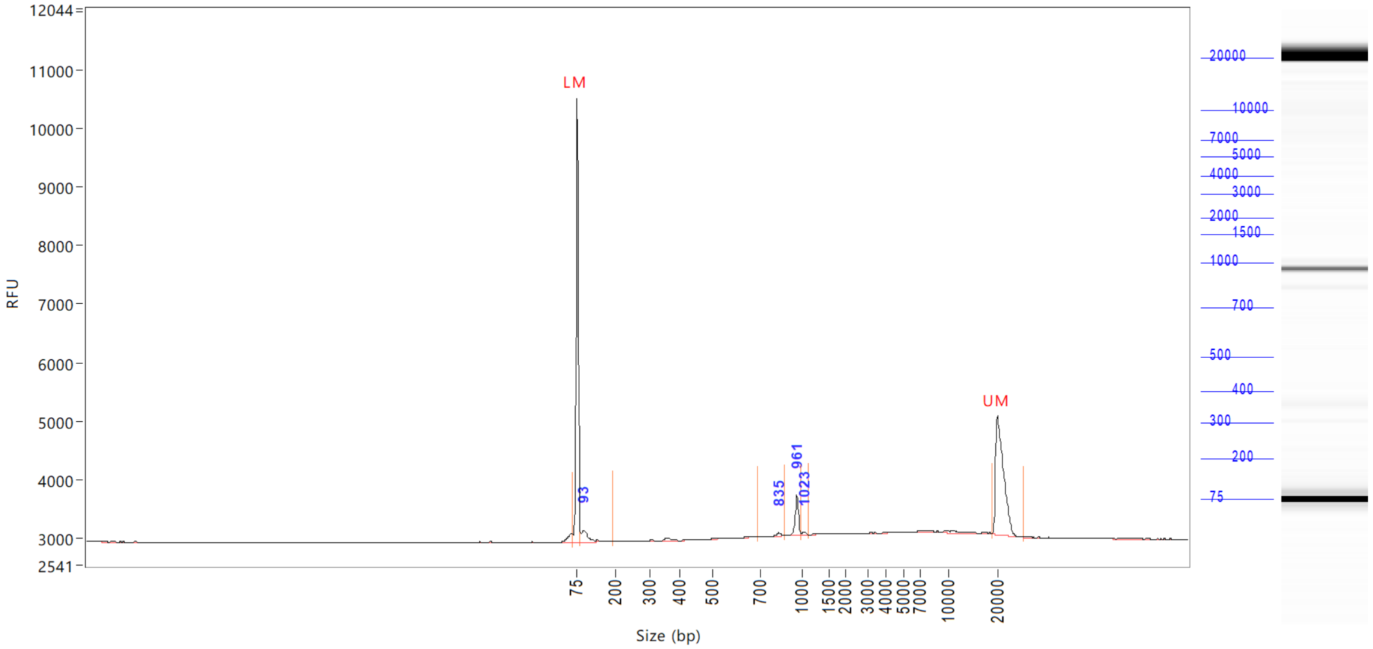

Peak	Size	Concentration	Relative concentration	Molarity	From	To	CV%	RFU
	(bp)	(ng/uL)	Percent	(nmole/L)	(bp)	(bp)		
1	75 (LM)	0.5000		11.2364	64	86	4.46	7590
2	93	0.5179	43.1	7.9496	86	187	20.56	203
3	835	0.0744	6.2	0.1507	690	874	5.61	55
4	961	0.5626	46.9	0.9697	874	996	1.66	680
5	1023	0.0455	3.8	0.0731	996	1123	2.50	51
6	20000 (UM)	0.2506		0.0201	18903	25199	4.10	2033

TIC:	1.2004	ng/uL
TIM:	9.1431	nmole/L
Total concentration:	1.8730	ng/uL

```
Sample peak width (sec): 5    Sample min peak height: 50    Sample baseline V to V?: Y    Sample baseline V to V points: 3
Sample filter: Binomial       Number of points for filter: 3  Sample start region (min): 0  Sample end region (min): 30
Marker peak width (sec): 5    Marker min peak height: 200   Marker baseline V to V?: Y    Marker baseline V to V points: 3
Lower marker selection: First peak > 500 RFU                Upper marker selection: Last peak > 200 RFU
Ladder size (bp)75, 200, 300, 400, 500, 700, 1000, 1500, 2000, 3000, 4000, 5000, 7000, 10000, 20000
Quantification using: Lower Marker          Final concentration (ng/uL): 0.5000          Dilution factor: 12.0
```

Sample: SampC3
Well location: C3
Created: Thursday, October 21, 2021 7:28:10 PM

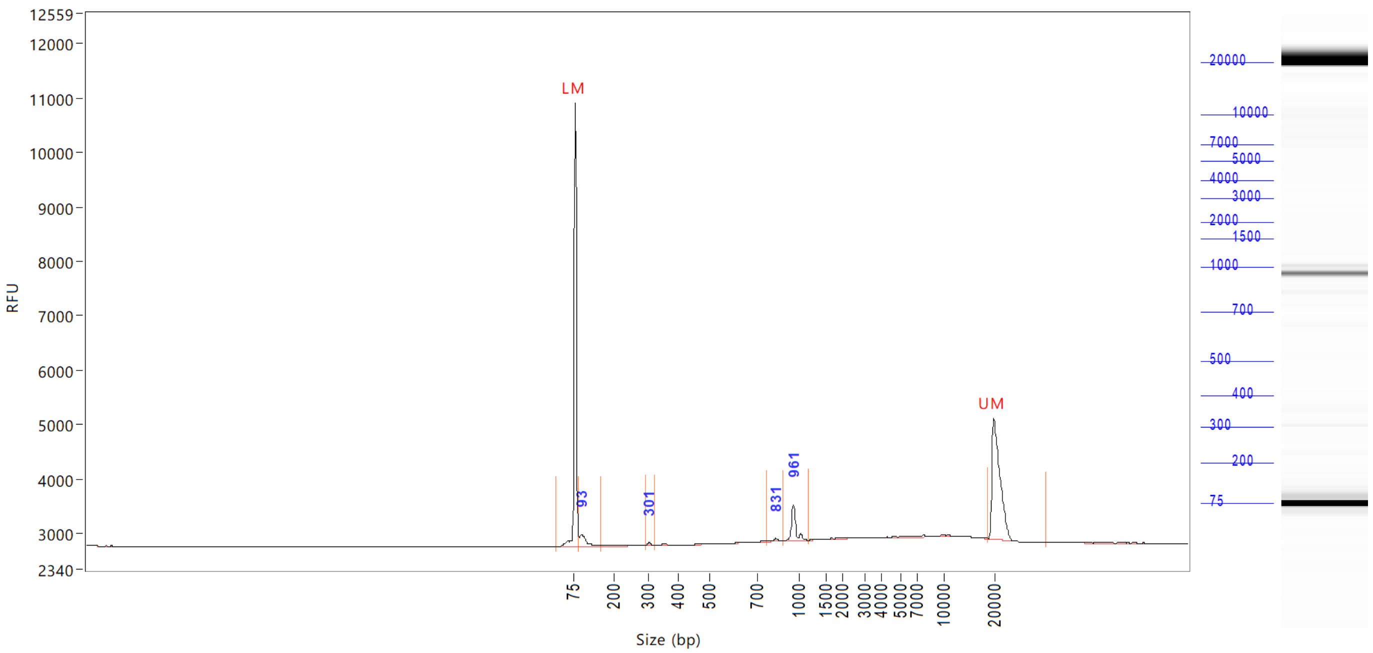

Peak	Size	Concentration	Relative concentration	Molarity	From	To	CV%	RFU
	(bp)	(ng/uL)	Percent	(nmole/L)	(bp)	(bp)		
1	75 (LM)	0.5000		11.3919	16	86	7.89	8167
2	93	0.4562	39.7	7.2741	86	158	15.43	222
3	301	0.0385	3.4	0.2099	293	321	1.68	52
4	831	0.0596	5.2	0.1183	762	883	3.00	52
5	961	0.5956	51.8	1.0190	883	1156	2.89	672
6	20000 (UM)	0.2459		0.0196	18903	30519	4.59	2224

TIC:	1.1499	ng/uL
TIM:	8.6213	nmole/L
Total concentration:	1.6857	ng/uL

```
Sample peak width (sec): 5    Sample min peak height: 50    Sample baseline V to V?: Y    Sample baseline V to V points: 3
Sample filter: Binomial       Number of points for filter: 3  Sample start region (min): 0  Sample end region (min): 30
Marker peak width (sec): 5    Marker min peak height: 200   Marker baseline V to V?: Y    Marker baseline V to V points: 3
Lower marker selection: First peak > 500 RFU                Upper marker selection: Last peak > 200 RFU
Ladder size (bp)75, 200, 300, 400, 500, 700, 1000, 1500, 2000, 3000, 4000, 5000, 7000, 10000, 20000
Quantification using: Lower Marker          Final concentration (ng/uL): 0.5000          Dilution factor: 12.0
```

Sample: SampD3
Well location: D3
Created: Thursday, October 21, 2021 7:28:10 PM

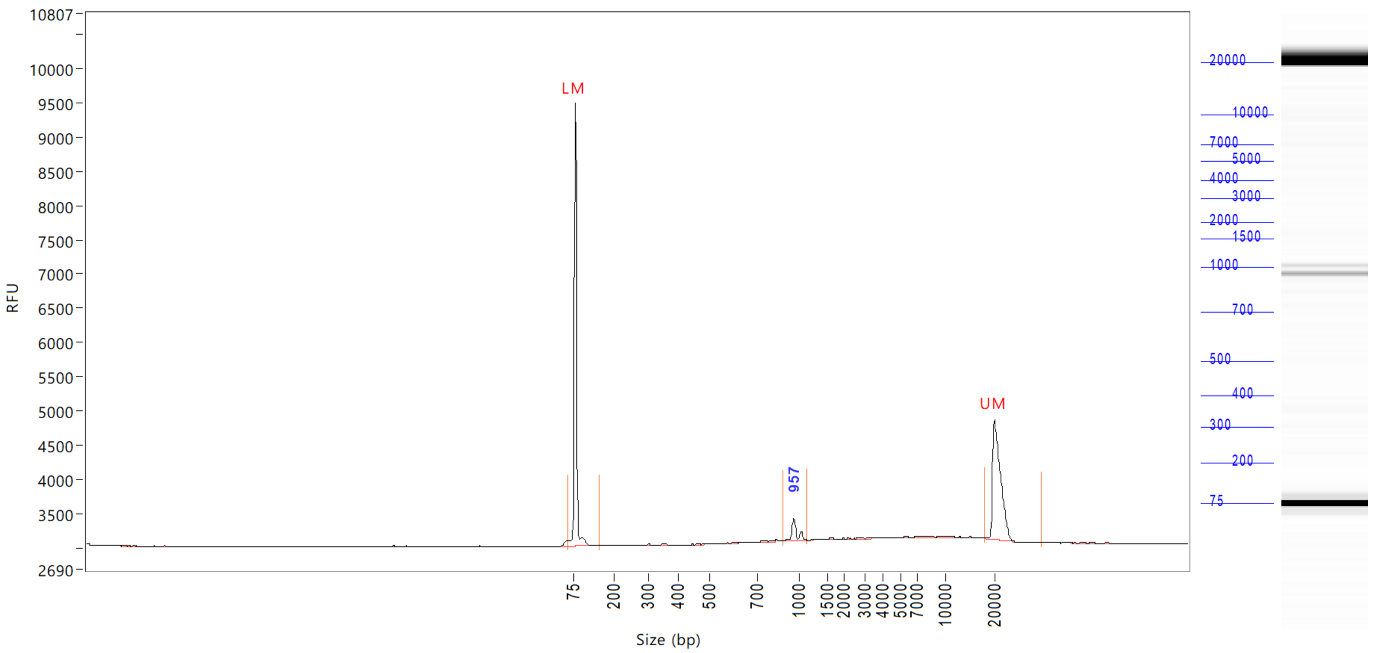

Peak	Size	Concentration	Relative concentration	Molarity	From	To	CV%	RFU
	(bp)	(ng/uL)	Percent	(nmole/L)	(bp)	(bp)		
1	75 (LM)	0.5000		11.0851	56	154	8.72	6480
2	957	0.4305	100.0	0.7327	879	1145	3.86	321
3	20000 (UM)	0.2417		0.0193	18171	29672	4.46	1754

TIC:	0.4305	ng/uL
TIM:	0.7327	nmole/L
Total concentration:	1.0465	ng/uL

Sample peak width (sec): 5   Sample min peak height: 50   Sample baseline V to V?: Y   Sample baseline V to V points: 3
Sample filter: Binomial   Number of points for filter: 3   Sample start region (min): 0   Sample end region (min): 30
Marker peak width (sec): 5   Marker min peak height: 200   Marker baseline V to V?: Y   Marker baseline V to V points: 3
Lower marker selection: First peak > 500 RFU   Upper marker selection: Last peak > 200 RFU
Ladder size (bp)75, 200, 300, 400, 500, 700, 1000, 1500, 2000, 3000, 4000, 5000, 7000, 10000, 20000
Quantification using: Lower Marker   Final concentration (ng/uL): 0.5000   Dilution factor: 12.0

Sample: SampE3
Well location: E3
Created: Thursday, October 21, 2021 7:28:10 PM

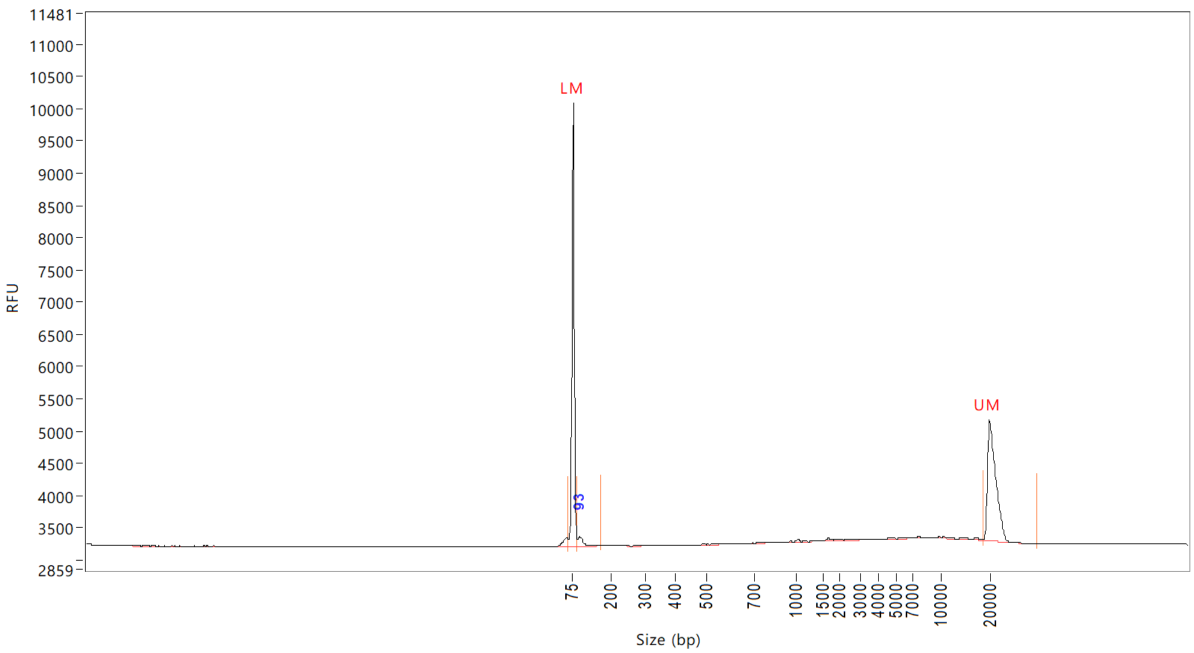

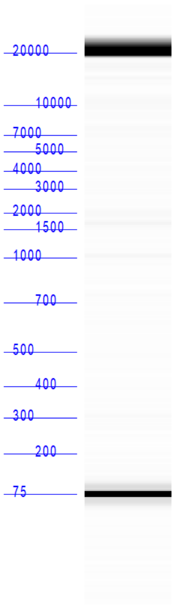

Peak	Size	Concentration	Relative concentration	Molarity	From	To	CV%	RFU
	(bp)	(ng/uL)	Percent	(nmole/L)	(bp)	(bp)		
1	75 (LM)	0.5000		11.2364	62	87	4.61	6888
2	93	0.3268	100.0	5.2108	87	166	15.04	143
3	20000 (UM)	0.2622		0.0209	18903	29672	4.65	1875

TIC:	0.3268	ng/uL
TIM:	5.2108	nmole/L
Total concentration:	0.9951	ng/uL

```
Sample peak width (sec): 5    Sample min peak height: 50    Sample baseline V to V?: Y    Sample baseline V to V points: 3
Sample filter: Binomial       Number of points for filter: 3  Sample start region (min): 0  Sample end region (min): 30
Marker peak width (sec): 5    Marker min peak height: 200   Marker baseline V to V?: Y    Marker baseline V to V points: 3
Lower marker selection: First peak > 500 RFU                Upper marker selection: Last peak > 200 RFU
Ladder size (bp)75, 200, 300, 400, 500, 700, 1000, 1500, 2000, 3000, 4000, 5000, 7000, 10000, 20000
Quantification using: Lower Marker          Final concentration (ng/uL): 0.5000          Dilution factor: 12.0
```

Sample: SampF3
Well location: F3
Created: Thursday, October 21, 2021 7:28:10 PM

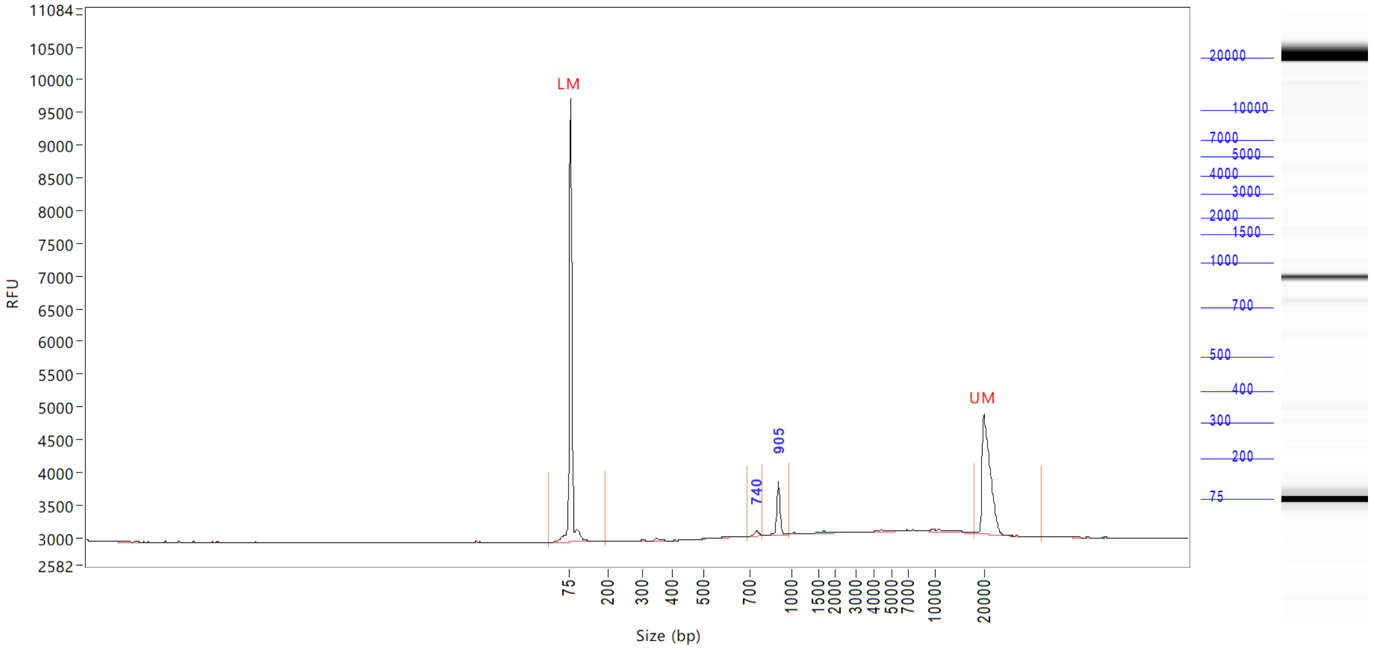

Peak	Size	Concentration	Relative concentration	Molarity	From	To	CV%	RFU
	(bp)	(ng/uL)	Percent	(nmole/L)	(bp)	(bp)		
1	75 (LM)	0.5000		11.0851	7	189	14.18	6787
2	740	0.0891	11.8	0.1983	687	788	2.79	74
3	905	0.6681	88.2	1.2246	788	987	2.39	829
4	20000 (UM)	0.2299		0.0183	18049	31728	5.29	1829

TIC:	0.7572	ng/uL
TIM:	1.4229	nmole/L
Total concentration:	1.3835	ng/uL

Sample peak width (sec): 5 Sample min peak height: 50 Sample baseline V to V?: Y Sample baseline V to V points: 3
Sample filter: Binomial Number of points for filter: 3 Sample start region (min): 0 Sample end region (min): 30
Marker peak width (sec): 5 Marker min peak height: 200 Marker baseline V to V?: Y Marker baseline V to V points: 3
Lower marker selection: First peak > 500 RFU Upper marker selection: Last peak > 200 RFU
Ladder size (bp)75, 200, 300, 400, 500, 700, 1000, 1500, 2000, 3000, 4000, 5000, 7000, 10000, 20000
Quantification using: Lower Marker Final concentration (ng/uL): 0.5000 Dilution factor: 12.0

Sample: SampG3
Well location: G3
Created: Thursday, October 21, 2021 7:28:10 PM

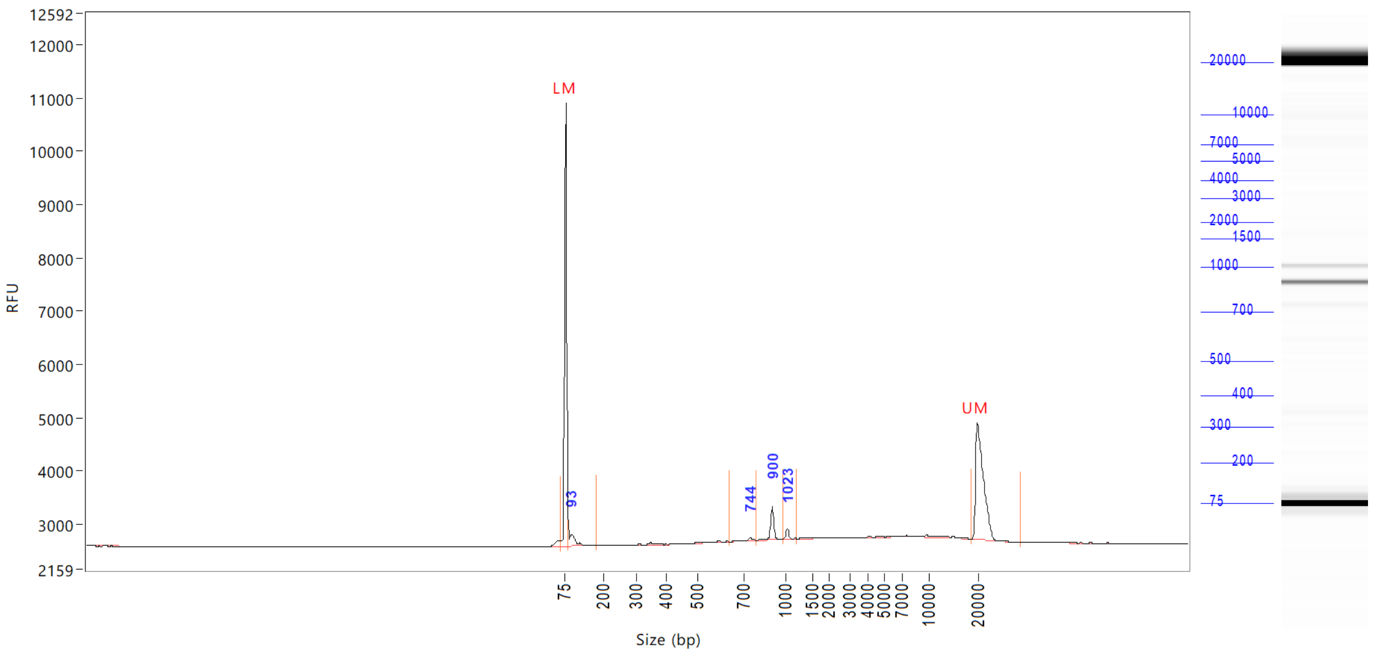

Peak	Size	Concentration	Relative concentration	Molarity	From	To	CV%	RFU
	(bp)	(ng/uL)	Percent	(nmole/L)	(bp)	(bp)		
1	75 (LM)	0.5000		11.2364	60	86	4.46	8332
2	93	0.3993	36.8	6.4928	86	173	14.20	221
3	744	0.0717	6.6	0.1631	636	783	4.54	53
4	900	0.4791	44.1	0.8820	783	978	2.43	640
5	1023	0.1364	12.6	0.2199	978	1212	3.67	190
6	20000 (UM)	0.2481		0.0198	18781	28947	4.48	2179

TIC:	1.0866	ng/uL
TIM:	7.7578	nmole/L
Total concentration:	1.5318	ng/uL

Sample peak width (sec): 5 Sample min peak height: 50 Sample baseline V to V?: Y Sample baseline V to V points: 3
Sample filter: Binomial Number of points for filter: 3 Sample start region (min): 0 Sample end region (min): 30
Marker peak width (sec): 5 Marker min peak height: 200 Marker baseline V to V?: Y Marker baseline V to V points: 3
Lower marker selection: First peak > 500 RFU Upper marker selection: Last peak > 200 RFU
Ladder size (bp)75, 200, 300, 400, 500, 700, 1000, 1500, 2000, 3000, 4000, 5000, 7000, 10000, 20000
Quantification using: Lower Marker Final concentration (ng/uL): 0.5000 Dilution factor: 12.0

Sample: SampH3
Well location: H3
Created: Thursday, October 21, 2021 7:28:10 PM

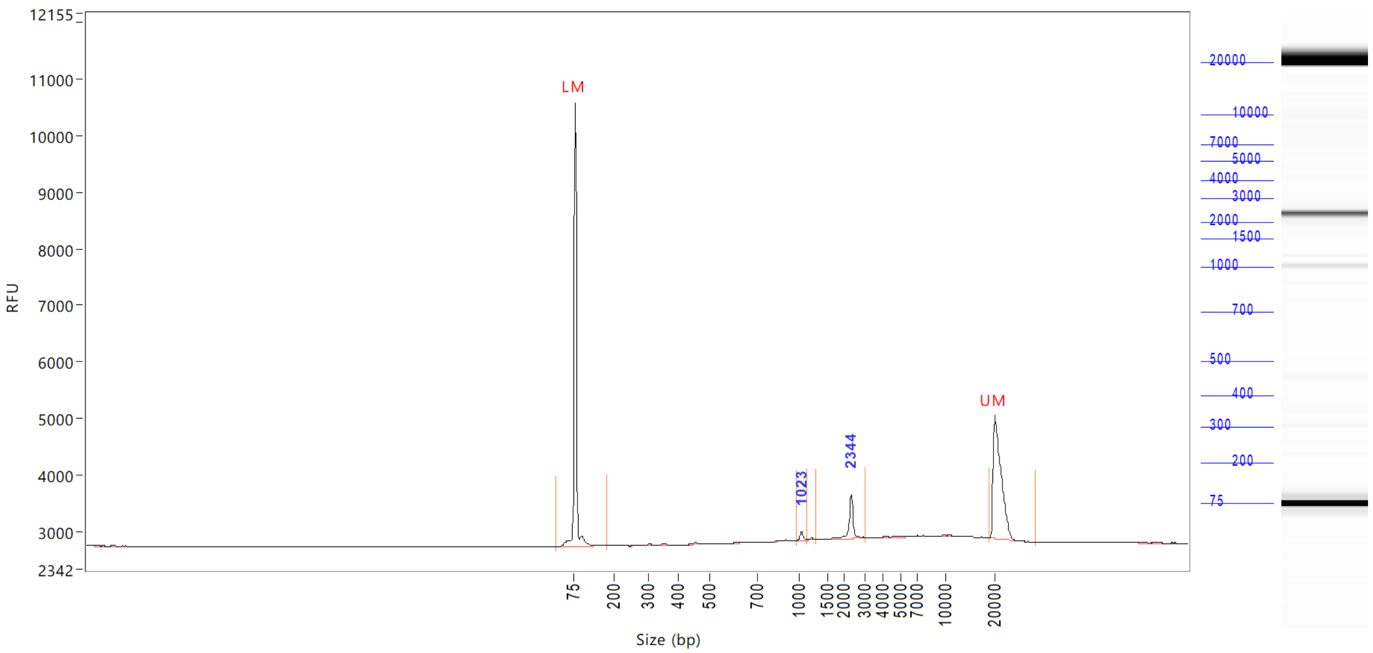

Peak	Size	Concentration	Relative concentration	Molarity	From	To	CV%	RFU
	(bp)	(ng/uL)	Percent	(nmole/L)	(bp)	(bp)		
1	75 (LM)	0.5000		11.2364	16	175	12.82	7839
2	1023	0.0957	13.0	0.1550	978	1112	2.13	146
3	2344	0.6420	87.0	0.4668	1278	3000	10.03	775
4	20000 (UM)	0.2313		0.0185	18903	28221	4.31	2164

TIC:	0.7377	ng/uL
TIM:	0.6218	nmole/L
Total concentration:	1.2323	ng/uL

Sample peak width (sec): 5 Sample min peak height: 50 Sample baseline V to V?: Y Sample baseline V to V points: 3
Sample filter: Binomial Number of points for filter: 3 Sample start region (min): 0 Sample end region (min): 30
Marker peak width (sec): 5 Marker min peak height: 200 Marker baseline V to V?: Y Marker baseline V to V points: 3
Lower marker selection: First peak > 500 RFU Upper marker selection: Last peak > 200 RFU
Ladder size (bp)75, 200, 300, 400, 500, 700, 1000, 1500, 2000, 3000, 4000, 5000, 7000, 10000, 20000
Quantification using: Lower Marker Final concentration (ng/uL): 0.5000 Dilution factor: 12.0

Sample: SampA4
Well location: A4
Created: Thursday, October 21, 2021 7:28:10 PM

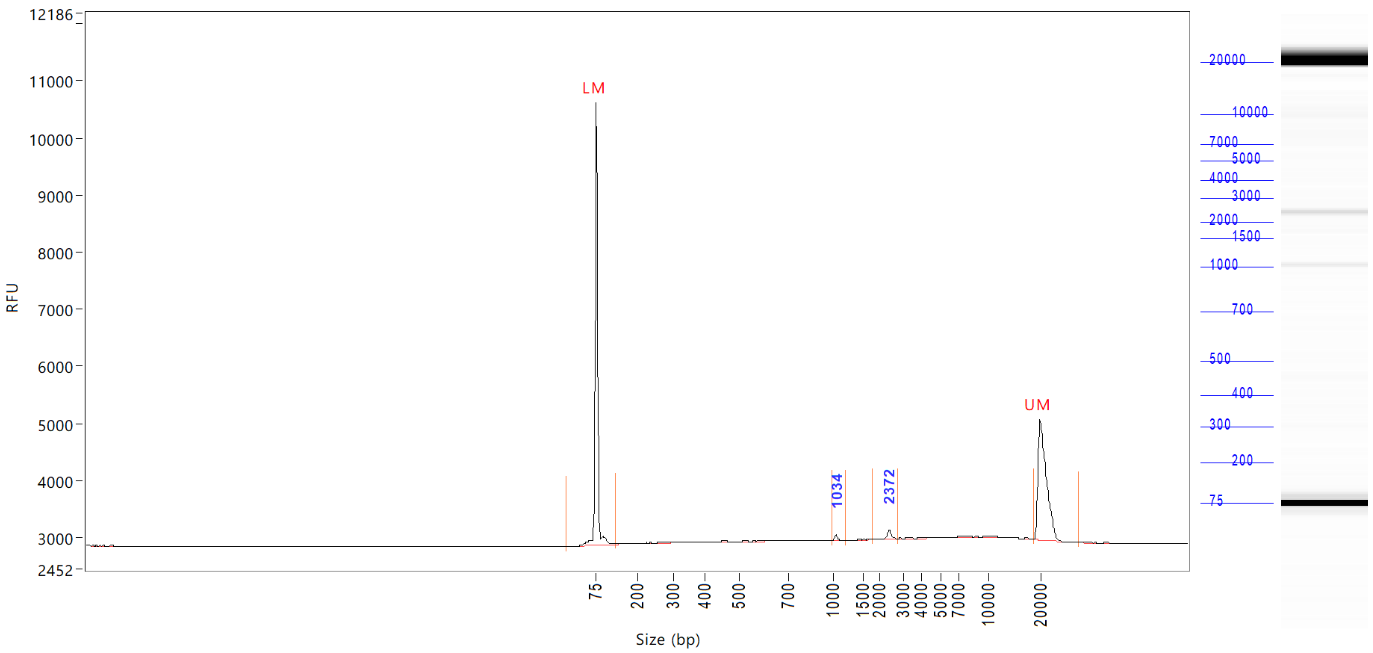

Peak	Size	Concentration	Relative concentration	Molarity	From	To	CV%	RFU
	(bp)	(ng/uL)	Percent	(nmole/L)	(bp)	(bp)		
1	75 (LM)	0.5000		11.2364	0	133	10.95	7762
2	1034	0.0651	28.2	0.1041	991	1190	3.18	94
3	2372	0.1660	71.8	0.1186	1789	2743	8.31	166
4	20000 (UM)	0.2455		0.0197	18781	27496	4.27	2117

TIC:	0.2312	ng/uL
TIM:	0.2226	nmole/L
Total concentration:	0.8060	ng/uL

```
Sample peak width (sec): 5    Sample min peak height: 50    Sample baseline V to V?: Y    Sample baseline V to V points: 3
Sample filter: Binomial       Number of points for filter: 3  Sample start region (min): 0  Sample end region (min): 30
Marker peak width (sec): 5    Marker min peak height: 200   Marker baseline V to V?: Y    Marker baseline V to V points: 3
Lower marker selection: First peak > 500 RFU                Upper marker selection: Last peak > 200 RFU
Ladder size (bp)75, 200, 300, 400, 500, 700, 1000, 1500, 2000, 3000, 4000, 5000, 7000, 10000, 20000
Quantification using: Lower Marker          Final concentration (ng/uL): 0.5000          Dilution factor: 12.0
```

Sample: SampB4
Well location: B4
Created: Thursday, October 21, 2021 7:28:10 PM

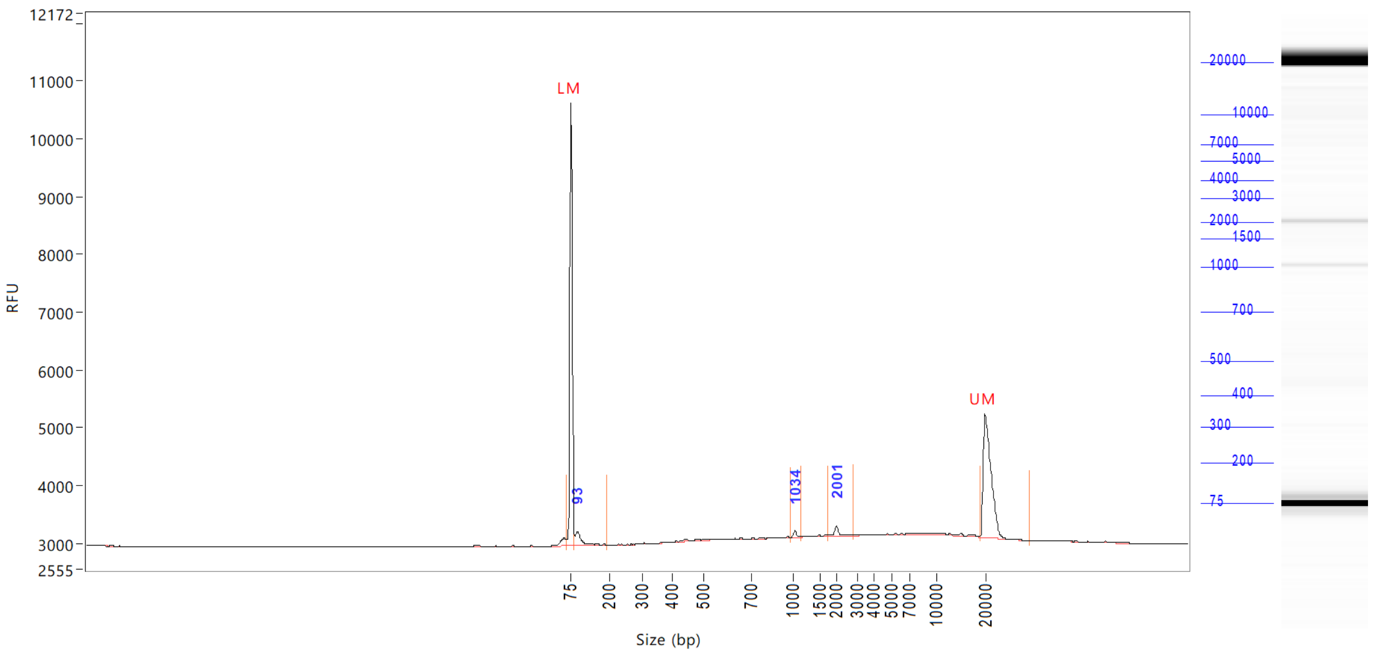

Peak	Size	Concentration	Relative concentration	Molarity	From	To	CV%	RFU
	(bp)	(ng/uL)	Percent	(nmole/L)	(bp)	(bp)		
1	75 (LM)	0.5000		11.2364	62	86	4.59	7673
2	93	0.5329	66.6	8.3350	86	192	19.75	247
3	1034	0.0759	9.5	0.1215	991	1145	2.61	117
4	2001	0.1914	23.9	0.1530	1731	2800	10.31	186
5	20000 (UM)	0.2562		0.0205	18903	29068	4.38	2147

TIC:	0.8001	ng/uL
TIM:	8.6095	nmole/L
Total concentration:	1.4925	ng/uL

```
Sample peak width (sec): 5    Sample min peak height: 50    Sample baseline V to V?: Y    Sample baseline V to V points: 3
Sample filter: Binomial       Number of points for filter: 3  Sample start region (min): 0  Sample end region (min): 30
Marker peak width (sec): 5    Marker min peak height: 200   Marker baseline V to V?: Y    Marker baseline V to V points: 3
Lower marker selection: First peak > 500 RFU                Upper marker selection: Last peak > 200 RFU
Ladder size (bp)75, 200, 300, 400, 500, 700, 1000, 1500, 2000, 3000, 4000, 5000, 7000, 10000, 20000
Quantification using: Lower Marker          Final concentration (ng/uL): 0.5000          Dilution factor: 12.0
```

Sample: SampC4
Well location: C4
Created: Thursday, October 21, 2021 7:28:10 PM

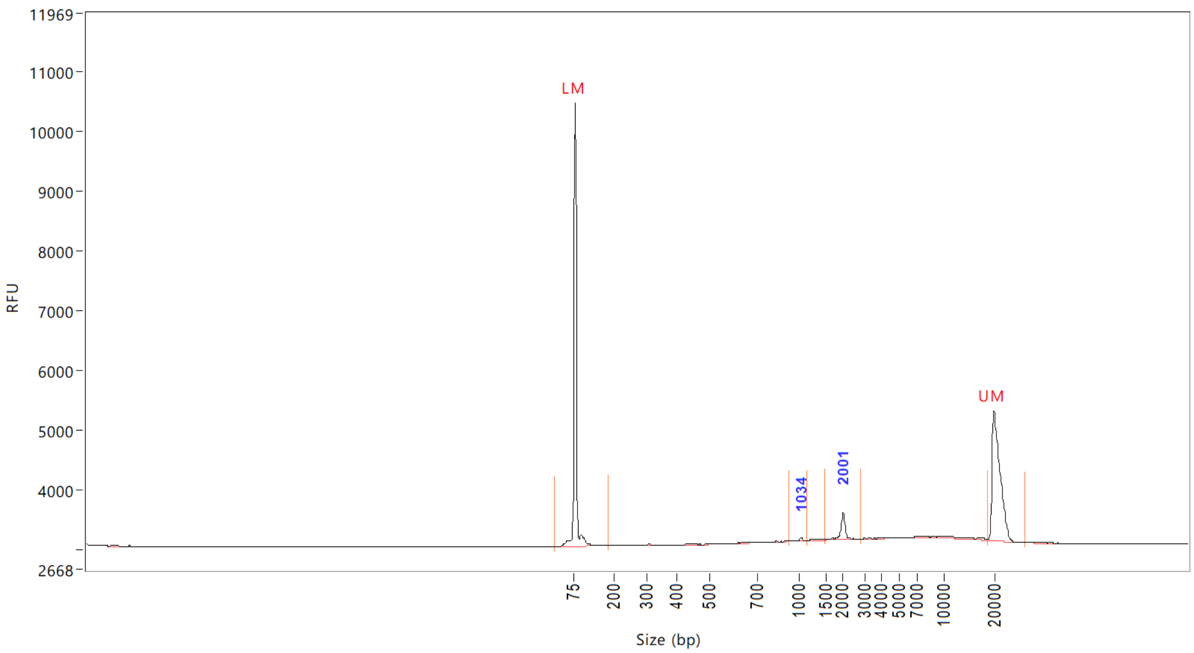

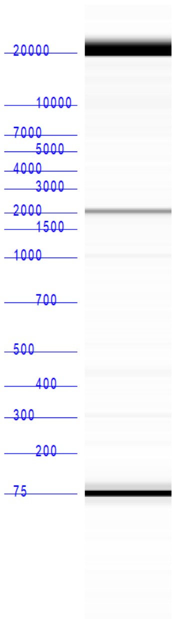

Peak	Size	Concentration	Relative concentration	Molarity	From	To	CV%	RFU
	(bp)	(ng/uL)	Percent	(nmole/L)	(bp)	(bp)		
1	75 (LM)	0.5000		10.9378	14	181	12.36	7428
2	1034	0.0498	10.7	0.0812	926	1134	4.15	50
3	2001	0.4139	89.3	0.3425	1478	2886	9.29	462
4	20000 (UM)	0.2476		0.0198	18903	26408	4.24	2176

TIC:	0.4637	ng/uL
TIM:	0.4237	nmole/L
Total concentration:	1.1030	ng/uL

```
Sample peak width (sec): 5    Sample min peak height: 50    Sample baseline V to V?: Y    Sample baseline V to V points: 3
Sample filter: Binomial       Number of points for filter: 3  Sample start region (min): 0  Sample end region (min): 30
Marker peak width (sec): 5    Marker min peak height: 200   Marker baseline V to V?: Y    Marker baseline V to V points: 3
Lower marker selection: First peak > 500 RFU                Upper marker selection: Last peak > 200 RFU
Ladder size (bp)75, 200, 300, 400, 500, 700, 1000, 1500, 2000, 3000, 4000, 5000, 7000, 10000, 20000
Quantification using: Lower Marker          Final concentration (ng/uL): 0.5000          Dilution factor: 12.0
```

Sample: SampD4
Well location: D4
Created: Thursday, October 21, 2021 7:28:10 PM

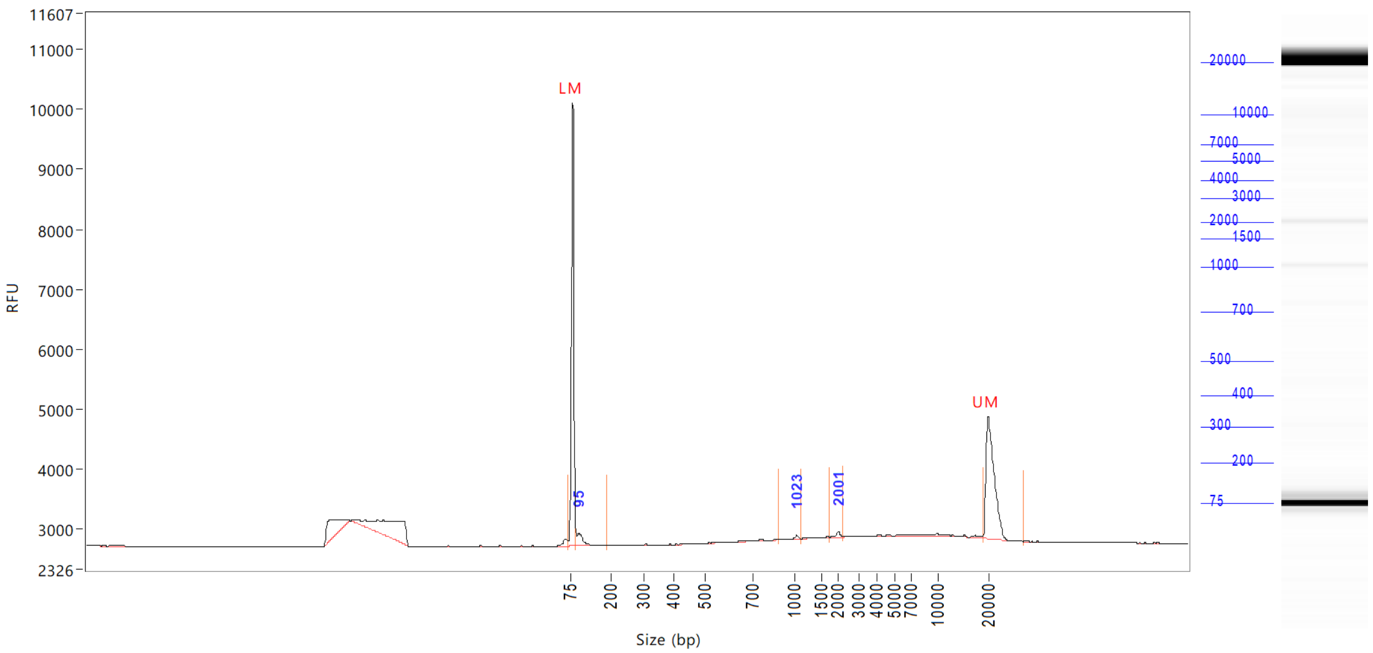

Peak	Size	Concentration	Relative concentration	Molarity	From	To	CV%	RFU
	(bp)	(ng/uL)	Percent	(nmole/L)	(bp)	(bp)		
1	75 (LM)	0.5000		11.0851	62	87	4.54	7409
2	95	0.4741	77.1	7.3462	87	189	17.81	206
3	1023	0.0584	9.5	0.0953	887	1112	3.59	63
4	2001	0.0824	13.4	0.0686	1731	2229	4.57	85
5	20000 (UM)	0.2542		0.0203	19024	27254	4.32	2047

TIC:	0.6149	ng/uL
TIM:	7.5101	nmole/L
Total concentration:	1.1193	ng/uL

```
Sample peak width (sec): 5    Sample min peak height: 50    Sample baseline V to V?: Y    Sample baseline V to V points: 3
Sample filter: Binomial       Number of points for filter: 3  Sample start region (min): 0  Sample end region (min): 30
Marker peak width (sec): 5    Marker min peak height: 200   Marker baseline V to V?: Y    Marker baseline V to V points: 3
Lower marker selection: First peak > 500 RFU                Upper marker selection: Last peak > 200 RFU
Ladder size (bp)75, 200, 300, 400, 500, 700, 1000, 1500, 2000, 3000, 4000, 5000, 7000, 10000, 20000
Quantification using: Lower Marker          Final concentration (ng/uL): 0.5000          Dilution factor: 12.0
```

Sample: SampE4
Well location: E4
Created: Thursday, October 21, 2021 7:28:10 PM

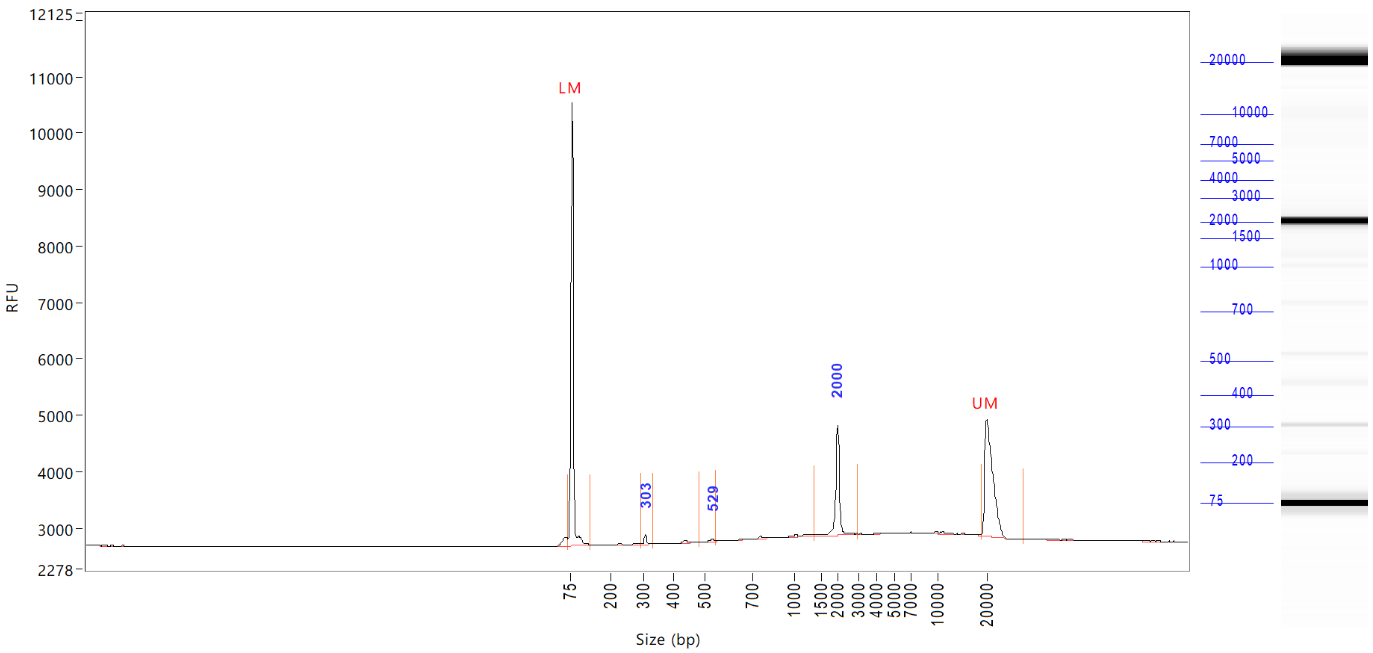

Peak	Size	Concentration	Relative concentration	Molarity	From	To	CV%	RFU
	(bp)	(ng/uL)	Percent	(nmole/L)	(bp)	(bp)		
1	75 (LM)	0.5000		11.0851	62	133	9.38	7861
2	303	0.1121	6.4	0.6105	293	331	1.34	161
3	529	0.0514	2.9	0.1622	481	544	2.06	56
4	2000	1.5905	90.7	1.3143	1378	2971	7.84	1955
5	20000 (UM)	0.2387		0.0191	18903	27496	4.42	2071

TIC:	1.7540	ng/uL
TIM:	2.0870	nmole/L
Total concentration:	2.3536	ng/uL

```
Sample peak width (sec): 5    Sample min peak height: 50    Sample baseline V to V?: Y    Sample baseline V to V points: 3
Sample filter: Binomial       Number of points for filter: 3  Sample start region (min): 0  Sample end region (min): 30
Marker peak width (sec): 5    Marker min peak height: 200   Marker baseline V to V?: Y    Marker baseline V to V points: 3
Lower marker selection: First peak > 500 RFU                Upper marker selection: Last peak > 200 RFU
Ladder size (bp)75, 200, 300, 400, 500, 700, 1000, 1500, 2000, 3000, 4000, 5000, 7000, 10000, 20000
Quantification using: Lower Marker          Final concentration (ng/uL): 0.5000          Dilution factor: 12.0
```

Sample: SampF4
Well location: F4
Created: Thursday, October 21, 2021 7:28:10 PM

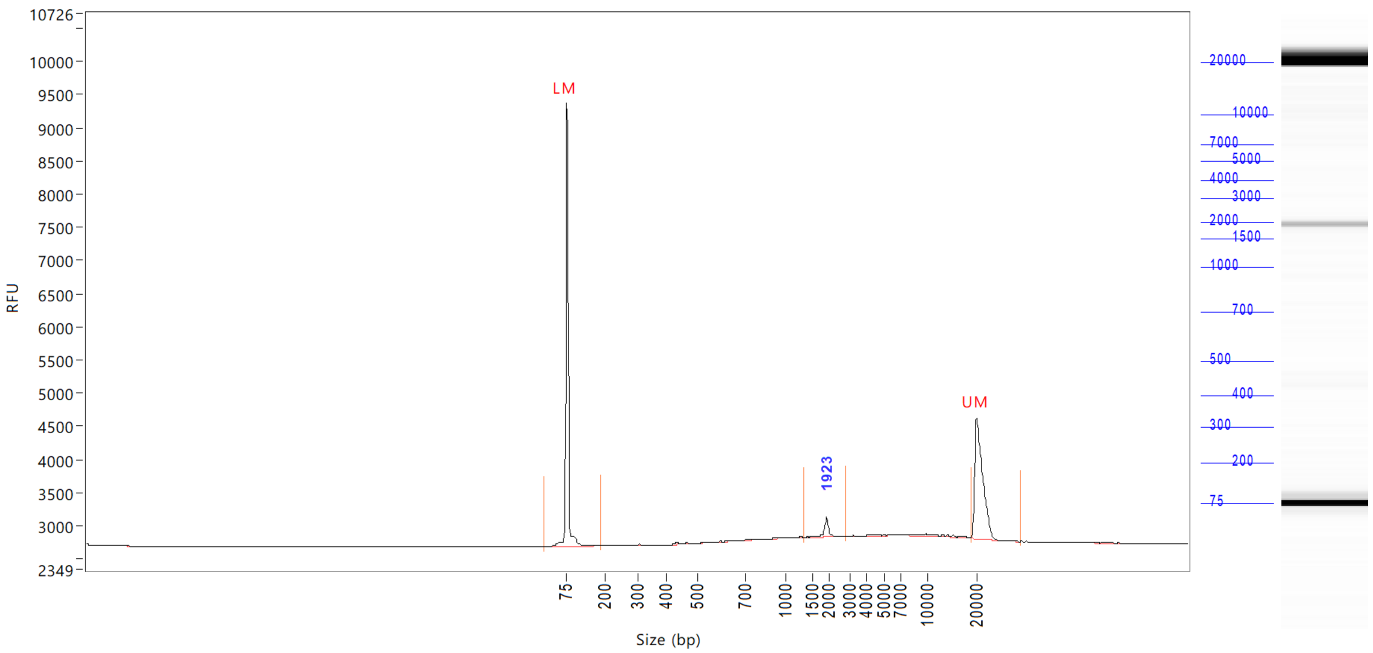

Peak	Size	Concentration	Relative concentration	Molarity	From	To	CV%	RFU
	(bp)	(ng/uL)	Percent	(nmole/L)	(bp)	(bp)		
1	75 (LM)	0.5000		10.9378	1	185	15.05	6694
2	1923	0.3292	100.0	0.2860	1334	2829	10.37	294
3	20000 (UM)	0.2350		0.0188	18903	29068	4.65	1814

TIC:	0.3292	ng/uL
TIM:	0.2860	nmole/L
Total concentration:	0.8841	ng/uL

Sample peak width (sec): 5   Sample min peak height: 50   Sample baseline V to V?: Y   Sample baseline V to V points: 3
Sample filter: Binomial   Number of points for filter: 3   Sample start region (min): 0   Sample end region (min): 30
Marker peak width (sec): 5   Marker min peak height: 200   Marker baseline V to V?: Y   Marker baseline V to V points: 3
Lower marker selection: First peak > 500 RFU   Upper marker selection: Last peak > 200 RFU
Ladder size (bp)75, 200, 300, 400, 500, 700, 1000, 1500, 2000, 3000, 4000, 5000, 7000, 10000, 20000
Quantification using: Lower Marker   Final concentration (ng/uL): 0.5000   Dilution factor: 12.0

Sample: SampG4
Well location: G4
Created: Thursday, October 21, 2021 7:28:10 PM

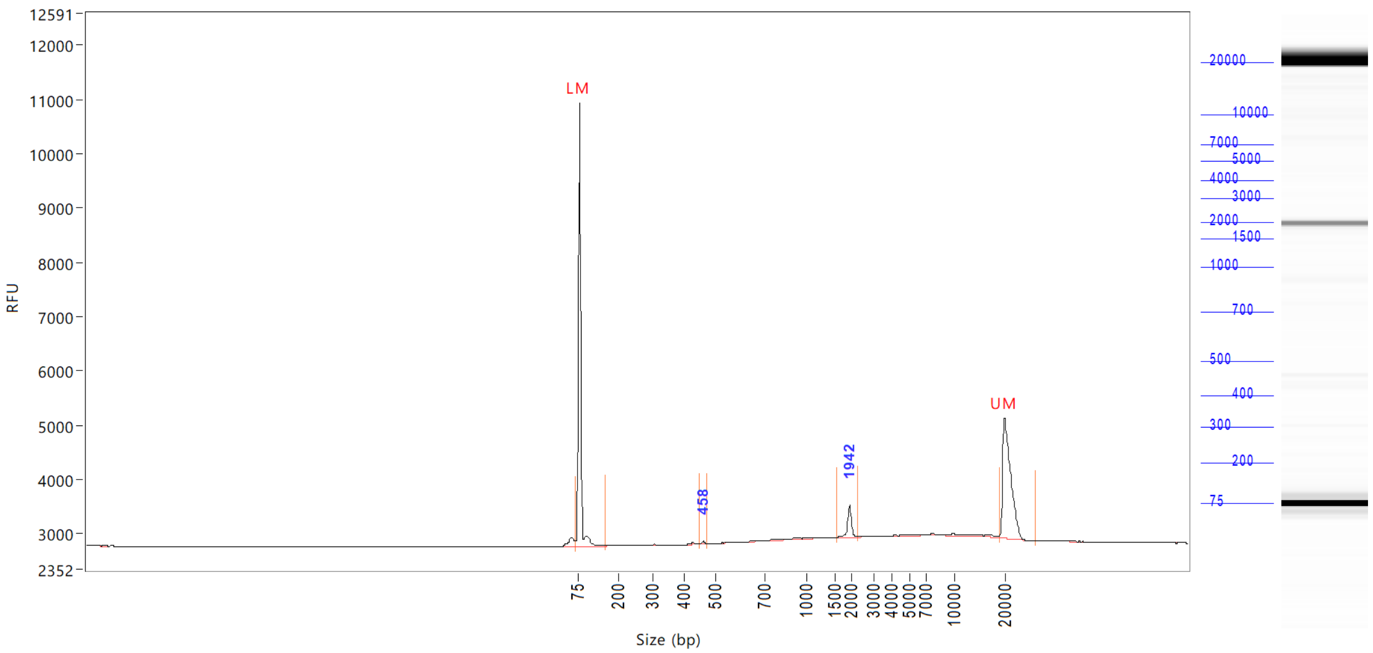

Peak	Size	Concentration	Relative concentration	Molarity	From	To	CV%	RFU
	(bp)	(ng/uL)	Percent	(nmole/L)	(bp)	(bp)		
1	75 (LM)	0.5000		11.0851	62	158	11.22	8181
2	458	0.0337	7.0	0.1216	449	470	0.83	51
3	1942	0.4507	93.0	0.3879	1520	2258	4.85	598
4	20000 (UM)	0.2422		0.0194	18903	25924	4.20	2205

TIC:	0.4844	ng/uL
TIM:	0.5094	nmole/L
Total concentration:	1.1355	ng/uL

```
Sample peak width (sec): 5    Sample min peak height: 50    Sample baseline V to V?: Y    Sample baseline V to V points: 3
Sample filter: Binomial       Number of points for filter: 3  Sample start region (min): 0  Sample end region (min): 30
Marker peak width (sec): 5    Marker min peak height: 200   Marker baseline V to V?: Y    Marker baseline V to V points: 3
Lower marker selection: First peak > 500 RFU                Upper marker selection: Last peak > 200 RFU
Ladder size (bp)75, 200, 300, 400, 500, 700, 1000, 1500, 2000, 3000, 4000, 5000, 7000, 10000, 20000
Quantification using: Lower Marker          Final concentration (ng/uL): 0.5000          Dilution factor: 12.0
```

Sample: SampH4
Well location: H4
Created: Thursday, October 21, 2021 7:28:10 PM

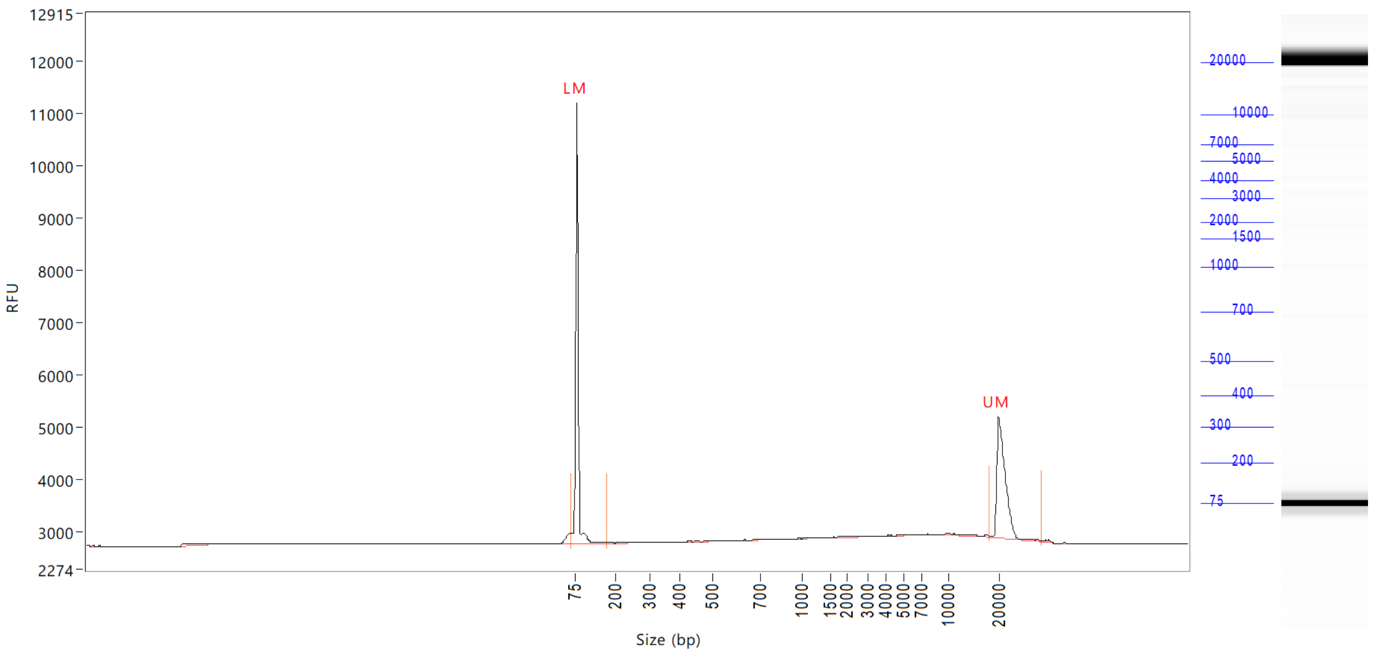

Peak	Size	Concentration	Relative concentration	Molarity	From	To	CV%	RFU
	(bp)	(ng/uL)	Percent	(nmole/L)	(bp)	(bp)		
1	75 (LM)	0.5000		10.9378	60	171	12.38	8448
2	20000 (UM)	0.2411		0.0191	18171	28584	5.85	2316

TIC:	0.0000	ng/uL
TIM:	0.0000	nmole/L
Total concentration:	0.4844	ng/uL

```
Sample peak width (sec): 5    Sample min peak height: 50    Sample baseline V to V?: Y    Sample baseline V to V points: 3
Sample filter: Binomial       Number of points for filter: 3  Sample start region (min): 0  Sample end region (min): 30
Marker peak width (sec): 5    Marker min peak height: 200   Marker baseline V to V?: Y    Marker baseline V to V points: 3
Lower marker selection: First peak > 500 RFU                Upper marker selection: Last peak > 200 RFU
Ladder size (bp)75, 200, 300, 400, 500, 700, 1000, 1500, 2000, 3000, 4000, 5000, 7000, 10000, 20000
Quantification using: Lower Marker          Final concentration (ng/uL): 0.5000          Dilution factor: 12.0
```

Sample: SampA5
Well location: A5
Created: Thursday, October 21, 2021 7:28:10 PM

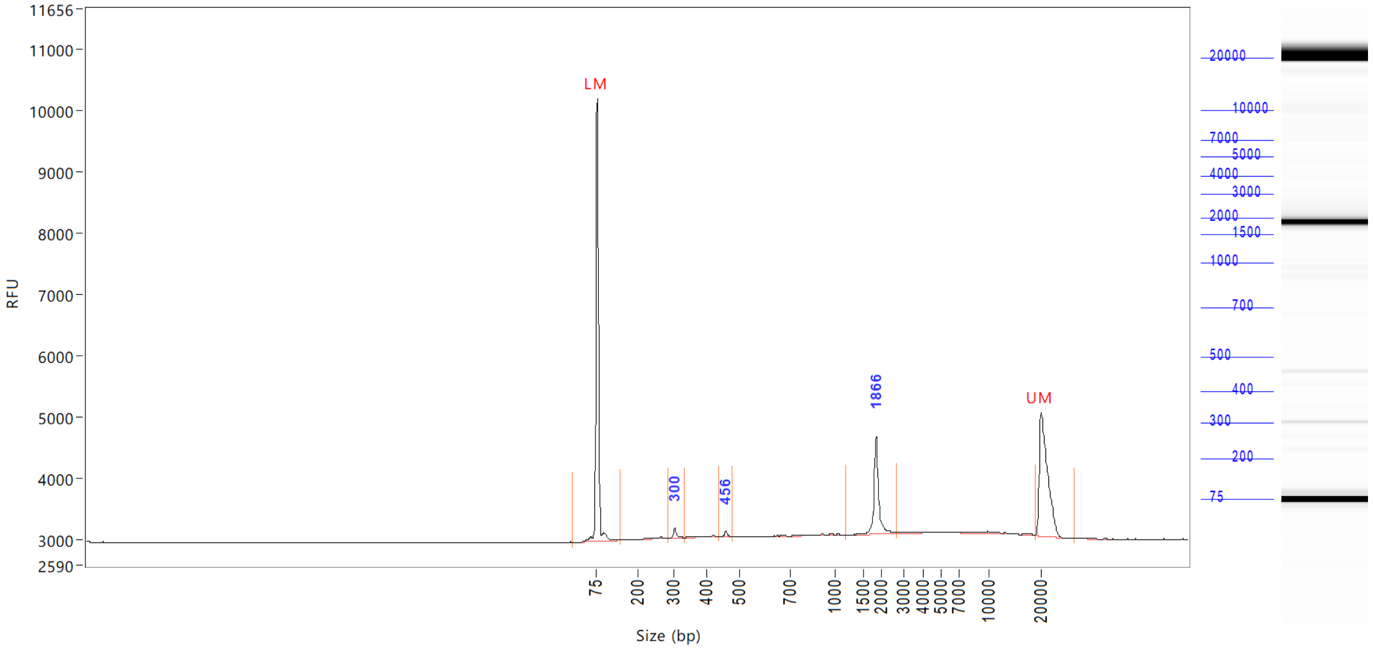

Peak	Size	Concentration	Relative concentration	Molarity	From	To	CV%	RFU
	(bp)	(ng/uL)	Percent	(nmole/L)	(bp)	(bp)		
1	75 (LM)	0.5000		11.2364	0	143	9.80	7228
2	300	0.1457	7.9	0.8018	280	329	2.57	160
3	456	0.0700	3.8	0.2532	437	479	1.17	87
4	1866	1.6368	88.4	1.4486	1190	2657	8.54	1593
5	20000 (UM)	0.2497		0.0199	18903	26408	4.20	2003

TIC:	1.8526	ng/uL
TIM:	2.5037	nmole/L
Total concentration:	2.4709	ng/uL

```
Sample peak width (sec): 5    Sample min peak height: 50    Sample baseline V to V?: Y    Sample baseline V to V points: 3
Sample filter: Binomial       Number of points for filter: 3  Sample start region (min): 0  Sample end region (min): 30
Marker peak width (sec): 5    Marker min peak height: 200   Marker baseline V to V?: Y    Marker baseline V to V points: 3
Lower marker selection: First peak > 500 RFU                Upper marker selection: Last peak > 200 RFU
Ladder size (bp)75, 200, 300, 400, 500, 700, 1000, 1500, 2000, 3000, 4000, 5000, 7000, 10000, 20000
Quantification using: Lower Marker          Final concentration (ng/uL): 0.5000          Dilution factor: 12.0
```

Sample: SampB5
Well location: B5
Created: Thursday, October 21, 2021 7:28:10 PM

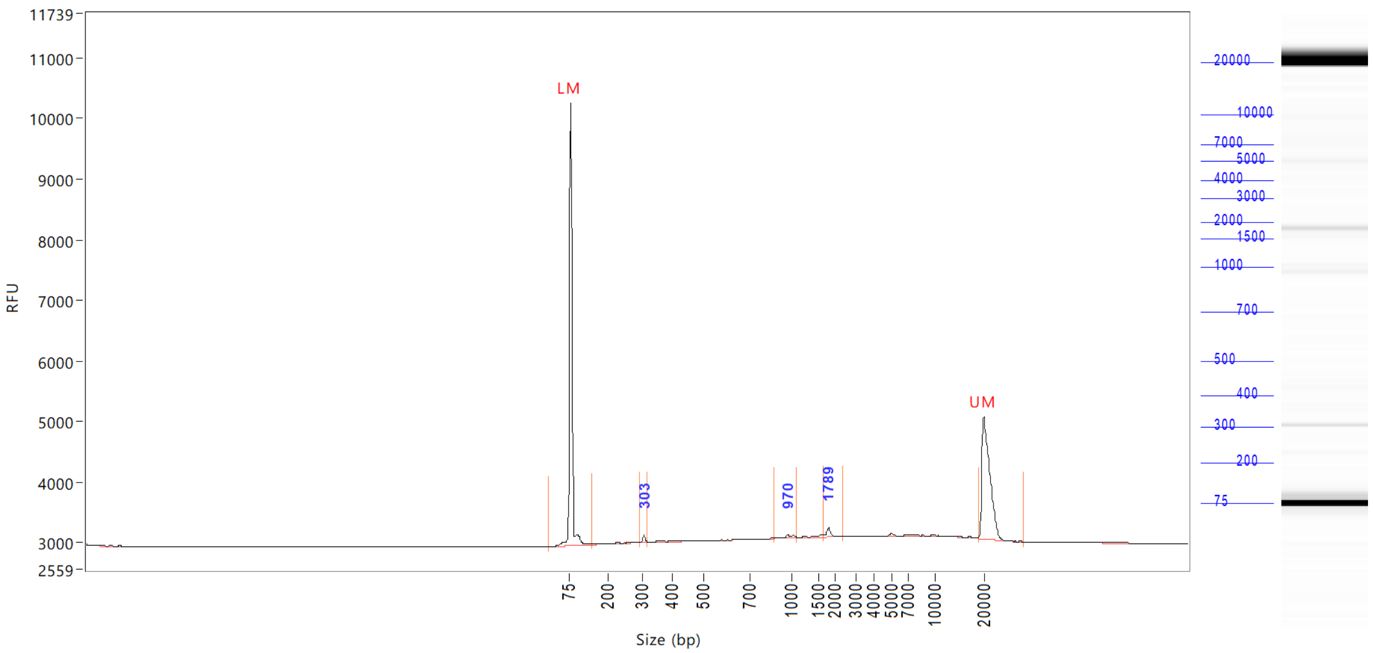

Peak	Size	Concentration	Relative concentration	Molarity	From	To	CV%	RFU
	(bp)	(ng/uL)	Percent	(nmole/L)	(bp)	(bp)		
1	75 (LM)	0.5000		10.9378	5	147	11.96	7324
2	303	0.0936	29.9	0.5098	291	317	1.38	130
3	970	0.0771	24.7	0.1287	874	1101	3.65	57
4	1789	0.1420	45.4	0.1293	1635	2372	6.75	148
5	20000 (UM)	0.2354		0.0188	18903	28100	4.50	2018

TIC:	0.3127	ng/uL
TIM:	0.7678	nmole/L
Total concentration:	0.8659	ng/uL

```
Sample peak width (sec): 5    Sample min peak height: 50    Sample baseline V to V?: Y    Sample baseline V to V points: 3
Sample filter: Binomial       Number of points for filter: 3  Sample start region (min): 0  Sample end region (min): 30
Marker peak width (sec): 5    Marker min peak height: 200   Marker baseline V to V?: Y    Marker baseline V to V points: 3
Lower marker selection: First peak > 500 RFU                Upper marker selection: Last peak > 200 RFU
Ladder size (bp)75, 200, 300, 400, 500, 700, 1000, 1500, 2000, 3000, 4000, 5000, 7000, 10000, 20000
Quantification using: Lower Marker          Final concentration (ng/uL): 0.5000          Dilution factor: 12.0
```

Sample: SampC5
Well location: C5
Created: Thursday, October 21, 2021 7:28:10 PM

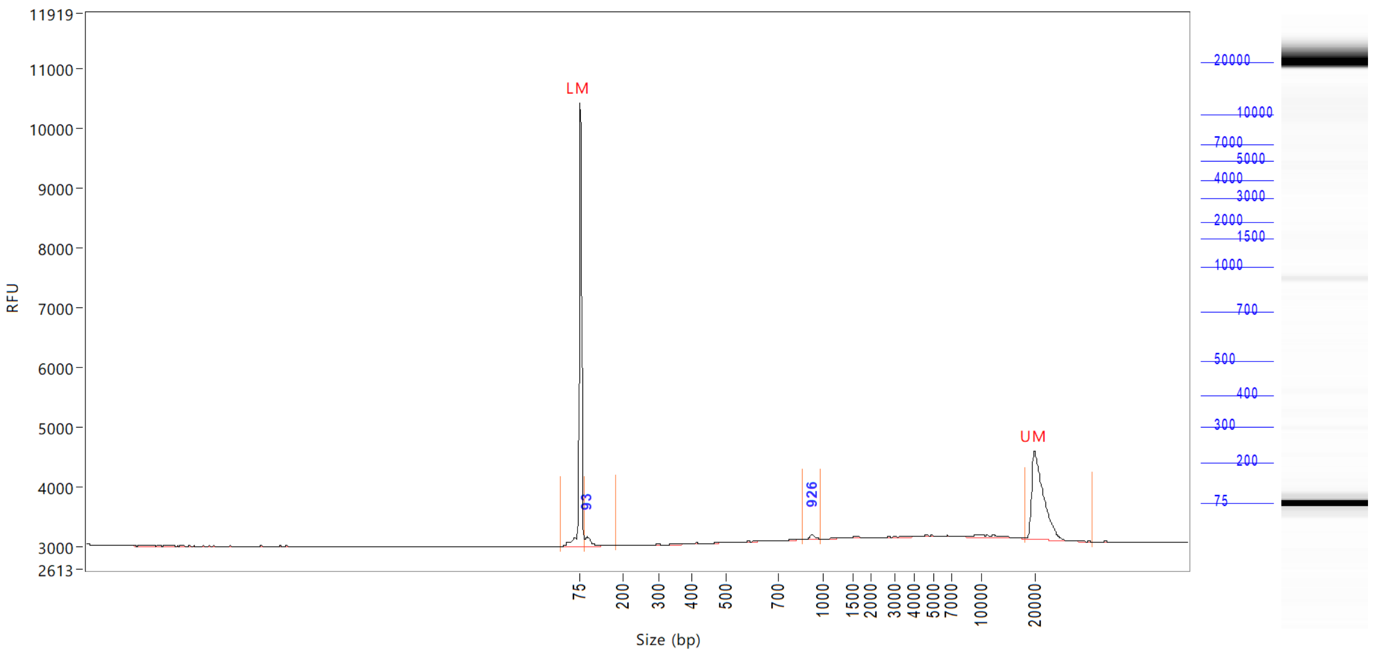

Peak	Size	Concentration	Relative concentration	Molarity	From	To	CV%	RFU
	(bp)	(ng/uL)	Percent	(nmole/L)	(bp)	(bp)		
1	75 (LM)	0.5000		11.3919	20	87	9.13	7434
2	93	0.3423	78.0	5.3040	87	181	19.25	154
3	926	0.0967	22.0	0.1726	861	987	2.17	89
4	20000 (UM)	0.2407		0.0190	18171	30760	6.46	1485

TIC:	0.4390	ng/uL
TIM:	5.4765	nmole/L
Total concentration:	1.1103	ng/uL

Sample peak width (sec): 5   Sample min peak height: 50   Sample baseline V to V?: Y   Sample baseline V to V points: 3
Sample filter: Binomial   Number of points for filter: 3   Sample start region (min): 0   Sample end region (min): 30
Marker peak width (sec): 5   Marker min peak height: 200   Marker baseline V to V?: Y   Marker baseline V to V points: 3
Lower marker selection: First peak > 500 RFU   Upper marker selection: Last peak > 200 RFU
Ladder size (bp)75, 200, 300, 400, 500, 700, 1000, 1500, 2000, 3000, 4000, 5000, 7000, 10000, 20000
Quantification using: Lower Marker   Final concentration (ng/uL): 0.5000   Dilution factor: 12.0

Sample: SampD5
Well location: D5
Created: Thursday, October 21, 2021 7:28:10 PM

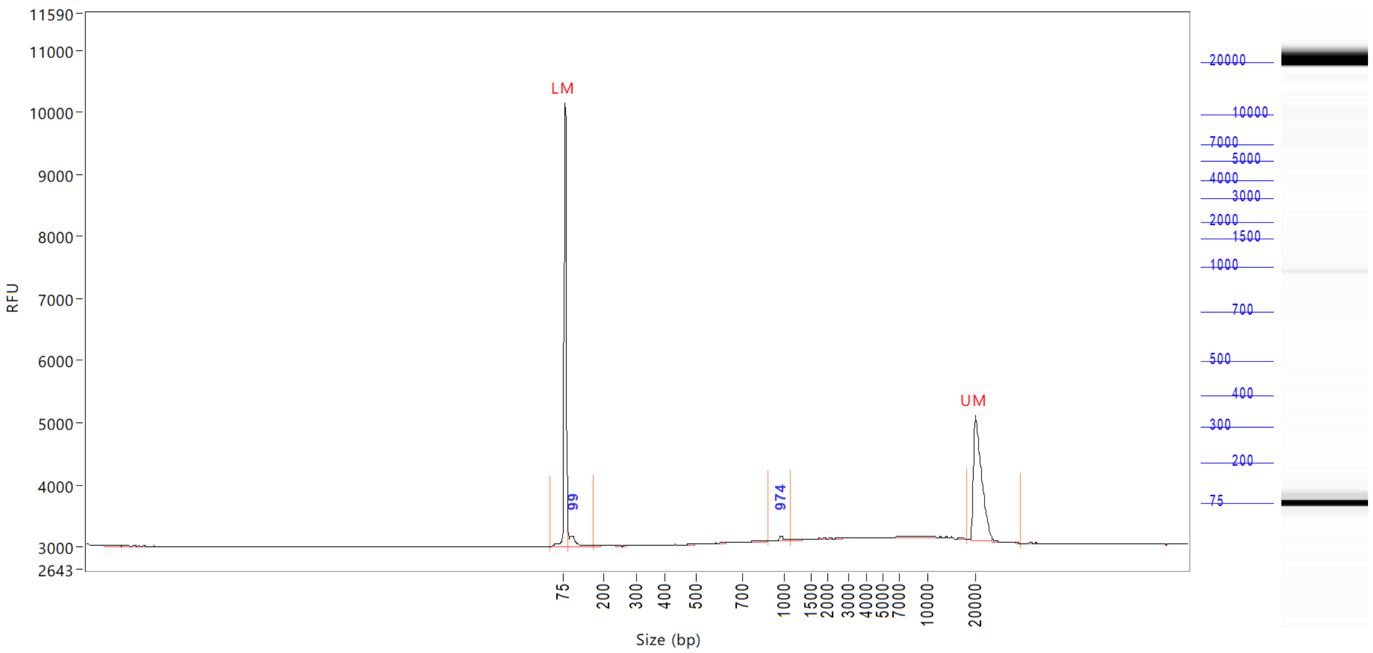

Peak	Size	Concentration	Relative concentration	Molarity	From	To	CV%	RFU
	(bp)	(ng/uL)	Percent	(nmole/L)	(bp)	(bp)		
1	75 (LM)	0.5000		11.2364	28	87	7.13	7149
2	99	0.3755	78.2	5.9289	87	169	16.27	163
3	974	0.1046	21.8	0.1754	883	1112	4.17	79
4	20000 (UM)	0.2614		0.0209	18293	29430	4.78	2022

TIC:	0.4801	ng/uL
TIM:	6.1043	nmole/L
Total concentration:	1.0539	ng/uL

Sample peak width (sec): 5   Sample min peak height: 50   Sample baseline V to V?: Y   Sample baseline V to V points: 3
Sample filter: Binomial   Number of points for filter: 3 Sample start region (min): 0 Sample end region (min): 30
Marker peak width (sec): 5   Marker min peak height: 200   Marker baseline V to V?: Y   Marker baseline V to V points: 3
Lower marker selection: First peak > 500 RFU   Upper marker selection: Last peak > 200 RFU
Ladder size (bp)75, 200, 300, 400, 500, 700, 1000, 1500, 2000, 3000, 4000, 5000, 7000, 10000, 20000
Quantification using: Lower Marker   Final concentration (ng/uL): 0.5000   Dilution factor: 12.0

Sample: SampE5
Well location: E5
Created: Thursday, October 21, 2021 7:28:10 PM

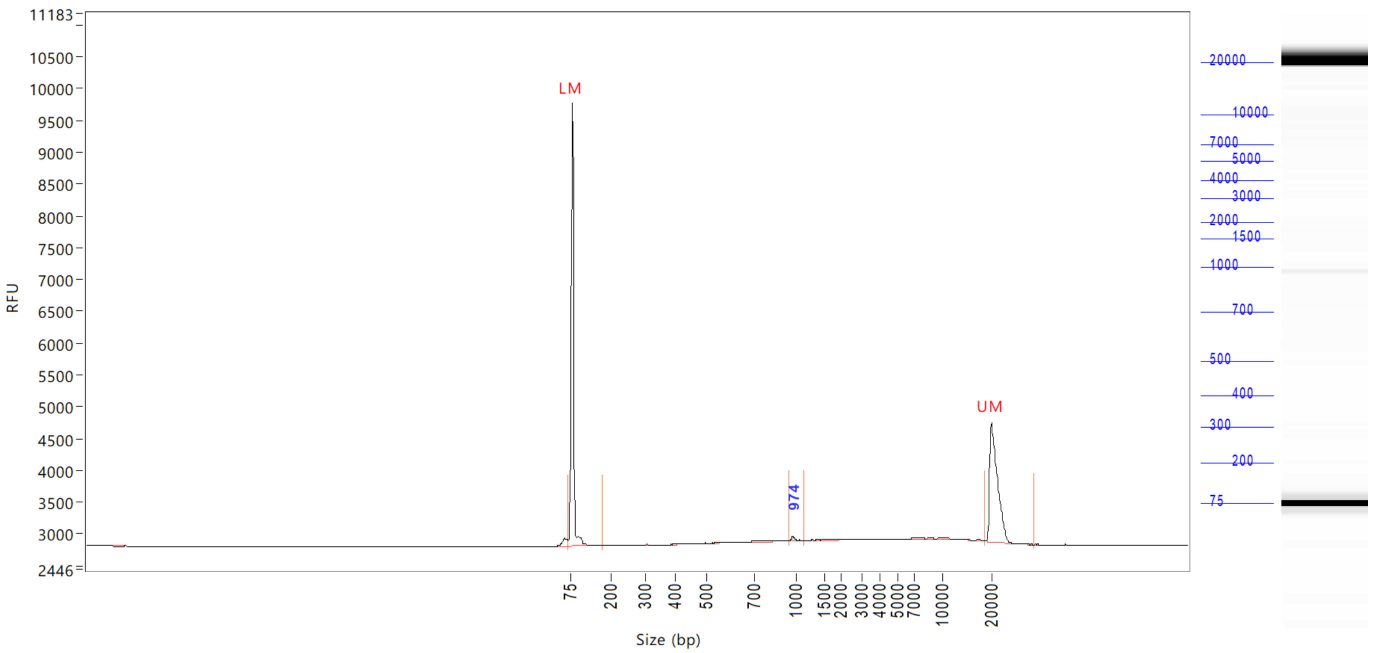

Peak	Size	Concentration	Relative concentration	Molarity	From	To	CV%	RFU
	(bp)	(ng/uL)	Percent	(nmole/L)	(bp)	(bp)		
1	75 (LM)	0.5000		11.0851	62	171	9.99	6975
2	974	0.0634	100.0	0.1064	944	1134	3.09	69
3	20000 (UM)	0.2402		0.0192	18659	28463	4.43	1871

TIC:	0.0634	ng/uL
TIM:	0.1064	nmole/L
Total concentration:	0.5630	ng/uL

Sample peak width (sec): 5 Sample min peak height: 50 Sample baseline V to V?: Y Sample baseline V to V points: 3
Sample filter: Binomial Number of points for filter: 3 Sample start region (min): 0 Sample end region (min): 30
Marker peak width (sec): 5 Marker min peak height: 200 Marker baseline V to V?: Y Marker baseline V to V points: 3
Lower marker selection: First peak > 500 RFU Upper marker selection: Last peak > 200 RFU
Ladder size (bp)75, 200, 300, 400, 500, 700, 1000, 1500, 2000, 3000, 4000, 5000, 7000, 10000, 20000
Quantification using: Lower Marker Final concentration (ng/uL): 0.5000 Dilution factor: 12.0

Sample: SampF5
Well location: F5
Created: Thursday, October 21, 2021 7:28:10 PM

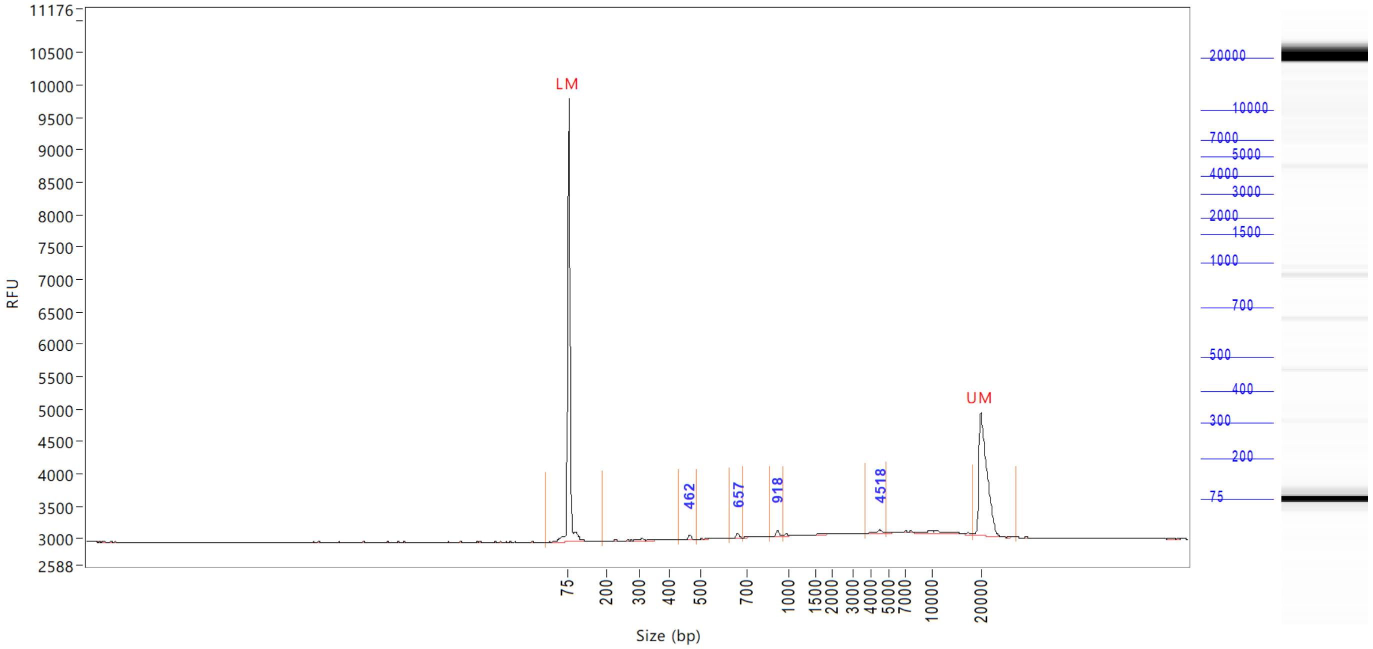

Peak	Size	Concentration	Relative concentration	Molarity	From	To	CV%	RFU
	(bp)	(ng/uL)	Percent	(nmole/L)	(bp)	(bp)		
1	75 (LM)	0.5000		10.9378	3	183	13.25	6854
2	462	0.0574	22.6	0.2049	428	487	1.74	66
3	657	0.0543	21.4	0.1371	623	680	1.22	67
4	918	0.0767	30.2	0.1382	857	952	1.49	98
5	4518	0.0657	25.8	0.0247	3643	4897	5.78	55
6	20000 (UM)	0.2345		0.0188	18171	27133	4.56	1893

TIC:	0.2541	ng/uL
TIM:	0.5049	nmole/L
Total concentration:	0.8125	ng/uL

Sample peak width (sec): 5   Sample min peak height: 50   Sample baseline V to V?: Y   Sample baseline V to V points: 3
Sample filter: Binomial   Number of points for filter: 3 Sample start region (min): 0 Sample end region (min): 30
Marker peak width (sec): 5   Marker min peak height: 200   Marker baseline V to V?: Y   Marker baseline V to V points: 3
Lower marker selection: First peak > 500 RFU   Upper marker selection: Last peak > 200 RFU
Ladder size (bp)75, 200, 300, 400, 500, 700, 1000, 1500, 2000, 3000, 4000, 5000, 7000, 10000, 20000
Quantification using: Lower Marker   Final concentration (ng/uL): 0.5000   Dilution factor: 12.0

Sample: SampG5
Well location: G5
Created: Thursday, October 21, 2021 7:28:10 PM

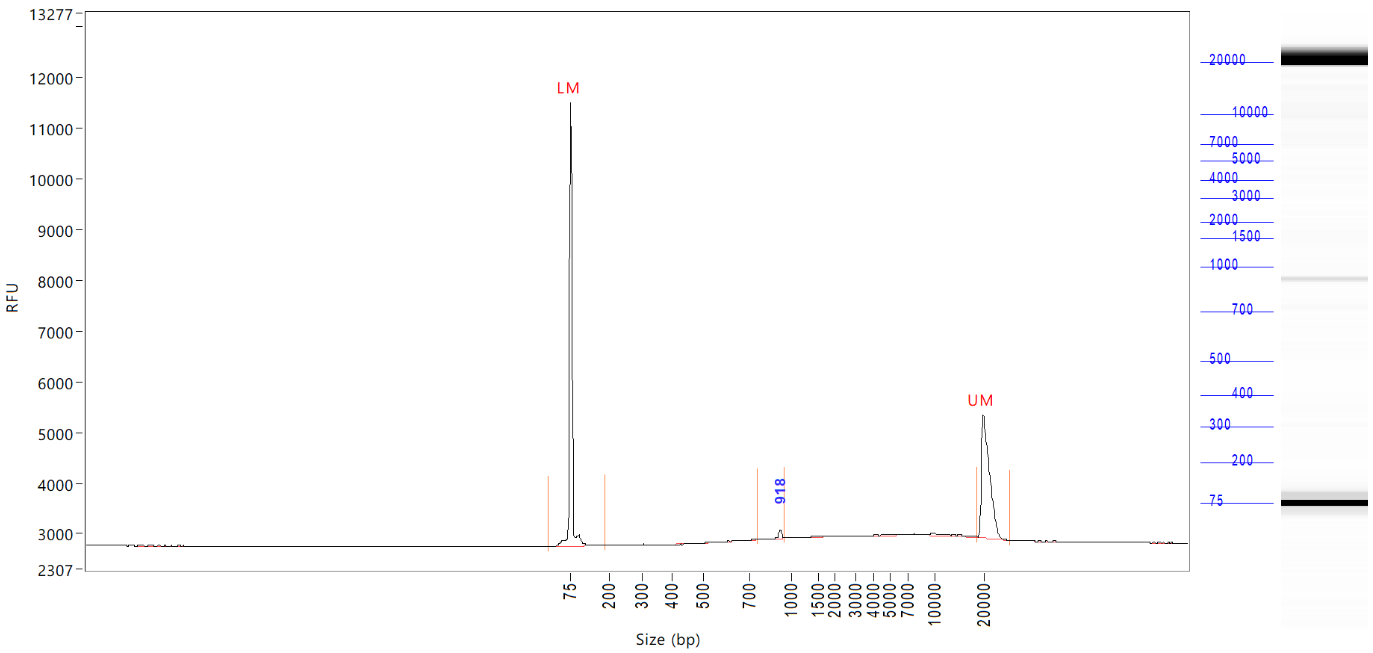

Peak	Size	Concentration	Relative concentration	Molarity	From	To	CV%	RFU
	(bp)	(ng/uL)	Percent	(nmole/L)	(bp)	(bp)		
1	75 (LM)	0.5000		11.0851	3	187	13.98	8762
2	918	0.1121	100.0	0.2048	753	948	4.40	171
3	20000 (UM)	0.2388		0.0190	18903	25561	4.29	2421

TIC:	0.1121	ng/uL
TIM:	0.2048	nmole/L
Total concentration:	0.5767	ng/uL

Sample peak width (sec): 5 Sample min peak height: 50 Sample baseline V to V?: Y Sample baseline V to V points: 3
Sample filter: Binomial Number of points for filter: 3 Sample start region (min): 0 Sample end region (min): 30
Marker peak width (sec): 5 Marker min peak height: 200 Marker baseline V to V?: Y Marker baseline V to V points: 3
Lower marker selection: First peak > 500 RFU Upper marker selection: Last peak > 200 RFU
Ladder size (bp)75, 200, 300, 400, 500, 700, 1000, 1500, 2000, 3000, 4000, 5000, 7000, 10000, 20000
Quantification using: Lower Marker Final concentration (ng/uL): 0.5000 Dilution factor: 12.0

Sample: SampH5
Well location: H5
Created: Thursday, October 21, 2021 7:28:10 PM

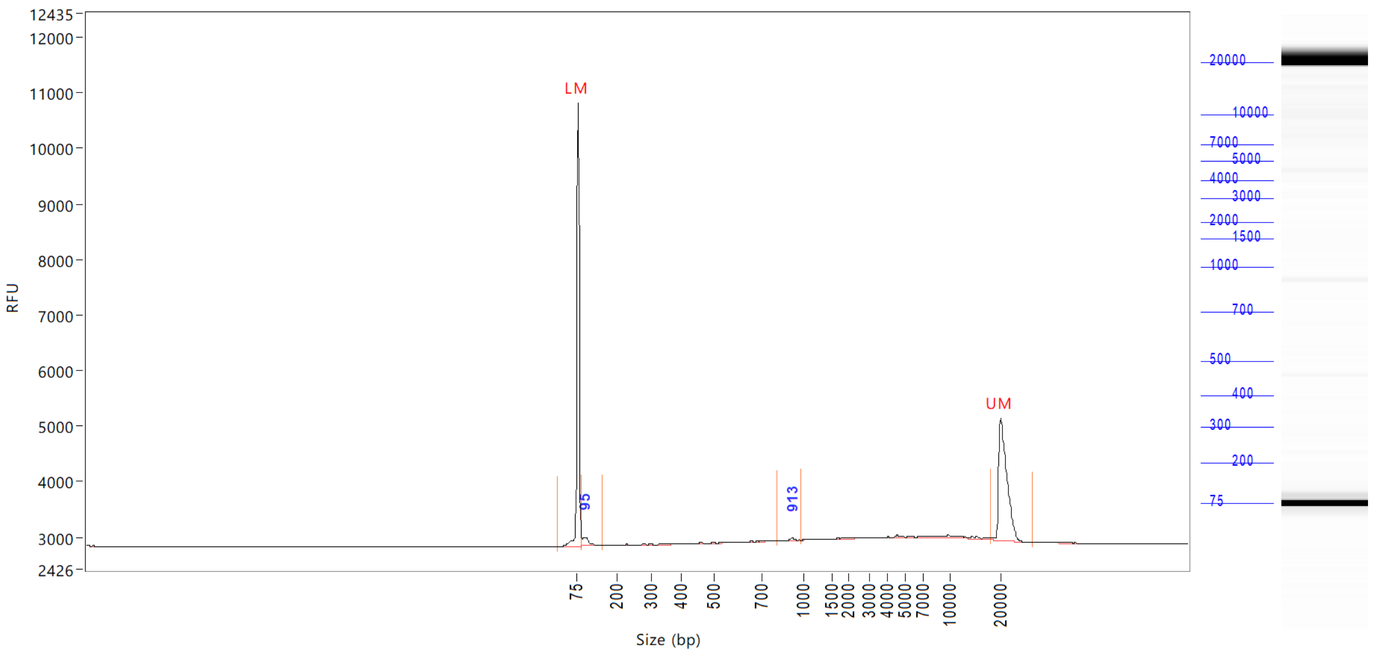

Peak	Size	Concentration	Relative concentration	Molarity	From	To	CV%	RFU
	(bp)	(ng/uL)	Percent	(nmole/L)	(bp)	(bp)		
1	75 (LM)	0.5000		11.2364	12	87	7.82	7992
2	95	0.3161	86.5	5.0398	87	150	13.95	157
3	913	0.0491	13.5	0.0893	809	978	3.19	51
4	20000 (UM)	0.2480		0.0198	18171	26529	4.34	2197

TIC:	0.3652	ng/uL
TIM:	5.1292	nmole/L
Total concentration:	1.0628	ng/uL

```
Sample peak width (sec): 5    Sample min peak height: 50    Sample baseline V to V?: Y    Sample baseline V to V points: 3
Sample filter: Binomial       Number of points for filter: 3  Sample start region (min): 0  Sample end region (min): 30
Marker peak width (sec): 5    Marker min peak height: 200   Marker baseline V to V?: Y    Marker baseline V to V points: 3
Lower marker selection: First peak > 500 RFU                Upper marker selection: Last peak > 200 RFU
Ladder size (bp)75, 200, 300, 400, 500, 700, 1000, 1500, 2000, 3000, 4000, 5000, 7000, 10000, 20000
Quantification using: Lower Marker          Final concentration (ng/uL): 0.5000          Dilution factor: 12.0
```

Sample: SampA6
Well location: A6
Created: Thursday, October 21, 2021 7:28:10 PM

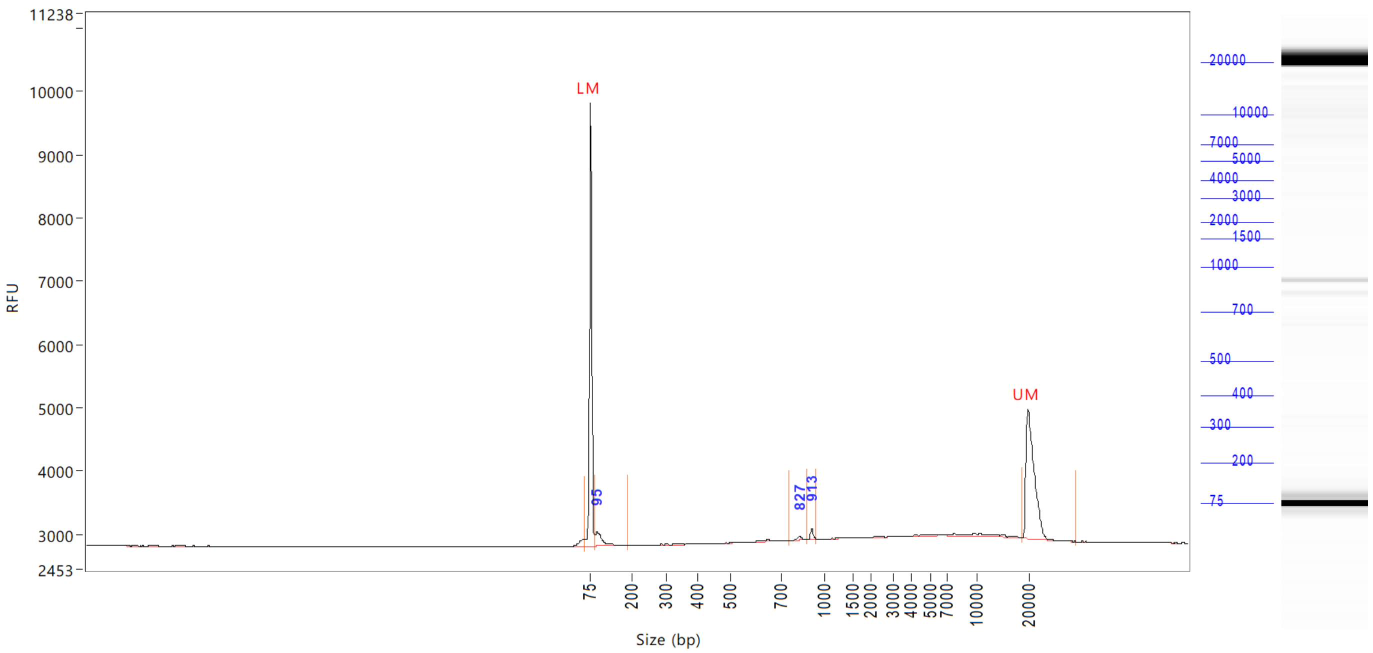

Peak	Size	Concentration	Relative concentration	Molarity	From	To	CV%	RFU
	(bp)	(ng/uL)	Percent	(nmole/L)	(bp)	(bp)		
1	75 (LM)	0.5000		11.2364	58	87	5.11	7015
2	95	0.5578	73.6	8.6429	87	190	18.35	221
3	827	0.0692	9.1	0.1392	757	883	2.22	65
4	913	0.1314	17.3	0.2379	883	944	1.08	178
5	20000 (UM)	0.2701		0.0216	18903	29309	4.55	2054

TIC:	0.7584	ng/uL
TIM:	9.0199	nmole/L
Total concentration:	1.4576	ng/uL

Sample peak width (sec): 5   Sample min peak height: 50   Sample baseline V to V?: Y   Sample baseline V to V points: 3
Sample filter: Binomial   Number of points for filter: 3   Sample start region (min): 0   Sample end region (min): 30
Marker peak width (sec): 5   Marker min peak height: 200   Marker baseline V to V?: Y   Marker baseline V to V points: 3
Lower marker selection: First peak > 500 RFU   Upper marker selection: Last peak > 200 RFU
Ladder size (bp)75, 200, 300, 400, 500, 700, 1000, 1500, 2000, 3000, 4000, 5000, 7000, 10000, 20000
Quantification using: Lower Marker   Final concentration (ng/uL): 0.5000   Dilution factor: 12.0

Sample: SampB6
Well location: B6
Created: Thursday, October 21, 2021 7:28:10 PM

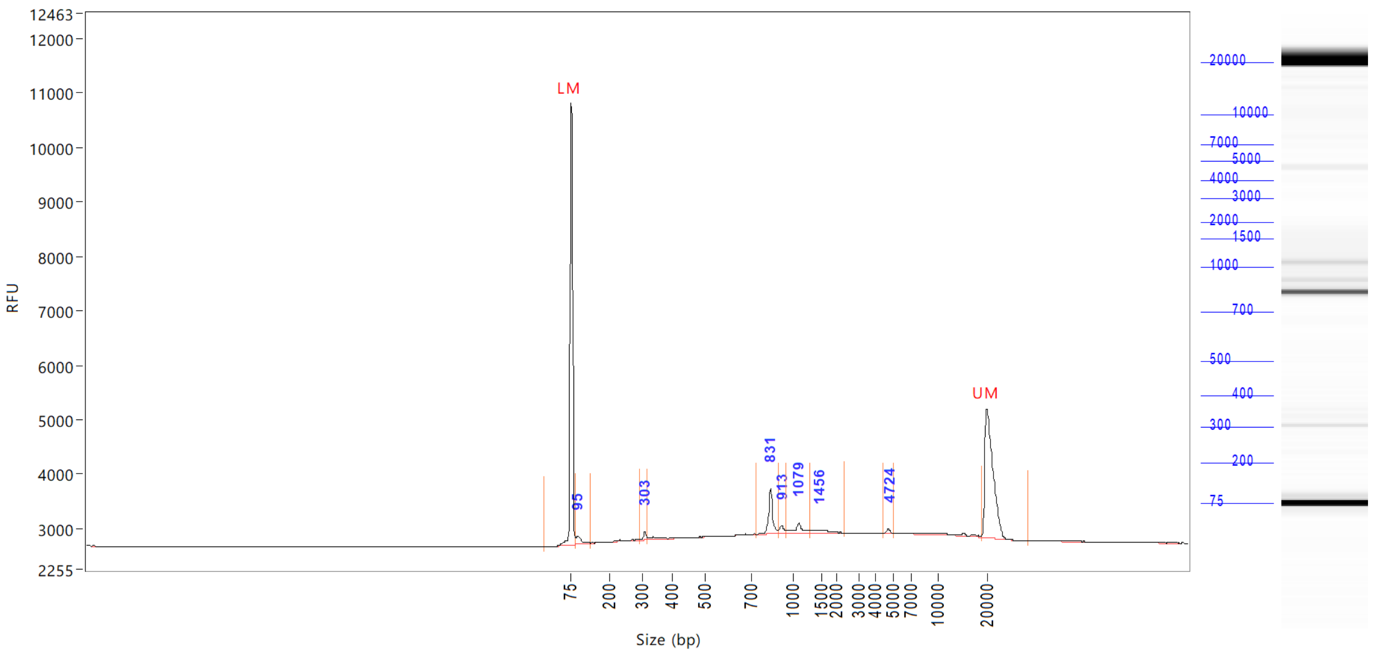

Peak	Size	Concentration	Relative concentration	Molarity	From	To	CV%	RFU
	(bp)	(ng/uL)	Percent	(nmole/L)	(bp)	(bp)		
1	75 (LM)	0.5000		11.2364	0	87	7.69	8128
2	95	0.2543	13.6	4.2183	87	137	10.60	151
3	303	0.1110	5.9	0.6064	289	311	1.46	142
4	831	0.7565	40.5	1.5020	727	887	2.46	832
5	913	0.1583	8.5	0.2845	887	952	1.92	152
6	1079	0.3340	17.9	0.5054	952	1300	8.54	193
7	1456	0.1929	10.3	0.2051	1300	2372	12.73	52
8	4724	0.0609	3.3	0.0215	4380	5001	2.40	81
9	20000 (UM)	0.2676		0.0214	18903	28342	4.41	2372

TIC:	1.8679	ng/uL
TIM:	7.3431	nmole/L
Total concentration:	2.5135	ng/uL

Sample peak width (sec): 5 Sample min peak height: 50 Sample baseline V to V?: Y Sample baseline V to V points: 3
Sample filter: Binomial Number of points for filter: 3 Sample start region (min): 0 Sample end region (min): 30
Marker peak width (sec): 5 Marker min peak height: 200 Marker baseline V to V?: Y Marker baseline V to V points: 3
Lower marker selection: First peak > 500 RFU Upper marker selection: Last peak > 200 RFU
Ladder size (bp)75, 200, 300, 400, 500, 700, 1000, 1500, 2000, 3000, 4000, 5000, 7000, 10000, 20000
Quantification using: Lower Marker Final concentration (ng/uL): 0.5000 Dilution factor: 12.0

Sample: SampC6
Well location: C6
Created: Thursday, October 21, 2021 7:28:10 PM

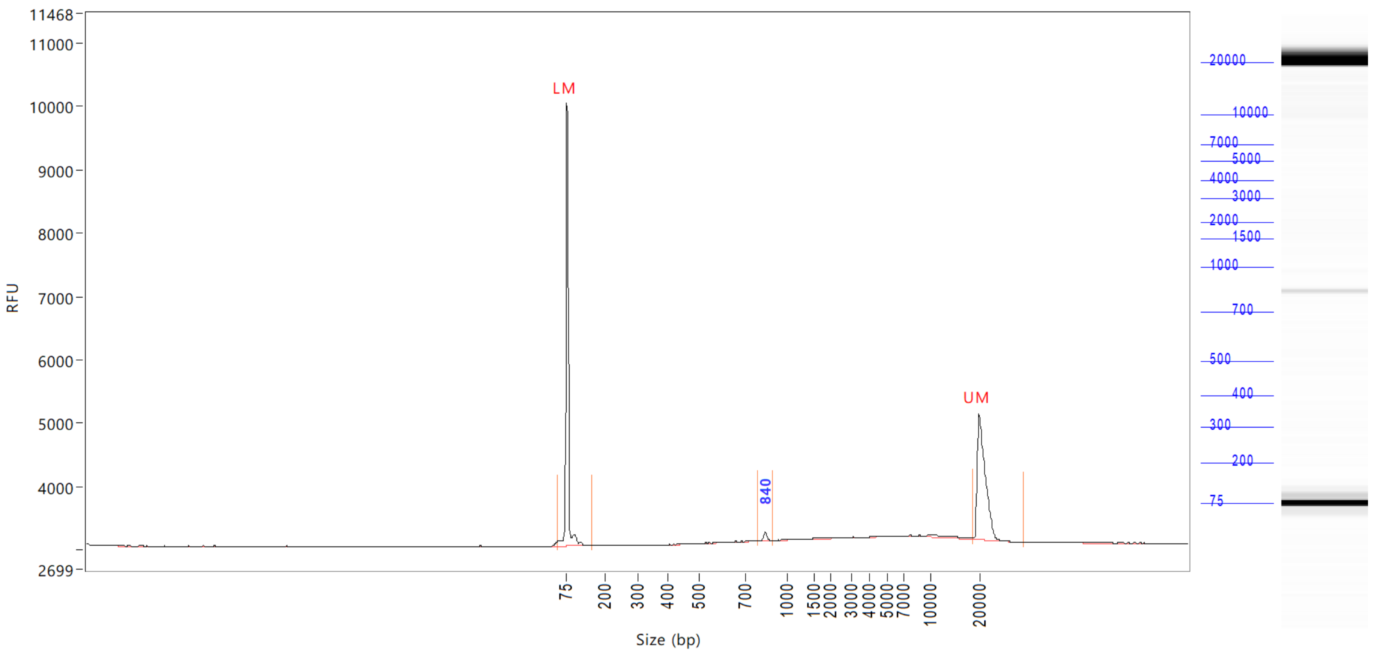

Peak	Size	Concentration	Relative concentration	Molarity	From	To	CV%	RFU
	(bp)	(ng/uL)	Percent	(nmole/L)	(bp)	(bp)		
1	75 (LM)	0.5000		10.9378	47	158	11.43	7000
2	840	0.1239	100.0	0.2444	783	896	1.83	152
3	20000 (UM)	0.2446		0.0195	18659	29189	4.73	1982

TIC:	0.1239	ng/uL
TIM:	0.2444	nmole/L
Total concentration:	0.6691	ng/uL

Sample peak width (sec): 5 Sample min peak height: 50 Sample baseline V to V?: Y Sample baseline V to V points: 3
Sample filter: Binomial Number of points for filter: 3 Sample start region (min): 0 Sample end region (min): 30
Marker peak width (sec): 5 Marker min peak height: 200 Marker baseline V to V?: Y Marker baseline V to V points: 3
Lower marker selection: First peak > 500 RFU Upper marker selection: Last peak > 200 RFU
Ladder size (bp)75, 200, 300, 400, 500, 700, 1000, 1500, 2000, 3000, 4000, 5000, 7000, 10000, 20000
Quantification using: Lower Marker Final concentration (ng/uL): 0.5000 Dilution factor: 12.0

Sample: SampD6
Well location: D6
Created: Thursday, October 21, 2021 7:28:10 PM

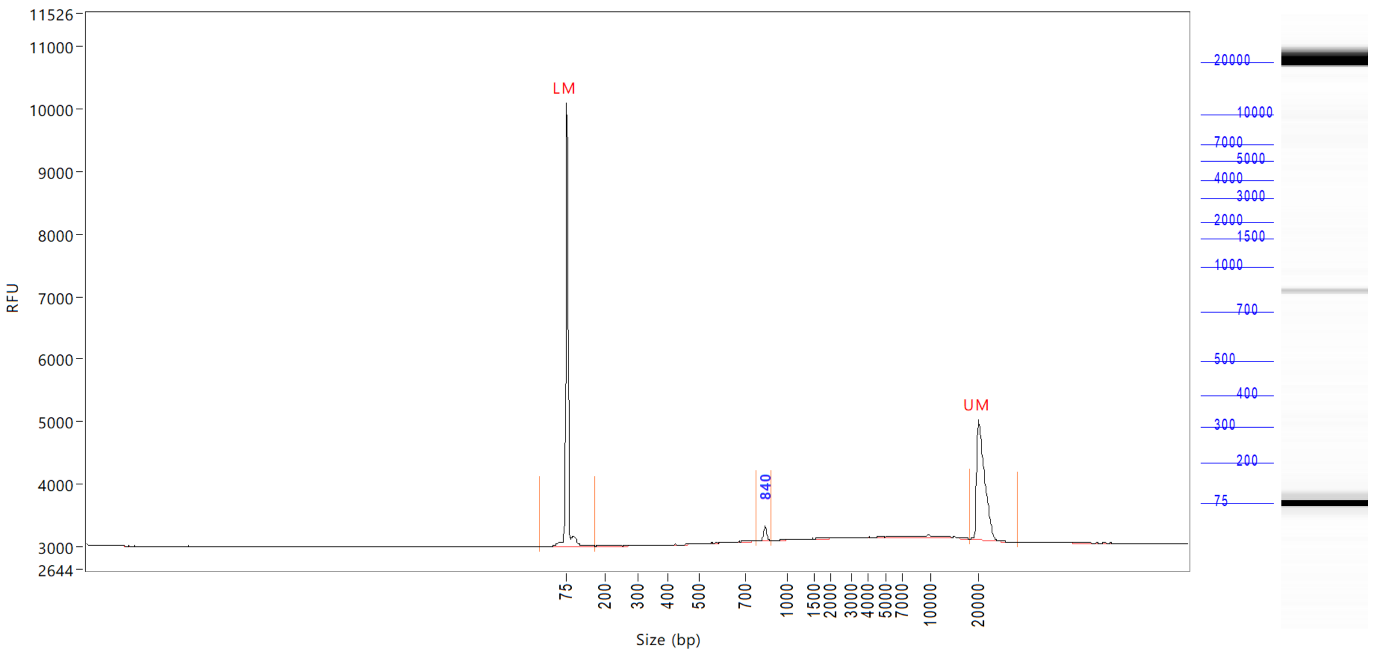

Peak	Size	Concentration	Relative concentration	Molarity	From	To	CV%	RFU
	(bp)	(ng/uL)	Percent	(nmole/L)	(bp)	(bp)		
1	75 (LM)	0.5000		11.0851	0	168	14.57	7100
2	840	0.1797	100.0	0.3546	770	883	1.61	238
3	20000 (UM)	0.2341		0.0187	18049	27979	4.50	1921

TIC:	0.1797	ng/uL
TIM:	0.3546	nmole/L
Total concentration:	0.6883	ng/uL

```
Sample peak width (sec): 5    Sample min peak height: 50    Sample baseline V to V?: Y    Sample baseline V to V points: 3
Sample filter: Binomial       Number of points for filter: 3  Sample start region (min): 0  Sample end region (min): 30
Marker peak width (sec): 5    Marker min peak height: 200   Marker baseline V to V?: Y    Marker baseline V to V points: 3
Lower marker selection: First peak > 500 RFU                Upper marker selection: Last peak > 200 RFU
Ladder size (bp)75, 200, 300, 400, 500, 700, 1000, 1500, 2000, 3000, 4000, 5000, 7000, 10000, 20000
Quantification using: Lower Marker          Final concentration (ng/uL): 0.5000          Dilution factor: 12.0
```

Sample: SampE6
Well location: E6
Created: Thursday, October 21, 2021 7:28:10 PM

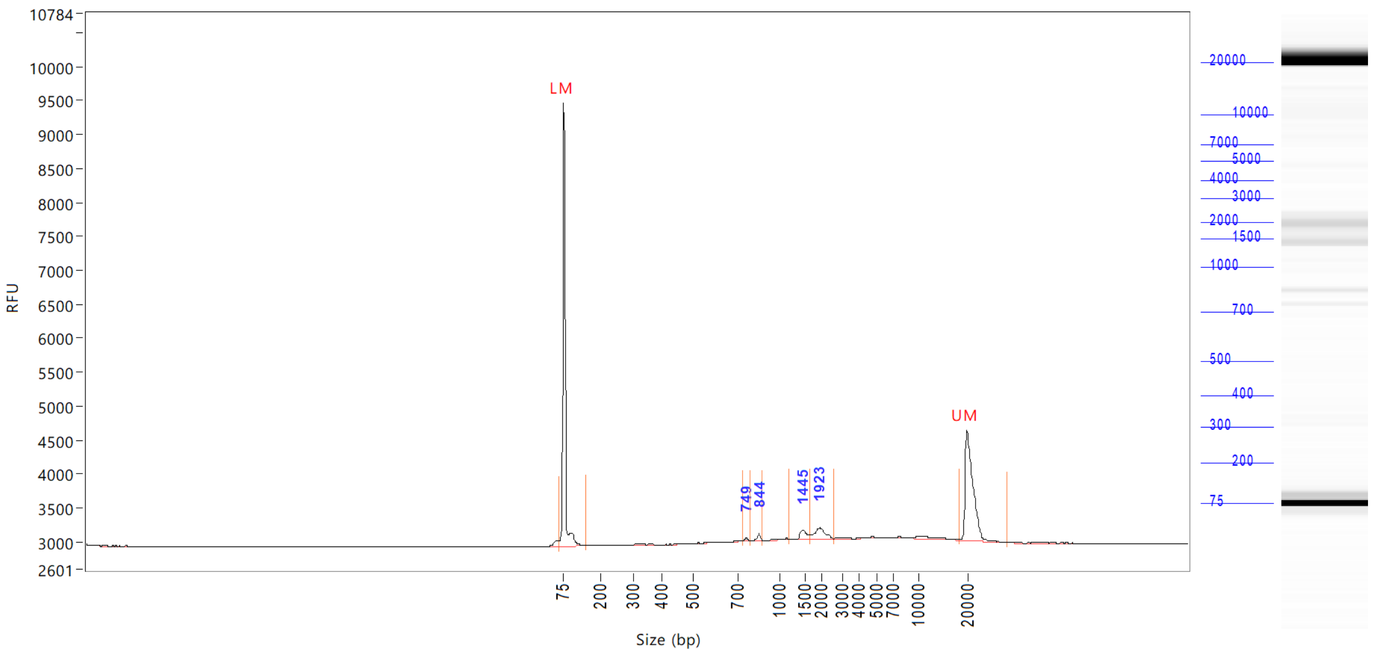

Peak	Size	Concentration	Relative concentration	Molarity	From	To	CV%	RFU
	(bp)	(ng/uL)	Percent	(nmole/L)	(bp)	(bp)		
1	75 (LM)	0.5000		10.7944	64	147	11.55	6536
2	749	0.0460	5.7	0.1016	723	783	1.39	56
3	844	0.0867	10.7	0.1706	783	874	1.98	97
4	1445	0.2572	31.8	0.2940	1178	1616	5.95	135
5	1923	0.4179	51.7	0.3522	1616	2572	9.87	164
6	20000 (UM)	0.2152		0.0171	18293	28584	5.50	1621

TIC:	0.8077	ng/uL
TIM:	0.9184	nmole/L
Total concentration:	1.3922	ng/uL

Sample peak width (sec): 5   Sample min peak height: 50   Sample baseline V to V?: Y   Sample baseline V to V points: 3
Sample filter: Binomial   Number of points for filter: 3 Sample start region (min): 0 Sample end region (min): 30
Marker peak width (sec): 5   Marker min peak height: 200   Marker baseline V to V?: Y   Marker baseline V to V points: 3
Lower marker selection: First peak > 500 RFU   Upper marker selection: Last peak > 200 RFU
Ladder size (bp)75, 200, 300, 400, 500, 700, 1000, 1500, 2000, 3000, 4000, 5000, 7000, 10000, 20000
Quantification using: Lower Marker   Final concentration (ng/uL): 0.5000   Dilution factor: 12.0

Sample: SampF6
Well location: F6
Created: Thursday, October 21, 2021 7:28:10 PM

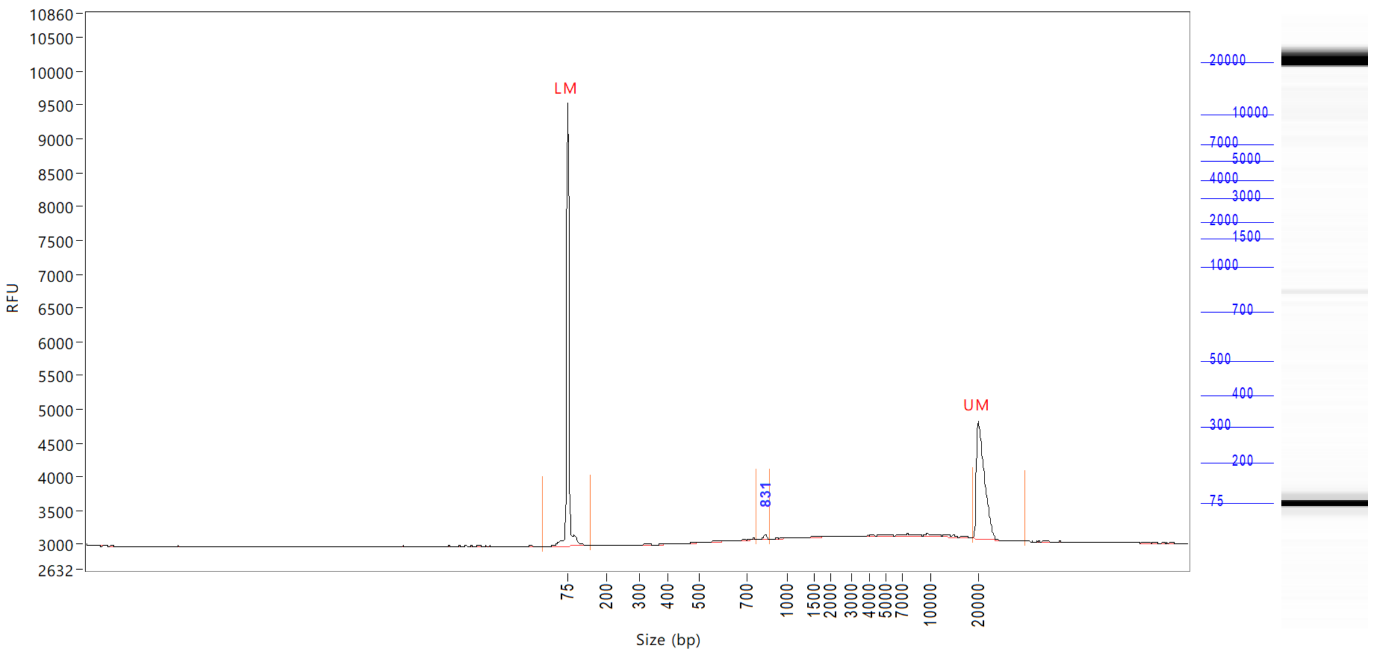

Peak	Size	Concentration	Relative concentration	Molarity	From	To	CV%	RFU
	(bp)	(ng/uL)	Percent	(nmole/L)	(bp)	(bp)		
1	75 (LM)	0.5000		11.2364	0	148	13.36	6569
2	831	0.0628	100.0	0.1248	775	870	1.67	77
3	20000 (UM)	0.2275		0.0182	18781	29551	4.65	1751

TIC:	0.0628	ng/uL
TIM:	0.1248	nmole/L
Total concentration:	0.6473	ng/uL

```
Sample peak width (sec): 5    Sample min peak height: 50    Sample baseline V to V?: Y    Sample baseline V to V points: 3
Sample filter: Binomial       Number of points for filter: 3  Sample start region (min): 0  Sample end region (min): 30
Marker peak width (sec): 5    Marker min peak height: 200   Marker baseline V to V?: Y    Marker baseline V to V points: 3
Lower marker selection: First peak > 500 RFU                Upper marker selection: Last peak > 200 RFU
Ladder size (bp)75, 200, 300, 400, 500, 700, 1000, 1500, 2000, 3000, 4000, 5000, 7000, 10000, 20000
Quantification using: Lower Marker          Final concentration (ng/uL): 0.5000          Dilution factor: 12.0
```

Sample: SampG6
Well location: G6
Created: Thursday, October 21, 2021 7:28:10 PM

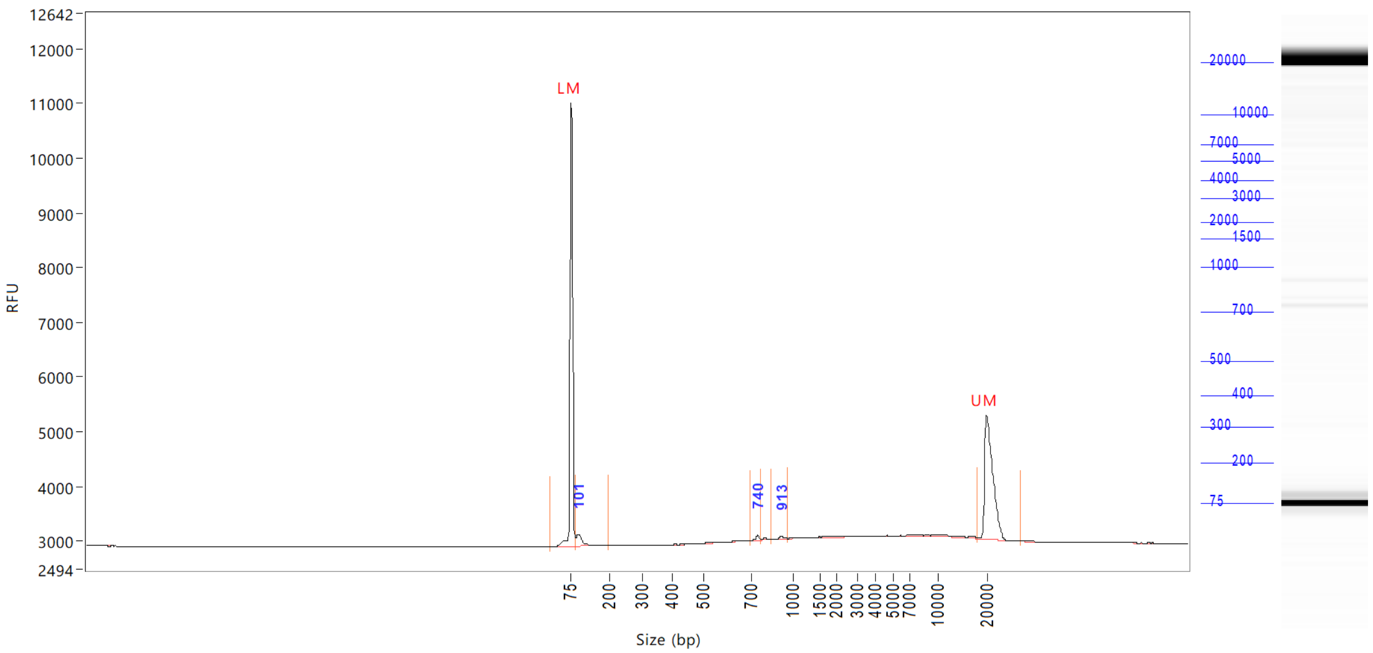

Peak	Size	Concentration	Relative concentration	Molarity	From	To	CV%	RFU
	(bp)	(ng/uL)	Percent	(nmole/L)	(bp)	(bp)		
1	75 (LM)	0.5000		11.2364	9	87	8.43	8105
2	101	0.4383	78.8	6.8557	87	194	15.99	211
3	740	0.0674	12.1	0.1514	695	766	1.78	89
4	913	0.0506	9.1	0.0922	844	961	2.46	56
5	20000 (UM)	0.2547		0.0203	18171	26891	4.54	2271

TIC:	0.5562	ng/uL
TIM:	7.0993	nmole/L
Total concentration:	1.1641	ng/uL

Sample peak width (sec): 5   Sample min peak height: 50   Sample baseline V to V?: Y   Sample baseline V to V points: 3
Sample filter: Binomial   Number of points for filter: 3   Sample start region (min): 0   Sample end region (min): 30
Marker peak width (sec): 5   Marker min peak height: 200   Marker baseline V to V?: Y   Marker baseline V to V points: 3
Lower marker selection: First peak > 500 RFU   Upper marker selection: Last peak > 200 RFU
Ladder size (bp)75, 200, 300, 400, 500, 700, 1000, 1500, 2000, 3000, 4000, 5000, 7000, 10000, 20000
Quantification using: Lower Marker   Final concentration (ng/uL): 0.5000   Dilution factor: 12.0

Sample: SampH6
Well location: H6
Created: Thursday, October 21, 2021 7:28:10 PM

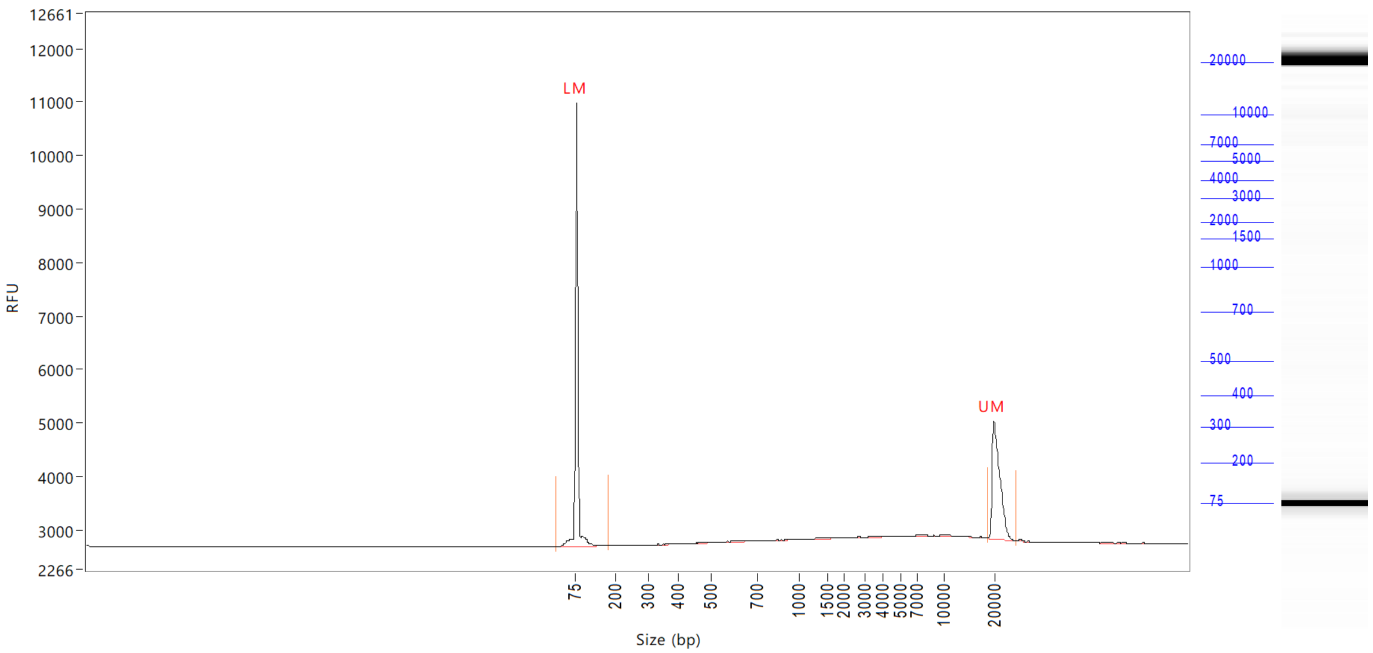

Peak	Size	Concentration	Relative concentration	Molarity	From	To	CV%	RFU
	(bp)	(ng/uL)	Percent	(nmole/L)	(bp)	(bp)		
1	75 (LM)	0.5000		11.0851	12	175	15.31	8304
2	20000 (UM)	0.2185		0.0175	18903	24594	4.05	2192

TIC:	0.0000	ng/uL
TIM:	0.0000	nmole/L
Total concentration:	0.5213	ng/uL

Sample peak width (sec): 5 Sample min peak height: 50 Sample baseline V to V?: Y Sample baseline V to V points: 3
Sample filter: Binomial Number of points for filter: 3 Sample start region (min): 0 Sample end region (min): 30
Marker peak width (sec): 5 Marker min peak height: 200 Marker baseline V to V?: Y Marker baseline V to V points: 3
Lower marker selection: First peak > 500 RFU Upper marker selection: Last peak > 200 RFU
Ladder size (bp)75, 200, 300, 400, 500, 700, 1000, 1500, 2000, 3000, 4000, 5000, 7000, 10000, 20000
Quantification using: Lower Marker Final concentration (ng/uL): 0.5000 Dilution factor: 12.0

Sample: SampA7
Well location: A7
Created: Thursday, October 21, 2021 7:28:10 PM

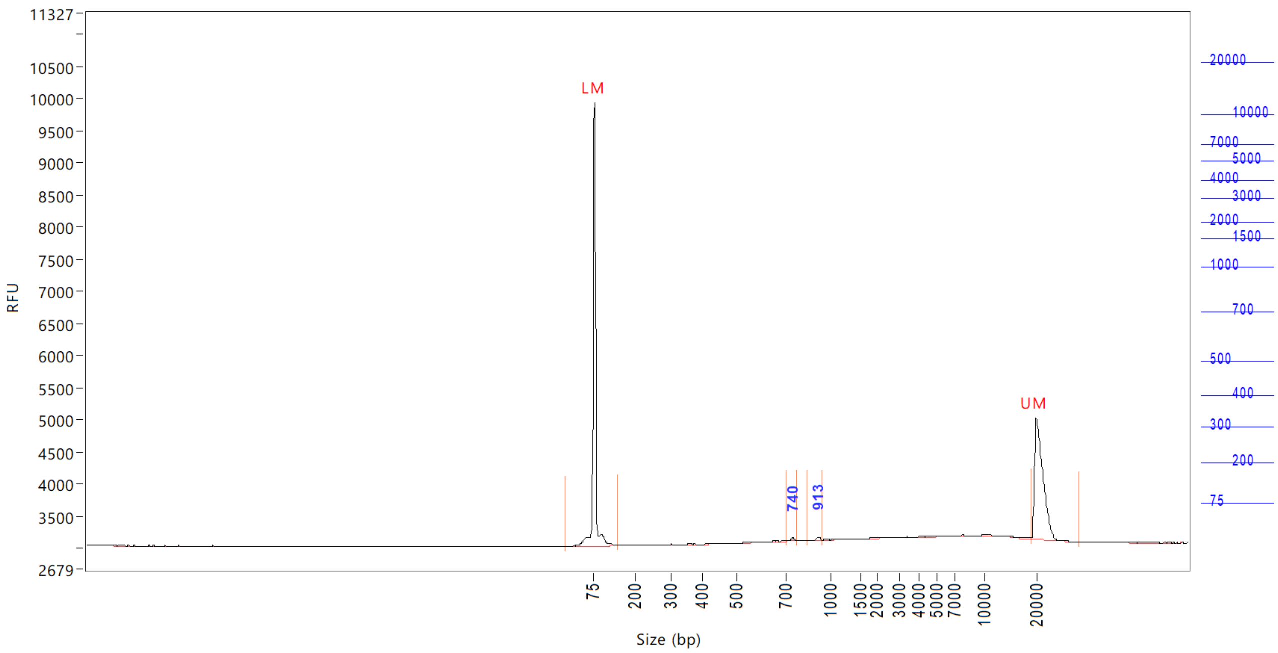

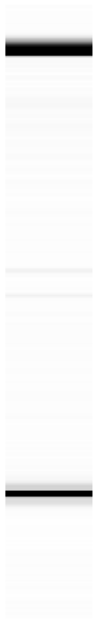

Peak	Size	Concentration	Relative concentration	Molarity	From	To	CV%	RFU
	(bp)	(ng/uL)	Percent	(nmole/L)	(bp)	(bp)		
1	75 (LM)	0.5000		11.2364	0	145	14.19	6907
2	740	0.0418	47.9	0.0937	697	770	1.83	53
3	913	0.0454	52.1	0.0828	840	939	1.72	52
4	20000 (UM)	0.2276		0.0182	18903	28100	4.42	1880

TIC:	0.0872	ng/uL
TIM:	0.1764	nmole/L
Total concentration:	0.5860	ng/uL

```
Sample peak width (sec): 5    Sample min peak height: 50    Sample baseline V to V?: Y    Sample baseline V to V points: 3
Sample filter: Binomial       Number of points for filter: 3  Sample start region (min): 0  Sample end region (min): 30
Marker peak width (sec): 5    Marker min peak height: 200   Marker baseline V to V?: Y    Marker baseline V to V points: 3
Lower marker selection: First peak > 500 RFU                Upper marker selection: Last peak > 200 RFU
Ladder size (bp)75, 200, 300, 400, 500, 700, 1000, 1500, 2000, 3000, 4000, 5000, 7000, 10000, 20000
Quantification using: Lower Marker          Final concentration (ng/uL): 0.5000          Dilution factor: 12.0
```

Sample: SampB7
Well location: B7
Created: Thursday, October 21, 2021 7:28:10 PM

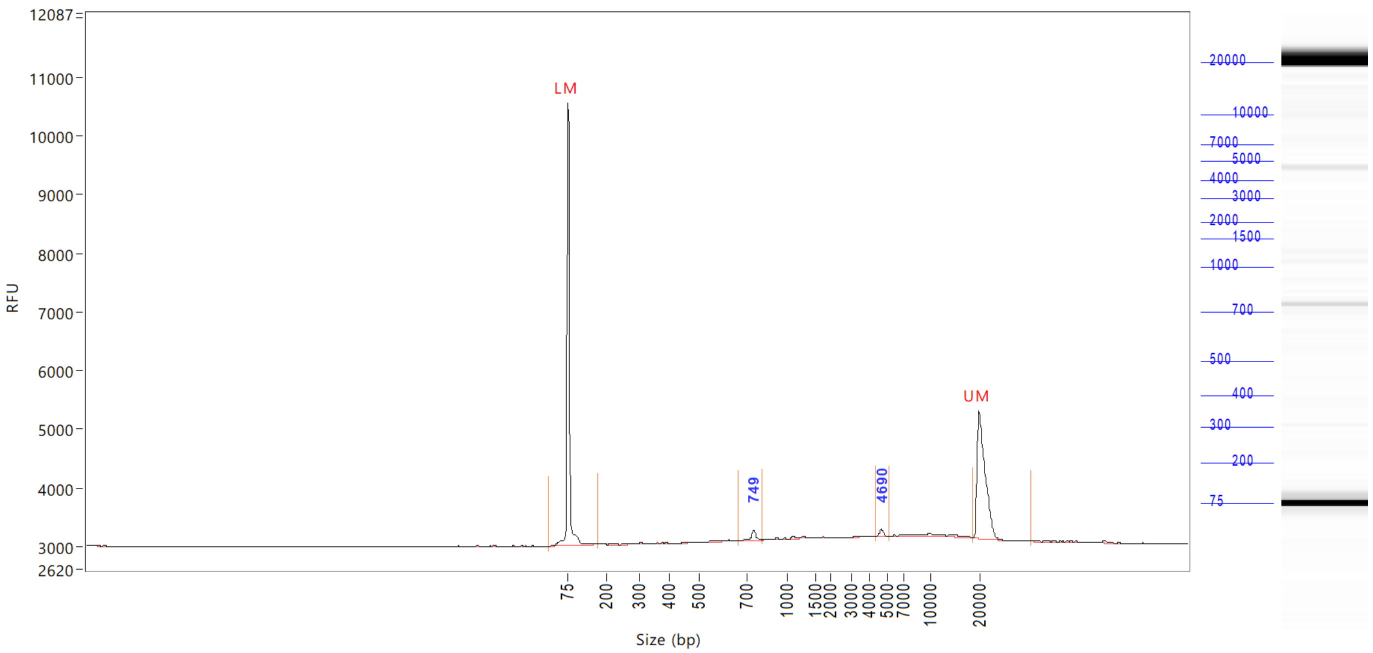

Peak	Size	Concentration	Relative concentration	Molarity	From	To	CV%	RFU
	(bp)	(ng/uL)	Percent	(nmole/L)	(bp)	(bp)		
1	75 (LM)	0.5000		11.0851	12	171	12.57	7549
2	749	0.1959	69.2	0.4380	662	809	3.61	181
3	4690	0.0872	30.8	0.0308	4380	5223	2.93	116
4	20000 (UM)	0.2498		0.0200	18781	30519	4.87	2165

TIC:	0.2831	ng/uL
TIM:	0.4688	nmole/L
Total concentration:	0.9053	ng/uL

Sample peak width (sec): 5   Sample min peak height: 50   Sample baseline V to V?: Y   Sample baseline V to V points: 3
Sample filter: Binomial   Number of points for filter: 3 Sample start region (min): 0 Sample end region (min): 30
Marker peak width (sec): 5   Marker min peak height: 200   Marker baseline V to V?: Y   Marker baseline V to V points: 3
Lower marker selection: First peak > 500 RFU   Upper marker selection: Last peak > 200 RFU
Ladder size (bp)75, 200, 300, 400, 500, 700, 1000, 1500, 2000, 3000, 4000, 5000, 7000, 10000, 20000
Quantification using: Lower Marker   Final concentration (ng/uL): 0.5000   Dilution factor: 12.0

Sample: SampC7
Well location: C7
Created: Thursday, October 21, 2021 7:28:10 PM

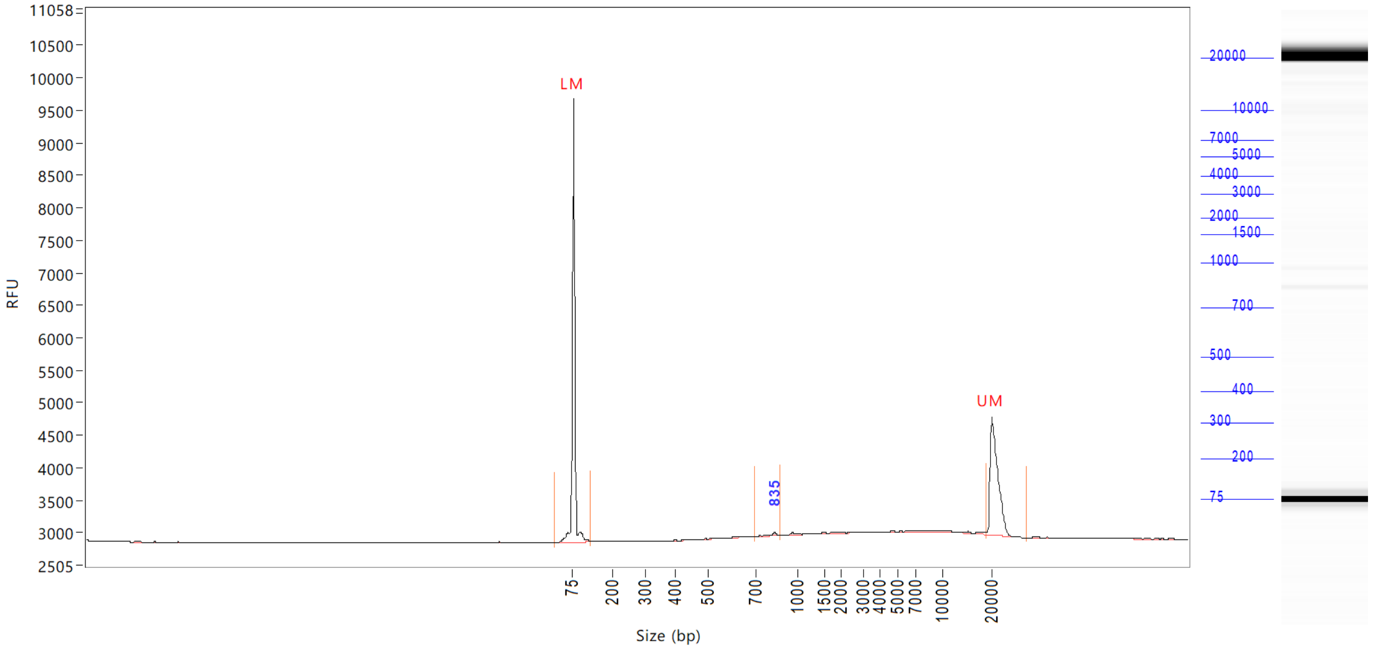

Peak	Size	Concentration	Relative concentration	Molarity	From	To	CV%	RFU
	(bp)	(ng/uL)	Percent	(nmole/L)	(bp)	(bp)		
1	75 (LM)	0.5000		11.2364	16	129	11.26	6832
2	835	0.0653	100.0	0.1333	695	870	5.34	56
3	20000 (UM)	0.2249		0.0180	18903	26891	4.19	1804

TIC:	0.0653	ng/uL
TIM:	0.1333	nmole/L
Total concentration:	0.6383	ng/uL

Sample peak width (sec): 5   Sample min peak height: 50   Sample baseline V to V?: Y   Sample baseline V to V points: 3
Sample filter: Binomial   Number of points for filter: 3   Sample start region (min): 0   Sample end region (min): 30
Marker peak width (sec): 5   Marker min peak height: 200   Marker baseline V to V?: Y   Marker baseline V to V points: 3
Lower marker selection: First peak > 500 RFU   Upper marker selection: Last peak > 200 RFU
Ladder size (bp)75, 200, 300, 400, 500, 700, 1000, 1500, 2000, 3000, 4000, 5000, 7000, 10000, 20000
Quantification using: Lower Marker   Final concentration (ng/uL): 0.5000   Dilution factor: 12.0

Sample: SampD7
Well location: D7
Created: Thursday, October 21, 2021 7:28:10 PM

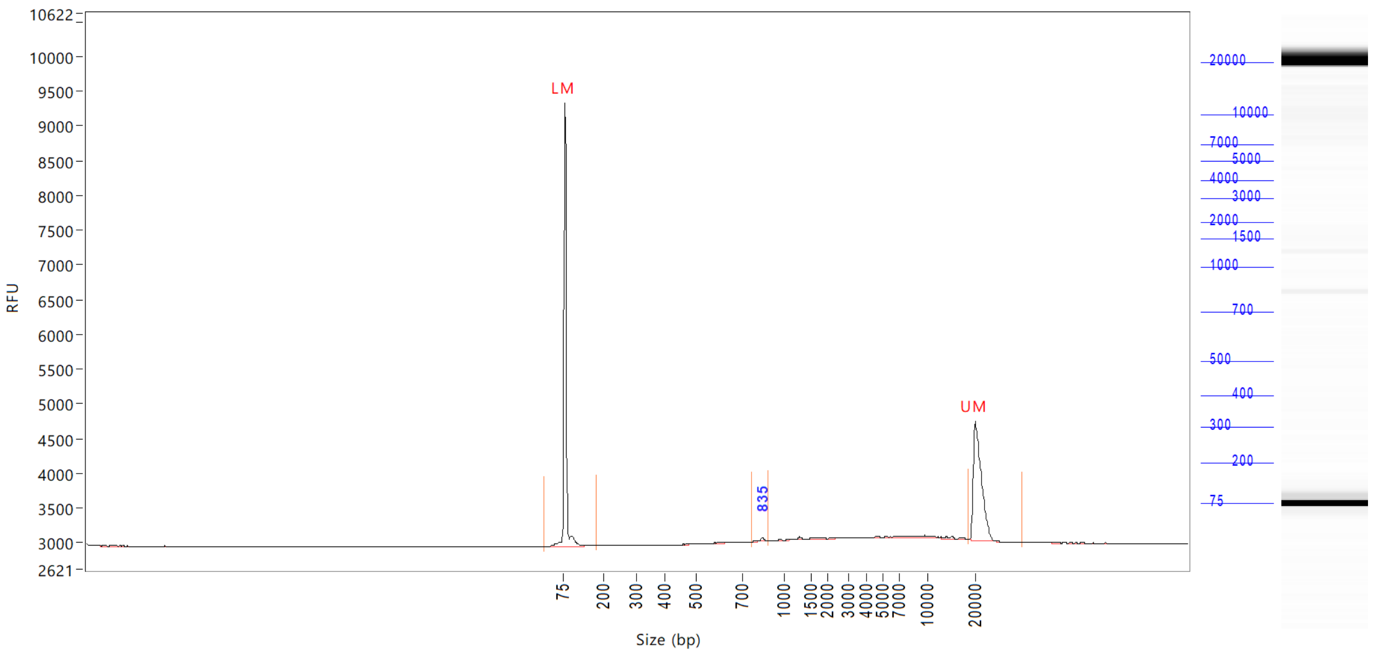

Peak	Size	Concentration	Relative concentration	Molarity	From	To	CV%	RFU
	(bp)	(ng/uL)	Percent	(nmole/L)	(bp)	(bp)		
1	75 (LM)	0.5000		11.0851	9	175	13.23	6393
2	835	0.0551	100.0	0.1091	762	879	2.29	56
3	20000 (UM)	0.2373		0.0190	18659	29672	4.71	1716

TIC:	0.0551	ng/uL
TIM:	0.1091	nmole/L
Total concentration:	0.7067	ng/uL

Sample peak width (sec): 5   Sample min peak height: 50   Sample baseline V to V?: Y   Sample baseline V to V points: 3
Sample filter: Binomial   Number of points for filter: 3 Sample start region (min): 0 Sample end region (min): 30
Marker peak width (sec): 5   Marker min peak height: 200   Marker baseline V to V?: Y   Marker baseline V to V points: 3
Lower marker selection: First peak > 500 RFU   Upper marker selection: Last peak > 200 RFU
Ladder size (bp)75, 200, 300, 400, 500, 700, 1000, 1500, 2000, 3000, 4000, 5000, 7000, 10000, 20000
Quantification using: Lower Marker   Final concentration (ng/uL): 0.5000   Dilution factor: 12.0

Sample: SampE7
Well location: E7
Created: Thursday, October 21, 2021 7:28:10 PM

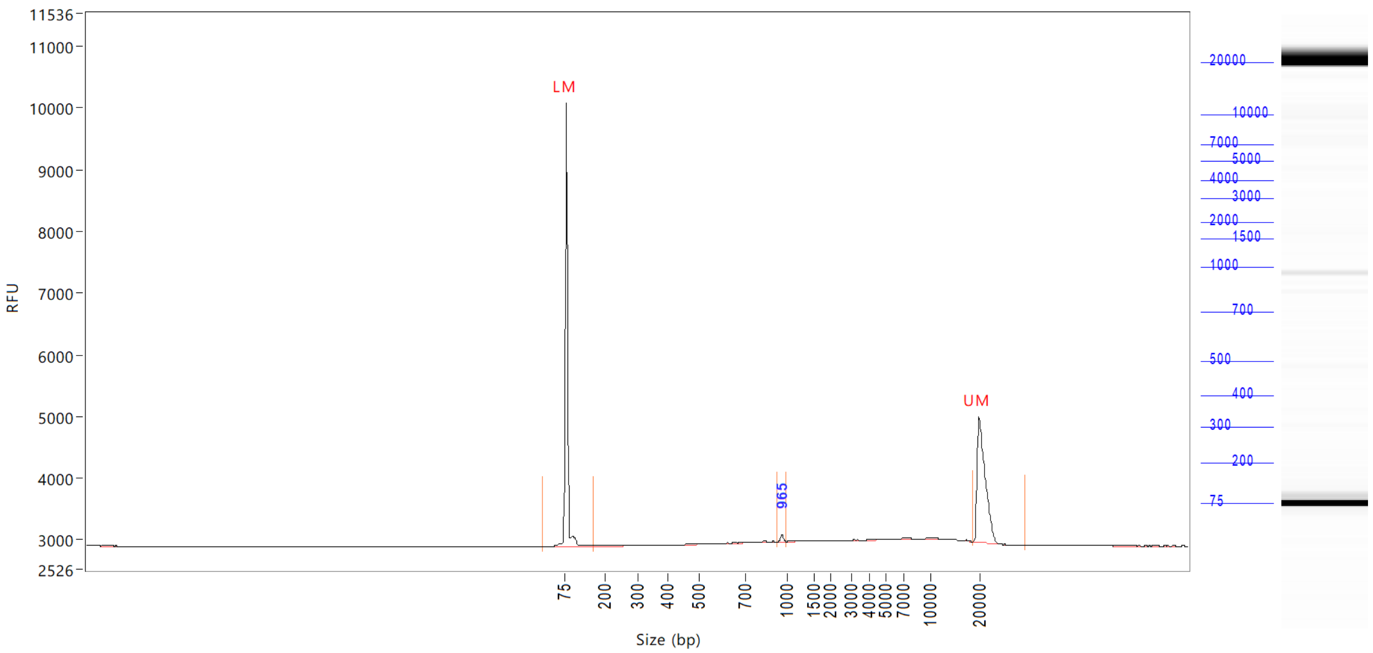

Peak	Size	Concentration	Relative concentration	Molarity	From	To	CV%	RFU
	(bp)	(ng/uL)	Percent	(nmole/L)	(bp)	(bp)		
1	75 (LM)	0.5000		10.9378	3	166	13.16	7204
2	965	0.0793	100.0	0.1361	926	996	1.17	110
3	20000 (UM)	0.2535		0.0203	18659	29309	4.61	2036

TIC:	0.0793	ng/uL
TIM:	0.1361	nmole/L
Total concentration:	0.6218	ng/uL

```
Sample peak width (sec): 5    Sample min peak height: 50    Sample baseline V to V?: Y    Sample baseline V to V points: 3
Sample filter: Binomial       Number of points for filter: 3  Sample start region (min): 0  Sample end region (min): 30
Marker peak width (sec): 5    Marker min peak height: 200   Marker baseline V to V?: Y    Marker baseline V to V points: 3
Lower marker selection: First peak > 500 RFU                Upper marker selection: Last peak > 200 RFU
Ladder size (bp)75, 200, 300, 400, 500, 700, 1000, 1500, 2000, 3000, 4000, 5000, 7000, 10000, 20000
Quantification using: Lower Marker          Final concentration (ng/uL): 0.5000          Dilution factor: 12.0
```

Sample: SampF7
Well location: F7
Created: Thursday, October 21, 2021 7:28:10 PM

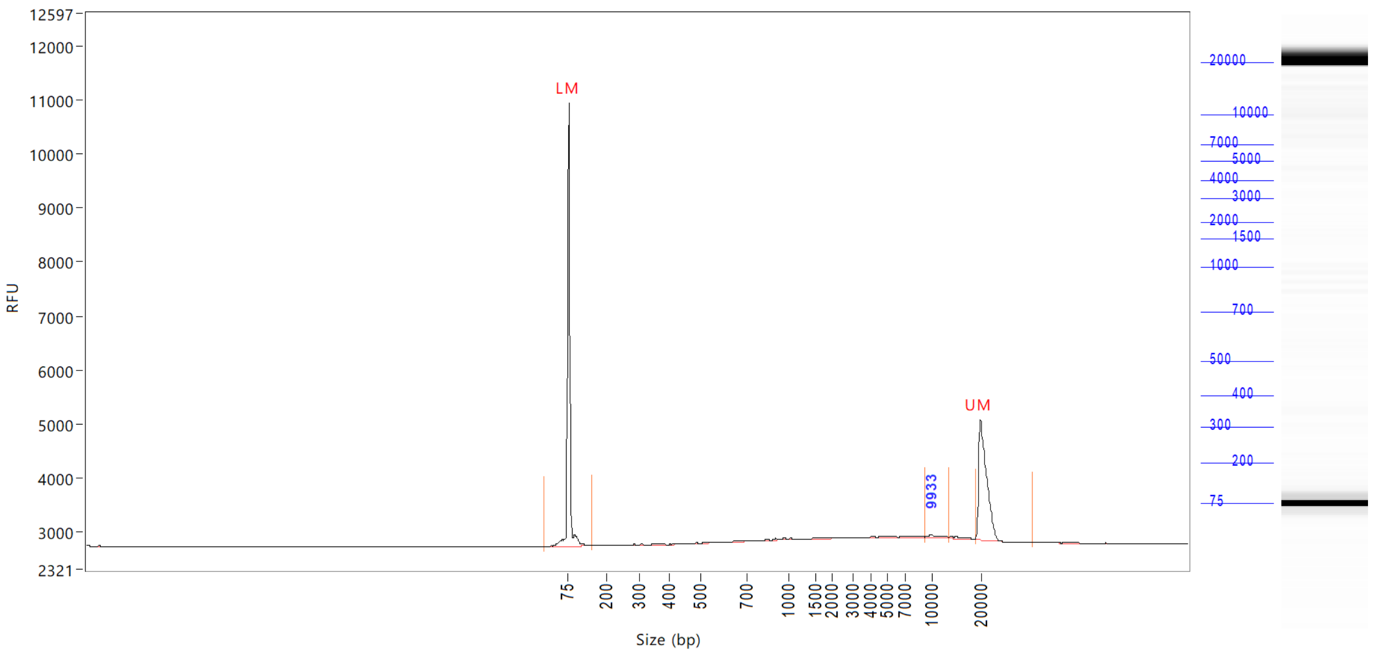

Peak	Size	Concentration	Relative concentration	Molarity	From	To	CV%	RFU
	(bp)	(ng/uL)	Percent	(nmole/L)	(bp)	(bp)		
1	75 (LM)	0.5000		11.0851	0	148	13.98	8208
2	9933	0.1251	100.0	0.0189	9200	13415	10.93	55
3	20000 (UM)	0.2326		0.0185	19024	30639	4.66	2216

TIC:	0.1251	ng/uL
TIM:	0.0189	nmole/L
Total concentration:	0.7566	ng/uL

Sample peak width (sec): 5   Sample min peak height: 50   Sample baseline V to V?: Y   Sample baseline V to V points: 3
Sample filter: Binomial   Number of points for filter: 3   Sample start region (min): 0   Sample end region (min): 30
Marker peak width (sec): 5   Marker min peak height: 200   Marker baseline V to V?: Y   Marker baseline V to V points: 3
Lower marker selection: First peak > 500 RFU   Upper marker selection: Last peak > 200 RFU
Ladder size (bp)75, 200, 300, 400, 500, 700, 1000, 1500, 2000, 3000, 4000, 5000, 7000, 10000, 20000
Quantification using: Lower Marker   Final concentration (ng/uL): 0.5000   Dilution factor: 12.0

Sample: SampG7
Well location: G7
Created: Thursday, October 21, 2021 7:28:10 PM

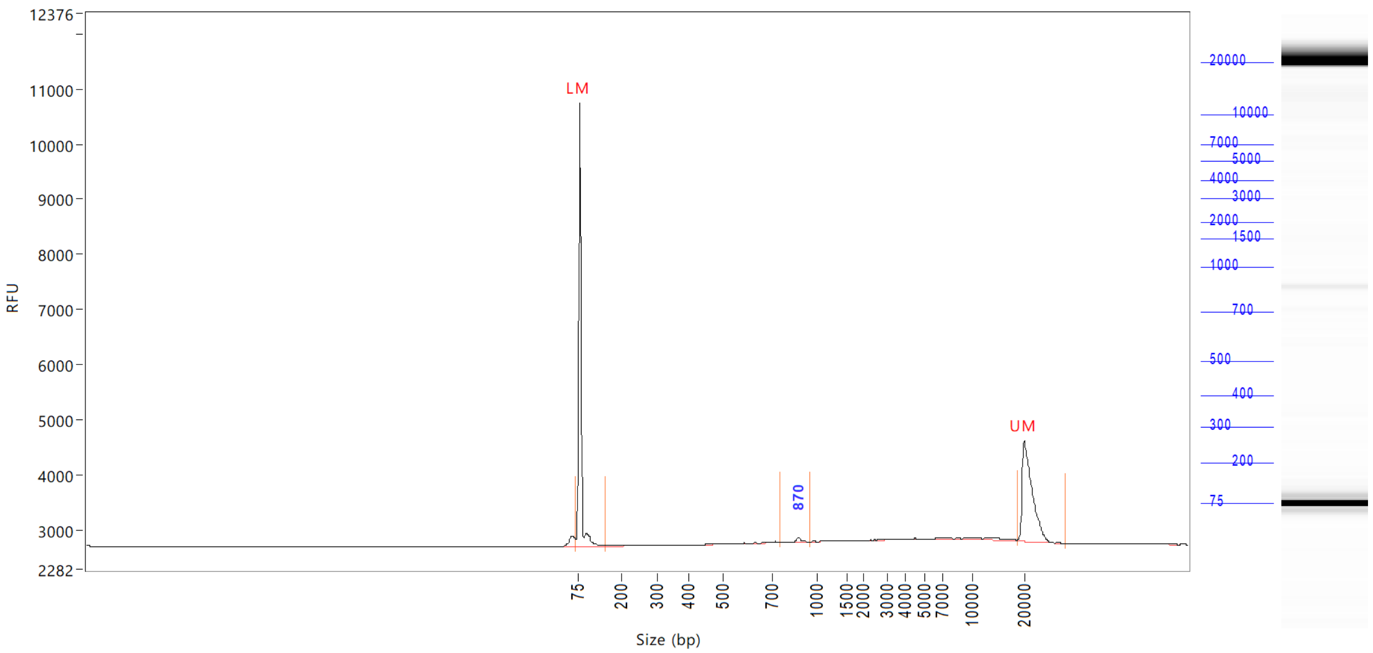

Peak	Size	Concentration	Relative concentration	Molarity	From	To	CV%	RFU
	(bp)	(ng/uL)	Percent	(nmole/L)	(bp)	(bp)		
1	75 (LM)	0.5000		10.7944	64	152	11.81	8067
2	870	0.0915	100.0	0.1757	740	948	4.04	89
3	20000 (UM)	0.2330		0.0184	18537	27738	5.44	1830

TIC:	0.0915	ng/uL
TIM:	0.1757	nmole/L
Total concentration:	0.6375	ng/uL

```
Sample peak width (sec): 5    Sample min peak height: 50    Sample baseline V to V?: Y    Sample baseline V to V points: 3
Sample filter: Binomial       Number of points for filter: 3  Sample start region (min): 0  Sample end region (min): 30
Marker peak width (sec): 5    Marker min peak height: 200   Marker baseline V to V?: Y    Marker baseline V to V points: 3
Lower marker selection: First peak > 500 RFU                Upper marker selection: Last peak > 200 RFU
Ladder size (bp)75, 200, 300, 400, 500, 700, 1000, 1500, 2000, 3000, 4000, 5000, 7000, 10000, 20000
Quantification using: Lower Marker          Final concentration (ng/uL): 0.5000          Dilution factor: 12.0
```

Sample: SampH7
Well location: H7
Created: Thursday, October 21, 2021 7:28:10 PM

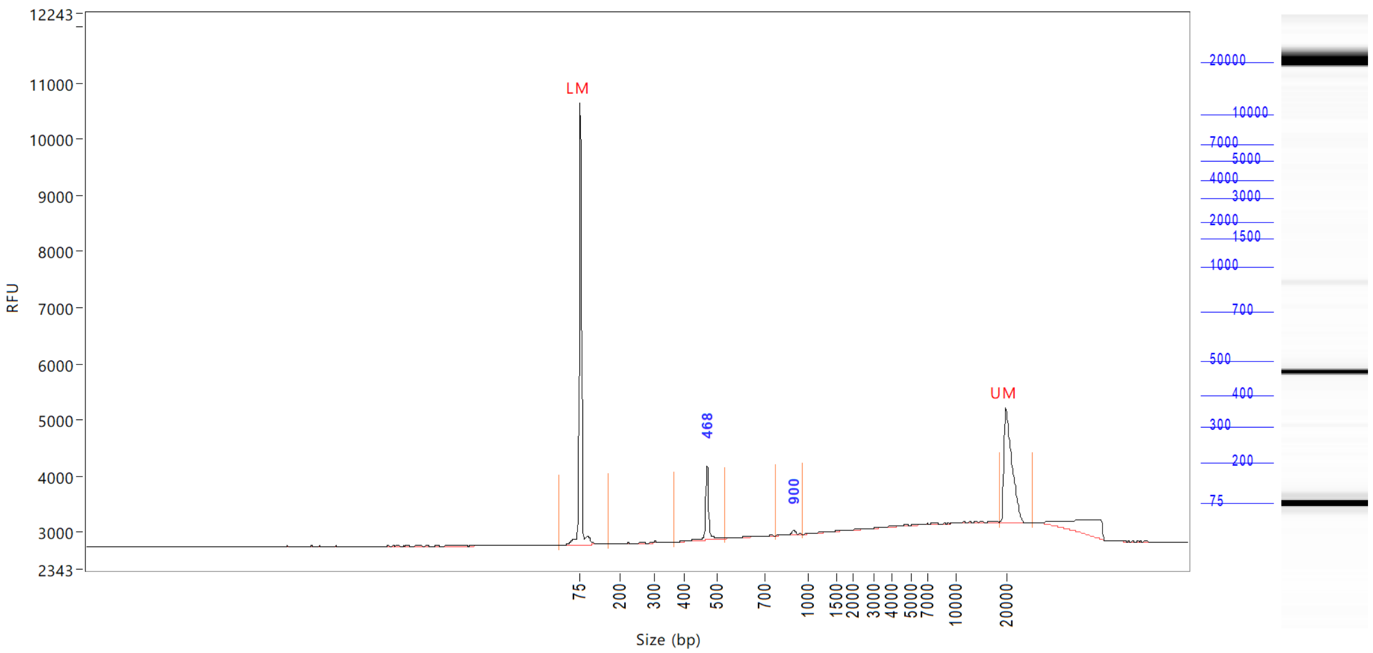

Peak	Size	Concentration	Relative concentration	Molarity	From	To	CV%	RFU
	(bp)	(ng/uL)	Percent	(nmole/L)	(bp)	(bp)		
1	75 (LM)	0.5000		11.0851	11	164	12.25	7882
2	468	1.0705	93.0	3.7800	368	529	3.50	1317
3	900	0.0802	7.0	0.1494	766	965	3.86	80
4	20000 (UM)	0.2287		0.0183	18659	25199	4.21	2047

TIC:	1.1508	ng/uL
TIM:	3.9294	nmole/L
Total concentration:	1.6571	ng/uL

Sample peak width (sec): 5 Sample min peak height: 50 Sample baseline V to V?: Y Sample baseline V to V points: 3
Sample filter: Binomial Number of points for filter: 3 Sample start region (min): 0 Sample end region (min): 30
Marker peak width (sec): 5 Marker min peak height: 200 Marker baseline V to V?: Y Marker baseline V to V points: 3
Lower marker selection: First peak > 500 RFU Upper marker selection: Last peak > 200 RFU
Ladder size (bp)75, 200, 300, 400, 500, 700, 1000, 1500, 2000, 3000, 4000, 5000, 7000, 10000, 20000
Quantification using: Lower Marker Final concentration (ng/uL): 0.5000 Dilution factor: 12.0

Sample: SampA8
Well location: A8
Created: Thursday, October 21, 2021 7:28:10 PM

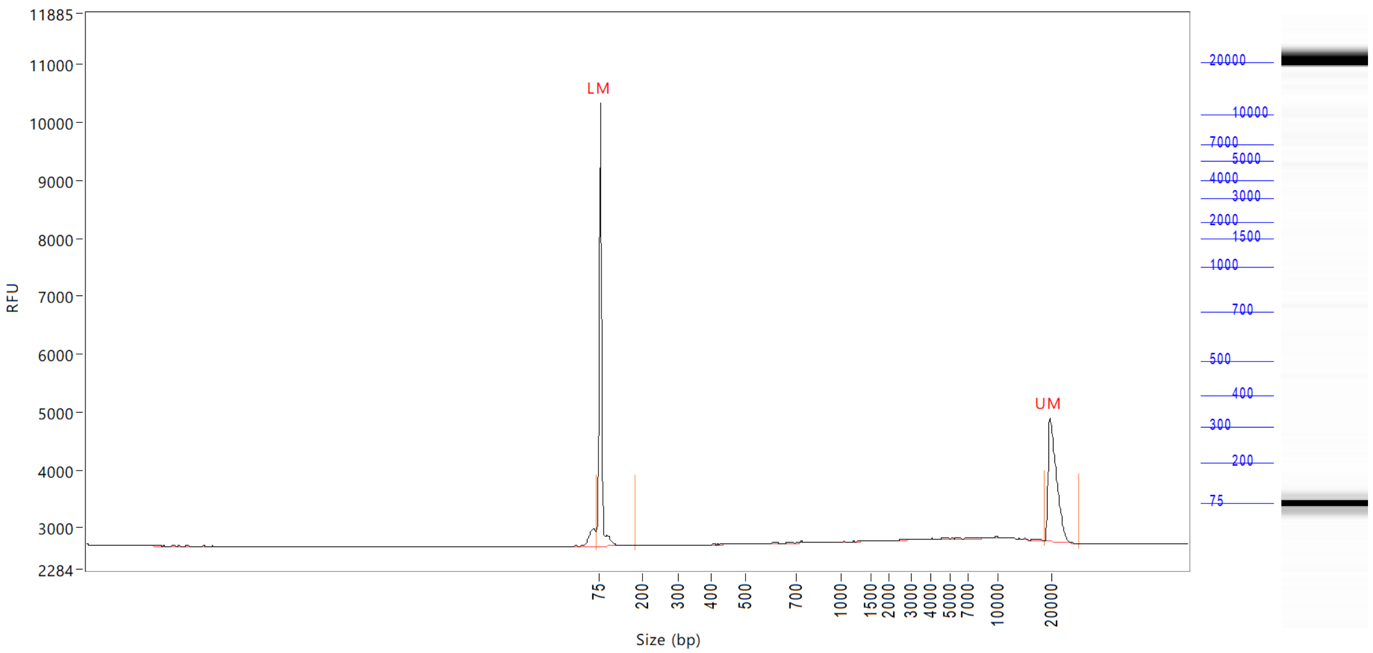

Peak	Size	Concentration	Relative concentration	Molarity	From	To	CV%	RFU
	(bp)	(ng/uL)	Percent	(nmole/L)	(bp)	(bp)		
1	75 (LM)	0.5000		11.0851	62	177	11.25	7666
2	20000 (UM)	0.2388		0.0191	18903	25561	4.10	2124

TIC:	0.0000	ng/uL
TIM:	0.0000	nmole/L
Total concentration:	0.5291	ng/uL

Sample peak width (sec): 5 Sample min peak height: 50 Sample baseline V to V?: Y Sample baseline V to V points: 3
Sample filter: Binomial Number of points for filter: 3 Sample start region (min): 0 Sample end region (min): 30
Marker peak width (sec): 5 Marker min peak height: 200 Marker baseline V to V?: Y Marker baseline V to V points: 3
Lower marker selection: First peak > 500 RFU Upper marker selection: Last peak > 200 RFU
Ladder size (bp)75, 200, 300, 400, 500, 700, 1000, 1500, 2000, 3000, 4000, 5000, 7000, 10000, 20000
Quantification using: Lower Marker Final concentration (ng/uL): 0.5000 Dilution factor: 12.0

Sample: SampB8
Well location: B8
Created: Thursday, October 21, 2021 7:28:10 PM

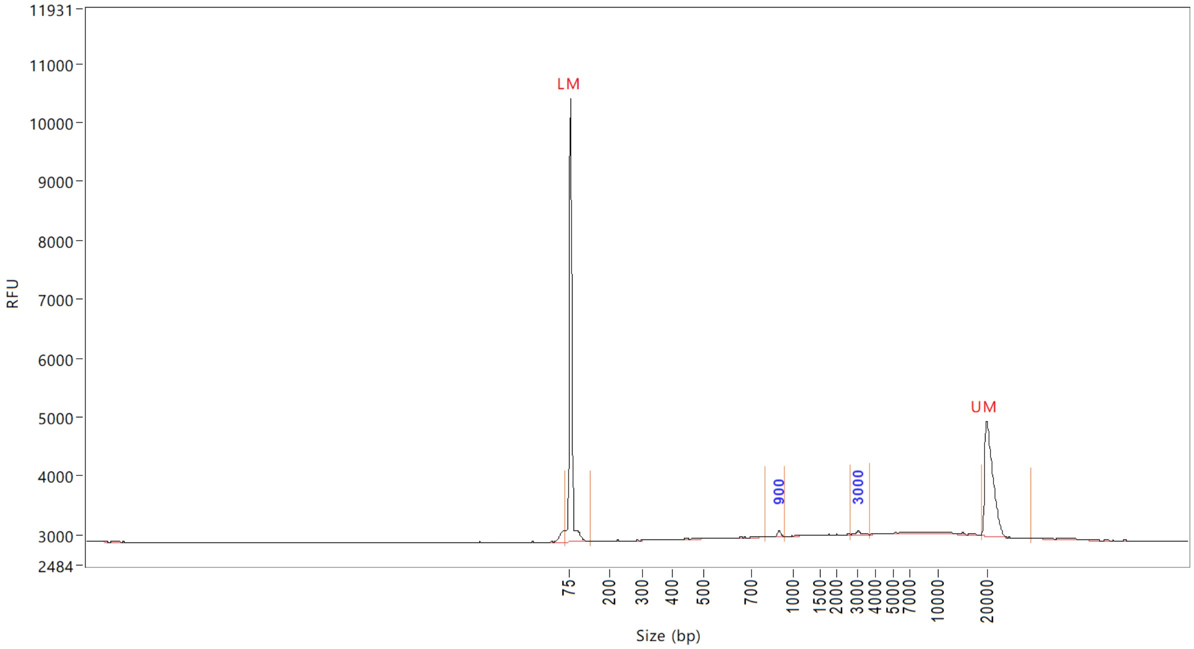

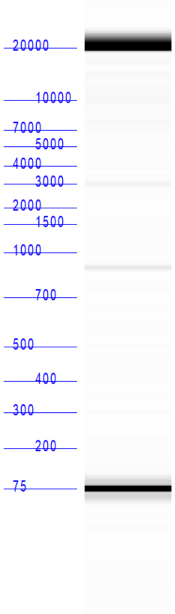

Peak	Size	Concentration	Relative concentration	Molarity	From	To	CV%	RFU
	(bp)	(ng/uL)	Percent	(nmole/L)	(bp)	(bp)		
1	75 (LM)	0.5000		10.9378	62	139	10.21	7541
2	900	0.0920	59.1	0.1699	801	944	2.61	102
3	3000	0.0638	40.9	0.0343	2686	3679	6.36	57
4	20000 (UM)	0.2284		0.0182	19024	29068	4.70	1952

TIC:	0.1557	ng/uL
TIM:	0.2043	nmole/L
Total concentration:	0.7615	ng/uL

```
Sample peak width (sec): 5    Sample min peak height: 50    Sample baseline V to V?: Y    Sample baseline V to V points: 3
Sample filter: Binomial       Number of points for filter: 3  Sample start region (min): 0  Sample end region (min): 30
Marker peak width (sec): 5    Marker min peak height: 200   Marker baseline V to V?: Y    Marker baseline V to V points: 3
Lower marker selection: First peak > 500 RFU                Upper marker selection: Last peak > 200 RFU
Ladder size (bp)75, 200, 300, 400, 500, 700, 1000, 1500, 2000, 3000, 4000, 5000, 7000, 10000, 20000
Quantification using: Lower Marker          Final concentration (ng/uL): 0.5000          Dilution factor: 12.0
```

Sample: SampC8
Well location: C8
Created: Thursday, October 21, 2021 7:28:10 PM

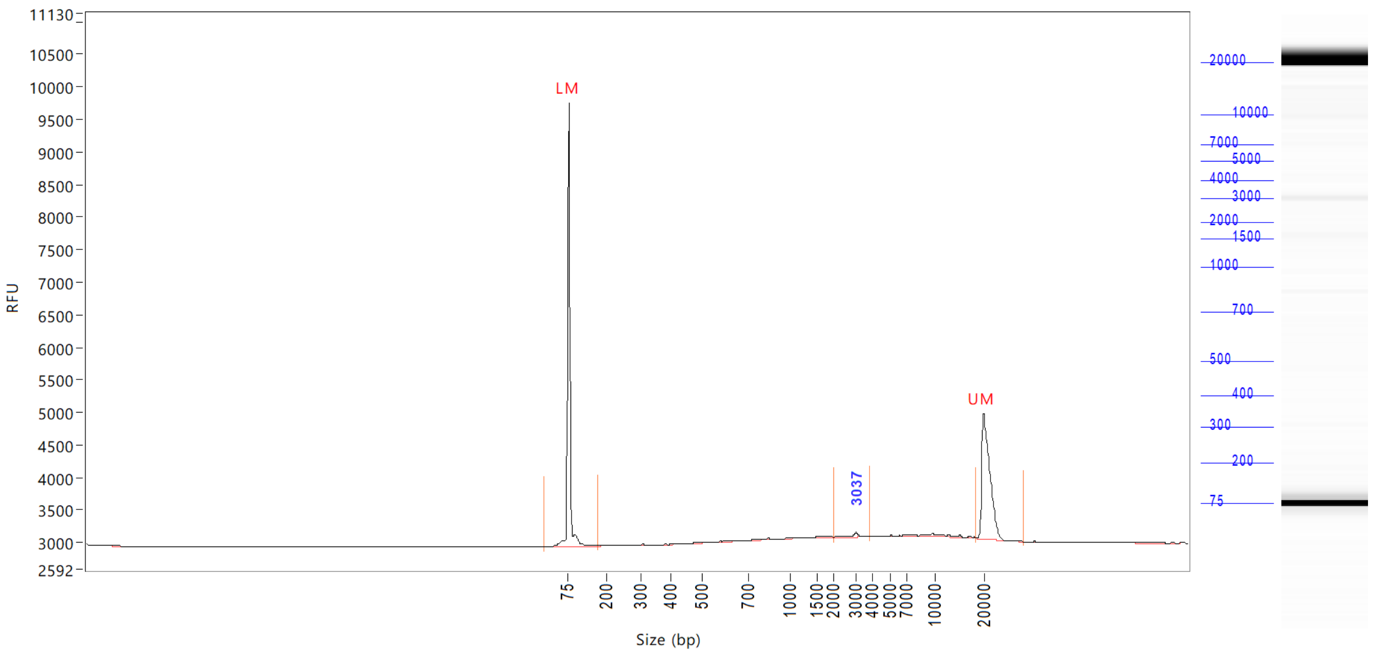

Peak	Size	Concentration	Relative concentration	Molarity	From	To	CV%	RFU
	(bp)	(ng/uL)	Percent	(nmole/L)	(bp)	(bp)		
1	75 (LM)	0.5000		10.9378	0	168	14.86	6824
2	3037	0.1088	100.0	0.0627	1981	3822	13.19	70
3	20000 (UM)	0.2409		0.0192	18537	28221	4.47	1925

TIC:	0.1088	ng/uL
TIM:	0.0627	nmole/L
Total concentration:	0.7820	ng/uL

Sample peak width (sec): 5   Sample min peak height: 50   Sample baseline V to V?: Y   Sample baseline V to V points: 3
Sample filter: Binomial   Number of points for filter: 3 Sample start region (min): 0 Sample end region (min): 30
Marker peak width (sec): 5   Marker min peak height: 200   Marker baseline V to V?: Y   Marker baseline V to V points: 3
Lower marker selection: First peak > 500 RFU   Upper marker selection: Last peak > 200 RFU
Ladder size (bp)75, 200, 300, 400, 500, 700, 1000, 1500, 2000, 3000, 4000, 5000, 7000, 10000, 20000
Quantification using: Lower Marker   Final concentration (ng/uL): 0.5000   Dilution factor: 12.0

Sample: SampD8
Well location: D8
Created: Thursday, October 21, 2021 7:28:10 PM

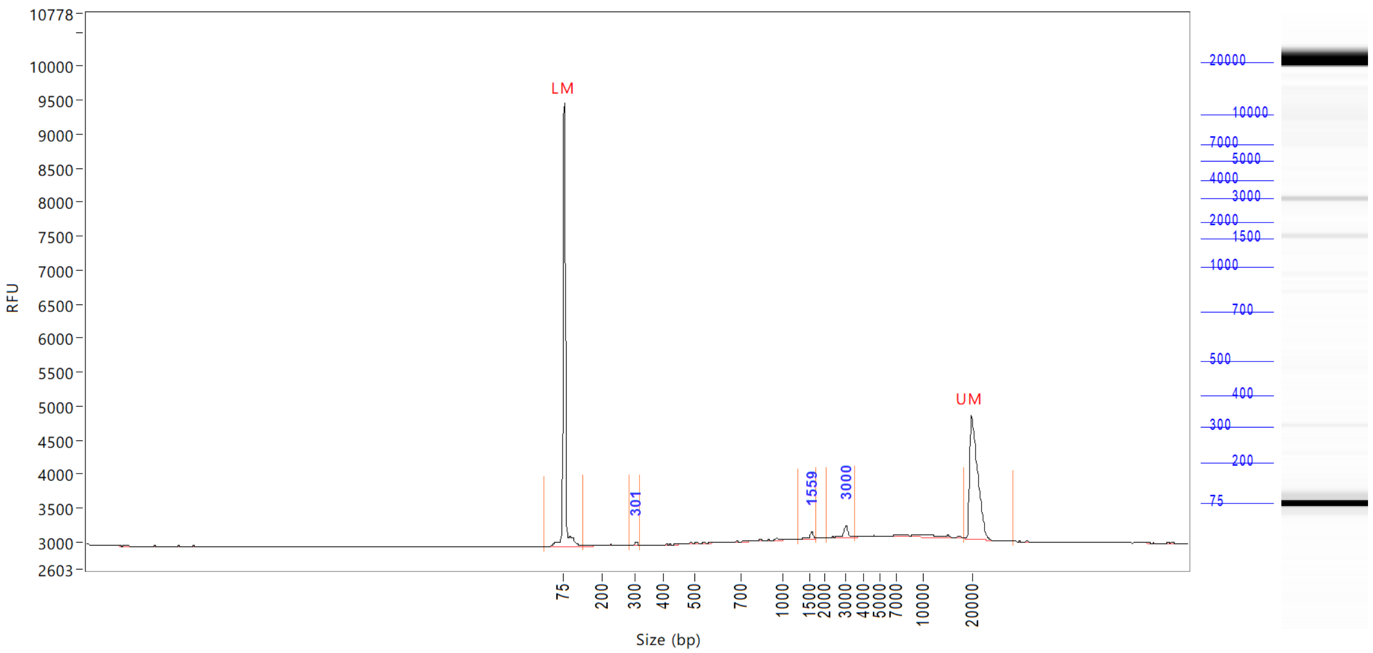

Peak	Size	Concentration	Relative concentration	Molarity	From	To	CV%	RFU
	(bp)	(ng/uL)	Percent	(nmole/L)	(bp)	(bp)		
1	75 (LM)	0.5000		11.2364	9	137	12.48	6534
2	301	0.0517	15.4	0.2845	282	315	1.94	53
3	1559	0.0928	27.6	0.1003	1289	1693	5.43	94
4	3000	0.1917	57.0	0.1090	2030	3572	8.15	175
5	20000 (UM)	0.2367		0.0190	18293	28584	4.36	1828

TIC:	0.3362	ng/uL
TIM:	0.4938	nmole/L
Total concentration:	1.0068	ng/uL

```
Sample peak width (sec): 5    Sample min peak height: 50    Sample baseline V to V?: Y    Sample baseline V to V points: 3
Sample filter: Binomial       Number of points for filter: 3  Sample start region (min): 0  Sample end region (min): 30
Marker peak width (sec): 5    Marker min peak height: 200   Marker baseline V to V?: Y    Marker baseline V to V points: 3
Lower marker selection: First peak > 500 RFU                Upper marker selection: Last peak > 200 RFU
Ladder size (bp)75, 200, 300, 400, 500, 700, 1000, 1500, 2000, 3000, 4000, 5000, 7000, 10000, 20000
Quantification using: Lower Marker          Final concentration (ng/uL): 0.5000          Dilution factor: 12.0
```

Sample: SampE8
Well location: E8
Created: Thursday, October 21, 2021 7:28:10 PM

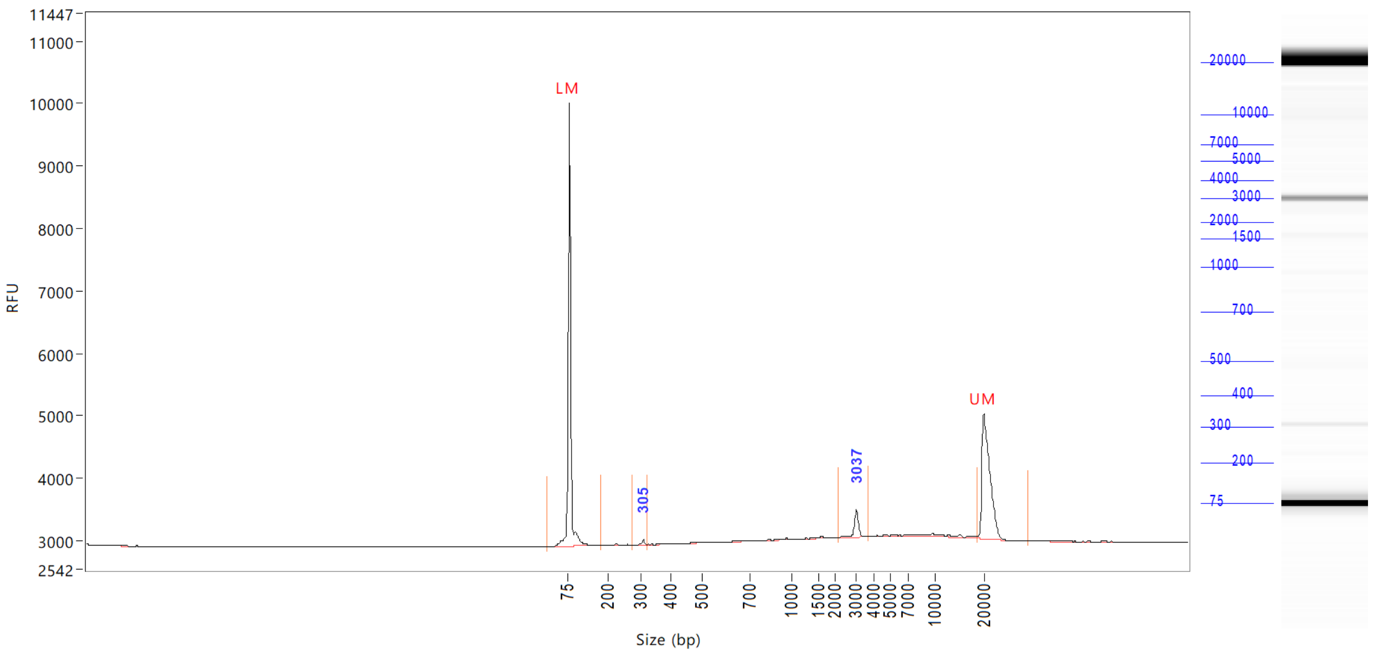

Peak	Size	Concentration	Relative concentration	Molarity	From	To	CV%	RFU
	(bp)	(ng/uL)	Percent	(nmole/L)	(bp)	(bp)		
1	75 (LM)	0.5000		10.9378	5	179	14.23	7111
2	305	0.0732	15.8	0.4002	273	317	2.54	91
3	3037	0.3904	84.2	0.2174	2201	3750	6.55	447
4	20000 (UM)	0.2327		0.0186	18659	29068	4.40	1997

TIC:	0.4637	ng/uL
TIM:	0.6176	nmole/L
Total concentration:	1.0559	ng/uL

Sample peak width (sec): 5 Sample min peak height: 50 Sample baseline V to V?: Y Sample baseline V to V points: 3
Sample filter: Binomial Number of points for filter: 3 Sample start region (min): 0 Sample end region (min): 30
Marker peak width (sec): 5 Marker min peak height: 200 Marker baseline V to V?: Y Marker baseline V to V points: 3
Lower marker selection: First peak > 500 RFU Upper marker selection: Last peak > 200 RFU
Ladder size (bp)75, 200, 300, 400, 500, 700, 1000, 1500, 2000, 3000, 4000, 5000, 7000, 10000, 20000
Quantification using: Lower Marker Final concentration (ng/uL): 0.5000 Dilution factor: 12.0

Sample: SampF8
Well location: F8
Created: Thursday, October 21, 2021 7:28:10 PM

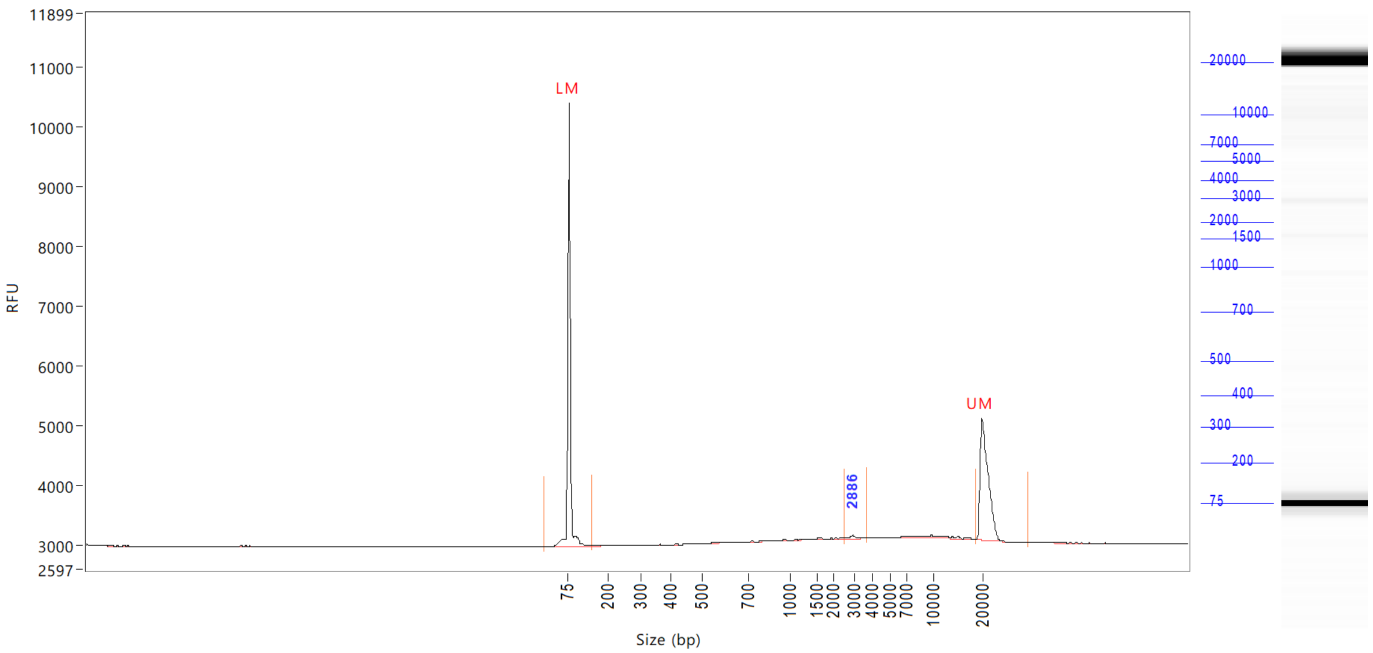

Peak	Size	Concentration	Relative concentration	Molarity	From	To	CV%	RFU
	(bp)	(ng/uL)	Percent	(nmole/L)	(bp)	(bp)		
1	75 (LM)	0.5000		11.2364	0	147	14.21	7435
2	2886	0.0715	100.0	0.0404	2486	3679	7.59	57
3	20000 (UM)	0.2342		0.0187	18659	29309	4.51	2033

TIC:	0.0715	ng/uL
TIM:	0.0404	nmole/L
Total concentration:	0.7350	ng/uL

Sample peak width (sec): 5   Sample min peak height: 50   Sample baseline V to V?: Y   Sample baseline V to V points: 3
Sample filter: Binomial   Number of points for filter: 3   Sample start region (min): 0   Sample end region (min): 30
Marker peak width (sec): 5   Marker min peak height: 200   Marker baseline V to V?: Y   Marker baseline V to V points: 3
Lower marker selection: First peak > 500 RFU   Upper marker selection: Last peak > 200 RFU
Ladder size (bp)75, 200, 300, 400, 500, 700, 1000, 1500, 2000, 3000, 4000, 5000, 7000, 10000, 20000
Quantification using: Lower Marker   Final concentration (ng/uL): 0.5000   Dilution factor: 12.0

Sample: SampG8
Well location: G8
Created: Thursday, October 21, 2021 7:28:10 PM

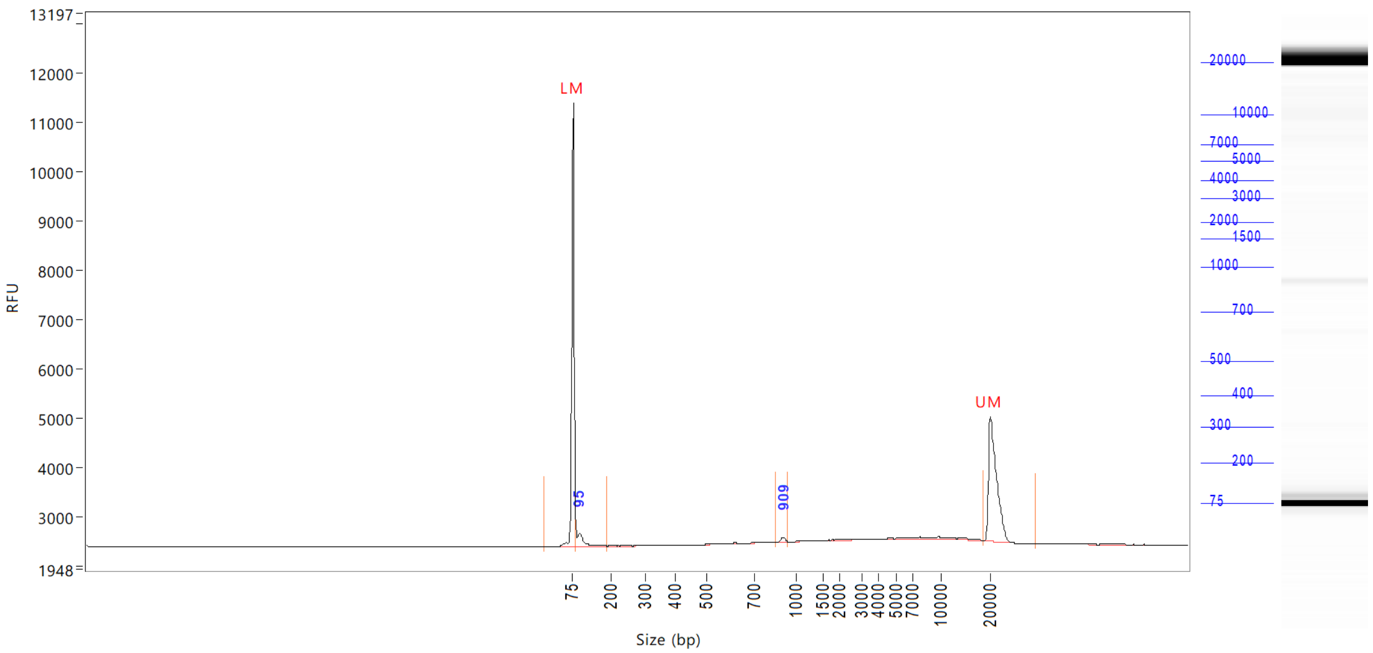

Peak	Size	Concentration	Relative concentration	Molarity	From	To	CV%	RFU
	(bp)	(ng/uL)	Percent	(nmole/L)	(bp)	(bp)		
1	75 (LM)	0.5000		11.3919	0	86	8.18	8991
2	95	0.4853	85.5	7.6636	86	183	17.91	261
3	909	0.0826	14.5	0.1511	848	944	1.78	96
4	20000 (UM)	0.2604		0.0207	18659	29189	4.54	2518

TIC:	0.5679	ng/uL
TIM:	7.8146	nmole/L
Total concentration:	1.1407	ng/uL

```
Sample peak width (sec): 5    Sample min peak height: 50    Sample baseline V to V?: Y    Sample baseline V to V points: 3
Sample filter: Binomial       Number of points for filter: 3  Sample start region (min): 0  Sample end region (min): 30
Marker peak width (sec): 5    Marker min peak height: 200   Marker baseline V to V?: Y    Marker baseline V to V points: 3
Lower marker selection: First peak > 500 RFU                Upper marker selection: Last peak > 200 RFU
Ladder size (bp)75, 200, 300, 400, 500, 700, 1000, 1500, 2000, 3000, 4000, 5000, 7000, 10000, 20000
Quantification using: Lower Marker          Final concentration (ng/uL): 0.5000          Dilution factor: 12.0
```

Sample: SampH8
Well location: H8
Created: Thursday, October 21, 2021 7:28:10 PM

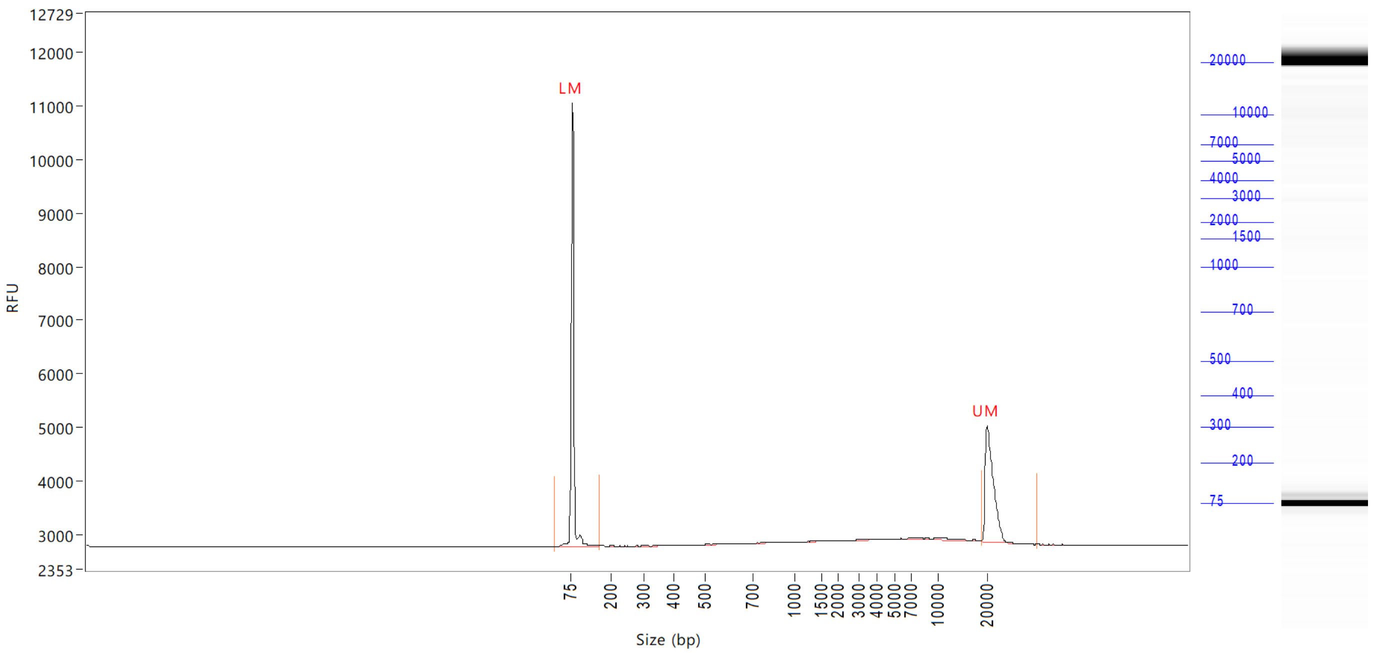

Peak	Size	Concentration	Relative concentration	Molarity	From	To	CV%	RFU
	(bp)	(ng/uL)	Percent	(nmole/L)	(bp)	(bp)		
1	75 (LM)	0.5000		11.0851	18	162	11.37	8290
2	20000 (UM)	0.2343		0.0187	18903	30156	4.67	2164

TIC:	0.0000	ng/uL
TIM:	0.0000	nmole/L
Total concentration:	0.5606	ng/uL

Sample peak width (sec): 5 Sample min peak height: 50 Sample baseline V to V?: Y Sample baseline V to V points: 3
Sample filter: Binomial Number of points for filter: 3 Sample start region (min): 0 Sample end region (min): 30
Marker peak width (sec): 5 Marker min peak height: 200 Marker baseline V to V?: Y Marker baseline V to V points: 3
Lower marker selection: First peak > 500 RFU Upper marker selection: Last peak > 200 RFU
Ladder size (bp)75, 200, 300, 400, 500, 700, 1000, 1500, 2000, 3000, 4000, 5000, 7000, 10000, 20000
Quantification using: Lower Marker Final concentration (ng/uL): 0.5000 Dilution factor: 12.0

Sample: SampA9
Well location: A9
Created: Thursday, October 21, 2021 7:28:10 PM

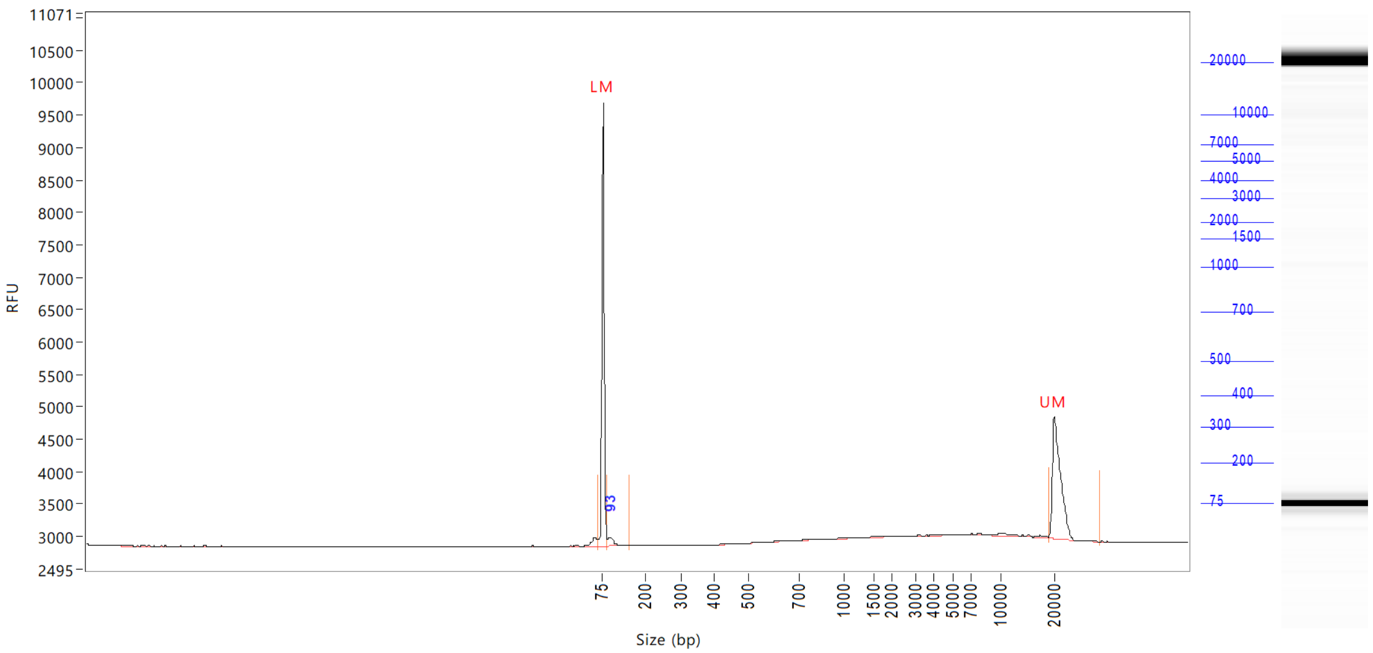

Peak	Size	Concentration	Relative concentration	Molarity	From	To	CV%	RFU
	(bp)	(ng/uL)	Percent	(nmole/L)	(bp)	(bp)		
1	75 (LM)	0.5000		11.2364	60	87	4.72	6848
2	93	0.3239	100.0	5.2658	87	154	13.21	133
3	20000 (UM)	0.2563		0.0205	18903	28463	4.39	1879

TIC:	0.3239	ng/uL
TIM:	5.2658	nmole/L
Total concentration:	0.9567	ng/uL

```
Sample peak width (sec): 5    Sample min peak height: 50    Sample baseline V to V?: Y    Sample baseline V to V points: 3
Sample filter: Binomial       Number of points for filter: 3  Sample start region (min): 0  Sample end region (min): 30
Marker peak width (sec): 5    Marker min peak height: 200   Marker baseline V to V?: Y    Marker baseline V to V points: 3
Lower marker selection: First peak > 500 RFU                Upper marker selection: Last peak > 200 RFU
Ladder size (bp)75, 200, 300, 400, 500, 700, 1000, 1500, 2000, 3000, 4000, 5000, 7000, 10000, 20000
Quantification using: Lower Marker         Final concentration (ng/uL): 0.5000         Dilution factor: 12.0
```

Sample: SampB9
Well location: B9
Created: Thursday, October 21, 2021 7:28:10 PM

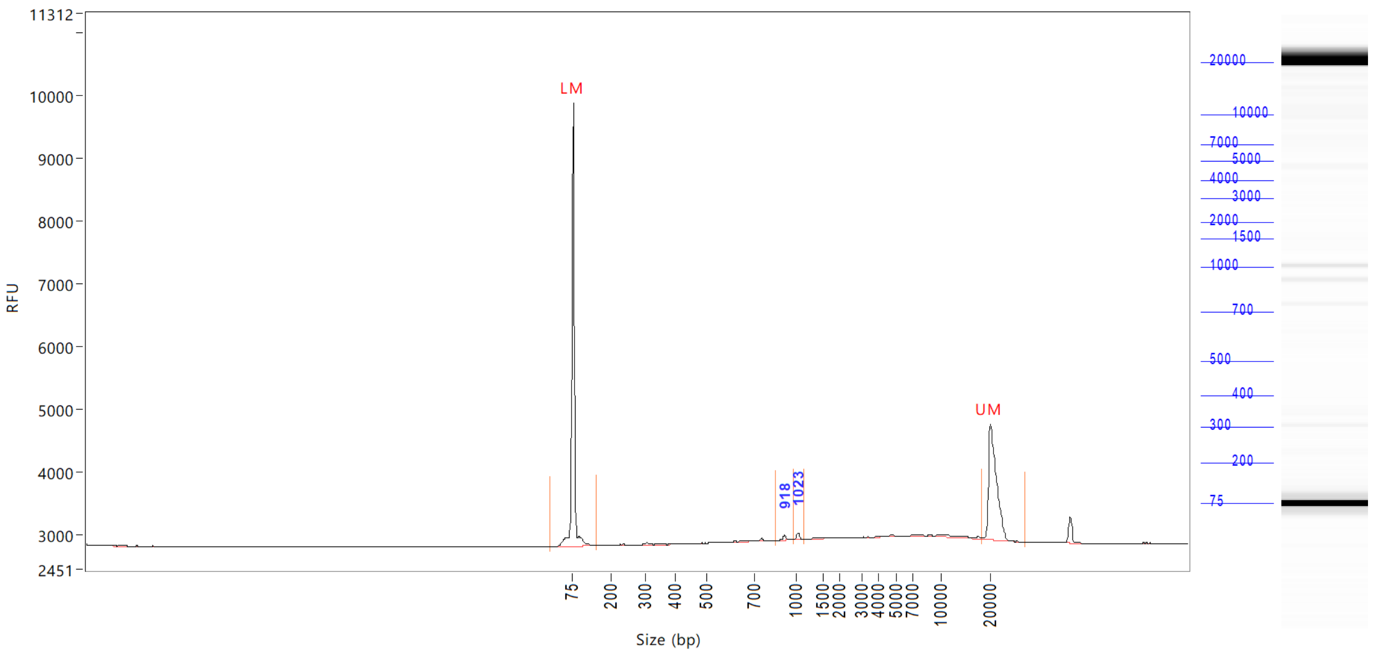

Peak	Size	Concentration	Relative concentration	Molarity	From	To	CV%	RFU
	(bp)	(ng/uL)	Percent	(nmole/L)	(bp)	(bp)		
1	75 (LM)	0.5000		11.0851	5	152	14.25	7077
2	918	0.0636	45.9	0.1149	848	983	2.13	74
3	1023	0.0750	54.1	0.1210	983	1145	2.38	109
4	20000 (UM)	0.2179		0.0174	18293	27133	4.32	1842

TIC:	0.1387	ng/uL
TIM:	0.2359	nmole/L
Total concentration:	0.7940	ng/uL

Sample peak width (sec): 5 Sample min peak height: 50 Sample baseline V to V?: Y Sample baseline V to V points: 3
Sample filter: Binomial Number of points for filter: 3 Sample start region (min): 0 Sample end region (min): 30
Marker peak width (sec): 5 Marker min peak height: 200 Marker baseline V to V?: Y Marker baseline V to V points: 3
Lower marker selection: First peak > 500 RFU Upper marker selection: Last peak > 200 RFU
Ladder size (bp)75, 200, 300, 400, 500, 700, 1000, 1500, 2000, 3000, 4000, 5000, 7000, 10000, 20000
Quantification using: Lower Marker Final concentration (ng/uL): 0.5000 Dilution factor: 12.0

Sample: SampC9
Well location: C9
Created: Thursday, October 21, 2021 7:28:10 PM

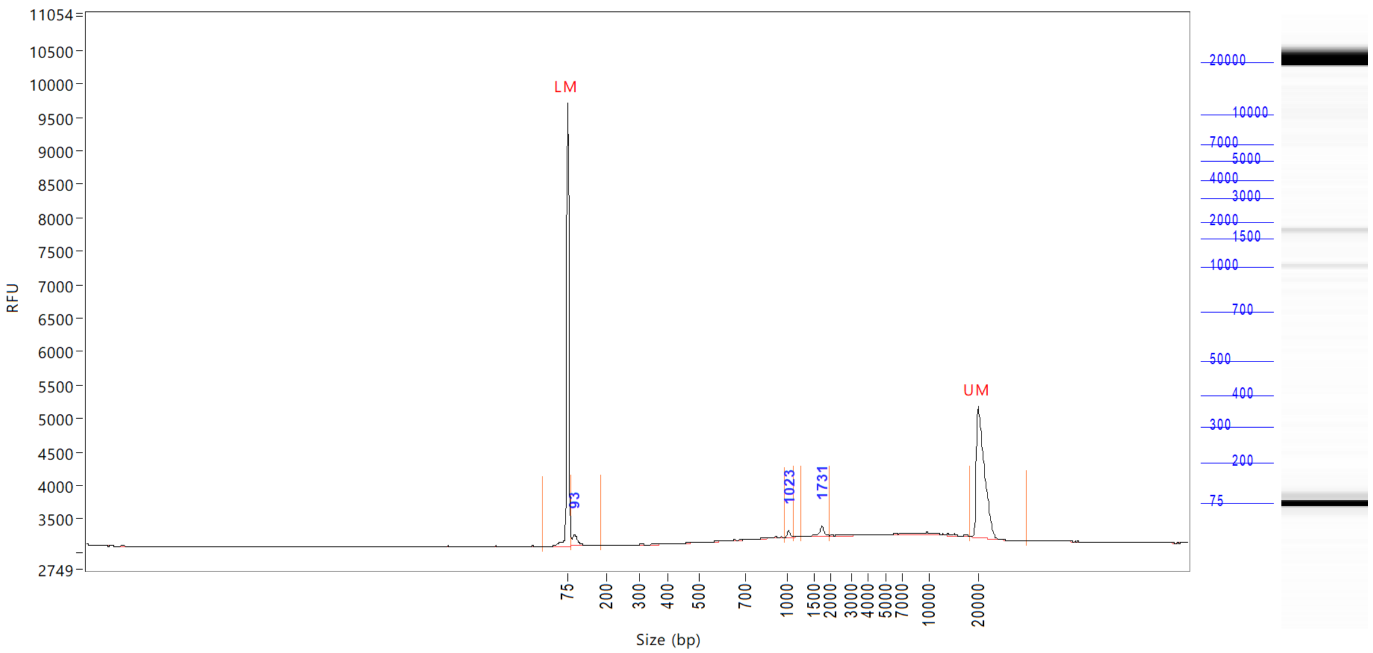

Peak	Size	Concentration	Relative concentration	Molarity	From	To	CV%	RFU
	(bp)	(ng/uL)	Percent	(nmole/L)	(bp)	(bp)		
1	75 (LM)	0.5000		11.3919	0	86	8.19	6631
2	93	0.3905	57.8	6.2265	86	181	17.38	167
3	1023	0.0842	12.5	0.1363	978	1123	2.59	103
4	1731	0.2014	29.8	0.2011	1267	1962	8.66	146
5	20000 (UM)	0.2729		0.0217	18171	30035	4.73	1956

TIC:	0.6762	ng/uL
TIM:	6.5639	nmole/L
Total concentration:	1.2212	ng/uL

Sample peak width (sec): 5   Sample min peak height: 50   Sample baseline V to V?: Y   Sample baseline V to V points: 3
Sample filter: Binomial   Number of points for filter: 3 Sample start region (min): 0 Sample end region (min): 30
Marker peak width (sec): 5   Marker min peak height: 200   Marker baseline V to V?: Y   Marker baseline V to V points: 3
Lower marker selection: First peak > 500 RFU   Upper marker selection: Last peak > 200 RFU
Ladder size (bp)75, 200, 300, 400, 500, 700, 1000, 1500, 2000, 3000, 4000, 5000, 7000, 10000, 20000
Quantification using: Lower Marker   Final concentration (ng/uL): 0.5000   Dilution factor: 12.0

Sample: SampD9
Well location: D9
Created: Thursday, October 21, 2021 7:28:10 PM

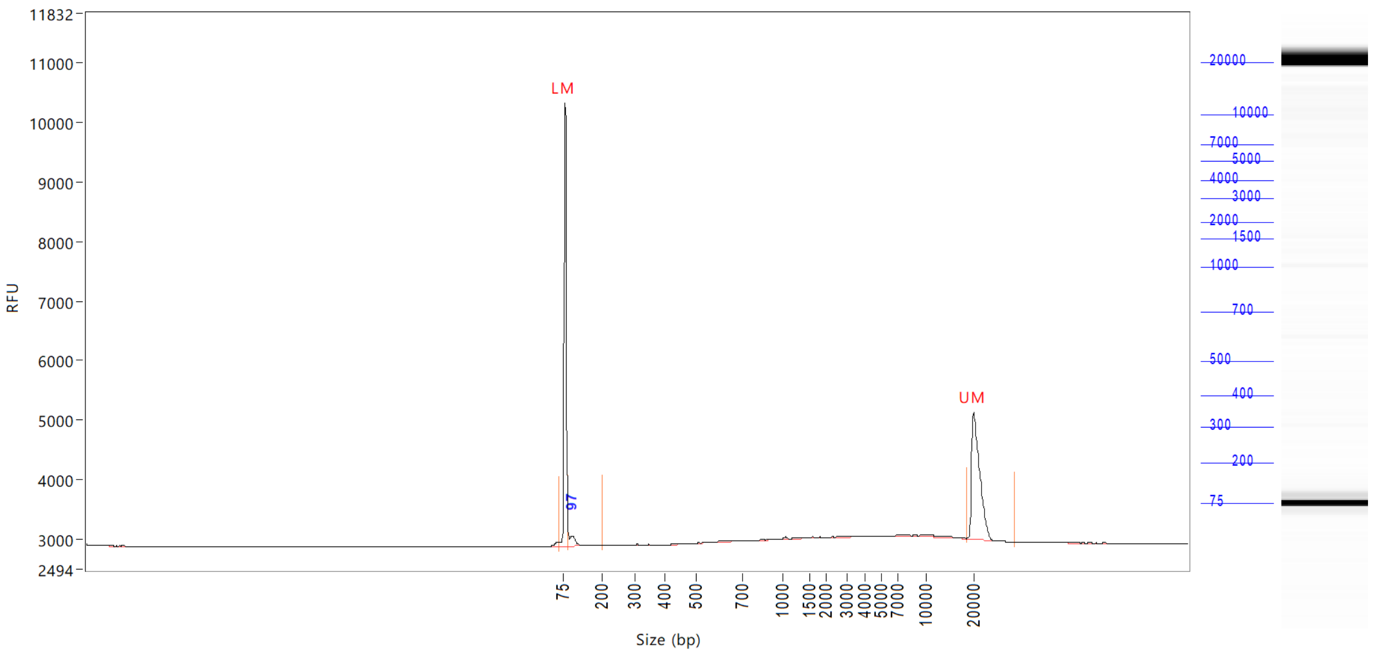

Peak	Size	Concentration	Relative concentration	Molarity	From	To	CV%	RFU
	(bp)	(ng/uL)	Percent	(nmole/L)	(bp)	(bp)		
1	75 (LM)	0.5000		11.0851	60	87	4.47	7458
2	97	0.3578	100.0	5.5957	87	196	17.53	163
3	20000 (UM)	0.2654		0.0212	18415	28463	4.32	2122

TIC:	0.3578	ng/uL
TIM:	5.5957	nmole/L
Total concentration:	1.0184	ng/uL

Sample peak width (sec): 5   Sample min peak height: 50   Sample baseline V to V?: Y   Sample baseline V to V points: 3
Sample filter: Binomial   Number of points for filter: 3 Sample start region (min): 0 Sample end region (min): 30
Marker peak width (sec): 5   Marker min peak height: 200   Marker baseline V to V?: Y   Marker baseline V to V points: 3
Lower marker selection: First peak > 500 RFU   Upper marker selection: Last peak > 200 RFU
Ladder size (bp)75, 200, 300, 400, 500, 700, 1000, 1500, 2000, 3000, 4000, 5000, 7000, 10000, 20000
Quantification using: Lower Marker   Final concentration (ng/uL): 0.5000   Dilution factor: 12.0

Sample: SampE9
Well location: E9
Created: Thursday, October 21, 2021 7:28:10 PM

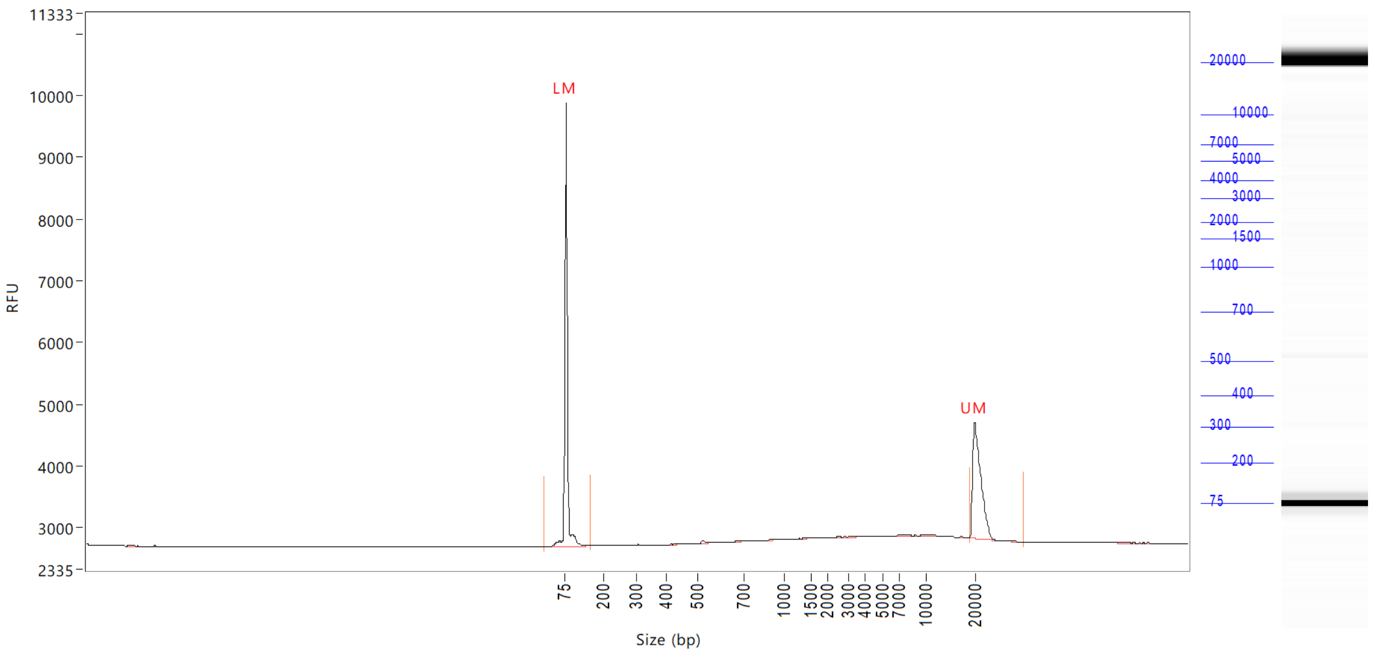

Peak	Size	Concentration	Relative concentration	Molarity	From	To	CV%	RFU
	(bp)	(ng/uL)	Percent	(nmole/L)	(bp)	(bp)		
1	75 (LM)	0.5000		11.0851	3	154	13.63	7189
2	20000 (UM)	0.2298		0.0183	18903	30156	4.96	1870

TIC:	0.0000	ng/uL
TIM:	0.0000	nmole/L
Total concentration:	0.4384	ng/uL

Sample peak width (sec): 5 Sample min peak height: 50 Sample baseline V to V?: Y Sample baseline V to V points: 3
Sample filter: Binomial Number of points for filter: 3 Sample start region (min): 0 Sample end region (min): 30
Marker peak width (sec): 5 Marker min peak height: 200 Marker baseline V to V?: Y Marker baseline V to V points: 3
Lower marker selection: First peak > 500 RFU Upper marker selection: Last peak > 200 RFU
Ladder size (bp)75, 200, 300, 400, 500, 700, 1000, 1500, 2000, 3000, 4000, 5000, 7000, 10000, 20000
Quantification using: Lower Marker Final concentration (ng/uL): 0.5000 Dilution factor: 12.0

Sample: SampF9
Well location: F9
Created: Thursday, October 21, 2021 7:28:10 PM

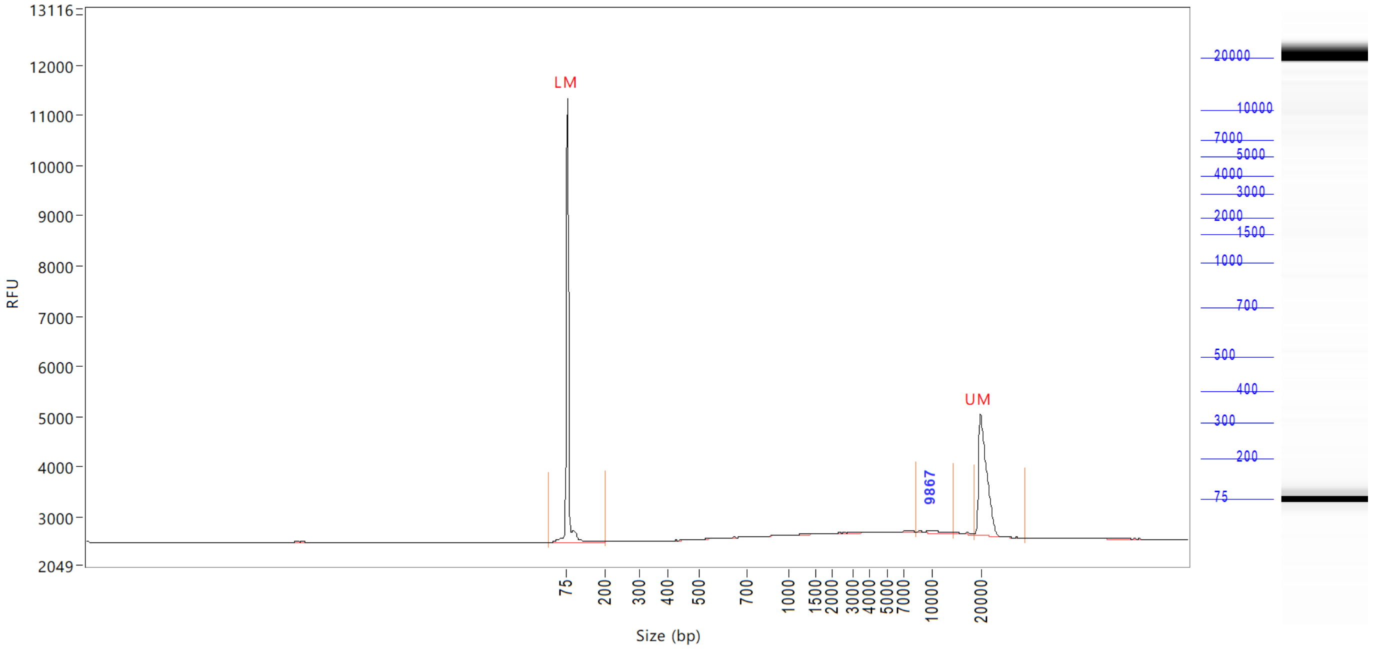

Peak	Size	Concentration	Relative concentration	Molarity	From	To	CV%	RFU
	(bp)	(ng/uL)	Percent	(nmole/L)	(bp)	(bp)		
1	75 (LM)	0.5000		10.9378	18	198	16.29	8844
2	9867	0.1670	100.0	0.0254	8334	14269	15.40	53
3	20000 (UM)	0.2392		0.0191	18903	29189	4.46	2415

TIC:	0.1670	ng/uL
TIM:	0.0254	nmole/L
Total concentration:	0.6313	ng/uL

Sample peak width (sec): 5   Sample min peak height: 50   Sample baseline V to V?: Y   Sample baseline V to V points: 3
Sample filter: Binomial   Number of points for filter: 3 Sample start region (min): 0 Sample end region (min): 30
Marker peak width (sec): 5   Marker min peak height: 200   Marker baseline V to V?: Y   Marker baseline V to V points: 3
Lower marker selection: First peak > 500 RFU   Upper marker selection: Last peak > 200 RFU
Ladder size (bp)75, 200, 300, 400, 500, 700, 1000, 1500, 2000, 3000, 4000, 5000, 7000, 10000, 20000
Quantification using: Lower Marker   Final concentration (ng/uL): 0.5000   Dilution factor: 12.0

Sample: SampG9
Well location: G9
Created: Thursday, October 21, 2021 7:28:10 PM

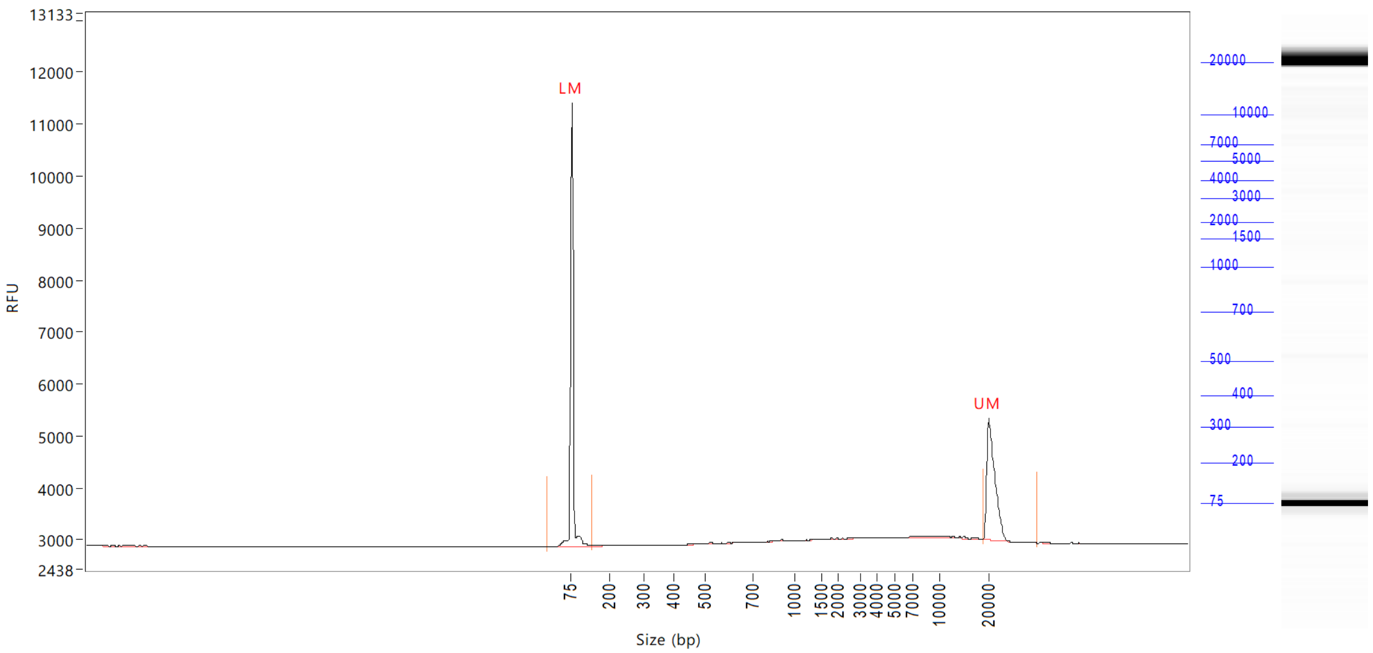

Peak	Size	Concentration	Relative concentration	Molarity	From	To	CV%	RFU
	(bp)	(ng/uL)	Percent	(nmole/L)	(bp)	(bp)		
1	75 (LM)	0.5000		11.0851	0	141	12.54	8547
2	20000 (UM)	0.2351		0.0188	18781	29793	4.77	2328

TIC:	0.0000	ng/uL
TIM:	0.0000	nmole/L
Total concentration:	0.4792	ng/uL

```
Sample peak width (sec): 5    Sample min peak height: 50    Sample baseline V to V?: Y    Sample baseline V to V points: 3
Sample filter: Binomial       Number of points for filter: 3  Sample start region (min): 0  Sample end region (min): 30
Marker peak width (sec): 5    Marker min peak height: 200   Marker baseline V to V?: Y    Marker baseline V to V points: 3
Lower marker selection: First peak > 500 RFU                Upper marker selection: Last peak > 200 RFU
Ladder size (bp)75, 200, 300, 400, 500, 700, 1000, 1500, 2000, 3000, 4000, 5000, 7000, 10000, 20000
Quantification using: Lower Marker          Final concentration (ng/uL): 0.5000          Dilution factor: 12.0
```

Sample: SampH9
Well location: H9
Created: Thursday, October 21, 2021 7:28:10 PM

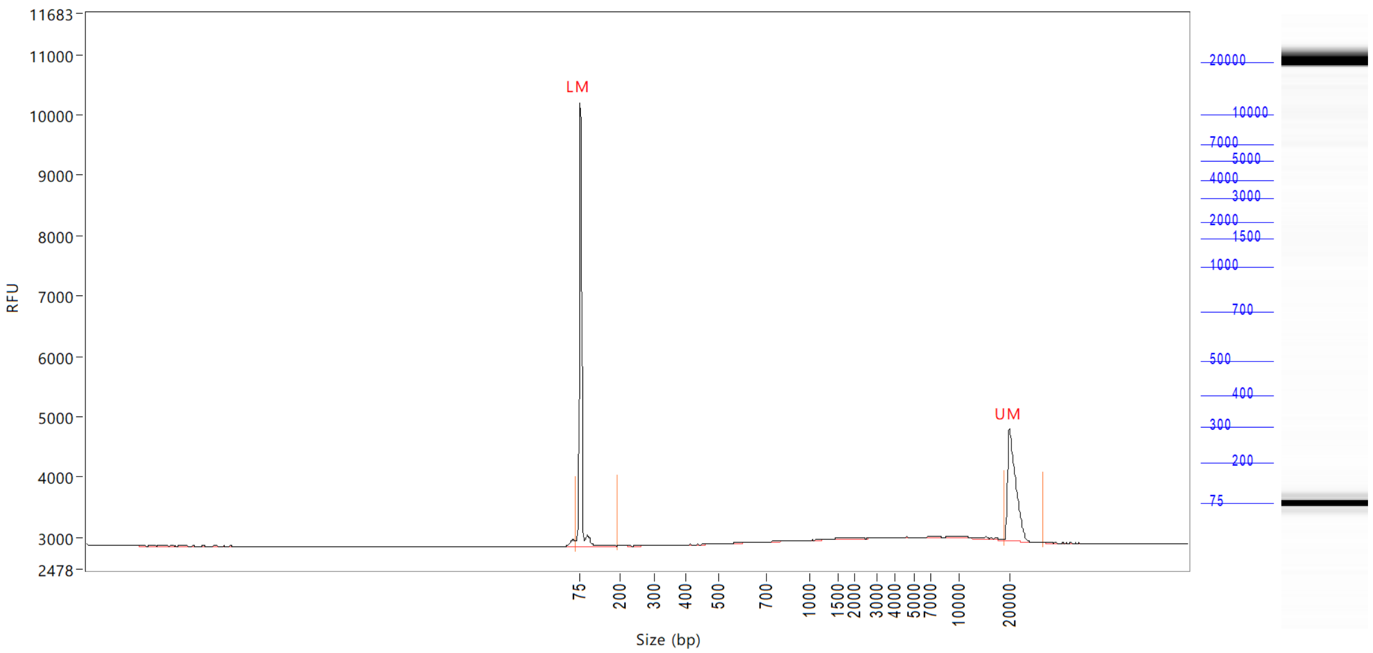

Peak	Size	Concentration	Relative concentration	Molarity	From	To	CV%	RFU
	(bp)	(ng/uL)	Percent	(nmole/L)	(bp)	(bp)		
1	75 (LM)	0.5000		10.9378	60	190	11.76	7354
2	20000 (UM)	0.2193		0.0176	18781	26649	4.33	1852

TIC:	0.0000	ng/uL
TIM:	0.0000	nmole/L
Total concentration:	0.6277	ng/uL

Sample peak width (sec): 5   Sample min peak height: 50   Sample baseline V to V?: Y   Sample baseline V to V points: 3
Sample filter: Binomial   Number of points for filter: 3 Sample start region (min): 0 Sample end region (min): 30
Marker peak width (sec): 5   Marker min peak height: 200   Marker baseline V to V?: Y   Marker baseline V to V points: 3
Lower marker selection: First peak > 500 RFU   Upper marker selection: Last peak > 200 RFU
Ladder size (bp)75, 200, 300, 400, 500, 700, 1000, 1500, 2000, 3000, 4000, 5000, 7000, 10000, 20000
Quantification using: Lower Marker   Final concentration (ng/uL): 0.5000   Dilution factor: 12.0

Sample: SampA10
Well location: A10
Created: Thursday, October 21, 2021 7:28:10 PM

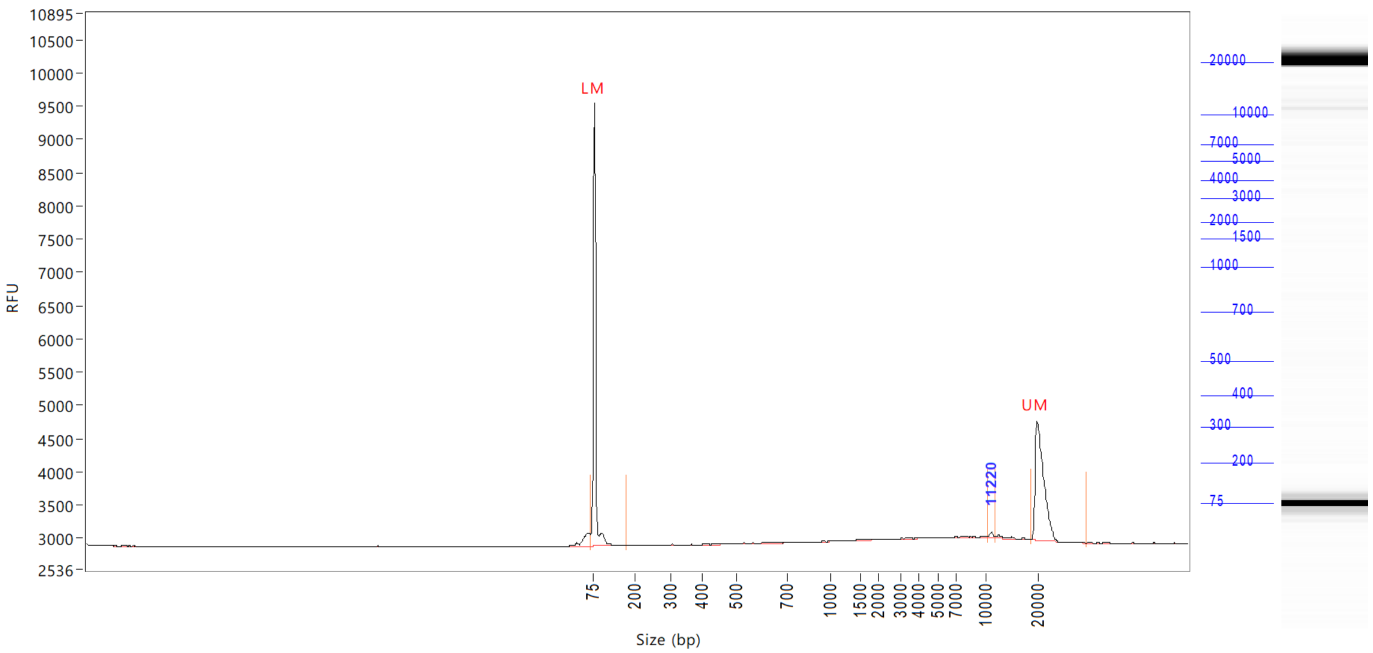

Peak	Size	Concentration	Relative concentration	Molarity	From	To	CV%	RFU
	(bp)	(ng/uL)	Percent	(nmole/L)	(bp)	(bp)		
1	75 (LM)	0.5000		10.9378	64	169	10.45	6673
2	11220	0.0678	100.0	0.0101	10489	11830	2.96	90
3	20000 (UM)	0.2383		0.0191	18781	29430	4.60	1791

TIC:	0.0678	ng/uL
TIM:	0.0101	nmole/L
Total concentration:	0.6582	ng/uL

Sample peak width (sec): 5   Sample min peak height: 50   Sample baseline V to V?: Y   Sample baseline V to V points: 3
Sample filter: Binomial   Number of points for filter: 3 Sample start region (min): 0 Sample end region (min): 30
Marker peak width (sec): 5   Marker min peak height: 200   Marker baseline V to V?: Y   Marker baseline V to V points: 3
Lower marker selection: First peak > 500 RFU   Upper marker selection: Last peak > 200 RFU
Ladder size (bp)75, 200, 300, 400, 500, 700, 1000, 1500, 2000, 3000, 4000, 5000, 7000, 10000, 20000
Quantification using: Lower Marker   Final concentration (ng/uL): 0.5000   Dilution factor: 12.0

Sample: SampB10
Well location: B10
Created: Thursday, October 21, 2021 7:28:10 PM

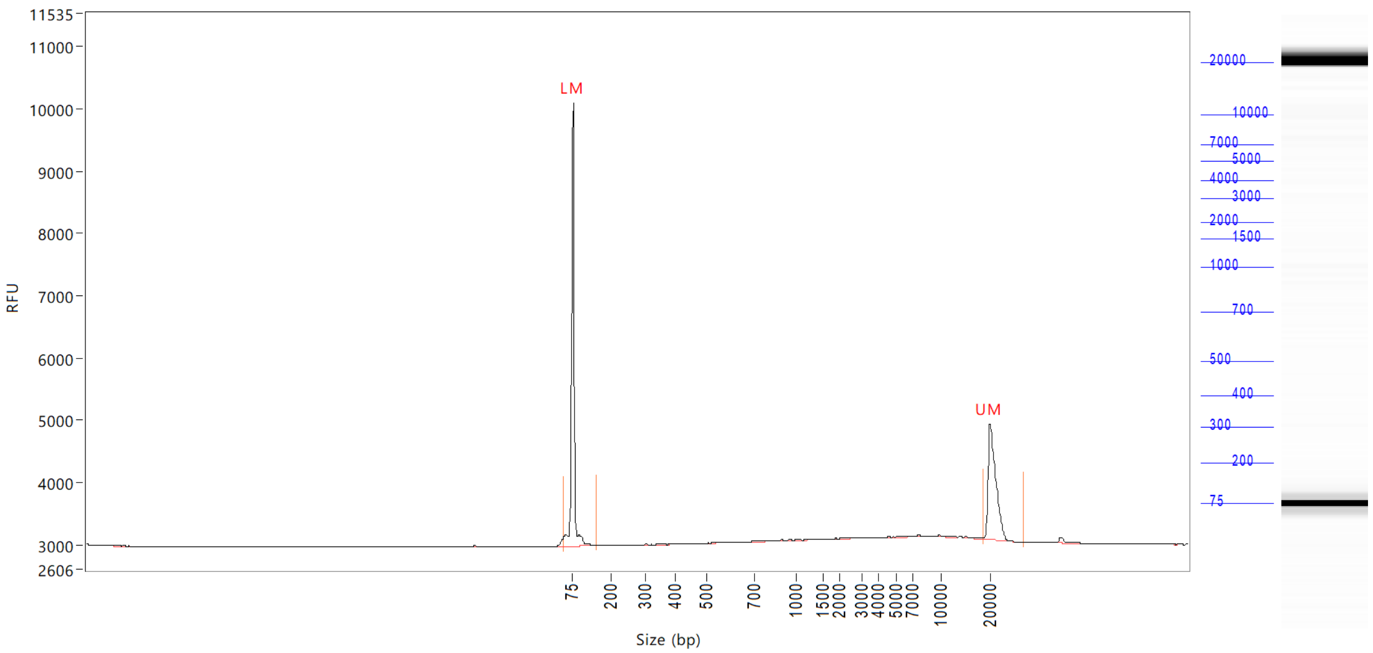

Peak	Size	Concentration	Relative concentration	Molarity	From	To	CV%	RFU
	(bp)	(ng/uL)	Percent	(nmole/L)	(bp)	(bp)		
1	75 (LM)	0.5000		11.0851	48	152	12.33	7131
2	20000 (UM)	0.2101		0.0169	18781	26649	4.14	1862

TIC:	0.0000	ng/uL
TIM:	0.0000	nmole/L
Total concentration:	0.4694	ng/uL

Sample peak width (sec): 5 Sample min peak height: 50 Sample baseline V to V?: Y Sample baseline V to V points: 3
Sample filter: Binomial Number of points for filter: 3 Sample start region (min): 0 Sample end region (min): 30
Marker peak width (sec): 5 Marker min peak height: 200 Marker baseline V to V?: Y Marker baseline V to V points: 3
Lower marker selection: First peak > 500 RFU Upper marker selection: Last peak > 200 RFU
Ladder size (bp)75, 200, 300, 400, 500, 700, 1000, 1500, 2000, 3000, 4000, 5000, 7000, 10000, 20000
Quantification using: Lower Marker Final concentration (ng/uL): 0.5000 Dilution factor: 12.0

Sample: SampC10
Well location: C10
Created: Thursday, October 21, 2021 7:28:10 PM

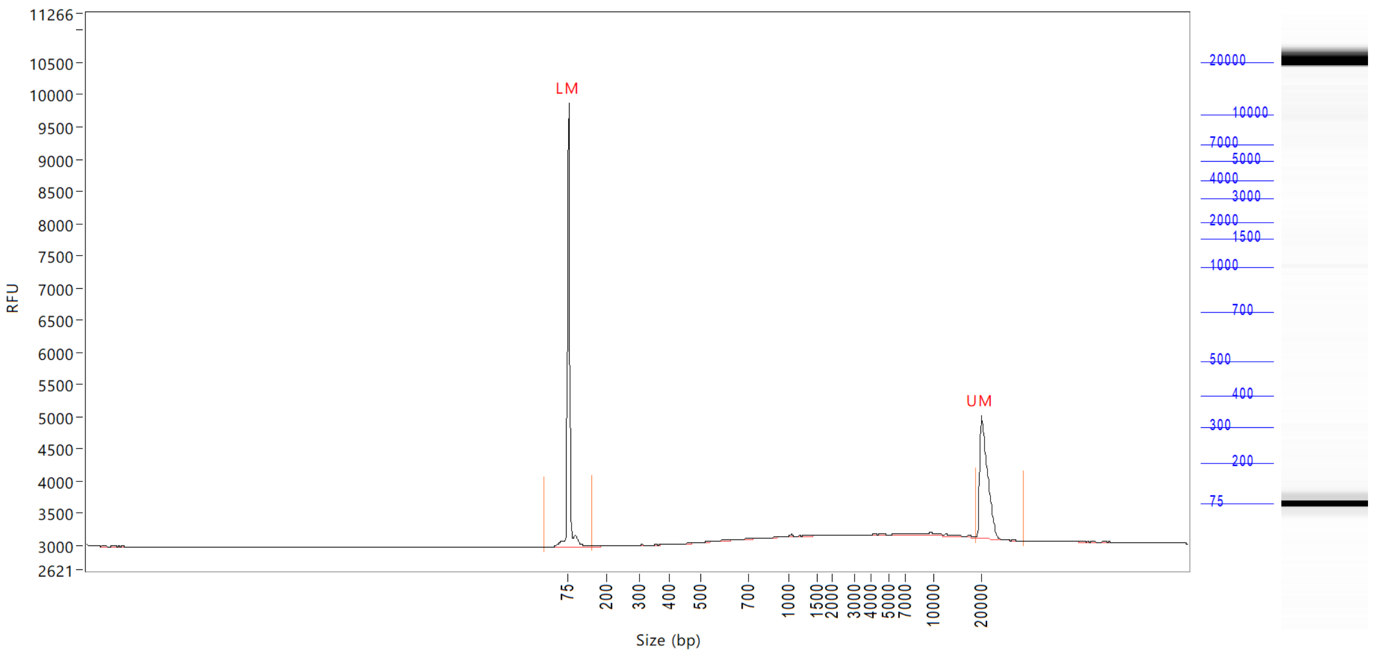

Peak	Size	Concentration	Relative concentration	Molarity	From	To	CV%	RFU
	(bp)	(ng/uL)	Percent	(nmole/L)	(bp)	(bp)		
1	75 (LM)	0.5000		11.0851	0	152	14.07	6908
2	20000 (UM)	0.2342		0.0187	18781	28463	4.54	1898

TIC:	0.0000	ng/uL
TIM:	0.0000	nmole/L
Total concentration:	0.7526	ng/uL

Sample peak width (sec): 5 Sample min peak height: 50 Sample baseline V to V?: Y Sample baseline V to V points: 3
Sample filter: Binomial Number of points for filter: 3 Sample start region (min): 0 Sample end region (min): 30
Marker peak width (sec): 5 Marker min peak height: 200 Marker baseline V to V?: Y Marker baseline V to V points: 3
Lower marker selection: First peak > 500 RFU Upper marker selection: Last peak > 200 RFU
Ladder size (bp)75, 200, 300, 400, 500, 700, 1000, 1500, 2000, 3000, 4000, 5000, 7000, 10000, 20000
Quantification using: Lower Marker Final concentration (ng/uL): 0.5000 Dilution factor: 12.0

Sample: SampD10
Well location: D10
Created: Thursday, October 21, 2021 7:28:10 PM

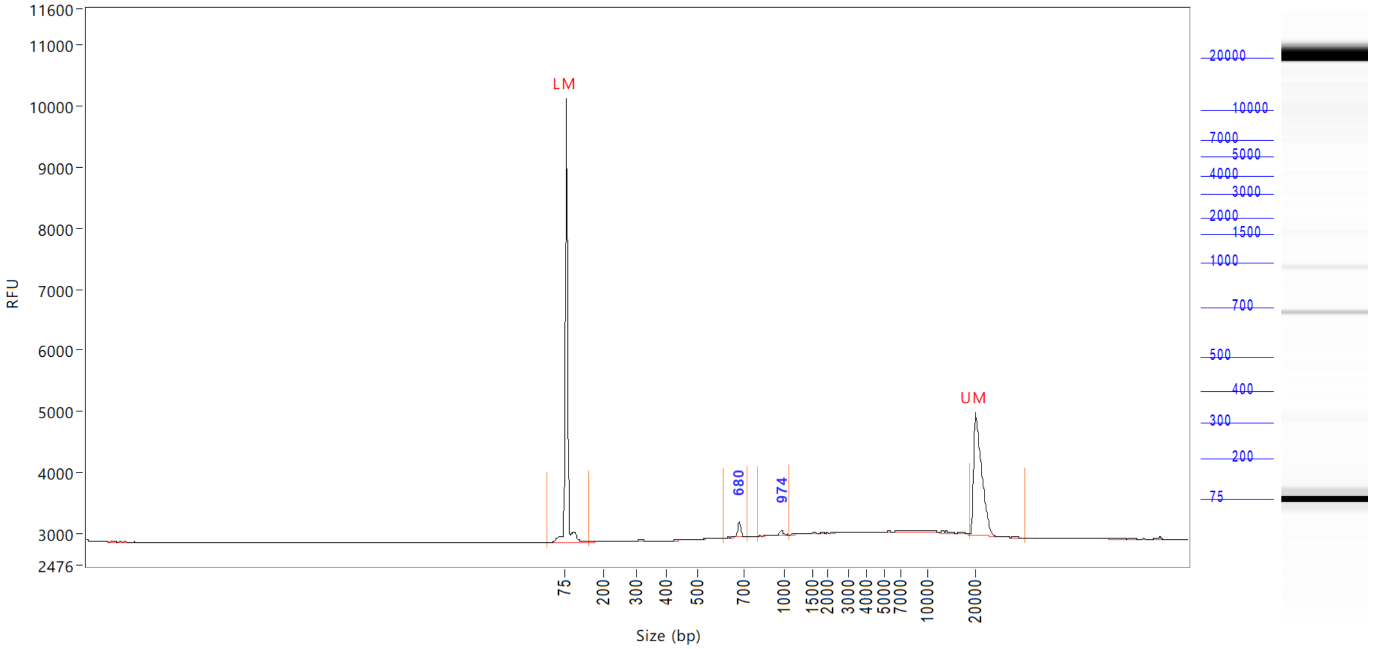

Peak	Size	Concentration	Relative concentration	Molarity	From	To	CV%	RFU
	(bp)	(ng/uL)	Percent	(nmole/L)	(bp)	(bp)		
1	75 (LM)	0.5000		11.0851	16	150	13.17	7292
2	680	0.1948	71.7	0.4743	613	723	1.70	250
3	974	0.0768	28.3	0.1328	796	1056	5.06	83
4	20000 (UM)	0.2378		0.0189	18903	30156	4.81	2003

TIC:	0.2716	ng/uL
TIM:	0.6071	nmole/L
Total concentration:	0.8436	ng/uL

```
Sample peak width (sec): 5    Sample min peak height: 50    Sample baseline V to V?: Y    Sample baseline V to V points: 3
Sample filter: Binomial       Number of points for filter: 3  Sample start region (min): 0  Sample end region (min): 30
Marker peak width (sec): 5    Marker min peak height: 200   Marker baseline V to V?: Y    Marker baseline V to V points: 3
Lower marker selection: First peak > 500 RFU                Upper marker selection: Last peak > 200 RFU
Ladder size (bp)75, 200, 300, 400, 500, 700, 1000, 1500, 2000, 3000, 4000, 5000, 7000, 10000, 20000
Quantification using: Lower Marker          Final concentration (ng/uL): 0.5000          Dilution factor: 12.0
```

Sample: SampE10
Well location: E10
Created: Thursday, October 21, 2021 7:28:10 PM

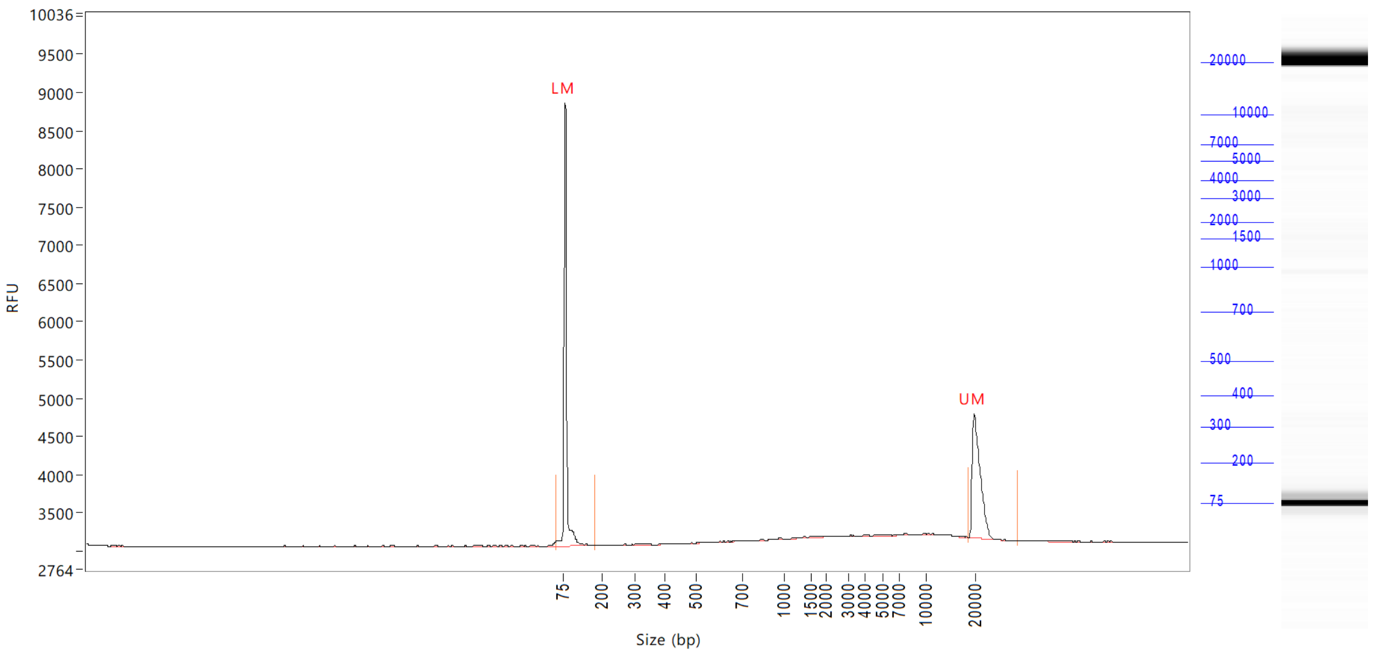

Peak	Size	Concentration	Relative concentration	Molarity	From	To	CV%	RFU
	(bp)	(ng/uL)	Percent	(nmole/L)	(bp)	(bp)		
1	75 (LM)	0.5000		10.7944	48	173	14.56	5806
2	20000 (UM)	0.2267		0.0181	18903	28947	4.66	1629

TIC:	0.0000	ng/uL
TIM:	0.0000	nmole/L
Total concentration:	0.6039	ng/uL

```
Sample peak width (sec): 5    Sample min peak height: 50    Sample baseline V to V?: Y    Sample baseline V to V points: 3
Sample filter: Binomial       Number of points for filter: 3  Sample start region (min): 0  Sample end region (min): 30
Marker peak width (sec): 5    Marker min peak height: 200   Marker baseline V to V?: Y    Marker baseline V to V points: 3
Lower marker selection: First peak > 500 RFU                Upper marker selection: Last peak > 200 RFU
Ladder size (bp)75, 200, 300, 400, 500, 700, 1000, 1500, 2000, 3000, 4000, 5000, 7000, 10000, 20000
Quantification using: Lower Marker          Final concentration (ng/uL): 0.5000          Dilution factor: 12.0
```

Sample: SampF10
Well location: F10
Created: Thursday, October 21, 2021 7:28:10 PM

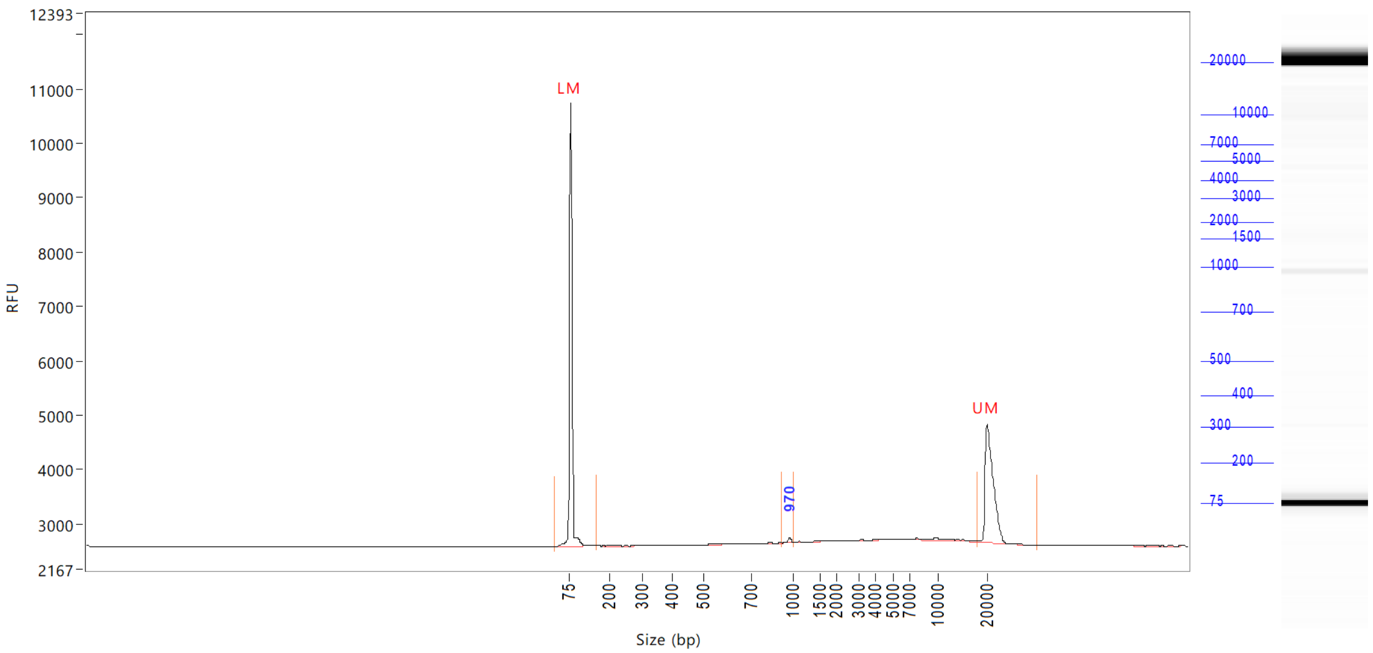

Peak	Size	Concentration	Relative concentration	Molarity	From	To	CV%	RFU
	(bp)	(ng/uL)	Percent	(nmole/L)	(bp)	(bp)		
1	75 (LM)	0.5000		11.0851	28	160	9.69	8168
2	970	0.0651	100.0	0.1106	918	1012	1.42	90
3	20000 (UM)	0.2341		0.0187	18049	30156	4.66	2188

TIC:	0.0651	ng/uL
TIM:	0.1106	nmole/L
Total concentration:	0.6031	ng/uL

Sample peak width (sec): 5 Sample min peak height: 50 Sample baseline V to V?: Y Sample baseline V to V points: 3
Sample filter: Binomial Number of points for filter: 3 Sample start region (min): 0 Sample end region (min): 30
Marker peak width (sec): 5 Marker min peak height: 200 Marker baseline V to V?: Y Marker baseline V to V points: 3
Lower marker selection: First peak > 500 RFU Upper marker selection: Last peak > 200 RFU
Ladder size (bp)75, 200, 300, 400, 500, 700, 1000, 1500, 2000, 3000, 4000, 5000, 7000, 10000, 20000
Quantification using: Lower Marker Final concentration (ng/uL): 0.5000 Dilution factor: 12.0

Sample: SampG10
Well location: G10
Created: Thursday, October 21, 2021 7:28:10 PM

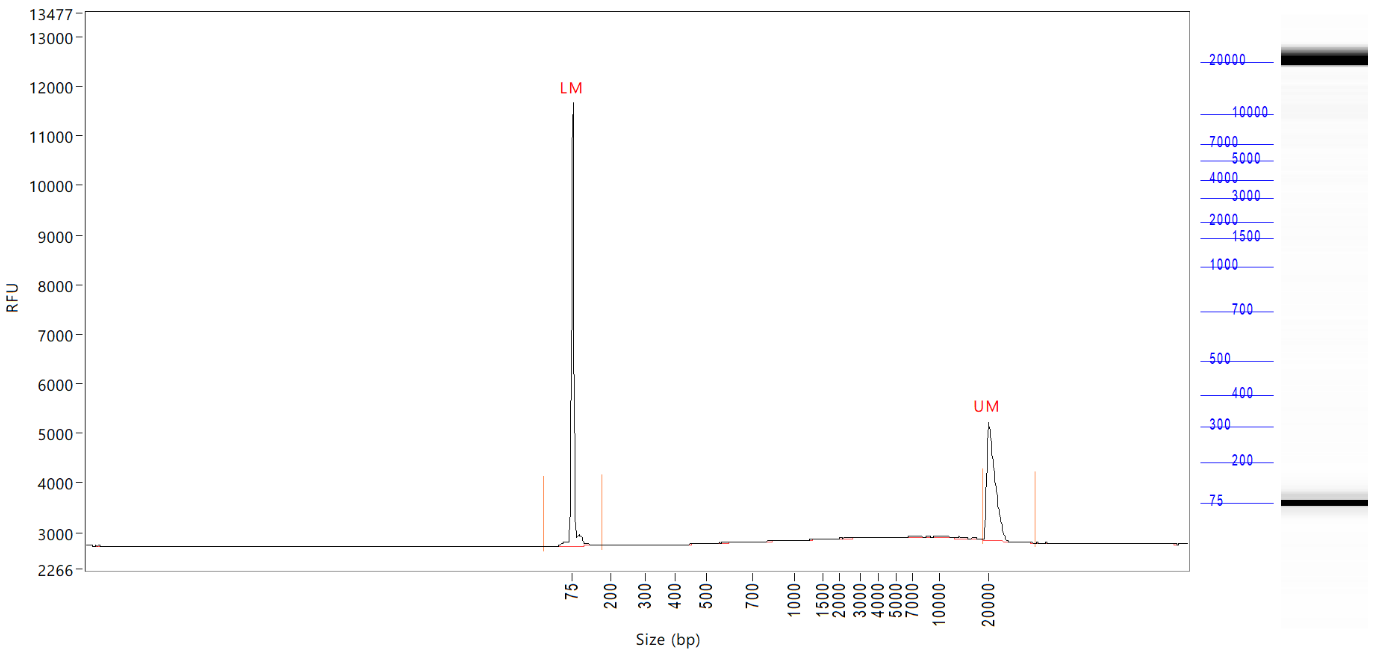

Peak	Size	Concentration	Relative concentration	Molarity	From	To	CV%	RFU
	(bp)	(ng/uL)	Percent	(nmole/L)	(bp)	(bp)		
1	75 (LM)	0.5000		11.0851	0	169	13.13	8955
2	20000 (UM)	0.2334		0.0186	18903	29430	4.61	2387

TIC:	0.0000	ng/uL
TIM:	0.0000	nmole/L
Total concentration:	0.4726	ng/uL

Sample peak width (sec): 5 Sample min peak height: 50 Sample baseline V to V?: Y Sample baseline V to V points: 3
Sample filter: Binomial Number of points for filter: 3 Sample start region (min): 0 Sample end region (min): 30
Marker peak width (sec): 5 Marker min peak height: 200 Marker baseline V to V?: Y Marker baseline V to V points: 3
Lower marker selection: First peak > 500 RFU Upper marker selection: Last peak > 200 RFU
Ladder size (bp)75, 200, 300, 400, 500, 700, 1000, 1500, 2000, 3000, 4000, 5000, 7000, 10000, 20000
Quantification using: Lower Marker Final concentration (ng/uL): 0.5000 Dilution factor: 12.0

Sample: SampH10
Well location: H10
Created: Thursday, October 21, 2021 7:28:10 PM

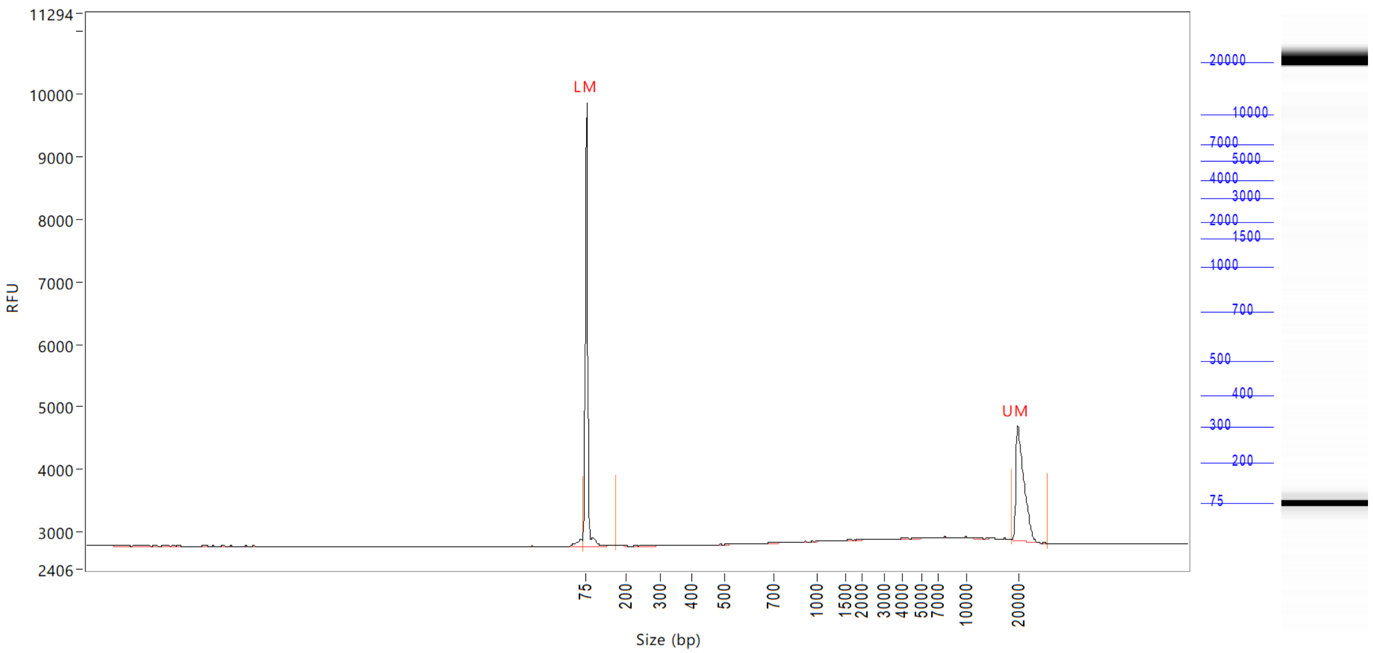

Peak	Size	Concentration	Relative concentration	Molarity	From	To	CV%	RFU
	(bp)	(ng/uL)	Percent	(nmole/L)	(bp)	(bp)		
1	75 (LM)	0.5000		11.0851	64	166	9.03	7100
2	20000 (UM)	0.2325		0.0186	18781	25924	4.24	1842

TIC:	0.0000	ng/uL
TIM:	0.0000	nmole/L
Total concentration:	0.4377	ng/uL

Sample peak width (sec): 5 Sample min peak height: 50 Sample baseline V to V?: Y Sample baseline V to V points: 3
Sample filter: Binomial Number of points for filter: 3 Sample start region (min): 0 Sample end region (min): 30
Marker peak width (sec): 5 Marker min peak height: 200 Marker baseline V to V?: Y Marker baseline V to V points: 3
Lower marker selection: First peak > 500 RFU Upper marker selection: Last peak > 200 RFU
Ladder size (bp)75, 200, 300, 400, 500, 700, 1000, 1500, 2000, 3000, 4000, 5000, 7000, 10000, 20000
Quantification using: Lower Marker Final concentration (ng/uL): 0.5000 Dilution factor: 12.0

Sample: SampA11
Well location: A11
Created: Thursday, October 21, 2021 7:28:10 PM

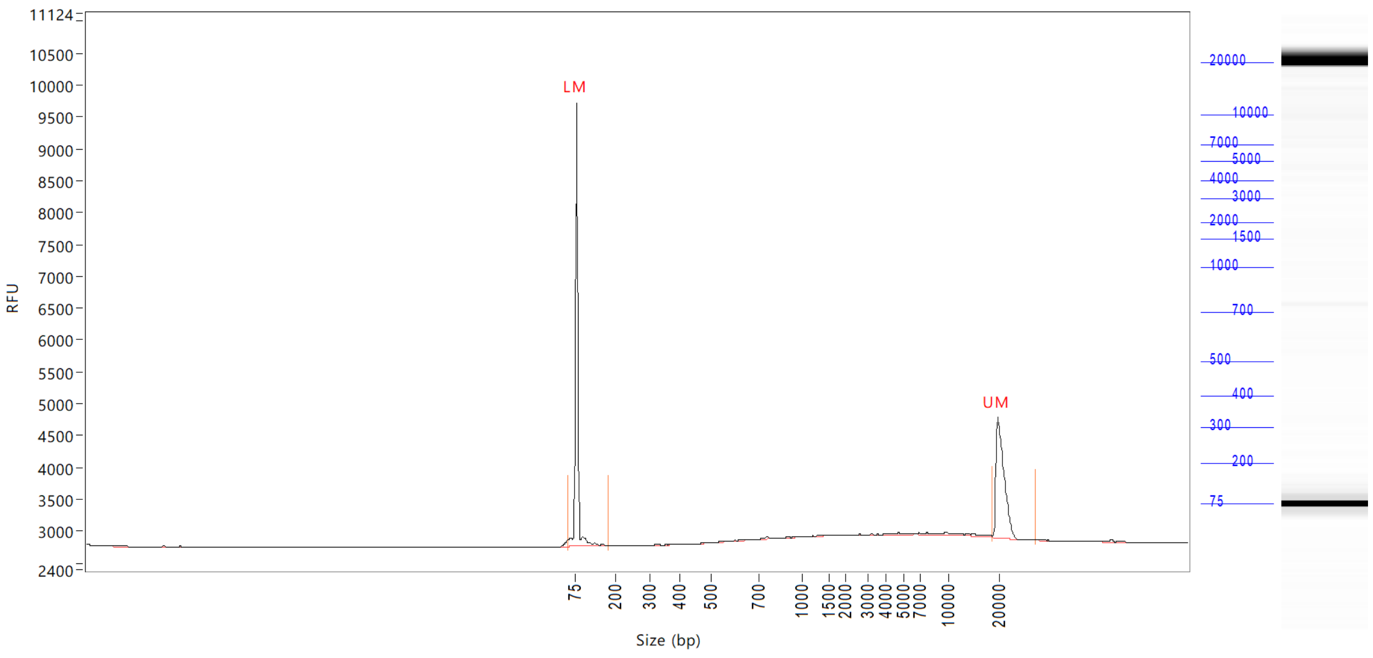

Peak	Size	Concentration	Relative concentration	Molarity	From	To	CV%	RFU
	(bp)	(ng/uL)	Percent	(nmole/L)	(bp)	(bp)		
1	75 (LM)	0.5000		10.9378	50	175	14.15	6963
2	20000 (UM)	0.2344		0.0188	18903	27496	4.24	1906

TIC:	0.0000	ng/uL
TIM:	0.0000	nmole/L
Total concentration:	0.7051	ng/uL

Sample peak width (sec): 5 Sample min peak height: 50 Sample baseline V to V?: Y Sample baseline V to V points: 3
Sample filter: Binomial Number of points for filter: 3 Sample start region (min): 0 Sample end region (min): 30
Marker peak width (sec): 5 Marker min peak height: 200 Marker baseline V to V?: Y Marker baseline V to V points: 3
Lower marker selection: First peak > 500 RFU Upper marker selection: Last peak > 200 RFU
Ladder size (bp)75, 200, 300, 400, 500, 700, 1000, 1500, 2000, 3000, 4000, 5000, 7000, 10000, 20000
Quantification using: Lower Marker Final concentration (ng/uL): 0.5000 Dilution factor: 12.0

Sample: SampB11
Well location: B11
Created: Thursday, October 21, 2021 7:28:10 PM

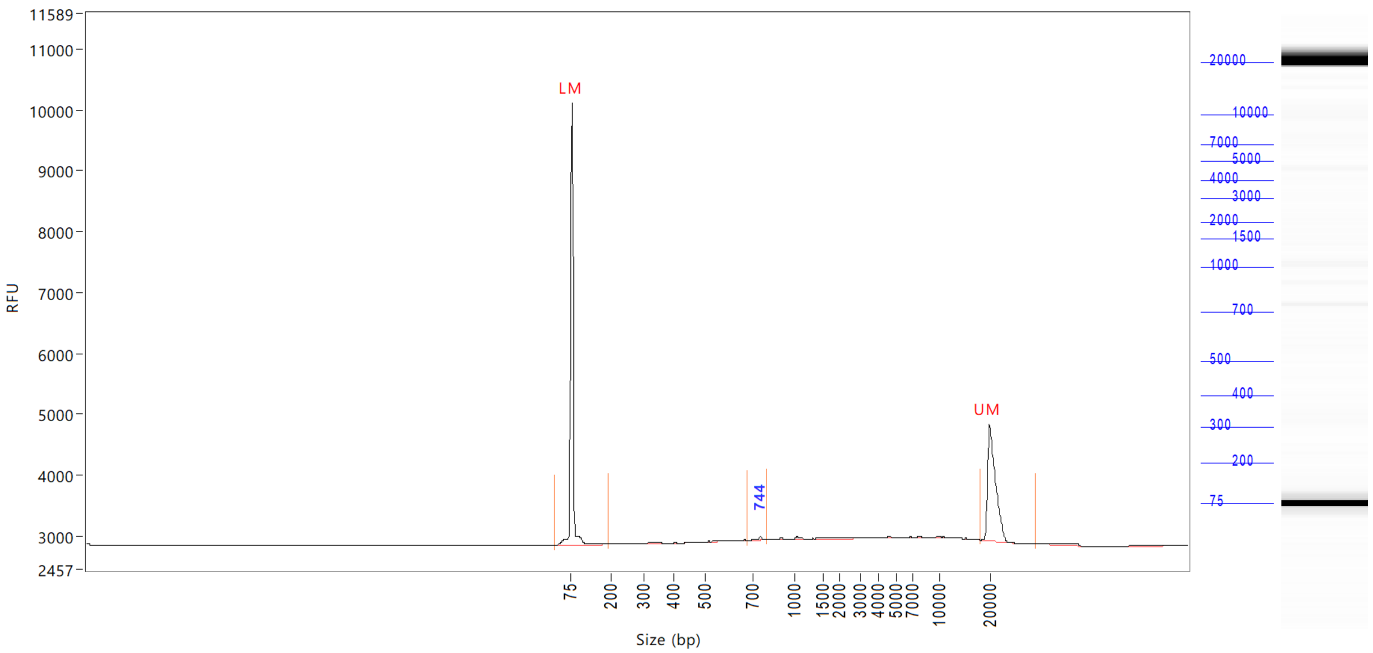

Peak	Size	Concentration	Relative concentration	Molarity	From	To	CV%	RFU
	(bp)	(ng/uL)	Percent	(nmole/L)	(bp)	(bp)		
1	75 (LM)	0.5000		11.0851	22	190	14.21	7274
2	744	0.0523	100.0	0.1174	677	788	2.95	54
3	20000 (UM)	0.2223		0.0178	18171	29309	4.50	1907

TIC:	0.0523	ng/uL
TIM:	0.1174	nmole/L
Total concentration:	0.5958	ng/uL

Sample peak width (sec): 5 Sample min peak height: 50 Sample baseline V to V?: Y Sample baseline V to V points: 3
Sample filter: Binomial Number of points for filter: 3 Sample start region (min): 0 Sample end region (min): 30
Marker peak width (sec): 5 Marker min peak height: 200 Marker baseline V to V?: Y Marker baseline V to V points: 3
Lower marker selection: First peak > 500 RFU Upper marker selection: Last peak > 200 RFU
Ladder size (bp)75, 200, 300, 400, 500, 700, 1000, 1500, 2000, 3000, 4000, 5000, 7000, 10000, 20000
Quantification using: Lower Marker Final concentration (ng/uL): 0.5000 Dilution factor: 12.0

Sample: SampC11
Well location: C11
Created: Thursday, October 21, 2021 7:28:10 PM

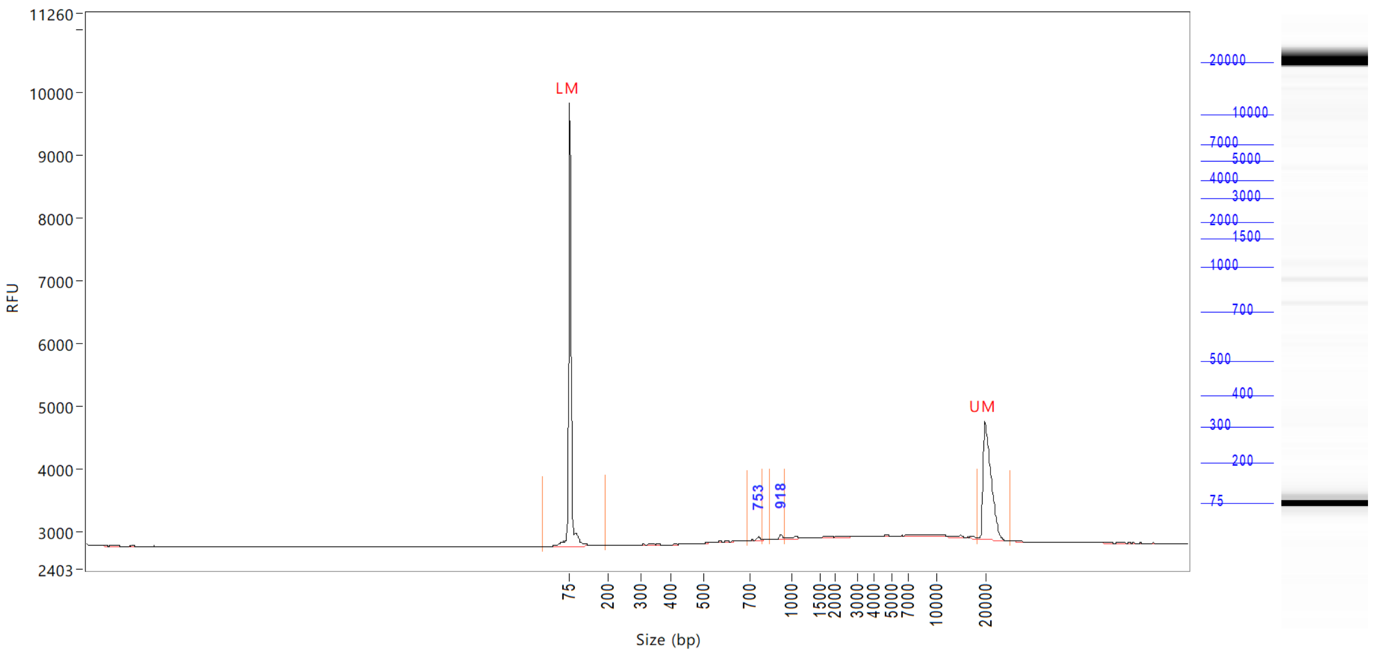

Peak	Size	Concentration	Relative concentration	Molarity	From	To	CV%	RFU
	(bp)	(ng/uL)	Percent	(nmole/L)	(bp)	(bp)		
1	75 (LM)	0.5000		11.0851	0	192	15.39	7074
2	753	0.0563	46.2	0.1244	690	788	2.77	56
3	918	0.0656	53.8	0.1186	840	944	1.97	78
4	20000 (UM)	0.2273		0.0182	18537	25078	4.29	1880

TIC:	0.1219	ng/uL
TIM:	0.2430	nmole/L
Total concentration:	0.7383	ng/uL

Sample peak width (sec): 5 Sample min peak height: 50 Sample baseline V to V?: Y Sample baseline V to V points: 3
Sample filter: Binomial Number of points for filter: 3 Sample start region (min): 0 Sample end region (min): 30
Marker peak width (sec): 5 Marker min peak height: 200 Marker baseline V to V?: Y Marker baseline V to V points: 3
Lower marker selection: First peak > 500 RFU Upper marker selection: Last peak > 200 RFU
Ladder size (bp)75, 200, 300, 400, 500, 700, 1000, 1500, 2000, 3000, 4000, 5000, 7000, 10000, 20000
Quantification using: Lower Marker Final concentration (ng/uL): 0.5000 Dilution factor: 12.0

Sample: SampD11
Well location: D11
Created: Thursday, October 21, 2021 7:28:10 PM

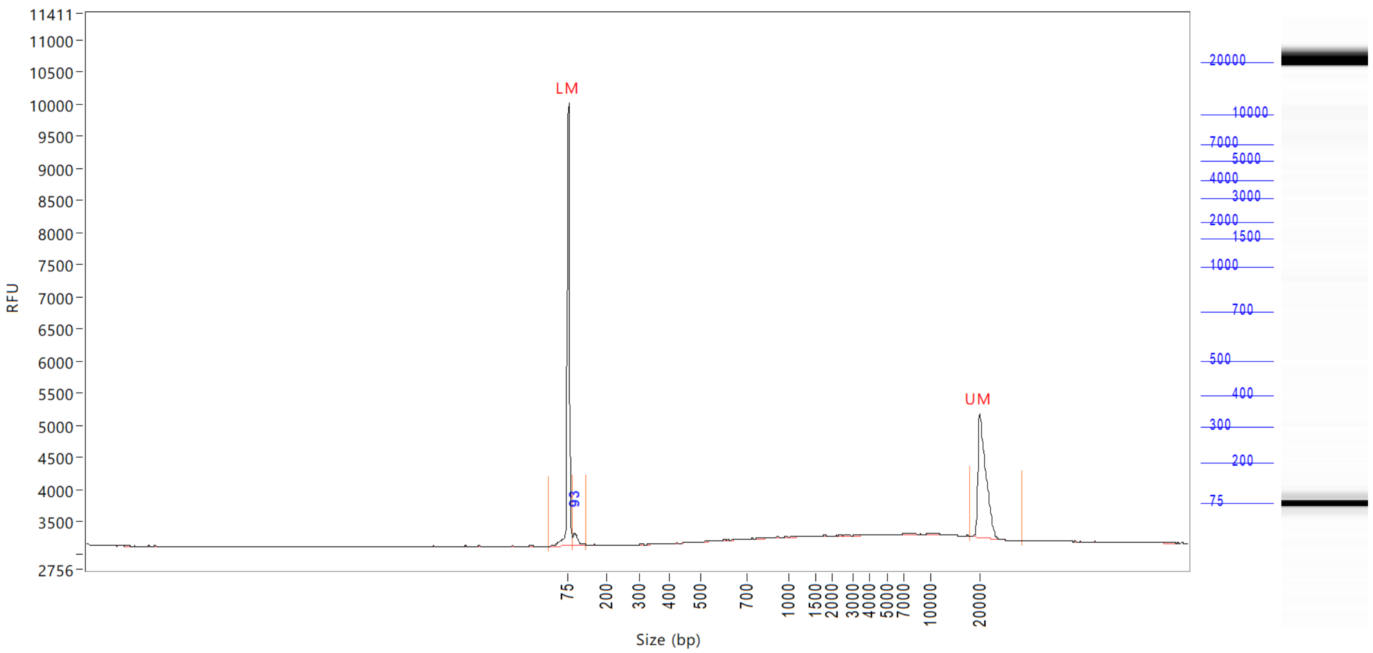

Peak	Size	Concentration	Relative concentration	Molarity	From	To	CV%	RFU
	(bp)	(ng/uL)	Percent	(nmole/L)	(bp)	(bp)		
1	75 (LM)	0.5000		11.5518	12	86	8.75	6908
2	93	0.3589	100.0	6.0133	86	133	10.61	185
3	20000 (UM)	0.2441		0.0195	18049	28705	4.48	1922

TIC:	0.3589	ng/uL
TIM:	6.0133	nmole/L
Total concentration:	0.9183	ng/uL

```
Sample peak width (sec): 5     Sample min peak height: 50    Sample baseline V to V?: Y    Sample baseline V to V points: 3
Sample filter: Binomial        Number of points for filter: 3  Sample start region (min): 0  Sample end region (min): 30
Marker peak width (sec): 5     Marker min peak height: 200   Marker baseline V to V?: Y    Marker baseline V to V points: 3
Lower marker selection: First peak > 500 RFU                 Upper marker selection: Last peak > 200 RFU
Ladder size (bp)75, 200, 300, 400, 500, 700, 1000, 1500, 2000, 3000, 4000, 5000, 7000, 10000, 20000
Quantification using: Lower Marker          Final concentration (ng/uL): 0.5000          Dilution factor: 12.0
```

Sample: SampE11
Well location: E11
Created: Thursday, October 21, 2021 7:28:10 PM

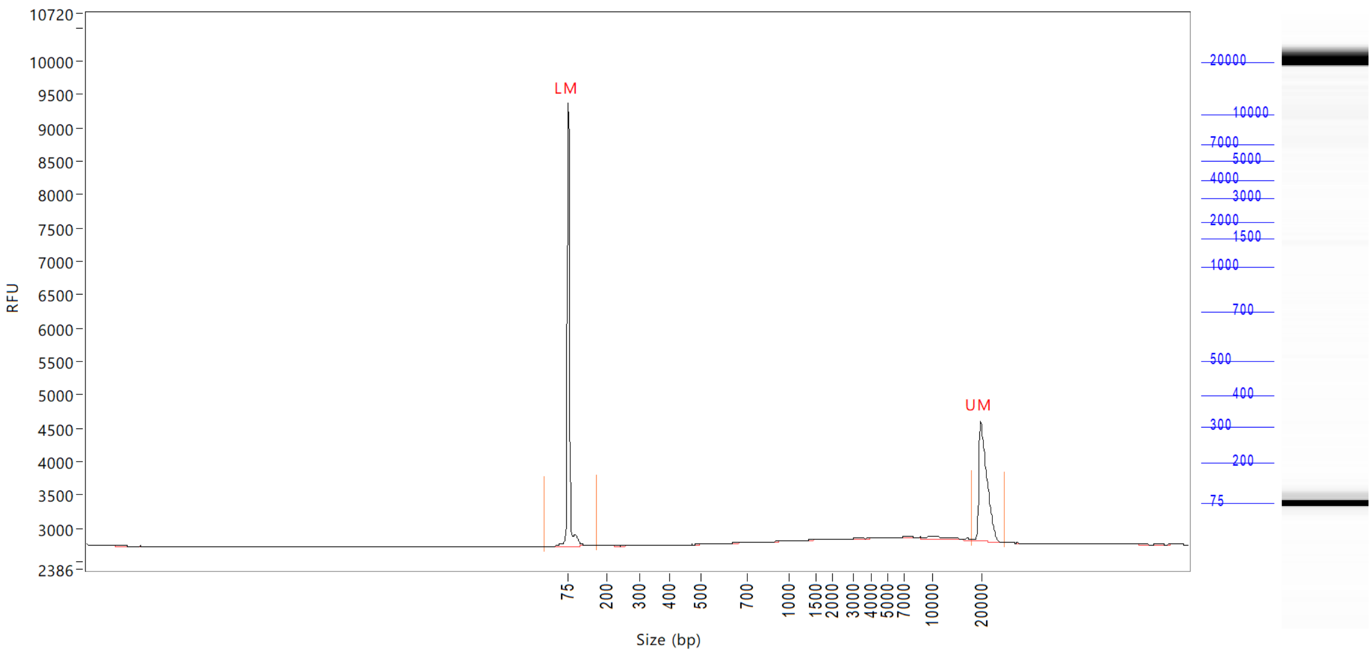

Peak	Size	Concentration	Relative concentration	Molarity	From	To	CV%	RFU
	(bp)	(ng/uL)	Percent	(nmole/L)	(bp)	(bp)		
1	75 (LM)	0.5000		10.9378	0	168	12.10	6657
2	20000 (UM)	0.2340		0.0187	18171	24957	4.34	1795

TIC:	0.0000	ng/uL
TIM:	0.0000	nmole/L
Total concentration:	0.6022	ng/uL

Sample peak width (sec): 5 Sample min peak height: 50 Sample baseline V to V?: Y Sample baseline V to V points: 3
Sample filter: Binomial Number of points for filter: 3 Sample start region (min): 0 Sample end region (min): 30
Marker peak width (sec): 5 Marker min peak height: 200 Marker baseline V to V?: Y Marker baseline V to V points: 3
Lower marker selection: First peak > 500 RFU Upper marker selection: Last peak > 200 RFU
Ladder size (bp)75, 200, 300, 400, 500, 700, 1000, 1500, 2000, 3000, 4000, 5000, 7000, 10000, 20000
Quantification using: Lower Marker Final concentration (ng/uL): 0.5000 Dilution factor: 12.0

Sample: SampF11
Well location: F11
Created: Thursday, October 21, 2021 7:28:10 PM

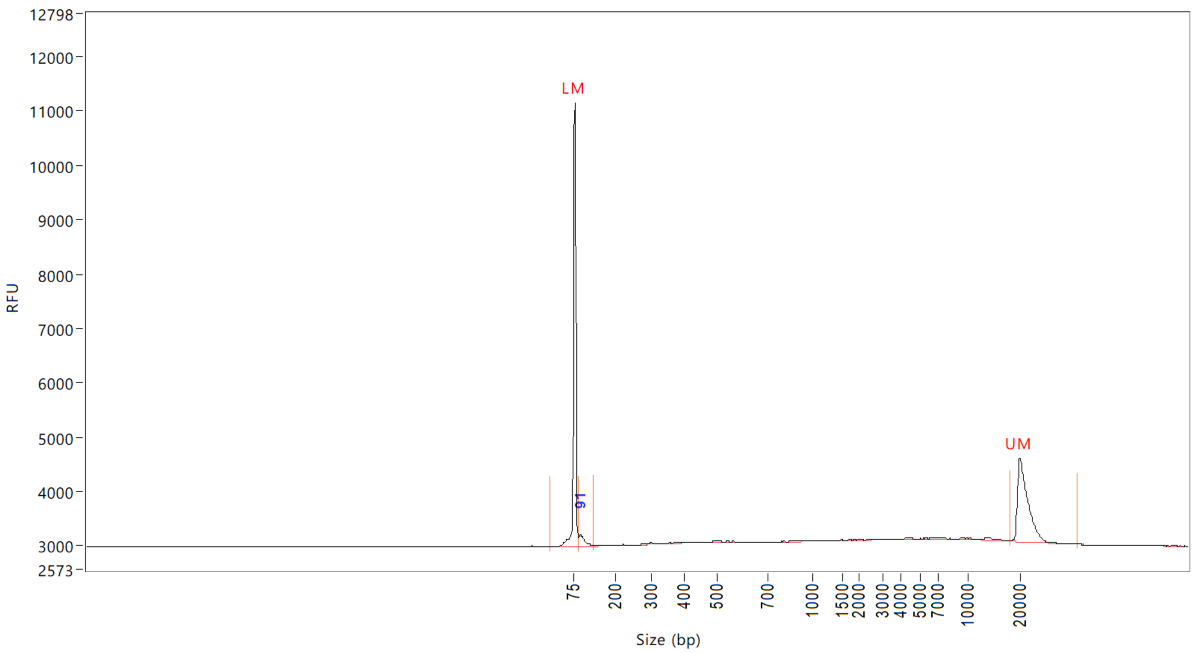

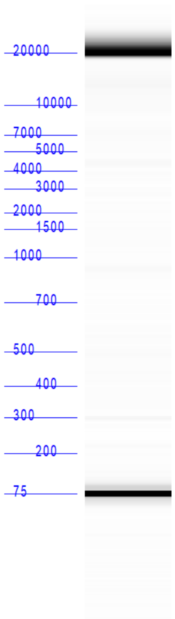

Peak	Size	Concentration	Relative concentration	Molarity	From	To	CV%	RFU
	(bp)	(ng/uL)	Percent	(nmole/L)	(bp)	(bp)		
1	75 (LM)	0.5000		11.5518	5	86	8.80	8169
2	91	0.3760	100.0	6.3002	86	133	11.07	211
3	20000 (UM)	0.2087		0.0165	18171	30881	6.23	1538

TIC:	0.3760	ng/uL
TIM:	6.3002	nmole/L
Total concentration:	1.0606	ng/uL

```
Sample peak width (sec): 5    Sample min peak height: 50    Sample baseline V to V?: Y    Sample baseline V to V points: 3
Sample filter: Binomial       Number of points for filter: 3  Sample start region (min): 0  Sample end region (min): 30
Marker peak width (sec): 5    Marker min peak height: 200   Marker baseline V to V?: Y    Marker baseline V to V points: 3
Lower marker selection: First peak > 500 RFU                Upper marker selection: Last peak > 200 RFU
Ladder size (bp)75, 200, 300, 400, 500, 700, 1000, 1500, 2000, 3000, 4000, 5000, 7000, 10000, 20000
Quantification using: Lower Marker          Final concentration (ng/uL): 0.5000          Dilution factor: 12.0
```

Sample: SampG11
Well location: G11
Created: Thursday, October 21, 2021 7:28:10 PM

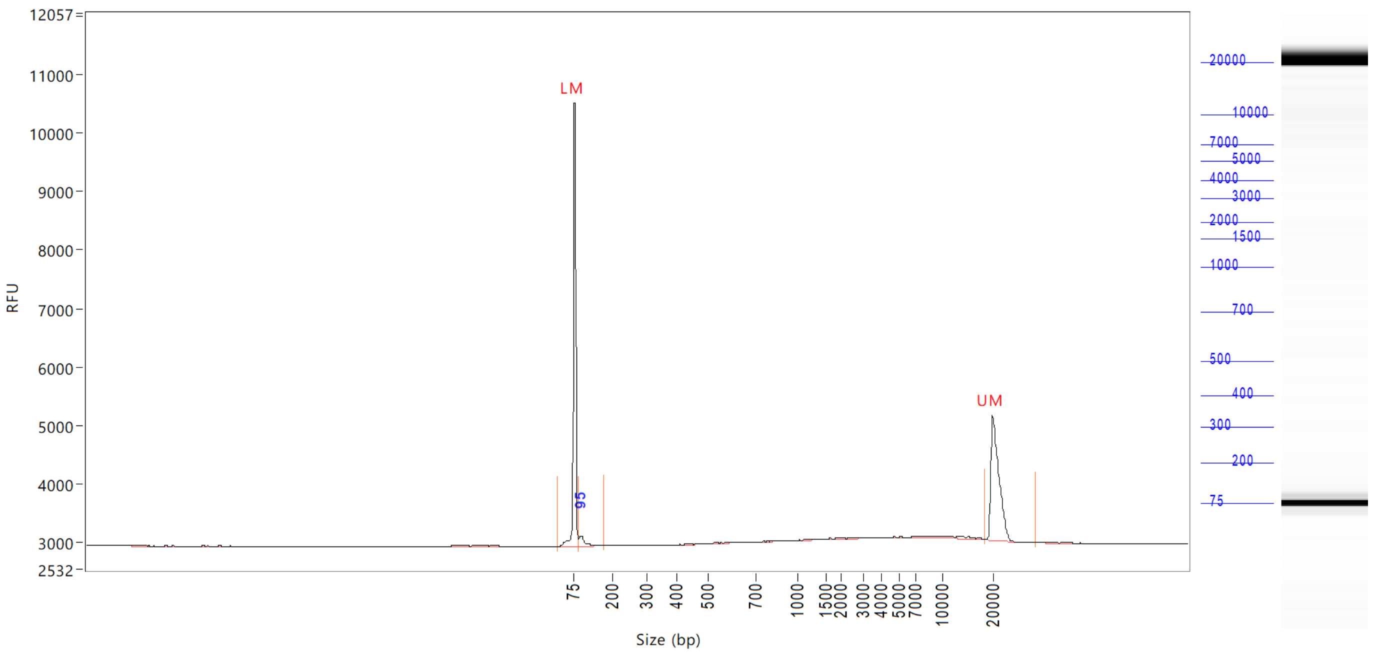

Peak	Size	Concentration	Relative concentration	Molarity	From	To	CV%	RFU
	(bp)	(ng/uL)	Percent	(nmole/L)	(bp)	(bp)		
1	75 (LM)	0.5000		11.2364	22	87	7.82	7609
2	95	0.3583	100.0	5.5507	87	171	18.04	179
3	20000 (UM)	0.2488		0.0199	18537	28705	4.47	2133

TIC:	0.3583	ng/uL
TIM:	5.5507	nmole/L
Total concentration:	1.0351	ng/uL

```
Sample peak width (sec): 5    Sample min peak height: 50    Sample baseline V to V?: Y    Sample baseline V to V points: 3
Sample filter: Binomial       Number of points for filter: 3  Sample start region (min): 0  Sample end region (min): 30
Marker peak width (sec): 5    Marker min peak height: 200   Marker baseline V to V?: Y    Marker baseline V to V points: 3
Lower marker selection: First peak > 500 RFU                Upper marker selection: Last peak > 200 RFU
Ladder size (bp)75, 200, 300, 400, 500, 700, 1000, 1500, 2000, 3000, 4000, 5000, 7000, 10000, 20000
Quantification using: Lower Marker          Final concentration (ng/uL): 0.5000          Dilution factor: 12.0
```

Sample: SampH11
Well location: H11
Created: Thursday, October 21, 2021 7:28:10 PM

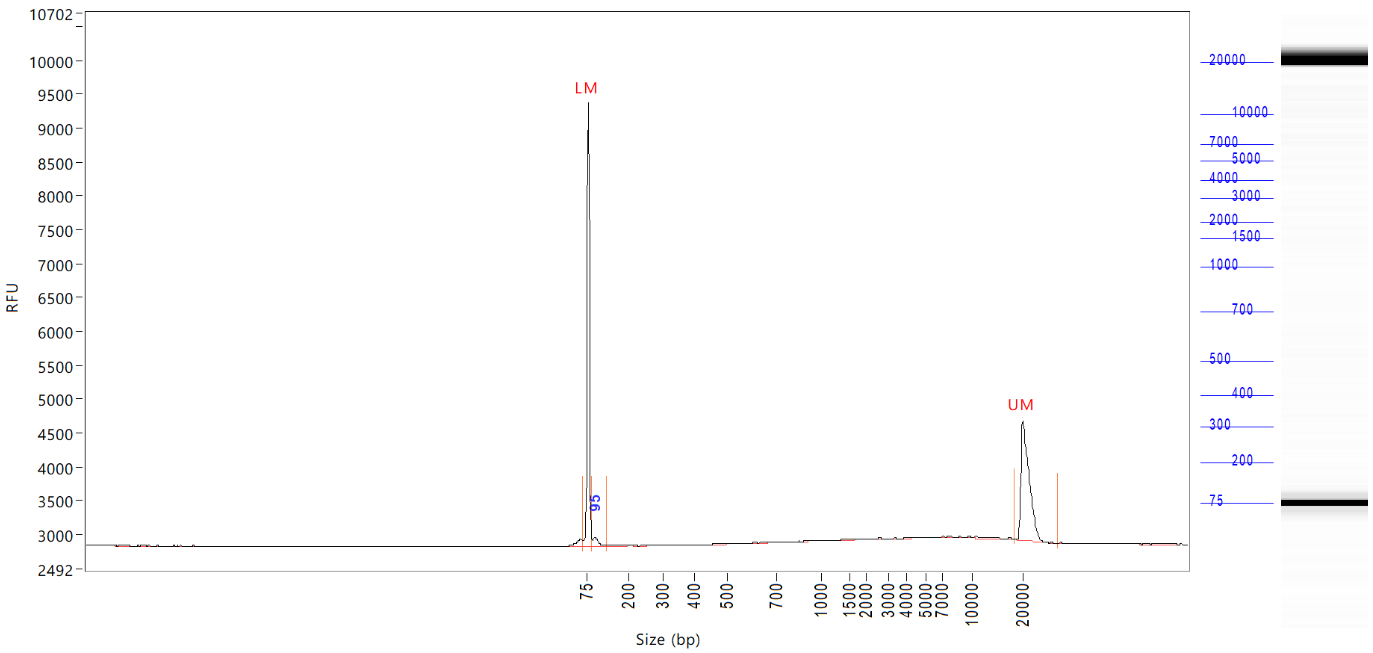

Peak	Size	Concentration	Relative concentration	Molarity	From	To	CV%	RFU
	(bp)	(ng/uL)	Percent	(nmole/L)	(bp)	(bp)		
1	75 (LM)	0.5000		11.0851	60	87	4.65	6561
2	95	0.2856	100.0	4.6894	87	131	9.77	125
3	20000 (UM)	0.2445		0.0196	18171	26770	4.28	1756

TIC:	0.2856	ng/uL
TIM:	4.6894	nmole/L
Total concentration:	0.9047	ng/uL

Sample peak width (sec): 5   Sample min peak height: 50   Sample baseline V to V?: Y   Sample baseline V to V points: 3
Sample filter: Binomial   Number of points for filter: 3   Sample start region (min): 0   Sample end region (min): 30
Marker peak width (sec): 5   Marker min peak height: 200   Marker baseline V to V?: Y   Marker baseline V to V points: 3
Lower marker selection: First peak > 500 RFU   Upper marker selection: Last peak > 200 RFU
Ladder size (bp)75, 200, 300, 400, 500, 700, 1000, 1500, 2000, 3000, 4000, 5000, 7000, 10000, 20000
Quantification using: Lower Marker   Final concentration (ng/uL): 0.5000   Dilution factor: 12.0

Sample: SampA12
Well location: A12
Created: Thursday, October 21, 2021 7:28:10 PM

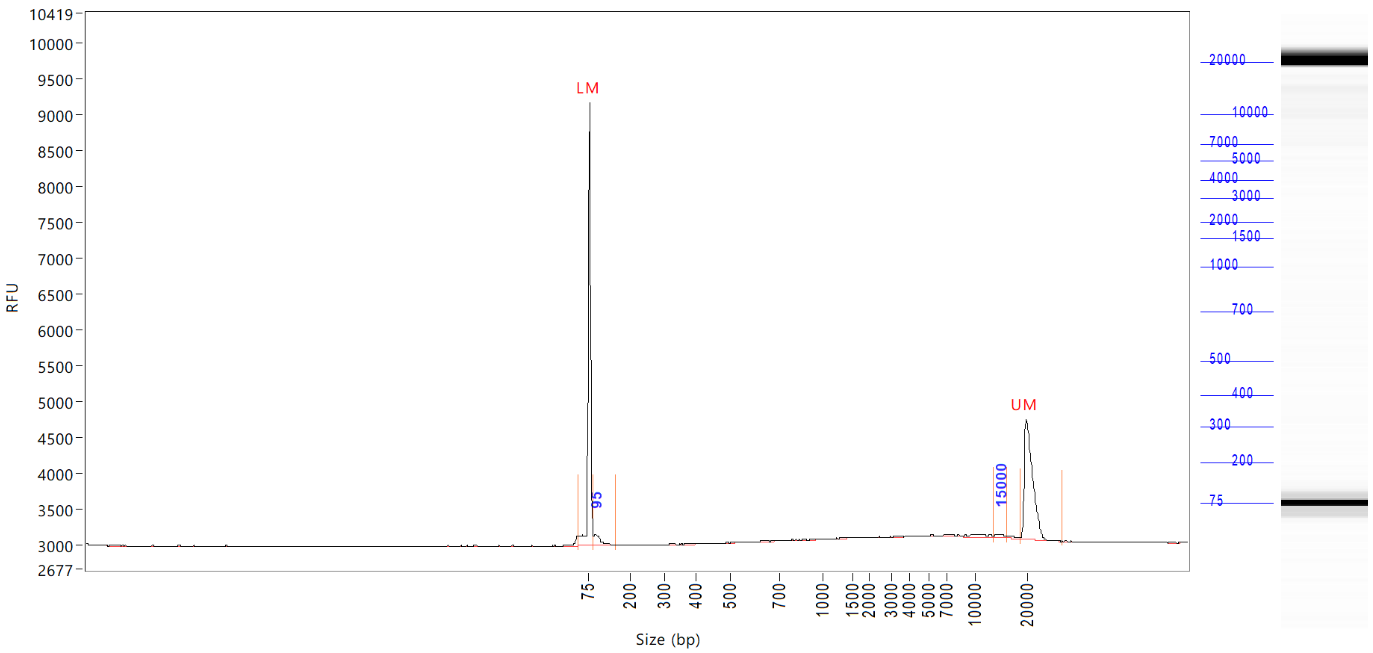

Peak	Size	Concentration	Relative concentration	Molarity	From	To	CV%	RFU
	(bp)	(ng/uL)	Percent	(nmole/L)	(bp)	(bp)		
1	75 (LM)	0.5000		11.2364	45	87	7.66	6177
2	95	0.4018	80.2	6.4682	87	154	13.21	153
3	15000	0.0990	19.8	0.0111	13415	16220	4.99	54
4	20000 (UM)	0.2364		0.0190	18781	26770	4.29	1673

TIC:	0.5007	ng/uL
TIM:	6.4793	nmole/L
Total concentration:	1.2786	ng/uL

```
Sample peak width (sec): 5    Sample min peak height: 50    Sample baseline V to V?: Y    Sample baseline V to V points: 3
Sample filter: Binomial       Number of points for filter: 3  Sample start region (min): 0  Sample end region (min): 30
Marker peak width (sec): 5    Marker min peak height: 200   Marker baseline V to V?: Y    Marker baseline V to V points: 3
Lower marker selection: First peak > 500 RFU                Upper marker selection: Last peak > 200 RFU
Ladder size (bp)75, 200, 300, 400, 500, 700, 1000, 1500, 2000, 3000, 4000, 5000, 7000, 10000, 20000
Quantification using: Lower Marker          Final concentration (ng/uL): 0.5000         Dilution factor: 12.0
```

Sample: SampB12
Well location: B12
Created: Thursday, October 21, 2021 7:28:10 PM

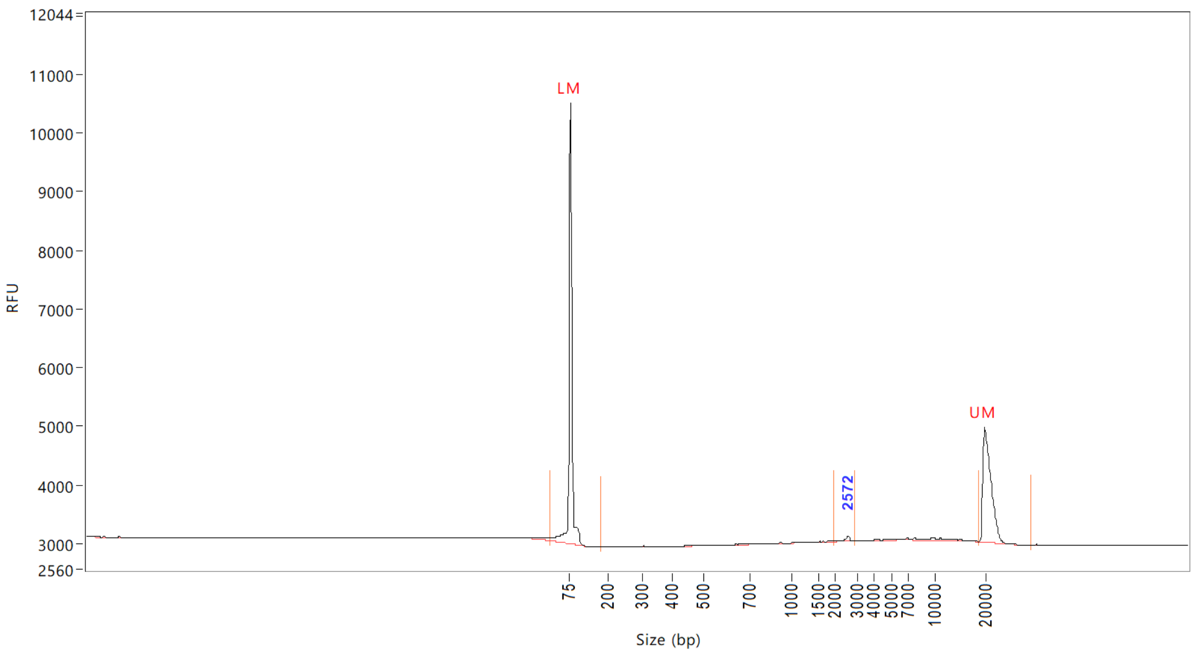

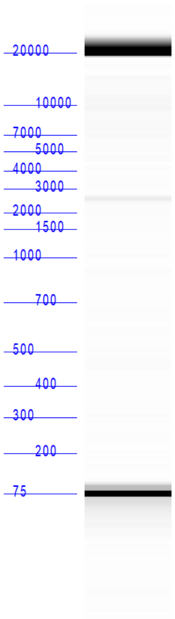

Peak	Size	Concentration	Relative concentration	Molarity	From	To	CV%	RFU
	(bp)	(ng/uL)	Percent	(nmole/L)	(bp)	(bp)		
1	75 (LM)	0.5000		11.3919	11	173	16.85	7534
2	2572	0.0808	100.0	0.0542	1962	2914	8.01	85
3	20000 (UM)	0.2110		0.0168	18781	29430	4.52	1963

TIC:	0.0808	ng/uL
TIM:	0.0542	nmole/L
Total concentration:	0.5756	ng/uL

Sample peak width (sec): 5 Sample min peak height: 50 Sample baseline V to V?: Y Sample baseline V to V points: 3
Sample filter: Binomial Number of points for filter: 3 Sample start region (min): 0 Sample end region (min): 30
Marker peak width (sec): 5 Marker min peak height: 200 Marker baseline V to V?: Y Marker baseline V to V points: 3
Lower marker selection: First peak > 500 RFU Upper marker selection: Last peak > 200 RFU
Ladder size (bp)75, 200, 300, 400, 500, 700, 1000, 1500, 2000, 3000, 4000, 5000, 7000, 10000, 20000
Quantification using: Lower Marker Final concentration (ng/uL): 0.5000 Dilution factor: 12.0

Sample: SampC12
Well location: C12
Created: Thursday, October 21, 2021 7:28:10 PM

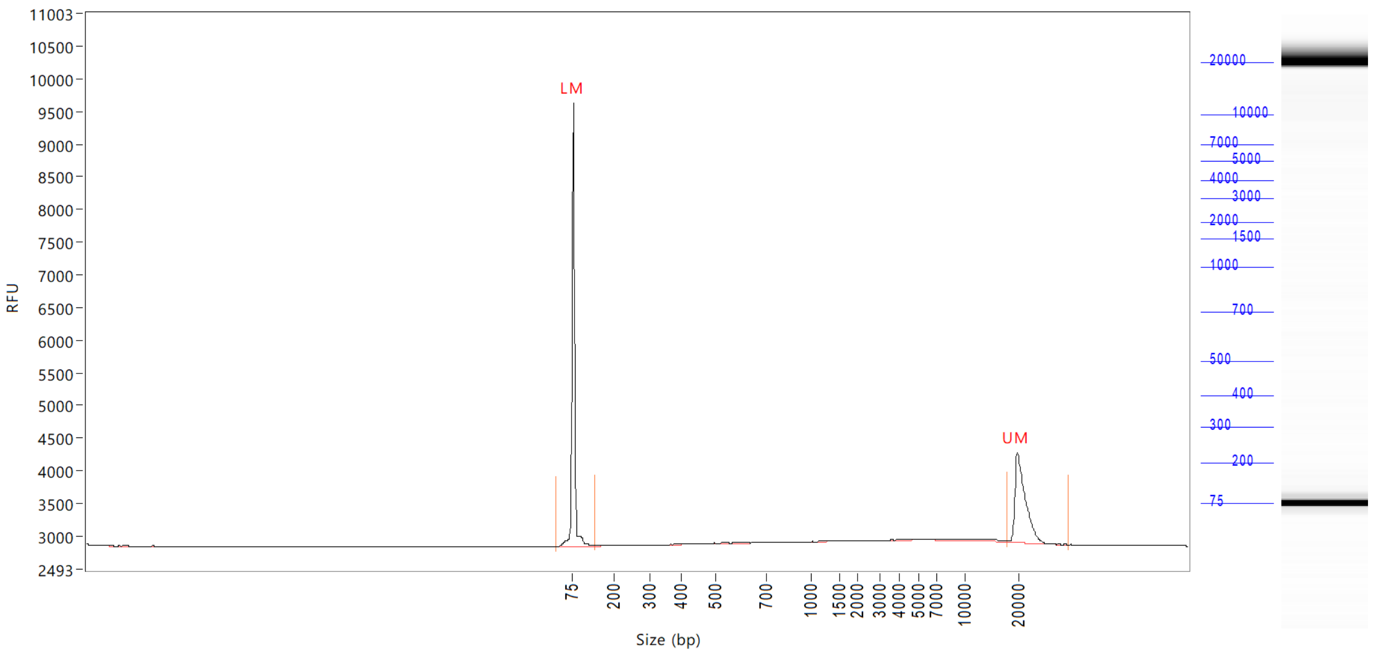

Peak	Size	Concentration	Relative concentration	Molarity	From	To	CV%	RFU
	(bp)	(ng/uL)	Percent	(nmole/L)	(bp)	(bp)		
1	75 (LM)	0.5000		10.9378	28	141	11.44	6799
2	20000 (UM)	0.2047		0.0162	18049	29793	5.96	1384

TIC:	0.0000	ng/uL
TIM:	0.0000	nmole/L
Total concentration:	0.6659	ng/uL

Sample peak width (sec): 5 Sample min peak height: 50 Sample baseline V to V?: Y Sample baseline V to V points: 3
Sample filter: Binomial Number of points for filter: 3 Sample start region (min): 0 Sample end region (min): 30
Marker peak width (sec): 5 Marker min peak height: 200 Marker baseline V to V?: Y Marker baseline V to V points: 3
Lower marker selection: First peak > 500 RFU Upper marker selection: Last peak > 200 RFU
Ladder size (bp)75, 200, 300, 400, 500, 700, 1000, 1500, 2000, 3000, 4000, 5000, 7000, 10000, 20000
Quantification using: Lower Marker Final concentration (ng/uL): 0.5000 Dilution factor: 12.0

Sample: SampD12
Well location: D12
Created: Thursday, October 21, 2021 7:28:10 PM

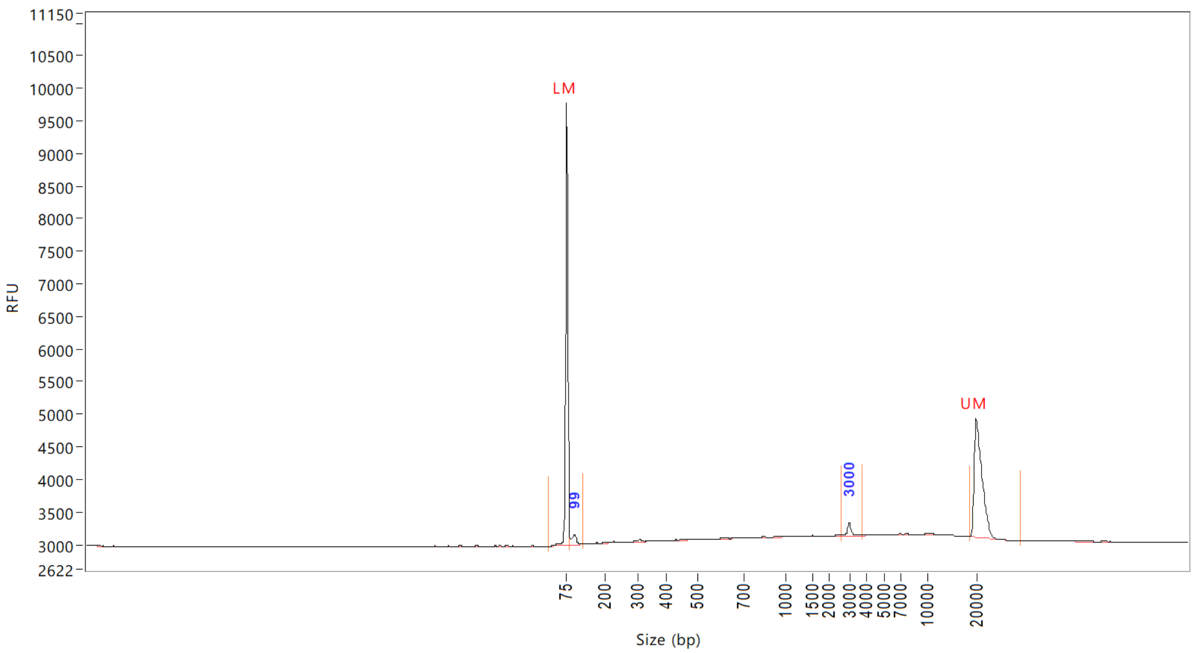

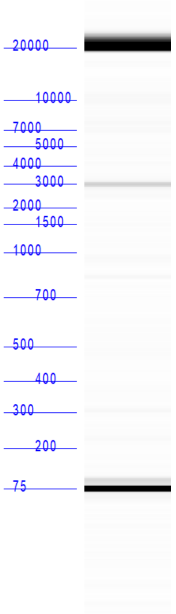

Peak	Size	Concentration	Relative concentration	Molarity	From	To	CV%	RFU
	(bp)	(ng/uL)	Percent	(nmole/L)	(bp)	(bp)		
1	75 (LM)	0.5000		11.3919	20	86	6.55	6796
2	99	0.2896	57.6	4.9028	86	126	7.78	155
3	3000	0.2128	42.4	0.1171	2629	3750	6.03	197
4	20000 (UM)	0.2505		0.0200	18903	29309	4.57	1832

TIC:	0.5024	ng/uL
TIM:	5.0200	nmole/L
Total concentration:	1.1612	ng/uL

```
Sample peak width (sec): 5    Sample min peak height: 50    Sample baseline V to V?: Y    Sample baseline V to V points: 3
Sample filter: Binomial       Number of points for filter: 3  Sample start region (min): 0  Sample end region (min): 30
Marker peak width (sec): 5    Marker min peak height: 200   Marker baseline V to V?: Y    Marker baseline V to V points: 3
Lower marker selection: First peak > 500 RFU                Upper marker selection: Last peak > 200 RFU
Ladder size (bp)75, 200, 300, 400, 500, 700, 1000, 1500, 2000, 3000, 4000, 5000, 7000, 10000, 20000
Quantification using: Lower Marker          Final concentration (ng/uL): 0.5000          Dilution factor: 12.0
```

Sample: SampE12
Well location: E12
Created: Thursday, October 21, 2021 7:28:10 PM

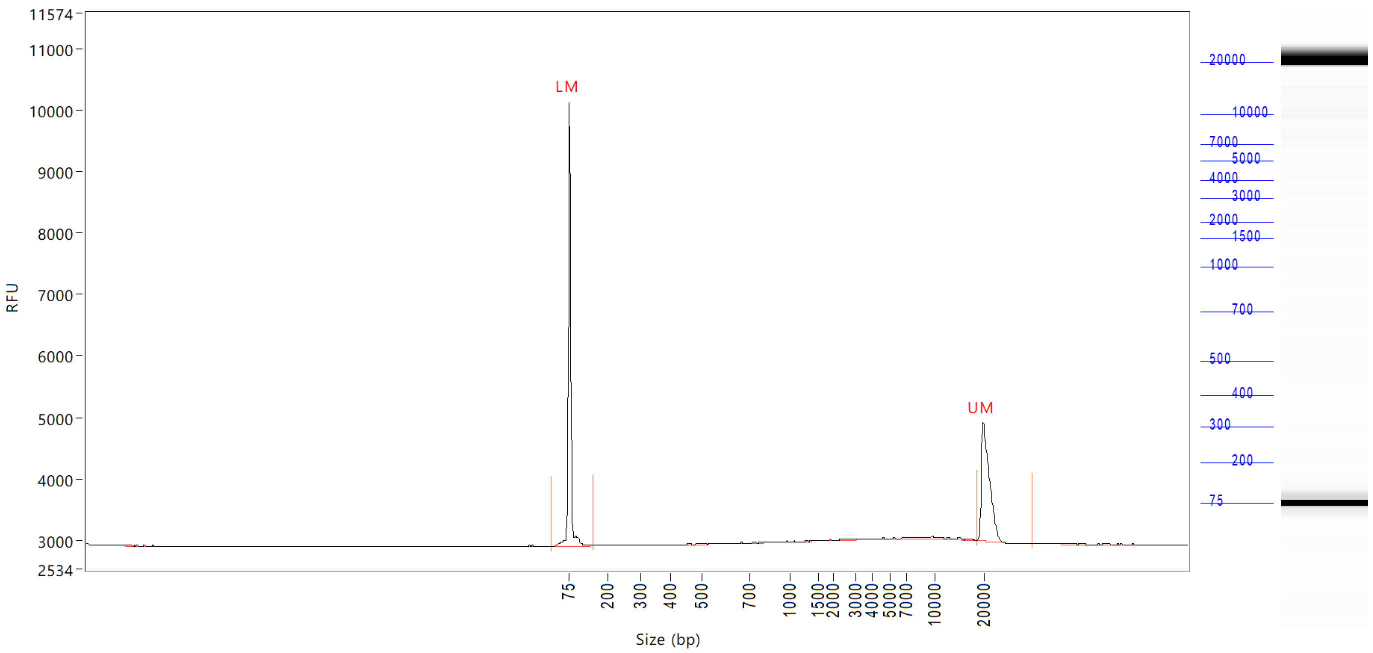

Peak	Size	Concentration	Relative concentration	Molarity	From	To	CV%	RFU
	(bp)	(ng/uL)	Percent	(nmole/L)	(bp)	(bp)		
1	75 (LM)	0.5000		11.2364	20	150	12.42	7220
2	20000 (UM)	0.2329		0.0186	18659	30156	4.66	1922

TIC:	0.0000	ng/uL
TIM:	0.0000	nmole/L
Total concentration:	0.5610	ng/uL

Sample peak width (sec): 5 Sample min peak height: 50 Sample baseline V to V?: Y Sample baseline V to V points: 3
Sample filter: Binomial Number of points for filter: 3 Sample start region (min): 0 Sample end region (min): 30
Marker peak width (sec): 5 Marker min peak height: 200 Marker baseline V to V?: Y Marker baseline V to V points: 3
Lower marker selection: First peak > 500 RFU Upper marker selection: Last peak > 200 RFU
Ladder size (bp)75, 200, 300, 400, 500, 700, 1000, 1500, 2000, 3000, 4000, 5000, 7000, 10000, 20000
Quantification using: Lower Marker Final concentration (ng/uL): 0.5000 Dilution factor: 12.0

Sample: SampF12
Well location: F12
Created: Thursday, October 21, 2021 7:28:10 PM

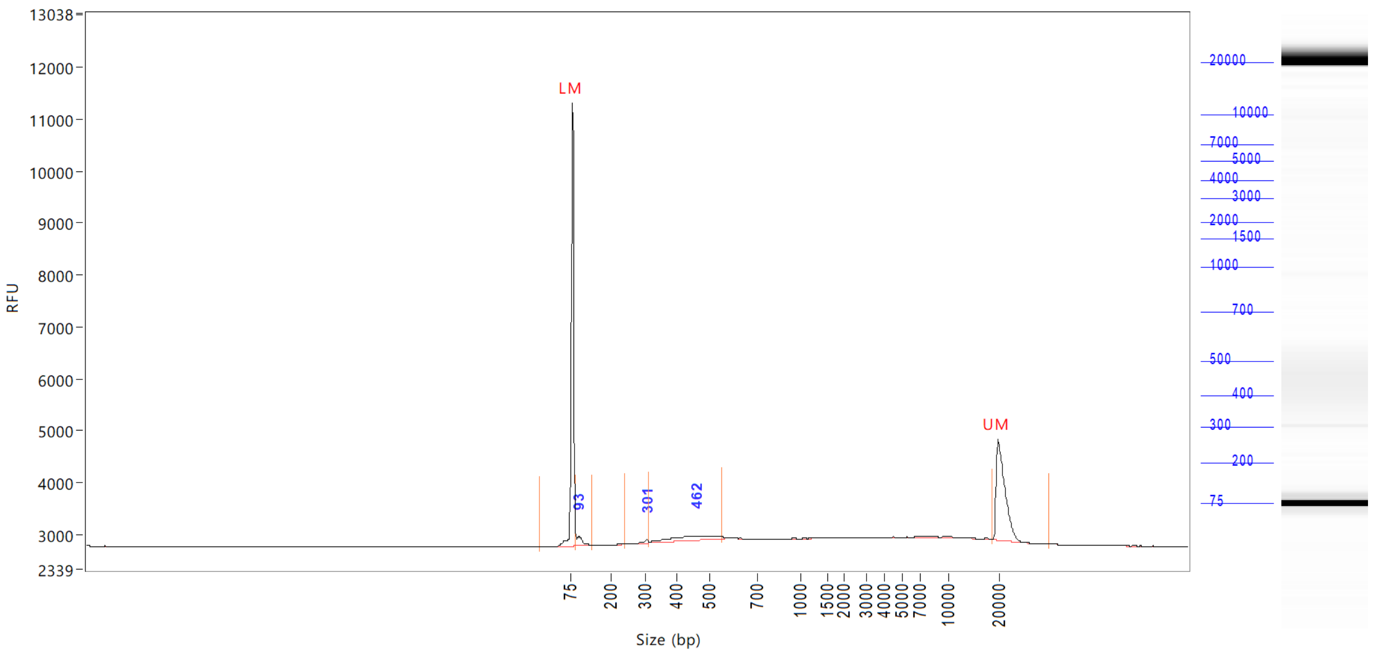

Peak	Size	Concentration	Relative concentration	Molarity	From	To	CV%	RFU
	(bp)	(ng/uL)	Percent	(nmole/L)	(bp)	(bp)		
1	75 (LM)	0.5000		11.2364	0	87	8.97	8543
2	93	0.2987	23.0	4.9050	87	135	10.59	173
3	301	0.0901	7.0	0.5094	237	309	4.90	83
4	462	0.9076	70.0	3.4568	309	552	13.53	89
5	20000 (UM)	0.2182		0.0173	18903	30156	4.88	1946

TIC:	1.2964	ng/uL
TIM:	8.8711	nmole/L
Total concentration:	1.7277	ng/uL

Sample peak width (sec): 5   Sample min peak height: 50   Sample baseline V to V?: Y   Sample baseline V to V points: 3
Sample filter: Binomial   Number of points for filter: 3 Sample start region (min): 0 Sample end region (min): 30
Marker peak width (sec): 5   Marker min peak height: 200   Marker baseline V to V?: Y   Marker baseline V to V points: 3
Lower marker selection: First peak > 500 RFU   Upper marker selection: Last peak > 200 RFU
Ladder size (bp)75, 200, 300, 400, 500, 700, 1000, 1500, 2000, 3000, 4000, 5000, 7000, 10000, 20000
Quantification using: Lower Marker   Final concentration (ng/uL): 0.5000   Dilution factor: 12.0

Sample: SampG12
Well location: G12
Created: Thursday, October 21, 2021 7:28:10 PM

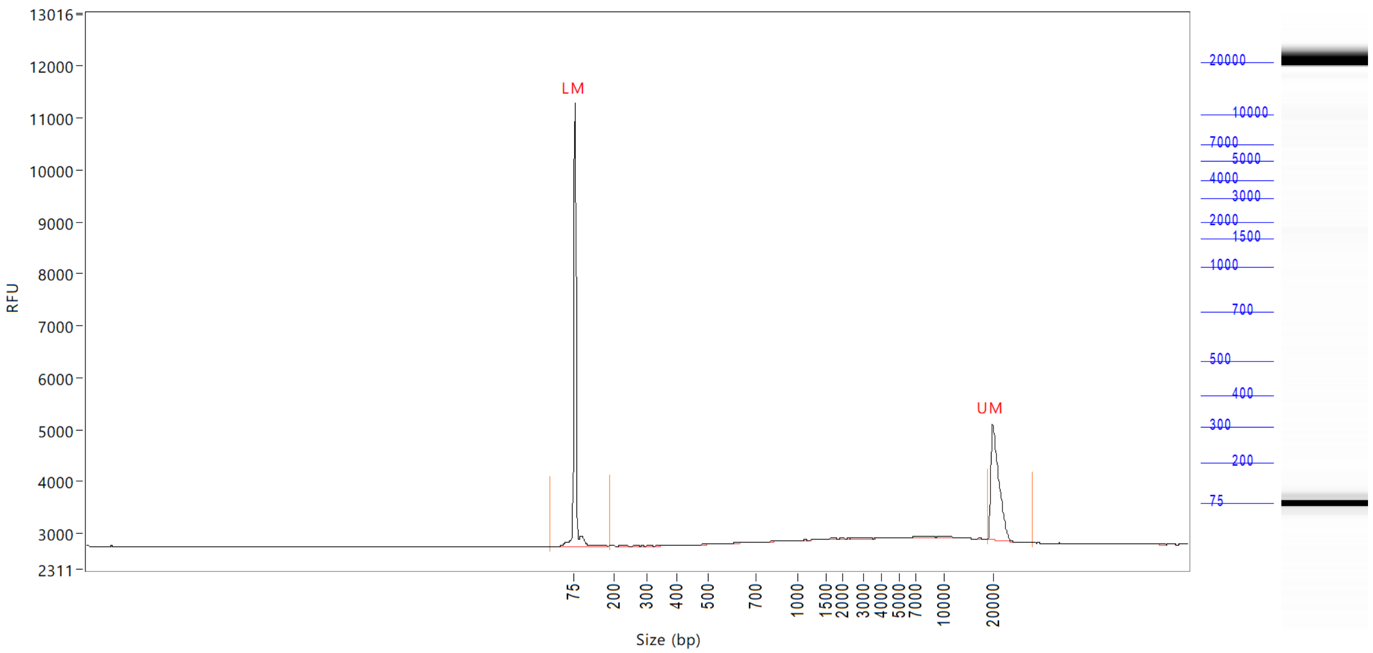

Peak	Size	Concentration	Relative concentration	Molarity	From	To	CV%	RFU
	(bp)	(ng/uL)	Percent	(nmole/L)	(bp)	(bp)		
1	75 (LM)	0.5000		11.0851	0	185	13.01	8554
2	20000 (UM)	0.2278		0.0182	18903	28100	4.33	2236

TIC:	0.0000	ng/uL
TIM:	0.0000	nmole/L
Total concentration:	0.4329	ng/uL

Sample peak width (sec): 5   Sample min peak height: 50   Sample baseline V to V?: Y   Sample baseline V to V points: 3
Sample filter: Binomial   Number of points for filter: 3 Sample start region (min): 0 Sample end region (min): 30
Marker peak width (sec): 5   Marker min peak height: 200   Marker baseline V to V?: Y   Marker baseline V to V points: 3
Lower marker selection: First peak > 500 RFU   Upper marker selection: Last peak > 200 RFU
Ladder size (bp)75, 200, 300, 400, 500, 700, 1000, 1500, 2000, 3000, 4000, 5000, 7000, 10000, 20000
Quantification using: Lower Marker   Final concentration (ng/uL): 0.5000   Dilution factor: 12.0

Sample: SampH12
Well location: H12
Created: Thursday, October 21, 2021 7:28:10 PM

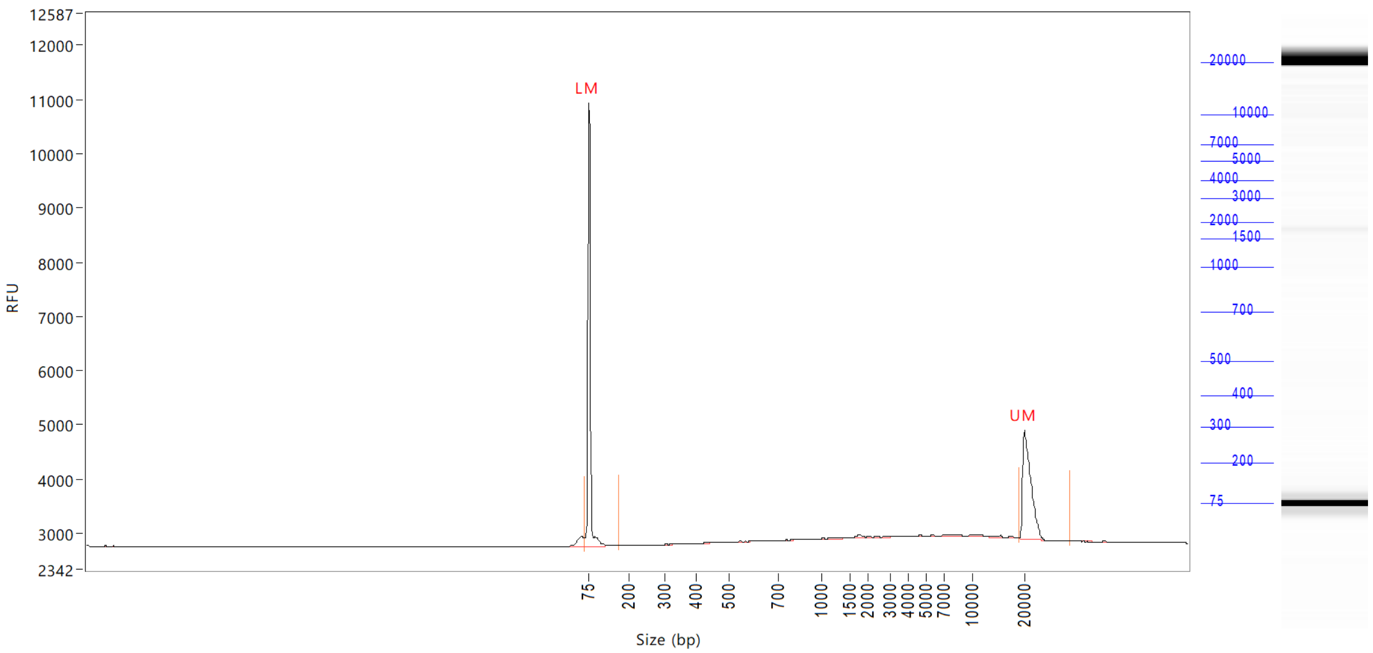

Peak	Size	Concentration	Relative concentration	Molarity	From	To	CV%	RFU
	(bp)	(ng/uL)	Percent	(nmole/L)	(bp)	(bp)		
1	75 (LM)	0.5000		10.9378	64	168	11.48	8183
2	20000 (UM)	0.2123		0.0169	18903	28947	4.32	1991

TIC:	0.0000	ng/uL
TIM:	0.0000	nmole/L
Total concentration:	0.6571	ng/uL

Sample peak width (sec): 5 Sample min peak height: 200 Sample baseline V to V?: Y Sample baseline V to V points: 3
Sample filter: Binomial Number of points for filter: 3 Sample start region (min): 0 Sample end region (min): 30
Marker peak width (sec): 5 Marker min peak height: 200 Marker baseline V to V?: Y Marker baseline V to V points: 3
Lower marker selection: First peak > 500 RFU Upper marker selection: Last peak > 200 RFU
Ladder size (bp)75, 200, 300, 400, 500, 700, 1000, 1500, 2000, 3000, 4000, 5000, 7000, 10000, 20000
Quantification using: Lower Marker Final concentration (ng/uL): 0.5000 Dilution factor: 12.0

Sample: DNA size ladder
Well location: A7
Created: Thursday, October 21, 2021 7:28:10 PM
Import from: C:\Users\admin\Desktop\210810_alberto_ladder.SCAL
Fit type: Point to point

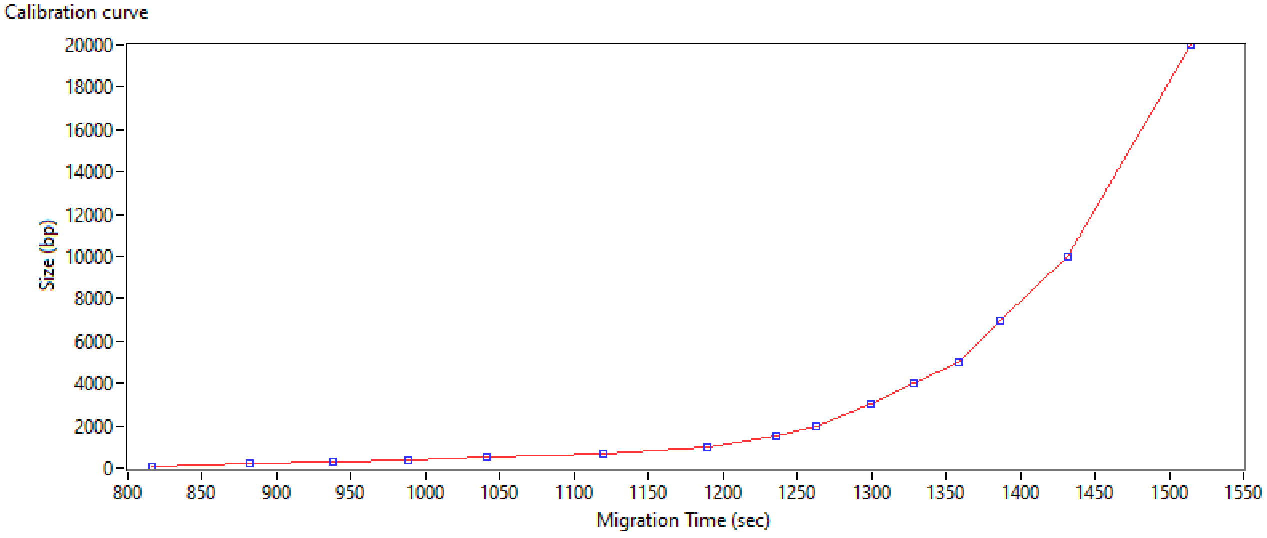

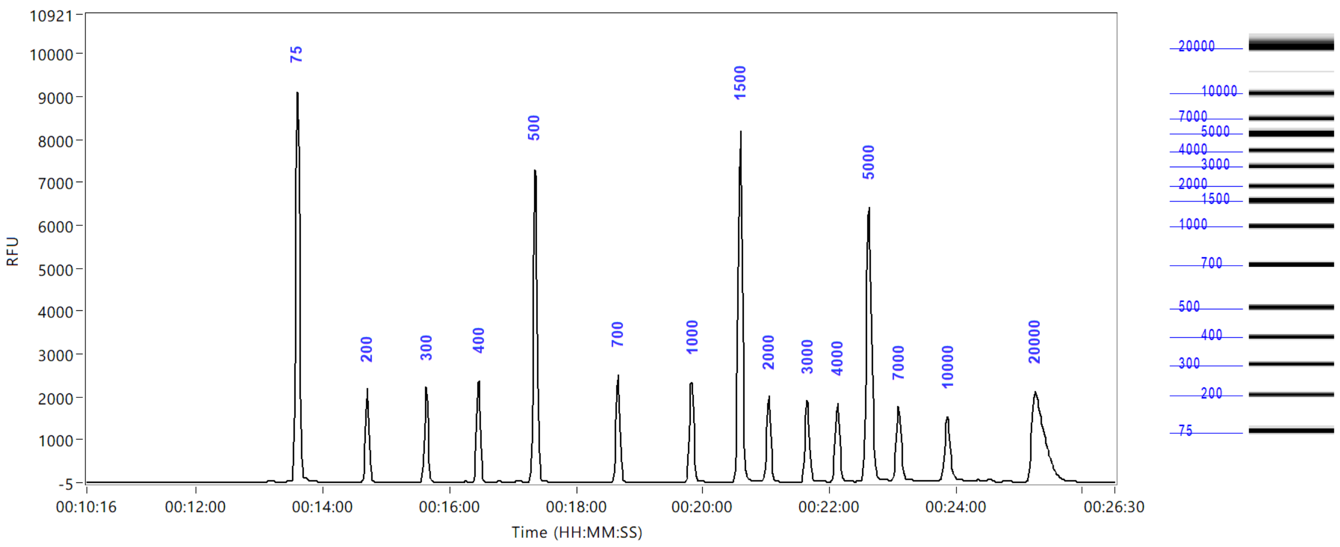

Supplement: Supplementary file 2 — sb3c00292_si_002.zip [file sb3c00292_si_002.zip › dnada_supplementary_material_pks_library_build/colony_pcr/ZAG/19-18-30_plate8/colonypcrplate_8.pdf]
