# Supplementary material for: Automated Platform for the Plasmid Construction Process
Source: ACS Synth Biol. 2023 Nov 10;12(12):3506–13. doi: 10.1021/acssynbio.3c00292 (PMC10729297; doi:10.1021/acssynbio.3c00292)

Instrument controller software run summary:

Filename and data path: C:\AATI\Data\2021 10 20\18-28-55\2021 10 20 18H 28M..raw
Created: Wednesday, October 20, 2021 6:38:33 PM
Number of capillaries: 96
Array serial number: 111920-09SFS
Effect length: 33 cm
Array usage count: 264
Instrument type: ZAG DNA Analyzer
Instrument controller software version: 1.0.1.9
Device serial number: Z7597

Method Information

Method name: ZAG130GP33 - DNA 75-20000bp - Gel Prime Only.mthds
Gel prime: Yes
Full conditioning: No
Gel prime to buffer: No
Gel selection: Gel 1
Perform prerun: 8.0 kV, 30 sec.
Rinse: No
Marker 1: Row: A, 5.0 kV, 5 sec.
Rinse: No
Sample injection: 5.0 kV, 5 sec.
Separation: 8.0 kV, 30.0 min.
Tray name: Sample Tray-4

Analysis mode: DNA

Notes

Gel Image

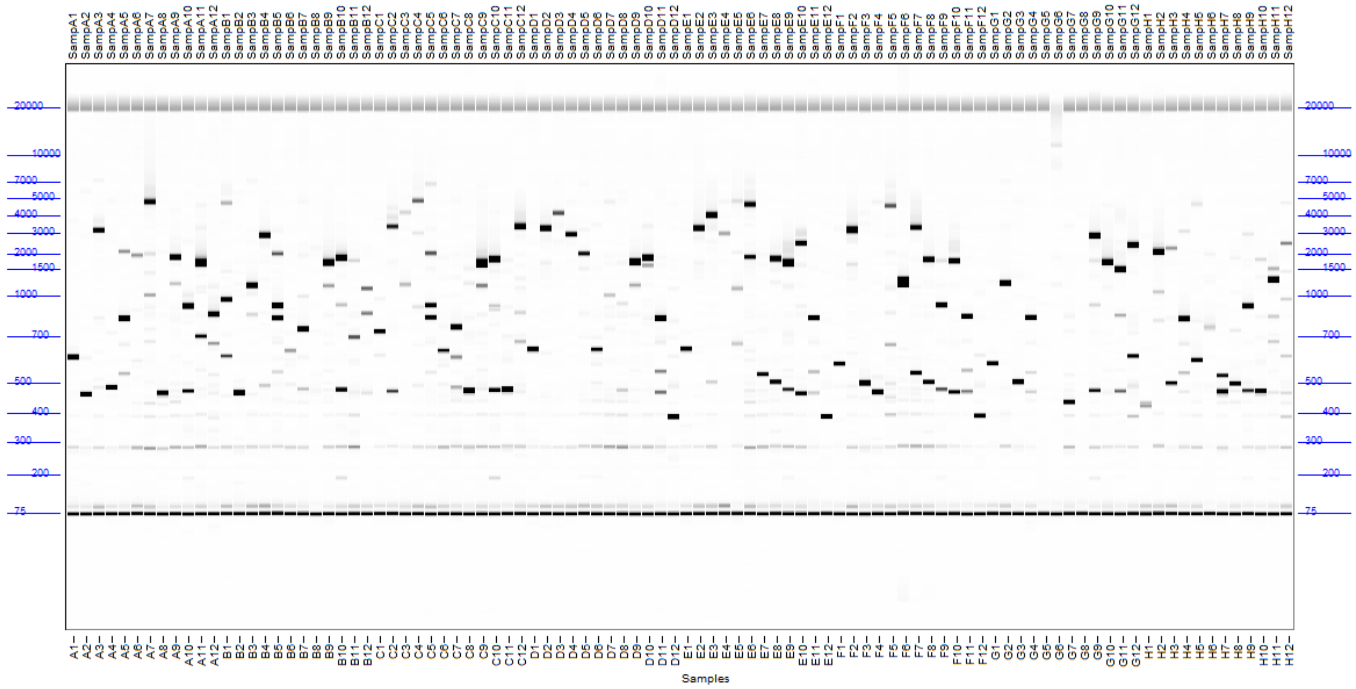

Filename and data path: C:\AATI\Data\2021 10 20\18-28-55\2021 10 20 18H 28M..raw

A1: SampA1

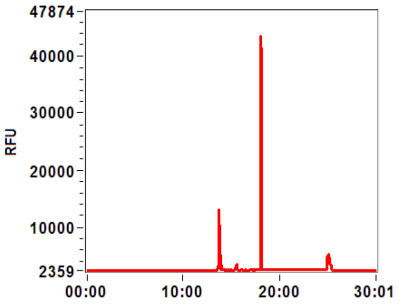

A2: SampA2

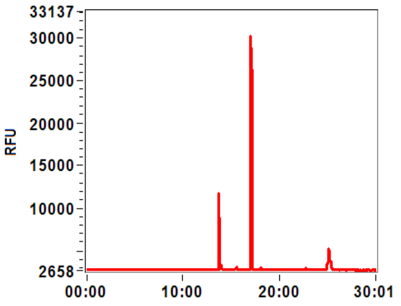

A3: SampA3

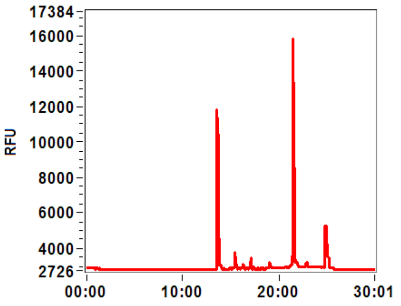

A4: SampA4

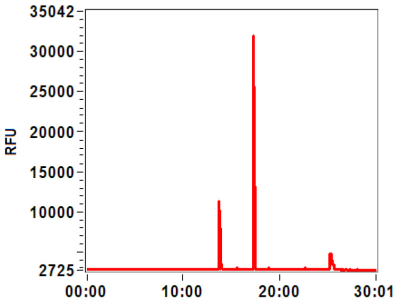

A5: SampA5

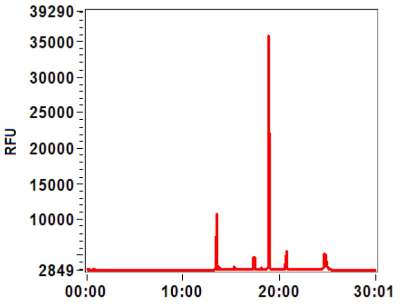

A6: SampA6

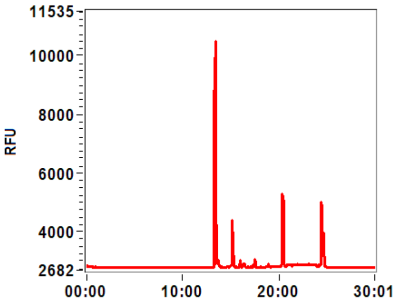

A7: SampA7

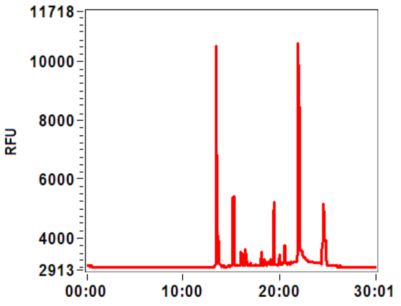

A8: SampA8

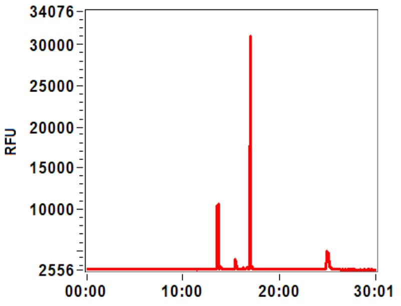

A9: SampA9

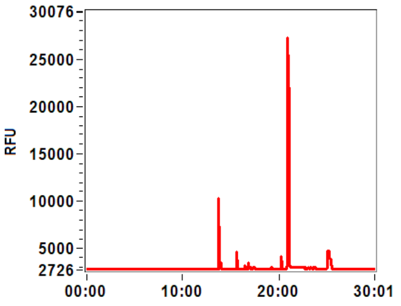

A10: SampA10

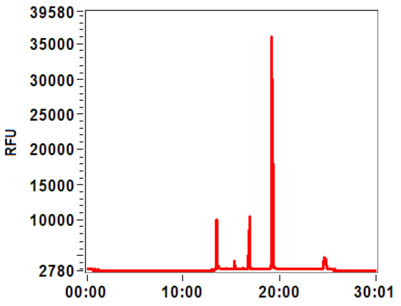

A11: SampA11

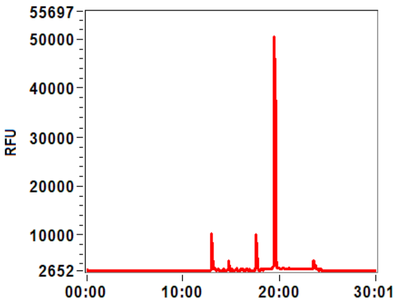

A12: SampA12

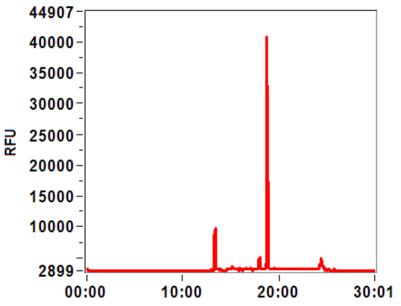

Filename and data path: C:\AATI\Data\2021 10 20\18-28-55\2021 10 20 18H 28M..raw

B1: SampB1

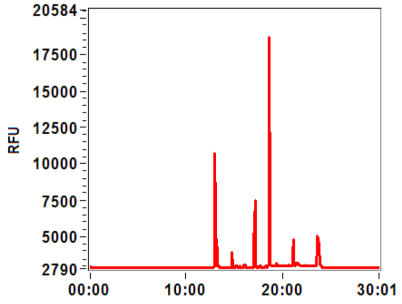

B2: SampB2

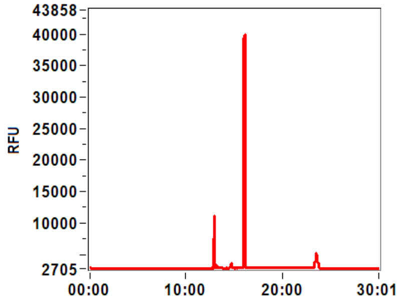

B3: SampB3

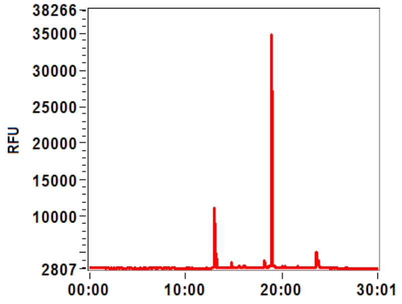

B4: SampB4

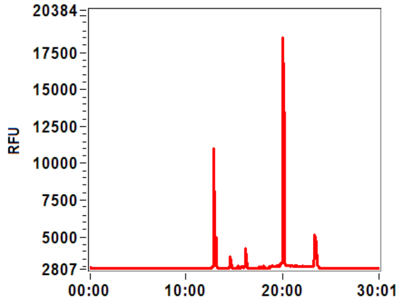

B5: SampB5

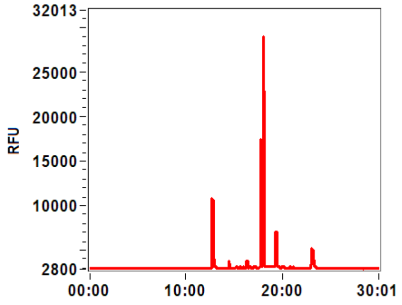

B6: SampB6

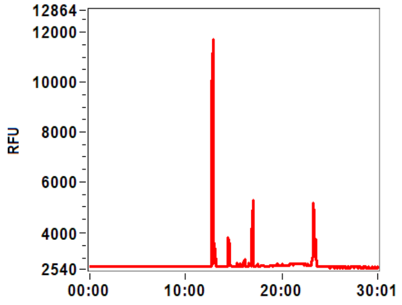

B7: SampB7

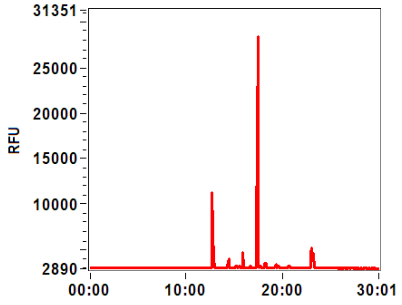

B8: SampB8

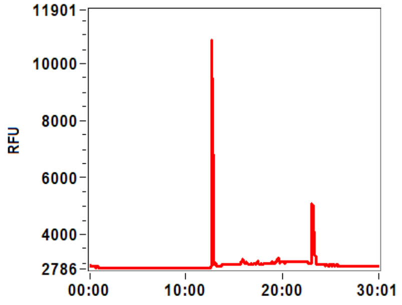

B9: SampB9

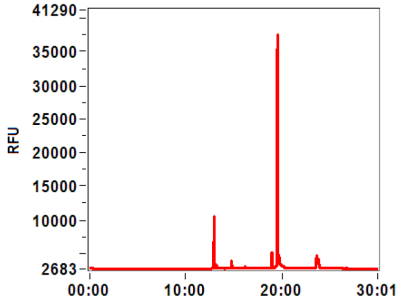

B10: SampB10

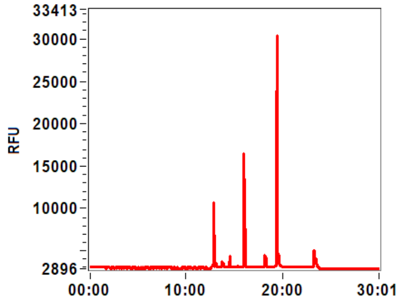

B11: SampB11

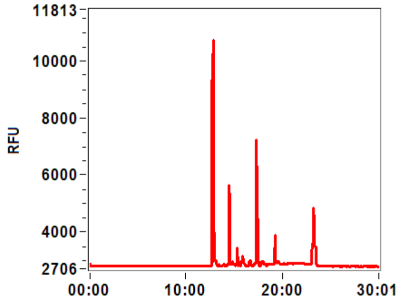

B12: SampB12

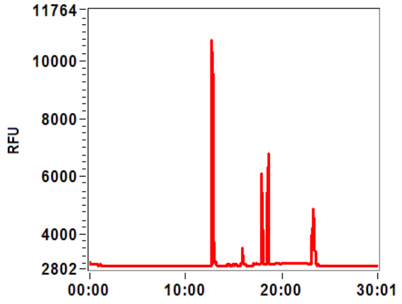

Filename and data path: C:\AATI\Data\2021 10 20\18-28-55\2021 10 20 18H 28M..raw

C1: SampC1

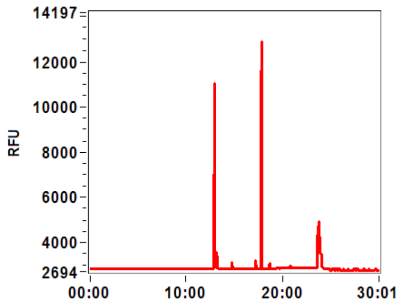

C2: SampC2

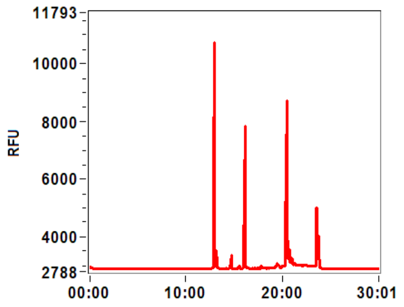

C3: SampC3

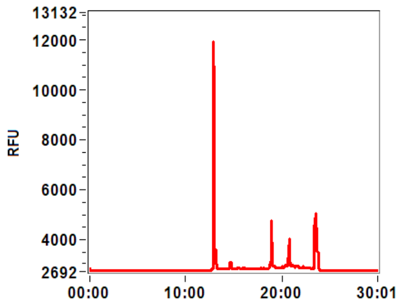

C4: SampC4

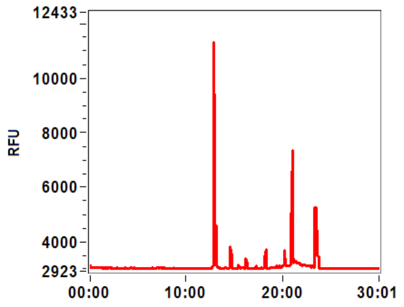

C5: SampC5

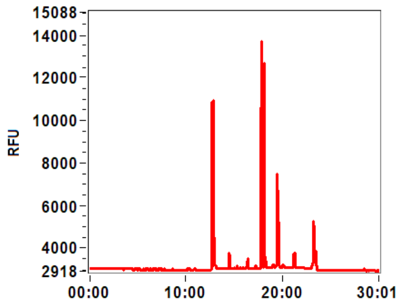

C6: SampC6

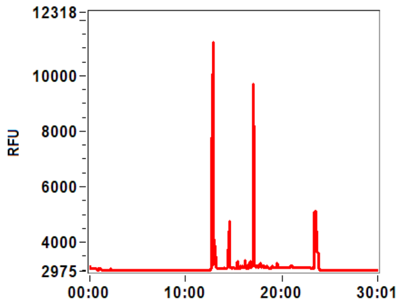

C7: SampC7

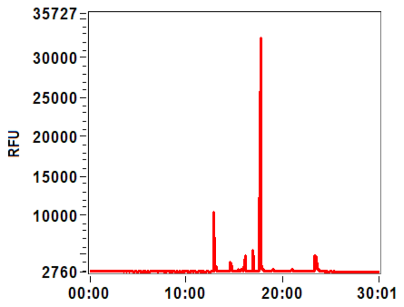

C8: SampC8

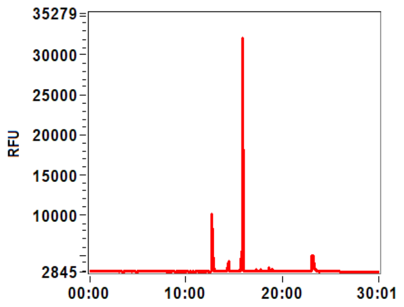

C9: SampC9

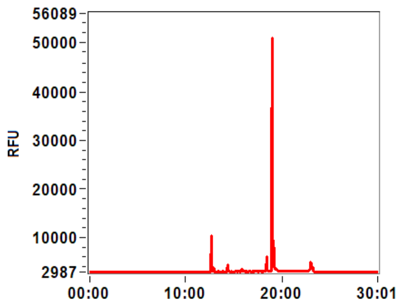

C10: SampC10

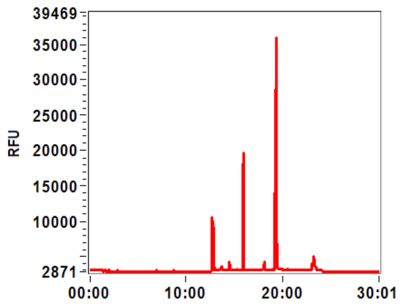

C11: SampC11

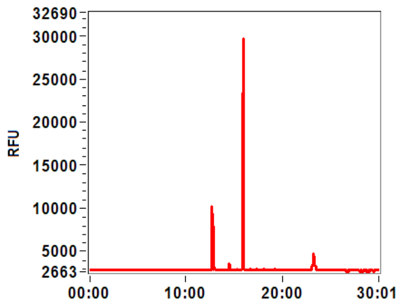

C12: SampC12

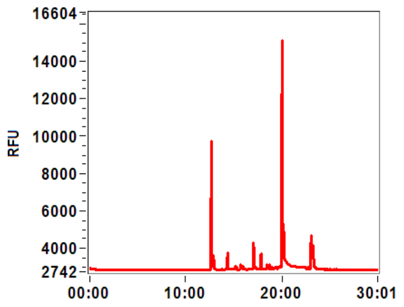

Filename and data path: C:\AATI\Data\2021 10 20\18-28-55\2021 10 20 18H 28M..raw

D1: SampD1

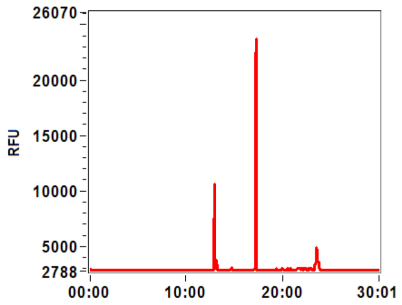

D2: SampD2

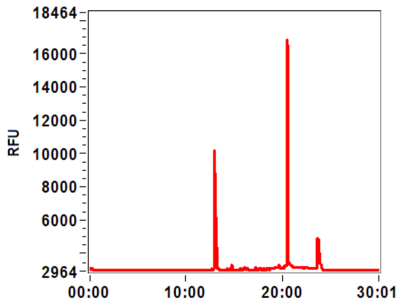

D3: SampD3

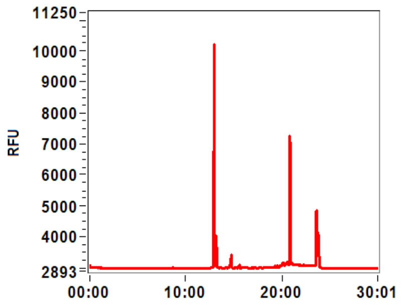

D4: SampD4

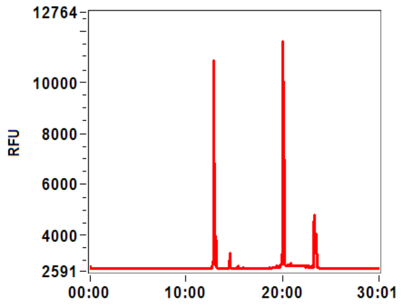

D5: SampD5

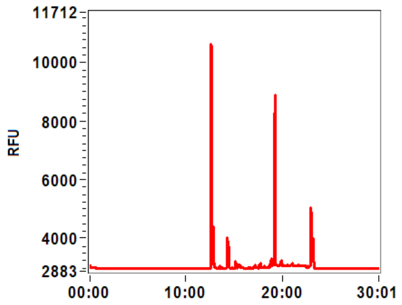

D6: SampD6

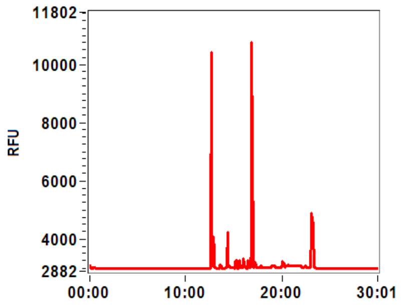

D7: SampD7

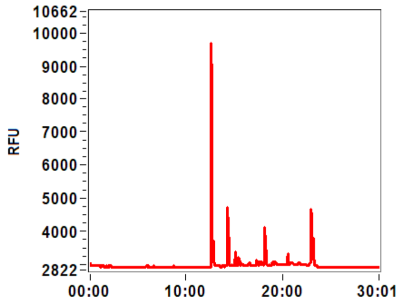

D8: SampD8

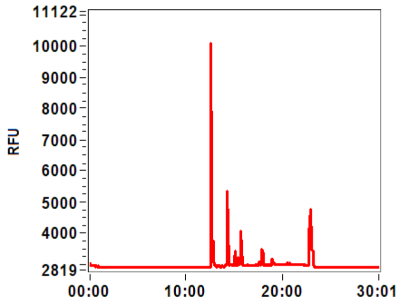

D9: SampD9

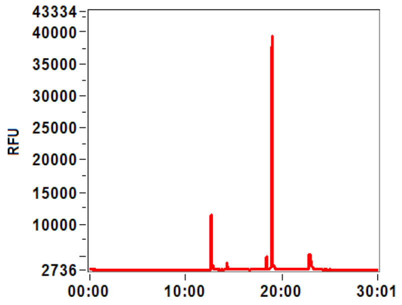

D10: SampD10

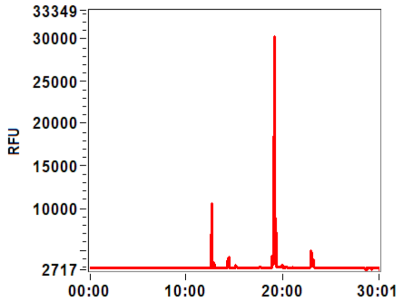

D11: SampD11

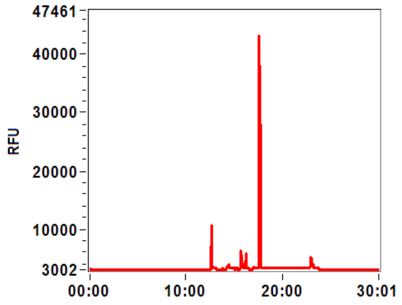

D12: SampD12

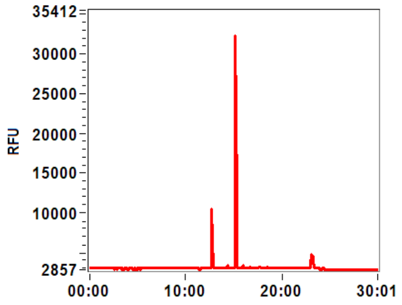

Filename and data path: C:\AATI\Data\2021 10 20\18-28-55\2021 10 20 18H 28M..raw

E1: SampE1

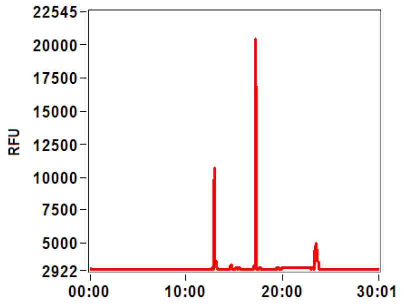

E2: SampE2

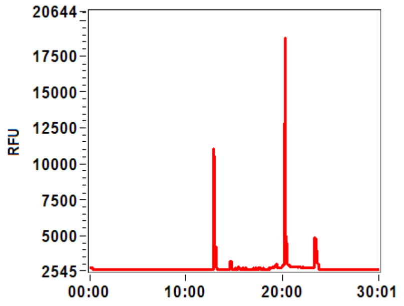

E3: SampE3

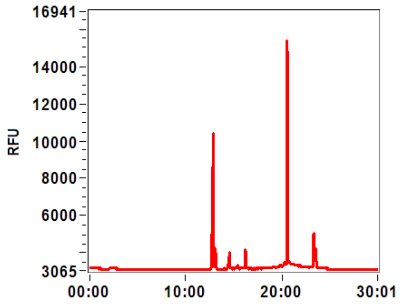

E4: SampE4

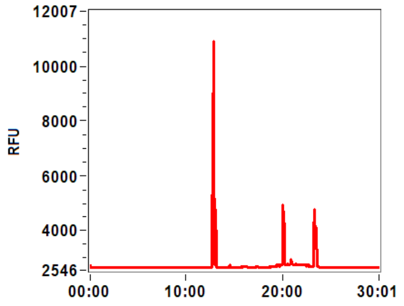

E5: SampE5

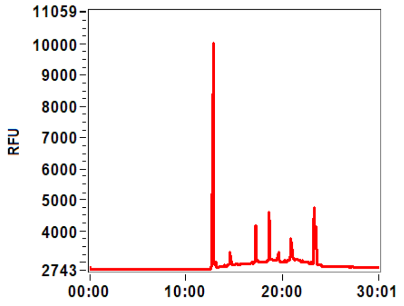

E6: SampE6

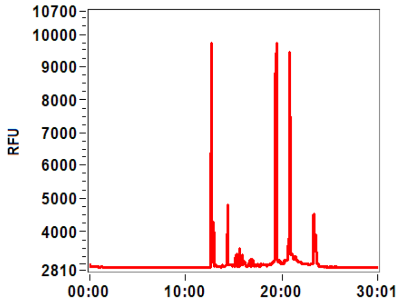

E7: SampE7

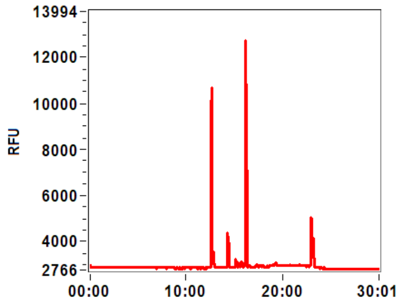

E8: SampE8

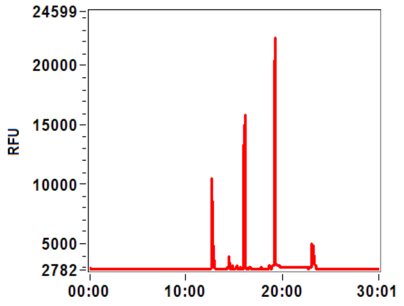

E9: SampE9

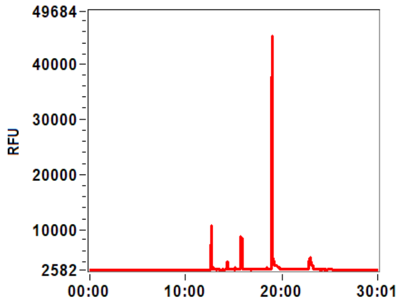

E10: SampE10

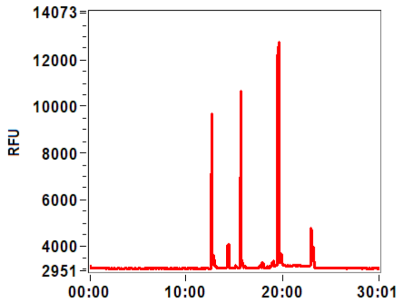

E11: SampE11

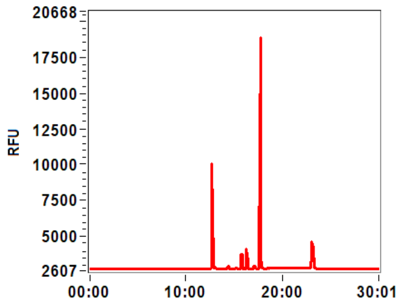

E12: SampE12

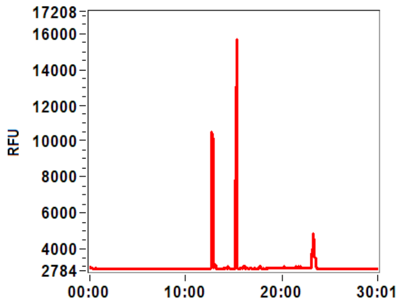

Filename and data path: C:\AATI\Data\2021 10 20\18-28-55\2021 10 20 18H 28M..raw

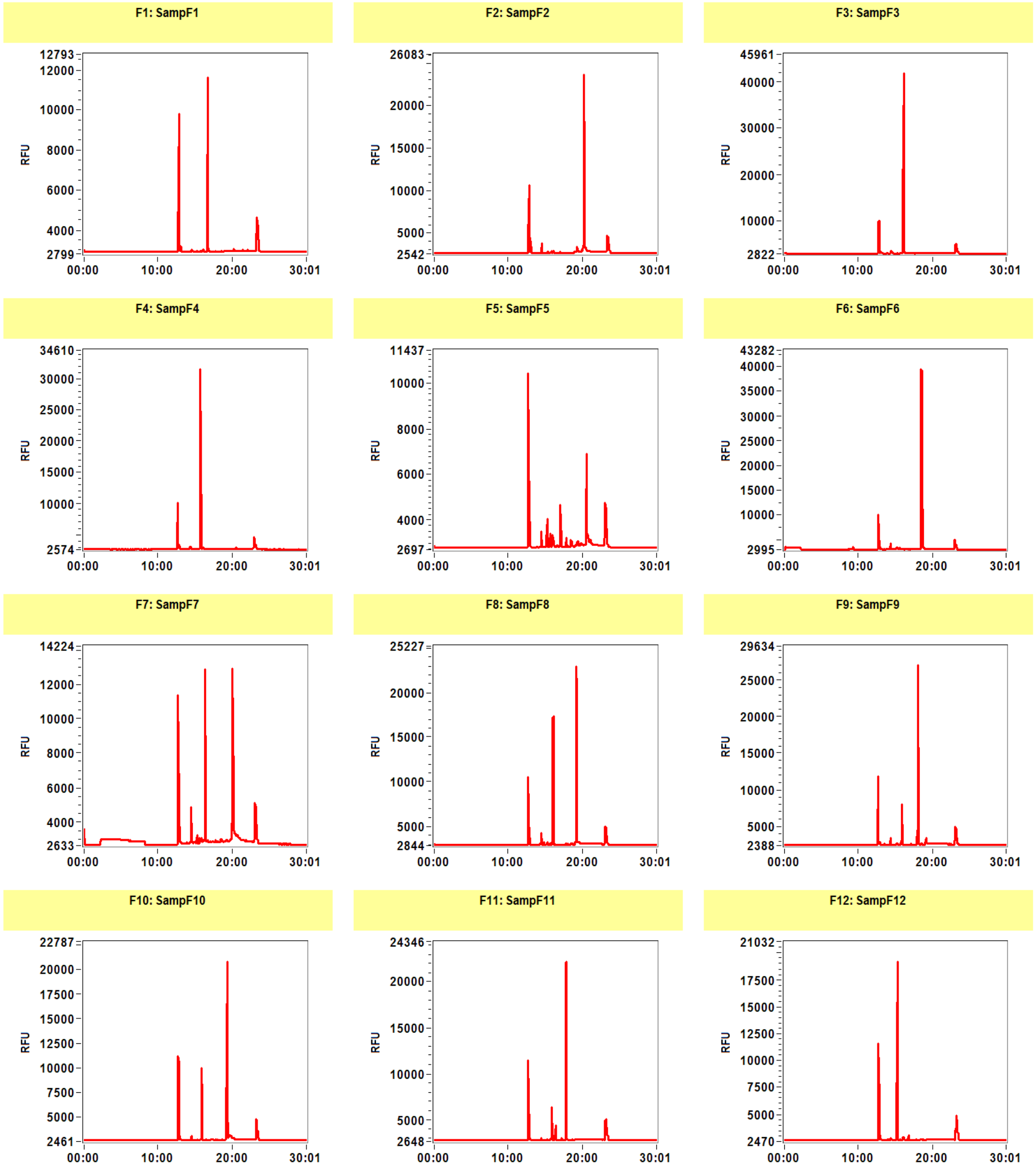

Filename and data path: C:\AATI\Data\2021 10 20\18-28-55\2021 10 20 18H 28M..raw

G1: SampG1

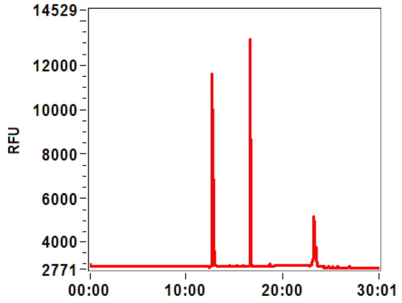

G2: SampG2

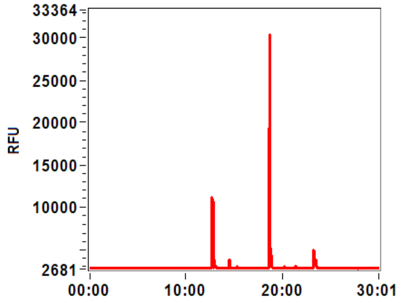

G3: SampG3

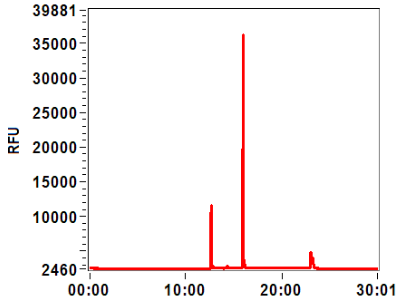

G4: SampG4

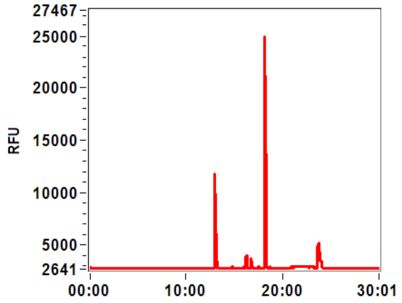

G5: SampG5

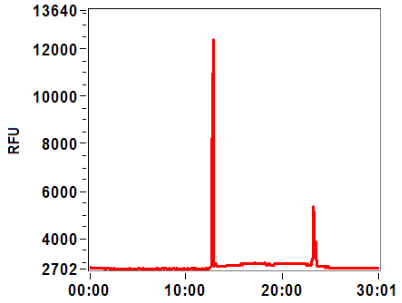

G6: SampG6

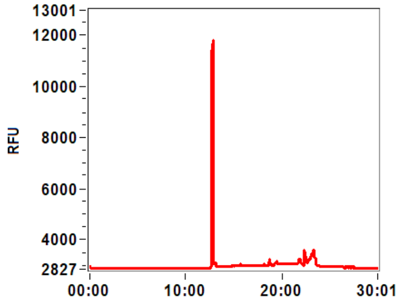

G7: SampG7

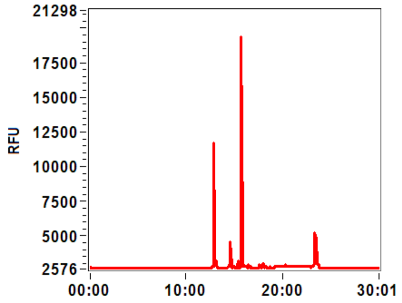

G8: SampG8

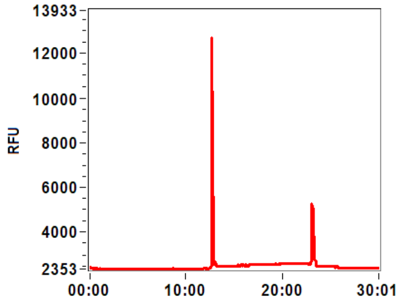

G9: SampG9

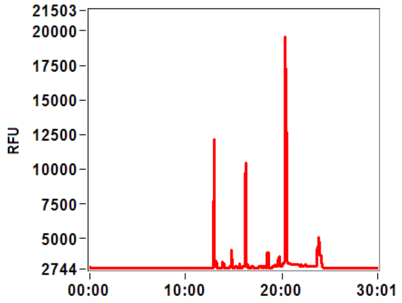

G10: SampG10

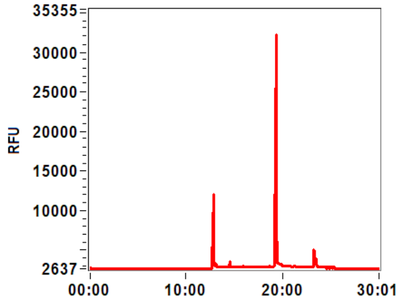

G11: SampG11

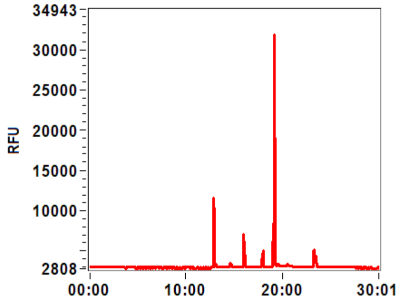

G12: SampG12

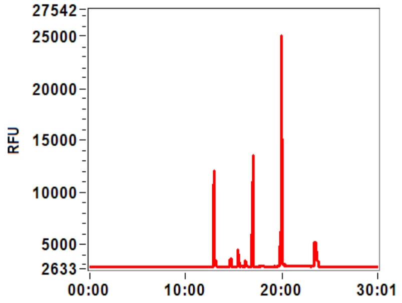

Filename and data path: C:\AATI\Data\2021 10 20\18-28-55\2021 10 20 18H 28M..raw

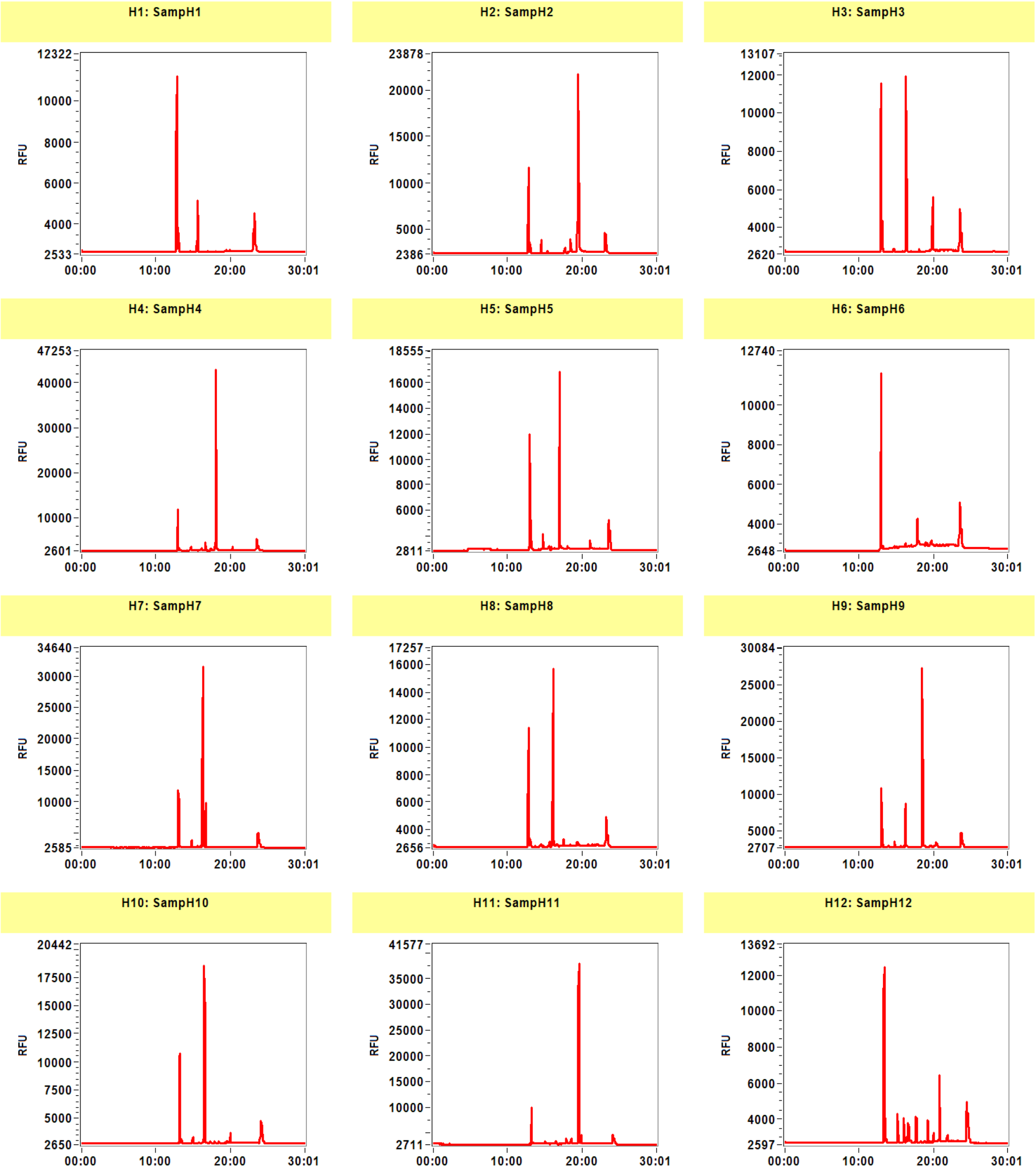

Sample: SampA1
Well location: A1
Created: Wednesday, October 20, 2021 6:38:33 PM

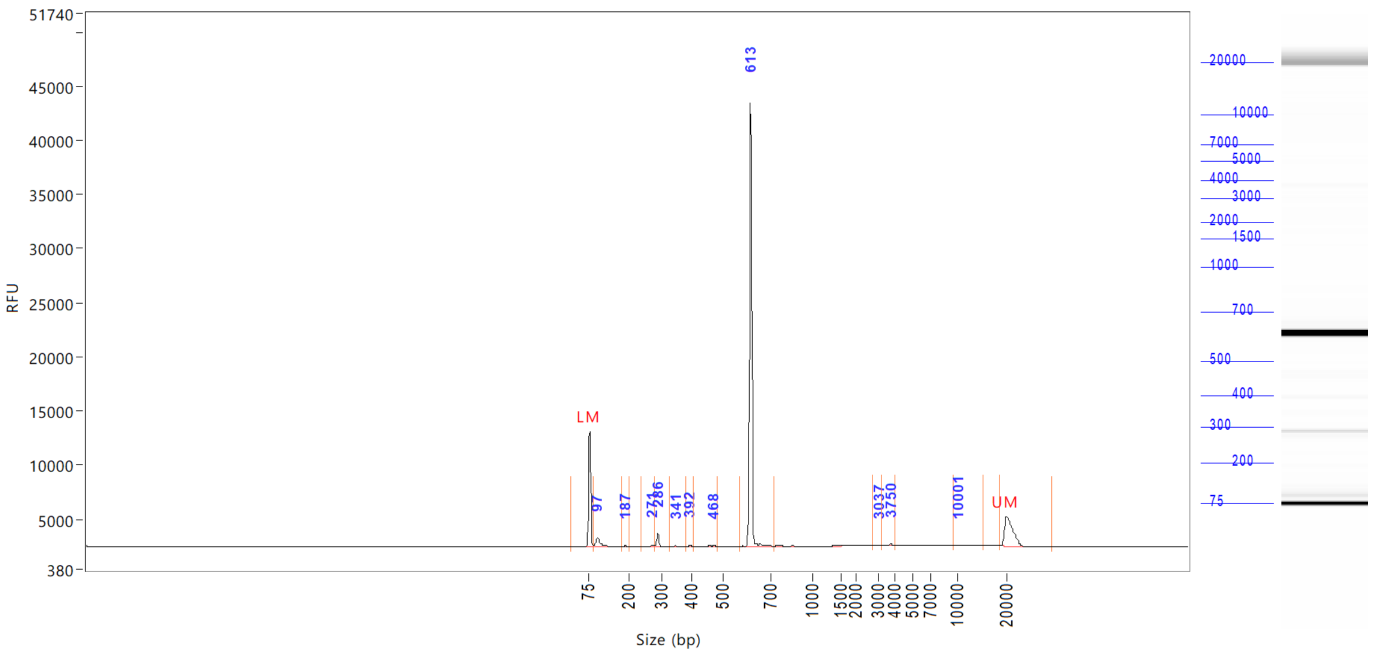

Peak	Size (bp)	Concentration (ng/uL)	From (bp)	To (bp)	Average size (bp)	CV%	RFU	Corrected peak area
1	75 (LM)	0.5000	16	86	72	5.00	10652	55.380
2	97	0.9789	86	177	105	15.56	807	9.035
3	187	0.0443	177	201	185	2.33	78	0.409
4	271	0.1450	235	278	268	2.88	191	1.338
5	286	0.6317	278	325	286	1.82	1177	5.831
6	341	0.0603	325	382	345	3.58	78	0.556
7	392	0.0878	382	403	391	0.83	188	0.810
8	468	0.1288	403	481	457	2.60	111	1.189
9	613	19.0261	565	718	611	1.32	41043	175.612
10	3037	0.0431	2743	3215	2973	3.51	59	0.398
11	3750	0.0785	3215	3964	3657	4.03	145	0.725
12	10001	0.0761	9667	15244	11258	12.58	53	0.702
13	20000 (UM)	0.2570	18537	29068	20531	4.19	2764	28.461

TIC:	21.3006	ng/uL
TIM:	72.6633	nmole/L
Total concentration:	21.7362	ng/uL

```
Sample peak width (sec): 5     Sample min peak height: 50    Sample baseline V to V?: Y    Sample baseline V to V points: 3
Sample filter: Binomial        Number of points for filter: 3  Sample start region (min): 0  Sample end region (min): 30
Marker peak width (sec): 5     Marker min peak height: 200   Marker baseline V to V?: Y    Marker baseline V to V points: 3
Lower marker selection: First peak > 500 RFU                 Upper marker selection: Last peak > 200 RFU
Ladder size (bp)75, 200, 300, 400, 500, 700, 1000, 1500, 2000, 3000, 4000, 5000, 7000, 10000, 20000
Quantification using: Lower Marker          Final concentration (ng/uL): 0.5000          Dilution factor: 12.0
```

Sample: SampA2
Well location: A2
Created: Wednesday, October 20, 2021 6:38:33 PM

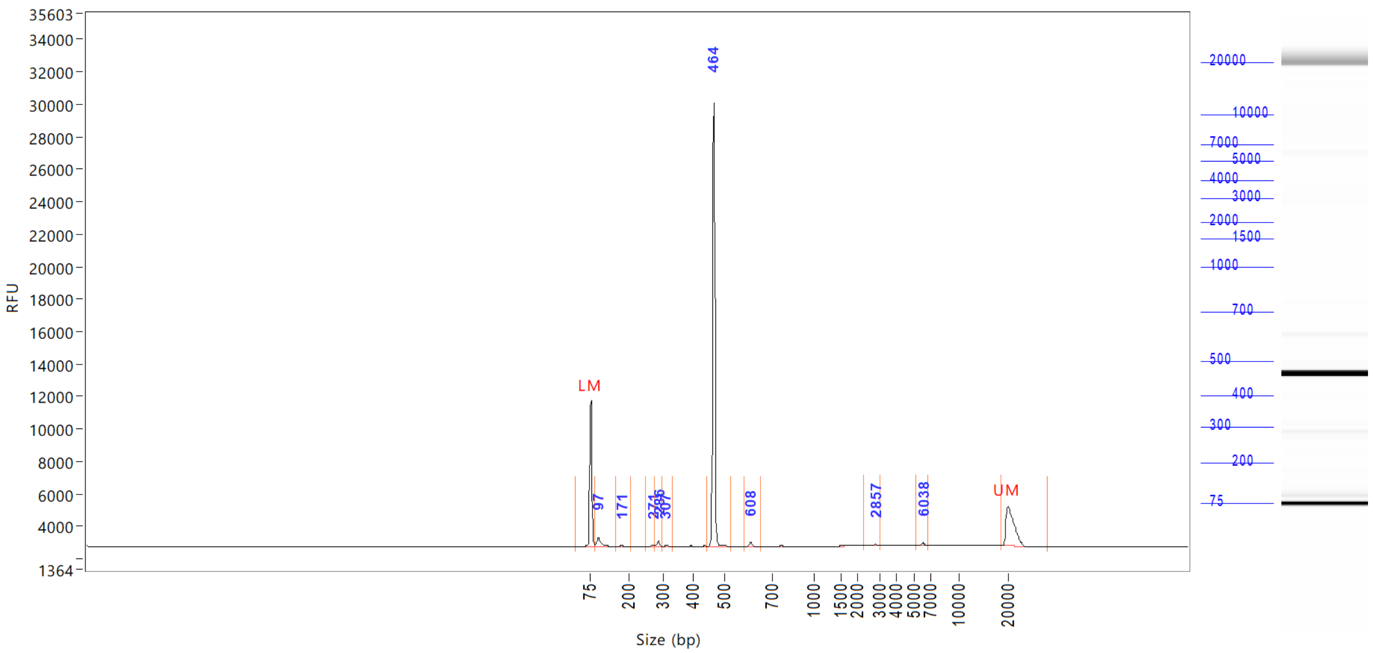

Peak	Size (bp)	Concentration (ng/uL)	From (bp)	To (bp)	Average size (bp)	CV%	RFU	Corrected peak area
1	75 (LM)	0.5000	28	86	72	4.95	8988	47.294
2	97	0.7397	86	154	102	12.11	577	5.831
3	171	0.0753	154	201	171	4.71	88	0.594
4	271	0.1178	244	277	267	2.30	109	0.929
5	286	0.2257	277	296	283	1.19	337	1.779
6	307	0.0684	296	331	307	1.99	83	0.539
7	464	14.9226	443	524	462	0.96	27361	117.625
8	608	0.1650	580	654	606	1.34	254	1.301
9	2857	0.0564	2258	3037	2811	4.88	74	0.444
10	6038	0.0805	5223	6704	5900	3.80	140	0.634
11	20000 (UM)	0.2618	18781	27979	20566	4.15	2455	24.763

TIC:	16.4514	ng/uL
TIM:	68.6887	nmole/L
Total concentration:	16.9397	ng/uL

```
Sample peak width (sec): 5    Sample min peak height: 50    Sample baseline V to V?: Y    Sample baseline V to V points: 3
Sample filter: Binomial       Number of points for filter: 3  Sample start region (min): 0  Sample end region (min): 30
Marker peak width (sec): 5    Marker min peak height: 200   Marker baseline V to V?: Y    Marker baseline V to V points: 3
Lower marker selection: First peak > 500 RFU                Upper marker selection: Last peak > 200 RFU
Ladder size (bp)75, 200, 300, 400, 500, 700, 1000, 1500, 2000, 3000, 4000, 5000, 7000, 10000, 20000
Quantification using: Lower Marker          Final concentration (ng/uL): 0.5000          Dilution factor: 12.0
```

Sample: SampA3
Well location: A3
Created: Wednesday, October 20, 2021 6:38:33 PM

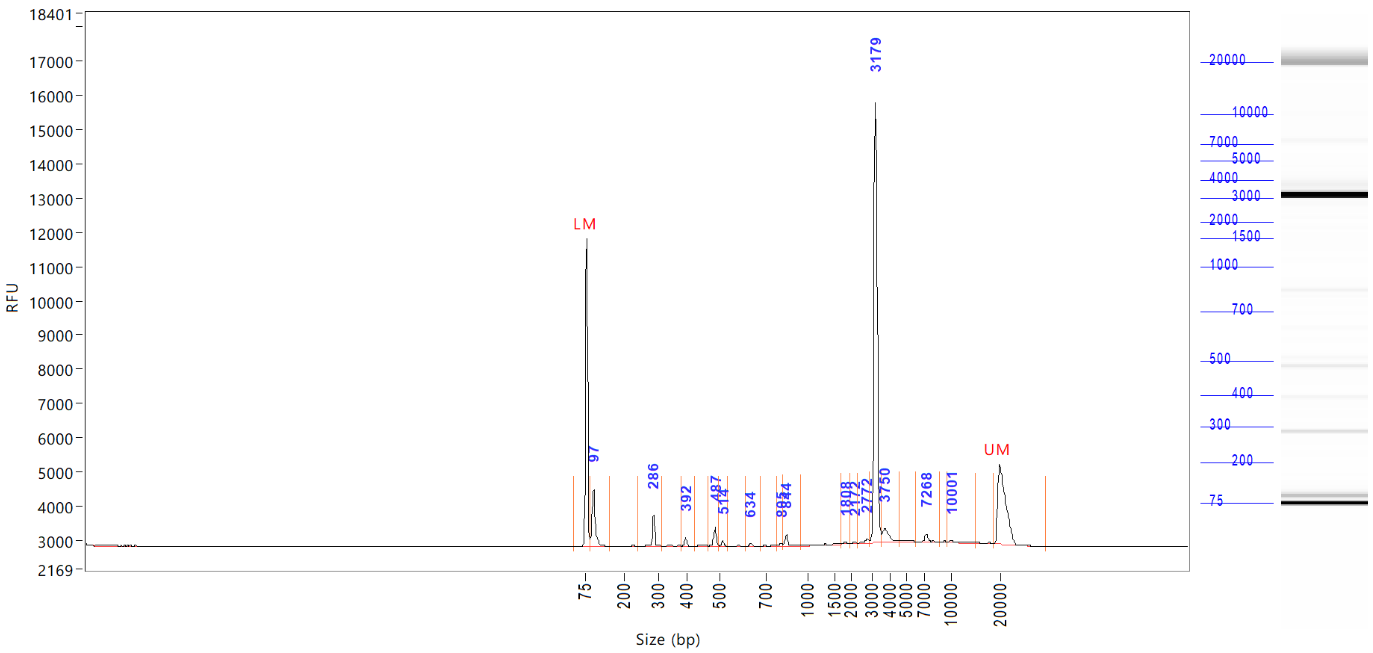

Peak	Size (bp)	Concentration (ng/uL)	From (bp)	To (bp)	Average size (bp)	CV%	RFU	Corrected peak area
1	75 (LM)	0.5000	35	86	73	4.72	9007	46.099
2	97	1.7119	86	148	97	8.66	1678	13.152
3	286	0.6469	239	313	282	3.14	910	4.970
4	392	0.1597	376	420	389	1.31	253	1.227
5	487	0.3845	464	498	483	1.05	563	2.954
6	514	0.1033	498	537	510	1.11	167	0.794
7	634	0.0662	613	677	634	1.78	85	0.509
8	805	0.0502	779	818	802	1.23	78	0.386
9	844	0.2324	818	948	847	2.80	336	1.786
10	1808	0.0360	1712	1981	1803	2.79	55	0.276
11	2172	0.0327	1981	2315	2139	3.10	58	0.252
12	2772	0.1178	2315	2914	2705	5.55	111	0.905
13	3179	7.8084	2914	3536	3151	2.93	12869	59.992
14	3750	0.5149	3536	4518	3839	5.88	410	3.956
15	7268	0.1699	6112	8734	7371	5.99	233	1.306
16	10001	0.1333	9667	15122	11787	13.95	56	1.024
17	20000 (UM)	0.2574	18781	29430	20542	4.39	2318	23.734

TIC:	12.1682	ng/uL
TIM:	40.2828	nmole/L
Total concentration:	12.6397	ng/uL

```
Sample peak width (sec): 5    Sample min peak height: 50    Sample baseline V to V?: Y    Sample baseline V to V points: 3
Sample filter: Binomial       Number of points for filter: 3  Sample start region (min): 0  Sample end region (min): 30
Marker peak width (sec): 5    Marker min peak height: 200   Marker baseline V to V?: Y    Marker baseline V to V points: 3
Lower marker selection: First peak > 500 RFU                Upper marker selection: Last peak > 200 RFU
Ladder size (bp)75, 200, 300, 400, 500, 700, 1000, 1500, 2000, 3000, 4000, 5000, 7000, 10000, 20000
Quantification using: Lower Marker          Final concentration (ng/uL): 0.5000          Dilution factor: 12.0
```

Sample: SampA4
Well location: A4
Created: Wednesday, October 20, 2021 6:38:33 PM

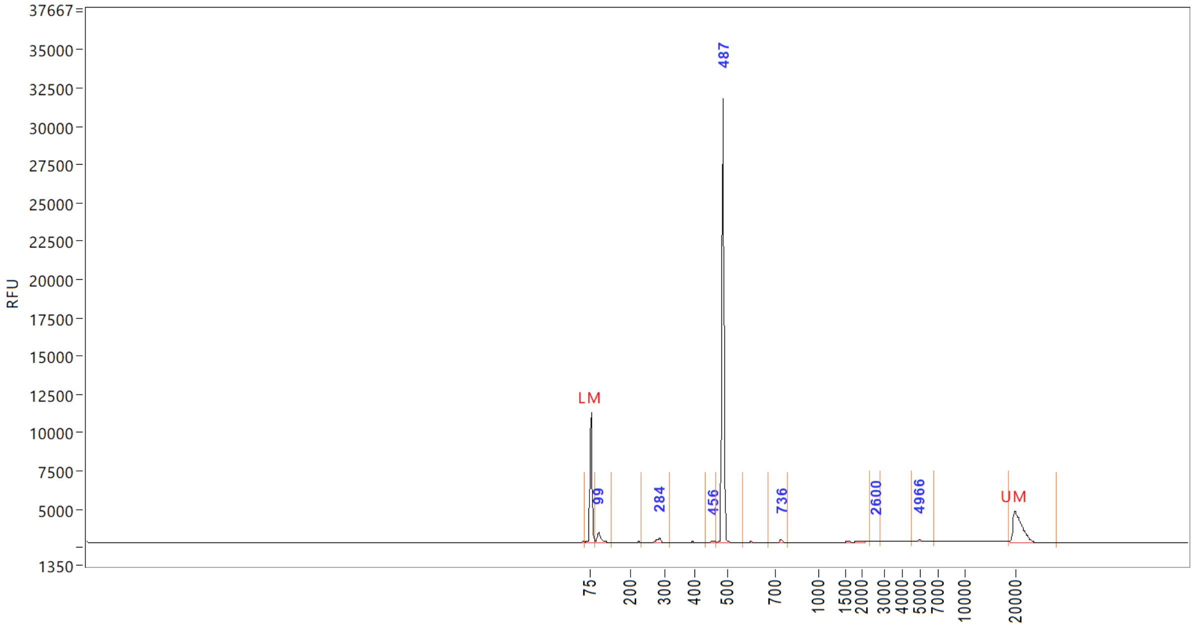

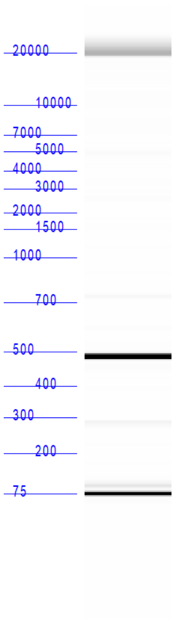

Peak	Size (bp)	Concentration (ng/uL)	From (bp)	To (bp)	Average size (bp)	CV%	RFU	Corrected peak area
1	75 (LM)	0.5000	56	87	73	4.50	8467	43.225
2	99	0.7990	87	139	99	7.44	693	5.756
3	284	0.3202	230	313	278	3.57	293	2.307
4	456	0.0912	430	466	451	1.92	74	0.657
5	487	16.6960	466	565	484	0.81	29024	120.280
6	736	0.1318	672	783	729	1.83	190	0.950
7	2600	0.0513	2286	2800	2582	3.64	64	0.369
8	4966	0.1014	4518	6704	5087	8.19	104	0.730
9	20000 (UM)	0.2573	18659	28221	20609	4.66	2030	22.240

TIC:	18.1910	ng/uL
TIM:	72.6057	nmole/L
Total concentration:	18.7524	ng/uL

Sample peak width (sec): 5 Sample min peak height: 50 Sample baseline V to V?: Y Sample baseline V to V points: 3
Sample filter: Binomial Number of points for filter: 3 Sample start region (min): 0 Sample end region (min): 30
Marker peak width (sec): 5 Marker min peak height: 200 Marker baseline V to V?: Y Marker baseline V to V points: 3
Lower marker selection: First peak > 500 RFU Upper marker selection: Last peak > 200 RFU
Ladder size (bp)75, 200, 300, 400, 500, 700, 1000, 1500, 2000, 3000, 4000, 5000, 7000, 10000, 20000
Quantification using: Lower Marker Final concentration (ng/uL): 0.5000 Dilution factor: 12.0

Sample: SampA5
Well location: A5
Created: Wednesday, October 20, 2021 6:38:33 PM

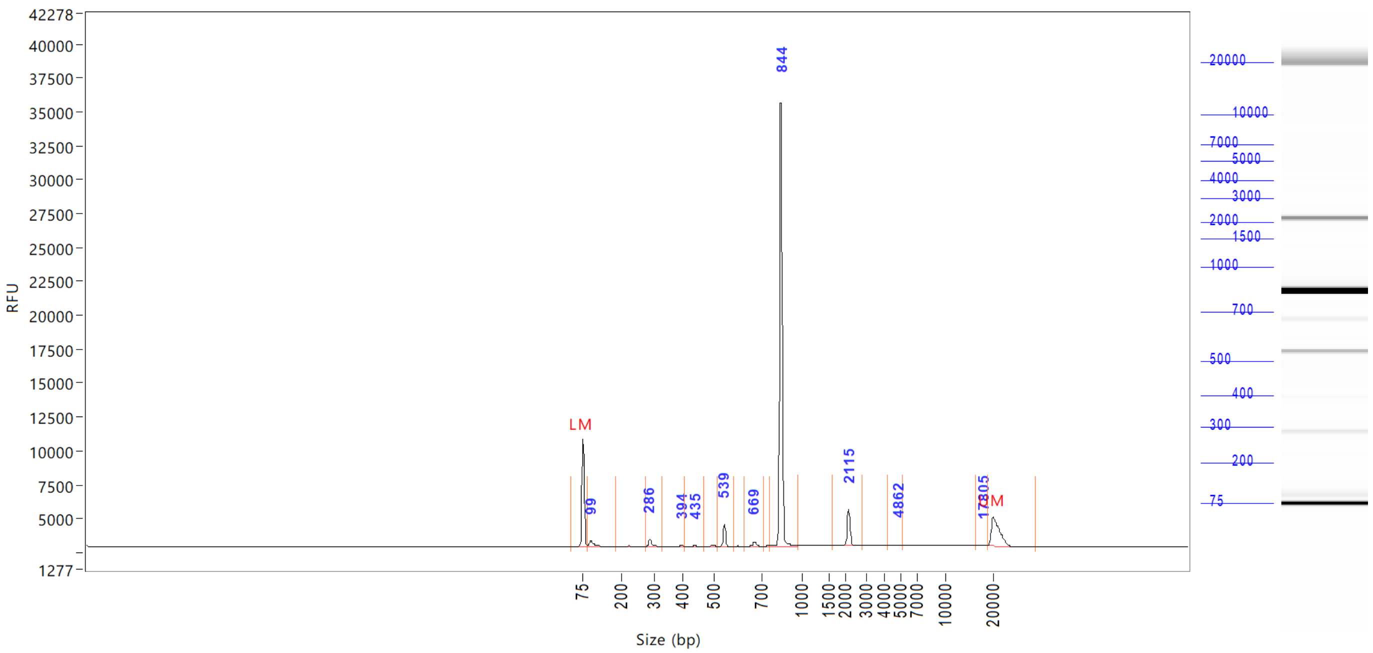

Peak	Size (bp)	Concentration (ng/uL)	From (bp)	To (bp)	Average size (bp)	CV%	RFU	Corrected peak area
1	75 (LM)	0.5000	37	87	73	5.08	7881	40.928
2	99	0.7138	87	181	104	14.70	408	4.869
3	286	0.4686	273	323	286	2.47	535	3.196
4	394	0.1066	323	403	382	4.51	119	0.727
5	435	0.0903	403	466	436	3.00	55	0.616
6	539	1.0854	511	583	537	1.16	1626	7.404
7	669	0.2986	623	714	668	1.40	349	2.037
8	844	20.4019	762	970	836	1.43	32744	139.168
9	2115	1.7732	1559	2829	2104	5.27	2629	12.096
10	4862	0.0828	4242	5149	4737	4.68	54	0.565
11	17805	0.0705	16342	18903	17753	3.71	56	0.481
12	20000 (UM)	0.2683	18903	28584	20615	4.41	2106	21.964

TIC:	25.0918	ng/uL
TIM:	60.4166	nmole/L
Total concentration:	25.7241	ng/uL

```
Sample peak width (sec): 5    Sample min peak height: 50    Sample baseline V to V?: Y    Sample baseline V to V points: 3
Sample filter: Binomial       Number of points for filter: 3  Sample start region (min): 0  Sample end region (min): 30
Marker peak width (sec): 5    Marker min peak height: 200   Marker baseline V to V?: Y    Marker baseline V to V points: 3
Lower marker selection: First peak > 500 RFU                Upper marker selection: Last peak > 200 RFU
Ladder size (bp)75, 200, 300, 400, 500, 700, 1000, 1500, 2000, 3000, 4000, 5000, 7000, 10000, 20000
Quantification using: Lower Marker          Final concentration (ng/uL): 0.5000          Dilution factor: 12.0
```

Sample: SampA6
Well location: A6
Created: Wednesday, October 20, 2021 6:38:33 PM

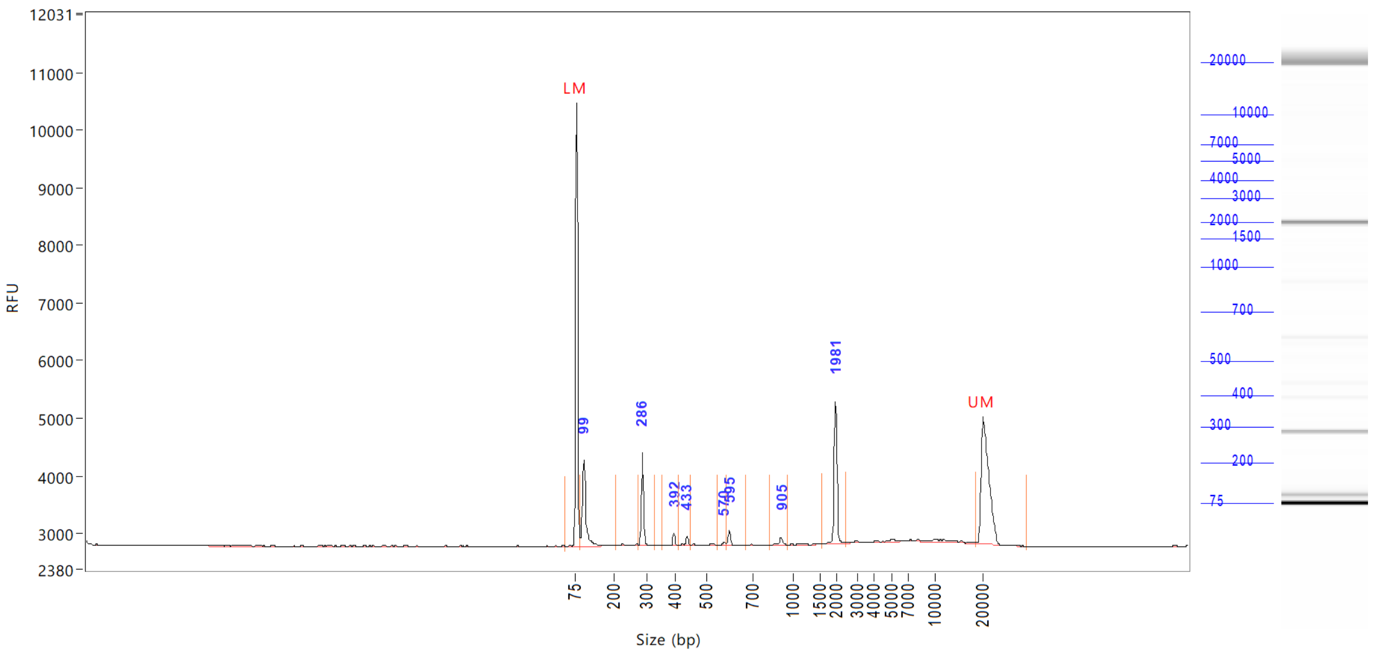

Peak	Size (bp)	Concentration (ng/uL)	From (bp)	To (bp)	Average size (bp)	CV%	RFU	Corrected peak area
1	75 (LM)	0.5000	41	87	74	4.45	7708	41.243
2	99	1.7654	87	203	102	14.42	1489	12.135
3	286	1.1215	273	327	284	1.94	1610	7.709
4	392	0.1592	352	411	389	2.07	218	1.094
5	433	0.1393	411	449	431	1.82	152	0.958
6	570	0.0663	547	583	569	1.35	70	0.455
7	595	0.2133	583	667	597	1.93	282	1.466
8	905	0.1368	822	961	893	2.81	139	0.940
9	1981	1.5991	1539	2458	1958	3.63	2457	10.992
10	20000 (UM)	0.2659	18415	29068	20576	4.18	2192	21.930

TIC:	5.2009	ng/uL
TIM:	38.5000	nmole/L
Total concentration:	5.9218	ng/uL

Sample peak width (sec): 5 Sample min peak height: 50 Sample baseline V to V?: Y Sample baseline V to V points: 3
Sample filter: Binomial Number of points for filter: 3 Sample start region (min): 0 Sample end region (min): 30
Marker peak width (sec): 5 Marker min peak height: 200 Marker baseline V to V?: Y Marker baseline V to V points: 3
Lower marker selection: First peak > 500 RFU Upper marker selection: Last peak > 200 RFU
Ladder size (bp)75, 200, 300, 400, 500, 700, 1000, 1500, 2000, 3000, 4000, 5000, 7000, 10000, 20000
Quantification using: Lower Marker Final concentration (ng/uL): 0.5000 Dilution factor: 12.0

Sample: SampA7
Well location: A7
Created: Wednesday, October 20, 2021 6:38:33 PM

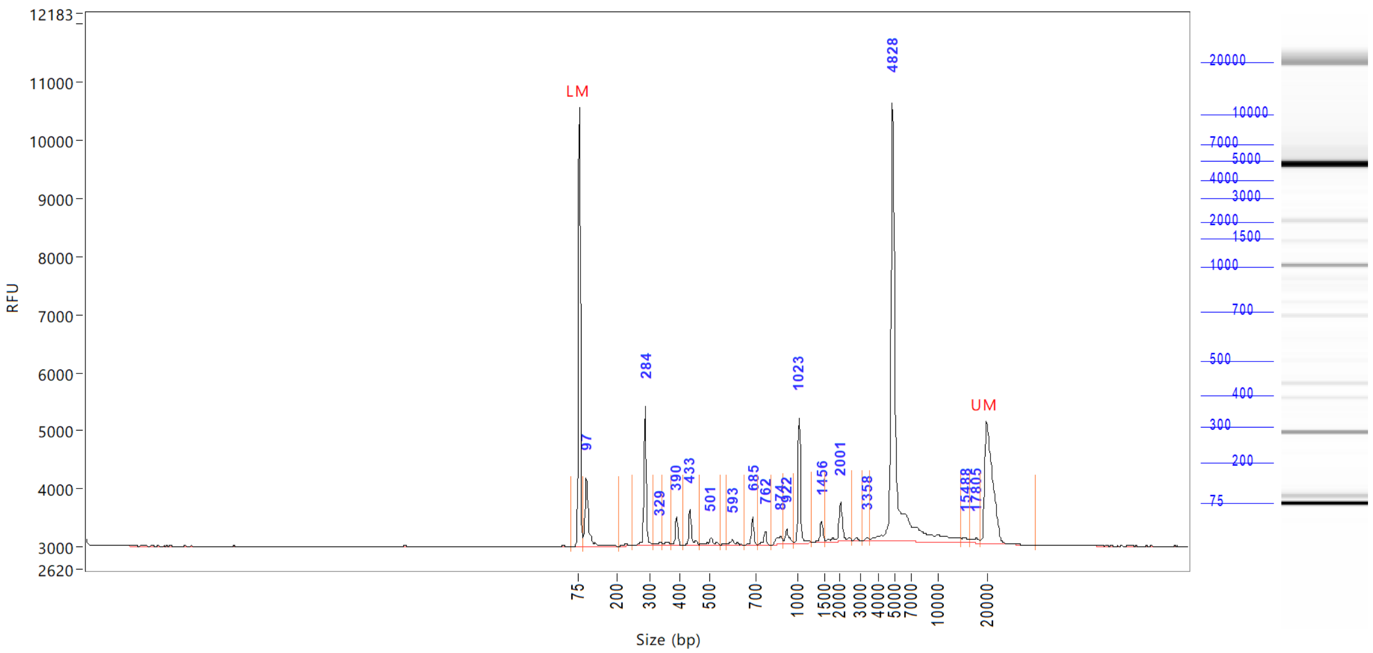

Peak	Size (bp)	Concentration (ng/uL)	From (bp)	To (bp)	Average size (bp)	CV%	RFU	Corrected peak area
1	75 (LM)	0.5000	47	86	73	4.47	7556	39.039
2	97	1.6195	86	201	101	16.16	1186	10.537
3	284	1.8853	242	309	281	2.11	2404	12.267
4	329	0.1012	309	343	327	3.00	51	0.659
5	390	0.3622	370	409	387	1.28	488	2.357
6	433	0.4872	409	464	435	1.89	602	3.170
7	501	0.1958	464	542	497	3.37	116	1.274
8	593	0.1364	567	644	596	2.66	90	0.888
9	685	0.3405	644	701	680	0.99	490	2.215
10	762	0.1941	701	809	753	2.05	238	1.263
11	874	0.2134	809	900	862	2.27	136	1.388
12	922	0.2459	900	970	926	1.68	247	1.600
13	1023	1.4726	970	1278	1026	3.64	2147	9.582
14	1456	0.2631	1278	1520	1434	2.74	360	1.712
15	2001	0.7058	1520	2600	1993	8.59	680	4.592
16	3358	0.0484	3072	3572	3343	3.41	60	0.315
17	4828	8.7429	3572	14757	5552	31.20	7549	56.886
18	15488	0.1120	14757	16586	15619	3.49	83	0.728
19	17805	0.1179	16586	18537	17549	3.24	80	0.767
20	20000 (UM)	0.2800	18537	30156	20584	4.72	2105	21.866

TIC:	17.2441	ng/uL
TIM:	50.2629	nmole/L
Total concentration:	17.4015	ng/uL

```
Sample peak width (sec): 5    Sample min peak height: 50    Sample baseline V to V?: Y    Sample baseline V to V points: 3
Sample filter: Binomial       Number of points for filter: 3  Sample start region (min): 0  Sample end region (min): 30
Marker peak width (sec): 5    Marker min peak height: 200   Marker baseline V to V?: Y    Marker baseline V to V points: 3
Lower marker selection: First peak > 500 RFU                Upper marker selection: Last peak > 200 RFU
Ladder size (bp)75, 200, 300, 400, 500, 700, 1000, 1500, 2000, 3000, 4000, 5000, 7000, 10000, 20000
Quantification using: Lower Marker          Final concentration (ng/uL): 0.5000          Dilution factor: 12.0
```

Sample: SampA8
Well location: A8
Created: Wednesday, October 20, 2021 6:38:33 PM

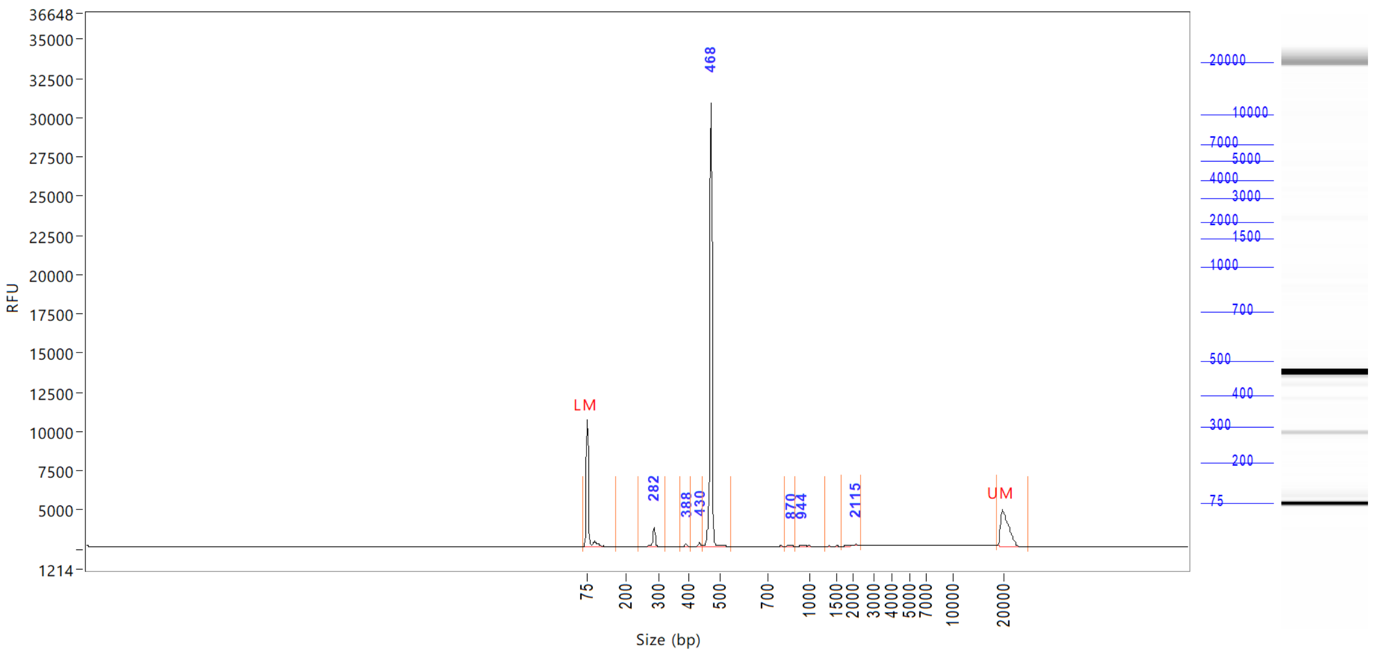

Peak	Size (bp)	Concentration (ng/uL)	From (bp)	To (bp)	Average size (bp)	CV%	RFU	Corrected peak area
1	75 (LM)	0.5000	62	166	75	13.57	8065	45.952
2	282	0.8823	239	317	280	2.51	1194	6.757
3	388	0.1097	372	403	386	1.09	179	0.840
4	430	0.1720	403	441	429	1.23	273	1.317
5	468	16.5045	441	544	466	1.18	28321	126.403
6	870	0.0774	822	900	857	1.84	67	0.593
7	944	0.0896	900	1278	986	7.64	55	0.686
8	2115	0.0846	1616	2372	2064	6.60	98	0.648
9	20000 (UM)	0.2561	18781	25320	20564	4.08	2311	23.538

TIC:	17.9200	ng/uL
TIM:	64.9531	nmole/L
Total concentration:	18.4932	ng/uL

```
Sample peak width (sec): 5    Sample min peak height: 50    Sample baseline V to V?: Y    Sample baseline V to V points: 3
Sample filter: Binomial       Number of points for filter: 3  Sample start region (min): 0  Sample end region (min): 30
Marker peak width (sec): 5    Marker min peak height: 200   Marker baseline V to V?: Y    Marker baseline V to V points: 3
Lower marker selection: First peak > 500 RFU                Upper marker selection: Last peak > 200 RFU
Ladder size (bp)75, 200, 300, 400, 500, 700, 1000, 1500, 2000, 3000, 4000, 5000, 7000, 10000, 20000
Quantification using: Lower Marker          Final concentration (ng/uL): 0.5000          Dilution factor: 12.0
```

Sample: SampA9
Well location: A9
Created: Wednesday, October 20, 2021 6:38:33 PM

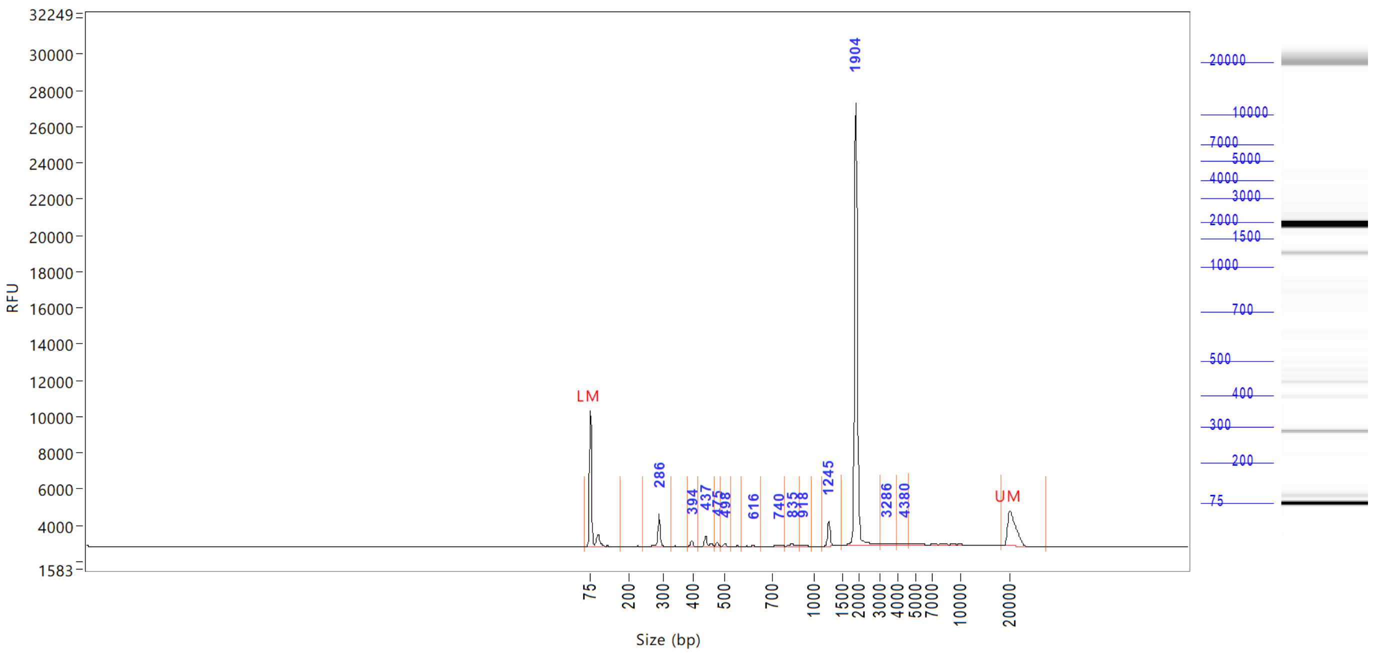

Peak	Size (bp)	Concentration (ng/uL)	From (bp)	To (bp)	Average size (bp)	CV%	RFU	Corrected peak area
1	75 (LM)	0.5000	58	171	77	13.05	7517	44.044
2	286	1.2530	241	325	284	2.97	1771	9.198
3	394	0.2095	380	414	391	1.00	343	1.538
4	437	0.4793	414	466	441	2.12	566	3.518
5	475	0.1442	466	487	473	0.93	212	1.059
6	498	0.1130	487	521	496	1.05	150	0.830
7	616	0.0808	570	649	605	2.69	68	0.593
8	740	0.1346	649	788	727	4.15	68	0.988
9	835	0.2109	788	896	842	3.08	152	1.548
10	918	0.1002	896	978	919	1.69	94	0.735
11	1245	0.8062	1112	1500	1244	3.28	1332	5.918
12	1904	15.6108	1500	3000	1908	5.90	24454	114.593
13	3286	0.1259	3000	3929	3387	7.99	55	0.924
14	4380	0.0769	3929	4587	4266	4.03	65	0.565
15	20000 (UM)	0.2235	18293	27133	20479	4.05	1961	19.683

TIC:	19.3453	ng/uL
TIM:	26.5458	nmole/L
Total concentration:	19.7060	ng/uL

```
Sample peak width (sec): 5    Sample min peak height: 50    Sample baseline V to V?: Y    Sample baseline V to V points: 3
Sample filter: Binomial       Number of points for filter: 3  Sample start region (min): 0  Sample end region (min): 30
Marker peak width (sec): 5    Marker min peak height: 200   Marker baseline V to V?: Y    Marker baseline V to V points: 3
Lower marker selection: First peak > 500 RFU                Upper marker selection: Last peak > 200 RFU
Ladder size (bp)75, 200, 300, 400, 500, 700, 1000, 1500, 2000, 3000, 4000, 5000, 7000, 10000, 20000
Quantification using: Lower Marker          Final concentration (ng/uL): 0.5000          Dilution factor: 12.0
```

Sample: SampA10
Well location: A10
Created: Wednesday, October 20, 2021 6:38:33 PM

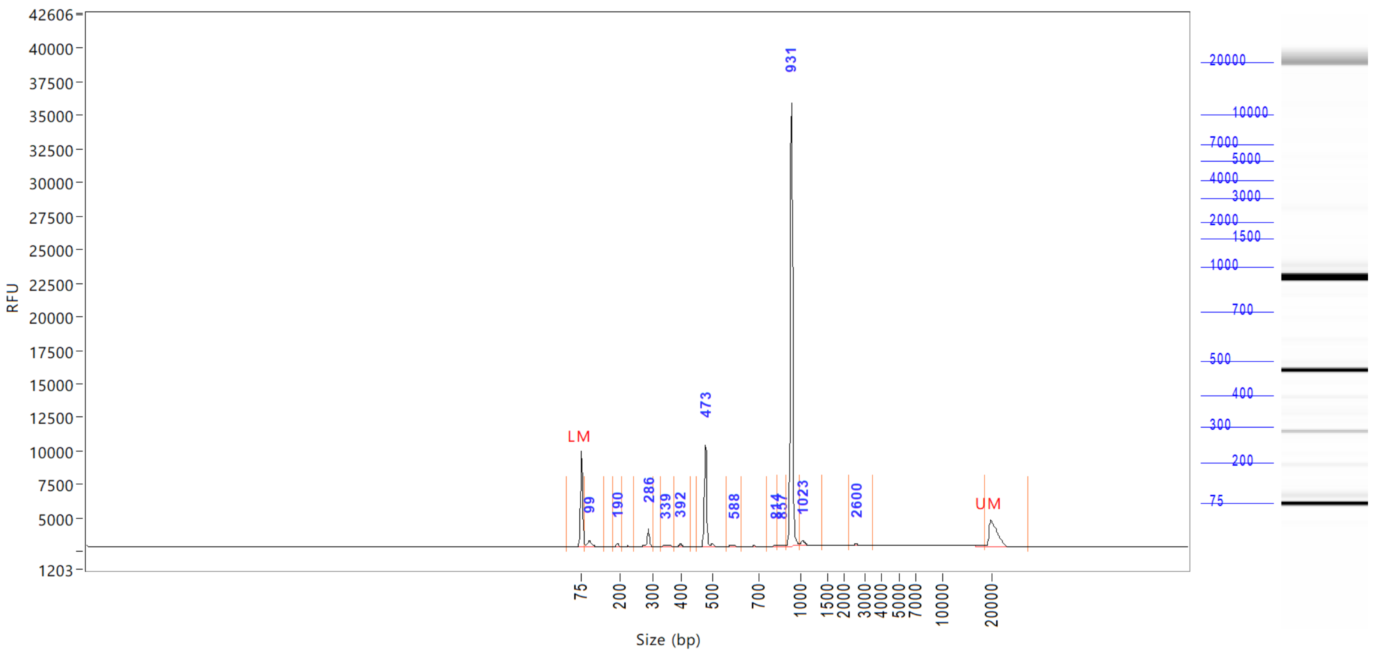

Peak	Size (bp)	Concentration (ng/uL)	From (bp)	To (bp)	Average size (bp)	CV%	RFU	Corrected peak area
1	75 (LM)	0.5000	26	86	72	4.92	7114	38.001
2	99	0.7766	86	148	100	10.43	482	4.919
3	190	0.2074	177	206	188	2.22	244	1.313
4	286	1.0233	242	301	282	2.47	1267	6.481
5	339	0.2174	325	372	345	2.95	125	1.377
6	392	0.1888	372	428	390	1.97	215	1.195
7	473	5.3684	445	557	473	1.60	7578	34.001
8	588	0.1342	557	623	582	1.80	118	0.850
9	814	0.0713	757	831	807	1.64	84	0.452
10	857	0.0744	831	892	858	1.91	61	0.471
11	931	21.6543	892	991	929	1.14	33054	137.147
12	1023	0.5105	991	1411	1058	7.15	359	3.233
13	2600	0.1886	2229	3429	2696	9.71	110	1.194
14	20000 (UM)	0.2557	18781	27738	20624	4.20	1918	19.437

TIC:	30.4152	ng/uL
TIM:	80.9853	nmole/L
Total concentration:	30.9693	ng/uL

```
Sample peak width (sec): 5    Sample min peak height: 50    Sample baseline V to V?: Y    Sample baseline V to V points: 3
Sample filter: Binomial       Number of points for filter: 3  Sample start region (min): 0  Sample end region (min): 30
Marker peak width (sec): 5    Marker min peak height: 200   Marker baseline V to V?: Y    Marker baseline V to V points: 3
Lower marker selection: First peak > 500 RFU                Upper marker selection: Last peak > 200 RFU
Ladder size (bp)75, 200, 300, 400, 500, 700, 1000, 1500, 2000, 3000, 4000, 5000, 7000, 10000, 20000
Quantification using: Lower Marker          Final concentration (ng/uL): 0.5000          Dilution factor: 12.0
```

Sample: SampA11
Well location: A11
Created: Wednesday, October 20, 2021 6:38:33 PM

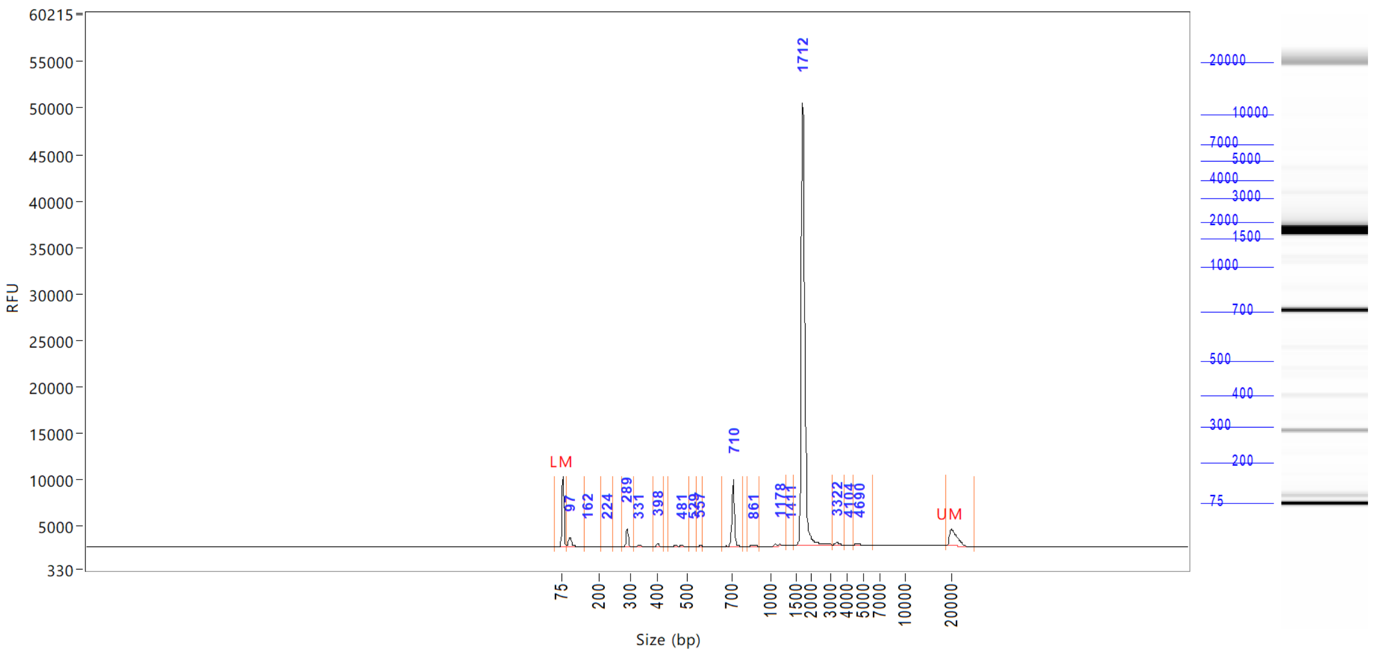

Peak	Size (bp)	Concentration (ng/uL)	From (bp)	To (bp)	Average size (bp)	CV%	RFU	Corrected peak area
1	75 (LM)	0.5000	50	87	73	4.59	7545	39.629
2	97	1.3797	87	152	102	12.13	1011	9.113
3	162	0.1206	152	205	168	6.41	65	0.797
4	224	0.0770	205	244	226	3.02	65	0.509
5	289	1.4172	275	313	287	1.66	1911	9.360
6	331	0.2226	313	384	337	5.05	96	1.470
7	398	0.2799	384	418	396	1.10	389	1.849
8	481	0.2797	433	504	470	2.95	160	1.848
9	529	0.0545	504	539	520	1.63	53	0.360
10	557	0.1448	539	570	553	1.03	214	0.956
11	710	4.9668	657	783	705	1.35	7180	32.805
12	861	0.1305	827	909	862	1.83	108	0.862
13	1178	0.3393	909	1311	1121	6.12	223	2.241
14	1411	0.0638	1311	1456	1393	2.19	90	0.421
15	1712	42.9838	1456	3037	1720	7.04	47764	283.899
16	3322	0.3040	3037	3786	3346	5.27	280	2.008
17	4104	0.1102	3786	4345	4064	3.93	64	0.728
18	4690	0.1975	4345	6038	4668	5.03	178	1.304
19	20000 (UM)	0.2274	18903	25199	20596	3.99	1824	18.022

TIC:	53.0719	ng/uL
TIM:	89.7267	nmole/L
Total concentration:	53.3985	ng/uL

```
Sample peak width (sec): 5    Sample min peak height: 50    Sample baseline V to V?: Y    Sample baseline V to V points: 3
Sample filter: Binomial       Number of points for filter: 3  Sample start region (min): 0  Sample end region (min): 30
Marker peak width (sec): 5    Marker min peak height: 200   Marker baseline V to V?: Y    Marker baseline V to V points: 3
Lower marker selection: First peak > 500 RFU                Upper marker selection: Last peak > 200 RFU
Ladder size (bp)75, 200, 300, 400, 500, 700, 1000, 1500, 2000, 3000, 4000, 5000, 7000, 10000, 20000
Quantification using: Lower Marker          Final concentration (ng/uL): 0.5000          Dilution factor: 12.0
```

Sample: SampA12
Well location: A12
Created: Wednesday, October 20, 2021 6:38:33 PM

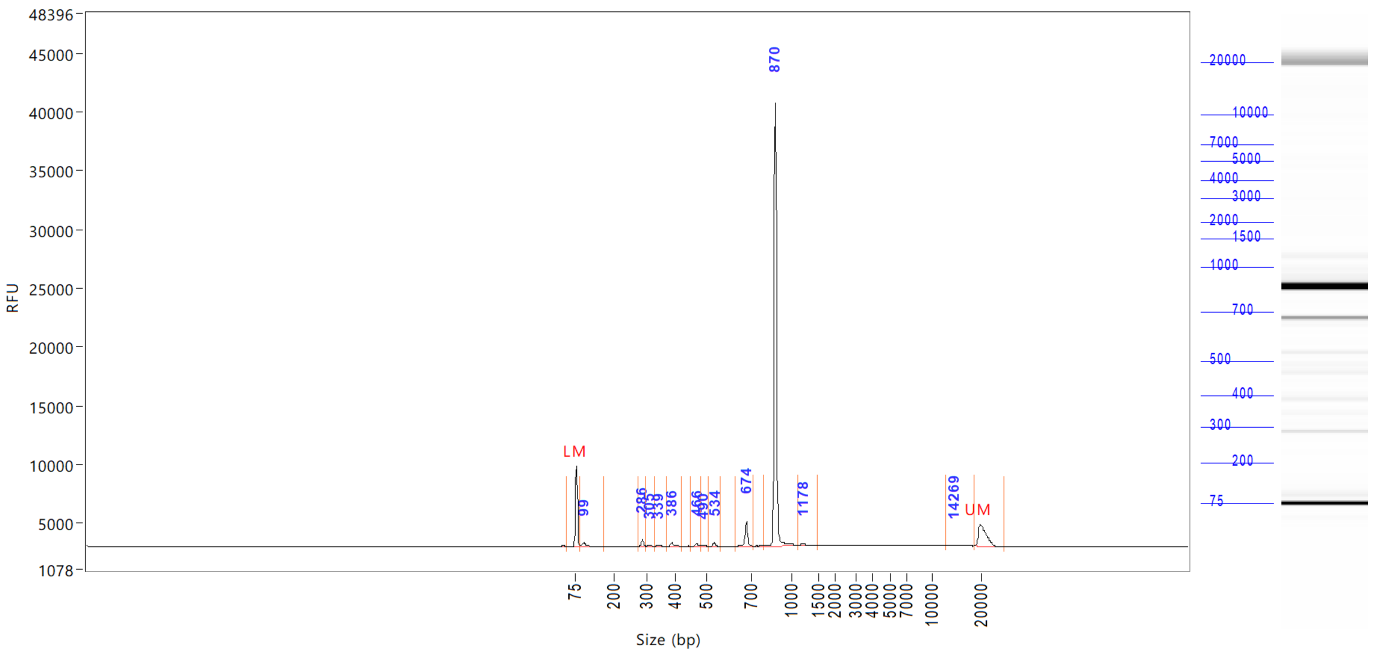

Peak	Size (bp)	Concentration (ng/uL)	From (bp)	To (bp)	Average size (bp)	CV%	RFU	Corrected peak area
1	75 (LM)	0.5000	43	86	73	4.61	6849	35.420
2	99	0.6969	86	168	105	14.38	317	4.114
3	286	0.5170	273	298	284	1.39	635	3.052
4	305	0.1135	298	325	309	2.58	67	0.670
5	339	0.2102	325	370	341	2.33	141	1.241
6	386	0.4171	370	418	389	2.22	300	2.462
7	466	0.3172	449	483	468	1.58	280	1.873
8	490	0.1975	483	506	491	1.14	141	1.166
9	534	0.3000	506	560	530	1.47	337	1.771
10	674	1.7082	629	705	670	1.30	2180	10.084
11	870	28.1095	788	1101	870	2.02	37780	165.941
12	1178	0.2936	1101	1467	1194	4.53	238	1.733
13	14269	0.1518	12684	18537	14810	8.76	51	0.896
14	20000 (UM)	0.2570	18781	24957	20528	3.91	1822	18.207

TIC:	33.0326	ng/uL
TIM:	77.7817	nmole/L
Total concentration:	33.6882	ng/uL

```
Sample peak width (sec): 5    Sample min peak height: 50    Sample baseline V to V?: Y    Sample baseline V to V points: 3
Sample filter: Binomial       Number of points for filter: 3  Sample start region (min): 0  Sample end region (min): 30
Marker peak width (sec): 5    Marker min peak height: 200   Marker baseline V to V?: Y    Marker baseline V to V points: 3
Lower marker selection: First peak > 500 RFU                Upper marker selection: Last peak > 200 RFU
Ladder size (bp)75, 200, 300, 400, 500, 700, 1000, 1500, 2000, 3000, 4000, 5000, 7000, 10000, 20000
Quantification using: Lower Marker          Final concentration (ng/uL): 0.5000          Dilution factor: 12.0
```

Sample: SampB1
Well location: B1
Created: Wednesday, October 20, 2021 6:38:33 PM

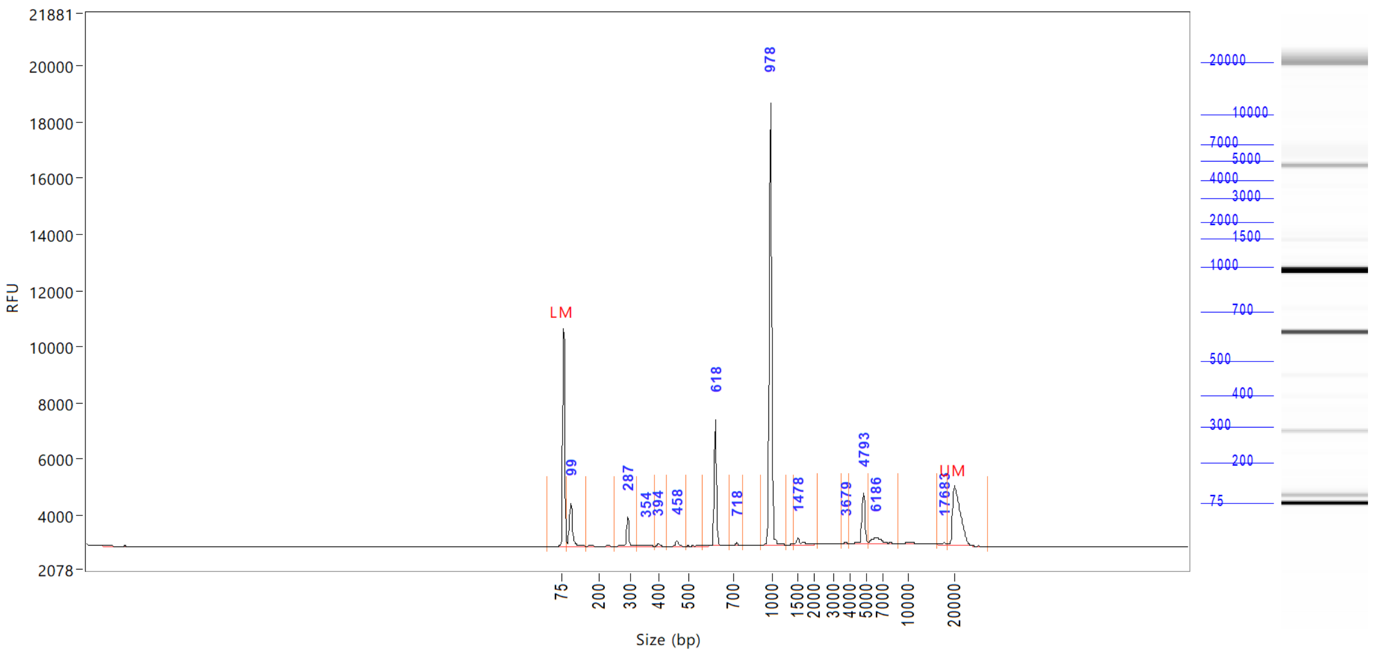

Peak	Size (bp)	Concentration (ng/uL)	From (bp)	To (bp)	Average size (bp)	CV%	RFU	Corrected peak area
1	75 (LM)	0.5000	24	87	73	5.34	7778	41.950
2	99	1.7220	87	150	100	9.19	1541	12.039
3	287	0.8004	246	317	285	2.84	1036	5.596
4	354	0.1196	317	382	343	4.14	58	0.836
5	394	0.0794	382	424	394	1.41	109	0.555
6	458	0.1570	424	487	456	1.47	199	1.097
7	618	2.8987	560	685	615	1.26	4514	20.267
8	718	0.0858	685	770	716	1.92	107	0.600
9	978	9.9164	913	1267	979	1.83	15783	69.333
10	1478	0.2669	1411	2144	1554	8.44	257	1.866
11	3679	0.0460	3465	3893	3664	2.62	68	0.321
12	4793	1.1936	3893	5075	4735	3.26	1800	8.345
13	6186	0.4910	5075	8867	6318	12.23	201	3.433
14	17683	0.0803	16220	18415	17426	3.49	64	0.562
15	20000 (UM)	0.2613	18415	27254	20520	4.29	2128	21.922

TIC:	17.8569	ng/uL
TIM:	59.8462	nmole/L
Total concentration:	18.2344	ng/uL

Sample peak width (sec): 5 Sample min peak height: 50 Sample baseline V to V?: Y Sample baseline V to V points: 3
Sample filter: Binomial Number of points for filter: 3 Sample start region (min): 0 Sample end region (min): 30
Marker peak width (sec): 5 Marker min peak height: 200 Marker baseline V to V?: Y Marker baseline V to V points: 3
Lower marker selection: First peak > 500 RFU Upper marker selection: Last peak > 200 RFU
Ladder size (bp)75, 200, 300, 400, 500, 700, 1000, 1500, 2000, 3000, 4000, 5000, 7000, 10000, 20000
Quantification using: Lower Marker Final concentration (ng/uL): 0.5000 Dilution factor: 12.0

Sample: SampB2
Well location: B2
Created: Wednesday, October 20, 2021 6:38:33 PM

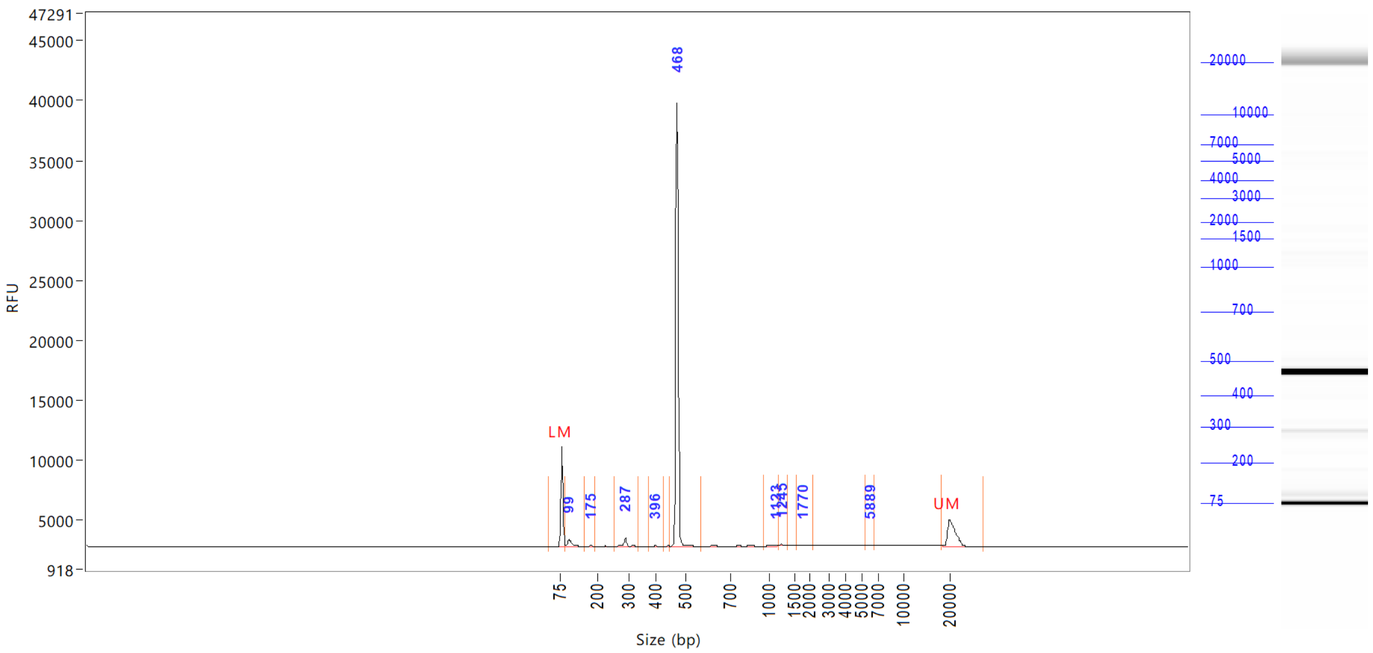

Peak	Size (bp)	Concentration (ng/uL)	From (bp)	To (bp)	Average size (bp)	CV%	RFU	Corrected peak area
1	75 (LM)	0.5000	31	89	74	5.22	8316	44.975
2	99	0.8466	89	154	106	12.93	552	6.346
3	175	0.1302	154	192	171	3.91	143	0.976
4	287	0.7237	253	333	284	4.23	686	5.425
5	396	0.0744	370	422	398	2.62	64	0.558
6	468	23.4970	445	567	466	1.20	37050	176.131
7	1123	0.0981	961	1178	1067	5.23	71	0.735
8	1245	0.0935	1178	1378	1237	2.48	137	0.701
9	1770	0.0624	1539	2172	1779	5.50	54	0.467
10	5889	0.0404	5371	6482	5749	3.25	66	0.303
11	20000 (UM)	0.2584	18415	27496	20631	4.30	2243	23.245

TIC:	25.5662	ng/uL
TIM:	102.1806	nmole/L
Total concentration:	26.1844	ng/uL

```
Sample peak width (sec): 5    Sample min peak height: 50    Sample baseline V to V?: Y    Sample baseline V to V points: 3
Sample filter: Binomial       Number of points for filter: 3  Sample start region (min): 0  Sample end region (min): 30
Marker peak width (sec): 5    Marker min peak height: 200   Marker baseline V to V?: Y    Marker baseline V to V points: 3
Lower marker selection: First peak > 500 RFU                Upper marker selection: Last peak > 200 RFU
Ladder size (bp)75, 200, 300, 400, 500, 700, 1000, 1500, 2000, 3000, 4000, 5000, 7000, 10000, 20000
Quantification using: Lower Marker          Final concentration (ng/uL): 0.5000          Dilution factor: 12.0
```

Sample: SampB3
Well location: B3
Created: Wednesday, October 20, 2021 6:38:33 PM

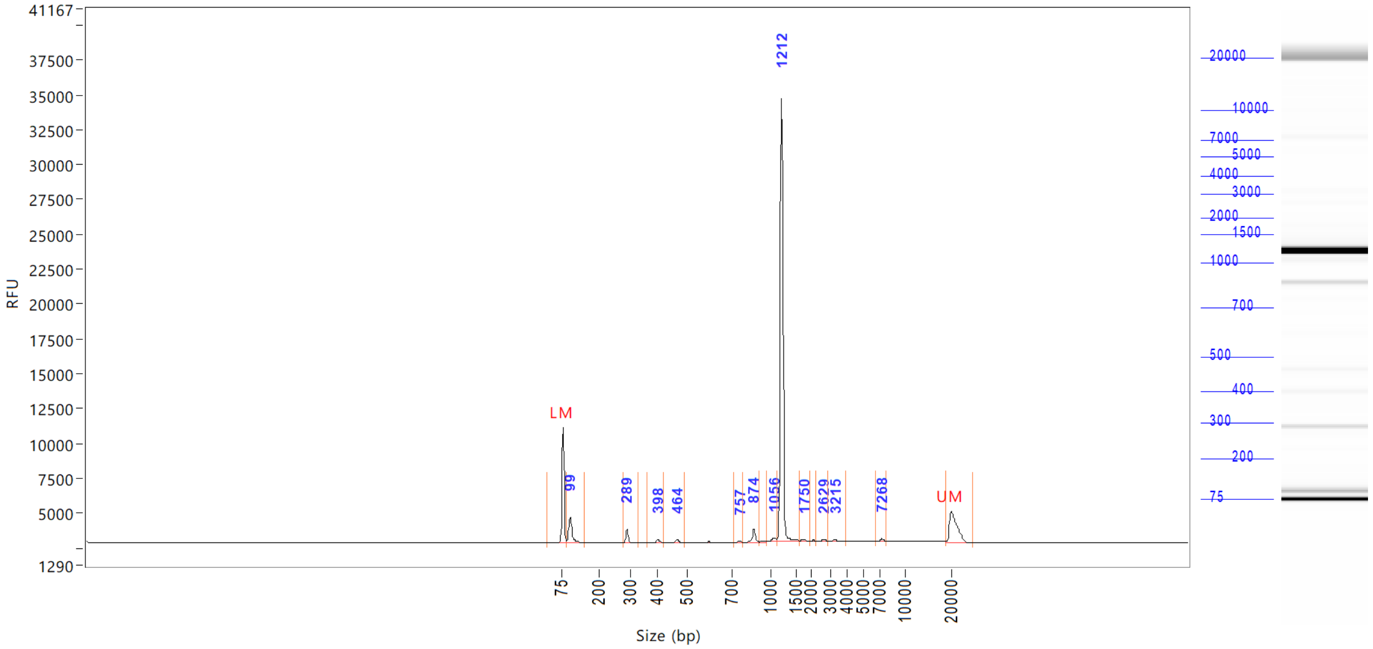

Peak	Size (bp)	Concentration (ng/uL)	From (bp)	To (bp)	Average size (bp)	CV%	RFU	Corrected peak area
1	75 (LM)	0.5000	26	87	73	5.67	8206	44.099
2	99	1.8962	87	152	100	9.32	1777	13.937
3	289	0.6530	277	327	287	2.02	927	4.799
4	398	0.2144	362	420	393	2.37	222	1.576
5	464	0.1900	420	489	461	1.99	223	1.396
6	757	0.0489	718	788	752	1.49	62	0.360
7	874	0.6201	788	909	864	1.44	927	4.558
8	1056	0.2792	970	1123	1056	3.81	271	2.052
9	1212	19.7711	1123	1597	1204	3.09	31810	145.315
10	1750	0.0885	1597	1962	1733	5.20	68	0.651
11	2629	0.0544	2258	2829	2590	4.13	68	0.400
12	3215	0.0808	2829	3929	3160	5.35	89	0.594
13	7268	0.1210	6630	7734	7205	2.94	176	0.889
14	20000 (UM)	0.2469	18903	24715	20588	4.00	2149	21.778

TIC:	24.0176	ng/uL
TIM:	65.3974	nmole/L
Total concentration:	24.6088	ng/uL

Sample peak width (sec): 5   Sample min peak height: 50   Sample baseline V to V?: Y   Sample baseline V to V points: 3
Sample filter: Binomial   Number of points for filter: 3 Sample start region (min): 0 Sample end region (min): 30
Marker peak width (sec): 5   Marker min peak height: 200   Marker baseline V to V?: Y   Marker baseline V to V points: 3
Lower marker selection: First peak > 500 RFU   Upper marker selection: Last peak > 200 RFU
Ladder size (bp)75, 200, 300, 400, 500, 700, 1000, 1500, 2000, 3000, 4000, 5000, 7000, 10000, 20000
Quantification using: Lower Marker   Final concentration (ng/uL): 0.5000   Dilution factor: 12.0

Sample: SampB4
Well location: B4
Created: Wednesday, October 20, 2021 6:38:33 PM

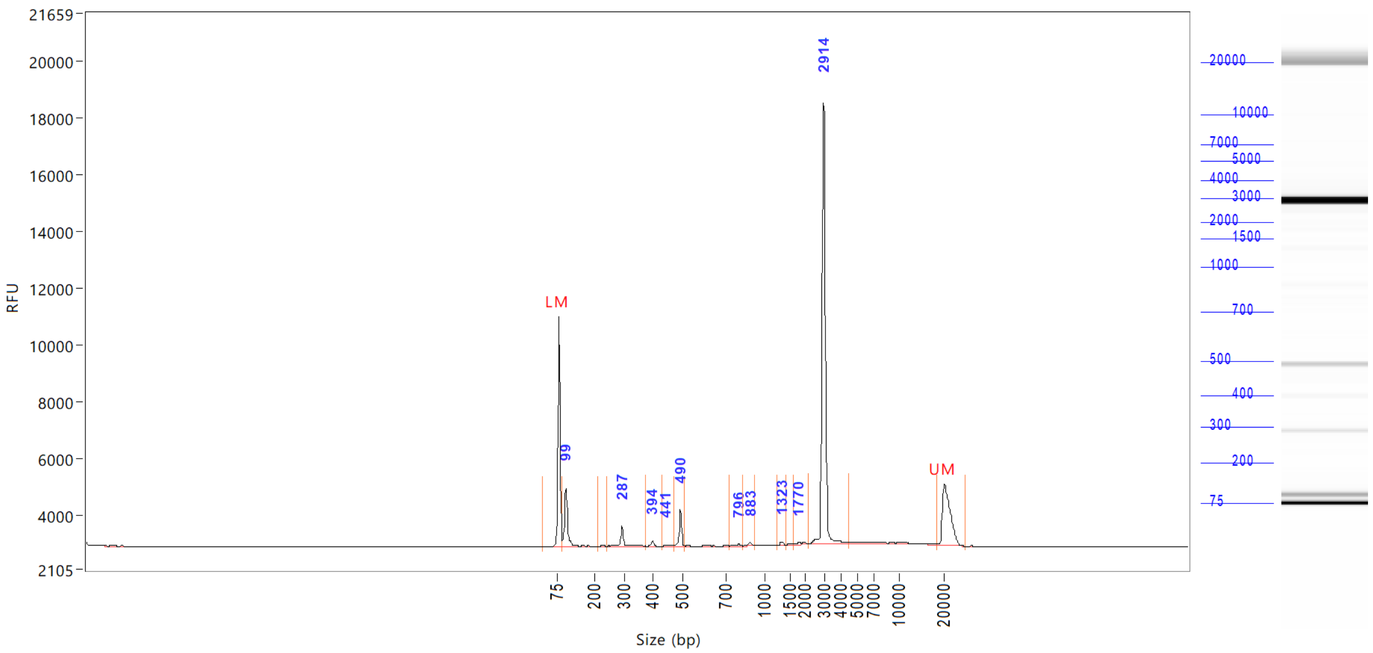

Peak	Size (bp)	Concentration (ng/uL)	From (bp)	To (bp)	Average size (bp)	CV%	RFU	Corrected peak area
1	75 (LM)	0.5000	20	87	74	5.38	8062	44.552
2	99	2.1734	87	208	101	13.05	2058	16.138
3	287	0.6068	239	376	287	5.64	702	4.505
4	394	0.1268	376	430	395	1.59	185	0.941
5	441	0.0986	430	470	450	2.16	55	0.732
6	490	0.8314	470	509	489	0.99	1275	6.173
7	796	0.0702	723	831	773	3.17	64	0.521
8	883	0.0969	831	926	877	1.64	122	0.719
9	1323	0.0759	1256	1411	1320	2.03	119	0.564
10	1770	0.1372	1578	2172	1859	6.88	89	1.018
11	2914	10.8621	2172	4483	2918	6.16	15544	80.654
12	20000 (UM)	0.2525	18537	24594	20595	4.15	2164	22.498

TIC:	15.0792	ng/uL
TIM:	49.1763	nmole/L
Total concentration:	15.6523	ng/uL

```
Sample peak width (sec): 5    Sample min peak height: 50    Sample baseline V to V?: Y    Sample baseline V to V points: 3
Sample filter: Binomial       Number of points for filter: 3  Sample start region (min): 0  Sample end region (min): 30
Marker peak width (sec): 5    Marker min peak height: 200   Marker baseline V to V?: Y    Marker baseline V to V points: 3
Lower marker selection: First peak > 500 RFU                Upper marker selection: Last peak > 200 RFU
Ladder size (bp)75, 200, 300, 400, 500, 700, 1000, 1500, 2000, 3000, 4000, 5000, 7000, 10000, 20000
Quantification using: Lower Marker          Final concentration (ng/uL): 0.5000          Dilution factor: 12.0
```

Sample: SampB5
Well location: B5
Created: Wednesday, October 20, 2021 6:38:33 PM

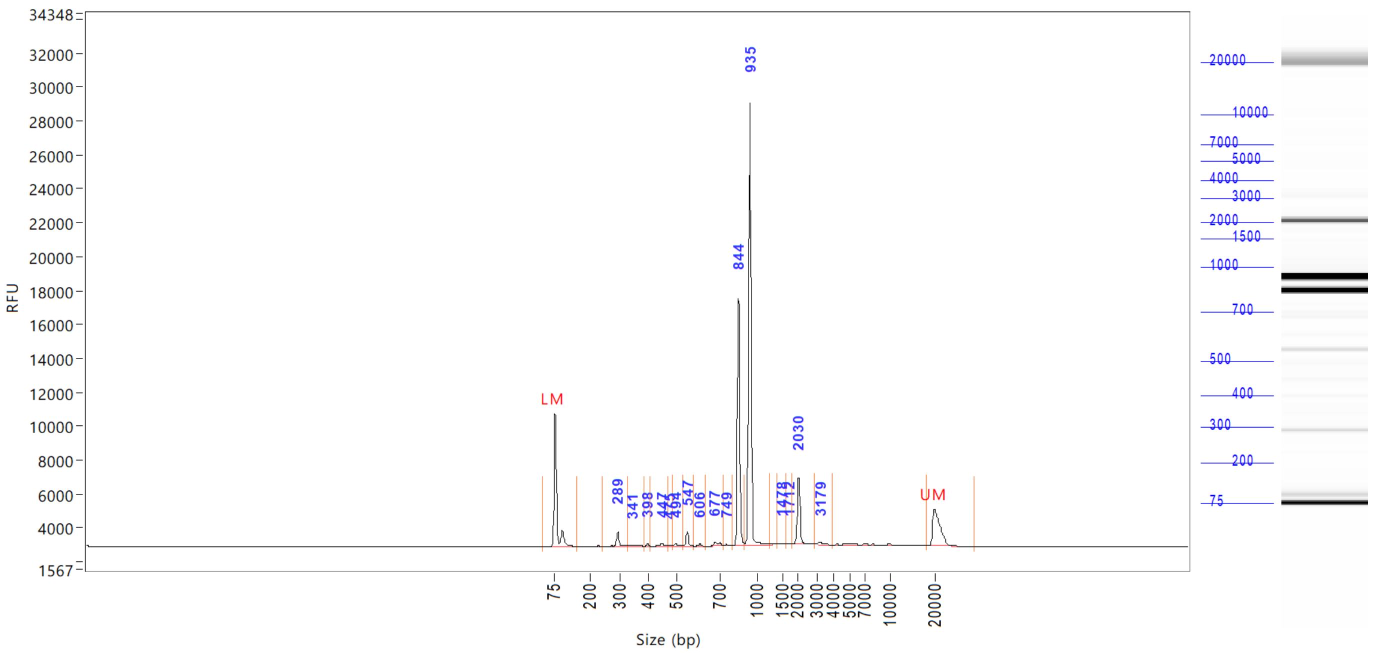

Peak	Size (bp)	Concentration (ng/uL)	From (bp)	To (bp)	Average size (bp)	CV%	RFU	Corrected peak area
1	75 (LM)	0.5000	35	152	79	14.88	7824	52.355
2	289	0.6354	241	325	288	3.87	893	5.544
3	341	0.1457	325	384	350	4.07	83	1.271
4	398	0.0826	384	409	395	0.98	154	0.721
5	447	0.1690	409	468	445	2.29	168	1.474
6	475	0.0372	468	483	475	0.98	51	0.325
7	494	0.1255	483	527	496	1.80	126	1.095
8	547	0.4391	527	578	545	1.06	826	3.831
9	606	0.0732	578	631	603	1.23	121	0.639
10	677	0.2540	631	723	685	1.92	235	2.217
11	749	0.0636	723	792	746	1.79	74	0.555
12	844	7.5169	792	892	841	1.21	14573	65.591
13	935	13.6072	892	1223	934	2.27	26121	118.734
14	1478	0.0502	1400	1616	1472	3.08	63	0.438
15	1712	0.0467	1616	1827	1730	3.33	67	0.407
16	2030	2.1647	1827	2857	2024	4.11	3941	18.889
17	3179	0.1315	2857	3929	3190	4.81	162	1.147
18	20000 (UM)	0.2092	18293	28947	20546	4.42	2131	21.910

TIC:	25.5425	ng/uL
TIM:	48.7225	nmole/L
Total concentration:	25.7700	ng/uL

```
Sample peak width (sec): 5    Sample min peak height: 50    Sample baseline V to V?: Y    Sample baseline V to V points: 3
Sample filter: Binomial       Number of points for filter: 3  Sample start region (min): 0  Sample end region (min): 30
Marker peak width (sec): 5    Marker min peak height: 200   Marker baseline V to V?: Y    Marker baseline V to V points: 3
Lower marker selection: First peak > 500 RFU                Upper marker selection: Last peak > 200 RFU
Ladder size (bp)75, 200, 300, 400, 500, 700, 1000, 1500, 2000, 3000, 4000, 5000, 7000, 10000, 20000
Quantification using: Lower Marker          Final concentration (ng/uL): 0.5000          Dilution factor: 12.0
```

Sample: SampB6
Well location: B6
Created: Wednesday, October 20, 2021 6:38:33 PM

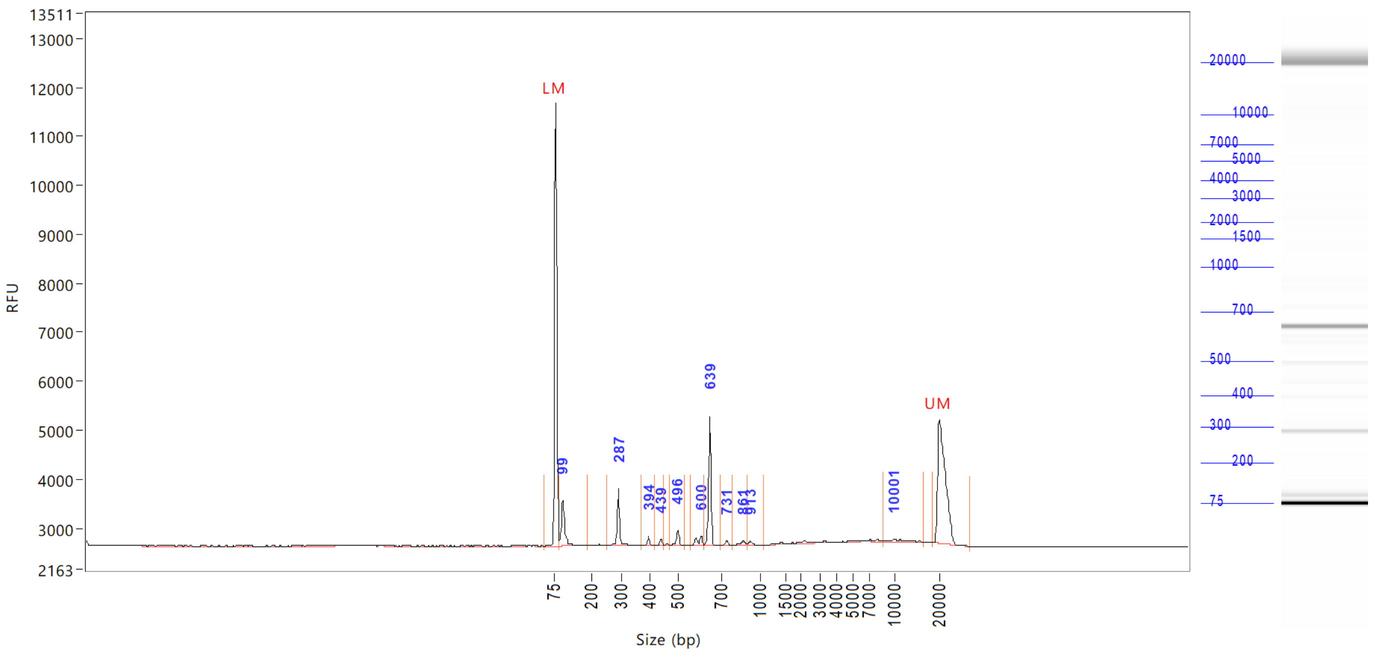

Peak	Size (bp)	Concentration (ng/uL)	From (bp)	To (bp)	Average size (bp)	CV%	RFU	Corrected peak area
1	75 (LM)	0.5000	37	87	73	4.53	9060	48.358
2	99	1.0174	87	185	102	13.63	930	8.200
3	287	0.8338	248	372	287	4.48	1166	6.720
4	394	0.1040	372	414	392	1.18	179	0.838
5	439	0.0847	414	447	436	0.99	141	0.683
6	496	0.2078	468	524	492	1.49	307	1.675
7	600	0.2190	552	613	587	2.30	186	1.765
8	639	1.5132	613	692	638	0.94	2642	12.196
9	731	0.0683	692	775	726	1.62	99	0.550
10	861	0.0893	775	896	850	2.83	75	0.720
11	913	0.0582	896	1056	921	2.63	73	0.469
12	10001	0.1879	8800	16586	11696	17.56	58	1.514
13	20000 (UM)	0.2744	18537	26770	20600	4.24	2522	26.540

TIC:	4.3837	ng/uL
TIM:	27.5859	nmole/L
Total concentration:	4.7658	ng/uL

Sample peak width (sec): 5   Sample min peak height: 50   Sample baseline V to V?: Y   Sample baseline V to V points: 3
Sample filter: Binomial   Number of points for filter: 3 Sample start region (min): 0 Sample end region (min): 30
Marker peak width (sec): 5   Marker min peak height: 200   Marker baseline V to V?: Y   Marker baseline V to V points: 3
Lower marker selection: First peak > 500 RFU   Upper marker selection: Last peak > 200 RFU
Ladder size (bp)75, 200, 300, 400, 500, 700, 1000, 1500, 2000, 3000, 4000, 5000, 7000, 10000, 20000
Quantification using: Lower Marker   Final concentration (ng/uL): 0.5000   Dilution factor: 12.0

Sample: SampB7
Well location: B7
Created: Wednesday, October 20, 2021 6:38:33 PM

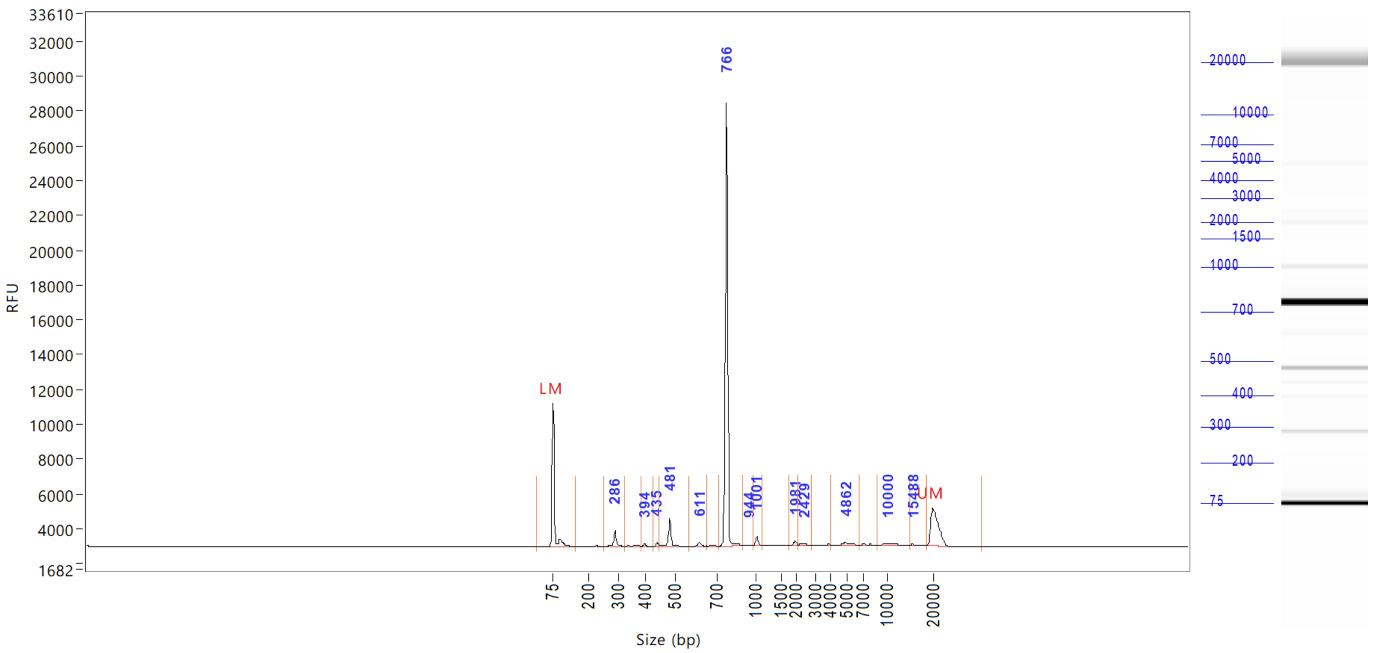

Peak	Size (bp)	Concentration (ng/uL)	From (bp)	To (bp)	Average size (bp)	CV%	RFU	Corrected peak area
1	75 (LM)	0.5000	22	154	77	14.32	8222	49.641
2	286	0.6173	251	321	285	2.63	895	5.107
3	394	0.1012	382	426	393	1.72	169	0.837
4	435	0.1126	426	447	435	0.96	196	0.931
5	481	1.0357	447	565	479	1.86	1646	8.569
6	611	0.1763	565	652	611	1.87	221	1.459
7	766	13.9443	701	896	760	1.85	25492	115.369
8	944	0.0871	896	974	931	2.21	64	0.721
9	1001	0.2778	974	1112	1003	1.44	519	2.299
10	1981	0.1447	1789	2087	1950	2.89	233	1.197
11	2429	0.0969	2087	2772	2347	6.29	81	0.801
12	4862	0.2108	4000	6630	4980	11.34	154	1.744
13	10000	0.2172	8800	14757	11431	15.42	59	1.797
14	15488	0.0885	14757	18659	16347	6.84	61	0.732
15	20000 (UM)	0.2332	18659	30881	20539	4.77	2210	23.157

TIC:	17.1104	ng/uL
TIM:	39.5516	nmole/L
Total concentration:	17.4452	ng/uL

```
Sample peak width (sec): 5     Sample min peak height: 50     Sample baseline V to V?: Y     Sample baseline V to V points: 3
Sample filter: Binomial        Number of points for filter: 3  Sample start region (min): 0  Sample end region (min): 30
Marker peak width (sec): 5     Marker min peak height: 200    Marker baseline V to V?: Y     Marker baseline V to V points: 3
Lower marker selection: First peak > 500 RFU                  Upper marker selection: Last peak > 200 RFU
Ladder size (bp)75, 200, 300, 400, 500, 700, 1000, 1500, 2000, 3000, 4000, 5000, 7000, 10000, 20000
Quantification using: Lower Marker          Final concentration (ng/uL): 0.5000          Dilution factor: 12.0
```

Sample: SampB8
Well location: B8
Created: Wednesday, October 20, 2021 6:38:33 PM

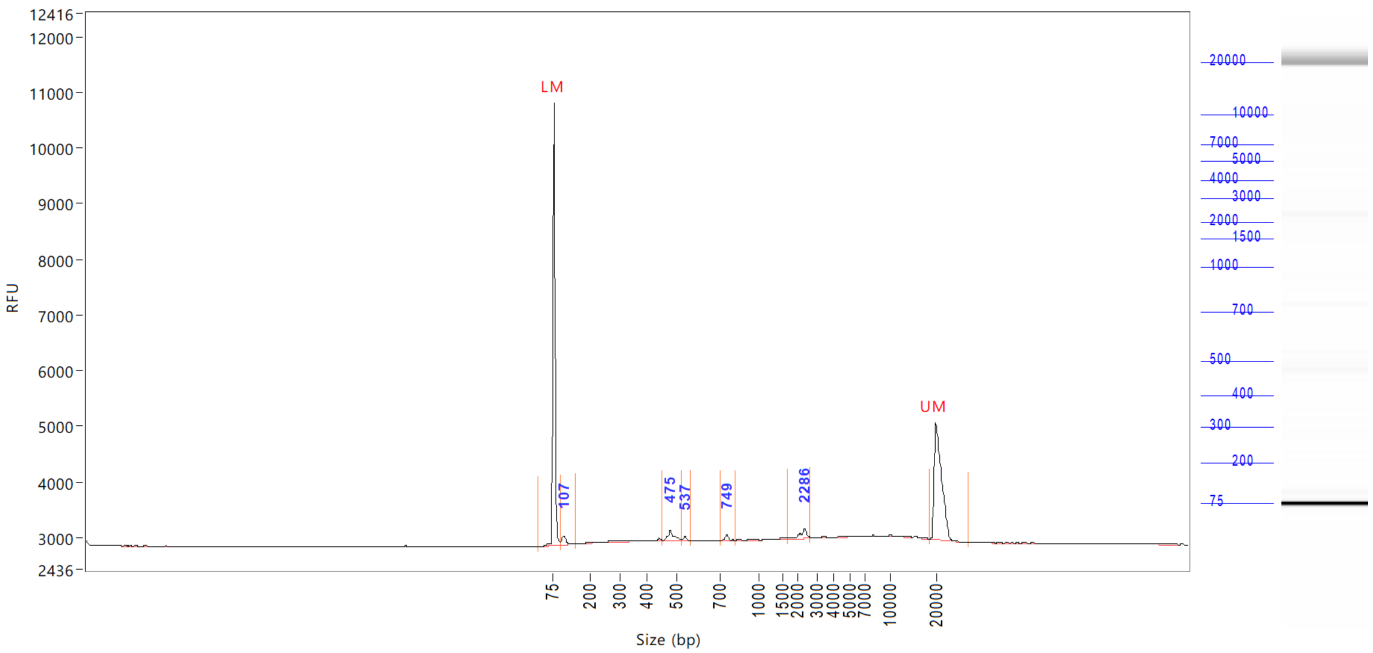

Peak	Size (bp)	Concentration (ng/uL)	From (bp)	To (bp)	Average size (bp)	CV%	RFU	Corrected peak area
1	75 (LM)	0.5000	20	95	72	6.15	7958	45.053
2	107	0.2323	95	148	107	8.23	162	1.744
3	475	0.3238	450	521	479	3.14	200	2.432
4	537	0.0575	521	562	532	1.18	76	0.432
5	749	0.0779	701	818	744	2.29	104	0.585
6	2286	0.2266	1655	2600	2181	8.86	171	1.701
7	20000 (UM)	0.2378	18537	27254	20590	4.20	2095	21.425

TIC:	0.9182	ng/uL
TIM:	5.1994	nmole/L
Total concentration:	1.4999	ng/uL

Sample peak width (sec): 5 Sample min peak height: 50 Sample baseline V to V?: Y Sample baseline V to V points: 3
Sample filter: Binomial Number of points for filter: 3 Sample start region (min): 0 Sample end region (min): 30
Marker peak width (sec): 5 Marker min peak height: 200 Marker baseline V to V?: Y Marker baseline V to V points: 3
Lower marker selection: First peak > 500 RFU Upper marker selection: Last peak > 200 RFU
Ladder size (bp)75, 200, 300, 400, 500, 700, 1000, 1500, 2000, 3000, 4000, 5000, 7000, 10000, 20000
Quantification using: Lower Marker Final concentration (ng/uL): 0.5000 Dilution factor: 12.0

Sample: SampB9
Well location: B9
Created: Wednesday, October 20, 2021 6:38:33 PM

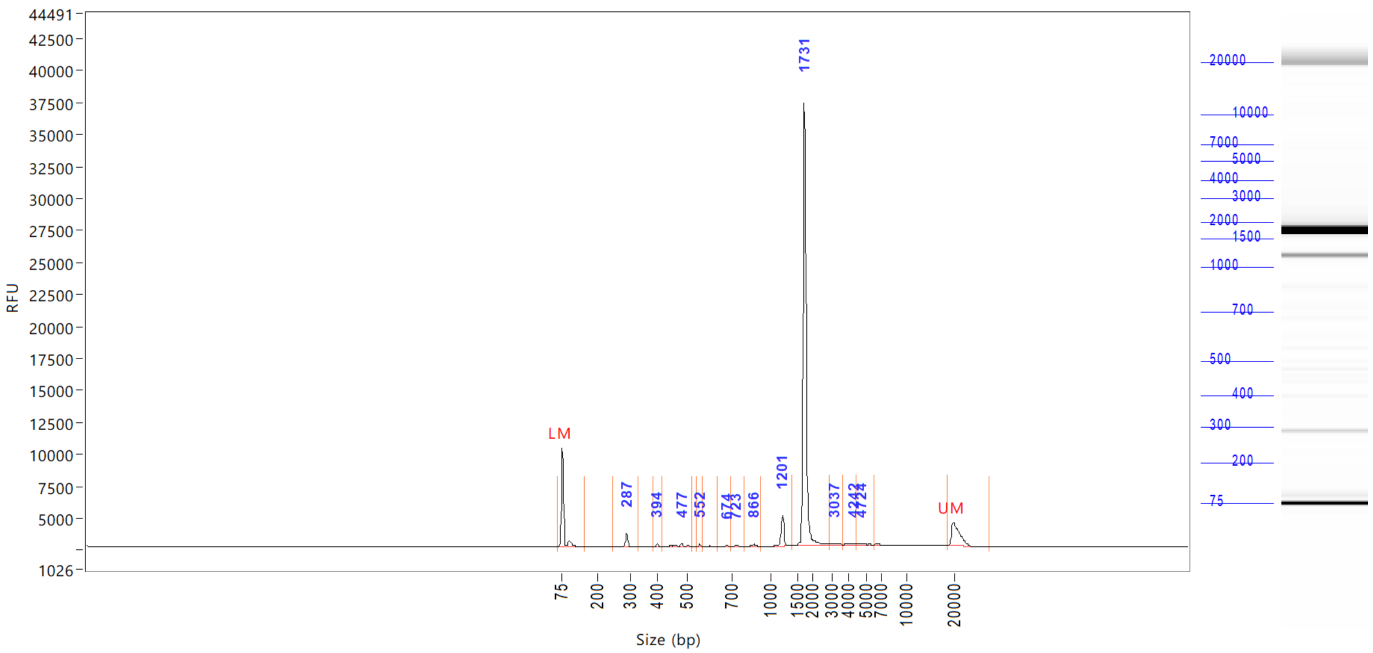

Peak	Size (bp)	Concentration (ng/uL)	From (bp)	To (bp)	Average size (bp)	CV%	RFU	Corrected peak area
1	75 (LM)	0.5000	62	154	76	13.43	7759	45.496
2	287	0.7255	244	325	285	2.89	1052	5.501
3	394	0.1155	384	414	394	1.11	184	0.876
4	477	0.3625	414	519	465	4.46	237	2.748
5	552	0.0813	537	565	549	0.83	156	0.616
6	674	0.0705	631	692	670	1.52	90	0.535
7	723	0.0869	692	783	727	2.63	83	0.659
8	866	0.1296	783	913	857	2.11	165	0.982
9	1201	1.4418	913	1400	1192	3.33	2415	10.933
10	1731	23.4494	1400	2857	1730	6.42	34670	177.807
11	3037	0.1591	2857	3608	3171	6.71	83	1.206
12	4242	0.1065	3608	4414	4023	5.70	52	0.808
13	4724	0.0931	4414	6038	4910	8.58	50	0.706
14	20000 (UM)	0.2115	18659	27738	20626	4.46	1814	19.246

TIC:	26.8216	ng/uL
TIM:	31.2752	nmole/L
Total concentration:	27.1672	ng/uL

Sample peak width (sec): 5 Sample min peak height: 50 Sample baseline V to V?: Y Sample baseline V to V points: 3
Sample filter: Binomial Number of points for filter: 3 Sample start region (min): 0 Sample end region (min): 30
Marker peak width (sec): 5 Marker min peak height: 200 Marker baseline V to V?: Y Marker baseline V to V points: 3
Lower marker selection: First peak > 500 RFU Upper marker selection: Last peak > 200 RFU
Ladder size (bp)75, 200, 300, 400, 500, 700, 1000, 1500, 2000, 3000, 4000, 5000, 7000, 10000, 20000
Quantification using: Lower Marker Final concentration (ng/uL): 0.5000 Dilution factor: 12.0

Sample: SampB10
Well location: B10
Created: Wednesday, October 20, 2021 6:38:33 PM

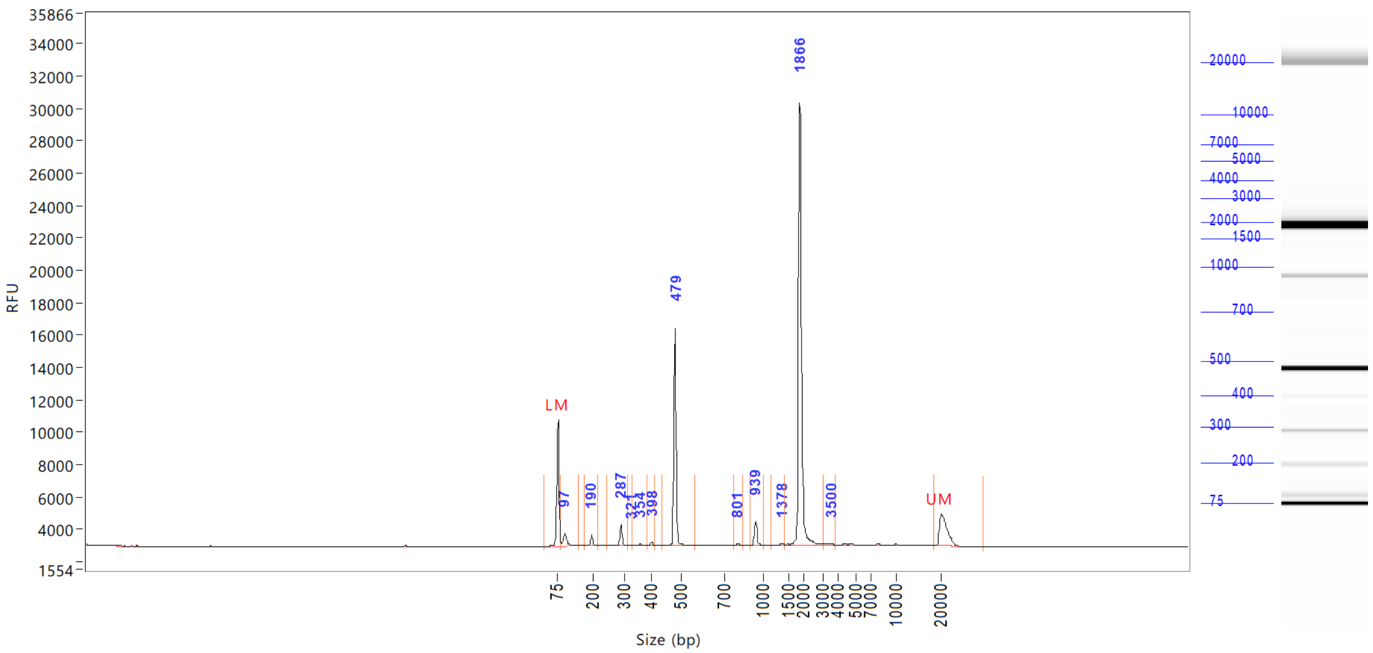

Peak	Size (bp)	Concentration (ng/uL)	From (bp)	To (bp)	Average size (bp)	CV%	RFU	Corrected peak area
1	75 (LM)	0.5000	26	86	73	5.12	7827	42.249
2	97	1.0537	86	147	99	9.67	786	7.420
3	190	0.4982	168	215	190	2.30	659	3.508
4	287	1.0359	241	311	285	2.46	1335	7.294
5	321	0.0473	311	329	319	1.50	58	0.333
6	354	0.1508	329	384	351	3.27	70	1.062
7	398	0.1364	384	407	395	0.96	215	0.961
8	479	9.0100	433	565	477	1.20	13414	63.444
9	801	0.0954	766	840	801	1.99	113	0.672
10	939	0.9533	905	1023	939	1.24	1452	6.713
11	1378	0.1077	1167	1445	1349	4.82	97	0.758
12	1866	20.2933	1445	3037	1887	5.82	27335	142.896
13	3500	0.0822	3037	3786	3360	5.55	57	0.579
14	20000 (UM)	0.2399	18537	29309	20602	4.45	2010	20.268

TIC:	33.4643	ng/uL
TIM:	80.1088	nmole/L
Total concentration:	34.0482	ng/uL

Sample peak width (sec): 5   Sample min peak height: 50   Sample baseline V to V?: Y   Sample baseline V to V points: 3
Sample filter: Binomial   Number of points for filter: 3   Sample start region (min): 0   Sample end region (min): 30
Marker peak width (sec): 5   Marker min peak height: 200   Marker baseline V to V?: Y   Marker baseline V to V points: 3
Lower marker selection: First peak > 500 RFU   Upper marker selection: Last peak > 200 RFU
Ladder size (bp)75, 200, 300, 400, 500, 700, 1000, 1500, 2000, 3000, 4000, 5000, 7000, 10000, 20000
Quantification using: Lower Marker   Final concentration (ng/uL): 0.5000   Dilution factor: 12.0

Sample: SampB11
Well location: B11
Created: Wednesday, October 20, 2021 6:38:33 PM

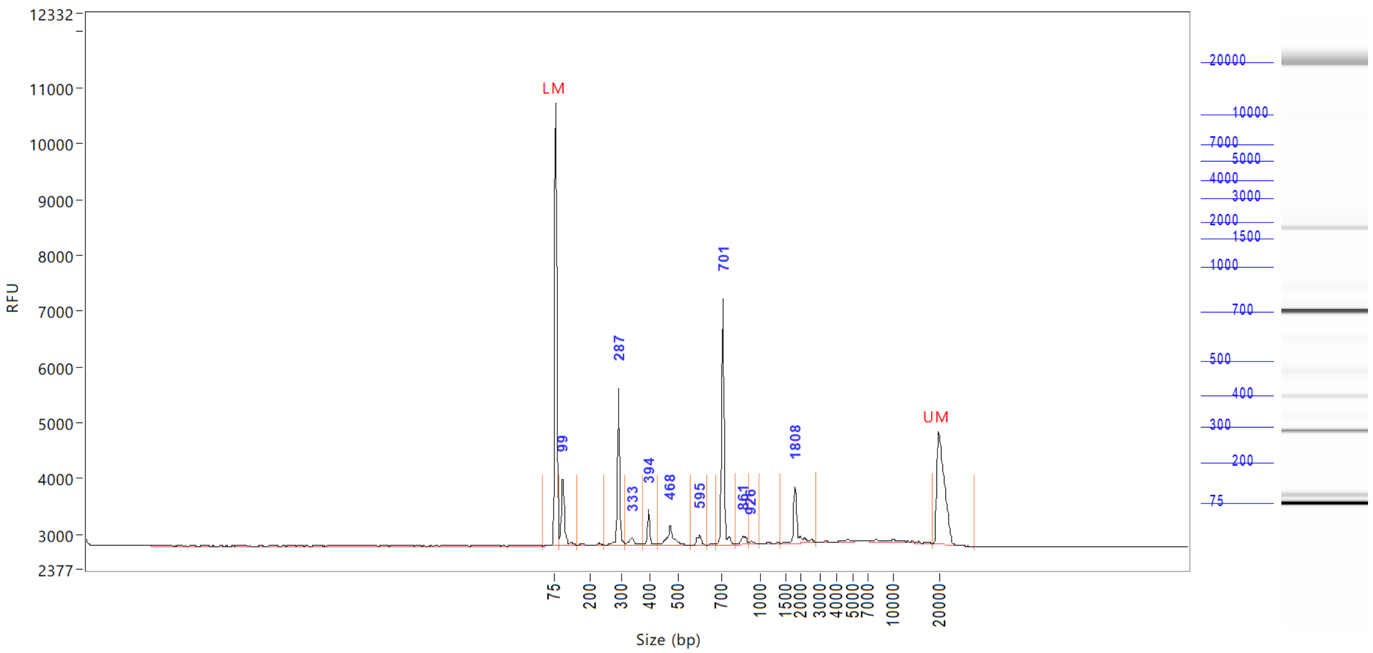

Peak	Size (bp)	Concentration (ng/uL)	From (bp)	To (bp)	Average size (bp)	CV%	RFU	Corrected peak area
1	75 (LM)	0.5000	31	87	74	4.81	7943	42.608
2	99	1.4052	87	152	100	9.05	1195	9.979
3	287	2.0116	241	311	285	2.14	2809	14.285
4	333	0.2360	311	374	335	4.17	125	1.676
5	394	0.4680	374	428	395	1.81	642	3.323
6	468	0.5923	428	555	470	3.56	348	4.206
7	595	0.2128	555	626	589	1.73	178	1.511
8	701	2.9561	674	801	702	1.74	4399	20.992
9	861	0.2020	801	905	862	2.30	143	1.435
10	926	0.0644	905	991	934	2.08	55	0.457
11	1808	0.8749	1389	2772	1872	11.58	1019	6.213
12	20000 (UM)	0.2437	18659	27738	20523	4.19	2016	20.764

TIC:	9.0233	ng/uL
TIM:	48.6590	nmole/L
Total concentration:	9.5337	ng/uL

```
Sample peak width (sec): 5     Sample min peak height: 50     Sample baseline V to V?: Y     Sample baseline V to V points: 3
Sample filter: Binomial        Number of points for filter: 3  Sample start region (min): 0  Sample end region (min): 30
Marker peak width (sec): 5     Marker min peak height: 200    Marker baseline V to V?: Y     Marker baseline V to V points: 3
Lower marker selection: First peak > 500 RFU                  Upper marker selection: Last peak > 200 RFU
Ladder size (bp)75, 200, 300, 400, 500, 700, 1000, 1500, 2000, 3000, 4000, 5000, 7000, 10000, 20000
Quantification using: Lower Marker          Final concentration (ng/uL): 0.5000          Dilution factor: 12.0
```

Sample: SampB12
Well location: B12
Created: Wednesday, October 20, 2021 6:38:33 PM

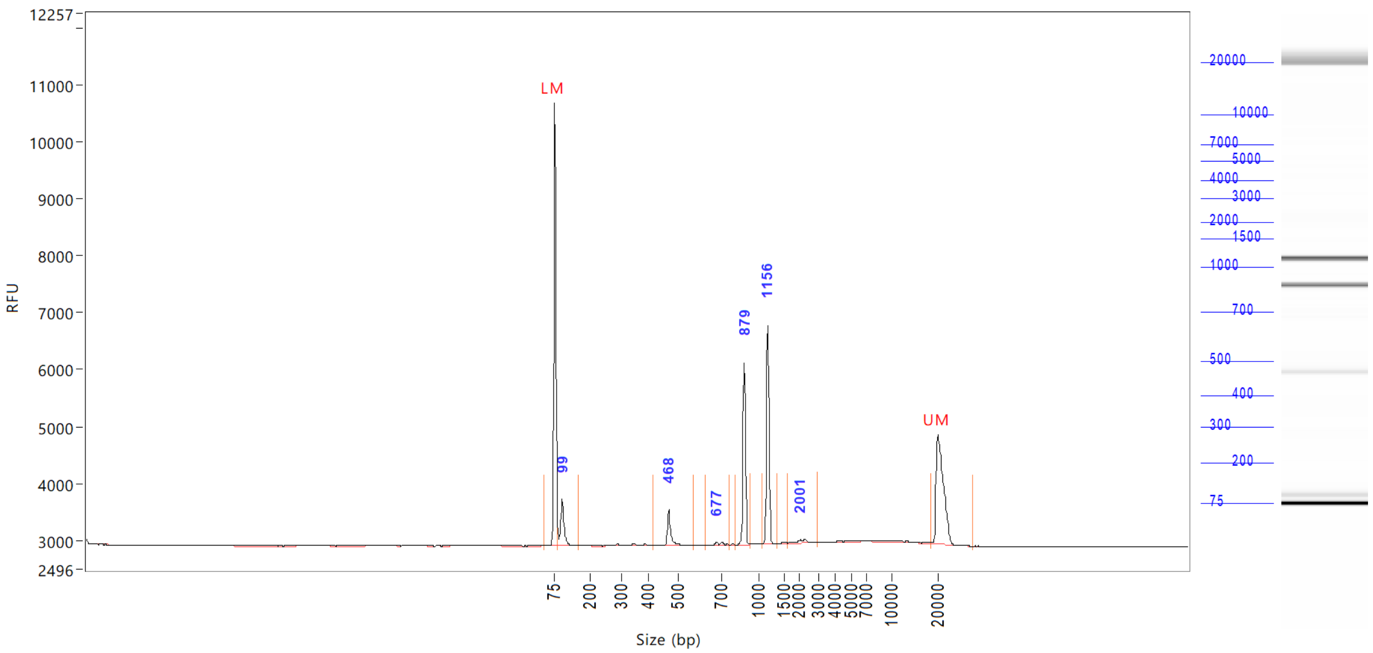

Peak	Size (bp)	Concentration (ng/uL)	From (bp)	To (bp)	Average size (bp)	CV%	RFU	Corrected peak area
1	75 (LM)	0.5000	41	87	73	4.72	7791	41.441
2	99	1.0257	87	160	100	9.29	820	7.085
3	468	0.6313	416	570	471	3.30	634	4.361
4	677	0.1081	629	762	688	3.57	62	0.747
5	879	2.0099	805	931	874	1.14	3195	13.882
6	1156	2.3509	1045	1345	1142	2.27	3837	16.237
7	2001	0.1351	1578	2886	2116	11.79	57	0.933
8	20000 (UM)	0.2338	18537	27859	20618	4.11	1927	19.380

TIC:	6.2611	ng/uL
TIM:	26.5860	nmole/L
Total concentration:	6.8517	ng/uL

```
Sample peak width (sec): 5    Sample min peak height: 50    Sample baseline V to V?: Y    Sample baseline V to V points: 3
Sample filter: Binomial       Number of points for filter: 3  Sample start region (min): 0  Sample end region (min): 30
Marker peak width (sec): 5    Marker min peak height: 200   Marker baseline V to V?: Y    Marker baseline V to V points: 3
Lower marker selection: First peak > 500 RFU                Upper marker selection: Last peak > 200 RFU
Ladder size (bp)75, 200, 300, 400, 500, 700, 1000, 1500, 2000, 3000, 4000, 5000, 7000, 10000, 20000
Quantification using: Lower Marker          Final concentration (ng/uL): 0.5000          Dilution factor: 12.0
```

Sample: SampC1
Well location: C1
Created: Wednesday, October 20, 2021 6:38:33 PM

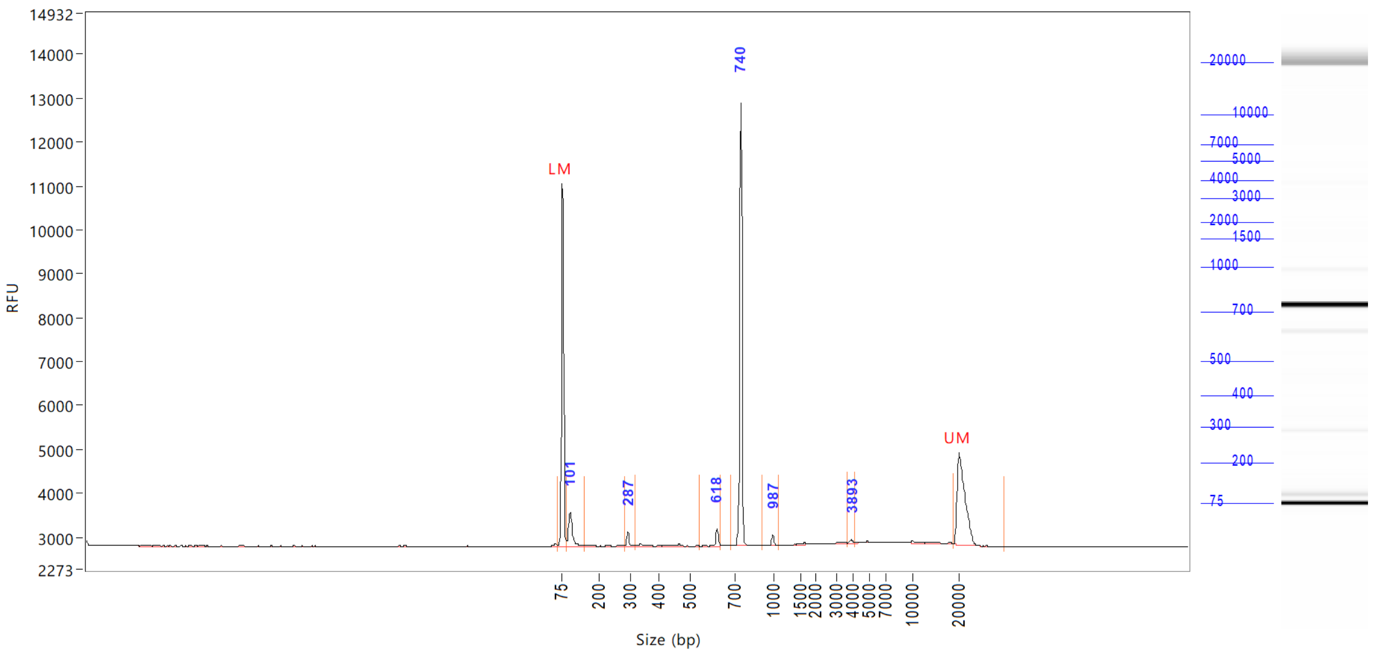

Peak	Size (bp)	Concentration (ng/uL)	From (bp)	To (bp)	Average size (bp)	CV%	RFU	Corrected peak area
1	75 (LM)	0.5000	58	89	74	4.51	8261	45.031
2	101	0.9545	89	152	103	10.10	771	7.163
3	287	0.2218	278	313	288	1.97	325	1.665
4	618	0.2387	544	636	616	1.67	398	1.791
5	740	5.7642	682	909	740	1.59	10103	43.261
6	987	0.1399	909	1068	980	1.44	239	1.050
7	3893	0.0481	3679	4070	3872	2.22	83	0.361
8	20000 (UM)	0.2417	18781	29793	20608	4.55	2086	21.764

TIC:	7.3671	ng/uL
TIM:	30.1975	nmole/L
Total concentration:	8.1105	ng/uL

Sample peak width (sec): 5 Sample min peak height: 50 Sample baseline V to V?: Y Sample baseline V to V points: 3
Sample filter: Binomial Number of points for filter: 3 Sample start region (min): 0 Sample end region (min): 30
Marker peak width (sec): 5 Marker min peak height: 200 Marker baseline V to V?: Y Marker baseline V to V points: 3
Lower marker selection: First peak > 500 RFU Upper marker selection: Last peak > 200 RFU
Ladder size (bp)75, 200, 300, 400, 500, 700, 1000, 1500, 2000, 3000, 4000, 5000, 7000, 10000, 20000
Quantification using: Lower Marker Final concentration (ng/uL): 0.5000 Dilution factor: 12.0

Sample: SampC2
Well location: C2
Created: Wednesday, October 20, 2021 6:38:33 PM

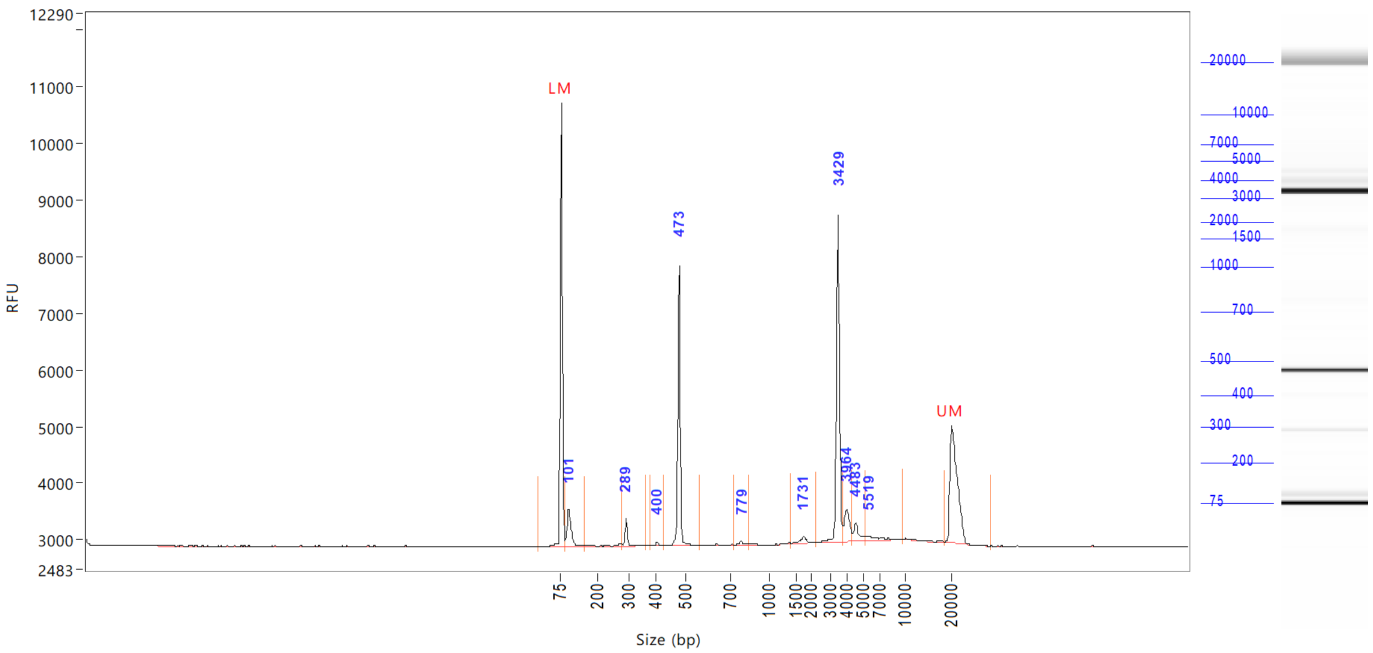

Peak	Size (bp)	Concentration (ng/uL)	From (bp)	To (bp)	Average size (bp)	CV%	RFU	Corrected peak area
1	75 (LM)	0.5000	1	89	74	7.13	7839	43.931
2	101	0.8948	89	158	103	10.64	651	6.552
3	289	0.3589	278	362	291	3.79	480	2.628
4	400	0.0643	378	422	398	2.02	75	0.471
5	473	3.2143	422	555	472	1.61	4943	23.535
6	779	0.1057	727	840	777	3.46	76	0.774
7	1731	0.1761	1411	2229	1698	8.21	127	1.289
8	3429	3.7637	2229	3715	3369	4.01	5759	27.558
9	3964	0.6825	3715	4311	3968	3.67	578	4.997
10	4483	0.3529	4311	5223	4606	4.96	315	2.584
11	5519	0.2112	5223	9600	6596	15.52	75	1.546
12	20000 (UM)	0.2342	18537	28463	20568	4.11	2068	20.579

TIC:	9.8243	ng/uL
TIM:	30.4623	nmole/L
Total concentration:	10.1084	ng/uL

```
Sample peak width (sec): 5     Sample min peak height: 50    Sample baseline V to V?: Y    Sample baseline V to V points: 3
Sample filter: Binomial        Number of points for filter: 3  Sample start region (min): 0  Sample end region (min): 30
Marker peak width (sec): 5     Marker min peak height: 200   Marker baseline V to V?: Y    Marker baseline V to V points: 3
Lower marker selection: First peak > 500 RFU                 Upper marker selection: Last peak > 200 RFU
Ladder size (bp)75, 200, 300, 400, 500, 700, 1000, 1500, 2000, 3000, 4000, 5000, 7000, 10000, 20000
Quantification using: Lower Marker          Final concentration (ng/uL): 0.5000         Dilution factor: 12.0
```

Sample: SampC3
Well location: C3
Created: Wednesday, October 20, 2021 6:38:33 PM

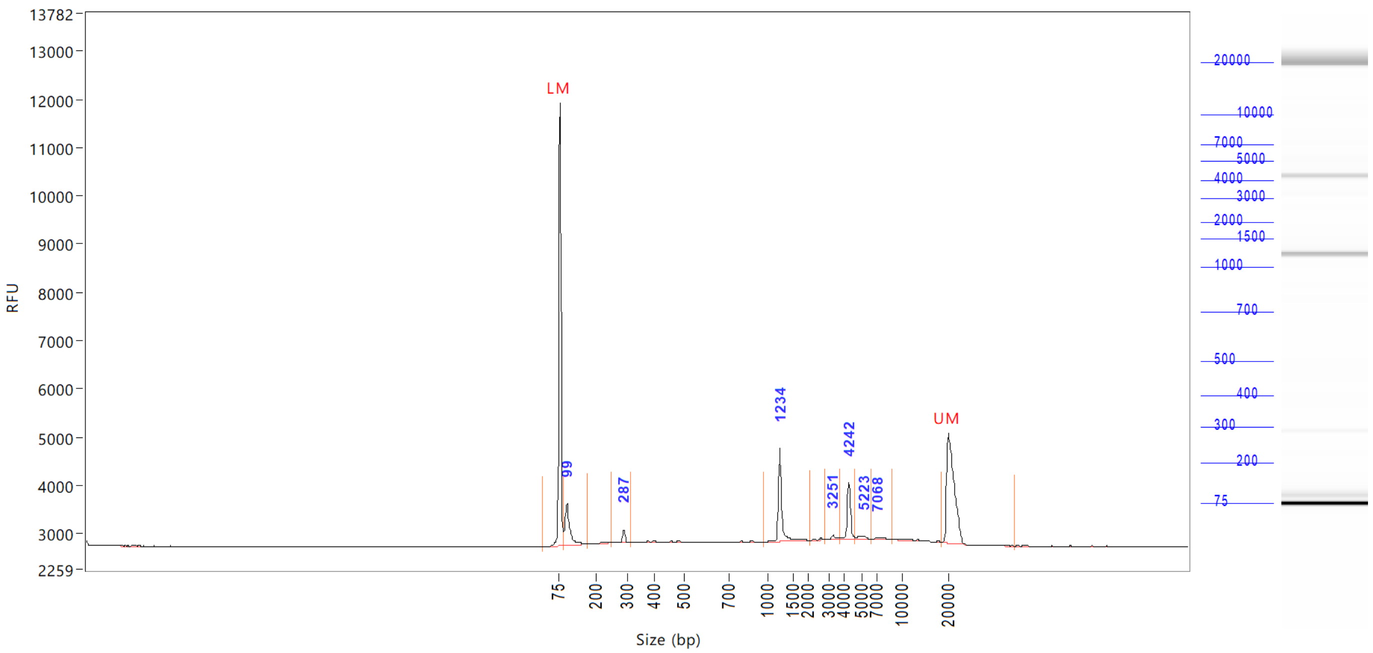

Peak	Size (bp)	Concentration (ng/uL)	From (bp)	To (bp)	Average size (bp)	CV%	RFU	Corrected peak area
1	75 (LM)	0.5000	16	87	73	5.90	9196	50.983
2	99	1.2112	87	168	103	14.02	858	10.291
3	287	0.1763	251	313	285	2.67	269	1.498
4	1234	1.3036	974	2058	1254	9.39	1928	11.077
5	3251	0.0841	2857	3715	3325	5.87	89	0.714
6	4242	0.7175	3715	4656	4199	3.25	1181	6.097
7	5223	0.1342	4656	6186	5142	7.36	80	1.140
8	7068	0.0887	6186	8867	7305	8.13	51	0.754
9	20000 (UM)	0.2291	18415	34267	20551	5.29	2265	23.359

TIC:	3.7154	ng/uL
TIM:	22.4248	nmole/L
Total concentration:	4.1721	ng/uL

Sample peak width (sec): 5   Sample min peak height: 50   Sample baseline V to V?: Y   Sample baseline V to V points: 3
Sample filter: Binomial   Number of points for filter: 3 Sample start region (min): 0 Sample end region (min): 30
Marker peak width (sec): 5   Marker min peak height: 200   Marker baseline V to V?: Y   Marker baseline V to V points: 3
Lower marker selection: First peak > 500 RFU   Upper marker selection: Last peak > 200 RFU
Ladder size (bp)75, 200, 300, 400, 500, 700, 1000, 1500, 2000, 3000, 4000, 5000, 7000, 10000, 20000
Quantification using: Lower Marker   Final concentration (ng/uL): 0.5000   Dilution factor: 12.0

Sample: SampC4
Well location: C4
Created: Wednesday, October 20, 2021 6:38:33 PM

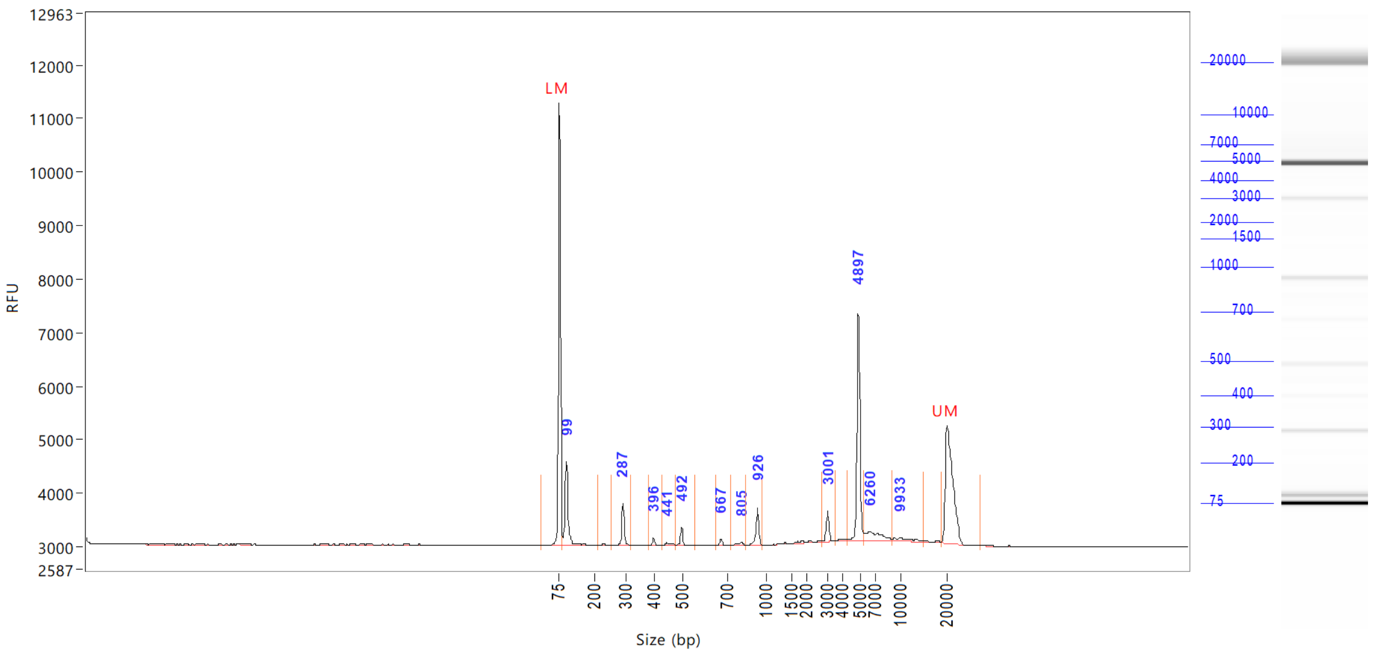

Peak	Size (bp)	Concentration (ng/uL)	From (bp)	To (bp)	Average size (bp)	CV%	RFU	Corrected peak area
1	75 (LM)	0.5000	16	87	73	5.99	8273	44.892
2	99	1.6850	87	205	102	15.23	1562	12.607
3	287	0.5569	251	313	285	2.12	788	4.166
4	396	0.0927	378	424	394	1.41	137	0.693
5	441	0.0925	424	470	448	2.24	54	0.692
6	492	0.2406	470	549	490	1.63	343	1.800
7	667	0.0900	646	723	667	1.53	121	0.673
8	805	0.0720	723	835	784	2.98	62	0.538
9	926	0.4593	835	970	920	1.66	700	3.437
10	3001	0.3408	2743	3465	2993	2.57	561	2.550
11	4897	2.7595	4208	5519	4897	2.80	4256	20.646
12	6260	0.5389	5519	8934	6903	12.87	175	4.032
13	9933	0.2152	8934	15000	11129	15.15	61	1.610
14	20000 (UM)	0.2513	18781	27254	20610	4.17	2185	22.563

TIC:	7.1433	ng/uL
TIM:	34.3472	nmole/L
Total concentration:	7.4940	ng/uL

```
Sample peak width (sec): 5     Sample min peak height: 50     Sample baseline V to V?: Y     Sample baseline V to V points: 3
Sample filter: Binomial        Number of points for filter: 3  Sample start region (min): 0  Sample end region (min): 30
Marker peak width (sec): 5     Marker min peak height: 200    Marker baseline V to V?: Y     Marker baseline V to V points: 3
Lower marker selection: First peak > 500 RFU                  Upper marker selection: Last peak > 200 RFU
Ladder size (bp)75, 200, 300, 400, 500, 700, 1000, 1500, 2000, 3000, 4000, 5000, 7000, 10000, 20000
Quantification using: Lower Marker          Final concentration (ng/uL): 0.5000          Dilution factor: 12.0
```

Sample: SampC5
Well location: C5
Created: Wednesday, October 20, 2021 6:38:33 PM

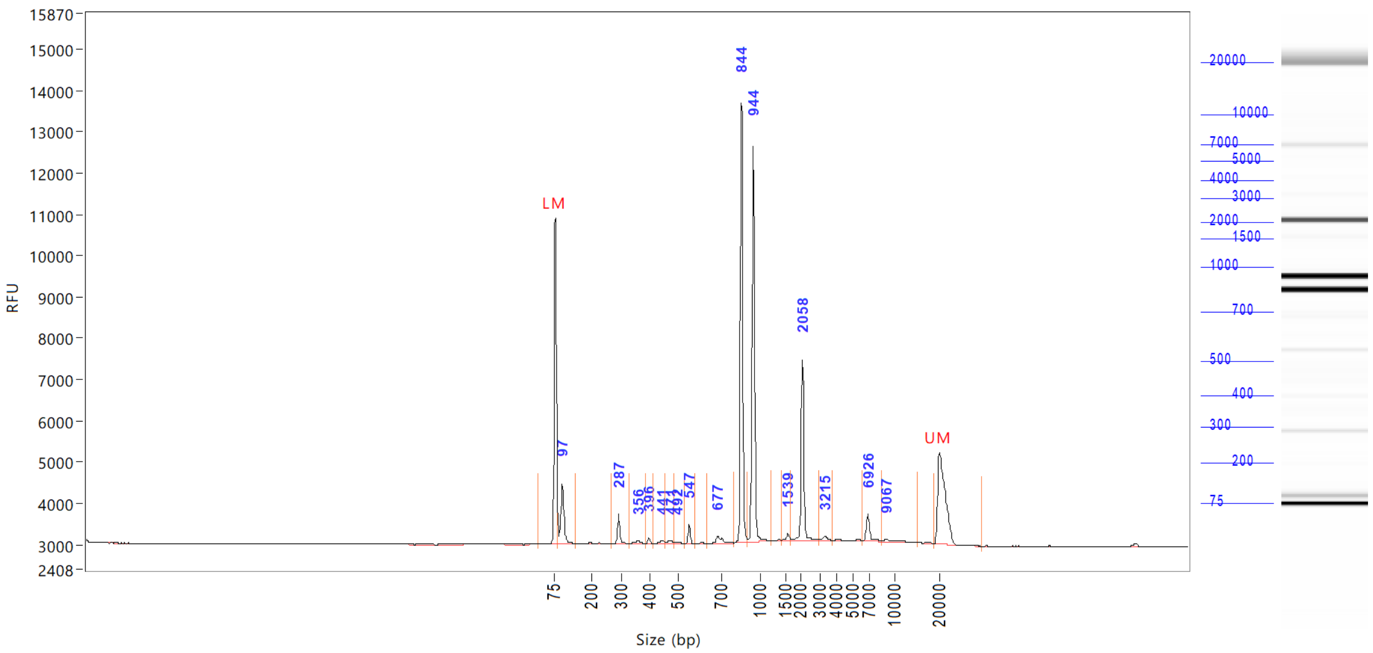

Peak	Size (bp)	Concentration (ng/uL)	From (bp)	To (bp)	Average size (bp)	CV%	RFU	Corrected peak area
1	75 (LM)	0.5000	16	86	72	4.91	7914	43.917
2	97	1.6453	86	147	97	8.15	1463	12.043
3	287	0.5335	268	325	286	2.28	716	3.905
4	356	0.1274	325	384	351	3.32	64	0.933
5	396	0.0984	384	411	394	1.00	153	0.720
6	441	0.1137	411	454	438	2.13	81	0.832
7	471	0.1101	454	483	468	1.63	79	0.806
8	492	0.0715	483	527	494	1.64	53	0.523
9	547	0.2879	527	572	543	1.00	469	2.108
10	677	0.2548	629	788	688	3.33	165	1.865
11	844	6.4539	788	900	844	1.21	10664	47.239
12	944	6.0303	900	1201	937	1.67	9591	44.139
13	1539	0.1348	1411	1674	1526	3.84	162	0.987
14	2058	2.8315	1674	2914	2040	5.72	4370	20.725
15	3215	0.1243	2914	3715	3226	5.56	119	0.910
16	6926	0.4973	6112	8467	6940	5.92	667	3.640
17	9067	0.1484	8467	15122	10264	14.83	53	1.086
18	20000 (UM)	0.2604	18659	29309	20599	4.53	2216	22.873

TIC:	19.4630	ng/uL
TIM:	60.2743	nmole/L
Total concentration:	19.7405	ng/uL

```
Sample peak width (sec): 5    Sample min peak height: 50    Sample baseline V to V?: Y    Sample baseline V to V points: 3
Sample filter: Binomial       Number of points for filter: 3  Sample start region (min): 0  Sample end region (min): 30
Marker peak width (sec): 5    Marker min peak height: 200   Marker baseline V to V?: Y    Marker baseline V to V points: 3
Lower marker selection: First peak > 500 RFU                Upper marker selection: Last peak > 200 RFU
Ladder size (bp)75, 200, 300, 400, 500, 700, 1000, 1500, 2000, 3000, 4000, 5000, 7000, 10000, 20000
Quantification using: Lower Marker          Final concentration (ng/uL): 0.5000          Dilution factor: 12.0
```

Sample: SampC6
Well location: C6
Created: Wednesday, October 20, 2021 6:38:33 PM

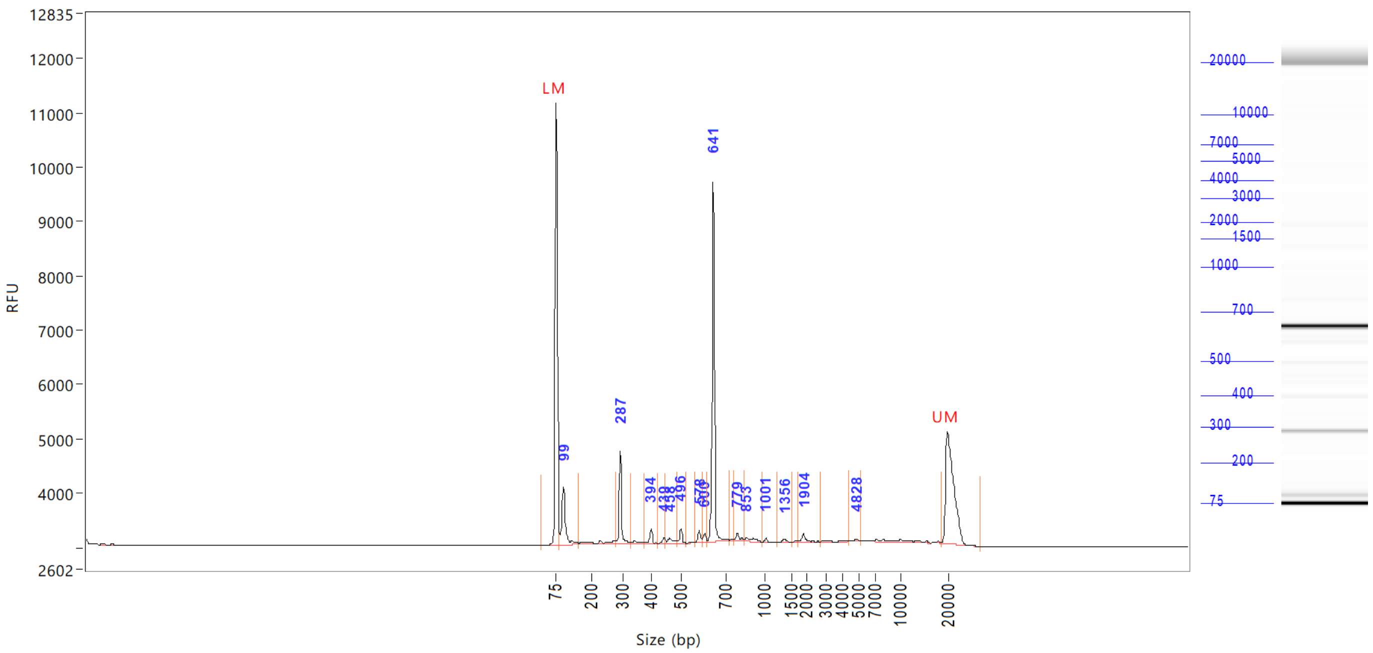

Peak	Size (bp)	Concentration (ng/uL)	From (bp)	To (bp)	Average size (bp)	CV%	RFU	Corrected peak area
1	75 (LM)	0.5000	28	87	73	5.16	8154	44.352
2	99	1.3370	87	150	101	11.30	1060	9.883
3	287	1.1657	275	323	286	2.14	1705	8.616
4	394	0.1750	372	418	393	1.51	259	1.294
5	439	0.0777	418	447	437	1.27	103	0.575
6	458	0.1402	447	483	462	2.20	98	1.036
7	496	0.1848	483	521	494	0.98	276	1.366
8	578	0.1611	557	590	575	1.19	225	1.191
9	600	0.1109	590	616	600	1.13	154	0.819
10	641	4.0283	616	723	639	1.43	6608	29.777
11	779	0.1363	757	831	788	2.37	141	1.008
12	853	0.1338	831	974	885	3.83	59	0.989
13	1001	0.0527	974	1234	1019	4.97	75	0.390
14	1356	0.0813	1234	1539	1377	4.41	72	0.601
15	1904	0.1568	1750	2686	1980	10.37	160	1.159
16	4828	0.0454	4311	5075	4756	3.44	50	0.336
17	20000 (UM)	0.2571	18659	27133	20651	4.47	2063	22.802

TIC:	7.9871	ng/uL
TIM:	42.5848	nmole/L
Total concentration:	8.6238	ng/uL

```
Sample peak width (sec): 5    Sample min peak height: 50    Sample baseline V to V?: Y    Sample baseline V to V points: 3
Sample filter: Binomial       Number of points for filter: 3  Sample start region (min): 0  Sample end region (min): 30
Marker peak width (sec): 5    Marker min peak height: 200   Marker baseline V to V?: Y    Marker baseline V to V points: 3
Lower marker selection: First peak > 500 RFU                Upper marker selection: Last peak > 200 RFU
Ladder size (bp)75, 200, 300, 400, 500, 700, 1000, 1500, 2000, 3000, 4000, 5000, 7000, 10000, 20000
Quantification using: Lower Marker         Final concentration (ng/uL): 0.5000         Dilution factor: 12.0
```

Sample: SampC7
Well location: C7
Created: Wednesday, October 20, 2021 6:38:33 PM

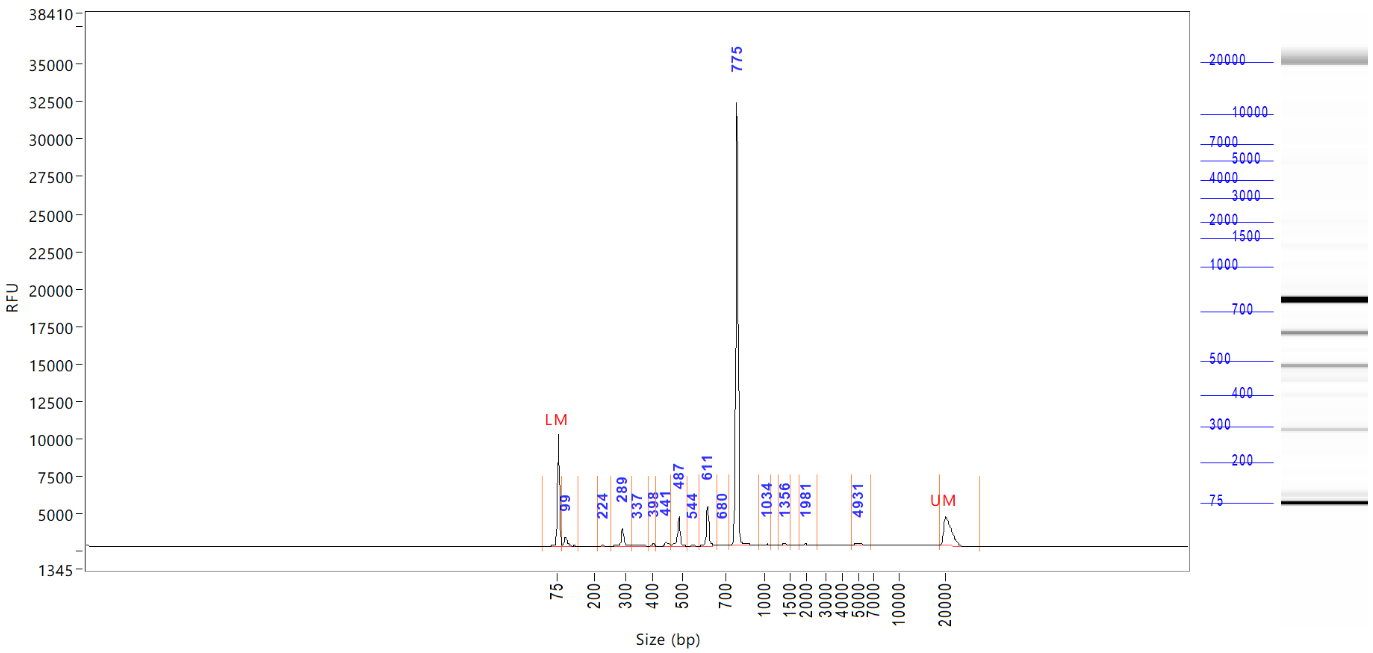

Peak	Size (bp)	Concentration (ng/uL)	From (bp)	To (bp)	Average size (bp)	CV%	RFU	Corrected peak area
1	75 (LM)	0.5000	20	87	73	6.02	7472	40.485
2	99	0.8111	87	147	101	9.68	561	5.473
3	224	0.0839	208	255	229	4.53	50	0.566
4	289	1.0605	255	321	288	3.14	1126	7.155
5	337	0.1376	321	382	346	4.46	52	0.929
6	398	0.1254	382	411	396	1.11	173	0.846
7	441	0.3337	411	460	444	1.81	284	2.252
8	487	1.5531	460	521	483	1.38	1992	10.480
9	544	0.0507	521	572	544	1.60	62	0.342
10	611	1.9641	572	659	611	1.40	2622	13.253
11	680	0.0650	659	718	678	1.50	55	0.439
12	775	19.3581	718	952	774	1.86	29580	130.620
13	1034	0.0558	952	1112	1026	2.94	71	0.377
14	1356	0.1016	1267	1489	1364	2.87	113	0.686
15	1981	0.1006	1789	2543	2024	7.55	84	0.679
16	4931	0.1423	4552	6556	5147	8.64	82	0.960
17	20000 (UM)	0.2454	18903	27617	20632	4.24	1961	19.872

TIC:	25.9436	ng/uL
TIM:	74.6530	nmole/L
Total concentration:	26.2822	ng/uL

```
Sample peak width (sec): 5    Sample min peak height: 50    Sample baseline V to V?: Y    Sample baseline V to V points: 3
Sample filter: Binomial       Number of points for filter: 3  Sample start region (min): 0  Sample end region (min): 30
Marker peak width (sec): 5    Marker min peak height: 200   Marker baseline V to V?: Y    Marker baseline V to V points: 3
Lower marker selection: First peak > 500 RFU                Upper marker selection: Last peak > 200 RFU
Ladder size (bp)75, 200, 300, 400, 500, 700, 1000, 1500, 2000, 3000, 4000, 5000, 7000, 10000, 20000
Quantification using: Lower Marker          Final concentration (ng/uL): 0.5000         Dilution factor: 12.0
```

Sample: SampC8
Well location: C8
Created: Wednesday, October 20, 2021 6:38:33 PM

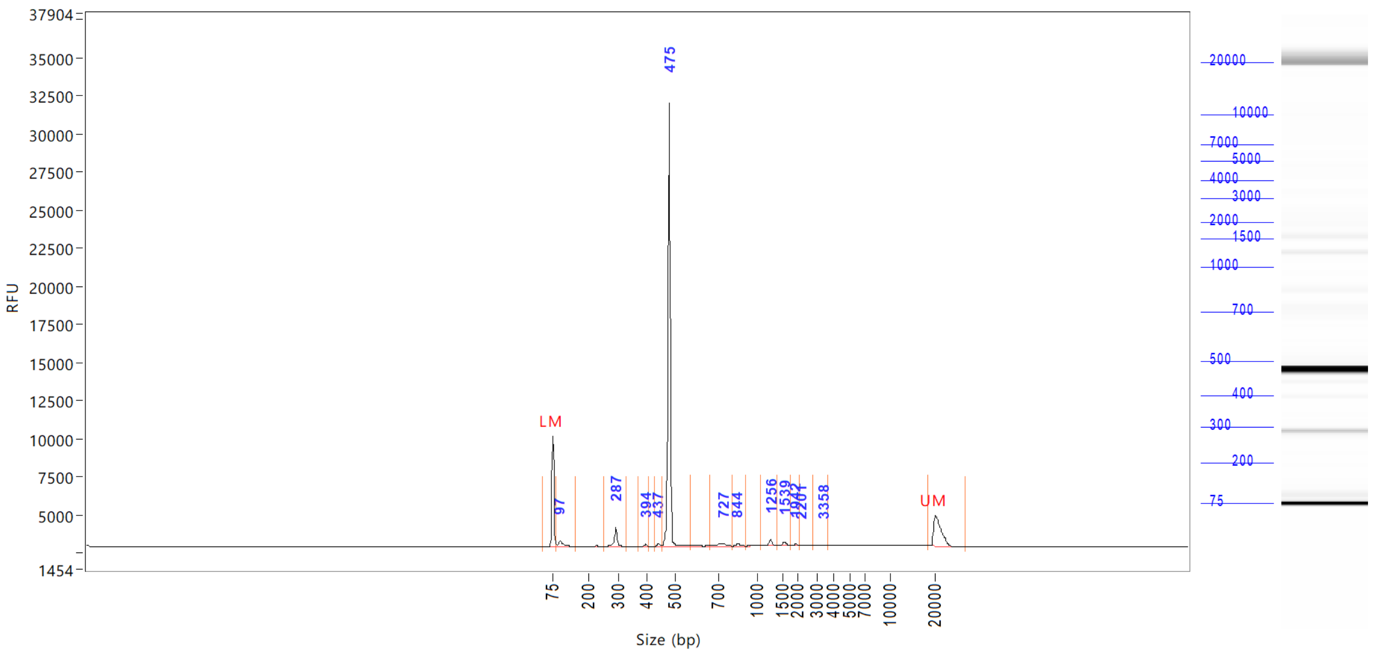

Peak	Size (bp)	Concentration (ng/uL)	From (bp)	To (bp)	Average size (bp)	CV%	RFU	Corrected peak area
1	75 (LM)	0.5000	39	87	73	4.80	7198	39.602
2	97	0.6562	87	154	102	11.35	347	4.331
3	287	1.1983	248	323	284	2.61	1217	7.909
4	394	0.1124	370	407	392	1.28	150	0.742
5	437	0.1464	426	450	438	1.13	184	0.967
6	475	21.0121	450	567	474	1.25	29112	138.689
7	727	0.3564	654	805	708	3.63	145	2.353
8	844	0.1735	805	905	847	1.95	157	1.145
9	1256	0.3124	1068	1389	1237	3.20	404	2.062
10	1539	0.2798	1389	1750	1553	4.53	268	1.847
11	1942	0.1355	1750	2115	1934	4.99	90	0.895
12	2201	0.1236	2115	2800	2358	7.23	64	0.816
13	3358	0.0623	2800	3679	3256	5.76	52	0.411
14	20000 (UM)	0.2608	18537	26649	20631	4.19	2014	20.659

TIC:	24.5692	ng/uL
TIM:	93.5806	nmole/L
Total concentration:	24.9840	ng/uL

```
Sample peak width (sec): 5    Sample min peak height: 50    Sample baseline V to V?: Y    Sample baseline V to V points: 3
Sample filter: Binomial       Number of points for filter: 3  Sample start region (min): 0  Sample end region (min): 30
Marker peak width (sec): 5    Marker min peak height: 200   Marker baseline V to V?: Y    Marker baseline V to V points: 3
Lower marker selection: First peak > 500 RFU                Upper marker selection: Last peak > 200 RFU
Ladder size (bp)75, 200, 300, 400, 500, 700, 1000, 1500, 2000, 3000, 4000, 5000, 7000, 10000, 20000
Quantification using: Lower Marker          Final concentration (ng/uL): 0.5000          Dilution factor: 12.0
```

Sample: SampC9
Well location: C9
Created: Wednesday, October 20, 2021 6:38:33 PM

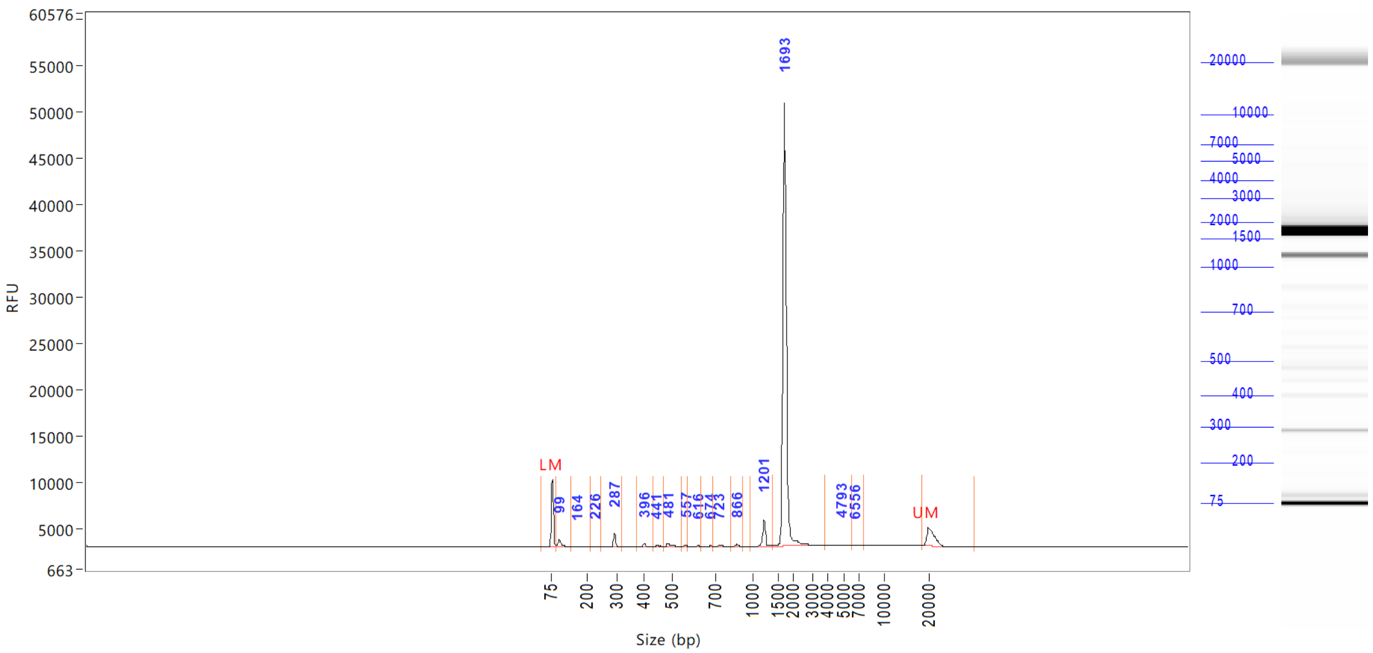

Peak	Size (bp)	Concentration (ng/uL)	From (bp)	To (bp)	Average size (bp)	CV%	RFU	Corrected peak area
1	75 (LM)	0.5000	37	87	73	5.18	7311	39.913
2	99	1.0941	87	143	100	9.95	781	7.278
3	164	0.1248	143	206	168	8.84	54	0.830
4	226	0.0676	206	244	225	2.95	60	0.450
5	287	1.1512	244	317	286	2.69	1364	7.658
6	396	0.2420	370	431	395	1.94	307	1.610
7	441	0.1813	431	468	445	1.97	184	1.206
8	481	0.5302	468	539	490	3.22	361	3.527
9	557	0.1581	539	567	553	0.99	218	1.052
10	616	0.1123	567	631	601	2.43	81	0.747
11	674	0.0794	631	687	670	1.59	88	0.528
12	723	0.1837	687	822	727	3.29	138	1.222
13	866	0.1916	822	918	863	1.59	216	1.275
14	1201	2.1334	970	1378	1194	3.24	2887	14.192
15	1693	43.4138	1378	3822	1716	10.46	47829	288.796
16	4793	0.3367	3822	6038	4604	11.37	92	2.240
17	6556	0.0935	6038	7534	6700	5.88	68	0.622
18	20000 (UM)	0.2452	18415	30398	20638	4.49	1927	19.575

TIC:	50.0939	ng/uL
TIM:	76.2460	nmole/L
Total concentration:	50.2734	ng/uL

```
Sample peak width (sec): 5    Sample min peak height: 50    Sample baseline V to V?: Y    Sample baseline V to V points: 3
Sample filter: Binomial       Number of points for filter: 3  Sample start region (min): 0  Sample end region (min): 30
Marker peak width (sec): 5    Marker min peak height: 200   Marker baseline V to V?: Y    Marker baseline V to V points: 3
Lower marker selection: First peak > 500 RFU                Upper marker selection: Last peak > 200 RFU
Ladder size (bp)75, 200, 300, 400, 500, 700, 1000, 1500, 2000, 3000, 4000, 5000, 7000, 10000, 20000
Quantification using: Lower Marker          Final concentration (ng/uL): 0.5000          Dilution factor: 12.0
```

Sample: SampC10
Well location: C10
Created: Wednesday, October 20, 2021 6:38:33 PM

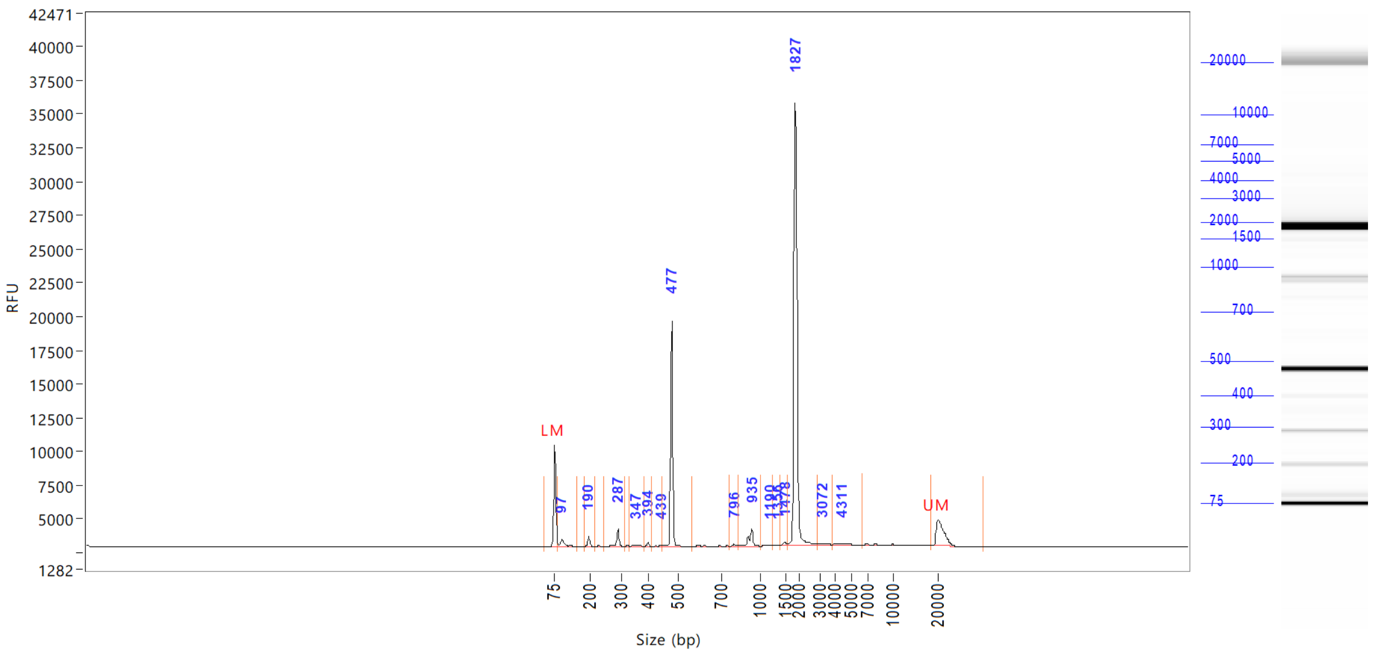

Peak	Size (bp)	Concentration (ng/uL)	From (bp)	To (bp)	Average size (bp)	CV%	RFU	Corrected peak area
1	75 (LM)	0.5000	41	87	73	4.90	7522	40.568
2	97	0.8419	87	154	102	11.55	554	5.692
3	190	0.5985	179	214	190	2.14	770	4.047
4	287	1.0029	242	309	284	2.43	1307	6.781
5	347	0.1947	327	384	351	3.27	87	1.316
6	394	0.1614	384	407	394	0.92	245	1.091
7	439	0.0712	407	447	431	2.45	55	0.481
8	477	10.6006	447	565	476	1.07	16645	71.674
9	796	0.1177	762	835	798	2.04	135	0.796
10	935	1.2995	835	1001	918	2.49	1261	8.786
11	1190	0.0346	1001	1256	1176	3.54	52	0.234
12	1356	0.0420	1256	1400	1342	2.01	63	0.284
13	1478	0.1980	1400	1559	1481	2.36	226	1.339
14	1827	24.7857	1559	2914	1841	5.92	32833	167.584
15	3072	0.1977	2914	3750	3229	7.24	100	1.336
16	4311	0.2058	3750	6408	4436	10.31	89	1.392
17	20000 (UM)	0.2482	18415	29914	20565	4.36	1971	20.140

TIC:	40.3521	ng/uL
TIM:	88.2766	nmole/L
Total concentration:	40.7055	ng/uL

```
Sample peak width (sec): 5     Sample min peak height: 50    Sample baseline V to V?: Y    Sample baseline V to V points: 3
Sample filter: Binomial        Number of points for filter: 3  Sample start region (min): 0  Sample end region (min): 30
Marker peak width (sec): 5     Marker min peak height: 200   Marker baseline V to V?: Y    Marker baseline V to V points: 3
Lower marker selection: First peak > 500 RFU                 Upper marker selection: Last peak > 200 RFU
Ladder size (bp)75, 200, 300, 400, 500, 700, 1000, 1500, 2000, 3000, 4000, 5000, 7000, 10000, 20000
Quantification using: Lower Marker          Final concentration (ng/uL): 0.5000          Dilution factor: 12.0
```

Sample: SampC11
Well location: C11
Created: Wednesday, October 20, 2021 6:38:33 PM

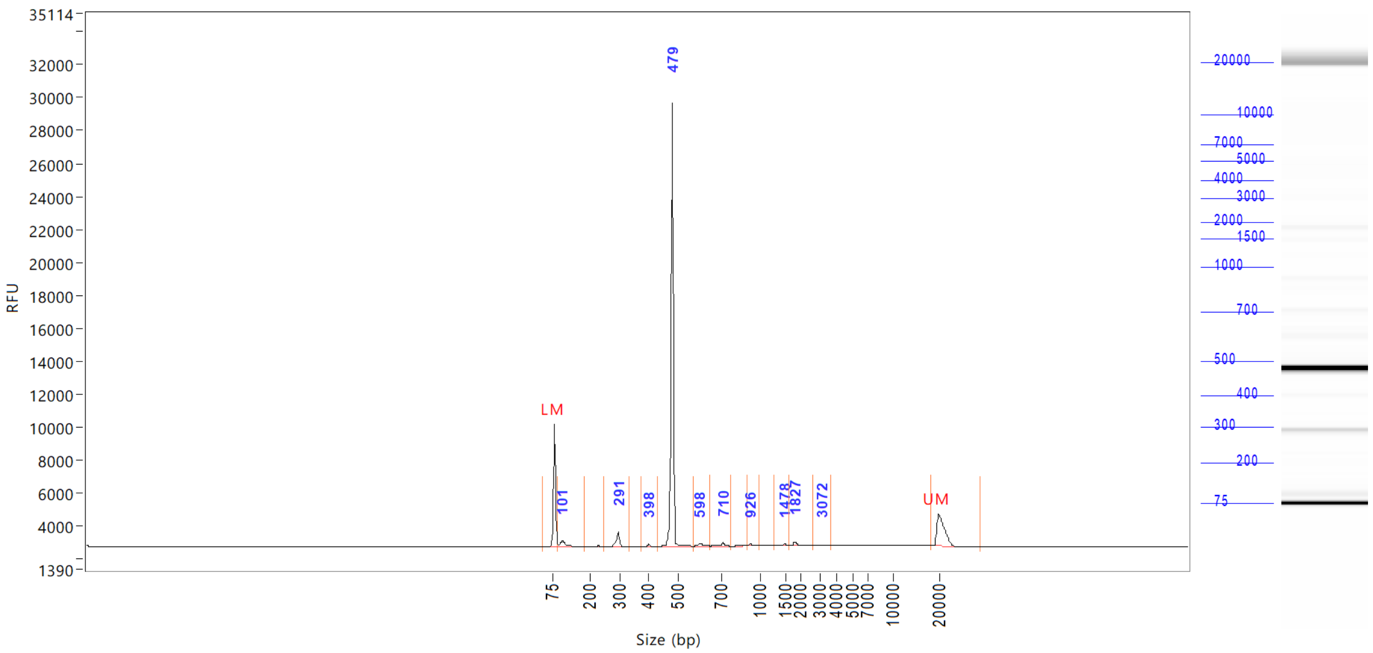

Peak	Size (bp)	Concentration (ng/uL)	From (bp)	To (bp)	Average size (bp)	CV%	RFU	Corrected peak area
1	75 (LM)	0.5000	35	89	74	4.89	7466	40.937
2	101	0.6888	89	181	107	15.06	377	4.700
3	291	0.9258	242	331	285	2.83	905	6.316
4	398	0.1065	376	431	399	2.08	131	0.726
5	479	18.5411	431	570	478	1.25	26940	126.503
6	598	0.2924	570	649	606	2.72	157	1.995
7	710	0.2232	649	770	698	2.46	215	1.523
8	926	0.0976	896	996	921	1.51	137	0.666
9	1478	0.0908	1289	1635	1482	3.32	93	0.619
10	1827	0.2228	1635	2657	1852	8.14	241	1.520
11	3072	0.0726	2657	3679	3140	5.95	54	0.496
12	20000 (UM)	0.2397	18415	28947	20521	4.27	1945	19.626

TIC:	21.2616	ng/uL
TIM:	82.0121	nmole/L
Total concentration:	21.6641	ng/uL

Sample peak width (sec): 5 Sample min peak height: 50 Sample baseline V to V?: Y Sample baseline V to V points: 3
Sample filter: Binomial Number of points for filter: 3 Sample start region (min): 0 Sample end region (min): 30
Marker peak width (sec): 5 Marker min peak height: 200 Marker baseline V to V?: Y Marker baseline V to V points: 3
Lower marker selection: First peak > 500 RFU Upper marker selection: Last peak > 200 RFU
Ladder size (bp)75, 200, 300, 400, 500, 700, 1000, 1500, 2000, 3000, 4000, 5000, 7000, 10000, 20000
Quantification using: Lower Marker Final concentration (ng/uL): 0.5000 Dilution factor: 12.0

Sample: SampC12
Well location: C12
Created: Wednesday, October 20, 2021 6:38:33 PM

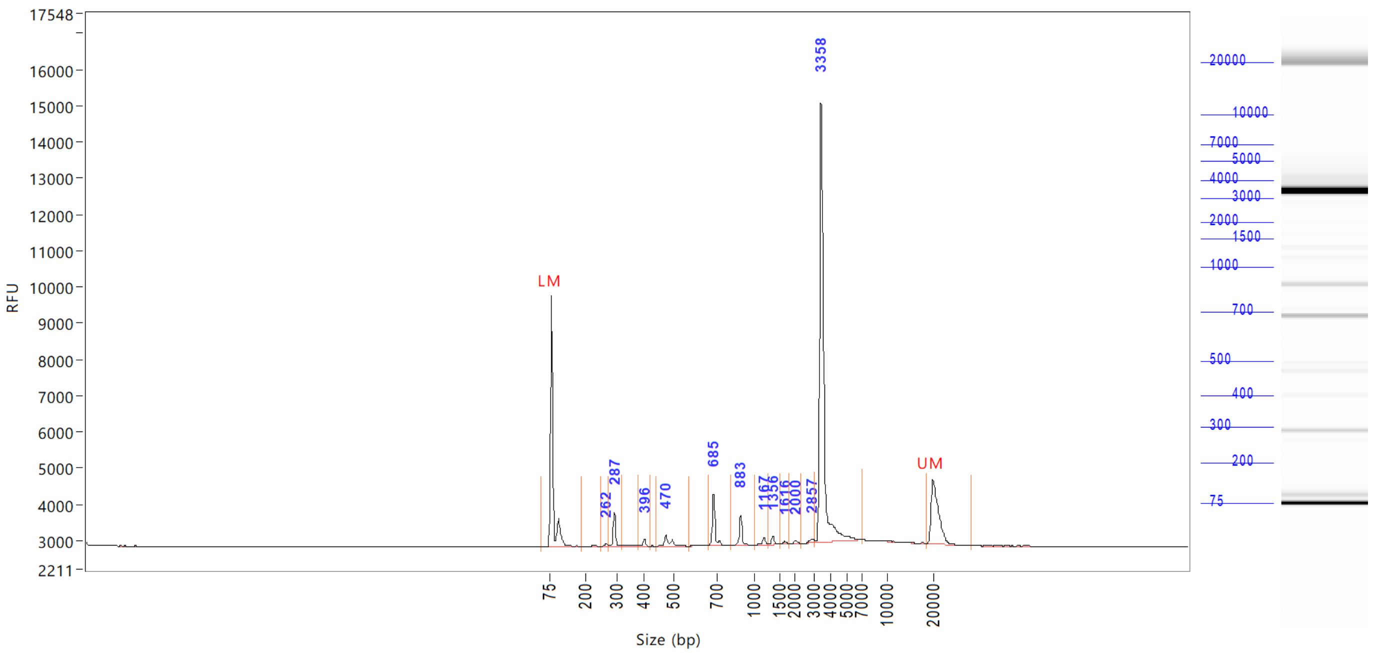

Peak	Size (bp)	Concentration (ng/uL)	From (bp)	To (bp)	Average size (bp)	CV%	RFU	Corrected peak area
1	75 (LM)	0.5000	41	181	79	16.46	6906	46.390
2	262	0.0683	244	271	260	2.05	81	0.528
3	287	0.6700	271	317	288	2.06	946	5.180
4	396	0.1251	374	416	394	1.52	177	0.967
5	470	0.4663	439	567	483	4.38	298	3.605
6	685	1.0120	659	809	684	2.07	1424	7.824
7	883	0.5815	809	996	883	2.34	848	4.496
8	1167	0.1624	996	1256	1139	4.27	208	1.256
9	1356	0.1506	1256	1500	1338	2.63	222	1.165
10	1616	0.0620	1500	1808	1610	3.51	85	0.480
11	2000	0.0645	1808	2315	2002	3.75	79	0.499
12	2857	0.1035	2315	3001	2813	4.22	106	0.800
13	3358	9.8050	3001	6926	3494	10.43	12138	75.809
14	20000 (UM)	0.1981	18659	28584	20655	4.86	1784	18.375

TIC:	13.2712	ng/uL
TIM:	15.1050	nmole/L
Total concentration:	13.5120	ng/uL

```
Sample peak width (sec): 5    Sample min peak height: 50    Sample baseline V to V?: Y    Sample baseline V to V points: 3
Sample filter: Binomial       Number of points for filter: 3  Sample start region (min): 0  Sample end region (min): 30
Marker peak width (sec): 5    Marker min peak height: 200   Marker baseline V to V?: Y    Marker baseline V to V points: 3
Lower marker selection: First peak > 500 RFU                Upper marker selection: Last peak > 200 RFU
Ladder size (bp)75, 200, 300, 400, 500, 700, 1000, 1500, 2000, 3000, 4000, 5000, 7000, 10000, 20000
Quantification using: Lower Marker          Final concentration (ng/uL): 0.5000          Dilution factor: 12.0
```

Sample: SampD1
Well location: D1
Created: Wednesday, October 20, 2021 6:38:33 PM

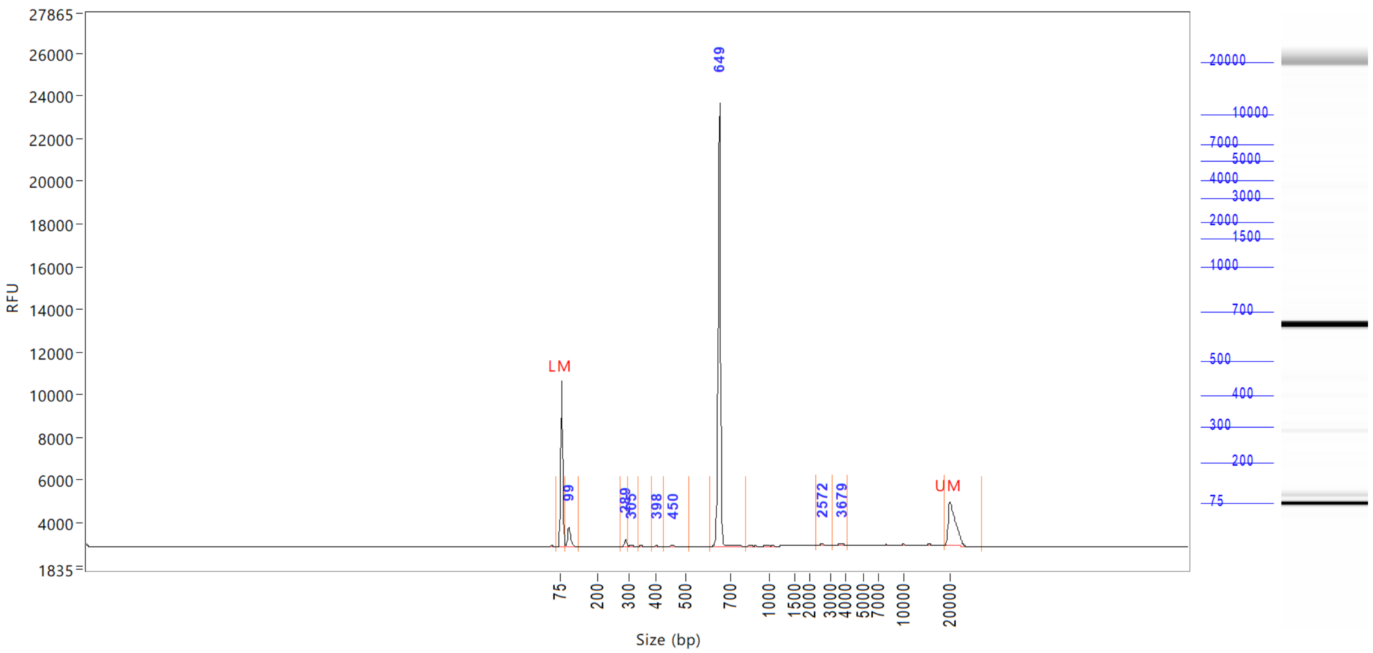

Peak	Size (bp)	Concentration (ng/uL)	From (bp)	To (bp)	Average size (bp)	CV%	RFU	Corrected peak area
1	75 (LM)	0.5000	58	87	73	4.58	7778	41.219
2	99	0.9978	87	133	99	6.72	895	6.855
3	289	0.2359	273	296	286	1.34	319	1.621
4	305	0.0701	296	331	305	2.18	64	0.482
5	398	0.0542	384	422	396	1.50	72	0.372
6	450	0.1123	422	511	454	3.39	84	0.771
7	649	13.3897	603	814	646	1.46	20801	91.984
8	2572	0.0682	2286	3144	2664	7.08	58	0.468
9	3679	0.0844	3144	4035	3606	5.29	74	0.580
10	20000 (UM)	0.2501	18781	27012	20569	4.13	2017	20.618

TIC:	15.0126	ng/uL
TIM:	53.1081	nmole/L
Total concentration:	15.5772	ng/uL

Sample peak width (sec): 5   Sample min peak height: 50   Sample baseline V to V?: Y   Sample baseline V to V points: 3
Sample filter: Binomial   Number of points for filter: 3 Sample start region (min): 0 Sample end region (min): 30
Marker peak width (sec): 5   Marker min peak height: 200   Marker baseline V to V?: Y   Marker baseline V to V points: 3
Lower marker selection: First peak > 500 RFU   Upper marker selection: Last peak > 200 RFU
Ladder size (bp)75, 200, 300, 400, 500, 700, 1000, 1500, 2000, 3000, 4000, 5000, 7000, 10000, 20000
Quantification using: Lower Marker   Final concentration (ng/uL): 0.5000   Dilution factor: 12.0

Sample: SampD2
Well location: D2
Created: Wednesday, October 20, 2021 6:38:33 PM

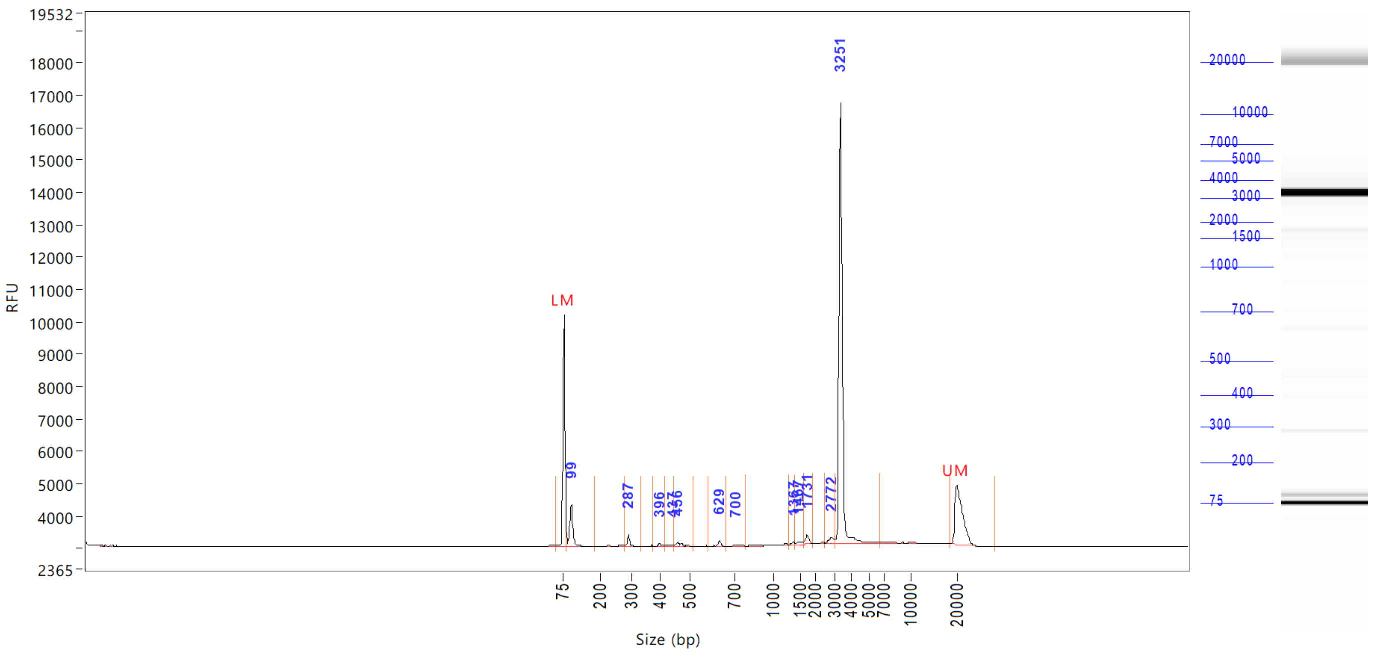

Peak	Size (bp)	Concentration (ng/uL)	From (bp)	To (bp)	Average size (bp)	CV%	RFU	Corrected peak area
1	75 (LM)	0.5000	48	87	73	4.94	7154	38.556
2	99	1.5530	87	183	100	10.71	1283	9.980
3	287	0.2879	277	329	287	2.68	340	1.850
4	396	0.0787	376	418	397	2.24	69	0.506
5	437	0.0704	418	447	435	1.61	56	0.452
6	456	0.1859	447	516	466	2.97	116	1.194
7	629	0.1545	580	662	624	2.12	178	0.993
8	700	0.0653	662	779	710	3.42	50	0.420
9	1367	0.0694	1278	1411	1359	2.37	78	0.446
10	1467	0.1448	1411	1597	1491	3.43	98	0.931
11	1731	0.2640	1597	1942	1710	3.53	286	1.697
12	2772	0.2472	2486	2971	2767	4.43	180	1.589
13	3251	11.2899	2971	6408	3312	8.02	13636	72.549
14	20000 (UM)	0.2398	18415	28221	20571	4.37	1807	18.495

TIC:	14.4109	ng/uL
TIM:	35.2158	nmole/L
Total concentration:	14.7820	ng/uL

```
Sample peak width (sec): 5    Sample min peak height: 50    Sample baseline V to V?: Y    Sample baseline V to V points: 3
Sample filter: Binomial       Number of points for filter: 3  Sample start region (min): 0  Sample end region (min): 30
Marker peak width (sec): 5    Marker min peak height: 200   Marker baseline V to V?: Y    Marker baseline V to V points: 3
Lower marker selection: First peak > 500 RFU                Upper marker selection: Last peak > 200 RFU
Ladder size (bp)75, 200, 300, 400, 500, 700, 1000, 1500, 2000, 3000, 4000, 5000, 7000, 10000, 20000
Quantification using: Lower Marker          Final concentration (ng/uL): 0.5000          Dilution factor: 12.0
```

Sample: SampD3
Well location: D3
Created: Wednesday, October 20, 2021 6:38:33 PM

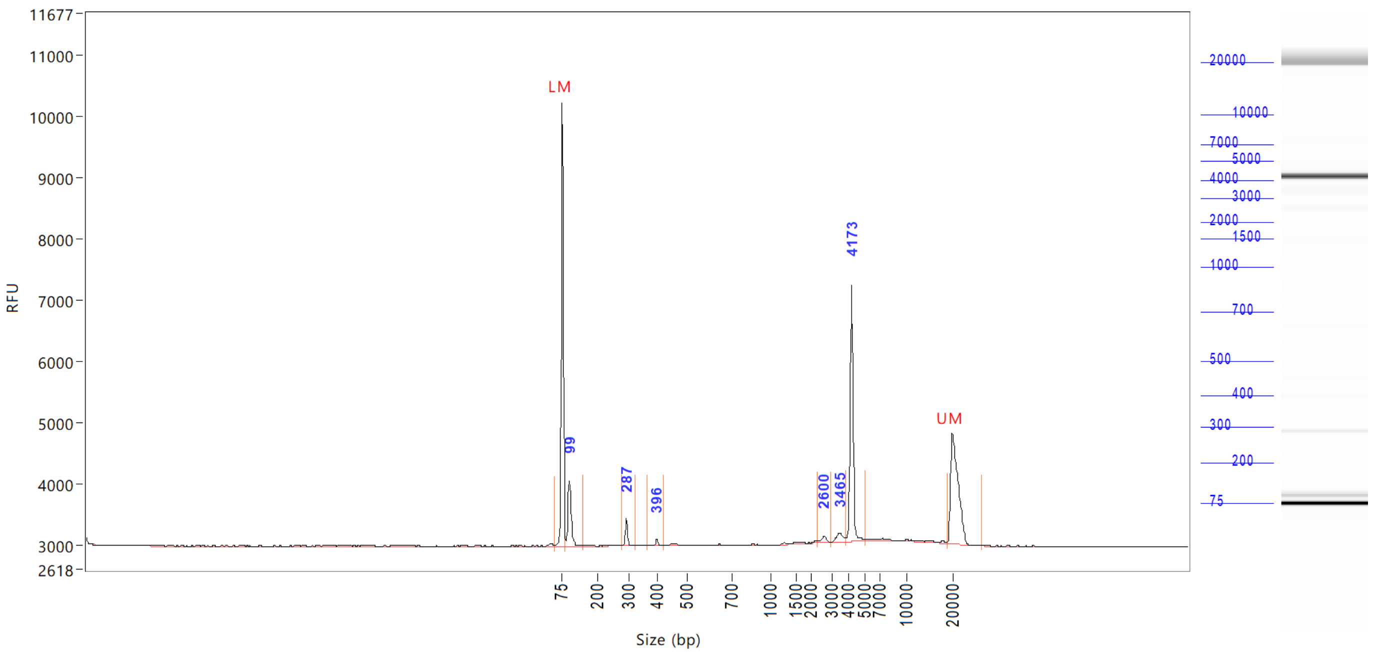

Peak	Size (bp)	Concentration (ng/uL)	From (bp)	To (bp)	Average size (bp)	CV%	RFU	Corrected peak area
1	75 (LM)	0.5000	50	87	73	4.97	7235	38.650
2	99	1.2993	87	147	99	8.08	1065	8.369
3	287	0.3470	275	321	286	1.71	446	2.235
4	396	0.0827	360	418	391	2.06	107	0.533
5	2600	0.1263	2258	2943	2545	5.09	111	0.814
6	3465	0.2365	2943	3893	3475	5.87	142	1.523
7	4173	3.0096	3893	5149	4184	3.25	4184	19.387
8	20000 (UM)	0.2395	18781	26408	20589	4.04	1812	18.510

TIC:	5.1014	ng/uL
TIM:	25.2715	nmole/L
Total concentration:	5.9215	ng/uL

```
Sample peak width (sec): 5    Sample min peak height: 50    Sample baseline V to V?: Y    Sample baseline V to V points: 3
Sample filter: Binomial       Number of points for filter: 3  Sample start region (min): 0  Sample end region (min): 30
Marker peak width (sec): 5    Marker min peak height: 200   Marker baseline V to V?: Y    Marker baseline V to V points: 3
Lower marker selection: First peak > 500 RFU                Upper marker selection: Last peak > 200 RFU
Ladder size (bp)75, 200, 300, 400, 500, 700, 1000, 1500, 2000, 3000, 4000, 5000, 7000, 10000, 20000
Quantification using: Lower Marker          Final concentration (ng/uL): 0.5000          Dilution factor: 12.0
```

Sample: SampD4
Well location: D4
Created: Wednesday, October 20, 2021 6:38:33 PM

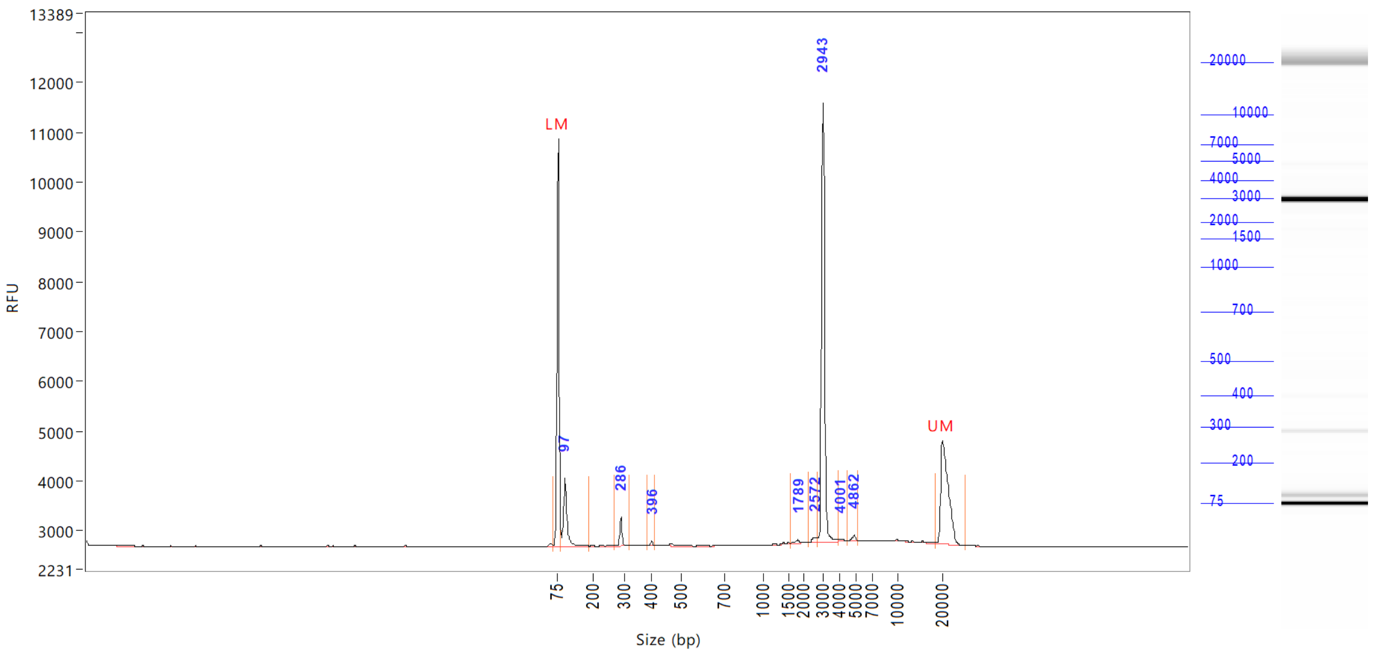

Peak	Size (bp)	Concentration (ng/uL)	From (bp)	To (bp)	Average size (bp)	CV%	RFU	Corrected peak area
1	75 (LM)	0.5000	60	87	73	4.72	8179	43.561
2	97	1.6270	87	185	101	12.90	1359	11.813
3	286	0.4213	266	317	286	2.21	580	3.058
4	396	0.0627	380	411	394	1.18	93	0.455
5	1789	0.0871	1539	2201	1775	7.35	67	0.632
6	2572	0.1264	2201	2715	2526	4.77	97	0.918
7	2943	5.7728	2715	3893	2956	3.78	8828	41.912
8	4001	0.0587	3893	4483	4067	3.23	54	0.426
9	4862	0.0881	4483	5223	4799	2.47	117	0.640
10	20000 (UM)	0.2416	18537	24957	20589	4.16	2069	21.052

TIC:	8.2441	ng/uL
TIM:	32.5702	nmole/L
Total concentration:	8.7650	ng/uL

Sample peak width (sec): 5 Sample min peak height: 50 Sample baseline V to V?: Y Sample baseline V to V points: 3
Sample filter: Binomial Number of points for filter: 3 Sample start region (min): 0 Sample end region (min): 30
Marker peak width (sec): 5 Marker min peak height: 200 Marker baseline V to V?: Y Marker baseline V to V points: 3
Lower marker selection: First peak > 500 RFU Upper marker selection: Last peak > 200 RFU
Ladder size (bp)75, 200, 300, 400, 500, 700, 1000, 1500, 2000, 3000, 4000, 5000, 7000, 10000, 20000
Quantification using: Lower Marker Final concentration (ng/uL): 0.5000 Dilution factor: 12.0

Sample: SampD5
Well location: D5
Created: Wednesday, October 20, 2021 6:38:33 PM

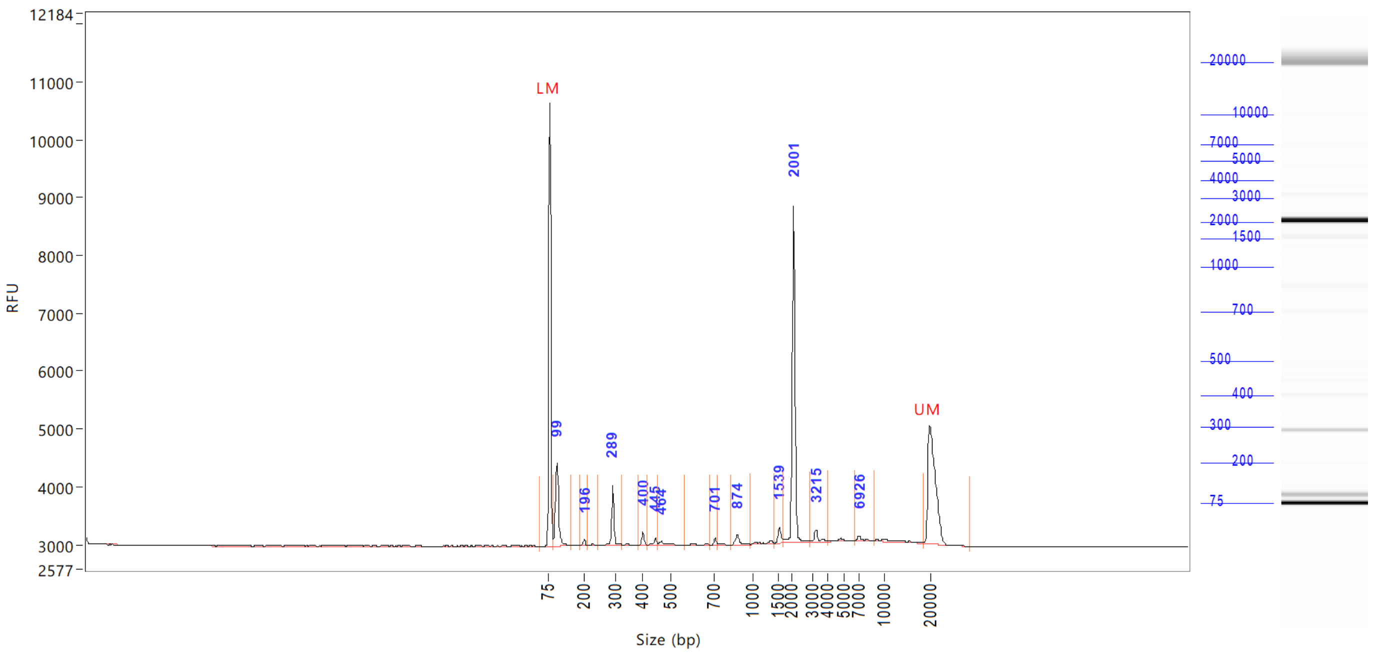

Peak	Size (bp)	Concentration (ng/uL)	From (bp)	To (bp)	Average size (bp)	CV%	RFU	Corrected peak area
1	75 (LM)	0.5000	43	87	74	4.41	7673	42.974
2	99	1.5925	87	154	100	8.32	1432	11.406
3	196	0.0720	183	206	194	1.70	104	0.516
4	289	0.7815	242	325	287	2.71	1039	5.597
5	400	0.1595	384	416	397	1.10	230	1.142
6	445	0.1097	416	452	440	1.71	120	0.785
7	464	0.1613	452	557	481	4.49	66	1.156
8	701	0.0796	680	723	700	1.06	117	0.570
9	874	0.1847	827	978	879	2.90	177	1.323
10	1539	0.2017	1433	1655	1535	3.04	282	1.445
11	2001	3.8482	1655	2914	2022	4.71	5826	27.562
12	3215	0.1959	2914	3964	3290	7.32	213	1.403
13	6926	0.1109	6334	8734	7239	8.33	94	0.794
14	20000 (UM)	0.2446	18537	28826	20577	4.27	2052	21.025

TIC:	7.4975	ng/uL
TIM:	36.8678	nmole/L
Total concentration:	8.0332	ng/uL

```
Sample peak width (sec): 5     Sample min peak height: 50     Sample baseline V to V?: Y     Sample baseline V to V points: 3
Sample filter: Binomial        Number of points for filter: 3  Sample start region (min): 0  Sample end region (min): 30
Marker peak width (sec): 5     Marker min peak height: 200    Marker baseline V to V?: Y     Marker baseline V to V points: 3
Lower marker selection: First peak > 500 RFU                  Upper marker selection: Last peak > 200 RFU
Ladder size (bp)75, 200, 300, 400, 500, 700, 1000, 1500, 2000, 3000, 4000, 5000, 7000, 10000, 20000
Quantification using: Lower Marker          Final concentration (ng/uL): 0.5000          Dilution factor: 12.0
```

Sample: SampD6
Well location: D6
Created: Wednesday, October 20, 2021 6:38:33 PM

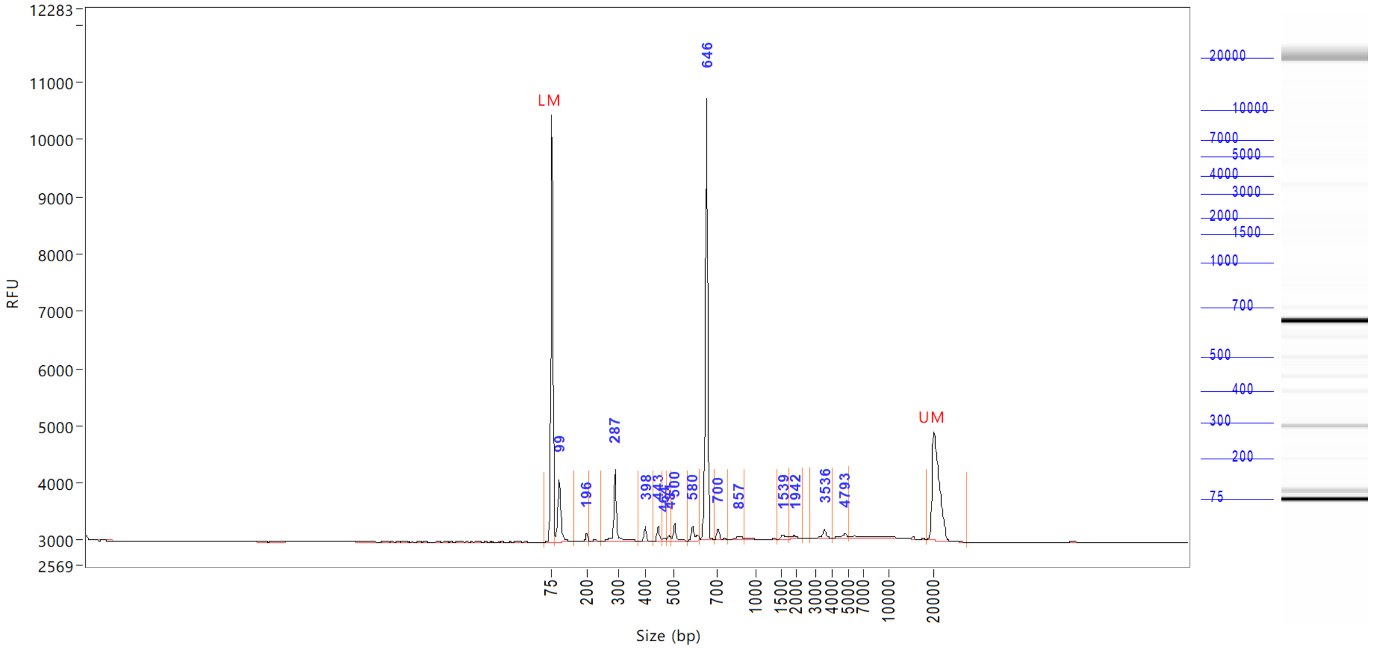

Peak	Size (bp)	Concentration (ng/uL)	From (bp)	To (bp)	Average size (bp)	CV%	RFU	Corrected peak area
1	75 (LM)	0.5000	47	87	73	4.83	7457	40.506
2	99	1.3325	87	152	100	9.01	1096	8.996
3	196	0.1616	152	205	187	6.62	144	1.091
4	287	1.1983	246	374	291	5.50	1253	8.090
5	398	0.1966	374	428	396	1.54	260	1.327
6	443	0.1990	428	454	441	1.13	256	1.343
7	464	0.0551	454	471	462	1.09	57	0.372
8	481	0.0883	471	489	480	0.99	103	0.596
9	500	0.2563	489	560	501	2.21	301	1.730
10	580	0.2813	560	618	587	2.18	246	1.899
11	646	5.1766	618	682	643	0.93	7723	34.947
12	700	0.1481	682	775	705	2.54	182	0.999
13	857	0.1170	775	909	857	3.26	59	0.790
14	1539	0.0967	1433	1770	1579	5.68	70	0.653
15	1942	0.0770	1770	2315	1936	5.93	51	0.520
16	3536	0.1410	2715	4035	3431	5.97	156	0.952
17	4793	0.0741	4035	5075	4683	4.36	69	0.500
18	20000 (UM)	0.2326	18171	27133	20503	4.19	1879	18.844

TIC:	9.5995	ng/uL
TIM:	47.8438	nmole/L
Total concentration:	9.9242	ng/uL

```
Sample peak width (sec): 5    Sample min peak height: 50    Sample baseline V to V?: Y    Sample baseline V to V points: 3
Sample filter: Binomial       Number of points for filter: 3  Sample start region (min): 0  Sample end region (min): 30
Marker peak width (sec): 5    Marker min peak height: 200   Marker baseline V to V?: Y    Marker baseline V to V points: 3
Lower marker selection: First peak > 500 RFU                Upper marker selection: Last peak > 200 RFU
Ladder size (bp)75, 200, 300, 400, 500, 700, 1000, 1500, 2000, 3000, 4000, 5000, 7000, 10000, 20000
Quantification using: Lower Marker          Final concentration (ng/uL): 0.5000          Dilution factor: 12.0
```

Sample: SampD7
Well location: D7
Created: Wednesday, October 20, 2021 6:38:33 PM

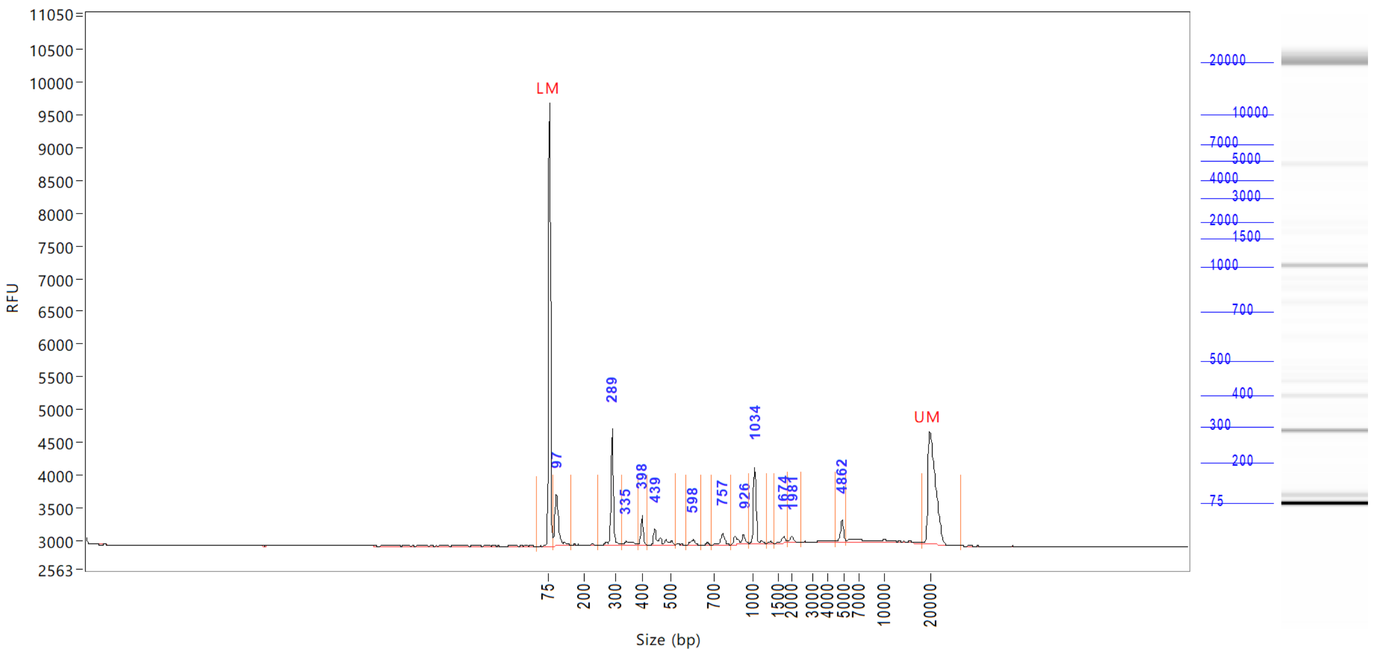

Peak	Size (bp)	Concentration (ng/uL)	From (bp)	To (bp)	Average size (bp)	CV%	RFU	Corrected peak area
1	75 (LM)	0.5000	30	87	73	4.80	6777	36.654
2	97	1.1874	87	154	101	11.10	778	7.254
3	289	1.6227	242	323	286	2.63	1775	9.913
4	335	0.1731	323	382	349	4.45	60	1.058
5	398	0.3711	382	416	395	1.15	443	2.267
6	439	0.4569	416	519	456	4.51	247	2.791
7	598	0.1269	565	639	594	1.97	86	0.775
8	757	0.2318	685	827	754	2.85	187	1.416
9	926	0.2943	827	970	890	3.95	136	1.798
10	1034	0.8840	970	1267	1024	3.37	1164	5.400
11	1674	0.0978	1411	1808	1666	4.23	93	0.597
12	1981	0.1148	1808	2429	1976	5.70	91	0.702
13	4862	0.2688	4449	5223	4821	2.38	326	1.642
14	20000 (UM)	0.2423	18415	26770	20599	4.19	1724	17.765

TIC:	5.8296	ng/uL
TIM:	35.7552	nmole/L
Total concentration:	6.4456	ng/uL

Sample peak width (sec): 5 Sample min peak height: 50 Sample baseline V to V?: Y Sample baseline V to V points: 3
Sample filter: Binomial Number of points for filter: 3 Sample start region (min): 0 Sample end region (min): 30
Marker peak width (sec): 5 Marker min peak height: 200 Marker baseline V to V?: Y Marker baseline V to V points: 3
Lower marker selection: First peak > 500 RFU Upper marker selection: Last peak > 200 RFU
Ladder size (bp)75, 200, 300, 400, 500, 700, 1000, 1500, 2000, 3000, 4000, 5000, 7000, 10000, 20000
Quantification using: Lower Marker Final concentration (ng/uL): 0.5000 Dilution factor: 12.0

Sample: SampD8
Well location: D8
Created: Wednesday, October 20, 2021 6:38:33 PM

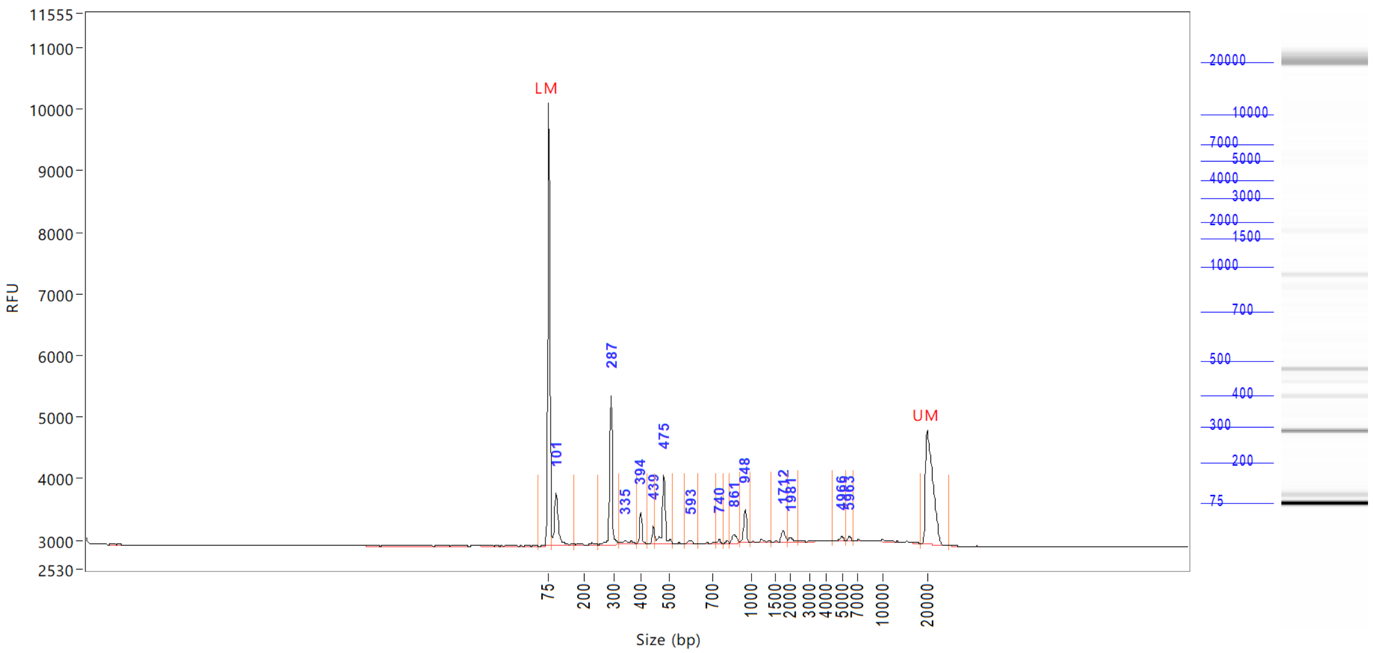

Peak	Size (bp)	Concentration (ng/uL)	From (bp)	To (bp)	Average size (bp)	CV%	RFU	Corrected peak area
1	75 (LM)	0.5000	41	87	73	4.96	7203	39.388
2	101	1.2631	87	168	104	13.69	846	8.292
3	287	2.0634	244	317	285	2.44	2412	13.546
4	335	0.1784	317	382	346	4.84	66	1.171
5	394	0.3949	382	418	394	1.23	519	2.593
6	439	0.2193	418	449	438	1.08	290	1.439
7	475	0.9949	449	511	474	1.89	1109	6.531
8	593	0.0936	567	629	591	2.07	53	0.614
9	740	0.0558	718	775	739	1.47	70	0.366
10	861	0.1908	827	905	864	1.86	147	1.253
11	948	0.4141	905	996	946	1.26	544	2.719
12	1712	0.1778	1411	1866	1704	4.68	184	1.167
13	1981	0.0822	1866	2372	2014	5.50	73	0.539
14	4966	0.0736	4380	5445	4923	3.70	75	0.483
15	5963	0.0585	5445	6408	5847	3.44	78	0.384
16	20000 (UM)	0.2376	18659	24957	20563	3.98	1853	18.715

TIC:	6.2604	ng/uL
TIM:	40.3784	nmole/L
Total concentration:	6.7112	ng/uL

```
Sample peak width (sec): 5    Sample min peak height: 50    Sample baseline V to V?: Y    Sample baseline V to V points: 3
Sample filter: Binomial       Number of points for filter: 3  Sample start region (min): 0  Sample end region (min): 30
Marker peak width (sec): 5    Marker min peak height: 200   Marker baseline V to V?: Y    Marker baseline V to V points: 3
Lower marker selection: First peak > 500 RFU                Upper marker selection: Last peak > 200 RFU
Ladder size (bp)75, 200, 300, 400, 500, 700, 1000, 1500, 2000, 3000, 4000, 5000, 7000, 10000, 20000
Quantification using: Lower Marker          Final concentration (ng/uL): 0.5000          Dilution factor: 12.0
```

Sample: SampD9
Well location: D9
Created: Wednesday, October 20, 2021 6:38:33 PM

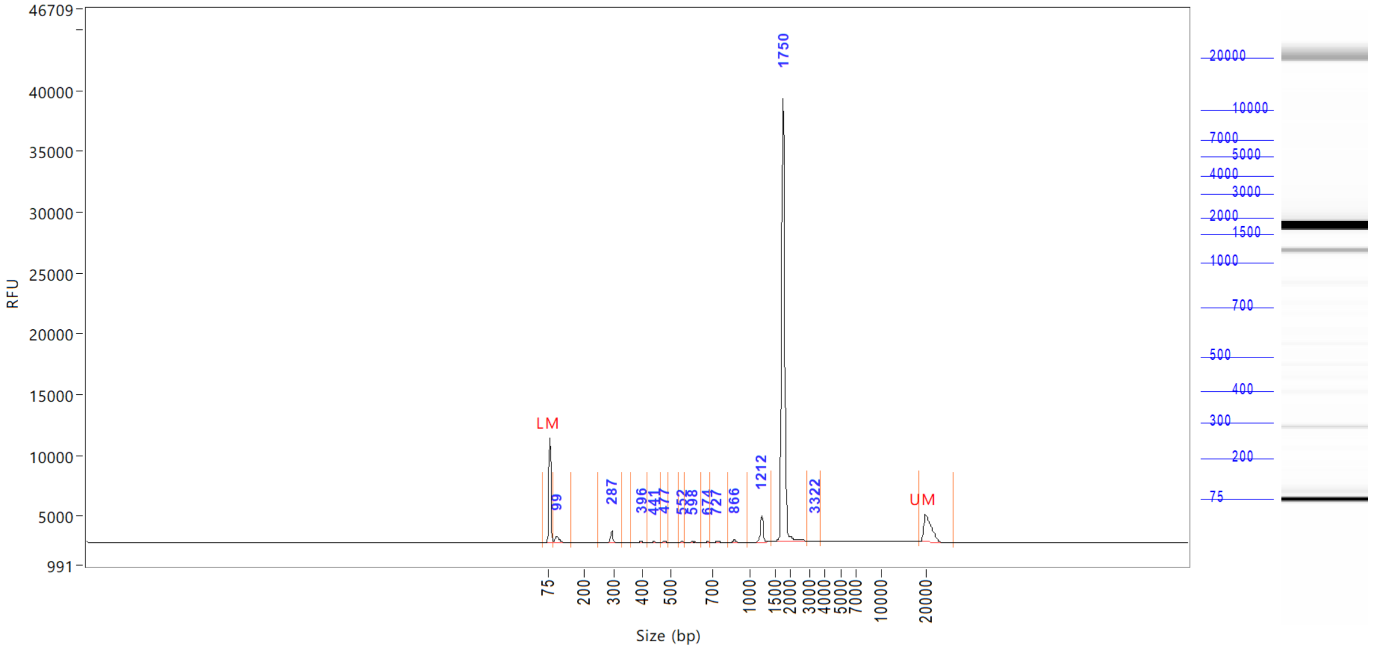

Peak	Size (bp)	Concentration (ng/uL)	From (bp)	To (bp)	Average size (bp)	CV%	RFU	Corrected peak area
1	75 (LM)	0.5000	48	87	73	5.01	8561	47.791
2	99	0.7468	87	152	102	12.20	537	5.949
3	287	0.6627	242	323	285	3.22	926	5.278
4	396	0.1377	358	416	391	2.63	171	1.097
5	441	0.1009	416	466	443	2.24	103	0.804
6	477	0.1032	466	492	476	1.01	163	0.822
7	552	0.0814	537	567	551	0.87	149	0.648
8	598	0.0827	567	641	598	2.12	63	0.659
9	674	0.0402	641	687	671	1.17	62	0.320
10	727	0.0904	687	818	726	3.03	83	0.720
11	866	0.1537	818	970	867	2.39	195	1.224
12	1212	1.3236	970	1411	1205	3.09	2126	10.543
13	1750	23.3107	1411	2886	1747	5.89	36479	185.673
14	3322	0.1154	2886	3715	3211	7.09	59	0.919
15	20000 (UM)	0.2411	18659	26529	20623	4.16	2221	23.042

TIC:	26.9495	ng/uL
TIM:	42.0574	nmole/L
Total concentration:	27.4512	ng/uL

Sample peak width (sec): 5 Sample min peak height: 50 Sample baseline V to V?: Y Sample baseline V to V points: 3
Sample filter: Binomial Number of points for filter: 3 Sample start region (min): 0 Sample end region (min): 30
Marker peak width (sec): 5 Marker min peak height: 200 Marker baseline V to V?: Y Marker baseline V to V points: 3
Lower marker selection: First peak > 500 RFU Upper marker selection: Last peak > 200 RFU
Ladder size (bp)75, 200, 300, 400, 500, 700, 1000, 1500, 2000, 3000, 4000, 5000, 7000, 10000, 20000
Quantification using: Lower Marker Final concentration (ng/uL): 0.5000 Dilution factor: 12.0

Sample: SampD10
Well location: D10
Created: Wednesday, October 20, 2021 6:38:33 PM

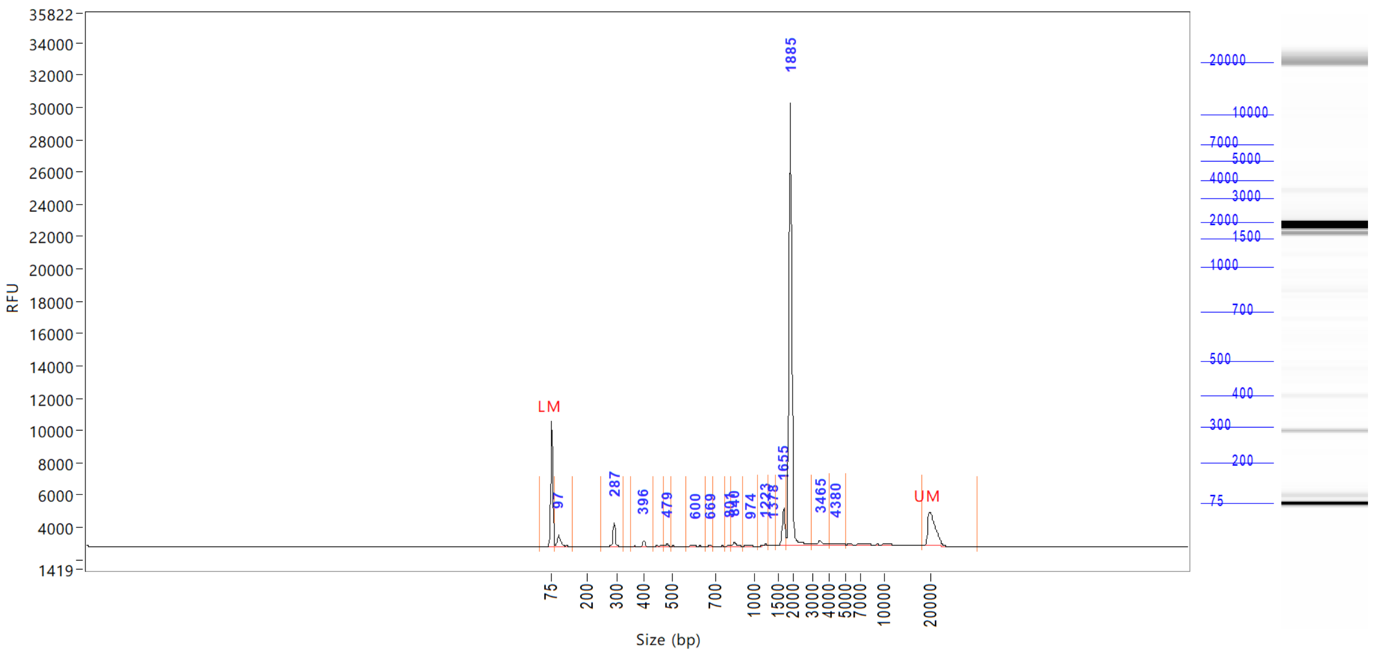

Peak	Size (bp)	Concentration (ng/uL)	From (bp)	To (bp)	Average size (bp)	CV%	RFU	Corrected peak area
1	75 (LM)	0.5000	35	87	72	5.16	7797	42.515
2	97	0.9520	87	147	100	9.29	739	6.746
3	287	1.0835	246	321	286	2.32	1438	7.678
4	396	0.2654	349	431	391	3.14	316	1.881
5	479	0.1087	468	492	477	1.24	132	0.771
6	600	0.1292	560	649	596	3.28	60	0.916
7	669	0.0592	649	687	667	0.77	99	0.420
8	801	0.0663	770	814	794	1.14	107	0.470
9	840	0.2339	814	909	848	2.18	225	1.658
10	974	0.0903	909	1068	954	2.33	70	0.640
11	1223	0.0670	1068	1300	1206	2.87	91	0.475
12	1378	0.0360	1300	1433	1371	2.26	51	0.255
13	1655	1.5640	1433	1750	1637	3.55	2351	11.082
14	1885	19.1413	1750	2971	1890	5.31	27413	135.633
15	3465	0.3088	2971	4070	3413	6.90	295	2.188
16	4380	0.0849	4070	5149	4443	5.36	53	0.601
17	20000 (UM)	0.2513	18293	30519	20546	4.52	2100	21.367

TIC:	24.1905	ng/uL
TIM:	43.1662	nmole/L
Total concentration:	24.5959	ng/uL

```
Sample peak width (sec): 5     Sample min peak height: 50     Sample baseline V to V?: Y     Sample baseline V to V points: 3
Sample filter: Binomial        Number of points for filter: 3  Sample start region (min): 0  Sample end region (min): 30
Marker peak width (sec): 5     Marker min peak height: 200    Marker baseline V to V?: Y     Marker baseline V to V points: 3
Lower marker selection: First peak > 500 RFU                  Upper marker selection: Last peak > 200 RFU
Ladder size (bp)75, 200, 300, 400, 500, 700, 1000, 1500, 2000, 3000, 4000, 5000, 7000, 10000, 20000
Quantification using: Lower Marker          Final concentration (ng/uL): 0.5000          Dilution factor: 12.0
```

Sample: SampD11
Well location: D11
Created: Wednesday, October 20, 2021 6:38:33 PM

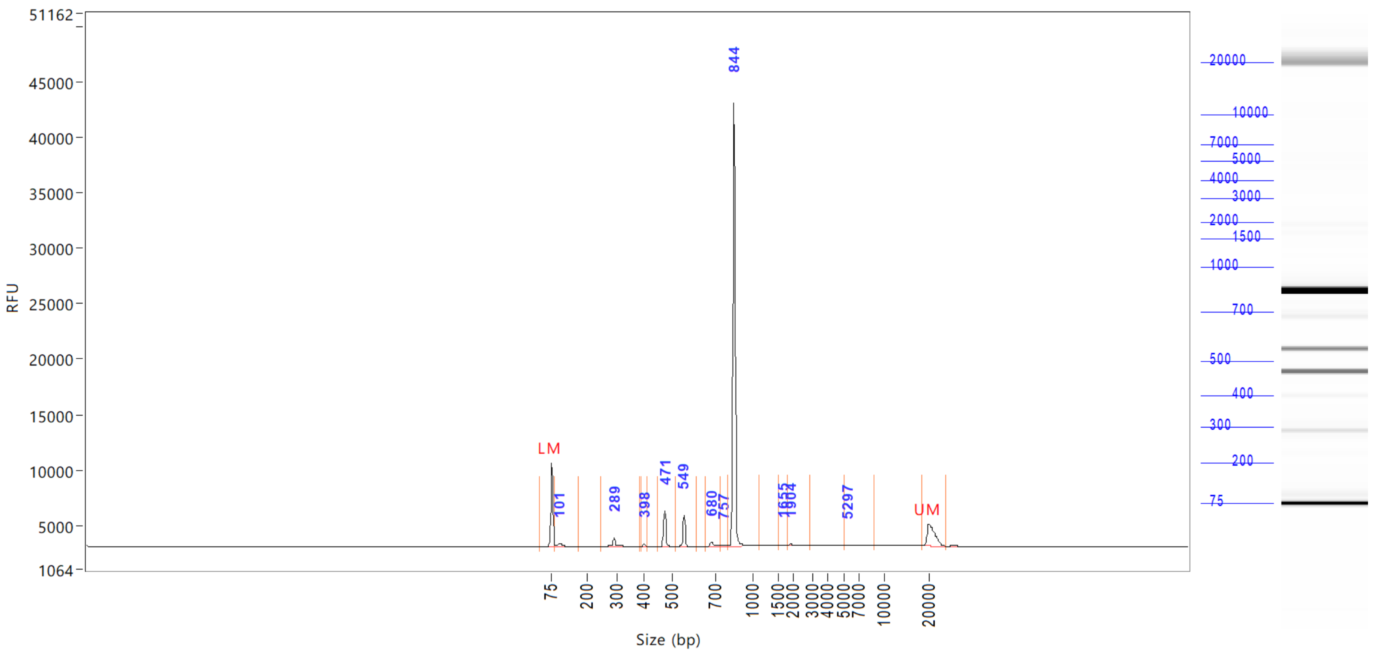

Peak	Size (bp)	Concentration (ng/uL)	From (bp)	To (bp)	Average size (bp)	CV%	RFU	Corrected peak area
1	75 (LM)	0.5000	37	87	73	5.08	7518	40.486
2	101	0.6537	87	169	107	15.24	295	4.411
3	289	0.9128	248	382	291	6.05	761	6.159
4	398	0.1586	386	411	395	0.93	218	1.070
5	471	2.2904	445	509	468	1.21	3206	15.455
6	549	1.9129	509	613	547	1.32	2824	12.908
7	680	0.4422	652	736	686	2.19	364	2.984
8	757	0.0859	736	792	760	1.97	70	0.580
9	844	26.3961	792	1112	838	1.58	40003	178.113
10	1655	0.0887	1500	1789	1643	3.99	94	0.598
11	1904	0.1812	1789	2886	2053	12.54	146	1.223
12	5297	0.1607	5000	8734	6553	17.40	53	1.084
13	20000 (UM)	0.2532	18293	23869	20546	4.17	1994	20.503

TIC:	33.2831	ng/uL
TIM:	83.0258	nmole/L
Total concentration:	33.6716	ng/uL

Sample peak width (sec): 5 Sample min peak height: 50 Sample baseline V to V?: Y Sample baseline V to V points: 3
Sample filter: Binomial Number of points for filter: 3 Sample start region (min): 0 Sample end region (min): 30
Marker peak width (sec): 5 Marker min peak height: 200 Marker baseline V to V?: Y Marker baseline V to V points: 3
Lower marker selection: First peak > 500 RFU Upper marker selection: Last peak > 200 RFU
Ladder size (bp)75, 200, 300, 400, 500, 700, 1000, 1500, 2000, 3000, 4000, 5000, 7000, 10000, 20000
Quantification using: Lower Marker Final concentration (ng/uL): 0.5000 Dilution factor: 12.0

Sample: SampD12
Well location: D12
Created: Wednesday, October 20, 2021 6:38:33 PM

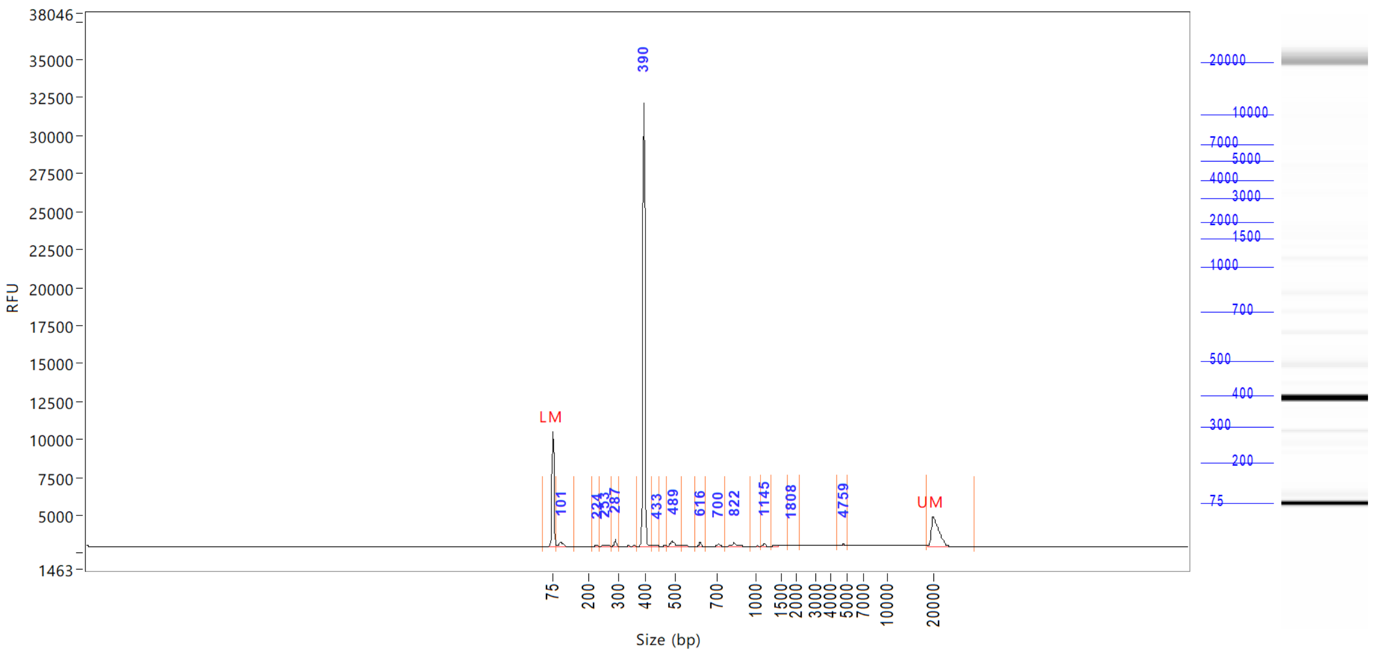

Peak	Size (bp)	Concentration (ng/uL)	From (bp)	To (bp)	Average size (bp)	CV%	RFU	Corrected peak area
1	75 (LM)	0.5000	39	87	73	4.99	7541	40.751
2	101	0.4725	87	152	102	9.79	253	3.209
3	224	0.0577	212	235	223	1.71	73	0.392
4	253	0.2049	235	275	252	3.38	113	1.391
5	287	0.3017	275	296	285	1.22	420	2.049
6	390	20.3737	366	420	388	1.02	29219	138.374
7	433	0.0730	420	447	431	1.12	83	0.496
8	489	0.4690	471	527	492	2.05	335	3.186
9	616	0.1568	590	641	613	0.92	234	1.065
10	700	0.1196	641	753	699	2.00	142	0.812
11	822	0.2488	753	957	827	3.59	223	1.689
12	1145	0.1219	1079	1289	1142	2.20	197	0.828
13	1808	0.0603	1693	2201	1853	6.30	51	0.409
14	4759	0.0635	4345	5001	4682	2.99	69	0.431
15	20000 (UM)	0.2415	18659	29309	20679	4.47	1877	19.685

TIC:	22.7233	ng/uL
TIM:	100.8000	nmole/L
Total concentration:	23.2845	ng/uL

Sample peak width (sec): 5 Sample min peak height: 50 Sample baseline V to V?: Y Sample baseline V to V points: 3
Sample filter: Binomial Number of points for filter: 3 Sample start region (min): 0 Sample end region (min): 30
Marker peak width (sec): 5 Marker min peak height: 200 Marker baseline V to V?: Y Marker baseline V to V points: 3
Lower marker selection: First peak > 500 RFU Upper marker selection: Last peak > 200 RFU
Ladder size (bp)75, 200, 300, 400, 500, 700, 1000, 1500, 2000, 3000, 4000, 5000, 7000, 10000, 20000
Quantification using: Lower Marker Final concentration (ng/uL): 0.5000 Dilution factor: 12.0

Sample: SampE1
Well location: E1
Created: Wednesday, October 20, 2021 6:38:33 PM

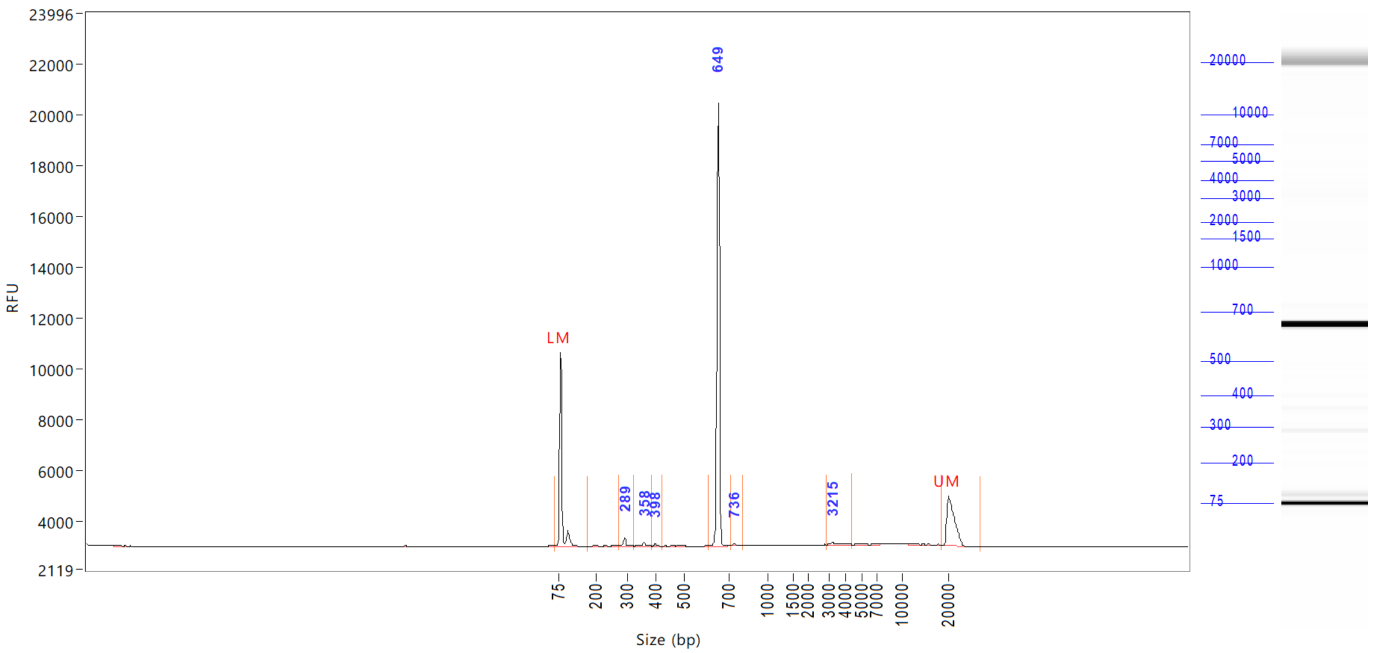

Peak	Size (bp)	Concentration (ng/uL)	From (bp)	To (bp)	Average size (bp)	CV%	RFU	Corrected peak area
1	75 (LM)	0.5000	58	166	77	12.98	7617	45.693
2	289	0.2519	271	321	289	2.81	324	1.919
3	358	0.1464	321	386	353	2.99	149	1.115
4	398	0.0547	386	420	396	1.36	80	0.416
5	649	10.0679	606	710	647	0.97	17466	76.672
6	736	0.0730	710	805	735	2.27	88	0.556
7	3215	0.1395	2886	4449	3465	10.69	82	1.062
8	20000 (UM)	0.2209	18415	27012	20546	4.16	1947	20.187

TIC:	10.7333	ng/uL
TIM:	28.1814	nmole/L
Total concentration:	11.2038	ng/uL

```
Sample peak width (sec): 5    Sample min peak height: 50    Sample baseline V to V?: Y    Sample baseline V to V points: 3
Sample filter: Binomial       Number of points for filter: 3  Sample start region (min): 0  Sample end region (min): 30
Marker peak width (sec): 5    Marker min peak height: 200   Marker baseline V to V?: Y    Marker baseline V to V points: 3
Lower marker selection: First peak > 500 RFU                Upper marker selection: Last peak > 200 RFU
Ladder size (bp)75, 200, 300, 400, 500, 700, 1000, 1500, 2000, 3000, 4000, 5000, 7000, 10000, 20000
Quantification using: Lower Marker          Final concentration (ng/uL): 0.5000          Dilution factor: 12.0
```

Sample: SampE2
Well location: E2
Created: Wednesday, October 20, 2021 6:38:33 PM

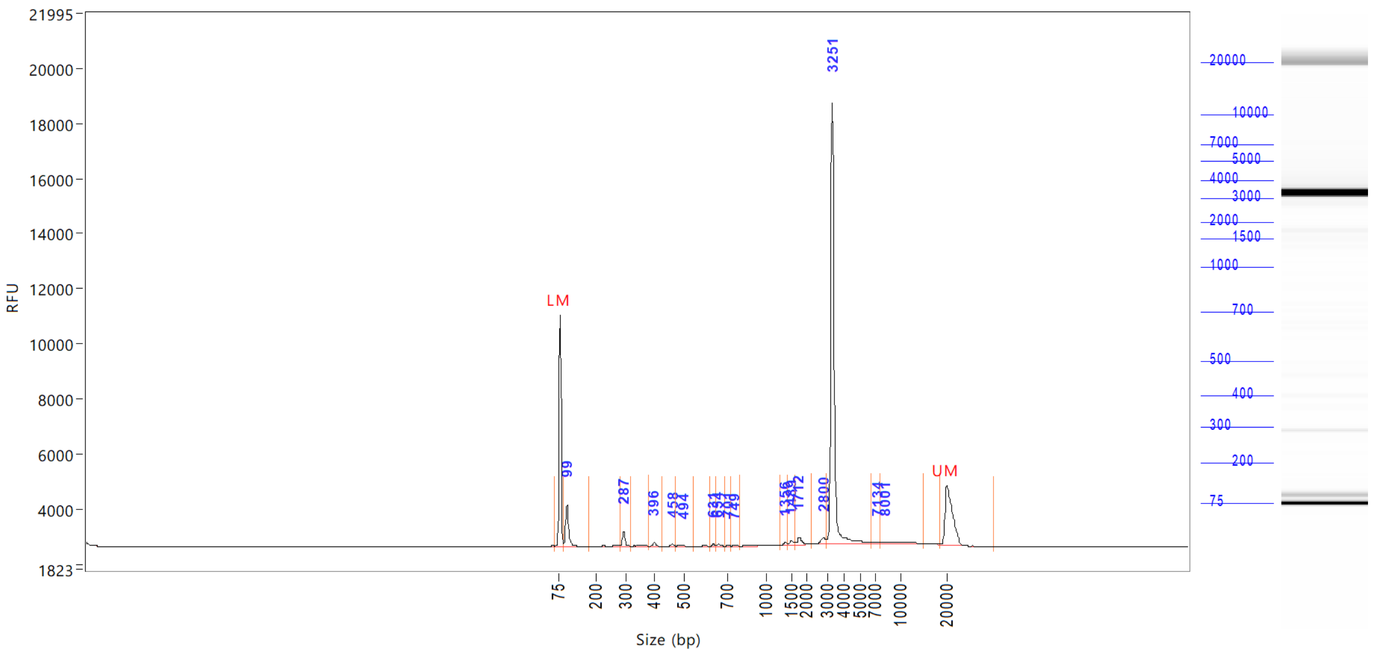

Peak	Size (bp)	Concentration (ng/uL)	From (bp)	To (bp)	Average size (bp)	CV%	RFU	Corrected peak area
1	75 (LM)	0.5000	58	87	74	4.67	8416	45.046
2	99	1.6203	87	175	100	10.33	1537	12.164
3	287	0.3779	277	313	287	1.66	544	2.837
4	396	0.0985	378	428	396	1.47	134	0.739
5	458	0.0730	428	471	455	1.52	95	0.548
6	494	0.0571	471	544	492	2.61	51	0.429
7	631	0.0474	616	644	630	0.88	82	0.356
8	654	0.0627	644	685	658	1.25	68	0.471
9	701	0.0322	685	723	700	0.92	57	0.242
10	749	0.0407	723	792	747	1.70	53	0.306
11	1356	0.0815	1267	1411	1351	2.43	108	0.612
12	1489	0.1607	1411	1597	1494	3.31	136	1.206
13	1712	0.2593	1597	2201	1736	5.33	272	1.947
14	2800	0.2872	2201	3000	2772	5.63	229	2.156
15	3251	11.9485	3000	6482	3354	10.72	16037	89.705
16	7134	0.0997	6482	7601	7047	4.40	77	0.748
17	8001	0.3130	7601	15000	10204	19.11	64	2.350
18	20000 (UM)	0.2442	18537	30156	20584	4.55	2163	21.998

TIC:	15.5596	ng/uL
TIM:	36.7132	nmole/L
Total concentration:	15.8222	ng/uL

```
Sample peak width (sec): 5    Sample min peak height: 50    Sample baseline V to V?: Y    Sample baseline V to V points: 3
Sample filter: Binomial       Number of points for filter: 3  Sample start region (min): 0  Sample end region (min): 30
Marker peak width (sec): 5    Marker min peak height: 200   Marker baseline V to V?: Y    Marker baseline V to V points: 3
Lower marker selection: First peak > 500 RFU                Upper marker selection: Last peak > 200 RFU
Ladder size (bp)75, 200, 300, 400, 500, 700, 1000, 1500, 2000, 3000, 4000, 5000, 7000, 10000, 20000
Quantification using: Lower Marker          Final concentration (ng/uL): 0.5000          Dilution factor: 12.0
```

Sample: SampE3
Well location: E3
Created: Wednesday, October 20, 2021 6:38:33 PM

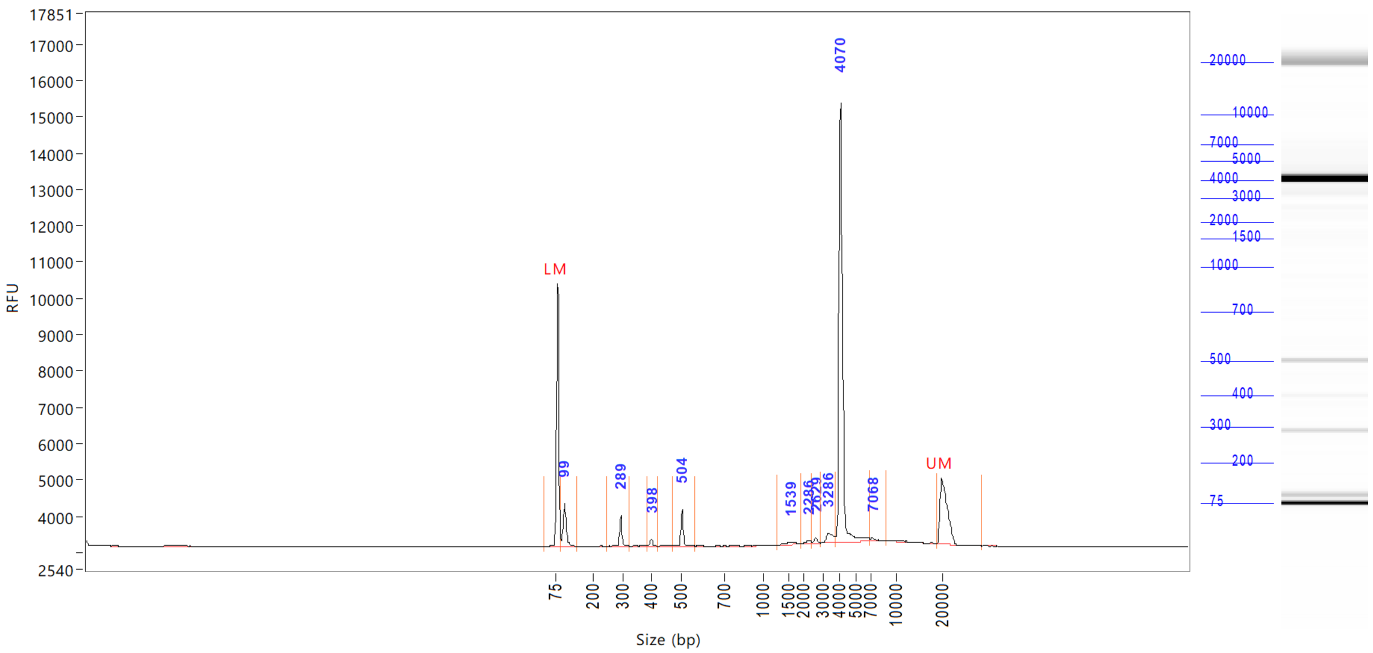

Peak	Size (bp)	Concentration (ng/uL)	From (bp)	To (bp)	Average size (bp)	CV%	RFU	Corrected peak area
1	75 (LM)	0.5000	33	87	74	5.43	7234	39.878
2	99	1.4347	87	145	99	7.34	1187	9.535
3	289	0.7118	242	317	285	2.82	855	4.731
4	398	0.1525	384	420	396	1.29	198	1.013
5	504	0.7344	473	562	502	1.93	1012	4.881
6	1539	0.1781	1278	1962	1543	8.63	68	1.184
7	2286	0.1011	1962	2429	2212	5.57	70	0.672
8	2629	0.1206	2429	2886	2592	3.23	144	0.801
9	3286	0.4521	2886	3786	3408	6.14	269	3.005
10	4070	10.5526	3786	6852	4119	8.10	12121	70.135
11	7068	0.1045	6852	8867	7295	5.35	82	0.694
12	20000 (UM)	0.2273	18903	28705	20538	4.28	1794	18.128

TIC:	14.5423	ng/uL
TIM:	35.7464	nmole/L
Total concentration:	15.0114	ng/uL

```
Sample peak width (sec): 5    Sample min peak height: 50    Sample baseline V to V?: Y    Sample baseline V to V points: 3
Sample filter: Binomial       Number of points for filter: 3  Sample start region (min): 0  Sample end region (min): 30
Marker peak width (sec): 5    Marker min peak height: 200   Marker baseline V to V?: Y    Marker baseline V to V points: 3
Lower marker selection: First peak > 500 RFU                Upper marker selection: Last peak > 200 RFU
Ladder size (bp)75, 200, 300, 400, 500, 700, 1000, 1500, 2000, 3000, 4000, 5000, 7000, 10000, 20000
Quantification using: Lower Marker          Final concentration (ng/uL): 0.5000         Dilution factor: 12.0
```

Sample: SampE4
Well location: E4
Created: Wednesday, October 20, 2021 6:38:33 PM

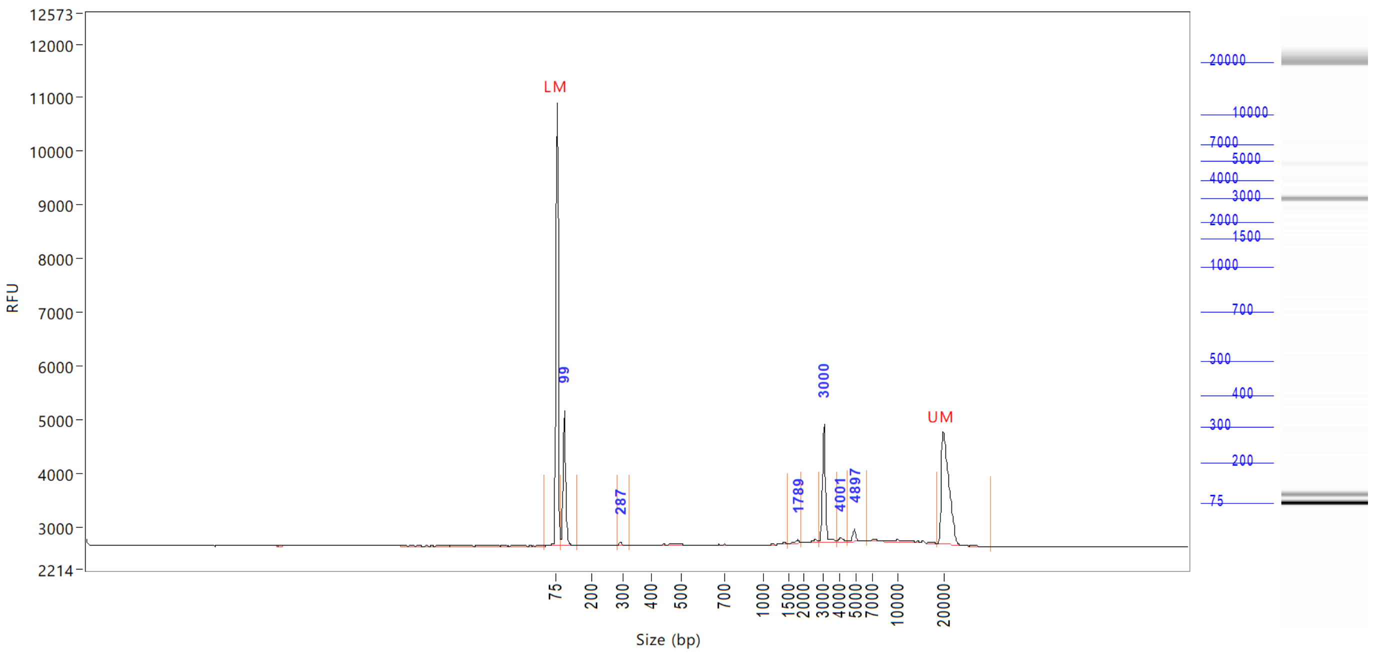

Peak	Size (bp)	Concentration (ng/uL)	From (bp)	To (bp)	Average size (bp)	CV%	RFU	Corrected peak area
1	75 (LM)	0.5000	35	87	74	4.98	8270	45.596
2	99	2.2448	87	147	99	6.01	2531	17.059
3	287	0.0687	277	321	290	3.19	80	0.522
4	1789	0.0546	1467	1942	1739	5.89	58	0.415
5	3000	1.3347	2743	3822	3001	4.22	2185	10.143
6	4001	0.0666	3822	4414	4057	3.23	72	0.506
7	4897	0.1749	4414	6408	4912	6.32	231	1.329
8	20000 (UM)	0.2338	18537	30519	20609	4.47	2087	21.324

TIC:	3.9444	ng/uL
TIM:	38.4919	nmole/L
Total concentration:	4.5779	ng/uL

Sample peak width (sec): 5 Sample min peak height: 50 Sample baseline V to V?: Y Sample baseline V to V points: 3
Sample filter: Binomial Number of points for filter: 3 Sample start region (min): 0 Sample end region (min): 30
Marker peak width (sec): 5 Marker min peak height: 200 Marker baseline V to V?: Y Marker baseline V to V points: 3
Lower marker selection: First peak > 500 RFU Upper marker selection: Last peak > 200 RFU
Ladder size (bp)75, 200, 300, 400, 500, 700, 1000, 1500, 2000, 3000, 4000, 5000, 7000, 10000, 20000
Quantification using: Lower Marker Final concentration (ng/uL): 0.5000 Dilution factor: 12.0

Sample: SampE5
Well location: E5
Created: Wednesday, October 20, 2021 6:38:33 PM

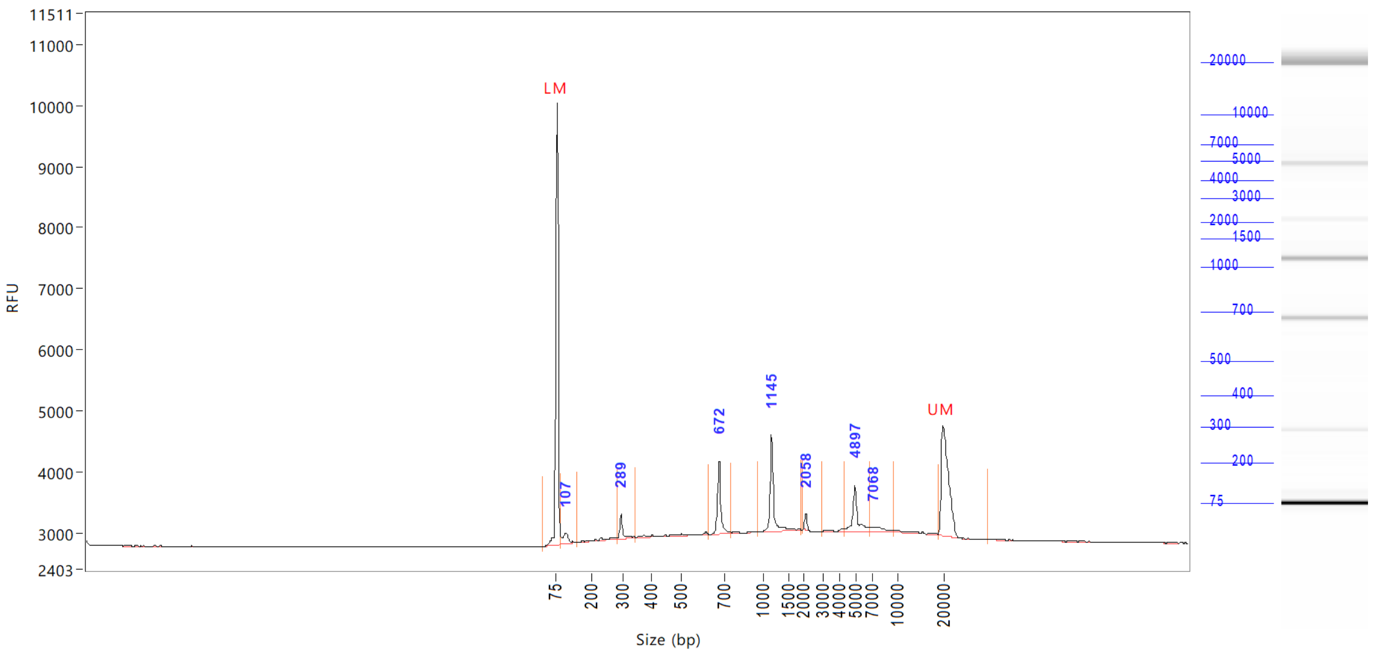

Peak	Size (bp)	Concentration (ng/uL)	From (bp)	To (bp)	Average size (bp)	CV%	RFU	Corrected peak area
1	75 (LM)	0.5000	30	91	74	6.85	7252	41.585
2	107	0.3575	91	143	105	9.38	172	2.478
3	289	0.4005	277	343	294	4.40	408	2.776
4	672	1.0457	626	749	674	1.97	1198	7.248
5	1145	1.4035	952	1942	1182	10.31	1576	9.728
6	2058	0.1522	1981	2886	2072	4.62	250	1.055
7	4897	0.8490	4242	6630	5048	9.72	764	5.884
8	7068	0.2459	6630	9533	7785	9.76	75	1.705
9	20000 (UM)	0.2137	18903	29914	20543	4.39	1809	17.771

TIC:	4.4544	ng/uL
TIM:	12.7903	nmole/L
Total concentration:	5.1036	ng/uL

Sample peak width (sec): 5 Sample min peak height: 50 Sample baseline V to V?: Y Sample baseline V to V points: 3
Sample filter: Binomial Number of points for filter: 3 Sample start region (min): 0 Sample end region (min): 30
Marker peak width (sec): 5 Marker min peak height: 200 Marker baseline V to V?: Y Marker baseline V to V points: 3
Lower marker selection: First peak > 500 RFU Upper marker selection: Last peak > 200 RFU
Ladder size (bp)75, 200, 300, 400, 500, 700, 1000, 1500, 2000, 3000, 4000, 5000, 7000, 10000, 20000
Quantification using: Lower Marker Final concentration (ng/uL): 0.5000 Dilution factor: 12.0

Sample: SampE6
Well location: E6
Created: Wednesday, October 20, 2021 6:38:33 PM

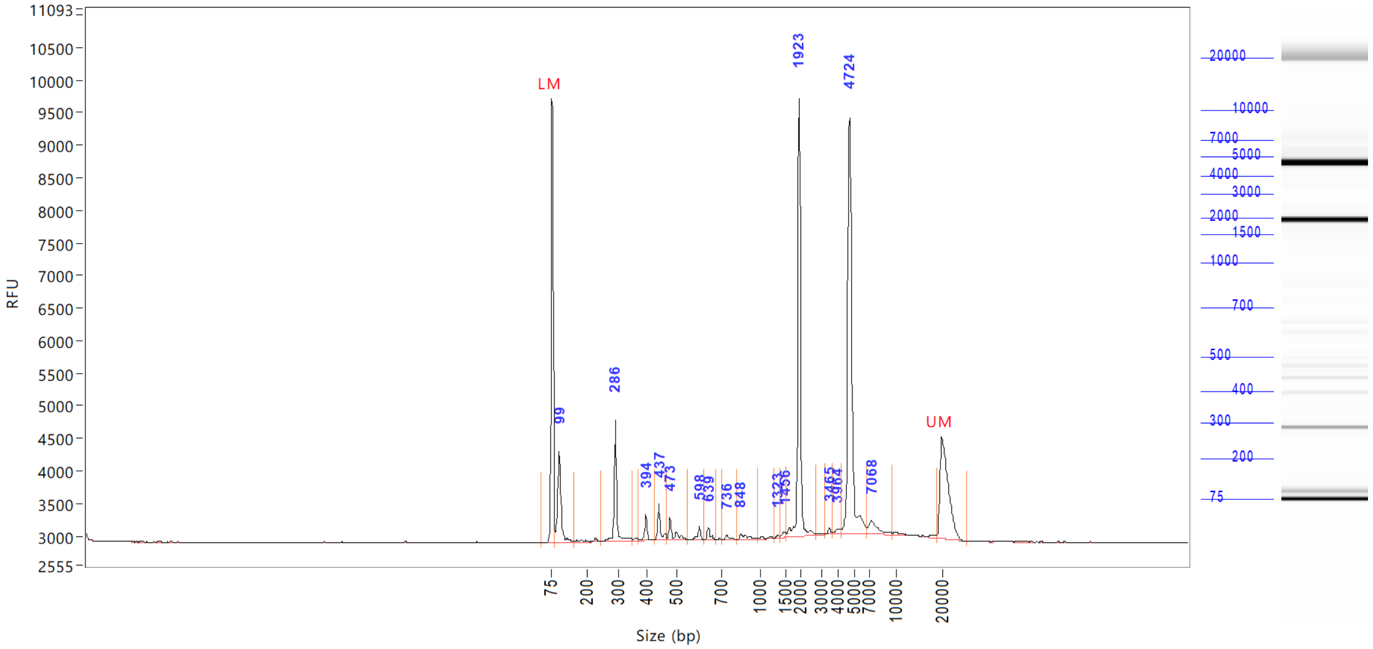

Peak	Size (bp)	Concentration (ng/uL)	From (bp)	To (bp)	Average size (bp)	CV%	RFU	Corrected peak area
1	75 (LM)	0.5000	41	87	74	4.55	6809	37.761
2	99	1.9021	87	148	100	9.35	1392	11.971
3	286	1.7284	241	347	286	4.94	1870	10.878
4	394	0.3237	370	422	390	1.52	391	2.037
5	437	0.4996	422	466	440	2.19	549	3.144
6	473	0.4551	466	547	486	3.75	355	2.864
7	598	0.2070	547	623	591	2.19	201	1.303
8	639	0.1726	623	674	641	1.43	193	1.086
9	736	0.0869	705	814	745	2.84	77	0.547
10	848	0.1744	814	974	880	4.12	78	1.097
11	1323	0.0384	1256	1378	1325	2.07	52	0.242
12	1456	0.0958	1378	1501	1449	2.35	90	0.603
13	1923	5.0881	1501	2715	1910	5.75	6731	32.022
14	3465	0.0680	3251	3679	3472	2.62	98	0.428
15	3964	0.1114	3679	4173	3967	3.27	81	0.701
16	4724	6.3584	4173	6556	4758	6.50	6383	40.016
17	7068	0.4535	6556	9600	7534	9.76	202	2.854
18	20000 (UM)	0.2310	18781	25320	20686	4.63	1565	17.444

TIC:	17.7634	ng/uL
TIM:	54.4069	nmole/L
Total concentration:	18.1796	ng/uL

```
Sample peak width (sec): 5     Sample min peak height: 50    Sample baseline V to V?: Y    Sample baseline V to V points: 3
Sample filter: Binomial        Number of points for filter: 3  Sample start region (min): 0  Sample end region (min): 30
Marker peak width (sec): 5     Marker min peak height: 200   Marker baseline V to V?: Y    Marker baseline V to V points: 3
Lower marker selection: First peak > 500 RFU                 Upper marker selection: Last peak > 200 RFU
Ladder size (bp)75, 200, 300, 400, 500, 700, 1000, 1500, 2000, 3000, 4000, 5000, 7000, 10000, 20000
Quantification using: Lower Marker          Final concentration (ng/uL): 0.5000          Dilution factor: 12.0
```

Sample: SampE7
Well location: E7
Created: Wednesday, October 20, 2021 6:38:33 PM

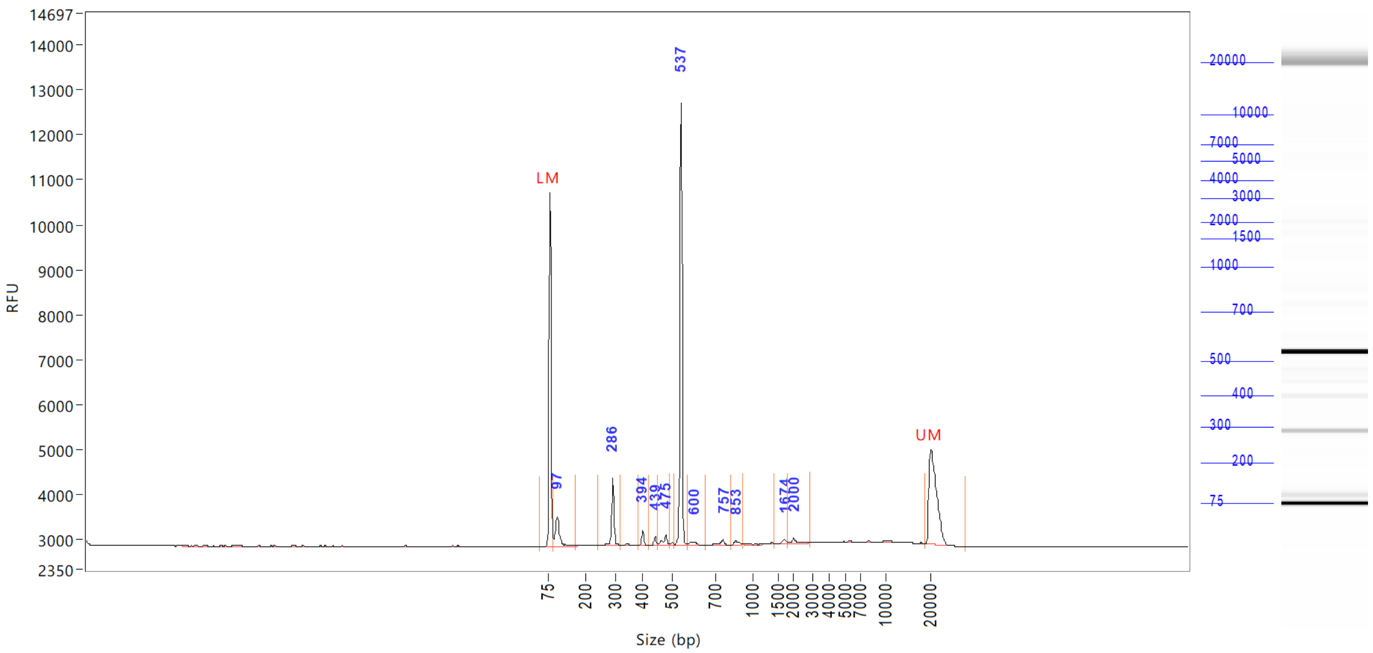

Peak	Size (bp)	Concentration (ng/uL)	From (bp)	To (bp)	Average size (bp)	CV%	RFU	Corrected peak area
1	75 (LM)	0.5000	39	87	73	4.62	7857	42.208
2	97	0.9338	87	166	101	11.56	666	6.569
3	286	1.1916	241	313	285	2.35	1502	8.382
4	394	0.2463	382	416	394	1.08	348	1.732
5	439	0.1294	416	450	437	1.12	180	0.910
6	475	0.2503	450	489	469	1.86	213	1.761
7	537	6.1932	509	567	535	0.98	9828	43.567
8	600	0.1214	567	654	594	2.54	70	0.854
9	757	0.1463	654	818	732	3.93	109	1.029
10	853	0.0979	818	909	863	2.08	74	0.688
11	1674	0.0989	1422	1827	1636	5.56	92	0.696
12	2000	0.1515	1827	2857	2097	9.87	117	1.065
13	20000 (UM)	0.2626	18659	27738	20565	4.30	2113	22.164

TIC:	9.5605	ng/uL
TIM:	44.5722	nmole/L
Total concentration:	10.0563	ng/uL

```
Sample peak width (sec): 5    Sample min peak height: 50    Sample baseline V to V?: Y    Sample baseline V to V points: 3
Sample filter: Binomial       Number of points for filter: 3  Sample start region (min): 0  Sample end region (min): 30
Marker peak width (sec): 5    Marker min peak height: 200   Marker baseline V to V?: Y    Marker baseline V to V points: 3
Lower marker selection: First peak > 500 RFU                Upper marker selection: Last peak > 200 RFU
Ladder size (bp)75, 200, 300, 400, 500, 700, 1000, 1500, 2000, 3000, 4000, 5000, 7000, 10000, 20000
Quantification using: Lower Marker          Final concentration (ng/uL): 0.5000          Dilution factor: 12.0
```

Sample: SampE8
Well location: E8
Created: Wednesday, October 20, 2021 6:38:33 PM

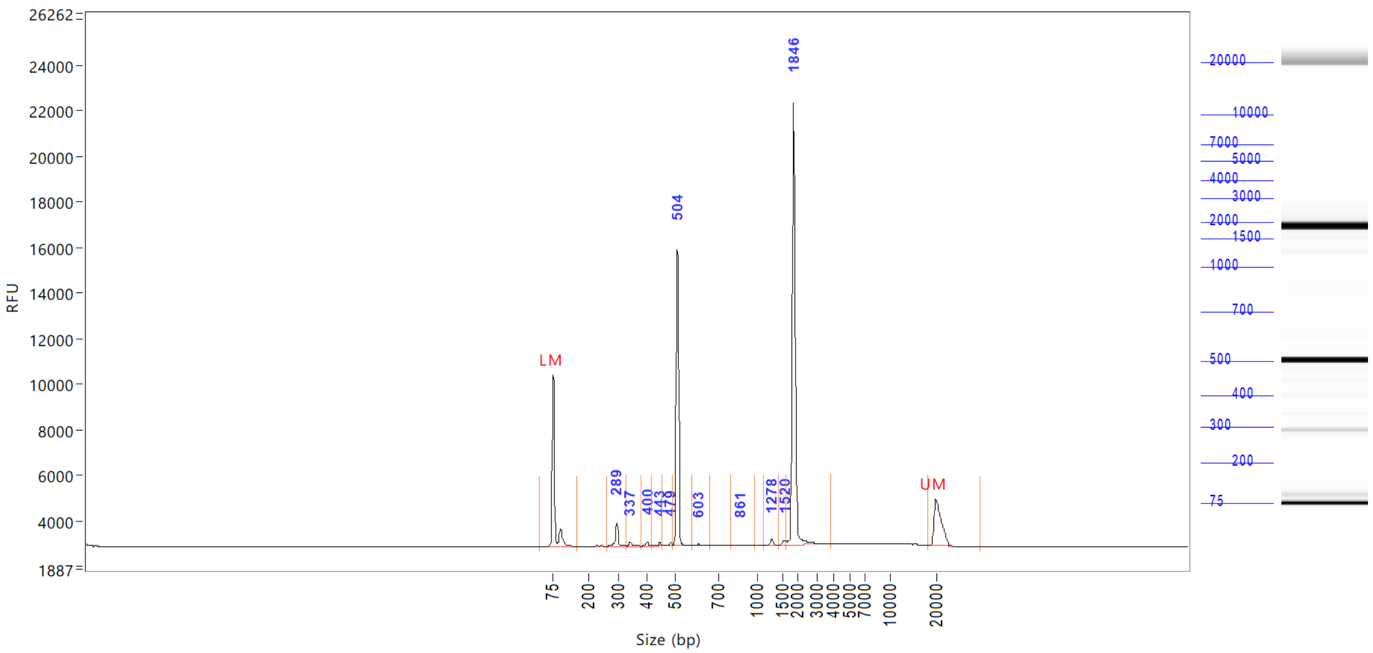

Peak	Size (bp)	Concentration (ng/uL)	From (bp)	To (bp)	Average size (bp)	CV%	RFU	Corrected peak area
1	75 (LM)	0.5000	28	158	79	15.07	7554	49.753
2	289	0.7885	257	325	287	2.78	1053	6.538
3	337	0.1337	325	378	339	2.52	189	1.109
4	400	0.1299	378	418	399	1.45	190	1.077
5	443	0.1129	418	452	440	1.55	163	0.936
6	479	0.1375	452	489	473	2.05	147	1.140
7	504	7.3430	489	570	503	1.13	12996	60.889
8	603	0.0952	570	657	602	2.63	82	0.789
9	861	0.0945	792	983	861	4.26	51	0.784
10	1278	0.1811	1123	1411	1266	3.46	254	1.502
11	1520	0.1685	1411	1616	1526	3.20	215	1.397
12	1846	11.7101	1616	3822	1865	7.66	19408	97.102
13	20000 (UM)	0.2061	18171	29914	20578	4.54	2048	20.508

TIC:	20.8948	ng/uL
TIM:	41.8198	nmole/L
Total concentration:	21.1484	ng/uL

```
Sample peak width (sec): 5    Sample min peak height: 50    Sample baseline V to V?: Y    Sample baseline V to V points: 3
Sample filter: Binomial       Number of points for filter: 3  Sample start region (min): 0  Sample end region (min): 30
Marker peak width (sec): 5    Marker min peak height: 200   Marker baseline V to V?: Y    Marker baseline V to V points: 3
Lower marker selection: First peak > 500 RFU                Upper marker selection: Last peak > 200 RFU
Ladder size (bp)75, 200, 300, 400, 500, 700, 1000, 1500, 2000, 3000, 4000, 5000, 7000, 10000, 20000
Quantification using: Lower Marker          Final concentration (ng/uL): 0.5000          Dilution factor: 12.0
```

Sample: SampE9
Well location: E9
Created: Wednesday, October 20, 2021 6:38:33 PM

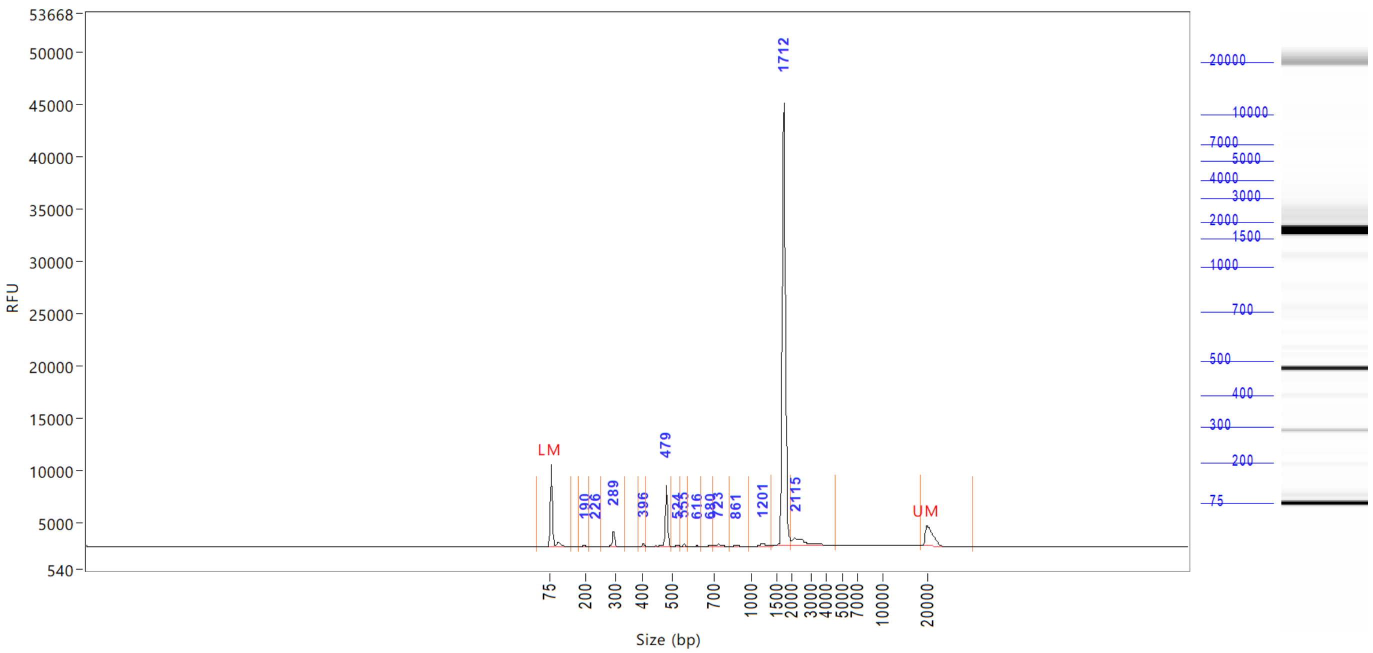

Peak	Size (bp)	Concentration (ng/uL)	From (bp)	To (bp)	Average size (bp)	CV%	RFU	Corrected peak area
1	75 (LM)	0.5000	28	143	77	13.72	7878	47.498
2	190	0.1561	171	206	189	2.88	194	1.235
3	226	0.0545	206	250	227	3.31	59	0.432
4	289	1.0155	250	327	287	2.74	1456	8.039
5	396	0.1518	384	407	396	0.99	261	1.202
6	479	3.5287	407	498	476	1.88	5892	27.935
7	524	0.1124	498	539	519	2.01	111	0.890
8	555	0.1244	539	570	552	1.00	203	0.985
9	616	0.0597	570	639	606	2.22	83	0.473
10	680	0.1215	639	690	677	1.30	132	0.962
11	723	0.3196	690	822	728	3.82	196	2.530
12	861	0.1088	822	974	872	3.17	90	0.861
13	1201	0.2879	974	1389	1193	4.82	270	2.279
14	1712	28.5737	1389	2000	1708	3.66	42399	226.200
15	2115	1.8432	2000	4552	2506	18.74	689	14.592
16	20000 (UM)	0.2083	18415	30156	20591	4.47	1961	19.792

TIC:	36.4580	ng/uL
TIM:	51.6611	nmole/L
Total concentration:	36.6406	ng/uL

Sample peak width (sec): 5 Sample min peak height: 50 Sample baseline V to V?: Y Sample baseline V to V points: 3
Sample filter: Binomial Number of points for filter: 3 Sample start region (min): 0 Sample end region (min): 30
Marker peak width (sec): 5 Marker min peak height: 200 Marker baseline V to V?: Y Marker baseline V to V points: 3
Lower marker selection: First peak > 500 RFU Upper marker selection: Last peak > 200 RFU
Ladder size (bp)75, 200, 300, 400, 500, 700, 1000, 1500, 2000, 3000, 4000, 5000, 7000, 10000, 20000
Quantification using: Lower Marker Final concentration (ng/uL): 0.5000 Dilution factor: 12.0

Sample: SampE10
Well location: E10
Created: Wednesday, October 20, 2021 6:38:33 PM

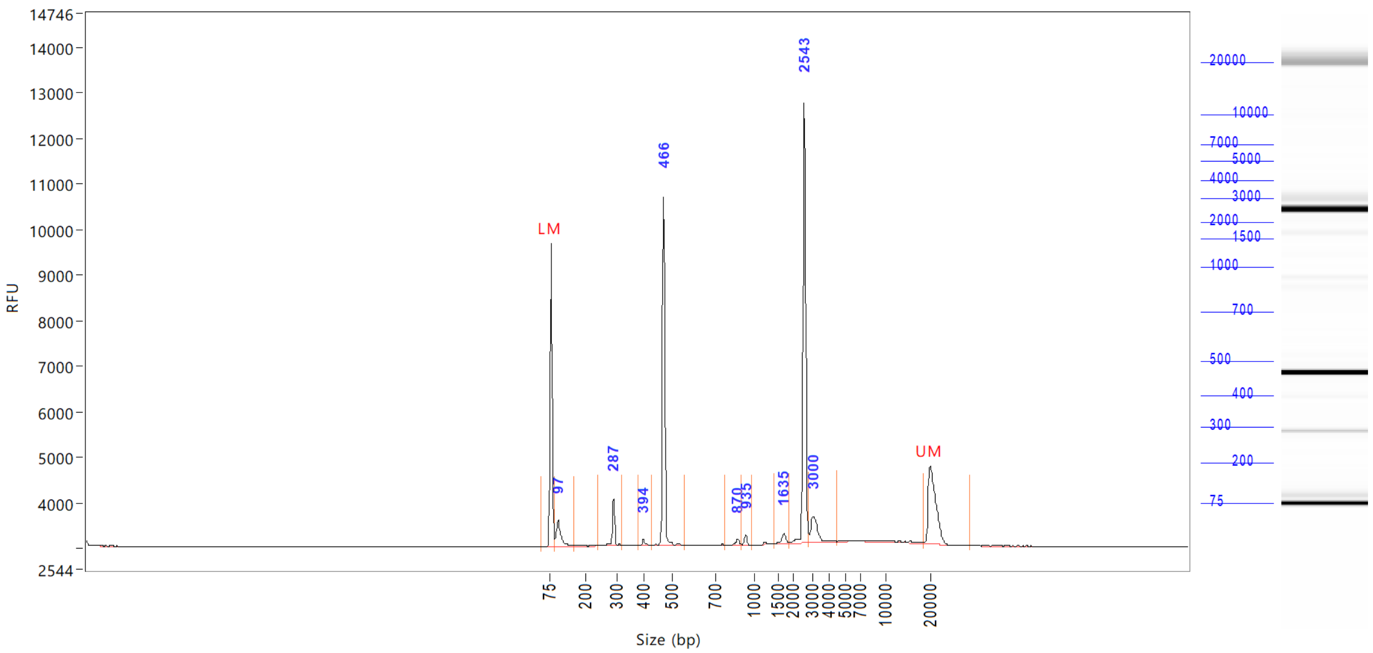

Peak	Size (bp)	Concentration (ng/uL)	From (bp)	To (bp)	Average size (bp)	CV%	RFU	Corrected peak area
1	75 (LM)	0.5000	41	87	73	5.19	6658	36.537
2	97	1.0962	87	154	103	12.35	583	6.675
3	287	0.9699	239	317	285	2.82	1041	5.906
4	394	0.1608	378	428	397	2.30	143	0.979
5	466	6.0453	428	552	465	1.82	7641	36.813
6	870	0.1859	766	905	853	3.48	131	1.132
7	935	0.1719	905	987	929	1.24	217	1.047
8	1635	0.2613	1389	1866	1633	5.12	240	1.591
9	2543	7.3229	1866	2800	2504	3.88	9674	44.593
10	3000	1.0523	2800	4483	3112	9.09	576	6.408
11	20000 (UM)	0.2456	18537	28826	20550	4.52	1705	17.945

TIC:	17.2665	ng/uL
TIM:	51.4306	nmole/L
Total concentration:	17.8688	ng/uL

Sample peak width (sec): 5 Sample min peak height: 50 Sample baseline V to V?: Y Sample baseline V to V points: 3
Sample filter: Binomial Number of points for filter: 3 Sample start region (min): 0 Sample end region (min): 30
Marker peak width (sec): 5 Marker min peak height: 200 Marker baseline V to V?: Y Marker baseline V to V points: 3
Lower marker selection: First peak > 500 RFU Upper marker selection: Last peak > 200 RFU
Ladder size (bp)75, 200, 300, 400, 500, 700, 1000, 1500, 2000, 3000, 4000, 5000, 7000, 10000, 20000
Quantification using: Lower Marker Final concentration (ng/uL): 0.5000 Dilution factor: 12.0

Sample: SampE11
Well location: E11
Created: Wednesday, October 20, 2021 6:38:33 PM

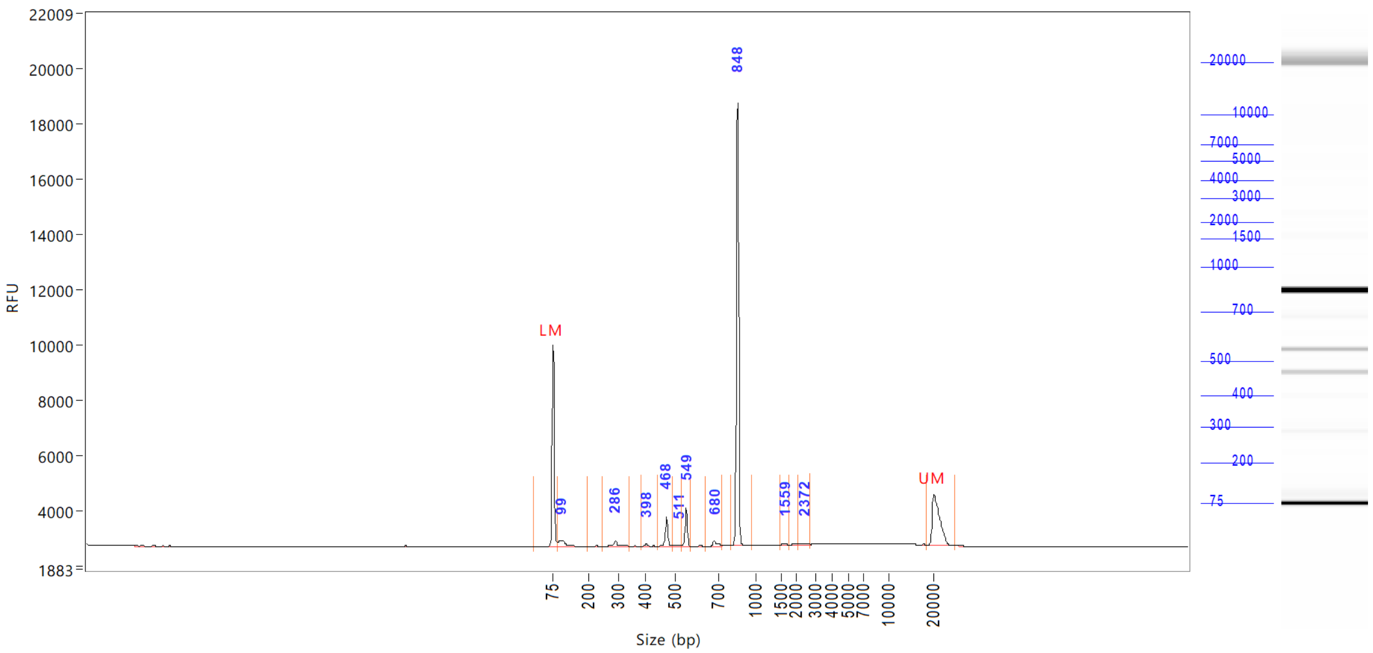

Peak	Size (bp)	Concentration (ng/uL)	From (bp)	To (bp)	Average size (bp)	CV%	RFU	Corrected peak area
1	75 (LM)	0.5000	11	89	74	5.01	7311	39.898
2	99	0.6163	89	198	111	18.78	220	4.098
3	286	0.3027	244	337	287	5.01	225	2.013
4	398	0.0744	384	439	400	2.69	72	0.495
5	468	0.8253	439	489	466	1.10	1081	5.488
6	511	0.0688	489	527	508	1.97	53	0.457
7	549	0.9395	527	572	545	0.95	1406	6.247
8	680	0.2097	636	727	683	1.78	182	1.395
9	848	10.3026	792	965	842	1.20	16060	68.509
10	1559	0.0696	1456	1731	1556	3.87	73	0.463
11	2372	0.0561	2058	2686	2339	5.20	54	0.373
12	20000 (UM)	0.2393	18537	24715	20564	4.18	1845	19.099

TIC:	13.4649	ng/uL
TIM:	37.8909	nmole/L
Total concentration:	13.8893	ng/uL

```
Sample peak width (sec): 5    Sample min peak height: 50    Sample baseline V to V?: Y    Sample baseline V to V points: 3
Sample filter: Binomial       Number of points for filter: 3  Sample start region (min): 0  Sample end region (min): 30
Marker peak width (sec): 5    Marker min peak height: 200   Marker baseline V to V?: Y    Marker baseline V to V points: 3
Lower marker selection: First peak > 500 RFU                Upper marker selection: Last peak > 200 RFU
Ladder size (bp)75, 200, 300, 400, 500, 700, 1000, 1500, 2000, 3000, 4000, 5000, 7000, 10000, 20000
Quantification using: Lower Marker          Final concentration (ng/uL): 0.5000          Dilution factor: 12.0
```

Sample: SampE12
Well location: E12
Created: Wednesday, October 20, 2021 6:38:33 PM

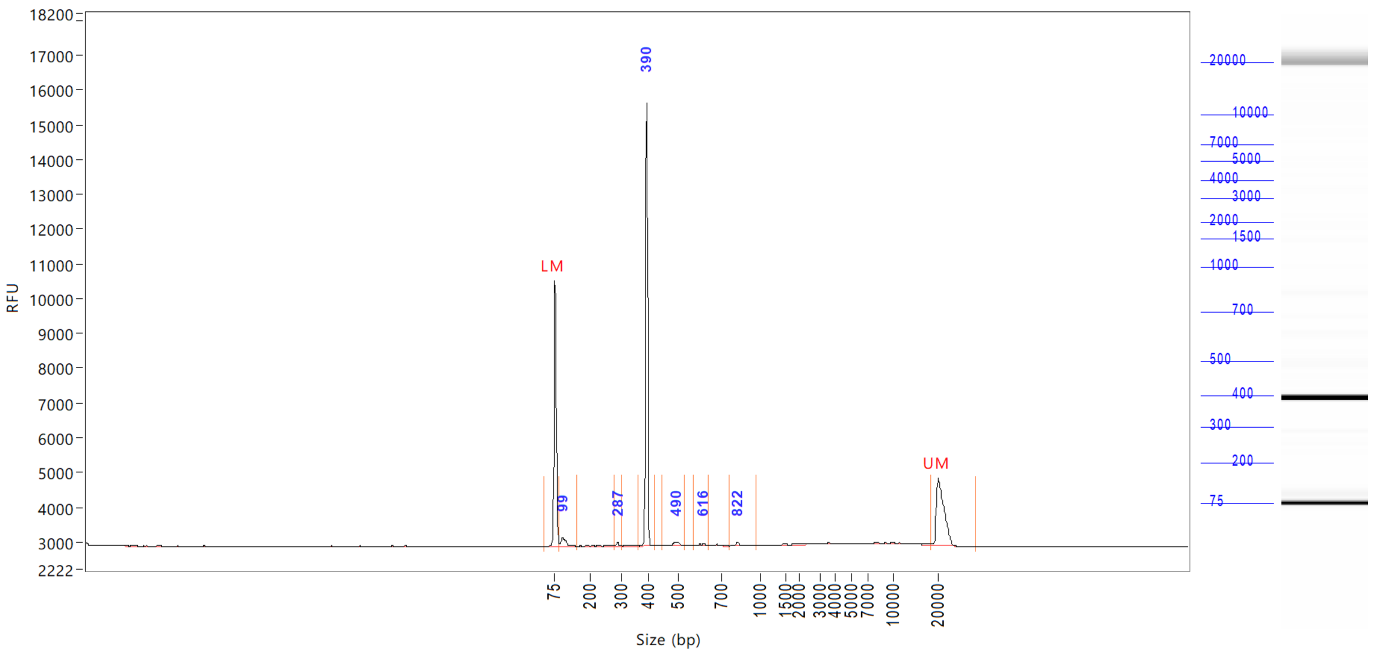

Peak	Size (bp)	Concentration (ng/uL)	From (bp)	To (bp)	Average size (bp)	CV%	RFU	Corrected peak area
1	75 (LM)	0.5000	39	89	74	4.95	7635	42.038
2	99	0.4695	89	154	104	9.62	251	3.289
3	287	0.0806	277	303	285	1.39	116	0.565
4	390	8.1589	364	420	389	1.00	12756	57.164
5	490	0.2246	443	527	486	3.66	98	1.573
6	616	0.0802	570	639	606	1.76	81	0.562
7	822	0.1148	762	970	833	4.50	101	0.804
8	20000 (UM)	0.2395	18415	28221	20589	4.25	1933	20.133

TIC:	9.1285	ng/uL
TIM:	43.5910	nmole/L
Total concentration:	9.7579	ng/uL

Sample peak width (sec): 5 Sample min peak height: 50 Sample baseline V to V?: Y Sample baseline V to V points: 3
Sample filter: Binomial Number of points for filter: 3 Sample start region (min): 0 Sample end region (min): 30
Marker peak width (sec): 5 Marker min peak height: 200 Marker baseline V to V?: Y Marker baseline V to V points: 3
Lower marker selection: First peak > 500 RFU Upper marker selection: Last peak > 200 RFU
Ladder size (bp)75, 200, 300, 400, 500, 700, 1000, 1500, 2000, 3000, 4000, 5000, 7000, 10000, 20000
Quantification using: Lower Marker Final concentration (ng/uL): 0.5000 Dilution factor: 12.0

Sample: SampF1
Well location: F1
Created: Wednesday, October 20, 2021 6:38:33 PM

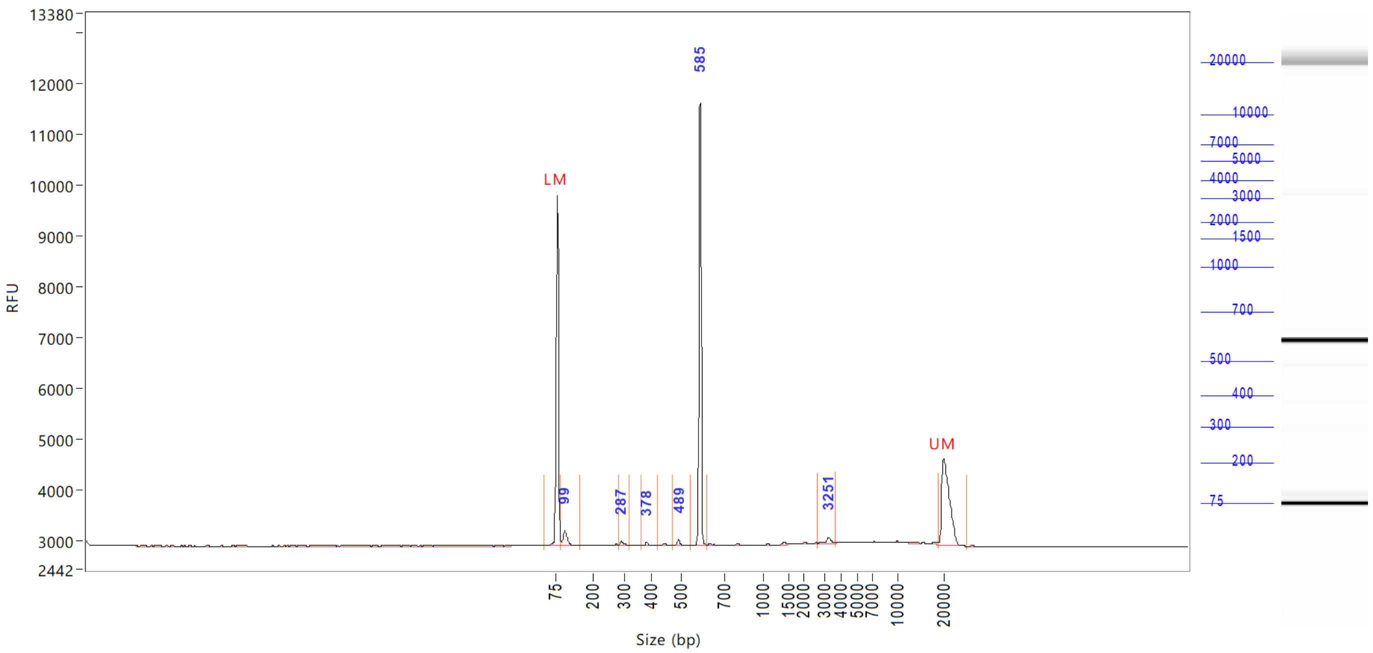

Peak	Size (bp)	Concentration (ng/uL)	From (bp)	To (bp)	Average size (bp)	CV%	RFU	Corrected peak area
1	75 (LM)	0.5000	33	89	73	5.76	6903	37.466
2	99	0.5326	89	154	103	10.76	313	3.326
3	287	0.1386	280	319	292	2.81	92	0.865
4	378	0.0772	362	416	381	2.66	68	0.482
5	489	0.1250	468	542	490	2.19	116	0.780
6	585	6.1807	542	616	581	0.89	8725	38.594
7	3251	0.1306	2686	3715	3220	5.25	119	0.816
8	20000 (UM)	0.2339	18781	25199	20544	4.08	1703	17.526

TIC:	7.1847	ng/uL
TIM:	27.5982	nmole/L
Total concentration:	7.9200	ng/uL

```
Sample peak width (sec): 5    Sample min peak height: 50    Sample baseline V to V?: Y    Sample baseline V to V points: 3
Sample filter: Binomial       Number of points for filter: 3  Sample start region (min): 0  Sample end region (min): 30
Marker peak width (sec): 5    Marker min peak height: 200   Marker baseline V to V?: Y    Marker baseline V to V points: 3
Lower marker selection: First peak > 500 RFU                Upper marker selection: Last peak > 200 RFU
Ladder size (bp)75, 200, 300, 400, 500, 700, 1000, 1500, 2000, 3000, 4000, 5000, 7000, 10000, 20000
Quantification using: Lower Marker          Final concentration (ng/uL): 0.5000          Dilution factor: 12.0
```

Sample: SampF2
Well location: F2
Created: Wednesday, October 20, 2021 6:38:33 PM

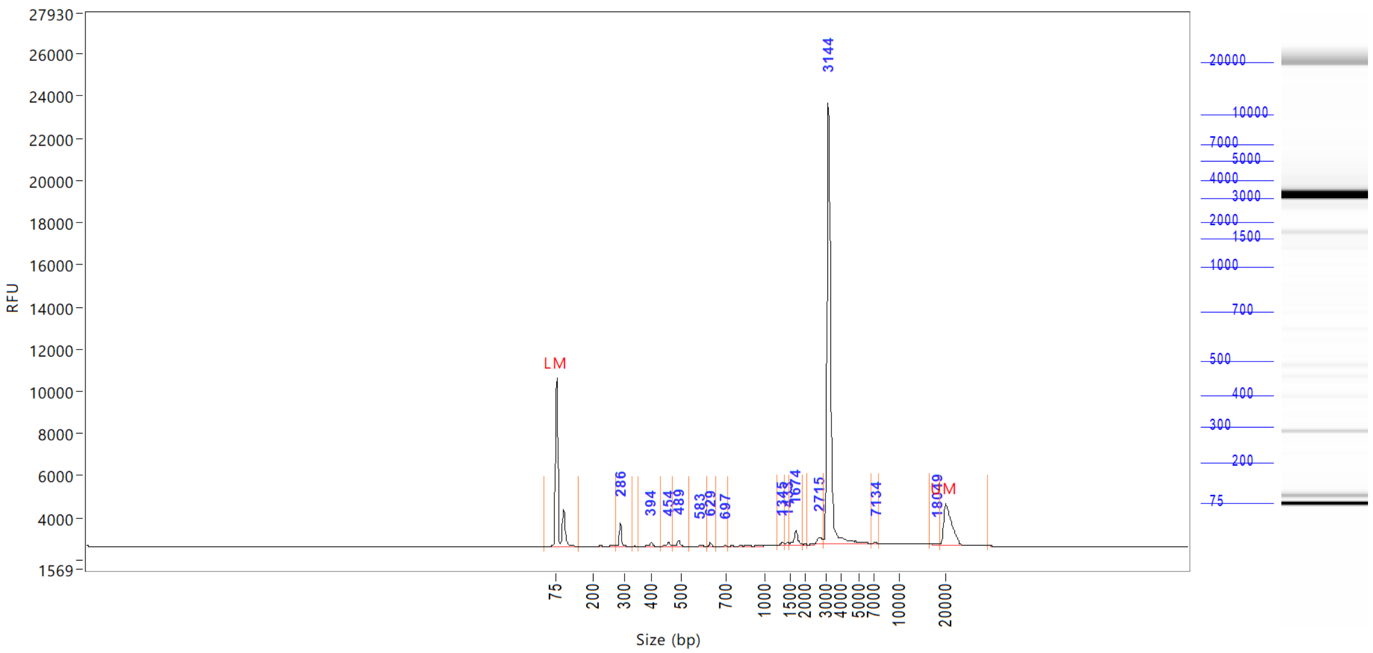

Peak	Size (bp)	Concentration (ng/uL)	From (bp)	To (bp)	Average size (bp)	CV%	RFU	Corrected peak area
1	75 (LM)	0.5000	33	150	79	15.90	7991	57.189
2	286	0.5791	275	325	286	2.06	1100	5.519
3	394	0.1444	349	430	390	3.29	201	1.377
4	454	0.1506	430	468	450	1.79	217	1.436
5	489	0.1966	468	537	487	1.89	284	1.874
6	583	0.0515	537	611	581	2.39	54	0.491
7	629	0.0822	611	659	627	1.06	172	0.783
8	697	0.0345	659	718	692	1.52	69	0.329
9	1345	0.0745	1256	1389	1333	2.11	148	0.710
10	1433	0.0796	1389	1489	1441	2.02	134	0.759
11	1674	0.4144	1489	1923	1654	4.69	669	3.950
12	2715	0.3005	2087	2886	2682	4.98	318	2.864
13	3144	13.1989	2886	6704	3228	10.41	20965	125.804
14	7134	0.0366	6704	7601	7057	2.92	60	0.349
15	18049	0.0431	16342	18781	17783	3.52	54	0.410
16	20000 (UM)	0.1822	18781	29068	20625	4.42	1975	20.843

TIC:	15.3863	ng/uL
TIM:	13.1217	nmole/L
Total concentration:	15.7420	ng/uL

```
Sample peak width (sec): 5     Sample min peak height: 50    Sample baseline V to V?: Y    Sample baseline V to V points: 3
Sample filter: Binomial        Number of points for filter: 3  Sample start region (min): 0  Sample end region (min): 30
Marker peak width (sec): 5     Marker min peak height: 200   Marker baseline V to V?: Y    Marker baseline V to V points: 3
Lower marker selection: First peak > 500 RFU                 Upper marker selection: Last peak > 200 RFU
Ladder size (bp)75, 200, 300, 400, 500, 700, 1000, 1500, 2000, 3000, 4000, 5000, 7000, 10000, 20000
Quantification using: Lower Marker          Final concentration (ng/uL): 0.5000          Dilution factor: 12.0
```

Sample: SampF3
Well location: F3
Created: Wednesday, October 20, 2021 6:38:33 PM

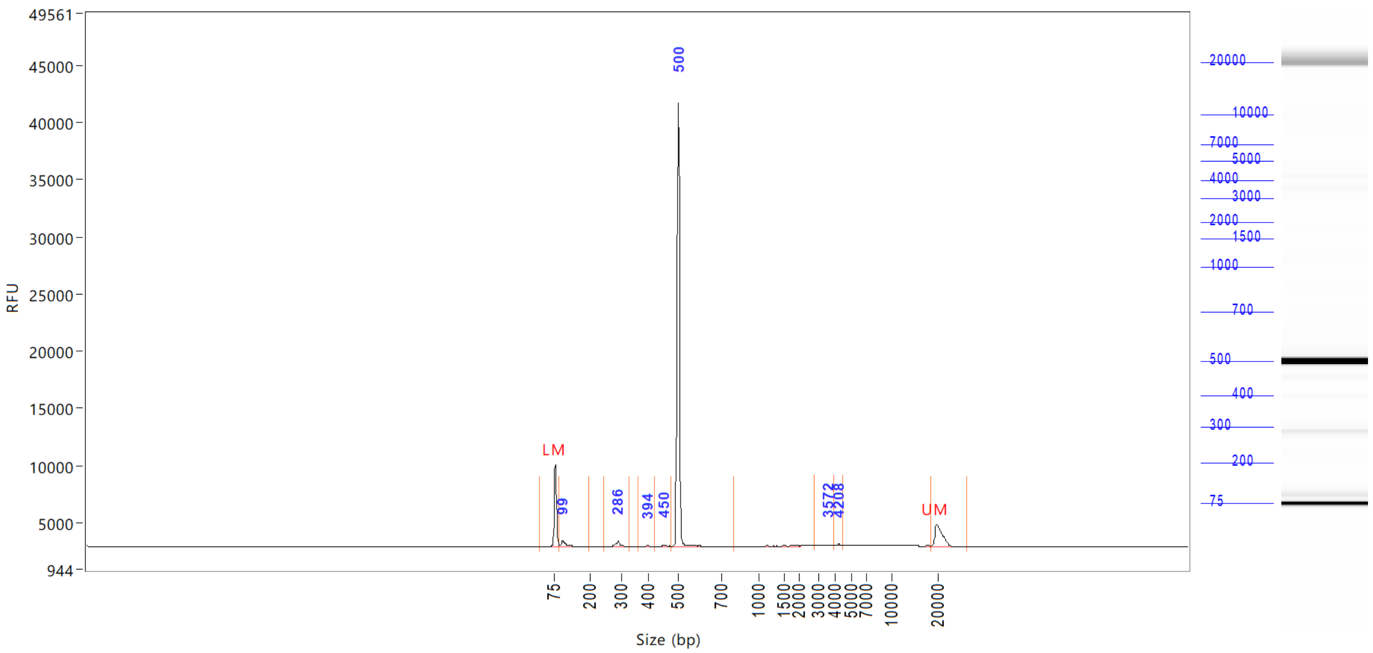

Peak	Size (bp)	Concentration (ng/uL)	From (bp)	To (bp)	Average size (bp)	CV%	RFU	Corrected peak area
1	75 (LM)	0.5000	22	87	72	5.42	7144	39.799
2	99	0.9168	87	192	106	15.91	514	6.081
3	286	0.6782	239	327	284	3.74	505	4.499
4	394	0.0710	360	416	391	2.03	79	0.471
5	450	0.1892	416	477	453	2.34	164	1.255
6	500	27.5850	477	792	500	2.70	38852	182.975
7	3572	0.1724	2743	3964	3454	7.34	121	1.143
8	4208	0.1153	3964	4518	4183	2.98	128	0.765
9	20000 (UM)	0.2452	18659	26529	20513	4.27	1865	19.514

TIC:	29.7279	ng/uL
TIM:	110.0286	nmole/L
Total concentration:	30.2730	ng/uL

Sample peak width (sec): 5   Sample min peak height: 50   Sample baseline V to V?: Y   Sample baseline V to V points: 3
Sample filter: Binomial   Number of points for filter: 3   Sample start region (min): 0   Sample end region (min): 30
Marker peak width (sec): 5   Marker min peak height: 200   Marker baseline V to V?: Y   Marker baseline V to V points: 3
Lower marker selection: First peak > 500 RFU   Upper marker selection: Last peak > 200 RFU
Ladder size (bp)75, 200, 300, 400, 500, 700, 1000, 1500, 2000, 3000, 4000, 5000, 7000, 10000, 20000
Quantification using: Lower Marker   Final concentration (ng/uL): 0.5000   Dilution factor: 12.0

Sample: SampF4
Well location: F4
Created: Wednesday, October 20, 2021 6:38:33 PM

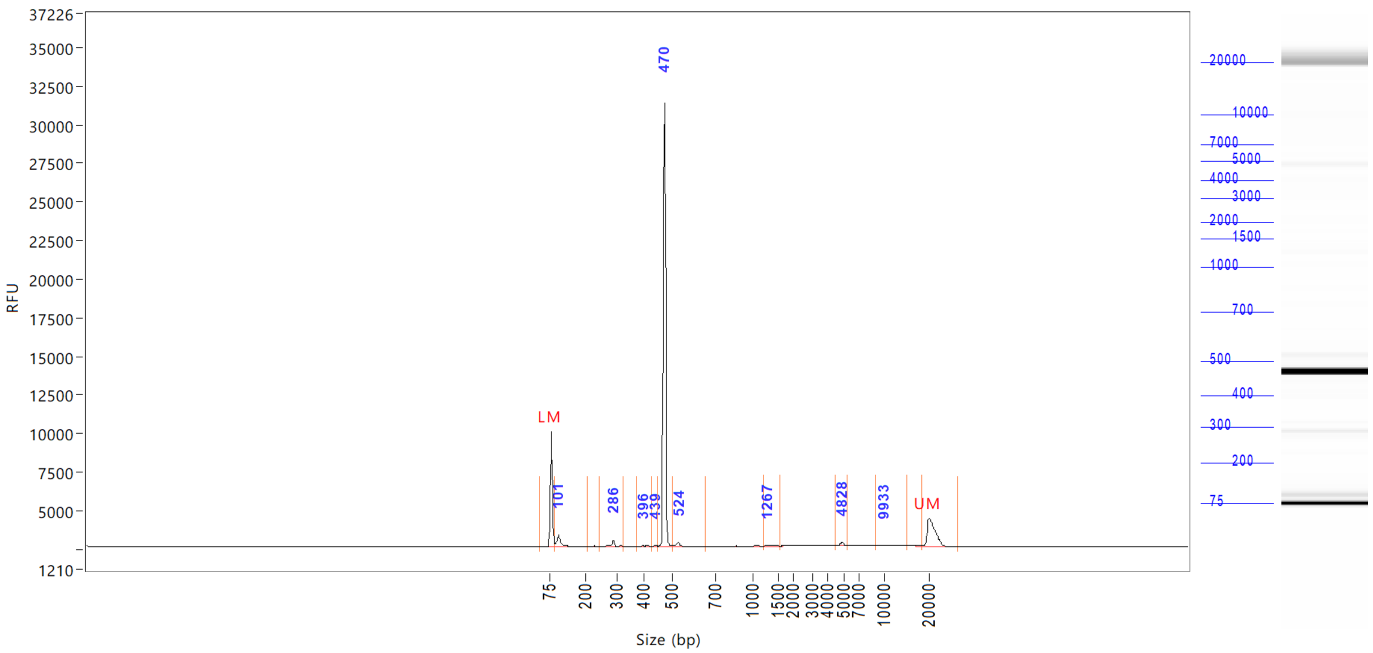

Peak	Size (bp)	Concentration (ng/uL)	From (bp)	To (bp)	Average size (bp)	CV%	RFU	Corrected peak area
1	75 (LM)	0.5000	35	87	73	5.11	7443	40.186
2	101	1.0997	87	201	103	15.36	732	7.365
3	286	0.5172	241	321	284	4.41	417	3.464
4	396	0.0943	372	424	402	2.73	51	0.631
5	439	0.0637	424	449	437	1.39	61	0.427
6	470	19.5638	449	500	469	0.89	28780	131.033
7	524	0.3544	500	654	530	5.57	250	2.373
8	1267	0.0862	1190	1559	1305	5.77	74	0.578
9	4828	0.1702	4449	5445	4833	2.40	235	1.140
10	9933	0.1547	8867	14879	11071	15.09	53	1.036
11	20000 (UM)	0.2419	18537	26529	20630	4.36	1850	19.442

TIC:	22.1042	ng/uL
TIM:	91.0825	nmole/L
Total concentration:	22.5199	ng/uL

```
Sample peak width (sec): 5     Sample min peak height: 50     Sample baseline V to V?: Y     Sample baseline V to V points: 3
Sample filter: Binomial        Number of points for filter: 3  Sample start region (min): 0  Sample end region (min): 30
Marker peak width (sec): 5     Marker min peak height: 200    Marker baseline V to V?: Y     Marker baseline V to V points: 3
Lower marker selection: First peak > 500 RFU                  Upper marker selection: Last peak > 200 RFU
Ladder size (bp)75, 200, 300, 400, 500, 700, 1000, 1500, 2000, 3000, 4000, 5000, 7000, 10000, 20000
Quantification using: Lower Marker          Final concentration (ng/uL): 0.5000          Dilution factor: 12.0
```

Sample: SampF5
Well location: F5
Created: Wednesday, October 20, 2021 6:38:33 PM

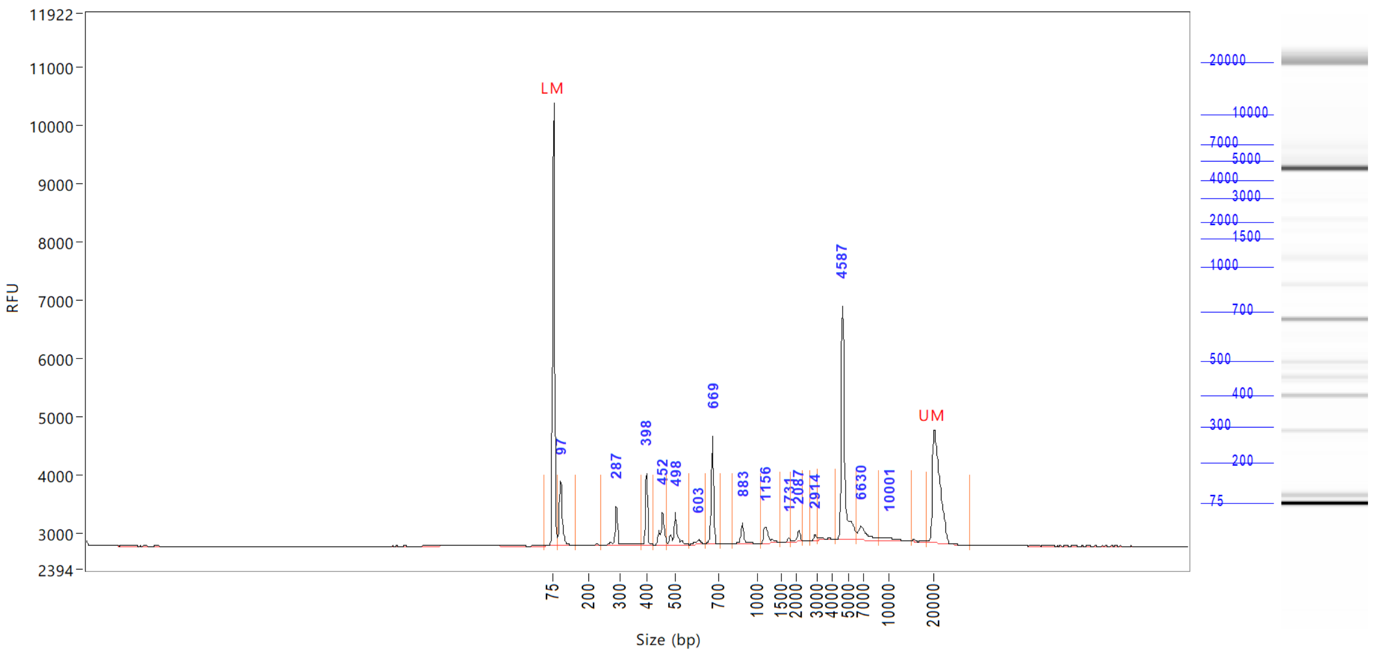

Peak	Size (bp)	Concentration (ng/uL)	From (bp)	To (bp)	Average size (bp)	CV%	RFU	Corrected peak area
1	75 (LM)	0.5000	45	87	73	4.82	7606	40.842
2	97	1.3352	87	154	99	8.58	1107	9.088
3	287	0.6985	241	378	292	7.41	678	4.755
4	398	0.8510	378	422	395	1.15	1228	5.793
5	452	0.6390	422	470	449	1.85	568	4.349
6	498	0.6409	470	560	496	2.87	551	4.362
7	603	0.1295	560	636	599	2.58	77	0.881
8	669	1.2561	636	714	666	1.02	1858	8.550
9	883	0.3241	801	1068	886	3.50	367	2.206
10	1156	0.3797	1068	1467	1183	5.96	278	2.585
11	1731	0.0495	1467	1808	1688	4.47	63	0.337
12	2087	0.1488	1808	2258	2024	4.12	183	1.013
13	2914	0.0612	2657	3037	2901	2.58	87	0.416
14	4587	3.2874	4173	6038	4700	5.99	3996	22.377
15	6630	0.4528	6038	8867	7045	10.17	233	3.082
16	10001	0.1923	8867	14879	10884	15.34	58	1.309
17	20000 (UM)	0.2440	18415	27979	20627	4.24	1929	19.933

TIC:	10.4459	ng/uL
TIM:	40.1734	nmole/L
Total concentration:	10.6218	ng/uL

Sample peak width (sec): 5   Sample min peak height: 50   Sample baseline V to V?: Y   Sample baseline V to V points: 3
Sample filter: Binomial   Number of points for filter: 3 Sample start region (min): 0 Sample end region (min): 30
Marker peak width (sec): 5   Marker min peak height: 200   Marker baseline V to V?: Y   Marker baseline V to V points: 3
Lower marker selection: First peak > 500 RFU   Upper marker selection: Last peak > 200 RFU
Ladder size (bp)75, 200, 300, 400, 500, 700, 1000, 1500, 2000, 3000, 4000, 5000, 7000, 10000, 20000
Quantification using: Lower Marker   Final concentration (ng/uL): 0.5000   Dilution factor: 12.0

Sample: SampF6
Well location: F6
Created: Wednesday, October 20, 2021 6:38:33 PM

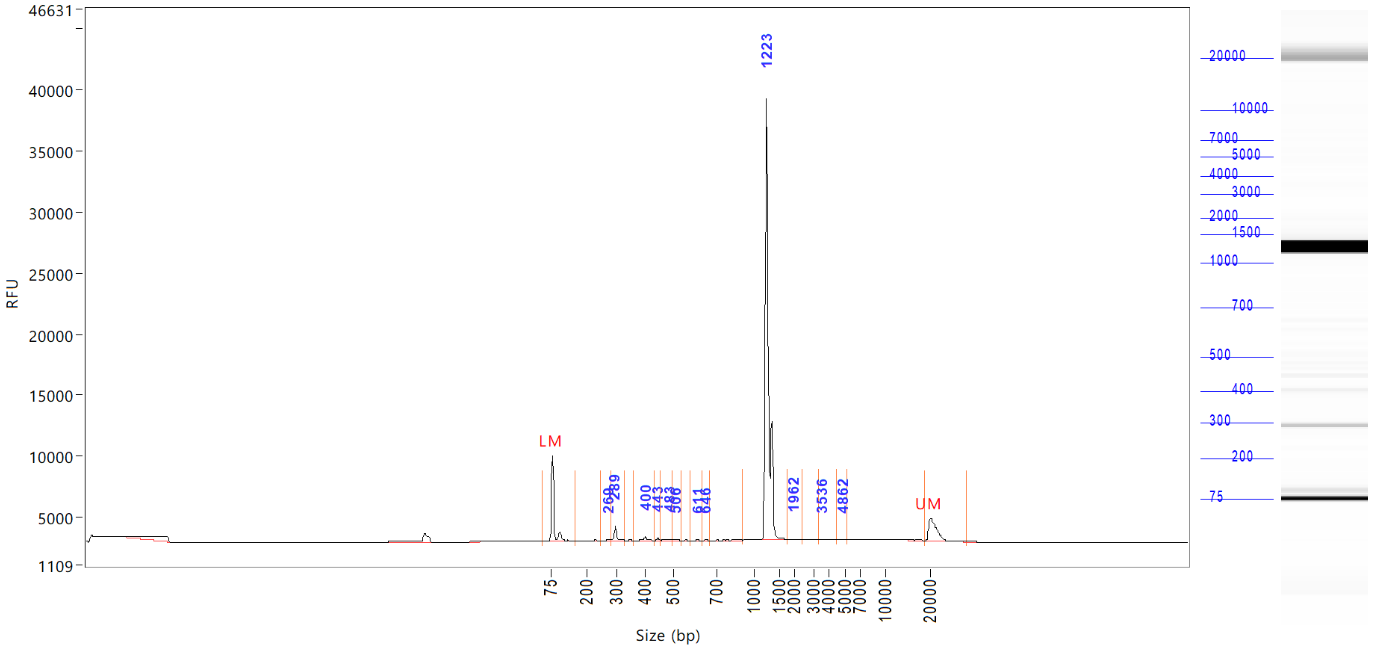

Peak	Size (bp)	Concentration (ng/uL)	From (bp)	To (bp)	Average size (bp)	CV%	RFU	Corrected peak area
1	75 (LM)	0.5000	41	158	78	14.01	7013	44.904
2	269	0.0739	242	278	266	3.12	54	0.553
3	289	0.8577	278	327	290	2.24	1204	6.419
4	400	0.3171	356	433	396	3.11	299	2.373
5	443	0.1507	433	454	443	0.90	242	1.128
6	483	0.1712	454	492	474	2.06	141	1.282
7	506	0.1101	492	534	508	1.63	86	0.824
8	611	0.0646	578	631	604	1.63	77	0.484
9	646	0.0614	631	664	646	0.79	103	0.459
10	1223	28.7741	905	1789	1248	4.70	36200	215.346
11	1962	0.1057	1789	2401	1973	6.50	78	0.791
12	3536	0.0549	3322	4414	3711	7.47	50	0.411
13	4862	0.0502	4414	5149	4804	2.98	52	0.375
14	20000 (UM)	0.2295	18781	27859	20920	7.38	1831	20.614

TIC:	30.7916	ng/uL
TIM:	46.5630	nmole/L
Total concentration:	31.4340	ng/uL

```
Sample peak width (sec): 5    Sample min peak height: 50    Sample baseline V to V?: Y    Sample baseline V to V points: 3
Sample filter: Binomial       Number of points for filter: 3  Sample start region (min): 0  Sample end region (min): 30
Marker peak width (sec): 5    Marker min peak height: 200   Marker baseline V to V?: Y    Marker baseline V to V points: 3
Lower marker selection: First peak > 500 RFU                Upper marker selection: Last peak > 200 RFU
Ladder size (bp)75, 200, 300, 400, 500, 700, 1000, 1500, 2000, 3000, 4000, 5000, 7000, 10000, 20000
Quantification using: Lower Marker          Final concentration (ng/uL): 0.5000          Dilution factor: 12.0
```

Sample: SampF7
Well location: F7
Created: Wednesday, October 20, 2021 6:38:33 PM

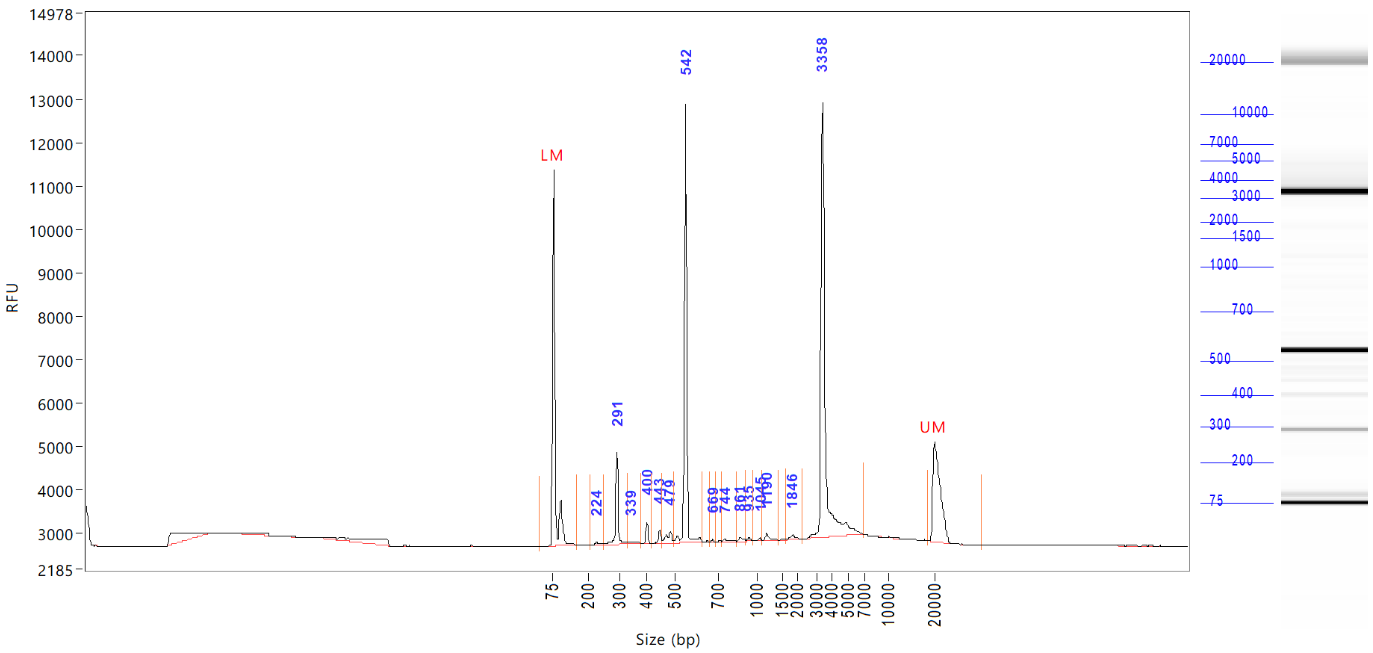

Peak	Size (bp)	Concentration (ng/uL)	From (bp)	To (bp)	Average size (bp)	CV%	RFU	Corrected peak area
1	75 (LM)	0.5000	26	160	79	14.31	8666	57.211
2	224	0.0627	206	248	226	4.26	57	0.598
3	291	1.2817	248	331	288	3.27	2103	12.221
4	339	0.0788	331	380	349	3.46	50	0.751
5	400	0.2397	380	414	397	1.00	490	2.285
6	443	0.1602	414	452	441	1.30	291	1.527
7	479	0.2927	452	492	472	2.10	265	2.791
8	542	4.9608	492	626	541	2.17	10088	47.303
9	669	0.0297	657	685	667	0.78	60	0.283
10	744	0.0709	718	840	759	3.77	61	0.676
11	861	0.0841	840	909	871	1.97	97	0.802
12	935	0.0513	909	970	933	1.44	86	0.489
13	1045	0.0432	970	1101	1022	2.76	75	0.412
14	1190	0.1379	1101	1433	1223	6.15	160	1.315
15	1846	0.0933	1616	2258	1840	5.45	108	0.889
16	3358	6.7296	2258	7000	3521	14.24	10018	64.169
17	20000 (UM)	0.2073	18659	30639	20648	4.43	2291	23.721

TIC:	14.3166	ng/uL
TIM:	29.8104	nmole/L
Total concentration:	14.4067	ng/uL

```
Sample peak width (sec): 5    Sample min peak height: 50    Sample baseline V to V?: Y    Sample baseline V to V points: 3
Sample filter: Binomial       Number of points for filter: 3  Sample start region (min): 0  Sample end region (min): 30
Marker peak width (sec): 5    Marker min peak height: 200   Marker baseline V to V?: Y    Marker baseline V to V points: 3
Lower marker selection: First peak > 500 RFU                Upper marker selection: Last peak > 200 RFU
Ladder size (bp)75, 200, 300, 400, 500, 700, 1000, 1500, 2000, 3000, 4000, 5000, 7000, 10000, 20000
Quantification using: Lower Marker          Final concentration (ng/uL): 0.5000          Dilution factor: 12.0
```

Sample: SampF8
Well location: F8
Created: Wednesday, October 20, 2021 6:38:33 PM

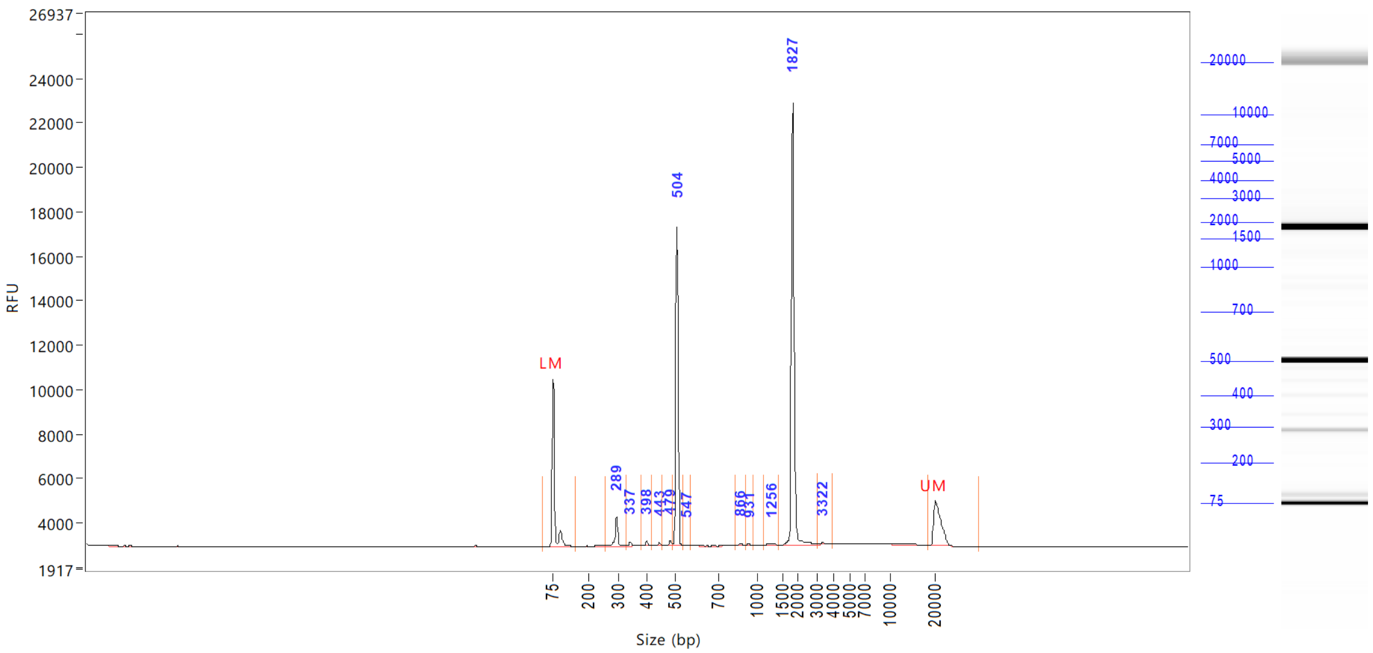

Peak	Size (bp)	Concentration (ng/uL)	From (bp)	To (bp)	Average size (bp)	CV%	RFU	Corrected peak area
1	75 (LM)	0.5000	39	154	78	13.83	7551	47.553
2	289	1.0128	253	323	286	2.70	1299	8.027
3	337	0.1571	323	380	339	2.83	196	1.245
4	398	0.1358	380	414	397	1.10	212	1.076
5	443	0.1210	414	450	437	1.63	158	0.959
6	479	0.1878	450	487	473	1.91	220	1.489
7	504	8.2577	487	532	501	0.82	14335	65.446
8	547	0.0543	532	570	545	1.40	59	0.430
9	866	0.1113	827	905	864	1.87	116	0.882
10	931	0.0699	905	974	930	1.43	104	0.554
11	1256	0.1447	1112	1433	1249	5.55	89	1.147
12	1827	12.0077	1433	3001	1836	6.47	19931	95.167
13	3322	0.1050	3001	3964	3394	7.09	73	0.832
14	20000 (UM)	0.2137	18415	29551	20623	4.31	2014	20.321

TIC:	22.3651	ng/uL
TIM:	46.8883	nmole/L
Total concentration:	22.8681	ng/uL

Sample peak width (sec): 5   Sample min peak height: 50   Sample baseline V to V?: Y   Sample baseline V to V points: 3
Sample filter: Binomial   Number of points for filter: 3   Sample start region (min): 0   Sample end region (min): 30
Marker peak width (sec): 5   Marker min peak height: 200   Marker baseline V to V?: Y   Marker baseline V to V points: 3
Lower marker selection: First peak > 500 RFU   Upper marker selection: Last peak > 200 RFU
Ladder size (bp)75, 200, 300, 400, 500, 700, 1000, 1500, 2000, 3000, 4000, 5000, 7000, 10000, 20000
Quantification using: Lower Marker   Final concentration (ng/uL): 0.5000   Dilution factor: 12.0

Sample: SampF9
Well location: F9
Created: Wednesday, October 20, 2021 6:38:33 PM

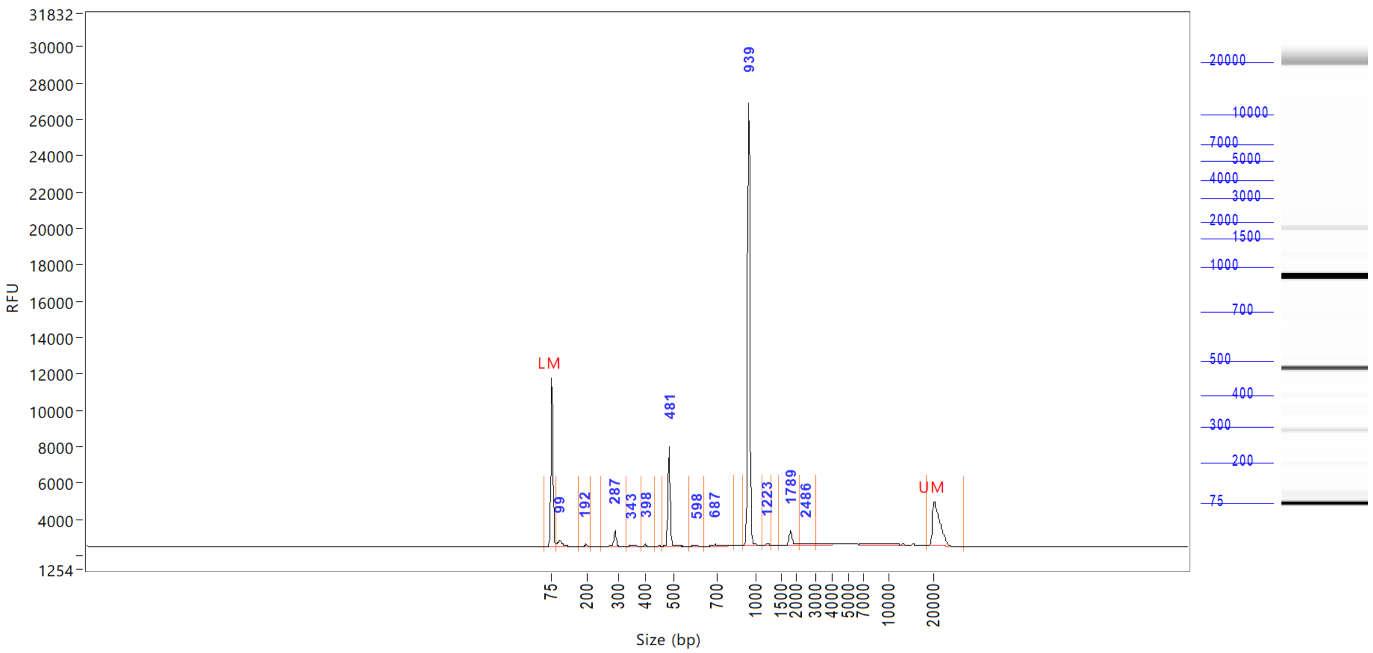

Peak	Size (bp)	Concentration (ng/uL)	From (bp)	To (bp)	Average size (bp)	CV%	RFU	Corrected peak area
1	75 (LM)	0.5000	50	89	73	4.85	9311	50.276
2	99	0.6075	89	168	106	13.90	375	5.090
3	192	0.1281	168	206	189	3.42	165	1.073
4	287	0.6229	246	327	286	2.77	890	5.219
5	343	0.1362	327	388	351	3.54	101	1.141
6	398	0.0810	388	430	398	1.48	134	0.679
7	481	3.1497	456	567	479	1.78	5506	26.392
8	598	0.0590	567	634	592	1.81	68	0.494
9	687	0.1400	634	827	702	4.38	95	1.173
10	939	12.6097	896	1112	937	1.27	24397	105.661
11	1223	0.1017	1112	1300	1201	3.45	133	0.852
12	1789	0.5709	1445	2144	1808	5.68	834	4.784
13	2486	0.1582	2144	3001	2533	9.54	72	1.326
14	20000 (UM)	0.2506	18537	26770	20640	4.21	2416	25.198

TIC:	18.3649	ng/uL
TIM:	49.3060	nmole/L
Total concentration:	18.7599	ng/uL

```
Sample peak width (sec): 5    Sample min peak height: 50    Sample baseline V to V?: Y    Sample baseline V to V points: 3
Sample filter: Binomial       Number of points for filter: 3  Sample start region (min): 0  Sample end region (min): 30
Marker peak width (sec): 5    Marker min peak height: 200   Marker baseline V to V?: Y    Marker baseline V to V points: 3
Lower marker selection: First peak > 500 RFU                Upper marker selection: Last peak > 200 RFU
Ladder size (bp)75, 200, 300, 400, 500, 700, 1000, 1500, 2000, 3000, 4000, 5000, 7000, 10000, 20000
Quantification using: Lower Marker          Final concentration (ng/uL): 0.5000          Dilution factor: 12.0
```

Sample: SampF10
Well location: F10
Created: Wednesday, October 20, 2021 6:38:33 PM

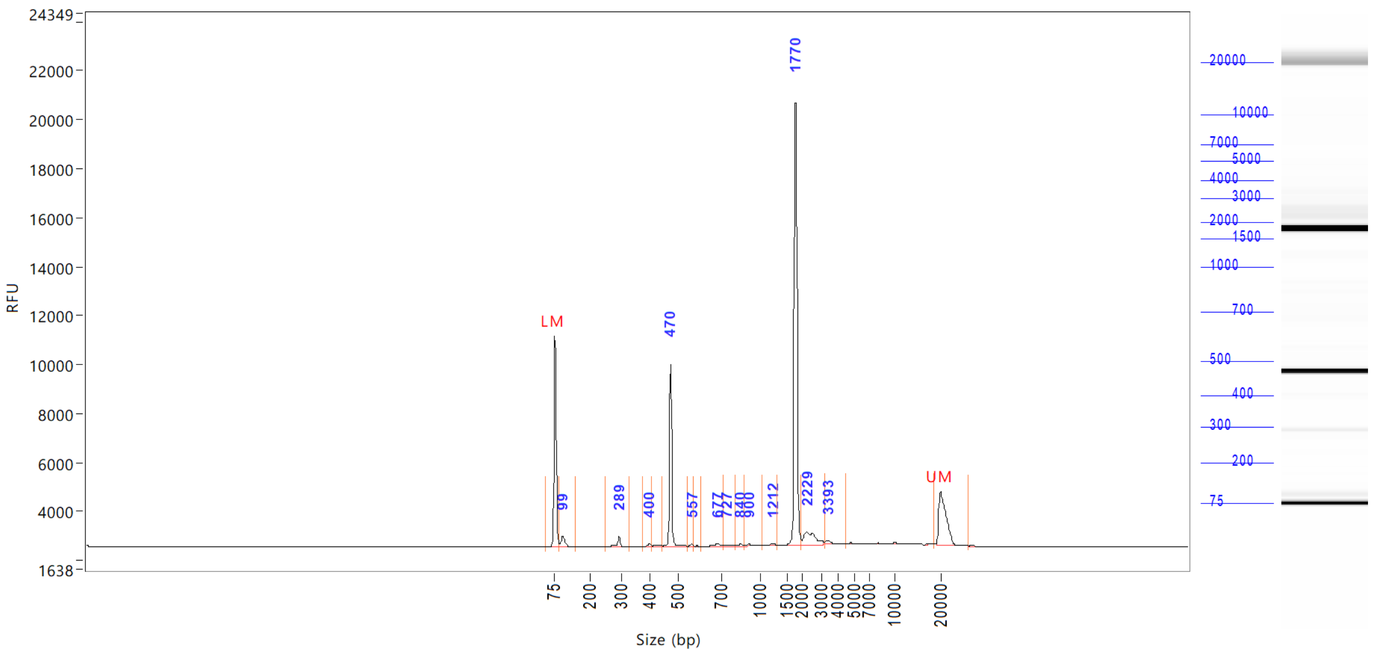

Peak	Size (bp)	Concentration (ng/uL)	From (bp)	To (bp)	Average size (bp)	CV%	RFU	Corrected peak area
1	75 (LM)	0.5000	43	89	74	4.79	8579	46.819
2	99	0.5622	89	147	102	9.26	422	4.387
3	289	0.3619	246	327	285	3.43	435	2.824
4	400	0.0796	376	407	395	1.52	100	0.621
5	470	4.4418	445	539	469	1.28	7435	34.660
6	557	0.0517	539	570	554	0.98	86	0.404
7	677	0.1634	600	710	676	2.75	91	1.275
8	727	0.0977	710	801	744	3.38	67	0.762
9	840	0.0780	801	874	836	2.05	89	0.609
10	900	0.0633	874	1012	903	2.46	79	0.494
11	1212	0.1067	1012	1323	1190	4.40	90	0.833
12	1770	10.8431	1323	2001	1774	3.01	18094	84.611
13	2229	1.2476	2001	3179	2449	11.84	514	9.735
14	3393	0.2655	3179	4483	3636	10.10	164	2.072
15	20000 (UM)	0.2309	18415	26166	20531	3.97	2148	21.625

TIC:	18.3626	ng/uL
TIM:	39.2605	nmole/L
Total concentration:	18.6714	ng/uL

```
Sample peak width (sec): 5    Sample min peak height: 50    Sample baseline V to V?: Y    Sample baseline V to V points: 3
Sample filter: Binomial       Number of points for filter: 3  Sample start region (min): 0  Sample end region (min): 30
Marker peak width (sec): 5    Marker min peak height: 200   Marker baseline V to V?: Y    Marker baseline V to V points: 3
Lower marker selection: First peak > 500 RFU                Upper marker selection: Last peak > 200 RFU
Ladder size (bp)75, 200, 300, 400, 500, 700, 1000, 1500, 2000, 3000, 4000, 5000, 7000, 10000, 20000
Quantification using: Lower Marker          Final concentration (ng/uL): 0.5000          Dilution factor: 12.0
```

Sample: SampF11
Well location: F11
Created: Wednesday, October 20, 2021 6:38:33 PM

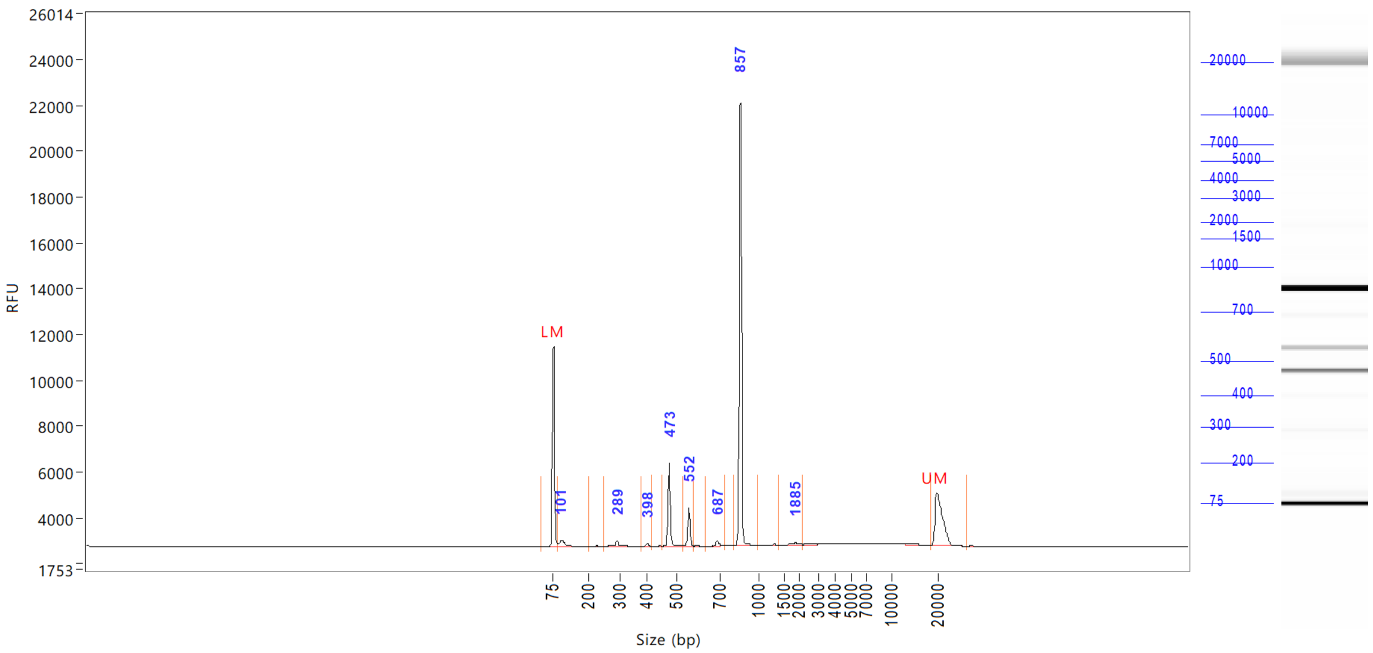

Peak	Size (bp)	Concentration (ng/uL)	From (bp)	To (bp)	Average size (bp)	CV%	RFU	Corrected peak area
1	75 (LM)	0.5000	35	89	72	5.03	8709	48.934
2	101	0.5162	89	198	111	19.29	273	4.210
3	289	0.3269	244	378	290	6.93	254	2.666
4	398	0.0682	378	411	395	1.12	107	0.556
5	473	2.1038	447	524	471	1.41	3619	17.158
6	552	0.9231	524	575	551	1.01	1662	7.529
7	687	0.1610	629	731	685	1.66	204	1.313
8	857	10.3518	809	996	852	1.31	19354	84.427
9	1885	0.1100	1389	2172	1793	9.47	87	0.897
10	20000 (UM)	0.2420	18659	26649	20536	4.13	2314	23.684

TIC:	14.5611	ng/uL
TIM:	40.3681	nmole/L
Total concentration:	14.9958	ng/uL

```
Sample peak width (sec): 5    Sample min peak height: 50    Sample baseline V to V?: Y    Sample baseline V to V points: 3
Sample filter: Binomial       Number of points for filter: 3  Sample start region (min): 0  Sample end region (min): 30
Marker peak width (sec): 5    Marker min peak height: 200   Marker baseline V to V?: Y    Marker baseline V to V points: 3
Lower marker selection: First peak > 500 RFU                Upper marker selection: Last peak > 200 RFU
Ladder size (bp)75, 200, 300, 400, 500, 700, 1000, 1500, 2000, 3000, 4000, 5000, 7000, 10000, 20000
Quantification using: Lower Marker          Final concentration (ng/uL): 0.5000          Dilution factor: 12.0
```

Sample: SampF12
Well location: F12
Created: Wednesday, October 20, 2021 6:38:33 PM

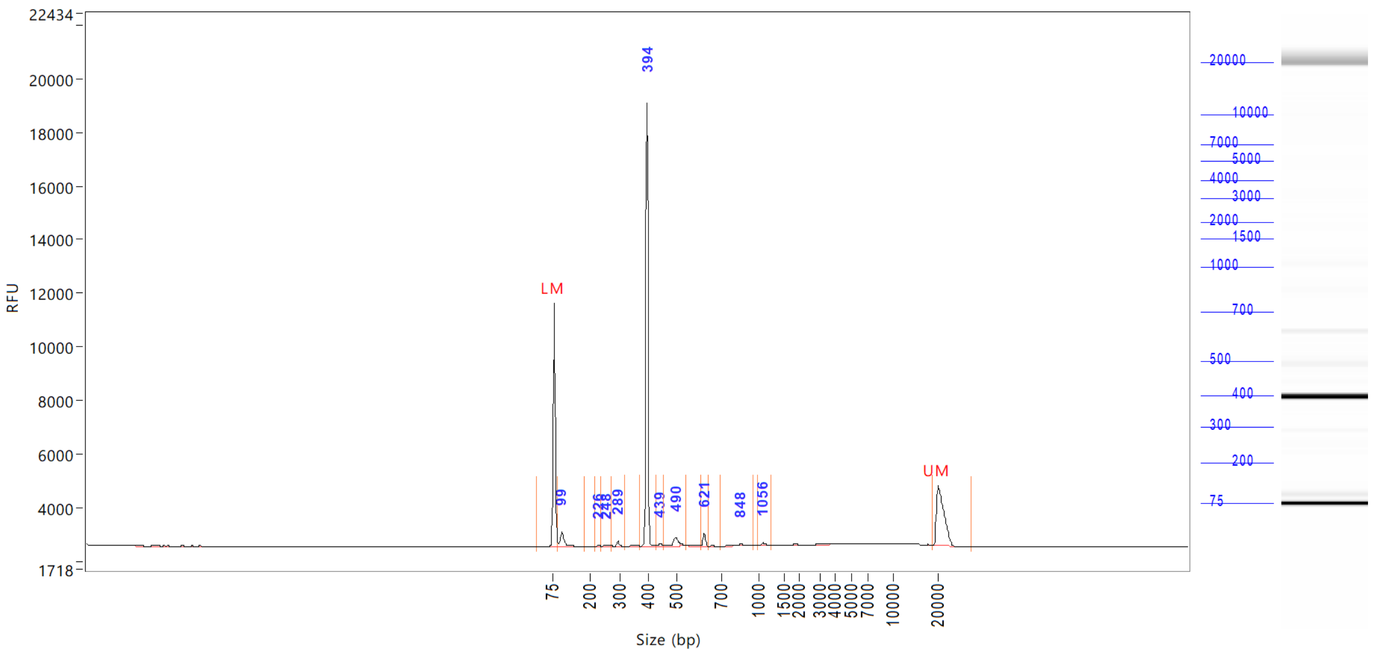

Peak	Size (bp)	Concentration (ng/uL)	From (bp)	To (bp)	Average size (bp)	CV%	RFU	Corrected peak area
1	75 (LM)	0.5000	14	89	73	5.00	9072	48.718
2	99	0.6469	89	183	103	12.58	545	5.252
3	226	0.0338	217	237	225	1.64	54	0.275
4	248	0.0781	237	271	251	2.99	56	0.634
5	289	0.1178	271	311	287	1.72	185	0.956
6	394	9.5911	370	426	391	0.98	16543	77.876
7	439	0.0618	426	450	436	1.04	89	0.502
8	490	0.4282	450	534	492	2.67	321	3.477
9	621	0.2429	606	644	618	0.81	458	1.972
10	848	0.1313	697	948	841	4.77	99	1.066
11	1056	0.0939	991	1212	1059	3.84	107	0.763
12	20000 (UM)	0.2333	18659	27133	20554	4.02	2239	22.729

TIC:	11.4260	ng/uL
TIM:	54.8213	nmole/L
Total concentration:	11.8976	ng/uL

```
Sample peak width (sec): 5    Sample min peak height: 50    Sample baseline V to V?: Y    Sample baseline V to V points: 3
Sample filter: Binomial       Number of points for filter: 3  Sample start region (min): 0  Sample end region (min): 30
Marker peak width (sec): 5    Marker min peak height: 200   Marker baseline V to V?: Y    Marker baseline V to V points: 3
Lower marker selection: First peak > 500 RFU                Upper marker selection: Last peak > 200 RFU
Ladder size (bp)75, 200, 300, 400, 500, 700, 1000, 1500, 2000, 3000, 4000, 5000, 7000, 10000, 20000
Quantification using: Lower Marker          Final concentration (ng/uL): 0.5000         Dilution factor: 12.0
```

Sample: SampG1
Well location: G1
Created: Wednesday, October 20, 2021 6:38:33 PM

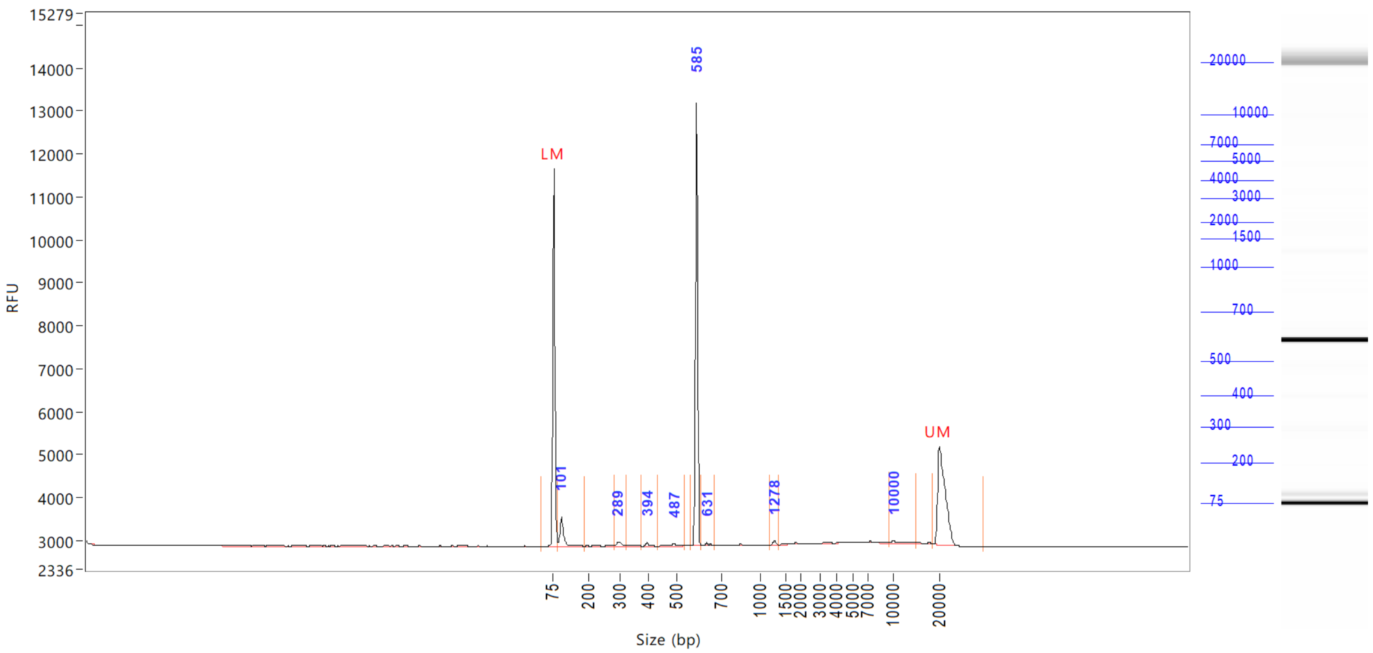

Peak	Size (bp)	Concentration (ng/uL)	From (bp)	To (bp)	Average size (bp)	CV%	RFU	Corrected peak area
1	75 (LM)	0.5000	35	89	74	4.86	8781	48.165
2	101	0.7668	89	179	103	11.72	693	6.156
3	289	0.1290	280	319	293	2.64	98	1.036
4	394	0.0606	376	431	394	1.84	76	0.487
5	487	0.0896	431	529	482	3.85	55	0.719
6	585	5.6762	557	608	582	0.87	10331	45.566
7	631	0.0782	608	669	631	1.98	72	0.628
8	1278	0.0664	1190	1378	1270	2.51	98	0.533
9	10000	0.1408	9400	14879	11675	14.16	52	1.131
10	20000 (UM)	0.2441	18537	29672	20607	4.49	2282	23.518

TIC:	7.0076	ng/uL
TIM:	29.8689	nmole/L
Total concentration:	7.4436	ng/uL

```
Sample peak width (sec): 5    Sample min peak height: 50    Sample baseline V to V?: Y    Sample baseline V to V points: 3
Sample filter: Binomial       Number of points for filter: 3  Sample start region (min): 0  Sample end region (min): 30
Marker peak width (sec): 5    Marker min peak height: 200   Marker baseline V to V?: Y    Marker baseline V to V points: 3
Lower marker selection: First peak > 500 RFU                Upper marker selection: Last peak > 200 RFU
Ladder size (bp)75, 200, 300, 400, 500, 700, 1000, 1500, 2000, 3000, 4000, 5000, 7000, 10000, 20000
Quantification using: Lower Marker          Final concentration (ng/uL): 0.5000          Dilution factor: 12.0
```

Sample: SampG2
Well location: G2
Created: Wednesday, October 20, 2021 6:38:33 PM

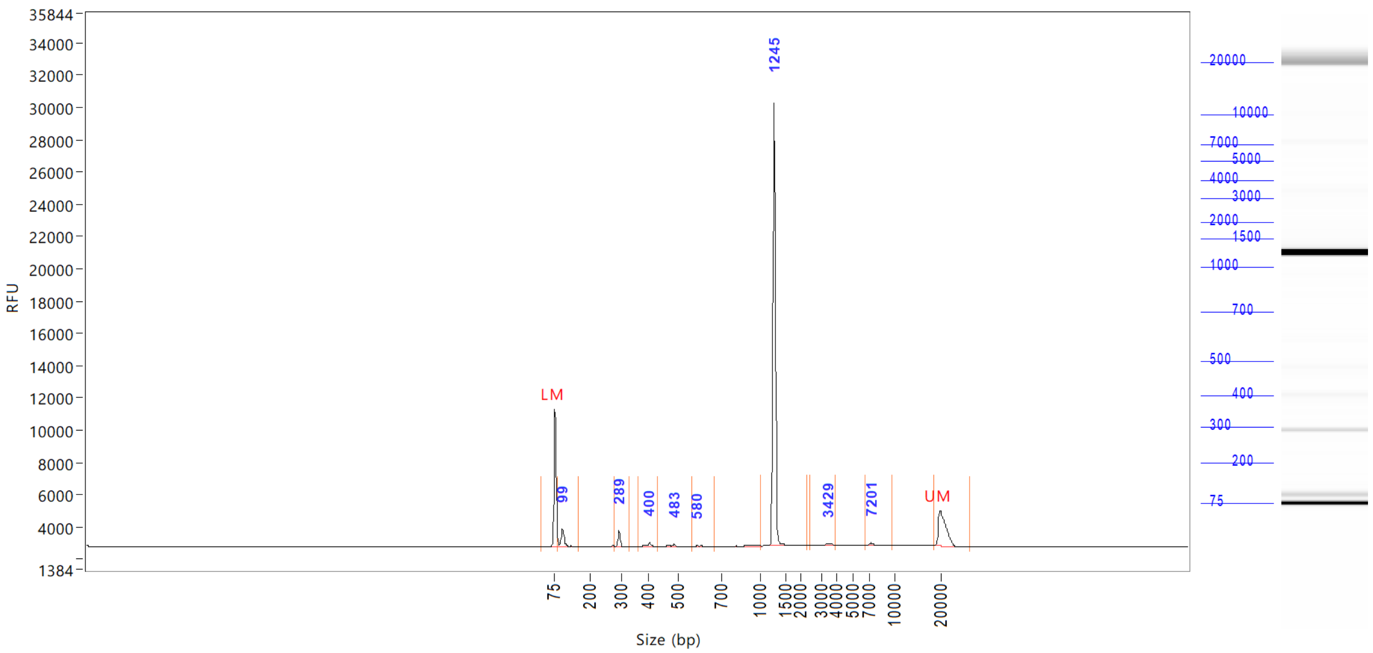

Peak	Size (bp)	Concentration (ng/uL)	From (bp)	To (bp)	Average size (bp)	CV%	RFU	Corrected peak area
1	75 (LM)	0.5000	30	87	74	4.86	8489	46.234
2	99	1.2118	87	156	101	8.67	1128	9.337
3	289	0.6624	278	327	289	2.10	991	5.104
4	400	0.2612	360	426	393	2.88	230	2.013
5	483	0.2167	426	565	479	4.21	140	1.670
6	580	0.0848	565	664	597	3.64	53	0.653
7	1245	16.4912	1001	2258	1250	4.58	27503	127.076
8	3429	0.1631	2458	3893	3246	9.79	112	1.257
9	7201	0.1733	6556	9667	7856	11.14	134	1.335
10	20000 (UM)	0.2411	18537	26529	20596	4.12	2194	22.293

TIC:	19.2645	ng/uL
TIM:	47.3784	nmole/L
Total concentration:	19.7443	ng/uL

```
Sample peak width (sec): 5     Sample min peak height: 50     Sample baseline V to V?: Y     Sample baseline V to V points: 3
Sample filter: Binomial        Number of points for filter: 3  Sample start region (min): 0  Sample end region (min): 30
Marker peak width (sec): 5     Marker min peak height: 200    Marker baseline V to V?: Y     Marker baseline V to V points: 3
Lower marker selection: First peak > 500 RFU                  Upper marker selection: Last peak > 200 RFU
Ladder size (bp)75, 200, 300, 400, 500, 700, 1000, 1500, 2000, 3000, 4000, 5000, 7000, 10000, 20000
Quantification using: Lower Marker          Final concentration (ng/uL): 0.5000          Dilution factor: 12.0
```

Sample: SampG3
Well location: G3
Created: Wednesday, October 20, 2021 6:38:33 PM

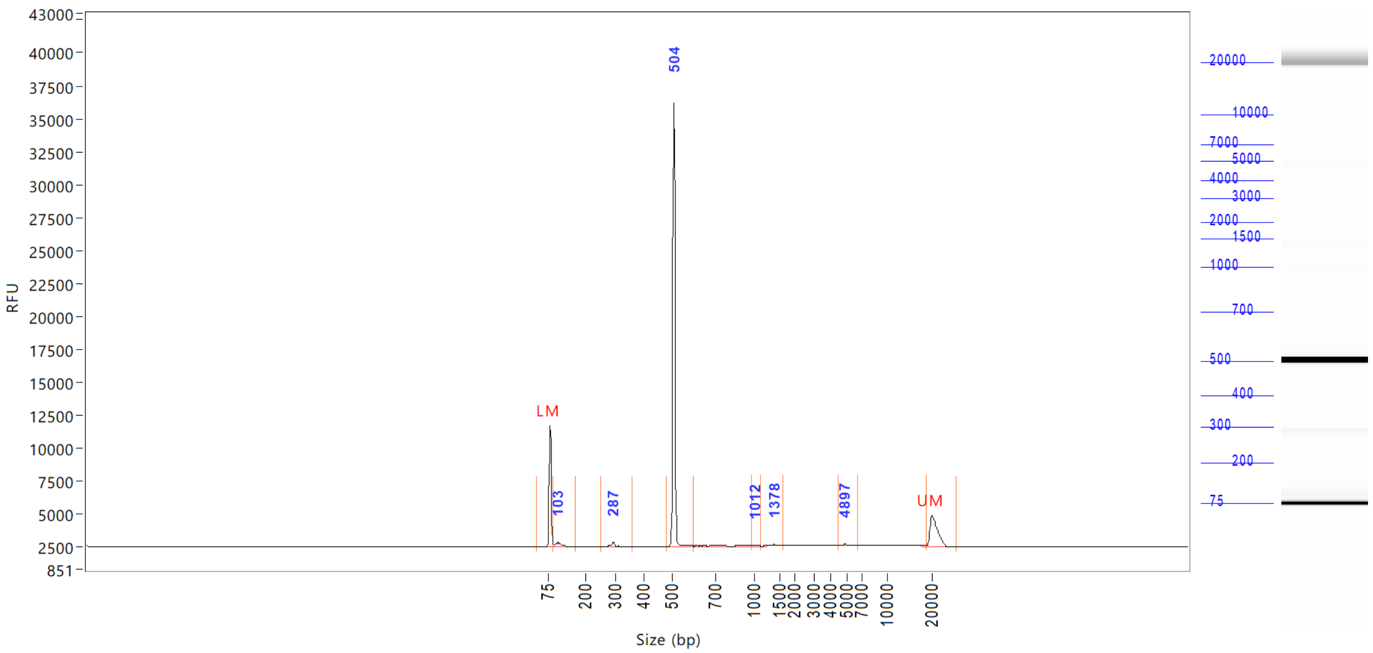

Peak	Size (bp)	Concentration (ng/uL)	From (bp)	To (bp)	Average size (bp)	CV%	RFU	Corrected peak area
1	75 (LM)	0.5000	28	87	74	4.50	9182	49.243
2	103	0.5443	87	166	105	12.92	301	4.467
3	287	0.3863	250	356	287	4.77	328	3.170
4	504	18.7947	481	595	502	1.24	33674	154.250
5	1012	0.0405	978	1134	1009	2.35	67	0.332
6	1378	0.1174	1134	1597	1389	5.32	117	0.963
7	4897	0.0870	4483	6556	4949	5.81	106	0.714
8	20000 (UM)	0.2383	18659	25320	20508	4.02	2319	23.469

TIC:	19.9702	ng/uL
TIM:	72.5685	nmole/L
Total concentration:	20.4905	ng/uL

Sample peak width (sec): 5 Sample min peak height: 50 Sample baseline V to V?: Y Sample baseline V to V points: 3
Sample filter: Binomial Number of points for filter: 3 Sample start region (min): 0 Sample end region (min): 30
Marker peak width (sec): 5 Marker min peak height: 200 Marker baseline V to V?: Y Marker baseline V to V points: 3
Lower marker selection: First peak > 500 RFU Upper marker selection: Last peak > 200 RFU
Ladder size (bp)75, 200, 300, 400, 500, 700, 1000, 1500, 2000, 3000, 4000, 5000, 7000, 10000, 20000
Quantification using: Lower Marker Final concentration (ng/uL): 0.5000 Dilution factor: 12.0

Sample: SampG4
Well location: G4
Created: Wednesday, October 20, 2021 6:38:33 PM

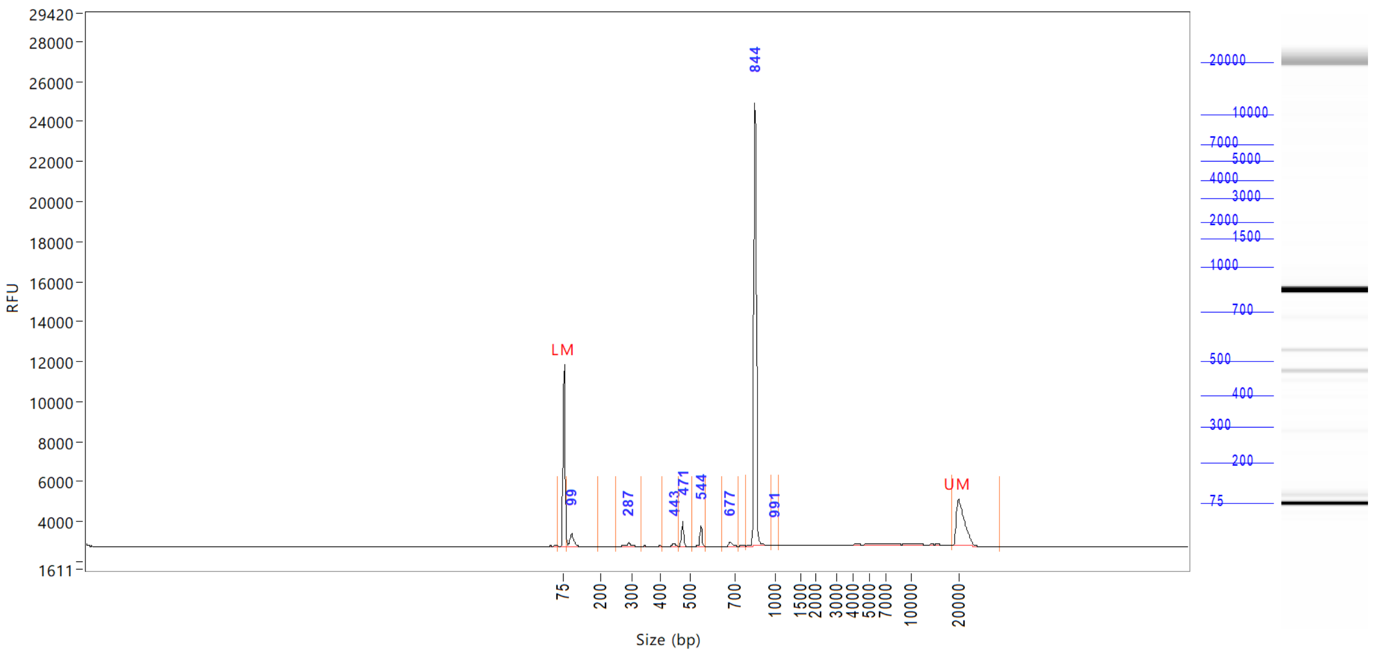

Peak	Size (bp)	Concentration (ng/uL)	From (bp)	To (bp)	Average size (bp)	CV%	RFU	Corrected peak area
1	75 (LM)	0.5000	56	87	73	4.45	9105	47.960
2	99	0.8437	87	190	102	11.89	699	6.744
3	287	0.2426	246	329	284	4.66	205	1.939
4	443	0.1356	403	458	438	2.36	165	1.084
5	471	0.6910	458	501	470	1.02	1219	5.523
6	544	0.5536	501	565	541	1.18	995	4.425
7	677	0.1711	639	718	677	1.61	205	1.368
8	844	11.6979	775	965	843	1.39	22199	93.505
9	991	0.0474	965	1068	990	1.61	58	0.379
10	20000 (UM)	0.2570	18537	28947	20612	4.60	2324	24.649

TIC:	14.3829	ng/uL
TIM:	42.9348	nmole/L
Total concentration:	14.8964	ng/uL

Sample peak width (sec): 5 Sample min peak height: 50 Sample baseline V to V?: Y Sample baseline V to V points: 3
Sample filter: Binomial Number of points for filter: 3 Sample start region (min): 0 Sample end region (min): 30
Marker peak width (sec): 5 Marker min peak height: 200 Marker baseline V to V?: Y Marker baseline V to V points: 3
Lower marker selection: First peak > 500 RFU Upper marker selection: Last peak > 200 RFU
Ladder size (bp)75, 200, 300, 400, 500, 700, 1000, 1500, 2000, 3000, 4000, 5000, 7000, 10000, 20000
Quantification using: Lower Marker Final concentration (ng/uL): 0.5000 Dilution factor: 12.0

Sample: SampG5
Well location: G5
Created: Wednesday, October 20, 2021 6:38:33 PM

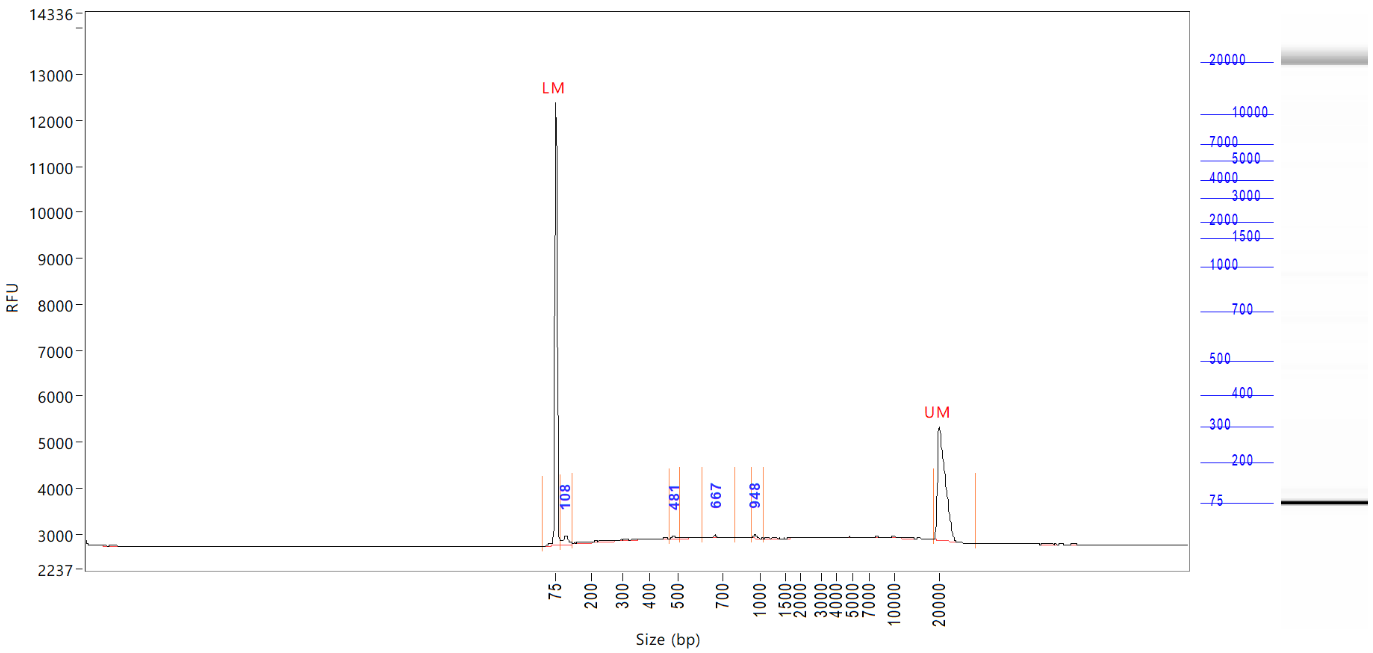

Peak	Size (bp)	Concentration (ng/uL)	From (bp)	To (bp)	Average size (bp)	CV%	RFU	Corrected peak area
1	75 (LM)	0.5000	31	93	73	5.93	9651	52.825
2	108	0.2907	93	133	108	8.55	194	2.559
3	481	0.0455	466	501	482	1.54	57	0.401
4	667	0.0946	611	796	676	5.55	75	0.833
5	948	0.0467	926	1045	950	1.41	77	0.411
6	20000 (UM)	0.2460	18781	28100	20661	4.46	2468	25.991

TIC:	0.4774	ng/uL
TIM:	4.8867	nmole/L
Total concentration:	1.1558	ng/uL

```
Sample peak width (sec): 5    Sample min peak height: 50    Sample baseline V to V?: Y    Sample baseline V to V points: 3
Sample filter: Binomial       Number of points for filter: 3  Sample start region (min): 0  Sample end region (min): 30
Marker peak width (sec): 5    Marker min peak height: 200   Marker baseline V to V?: Y    Marker baseline V to V points: 3
Lower marker selection: First peak > 500 RFU                Upper marker selection: Last peak > 200 RFU
Ladder size (bp)75, 200, 300, 400, 500, 700, 1000, 1500, 2000, 3000, 4000, 5000, 7000, 10000, 20000
Quantification using: Lower Marker          Final concentration (ng/uL): 0.5000          Dilution factor: 12.0
```

Sample: SampG6
Well location: G6
Created: Wednesday, October 20, 2021 6:38:33 PM

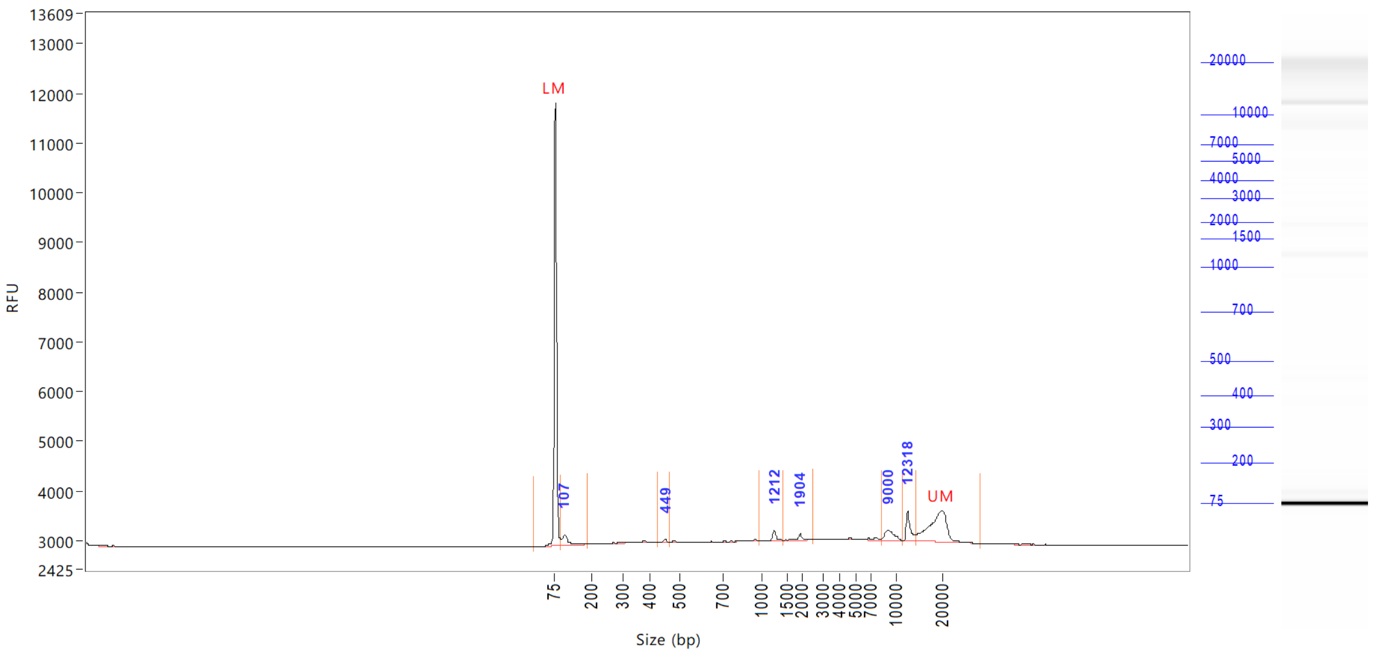

Peak	Size (bp)	Concentration (ng/uL)	From (bp)	To (bp)	Average size (bp)	CV%	RFU	Corrected peak area
1	75 (LM)	0.5000	0	95	73	6.75	8924	50.597
2	107	0.3724	95	183	114	16.23	216	3.140
3	449	0.0511	422	462	445	1.74	64	0.431
4	1212	0.1568	978	1411	1217	4.61	200	1.322
5	1904	0.1329	1411	2515	1871	11.07	126	1.121
6	9000	0.3467	8201	11342	9213	6.10	207	2.923
7	12318	0.4316	11342	14025	12585	4.78	610	3.639
8	20000 (UM)	0.1447	14025	28221	18607	10.91	635	14.639

TIC:	1.4913	ng/uL
TIM:	6.0015	nmole/L
Total concentration:	1.8955	ng/uL

Sample peak width (sec): 5   Sample min peak height: 50   Sample baseline V to V?: Y   Sample baseline V to V points: 3
Sample filter: Binomial   Number of points for filter: 3 Sample start region (min): 0 Sample end region (min): 30
Marker peak width (sec): 5   Marker min peak height: 200   Marker baseline V to V?: Y   Marker baseline V to V points: 3
Lower marker selection: First peak > 500 RFU   Upper marker selection: Last peak > 200 RFU
Ladder size (bp)75, 200, 300, 400, 500, 700, 1000, 1500, 2000, 3000, 4000, 5000, 7000, 10000, 20000
Quantification using: Lower Marker   Final concentration (ng/uL): 0.5000   Dilution factor: 12.0

Sample: SampG7
Well location: G7
Created: Wednesday, October 20, 2021 6:38:33 PM

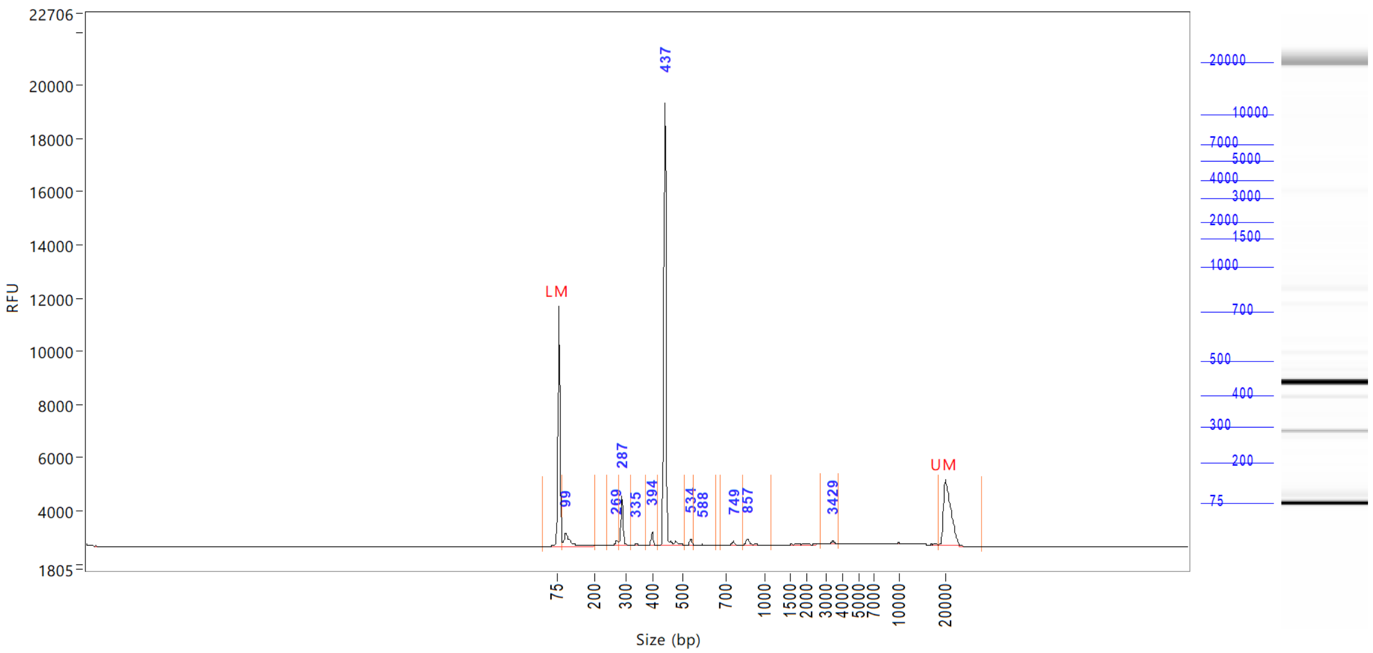

Peak	Size (bp)	Concentration (ng/uL)	From (bp)	To (bp)	Average size (bp)	CV%	RFU	Corrected peak area
1	75 (LM)	0.5000	20	87	74	5.33	9007	48.875
2	99	0.8450	87	200	108	17.88	482	6.883
3	269	0.1783	239	277	267	2.62	168	1.453
4	287	1.1657	277	315	286	1.70	1882	9.496
5	335	0.1031	315	372	340	4.01	65	0.840
6	394	0.2985	372	414	391	1.18	519	2.431
7	437	9.3925	414	509	437	1.74	16674	76.509
8	534	0.1330	509	549	531	1.09	238	1.083
9	588	0.0675	549	649	587	3.11	51	0.550
10	749	0.1300	674	818	738	2.93	165	1.059
11	857	0.2791	818	1090	876	4.71	255	2.273
12	3429	0.1004	2715	3786	3363	5.56	132	0.818
13	20000 (UM)	0.2553	18415	27859	20495	4.20	2453	24.957

TIC:	12.6930	ng/uL
TIM:	59.2374	nmole/L
Total concentration:	13.1226	ng/uL

```
Sample peak width (sec): 5    Sample min peak height: 50    Sample baseline V to V?: Y    Sample baseline V to V points: 3
Sample filter: Binomial       Number of points for filter: 3  Sample start region (min): 0  Sample end region (min): 30
Marker peak width (sec): 5    Marker min peak height: 200   Marker baseline V to V?: Y    Marker baseline V to V points: 3
Lower marker selection: First peak > 500 RFU                Upper marker selection: Last peak > 200 RFU
Ladder size (bp)75, 200, 300, 400, 500, 700, 1000, 1500, 2000, 3000, 4000, 5000, 7000, 10000, 20000
Quantification using: Lower Marker          Final concentration (ng/uL): 0.5000          Dilution factor: 12.0
```

Sample: SampG8
Well location: G8
Created: Wednesday, October 20, 2021 6:38:33 PM

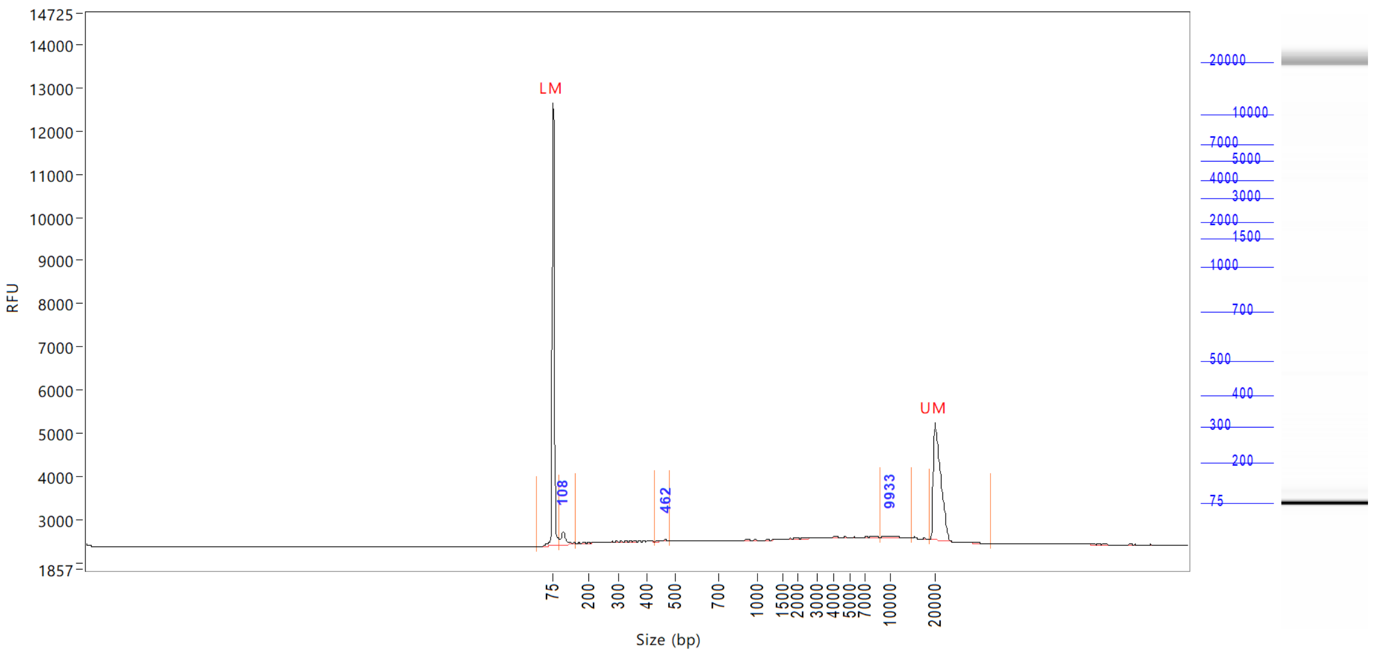

Peak	Size (bp)	Concentration (ng/uL)	From (bp)	To (bp)	Average size (bp)	CV%	RFU	Corrected peak area
1	75 (LM)	0.5000	20	97	74	6.55	10264	57.939
2	108	0.3996	97	154	113	10.99	296	3.858
3	462	0.0397	426	479	456	2.56	53	0.384
4	9933	0.0902	8800	14879	10659	12.89	51	0.871
5	20000 (UM)	0.2381	18781	32211	20581	4.71	2704	27.591

TIC:	0.5295	ng/uL
TIM:	5.9654	nmole/L
Total concentration:	0.9778	ng/uL

```
Sample peak width (sec): 5    Sample min peak height: 50    Sample baseline V to V?: Y    Sample baseline V to V points: 3
Sample filter: Binomial       Number of points for filter: 3  Sample start region (min): 0  Sample end region (min): 30
Marker peak width (sec): 5    Marker min peak height: 200   Marker baseline V to V?: Y    Marker baseline V to V points: 3
Lower marker selection: First peak > 500 RFU                Upper marker selection: Last peak > 200 RFU
Ladder size (bp)75, 200, 300, 400, 500, 700, 1000, 1500, 2000, 3000, 4000, 5000, 7000, 10000, 20000
Quantification using: Lower Marker          Final concentration (ng/uL): 0.5000          Dilution factor: 12.0
```

Sample: SampG9
Well location: G9
Created: Wednesday, October 20, 2021 6:38:33 PM

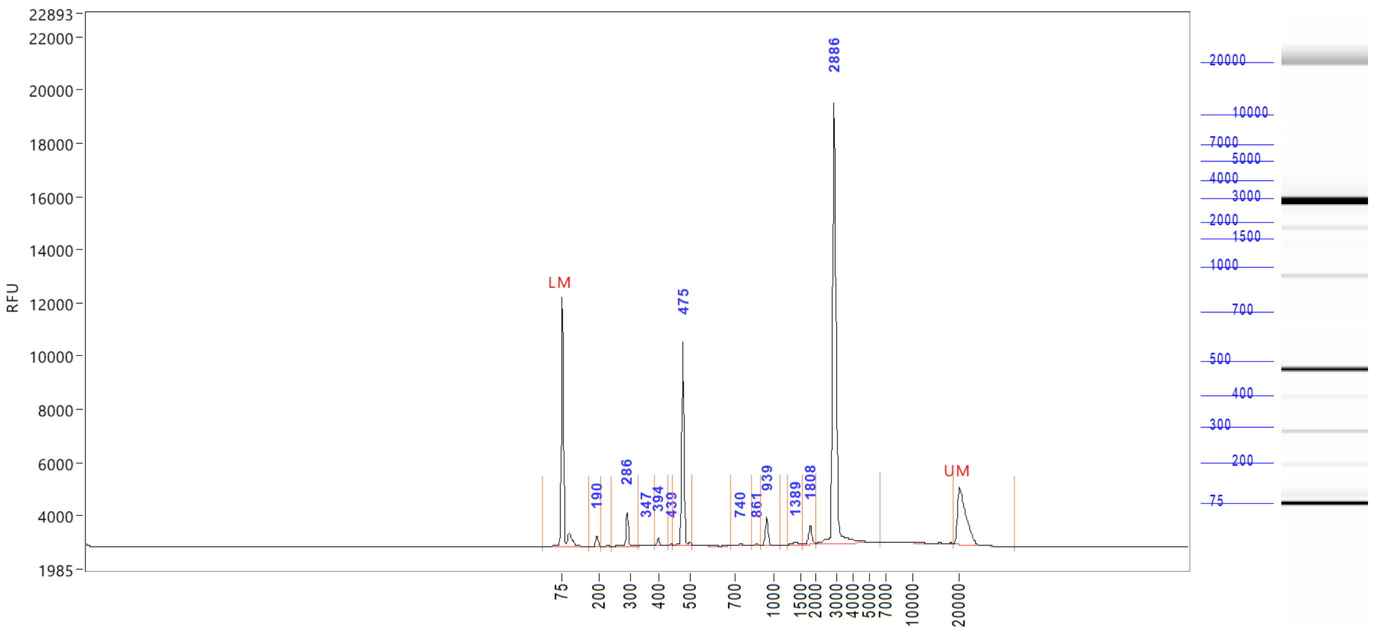

Peak	Size (bp)	Concentration (ng/uL)	From (bp)	To (bp)	Average size (bp)	CV%	RFU	Corrected peak area
1	75 (LM)	0.5000	11	166	76	14.22	9367	55.510
2	190	0.2340	166	206	188	2.47	403	2.164
3	286	0.7796	239	327	284	3.52	1282	7.212
4	347	0.0984	327	384	350	3.79	51	0.911
5	394	0.1721	384	428	395	2.05	309	1.593
6	439	0.0411	428	445	438	0.90	76	0.380
7	475	3.9021	445	511	474	1.20	7647	36.101
8	740	0.1271	685	827	738	4.64	93	1.176
9	861	0.0587	827	900	864	1.96	67	0.543
10	939	0.5679	900	1112	938	1.52	1060	5.254
11	1389	0.1337	1234	1559	1397	5.13	106	1.237
12	1808	0.4235	1559	2000	1791	3.95	697	3.918
13	2886	10.0928	2000	6334	2913	9.23	16606	93.375
14	20000 (UM)	0.2204	18781	31728	20801	5.18	2174	24.473

TIC:	16.6310	ng/uL
TIM:	29.0831	nmole/L
Total concentration:	16.9156	ng/uL

```
Sample peak width (sec): 5    Sample min peak height: 50    Sample baseline V to V?: Y    Sample baseline V to V points: 3
Sample filter: Binomial       Number of points for filter: 3  Sample start region (min): 0  Sample end region (min): 30
Marker peak width (sec): 5    Marker min peak height: 200   Marker baseline V to V?: Y    Marker baseline V to V points: 3
Lower marker selection: First peak > 500 RFU                Upper marker selection: Last peak > 200 RFU
Ladder size (bp)75, 200, 300, 400, 500, 700, 1000, 1500, 2000, 3000, 4000, 5000, 7000, 10000, 20000
Quantification using: Lower Marker          Final concentration (ng/uL): 0.5000          Dilution factor: 12.0
```

Sample: SampG10
Well location: G10
Created: Wednesday, October 20, 2021 6:38:33 PM

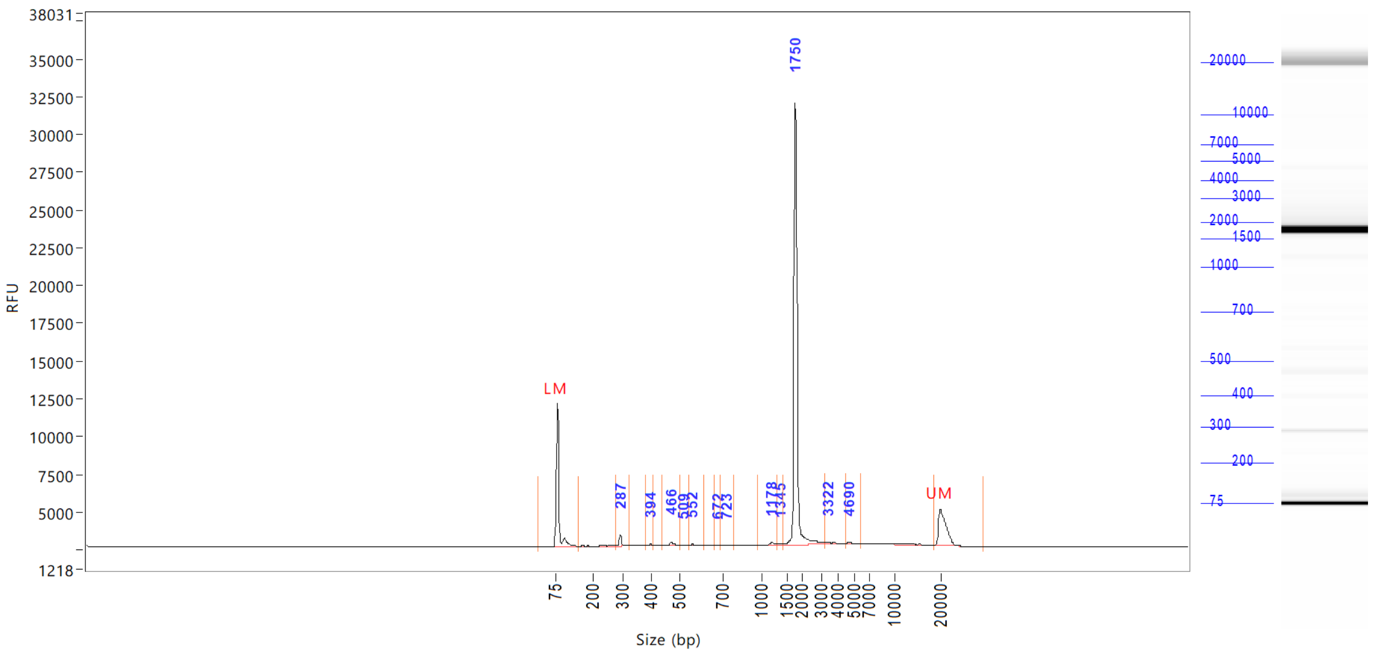

Peak	Size (bp)	Concentration (ng/uL)	From (bp)	To (bp)	Average size (bp)	CV%	RFU	Corrected peak area
1	75 (LM)	0.5000	12	148	77	14.77	9460	58.083
2	287	0.4135	275	319	287	2.32	765	4.003
3	394	0.0597	378	407	394	1.19	110	0.578
4	466	0.2531	437	498	468	2.23	279	2.451
5	509	0.0463	498	539	512	1.97	55	0.448
6	552	0.0710	539	608	556	2.23	123	0.687
7	672	0.0299	657	687	671	0.92	59	0.289
8	723	0.0515	687	779	722	2.37	66	0.498
9	1178	0.1532	965	1289	1164	5.63	183	1.483
10	1345	0.0470	1289	1400	1344	2.49	61	0.455
11	1750	15.5910	1400	3144	1774	9.91	29307	150.929
12	3322	0.2050	3144	4449	3642	9.74	128	1.984
13	4690	0.0888	4449	5741	4716	4.62	149	0.859
14	20000 (UM)	0.2096	18537	29309	20514	4.25	2368	24.350

TIC:	17.0098	ng/uL
TIM:	18.9237	nmole/L
Total concentration:	17.4657	ng/uL

```
Sample peak width (sec): 5    Sample min peak height: 50    Sample baseline V to V?: Y    Sample baseline V to V points: 3
Sample filter: Binomial       Number of points for filter: 3  Sample start region (min): 0  Sample end region (min): 30
Marker peak width (sec): 5    Marker min peak height: 200   Marker baseline V to V?: Y    Marker baseline V to V points: 3
Lower marker selection: First peak > 500 RFU                Upper marker selection: Last peak > 200 RFU
Ladder size (bp)75, 200, 300, 400, 500, 700, 1000, 1500, 2000, 3000, 4000, 5000, 7000, 10000, 20000
Quantification using: Lower Marker          Final concentration (ng/uL): 0.5000          Dilution factor: 12.0
```

Sample: SampG11
Well location: G11
Created: Wednesday, October 20, 2021 6:38:33 PM

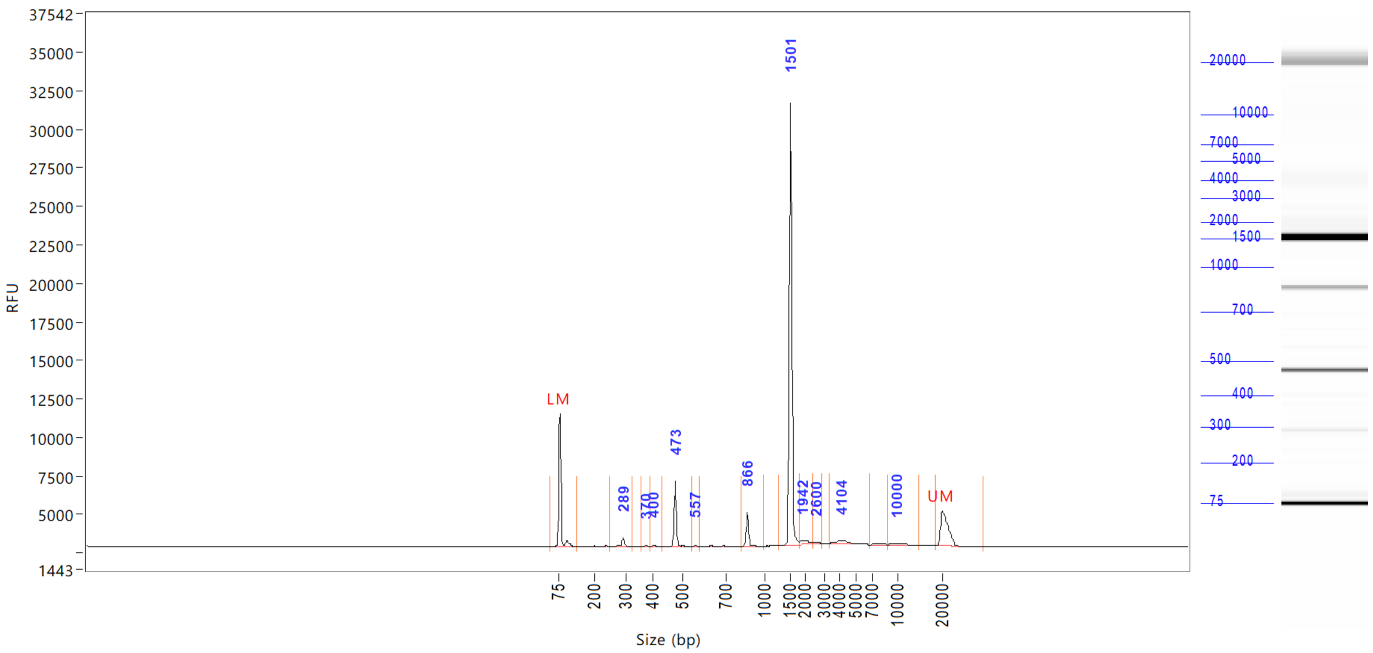

Peak	Size (bp)	Concentration (ng/uL)	From (bp)	To (bp)	Average size (bp)	CV%	RFU	Corrected peak area
1	75 (LM)	0.5000	41	135	75	12.10	8610	50.296
2	289	0.4360	248	323	286	3.65	541	3.655
3	370	0.0376	356	386	368	1.46	56	0.315
4	400	0.0748	386	431	401	2.26	111	0.627
5	473	2.2566	431	542	472	1.29	4212	18.916
6	557	0.0566	542	575	554	0.84	114	0.474
7	866	1.1640	822	996	862	1.80	2165	9.758
8	1501	15.7891	1289	1827	1513	2.93	28759	132.354
9	1942	0.4268	1827	2429	2028	7.43	253	3.578
10	2600	0.0978	2429	2857	2597	3.91	127	0.820
11	4104	0.4766	3286	6778	4115	10.69	193	3.995
12	10000	0.1765	8867	14879	11483	15.00	51	1.479
13	20000 (UM)	0.2265	18415	28947	20544	4.26	2244	22.780

TIC:	20.9923	ng/uL
TIM:	31.0423	nmole/L
Total concentration:	21.3420	ng/uL

Sample peak width (sec): 5   Sample min peak height: 50   Sample baseline V to V?: Y   Sample baseline V to V points: 3
Sample filter: Binomial   Number of points for filter: 3 Sample start region (min): 0 Sample end region (min): 30
Marker peak width (sec): 5   Marker min peak height: 200   Marker baseline V to V?: Y   Marker baseline V to V points: 3
Lower marker selection: First peak > 500 RFU   Upper marker selection: Last peak > 200 RFU
Ladder size (bp)75, 200, 300, 400, 500, 700, 1000, 1500, 2000, 3000, 4000, 5000, 7000, 10000, 20000
Quantification using: Lower Marker   Final concentration (ng/uL): 0.5000   Dilution factor: 12.0

Sample: SampG12
Well location: G12
Created: Wednesday, October 20, 2021 6:38:33 PM

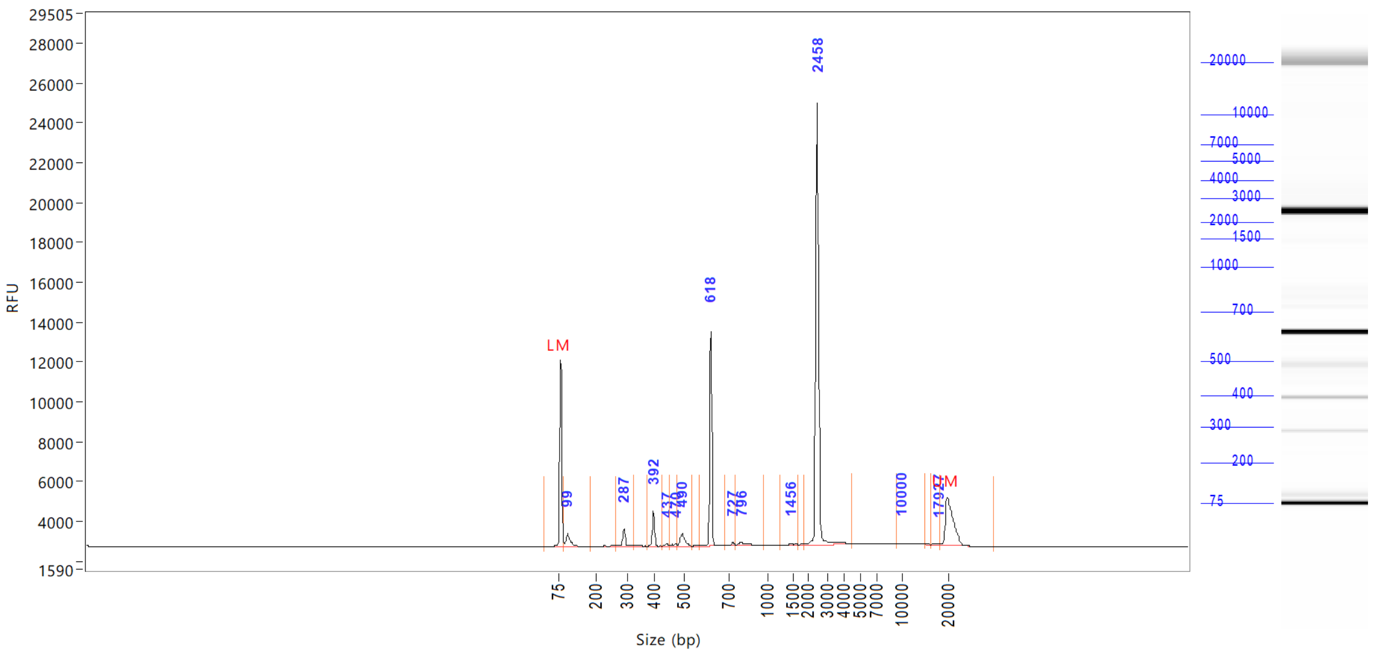

Peak	Size (bp)	Concentration (ng/uL)	From (bp)	To (bp)	Average size (bp)	CV%	RFU	Corrected peak area
1	75 (LM)	0.5000	18	87	74	5.38	9384	50.730
2	99	0.7943	87	181	103	13.04	660	6.716
3	287	0.5769	264	323	286	2.90	870	4.878
4	392	0.9480	370	420	390	1.02	1741	8.015
5	437	0.0850	420	449	436	1.38	97	0.719
6	470	0.1285	449	475	463	1.61	136	1.086
7	490	0.6892	475	529	491	1.79	623	5.827
8	618	5.3093	567	682	615	0.99	10794	44.890
9	727	0.1143	682	753	728	1.63	202	0.966
10	796	0.3578	753	970	820	4.84	171	3.025
11	1456	0.1227	1234	1693	1481	6.05	117	1.037
12	2458	13.2063	1846	4483	2459	8.96	22227	111.659
13	10000	0.1514	9467	15000	11786	13.69	55	1.280
14	17927	0.0457	16342	18537	17618	3.36	51	0.387
15	20000 (UM)	0.2511	18537	30035	20577	4.67	2381	25.471

TIC:	22.5294	ng/uL
TIM:	47.2552	nmole/L
Total concentration:	22.9030	ng/uL

Sample peak width (sec): 5 Sample min peak height: 50 Sample baseline V to V?: Y Sample baseline V to V points: 3
Sample filter: Binomial Number of points for filter: 3 Sample start region (min): 0 Sample end region (min): 30
Marker peak width (sec): 5 Marker min peak height: 200 Marker baseline V to V?: Y Marker baseline V to V points: 3
Lower marker selection: First peak > 500 RFU Upper marker selection: Last peak > 200 RFU
Ladder size (bp)75, 200, 300, 400, 500, 700, 1000, 1500, 2000, 3000, 4000, 5000, 7000, 10000, 20000
Quantification using: Lower Marker Final concentration (ng/uL): 0.5000 Dilution factor: 12.0

Sample: SampH1
Well location: H1
Created: Wednesday, October 20, 2021 6:38:33 PM

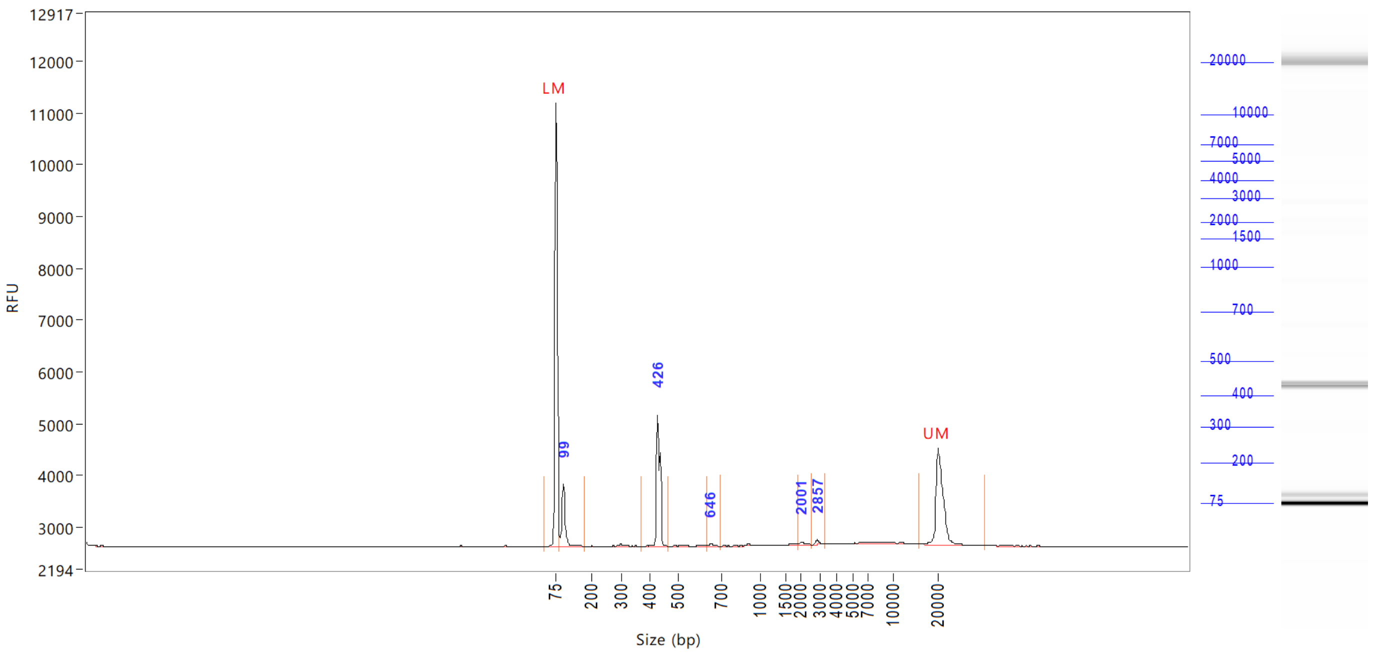

Peak	Size (bp)	Concentration (ng/uL)	From (bp)	To (bp)	Average size (bp)	CV%	RFU	Corrected peak area
1	75 (LM)	0.5000	35	87	73	5.26	8566	50.876
2	99	1.1713	87	171	100	9.51	1203	9.932
3	426	2.2467	370	464	427	1.60	2535	19.051
4	646	0.0381	629	692	647	1.60	53	0.323
5	2001	0.0521	1904	2600	2055	5.87	63	0.442
6	2857	0.0565	2600	3251	2836	3.47	87	0.479
7	20000 (UM)	0.1776	15732	30156	20382	5.39	1856	18.070

TIC:	3.5647	ng/uL
TIM:	28.0631	nmole/L
Total concentration:	4.0313	ng/uL

Sample peak width (sec): 5   Sample min peak height: 50   Sample baseline V to V?: Y   Sample baseline V to V points: 3
Sample filter: Binomial   Number of points for filter: 3   Sample start region (min): 0   Sample end region (min): 30
Marker peak width (sec): 5   Marker min peak height: 200   Marker baseline V to V?: Y   Marker baseline V to V points: 3
Lower marker selection: First peak > 500 RFU   Upper marker selection: Last peak > 200 RFU
Ladder size (bp)75, 200, 300, 400, 500, 700, 1000, 1500, 2000, 3000, 4000, 5000, 7000, 10000, 20000
Quantification using: Lower Marker   Final concentration (ng/uL): 0.5000   Dilution factor: 12.0

Sample: SampH2
Well location: H2
Created: Wednesday, October 20, 2021 6:38:33 PM

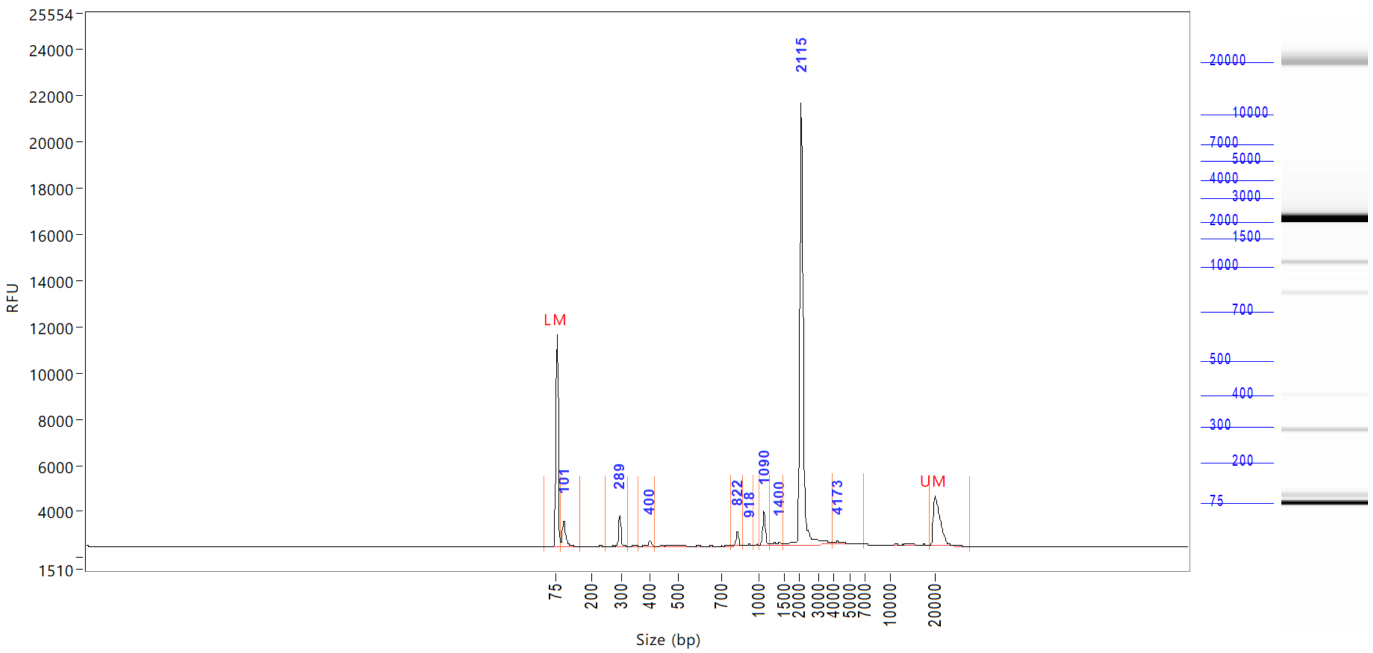

Peak	Size (bp)	Concentration (ng/uL)	From (bp)	To (bp)	Average size (bp)	CV%	RFU	Corrected peak area
1	75 (LM)	0.5000	31	87	74	4.89	9196	50.400
2	101	1.2192	87	156	100	9.80	1125	10.241
3	289	0.8503	244	317	288	2.22	1329	7.142
4	400	0.2028	360	418	395	2.42	253	1.703
5	822	0.3608	779	866	822	1.45	606	3.031
6	918	0.0556	866	948	901	2.05	62	0.467
7	1090	0.8246	1001	1201	1080	2.87	1457	6.927
8	1400	0.1652	1201	1489	1344	5.79	98	1.387
9	2115	13.2237	1489	3929	2144	11.59	19136	111.079
10	4173	0.2531	3929	6778	4508	11.05	127	2.126
11	20000 (UM)	0.2073	18659	27617	20535	4.66	2174	20.900

TIC:	17.1551	ng/uL
TIM:	38.2490	nmole/L
Total concentration:	17.6268	ng/uL

```
Sample peak width (sec): 5    Sample min peak height: 50    Sample baseline V to V?: Y    Sample baseline V to V points: 3
Sample filter: Binomial       Number of points for filter: 3  Sample start region (min): 0  Sample end region (min): 30
Marker peak width (sec): 5    Marker min peak height: 200   Marker baseline V to V?: Y    Marker baseline V to V points: 3
Lower marker selection: First peak > 500 RFU                Upper marker selection: Last peak > 200 RFU
Ladder size (bp)75, 200, 300, 400, 500, 700, 1000, 1500, 2000, 3000, 4000, 5000, 7000, 10000, 20000
Quantification using: Lower Marker          Final concentration (ng/uL): 0.5000          Dilution factor: 12.0
```

Sample: SampH3
Well location: H3
Created: Wednesday, October 20, 2021 6:38:33 PM

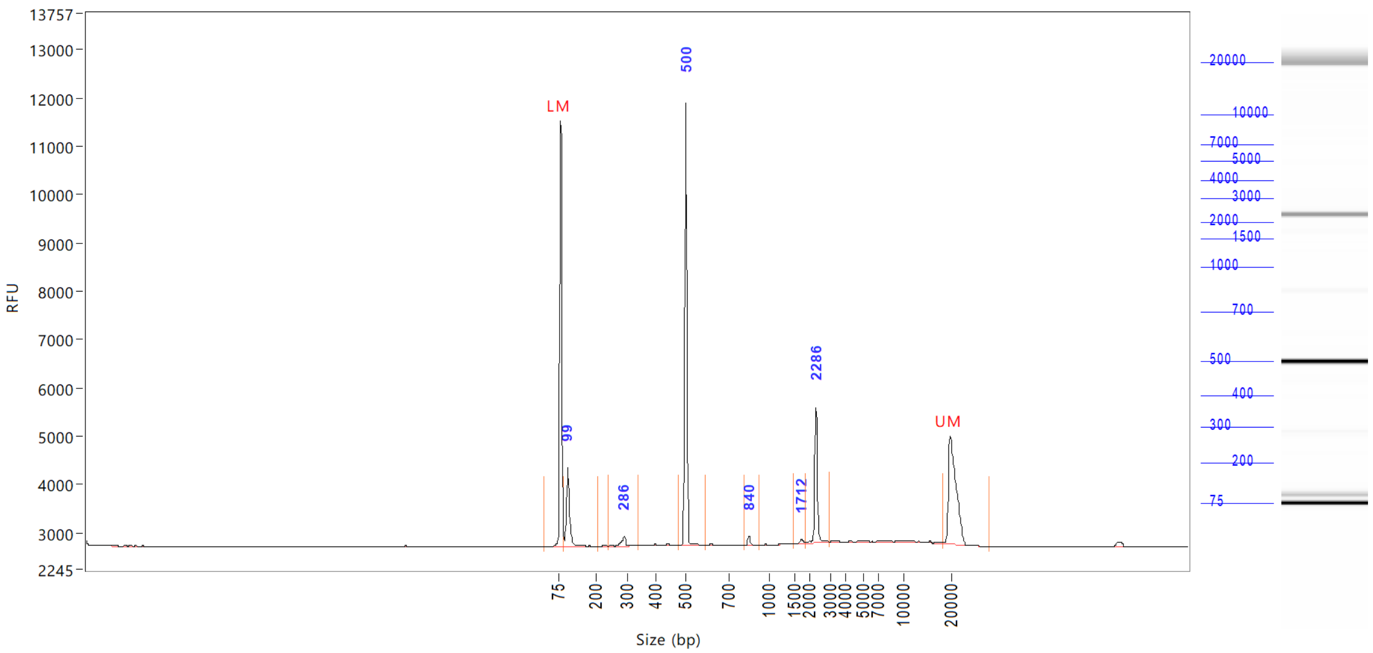

Peak	Size (bp)	Concentration (ng/uL)	From (bp)	To (bp)	Average size (bp)	CV%	RFU	Corrected peak area
1	75 (LM)	0.5000	22	87	74	5.10	8800	48.124
2	99	1.5397	87	201	101	10.43	1631	12.350
3	286	0.2171	233	335	281	3.98	203	1.741
4	500	4.9994	479	590	498	1.29	9183	40.099
5	840	0.1151	814	926	843	1.93	183	0.923
6	1712	0.0720	1489	1866	1699	4.93	79	0.578
7	2286	1.5788	1866	3000	2273	4.97	2814	12.663
8	20000 (UM)	0.2402	18415	28342	20543	4.16	2233	23.114

TIC:	8.5222	ng/uL
TIM:	44.2620	nmole/L
Total concentration:	8.9837	ng/uL

Sample peak width (sec): 5 Sample min peak height: 50 Sample baseline V to V?: Y Sample baseline V to V points: 3
Sample filter: Binomial Number of points for filter: 3 Sample start region (min): 0 Sample end region (min): 30
Marker peak width (sec): 5 Marker min peak height: 200 Marker baseline V to V?: Y Marker baseline V to V points: 3
Lower marker selection: First peak > 500 RFU Upper marker selection: Last peak > 200 RFU
Ladder size (bp)75, 200, 300, 400, 500, 700, 1000, 1500, 2000, 3000, 4000, 5000, 7000, 10000, 20000
Quantification using: Lower Marker Final concentration (ng/uL): 0.5000 Dilution factor: 12.0

Sample: SampH4
Well location: H4
Created: Wednesday, October 20, 2021 6:38:33 PM

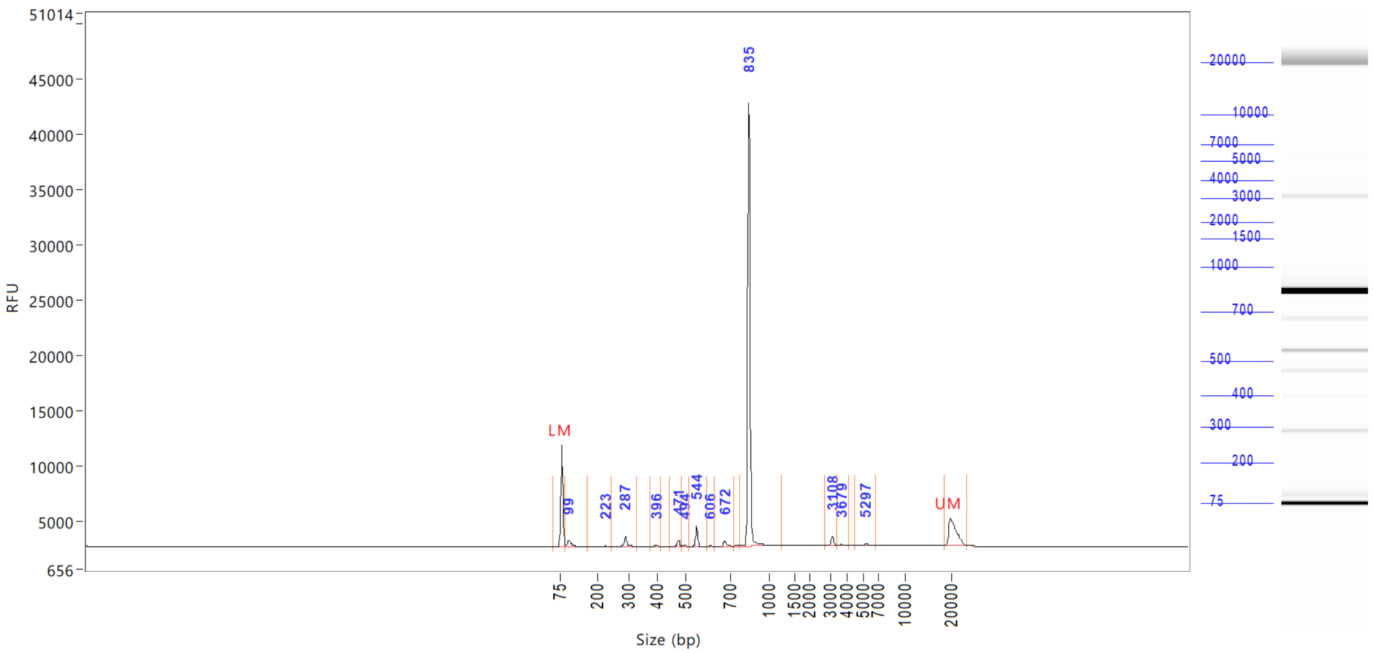

Peak	Size (bp)	Concentration (ng/uL)	From (bp)	To (bp)	Average size (bp)	CV%	RFU	Corrected peak area
1	75 (LM)	0.5000	47	87	73	4.61	9268	49.236
2	99	0.7660	87	166	103	12.38	577	6.286
3	223	0.0520	166	241	214	8.41	50	0.427
4	287	0.7168	241	327	286	3.37	910	5.882
5	396	0.0907	374	412	393	1.36	153	0.744
6	471	0.3168	445	481	469	1.25	543	2.600
7	494	0.0607	481	509	491	1.30	54	0.498
8	544	0.9848	509	588	541	1.22	1849	8.081
9	606	0.0421	588	626	604	1.07	62	0.346
10	672	0.3528	626	718	674	1.60	462	2.895
11	835	21.5614	770	1245	837	2.88	40206	176.932
12	3108	0.3831	2743	3429	3084	2.68	747	3.144
13	3679	0.0752	3429	4070	3745	4.39	61	0.617
14	5297	0.1018	4518	6704	5230	9.64	67	0.835
15	20000 (UM)	0.2492	18537	23748	20586	4.03	2402	24.536

TIC:	25.5043	ng/uL
TIM:	65.0684	nmole/L
Total concentration:	25.8942	ng/uL

```
Sample peak width (sec): 5    Sample min peak height: 50    Sample baseline V to V?: Y    Sample baseline V to V points: 3
Sample filter: Binomial       Number of points for filter: 3  Sample start region (min): 0  Sample end region (min): 30
Marker peak width (sec): 5    Marker min peak height: 200   Marker baseline V to V?: Y    Marker baseline V to V points: 3
Lower marker selection: First peak > 500 RFU                Upper marker selection: Last peak > 200 RFU
Ladder size (bp)75, 200, 300, 400, 500, 700, 1000, 1500, 2000, 3000, 4000, 5000, 7000, 10000, 20000
Quantification using: Lower Marker          Final concentration (ng/uL): 0.5000          Dilution factor: 12.0
```

Sample: SampH5
Well location: H5
Created: Wednesday, October 20, 2021 6:38:33 PM

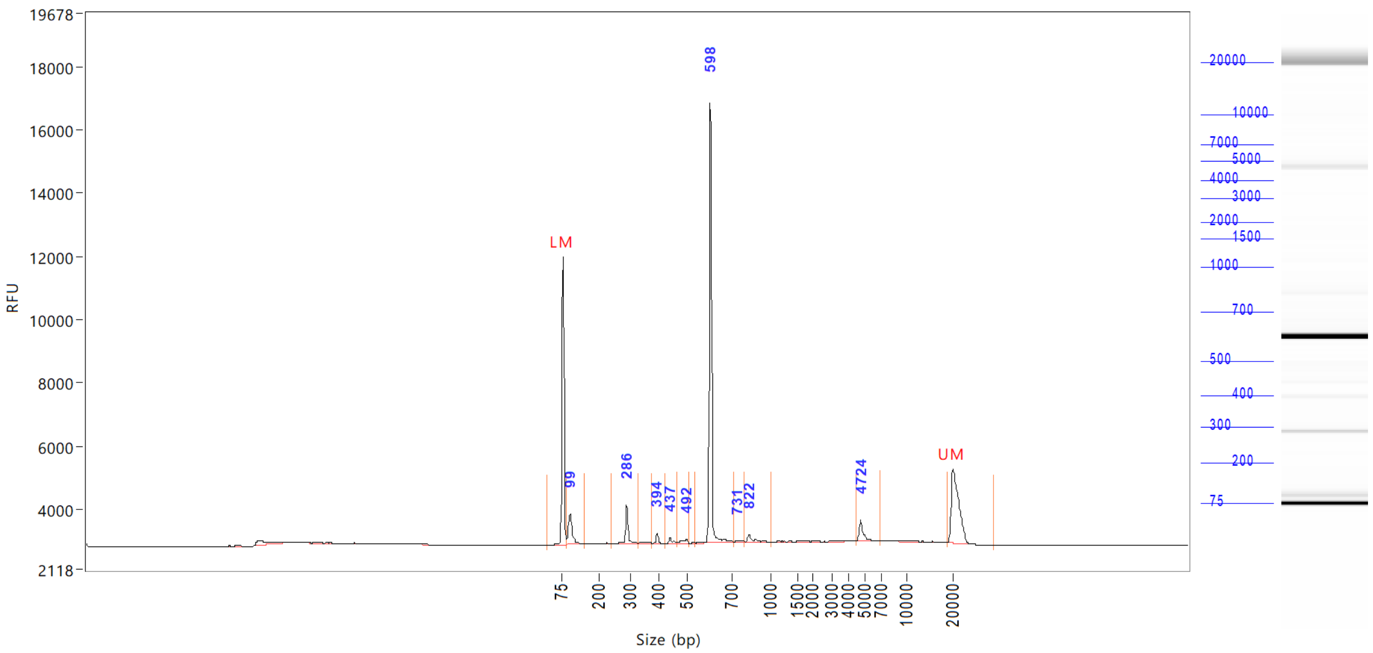

Peak	Size (bp)	Concentration (ng/uL)	From (bp)	To (bp)	Average size (bp)	CV%	RFU	Corrected peak area
1	75 (LM)	0.5000	26	87	73	5.14	9086	48.369
2	99	1.1203	87	147	101	10.19	948	9.031
3	286	0.8803	239	325	285	3.44	1229	7.097
4	394	0.2031	378	424	393	1.44	316	1.637
5	437	0.1562	424	464	441	1.96	184	1.260
6	492	0.1474	464	506	483	1.95	111	1.188
7	598	7.9234	532	710	599	2.31	13922	63.875
8	731	0.0902	710	792	748	3.14	53	0.727
9	822	0.2962	792	996	851	5.04	251	2.388
10	4724	0.5249	4449	6926	4865	8.34	680	4.232
11	20000 (UM)	0.2446	18659	28826	20614	4.06	2338	23.662

TIC:	11.3419	ng/uL
TIM:	47.9470	nmole/L
Total concentration:	11.8862	ng/uL

Sample peak width (sec): 5 Sample min peak height: 50 Sample baseline V to V?: Y Sample baseline V to V points: 3
Sample filter: Binomial Number of points for filter: 3 Sample start region (min): 0 Sample end region (min): 30
Marker peak width (sec): 5 Marker min peak height: 200 Marker baseline V to V?: Y Marker baseline V to V points: 3
Lower marker selection: First peak > 500 RFU Upper marker selection: Last peak > 200 RFU
Ladder size (bp)75, 200, 300, 400, 500, 700, 1000, 1500, 2000, 3000, 4000, 5000, 7000, 10000, 20000
Quantification using: Lower Marker Final concentration (ng/uL): 0.5000 Dilution factor: 12.0

Sample: SampH6
Well location: H6
Created: Wednesday, October 20, 2021 6:38:33 PM

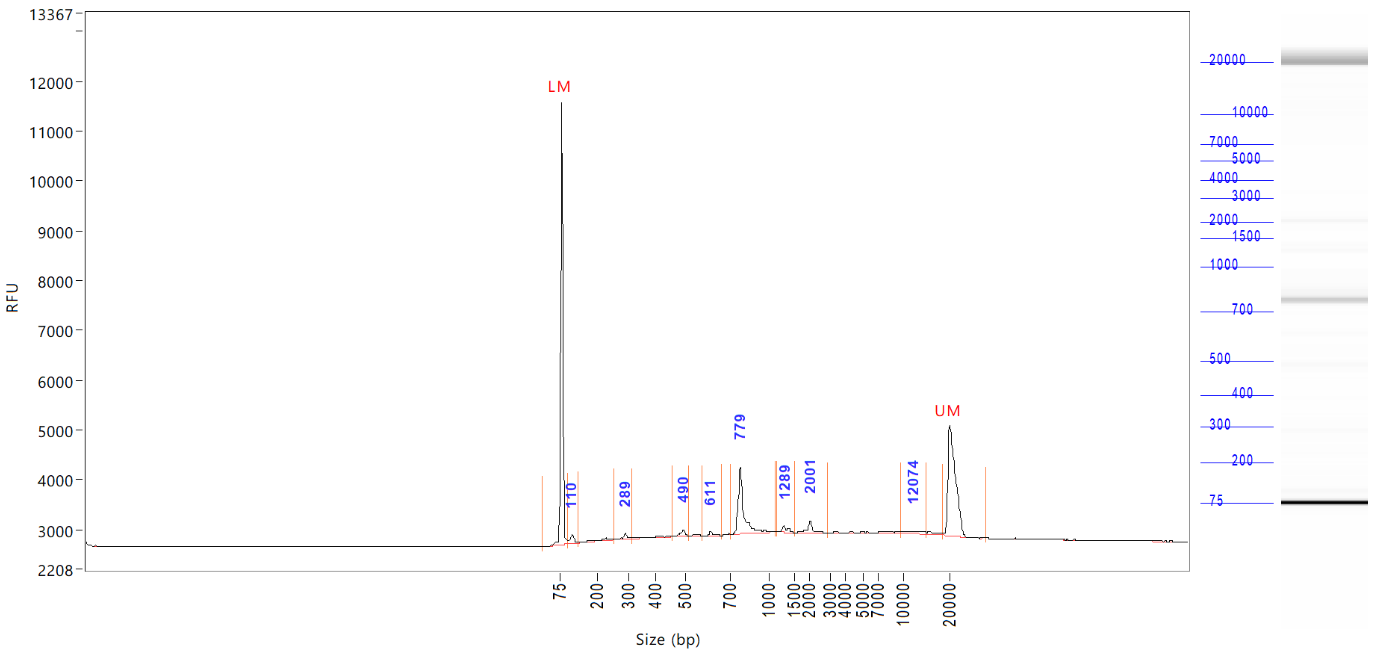

Peak	Size (bp)	Concentration (ng/uL)	From (bp)	To (bp)	Average size (bp)	CV%	RFU	Corrected peak area
1	75 (LM)	0.5000	11	97	74	6.28	8887	49.662
2	110	0.2232	97	133	110	7.22	175	1.847
3	289	0.1400	251	313	284	4.55	121	1.159
4	490	0.1644	454	509	484	2.15	139	1.361
5	611	0.0694	572	659	610	2.35	78	0.574
6	779	1.5754	705	1112	795	6.34	1338	13.040
7	1289	0.1201	1167	1520	1308	4.25	130	0.994
8	2001	0.1954	1520	2886	1999	8.36	240	1.617
9	12074	0.1569	9667	15000	12106	13.13	52	1.298
10	20000 (UM)	0.2306	18415	27859	20586	4.28	2218	22.906

TIC:	2.6447	ng/uL
TIM:	8.4843	nmole/L
Total concentration:	3.1575	ng/uL

Sample peak width (sec): 5 Sample min peak height: 50 Sample baseline V to V?: Y Sample baseline V to V points: 3
Sample filter: Binomial Number of points for filter: 3 Sample start region (min): 0 Sample end region (min): 30
Marker peak width (sec): 5 Marker min peak height: 200 Marker baseline V to V?: Y Marker baseline V to V points: 3
Lower marker selection: First peak > 500 RFU Upper marker selection: Last peak > 200 RFU
Ladder size (bp)75, 200, 300, 400, 500, 700, 1000, 1500, 2000, 3000, 4000, 5000, 7000, 10000, 20000
Quantification using: Lower Marker Final concentration (ng/uL): 0.5000 Dilution factor: 12.0

Sample: SampH7
Well location: H7
Created: Wednesday, October 20, 2021 6:38:33 PM

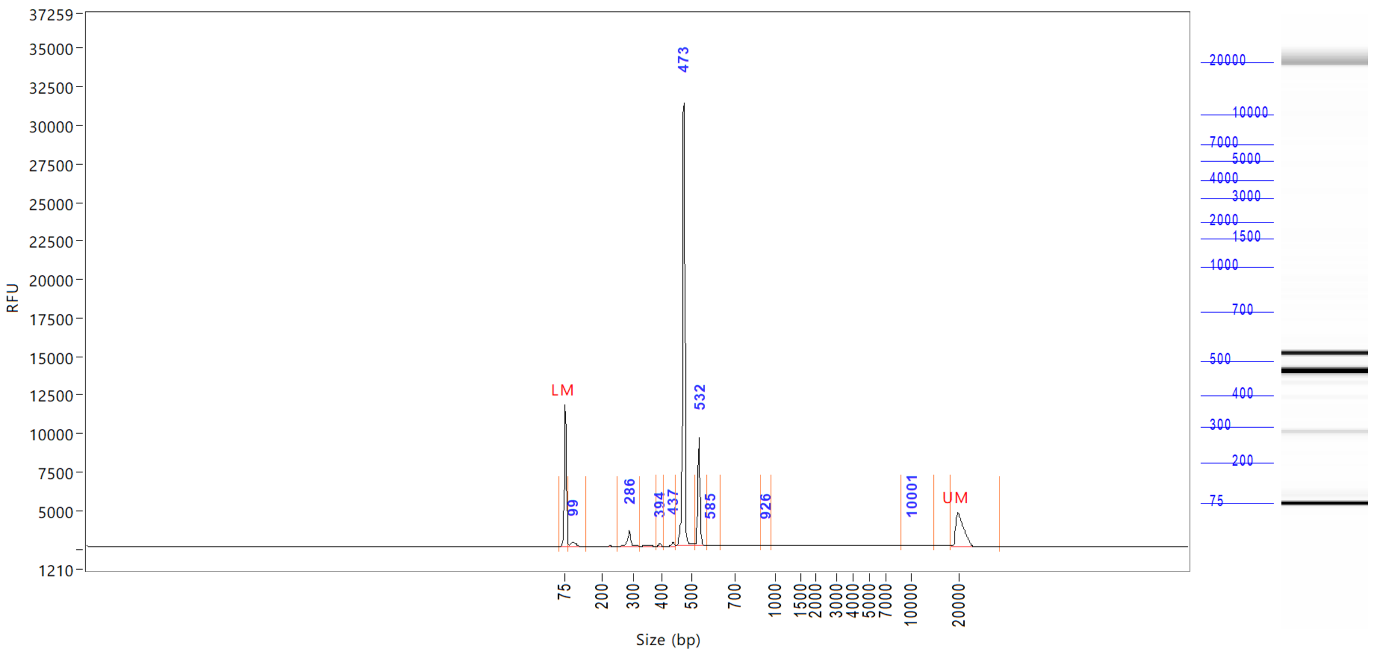

Peak	Size (bp)	Concentration (ng/uL)	From (bp)	To (bp)	Average size (bp)	CV%	RFU	Corrected peak area
1	75 (LM)	0.5000	54	87	73	4.53	9158	48.091
2	99	0.5065	87	145	102	10.12	310	4.060
3	286	0.9075	250	321	282	3.02	1018	7.274
4	394	0.1013	380	405	390	1.03	174	0.812
5	437	0.1743	405	447	434	1.16	290	1.397
6	473	16.9454	447	514	471	1.17	28773	135.821
7	532	3.9107	514	567	530	1.10	6992	31.345
8	585	0.0777	567	631	585	2.14	53	0.623
9	926	0.0416	896	965	922	1.24	61	0.334
10	10001	0.1395	8800	15000	11095	14.75	51	1.118
11	20000 (UM)	0.2322	18415	28947	20570	4.38	2198	22.336

TIC:	22.8045	ng/uL
TIM:	86.1905	nmole/L
Total concentration:	23.3288	ng/uL

```
Sample peak width (sec): 5    Sample min peak height: 50    Sample baseline V to V?: Y    Sample baseline V to V points: 3
Sample filter: Binomial       Number of points for filter: 3  Sample start region (min): 0  Sample end region (min): 30
Marker peak width (sec): 5    Marker min peak height: 200   Marker baseline V to V?: Y    Marker baseline V to V points: 3
Lower marker selection: First peak > 500 RFU                Upper marker selection: Last peak > 200 RFU
Ladder size (bp)75, 200, 300, 400, 500, 700, 1000, 1500, 2000, 3000, 4000, 5000, 7000, 10000, 20000
Quantification using: Lower Marker          Final concentration (ng/uL): 0.5000          Dilution factor: 12.0
```

Sample: SampH8
Well location: H8
Created: Wednesday, October 20, 2021 6:38:33 PM

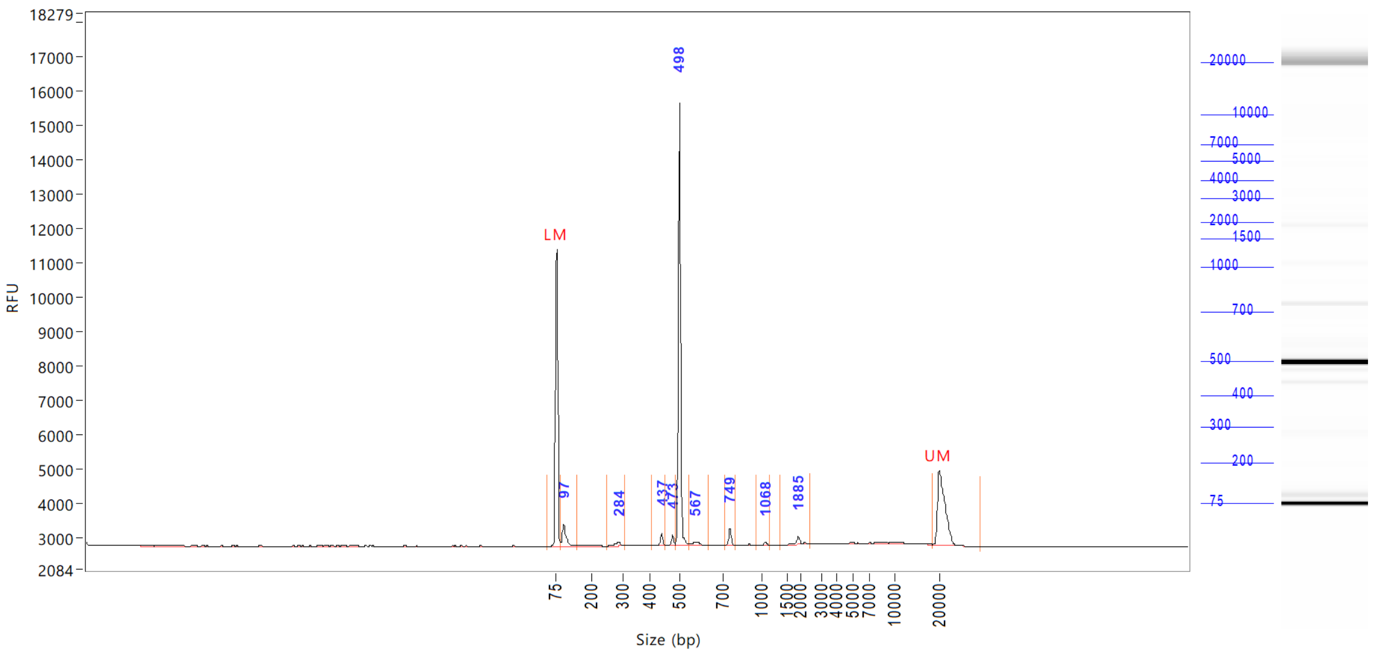

Peak	Size (bp)	Concentration (ng/uL)	From (bp)	To (bp)	Average size (bp)	CV%	RFU	Corrected peak area
1	75 (LM)	0.5000	45	87	73	4.57	8632	46.597
2	97	0.8080	87	148	101	10.04	623	6.275
3	284	0.1800	246	305	279	3.68	121	1.398
4	437	0.2532	403	452	434	1.39	383	1.967
5	473	0.2021	452	485	473	1.11	313	1.570
6	498	7.6261	485	544	497	1.07	12920	59.226
7	567	0.2030	544	631	572	2.79	101	1.577
8	749	0.2963	714	792	747	1.39	493	2.301
9	1068	0.0664	957	1156	1060	3.22	95	0.516
10	1885	0.2139	1378	2429	1916	8.63	232	1.661
11	20000 (UM)	0.2378	18415	29189	20592	4.59	2167	22.161

TIC:	9.8490	ng/uL
TIM:	42.6336	nmole/L
Total concentration:	10.2857	ng/uL

Sample peak width (sec): 5 Sample min peak height: 50 Sample baseline V to V?: Y Sample baseline V to V points: 3
Sample filter: Binomial Number of points for filter: 3 Sample start region (min): 0 Sample end region (min): 30
Marker peak width (sec): 5 Marker min peak height: 200 Marker baseline V to V?: Y Marker baseline V to V points: 3
Lower marker selection: First peak > 500 RFU Upper marker selection: Last peak > 200 RFU
Ladder size (bp)75, 200, 300, 400, 500, 700, 1000, 1500, 2000, 3000, 4000, 5000, 7000, 10000, 20000
Quantification using: Lower Marker Final concentration (ng/uL): 0.5000 Dilution factor: 12.0

Sample: SampH9
Well location: H9
Created: Wednesday, October 20, 2021 6:38:33 PM

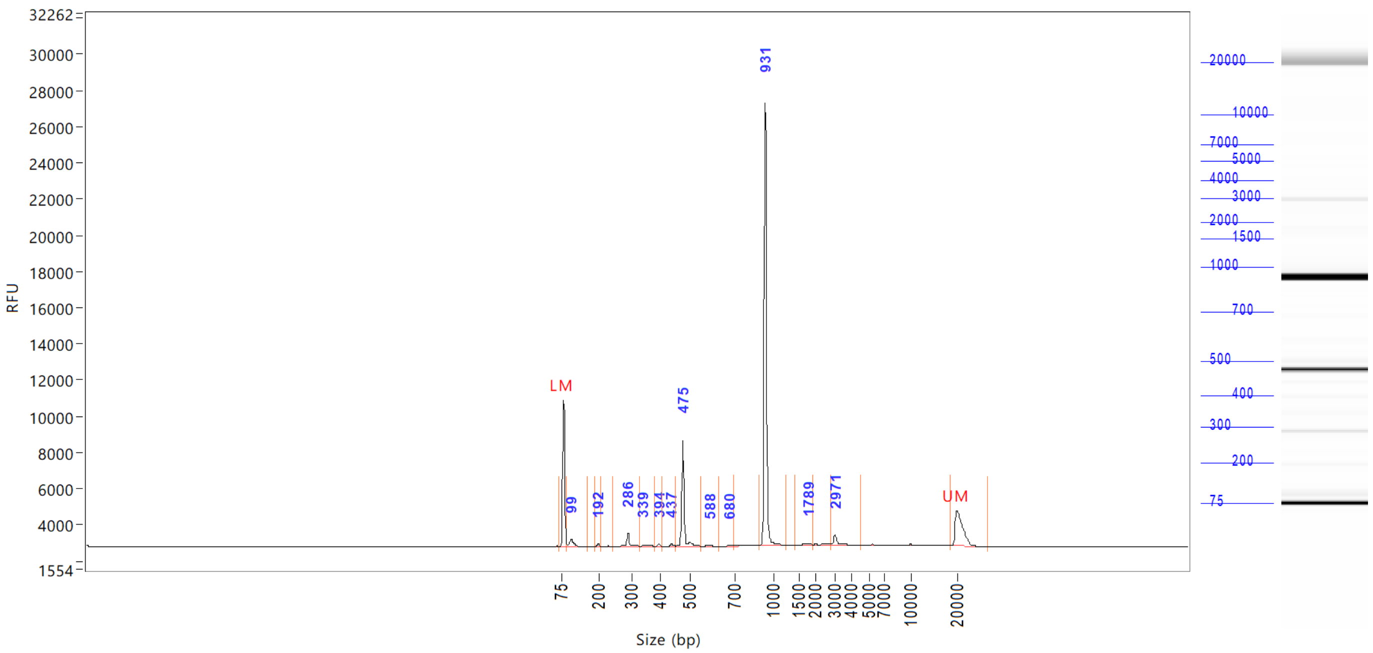

Peak	Size (bp)	Concentration (ng/uL)	From (bp)	To (bp)	Average size (bp)	CV%	RFU	Corrected peak area
1	75 (LM)	0.5000	60	89	74	4.33	8066	42.883
2	99	0.5823	89	160	103	9.75	373	4.162
3	192	0.1124	181	203	189	1.79	149	0.804
4	286	0.6179	239	327	285	3.81	712	4.417
5	339	0.1463	327	384	349	3.85	94	1.046
6	394	0.0975	384	409	392	1.00	152	0.697
7	437	0.0965	409	452	436	1.62	128	0.689
8	475	3.9257	452	549	475	1.94	5839	28.058
9	588	0.0895	549	629	583	2.17	73	0.640
10	680	0.0544	629	697	675	1.59	66	0.389
11	931	14.7181	887	1234	931	1.98	24493	105.193
12	1789	0.1396	1400	1923	1702	6.75	100	0.998
13	2971	0.4243	2743	4518	3092	10.49	524	3.032
14	20000 (UM)	0.2244	18415	26649	20534	4.19	1949	19.250

TIC:	21.0045	ng/uL
TIM:	55.6568	nmole/L
Total concentration:	21.4265	ng/uL

```
Sample peak width (sec): 5    Sample min peak height: 50    Sample baseline V to V?: Y    Sample baseline V to V points: 3
Sample filter: Binomial       Number of points for filter: 3  Sample start region (min): 0  Sample end region (min): 30
Marker peak width (sec): 5    Marker min peak height: 200   Marker baseline V to V?: Y    Marker baseline V to V points: 3
Lower marker selection: First peak > 500 RFU                Upper marker selection: Last peak > 200 RFU
Ladder size (bp)75, 200, 300, 400, 500, 700, 1000, 1500, 2000, 3000, 4000, 5000, 7000, 10000, 20000
Quantification using: Lower Marker          Final concentration (ng/uL): 0.5000          Dilution factor: 12.0
```

Sample: SampH10
Well location: H10
Created: Wednesday, October 20, 2021 6:38:33 PM

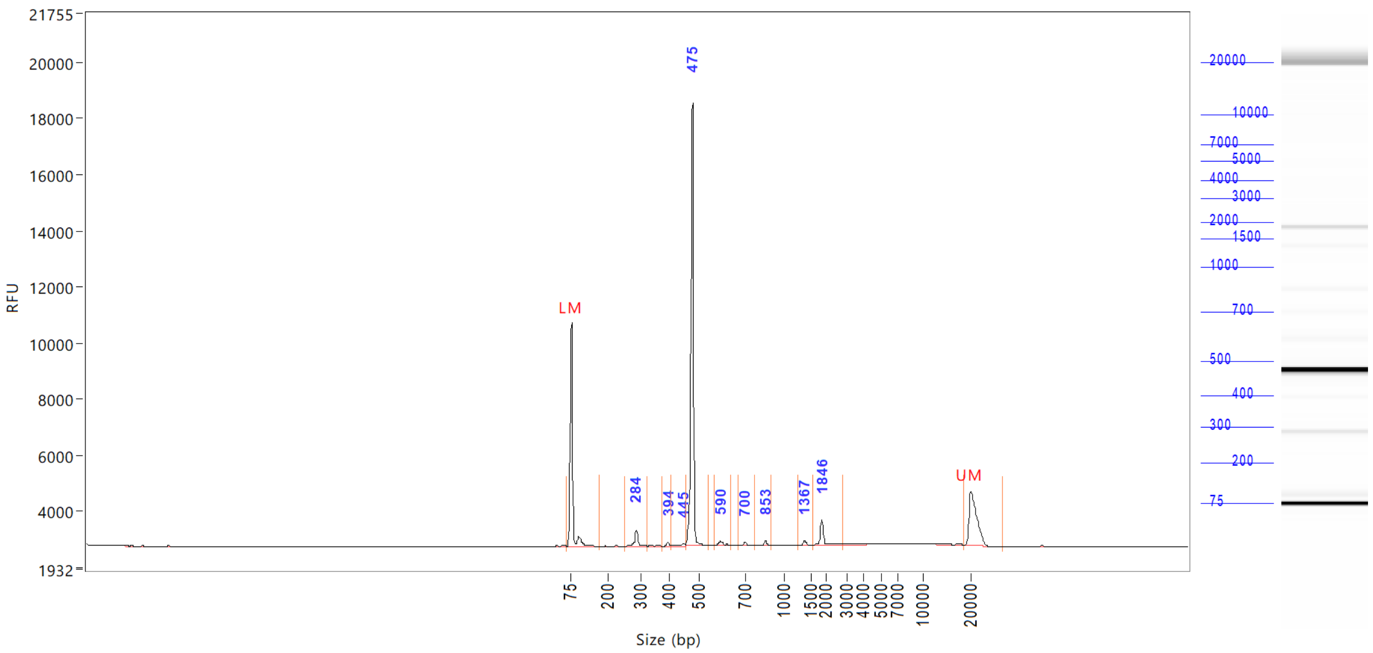

Peak	Size (bp)	Concentration (ng/uL)	From (bp)	To (bp)	Average size (bp)	CV%	RFU	Corrected peak area
1	75 (LM)	0.5000	62	169	75	12.30	7952	46.416
2	284	0.5099	250	323	283	3.05	587	3.945
3	394	0.0804	378	403	391	1.07	128	0.622
4	445	0.1165	403	452	437	2.74	85	0.901
5	475	9.8512	452	539	472	1.25	15825	76.209
6	590	0.2294	562	636	593	2.41	163	1.775
7	700	0.1062	669	770	702	2.20	129	0.822
8	853	0.1161	770	900	851	1.62	166	0.898
9	1367	0.1250	1245	1539	1372	3.68	165	0.967
10	1846	0.5711	1539	2772	1868	10.13	871	4.418
11	20000 (UM)	0.2137	18659	26891	20603	4.10	1947	19.841

TIC:	11.7059	ng/uL
TIM:	39.8467	nmole/L
Total concentration:	12.1161	ng/uL

```
Sample peak width (sec): 5    Sample min peak height: 50    Sample baseline V to V?: Y    Sample baseline V to V points: 3
Sample filter: Binomial       Number of points for filter: 3  Sample start region (min): 0  Sample end region (min): 30
Marker peak width (sec): 5    Marker min peak height: 200   Marker baseline V to V?: Y    Marker baseline V to V points: 3
Lower marker selection: First peak > 500 RFU                Upper marker selection: Last peak > 200 RFU
Ladder size (bp)75, 200, 300, 400, 500, 700, 1000, 1500, 2000, 3000, 4000, 5000, 7000, 10000, 20000
Quantification using: Lower Marker          Final concentration (ng/uL): 0.5000          Dilution factor: 12.0
```

Sample: SampH11
Well location: H11
Created: Wednesday, October 20, 2021 6:38:33 PM

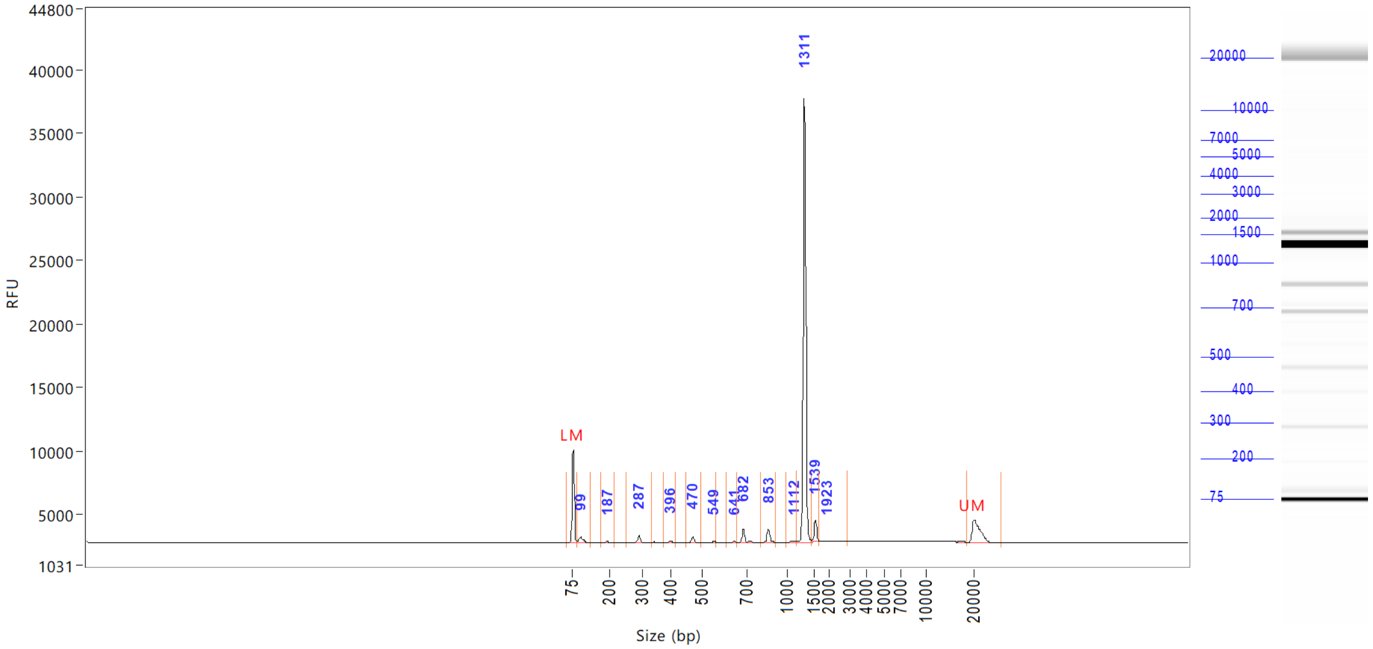

Peak	Size (bp)	Concentration (ng/uL)	From (bp)	To (bp)	Average size (bp)	CV%	RFU	Corrected peak area
1	75 (LM)	0.5000	56	86	72	4.21	7277	38.750
2	99	0.6366	86	131	100	7.93	408	4.112
3	187	0.0712	169	210	186	2.46	87	0.460
4	287	0.5523	248	329	287	4.36	558	3.567
5	396	0.1438	374	414	395	1.70	151	0.929
6	470	0.3951	447	496	467	1.23	499	2.551
7	549	0.1036	496	565	540	2.56	94	0.669
8	641	0.0748	611	657	634	1.58	86	0.483
9	682	0.8430	657	805	685	2.28	1093	5.444
10	853	0.7297	805	913	851	1.33	1068	4.713
11	1112	0.0945	991	1167	1097	3.90	77	0.610
12	1311	23.8017	1167	1456	1311	2.06	34949	153.719
13	1539	1.1607	1456	1674	1525	2.49	1723	7.496
14	1923	0.2639	1674	2857	1975	11.99	88	1.704
15	20000 (UM)	0.2345	18537	25682	20632	4.13	1774	18.173

TIC:	28.8709	ng/uL
TIM:	51.6853	nmole/L
Total concentration:	29.4211	ng/uL

```
Sample peak width (sec): 5    Sample min peak height: 50    Sample baseline V to V?: Y    Sample baseline V to V points: 3
Sample filter: Binomial       Number of points for filter: 3  Sample start region (min): 0  Sample end region (min): 30
Marker peak width (sec): 5    Marker min peak height: 200   Marker baseline V to V?: Y    Marker baseline V to V points: 3
Lower marker selection: First peak > 500 RFU                Upper marker selection: Last peak > 200 RFU
Ladder size (bp)75, 200, 300, 400, 500, 700, 1000, 1500, 2000, 3000, 4000, 5000, 7000, 10000, 20000
Quantification using: Lower Marker          Final concentration (ng/uL): 0.5000          Dilution factor: 12.0
```

Sample: SampH12
Well location: H12
Created: Wednesday, October 20, 2021 6:38:33 PM

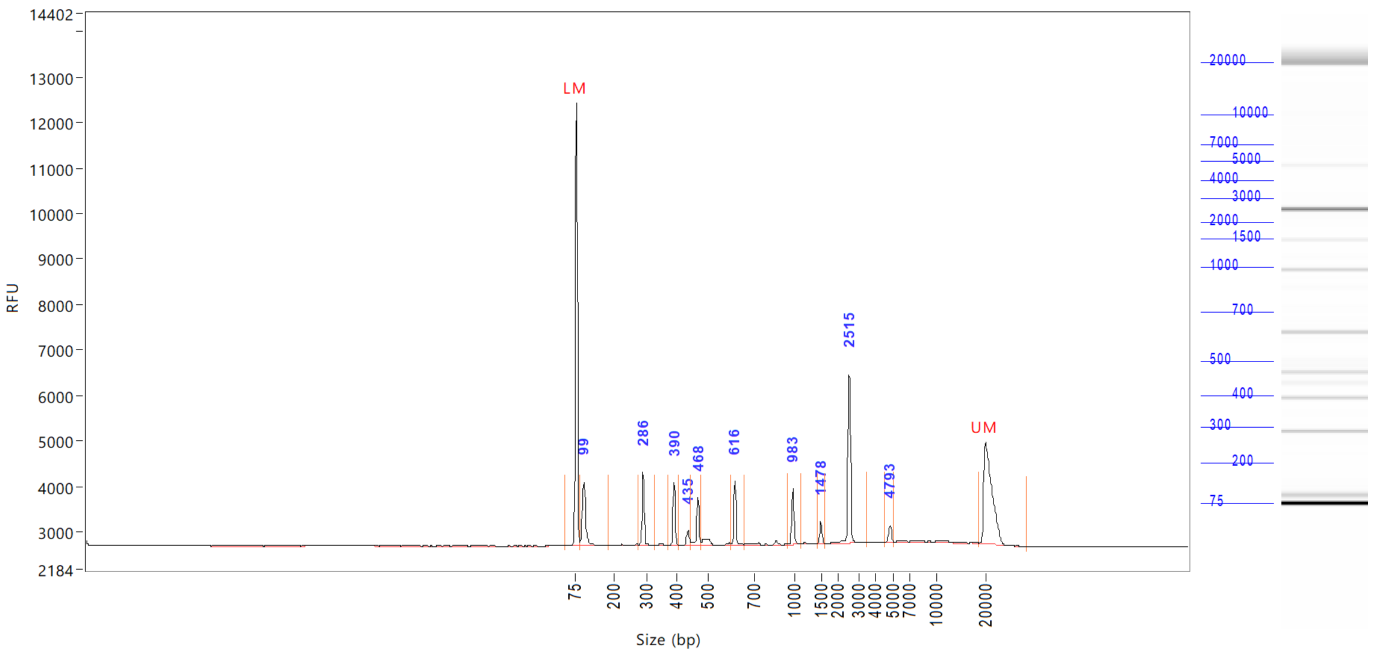

Peak	Size (bp)	Concentration (ng/uL)	From (bp)	To (bp)	Average size (bp)	CV%	RFU	Corrected peak area
1	75 (LM)	0.5000	41	86	73	4.16	9751	49.594
2	99	1.3624	86	181	99	10.71	1405	11.261
3	286	0.8960	273	325	285	1.63	1610	7.406
4	390	0.7538	372	409	388	0.95	1369	6.231
5	435	0.2432	409	445	433	1.25	329	2.010
6	468	0.5993	445	477	465	1.23	1049	4.953
7	616	0.7017	600	654	615	1.03	1399	5.800
8	983	0.5893	939	1112	981	1.19	1228	4.871
9	1478	0.2189	1411	1578	1468	1.49	481	1.809
10	2515	1.8360	1578	3465	2490	5.91	3686	15.175
11	4793	0.1691	4483	5001	4767	1.56	369	1.398
12	20000 (UM)	0.2421	18781	28584	20711	4.53	2229	24.018

TIC:	7.3696	ng/uL
TIM:	38.3930	nmole/L
Total concentration:	8.2417	ng/uL

```
Sample peak width (sec): 5    Sample min peak height: 200   Sample baseline V to V?: Y    Sample baseline V to V points: 3
Sample filter: Binomial       Number of points for filter: 3  Sample start region (min): 0  Sample end region (min): 30
Marker peak width (sec): 5    Marker min peak height: 200   Marker baseline V to V?: Y    Marker baseline V to V points: 3
Lower marker selection: First peak > 500 RFU                Upper marker selection: Last peak > 200 RFU
Ladder size (bp)75, 200, 300, 400, 500, 700, 1000, 1500, 2000, 3000, 4000, 5000, 7000, 10000, 20000
Quantification using: Lower Marker         Final concentration (ng/uL): 0.5000         Dilution factor: 12.0
```

Sample: DNA size ladder
Well location: A7
Created: Wednesday, October 20, 2021 6:38:33 PM
Import from: C:\Users\admin\Desktop\210810_alberto_ladder.SCAL
Fit type: Point to point

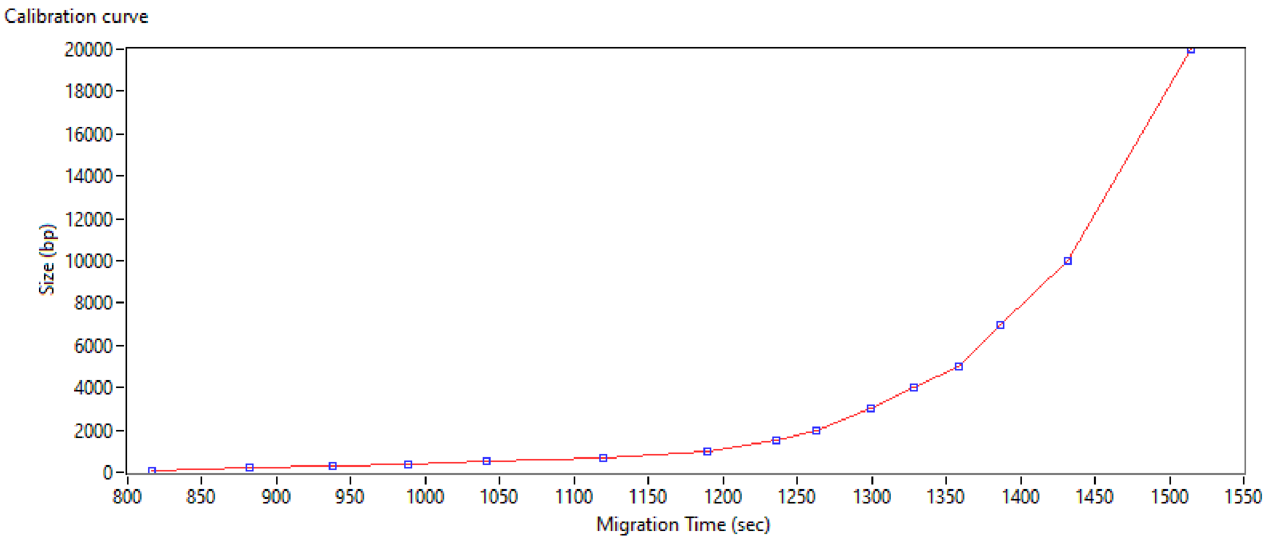

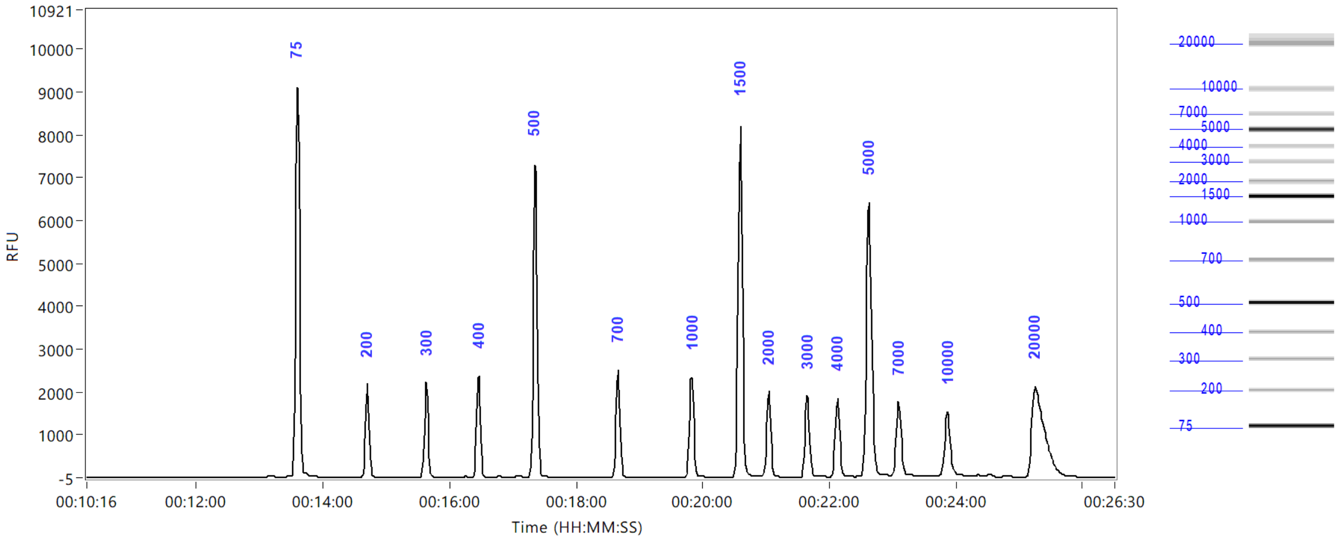

Supplement: Supplementary file 2 — sb3c00292_si_002.zip [file sb3c00292_si_002.zip › dnada_supplementary_material_pks_library_build/colony_pcr/ZAG/18-28-55_plate4/colonypcrplate_4.pdf]
