# Supplementary material for: Automated Platform for the Plasmid Construction Process
Source: ACS Synth Biol. 2023 Nov 10;12(12):3506–13. doi: 10.1021/acssynbio.3c00292 (PMC10729297; doi:10.1021/acssynbio.3c00292)

Instrument controller software run summary:

Filename and data path: C:\AATI\Data\2021 10 21\20-38-22\2021 10 21 20H 38M..raw
Created: Thursday, October 21, 2021 8:58:49 PM
Number of capillaries: 96
Array serial number: 111920-09SFS
Effect length: 33 cm
Array usage count: 270
Instrument type: ZAG DNA Analyzer
Instrument controller software version: 1.0.1.9
Device serial number: Z7597

Method Information

Method name: ZAG130FC33 - DNA 75-20000bp - Full Conditioning.mthds
Gel prime: No
Full conditioning: Yes
Gel prime to buffer: No
Gel selection: Gel 1
Perform prerun: 8.0 kV, 30 sec.
Rinse: No
Marker 1: Row: A, 5.0 kV, 5 sec.
Rinse: No
Sample injection: 5.0 kV, 5 sec.
Separation: 8.0 kV, 30.0 min.
Tray name: Sample Tray-6

Analysis mode: DNA

Notes

Gel Image

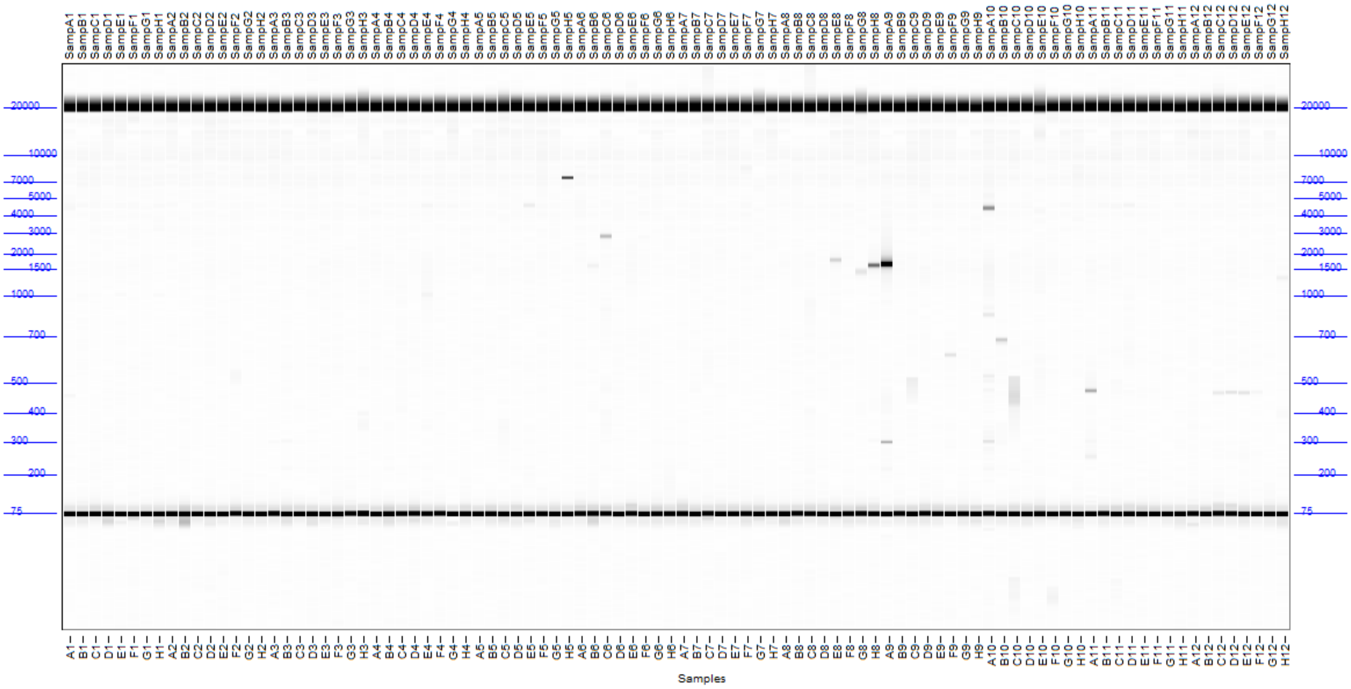

Filename and data path: C:\AATI\Data\2021 10 21\20-38-22\2021 10 21 20H 38M..raw

A1: SampA1

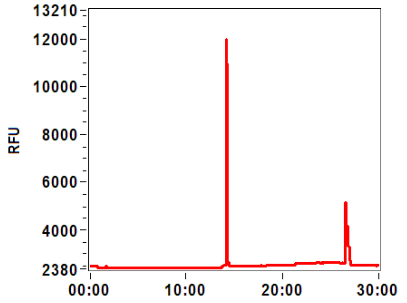

B1: SampB1

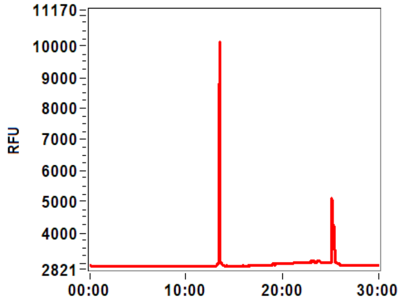

C1: SampC1

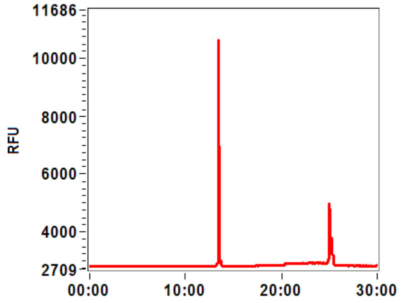

D1: SampD1

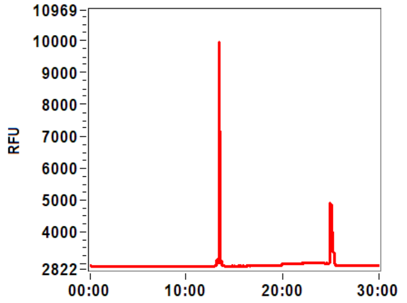

E1: SampE1

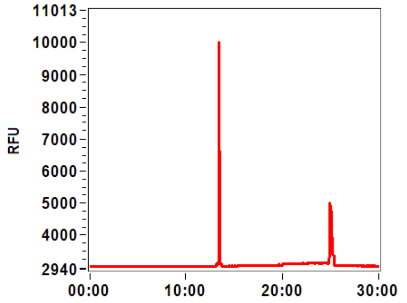

F1: SampF1

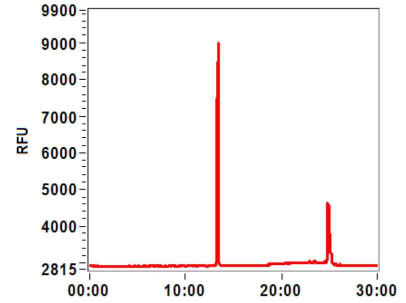

G1: SampG1

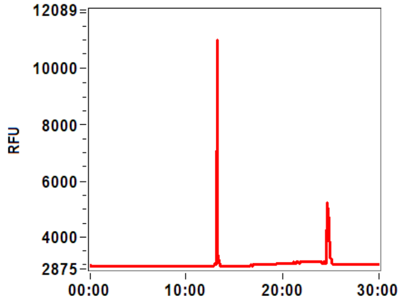

H1: SampH1

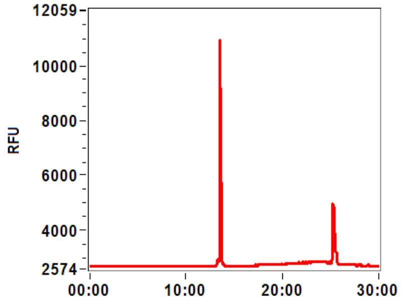

A2: SampA2

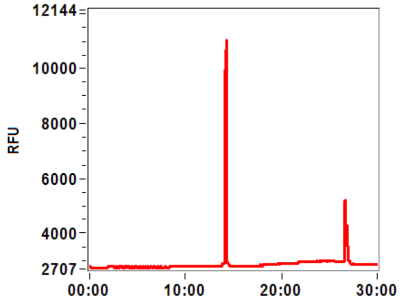

B2: SampB2

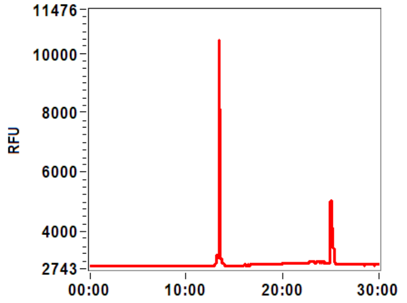

C2: SampC2

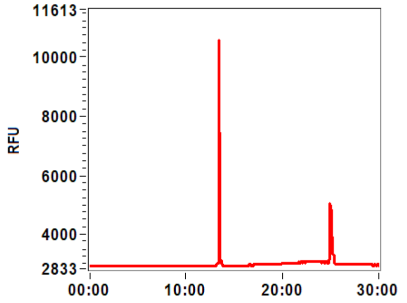

D2: SampD2

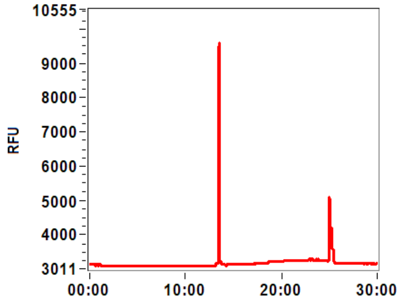

Filename and data path: C:\AATI\Data\2021 10 21\20-38-22\2021 10 21 20H 38M..raw

E2: SampE2

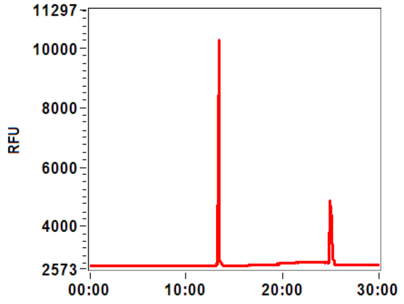

F2: SampF2

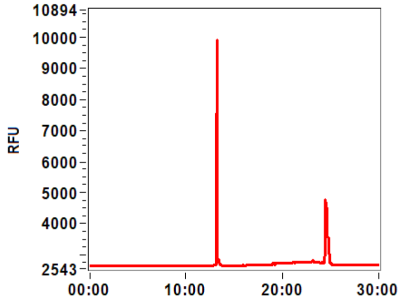

G2: SampG2

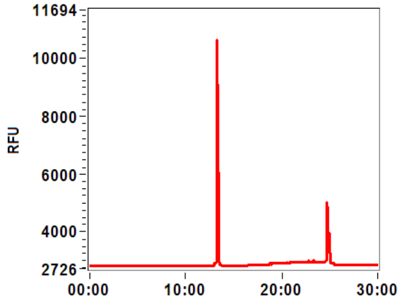

H2: SampH2

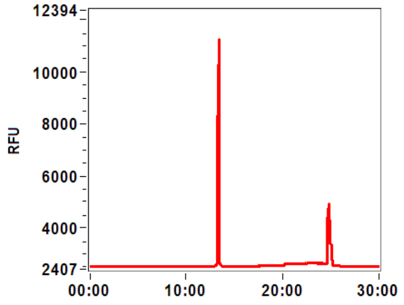

A3: SampA3

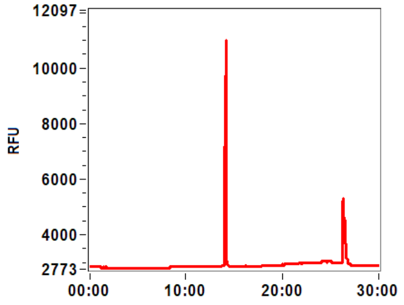

B3: SampB3

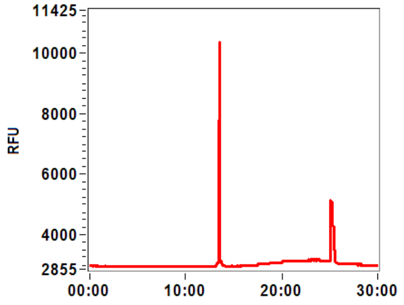

C3: SampC3

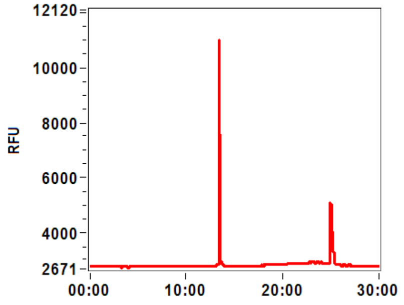

D3: SampD3

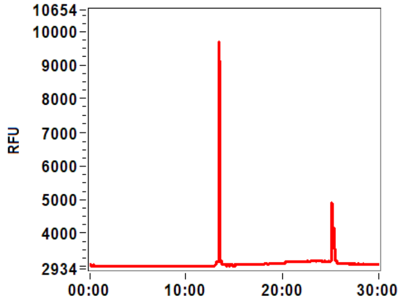

E3: SampE3

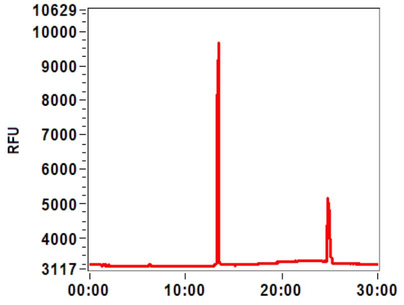

F3: SampF3

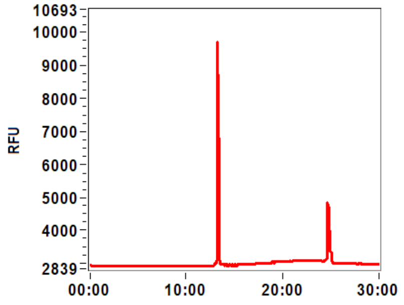

G3: SampG3

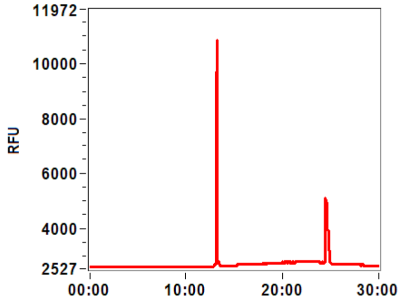

H3: SampH3

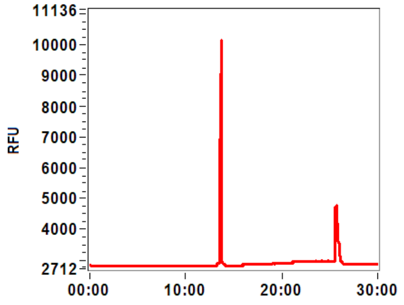

Filename and data path: C:\AATI\Data\2021 10 21\20-38-22\2021 10 21 20H 38M..raw

A4: SampA4

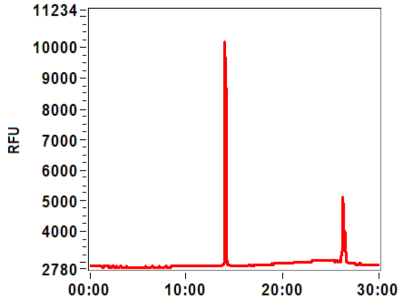

B4: SampB4

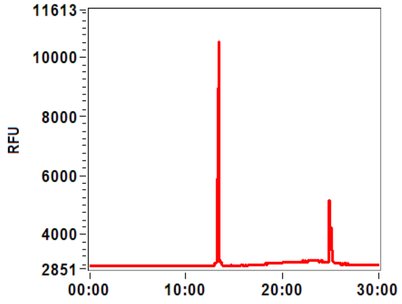

C4: SampC4

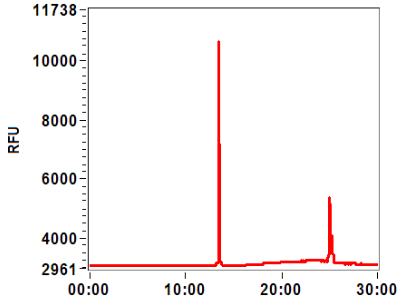

D4: SampD4

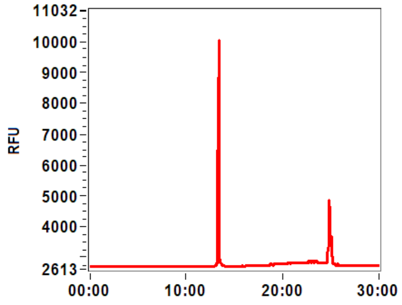

E4: SampE4

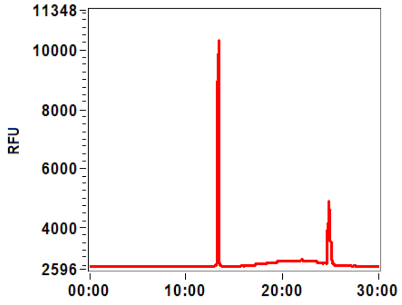

F4: SampF4

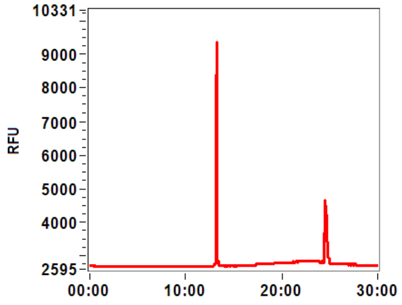

G4: SampG4

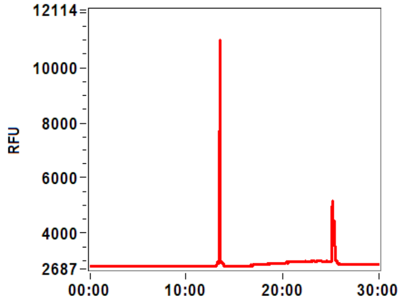

H4: SampH4

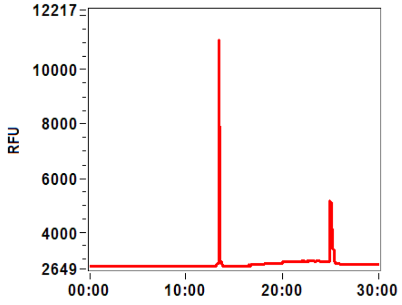

A5: SampA5

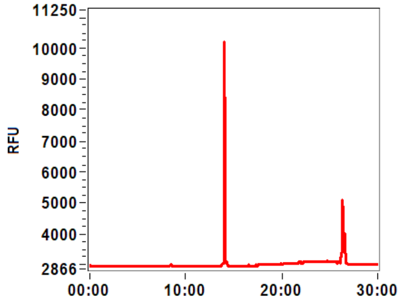

B5: SampB5

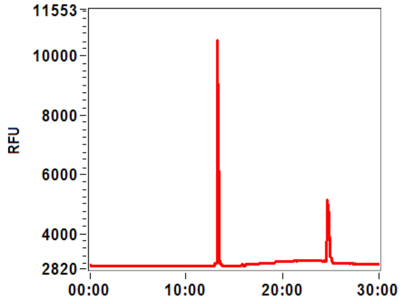

C5: SampC5

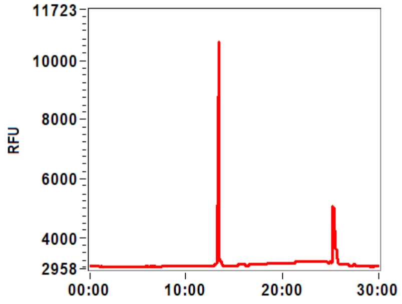

D5: SampD5

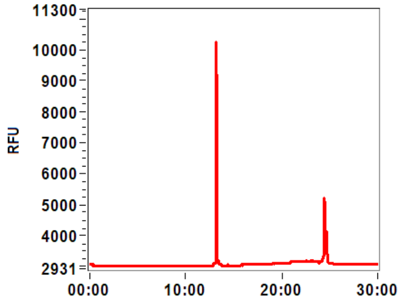

Filename and data path: C:\AATI\Data\2021 10 21\20-38-22\2021 10 21 20H 38M..raw

E5: SampE5

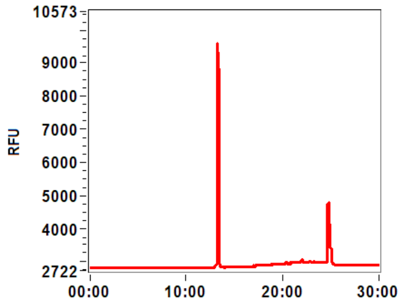

F5: SampF5

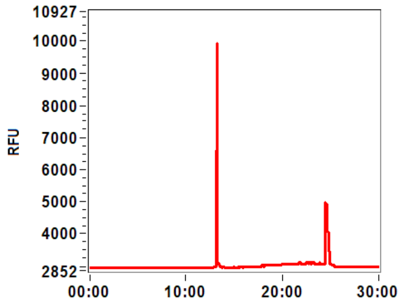

G5: SampG5

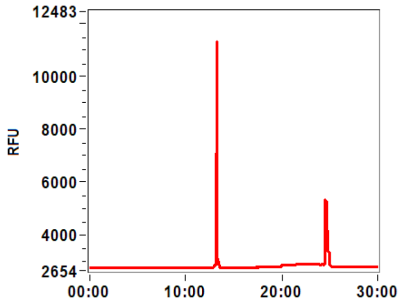

H5: SampH5

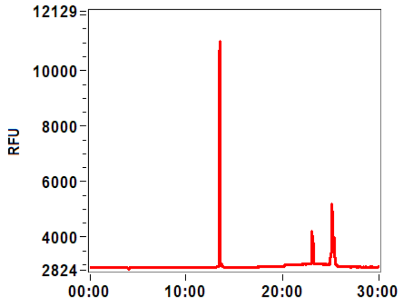

A6: SampA6

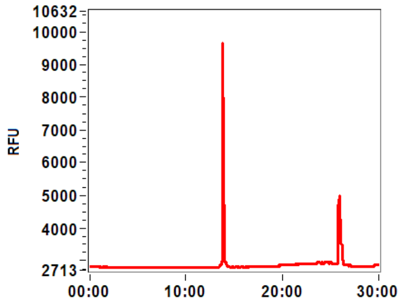

B6: SampB6

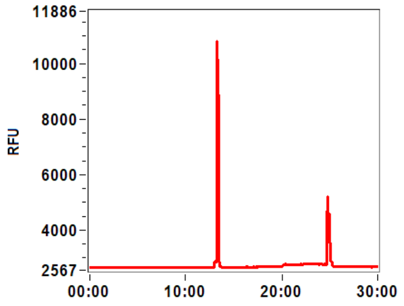

C6: SampC6

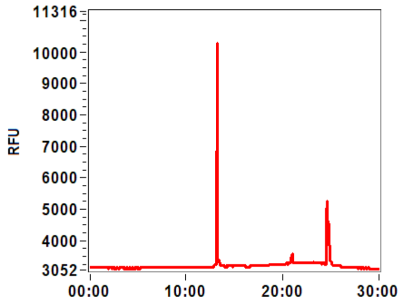

D6: SampD6

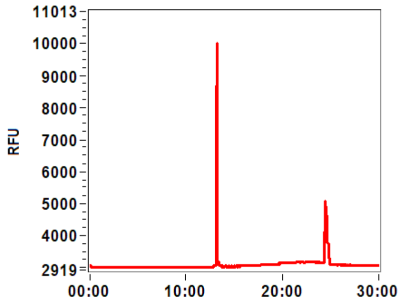

E6: SampE6

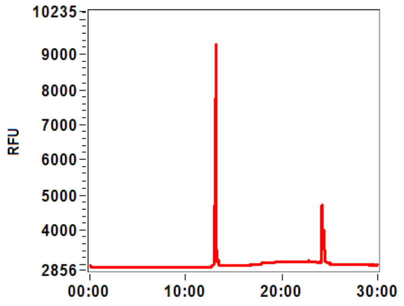

F6: SampF6

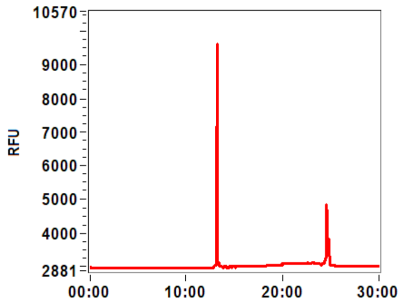

G6: SampG6

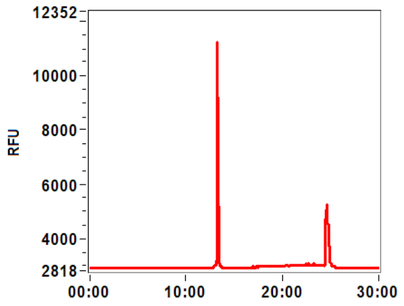

H6: SampH6

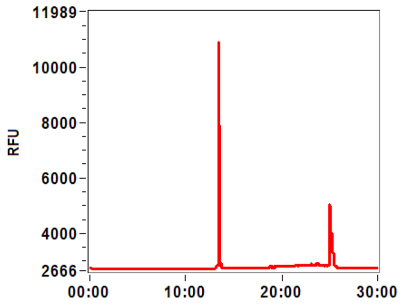

Filename and data path: C:\AATI\Data\2021 10 21\20-38-22\2021 10 21 20H 38M..raw

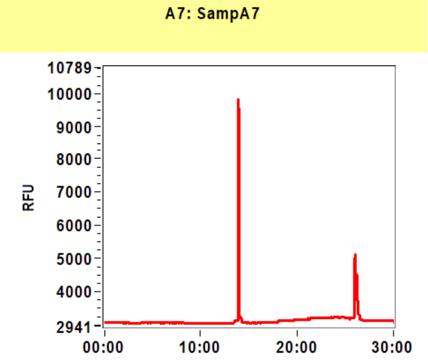

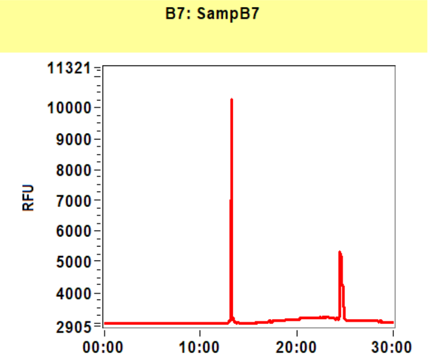

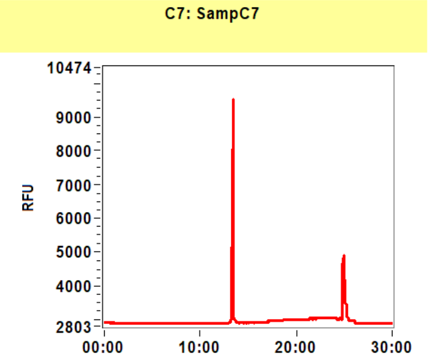

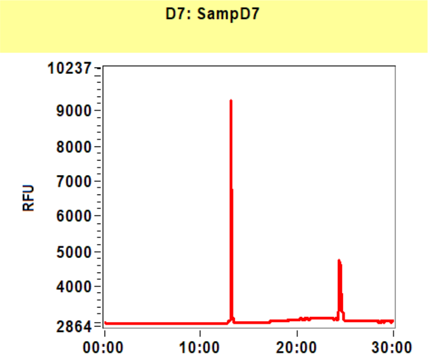

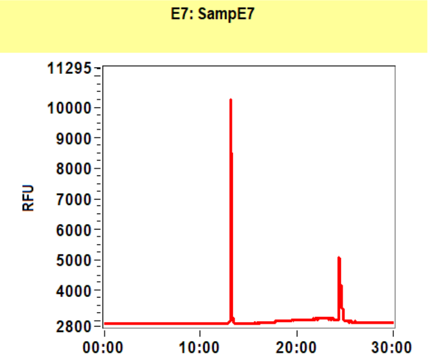

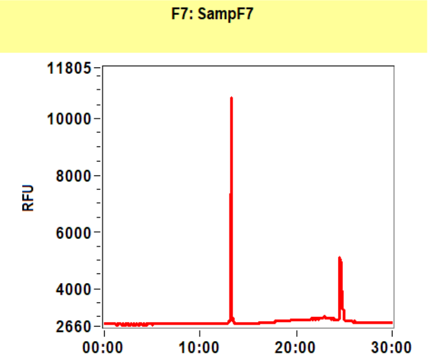

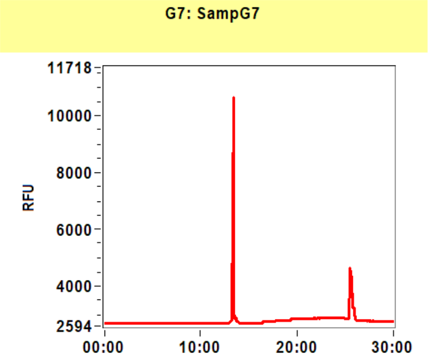

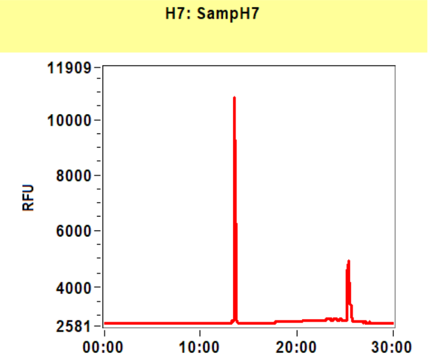

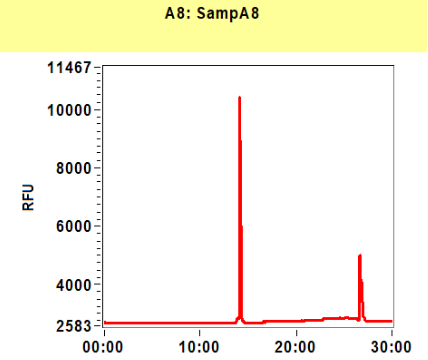

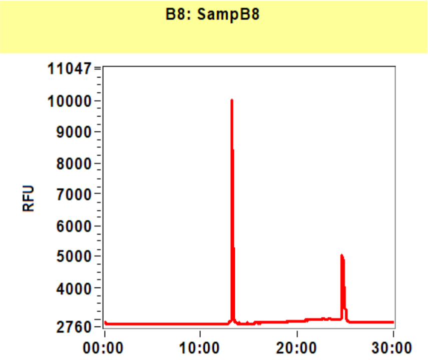

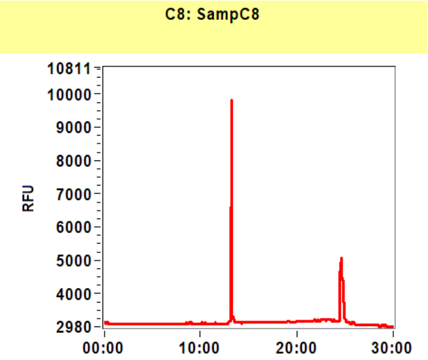

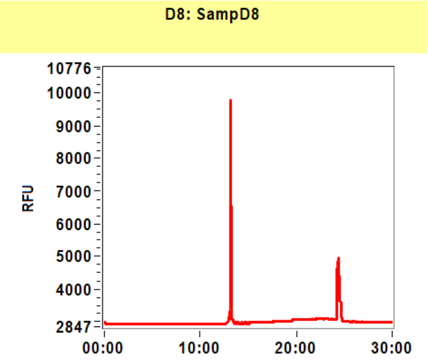

Filename and data path: C:\AATI\Data\2021 10 21\20-38-22\2021 10 21 20H 38M..raw

E8: SampE8

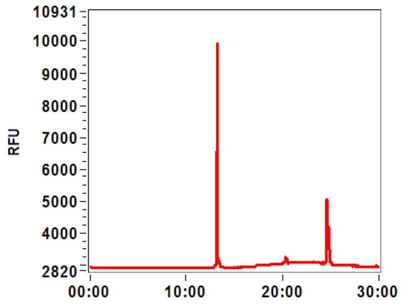

F8: SampF8

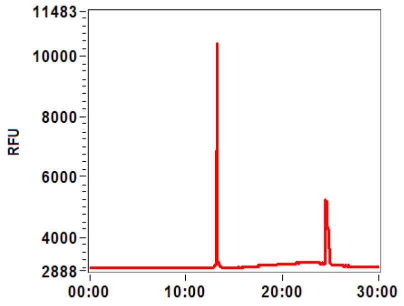

G8: SampG8

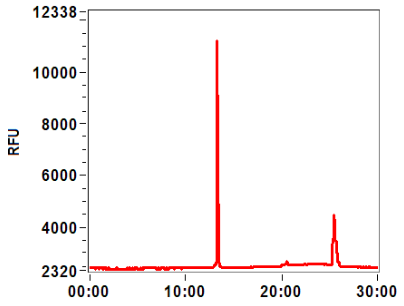

H8: SampH8

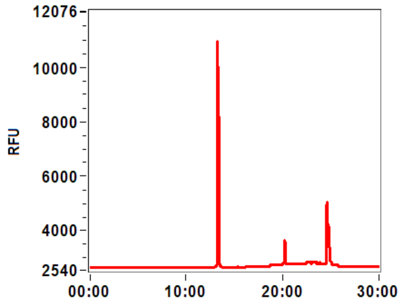

A9: SampA9

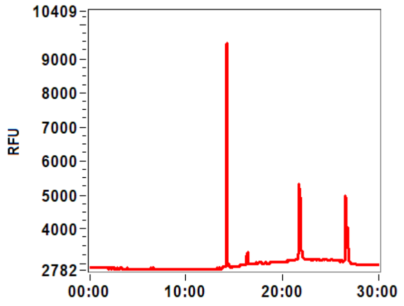

B9: SampB9

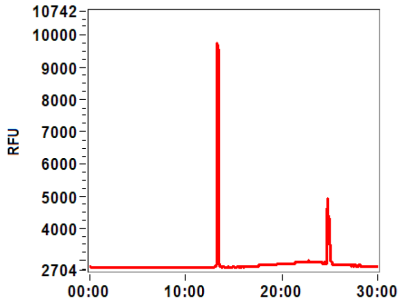

C9: SampC9

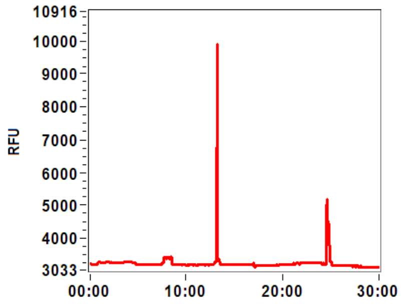

D9: SampD9

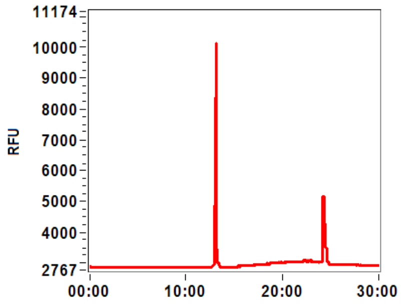

E9: SampE9

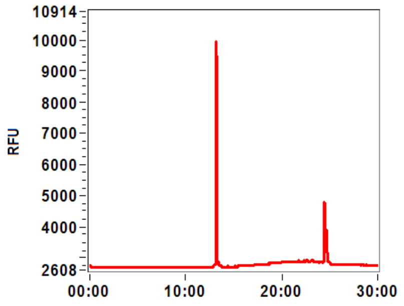

F9: SampF9

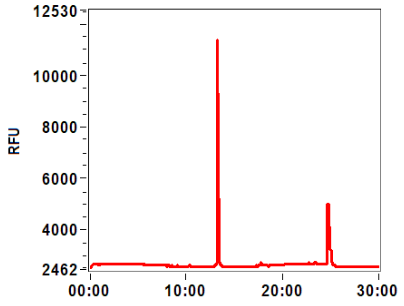

G9: SampG9

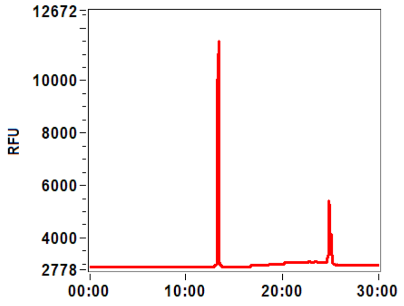

H9: SampH9

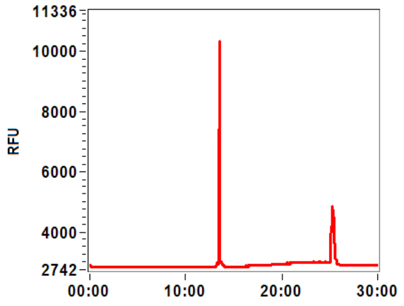

Filename and data path: C:\AATI\Data\2021 10 21\20-38-22\2021 10 21 20H 38M..raw

A10: SampA10

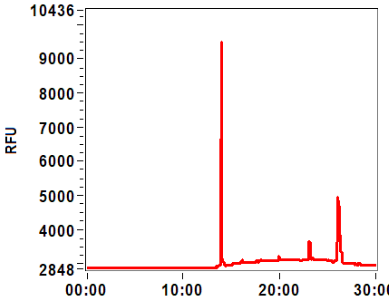

B10: SampB10

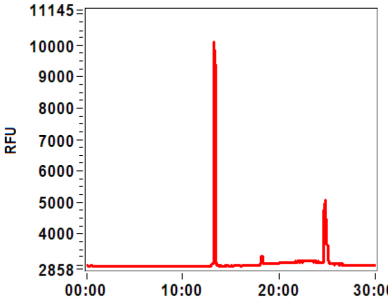

C10: SampC10

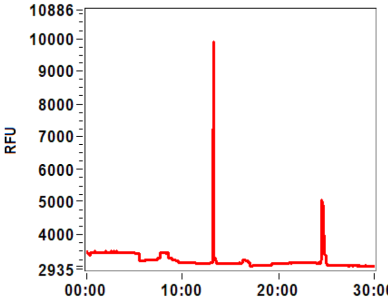

D10: SampD10

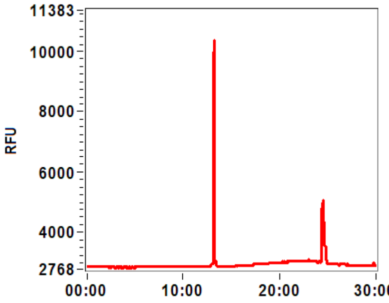

E10: SampE10

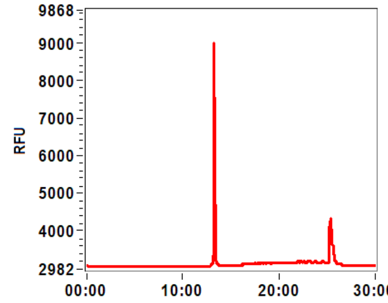

F10: SampF10

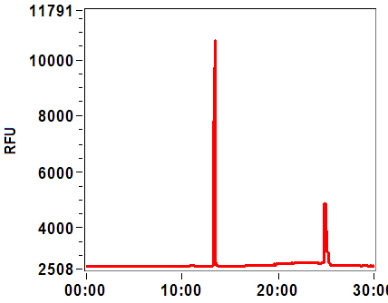

G10: SampG10

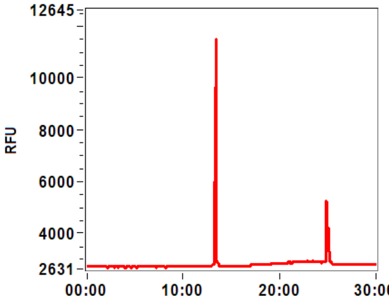

H10: SampH10

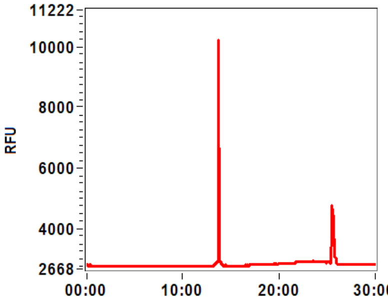

A11: SampA11

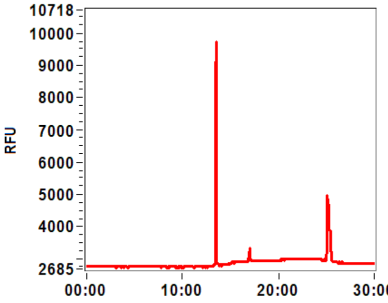

B11: SampB11

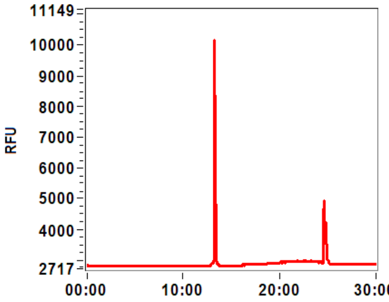

C11: SampC11

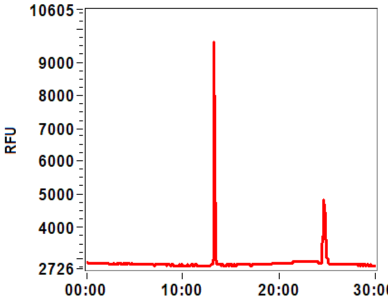

D11: SampD11

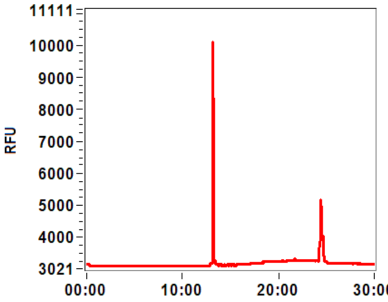

Filename and data path: C:\AATI\Data\2021 10 21\20-38-22\2021 10 21 20H 38M..raw

E11: SampE11

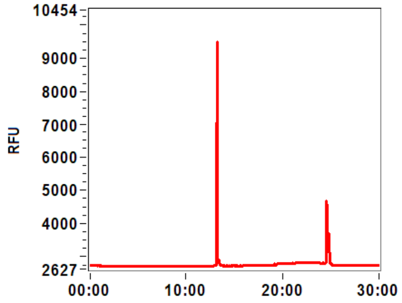

F11: SampF11

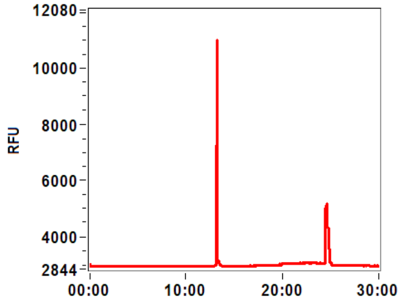

G11: SampG11

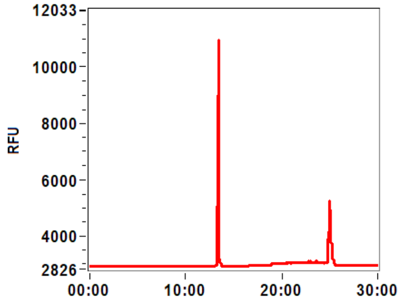

H11: SampH11

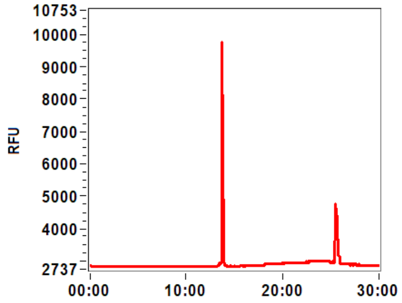

A12: SampA12

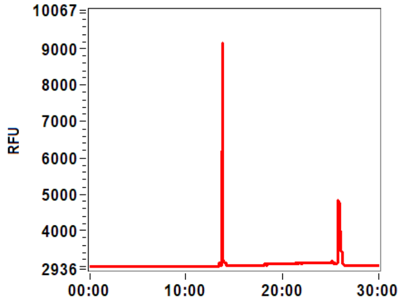

B12: SampB12

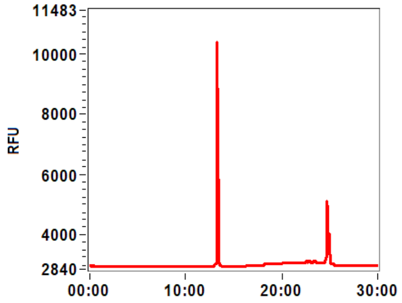

C12: SampC12

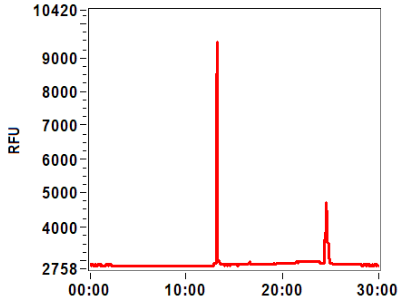

D12: SampD12

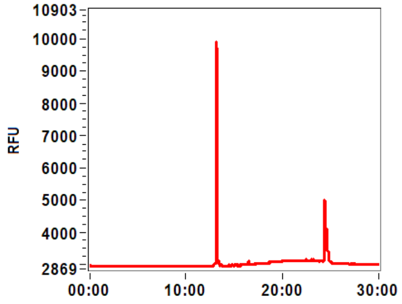

E12: SampE12

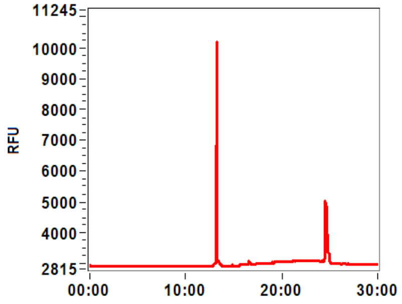

F12: SampF12

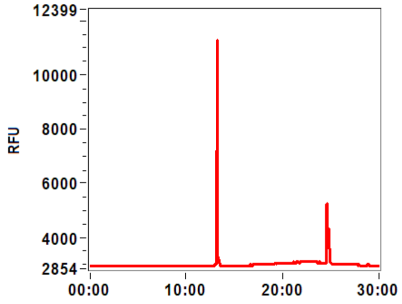

G12: SampG12

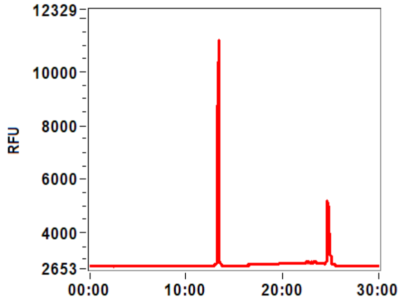

H12: SampH12

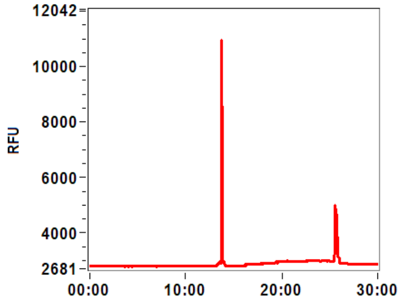

Sample: SampA1
Well location: A1
Created: Thursday, October 21, 2021 8:58:49 PM

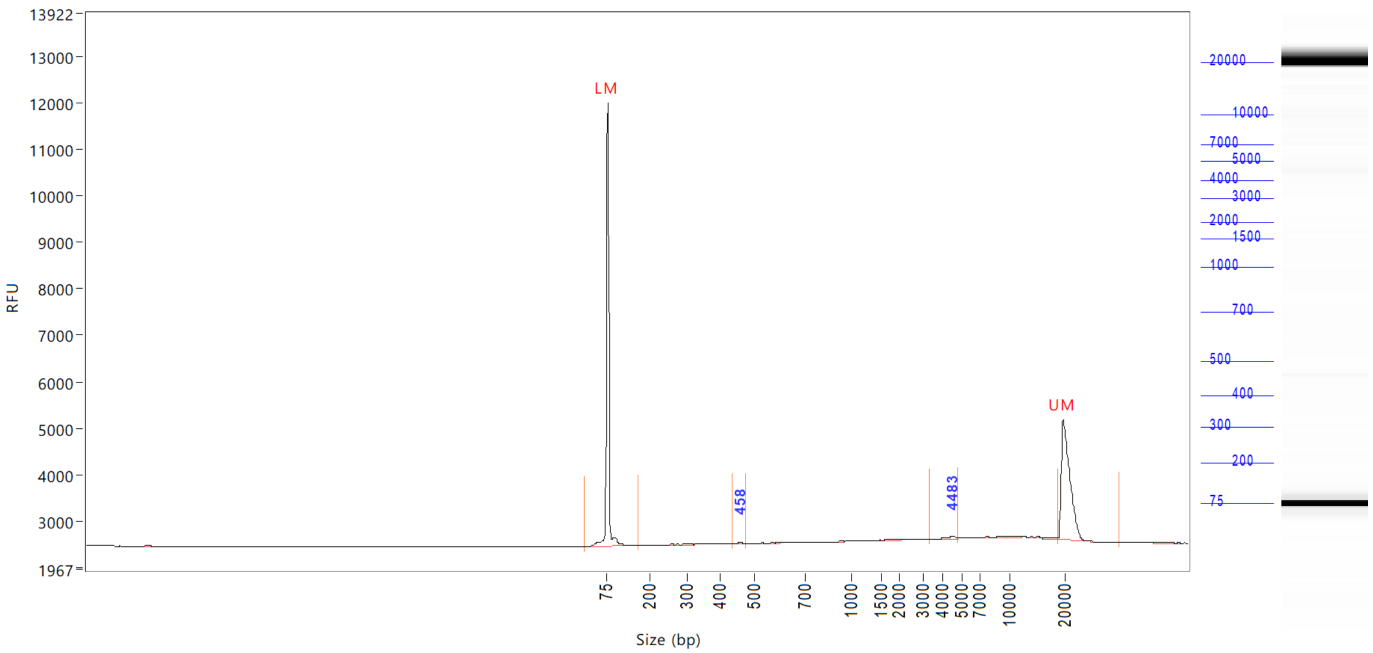

Peak	Size	Concentration	Relative concentration	Molarity	From	To	CV%	RFU
	(bp)	(ng/uL)	Percent	(nmole/L)	(bp)	(bp)		
1	75 (LM)	0.5000		11.2364	7	164	12.04	9547
2	458	0.0354	34.3	0.1279	437	475	1.52	50
3	4483	0.0676	65.7	0.0255	3358	4862	8.01	52
4	20000 (UM)	0.2434		0.0195	18903	30398	4.82	2572

TIC:	0.1030	ng/uL
TIM:	0.1534	nmole/L
Total concentration:	0.4972	ng/uL

```
Sample peak width (sec): 5    Sample min peak height: 50    Sample baseline V to V?: Y    Sample baseline V to V points: 3
Sample filter: Binomial       Number of points for filter: 3  Sample start region (min): 0  Sample end region (min): 30
Marker peak width (sec): 5    Marker min peak height: 200   Marker baseline V to V?: Y    Marker baseline V to V points: 3
Lower marker selection: First peak > 500 RFU                Upper marker selection: Last peak > 200 RFU
Ladder size (bp)75, 200, 300, 400, 500, 700, 1000, 1500, 2000, 3000, 4000, 5000, 7000, 10000, 20000
Quantification using: Lower Marker          Final concentration (ng/uL): 0.5000          Dilution factor: 12.0
```

Sample: SampB1
Well location: B1
Created: Thursday, October 21, 2021 8:58:49 PM

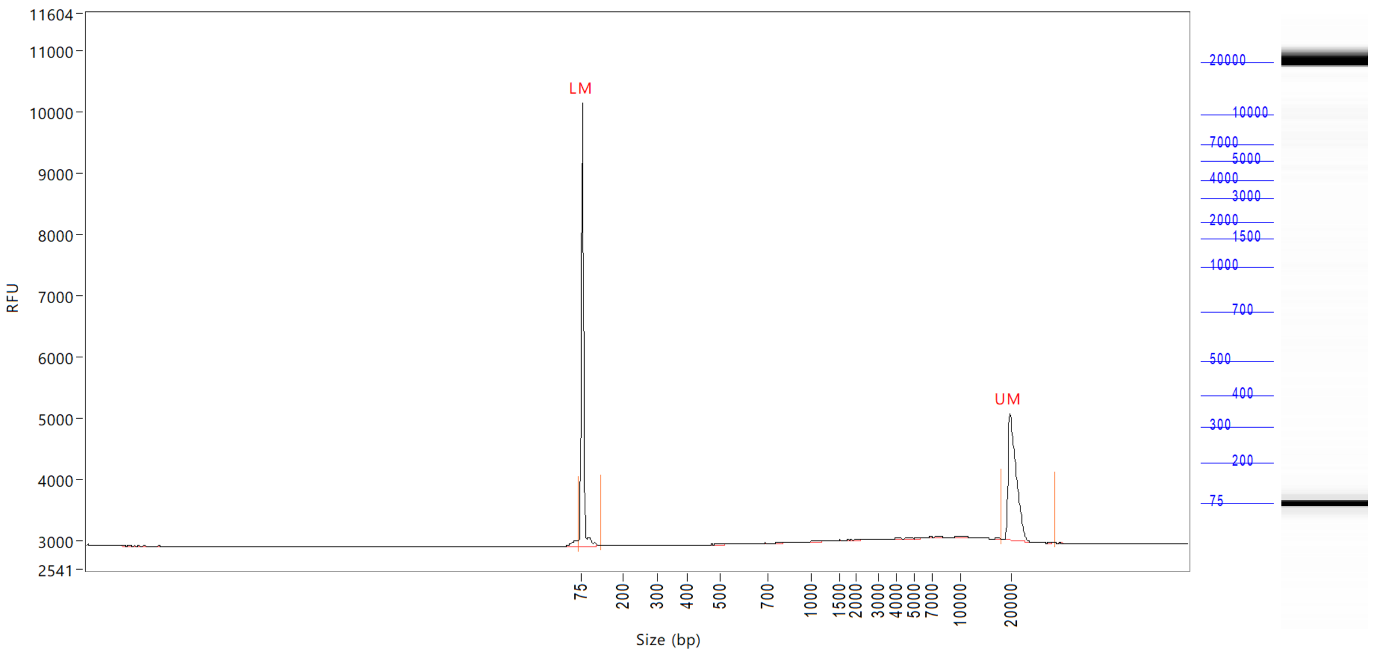

Peak	Size	Concentration	Relative concentration	Molarity	From	To	CV%	RFU
	(bp)	(ng/uL)	Percent	(nmole/L)	(bp)	(bp)		
1	75 (LM)	0.5000		11.0851	62	131	9.12	7238
2	20000 (UM)	0.2548		0.0204	18293	28826	4.43	2067

TIC:	0.0000	ng/uL
TIM:	0.0000	nmole/L
Total concentration:	0.5864	ng/uL

Sample peak width (sec): 5 Sample min peak height: 50 Sample baseline V to V?: Y Sample baseline V to V points: 3
Sample filter: Binomial Number of points for filter: 3 Sample start region (min): 0 Sample end region (min): 30
Marker peak width (sec): 5 Marker min peak height: 200 Marker baseline V to V?: Y Marker baseline V to V points: 3
Lower marker selection: First peak > 500 RFU Upper marker selection: Last peak > 200 RFU
Ladder size (bp)75, 200, 300, 400, 500, 700, 1000, 1500, 2000, 3000, 4000, 5000, 7000, 10000, 20000
Quantification using: Lower Marker Final concentration (ng/uL): 0.5000 Dilution factor: 12.0

Sample: SampC1
Well location: C1
Created: Thursday, October 21, 2021 8:58:49 PM

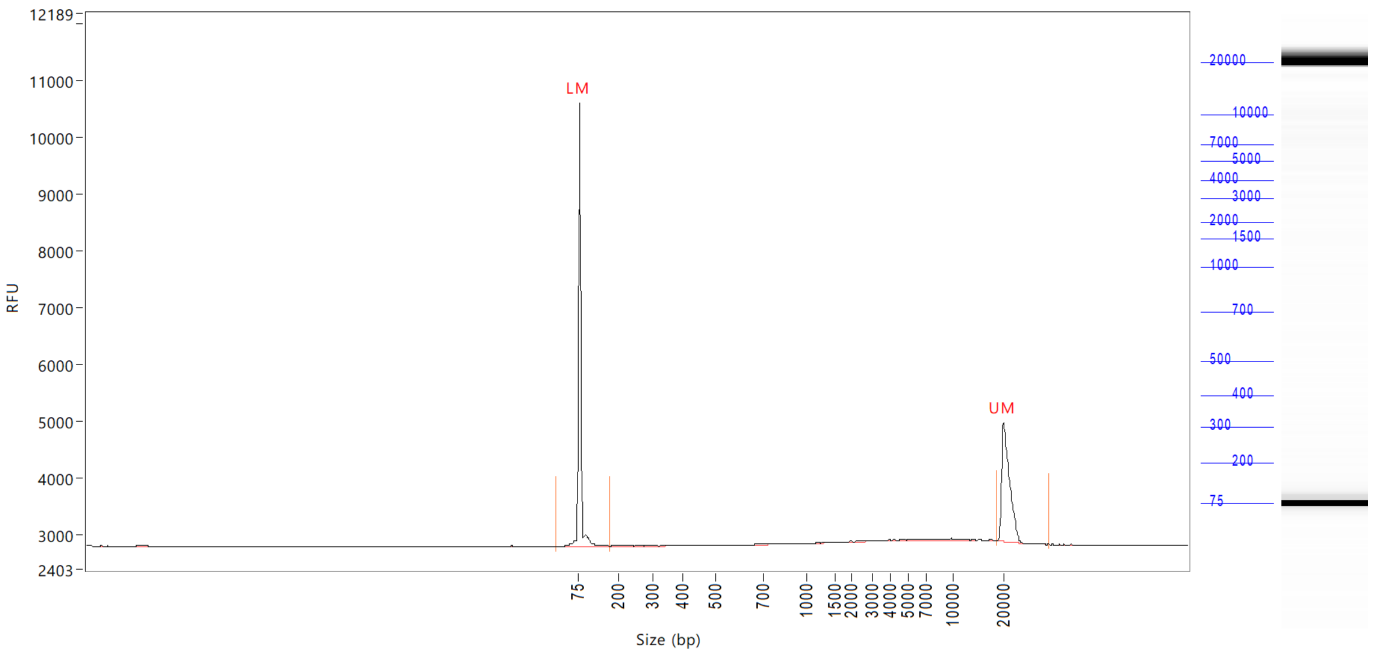

Peak	Size	Concentration	Relative concentration	Molarity	From	To	CV%	RFU
	(bp)	(ng/uL)	Percent	(nmole/L)	(bp)	(bp)		
1	75 (LM)	0.5000		11.0851	5	168	13.44	7819
2	20000 (UM)	0.2313		0.0185	18781	29068	4.27	2083

TIC:	0.0000	ng/uL
TIM:	0.0000	nmole/L
Total concentration:	0.5992	ng/uL

Sample peak width (sec): 5 Sample min peak height: 50 Sample baseline V to V?: Y Sample baseline V to V points: 3
Sample filter: Binomial Number of points for filter: 3 Sample start region (min): 0 Sample end region (min): 30
Marker peak width (sec): 5 Marker min peak height: 200 Marker baseline V to V?: Y Marker baseline V to V points: 3
Lower marker selection: First peak > 500 RFU Upper marker selection: Last peak > 200 RFU
Ladder size (bp)75, 200, 300, 400, 500, 700, 1000, 1500, 2000, 3000, 4000, 5000, 7000, 10000, 20000
Quantification using: Lower Marker Final concentration (ng/uL): 0.5000 Dilution factor: 12.0

Sample: SampD1
Well location: D1
Created: Thursday, October 21, 2021 8:58:49 PM

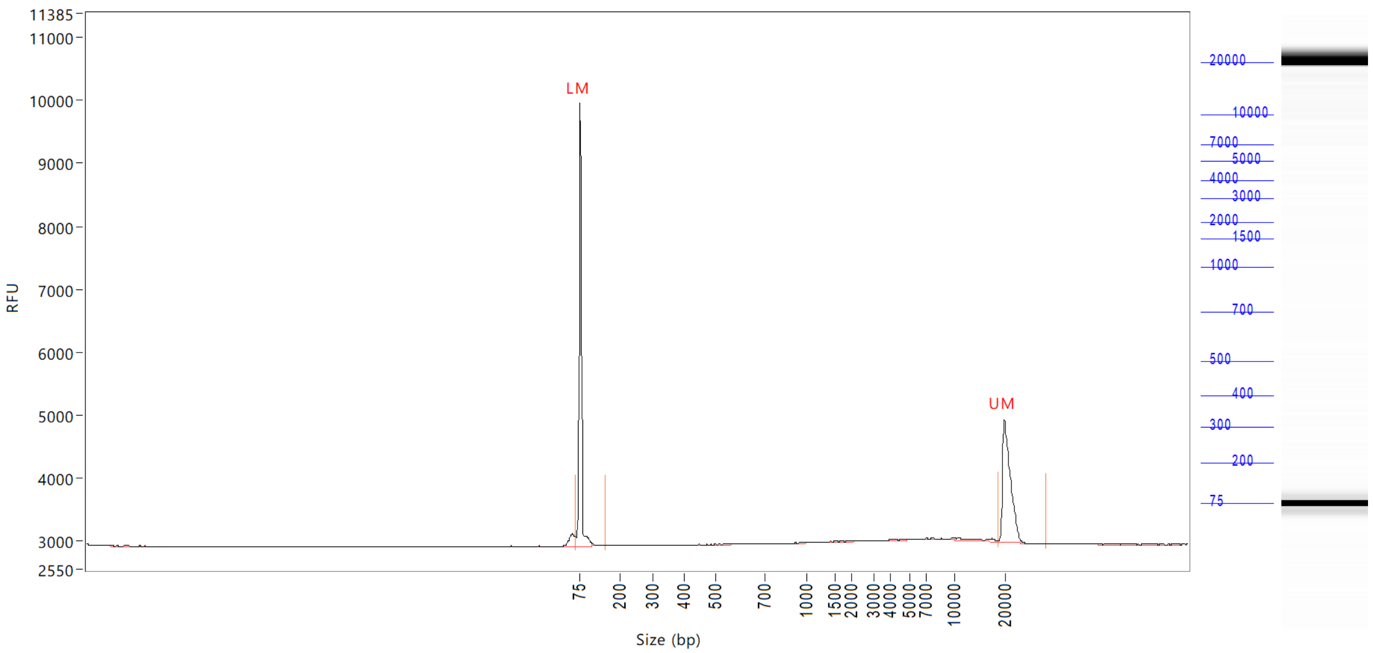

Peak	Size	Concentration	Relative concentration	Molarity	From	To	CV%	RFU
	(bp)	(ng/uL)	Percent	(nmole/L)	(bp)	(bp)		
1	75 (LM)	0.5000		11.0851	62	152	10.13	7055
2	20000 (UM)	0.2450		0.0196	18903	28463	4.38	1937

TIC:	0.0000	ng/uL
TIM:	0.0000	nmole/L
Total concentration:	0.6466	ng/uL

Sample peak width (sec): 5 Sample min peak height: 50 Sample baseline V to V?: Y Sample baseline V to V points: 3
Sample filter: Binomial Number of points for filter: 3 Sample start region (min): 0 Sample end region (min): 30
Marker peak width (sec): 5 Marker min peak height: 200 Marker baseline V to V?: Y Marker baseline V to V points: 3
Lower marker selection: First peak > 500 RFU Upper marker selection: Last peak > 200 RFU
Ladder size (bp)75, 200, 300, 400, 500, 700, 1000, 1500, 2000, 3000, 4000, 5000, 7000, 10000, 20000
Quantification using: Lower Marker Final concentration (ng/uL): 0.5000 Dilution factor: 12.0

Sample: SampE1
Well location: E1
Created: Thursday, October 21, 2021 8:58:49 PM

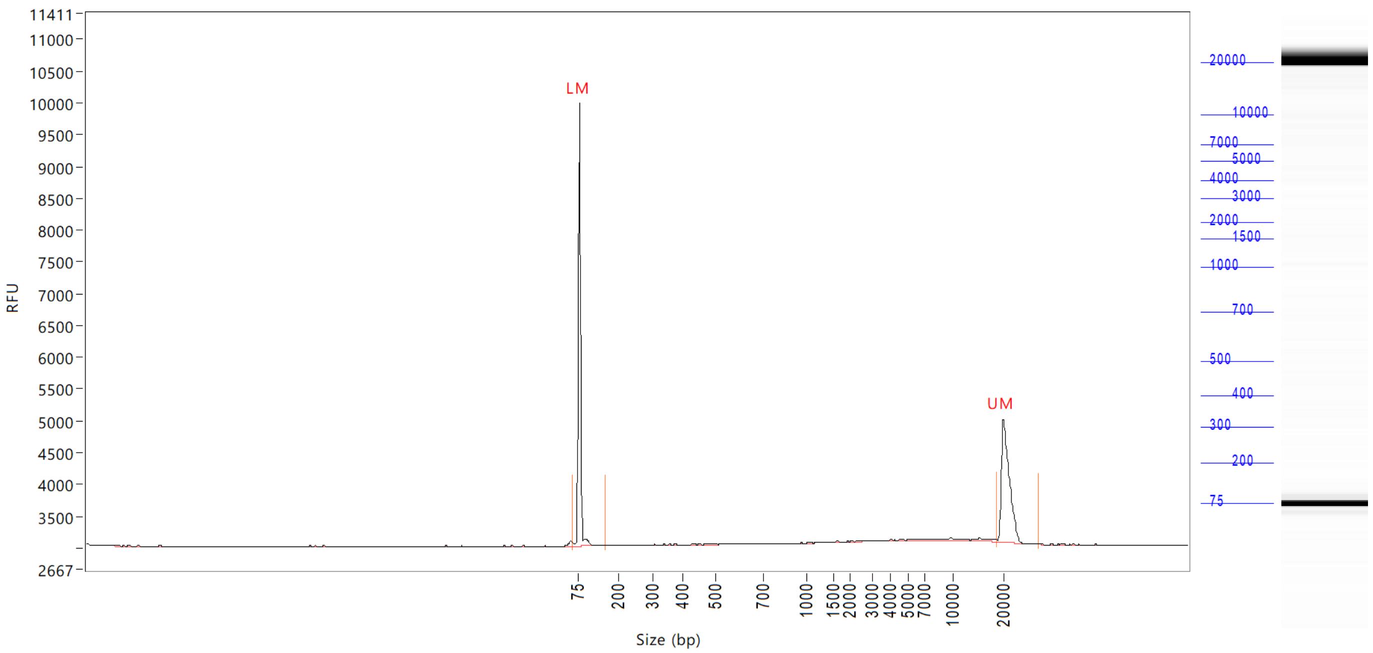

Peak	Size	Concentration	Relative concentration	Molarity	From	To	CV%	RFU
	(bp)	(ng/uL)	Percent	(nmole/L)	(bp)	(bp)		
1	75 (LM)	0.5000		11.2364	56	156	8.48	6981
2	20000 (UM)	0.2531		0.0202	18903	27254	4.15	1937

TIC:	0.0000	ng/uL
TIM:	0.0000	nmole/L
Total concentration:	0.6873	ng/uL

Sample peak width (sec): 5 Sample min peak height: 50 Sample baseline V to V?: Y Sample baseline V to V points: 3
Sample filter: Binomial Number of points for filter: 3 Sample start region (min): 0 Sample end region (min): 30
Marker peak width (sec): 5 Marker min peak height: 200 Marker baseline V to V?: Y Marker baseline V to V points: 3
Lower marker selection: First peak > 500 RFU Upper marker selection: Last peak > 200 RFU
Ladder size (bp)75, 200, 300, 400, 500, 700, 1000, 1500, 2000, 3000, 4000, 5000, 7000, 10000, 20000
Quantification using: Lower Marker Final concentration (ng/uL): 0.5000 Dilution factor: 12.0

Sample: SampF1
Well location: F1
Created: Thursday, October 21, 2021 8:58:49 PM

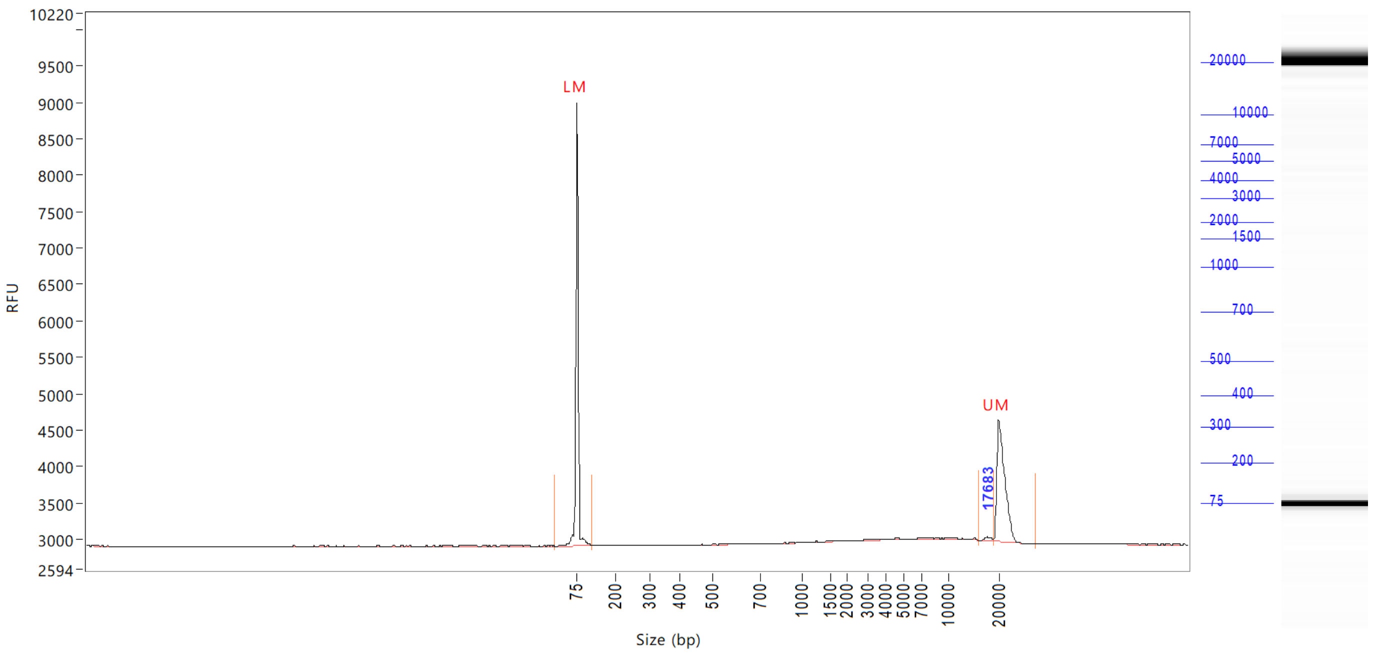

Peak	Size	Concentration	Relative concentration	Molarity	From	To	CV%	RFU
	(bp)	(ng/uL)	Percent	(nmole/L)	(bp)	(bp)		
1	75 (LM)	0.5000		11.3919	7	120	9.63	6088
2	17683	0.0735	100.0	0.0068	16098	18903	3.39	51
3	20000 (UM)	0.2334		0.0187	18903	27375	4.20	1665

TIC:	0.0735	ng/uL
TIM:	0.0068	nmole/L
Total concentration:	0.6392	ng/uL

```
Sample peak width (sec): 5    Sample min peak height: 50    Sample baseline V to V?: Y    Sample baseline V to V points: 3
Sample filter: Binomial       Number of points for filter: 3  Sample start region (min): 0  Sample end region (min): 30
Marker peak width (sec): 5    Marker min peak height: 200   Marker baseline V to V?: Y    Marker baseline V to V points: 3
Lower marker selection: First peak > 500 RFU                Upper marker selection: Last peak > 200 RFU
Ladder size (bp)75, 200, 300, 400, 500, 700, 1000, 1500, 2000, 3000, 4000, 5000, 7000, 10000, 20000
Quantification using: Lower Marker          Final concentration (ng/uL): 0.5000          Dilution factor: 12.0
```

Sample: SampG1
Well location: G1
Created: Thursday, October 21, 2021 8:58:49 PM

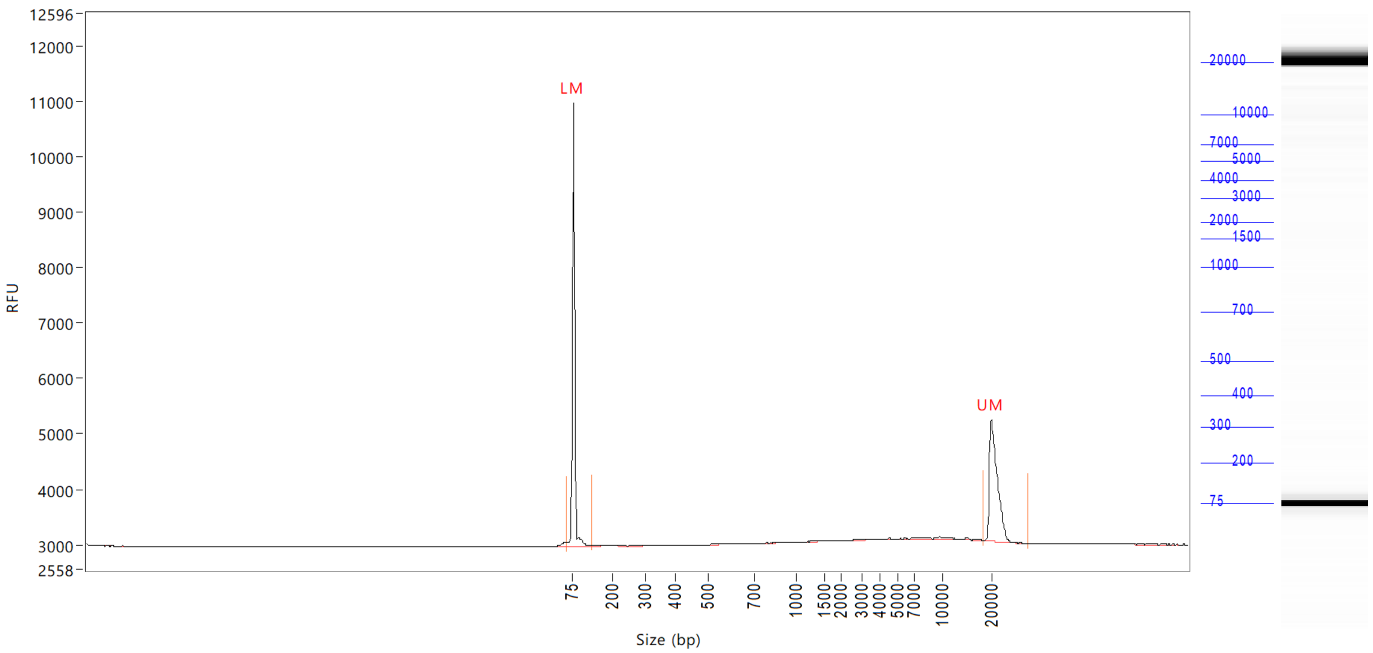

Peak	Size	Concentration	Relative concentration	Molarity	From	To	CV%	RFU
	(bp)	(ng/uL)	Percent	(nmole/L)	(bp)	(bp)		
1	75 (LM)	0.5000		11.0851	56	135	8.47	8017
2	20000 (UM)	0.2509		0.0201	18293	27254	4.44	2196

TIC:	0.0000	ng/uL
TIM:	0.0000	nmole/L
Total concentration:	0.5547	ng/uL

Sample peak width (sec): 5 Sample min peak height: 50 Sample baseline V to V?: Y Sample baseline V to V points: 3
Sample filter: Binomial Number of points for filter: 3 Sample start region (min): 0 Sample end region (min): 30
Marker peak width (sec): 5 Marker min peak height: 200 Marker baseline V to V?: Y Marker baseline V to V points: 3
Lower marker selection: First peak > 500 RFU Upper marker selection: Last peak > 200 RFU
Ladder size (bp)75, 200, 300, 400, 500, 700, 1000, 1500, 2000, 3000, 4000, 5000, 7000, 10000, 20000
Quantification using: Lower Marker Final concentration (ng/uL): 0.5000 Dilution factor: 12.0

Sample: SampH1
Well location: H1
Created: Thursday, October 21, 2021 8:58:49 PM

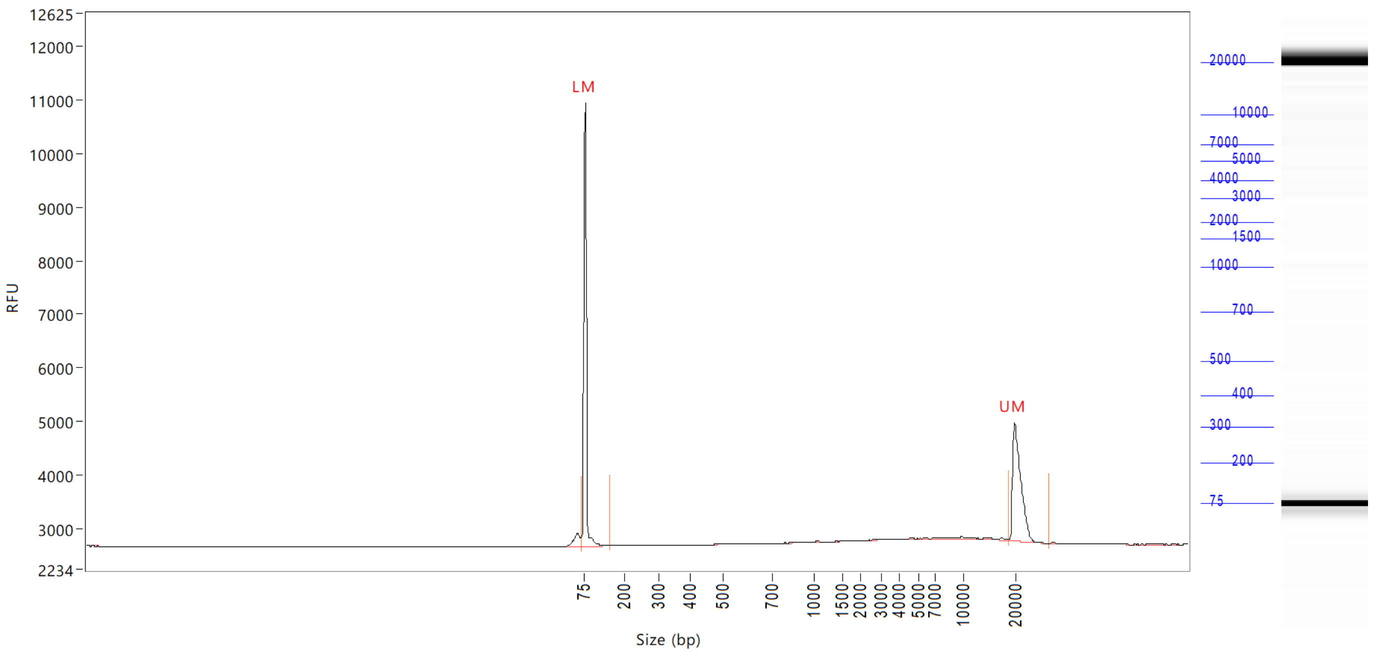

Peak	Size	Concentration	Relative concentration	Molarity	From	To	CV%	RFU
	(bp)	(ng/uL)	Percent	(nmole/L)	(bp)	(bp)		
1	75 (LM)	0.5000		11.0851	64	150	10.14	8300
2	20000 (UM)	0.2394		0.0191	18903	26891	4.27	2212

TIC:	0.0000	ng/uL
TIM:	0.0000	nmole/L
Total concentration:	0.5475	ng/uL

```
Sample peak width (sec): 5    Sample min peak height: 50    Sample baseline V to V?: Y    Sample baseline V to V points: 3
Sample filter: Binomial       Number of points for filter: 3  Sample start region (min): 0  Sample end region (min): 30
Marker peak width (sec): 5    Marker min peak height: 200   Marker baseline V to V?: Y    Marker baseline V to V points: 3
Lower marker selection: First peak > 500 RFU                Upper marker selection: Last peak > 200 RFU
Ladder size (bp)75, 200, 300, 400, 500, 700, 1000, 1500, 2000, 3000, 4000, 5000, 7000, 10000, 20000
Quantification using: Lower Marker          Final concentration (ng/uL): 0.5000          Dilution factor: 12.0
```

Sample: SampA2
Well location: A2
Created: Thursday, October 21, 2021 8:58:49 PM

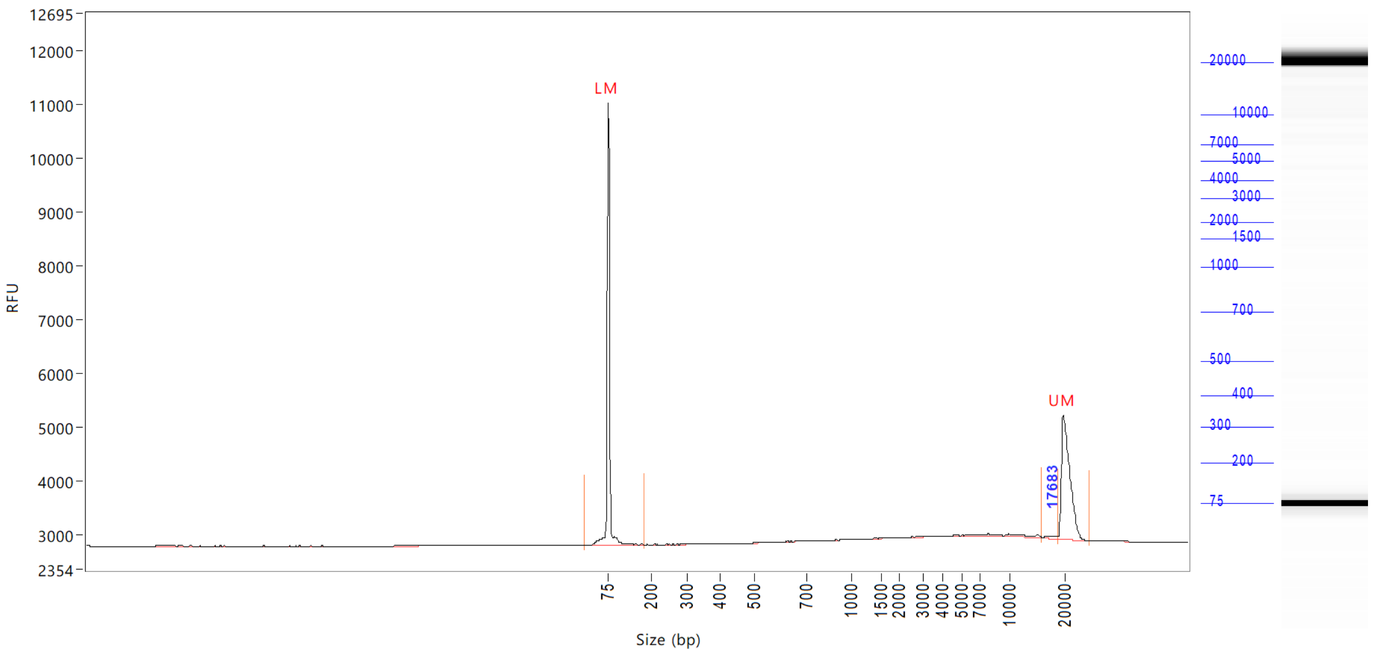

Peak	Size	Concentration	Relative concentration	Molarity	From	To	CV%	RFU
	(bp)	(ng/uL)	Percent	(nmole/L)	(bp)	(bp)		
1	75 (LM)	0.5000		11.2364	5	181	13.06	8239
2	17683	0.0875	100.0	0.0081	15854	19024	4.92	56
3	20000 (UM)	0.2457		0.0197	19024	24715	4.09	2316

TIC:	0.0875	ng/uL
TIM:	0.0081	nmole/L
Total concentration:	0.5919	ng/uL

```
Sample peak width (sec): 5    Sample min peak height: 50    Sample baseline V to V?: Y    Sample baseline V to V points: 3
Sample filter: Binomial       Number of points for filter: 3  Sample start region (min): 0  Sample end region (min): 30
Marker peak width (sec): 5    Marker min peak height: 200   Marker baseline V to V?: Y    Marker baseline V to V points: 3
Lower marker selection: First peak > 500 RFU                Upper marker selection: Last peak > 200 RFU
Ladder size (bp)75, 200, 300, 400, 500, 700, 1000, 1500, 2000, 3000, 4000, 5000, 7000, 10000, 20000
Quantification using: Lower Marker          Final concentration (ng/uL): 0.5000          Dilution factor: 12.0
```

Sample: SampB2
Well location: B2
Created: Thursday, October 21, 2021 8:58:49 PM

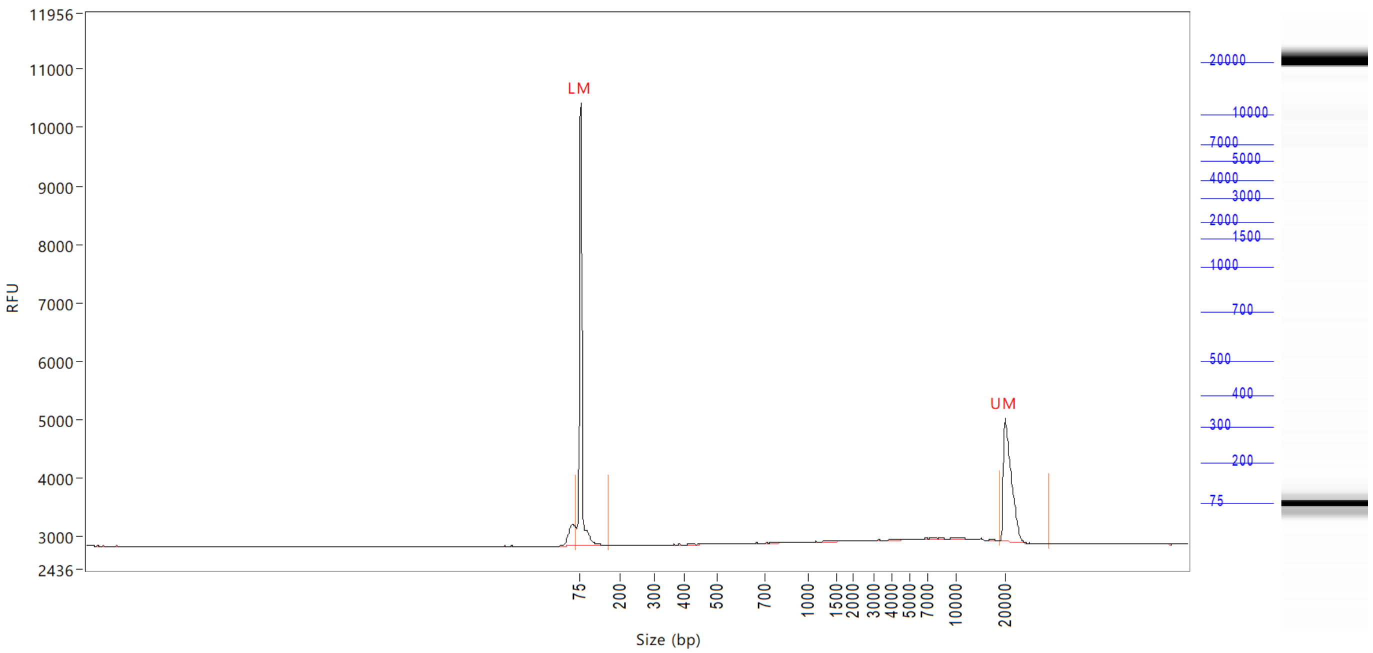

Peak	Size	Concentration	Relative concentration	Molarity	From	To	CV%	RFU
	(bp)	(ng/uL)	Percent	(nmole/L)	(bp)	(bp)		
1	75 (LM)	0.5000		11.0851	62	162	11.27	7594
2	20000 (UM)	0.2294		0.0184	18903	28584	4.44	2115

TIC:	0.0000	ng/uL
TIM:	0.0000	nmole/L
Total concentration:	0.4689	ng/uL

Sample peak width (sec): 5 Sample min peak height: 50 Sample baseline V to V?: Y Sample baseline V to V points: 3
Sample filter: Binomial Number of points for filter: 3 Sample start region (min): 0 Sample end region (min): 30
Marker peak width (sec): 5 Marker min peak height: 200 Marker baseline V to V?: Y Marker baseline V to V points: 3
Lower marker selection: First peak > 500 RFU Upper marker selection: Last peak > 200 RFU
Ladder size (bp)75, 200, 300, 400, 500, 700, 1000, 1500, 2000, 3000, 4000, 5000, 7000, 10000, 20000
Quantification using: Lower Marker Final concentration (ng/uL): 0.5000 Dilution factor: 12.0

Sample: SampC2
Well location: C2
Created: Thursday, October 21, 2021 8:58:49 PM

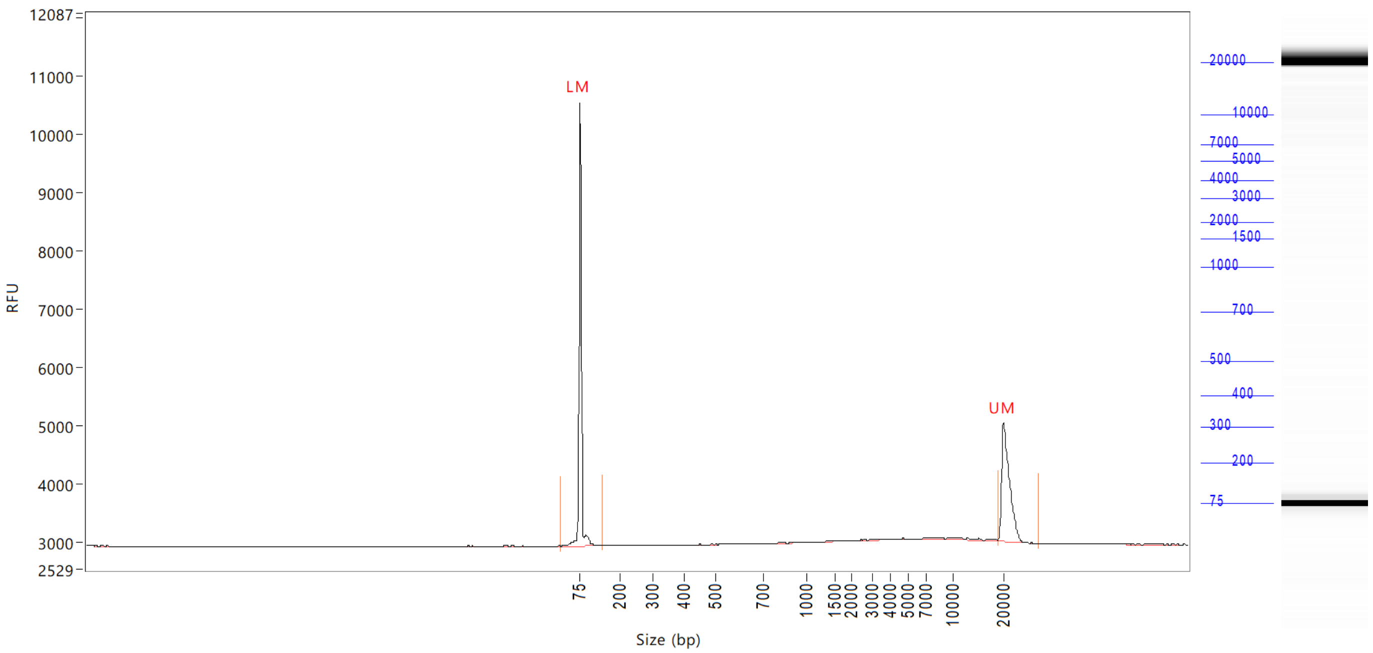

Peak	Size	Concentration	Relative concentration	Molarity	From	To	CV%	RFU
	(bp)	(ng/uL)	Percent	(nmole/L)	(bp)	(bp)		
1	75 (LM)	0.5000		11.2364	14	143	11.00	7631
2	20000 (UM)	0.2340		0.0188	18903	27012	4.29	2029

TIC:	0.0000	ng/uL
TIM:	0.0000	nmole/L
Total concentration:	0.5666	ng/uL

Sample peak width (sec): 5 Sample min peak height: 50 Sample baseline V to V?: Y Sample baseline V to V points: 3
Sample filter: Binomial Number of points for filter: 3 Sample start region (min): 0 Sample end region (min): 30
Marker peak width (sec): 5 Marker min peak height: 200 Marker baseline V to V?: Y Marker baseline V to V points: 3
Lower marker selection: First peak > 500 RFU Upper marker selection: Last peak > 200 RFU
Ladder size (bp)75, 200, 300, 400, 500, 700, 1000, 1500, 2000, 3000, 4000, 5000, 7000, 10000, 20000
Quantification using: Lower Marker Final concentration (ng/uL): 0.5000 Dilution factor: 12.0

Sample: SampD2
Well location: D2
Created: Thursday, October 21, 2021 8:58:49 PM

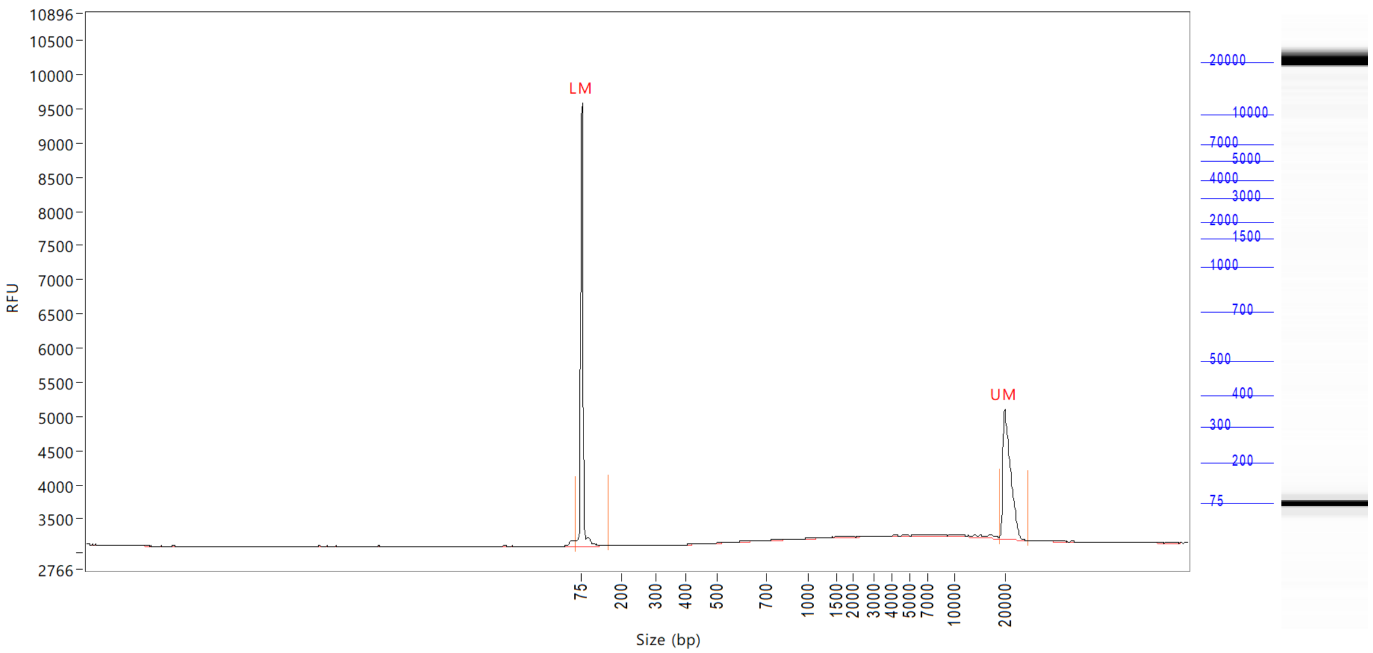

Peak	Size	Concentration	Relative concentration	Molarity	From	To	CV%	RFU
	(bp)	(ng/uL)	Percent	(nmole/L)	(bp)	(bp)		
1	75 (LM)	0.5000		11.0851	56	160	11.13	6494
2	20000 (UM)	0.2503		0.0201	18903	24594	4.00	1896

TIC:	0.0000	ng/uL
TIM:	0.0000	nmole/L
Total concentration:	0.7832	ng/uL

Sample peak width (sec): 5 Sample min peak height: 50 Sample baseline V to V?: Y Sample baseline V to V points: 3
Sample filter: Binomial Number of points for filter: 3 Sample start region (min): 0 Sample end region (min): 30
Marker peak width (sec): 5 Marker min peak height: 200 Marker baseline V to V?: Y Marker baseline V to V points: 3
Lower marker selection: First peak > 500 RFU Upper marker selection: Last peak > 200 RFU
Ladder size (bp)75, 200, 300, 400, 500, 700, 1000, 1500, 2000, 3000, 4000, 5000, 7000, 10000, 20000
Quantification using: Lower Marker Final concentration (ng/uL): 0.5000 Dilution factor: 12.0

Sample: SampE2
Well location: E2
Created: Thursday, October 21, 2021 8:58:49 PM

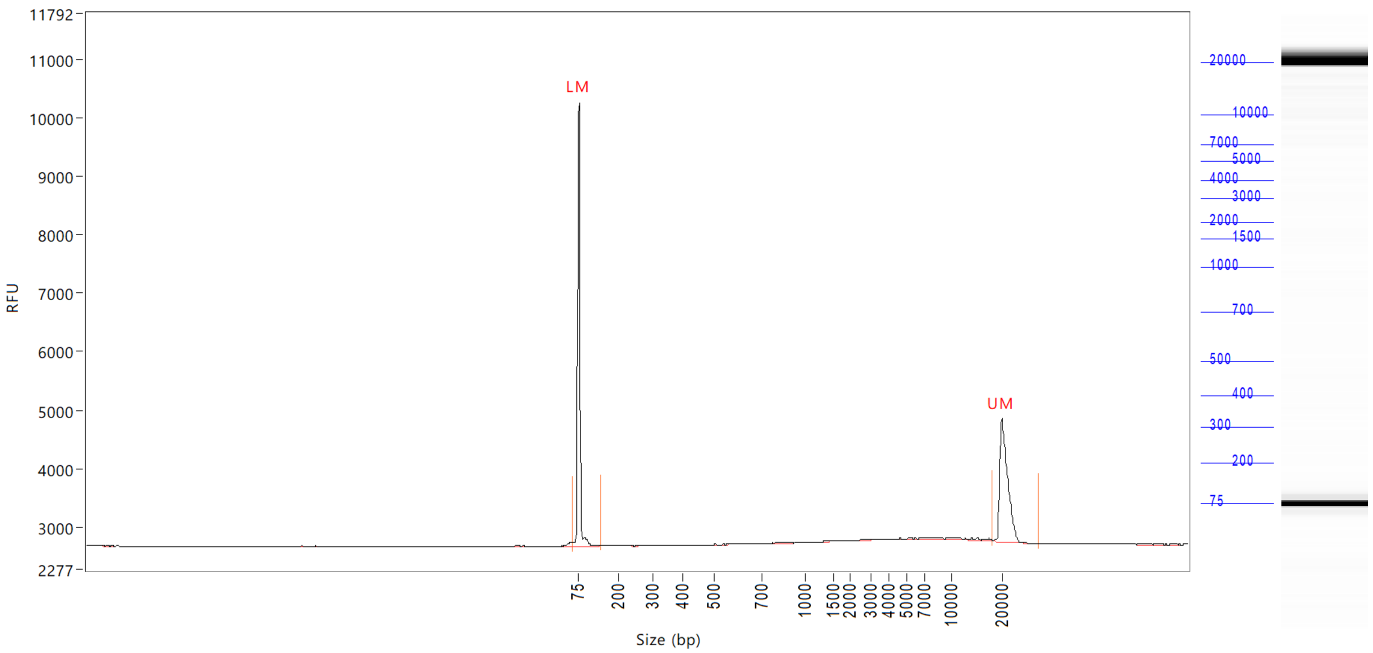

Peak	Size	Concentration	Relative concentration	Molarity	From	To	CV%	RFU
	(bp)	(ng/uL)	Percent	(nmole/L)	(bp)	(bp)		
1	75 (LM)	0.5000		11.2364	56	145	8.93	7600
2	20000 (UM)	0.2403		0.0193	18171	27254	4.27	2103

TIC:	0.0000	ng/uL
TIM:	0.0000	nmole/L
Total concentration:	0.5820	ng/uL

```
Sample peak width (sec): 5    Sample min peak height: 50    Sample baseline V to V?: Y    Sample baseline V to V points: 3
Sample filter: Binomial       Number of points for filter: 3  Sample start region (min): 0  Sample end region (min): 30
Marker peak width (sec): 5    Marker min peak height: 200   Marker baseline V to V?: Y    Marker baseline V to V points: 3
Lower marker selection: First peak > 500 RFU                Upper marker selection: Last peak > 200 RFU
Ladder size (bp)75, 200, 300, 400, 500, 700, 1000, 1500, 2000, 3000, 4000, 5000, 7000, 10000, 20000
Quantification using: Lower Marker          Final concentration (ng/uL): 0.5000          Dilution factor: 12.0
```

Sample: SampF2
Well location: F2
Created: Thursday, October 21, 2021 8:58:49 PM

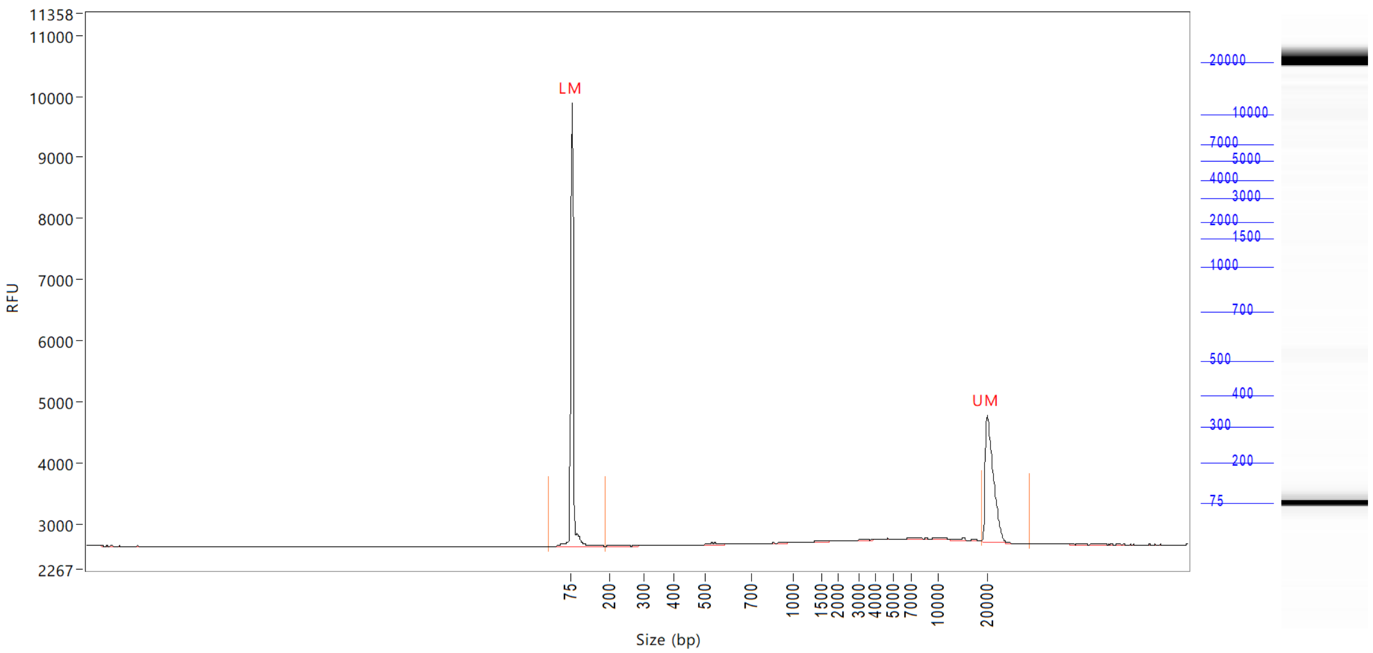

Peak	Size	Concentration	Relative concentration	Molarity	From	To	CV%	RFU
	(bp)	(ng/uL)	Percent	(nmole/L)	(bp)	(bp)		
1	75 (LM)	0.5000		10.9378	5	183	13.76	7265
2	20000 (UM)	0.2452		0.0195	18903	28705	4.63	2066

TIC:	0.0000	ng/uL
TIM:	0.0000	nmole/L
Total concentration:	0.6624	ng/uL

Sample peak width (sec): 5 Sample min peak height: 50 Sample baseline V to V?: Y Sample baseline V to V points: 3
Sample filter: Binomial Number of points for filter: 3 Sample start region (min): 0 Sample end region (min): 30
Marker peak width (sec): 5 Marker min peak height: 200 Marker baseline V to V?: Y Marker baseline V to V points: 3
Lower marker selection: First peak > 500 RFU Upper marker selection: Last peak > 200 RFU
Ladder size (bp)75, 200, 300, 400, 500, 700, 1000, 1500, 2000, 3000, 4000, 5000, 7000, 10000, 20000
Quantification using: Lower Marker Final concentration (ng/uL): 0.5000 Dilution factor: 12.0

Sample: SampG2
Well location: G2
Created: Thursday, October 21, 2021 8:58:49 PM

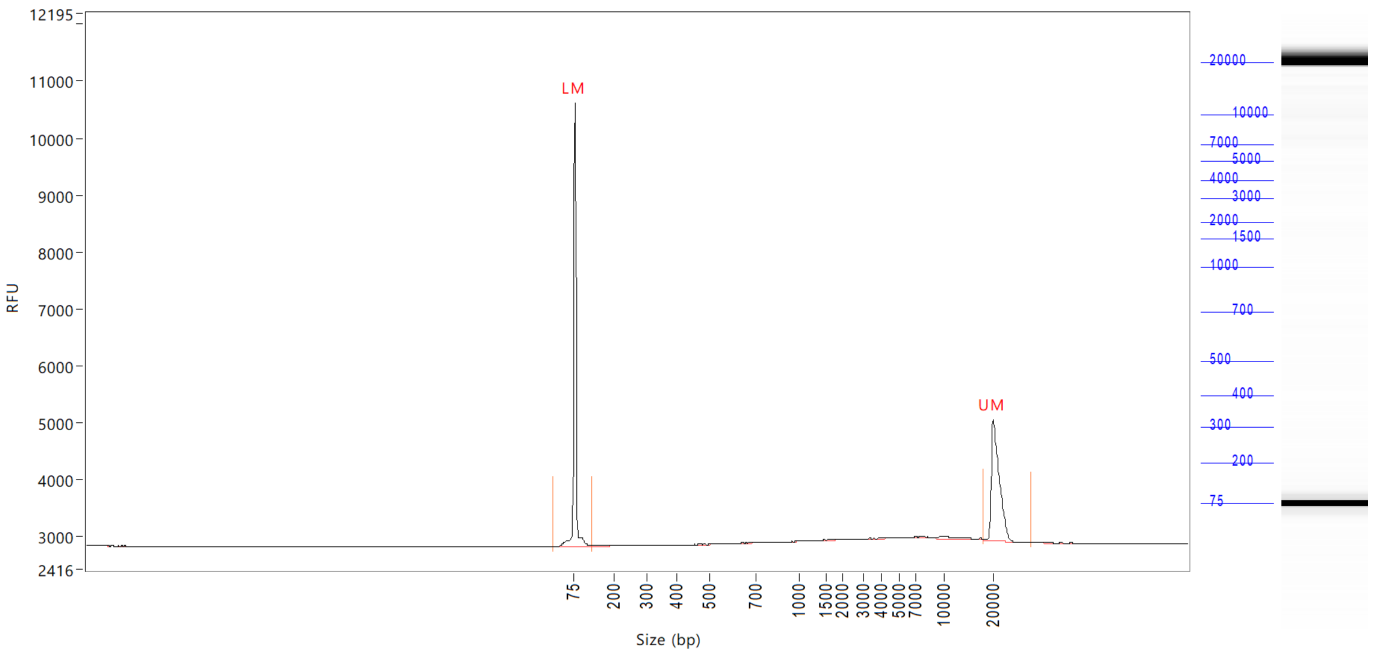

Peak	Size	Concentration	Relative concentration	Molarity	From	To	CV%	RFU
	(bp)	(ng/uL)	Percent	(nmole/L)	(bp)	(bp)		
1	75 (LM)	0.5000		11.2364	7	131	12.06	7814
2	20000 (UM)	0.2375		0.0190	18171	27617	4.41	2118

TIC:	0.0000	ng/uL
TIM:	0.0000	nmole/L
Total concentration:	0.5743	ng/uL

Sample peak width (sec): 5   Sample min peak height: 50   Sample baseline V to V?: Y   Sample baseline V to V points: 3
Sample filter: Binomial   Number of points for filter: 3   Sample start region (min): 0   Sample end region (min): 30
Marker peak width (sec): 5   Marker min peak height: 200   Marker baseline V to V?: Y   Marker baseline V to V points: 3
Lower marker selection: First peak > 500 RFU   Upper marker selection: Last peak > 200 RFU
Ladder size (bp)75, 200, 300, 400, 500, 700, 1000, 1500, 2000, 3000, 4000, 5000, 7000, 10000, 20000
Quantification using: Lower Marker   Final concentration (ng/uL): 0.5000   Dilution factor: 12.0

Sample: SampH2
Well location: H2
Created: Thursday, October 21, 2021 8:58:49 PM

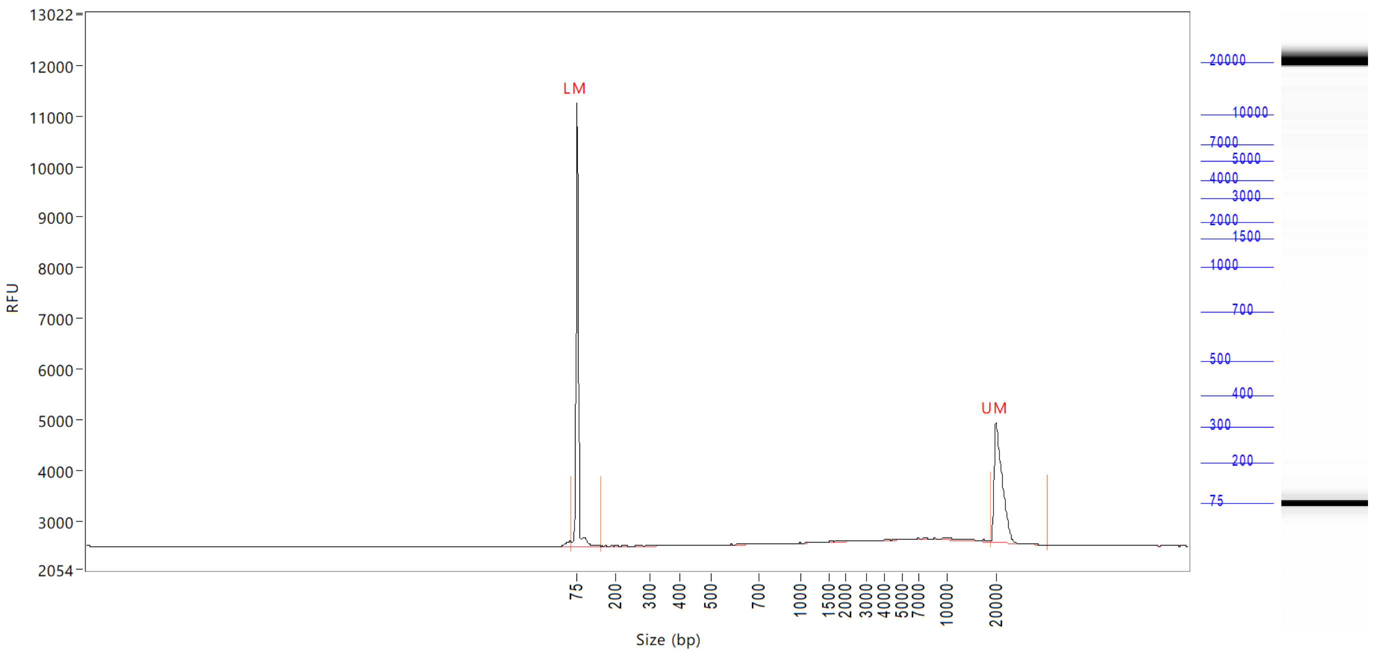

Peak	Size	Concentration	Relative concentration	Molarity	From	To	CV%	RFU
	(bp)	(ng/uL)	Percent	(nmole/L)	(bp)	(bp)		
1	75 (LM)	0.5000		11.0851	58	150	10.31	8768
2	20000 (UM)	0.2468		0.0197	18781	30398	4.74	2357

TIC:	0.0000	ng/uL
TIM:	0.0000	nmole/L
Total concentration:	0.5081	ng/uL

```
Sample peak width (sec): 5    Sample min peak height: 50    Sample baseline V to V?: Y    Sample baseline V to V points: 3
Sample filter: Binomial       Number of points for filter: 3  Sample start region (min): 0  Sample end region (min): 30
Marker peak width (sec): 5    Marker min peak height: 200   Marker baseline V to V?: Y    Marker baseline V to V points: 3
Lower marker selection: First peak > 500 RFU                Upper marker selection: Last peak > 200 RFU
Ladder size (bp)75, 200, 300, 400, 500, 700, 1000, 1500, 2000, 3000, 4000, 5000, 7000, 10000, 20000
Quantification using: Lower Marker          Final concentration (ng/uL): 0.5000          Dilution factor: 12.0
```

Sample: SampA3
Well location: A3
Created: Thursday, October 21, 2021 8:58:49 PM

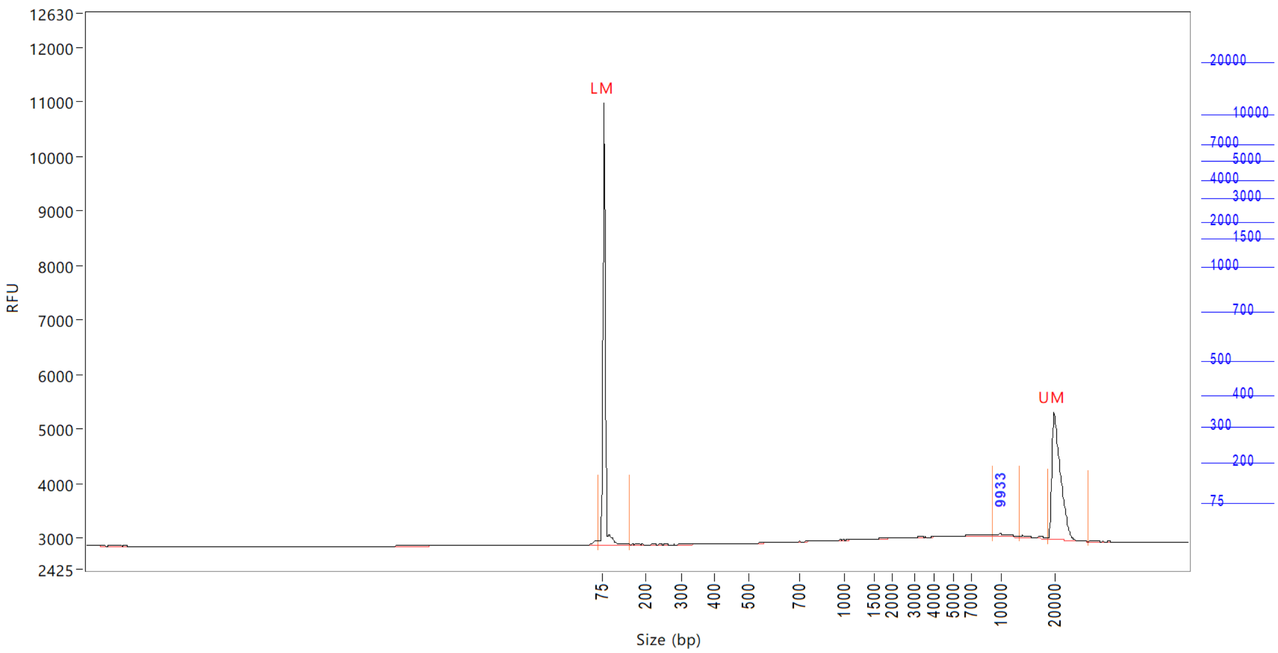

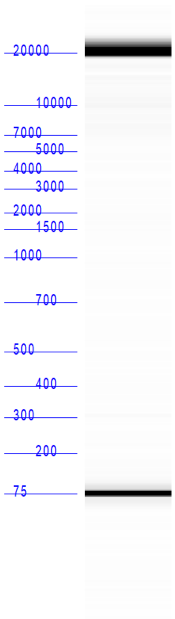

Peak	Size	Concentration	Relative concentration	Molarity	From	To	CV%	RFU
	(bp)	(ng/uL)	Percent	(nmole/L)	(bp)	(bp)		
1	75 (LM)	0.5000		10.9378	58	154	10.19	8136
2	9933	0.1194	100.0	0.0180	9200	13537	11.01	52
3	20000 (UM)	0.2614		0.0210	18903	26287	4.40	2323

TIC:	0.1194	ng/uL
TIM:	0.0180	nmole/L
Total concentration:	0.6449	ng/uL

```
Sample peak width (sec): 5    Sample min peak height: 50    Sample baseline V to V?: Y    Sample baseline V to V points: 3
Sample filter: Binomial       Number of points for filter: 3  Sample start region (min): 0  Sample end region (min): 30
Marker peak width (sec): 5    Marker min peak height: 200   Marker baseline V to V?: Y    Marker baseline V to V points: 3
Lower marker selection: First peak > 500 RFU                Upper marker selection: Last peak > 200 RFU
Ladder size (bp)75, 200, 300, 400, 500, 700, 1000, 1500, 2000, 3000, 4000, 5000, 7000, 10000, 20000
Quantification using: Lower Marker          Final concentration (ng/uL): 0.5000          Dilution factor: 12.0
```

Sample: SampB3
Well location: B3
Created: Thursday, October 21, 2021 8:58:49 PM

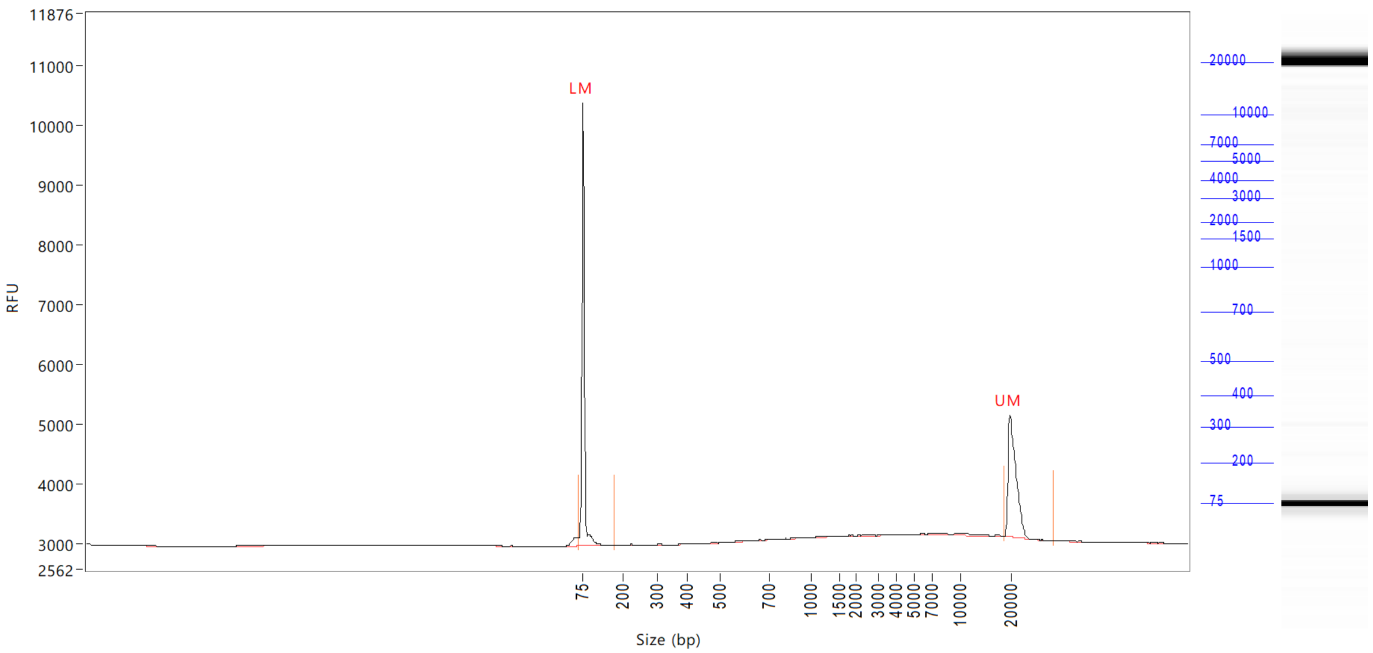

Peak	Size	Concentration	Relative concentration	Molarity	From	To	CV%	RFU
	(bp)	(ng/uL)	Percent	(nmole/L)	(bp)	(bp)		
1	75 (LM)	0.5000		11.0851	62	171	11.41	7434
2	20000 (UM)	0.2338		0.0188	18781	28584	4.29	2037

TIC:	0.0000	ng/uL
TIM:	0.0000	nmole/L
Total concentration:	0.6284	ng/uL

Sample peak width (sec): 5 Sample min peak height: 50 Sample baseline V to V?: Y Sample baseline V to V points: 3
Sample filter: Binomial Number of points for filter: 3 Sample start region (min): 0 Sample end region (min): 30
Marker peak width (sec): 5 Marker min peak height: 200 Marker baseline V to V?: Y Marker baseline V to V points: 3
Lower marker selection: First peak > 500 RFU Upper marker selection: Last peak > 200 RFU
Ladder size (bp)75, 200, 300, 400, 500, 700, 1000, 1500, 2000, 3000, 4000, 5000, 7000, 10000, 20000
Quantification using: Lower Marker Final concentration (ng/uL): 0.5000 Dilution factor: 12.0

Sample: SampC3
Well location: C3
Created: Thursday, October 21, 2021 8:58:49 PM

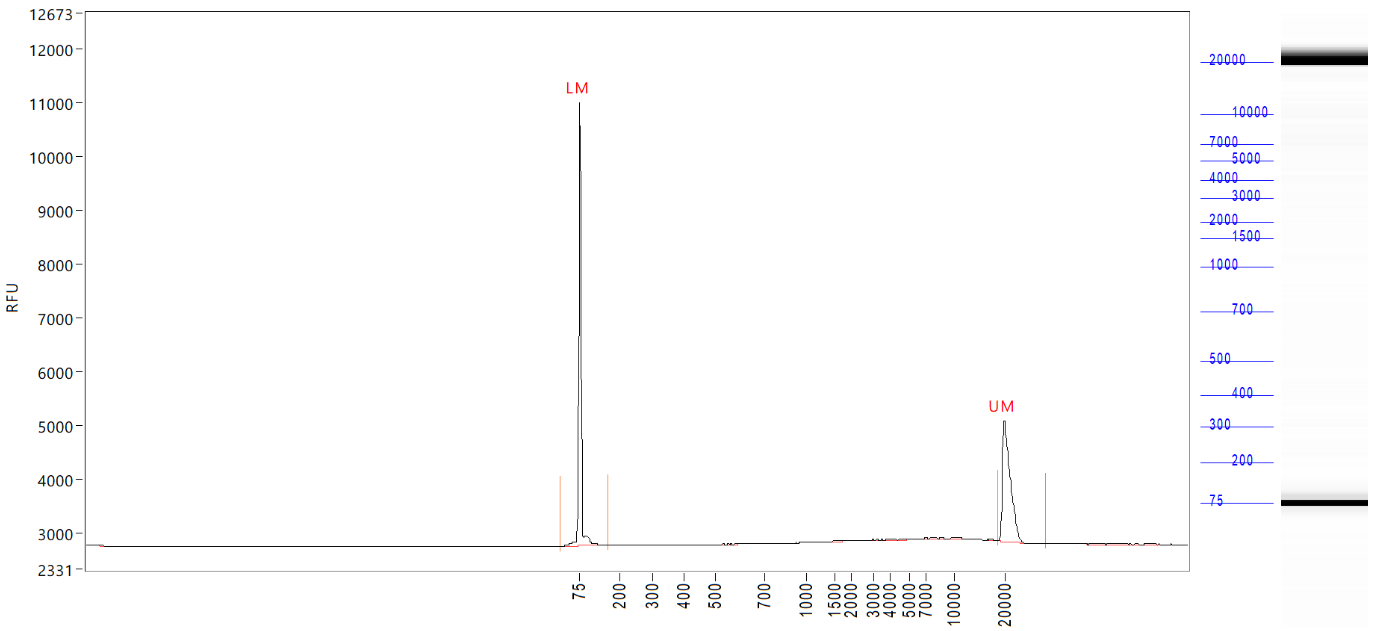

Peak	Size	Concentration	Relative concentration	Molarity	From	To	CV%	RFU
	(bp)	(ng/uL)	Percent	(nmole/L)	(bp)	(bp)		
1	75 (LM)	0.5000		11.0851	14	162	11.42	8256
2	20000 (UM)	0.2394		0.0191	18903	28463	4.44	2243

TIC:	0.0000	ng/uL
TIM:	0.0000	nmole/L
Total concentration:	0.4609	ng/uL

```
Sample peak width (sec): 5    Sample min peak height: 50    Sample baseline V to V?: Y    Sample baseline V to V points: 3
Sample filter: Binomial       Number of points for filter: 3  Sample start region (min): 0  Sample end region (min): 30
Marker peak width (sec): 5    Marker min peak height: 200   Marker baseline V to V?: Y    Marker baseline V to V points: 3
Lower marker selection: First peak > 500 RFU                Upper marker selection: Last peak > 200 RFU
Ladder size (bp)75, 200, 300, 400, 500, 700, 1000, 1500, 2000, 3000, 4000, 5000, 7000, 10000, 20000
Quantification using: Lower Marker          Final concentration (ng/uL): 0.5000          Dilution factor: 12.0
```

Sample: SampD3
Well location: D3
Created: Thursday, October 21, 2021 8:58:49 PM

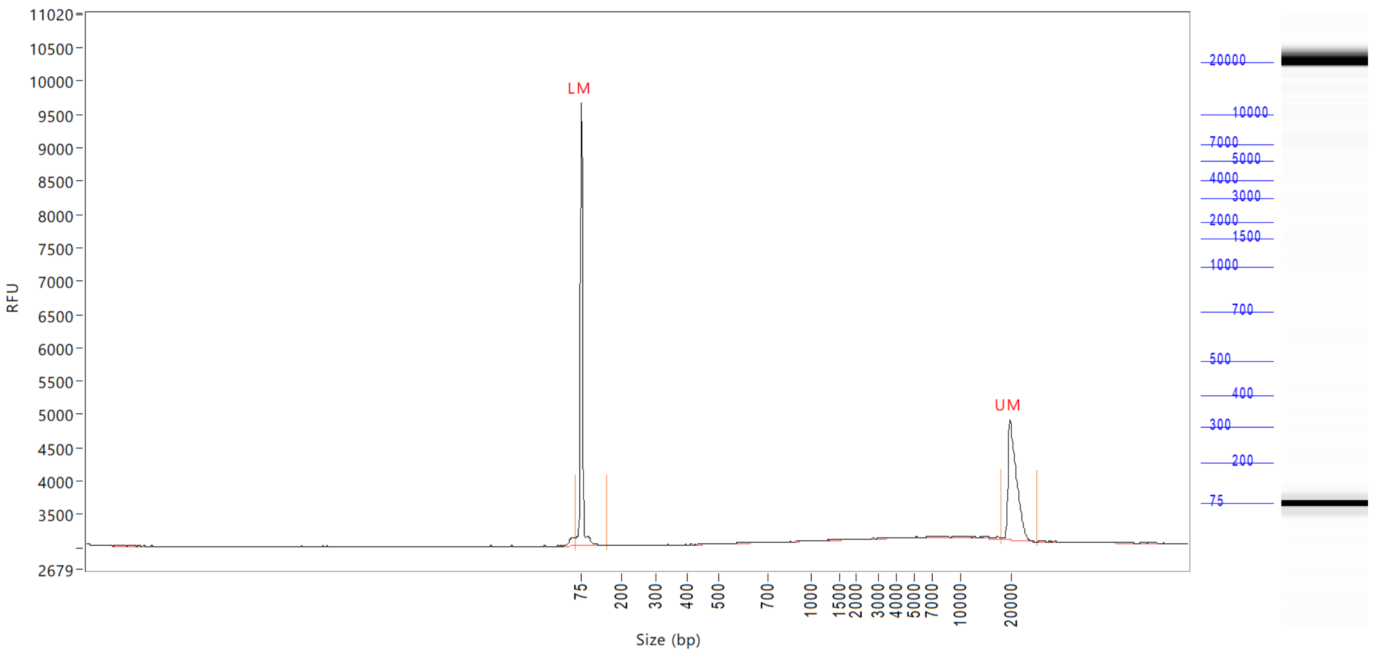

Peak	Size	Concentration	Relative concentration	Molarity	From	To	CV%	RFU
	(bp)	(ng/uL)	Percent	(nmole/L)	(bp)	(bp)		
1	75 (LM)	0.5000		11.0851	56	154	9.39	6656
2	20000 (UM)	0.2487		0.0199	18171	25199	4.41	1797

TIC:	0.0000	ng/uL
TIM:	0.0000	nmole/L
Total concentration:	0.5961	ng/uL

Sample peak width (sec): 5 Sample min peak height: 50 Sample baseline V to V?: Y Sample baseline V to V points: 3
Sample filter: Binomial Number of points for filter: 3 Sample start region (min): 0 Sample end region (min): 30
Marker peak width (sec): 5 Marker min peak height: 200 Marker baseline V to V?: Y Marker baseline V to V points: 3
Lower marker selection: First peak > 500 RFU Upper marker selection: Last peak > 200 RFU
Ladder size (bp)75, 200, 300, 400, 500, 700, 1000, 1500, 2000, 3000, 4000, 5000, 7000, 10000, 20000
Quantification using: Lower Marker Final concentration (ng/uL): 0.5000 Dilution factor: 12.0

Sample: SampE3
Well location: E3
Created: Thursday, October 21, 2021 8:58:49 PM

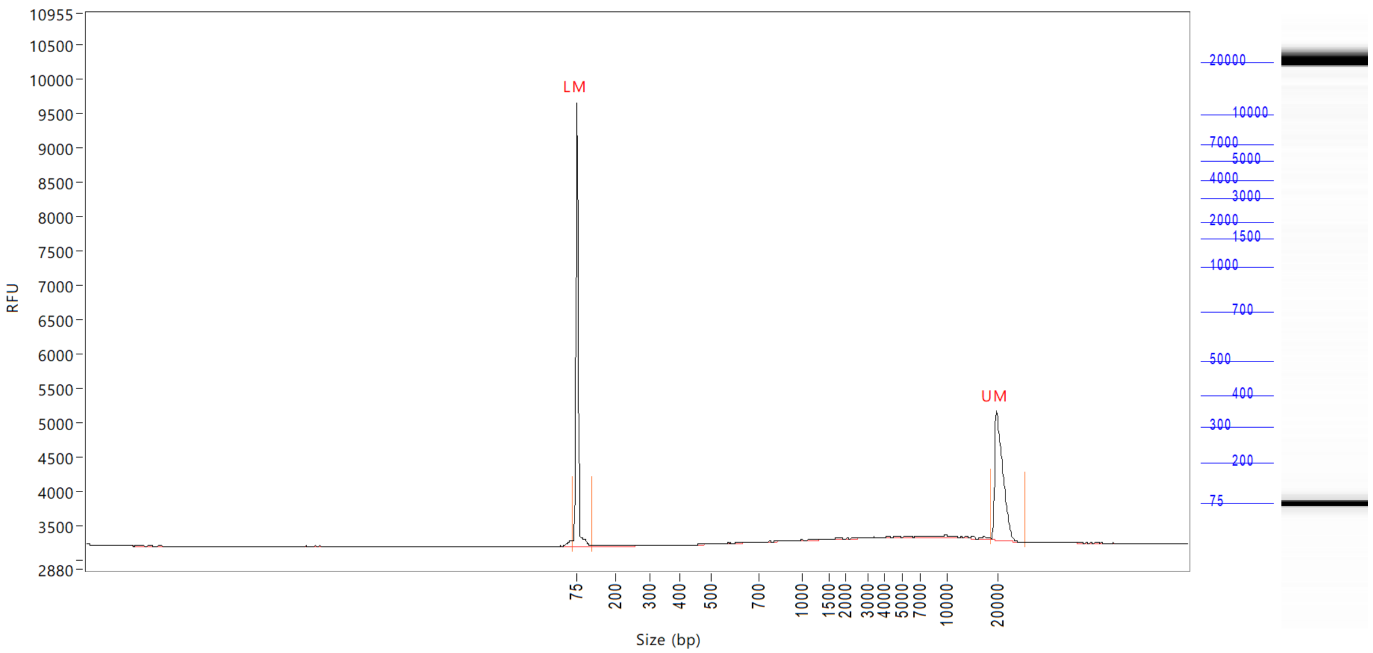

Peak	Size	Concentration	Relative concentration	Molarity	From	To	CV%	RFU
	(bp)	(ng/uL)	Percent	(nmole/L)	(bp)	(bp)		
1	75 (LM)	0.5000		11.2364	62	124	7.80	6451
2	20000 (UM)	0.2590		0.0208	18903	25682	4.25	1874

TIC:	0.0000	ng/uL
TIM:	0.0000	nmole/L
Total concentration:	0.6801	ng/uL

```
Sample peak width (sec): 5    Sample min peak height: 50    Sample baseline V to V?: Y    Sample baseline V to V points: 3
Sample filter: Binomial       Number of points for filter: 3  Sample start region (min): 0  Sample end region (min): 30
Marker peak width (sec): 5    Marker min peak height: 200   Marker baseline V to V?: Y    Marker baseline V to V points: 3
Lower marker selection: First peak > 500 RFU                Upper marker selection: Last peak > 200 RFU
Ladder size (bp)75, 200, 300, 400, 500, 700, 1000, 1500, 2000, 3000, 4000, 5000, 7000, 10000, 20000
Quantification using: Lower Marker          Final concentration (ng/uL): 0.5000          Dilution factor: 12.0
```

Sample: SampF3
Well location: F3
Created: Thursday, October 21, 2021 8:58:49 PM

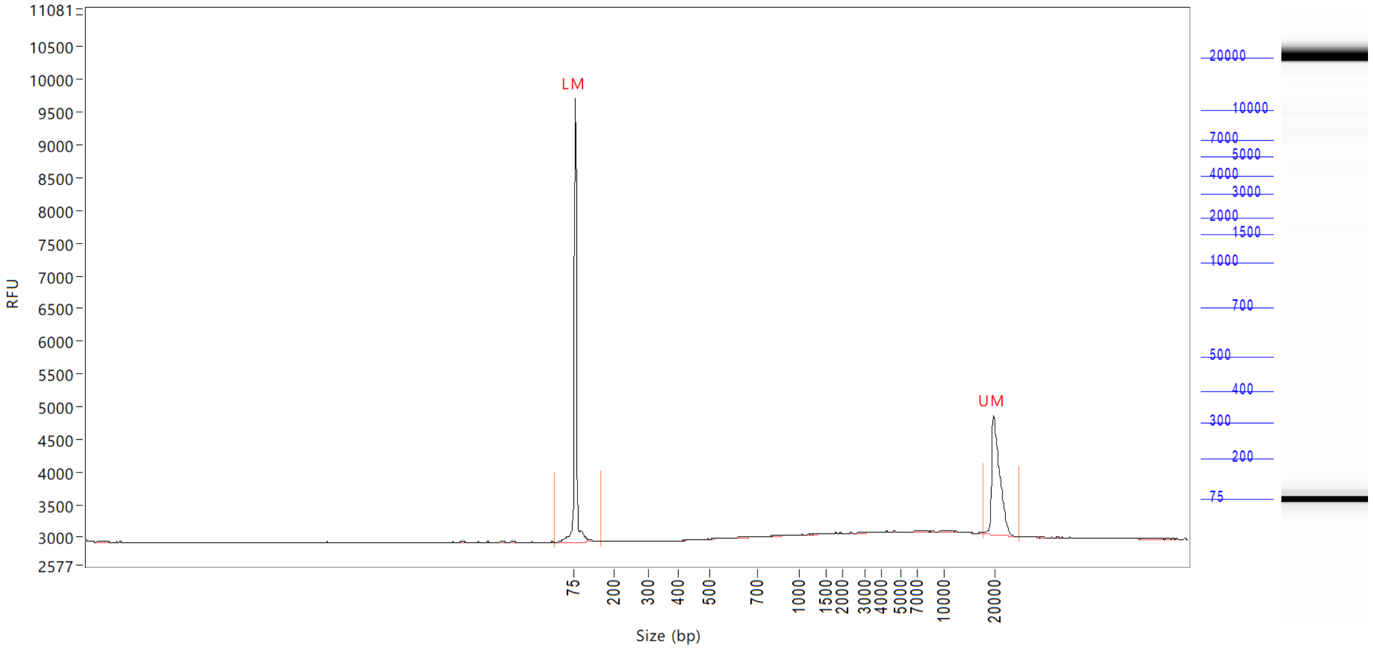

Peak	Size	Concentration	Relative concentration	Molarity	From	To	CV%	RFU
	(bp)	(ng/uL)	Percent	(nmole/L)	(bp)	(bp)		
1	75 (LM)	0.5000		11.2364	12	156	14.17	6791
2	20000 (UM)	0.2284		0.0183	17927	25078	4.32	1826

TIC:	0.0000	ng/uL
TIM:	0.0000	nmole/L
Total concentration:	0.5967	ng/uL

Sample peak width (sec): 5 Sample min peak height: 50 Sample baseline V to V?: Y Sample baseline V to V points: 3
Sample filter: Binomial Number of points for filter: 3 Sample start region (min): 0 Sample end region (min): 30
Marker peak width (sec): 5 Marker min peak height: 200 Marker baseline V to V?: Y Marker baseline V to V points: 3
Lower marker selection: First peak > 500 RFU Upper marker selection: Last peak > 200 RFU
Ladder size (bp)75, 200, 300, 400, 500, 700, 1000, 1500, 2000, 3000, 4000, 5000, 7000, 10000, 20000
Quantification using: Lower Marker Final concentration (ng/uL): 0.5000 Dilution factor: 12.0

Sample: SampG3
Well location: G3
Created: Thursday, October 21, 2021 8:58:49 PM

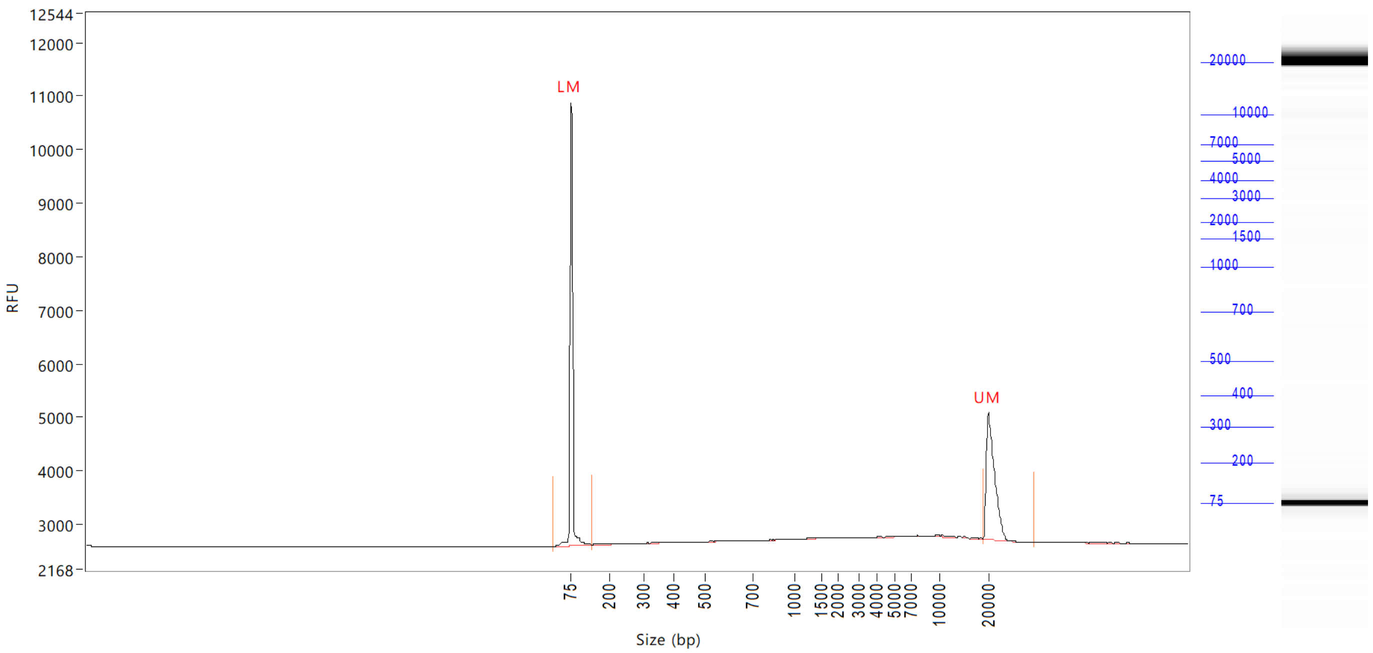

Peak	Size	Concentration	Relative concentration	Molarity	From	To	CV%	RFU
	(bp)	(ng/uL)	Percent	(nmole/L)	(bp)	(bp)		
1	75 (LM)	0.5000		11.0851	20	141	11.41	8282
2	20000 (UM)	0.2544		0.0203	18903	29309	4.74	2366

TIC:	0.0000	ng/uL
TIM:	0.0000	nmole/L
Total concentration:	0.4620	ng/uL

```
Sample peak width (sec): 5    Sample min peak height: 50    Sample baseline V to V?: Y    Sample baseline V to V points: 3
Sample filter: Binomial       Number of points for filter: 3  Sample start region (min): 0  Sample end region (min): 30
Marker peak width (sec): 5    Marker min peak height: 200   Marker baseline V to V?: Y    Marker baseline V to V points: 3
Lower marker selection: First peak > 500 RFU                Upper marker selection: Last peak > 200 RFU
Ladder size (bp)75, 200, 300, 400, 500, 700, 1000, 1500, 2000, 3000, 4000, 5000, 7000, 10000, 20000
Quantification using: Lower Marker          Final concentration (ng/uL): 0.5000          Dilution factor: 12.0
```

Sample: SampH3
Well location: H3
Created: Thursday, October 21, 2021 8:58:49 PM

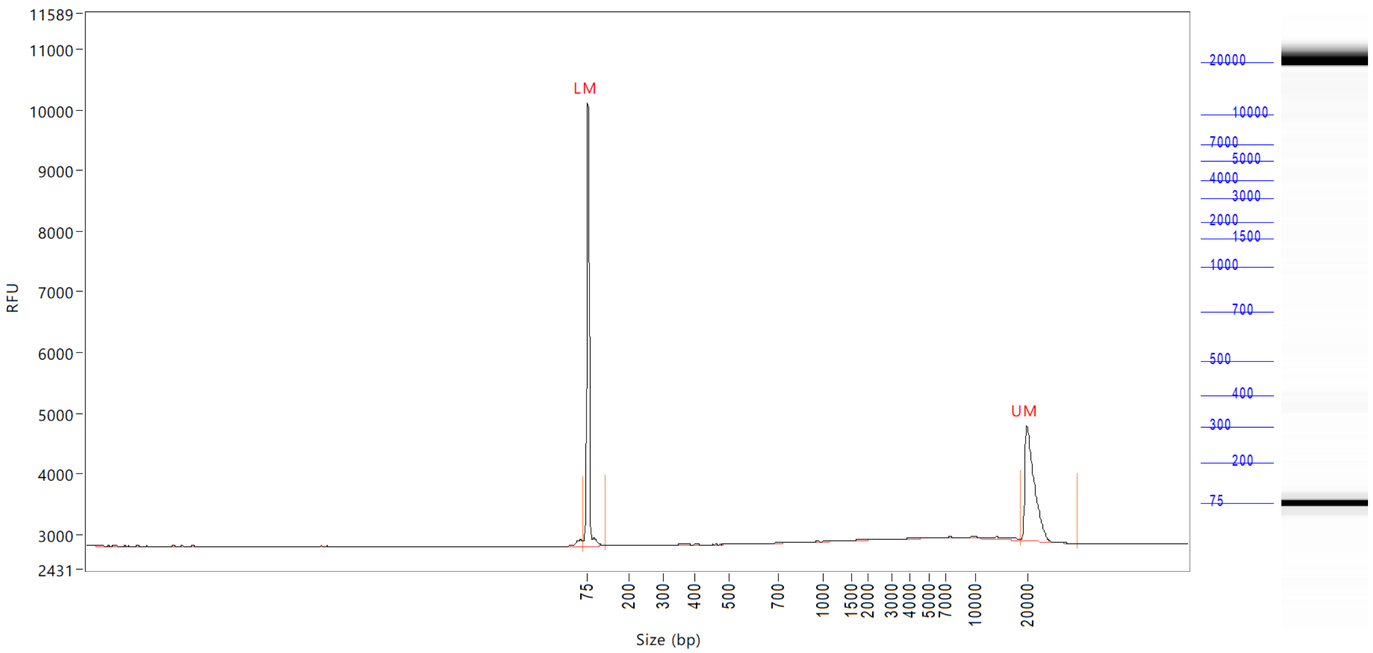

Peak	Size	Concentration	Relative concentration	Molarity	From	To	CV%	RFU
	(bp)	(ng/uL)	Percent	(nmole/L)	(bp)	(bp)		
1	75 (LM)	0.5000		10.9378	62	129	7.63	7314
2	20000 (UM)	0.2448		0.0194	18903	29793	5.09	1900

TIC:	0.0000	ng/uL
TIM:	0.0000	nmole/L
Total concentration:	0.7023	ng/uL

```
Sample peak width (sec): 5    Sample min peak height: 50    Sample baseline V to V?: Y    Sample baseline V to V points: 3
Sample filter: Binomial       Number of points for filter: 3  Sample start region (min): 0  Sample end region (min): 30
Marker peak width (sec): 5    Marker min peak height: 200   Marker baseline V to V?: Y    Marker baseline V to V points: 3
Lower marker selection: First peak > 500 RFU                Upper marker selection: Last peak > 200 RFU
Ladder size (bp)75, 200, 300, 400, 500, 700, 1000, 1500, 2000, 3000, 4000, 5000, 7000, 10000, 20000
Quantification using: Lower Marker          Final concentration (ng/uL): 0.5000          Dilution factor: 12.0
```

Sample: SampA4
Well location: A4
Created: Thursday, October 21, 2021 8:58:49 PM

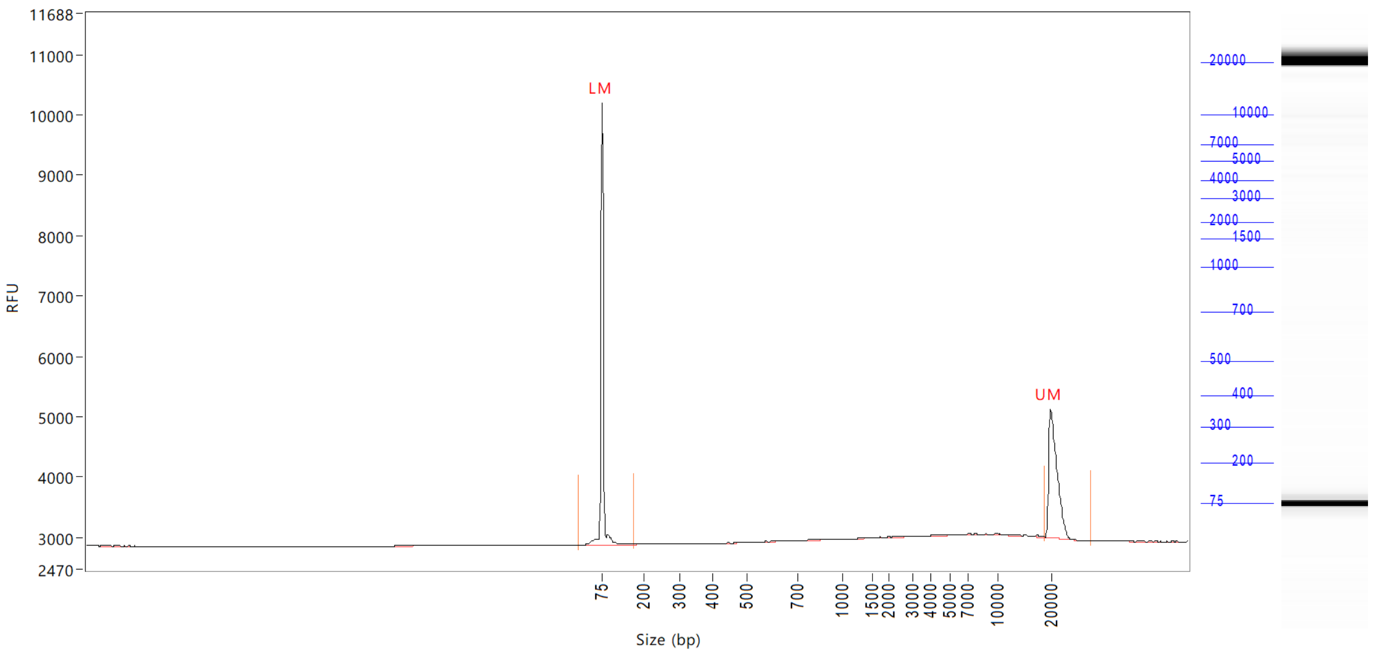

Peak	Size	Concentration	Relative concentration	Molarity	From	To	CV%	RFU
	(bp)	(ng/uL)	Percent	(nmole/L)	(bp)	(bp)		
1	75 (LM)	0.5000		11.2364	9	169	13.30	7340
2	20000 (UM)	0.2460		0.0197	18903	27617	4.20	2119

TIC:	0.0000	ng/uL
TIM:	0.0000	nmole/L
Total concentration:	0.4942	ng/uL

Sample peak width (sec): 5 Sample min peak height: 50 Sample baseline V to V?: Y Sample baseline V to V points: 3
Sample filter: Binomial Number of points for filter: 3 Sample start region (min): 0 Sample end region (min): 30
Marker peak width (sec): 5 Marker min peak height: 200 Marker baseline V to V?: Y Marker baseline V to V points: 3
Lower marker selection: First peak > 500 RFU Upper marker selection: Last peak > 200 RFU
Ladder size (bp)75, 200, 300, 400, 500, 700, 1000, 1500, 2000, 3000, 4000, 5000, 7000, 10000, 20000
Quantification using: Lower Marker Final concentration (ng/uL): 0.5000 Dilution factor: 12.0

Sample: SampB4
Well location: B4
Created: Thursday, October 21, 2021 8:58:49 PM

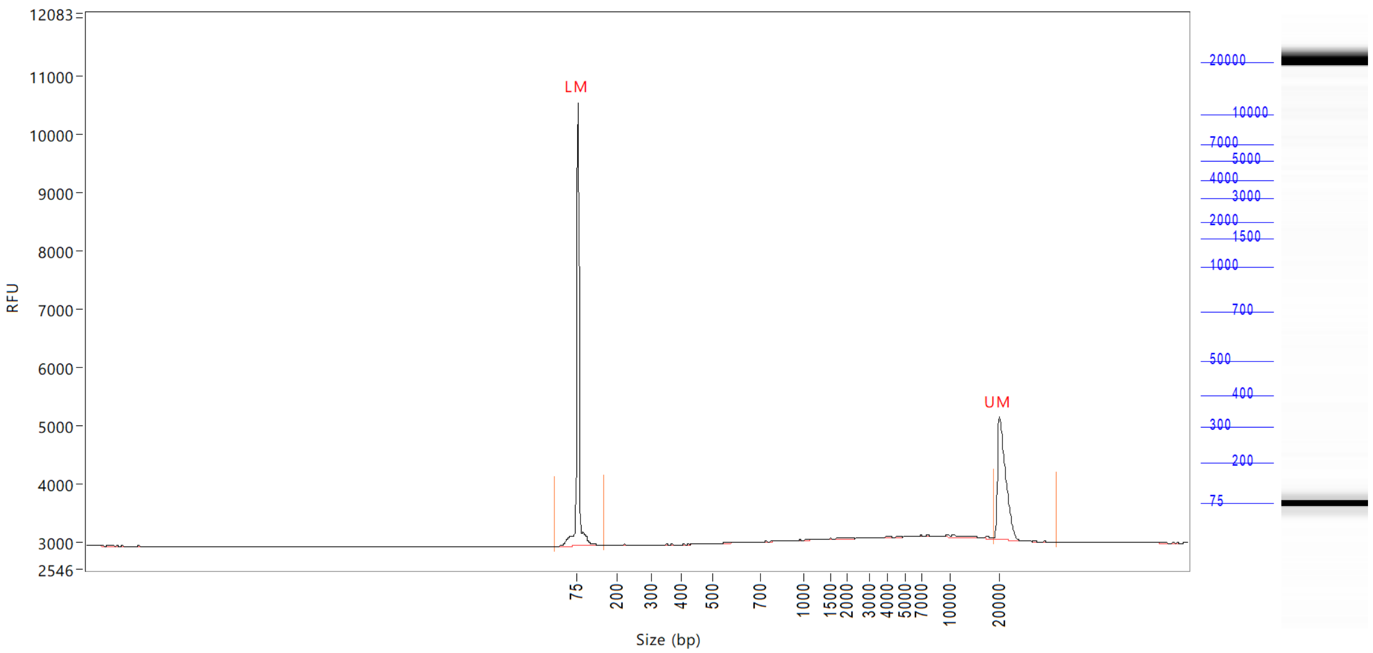

Peak	Size	Concentration	Relative concentration	Molarity	From	To	CV%	RFU
	(bp)	(ng/uL)	Percent	(nmole/L)	(bp)	(bp)		
1	75 (LM)	0.5000		11.2364	3	160	14.30	7612
2	20000 (UM)	0.2277		0.0182	18903	31486	4.75	2108

TIC:	0.0000	ng/uL
TIM:	0.0000	nmole/L
Total concentration:	0.5329	ng/uL

Sample peak width (sec): 5 Sample min peak height: 50 Sample baseline V to V?: Y Sample baseline V to V points: 3
Sample filter: Binomial Number of points for filter: 3 Sample start region (min): 0 Sample end region (min): 30
Marker peak width (sec): 5 Marker min peak height: 200 Marker baseline V to V?: Y Marker baseline V to V points: 3
Lower marker selection: First peak > 500 RFU Upper marker selection: Last peak > 200 RFU
Ladder size (bp)75, 200, 300, 400, 500, 700, 1000, 1500, 2000, 3000, 4000, 5000, 7000, 10000, 20000
Quantification using: Lower Marker Final concentration (ng/uL): 0.5000 Dilution factor: 12.0

Sample: SampC4
Well location: C4
Created: Thursday, October 21, 2021 8:58:49 PM

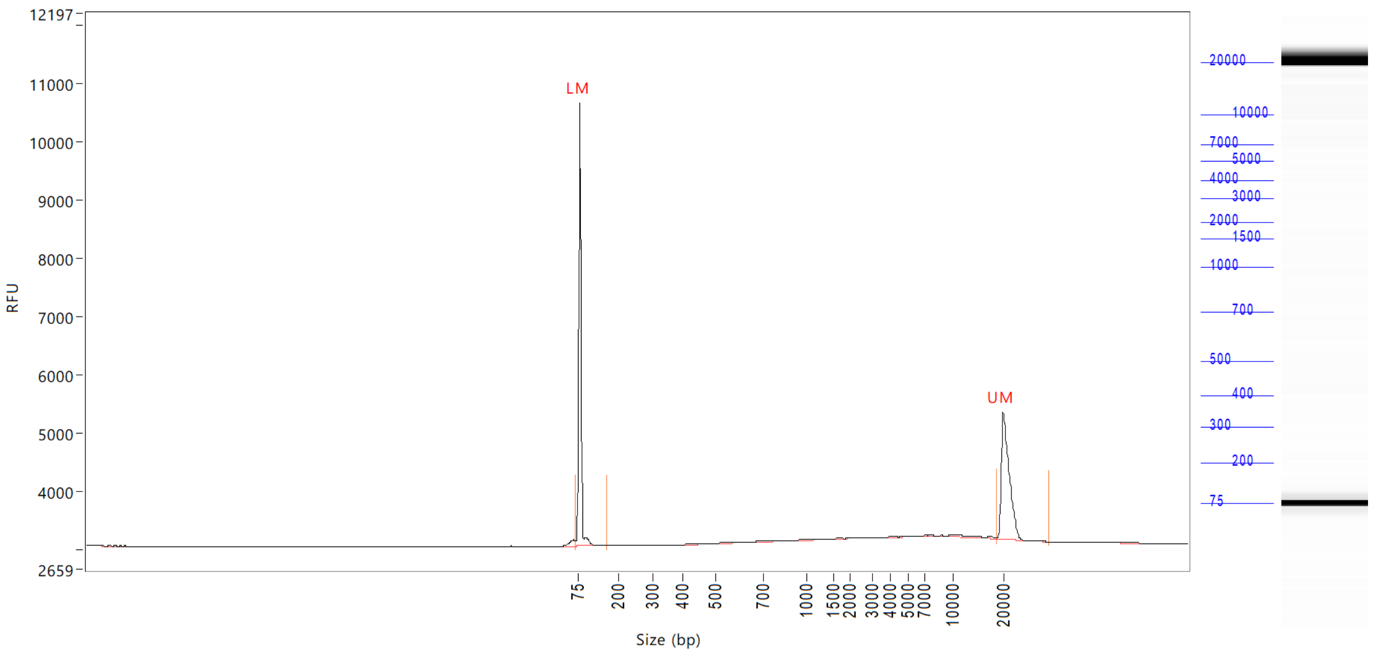

Peak	Size	Concentration	Relative concentration	Molarity	From	To	CV%	RFU
	(bp)	(ng/uL)	Percent	(nmole/L)	(bp)	(bp)		
1	75 (LM)	0.5000		10.9378	64	162	9.31	7614
2	20000 (UM)	0.2557		0.0204	18903	29068	4.27	2173

TIC:	0.0000	ng/uL
TIM:	0.0000	nmole/L
Total concentration:	0.7195	ng/uL

Sample peak width (sec): 5 Sample min peak height: 50 Sample baseline V to V?: Y Sample baseline V to V points: 3
Sample filter: Binomial Number of points for filter: 3 Sample start region (min): 0 Sample end region (min): 30
Marker peak width (sec): 5 Marker min peak height: 200 Marker baseline V to V?: Y Marker baseline V to V points: 3
Lower marker selection: First peak > 500 RFU Upper marker selection: Last peak > 200 RFU
Ladder size (bp)75, 200, 300, 400, 500, 700, 1000, 1500, 2000, 3000, 4000, 5000, 7000, 10000, 20000
Quantification using: Lower Marker Final concentration (ng/uL): 0.5000 Dilution factor: 12.0

Sample: SampD4
Well location: D4
Created: Thursday, October 21, 2021 8:58:49 PM

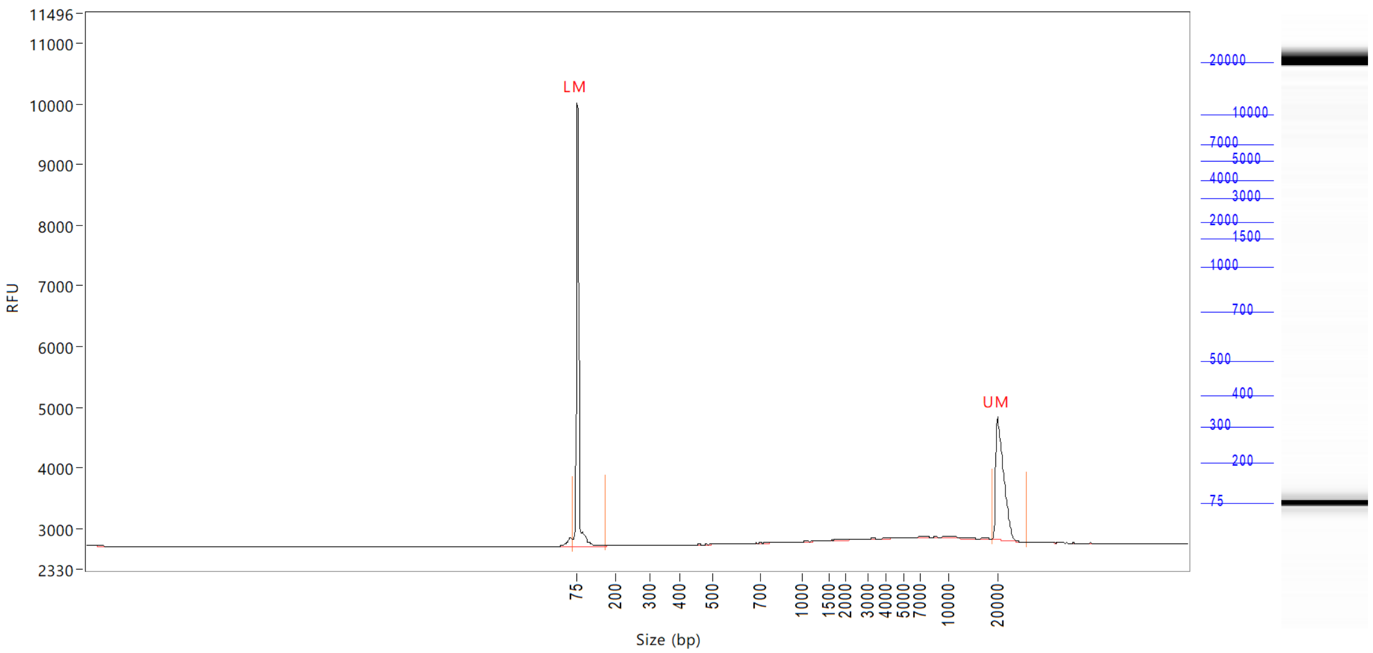

Peak	Size	Concentration	Relative concentration	Molarity	From	To	CV%	RFU
	(bp)	(ng/uL)	Percent	(nmole/L)	(bp)	(bp)		
1	75 (LM)	0.5000		10.7944	64	168	11.71	7324
2	20000 (UM)	0.2327		0.0186	18903	25924	4.11	2026

TIC:	0.0000	ng/uL
TIM:	0.0000	nmole/L
Total concentration:	0.5084	ng/uL

Sample peak width (sec): 5 Sample min peak height: 50 Sample baseline V to V?: Y Sample baseline V to V points: 3
Sample filter: Binomial Number of points for filter: 3 Sample start region (min): 0 Sample end region (min): 30
Marker peak width (sec): 5 Marker min peak height: 200 Marker baseline V to V?: Y Marker baseline V to V points: 3
Lower marker selection: First peak > 500 RFU Upper marker selection: Last peak > 200 RFU
Ladder size (bp)75, 200, 300, 400, 500, 700, 1000, 1500, 2000, 3000, 4000, 5000, 7000, 10000, 20000
Quantification using: Lower Marker Final concentration (ng/uL): 0.5000 Dilution factor: 12.0

Sample: SampE4
Well location: E4
Created: Thursday, October 21, 2021 8:58:49 PM

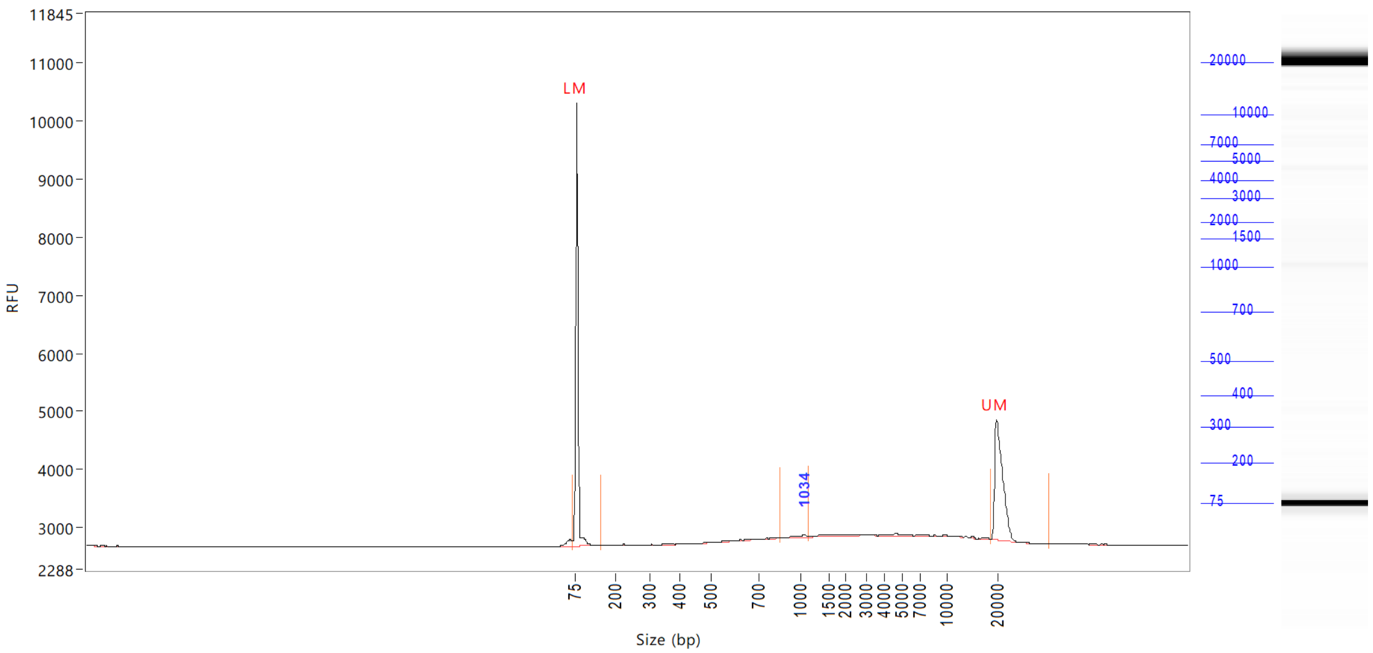

Peak	Size	Concentration	Relative concentration	Molarity	From	To	CV%	RFU
	(bp)	(ng/uL)	Percent	(nmole/L)	(bp)	(bp)		
1	75 (LM)	0.5000		10.9378	64	152	9.59	7631
2	1034	0.1179	100.0	0.1972	844	1134	7.29	55
3	20000 (UM)	0.2454		0.0197	18903	30639	4.44	2078

TIC:	0.1179	ng/uL
TIM:	0.1972	nmole/L
Total concentration:	0.7529	ng/uL

```
Sample peak width (sec): 5    Sample min peak height: 50    Sample baseline V to V?: Y    Sample baseline V to V points: 3
Sample filter: Binomial       Number of points for filter: 3  Sample start region (min): 0  Sample end region (min): 30
Marker peak width (sec): 5    Marker min peak height: 200   Marker baseline V to V?: Y    Marker baseline V to V points: 3
Lower marker selection: First peak > 500 RFU                Upper marker selection: Last peak > 200 RFU
Ladder size (bp)75, 200, 300, 400, 500, 700, 1000, 1500, 2000, 3000, 4000, 5000, 7000, 10000, 20000
Quantification using: Lower Marker          Final concentration (ng/uL): 0.5000          Dilution factor: 12.0
```

Sample: SampF4
Well location: F4
Created: Thursday, October 21, 2021 8:58:49 PM

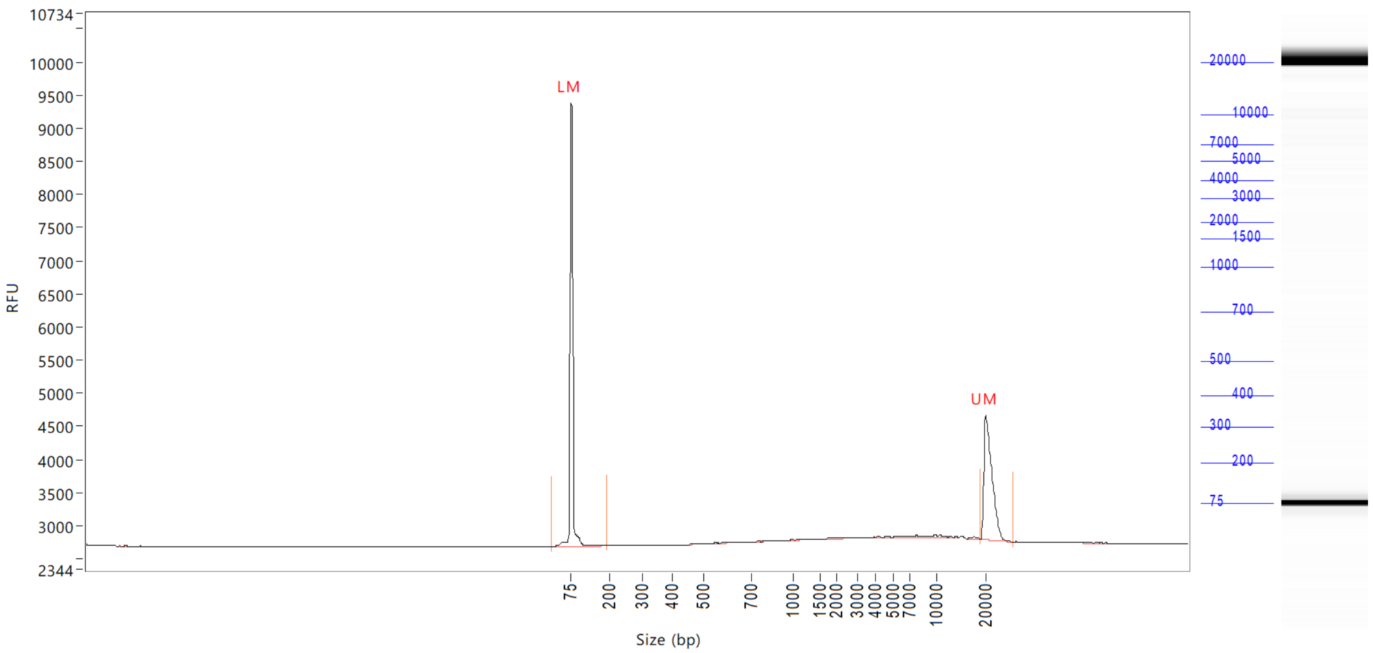

Peak	Size	Concentration	Relative concentration	Molarity	From	To	CV%	RFU
	(bp)	(ng/uL)	Percent	(nmole/L)	(bp)	(bp)		
1	75 (LM)	0.5000		10.9378	16	190	13.45	6703
2	20000 (UM)	0.2368		0.0189	18903	25440	4.34	1876

TIC:	0.0000	ng/uL
TIM:	0.0000	nmole/L
Total concentration:	0.5033	ng/uL

Sample peak width (sec): 5   Sample min peak height: 50   Sample baseline V to V?: Y   Sample baseline V to V points: 3
Sample filter: Binomial   Number of points for filter: 3 Sample start region (min): 0 Sample end region (min): 30
Marker peak width (sec): 5   Marker min peak height: 200   Marker baseline V to V?: Y   Marker baseline V to V points: 3
Lower marker selection: First peak > 500 RFU   Upper marker selection: Last peak > 200 RFU
Ladder size (bp)75, 200, 300, 400, 500, 700, 1000, 1500, 2000, 3000, 4000, 5000, 7000, 10000, 20000
Quantification using: Lower Marker   Final concentration (ng/uL): 0.5000   Dilution factor: 12.0

Sample: SampG4
Well location: G4
Created: Thursday, October 21, 2021 8:58:49 PM

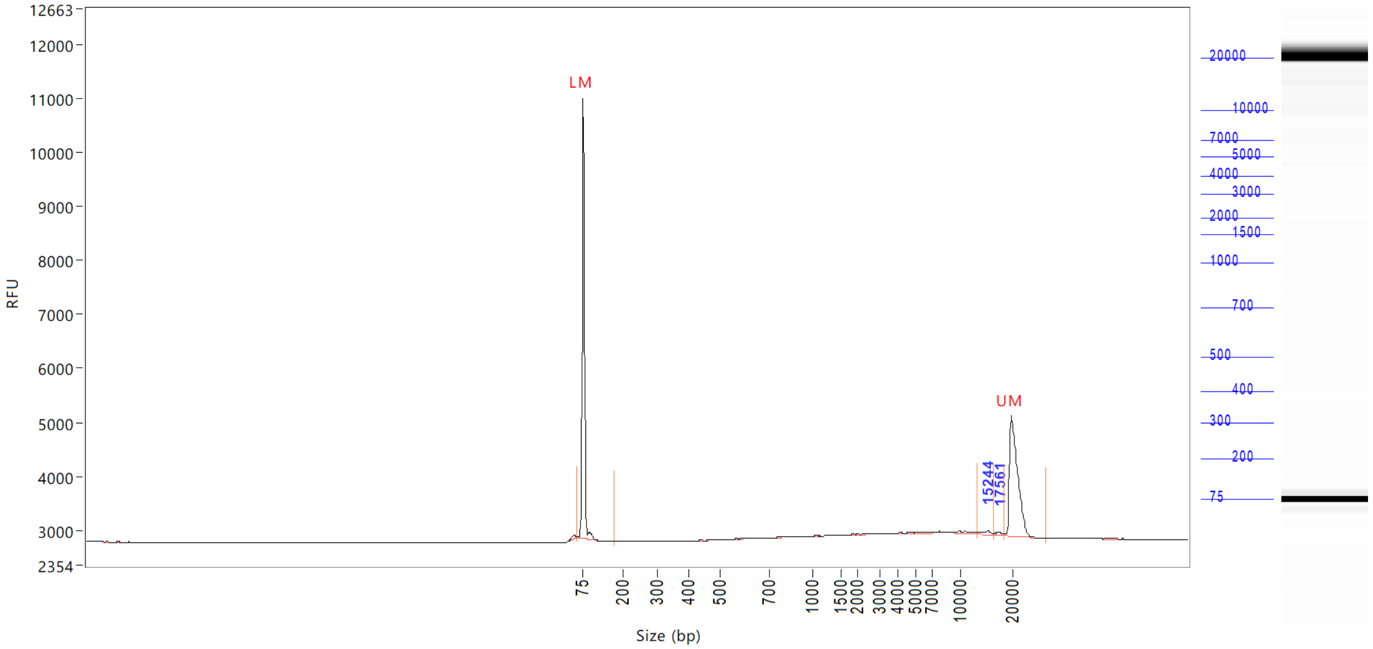

Peak	Size	Concentration	Relative concentration	Molarity	From	To	CV%	RFU
	(bp)	(ng/uL)	Percent	(nmole/L)	(bp)	(bp)		
1	75 (LM)	0.5000		11.0851	56	173	7.36	8163
2	15244	0.1001	57.6	0.0111	13171	16342	5.83	66
3	17561	0.0738	42.4	0.0070	16342	18659	3.74	52
4	20000 (UM)	0.2624		0.0210	18659	26891	4.37	2246

TIC:	0.1739	ng/uL
TIM:	0.0181	nmole/L
Total concentration:	0.6056	ng/uL

```
Sample peak width (sec): 5    Sample min peak height: 50    Sample baseline V to V?: Y    Sample baseline V to V points: 3
Sample filter: Binomial       Number of points for filter: 3  Sample start region (min): 0  Sample end region (min): 30
Marker peak width (sec): 5    Marker min peak height: 200   Marker baseline V to V?: Y    Marker baseline V to V points: 3
Lower marker selection: First peak > 500 RFU                Upper marker selection: Last peak > 200 RFU
Ladder size (bp)75, 200, 300, 400, 500, 700, 1000, 1500, 2000, 3000, 4000, 5000, 7000, 10000, 20000
Quantification using: Lower Marker          Final concentration (ng/uL): 0.5000          Dilution factor: 12.0
```

Sample: SampH4
Well location: H4
Created: Thursday, October 21, 2021 8:58:49 PM

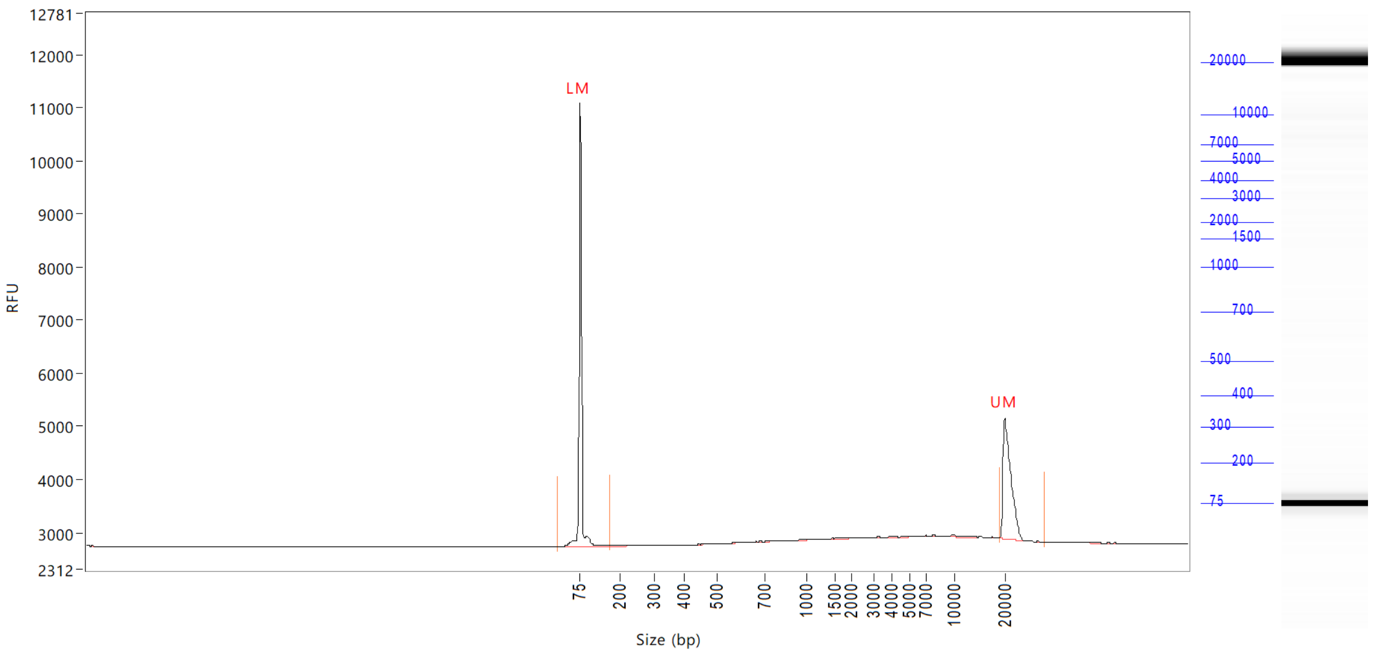

Peak	Size	Concentration	Relative concentration	Molarity	From	To	CV%	RFU
	(bp)	(ng/uL)	Percent	(nmole/L)	(bp)	(bp)		
1	75 (LM)	0.5000		11.0851	7	169	13.89	8365
2	20000 (UM)	0.2340		0.0187	18903	27859	4.24	2280

TIC:	0.0000	ng/uL
TIM:	0.0000	nmole/L
Total concentration:	0.4896	ng/uL

Sample peak width (sec): 5   Sample min peak height: 50   Sample baseline V to V?: Y   Sample baseline V to V points: 3
Sample filter: Binomial   Number of points for filter: 3   Sample start region (min): 0   Sample end region (min): 30
Marker peak width (sec): 5   Marker min peak height: 200   Marker baseline V to V?: Y   Marker baseline V to V points: 3
Lower marker selection: First peak > 500 RFU   Upper marker selection: Last peak > 200 RFU
Ladder size (bp)75, 200, 300, 400, 500, 700, 1000, 1500, 2000, 3000, 4000, 5000, 7000, 10000, 20000
Quantification using: Lower Marker   Final concentration (ng/uL): 0.5000   Dilution factor: 12.0

Sample: SampA5
Well location: A5
Created: Thursday, October 21, 2021 8:58:49 PM

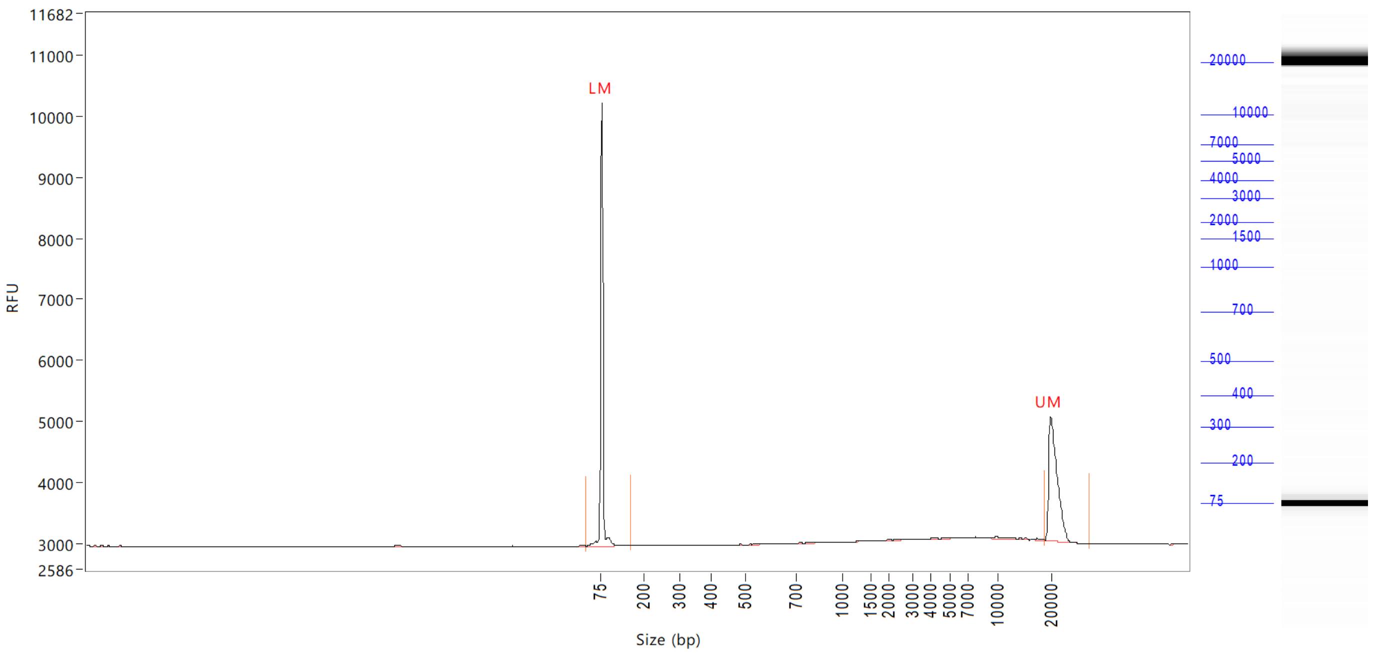

Peak	Size	Concentration	Relative concentration	Molarity	From	To	CV%	RFU
	(bp)	(ng/uL)	Percent	(nmole/L)	(bp)	(bp)		
1	75 (LM)	0.5000		11.2364	28	162	10.04	7264
2	20000 (UM)	0.2555		0.0204	18903	27133	4.24	2045

TIC:	0.0000	ng/uL
TIM:	0.0000	nmole/L
Total concentration:	0.5799	ng/uL

```
Sample peak width (sec): 5    Sample min peak height: 50    Sample baseline V to V?: Y    Sample baseline V to V points: 3
Sample filter: Binomial       Number of points for filter: 3  Sample start region (min): 0  Sample end region (min): 30
Marker peak width (sec): 5    Marker min peak height: 200   Marker baseline V to V?: Y    Marker baseline V to V points: 3
Lower marker selection: First peak > 500 RFU                Upper marker selection: Last peak > 200 RFU
Ladder size (bp)75, 200, 300, 400, 500, 700, 1000, 1500, 2000, 3000, 4000, 5000, 7000, 10000, 20000
Quantification using: Lower Marker          Final concentration (ng/uL): 0.5000          Dilution factor: 12.0
```

Sample: SampB5
Well location: B5
Created: Thursday, October 21, 2021 8:58:49 PM

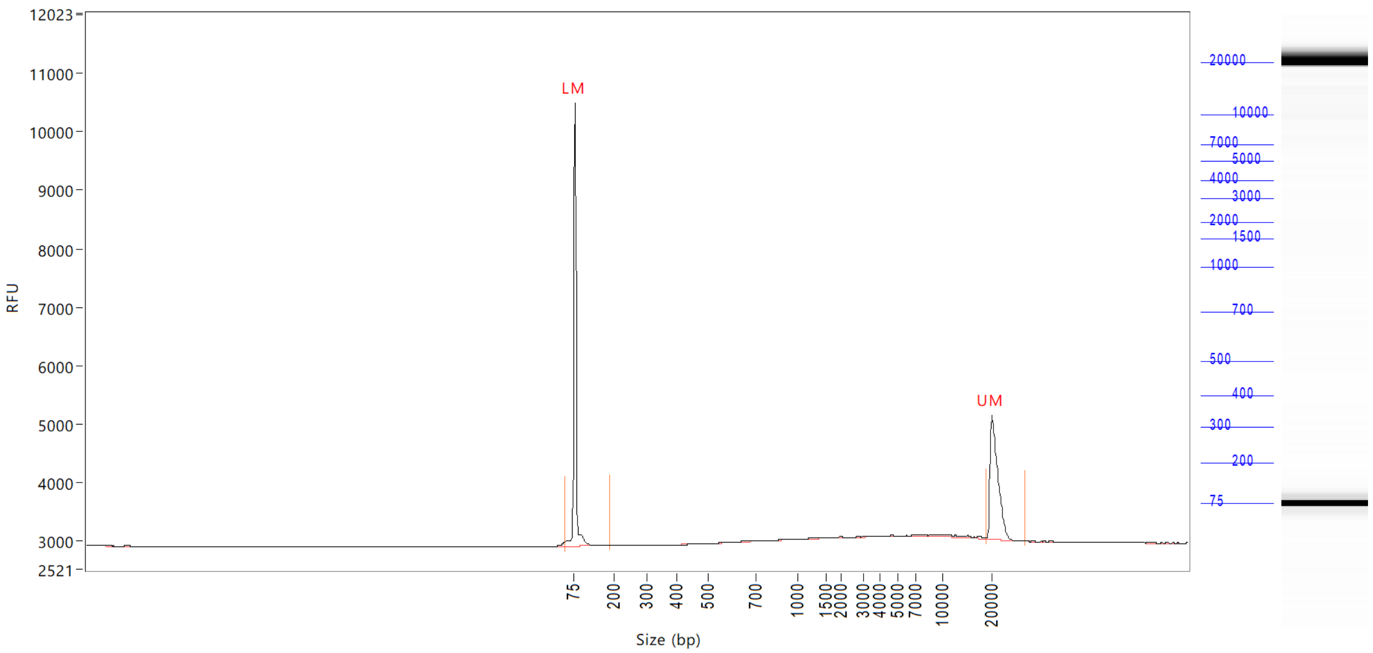

Peak	Size	Concentration	Relative concentration	Molarity	From	To	CV%	RFU
	(bp)	(ng/uL)	Percent	(nmole/L)	(bp)	(bp)		
1	75 (LM)	0.5000		10.9378	48	189	12.16	7587
2	20000 (UM)	0.2424		0.0194	18903	26529	4.36	2128

TIC:	0.0000	ng/uL
TIM:	0.0000	nmole/L
Total concentration:	0.5995	ng/uL

```
Sample peak width (sec): 5    Sample min peak height: 50    Sample baseline V to V?: Y    Sample baseline V to V points: 3
Sample filter: Binomial       Number of points for filter: 3  Sample start region (min): 0  Sample end region (min): 30
Marker peak width (sec): 5    Marker min peak height: 200   Marker baseline V to V?: Y    Marker baseline V to V points: 3
Lower marker selection: First peak > 500 RFU                Upper marker selection: Last peak > 200 RFU
Ladder size (bp)75, 200, 300, 400, 500, 700, 1000, 1500, 2000, 3000, 4000, 5000, 7000, 10000, 20000
Quantification using: Lower Marker          Final concentration (ng/uL): 0.5000          Dilution factor: 12.0
```

Sample: SampC5
Well location: C5
Created: Thursday, October 21, 2021 8:58:49 PM

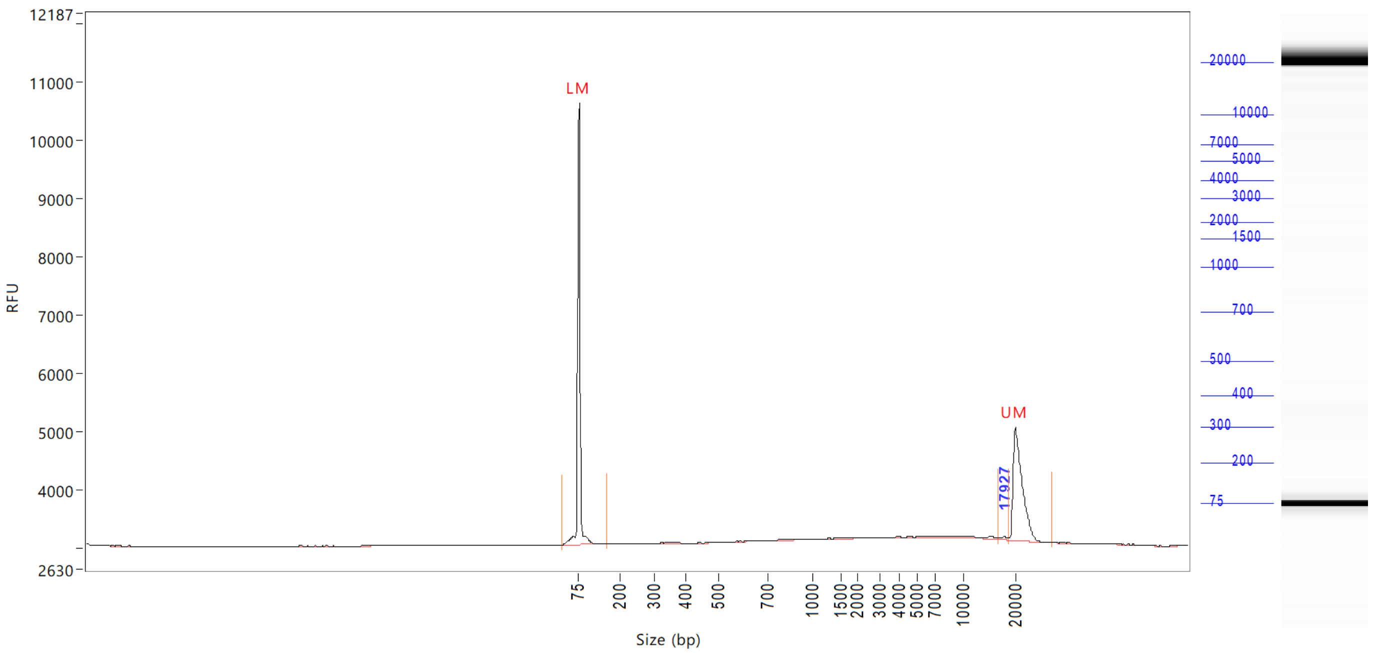

Peak	Size	Concentration	Relative concentration	Molarity	From	To	CV%	RFU
	(bp)	(ng/uL)	Percent	(nmole/L)	(bp)	(bp)		
1	75 (LM)	0.5000		11.3919	24	160	11.97	7604
2	17927	0.0622	100.0	0.0057	16708	18781	3.21	53
3	20000 (UM)	0.2390		0.0190	18781	27254	4.93	1957

TIC:	0.0622	ng/uL
TIM:	0.0057	nmole/L
Total concentration:	0.7467	ng/uL

Sample peak width (sec): 5   Sample min peak height: 50   Sample baseline V to V?: Y   Sample baseline V to V points: 3
Sample filter: Binomial   Number of points for filter: 3 Sample start region (min): 0 Sample end region (min): 30
Marker peak width (sec): 5   Marker min peak height: 200   Marker baseline V to V?: Y   Marker baseline V to V points: 3
Lower marker selection: First peak > 500 RFU   Upper marker selection: Last peak > 200 RFU
Ladder size (bp)75, 200, 300, 400, 500, 700, 1000, 1500, 2000, 3000, 4000, 5000, 7000, 10000, 20000
Quantification using: Lower Marker   Final concentration (ng/uL): 0.5000   Dilution factor: 12.0

Sample: SampD5
Well location: D5
Created: Thursday, October 21, 2021 8:58:49 PM

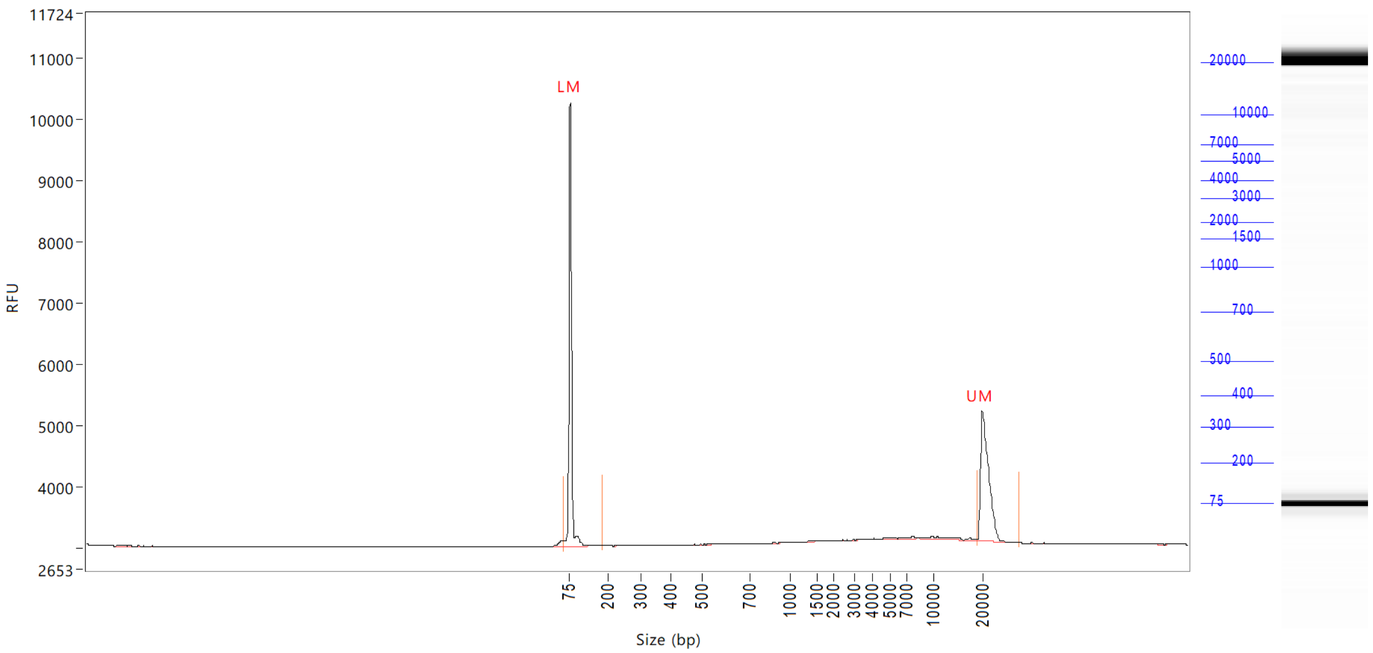

Peak	Size	Concentration	Relative concentration	Molarity	From	To	CV%	RFU
	(bp)	(ng/uL)	Percent	(nmole/L)	(bp)	(bp)		
1	75 (LM)	0.5000		11.0851	56	181	11.51	7245
2	20000 (UM)	0.2555		0.0203	18903	27617	4.51	2112

TIC:	0.0000	ng/uL
TIM:	0.0000	nmole/L
Total concentration:	0.5746	ng/uL

Sample peak width (sec): 5   Sample min peak height: 50   Sample baseline V to V?: Y   Sample baseline V to V points: 3
Sample filter: Binomial   Number of points for filter: 3 Sample start region (min): 0 Sample end region (min): 30
Marker peak width (sec): 5   Marker min peak height: 200   Marker baseline V to V?: Y   Marker baseline V to V points: 3
Lower marker selection: First peak > 500 RFU   Upper marker selection: Last peak > 200 RFU
Ladder size (bp)75, 200, 300, 400, 500, 700, 1000, 1500, 2000, 3000, 4000, 5000, 7000, 10000, 20000
Quantification using: Lower Marker   Final concentration (ng/uL): 0.5000   Dilution factor: 12.0

Sample: SampE5
Well location: E5
Created: Thursday, October 21, 2021 8:58:49 PM

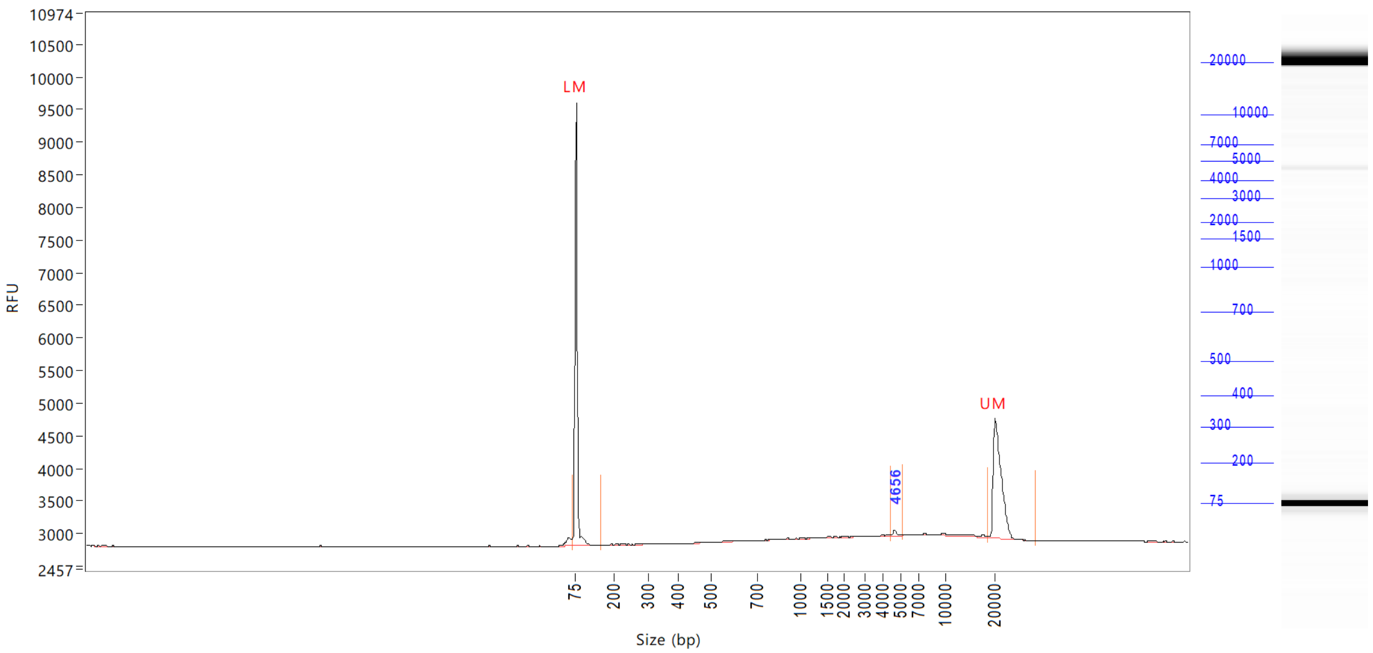

Peak	Size	Concentration	Relative concentration	Molarity	From	To	CV%	RFU
	(bp)	(ng/uL)	Percent	(nmole/L)	(bp)	(bp)		
1	75 (LM)	0.5000		11.0851	64	154	8.94	6796
2	4656	0.0734	100.0	0.0259	4414	5223	3.01	85
3	20000 (UM)	0.2482		0.0198	18537	28100	4.37	1847

TIC:	0.0734	ng/uL
TIM:	0.0259	nmole/L
Total concentration:	0.7163	ng/uL

```
Sample peak width (sec): 5    Sample min peak height: 50    Sample baseline V to V?: Y    Sample baseline V to V points: 3
Sample filter: Binomial       Number of points for filter: 3  Sample start region (min): 0  Sample end region (min): 30
Marker peak width (sec): 5    Marker min peak height: 200   Marker baseline V to V?: Y    Marker baseline V to V points: 3
Lower marker selection: First peak > 500 RFU                Upper marker selection: Last peak > 200 RFU
Ladder size (bp)75, 200, 300, 400, 500, 700, 1000, 1500, 2000, 3000, 4000, 5000, 7000, 10000, 20000
Quantification using: Lower Marker          Final concentration (ng/uL): 0.5000          Dilution factor: 12.0
```

Sample: SampF5
Well location: F5
Created: Thursday, October 21, 2021 8:58:49 PM

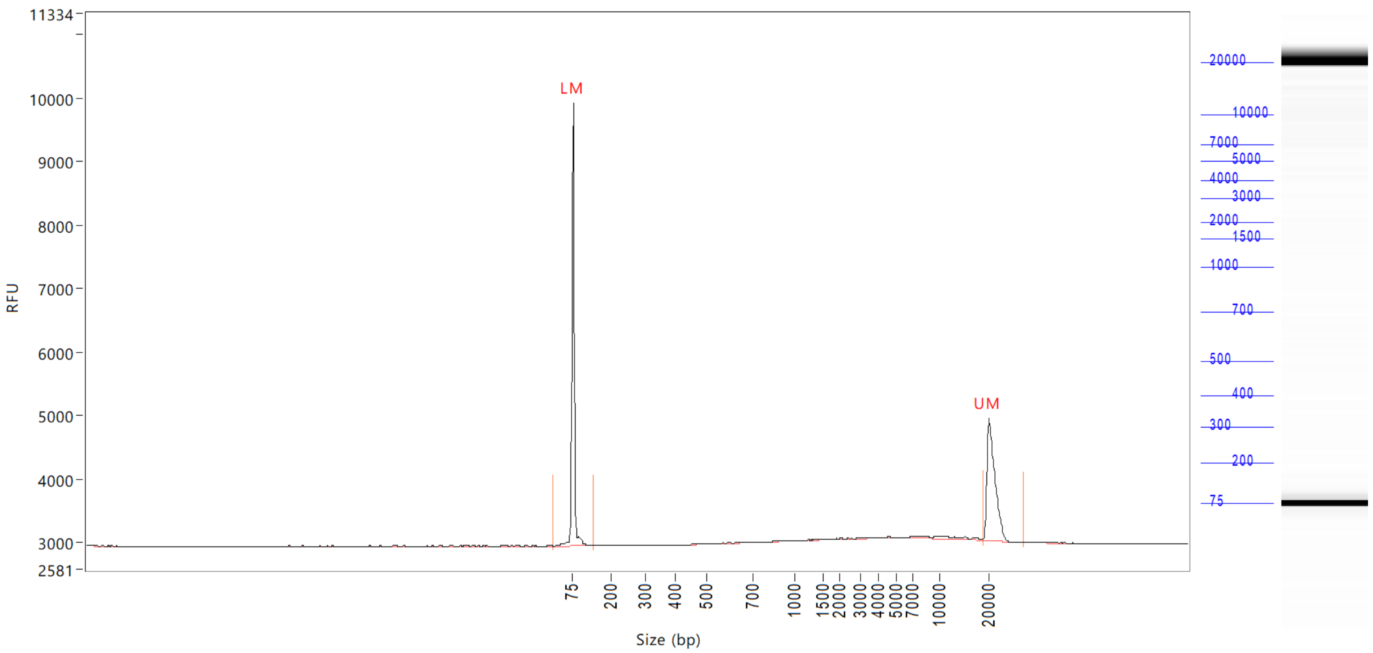

Peak	Size	Concentration	Relative concentration	Molarity	From	To	CV%	RFU
	(bp)	(ng/uL)	Percent	(nmole/L)	(bp)	(bp)		
1	75 (LM)	0.5000		11.0851	14	141	10.91	6987
2	20000 (UM)	0.2445		0.0196	18781	27012	4.32	1935

TIC:	0.0000	ng/uL
TIM:	0.0000	nmole/L
Total concentration:	0.7513	ng/uL

Sample peak width (sec): 5   Sample min peak height: 50   Sample baseline V to V?: Y   Sample baseline V to V points: 3
Sample filter: Binomial   Number of points for filter: 3 Sample start region (min): 0 Sample end region (min): 30
Marker peak width (sec): 5   Marker min peak height: 200   Marker baseline V to V?: Y   Marker baseline V to V points: 3
Lower marker selection: First peak > 500 RFU   Upper marker selection: Last peak > 200 RFU
Ladder size (bp)75, 200, 300, 400, 500, 700, 1000, 1500, 2000, 3000, 4000, 5000, 7000, 10000, 20000
Quantification using: Lower Marker   Final concentration (ng/uL): 0.5000   Dilution factor: 12.0

Sample: SampG5
Well location: G5
Created: Thursday, October 21, 2021 8:58:49 PM

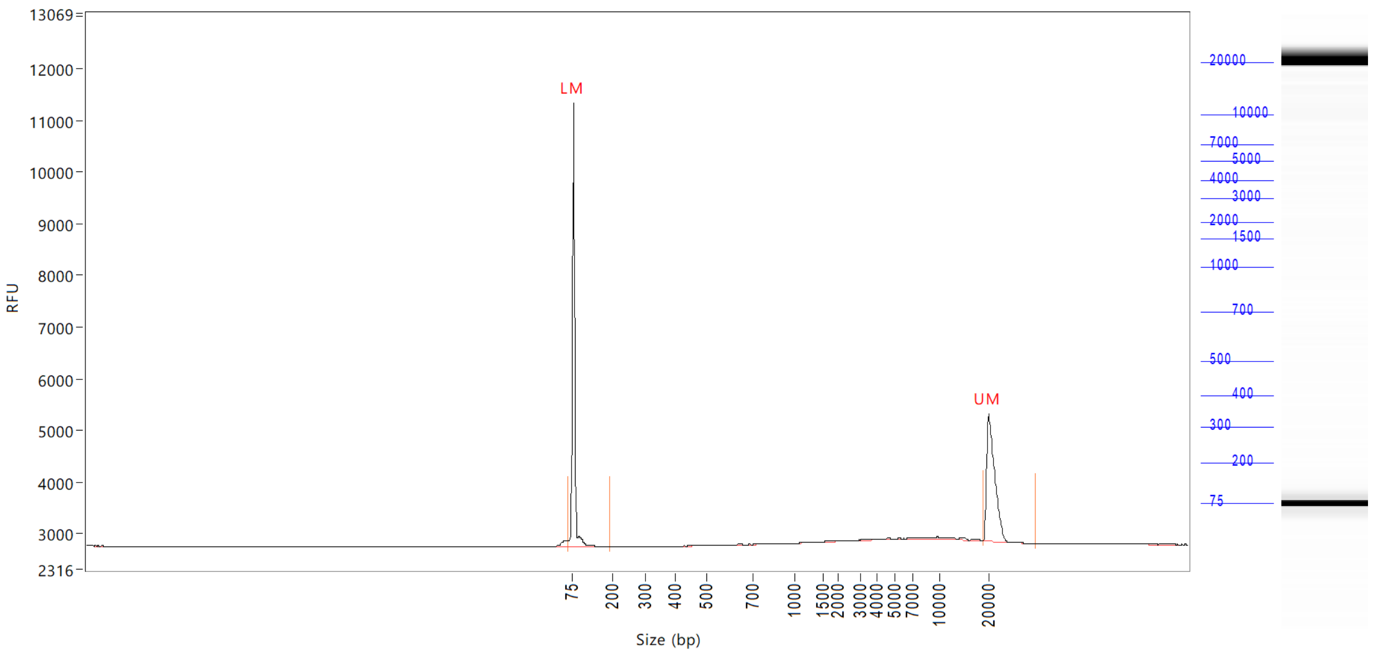

Peak	Size	Concentration	Relative concentration	Molarity	From	To	CV%	RFU
	(bp)	(ng/uL)	Percent	(nmole/L)	(bp)	(bp)		
1	75 (LM)	0.5000		11.0851	62	190	11.39	8596
2	20000 (UM)	0.2588		0.0206	18903	29551	4.44	2470

TIC:	0.0000	ng/uL
TIM:	0.0000	nmole/L
Total concentration:	0.5266	ng/uL

Sample peak width (sec): 5   Sample min peak height: 50   Sample baseline V to V?: Y   Sample baseline V to V points: 3
Sample filter: Binomial   Number of points for filter: 3 Sample start region (min): 0 Sample end region (min): 30
Marker peak width (sec): 5   Marker min peak height: 200   Marker baseline V to V?: Y   Marker baseline V to V points: 3
Lower marker selection: First peak > 500 RFU   Upper marker selection: Last peak > 200 RFU
Ladder size (bp)75, 200, 300, 400, 500, 700, 1000, 1500, 2000, 3000, 4000, 5000, 7000, 10000, 20000
Quantification using: Lower Marker   Final concentration (ng/uL): 0.5000   Dilution factor: 12.0

Sample: SampH5
Well location: H5
Created: Thursday, October 21, 2021 8:58:49 PM

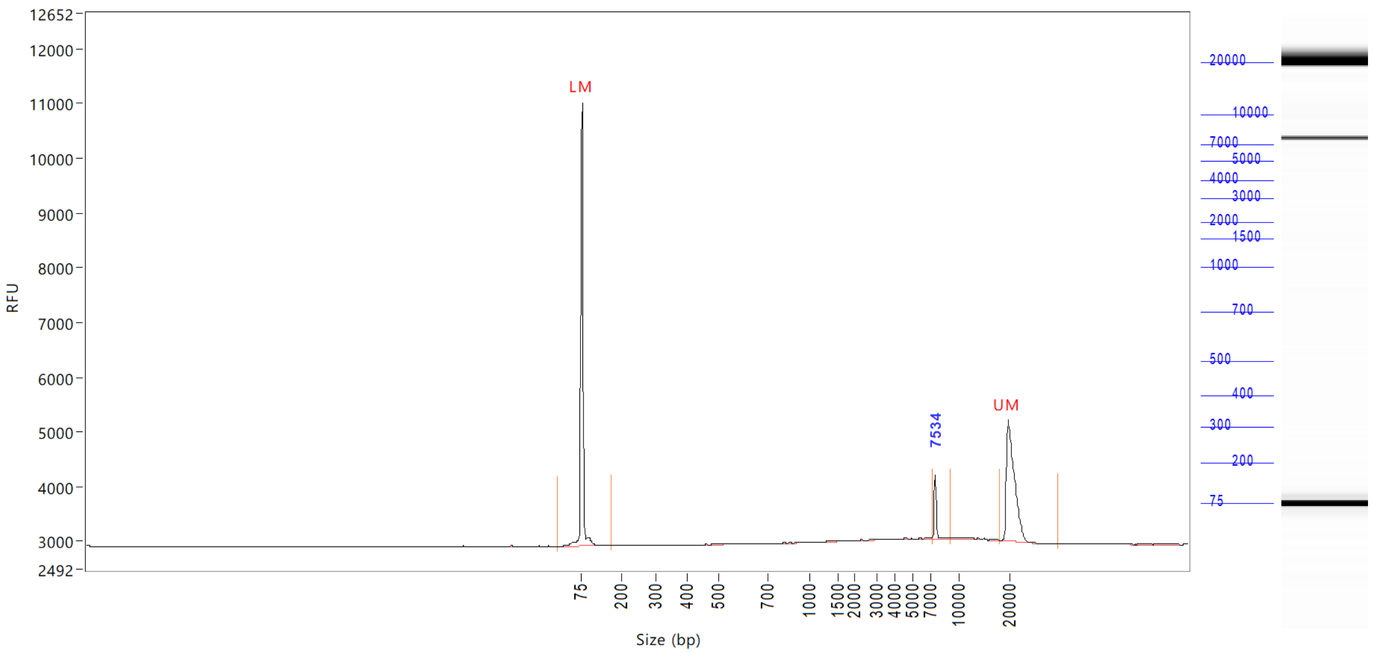

Peak	Size	Concentration	Relative concentration	Molarity	From	To	CV%	RFU
	(bp)	(ng/uL)	Percent	(nmole/L)	(bp)	(bp)		
1	75 (LM)	0.5000		11.2364	1	168	11.90	8110
2	7534	0.4268	100.0	0.0930	7201	9200	3.72	1179
3	20000 (UM)	0.2397		0.0192	18293	29793	4.62	2204

TIC:	0.4268	ng/uL
TIM:	0.0930	nmole/L
Total concentration:	0.8169	ng/uL

Sample peak width (sec): 5   Sample min peak height: 50   Sample baseline V to V?: Y   Sample baseline V to V points: 3
Sample filter: Binomial   Number of points for filter: 3 Sample start region (min): 0 Sample end region (min): 30
Marker peak width (sec): 5   Marker min peak height: 200   Marker baseline V to V?: Y   Marker baseline V to V points: 3
Lower marker selection: First peak > 500 RFU   Upper marker selection: Last peak > 200 RFU
Ladder size (bp)75, 200, 300, 400, 500, 700, 1000, 1500, 2000, 3000, 4000, 5000, 7000, 10000, 20000
Quantification using: Lower Marker   Final concentration (ng/uL): 0.5000   Dilution factor: 12.0

Sample: SampA6
Well location: A6
Created: Thursday, October 21, 2021 8:58:49 PM

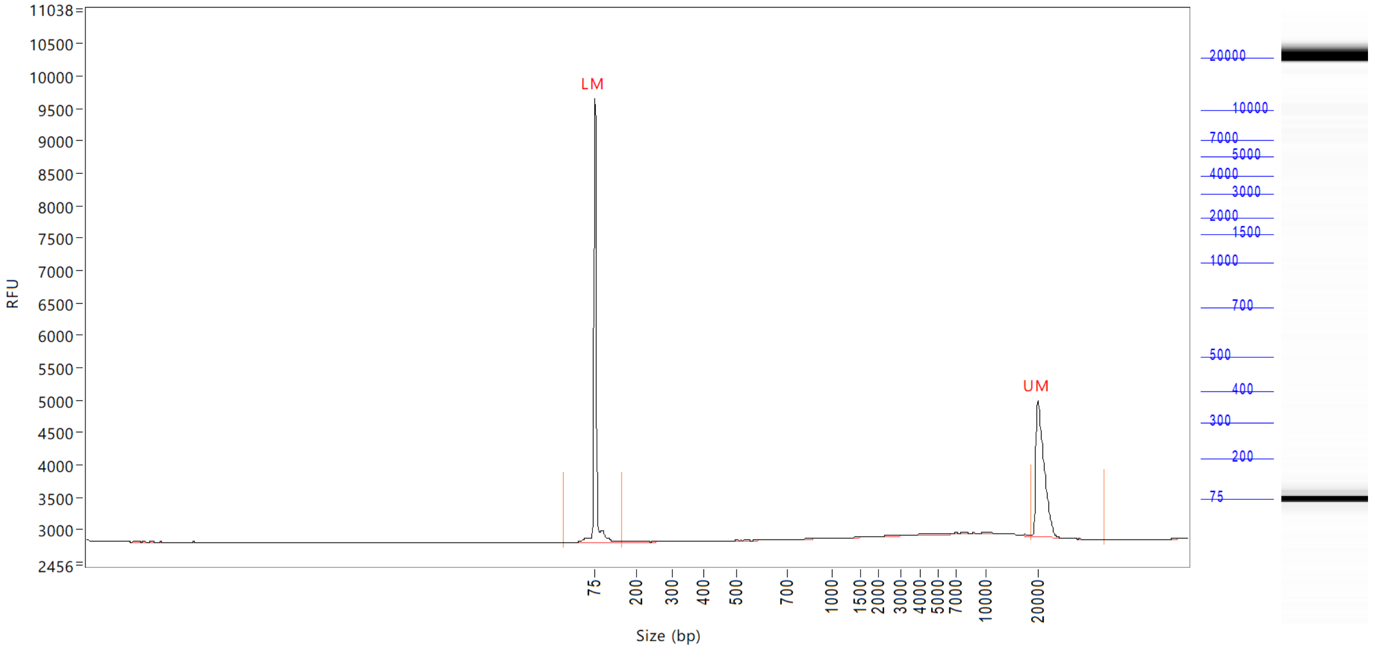

Peak	Size	Concentration	Relative concentration	Molarity	From	To	CV%	RFU
	(bp)	(ng/uL)	Percent	(nmole/L)	(bp)	(bp)		
1	75 (LM)	0.5000		10.9378	0	156	14.43	6859
2	20000 (UM)	0.2551		0.0204	18659	32574	5.03	2100

TIC:	0.0000	ng/uL
TIM:	0.0000	nmole/L
Total concentration:	0.5846	ng/uL

```
Sample peak width (sec): 5    Sample min peak height: 50    Sample baseline V to V?: Y    Sample baseline V to V points: 3
Sample filter: Binomial       Number of points for filter: 3  Sample start region (min): 0  Sample end region (min): 30
Marker peak width (sec): 5    Marker min peak height: 200   Marker baseline V to V?: Y    Marker baseline V to V points: 3
Lower marker selection: First peak > 500 RFU                Upper marker selection: Last peak > 200 RFU
Ladder size (bp)75, 200, 300, 400, 500, 700, 1000, 1500, 2000, 3000, 4000, 5000, 7000, 10000, 20000
Quantification using: Lower Marker          Final concentration (ng/uL): 0.5000          Dilution factor: 12.0
```

Sample: SampB6
Well location: B6
Created: Thursday, October 21, 2021 8:58:49 PM

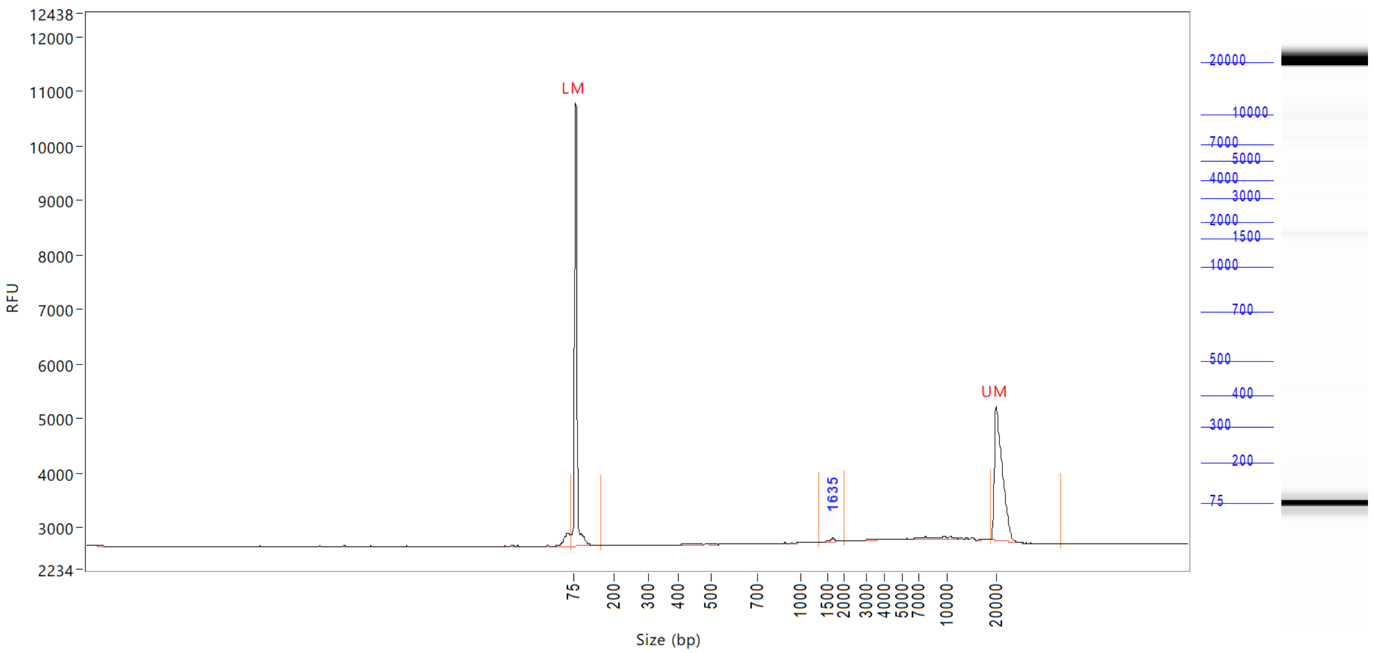

Peak	Size	Concentration	Relative concentration	Molarity	From	To	CV%	RFU
	(bp)	(ng/uL)	Percent	(nmole/L)	(bp)	(bp)		
1	75 (LM)	0.5000		10.9378	62	158	11.15	8146
2	1635	0.0759	100.0	0.0773	1334	2001	7.96	73
3	20000 (UM)	0.2531		0.0202	18903	33058	4.67	2456

TIC:	0.0759	ng/uL
TIM:	0.0773	nmole/L
Total concentration:	0.4993	ng/uL

```
Sample peak width (sec): 5    Sample min peak height: 50    Sample baseline V to V?: Y    Sample baseline V to V points: 3
Sample filter: Binomial       Number of points for filter: 3  Sample start region (min): 0  Sample end region (min): 30
Marker peak width (sec): 5    Marker min peak height: 200   Marker baseline V to V?: Y    Marker baseline V to V points: 3
Lower marker selection: First peak > 500 RFU                Upper marker selection: Last peak > 200 RFU
Ladder size (bp)75, 200, 300, 400, 500, 700, 1000, 1500, 2000, 3000, 4000, 5000, 7000, 10000, 20000
Quantification using: Lower Marker          Final concentration (ng/uL): 0.5000          Dilution factor: 12.0
```

Sample: SampC6
Well location: C6
Created: Thursday, October 21, 2021 8:58:49 PM

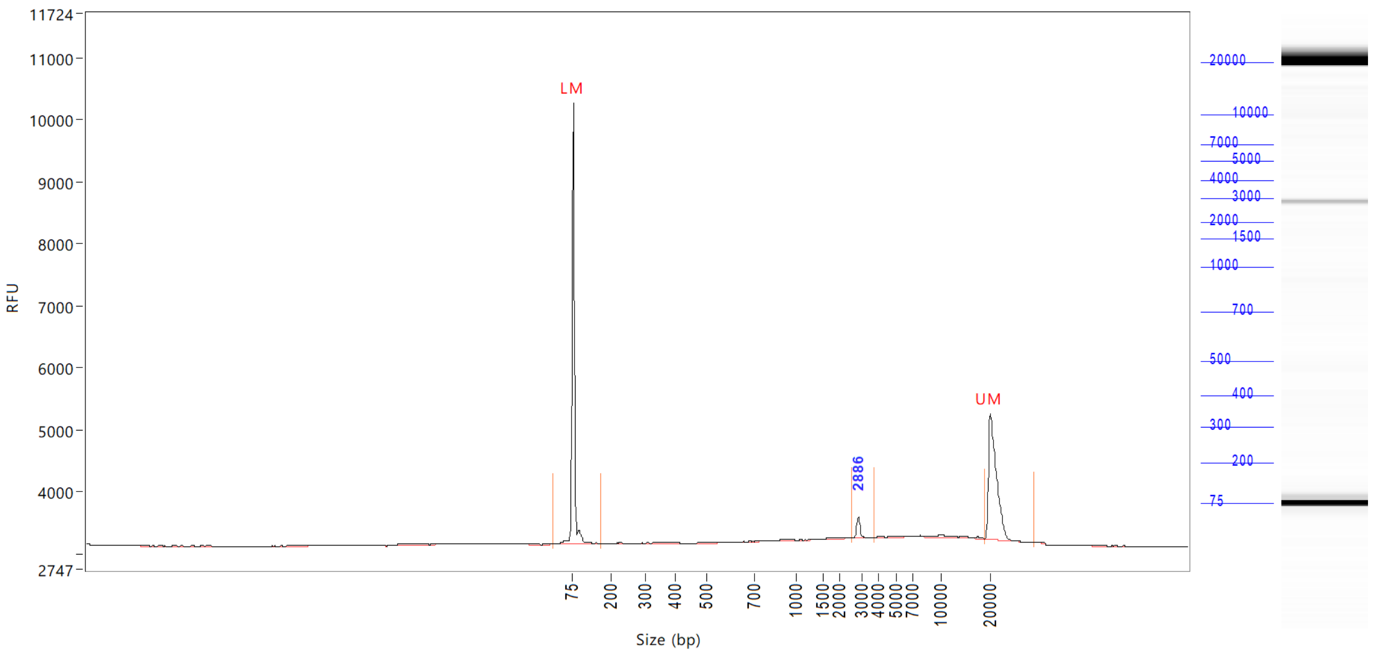

Peak	Size	Concentration	Relative concentration	Molarity	From	To	CV%	RFU
	(bp)	(ng/uL)	Percent	(nmole/L)	(bp)	(bp)		
1	75 (LM)	0.5000		10.9378	12	164	12.05	7136
2	2886	0.2512	100.0	0.1438	2572	3750	5.30	331
3	20000 (UM)	0.2464		0.0196	18903	28826	4.56	2026

TIC:	0.2512	ng/uL
TIM:	0.1438	nmole/L
Total concentration:	1.0150	ng/uL

Sample peak width (sec): 5 Sample min peak height: 50 Sample baseline V to V?: Y Sample baseline V to V points: 3
Sample filter: Binomial Number of points for filter: 3 Sample start region (min): 0 Sample end region (min): 30
Marker peak width (sec): 5 Marker min peak height: 200 Marker baseline V to V?: Y Marker baseline V to V points: 3
Lower marker selection: First peak > 500 RFU Upper marker selection: Last peak > 200 RFU
Ladder size (bp)75, 200, 300, 400, 500, 700, 1000, 1500, 2000, 3000, 4000, 5000, 7000, 10000, 20000
Quantification using: Lower Marker Final concentration (ng/uL): 0.5000 Dilution factor: 12.0

Sample: SampD6
Well location: D6
Created: Thursday, October 21, 2021 8:58:49 PM

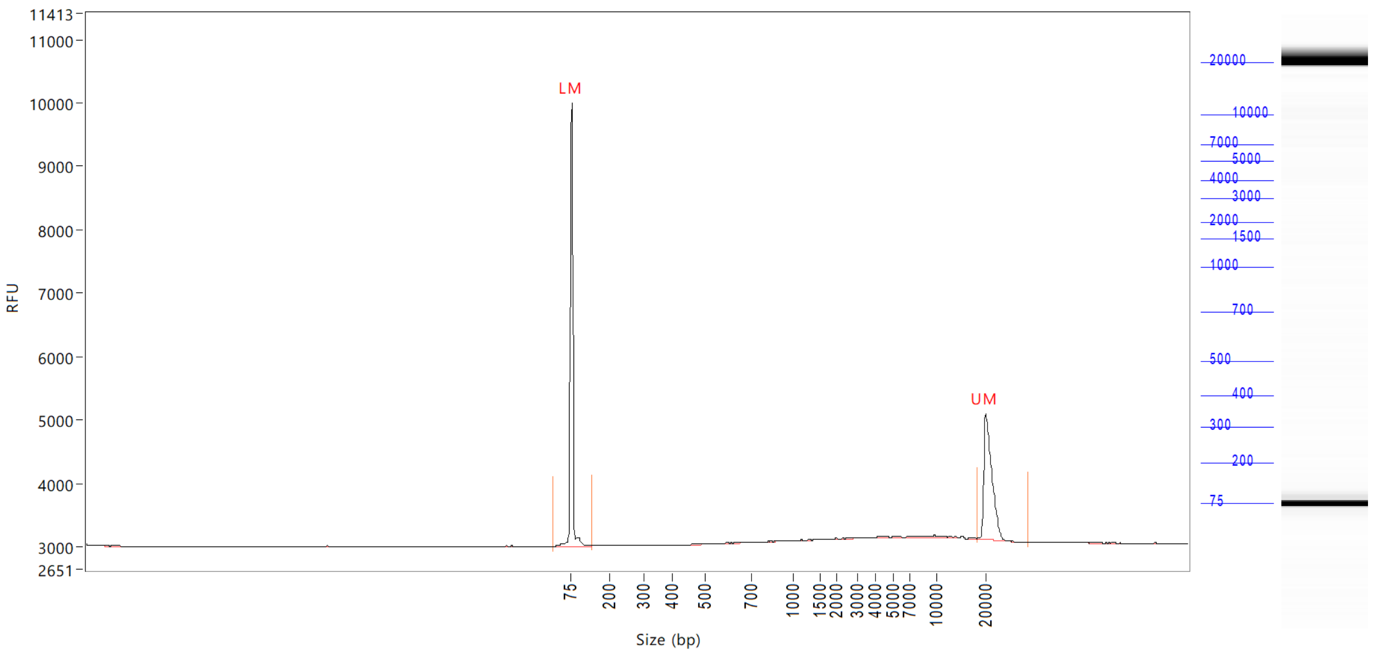

Peak	Size	Concentration	Relative concentration	Molarity	From	To	CV%	RFU
	(bp)	(ng/uL)	Percent	(nmole/L)	(bp)	(bp)		
1	75 (LM)	0.5000		11.2364	16	141	11.24	6999
2	20000 (UM)	0.2461		0.0197	18171	28463	4.46	1981

TIC:	0.0000	ng/uL
TIM:	0.0000	nmole/L
Total concentration:	0.5828	ng/uL

Sample peak width (sec): 5 Sample min peak height: 50 Sample baseline V to V?: Y Sample baseline V to V points: 3
Sample filter: Binomial Number of points for filter: 3 Sample start region (min): 0 Sample end region (min): 30
Marker peak width (sec): 5 Marker min peak height: 200 Marker baseline V to V?: Y Marker baseline V to V points: 3
Lower marker selection: First peak > 500 RFU Upper marker selection: Last peak > 200 RFU
Ladder size (bp)75, 200, 300, 400, 500, 700, 1000, 1500, 2000, 3000, 4000, 5000, 7000, 10000, 20000
Quantification using: Lower Marker Final concentration (ng/uL): 0.5000 Dilution factor: 12.0

Sample: SampE6
Well location: E6
Created: Thursday, October 21, 2021 8:58:49 PM

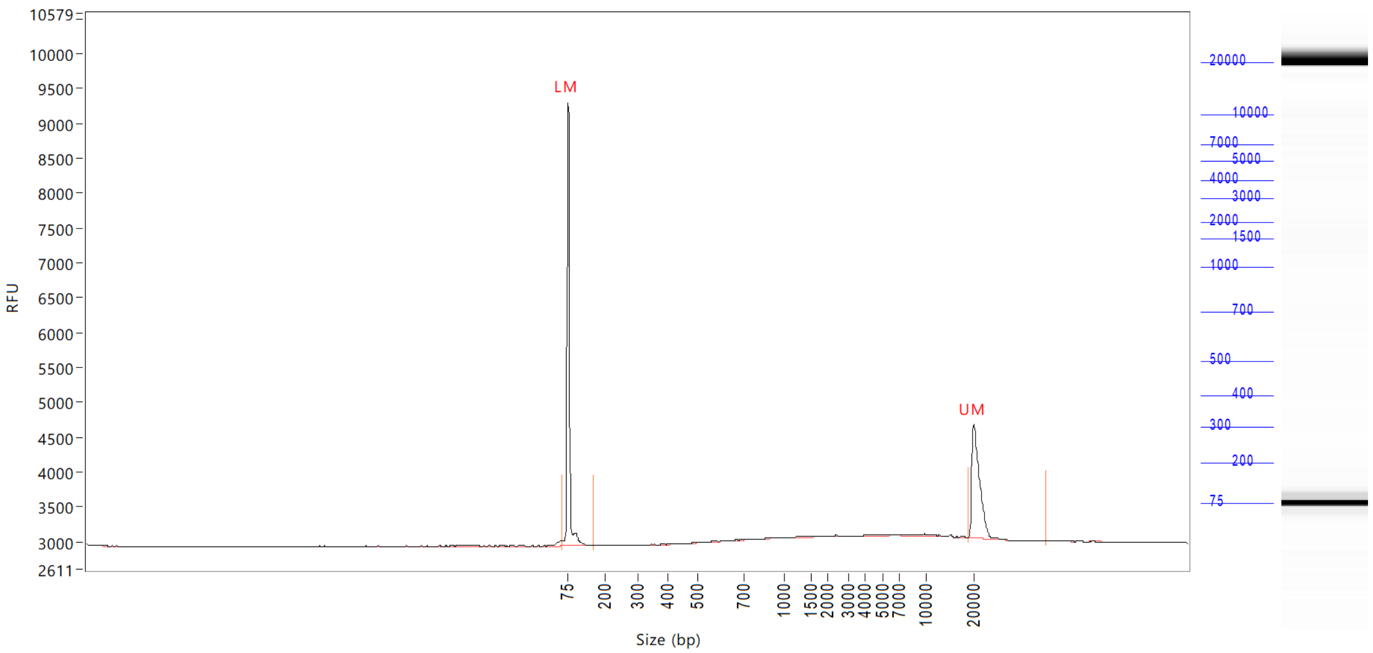

Peak	Size	Concentration	Relative concentration	Molarity	From	To	CV%	RFU
	(bp)	(ng/uL)	Percent	(nmole/L)	(bp)	(bp)		
1	75 (LM)	0.5000		10.7944	60	158	11.74	6359
2	20000 (UM)	0.2153		0.0171	18903	35113	6.23	1636

TIC:	0.0000	ng/uL
TIM:	0.0000	nmole/L
Total concentration:	0.5108	ng/uL

Sample peak width (sec): 5   Sample min peak height: 50   Sample baseline V to V?: Y   Sample baseline V to V points: 3
Sample filter: Binomial   Number of points for filter: 3   Sample start region (min): 0   Sample end region (min): 30
Marker peak width (sec): 5   Marker min peak height: 200   Marker baseline V to V?: Y   Marker baseline V to V points: 3
Lower marker selection: First peak > 500 RFU   Upper marker selection: Last peak > 200 RFU
Ladder size (bp)75, 200, 300, 400, 500, 700, 1000, 1500, 2000, 3000, 4000, 5000, 7000, 10000, 20000
Quantification using: Lower Marker   Final concentration (ng/uL): 0.5000   Dilution factor: 12.0

Sample: SampF6
Well location: F6
Created: Thursday, October 21, 2021 8:58:49 PM

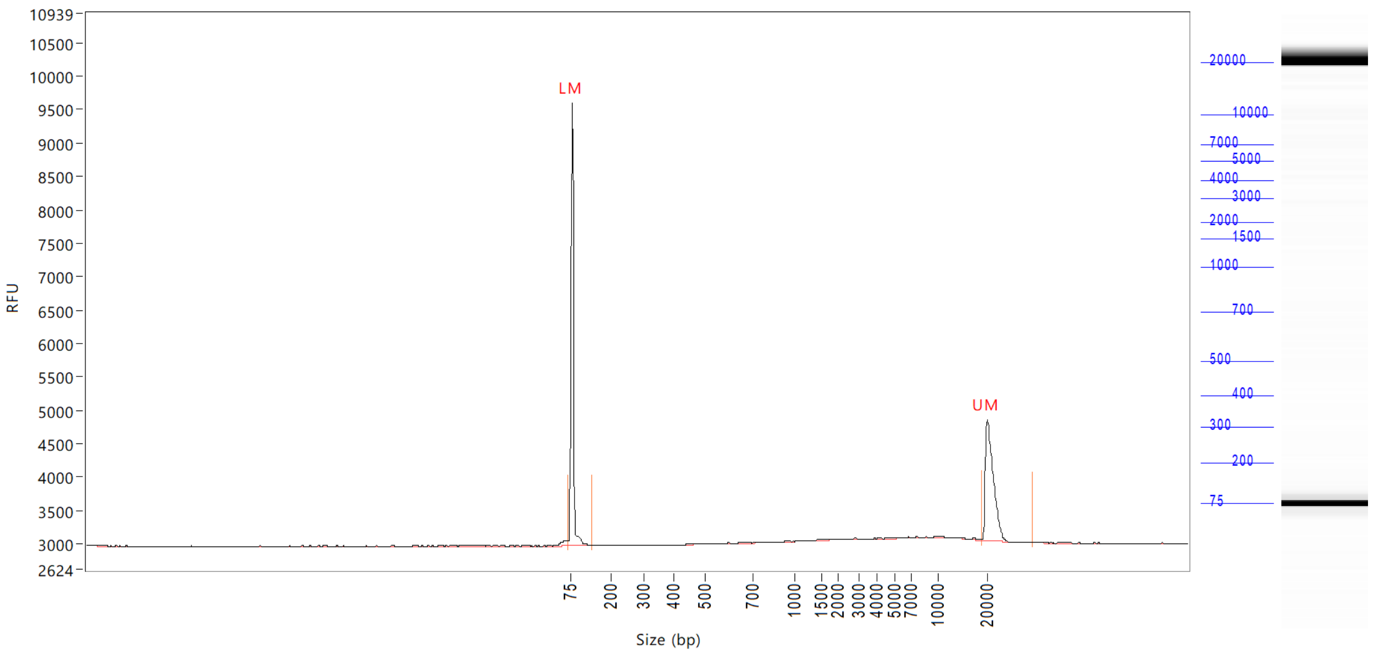

Peak	Size	Concentration	Relative concentration	Molarity	From	To	CV%	RFU
	(bp)	(ng/uL)	Percent	(nmole/L)	(bp)	(bp)		
1	75 (LM)	0.5000		11.0851	62	141	9.52	6636
2	20000 (UM)	0.2422		0.0193	18903	29189	4.53	1803

TIC:	0.0000	ng/uL
TIM:	0.0000	nmole/L
Total concentration:	0.5712	ng/uL

Sample peak width (sec): 5 Sample min peak height: 50 Sample baseline V to V?: Y Sample baseline V to V points: 3
Sample filter: Binomial Number of points for filter: 3 Sample start region (min): 0 Sample end region (min): 30
Marker peak width (sec): 5 Marker min peak height: 200 Marker baseline V to V?: Y Marker baseline V to V points: 3
Lower marker selection: First peak > 500 RFU Upper marker selection: Last peak > 200 RFU
Ladder size (bp)75, 200, 300, 400, 500, 700, 1000, 1500, 2000, 3000, 4000, 5000, 7000, 10000, 20000
Quantification using: Lower Marker Final concentration (ng/uL): 0.5000 Dilution factor: 12.0

Sample: SampG6
Well location: G6
Created: Thursday, October 21, 2021 8:58:49 PM

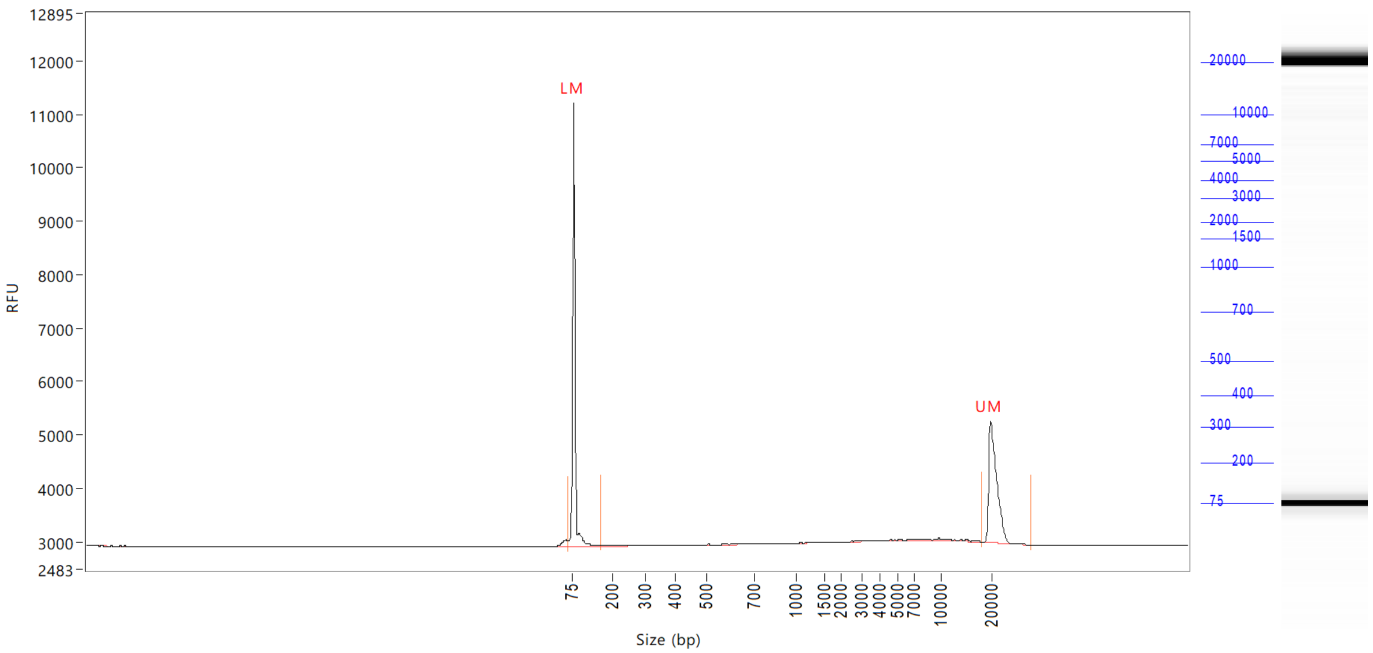

Peak	Size	Concentration	Relative concentration	Molarity	From	To	CV%	RFU
	(bp)	(ng/uL)	Percent	(nmole/L)	(bp)	(bp)		
1	75 (LM)	0.5000		10.9378	62	164	12.48	8319
2	20000 (UM)	0.2422		0.0194	18171	28221	4.42	2275

TIC:	0.0000	ng/uL
TIM:	0.0000	nmole/L
Total concentration:	0.5676	ng/uL

Sample peak width (sec): 5   Sample min peak height: 50   Sample baseline V to V?: Y   Sample baseline V to V points: 3
Sample filter: Binomial   Number of points for filter: 3 Sample start region (min): 0 Sample end region (min): 30
Marker peak width (sec): 5   Marker min peak height: 200   Marker baseline V to V?: Y   Marker baseline V to V points: 3
Lower marker selection: First peak > 500 RFU   Upper marker selection: Last peak > 200 RFU
Ladder size (bp)75, 200, 300, 400, 500, 700, 1000, 1500, 2000, 3000, 4000, 5000, 7000, 10000, 20000
Quantification using: Lower Marker   Final concentration (ng/uL): 0.5000   Dilution factor: 12.0

Sample: SampH6
Well location: H6
Created: Thursday, October 21, 2021 8:58:49 PM

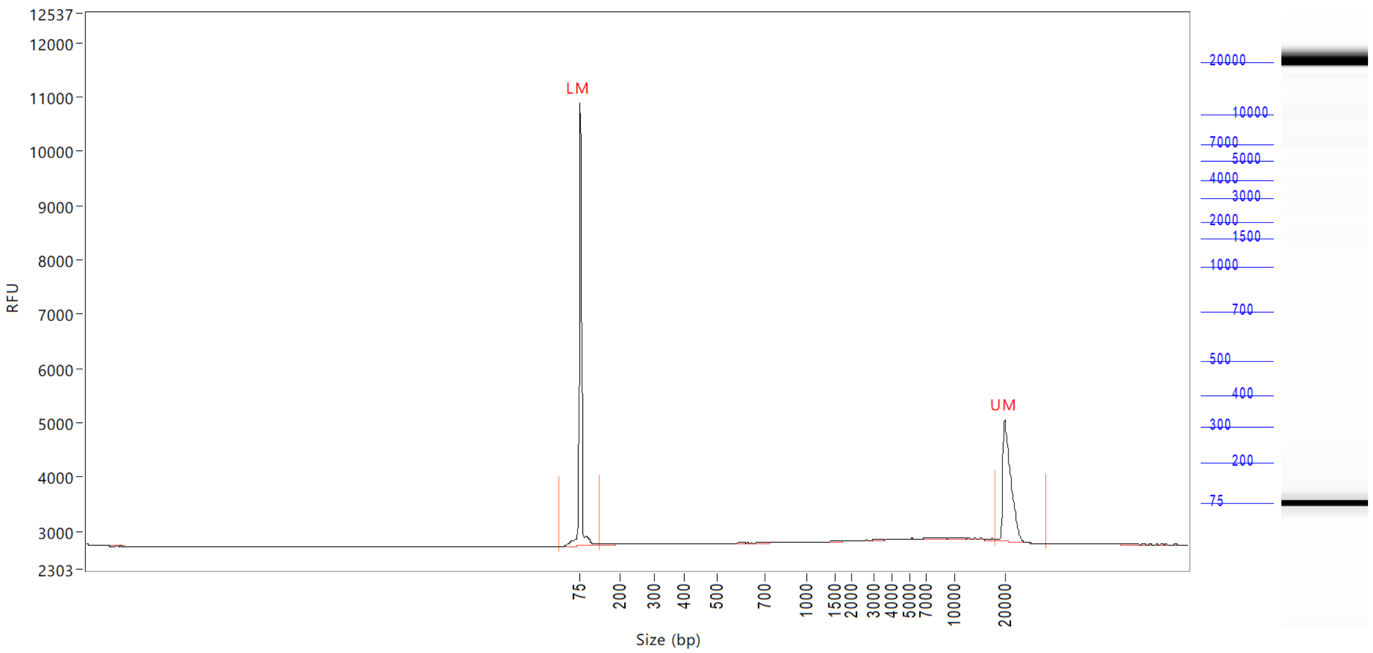

Peak	Size	Concentration	Relative concentration	Molarity	From	To	CV%	RFU
	(bp)	(ng/uL)	Percent	(nmole/L)	(bp)	(bp)		
1	75 (LM)	0.5000		11.0851	11	135	12.33	8168
2	20000 (UM)	0.2373		0.0190	18171	28221	4.45	2233

TIC:	0.0000	ng/uL
TIM:	0.0000	nmole/L
Total concentration:	0.6528	ng/uL

```
Sample peak width (sec): 5    Sample min peak height: 50    Sample baseline V to V?: Y    Sample baseline V to V points: 3
Sample filter: Binomial       Number of points for filter: 3  Sample start region (min): 0  Sample end region (min): 30
Marker peak width (sec): 5    Marker min peak height: 200   Marker baseline V to V?: Y    Marker baseline V to V points: 3
Lower marker selection: First peak > 500 RFU                Upper marker selection: Last peak > 200 RFU
Ladder size (bp)75, 200, 300, 400, 500, 700, 1000, 1500, 2000, 3000, 4000, 5000, 7000, 10000, 20000
Quantification using: Lower Marker          Final concentration (ng/uL): 0.5000          Dilution factor: 12.0
```

Sample: SampA7
Well location: A7
Created: Thursday, October 21, 2021 8:58:49 PM

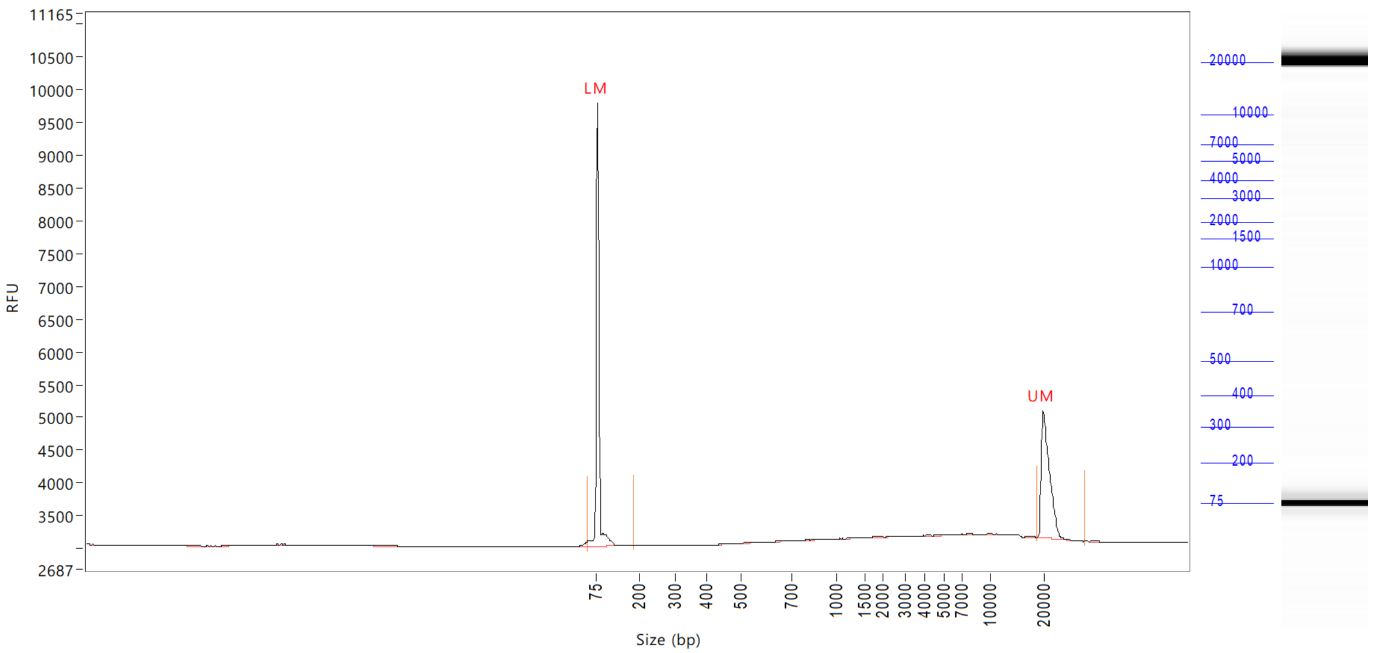

Peak	Size	Concentration	Relative concentration	Molarity	From	To	CV%	RFU
	(bp)	(ng/uL)	Percent	(nmole/L)	(bp)	(bp)		
1	75 (LM)	0.5000		10.9378	48	183	12.77	6770
2	20000 (UM)	0.2449		0.0196	18659	27859	4.34	1943

TIC:	0.0000	ng/uL
TIM:	0.0000	nmole/L
Total concentration:	0.4576	ng/uL

Sample peak width (sec): 5 Sample min peak height: 50 Sample baseline V to V?: Y Sample baseline V to V points: 3
Sample filter: Binomial Number of points for filter: 3 Sample start region (min): 0 Sample end region (min): 30
Marker peak width (sec): 5 Marker min peak height: 200 Marker baseline V to V?: Y Marker baseline V to V points: 3
Lower marker selection: First peak > 500 RFU Upper marker selection: Last peak > 200 RFU
Ladder size (bp)75, 200, 300, 400, 500, 700, 1000, 1500, 2000, 3000, 4000, 5000, 7000, 10000, 20000
Quantification using: Lower Marker Final concentration (ng/uL): 0.5000 Dilution factor: 12.0

Sample: SampB7
Well location: B7
Created: Thursday, October 21, 2021 8:58:49 PM

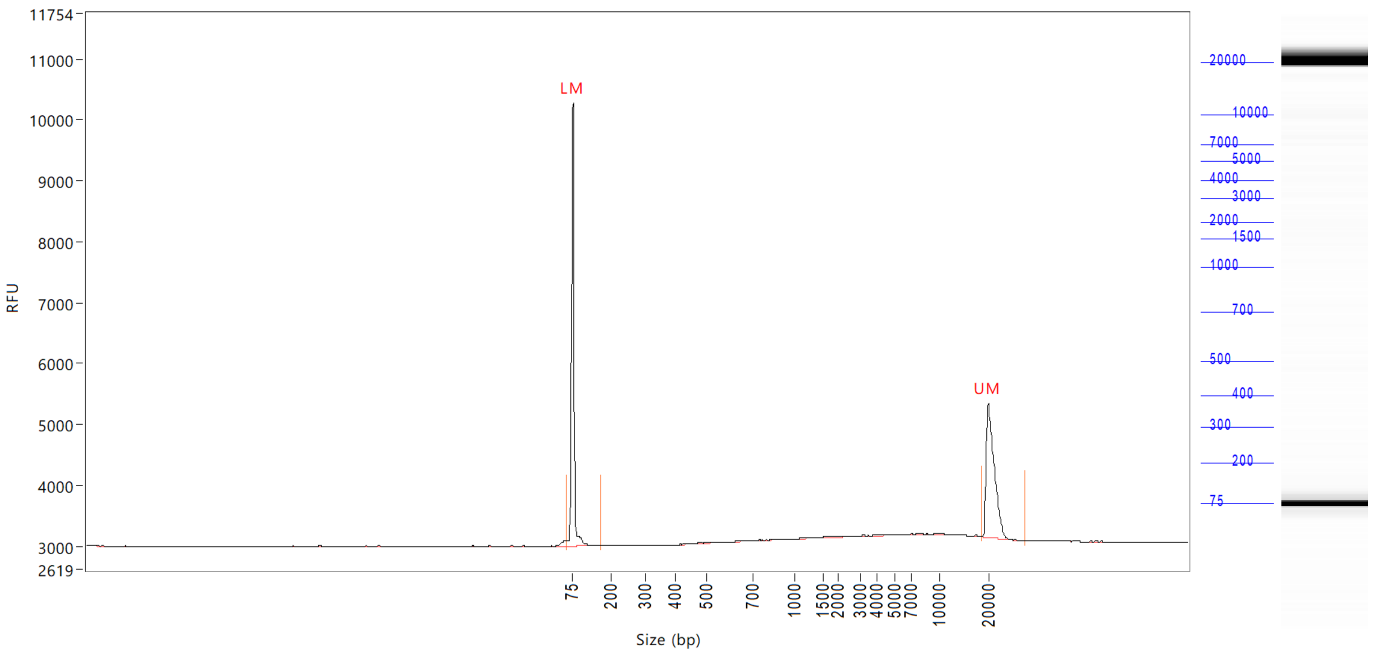

Peak	Size	Concentration	Relative concentration	Molarity	From	To	CV%	RFU
	(bp)	(ng/uL)	Percent	(nmole/L)	(bp)	(bp)		
1	75 (LM)	0.5000		11.0851	58	166	12.50	7293
2	20000 (UM)	0.2599		0.0208	18781	27375	4.43	2206

TIC:	0.0000	ng/uL
TIM:	0.0000	nmole/L
Total concentration:	0.5321	ng/uL

```
Sample peak width (sec): 5    Sample min peak height: 50    Sample baseline V to V?: Y    Sample baseline V to V points: 3
Sample filter: Binomial       Number of points for filter: 3  Sample start region (min): 0  Sample end region (min): 30
Marker peak width (sec): 5    Marker min peak height: 200   Marker baseline V to V?: Y    Marker baseline V to V points: 3
Lower marker selection: First peak > 500 RFU                Upper marker selection: Last peak > 200 RFU
Ladder size (bp)75, 200, 300, 400, 500, 700, 1000, 1500, 2000, 3000, 4000, 5000, 7000, 10000, 20000
Quantification using: Lower Marker          Final concentration (ng/uL): 0.5000          Dilution factor: 12.0
```

Sample: SampC7
Well location: C7
Created: Thursday, October 21, 2021 8:58:49 PM

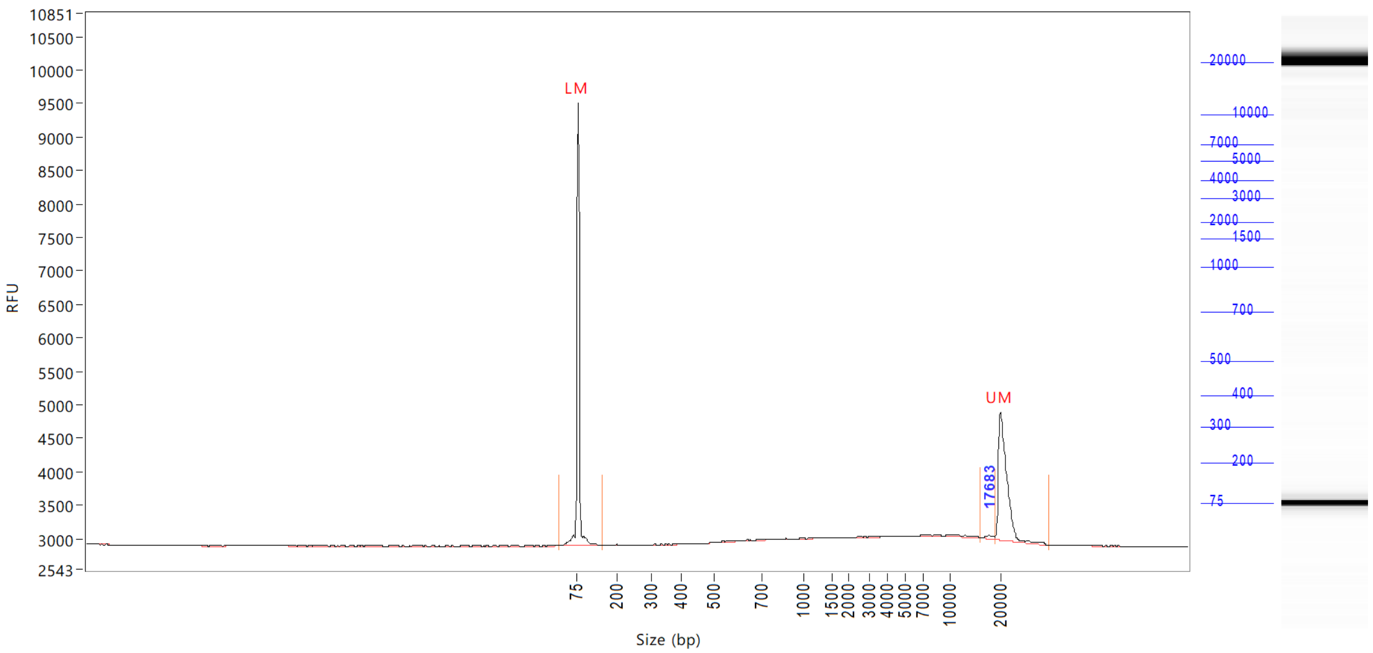

Peak	Size	Concentration	Relative concentration	Molarity	From	To	CV%	RFU
	(bp)	(ng/uL)	Percent	(nmole/L)	(bp)	(bp)		
1	75 (LM)	0.5000		11.0851	16	152	12.56	6628
2	17683	0.0772	100.0	0.0071	15976	18903	3.85	51
3	20000 (UM)	0.2530		0.0200	18903	29551	7.27	1903

TIC:	0.0772	ng/uL
TIM:	0.0071	nmole/L
Total concentration:	0.6968	ng/uL

Sample peak width (sec): 5   Sample min peak height: 50   Sample baseline V to V?: Y   Sample baseline V to V points: 3
Sample filter: Binomial   Number of points for filter: 3   Sample start region (min): 0   Sample end region (min): 30
Marker peak width (sec): 5   Marker min peak height: 200   Marker baseline V to V?: Y   Marker baseline V to V points: 3
Lower marker selection: First peak > 500 RFU   Upper marker selection: Last peak > 200 RFU
Ladder size (bp)75, 200, 300, 400, 500, 700, 1000, 1500, 2000, 3000, 4000, 5000, 7000, 10000, 20000
Quantification using: Lower Marker   Final concentration (ng/uL): 0.5000   Dilution factor: 12.0

Sample: SampD7
Well location: D7
Created: Thursday, October 21, 2021 8:58:49 PM

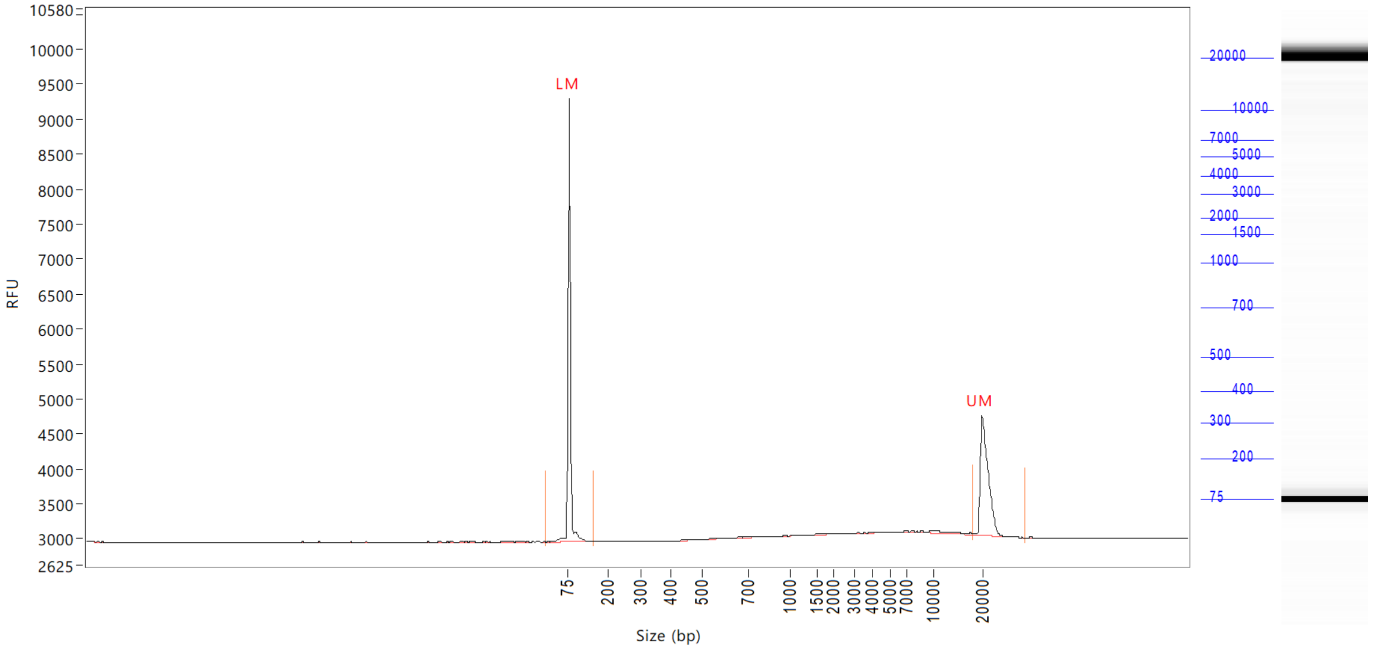

Peak	Size	Concentration	Relative concentration	Molarity	From	To	CV%	RFU
	(bp)	(ng/uL)	Percent	(nmole/L)	(bp)	(bp)		
1	75 (LM)	0.5000		11.0851	1	152	12.87	6349
2	20000 (UM)	0.2360		0.0189	18049	28584	4.61	1709

TIC:	0.0000	ng/uL
TIM:	0.0000	nmole/L
Total concentration:	0.6804	ng/uL

```
Sample peak width (sec): 5    Sample min peak height: 50    Sample baseline V to V?: Y    Sample baseline V to V points: 3
Sample filter: Binomial       Number of points for filter: 3  Sample start region (min): 0  Sample end region (min): 30
Marker peak width (sec): 5    Marker min peak height: 200   Marker baseline V to V?: Y    Marker baseline V to V points: 3
Lower marker selection: First peak > 500 RFU                Upper marker selection: Last peak > 200 RFU
Ladder size (bp)75, 200, 300, 400, 500, 700, 1000, 1500, 2000, 3000, 4000, 5000, 7000, 10000, 20000
Quantification using: Lower Marker          Final concentration (ng/uL): 0.5000          Dilution factor: 12.0
```

Sample: SampE7
Well location: E7
Created: Thursday, October 21, 2021 8:58:49 PM

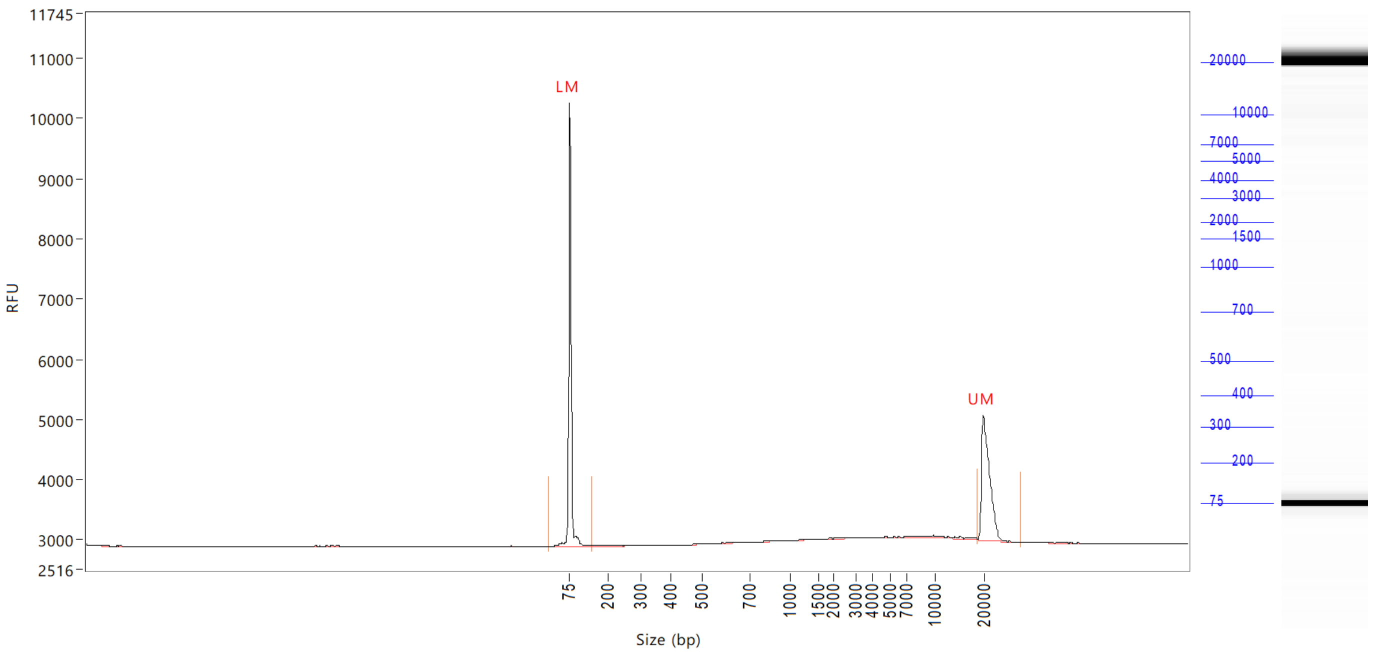

Peak	Size	Concentration	Relative concentration	Molarity	From	To	CV%	RFU
	(bp)	(ng/uL)	Percent	(nmole/L)	(bp)	(bp)		
1	75 (LM)	0.5000		11.0851	11	148	11.93	7374
2	20000 (UM)	0.2517		0.0201	18903	27617	4.57	2078

TIC:	0.0000	ng/uL
TIM:	0.0000	nmole/L
Total concentration:	0.6141	ng/uL

```
Sample peak width (sec): 5    Sample min peak height: 50    Sample baseline V to V?: Y    Sample baseline V to V points: 3
Sample filter: Binomial       Number of points for filter: 3  Sample start region (min): 0  Sample end region (min): 30
Marker peak width (sec): 5    Marker min peak height: 200   Marker baseline V to V?: Y    Marker baseline V to V points: 3
Lower marker selection: First peak > 500 RFU                Upper marker selection: Last peak > 200 RFU
Ladder size (bp)75, 200, 300, 400, 500, 700, 1000, 1500, 2000, 3000, 4000, 5000, 7000, 10000, 20000
Quantification using: Lower Marker          Final concentration (ng/uL): 0.5000          Dilution factor: 12.0
```

Sample: SampF7
Well location: F7
Created: Thursday, October 21, 2021 8:58:49 PM

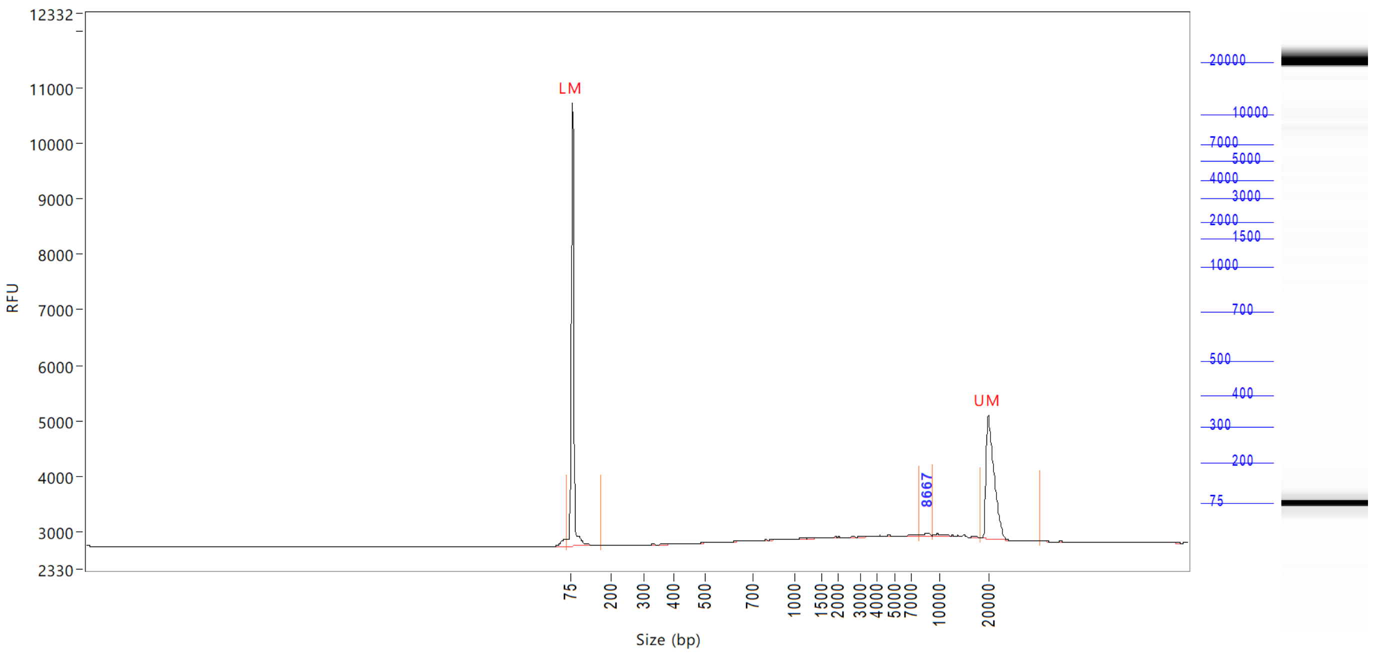

Peak	Size	Concentration	Relative concentration	Molarity	From	To	CV%	RFU
	(bp)	(ng/uL)	Percent	(nmole/L)	(bp)	(bp)		
1	75 (LM)	0.5000		10.9378	60	168	11.68	7984
2	8667	0.0581	100.0	0.0113	7867	9200	3.69	51
3	20000 (UM)	0.2417		0.0194	18293	30519	4.53	2224

TIC:	0.0581	ng/uL
TIM:	0.0113	nmole/L
Total concentration:	0.4918	ng/uL

```
Sample peak width (sec): 5    Sample min peak height: 50    Sample baseline V to V?: Y    Sample baseline V to V points: 3
Sample filter: Binomial       Number of points for filter: 3  Sample start region (min): 0  Sample end region (min): 30
Marker peak width (sec): 5    Marker min peak height: 200   Marker baseline V to V?: Y    Marker baseline V to V points: 3
Lower marker selection: First peak > 500 RFU                Upper marker selection: Last peak > 200 RFU
Ladder size (bp)75, 200, 300, 400, 500, 700, 1000, 1500, 2000, 3000, 4000, 5000, 7000, 10000, 20000
Quantification using: Lower Marker          Final concentration (ng/uL): 0.5000          Dilution factor: 12.0
```

Sample: SampG7
Well location: G7
Created: Thursday, October 21, 2021 8:58:49 PM

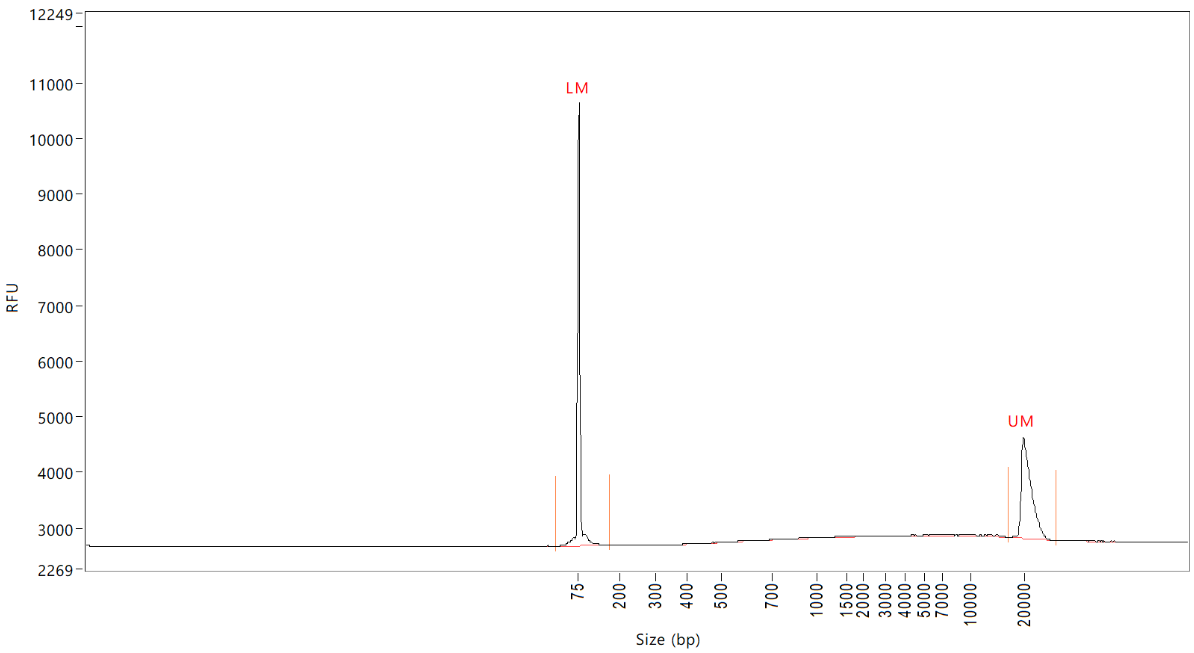

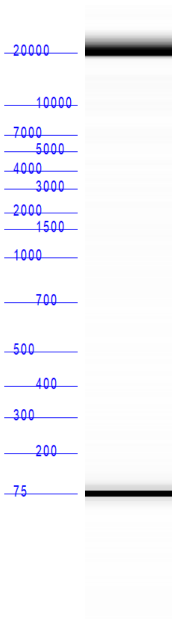

Peak	Size	Concentration	Relative concentration	Molarity	From	To	CV%	RFU
	(bp)	(ng/uL)	Percent	(nmole/L)	(bp)	(bp)		
1	75 (LM)	0.5000		11.0851	7	166	13.97	7968
2	20000 (UM)	0.2296		0.0183	17073	26166	5.27	1809

TIC:	0.0000	ng/uL
TIM:	0.0000	nmole/L
Total concentration:	0.4178	ng/uL

Sample peak width (sec): 5 Sample min peak height: 50 Sample baseline V to V?: Y Sample baseline V to V points: 3
Sample filter: Binomial Number of points for filter: 3 Sample start region (min): 0 Sample end region (min): 30
Marker peak width (sec): 5 Marker min peak height: 200 Marker baseline V to V?: Y Marker baseline V to V points: 3
Lower marker selection: First peak > 500 RFU Upper marker selection: Last peak > 200 RFU
Ladder size (bp)75, 200, 300, 400, 500, 700, 1000, 1500, 2000, 3000, 4000, 5000, 7000, 10000, 20000
Quantification using: Lower Marker Final concentration (ng/uL): 0.5000 Dilution factor: 12.0

Sample: SampH7
Well location: H7
Created: Thursday, October 21, 2021 8:58:49 PM

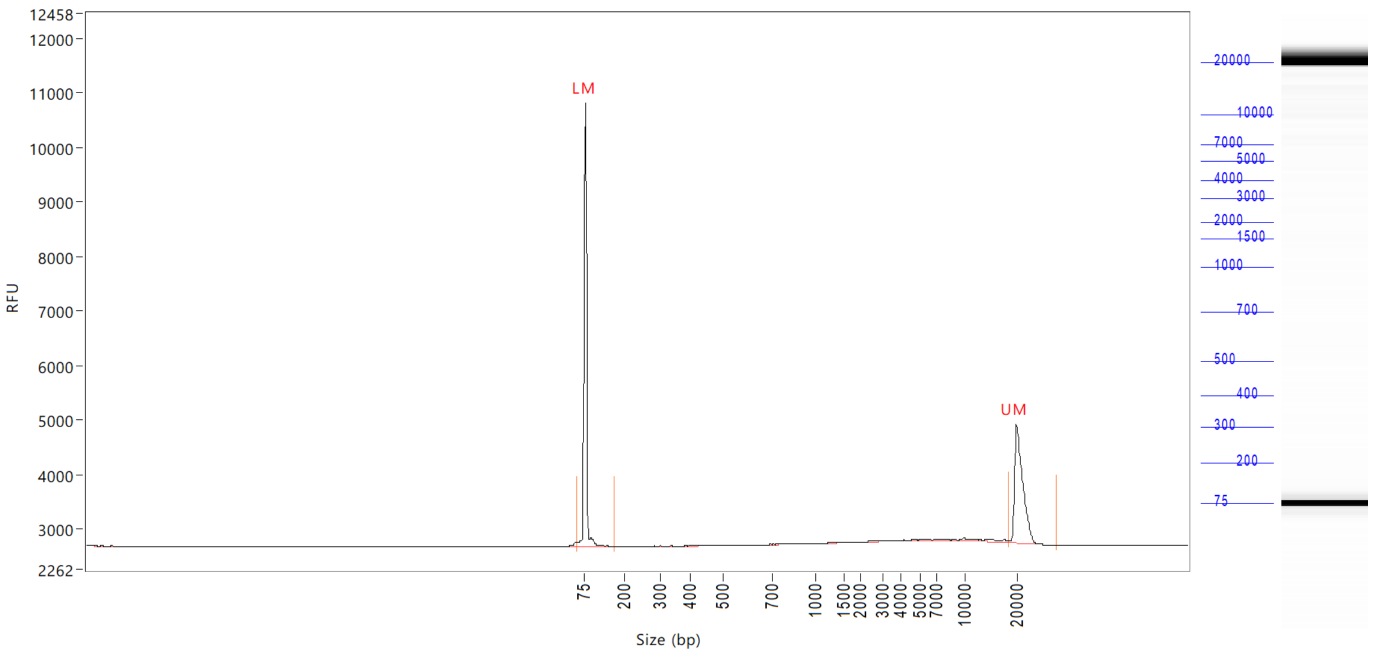

Peak	Size	Concentration	Relative concentration	Molarity	From	To	CV%	RFU
	(bp)	(ng/uL)	Percent	(nmole/L)	(bp)	(bp)		
1	75 (LM)	0.5000		11.0851	52	168	10.49	8150
2	20000 (UM)	0.2417		0.0193	18537	27859	4.35	2181

TIC:	0.0000	ng/uL
TIM:	0.0000	nmole/L
Total concentration:	0.5877	ng/uL

Sample peak width (sec): 5 Sample min peak height: 50 Sample baseline V to V?: Y Sample baseline V to V points: 3
Sample filter: Binomial Number of points for filter: 3 Sample start region (min): 0 Sample end region (min): 30
Marker peak width (sec): 5 Marker min peak height: 200 Marker baseline V to V?: Y Marker baseline V to V points: 3
Lower marker selection: First peak > 500 RFU Upper marker selection: Last peak > 200 RFU
Ladder size (bp)75, 200, 300, 400, 500, 700, 1000, 1500, 2000, 3000, 4000, 5000, 7000, 10000, 20000
Quantification using: Lower Marker Final concentration (ng/uL): 0.5000 Dilution factor: 12.0

Sample: SampA8
Well location: A8
Created: Thursday, October 21, 2021 8:58:49 PM

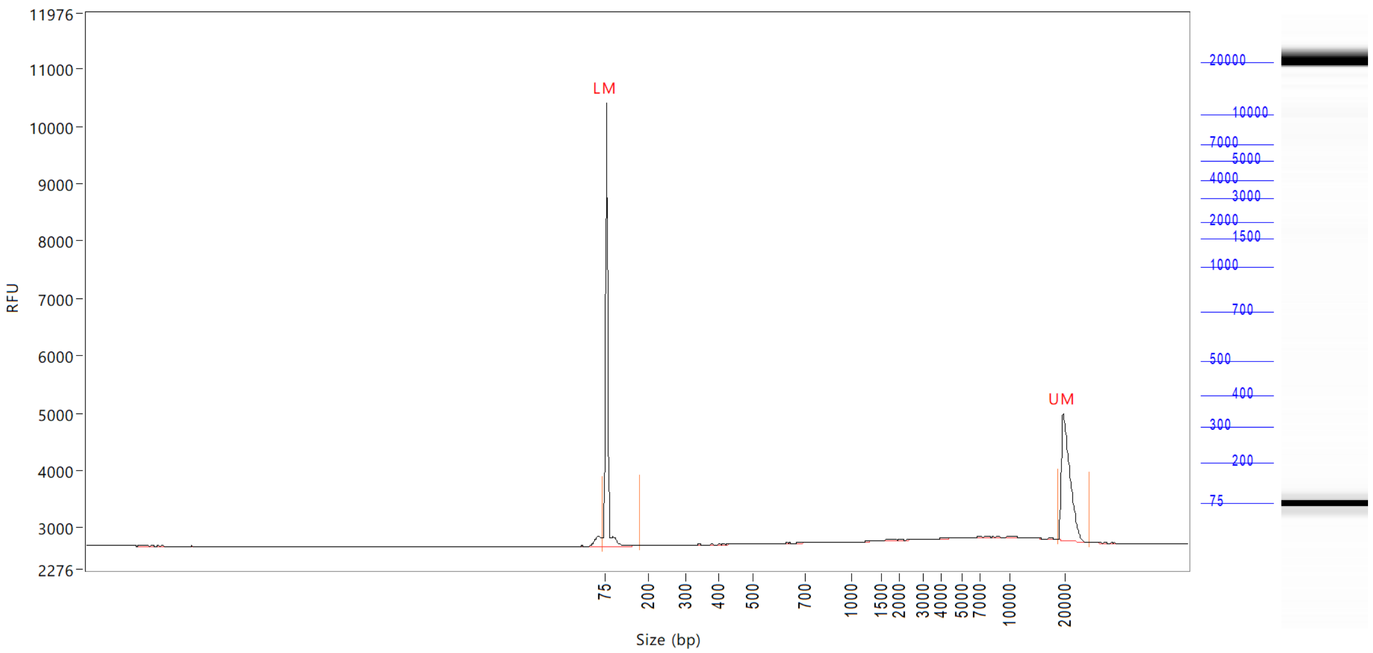

Peak	Size	Concentration	Relative concentration	Molarity	From	To	CV%	RFU
	(bp)	(ng/uL)	Percent	(nmole/L)	(bp)	(bp)		
1	75 (LM)	0.5000		10.9378	64	173	10.96	7749
2	20000 (UM)	0.2629		0.0211	18903	24715	4.19	2202

TIC:	0.0000	ng/uL
TIM:	0.0000	nmole/L
Total concentration:	0.4880	ng/uL

Sample peak width (sec): 5 Sample min peak height: 50 Sample baseline V to V?: Y Sample baseline V to V points: 3
Sample filter: Binomial Number of points for filter: 3 Sample start region (min): 0 Sample end region (min): 30
Marker peak width (sec): 5 Marker min peak height: 200 Marker baseline V to V?: Y Marker baseline V to V points: 3
Lower marker selection: First peak > 500 RFU Upper marker selection: Last peak > 200 RFU
Ladder size (bp)75, 200, 300, 400, 500, 700, 1000, 1500, 2000, 3000, 4000, 5000, 7000, 10000, 20000
Quantification using: Lower Marker Final concentration (ng/uL): 0.5000 Dilution factor: 12.0

Sample: SampB8
Well location: B8
Created: Thursday, October 21, 2021 8:58:49 PM

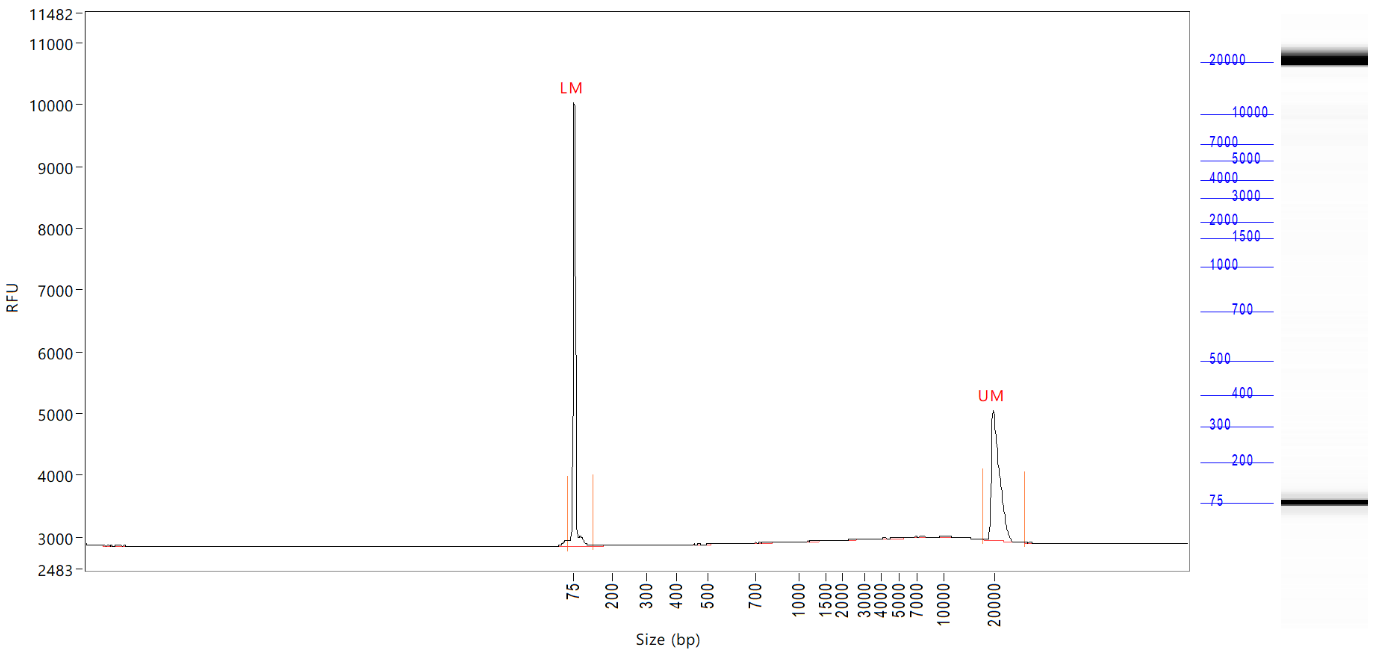

Peak	Size	Concentration	Relative concentration	Molarity	From	To	CV%	RFU
	(bp)	(ng/uL)	Percent	(nmole/L)	(bp)	(bp)		
1	75 (LM)	0.5000		10.9378	60	137	9.05	7191
2	20000 (UM)	0.2573		0.0206	18049	26166	4.30	2115

TIC:	0.0000	ng/uL
TIM:	0.0000	nmole/L
Total concentration:	0.5362	ng/uL

Sample peak width (sec): 5   Sample min peak height: 50   Sample baseline V to V?: Y   Sample baseline V to V points: 3
Sample filter: Binomial   Number of points for filter: 3 Sample start region (min): 0 Sample end region (min): 30
Marker peak width (sec): 5   Marker min peak height: 200   Marker baseline V to V?: Y   Marker baseline V to V points: 3
Lower marker selection: First peak > 500 RFU   Upper marker selection: Last peak > 200 RFU
Ladder size (bp)75, 200, 300, 400, 500, 700, 1000, 1500, 2000, 3000, 4000, 5000, 7000, 10000, 20000
Quantification using: Lower Marker   Final concentration (ng/uL): 0.5000   Dilution factor: 12.0

Sample: SampC8
Well location: C8
Created: Thursday, October 21, 2021 8:58:49 PM

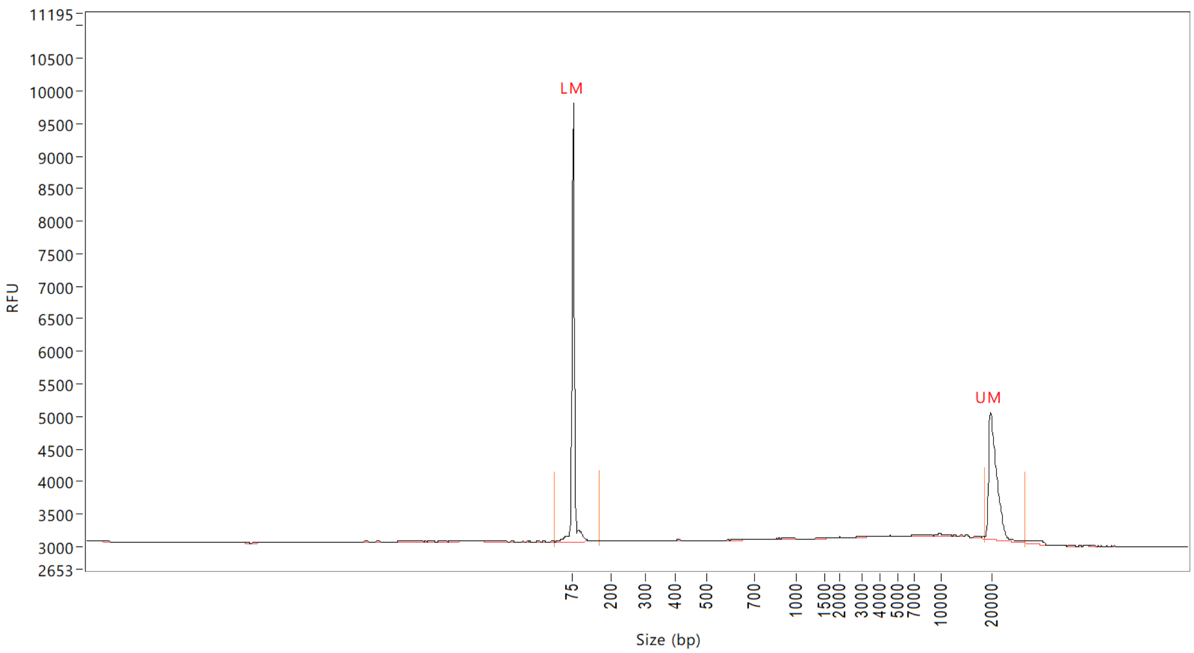

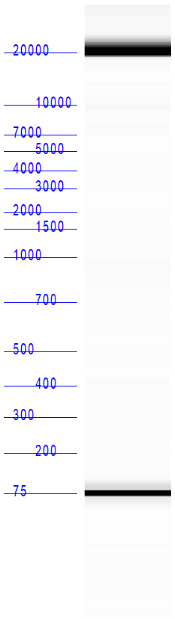

Peak	Size	Concentration	Relative concentration	Molarity	From	To	CV%	RFU
	(bp)	(ng/uL)	Percent	(nmole/L)	(bp)	(bp)		
1	75 (LM)	0.5000		11.0851	18	160	13.71	6752
2	20000 (UM)	0.2567		0.0205	18781	26770	5.50	1952

TIC:	0.0000	ng/uL
TIM:	0.0000	nmole/L
Total concentration:	0.7537	ng/uL

Sample peak width (sec): 5   Sample min peak height: 50   Sample baseline V to V?: Y   Sample baseline V to V points: 3
Sample filter: Binomial   Number of points for filter: 3 Sample start region (min): 0 Sample end region (min): 30
Marker peak width (sec): 5   Marker min peak height: 200   Marker baseline V to V?: Y   Marker baseline V to V points: 3
Lower marker selection: First peak > 500 RFU   Upper marker selection: Last peak > 200 RFU
Ladder size (bp)75, 200, 300, 400, 500, 700, 1000, 1500, 2000, 3000, 4000, 5000, 7000, 10000, 20000
Quantification using: Lower Marker   Final concentration (ng/uL): 0.5000   Dilution factor: 12.0

Sample: SampD8
Well location: D8
Created: Thursday, October 21, 2021 8:58:49 PM

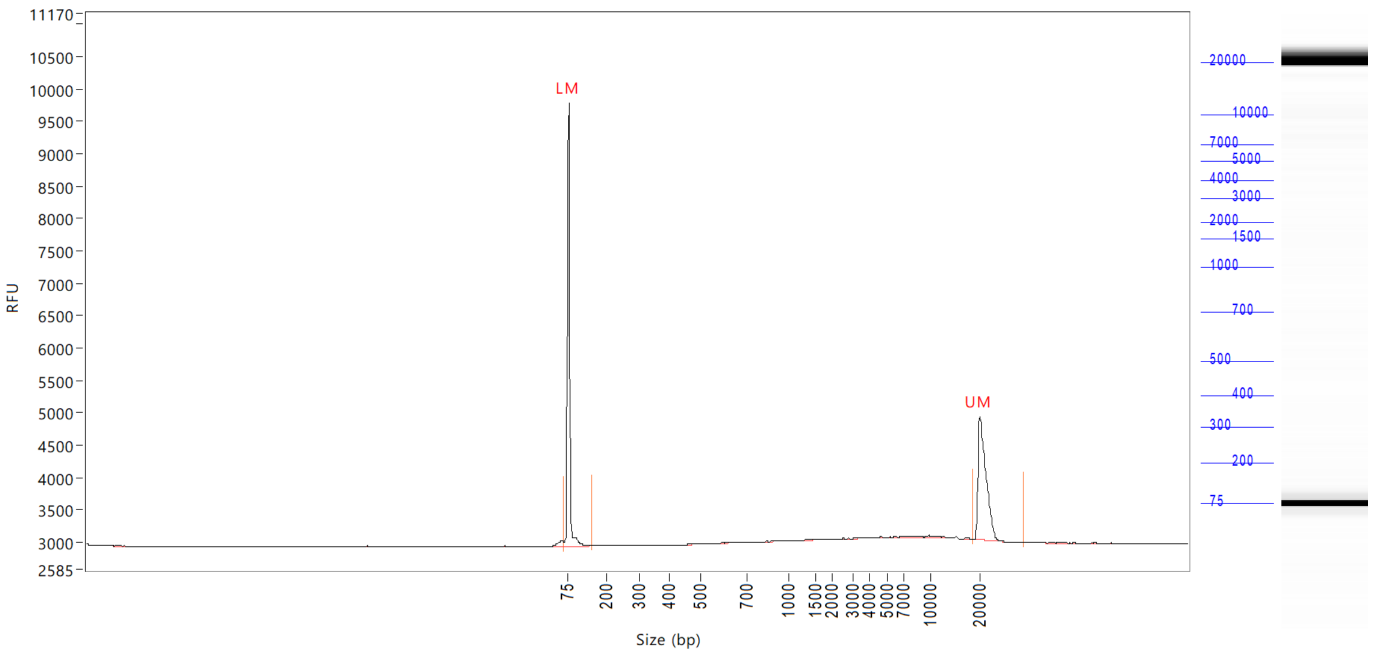

Peak	Size	Concentration	Relative concentration	Molarity	From	To	CV%	RFU
	(bp)	(ng/uL)	Percent	(nmole/L)	(bp)	(bp)		
1	75 (LM)	0.5000		11.0851	62	152	10.55	6857
2	20000 (UM)	0.2502		0.0200	18659	28947	4.36	1908

TIC:	0.0000	ng/uL
TIM:	0.0000	nmole/L
Total concentration:	0.5086	ng/uL

Sample peak width (sec): 5 Sample min peak height: 50 Sample baseline V to V?: Y Sample baseline V to V points: 3
Sample filter: Binomial Number of points for filter: 3 Sample start region (min): 0 Sample end region (min): 30
Marker peak width (sec): 5 Marker min peak height: 200 Marker baseline V to V?: Y Marker baseline V to V points: 3
Lower marker selection: First peak > 500 RFU Upper marker selection: Last peak > 200 RFU
Ladder size (bp)75, 200, 300, 400, 500, 700, 1000, 1500, 2000, 3000, 4000, 5000, 7000, 10000, 20000
Quantification using: Lower Marker Final concentration (ng/uL): 0.5000 Dilution factor: 12.0

Sample: SampE8
Well location: E8
Created: Thursday, October 21, 2021 8:58:49 PM

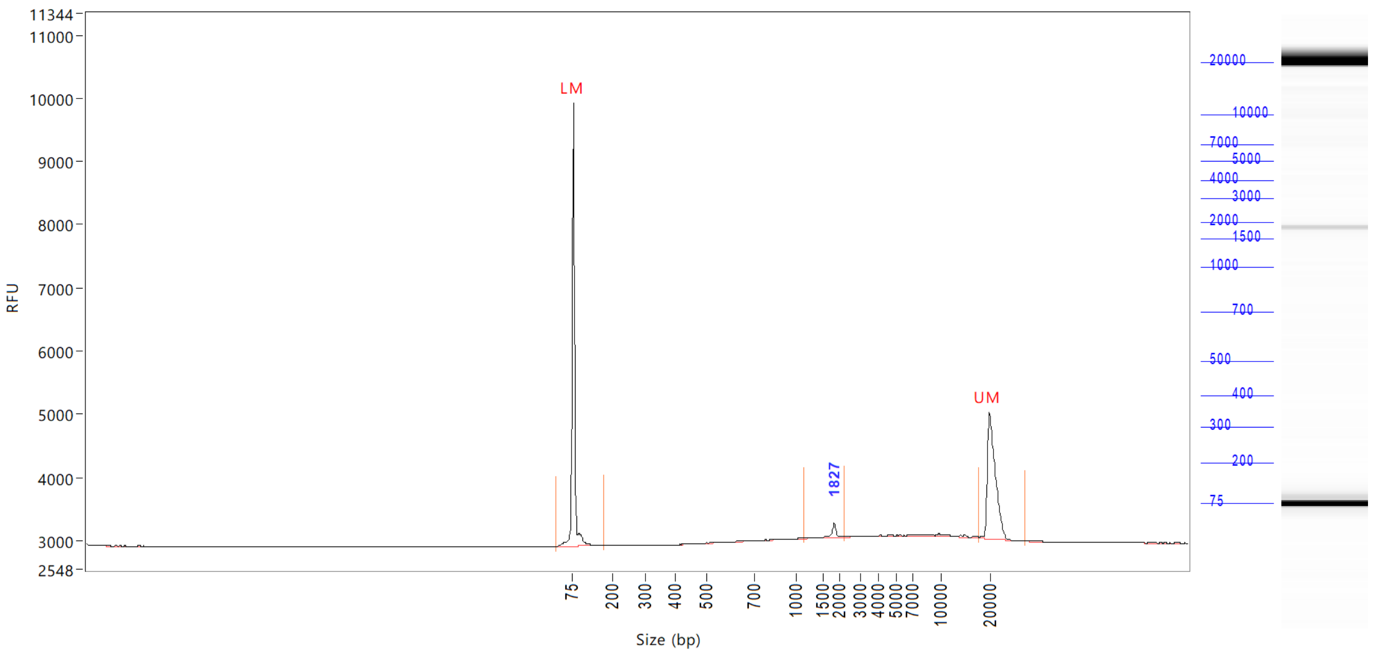

Peak	Size	Concentration	Relative concentration	Molarity	From	To	CV%	RFU
	(bp)	(ng/uL)	Percent	(nmole/L)	(bp)	(bp)		
1	75 (LM)	0.5000		11.2364	20	173	13.08	7024
2	1827	0.2518	100.0	0.2403	1145	2229	11.91	225
3	20000 (UM)	0.2402		0.0193	17927	27133	4.45	2007

TIC:	0.2518	ng/uL
TIM:	0.2403	nmole/L
Total concentration:	0.7582	ng/uL

```
Sample peak width (sec): 5    Sample min peak height: 50    Sample baseline V to V?: Y    Sample baseline V to V points: 3
Sample filter: Binomial       Number of points for filter: 3  Sample start region (min): 0  Sample end region (min): 30
Marker peak width (sec): 5    Marker min peak height: 200   Marker baseline V to V?: Y    Marker baseline V to V points: 3
Lower marker selection: First peak > 500 RFU                Upper marker selection: Last peak > 200 RFU
Ladder size (bp)75, 200, 300, 400, 500, 700, 1000, 1500, 2000, 3000, 4000, 5000, 7000, 10000, 20000
Quantification using: Lower Marker          Final concentration (ng/uL): 0.5000          Dilution factor: 12.0
```

Sample: SampF8
Well location: F8
Created: Thursday, October 21, 2021 8:58:49 PM

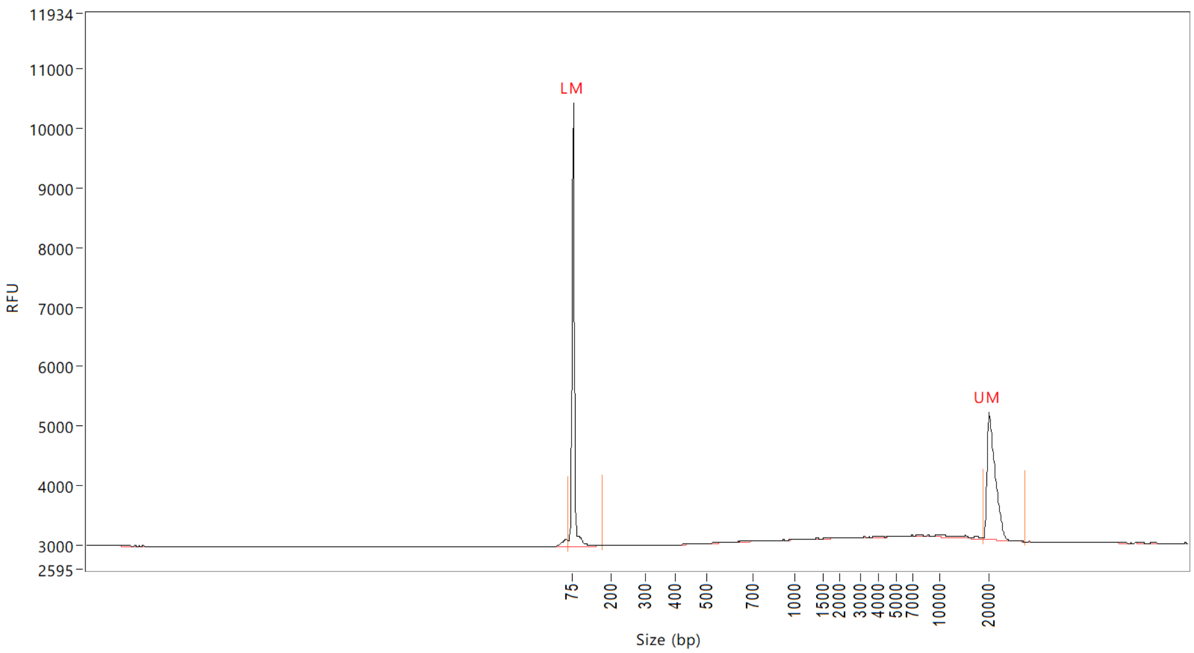

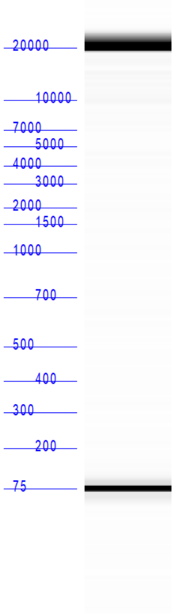

Peak	Size	Concentration	Relative concentration	Molarity	From	To	CV%	RFU
	(bp)	(ng/uL)	Percent	(nmole/L)	(bp)	(bp)		
1	75 (LM)	0.5000		10.9378	60	169	11.89	7462
2	20000 (UM)	0.2575		0.0205	18903	27254	4.47	2146

TIC:	0.0000	ng/uL
TIM:	0.0000	nmole/L
Total concentration:	0.6389	ng/uL

Sample peak width (sec): 5 Sample min peak height: 50 Sample baseline V to V?: Y Sample baseline V to V points: 3
Sample filter: Binomial Number of points for filter: 3 Sample start region (min): 0 Sample end region (min): 30
Marker peak width (sec): 5 Marker min peak height: 200 Marker baseline V to V?: Y Marker baseline V to V points: 3
Lower marker selection: First peak > 500 RFU Upper marker selection: Last peak > 200 RFU
Ladder size (bp)75, 200, 300, 400, 500, 700, 1000, 1500, 2000, 3000, 4000, 5000, 7000, 10000, 20000
Quantification using: Lower Marker Final concentration (ng/uL): 0.5000 Dilution factor: 12.0

Sample: SampG8
Well location: G8
Created: Thursday, October 21, 2021 8:58:49 PM

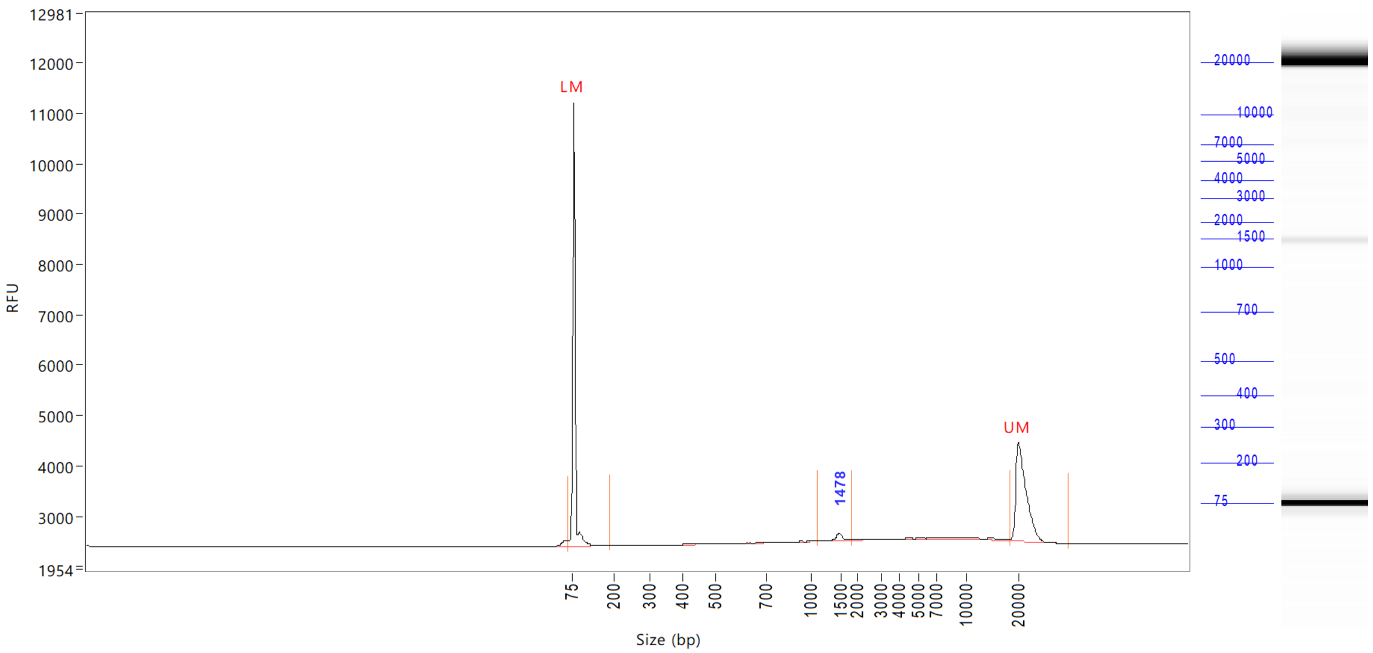

Peak	Size	Concentration	Relative concentration	Molarity	From	To	CV%	RFU
	(bp)	(ng/uL)	Percent	(nmole/L)	(bp)	(bp)		
1	75 (LM)	0.5000		10.7944	60	185	11.90	8807
2	1478	0.1725	100.0	0.1945	1112	1827	6.54	159
3	20000 (UM)	0.2364		0.0188	18415	29672	5.44	1974

TIC:	0.1725	ng/uL
TIM:	0.1945	nmole/L
Total concentration:	0.6760	ng/uL

```
Sample peak width (sec): 5    Sample min peak height: 50    Sample baseline V to V?: Y    Sample baseline V to V points: 3
Sample filter: Binomial       Number of points for filter: 3  Sample start region (min): 0  Sample end region (min): 30
Marker peak width (sec): 5    Marker min peak height: 200   Marker baseline V to V?: Y    Marker baseline V to V points: 3
Lower marker selection: First peak > 500 RFU                Upper marker selection: Last peak > 200 RFU
Ladder size (bp)75, 200, 300, 400, 500, 700, 1000, 1500, 2000, 3000, 4000, 5000, 7000, 10000, 20000
Quantification using: Lower Marker          Final concentration (ng/uL): 0.5000          Dilution factor: 12.0
```

Sample: SampH8
Well location: H8
Created: Thursday, October 21, 2021 8:58:49 PM

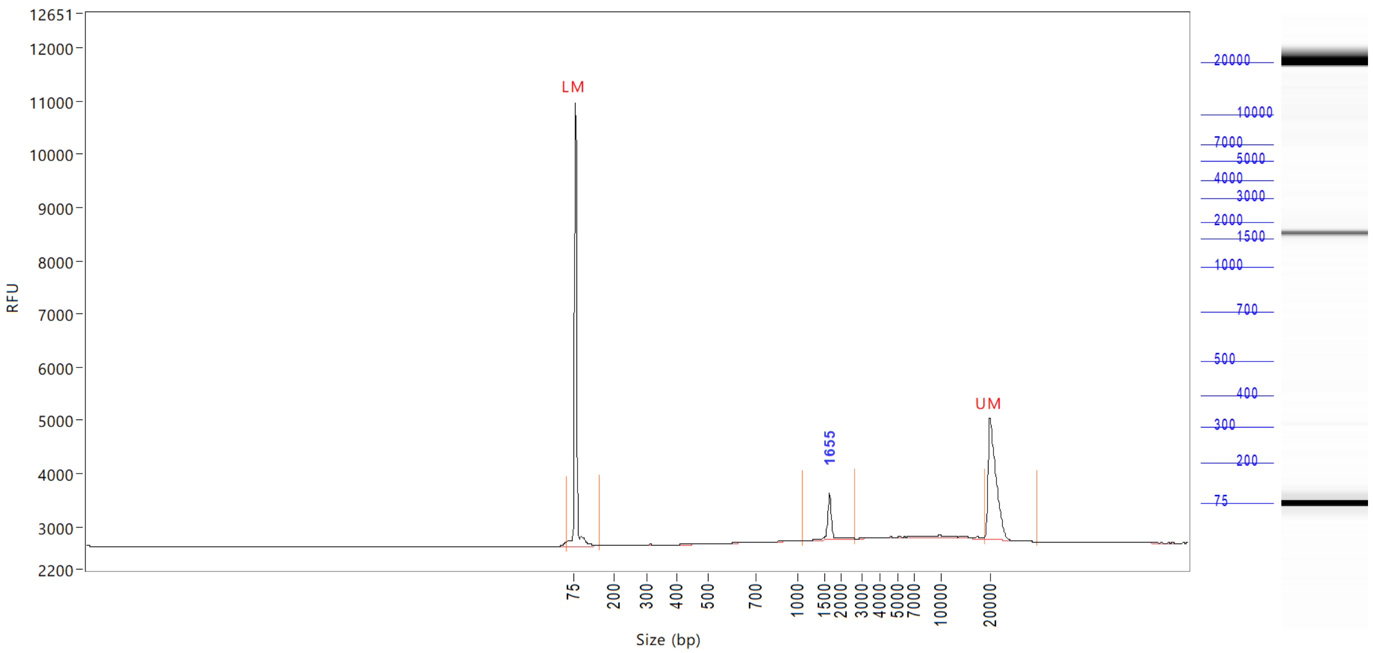

Peak	Size	Concentration	Relative concentration	Molarity	From	To	CV%	RFU
	(bp)	(ng/uL)	Percent	(nmole/L)	(bp)	(bp)		
1	75 (LM)	0.5000		10.9378	48	154	11.28	8348
2	1655	0.7114	100.0	0.7084	1101	2686	11.33	886
3	20000 (UM)	0.2426		0.0194	18903	29551	4.66	2281

TIC:	0.7114	ng/uL
TIM:	0.7084	nmole/L
Total concentration:	1.2384	ng/uL

Sample peak width (sec): 5   Sample min peak height: 50   Sample baseline V to V?: Y   Sample baseline V to V points: 3
Sample filter: Binomial   Number of points for filter: 3   Sample start region (min): 0   Sample end region (min): 30
Marker peak width (sec): 5   Marker min peak height: 200   Marker baseline V to V?: Y   Marker baseline V to V points: 3
Lower marker selection: First peak > 500 RFU   Upper marker selection: Last peak > 200 RFU
Ladder size (bp)75, 200, 300, 400, 500, 700, 1000, 1500, 2000, 3000, 4000, 5000, 7000, 10000, 20000
Quantification using: Lower Marker   Final concentration (ng/uL): 0.5000   Dilution factor: 12.0

Sample: SampA9
Well location: A9
Created: Thursday, October 21, 2021 8:58:49 PM

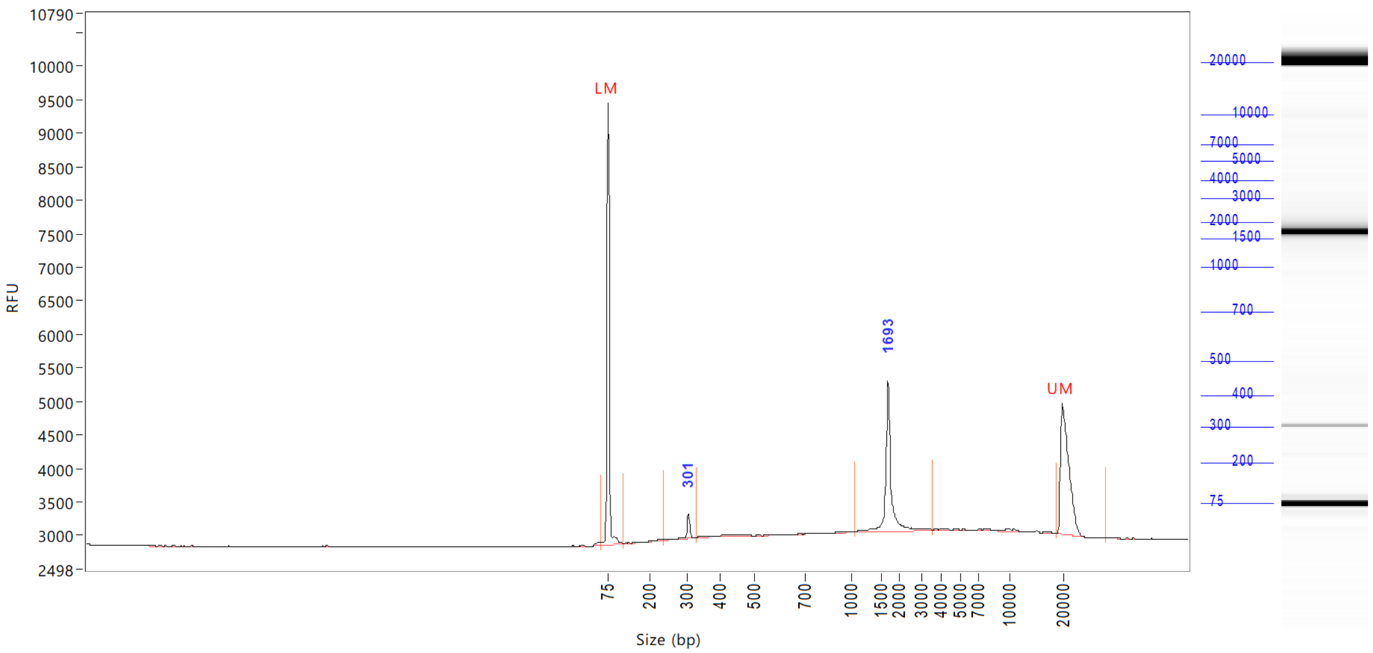

Peak	Size	Concentration	Relative concentration	Molarity	From	To	CV%	RFU
	(bp)	(ng/uL)	Percent	(nmole/L)	(bp)	(bp)		
1	75 (LM)	0.5000		11.2364	56	120	7.26	6602
2	301	0.3647	11.1	2.0336	237	327	5.59	369
3	1693	2.9256	88.9	2.7472	1068	3536	18.33	2244
4	20000 (UM)	0.2640		0.0211	19024	28100	4.21	1950

TIC:	3.2903	ng/uL
TIM:	4.7808	nmole/L
Total concentration:	4.0299	ng/uL

```
Sample peak width (sec): 5    Sample min peak height: 50    Sample baseline V to V?: Y    Sample baseline V to V points: 3
Sample filter: Binomial       Number of points for filter: 3  Sample start region (min): 0  Sample end region (min): 30
Marker peak width (sec): 5    Marker min peak height: 200   Marker baseline V to V?: Y    Marker baseline V to V points: 3
Lower marker selection: First peak > 500 RFU                Upper marker selection: Last peak > 200 RFU
Ladder size (bp)75, 200, 300, 400, 500, 700, 1000, 1500, 2000, 3000, 4000, 5000, 7000, 10000, 20000
Quantification using: Lower Marker          Final concentration (ng/uL): 0.5000          Dilution factor: 12.0
```

Sample: SampB9
Well location: B9
Created: Thursday, October 21, 2021 8:58:49 PM

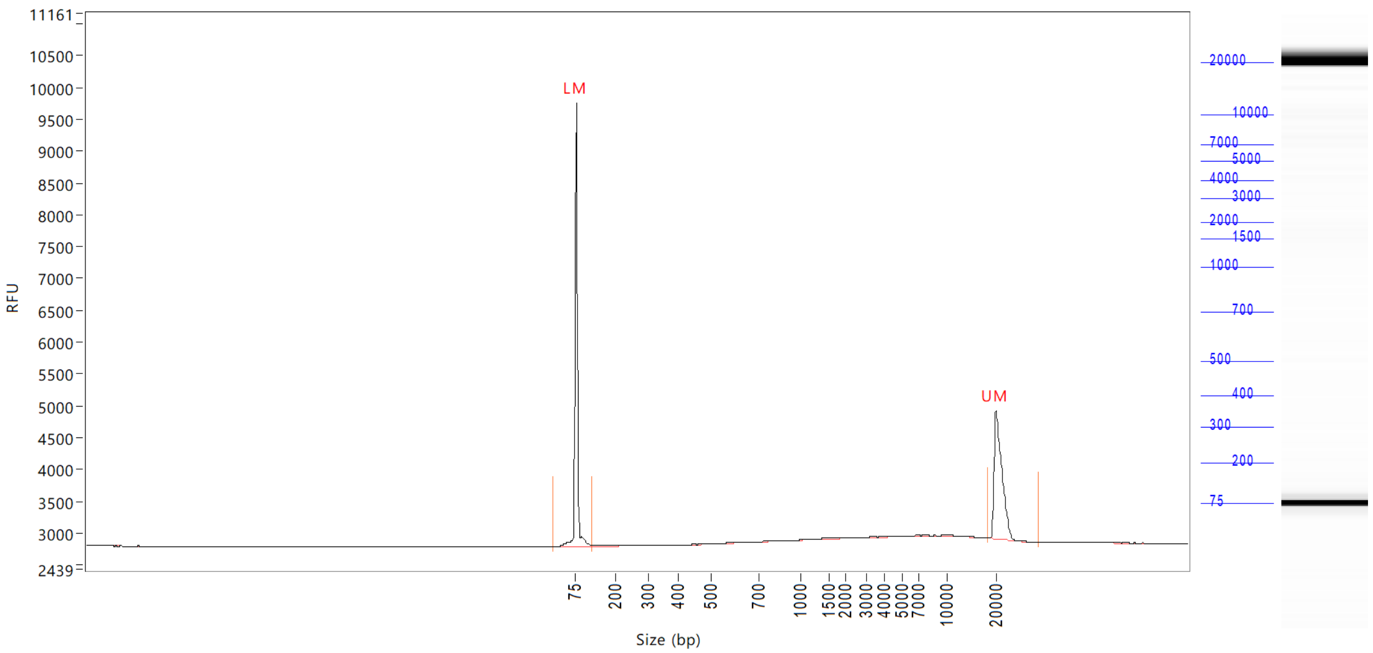

Peak	Size	Concentration	Relative concentration	Molarity	From	To	CV%	RFU
	(bp)	(ng/uL)	Percent	(nmole/L)	(bp)	(bp)		
1	75 (LM)	0.5000		11.0851	5	128	10.87	6969
2	20000 (UM)	0.2434		0.0195	18171	28342	4.31	2006

TIC:	0.0000	ng/uL
TIM:	0.0000	nmole/L
Total concentration:	0.5458	ng/uL

Sample peak width (sec): 5 Sample min peak height: 50 Sample baseline V to V?: Y Sample baseline V to V points: 3
Sample filter: Binomial Number of points for filter: 3 Sample start region (min): 0 Sample end region (min): 30
Marker peak width (sec): 5 Marker min peak height: 200 Marker baseline V to V?: Y Marker baseline V to V points: 3
Lower marker selection: First peak > 500 RFU Upper marker selection: Last peak > 200 RFU
Ladder size (bp)75, 200, 300, 400, 500, 700, 1000, 1500, 2000, 3000, 4000, 5000, 7000, 10000, 20000
Quantification using: Lower Marker Final concentration (ng/uL): 0.5000 Dilution factor: 12.0

Sample: SampC9
Well location: C9
Created: Thursday, October 21, 2021 8:58:49 PM

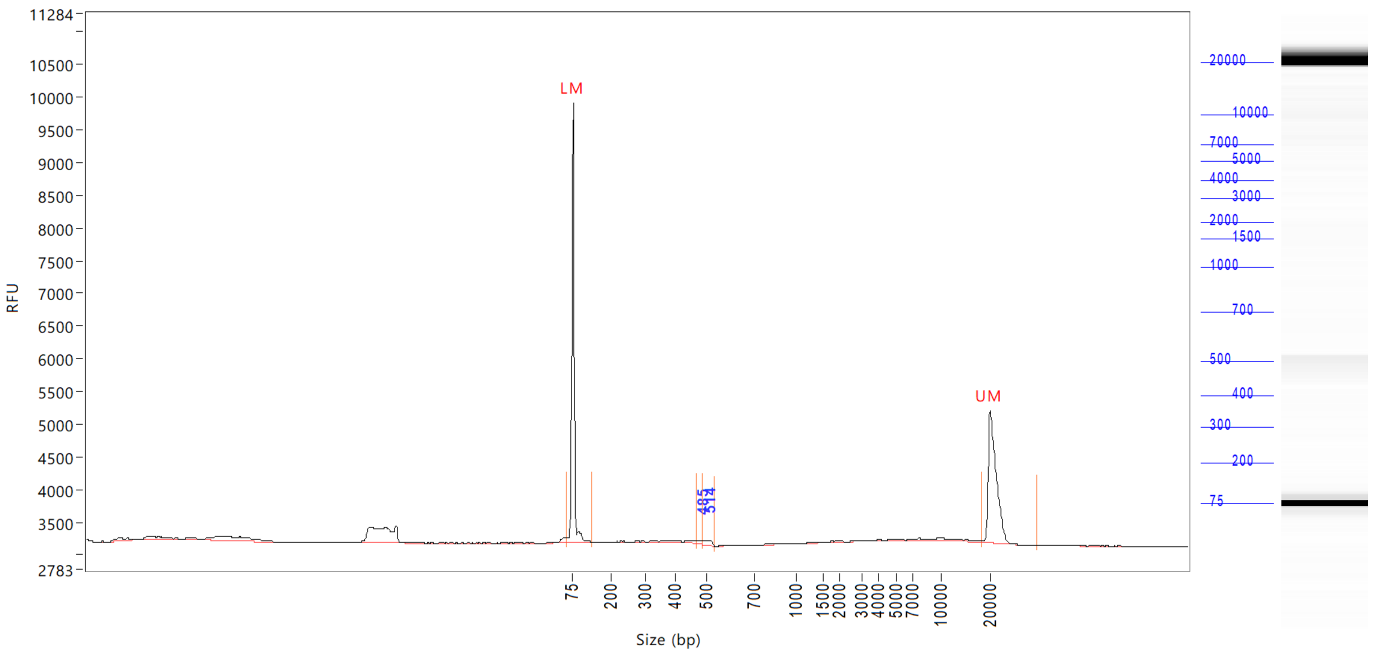

Peak	Size	Concentration	Relative concentration	Molarity	From	To	CV%	RFU
	(bp)	(ng/uL)	Percent	(nmole/L)	(bp)	(bp)		
1	75 (LM)	0.5000		11.0851	58	139	9.14	6734
2	485	0.0917	34.2	0.3162	466	487	1.36	55
3	514	0.1766	65.8	0.5779	487	529	1.97	85
4	20000 (UM)	0.2726		0.0218	18415	29551	4.73	2009

TIC:	0.2683	ng/uL
TIM:	0.8941	nmole/L
Total concentration:	1.1069	ng/uL

Sample peak width (sec): 5 Sample min peak height: 50 Sample baseline V to V?: Y Sample baseline V to V points: 3
Sample filter: Binomial Number of points for filter: 3 Sample start region (min): 0 Sample end region (min): 30
Marker peak width (sec): 5 Marker min peak height: 200 Marker baseline V to V?: Y Marker baseline V to V points: 3
Lower marker selection: First peak > 500 RFU Upper marker selection: Last peak > 200 RFU
Ladder size (bp)75, 200, 300, 400, 500, 700, 1000, 1500, 2000, 3000, 4000, 5000, 7000, 10000, 20000
Quantification using: Lower Marker Final concentration (ng/uL): 0.5000 Dilution factor: 12.0

Sample: SampD9
Well location: D9
Created: Thursday, October 21, 2021 8:58:49 PM

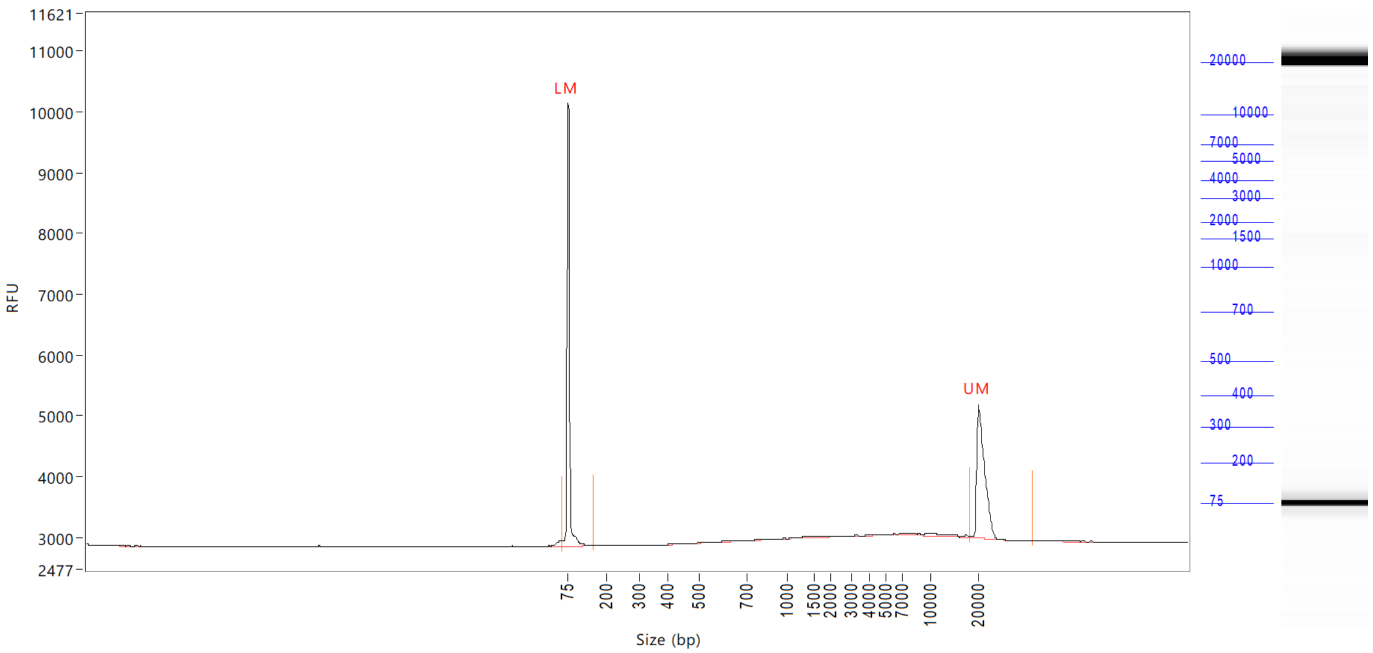

Peak	Size	Concentration	Relative concentration	Molarity	From	To	CV%	RFU
	(bp)	(ng/uL)	Percent	(nmole/L)	(bp)	(bp)		
1	75 (LM)	0.5000		10.7944	60	158	11.51	7303
2	20000 (UM)	0.2551		0.0203	18171	31244	4.92	2182

TIC:	0.0000	ng/uL
TIM:	0.0000	nmole/L
Total concentration:	0.7070	ng/uL

Sample peak width (sec): 5 Sample min peak height: 50 Sample baseline V to V?: Y Sample baseline V to V points: 3
Sample filter: Binomial Number of points for filter: 3 Sample start region (min): 0 Sample end region (min): 30
Marker peak width (sec): 5 Marker min peak height: 200 Marker baseline V to V?: Y Marker baseline V to V points: 3
Lower marker selection: First peak > 500 RFU Upper marker selection: Last peak > 200 RFU
Ladder size (bp)75, 200, 300, 400, 500, 700, 1000, 1500, 2000, 3000, 4000, 5000, 7000, 10000, 20000
Quantification using: Lower Marker Final concentration (ng/uL): 0.5000 Dilution factor: 12.0

Sample: SampE9
Well location: E9
Created: Thursday, October 21, 2021 8:58:49 PM

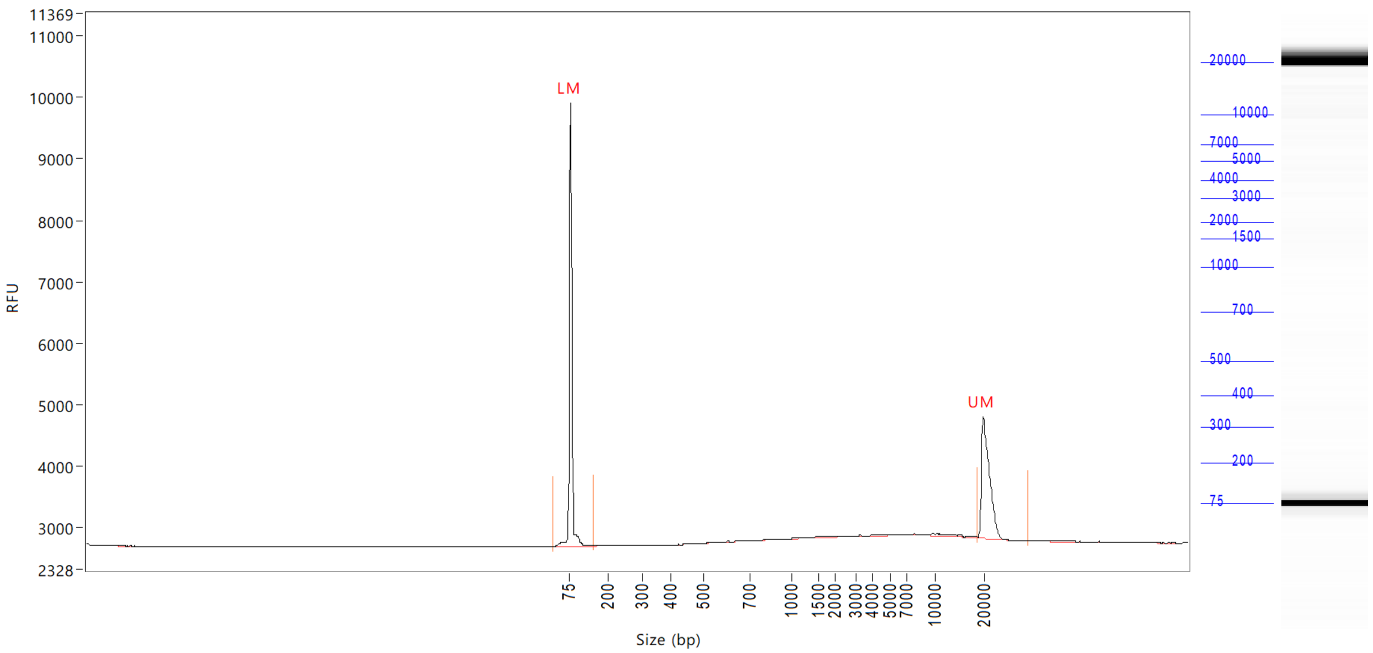

Peak	Size	Concentration	Relative concentration	Molarity	From	To	CV%	RFU
	(bp)	(ng/uL)	Percent	(nmole/L)	(bp)	(bp)		
1	75 (LM)	0.5000		11.0851	20	150	12.92	7223
2	20000 (UM)	0.2371		0.0189	18903	29189	4.53	1976

TIC:	0.0000	ng/uL
TIM:	0.0000	nmole/L
Total concentration:	0.5729	ng/uL

Sample peak width (sec): 5 Sample min peak height: 50 Sample baseline V to V?: Y Sample baseline V to V points: 3
Sample filter: Binomial Number of points for filter: 3 Sample start region (min): 0 Sample end region (min): 30
Marker peak width (sec): 5 Marker min peak height: 200 Marker baseline V to V?: Y Marker baseline V to V points: 3
Lower marker selection: First peak > 500 RFU Upper marker selection: Last peak > 200 RFU
Ladder size (bp)75, 200, 300, 400, 500, 700, 1000, 1500, 2000, 3000, 4000, 5000, 7000, 10000, 20000
Quantification using: Lower Marker Final concentration (ng/uL): 0.5000 Dilution factor: 12.0

Sample: SampF9
Well location: F9
Created: Thursday, October 21, 2021 8:58:49 PM

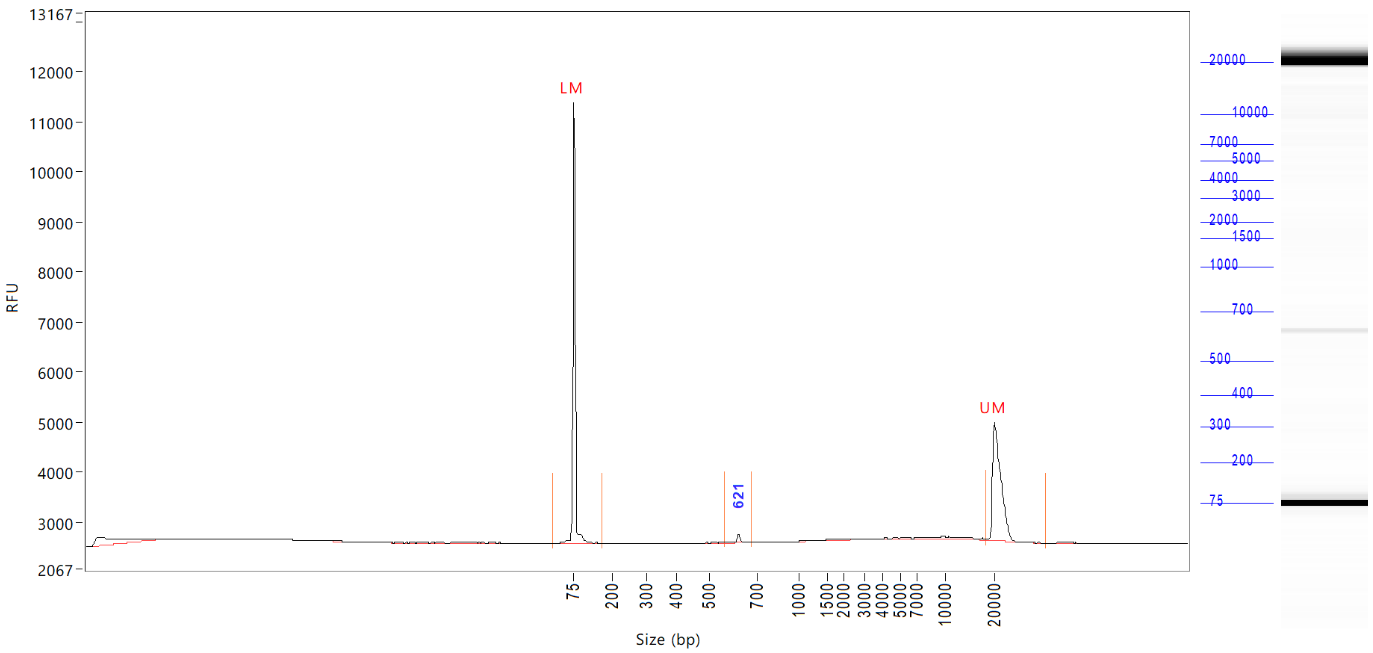

Peak	Size	Concentration	Relative concentration	Molarity	From	To	CV%	RFU
	(bp)	(ng/uL)	Percent	(nmole/L)	(bp)	(bp)		
1	75 (LM)	0.5000		11.0851	11	164	10.99	8812
2	621	0.1484	100.0	0.3940	562	680	3.48	168
3	20000 (UM)	0.2484		0.0199	18171	30277	4.73	2372

TIC:	0.1484	ng/uL
TIM:	0.3940	nmole/L
Total concentration:	0.7110	ng/uL

Sample peak width (sec): 5 Sample min peak height: 50 Sample baseline V to V?: Y Sample baseline V to V points: 3
Sample filter: Binomial Number of points for filter: 3 Sample start region (min): 0 Sample end region (min): 30
Marker peak width (sec): 5 Marker min peak height: 200 Marker baseline V to V?: Y Marker baseline V to V points: 3
Lower marker selection: First peak > 500 RFU Upper marker selection: Last peak > 200 RFU
Ladder size (bp)75, 200, 300, 400, 500, 700, 1000, 1500, 2000, 3000, 4000, 5000, 7000, 10000, 20000
Quantification using: Lower Marker Final concentration (ng/uL): 0.5000 Dilution factor: 12.0

Sample: SampG9
Well location: G9
Created: Thursday, October 21, 2021 8:58:49 PM

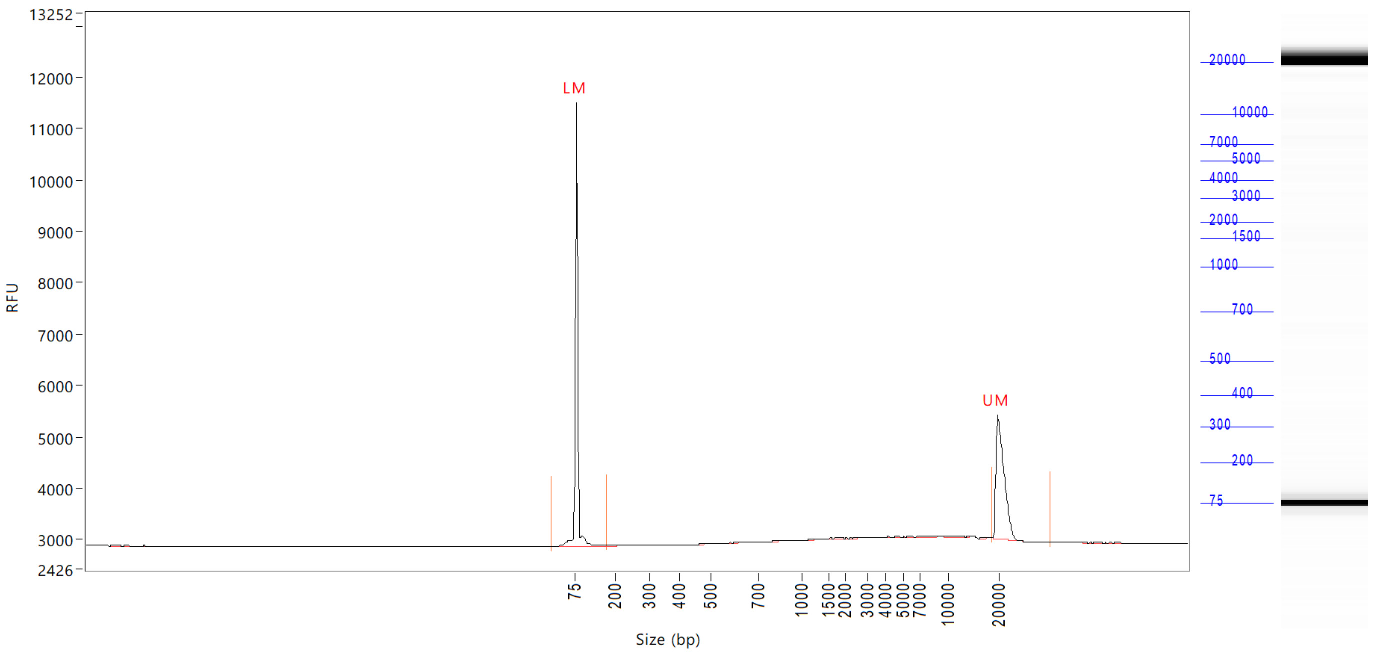

Peak	Size	Concentration	Relative concentration	Molarity	From	To	CV%	RFU
	(bp)	(ng/uL)	Percent	(nmole/L)	(bp)	(bp)		
1	75 (LM)	0.5000		11.0851	0	171	14.66	8650
2	20000 (UM)	0.2388		0.0191	18781	30519	4.64	2402

TIC:	0.0000	ng/uL
TIM:	0.0000	nmole/L
Total concentration:	0.4547	ng/uL

Sample peak width (sec): 5   Sample min peak height: 50   Sample baseline V to V?: Y   Sample baseline V to V points: 3
Sample filter: Binomial   Number of points for filter: 3 Sample start region (min): 0 Sample end region (min): 30
Marker peak width (sec): 5   Marker min peak height: 200   Marker baseline V to V?: Y   Marker baseline V to V points: 3
Lower marker selection: First peak > 500 RFU   Upper marker selection: Last peak > 200 RFU
Ladder size (bp)75, 200, 300, 400, 500, 700, 1000, 1500, 2000, 3000, 4000, 5000, 7000, 10000, 20000
Quantification using: Lower Marker   Final concentration (ng/uL): 0.5000   Dilution factor: 12.0

Sample: SampH9
Well location: H9
Created: Thursday, October 21, 2021 8:58:49 PM

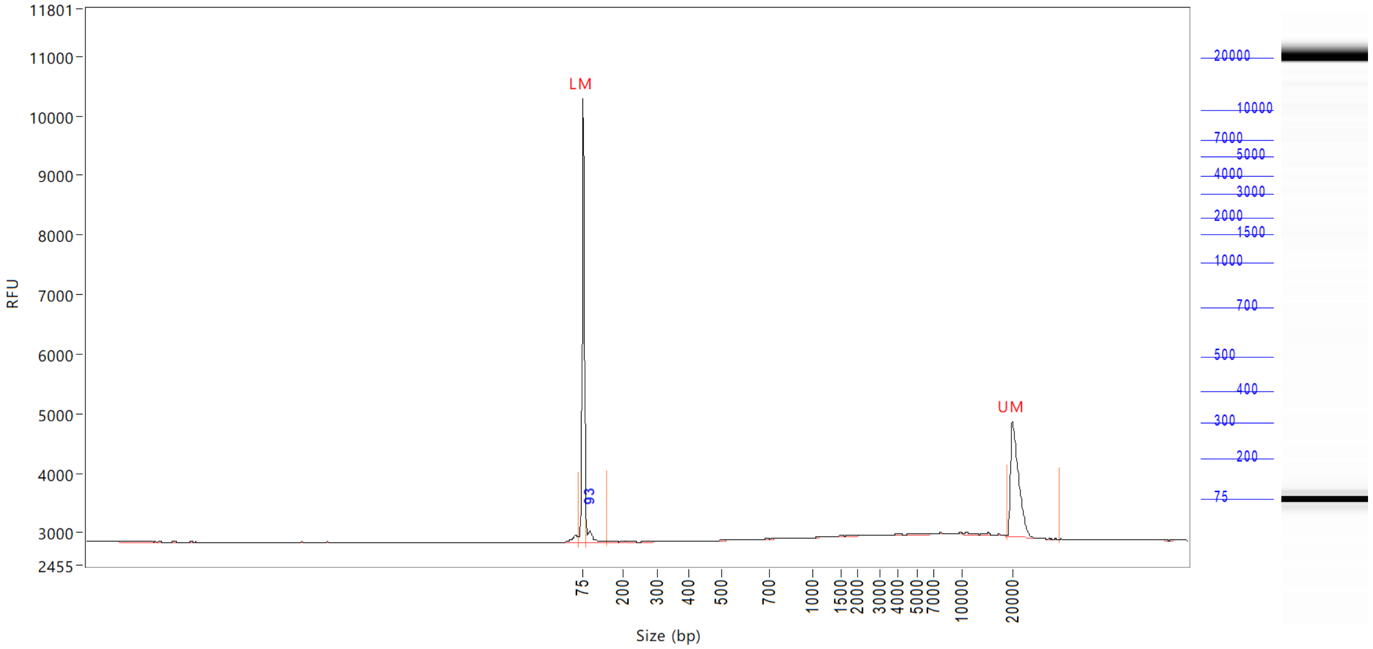

Peak	Size	Concentration	Relative concentration	Molarity	From	To	CV%	RFU
	(bp)	(ng/uL)	Percent	(nmole/L)	(bp)	(bp)		
1	75 (LM)	0.5000		11.2364	60	86	4.57	7466
2	93	0.3590	100.0	5.9550	86	148	13.42	181
3	20000 (UM)	0.2421		0.0194	18903	29309	4.50	1923

TIC:	0.3590	ng/uL
TIM:	5.9550	nmole/L
Total concentration:	0.9520	ng/uL

Sample peak width (sec): 5 Sample min peak height: 50 Sample baseline V to V?: Y Sample baseline V to V points: 3
Sample filter: Binomial Number of points for filter: 3 Sample start region (min): 0 Sample end region (min): 30
Marker peak width (sec): 5 Marker min peak height: 200 Marker baseline V to V?: Y Marker baseline V to V points: 3
Lower marker selection: First peak > 500 RFU Upper marker selection: Last peak > 200 RFU
Ladder size (bp)75, 200, 300, 400, 500, 700, 1000, 1500, 2000, 3000, 4000, 5000, 7000, 10000, 20000
Quantification using: Lower Marker Final concentration (ng/uL): 0.5000 Dilution factor: 12.0

Sample: SampA10
Well location: A10
Created: Thursday, October 21, 2021 8:58:49 PM

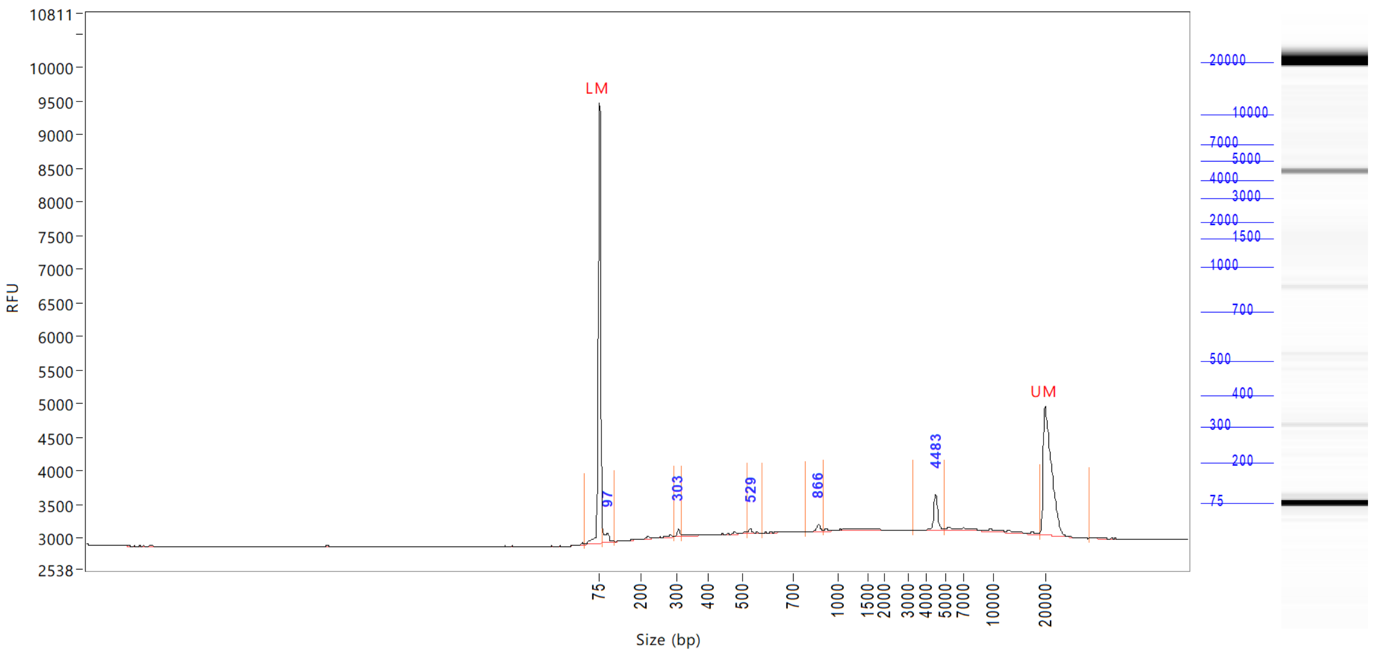

Peak	Size	Concentration	Relative concentration	Molarity	From	To	CV%	RFU
	(bp)	(ng/uL)	Percent	(nmole/L)	(bp)	(bp)		
1	75 (LM)	0.5000		11.2364	31	87	7.09	6563
2	97	0.2536	23.7	4.3372	87	122	6.74	126
3	303	0.1170	10.9	0.6372	291	311	1.31	127
4	529	0.0877	8.2	0.2707	516	575	2.42	72
5	866	0.1026	9.6	0.1956	783	900	2.16	102
6	4483	0.5080	47.5	0.1884	3286	4966	4.90	537
7	20000 (UM)	0.2674		0.0214	18903	28463	4.35	1925

TIC:	1.0689	ng/uL
TIM:	5.6292	nmole/L
Total concentration:	2.3309	ng/uL

```
Sample peak width (sec): 5    Sample min peak height: 50    Sample baseline V to V?: Y    Sample baseline V to V points: 3
Sample filter: Binomial       Number of points for filter: 3  Sample start region (min): 0  Sample end region (min): 30
Marker peak width (sec): 5    Marker min peak height: 200   Marker baseline V to V?: Y    Marker baseline V to V points: 3
Lower marker selection: First peak > 500 RFU                Upper marker selection: Last peak > 200 RFU
Ladder size (bp)75, 200, 300, 400, 500, 700, 1000, 1500, 2000, 3000, 4000, 5000, 7000, 10000, 20000
Quantification using: Lower Marker          Final concentration (ng/uL): 0.5000          Dilution factor: 12.0
```

Sample: SampB10
Well location: B10
Created: Thursday, October 21, 2021 8:58:49 PM

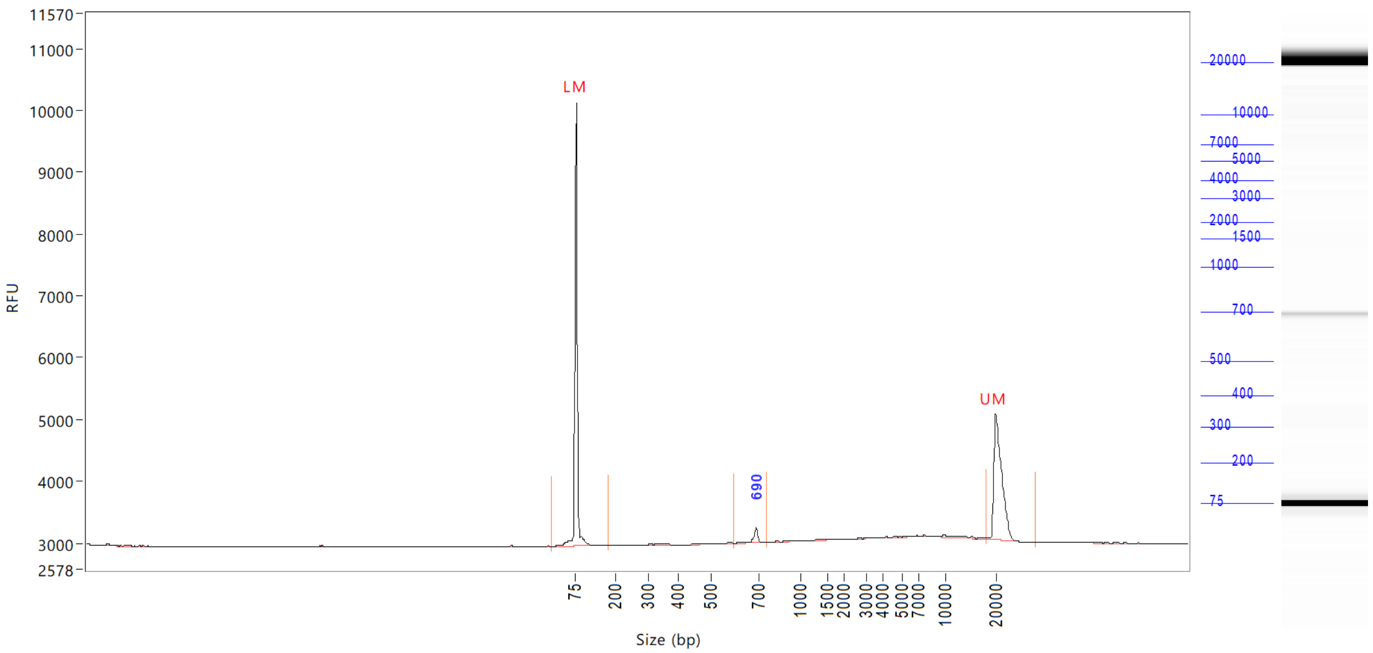

Peak	Size	Concentration	Relative concentration	Molarity	From	To	CV%	RFU
	(bp)	(ng/uL)	Percent	(nmole/L)	(bp)	(bp)		
1	75 (LM)	0.5000		11.2364	1	177	12.55	7178
2	690	0.2596	100.0	0.6273	598	753	2.99	258
3	20000 (UM)	0.2449		0.0196	18171	27979	4.27	2031

TIC:	0.2596	ng/uL
TIM:	0.6273	nmole/L
Total concentration:	0.8453	ng/uL

Sample peak width (sec): 5   Sample min peak height: 50   Sample baseline V to V?: Y   Sample baseline V to V points: 3
Sample filter: Binomial   Number of points for filter: 3 Sample start region (min): 0 Sample end region (min): 30
Marker peak width (sec): 5   Marker min peak height: 200   Marker baseline V to V?: Y   Marker baseline V to V points: 3
Lower marker selection: First peak > 500 RFU   Upper marker selection: Last peak > 200 RFU
Ladder size (bp)75, 200, 300, 400, 500, 700, 1000, 1500, 2000, 3000, 4000, 5000, 7000, 10000, 20000
Quantification using: Lower Marker   Final concentration (ng/uL): 0.5000   Dilution factor: 12.0

Sample: SampC10
Well location: C10
Created: Thursday, October 21, 2021 8:58:49 PM

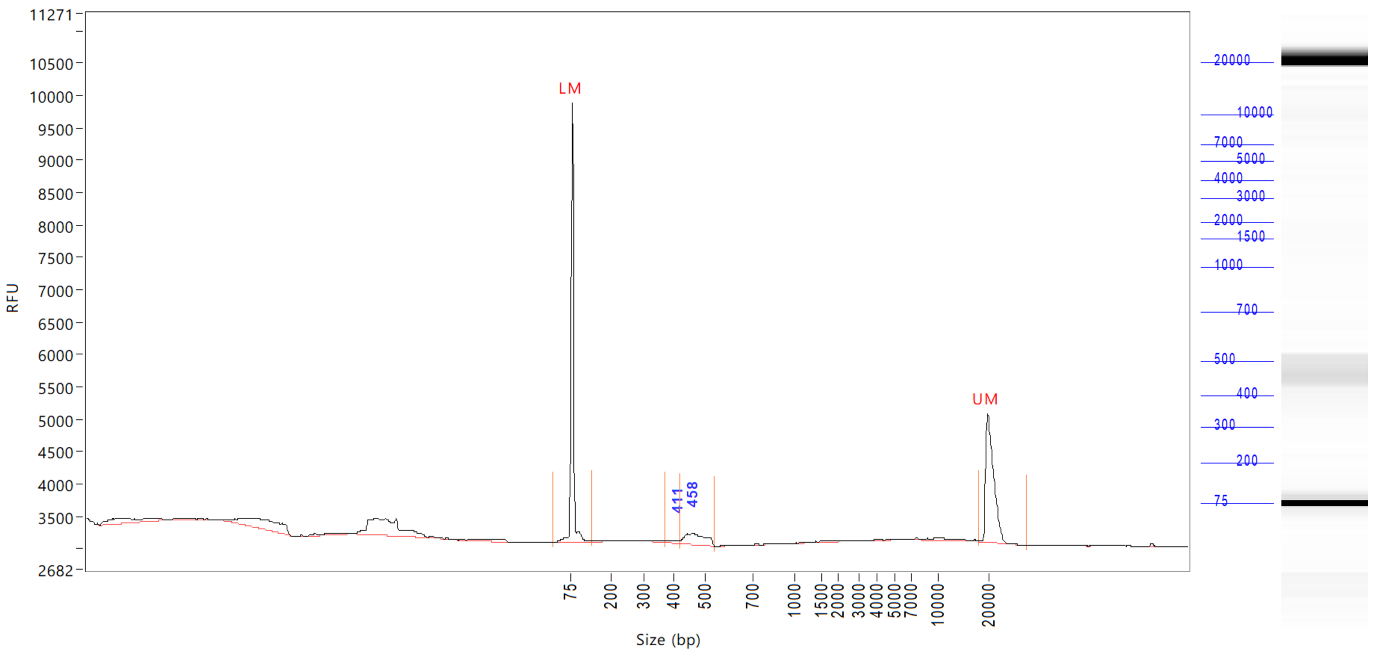

Peak	Size	Concentration	Relative concentration	Molarity	From	To	CV%	RFU
	(bp)	(ng/uL)	Percent	(nmole/L)	(bp)	(bp)		
1	75 (LM)	0.5000		11.2364	16	139	12.23	6796
2	411	0.1566	12.9	0.6489	372	418	3.39	51
3	458	1.0614	87.1	3.7240	418	537	6.00	172
4	20000 (UM)	0.2536		0.0203	18171	27979	4.32	1978

TIC:	1.2180	ng/uL
TIM:	4.3729	nmole/L
Total concentration:	1.8870	ng/uL

Sample peak width (sec): 5 Sample min peak height: 50 Sample baseline V to V?: Y Sample baseline V to V points: 3
Sample filter: Binomial Number of points for filter: 3 Sample start region (min): 0 Sample end region (min): 30
Marker peak width (sec): 5 Marker min peak height: 200 Marker baseline V to V?: Y Marker baseline V to V points: 3
Lower marker selection: First peak > 500 RFU Upper marker selection: Last peak > 200 RFU
Ladder size (bp)75, 200, 300, 400, 500, 700, 1000, 1500, 2000, 3000, 4000, 5000, 7000, 10000, 20000
Quantification using: Lower Marker Final concentration (ng/uL): 0.5000 Dilution factor: 12.0

Sample: SampD10
Well location: D10
Created: Thursday, October 21, 2021 8:58:49 PM

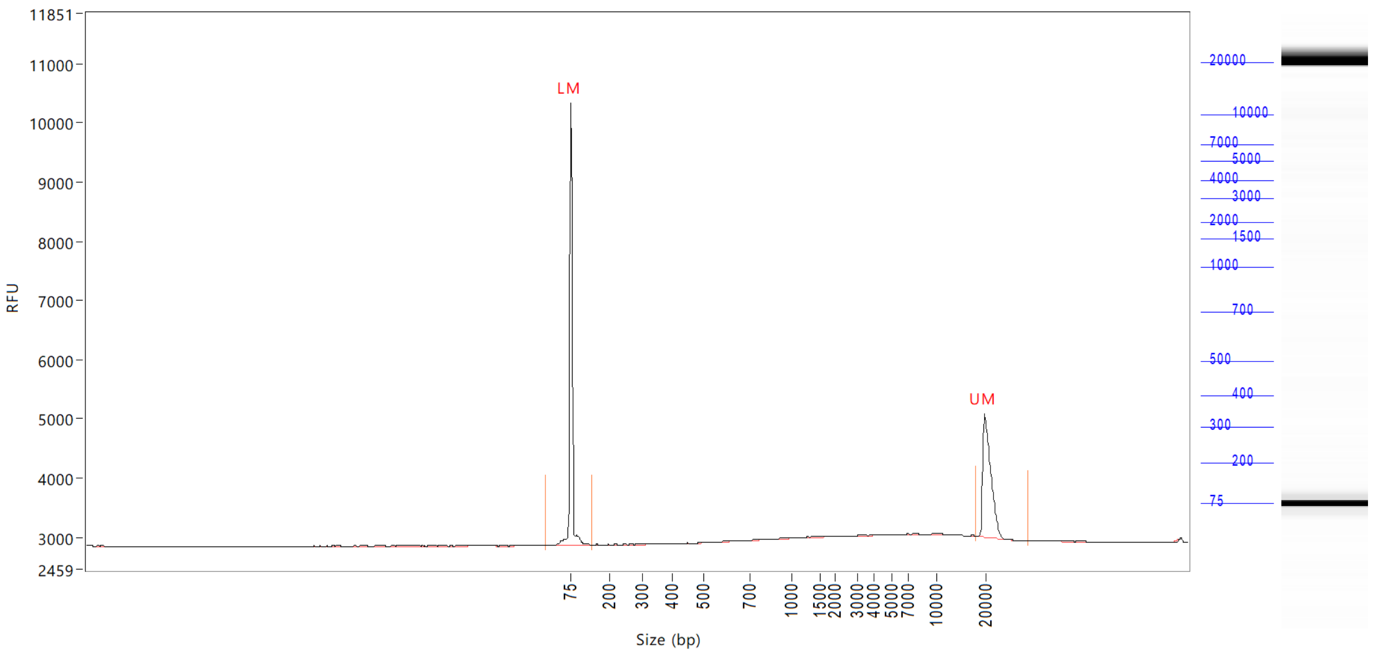

Peak	Size	Concentration	Relative concentration	Molarity	From	To	CV%	RFU
	(bp)	(ng/uL)	Percent	(nmole/L)	(bp)	(bp)		
1	75 (LM)	0.5000		11.2364	0	145	12.94	7489
2	20000 (UM)	0.2403		0.0192	18293	28705	4.39	2090

TIC:	0.0000	ng/uL
TIM:	0.0000	nmole/L
Total concentration:	0.5415	ng/uL

Sample peak width (sec): 5 Sample min peak height: 50 Sample baseline V to V?: Y Sample baseline V to V points: 3
Sample filter: Binomial Number of points for filter: 3 Sample start region (min): 0 Sample end region (min): 30
Marker peak width (sec): 5 Marker min peak height: 200 Marker baseline V to V?: Y Marker baseline V to V points: 3
Lower marker selection: First peak > 500 RFU Upper marker selection: Last peak > 200 RFU
Ladder size (bp)75, 200, 300, 400, 500, 700, 1000, 1500, 2000, 3000, 4000, 5000, 7000, 10000, 20000
Quantification using: Lower Marker Final concentration (ng/uL): 0.5000 Dilution factor: 12.0

Sample: SampE10
Well location: E10
Created: Thursday, October 21, 2021 8:58:49 PM

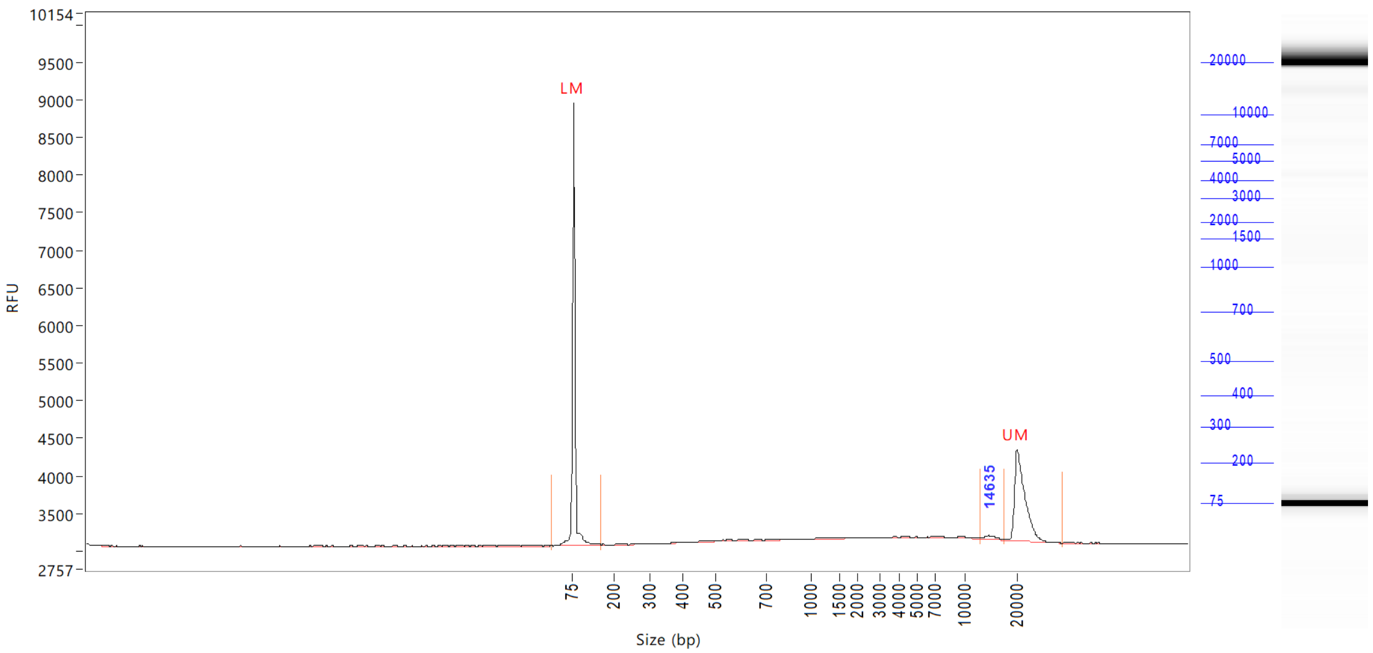

Peak	Size	Concentration	Relative concentration	Molarity	From	To	CV%	RFU
	(bp)	(ng/uL)	Percent	(nmole/L)	(bp)	(bp)		
1	75 (LM)	0.5000		11.0851	11	156	13.25	5899
2	14635	0.1353	100.0	0.0149	12806	17561	7.80	52
3	20000 (UM)	0.2151		0.0171	17561	28584	5.89	1222

TIC:	0.1353	ng/uL
TIM:	0.0149	nmole/L
Total concentration:	0.8174	ng/uL

Sample peak width (sec): 5 Sample min peak height: 50 Sample baseline V to V?: Y Sample baseline V to V points: 3
Sample filter: Binomial Number of points for filter: 3 Sample start region (min): 0 Sample end region (min): 30
Marker peak width (sec): 5 Marker min peak height: 200 Marker baseline V to V?: Y Marker baseline V to V points: 3
Lower marker selection: First peak > 500 RFU Upper marker selection: Last peak > 200 RFU
Ladder size (bp)75, 200, 300, 400, 500, 700, 1000, 1500, 2000, 3000, 4000, 5000, 7000, 10000, 20000
Quantification using: Lower Marker Final concentration (ng/uL): 0.5000 Dilution factor: 12.0

Sample: SampF10
Well location: F10
Created: Thursday, October 21, 2021 8:58:49 PM

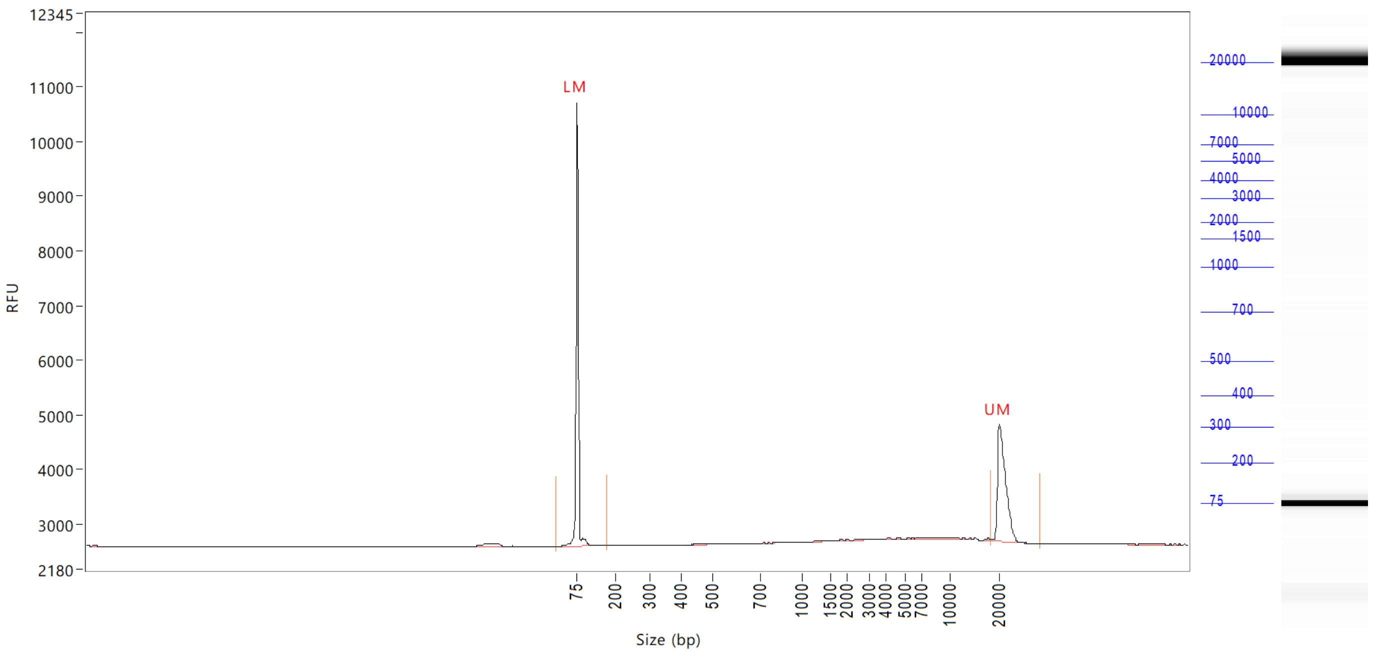

Peak	Size	Concentration	Relative concentration	Molarity	From	To	CV%	RFU
	(bp)	(ng/uL)	Percent	(nmole/L)	(bp)	(bp)		
1	75 (LM)	0.5000		11.2364	12	169	10.16	8116
2	20000 (UM)	0.2405		0.0192	18415	28221	4.25	2153

TIC:	0.0000	ng/uL
TIM:	0.0000	nmole/L
Total concentration:	0.4914	ng/uL

Sample peak width (sec): 5   Sample min peak height: 50   Sample baseline V to V?: Y   Sample baseline V to V points: 3
Sample filter: Binomial   Number of points for filter: 3 Sample start region (min): 0 Sample end region (min): 30
Marker peak width (sec): 5   Marker min peak height: 200   Marker baseline V to V?: Y   Marker baseline V to V points: 3
Lower marker selection: First peak > 500 RFU   Upper marker selection: Last peak > 200 RFU
Ladder size (bp)75, 200, 300, 400, 500, 700, 1000, 1500, 2000, 3000, 4000, 5000, 7000, 10000, 20000
Quantification using: Lower Marker   Final concentration (ng/uL): 0.5000   Dilution factor: 12.0

Sample: SampG10
Well location: G10
Created: Thursday, October 21, 2021 8:58:49 PM

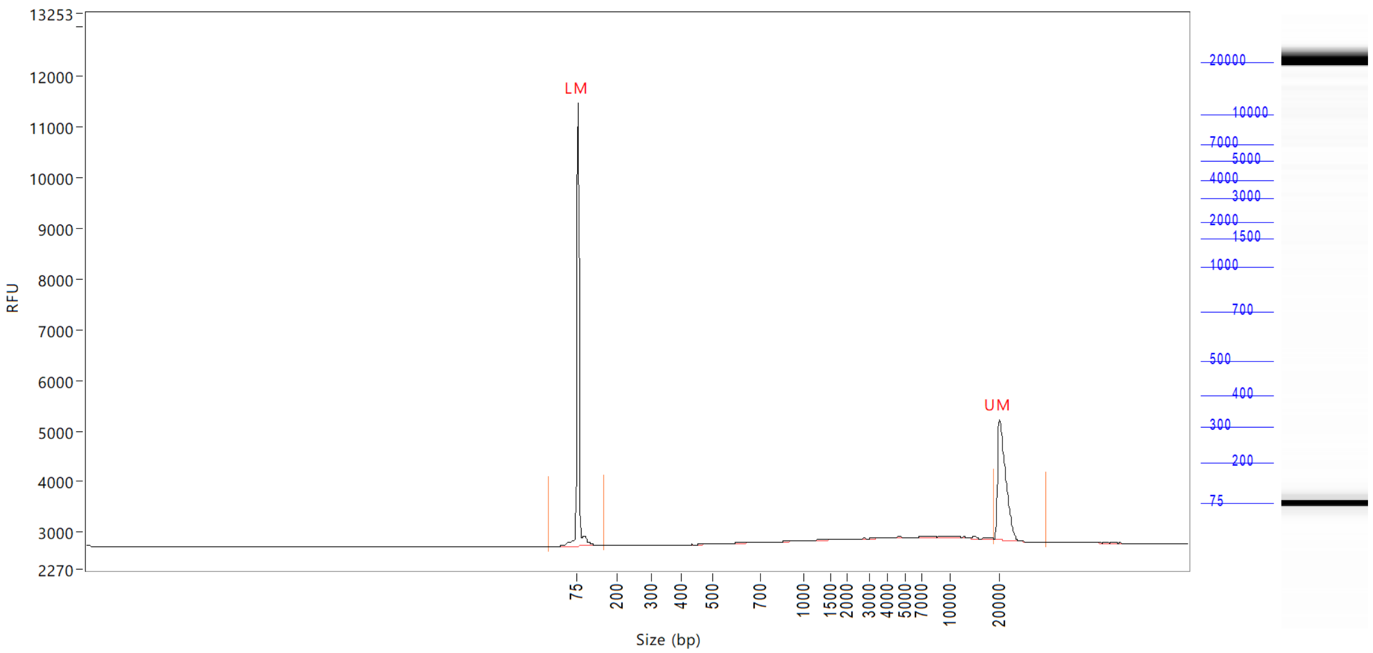

Peak	Size	Concentration	Relative concentration	Molarity	From	To	CV%	RFU
	(bp)	(ng/uL)	Percent	(nmole/L)	(bp)	(bp)		
1	75 (LM)	0.5000		11.0851	0	160	12.17	8768
2	20000 (UM)	0.2373		0.0189	18903	29430	4.34	2379

TIC:	0.0000	ng/uL
TIM:	0.0000	nmole/L
Total concentration:	0.5057	ng/uL

Sample peak width (sec): 5   Sample min peak height: 50   Sample baseline V to V?: Y   Sample baseline V to V points: 3
Sample filter: Binomial   Number of points for filter: 3 Sample start region (min): 0 Sample end region (min): 30
Marker peak width (sec): 5   Marker min peak height: 200   Marker baseline V to V?: Y   Marker baseline V to V points: 3
Lower marker selection: First peak > 500 RFU   Upper marker selection: Last peak > 200 RFU
Ladder size (bp)75, 200, 300, 400, 500, 700, 1000, 1500, 2000, 3000, 4000, 5000, 7000, 10000, 20000
Quantification using: Lower Marker   Final concentration (ng/uL): 0.5000   Dilution factor: 12.0

Sample: SampH10
Well location: H10
Created: Thursday, October 21, 2021 8:58:49 PM

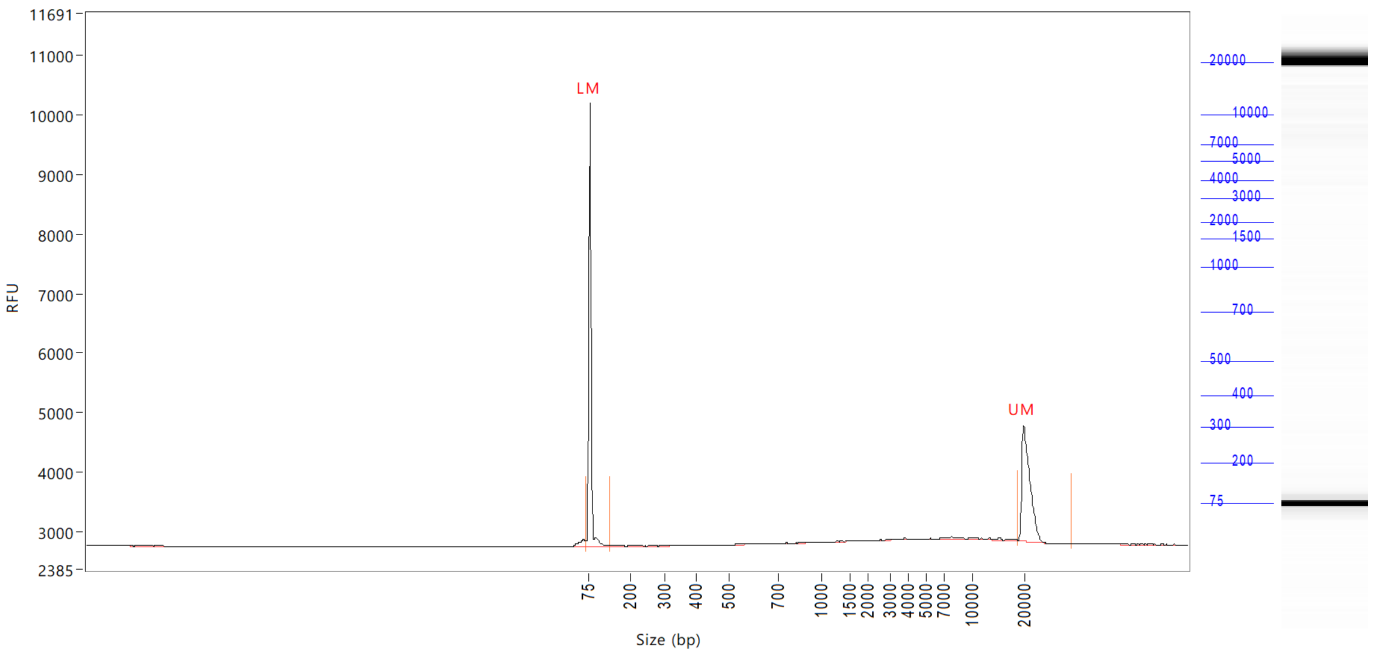

Peak	Size	Concentration	Relative concentration	Molarity	From	To	CV%	RFU
	(bp)	(ng/uL)	Percent	(nmole/L)	(bp)	(bp)		
1	75 (LM)	0.5000		11.0851	62	135	9.00	7437
2	20000 (UM)	0.2355		0.0188	18903	29189	4.58	1941

TIC:	0.0000	ng/uL
TIM:	0.0000	nmole/L
Total concentration:	0.6918	ng/uL

Sample peak width (sec): 5 Sample min peak height: 50 Sample baseline V to V?: Y Sample baseline V to V points: 3
Sample filter: Binomial Number of points for filter: 3 Sample start region (min): 0 Sample end region (min): 30
Marker peak width (sec): 5 Marker min peak height: 200 Marker baseline V to V?: Y Marker baseline V to V points: 3
Lower marker selection: First peak > 500 RFU Upper marker selection: Last peak > 200 RFU
Ladder size (bp)75, 200, 300, 400, 500, 700, 1000, 1500, 2000, 3000, 4000, 5000, 7000, 10000, 20000
Quantification using: Lower Marker Final concentration (ng/uL): 0.5000 Dilution factor: 12.0

Sample: SampA11
Well location: A11
Created: Thursday, October 21, 2021 8:58:49 PM

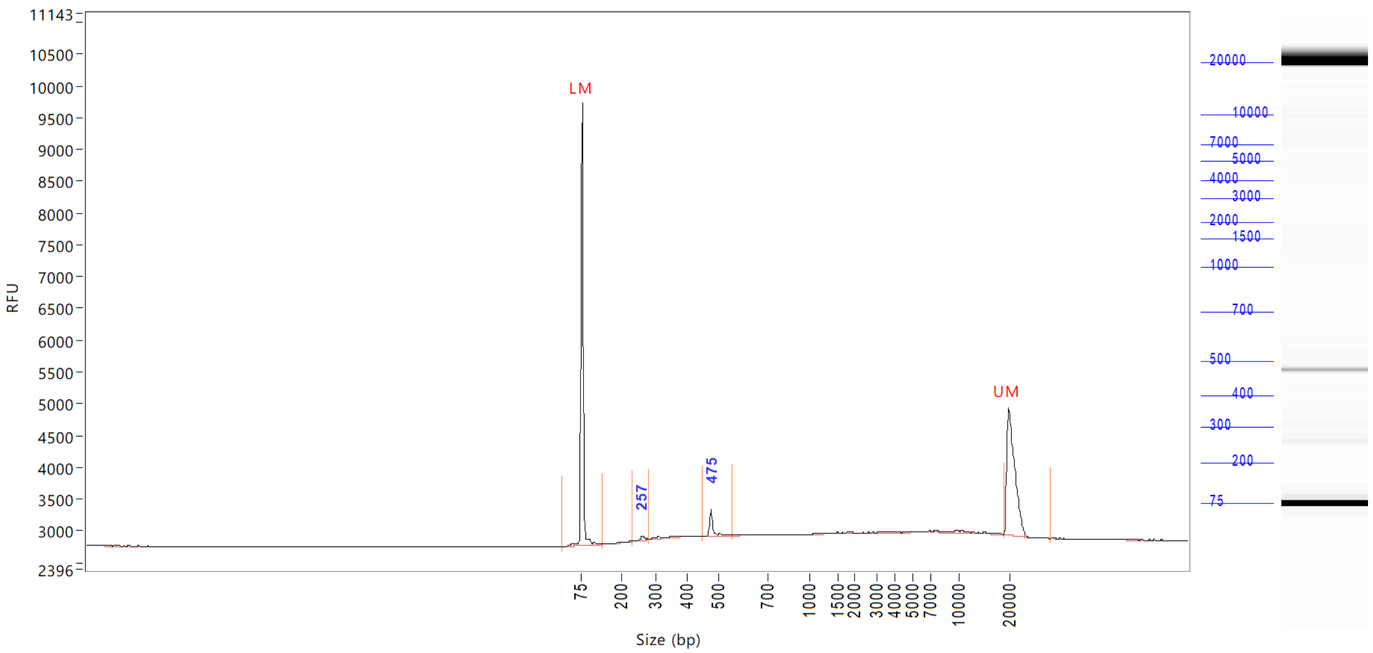

Peak	Size	Concentration	Relative concentration	Molarity	From	To	CV%	RFU
	(bp)	(ng/uL)	Percent	(nmole/L)	(bp)	(bp)		
1	75 (LM)	0.5000		11.0851	16	141	9.67	6976
2	257	0.1165	21.0	0.7396	230	278	3.49	70
3	475	0.4370	79.0	1.4918	450	555	3.97	412
4	20000 (UM)	0.2626		0.0210	19024	28221	4.43	2012

TIC:	0.5535	ng/uL
TIM:	2.2314	nmole/L
Total concentration:	1.2495	ng/uL

```
Sample peak width (sec): 5    Sample min peak height: 50    Sample baseline V to V?: Y    Sample baseline V to V points: 3
Sample filter: Binomial       Number of points for filter: 3  Sample start region (min): 0  Sample end region (min): 30
Marker peak width (sec): 5    Marker min peak height: 200   Marker baseline V to V?: Y    Marker baseline V to V points: 3
Lower marker selection: First peak > 500 RFU                Upper marker selection: Last peak > 200 RFU
Ladder size (bp)75, 200, 300, 400, 500, 700, 1000, 1500, 2000, 3000, 4000, 5000, 7000, 10000, 20000
Quantification using: Lower Marker          Final concentration (ng/uL): 0.5000          Dilution factor: 12.0
```

Sample: SampB11
Well location: B11
Created: Thursday, October 21, 2021 8:58:49 PM

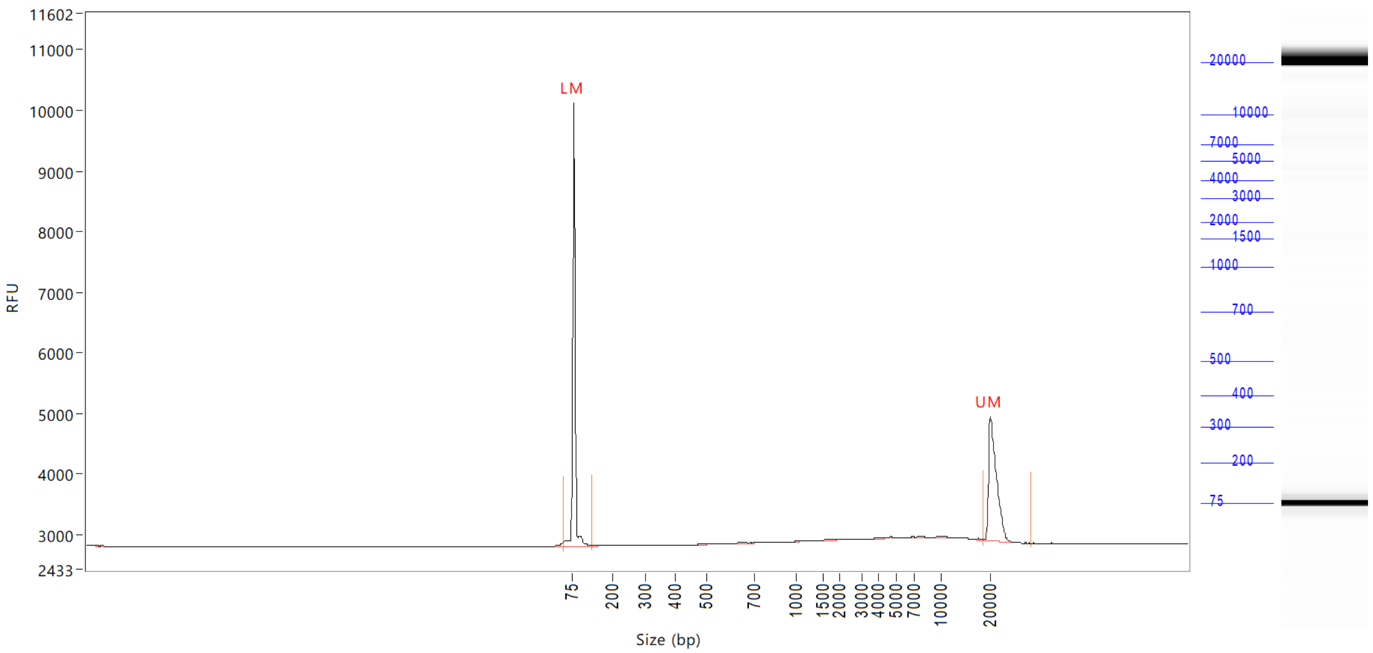

Peak	Size	Concentration	Relative concentration	Molarity	From	To	CV%	RFU
	(bp)	(ng/uL)	Percent	(nmole/L)	(bp)	(bp)		
1	75 (LM)	0.5000		11.0851	47	133	9.51	7324
2	20000 (UM)	0.2434		0.0194	18781	28342	4.44	2041

TIC:	0.0000	ng/uL
TIM:	0.0000	nmole/L
Total concentration:	0.5115	ng/uL

Sample peak width (sec): 5 Sample min peak height: 50 Sample baseline V to V?: Y Sample baseline V to V points: 3
Sample filter: Binomial Number of points for filter: 3 Sample start region (min): 0 Sample end region (min): 30
Marker peak width (sec): 5 Marker min peak height: 200 Marker baseline V to V?: Y Marker baseline V to V points: 3
Lower marker selection: First peak > 500 RFU Upper marker selection: Last peak > 200 RFU
Ladder size (bp)75, 200, 300, 400, 500, 700, 1000, 1500, 2000, 3000, 4000, 5000, 7000, 10000, 20000
Quantification using: Lower Marker Final concentration (ng/uL): 0.5000 Dilution factor: 12.0

Sample: SampC11
Well location: C11
Created: Thursday, October 21, 2021 8:58:49 PM

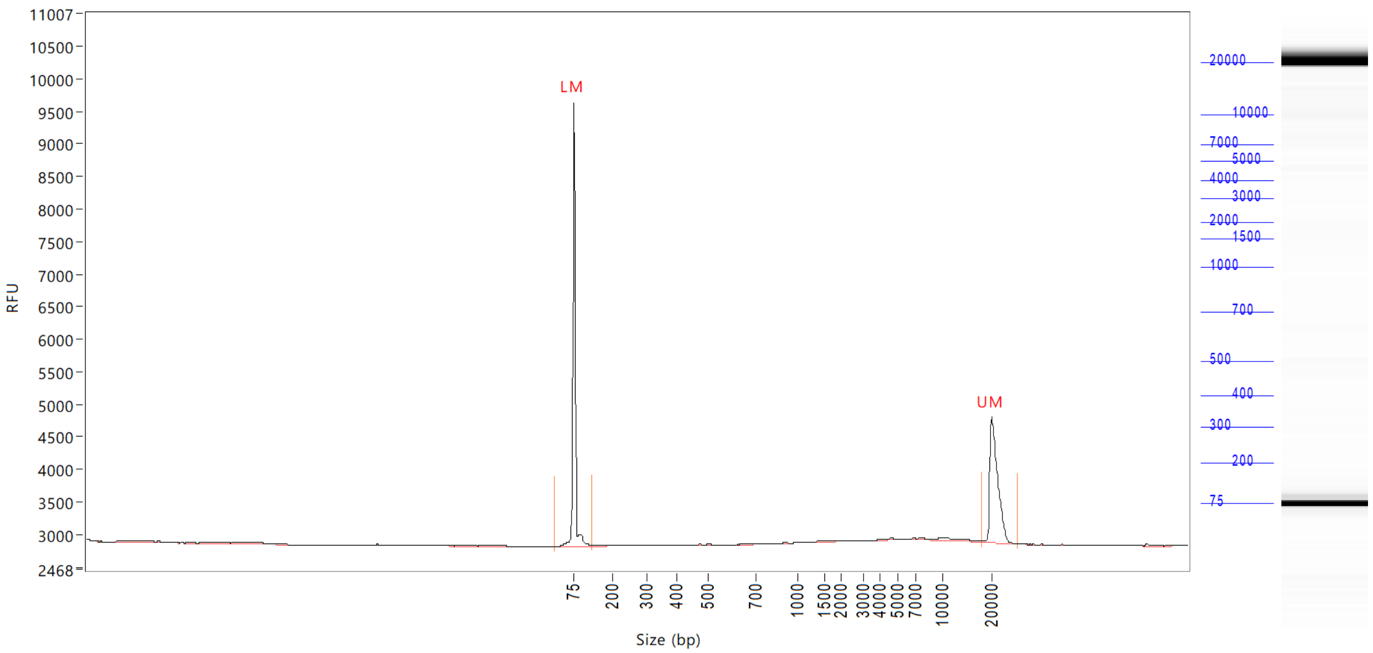

Peak	Size	Concentration	Relative concentration	Molarity	From	To	CV%	RFU
	(bp)	(ng/uL)	Percent	(nmole/L)	(bp)	(bp)		
1	75 (LM)	0.5000		11.2364	14	135	11.81	6824
2	20000 (UM)	0.2436		0.0196	18049	25320	4.40	1946

TIC:	0.0000	ng/uL
TIM:	0.0000	nmole/L
Total concentration:	0.7578	ng/uL

Sample peak width (sec): 5   Sample min peak height: 50   Sample baseline V to V?: Y   Sample baseline V to V points: 3
Sample filter: Binomial   Number of points for filter: 3 Sample start region (min): 0 Sample end region (min): 30
Marker peak width (sec): 5   Marker min peak height: 200   Marker baseline V to V?: Y   Marker baseline V to V points: 3
Lower marker selection: First peak > 500 RFU   Upper marker selection: Last peak > 200 RFU
Ladder size (bp)75, 200, 300, 400, 500, 700, 1000, 1500, 2000, 3000, 4000, 5000, 7000, 10000, 20000
Quantification using: Lower Marker   Final concentration (ng/uL): 0.5000   Dilution factor: 12.0

Sample: SampD11
Well location: D11
Created: Thursday, October 21, 2021 8:58:49 PM

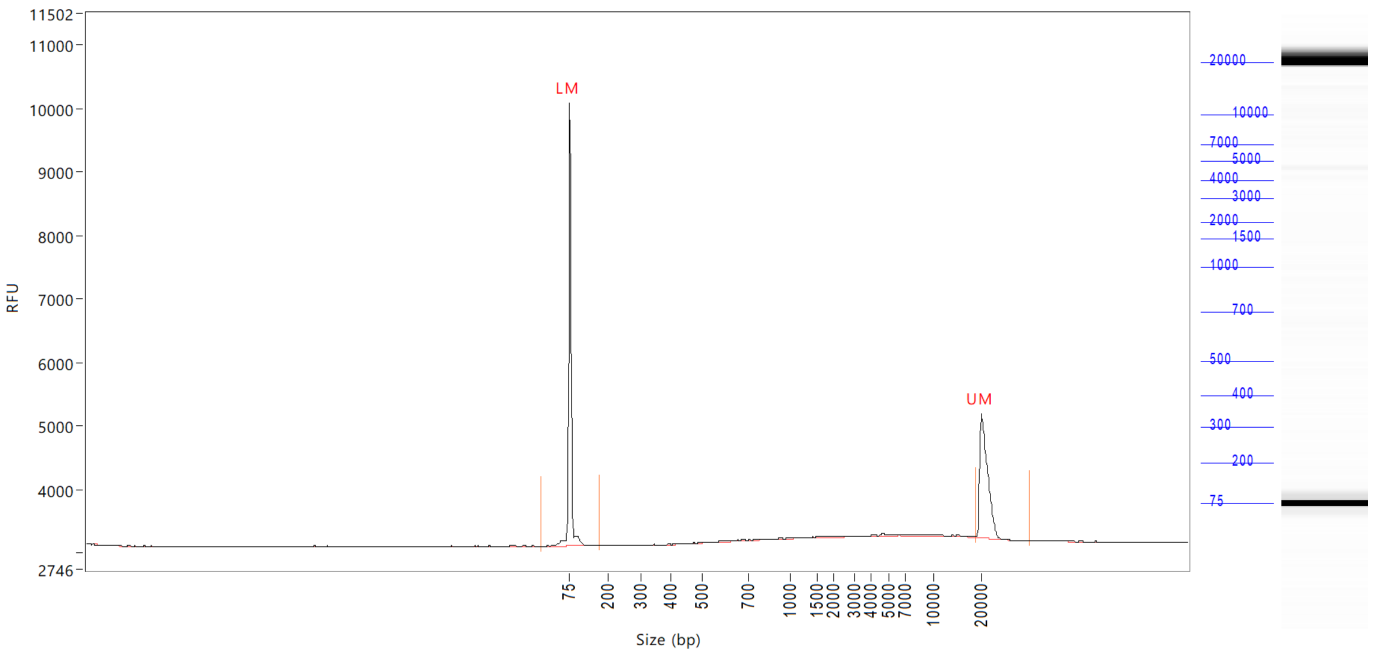

Peak	Size	Concentration	Relative concentration	Molarity	From	To	CV%	RFU
	(bp)	(ng/uL)	Percent	(nmole/L)	(bp)	(bp)		
1	75 (LM)	0.5000		11.0851	0	173	14.79	6989
2	20000 (UM)	0.2407		0.0192	18903	29914	4.55	1957

TIC:	0.0000	ng/uL
TIM:	0.0000	nmole/L
Total concentration:	0.6458	ng/uL

Sample peak width (sec): 5   Sample min peak height: 50   Sample baseline V to V?: Y   Sample baseline V to V points: 3
Sample filter: Binomial   Number of points for filter: 3 Sample start region (min): 0 Sample end region (min): 30
Marker peak width (sec): 5   Marker min peak height: 200   Marker baseline V to V?: Y   Marker baseline V to V points: 3
Lower marker selection: First peak > 500 RFU   Upper marker selection: Last peak > 200 RFU
Ladder size (bp)75, 200, 300, 400, 500, 700, 1000, 1500, 2000, 3000, 4000, 5000, 7000, 10000, 20000
Quantification using: Lower Marker   Final concentration (ng/uL): 0.5000   Dilution factor: 12.0

Sample: SampE11
Well location: E11
Created: Thursday, October 21, 2021 8:58:49 PM

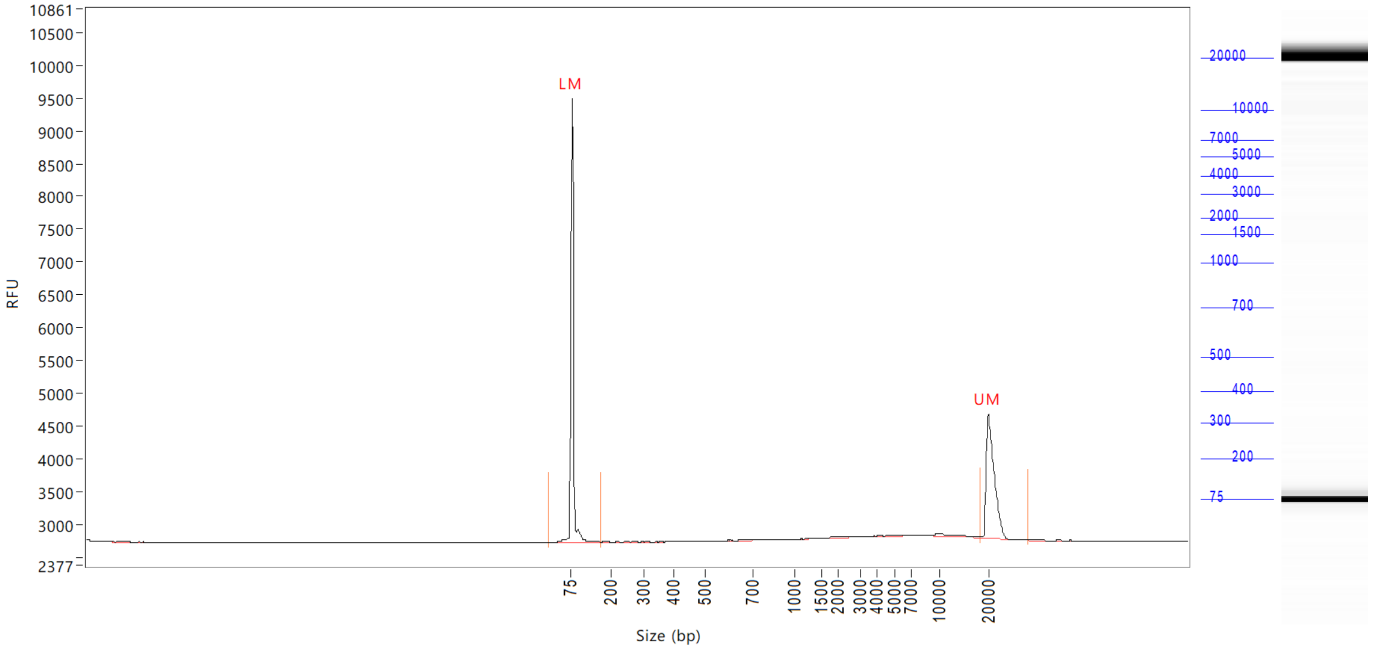

Peak	Size	Concentration	Relative concentration	Molarity	From	To	CV%	RFU
	(bp)	(ng/uL)	Percent	(nmole/L)	(bp)	(bp)		
1	75 (LM)	0.5000		11.0851	3	166	13.18	6781
2	20000 (UM)	0.2366		0.0189	18293	28100	4.50	1892

TIC:	0.0000	ng/uL
TIM:	0.0000	nmole/L
Total concentration:	0.5619	ng/uL

Sample peak width (sec): 5 Sample min peak height: 50 Sample baseline V to V?: Y Sample baseline V to V points: 3
Sample filter: Binomial Number of points for filter: 3 Sample start region (min): 0 Sample end region (min): 30
Marker peak width (sec): 5 Marker min peak height: 200 Marker baseline V to V?: Y Marker baseline V to V points: 3
Lower marker selection: First peak > 500 RFU Upper marker selection: Last peak > 200 RFU
Ladder size (bp)75, 200, 300, 400, 500, 700, 1000, 1500, 2000, 3000, 4000, 5000, 7000, 10000, 20000
Quantification using: Lower Marker Final concentration (ng/uL): 0.5000 Dilution factor: 12.0

Sample: SampF11
Well location: F11
Created: Thursday, October 21, 2021 8:58:49 PM

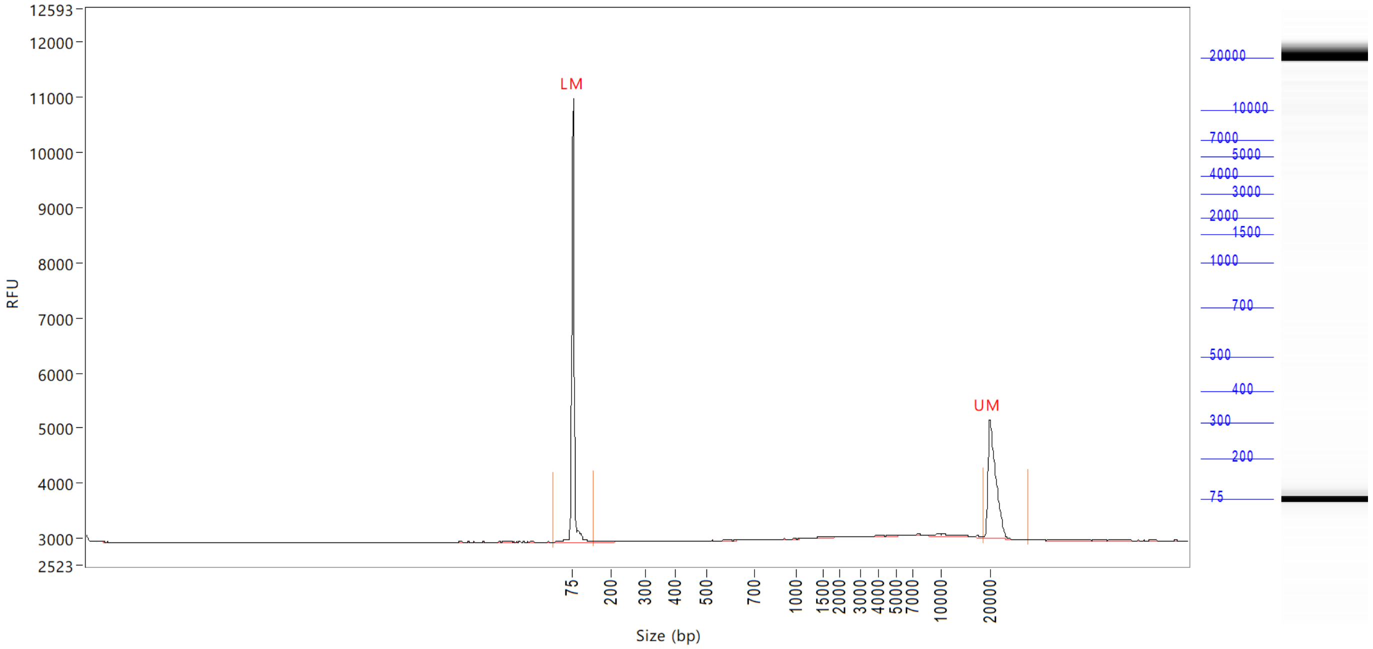

Peak	Size	Concentration	Relative concentration	Molarity	From	To	CV%	RFU
	(bp)	(ng/uL)	Percent	(nmole/L)	(bp)	(bp)		
1	75 (LM)	0.5000		11.0851	12	141	11.46	8043
2	20000 (UM)	0.2354		0.0188	18903	27738	4.36	2137

TIC:	0.0000	ng/uL
TIM:	0.0000	nmole/L
Total concentration:	0.6268	ng/uL

```
Sample peak width (sec): 5    Sample min peak height: 50    Sample baseline V to V?: Y    Sample baseline V to V points: 3
Sample filter: Binomial       Number of points for filter: 3  Sample start region (min): 0  Sample end region (min): 30
Marker peak width (sec): 5    Marker min peak height: 200   Marker baseline V to V?: Y    Marker baseline V to V points: 3
Lower marker selection: First peak > 500 RFU                Upper marker selection: Last peak > 200 RFU
Ladder size (bp)75, 200, 300, 400, 500, 700, 1000, 1500, 2000, 3000, 4000, 5000, 7000, 10000, 20000
Quantification using: Lower Marker          Final concentration (ng/uL): 0.5000          Dilution factor: 12.0
```

Sample: SampG11
Well location: G11
Created: Thursday, October 21, 2021 8:58:49 PM

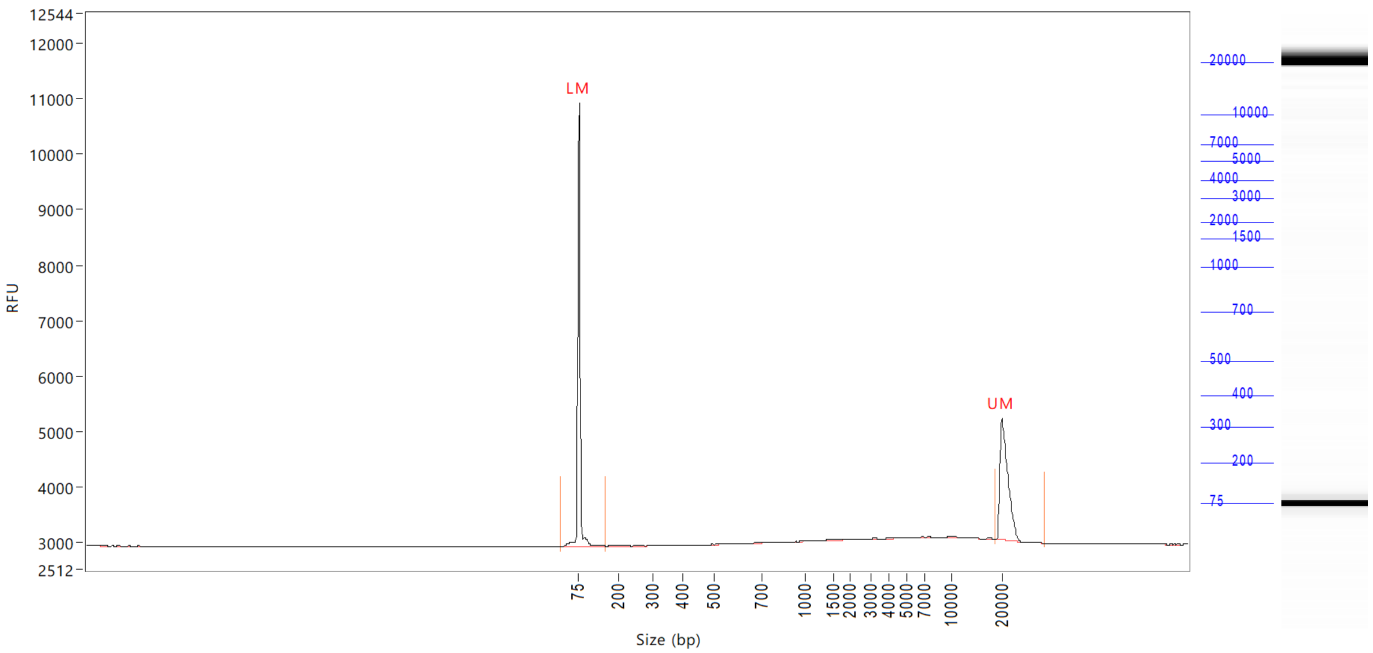

Peak	Size	Concentration	Relative concentration	Molarity	From	To	CV%	RFU
	(bp)	(ng/uL)	Percent	(nmole/L)	(bp)	(bp)		
1	75 (LM)	0.5000		11.2364	20	162	11.81	8017
2	20000 (UM)	0.2385		0.0191	18781	28584	4.19	2188

TIC:	0.0000	ng/uL
TIM:	0.0000	nmole/L
Total concentration:	0.4335	ng/uL

Sample peak width (sec): 5 Sample min peak height: 50 Sample baseline V to V?: Y Sample baseline V to V points: 3
Sample filter: Binomial Number of points for filter: 3 Sample start region (min): 0 Sample end region (min): 30
Marker peak width (sec): 5 Marker min peak height: 200 Marker baseline V to V?: Y Marker baseline V to V points: 3
Lower marker selection: First peak > 500 RFU Upper marker selection: Last peak > 200 RFU
Ladder size (bp)75, 200, 300, 400, 500, 700, 1000, 1500, 2000, 3000, 4000, 5000, 7000, 10000, 20000
Quantification using: Lower Marker Final concentration (ng/uL): 0.5000 Dilution factor: 12.0

Sample: SampH11
Well location: H11
Created: Thursday, October 21, 2021 8:58:49 PM

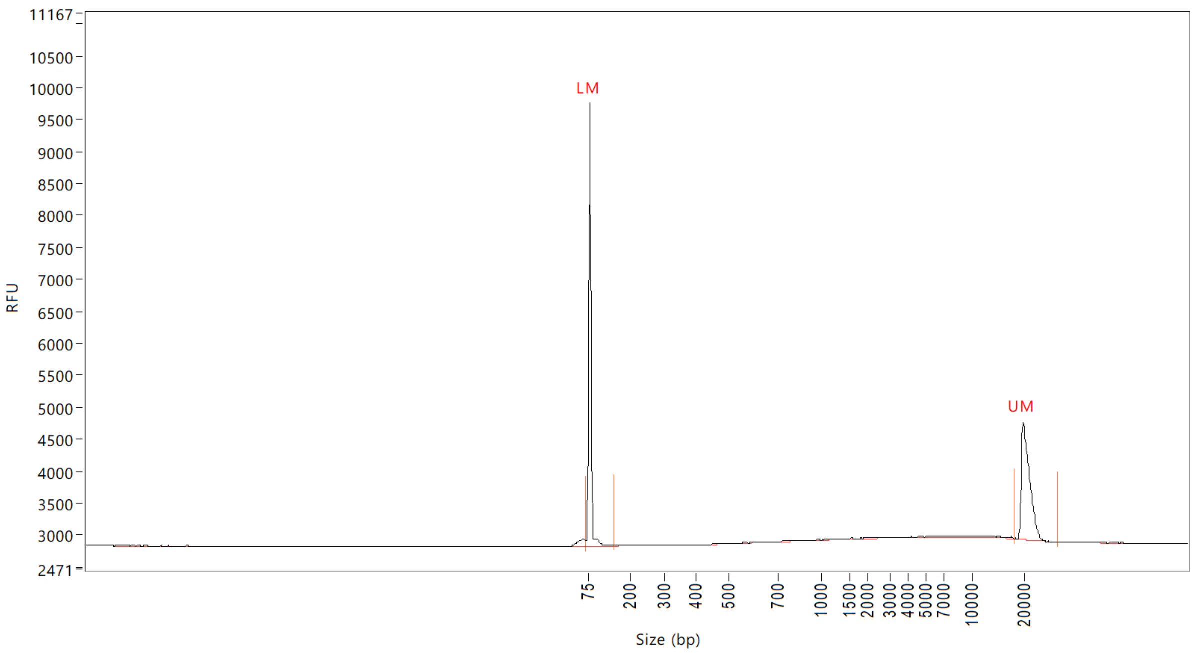

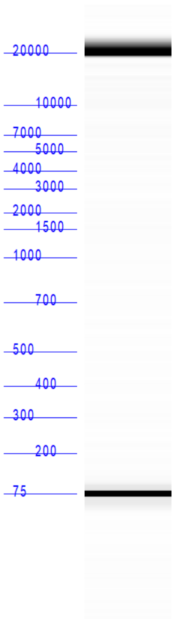

Peak	Size	Concentration	Relative concentration	Molarity	From	To	CV%	RFU
	(bp)	(ng/uL)	Percent	(nmole/L)	(bp)	(bp)		
1	75 (LM)	0.5000		11.0851	64	150	9.54	6947
2	20000 (UM)	0.2446		0.0196	18171	26529	4.31	1829

TIC:	0.0000	ng/uL
TIM:	0.0000	nmole/L
Total concentration:	0.5754	ng/uL

Sample peak width (sec): 5 Sample min peak height: 50 Sample baseline V to V?: Y Sample baseline V to V points: 3
Sample filter: Binomial Number of points for filter: 3 Sample start region (min): 0 Sample end region (min): 30
Marker peak width (sec): 5 Marker min peak height: 200 Marker baseline V to V?: Y Marker baseline V to V points: 3
Lower marker selection: First peak > 500 RFU Upper marker selection: Last peak > 200 RFU
Ladder size (bp)75, 200, 300, 400, 500, 700, 1000, 1500, 2000, 3000, 4000, 5000, 7000, 10000, 20000
Quantification using: Lower Marker Final concentration (ng/uL): 0.5000 Dilution factor: 12.0

Sample: SampA12
Well location: A12
Created: Thursday, October 21, 2021 8:58:49 PM

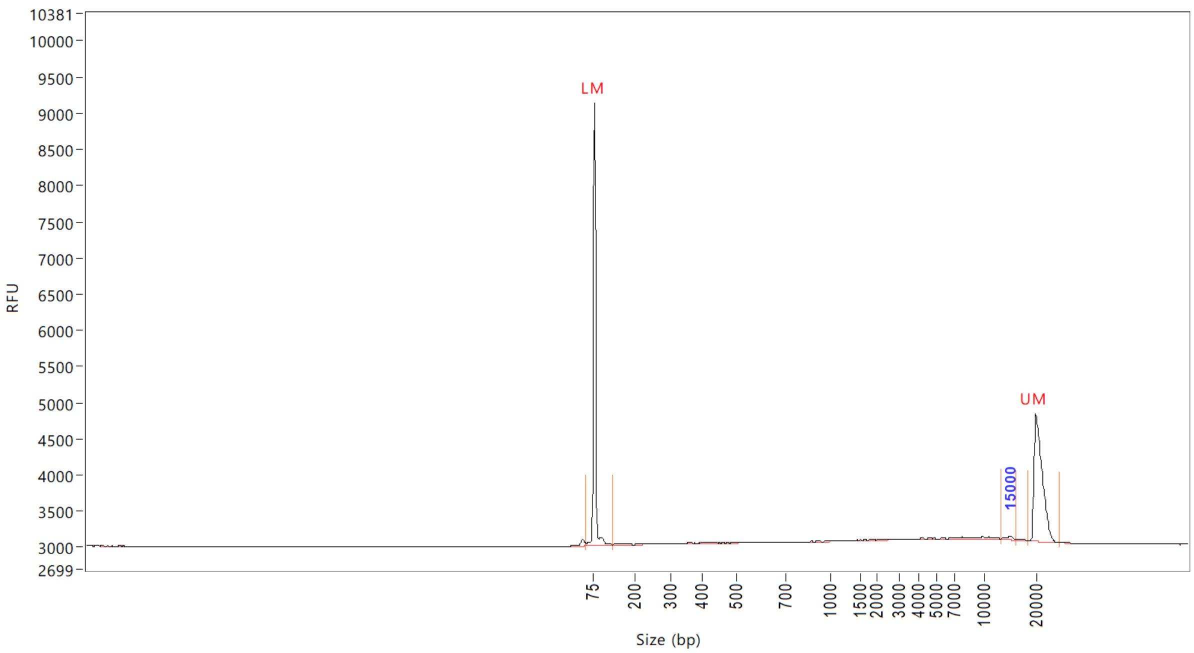

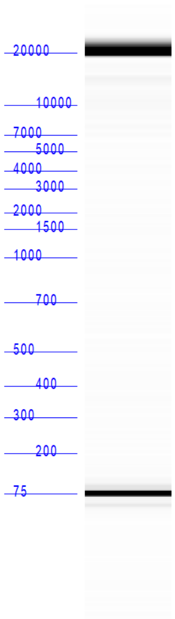

Peak	Size	Concentration	Relative concentration	Molarity	From	To	CV%	RFU
	(bp)	(ng/uL)	Percent	(nmole/L)	(bp)	(bp)		
1	75 (LM)	0.5000		11.0851	48	129	8.83	6129
2	15000	0.0873	100.0	0.0098	13171	16098	4.66	53
3	20000 (UM)	0.2557		0.0205	18415	24473	4.03	1769

TIC:	0.0873	ng/uL
TIM:	0.0098	nmole/L
Total concentration:	0.7952	ng/uL

Sample peak width (sec): 5 Sample min peak height: 50 Sample baseline V to V?: Y Sample baseline V to V points: 3
Sample filter: Binomial Number of points for filter: 3 Sample start region (min): 0 Sample end region (min): 30
Marker peak width (sec): 5 Marker min peak height: 200 Marker baseline V to V?: Y Marker baseline V to V points: 3
Lower marker selection: First peak > 500 RFU Upper marker selection: Last peak > 200 RFU
Ladder size (bp)75, 200, 300, 400, 500, 700, 1000, 1500, 2000, 3000, 4000, 5000, 7000, 10000, 20000
Quantification using: Lower Marker Final concentration (ng/uL): 0.5000 Dilution factor: 12.0

Sample: SampB12
Well location: B12
Created: Thursday, October 21, 2021 8:58:49 PM

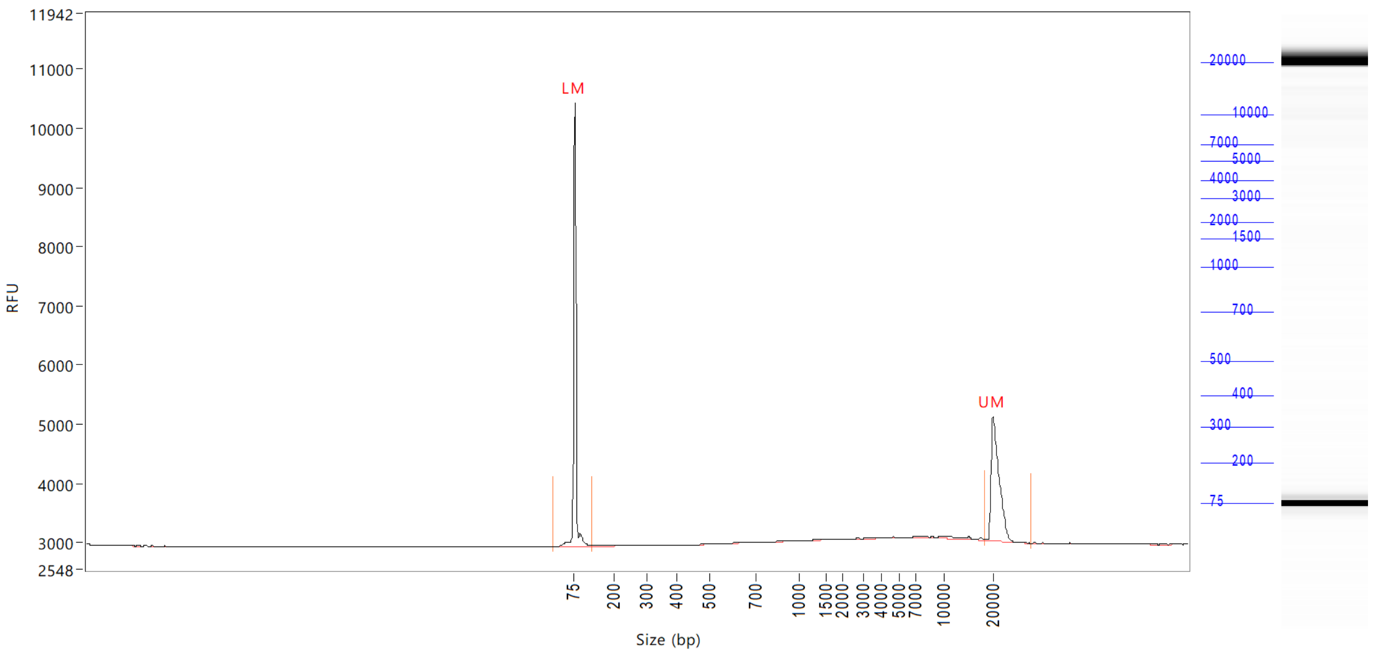

Peak	Size	Concentration	Relative concentration	Molarity	From	To	CV%	RFU
	(bp)	(ng/uL)	Percent	(nmole/L)	(bp)	(bp)		
1	75 (LM)	0.5000		11.2364	7	133	11.52	7507
2	20000 (UM)	0.2488		0.0199	18293	27617	4.51	2090

TIC:	0.0000	ng/uL
TIM:	0.0000	nmole/L
Total concentration:	0.5993	ng/uL

```
Sample peak width (sec): 5    Sample min peak height: 50    Sample baseline V to V?: Y    Sample baseline V to V points: 3
Sample filter: Binomial       Number of points for filter: 3  Sample start region (min): 0  Sample end region (min): 30
Marker peak width (sec): 5    Marker min peak height: 200   Marker baseline V to V?: Y    Marker baseline V to V points: 3
Lower marker selection: First peak > 500 RFU                Upper marker selection: Last peak > 200 RFU
Ladder size (bp)75, 200, 300, 400, 500, 700, 1000, 1500, 2000, 3000, 4000, 5000, 7000, 10000, 20000
Quantification using: Lower Marker          Final concentration (ng/uL): 0.5000          Dilution factor: 12.0
```

Sample: SampC12
Well location: C12
Created: Thursday, October 21, 2021 8:58:49 PM

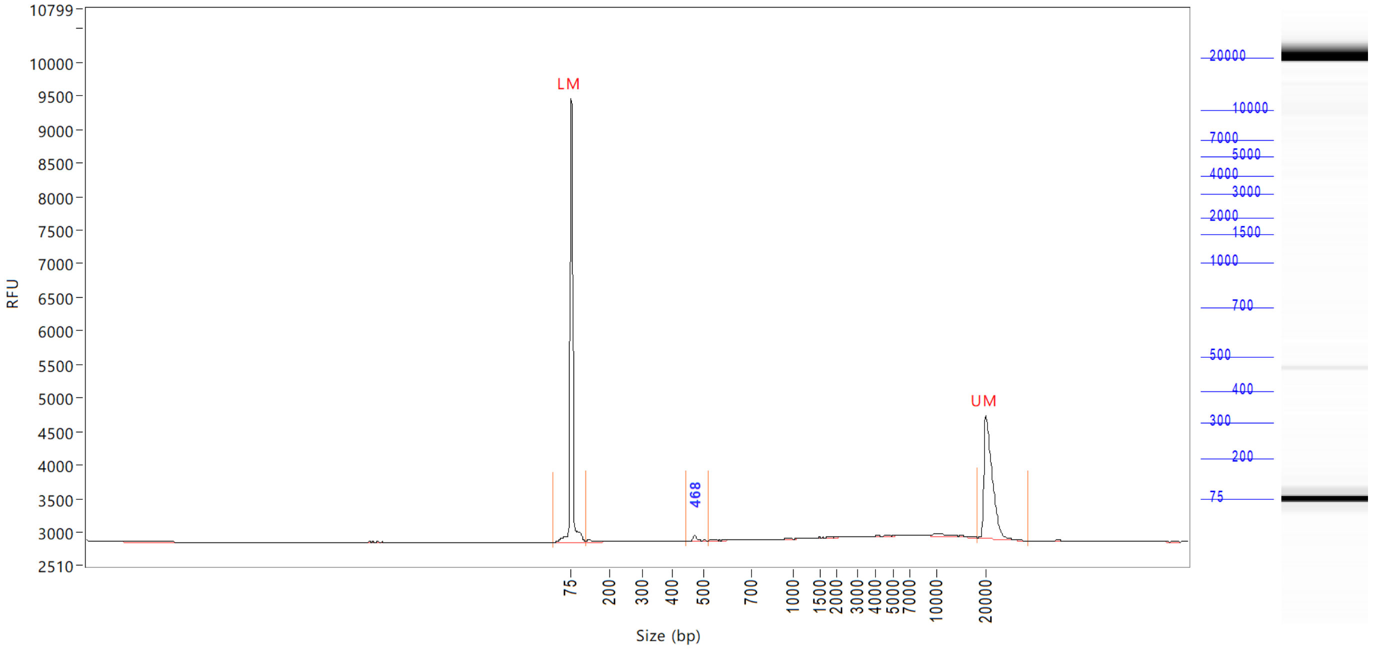

Peak	Size	Concentration	Relative concentration	Molarity	From	To	CV%	RFU
	(bp)	(ng/uL)	Percent	(nmole/L)	(bp)	(bp)		
1	75 (LM)	0.5000		11.0851	20	122	11.66	6623
2	468	0.0971	100.0	0.3405	443	514	2.61	96
3	20000 (UM)	0.2341		0.0186	18415	28463	5.10	1838

TIC:	0.0971	ng/uL
TIM:	0.3405	nmole/L
Total concentration:	0.7293	ng/uL

```
Sample peak width (sec): 5    Sample min peak height: 50    Sample baseline V to V?: Y    Sample baseline V to V points: 3
Sample filter: Binomial       Number of points for filter: 3  Sample start region (min): 0  Sample end region (min): 30
Marker peak width (sec): 5    Marker min peak height: 200   Marker baseline V to V?: Y    Marker baseline V to V points: 3
Lower marker selection: First peak > 500 RFU                Upper marker selection: Last peak > 200 RFU
Ladder size (bp)75, 200, 300, 400, 500, 700, 1000, 1500, 2000, 3000, 4000, 5000, 7000, 10000, 20000
Quantification using: Lower Marker          Final concentration (ng/uL): 0.5000          Dilution factor: 12.0
```

Sample: SampD12
Well location: D12
Created: Thursday, October 21, 2021 8:58:49 PM

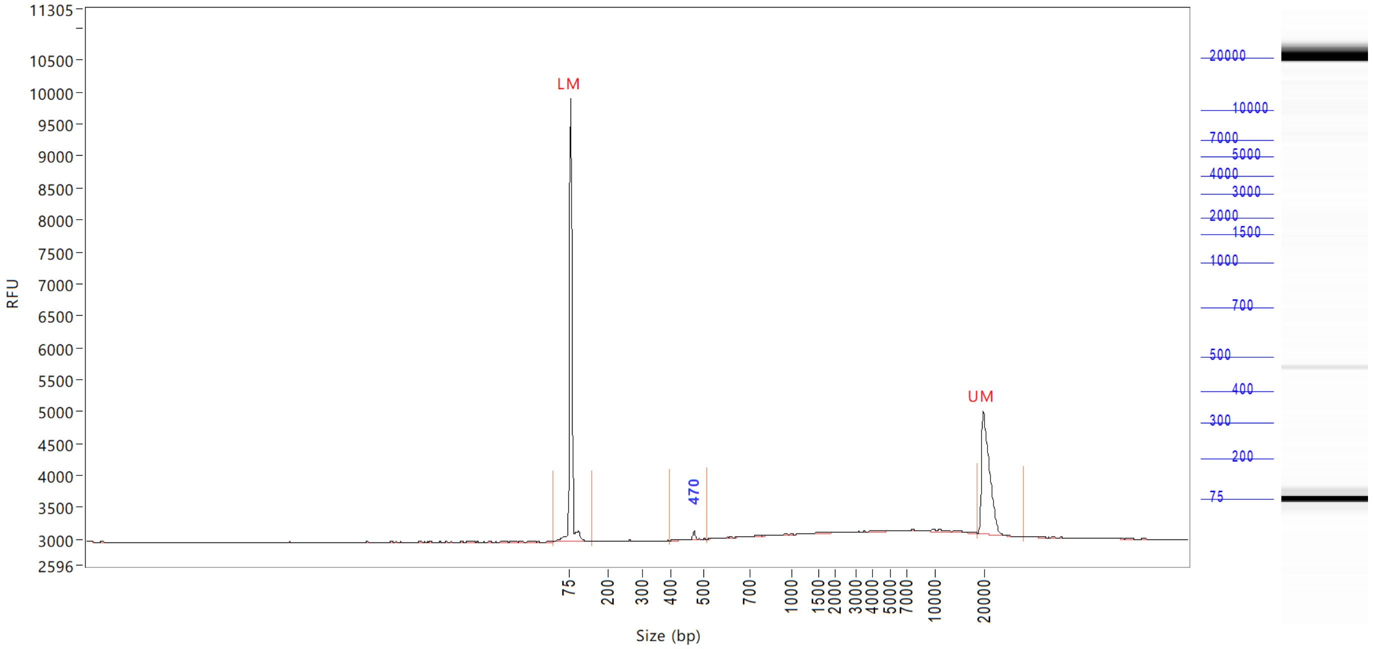

Peak	Size	Concentration	Relative concentration	Molarity	From	To	CV%	RFU
	(bp)	(ng/uL)	Percent	(nmole/L)	(bp)	(bp)		
1	75 (LM)	0.5000		11.0851	20	145	12.09	6950
2	470	0.1230	100.0	0.4344	392	514	3.70	138
3	20000 (UM)	0.2381		0.0190	18903	28100	4.54	1927

TIC:	0.1230	ng/uL
TIM:	0.4344	nmole/L
Total concentration:	0.7056	ng/uL

```
Sample peak width (sec): 5    Sample min peak height: 50    Sample baseline V to V?: Y    Sample baseline V to V points: 3
Sample filter: Binomial       Number of points for filter: 3  Sample start region (min): 0  Sample end region (min): 30
Marker peak width (sec): 5    Marker min peak height: 200   Marker baseline V to V?: Y    Marker baseline V to V points: 3
Lower marker selection: First peak > 500 RFU                Upper marker selection: Last peak > 200 RFU
Ladder size (bp)75, 200, 300, 400, 500, 700, 1000, 1500, 2000, 3000, 4000, 5000, 7000, 10000, 20000
Quantification using: Lower Marker          Final concentration (ng/uL): 0.5000          Dilution factor: 12.0
```

Sample: SampE12
Well location: E12
Created: Thursday, October 21, 2021 8:58:49 PM

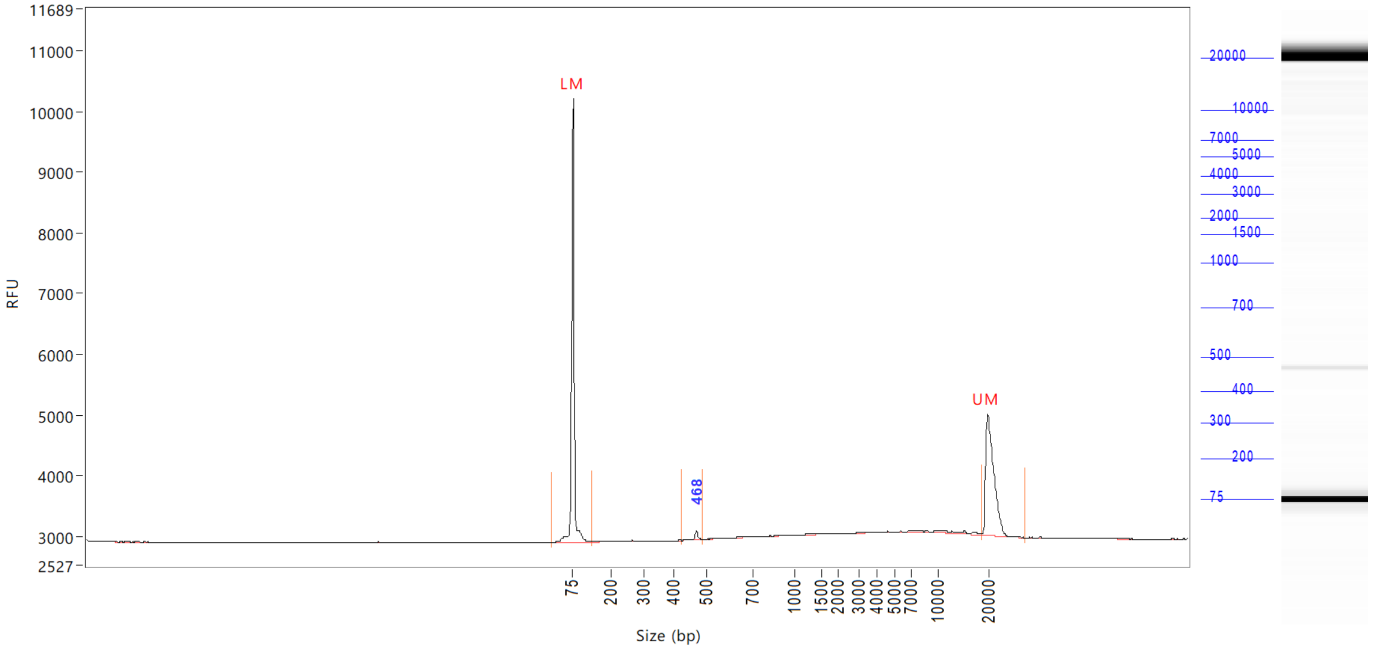

Peak	Size	Concentration	Relative concentration	Molarity	From	To	CV%	RFU
	(bp)	(ng/uL)	Percent	(nmole/L)	(bp)	(bp)		
1	75 (LM)	0.5000		11.2364	7	135	13.04	7320
2	468	0.1162	100.0	0.4112	424	490	2.04	141
3	20000 (UM)	0.2348		0.0188	18903	27496	4.47	1996

TIC:	0.1162	ng/uL
TIM:	0.4112	nmole/L
Total concentration:	0.7751	ng/uL

```
Sample peak width (sec): 5    Sample min peak height: 50    Sample baseline V to V?: Y    Sample baseline V to V points: 3
Sample filter: Binomial       Number of points for filter: 3  Sample start region (min): 0  Sample end region (min): 30
Marker peak width (sec): 5    Marker min peak height: 200   Marker baseline V to V?: Y    Marker baseline V to V points: 3
Lower marker selection: First peak > 500 RFU                Upper marker selection: Last peak > 200 RFU
Ladder size (bp)75, 200, 300, 400, 500, 700, 1000, 1500, 2000, 3000, 4000, 5000, 7000, 10000, 20000
Quantification using: Lower Marker          Final concentration (ng/uL): 0.5000          Dilution factor: 12.0
```

Sample: SampF12
Well location: F12
Created: Thursday, October 21, 2021 8:58:49 PM

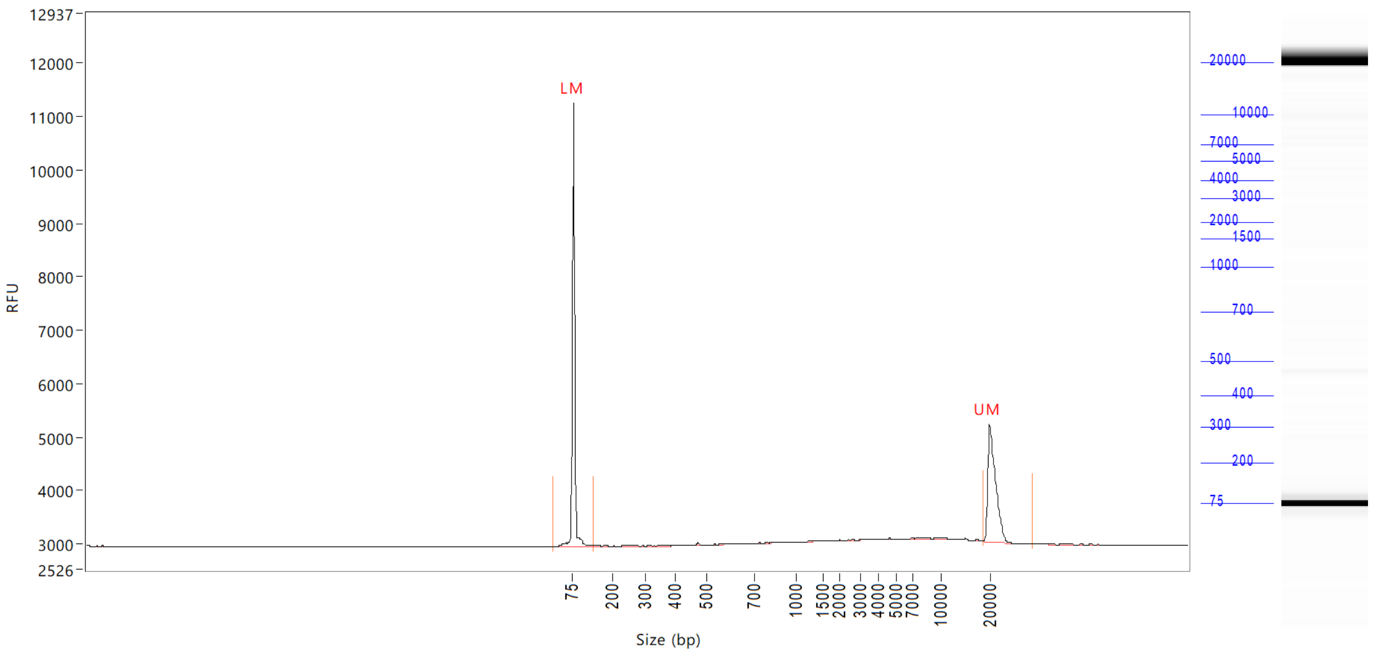

Peak	Size	Concentration	Relative concentration	Molarity	From	To	CV%	RFU
	(bp)	(ng/uL)	Percent	(nmole/L)	(bp)	(bp)		
1	75 (LM)	0.5000		11.2364	11	139	11.49	8323
2	20000 (UM)	0.2299		0.0184	18781	28705	4.46	2201

TIC:	0.0000	ng/uL
TIM:	0.0000	nmole/L
Total concentration:	0.4821	ng/uL

Sample peak width (sec): 5   Sample min peak height: 50   Sample baseline V to V?: Y   Sample baseline V to V points: 3
Sample filter: Binomial   Number of points for filter: 3   Sample start region (min): 0   Sample end region (min): 30
Marker peak width (sec): 5   Marker min peak height: 200   Marker baseline V to V?: Y   Marker baseline V to V points: 3
Lower marker selection: First peak > 500 RFU   Upper marker selection: Last peak > 200 RFU
Ladder size (bp)75, 200, 300, 400, 500, 700, 1000, 1500, 2000, 3000, 4000, 5000, 7000, 10000, 20000
Quantification using: Lower Marker   Final concentration (ng/uL): 0.5000   Dilution factor: 12.0

Sample: SampG12
Well location: G12
Created: Thursday, October 21, 2021 8:58:49 PM

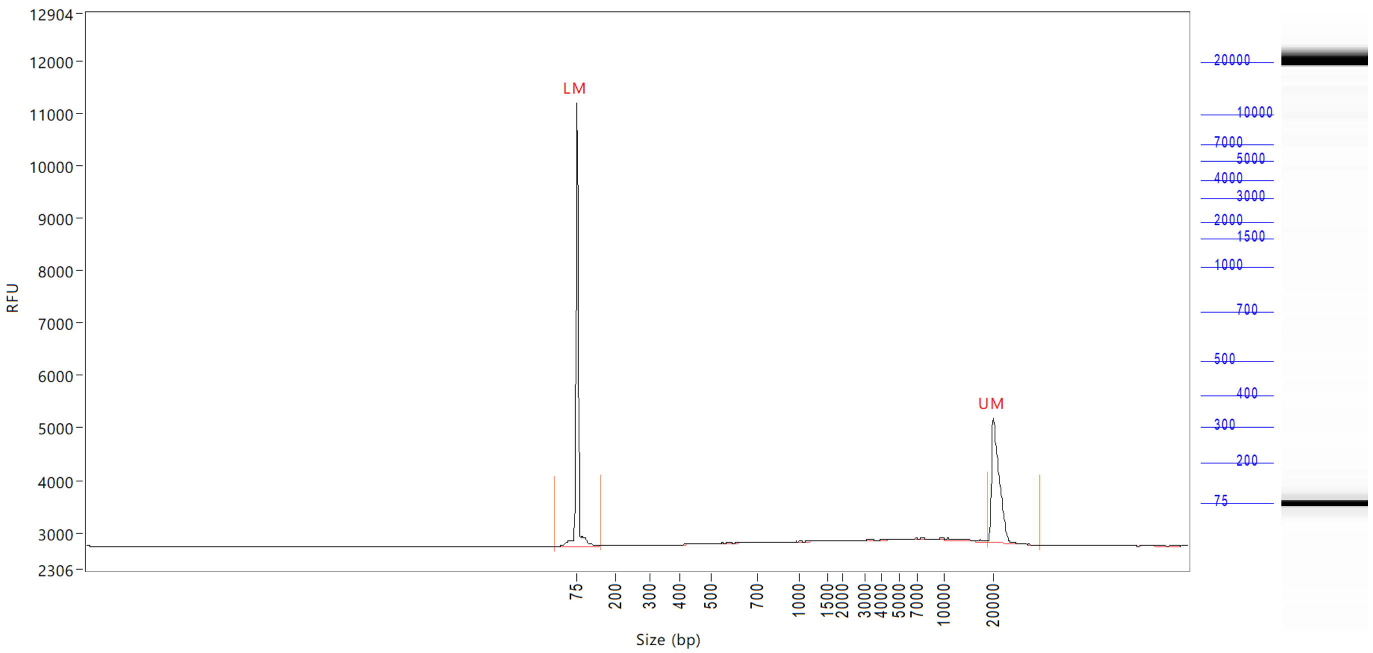

Peak	Size	Concentration	Relative concentration	Molarity	From	To	CV%	RFU
	(bp)	(ng/uL)	Percent	(nmole/L)	(bp)	(bp)		
1	75 (LM)	0.5000		11.2364	3	152	14.61	8466
2	20000 (UM)	0.2320		0.0185	18781	29430	4.47	2360

TIC:	0.0000	ng/uL
TIM:	0.0000	nmole/L
Total concentration:	0.5179	ng/uL

Sample peak width (sec): 5 Sample min peak height: 50 Sample baseline V to V?: Y Sample baseline V to V points: 3
Sample filter: Binomial Number of points for filter: 3 Sample start region (min): 0 Sample end region (min): 30
Marker peak width (sec): 5 Marker min peak height: 200 Marker baseline V to V?: Y Marker baseline V to V points: 3
Lower marker selection: First peak > 500 RFU Upper marker selection: Last peak > 200 RFU
Ladder size (bp)75, 200, 300, 400, 500, 700, 1000, 1500, 2000, 3000, 4000, 5000, 7000, 10000, 20000
Quantification using: Lower Marker Final concentration (ng/uL): 0.5000 Dilution factor: 12.0

Sample: SampH12
Well location: H12
Created: Thursday, October 21, 2021 8:58:49 PM

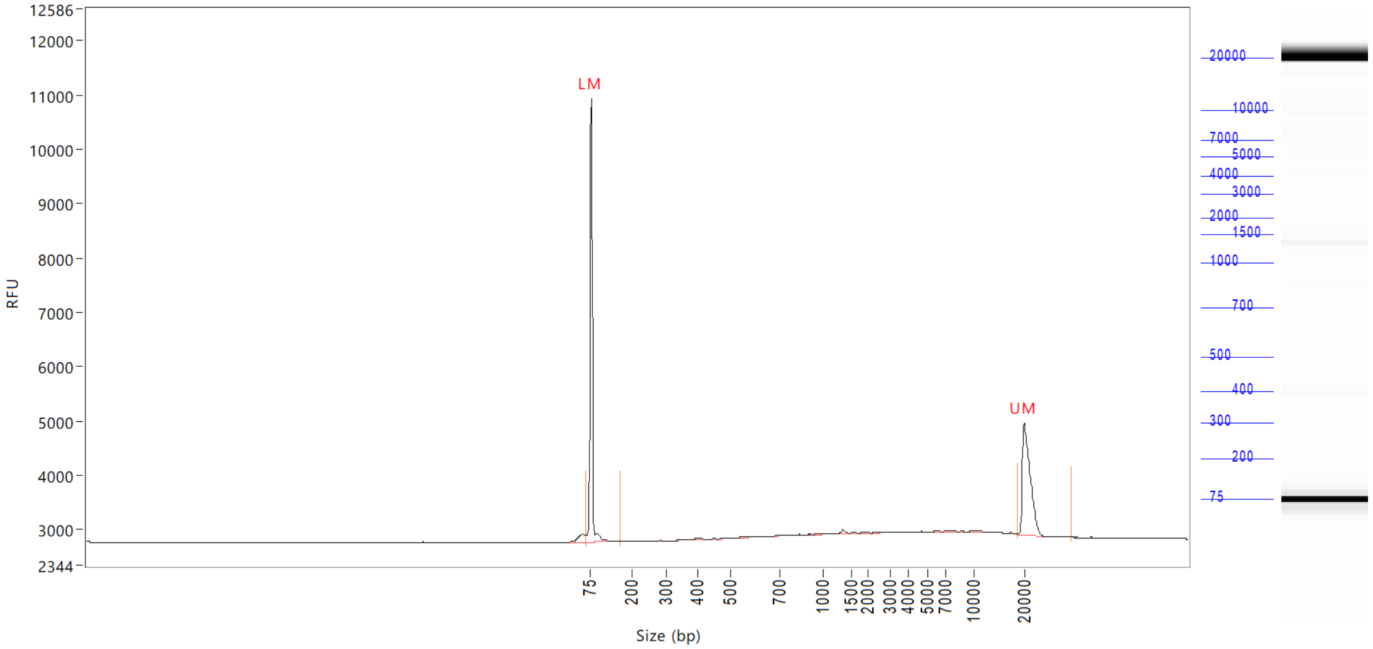

Peak	Size	Concentration	Relative concentration	Molarity	From	To	CV%	RFU
	(bp)	(ng/uL)	Percent	(nmole/L)	(bp)	(bp)		
1	75 (LM)	0.5000		11.0851	62	162	10.06	8178
2	20000 (UM)	0.2194		0.0176	18659	29068	4.29	2072

TIC:	0.0000	ng/uL
TIM:	0.0000	nmole/L
Total concentration:	0.5882	ng/uL

```
Sample peak width (sec): 5    Sample min peak height: 200   Sample baseline V to V?: Y    Sample baseline V to V points: 3
Sample filter: Binomial       Number of points for filter: 3  Sample start region (min): 0  Sample end region (min): 30
Marker peak width (sec): 5    Marker min peak height: 200   Marker baseline V to V?: Y    Marker baseline V to V points: 3
Lower marker selection: First peak > 500 RFU                Upper marker selection: Last peak > 200 RFU
Ladder size (bp)75, 200, 300, 400, 500, 700, 1000, 1500, 2000, 3000, 4000, 5000, 7000, 10000, 20000
Quantification using: Lower Marker          Final concentration (ng/uL): 0.5000          Dilution factor: 12.0
```

Sample: DNA size ladder
Well location: A7
Created: Thursday, October 21, 2021 8:58:49 PM
Import from: C:\Users\admin\Desktop\210810_alberto_ladder.SCAL
Fit type: Point to point

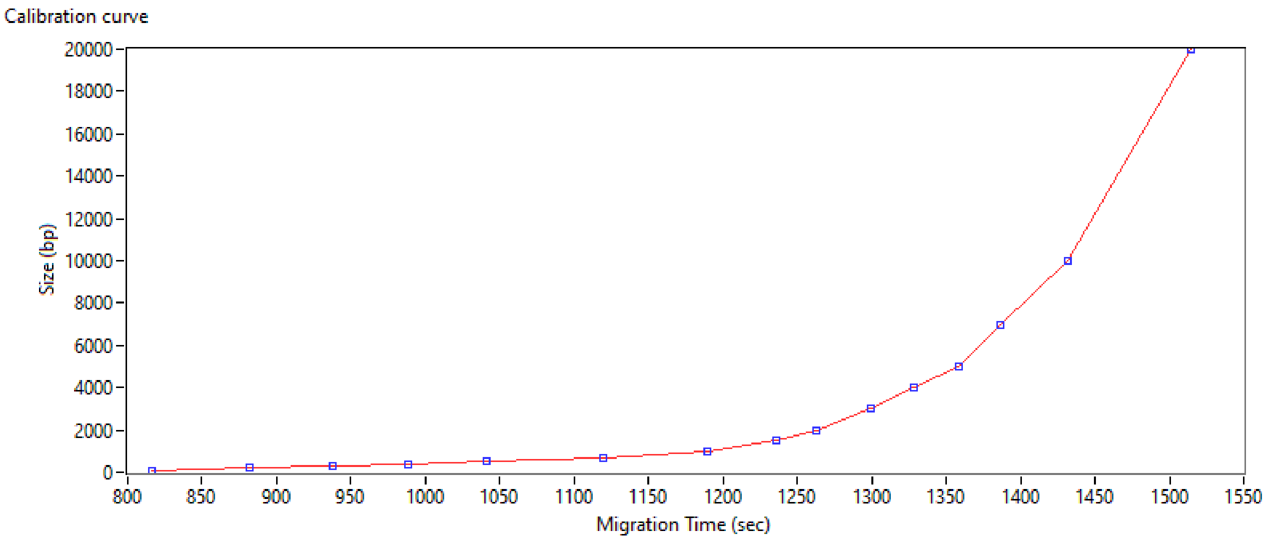

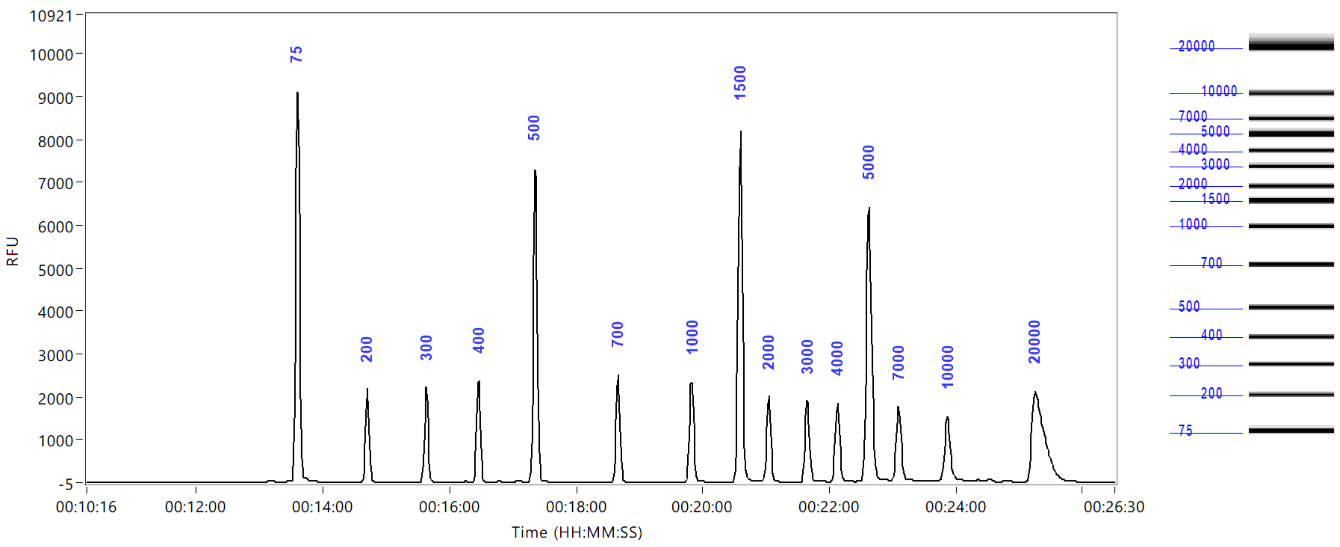

Supplement: Supplementary file 2 — sb3c00292_si_002.zip [file sb3c00292_si_002.zip › dnada_supplementary_material_pks_library_build/colony_pcr/ZAG/20-38-22_plate10/colonypcrplate_10.pdf]
