# Supplementary material for: Automated Platform for the Plasmid Construction Process
Source: ACS Synth Biol. 2023 Nov 10;12(12):3506–13. doi: 10.1021/acssynbio.3c00292 (PMC10729297; doi:10.1021/acssynbio.3c00292)

Instrument controller software run summary:

Filename and data path: C:\AATI\Data\2021 10 21\19-58-21\2021 10 21 19H 58M..raw
Created: Thursday, October 21, 2021 8:08:12 PM
Number of capillaries: 96
Array serial number: 111920-09SFS
Effect length: 33 cm
Array usage count: 269
Instrument type: ZAG DNA Analyzer
Instrument controller software version: 1.0.1.9
Device serial number: Z7597

Method Information

Method name: ZAG130GP33 - DNA 75-20000bp - Gel Prime Only.mthds
Gel prime: Yes
Full conditioning: No
Gel prime to buffer: No
Gel selection: Gel 1
Perform prerun: 8.0 kV, 30 sec.
Rinse: No
Marker 1: Row: A, 5.0 kV, 5 sec.
Rinse: No
Sample injection: 5.0 kV, 5 sec.
Separation: 8.0 kV, 30.0 min.
Tray name: Sample Tray-5

Analysis mode: DNA

Notes

Gel Image

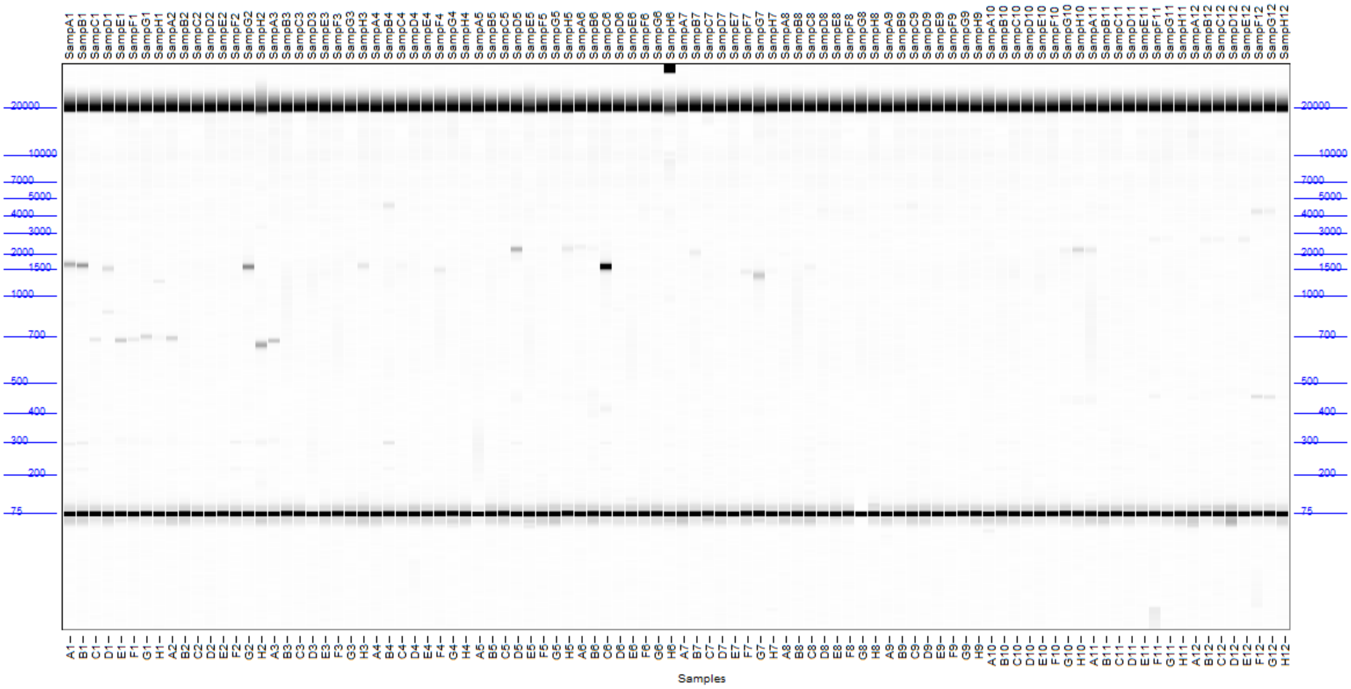
Samples
20000
10000
7000
5000
4000
3000
2000
1500
1000
700
500
400
300
200
75

Filename and data path: C:\AATI\Data\2021 10 21\19-58-21\2021 10 21 19H 58M..raw

A1: SampA1

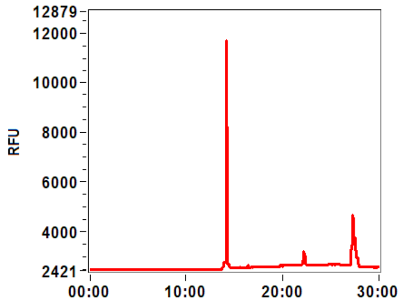

B1: SampB1

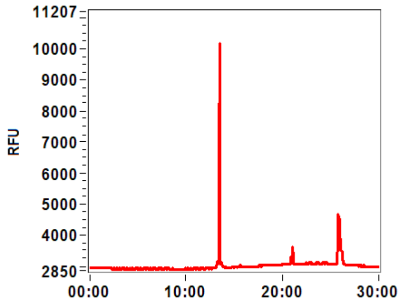

C1: SampC1

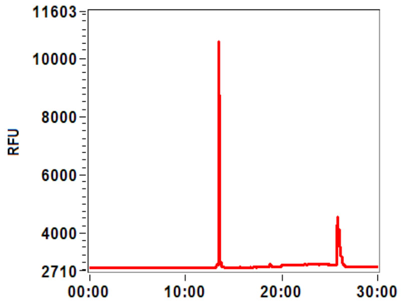

D1: SampD1

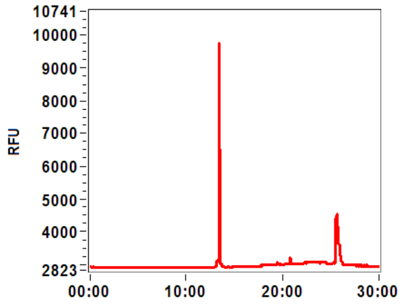

E1: SampE1

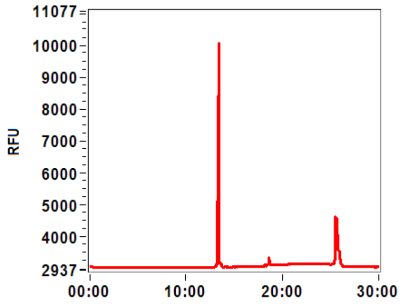

F1: SampF1

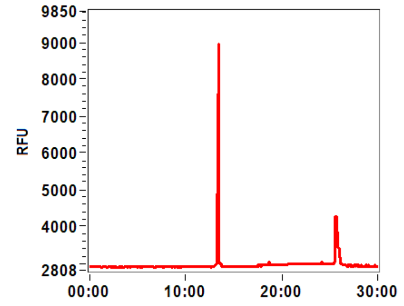

G1: SampG1

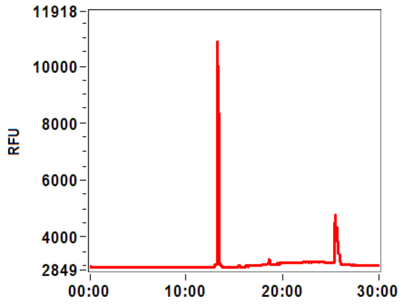

H1: SampH1

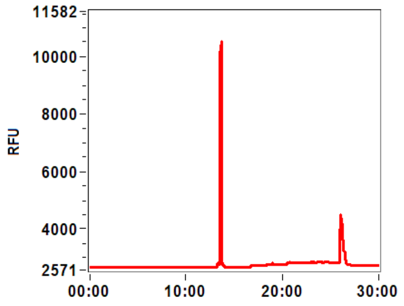

A2: SampA2

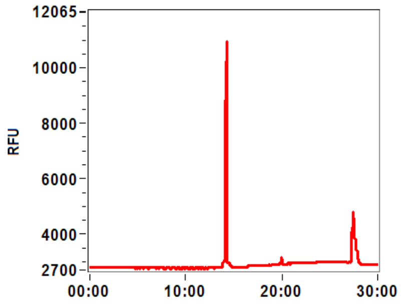

B2: SampB2

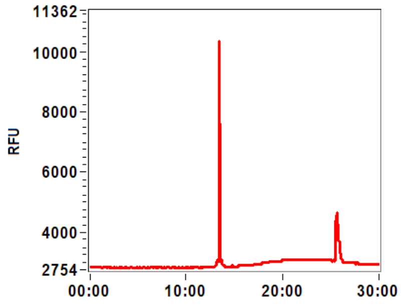

C2: SampC2

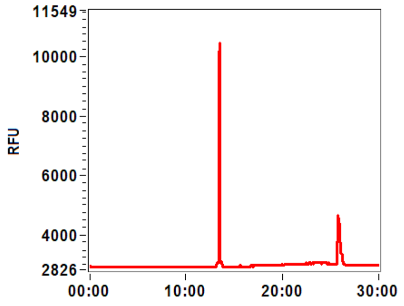

D2: SampD2

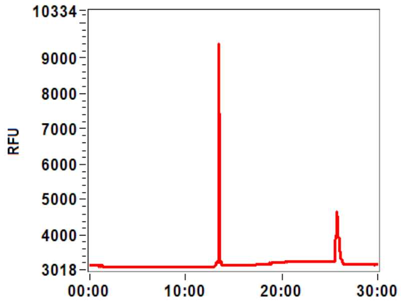

Filename and data path: C:\AATI\Data\2021 10 21\19-58-21\2021 10 21 19H 58M..raw

E2: SampE2

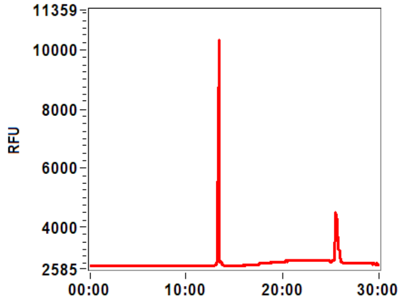

F2: SampF2

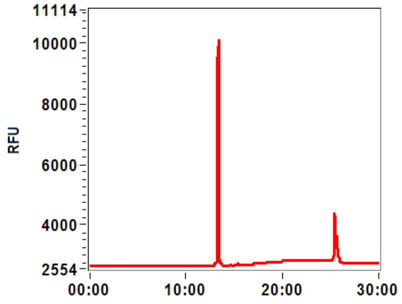

G2: SampG2

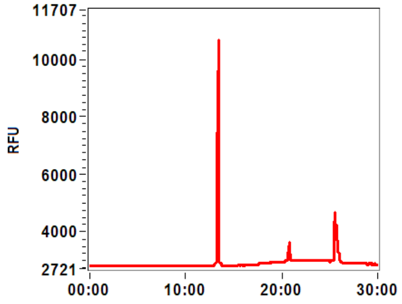

H2: SampH2

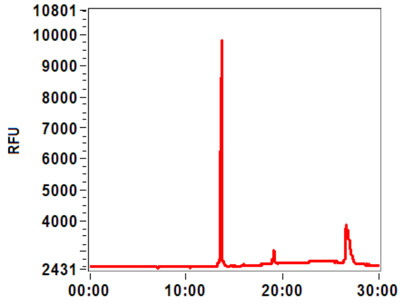

A3: SampA3

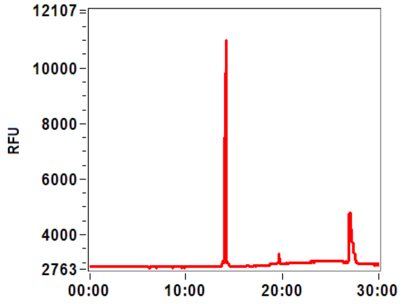

B3: SampB3

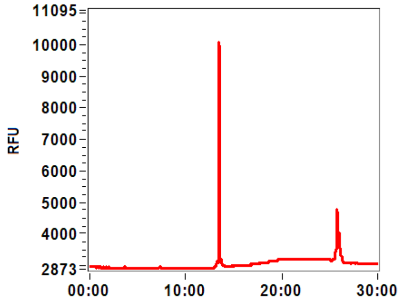

C3: SampC3

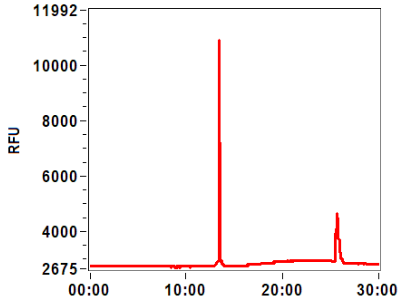

D3: SampD3

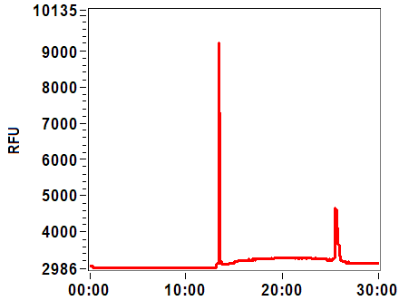

E3: SampE3

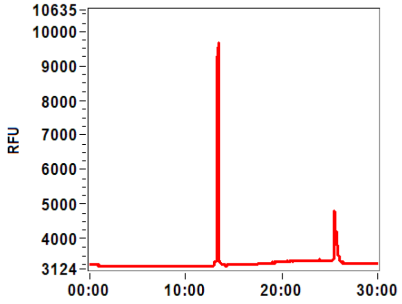

F3: SampF3

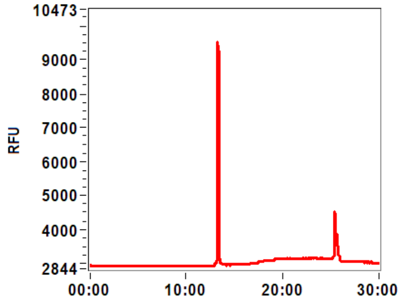

G3: SampG3

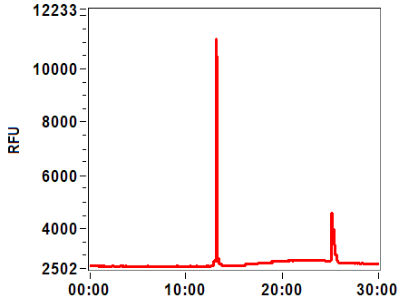

H3: SampH3

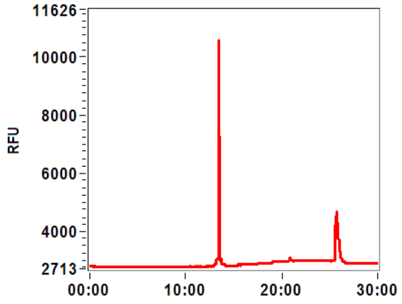

Filename and data path: C:\AATI\Data\2021 10 21\19-58-21\2021 10 21 19H 58M..raw

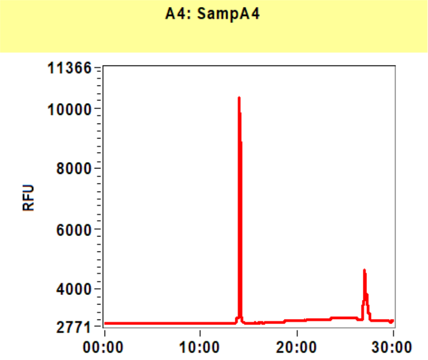

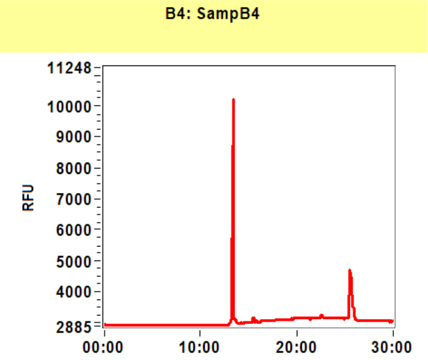

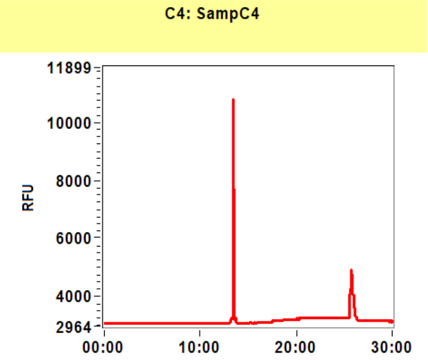

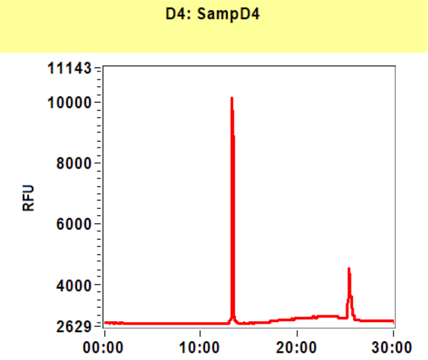

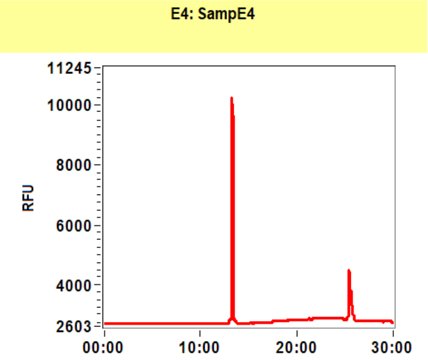

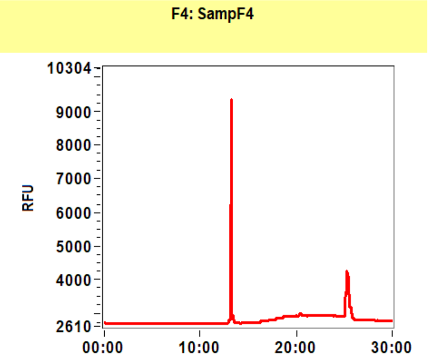

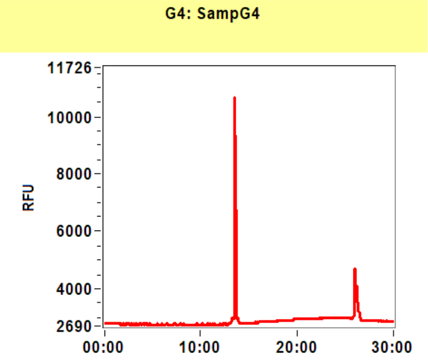

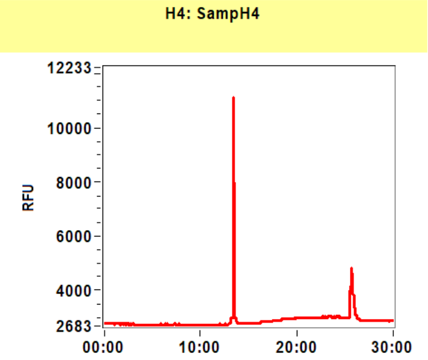

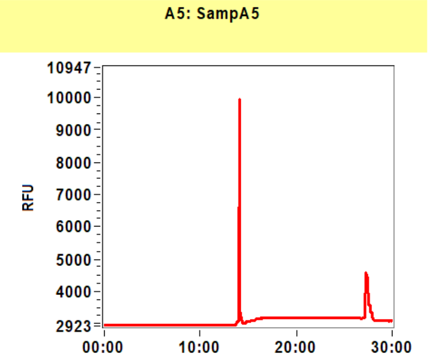

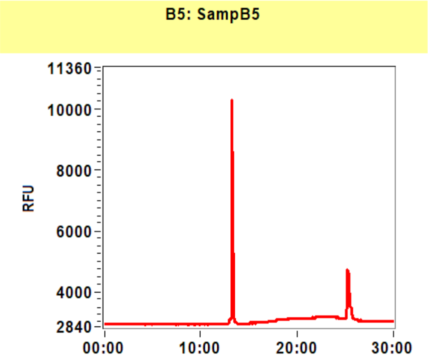

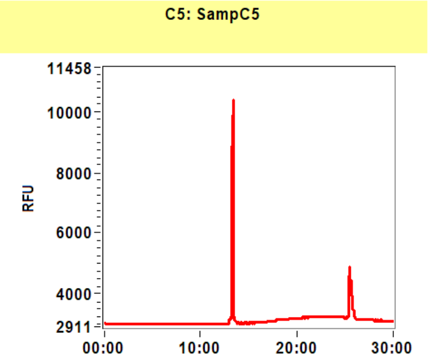

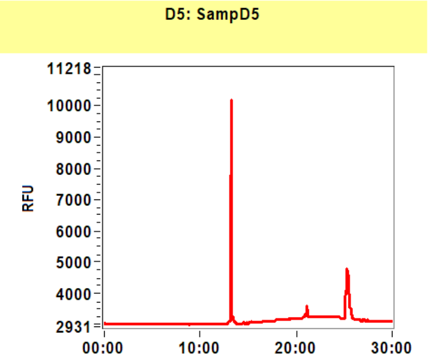

Filename and data path: C:\AATI\Data\2021 10 21\19-58-21\2021 10 21 19H 58M..raw

E5: SampE5

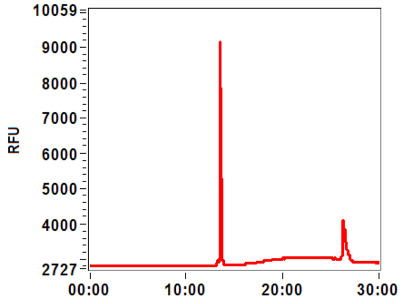

F5: SampF5

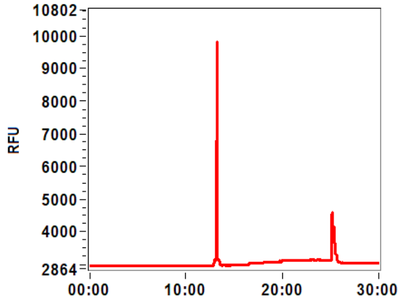

G5: SampG5

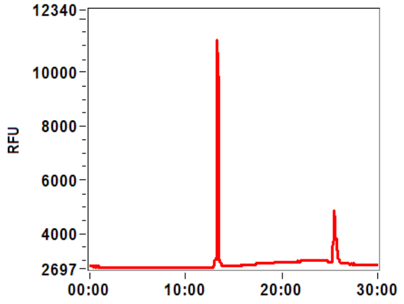

H5: SampH5

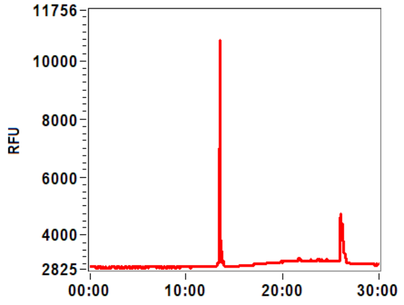

A6: SampA6

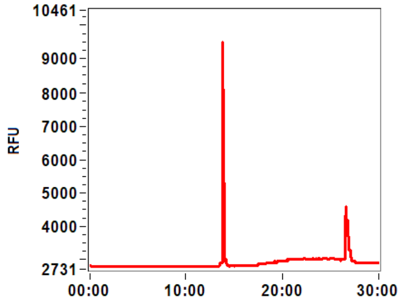

B6: SampB6

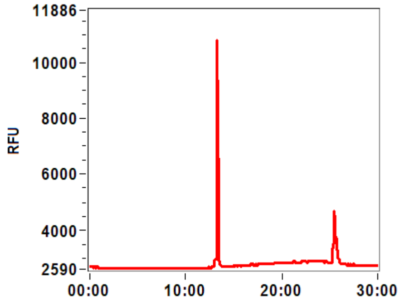

C6: SampC6

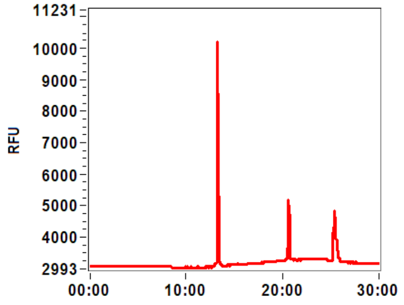

D6: SampD6

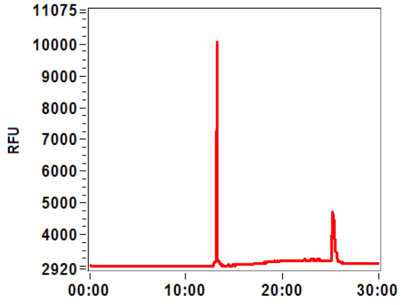

E6: SampE6

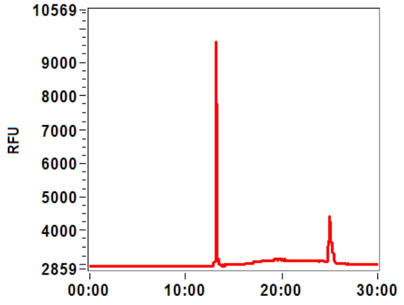

F6: SampF6

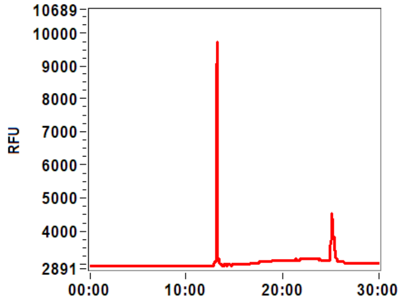

G6: SampG6

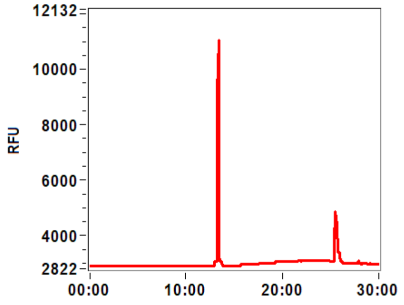

H6: SampH6

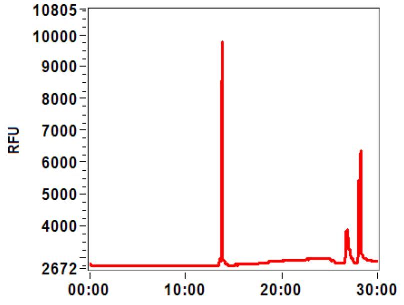

Filename and data path: C:\AATI\Data\2021 10 21\19-58-21\2021 10 21 19H 58M..raw

A7: SampA7

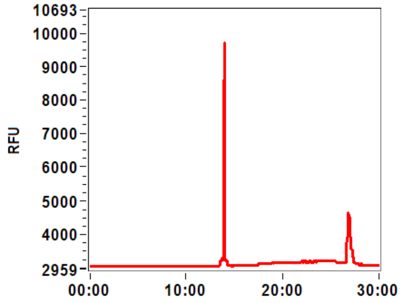

B7: SampB7

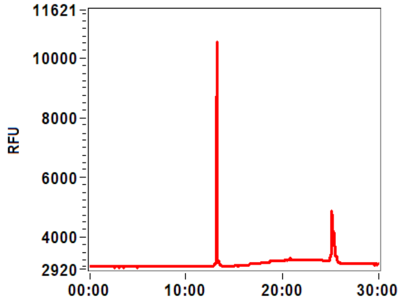

C7: SampC7

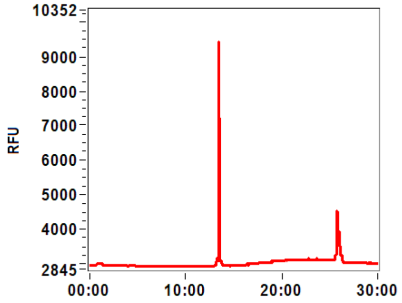

D7: SampD7

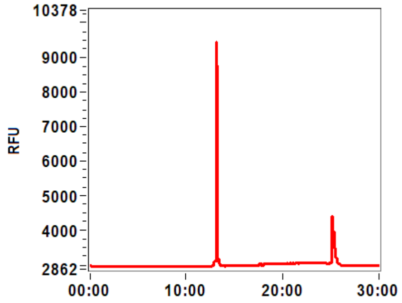

E7: SampE7

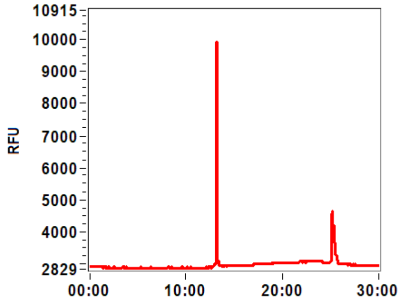

F7: SampF7

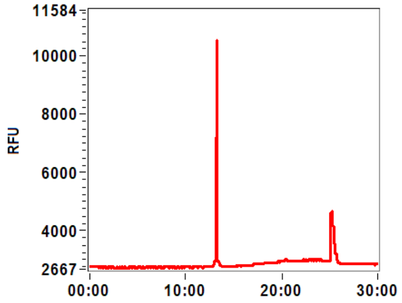

G7: SampG7

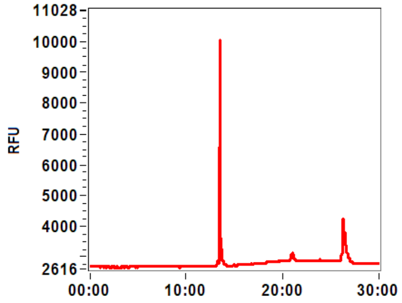

H7: SampH7

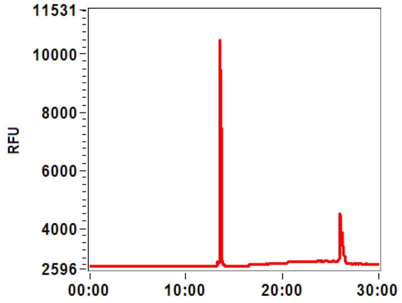

A8: SampA8

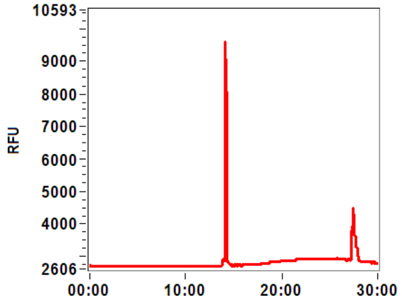

B8: SampB8

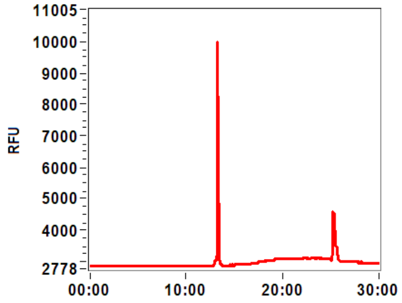

C8: SampC8

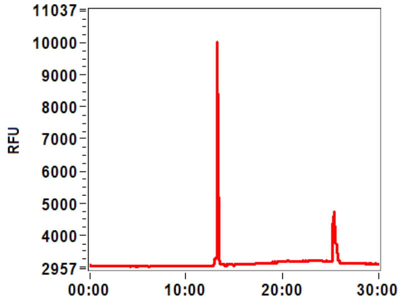

D8: SampD8

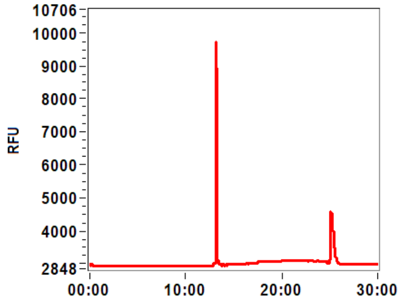

Filename and data path: C:\AATI\Data\2021 10 21\19-58-21\2021 10 21 19H 58M..raw

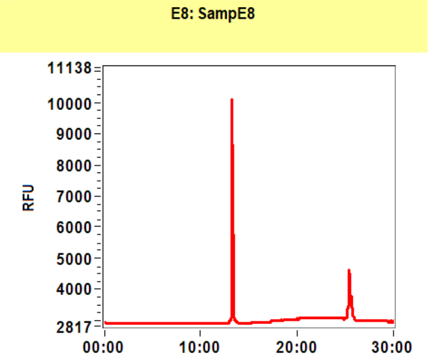

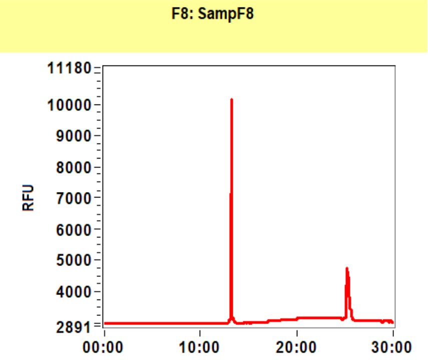

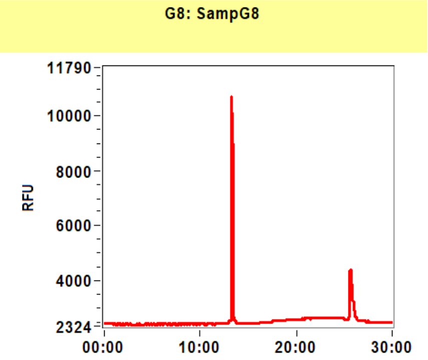

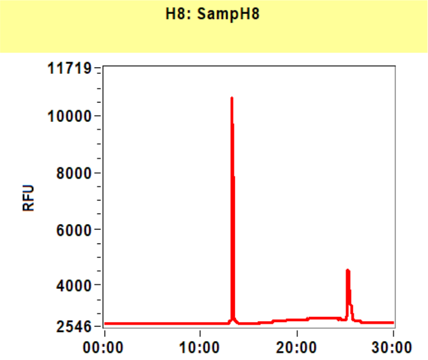

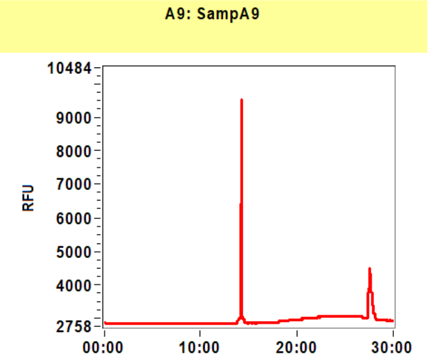

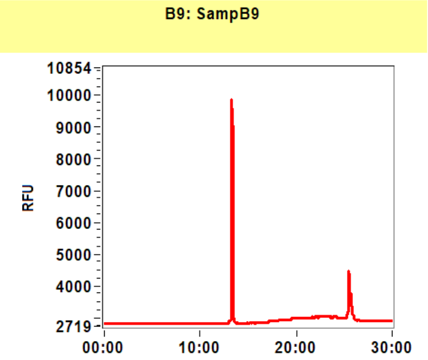

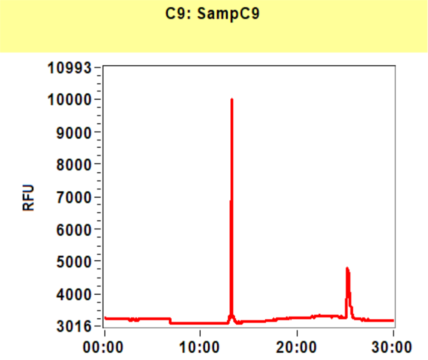

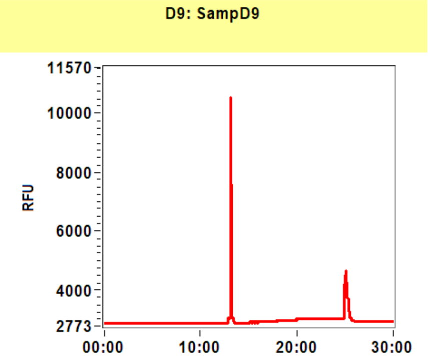

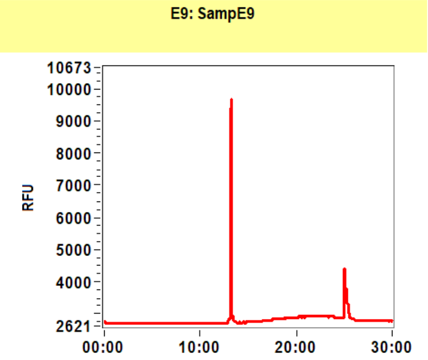

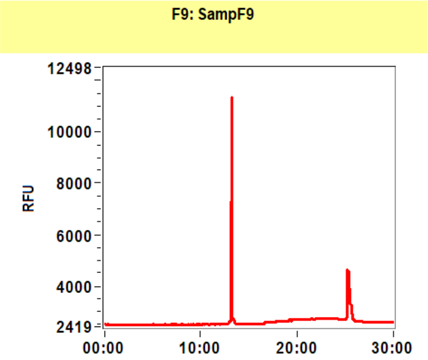

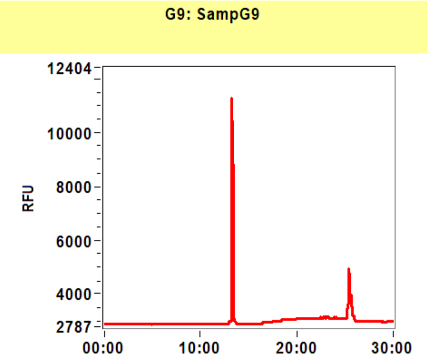

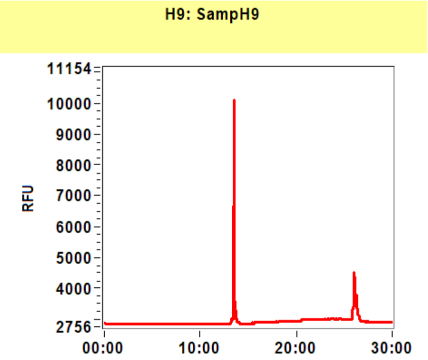

Filename and data path: C:\AATI\Data\2021 10 21\19-58-21\2021 10 21 19H 58M..raw

A10: SampA10

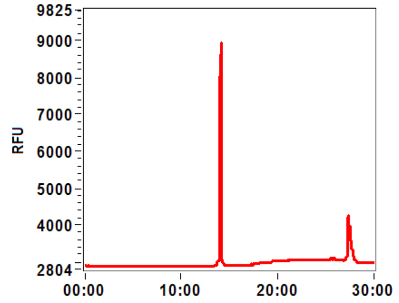

B10: SampB10

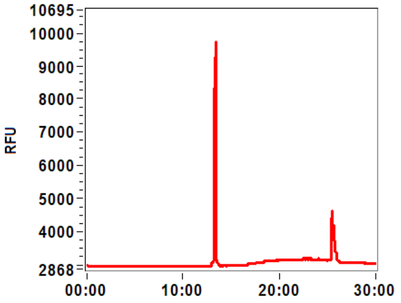

C10: SampC10

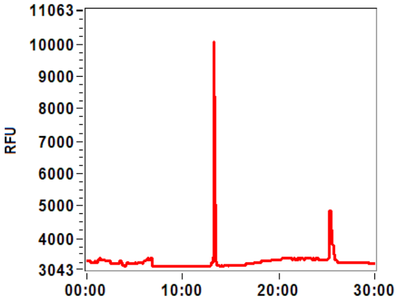

D10: SampD10

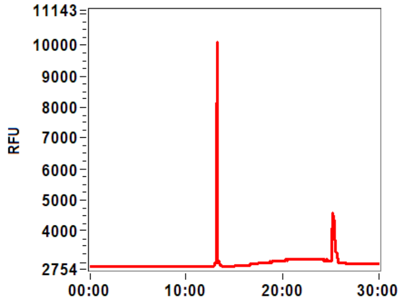

E10: SampE10

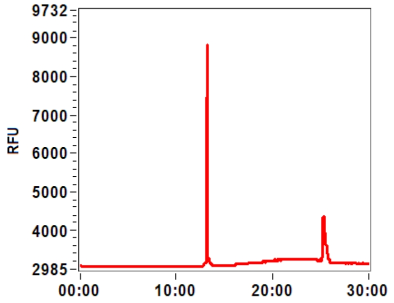

F10: SampF10

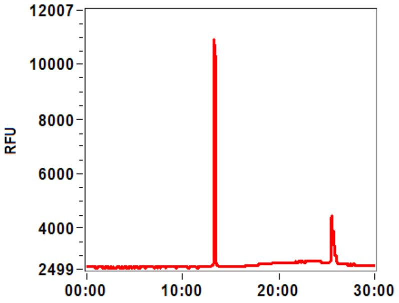

G10: SampG10

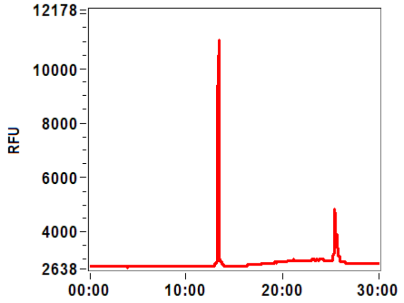

H10: SampH10

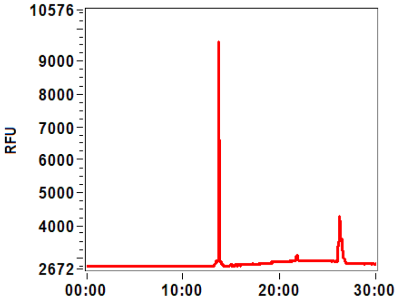

A11: SampA11

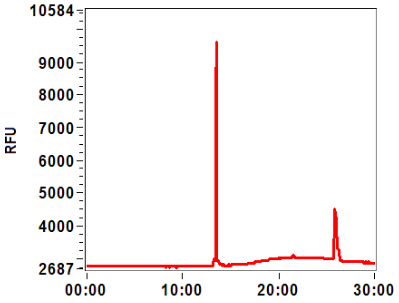

B11: SampB11

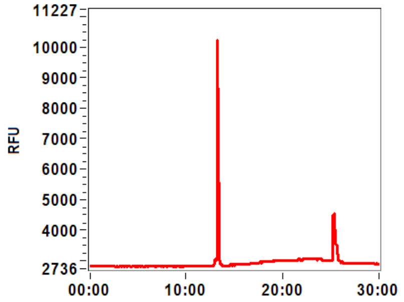

C11: SampC11

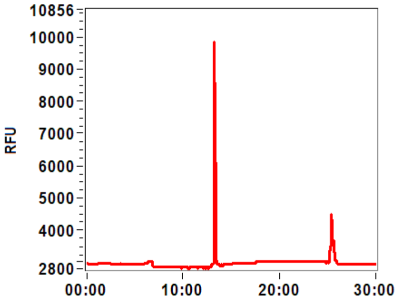

D11: SampD11

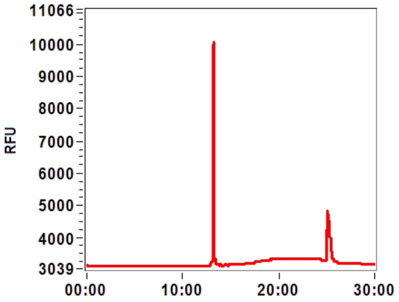

Filename and data path: C:\AATI\Data\2021 10 21\19-58-21\2021 10 21 19H 58M..raw

E11: SampE11

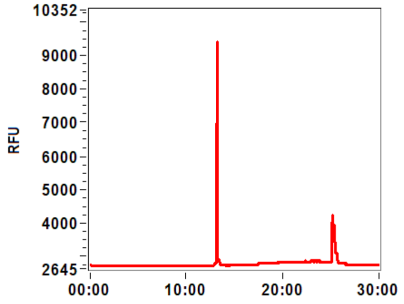

F11: SampF11

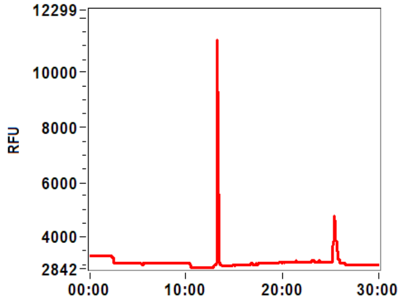

G11: SampG11

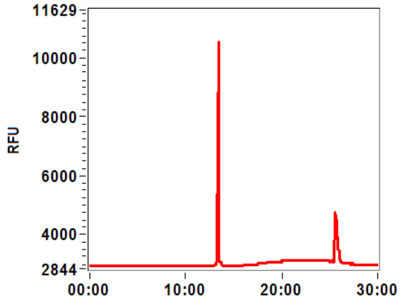

H11: SampH11

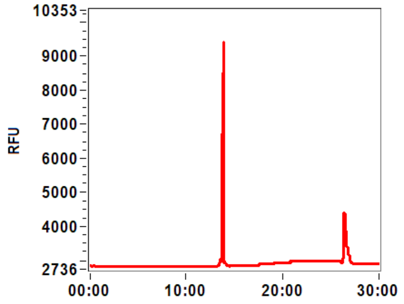

A12: SampA12

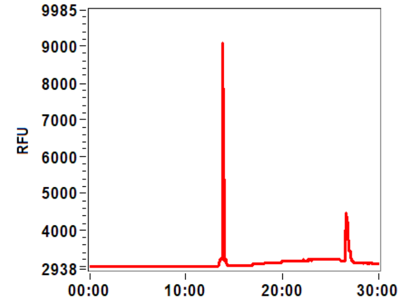

B12: SampB12

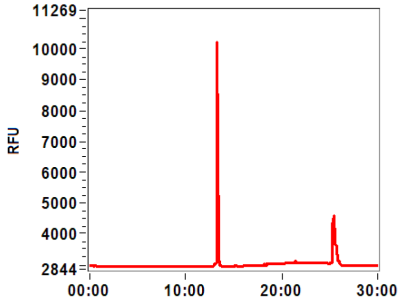

C12: SampC12

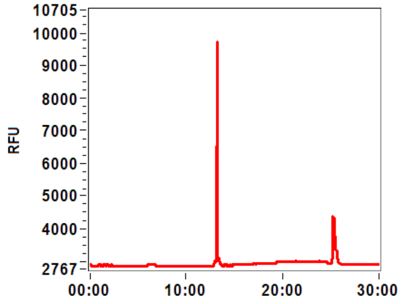

D12: SampD12

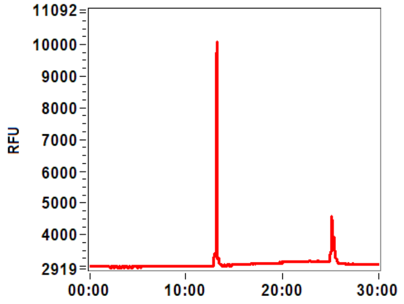

E12: SampE12

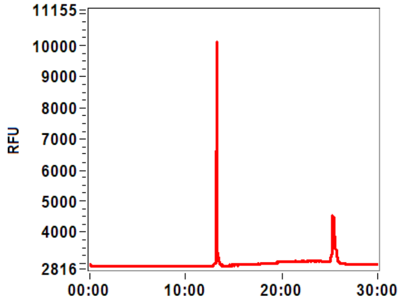

F12: SampF12

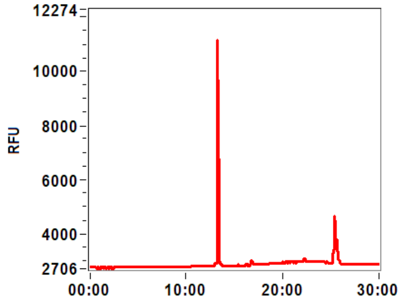

G12: SampG12

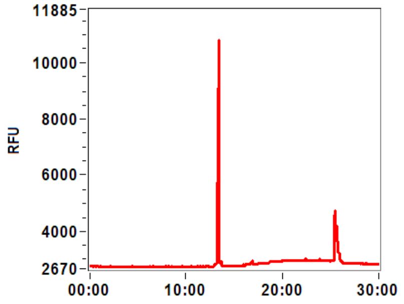

H12: SampH12

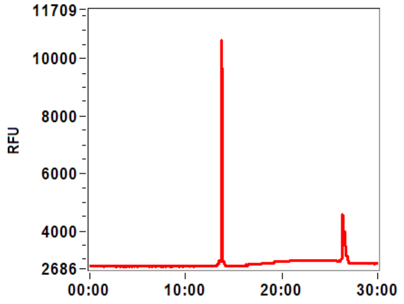

Sample: SampA1
Well location: A1
Created: Thursday, October 21, 2021 8:08:12 PM

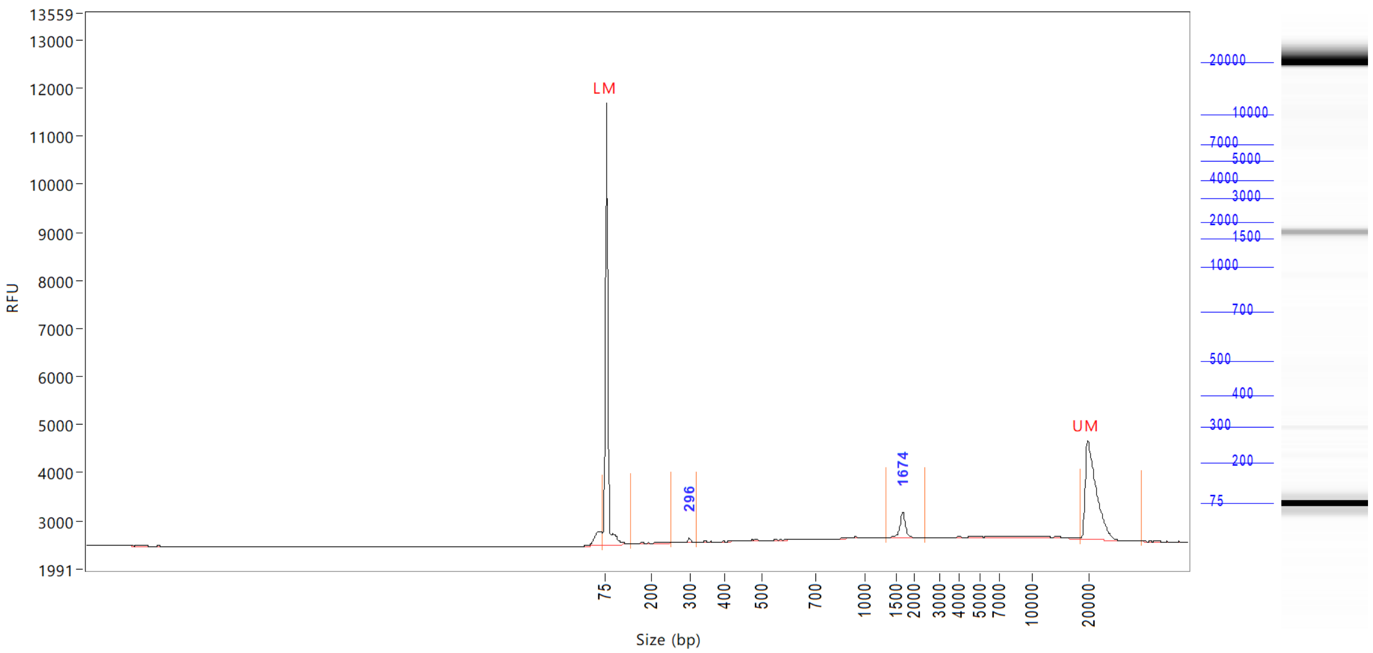

Peak	Size	Concentration	Relative concentration	Molarity	From	To	CV%	RFU
	(bp)	(ng/uL)	Percent	(nmole/L)	(bp)	(bp)		
1	75 (LM)	0.5000		11.0851	64	143	10.06	9219
2	296	0.0824	13.7	0.4706	248	317	5.34	90
3	1674	0.5207	86.3	0.5167	1356	2429	7.71	537
4	20000 (UM)	0.2452		0.0194	18537	29309	5.55	2050

TIC:	0.6031	ng/uL
TIM:	0.9872	nmole/L
Total concentration:	1.2283	ng/uL

```
Sample peak width (sec): 5    Sample min peak height: 50    Sample baseline V to V?: Y    Sample baseline V to V points: 3
Sample filter: Binomial       Number of points for filter: 3  Sample start region (min): 0  Sample end region (min): 30
Marker peak width (sec): 5    Marker min peak height: 200   Marker baseline V to V?: Y    Marker baseline V to V points: 3
Lower marker selection: First peak > 500 RFU                Upper marker selection: Last peak > 200 RFU
Ladder size (bp)75, 200, 300, 400, 500, 700, 1000, 1500, 2000, 3000, 4000, 5000, 7000, 10000, 20000
Quantification using: Lower Marker          Final concentration (ng/uL): 0.5000          Dilution factor: 12.0
```

Sample: SampB1
Well location: B1
Created: Thursday, October 21, 2021 8:08:12 PM

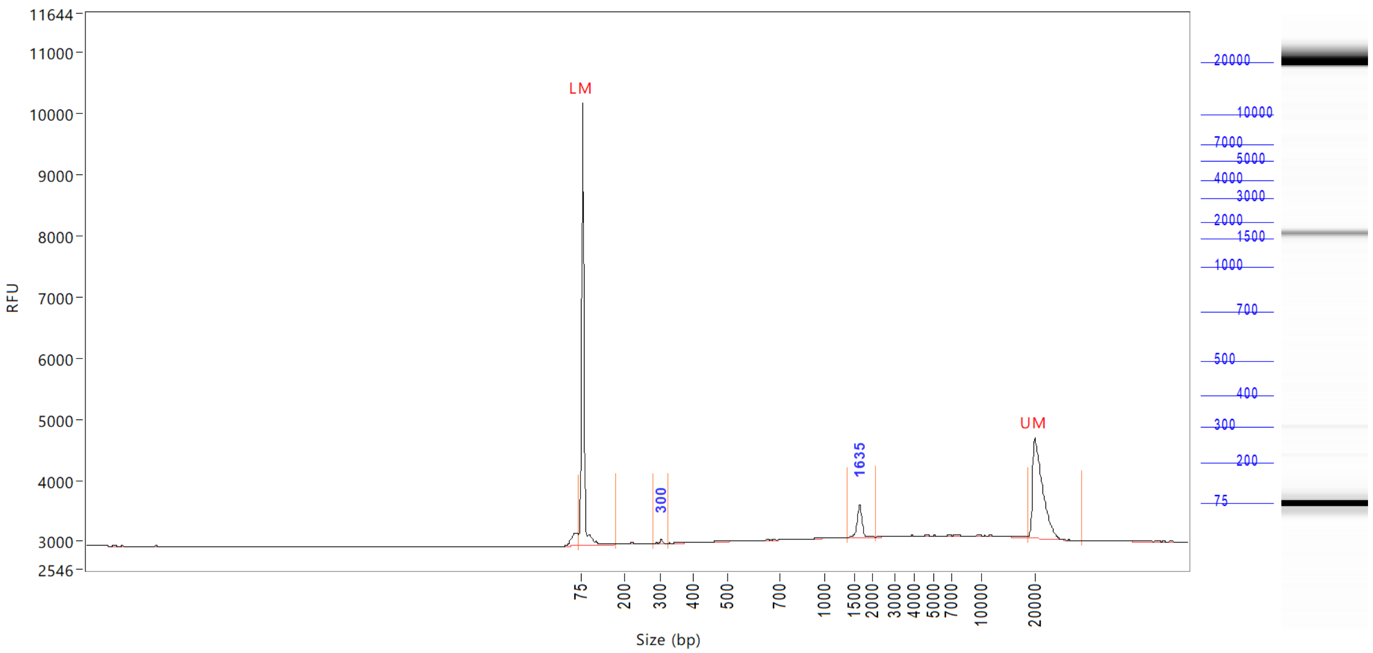

Peak	Size	Concentration	Relative concentration	Molarity	From	To	CV%	RFU
	(bp)	(ng/uL)	Percent	(nmole/L)	(bp)	(bp)		
1	75 (LM)	0.5000		10.9378	62	173	11.98	7256
2	300	0.0687	11.1	0.3820	278	317	2.45	71
3	1635	0.5501	88.9	0.5583	1378	2144	5.52	545
4	20000 (UM)	0.2341		0.0186	18659	28947	5.31	1640

TIC:	0.6188	ng/uL
TIM:	0.9403	nmole/L
Total concentration:	1.0933	ng/uL

```
Sample peak width (sec): 5    Sample min peak height: 50    Sample baseline V to V?: Y    Sample baseline V to V points: 3
Sample filter: Binomial       Number of points for filter: 3  Sample start region (min): 0  Sample end region (min): 30
Marker peak width (sec): 5    Marker min peak height: 200   Marker baseline V to V?: Y    Marker baseline V to V points: 3
Lower marker selection: First peak > 500 RFU                Upper marker selection: Last peak > 200 RFU
Ladder size (bp)75, 200, 300, 400, 500, 700, 1000, 1500, 2000, 3000, 4000, 5000, 7000, 10000, 20000
Quantification using: Lower Marker          Final concentration (ng/uL): 0.5000          Dilution factor: 12.0
```

Sample: SampC1
Well location: C1
Created: Thursday, October 21, 2021 8:08:12 PM

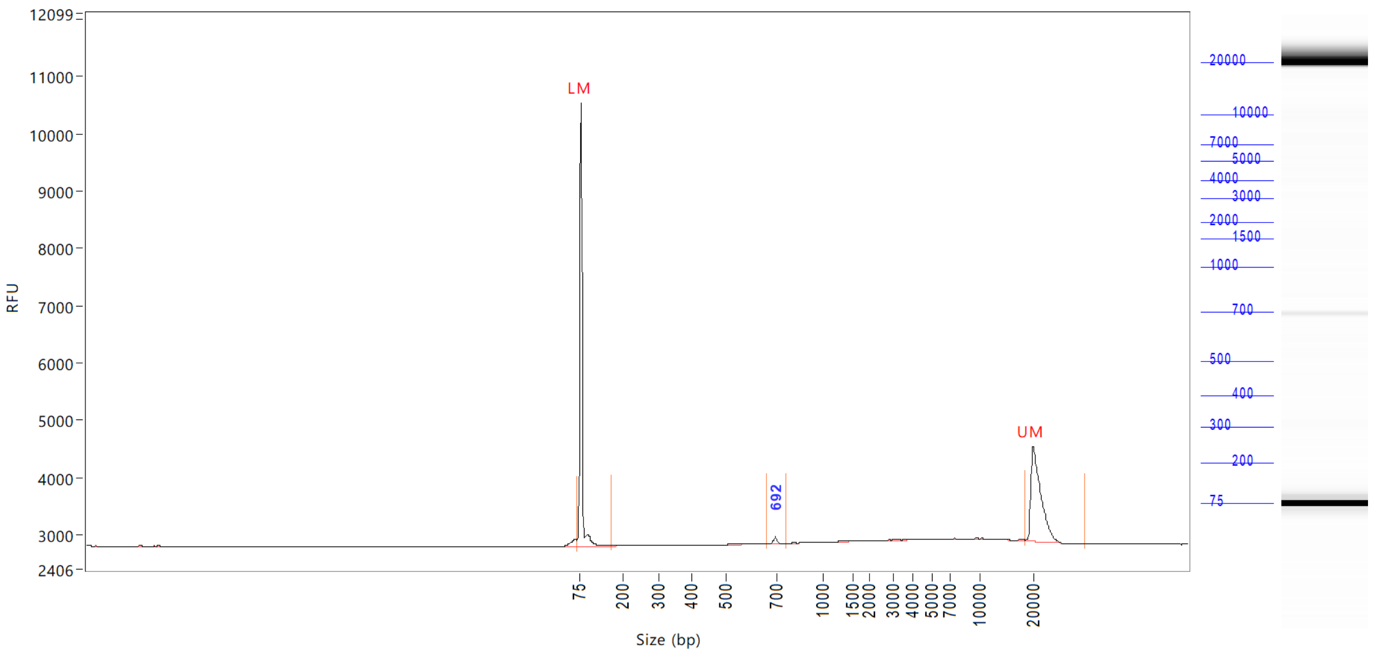

Peak	Size	Concentration	Relative concentration	Molarity	From	To	CV%	RFU
	(bp)	(ng/uL)	Percent	(nmole/L)	(bp)	(bp)		
1	75 (LM)	0.5000		10.9378	64	166	11.09	7746
2	692	0.1056	100.0	0.2519	657	762	2.00	116
3	20000 (UM)	0.2266		0.0179	18659	29672	5.68	1658

TIC:	0.1056	ng/uL
TIM:	0.2519	nmole/L
Total concentration:	0.6132	ng/uL

Sample peak width (sec): 5   Sample min peak height: 50   Sample baseline V to V?: Y   Sample baseline V to V points: 3
Sample filter: Binomial   Number of points for filter: 3   Sample start region (min): 0   Sample end region (min): 30
Marker peak width (sec): 5   Marker min peak height: 200   Marker baseline V to V?: Y   Marker baseline V to V points: 3
Lower marker selection: First peak > 500 RFU   Upper marker selection: Last peak > 200 RFU
Ladder size (bp)75, 200, 300, 400, 500, 700, 1000, 1500, 2000, 3000, 4000, 5000, 7000, 10000, 20000
Quantification using: Lower Marker   Final concentration (ng/uL): 0.5000   Dilution factor: 12.0

Sample: SampD1
Well location: D1
Created: Thursday, October 21, 2021 8:08:12 PM

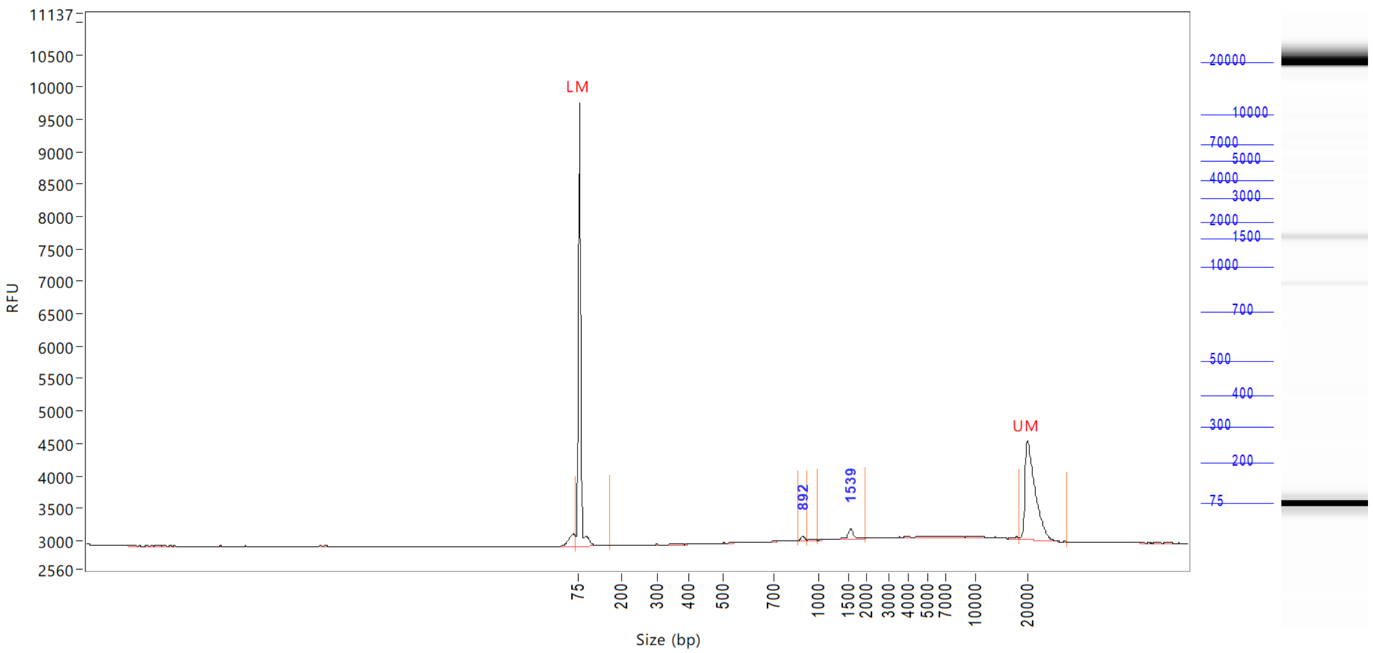

Peak	Size	Concentration	Relative concentration	Molarity	From	To	CV%	RFU
	(bp)	(ng/uL)	Percent	(nmole/L)	(bp)	(bp)		
1	75 (LM)	0.5000		11.0851	64	168	10.16	6850
2	892	0.0647	21.8	0.1202	857	918	1.33	73
3	1539	0.2318	78.2	0.2523	991	1981	9.61	162
4	20000 (UM)	0.2363		0.0187	18537	27496	5.48	1524

TIC:	0.2965	ng/uL
TIM:	0.3726	nmole/L
Total concentration:	0.7111	ng/uL

```
Sample peak width (sec): 5    Sample min peak height: 50    Sample baseline V to V?: Y    Sample baseline V to V points: 3
Sample filter: Binomial       Number of points for filter: 3  Sample start region (min): 0  Sample end region (min): 30
Marker peak width (sec): 5    Marker min peak height: 200   Marker baseline V to V?: Y    Marker baseline V to V points: 3
Lower marker selection: First peak > 500 RFU                Upper marker selection: Last peak > 200 RFU
Ladder size (bp)75, 200, 300, 400, 500, 700, 1000, 1500, 2000, 3000, 4000, 5000, 7000, 10000, 20000
Quantification using: Lower Marker          Final concentration (ng/uL): 0.5000          Dilution factor: 12.0
```

Sample: SampE1
Well location: E1
Created: Thursday, October 21, 2021 8:08:12 PM

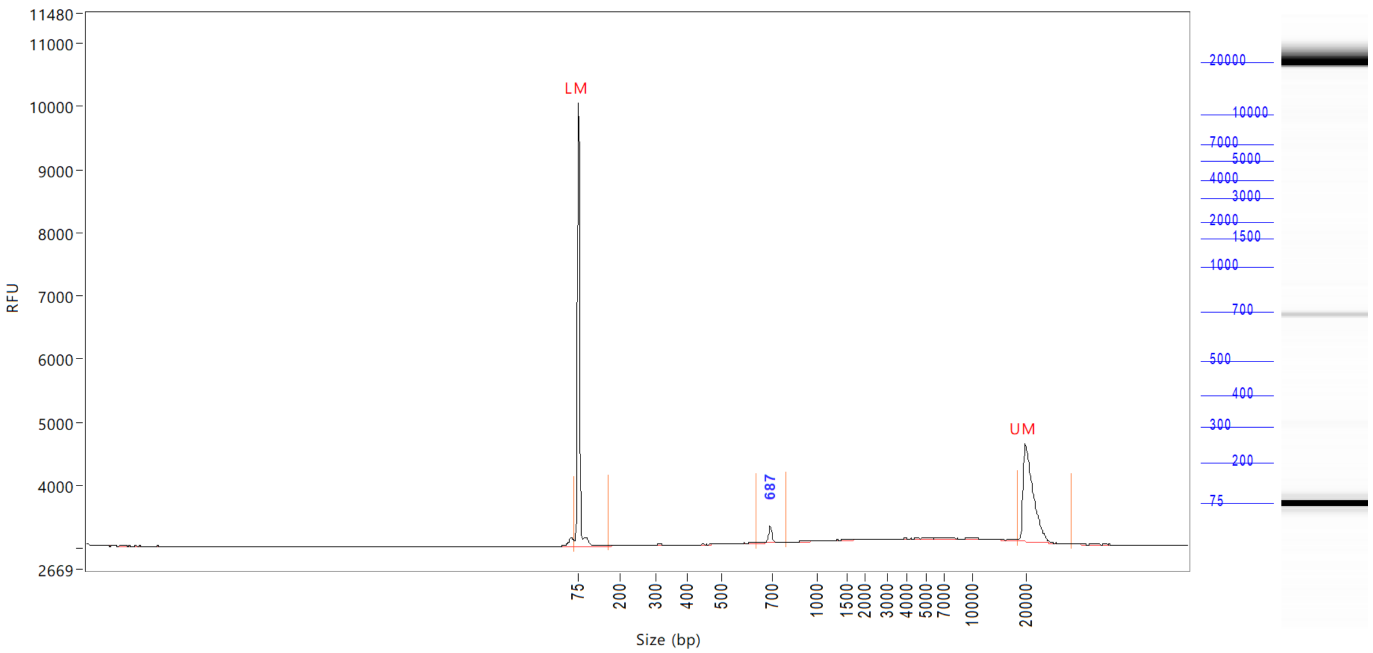

Peak	Size	Concentration	Relative concentration	Molarity	From	To	CV%	RFU
	(bp)	(ng/uL)	Percent	(nmole/L)	(bp)	(bp)		
1	75 (LM)	0.5000		10.9378	62	166	10.39	7039
2	687	0.2683	100.0	0.6427	631	788	2.79	258
3	20000 (UM)	0.2365		0.0188	18537	28584	5.51	1549

TIC:	0.2683	ng/uL
TIM:	0.6427	nmole/L
Total concentration:	0.7923	ng/uL

```
Sample peak width (sec): 5    Sample min peak height: 50    Sample baseline V to V?: Y    Sample baseline V to V points: 3
Sample filter: Binomial       Number of points for filter: 3  Sample start region (min): 0  Sample end region (min): 30
Marker peak width (sec): 5    Marker min peak height: 200   Marker baseline V to V?: Y    Marker baseline V to V points: 3
Lower marker selection: First peak > 500 RFU                Upper marker selection: Last peak > 200 RFU
Ladder size (bp)75, 200, 300, 400, 500, 700, 1000, 1500, 2000, 3000, 4000, 5000, 7000, 10000, 20000
Quantification using: Lower Marker          Final concentration (ng/uL): 0.5000          Dilution factor: 12.0
```

Sample: SampF1
Well location: F1
Created: Thursday, October 21, 2021 8:08:12 PM

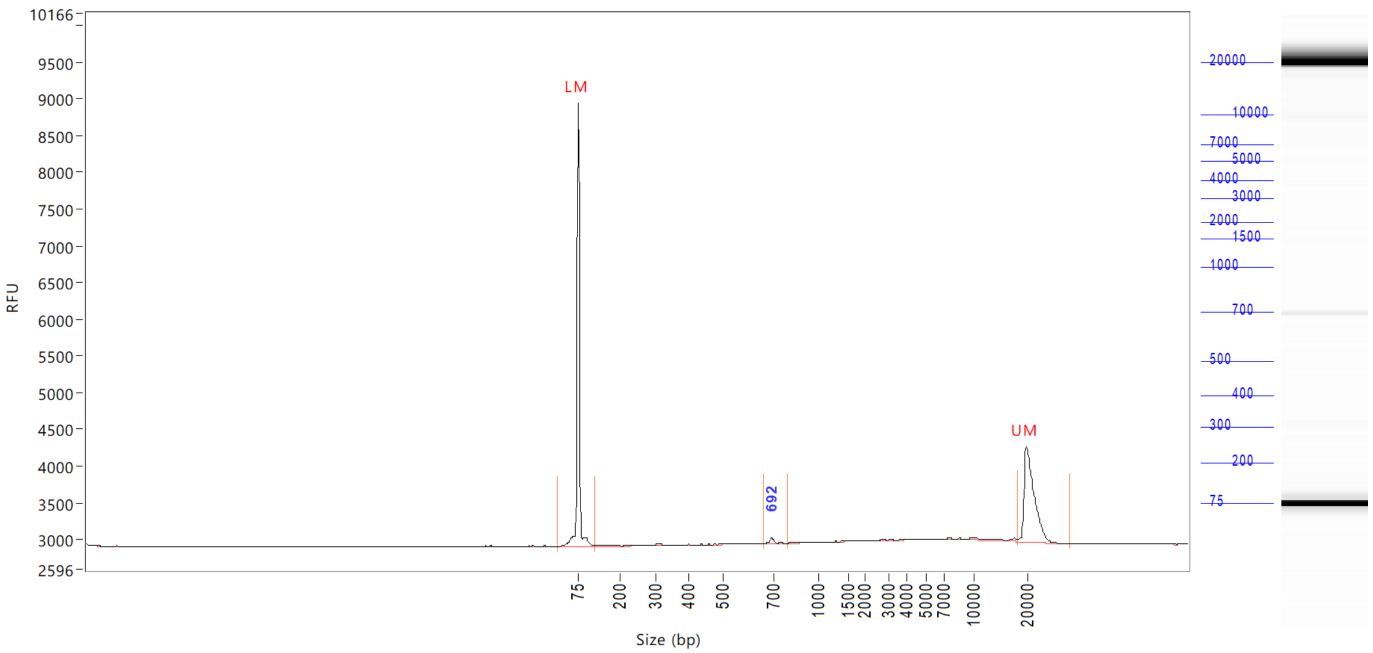

Peak	Size	Concentration	Relative concentration	Molarity	From	To	CV%	RFU
	(bp)	(ng/uL)	Percent	(nmole/L)	(bp)	(bp)		
1	75 (LM)	0.5000		11.3919	14	126	11.50	6050
2	692	0.1098	100.0	0.2592	664	792	3.09	85
3	20000 (UM)	0.2171		0.0173	18171	28100	5.53	1301

TIC:	0.1098	ng/uL
TIM:	0.2592	nmole/L
Total concentration:	0.7785	ng/uL

Sample peak width (sec): 5   Sample min peak height: 50   Sample baseline V to V?: Y   Sample baseline V to V points: 3
Sample filter: Binomial   Number of points for filter: 3 Sample start region (min): 0 Sample end region (min): 30
Marker peak width (sec): 5   Marker min peak height: 200   Marker baseline V to V?: Y   Marker baseline V to V points: 3
Lower marker selection: First peak > 500 RFU   Upper marker selection: Last peak > 200 RFU
Ladder size (bp)75, 200, 300, 400, 500, 700, 1000, 1500, 2000, 3000, 4000, 5000, 7000, 10000, 20000
Quantification using: Lower Marker   Final concentration (ng/uL): 0.5000   Dilution factor: 12.0

Sample: SampG1
Well location: G1
Created: Thursday, October 21, 2021 8:08:12 PM

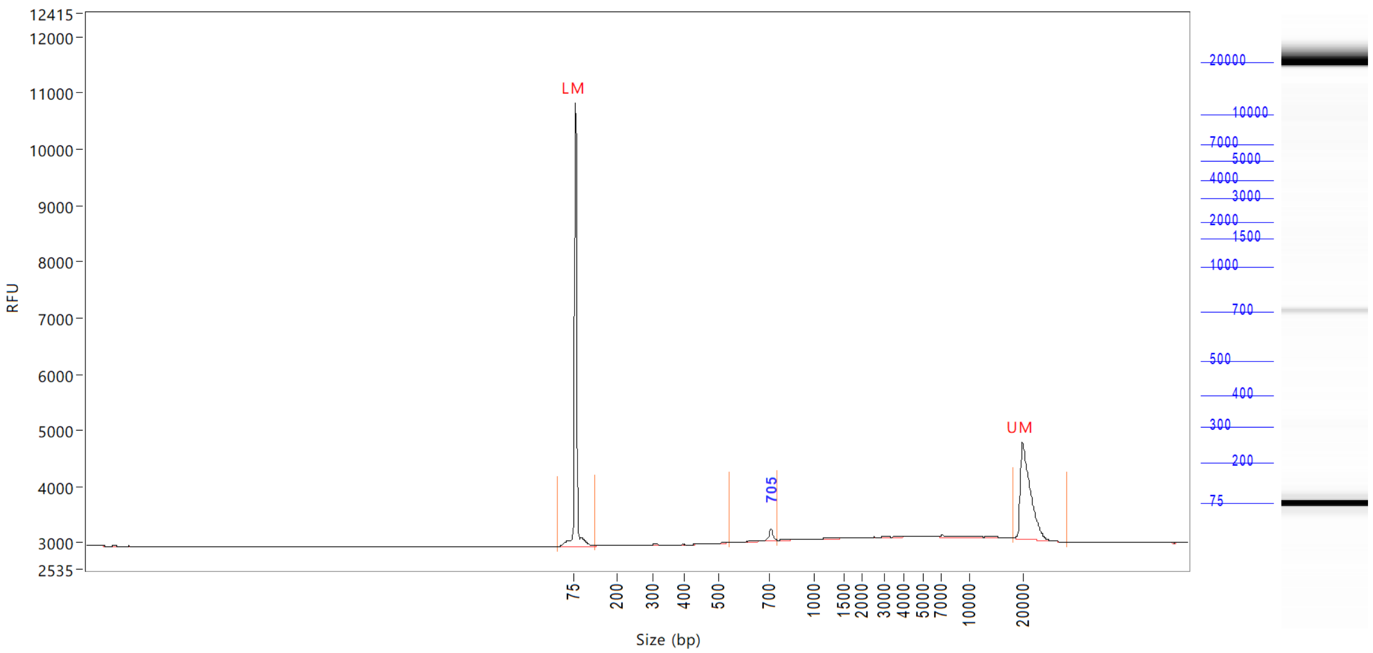

Peak	Size	Concentration	Relative concentration	Molarity	From	To	CV%	RFU
	(bp)	(ng/uL)	Percent	(nmole/L)	(bp)	(bp)		
1	75 (LM)	0.5000		11.2364	22	133	10.95	7892
2	705	0.2527	100.0	0.6071	542	753	5.94	217
3	20000 (UM)	0.2276		0.0180	18293	28342	5.44	1717

TIC:	0.2527	ng/uL
TIM:	0.6071	nmole/L
Total concentration:	0.8494	ng/uL

```
Sample peak width (sec): 5    Sample min peak height: 50    Sample baseline V to V?: Y    Sample baseline V to V points: 3
Sample filter: Binomial       Number of points for filter: 3  Sample start region (min): 0  Sample end region (min): 30
Marker peak width (sec): 5    Marker min peak height: 200   Marker baseline V to V?: Y    Marker baseline V to V points: 3
Lower marker selection: First peak > 500 RFU                Upper marker selection: Last peak > 200 RFU
Ladder size (bp)75, 200, 300, 400, 500, 700, 1000, 1500, 2000, 3000, 4000, 5000, 7000, 10000, 20000
Quantification using: Lower Marker          Final concentration (ng/uL): 0.5000          Dilution factor: 12.0
```

Sample: SampH1
Well location: H1
Created: Thursday, October 21, 2021 8:08:12 PM

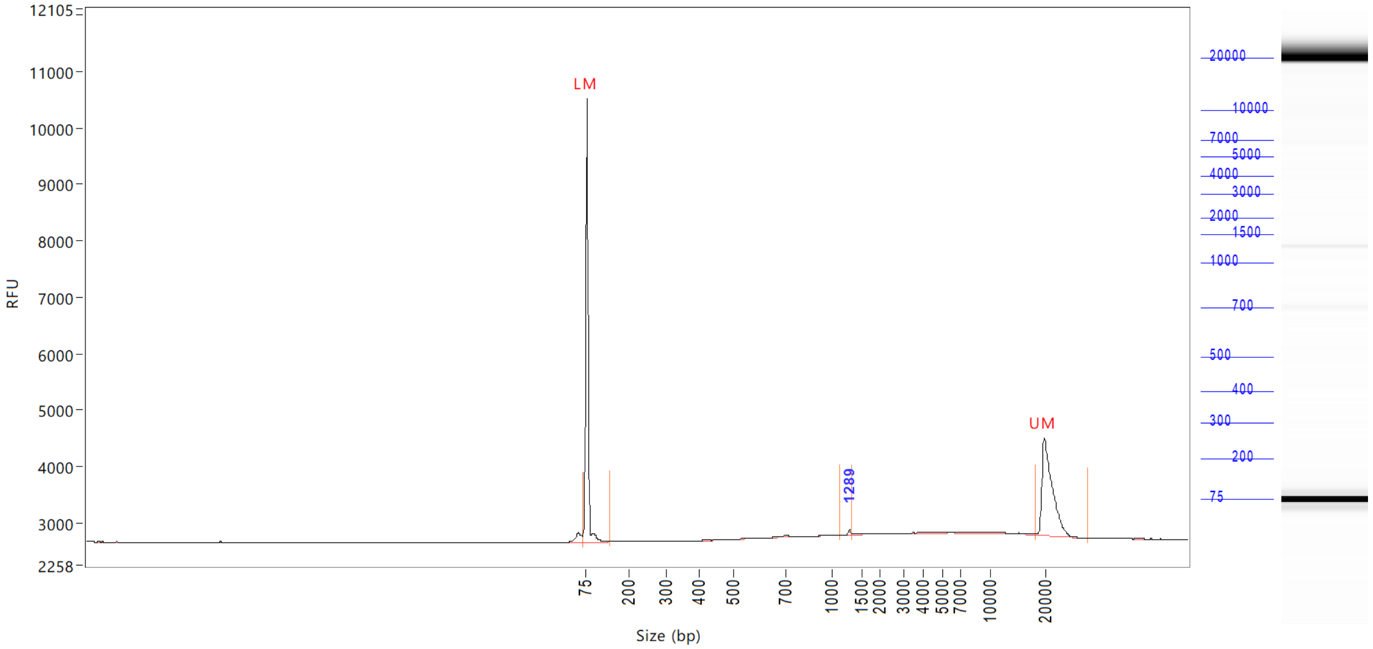

Peak	Size	Concentration	Relative concentration	Molarity	From	To	CV%	RFU
	(bp)	(ng/uL)	Percent	(nmole/L)	(bp)	(bp)		
1	75 (LM)	0.5000		11.0851	62	143	9.18	7865
2	1289	0.0576	100.0	0.0749	1123	1345	3.68	90
3	20000 (UM)	0.2378		0.0189	18171	27859	5.65	1716

TIC:	0.0576	ng/uL
TIM:	0.0749	nmole/L
Total concentration:	0.6022	ng/uL

```
Sample peak width (sec): 5    Sample min peak height: 50    Sample baseline V to V?: Y    Sample baseline V to V points: 3
Sample filter: Binomial       Number of points for filter: 3  Sample start region (min): 0  Sample end region (min): 30
Marker peak width (sec): 5    Marker min peak height: 200   Marker baseline V to V?: Y    Marker baseline V to V points: 3
Lower marker selection: First peak > 500 RFU                Upper marker selection: Last peak > 200 RFU
Ladder size (bp)75, 200, 300, 400, 500, 700, 1000, 1500, 2000, 3000, 4000, 5000, 7000, 10000, 20000
Quantification using: Lower Marker          Final concentration (ng/uL): 0.5000          Dilution factor: 12.0
```

Sample: SampA2
Well location: A2
Created: Thursday, October 21, 2021 8:08:12 PM

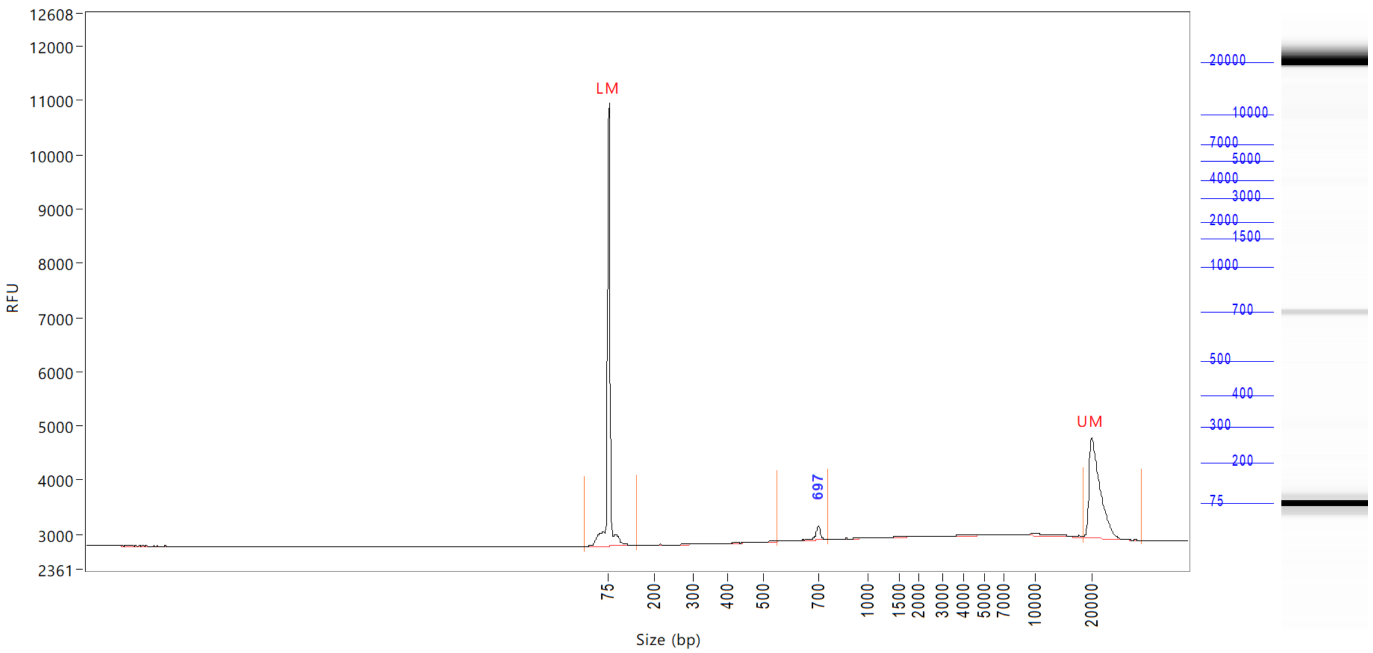

Peak	Size	Concentration	Relative concentration	Molarity	From	To	CV%	RFU
	(bp)	(ng/uL)	Percent	(nmole/L)	(bp)	(bp)		
1	75 (LM)	0.5000		11.3919	9	150	14.80	8181
2	697	0.2468	100.0	0.5964	547	753	5.29	246
3	20000 (UM)	0.2238		0.0177	18537	28705	5.49	1850

TIC:	0.2468	ng/uL
TIM:	0.5964	nmole/L
Total concentration:	0.7923	ng/uL

```
Sample peak width (sec): 5    Sample min peak height: 50    Sample baseline V to V?: Y    Sample baseline V to V points: 3
Sample filter: Binomial       Number of points for filter: 3  Sample start region (min): 0  Sample end region (min): 30
Marker peak width (sec): 5    Marker min peak height: 200   Marker baseline V to V?: Y    Marker baseline V to V points: 3
Lower marker selection: First peak > 500 RFU                Upper marker selection: Last peak > 200 RFU
Ladder size (bp)75, 200, 300, 400, 500, 700, 1000, 1500, 2000, 3000, 4000, 5000, 7000, 10000, 20000
Quantification using: Lower Marker          Final concentration (ng/uL): 0.5000          Dilution factor: 12.0
```

Sample: SampB2
Well location: B2
Created: Thursday, October 21, 2021 8:08:12 PM

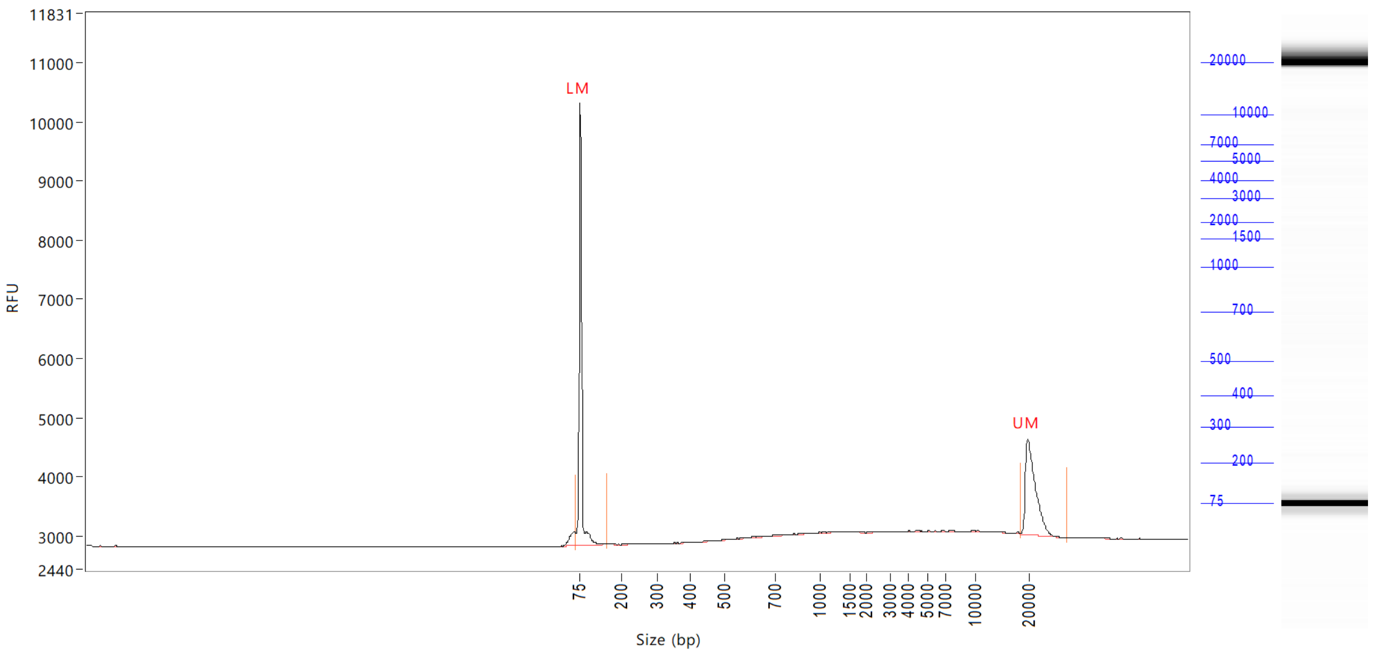

Peak	Size	Concentration	Relative concentration	Molarity	From	To	CV%	RFU
	(bp)	(ng/uL)	Percent	(nmole/L)	(bp)	(bp)		
1	75 (LM)	0.5000		10.9378	64	154	10.15	7486
2	20000 (UM)	0.2202		0.0175	18659	27254	5.33	1609

TIC:	0.0000	ng/uL
TIM:	0.0000	nmole/L
Total concentration:	0.5270	ng/uL

Sample peak width (sec): 5 Sample min peak height: 50 Sample baseline V to V?: Y Sample baseline V to V points: 3
Sample filter: Binomial Number of points for filter: 3 Sample start region (min): 0 Sample end region (min): 30
Marker peak width (sec): 5 Marker min peak height: 200 Marker baseline V to V?: Y Marker baseline V to V points: 3
Lower marker selection: First peak > 500 RFU Upper marker selection: Last peak > 200 RFU
Ladder size (bp)75, 200, 300, 400, 500, 700, 1000, 1500, 2000, 3000, 4000, 5000, 7000, 10000, 20000
Quantification using: Lower Marker Final concentration (ng/uL): 0.5000 Dilution factor: 12.0

Sample: SampC2
Well location: C2
Created: Thursday, October 21, 2021 8:08:12 PM

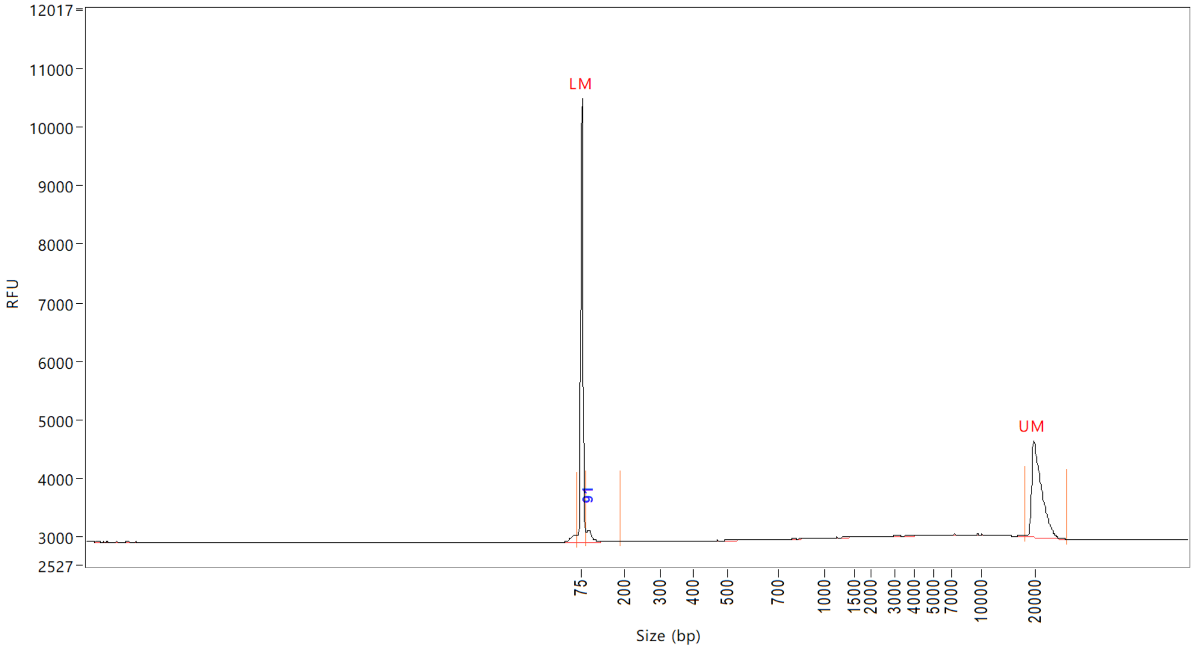

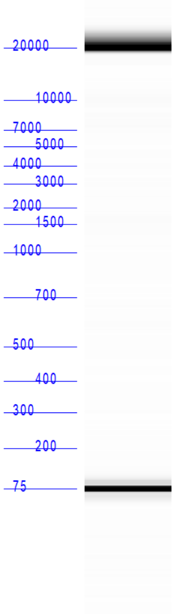

Peak	Size	Concentration	Relative concentration	Molarity	From	To	CV%	RFU
	(bp)	(ng/uL)	Percent	(nmole/L)	(bp)	(bp)		
1	75 (LM)	0.5000		11.2364	60	86	4.77	7580
2	91	0.4385	100.0	7.1287	86	187	17.76	204
3	20000 (UM)	0.2405		0.0191	18415	26045	5.26	1638

TIC:	0.4385	ng/uL
TIM:	7.1287	nmole/L
Total concentration:	0.9057	ng/uL

Sample peak width (sec): 5 Sample min peak height: 50 Sample baseline V to V?: Y Sample baseline V to V points: 3
Sample filter: Binomial Number of points for filter: 3 Sample start region (min): 0 Sample end region (min): 30
Marker peak width (sec): 5 Marker min peak height: 200 Marker baseline V to V?: Y Marker baseline V to V points: 3
Lower marker selection: First peak > 500 RFU Upper marker selection: Last peak > 200 RFU
Ladder size (bp)75, 200, 300, 400, 500, 700, 1000, 1500, 2000, 3000, 4000, 5000, 7000, 10000, 20000
Quantification using: Lower Marker Final concentration (ng/uL): 0.5000 Dilution factor: 12.0

Sample: SampD2
Well location: D2
Created: Thursday, October 21, 2021 8:08:12 PM

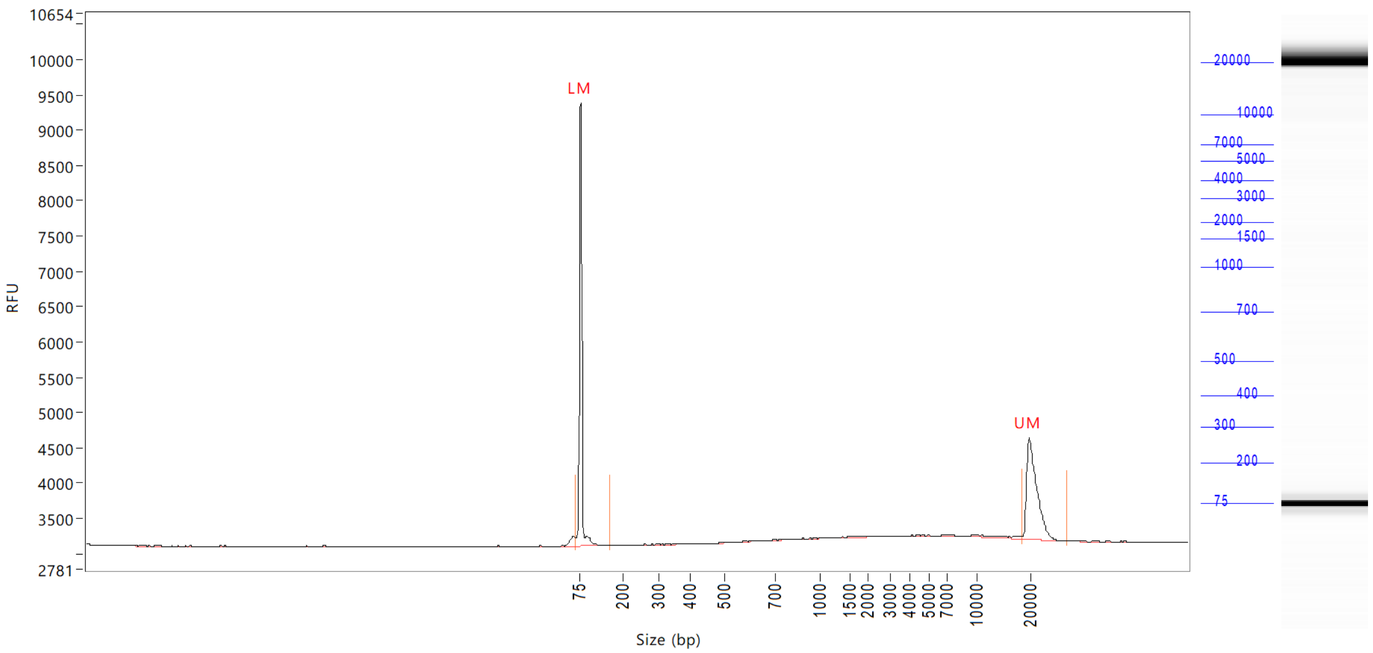

Peak	Size	Concentration	Relative concentration	Molarity	From	To	CV%	RFU
	(bp)	(ng/uL)	Percent	(nmole/L)	(bp)	(bp)		
1	75 (LM)	0.5000		11.0851	62	162	11.28	6286
2	20000 (UM)	0.2378		0.0189	18659	27133	5.45	1449

TIC:	0.0000	ng/uL
TIM:	0.0000	nmole/L
Total concentration:	0.6519	ng/uL

```
Sample peak width (sec): 5    Sample min peak height: 50    Sample baseline V to V?: Y    Sample baseline V to V points: 3
Sample filter: Binomial       Number of points for filter: 3  Sample start region (min): 0  Sample end region (min): 30
Marker peak width (sec): 5    Marker min peak height: 200   Marker baseline V to V?: Y    Marker baseline V to V points: 3
Lower marker selection: First peak > 500 RFU                Upper marker selection: Last peak > 200 RFU
Ladder size (bp)75, 200, 300, 400, 500, 700, 1000, 1500, 2000, 3000, 4000, 5000, 7000, 10000, 20000
Quantification using: Lower Marker          Final concentration (ng/uL): 0.5000          Dilution factor: 12.0
```

Sample: SampE2
Well location: E2
Created: Thursday, October 21, 2021 8:08:12 PM

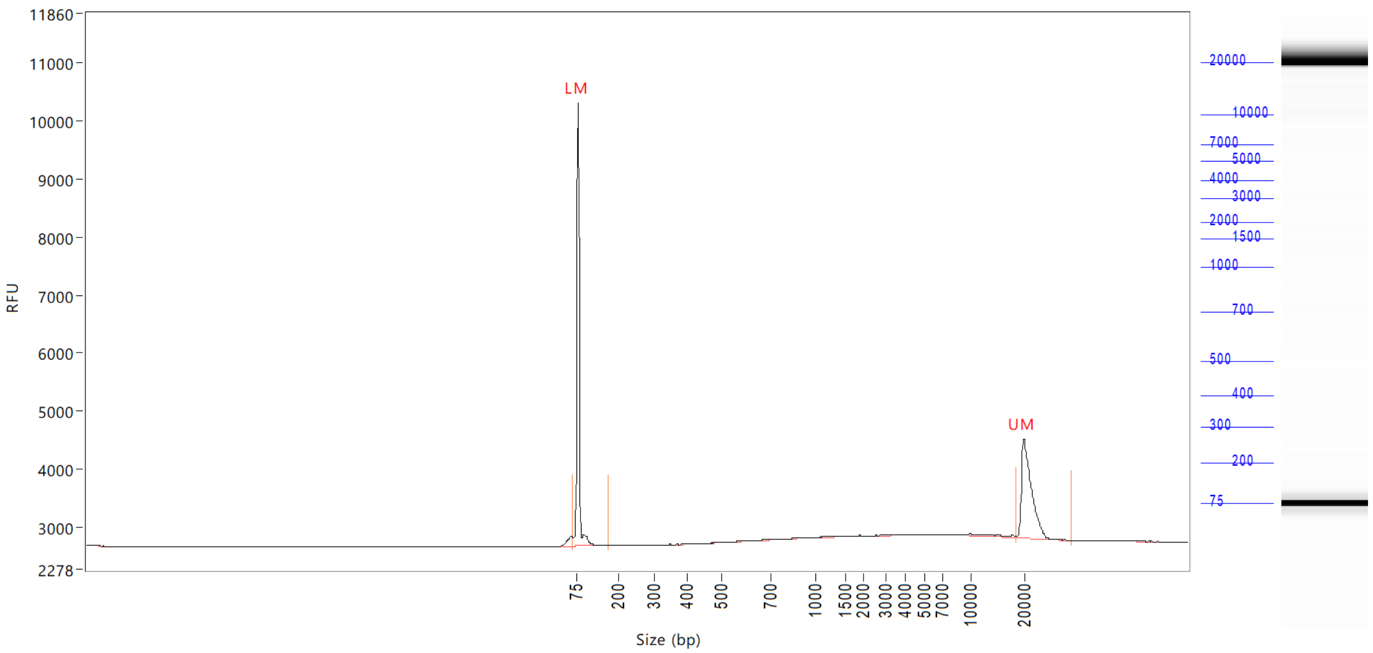

Peak	Size	Concentration	Relative concentration	Molarity	From	To	CV%	RFU
	(bp)	(ng/uL)	Percent	(nmole/L)	(bp)	(bp)		
1	75 (LM)	0.5000		10.9378	62	168	10.12	7649
2	20000 (UM)	0.2327		0.0184	18537	28947	5.60	1720

TIC:	0.0000	ng/uL
TIM:	0.0000	nmole/L
Total concentration:	0.5812	ng/uL

Sample peak width (sec): 5 Sample min peak height: 50 Sample baseline V to V?: Y Sample baseline V to V points: 3
Sample filter: Binomial Number of points for filter: 3 Sample start region (min): 0 Sample end region (min): 30
Marker peak width (sec): 5 Marker min peak height: 200 Marker baseline V to V?: Y Marker baseline V to V points: 3
Lower marker selection: First peak > 500 RFU Upper marker selection: Last peak > 200 RFU
Ladder size (bp)75, 200, 300, 400, 500, 700, 1000, 1500, 2000, 3000, 4000, 5000, 7000, 10000, 20000
Quantification using: Lower Marker Final concentration (ng/uL): 0.5000 Dilution factor: 12.0

Sample: SampF2
Well location: F2
Created: Thursday, October 21, 2021 8:08:12 PM

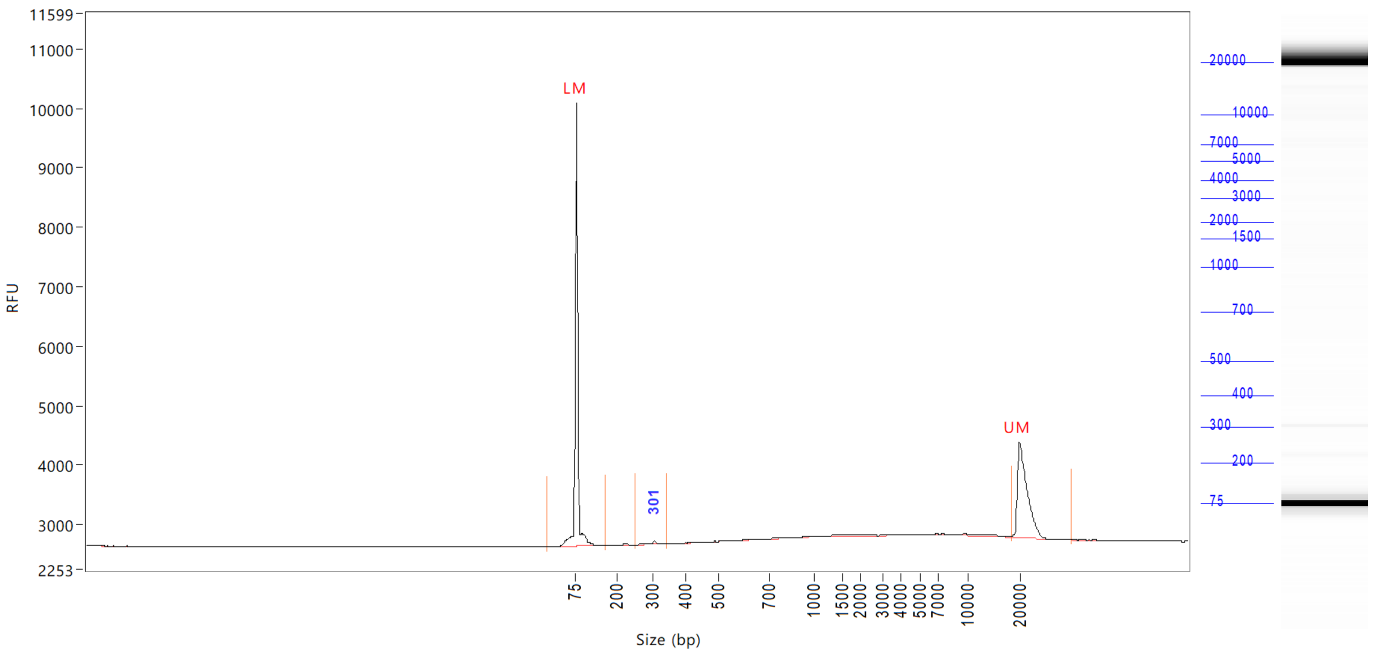

Peak	Size	Concentration	Relative concentration	Molarity	From	To	CV%	RFU
	(bp)	(ng/uL)	Percent	(nmole/L)	(bp)	(bp)		
1	75 (LM)	0.5000		11.2364	0	164	13.94	7461
2	301	0.0637	100.0	0.3530	250	339	5.15	61
3	20000 (UM)	0.2082		0.0165	18293	29793	5.37	1612

TIC:	0.0637	ng/uL
TIM:	0.3530	nmole/L
Total concentration:	0.5867	ng/uL

Sample peak width (sec): 5   Sample min peak height: 50   Sample baseline V to V?: Y   Sample baseline V to V points: 3
Sample filter: Binomial   Number of points for filter: 3 Sample start region (min): 0 Sample end region (min): 30
Marker peak width (sec): 5   Marker min peak height: 200   Marker baseline V to V?: Y   Marker baseline V to V points: 3
Lower marker selection: First peak > 500 RFU   Upper marker selection: Last peak > 200 RFU
Ladder size (bp)75, 200, 300, 400, 500, 700, 1000, 1500, 2000, 3000, 4000, 5000, 7000, 10000, 20000
Quantification using: Lower Marker   Final concentration (ng/uL): 0.5000   Dilution factor: 12.0

Sample: SampG2
Well location: G2
Created: Thursday, October 21, 2021 8:08:12 PM

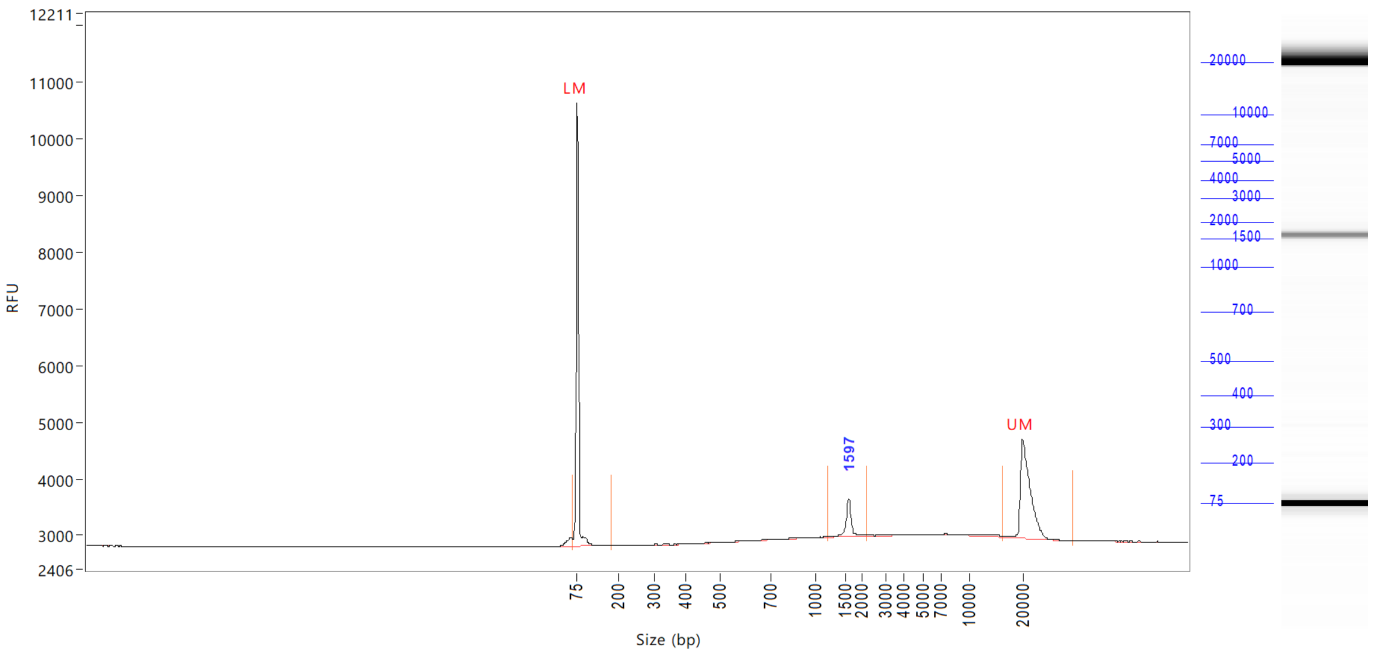

Peak	Size	Concentration	Relative concentration	Molarity	From	To	CV%	RFU
	(bp)	(ng/uL)	Percent	(nmole/L)	(bp)	(bp)		
1	75 (LM)	0.5000		11.0851	62	177	9.85	7829
2	1597	0.7360	100.0	0.7605	1212	2172	7.95	677
3	20000 (UM)	0.2381		0.0189	16220	29672	5.98	1753

TIC:	0.7360	ng/uL
TIM:	0.7605	nmole/L
Total concentration:	1.2699	ng/uL

Sample peak width (sec): 5 Sample min peak height: 50 Sample baseline V to V?: Y Sample baseline V to V points: 3
Sample filter: Binomial Number of points for filter: 3 Sample start region (min): 0 Sample end region (min): 30
Marker peak width (sec): 5 Marker min peak height: 200 Marker baseline V to V?: Y Marker baseline V to V points: 3
Lower marker selection: First peak > 500 RFU Upper marker selection: Last peak > 200 RFU
Ladder size (bp)75, 200, 300, 400, 500, 700, 1000, 1500, 2000, 3000, 4000, 5000, 7000, 10000, 20000
Quantification using: Lower Marker Final concentration (ng/uL): 0.5000 Dilution factor: 12.0

Sample: SampH2
Well location: H2
Created: Thursday, October 21, 2021 8:08:12 PM

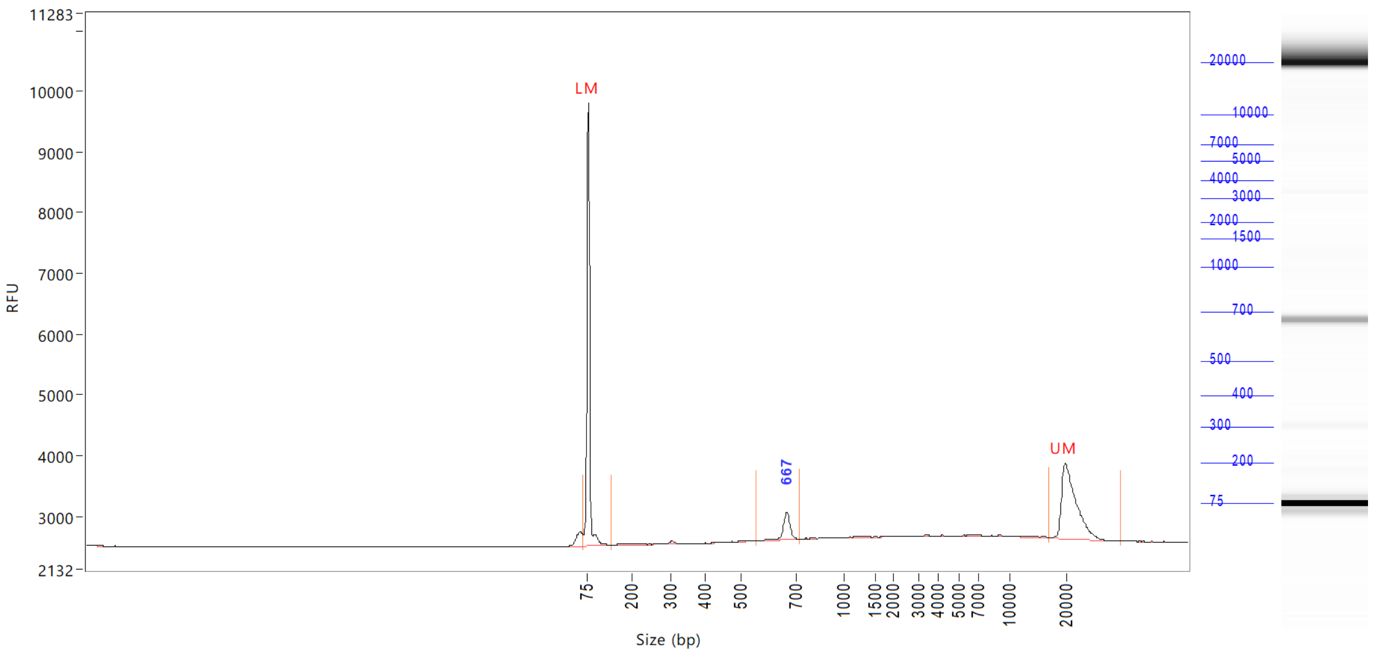

Peak	Size	Concentration	Relative concentration	Molarity	From	To	CV%	RFU
	(bp)	(ng/uL)	Percent	(nmole/L)	(bp)	(bp)		
1	75 (LM)	0.5000		11.0851	62	141	9.52	7305
2	667	0.6682	100.0	1.6662	552	723	3.29	455
3	20000 (UM)	0.2230		0.0175	16952	29914	7.40	1239

TIC:	0.6682	ng/uL
TIM:	1.6662	nmole/L
Total concentration:	1.2604	ng/uL

Sample peak width (sec): 5 Sample min peak height: 50 Sample baseline V to V?: Y Sample baseline V to V points: 3
Sample filter: Binomial Number of points for filter: 3 Sample start region (min): 0 Sample end region (min): 30
Marker peak width (sec): 5 Marker min peak height: 200 Marker baseline V to V?: Y Marker baseline V to V points: 3
Lower marker selection: First peak > 500 RFU Upper marker selection: Last peak > 200 RFU
Ladder size (bp)75, 200, 300, 400, 500, 700, 1000, 1500, 2000, 3000, 4000, 5000, 7000, 10000, 20000
Quantification using: Lower Marker Final concentration (ng/uL): 0.5000 Dilution factor: 12.0

Sample: SampA3
Well location: A3
Created: Thursday, October 21, 2021 8:08:12 PM

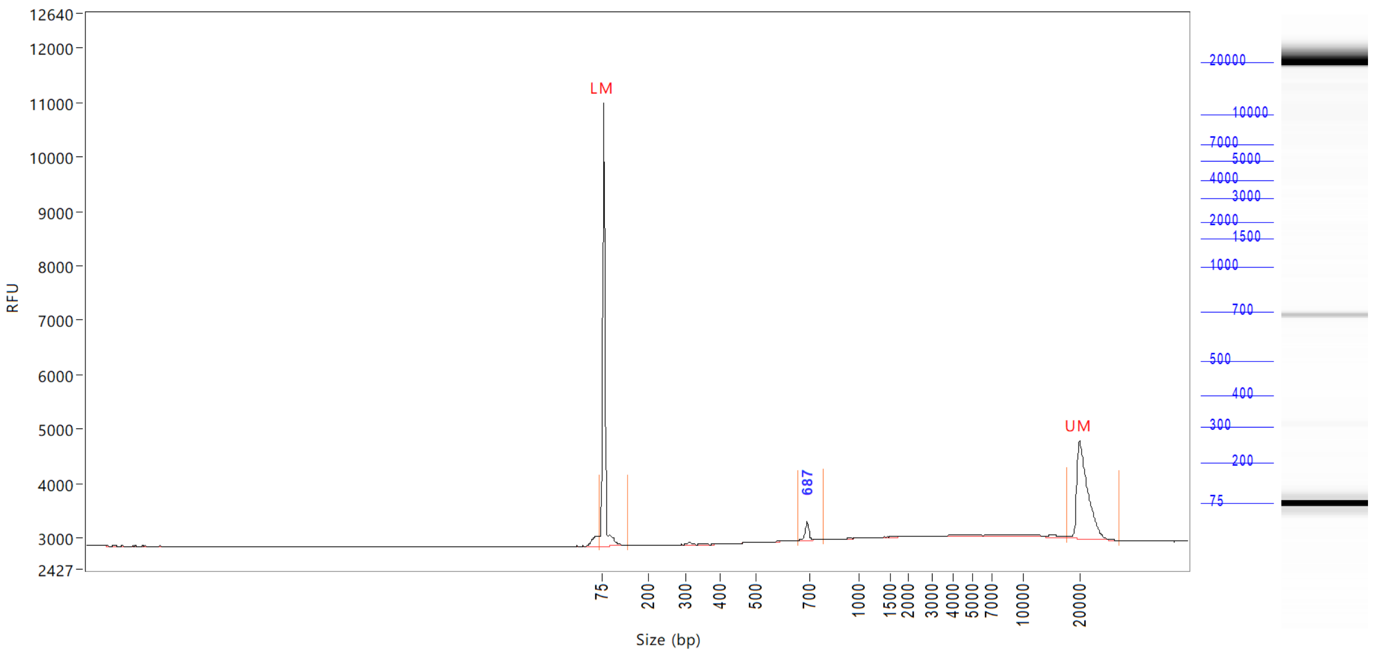

Peak	Size	Concentration	Relative concentration	Molarity	From	To	CV%	RFU
	(bp)	(ng/uL)	Percent	(nmole/L)	(bp)	(bp)		
1	75 (LM)	0.5000		10.9378	66	143	9.70	8155
2	687	0.2796	100.0	0.6698	657	779	2.15	337
3	20000 (UM)	0.2436		0.0193	17683	27012	5.60	1803

TIC:	0.2796	ng/uL
TIM:	0.6698	nmole/L
Total concentration:	0.9493	ng/uL

Sample peak width (sec): 5   Sample min peak height: 50   Sample baseline V to V?: Y   Sample baseline V to V points: 3
Sample filter: Binomial   Number of points for filter: 3 Sample start region (min): 0 Sample end region (min): 30
Marker peak width (sec): 5   Marker min peak height: 200   Marker baseline V to V?: Y   Marker baseline V to V points: 3
Lower marker selection: First peak > 500 RFU   Upper marker selection: Last peak > 200 RFU
Ladder size (bp)75, 200, 300, 400, 500, 700, 1000, 1500, 2000, 3000, 4000, 5000, 7000, 10000, 20000
Quantification using: Lower Marker   Final concentration (ng/uL): 0.5000   Dilution factor: 12.0

Sample: SampB3
Well location: B3
Created: Thursday, October 21, 2021 8:08:12 PM

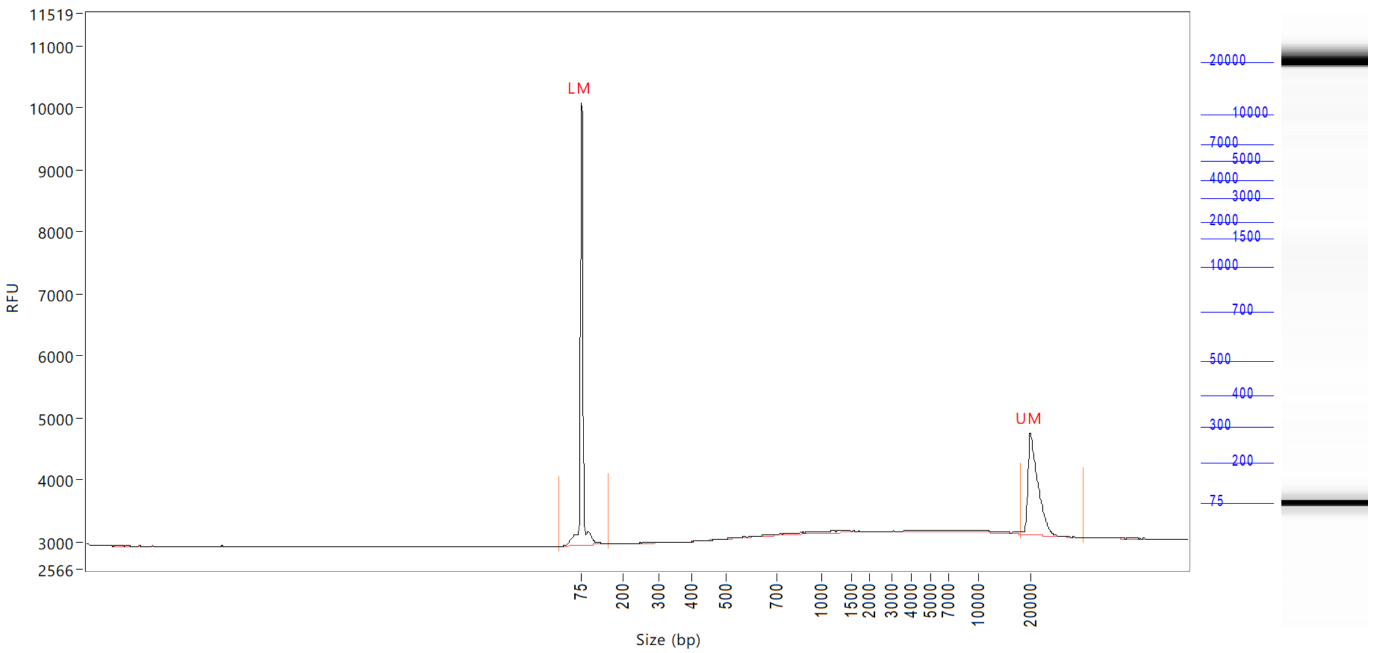

Peak	Size	Concentration	Relative concentration	Molarity	From	To	CV%	RFU
	(bp)	(ng/uL)	Percent	(nmole/L)	(bp)	(bp)		
1	75 (LM)	0.5000		11.0851	12	156	13.73	7134
2	20000 (UM)	0.2194		0.0175	18171	29914	5.57	1638

TIC:	0.0000	ng/uL
TIM:	0.0000	nmole/L
Total concentration:	0.8865	ng/uL

Sample peak width (sec): 5 Sample min peak height: 50 Sample baseline V to V?: Y Sample baseline V to V points: 3
Sample filter: Binomial Number of points for filter: 3 Sample start region (min): 0 Sample end region (min): 30
Marker peak width (sec): 5 Marker min peak height: 200 Marker baseline V to V?: Y Marker baseline V to V points: 3
Lower marker selection: First peak > 500 RFU Upper marker selection: Last peak > 200 RFU
Ladder size (bp)75, 200, 300, 400, 500, 700, 1000, 1500, 2000, 3000, 4000, 5000, 7000, 10000, 20000
Quantification using: Lower Marker Final concentration (ng/uL): 0.5000 Dilution factor: 12.0

Sample: SampC3
Well location: C3
Created: Thursday, October 21, 2021 8:08:12 PM

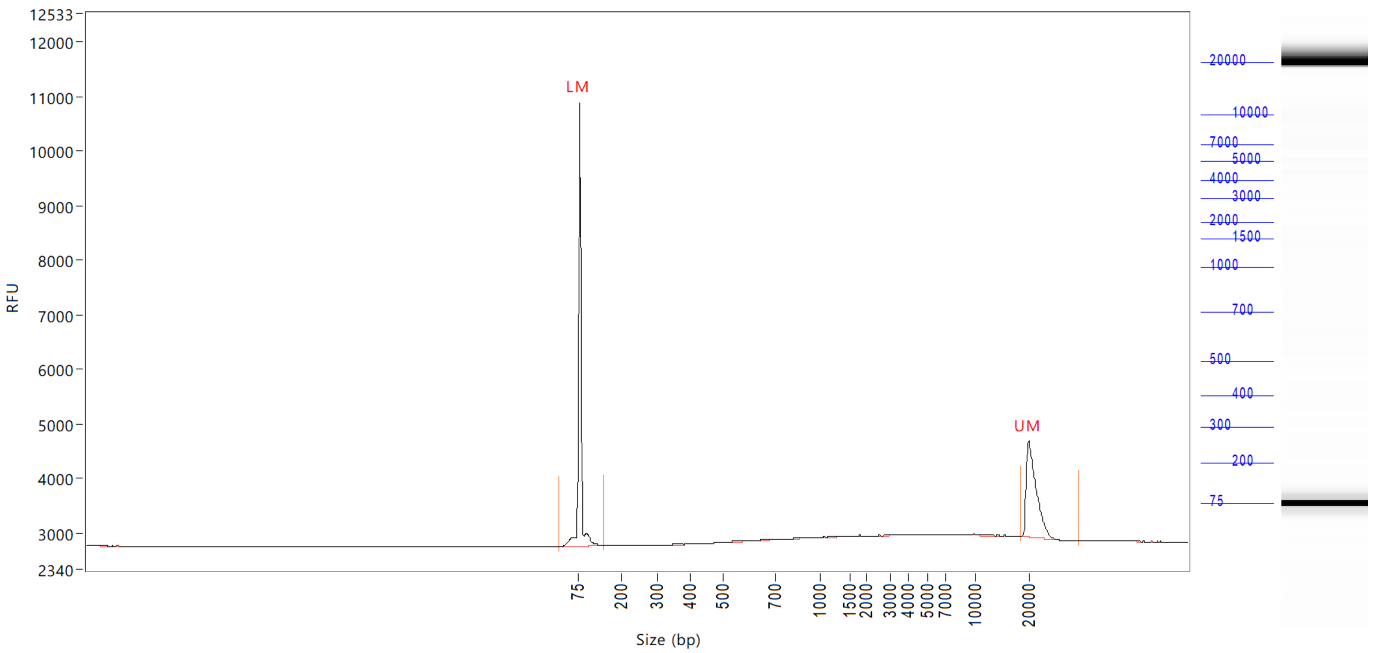

Peak	Size	Concentration	Relative concentration	Molarity	From	To	CV%	RFU
	(bp)	(ng/uL)	Percent	(nmole/L)	(bp)	(bp)		
1	75 (LM)	0.5000		11.0851	18	147	13.50	8141
2	20000 (UM)	0.2093		0.0166	18659	29551	5.36	1756

TIC:	0.0000	ng/uL
TIM:	0.0000	nmole/L
Total concentration:	0.4351	ng/uL

Sample peak width (sec): 5   Sample min peak height: 50   Sample baseline V to V?: Y   Sample baseline V to V points: 3
Sample filter: Binomial   Number of points for filter: 3   Sample start region (min): 0   Sample end region (min): 30
Marker peak width (sec): 5   Marker min peak height: 200   Marker baseline V to V?: Y   Marker baseline V to V points: 3
Lower marker selection: First peak > 500 RFU   Upper marker selection: Last peak > 200 RFU
Ladder size (bp)75, 200, 300, 400, 500, 700, 1000, 1500, 2000, 3000, 4000, 5000, 7000, 10000, 20000
Quantification using: Lower Marker   Final concentration (ng/uL): 0.5000   Dilution factor: 12.0

Sample: SampD3
Well location: D3
Created: Thursday, October 21, 2021 8:08:12 PM

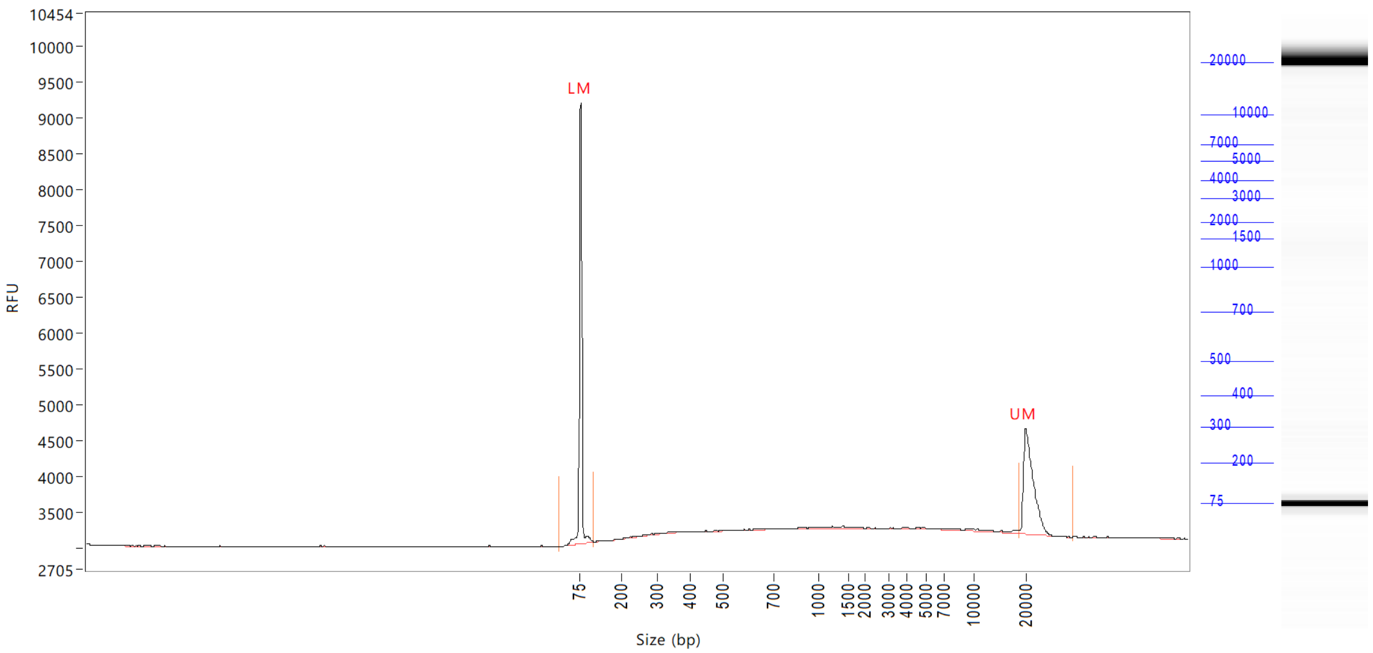

Peak	Size	Concentration	Relative concentration	Molarity	From	To	CV%	RFU
	(bp)	(ng/uL)	Percent	(nmole/L)	(bp)	(bp)		
1	75 (LM)	0.5000		11.3919	12	114	8.91	6154
2	20000 (UM)	0.2409		0.0191	18781	29068	5.34	1467

TIC:	0.0000	ng/uL
TIM:	0.0000	nmole/L
Total concentration:	0.9349	ng/uL

Sample peak width (sec): 5 Sample min peak height: 50 Sample baseline V to V?: Y Sample baseline V to V points: 3
Sample filter: Binomial Number of points for filter: 3 Sample start region (min): 0 Sample end region (min): 30
Marker peak width (sec): 5 Marker min peak height: 200 Marker baseline V to V?: Y Marker baseline V to V points: 3
Lower marker selection: First peak > 500 RFU Upper marker selection: Last peak > 200 RFU
Ladder size (bp)75, 200, 300, 400, 500, 700, 1000, 1500, 2000, 3000, 4000, 5000, 7000, 10000, 20000
Quantification using: Lower Marker Final concentration (ng/uL): 0.5000 Dilution factor: 12.0

Sample: SampE3
Well location: E3
Created: Thursday, October 21, 2021 8:08:12 PM

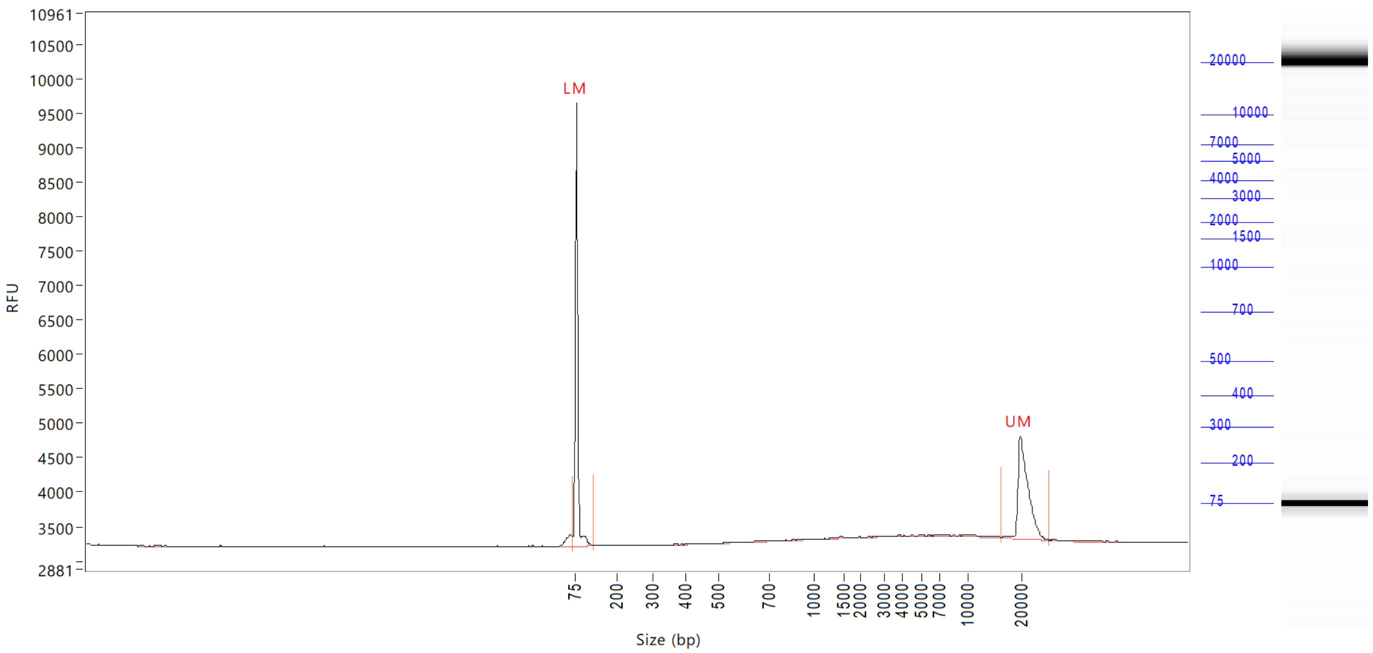

Peak	Size	Concentration	Relative concentration	Molarity	From	To	CV%	RFU
	(bp)	(ng/uL)	Percent	(nmole/L)	(bp)	(bp)		
1	75 (LM)	0.5000		11.0851	64	128	8.88	6453
2	20000 (UM)	0.2465		0.0197	16220	25440	5.61	1494

TIC:	0.0000	ng/uL
TIM:	0.0000	nmole/L
Total concentration:	0.6522	ng/uL

```
Sample peak width (sec): 5    Sample min peak height: 50    Sample baseline V to V?: Y    Sample baseline V to V points: 3
Sample filter: Binomial       Number of points for filter: 3  Sample start region (min): 0  Sample end region (min): 30
Marker peak width (sec): 5    Marker min peak height: 200   Marker baseline V to V?: Y    Marker baseline V to V points: 3
Lower marker selection: First peak > 500 RFU                Upper marker selection: Last peak > 200 RFU
Ladder size (bp)75, 200, 300, 400, 500, 700, 1000, 1500, 2000, 3000, 4000, 5000, 7000, 10000, 20000
Quantification using: Lower Marker          Final concentration (ng/uL): 0.5000          Dilution factor: 12.0
```

Sample: SampF3
Well location: F3
Created: Thursday, October 21, 2021 8:08:12 PM

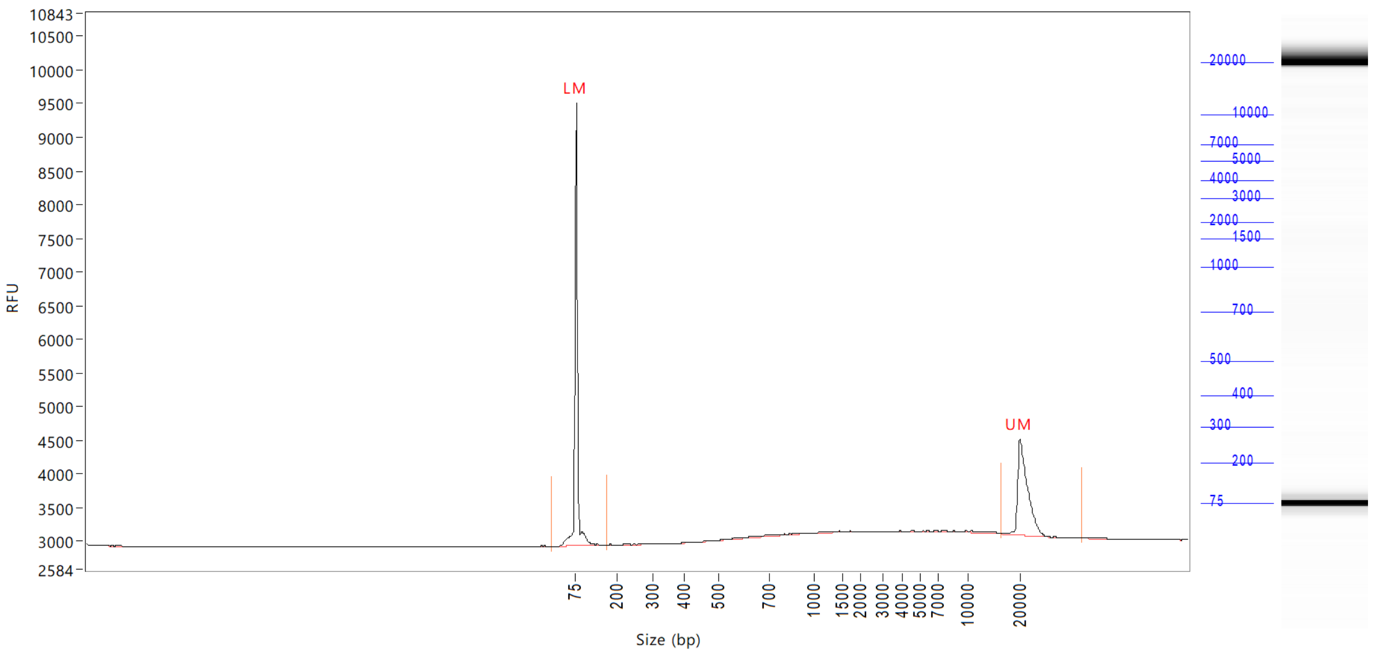

Peak	Size	Concentration	Relative concentration	Molarity	From	To	CV%	RFU
	(bp)	(ng/uL)	Percent	(nmole/L)	(bp)	(bp)		
1	75 (LM)	0.5000		11.0851	1	169	13.82	6589
2	20000 (UM)	0.2112		0.0168	16342	31607	6.53	1424

TIC:	0.0000	ng/uL
TIM:	0.0000	nmole/L
Total concentration:	0.6888	ng/uL

Sample peak width (sec): 5 Sample min peak height: 50 Sample baseline V to V?: Y Sample baseline V to V points: 3
Sample filter: Binomial Number of points for filter: 3 Sample start region (min): 0 Sample end region (min): 30
Marker peak width (sec): 5 Marker min peak height: 200 Marker baseline V to V?: Y Marker baseline V to V points: 3
Lower marker selection: First peak > 500 RFU Upper marker selection: Last peak > 200 RFU
Ladder size (bp)75, 200, 300, 400, 500, 700, 1000, 1500, 2000, 3000, 4000, 5000, 7000, 10000, 20000
Quantification using: Lower Marker Final concentration (ng/uL): 0.5000 Dilution factor: 12.0

Sample: SampG3
Well location: G3
Created: Thursday, October 21, 2021 8:08:12 PM

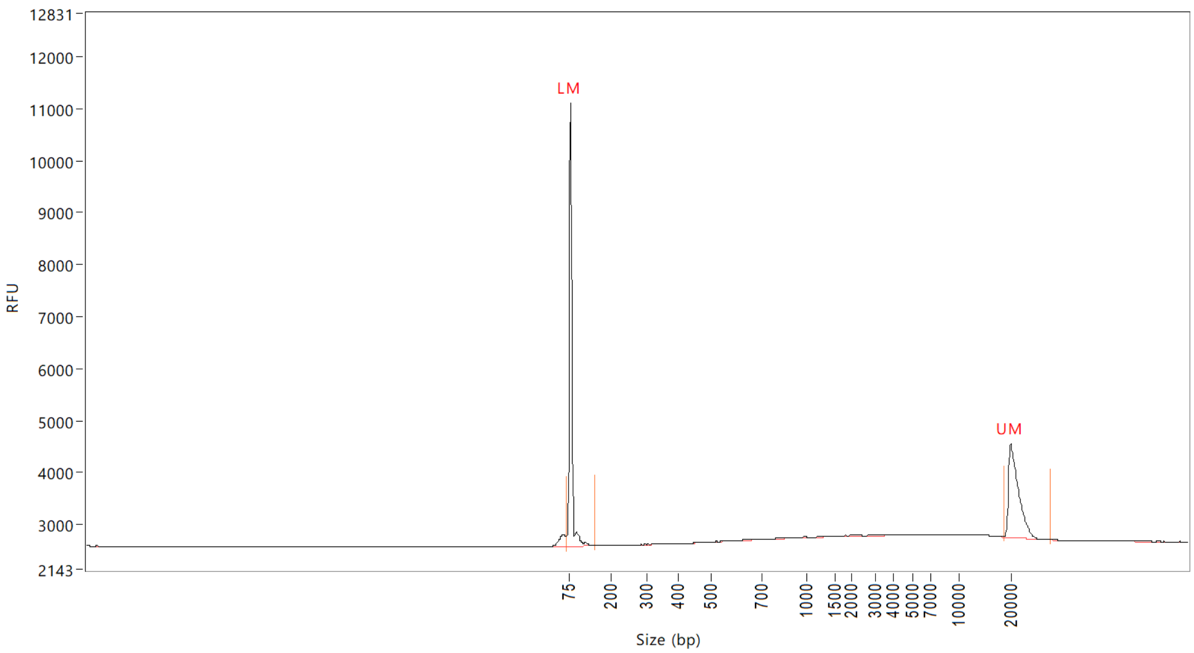

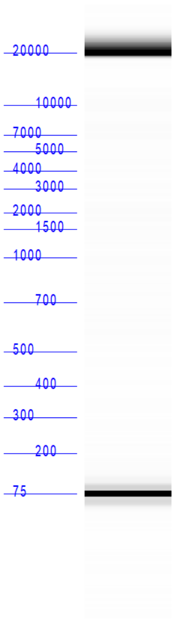

Peak	Size	Concentration	Relative concentration	Molarity	From	To	CV%	RFU
	(bp)	(ng/uL)	Percent	(nmole/L)	(bp)	(bp)		
1	75 (LM)	0.5000		10.9378	64	150	11.59	8538
2	20000 (UM)	0.2214		0.0176	18537	27617	5.39	1814

TIC:	0.0000	ng/uL
TIM:	0.0000	nmole/L
Total concentration:	0.4713	ng/uL

Sample peak width (sec): 5 Sample min peak height: 50 Sample baseline V to V?: Y Sample baseline V to V points: 3
Sample filter: Binomial Number of points for filter: 3 Sample start region (min): 0 Sample end region (min): 30
Marker peak width (sec): 5 Marker min peak height: 200 Marker baseline V to V?: Y Marker baseline V to V points: 3
Lower marker selection: First peak > 500 RFU Upper marker selection: Last peak > 200 RFU
Ladder size (bp)75, 200, 300, 400, 500, 700, 1000, 1500, 2000, 3000, 4000, 5000, 7000, 10000, 20000
Quantification using: Lower Marker Final concentration (ng/uL): 0.5000 Dilution factor: 12.0

Sample: SampH3
Well location: H3
Created: Thursday, October 21, 2021 8:08:12 PM

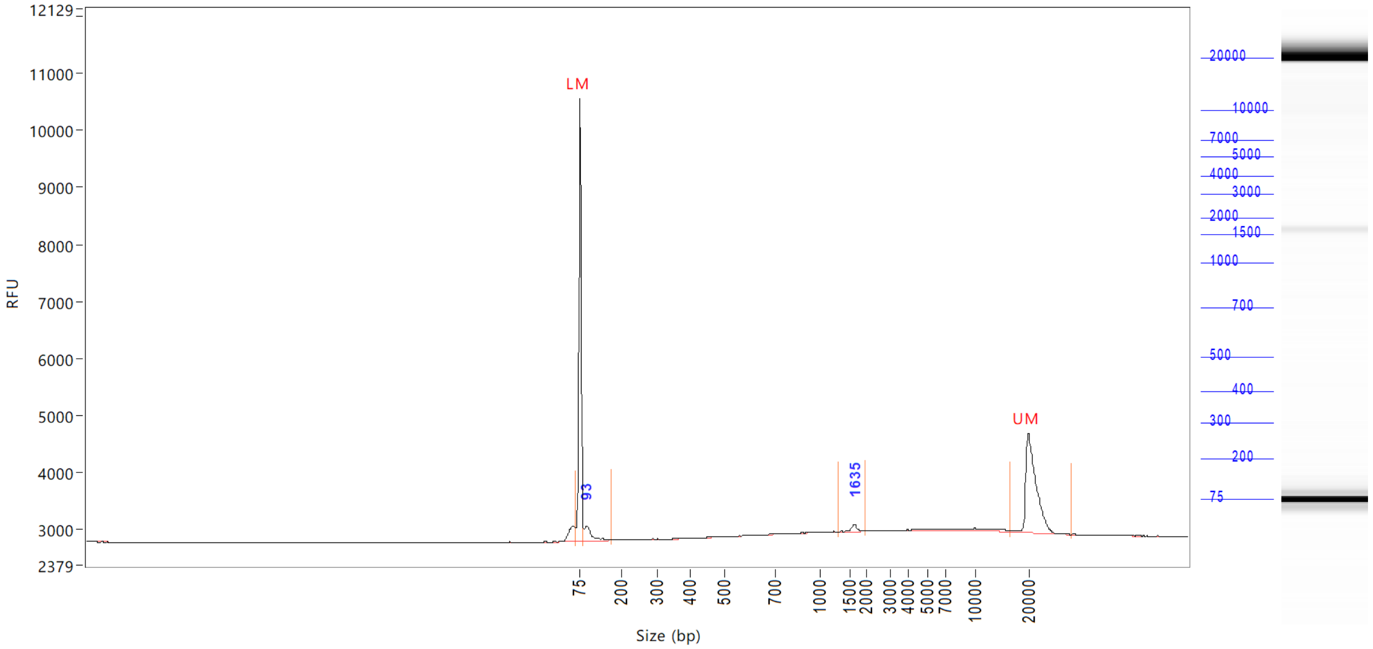

Peak	Size	Concentration	Relative concentration	Molarity	From	To	CV%	RFU
	(bp)	(ng/uL)	Percent	(nmole/L)	(bp)	(bp)		
1	75 (LM)	0.5000		11.3919	62	86	4.95	7772
2	93	0.6115	78.8	9.7498	86	169	15.80	267
3	1635	0.1641	21.2	0.1686	1311	1942	6.61	133
4	20000 (UM)	0.2455		0.0195	16464	28100	5.75	1741

TIC:	0.7756	ng/uL
TIM:	9.9184	nmole/L
Total concentration:	1.3292	ng/uL

Sample peak width (sec): 5   Sample min peak height: 50   Sample baseline V to V?: Y   Sample baseline V to V points: 3
Sample filter: Binomial   Number of points for filter: 3 Sample start region (min): 0 Sample end region (min): 30
Marker peak width (sec): 5   Marker min peak height: 200   Marker baseline V to V?: Y   Marker baseline V to V points: 3
Lower marker selection: First peak > 500 RFU   Upper marker selection: Last peak > 200 RFU
Ladder size (bp)75, 200, 300, 400, 500, 700, 1000, 1500, 2000, 3000, 4000, 5000, 7000, 10000, 20000
Quantification using: Lower Marker   Final concentration (ng/uL): 0.5000   Dilution factor: 12.0

Sample: SampA4
Well location: A4
Created: Thursday, October 21, 2021 8:08:12 PM

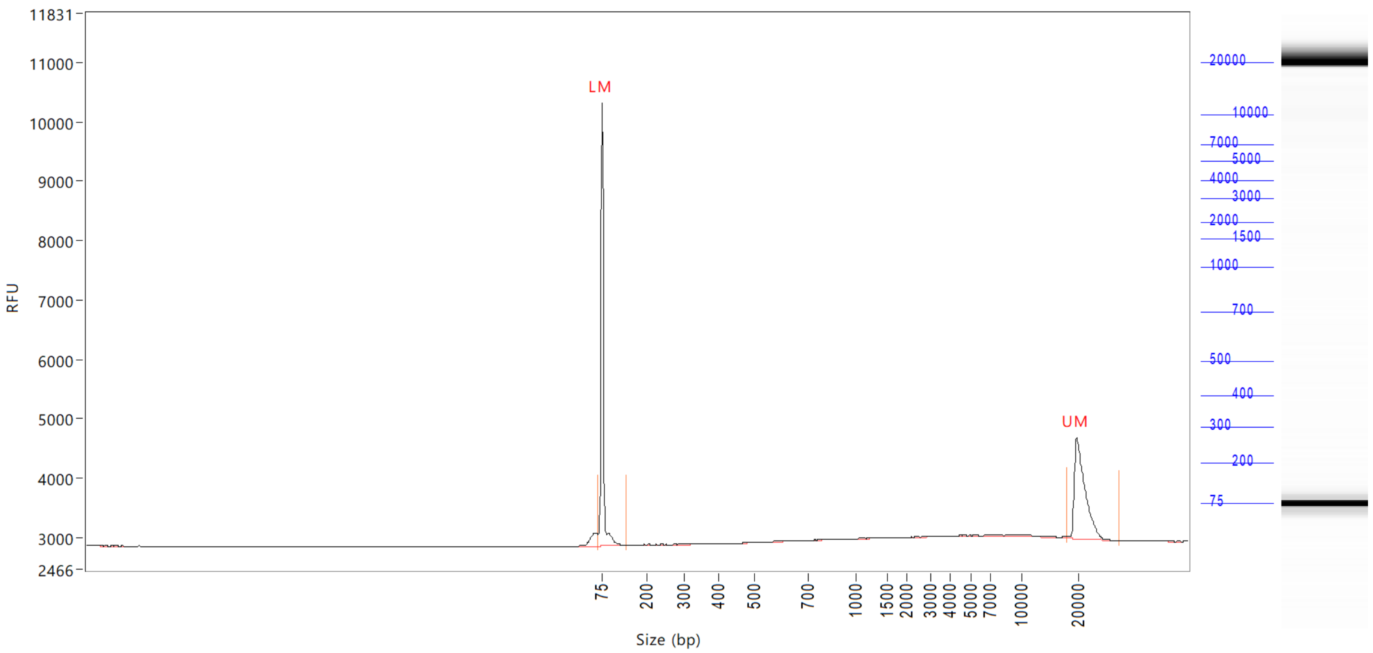

Peak	Size	Concentration	Relative concentration	Molarity	From	To	CV%	RFU
	(bp)	(ng/uL)	Percent	(nmole/L)	(bp)	(bp)		
1	75 (LM)	0.5000		11.0851	64	143	10.12	7475
2	20000 (UM)	0.2370		0.0188	18293	27375	5.52	1692

TIC:	0.0000	ng/uL
TIM:	0.0000	nmole/L
Total concentration:	0.6058	ng/uL

Sample peak width (sec): 5   Sample min peak height: 50   Sample baseline V to V?: Y   Sample baseline V to V points: 3
Sample filter: Binomial   Number of points for filter: 3 Sample start region (min): 0 Sample end region (min): 30
Marker peak width (sec): 5   Marker min peak height: 200   Marker baseline V to V?: Y   Marker baseline V to V points: 3
Lower marker selection: First peak > 500 RFU   Upper marker selection: Last peak > 200 RFU
Ladder size (bp)75, 200, 300, 400, 500, 700, 1000, 1500, 2000, 3000, 4000, 5000, 7000, 10000, 20000
Quantification using: Lower Marker   Final concentration (ng/uL): 0.5000   Dilution factor: 12.0

Sample: SampB4
Well location: B4
Created: Thursday, October 21, 2021 8:08:12 PM

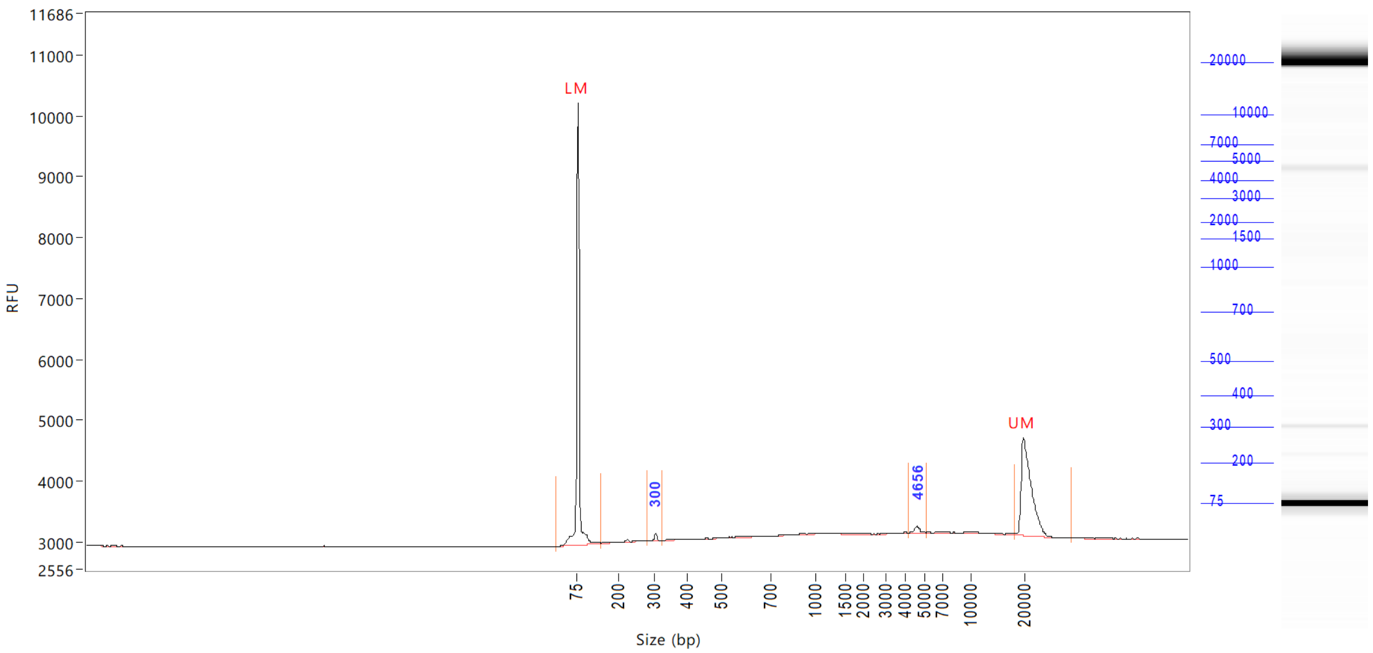

Peak	Size	Concentration	Relative concentration	Molarity	From	To	CV%	RFU
	(bp)	(ng/uL)	Percent	(nmole/L)	(bp)	(bp)		
1	75 (LM)	0.5000		11.0851	11	143	13.09	7274
2	300	0.1023	45.9	0.5667	277	321	2.06	130
3	4656	0.1208	54.1	0.0432	4173	5223	3.76	114
4	20000 (UM)	0.2182		0.0173	18415	29068	5.68	1607

TIC:	0.2232	ng/uL
TIM:	0.6099	nmole/L
Total concentration:	0.8415	ng/uL

```
Sample peak width (sec): 5    Sample min peak height: 50    Sample baseline V to V?: Y    Sample baseline V to V points: 3
Sample filter: Binomial       Number of points for filter: 3  Sample start region (min): 0  Sample end region (min): 30
Marker peak width (sec): 5    Marker min peak height: 200   Marker baseline V to V?: Y    Marker baseline V to V points: 3
Lower marker selection: First peak > 500 RFU                Upper marker selection: Last peak > 200 RFU
Ladder size (bp)75, 200, 300, 400, 500, 700, 1000, 1500, 2000, 3000, 4000, 5000, 7000, 10000, 20000
Quantification using: Lower Marker          Final concentration (ng/uL): 0.5000          Dilution factor: 12.0
```

Sample: SampC4
Well location: C4
Created: Thursday, October 21, 2021 8:08:12 PM

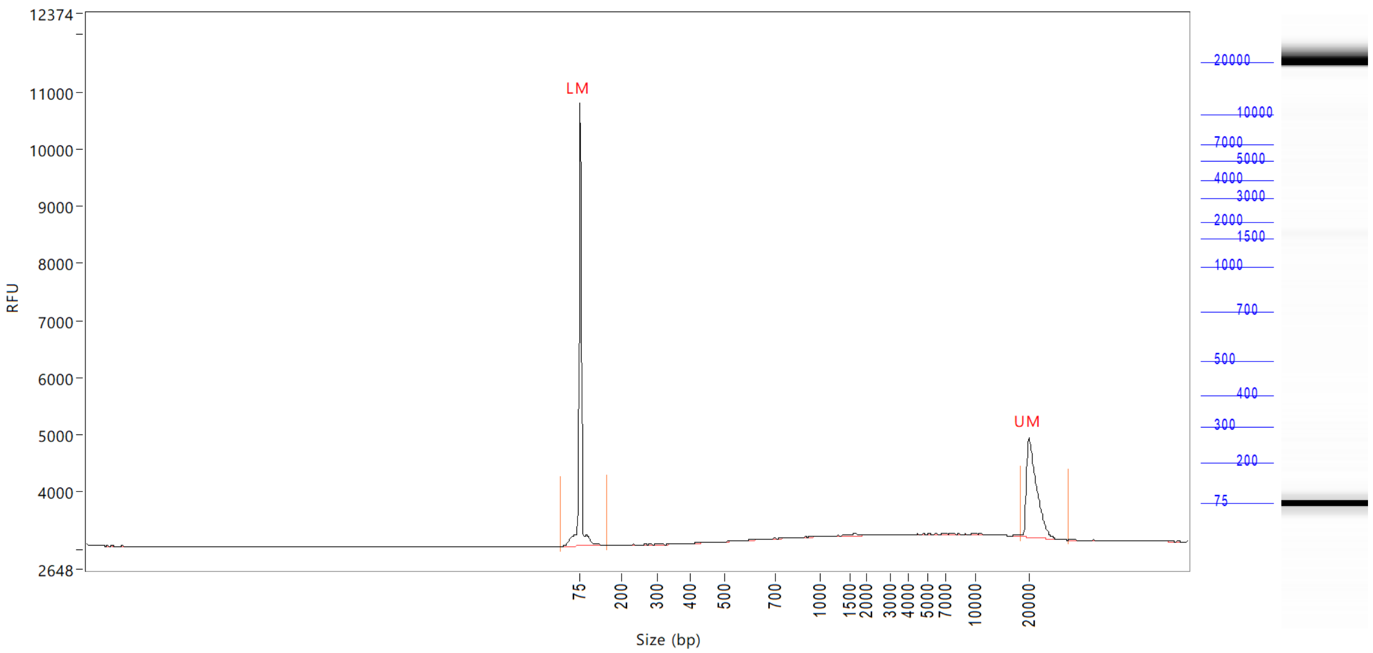

Peak	Size	Concentration	Relative concentration	Molarity	From	To	CV%	RFU
	(bp)	(ng/uL)	Percent	(nmole/L)	(bp)	(bp)		
1	75 (LM)	0.5000		11.2364	16	156	13.17	7763
2	20000 (UM)	0.2241		0.0178	18537	27375	5.30	1738

TIC:	0.0000	ng/uL
TIM:	0.0000	nmole/L
Total concentration:	0.4982	ng/uL

Sample peak width (sec): 5   Sample min peak height: 50   Sample baseline V to V?: Y   Sample baseline V to V points: 3
Sample filter: Binomial   Number of points for filter: 3   Sample start region (min): 0   Sample end region (min): 30
Marker peak width (sec): 5   Marker min peak height: 200   Marker baseline V to V?: Y   Marker baseline V to V points: 3
Lower marker selection: First peak > 500 RFU   Upper marker selection: Last peak > 200 RFU
Ladder size (bp)75, 200, 300, 400, 500, 700, 1000, 1500, 2000, 3000, 4000, 5000, 7000, 10000, 20000
Quantification using: Lower Marker   Final concentration (ng/uL): 0.5000   Dilution factor: 12.0

Sample: SampD4
Well location: D4
Created: Thursday, October 21, 2021 8:08:12 PM

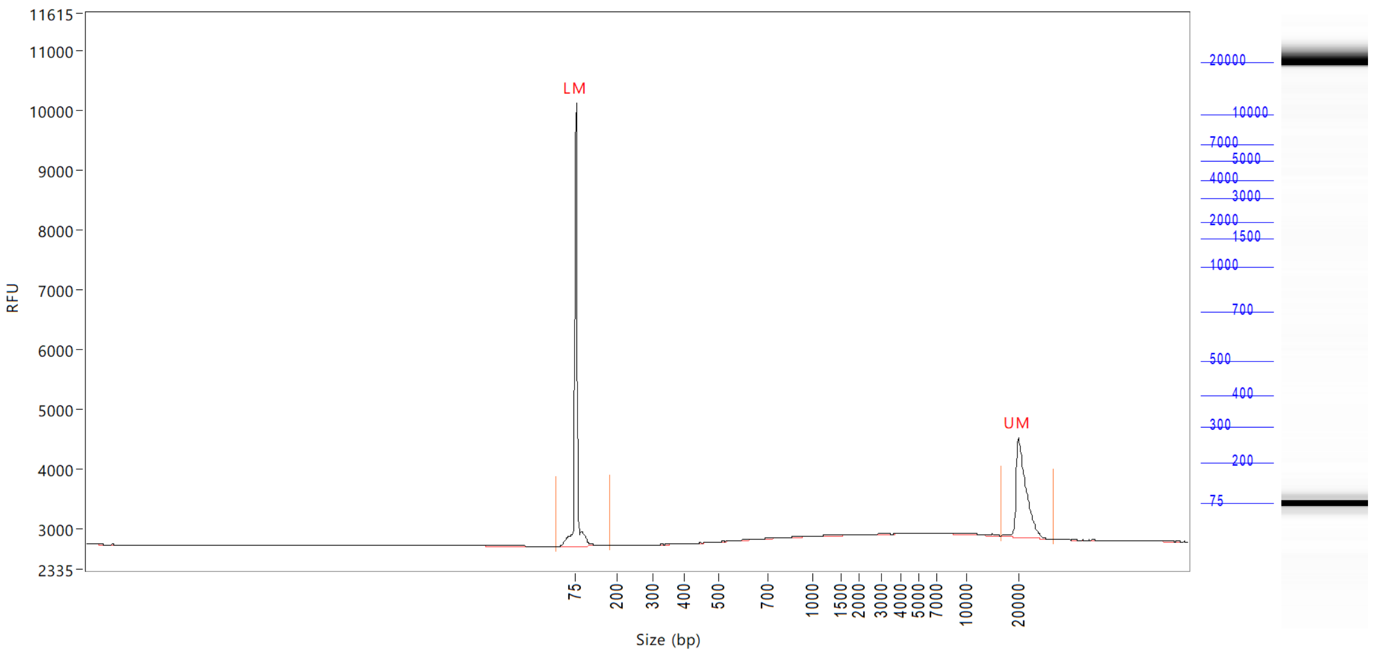

Peak	Size	Concentration	Relative concentration	Molarity	From	To	CV%	RFU
	(bp)	(ng/uL)	Percent	(nmole/L)	(bp)	(bp)		
1	75 (LM)	0.5000		11.2364	18	175	14.80	7413
2	20000 (UM)	0.2164		0.0172	16586	26770	5.53	1666

TIC:	0.0000	ng/uL
TIM:	0.0000	nmole/L
Total concentration:	0.5735	ng/uL

```
Sample peak width (sec): 5    Sample min peak height: 50    Sample baseline V to V?: Y    Sample baseline V to V points: 3
Sample filter: Binomial       Number of points for filter: 3  Sample start region (min): 0  Sample end region (min): 30
Marker peak width (sec): 5    Marker min peak height: 200   Marker baseline V to V?: Y    Marker baseline V to V points: 3
Lower marker selection: First peak > 500 RFU                Upper marker selection: Last peak > 200 RFU
Ladder size (bp)75, 200, 300, 400, 500, 700, 1000, 1500, 2000, 3000, 4000, 5000, 7000, 10000, 20000
Quantification using: Lower Marker          Final concentration (ng/uL): 0.5000          Dilution factor: 12.0
```

Sample: SampE4
Well location: E4
Created: Thursday, October 21, 2021 8:08:12 PM

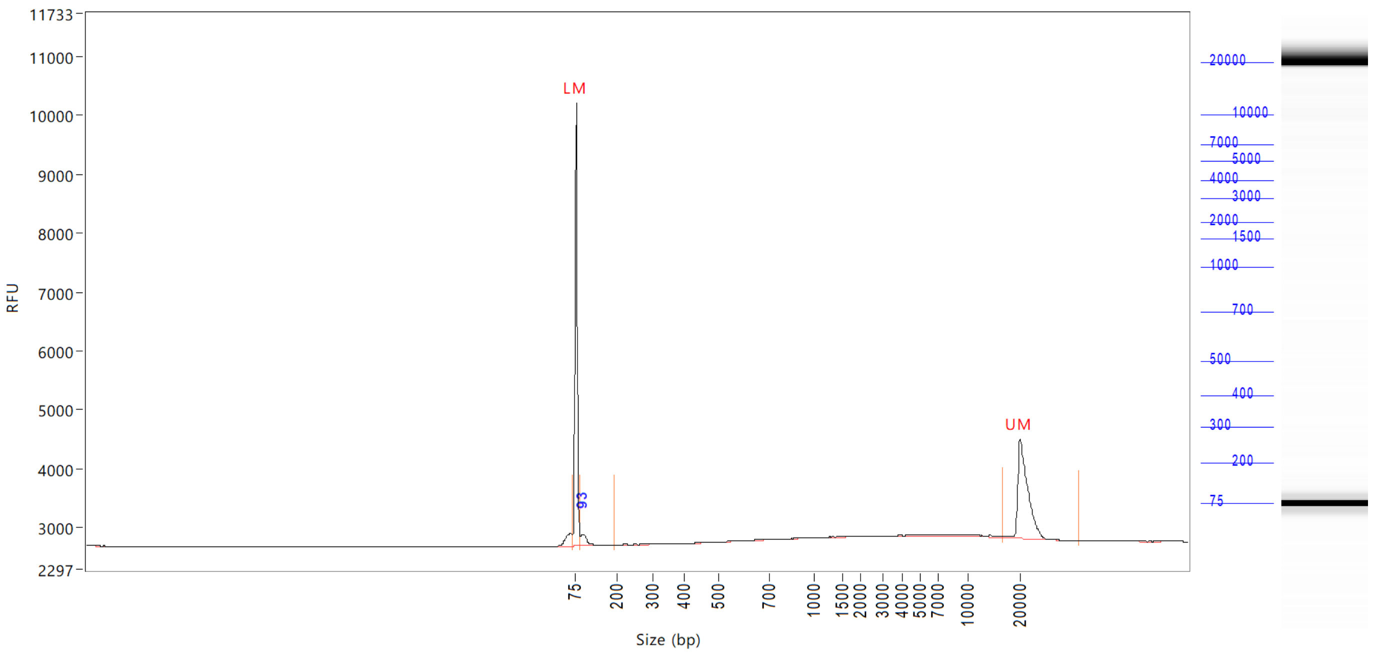

Peak	Size	Concentration	Relative concentration	Molarity	From	To	CV%	RFU
	(bp)	(ng/uL)	Percent	(nmole/L)	(bp)	(bp)		
1	75 (LM)	0.5000		11.2364	64	87	4.69	7532
2	93	0.4438	100.0	7.0080	87	190	17.64	193
3	20000 (UM)	0.2502		0.0199	16708	31244	6.00	1681

TIC:	0.4438	ng/uL
TIM:	7.0080	nmole/L
Total concentration:	0.9688	ng/uL

```
Sample peak width (sec): 5    Sample min peak height: 50    Sample baseline V to V?: Y    Sample baseline V to V points: 3
Sample filter: Binomial       Number of points for filter: 3  Sample start region (min): 0  Sample end region (min): 30
Marker peak width (sec): 5    Marker min peak height: 200   Marker baseline V to V?: Y    Marker baseline V to V points: 3
Lower marker selection: First peak > 500 RFU                Upper marker selection: Last peak > 200 RFU
Ladder size (bp)75, 200, 300, 400, 500, 700, 1000, 1500, 2000, 3000, 4000, 5000, 7000, 10000, 20000
Quantification using: Lower Marker          Final concentration (ng/uL): 0.5000          Dilution factor: 12.0
```

Sample: SampF4
Well location: F4
Created: Thursday, October 21, 2021 8:08:12 PM

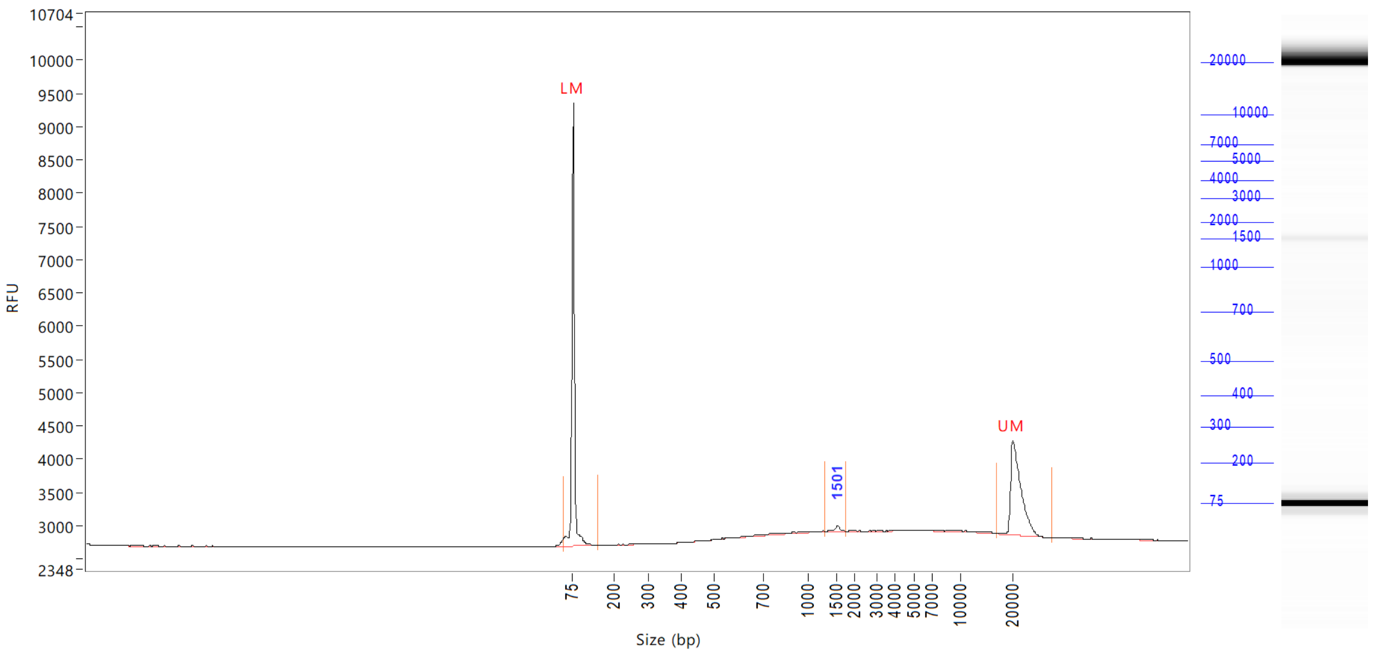

Peak	Size	Concentration	Relative concentration	Molarity	From	To	CV%	RFU
	(bp)	(ng/uL)	Percent	(nmole/L)	(bp)	(bp)		
1	75 (LM)	0.5000		11.0851	48	150	11.73	6671
2	1501	0.1307	100.0	0.1447	1300	1770	6.05	86
3	20000 (UM)	0.2174		0.0172	16830	27375	5.71	1411

TIC:	0.1307	ng/uL
TIM:	0.1447	nmole/L
Total concentration:	0.7375	ng/uL

```
Sample peak width (sec): 5    Sample min peak height: 50    Sample baseline V to V?: Y    Sample baseline V to V points: 3
Sample filter: Binomial       Number of points for filter: 3  Sample start region (min): 0  Sample end region (min): 30
Marker peak width (sec): 5    Marker min peak height: 200   Marker baseline V to V?: Y    Marker baseline V to V points: 3
Lower marker selection: First peak > 500 RFU                Upper marker selection: Last peak > 200 RFU
Ladder size (bp)75, 200, 300, 400, 500, 700, 1000, 1500, 2000, 3000, 4000, 5000, 7000, 10000, 20000
Quantification using: Lower Marker          Final concentration (ng/uL): 0.5000          Dilution factor: 12.0
```

Sample: SampG4
Well location: G4
Created: Thursday, October 21, 2021 8:08:12 PM

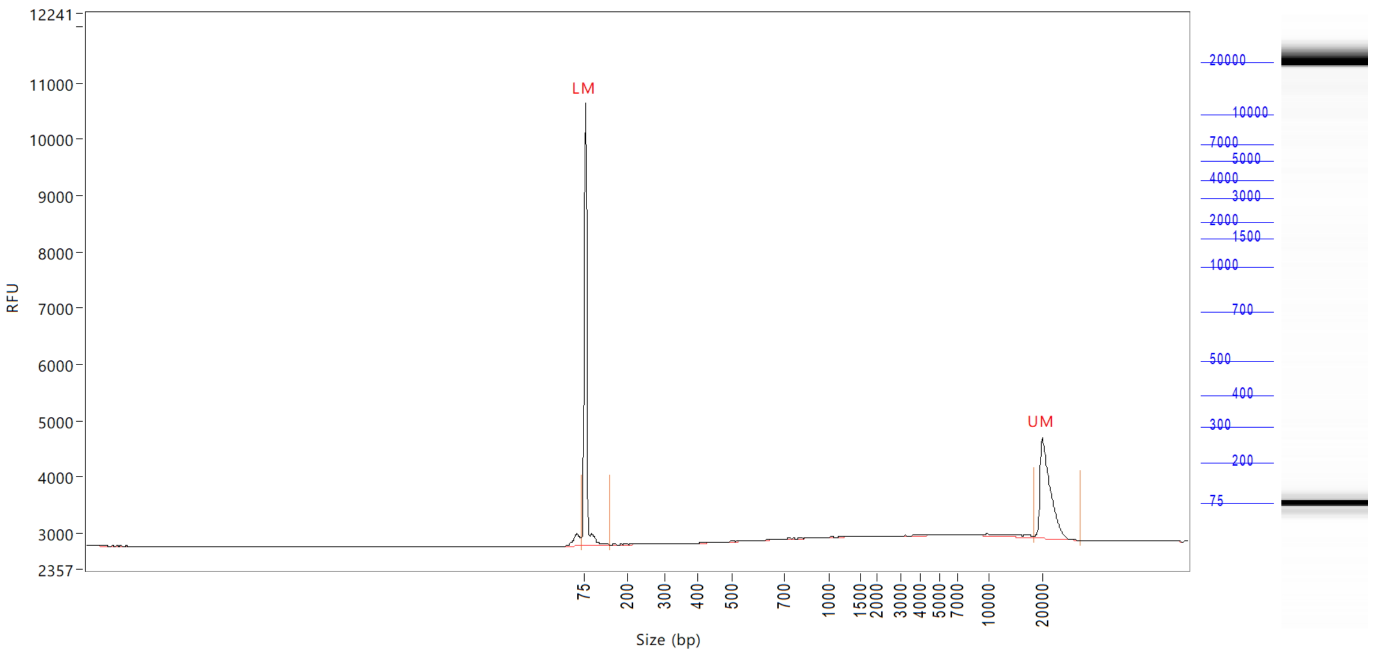

Peak	Size	Concentration	Relative concentration	Molarity	From	To	CV%	RFU
	(bp)	(ng/uL)	Percent	(nmole/L)	(bp)	(bp)		
1	75 (LM)	0.5000		10.9378	64	148	9.98	7886
2	20000 (UM)	0.2388		0.0189	18537	27012	5.39	1791

TIC:	0.0000	ng/uL
TIM:	0.0000	nmole/L
Total concentration:	0.6455	ng/uL

```
Sample peak width (sec): 5    Sample min peak height: 50    Sample baseline V to V?: Y    Sample baseline V to V points: 3
Sample filter: Binomial       Number of points for filter: 3  Sample start region (min): 0  Sample end region (min): 30
Marker peak width (sec): 5    Marker min peak height: 200   Marker baseline V to V?: Y    Marker baseline V to V points: 3
Lower marker selection: First peak > 500 RFU                Upper marker selection: Last peak > 200 RFU
Ladder size (bp)75, 200, 300, 400, 500, 700, 1000, 1500, 2000, 3000, 4000, 5000, 7000, 10000, 20000
Quantification using: Lower Marker          Final concentration (ng/uL): 0.5000          Dilution factor: 12.0
```

Sample: SampH4
Well location: H4
Created: Thursday, October 21, 2021 8:08:12 PM

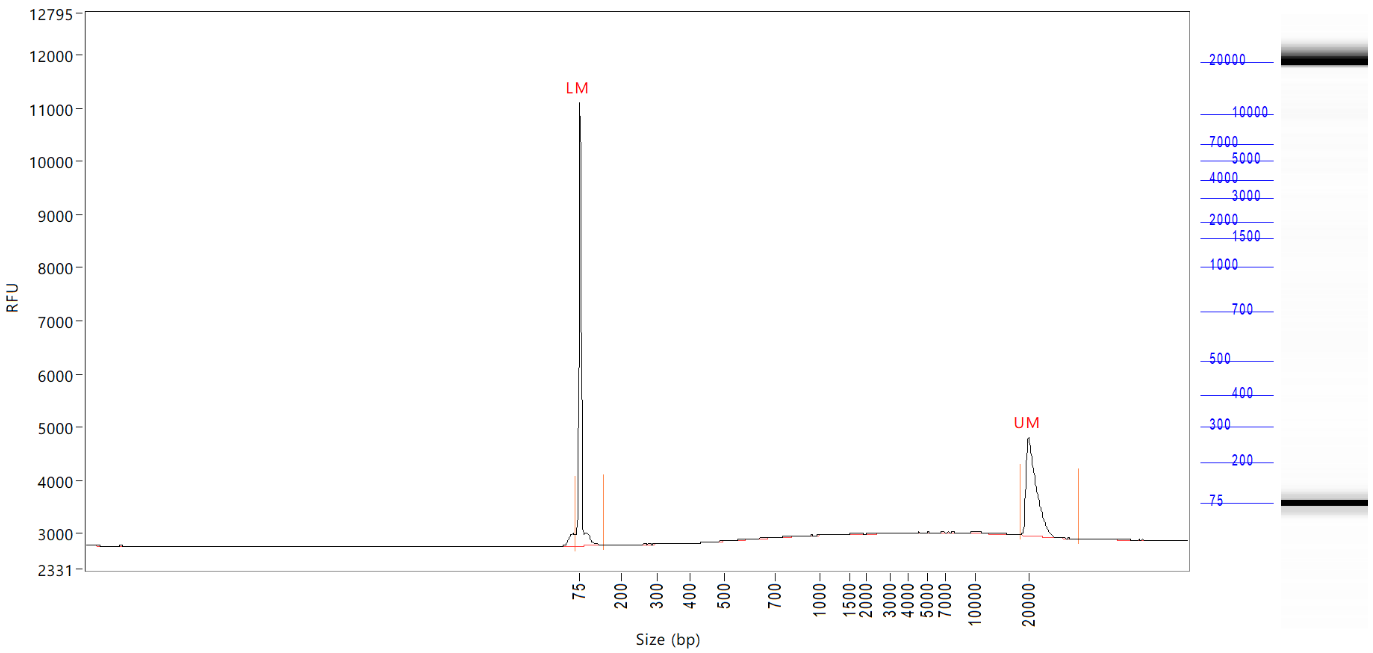

Peak	Size	Concentration	Relative concentration	Molarity	From	To	CV%	RFU
	(bp)	(ng/uL)	Percent	(nmole/L)	(bp)	(bp)		
1	75 (LM)	0.5000		10.9378	64	145	10.51	8356
2	20000 (UM)	0.2327		0.0184	18415	29551	5.72	1860

TIC:	0.0000	ng/uL
TIM:	0.0000	nmole/L
Total concentration:	0.5337	ng/uL

Sample peak width (sec): 5 Sample min peak height: 50 Sample baseline V to V?: Y Sample baseline V to V points: 3
Sample filter: Binomial Number of points for filter: 3 Sample start region (min): 0 Sample end region (min): 30
Marker peak width (sec): 5 Marker min peak height: 200 Marker baseline V to V?: Y Marker baseline V to V points: 3
Lower marker selection: First peak > 500 RFU Upper marker selection: Last peak > 200 RFU
Ladder size (bp)75, 200, 300, 400, 500, 700, 1000, 1500, 2000, 3000, 4000, 5000, 7000, 10000, 20000
Quantification using: Lower Marker Final concentration (ng/uL): 0.5000 Dilution factor: 12.0

Sample: SampA5
Well location: A5
Created: Thursday, October 21, 2021 8:08:12 PM

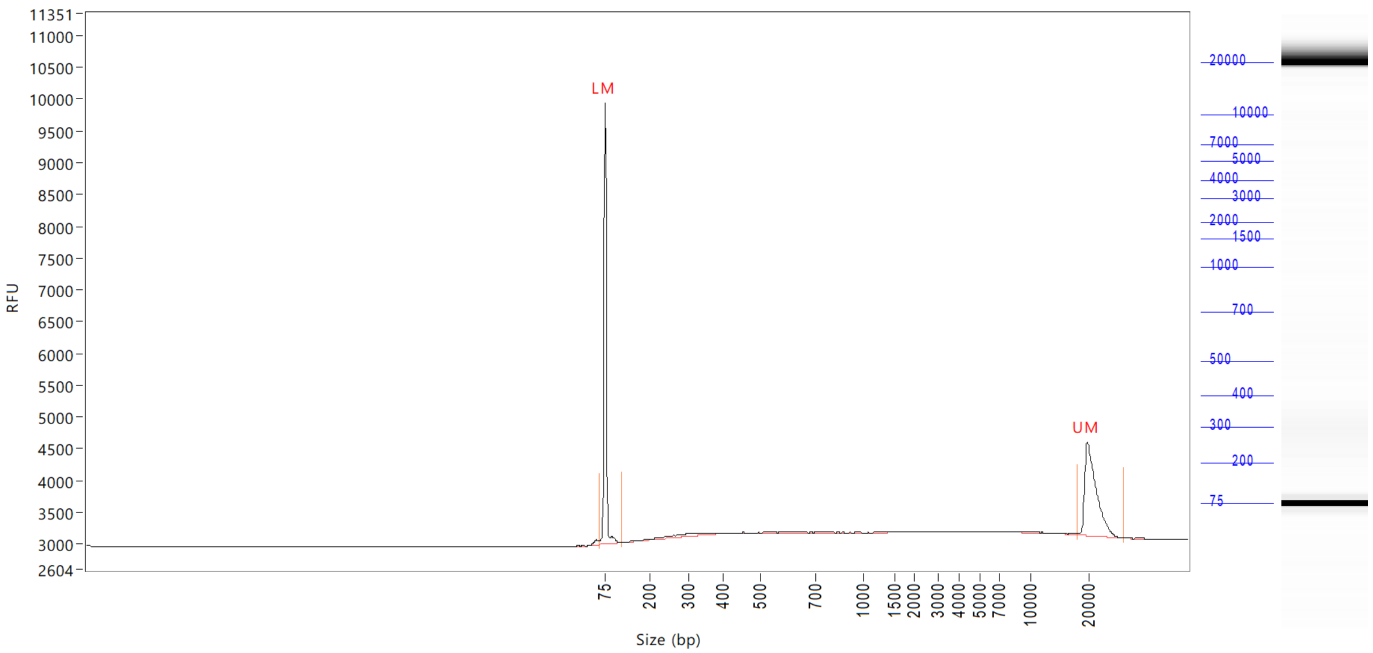

Peak	Size	Concentration	Relative concentration	Molarity	From	To	CV%	RFU
	(bp)	(ng/uL)	Percent	(nmole/L)	(bp)	(bp)		
1	75 (LM)	0.5000		11.2364	58	122	6.61	6952
2	20000 (UM)	0.2497		0.0198	18171	26166	5.68	1477

TIC:	0.0000	ng/uL
TIM:	0.0000	nmole/L
Total concentration:	0.9756	ng/uL

Sample peak width (sec): 5 Sample min peak height: 50 Sample baseline V to V?: Y Sample baseline V to V points: 3
Sample filter: Binomial Number of points for filter: 3 Sample start region (min): 0 Sample end region (min): 30
Marker peak width (sec): 5 Marker min peak height: 200 Marker baseline V to V?: Y Marker baseline V to V points: 3
Lower marker selection: First peak > 500 RFU Upper marker selection: Last peak > 200 RFU
Ladder size (bp)75, 200, 300, 400, 500, 700, 1000, 1500, 2000, 3000, 4000, 5000, 7000, 10000, 20000
Quantification using: Lower Marker Final concentration (ng/uL): 0.5000 Dilution factor: 12.0

Sample: SampB5
Well location: B5
Created: Thursday, October 21, 2021 8:08:12 PM

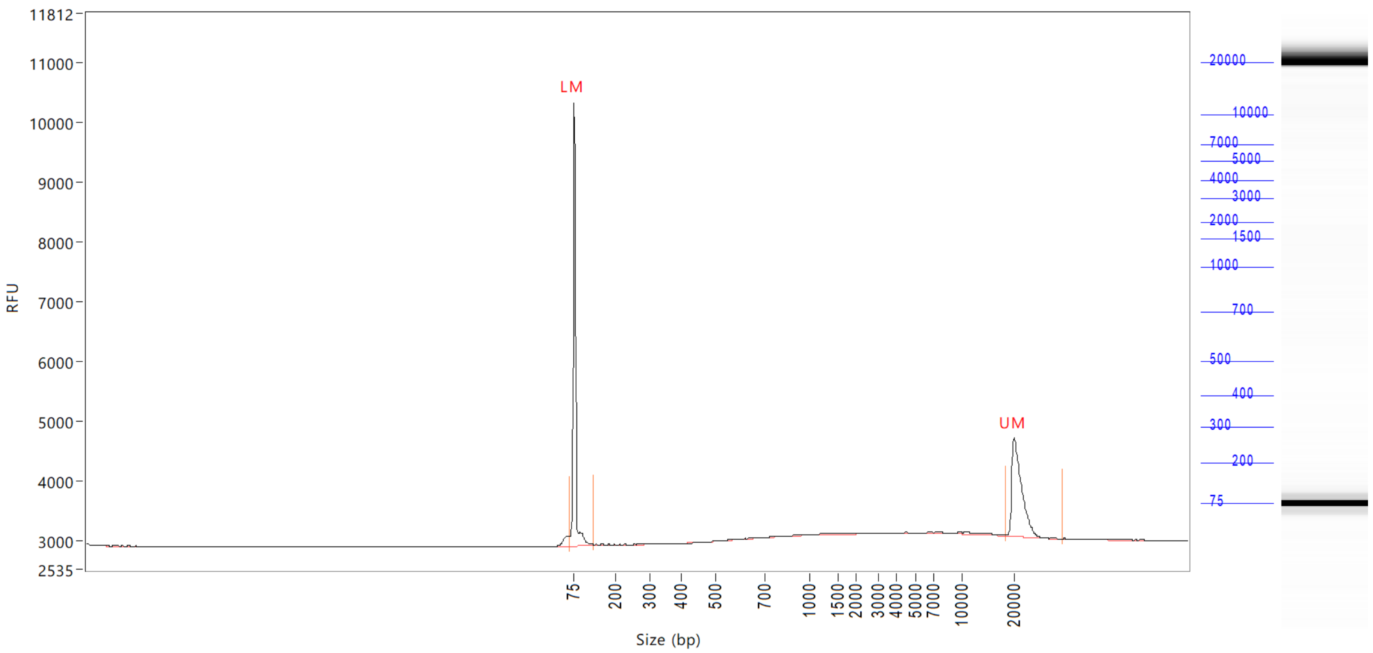

Peak	Size	Concentration	Relative concentration	Molarity	From	To	CV%	RFU
	(bp)	(ng/uL)	Percent	(nmole/L)	(bp)	(bp)		
1	75 (LM)	0.5000		10.9378	62	133	10.51	7407
2	20000 (UM)	0.2244		0.0178	18293	29189	5.45	1642

TIC:	0.0000	ng/uL
TIM:	0.0000	nmole/L
Total concentration:	0.6816	ng/uL

Sample peak width (sec): 5 Sample min peak height: 50 Sample baseline V to V?: Y Sample baseline V to V points: 3
Sample filter: Binomial Number of points for filter: 3 Sample start region (min): 0 Sample end region (min): 30
Marker peak width (sec): 5 Marker min peak height: 200 Marker baseline V to V?: Y Marker baseline V to V points: 3
Lower marker selection: First peak > 500 RFU Upper marker selection: Last peak > 200 RFU
Ladder size (bp)75, 200, 300, 400, 500, 700, 1000, 1500, 2000, 3000, 4000, 5000, 7000, 10000, 20000
Quantification using: Lower Marker Final concentration (ng/uL): 0.5000 Dilution factor: 12.0

Sample: SampC5
Well location: C5
Created: Thursday, October 21, 2021 8:08:12 PM

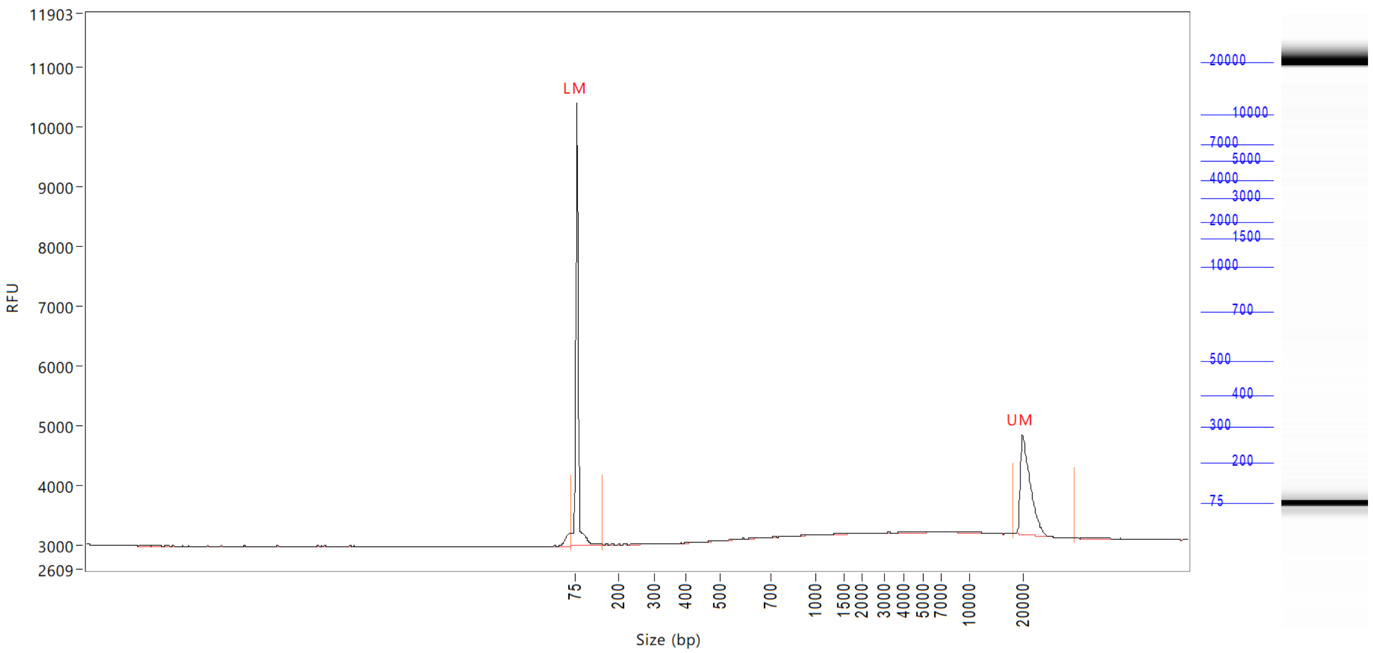

Peak	Size	Concentration	Relative concentration	Molarity	From	To	CV%	RFU
	(bp)	(ng/uL)	Percent	(nmole/L)	(bp)	(bp)		
1	75 (LM)	0.5000		10.7944	60	154	12.17	7418
2	20000 (UM)	0.2214		0.0175	18171	29793	5.43	1672

TIC:	0.0000	ng/uL
TIM:	0.0000	nmole/L
Total concentration:	0.5805	ng/uL

Sample peak width (sec): 5 Sample min peak height: 50 Sample baseline V to V?: Y Sample baseline V to V points: 3
Sample filter: Binomial Number of points for filter: 3 Sample start region (min): 0 Sample end region (min): 30
Marker peak width (sec): 5 Marker min peak height: 200 Marker baseline V to V?: Y Marker baseline V to V points: 3
Lower marker selection: First peak > 500 RFU Upper marker selection: Last peak > 200 RFU
Ladder size (bp)75, 200, 300, 400, 500, 700, 1000, 1500, 2000, 3000, 4000, 5000, 7000, 10000, 20000
Quantification using: Lower Marker Final concentration (ng/uL): 0.5000 Dilution factor: 12.0

Sample: SampD5
Well location: D5
Created: Thursday, October 21, 2021 8:08:12 PM

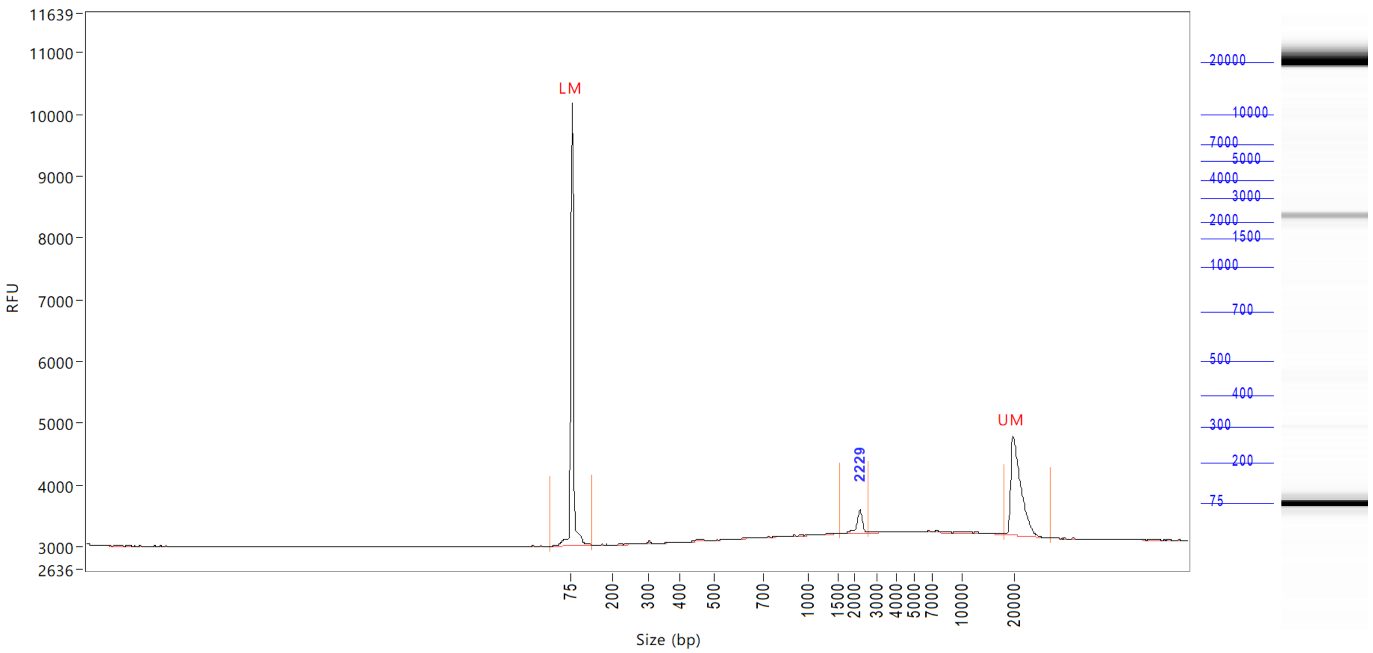

Peak	Size	Concentration	Relative concentration	Molarity	From	To	CV%	RFU
	(bp)	(ng/uL)	Percent	(nmole/L)	(bp)	(bp)		
1	75 (LM)	0.5000		11.2364	11	135	12.27	7184
2	2229	0.4198	100.0	0.3191	1578	2629	7.38	393
3	20000 (UM)	0.2204		0.0175	18293	27012	5.33	1607

TIC:	0.4198	ng/uL
TIM:	0.3191	nmole/L
Total concentration:	0.9615	ng/uL

```
Sample peak width (sec): 5    Sample min peak height: 50    Sample baseline V to V?: Y    Sample baseline V to V points: 3
Sample filter: Binomial       Number of points for filter: 3  Sample start region (min): 0  Sample end region (min): 30
Marker peak width (sec): 5    Marker min peak height: 200   Marker baseline V to V?: Y    Marker baseline V to V points: 3
Lower marker selection: First peak > 500 RFU                Upper marker selection: Last peak > 200 RFU
Ladder size (bp)75, 200, 300, 400, 500, 700, 1000, 1500, 2000, 3000, 4000, 5000, 7000, 10000, 20000
Quantification using: Lower Marker          Final concentration (ng/uL): 0.5000          Dilution factor: 12.0
```

Sample: SampE5
Well location: E5
Created: Thursday, October 21, 2021 8:08:12 PM

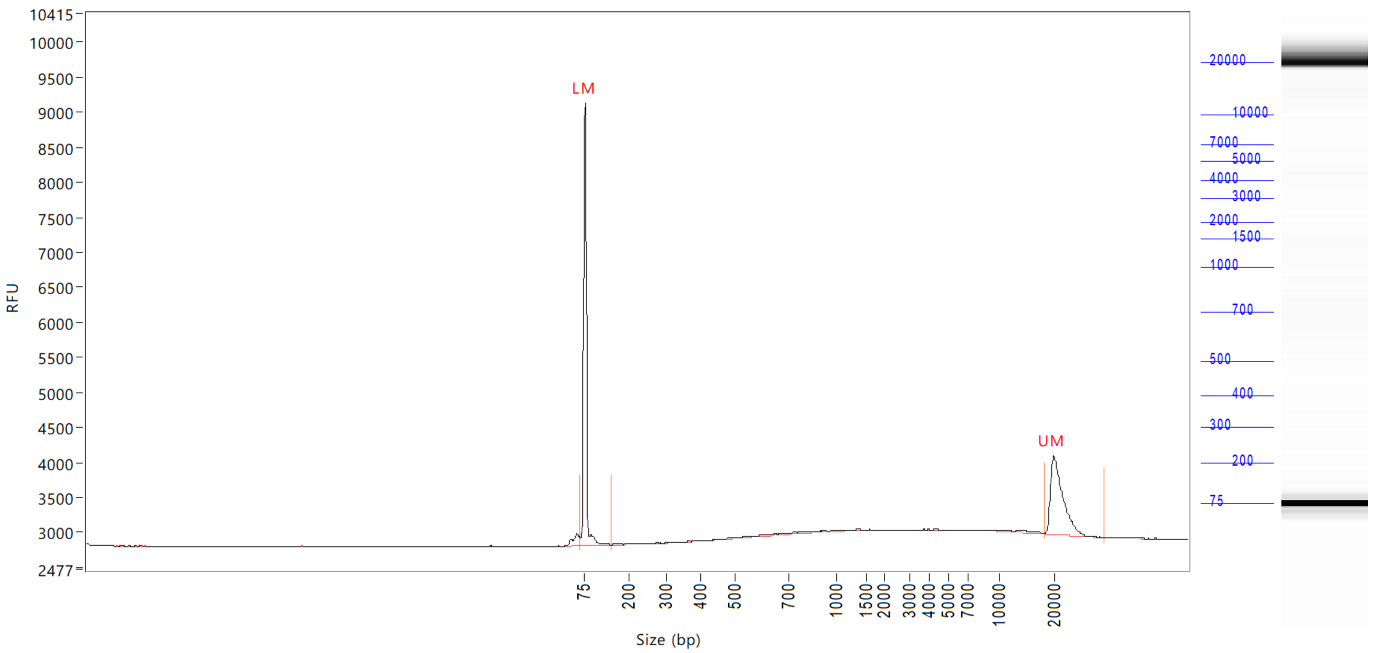

Peak	Size	Concentration	Relative concentration	Molarity	From	To	CV%	RFU
	(bp)	(ng/uL)	Percent	(nmole/L)	(bp)	(bp)		
1	75 (LM)	0.5000		11.0851	62	148	10.12	6332
2	20000 (UM)	0.2104		0.0166	18171	29068	6.42	1119

TIC:	0.0000	ng/uL
TIM:	0.0000	nmole/L
Total concentration:	0.8011	ng/uL

```
Sample peak width (sec): 5    Sample min peak height: 50    Sample baseline V to V?: Y    Sample baseline V to V points: 3
Sample filter: Binomial       Number of points for filter: 3  Sample start region (min): 0  Sample end region (min): 30
Marker peak width (sec): 5    Marker min peak height: 200   Marker baseline V to V?: Y    Marker baseline V to V points: 3
Lower marker selection: First peak > 500 RFU                Upper marker selection: Last peak > 200 RFU
Ladder size (bp)75, 200, 300, 400, 500, 700, 1000, 1500, 2000, 3000, 4000, 5000, 7000, 10000, 20000
Quantification using: Lower Marker          Final concentration (ng/uL): 0.5000          Dilution factor: 12.0
```

Sample: SampF5
Well location: F5
Created: Thursday, October 21, 2021 8:08:12 PM

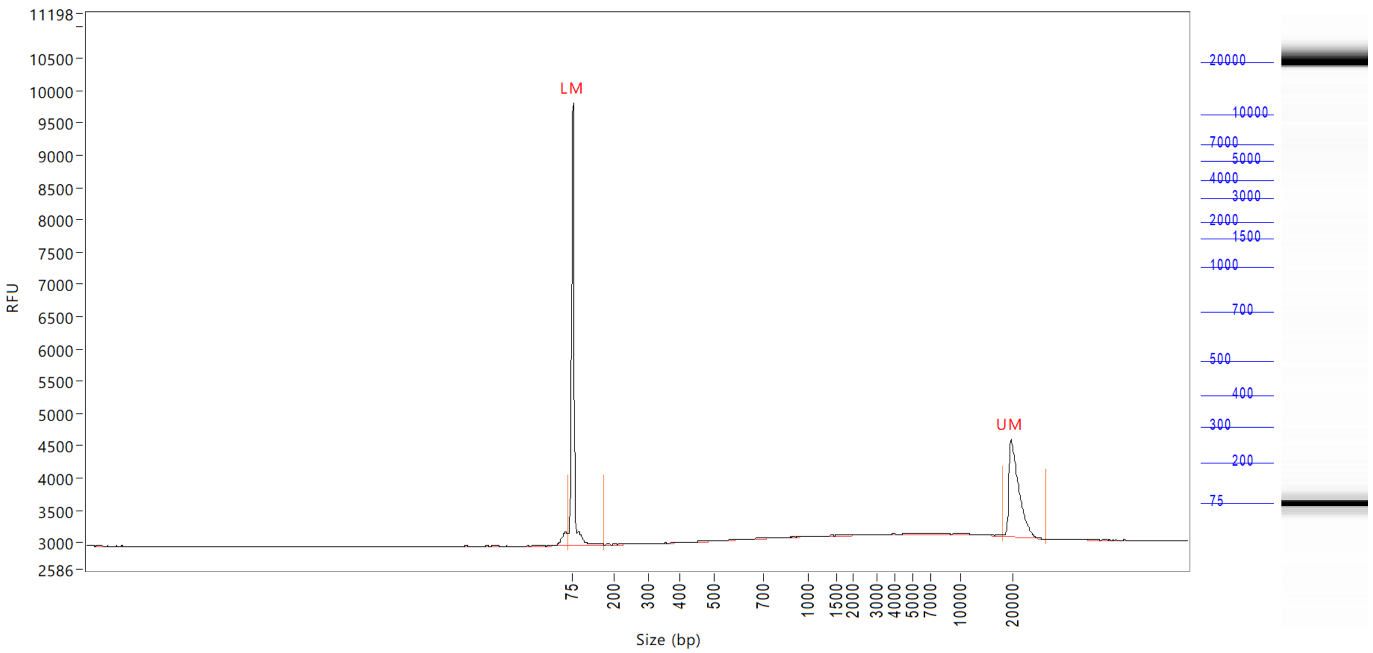

Peak	Size	Concentration	Relative concentration	Molarity	From	To	CV%	RFU
	(bp)	(ng/uL)	Percent	(nmole/L)	(bp)	(bp)		
1	75 (LM)	0.5000		10.9378	62	171	13.49	6869
2	20000 (UM)	0.2172		0.0173	18293	26770	5.36	1509

TIC:	0.0000	ng/uL
TIM:	0.0000	nmole/L
Total concentration:	0.5848	ng/uL

```
Sample peak width (sec): 5    Sample min peak height: 50    Sample baseline V to V?: Y    Sample baseline V to V points: 3
Sample filter: Binomial       Number of points for filter: 3  Sample start region (min): 0  Sample end region (min): 30
Marker peak width (sec): 5    Marker min peak height: 200   Marker baseline V to V?: Y    Marker baseline V to V points: 3
Lower marker selection: First peak > 500 RFU                Upper marker selection: Last peak > 200 RFU
Ladder size (bp)75, 200, 300, 400, 500, 700, 1000, 1500, 2000, 3000, 4000, 5000, 7000, 10000, 20000
Quantification using: Lower Marker          Final concentration (ng/uL): 0.5000          Dilution factor: 12.0
```

Sample: SampG5
Well location: G5
Created: Thursday, October 21, 2021 8:08:12 PM

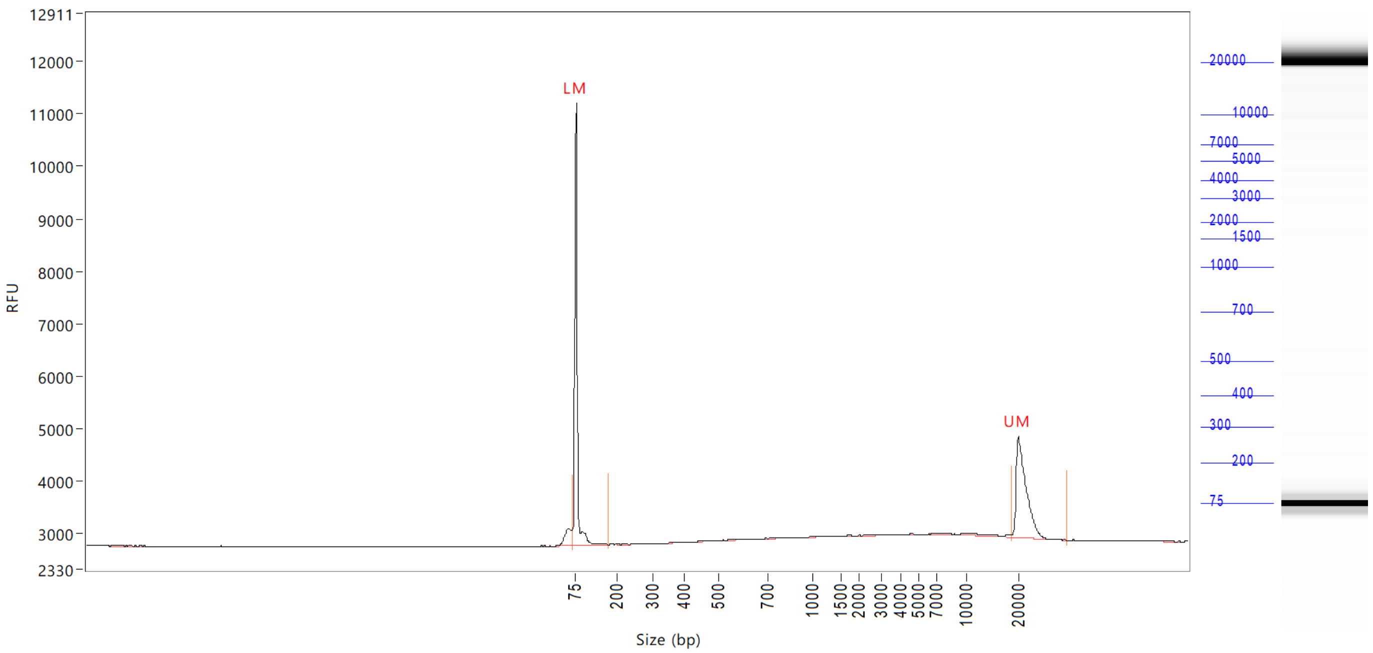

Peak	Size	Concentration	Relative concentration	Molarity	From	To	CV%	RFU
	(bp)	(ng/uL)	Percent	(nmole/L)	(bp)	(bp)		
1	75 (LM)	0.5000		10.9378	64	173	12.64	8436
2	20000 (UM)	0.2314		0.0183	18537	29189	5.70	1920

TIC:	0.0000	ng/uL
TIM:	0.0000	nmole/L
Total concentration:	0.5378	ng/uL

Sample peak width (sec): 5 Sample min peak height: 50 Sample baseline V to V?: Y Sample baseline V to V points: 3
Sample filter: Binomial Number of points for filter: 3 Sample start region (min): 0 Sample end region (min): 30
Marker peak width (sec): 5 Marker min peak height: 200 Marker baseline V to V?: Y Marker baseline V to V points: 3
Lower marker selection: First peak > 500 RFU Upper marker selection: Last peak > 200 RFU
Ladder size (bp)75, 200, 300, 400, 500, 700, 1000, 1500, 2000, 3000, 4000, 5000, 7000, 10000, 20000
Quantification using: Lower Marker Final concentration (ng/uL): 0.5000 Dilution factor: 12.0

Sample: SampH5
Well location: H5
Created: Thursday, October 21, 2021 8:08:12 PM

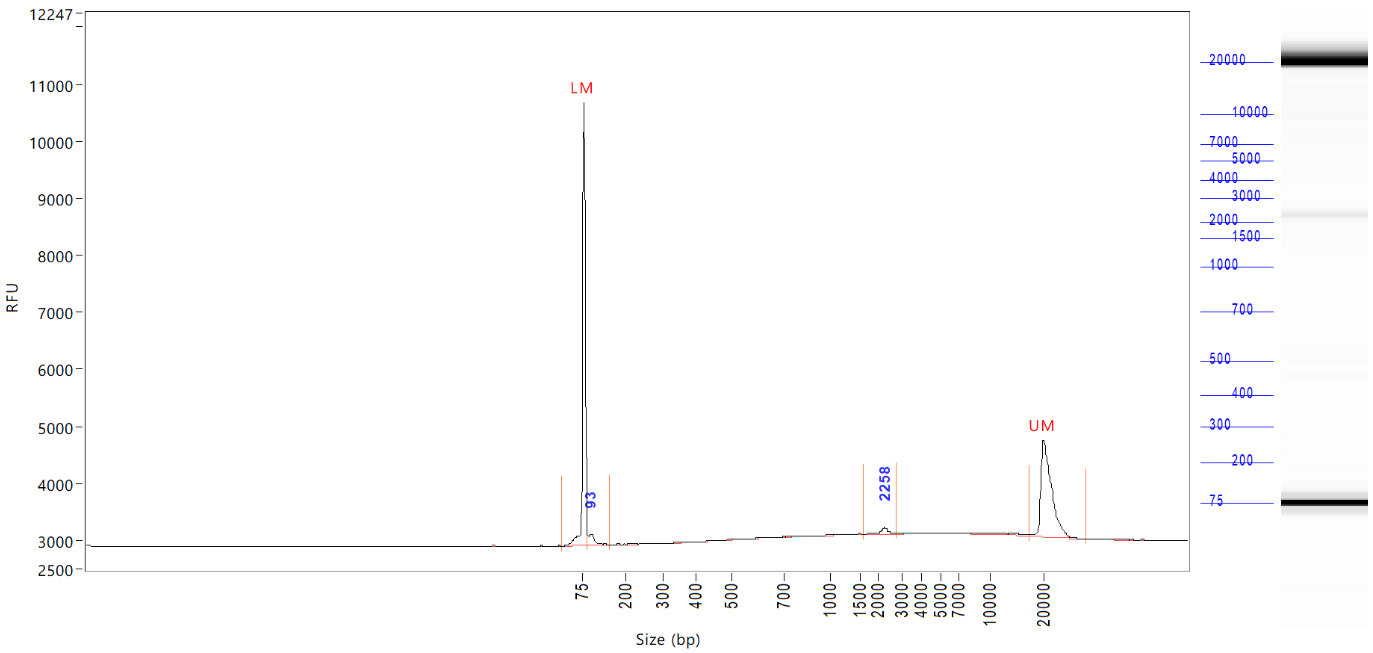

Peak	Size	Concentration	Relative concentration	Molarity	From	To	CV%	RFU
	(bp)	(ng/uL)	Percent	(nmole/L)	(bp)	(bp)		
1	75 (LM)	0.5000		11.2364	12	87	9.32	7777
2	93	0.3852	68.1	6.2636	87	148	12.46	189
3	2258	0.1806	31.9	0.1363	1578	2800	11.11	115
4	20000 (UM)	0.2350		0.0187	17317	27859	5.82	1694

TIC:	0.5658	ng/uL
TIM:	6.3999	nmole/L
Total concentration:	1.1600	ng/uL

Sample peak width (sec): 5 Sample min peak height: 50 Sample baseline V to V?: Y Sample baseline V to V points: 3
Sample filter: Binomial Number of points for filter: 3 Sample start region (min): 0 Sample end region (min): 30
Marker peak width (sec): 5 Marker min peak height: 200 Marker baseline V to V?: Y Marker baseline V to V points: 3
Lower marker selection: First peak > 500 RFU Upper marker selection: Last peak > 200 RFU
Ladder size (bp)75, 200, 300, 400, 500, 700, 1000, 1500, 2000, 3000, 4000, 5000, 7000, 10000, 20000
Quantification using: Lower Marker Final concentration (ng/uL): 0.5000 Dilution factor: 12.0

Sample: SampA6
Well location: A6
Created: Thursday, October 21, 2021 8:08:12 PM

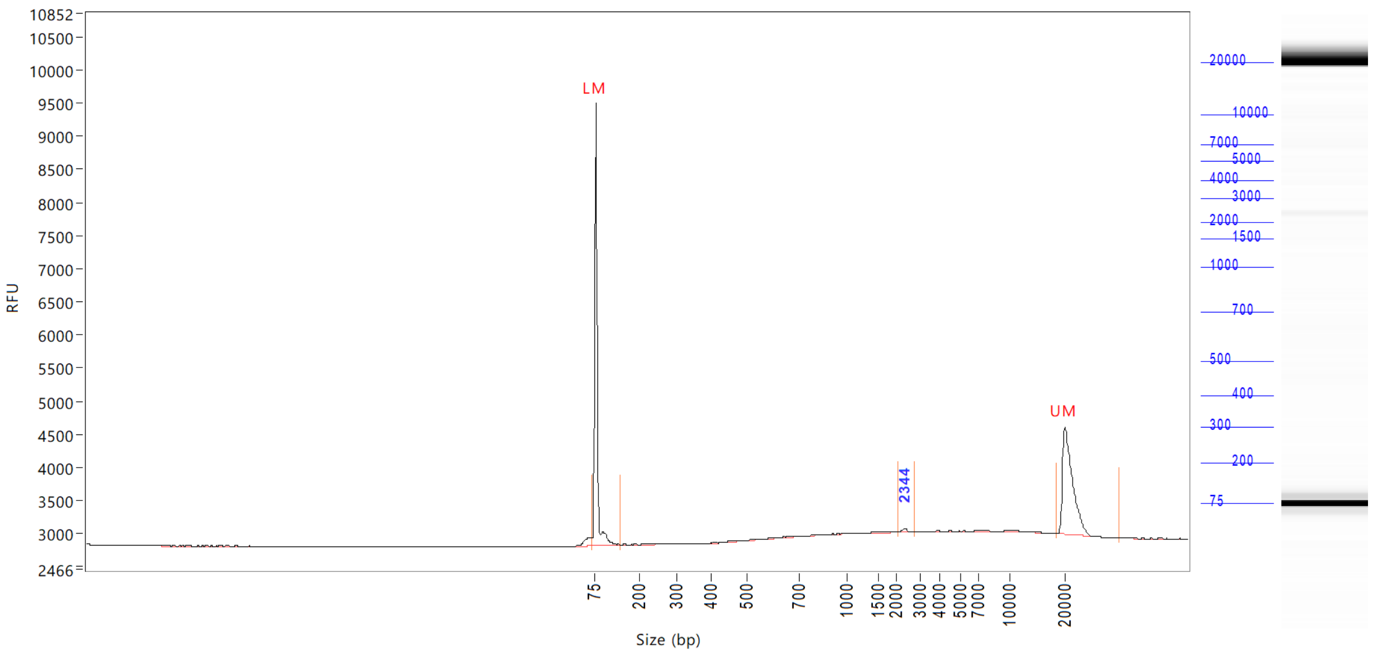

Peak	Size	Concentration	Relative concentration	Molarity	From	To	CV%	RFU
	(bp)	(ng/uL)	Percent	(nmole/L)	(bp)	(bp)		
1	75 (LM)	0.5000		11.0851	64	145	11.27	6691
2	2344	0.0645	100.0	0.0455	2087	2772	5.55	58
3	20000 (UM)	0.2385		0.0189	18537	29672	5.22	1610

TIC:	0.0645	ng/uL
TIM:	0.0455	nmole/L
Total concentration:	0.6397	ng/uL

Sample peak width (sec): 5 Sample min peak height: 50 Sample baseline V to V?: Y Sample baseline V to V points: 3
Sample filter: Binomial Number of points for filter: 3 Sample start region (min): 0 Sample end region (min): 30
Marker peak width (sec): 5 Marker min peak height: 200 Marker baseline V to V?: Y Marker baseline V to V points: 3
Lower marker selection: First peak > 500 RFU Upper marker selection: Last peak > 200 RFU
Ladder size (bp)75, 200, 300, 400, 500, 700, 1000, 1500, 2000, 3000, 4000, 5000, 7000, 10000, 20000
Quantification using: Lower Marker Final concentration (ng/uL): 0.5000 Dilution factor: 12.0

Sample: SampB6
Well location: B6
Created: Thursday, October 21, 2021 8:08:12 PM

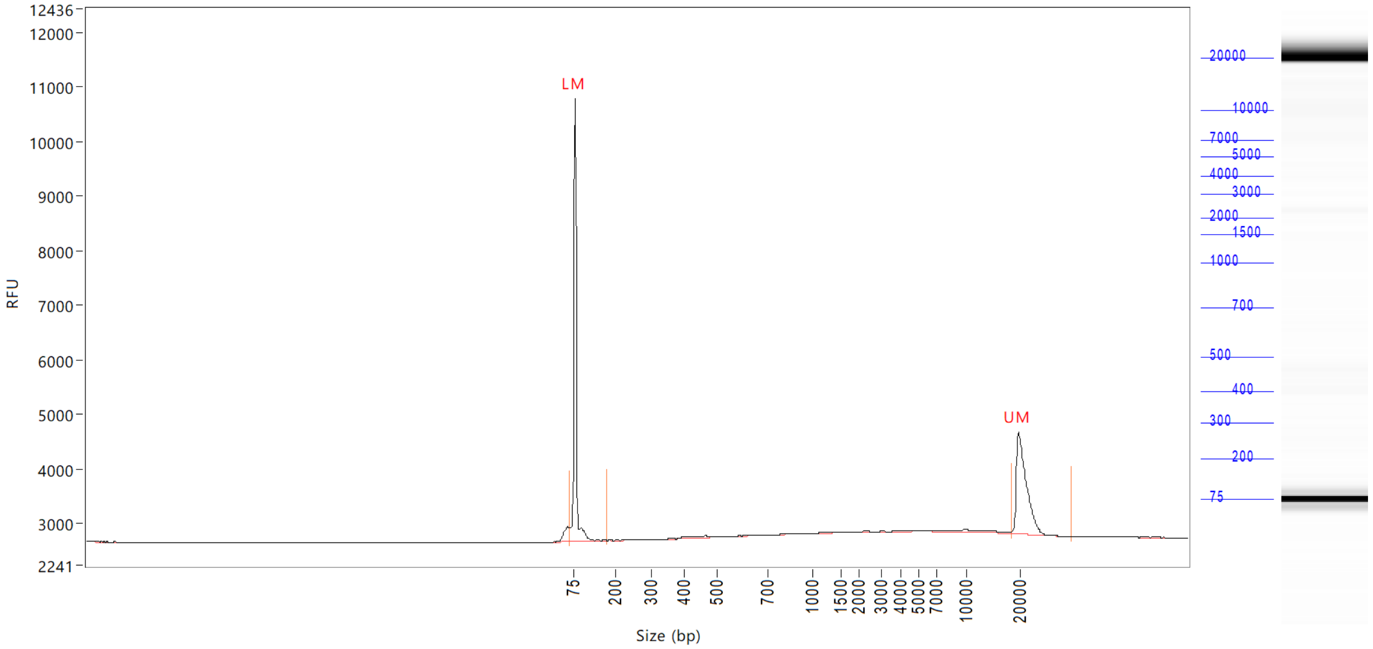

Peak	Size	Concentration	Relative concentration	Molarity	From	To	CV%	RFU
	(bp)	(ng/uL)	Percent	(nmole/L)	(bp)	(bp)		
1	75 (LM)	0.5000		10.9378	60	171	11.59	8129
2	20000 (UM)	0.2318		0.0184	18415	29914	5.56	1860

TIC:	0.0000	ng/uL
TIM:	0.0000	nmole/L
Total concentration:	0.8166	ng/uL

Sample peak width (sec): 5   Sample min peak height: 50   Sample baseline V to V?: Y   Sample baseline V to V points: 3
Sample filter: Binomial   Number of points for filter: 3   Sample start region (min): 0   Sample end region (min): 30
Marker peak width (sec): 5   Marker min peak height: 200   Marker baseline V to V?: Y   Marker baseline V to V points: 3
Lower marker selection: First peak > 500 RFU   Upper marker selection: Last peak > 200 RFU
Ladder size (bp)75, 200, 300, 400, 500, 700, 1000, 1500, 2000, 3000, 4000, 5000, 7000, 10000, 20000
Quantification using: Lower Marker   Final concentration (ng/uL): 0.5000   Dilution factor: 12.0

Sample: SampC6
Well location: C6
Created: Thursday, October 21, 2021 8:08:12 PM

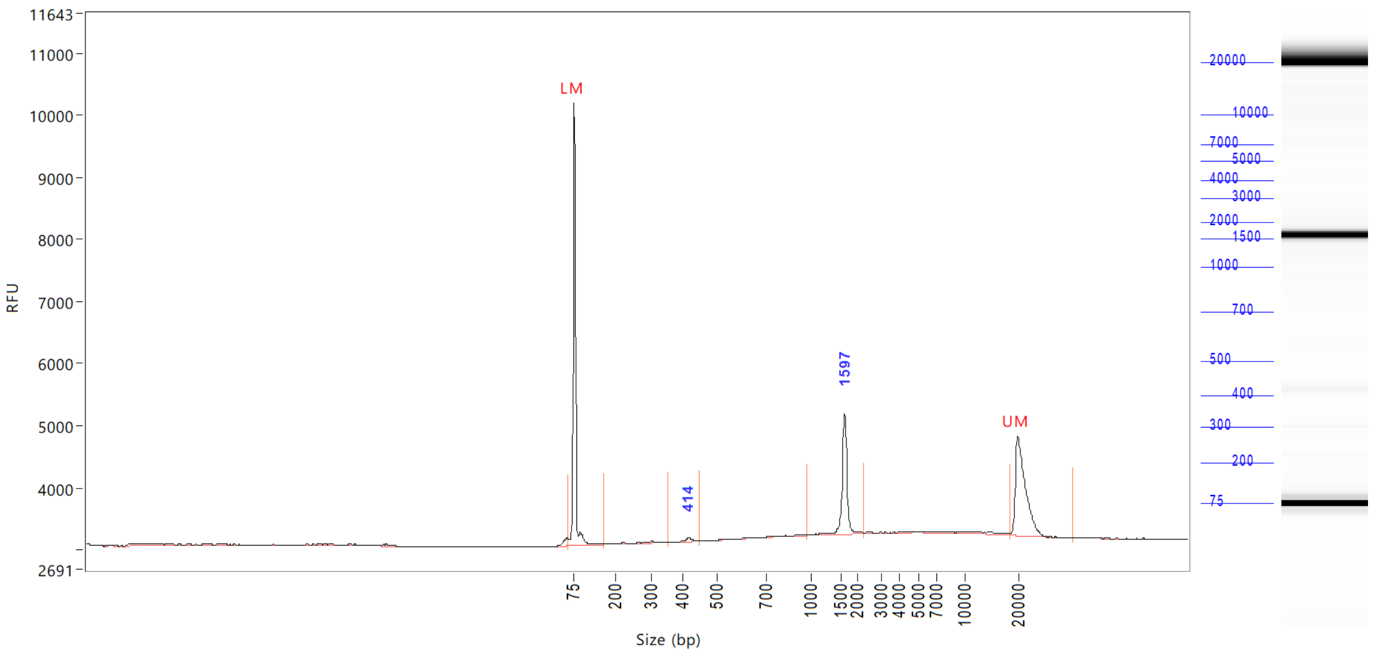

Peak	Size	Concentration	Relative concentration	Molarity	From	To	CV%	RFU
	(bp)	(ng/uL)	Percent	(nmole/L)	(bp)	(bp)		
1	75 (LM)	0.5000		10.9378	60	164	11.68	7141
2	414	0.1158	5.3	0.4613	352	450	3.89	55
3	1597	2.0862	94.7	2.1803	970	2258	8.75	1936
4	20000 (UM)	0.2380		0.0188	18537	30398	5.70	1605

TIC:	2.2020	ng/uL
TIM:	2.6416	nmole/L
Total concentration:	2.8598	ng/uL

```
Sample peak width (sec): 5    Sample min peak height: 50    Sample baseline V to V?: Y    Sample baseline V to V points: 3
Sample filter: Binomial       Number of points for filter: 3  Sample start region (min): 0  Sample end region (min): 30
Marker peak width (sec): 5    Marker min peak height: 200   Marker baseline V to V?: Y    Marker baseline V to V points: 3
Lower marker selection: First peak > 500 RFU                Upper marker selection: Last peak > 200 RFU
Ladder size (bp)75, 200, 300, 400, 500, 700, 1000, 1500, 2000, 3000, 4000, 5000, 7000, 10000, 20000
Quantification using: Lower Marker          Final concentration (ng/uL): 0.5000          Dilution factor: 12.0
```

Sample: SampD6
Well location: D6
Created: Thursday, October 21, 2021 8:08:12 PM

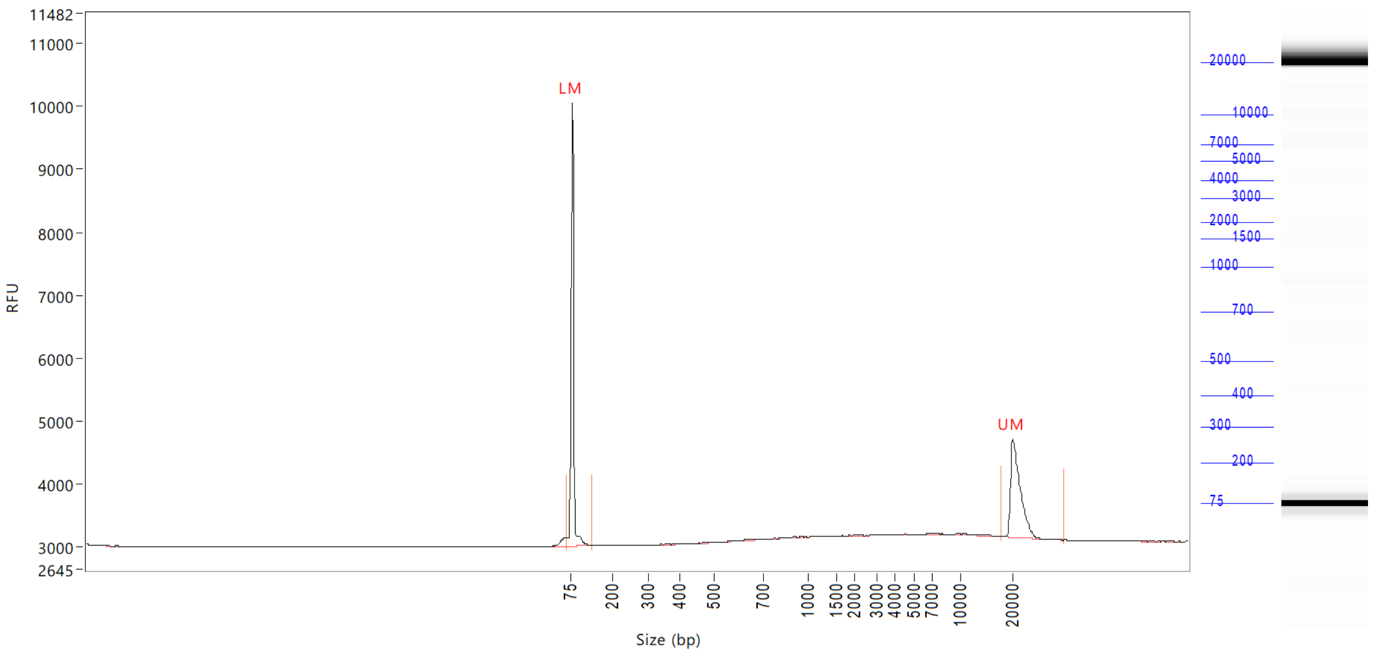

Peak	Size	Concentration	Relative concentration	Molarity	From	To	CV%	RFU
	(bp)	(ng/uL)	Percent	(nmole/L)	(bp)	(bp)		
1	75 (LM)	0.5000		11.0851	60	133	9.07	7056
2	20000 (UM)	0.2282		0.0181	17683	29914	5.54	1556

TIC:	0.0000	ng/uL
TIM:	0.0000	nmole/L
Total concentration:	0.5246	ng/uL

Sample peak width (sec): 5 Sample min peak height: 50 Sample baseline V to V?: Y Sample baseline V to V points: 3
Sample filter: Binomial Number of points for filter: 3 Sample start region (min): 0 Sample end region (min): 30
Marker peak width (sec): 5 Marker min peak height: 200 Marker baseline V to V?: Y Marker baseline V to V points: 3
Lower marker selection: First peak > 500 RFU Upper marker selection: Last peak > 200 RFU
Ladder size (bp)75, 200, 300, 400, 500, 700, 1000, 1500, 2000, 3000, 4000, 5000, 7000, 10000, 20000
Quantification using: Lower Marker Final concentration (ng/uL): 0.5000 Dilution factor: 12.0

Sample: SampE6
Well location: E6
Created: Thursday, October 21, 2021 8:08:12 PM

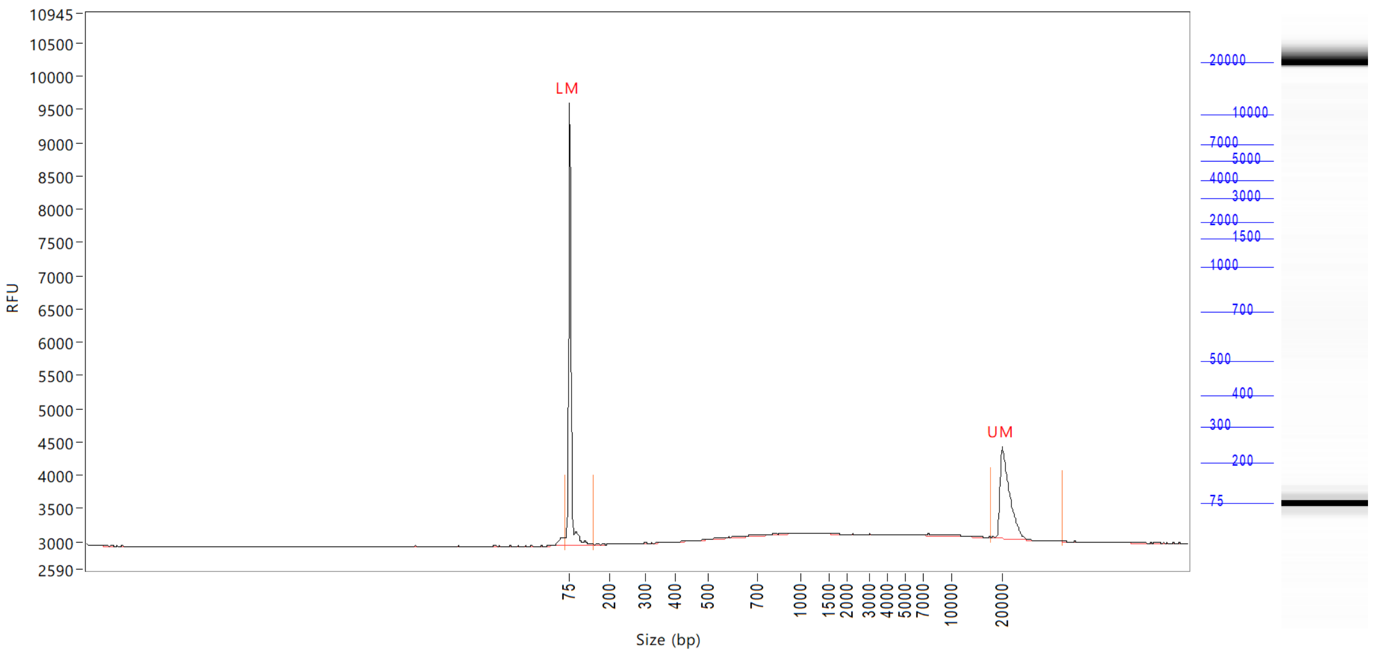

Peak	Size	Concentration	Relative concentration	Molarity	From	To	CV%	RFU
	(bp)	(ng/uL)	Percent	(nmole/L)	(bp)	(bp)		
1	75 (LM)	0.5000		10.9378	60	150	11.80	6662
2	20000 (UM)	0.2088		0.0165	17805	31607	6.51	1373

TIC:	0.0000	ng/uL
TIM:	0.0000	nmole/L
Total concentration:	0.7658	ng/uL

Sample peak width (sec): 5 Sample min peak height: 50 Sample baseline V to V?: Y Sample baseline V to V points: 3
Sample filter: Binomial Number of points for filter: 3 Sample start region (min): 0 Sample end region (min): 30
Marker peak width (sec): 5 Marker min peak height: 200 Marker baseline V to V?: Y Marker baseline V to V points: 3
Lower marker selection: First peak > 500 RFU Upper marker selection: Last peak > 200 RFU
Ladder size (bp)75, 200, 300, 400, 500, 700, 1000, 1500, 2000, 3000, 4000, 5000, 7000, 10000, 20000
Quantification using: Lower Marker Final concentration (ng/uL): 0.5000 Dilution factor: 12.0

Sample: SampF6
Well location: F6
Created: Thursday, October 21, 2021 8:08:12 PM

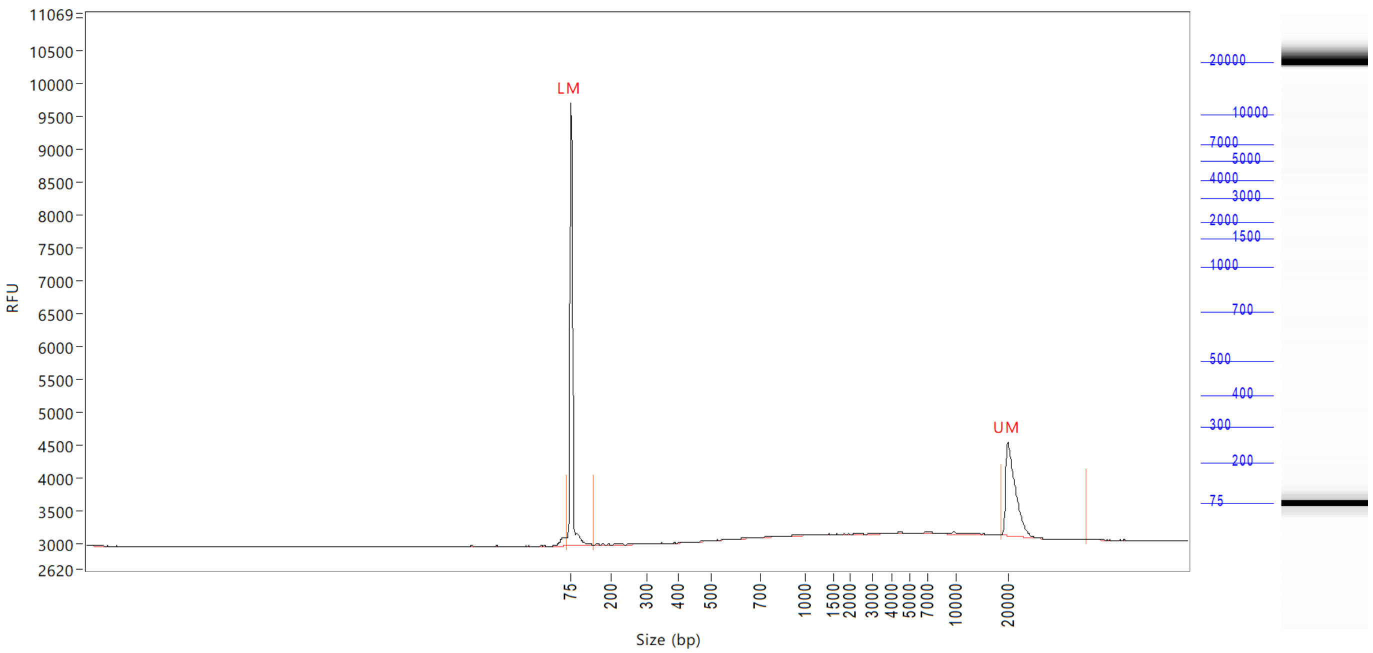

Peak	Size	Concentration	Relative concentration	Molarity	From	To	CV%	RFU
	(bp)	(ng/uL)	Percent	(nmole/L)	(bp)	(bp)		
1	75 (LM)	0.5000		10.9378	64	143	10.66	6742
2	20000 (UM)	0.2161		0.0171	18537	35113	6.64	1426

TIC:	0.0000	ng/uL
TIM:	0.0000	nmole/L
Total concentration:	0.6372	ng/uL

Sample peak width (sec): 5 Sample min peak height: 50 Sample baseline V to V?: Y Sample baseline V to V points: 3
Sample filter: Binomial Number of points for filter: 3 Sample start region (min): 0 Sample end region (min): 30
Marker peak width (sec): 5 Marker min peak height: 200 Marker baseline V to V?: Y Marker baseline V to V points: 3
Lower marker selection: First peak > 500 RFU Upper marker selection: Last peak > 200 RFU
Ladder size (bp)75, 200, 300, 400, 500, 700, 1000, 1500, 2000, 3000, 4000, 5000, 7000, 10000, 20000
Quantification using: Lower Marker Final concentration (ng/uL): 0.5000 Dilution factor: 12.0

Sample: SampG6
Well location: G6
Created: Thursday, October 21, 2021 8:08:12 PM

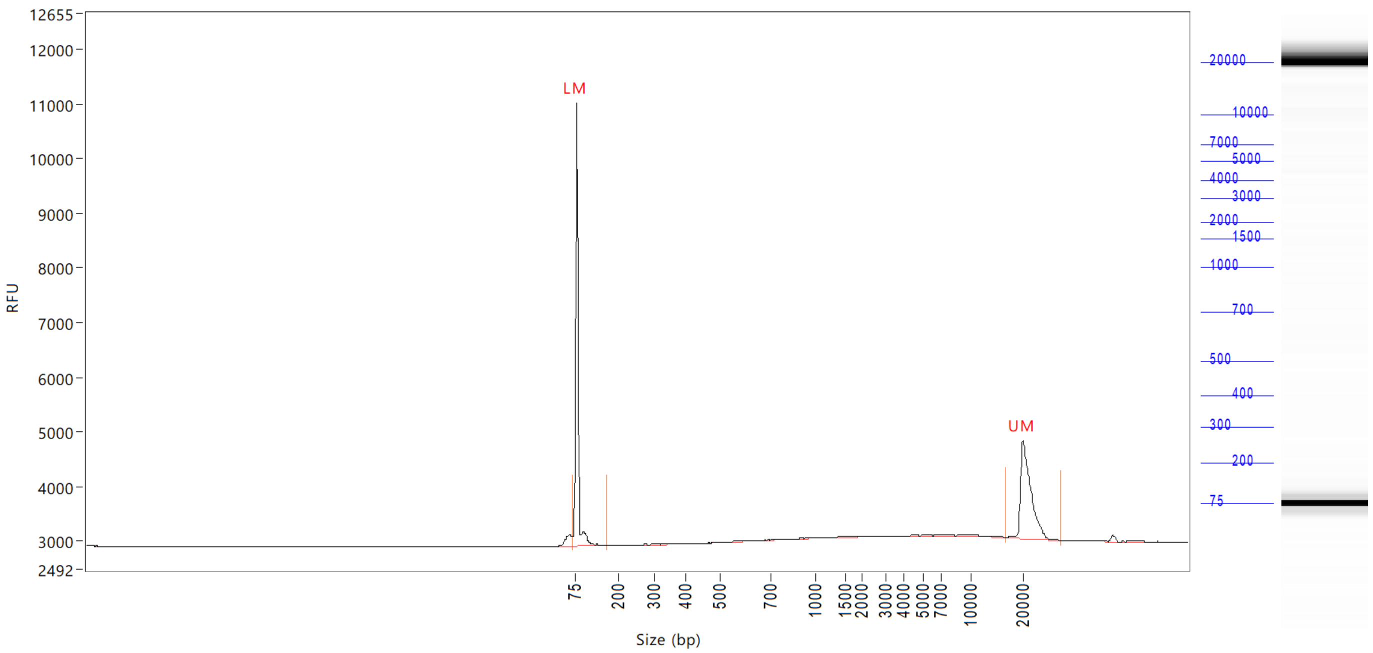

Peak	Size	Concentration	Relative concentration	Molarity	From	To	CV%	RFU
	(bp)	(ng/uL)	Percent	(nmole/L)	(bp)	(bp)		
1	75 (LM)	0.5000		10.7944	64	166	12.07	8114
2	20000 (UM)	0.2312		0.0184	16586	27012	5.87	1794

TIC:	0.0000	ng/uL
TIM:	0.0000	nmole/L
Total concentration:	0.5572	ng/uL

Sample peak width (sec): 5 Sample min peak height: 50 Sample baseline V to V?: Y Sample baseline V to V points: 3
Sample filter: Binomial Number of points for filter: 3 Sample start region (min): 0 Sample end region (min): 30
Marker peak width (sec): 5 Marker min peak height: 200 Marker baseline V to V?: Y Marker baseline V to V points: 3
Lower marker selection: First peak > 500 RFU Upper marker selection: Last peak > 200 RFU
Ladder size (bp)75, 200, 300, 400, 500, 700, 1000, 1500, 2000, 3000, 4000, 5000, 7000, 10000, 20000
Quantification using: Lower Marker Final concentration (ng/uL): 0.5000 Dilution factor: 12.0

Sample: SampH6
Well location: H6
Created: Thursday, October 21, 2021 8:08:12 PM

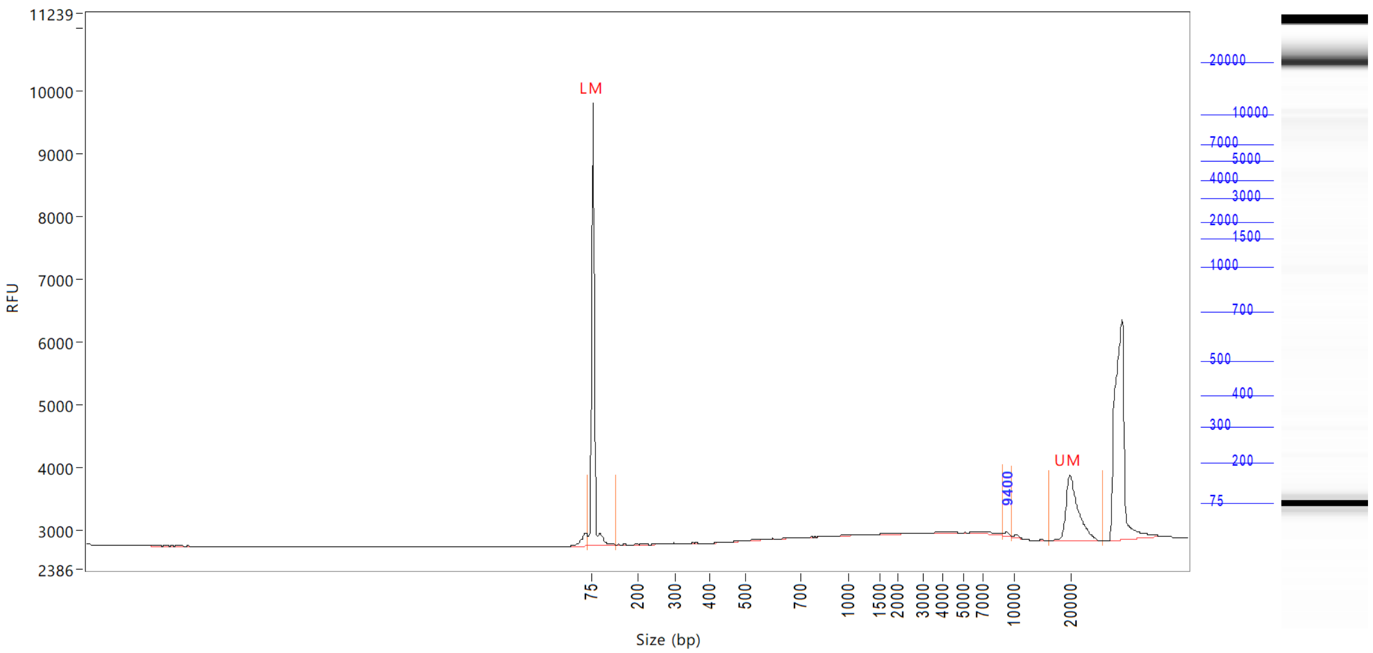

Peak	Size	Concentration	Relative concentration	Molarity	From	To	CV%	RFU
	(bp)	(ng/uL)	Percent	(nmole/L)	(bp)	(bp)		
1	75 (LM)	0.5000		11.0851	62	137	9.61	7064
2	9400	0.0654	100.0	0.0115	8934	9933	2.70	61
3	20000 (UM)	0.1663		0.0132	16220	25803	6.28	1049

TIC:	0.0654	ng/uL
TIM:	0.0115	nmole/L
Total concentration:	0.6766	ng/uL

```
Sample peak width (sec): 5    Sample min peak height: 50    Sample baseline V to V?: Y    Sample baseline V to V points: 3
Sample filter: Binomial       Number of points for filter: 3  Sample start region (min): 0  Sample end region (min): 30
Marker peak width (sec): 5    Marker min peak height: 200   Marker baseline V to V?: Y    Marker baseline V to V points: 3
Lower marker selection: First peak > 500 RFU                Upper marker selection: Last peak > 200 RFU
Ladder size (bp)75, 200, 300, 400, 500, 700, 1000, 1500, 2000, 3000, 4000, 5000, 7000, 10000, 20000
Quantification using: Lower Marker          Final concentration (ng/uL): 0.5000         Dilution factor: 12.0
```

Sample: SampA7
Well location: A7
Created: Thursday, October 21, 2021 8:08:12 PM

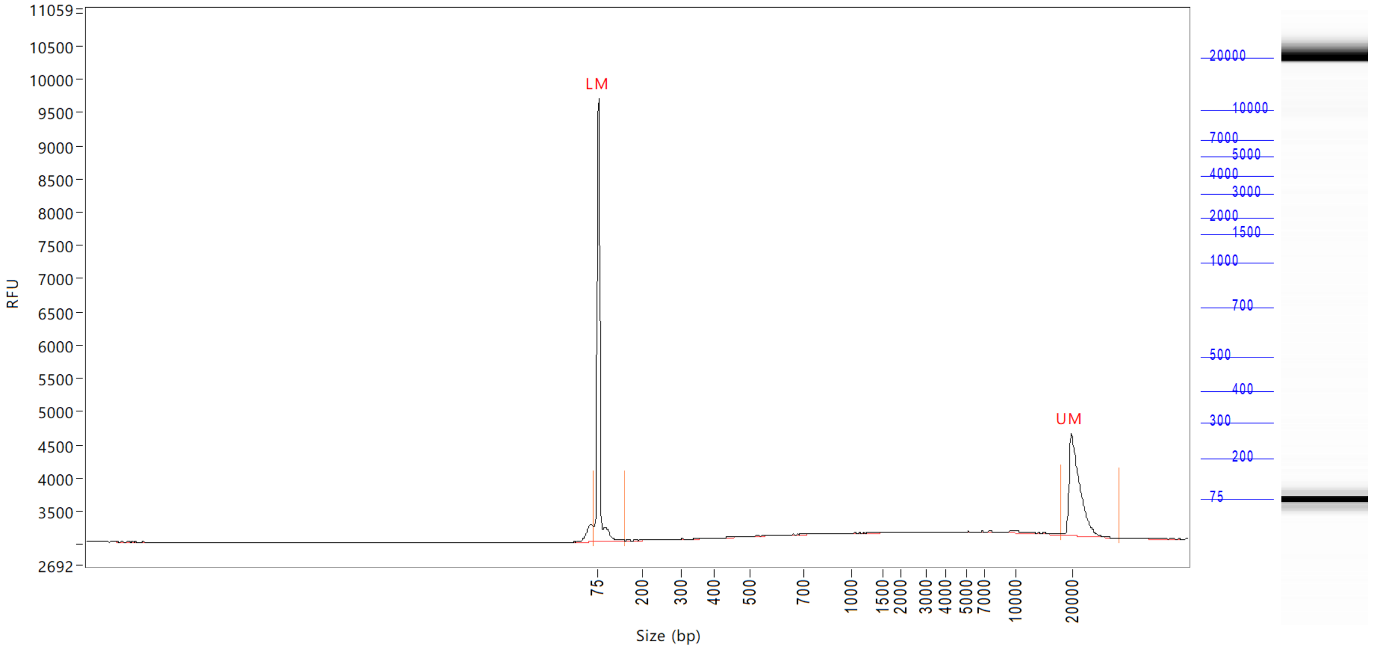

Peak	Size	Concentration	Relative concentration	Molarity	From	To	CV%	RFU
	(bp)	(ng/uL)	Percent	(nmole/L)	(bp)	(bp)		
1	75 (LM)	0.5000		11.0851	62	150	11.42	6675
2	20000 (UM)	0.2345		0.0186	18049	28463	5.82	1525

TIC:	0.0000	ng/uL
TIM:	0.0000	nmole/L
Total concentration:	0.5794	ng/uL

```
Sample peak width (sec): 5    Sample min peak height: 50    Sample baseline V to V?: Y    Sample baseline V to V points: 3
Sample filter: Binomial       Number of points for filter: 3  Sample start region (min): 0  Sample end region (min): 30
Marker peak width (sec): 5    Marker min peak height: 200   Marker baseline V to V?: Y    Marker baseline V to V points: 3
Lower marker selection: First peak > 500 RFU                Upper marker selection: Last peak > 200 RFU
Ladder size (bp)75, 200, 300, 400, 500, 700, 1000, 1500, 2000, 3000, 4000, 5000, 7000, 10000, 20000
Quantification using: Lower Marker          Final concentration (ng/uL): 0.5000          Dilution factor: 12.0
```

Sample: SampB7
Well location: B7
Created: Thursday, October 21, 2021 8:08:12 PM

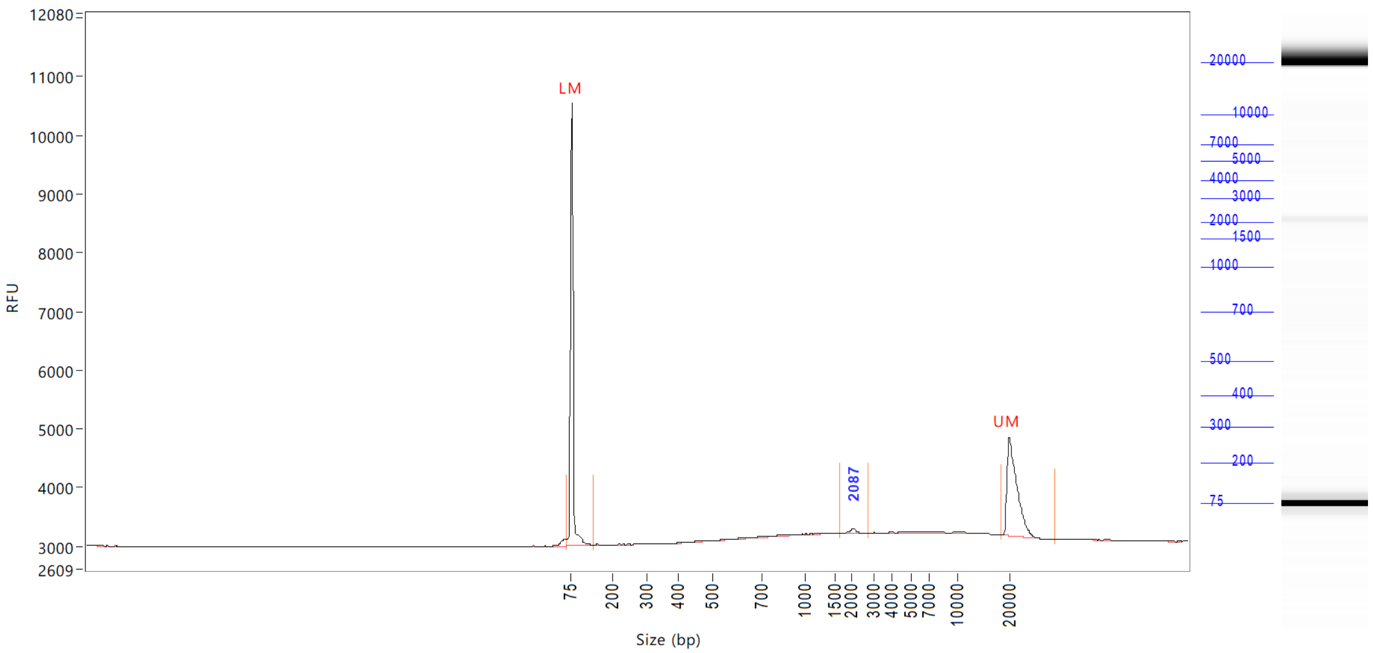

Peak	Size	Concentration	Relative concentration	Molarity	From	To	CV%	RFU
	(bp)	(ng/uL)	Percent	(nmole/L)	(bp)	(bp)		
1	75 (LM)	0.5000		10.9378	62	143	10.44	7558
2	2087	0.1204	100.0	0.0967	1655	2743	8.82	89
3	20000 (UM)	0.2340		0.0185	18415	28947	5.48	1679

TIC:	0.1204	ng/uL
TIM:	0.0967	nmole/L
Total concentration:	0.6680	ng/uL

```
Sample peak width (sec): 5    Sample min peak height: 50    Sample baseline V to V?: Y    Sample baseline V to V points: 3
Sample filter: Binomial       Number of points for filter: 3  Sample start region (min): 0  Sample end region (min): 30
Marker peak width (sec): 5    Marker min peak height: 200   Marker baseline V to V?: Y    Marker baseline V to V points: 3
Lower marker selection: First peak > 500 RFU                Upper marker selection: Last peak > 200 RFU
Ladder size (bp)75, 200, 300, 400, 500, 700, 1000, 1500, 2000, 3000, 4000, 5000, 7000, 10000, 20000
Quantification using: Lower Marker          Final concentration (ng/uL): 0.5000          Dilution factor: 12.0
```

Sample: SampC7
Well location: C7
Created: Thursday, October 21, 2021 8:08:12 PM

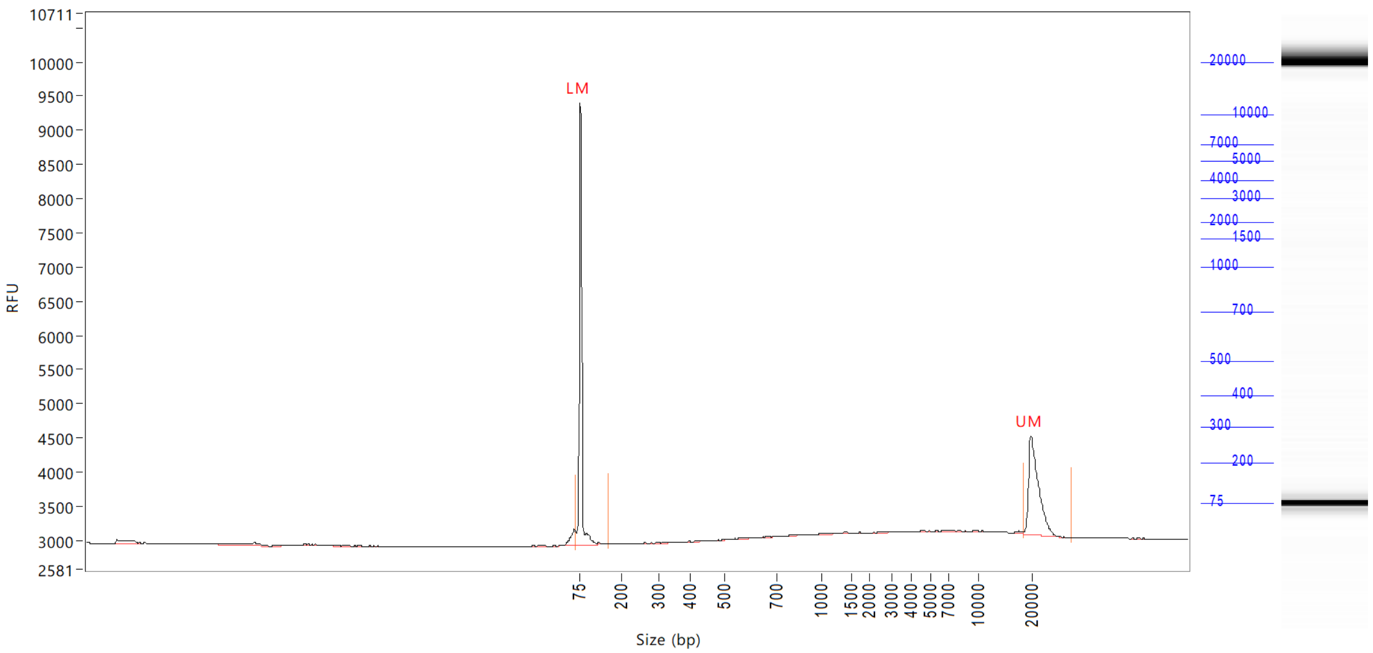

Peak	Size	Concentration	Relative concentration	Molarity	From	To	CV%	RFU
	(bp)	(ng/uL)	Percent	(nmole/L)	(bp)	(bp)		
1	75 (LM)	0.5000		10.9378	64	160	10.85	6477
2	20000 (UM)	0.2289		0.0182	18659	27496	5.36	1450

TIC:	0.0000	ng/uL
TIM:	0.0000	nmole/L
Total concentration:	0.6910	ng/uL

Sample peak width (sec): 5   Sample min peak height: 50   Sample baseline V to V?: Y   Sample baseline V to V points: 3
Sample filter: Binomial   Number of points for filter: 3 Sample start region (min): 0 Sample end region (min): 30
Marker peak width (sec): 5   Marker min peak height: 200   Marker baseline V to V?: Y   Marker baseline V to V points: 3
Lower marker selection: First peak > 500 RFU   Upper marker selection: Last peak > 200 RFU
Ladder size (bp)75, 200, 300, 400, 500, 700, 1000, 1500, 2000, 3000, 4000, 5000, 7000, 10000, 20000
Quantification using: Lower Marker   Final concentration (ng/uL): 0.5000   Dilution factor: 12.0

Sample: SampD7
Well location: D7
Created: Thursday, October 21, 2021 8:08:12 PM

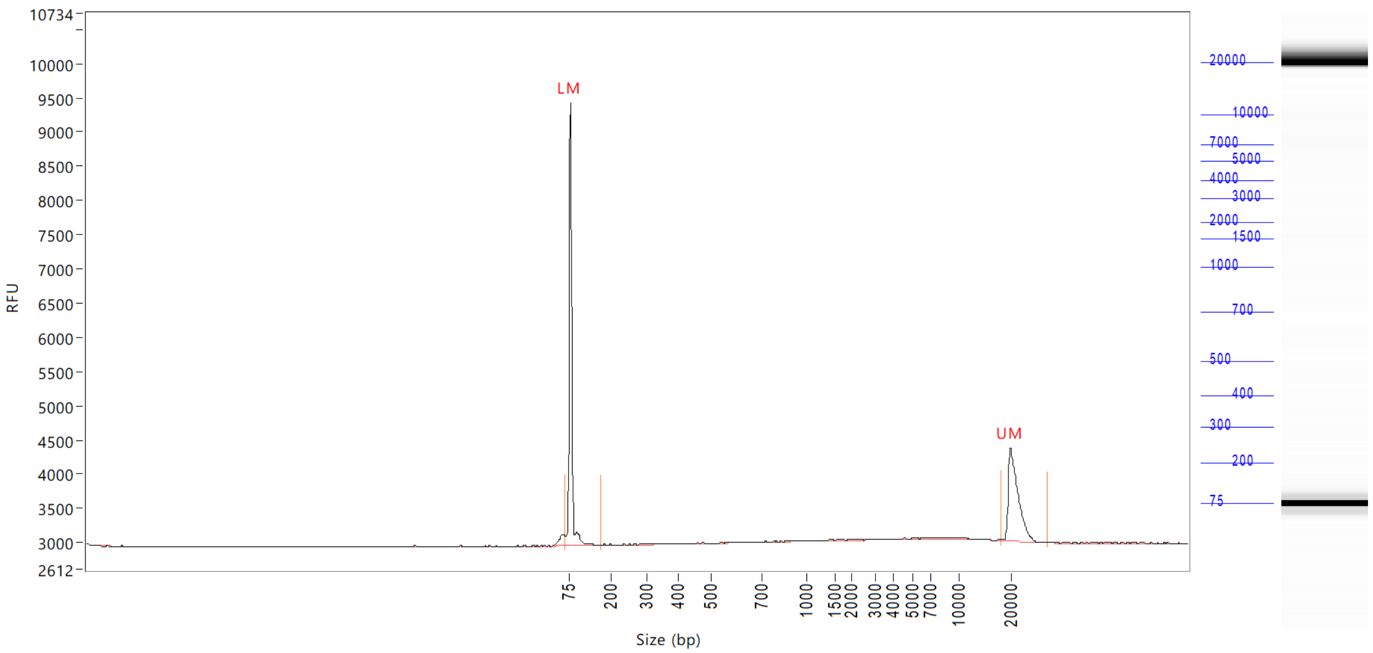

Peak	Size	Concentration	Relative concentration	Molarity	From	To	CV%	RFU
	(bp)	(ng/uL)	Percent	(nmole/L)	(bp)	(bp)		
1	75 (LM)	0.5000		10.9378	62	169	12.09	6480
2	20000 (UM)	0.2130		0.0170	18049	26891	5.36	1358

TIC:	0.0000	ng/uL
TIM:	0.0000	nmole/L
Total concentration:	0.5258	ng/uL

```
Sample peak width (sec): 5    Sample min peak height: 50    Sample baseline V to V?: Y    Sample baseline V to V points: 3
Sample filter: Binomial       Number of points for filter: 3  Sample start region (min): 0  Sample end region (min): 30
Marker peak width (sec): 5    Marker min peak height: 200   Marker baseline V to V?: Y    Marker baseline V to V points: 3
Lower marker selection: First peak > 500 RFU                Upper marker selection: Last peak > 200 RFU
Ladder size (bp)75, 200, 300, 400, 500, 700, 1000, 1500, 2000, 3000, 4000, 5000, 7000, 10000, 20000
Quantification using: Lower Marker          Final concentration (ng/uL): 0.5000          Dilution factor: 12.0
```

Sample: SampE7
Well location: E7
Created: Thursday, October 21, 2021 8:08:12 PM

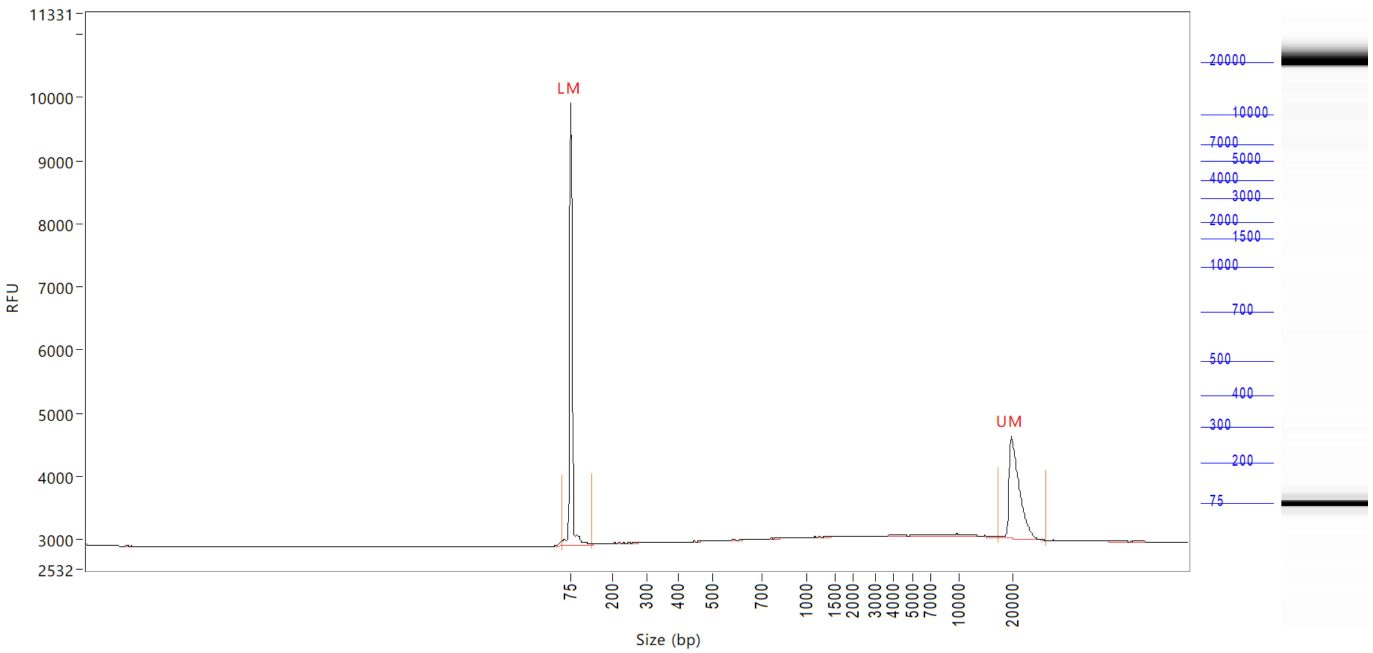

Peak	Size	Concentration	Relative concentration	Molarity	From	To	CV%	RFU
	(bp)	(ng/uL)	Percent	(nmole/L)	(bp)	(bp)		
1	75 (LM)	0.5000		11.2364	48	141	10.42	7015
2	20000 (UM)	0.2407		0.0191	17439	26529	5.57	1628

TIC:	0.0000	ng/uL
TIM:	0.0000	nmole/L
Total concentration:	0.6176	ng/uL

Sample peak width (sec): 5 Sample min peak height: 50 Sample baseline V to V?: Y Sample baseline V to V points: 3
Sample filter: Binomial Number of points for filter: 3 Sample start region (min): 0 Sample end region (min): 30
Marker peak width (sec): 5 Marker min peak height: 200 Marker baseline V to V?: Y Marker baseline V to V points: 3
Lower marker selection: First peak > 500 RFU Upper marker selection: Last peak > 200 RFU
Ladder size (bp)75, 200, 300, 400, 500, 700, 1000, 1500, 2000, 3000, 4000, 5000, 7000, 10000, 20000
Quantification using: Lower Marker Final concentration (ng/uL): 0.5000 Dilution factor: 12.0

Sample: SampF7
Well location: F7
Created: Thursday, October 21, 2021 8:08:12 PM

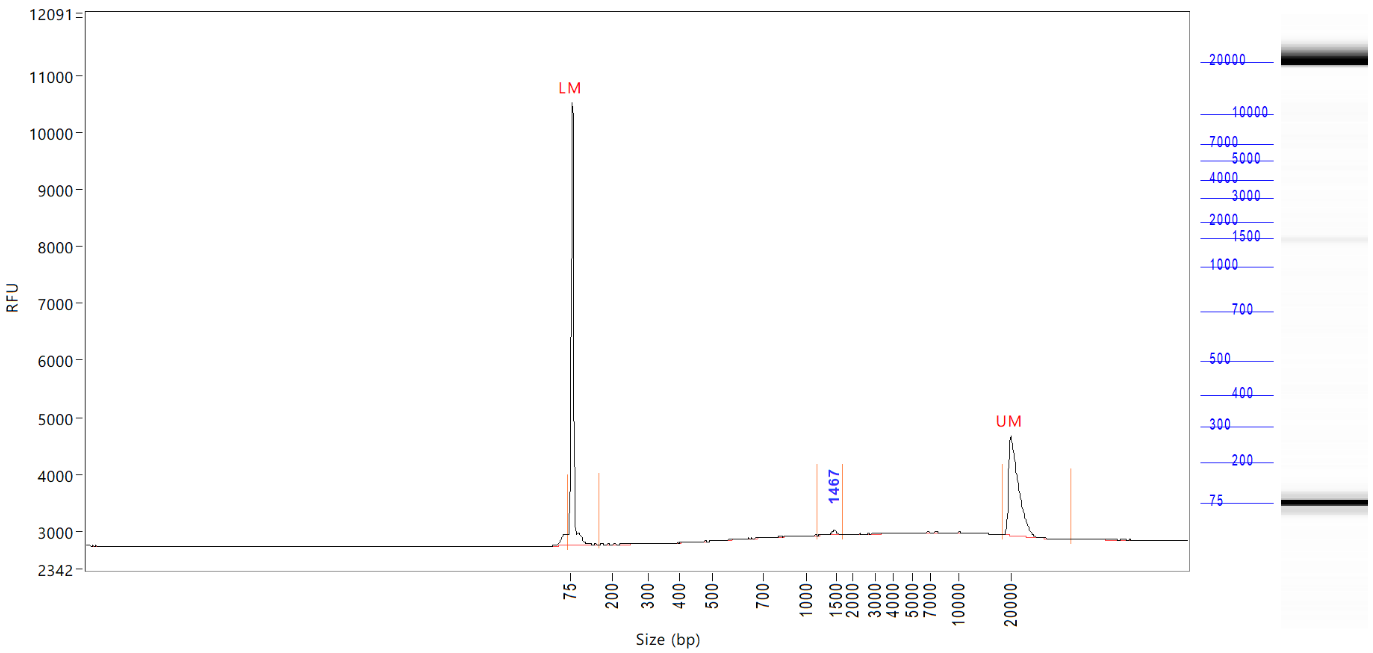

Peak	Size	Concentration	Relative concentration	Molarity	From	To	CV%	RFU
	(bp)	(ng/uL)	Percent	(nmole/L)	(bp)	(bp)		
1	75 (LM)	0.5000		10.9378	64	160	9.71	7772
2	1467	0.0955	100.0	0.1081	1178	1693	5.76	83
3	20000 (UM)	0.2266		0.0179	18293	31607	5.77	1733

TIC:	0.0955	ng/uL
TIM:	0.1081	nmole/L
Total concentration:	0.4758	ng/uL

Sample peak width (sec): 5 Sample min peak height: 50 Sample baseline V to V?: Y Sample baseline V to V points: 3
Sample filter: Binomial Number of points for filter: 3 Sample start region (min): 0 Sample end region (min): 30
Marker peak width (sec): 5 Marker min peak height: 200 Marker baseline V to V?: Y Marker baseline V to V points: 3
Lower marker selection: First peak > 500 RFU Upper marker selection: Last peak > 200 RFU
Ladder size (bp)75, 200, 300, 400, 500, 700, 1000, 1500, 2000, 3000, 4000, 5000, 7000, 10000, 20000
Quantification using: Lower Marker Final concentration (ng/uL): 0.5000 Dilution factor: 12.0

Sample: SampG7
Well location: G7
Created: Thursday, October 21, 2021 8:08:12 PM

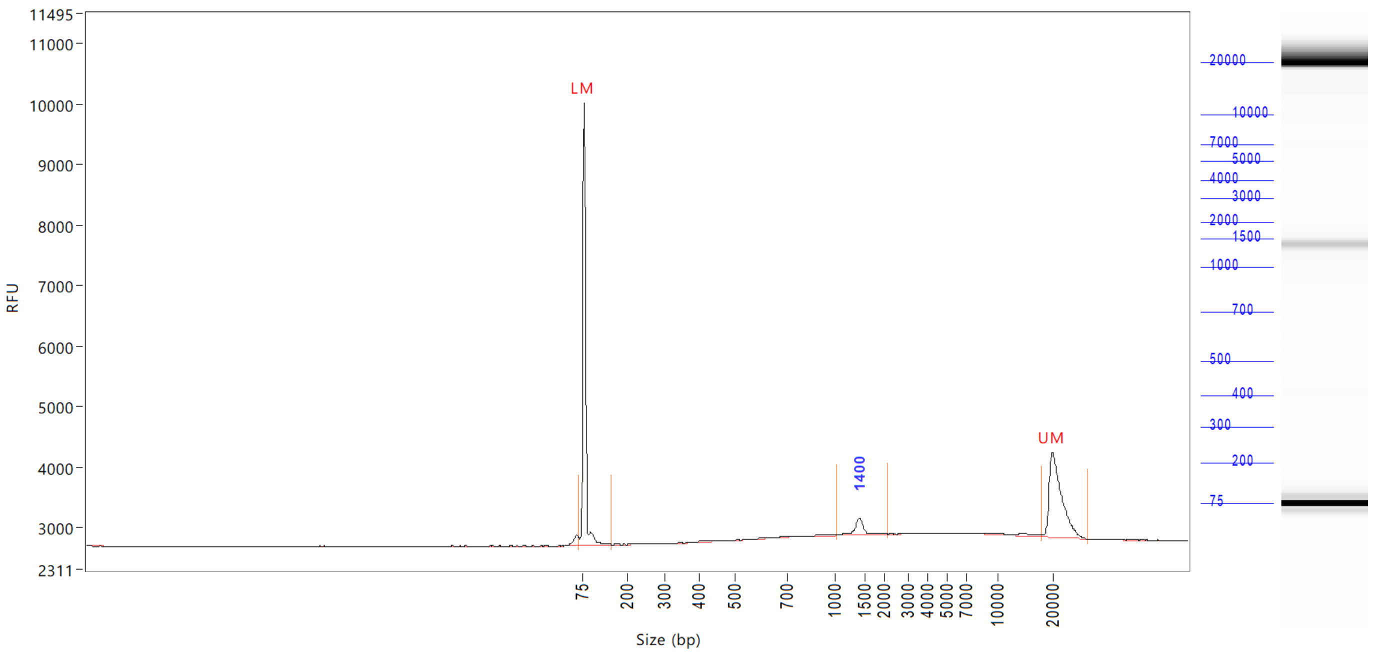

Peak	Size	Concentration	Relative concentration	Molarity	From	To	CV%	RFU
	(bp)	(ng/uL)	Percent	(nmole/L)	(bp)	(bp)		
1	75 (LM)	0.5000		10.9378	64	154	10.93	7324
2	1400	0.5096	100.0	0.6013	1034	2172	11.02	284
3	20000 (UM)	0.2245		0.0178	17927	26408	6.20	1410

TIC:	0.5096	ng/uL
TIM:	0.6013	nmole/L
Total concentration:	1.0826	ng/uL

Sample peak width (sec): 5 Sample min peak height: 50 Sample baseline V to V?: Y Sample baseline V to V points: 3
Sample filter: Binomial Number of points for filter: 3 Sample start region (min): 0 Sample end region (min): 30
Marker peak width (sec): 5 Marker min peak height: 200 Marker baseline V to V?: Y Marker baseline V to V points: 3
Lower marker selection: First peak > 500 RFU Upper marker selection: Last peak > 200 RFU
Ladder size (bp)75, 200, 300, 400, 500, 700, 1000, 1500, 2000, 3000, 4000, 5000, 7000, 10000, 20000
Quantification using: Lower Marker Final concentration (ng/uL): 0.5000 Dilution factor: 12.0

Sample: SampH7
Well location: H7
Created: Thursday, October 21, 2021 8:08:12 PM

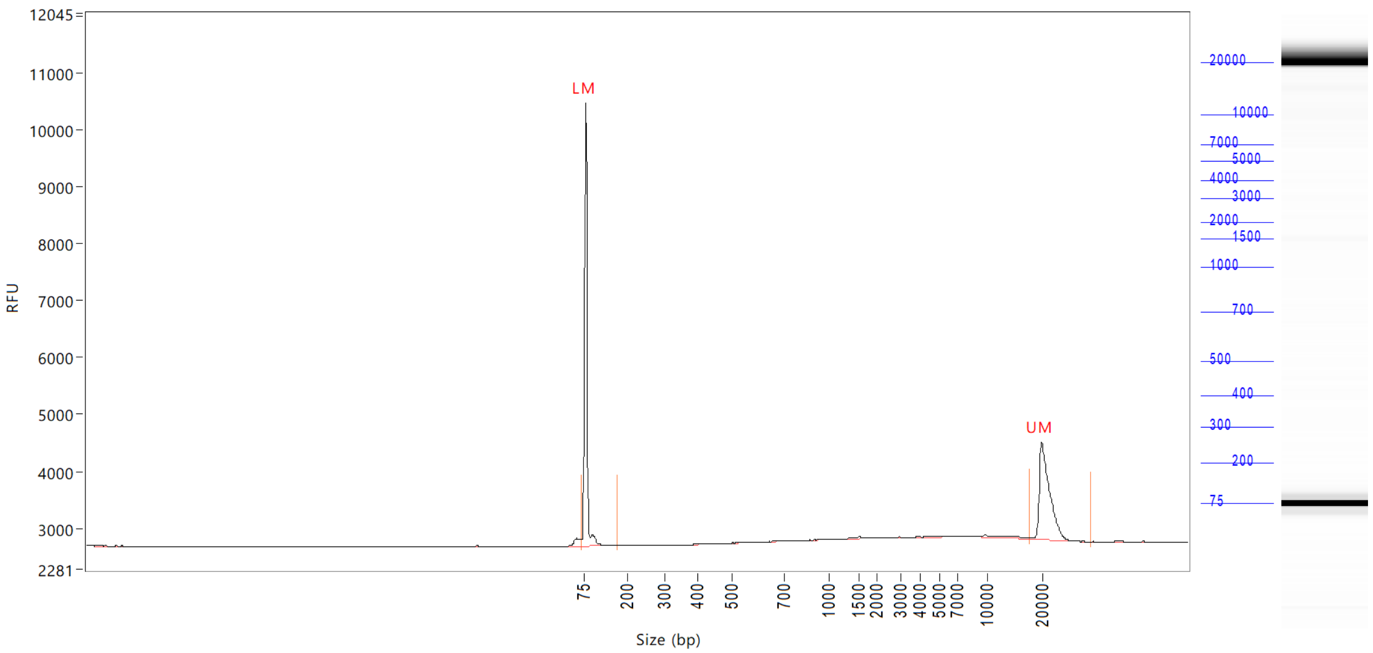

Peak	Size	Concentration	Relative concentration	Molarity	From	To	CV%	RFU
	(bp)	(ng/uL)	Percent	(nmole/L)	(bp)	(bp)		
1	75 (LM)	0.5000		11.0851	62	168	10.18	7796
2	20000 (UM)	0.2380		0.0189	17805	29068	5.63	1702

TIC:	0.0000	ng/uL
TIM:	0.0000	nmole/L
Total concentration:	0.5170	ng/uL

```
Sample peak width (sec): 5    Sample min peak height: 50    Sample baseline V to V?: Y    Sample baseline V to V points: 3
Sample filter: Binomial       Number of points for filter: 3  Sample start region (min): 0  Sample end region (min): 30
Marker peak width (sec): 5    Marker min peak height: 200   Marker baseline V to V?: Y    Marker baseline V to V points: 3
Lower marker selection: First peak > 500 RFU                Upper marker selection: Last peak > 200 RFU
Ladder size (bp)75, 200, 300, 400, 500, 700, 1000, 1500, 2000, 3000, 4000, 5000, 7000, 10000, 20000
Quantification using: Lower Marker          Final concentration (ng/uL): 0.5000          Dilution factor: 12.0
```

Sample: SampA8
Well location: A8
Created: Thursday, October 21, 2021 8:08:12 PM

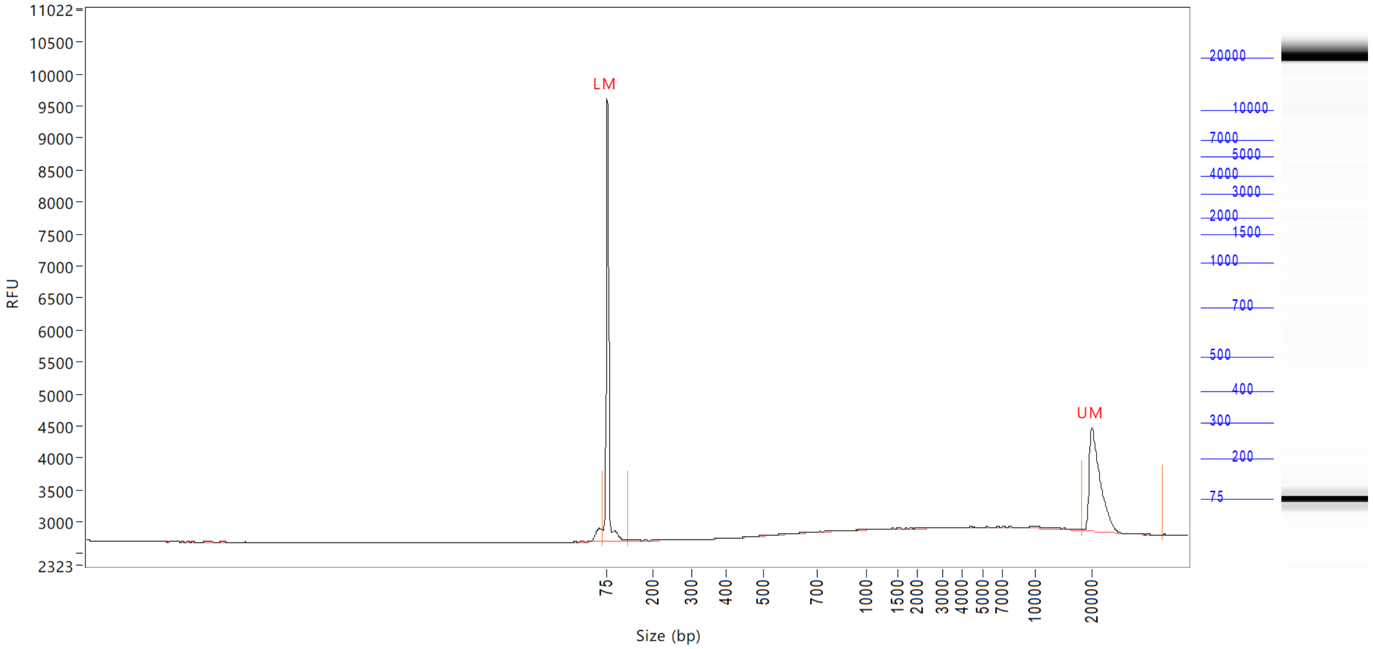

Peak	Size	Concentration	Relative concentration	Molarity	From	To	CV%	RFU
	(bp)	(ng/uL)	Percent	(nmole/L)	(bp)	(bp)		
1	75 (LM)	0.5000		10.9378	66	133	9.30	6938
2	20000 (UM)	0.2513		0.0199	18171	32211	6.09	1613

TIC:	0.0000	ng/uL
TIM:	0.0000	nmole/L
Total concentration:	0.6236	ng/uL

Sample peak width (sec): 5   Sample min peak height: 50   Sample baseline V to V?: Y   Sample baseline V to V points: 3
Sample filter: Binomial   Number of points for filter: 3   Sample start region (min): 0   Sample end region (min): 30
Marker peak width (sec): 5   Marker min peak height: 200   Marker baseline V to V?: Y   Marker baseline V to V points: 3
Lower marker selection: First peak > 500 RFU   Upper marker selection: Last peak > 200 RFU
Ladder size (bp)75, 200, 300, 400, 500, 700, 1000, 1500, 2000, 3000, 4000, 5000, 7000, 10000, 20000
Quantification using: Lower Marker   Final concentration (ng/uL): 0.5000   Dilution factor: 12.0

Sample: SampB8
Well location: B8
Created: Thursday, October 21, 2021 8:08:12 PM

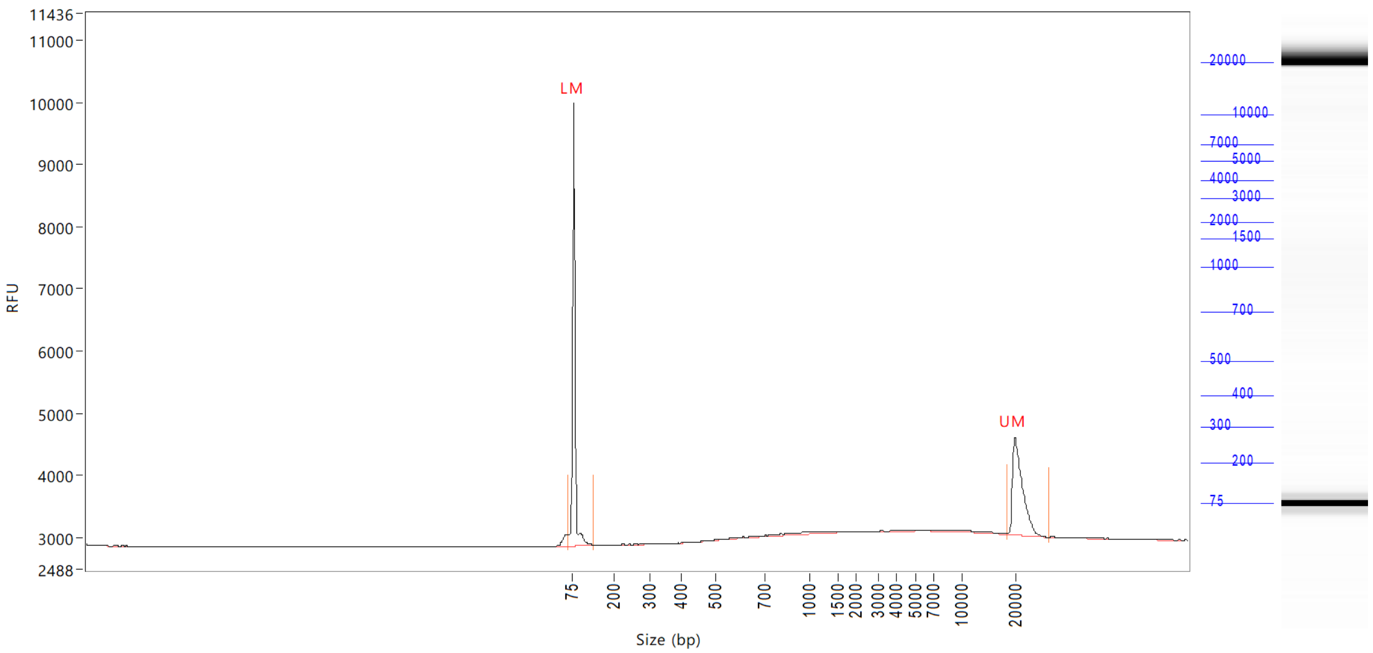

Peak	Size	Concentration	Relative concentration	Molarity	From	To	CV%	RFU
	(bp)	(ng/uL)	Percent	(nmole/L)	(bp)	(bp)		
1	75 (LM)	0.5000		10.9378	62	135	10.13	7144
2	20000 (UM)	0.2257		0.0179	18415	26529	5.33	1566

TIC:	0.0000	ng/uL
TIM:	0.0000	nmole/L
Total concentration:	0.9305	ng/uL

Sample peak width (sec): 5 Sample min peak height: 50 Sample baseline V to V?: Y Sample baseline V to V points: 3
Sample filter: Binomial Number of points for filter: 3 Sample start region (min): 0 Sample end region (min): 30
Marker peak width (sec): 5 Marker min peak height: 200 Marker baseline V to V?: Y Marker baseline V to V points: 3
Lower marker selection: First peak > 500 RFU Upper marker selection: Last peak > 200 RFU
Ladder size (bp)75, 200, 300, 400, 500, 700, 1000, 1500, 2000, 3000, 4000, 5000, 7000, 10000, 20000
Quantification using: Lower Marker Final concentration (ng/uL): 0.5000 Dilution factor: 12.0

Sample: SampC8
Well location: C8
Created: Thursday, October 21, 2021 8:08:12 PM

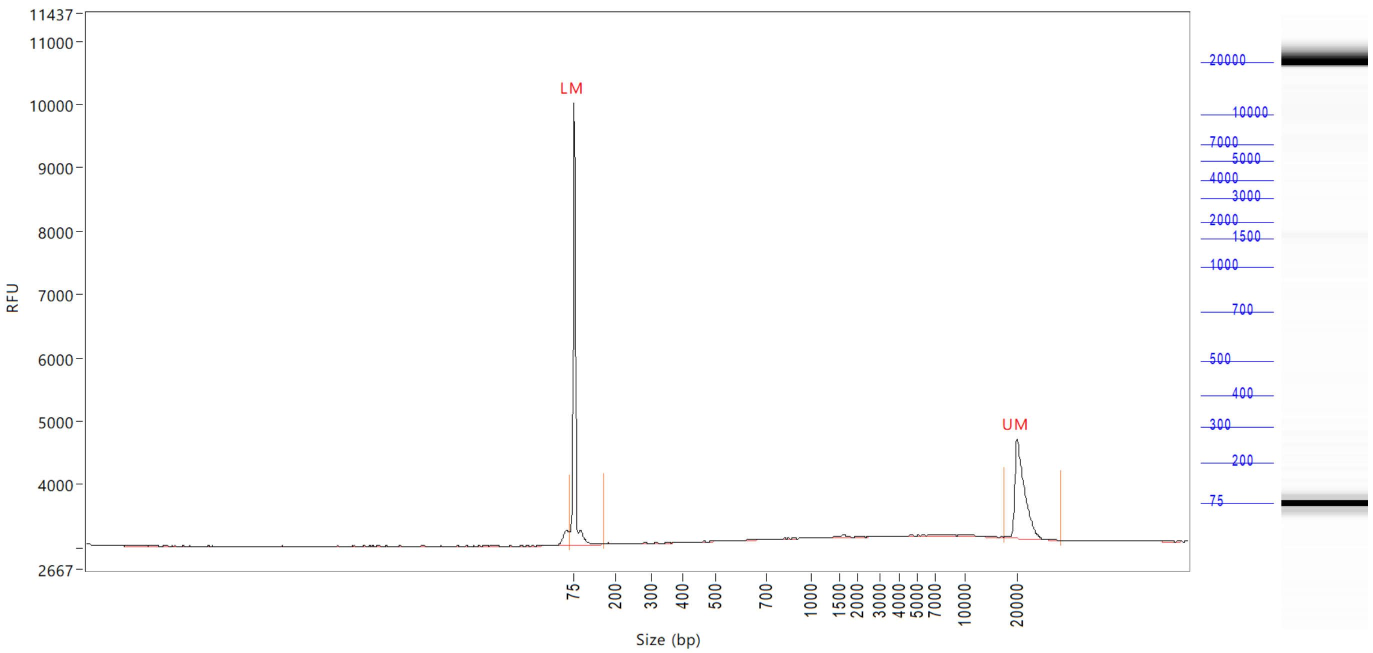

Peak	Size	Concentration	Relative concentration	Molarity	From	To	CV%	RFU
	(bp)	(ng/uL)	Percent	(nmole/L)	(bp)	(bp)		
1	75 (LM)	0.5000		10.9378	62	164	12.00	6990
2	20000 (UM)	0.2243		0.0178	17439	28463	5.58	1572

TIC:	0.0000	ng/uL
TIM:	0.0000	nmole/L
Total concentration:	0.6821	ng/uL

Sample peak width (sec): 5 Sample min peak height: 50 Sample baseline V to V?: Y Sample baseline V to V points: 3
Sample filter: Binomial Number of points for filter: 3 Sample start region (min): 0 Sample end region (min): 30
Marker peak width (sec): 5 Marker min peak height: 200 Marker baseline V to V?: Y Marker baseline V to V points: 3
Lower marker selection: First peak > 500 RFU Upper marker selection: Last peak > 200 RFU
Ladder size (bp)75, 200, 300, 400, 500, 700, 1000, 1500, 2000, 3000, 4000, 5000, 7000, 10000, 20000
Quantification using: Lower Marker Final concentration (ng/uL): 0.5000 Dilution factor: 12.0

Sample: SampD8
Well location: D8
Created: Thursday, October 21, 2021 8:08:12 PM

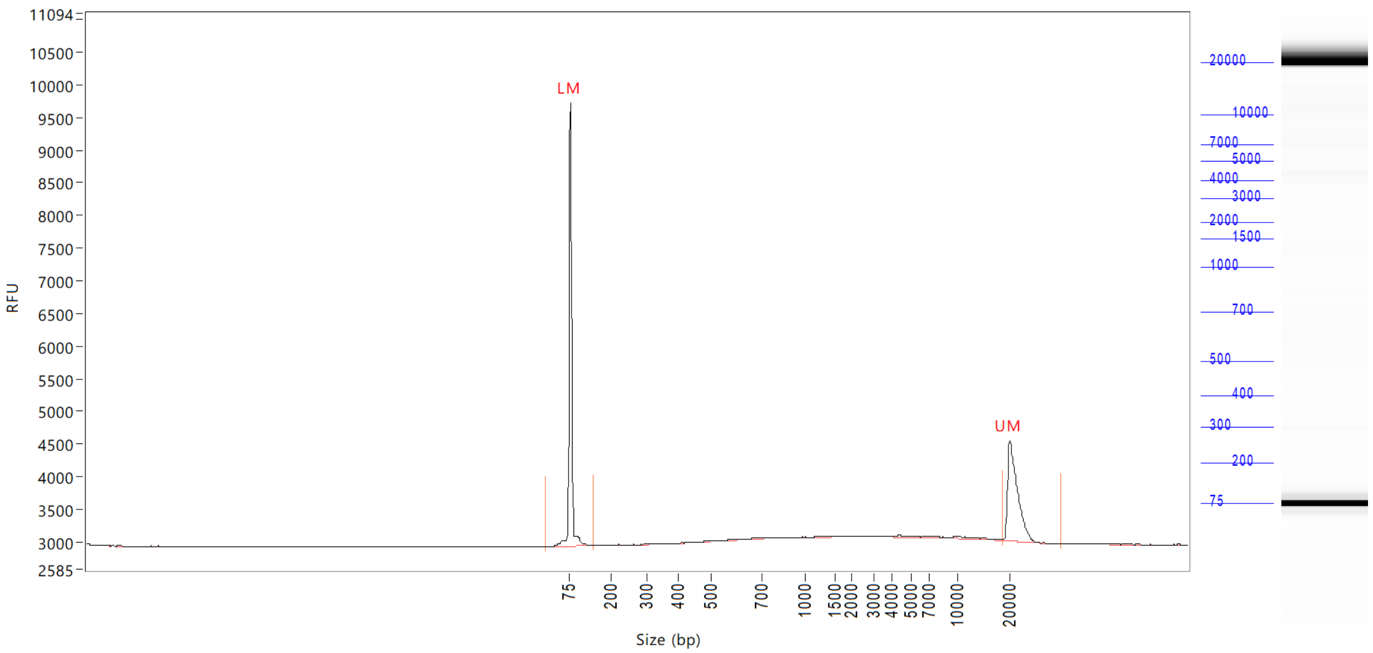

Peak	Size	Concentration	Relative concentration	Molarity	From	To	CV%	RFU
	(bp)	(ng/uL)	Percent	(nmole/L)	(bp)	(bp)		
1	75 (LM)	0.5000		11.2364	3	147	12.09	6794
2	20000 (UM)	0.2318		0.0183	18537	29793	5.59	1538

TIC:	0.0000	ng/uL
TIM:	0.0000	nmole/L
Total concentration:	0.7072	ng/uL

```
Sample peak width (sec): 5    Sample min peak height: 50    Sample baseline V to V?: Y    Sample baseline V to V points: 3
Sample filter: Binomial       Number of points for filter: 3  Sample start region (min): 0  Sample end region (min): 30
Marker peak width (sec): 5    Marker min peak height: 200   Marker baseline V to V?: Y    Marker baseline V to V points: 3
Lower marker selection: First peak > 500 RFU                Upper marker selection: Last peak > 200 RFU
Ladder size (bp)75, 200, 300, 400, 500, 700, 1000, 1500, 2000, 3000, 4000, 5000, 7000, 10000, 20000
Quantification using: Lower Marker          Final concentration (ng/uL): 0.5000          Dilution factor: 12.0
```

Sample: SampE8
Well location: E8
Created: Thursday, October 21, 2021 8:08:12 PM

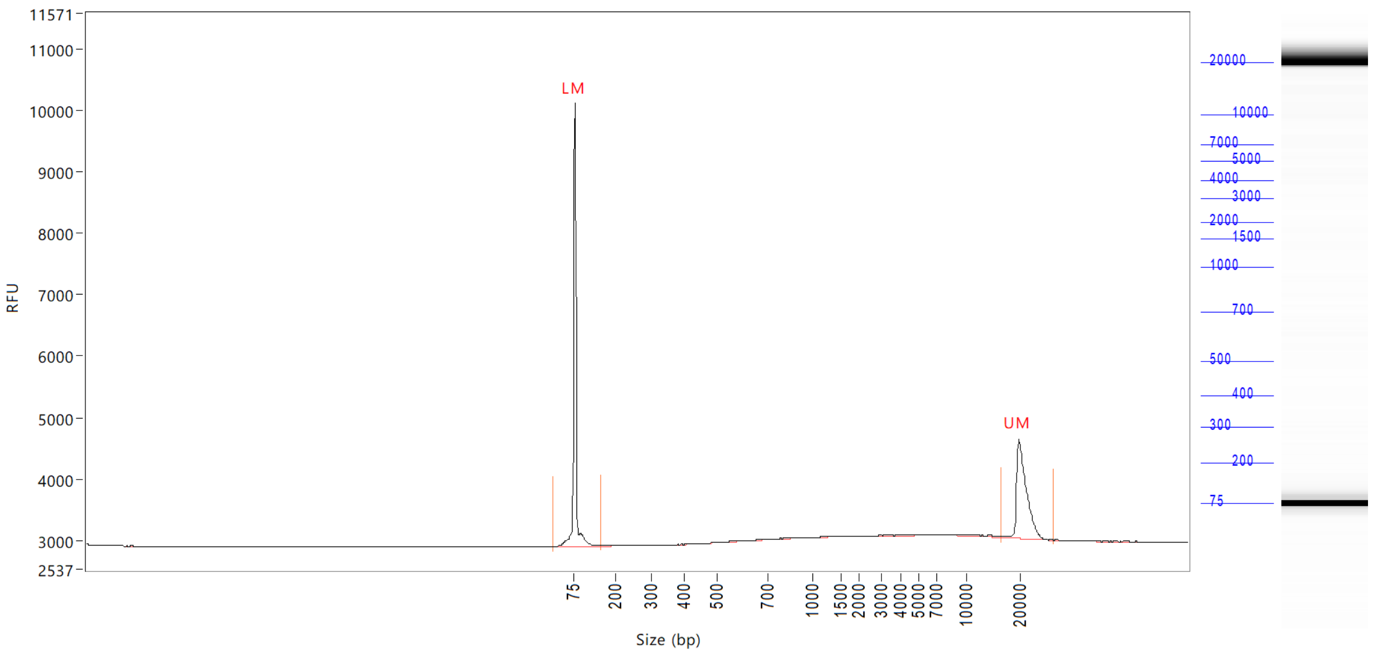

Peak	Size	Concentration	Relative concentration	Molarity	From	To	CV%	RFU
	(bp)	(ng/uL)	Percent	(nmole/L)	(bp)	(bp)		
1	75 (LM)	0.5000		11.0851	11	152	14.41	7220
2	20000 (UM)	0.2212		0.0176	16464	26649	5.51	1616

TIC:	0.0000	ng/uL
TIM:	0.0000	nmole/L
Total concentration:	0.5525	ng/uL

Sample peak width (sec): 5 Sample min peak height: 50 Sample baseline V to V?: Y Sample baseline V to V points: 3
Sample filter: Binomial Number of points for filter: 3 Sample start region (min): 0 Sample end region (min): 30
Marker peak width (sec): 5 Marker min peak height: 200 Marker baseline V to V?: Y Marker baseline V to V points: 3
Lower marker selection: First peak > 500 RFU Upper marker selection: Last peak > 200 RFU
Ladder size (bp)75, 200, 300, 400, 500, 700, 1000, 1500, 2000, 3000, 4000, 5000, 7000, 10000, 20000
Quantification using: Lower Marker Final concentration (ng/uL): 0.5000 Dilution factor: 12.0

Sample: SampF8
Well location: F8
Created: Thursday, October 21, 2021 8:08:12 PM

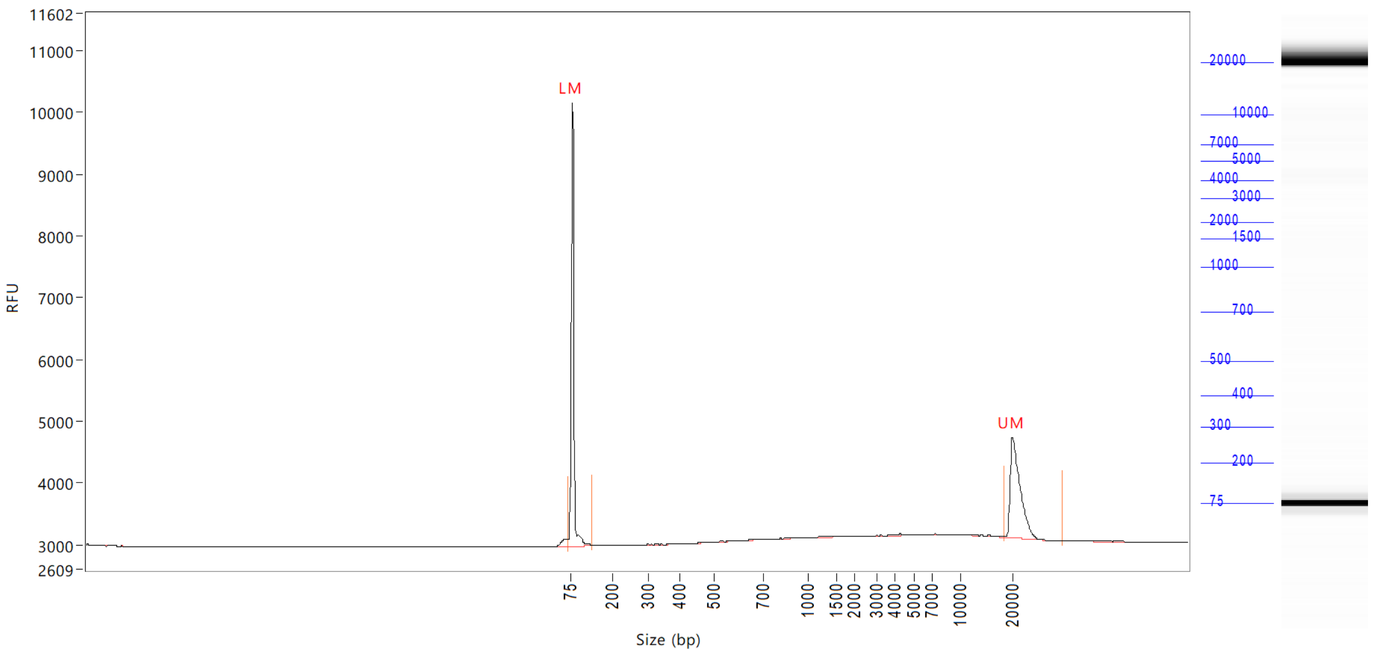

Peak	Size	Concentration	Relative concentration	Molarity	From	To	CV%	RFU
	(bp)	(ng/uL)	Percent	(nmole/L)	(bp)	(bp)		
1	75 (LM)	0.5000		10.9378	64	137	9.73	7184
2	20000 (UM)	0.2372		0.0188	18171	29551	5.65	1628

TIC:	0.0000	ng/uL
TIM:	0.0000	nmole/L
Total concentration:	0.5078	ng/uL

Sample peak width (sec): 5   Sample min peak height: 50   Sample baseline V to V?: Y   Sample baseline V to V points: 3
Sample filter: Binomial   Number of points for filter: 3 Sample start region (min): 0 Sample end region (min): 30
Marker peak width (sec): 5   Marker min peak height: 200   Marker baseline V to V?: Y   Marker baseline V to V points: 3
Lower marker selection: First peak > 500 RFU   Upper marker selection: Last peak > 200 RFU
Ladder size (bp)75, 200, 300, 400, 500, 700, 1000, 1500, 2000, 3000, 4000, 5000, 7000, 10000, 20000
Quantification using: Lower Marker   Final concentration (ng/uL): 0.5000   Dilution factor: 12.0

Sample: SampG8
Well location: G8
Created: Thursday, October 21, 2021 8:08:12 PM

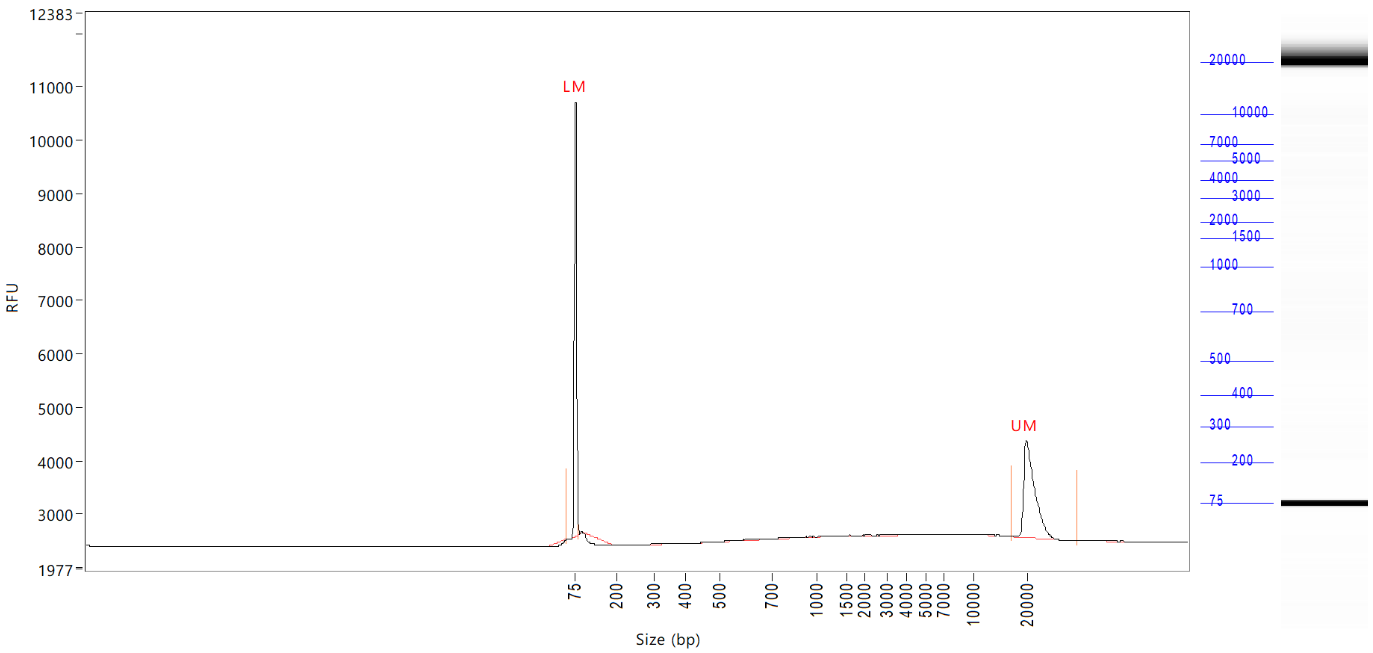

Peak	Size	Concentration	Relative concentration	Molarity	From	To	CV%	RFU
	(bp)	(ng/uL)	Percent	(nmole/L)	(bp)	(bp)		
1	75 (LM)	0.5000		11.3919	48	86	4.29	8132
2	20000 (UM)	0.2665		0.0211	16952	29430	6.00	1832

TIC:	0.0000	ng/uL
TIM:	0.0000	nmole/L
Total concentration:	0.5535	ng/uL

Sample peak width (sec): 5 Sample min peak height: 50 Sample baseline V to V?: Y Sample baseline V to V points: 3
Sample filter: Binomial Number of points for filter: 3 Sample start region (min): 0 Sample end region (min): 30
Marker peak width (sec): 5 Marker min peak height: 200 Marker baseline V to V?: Y Marker baseline V to V points: 3
Lower marker selection: First peak > 500 RFU Upper marker selection: Last peak > 200 RFU
Ladder size (bp)75, 200, 300, 400, 500, 700, 1000, 1500, 2000, 3000, 4000, 5000, 7000, 10000, 20000
Quantification using: Lower Marker Final concentration (ng/uL): 0.5000 Dilution factor: 12.0

Sample: SampH8
Well location: H8
Created: Thursday, October 21, 2021 8:08:12 PM

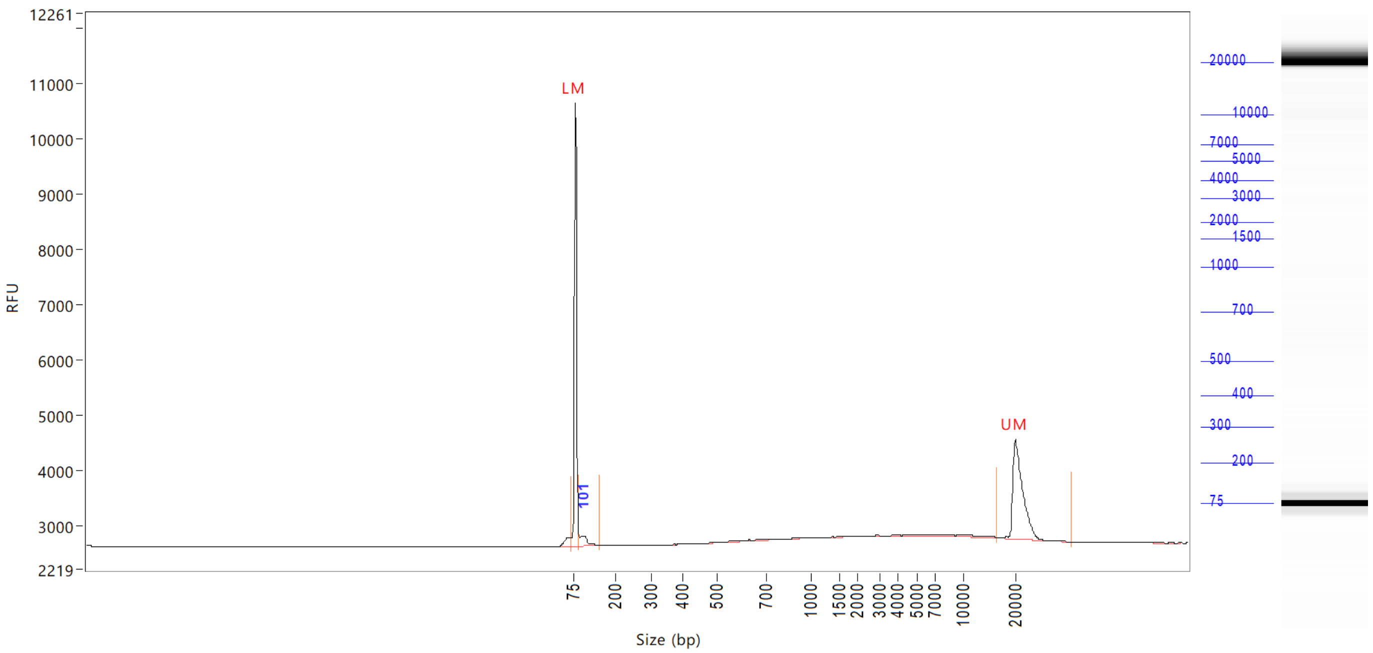

Peak	Size	Concentration	Relative concentration	Molarity	From	To	CV%	RFU
	(bp)	(ng/uL)	Percent	(nmole/L)	(bp)	(bp)		
1	75 (LM)	0.5000		11.2364	62	87	4.67	8019
2	101	0.4387	100.0	6.9943	87	150	12.92	185
3	20000 (UM)	0.2525		0.0200	16586	30639	6.17	1793

TIC:	0.4387	ng/uL
TIM:	6.9943	nmole/L
Total concentration:	1.0984	ng/uL

```
Sample peak width (sec): 5    Sample min peak height: 50    Sample baseline V to V?: Y    Sample baseline V to V points: 3
Sample filter: Binomial       Number of points for filter: 3  Sample start region (min): 0  Sample end region (min): 30
Marker peak width (sec): 5    Marker min peak height: 200   Marker baseline V to V?: Y    Marker baseline V to V points: 3
Lower marker selection: First peak > 500 RFU                Upper marker selection: Last peak > 200 RFU
Ladder size (bp)75, 200, 300, 400, 500, 700, 1000, 1500, 2000, 3000, 4000, 5000, 7000, 10000, 20000
Quantification using: Lower Marker          Final concentration (ng/uL): 0.5000          Dilution factor: 12.0
```

Sample: SampA9
Well location: A9
Created: Thursday, October 21, 2021 8:08:12 PM

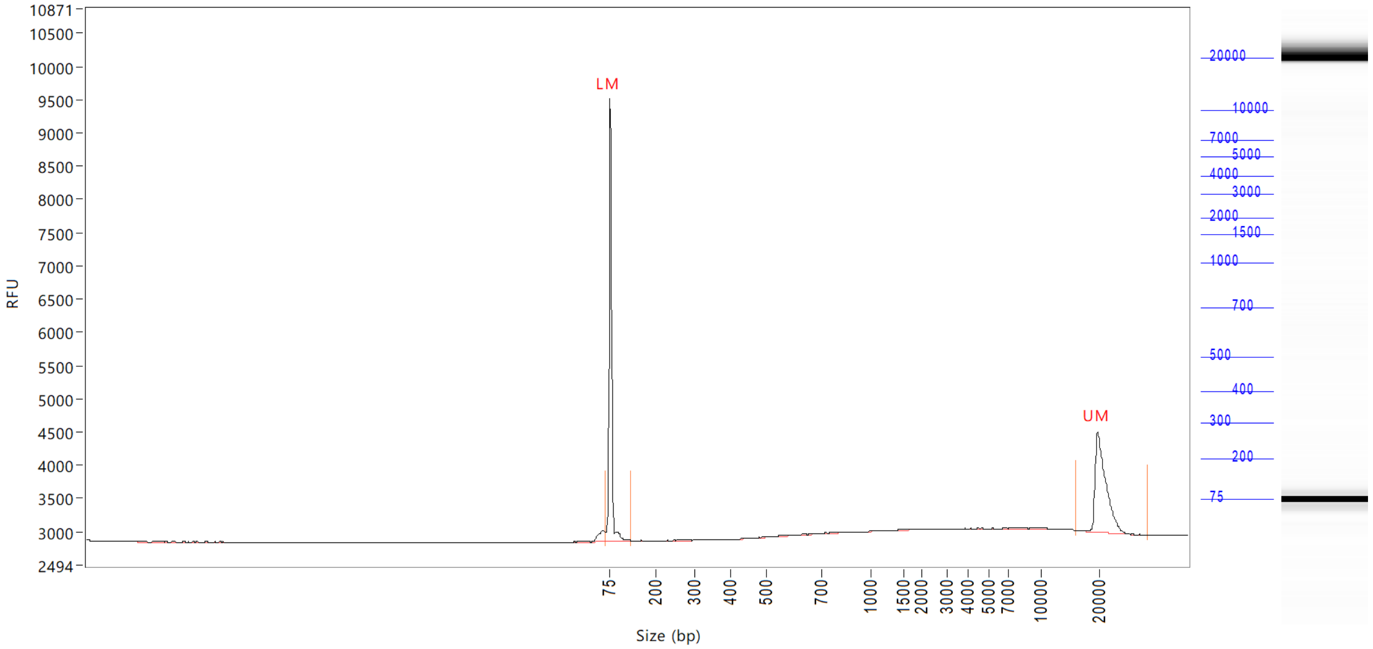

Peak	Size	Concentration	Relative concentration	Molarity	From	To	CV%	RFU
	(bp)	(ng/uL)	Percent	(nmole/L)	(bp)	(bp)		
1	75 (LM)	0.5000		11.0851	62	133	8.75	6683
2	20000 (UM)	0.2492		0.0198	16220	28463	5.55	1508

TIC:	0.0000	ng/uL
TIM:	0.0000	nmole/L
Total concentration:	0.5252	ng/uL

```
Sample peak width (sec): 5    Sample min peak height: 50    Sample baseline V to V?: Y    Sample baseline V to V points: 3
Sample filter: Binomial       Number of points for filter: 3  Sample start region (min): 0  Sample end region (min): 30
Marker peak width (sec): 5    Marker min peak height: 200   Marker baseline V to V?: Y    Marker baseline V to V points: 3
Lower marker selection: First peak > 500 RFU                Upper marker selection: Last peak > 200 RFU
Ladder size (bp)75, 200, 300, 400, 500, 700, 1000, 1500, 2000, 3000, 4000, 5000, 7000, 10000, 20000
Quantification using: Lower Marker          Final concentration (ng/uL): 0.5000          Dilution factor: 12.0
```

Sample: SampB9
Well location: B9
Created: Thursday, October 21, 2021 8:08:12 PM

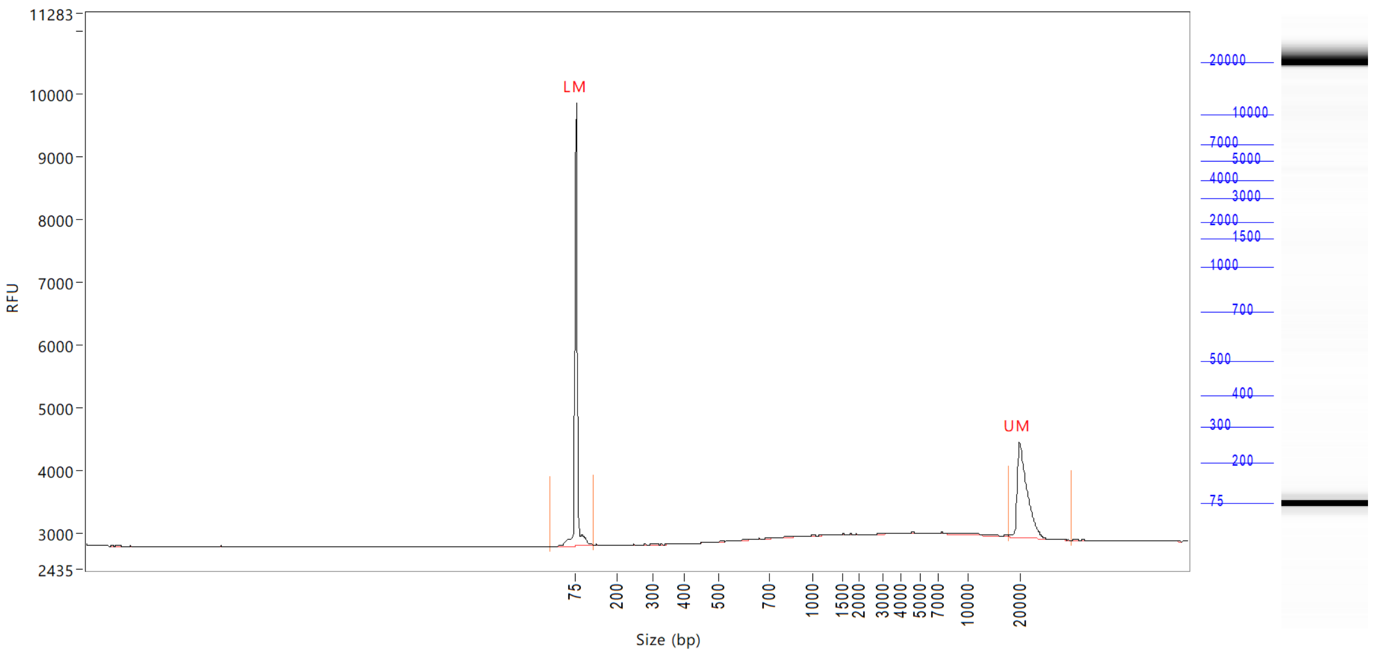

Peak	Size	Concentration	Relative concentration	Molarity	From	To	CV%	RFU
	(bp)	(ng/uL)	Percent	(nmole/L)	(bp)	(bp)		
1	75 (LM)	0.5000		11.2364	0	129	11.68	7063
2	20000 (UM)	0.2196		0.0174	17927	29793	5.88	1525

TIC:	0.0000	ng/uL
TIM:	0.0000	nmole/L
Total concentration:	0.6521	ng/uL

Sample peak width (sec): 5 Sample min peak height: 50 Sample baseline V to V?: Y Sample baseline V to V points: 3
Sample filter: Binomial Number of points for filter: 3 Sample start region (min): 0 Sample end region (min): 30
Marker peak width (sec): 5 Marker min peak height: 200 Marker baseline V to V?: Y Marker baseline V to V points: 3
Lower marker selection: First peak > 500 RFU Upper marker selection: Last peak > 200 RFU
Ladder size (bp)75, 200, 300, 400, 500, 700, 1000, 1500, 2000, 3000, 4000, 5000, 7000, 10000, 20000
Quantification using: Lower Marker Final concentration (ng/uL): 0.5000 Dilution factor: 12.0

Sample: SampC9
Well location: C9
Created: Thursday, October 21, 2021 8:08:12 PM

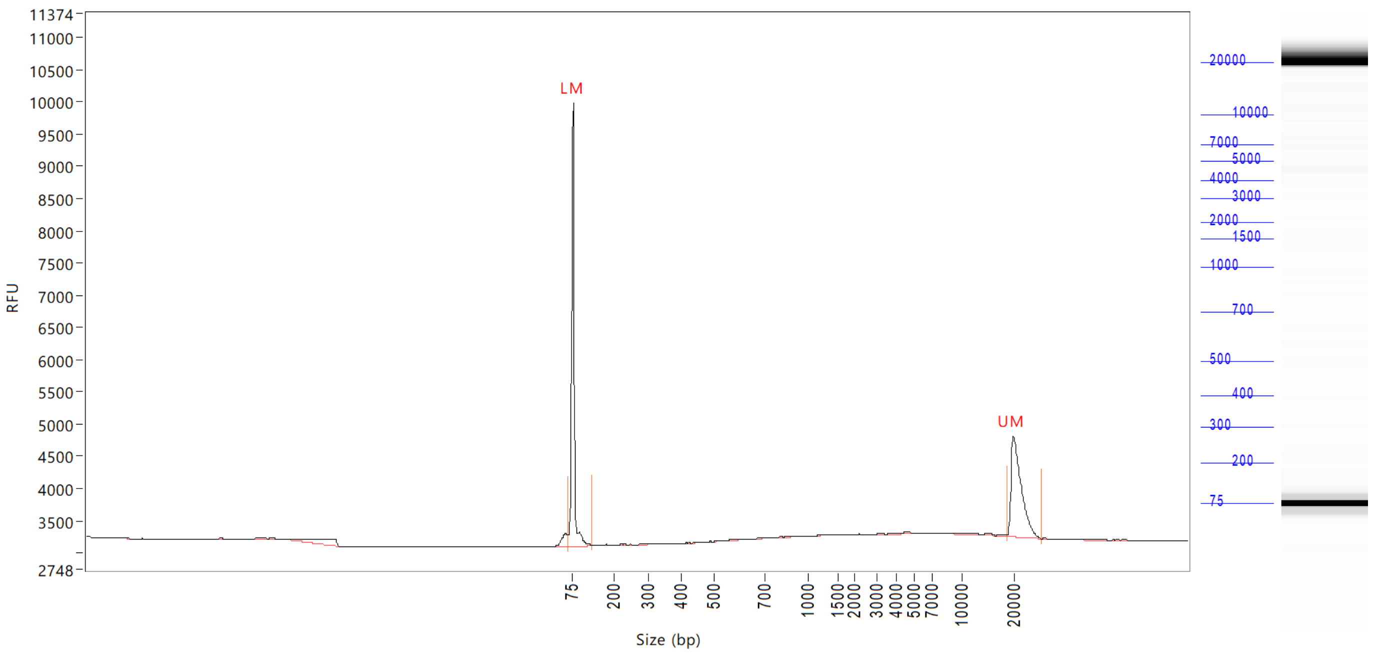

Peak	Size	Concentration	Relative concentration	Molarity	From	To	CV%	RFU
	(bp)	(ng/uL)	Percent	(nmole/L)	(bp)	(bp)		
1	75 (LM)	0.5000		11.0851	62	131	10.37	6892
2	20000 (UM)	0.2328		0.0184	18659	25440	5.28	1569

TIC:	0.0000	ng/uL
TIM:	0.0000	nmole/L
Total concentration:	0.6868	ng/uL

Sample peak width (sec): 5 Sample min peak height: 50 Sample baseline V to V?: Y Sample baseline V to V points: 3
Sample filter: Binomial Number of points for filter: 3 Sample start region (min): 0 Sample end region (min): 30
Marker peak width (sec): 5 Marker min peak height: 200 Marker baseline V to V?: Y Marker baseline V to V points: 3
Lower marker selection: First peak > 500 RFU Upper marker selection: Last peak > 200 RFU
Ladder size (bp)75, 200, 300, 400, 500, 700, 1000, 1500, 2000, 3000, 4000, 5000, 7000, 10000, 20000
Quantification using: Lower Marker Final concentration (ng/uL): 0.5000 Dilution factor: 12.0

Sample: SampD9
Well location: D9
Created: Thursday, October 21, 2021 8:08:12 PM

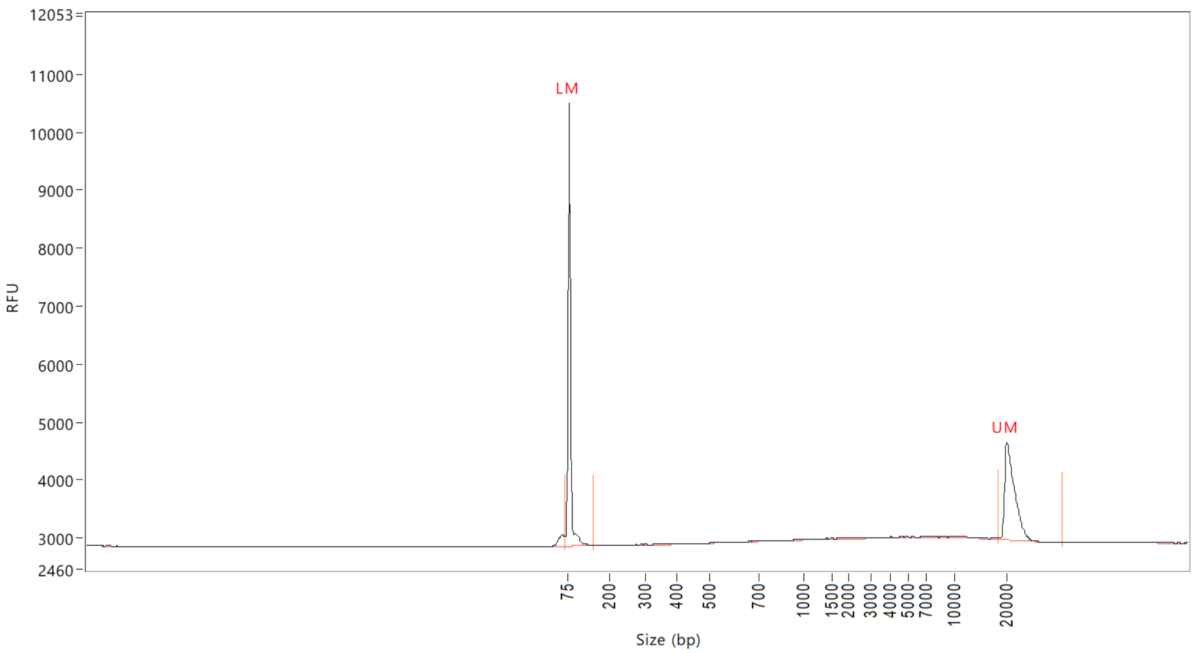

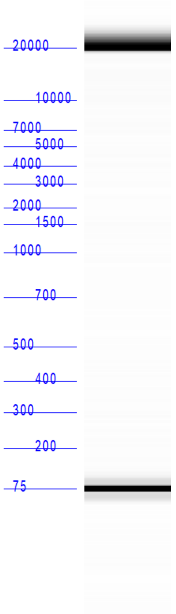

Peak	Size	Concentration	Relative concentration	Molarity	From	To	CV%	RFU
	(bp)	(ng/uL)	Percent	(nmole/L)	(bp)	(bp)		
1	75 (LM)	0.5000		10.9378	64	150	10.80	7659
2	20000 (UM)	0.2305		0.0182	18293	30639	5.54	1698

TIC:	0.0000	ng/uL
TIM:	0.0000	nmole/L
Total concentration:	0.5748	ng/uL

```
Sample peak width (sec): 5    Sample min peak height: 50    Sample baseline V to V?: Y    Sample baseline V to V points: 3
Sample filter: Binomial       Number of points for filter: 3  Sample start region (min): 0  Sample end region (min): 30
Marker peak width (sec): 5    Marker min peak height: 200   Marker baseline V to V?: Y    Marker baseline V to V points: 3
Lower marker selection: First peak > 500 RFU                Upper marker selection: Last peak > 200 RFU
Ladder size (bp)75, 200, 300, 400, 500, 700, 1000, 1500, 2000, 3000, 4000, 5000, 7000, 10000, 20000
Quantification using: Lower Marker          Final concentration (ng/uL): 0.5000          Dilution factor: 12.0
```

Sample: SampE9
Well location: E9
Created: Thursday, October 21, 2021 8:08:12 PM

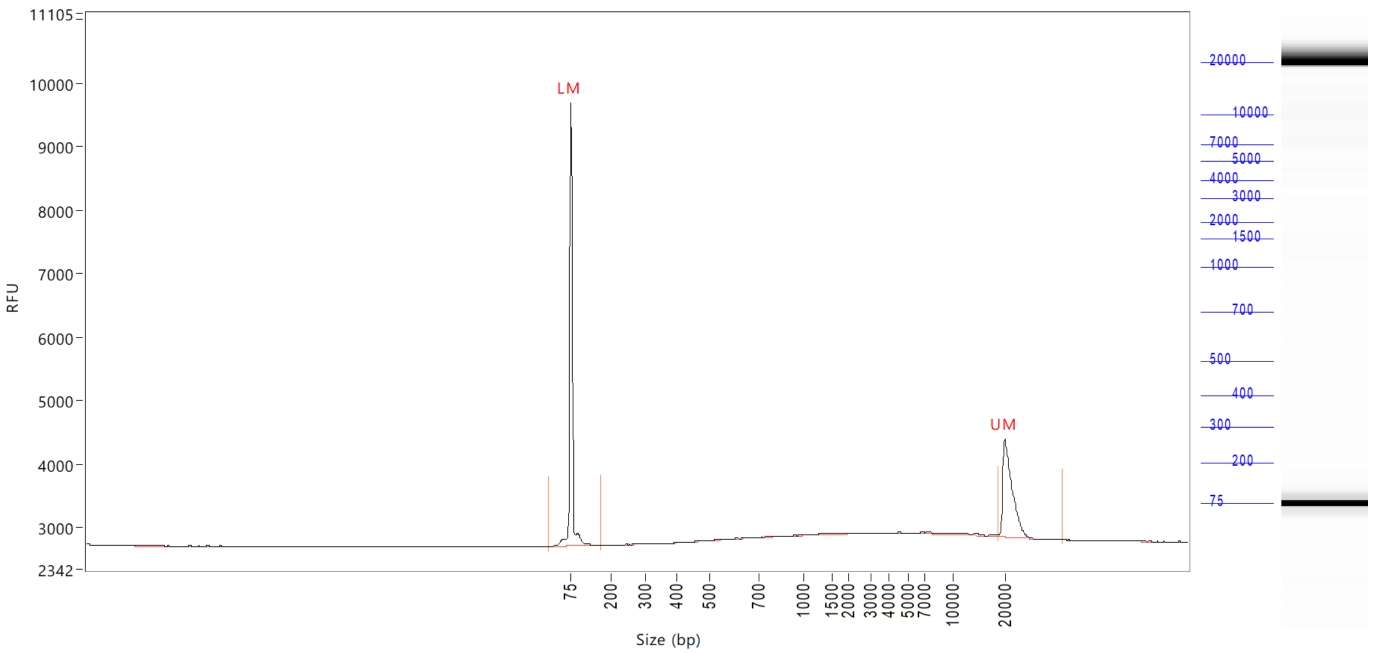

Peak	Size	Concentration	Relative concentration	Molarity	From	To	CV%	RFU
	(bp)	(ng/uL)	Percent	(nmole/L)	(bp)	(bp)		
1	75 (LM)	0.5000		11.2364	7	166	14.67	6994
2	20000 (UM)	0.2117		0.0168	18659	31244	5.63	1552

TIC:	0.0000	ng/uL
TIM:	0.0000	nmole/L
Total concentration:	0.6184	ng/uL

Sample peak width (sec): 5 Sample min peak height: 50 Sample baseline V to V?: Y Sample baseline V to V points: 3
Sample filter: Binomial Number of points for filter: 3 Sample start region (min): 0 Sample end region (min): 30
Marker peak width (sec): 5 Marker min peak height: 200 Marker baseline V to V?: Y Marker baseline V to V points: 3
Lower marker selection: First peak > 500 RFU Upper marker selection: Last peak > 200 RFU
Ladder size (bp)75, 200, 300, 400, 500, 700, 1000, 1500, 2000, 3000, 4000, 5000, 7000, 10000, 20000
Quantification using: Lower Marker Final concentration (ng/uL): 0.5000 Dilution factor: 12.0

Sample: SampF9
Well location: F9
Created: Thursday, October 21, 2021 8:08:12 PM

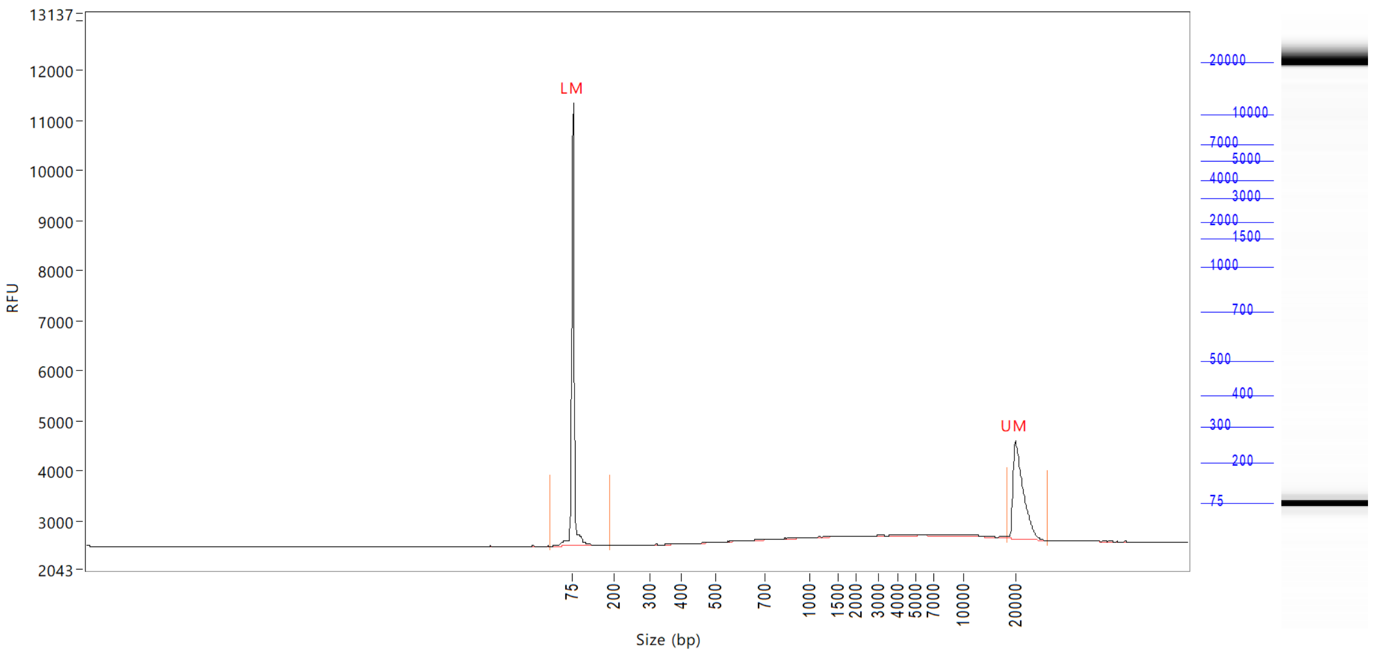

Peak	Size	Concentration	Relative concentration	Molarity	From	To	CV%	RFU
	(bp)	(ng/uL)	Percent	(nmole/L)	(bp)	(bp)		
1	75 (LM)	0.5000		11.0851	7	189	14.33	8854
2	20000 (UM)	0.2285		0.0181	18415	26045	5.32	1955

TIC:	0.0000	ng/uL
TIM:	0.0000	nmole/L
Total concentration:	0.5506	ng/uL

Sample peak width (sec): 5 Sample min peak height: 50 Sample baseline V to V?: Y Sample baseline V to V points: 3
Sample filter: Binomial Number of points for filter: 3 Sample start region (min): 0 Sample end region (min): 30
Marker peak width (sec): 5 Marker min peak height: 200 Marker baseline V to V?: Y Marker baseline V to V points: 3
Lower marker selection: First peak > 500 RFU Upper marker selection: Last peak > 200 RFU
Ladder size (bp)75, 200, 300, 400, 500, 700, 1000, 1500, 2000, 3000, 4000, 5000, 7000, 10000, 20000
Quantification using: Lower Marker Final concentration (ng/uL): 0.5000 Dilution factor: 12.0

Sample: SampG9
Well location: G9
Created: Thursday, October 21, 2021 8:08:12 PM

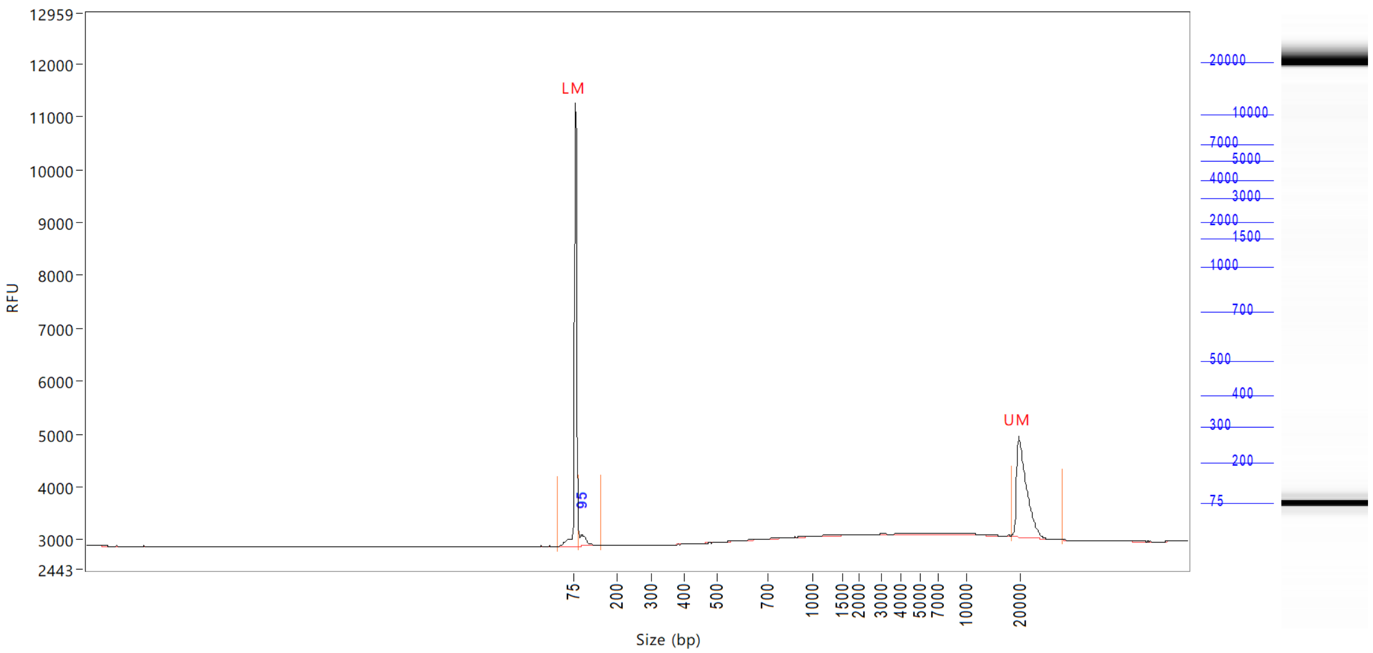

Peak	Size	Concentration	Relative concentration	Molarity	From	To	CV%	RFU
	(bp)	(ng/uL)	Percent	(nmole/L)	(bp)	(bp)		
1	75 (LM)	0.5000		11.2364	22	87	8.24	8396
2	95	0.4003	100.0	6.5079	87	150	12.65	208
3	20000 (UM)	0.2422		0.0192	18415	28221	5.49	1897

TIC:	0.4003	ng/uL
TIM:	6.5079	nmole/L
Total concentration:	1.0105	ng/uL

```
Sample peak width (sec): 5    Sample min peak height: 50    Sample baseline V to V?: Y    Sample baseline V to V points: 3
Sample filter: Binomial       Number of points for filter: 3  Sample start region (min): 0  Sample end region (min): 30
Marker peak width (sec): 5    Marker min peak height: 200   Marker baseline V to V?: Y    Marker baseline V to V points: 3
Lower marker selection: First peak > 500 RFU                Upper marker selection: Last peak > 200 RFU
Ladder size (bp)75, 200, 300, 400, 500, 700, 1000, 1500, 2000, 3000, 4000, 5000, 7000, 10000, 20000
Quantification using: Lower Marker          Final concentration (ng/uL): 0.5000          Dilution factor: 12.0
```

Sample: SampH9
Well location: H9
Created: Thursday, October 21, 2021 8:08:12 PM

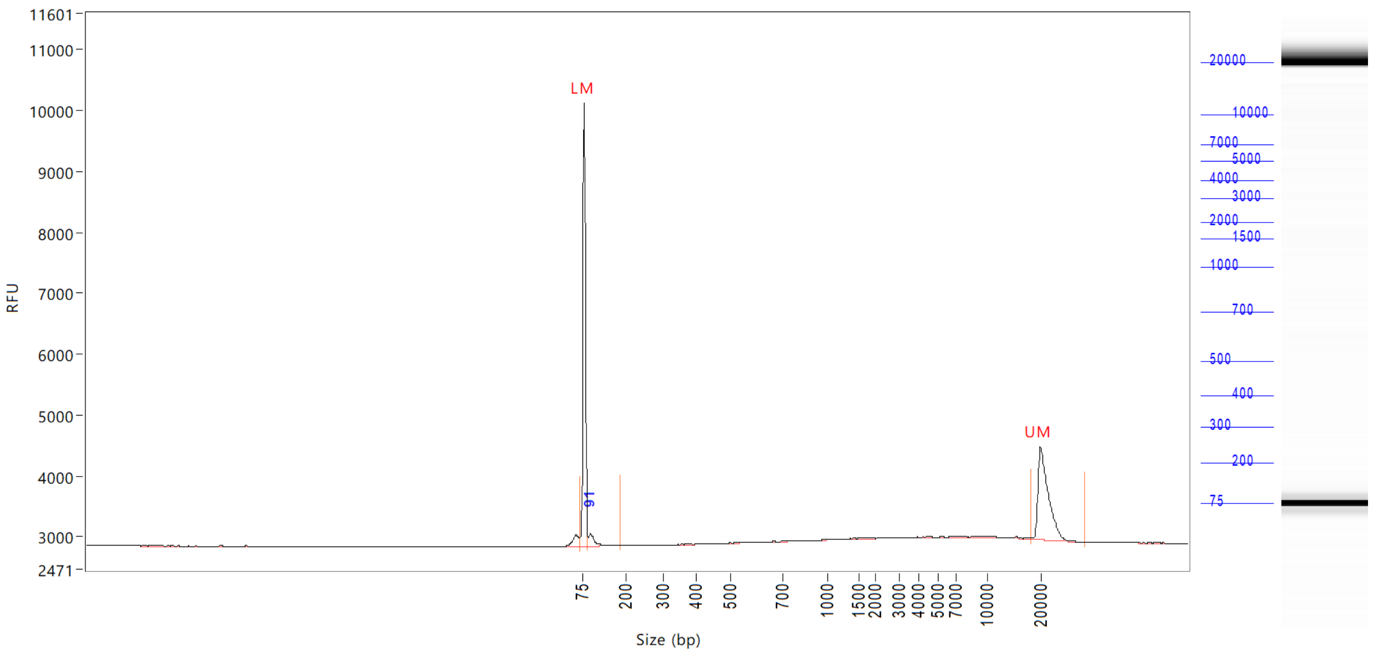

Peak	Size	Concentration	Relative concentration	Molarity	From	To	CV%	RFU
	(bp)	(ng/uL)	Percent	(nmole/L)	(bp)	(bp)		
1	75 (LM)	0.5000		11.0851	64	87	4.63	7292
2	91	0.4326	100.0	6.8980	87	181	16.92	200
3	20000 (UM)	0.2332		0.0185	18171	28100	5.67	1532

TIC:	0.4326	ng/uL
TIM:	6.8980	nmole/L
Total concentration:	1.0110	ng/uL

```
Sample peak width (sec): 5    Sample min peak height: 50    Sample baseline V to V?: Y    Sample baseline V to V points: 3
Sample filter: Binomial       Number of points for filter: 3  Sample start region (min): 0  Sample end region (min): 30
Marker peak width (sec): 5    Marker min peak height: 200   Marker baseline V to V?: Y    Marker baseline V to V points: 3
Lower marker selection: First peak > 500 RFU                Upper marker selection: Last peak > 200 RFU
Ladder size (bp)75, 200, 300, 400, 500, 700, 1000, 1500, 2000, 3000, 4000, 5000, 7000, 10000, 20000
Quantification using: Lower Marker          Final concentration (ng/uL): 0.5000          Dilution factor: 12.0
```

Sample: SampA10
Well location: A10
Created: Thursday, October 21, 2021 8:08:12 PM

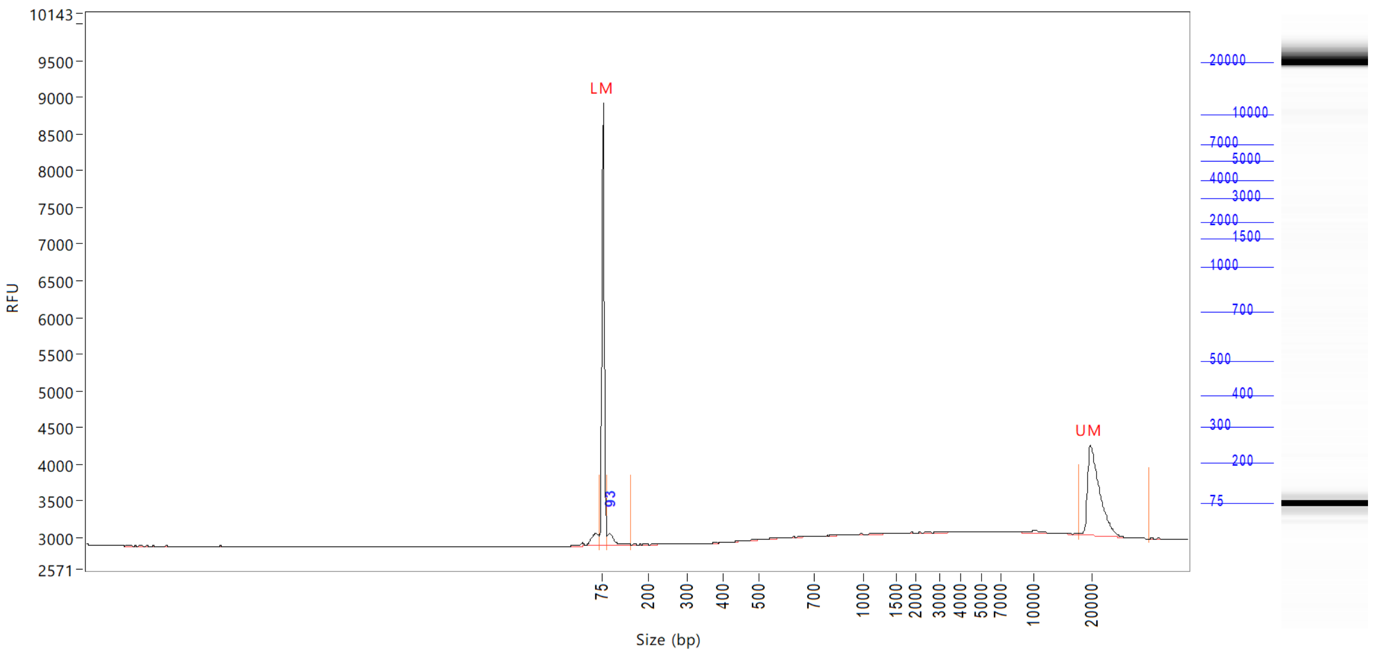

Peak	Size	Concentration	Relative concentration	Molarity	From	To	CV%	RFU
	(bp)	(ng/uL)	Percent	(nmole/L)	(bp)	(bp)		
1	75 (LM)	0.5000		11.2364	66	86	4.33	6038
2	93	0.4153	100.0	6.8890	86	152	13.01	163
3	20000 (UM)	0.2530		0.0200	18049	30156	5.96	1222

TIC:	0.4153	ng/uL
TIM:	6.8890	nmole/L
Total concentration:	1.0687	ng/uL

Sample peak width (sec): 5   Sample min peak height: 50   Sample baseline V to V?: Y   Sample baseline V to V points: 3
Sample filter: Binomial   Number of points for filter: 3 Sample start region (min): 0 Sample end region (min): 30
Marker peak width (sec): 5   Marker min peak height: 200   Marker baseline V to V?: Y   Marker baseline V to V points: 3
Lower marker selection: First peak > 500 RFU   Upper marker selection: Last peak > 200 RFU
Ladder size (bp)75, 200, 300, 400, 500, 700, 1000, 1500, 2000, 3000, 4000, 5000, 7000, 10000, 20000
Quantification using: Lower Marker   Final concentration (ng/uL): 0.5000   Dilution factor: 12.0

Sample: SampB10
Well location: B10
Created: Thursday, October 21, 2021 8:08:12 PM

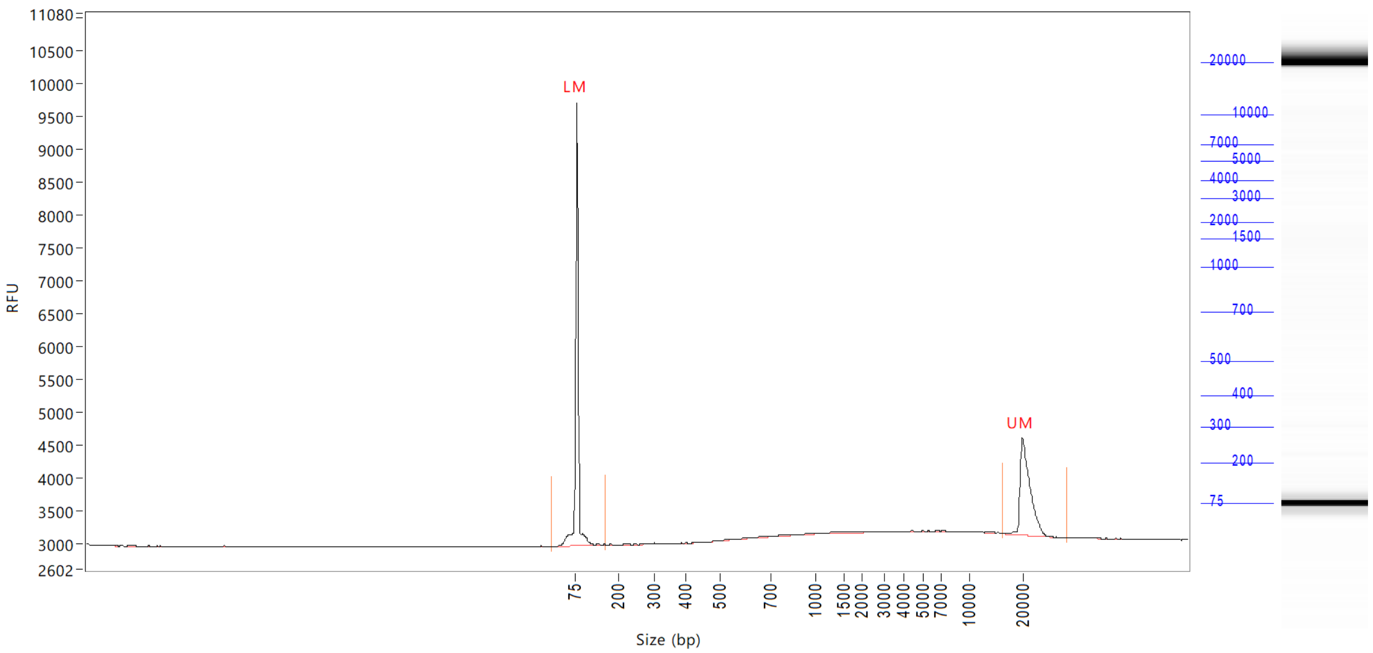

Peak	Size	Concentration	Relative concentration	Molarity	From	To	CV%	RFU
	(bp)	(ng/uL)	Percent	(nmole/L)	(bp)	(bp)		
1	75 (LM)	0.5000		11.0851	1	164	15.05	6766
2	20000 (UM)	0.2043		0.0163	16220	28463	5.56	1499

TIC:	0.0000	ng/uL
TIM:	0.0000	nmole/L
Total concentration:	0.5558	ng/uL

Sample peak width (sec): 5 Sample min peak height: 50 Sample baseline V to V?: Y Sample baseline V to V points: 3
Sample filter: Binomial Number of points for filter: 3 Sample start region (min): 0 Sample end region (min): 30
Marker peak width (sec): 5 Marker min peak height: 200 Marker baseline V to V?: Y Marker baseline V to V points: 3
Lower marker selection: First peak > 500 RFU Upper marker selection: Last peak > 200 RFU
Ladder size (bp)75, 200, 300, 400, 500, 700, 1000, 1500, 2000, 3000, 4000, 5000, 7000, 10000, 20000
Quantification using: Lower Marker Final concentration (ng/uL): 0.5000 Dilution factor: 12.0

Sample: SampC10
Well location: C10
Created: Thursday, October 21, 2021 8:08:12 PM

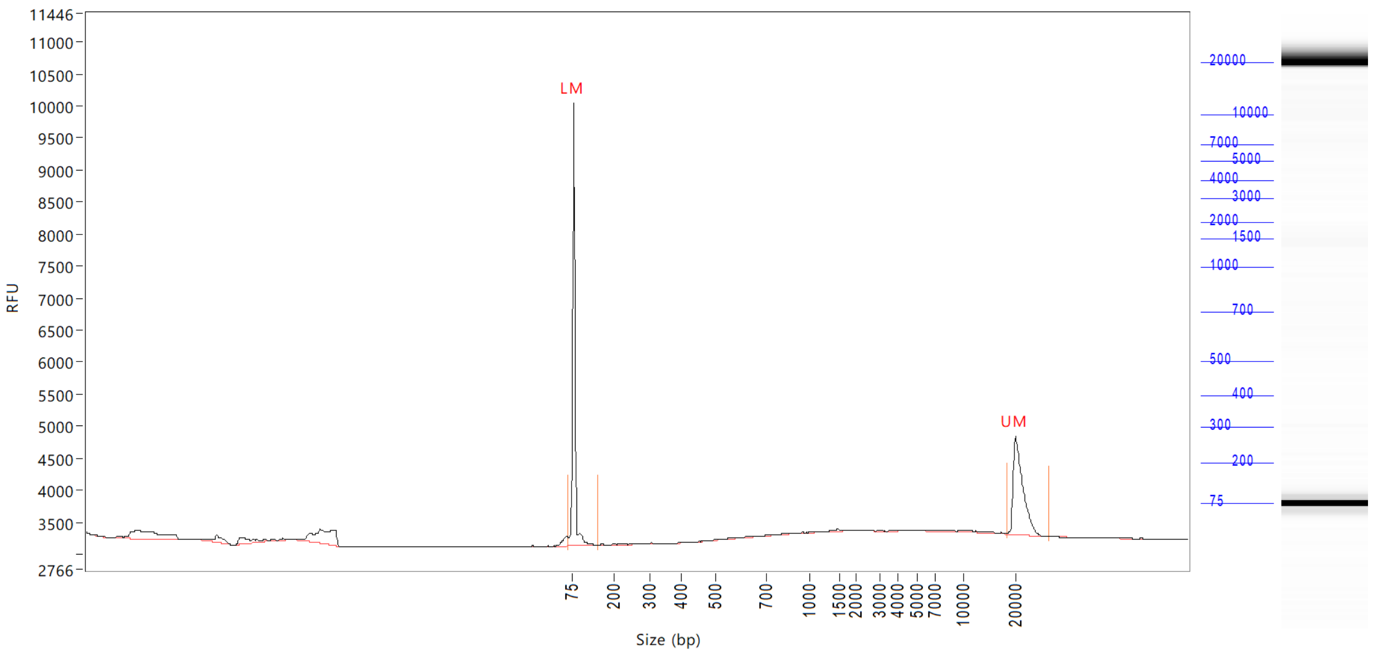

Peak	Size	Concentration	Relative concentration	Molarity	From	To	CV%	RFU
	(bp)	(ng/uL)	Percent	(nmole/L)	(bp)	(bp)		
1	75 (LM)	0.5000		10.9378	62	148	10.02	6927
2	20000 (UM)	0.2251		0.0179	18415	26408	5.22	1529

TIC:	0.0000	ng/uL
TIM:	0.0000	nmole/L
Total concentration:	0.7516	ng/uL

Sample peak width (sec): 5 Sample min peak height: 50 Sample baseline V to V?: Y Sample baseline V to V points: 3
Sample filter: Binomial Number of points for filter: 3 Sample start region (min): 0 Sample end region (min): 30
Marker peak width (sec): 5 Marker min peak height: 200 Marker baseline V to V?: Y Marker baseline V to V points: 3
Lower marker selection: First peak > 500 RFU Upper marker selection: Last peak > 200 RFU
Ladder size (bp)75, 200, 300, 400, 500, 700, 1000, 1500, 2000, 3000, 4000, 5000, 7000, 10000, 20000
Quantification using: Lower Marker Final concentration (ng/uL): 0.5000 Dilution factor: 12.0

Sample: SampD10
Well location: D10
Created: Thursday, October 21, 2021 8:08:12 PM

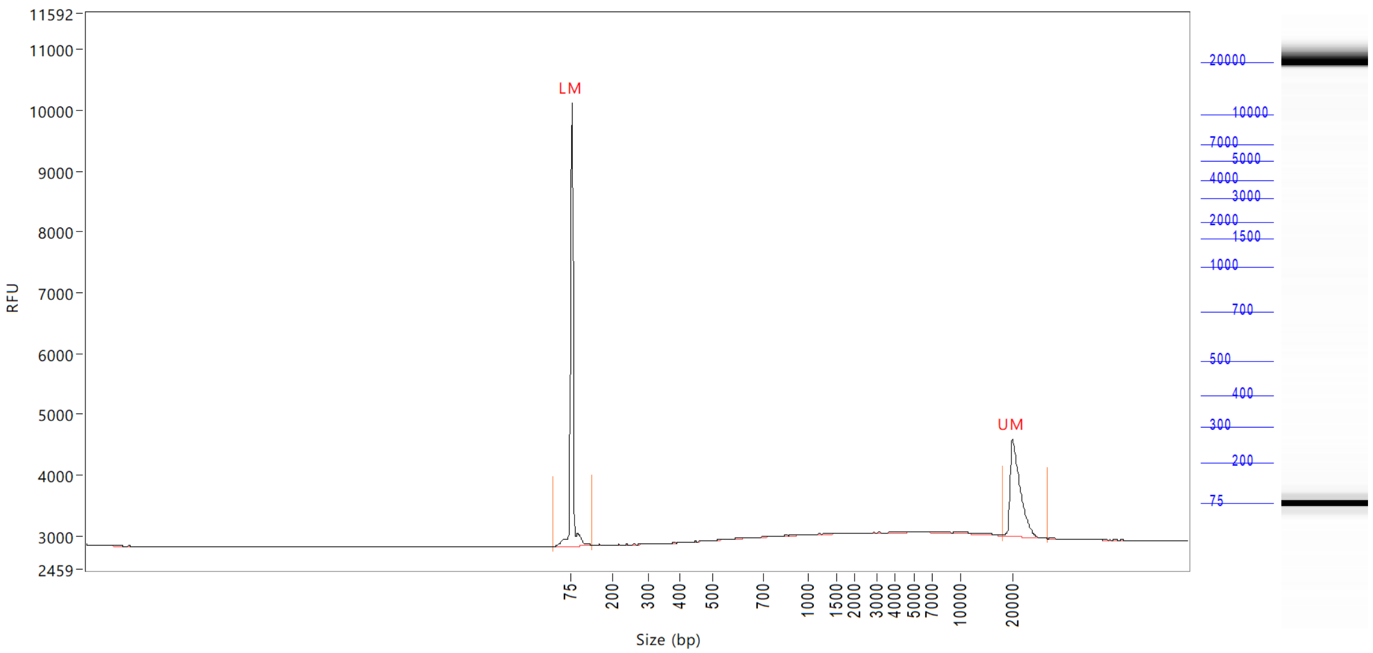

Peak	Size	Concentration	Relative concentration	Molarity	From	To	CV%	RFU
	(bp)	(ng/uL)	Percent	(nmole/L)	(bp)	(bp)		
1	75 (LM)	0.5000		11.0851	20	135	12.83	7294
2	20000 (UM)	0.2189		0.0174	18049	26649	5.33	1594

TIC:	0.0000	ng/uL
TIM:	0.0000	nmole/L
Total concentration:	0.6018	ng/uL

Sample peak width (sec): 5 Sample min peak height: 50 Sample baseline V to V?: Y Sample baseline V to V points: 3
Sample filter: Binomial Number of points for filter: 3 Sample start region (min): 0 Sample end region (min): 30
Marker peak width (sec): 5 Marker min peak height: 200 Marker baseline V to V?: Y Marker baseline V to V points: 3
Lower marker selection: First peak > 500 RFU Upper marker selection: Last peak > 200 RFU
Ladder size (bp)75, 200, 300, 400, 500, 700, 1000, 1500, 2000, 3000, 4000, 5000, 7000, 10000, 20000
Quantification using: Lower Marker Final concentration (ng/uL): 0.5000 Dilution factor: 12.0

Sample: SampE10
Well location: E10
Created: Thursday, October 21, 2021 8:08:12 PM

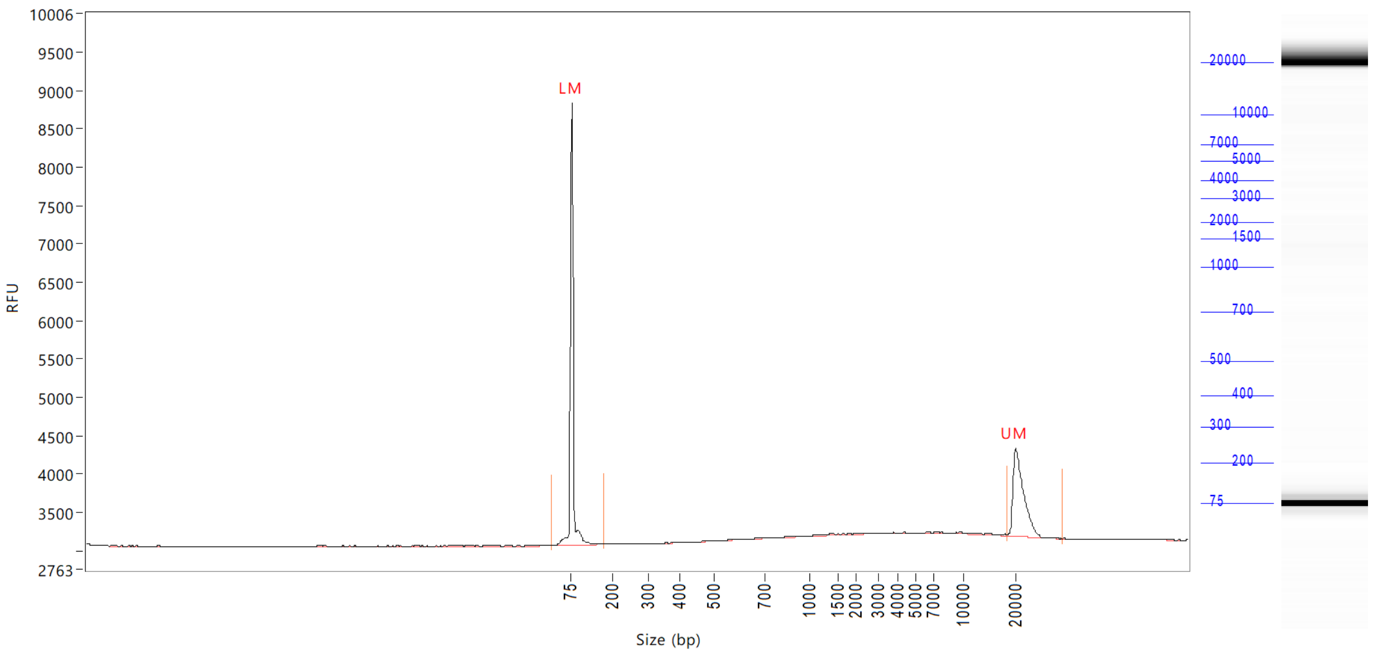

Peak	Size	Concentration	Relative concentration	Molarity	From	To	CV%	RFU
	(bp)	(ng/uL)	Percent	(nmole/L)	(bp)	(bp)		
1	75 (LM)	0.5000		11.0851	18	173	14.85	5773
2	20000 (UM)	0.2026		0.0161	18415	28826	5.80	1156

TIC:	0.0000	ng/uL
TIM:	0.0000	nmole/L
Total concentration:	0.5825	ng/uL

```
Sample peak width (sec): 5    Sample min peak height: 50    Sample baseline V to V?: Y    Sample baseline V to V points: 3
Sample filter: Binomial       Number of points for filter: 3  Sample start region (min): 0  Sample end region (min): 30
Marker peak width (sec): 5    Marker min peak height: 200   Marker baseline V to V?: Y    Marker baseline V to V points: 3
Lower marker selection: First peak > 500 RFU                Upper marker selection: Last peak > 200 RFU
Ladder size (bp)75, 200, 300, 400, 500, 700, 1000, 1500, 2000, 3000, 4000, 5000, 7000, 10000, 20000
Quantification using: Lower Marker          Final concentration (ng/uL): 0.5000          Dilution factor: 12.0
```

Sample: SampF10
Well location: F10
Created: Thursday, October 21, 2021 8:08:12 PM

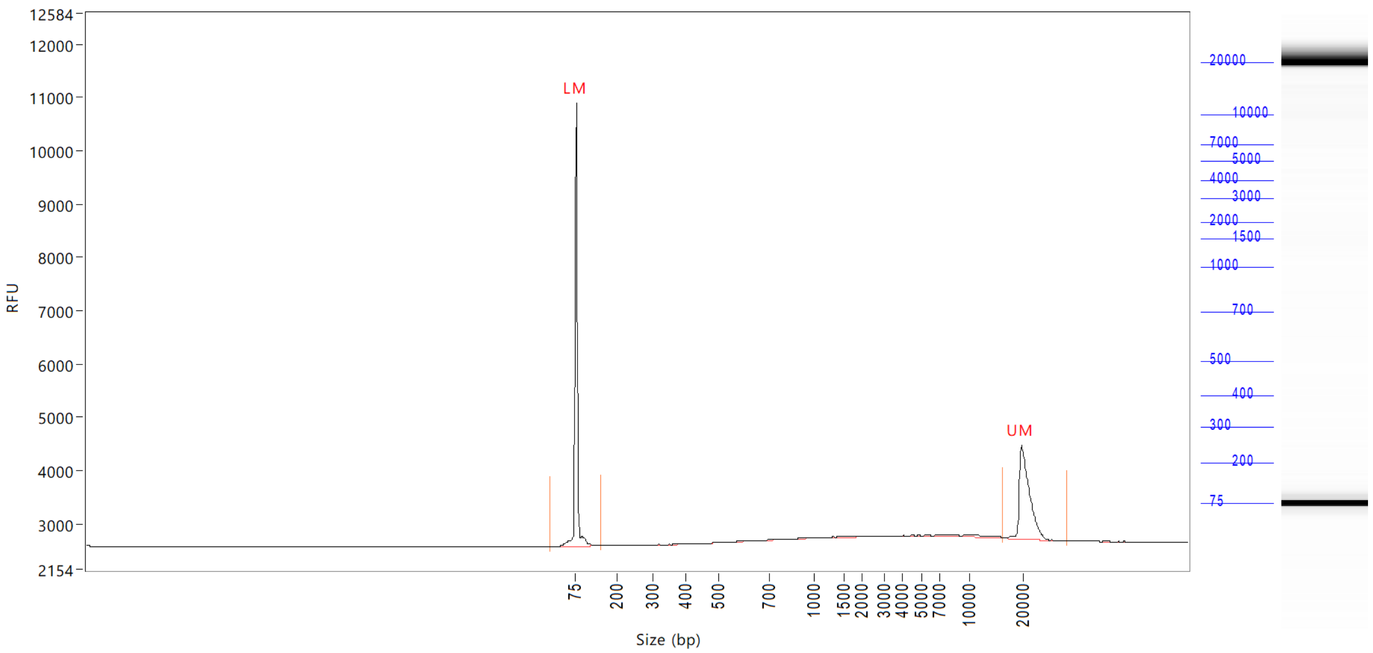

Peak	Size	Concentration	Relative concentration	Molarity	From	To	CV%	RFU
	(bp)	(ng/uL)	Percent	(nmole/L)	(bp)	(bp)		
1	75 (LM)	0.5000		11.0851	1	148	12.20	8331
2	20000 (UM)	0.2228		0.0177	16342	28463	5.79	1763

TIC:	0.0000	ng/uL
TIM:	0.0000	nmole/L
Total concentration:	0.6065	ng/uL

Sample peak width (sec): 5 Sample min peak height: 50 Sample baseline V to V?: Y Sample baseline V to V points: 3
Sample filter: Binomial Number of points for filter: 3 Sample start region (min): 0 Sample end region (min): 30
Marker peak width (sec): 5 Marker min peak height: 200 Marker baseline V to V?: Y Marker baseline V to V points: 3
Lower marker selection: First peak > 500 RFU Upper marker selection: Last peak > 200 RFU
Ladder size (bp)75, 200, 300, 400, 500, 700, 1000, 1500, 2000, 3000, 4000, 5000, 7000, 10000, 20000
Quantification using: Lower Marker Final concentration (ng/uL): 0.5000 Dilution factor: 12.0

Sample: SampG10
Well location: G10
Created: Thursday, October 21, 2021 8:08:12 PM

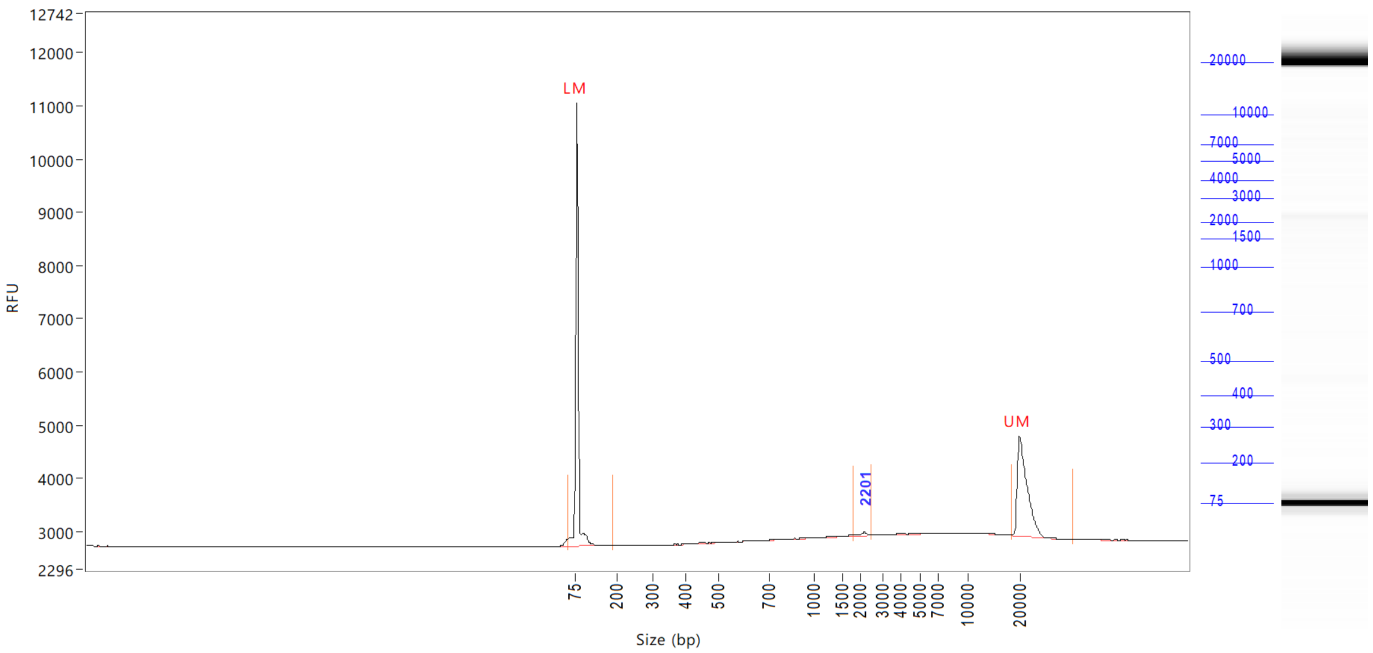

Peak	Size	Concentration	Relative concentration	Molarity	From	To	CV%	RFU
	(bp)	(ng/uL)	Percent	(nmole/L)	(bp)	(bp)		
1	75 (LM)	0.5000		10.9378	48	183	12.43	8340
2	2201	0.0757	100.0	0.0597	1789	2515	7.34	63
3	20000 (UM)	0.2155		0.0171	18537	30035	5.49	1890

TIC:	0.0757	ng/uL
TIM:	0.0597	nmole/L
Total concentration:	0.4775	ng/uL

Sample peak width (sec): 5   Sample min peak height: 50   Sample baseline V to V?: Y   Sample baseline V to V points: 3
Sample filter: Binomial   Number of points for filter: 3 Sample start region (min): 0 Sample end region (min): 30
Marker peak width (sec): 5   Marker min peak height: 200   Marker baseline V to V?: Y   Marker baseline V to V points: 3
Lower marker selection: First peak > 500 RFU   Upper marker selection: Last peak > 200 RFU
Ladder size (bp)75, 200, 300, 400, 500, 700, 1000, 1500, 2000, 3000, 4000, 5000, 7000, 10000, 20000
Quantification using: Lower Marker   Final concentration (ng/uL): 0.5000   Dilution factor: 12.0

Sample: SampH10
Well location: H10
Created: Thursday, October 21, 2021 8:08:12 PM

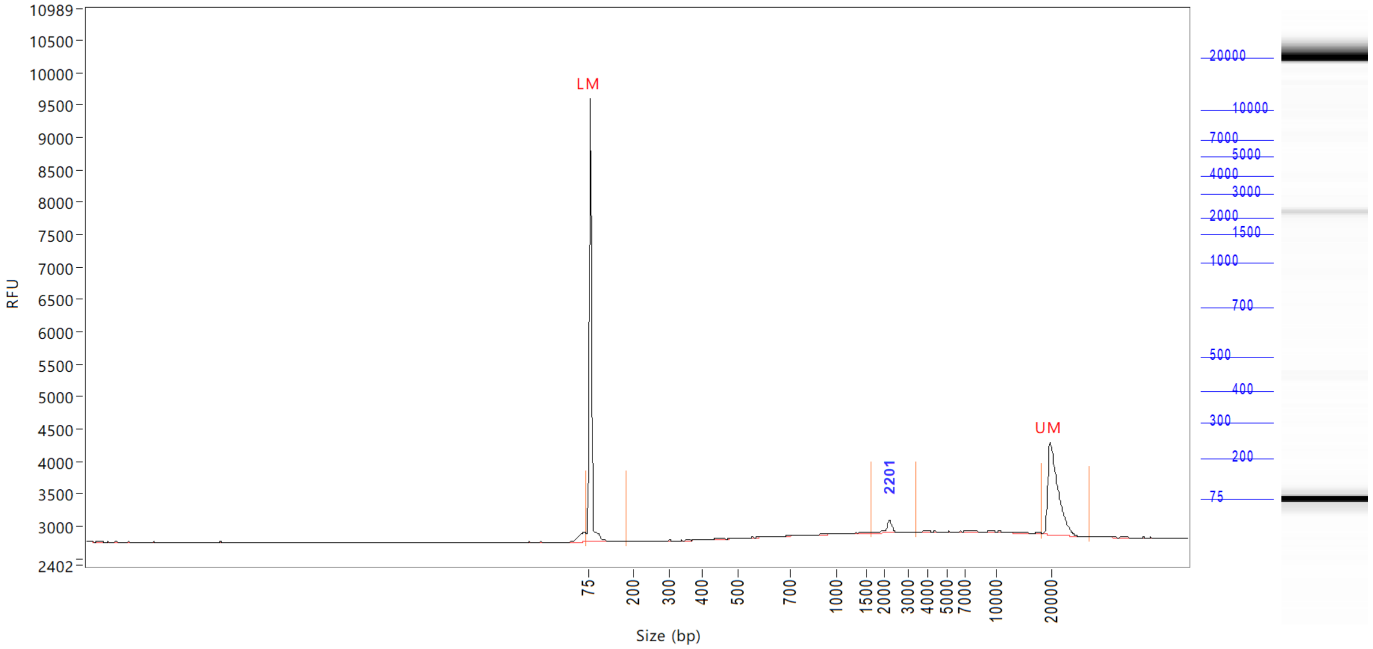

Peak	Size	Concentration	Relative concentration	Molarity	From	To	CV%	RFU
	(bp)	(ng/uL)	Percent	(nmole/L)	(bp)	(bp)		
1	75 (LM)	0.5000		10.9378	64	181	9.84	6853
2	2201	0.2731	100.0	0.2049	1635	3393	12.09	197
3	20000 (UM)	0.2143		0.0170	18537	27254	5.25	1416

TIC:	0.2731	ng/uL
TIM:	0.2049	nmole/L
Total concentration:	0.7628	ng/uL

Sample peak width (sec): 5   Sample min peak height: 50   Sample baseline V to V?: Y   Sample baseline V to V points: 3
Sample filter: Binomial   Number of points for filter: 3   Sample start region (min): 0   Sample end region (min): 30
Marker peak width (sec): 5   Marker min peak height: 200   Marker baseline V to V?: Y   Marker baseline V to V points: 3
Lower marker selection: First peak > 500 RFU   Upper marker selection: Last peak > 200 RFU
Ladder size (bp)75, 200, 300, 400, 500, 700, 1000, 1500, 2000, 3000, 4000, 5000, 7000, 10000, 20000
Quantification using: Lower Marker   Final concentration (ng/uL): 0.5000   Dilution factor: 12.0

Sample: SampA11
Well location: A11
Created: Thursday, October 21, 2021 8:08:12 PM

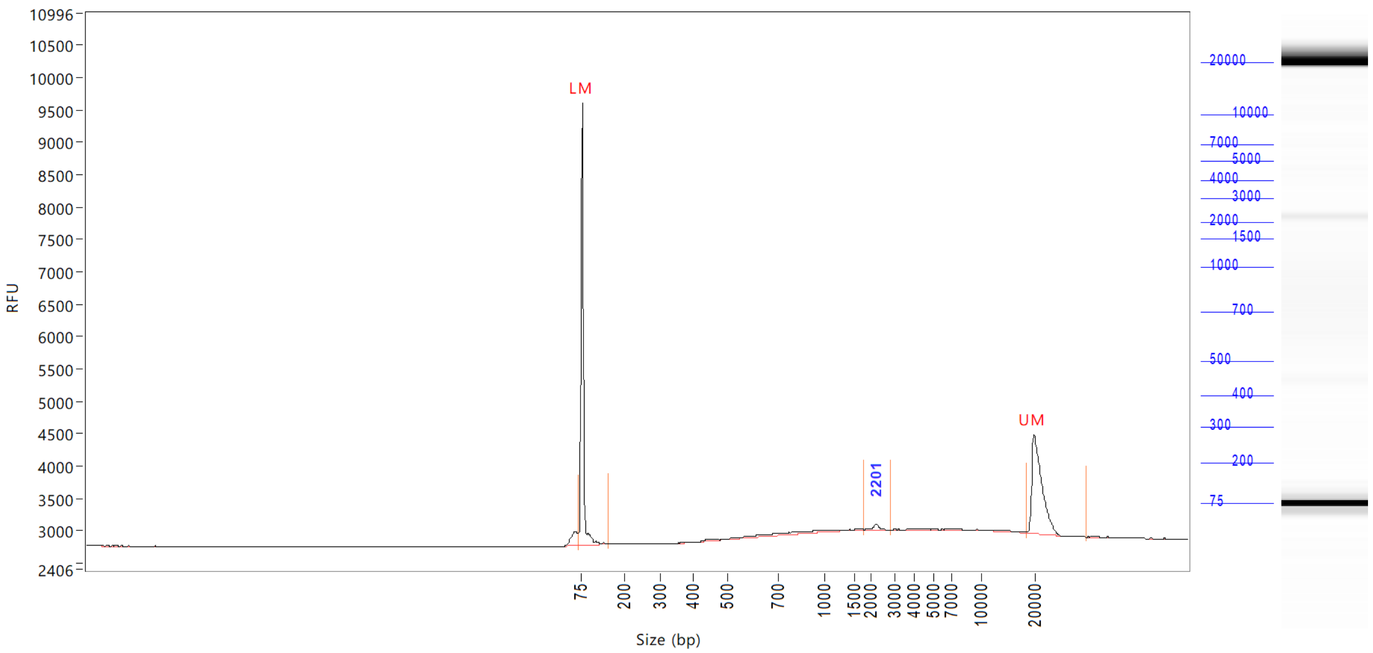

Peak	Size	Concentration	Relative concentration	Molarity	From	To	CV%	RFU
	(bp)	(ng/uL)	Percent	(nmole/L)	(bp)	(bp)		
1	75 (LM)	0.5000		10.9378	64	154	10.52	6843
2	2201	0.1341	100.0	0.1021	1789	2857	8.14	95
3	20000 (UM)	0.2324		0.0184	18537	29793	5.50	1540

TIC:	0.1341	ng/uL
TIM:	0.1021	nmole/L
Total concentration:	1.1267	ng/uL

Sample peak width (sec): 5   Sample min peak height: 50   Sample baseline V to V?: Y   Sample baseline V to V points: 3
Sample filter: Binomial   Number of points for filter: 3 Sample start region (min): 0 Sample end region (min): 30
Marker peak width (sec): 5   Marker min peak height: 200   Marker baseline V to V?: Y   Marker baseline V to V points: 3
Lower marker selection: First peak > 500 RFU   Upper marker selection: Last peak > 200 RFU
Ladder size (bp)75, 200, 300, 400, 500, 700, 1000, 1500, 2000, 3000, 4000, 5000, 7000, 10000, 20000
Quantification using: Lower Marker   Final concentration (ng/uL): 0.5000   Dilution factor: 12.0

Sample: SampB11
Well location: B11
Created: Thursday, October 21, 2021 8:08:12 PM

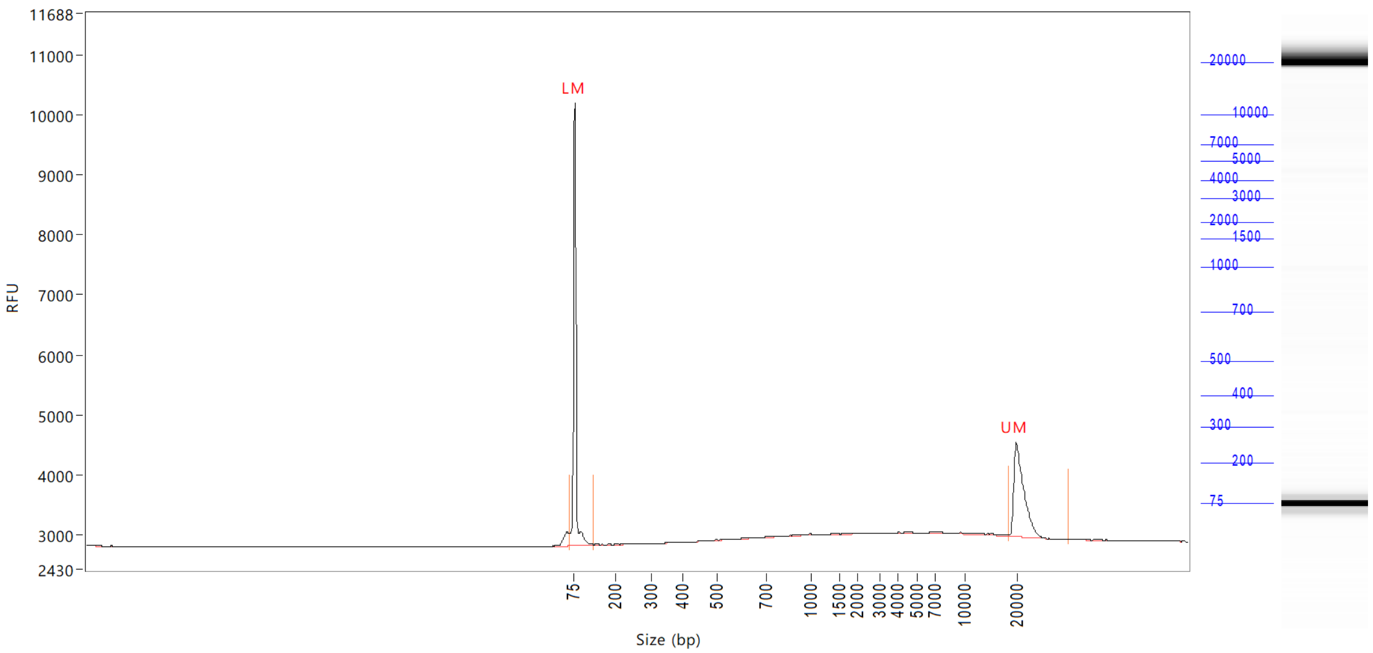

Peak	Size	Concentration	Relative concentration	Molarity	From	To	CV%	RFU
	(bp)	(ng/uL)	Percent	(nmole/L)	(bp)	(bp)		
1	75 (LM)	0.5000		11.0851	62	133	10.03	7387
2	20000 (UM)	0.2161		0.0172	18415	29914	5.63	1582

TIC:	0.0000	ng/uL
TIM:	0.0000	nmole/L
Total concentration:	0.6336	ng/uL

Sample peak width (sec): 5 Sample min peak height: 50 Sample baseline V to V?: Y Sample baseline V to V points: 3
Sample filter: Binomial Number of points for filter: 3 Sample start region (min): 0 Sample end region (min): 30
Marker peak width (sec): 5 Marker min peak height: 200 Marker baseline V to V?: Y Marker baseline V to V points: 3
Lower marker selection: First peak > 500 RFU Upper marker selection: Last peak > 200 RFU
Ladder size (bp)75, 200, 300, 400, 500, 700, 1000, 1500, 2000, 3000, 4000, 5000, 7000, 10000, 20000
Quantification using: Lower Marker Final concentration (ng/uL): 0.5000 Dilution factor: 12.0

Sample: SampC11
Well location: C11
Created: Thursday, October 21, 2021 8:08:12 PM

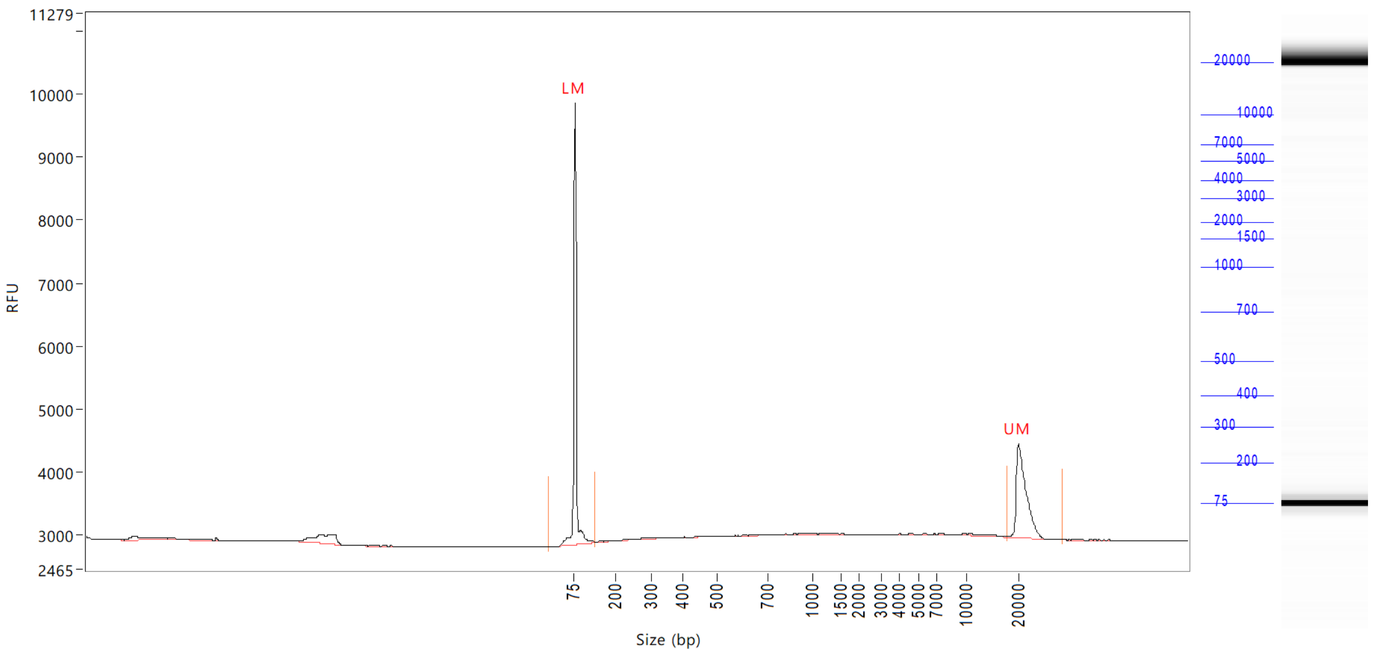

Peak	Size	Concentration	Relative concentration	Molarity	From	To	CV%	RFU
	(bp)	(ng/uL)	Percent	(nmole/L)	(bp)	(bp)		
1	75 (LM)	0.5000		11.0851	0	135	12.52	7015
2	20000 (UM)	0.2107		0.0167	17927	28342	5.42	1503

TIC:	0.0000	ng/uL
TIM:	0.0000	nmole/L
Total concentration:	0.6678	ng/uL

Sample peak width (sec): 5   Sample min peak height: 50   Sample baseline V to V?: Y   Sample baseline V to V points: 3
Sample filter: Binomial   Number of points for filter: 3   Sample start region (min): 0   Sample end region (min): 30
Marker peak width (sec): 5   Marker min peak height: 200   Marker baseline V to V?: Y   Marker baseline V to V points: 3
Lower marker selection: First peak > 500 RFU   Upper marker selection: Last peak > 200 RFU
Ladder size (bp)75, 200, 300, 400, 500, 700, 1000, 1500, 2000, 3000, 4000, 5000, 7000, 10000, 20000
Quantification using: Lower Marker   Final concentration (ng/uL): 0.5000   Dilution factor: 12.0

Sample: SampD11
Well location: D11
Created: Thursday, October 21, 2021 8:08:12 PM

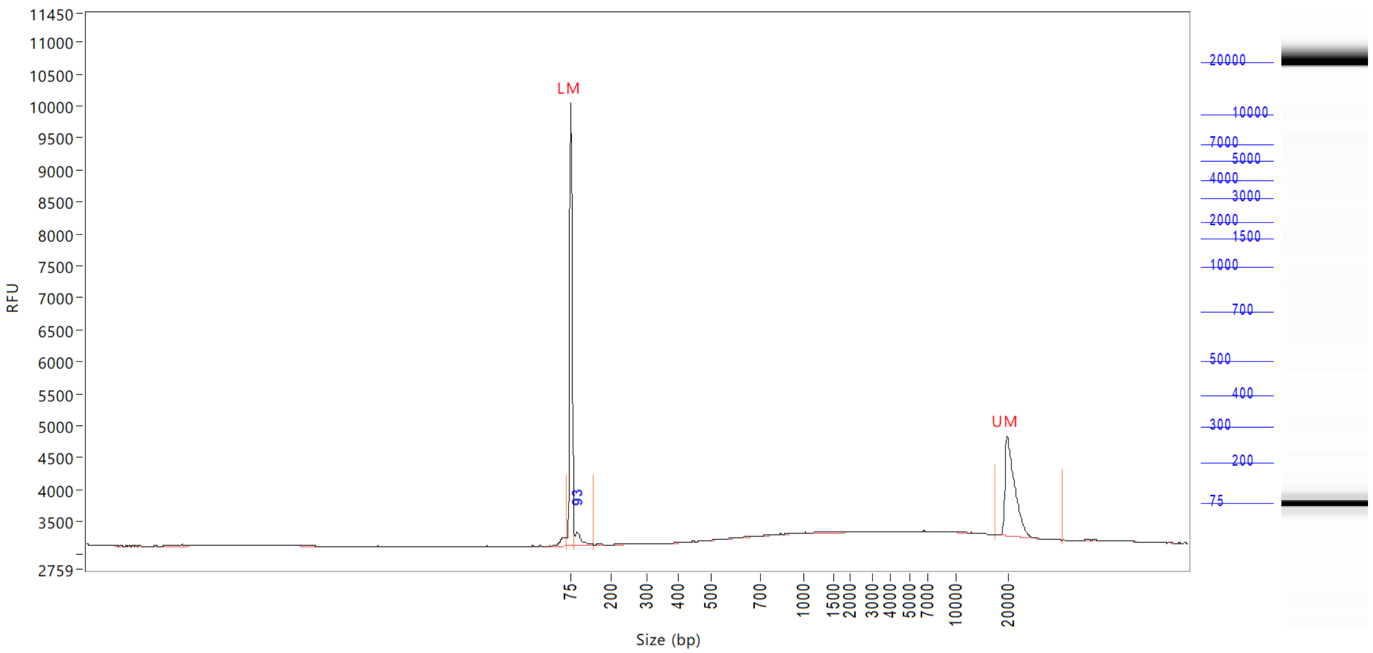

Peak	Size	Concentration	Relative concentration	Molarity	From	To	CV%	RFU
	(bp)	(ng/uL)	Percent	(nmole/L)	(bp)	(bp)		
1	75 (LM)	0.5000		11.3919	62	86	4.76	6936
2	93	0.4794	100.0	7.8724	86	145	12.89	205
3	20000 (UM)	0.2384		0.0189	17561	30519	5.67	1566

TIC:	0.4794	ng/uL
TIM:	7.8724	nmole/L
Total concentration:	1.0745	ng/uL

Sample peak width (sec): 5 Sample min peak height: 50 Sample baseline V to V?: Y Sample baseline V to V points: 3
Sample filter: Binomial Number of points for filter: 3 Sample start region (min): 0 Sample end region (min): 30
Marker peak width (sec): 5 Marker min peak height: 200 Marker baseline V to V?: Y Marker baseline V to V points: 3
Lower marker selection: First peak > 500 RFU Upper marker selection: Last peak > 200 RFU
Ladder size (bp)75, 200, 300, 400, 500, 700, 1000, 1500, 2000, 3000, 4000, 5000, 7000, 10000, 20000
Quantification using: Lower Marker Final concentration (ng/uL): 0.5000 Dilution factor: 12.0

Sample: SampE11
Well location: E11
Created: Thursday, October 21, 2021 8:08:12 PM

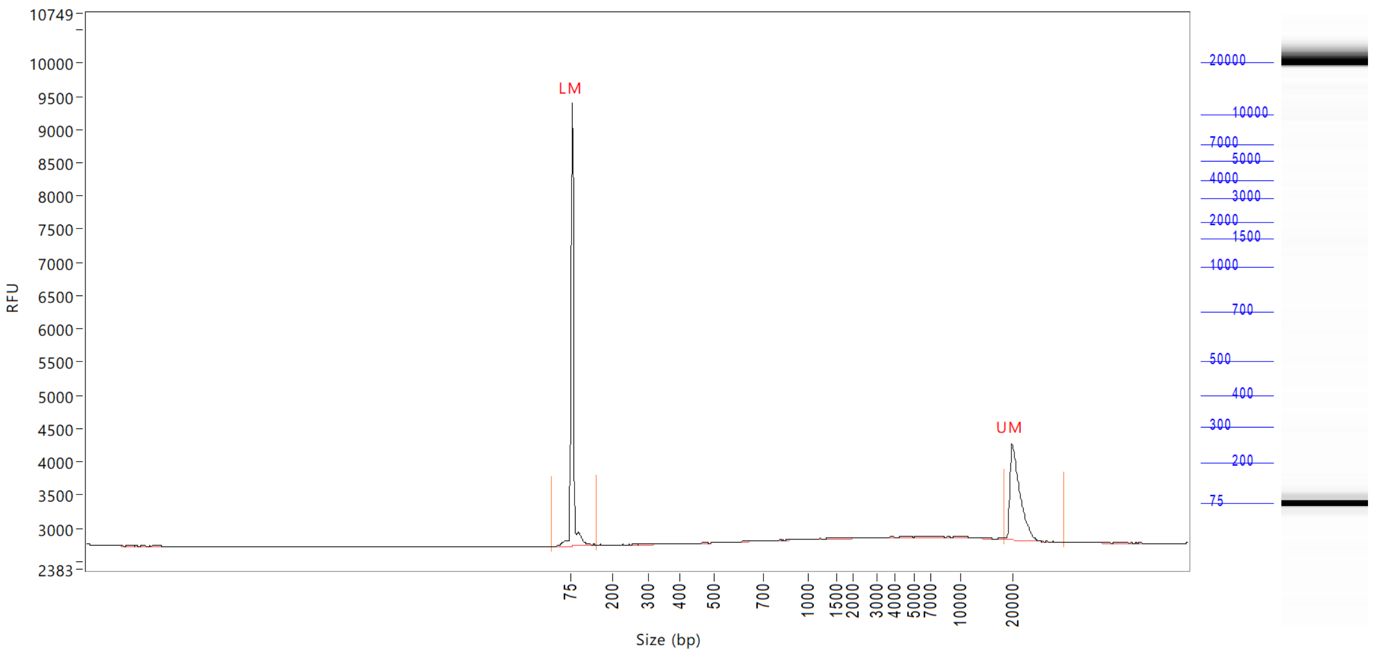

Peak	Size	Concentration	Relative concentration	Molarity	From	To	CV%	RFU
	(bp)	(ng/uL)	Percent	(nmole/L)	(bp)	(bp)		
1	75 (LM)	0.5000		11.0851	12	150	13.08	6679
2	20000 (UM)	0.2161		0.0171	18415	29914	5.69	1440

TIC:	0.0000	ng/uL
TIM:	0.0000	nmole/L
Total concentration:	0.5254	ng/uL

Sample peak width (sec): 5 Sample min peak height: 50 Sample baseline V to V?: Y Sample baseline V to V points: 3
Sample filter: Binomial Number of points for filter: 3 Sample start region (min): 0 Sample end region (min): 30
Marker peak width (sec): 5 Marker min peak height: 200 Marker baseline V to V?: Y Marker baseline V to V points: 3
Lower marker selection: First peak > 500 RFU Upper marker selection: Last peak > 200 RFU
Ladder size (bp)75, 200, 300, 400, 500, 700, 1000, 1500, 2000, 3000, 4000, 5000, 7000, 10000, 20000
Quantification using: Lower Marker Final concentration (ng/uL): 0.5000 Dilution factor: 12.0

Sample: SampF11
Well location: F11
Created: Thursday, October 21, 2021 8:08:12 PM

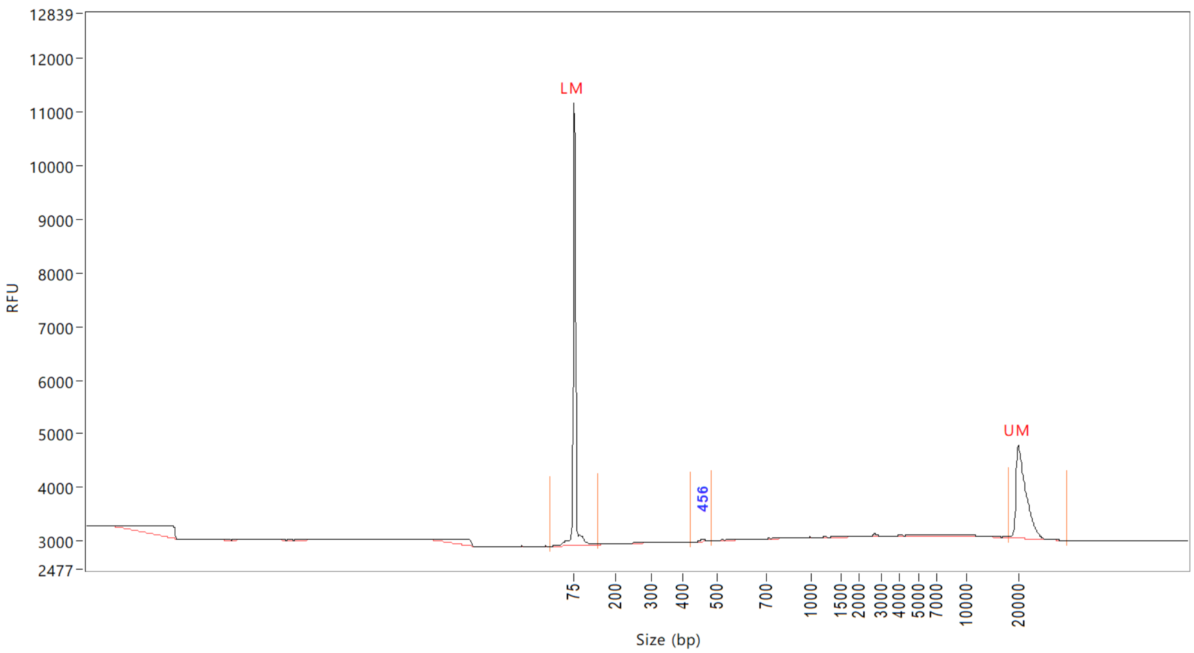

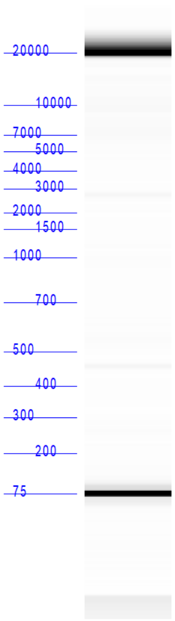

Peak	Size	Concentration	Relative concentration	Molarity	From	To	CV%	RFU
	(bp)	(ng/uL)	Percent	(nmole/L)	(bp)	(bp)		
1	75 (LM)	0.5000		11.0851	5	147	12.25	8264
2	456	0.0477	100.0	0.1726	424	483	1.81	54
3	20000 (UM)	0.2166		0.0172	18293	29189	5.62	1732

TIC:	0.0477	ng/uL
TIM:	0.1726	nmole/L
Total concentration:	0.6656	ng/uL

```
Sample peak width (sec): 5    Sample min peak height: 50    Sample baseline V to V?: Y    Sample baseline V to V points: 3
Sample filter: Binomial       Number of points for filter: 3  Sample start region (min): 0  Sample end region (min): 30
Marker peak width (sec): 5    Marker min peak height: 200   Marker baseline V to V?: Y    Marker baseline V to V points: 3
Lower marker selection: First peak > 500 RFU                Upper marker selection: Last peak > 200 RFU
Ladder size (bp)75, 200, 300, 400, 500, 700, 1000, 1500, 2000, 3000, 4000, 5000, 7000, 10000, 20000
Quantification using: Lower Marker          Final concentration (ng/uL): 0.5000          Dilution factor: 12.0
```

Sample: SampG11
Well location: G11
Created: Thursday, October 21, 2021 8:08:12 PM

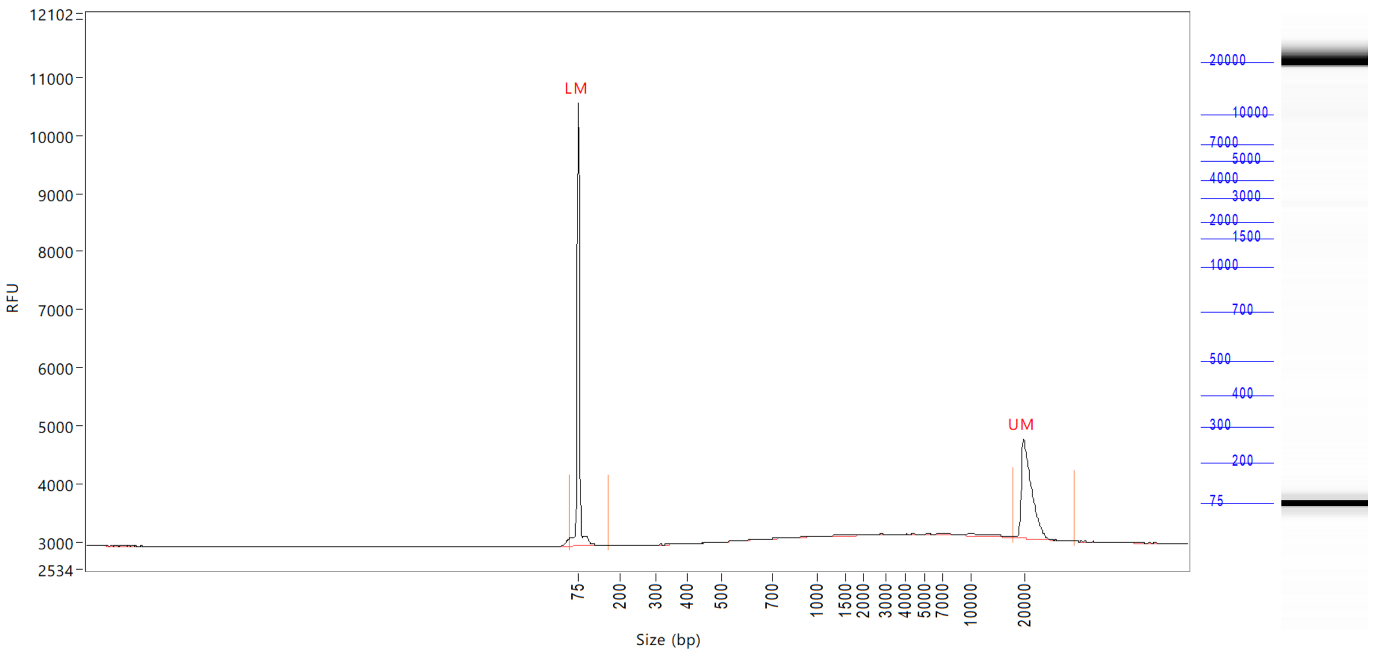

Peak	Size	Concentration	Relative concentration	Molarity	From	To	CV%	RFU
	(bp)	(ng/uL)	Percent	(nmole/L)	(bp)	(bp)		
1	75 (LM)	0.5000		11.0851	50	166	11.18	7637
2	20000 (UM)	0.2303		0.0183	18049	29551	5.57	1718

TIC:	0.0000	ng/uL
TIM:	0.0000	nmole/L
Total concentration:	0.5525	ng/uL

```
Sample peak width (sec): 5    Sample min peak height: 50    Sample baseline V to V?: Y    Sample baseline V to V points: 3
Sample filter: Binomial       Number of points for filter: 3  Sample start region (min): 0  Sample end region (min): 30
Marker peak width (sec): 5    Marker min peak height: 200   Marker baseline V to V?: Y    Marker baseline V to V points: 3
Lower marker selection: First peak > 500 RFU                Upper marker selection: Last peak > 200 RFU
Ladder size (bp)75, 200, 300, 400, 500, 700, 1000, 1500, 2000, 3000, 4000, 5000, 7000, 10000, 20000
Quantification using: Lower Marker          Final concentration (ng/uL): 0.5000          Dilution factor: 12.0
```

Sample: SampH11
Well location: H11
Created: Thursday, October 21, 2021 8:08:12 PM

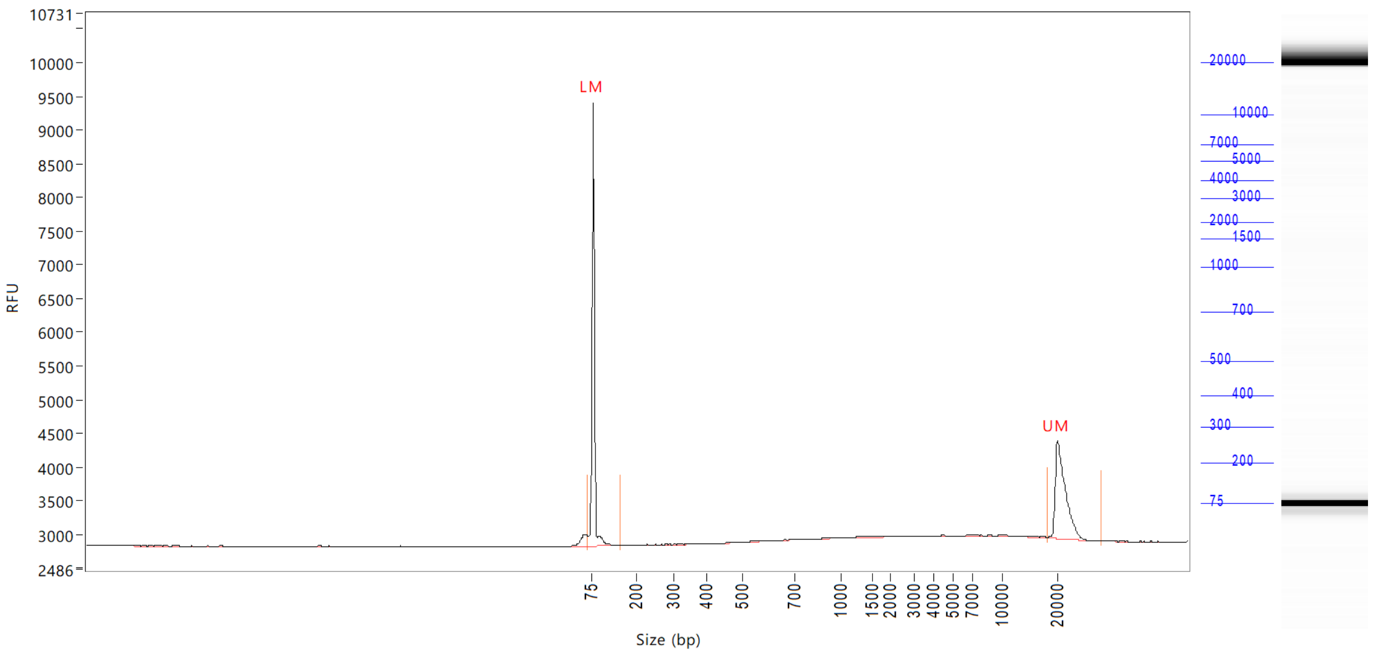

Peak	Size	Concentration	Relative concentration	Molarity	From	To	CV%	RFU
	(bp)	(ng/uL)	Percent	(nmole/L)	(bp)	(bp)		
1	75 (LM)	0.5000		10.9378	62	152	9.83	6584
2	20000 (UM)	0.2315		0.0184	18049	27979	5.37	1454

TIC:	0.0000	ng/uL
TIM:	0.0000	nmole/L
Total concentration:	0.6009	ng/uL

Sample peak width (sec): 5 Sample min peak height: 50 Sample baseline V to V?: Y Sample baseline V to V points: 3
Sample filter: Binomial Number of points for filter: 3 Sample start region (min): 0 Sample end region (min): 30
Marker peak width (sec): 5 Marker min peak height: 200 Marker baseline V to V?: Y Marker baseline V to V points: 3
Lower marker selection: First peak > 500 RFU Upper marker selection: Last peak > 200 RFU
Ladder size (bp)75, 200, 300, 400, 500, 700, 1000, 1500, 2000, 3000, 4000, 5000, 7000, 10000, 20000
Quantification using: Lower Marker Final concentration (ng/uL): 0.5000 Dilution factor: 12.0

Sample: SampA12
Well location: A12
Created: Thursday, October 21, 2021 8:08:12 PM

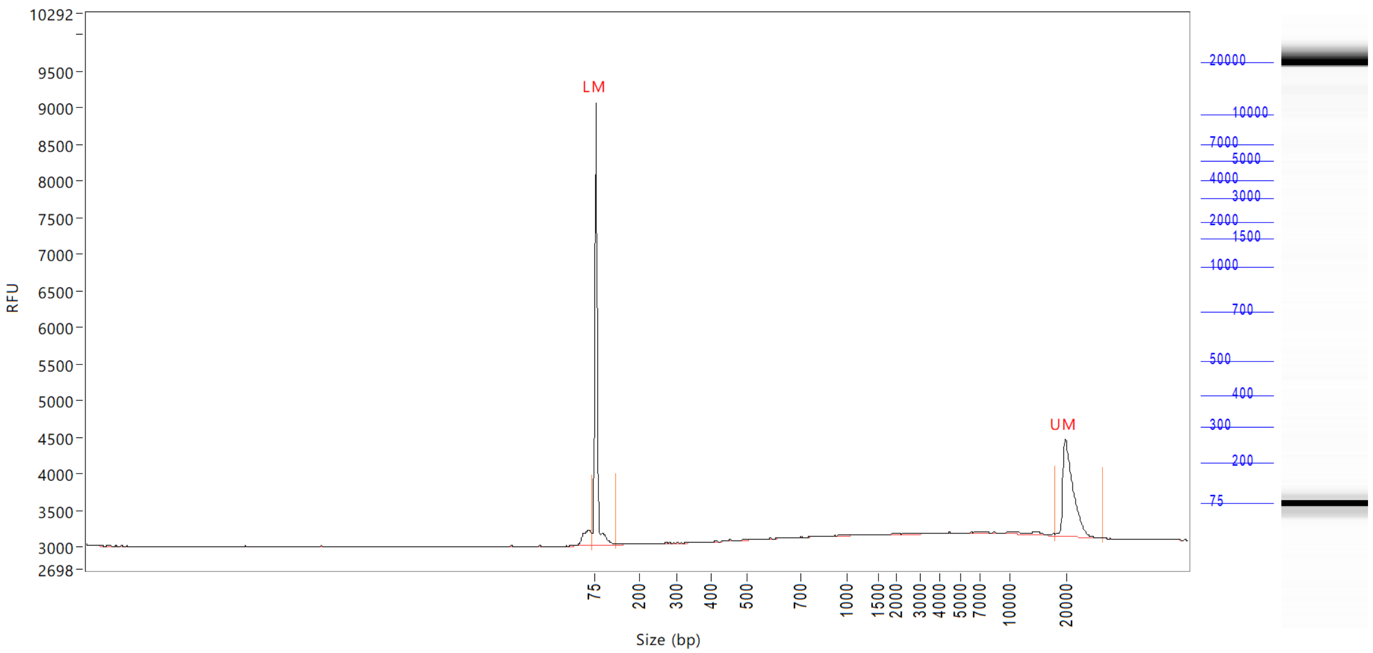

Peak	Size	Concentration	Relative concentration	Molarity	From	To	CV%	RFU
	(bp)	(ng/uL)	Percent	(nmole/L)	(bp)	(bp)		
1	75 (LM)	0.5000		11.0851	64	133	9.82	6053
2	20000 (UM)	0.2239		0.0178	18171	26649	5.23	1337

TIC:	0.0000	ng/uL
TIM:	0.0000	nmole/L
Total concentration:	0.7393	ng/uL

Sample peak width (sec): 5   Sample min peak height: 50   Sample baseline V to V?: Y   Sample baseline V to V points: 3
Sample filter: Binomial   Number of points for filter: 3 Sample start region (min): 0 Sample end region (min): 30
Marker peak width (sec): 5   Marker min peak height: 200   Marker baseline V to V?: Y   Marker baseline V to V points: 3
Lower marker selection: First peak > 500 RFU   Upper marker selection: Last peak > 200 RFU
Ladder size (bp)75, 200, 300, 400, 500, 700, 1000, 1500, 2000, 3000, 4000, 5000, 7000, 10000, 20000
Quantification using: Lower Marker   Final concentration (ng/uL): 0.5000   Dilution factor: 12.0

Sample: SampB12
Well location: B12
Created: Thursday, October 21, 2021 8:08:12 PM

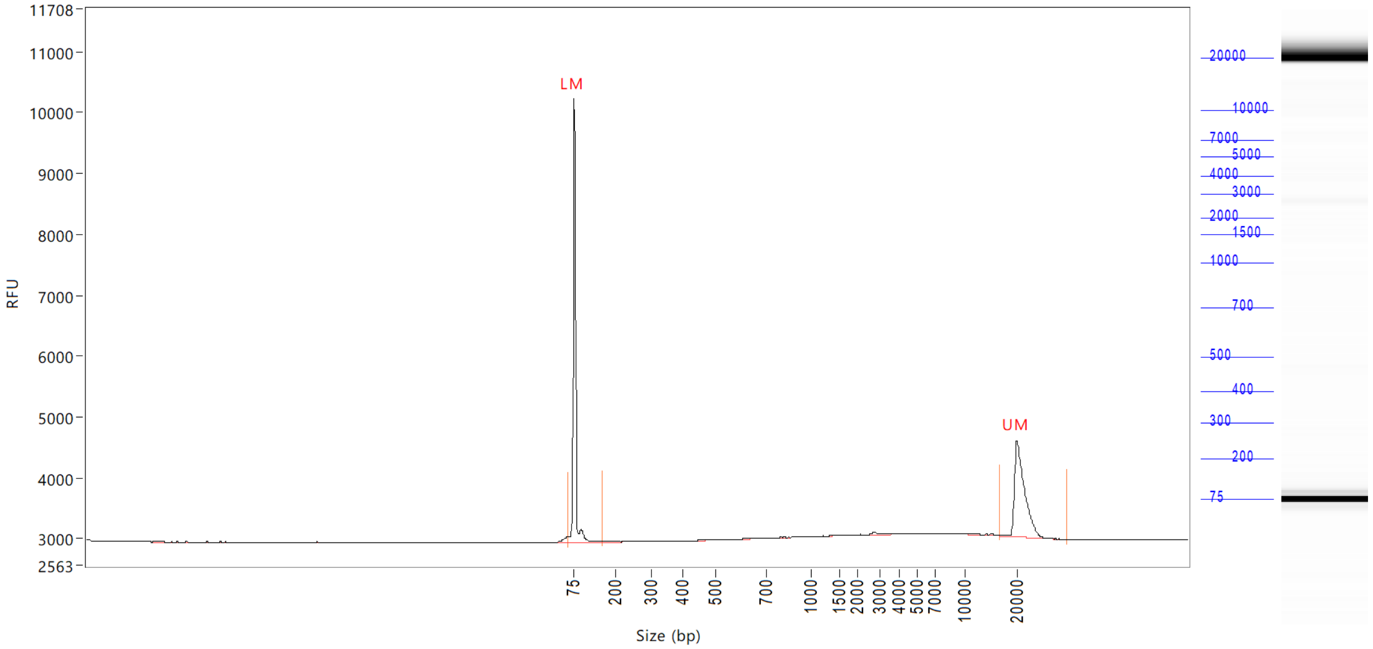

Peak	Size	Concentration	Relative concentration	Molarity	From	To	CV%	RFU
	(bp)	(ng/uL)	Percent	(nmole/L)	(bp)	(bp)		
1	75 (LM)	0.5000		10.9378	60	162	11.16	7308
2	20000 (UM)	0.2307		0.0183	16586	29551	5.70	1579

TIC:	0.0000	ng/uL
TIM:	0.0000	nmole/L
Total concentration:	0.4780	ng/uL

```
Sample peak width (sec): 5    Sample min peak height: 50    Sample baseline V to V?: Y    Sample baseline V to V points: 3
Sample filter: Binomial       Number of points for filter: 3  Sample start region (min): 0  Sample end region (min): 30
Marker peak width (sec): 5    Marker min peak height: 200   Marker baseline V to V?: Y    Marker baseline V to V points: 3
Lower marker selection: First peak > 500 RFU                Upper marker selection: Last peak > 200 RFU
Ladder size (bp)75, 200, 300, 400, 500, 700, 1000, 1500, 2000, 3000, 4000, 5000, 7000, 10000, 20000
Quantification using: Lower Marker          Final concentration (ng/uL): 0.5000          Dilution factor: 12.0
```

Sample: SampC12
Well location: C12
Created: Thursday, October 21, 2021 8:08:12 PM

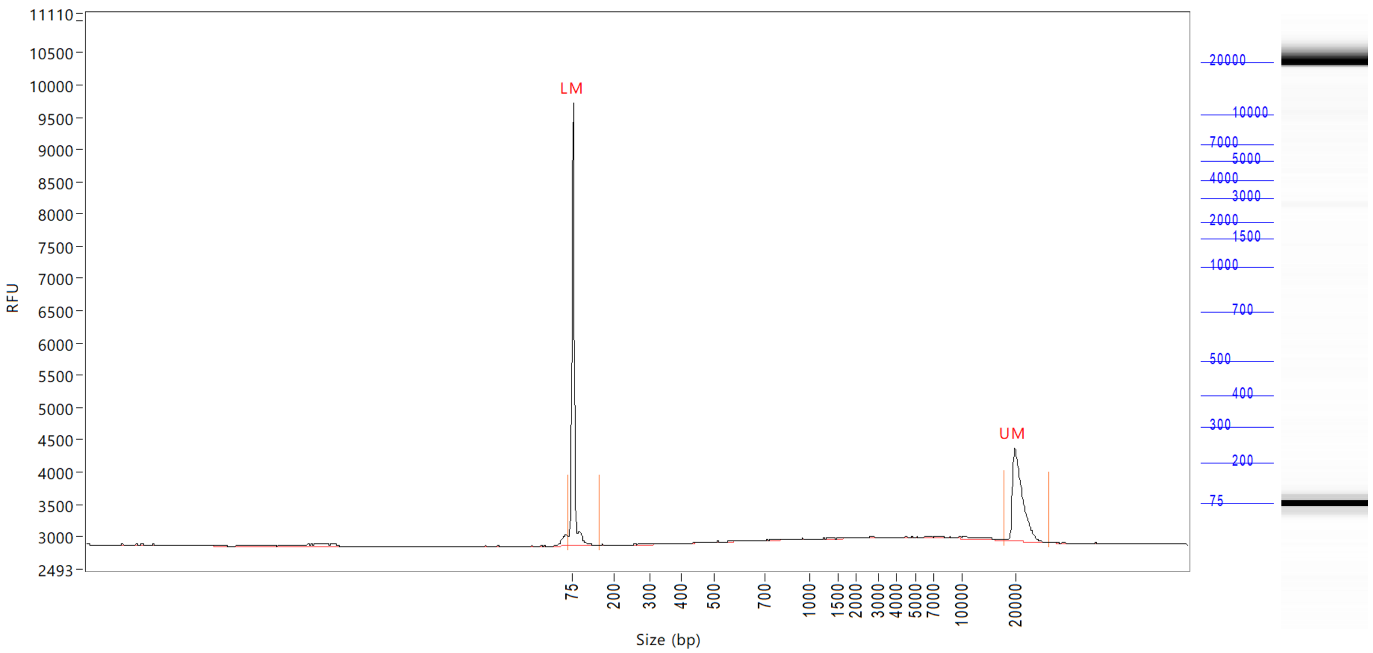

Peak	Size	Concentration	Relative concentration	Molarity	From	To	CV%	RFU
	(bp)	(ng/uL)	Percent	(nmole/L)	(bp)	(bp)		
1	75 (LM)	0.5000		10.9378	64	156	11.37	6877
2	20000 (UM)	0.2144		0.0170	17927	26408	5.52	1431

TIC:	0.0000	ng/uL
TIM:	0.0000	nmole/L
Total concentration:	0.6399	ng/uL

```
Sample peak width (sec): 5    Sample min peak height: 50    Sample baseline V to V?: Y    Sample baseline V to V points: 3
Sample filter: Binomial       Number of points for filter: 3  Sample start region (min): 0  Sample end region (min): 30
Marker peak width (sec): 5    Marker min peak height: 200   Marker baseline V to V?: Y    Marker baseline V to V points: 3
Lower marker selection: First peak > 500 RFU                Upper marker selection: Last peak > 200 RFU
Ladder size (bp)75, 200, 300, 400, 500, 700, 1000, 1500, 2000, 3000, 4000, 5000, 7000, 10000, 20000
Quantification using: Lower Marker          Final concentration (ng/uL): 0.5000          Dilution factor: 12.0
```

Sample: SampD12
Well location: D12
Created: Thursday, October 21, 2021 8:08:12 PM

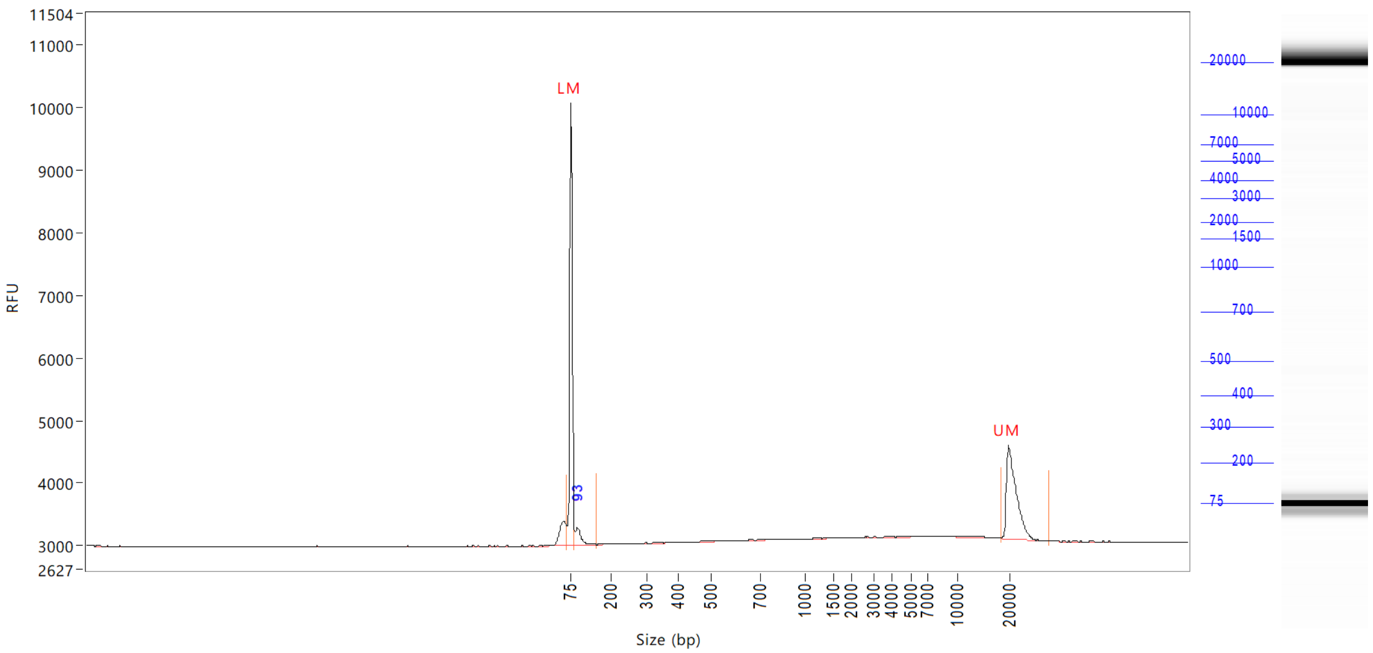

Peak	Size	Concentration	Relative concentration	Molarity	From	To	CV%	RFU
	(bp)	(ng/uL)	Percent	(nmole/L)	(bp)	(bp)		
1	75 (LM)	0.5000		11.2364	64	86	4.84	7078
2	93	0.5887	100.0	9.7650	86	154	12.26	275
3	20000 (UM)	0.2273		0.0180	18415	27738	5.40	1491

TIC:	0.5887	ng/uL
TIM:	9.7650	nmole/L
Total concentration:	1.1615	ng/uL

Sample peak width (sec): 5 Sample min peak height: 50 Sample baseline V to V?: Y Sample baseline V to V points: 3
Sample filter: Binomial Number of points for filter: 3 Sample start region (min): 0 Sample end region (min): 30
Marker peak width (sec): 5 Marker min peak height: 200 Marker baseline V to V?: Y Marker baseline V to V points: 3
Lower marker selection: First peak > 500 RFU Upper marker selection: Last peak > 200 RFU
Ladder size (bp)75, 200, 300, 400, 500, 700, 1000, 1500, 2000, 3000, 4000, 5000, 7000, 10000, 20000
Quantification using: Lower Marker Final concentration (ng/uL): 0.5000 Dilution factor: 12.0

Sample: SampE12
Well location: E12
Created: Thursday, October 21, 2021 8:08:12 PM

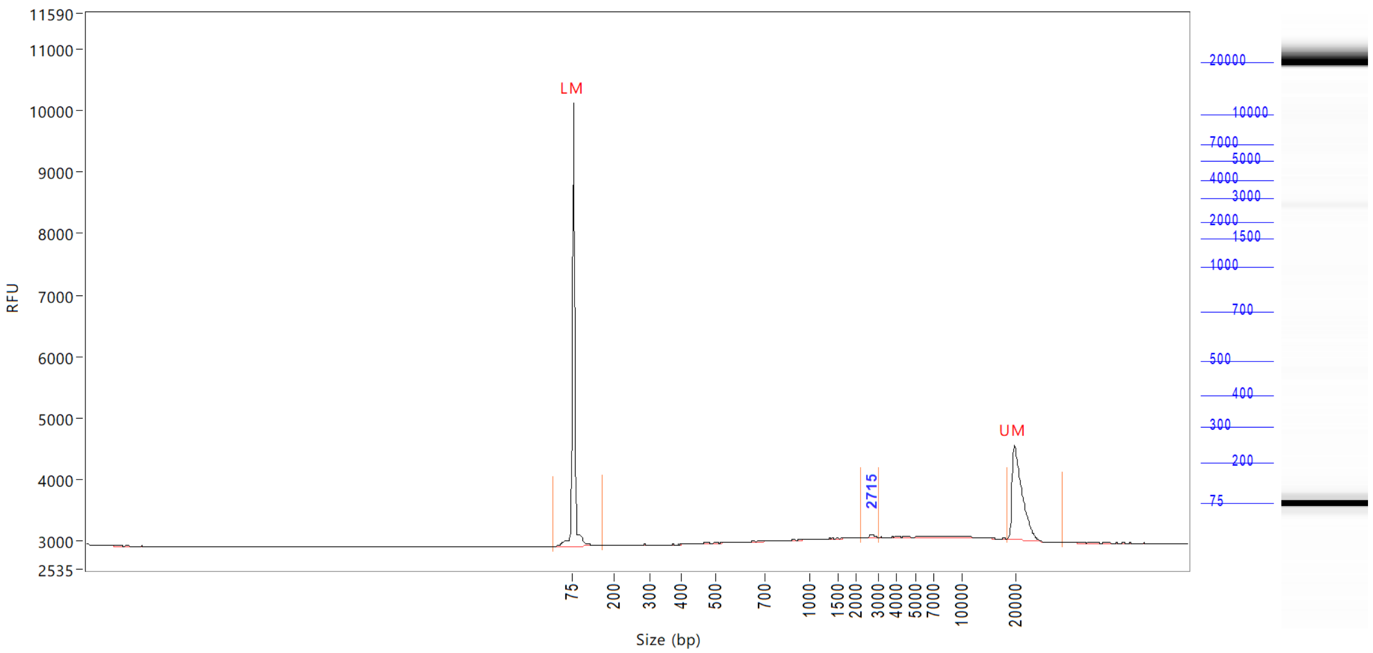

Peak	Size	Concentration	Relative concentration	Molarity	From	To	CV%	RFU
	(bp)	(ng/uL)	Percent	(nmole/L)	(bp)	(bp)		
1	75 (LM)	0.5000		11.0851	16	166	13.11	7231
2	2715	0.0684	100.0	0.0422	2229	3000	5.95	57
3	20000 (UM)	0.2133		0.0169	18415	29189	5.45	1532

TIC:	0.0684	ng/uL
TIM:	0.0422	nmole/L
Total concentration:	0.5144	ng/uL

Sample peak width (sec): 5   Sample min peak height: 50   Sample baseline V to V?: Y   Sample baseline V to V points: 3
Sample filter: Binomial   Number of points for filter: 3   Sample start region (min): 0   Sample end region (min): 30
Marker peak width (sec): 5   Marker min peak height: 200   Marker baseline V to V?: Y   Marker baseline V to V points: 3
Lower marker selection: First peak > 500 RFU   Upper marker selection: Last peak > 200 RFU
Ladder size (bp)75, 200, 300, 400, 500, 700, 1000, 1500, 2000, 3000, 4000, 5000, 7000, 10000, 20000
Quantification using: Lower Marker   Final concentration (ng/uL): 0.5000   Dilution factor: 12.0

Sample: SampF12
Well location: F12
Created: Thursday, October 21, 2021 8:08:12 PM

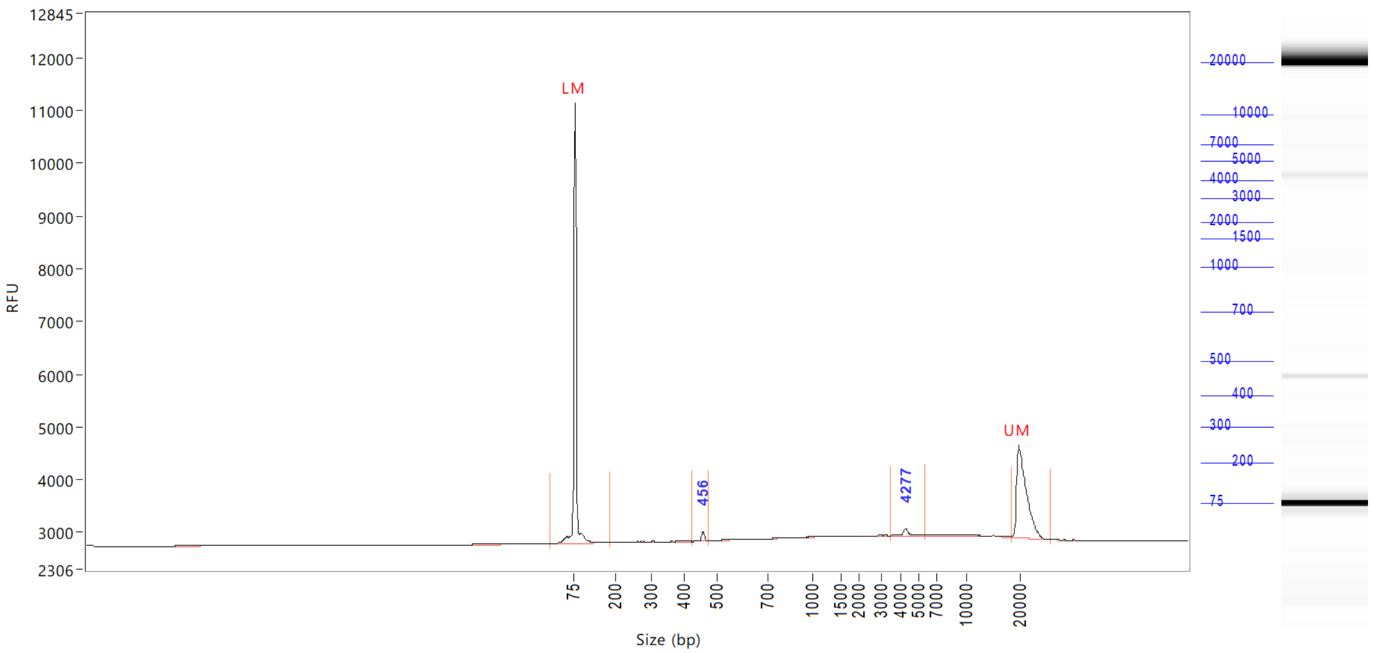

Peak	Size	Concentration	Relative concentration	Molarity	From	To	CV%	RFU
	(bp)	(ng/uL)	Percent	(nmole/L)	(bp)	(bp)		
1	75 (LM)	0.5000		11.0851	3	183	14.28	8363
2	456	0.1144	44.0	0.4154	426	471	1.26	175
3	4277	0.1457	56.0	0.0557	3465	5593	8.36	129
4	20000 (UM)	0.2079		0.0165	18537	26045	5.20	1748

TIC:	0.2600	ng/uL
TIM:	0.4710	nmole/L
Total concentration:	0.6603	ng/uL

```
Sample peak width (sec): 5    Sample min peak height: 50    Sample baseline V to V?: Y    Sample baseline V to V points: 3
Sample filter: Binomial       Number of points for filter: 3  Sample start region (min): 0  Sample end region (min): 30
Marker peak width (sec): 5    Marker min peak height: 200   Marker baseline V to V?: Y    Marker baseline V to V points: 3
Lower marker selection: First peak > 500 RFU                Upper marker selection: Last peak > 200 RFU
Ladder size (bp)75, 200, 300, 400, 500, 700, 1000, 1500, 2000, 3000, 4000, 5000, 7000, 10000, 20000
Quantification using: Lower Marker          Final concentration (ng/uL): 0.5000          Dilution factor: 12.0
```

Sample: SampG12
Well location: G12
Created: Thursday, October 21, 2021 8:08:12 PM

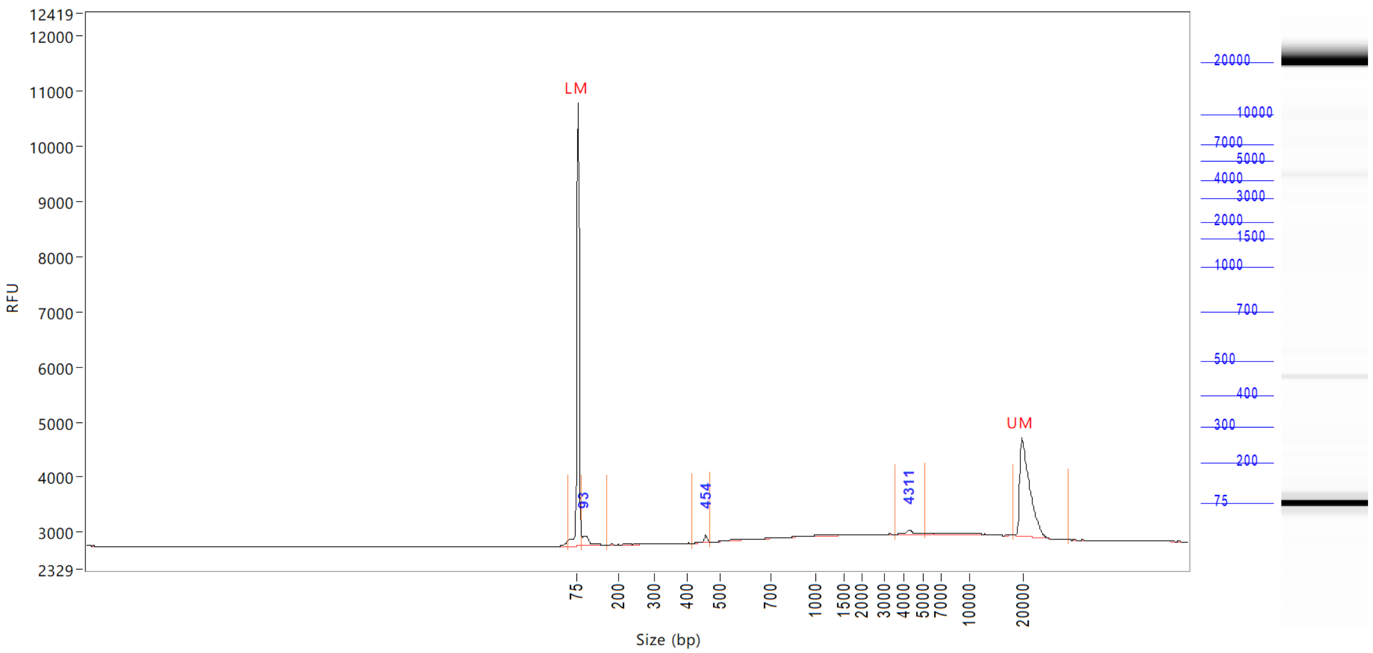

Peak	Size	Concentration	Relative concentration	Molarity	From	To	CV%	RFU
	(bp)	(ng/uL)	Percent	(nmole/L)	(bp)	(bp)		
1	75 (LM)	0.5000		11.2364	50	87	5.91	8054
2	93	0.3948	65.5	6.2952	87	162	15.50	183
3	454	0.1026	17.0	0.3745	416	470	1.60	131
4	4311	0.1055	17.5	0.0407	3608	5297	6.94	75
5	20000 (UM)	0.2359		0.0187	18293	28705	5.38	1795

TIC:	0.6030	ng/uL
TIM:	6.7103	nmole/L
Total concentration:	1.0829	ng/uL

```
Sample peak width (sec): 5     Sample min peak height: 50     Sample baseline V to V?: Y     Sample baseline V to V points: 3
Sample filter: Binomial        Number of points for filter: 3  Sample start region (min): 0  Sample end region (min): 30
Marker peak width (sec): 5     Marker min peak height: 200    Marker baseline V to V?: Y     Marker baseline V to V points: 3
Lower marker selection: First peak > 500 RFU                  Upper marker selection: Last peak > 200 RFU
Ladder size (bp)75, 200, 300, 400, 500, 700, 1000, 1500, 2000, 3000, 4000, 5000, 7000, 10000, 20000
Quantification using: Lower Marker          Final concentration (ng/uL): 0.5000          Dilution factor: 12.0
```

Sample: SampH12
Well location: H12
Created: Thursday, October 21, 2021 8:08:12 PM

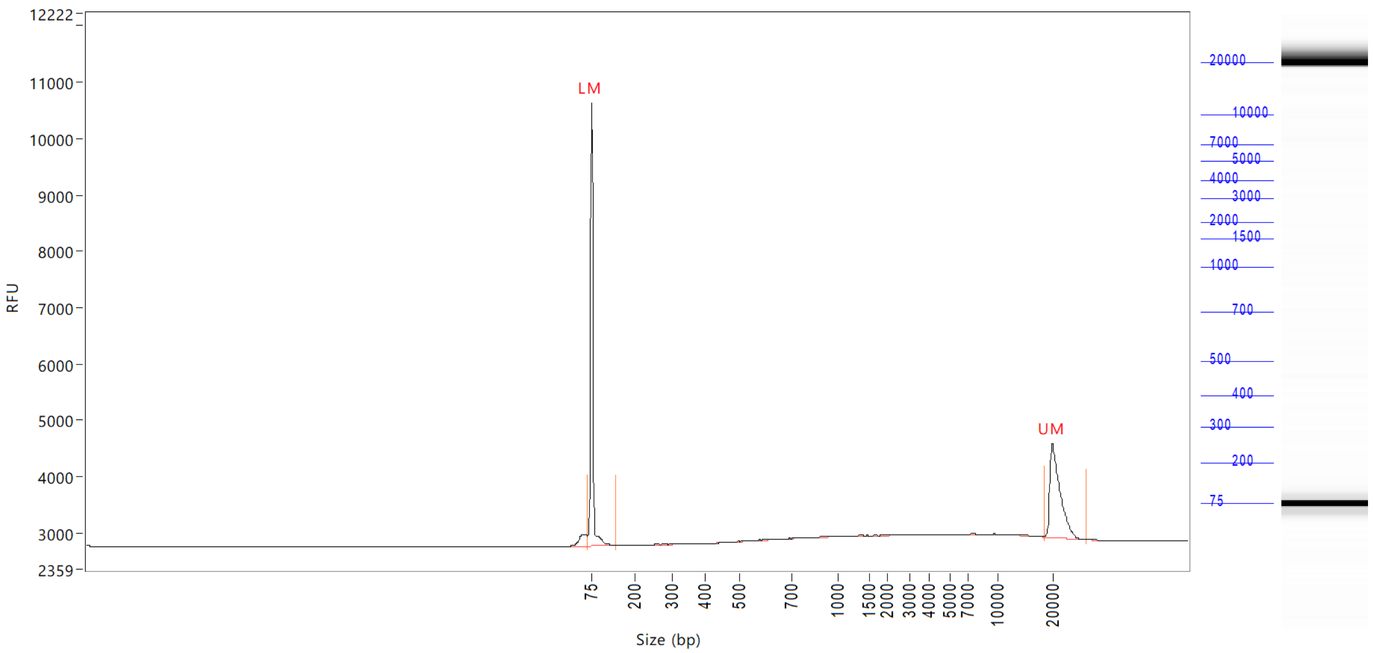

Peak	Size	Concentration	Relative concentration	Molarity	From	To	CV%	RFU
	(bp)	(ng/uL)	Percent	(nmole/L)	(bp)	(bp)		
1	75 (LM)	0.5000		11.0851	64	147	10.12	7873
2	20000 (UM)	0.2154		0.0171	18415	26166	5.27	1661

TIC:	0.0000	ng/uL
TIM:	0.0000	nmole/L
Total concentration:	0.4519	ng/uL

Sample peak width (sec): 5   Sample min peak height: 200   Sample baseline V to V?: Y   Sample baseline V to V points: 3
Sample filter: Binomial   Number of points for filter: 3   Sample start region (min): 0   Sample end region (min): 30
Marker peak width (sec): 5   Marker min peak height: 200   Marker baseline V to V?: Y   Marker baseline V to V points: 3
Lower marker selection: First peak > 500 RFU   Upper marker selection: Last peak > 200 RFU
Ladder size (bp)75, 200, 300, 400, 500, 700, 1000, 1500, 2000, 3000, 4000, 5000, 7000, 10000, 20000
Quantification using: Lower Marker   Final concentration (ng/uL): 0.5000   Dilution factor: 12.0

Sample: DNA size ladder
Well location: A7
Created: Thursday, October 21, 2021 8:08:12 PM
Import from: C:\Users\admin\Desktop\210810_alberto_ladder.SCAL
Fit type: Point to point

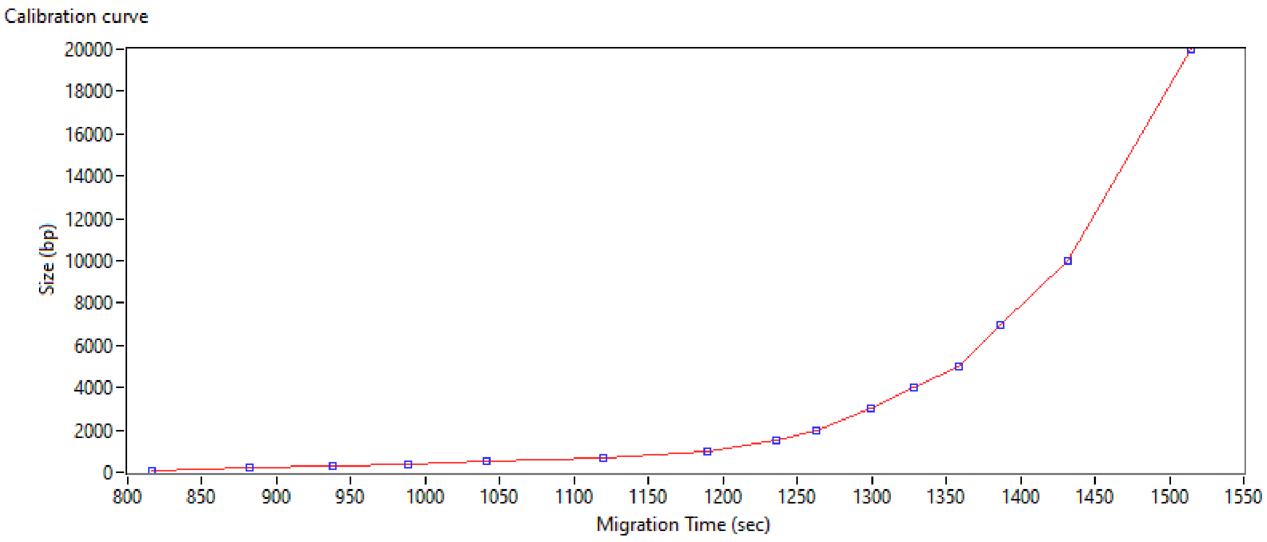

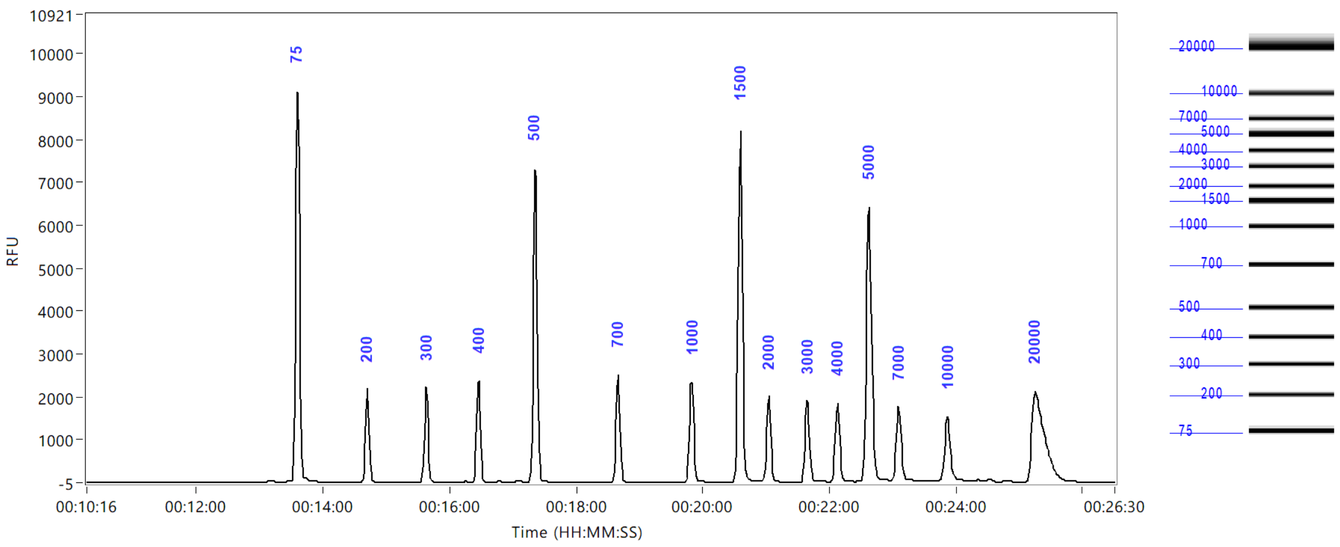

Supplement: Supplementary file 2 — sb3c00292_si_002.zip [file sb3c00292_si_002.zip › dnada_supplementary_material_pks_library_build/colony_pcr/ZAG/19-58-21_plate9/colonypcrplate_9.pdf]
