# Supplementary material for: Automated Platform for the Plasmid Construction Process
Source: ACS Synth Biol. 2023 Nov 10;12(12):3506–13. doi: 10.1021/acssynbio.3c00292 (PMC10729297; doi:10.1021/acssynbio.3c00292)

Instrument controller software run summary:

Filename and data path: C:\AATI\Data\2021 10 21\17-58-41\2021 10 21 17H 58M..raw
Created: Thursday, October 21, 2021 6:08:16 PM
Number of capillaries: 96
Array serial number: 111920-09SFS
Effect length: 33 cm
Array usage count: 266
Instrument type: ZAG DNA Analyzer
Instrument controller software version: 1.0.1.9
Device serial number: Z7597

Method Information

Method name: ZAG130GP33 - DNA 75-20000bp - Gel Prime Only.mthds
Gel prime: Yes
Full conditioning: No
Gel prime to buffer: No
Gel selection: Gel 1
Perform prerun: 8.0 kV, 30 sec.
Rinse: No
Marker 1: Row: A, 5.0 kV, 5 sec.
Rinse: No
Sample injection: 5.0 kV, 5 sec.
Separation: 8.0 kV, 30.0 min.
Tray name: Sample Tray-2

Analysis mode: DNA

Notes

Gel Image

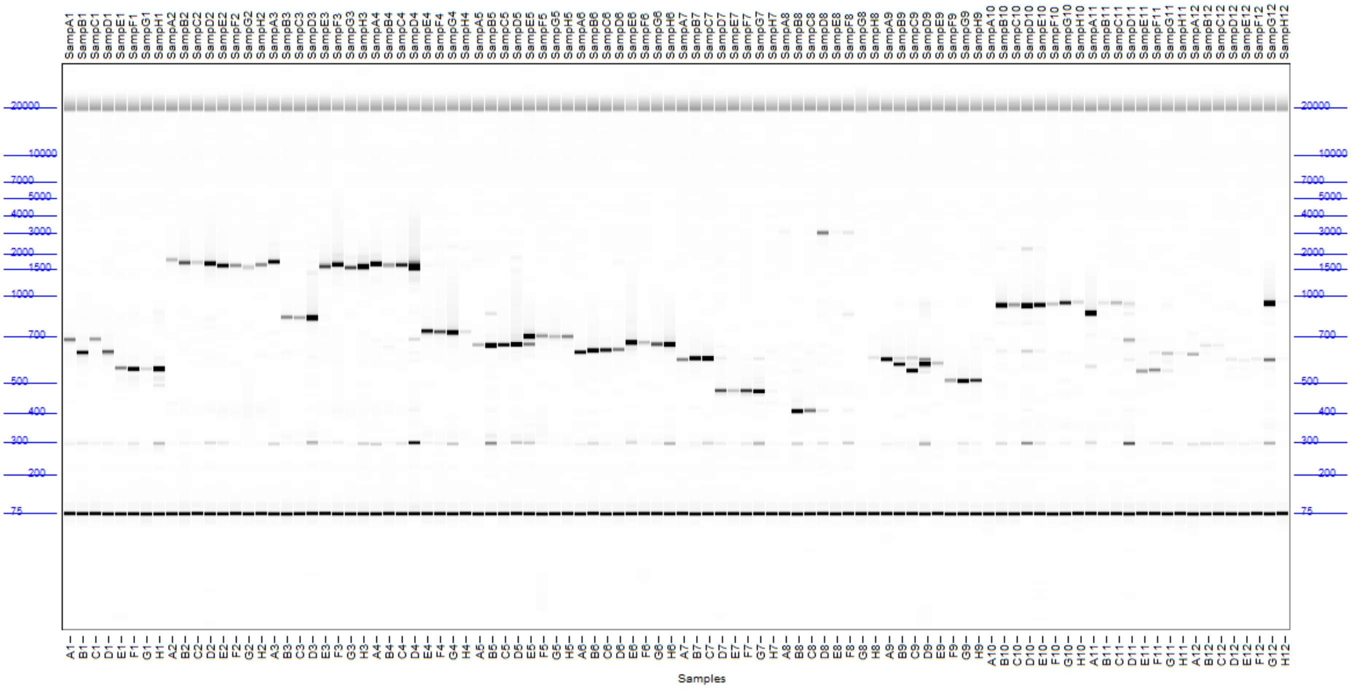

Filename and data path: C:\AATI\Data\2021 10 21\17-58-41\2021 10 21 17H 58M..raw

A1: SampA1

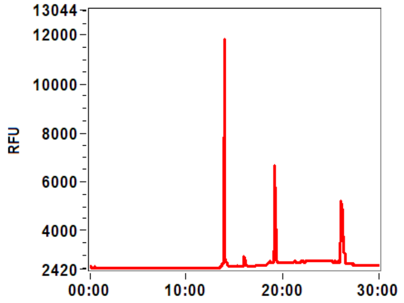

B1: SampB1

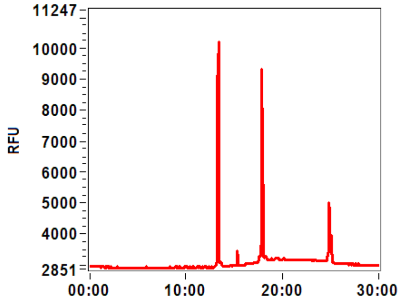

C1: SampC1

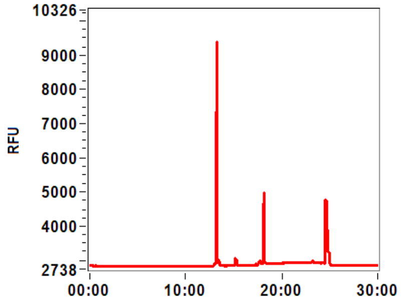

D1: SampD1

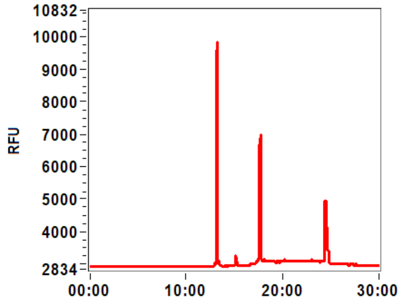

E1: SampE1

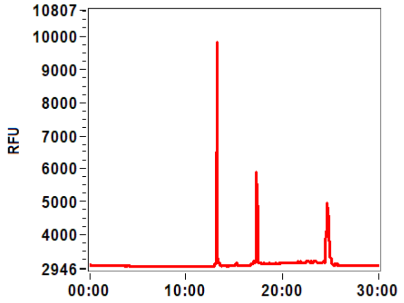

F1: SampF1

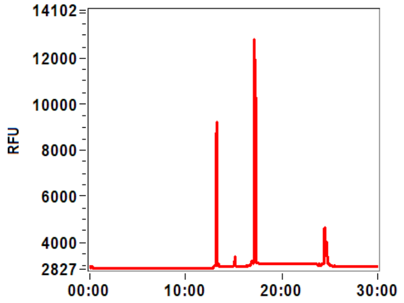

G1: SampG1

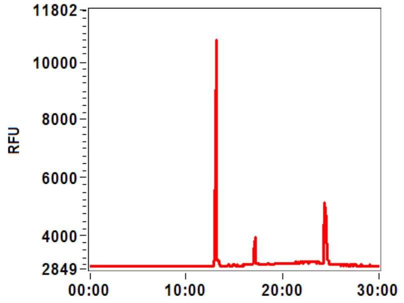

H1: SampH1

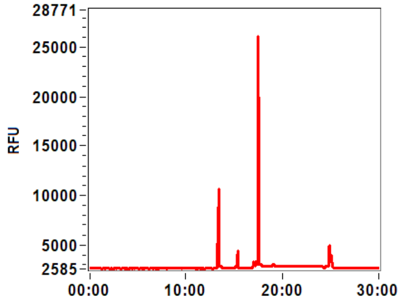

A2: SampA2

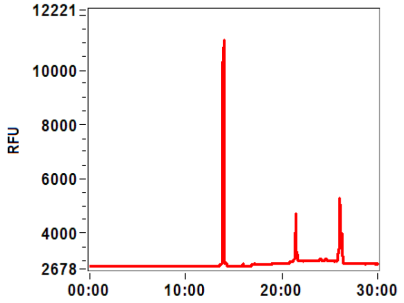

B2: SampB2

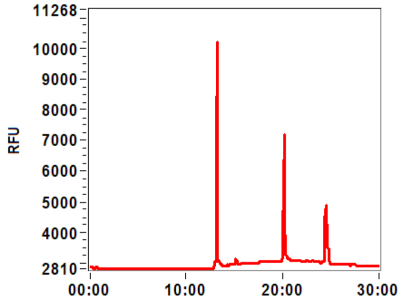

C2: SampC2

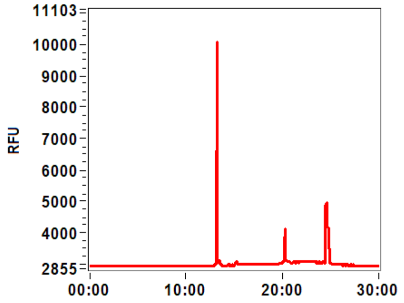

D2: SampD2

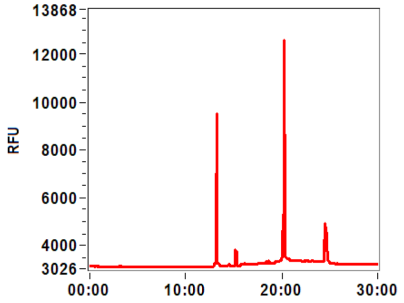

Filename and data path: C:\AATI\Data\2021 10 21\17-58-41\2021 10 21 17H 58M..raw

E2: SampE2

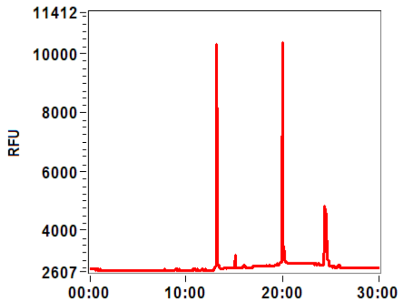

F2: SampF2

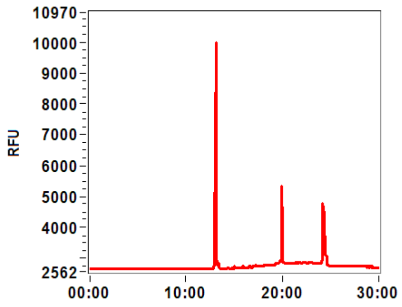

G2: SampG2

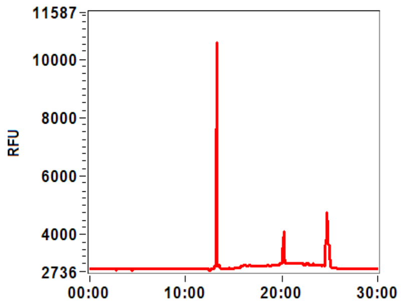

H2: SampH2

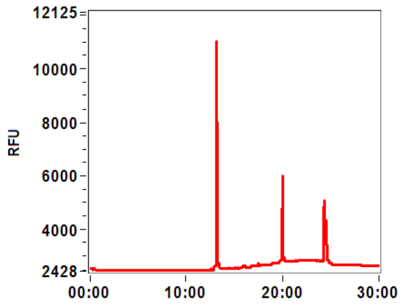

A3: SampA3

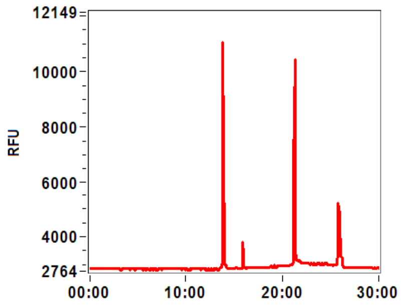

B3: SampB3

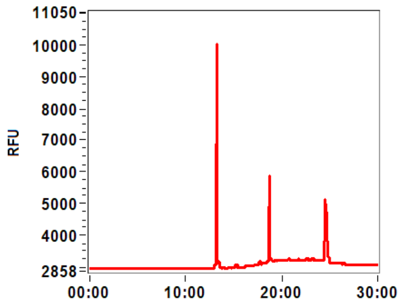

C3: SampC3

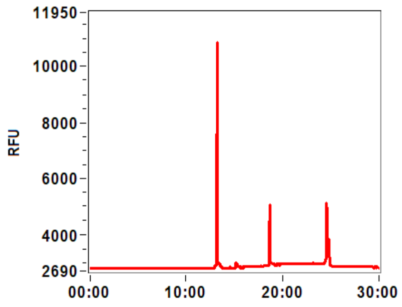

D3: SampD3

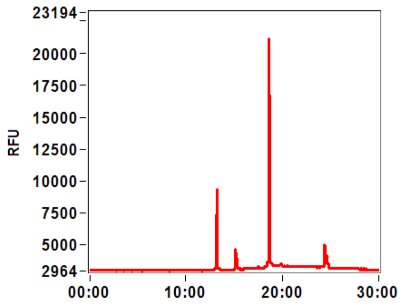

E3: SampE3

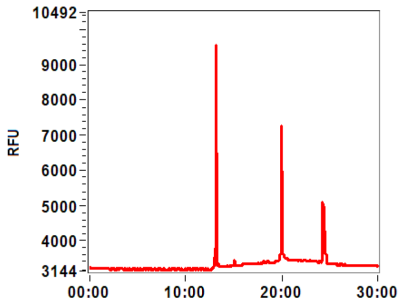

F3: SampF3

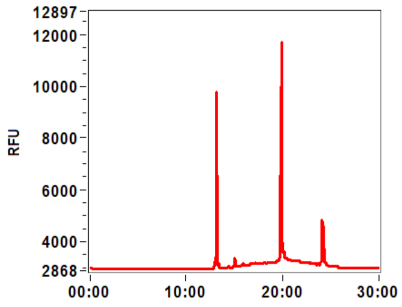

G3: SampG3

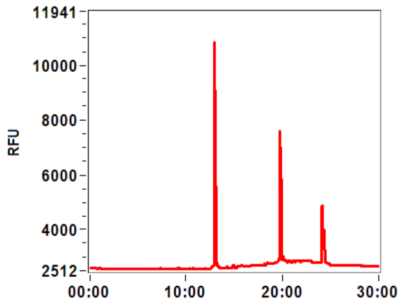

H3: SampH3

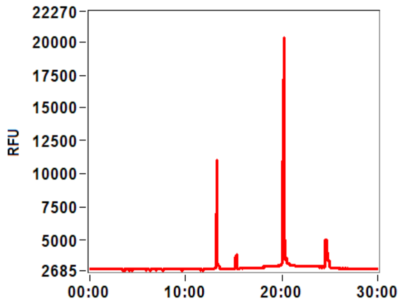

Filename and data path: C:\AATI\Data\2021 10 21\17-58-41\2021 10 21 17H 58M..raw

A4: SampA4

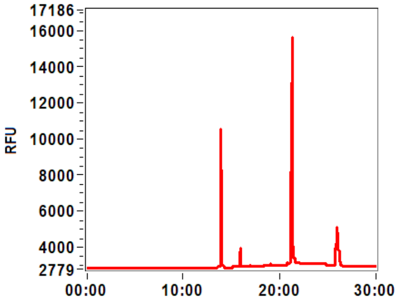

B4: SampB4

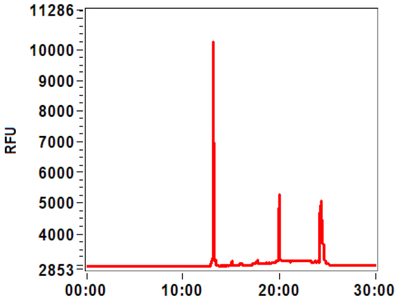

C4: SampC4

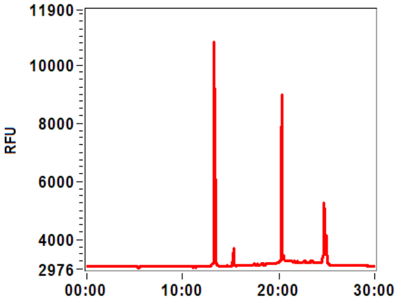

D4: SampD4

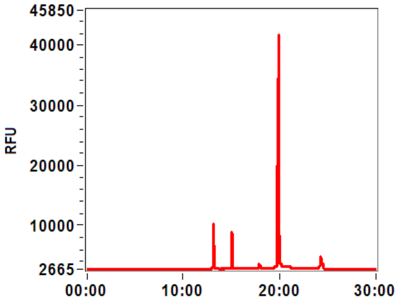

E4: SampE4

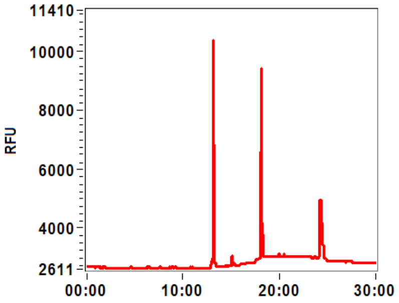

F4: SampF4

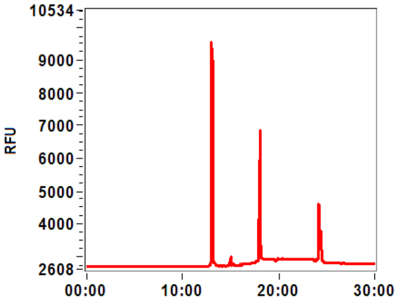

G4: SampG4

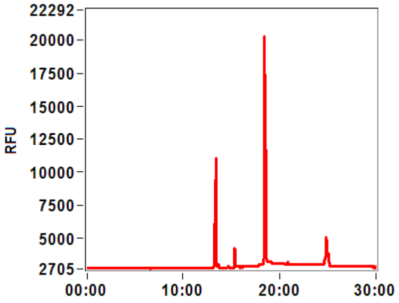

H4: SampH4

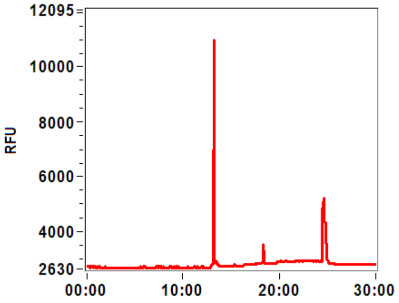

A5: SampA5

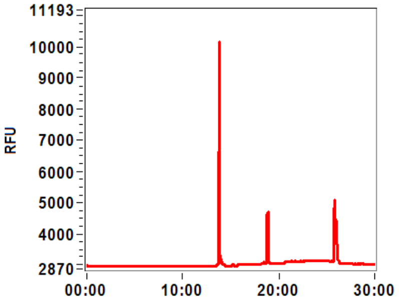

B5: SampB5

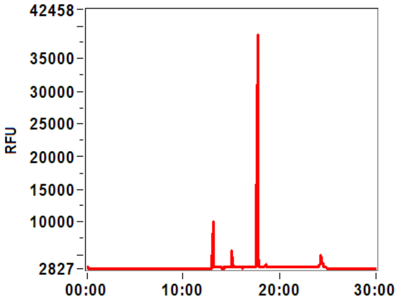

C5: SampC5

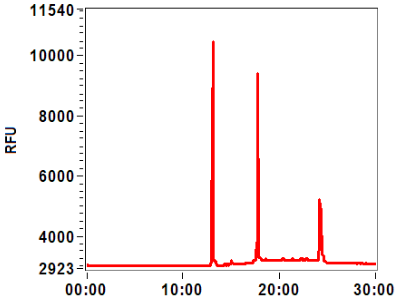

D5: SampD5

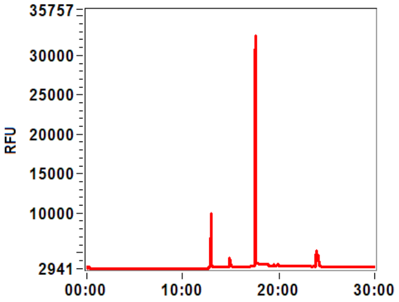

Filename and data path: C:\AATI\Data\2021 10 21\17-58-41\2021 10 21 17H 58M..raw

E5: SampE5

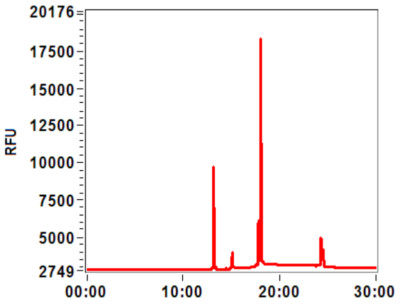

F5: SampF5

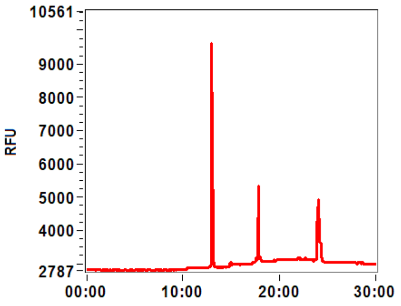

G5: SampG5

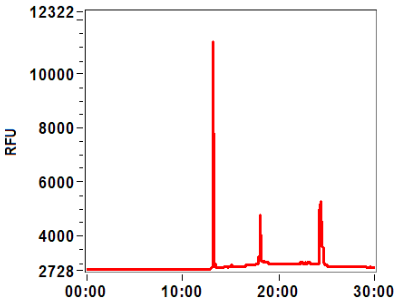

H5: SampH5

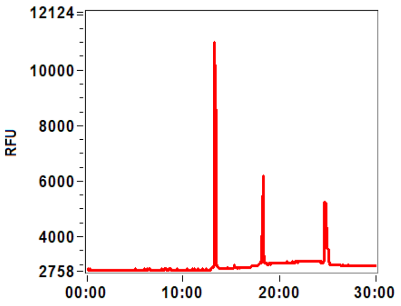

A6: SampA6

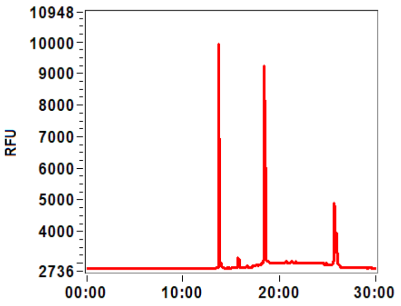

B6: SampB6

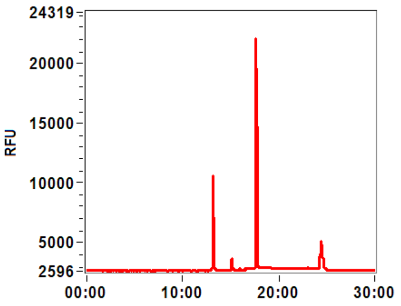

C6: SampC6

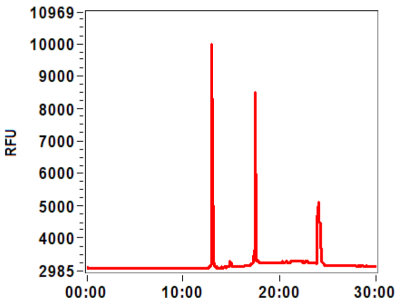

D6: SampD6

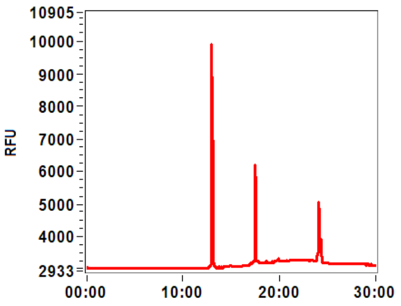

E6: SampE6

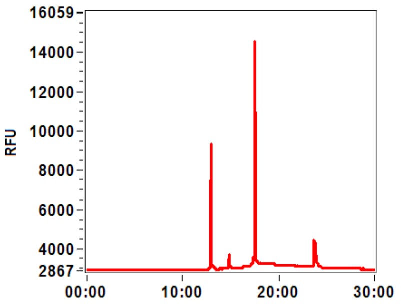

F6: SampF6

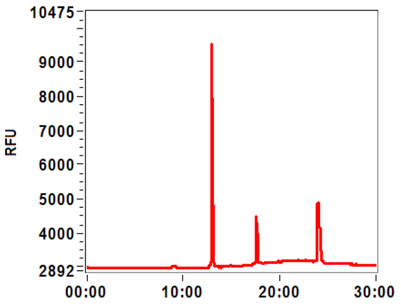

G6: SampG6

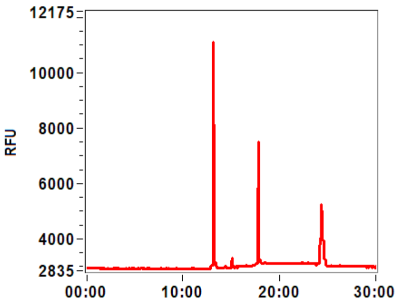

H6: SampH6

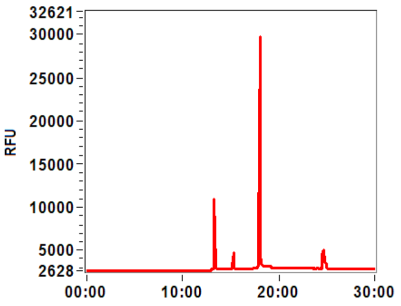

Filename and data path: C:\AATI\Data\2021 10 21\17-58-41\2021 10 21 17H 58M..raw

A7: SampA7

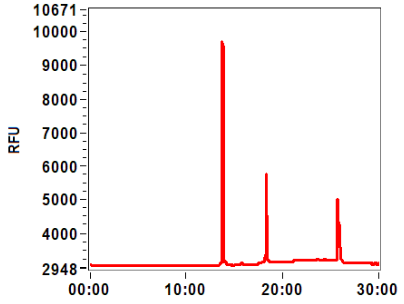

B7: SampB7

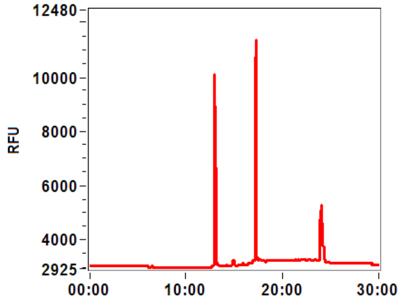

C7: SampC7

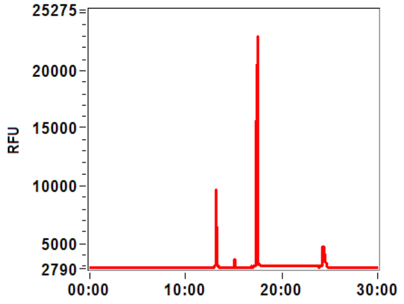

D7: SampD7

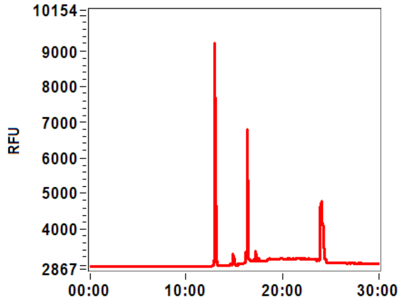

E7: SampE7

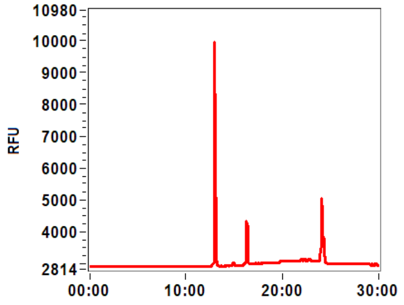

F7: SampF7

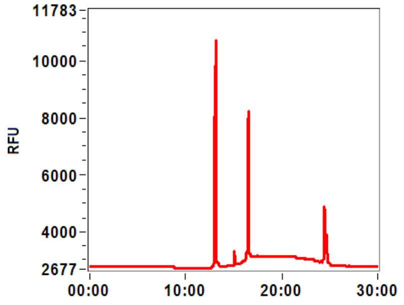

G7: SampG7

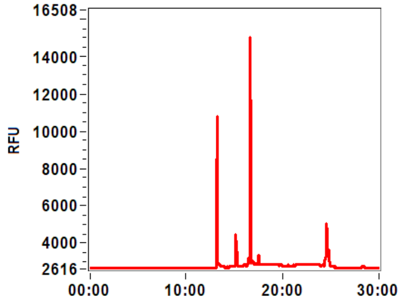

H7: SampH7

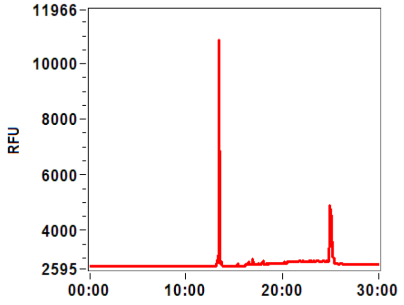

A8: SampA8

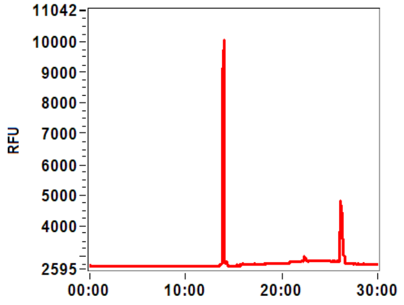

B8: SampB8

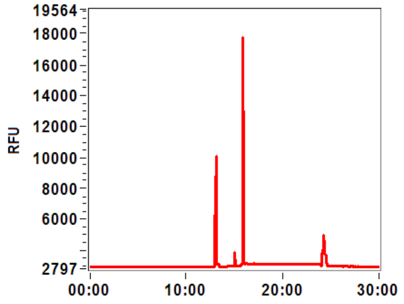

C8: SampC8

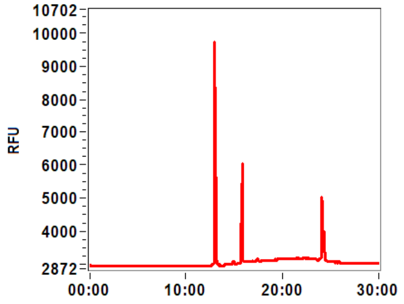

D8: SampD8

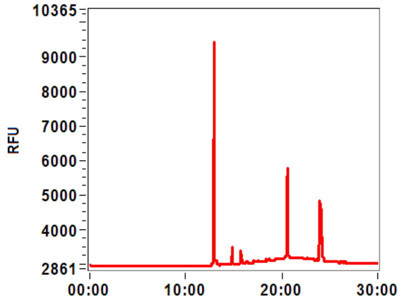

Filename and data path: C:\AATI\Data\2021 10 21\17-58-41\2021 10 21 17H 58M..raw

E8: SampE8

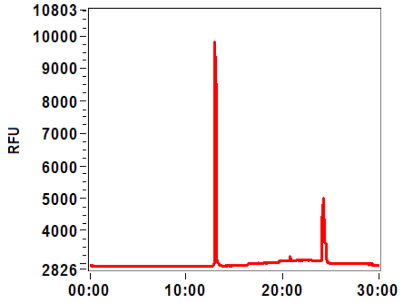

F8: SampF8

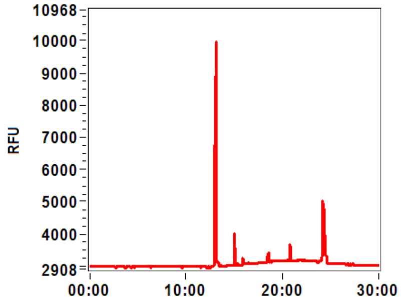

G8: SampG8

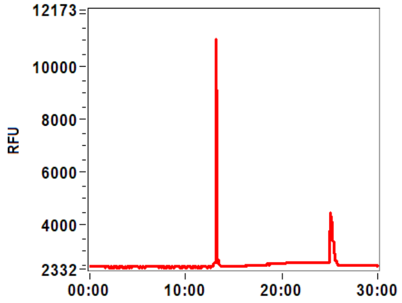

H8: SampH8

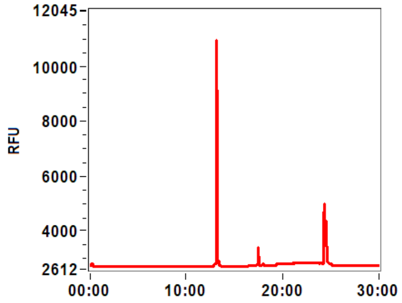

A9: SampA9

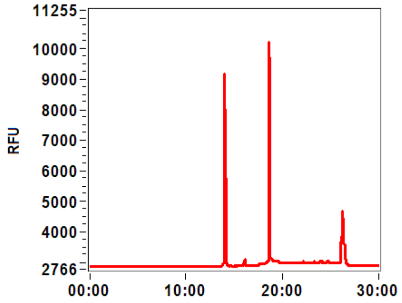

B9: SampB9

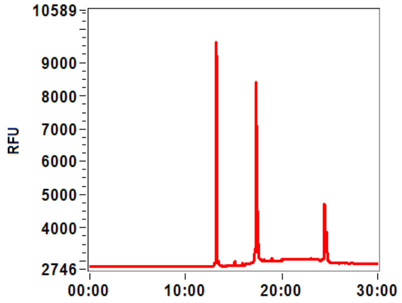

C9: SampC9

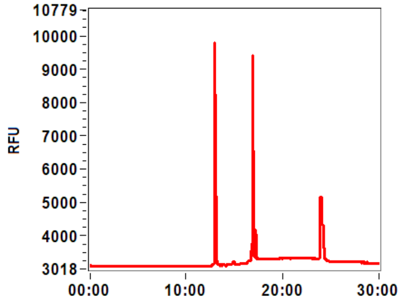

D9: SampD9

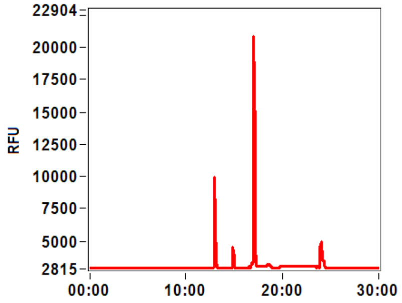

E9: SampE9

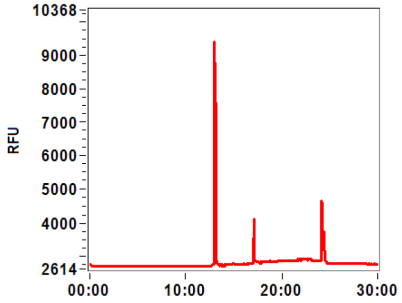

F9: SampF9

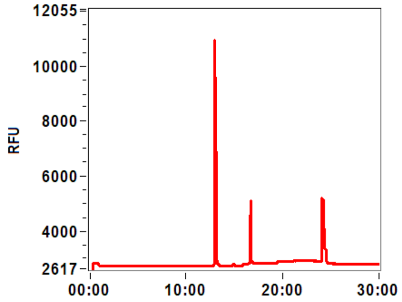

G9: SampG9

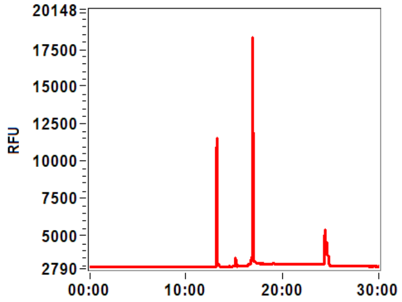

H9: SampH9

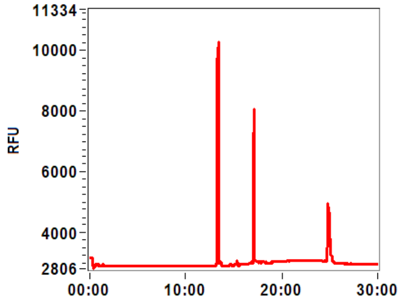

Filename and data path: C:\AATI\Data\2021 10 21\17-58-41\2021 10 21 17H 58M..raw

A10: SampA10

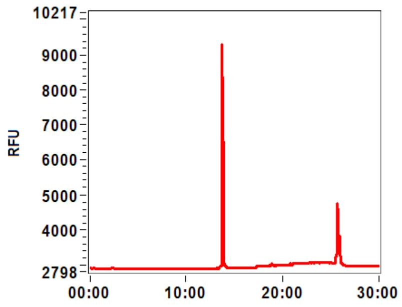

B10: SampB10

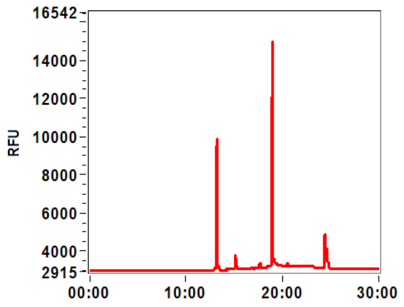

C10: SampC10

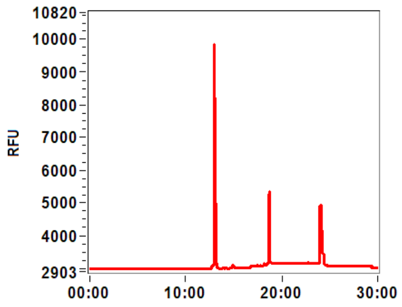

D10: SampD10

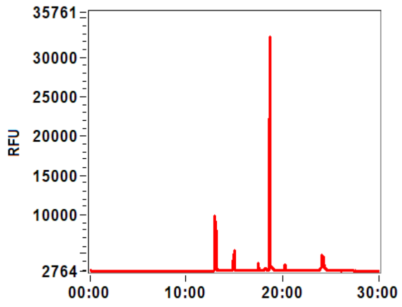

E10: SampE10

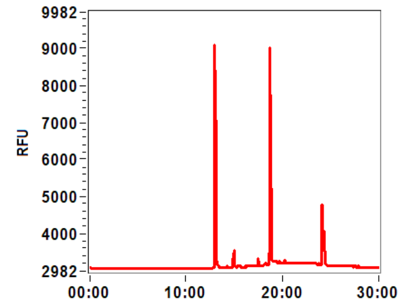

F10: SampF10

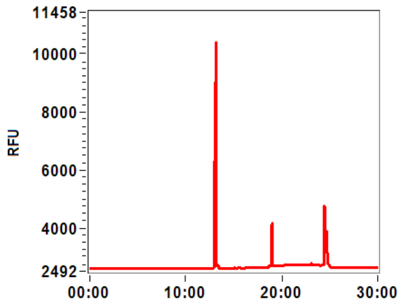

G10: SampG10

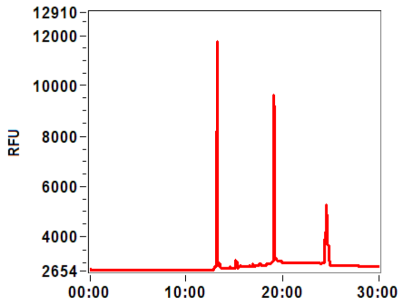

H10: SampH10

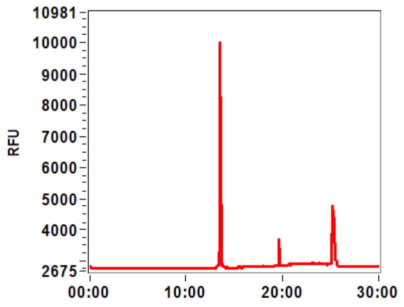

A11: SampA11

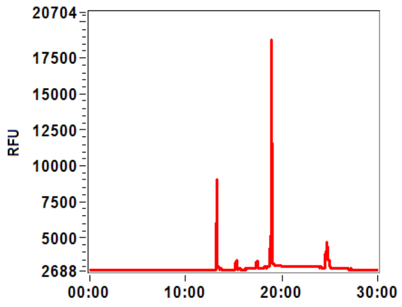

B11: SampB11

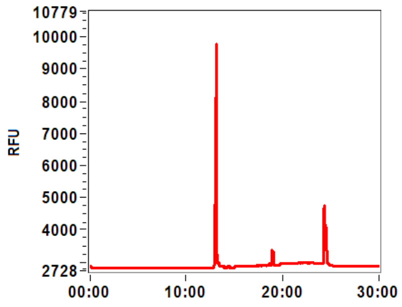

C11: SampC11

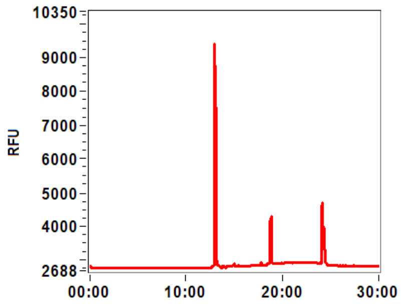

D11: SampD11

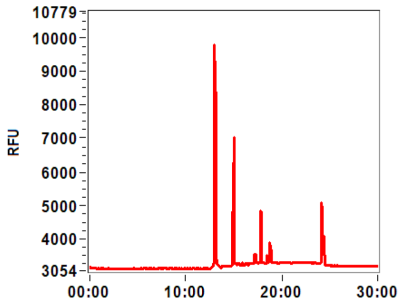

Filename and data path: C:\AATI\Data\2021 10 21\17-58-41\2021 10 21 17H 58M..raw

E11: SampE11

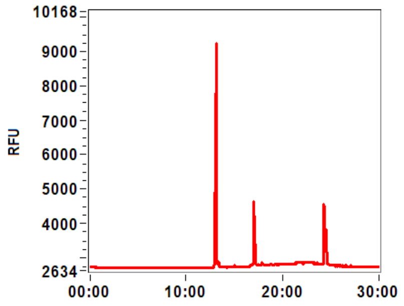

F11: SampF11

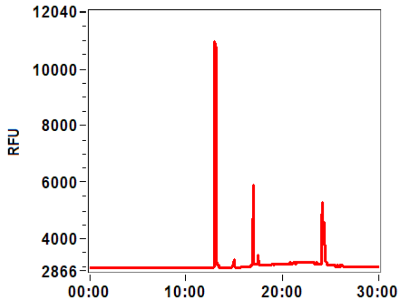

G11: SampG11

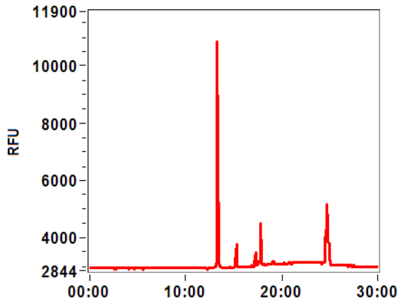

H11: SampH11

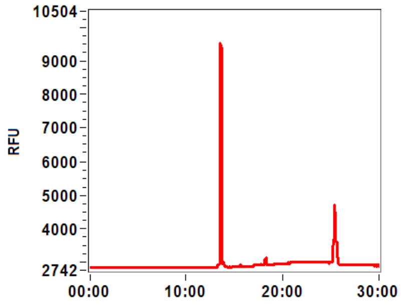

A12: SampA12

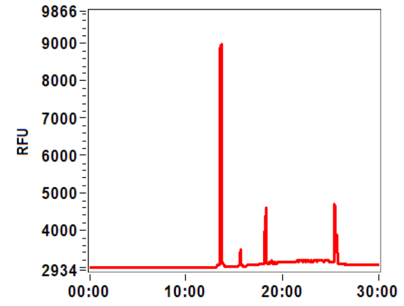

B12: SampB12

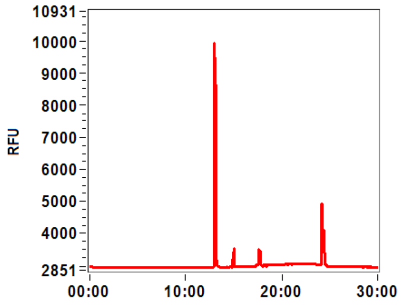

C12: SampC12

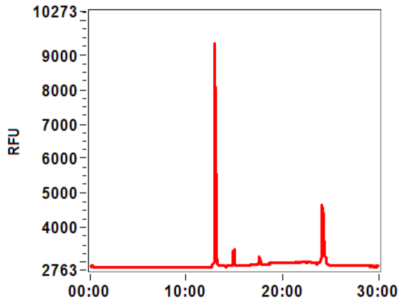

D12: SampD12

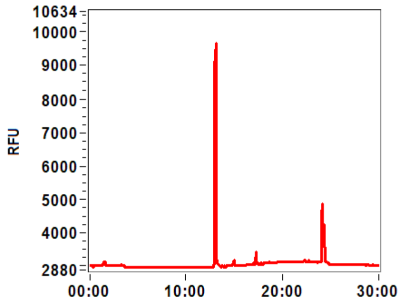

E12: SampE12

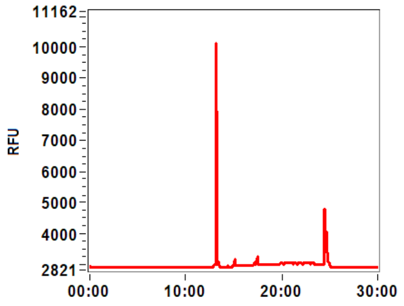

F12: SampF12

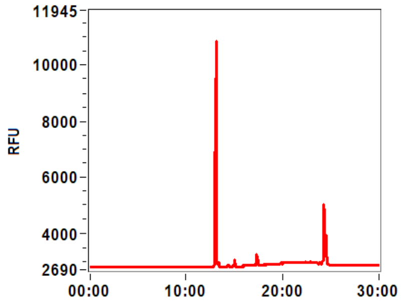

G12: SampG12

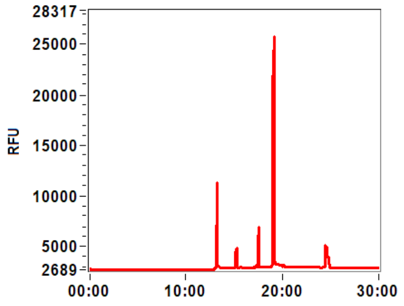

H12: SampH12

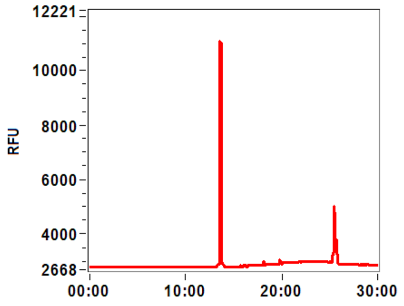

Sample: SampA1
Well location: A1
Created: Thursday, October 21, 2021 6:08:16 PM

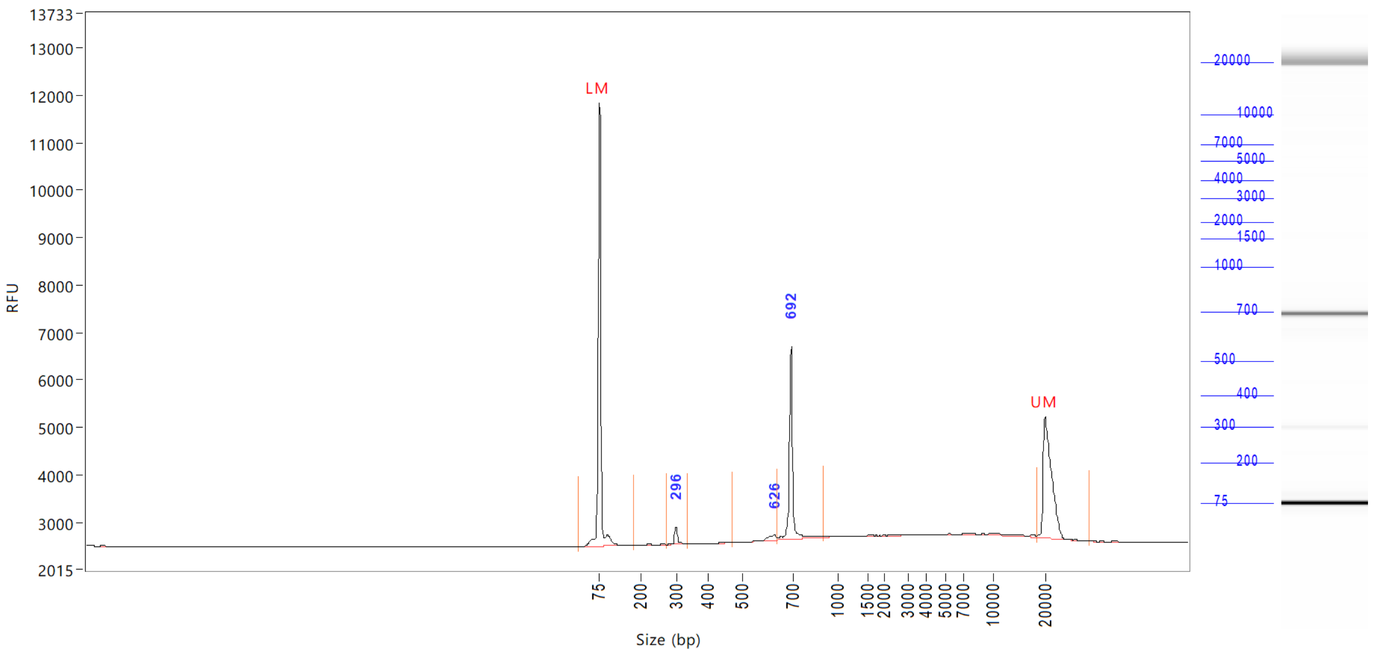

Peak	Size	Concentration	Relative concentration	Molarity	From	To	CV%	RFU
	(bp)	(ng/uL)	Percent	(nmole/L)	(bp)	(bp)		
1	75 (LM)	0.5000		10.9378	14	181	13.51	9357
2	296	0.2410	8.5	1.3349	269	331	2.35	372
3	626	0.1815	6.4	0.4945	470	636	4.66	115
4	692	2.4287	85.2	5.7593	636	909	4.23	4059
5	20000 (UM)	0.2357		0.0189	18415	28342	4.87	2551

TIC:	2.8512	ng/uL
TIM:	7.5887	nmole/L
Total concentration:	3.2999	ng/uL

Sample peak width (sec): 5   Sample min peak height: 50   Sample baseline V to V?: Y   Sample baseline V to V points: 3
Sample filter: Binomial   Number of points for filter: 3 Sample start region (min): 0 Sample end region (min): 30
Marker peak width (sec): 5   Marker min peak height: 200   Marker baseline V to V?: Y   Marker baseline V to V points: 3
Lower marker selection: First peak > 500 RFU   Upper marker selection: Last peak > 200 RFU
Ladder size (bp)75, 200, 300, 400, 500, 700, 1000, 1500, 2000, 3000, 4000, 5000, 7000, 10000, 20000
Quantification using: Lower Marker   Final concentration (ng/uL): 0.5000   Dilution factor: 12.0

Sample: SampB1
Well location: B1
Created: Thursday, October 21, 2021 6:08:16 PM

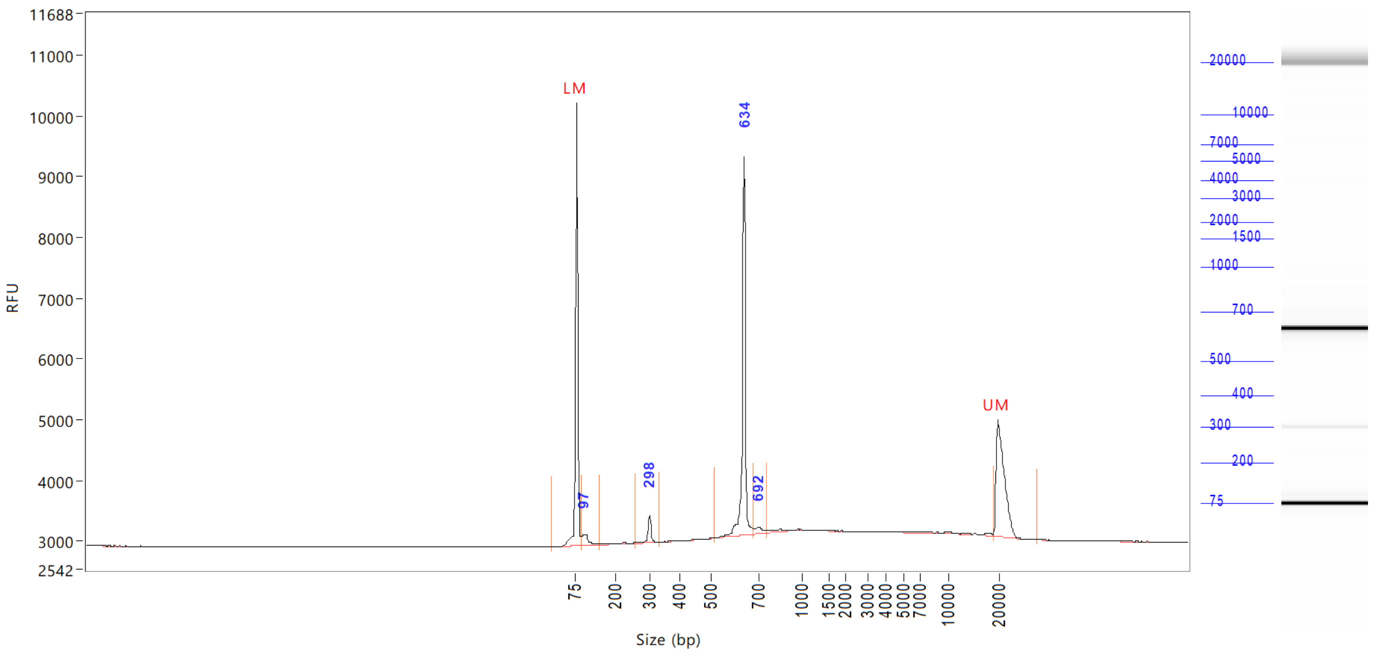

Peak	Size	Concentration	Relative concentration	Molarity	From	To	CV%	RFU
	(bp)	(ng/uL)	Percent	(nmole/L)	(bp)	(bp)		
1	75 (LM)	0.5000		11.3919	0	89	8.23	7297
2	97	0.4153	6.8	6.5575	89	148	12.82	179
3	298	0.3996	6.6	2.2208	257	331	2.77	461
4	634	5.0445	83.1	13.2402	509	674	2.30	6223
5	692	0.2077	3.4	0.4912	674	740	2.38	107
6	20000 (UM)	0.2426		0.0194	19024	27859	4.49	1923

TIC:	6.0671	ng/uL
TIM:	22.5098	nmole/L
Total concentration:	6.6670	ng/uL

```
Sample peak width (sec): 5    Sample min peak height: 50    Sample baseline V to V?: Y    Sample baseline V to V points: 3
Sample filter: Binomial       Number of points for filter: 3  Sample start region (min): 0  Sample end region (min): 30
Marker peak width (sec): 5    Marker min peak height: 200   Marker baseline V to V?: Y    Marker baseline V to V points: 3
Lower marker selection: First peak > 500 RFU                Upper marker selection: Last peak > 200 RFU
Ladder size (bp)75, 200, 300, 400, 500, 700, 1000, 1500, 2000, 3000, 4000, 5000, 7000, 10000, 20000
Quantification using: Lower Marker          Final concentration (ng/uL): 0.5000          Dilution factor: 12.0
```

Sample: SampC1
Well location: C1
Created: Thursday, October 21, 2021 6:08:16 PM

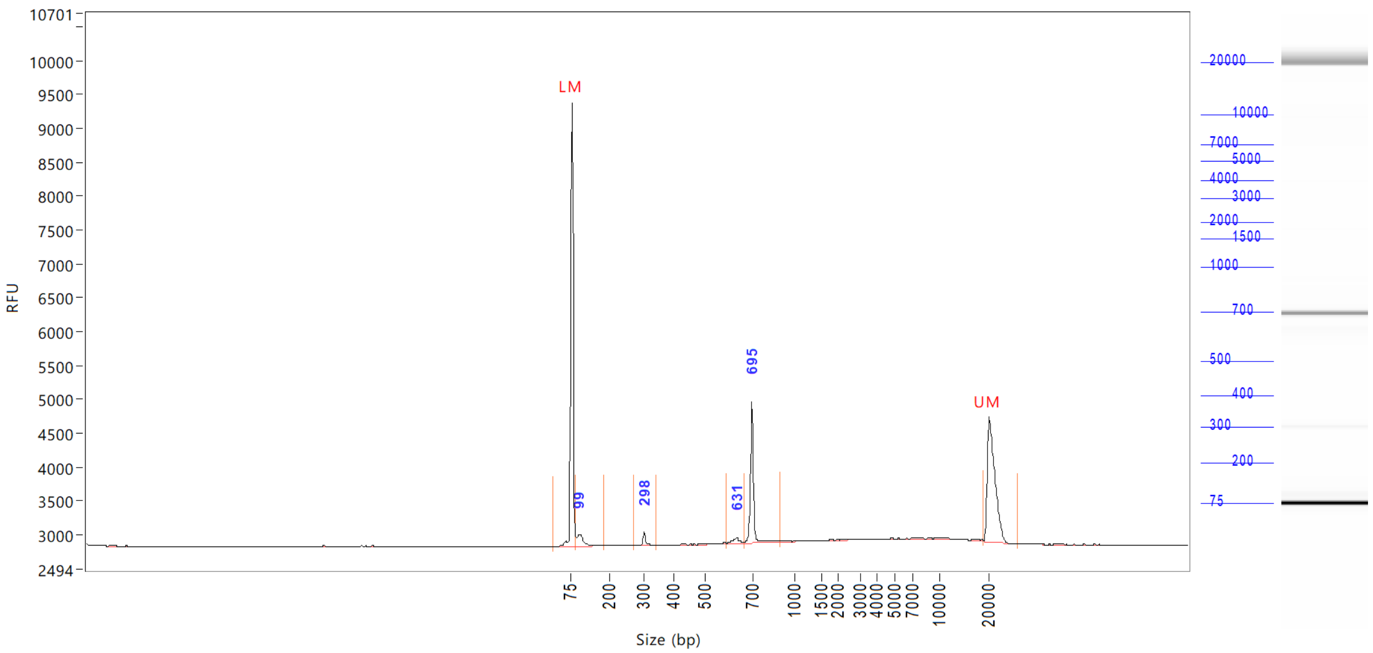

Peak	Size	Concentration	Relative concentration	Molarity	From	To	CV%	RFU
	(bp)	(ng/uL)	Percent	(nmole/L)	(bp)	(bp)		
1	75 (LM)	0.5000		11.2364	16	89	6.61	6555
2	99	0.4402	16.7	6.8210	89	179	15.50	174
3	298	0.2026	7.7	1.1145	271	339	2.85	206
4	631	0.1887	7.2	0.4976	585	662	2.85	95
5	695	1.7997	68.4	4.2494	662	887	3.91	2093
6	20000 (UM)	0.2451		0.0196	19024	25682	3.95	1855

TIC:	2.6312	ng/uL
TIM:	12.6825	nmole/L
Total concentration:	3.0997	ng/uL

```
Sample peak width (sec): 5    Sample min peak height: 50    Sample baseline V to V?: Y    Sample baseline V to V points: 3
Sample filter: Binomial       Number of points for filter: 3  Sample start region (min): 0  Sample end region (min): 30
Marker peak width (sec): 5    Marker min peak height: 200   Marker baseline V to V?: Y    Marker baseline V to V points: 3
Lower marker selection: First peak > 500 RFU                Upper marker selection: Last peak > 200 RFU
Ladder size (bp)75, 200, 300, 400, 500, 700, 1000, 1500, 2000, 3000, 4000, 5000, 7000, 10000, 20000
Quantification using: Lower Marker          Final concentration (ng/uL): 0.5000          Dilution factor: 12.0
```

Sample: SampD1
Well location: D1
Created: Thursday, October 21, 2021 6:08:16 PM

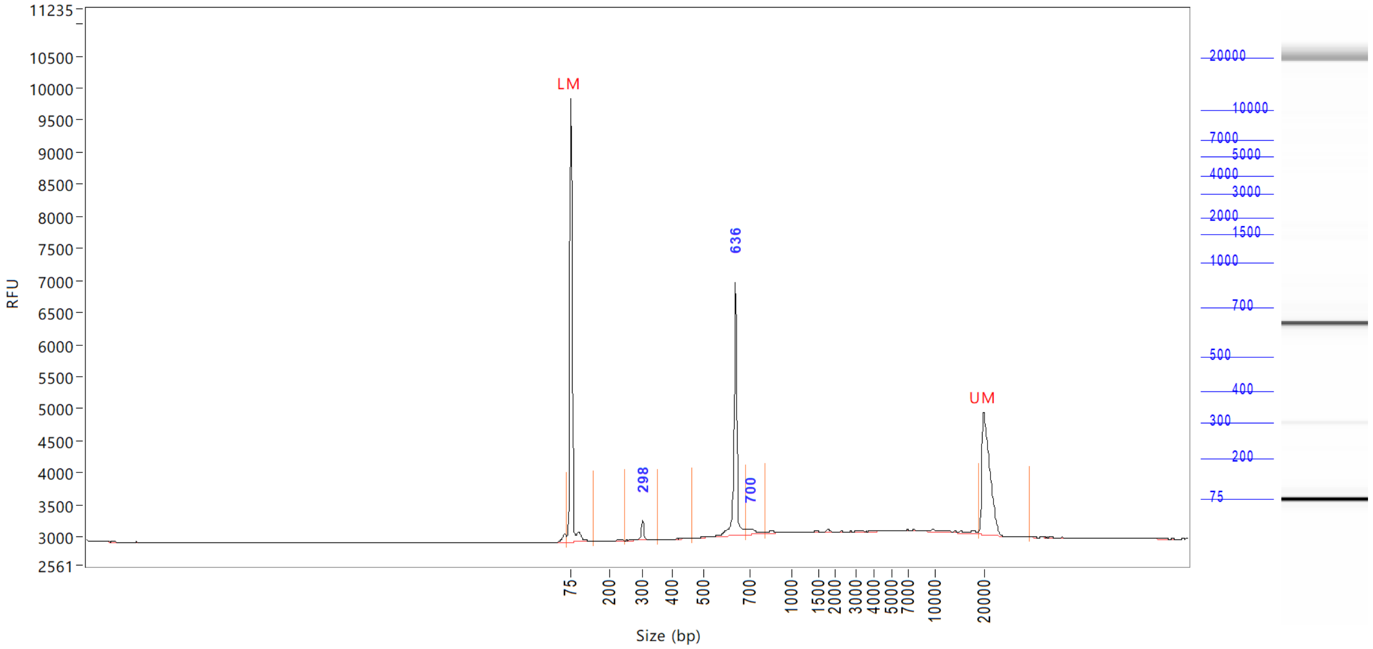

Peak	Size	Concentration	Relative concentration	Molarity	From	To	CV%	RFU
	(bp)	(ng/uL)	Percent	(nmole/L)	(bp)	(bp)		
1	75 (LM)	0.5000		11.0851	62	147	10.44	6926
2	298	0.2950	7.7	1.6392	244	347	3.72	322
3	636	3.3104	86.0	8.6613	462	677	2.96	3954
4	700	0.2454	6.4	0.5633	677	805	4.82	87
5	20000 (UM)	0.2449		0.0196	18903	29309	4.45	1914

TIC:	3.8508	ng/uL
TIM:	10.8639	nmole/L
Total concentration:	4.4974	ng/uL

```
Sample peak width (sec): 5    Sample min peak height: 50    Sample baseline V to V?: Y    Sample baseline V to V points: 3
Sample filter: Binomial       Number of points for filter: 3  Sample start region (min): 0  Sample end region (min): 30
Marker peak width (sec): 5    Marker min peak height: 200   Marker baseline V to V?: Y    Marker baseline V to V points: 3
Lower marker selection: First peak > 500 RFU                Upper marker selection: Last peak > 200 RFU
Ladder size (bp)75, 200, 300, 400, 500, 700, 1000, 1500, 2000, 3000, 4000, 5000, 7000, 10000, 20000
Quantification using: Lower Marker          Final concentration (ng/uL): 0.5000          Dilution factor: 12.0
```

Sample: SampE1
Well location: E1
Created: Thursday, October 21, 2021 6:08:16 PM

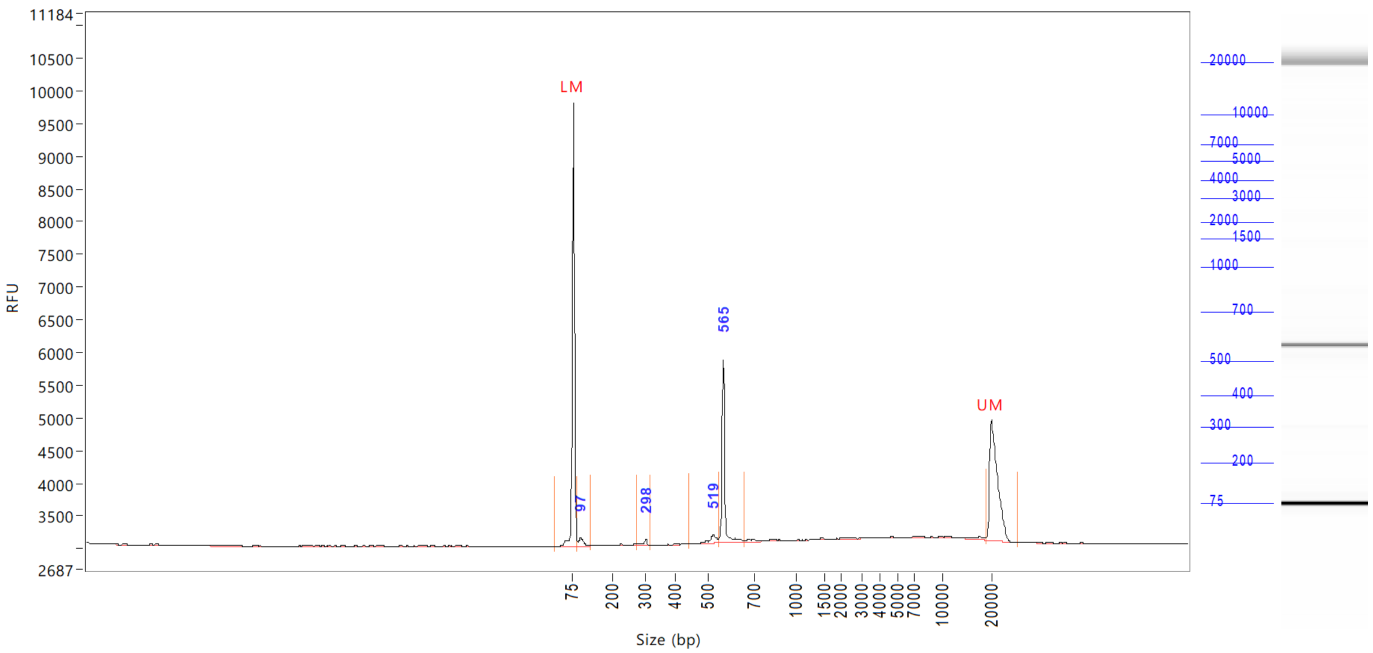

Peak	Size	Concentration	Relative concentration	Molarity	From	To	CV%	RFU
	(bp)	(ng/uL)	Percent	(nmole/L)	(bp)	(bp)		
1	75 (LM)	0.5000		11.3919	16	87	7.57	6788
2	97	0.2413	8.2	4.0020	87	129	9.09	119
3	298	0.1277	4.3	0.7271	269	313	3.42	88
4	519	0.2745	9.3	0.8823	441	544	4.38	120
5	565	2.3047	78.2	6.7008	544	654	2.82	2797
6	20000 (UM)	0.2521		0.0201	18903	25440	4.30	1837

TIC:	2.9483	ng/uL
TIM:	12.3122	nmole/L
Total concentration:	3.6877	ng/uL

```
Sample peak width (sec): 5    Sample min peak height: 50    Sample baseline V to V?: Y    Sample baseline V to V points: 3
Sample filter: Binomial       Number of points for filter: 3  Sample start region (min): 0  Sample end region (min): 30
Marker peak width (sec): 5    Marker min peak height: 200   Marker baseline V to V?: Y    Marker baseline V to V points: 3
Lower marker selection: First peak > 500 RFU                Upper marker selection: Last peak > 200 RFU
Ladder size (bp)75, 200, 300, 400, 500, 700, 1000, 1500, 2000, 3000, 4000, 5000, 7000, 10000, 20000
Quantification using: Lower Marker          Final concentration (ng/uL): 0.5000          Dilution factor: 12.0
```

Sample: SampF1
Well location: F1
Created: Thursday, October 21, 2021 6:08:16 PM

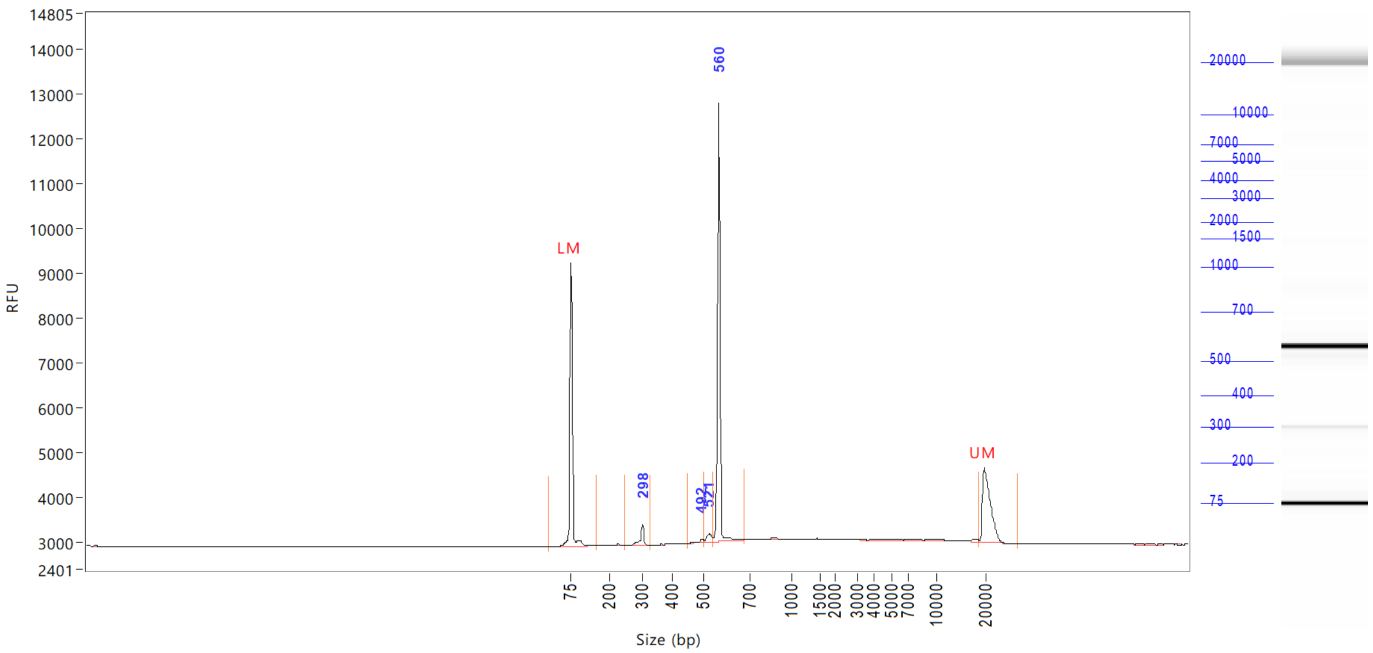

Peak	Size	Concentration	Relative concentration	Molarity	From	To	CV%	RFU
	(bp)	(ng/uL)	Percent	(nmole/L)	(bp)	(bp)		
1	75 (LM)	0.5000		11.0851	7	156	12.93	6311
2	298	0.5186	6.1	2.9315	246	321	3.41	457
3	492	0.1063	1.3	0.3598	445	500	2.41	83
4	521	0.3159	3.7	1.0017	500	539	2.05	181
5	560	7.4942	88.8	22.0615	539	674	1.76	9802
6	20000 (UM)	0.2290		0.0184	18903	26891	4.27	1661

TIC:	8.4350	ng/uL
TIM:	26.3545	nmole/L
Total concentration:	9.0463	ng/uL

```
Sample peak width (sec): 5    Sample min peak height: 50    Sample baseline V to V?: Y    Sample baseline V to V points: 3
Sample filter: Binomial       Number of points for filter: 3  Sample start region (min): 0  Sample end region (min): 30
Marker peak width (sec): 5    Marker min peak height: 200   Marker baseline V to V?: Y    Marker baseline V to V points: 3
Lower marker selection: First peak > 500 RFU                Upper marker selection: Last peak > 200 RFU
Ladder size (bp)75, 200, 300, 400, 500, 700, 1000, 1500, 2000, 3000, 4000, 5000, 7000, 10000, 20000
Quantification using: Lower Marker          Final concentration (ng/uL): 0.5000          Dilution factor: 12.0
```

Sample: SampG1
Well location: G1
Created: Thursday, October 21, 2021 6:08:16 PM

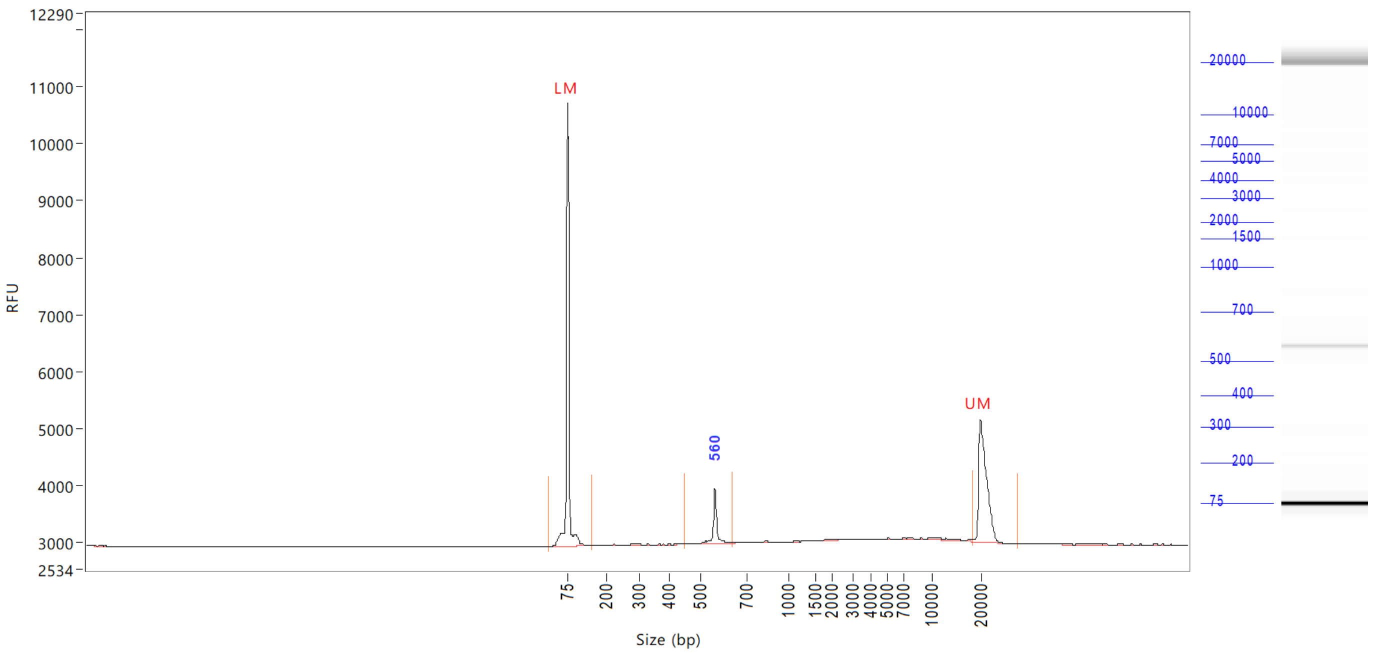

Peak	Size	Concentration	Relative concentration	Molarity	From	To	CV%	RFU
	(bp)	(ng/uL)	Percent	(nmole/L)	(bp)	(bp)		
1	75 (LM)	0.5000		11.3919	14	152	15.28	7792
2	560	0.7656	100.0	2.2579	449	639	4.44	969
3	20000 (UM)	0.2186		0.0174	18415	27738	4.38	2142

TIC:	0.7656	ng/uL
TIM:	2.2579	nmole/L
Total concentration:	1.3075	ng/uL

Sample peak width (sec): 5   Sample min peak height: 50   Sample baseline V to V?: Y   Sample baseline V to V points: 3
Sample filter: Binomial   Number of points for filter: 3 Sample start region (min): 0 Sample end region (min): 30
Marker peak width (sec): 5   Marker min peak height: 200   Marker baseline V to V?: Y   Marker baseline V to V points: 3
Lower marker selection: First peak > 500 RFU   Upper marker selection: Last peak > 200 RFU
Ladder size (bp)75, 200, 300, 400, 500, 700, 1000, 1500, 2000, 3000, 4000, 5000, 7000, 10000, 20000
Quantification using: Lower Marker   Final concentration (ng/uL): 0.5000   Dilution factor: 12.0

Sample: SampH1
Well location: H1
Created: Thursday, October 21, 2021 6:08:16 PM

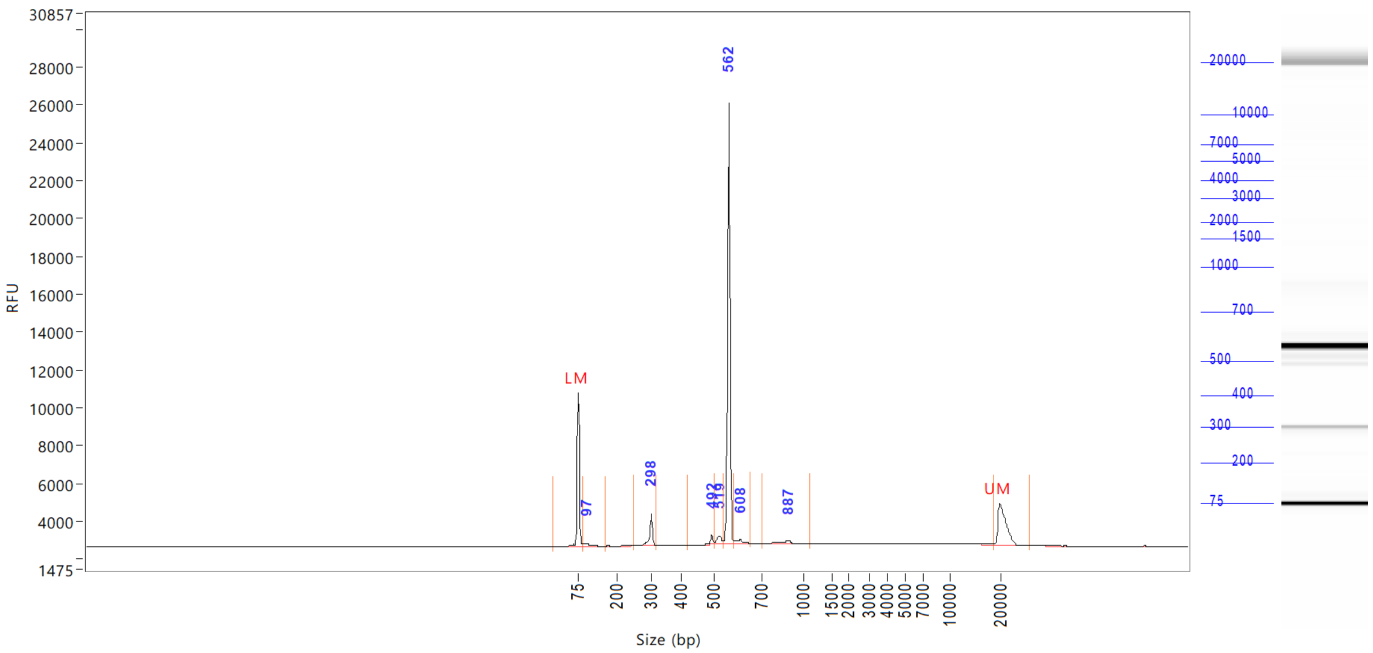

Peak	Size	Concentration	Relative concentration	Molarity	From	To	CV%	RFU
	(bp)	(ng/uL)	Percent	(nmole/L)	(bp)	(bp)		
1	75 (LM)	0.5000		11.3919	0	87	6.98	8091
2	97	0.3783	2.2	5.9734	87	160	15.18	183
3	298	1.3572	7.8	7.6193	246	315	2.78	1700
4	492	0.3603	2.1	1.2174	416	500	2.67	528
5	519	0.5576	3.2	1.7748	500	537	1.81	446
6	562	14.0944	80.8	41.4913	537	585	0.99	23344
7	608	0.3061	1.8	0.8354	585	652	2.15	197
8	887	0.3973	2.3	0.7658	701	1090	6.25	165
9	20000 (UM)	0.2446		0.0196	18903	26045	4.08	2169

TIC:	17.4512	ng/uL
TIM:	59.6775	nmole/L
Total concentration:	17.8698	ng/uL

```
Sample peak width (sec): 5    Sample min peak height: 50    Sample baseline V to V?: Y    Sample baseline V to V points: 3
Sample filter: Binomial       Number of points for filter: 3  Sample start region (min): 0  Sample end region (min): 30
Marker peak width (sec): 5    Marker min peak height: 200   Marker baseline V to V?: Y    Marker baseline V to V points: 3
Lower marker selection: First peak > 500 RFU                Upper marker selection: Last peak > 200 RFU
Ladder size (bp)75, 200, 300, 400, 500, 700, 1000, 1500, 2000, 3000, 4000, 5000, 7000, 10000, 20000
Quantification using: Lower Marker          Final concentration (ng/uL): 0.5000          Dilution factor: 12.0
```

Sample: SampA2
Well location: A2
Created: Thursday, October 21, 2021 6:08:16 PM

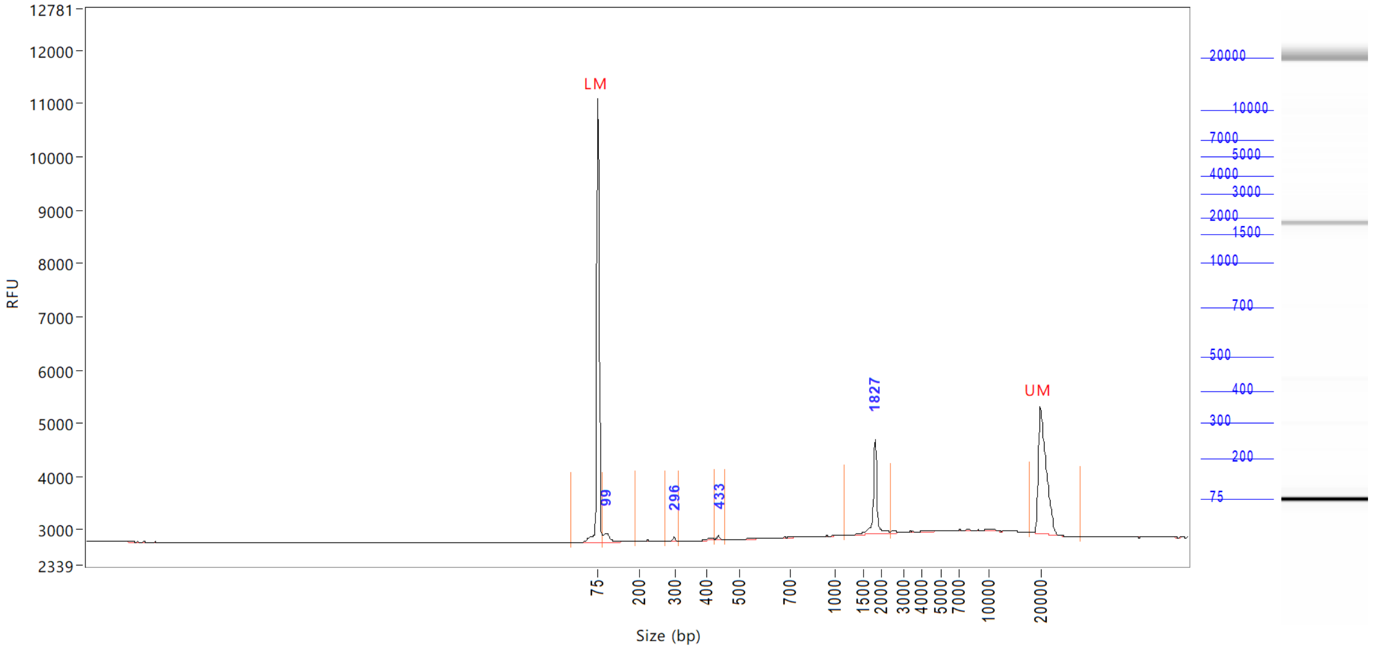

Peak	Size	Concentration	Relative concentration	Molarity	From	To	CV%	RFU
	(bp)	(ng/uL)	Percent	(nmole/L)	(bp)	(bp)		
1	75 (LM)	0.5000		11.3919	0	89	8.45	8342
2	99	0.3487	19.3	5.3519	89	189	17.60	170
3	296	0.0541	3.0	0.3029	271	313	1.88	79
4	433	0.0688	3.8	0.2614	426	454	1.09	96
5	1827	1.3358	73.9	1.2196	1178	2401	7.61	1772
6	20000 (UM)	0.2489		0.0200	18049	27617	3.90	2372

TIC:	1.8074	ng/uL
TIM:	7.1358	nmole/L
Total concentration:	2.3094	ng/uL

```
Sample peak width (sec): 5    Sample min peak height: 50    Sample baseline V to V?: Y    Sample baseline V to V points: 3
Sample filter: Binomial       Number of points for filter: 3  Sample start region (min): 0  Sample end region (min): 30
Marker peak width (sec): 5    Marker min peak height: 200   Marker baseline V to V?: Y    Marker baseline V to V points: 3
Lower marker selection: First peak > 500 RFU                Upper marker selection: Last peak > 200 RFU
Ladder size (bp)75, 200, 300, 400, 500, 700, 1000, 1500, 2000, 3000, 4000, 5000, 7000, 10000, 20000
Quantification using: Lower Marker          Final concentration (ng/uL): 0.5000          Dilution factor: 12.0
```

Sample: SampB2
Well location: B2
Created: Thursday, October 21, 2021 6:08:16 PM

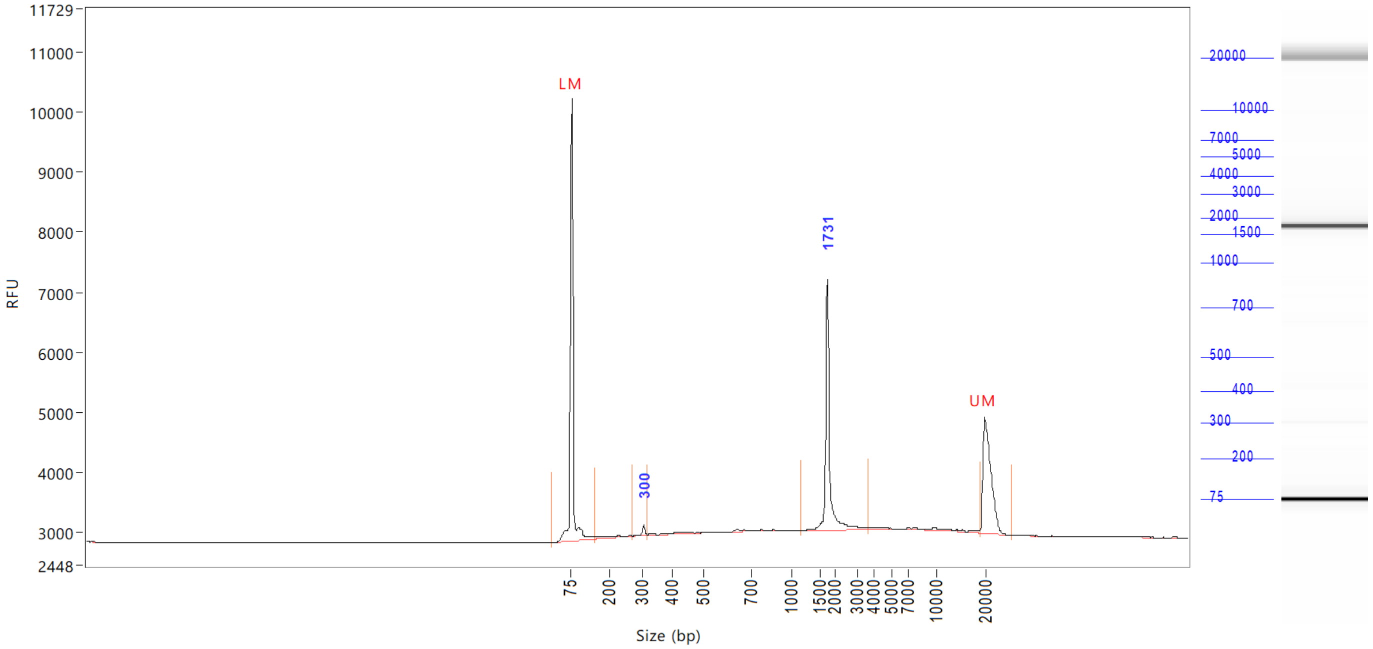

Peak	Size	Concentration	Relative concentration	Molarity	From	To	CV%	RFU
	(bp)	(ng/uL)	Percent	(nmole/L)	(bp)	(bp)		
1	75 (LM)	0.5000		10.9378	14	150	17.03	7394
2	300	0.1372	3.9	0.7627	266	311	2.27	183
3	1731	3.3950	96.1	3.0893	1145	3608	17.08	4188
4	20000 (UM)	0.2120		0.0169	19024	25561	4.20	1930

TIC:	3.5323	ng/uL
TIM:	3.8520	nmole/L
Total concentration:	4.3769	ng/uL

```
Sample peak width (sec): 5    Sample min peak height: 50    Sample baseline V to V?: Y    Sample baseline V to V points: 3
Sample filter: Binomial       Number of points for filter: 3  Sample start region (min): 0  Sample end region (min): 30
Marker peak width (sec): 5    Marker min peak height: 200   Marker baseline V to V?: Y    Marker baseline V to V points: 3
Lower marker selection: First peak > 500 RFU                Upper marker selection: Last peak > 200 RFU
Ladder size (bp)75, 200, 300, 400, 500, 700, 1000, 1500, 2000, 3000, 4000, 5000, 7000, 10000, 20000
Quantification using: Lower Marker          Final concentration (ng/uL): 0.5000          Dilution factor: 12.0
```

Sample: SampC2
Well location: C2
Created: Thursday, October 21, 2021 6:08:16 PM

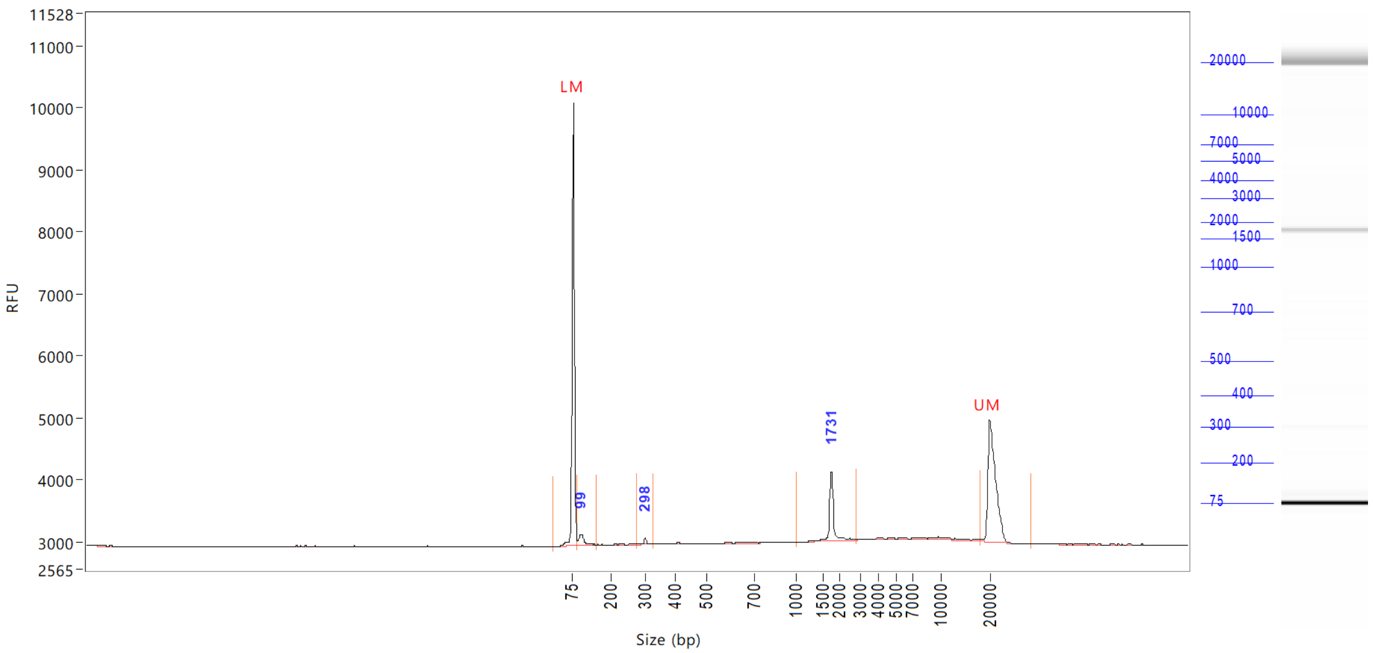

Peak	Size	Concentration	Relative concentration	Molarity	From	To	CV%	RFU
	(bp)	(ng/uL)	Percent	(nmole/L)	(bp)	(bp)		
1	75 (LM)	0.5000		11.2364	12	89	6.46	7152
2	99	0.3539	23.8	5.5884	89	148	11.92	178
3	298	0.0860	5.8	0.4763	275	325	2.16	105
4	1731	1.0449	70.4	0.9745	1001	2800	12.15	1114
5	20000 (UM)	0.2467		0.0197	18293	28463	4.44	1961

TIC:	1.4848	ng/uL
TIM:	7.0393	nmole/L
Total concentration:	2.1992	ng/uL

```
Sample peak width (sec): 5    Sample min peak height: 50    Sample baseline V to V?: Y    Sample baseline V to V points: 3
Sample filter: Binomial       Number of points for filter: 3  Sample start region (min): 0  Sample end region (min): 30
Marker peak width (sec): 5    Marker min peak height: 200   Marker baseline V to V?: Y    Marker baseline V to V points: 3
Lower marker selection: First peak > 500 RFU                Upper marker selection: Last peak > 200 RFU
Ladder size (bp)75, 200, 300, 400, 500, 700, 1000, 1500, 2000, 3000, 4000, 5000, 7000, 10000, 20000
Quantification using: Lower Marker          Final concentration (ng/uL): 0.5000          Dilution factor: 12.0
```

Sample: SampD2
Well location: D2
Created: Thursday, October 21, 2021 6:08:16 PM

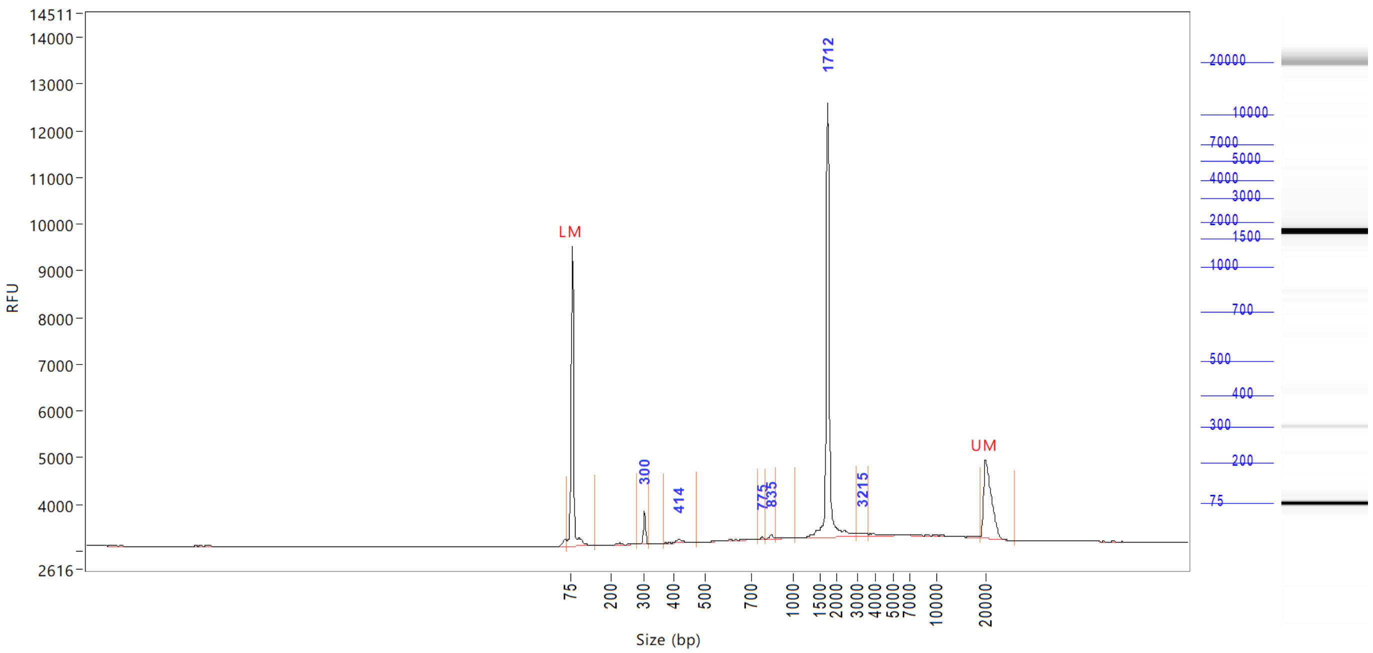

Peak	Size	Concentration	Relative concentration	Molarity	From	To	CV%	RFU
	(bp)	(ng/uL)	Percent	(nmole/L)	(bp)	(bp)		
1	75 (LM)	0.5000		10.9378	60	147	11.26	6413
2	300	0.5460	6.0	3.0140	278	313	1.31	710
3	414	0.1692	1.9	0.6692	366	473	4.34	68
4	775	0.0388	0.4	0.0832	736	796	1.34	52
5	835	0.0896	1.0	0.1772	796	874	1.66	99
6	1712	8.1186	89.1	7.7744	1012	2943	11.24	9308
7	3215	0.1531	1.7	0.0776	2943	3643	6.50	67
8	20000 (UM)	0.2211		0.0178	18903	25924	4.08	1680

TIC:	9.1154	ng/uL
TIM:	11.7956	nmole/L
Total concentration:	9.6797	ng/uL

```
Sample peak width (sec): 5    Sample min peak height: 50    Sample baseline V to V?: Y    Sample baseline V to V points: 3
Sample filter: Binomial       Number of points for filter: 3  Sample start region (min): 0  Sample end region (min): 30
Marker peak width (sec): 5    Marker min peak height: 200   Marker baseline V to V?: Y    Marker baseline V to V points: 3
Lower marker selection: First peak > 500 RFU                Upper marker selection: Last peak > 200 RFU
Ladder size (bp)75, 200, 300, 400, 500, 700, 1000, 1500, 2000, 3000, 4000, 5000, 7000, 10000, 20000
Quantification using: Lower Marker          Final concentration (ng/uL): 0.5000          Dilution factor: 12.0
```

Sample: SampE2
Well location: E2
Created: Thursday, October 21, 2021 6:08:16 PM

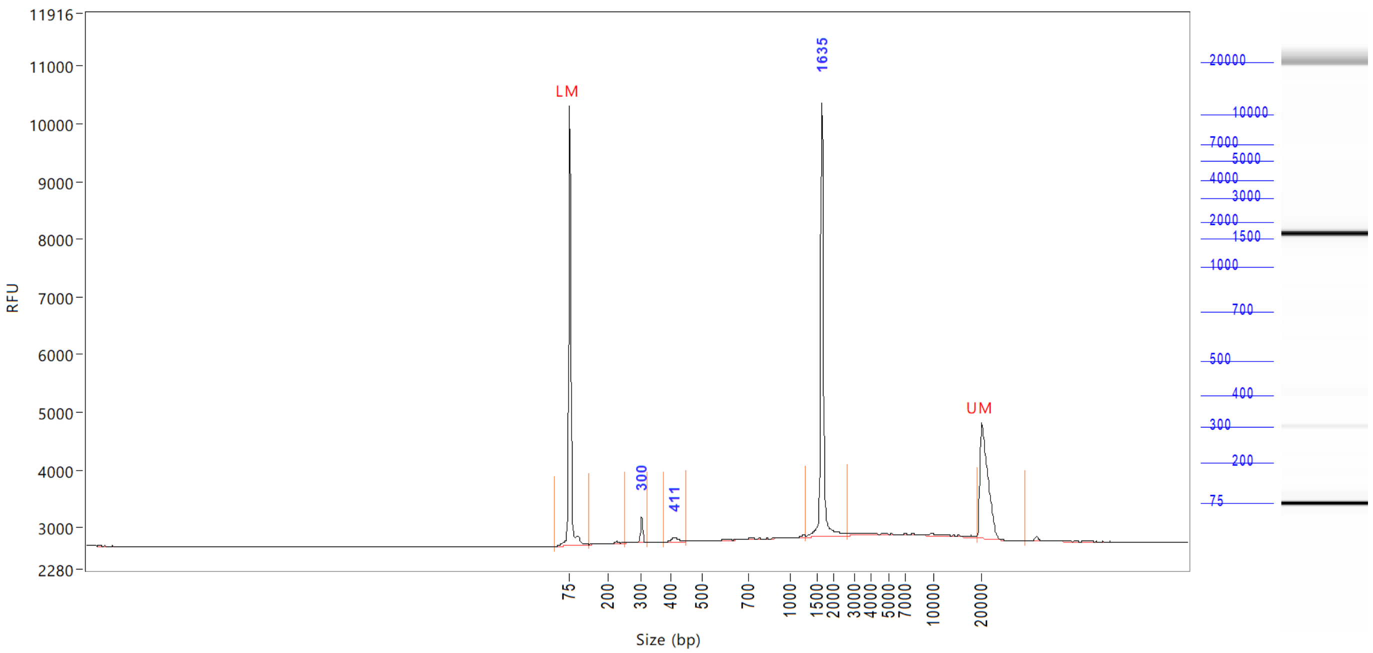

Peak	Size	Concentration	Relative concentration	Molarity	From	To	CV%	RFU
	(bp)	(ng/uL)	Percent	(nmole/L)	(bp)	(bp)		
1	75 (LM)	0.5000		11.0851	26	135	10.50	7630
2	300	0.2982	5.3	1.6516	251	321	1.84	445
3	411	0.1406	2.5	0.5644	378	449	2.95	66
4	1635	5.1352	92.1	5.1449	1289	2715	8.85	7530
5	20000 (UM)	0.2344		0.0187	19024	28826	4.37	1996

TIC:	5.5741	ng/uL
TIM:	7.3609	nmole/L
Total concentration:	6.3009	ng/uL

```
Sample peak width (sec): 5     Sample min peak height: 50    Sample baseline V to V?: Y    Sample baseline V to V points: 3
Sample filter: Binomial        Number of points for filter: 3  Sample start region (min): 0  Sample end region (min): 30
Marker peak width (sec): 5     Marker min peak height: 200   Marker baseline V to V?: Y    Marker baseline V to V points: 3
Lower marker selection: First peak > 500 RFU                 Upper marker selection: Last peak > 200 RFU
Ladder size (bp)75, 200, 300, 400, 500, 700, 1000, 1500, 2000, 3000, 4000, 5000, 7000, 10000, 20000
Quantification using: Lower Marker          Final concentration (ng/uL): 0.5000          Dilution factor: 12.0
```

Sample: SampF2
Well location: F2
Created: Thursday, October 21, 2021 6:08:16 PM

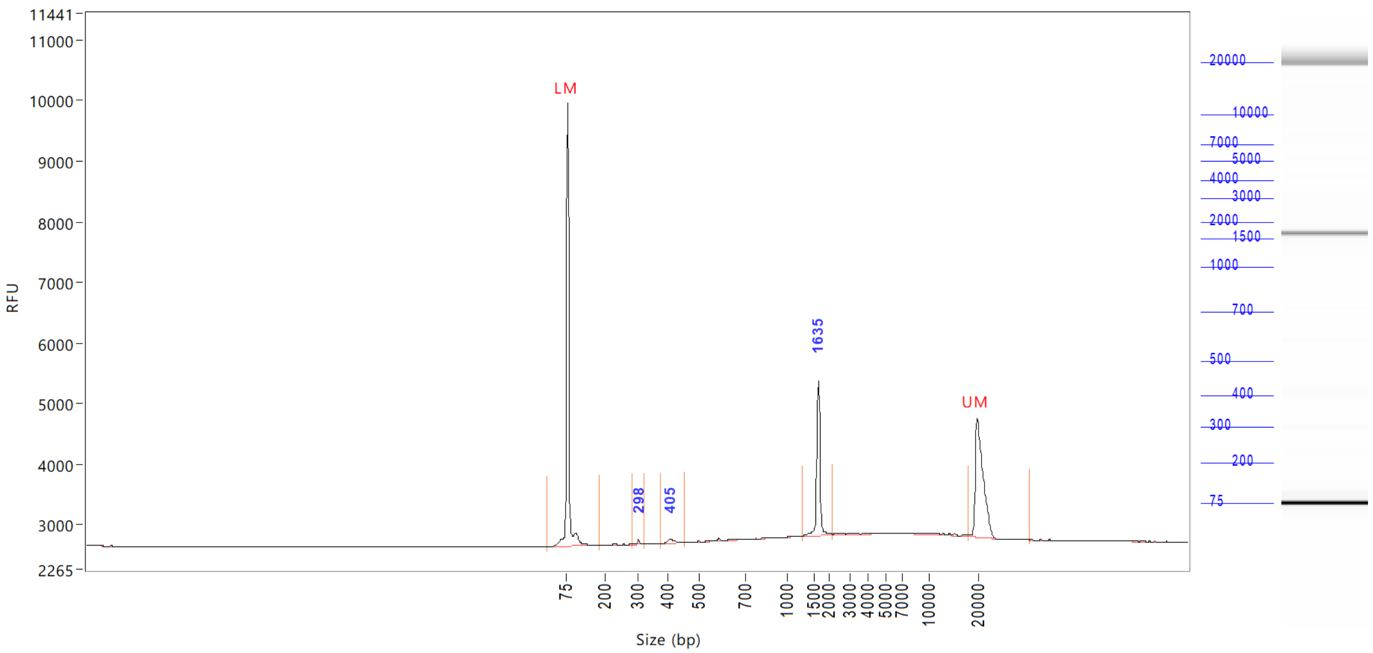

Peak	Size	Concentration	Relative concentration	Molarity	From	To	CV%	RFU
	(bp)	(ng/uL)	Percent	(nmole/L)	(bp)	(bp)		
1	75 (LM)	0.5000		10.9378	12	179	14.14	7328
2	298	0.0547	2.9	0.3028	282	317	1.54	79
3	405	0.1253	6.6	0.5041	378	454	3.21	62
4	1635	1.7183	90.5	1.7438	1289	2144	5.32	2553
5	20000 (UM)	0.2209		0.0177	18171	30760	4.76	1958

TIC:	1.8983	ng/uL
TIM:	2.5508	nmole/L
Total concentration:	2.4105	ng/uL

Sample peak width (sec): 5   Sample min peak height: 50   Sample baseline V to V?: Y   Sample baseline V to V points: 3
Sample filter: Binomial   Number of points for filter: 3 Sample start region (min): 0 Sample end region (min): 30
Marker peak width (sec): 5   Marker min peak height: 200   Marker baseline V to V?: Y   Marker baseline V to V points: 3
Lower marker selection: First peak > 500 RFU   Upper marker selection: Last peak > 200 RFU
Ladder size (bp)75, 200, 300, 400, 500, 700, 1000, 1500, 2000, 3000, 4000, 5000, 7000, 10000, 20000
Quantification using: Lower Marker   Final concentration (ng/uL): 0.5000   Dilution factor: 12.0

Sample: SampG2
Well location: G2
Created: Thursday, October 21, 2021 6:08:16 PM

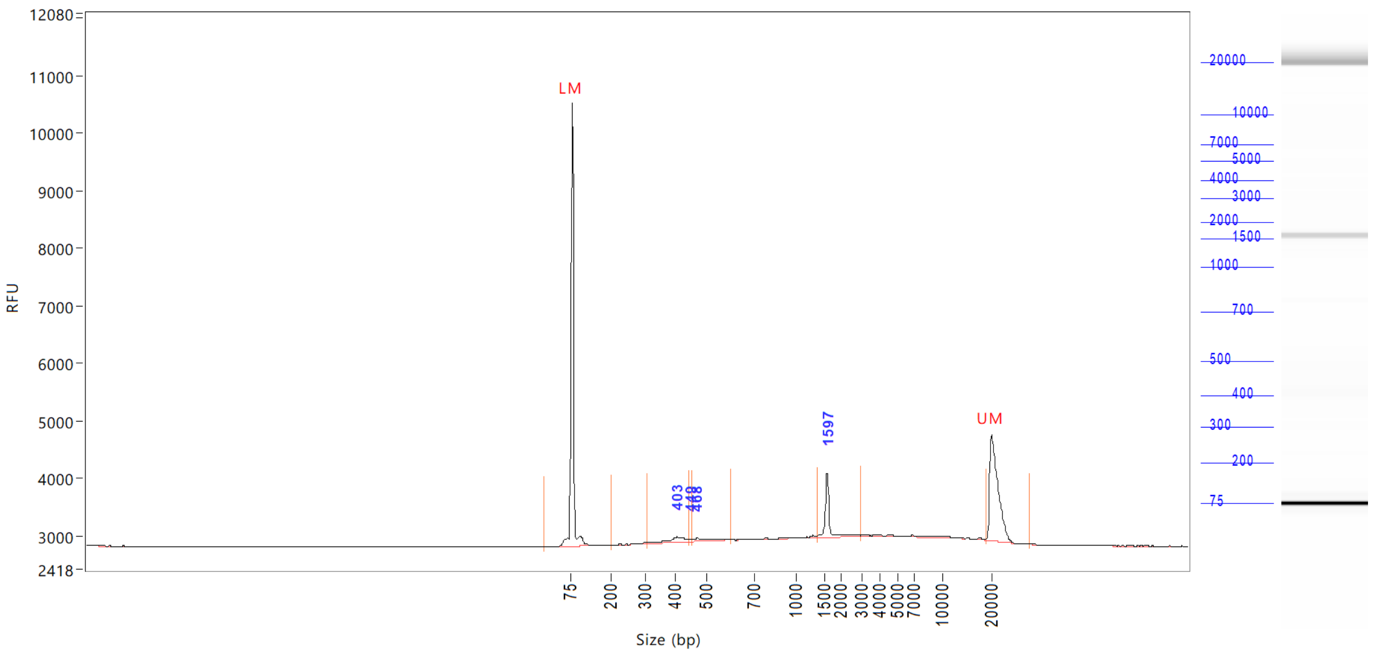

Peak	Size	Concentration	Relative concentration	Molarity	From	To	CV%	RFU
	(bp)	(ng/uL)	Percent	(nmole/L)	(bp)	(bp)		
1	75 (LM)	0.5000		11.0851	0	200	15.87	7716
2	403	0.3974	24.5	1.6595	303	443	8.40	92
3	449	0.0398	2.5	0.1461	443	452	0.72	51
4	468	0.2324	14.3	0.7725	452	598	6.53	51
5	1597	0.9535	58.7	0.9513	1367	2943	16.44	1119
6	20000 (UM)	0.2176		0.0173	18903	27738	4.84	1852

TIC:	1.6231	ng/uL
TIM:	3.5295	nmole/L
Total concentration:	2.0858	ng/uL

Sample peak width (sec): 5 Sample min peak height: 50 Sample baseline V to V?: Y Sample baseline V to V points: 3
Sample filter: Binomial Number of points for filter: 3 Sample start region (min): 0 Sample end region (min): 30
Marker peak width (sec): 5 Marker min peak height: 200 Marker baseline V to V?: Y Marker baseline V to V points: 3
Lower marker selection: First peak > 500 RFU Upper marker selection: Last peak > 200 RFU
Ladder size (bp)75, 200, 300, 400, 500, 700, 1000, 1500, 2000, 3000, 4000, 5000, 7000, 10000, 20000
Quantification using: Lower Marker Final concentration (ng/uL): 0.5000 Dilution factor: 12.0

Sample: SampH2
Well location: H2
Created: Thursday, October 21, 2021 6:08:16 PM

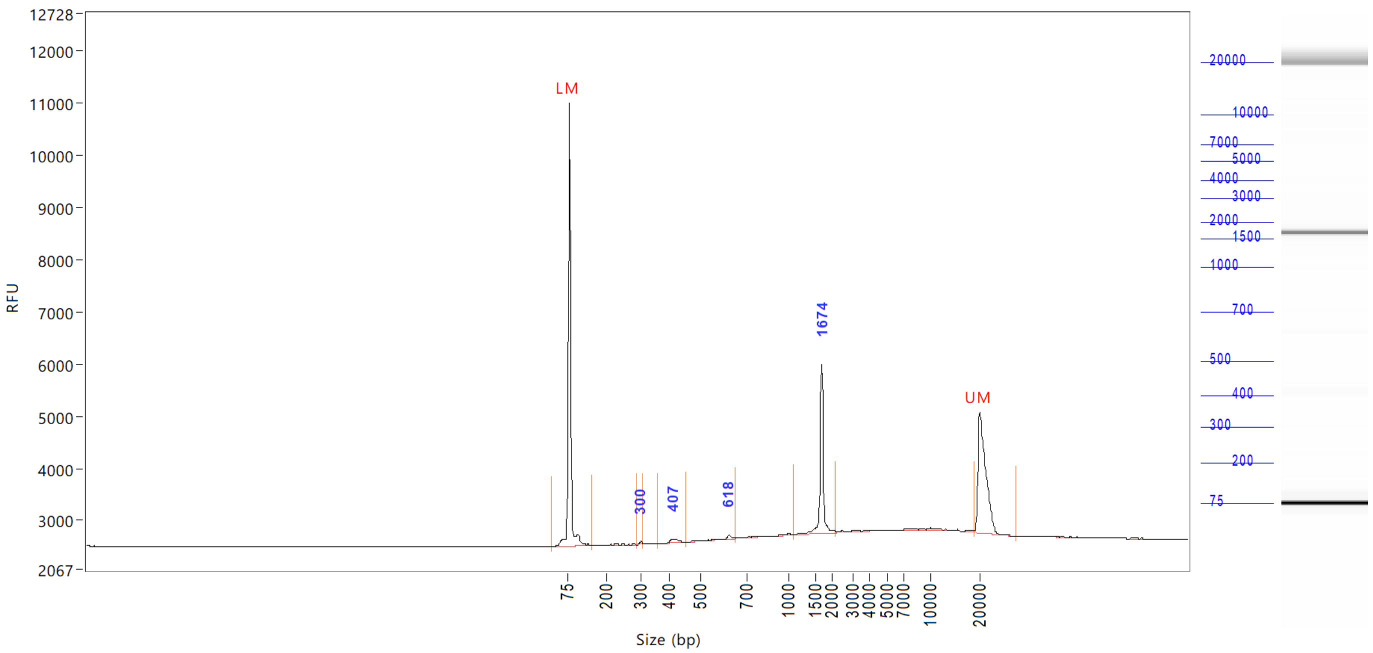

Peak	Size	Concentration	Relative concentration	Molarity	From	To	CV%	RFU
	(bp)	(ng/uL)	Percent	(nmole/L)	(bp)	(bp)		
1	75 (LM)	0.5000		10.9378	22	148	13.19	8514
2	300	0.0357	1.6	0.1972	287	307	1.12	63
3	407	0.1420	6.3	0.5699	356	450	3.00	79
4	618	0.0934	4.1	0.2623	450	646	8.27	94
5	1674	1.9936	88.0	1.9985	1068	2172	6.73	3240
6	20000 (UM)	0.2247		0.0179	19024	27617	4.37	2302

TIC:	2.2647	ng/uL
TIM:	3.0280	nmole/L
Total concentration:	2.7127	ng/uL

```
Sample peak width (sec): 5    Sample min peak height: 50    Sample baseline V to V?: Y    Sample baseline V to V points: 3
Sample filter: Binomial       Number of points for filter: 3  Sample start region (min): 0  Sample end region (min): 30
Marker peak width (sec): 5    Marker min peak height: 200   Marker baseline V to V?: Y    Marker baseline V to V points: 3
Lower marker selection: First peak > 500 RFU                Upper marker selection: Last peak > 200 RFU
Ladder size (bp)75, 200, 300, 400, 500, 700, 1000, 1500, 2000, 3000, 4000, 5000, 7000, 10000, 20000
Quantification using: Lower Marker          Final concentration (ng/uL): 0.5000          Dilution factor: 12.0
```

Sample: SampA3
Well location: A3
Created: Thursday, October 21, 2021 6:08:16 PM

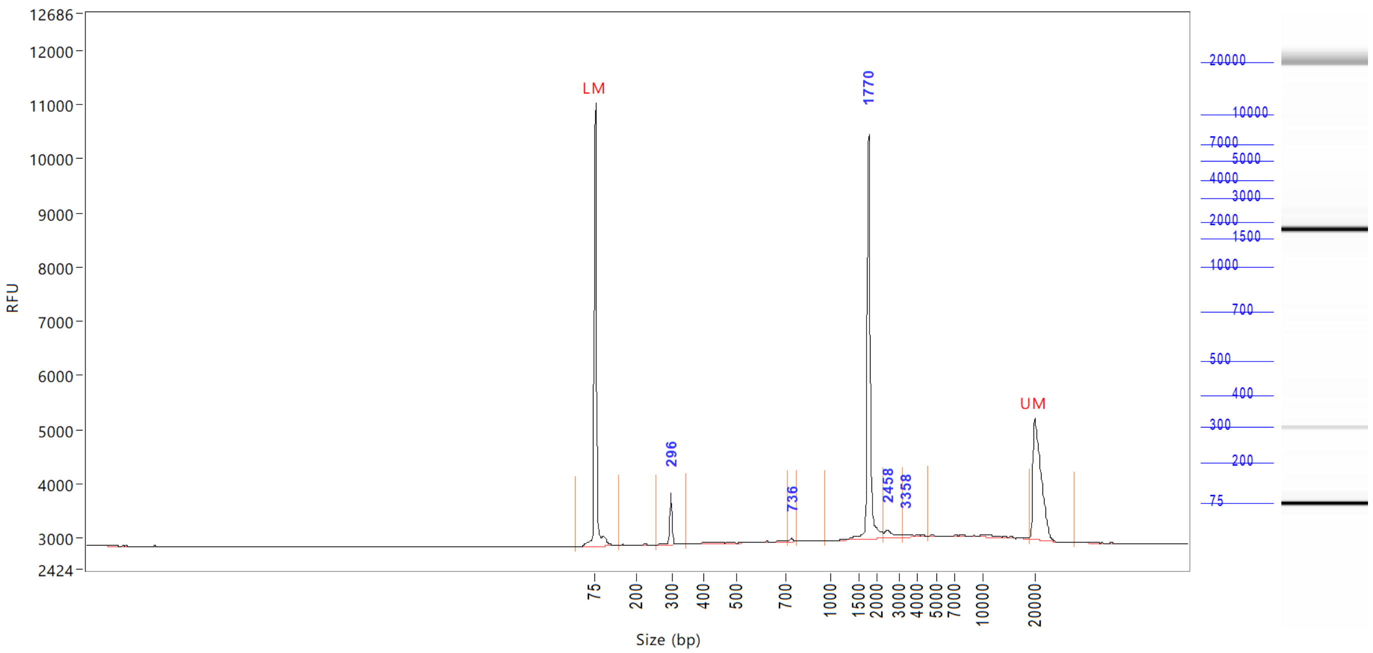

Peak	Size	Concentration	Relative concentration	Molarity	From	To	CV%	RFU
	(bp)	(ng/uL)	Percent	(nmole/L)	(bp)	(bp)		
1	75 (LM)	0.5000		11.0851	16	147	12.19	8197
2	296	0.5962	10.5	3.3241	255	343	2.72	950
3	736	0.0422	0.7	0.0955	705	762	1.23	68
4	1770	4.6677	82.4	4.3856	961	2315	6.52	7466
5	2458	0.2480	4.4	0.1553	2315	3144	9.05	156
6	3358	0.1125	2.0	0.0509	3144	4552	9.94	51
7	20000 (UM)	0.2349		0.0188	18903	27496	4.11	2246

TIC:	5.6666	ng/uL
TIM:	8.0114	nmole/L
Total concentration:	6.1772	ng/uL

```
Sample peak width (sec): 5    Sample min peak height: 50    Sample baseline V to V?: Y    Sample baseline V to V points: 3
Sample filter: Binomial       Number of points for filter: 3  Sample start region (min): 0  Sample end region (min): 30
Marker peak width (sec): 5    Marker min peak height: 200   Marker baseline V to V?: Y    Marker baseline V to V points: 3
Lower marker selection: First peak > 500 RFU                Upper marker selection: Last peak > 200 RFU
Ladder size (bp)75, 200, 300, 400, 500, 700, 1000, 1500, 2000, 3000, 4000, 5000, 7000, 10000, 20000
Quantification using: Lower Marker          Final concentration (ng/uL): 0.5000          Dilution factor: 12.0
```

Sample: SampB3
Well location: B3
Created: Thursday, October 21, 2021 6:08:16 PM

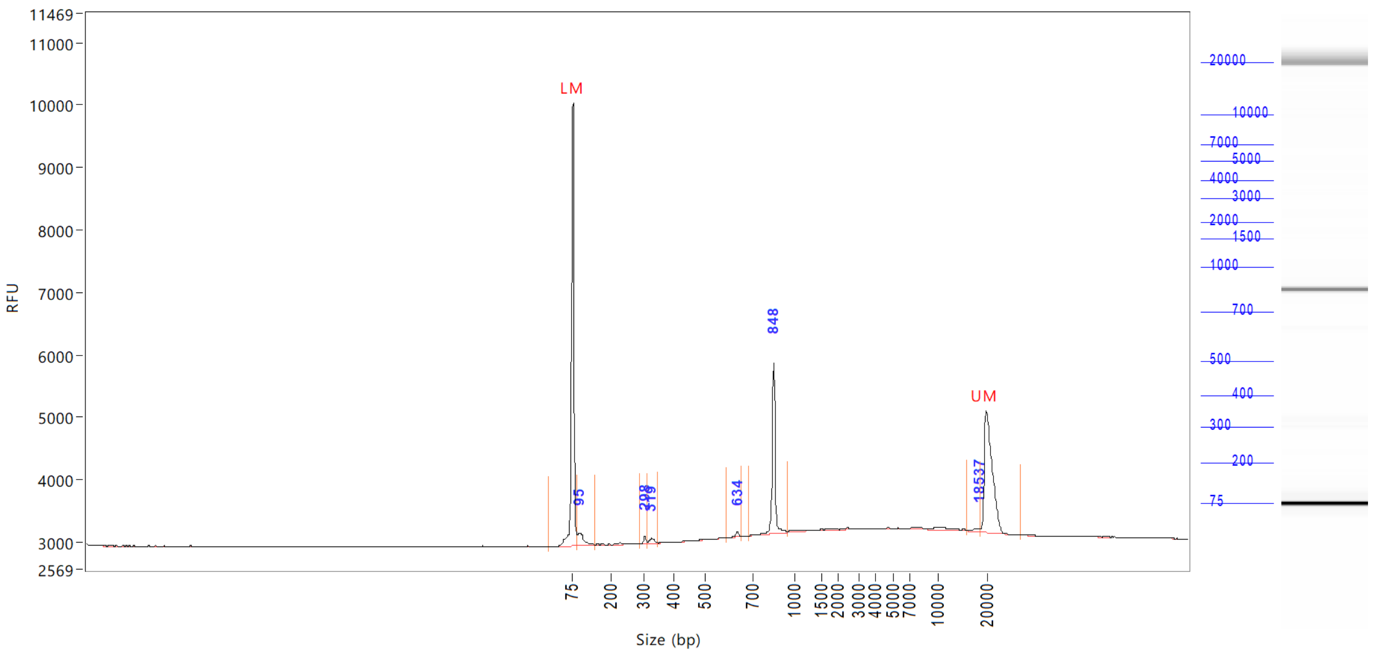

Peak	Size	Concentration	Relative concentration	Molarity	From	To	CV%	RFU
	(bp)	(ng/uL)	Percent	(nmole/L)	(bp)	(bp)		
1	75 (LM)	0.5000		11.5518	1	87	9.13	7104
2	95	0.4638	16.4	7.4666	87	145	12.28	207
3	298	0.0820	2.9	0.4525	287	307	1.26	112
4	319	0.1136	4.0	0.5837	307	345	2.35	82
5	634	0.0659	2.3	0.1727	590	649	1.32	75
6	848	2.0318	71.9	3.9529	682	952	2.59	2746
7	18537	0.0689	2.4	0.0064	16098	18903	4.19	50
8	20000 (UM)	0.2332		0.0187	18903	27012	4.22	1959

TIC:	2.8260	ng/uL
TIM:	12.6348	nmole/L
Total concentration:	3.4925	ng/uL

```
Sample peak width (sec): 5    Sample min peak height: 50    Sample baseline V to V?: Y    Sample baseline V to V points: 3
Sample filter: Binomial       Number of points for filter: 3  Sample start region (min): 0  Sample end region (min): 30
Marker peak width (sec): 5    Marker min peak height: 200   Marker baseline V to V?: Y    Marker baseline V to V points: 3
Lower marker selection: First peak > 500 RFU                Upper marker selection: Last peak > 200 RFU
Ladder size (bp)75, 200, 300, 400, 500, 700, 1000, 1500, 2000, 3000, 4000, 5000, 7000, 10000, 20000
Quantification using: Lower Marker          Final concentration (ng/uL): 0.5000          Dilution factor: 12.0
```

Sample: SampC3
Well location: C3
Created: Thursday, October 21, 2021 6:08:16 PM

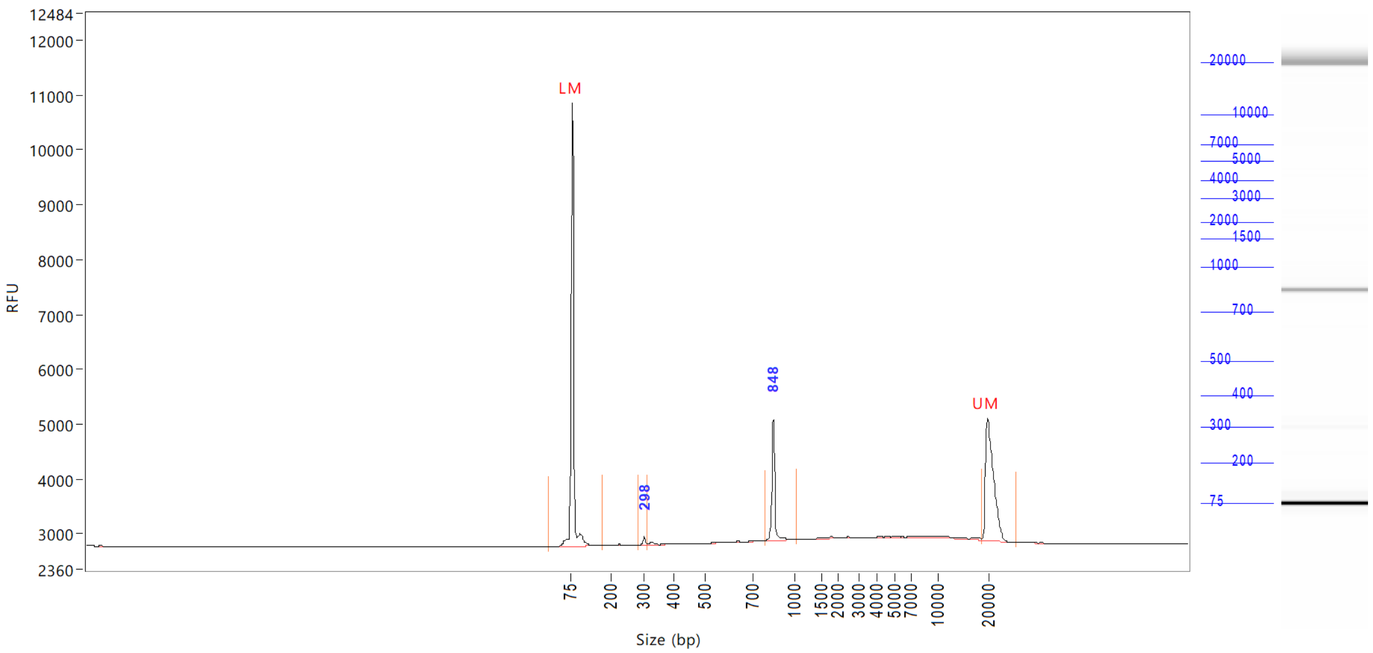

Peak	Size	Concentration	Relative concentration	Molarity	From	To	CV%	RFU
	(bp)	(ng/uL)	Percent	(nmole/L)	(bp)	(bp)		
1	75 (LM)	0.5000		10.9378	0	175	14.94	8086
2	298	0.0999	6.5	0.5554	280	307	1.31	160
3	848	1.4354	93.5	2.7858	788	1034	3.15	2208
4	20000 (UM)	0.2344		0.0188	18781	25803	4.26	2227

TIC:	1.5353	ng/uL
TIM:	3.3412	nmole/L
Total concentration:	2.0951	ng/uL

```
Sample peak width (sec): 5    Sample min peak height: 50    Sample baseline V to V?: Y    Sample baseline V to V points: 3
Sample filter: Binomial       Number of points for filter: 3  Sample start region (min): 0  Sample end region (min): 30
Marker peak width (sec): 5    Marker min peak height: 200   Marker baseline V to V?: Y    Marker baseline V to V points: 3
Lower marker selection: First peak > 500 RFU                Upper marker selection: Last peak > 200 RFU
Ladder size (bp)75, 200, 300, 400, 500, 700, 1000, 1500, 2000, 3000, 4000, 5000, 7000, 10000, 20000
Quantification using: Lower Marker          Final concentration (ng/uL): 0.5000          Dilution factor: 12.0
```

Sample: SampD3
Well location: D3
Created: Thursday, October 21, 2021 6:08:16 PM

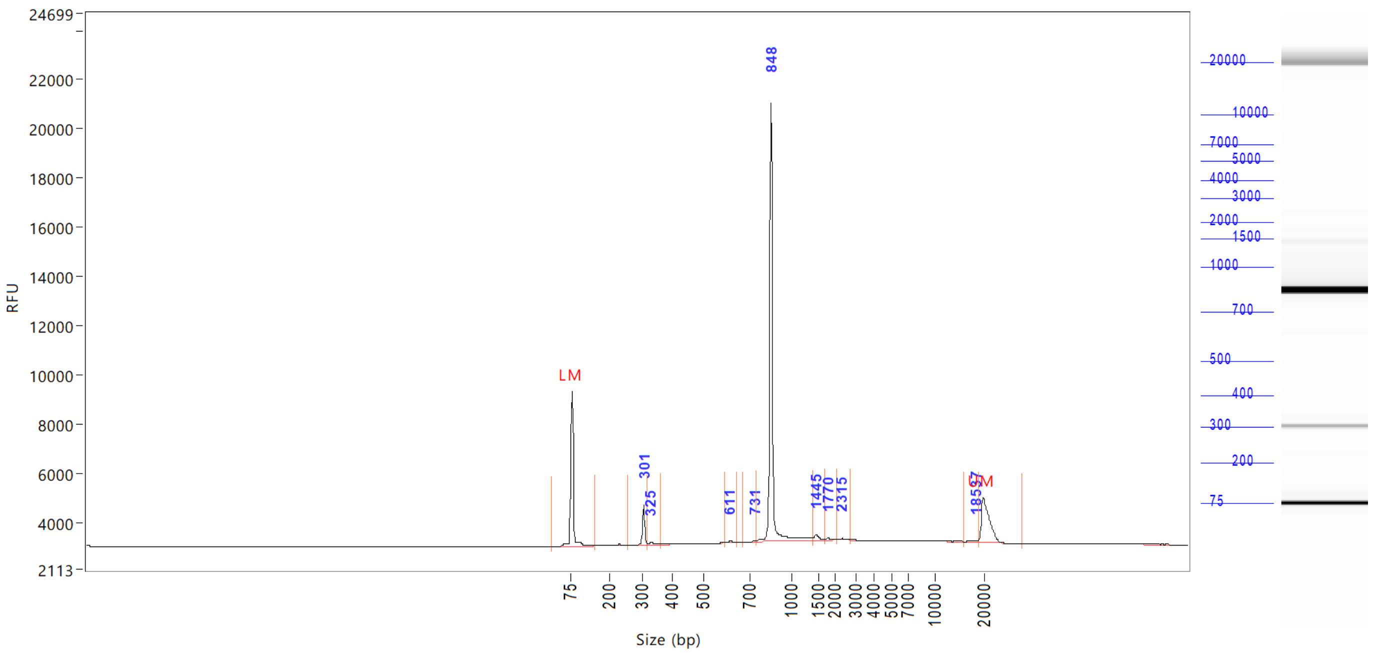

Peak	Size	Concentration	Relative concentration	Molarity	From	To	CV%	RFU
	(bp)	(ng/uL)	Percent	(nmole/L)	(bp)	(bp)		
1	75 (LM)	0.5000		10.9378	12	148	13.45	6300
2	301	1.2324	7.7	6.8025	253	311	1.71	1609
3	325	0.1815	1.1	0.9131	311	360	3.17	92
4	611	0.0709	0.4	0.1908	590	641	1.35	81
5	731	0.0443	0.3	0.1019	669	736	2.94	52
6	848	14.0176	87.2	26.9208	736	1378	7.87	17841
7	1445	0.2872	1.8	0.3234	1378	1674	4.38	235
8	1770	0.0879	0.5	0.0798	1674	2115	5.95	71
9	2315	0.0704	0.4	0.0495	2115	2743	5.06	66
10	18537	0.0900	0.6	0.0083	16098	19024	4.22	54
11	20000 (UM)	0.2373		0.0190	19024	27859	4.40	1812

TIC:	16.0821	ng/uL
TIM:	35.3901	nmole/L
Total concentration:	16.4567	ng/uL

```
Sample peak width (sec): 5    Sample min peak height: 50    Sample baseline V to V?: Y    Sample baseline V to V points: 3
Sample filter: Binomial       Number of points for filter: 3  Sample start region (min): 0  Sample end region (min): 30
Marker peak width (sec): 5    Marker min peak height: 200   Marker baseline V to V?: Y    Marker baseline V to V points: 3
Lower marker selection: First peak > 500 RFU                Upper marker selection: Last peak > 200 RFU
Ladder size (bp)75, 200, 300, 400, 500, 700, 1000, 1500, 2000, 3000, 4000, 5000, 7000, 10000, 20000
Quantification using: Lower Marker           Final concentration (ng/uL): 0.5000          Dilution factor: 12.0
```

Sample: SampE3
Well location: E3
Created: Thursday, October 21, 2021 6:08:16 PM

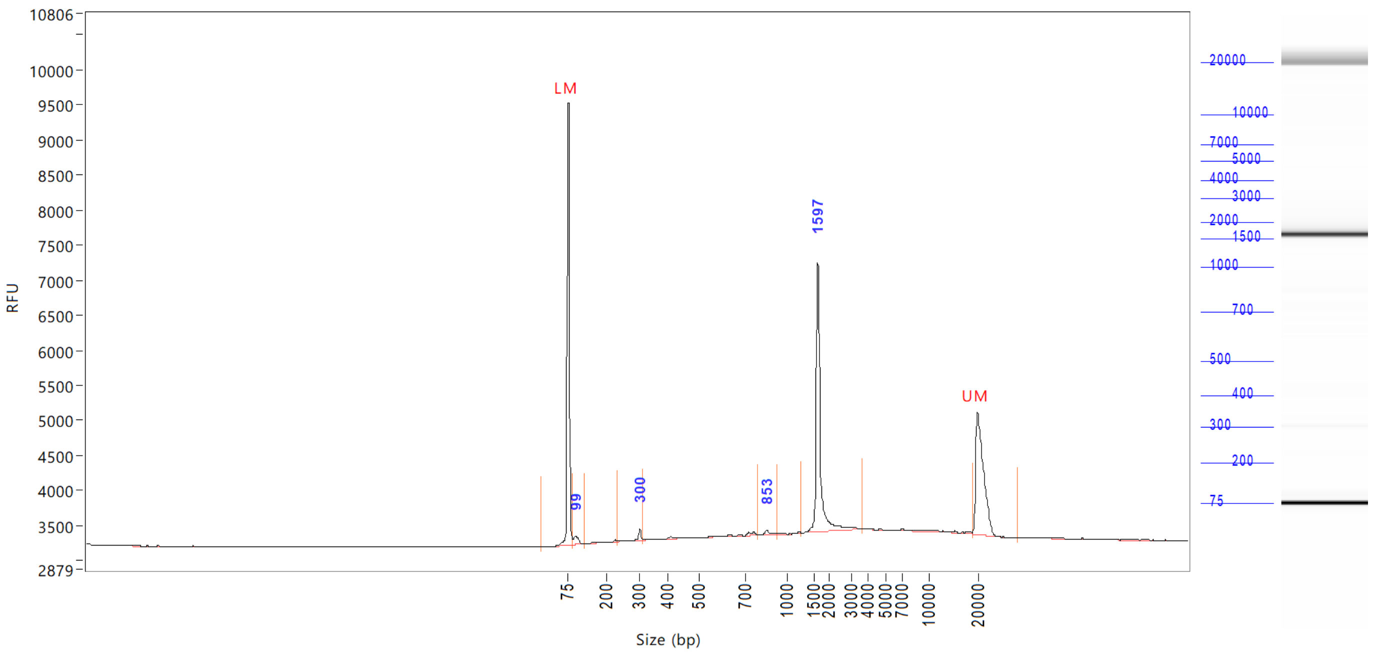

Peak	Size	Concentration	Relative concentration	Molarity	From	To	CV%	RFU
	(bp)	(ng/uL)	Percent	(nmole/L)	(bp)	(bp)		
1	75 (LM)	0.5000		11.2364	0	89	6.68	6316
2	99	0.2326	5.1	3.8189	89	129	7.48	117
3	300	0.1547	3.4	0.8683	235	313	5.30	156
4	853	0.0979	2.2	0.1880	788	926	3.51	61
5	1597	4.0337	89.3	3.9477	1267	3715	15.51	3845
6	20000 (UM)	0.2486		0.0198	19024	28463	4.28	1740

TIC:	4.5189	ng/uL
TIM:	8.8228	nmole/L
Total concentration:	5.1796	ng/uL

```
Sample peak width (sec): 5    Sample min peak height: 50    Sample baseline V to V?: Y    Sample baseline V to V points: 3
Sample filter: Binomial       Number of points for filter: 3  Sample start region (min): 0  Sample end region (min): 30
Marker peak width (sec): 5    Marker min peak height: 200   Marker baseline V to V?: Y    Marker baseline V to V points: 3
Lower marker selection: First peak > 500 RFU                Upper marker selection: Last peak > 200 RFU
Ladder size (bp)75, 200, 300, 400, 500, 700, 1000, 1500, 2000, 3000, 4000, 5000, 7000, 10000, 20000
Quantification using: Lower Marker          Final concentration (ng/uL): 0.5000          Dilution factor: 12.0
```

Sample: SampF3
Well location: F3
Created: Thursday, October 21, 2021 6:08:16 PM

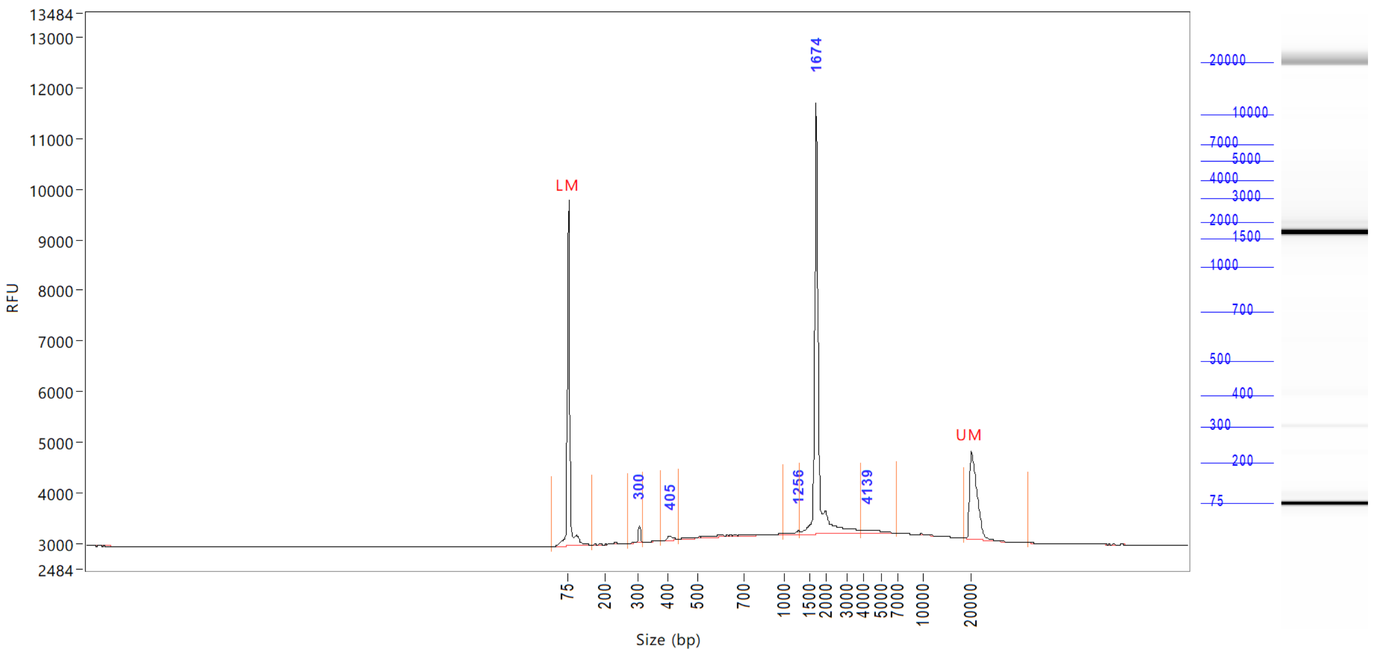

Peak	Size	Concentration	Relative concentration	Molarity	From	To	CV%	RFU
	(bp)	(ng/uL)	Percent	(nmole/L)	(bp)	(bp)		
1	75 (LM)	0.5000		11.0851	20	152	14.20	6841
2	300	0.2405	3.0	1.3278	266	315	1.58	336
3	405	0.1549	1.9	0.6231	376	435	2.68	80
4	1256	0.1105	1.4	0.1520	1000	1311	7.22	66
5	1674	7.3799	90.7	6.8287	1311	3857	20.77	8544
6	4139	0.2475	3.0	0.0896	3857	6926	12.04	65
7	20000 (UM)	0.2012		0.0160	18537	31849	4.98	1745

TIC:	8.1334	ng/uL
TIM:	9.0211	nmole/L
Total concentration:	8.6785	ng/uL

```
Sample peak width (sec): 5    Sample min peak height: 50    Sample baseline V to V?: Y    Sample baseline V to V points: 3
Sample filter: Binomial       Number of points for filter: 3  Sample start region (min): 0  Sample end region (min): 30
Marker peak width (sec): 5    Marker min peak height: 200   Marker baseline V to V?: Y    Marker baseline V to V points: 3
Lower marker selection: First peak > 500 RFU                Upper marker selection: Last peak > 200 RFU
Ladder size (bp)75, 200, 300, 400, 500, 700, 1000, 1500, 2000, 3000, 4000, 5000, 7000, 10000, 20000
Quantification using: Lower Marker          Final concentration (ng/uL): 0.5000          Dilution factor: 12.0
```

Sample: SampG3
Well location: G3
Created: Thursday, October 21, 2021 6:08:16 PM

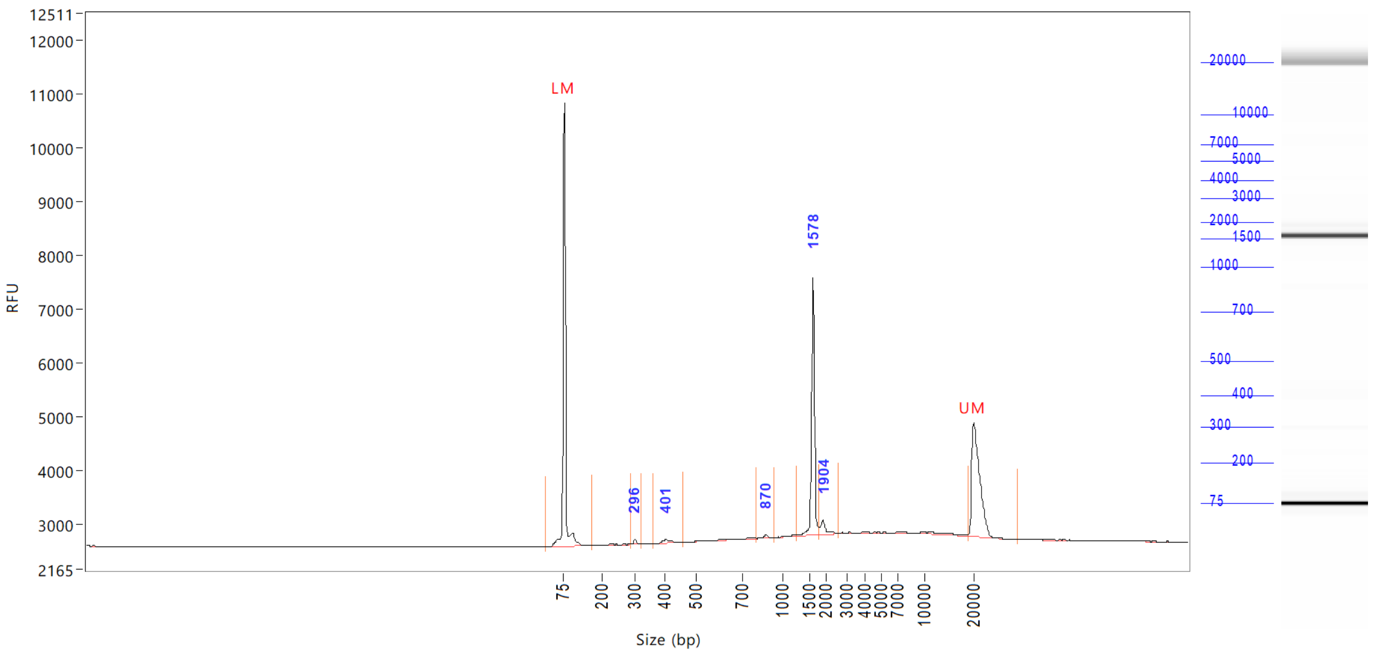

Peak	Size	Concentration	Relative concentration	Molarity	From	To	CV%	RFU
	(bp)	(ng/uL)	Percent	(nmole/L)	(bp)	(bp)		
1	75 (LM)	0.5000		11.0851	16	164	15.03	8265
2	296	0.0551	1.7	0.3075	287	321	1.36	95
3	401	0.1301	3.9	0.5258	360	460	4.18	59
4	870	0.0485	1.5	0.0921	805	935	2.34	60
5	1578	2.8006	84.7	2.9666	1234	1789	3.98	4801
6	1904	0.2719	8.2	0.2272	1789	2572	9.11	270
7	20000 (UM)	0.2179		0.0174	18903	29189	4.51	2117

TIC:	3.3062	ng/uL
TIM:	4.1192	nmole/L
Total concentration:	3.7869	ng/uL

Sample peak width (sec): 5   Sample min peak height: 50   Sample baseline V to V?: Y   Sample baseline V to V points: 3
Sample filter: Binomial   Number of points for filter: 3   Sample start region (min): 0   Sample end region (min): 30
Marker peak width (sec): 5   Marker min peak height: 200   Marker baseline V to V?: Y   Marker baseline V to V points: 3
Lower marker selection: First peak > 500 RFU   Upper marker selection: Last peak > 200 RFU
Ladder size (bp)75, 200, 300, 400, 500, 700, 1000, 1500, 2000, 3000, 4000, 5000, 7000, 10000, 20000
Quantification using: Lower Marker   Final concentration (ng/uL): 0.5000   Dilution factor: 12.0

Sample: SampH3
Well location: H3
Created: Thursday, October 21, 2021 6:08:16 PM

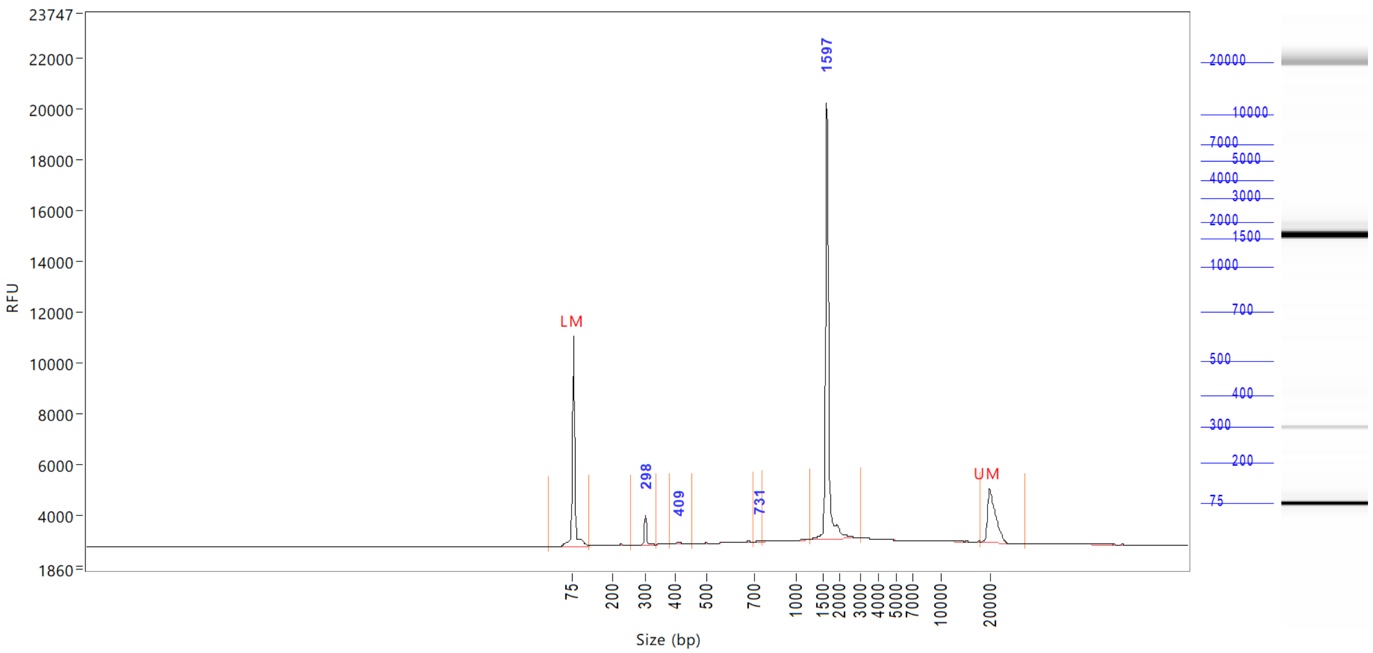

Peak	Size	Concentration	Relative concentration	Molarity	From	To	CV%	RFU
	(bp)	(ng/uL)	Percent	(nmole/L)	(bp)	(bp)		
1	75 (LM)	0.5000		11.0851	0	126	13.31	8259
2	298	0.6735	6.0	3.7553	257	333	2.05	1144
3	409	0.1319	1.2	0.5241	380	450	3.81	60
4	731	0.0405	0.4	0.0917	695	762	2.10	53
5	1597	10.4143	92.5	10.6083	1267	3001	8.47	17204
6	20000 (UM)	0.2118		0.0170	18293	27254	4.31	2120

TIC:	11.2602	ng/uL
TIM:	14.9794	nmole/L
Total concentration:	11.8033	ng/uL

Sample peak width (sec): 5   Sample min peak height: 50   Sample baseline V to V?: Y   Sample baseline V to V points: 3
Sample filter: Binomial   Number of points for filter: 3 Sample start region (min): 0 Sample end region (min): 30
Marker peak width (sec): 5   Marker min peak height: 200   Marker baseline V to V?: Y   Marker baseline V to V points: 3
Lower marker selection: First peak > 500 RFU   Upper marker selection: Last peak > 200 RFU
Ladder size (bp)75, 200, 300, 400, 500, 700, 1000, 1500, 2000, 3000, 4000, 5000, 7000, 10000, 20000
Quantification using: Lower Marker   Final concentration (ng/uL): 0.5000   Dilution factor: 12.0

Sample: SampA4
Well location: A4
Created: Thursday, October 21, 2021 6:08:16 PM

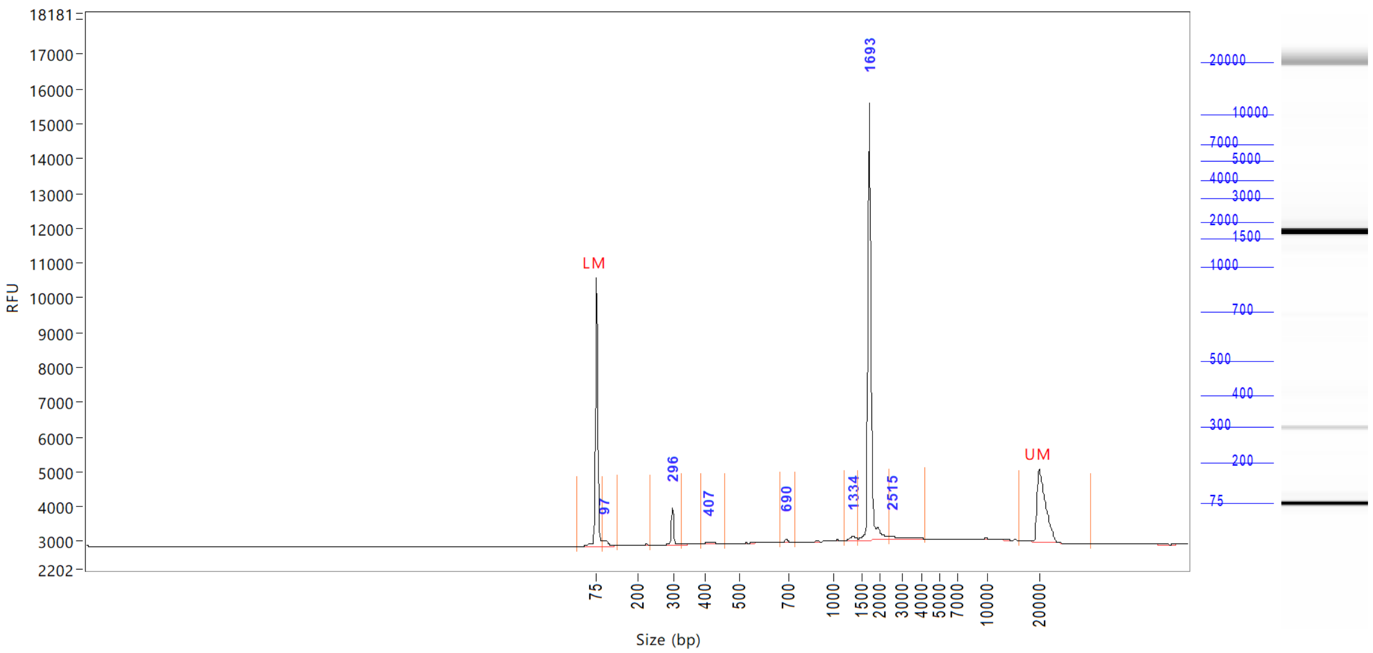

Peak	Size	Concentration	Relative concentration	Molarity	From	To	CV%	RFU
	(bp)	(ng/uL)	Percent	(nmole/L)	(bp)	(bp)		
1	75 (LM)	0.5000		11.3919	16	91	7.50	7714
2	97	0.3206	3.1	5.1110	91	137	10.00	167
3	296	0.7594	7.3	4.2631	232	323	3.17	1061
4	407	0.1555	1.5	0.6197	384	456	3.57	65
5	690	0.0724	0.7	0.1735	667	736	1.34	100
6	1334	0.1646	1.6	0.2050	1190	1422	4.09	119
7	1693	8.6662	83.2	8.4361	1422	2372	5.82	12586
8	2515	0.2792	2.7	0.1594	2372	4173	14.31	103
9	20000 (UM)	0.2606		0.0209	16220	29793	4.83	2097

TIC:	10.4179	ng/uL
TIM:	18.9678	nmole/L
Total concentration:	10.8774	ng/uL

Sample peak width (sec): 5 Sample min peak height: 50 Sample baseline V to V?: Y Sample baseline V to V points: 3
Sample filter: Binomial Number of points for filter: 3 Sample start region (min): 0 Sample end region (min): 30
Marker peak width (sec): 5 Marker min peak height: 200 Marker baseline V to V?: Y Marker baseline V to V points: 3
Lower marker selection: First peak > 500 RFU Upper marker selection: Last peak > 200 RFU
Ladder size (bp)75, 200, 300, 400, 500, 700, 1000, 1500, 2000, 3000, 4000, 5000, 7000, 10000, 20000
Quantification using: Lower Marker Final concentration (ng/uL): 0.5000 Dilution factor: 12.0

Sample: SampB4
Well location: B4
Created: Thursday, October 21, 2021 6:08:16 PM

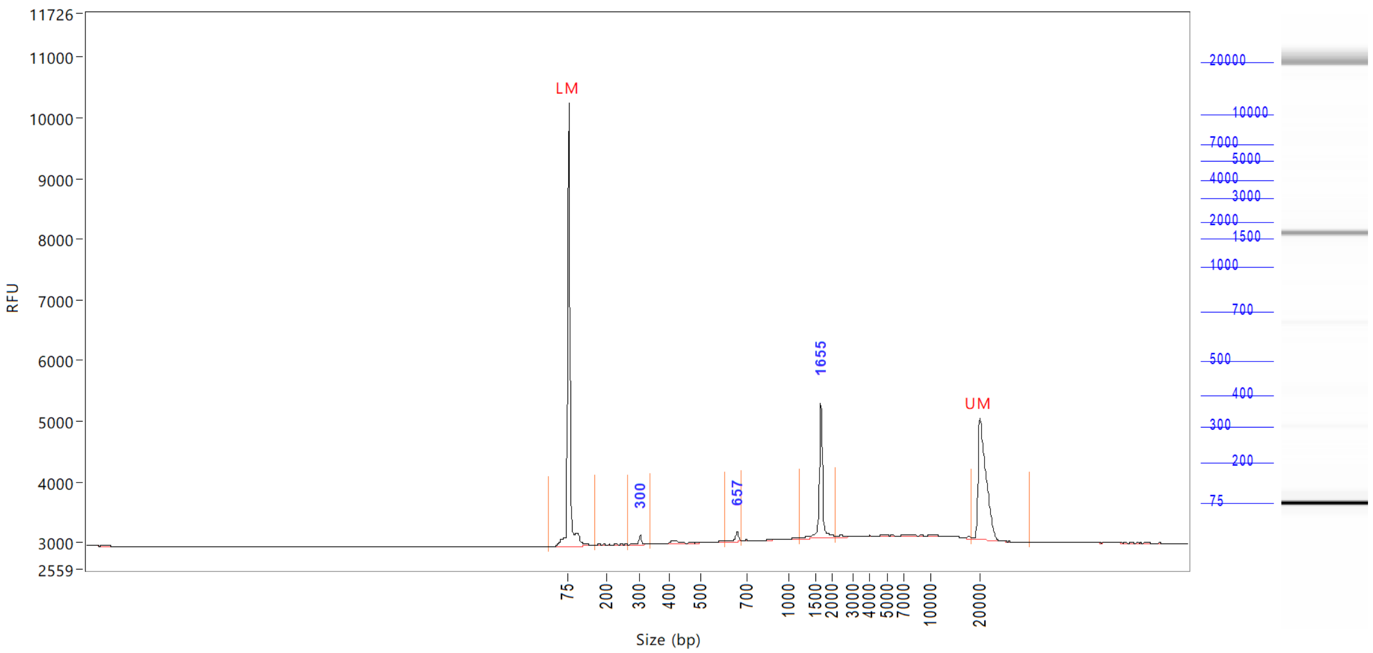

Peak	Size	Concentration	Relative concentration	Molarity	From	To	CV%	RFU
	(bp)	(ng/uL)	Percent	(nmole/L)	(bp)	(bp)		
1	75 (LM)	0.5000		10.9378	11	160	15.83	7324
2	300	0.1403	7.5	0.7771	264	331	3.53	163
3	657	0.1366	7.3	0.3470	603	674	2.00	162
4	1655	1.5943	85.2	1.6051	1212	2172	6.68	2233
5	20000 (UM)	0.2234		0.0178	18293	30277	4.90	1993

TIC:	1.8713	ng/uL
TIM:	2.7293	nmole/L
Total concentration:	2.5292	ng/uL

```
Sample peak width (sec): 5    Sample min peak height: 50    Sample baseline V to V?: Y    Sample baseline V to V points: 3
Sample filter: Binomial       Number of points for filter: 3  Sample start region (min): 0  Sample end region (min): 30
Marker peak width (sec): 5    Marker min peak height: 200   Marker baseline V to V?: Y    Marker baseline V to V points: 3
Lower marker selection: First peak > 500 RFU                Upper marker selection: Last peak > 200 RFU
Ladder size (bp)75, 200, 300, 400, 500, 700, 1000, 1500, 2000, 3000, 4000, 5000, 7000, 10000, 20000
Quantification using: Lower Marker          Final concentration (ng/uL): 0.5000          Dilution factor: 12.0
```

Sample: SampC4
Well location: C4
Created: Thursday, October 21, 2021 6:08:16 PM

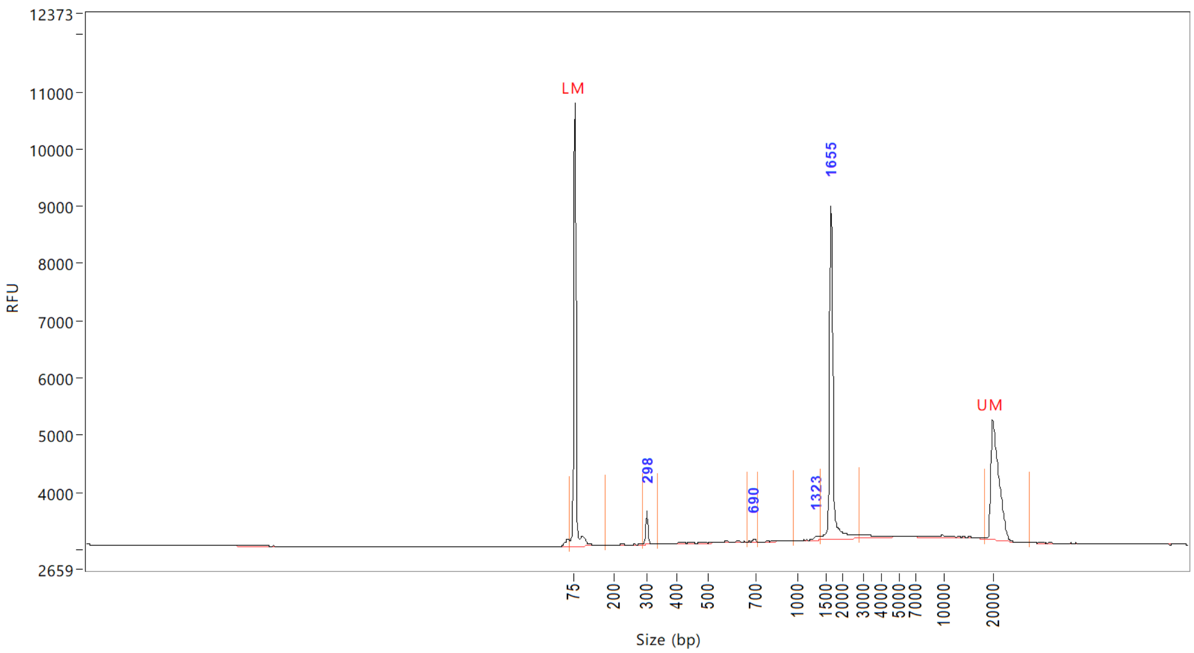

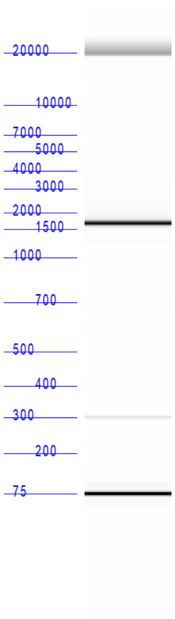

Peak	Size	Concentration	Relative concentration	Molarity	From	To	CV%	RFU
	(bp)	(ng/uL)	Percent	(nmole/L)	(bp)	(bp)		
1	75 (LM)	0.5000		10.9378	60	171	13.09	7759
2	298	0.3883	8.2	2.1504	286	331	1.51	597
3	690	0.0444	0.9	0.1066	662	710	1.29	56
4	1323	0.1160	2.4	0.1507	961	1400	9.17	62
5	1655	4.1905	88.4	4.0816	1400	2829	11.18	5829
6	20000 (UM)	0.2463		0.0197	18415	27496	4.53	2101

TIC:	4.7391	ng/uL
TIM:	6.4894	nmole/L
Total concentration:	5.3654	ng/uL

Sample peak width (sec): 5   Sample min peak height: 50   Sample baseline V to V?: Y   Sample baseline V to V points: 3
Sample filter: Binomial   Number of points for filter: 3 Sample start region (min): 0 Sample end region (min): 30
Marker peak width (sec): 5   Marker min peak height: 200   Marker baseline V to V?: Y   Marker baseline V to V points: 3
Lower marker selection: First peak > 500 RFU   Upper marker selection: Last peak > 200 RFU
Ladder size (bp)75, 200, 300, 400, 500, 700, 1000, 1500, 2000, 3000, 4000, 5000, 7000, 10000, 20000
Quantification using: Lower Marker   Final concentration (ng/uL): 0.5000   Dilution factor: 12.0

Sample: SampD4
Well location: D4
Created: Thursday, October 21, 2021 6:08:16 PM

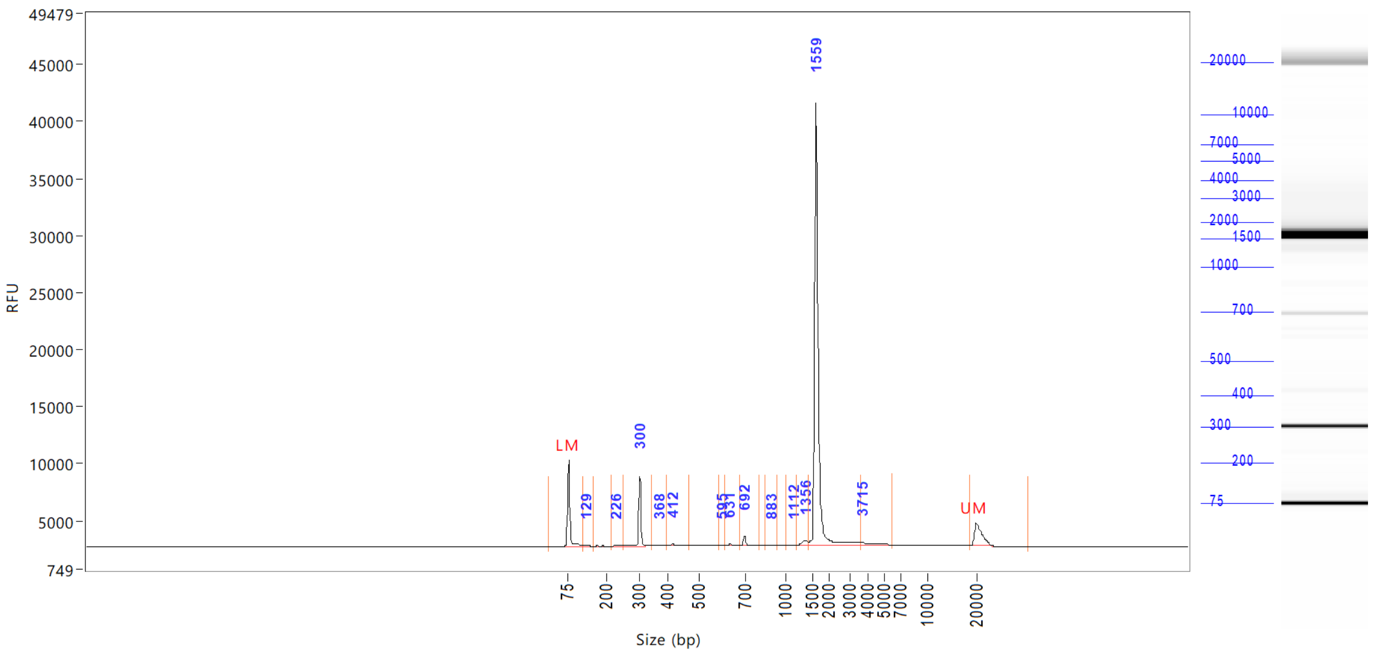

Peak	Size	Concentration	Relative concentration	Molarity	From	To	CV%	RFU
	(bp)	(ng/uL)	Percent	(nmole/L)	(bp)	(bp)		
1	75 (LM)	0.5000		10.9378	9	122	11.61	7567
2	129	0.1269	0.4	1.5446	122	158	7.34	73
3	226	0.0790	0.2	0.5645	212	250	4.01	62
4	300	3.9777	11.0	21.9564	250	343	2.20	6134
5	368	0.0971	0.3	0.4378	343	394	3.61	51
6	412	0.2576	0.7	1.0090	394	466	3.71	197
7	595	0.0521	0.1	0.1439	585	611	0.90	90
8	631	0.1449	0.4	0.3743	611	677	2.47	146
9	692	0.5269	1.5	1.2495	677	796	1.98	855
10	883	0.0594	0.2	0.1105	840	931	1.65	72
11	1112	0.0913	0.3	0.1353	1001	1178	3.67	80
12	1356	0.6115	1.7	0.7603	1178	1411	4.23	401
13	1559	29.8183	82.3	30.0943	1411	3608	16.63	38853
14	3715	0.3813	1.1	0.1539	3608	5889	9.54	186
15	20000 (UM)	0.2147		0.0171	18903	30881	4.98	1944

TIC:	36.2240	ng/uL
TIM:	58.5344	nmole/L
Total concentration:	36.4201	ng/uL

```
Sample peak width (sec): 5    Sample min peak height: 50    Sample baseline V to V?: Y    Sample baseline V to V points: 3
Sample filter: Binomial       Number of points for filter: 3  Sample start region (min): 0  Sample end region (min): 30
Marker peak width (sec): 5    Marker min peak height: 200   Marker baseline V to V?: Y    Marker baseline V to V points: 3
Lower marker selection: First peak > 500 RFU                Upper marker selection: Last peak > 200 RFU
Ladder size (bp)75, 200, 300, 400, 500, 700, 1000, 1500, 2000, 3000, 4000, 5000, 7000, 10000, 20000
Quantification using: Lower Marker          Final concentration (ng/uL): 0.5000          Dilution factor: 12.0
```

Sample: SampE4
Well location: E4
Created: Thursday, October 21, 2021 6:08:16 PM

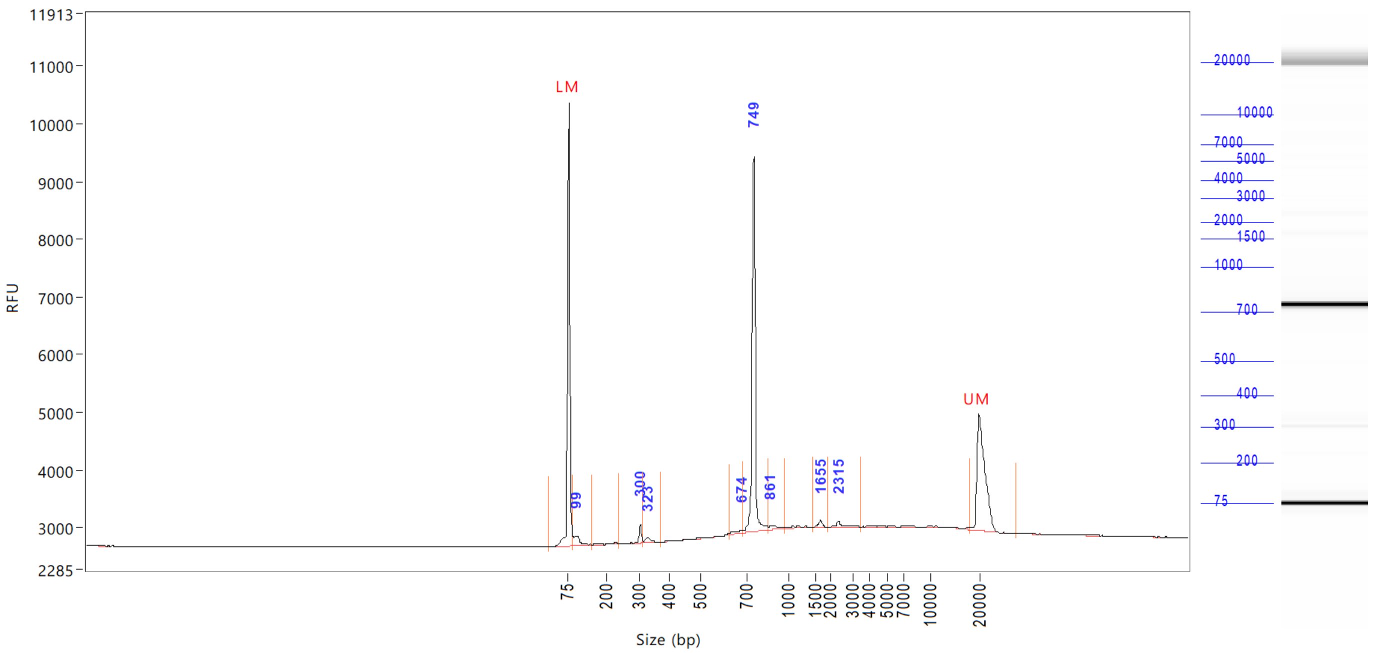

Peak	Size	Concentration	Relative concentration	Molarity	From	To	CV%	RFU
	(bp)	(ng/uL)	Percent	(nmole/L)	(bp)	(bp)		
1	75 (LM)	0.5000		11.2364	12	89	8.51	7687
2	99	0.3554	6.0	5.6117	89	150	12.25	176
3	300	0.2443	4.1	1.3620	233	309	4.48	335
4	323	0.1469	2.5	0.7391	309	368	3.54	88
5	674	0.1028	1.7	0.2570	626	685	2.82	56
6	749	4.7126	79.6	10.4387	685	857	2.67	6487
7	861	0.1467	2.5	0.2660	857	974	3.76	57
8	1655	0.1004	1.7	0.1016	1478	1923	4.38	119
9	2315	0.1143	1.9	0.0765	1923	3465	12.74	104
10	20000 (UM)	0.2381		0.0191	18049	27617	4.49	2024

TIC:	5.9233	ng/uL
TIM:	18.8526	nmole/L
Total concentration:	6.3463	ng/uL

```
Sample peak width (sec): 5     Sample min peak height: 50    Sample baseline V to V?: Y    Sample baseline V to V points: 3
Sample filter: Binomial        Number of points for filter: 3  Sample start region (min): 0  Sample end region (min): 30
Marker peak width (sec): 5     Marker min peak height: 200   Marker baseline V to V?: Y    Marker baseline V to V points: 3
Lower marker selection: First peak > 500 RFU                 Upper marker selection: Last peak > 200 RFU
Ladder size (bp)75, 200, 300, 400, 500, 700, 1000, 1500, 2000, 3000, 4000, 5000, 7000, 10000, 20000
Quantification using: Lower Marker          Final concentration (ng/uL): 0.5000          Dilution factor: 12.0
```

Sample: SampF4
Well location: F4
Created: Thursday, October 21, 2021 6:08:16 PM

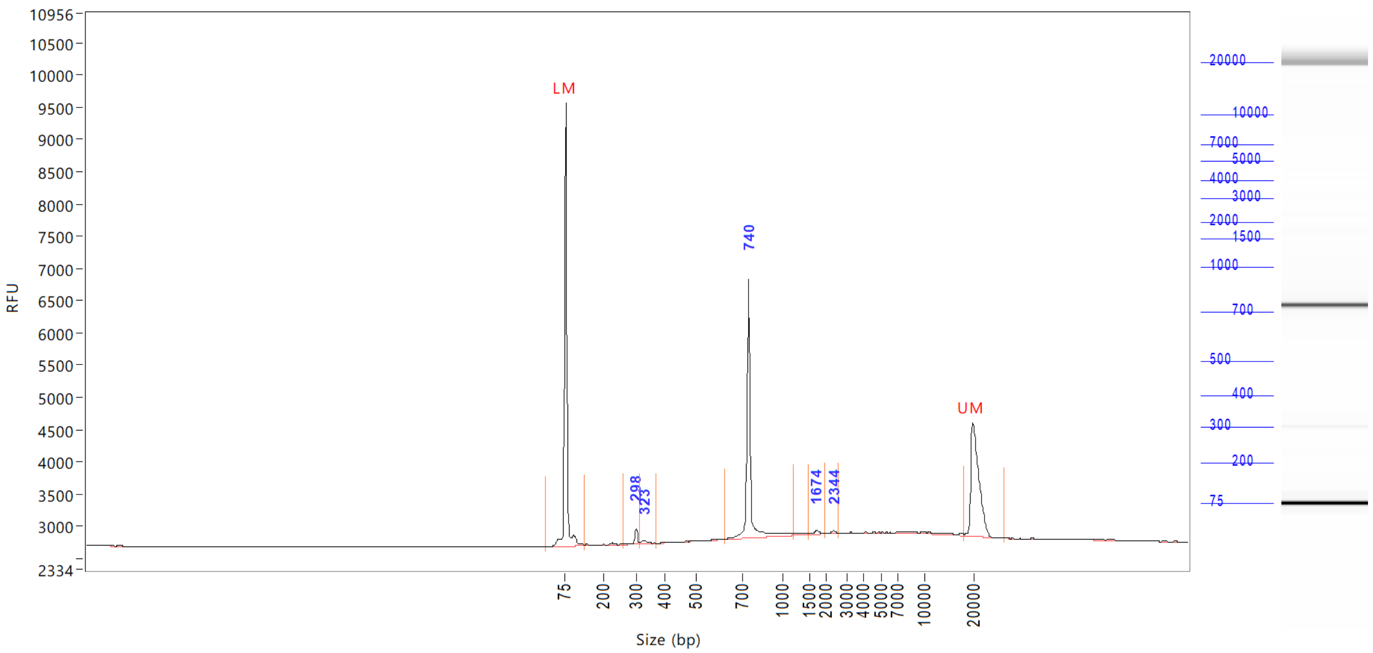

Peak	Size	Concentration	Relative concentration	Molarity	From	To	CV%	RFU
	(bp)	(ng/uL)	Percent	(nmole/L)	(bp)	(bp)		
1	75 (LM)	0.5000		11.0851	12	137	12.99	6888
2	298	0.1963	4.8	1.0908	262	309	2.59	246
3	323	0.0991	2.4	0.4983	309	368	3.85	55
4	740	3.6553	89.5	7.9364	621	1201	9.89	4018
5	1674	0.0819	2.0	0.0801	1478	1981	6.96	58
6	2344	0.0499	1.2	0.0359	1981	2572	5.72	52
7	20000 (UM)	0.2199		0.0176	18171	26287	4.30	1772

TIC:	4.0824	ng/uL
TIM:	9.6415	nmole/L
Total concentration:	4.6537	ng/uL

Sample peak width (sec): 5   Sample min peak height: 50   Sample baseline V to V?: Y   Sample baseline V to V points: 3
Sample filter: Binomial   Number of points for filter: 3   Sample start region (min): 0   Sample end region (min): 30
Marker peak width (sec): 5   Marker min peak height: 200   Marker baseline V to V?: Y   Marker baseline V to V points: 3
Lower marker selection: First peak > 500 RFU   Upper marker selection: Last peak > 200 RFU
Ladder size (bp)75, 200, 300, 400, 500, 700, 1000, 1500, 2000, 3000, 4000, 5000, 7000, 10000, 20000
Quantification using: Lower Marker   Final concentration (ng/uL): 0.5000   Dilution factor: 12.0

Sample: SampG4
Well location: G4
Created: Thursday, October 21, 2021 6:08:16 PM

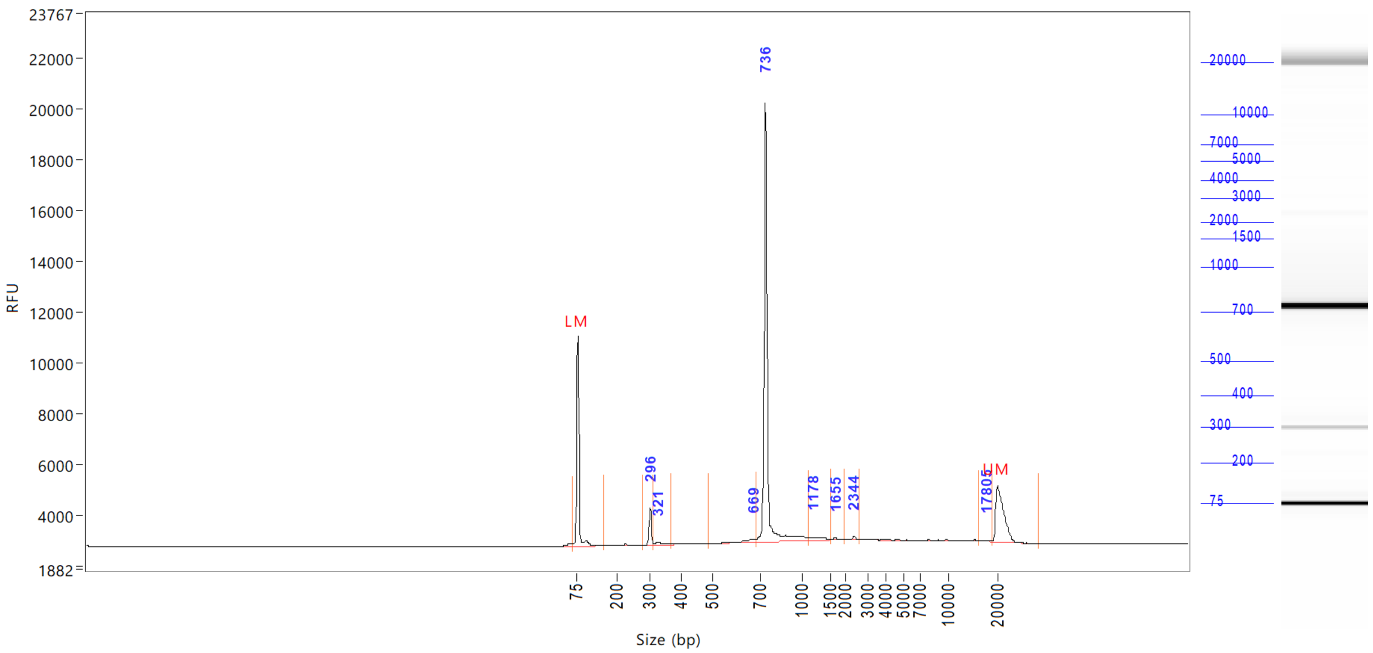

Peak	Size	Concentration	Relative concentration	Molarity	From	To	CV%	RFU
	(bp)	(ng/uL)	Percent	(nmole/L)	(bp)	(bp)		
1	75 (LM)	0.5000		10.9378	62	160	12.11	8266
2	296	0.8621	6.2	4.8072	275	307	1.18	1442
3	321	0.2005	1.4	1.0055	307	368	4.16	115
4	669	0.3223	2.3	0.8314	489	682	6.26	130
5	736	11.9767	85.6	26.2465	682	1101	7.98	17311
6	1178	0.3796	2.7	0.4908	1101	1539	9.41	135
7	1655	0.0783	0.6	0.0770	1539	1962	5.86	67
8	2344	0.0912	0.7	0.0651	1962	2629	4.48	145
9	17805	0.0785	0.6	0.0073	16220	19024	4.45	54
10	20000 (UM)	0.2386		0.0191	19024	28100	4.30	2193

TIC:	13.9891	ng/uL
TIM:	33.5308	nmole/L
Total concentration:	14.2634	ng/uL

```
Sample peak width (sec): 5    Sample min peak height: 50    Sample baseline V to V?: Y    Sample baseline V to V points: 3
Sample filter: Binomial       Number of points for filter: 3  Sample start region (min): 0  Sample end region (min): 30
Marker peak width (sec): 5    Marker min peak height: 200   Marker baseline V to V?: Y    Marker baseline V to V points: 3
Lower marker selection: First peak > 500 RFU                Upper marker selection: Last peak > 200 RFU
Ladder size (bp)75, 200, 300, 400, 500, 700, 1000, 1500, 2000, 3000, 4000, 5000, 7000, 10000, 20000
Quantification using: Lower Marker          Final concentration (ng/uL): 0.5000          Dilution factor: 12.0
```

Sample: SampH4
Well location: H4
Created: Thursday, October 21, 2021 6:08:16 PM

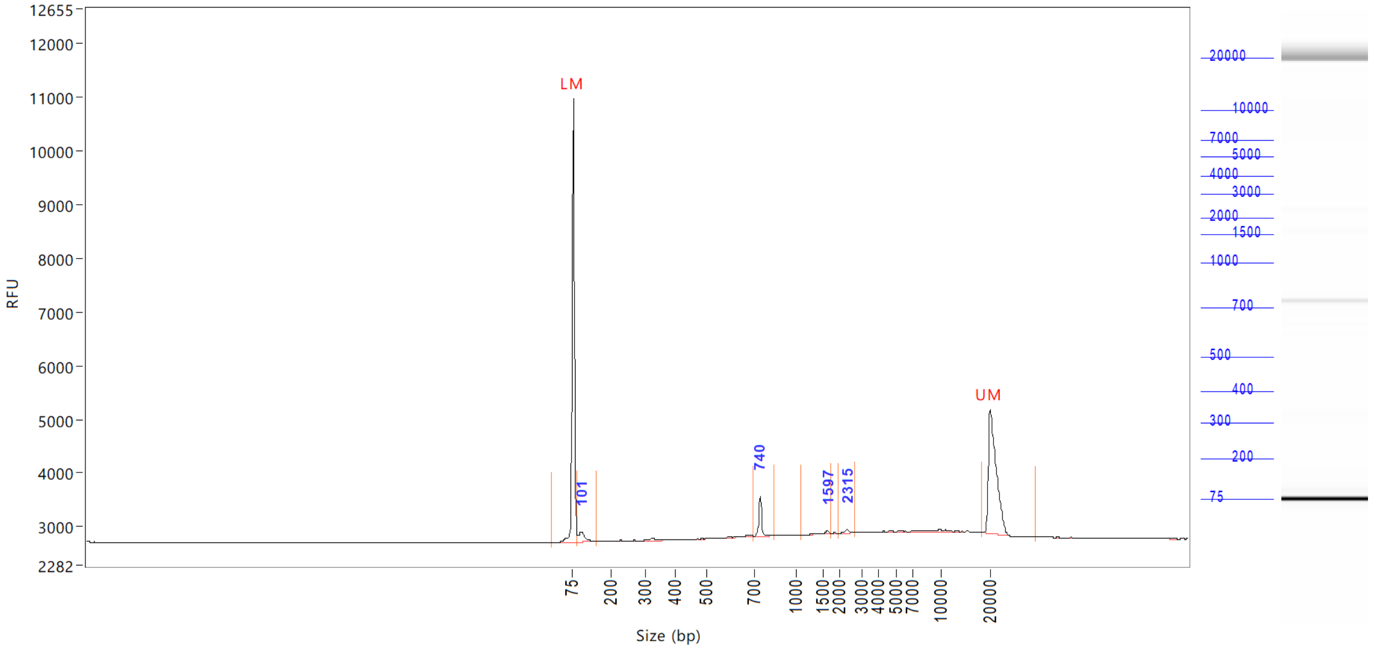

Peak	Size	Concentration	Relative concentration	Molarity	From	To	CV%	RFU
	(bp)	(ng/uL)	Percent	(nmole/L)	(bp)	(bp)		
1	75 (LM)	0.5000		11.2364	11	89	6.69	8285
2	101	0.3753	38.1	5.8142	89	152	13.62	190
3	740	0.4885	49.6	1.0894	697	844	2.16	743
4	1597	0.0643	6.5	0.0698	1079	1731	9.04	66
5	2315	0.0563	5.7	0.0405	1981	2686	5.66	72
6	20000 (UM)	0.2520		0.0201	18293	29309	4.48	2321

TIC:	0.9844	ng/uL
TIM:	7.0139	nmole/L
Total concentration:	1.5275	ng/uL

Sample peak width (sec): 5 Sample min peak height: 50 Sample baseline V to V?: Y Sample baseline V to V points: 3
Sample filter: Binomial Number of points for filter: 3 Sample start region (min): 0 Sample end region (min): 30
Marker peak width (sec): 5 Marker min peak height: 200 Marker baseline V to V?: Y Marker baseline V to V points: 3
Lower marker selection: First peak > 500 RFU Upper marker selection: Last peak > 200 RFU
Ladder size (bp)75, 200, 300, 400, 500, 700, 1000, 1500, 2000, 3000, 4000, 5000, 7000, 10000, 20000
Quantification using: Lower Marker Final concentration (ng/uL): 0.5000 Dilution factor: 12.0

Sample: SampA5
Well location: A5
Created: Thursday, October 21, 2021 6:08:16 PM

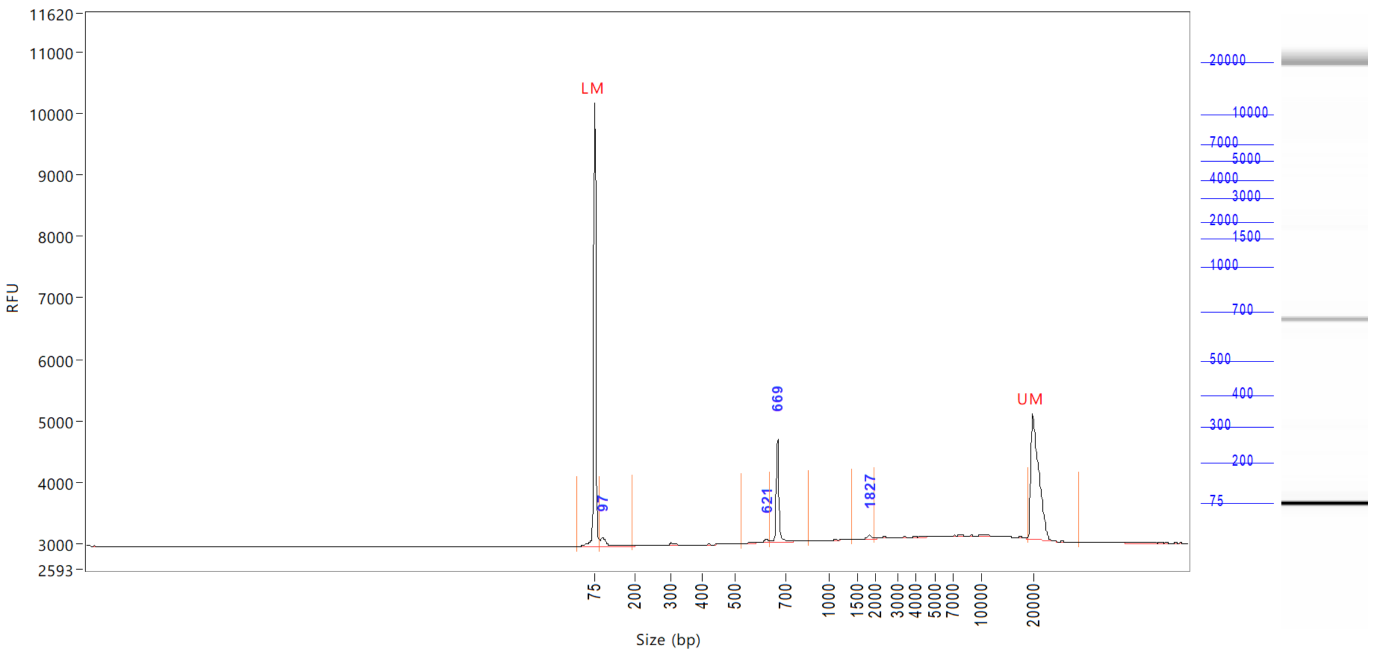

Peak	Size	Concentration	Relative concentration	Molarity	From	To	CV%	RFU
	(bp)	(ng/uL)	Percent	(nmole/L)	(bp)	(bp)		
1	75 (LM)	0.5000		11.3919	24	89	6.83	7213
2	97	0.3376	18.9	5.0409	89	189	20.04	136
3	621	0.0958	5.4	0.2611	521	636	4.48	54
4	669	1.2804	71.7	3.1452	636	857	2.85	1668
5	1827	0.0708	4.0	0.0653	1400	1962	6.11	69
6	20000 (UM)	0.2589		0.0208	18903	28826	4.44	2027

TIC:	1.7847	ng/uL
TIM:	8.5126	nmole/L
Total concentration:	2.2803	ng/uL

Sample peak width (sec): 5   Sample min peak height: 50   Sample baseline V to V?: Y   Sample baseline V to V points: 3
Sample filter: Binomial   Number of points for filter: 3   Sample start region (min): 0   Sample end region (min): 30
Marker peak width (sec): 5   Marker min peak height: 200   Marker baseline V to V?: Y   Marker baseline V to V points: 3
Lower marker selection: First peak > 500 RFU   Upper marker selection: Last peak > 200 RFU
Ladder size (bp)75, 200, 300, 400, 500, 700, 1000, 1500, 2000, 3000, 4000, 5000, 7000, 10000, 20000
Quantification using: Lower Marker   Final concentration (ng/uL): 0.5000   Dilution factor: 12.0

Sample: SampB5
Well location: B5
Created: Thursday, October 21, 2021 6:08:16 PM

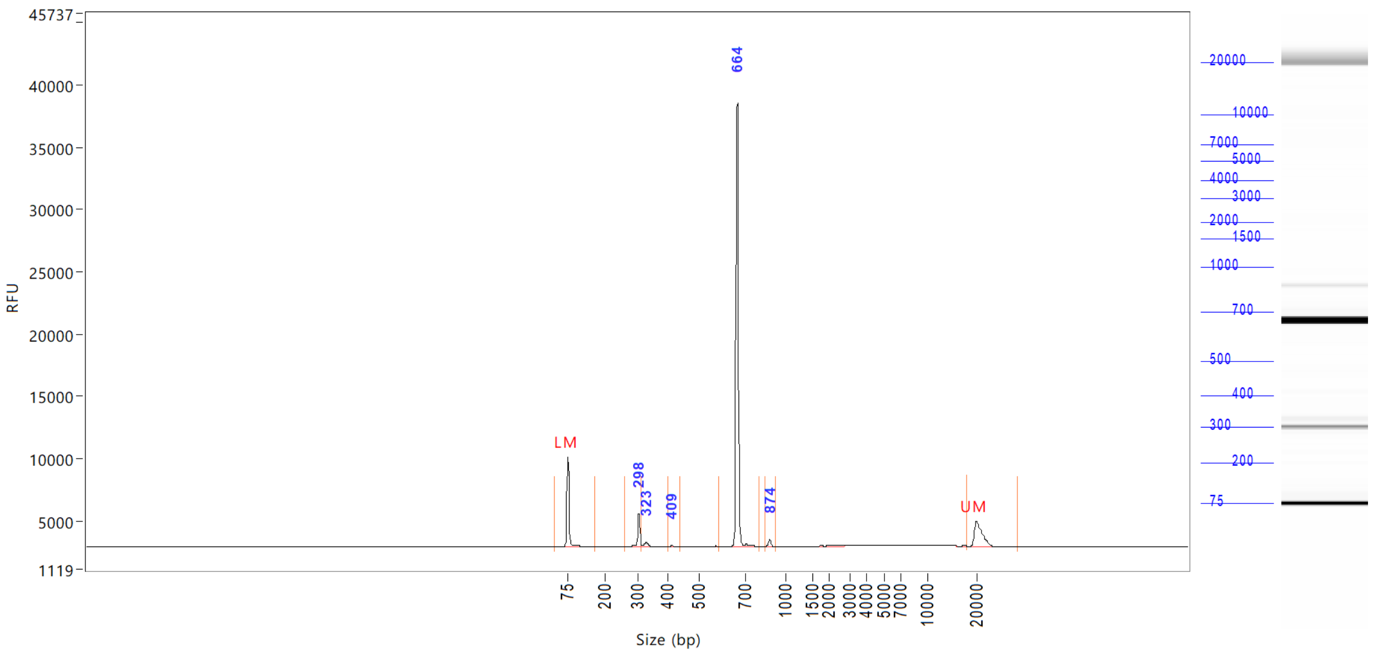

Peak	Size	Concentration	Relative concentration	Molarity	From	To	CV%	RFU
	(bp)	(ng/uL)	Percent	(nmole/L)	(bp)	(bp)		
1	75 (LM)	0.5000		10.9378	31	162	12.23	7187
2	298	1.9517	7.4	10.8460	255	309	1.95	2605
3	323	0.4979	1.9	2.4823	309	398	5.88	332
4	409	0.0729	0.3	0.2926	398	437	1.75	74
5	664	23.3635	88.9	58.1690	583	796	1.29	35655
6	874	0.3853	1.5	0.7264	844	926	1.06	593
7	20000 (UM)	0.2475		0.0198	18171	28584	4.32	1992

TIC:	26.2714	ng/uL
TIM:	72.5163	nmole/L
Total concentration:	26.8055	ng/uL

```
Sample peak width (sec): 5    Sample min peak height: 50    Sample baseline V to V?: Y    Sample baseline V to V points: 3
Sample filter: Binomial       Number of points for filter: 3  Sample start region (min): 0  Sample end region (min): 30
Marker peak width (sec): 5    Marker min peak height: 200   Marker baseline V to V?: Y    Marker baseline V to V points: 3
Lower marker selection: First peak > 500 RFU                Upper marker selection: Last peak > 200 RFU
Ladder size (bp)75, 200, 300, 400, 500, 700, 1000, 1500, 2000, 3000, 4000, 5000, 7000, 10000, 20000
Quantification using: Lower Marker          Final concentration (ng/uL): 0.5000          Dilution factor: 12.0
```

Sample: SampC5
Well location: C5
Created: Thursday, October 21, 2021 6:08:16 PM

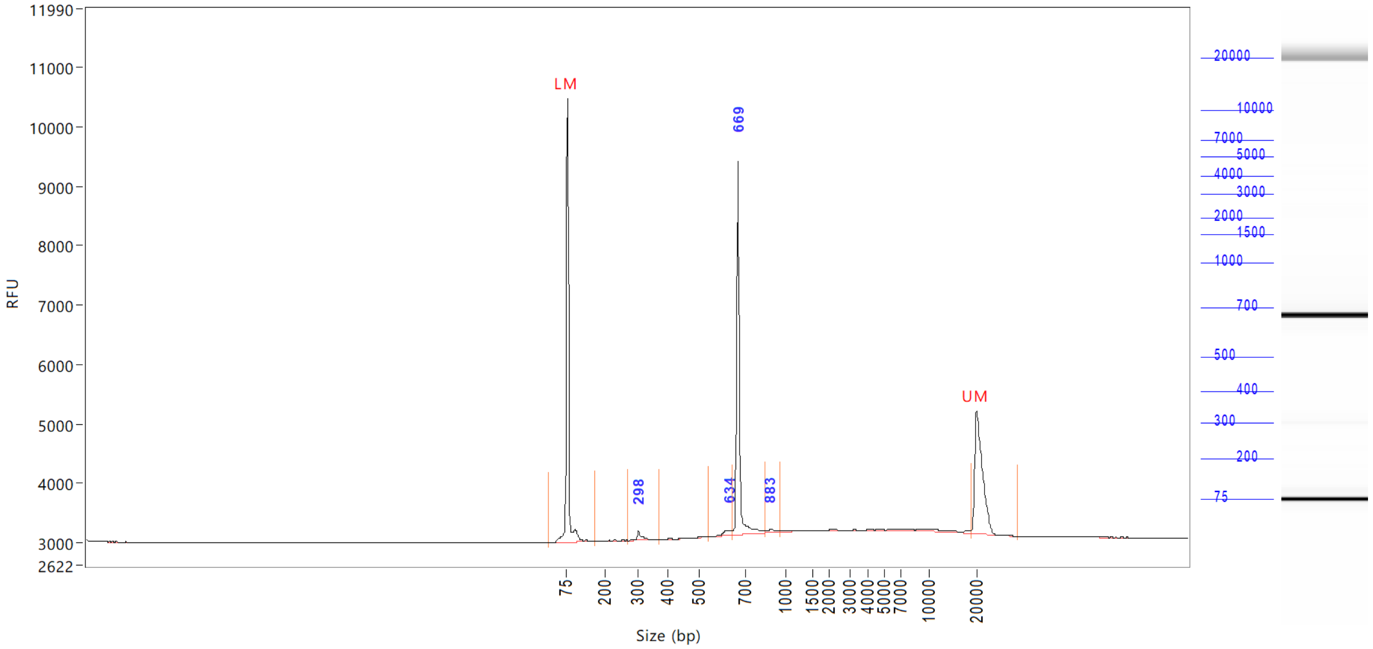

Peak	Size	Concentration	Relative concentration	Molarity	From	To	CV%	RFU
	(bp)	(ng/uL)	Percent	(nmole/L)	(bp)	(bp)		
1	75 (LM)	0.5000		10.9378	14	164	14.32	7482
2	298	0.2290	4.7	1.2310	268	370	5.44	167
3	634	0.1924	4.0	0.5133	539	644	3.01	88
4	669	4.2953	88.7	10.5192	644	840	3.71	6293
5	883	0.1233	2.5	0.2282	840	957	3.63	56
6	20000 (UM)	0.2363		0.0189	18903	28342	4.38	2058

TIC:	4.8400	ng/uL
TIM:	12.4916	nmole/L
Total concentration:	5.4350	ng/uL

```
Sample peak width (sec): 5    Sample min peak height: 50    Sample baseline V to V?: Y    Sample baseline V to V points: 3
Sample filter: Binomial       Number of points for filter: 3  Sample start region (min): 0  Sample end region (min): 30
Marker peak width (sec): 5    Marker min peak height: 200   Marker baseline V to V?: Y    Marker baseline V to V points: 3
Lower marker selection: First peak > 500 RFU                Upper marker selection: Last peak > 200 RFU
Ladder size (bp)75, 200, 300, 400, 500, 700, 1000, 1500, 2000, 3000, 4000, 5000, 7000, 10000, 20000
Quantification using: Lower Marker          Final concentration (ng/uL): 0.5000          Dilution factor: 12.0
```

Sample: SampD5
Well location: D5
Created: Thursday, October 21, 2021 6:08:16 PM

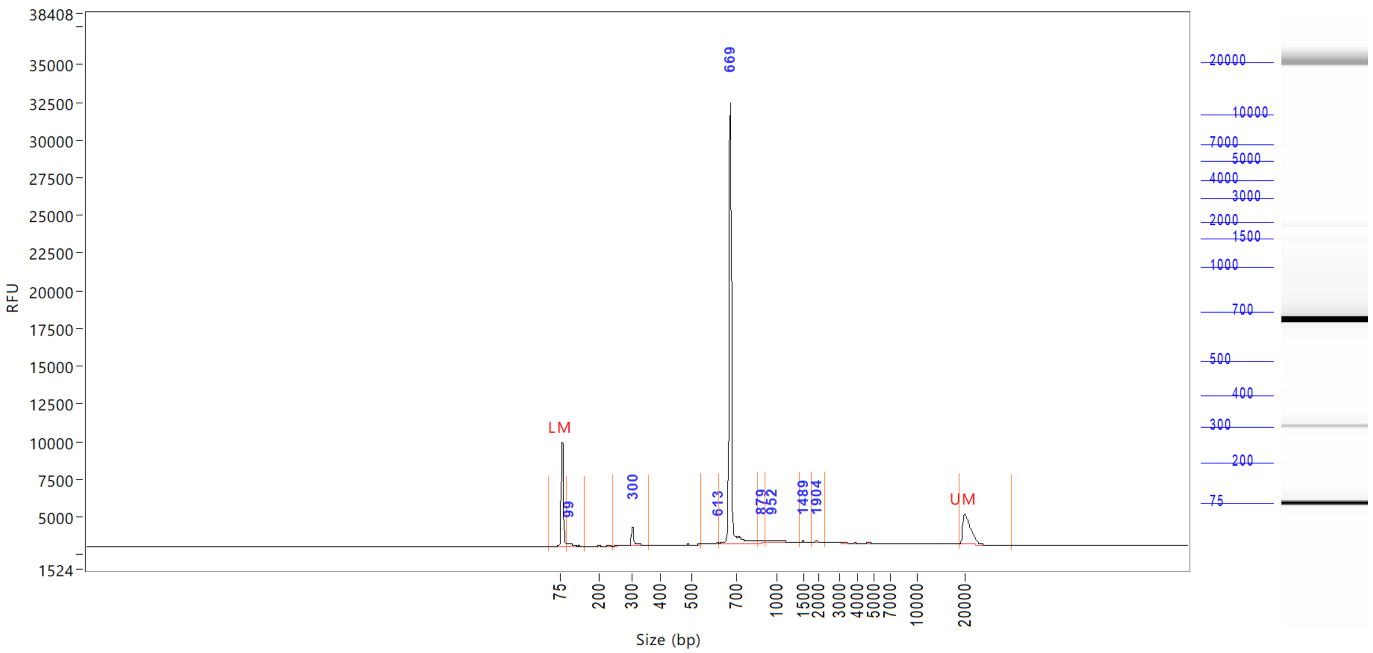

Peak	Size	Concentration	Relative concentration	Molarity	From	To	CV%	RFU
	(bp)	(ng/uL)	Percent	(nmole/L)	(bp)	(bp)		
1	75 (LM)	0.5000		11.0851	31	89	5.87	6955
2	99	0.4492	1.9	7.0939	89	150	12.28	203
3	300	1.3198	5.5	7.1887	241	358	3.99	1269
4	613	0.1518	0.6	0.4156	544	623	2.67	79
5	669	20.9254	87.0	51.2461	623	857	3.64	29297
6	879	0.2296	1.0	0.4270	857	913	1.96	134
7	952	0.7303	3.0	1.1234	913	1422	13.23	153
8	1489	0.1114	0.5	0.1235	1422	1750	3.30	117
9	1904	0.1366	0.6	0.1183	1750	2258	3.77	161
10	20000 (UM)	0.2646		0.0211	19024	29793	4.48	2047

TIC:	24.0540	ng/uL
TIM:	67.7365	nmole/L
Total concentration:	24.5856	ng/uL

```
Sample peak width (sec): 5     Sample min peak height: 50     Sample baseline V to V?: Y     Sample baseline V to V points: 3
Sample filter: Binomial        Number of points for filter: 3  Sample start region (min): 0  Sample end region (min): 30
Marker peak width (sec): 5     Marker min peak height: 200    Marker baseline V to V?: Y     Marker baseline V to V points: 3
Lower marker selection: First peak > 500 RFU                  Upper marker selection: Last peak > 200 RFU
Ladder size (bp)75, 200, 300, 400, 500, 700, 1000, 1500, 2000, 3000, 4000, 5000, 7000, 10000, 20000
Quantification using: Lower Marker          Final concentration (ng/uL): 0.5000          Dilution factor: 12.0
```

Sample: SampE5
Well location: E5
Created: Thursday, October 21, 2021 6:08:16 PM

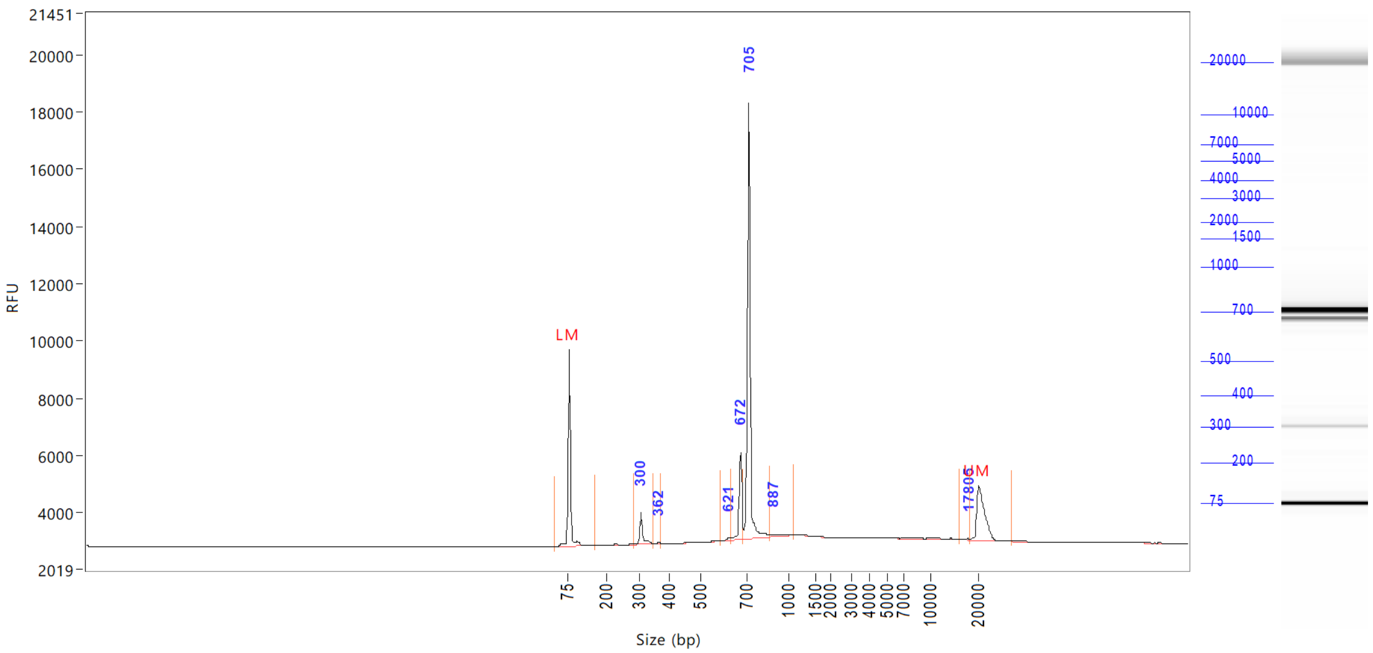

Peak	Size	Concentration	Relative concentration	Molarity	From	To	CV%	RFU
	(bp)	(ng/uL)	Percent	(nmole/L)	(bp)	(bp)		
1	75 (LM)	0.5000		11.0851	28	160	12.92	6898
2	300	1.0554	6.8	5.7298	278	345	3.43	1119
3	362	0.0575	0.4	0.2651	345	372	1.48	64
4	621	0.1090	0.7	0.2908	585	631	1.45	105
5	672	2.1859	14.2	5.3852	631	685	1.28	3049
6	705	11.6633	75.6	27.0352	685	861	3.16	15263
7	887	0.2819	1.8	0.5055	861	1090	4.54	95
8	17805	0.0675	0.4	0.0064	16220	18171	3.19	54
9	20000 (UM)	0.2424		0.0194	18171	26770	4.50	1923

TIC:	15.4206	ng/uL
TIM:	39.2180	nmole/L
Total concentration:	15.9216	ng/uL

```
Sample peak width (sec): 5    Sample min peak height: 50    Sample baseline V to V?: Y    Sample baseline V to V points: 3
Sample filter: Binomial       Number of points for filter: 3  Sample start region (min): 0  Sample end region (min): 30
Marker peak width (sec): 5    Marker min peak height: 200   Marker baseline V to V?: Y    Marker baseline V to V points: 3
Lower marker selection: First peak > 500 RFU                Upper marker selection: Last peak > 200 RFU
Ladder size (bp)75, 200, 300, 400, 500, 700, 1000, 1500, 2000, 3000, 4000, 5000, 7000, 10000, 20000
Quantification using: Lower Marker          Final concentration (ng/uL): 0.5000          Dilution factor: 12.0
```

Sample: SampF5
Well location: F5
Created: Thursday, October 21, 2021 6:08:16 PM

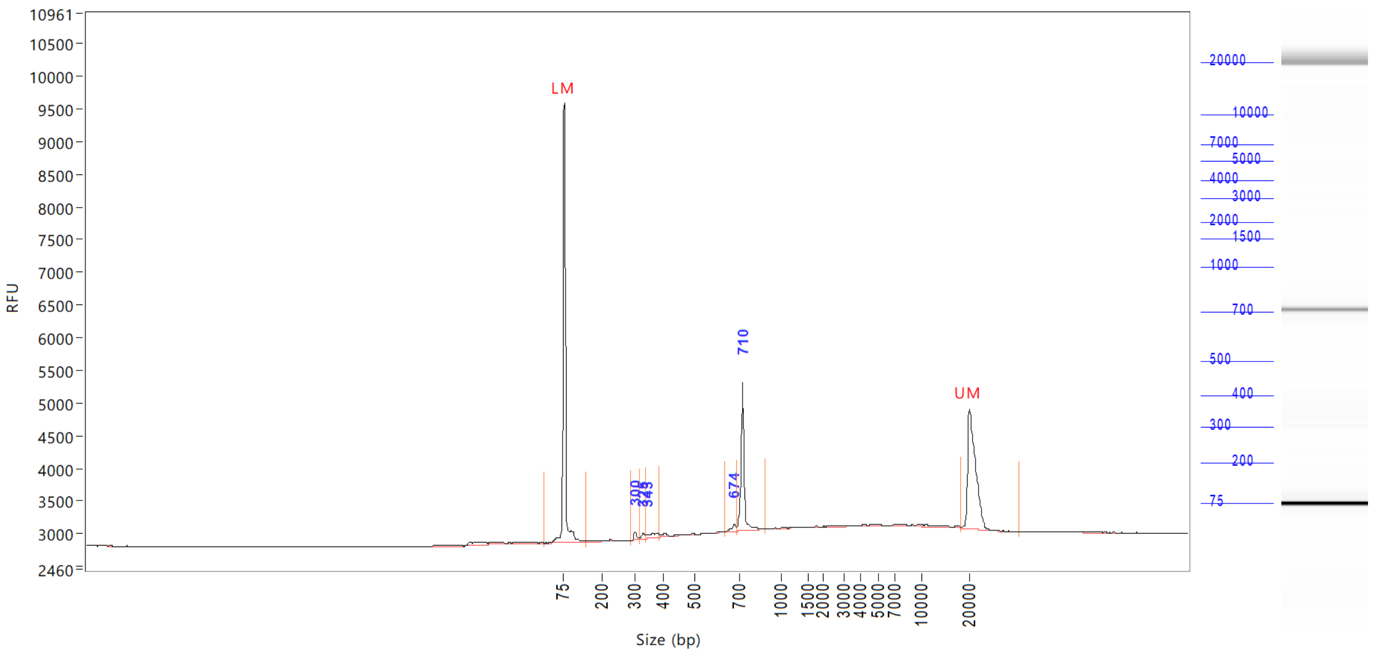

Peak	Size	Concentration	Relative concentration	Molarity	From	To	CV%	RFU
	(bp)	(ng/uL)	Percent	(nmole/L)	(bp)	(bp)		
1	75 (LM)	0.5000		11.0851	11	145	13.32	6735
2	300	0.1165	4.9	0.6364	287	317	2.07	122
3	325	0.1089	4.6	0.5460	317	337	1.81	80
4	343	0.2071	8.8	0.9465	337	386	4.00	67
5	674	0.1286	5.4	0.3172	634	685	1.86	110
6	710	1.8040	76.3	4.1582	685	879	3.67	2274
7	20000 (UM)	0.2289		0.0182	18293	30519	4.90	1834

TIC:	2.3650	ng/uL
TIM:	6.6043	nmole/L
Total concentration:	3.0925	ng/uL

Sample peak width (sec): 5 Sample min peak height: 50 Sample baseline V to V?: Y Sample baseline V to V points: 3
Sample filter: Binomial Number of points for filter: 3 Sample start region (min): 0 Sample end region (min): 30
Marker peak width (sec): 5 Marker min peak height: 200 Marker baseline V to V?: Y Marker baseline V to V points: 3
Lower marker selection: First peak > 500 RFU Upper marker selection: Last peak > 200 RFU
Ladder size (bp)75, 200, 300, 400, 500, 700, 1000, 1500, 2000, 3000, 4000, 5000, 7000, 10000, 20000
Quantification using: Lower Marker Final concentration (ng/uL): 0.5000 Dilution factor: 12.0

Sample: SampG5
Well location: G5
Created: Thursday, October 21, 2021 6:08:16 PM

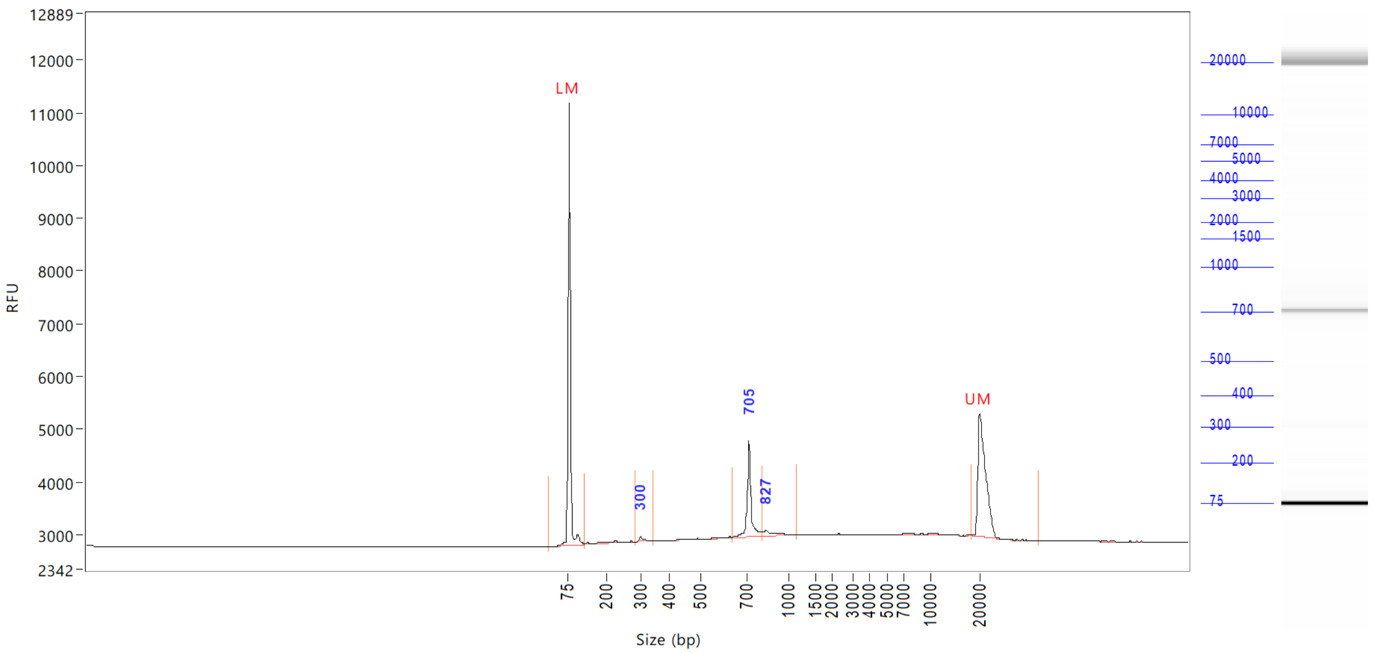

Peak	Size	Concentration	Relative concentration	Molarity	From	To	CV%	RFU
	(bp)	(ng/uL)	Percent	(nmole/L)	(bp)	(bp)		
1	75 (LM)	0.5000		11.0851	11	126	10.15	8411
2	300	0.1003	5.2	0.5392	286	341	3.91	87
3	705	1.5859	82.6	3.6865	631	801	3.59	1813
4	827	0.2339	12.2	0.4425	801	1123	6.69	116
5	20000 (UM)	0.2507		0.0200	18171	31969	5.43	2330

TIC:	1.9201	ng/uL
TIM:	4.6682	nmole/L
Total concentration:	2.4316	ng/uL

```
Sample peak width (sec): 5    Sample min peak height: 50    Sample baseline V to V?: Y    Sample baseline V to V points: 3
Sample filter: Binomial       Number of points for filter: 3  Sample start region (min): 0  Sample end region (min): 30
Marker peak width (sec): 5    Marker min peak height: 200   Marker baseline V to V?: Y    Marker baseline V to V points: 3
Lower marker selection: First peak > 500 RFU                Upper marker selection: Last peak > 200 RFU
Ladder size (bp)75, 200, 300, 400, 500, 700, 1000, 1500, 2000, 3000, 4000, 5000, 7000, 10000, 20000
Quantification using: Lower Marker          Final concentration (ng/uL): 0.5000          Dilution factor: 12.0
```

Sample: SampH5
Well location: H5
Created: Thursday, October 21, 2021 6:08:16 PM

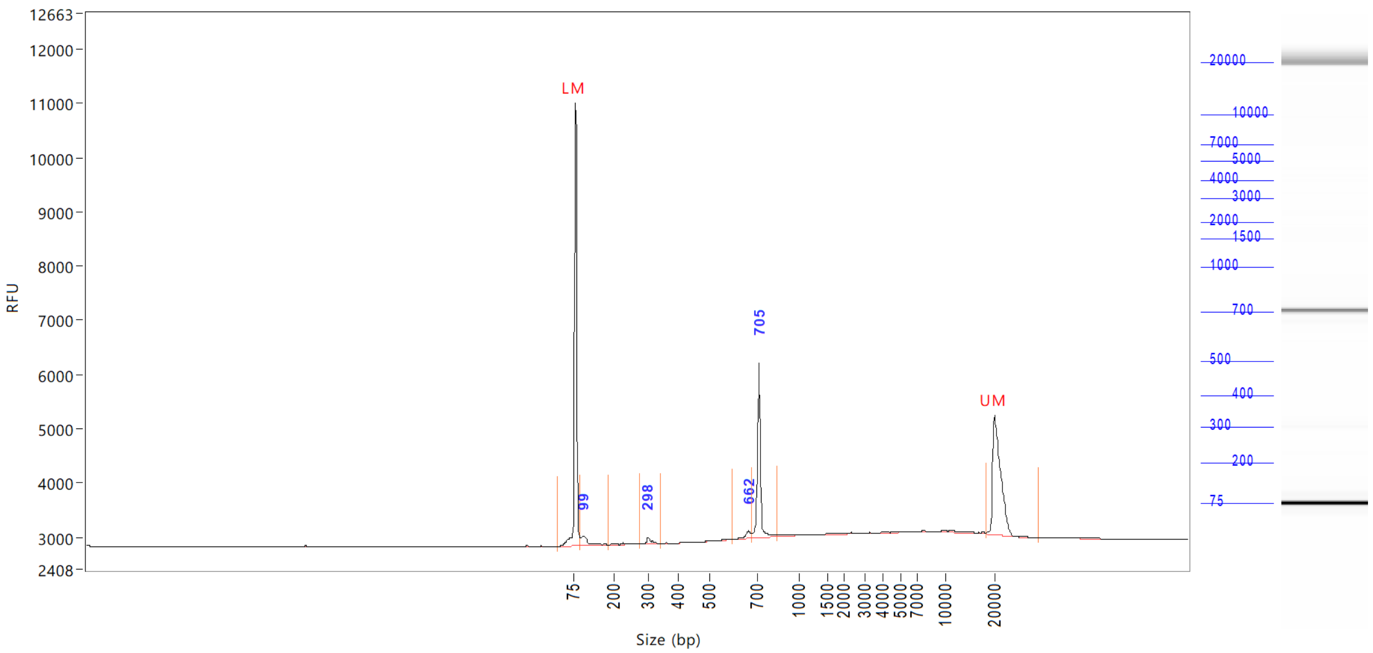

Peak	Size	Concentration	Relative concentration	Molarity	From	To	CV%	RFU
	(bp)	(ng/uL)	Percent	(nmole/L)	(bp)	(bp)		
1	75 (LM)	0.5000		11.2364	18	91	8.51	8185
2	99	0.3556	12.4	5.3100	91	179	17.57	181
3	298	0.1461	5.1	0.7934	275	339	3.45	126
4	662	0.1713	6.0	0.4325	593	674	2.68	139
5	705	2.1957	76.5	5.1111	674	840	2.89	3218
6	20000 (UM)	0.2408		0.0192	18293	28826	4.70	2210

TIC:	2.8688	ng/uL
TIM:	11.6470	nmole/L
Total concentration:	3.4163	ng/uL

Sample peak width (sec): 5   Sample min peak height: 50   Sample baseline V to V?: Y   Sample baseline V to V points: 3
Sample filter: Binomial   Number of points for filter: 3 Sample start region (min): 0 Sample end region (min): 30
Marker peak width (sec): 5   Marker min peak height: 200   Marker baseline V to V?: Y   Marker baseline V to V points: 3
Lower marker selection: First peak > 500 RFU   Upper marker selection: Last peak > 200 RFU
Ladder size (bp)75, 200, 300, 400, 500, 700, 1000, 1500, 2000, 3000, 4000, 5000, 7000, 10000, 20000
Quantification using: Lower Marker   Final concentration (ng/uL): 0.5000   Dilution factor: 12.0

Sample: SampA6
Well location: A6
Created: Thursday, October 21, 2021 6:08:16 PM

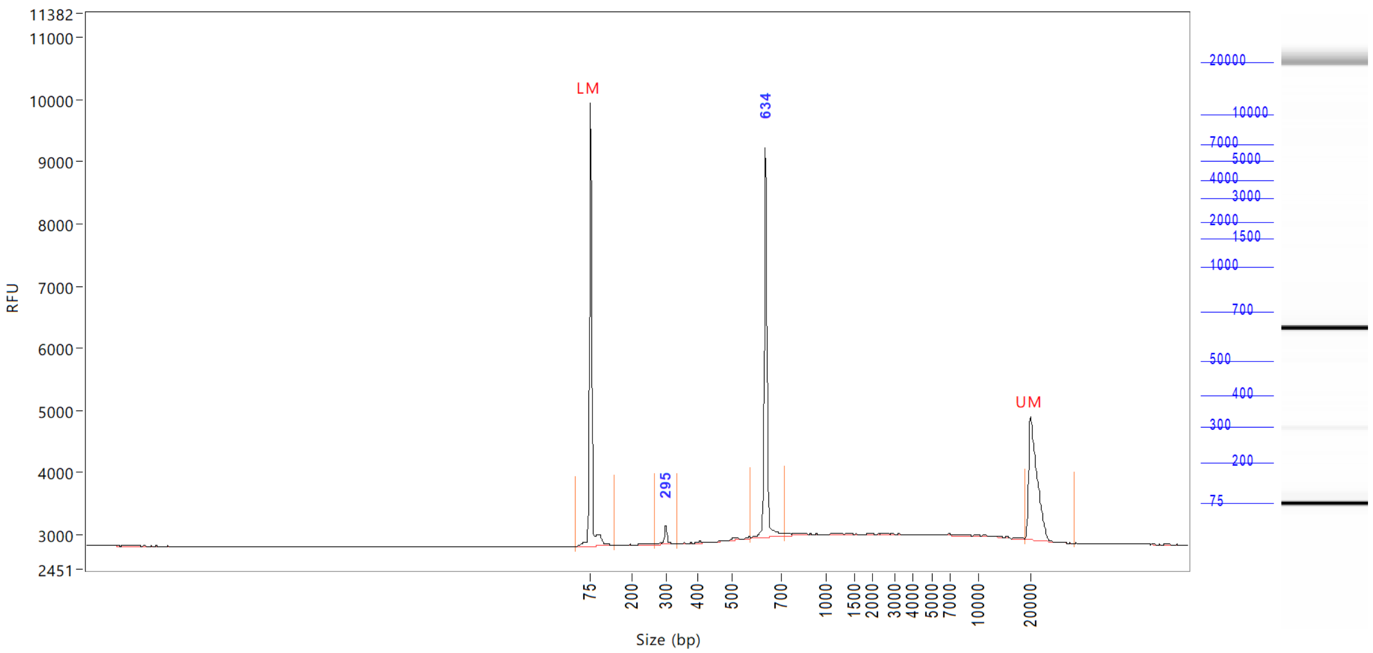

Peak	Size	Concentration	Relative concentration	Molarity	From	To	CV%	RFU
	(bp)	(ng/uL)	Percent	(nmole/L)	(bp)	(bp)		
1	75 (LM)	0.5000		11.0851	31	148	12.05	7132
2	295	0.2919	6.3	1.6389	264	331	3.08	308
3	634	4.3443	93.7	11.2945	575	714	1.93	6254
4	20000 (UM)	0.2460		0.0196	18903	28463	4.39	1970

TIC:	4.6363	ng/uL
TIM:	12.9334	nmole/L
Total concentration:	5.3596	ng/uL

Sample peak width (sec): 5 Sample min peak height: 50 Sample baseline V to V?: Y Sample baseline V to V points: 3
Sample filter: Binomial Number of points for filter: 3 Sample start region (min): 0 Sample end region (min): 30
Marker peak width (sec): 5 Marker min peak height: 200 Marker baseline V to V?: Y Marker baseline V to V points: 3
Lower marker selection: First peak > 500 RFU Upper marker selection: Last peak > 200 RFU
Ladder size (bp)75, 200, 300, 400, 500, 700, 1000, 1500, 2000, 3000, 4000, 5000, 7000, 10000, 20000
Quantification using: Lower Marker Final concentration (ng/uL): 0.5000 Dilution factor: 12.0

Sample: SampB6
Well location: B6
Created: Thursday, October 21, 2021 6:08:16 PM

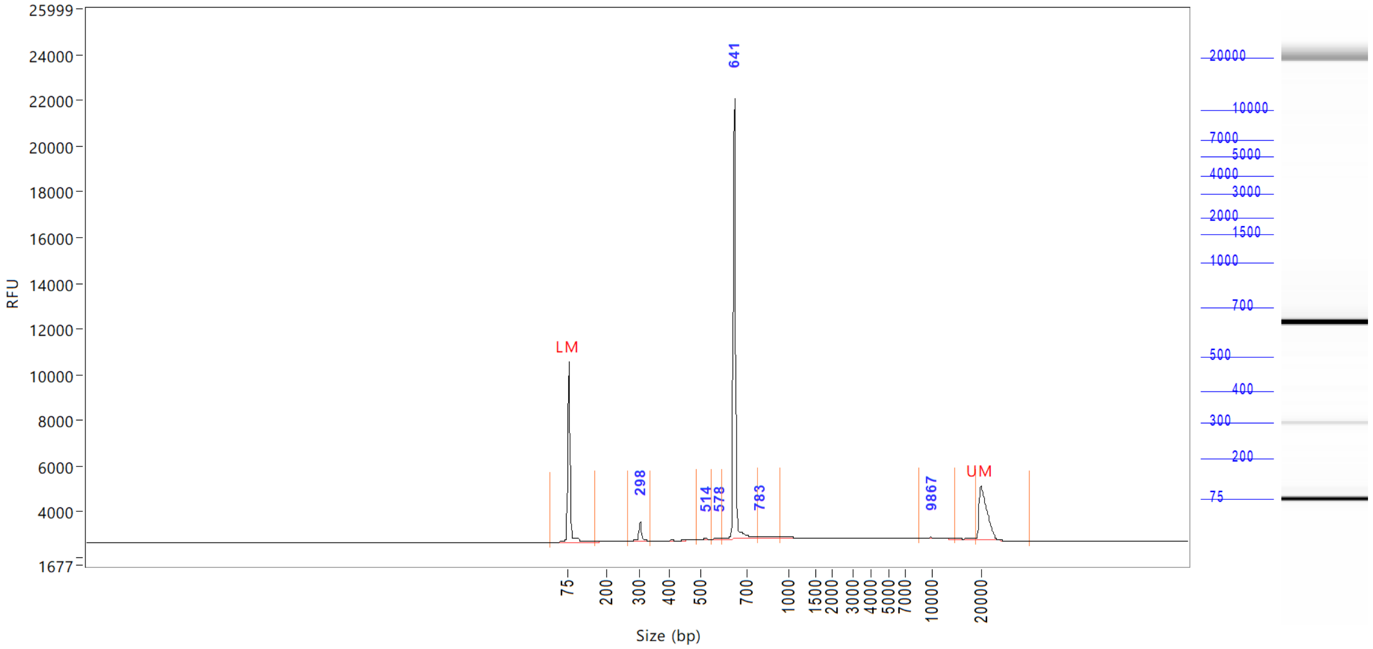

Peak	Size	Concentration	Relative concentration	Molarity	From	To	CV%	RFU
	(bp)	(ng/uL)	Percent	(nmole/L)	(bp)	(bp)		
1	75 (LM)	0.5000		10.7944	16	160	13.57	7913
2	298	0.6837	5.6	3.7866	264	333	2.89	869
3	514	0.0671	0.6	0.2137	485	542	2.40	50
4	578	0.0942	0.8	0.2728	542	590	2.32	61
5	641	10.9480	89.9	28.1077	590	766	2.20	19285
6	783	0.2464	2.0	0.4800	766	939	5.87	69
7	9867	0.1404	1.2	0.0207	8467	14635	15.34	52
8	20000 (UM)	0.2530		0.0202	18903	29914	4.53	2385

TIC:	12.1798	ng/uL
TIM:	32.8814	nmole/L
Total concentration:	12.6256	ng/uL

Sample peak width (sec): 5 Sample min peak height: 50 Sample baseline V to V?: Y Sample baseline V to V points: 3
Sample filter: Binomial Number of points for filter: 3 Sample start region (min): 0 Sample end region (min): 30
Marker peak width (sec): 5 Marker min peak height: 200 Marker baseline V to V?: Y Marker baseline V to V points: 3
Lower marker selection: First peak > 500 RFU Upper marker selection: Last peak > 200 RFU
Ladder size (bp)75, 200, 300, 400, 500, 700, 1000, 1500, 2000, 3000, 4000, 5000, 7000, 10000, 20000
Quantification using: Lower Marker Final concentration (ng/uL): 0.5000 Dilution factor: 12.0

Sample: SampC6
Well location: C6
Created: Thursday, October 21, 2021 6:08:16 PM

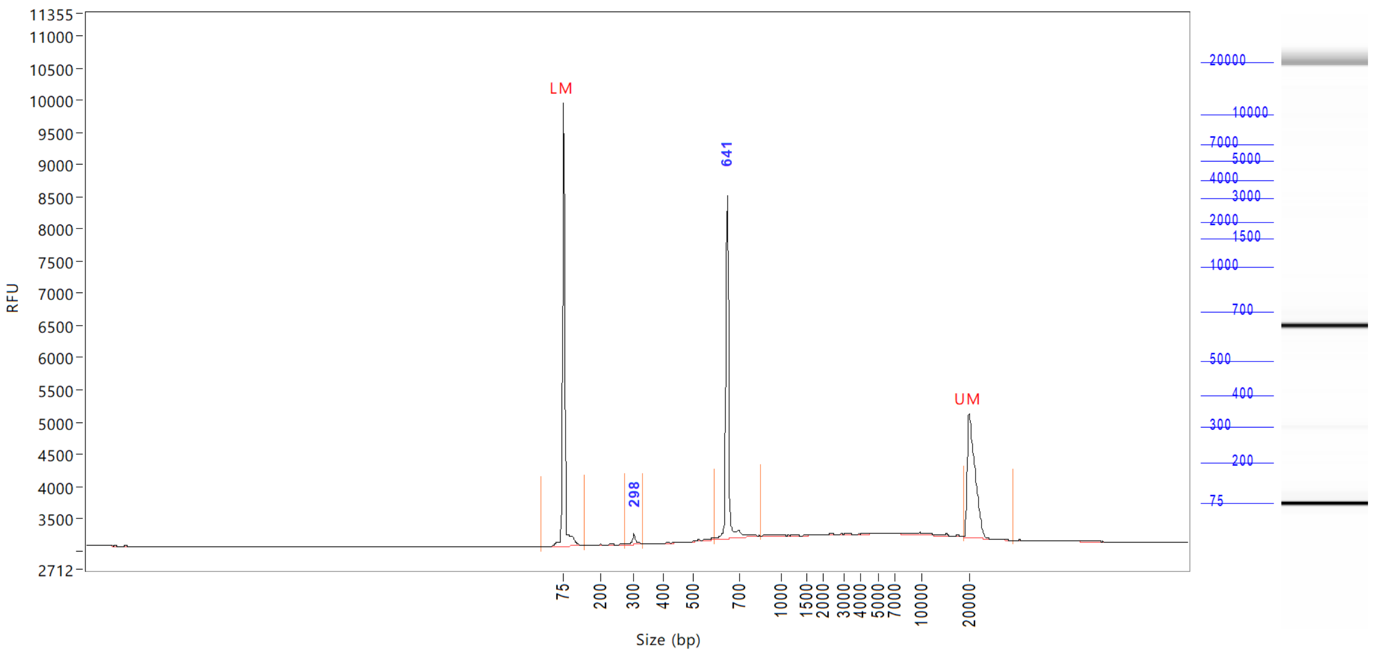

Peak	Size	Concentration	Relative concentration	Molarity	From	To	CV%	RFU
	(bp)	(ng/uL)	Percent	(nmole/L)	(bp)	(bp)		
1	75 (LM)	0.5000		11.0851	5	145	12.35	6903
2	298	0.1923	4.6	1.0648	271	329	3.03	169
3	641	3.9892	95.4	10.1942	585	857	3.17	5328
4	20000 (UM)	0.2385		0.0191	18903	29068	4.57	1921

TIC:	4.1815	ng/uL
TIM:	11.2590	nmole/L
Total concentration:	4.7908	ng/uL

```
Sample peak width (sec): 5    Sample min peak height: 50    Sample baseline V to V?: Y    Sample baseline V to V points: 3
Sample filter: Binomial       Number of points for filter: 3  Sample start region (min): 0  Sample end region (min): 30
Marker peak width (sec): 5    Marker min peak height: 200   Marker baseline V to V?: Y    Marker baseline V to V points: 3
Lower marker selection: First peak > 500 RFU                Upper marker selection: Last peak > 200 RFU
Ladder size (bp)75, 200, 300, 400, 500, 700, 1000, 1500, 2000, 3000, 4000, 5000, 7000, 10000, 20000
Quantification using: Lower Marker          Final concentration (ng/uL): 0.5000          Dilution factor: 12.0
```

Sample: SampD6
Well location: D6
Created: Thursday, October 21, 2021 6:08:16 PM

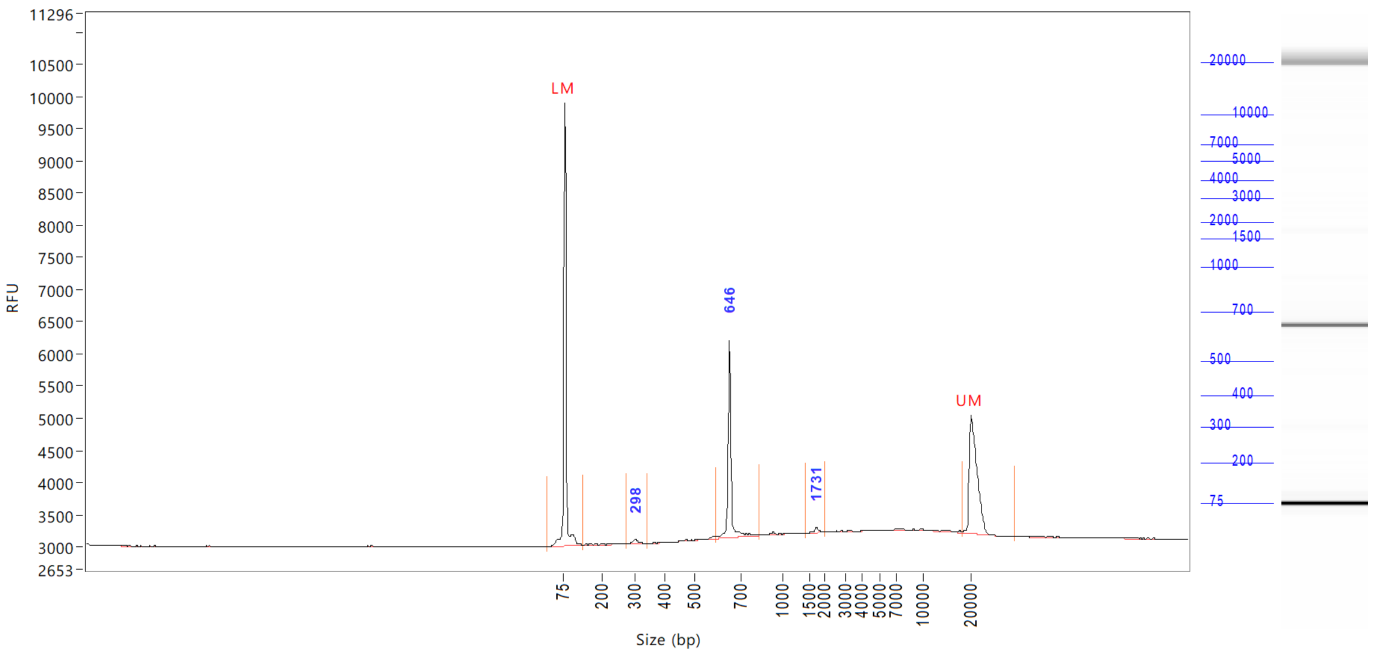

Peak	Size	Concentration	Relative concentration	Molarity	From	To	CV%	RFU
	(bp)	(ng/uL)	Percent	(nmole/L)	(bp)	(bp)		
1	75 (LM)	0.5000		11.0851	20	137	12.65	6900
2	298	0.1278	4.7	0.7029	273	341	4.06	73
3	646	2.4920	92.2	6.3192	590	827	4.16	3062
4	1731	0.0826	3.1	0.0791	1445	2000	5.90	79
5	20000 (UM)	0.2240		0.0179	18049	29068	4.42	1848

TIC:	2.7024	ng/uL
TIM:	7.1012	nmole/L
Total concentration:	3.2707	ng/uL

```
Sample peak width (sec): 5    Sample min peak height: 50    Sample baseline V to V?: Y    Sample baseline V to V points: 3
Sample filter: Binomial       Number of points for filter: 3  Sample start region (min): 0  Sample end region (min): 30
Marker peak width (sec): 5    Marker min peak height: 200   Marker baseline V to V?: Y    Marker baseline V to V points: 3
Lower marker selection: First peak > 500 RFU                Upper marker selection: Last peak > 200 RFU
Ladder size (bp)75, 200, 300, 400, 500, 700, 1000, 1500, 2000, 3000, 4000, 5000, 7000, 10000, 20000
Quantification using: Lower Marker          Final concentration (ng/uL): 0.5000          Dilution factor: 12.0
```

Sample: SampE6
Well location: E6
Created: Thursday, October 21, 2021 6:08:16 PM

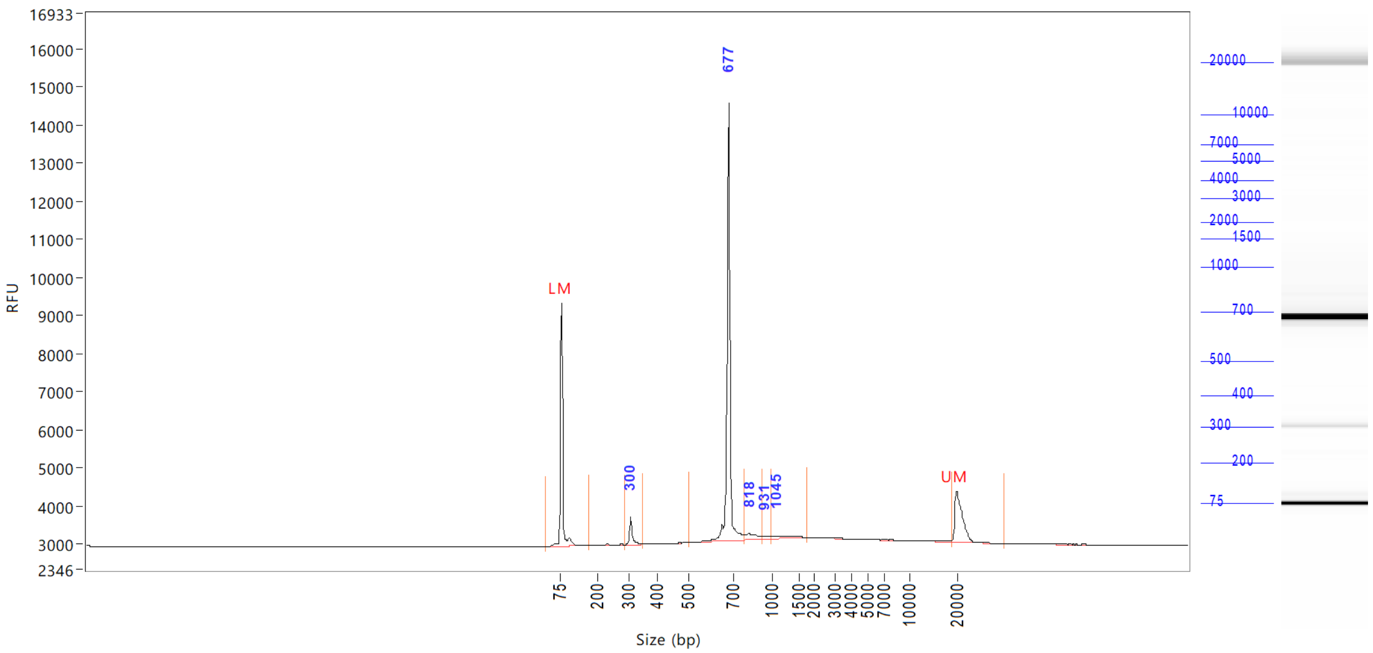

Peak	Size	Concentration	Relative concentration	Molarity	From	To	CV%	RFU
	(bp)	(ng/uL)	Percent	(nmole/L)	(bp)	(bp)		
1	75 (LM)	0.5000		10.9378	22	168	13.25	6394
2	300	0.7162	6.2	3.9011	284	343	2.57	735
3	677	9.8613	84.7	23.9721	501	788	2.91	11508
4	818	0.5062	4.4	0.9859	788	918	4.43	181
5	931	0.2027	1.7	0.3498	918	991	2.33	87
6	1045	0.3494	3.0	0.4797	991	1770	14.09	80
7	20000 (UM)	0.1776		0.0141	18903	30277	5.50	1337

TIC:	11.6359	ng/uL
TIM:	29.6886	nmole/L
Total concentration:	12.0350	ng/uL

Sample peak width (sec): 5 Sample min peak height: 50 Sample baseline V to V?: Y Sample baseline V to V points: 3
Sample filter: Binomial Number of points for filter: 3 Sample start region (min): 0 Sample end region (min): 30
Marker peak width (sec): 5 Marker min peak height: 200 Marker baseline V to V?: Y Marker baseline V to V points: 3
Lower marker selection: First peak > 500 RFU Upper marker selection: Last peak > 200 RFU
Ladder size (bp)75, 200, 300, 400, 500, 700, 1000, 1500, 2000, 3000, 4000, 5000, 7000, 10000, 20000
Quantification using: Lower Marker Final concentration (ng/uL): 0.5000 Dilution factor: 12.0

Sample: SampF6
Well location: F6
Created: Thursday, October 21, 2021 6:08:16 PM

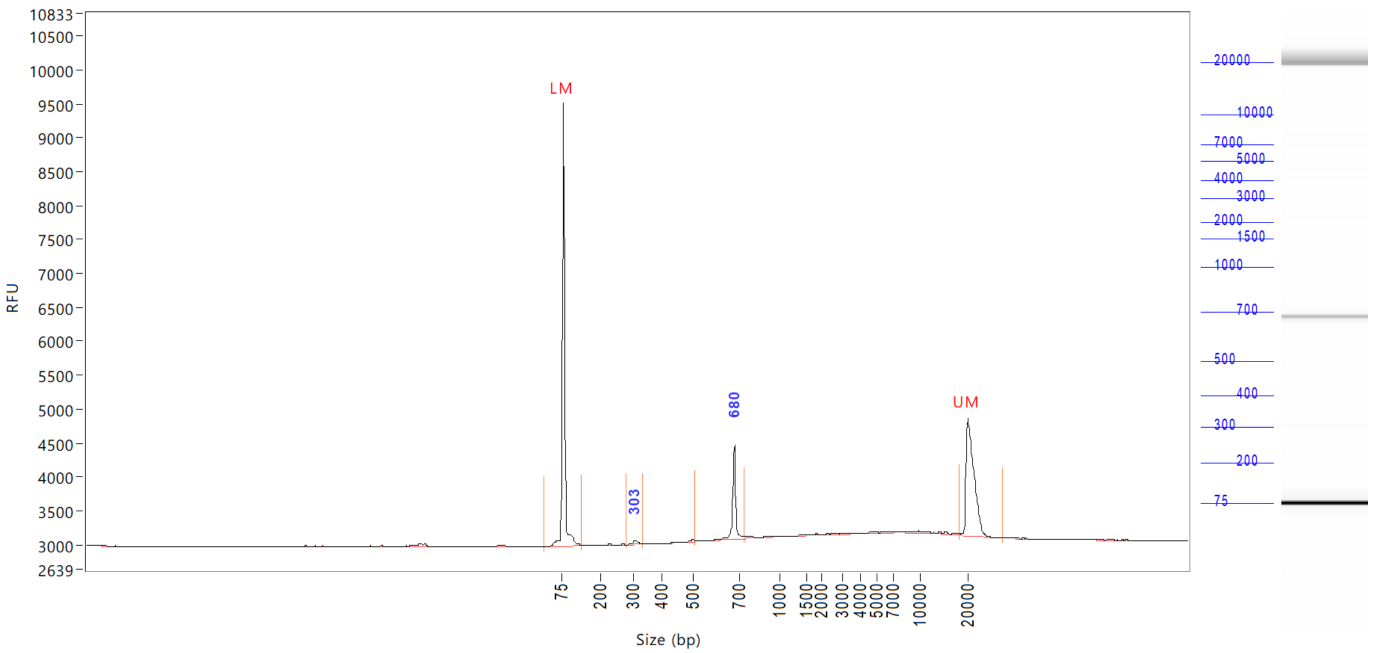

Peak	Size	Concentration	Relative concentration	Molarity	From	To	CV%	RFU
	(bp)	(ng/uL)	Percent	(nmole/L)	(bp)	(bp)		
1	75 (LM)	0.5000		10.9378	14	135	12.25	6546
2	303	0.0900	7.1	0.4853	280	329	2.69	60
3	680	1.1831	92.9	2.8932	509	740	3.40	1384
4	20000 (UM)	0.2269		0.0182	18293	27133	4.53	1740

TIC:	1.2731	ng/uL
TIM:	3.3785	nmole/L
Total concentration:	1.8594	ng/uL

Sample peak width (sec): 5   Sample min peak height: 50   Sample baseline V to V?: Y   Sample baseline V to V points: 3
Sample filter: Binomial   Number of points for filter: 3 Sample start region (min): 0 Sample end region (min): 30
Marker peak width (sec): 5   Marker min peak height: 200   Marker baseline V to V?: Y   Marker baseline V to V points: 3
Lower marker selection: First peak > 500 RFU   Upper marker selection: Last peak > 200 RFU
Ladder size (bp)75, 200, 300, 400, 500, 700, 1000, 1500, 2000, 3000, 4000, 5000, 7000, 10000, 20000
Quantification using: Lower Marker   Final concentration (ng/uL): 0.5000   Dilution factor: 12.0

Sample: SampG6
Well location: G6
Created: Thursday, October 21, 2021 6:08:16 PM

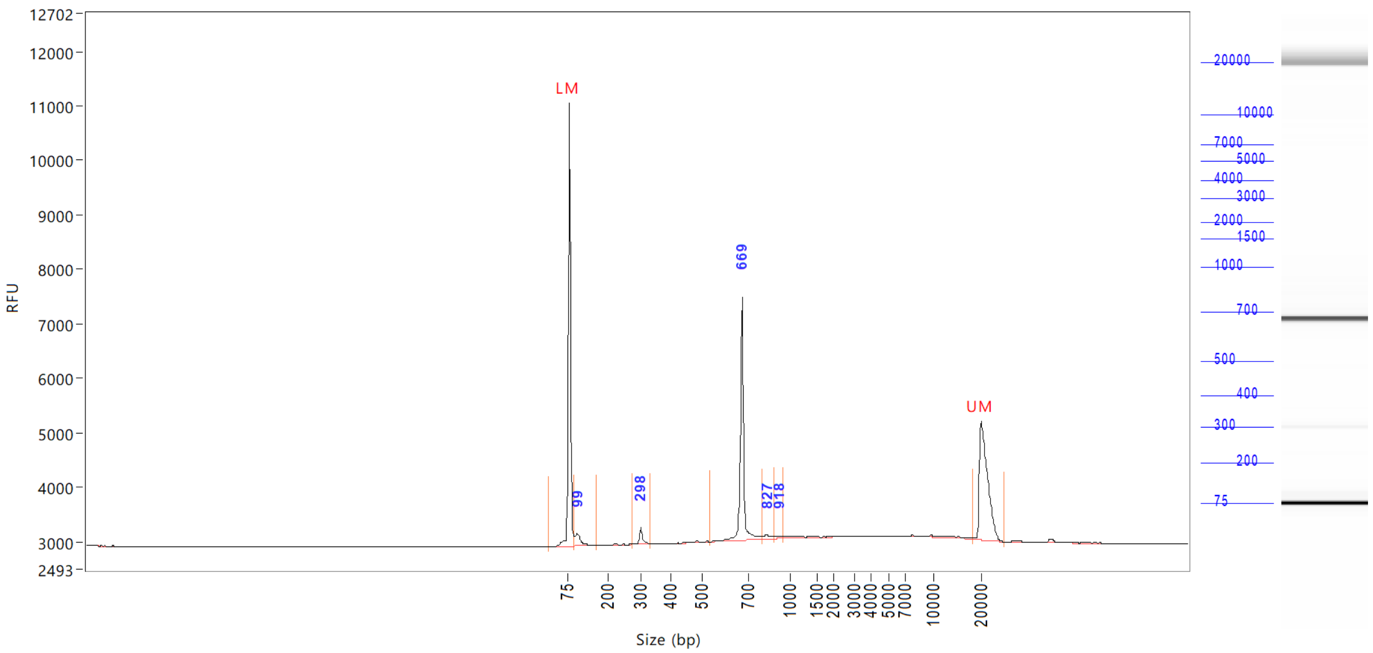

Peak	Size	Concentration	Relative concentration	Molarity	From	To	CV%	RFU
	(bp)	(ng/uL)	Percent	(nmole/L)	(bp)	(bp)		
1	75 (LM)	0.5000		11.2364	11	91	7.61	8153
2	99	0.4011	9.6	6.2139	91	164	13.89	221
3	298	0.2641	6.3	1.4528	275	331	2.53	312
4	669	3.3059	79.3	8.1083	537	796	3.26	4457
5	827	0.1292	3.1	0.2550	796	883	3.01	73
6	918	0.0690	1.7	0.1242	883	948	2.03	53
7	20000 (UM)	0.2520		0.0202	18415	24473	4.43	2192

TIC:	4.1692	ng/uL
TIM:	16.1541	nmole/L
Total concentration:	4.6491	ng/uL

```
Sample peak width (sec): 5    Sample min peak height: 50    Sample baseline V to V?: Y    Sample baseline V to V points: 3
Sample filter: Binomial       Number of points for filter: 3  Sample start region (min): 0  Sample end region (min): 30
Marker peak width (sec): 5    Marker min peak height: 200   Marker baseline V to V?: Y    Marker baseline V to V points: 3
Lower marker selection: First peak > 500 RFU                Upper marker selection: Last peak > 200 RFU
Ladder size (bp)75, 200, 300, 400, 500, 700, 1000, 1500, 2000, 3000, 4000, 5000, 7000, 10000, 20000
Quantification using: Lower Marker          Final concentration (ng/uL): 0.5000          Dilution factor: 12.0
```

Sample: SampH6
Well location: H6
Created: Thursday, October 21, 2021 6:08:16 PM

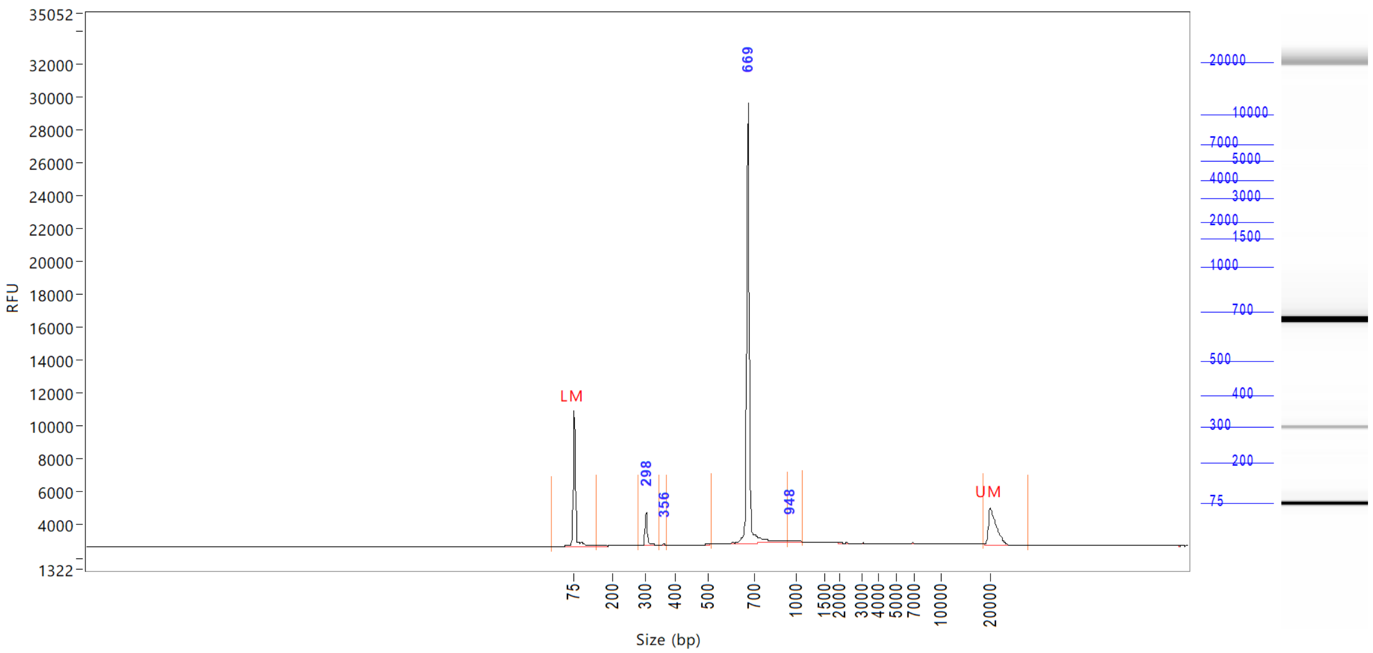

Peak	Size	Concentration	Relative concentration	Molarity	From	To	CV%	RFU
	(bp)	(ng/uL)	Percent	(nmole/L)	(bp)	(bp)		
1	75 (LM)	0.5000		10.9378	7	148	12.98	8243
2	298	1.3117	7.2	7.2406	277	341	2.50	1964
3	356	0.0568	0.3	0.2646	341	368	1.54	65
4	669	16.7956	91.9	41.0714	514	935	4.97	26765
5	948	0.1067	0.6	0.1813	935	1090	2.91	72
6	20000 (UM)	0.2343		0.0187	18537	27859	4.48	2191

TIC:	18.2709	ng/uL
TIM:	48.7578	nmole/L
Total concentration:	18.8173	ng/uL

```
Sample peak width (sec): 5    Sample min peak height: 50    Sample baseline V to V?: Y    Sample baseline V to V points: 3
Sample filter: Binomial       Number of points for filter: 3  Sample start region (min): 0  Sample end region (min): 30
Marker peak width (sec): 5    Marker min peak height: 200   Marker baseline V to V?: Y    Marker baseline V to V points: 3
Lower marker selection: First peak > 500 RFU                Upper marker selection: Last peak > 200 RFU
Ladder size (bp)75, 200, 300, 400, 500, 700, 1000, 1500, 2000, 3000, 4000, 5000, 7000, 10000, 20000
Quantification using: Lower Marker          Final concentration (ng/uL): 0.5000          Dilution factor: 12.0
```

Sample: SampA7
Well location: A7
Created: Thursday, October 21, 2021 6:08:16 PM

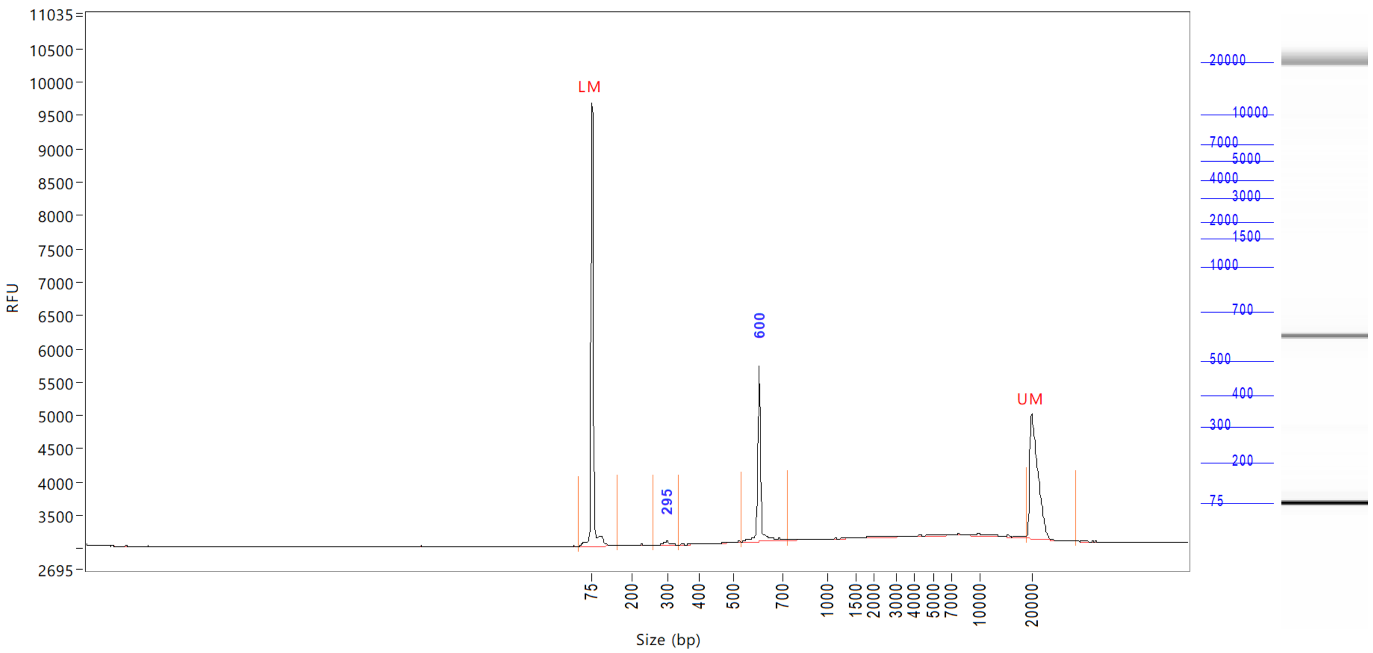

Peak	Size	Concentration	Relative concentration	Molarity	From	To	CV%	RFU
	(bp)	(ng/uL)	Percent	(nmole/L)	(bp)	(bp)		
1	75 (LM)	0.5000		10.9378	35	154	12.09	6661
2	295	0.1099	4.7	0.6235	259	333	4.39	54
3	600	2.2358	95.3	6.1018	532	731	3.73	2643
4	20000 (UM)	0.2439		0.0195	18903	28342	4.29	1880

TIC:	2.3458	ng/uL
TIM:	6.7253	nmole/L
Total concentration:	2.9733	ng/uL

```
Sample peak width (sec): 5    Sample min peak height: 50    Sample baseline V to V?: Y    Sample baseline V to V points: 3
Sample filter: Binomial       Number of points for filter: 3  Sample start region (min): 0  Sample end region (min): 30
Marker peak width (sec): 5    Marker min peak height: 200   Marker baseline V to V?: Y    Marker baseline V to V points: 3
Lower marker selection: First peak > 500 RFU                Upper marker selection: Last peak > 200 RFU
Ladder size (bp)75, 200, 300, 400, 500, 700, 1000, 1500, 2000, 3000, 4000, 5000, 7000, 10000, 20000
Quantification using: Lower Marker          Final concentration (ng/uL): 0.5000          Dilution factor: 12.0
```

Sample: SampB7
Well location: B7
Created: Thursday, October 21, 2021 6:08:16 PM

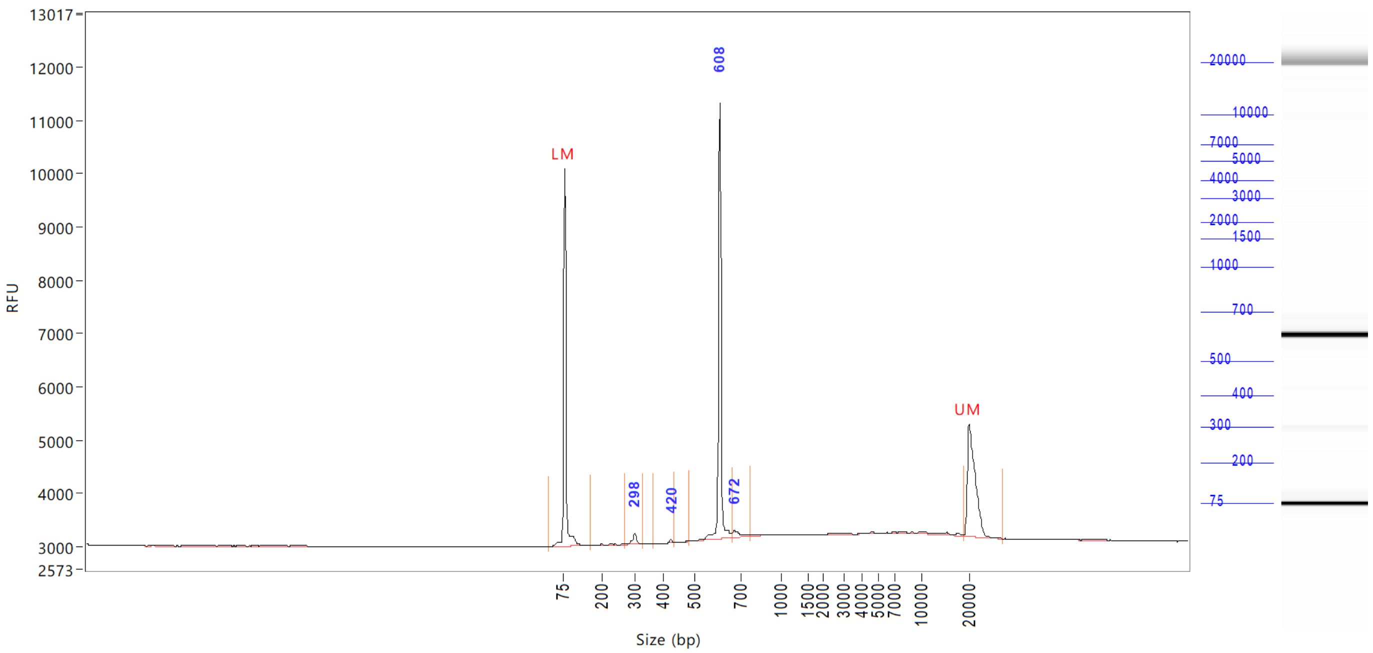

Peak	Size	Concentration	Relative concentration	Molarity	From	To	CV%	RFU
	(bp)	(ng/uL)	Percent	(nmole/L)	(bp)	(bp)		
1	75 (LM)	0.5000		11.0851	20	158	13.75	7087
2	298	0.2507	4.1	1.3980	269	325	2.97	216
3	420	0.0517	0.8	0.2061	360	433	3.38	68
4	608	5.6036	91.1	15.2676	483	662	2.24	8201
5	672	0.2457	4.0	0.5861	662	766	3.71	136
6	20000 (UM)	0.2488		0.0199	18781	27012	4.56	2103

TIC:	6.1518	ng/uL
TIM:	17.4577	nmole/L
Total concentration:	6.7203	ng/uL

Sample peak width (sec): 5 Sample min peak height: 50 Sample baseline V to V?: Y Sample baseline V to V points: 3
Sample filter: Binomial Number of points for filter: 3 Sample start region (min): 0 Sample end region (min): 30
Marker peak width (sec): 5 Marker min peak height: 200 Marker baseline V to V?: Y Marker baseline V to V points: 3
Lower marker selection: First peak > 500 RFU Upper marker selection: Last peak > 200 RFU
Ladder size (bp)75, 200, 300, 400, 500, 700, 1000, 1500, 2000, 3000, 4000, 5000, 7000, 10000, 20000
Quantification using: Lower Marker Final concentration (ng/uL): 0.5000 Dilution factor: 12.0

Sample: SampC7
Well location: C7
Created: Thursday, October 21, 2021 6:08:16 PM

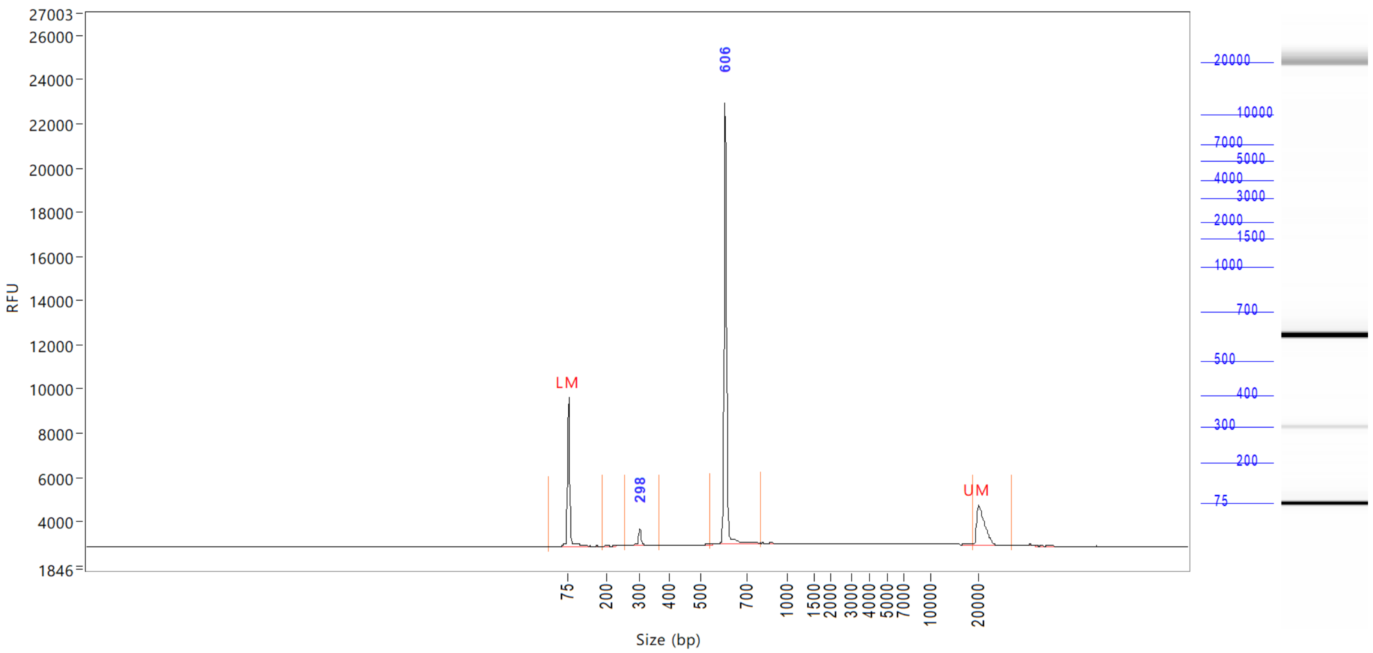

Peak	Size	Concentration	Relative concentration	Molarity	From	To	CV%	RFU
	(bp)	(ng/uL)	Percent	(nmole/L)	(bp)	(bp)		
1	75 (LM)	0.5000		10.9378	12	187	14.70	6726
2	298	0.7999	5.4	4.4301	255	366	3.48	775
3	606	13.9512	94.6	37.8860	539	805	2.32	19999
4	20000 (UM)	0.2369		0.0189	18903	26649	4.38	1817

TIC:	14.7511	ng/uL
TIM:	42.3161	nmole/L
Total concentration:	15.3249	ng/uL

```
Sample peak width (sec): 5    Sample min peak height: 50    Sample baseline V to V?: Y    Sample baseline V to V points: 3
Sample filter: Binomial       Number of points for filter: 3  Sample start region (min): 0  Sample end region (min): 30
Marker peak width (sec): 5    Marker min peak height: 200   Marker baseline V to V?: Y    Marker baseline V to V points: 3
Lower marker selection: First peak > 500 RFU                Upper marker selection: Last peak > 200 RFU
Ladder size (bp)75, 200, 300, 400, 500, 700, 1000, 1500, 2000, 3000, 4000, 5000, 7000, 10000, 20000
Quantification using: Lower Marker          Final concentration (ng/uL): 0.5000          Dilution factor: 12.0
```

Sample: SampD7
Well location: D7
Created: Thursday, October 21, 2021 6:08:16 PM

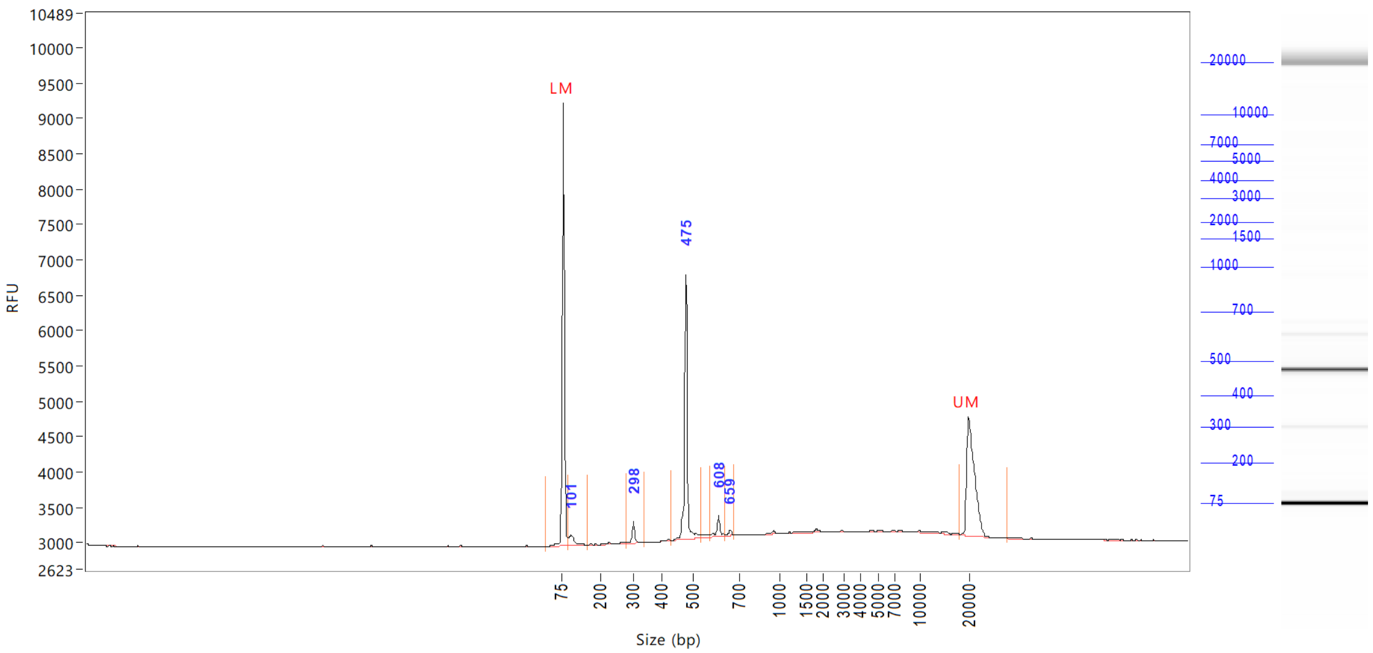

Peak	Size	Concentration	Relative concentration	Molarity	From	To	CV%	RFU
	(bp)	(ng/uL)	Percent	(nmole/L)	(bp)	(bp)		
1	75 (LM)	0.5000		11.2364	16	89	6.44	6278
2	101	0.3672	7.9	5.7981	89	154	12.83	143
3	298	0.3192	6.8	1.7676	280	335	2.33	314
4	475	3.5516	76.0	12.3291	430	534	2.21	3744
5	608	0.3523	7.5	0.9614	570	639	2.26	304
6	659	0.0816	1.7	0.2054	639	674	1.08	74
7	20000 (UM)	0.2490		0.0200	18171	27979	4.44	1682

TIC:	4.6718	ng/uL
TIM:	21.0617	nmole/L
Total concentration:	5.4670	ng/uL

Sample peak width (sec): 5 Sample min peak height: 50 Sample baseline V to V?: Y Sample baseline V to V points: 3
Sample filter: Binomial Number of points for filter: 3 Sample start region (min): 0 Sample end region (min): 30
Marker peak width (sec): 5 Marker min peak height: 200 Marker baseline V to V?: Y Marker baseline V to V points: 3
Lower marker selection: First peak > 500 RFU Upper marker selection: Last peak > 200 RFU
Ladder size (bp)75, 200, 300, 400, 500, 700, 1000, 1500, 2000, 3000, 4000, 5000, 7000, 10000, 20000
Quantification using: Lower Marker Final concentration (ng/uL): 0.5000 Dilution factor: 12.0

Sample: SampE7
Well location: E7
Created: Thursday, October 21, 2021 6:08:16 PM

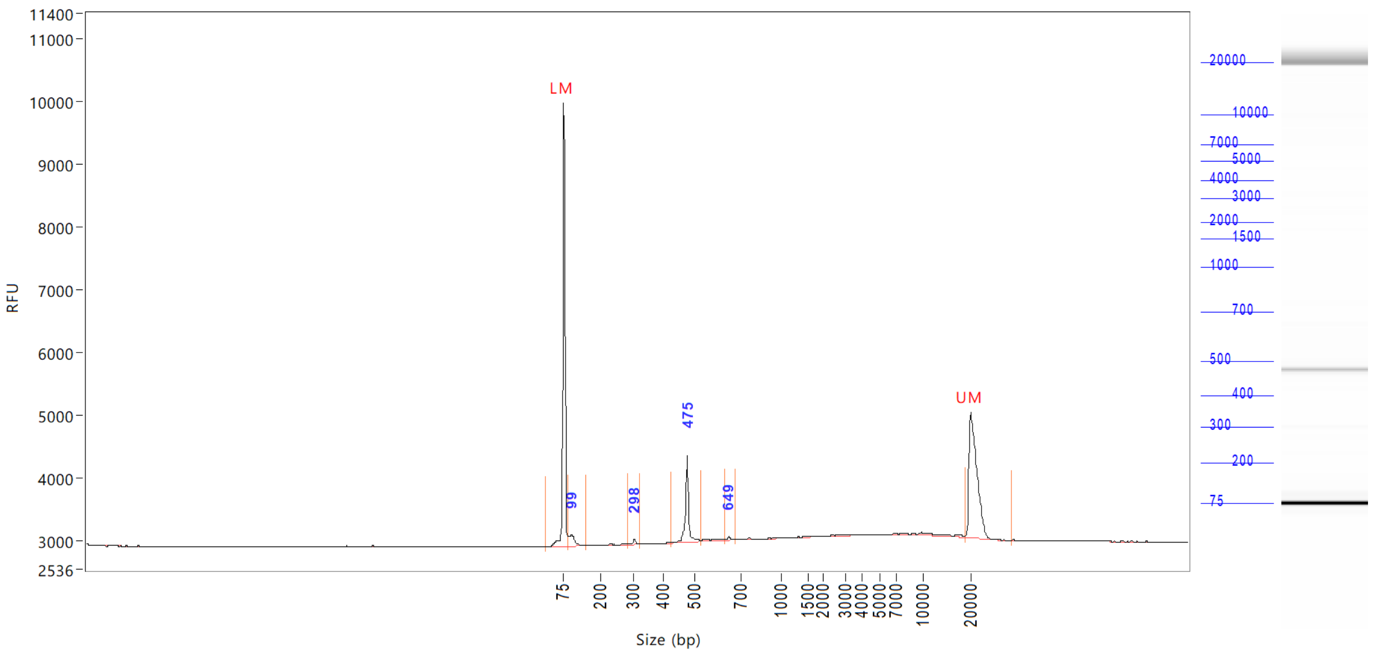

Peak	Size	Concentration	Relative concentration	Molarity	From	To	CV%	RFU
	(bp)	(ng/uL)	Percent	(nmole/L)	(bp)	(bp)		
1	75 (LM)	0.5000		11.2364	20	89	7.90	7082
2	99	0.3783	21.2	5.9731	89	150	12.50	173
3	298	0.0863	4.8	0.4777	278	317	2.30	90
4	475	1.2718	71.2	4.4245	424	524	2.35	1389
5	649	0.0491	2.8	0.1248	636	680	1.16	54
6	20000 (UM)	0.2603		0.0207	19024	28342	4.51	1996

TIC:	1.7855	ng/uL
TIM:	11.0000	nmole/L
Total concentration:	2.5133	ng/uL

Sample peak width (sec): 5 Sample min peak height: 50 Sample baseline V to V?: Y Sample baseline V to V points: 3
Sample filter: Binomial Number of points for filter: 3 Sample start region (min): 0 Sample end region (min): 30
Marker peak width (sec): 5 Marker min peak height: 200 Marker baseline V to V?: Y Marker baseline V to V points: 3
Lower marker selection: First peak > 500 RFU Upper marker selection: Last peak > 200 RFU
Ladder size (bp)75, 200, 300, 400, 500, 700, 1000, 1500, 2000, 3000, 4000, 5000, 7000, 10000, 20000
Quantification using: Lower Marker Final concentration (ng/uL): 0.5000 Dilution factor: 12.0

Sample: SampF7
Well location: F7
Created: Thursday, October 21, 2021 6:08:16 PM

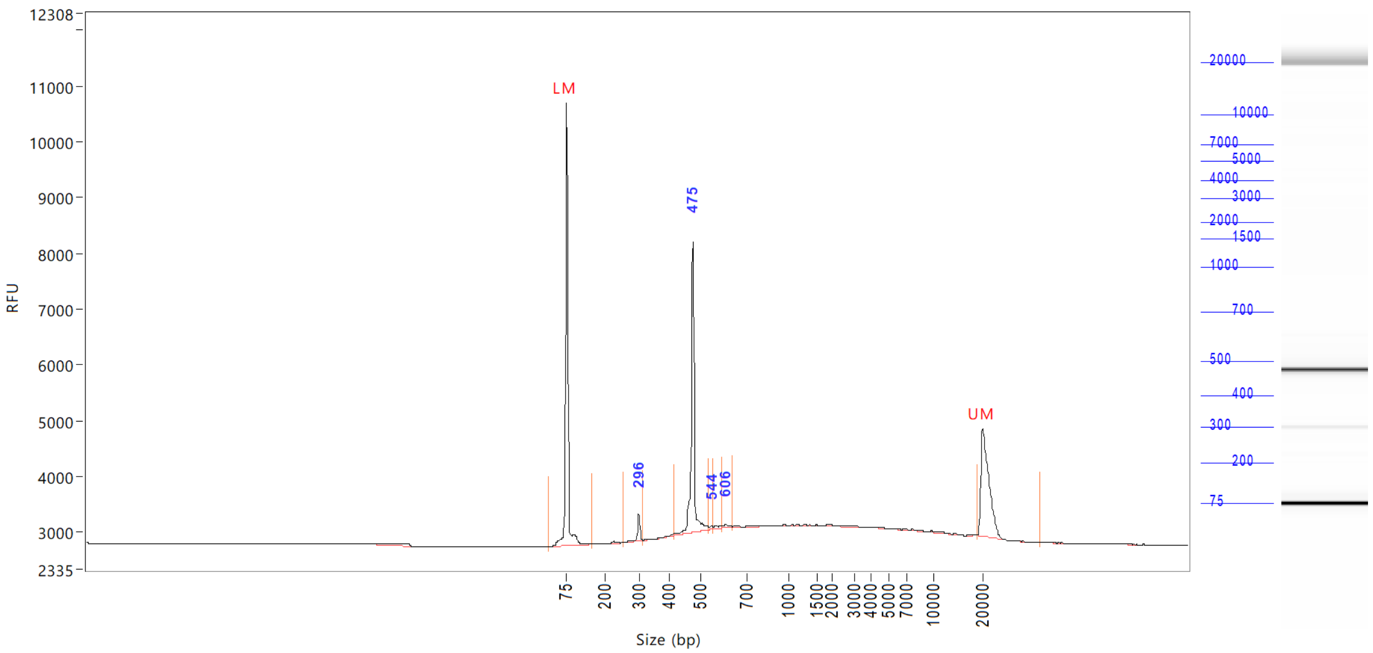

Peak	Size	Concentration	Relative concentration	Molarity	From	To	CV%	RFU
	(bp)	(ng/uL)	Percent	(nmole/L)	(bp)	(bp)		
1	75 (LM)	0.5000		11.2364	16	154	12.49	7956
2	296	0.3392	7.9	1.9043	255	311	3.23	474
3	475	3.8455	89.5	13.3493	418	537	2.56	5214
4	544	0.0475	1.1	0.1434	537	555	1.04	57
5	606	0.0634	1.5	0.1722	590	636	1.73	66
6	20000 (UM)	0.2078		0.0166	18903	31728	5.03	1928

TIC:	4.2956	ng/uL
TIM:	15.5693	nmole/L
Total concentration:	4.9517	ng/uL

```
Sample peak width (sec): 5    Sample min peak height: 50    Sample baseline V to V?: Y    Sample baseline V to V points: 3
Sample filter: Binomial       Number of points for filter: 3  Sample start region (min): 0  Sample end region (min): 30
Marker peak width (sec): 5    Marker min peak height: 200   Marker baseline V to V?: Y    Marker baseline V to V points: 3
Lower marker selection: First peak > 500 RFU                Upper marker selection: Last peak > 200 RFU
Ladder size (bp)75, 200, 300, 400, 500, 700, 1000, 1500, 2000, 3000, 4000, 5000, 7000, 10000, 20000
Quantification using: Lower Marker          Final concentration (ng/uL): 0.5000          Dilution factor: 12.0
```

Sample: SampG7
Well location: G7
Created: Thursday, October 21, 2021 6:08:16 PM

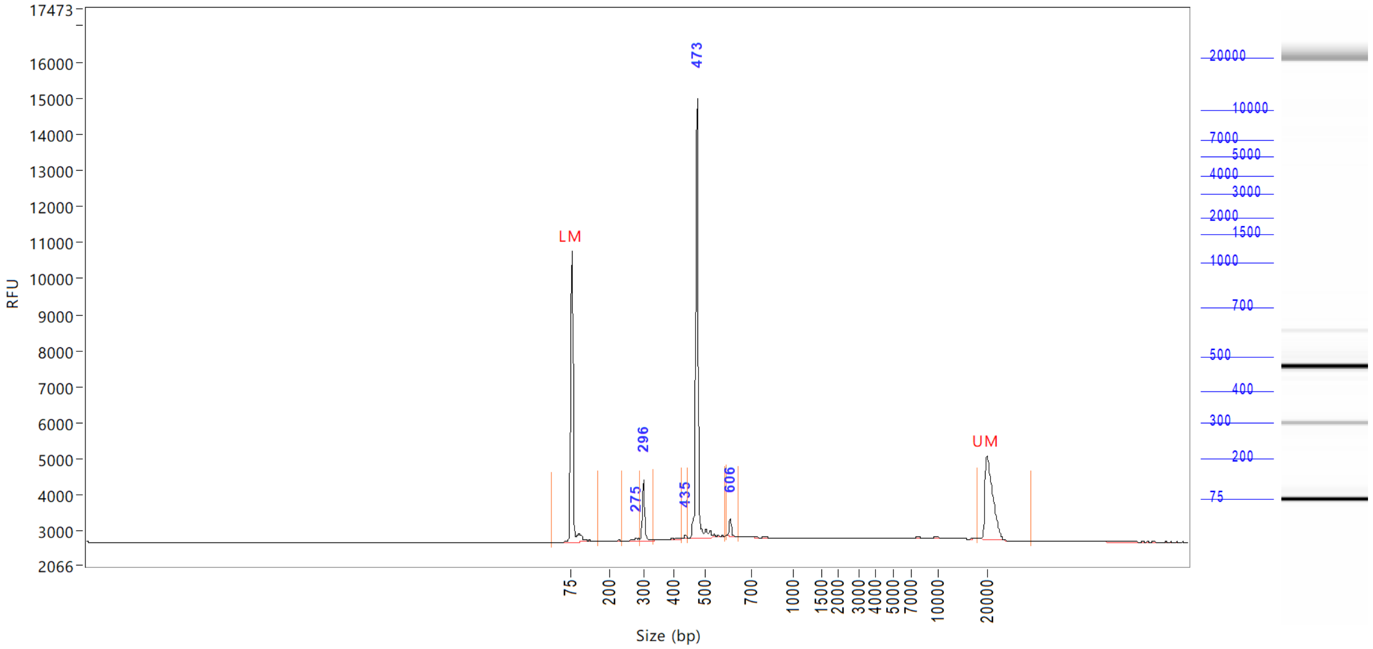

Peak	Size	Concentration	Relative concentration	Molarity	From	To	CV%	RFU
	(bp)	(ng/uL)	Percent	(nmole/L)	(bp)	(bp)		
1	75 (LM)	0.5000		10.9378	14	160	12.32	8077
2	275	0.1127	1.2	0.6868	233	286	4.42	55
3	296	1.1166	12.0	6.1842	286	333	1.61	1699
4	435	0.0676	0.7	0.2563	426	447	0.86	113
5	473	7.7452	83.3	26.9438	447	583	2.48	12195
6	606	0.2546	2.7	0.6938	588	644	1.09	466
7	20000 (UM)	0.2466		0.0198	18171	28947	4.23	2321

TIC:	9.2968	ng/uL
TIM:	34.7650	nmole/L
Total concentration:	9.6626	ng/uL

Sample peak width (sec): 5 Sample min peak height: 50 Sample baseline V to V?: Y Sample baseline V to V points: 3
Sample filter: Binomial Number of points for filter: 3 Sample start region (min): 0 Sample end region (min): 30
Marker peak width (sec): 5 Marker min peak height: 200 Marker baseline V to V?: Y Marker baseline V to V points: 3
Lower marker selection: First peak > 500 RFU Upper marker selection: Last peak > 200 RFU
Ladder size (bp)75, 200, 300, 400, 500, 700, 1000, 1500, 2000, 3000, 4000, 5000, 7000, 10000, 20000
Quantification using: Lower Marker Final concentration (ng/uL): 0.5000 Dilution factor: 12.0

Sample: SampH7
Well location: H7
Created: Thursday, October 21, 2021 6:08:16 PM

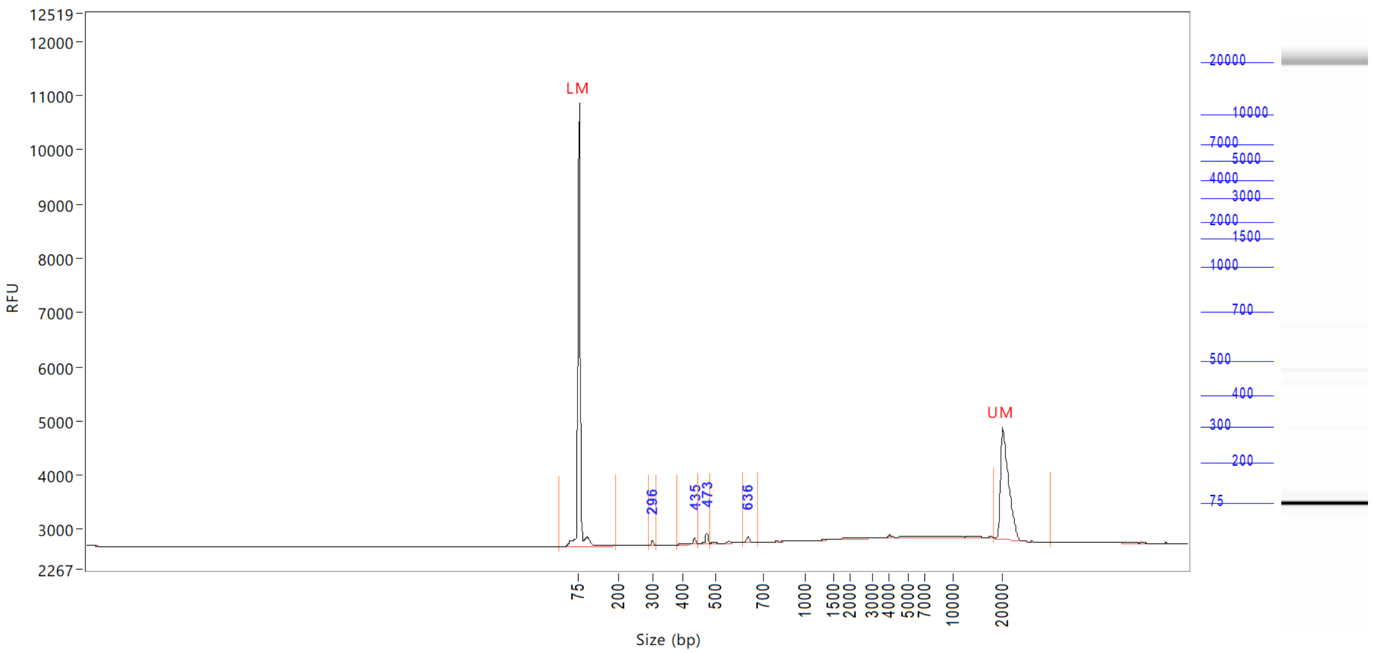

Peak	Size	Concentration	Relative concentration	Molarity	From	To	CV%	RFU
	(bp)	(ng/uL)	Percent	(nmole/L)	(bp)	(bp)		
1	75 (LM)	0.5000		11.0851	16	194	14.22	8193
2	296	0.0546	14.6	0.3043	287	311	1.21	86
3	435	0.1051	28.1	0.4058	384	447	3.22	132
4	473	0.1291	34.6	0.4518	447	485	1.09	200
5	636	0.0848	22.7	0.2195	616	677	1.37	119
6	20000 (UM)	0.2232		0.0178	18415	29672	4.62	2075

TIC:	0.3735	ng/uL
TIM:	1.3815	nmole/L
Total concentration:	0.9017	ng/uL

```
Sample peak width (sec): 5    Sample min peak height: 50    Sample baseline V to V?: Y    Sample baseline V to V points: 3
Sample filter: Binomial       Number of points for filter: 3  Sample start region (min): 0  Sample end region (min): 30
Marker peak width (sec): 5    Marker min peak height: 200   Marker baseline V to V?: Y    Marker baseline V to V points: 3
Lower marker selection: First peak > 500 RFU                Upper marker selection: Last peak > 200 RFU
Ladder size (bp)75, 200, 300, 400, 500, 700, 1000, 1500, 2000, 3000, 4000, 5000, 7000, 10000, 20000
Quantification using: Lower Marker          Final concentration (ng/uL): 0.5000          Dilution factor: 12.0
```

Sample: SampA8
Well location: A8
Created: Thursday, October 21, 2021 6:08:16 PM

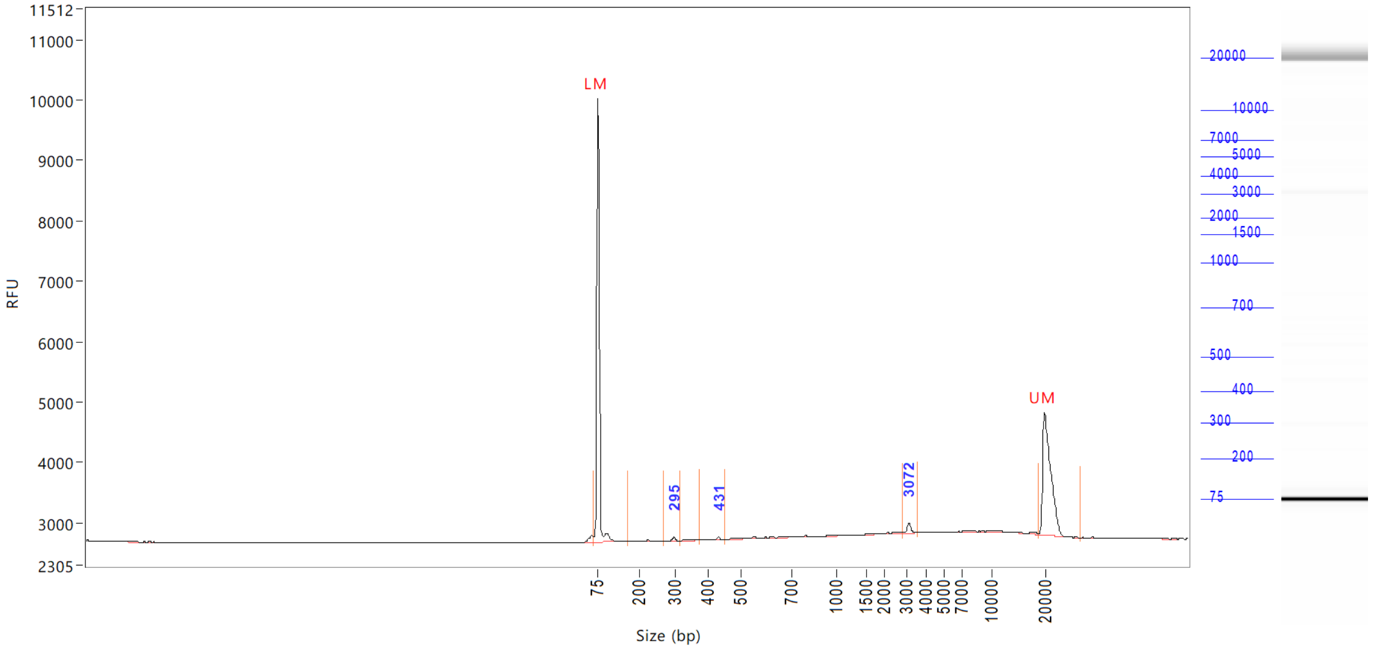

Peak	Size	Concentration	Relative concentration	Molarity	From	To	CV%	RFU
	(bp)	(ng/uL)	Percent	(nmole/L)	(bp)	(bp)		
1	75 (LM)	0.5000		11.0851	62	164	10.15	7352
2	295	0.0543	23.6	0.3068	268	311	2.32	64
3	431	0.0452	19.6	0.1749	376	450	2.66	51
4	3072	0.1308	56.8	0.0697	2857	3608	3.62	161
5	20000 (UM)	0.2476		0.0199	18903	26770	4.08	2024

TIC:	0.2303	ng/uL
TIM:	0.5515	nmole/L
Total concentration:	0.8996	ng/uL

```
Sample peak width (sec): 5    Sample min peak height: 50    Sample baseline V to V?: Y    Sample baseline V to V points: 3
Sample filter: Binomial       Number of points for filter: 3  Sample start region (min): 0  Sample end region (min): 30
Marker peak width (sec): 5    Marker min peak height: 200   Marker baseline V to V?: Y    Marker baseline V to V points: 3
Lower marker selection: First peak > 500 RFU                Upper marker selection: Last peak > 200 RFU
Ladder size (bp)75, 200, 300, 400, 500, 700, 1000, 1500, 2000, 3000, 4000, 5000, 7000, 10000, 20000
Quantification using: Lower Marker          Final concentration (ng/uL): 0.5000          Dilution factor: 12.0
```

Sample: SampB8
Well location: B8
Created: Thursday, October 21, 2021 6:08:16 PM

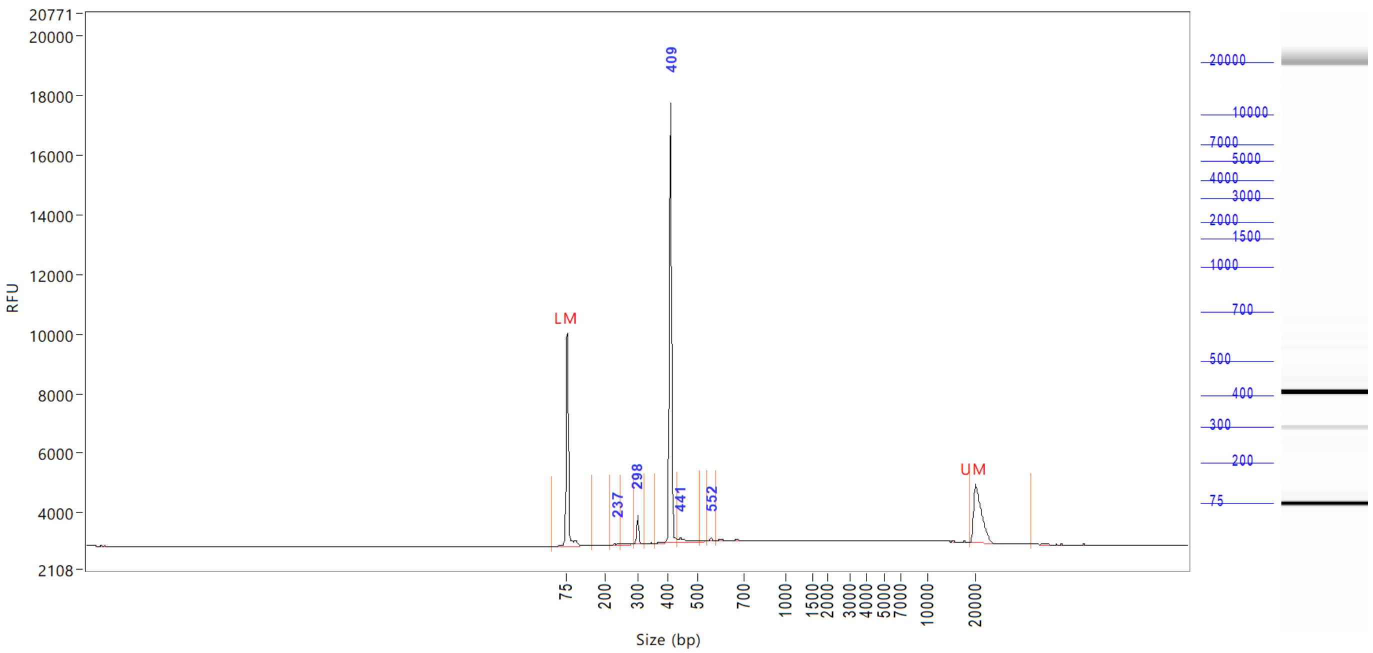

Peak	Size	Concentration	Relative concentration	Molarity	From	To	CV%	RFU
	(bp)	(ng/uL)	Percent	(nmole/L)	(bp)	(bp)		
1	75 (LM)	0.5000		11.0851	28	156	11.30	7152
2	237	0.0680	0.6	0.4818	214	244	3.20	52
3	298	0.6151	5.6	3.4297	284	317	1.18	950
4	409	9.9604	90.0	40.3645	358	433	1.16	14791
5	441	0.3305	3.0	1.1899	433	504	4.15	154
6	552	0.0894	0.8	0.2669	537	578	1.28	126
7	20000 (UM)	0.2342		0.0187	19024	31365	4.84	1966

TIC:	11.0633	ng/uL
TIM:	45.7328	nmole/L
Total concentration:	11.6524	ng/uL

Sample peak width (sec): 5 Sample min peak height: 50 Sample baseline V to V?: Y Sample baseline V to V points: 3
Sample filter: Binomial Number of points for filter: 3 Sample start region (min): 0 Sample end region (min): 30
Marker peak width (sec): 5 Marker min peak height: 200 Marker baseline V to V?: Y Marker baseline V to V points: 3
Lower marker selection: First peak > 500 RFU Upper marker selection: Last peak > 200 RFU
Ladder size (bp)75, 200, 300, 400, 500, 700, 1000, 1500, 2000, 3000, 4000, 5000, 7000, 10000, 20000
Quantification using: Lower Marker Final concentration (ng/uL): 0.5000 Dilution factor: 12.0

Sample: SampC8
Well location: C8
Created: Thursday, October 21, 2021 6:08:16 PM

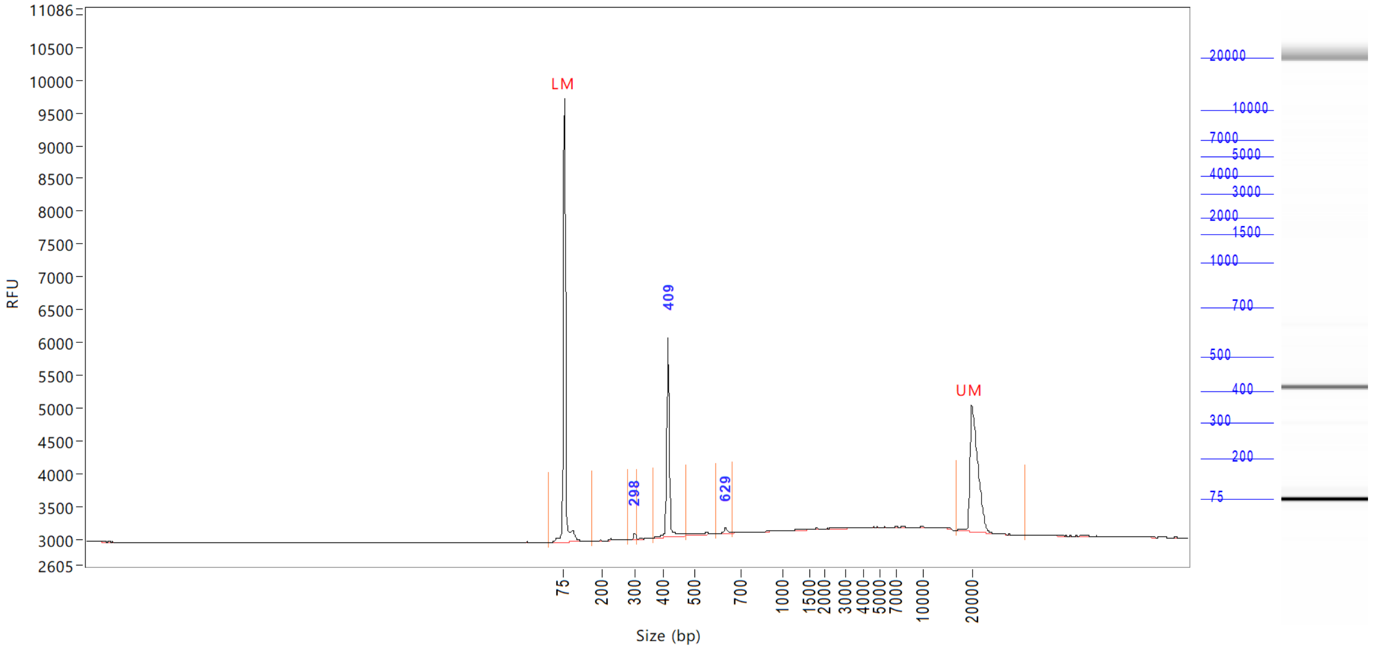

Peak	Size	Concentration	Relative concentration	Molarity	From	To	CV%	RFU
	(bp)	(ng/uL)	Percent	(nmole/L)	(bp)	(bp)		
1	75 (LM)	0.5000		11.0851	26	166	12.58	6774
2	298	0.0698	2.6	0.3876	277	307	1.26	98
3	409	2.5190	93.8	10.1086	360	470	2.61	3027
4	629	0.0971	3.6	0.2548	588	664	2.20	87
5	20000 (UM)	0.2462		0.0197	16830	31002	4.95	1934

TIC:	2.6858	ng/uL
TIM:	10.7511	nmole/L
Total concentration:	3.2113	ng/uL

```
Sample peak width (sec): 5    Sample min peak height: 50    Sample baseline V to V?: Y    Sample baseline V to V points: 3
Sample filter: Binomial       Number of points for filter: 3  Sample start region (min): 0  Sample end region (min): 30
Marker peak width (sec): 5    Marker min peak height: 200   Marker baseline V to V?: Y    Marker baseline V to V points: 3
Lower marker selection: First peak > 500 RFU                Upper marker selection: Last peak > 200 RFU
Ladder size (bp)75, 200, 300, 400, 500, 700, 1000, 1500, 2000, 3000, 4000, 5000, 7000, 10000, 20000
Quantification using: Lower Marker          Final concentration (ng/uL): 0.5000          Dilution factor: 12.0
```

Sample: SampD8
Well location: D8
Created: Thursday, October 21, 2021 6:08:16 PM

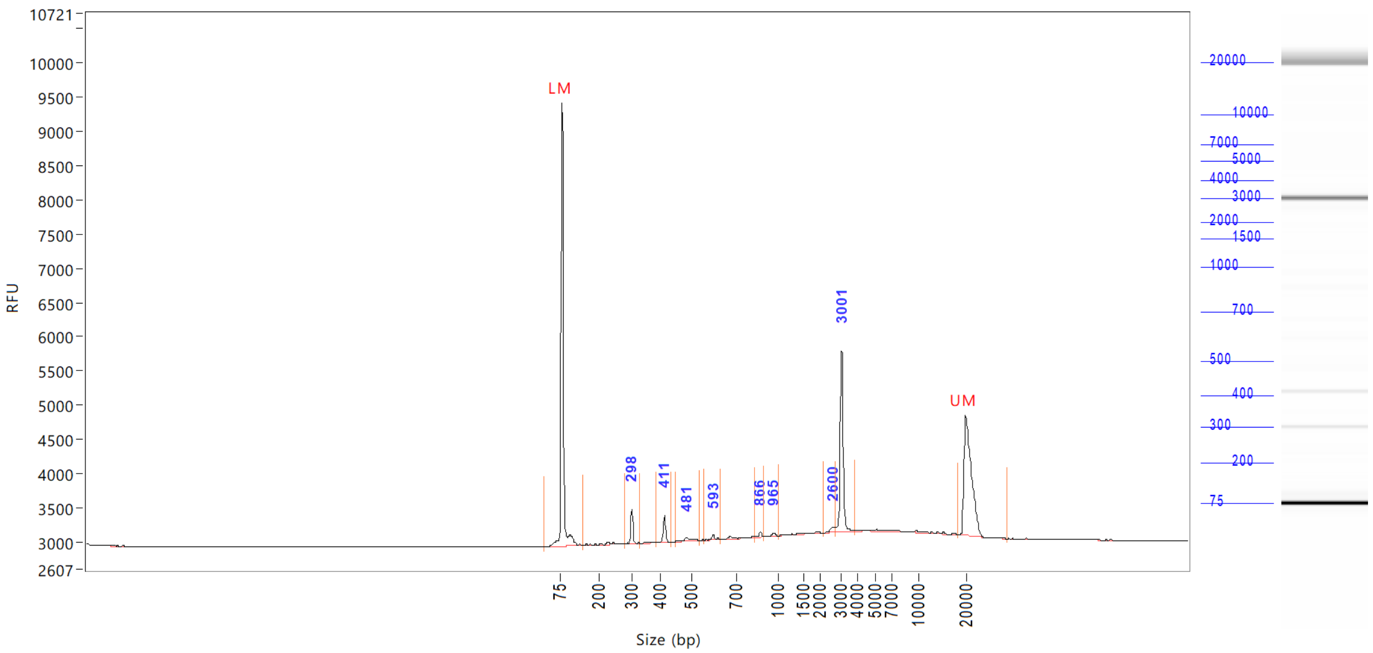

Peak	Size	Concentration	Relative concentration	Molarity	From	To	CV%	RFU
	(bp)	(ng/uL)	Percent	(nmole/L)	(bp)	(bp)		
1	75 (LM)	0.5000		10.9378	18	143	12.35	6480
2	298	0.3851	12.6	2.1330	277	327	1.65	508
3	411	0.3077	10.1	1.2408	382	431	1.07	403
4	481	0.1107	3.6	0.3739	447	532	3.18	52
5	593	0.0720	2.4	0.2000	555	626	1.99	70
6	866	0.0573	1.9	0.1097	827	900	1.41	70
7	965	0.0516	1.7	0.0883	900	1001	1.68	52
8	2600	0.0966	3.2	0.0625	2172	2715	4.62	77
9	3001	1.9787	64.7	1.0797	2715	3857	3.70	2639
10	20000 (UM)	0.2233		0.0179	18293	28705	4.20	1743

TIC:	3.0596	ng/uL
TIM:	5.2878	nmole/L
Total concentration:	3.6058	ng/uL

```
Sample peak width (sec): 5    Sample min peak height: 50    Sample baseline V to V?: Y    Sample baseline V to V points: 3
Sample filter: Binomial       Number of points for filter: 3  Sample start region (min): 0  Sample end region (min): 30
Marker peak width (sec): 5    Marker min peak height: 200   Marker baseline V to V?: Y    Marker baseline V to V points: 3
Lower marker selection: First peak > 500 RFU                Upper marker selection: Last peak > 200 RFU
Ladder size (bp)75, 200, 300, 400, 500, 700, 1000, 1500, 2000, 3000, 4000, 5000, 7000, 10000, 20000
Quantification using: Lower Marker          Final concentration (ng/uL): 0.5000          Dilution factor: 12.0
```

Sample: SampE8
Well location: E8
Created: Thursday, October 21, 2021 6:08:16 PM

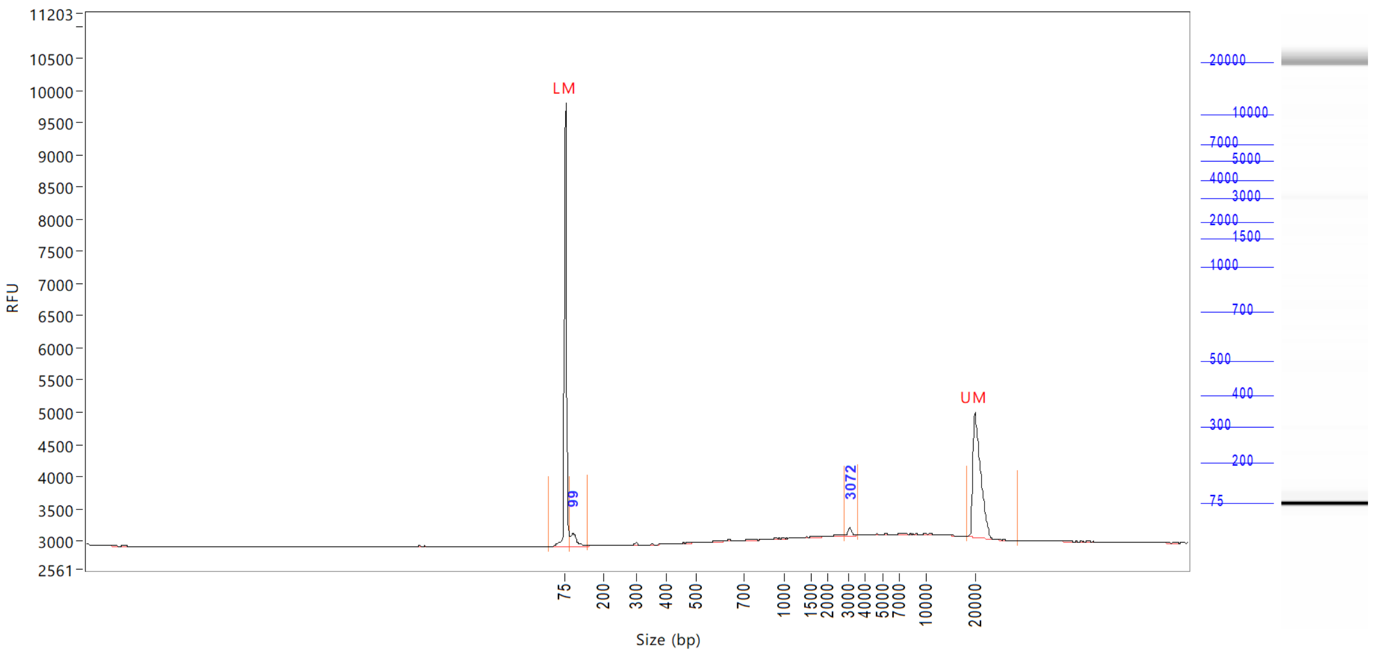

Peak	Size	Concentration	Relative concentration	Molarity	From	To	CV%	RFU
	(bp)	(ng/uL)	Percent	(nmole/L)	(bp)	(bp)		
1	75 (LM)	0.5000		11.3919	22	89	7.21	6906
2	99	0.4482	77.9	7.0780	89	148	12.18	206
3	3072	0.1270	22.1	0.0688	2772	3536	3.85	138
4	20000 (UM)	0.2503		0.0200	18171	28705	4.38	1939

TIC:	0.5753	ng/uL
TIM:	7.1468	nmole/L
Total concentration:	1.2286	ng/uL

```
Sample peak width (sec): 5    Sample min peak height: 50    Sample baseline V to V?: Y    Sample baseline V to V points: 3
Sample filter: Binomial       Number of points for filter: 3  Sample start region (min): 0  Sample end region (min): 30
Marker peak width (sec): 5    Marker min peak height: 200   Marker baseline V to V?: Y    Marker baseline V to V points: 3
Lower marker selection: First peak > 500 RFU                Upper marker selection: Last peak > 200 RFU
Ladder size (bp)75, 200, 300, 400, 500, 700, 1000, 1500, 2000, 3000, 4000, 5000, 7000, 10000, 20000
Quantification using: Lower Marker          Final concentration (ng/uL): 0.5000          Dilution factor: 12.0
```

Sample: SampF8
Well location: F8
Created: Thursday, October 21, 2021 6:08:16 PM

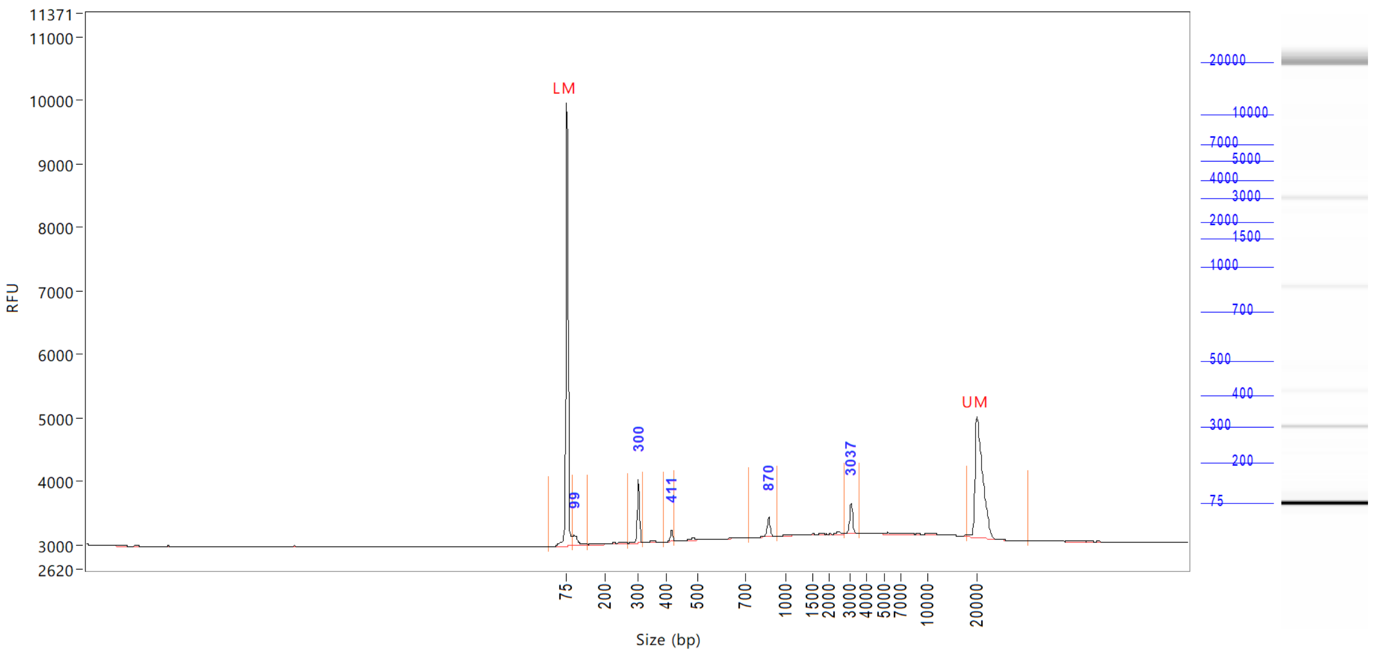

Peak	Size	Concentration	Relative concentration	Molarity	From	To	CV%	RFU
	(bp)	(ng/uL)	Percent	(nmole/L)	(bp)	(bp)		
1	75 (LM)	0.5000		11.2364	20	91	7.07	6986
2	99	0.3390	18.6	5.3021	91	145	11.90	165
3	300	0.7391	40.5	4.0935	268	315	1.49	1006
4	411	0.1332	7.3	0.5345	390	424	1.03	185
5	870	0.2495	13.7	0.4758	731	935	2.88	311
6	3037	0.3643	20.0	0.1983	2772	3572	2.89	493
7	20000 (UM)	0.2435		0.0195	18049	30639	4.76	1910

TIC:	1.8251	ng/uL
TIM:	10.6042	nmole/L
Total concentration:	2.5063	ng/uL

```
Sample peak width (sec): 5    Sample min peak height: 50    Sample baseline V to V?: Y    Sample baseline V to V points: 3
Sample filter: Binomial       Number of points for filter: 3  Sample start region (min): 0  Sample end region (min): 30
Marker peak width (sec): 5    Marker min peak height: 200   Marker baseline V to V?: Y    Marker baseline V to V points: 3
Lower marker selection: First peak > 500 RFU                Upper marker selection: Last peak > 200 RFU
Ladder size (bp)75, 200, 300, 400, 500, 700, 1000, 1500, 2000, 3000, 4000, 5000, 7000, 10000, 20000
Quantification using: Lower Marker          Final concentration (ng/uL): 0.5000          Dilution factor: 12.0
```

Sample: SampG8
Well location: G8
Created: Thursday, October 21, 2021 6:08:16 PM

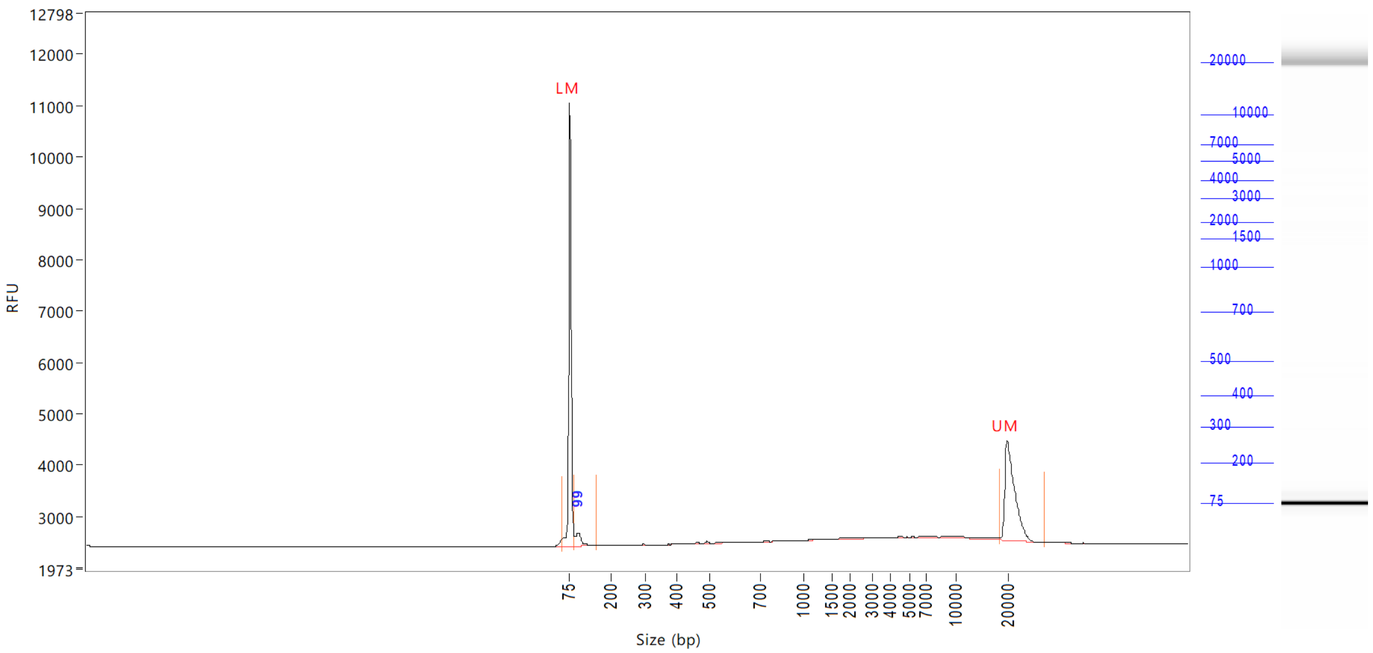

Peak	Size	Concentration	Relative concentration	Molarity	From	To	CV%	RFU
	(bp)	(ng/uL)	Percent	(nmole/L)	(bp)	(bp)		
1	75 (LM)	0.5000		11.2364	50	89	6.32	8647
2	99	0.4669	100.0	7.4439	89	154	11.41	256
3	20000 (UM)	0.2408		0.0191	18537	27375	5.26	1951

TIC:	0.4669	ng/uL
TIM:	7.4439	nmole/L
Total concentration:	1.1611	ng/uL

```
Sample peak width (sec): 5    Sample min peak height: 50    Sample baseline V to V?: Y    Sample baseline V to V points: 3
Sample filter: Binomial       Number of points for filter: 3  Sample start region (min): 0  Sample end region (min): 30
Marker peak width (sec): 5    Marker min peak height: 200   Marker baseline V to V?: Y    Marker baseline V to V points: 3
Lower marker selection: First peak > 500 RFU                Upper marker selection: Last peak > 200 RFU
Ladder size (bp)75, 200, 300, 400, 500, 700, 1000, 1500, 2000, 3000, 4000, 5000, 7000, 10000, 20000
Quantification using: Lower Marker          Final concentration (ng/uL): 0.5000          Dilution factor: 12.0
```

Sample: SampH8
Well location: H8
Created: Thursday, October 21, 2021 6:08:16 PM

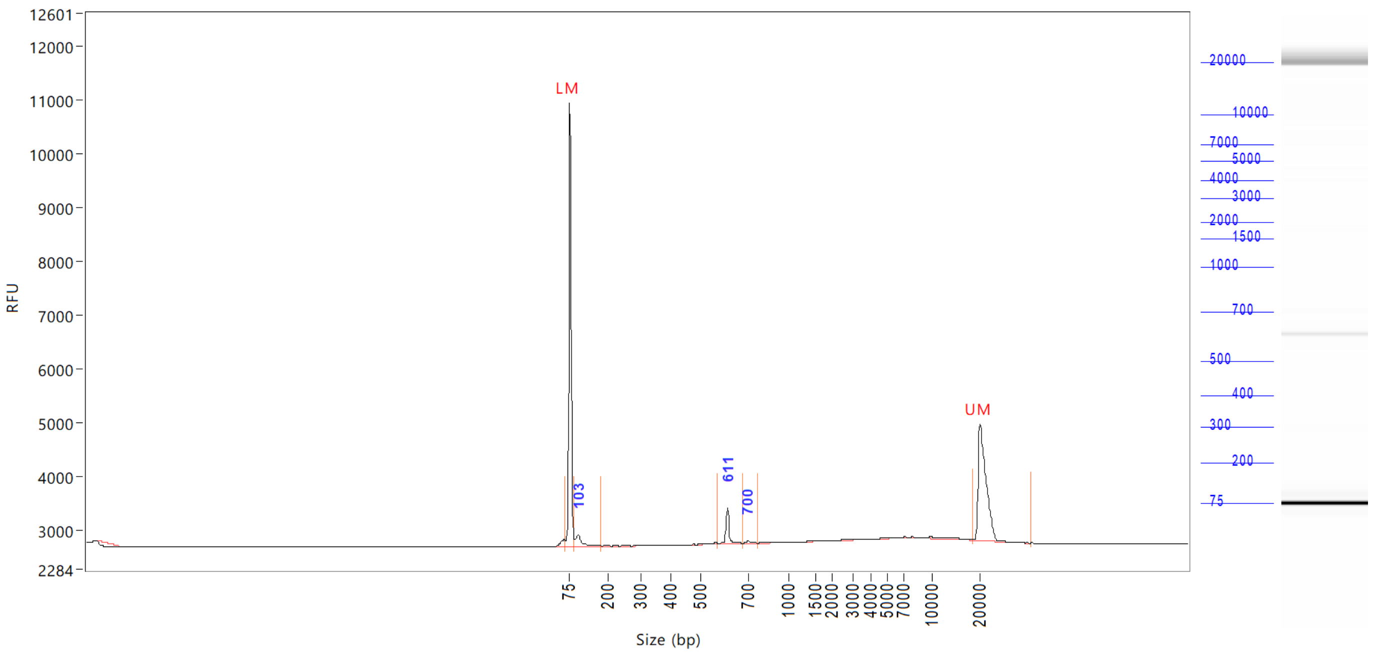

Peak	Size	Concentration	Relative concentration	Molarity	From	To	CV%	RFU
	(bp)	(ng/uL)	Percent	(nmole/L)	(bp)	(bp)		
1	75 (LM)	0.5000		11.0851	64	89	4.58	8246
2	103	0.4228	42.7	6.4889	89	177	15.60	205
3	611	0.5079	51.3	1.3703	567	677	2.39	656
4	700	0.0589	6.0	0.1375	677	766	2.70	55
5	20000 (UM)	0.2500		0.0199	18415	30639	4.67	2154

TIC:	0.9896	ng/uL
TIM:	7.9967	nmole/L
Total concentration:	1.5169	ng/uL

```
Sample peak width (sec): 5    Sample min peak height: 50    Sample baseline V to V?: Y    Sample baseline V to V points: 3
Sample filter: Binomial       Number of points for filter: 3  Sample start region (min): 0  Sample end region (min): 30
Marker peak width (sec): 5    Marker min peak height: 200   Marker baseline V to V?: Y    Marker baseline V to V points: 3
Lower marker selection: First peak > 500 RFU                Upper marker selection: Last peak > 200 RFU
Ladder size (bp)75, 200, 300, 400, 500, 700, 1000, 1500, 2000, 3000, 4000, 5000, 7000, 10000, 20000
Quantification using: Lower Marker          Final concentration (ng/uL): 0.5000          Dilution factor: 12.0
```

Sample: SampA9
Well location: A9
Created: Thursday, October 21, 2021 6:08:16 PM

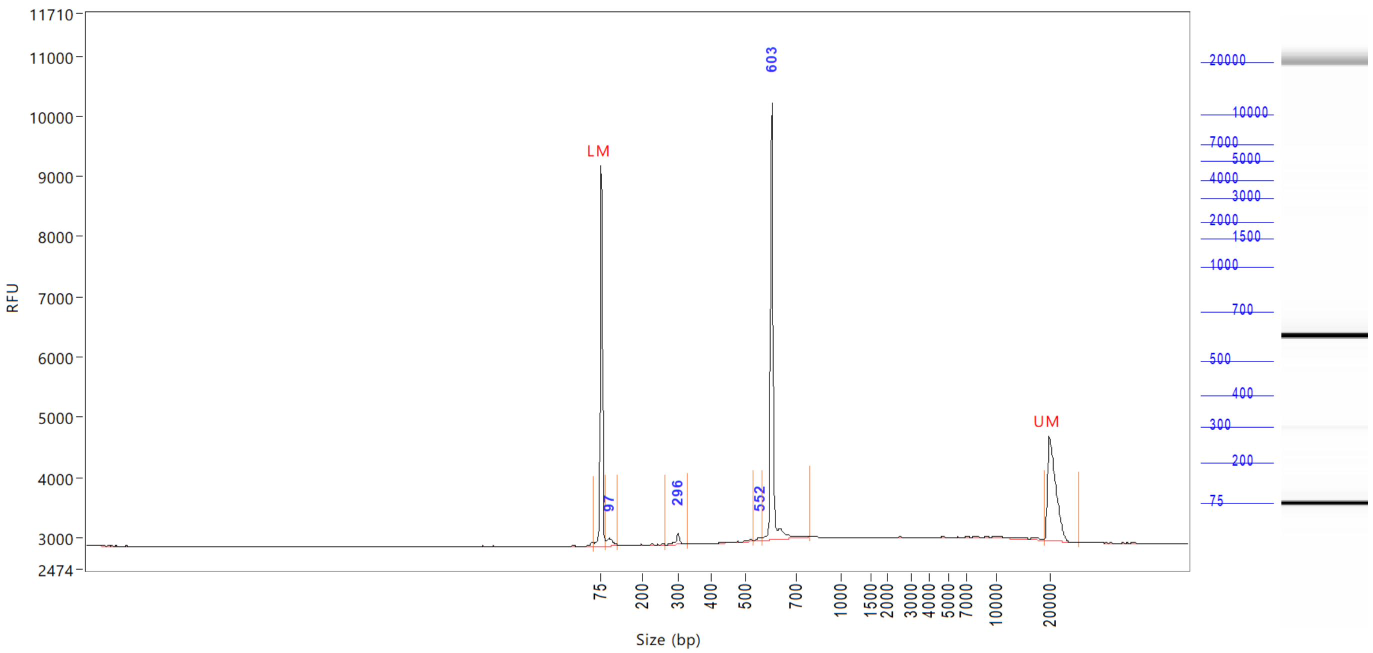

Peak	Size	Concentration	Relative concentration	Molarity	From	To	CV%	RFU
	(bp)	(ng/uL)	Percent	(nmole/L)	(bp)	(bp)		
1	75 (LM)	0.5000		11.0851	56	91	5.17	6315
2	97	0.3033	4.3	4.8831	91	126	8.40	128
3	296	0.2465	3.5	1.3839	262	325	3.07	196
4	552	0.0863	1.2	0.2581	532	565	1.66	54
5	603	6.3427	90.9	17.2811	565	792	3.03	7270
6	20000 (UM)	0.2520		0.0202	18903	25440	4.01	1730

TIC:	6.9787	ng/uL
TIM:	23.8062	nmole/L
Total concentration:	7.6827	ng/uL

```
Sample peak width (sec): 5    Sample min peak height: 50    Sample baseline V to V?: Y    Sample baseline V to V points: 3
Sample filter: Binomial       Number of points for filter: 3  Sample start region (min): 0  Sample end region (min): 30
Marker peak width (sec): 5    Marker min peak height: 200   Marker baseline V to V?: Y    Marker baseline V to V points: 3
Lower marker selection: First peak > 500 RFU                Upper marker selection: Last peak > 200 RFU
Ladder size (bp)75, 200, 300, 400, 500, 700, 1000, 1500, 2000, 3000, 4000, 5000, 7000, 10000, 20000
Quantification using: Lower Marker          Final concentration (ng/uL): 0.5000          Dilution factor: 12.0
```

Sample: SampB9
Well location: B9
Created: Thursday, October 21, 2021 6:08:16 PM

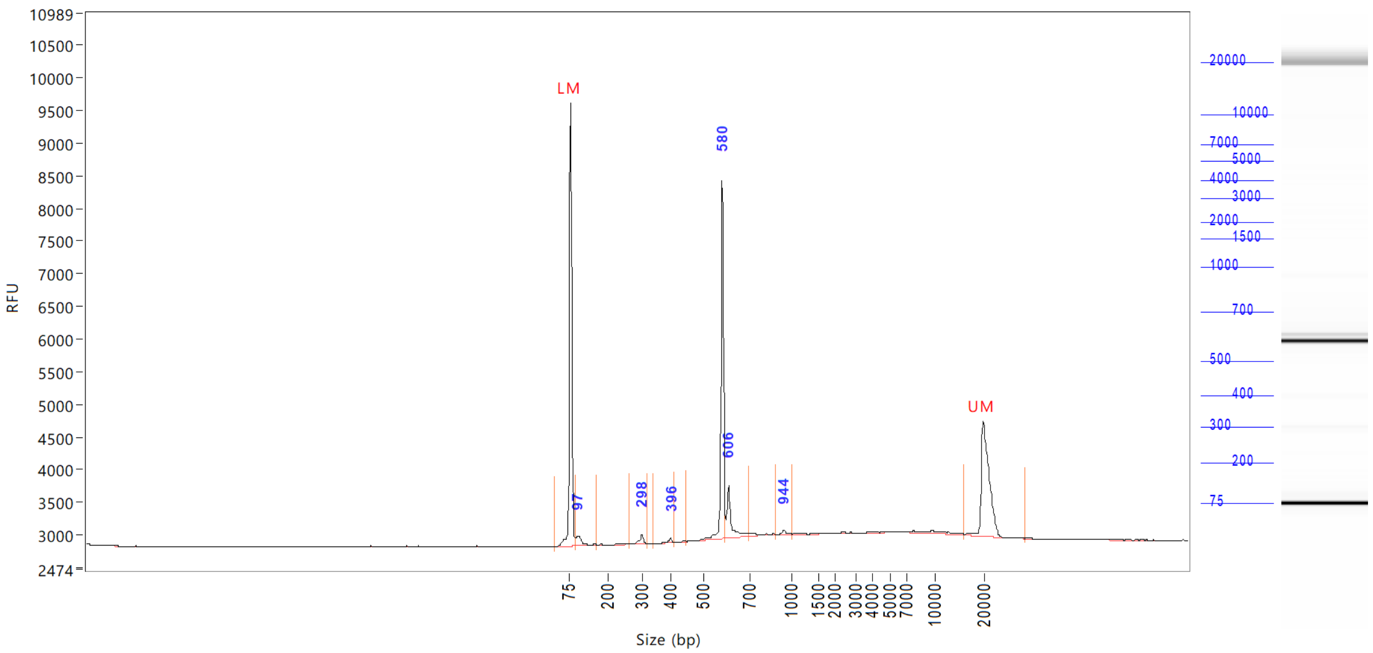

Peak	Size	Concentration	Relative concentration	Molarity	From	To	CV%	RFU
	(bp)	(ng/uL)	Percent	(nmole/L)	(bp)	(bp)		
1	75 (LM)	0.5000		11.2364	26	89	7.78	6798
2	97	0.3500	6.0	5.4234	89	162	15.20	152
3	298	0.1998	3.4	1.1255	259	319	2.94	148
4	396	0.0708	1.2	0.3004	335	405	2.97	71
5	580	4.1892	71.4	11.9685	447	595	2.24	5471
6	606	0.9946	16.9	2.6484	595	692	3.76	810
7	944	0.0659	1.1	0.1149	892	1012	2.38	62
8	20000 (UM)	0.2364		0.0190	16098	28463	5.07	1755

TIC:	5.8704	ng/uL
TIM:	21.5810	nmole/L
Total concentration:	6.4316	ng/uL

```
Sample peak width (sec): 5     Sample min peak height: 50     Sample baseline V to V?: Y     Sample baseline V to V points: 3
Sample filter: Binomial        Number of points for filter: 3  Sample start region (min): 0  Sample end region (min): 30
Marker peak width (sec): 5     Marker min peak height: 200    Marker baseline V to V?: Y     Marker baseline V to V points: 3
Lower marker selection: First peak > 500 RFU                  Upper marker selection: Last peak > 200 RFU
Ladder size (bp)75, 200, 300, 400, 500, 700, 1000, 1500, 2000, 3000, 4000, 5000, 7000, 10000, 20000
Quantification using: Lower Marker          Final concentration (ng/uL): 0.5000          Dilution factor: 12.0
```

Sample: SampC9
Well location: C9
Created: Thursday, October 21, 2021 6:08:16 PM

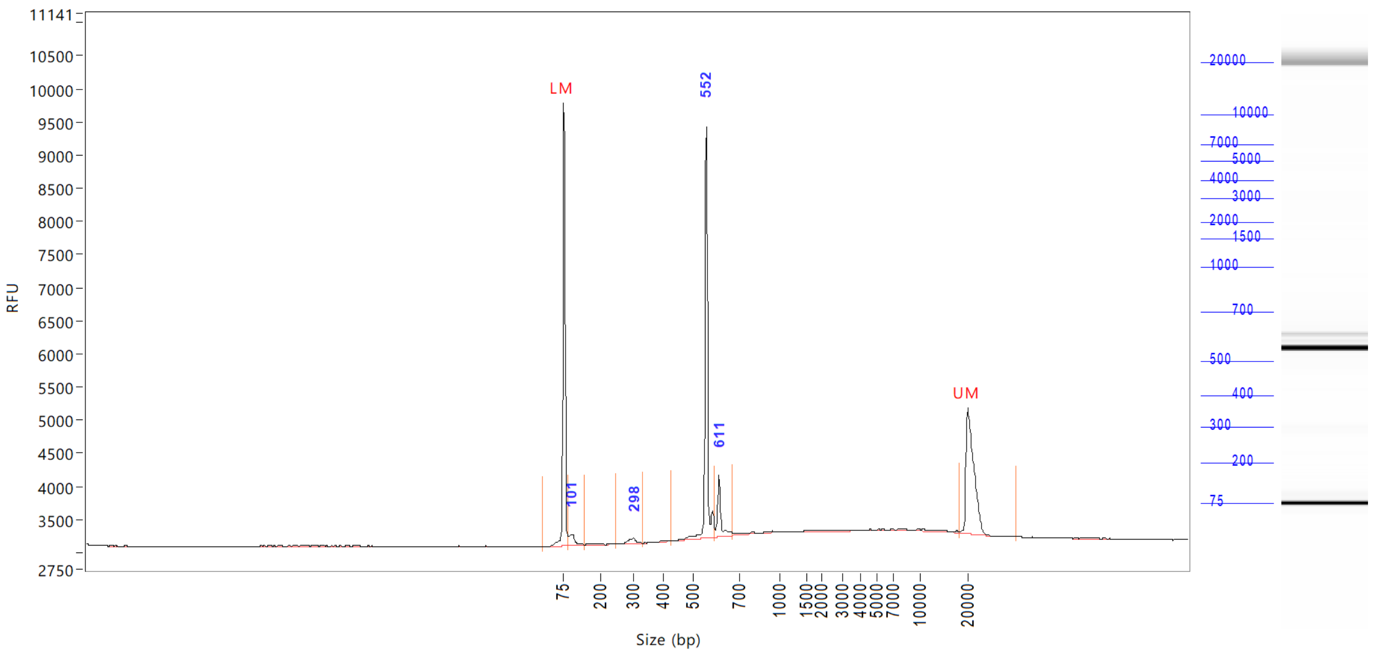

Peak	Size	Concentration	Relative concentration	Molarity	From	To	CV%	RFU
	(bp)	(ng/uL)	Percent	(nmole/L)	(bp)	(bp)		
1	75 (LM)	0.5000		11.2364	7	93	7.80	6698
2	101	0.3672	5.5	5.7439	93	143	10.49	175
3	298	0.2134	3.2	1.2187	244	327	4.30	84
4	552	5.0910	76.9	15.2322	428	593	2.75	6205
5	611	0.9485	14.3	2.5463	593	672	2.78	936
6	20000 (UM)	0.2609		0.0209	18171	30156	4.73	1895

TIC:	6.6201	ng/uL
TIM:	24.7411	nmole/L
Total concentration:	7.3959	ng/uL

Sample peak width (sec): 5 Sample min peak height: 50 Sample baseline V to V?: Y Sample baseline V to V points: 3
Sample filter: Binomial Number of points for filter: 3 Sample start region (min): 0 Sample end region (min): 30
Marker peak width (sec): 5 Marker min peak height: 200 Marker baseline V to V?: Y Marker baseline V to V points: 3
Lower marker selection: First peak > 500 RFU Upper marker selection: Last peak > 200 RFU
Ladder size (bp)75, 200, 300, 400, 500, 700, 1000, 1500, 2000, 3000, 4000, 5000, 7000, 10000, 20000
Quantification using: Lower Marker Final concentration (ng/uL): 0.5000 Dilution factor: 12.0

Sample: SampD9
Well location: D9
Created: Thursday, October 21, 2021 6:08:16 PM

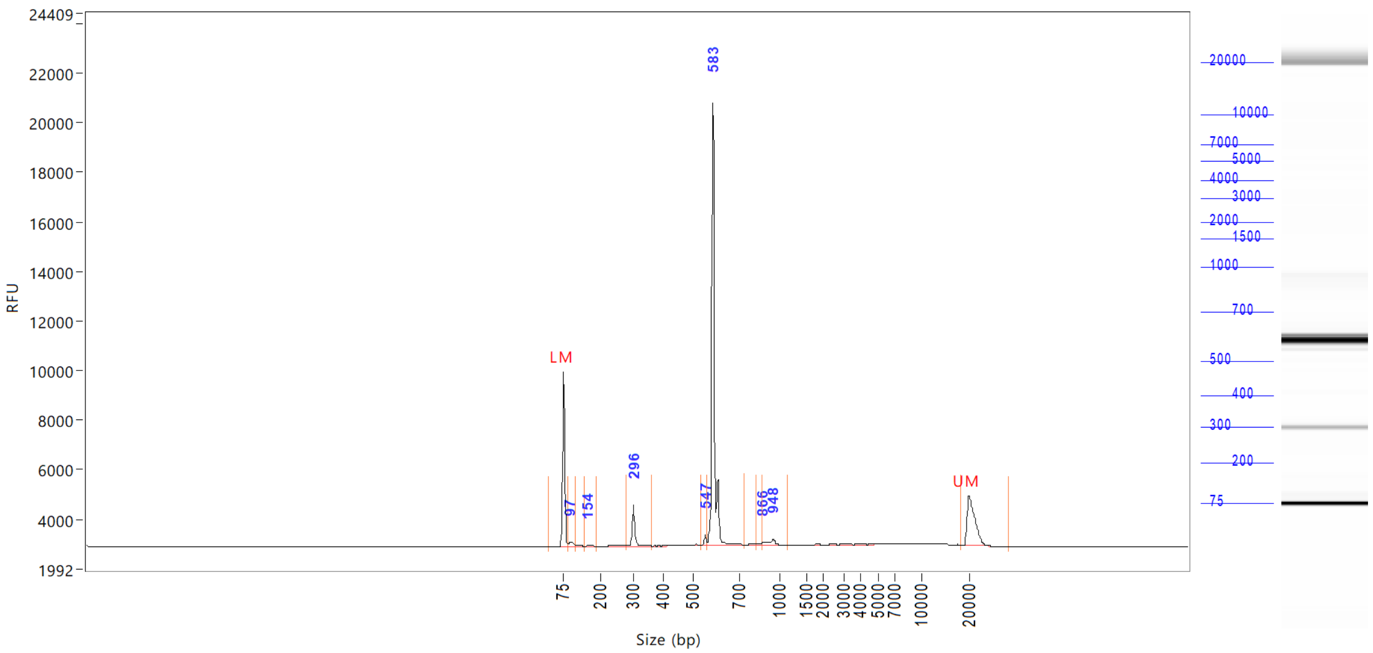

Peak	Size	Concentration	Relative concentration	Molarity	From	To	CV%	RFU
	(bp)	(ng/uL)	Percent	(nmole/L)	(bp)	(bp)		
1	75 (LM)	0.5000		11.2364	30	87	4.78	7049
2	97	0.3374	2.0	5.6528	87	116	7.00	181
3	154	0.0671	0.4	0.7065	145	181	4.79	53
4	296	1.4261	8.3	7.8986	278	360	2.53	1638
5	547	0.2946	1.7	0.8878	532	557	1.07	400
6	583	14.5812	84.6	41.1583	557	727	2.08	17864
7	866	0.0845	0.5	0.1629	822	870	1.52	93
8	948	0.4482	2.6	0.7984	870	1123	3.55	214
9	20000 (UM)	0.2630		0.0210	18415	28342	4.28	2002

TIC:	17.2391	ng/uL
TIM:	57.2653	nmole/L
Total concentration:	17.9655	ng/uL

Sample peak width (sec): 5 Sample min peak height: 50 Sample baseline V to V?: Y Sample baseline V to V points: 3
Sample filter: Binomial Number of points for filter: 3 Sample start region (min): 0 Sample end region (min): 30
Marker peak width (sec): 5 Marker min peak height: 200 Marker baseline V to V?: Y Marker baseline V to V points: 3
Lower marker selection: First peak > 500 RFU Upper marker selection: Last peak > 200 RFU
Ladder size (bp)75, 200, 300, 400, 500, 700, 1000, 1500, 2000, 3000, 4000, 5000, 7000, 10000, 20000
Quantification using: Lower Marker Final concentration (ng/uL): 0.5000 Dilution factor: 12.0

Sample: SampE9
Well location: E9
Created: Thursday, October 21, 2021 6:08:16 PM

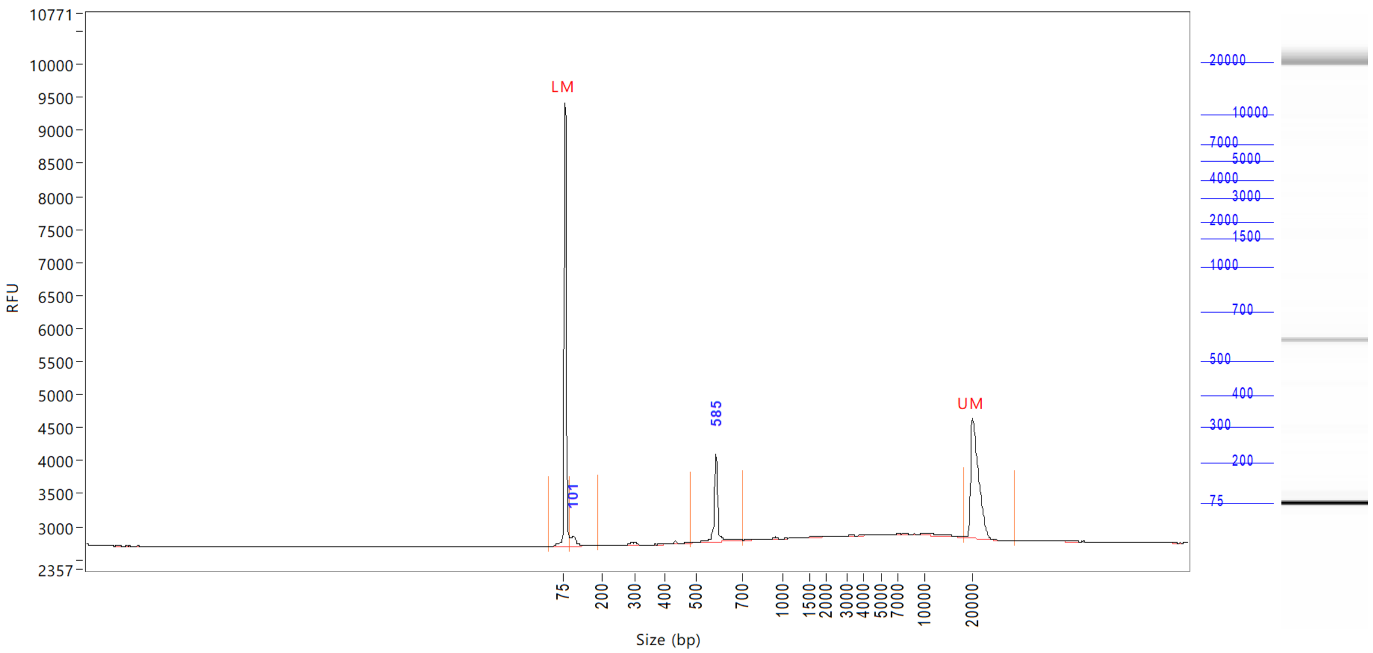

Peak	Size	Concentration	Relative concentration	Molarity	From	To	CV%	RFU
	(bp)	(ng/uL)	Percent	(nmole/L)	(bp)	(bp)		
1	75 (LM)	0.5000		11.2364	24	91	6.60	6723
2	101	0.3596	21.0	5.4178	91	185	17.92	159
3	585	1.3510	79.0	3.8005	483	701	4.39	1338
4	20000 (UM)	0.2462		0.0196	18293	28705	4.50	1819

TIC:	1.7106	ng/uL
TIM:	9.2184	nmole/L
Total concentration:	2.4194	ng/uL

Sample peak width (sec): 5 Sample min peak height: 50 Sample baseline V to V?: Y Sample baseline V to V points: 3
Sample filter: Binomial Number of points for filter: 3 Sample start region (min): 0 Sample end region (min): 30
Marker peak width (sec): 5 Marker min peak height: 200 Marker baseline V to V?: Y Marker baseline V to V points: 3
Lower marker selection: First peak > 500 RFU Upper marker selection: Last peak > 200 RFU
Ladder size (bp)75, 200, 300, 400, 500, 700, 1000, 1500, 2000, 3000, 4000, 5000, 7000, 10000, 20000
Quantification using: Lower Marker Final concentration (ng/uL): 0.5000 Dilution factor: 12.0

Sample: SampF9
Well location: F9
Created: Thursday, October 21, 2021 6:08:16 PM

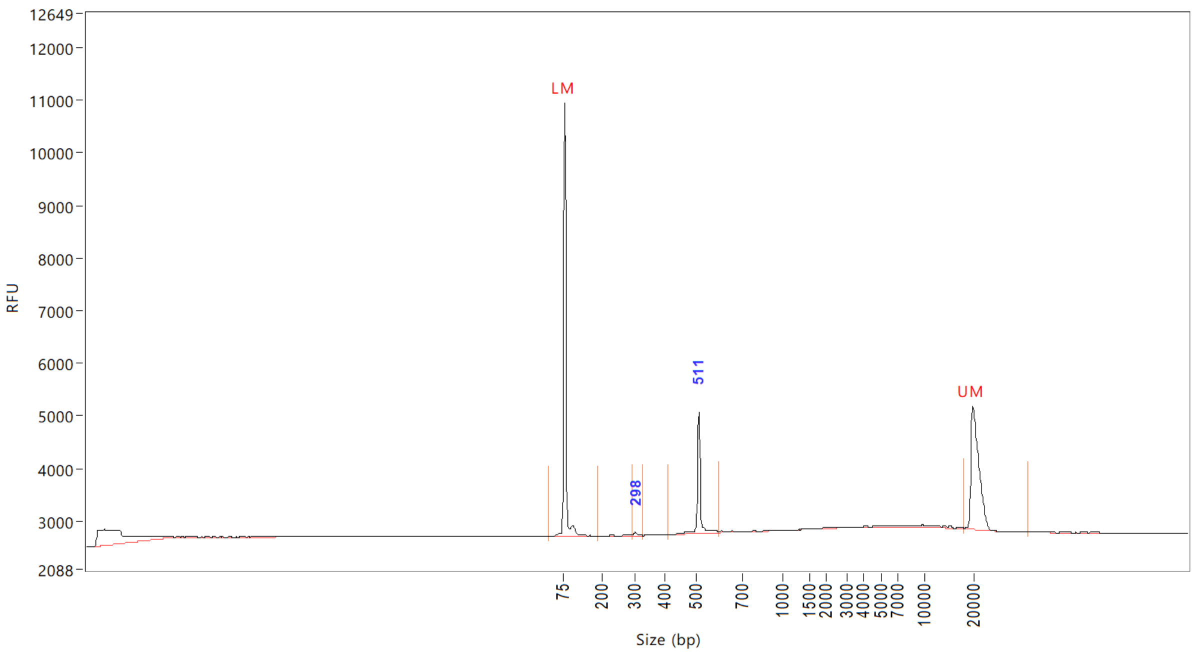

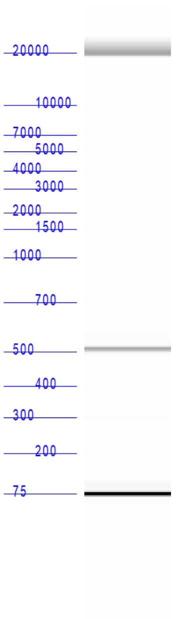

Peak	Size	Concentration	Relative concentration	Molarity	From	To	CV%	RFU
	(bp)	(ng/uL)	Percent	(nmole/L)	(bp)	(bp)		
1	75 (LM)	0.5000		11.0851	24	183	13.64	8250
2	298	0.0418	2.5	0.2313	287	323	1.87	60
3	511	1.6219	97.5	5.2331	409	595	3.79	2309
4	20000 (UM)	0.2418		0.0193	18171	31365	4.61	2331

TIC:	1.6637	ng/uL
TIM:	5.4644	nmole/L
Total concentration:	2.1845	ng/uL

```
Sample peak width (sec): 5    Sample min peak height: 50    Sample baseline V to V?: Y    Sample baseline V to V points: 3
Sample filter: Binomial       Number of points for filter: 3  Sample start region (min): 0  Sample end region (min): 30
Marker peak width (sec): 5    Marker min peak height: 200   Marker baseline V to V?: Y    Marker baseline V to V points: 3
Lower marker selection: First peak > 500 RFU                Upper marker selection: Last peak > 200 RFU
Ladder size (bp)75, 200, 300, 400, 500, 700, 1000, 1500, 2000, 3000, 4000, 5000, 7000, 10000, 20000
Quantification using: Lower Marker          Final concentration (ng/uL): 0.5000          Dilution factor: 12.0
```

Sample: SampG9
Well location: G9
Created: Thursday, October 21, 2021 6:08:16 PM

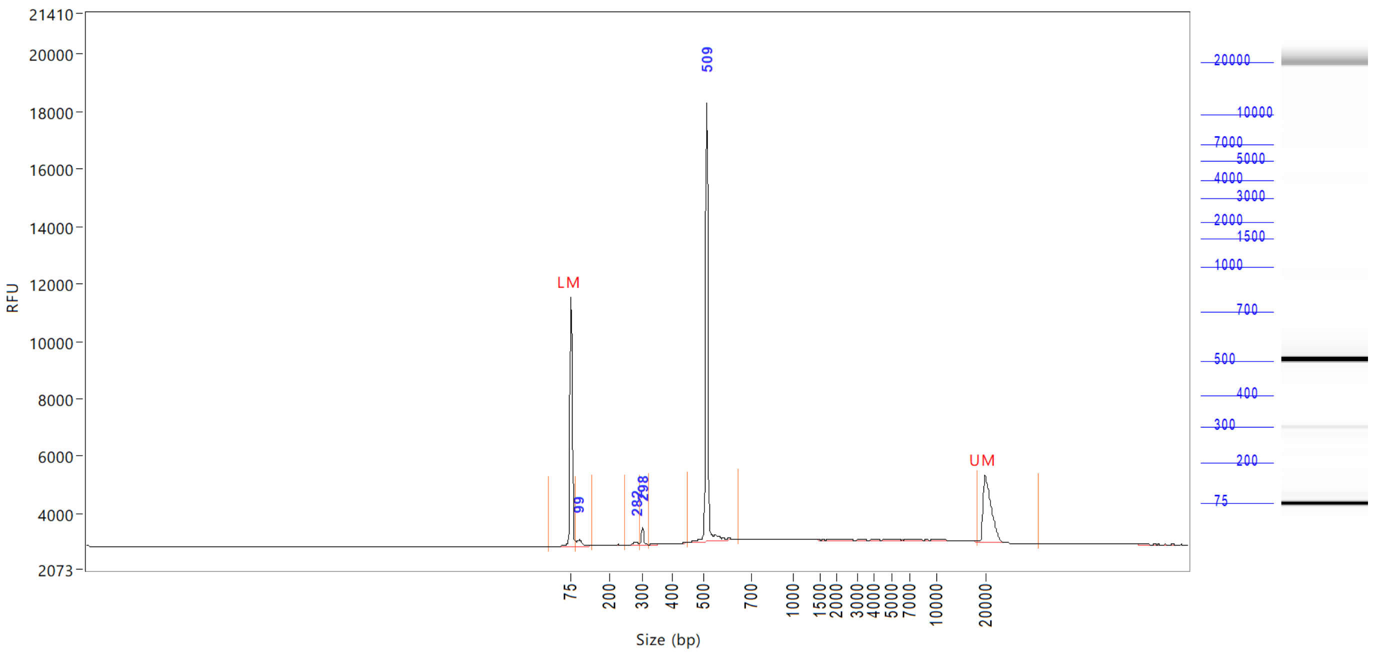

Peak	Size	Concentration	Relative concentration	Molarity	From	To	CV%	RFU
	(bp)	(ng/uL)	Percent	(nmole/L)	(bp)	(bp)		
1	75 (LM)	0.5000		11.2364	3	91	6.53	8680
2	99	0.3468	3.4	5.4764	91	145	10.59	215
3	282	0.1274	1.2	0.7623	246	289	3.06	74
4	298	0.3748	3.6	2.0831	289	317	1.21	602
5	509	9.4351	91.7	30.5624	449	646	2.88	15279
6	20000 (UM)	0.2545		0.0204	18293	31002	4.84	2334

TIC:	10.2842	ng/uL
TIM:	38.8842	nmole/L
Total concentration:	10.8304	ng/uL

Sample peak width (sec): 5 Sample min peak height: 50 Sample baseline V to V?: Y Sample baseline V to V points: 3
Sample filter: Binomial Number of points for filter: 3 Sample start region (min): 0 Sample end region (min): 30
Marker peak width (sec): 5 Marker min peak height: 200 Marker baseline V to V?: Y Marker baseline V to V points: 3
Lower marker selection: First peak > 500 RFU Upper marker selection: Last peak > 200 RFU
Ladder size (bp)75, 200, 300, 400, 500, 700, 1000, 1500, 2000, 3000, 4000, 5000, 7000, 10000, 20000
Quantification using: Lower Marker Final concentration (ng/uL): 0.5000 Dilution factor: 12.0

Sample: SampH9
Well location: H9
Created: Thursday, October 21, 2021 6:08:16 PM

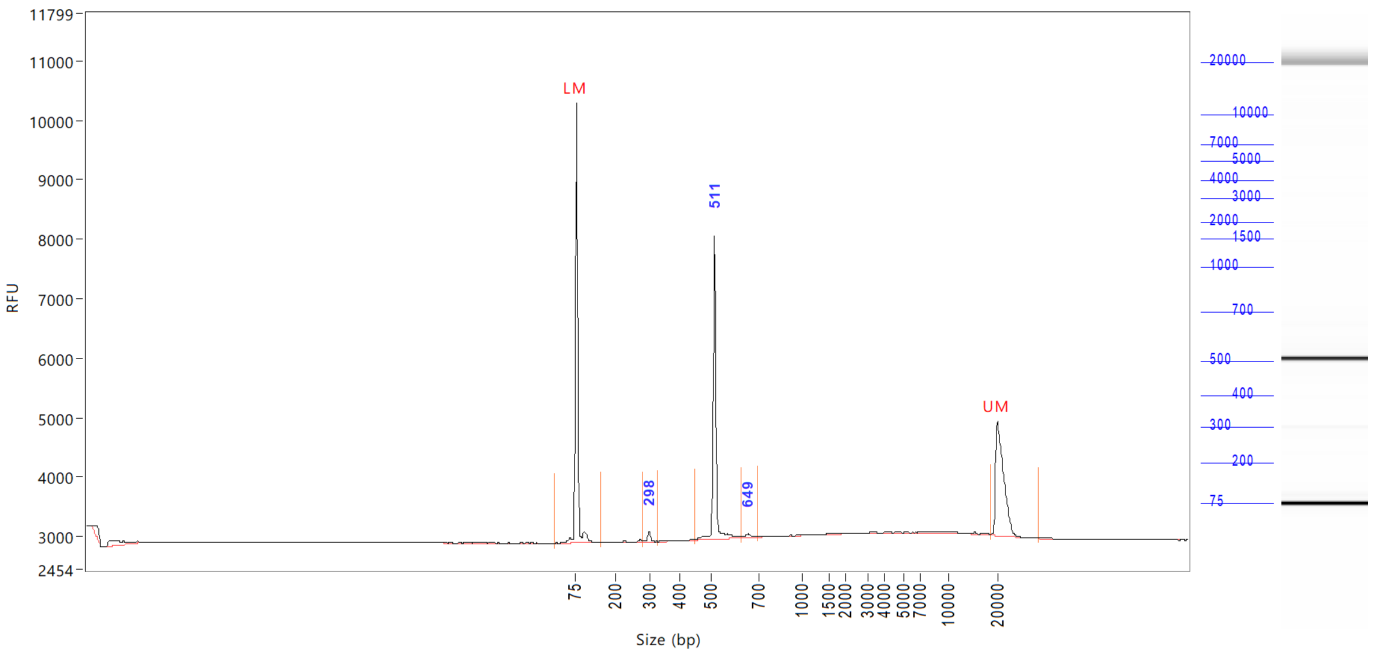

Peak	Size	Concentration	Relative concentration	Molarity	From	To	CV%	RFU
	(bp)	(ng/uL)	Percent	(nmole/L)	(bp)	(bp)		
1	75 (LM)	0.5000		11.0851	5	152	11.92	7408
2	298	0.1378	3.4	0.7686	280	325	2.03	168
3	511	3.8080	93.8	12.2625	447	623	3.76	5086
4	649	0.1149	2.8	0.2891	623	695	2.76	61
5	20000 (UM)	0.2304		0.0184	18537	28100	4.56	1929

TIC:	4.0607	ng/uL
TIM:	13.3201	nmole/L
Total concentration:	4.6152	ng/uL

Sample peak width (sec): 5 Sample min peak height: 50 Sample baseline V to V?: Y Sample baseline V to V points: 3
Sample filter: Binomial Number of points for filter: 3 Sample start region (min): 0 Sample end region (min): 30
Marker peak width (sec): 5 Marker min peak height: 200 Marker baseline V to V?: Y Marker baseline V to V points: 3
Lower marker selection: First peak > 500 RFU Upper marker selection: Last peak > 200 RFU
Ladder size (bp)75, 200, 300, 400, 500, 700, 1000, 1500, 2000, 3000, 4000, 5000, 7000, 10000, 20000
Quantification using: Lower Marker Final concentration (ng/uL): 0.5000 Dilution factor: 12.0

Sample: SampA10
Well location: A10
Created: Thursday, October 21, 2021 6:08:16 PM

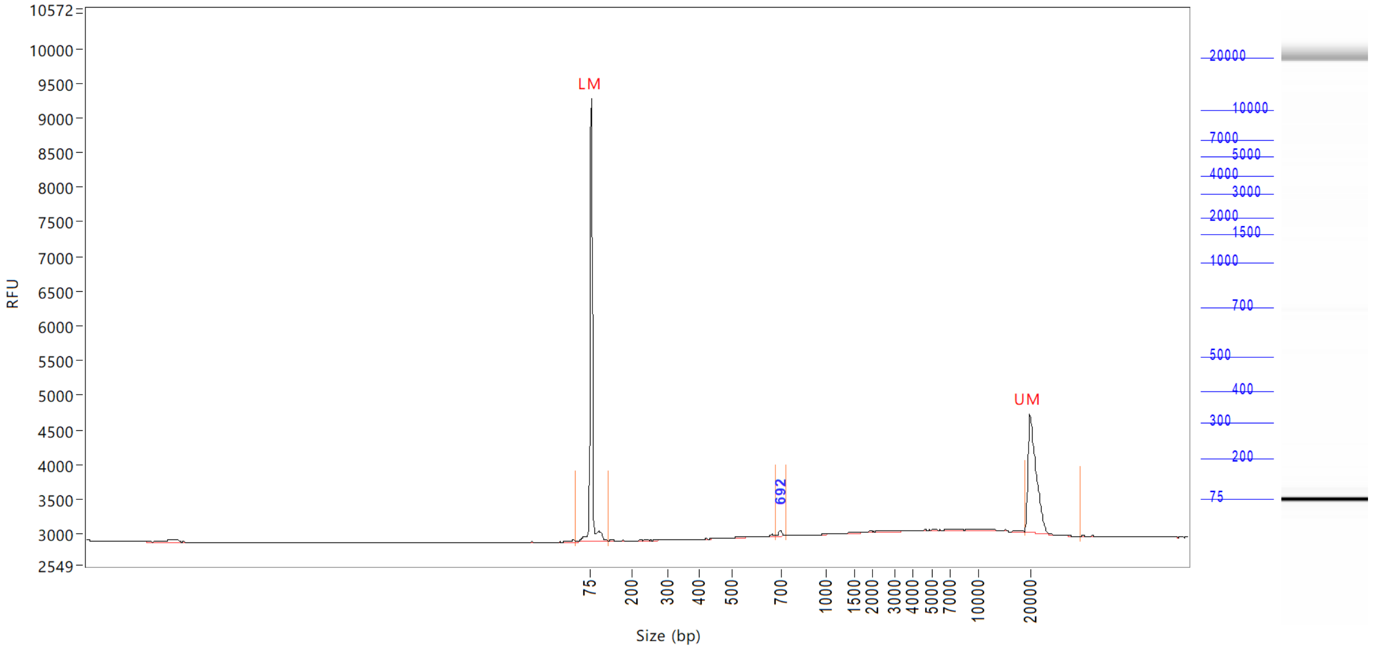

Peak	Size	Concentration	Relative concentration	Molarity	From	To	CV%	RFU
	(bp)	(ng/uL)	Percent	(nmole/L)	(bp)	(bp)		
1	75 (LM)	0.5000		11.2364	30	126	10.89	6401
2	692	0.0686	100.0	0.1634	677	731	1.18	75
3	20000 (UM)	0.2329		0.0186	18903	29672	4.47	1710

TIC:	0.0686	ng/uL
TIM:	0.1634	nmole/L
Total concentration:	0.7561	ng/uL

```
Sample peak width (sec): 5    Sample min peak height: 50    Sample baseline V to V?: Y    Sample baseline V to V points: 3
Sample filter: Binomial       Number of points for filter: 3  Sample start region (min): 0  Sample end region (min): 30
Marker peak width (sec): 5    Marker min peak height: 200   Marker baseline V to V?: Y    Marker baseline V to V points: 3
Lower marker selection: First peak > 500 RFU                Upper marker selection: Last peak > 200 RFU
Ladder size (bp)75, 200, 300, 400, 500, 700, 1000, 1500, 2000, 3000, 4000, 5000, 7000, 10000, 20000
Quantification using: Lower Marker          Final concentration (ng/uL): 0.5000          Dilution factor: 12.0
```

Sample: SampB10
Well location: B10
Created: Thursday, October 21, 2021 6:08:16 PM

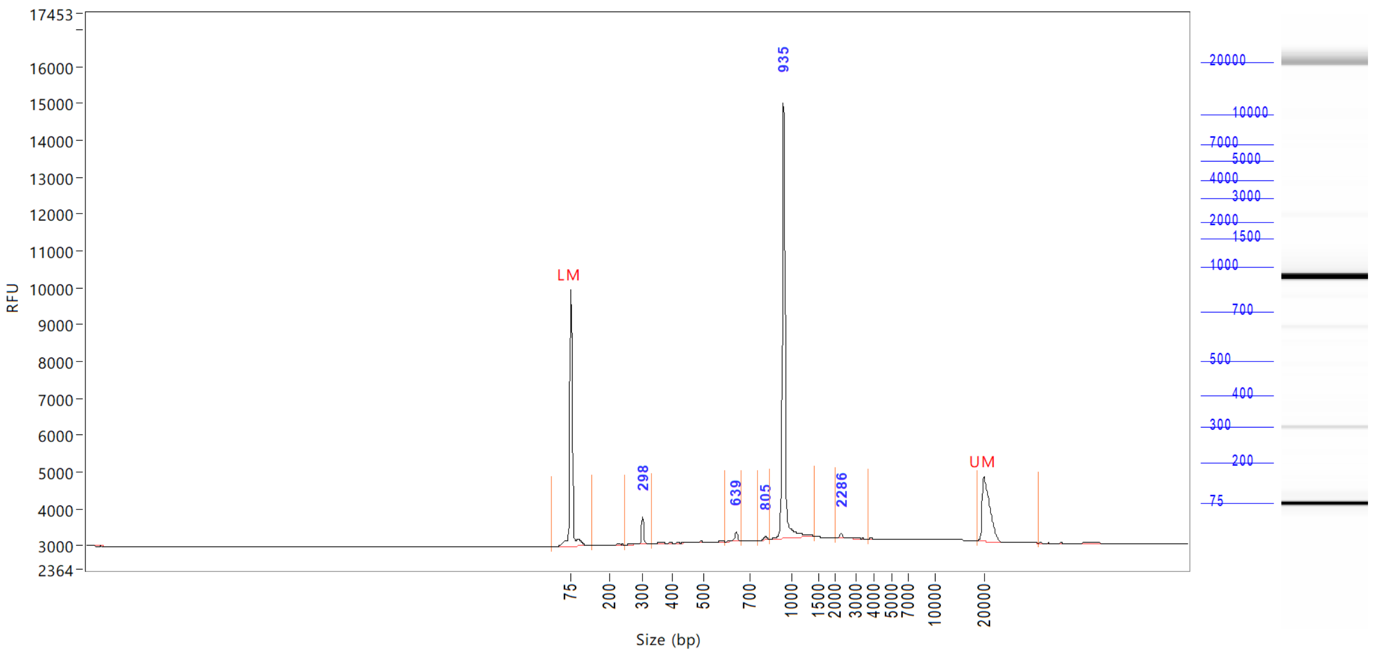

Peak	Size	Concentration	Relative concentration	Molarity	From	To	CV%	RFU
	(bp)	(ng/uL)	Percent	(nmole/L)	(bp)	(bp)		
1	75 (LM)	0.5000		11.0851	14	145	13.48	6955
2	298	0.5722	6.1	3.1800	244	331	3.16	750
3	639	0.1952	2.1	0.5067	593	662	1.36	261
4	805	0.0687	0.7	0.1412	749	840	1.57	81
5	935	8.4160	89.9	14.7048	840	1422	4.66	11840
6	2286	0.1072	1.1	0.0730	2000	3643	14.82	118
7	20000 (UM)	0.2048		0.0164	18537	31123	5.02	1768

TIC:	9.3593	ng/uL
TIM:	18.6057	nmole/L
Total concentration:	9.8004	ng/uL

Sample peak width (sec): 5   Sample min peak height: 50   Sample baseline V to V?: Y   Sample baseline V to V points: 3
Sample filter: Binomial   Number of points for filter: 3 Sample start region (min): 0 Sample end region (min): 30
Marker peak width (sec): 5   Marker min peak height: 200   Marker baseline V to V?: Y   Marker baseline V to V points: 3
Lower marker selection: First peak > 500 RFU   Upper marker selection: Last peak > 200 RFU
Ladder size (bp)75, 200, 300, 400, 500, 700, 1000, 1500, 2000, 3000, 4000, 5000, 7000, 10000, 20000
Quantification using: Lower Marker   Final concentration (ng/uL): 0.5000   Dilution factor: 12.0

Sample: SampC10
Well location: C10
Created: Thursday, October 21, 2021 6:08:16 PM

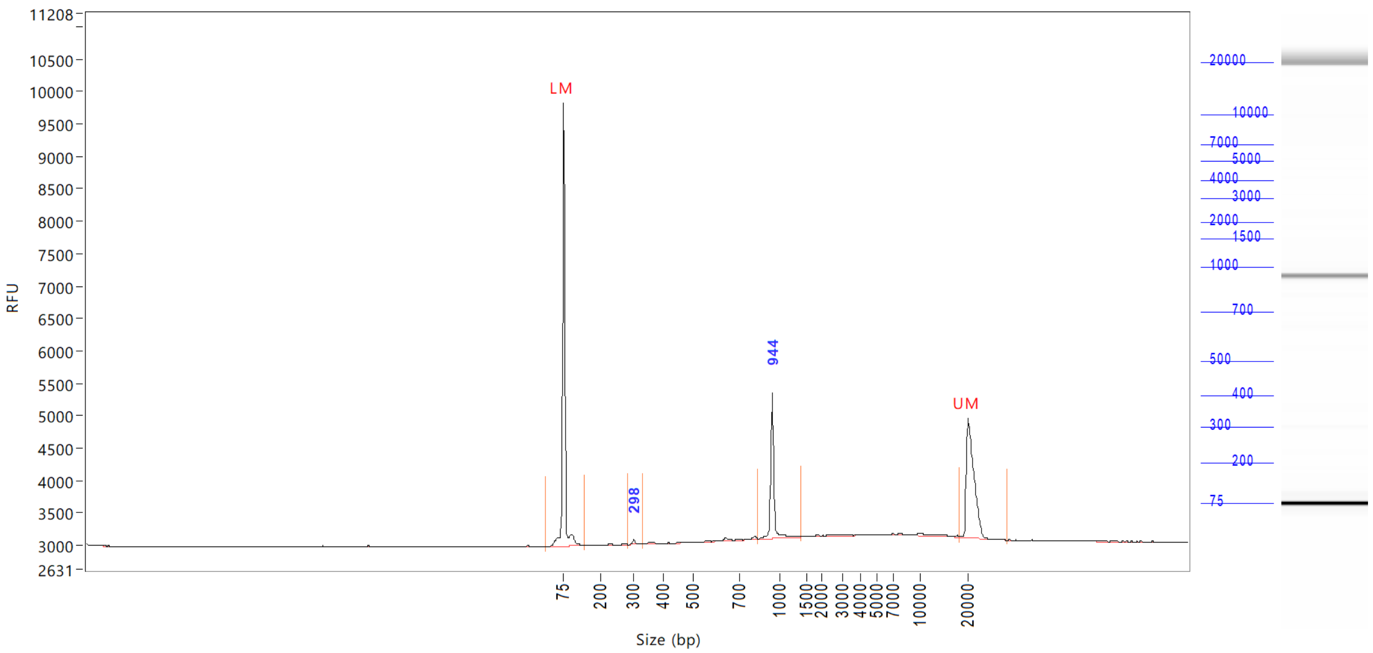

Peak	Size	Concentration	Relative concentration	Molarity	From	To	CV%	RFU
	(bp)	(ng/uL)	Percent	(nmole/L)	(bp)	(bp)		
1	75 (LM)	0.5000		11.0851	16	143	13.47	6849
2	298	0.0692	3.7	0.3817	282	327	2.10	87
3	944	1.8037	96.3	3.1249	835	1389	6.41	2247
4	20000 (UM)	0.2199		0.0176	18293	28221	4.23	1842

TIC:	1.8728	ng/uL
TIM:	3.5067	nmole/L
Total concentration:	2.5770	ng/uL

```
Sample peak width (sec): 5    Sample min peak height: 50    Sample baseline V to V?: Y    Sample baseline V to V points: 3
Sample filter: Binomial       Number of points for filter: 3  Sample start region (min): 0  Sample end region (min): 30
Marker peak width (sec): 5    Marker min peak height: 200   Marker baseline V to V?: Y    Marker baseline V to V points: 3
Lower marker selection: First peak > 500 RFU                Upper marker selection: Last peak > 200 RFU
Ladder size (bp)75, 200, 300, 400, 500, 700, 1000, 1500, 2000, 3000, 4000, 5000, 7000, 10000, 20000
Quantification using: Lower Marker          Final concentration (ng/uL): 0.5000          Dilution factor: 12.0
```

Sample: SampD10
Well location: D10
Created: Thursday, October 21, 2021 6:08:16 PM

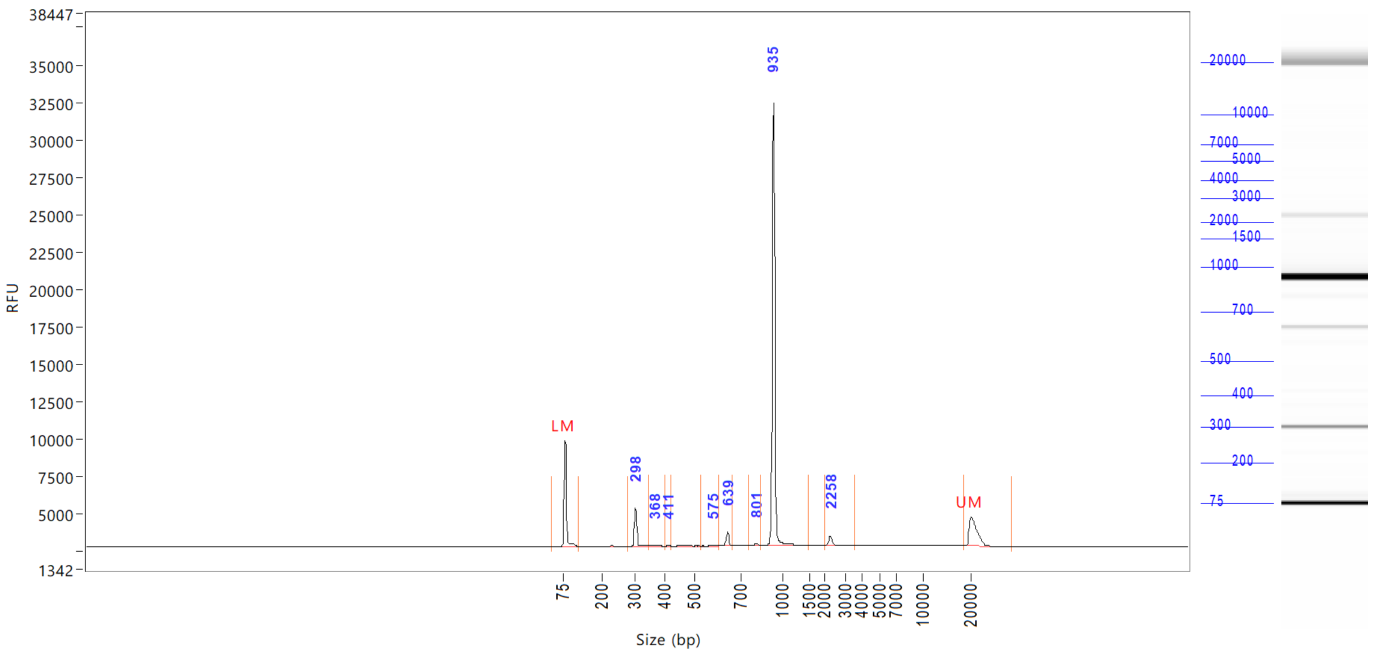

Peak	Size	Concentration	Relative concentration	Molarity	From	To	CV%	RFU
	(bp)	(ng/uL)	Percent	(nmole/L)	(bp)	(bp)		
1	75 (LM)	0.5000		10.9378	33	122	9.73	7054
2	298	1.8321	8.1	10.1132	275	343	2.34	2514
3	368	0.1009	0.4	0.4549	343	401	3.20	66
4	411	0.0547	0.2	0.2202	401	420	0.76	83
5	575	0.0728	0.3	0.2090	521	603	2.45	75
6	639	0.6469	2.8	1.6765	603	662	1.03	943
7	801	0.1320	0.6	0.2712	753	840	1.75	148
8	935	19.3972	85.2	34.1090	840	1500	4.38	29596
9	2258	0.5195	2.3	0.3726	2000	3536	9.33	640
10	20000 (UM)	0.2381		0.0191	18415	28342	4.37	1957

TIC:	22.7561	ng/uL
TIM:	47.4265	nmole/L
Total concentration:	23.3255	ng/uL

```
Sample peak width (sec): 5     Sample min peak height: 50     Sample baseline V to V?: Y     Sample baseline V to V points: 3
Sample filter: Binomial        Number of points for filter: 3  Sample start region (min): 0  Sample end region (min): 30
Marker peak width (sec): 5     Marker min peak height: 200    Marker baseline V to V?: Y     Marker baseline V to V points: 3
Lower marker selection: First peak > 500 RFU                  Upper marker selection: Last peak > 200 RFU
Ladder size (bp)75, 200, 300, 400, 500, 700, 1000, 1500, 2000, 3000, 4000, 5000, 7000, 10000, 20000
Quantification using: Lower Marker          Final concentration (ng/uL): 0.5000          Dilution factor: 12.0
```

Sample: SampE10
Well location: E10
Created: Thursday, October 21, 2021 6:08:16 PM

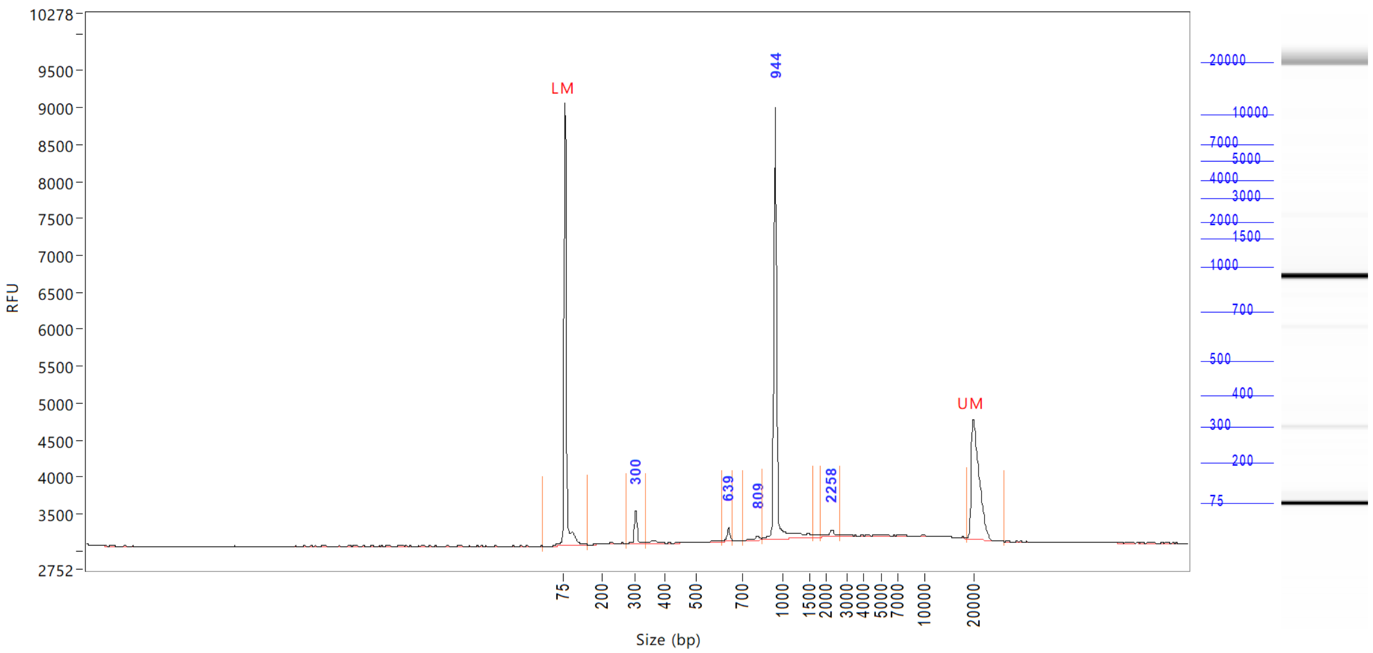

Peak	Size	Concentration	Relative concentration	Molarity	From	To	CV%	RFU
	(bp)	(ng/uL)	Percent	(nmole/L)	(bp)	(bp)		
1	75 (LM)	0.5000		10.9378	5	148	12.32	6005
2	300	0.4438	7.4	2.4499	269	333	2.88	444
3	639	0.1670	2.8	0.4322	611	657	1.22	182
4	809	0.0989	1.6	0.2056	705	840	3.92	61
5	944	5.1536	85.6	8.8543	840	1578	9.36	5842
6	2258	0.1565	2.6	0.1165	1846	2657	8.97	97
7	20000 (UM)	0.2316		0.0185	18781	26529	4.27	1623

TIC:	6.0199	ng/uL
TIM:	12.0584	nmole/L
Total concentration:	6.6340	ng/uL

Sample peak width (sec): 5   Sample min peak height: 50   Sample baseline V to V?: Y   Sample baseline V to V points: 3
Sample filter: Binomial   Number of points for filter: 3   Sample start region (min): 0   Sample end region (min): 30
Marker peak width (sec): 5   Marker min peak height: 200   Marker baseline V to V?: Y   Marker baseline V to V points: 3
Lower marker selection: First peak > 500 RFU   Upper marker selection: Last peak > 200 RFU
Ladder size (bp)75, 200, 300, 400, 500, 700, 1000, 1500, 2000, 3000, 4000, 5000, 7000, 10000, 20000
Quantification using: Lower Marker   Final concentration (ng/uL): 0.5000   Dilution factor: 12.0

Sample: SampF10
Well location: F10
Created: Thursday, October 21, 2021 6:08:16 PM

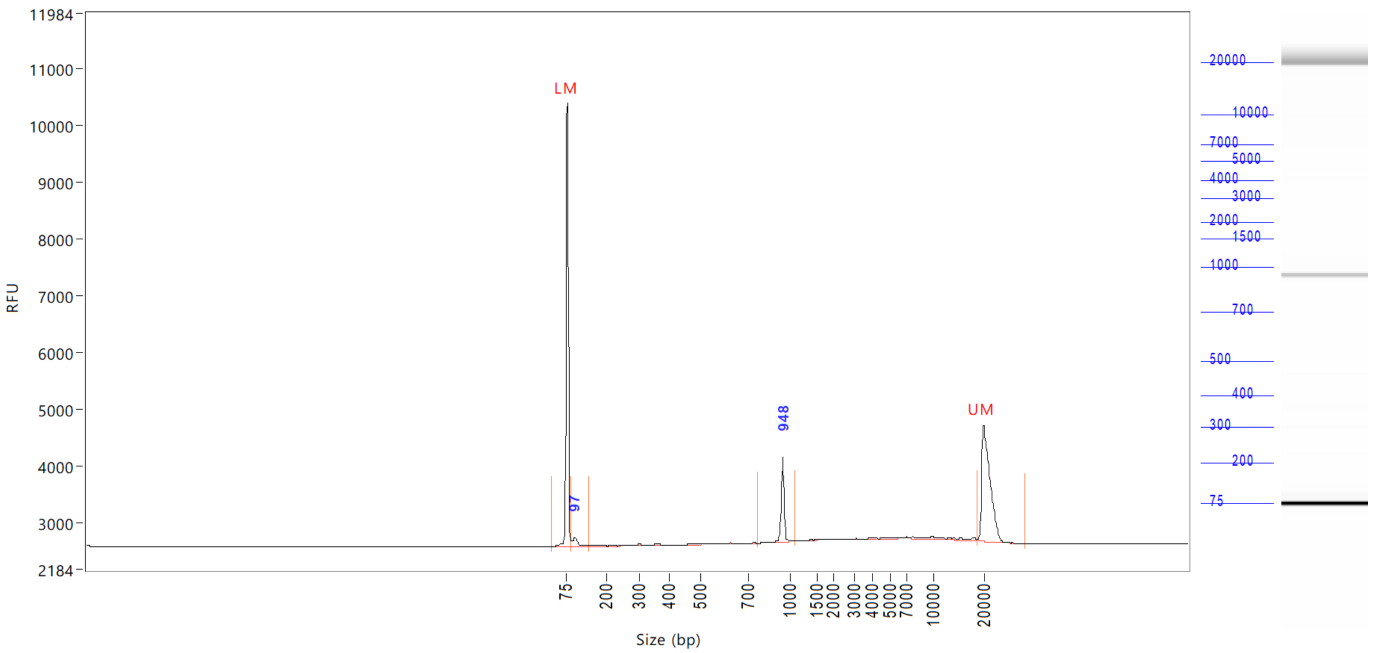

Peak	Size	Concentration	Relative concentration	Molarity	From	To	CV%	RFU
	(bp)	(ng/uL)	Percent	(nmole/L)	(bp)	(bp)		
1	75 (LM)	0.5000		11.3919	28	87	6.05	7834
2	97	0.2889	22.3	4.6506	87	143	12.22	152
3	948	1.0049	77.7	1.7520	770	1090	2.22	1486
4	20000 (UM)	0.2534		0.0201	18781	28584	4.71	2050

TIC:	1.2937	ng/uL
TIM:	6.4027	nmole/L
Total concentration:	2.0297	ng/uL

```
Sample peak width (sec): 5    Sample min peak height: 50    Sample baseline V to V?: Y    Sample baseline V to V points: 3
Sample filter: Binomial       Number of points for filter: 3  Sample start region (min): 0  Sample end region (min): 30
Marker peak width (sec): 5    Marker min peak height: 200   Marker baseline V to V?: Y    Marker baseline V to V points: 3
Lower marker selection: First peak > 500 RFU                Upper marker selection: Last peak > 200 RFU
Ladder size (bp)75, 200, 300, 400, 500, 700, 1000, 1500, 2000, 3000, 4000, 5000, 7000, 10000, 20000
Quantification using: Lower Marker          Final concentration (ng/uL): 0.5000          Dilution factor: 12.0
```

Sample: SampG10
Well location: G10
Created: Thursday, October 21, 2021 6:08:16 PM

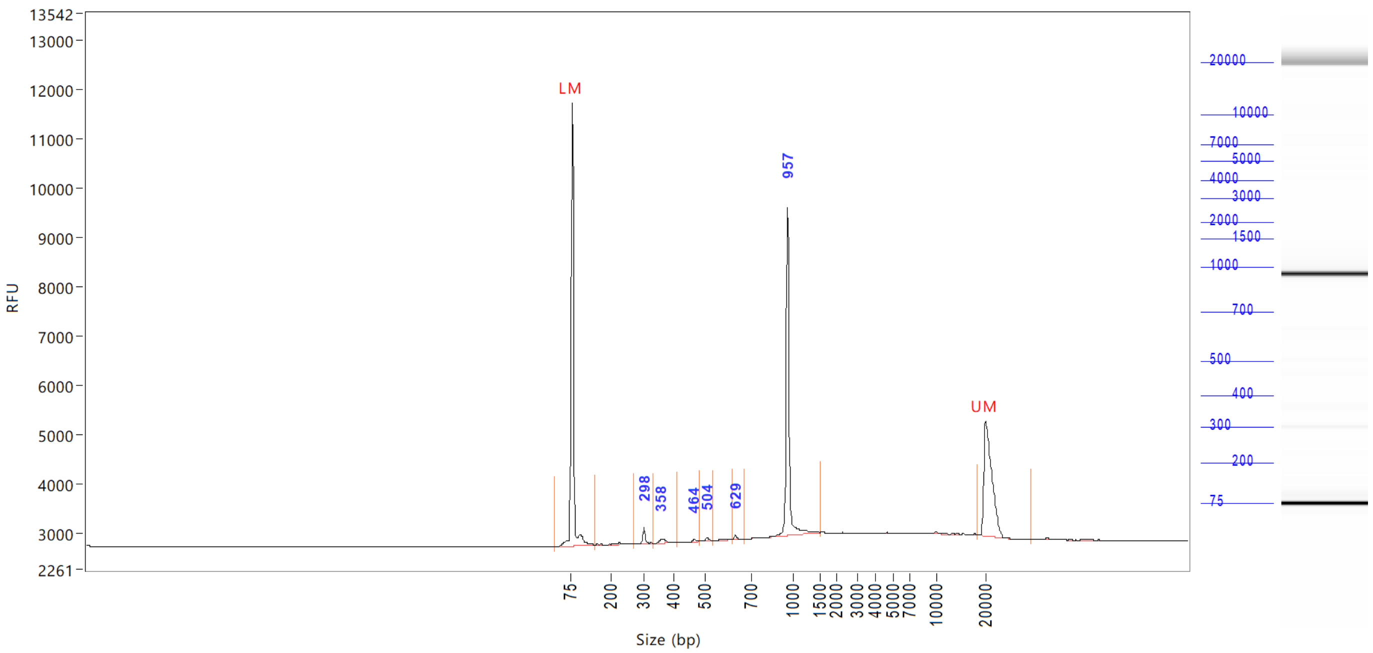

Peak	Size	Concentration	Relative concentration	Molarity	From	To	CV%	RFU
	(bp)	(ng/uL)	Percent	(nmole/L)	(bp)	(bp)		
1	75 (LM)	0.5000		11.0851	20	148	13.53	9006
2	298	0.1959	4.5	1.0848	266	327	2.12	325
3	358	0.1285	2.9	0.5856	327	412	3.66	89
4	464	0.0549	1.3	0.1971	412	483	3.02	59
5	504	0.0639	1.5	0.2082	483	534	1.84	79
6	629	0.0553	1.3	0.1447	616	667	1.43	87
7	957	3.8830	88.6	6.6298	667	1501	6.16	6652
8	20000 (UM)	0.2227		0.0178	18293	29068	4.44	2338

TIC:	4.3814	ng/uL
TIM:	8.8502	nmole/L
Total concentration:	4.6951	ng/uL

```
Sample peak width (sec): 5    Sample min peak height: 50    Sample baseline V to V?: Y    Sample baseline V to V points: 3
Sample filter: Binomial       Number of points for filter: 3  Sample start region (min): 0  Sample end region (min): 30
Marker peak width (sec): 5    Marker min peak height: 200   Marker baseline V to V?: Y    Marker baseline V to V points: 3
Lower marker selection: First peak > 500 RFU                Upper marker selection: Last peak > 200 RFU
Ladder size (bp)75, 200, 300, 400, 500, 700, 1000, 1500, 2000, 3000, 4000, 5000, 7000, 10000, 20000
Quantification using: Lower Marker         Final concentration (ng/uL): 0.5000         Dilution factor: 12.0
```

Sample: SampH10
Well location: H10
Created: Thursday, October 21, 2021 6:08:16 PM

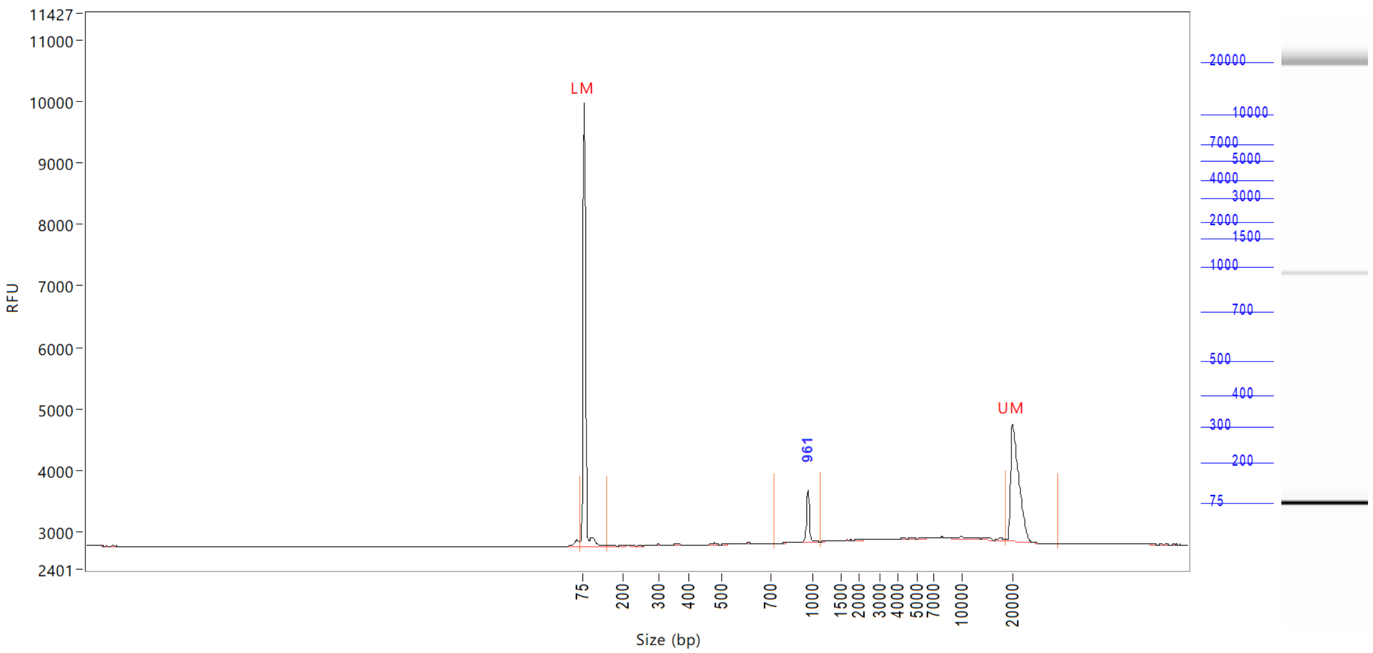

Peak	Size	Concentration	Relative concentration	Molarity	From	To	CV%	RFU
	(bp)	(ng/uL)	Percent	(nmole/L)	(bp)	(bp)		
1	75 (LM)	0.5000		10.9378	64	147	9.94	7214
2	961	0.6009	100.0	1.0367	723	1112	3.04	852
3	20000 (UM)	0.2348		0.0188	18659	28826	4.41	1907

TIC:	0.6009	ng/uL
TIM:	1.0367	nmole/L
Total concentration:	1.2681	ng/uL

```
Sample peak width (sec): 5    Sample min peak height: 50    Sample baseline V to V?: Y    Sample baseline V to V points: 3
Sample filter: Binomial       Number of points for filter: 3  Sample start region (min): 0  Sample end region (min): 30
Marker peak width (sec): 5    Marker min peak height: 200   Marker baseline V to V?: Y    Marker baseline V to V points: 3
Lower marker selection: First peak > 500 RFU                Upper marker selection: Last peak > 200 RFU
Ladder size (bp)75, 200, 300, 400, 500, 700, 1000, 1500, 2000, 3000, 4000, 5000, 7000, 10000, 20000
Quantification using: Lower Marker          Final concentration (ng/uL): 0.5000          Dilution factor: 12.0
```

Sample: SampA11
Well location: A11
Created: Thursday, October 21, 2021 6:08:16 PM

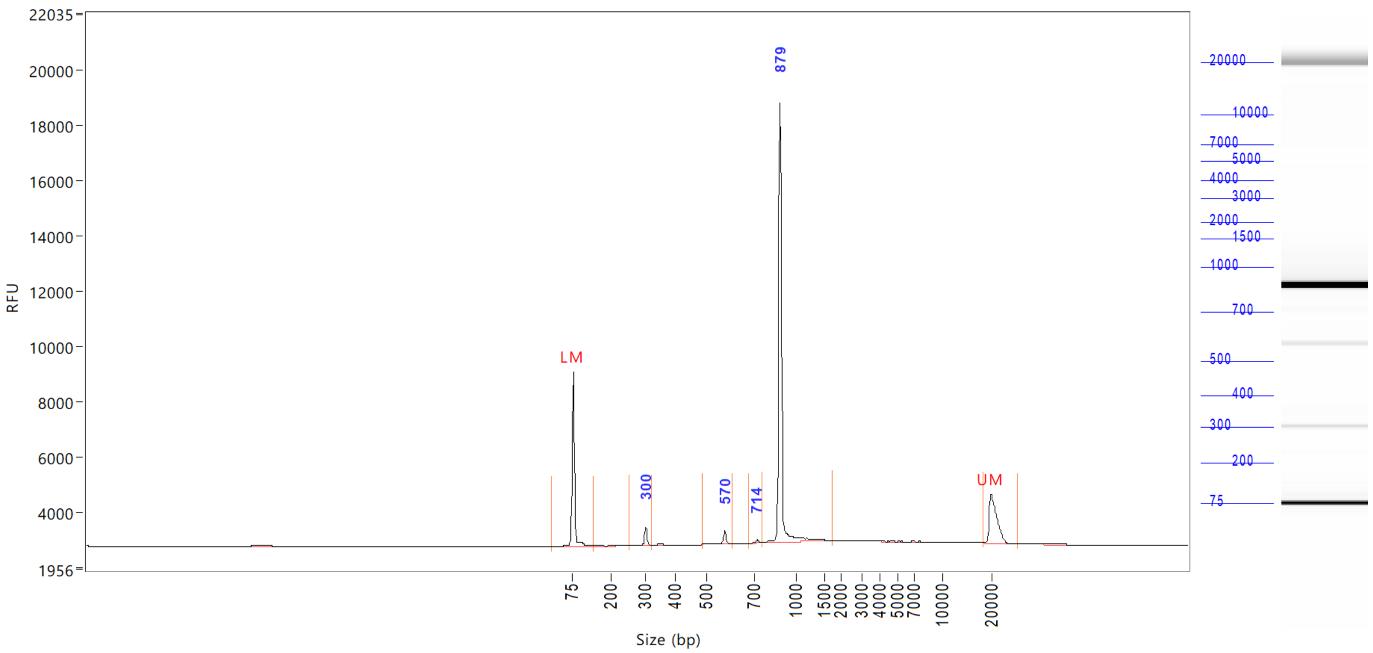

Peak	Size	Concentration	Relative concentration	Molarity	From	To	CV%	RFU
	(bp)	(ng/uL)	Percent	(nmole/L)	(bp)	(bp)		
1	75 (LM)	0.5000		10.7944	11	143	13.72	6295
2	300	0.6010	4.5	3.3175	251	321	2.34	682
3	570	0.4039	3.0	1.1702	487	606	2.22	505
4	714	0.1165	0.9	0.2692	672	753	1.95	112
5	879	12.2953	91.6	22.7378	753	1731	8.10	15892
6	20000 (UM)	0.2271		0.0183	18293	25199	3.99	1786

TIC:	13.4167	ng/uL
TIM:	27.4947	nmole/L
Total concentration:	14.0140	ng/uL

Sample peak width (sec): 5 Sample min peak height: 50 Sample baseline V to V?: Y Sample baseline V to V points: 3
Sample filter: Binomial Number of points for filter: 3 Sample start region (min): 0 Sample end region (min): 30
Marker peak width (sec): 5 Marker min peak height: 200 Marker baseline V to V?: Y Marker baseline V to V points: 3
Lower marker selection: First peak > 500 RFU Upper marker selection: Last peak > 200 RFU
Ladder size (bp)75, 200, 300, 400, 500, 700, 1000, 1500, 2000, 3000, 4000, 5000, 7000, 10000, 20000
Quantification using: Lower Marker Final concentration (ng/uL): 0.5000 Dilution factor: 12.0

Sample: SampB11
Well location: B11
Created: Thursday, October 21, 2021 6:08:16 PM

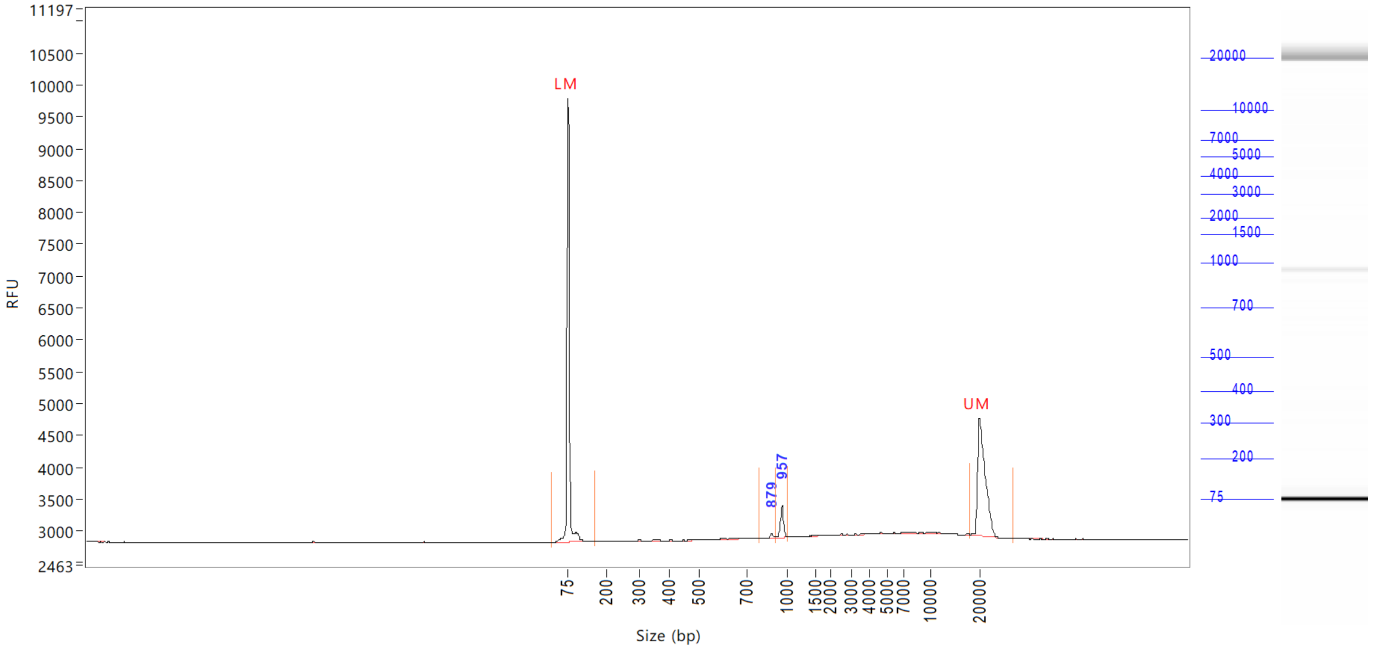

Peak	Size	Concentration	Relative concentration	Molarity	From	To	CV%	RFU
	(bp)	(ng/uL)	Percent	(nmole/L)	(bp)	(bp)		
1	75 (LM)	0.5000		10.9378	24	162	11.46	6973
2	879	0.0571	13.0	0.1081	788	909	2.67	66
3	957	0.3820	87.0	0.6604	909	1000	1.29	497
4	20000 (UM)	0.2229		0.0178	18171	27012	4.10	1846

TIC:	0.4390	ng/uL
TIM:	0.7685	nmole/L
Total concentration:	1.0113	ng/uL

Sample peak width (sec): 5 Sample min peak height: 50 Sample baseline V to V?: Y Sample baseline V to V points: 3
Sample filter: Binomial Number of points for filter: 3 Sample start region (min): 0 Sample end region (min): 30
Marker peak width (sec): 5 Marker min peak height: 200 Marker baseline V to V?: Y Marker baseline V to V points: 3
Lower marker selection: First peak > 500 RFU Upper marker selection: Last peak > 200 RFU
Ladder size (bp)75, 200, 300, 400, 500, 700, 1000, 1500, 2000, 3000, 4000, 5000, 7000, 10000, 20000
Quantification using: Lower Marker Final concentration (ng/uL): 0.5000 Dilution factor: 12.0

Sample: SampC11
Well location: C11
Created: Thursday, October 21, 2021 6:08:16 PM

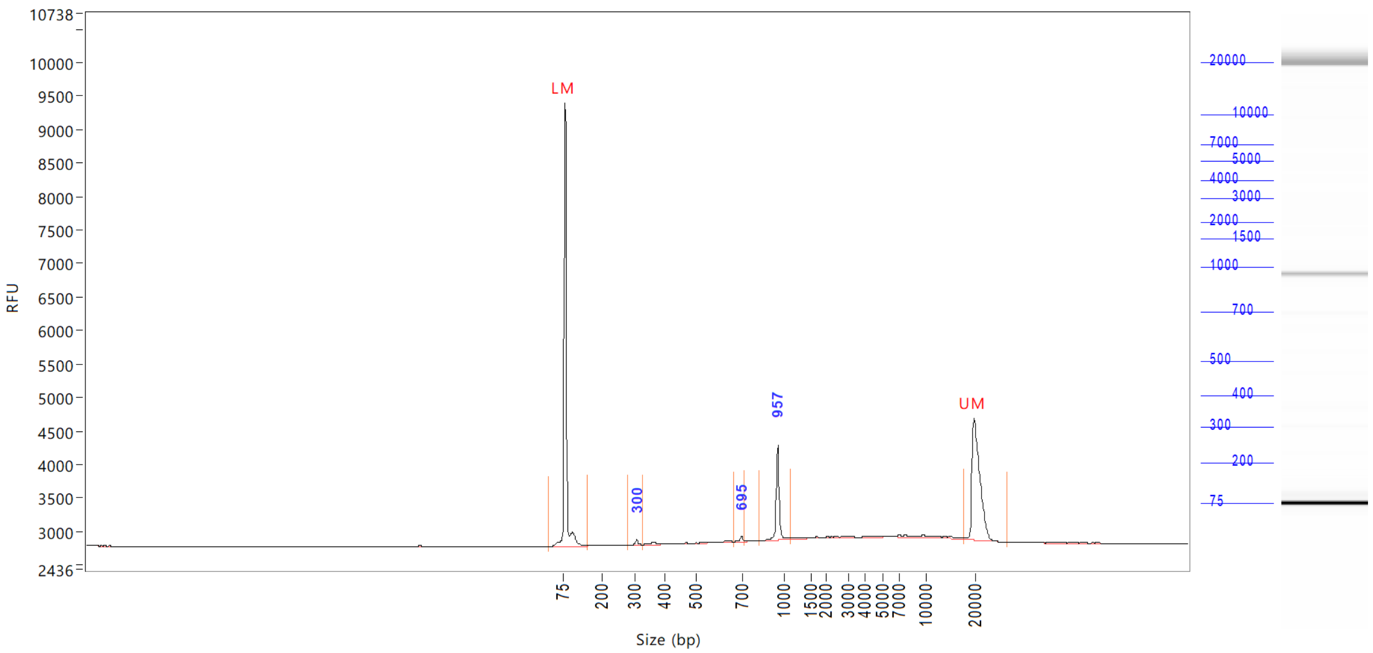

Peak	Size	Concentration	Relative concentration	Molarity	From	To	CV%	RFU
	(bp)	(ng/uL)	Percent	(nmole/L)	(bp)	(bp)		
1	75 (LM)	0.5000		10.9378	24	150	12.99	6632
2	300	0.0723	5.8	0.3975	275	321	2.48	77
3	695	0.0716	5.7	0.1712	659	710	1.40	83
4	957	1.1046	88.5	1.9078	818	1134	2.78	1418
5	20000 (UM)	0.2279		0.0183	17927	26770	4.33	1811

TIC:	1.2484	ng/uL
TIM:	2.4765	nmole/L
Total concentration:	2.0384	ng/uL

Sample peak width (sec): 5   Sample min peak height: 50   Sample baseline V to V?: Y   Sample baseline V to V points: 3
Sample filter: Binomial   Number of points for filter: 3 Sample start region (min): 0 Sample end region (min): 30
Marker peak width (sec): 5   Marker min peak height: 200   Marker baseline V to V?: Y   Marker baseline V to V points: 3
Lower marker selection: First peak > 500 RFU   Upper marker selection: Last peak > 200 RFU
Ladder size (bp)75, 200, 300, 400, 500, 700, 1000, 1500, 2000, 3000, 4000, 5000, 7000, 10000, 20000
Quantification using: Lower Marker   Final concentration (ng/uL): 0.5000   Dilution factor: 12.0

Sample: SampD11
Well location: D11
Created: Thursday, October 21, 2021 6:08:16 PM

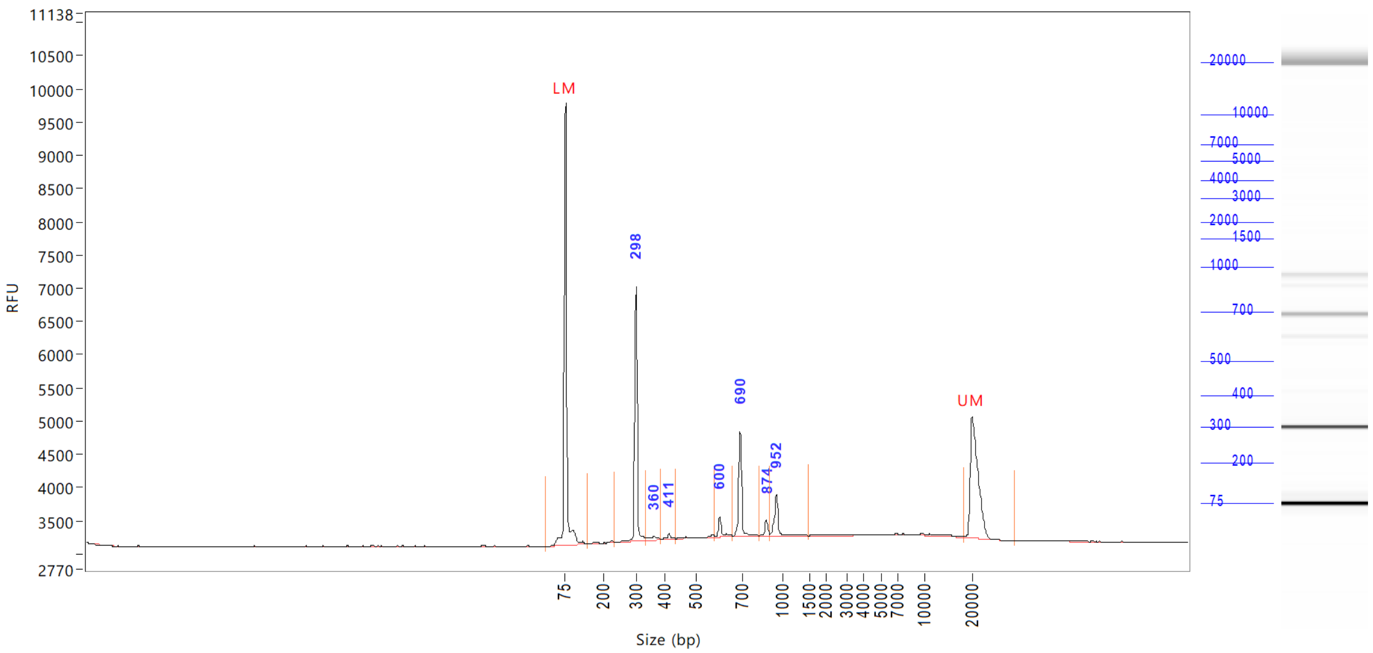

Peak	Size	Concentration	Relative concentration	Molarity	From	To	CV%	RFU
	(bp)	(ng/uL)	Percent	(nmole/L)	(bp)	(bp)		
1	75 (LM)	0.5000		11.0851	11	148	14.14	6670
2	298	2.7520	53.2	15.2418	233	333	2.57	3821
3	360	0.1401	2.7	0.6491	333	384	3.69	52
4	411	0.0929	1.8	0.3754	384	431	2.42	86
5	600	0.2813	5.4	0.7639	583	659	2.75	313
6	690	1.1538	22.3	2.7520	659	818	2.33	1583
7	874	0.1652	3.2	0.3121	818	900	1.54	236
8	952	0.5851	11.3	1.0021	900	1467	7.29	622
9	20000 (UM)	0.2175		0.0174	18171	28826	4.36	1819

TIC:	5.1703	ng/uL
TIM:	21.0964	nmole/L
Total concentration:	5.6274	ng/uL

```
Sample peak width (sec): 5    Sample min peak height: 50    Sample baseline V to V?: Y    Sample baseline V to V points: 3
Sample filter: Binomial       Number of points for filter: 3  Sample start region (min): 0  Sample end region (min): 30
Marker peak width (sec): 5    Marker min peak height: 200   Marker baseline V to V?: Y    Marker baseline V to V points: 3
Lower marker selection: First peak > 500 RFU                Upper marker selection: Last peak > 200 RFU
Ladder size (bp)75, 200, 300, 400, 500, 700, 1000, 1500, 2000, 3000, 4000, 5000, 7000, 10000, 20000
Quantification using: Lower Marker          Final concentration (ng/uL): 0.5000          Dilution factor: 12.0
```

Sample: SampE11
Well location: E11
Created: Thursday, October 21, 2021 6:08:16 PM

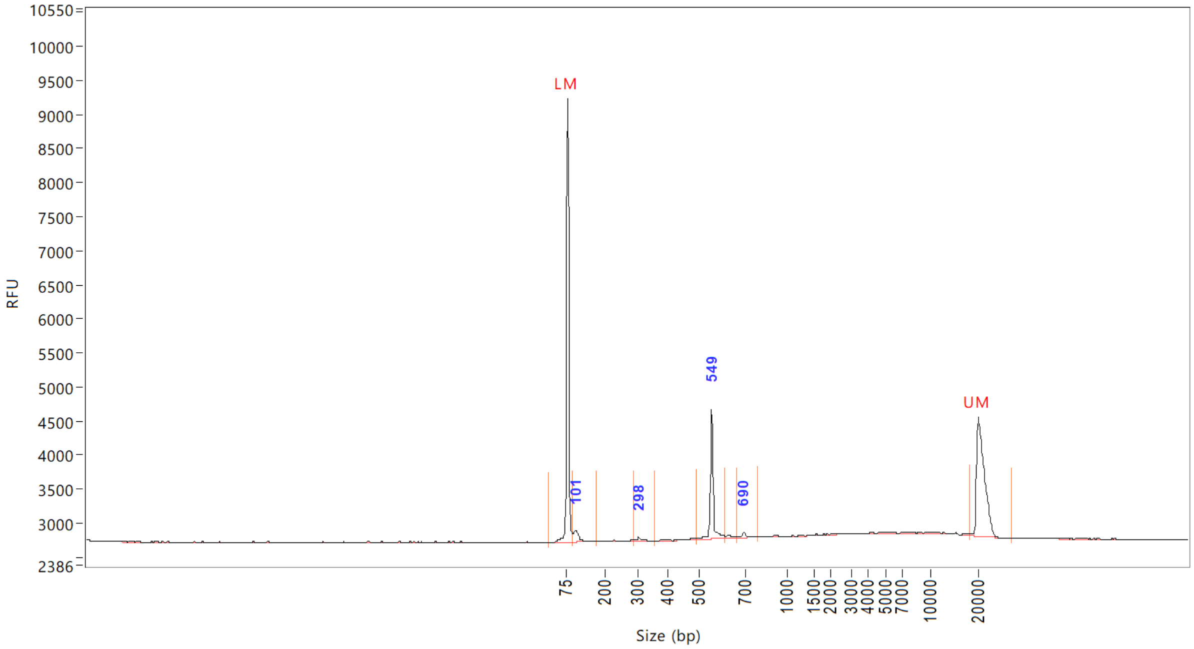

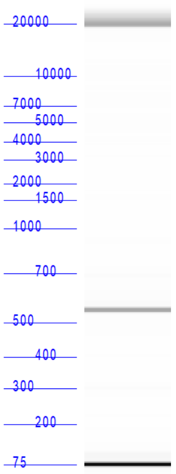

Peak	Size	Concentration	Relative concentration	Molarity	From	To	CV%	RFU
	(bp)	(ng/uL)	Percent	(nmole/L)	(bp)	(bp)		
1	75 (LM)	0.5000		11.2364	16	91	7.03	6519
2	101	0.3849	17.2	5.9077	91	169	14.36	171
3	298	0.0887	4.0	0.4802	284	354	5.06	54
4	549	1.6431	73.6	4.9160	492	611	2.51	1898
5	690	0.1171	5.2	0.2772	659	779	3.57	71
6	20000 (UM)	0.2388		0.0191	18171	26770	4.45	1753

TIC:	2.2337	ng/uL
TIM:	11.5811	nmole/L
Total concentration:	2.8672	ng/uL

```
Sample peak width (sec): 5    Sample min peak height: 50    Sample baseline V to V?: Y    Sample baseline V to V points: 3
Sample filter: Binomial       Number of points for filter: 3  Sample start region (min): 0  Sample end region (min): 30
Marker peak width (sec): 5    Marker min peak height: 200   Marker baseline V to V?: Y    Marker baseline V to V points: 3
Lower marker selection: First peak > 500 RFU                Upper marker selection: Last peak > 200 RFU
Ladder size (bp)75, 200, 300, 400, 500, 700, 1000, 1500, 2000, 3000, 4000, 5000, 7000, 10000, 20000
Quantification using: Lower Marker          Final concentration (ng/uL): 0.5000          Dilution factor: 12.0
```

Sample: SampF11
Well location: F11
Created: Thursday, October 21, 2021 6:08:16 PM

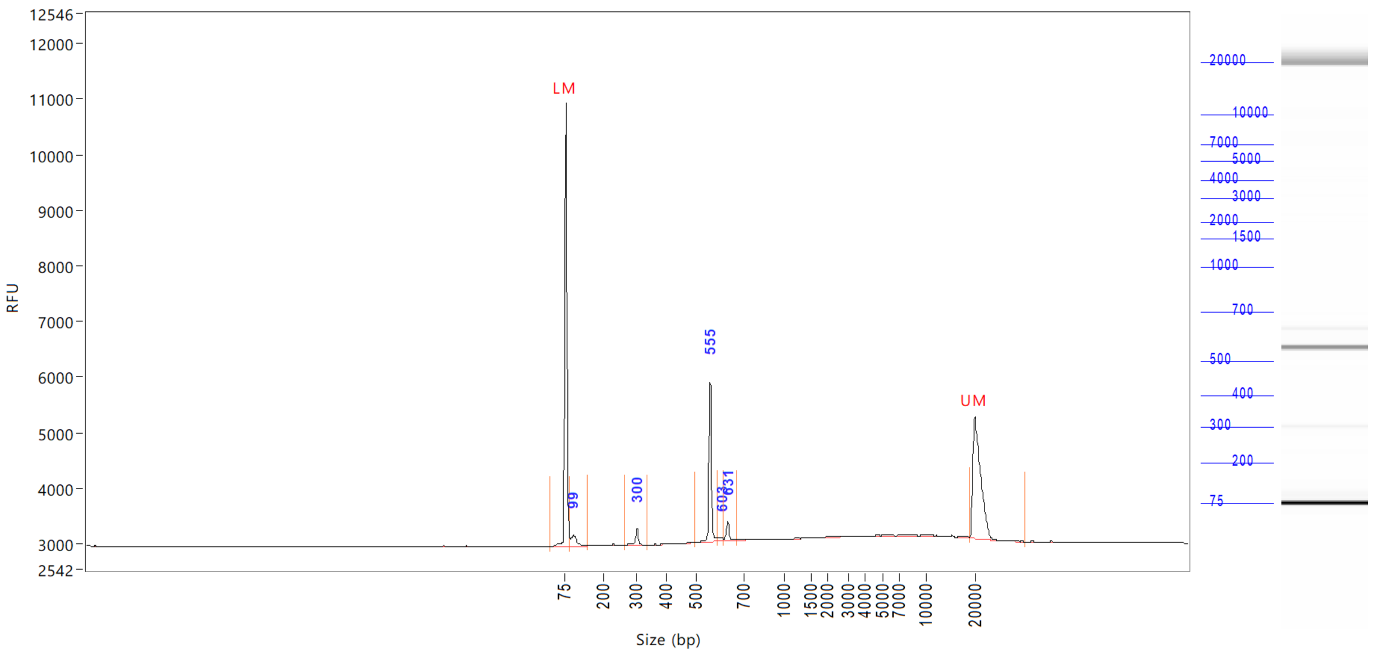

Peak	Size	Concentration	Relative concentration	Molarity	From	To	CV%	RFU
	(bp)	(ng/uL)	Percent	(nmole/L)	(bp)	(bp)		
1	75 (LM)	0.5000		11.0851	26	89	6.35	7993
2	99	0.3779	13.4	5.9677	89	148	12.32	197
3	300	0.2621	9.3	1.4612	264	337	3.15	300
4	555	1.8614	66.0	5.5391	494	585	1.54	2861
5	603	0.0787	2.8	0.2158	585	616	1.52	60
6	631	0.2395	8.5	0.6247	616	672	1.49	337
7	20000 (UM)	0.2465		0.0197	19024	30398	4.82	2179

TIC:	2.8196	ng/uL
TIM:	13.8085	nmole/L
Total concentration:	3.2601	ng/uL

```
Sample peak width (sec): 5    Sample min peak height: 50    Sample baseline V to V?: Y    Sample baseline V to V points: 3
Sample filter: Binomial       Number of points for filter: 3  Sample start region (min): 0  Sample end region (min): 30
Marker peak width (sec): 5    Marker min peak height: 200   Marker baseline V to V?: Y    Marker baseline V to V points: 3
Lower marker selection: First peak > 500 RFU                Upper marker selection: Last peak > 200 RFU
Ladder size (bp)75, 200, 300, 400, 500, 700, 1000, 1500, 2000, 3000, 4000, 5000, 7000, 10000, 20000
Quantification using: Lower Marker          Final concentration (ng/uL): 0.5000          Dilution factor: 12.0
```

Sample: SampG11
Well location: G11
Created: Thursday, October 21, 2021 6:08:16 PM

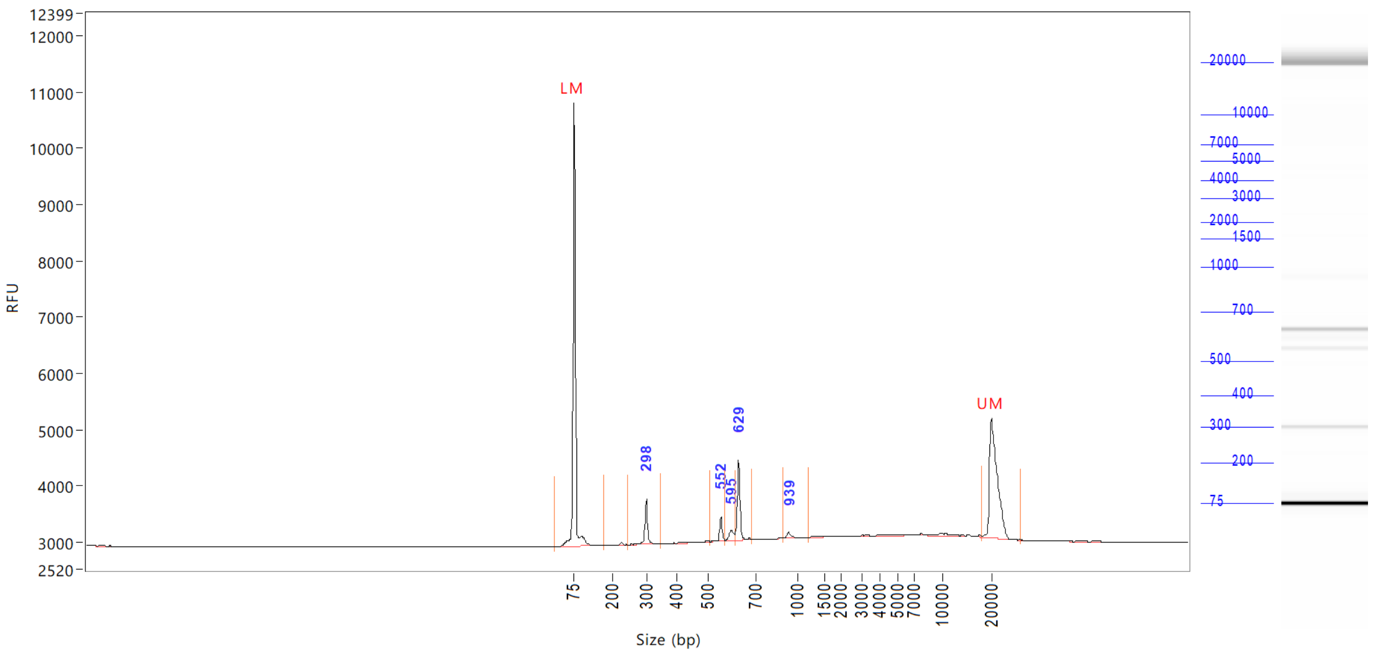

Peak	Size	Concentration	Relative concentration	Molarity	From	To	CV%	RFU
	(bp)	(ng/uL)	Percent	(nmole/L)	(bp)	(bp)		
1	75 (LM)	0.5000		11.0851	12	169	13.20	7889
2	298	0.5961	28.4	3.3125	242	345	2.66	816
3	552	0.2621	12.5	0.7857	506	570	1.17	441
4	595	0.2006	9.6	0.5547	570	613	1.74	176
5	629	0.9251	44.1	2.4282	613	685	1.50	1435
6	939	0.1157	5.5	0.2000	896	1201	5.10	113
7	20000 (UM)	0.2304		0.0185	18171	26045	4.23	2117

TIC:	2.0996	ng/uL
TIM:	7.2812	nmole/L
Total concentration:	2.5313	ng/uL

```
Sample peak width (sec): 5    Sample min peak height: 50    Sample baseline V to V?: Y    Sample baseline V to V points: 3
Sample filter: Binomial       Number of points for filter: 3  Sample start region (min): 0  Sample end region (min): 30
Marker peak width (sec): 5    Marker min peak height: 200   Marker baseline V to V?: Y    Marker baseline V to V points: 3
Lower marker selection: First peak > 500 RFU                Upper marker selection: Last peak > 200 RFU
Ladder size (bp)75, 200, 300, 400, 500, 700, 1000, 1500, 2000, 3000, 4000, 5000, 7000, 10000, 20000
Quantification using: Lower Marker          Final concentration (ng/uL): 0.5000          Dilution factor: 12.0
```

Sample: SampH11
Well location: H11
Created: Thursday, October 21, 2021 6:08:16 PM

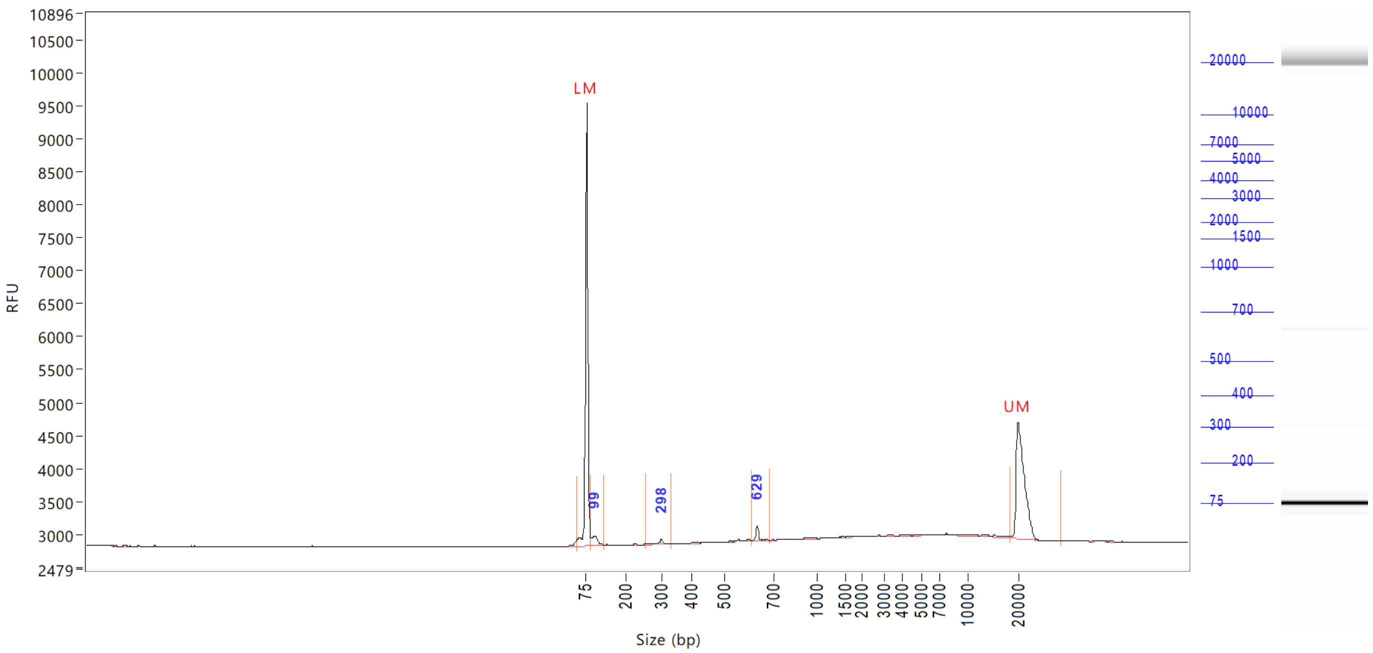

Peak	Size	Concentration	Relative concentration	Molarity	From	To	CV%	RFU
	(bp)	(ng/uL)	Percent	(nmole/L)	(bp)	(bp)		
1	75 (LM)	0.5000		11.2364	48	89	6.47	6719
2	99	0.3293	53.9	5.3544	89	129	8.48	150
3	298	0.1172	19.2	0.6555	255	333	4.75	72
4	629	0.1640	26.9	0.4297	606	682	1.50	213
5	20000 (UM)	0.2387		0.0191	18293	28221	4.39	1766

TIC:	0.6105	ng/uL
TIM:	6.4396	nmole/L
Total concentration:	1.2020	ng/uL

```
Sample peak width (sec): 5    Sample min peak height: 50    Sample baseline V to V?: Y    Sample baseline V to V points: 3
Sample filter: Binomial       Number of points for filter: 3  Sample start region (min): 0  Sample end region (min): 30
Marker peak width (sec): 5    Marker min peak height: 200   Marker baseline V to V?: Y    Marker baseline V to V points: 3
Lower marker selection: First peak > 500 RFU                Upper marker selection: Last peak > 200 RFU
Ladder size (bp)75, 200, 300, 400, 500, 700, 1000, 1500, 2000, 3000, 4000, 5000, 7000, 10000, 20000
Quantification using: Lower Marker          Final concentration (ng/uL): 0.5000          Dilution factor: 12.0
```

Sample: SampA12
Well location: A12
Created: Thursday, October 21, 2021 6:08:16 PM

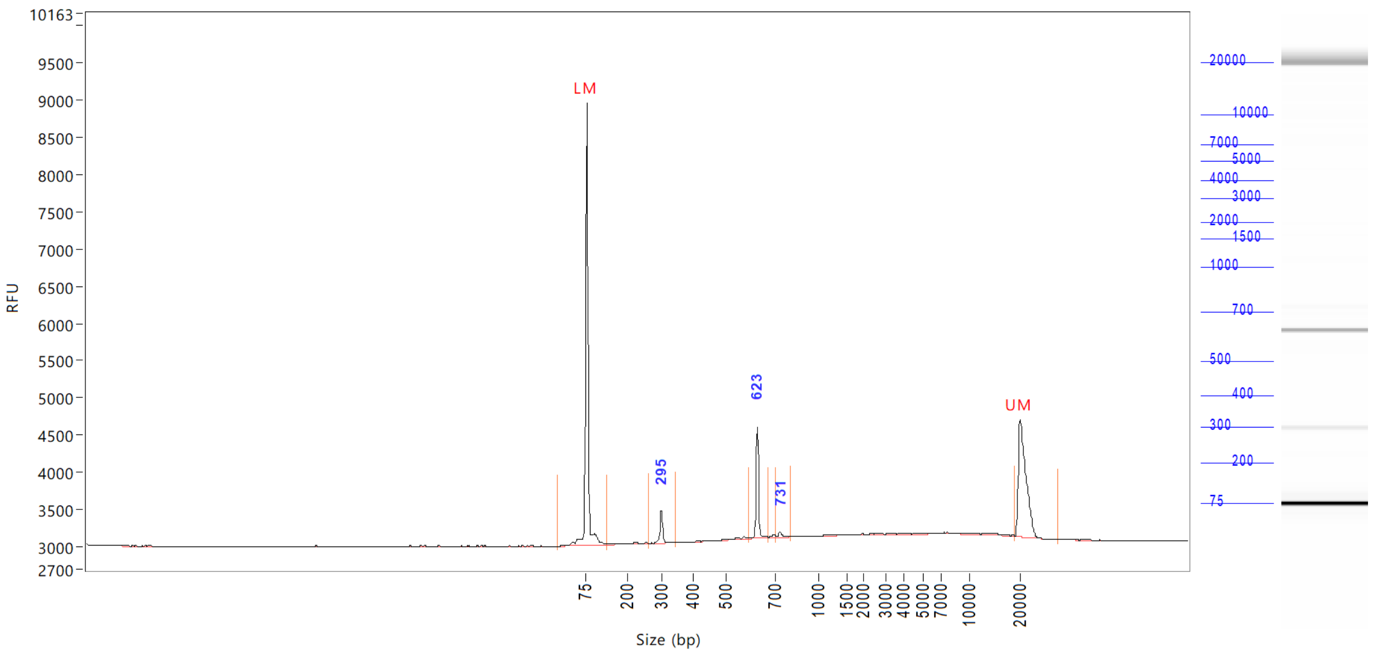

Peak	Size	Concentration	Relative concentration	Molarity	From	To	CV%	RFU
	(bp)	(ng/uL)	Percent	(nmole/L)	(bp)	(bp)		
1	75 (LM)	0.5000		11.2364	0	139	15.38	5950
2	295	0.4154	26.0	2.3239	262	341	2.66	428
3	623	1.1107	69.6	2.9339	595	672	1.15	1491
4	731	0.0688	4.3	0.1549	705	805	2.36	69
5	20000 (UM)	0.2053		0.0165	18903	27496	3.99	1578

TIC:	1.5948	ng/uL
TIM:	5.4127	nmole/L
Total concentration:	2.1902	ng/uL

```
Sample peak width (sec): 5     Sample min peak height: 50     Sample baseline V to V?: Y     Sample baseline V to V points: 3
Sample filter: Binomial        Number of points for filter: 3  Sample start region (min): 0  Sample end region (min): 30
Marker peak width (sec): 5     Marker min peak height: 200    Marker baseline V to V?: Y     Marker baseline V to V points: 3
Lower marker selection: First peak > 500 RFU                  Upper marker selection: Last peak > 200 RFU
Ladder size (bp)75, 200, 300, 400, 500, 700, 1000, 1500, 2000, 3000, 4000, 5000, 7000, 10000, 20000
Quantification using: Lower Marker          Final concentration (ng/uL): 0.5000          Dilution factor: 12.0
```

Sample: SampB12
Well location: B12
Created: Thursday, October 21, 2021 6:08:16 PM

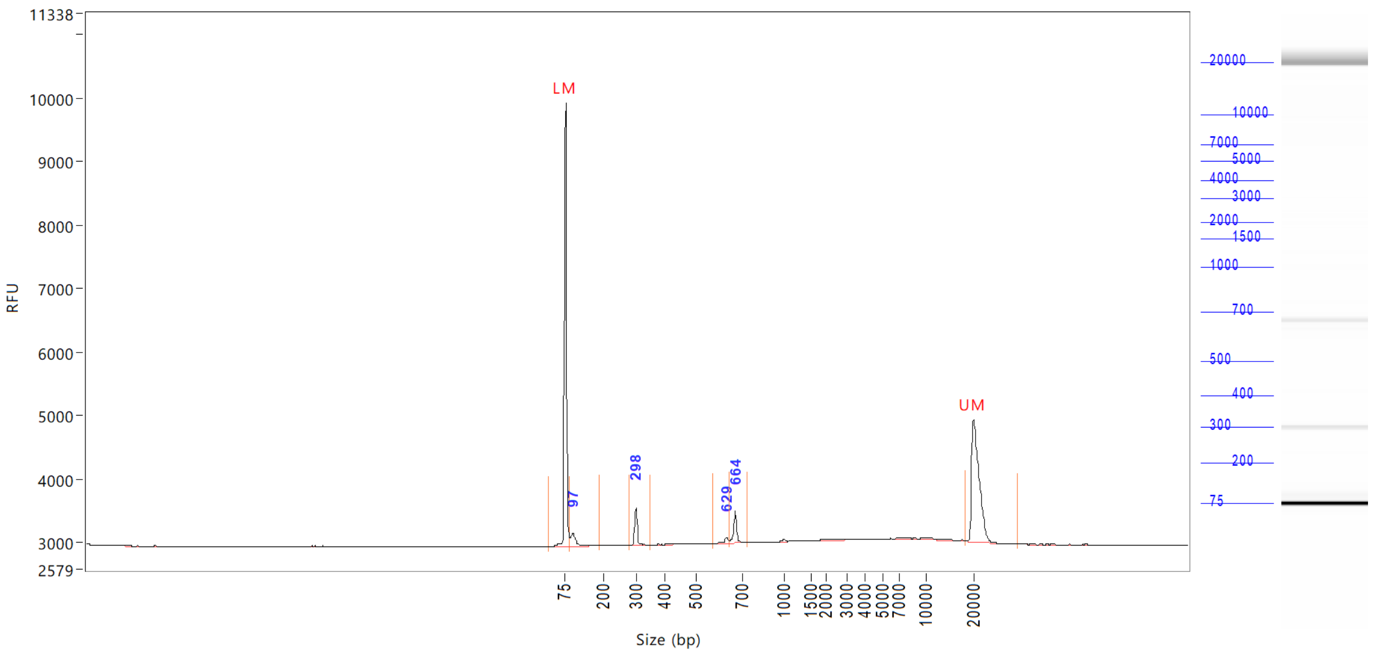

Peak	Size	Concentration	Relative concentration	Molarity	From	To	CV%	RFU
	(bp)	(ng/uL)	Percent	(nmole/L)	(bp)	(bp)		
1	75 (LM)	0.5000		11.3919	20	87	6.26	7000
2	97	0.4357	29.8	6.8147	87	185	18.47	200
3	298	0.4755	32.5	2.6424	277	345	2.10	593
4	629	0.1032	7.1	0.2731	570	641	2.33	82
5	664	0.4468	30.6	1.1073	641	731	1.73	499
6	20000 (UM)	0.2444		0.0196	18171	29309	4.32	1930

TIC:	1.4612	ng/uL
TIM:	10.8375	nmole/L
Total concentration:	1.9874	ng/uL

```
Sample peak width (sec): 5    Sample min peak height: 50    Sample baseline V to V?: Y    Sample baseline V to V points: 3
Sample filter: Binomial       Number of points for filter: 3  Sample start region (min): 0  Sample end region (min): 30
Marker peak width (sec): 5    Marker min peak height: 200   Marker baseline V to V?: Y    Marker baseline V to V points: 3
Lower marker selection: First peak > 500 RFU                Upper marker selection: Last peak > 200 RFU
Ladder size (bp)75, 200, 300, 400, 500, 700, 1000, 1500, 2000, 3000, 4000, 5000, 7000, 10000, 20000
Quantification using: Lower Marker          Final concentration (ng/uL): 0.5000          Dilution factor: 12.0
```

Sample: SampC12
Well location: C12
Created: Thursday, October 21, 2021 6:08:16 PM

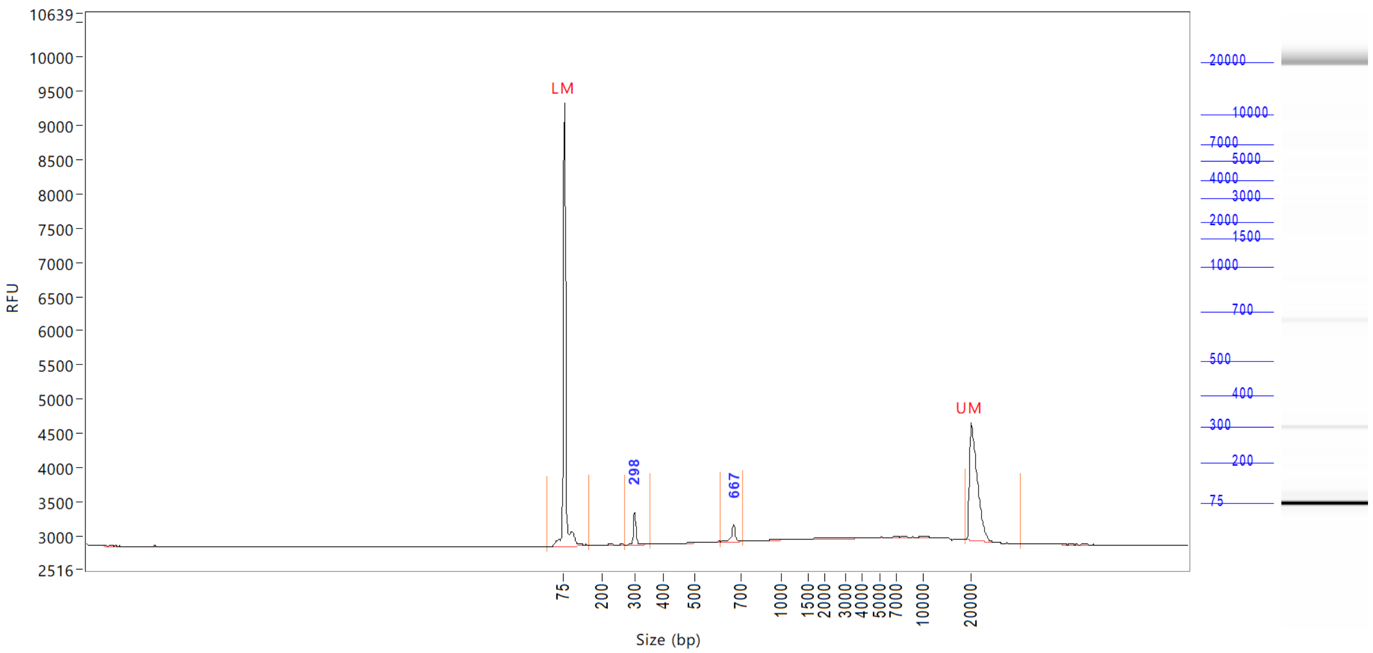

Peak	Size	Concentration	Relative concentration	Molarity	From	To	CV%	RFU
	(bp)	(ng/uL)	Percent	(nmole/L)	(bp)	(bp)		
1	75 (LM)	0.5000		10.9378	22	154	14.31	6489
2	298	0.4024	59.5	2.2211	269	349	2.97	477
3	667	0.2737	40.5	0.6826	608	705	2.44	243
4	20000 (UM)	0.2166		0.0172	18659	30277	4.72	1719

TIC:	0.6761	ng/uL
TIM:	2.9037	nmole/L
Total concentration:	1.2000	ng/uL

Sample peak width (sec): 5 Sample min peak height: 50 Sample baseline V to V?: Y Sample baseline V to V points: 3
Sample filter: Binomial Number of points for filter: 3 Sample start region (min): 0 Sample end region (min): 30
Marker peak width (sec): 5 Marker min peak height: 200 Marker baseline V to V?: Y Marker baseline V to V points: 3
Lower marker selection: First peak > 500 RFU Upper marker selection: Last peak > 200 RFU
Ladder size (bp)75, 200, 300, 400, 500, 700, 1000, 1500, 2000, 3000, 4000, 5000, 7000, 10000, 20000
Quantification using: Lower Marker Final concentration (ng/uL): 0.5000 Dilution factor: 12.0

Sample: SampD12
Well location: D12
Created: Thursday, October 21, 2021 6:08:16 PM

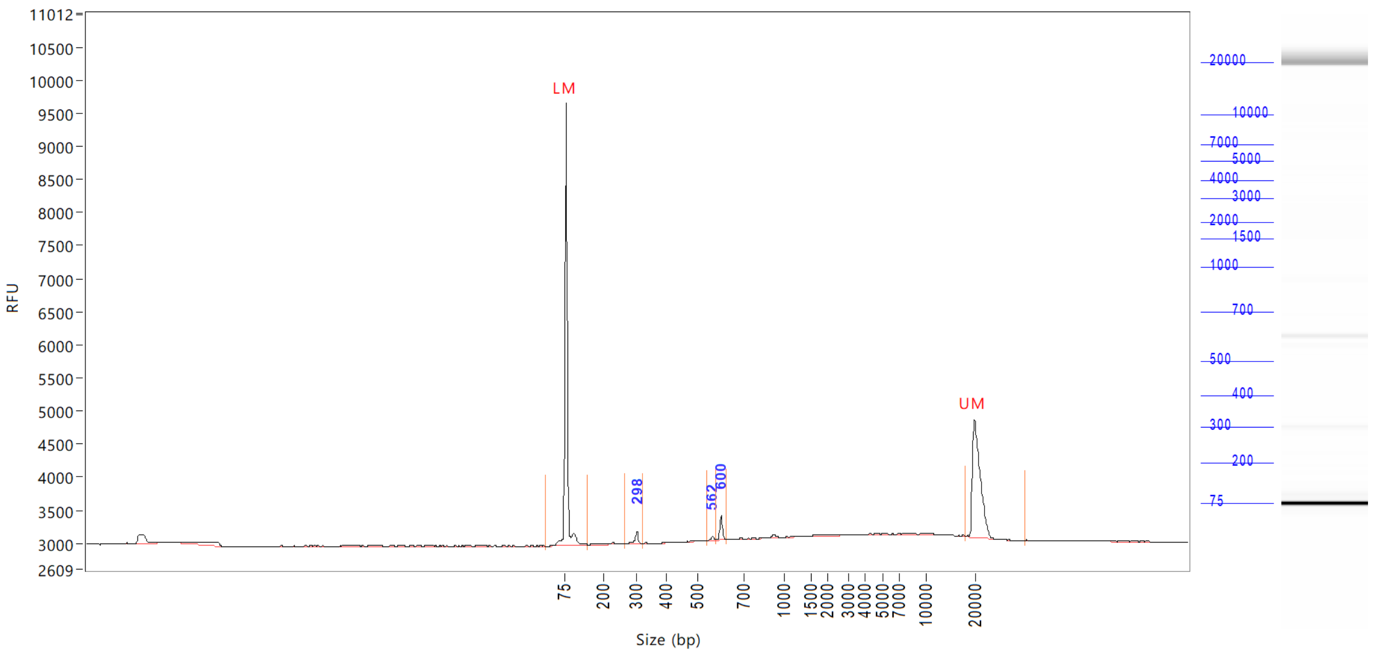

Peak	Size	Concentration	Relative concentration	Molarity	From	To	CV%	RFU
	(bp)	(ng/uL)	Percent	(nmole/L)	(bp)	(bp)		
1	75 (LM)	0.5000		11.0851	9	147	12.64	6703
2	298	0.2096	39.1	1.1765	262	317	3.06	191
3	562	0.0560	10.5	0.1641	539	580	1.42	62
4	600	0.2702	50.4	0.7425	580	626	1.05	364
5	20000 (UM)	0.2276		0.0182	18171	30519	4.74	1775

TIC:	0.5359	ng/uL
TIM:	2.0831	nmole/L
Total concentration:	1.1374	ng/uL

Sample peak width (sec): 5   Sample min peak height: 50   Sample baseline V to V?: Y   Sample baseline V to V points: 3
Sample filter: Binomial   Number of points for filter: 3 Sample start region (min): 0 Sample end region (min): 30
Marker peak width (sec): 5   Marker min peak height: 200   Marker baseline V to V?: Y   Marker baseline V to V points: 3
Lower marker selection: First peak > 500 RFU   Upper marker selection: Last peak > 200 RFU
Ladder size (bp)75, 200, 300, 400, 500, 700, 1000, 1500, 2000, 3000, 4000, 5000, 7000, 10000, 20000
Quantification using: Lower Marker   Final concentration (ng/uL): 0.5000   Dilution factor: 12.0

Sample: SampE12
Well location: E12
Created: Thursday, October 21, 2021 6:08:16 PM

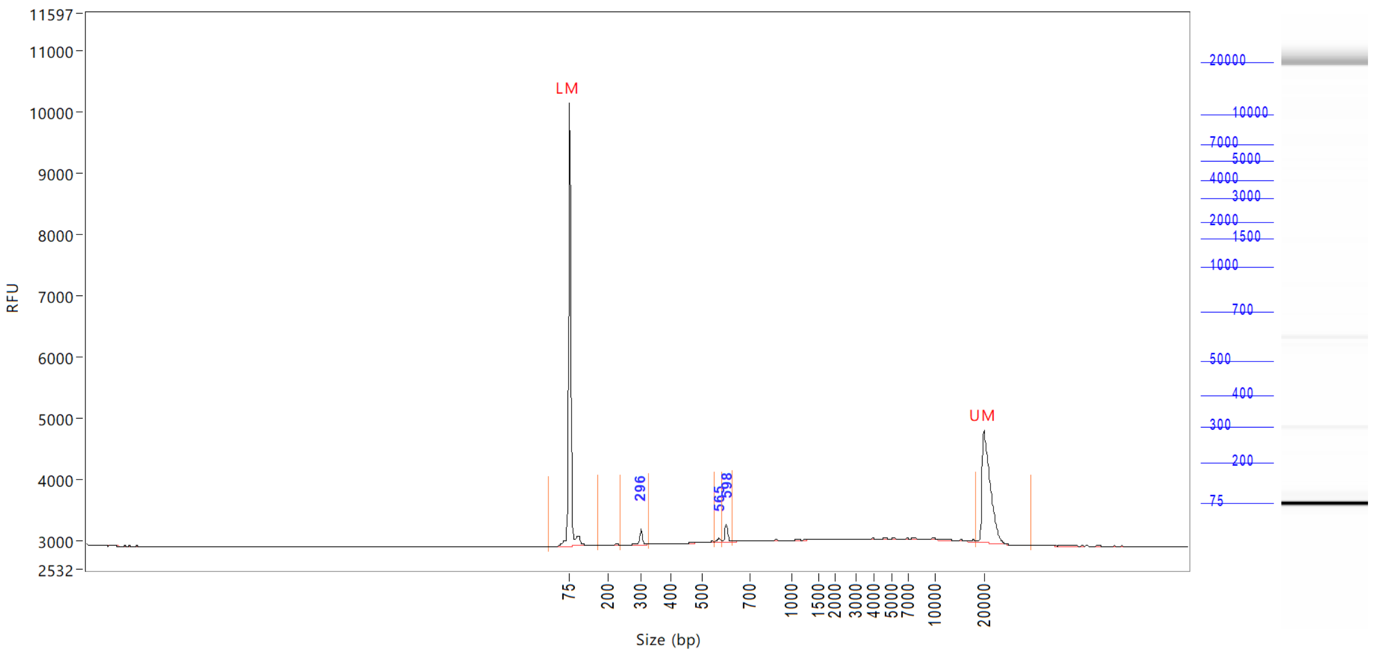

Peak	Size	Concentration	Relative concentration	Molarity	From	To	CV%	RFU
	(bp)	(ng/uL)	Percent	(nmole/L)	(bp)	(bp)		
1	75 (LM)	0.5000		11.0851	9	164	12.19	7238
2	296	0.2244	46.0	1.2640	233	321	3.66	267
3	565	0.0610	12.5	0.1783	547	580	1.35	66
4	598	0.2021	41.5	0.5590	580	623	1.10	288
5	20000 (UM)	0.2271		0.0181	18415	29672	4.90	1824

TIC:	0.4875	ng/uL
TIM:	2.0014	nmole/L
Total concentration:	0.9219	ng/uL

```
Sample peak width (sec): 5    Sample min peak height: 50    Sample baseline V to V?: Y    Sample baseline V to V points: 3
Sample filter: Binomial       Number of points for filter: 3  Sample start region (min): 0  Sample end region (min): 30
Marker peak width (sec): 5    Marker min peak height: 200   Marker baseline V to V?: Y    Marker baseline V to V points: 3
Lower marker selection: First peak > 500 RFU                Upper marker selection: Last peak > 200 RFU
Ladder size (bp)75, 200, 300, 400, 500, 700, 1000, 1500, 2000, 3000, 4000, 5000, 7000, 10000, 20000
Quantification using: Lower Marker          Final concentration (ng/uL): 0.5000          Dilution factor: 12.0
```

Sample: SampF12
Well location: F12
Created: Thursday, October 21, 2021 6:08:16 PM

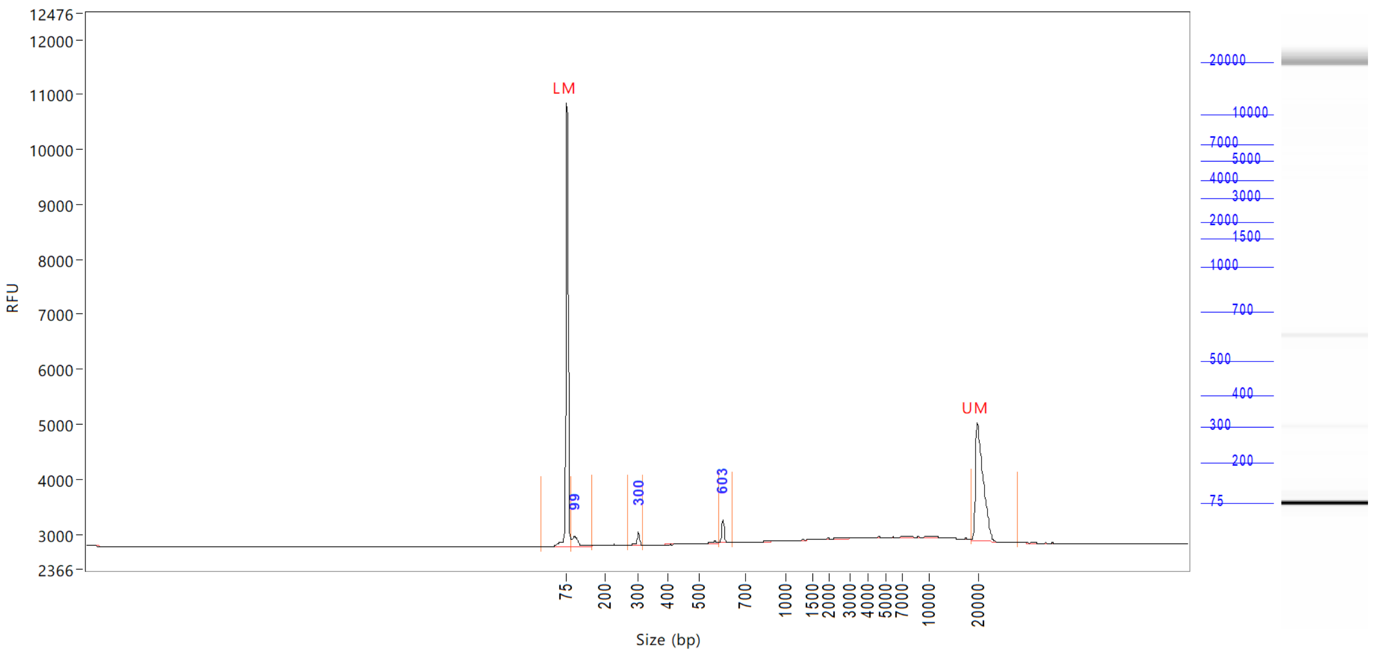

Peak	Size	Concentration	Relative concentration	Molarity	From	To	CV%	RFU
	(bp)	(ng/uL)	Percent	(nmole/L)	(bp)	(bp)		
1	75 (LM)	0.5000		11.2364	0	89	7.59	8079
2	99	0.3384	43.4	5.2928	89	158	13.74	176
3	300	0.1751	22.4	0.9761	268	313	2.36	233
4	603	0.2665	34.2	0.7286	585	644	1.34	411
5	20000 (UM)	0.2352		0.0188	18903	28463	4.42	2150

TIC:	0.7800	ng/uL
TIM:	6.9974	nmole/L
Total concentration:	1.2699	ng/uL

Sample peak width (sec): 5 Sample min peak height: 50 Sample baseline V to V?: Y Sample baseline V to V points: 3
Sample filter: Binomial Number of points for filter: 3 Sample start region (min): 0 Sample end region (min): 30
Marker peak width (sec): 5 Marker min peak height: 200 Marker baseline V to V?: Y Marker baseline V to V points: 3
Lower marker selection: First peak > 500 RFU Upper marker selection: Last peak > 200 RFU
Ladder size (bp)75, 200, 300, 400, 500, 700, 1000, 1500, 2000, 3000, 4000, 5000, 7000, 10000, 20000
Quantification using: Lower Marker Final concentration (ng/uL): 0.5000 Dilution factor: 12.0

Sample: SampG12
Well location: G12
Created: Thursday, October 21, 2021 6:08:16 PM

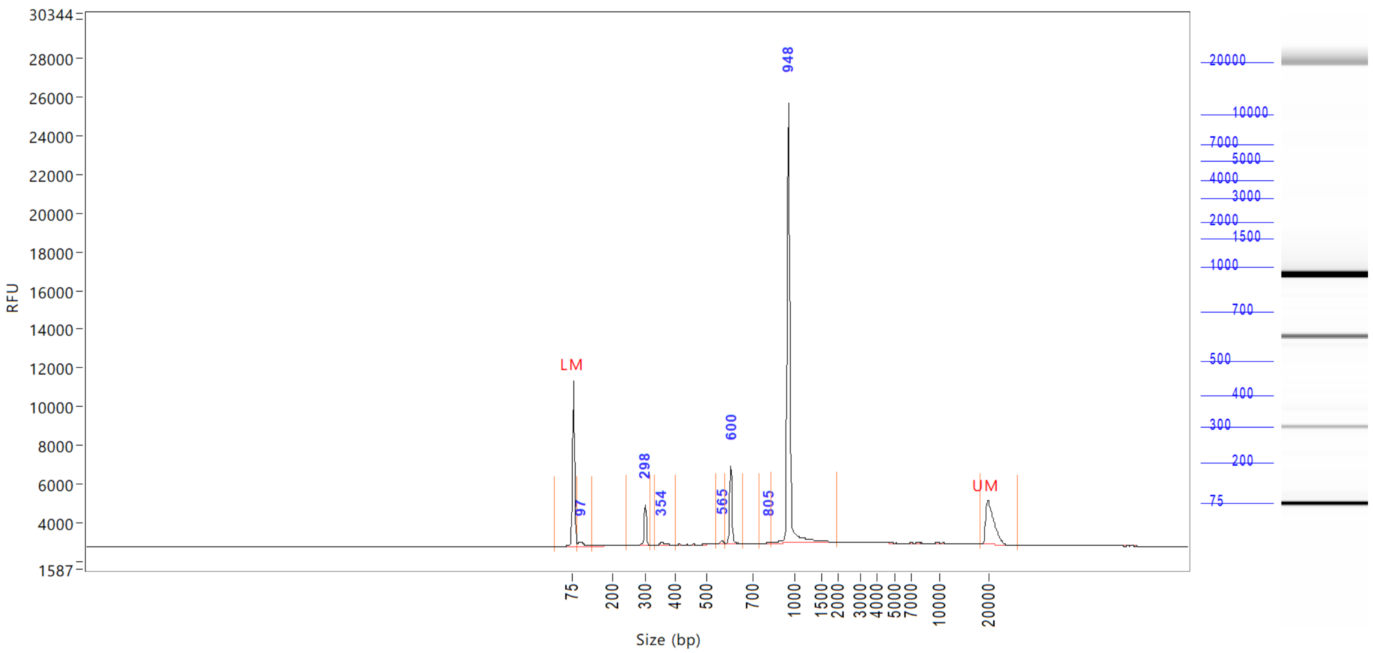

Peak	Size	Concentration	Relative concentration	Molarity	From	To	CV%	RFU
	(bp)	(ng/uL)	Percent	(nmole/L)	(bp)	(bp)		
1	75 (LM)	0.5000		11.3919	16	86	5.97	8589
2	97	0.4179	2.1	6.8620	86	137	11.70	204
3	298	1.3746	7.0	7.6388	244	313	2.25	2082
4	354	0.2148	1.1	0.9870	331	401	3.76	146
5	565	0.1762	0.9	0.5161	539	580	1.62	178
6	600	2.4059	12.3	6.6099	580	657	1.39	4017
7	805	0.0541	0.3	0.1110	744	831	1.92	73
8	948	14.8571	76.2	25.3405	831	1962	8.27	22800
9	20000 (UM)	0.2506		0.0200	18415	26045	4.22	2280

TIC:	19.5007	ng/uL
TIM:	48.0652	nmole/L
Total concentration:	19.9990	ng/uL

```
Sample peak width (sec): 5    Sample min peak height: 50    Sample baseline V to V?: Y    Sample baseline V to V points: 3
Sample filter: Binomial       Number of points for filter: 3  Sample start region (min): 0  Sample end region (min): 30
Marker peak width (sec): 5    Marker min peak height: 200   Marker baseline V to V?: Y    Marker baseline V to V points: 3
Lower marker selection: First peak > 500 RFU                Upper marker selection: Last peak > 200 RFU
Ladder size (bp)75, 200, 300, 400, 500, 700, 1000, 1500, 2000, 3000, 4000, 5000, 7000, 10000, 20000
Quantification using: Lower Marker          Final concentration (ng/uL): 0.5000          Dilution factor: 12.0
```

Sample: SampH12
Well location: H12
Created: Thursday, October 21, 2021 6:08:16 PM

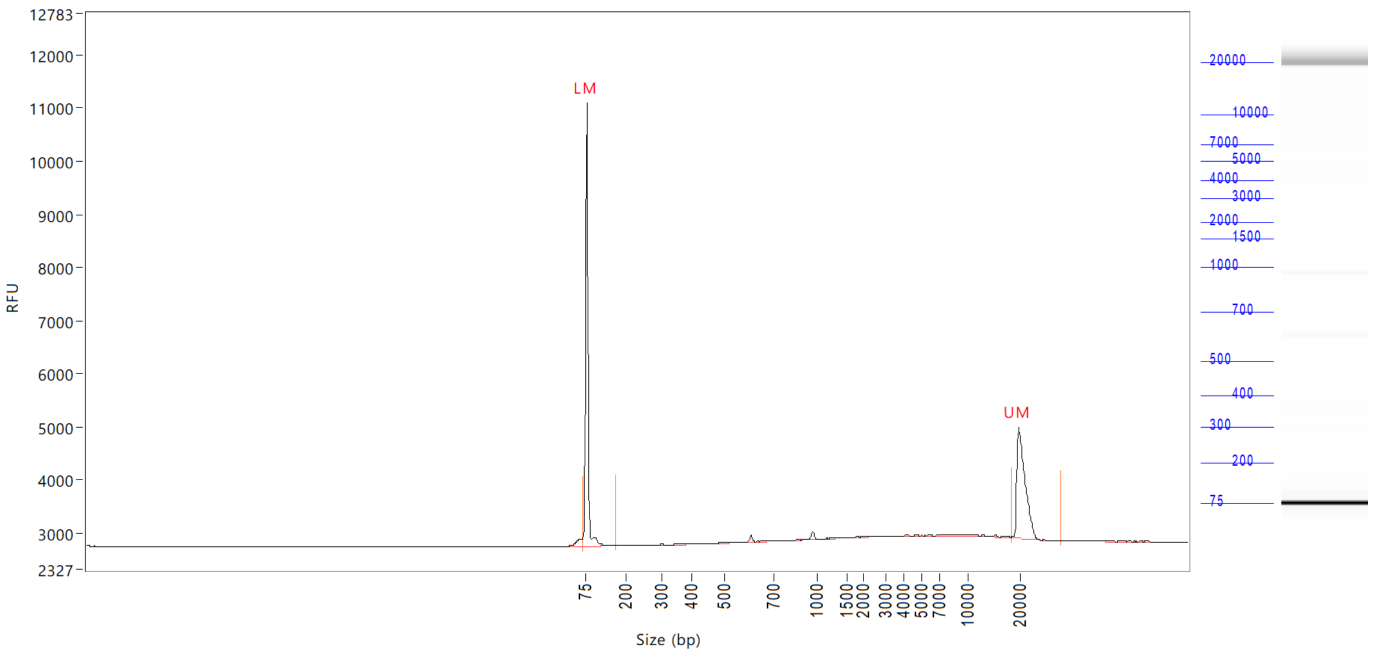

Peak	Size	Concentration	Relative concentration	Molarity	From	To	CV%	RFU
	(bp)	(ng/uL)	Percent	(nmole/L)	(bp)	(bp)		
1	75 (LM)	0.5000		10.9378	64	168	10.97	8350
2	20000 (UM)	0.2227		0.0178	18415	28221	4.42	2092

TIC:	0.0000	ng/uL
TIM:	0.0000	nmole/L
Total concentration:	0.7159	ng/uL

Sample peak width (sec): 5 Sample min peak height: 200 Sample baseline V to V?: Y Sample baseline V to V points: 3
Sample filter: Binomial Number of points for filter: 3 Sample start region (min): 0 Sample end region (min): 30
Marker peak width (sec): 5 Marker min peak height: 200 Marker baseline V to V?: Y Marker baseline V to V points: 3
Lower marker selection: First peak > 500 RFU Upper marker selection: Last peak > 200 RFU
Ladder size (bp)75, 200, 300, 400, 500, 700, 1000, 1500, 2000, 3000, 4000, 5000, 7000, 10000, 20000
Quantification using: Lower Marker Final concentration (ng/uL): 0.5000 Dilution factor: 12.0

Sample: DNA size ladder
Well location: A7
Created: Thursday, October 21, 2021 6:08:16 PM
Import from: C:\Users\admin\Desktop\210810_alberto_ladder.SCAL
Fit type: Point to point

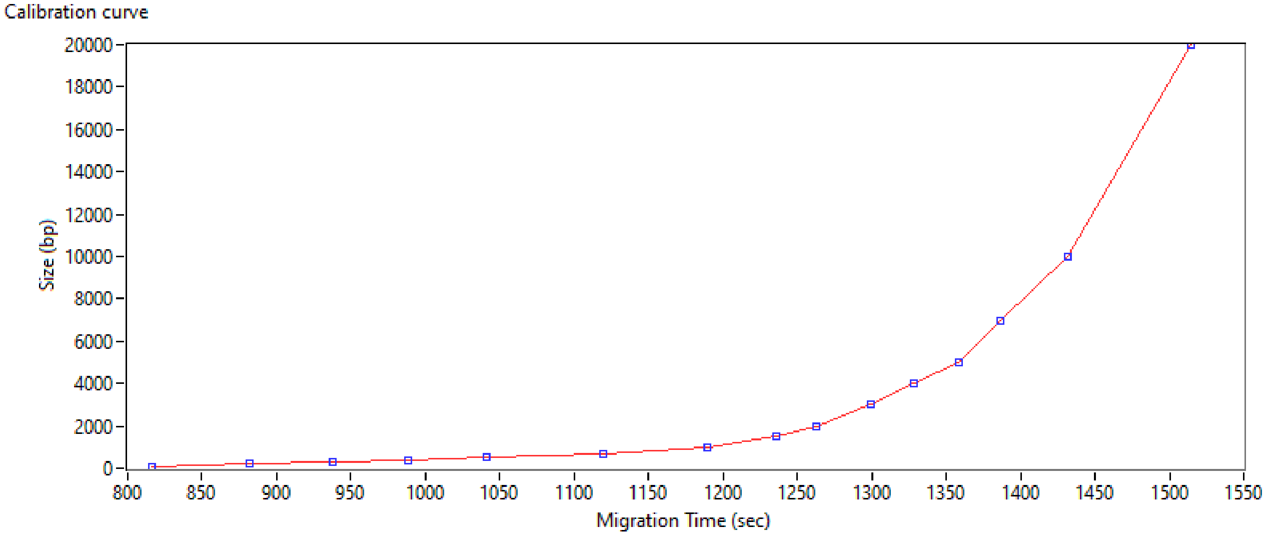

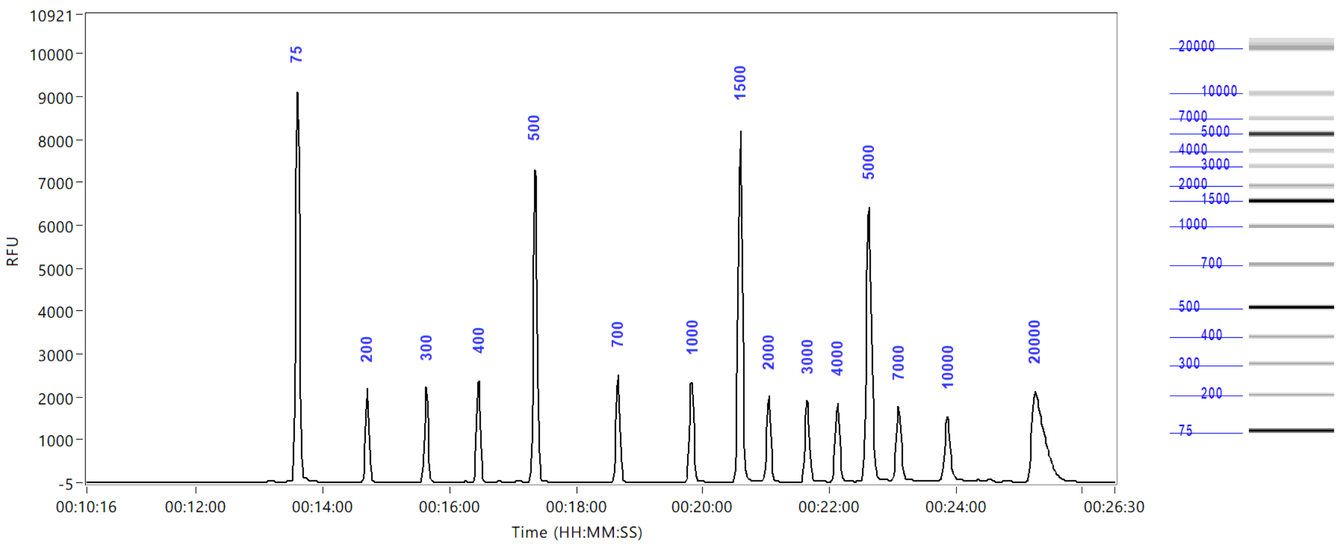

Supplement: Supplementary file 2 — sb3c00292_si_002.zip [file sb3c00292_si_002.zip › dnada_supplementary_material_pks_library_build/colony_pcr/ZAG/17-58-41_plate6/colonypcrplate_6.pdf]
