# Supplementary material for: Automated Platform for the Plasmid Construction Process
Source: ACS Synth Biol. 2023 Nov 10;12(12):3506–13. doi: 10.1021/acssynbio.3c00292 (PMC10729297; doi:10.1021/acssynbio.3c00292)

Instrument controller software run summary:

Filename and data path: C:\AATI\Data\2021 10 21\21-29-00\2021 10 21 21H 29M..raw
Created: Thursday, October 21, 2021 9:39:00 PM
Number of capillaries: 96
Array serial number: 111920-09SFS
Effect length: 33 cm
Array usage count: 271
Instrument type: ZAG DNA Analyzer
Instrument controller software version: 1.0.1.9
Device serial number: Z7597

Method Information

Method name: ZAG130GP33 - DNA 75-20000bp - Gel Prime Only.mthds
Gel prime: Yes
Full conditioning: No
Gel prime to buffer: No
Gel selection: Gel 1
Perform prerun: 8.0 kV, 30 sec.
Rinse: No
Marker 1: Row: A, 5.0 kV, 5 sec.
Rinse: No
Sample injection: 5.0 kV, 5 sec.
Separation: 8.0 kV, 30.0 min.
Tray name: Sample Tray-7

Analysis mode: DNA

Notes

Gel Image

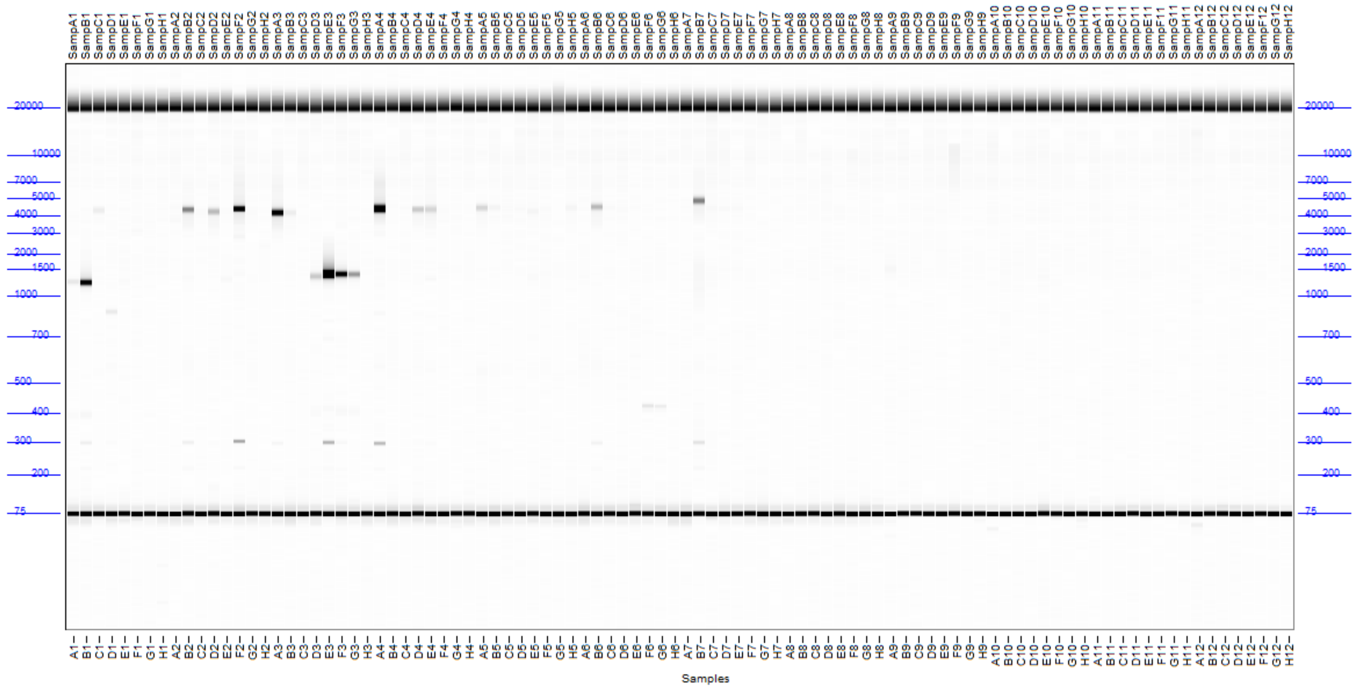

Filename and data path: C:\AATI\Data\2021 10 21\21-29-00\2021 10 21 21H 29M..raw

A1: SampA1

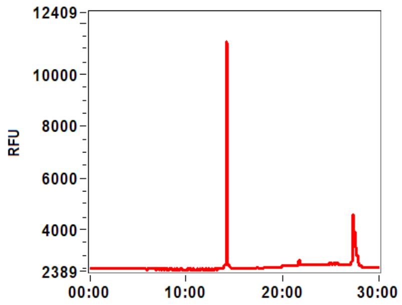

B1: SampB1

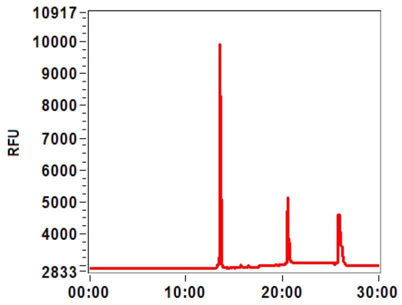

C1: SampC1

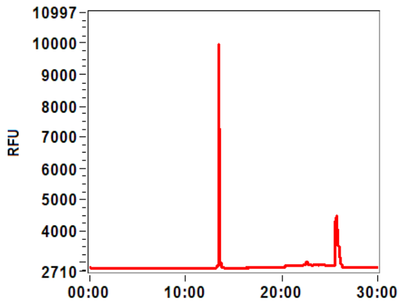

D1: SampD1

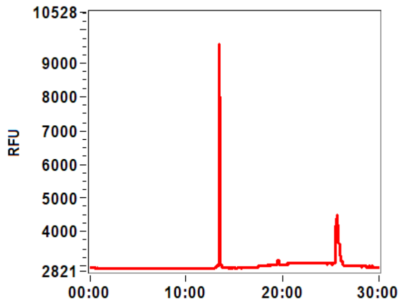

E1: SampE1

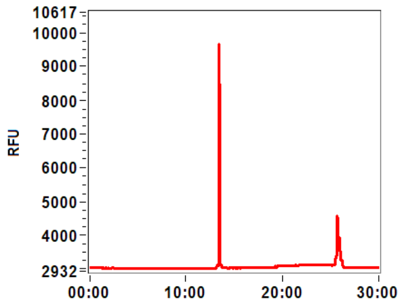

F1: SampF1

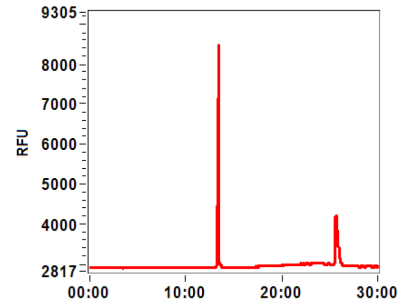

G1: SampG1

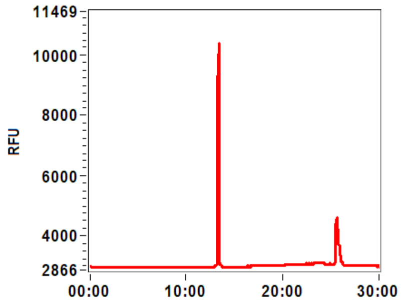

H1: SampH1

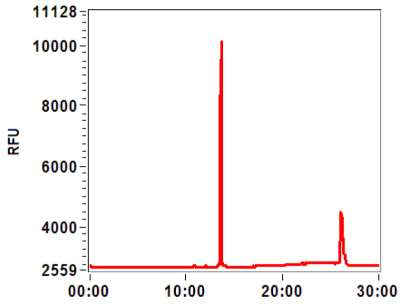

A2: SampA2

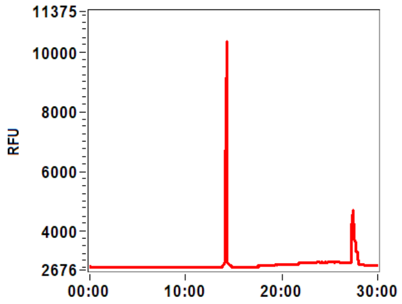

B2: SampB2

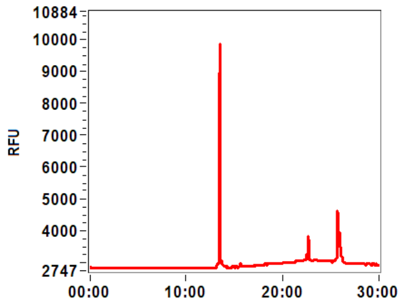

C2: SampC2

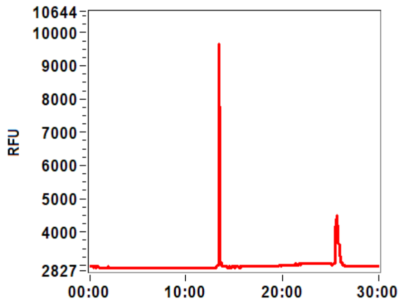

D2: SampD2

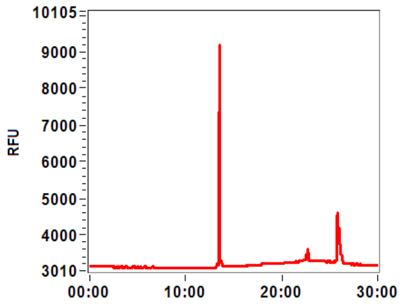

Filename and data path: C:\AATI\Data\2021 10 21\21-29-00\2021 10 21 21H 29M..raw

E2: SampE2

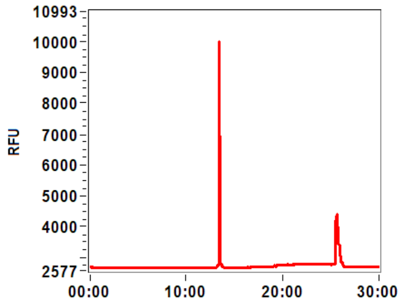

F2: SampF2

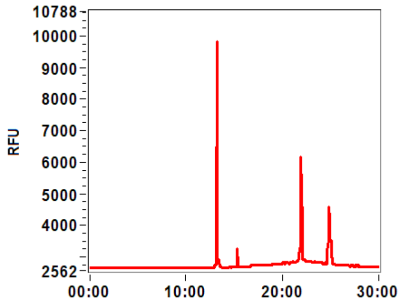

G2: SampG2

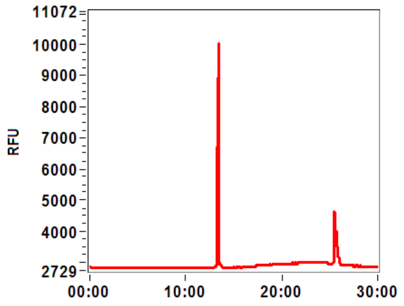

H2: SampH2

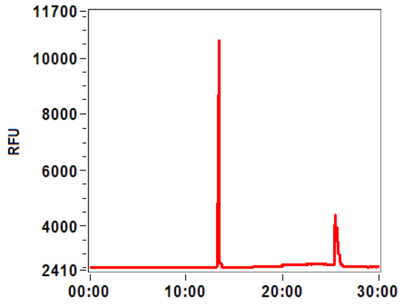

A3: SampA3

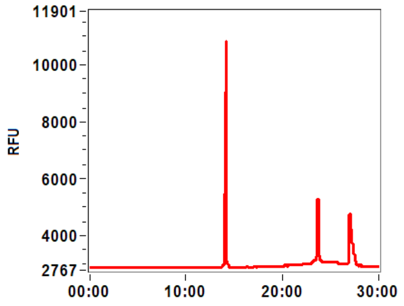

B3: SampB3

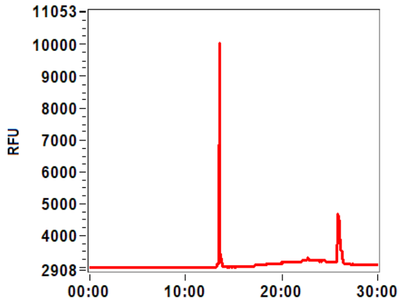

C3: SampC3

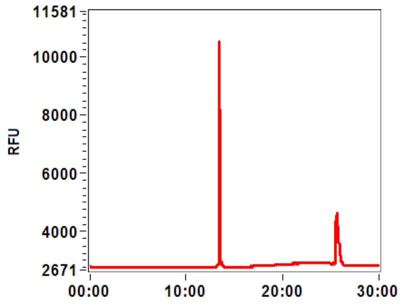

D3: SampD3

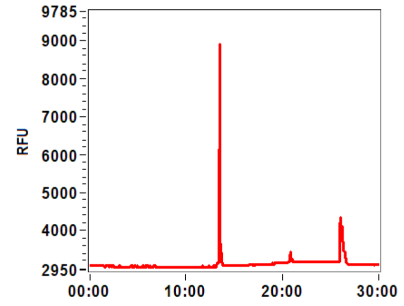

E3: SampE3

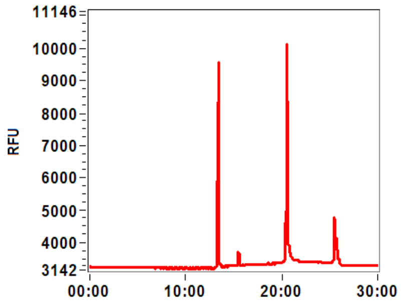

F3: SampF3

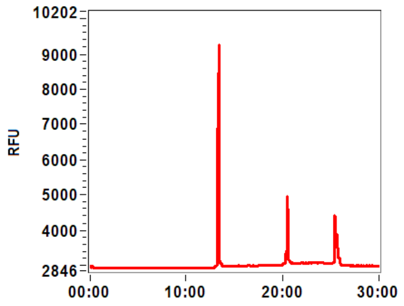

G3: SampG3

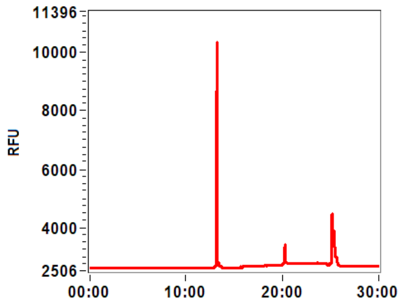

H3: SampH3

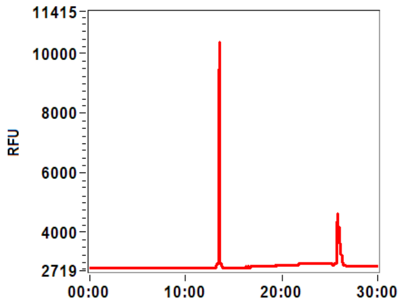

Filename and data path: C:\AATI\Data\2021 10 21\21-29-00\2021 10 21 21H 29M..raw

A4: SampA4

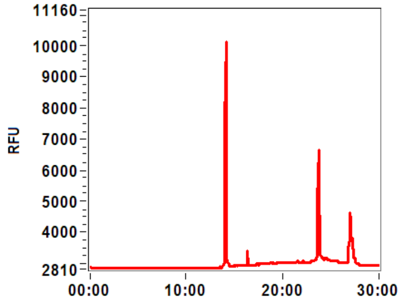

B4: SampB4

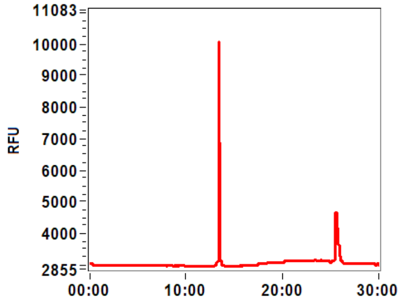

C4: SampC4

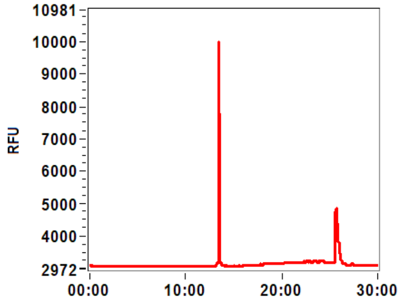

D4: SampD4

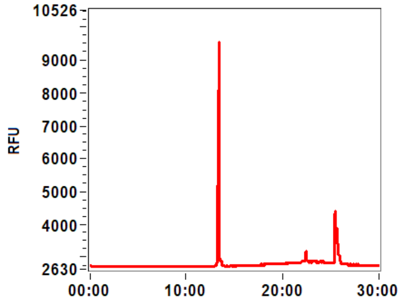

E4: SampE4

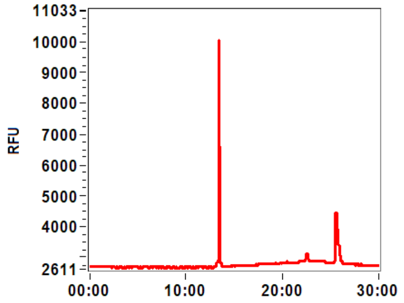

F4: SampF4

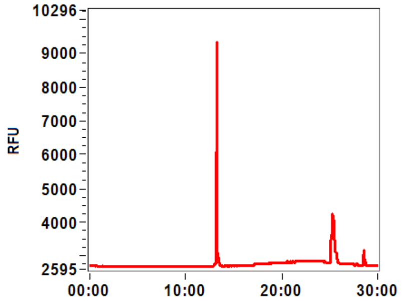

G4: SampG4

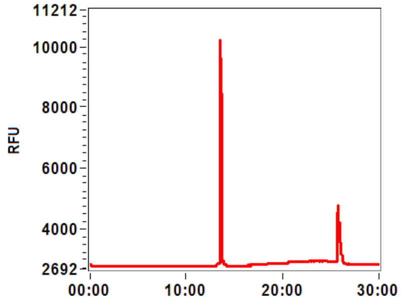

H4: SampH4

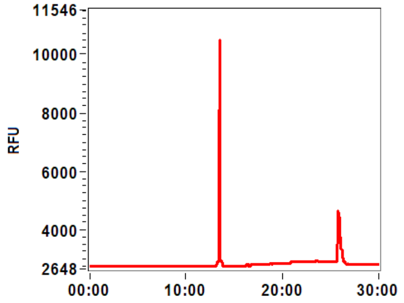

A5: SampA5

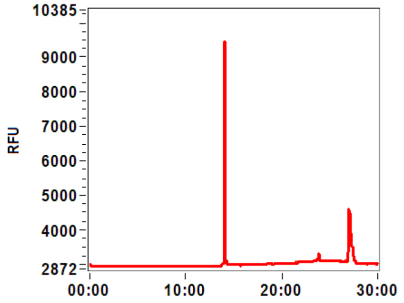

B5: SampB5

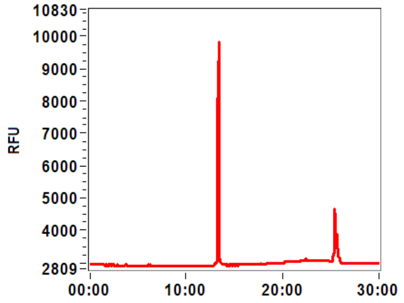

C5: SampC5

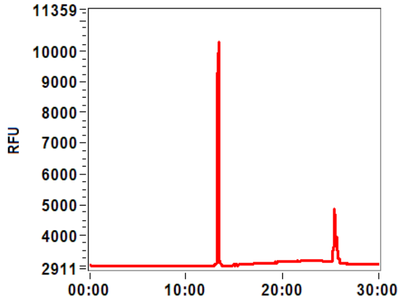

D5: SampD5

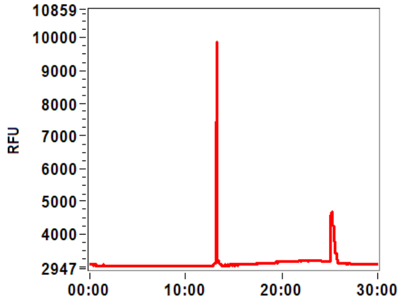

Filename and data path: C:\AATI\Data\2021 10 21\21-29-00\2021 10 21 21H 29M..raw

E5: SampE5

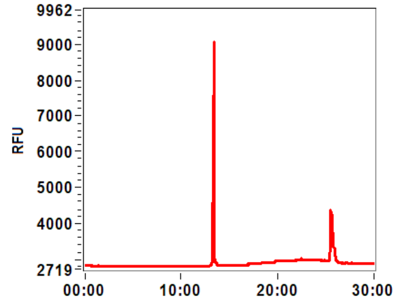

F5: SampF5

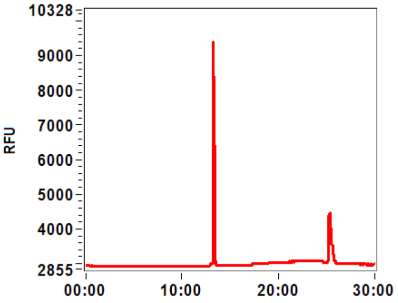

G5: SampG5

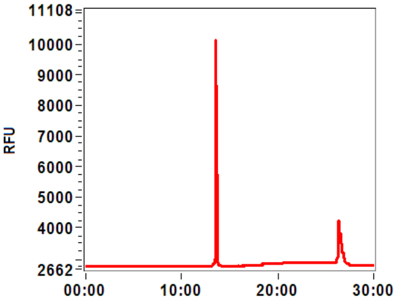

H5: SampH5

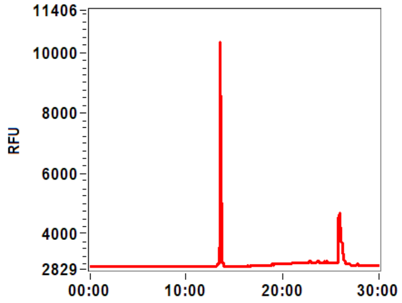

A6: SampA6

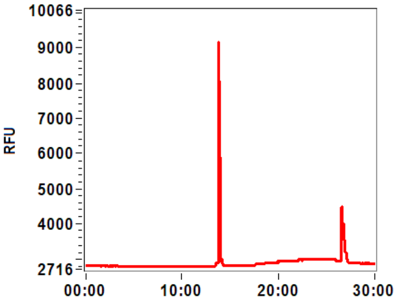

B6: SampB6

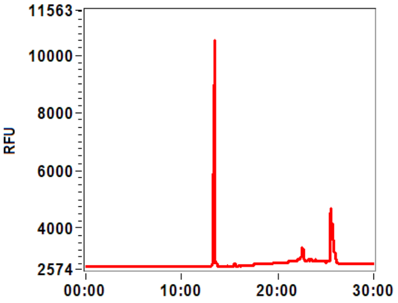

C6: SampC6

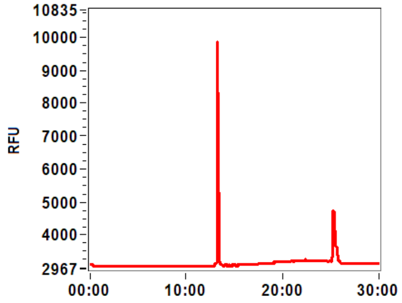

D6: SampD6

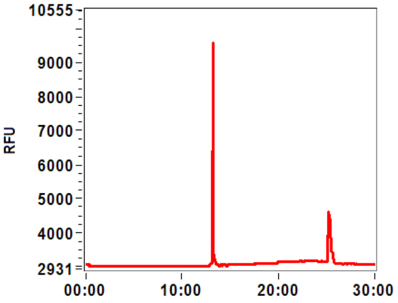

E6: SampE6

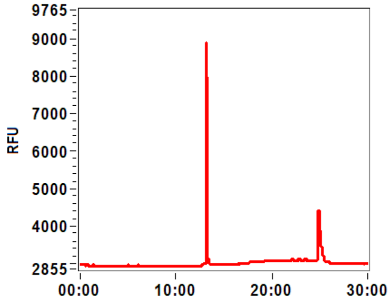

F6: SampF6

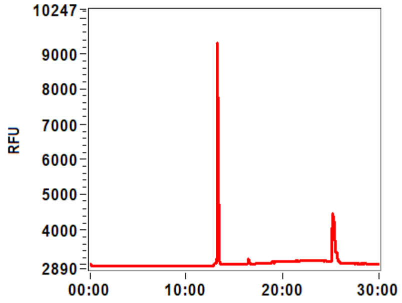

G6: SampG6

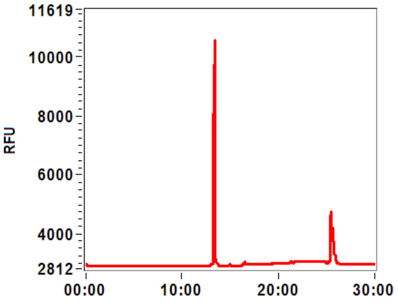

H6: SampH6

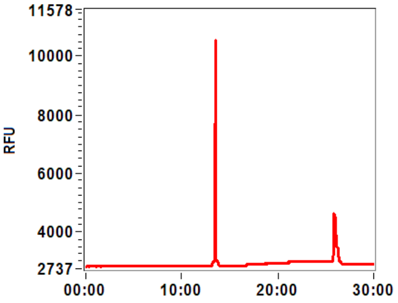

Filename and data path: C:\AATI\Data\2021 10 21\21-29-00\2021 10 21 21H 29M..raw

A7: SampA7

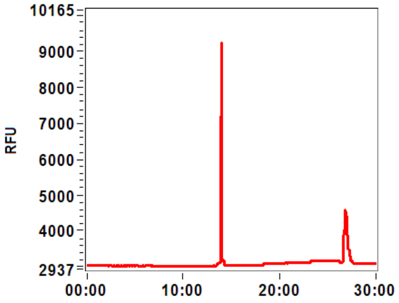

B7: SampB7

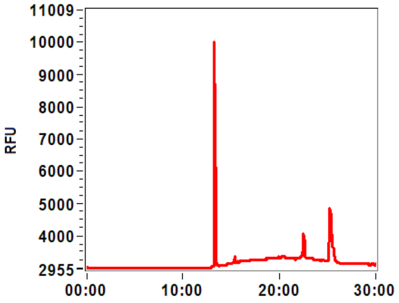

C7: SampC7

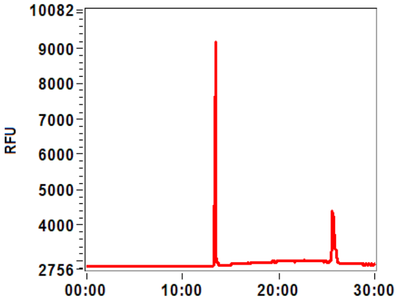

D7: SampD7

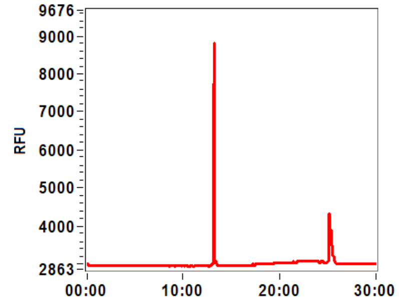

E7: SampE7

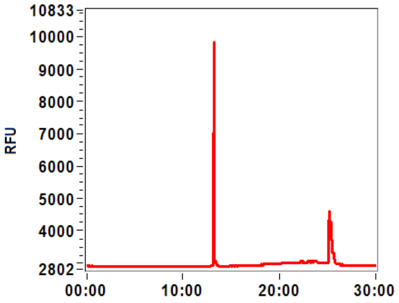

F7: SampF7

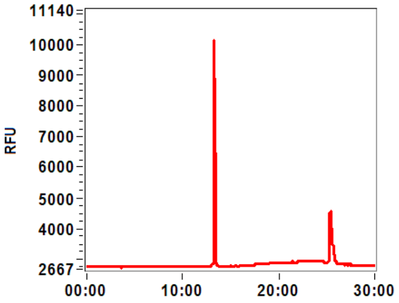

G7: SampG7

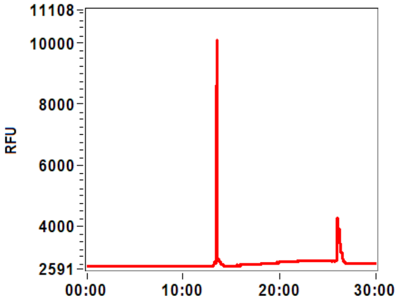

H7: SampH7

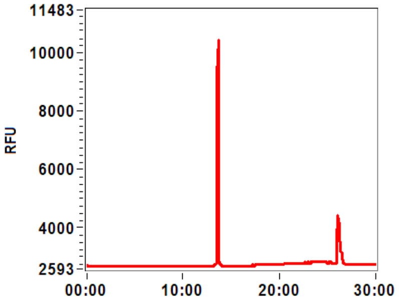

A8: SampA8

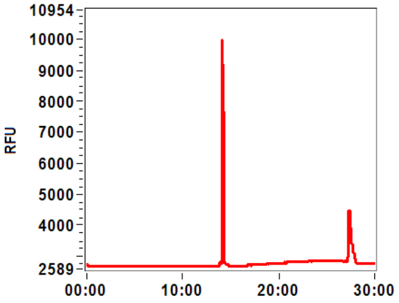

B8: SampB8

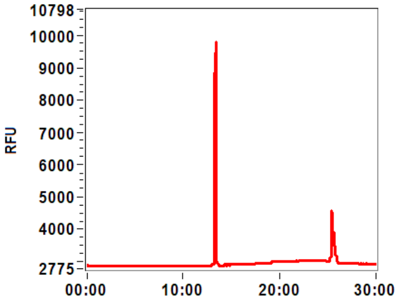

C8: SampC8

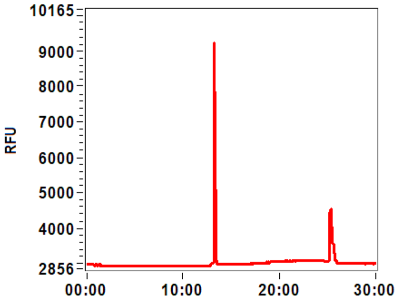

D8: SampD8

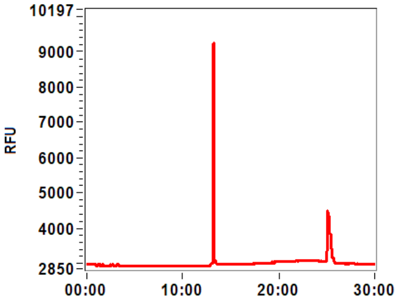

Filename and data path: C:\AATI\Data\2021 10 21\21-29-00\2021 10 21 21H 29M..raw

E8: SampE8

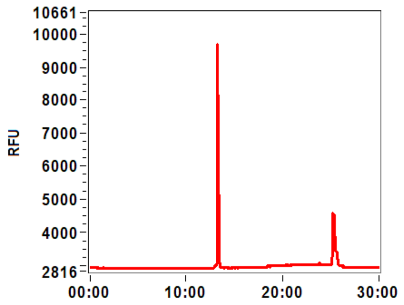

F8: SampF8

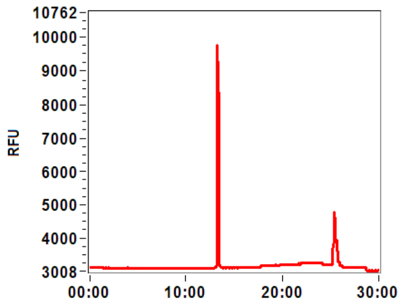

G8: SampG8

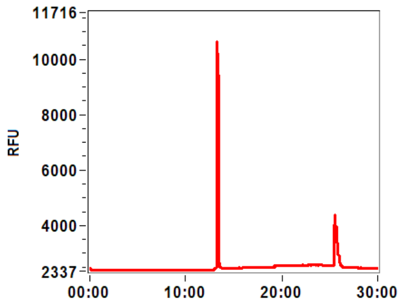

H8: SampH8

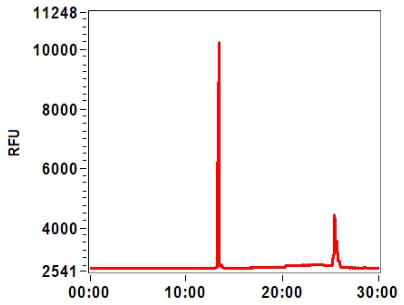

A9: SampA9

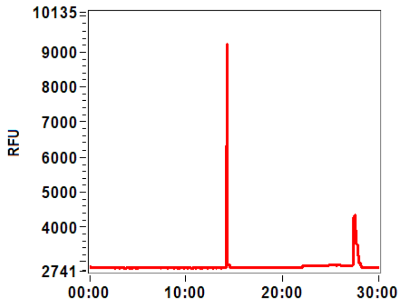

B9: SampB9

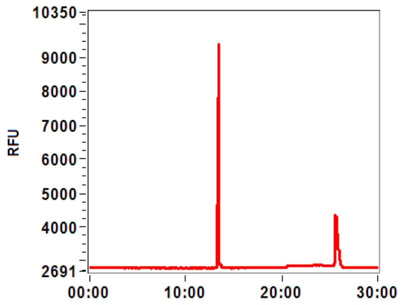

C9: SampC9

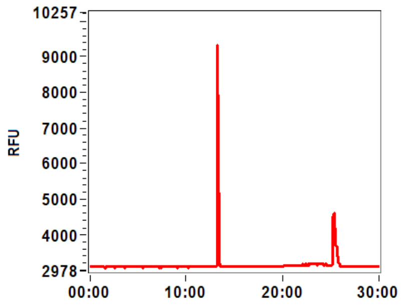

D9: SampD9

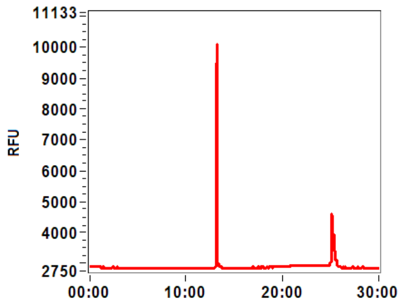

E9: SampE9

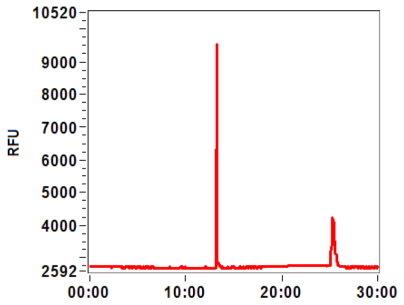

F9: SampF9

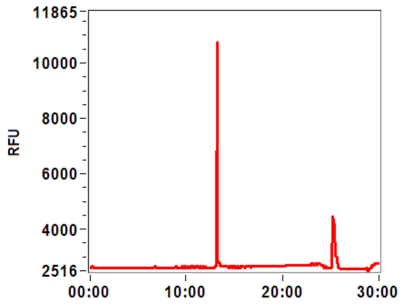

G9: SampG9

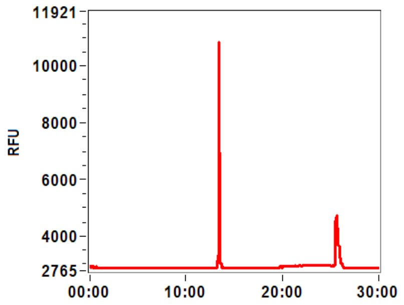

H9: SampH9

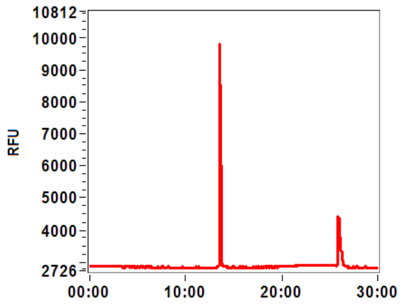

Filename and data path: C:\AATI\Data\2021 10 21\21-29-00\2021 10 21 21H 29M..raw

A10: SampA10

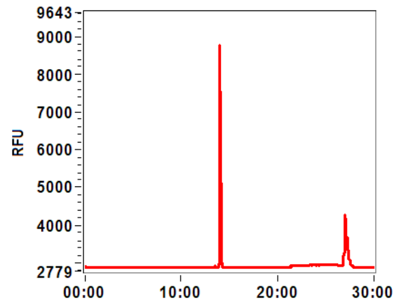

B10: SampB10

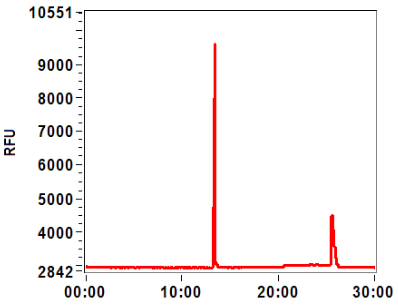

C10: SampC10

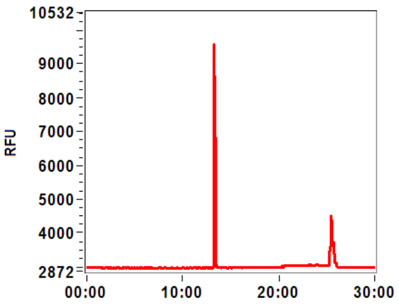

D10: SampD10

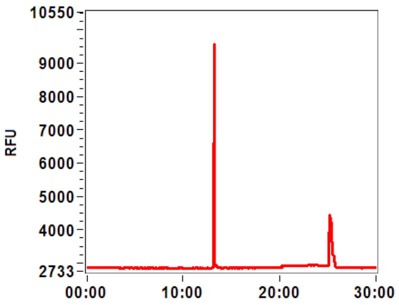

E10: SampE10

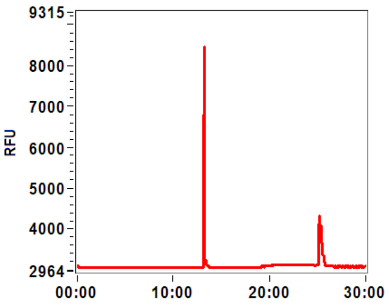

F10: SampF10

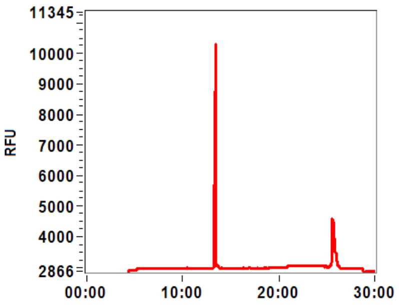

G10: SampG10

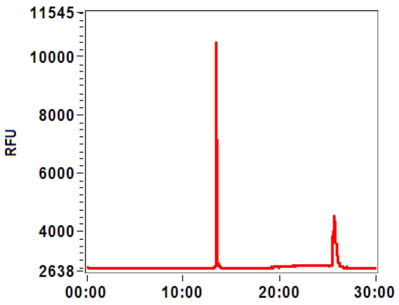

H10: SampH10

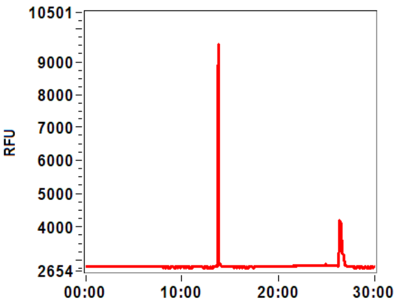

A11: SampA11

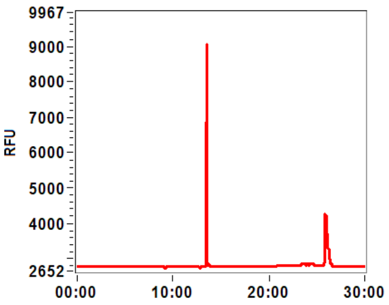

B11: SampB11

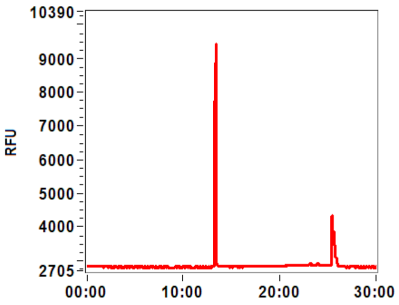

C11: SampC11

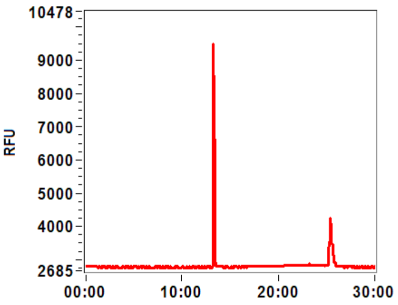

D11: SampD11

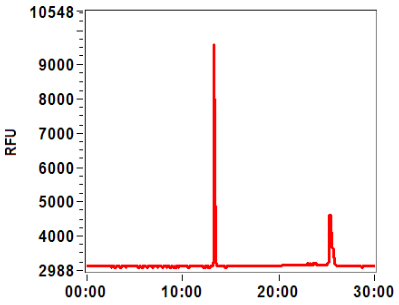

Filename and data path: C:\AATI\Data\2021 10 21\21-29-00\2021 10 21 21H 29M..raw

E11: SampE11

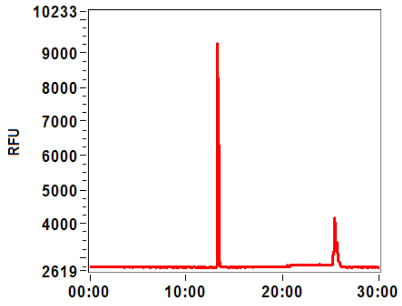

F11: SampF11

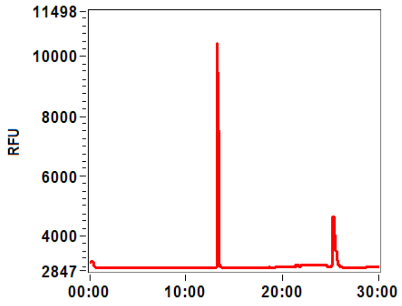

G11: SampG11

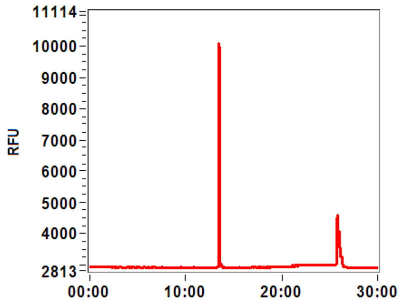

H11: SampH11

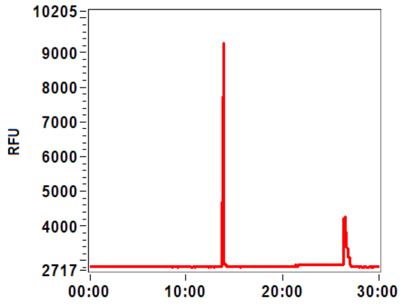

A12: SampA12

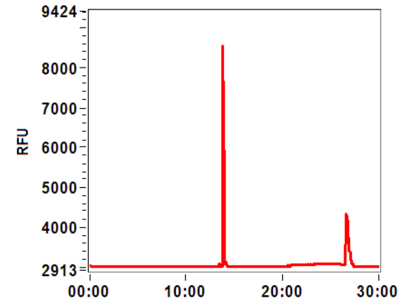

B12: SampB12

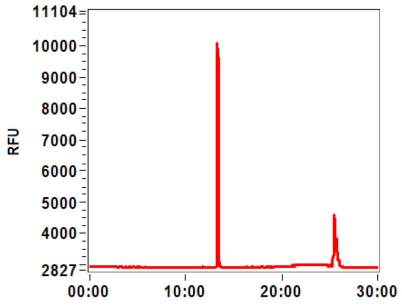

C12: SampC12

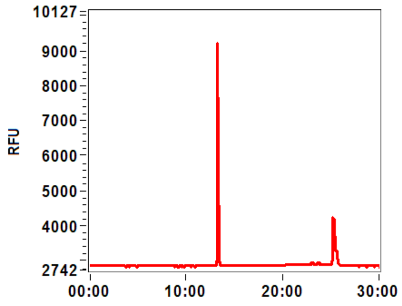

D12: SampD12

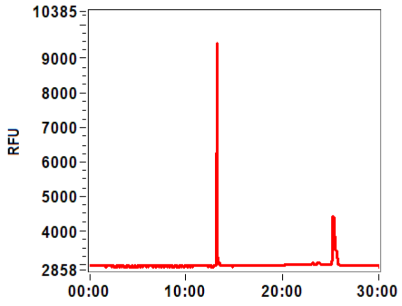

E12: SampE12

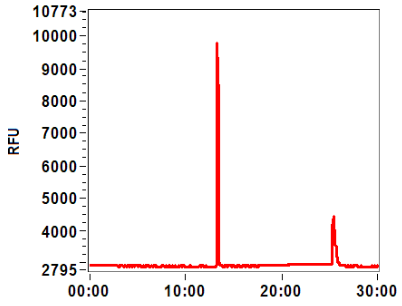

F12: SampF12

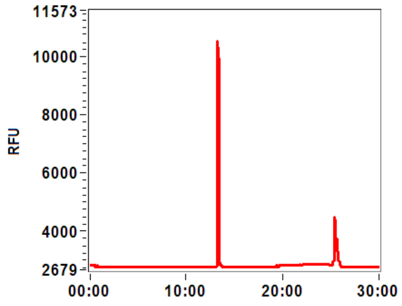

G12: SampG12

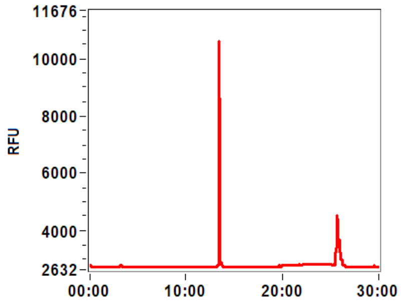

H12: SampH12

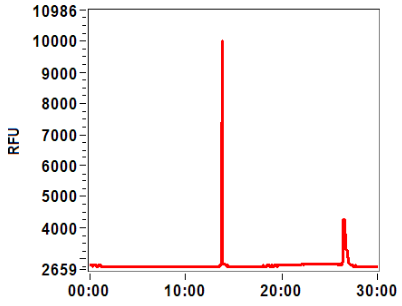

Sample: SampA1
Well location: A1
Created: Thursday, October 21, 2021 9:39:00 PM

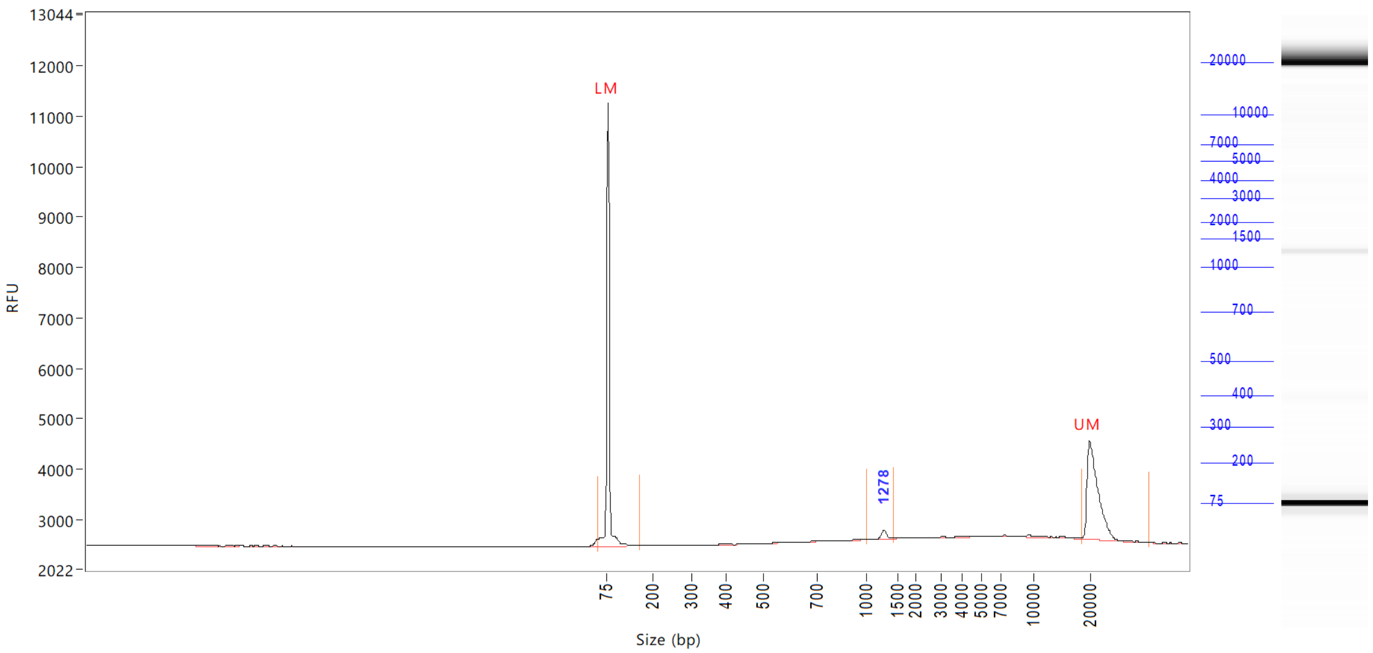

Peak	Size	Concentration	Relative concentration	Molarity	From	To	CV%	RFU
	(bp)	(ng/uL)	Percent	(nmole/L)	(bp)	(bp)		
1	75 (LM)	0.5000		10.9378	48	166	11.97	8805
2	1278	0.1651	100.0	0.2153	1012	1445	5.11	176
3	20000 (UM)	0.2322		0.0184	18537	30398	6.18	1957

TIC:	0.1651	ng/uL
TIM:	0.2153	nmole/L
Total concentration:	0.6225	ng/uL

Sample peak width (sec): 5 Sample min peak height: 50 Sample baseline V to V?: Y Sample baseline V to V points: 3
Sample filter: Binomial Number of points for filter: 3 Sample start region (min): 0 Sample end region (min): 30
Marker peak width (sec): 5 Marker min peak height: 200 Marker baseline V to V?: Y Marker baseline V to V points: 3
Lower marker selection: First peak > 500 RFU Upper marker selection: Last peak > 200 RFU
Ladder size (bp)75, 200, 300, 400, 500, 700, 1000, 1500, 2000, 3000, 4000, 5000, 7000, 10000, 20000
Quantification using: Lower Marker Final concentration (ng/uL): 0.5000 Dilution factor: 12.0

Sample: SampB1
Well location: B1
Created: Thursday, October 21, 2021 9:39:00 PM

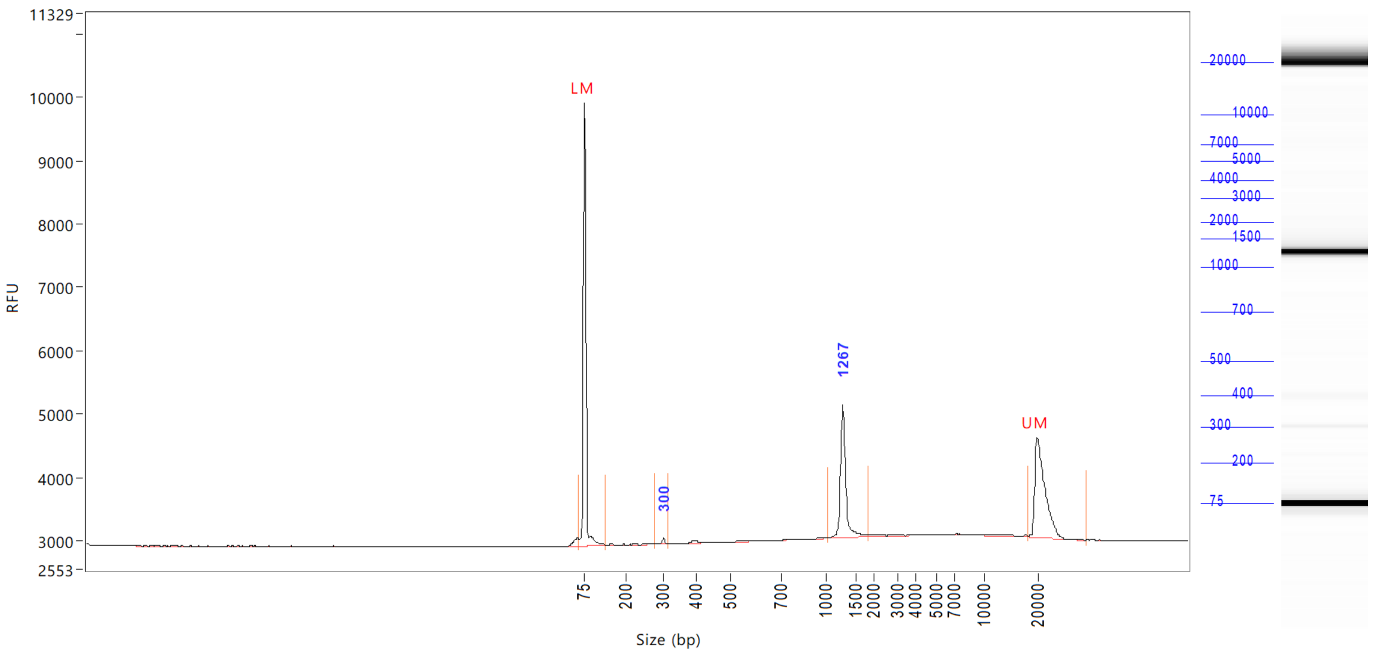

Peak	Size	Concentration	Relative concentration	Molarity	From	To	CV%	RFU
	(bp)	(ng/uL)	Percent	(nmole/L)	(bp)	(bp)		
1	75 (LM)	0.5000		11.0851	60	137	10.05	7007
2	300	0.0825	3.2	0.4571	277	311	1.81	95
3	1267	2.4788	96.8	3.1877	1012	1846	7.60	2090
4	20000 (UM)	0.2386		0.0189	18415	29189	5.77	1576

TIC:	2.5614	ng/uL
TIM:	3.6448	nmole/L
Total concentration:	3.2171	ng/uL

Sample peak width (sec): 5   Sample min peak height: 50   Sample baseline V to V?: Y   Sample baseline V to V points: 3
Sample filter: Binomial   Number of points for filter: 3 Sample start region (min): 0 Sample end region (min): 30
Marker peak width (sec): 5   Marker min peak height: 200   Marker baseline V to V?: Y   Marker baseline V to V points: 3
Lower marker selection: First peak > 500 RFU   Upper marker selection: Last peak > 200 RFU
Ladder size (bp)75, 200, 300, 400, 500, 700, 1000, 1500, 2000, 3000, 4000, 5000, 7000, 10000, 20000
Quantification using: Lower Marker   Final concentration (ng/uL): 0.5000   Dilution factor: 12.0

Sample: SampC1
Well location: C1
Created: Thursday, October 21, 2021 9:39:00 PM

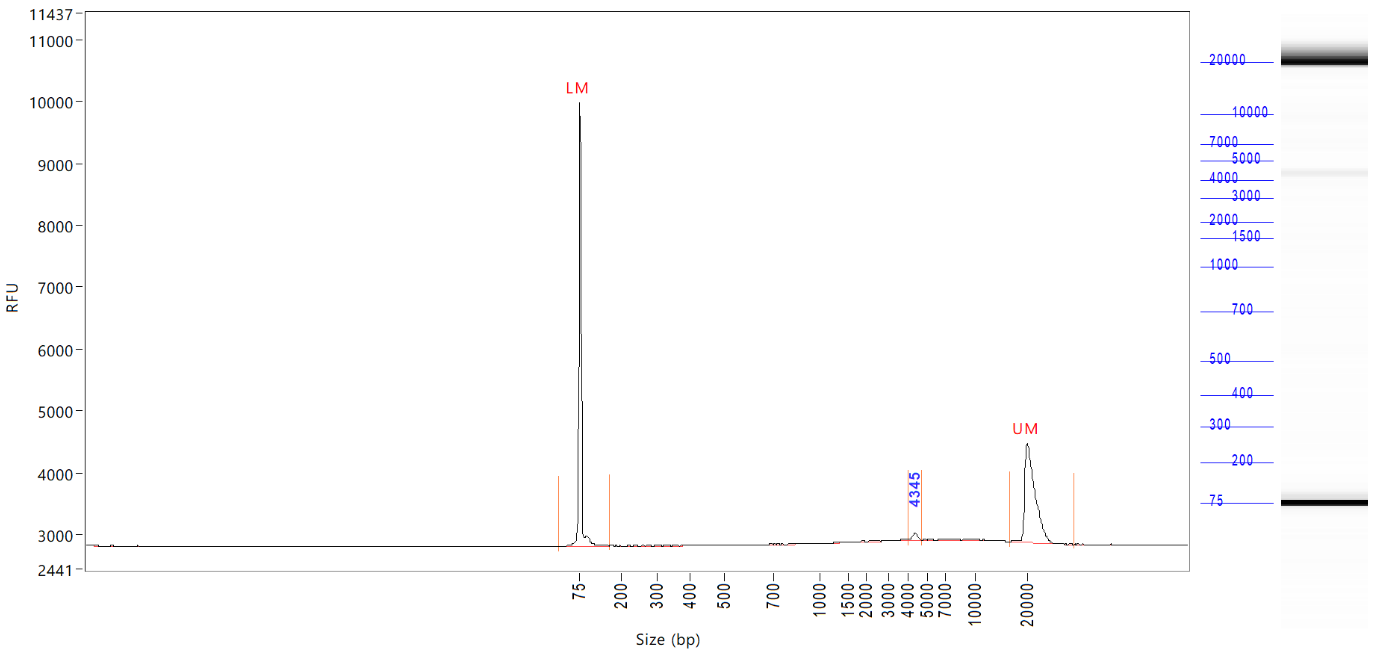

Peak	Size	Concentration	Relative concentration	Molarity	From	To	CV%	RFU
	(bp)	(ng/uL)	Percent	(nmole/L)	(bp)	(bp)		
1	75 (LM)	0.5000		11.0851	14	164	10.77	7189
2	4345	0.1228	100.0	0.0467	4001	4759	3.34	113
3	20000 (UM)	0.2375		0.0189	16586	28826	5.60	1610

TIC:	0.1228	ng/uL
TIM:	0.0467	nmole/L
Total concentration:	0.5744	ng/uL

```
Sample peak width (sec): 5    Sample min peak height: 50    Sample baseline V to V?: Y    Sample baseline V to V points: 3
Sample filter: Binomial       Number of points for filter: 3  Sample start region (min): 0  Sample end region (min): 30
Marker peak width (sec): 5    Marker min peak height: 200   Marker baseline V to V?: Y    Marker baseline V to V points: 3
Lower marker selection: First peak > 500 RFU                Upper marker selection: Last peak > 200 RFU
Ladder size (bp)75, 200, 300, 400, 500, 700, 1000, 1500, 2000, 3000, 4000, 5000, 7000, 10000, 20000
Quantification using: Lower Marker          Final concentration (ng/uL): 0.5000          Dilution factor: 12.0
```

Sample: SampD1
Well location: D1
Created: Thursday, October 21, 2021 9:39:00 PM

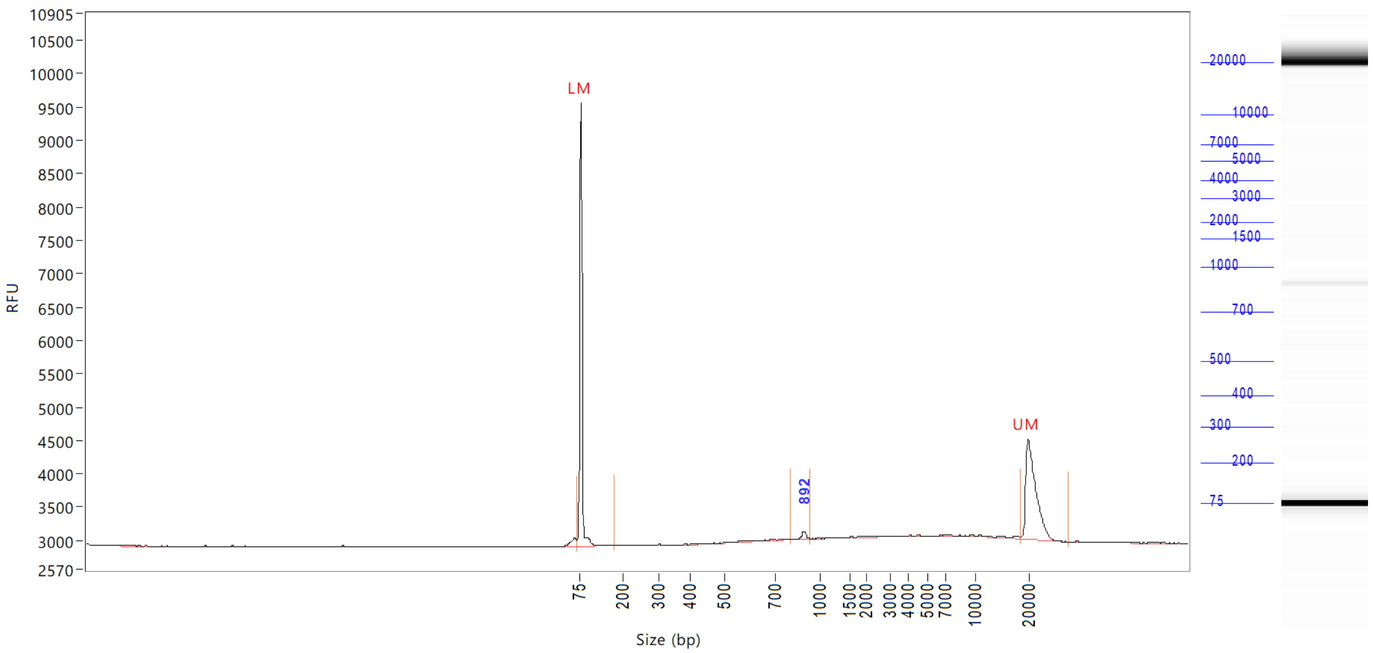

Peak	Size	Concentration	Relative concentration	Molarity	From	To	CV%	RFU
	(bp)	(ng/uL)	Percent	(nmole/L)	(bp)	(bp)		
1	75 (LM)	0.5000		11.0851	64	175	10.50	6657
2	892	0.1371	100.0	0.2559	801	935	2.55	119
3	20000 (UM)	0.2444		0.0194	18659	27738	5.35	1504

TIC:	0.1371	ng/uL
TIM:	0.2559	nmole/L
Total concentration:	0.7187	ng/uL

```
Sample peak width (sec): 5    Sample min peak height: 50    Sample baseline V to V?: Y    Sample baseline V to V points: 3
Sample filter: Binomial       Number of points for filter: 3  Sample start region (min): 0  Sample end region (min): 30
Marker peak width (sec): 5    Marker min peak height: 200   Marker baseline V to V?: Y    Marker baseline V to V points: 3
Lower marker selection: First peak > 500 RFU                Upper marker selection: Last peak > 200 RFU
Ladder size (bp)75, 200, 300, 400, 500, 700, 1000, 1500, 2000, 3000, 4000, 5000, 7000, 10000, 20000
Quantification using: Lower Marker          Final concentration (ng/uL): 0.5000          Dilution factor: 12.0
```

Sample: SampE1
Well location: E1
Created: Thursday, October 21, 2021 9:39:00 PM

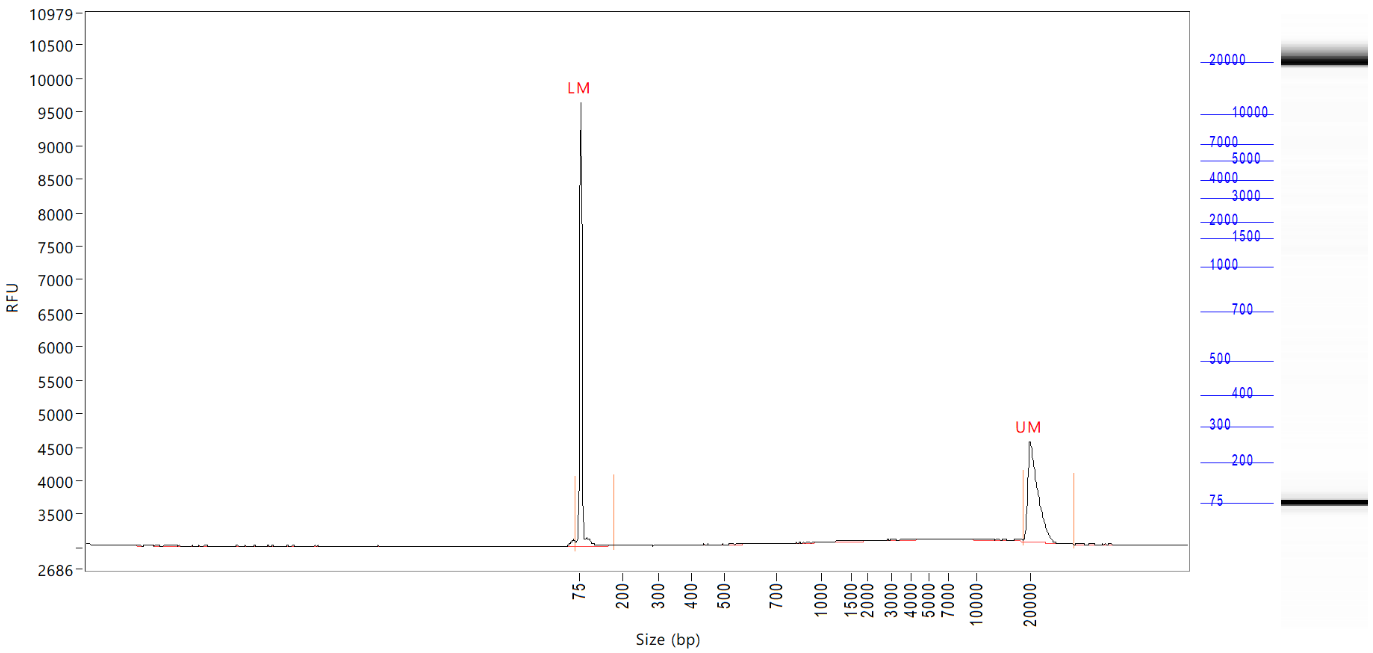

Peak	Size	Concentration	Relative concentration	Molarity	From	To	CV%	RFU
	(bp)	(ng/uL)	Percent	(nmole/L)	(bp)	(bp)		
1	75 (LM)	0.5000		10.9378	62	173	11.10	6626
2	20000 (UM)	0.2398		0.0191	18659	28221	5.37	1496

TIC:	0.0000	ng/uL
TIM:	0.0000	nmole/L
Total concentration:	0.5855	ng/uL

```
Sample peak width (sec): 5    Sample min peak height: 50    Sample baseline V to V?: Y    Sample baseline V to V points: 3
Sample filter: Binomial       Number of points for filter: 3  Sample start region (min): 0  Sample end region (min): 30
Marker peak width (sec): 5    Marker min peak height: 200   Marker baseline V to V?: Y    Marker baseline V to V points: 3
Lower marker selection: First peak > 500 RFU                Upper marker selection: Last peak > 200 RFU
Ladder size (bp)75, 200, 300, 400, 500, 700, 1000, 1500, 2000, 3000, 4000, 5000, 7000, 10000, 20000
Quantification using: Lower Marker          Final concentration (ng/uL): 0.5000          Dilution factor: 12.0
```

Sample: SampF1
Well location: F1
Created: Thursday, October 21, 2021 9:39:00 PM

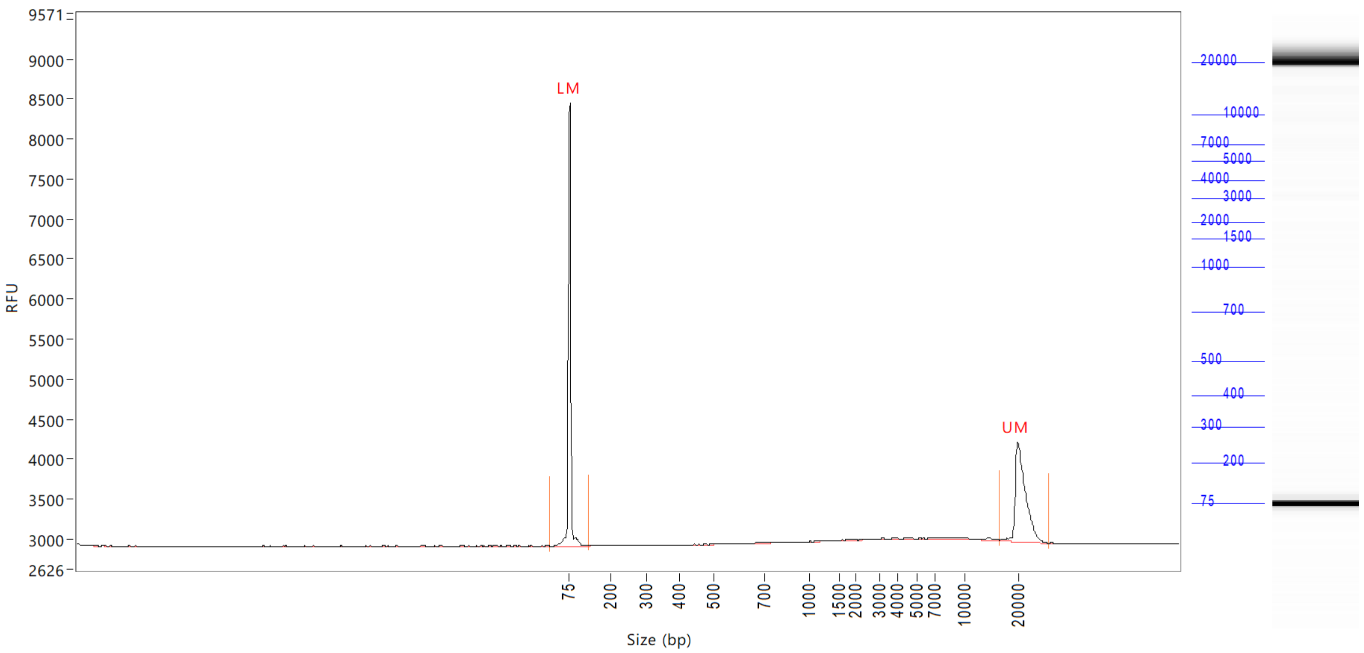

Peak	Size	Concentration	Relative concentration	Molarity	From	To	CV%	RFU
	(bp)	(ng/uL)	Percent	(nmole/L)	(bp)	(bp)		
1	75 (LM)	0.5000		11.3919	18	131	9.63	5546
2	20000 (UM)	0.2338		0.0186	16586	25803	5.67	1247

TIC:	0.0000	ng/uL
TIM:	0.0000	nmole/L
Total concentration:	0.6752	ng/uL

```
Sample peak width (sec): 5    Sample min peak height: 50    Sample baseline V to V?: Y    Sample baseline V to V points: 3
Sample filter: Binomial       Number of points for filter: 3  Sample start region (min): 0  Sample end region (min): 30
Marker peak width (sec): 5    Marker min peak height: 200   Marker baseline V to V?: Y    Marker baseline V to V points: 3
Lower marker selection: First peak > 500 RFU                Upper marker selection: Last peak > 200 RFU
Ladder size (bp)75, 200, 300, 400, 500, 700, 1000, 1500, 2000, 3000, 4000, 5000, 7000, 10000, 20000
Quantification using: Lower Marker          Final concentration (ng/uL): 0.5000          Dilution factor: 12.0
```

Sample: SampG1
Well location: G1
Created: Thursday, October 21, 2021 9:39:00 PM

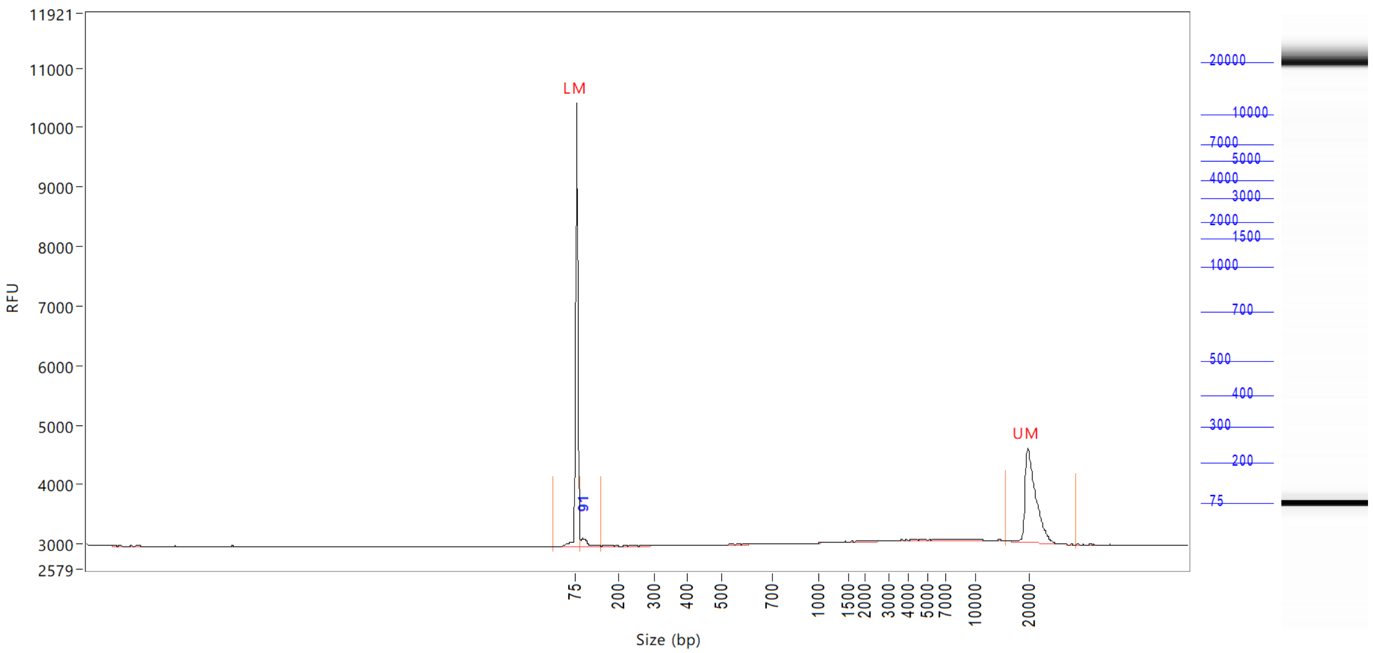

Peak	Size	Concentration	Relative concentration	Molarity	From	To	CV%	RFU
	(bp)	(ng/uL)	Percent	(nmole/L)	(bp)	(bp)		
1	75 (LM)	0.5000		11.2364	9	87	7.44	7467
2	91	0.2735	100.0	4.4919	87	148	12.83	134
3	20000 (UM)	0.2458		0.0196	15854	28826	5.93	1572

TIC:	0.2735	ng/uL
TIM:	4.4919	nmole/L
Total concentration:	0.7843	ng/uL

Sample peak width (sec): 5   Sample min peak height: 50   Sample baseline V to V?: Y   Sample baseline V to V points: 3
Sample filter: Binomial   Number of points for filter: 3 Sample start region (min): 0 Sample end region (min): 30
Marker peak width (sec): 5   Marker min peak height: 200   Marker baseline V to V?: Y   Marker baseline V to V points: 3
Lower marker selection: First peak > 500 RFU   Upper marker selection: Last peak > 200 RFU
Ladder size (bp)75, 200, 300, 400, 500, 700, 1000, 1500, 2000, 3000, 4000, 5000, 7000, 10000, 20000
Quantification using: Lower Marker   Final concentration (ng/uL): 0.5000   Dilution factor: 12.0

Sample: SampH1
Well location: H1
Created: Thursday, October 21, 2021 9:39:00 PM

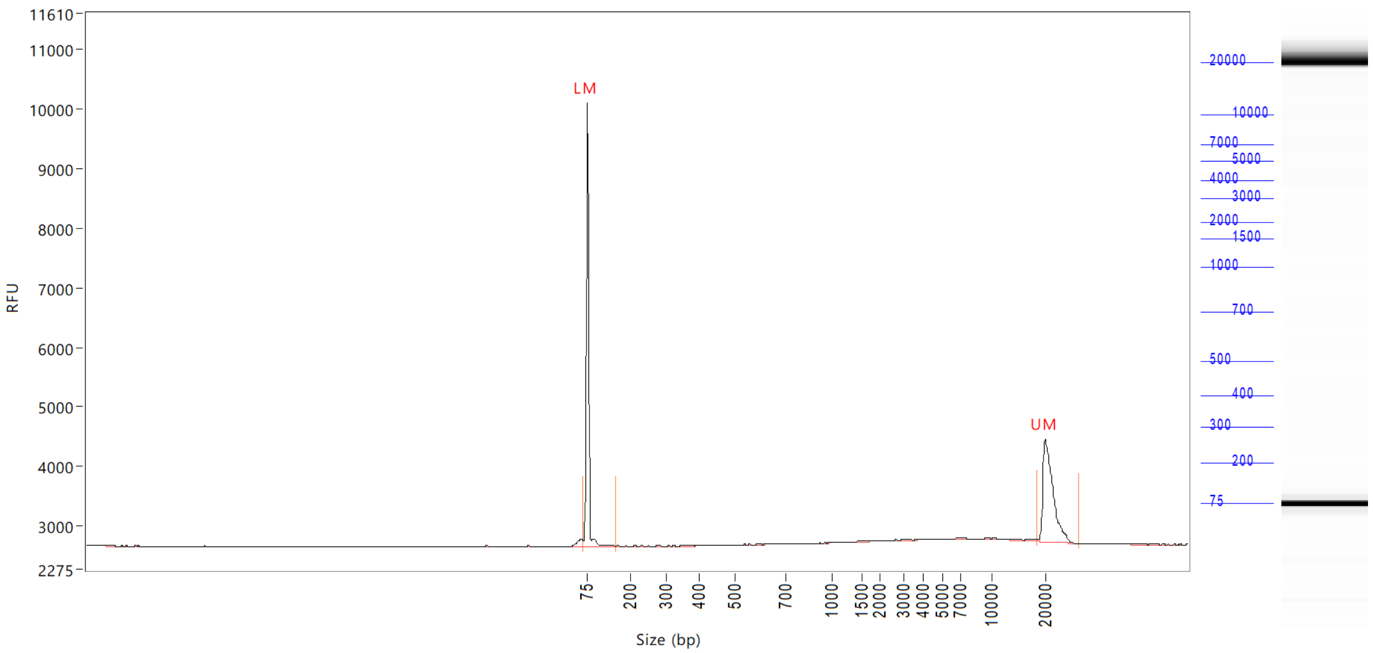

Peak	Size	Concentration	Relative concentration	Molarity	From	To	CV%	RFU
	(bp)	(ng/uL)	Percent	(nmole/L)	(bp)	(bp)		
1	75 (LM)	0.5000		11.2364	62	156	9.71	7462
2	20000 (UM)	0.2453		0.0195	18415	26166	5.32	1727

TIC:	0.0000	ng/uL
TIM:	0.0000	nmole/L
Total concentration:	0.5671	ng/uL

Sample peak width (sec): 5   Sample min peak height: 50   Sample baseline V to V?: Y   Sample baseline V to V points: 3
Sample filter: Binomial   Number of points for filter: 3 Sample start region (min): 0 Sample end region (min): 30
Marker peak width (sec): 5   Marker min peak height: 200   Marker baseline V to V?: Y   Marker baseline V to V points: 3
Lower marker selection: First peak > 500 RFU   Upper marker selection: Last peak > 200 RFU
Ladder size (bp)75, 200, 300, 400, 500, 700, 1000, 1500, 2000, 3000, 4000, 5000, 7000, 10000, 20000
Quantification using: Lower Marker   Final concentration (ng/uL): 0.5000   Dilution factor: 12.0

Sample: SampA2
Well location: A2
Created: Thursday, October 21, 2021 9:39:00 PM

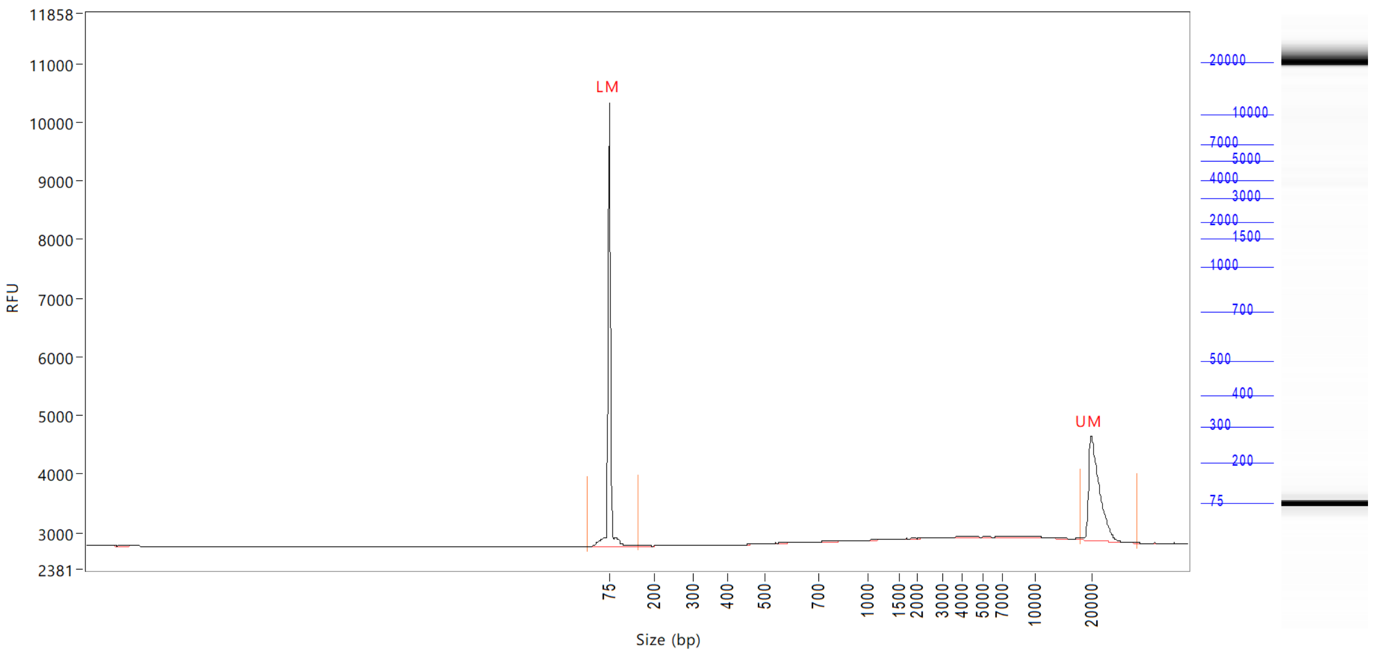

Peak	Size	Concentration	Relative concentration	Molarity	From	To	CV%	RFU
	(bp)	(ng/uL)	Percent	(nmole/L)	(bp)	(bp)		
1	75 (LM)	0.5000		11.3919	14	154	12.70	7572
2	20000 (UM)	0.2433		0.0192	18171	27979	5.89	1784

TIC:	0.0000	ng/uL
TIM:	0.0000	nmole/L
Total concentration:	0.6102	ng/uL

```
Sample peak width (sec): 5    Sample min peak height: 50    Sample baseline V to V?: Y    Sample baseline V to V points: 3
Sample filter: Binomial       Number of points for filter: 3  Sample start region (min): 0  Sample end region (min): 30
Marker peak width (sec): 5    Marker min peak height: 200   Marker baseline V to V?: Y    Marker baseline V to V points: 3
Lower marker selection: First peak > 500 RFU                Upper marker selection: Last peak > 200 RFU
Ladder size (bp)75, 200, 300, 400, 500, 700, 1000, 1500, 2000, 3000, 4000, 5000, 7000, 10000, 20000
Quantification using: Lower Marker          Final concentration (ng/uL): 0.5000          Dilution factor: 12.0
```

Sample: SampB2
Well location: B2
Created: Thursday, October 21, 2021 9:39:00 PM

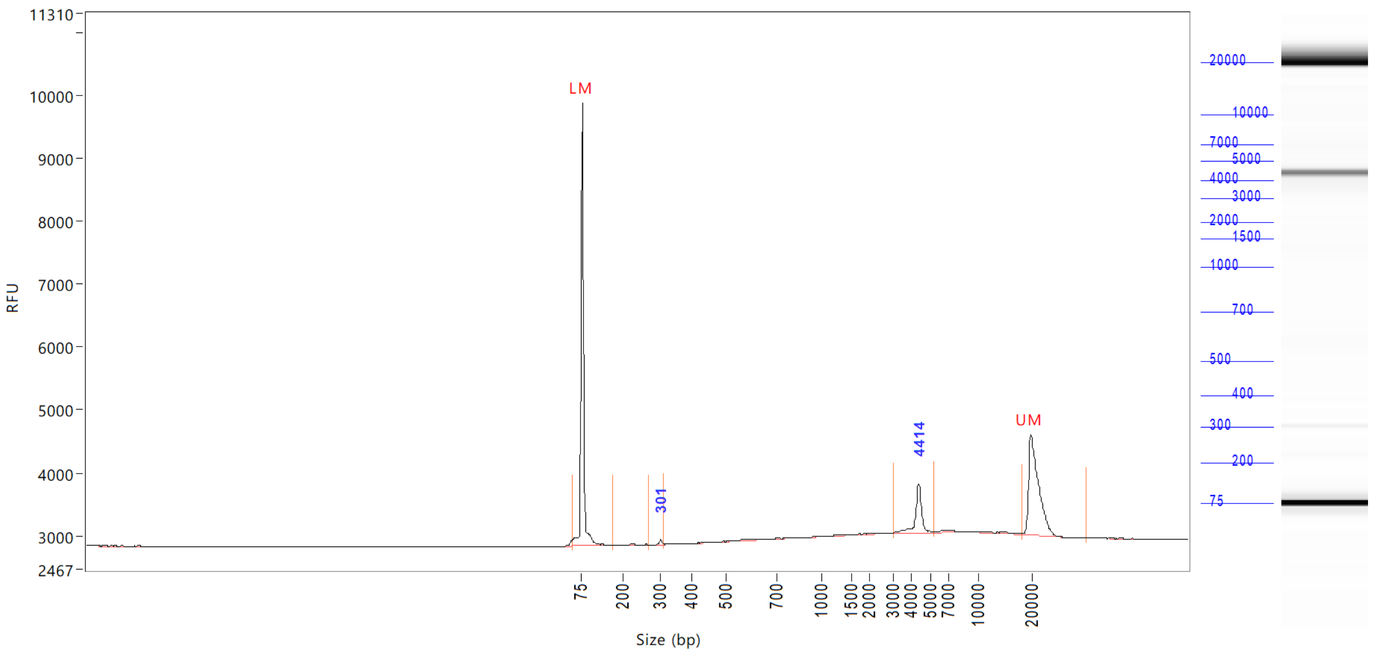

Peak	Size	Concentration	Relative concentration	Molarity	From	To	CV%	RFU
	(bp)	(ng/uL)	Percent	(nmole/L)	(bp)	(bp)		
1	75 (LM)	0.5000		10.9378	50	168	10.86	7054
2	301	0.0664	6.4	0.3655	269	313	1.49	88
3	4414	0.9719	93.6	0.3744	3037	5445	7.97	791
4	20000 (UM)	0.2229		0.0177	18415	30277	5.64	1594

TIC:	1.0383	ng/uL
TIM:	0.7398	nmole/L
Total concentration:	1.6107	ng/uL

```
Sample peak width (sec): 5    Sample min peak height: 50    Sample baseline V to V?: Y    Sample baseline V to V points: 3
Sample filter: Binomial       Number of points for filter: 3  Sample start region (min): 0  Sample end region (min): 30
Marker peak width (sec): 5    Marker min peak height: 200   Marker baseline V to V?: Y    Marker baseline V to V points: 3
Lower marker selection: First peak > 500 RFU                Upper marker selection: Last peak > 200 RFU
Ladder size (bp)75, 200, 300, 400, 500, 700, 1000, 1500, 2000, 3000, 4000, 5000, 7000, 10000, 20000
Quantification using: Lower Marker          Final concentration (ng/uL): 0.5000          Dilution factor: 12.0
```

Sample: SampC2
Well location: C2
Created: Thursday, October 21, 2021 9:39:00 PM

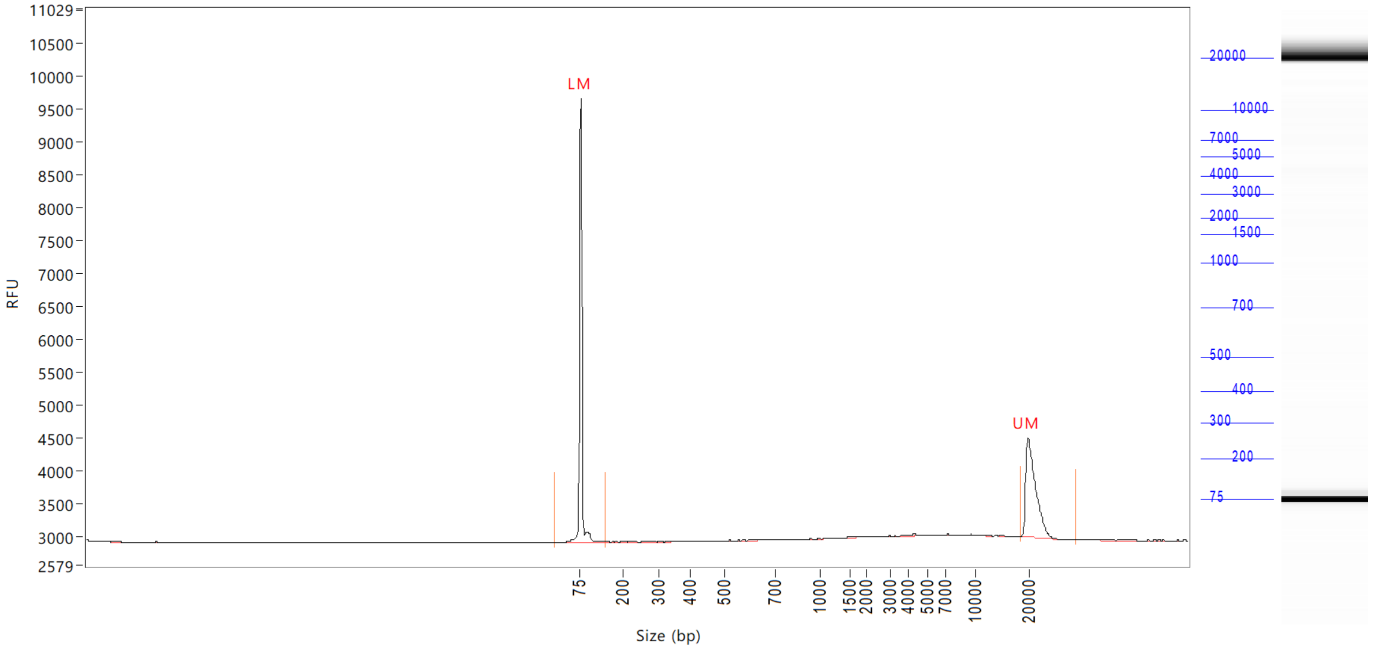

Peak	Size	Concentration	Relative concentration	Molarity	From	To	CV%	RFU
	(bp)	(ng/uL)	Percent	(nmole/L)	(bp)	(bp)		
1	75 (LM)	0.5000		11.2364	0	150	11.29	6752
2	20000 (UM)	0.2279		0.0181	18659	28947	5.34	1511

TIC:	0.0000	ng/uL
TIM:	0.0000	nmole/L
Total concentration:	0.5231	ng/uL

```
Sample peak width (sec): 5    Sample min peak height: 50    Sample baseline V to V?: Y    Sample baseline V to V points: 3
Sample filter: Binomial       Number of points for filter: 3  Sample start region (min): 0  Sample end region (min): 30
Marker peak width (sec): 5    Marker min peak height: 200   Marker baseline V to V?: Y    Marker baseline V to V points: 3
Lower marker selection: First peak > 500 RFU                Upper marker selection: Last peak > 200 RFU
Ladder size (bp)75, 200, 300, 400, 500, 700, 1000, 1500, 2000, 3000, 4000, 5000, 7000, 10000, 20000
Quantification using: Lower Marker          Final concentration (ng/uL): 0.5000          Dilution factor: 12.0
```

Sample: SampD2
Well location: D2
Created: Thursday, October 21, 2021 9:39:00 PM

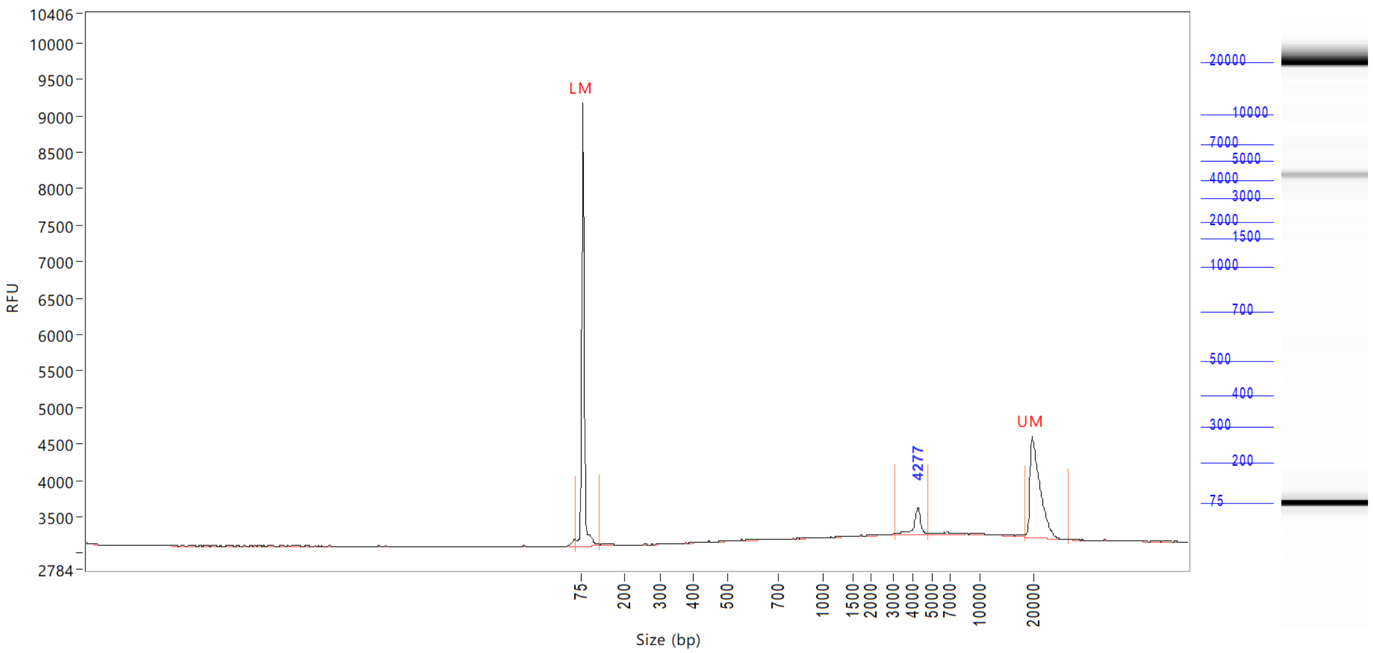

Peak	Size	Concentration	Relative concentration	Molarity	From	To	CV%	RFU
	(bp)	(ng/uL)	Percent	(nmole/L)	(bp)	(bp)		
1	75 (LM)	0.5000		10.9378	56	126	9.02	6087
2	4277	0.5915	100.0	0.2368	3037	4793	8.11	371
3	20000 (UM)	0.2318		0.0184	18537	26891	5.39	1390

TIC:	0.5915	ng/uL
TIM:	0.2368	nmole/L
Total concentration:	1.2345	ng/uL

Sample peak width (sec): 5 Sample min peak height: 50 Sample baseline V to V?: Y Sample baseline V to V points: 3
Sample filter: Binomial Number of points for filter: 3 Sample start region (min): 0 Sample end region (min): 30
Marker peak width (sec): 5 Marker min peak height: 200 Marker baseline V to V?: Y Marker baseline V to V points: 3
Lower marker selection: First peak > 500 RFU Upper marker selection: Last peak > 200 RFU
Ladder size (bp)75, 200, 300, 400, 500, 700, 1000, 1500, 2000, 3000, 4000, 5000, 7000, 10000, 20000
Quantification using: Lower Marker Final concentration (ng/uL): 0.5000 Dilution factor: 12.0

Sample: SampE2
Well location: E2
Created: Thursday, October 21, 2021 9:39:00 PM

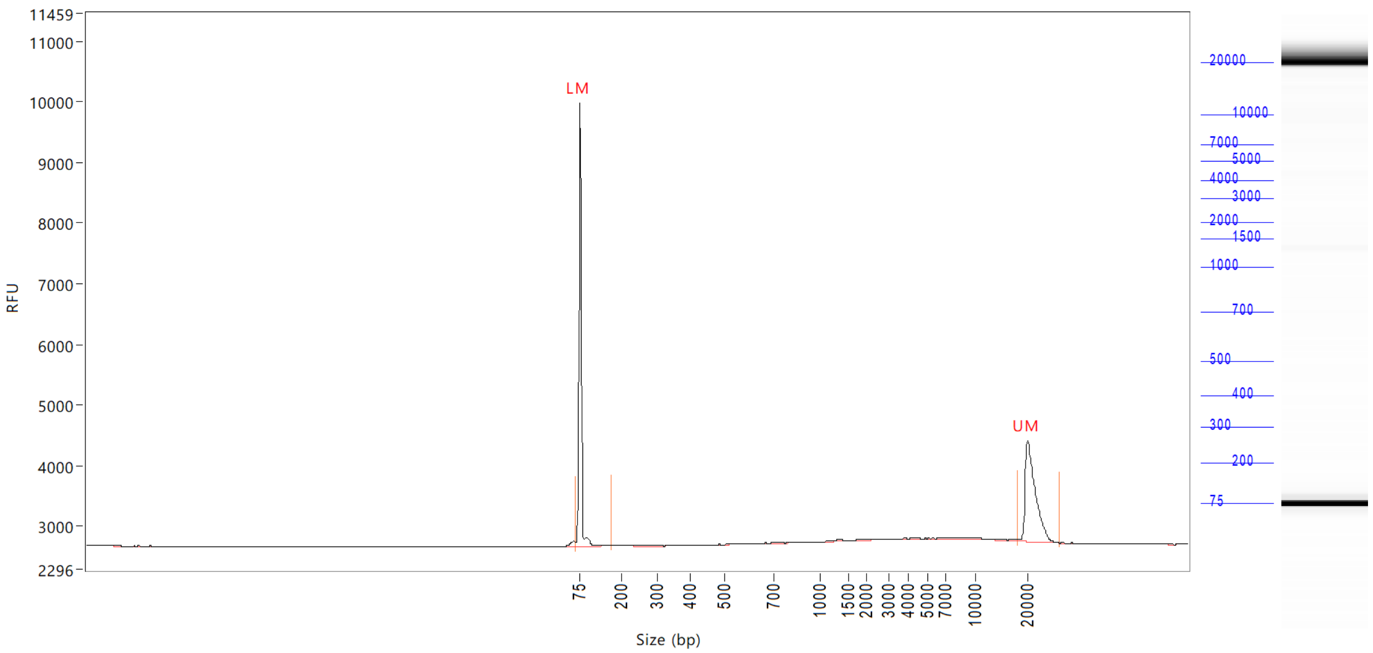

Peak	Size	Concentration	Relative concentration	Molarity	From	To	CV%	RFU
	(bp)	(ng/uL)	Percent	(nmole/L)	(bp)	(bp)		
1	75 (LM)	0.5000		11.0851	62	169	9.35	7320
2	20000 (UM)	0.2447		0.0194	18293	26166	5.39	1669

TIC:	0.0000	ng/uL
TIM:	0.0000	nmole/L
Total concentration:	0.5965	ng/uL

```
Sample peak width (sec): 5    Sample min peak height: 50    Sample baseline V to V?: Y    Sample baseline V to V points: 3
Sample filter: Binomial       Number of points for filter: 3  Sample start region (min): 0  Sample end region (min): 30
Marker peak width (sec): 5    Marker min peak height: 200   Marker baseline V to V?: Y    Marker baseline V to V points: 3
Lower marker selection: First peak > 500 RFU                Upper marker selection: Last peak > 200 RFU
Ladder size (bp)75, 200, 300, 400, 500, 700, 1000, 1500, 2000, 3000, 4000, 5000, 7000, 10000, 20000
Quantification using: Lower Marker          Final concentration (ng/uL): 0.5000          Dilution factor: 12.0
```

Sample: SampF2
Well location: F2
Created: Thursday, October 21, 2021 9:39:00 PM

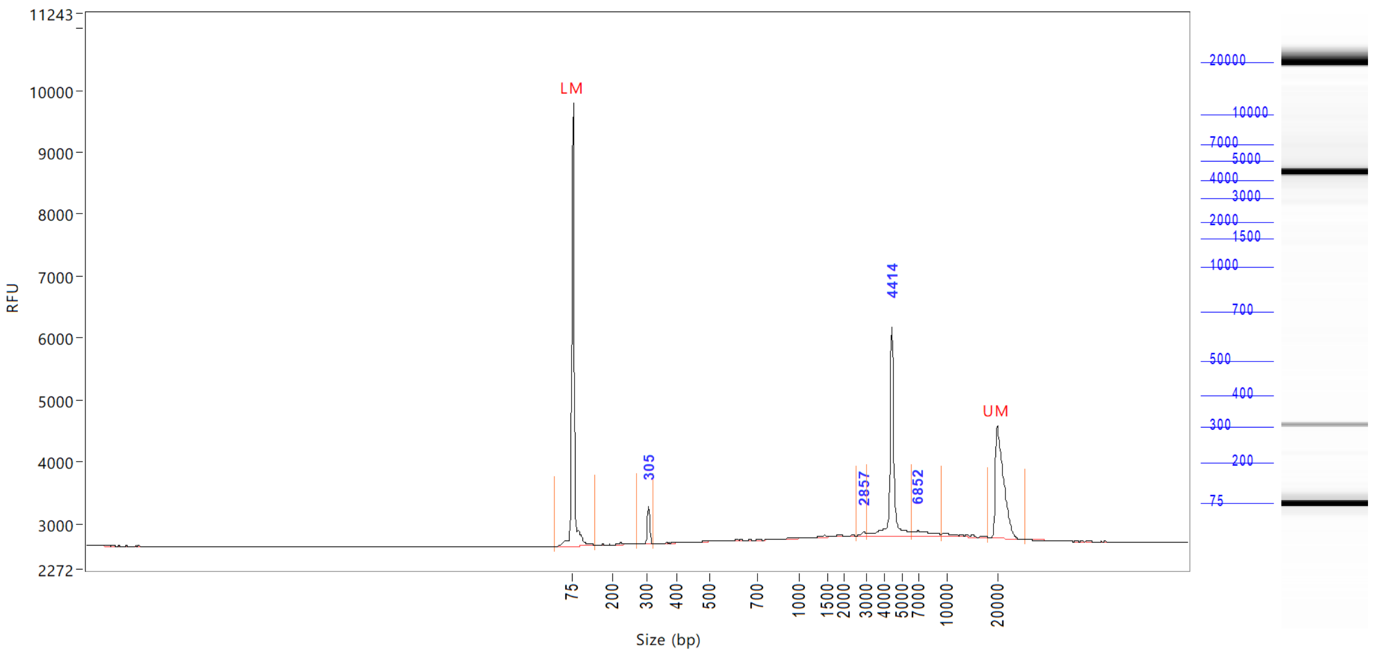

Peak	Size	Concentration	Relative concentration	Molarity	From	To	CV%	RFU
	(bp)	(ng/uL)	Percent	(nmole/L)	(bp)	(bp)		
1	75 (LM)	0.5000		11.0851	18	145	13.01	7166
2	305	0.4117	11.8	2.2423	271	321	1.67	596
3	2857	0.0603	1.7	0.0350	2543	3001	3.89	61
4	4414	2.7421	78.6	1.0223	3001	6112	8.01	3377
5	6852	0.2763	7.9	0.0608	6112	9334	11.86	81
6	20000 (UM)	0.2169		0.0174	18171	25320	4.40	1818

TIC:	3.4904	ng/uL
TIM:	3.3604	nmole/L
Total concentration:	4.0359	ng/uL

```
Sample peak width (sec): 5    Sample min peak height: 50    Sample baseline V to V?: Y    Sample baseline V to V points: 3
Sample filter: Binomial       Number of points for filter: 3  Sample start region (min): 0  Sample end region (min): 30
Marker peak width (sec): 5    Marker min peak height: 200   Marker baseline V to V?: Y    Marker baseline V to V points: 3
Lower marker selection: First peak > 500 RFU                Upper marker selection: Last peak > 200 RFU
Ladder size (bp)75, 200, 300, 400, 500, 700, 1000, 1500, 2000, 3000, 4000, 5000, 7000, 10000, 20000
Quantification using: Lower Marker          Final concentration (ng/uL): 0.5000          Dilution factor: 12.0
```

Sample: SampG2
Well location: G2
Created: Thursday, October 21, 2021 9:39:00 PM

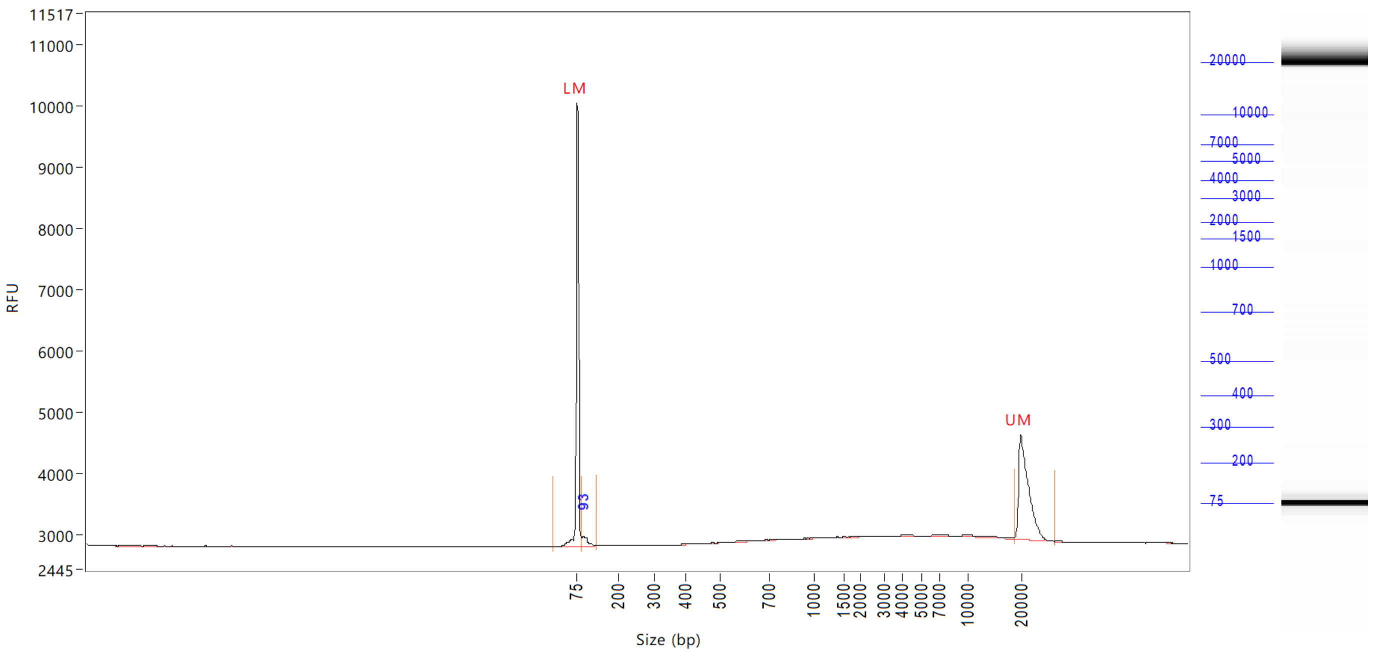

Peak	Size	Concentration	Relative concentration	Molarity	From	To	CV%	RFU
	(bp)	(ng/uL)	Percent	(nmole/L)	(bp)	(bp)		
1	75 (LM)	0.5000		11.2364	5	87	8.85	7249
2	93	0.3157	100.0	5.2361	87	133	10.13	159
3	20000 (UM)	0.2455		0.0194	18659	26408	5.36	1704

TIC:	0.3157	ng/uL
TIM:	5.2361	nmole/L
Total concentration:	0.9661	ng/uL

Sample peak width (sec): 5   Sample min peak height: 50   Sample baseline V to V?: Y   Sample baseline V to V points: 3
Sample filter: Binomial   Number of points for filter: 3 Sample start region (min): 0 Sample end region (min): 30
Marker peak width (sec): 5   Marker min peak height: 200   Marker baseline V to V?: Y   Marker baseline V to V points: 3
Lower marker selection: First peak > 500 RFU   Upper marker selection: Last peak > 200 RFU
Ladder size (bp)75, 200, 300, 400, 500, 700, 1000, 1500, 2000, 3000, 4000, 5000, 7000, 10000, 20000
Quantification using: Lower Marker   Final concentration (ng/uL): 0.5000   Dilution factor: 12.0

Sample: SampH2
Well location: H2
Created: Thursday, October 21, 2021 9:39:00 PM

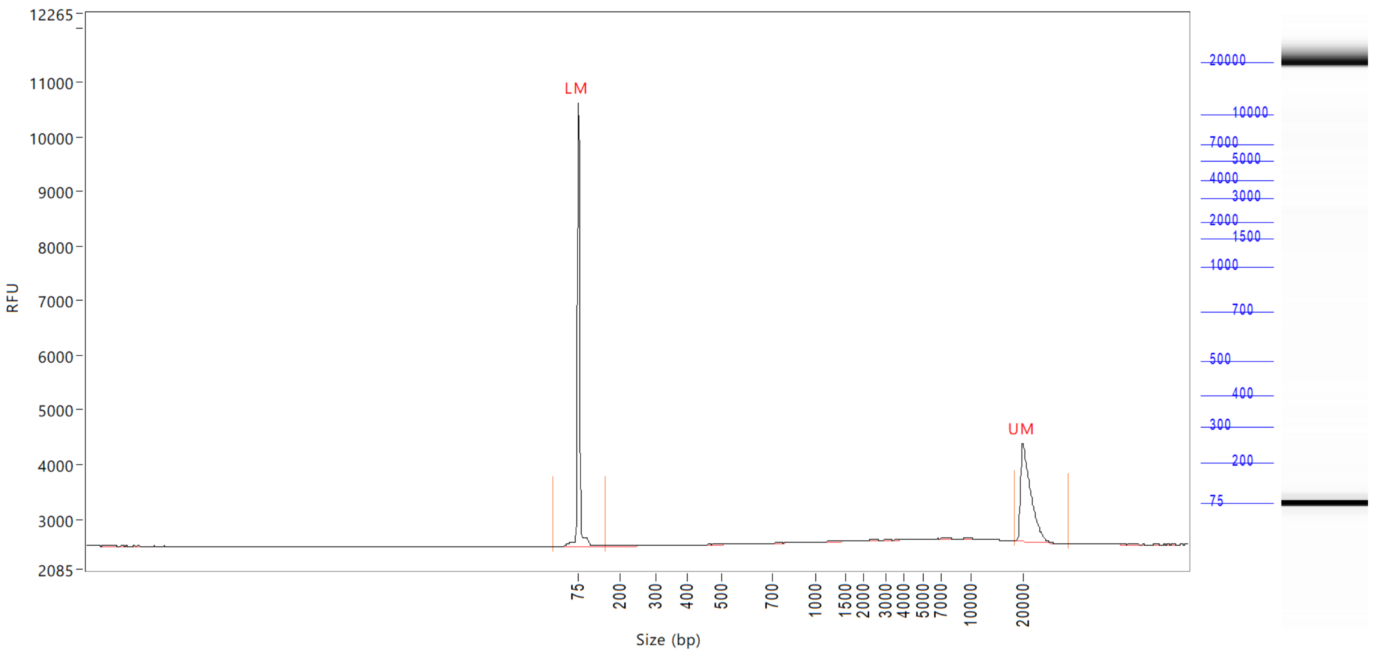

Peak	Size	Concentration	Relative concentration	Molarity	From	To	CV%	RFU
	(bp)	(ng/uL)	Percent	(nmole/L)	(bp)	(bp)		
1	75 (LM)	0.5000		11.0851	3	156	12.54	8139
2	20000 (UM)	0.2336		0.0185	18537	28584	5.54	1792

TIC:	0.0000	ng/uL
TIM:	0.0000	nmole/L
Total concentration:	0.5538	ng/uL

```
Sample peak width (sec): 5    Sample min peak height: 50    Sample baseline V to V?: Y    Sample baseline V to V points: 3
Sample filter: Binomial       Number of points for filter: 3  Sample start region (min): 0  Sample end region (min): 30
Marker peak width (sec): 5    Marker min peak height: 200   Marker baseline V to V?: Y    Marker baseline V to V points: 3
Lower marker selection: First peak > 500 RFU                Upper marker selection: Last peak > 200 RFU
Ladder size (bp)75, 200, 300, 400, 500, 700, 1000, 1500, 2000, 3000, 4000, 5000, 7000, 10000, 20000
Quantification using: Lower Marker          Final concentration (ng/uL): 0.5000          Dilution factor: 12.0
```

Sample: SampA3
Well location: A3
Created: Thursday, October 21, 2021 9:39:00 PM

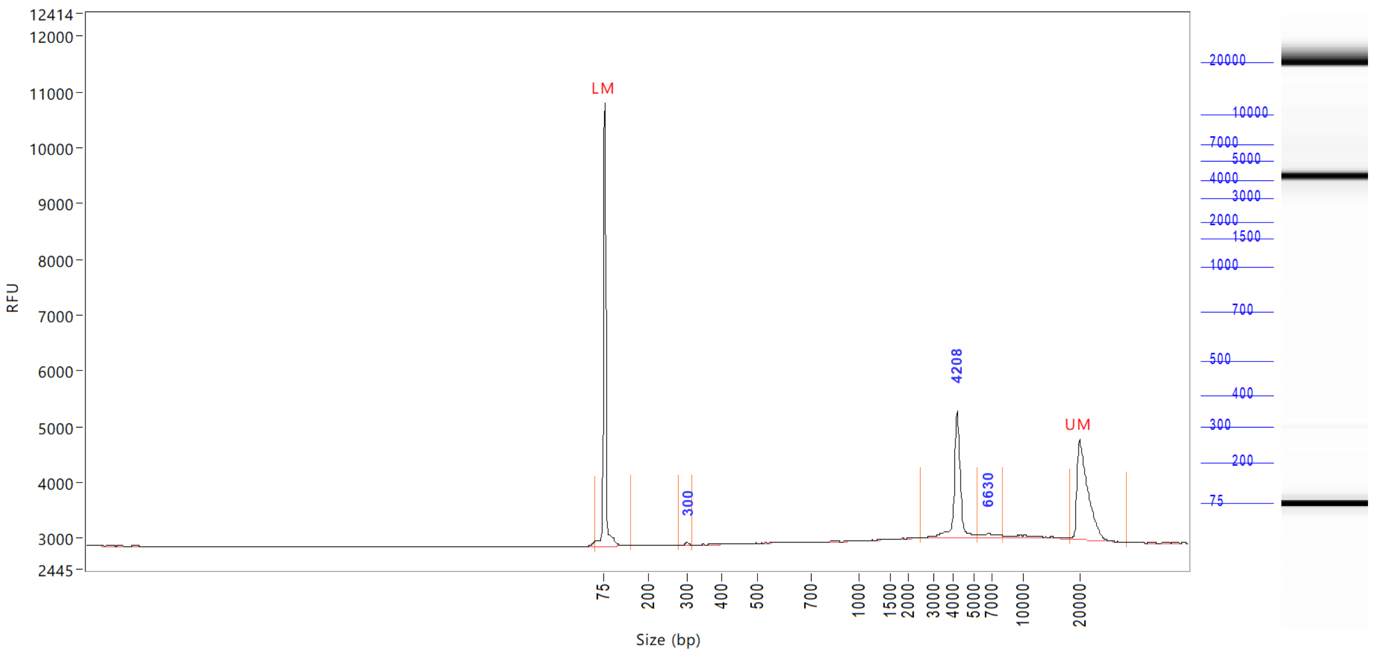

Peak	Size	Concentration	Relative concentration	Molarity	From	To	CV%	RFU
	(bp)	(ng/uL)	Percent	(nmole/L)	(bp)	(bp)		
1	75 (LM)	0.5000		11.2364	48	150	10.16	7963
2	300	0.0481	1.8	0.2673	278	315	1.78	56
3	4208	2.4777	90.6	0.9833	2515	5445	8.18	2274
4	6630	0.2103	7.7	0.0518	5445	8001	10.61	71
5	20000 (UM)	0.2424		0.0192	18293	28463	5.61	1790

TIC:	2.7361	ng/uL
TIM:	1.3024	nmole/L
Total concentration:	3.1932	ng/uL

Sample peak width (sec): 5   Sample min peak height: 50   Sample baseline V to V?: Y   Sample baseline V to V points: 3
Sample filter: Binomial   Number of points for filter: 3   Sample start region (min): 0   Sample end region (min): 30
Marker peak width (sec): 5   Marker min peak height: 200   Marker baseline V to V?: Y   Marker baseline V to V points: 3
Lower marker selection: First peak > 500 RFU   Upper marker selection: Last peak > 200 RFU
Ladder size (bp)75, 200, 300, 400, 500, 700, 1000, 1500, 2000, 3000, 4000, 5000, 7000, 10000, 20000
Quantification using: Lower Marker   Final concentration (ng/uL): 0.5000   Dilution factor: 12.0

Sample: SampB3
Well location: B3
Created: Thursday, October 21, 2021 9:39:00 PM

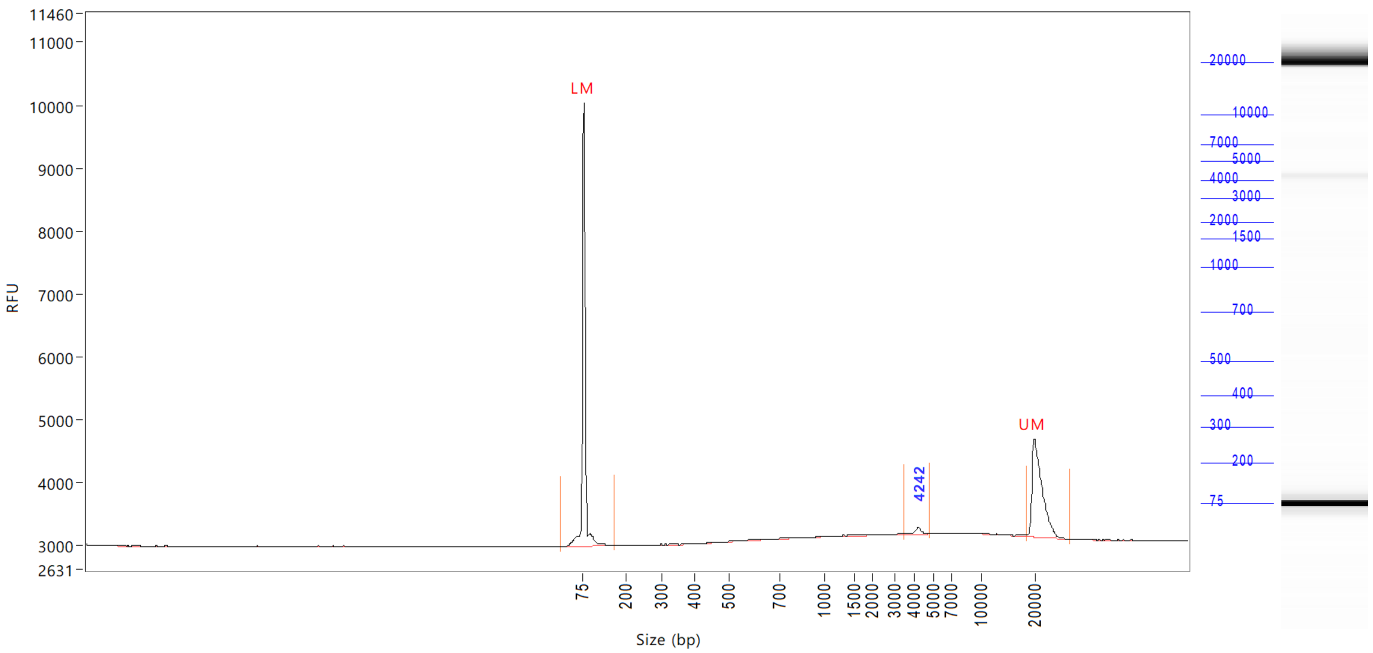

Peak	Size	Concentration	Relative concentration	Molarity	From	To	CV%	RFU
	(bp)	(ng/uL)	Percent	(nmole/L)	(bp)	(bp)		
1	75 (LM)	0.5000		11.2364	9	164	15.50	7051
2	4242	0.1476	100.0	0.0590	3500	4828	5.86	109
3	20000 (UM)	0.2165		0.0172	18659	26649	5.37	1559

TIC:	0.1476	ng/uL
TIM:	0.0590	nmole/L
Total concentration:	0.6421	ng/uL

Sample peak width (sec): 5 Sample min peak height: 50 Sample baseline V to V?: Y Sample baseline V to V points: 3
Sample filter: Binomial Number of points for filter: 3 Sample start region (min): 0 Sample end region (min): 30
Marker peak width (sec): 5 Marker min peak height: 200 Marker baseline V to V?: Y Marker baseline V to V points: 3
Lower marker selection: First peak > 500 RFU Upper marker selection: Last peak > 200 RFU
Ladder size (bp)75, 200, 300, 400, 500, 700, 1000, 1500, 2000, 3000, 4000, 5000, 7000, 10000, 20000
Quantification using: Lower Marker Final concentration (ng/uL): 0.5000 Dilution factor: 12.0

Sample: SampC3
Well location: C3
Created: Thursday, October 21, 2021 9:39:00 PM

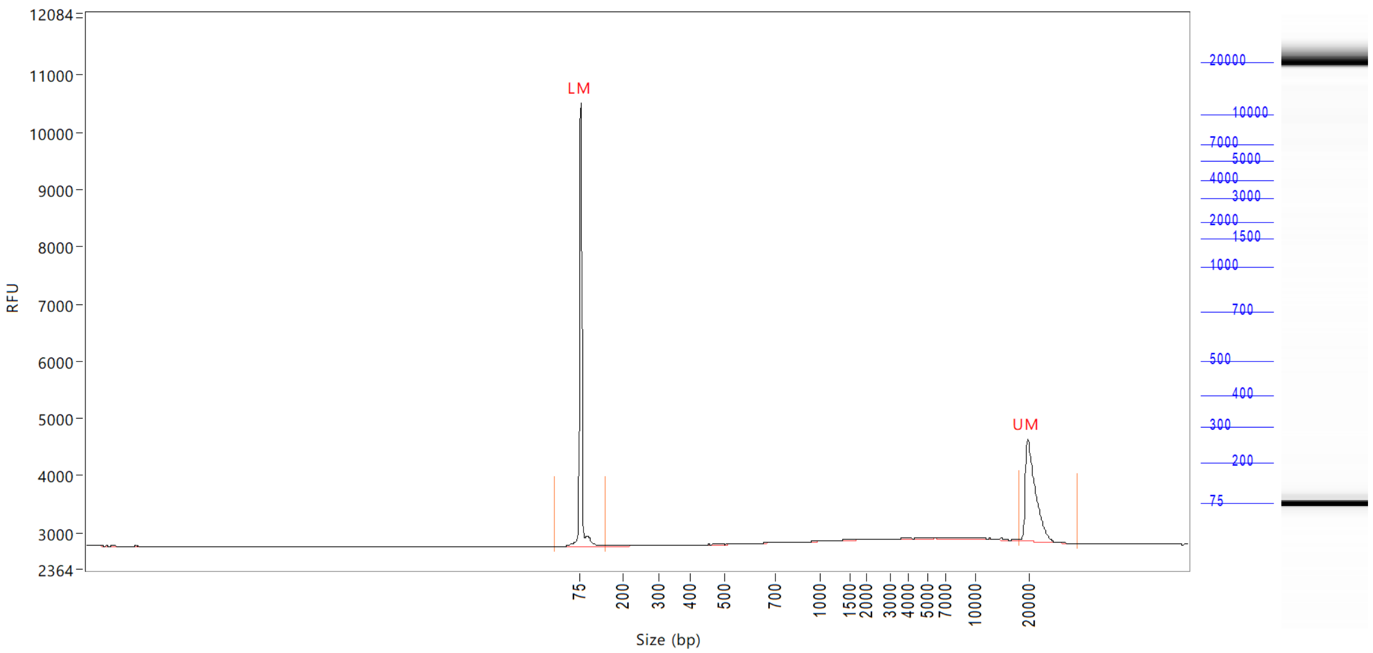

Peak	Size	Concentration	Relative concentration	Molarity	From	To	CV%	RFU
	(bp)	(ng/uL)	Percent	(nmole/L)	(bp)	(bp)		
1	75 (LM)	0.5000		11.2364	0	148	12.37	7767
2	20000 (UM)	0.2345		0.0186	18293	29309	5.62	1778

TIC:	0.0000	ng/uL
TIM:	0.0000	nmole/L
Total concentration:	0.6273	ng/uL

```
Sample peak width (sec): 5    Sample min peak height: 50    Sample baseline V to V?: Y    Sample baseline V to V points: 3
Sample filter: Binomial       Number of points for filter: 3  Sample start region (min): 0  Sample end region (min): 30
Marker peak width (sec): 5    Marker min peak height: 200   Marker baseline V to V?: Y    Marker baseline V to V points: 3
Lower marker selection: First peak > 500 RFU                Upper marker selection: Last peak > 200 RFU
Ladder size (bp)75, 200, 300, 400, 500, 700, 1000, 1500, 2000, 3000, 4000, 5000, 7000, 10000, 20000
Quantification using: Lower Marker          Final concentration (ng/uL): 0.5000          Dilution factor: 12.0
```

Sample: SampD3
Well location: D3
Created: Thursday, October 21, 2021 9:39:00 PM

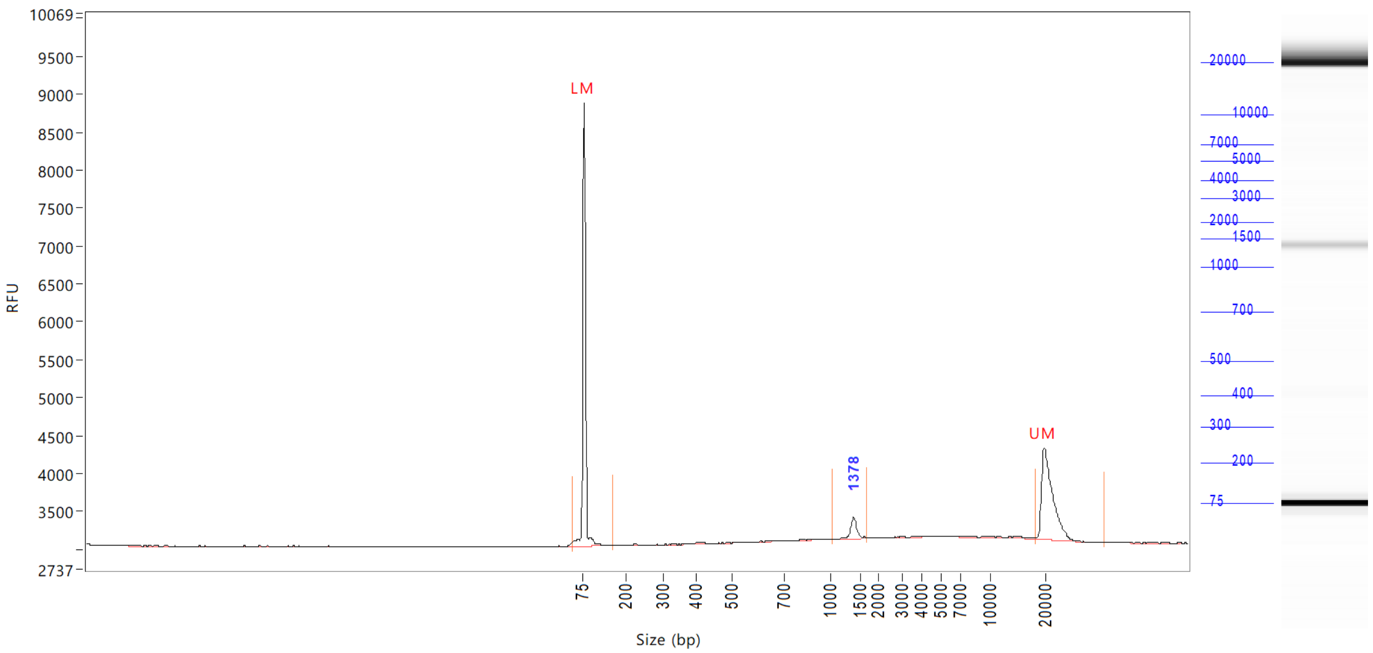

Peak	Size	Concentration	Relative concentration	Molarity	From	To	CV%	RFU
	(bp)	(ng/uL)	Percent	(nmole/L)	(bp)	(bp)		
1	75 (LM)	0.5000		11.0851	45	160	11.47	5855
2	1378	0.4601	100.0	0.5520	1034	1655	5.85	279
3	20000 (UM)	0.2297		0.0182	18415	30881	6.47	1216

TIC:	0.4601	ng/uL
TIM:	0.5520	nmole/L
Total concentration:	1.0481	ng/uL

Sample peak width (sec): 5   Sample min peak height: 50   Sample baseline V to V?: Y   Sample baseline V to V points: 3
Sample filter: Binomial   Number of points for filter: 3 Sample start region (min): 0 Sample end region (min): 30
Marker peak width (sec): 5   Marker min peak height: 200   Marker baseline V to V?: Y   Marker baseline V to V points: 3
Lower marker selection: First peak > 500 RFU   Upper marker selection: Last peak > 200 RFU
Ladder size (bp)75, 200, 300, 400, 500, 700, 1000, 1500, 2000, 3000, 4000, 5000, 7000, 10000, 20000
Quantification using: Lower Marker   Final concentration (ng/uL): 0.5000   Dilution factor: 12.0

Sample: SampE3
Well location: E3
Created: Thursday, October 21, 2021 9:39:00 PM

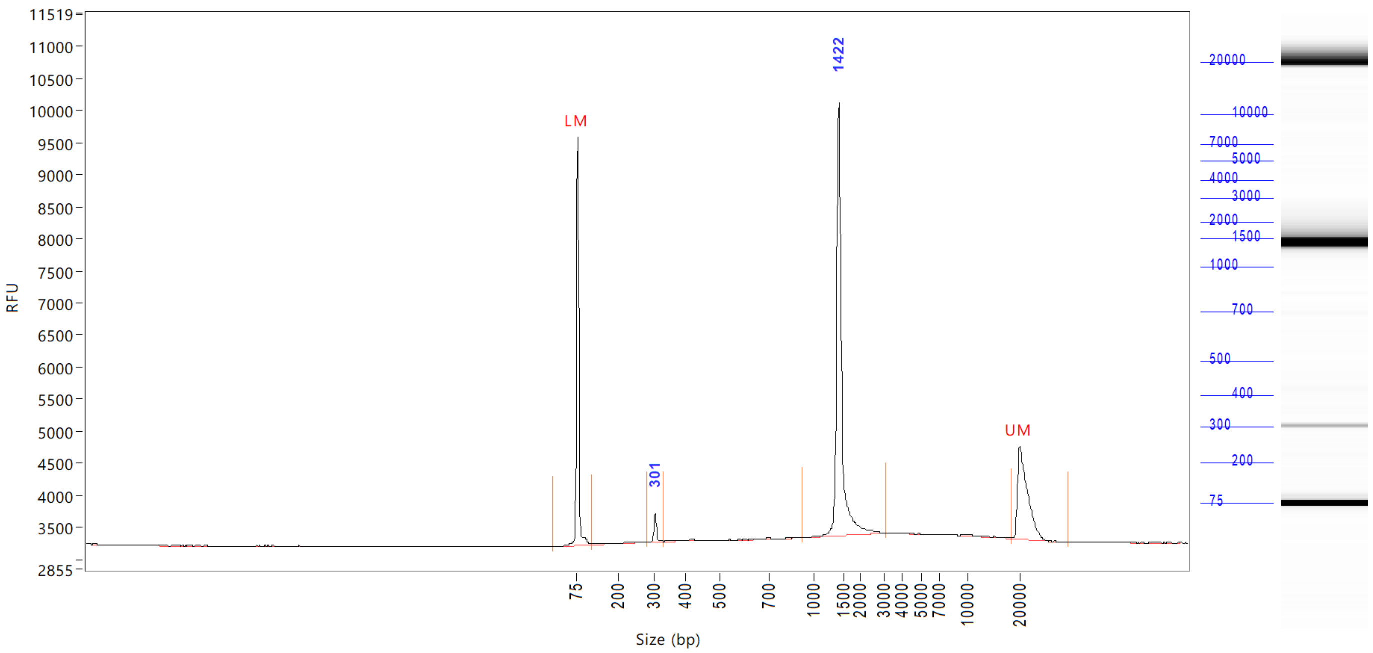

Peak	Size	Concentration	Relative concentration	Molarity	From	To	CV%	RFU
	(bp)	(ng/uL)	Percent	(nmole/L)	(bp)	(bp)		
1	75 (LM)	0.5000		11.2364	1	120	8.74	6364
2	301	0.3935	4.3	2.1645	278	327	1.59	433
3	1422	8.7942	95.7	9.9082	922	3108	12.22	6760
4	20000 (UM)	0.2439		0.0193	18415	29189	5.64	1437

TIC:	9.1876	ng/uL
TIM:	12.0727	nmole/L
Total concentration:	9.7731	ng/uL

```
Sample peak width (sec): 5    Sample min peak height: 50    Sample baseline V to V?: Y    Sample baseline V to V points: 3
Sample filter: Binomial       Number of points for filter: 3  Sample start region (min): 0  Sample end region (min): 30
Marker peak width (sec): 5    Marker min peak height: 200   Marker baseline V to V?: Y    Marker baseline V to V points: 3
Lower marker selection: First peak > 500 RFU                Upper marker selection: Last peak > 200 RFU
Ladder size (bp)75, 200, 300, 400, 500, 700, 1000, 1500, 2000, 3000, 4000, 5000, 7000, 10000, 20000
Quantification using: Lower Marker          Final concentration (ng/uL): 0.5000          Dilution factor: 12.0
```

Sample: SampF3
Well location: F3
Created: Thursday, October 21, 2021 9:39:00 PM

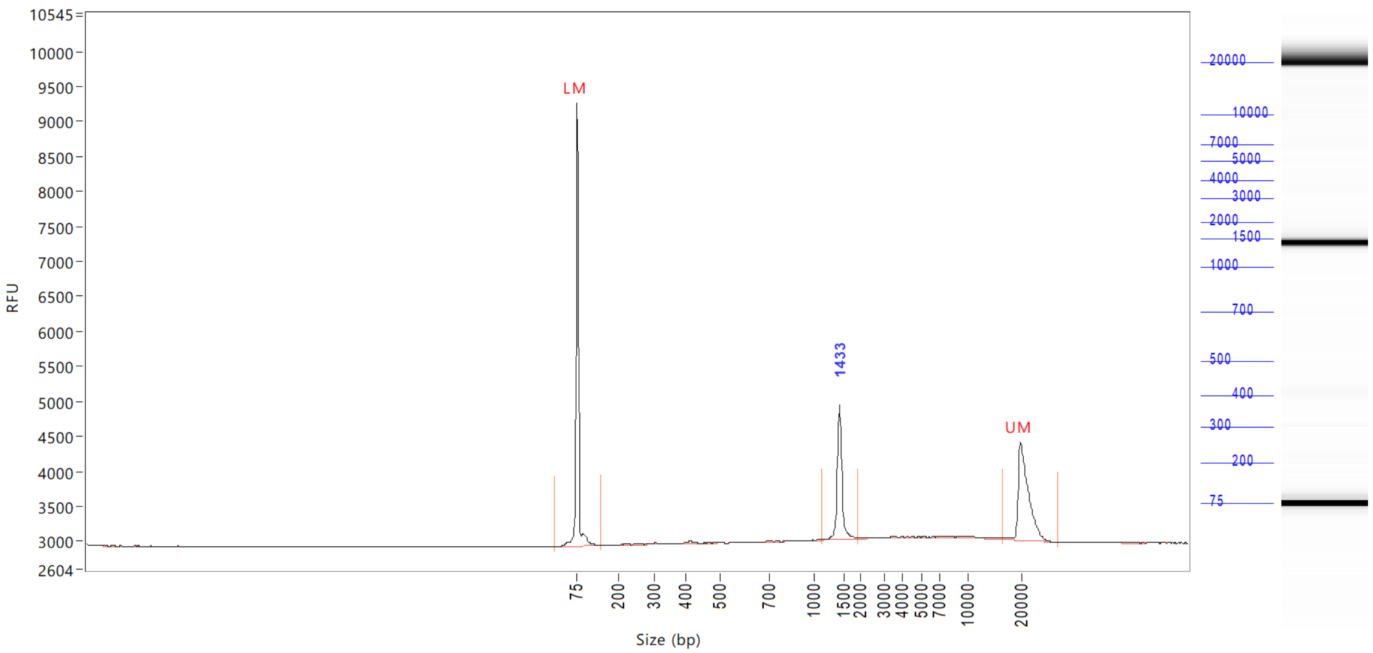

Peak	Size	Concentration	Relative concentration	Molarity	From	To	CV%	RFU
	(bp)	(ng/uL)	Percent	(nmole/L)	(bp)	(bp)		
1	75 (LM)	0.5000		11.0851	7	147	12.82	6341
2	1433	2.2424	100.0	2.5848	1123	1904	5.41	1929
3	20000 (UM)	0.2279		0.0181	16464	27133	5.76	1403

TIC:	2.2424	ng/uL
TIM:	2.5848	nmole/L
Total concentration:	2.9215	ng/uL

```
Sample peak width (sec): 5     Sample min peak height: 50     Sample baseline V to V?: Y     Sample baseline V to V points: 3
Sample filter: Binomial        Number of points for filter: 3  Sample start region (min): 0  Sample end region (min): 30
Marker peak width (sec): 5     Marker min peak height: 200    Marker baseline V to V?: Y     Marker baseline V to V points: 3
Lower marker selection: First peak > 500 RFU                  Upper marker selection: Last peak > 200 RFU
Ladder size (bp)75, 200, 300, 400, 500, 700, 1000, 1500, 2000, 3000, 4000, 5000, 7000, 10000, 20000
Quantification using: Lower Marker          Final concentration (ng/uL): 0.5000          Dilution factor: 12.0
```

Sample: SampG3
Well location: G3
Created: Thursday, October 21, 2021 9:39:00 PM

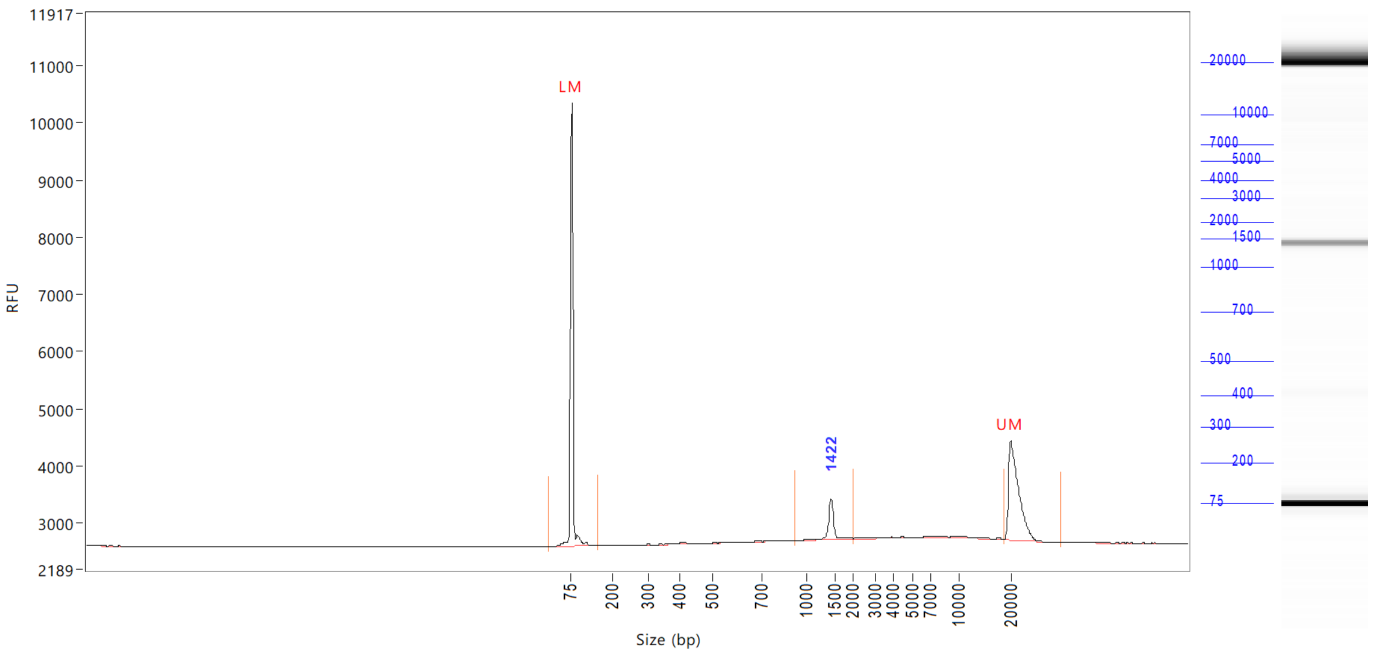

Peak	Size	Concentration	Relative concentration	Molarity	From	To	CV%	RFU
	(bp)	(ng/uL)	Percent	(nmole/L)	(bp)	(bp)		
1	75 (LM)	0.5000		11.2364	7	156	11.24	7767
2	1422	0.7584	100.0	0.8829	922	2030	8.61	716
3	20000 (UM)	0.2404		0.0190	18537	29551	5.68	1752

TIC:	0.7584	ng/uL
TIM:	0.8829	nmole/L
Total concentration:	1.2805	ng/uL

Sample peak width (sec): 5   Sample min peak height: 50   Sample baseline V to V?: Y   Sample baseline V to V points: 3
Sample filter: Binomial   Number of points for filter: 3   Sample start region (min): 0   Sample end region (min): 30
Marker peak width (sec): 5   Marker min peak height: 200   Marker baseline V to V?: Y   Marker baseline V to V points: 3
Lower marker selection: First peak > 500 RFU   Upper marker selection: Last peak > 200 RFU
Ladder size (bp)75, 200, 300, 400, 500, 700, 1000, 1500, 2000, 3000, 4000, 5000, 7000, 10000, 20000
Quantification using: Lower Marker   Final concentration (ng/uL): 0.5000   Dilution factor: 12.0

Sample: SampH3
Well location: H3
Created: Thursday, October 21, 2021 9:39:00 PM

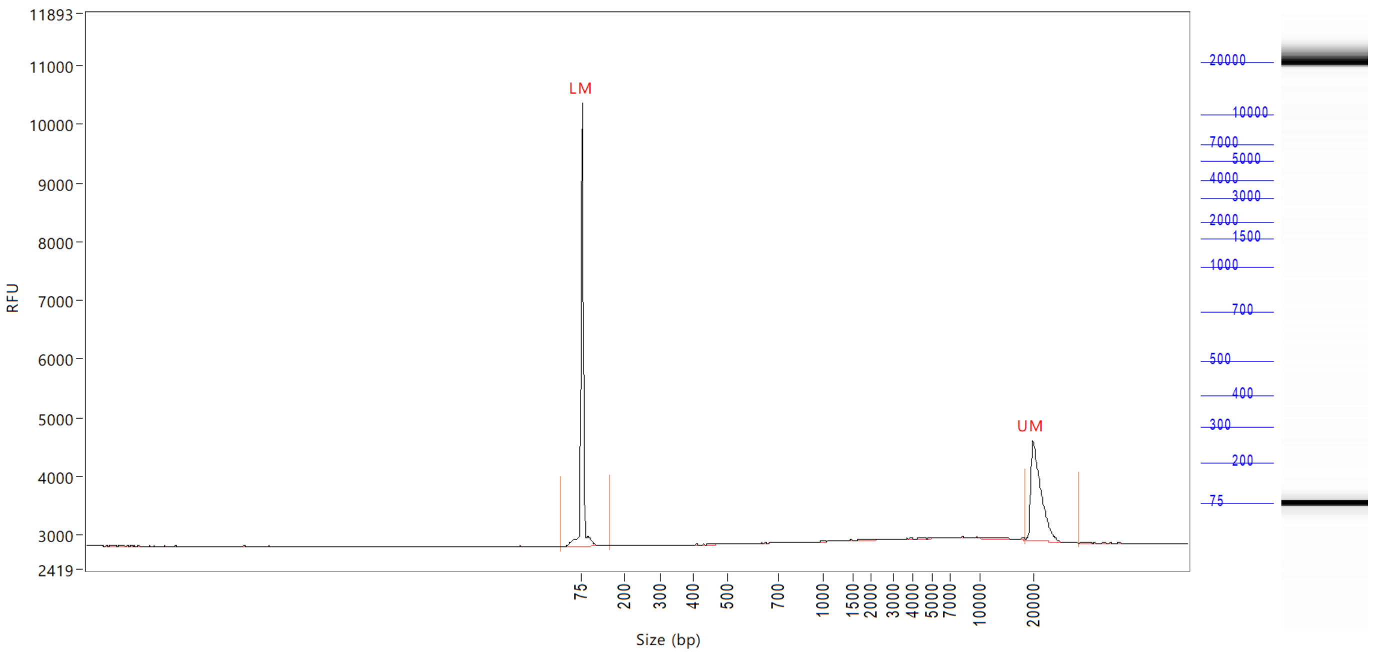

Peak	Size	Concentration	Relative concentration	Molarity	From	To	CV%	RFU
	(bp)	(ng/uL)	Percent	(nmole/L)	(bp)	(bp)		
1	75 (LM)	0.5000		11.2364	14	160	11.58	7567
2	20000 (UM)	0.2325		0.0184	18659	28584	5.58	1710

TIC:	0.0000	ng/uL
TIM:	0.0000	nmole/L
Total concentration:	0.4652	ng/uL

Sample peak width (sec): 5   Sample min peak height: 50   Sample baseline V to V?: Y   Sample baseline V to V points: 3
Sample filter: Binomial   Number of points for filter: 3   Sample start region (min): 0   Sample end region (min): 30
Marker peak width (sec): 5   Marker min peak height: 200   Marker baseline V to V?: Y   Marker baseline V to V points: 3
Lower marker selection: First peak > 500 RFU   Upper marker selection: Last peak > 200 RFU
Ladder size (bp)75, 200, 300, 400, 500, 700, 1000, 1500, 2000, 3000, 4000, 5000, 7000, 10000, 20000
Quantification using: Lower Marker   Final concentration (ng/uL): 0.5000   Dilution factor: 12.0

Sample: SampA4
Well location: A4
Created: Thursday, October 21, 2021 9:39:00 PM

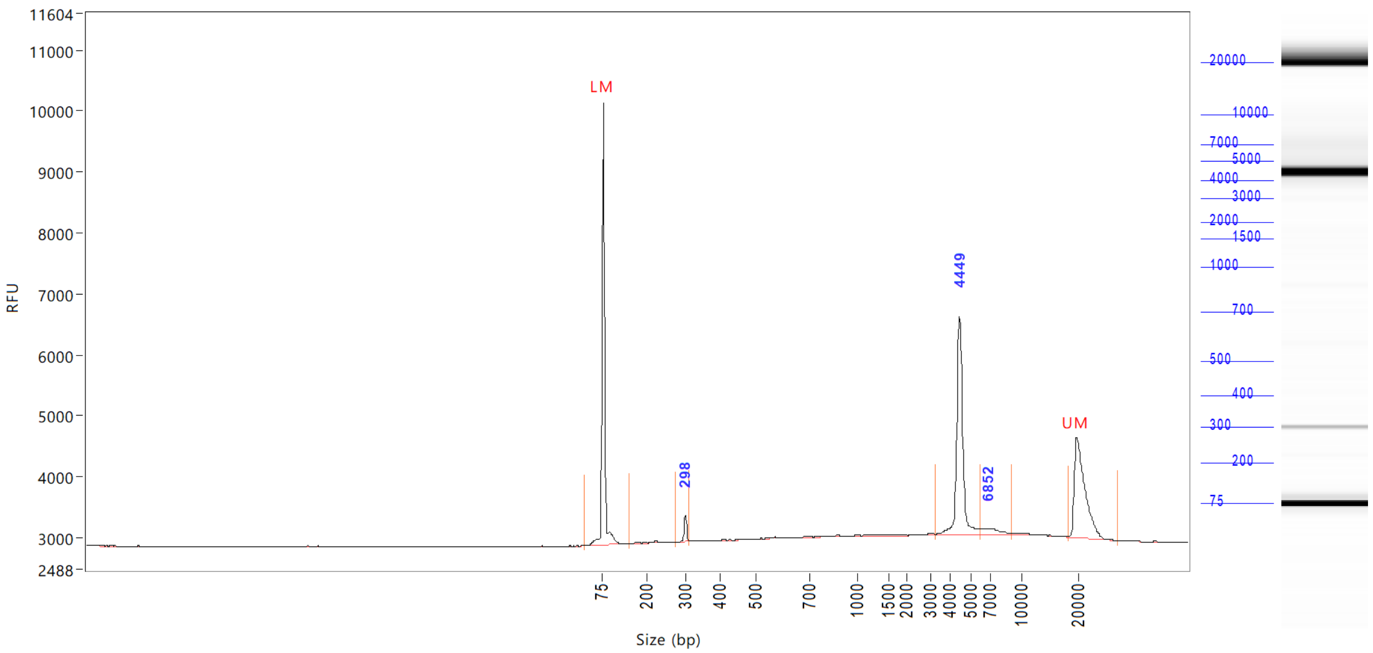

Peak	Size	Concentration	Relative concentration	Molarity	From	To	CV%	RFU
	(bp)	(ng/uL)	Percent	(nmole/L)	(bp)	(bp)		
1	75 (LM)	0.5000		11.2364	24	147	10.67	7257
2	298	0.3133	6.7	1.7410	273	307	1.31	438
3	4449	3.9699	85.3	1.4723	3215	5963	6.71	3594
4	6852	0.3727	8.0	0.0852	5963	9067	11.06	115
5	20000 (UM)	0.2345		0.0186	18293	27375	5.40	1652

TIC:	4.6559	ng/uL
TIM:	3.2985	nmole/L
Total concentration:	5.2102	ng/uL

```
Sample peak width (sec): 5    Sample min peak height: 50    Sample baseline V to V?: Y    Sample baseline V to V points: 3
Sample filter: Binomial       Number of points for filter: 3  Sample start region (min): 0  Sample end region (min): 30
Marker peak width (sec): 5    Marker min peak height: 200   Marker baseline V to V?: Y    Marker baseline V to V points: 3
Lower marker selection: First peak > 500 RFU                Upper marker selection: Last peak > 200 RFU
Ladder size (bp)75, 200, 300, 400, 500, 700, 1000, 1500, 2000, 3000, 4000, 5000, 7000, 10000, 20000
Quantification using: Lower Marker          Final concentration (ng/uL): 0.5000          Dilution factor: 12.0
```

Sample: SampB4
Well location: B4
Created: Thursday, October 21, 2021 9:39:00 PM

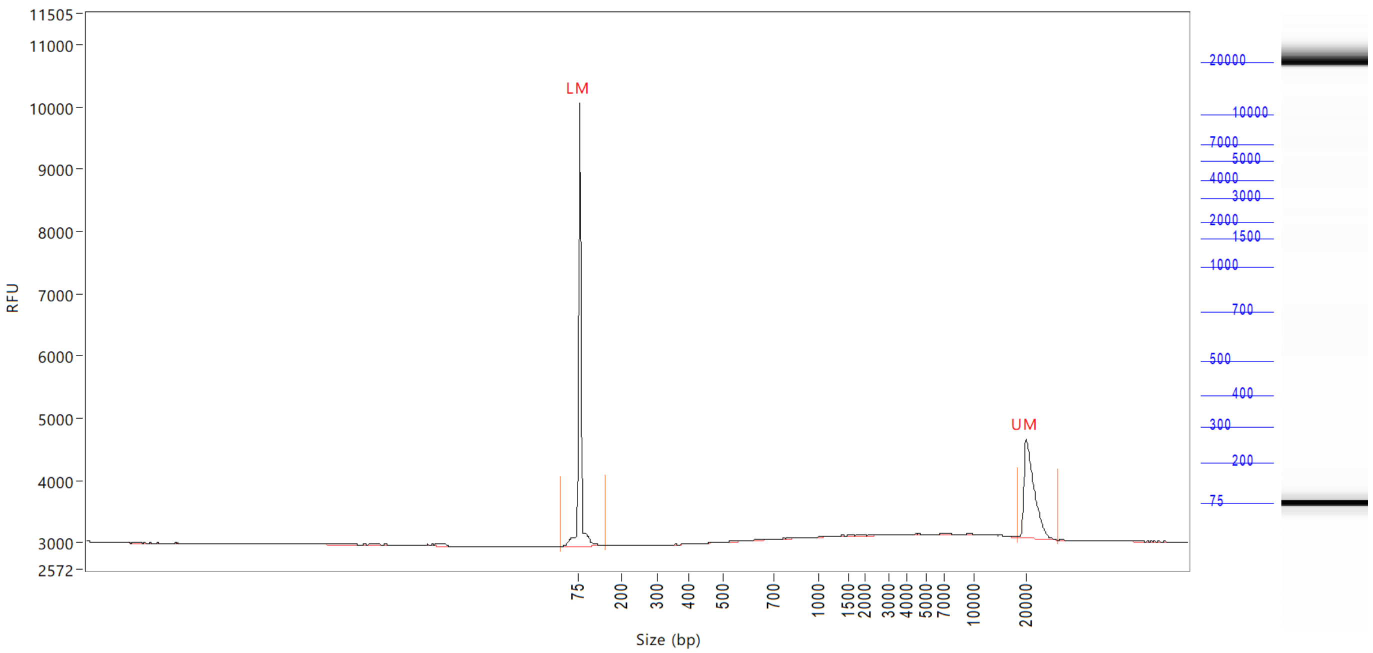

Peak	Size	Concentration	Relative concentration	Molarity	From	To	CV%	RFU
	(bp)	(ng/uL)	Percent	(nmole/L)	(bp)	(bp)		
1	75 (LM)	0.5000		11.0851	20	152	13.60	7135
2	20000 (UM)	0.2184		0.0173	18537	26045	5.37	1586

TIC:	0.0000	ng/uL
TIM:	0.0000	nmole/L
Total concentration:	0.5467	ng/uL

Sample peak width (sec): 5 Sample min peak height: 50 Sample baseline V to V?: Y Sample baseline V to V points: 3
Sample filter: Binomial Number of points for filter: 3 Sample start region (min): 0 Sample end region (min): 30
Marker peak width (sec): 5 Marker min peak height: 200 Marker baseline V to V?: Y Marker baseline V to V points: 3
Lower marker selection: First peak > 500 RFU Upper marker selection: Last peak > 200 RFU
Ladder size (bp)75, 200, 300, 400, 500, 700, 1000, 1500, 2000, 3000, 4000, 5000, 7000, 10000, 20000
Quantification using: Lower Marker Final concentration (ng/uL): 0.5000 Dilution factor: 12.0

Sample: SampC4
Well location: C4
Created: Thursday, October 21, 2021 9:39:00 PM

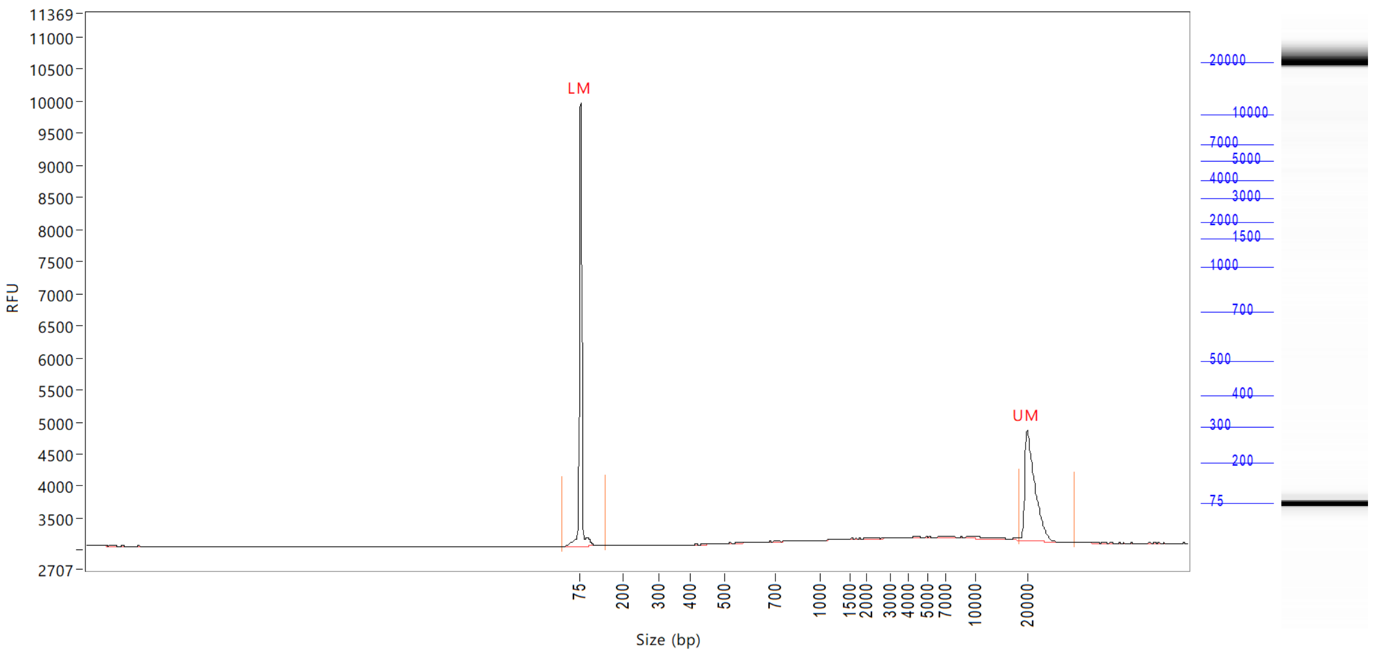

Peak	Size	Concentration	Relative concentration	Molarity	From	To	CV%	RFU
	(bp)	(ng/uL)	Percent	(nmole/L)	(bp)	(bp)		
1	75 (LM)	0.5000		11.3919	18	148	10.75	6918
2	20000 (UM)	0.2523		0.0200	18537	28826	5.55	1709

TIC:	0.0000	ng/uL
TIM:	0.0000	nmole/L
Total concentration:	0.6107	ng/uL

```
Sample peak width (sec): 5    Sample min peak height: 50    Sample baseline V to V?: Y    Sample baseline V to V points: 3
Sample filter: Binomial       Number of points for filter: 3  Sample start region (min): 0  Sample end region (min): 30
Marker peak width (sec): 5    Marker min peak height: 200   Marker baseline V to V?: Y    Marker baseline V to V points: 3
Lower marker selection: First peak > 500 RFU                Upper marker selection: Last peak > 200 RFU
Ladder size (bp)75, 200, 300, 400, 500, 700, 1000, 1500, 2000, 3000, 4000, 5000, 7000, 10000, 20000
Quantification using: Lower Marker          Final concentration (ng/uL): 0.5000          Dilution factor: 12.0
```

Sample: SampD4
Well location: D4
Created: Thursday, October 21, 2021 9:39:00 PM

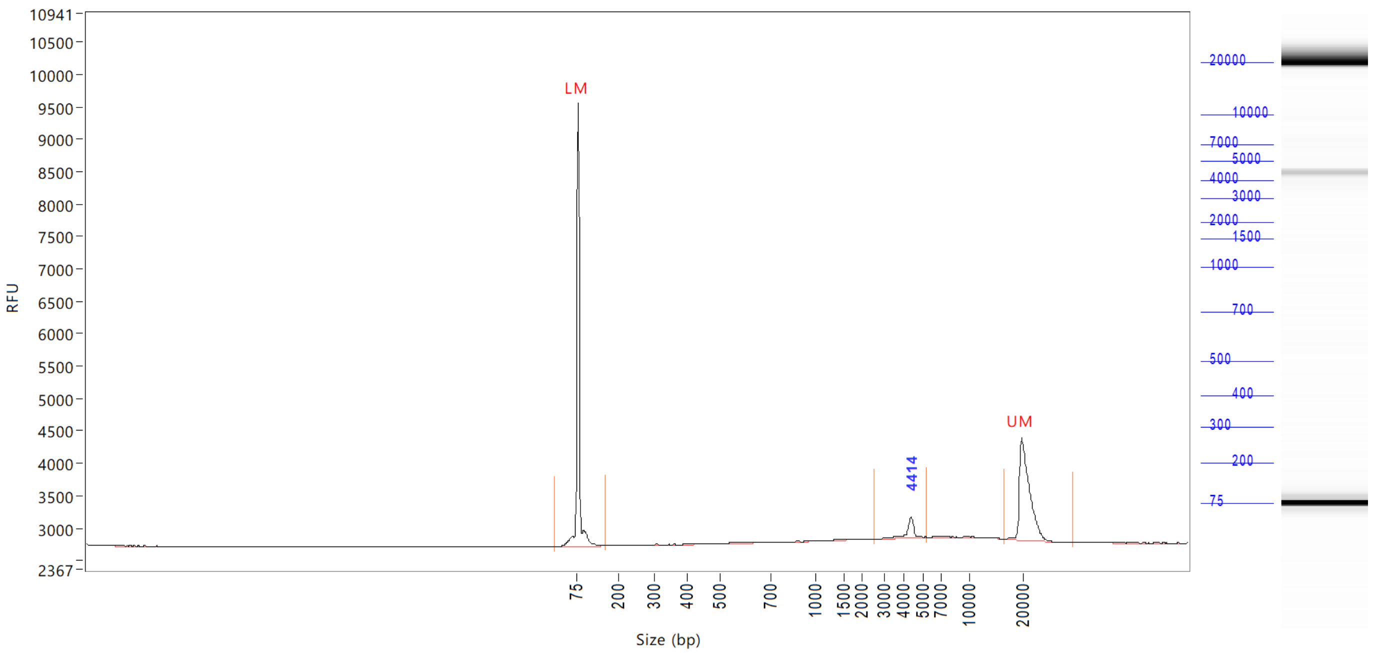

Peak	Size	Concentration	Relative concentration	Molarity	From	To	CV%	RFU
	(bp)	(ng/uL)	Percent	(nmole/L)	(bp)	(bp)		
1	75 (LM)	0.5000		10.9378	7	160	14.41	6851
2	4414	0.4427	100.0	0.1714	2572	5519	9.66	329
3	20000 (UM)	0.2245		0.0178	16464	29793	5.84	1583

TIC:	0.4427	ng/uL
TIM:	0.1714	nmole/L
Total concentration:	0.9130	ng/uL

```
Sample peak width (sec): 5    Sample min peak height: 50    Sample baseline V to V?: Y    Sample baseline V to V points: 3
Sample filter: Binomial       Number of points for filter: 3  Sample start region (min): 0  Sample end region (min): 30
Marker peak width (sec): 5    Marker min peak height: 200   Marker baseline V to V?: Y    Marker baseline V to V points: 3
Lower marker selection: First peak > 500 RFU                Upper marker selection: Last peak > 200 RFU
Ladder size (bp)75, 200, 300, 400, 500, 700, 1000, 1500, 2000, 3000, 4000, 5000, 7000, 10000, 20000
Quantification using: Lower Marker          Final concentration (ng/uL): 0.5000          Dilution factor: 12.0
```

Sample: SampE4
Well location: E4
Created: Thursday, October 21, 2021 9:39:00 PM

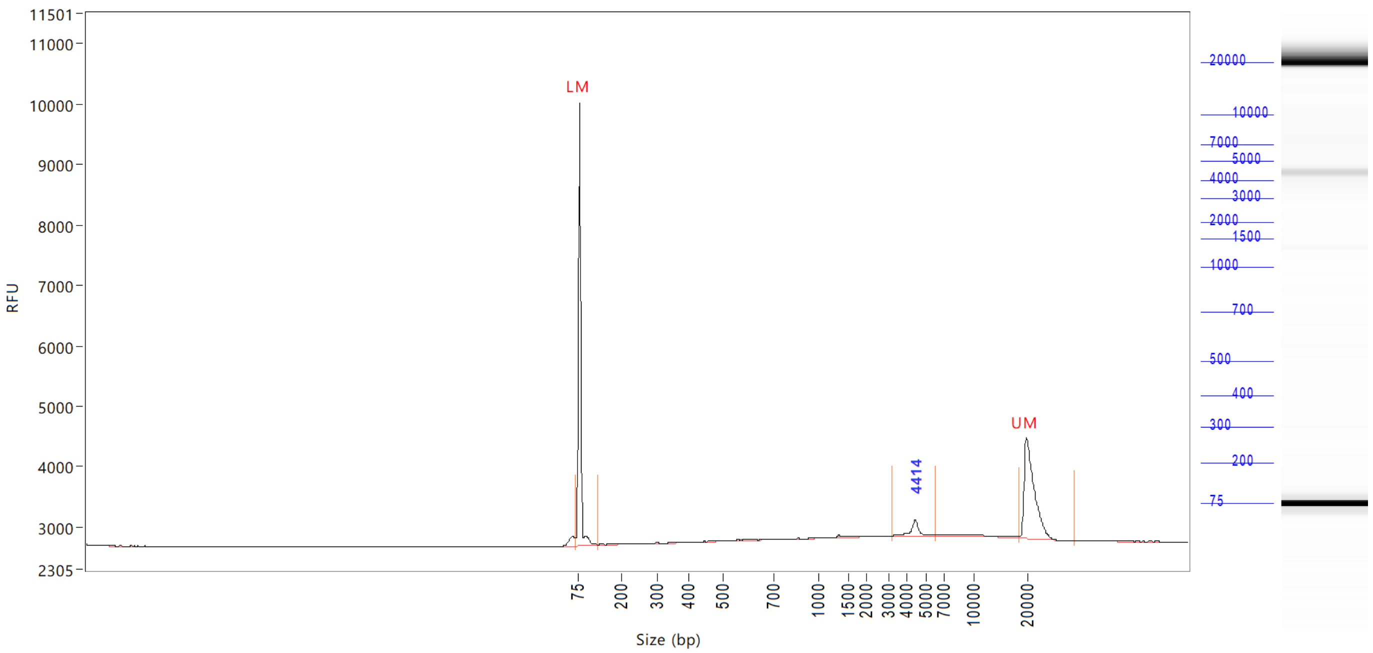

Peak	Size	Concentration	Relative concentration	Molarity	From	To	CV%	RFU
	(bp)	(ng/uL)	Percent	(nmole/L)	(bp)	(bp)		
1	75 (LM)	0.5000		11.0851	62	129	8.54	7341
2	4414	0.5051	100.0	0.1903	3215	6038	11.56	268
3	20000 (UM)	0.2408		0.0191	18537	28947	5.51	1657

TIC:	0.5051	ng/uL
TIM:	0.1903	nmole/L
Total concentration:	1.2142	ng/uL

Sample peak width (sec): 5 Sample min peak height: 50 Sample baseline V to V?: Y Sample baseline V to V points: 3
Sample filter: Binomial Number of points for filter: 3 Sample start region (min): 0 Sample end region (min): 30
Marker peak width (sec): 5 Marker min peak height: 200 Marker baseline V to V?: Y Marker baseline V to V points: 3
Lower marker selection: First peak > 500 RFU Upper marker selection: Last peak > 200 RFU
Ladder size (bp)75, 200, 300, 400, 500, 700, 1000, 1500, 2000, 3000, 4000, 5000, 7000, 10000, 20000
Quantification using: Lower Marker Final concentration (ng/uL): 0.5000 Dilution factor: 12.0

Sample: SampF4
Well location: F4
Created: Thursday, October 21, 2021 9:39:00 PM

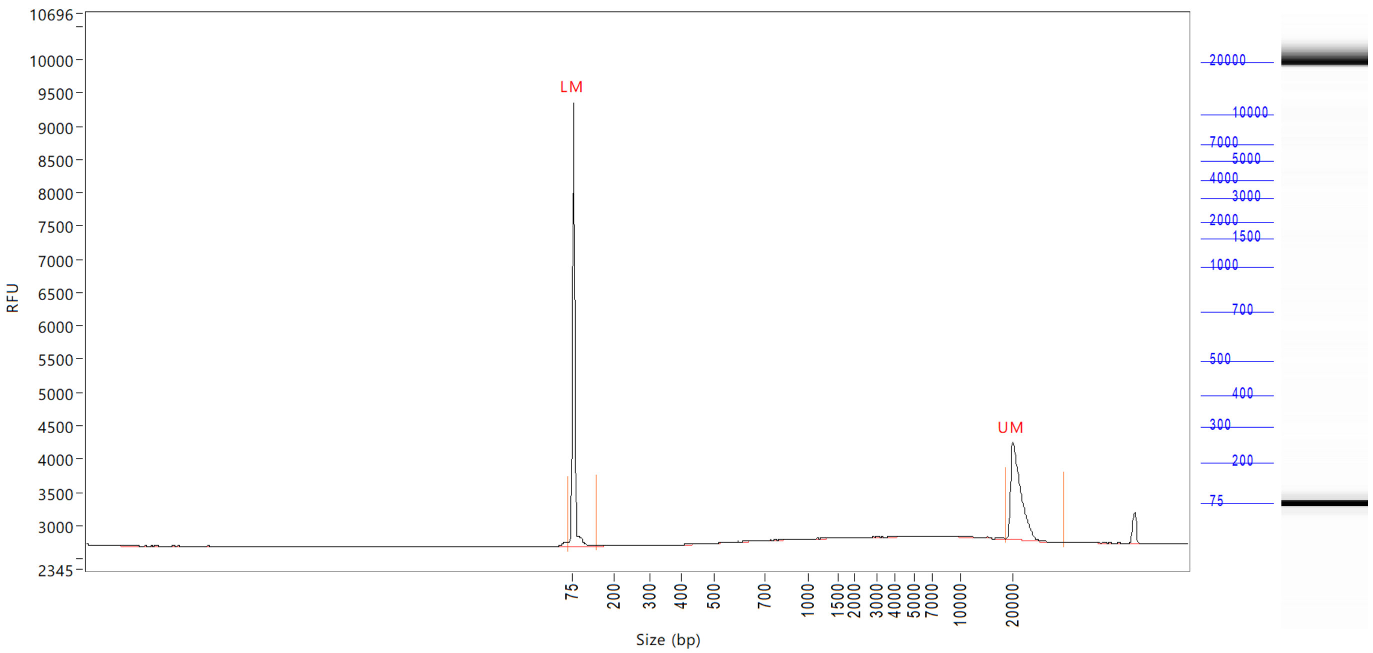

Peak	Size	Concentration	Relative concentration	Molarity	From	To	CV%	RFU
	(bp)	(ng/uL)	Percent	(nmole/L)	(bp)	(bp)		
1	75 (LM)	0.5000		11.0851	62	145	9.49	6675
2	20000 (UM)	0.2358		0.0186	18537	29793	5.62	1457

TIC:	0.0000	ng/uL
TIM:	0.0000	nmole/L
Total concentration:	0.4625	ng/uL

```
Sample peak width (sec): 5    Sample min peak height: 50    Sample baseline V to V?: Y    Sample baseline V to V points: 3
Sample filter: Binomial       Number of points for filter: 3  Sample start region (min): 0  Sample end region (min): 30
Marker peak width (sec): 5    Marker min peak height: 200   Marker baseline V to V?: Y    Marker baseline V to V points: 3
Lower marker selection: First peak > 500 RFU                Upper marker selection: Last peak > 200 RFU
Ladder size (bp)75, 200, 300, 400, 500, 700, 1000, 1500, 2000, 3000, 4000, 5000, 7000, 10000, 20000
Quantification using: Lower Marker          Final concentration (ng/uL): 0.5000          Dilution factor: 12.0
```

Sample: SampG4
Well location: G4
Created: Thursday, October 21, 2021 9:39:00 PM

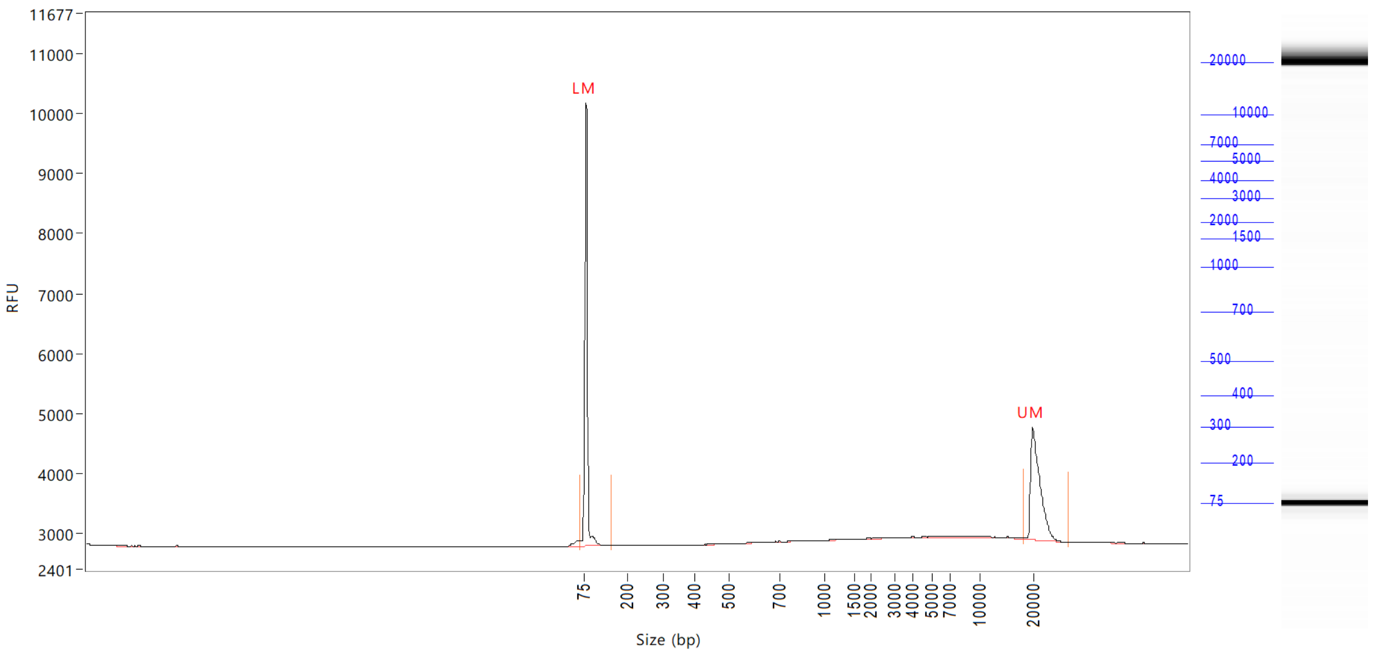

Peak	Size	Concentration	Relative concentration	Molarity	From	To	CV%	RFU
	(bp)	(ng/uL)	Percent	(nmole/L)	(bp)	(bp)		
1	75 (LM)	0.5000		10.9378	58	150	8.34	7404
2	20000 (UM)	0.2501		0.0198	18293	26770	5.01	1885

TIC:	0.0000	ng/uL
TIM:	0.0000	nmole/L
Total concentration:	0.5186	ng/uL

Sample peak width (sec): 5 Sample min peak height: 50 Sample baseline V to V?: Y Sample baseline V to V points: 3
Sample filter: Binomial Number of points for filter: 3 Sample start region (min): 0 Sample end region (min): 30
Marker peak width (sec): 5 Marker min peak height: 200 Marker baseline V to V?: Y Marker baseline V to V points: 3
Lower marker selection: First peak > 500 RFU Upper marker selection: Last peak > 200 RFU
Ladder size (bp)75, 200, 300, 400, 500, 700, 1000, 1500, 2000, 3000, 4000, 5000, 7000, 10000, 20000
Quantification using: Lower Marker Final concentration (ng/uL): 0.5000 Dilution factor: 12.0

Sample: SampH4
Well location: H4
Created: Thursday, October 21, 2021 9:39:00 PM

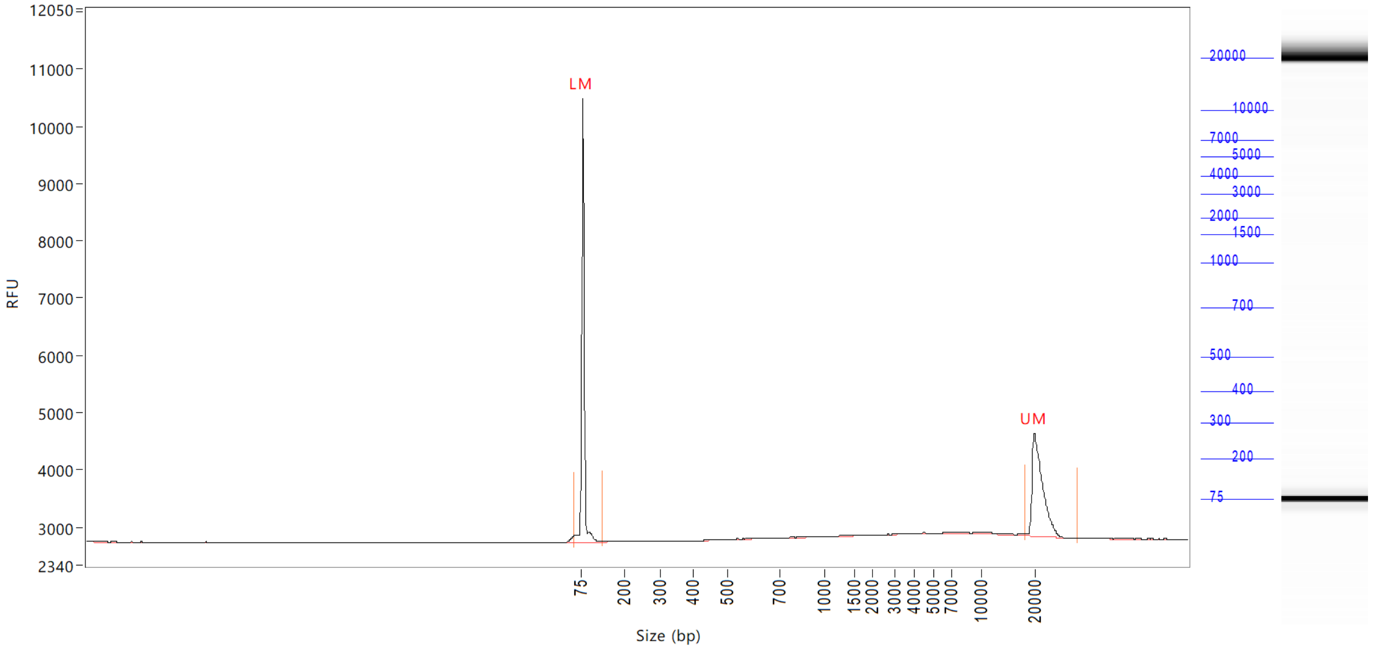

Peak	Size	Concentration	Relative concentration	Molarity	From	To	CV%	RFU
	(bp)	(ng/uL)	Percent	(nmole/L)	(bp)	(bp)		
1	75 (LM)	0.5000		11.0851	50	133	9.45	7758
2	20000 (UM)	0.2421		0.0192	18293	27979	5.60	1789

TIC:	0.0000	ng/uL
TIM:	0.0000	nmole/L
Total concentration:	0.6346	ng/uL

Sample peak width (sec): 5 Sample min peak height: 50 Sample baseline V to V?: Y Sample baseline V to V points: 3
Sample filter: Binomial Number of points for filter: 3 Sample start region (min): 0 Sample end region (min): 30
Marker peak width (sec): 5 Marker min peak height: 200 Marker baseline V to V?: Y Marker baseline V to V points: 3
Lower marker selection: First peak > 500 RFU Upper marker selection: Last peak > 200 RFU
Ladder size (bp)75, 200, 300, 400, 500, 700, 1000, 1500, 2000, 3000, 4000, 5000, 7000, 10000, 20000
Quantification using: Lower Marker Final concentration (ng/uL): 0.5000 Dilution factor: 12.0

Sample: SampA5
Well location: A5
Created: Thursday, October 21, 2021 9:39:00 PM

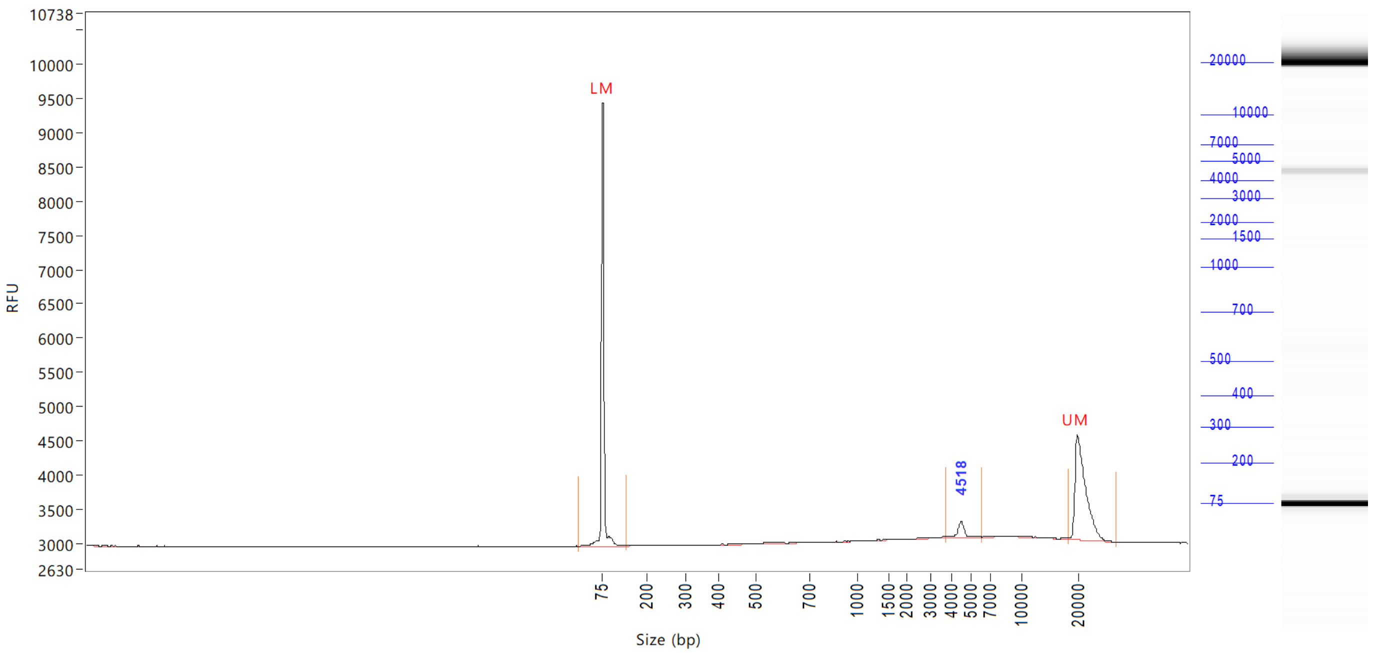

Peak	Size	Concentration	Relative concentration	Molarity	From	To	CV%	RFU
	(bp)	(ng/uL)	Percent	(nmole/L)	(bp)	(bp)		
1	75 (LM)	0.5000		11.2364	9	141	11.37	6476
2	4518	0.3479	100.0	0.1285	3715	6112	7.22	236
3	20000 (UM)	0.2436		0.0193	18415	26770	5.43	1525

TIC:	0.3479	ng/uL
TIM:	0.1285	nmole/L
Total concentration:	0.9231	ng/uL

```
Sample peak width (sec): 5    Sample min peak height: 50    Sample baseline V to V?: Y    Sample baseline V to V points: 3
Sample filter: Binomial       Number of points for filter: 3  Sample start region (min): 0  Sample end region (min): 30
Marker peak width (sec): 5    Marker min peak height: 200   Marker baseline V to V?: Y    Marker baseline V to V points: 3
Lower marker selection: First peak > 500 RFU                Upper marker selection: Last peak > 200 RFU
Ladder size (bp)75, 200, 300, 400, 500, 700, 1000, 1500, 2000, 3000, 4000, 5000, 7000, 10000, 20000
Quantification using: Lower Marker          Final concentration (ng/uL): 0.5000          Dilution factor: 12.0
```

Sample: SampB5
Well location: B5
Created: Thursday, October 21, 2021 9:39:00 PM

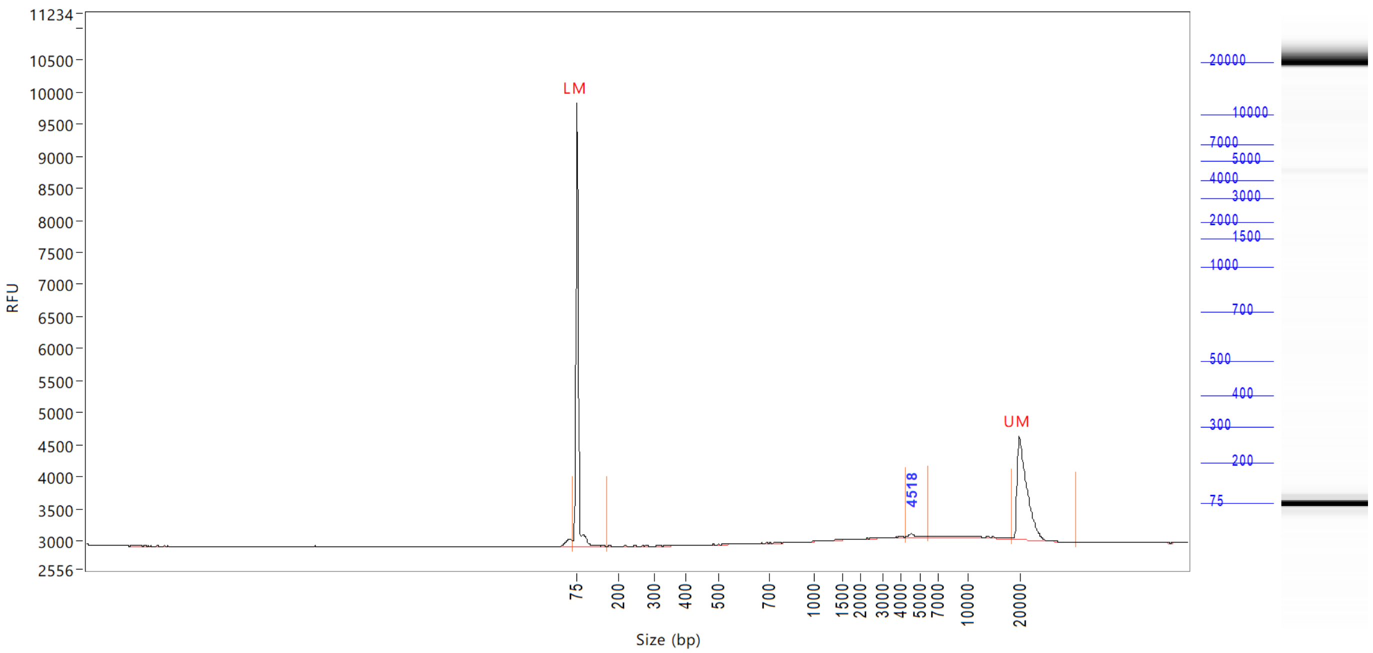

Peak	Size	Concentration	Relative concentration	Molarity	From	To	CV%	RFU
	(bp)	(ng/uL)	Percent	(nmole/L)	(bp)	(bp)		
1	75 (LM)	0.5000		11.0851	62	164	11.43	6937
2	4518	0.0868	100.0	0.0311	4173	5815	6.87	65
3	20000 (UM)	0.2373		0.0188	18415	30881	5.65	1615

TIC:	0.0868	ng/uL
TIM:	0.0311	nmole/L
Total concentration:	0.6779	ng/uL

Sample peak width (sec): 5 Sample min peak height: 50 Sample baseline V to V?: Y Sample baseline V to V points: 3
Sample filter: Binomial Number of points for filter: 3 Sample start region (min): 0 Sample end region (min): 30
Marker peak width (sec): 5 Marker min peak height: 200 Marker baseline V to V?: Y Marker baseline V to V points: 3
Lower marker selection: First peak > 500 RFU Upper marker selection: Last peak > 200 RFU
Ladder size (bp)75, 200, 300, 400, 500, 700, 1000, 1500, 2000, 3000, 4000, 5000, 7000, 10000, 20000
Quantification using: Lower Marker Final concentration (ng/uL): 0.5000 Dilution factor: 12.0

Sample: SampC5
Well location: C5
Created: Thursday, October 21, 2021 9:39:00 PM

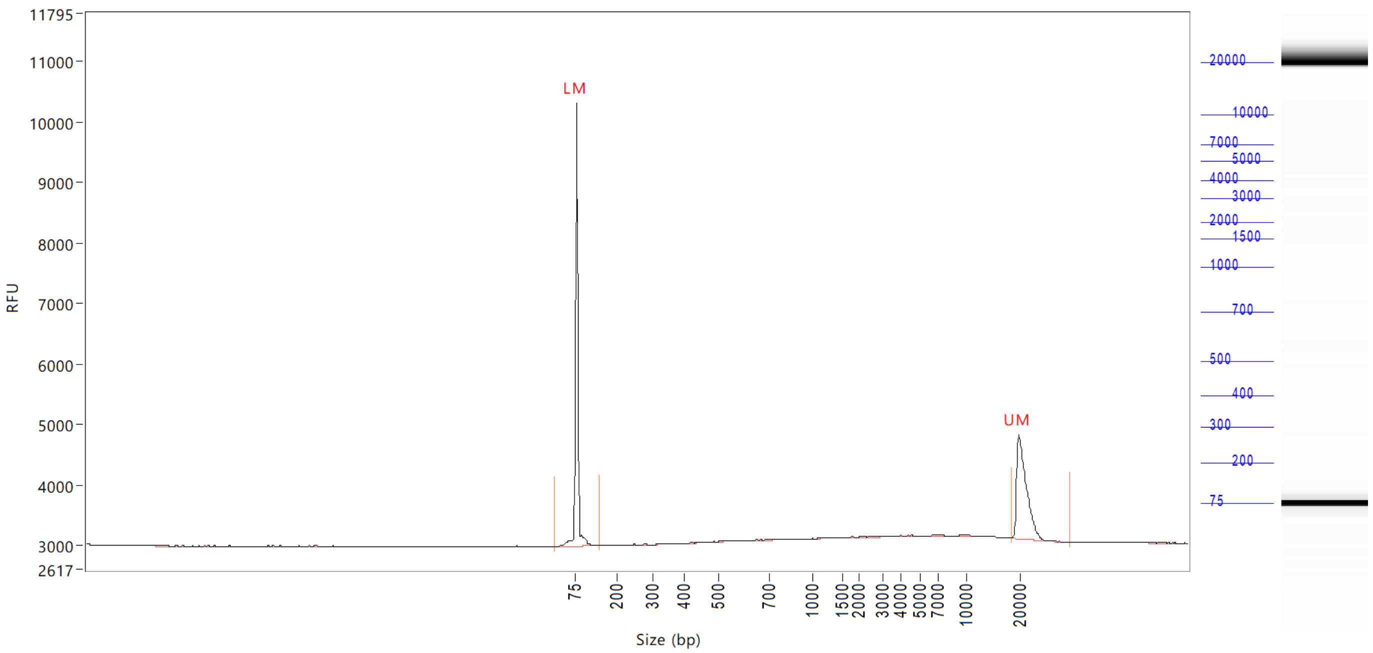

Peak	Size	Concentration	Relative concentration	Molarity	From	To	CV%	RFU
	(bp)	(ng/uL)	Percent	(nmole/L)	(bp)	(bp)		
1	75 (LM)	0.5000		11.2364	11	147	11.86	7330
2	20000 (UM)	0.2417		0.0192	18415	29672	5.37	1732

TIC:	0.0000	ng/uL
TIM:	0.0000	nmole/L
Total concentration:	0.6054	ng/uL

Sample peak width (sec): 5 Sample min peak height: 50 Sample baseline V to V?: Y Sample baseline V to V points: 3
Sample filter: Binomial Number of points for filter: 3 Sample start region (min): 0 Sample end region (min): 30
Marker peak width (sec): 5 Marker min peak height: 200 Marker baseline V to V?: Y Marker baseline V to V points: 3
Lower marker selection: First peak > 500 RFU Upper marker selection: Last peak > 200 RFU
Ladder size (bp)75, 200, 300, 400, 500, 700, 1000, 1500, 2000, 3000, 4000, 5000, 7000, 10000, 20000
Quantification using: Lower Marker Final concentration (ng/uL): 0.5000 Dilution factor: 12.0

Sample: SampD5
Well location: D5
Created: Thursday, October 21, 2021 9:39:00 PM

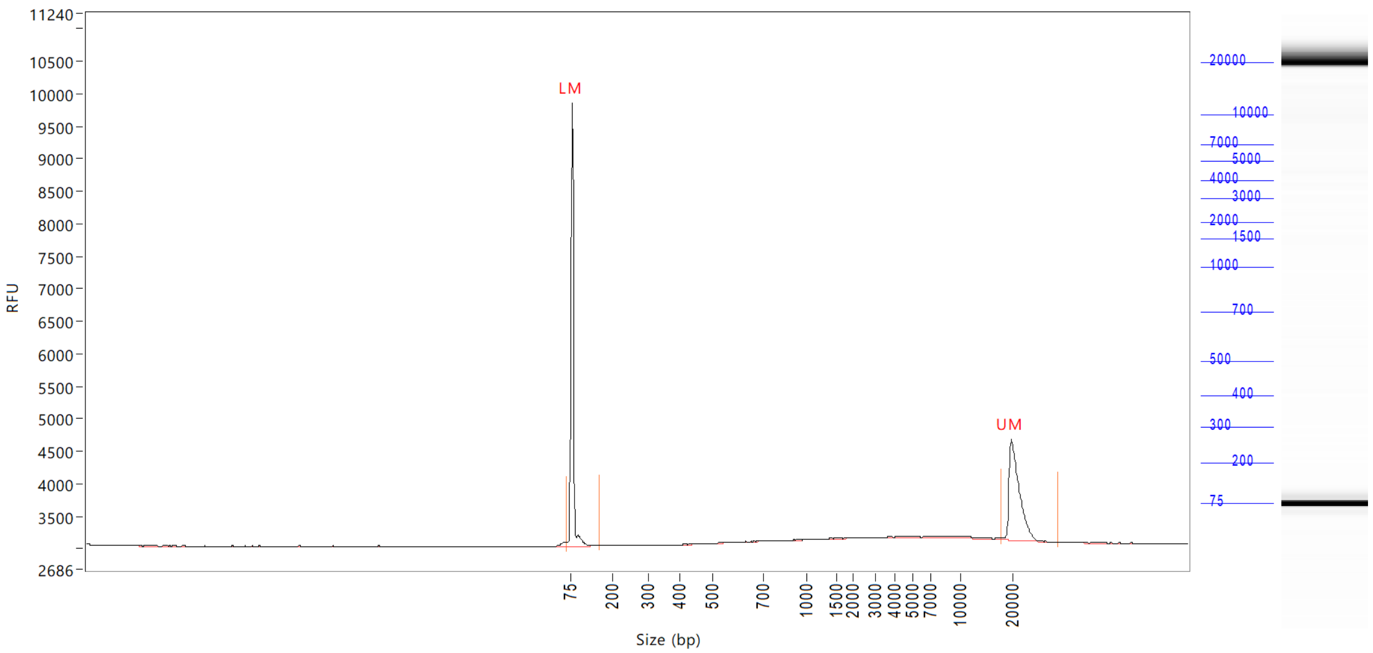

Peak	Size	Concentration	Relative concentration	Molarity	From	To	CV%	RFU
	(bp)	(ng/uL)	Percent	(nmole/L)	(bp)	(bp)		
1	75 (LM)	0.5000		11.0851	58	158	10.32	6834
2	20000 (UM)	0.2447		0.0194	17927	28826	5.69	1564

TIC:	0.0000	ng/uL
TIM:	0.0000	nmole/L
Total concentration:	0.6595	ng/uL

```
Sample peak width (sec): 5    Sample min peak height: 50    Sample baseline V to V?: Y    Sample baseline V to V points: 3
Sample filter: Binomial       Number of points for filter: 3  Sample start region (min): 0  Sample end region (min): 30
Marker peak width (sec): 5    Marker min peak height: 200   Marker baseline V to V?: Y    Marker baseline V to V points: 3
Lower marker selection: First peak > 500 RFU                Upper marker selection: Last peak > 200 RFU
Ladder size (bp)75, 200, 300, 400, 500, 700, 1000, 1500, 2000, 3000, 4000, 5000, 7000, 10000, 20000
Quantification using: Lower Marker          Final concentration (ng/uL): 0.5000          Dilution factor: 12.0
```

Sample: SampE5
Well location: E5
Created: Thursday, October 21, 2021 9:39:00 PM

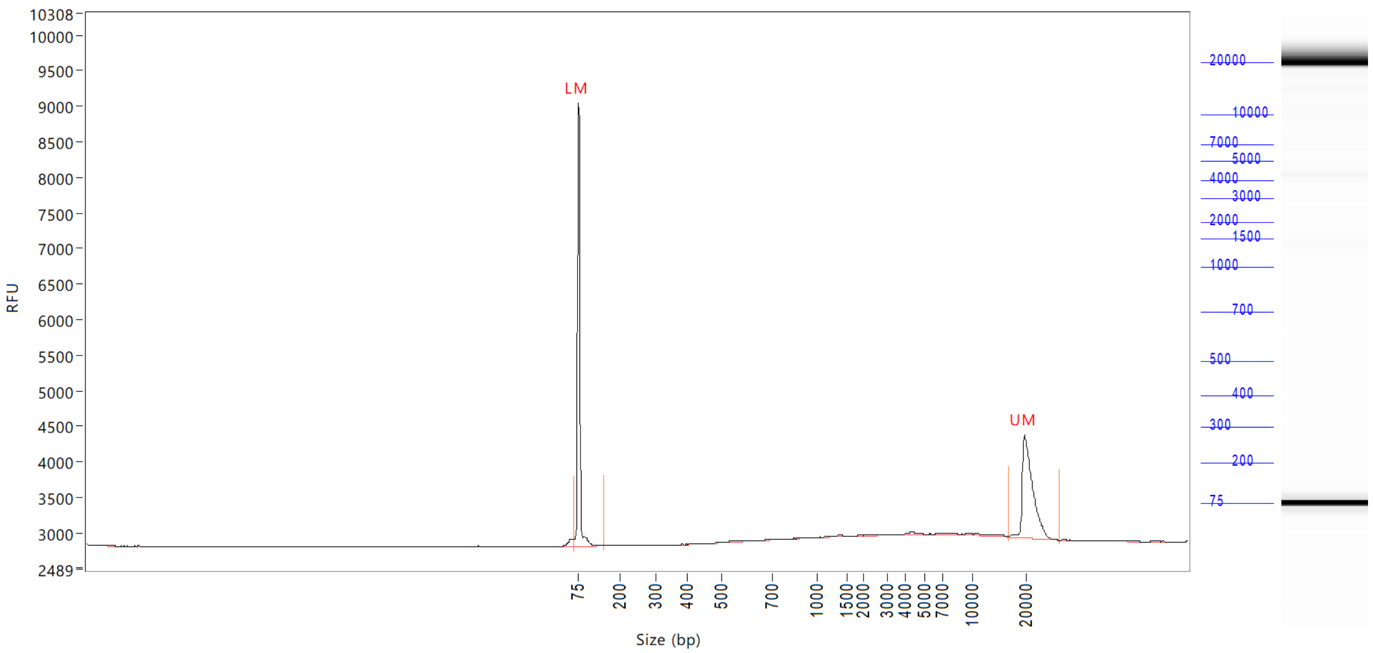

Peak	Size	Concentration	Relative concentration	Molarity	From	To	CV%	RFU
	(bp)	(ng/uL)	Percent	(nmole/L)	(bp)	(bp)		
1	75 (LM)	0.5000		10.9378	64	152	9.45	6246
2	20000 (UM)	0.2519		0.0201	16830	26649	5.73	1458

TIC:	0.0000	ng/uL
TIM:	0.0000	nmole/L
Total concentration:	0.7352	ng/uL

Sample peak width (sec): 5 Sample min peak height: 50 Sample baseline V to V?: Y Sample baseline V to V points: 3
Sample filter: Binomial Number of points for filter: 3 Sample start region (min): 0 Sample end region (min): 30
Marker peak width (sec): 5 Marker min peak height: 200 Marker baseline V to V?: Y Marker baseline V to V points: 3
Lower marker selection: First peak > 500 RFU Upper marker selection: Last peak > 200 RFU
Ladder size (bp)75, 200, 300, 400, 500, 700, 1000, 1500, 2000, 3000, 4000, 5000, 7000, 10000, 20000
Quantification using: Lower Marker Final concentration (ng/uL): 0.5000 Dilution factor: 12.0

Sample: SampF5
Well location: F5
Created: Thursday, October 21, 2021 9:39:00 PM

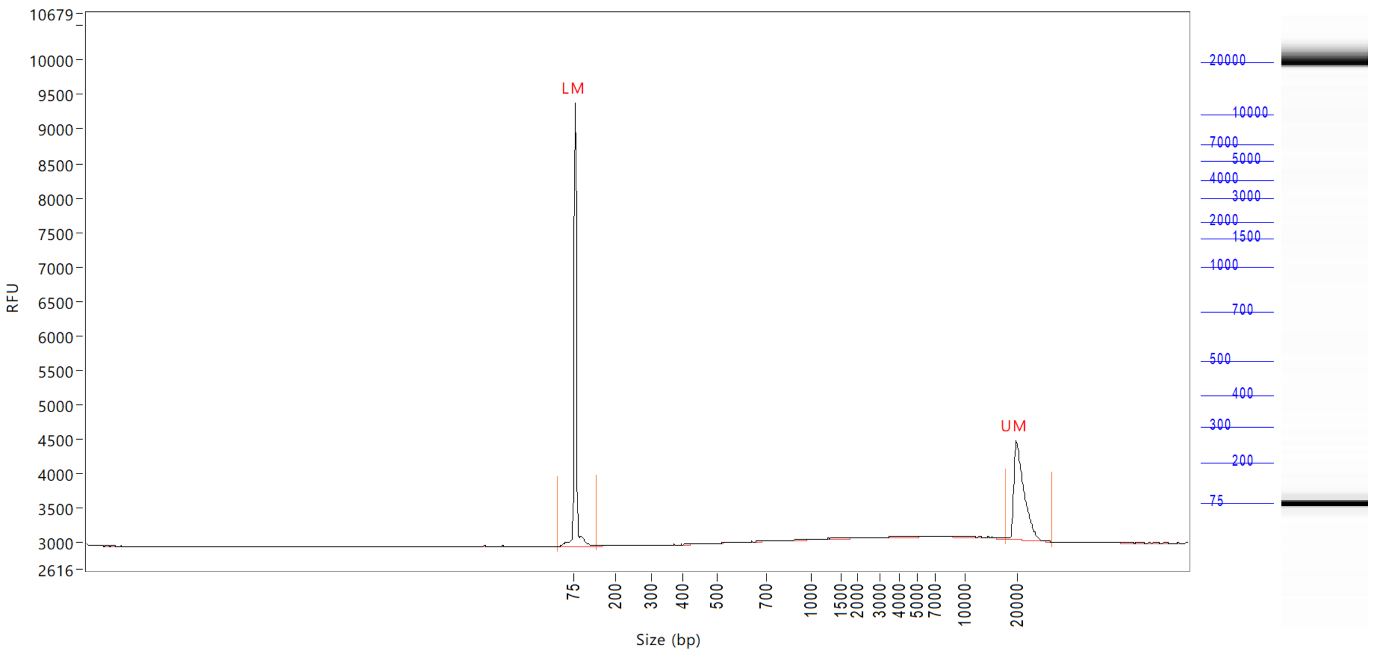

Peak	Size	Concentration	Relative concentration	Molarity	From	To	CV%	RFU
	(bp)	(ng/uL)	Percent	(nmole/L)	(bp)	(bp)		
1	75 (LM)	0.5000		11.2364	24	139	11.41	6441
2	20000 (UM)	0.2297		0.0183	17927	26891	5.47	1446

TIC:	0.0000	ng/uL
TIM:	0.0000	nmole/L
Total concentration:	0.6794	ng/uL

```
Sample peak width (sec): 5    Sample min peak height: 50    Sample baseline V to V?: Y    Sample baseline V to V points: 3
Sample filter: Binomial       Number of points for filter: 3  Sample start region (min): 0  Sample end region (min): 30
Marker peak width (sec): 5    Marker min peak height: 200   Marker baseline V to V?: Y    Marker baseline V to V points: 3
Lower marker selection: First peak > 500 RFU                Upper marker selection: Last peak > 200 RFU
Ladder size (bp)75, 200, 300, 400, 500, 700, 1000, 1500, 2000, 3000, 4000, 5000, 7000, 10000, 20000
Quantification using: Lower Marker          Final concentration (ng/uL): 0.5000          Dilution factor: 12.0
```

Sample: SampG5
Well location: G5
Created: Thursday, October 21, 2021 9:39:00 PM

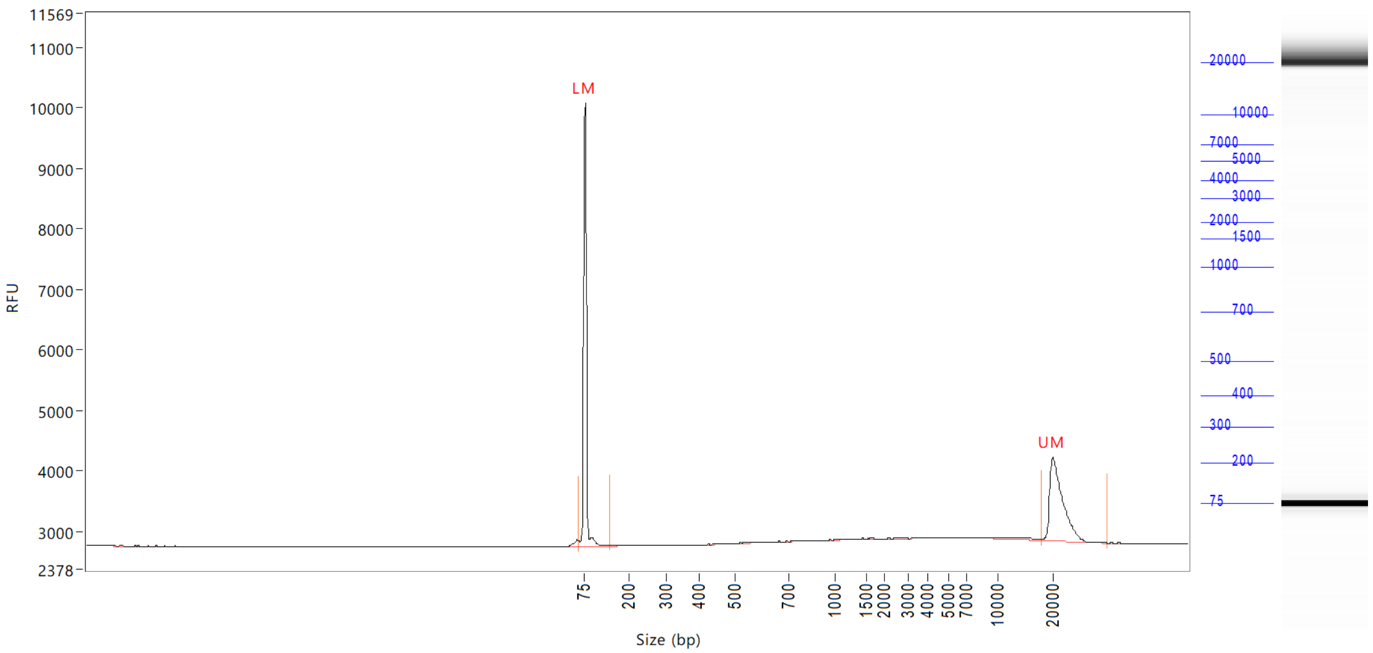

Peak	Size	Concentration	Relative concentration	Molarity	From	To	CV%	RFU
	(bp)	(ng/uL)	Percent	(nmole/L)	(bp)	(bp)		
1	75 (LM)	0.5000		11.2364	60	147	9.50	7346
2	20000 (UM)	0.2355		0.0184	17927	29914	6.77	1381

TIC:	0.0000	ng/uL
TIM:	0.0000	nmole/L
Total concentration:	0.5491	ng/uL

Sample peak width (sec): 5   Sample min peak height: 50   Sample baseline V to V?: Y   Sample baseline V to V points: 3
Sample filter: Binomial   Number of points for filter: 3   Sample start region (min): 0   Sample end region (min): 30
Marker peak width (sec): 5   Marker min peak height: 200   Marker baseline V to V?: Y   Marker baseline V to V points: 3
Lower marker selection: First peak > 500 RFU   Upper marker selection: Last peak > 200 RFU
Ladder size (bp)75, 200, 300, 400, 500, 700, 1000, 1500, 2000, 3000, 4000, 5000, 7000, 10000, 20000
Quantification using: Lower Marker   Final concentration (ng/uL): 0.5000   Dilution factor: 12.0

Sample: SampH5
Well location: H5
Created: Thursday, October 21, 2021 9:39:00 PM

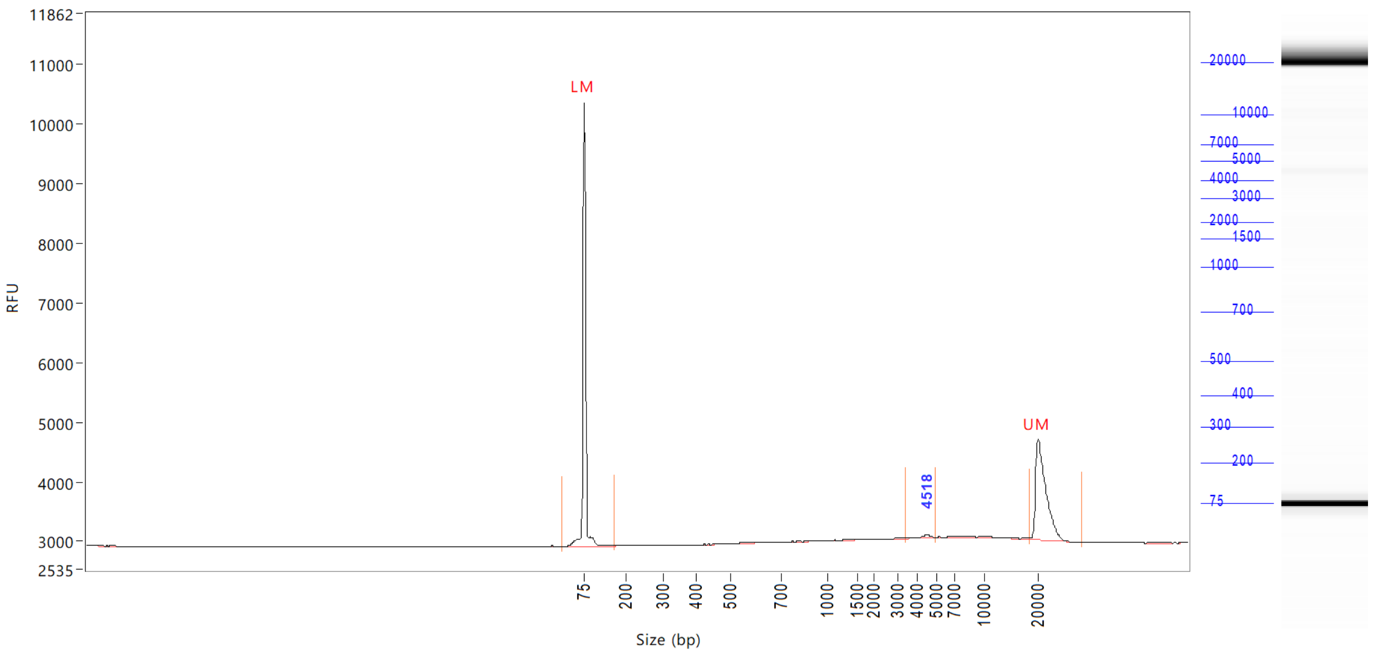

Peak	Size	Concentration	Relative concentration	Molarity	From	To	CV%	RFU
	(bp)	(ng/uL)	Percent	(nmole/L)	(bp)	(bp)		
1	75 (LM)	0.5000		11.2364	9	162	13.96	7452
2	4518	0.1144	100.0	0.0442	3358	4966	9.23	60
3	20000 (UM)	0.2285		0.0181	18537	28221	5.53	1699

TIC:	0.1144	ng/uL
TIM:	0.0442	nmole/L
Total concentration:	0.6938	ng/uL

```
Sample peak width (sec): 5    Sample min peak height: 50    Sample baseline V to V?: Y    Sample baseline V to V points: 3
Sample filter: Binomial       Number of points for filter: 3  Sample start region (min): 0  Sample end region (min): 30
Marker peak width (sec): 5    Marker min peak height: 200   Marker baseline V to V?: Y    Marker baseline V to V points: 3
Lower marker selection: First peak > 500 RFU                Upper marker selection: Last peak > 200 RFU
Ladder size (bp)75, 200, 300, 400, 500, 700, 1000, 1500, 2000, 3000, 4000, 5000, 7000, 10000, 20000
Quantification using: Lower Marker          Final concentration (ng/uL): 0.5000          Dilution factor: 12.0
```

Sample: SampA6
Well location: A6
Created: Thursday, October 21, 2021 9:39:00 PM

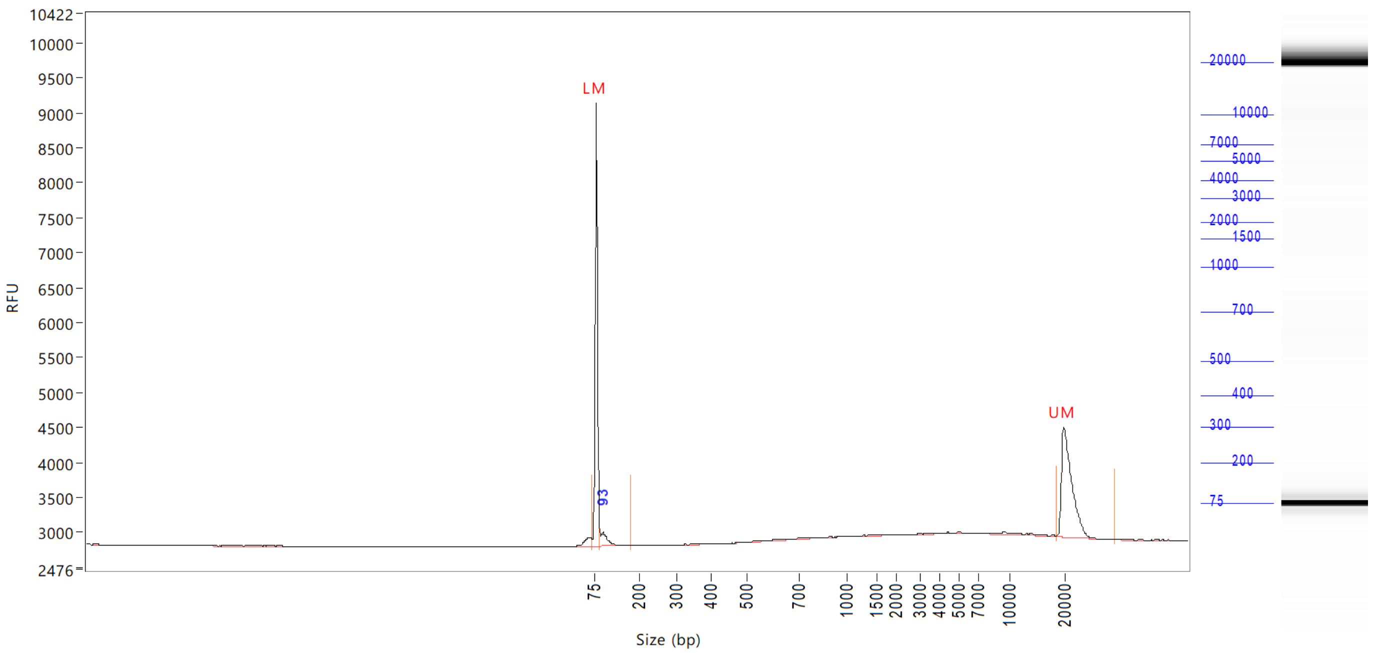

Peak	Size	Concentration	Relative concentration	Molarity	From	To	CV%	RFU
	(bp)	(ng/uL)	Percent	(nmole/L)	(bp)	(bp)		
1	75 (LM)	0.5000		11.0851	66	86	4.31	6345
2	93	0.5392	100.0	8.5967	86	175	16.80	192
3	20000 (UM)	0.2752		0.0219	18537	29068	5.59	1563

TIC:	0.5392	ng/uL
TIM:	8.5967	nmole/L
Total concentration:	1.3697	ng/uL

```
Sample peak width (sec): 5    Sample min peak height: 50    Sample baseline V to V?: Y    Sample baseline V to V points: 3
Sample filter: Binomial       Number of points for filter: 3  Sample start region (min): 0  Sample end region (min): 30
Marker peak width (sec): 5    Marker min peak height: 200    Marker baseline V to V?: Y    Marker baseline V to V points: 3
Lower marker selection: First peak > 500 RFU                 Upper marker selection: Last peak > 200 RFU
Ladder size (bp)75, 200, 300, 400, 500, 700, 1000, 1500, 2000, 3000, 4000, 5000, 7000, 10000, 20000
Quantification using: Lower Marker          Final concentration (ng/uL): 0.5000          Dilution factor: 12.0
```

Sample: SampB6
Well location: B6
Created: Thursday, October 21, 2021 9:39:00 PM

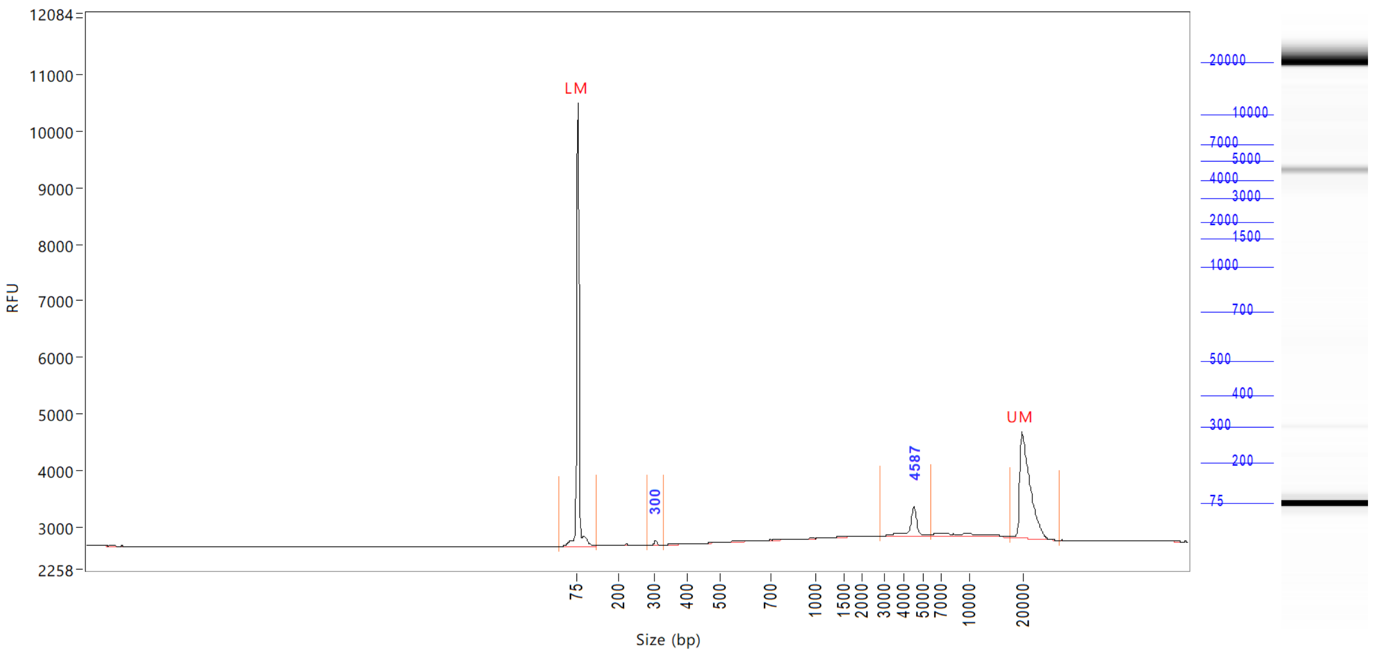

Peak	Size	Concentration	Relative concentration	Molarity	From	To	CV%	RFU
	(bp)	(ng/uL)	Percent	(nmole/L)	(bp)	(bp)		
1	75 (LM)	0.5000		11.2364	22	133	10.71	7850
2	300	0.0645	8.8	0.3560	280	325	2.01	79
3	4587	0.6713	91.2	0.2505	2800	5963	10.28	512
4	20000 (UM)	0.2577		0.0204	17805	27133	5.57	1888

TIC:	0.7358	ng/uL
TIM:	0.6065	nmole/L
Total concentration:	1.3866	ng/uL

```
Sample peak width (sec): 5    Sample min peak height: 50    Sample baseline V to V?: Y    Sample baseline V to V points: 3
Sample filter: Binomial       Number of points for filter: 3  Sample start region (min): 0  Sample end region (min): 30
Marker peak width (sec): 5    Marker min peak height: 200   Marker baseline V to V?: Y    Marker baseline V to V points: 3
Lower marker selection: First peak > 500 RFU                Upper marker selection: Last peak > 200 RFU
Ladder size (bp)75, 200, 300, 400, 500, 700, 1000, 1500, 2000, 3000, 4000, 5000, 7000, 10000, 20000
Quantification using: Lower Marker          Final concentration (ng/uL): 0.5000          Dilution factor: 12.0
```

Sample: SampC6
Well location: C6
Created: Thursday, October 21, 2021 9:39:00 PM

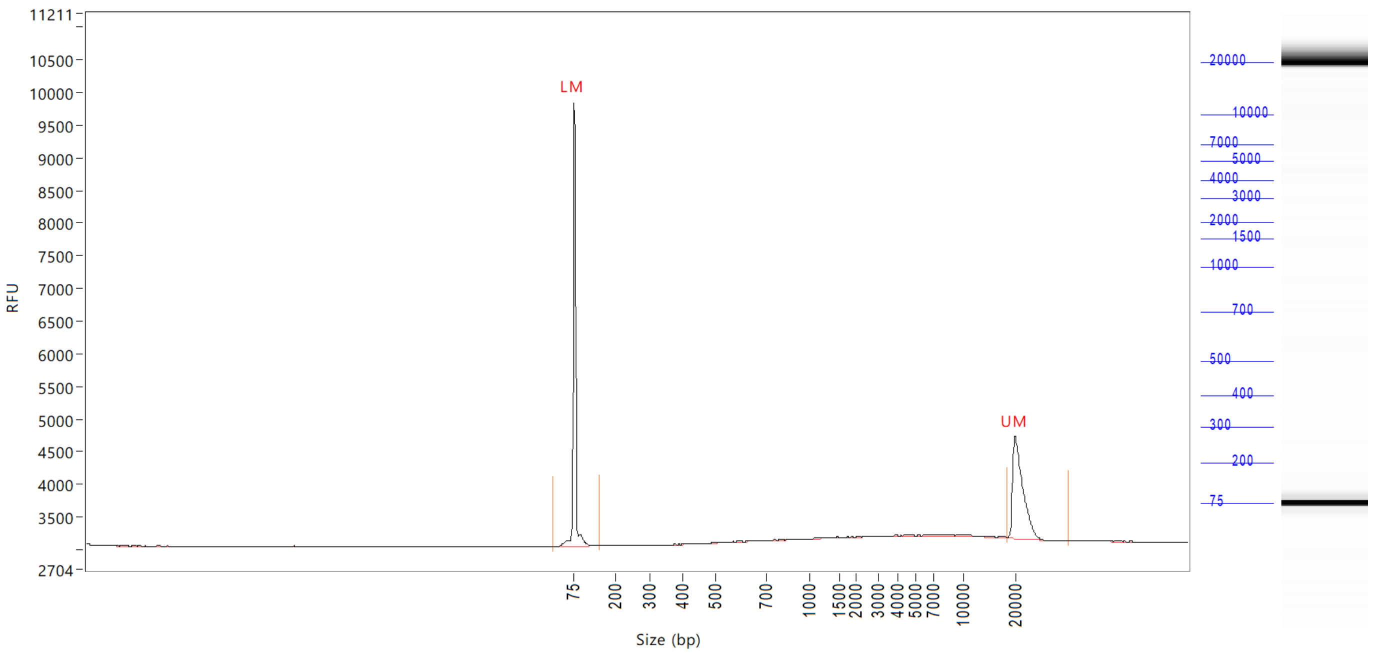

Peak	Size	Concentration	Relative concentration	Molarity	From	To	CV%	RFU
	(bp)	(ng/uL)	Percent	(nmole/L)	(bp)	(bp)		
1	75 (LM)	0.5000		11.0851	14	150	11.92	6796
2	20000 (UM)	0.2406		0.0191	18415	30156	5.84	1575

TIC:	0.0000	ng/uL
TIM:	0.0000	nmole/L
Total concentration:	0.5896	ng/uL

Sample peak width (sec): 5 Sample min peak height: 50 Sample baseline V to V?: Y Sample baseline V to V points: 3
Sample filter: Binomial Number of points for filter: 3 Sample start region (min): 0 Sample end region (min): 30
Marker peak width (sec): 5 Marker min peak height: 200 Marker baseline V to V?: Y Marker baseline V to V points: 3
Lower marker selection: First peak > 500 RFU Upper marker selection: Last peak > 200 RFU
Ladder size (bp)75, 200, 300, 400, 500, 700, 1000, 1500, 2000, 3000, 4000, 5000, 7000, 10000, 20000
Quantification using: Lower Marker Final concentration (ng/uL): 0.5000 Dilution factor: 12.0

Sample: SampD6
Well location: D6
Created: Thursday, October 21, 2021 9:39:00 PM

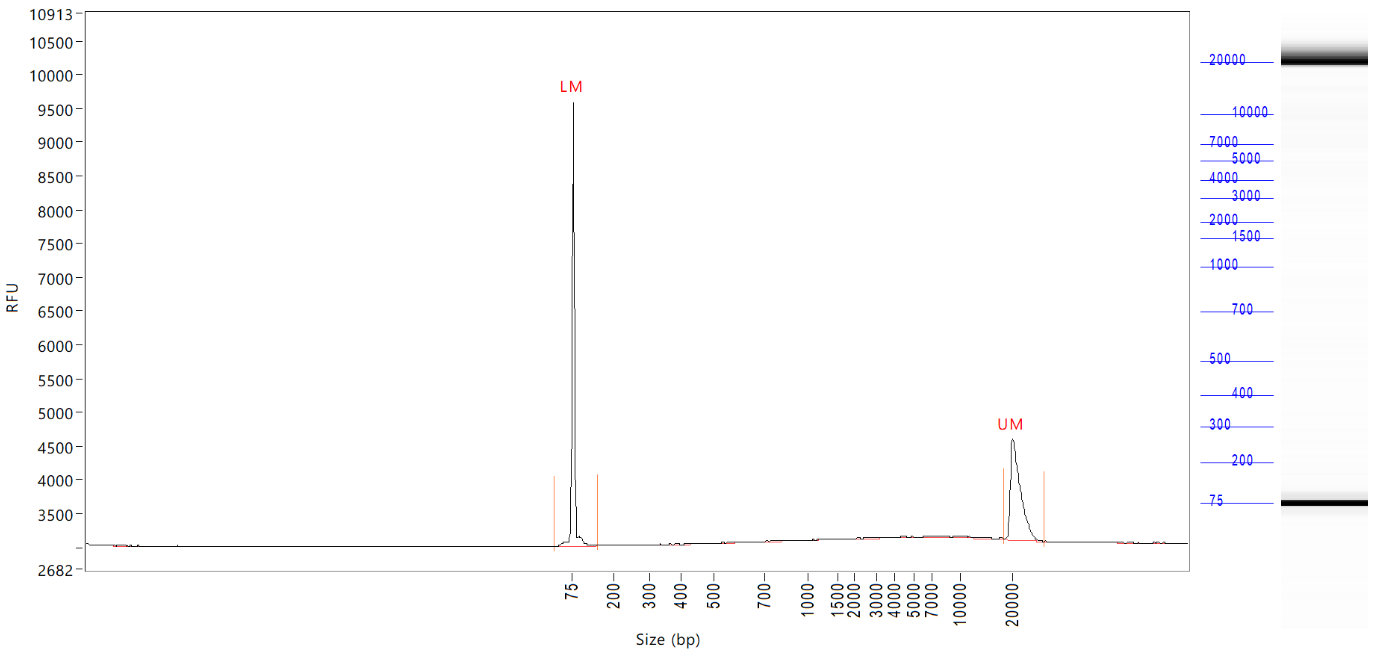

Peak	Size	Concentration	Relative concentration	Molarity	From	To	CV%	RFU
	(bp)	(ng/uL)	Percent	(nmole/L)	(bp)	(bp)		
1	75 (LM)	0.5000		11.2364	18	150	12.25	6577
2	20000 (UM)	0.2370		0.0187	18415	26045	5.36	1507

TIC:	0.0000	ng/uL
TIM:	0.0000	nmole/L
Total concentration:	0.7363	ng/uL

Sample peak width (sec): 5   Sample min peak height: 50   Sample baseline V to V?: Y   Sample baseline V to V points: 3
Sample filter: Binomial   Number of points for filter: 3 Sample start region (min): 0 Sample end region (min): 30
Marker peak width (sec): 5   Marker min peak height: 200   Marker baseline V to V?: Y   Marker baseline V to V points: 3
Lower marker selection: First peak > 500 RFU   Upper marker selection: Last peak > 200 RFU
Ladder size (bp)75, 200, 300, 400, 500, 700, 1000, 1500, 2000, 3000, 4000, 5000, 7000, 10000, 20000
Quantification using: Lower Marker   Final concentration (ng/uL): 0.5000   Dilution factor: 12.0

Sample: SampE6
Well location: E6
Created: Thursday, October 21, 2021 9:39:00 PM

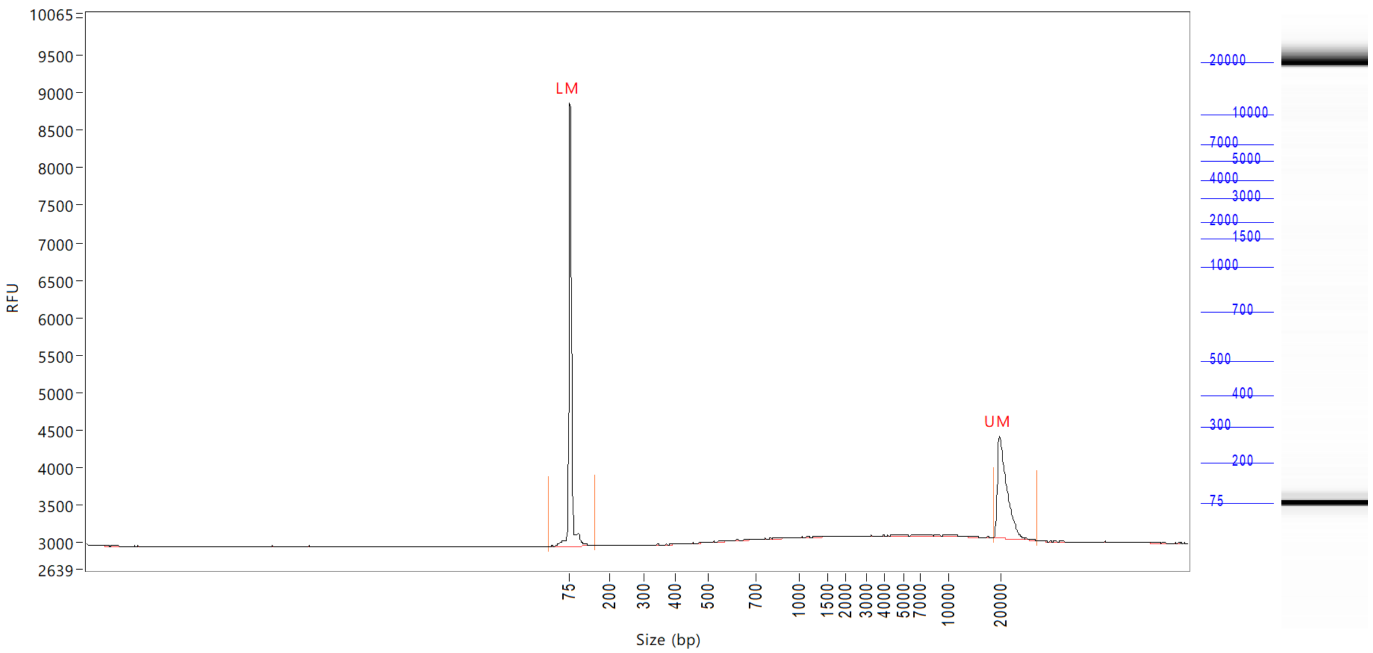

Peak	Size	Concentration	Relative concentration	Molarity	From	To	CV%	RFU
	(bp)	(ng/uL)	Percent	(nmole/L)	(bp)	(bp)		
1	75 (LM)	0.5000		10.9378	11	152	13.56	5931
2	20000 (UM)	0.2144		0.0170	18659	27375	5.60	1355

TIC:	0.0000	ng/uL
TIM:	0.0000	nmole/L
Total concentration:	0.5442	ng/uL

```
Sample peak width (sec): 5    Sample min peak height: 50    Sample baseline V to V?: Y    Sample baseline V to V points: 3
Sample filter: Binomial       Number of points for filter: 3  Sample start region (min): 0  Sample end region (min): 30
Marker peak width (sec): 5    Marker min peak height: 200   Marker baseline V to V?: Y    Marker baseline V to V points: 3
Lower marker selection: First peak > 500 RFU                Upper marker selection: Last peak > 200 RFU
Ladder size (bp)75, 200, 300, 400, 500, 700, 1000, 1500, 2000, 3000, 4000, 5000, 7000, 10000, 20000
Quantification using: Lower Marker          Final concentration (ng/uL): 0.5000          Dilution factor: 12.0
```

Sample: SampF6
Well location: F6
Created: Thursday, October 21, 2021 9:39:00 PM

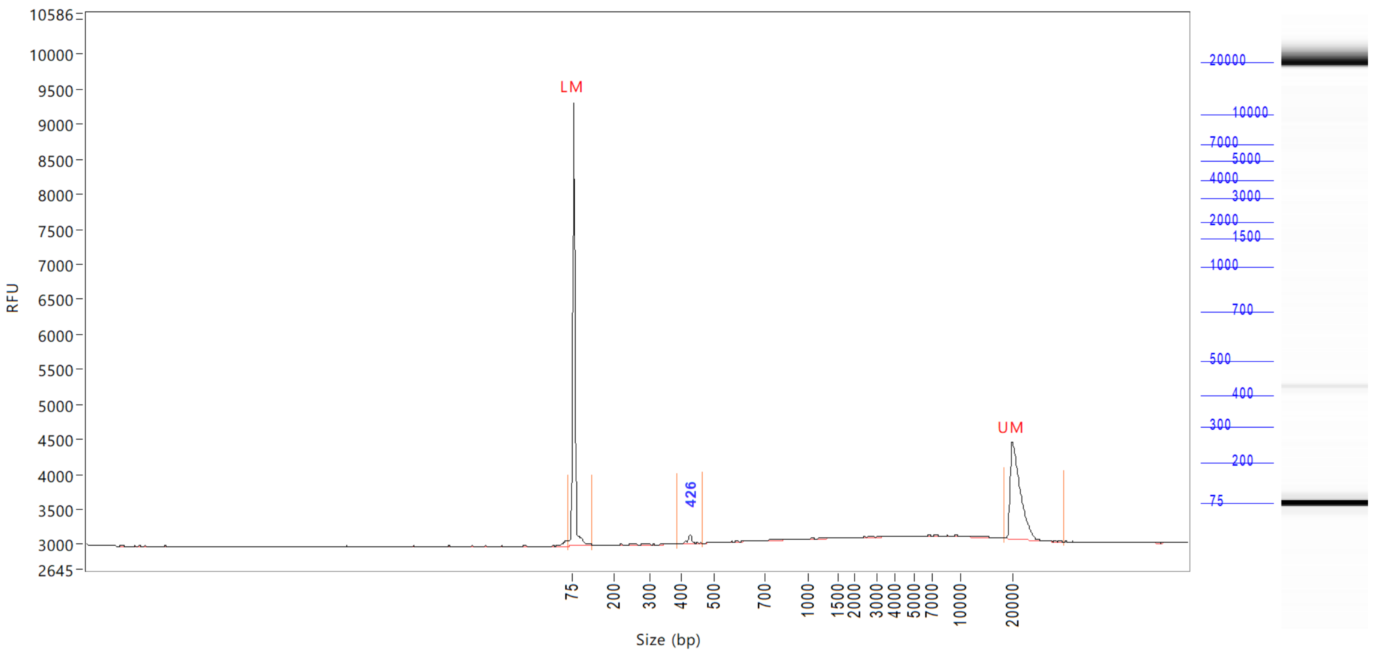

Peak	Size	Concentration	Relative concentration	Molarity	From	To	CV%	RFU
	(bp)	(ng/uL)	Percent	(nmole/L)	(bp)	(bp)		
1	75 (LM)	0.5000		11.0851	58	133	8.78	6338
2	426	0.1753	100.0	0.6851	386	462	2.31	136
3	20000 (UM)	0.2336		0.0185	18293	29793	5.69	1383

TIC:	0.1753	ng/uL
TIM:	0.6851	nmole/L
Total concentration:	0.7163	ng/uL

```
Sample peak width (sec): 5    Sample min peak height: 50    Sample baseline V to V?: Y    Sample baseline V to V points: 3
Sample filter: Binomial       Number of points for filter: 3  Sample start region (min): 0  Sample end region (min): 30
Marker peak width (sec): 5    Marker min peak height: 200   Marker baseline V to V?: Y    Marker baseline V to V points: 3
Lower marker selection: First peak > 500 RFU                Upper marker selection: Last peak > 200 RFU
Ladder size (bp)75, 200, 300, 400, 500, 700, 1000, 1500, 2000, 3000, 4000, 5000, 7000, 10000, 20000
Quantification using: Lower Marker          Final concentration (ng/uL): 0.5000          Dilution factor: 12.0
```

Sample: SampG6
Well location: G6
Created: Thursday, October 21, 2021 9:39:00 PM

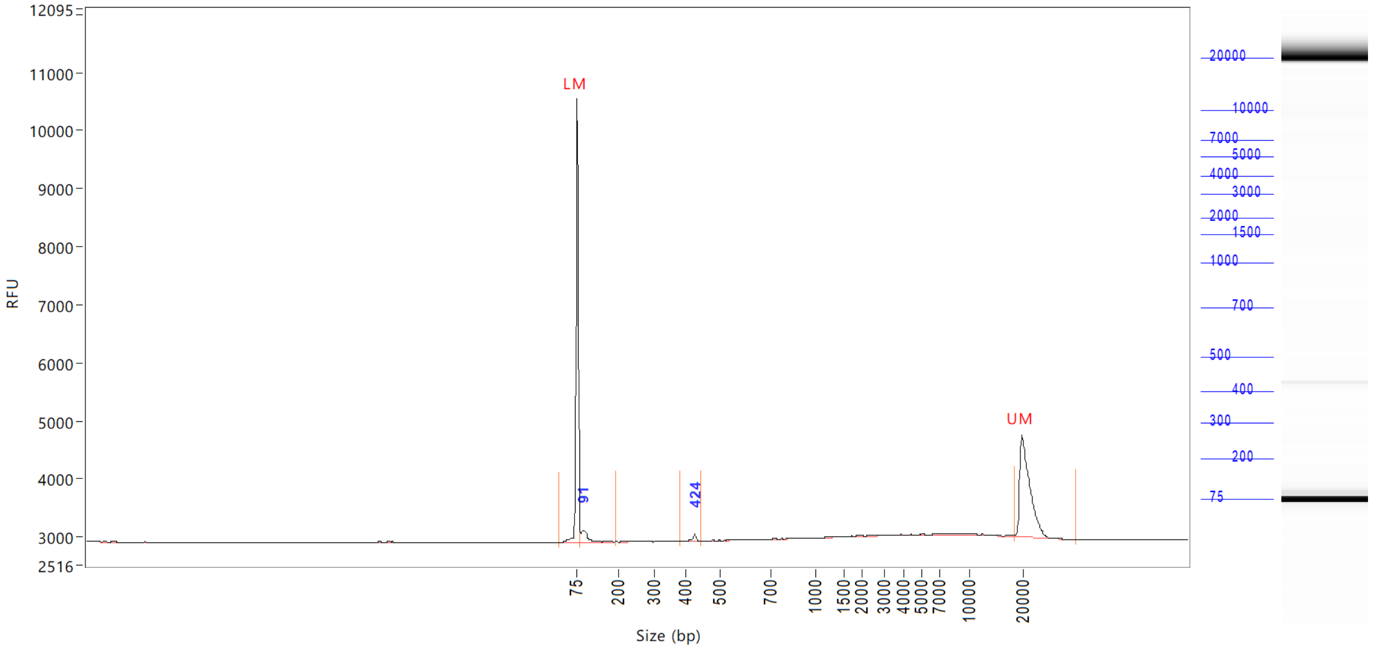

Peak	Size	Concentration	Relative concentration	Molarity	From	To	CV%	RFU
	(bp)	(ng/uL)	Percent	(nmole/L)	(bp)	(bp)		
1	75 (LM)	0.5000		11.3919	20	86	7.12	7656
2	91	0.4619	78.9	7.3645	86	190	19.69	210
3	424	0.1237	21.1	0.4869	380	445	2.07	117
4	20000 (UM)	0.2553		0.0202	18537	30277	5.73	1743

TIC:	0.5856	ng/uL
TIM:	7.8514	nmole/L
Total concentration:	1.0881	ng/uL

Sample peak width (sec): 5   Sample min peak height: 50   Sample baseline V to V?: Y   Sample baseline V to V points: 3
Sample filter: Binomial   Number of points for filter: 3 Sample start region (min): 0 Sample end region (min): 30
Marker peak width (sec): 5   Marker min peak height: 200   Marker baseline V to V?: Y   Marker baseline V to V points: 3
Lower marker selection: First peak > 500 RFU   Upper marker selection: Last peak > 200 RFU
Ladder size (bp)75, 200, 300, 400, 500, 700, 1000, 1500, 2000, 3000, 4000, 5000, 7000, 10000, 20000
Quantification using: Lower Marker   Final concentration (ng/uL): 0.5000   Dilution factor: 12.0

Sample: SampH6
Well location: H6
Created: Thursday, October 21, 2021 9:39:00 PM

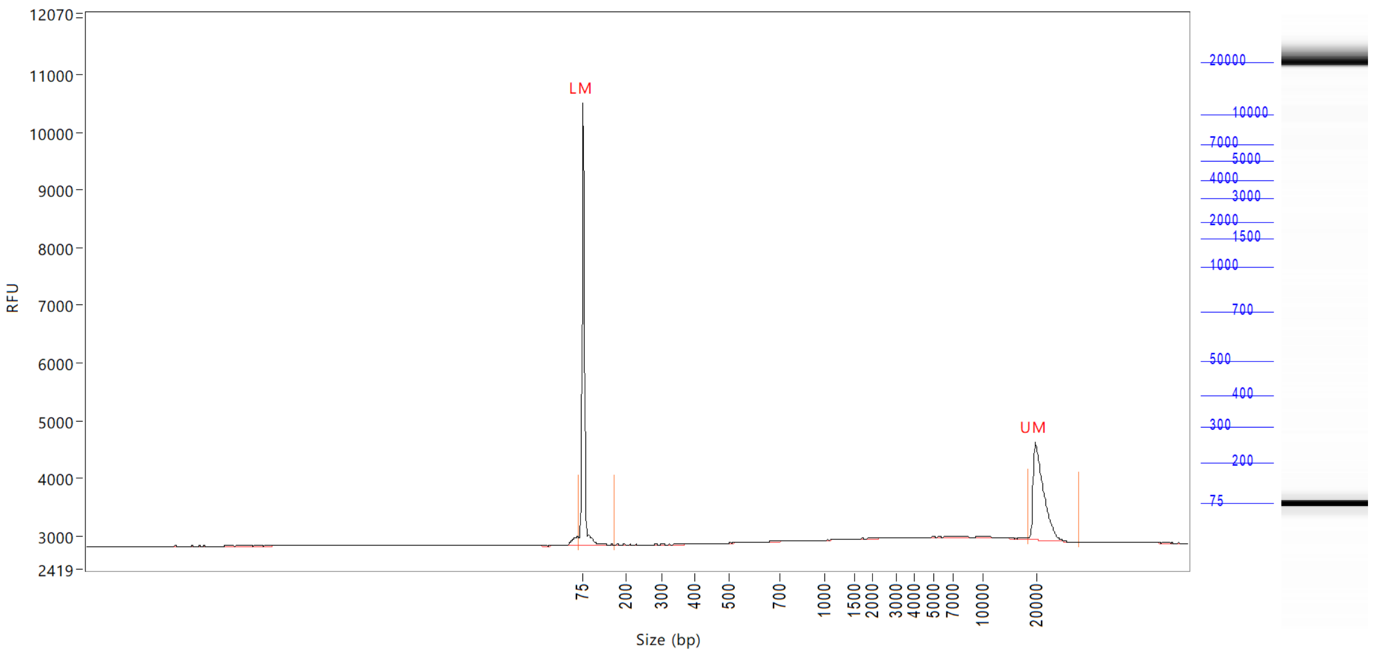

Peak	Size	Concentration	Relative concentration	Molarity	From	To	CV%	RFU
	(bp)	(ng/uL)	Percent	(nmole/L)	(bp)	(bp)		
1	75 (LM)	0.5000		11.0851	62	166	11.02	7696
2	20000 (UM)	0.2380		0.0188	18659	28221	5.54	1688

TIC:	0.0000	ng/uL
TIM:	0.0000	nmole/L
Total concentration:	0.4871	ng/uL

Sample peak width (sec): 5   Sample min peak height: 50   Sample baseline V to V?: Y   Sample baseline V to V points: 3
Sample filter: Binomial   Number of points for filter: 3   Sample start region (min): 0   Sample end region (min): 30
Marker peak width (sec): 5   Marker min peak height: 200   Marker baseline V to V?: Y   Marker baseline V to V points: 3
Lower marker selection: First peak > 500 RFU   Upper marker selection: Last peak > 200 RFU
Ladder size (bp)75, 200, 300, 400, 500, 700, 1000, 1500, 2000, 3000, 4000, 5000, 7000, 10000, 20000
Quantification using: Lower Marker   Final concentration (ng/uL): 0.5000   Dilution factor: 12.0

Sample: SampA7
Well location: A7
Created: Thursday, October 21, 2021 9:39:00 PM

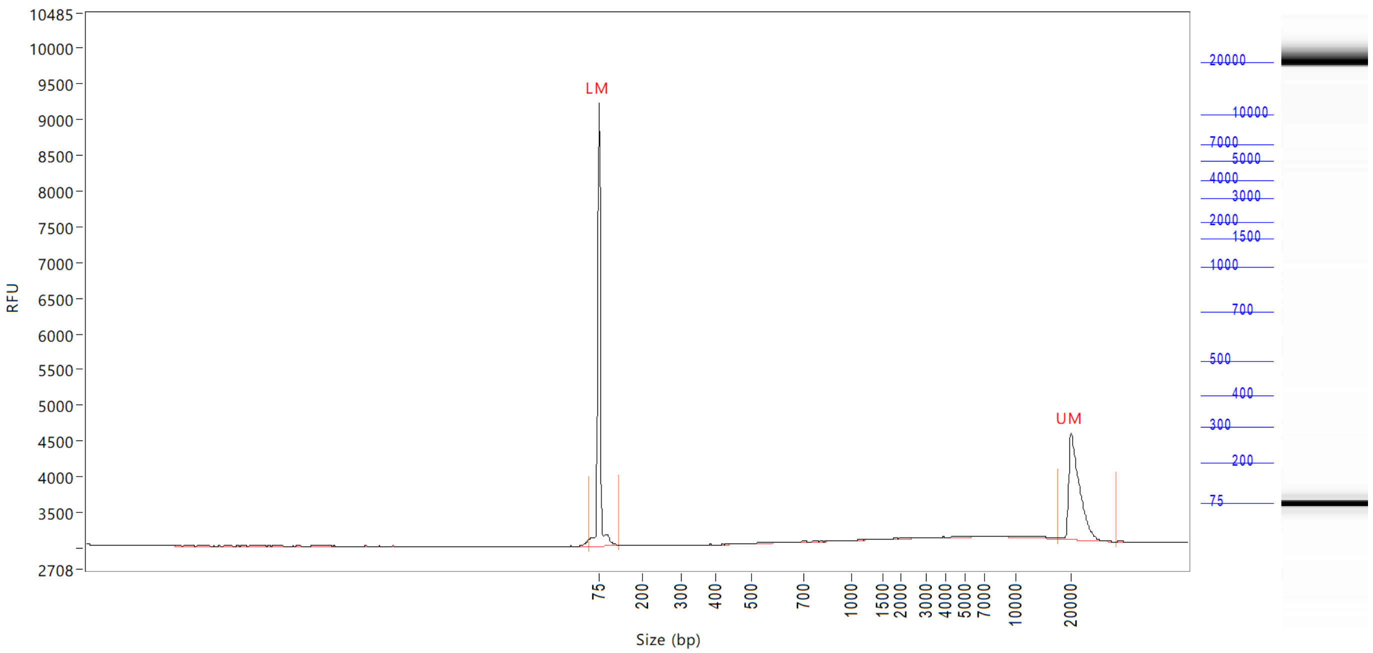

Peak	Size	Concentration	Relative concentration	Molarity	From	To	CV%	RFU
	(bp)	(ng/uL)	Percent	(nmole/L)	(bp)	(bp)		
1	75 (LM)	0.5000		11.2364	47	131	10.70	6209
2	20000 (UM)	0.2508		0.0198	17805	28100	5.78	1492

TIC:	0.0000	ng/uL
TIM:	0.0000	nmole/L
Total concentration:	0.6344	ng/uL

Sample peak width (sec): 5   Sample min peak height: 50   Sample baseline V to V?: Y   Sample baseline V to V points: 3
Sample filter: Binomial   Number of points for filter: 3 Sample start region (min): 0 Sample end region (min): 30
Marker peak width (sec): 5   Marker min peak height: 200   Marker baseline V to V?: Y   Marker baseline V to V points: 3
Lower marker selection: First peak > 500 RFU   Upper marker selection: Last peak > 200 RFU
Ladder size (bp)75, 200, 300, 400, 500, 700, 1000, 1500, 2000, 3000, 4000, 5000, 7000, 10000, 20000
Quantification using: Lower Marker   Final concentration (ng/uL): 0.5000   Dilution factor: 12.0

Sample: SampB7
Well location: B7
Created: Thursday, October 21, 2021 9:39:00 PM

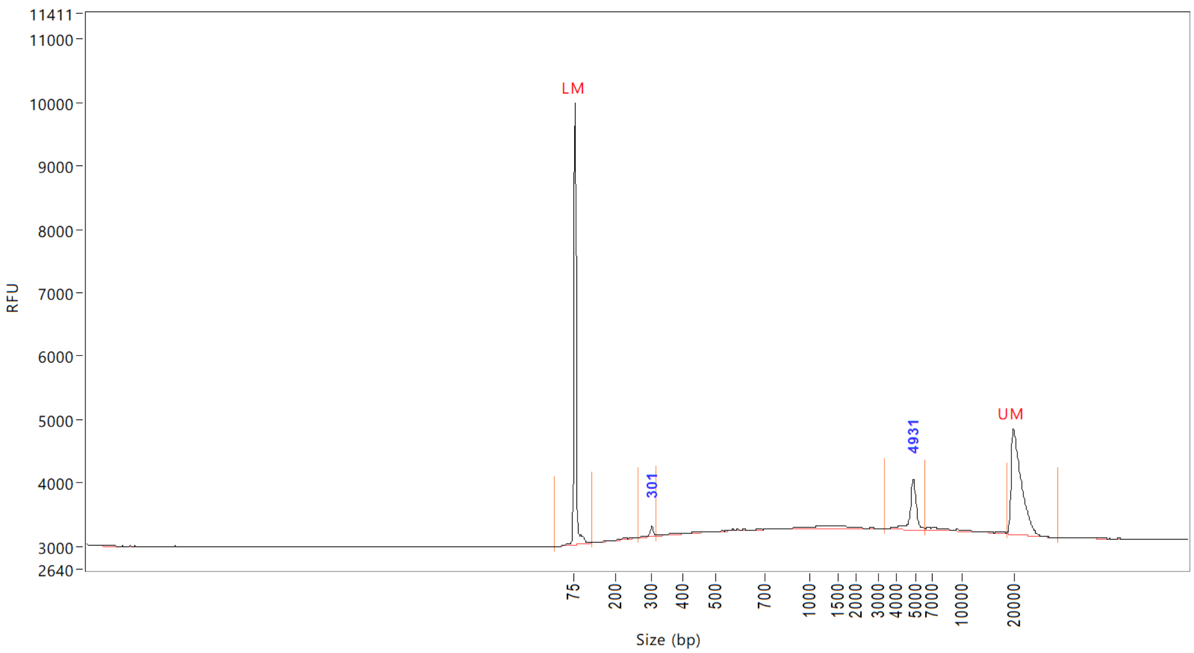

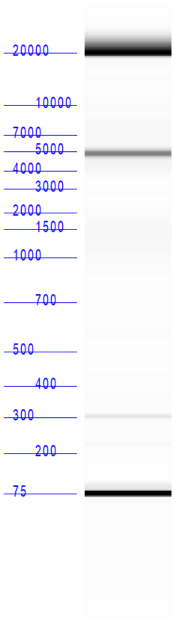

Peak	Size	Concentration	Relative concentration	Molarity	From	To	CV%	RFU
	(bp)	(ng/uL)	Percent	(nmole/L)	(bp)	(bp)		
1	75 (LM)	0.5000		11.0851	14	126	8.11	6982
2	301	0.1989	15.5	1.1053	264	317	3.51	177
3	4931	1.0873	84.5	0.3713	3393	6112	7.57	794
4	20000 (UM)	0.2614		0.0206	18659	28705	5.58	1657

TIC:	1.2862	ng/uL
TIM:	1.4766	nmole/L
Total concentration:	2.4813	ng/uL

Sample peak width (sec): 5   Sample min peak height: 50   Sample baseline V to V?: Y   Sample baseline V to V points: 3
Sample filter: Binomial   Number of points for filter: 3 Sample start region (min): 0 Sample end region (min): 30
Marker peak width (sec): 5   Marker min peak height: 200   Marker baseline V to V?: Y   Marker baseline V to V points: 3
Lower marker selection: First peak > 500 RFU   Upper marker selection: Last peak > 200 RFU
Ladder size (bp)75, 200, 300, 400, 500, 700, 1000, 1500, 2000, 3000, 4000, 5000, 7000, 10000, 20000
Quantification using: Lower Marker   Final concentration (ng/uL): 0.5000   Dilution factor: 12.0

Sample: SampC7
Well location: C7
Created: Thursday, October 21, 2021 9:39:00 PM

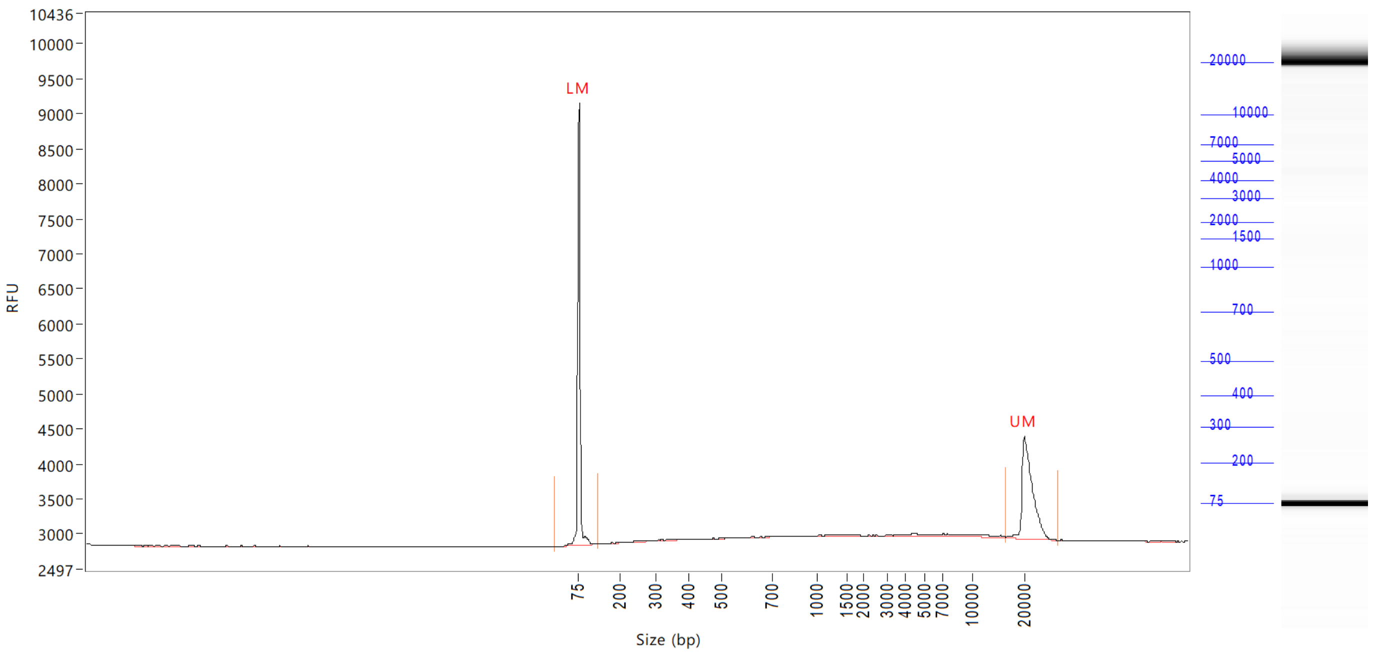

Peak	Size	Concentration	Relative concentration	Molarity	From	To	CV%	RFU
	(bp)	(ng/uL)	Percent	(nmole/L)	(bp)	(bp)		
1	75 (LM)	0.5000		11.2364	3	131	9.08	6327
2	20000 (UM)	0.2499		0.0199	16464	26408	5.82	1462

TIC:	0.0000	ng/uL
TIM:	0.0000	nmole/L
Total concentration:	0.8920	ng/uL

```
Sample peak width (sec): 5    Sample min peak height: 50    Sample baseline V to V?: Y    Sample baseline V to V points: 3
Sample filter: Binomial       Number of points for filter: 3  Sample start region (min): 0  Sample end region (min): 30
Marker peak width (sec): 5    Marker min peak height: 200   Marker baseline V to V?: Y    Marker baseline V to V points: 3
Lower marker selection: First peak > 500 RFU                Upper marker selection: Last peak > 200 RFU
Ladder size (bp)75, 200, 300, 400, 500, 700, 1000, 1500, 2000, 3000, 4000, 5000, 7000, 10000, 20000
Quantification using: Lower Marker          Final concentration (ng/uL): 0.5000          Dilution factor: 12.0
```

Sample: SampD7
Well location: D7
Created: Thursday, October 21, 2021 9:39:00 PM

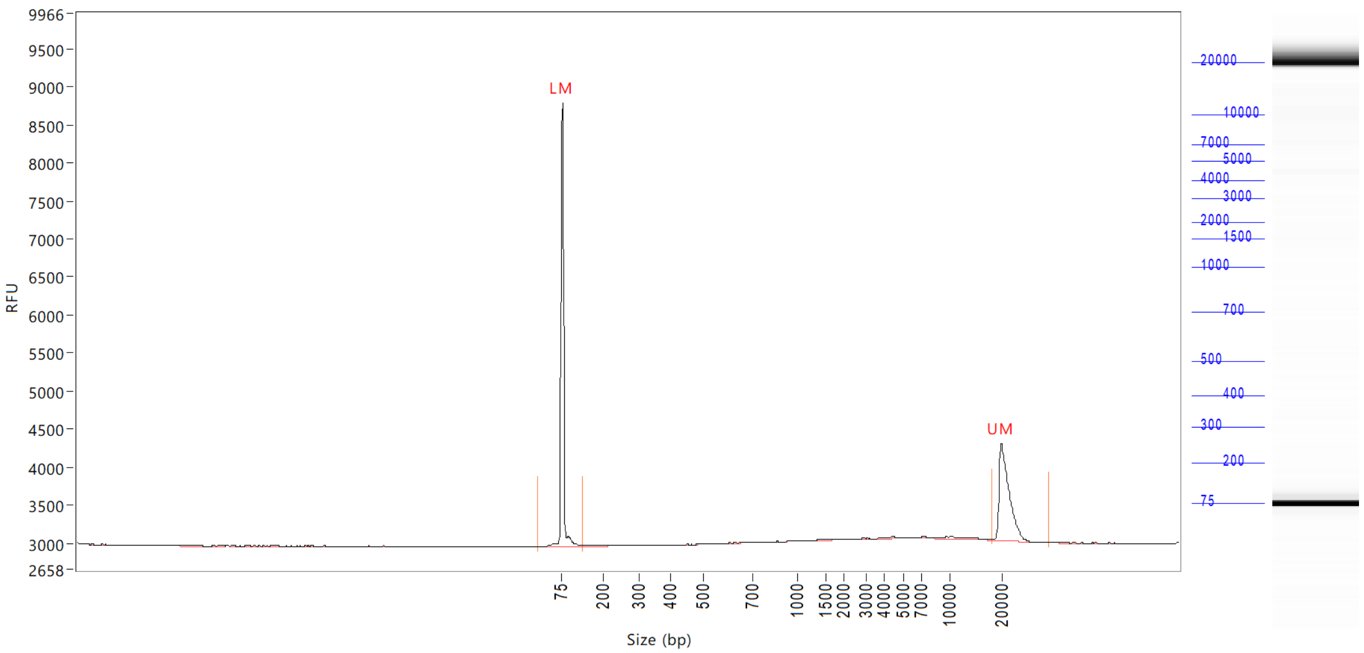

Peak	Size	Concentration	Relative concentration	Molarity	From	To	CV%	RFU
	(bp)	(ng/uL)	Percent	(nmole/L)	(bp)	(bp)		
1	75 (LM)	0.5000		11.2364	3	137	11.47	5841
2	20000 (UM)	0.2274		0.0181	18049	29189	5.66	1284

TIC:	0.0000	ng/uL
TIM:	0.0000	nmole/L
Total concentration:	0.6455	ng/uL

Sample peak width (sec): 5 Sample min peak height: 50 Sample baseline V to V?: Y Sample baseline V to V points: 3
Sample filter: Binomial Number of points for filter: 3 Sample start region (min): 0 Sample end region (min): 30
Marker peak width (sec): 5 Marker min peak height: 200 Marker baseline V to V?: Y Marker baseline V to V points: 3
Lower marker selection: First peak > 500 RFU Upper marker selection: Last peak > 200 RFU
Ladder size (bp)75, 200, 300, 400, 500, 700, 1000, 1500, 2000, 3000, 4000, 5000, 7000, 10000, 20000
Quantification using: Lower Marker Final concentration (ng/uL): 0.5000 Dilution factor: 12.0

Sample: SampE7
Well location: E7
Created: Thursday, October 21, 2021 9:39:00 PM

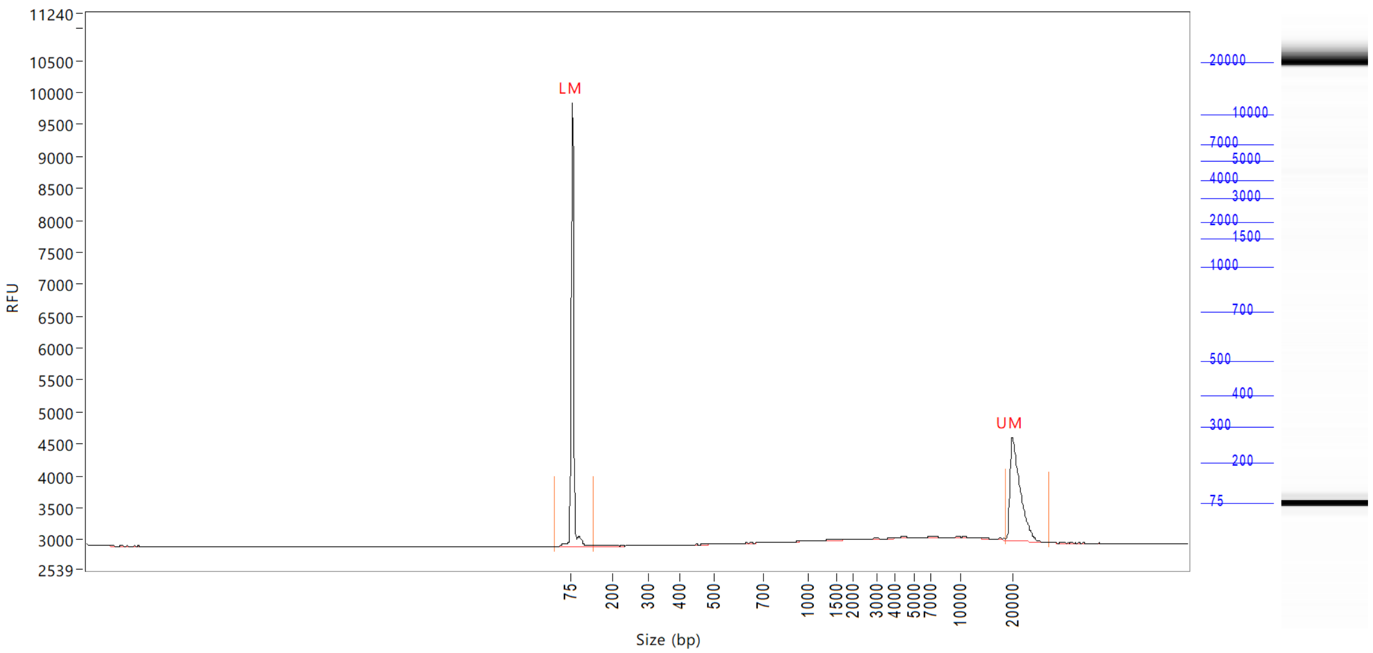

Peak	Size	Concentration	Relative concentration	Molarity	From	To	CV%	RFU
	(bp)	(ng/uL)	Percent	(nmole/L)	(bp)	(bp)		
1	75 (LM)	0.5000		11.0851	26	141	10.34	6955
2	20000 (UM)	0.2489		0.0197	18659	27133	5.52	1618

TIC:	0.0000	ng/uL
TIM:	0.0000	nmole/L
Total concentration:	0.5561	ng/uL

Sample peak width (sec): 5 Sample min peak height: 50 Sample baseline V to V?: Y Sample baseline V to V points: 3
Sample filter: Binomial Number of points for filter: 3 Sample start region (min): 0 Sample end region (min): 30
Marker peak width (sec): 5 Marker min peak height: 200 Marker baseline V to V?: Y Marker baseline V to V points: 3
Lower marker selection: First peak > 500 RFU Upper marker selection: Last peak > 200 RFU
Ladder size (bp)75, 200, 300, 400, 500, 700, 1000, 1500, 2000, 3000, 4000, 5000, 7000, 10000, 20000
Quantification using: Lower Marker Final concentration (ng/uL): 0.5000 Dilution factor: 12.0

Sample: SampF7
Well location: F7
Created: Thursday, October 21, 2021 9:39:00 PM

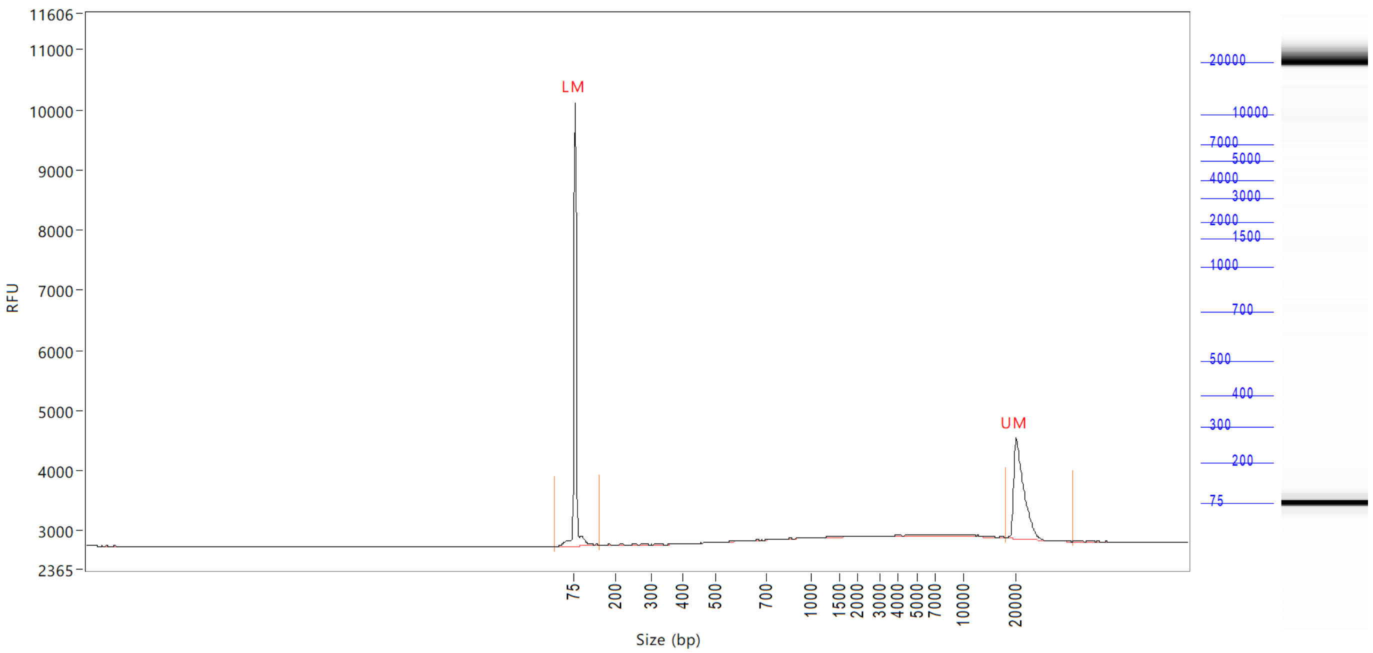

Peak	Size	Concentration	Relative concentration	Molarity	From	To	CV%	RFU
	(bp)	(ng/uL)	Percent	(nmole/L)	(bp)	(bp)		
1	75 (LM)	0.5000		11.0851	14	148	12.54	7379
2	20000 (UM)	0.2310		0.0182	18049	30760	6.01	1684

TIC:	0.0000	ng/uL
TIM:	0.0000	nmole/L
Total concentration:	0.6153	ng/uL

Sample peak width (sec): 5 Sample min peak height: 50 Sample baseline V to V?: Y Sample baseline V to V points: 3
Sample filter: Binomial Number of points for filter: 3 Sample start region (min): 0 Sample end region (min): 30
Marker peak width (sec): 5 Marker min peak height: 200 Marker baseline V to V?: Y Marker baseline V to V points: 3
Lower marker selection: First peak > 500 RFU Upper marker selection: Last peak > 200 RFU
Ladder size (bp)75, 200, 300, 400, 500, 700, 1000, 1500, 2000, 3000, 4000, 5000, 7000, 10000, 20000
Quantification using: Lower Marker Final concentration (ng/uL): 0.5000 Dilution factor: 12.0

Sample: SampG7
Well location: G7
Created: Thursday, October 21, 2021 9:39:00 PM

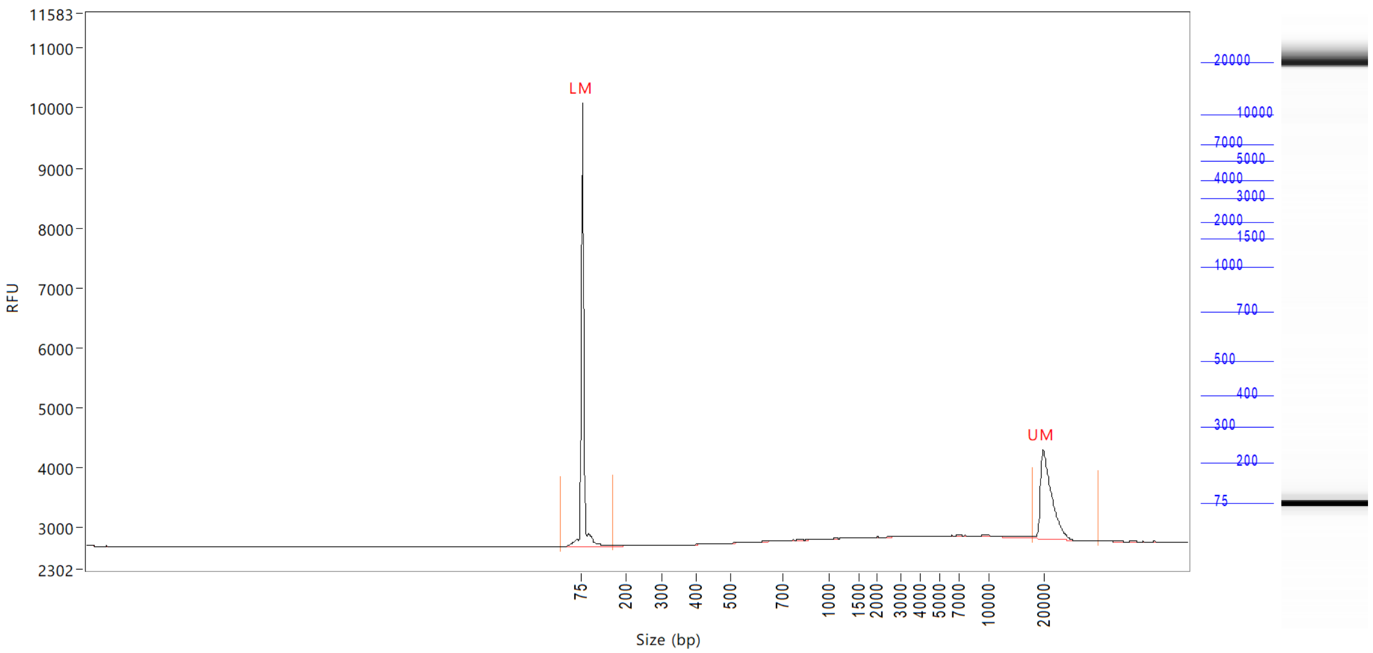

Peak	Size	Concentration	Relative concentration	Molarity	From	To	CV%	RFU
	(bp)	(ng/uL)	Percent	(nmole/L)	(bp)	(bp)		
1	75 (LM)	0.5000		11.0851	14	164	14.50	7418
2	20000 (UM)	0.2169		0.0172	18049	30035	5.88	1488

TIC:	0.0000	ng/uL
TIM:	0.0000	nmole/L
Total concentration:	0.4389	ng/uL

```
Sample peak width (sec): 5    Sample min peak height: 50    Sample baseline V to V?: Y    Sample baseline V to V points: 3
Sample filter: Binomial       Number of points for filter: 3  Sample start region (min): 0  Sample end region (min): 30
Marker peak width (sec): 5    Marker min peak height: 200   Marker baseline V to V?: Y    Marker baseline V to V points: 3
Lower marker selection: First peak > 500 RFU                Upper marker selection: Last peak > 200 RFU
Ladder size (bp)75, 200, 300, 400, 500, 700, 1000, 1500, 2000, 3000, 4000, 5000, 7000, 10000, 20000
Quantification using: Lower Marker          Final concentration (ng/uL): 0.5000          Dilution factor: 12.0
```

Sample: SampH7
Well location: H7
Created: Thursday, October 21, 2021 9:39:00 PM

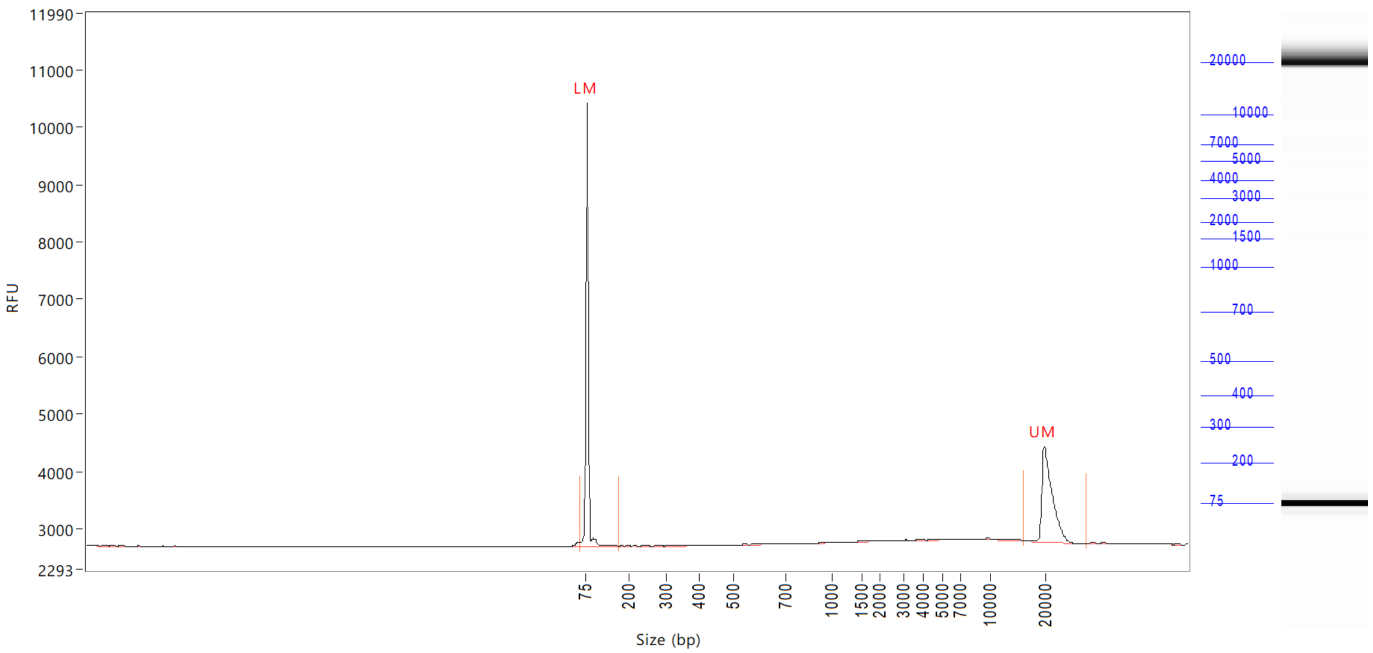

Peak	Size	Concentration	Relative concentration	Molarity	From	To	CV%	RFU
	(bp)	(ng/uL)	Percent	(nmole/L)	(bp)	(bp)		
1	75 (LM)	0.5000		11.0851	56	168	10.15	7751
2	20000 (UM)	0.2410		0.0192	15976	27738	5.77	1677

TIC:	0.0000	ng/uL
TIM:	0.0000	nmole/L
Total concentration:	0.4743	ng/uL

Sample peak width (sec): 5   Sample min peak height: 50   Sample baseline V to V?: Y   Sample baseline V to V points: 3
Sample filter: Binomial   Number of points for filter: 3   Sample start region (min): 0   Sample end region (min): 30
Marker peak width (sec): 5   Marker min peak height: 200   Marker baseline V to V?: Y   Marker baseline V to V points: 3
Lower marker selection: First peak > 500 RFU   Upper marker selection: Last peak > 200 RFU
Ladder size (bp)75, 200, 300, 400, 500, 700, 1000, 1500, 2000, 3000, 4000, 5000, 7000, 10000, 20000
Quantification using: Lower Marker   Final concentration (ng/uL): 0.5000   Dilution factor: 12.0

Sample: SampA8
Well location: A8
Created: Thursday, October 21, 2021 9:39:00 PM

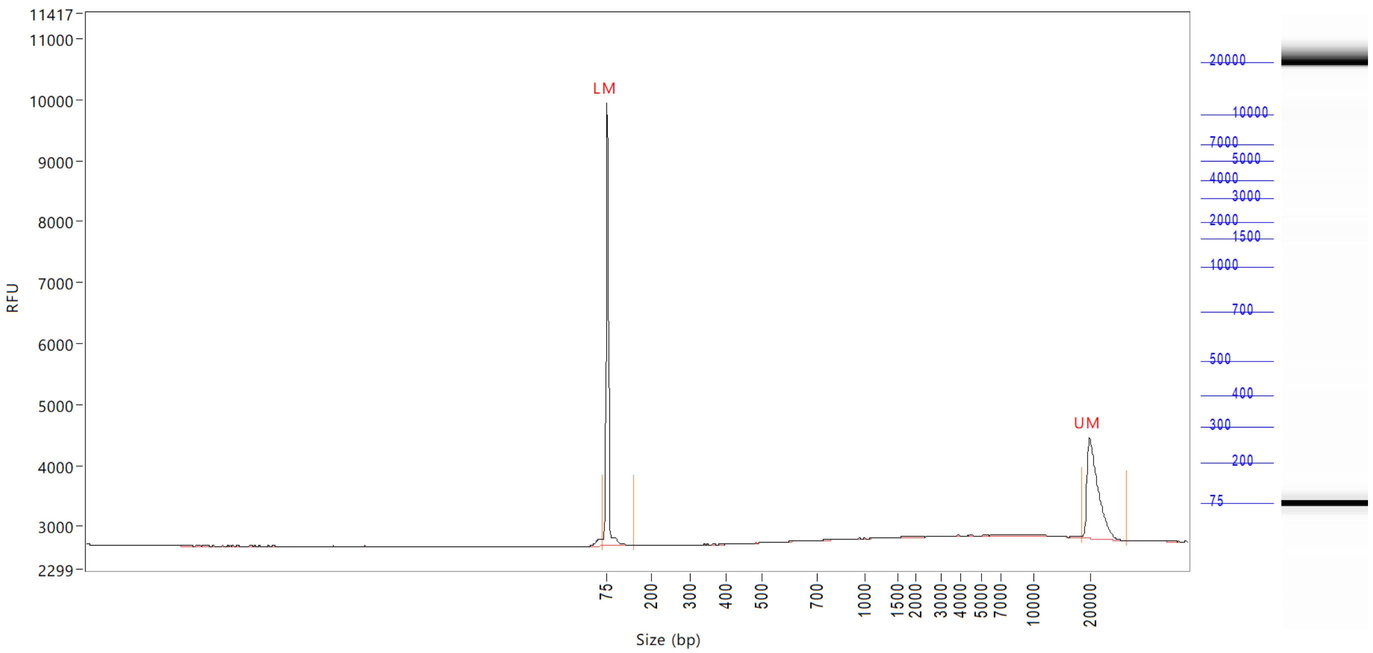

Peak	Size	Concentration	Relative concentration	Molarity	From	To	CV%	RFU
	(bp)	(ng/uL)	Percent	(nmole/L)	(bp)	(bp)		
1	75 (LM)	0.5000		11.0851	64	148	8.48	7277
2	20000 (UM)	0.2569		0.0204	18537	26529	5.40	1662

TIC:	0.0000	ng/uL
TIM:	0.0000	nmole/L
Total concentration:	0.4884	ng/uL

Sample peak width (sec): 5 Sample min peak height: 50 Sample baseline V to V?: Y Sample baseline V to V points: 3
Sample filter: Binomial Number of points for filter: 3 Sample start region (min): 0 Sample end region (min): 30
Marker peak width (sec): 5 Marker min peak height: 200 Marker baseline V to V?: Y Marker baseline V to V points: 3
Lower marker selection: First peak > 500 RFU Upper marker selection: Last peak > 200 RFU
Ladder size (bp)75, 200, 300, 400, 500, 700, 1000, 1500, 2000, 3000, 4000, 5000, 7000, 10000, 20000
Quantification using: Lower Marker Final concentration (ng/uL): 0.5000 Dilution factor: 12.0

Sample: SampB8
Well location: B8
Created: Thursday, October 21, 2021 9:39:00 PM

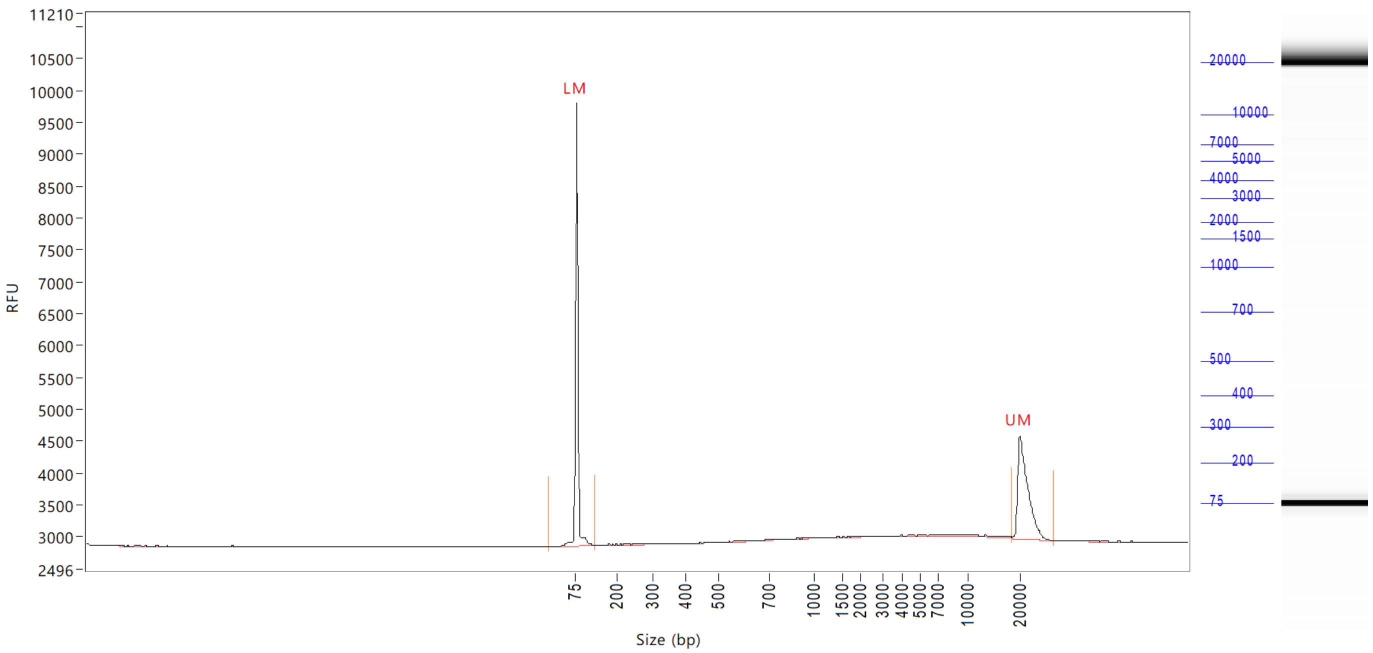

Peak	Size	Concentration	Relative concentration	Molarity	From	To	CV%	RFU
	(bp)	(ng/uL)	Percent	(nmole/L)	(bp)	(bp)		
1	75 (LM)	0.5000		11.2364	0	129	10.56	6958
2	20000 (UM)	0.2477		0.0196	18415	26287	5.53	1617

TIC:	0.0000	ng/uL
TIM:	0.0000	nmole/L
Total concentration:	0.6252	ng/uL

Sample peak width (sec): 5   Sample min peak height: 50   Sample baseline V to V?: Y   Sample baseline V to V points: 3
Sample filter: Binomial   Number of points for filter: 3 Sample start region (min): 0 Sample end region (min): 30
Marker peak width (sec): 5   Marker min peak height: 200   Marker baseline V to V?: Y   Marker baseline V to V points: 3
Lower marker selection: First peak > 500 RFU   Upper marker selection: Last peak > 200 RFU
Ladder size (bp)75, 200, 300, 400, 500, 700, 1000, 1500, 2000, 3000, 4000, 5000, 7000, 10000, 20000
Quantification using: Lower Marker   Final concentration (ng/uL): 0.5000   Dilution factor: 12.0

Sample: SampC8
Well location: C8
Created: Thursday, October 21, 2021 9:39:00 PM

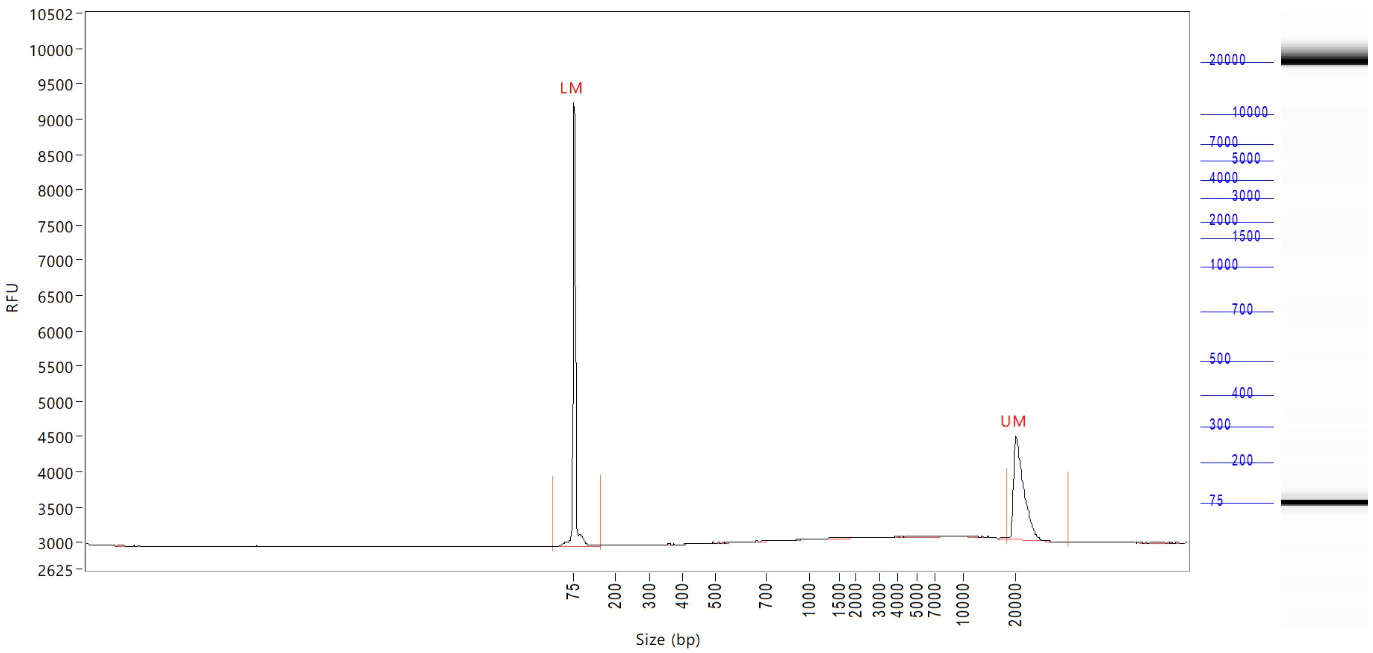

Peak	Size	Concentration	Relative concentration	Molarity	From	To	CV%	RFU
	(bp)	(ng/uL)	Percent	(nmole/L)	(bp)	(bp)		
1	75 (LM)	0.5000		10.9378	14	158	12.58	6293
2	20000 (UM)	0.2389		0.0189	18171	29914	5.72	1474

TIC:	0.0000	ng/uL
TIM:	0.0000	nmole/L
Total concentration:	0.5268	ng/uL

Sample peak width (sec): 5 Sample min peak height: 50 Sample baseline V to V?: Y Sample baseline V to V points: 3
Sample filter: Binomial Number of points for filter: 3 Sample start region (min): 0 Sample end region (min): 30
Marker peak width (sec): 5 Marker min peak height: 200 Marker baseline V to V?: Y Marker baseline V to V points: 3
Lower marker selection: First peak > 500 RFU Upper marker selection: Last peak > 200 RFU
Ladder size (bp)75, 200, 300, 400, 500, 700, 1000, 1500, 2000, 3000, 4000, 5000, 7000, 10000, 20000
Quantification using: Lower Marker Final concentration (ng/uL): 0.5000 Dilution factor: 12.0

Sample: SampD8
Well location: D8
Created: Thursday, October 21, 2021 9:39:00 PM

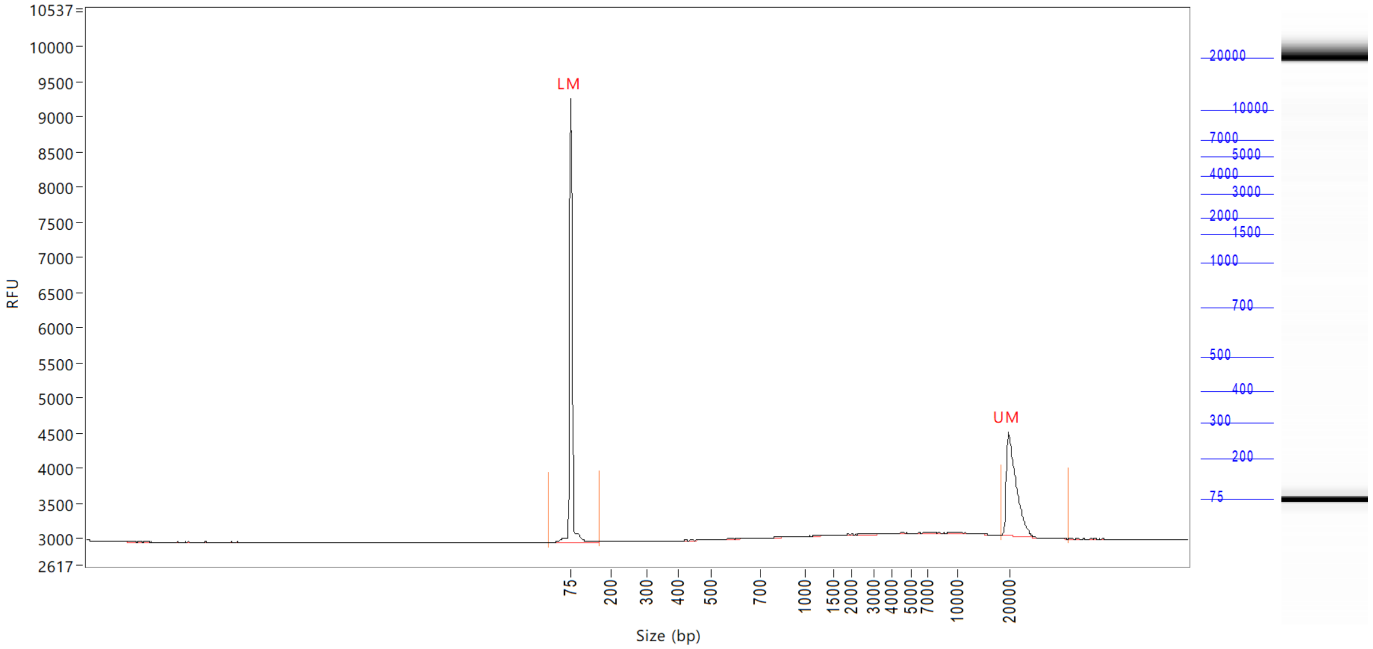

Peak	Size	Concentration	Relative concentration	Molarity	From	To	CV%	RFU
	(bp)	(ng/uL)	Percent	(nmole/L)	(bp)	(bp)		
1	75 (LM)	0.5000		11.2364	11	162	12.43	6329
2	20000 (UM)	0.2324		0.0184	18415	31486	5.72	1469

TIC:	0.0000	ng/uL
TIM:	0.0000	nmole/L
Total concentration:	0.5455	ng/uL

Sample peak width (sec): 5 Sample min peak height: 50 Sample baseline V to V?: Y Sample baseline V to V points: 3
Sample filter: Binomial Number of points for filter: 3 Sample start region (min): 0 Sample end region (min): 30
Marker peak width (sec): 5 Marker min peak height: 200 Marker baseline V to V?: Y Marker baseline V to V points: 3
Lower marker selection: First peak > 500 RFU Upper marker selection: Last peak > 200 RFU
Ladder size (bp)75, 200, 300, 400, 500, 700, 1000, 1500, 2000, 3000, 4000, 5000, 7000, 10000, 20000
Quantification using: Lower Marker Final concentration (ng/uL): 0.5000 Dilution factor: 12.0

Sample: SampE8
Well location: E8
Created: Thursday, October 21, 2021 9:39:00 PM

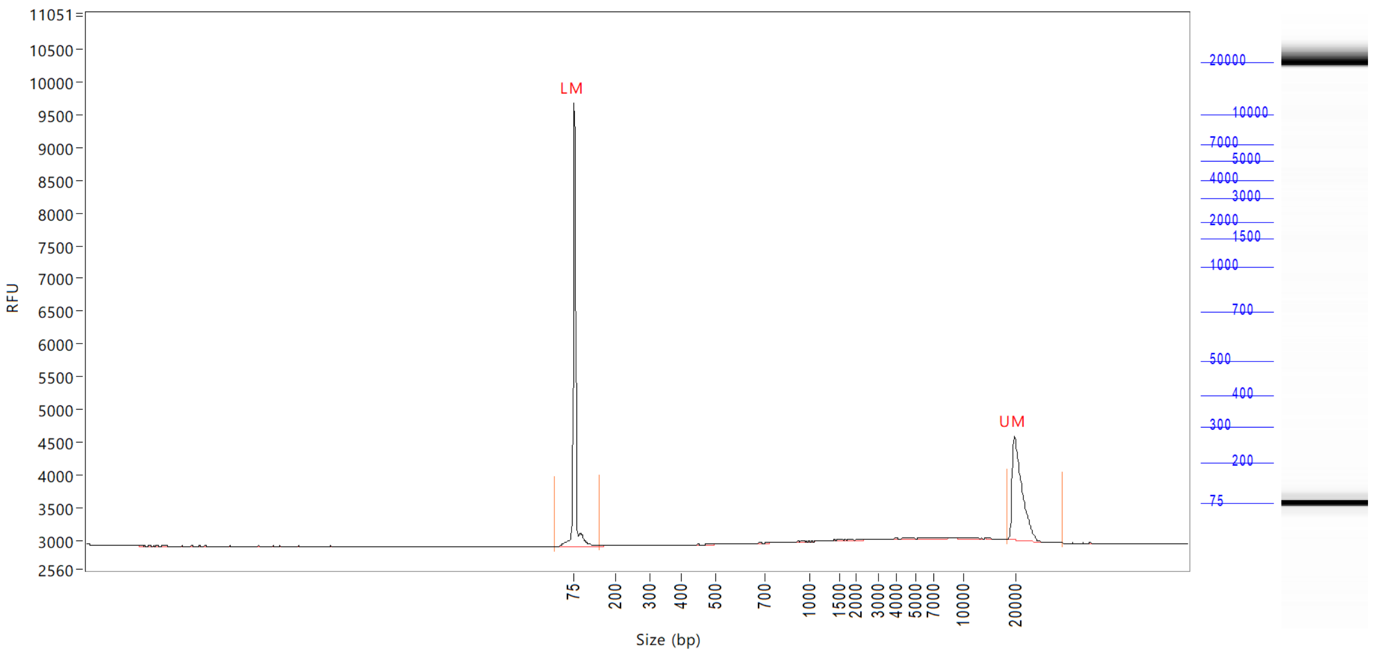

Peak	Size	Concentration	Relative concentration	Molarity	From	To	CV%	RFU
	(bp)	(ng/uL)	Percent	(nmole/L)	(bp)	(bp)		
1	75 (LM)	0.5000		11.0851	18	150	12.76	6785
2	20000 (UM)	0.2288		0.0182	18537	29068	5.32	1580

TIC:	0.0000	ng/uL
TIM:	0.0000	nmole/L
Total concentration:	0.5078	ng/uL

```
Sample peak width (sec): 5    Sample min peak height: 50    Sample baseline V to V?: Y    Sample baseline V to V points: 3
Sample filter: Binomial       Number of points for filter: 3  Sample start region (min): 0  Sample end region (min): 30
Marker peak width (sec): 5    Marker min peak height: 200   Marker baseline V to V?: Y    Marker baseline V to V points: 3
Lower marker selection: First peak > 500 RFU                Upper marker selection: Last peak > 200 RFU
Ladder size (bp)75, 200, 300, 400, 500, 700, 1000, 1500, 2000, 3000, 4000, 5000, 7000, 10000, 20000
Quantification using: Lower Marker          Final concentration (ng/uL): 0.5000          Dilution factor: 12.0
```

Sample: SampF8
Well location: F8
Created: Thursday, October 21, 2021 9:39:00 PM

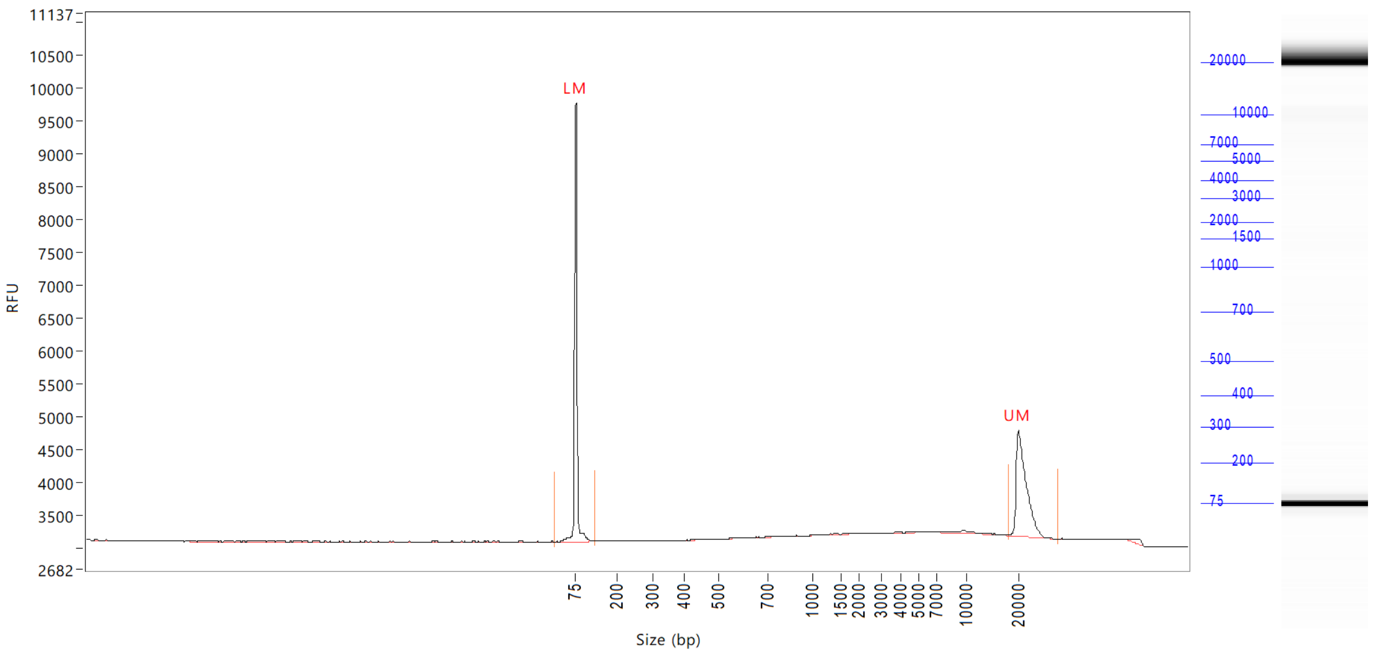

Peak	Size	Concentration	Relative concentration	Molarity	From	To	CV%	RFU
	(bp)	(ng/uL)	Percent	(nmole/L)	(bp)	(bp)		
1	75 (LM)	0.5000		11.2364	11	131	10.63	6683
2	20000 (UM)	0.2486		0.0197	18171	27496	5.47	1617

TIC:	0.0000	ng/uL
TIM:	0.0000	nmole/L
Total concentration:	0.5799	ng/uL

Sample peak width (sec): 5 Sample min peak height: 50 Sample baseline V to V?: Y Sample baseline V to V points: 3
Sample filter: Binomial Number of points for filter: 3 Sample start region (min): 0 Sample end region (min): 30
Marker peak width (sec): 5 Marker min peak height: 200 Marker baseline V to V?: Y Marker baseline V to V points: 3
Lower marker selection: First peak > 500 RFU Upper marker selection: Last peak > 200 RFU
Ladder size (bp)75, 200, 300, 400, 500, 700, 1000, 1500, 2000, 3000, 4000, 5000, 7000, 10000, 20000
Quantification using: Lower Marker Final concentration (ng/uL): 0.5000 Dilution factor: 12.0

Sample: SampG8
Well location: G8
Created: Thursday, October 21, 2021 9:39:00 PM

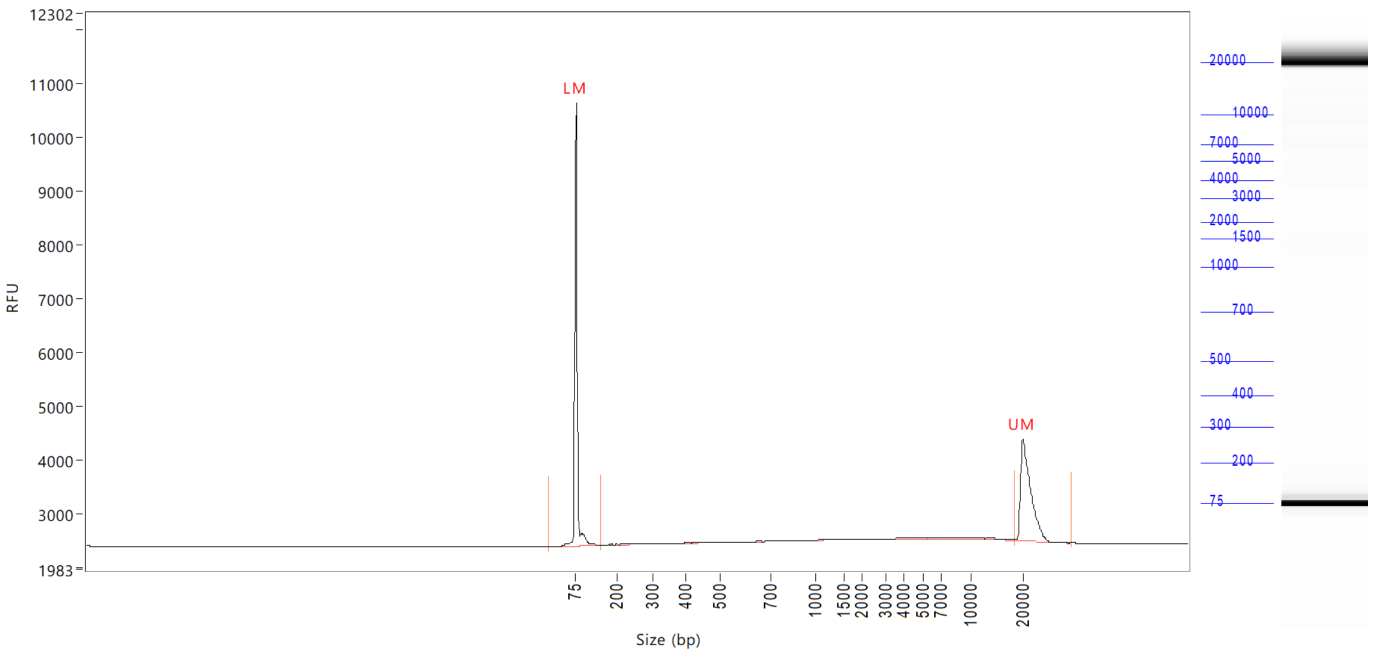

Peak	Size	Concentration	Relative concentration	Molarity	From	To	CV%	RFU
	(bp)	(ng/uL)	Percent	(nmole/L)	(bp)	(bp)		
1	75 (LM)	0.5000		11.0851	0	150	12.67	8240
2	20000 (UM)	0.2377		0.0189	18537	29068	5.54	1877

TIC:	0.0000	ng/uL
TIM:	0.0000	nmole/L
Total concentration:	0.5728	ng/uL

Sample peak width (sec): 5 Sample min peak height: 50 Sample baseline V to V?: Y Sample baseline V to V points: 3
Sample filter: Binomial Number of points for filter: 3 Sample start region (min): 0 Sample end region (min): 30
Marker peak width (sec): 5 Marker min peak height: 200 Marker baseline V to V?: Y Marker baseline V to V points: 3
Lower marker selection: First peak > 500 RFU Upper marker selection: Last peak > 200 RFU
Ladder size (bp)75, 200, 300, 400, 500, 700, 1000, 1500, 2000, 3000, 4000, 5000, 7000, 10000, 20000
Quantification using: Lower Marker Final concentration (ng/uL): 0.5000 Dilution factor: 12.0

Sample: SampH8
Well location: H8
Created: Thursday, October 21, 2021 9:39:00 PM

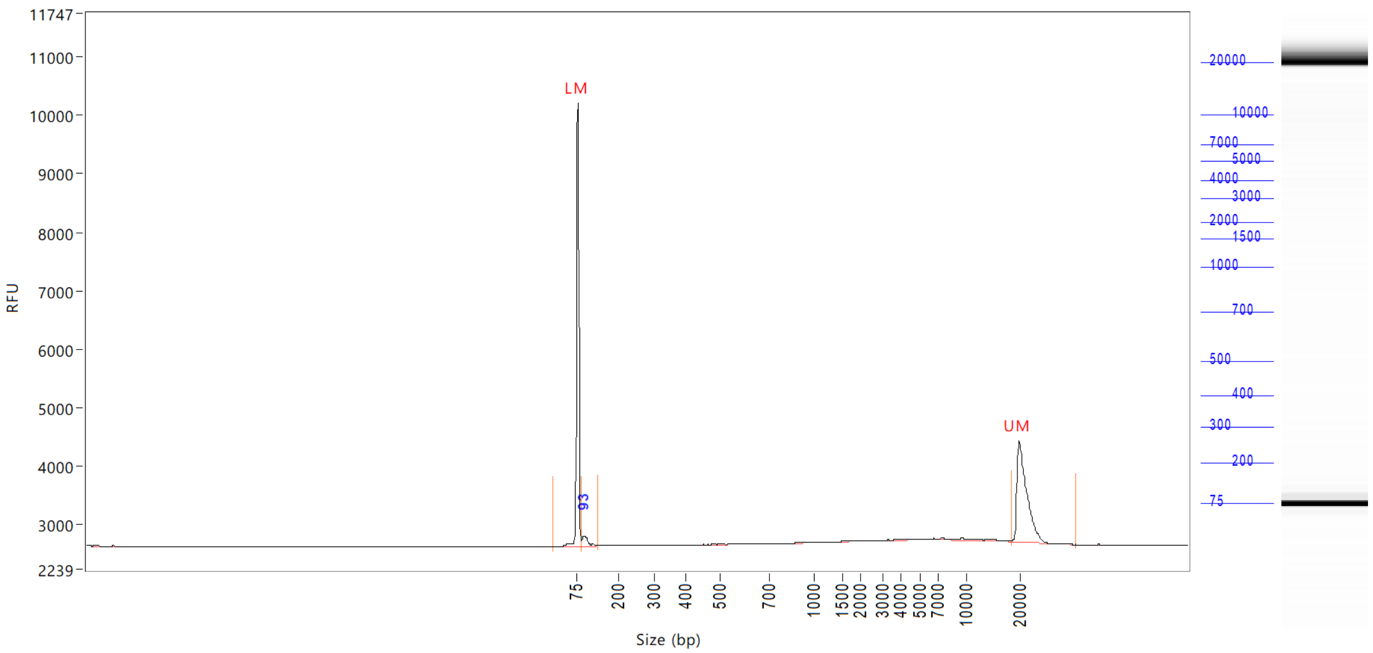

Peak	Size	Concentration	Relative concentration	Molarity	From	To	CV%	RFU
	(bp)	(ng/uL)	Percent	(nmole/L)	(bp)	(bp)		
1	75 (LM)	0.5000		11.3919	3	86	6.87	7595
2	93	0.3408	100.0	5.6533	86	137	11.41	162
3	20000 (UM)	0.2508		0.0198	18415	30881	5.65	1738

TIC:	0.3408	ng/uL
TIM:	5.6533	nmole/L
Total concentration:	0.9429	ng/uL

```
Sample peak width (sec): 5    Sample min peak height: 50    Sample baseline V to V?: Y    Sample baseline V to V points: 3
Sample filter: Binomial       Number of points for filter: 3  Sample start region (min): 0  Sample end region (min): 30
Marker peak width (sec): 5    Marker min peak height: 200   Marker baseline V to V?: Y    Marker baseline V to V points: 3
Lower marker selection: First peak > 500 RFU                Upper marker selection: Last peak > 200 RFU
Ladder size (bp)75, 200, 300, 400, 500, 700, 1000, 1500, 2000, 3000, 4000, 5000, 7000, 10000, 20000
Quantification using: Lower Marker          Final concentration (ng/uL): 0.5000          Dilution factor: 12.0
```

Sample: SampA9
Well location: A9
Created: Thursday, October 21, 2021 9:39:00 PM

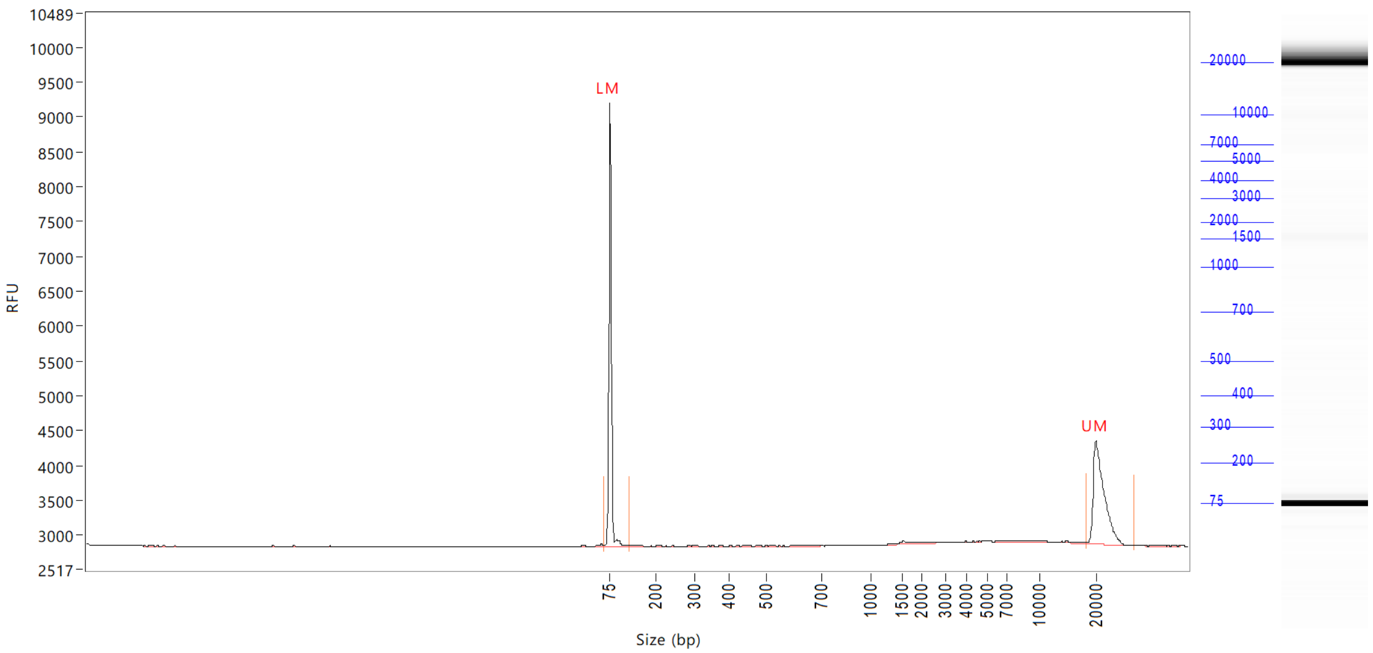

Peak	Size	Concentration	Relative concentration	Molarity	From	To	CV%	RFU
	(bp)	(ng/uL)	Percent	(nmole/L)	(bp)	(bp)		
1	75 (LM)	0.5000		11.2364	58	129	7.38	6371
2	20000 (UM)	0.2693		0.0214	18293	26649	5.41	1476

TIC:	0.0000	ng/uL
TIM:	0.0000	nmole/L
Total concentration:	0.7815	ng/uL

```
Sample peak width (sec): 5    Sample min peak height: 50    Sample baseline V to V?: Y    Sample baseline V to V points: 3
Sample filter: Binomial       Number of points for filter: 3  Sample start region (min): 0  Sample end region (min): 30
Marker peak width (sec): 5    Marker min peak height: 200   Marker baseline V to V?: Y    Marker baseline V to V points: 3
Lower marker selection: First peak > 500 RFU                Upper marker selection: Last peak > 200 RFU
Ladder size (bp)75, 200, 300, 400, 500, 700, 1000, 1500, 2000, 3000, 4000, 5000, 7000, 10000, 20000
Quantification using: Lower Marker          Final concentration (ng/uL): 0.5000          Dilution factor: 12.0
```

Sample: SampB9
Well location: B9
Created: Thursday, October 21, 2021 9:39:00 PM

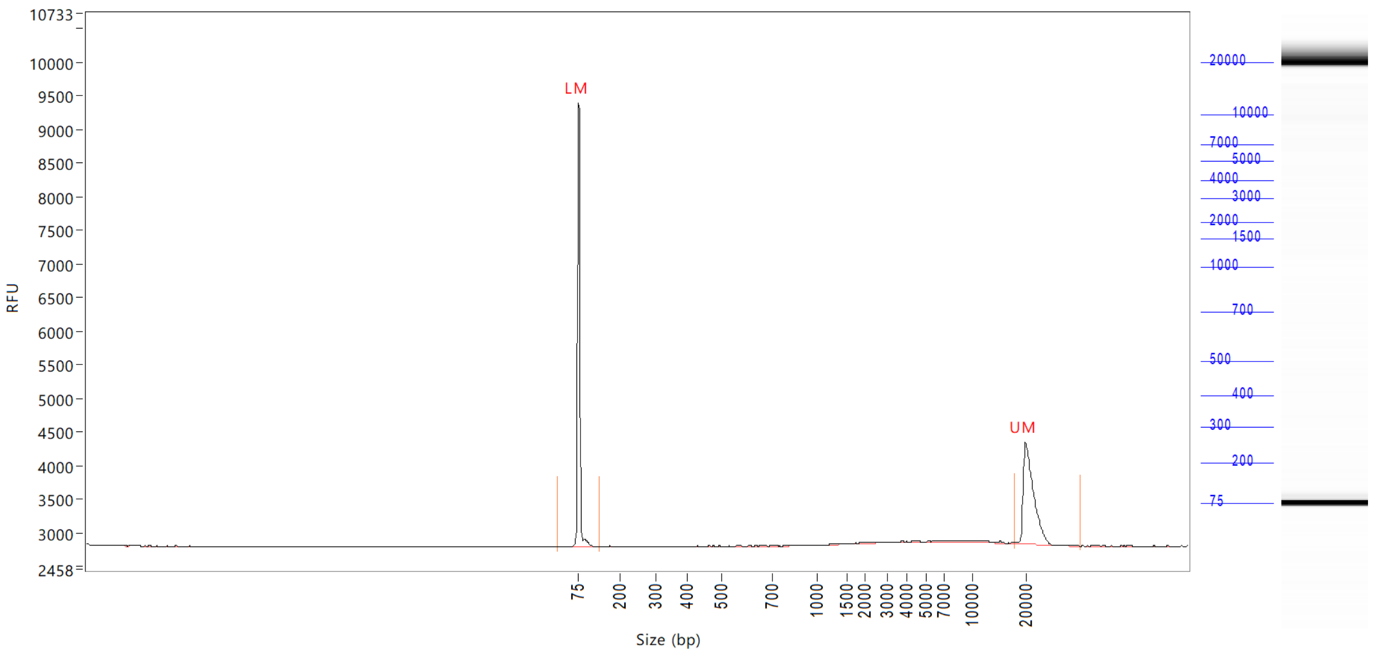

Peak	Size	Concentration	Relative concentration	Molarity	From	To	CV%	RFU
	(bp)	(ng/uL)	Percent	(nmole/L)	(bp)	(bp)		
1	75 (LM)	0.5000		11.0851	12	137	7.97	6618
2	20000 (UM)	0.2481		0.0197	18049	30398	5.63	1526

TIC:	0.0000	ng/uL
TIM:	0.0000	nmole/L
Total concentration:	0.6681	ng/uL

Sample peak width (sec): 5 Sample min peak height: 50 Sample baseline V to V?: Y Sample baseline V to V points: 3
Sample filter: Binomial Number of points for filter: 3 Sample start region (min): 0 Sample end region (min): 30
Marker peak width (sec): 5 Marker min peak height: 200 Marker baseline V to V?: Y Marker baseline V to V points: 3
Lower marker selection: First peak > 500 RFU Upper marker selection: Last peak > 200 RFU
Ladder size (bp)75, 200, 300, 400, 500, 700, 1000, 1500, 2000, 3000, 4000, 5000, 7000, 10000, 20000
Quantification using: Lower Marker Final concentration (ng/uL): 0.5000 Dilution factor: 12.0

Sample: SampC9
Well location: C9
Created: Thursday, October 21, 2021 9:39:00 PM

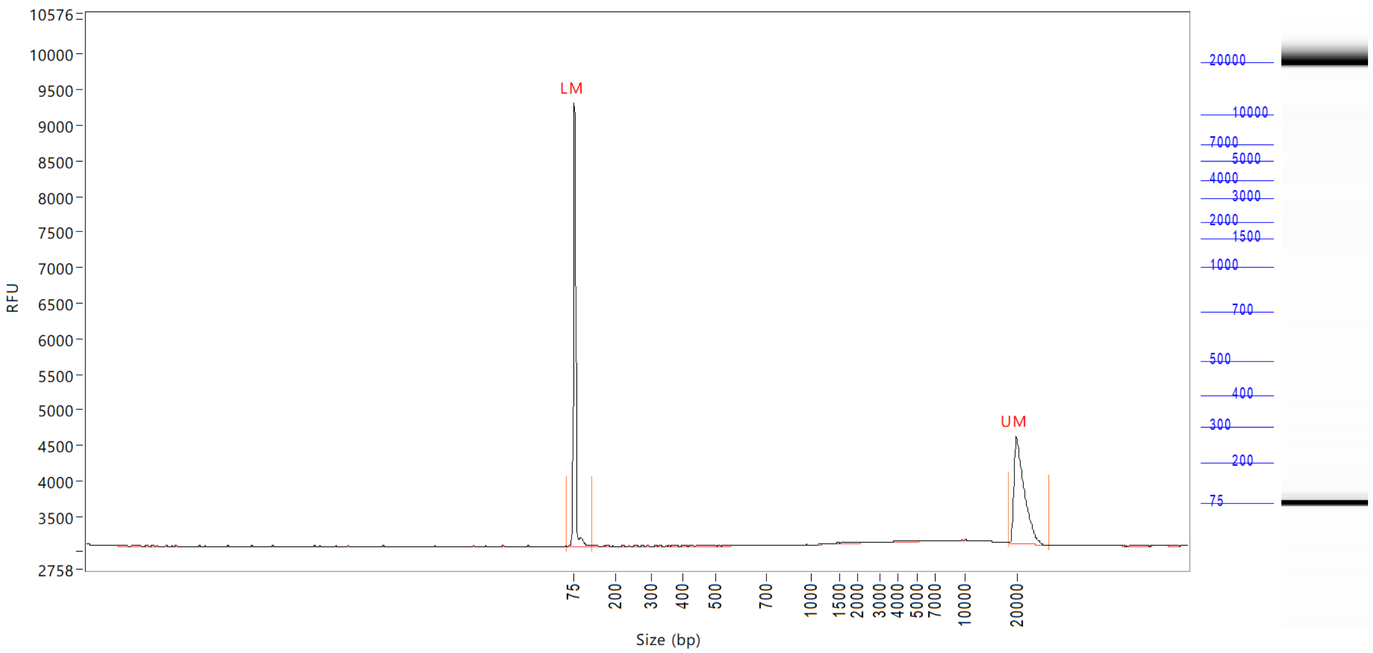

Peak	Size	Concentration	Relative concentration	Molarity	From	To	CV%	RFU
	(bp)	(ng/uL)	Percent	(nmole/L)	(bp)	(bp)		
1	75 (LM)	0.5000		10.9378	54	128	7.79	6248
2	20000 (UM)	0.2628		0.0208	18537	26045	5.41	1508

TIC:	0.0000	ng/uL
TIM:	0.0000	nmole/L
Total concentration:	0.6198	ng/uL

Sample peak width (sec): 5 Sample min peak height: 50 Sample baseline V to V?: Y Sample baseline V to V points: 3
Sample filter: Binomial Number of points for filter: 3 Sample start region (min): 0 Sample end region (min): 30
Marker peak width (sec): 5 Marker min peak height: 200 Marker baseline V to V?: Y Marker baseline V to V points: 3
Lower marker selection: First peak > 500 RFU Upper marker selection: Last peak > 200 RFU
Ladder size (bp)75, 200, 300, 400, 500, 700, 1000, 1500, 2000, 3000, 4000, 5000, 7000, 10000, 20000
Quantification using: Lower Marker Final concentration (ng/uL): 0.5000 Dilution factor: 12.0

Sample: SampD9
Well location: D9
Created: Thursday, October 21, 2021 9:39:00 PM

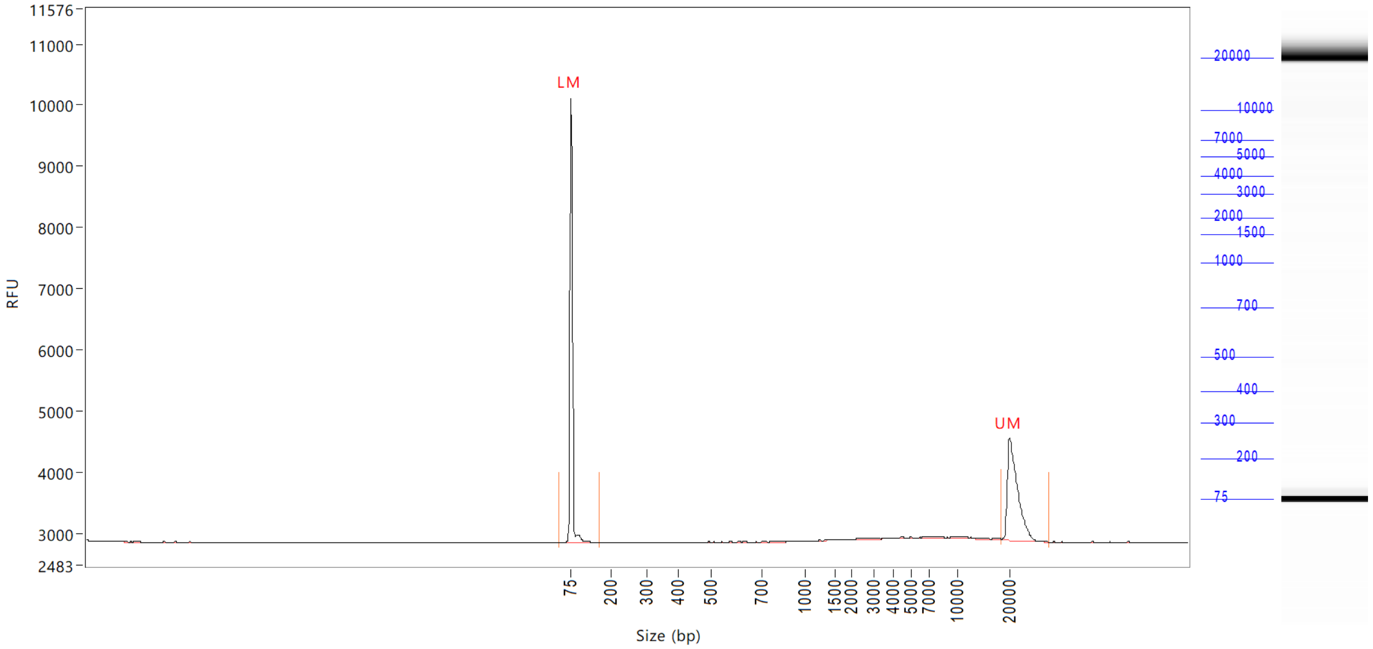

Peak	Size	Concentration	Relative concentration	Molarity	From	To	CV%	RFU
	(bp)	(ng/uL)	Percent	(nmole/L)	(bp)	(bp)		
1	75 (LM)	0.5000		11.0851	41	162	8.38	7270
2	20000 (UM)	0.2614		0.0207	18293	27496	5.56	1685

TIC:	0.0000	ng/uL
TIM:	0.0000	nmole/L
Total concentration:	0.6741	ng/uL

Sample peak width (sec): 5 Sample min peak height: 50 Sample baseline V to V?: Y Sample baseline V to V points: 3
Sample filter: Binomial Number of points for filter: 3 Sample start region (min): 0 Sample end region (min): 30
Marker peak width (sec): 5 Marker min peak height: 200 Marker baseline V to V?: Y Marker baseline V to V points: 3
Lower marker selection: First peak > 500 RFU Upper marker selection: Last peak > 200 RFU
Ladder size (bp)75, 200, 300, 400, 500, 700, 1000, 1500, 2000, 3000, 4000, 5000, 7000, 10000, 20000
Quantification using: Lower Marker Final concentration (ng/uL): 0.5000 Dilution factor: 12.0

Sample: SampE9
Well location: E9
Created: Thursday, October 21, 2021 9:39:00 PM

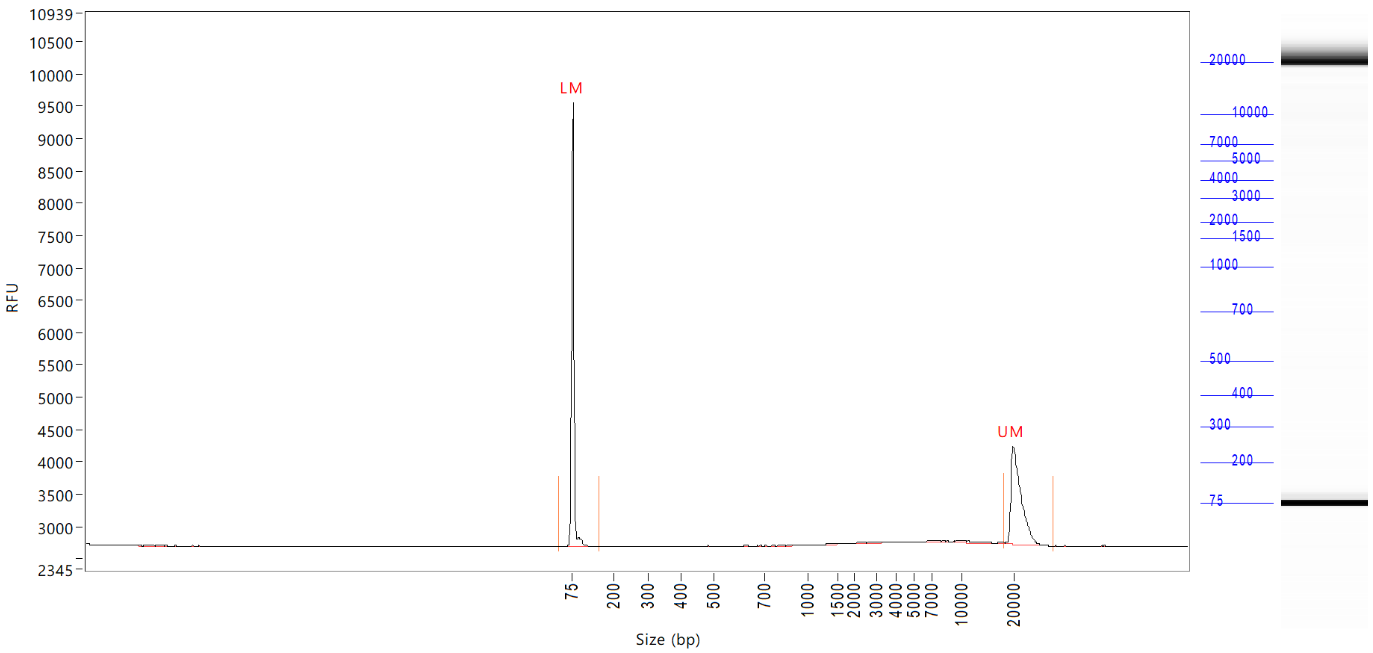

Peak	Size	Concentration	Relative concentration	Molarity	From	To	CV%	RFU
	(bp)	(ng/uL)	Percent	(nmole/L)	(bp)	(bp)		
1	75 (LM)	0.5000		11.0851	33	156	9.68	6874
2	20000 (UM)	0.2422		0.0192	18293	27617	5.50	1509

TIC:	0.0000	ng/uL
TIM:	0.0000	nmole/L
Total concentration:	0.6957	ng/uL

```
Sample peak width (sec): 5    Sample min peak height: 50    Sample baseline V to V?: Y    Sample baseline V to V points: 3
Sample filter: Binomial       Number of points for filter: 3  Sample start region (min): 0  Sample end region (min): 30
Marker peak width (sec): 5    Marker min peak height: 200   Marker baseline V to V?: Y    Marker baseline V to V points: 3
Lower marker selection: First peak > 500 RFU                Upper marker selection: Last peak > 200 RFU
Ladder size (bp)75, 200, 300, 400, 500, 700, 1000, 1500, 2000, 3000, 4000, 5000, 7000, 10000, 20000
Quantification using: Lower Marker          Final concentration (ng/uL): 0.5000          Dilution factor: 12.0
```

Sample: SampF9
Well location: F9
Created: Thursday, October 21, 2021 9:39:00 PM

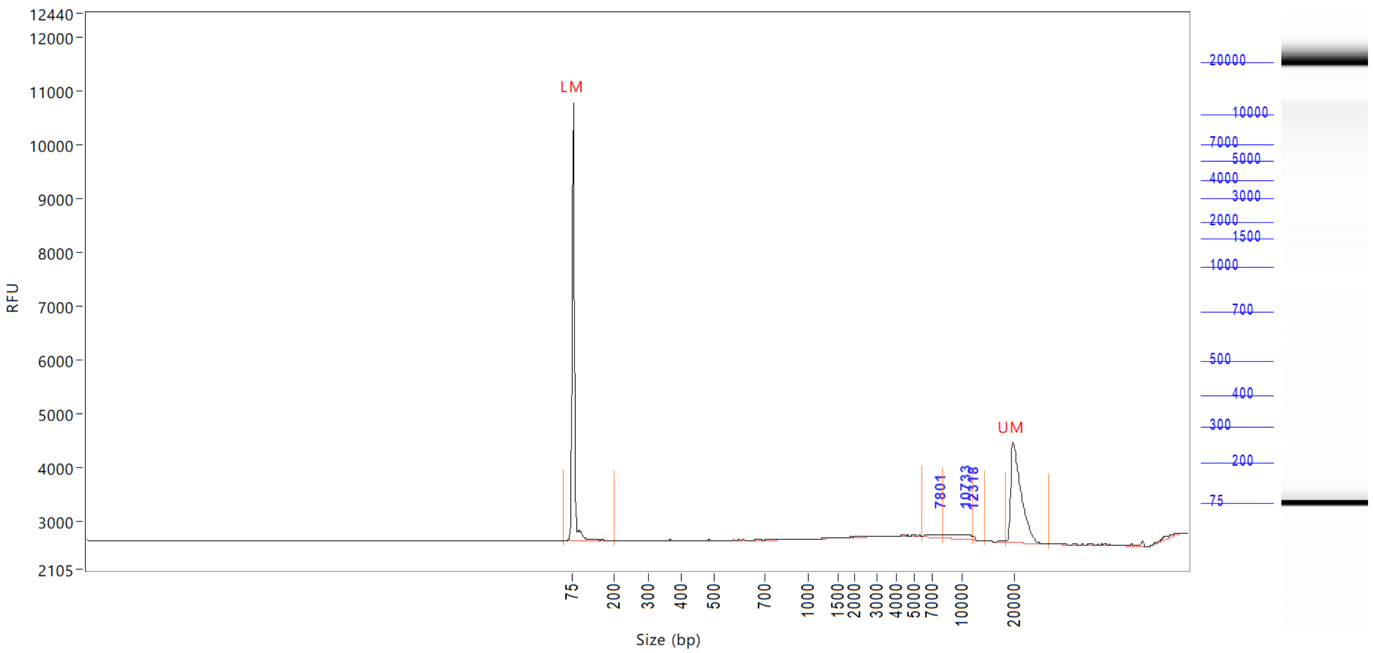

Peak	Size	Concentration	Relative concentration	Molarity	From	To	CV%	RFU
	(bp)	(ng/uL)	Percent	(nmole/L)	(bp)	(bp)		
1	75 (LM)	0.5000		10.9378	47	200	12.84	8150
2	7801	0.1256	26.1	0.0293	5741	8001	8.59	53
3	10733	0.3307	68.6	0.0545	8001	12318	11.97	87
4	12318	0.0256	5.3	0.0033	12318	14513	5.73	76
5	20000 (UM)	0.2489		0.0197	18415	26770	5.45	1875

TIC:	0.4819	ng/uL
TIM:	0.0871	nmole/L
Total concentration:	0.7658	ng/uL

```
Sample peak width (sec): 5    Sample min peak height: 50    Sample baseline V to V?: Y    Sample baseline V to V points: 3
Sample filter: Binomial       Number of points for filter: 3  Sample start region (min): 0  Sample end region (min): 30
Marker peak width (sec): 5    Marker min peak height: 200   Marker baseline V to V?: Y    Marker baseline V to V points: 3
Lower marker selection: First peak > 500 RFU                Upper marker selection: Last peak > 200 RFU
Ladder size (bp)75, 200, 300, 400, 500, 700, 1000, 1500, 2000, 3000, 4000, 5000, 7000, 10000, 20000
Quantification using: Lower Marker          Final concentration (ng/uL): 0.5000          Dilution factor: 12.0
```

Sample: SampG9
Well location: G9
Created: Thursday, October 21, 2021 9:39:00 PM

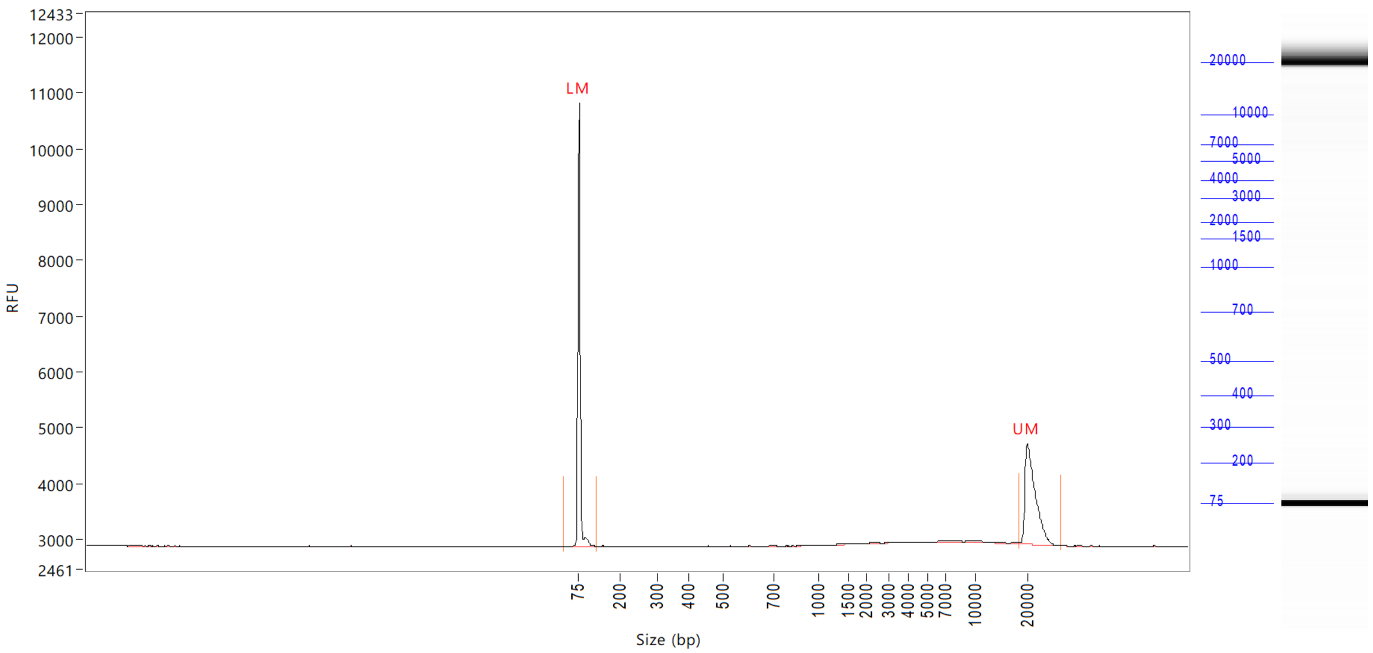

Peak	Size	Concentration	Relative concentration	Molarity	From	To	CV%	RFU
	(bp)	(ng/uL)	Percent	(nmole/L)	(bp)	(bp)		
1	75 (LM)	0.5000		11.0851	31	129	8.11	7975
2	20000 (UM)	0.2514		0.0199	18415	26166	5.37	1796

TIC:	0.0000	ng/uL
TIM:	0.0000	nmole/L
Total concentration:	0.6104	ng/uL

Sample peak width (sec): 5 Sample min peak height: 50 Sample baseline V to V?: Y Sample baseline V to V points: 3
Sample filter: Binomial Number of points for filter: 3 Sample start region (min): 0 Sample end region (min): 30
Marker peak width (sec): 5 Marker min peak height: 200 Marker baseline V to V?: Y Marker baseline V to V points: 3
Lower marker selection: First peak > 500 RFU Upper marker selection: Last peak > 200 RFU
Ladder size (bp)75, 200, 300, 400, 500, 700, 1000, 1500, 2000, 3000, 4000, 5000, 7000, 10000, 20000
Quantification using: Lower Marker Final concentration (ng/uL): 0.5000 Dilution factor: 12.0

Sample: SampH9
Well location: H9
Created: Thursday, October 21, 2021 9:39:00 PM

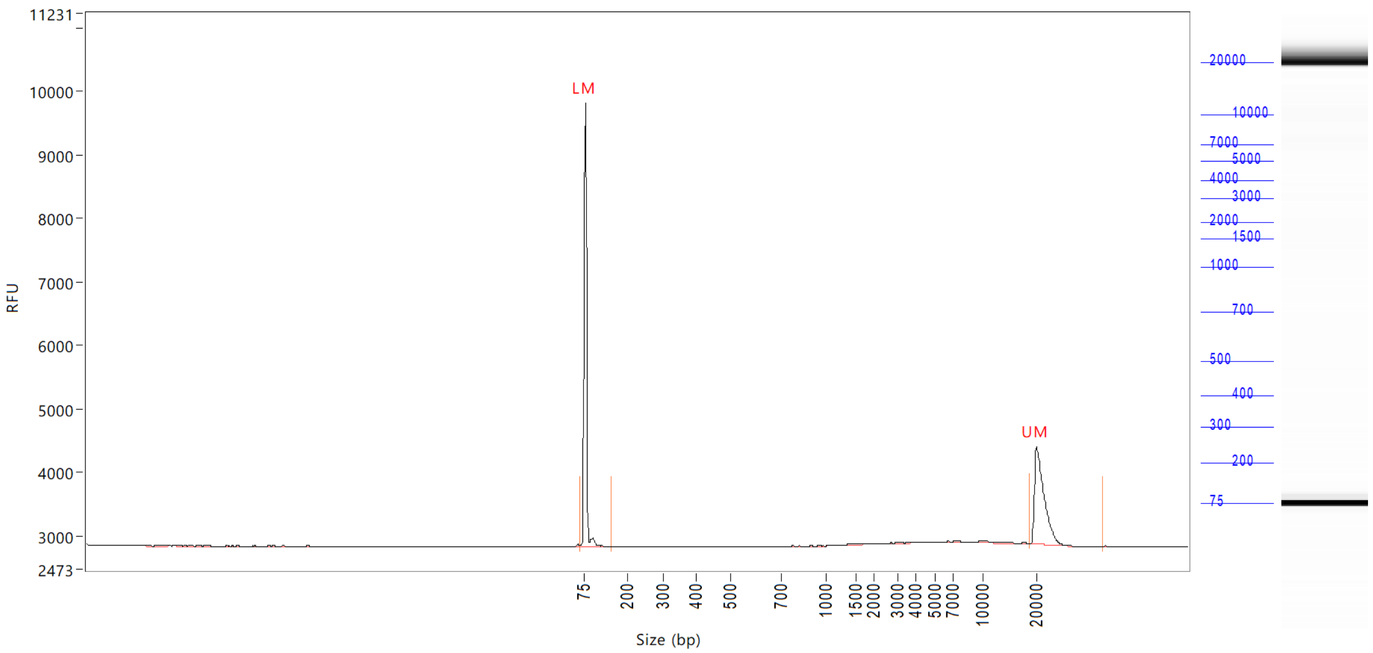

Peak	Size	Concentration	Relative concentration	Molarity	From	To	CV%	RFU
	(bp)	(ng/uL)	Percent	(nmole/L)	(bp)	(bp)		
1	75 (LM)	0.5000		11.0851	60	152	7.42	7001
2	20000 (UM)	0.2263		0.0179	18659	32453	5.75	1524

TIC:	0.0000	ng/uL
TIM:	0.0000	nmole/L
Total concentration:	0.5747	ng/uL

Sample peak width (sec): 5 Sample min peak height: 50 Sample baseline V to V?: Y Sample baseline V to V points: 3
Sample filter: Binomial Number of points for filter: 3 Sample start region (min): 0 Sample end region (min): 30
Marker peak width (sec): 5 Marker min peak height: 200 Marker baseline V to V?: Y Marker baseline V to V points: 3
Lower marker selection: First peak > 500 RFU Upper marker selection: Last peak > 200 RFU
Ladder size (bp)75, 200, 300, 400, 500, 700, 1000, 1500, 2000, 3000, 4000, 5000, 7000, 10000, 20000
Quantification using: Lower Marker Final concentration (ng/uL): 0.5000 Dilution factor: 12.0

Sample: SampA10
Well location: A10
Created: Thursday, October 21, 2021 9:39:00 PM

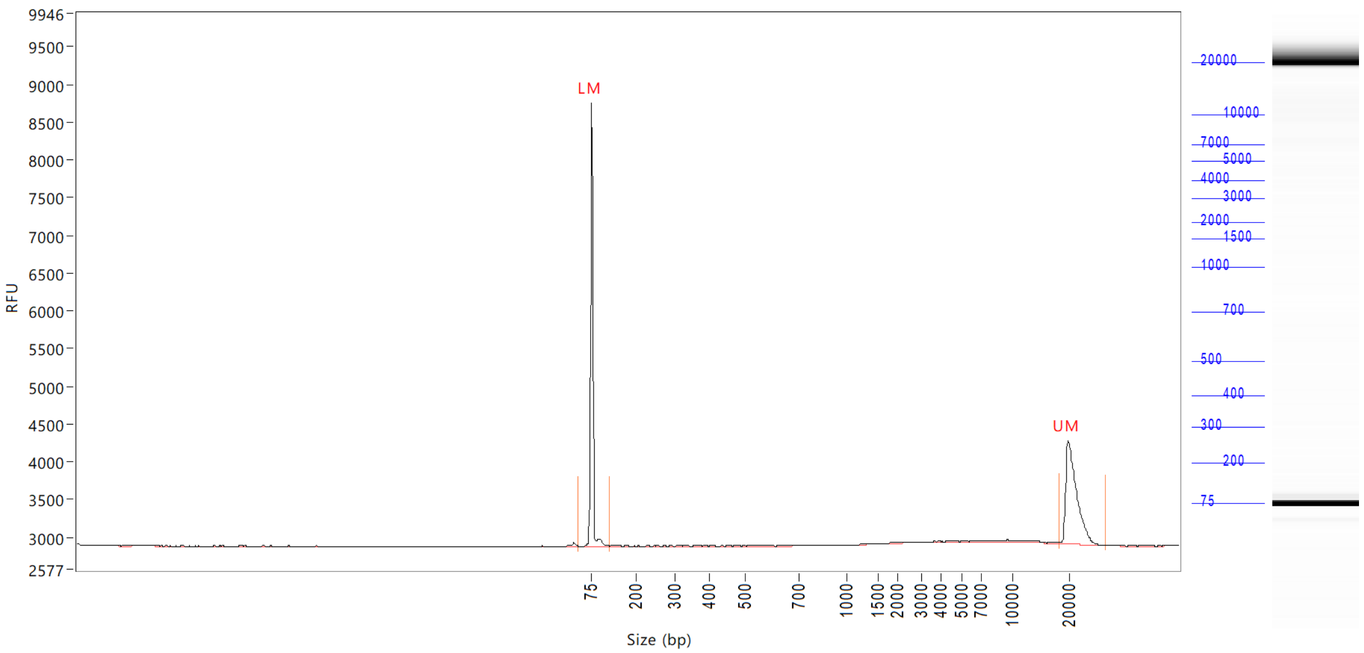

Peak	Size	Concentration	Relative concentration	Molarity	From	To	CV%	RFU
	(bp)	(ng/uL)	Percent	(nmole/L)	(bp)	(bp)		
1	75 (LM)	0.5000		11.2364	37	124	7.54	5888
2	20000 (UM)	0.2649		0.0210	18415	26649	5.47	1369

TIC:	0.0000	ng/uL
TIM:	0.0000	nmole/L
Total concentration:	0.8120	ng/uL

Sample peak width (sec): 5 Sample min peak height: 50 Sample baseline V to V?: Y Sample baseline V to V points: 3
Sample filter: Binomial Number of points for filter: 3 Sample start region (min): 0 Sample end region (min): 30
Marker peak width (sec): 5 Marker min peak height: 200 Marker baseline V to V?: Y Marker baseline V to V points: 3
Lower marker selection: First peak > 500 RFU Upper marker selection: Last peak > 200 RFU
Ladder size (bp)75, 200, 300, 400, 500, 700, 1000, 1500, 2000, 3000, 4000, 5000, 7000, 10000, 20000
Quantification using: Lower Marker Final concentration (ng/uL): 0.5000 Dilution factor: 12.0

Sample: SampB10
Well location: B10
Created: Thursday, October 21, 2021 9:39:00 PM

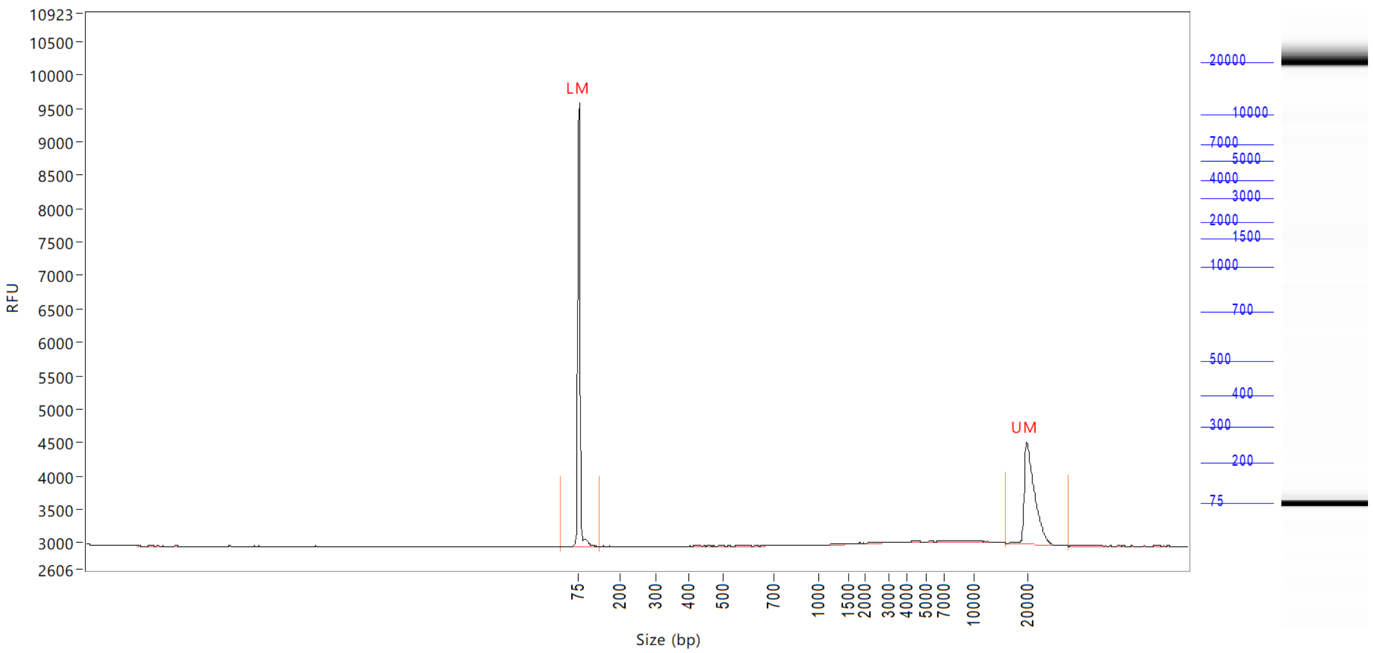

Peak	Size	Concentration	Relative concentration	Molarity	From	To	CV%	RFU
	(bp)	(ng/uL)	Percent	(nmole/L)	(bp)	(bp)		
1	75 (LM)	0.5000		11.2364	22	135	8.08	6650
2	20000 (UM)	0.2507		0.0199	16098	27979	5.60	1521

TIC:	0.0000	ng/uL
TIM:	0.0000	nmole/L
Total concentration:	0.5973	ng/uL

Sample peak width (sec): 5 Sample min peak height: 50 Sample baseline V to V?: Y Sample baseline V to V points: 3
Sample filter: Binomial Number of points for filter: 3 Sample start region (min): 0 Sample end region (min): 30
Marker peak width (sec): 5 Marker min peak height: 200 Marker baseline V to V?: Y Marker baseline V to V points: 3
Lower marker selection: First peak > 500 RFU Upper marker selection: Last peak > 200 RFU
Ladder size (bp)75, 200, 300, 400, 500, 700, 1000, 1500, 2000, 3000, 4000, 5000, 7000, 10000, 20000
Quantification using: Lower Marker Final concentration (ng/uL): 0.5000 Dilution factor: 12.0

Sample: SampC10
Well location: C10
Created: Thursday, October 21, 2021 9:39:00 PM

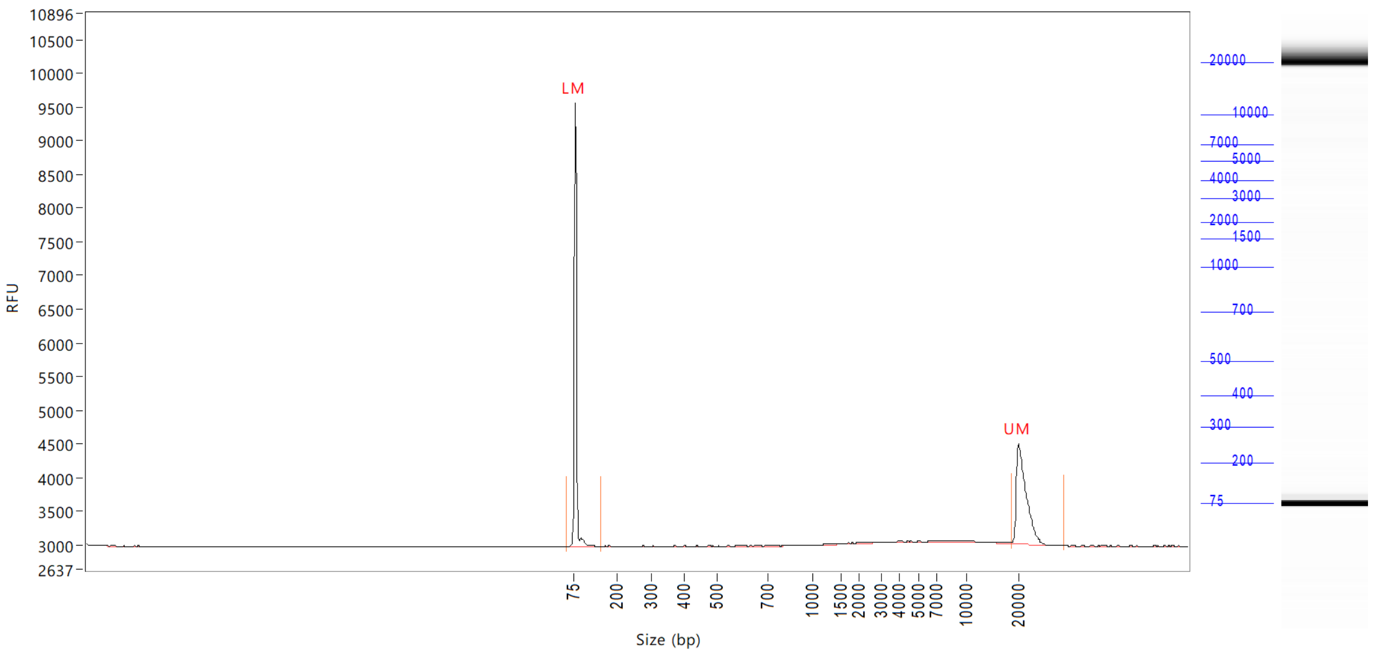

Peak	Size	Concentration	Relative concentration	Molarity	From	To	CV%	RFU
	(bp)	(ng/uL)	Percent	(nmole/L)	(bp)	(bp)		
1	75 (LM)	0.5000		11.0851	48	152	9.31	6603
2	20000 (UM)	0.2473		0.0195	18537	28705	5.46	1487

TIC:	0.0000	ng/uL
TIM:	0.0000	nmole/L
Total concentration:	0.6532	ng/uL

```
Sample peak width (sec): 5    Sample min peak height: 50    Sample baseline V to V?: Y    Sample baseline V to V points: 3
Sample filter: Binomial       Number of points for filter: 3  Sample start region (min): 0  Sample end region (min): 30
Marker peak width (sec): 5    Marker min peak height: 200   Marker baseline V to V?: Y    Marker baseline V to V points: 3
Lower marker selection: First peak > 500 RFU                Upper marker selection: Last peak > 200 RFU
Ladder size (bp)75, 200, 300, 400, 500, 700, 1000, 1500, 2000, 3000, 4000, 5000, 7000, 10000, 20000
Quantification using: Lower Marker          Final concentration (ng/uL): 0.5000          Dilution factor: 12.0
```

Sample: SampD10
Well location: D10
Created: Thursday, October 21, 2021 9:39:00 PM

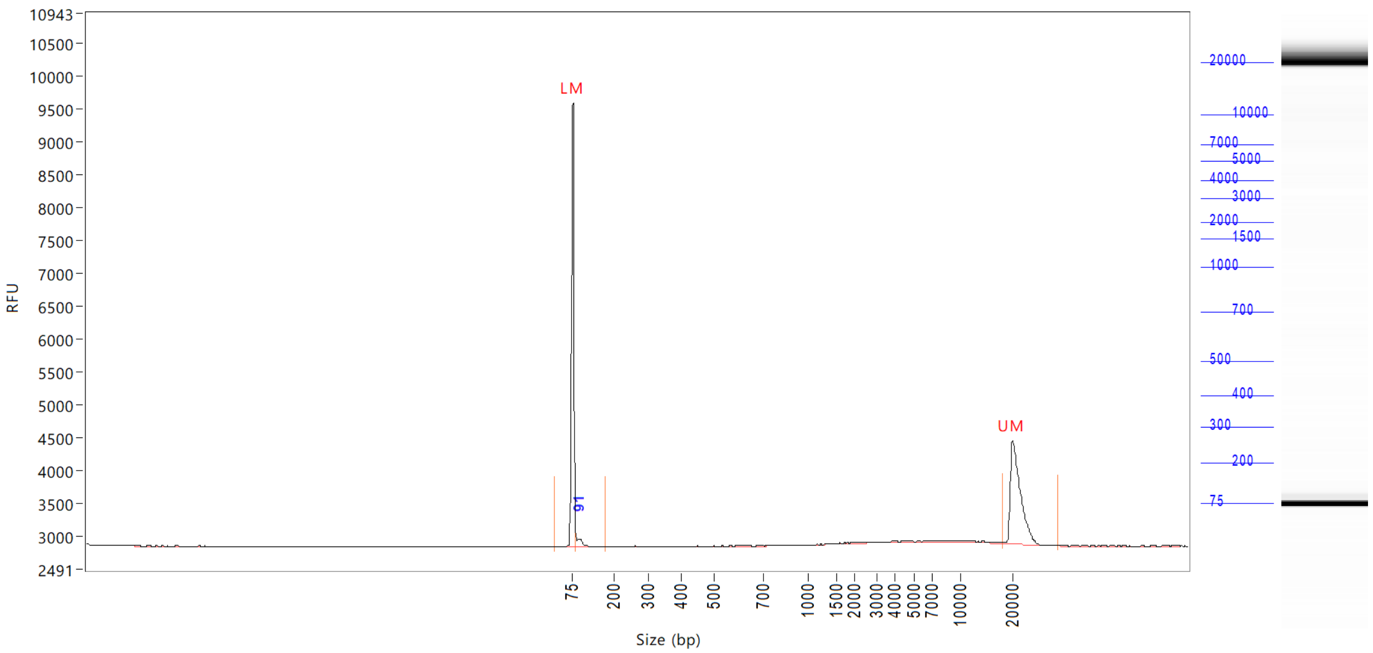

Peak	Size	Concentration	Relative concentration	Molarity	From	To	CV%	RFU
	(bp)	(ng/uL)	Percent	(nmole/L)	(bp)	(bp)		
1	75 (LM)	0.5000		11.3919	22	86	5.20	6759
2	91	0.2816	100.0	4.5786	86	173	16.56	121
3	20000 (UM)	0.2591		0.0205	17927	28705	5.48	1580

TIC:	0.2816	ng/uL
TIM:	4.5786	nmole/L
Total concentration:	0.9152	ng/uL

Sample peak width (sec): 5 Sample min peak height: 50 Sample baseline V to V?: Y Sample baseline V to V points: 3
Sample filter: Binomial Number of points for filter: 3 Sample start region (min): 0 Sample end region (min): 30
Marker peak width (sec): 5 Marker min peak height: 200 Marker baseline V to V?: Y Marker baseline V to V points: 3
Lower marker selection: First peak > 500 RFU Upper marker selection: Last peak > 200 RFU
Ladder size (bp)75, 200, 300, 400, 500, 700, 1000, 1500, 2000, 3000, 4000, 5000, 7000, 10000, 20000
Quantification using: Lower Marker Final concentration (ng/uL): 0.5000 Dilution factor: 12.0

Sample: SampE10
Well location: E10
Created: Thursday, October 21, 2021 9:39:00 PM

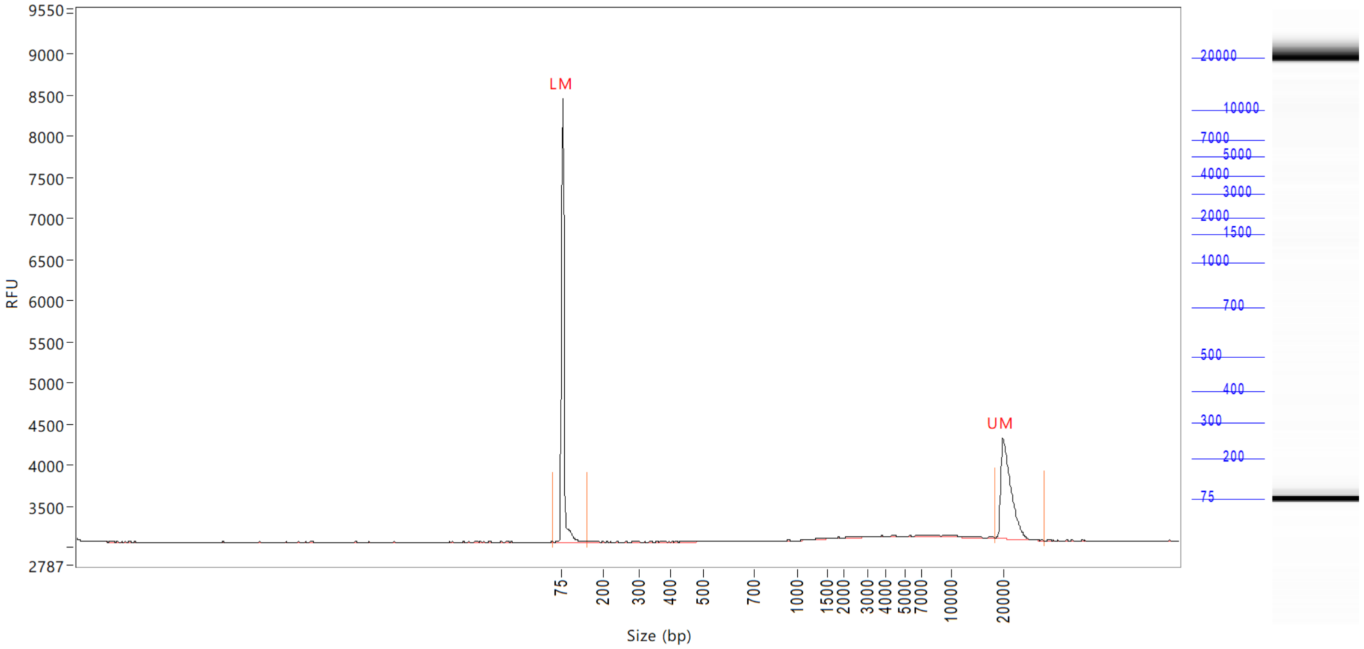

Peak	Size	Concentration	Relative concentration	Molarity	From	To	CV%	RFU
	(bp)	(ng/uL)	Percent	(nmole/L)	(bp)	(bp)		
1	75 (LM)	0.5000		10.7944	48	148	10.95	5404
2	20000 (UM)	0.2342		0.0185	18415	27979	5.41	1222

TIC:	0.0000	ng/uL
TIM:	0.0000	nmole/L
Total concentration:	0.6953	ng/uL

Sample peak width (sec): 5 Sample min peak height: 50 Sample baseline V to V?: Y Sample baseline V to V points: 3
Sample filter: Binomial Number of points for filter: 3 Sample start region (min): 0 Sample end region (min): 30
Marker peak width (sec): 5 Marker min peak height: 200 Marker baseline V to V?: Y Marker baseline V to V points: 3
Lower marker selection: First peak > 500 RFU Upper marker selection: Last peak > 200 RFU
Ladder size (bp)75, 200, 300, 400, 500, 700, 1000, 1500, 2000, 3000, 4000, 5000, 7000, 10000, 20000
Quantification using: Lower Marker Final concentration (ng/uL): 0.5000 Dilution factor: 12.0

Sample: SampF10
Well location: F10
Created: Thursday, October 21, 2021 9:39:00 PM

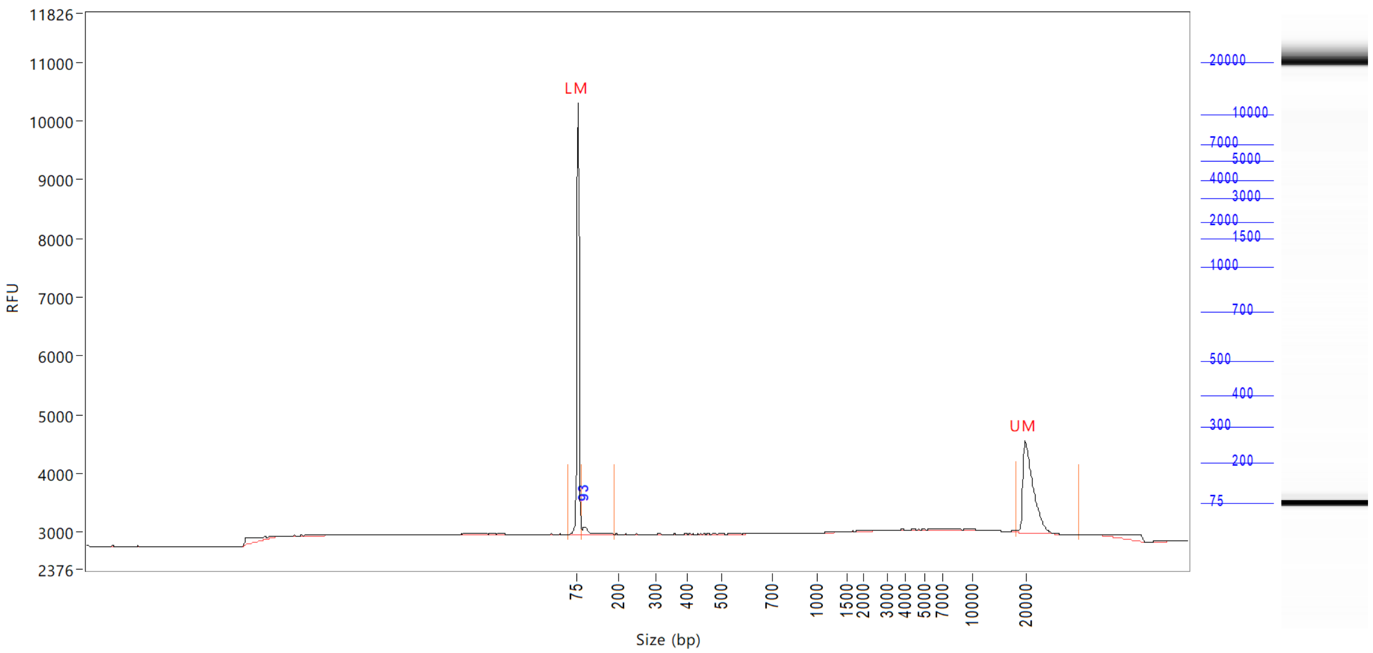

Peak	Size	Concentration	Relative concentration	Molarity	From	To	CV%	RFU
	(bp)	(ng/uL)	Percent	(nmole/L)	(bp)	(bp)		
1	75 (LM)	0.5000		11.2364	48	87	4.81	7346
2	93	0.2722	100.0	4.1393	87	183	21.60	120
3	20000 (UM)	0.2453		0.0194	18293	30277	5.94	1573

TIC:	0.2722	ng/uL
TIM:	4.1393	nmole/L
Total concentration:	0.8088	ng/uL

Sample peak width (sec): 5 Sample min peak height: 50 Sample baseline V to V?: Y Sample baseline V to V points: 3
Sample filter: Binomial Number of points for filter: 3 Sample start region (min): 0 Sample end region (min): 30
Marker peak width (sec): 5 Marker min peak height: 200 Marker baseline V to V?: Y Marker baseline V to V points: 3
Lower marker selection: First peak > 500 RFU Upper marker selection: Last peak > 200 RFU
Ladder size (bp)75, 200, 300, 400, 500, 700, 1000, 1500, 2000, 3000, 4000, 5000, 7000, 10000, 20000
Quantification using: Lower Marker Final concentration (ng/uL): 0.5000 Dilution factor: 12.0

Sample: SampG10
Well location: G10
Created: Thursday, October 21, 2021 9:39:00 PM

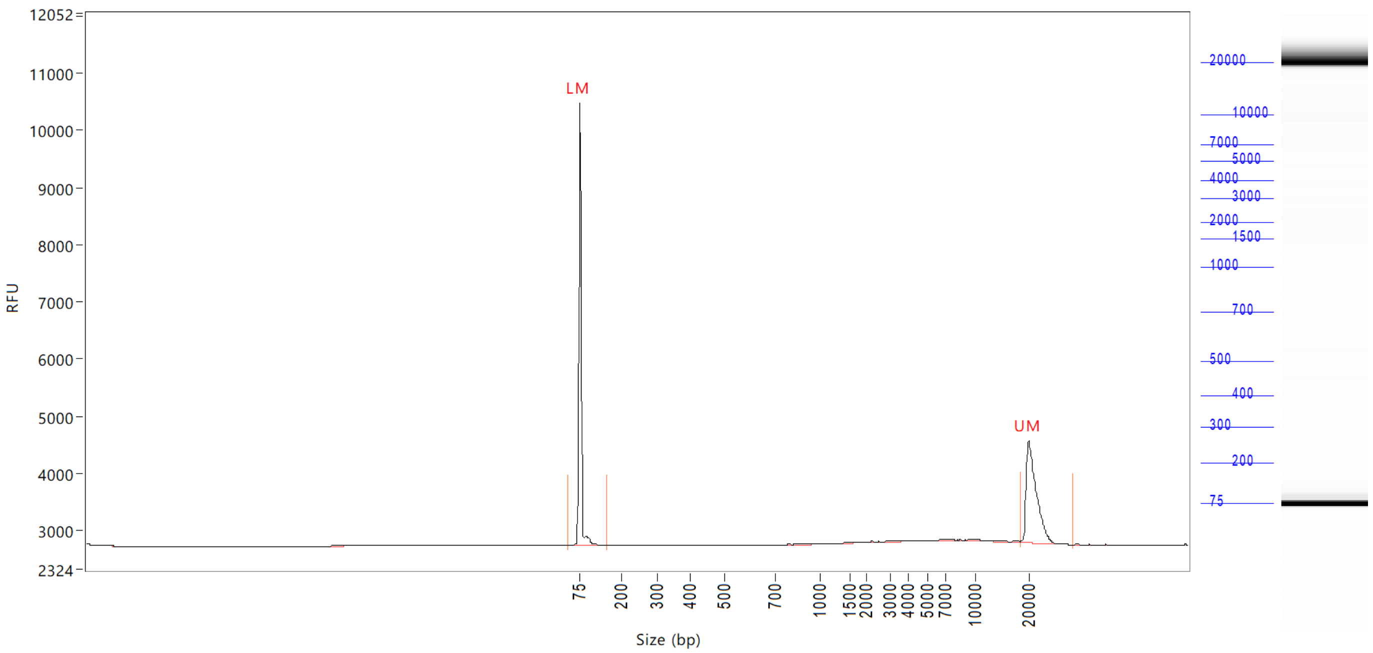

Peak	Size	Concentration	Relative concentration	Molarity	From	To	CV%	RFU
	(bp)	(ng/uL)	Percent	(nmole/L)	(bp)	(bp)		
1	75 (LM)	0.5000		11.0851	43	154	10.18	7760
2	20000 (UM)	0.2432		0.0193	18415	28463	5.43	1781

TIC:	0.0000	ng/uL
TIM:	0.0000	nmole/L
Total concentration:	0.5477	ng/uL

```
Sample peak width (sec): 5    Sample min peak height: 50    Sample baseline V to V?: Y    Sample baseline V to V points: 3
Sample filter: Binomial       Number of points for filter: 3  Sample start region (min): 0  Sample end region (min): 30
Marker peak width (sec): 5    Marker min peak height: 200   Marker baseline V to V?: Y    Marker baseline V to V points: 3
Lower marker selection: First peak > 500 RFU                Upper marker selection: Last peak > 200 RFU
Ladder size (bp)75, 200, 300, 400, 500, 700, 1000, 1500, 2000, 3000, 4000, 5000, 7000, 10000, 20000
Quantification using: Lower Marker          Final concentration (ng/uL): 0.5000          Dilution factor: 12.0
```

Sample: SampH10
Well location: H10
Created: Thursday, October 21, 2021 9:39:00 PM

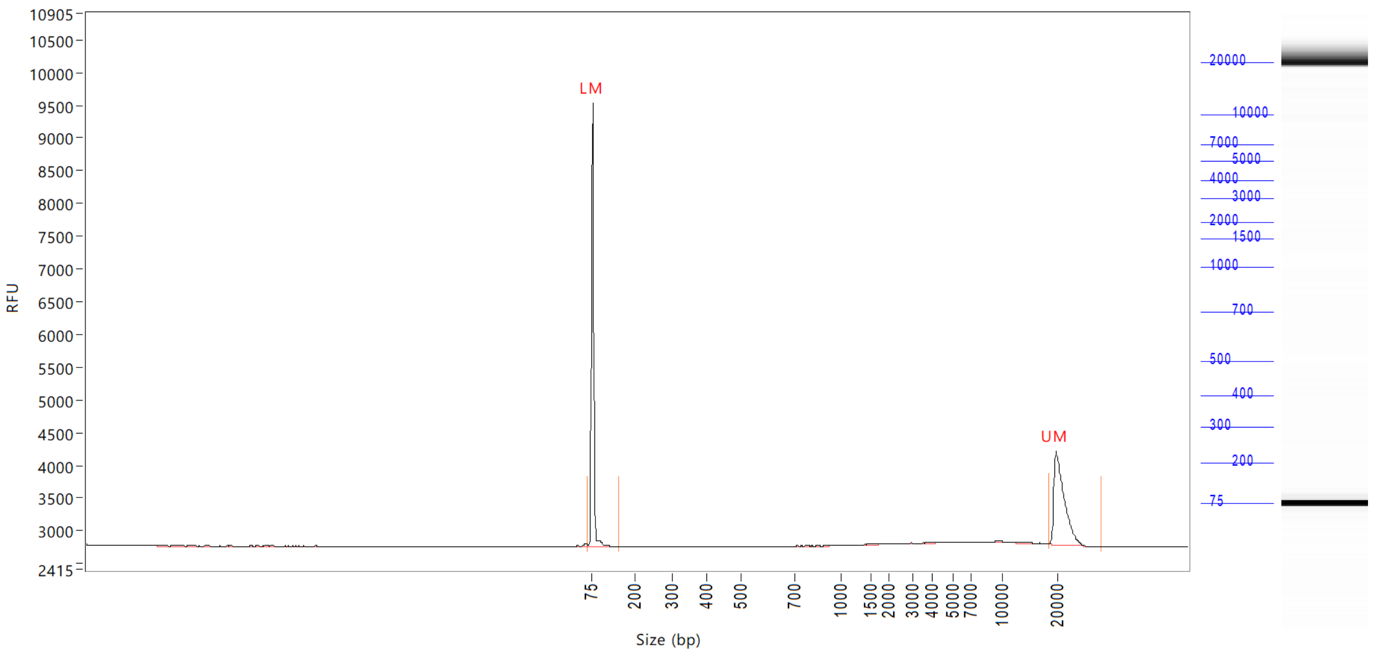

Peak	Size	Concentration	Relative concentration	Molarity	From	To	CV%	RFU
	(bp)	(ng/uL)	Percent	(nmole/L)	(bp)	(bp)		
1	75 (LM)	0.5000		11.0851	64	152	7.90	6789
2	20000 (UM)	0.2391		0.0190	18537	28221	5.35	1421

TIC:	0.0000	ng/uL
TIM:	0.0000	nmole/L
Total concentration:	0.5090	ng/uL

Sample peak width (sec): 5   Sample min peak height: 50   Sample baseline V to V?: Y   Sample baseline V to V points: 3
Sample filter: Binomial   Number of points for filter: 3 Sample start region (min): 0 Sample end region (min): 30
Marker peak width (sec): 5   Marker min peak height: 200   Marker baseline V to V?: Y   Marker baseline V to V points: 3
Lower marker selection: First peak > 500 RFU   Upper marker selection: Last peak > 200 RFU
Ladder size (bp)75, 200, 300, 400, 500, 700, 1000, 1500, 2000, 3000, 4000, 5000, 7000, 10000, 20000
Quantification using: Lower Marker   Final concentration (ng/uL): 0.5000   Dilution factor: 12.0

Sample: SampA11
Well location: A11
Created: Thursday, October 21, 2021 9:39:00 PM

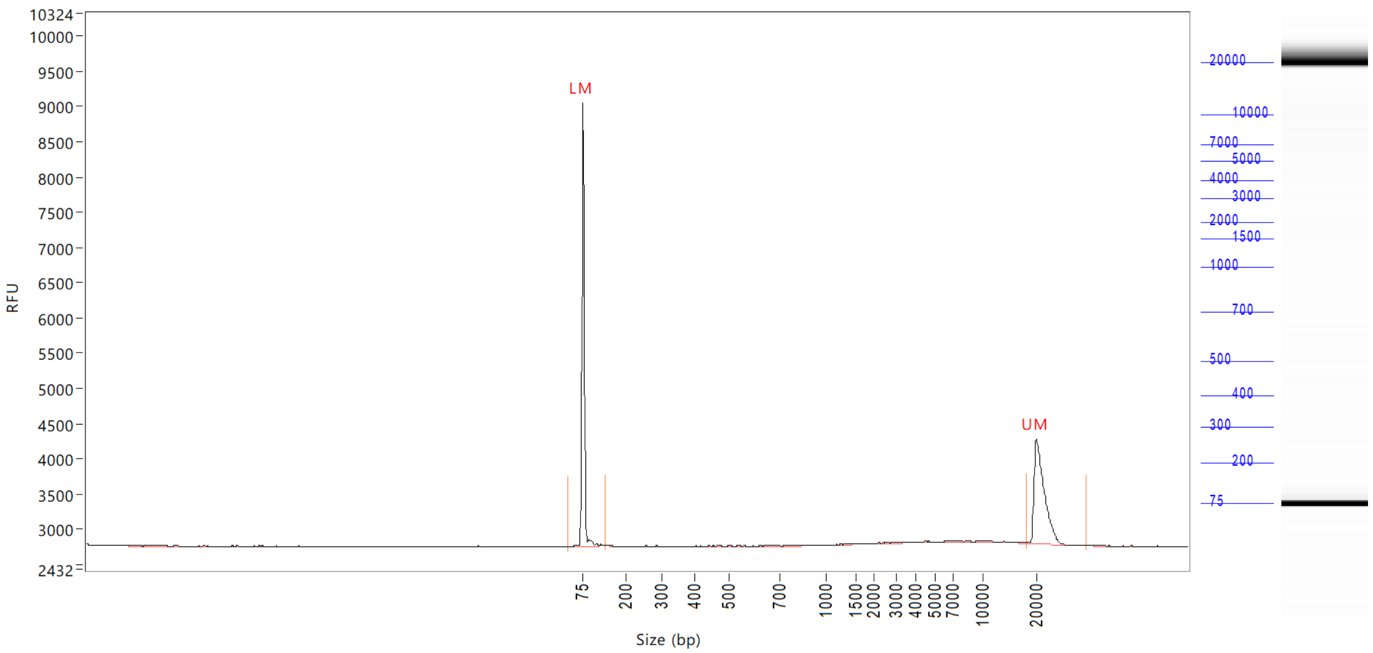

Peak	Size	Concentration	Relative concentration	Molarity	From	To	CV%	RFU
	(bp)	(ng/uL)	Percent	(nmole/L)	(bp)	(bp)		
1	75 (LM)	0.5000		11.2364	33	141	8.54	6303
2	20000 (UM)	0.2569		0.0204	18171	29068	5.58	1475

TIC:	0.0000	ng/uL
TIM:	0.0000	nmole/L
Total concentration:	0.6889	ng/uL

Sample peak width (sec): 5 Sample min peak height: 50 Sample baseline V to V?: Y Sample baseline V to V points: 3
Sample filter: Binomial Number of points for filter: 3 Sample start region (min): 0 Sample end region (min): 30
Marker peak width (sec): 5 Marker min peak height: 200 Marker baseline V to V?: Y Marker baseline V to V points: 3
Lower marker selection: First peak > 500 RFU Upper marker selection: Last peak > 200 RFU
Ladder size (bp)75, 200, 300, 400, 500, 700, 1000, 1500, 2000, 3000, 4000, 5000, 7000, 10000, 20000
Quantification using: Lower Marker Final concentration (ng/uL): 0.5000 Dilution factor: 12.0

Sample: SampB11
Well location: B11
Created: Thursday, October 21, 2021 9:39:00 PM

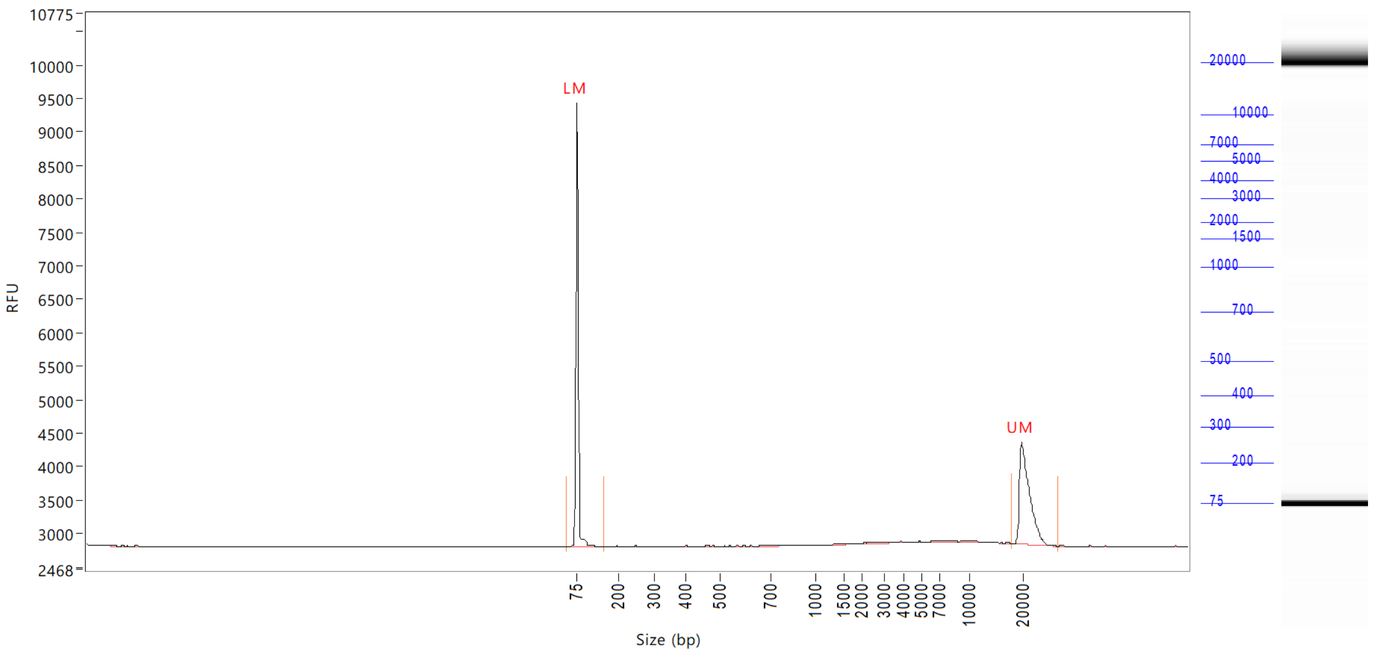

Peak	Size	Concentration	Relative concentration	Molarity	From	To	CV%	RFU
	(bp)	(ng/uL)	Percent	(nmole/L)	(bp)	(bp)		
1	75 (LM)	0.5000		11.2364	47	156	8.92	6642
2	20000 (UM)	0.2472		0.0196	18049	26770	5.39	1521

TIC:	0.0000	ng/uL
TIM:	0.0000	nmole/L
Total concentration:	0.5981	ng/uL

Sample peak width (sec): 5 Sample min peak height: 50 Sample baseline V to V?: Y Sample baseline V to V points: 3
Sample filter: Binomial Number of points for filter: 3 Sample start region (min): 0 Sample end region (min): 30
Marker peak width (sec): 5 Marker min peak height: 200 Marker baseline V to V?: Y Marker baseline V to V points: 3
Lower marker selection: First peak > 500 RFU Upper marker selection: Last peak > 200 RFU
Ladder size (bp)75, 200, 300, 400, 500, 700, 1000, 1500, 2000, 3000, 4000, 5000, 7000, 10000, 20000
Quantification using: Lower Marker Final concentration (ng/uL): 0.5000 Dilution factor: 12.0

Sample: SampC11
Well location: C11
Created: Thursday, October 21, 2021 9:39:00 PM

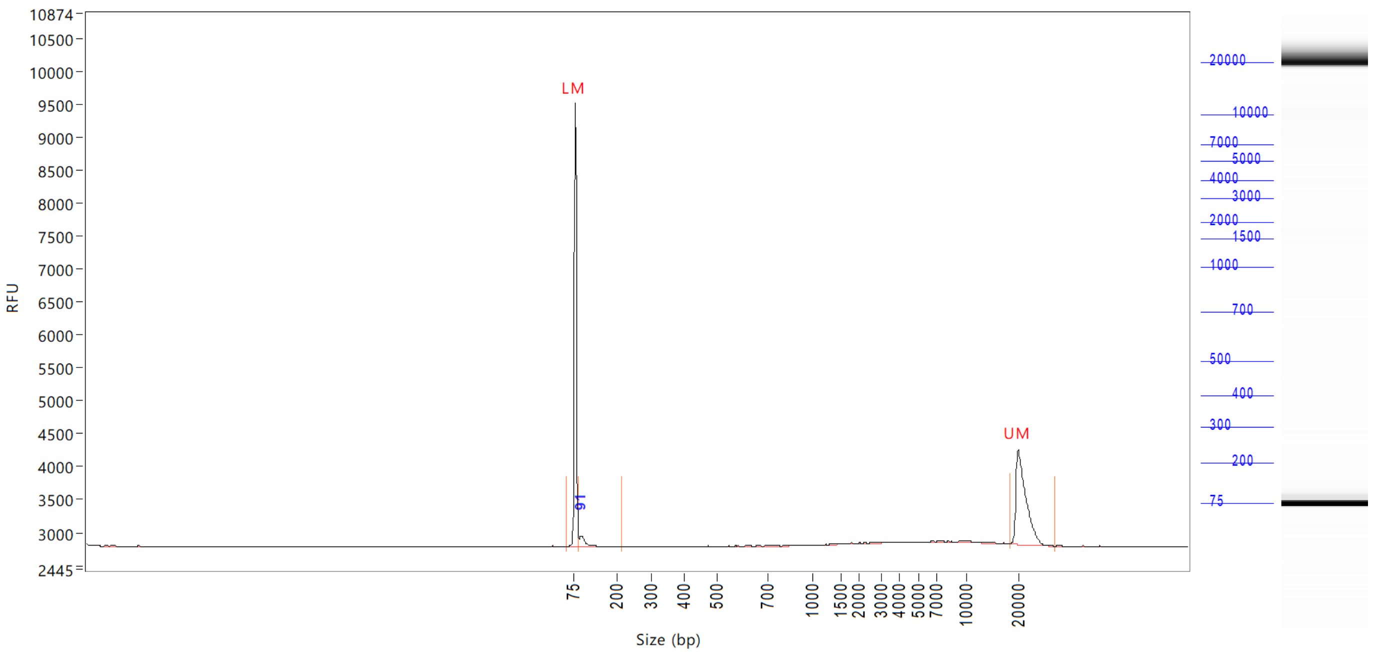

Peak	Size	Concentration	Relative concentration	Molarity	From	To	CV%	RFU
	(bp)	(ng/uL)	Percent	(nmole/L)	(bp)	(bp)		
1	75 (LM)	0.5000		11.2364	50	86	4.57	6739
2	91	0.4123	100.0	6.4490	86	212	23.51	163
3	20000 (UM)	0.2443		0.0194	18415	27012	5.46	1445

TIC:	0.4123	ng/uL
TIM:	6.4490	nmole/L
Total concentration:	0.9673	ng/uL

Sample peak width (sec): 5 Sample min peak height: 50 Sample baseline V to V?: Y Sample baseline V to V points: 3
Sample filter: Binomial Number of points for filter: 3 Sample start region (min): 0 Sample end region (min): 30
Marker peak width (sec): 5 Marker min peak height: 200 Marker baseline V to V?: Y Marker baseline V to V points: 3
Lower marker selection: First peak > 500 RFU Upper marker selection: Last peak > 200 RFU
Ladder size (bp)75, 200, 300, 400, 500, 700, 1000, 1500, 2000, 3000, 4000, 5000, 7000, 10000, 20000
Quantification using: Lower Marker Final concentration (ng/uL): 0.5000 Dilution factor: 12.0

Sample: SampD11
Well location: D11
Created: Thursday, October 21, 2021 9:39:00 PM

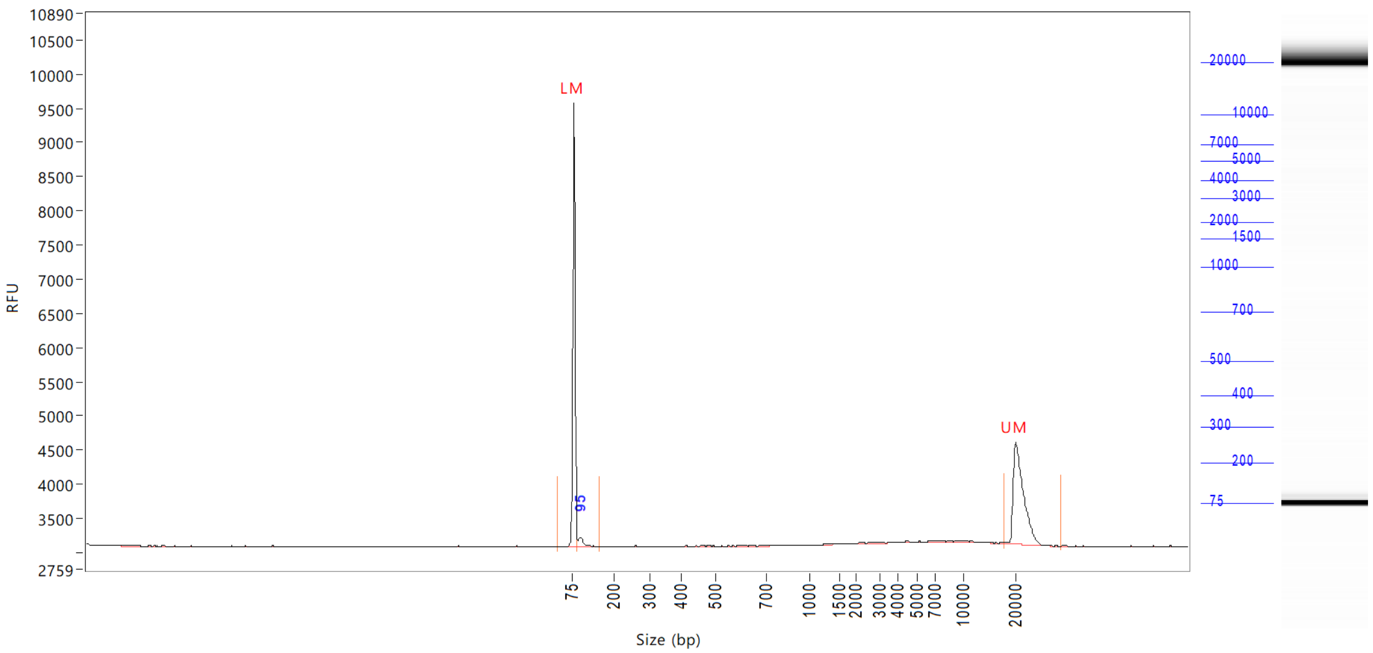

Peak	Size	Concentration	Relative concentration	Molarity	From	To	CV%	RFU
	(bp)	(ng/uL)	Percent	(nmole/L)	(bp)	(bp)		
1	75 (LM)	0.5000		11.0851	30	87	4.84	6501
2	95	0.2911	100.0	4.7337	87	156	14.17	132
3	20000 (UM)	0.2599		0.0206	17805	28584	5.55	1497

TIC:	0.2911	ng/uL
TIM:	4.7337	nmole/L
Total concentration:	0.9034	ng/uL

Sample peak width (sec): 5   Sample min peak height: 50   Sample baseline V to V?: Y   Sample baseline V to V points: 3
Sample filter: Binomial   Number of points for filter: 3 Sample start region (min): 0 Sample end region (min): 30
Marker peak width (sec): 5   Marker min peak height: 200   Marker baseline V to V?: Y   Marker baseline V to V points: 3
Lower marker selection: First peak > 500 RFU   Upper marker selection: Last peak > 200 RFU
Ladder size (bp)75, 200, 300, 400, 500, 700, 1000, 1500, 2000, 3000, 4000, 5000, 7000, 10000, 20000
Quantification using: Lower Marker   Final concentration (ng/uL): 0.5000   Dilution factor: 12.0

Sample: SampE11
Well location: E11
Created: Thursday, October 21, 2021 9:39:00 PM

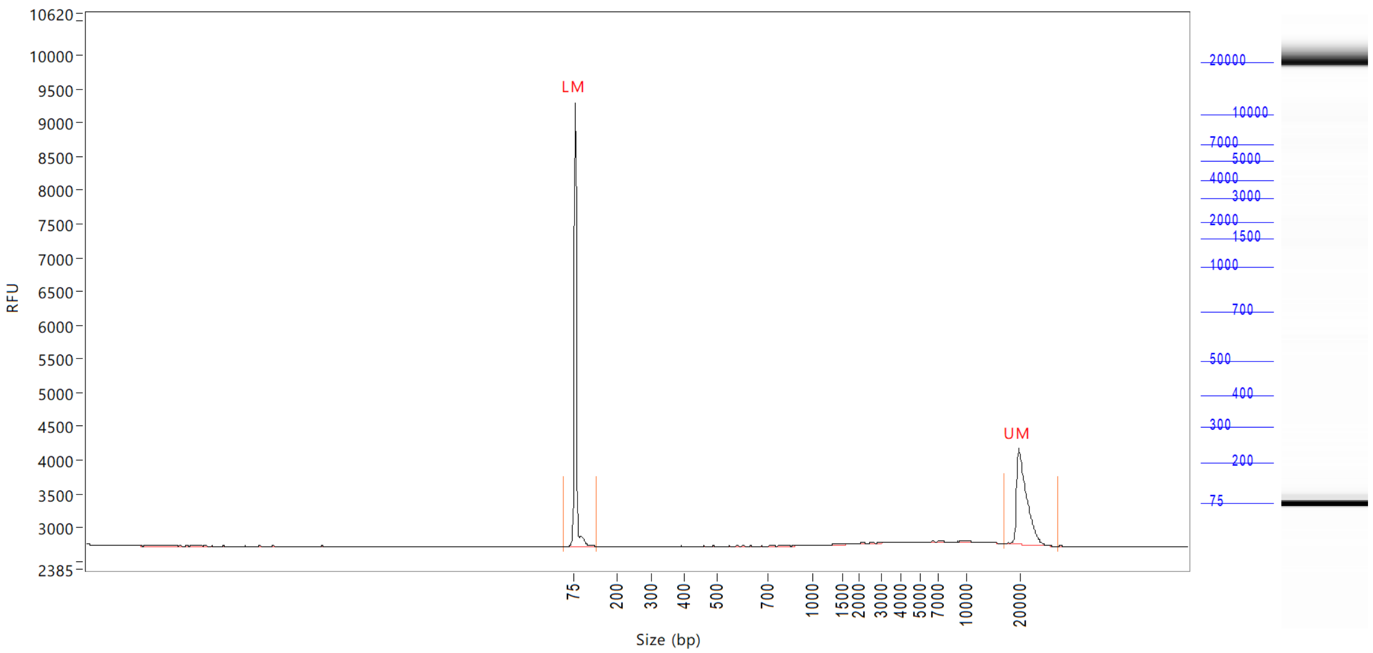

Peak	Size	Concentration	Relative concentration	Molarity	From	To	CV%	RFU
	(bp)	(ng/uL)	Percent	(nmole/L)	(bp)	(bp)		
1	75 (LM)	0.5000		11.0851	43	141	9.34	6586
2	20000 (UM)	0.2288		0.0181	17073	27496	5.47	1418

TIC:	0.0000	ng/uL
TIM:	0.0000	nmole/L
Total concentration:	0.4639	ng/uL

Sample peak width (sec): 5   Sample min peak height: 50   Sample baseline V to V?: Y   Sample baseline V to V points: 3
Sample filter: Binomial   Number of points for filter: 3 Sample start region (min): 0 Sample end region (min): 30
Marker peak width (sec): 5   Marker min peak height: 200   Marker baseline V to V?: Y   Marker baseline V to V points: 3
Lower marker selection: First peak > 500 RFU   Upper marker selection: Last peak > 200 RFU
Ladder size (bp)75, 200, 300, 400, 500, 700, 1000, 1500, 2000, 3000, 4000, 5000, 7000, 10000, 20000
Quantification using: Lower Marker   Final concentration (ng/uL): 0.5000   Dilution factor: 12.0

Sample: SampF11
Well location: F11
Created: Thursday, October 21, 2021 9:39:00 PM

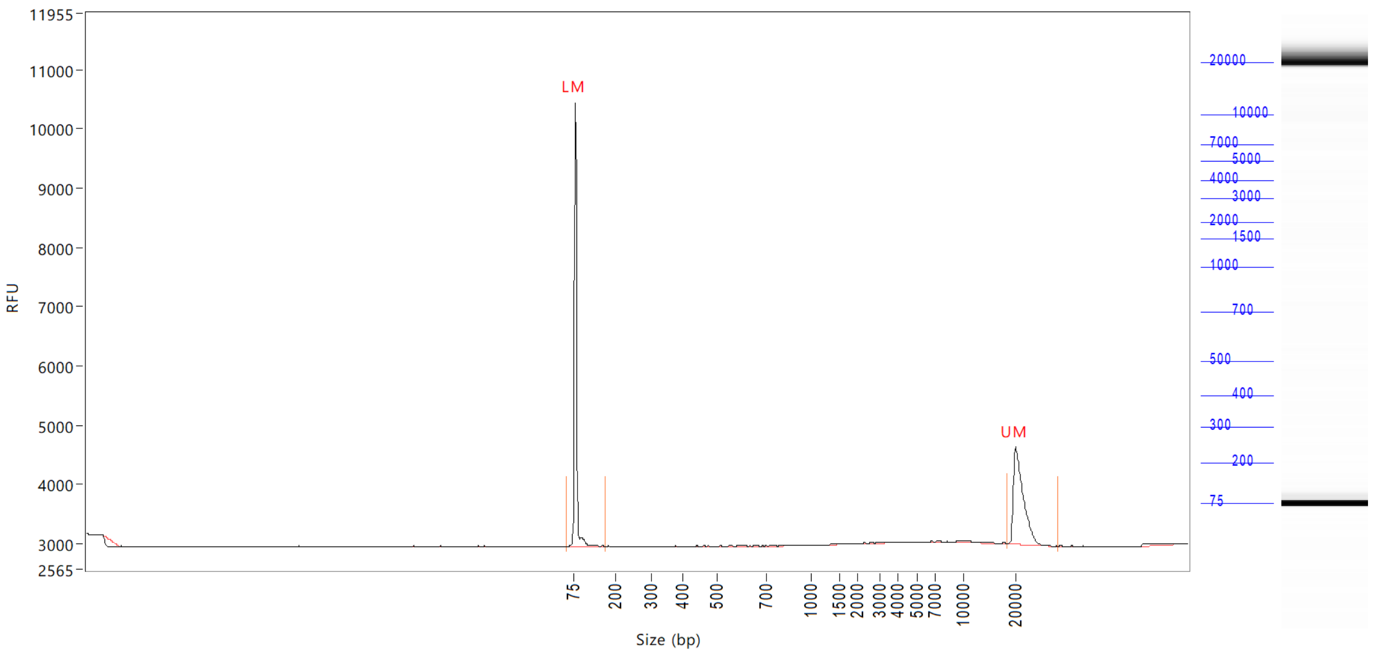

Peak	Size	Concentration	Relative concentration	Molarity	From	To	CV%	RFU
	(bp)	(ng/uL)	Percent	(nmole/L)	(bp)	(bp)		
1	75 (LM)	0.5000		11.0851	48	168	9.53	7502
2	20000 (UM)	0.2355		0.0187	18415	28221	5.33	1656

TIC:	0.0000	ng/uL
TIM:	0.0000	nmole/L
Total concentration:	0.4857	ng/uL

Sample peak width (sec): 5 Sample min peak height: 50 Sample baseline V to V?: Y Sample baseline V to V points: 3
Sample filter: Binomial Number of points for filter: 3 Sample start region (min): 0 Sample end region (min): 30
Marker peak width (sec): 5 Marker min peak height: 200 Marker baseline V to V?: Y Marker baseline V to V points: 3
Lower marker selection: First peak > 500 RFU Upper marker selection: Last peak > 200 RFU
Ladder size (bp)75, 200, 300, 400, 500, 700, 1000, 1500, 2000, 3000, 4000, 5000, 7000, 10000, 20000
Quantification using: Lower Marker Final concentration (ng/uL): 0.5000 Dilution factor: 12.0

Sample: SampG11
Well location: G11
Created: Thursday, October 21, 2021 9:39:00 PM

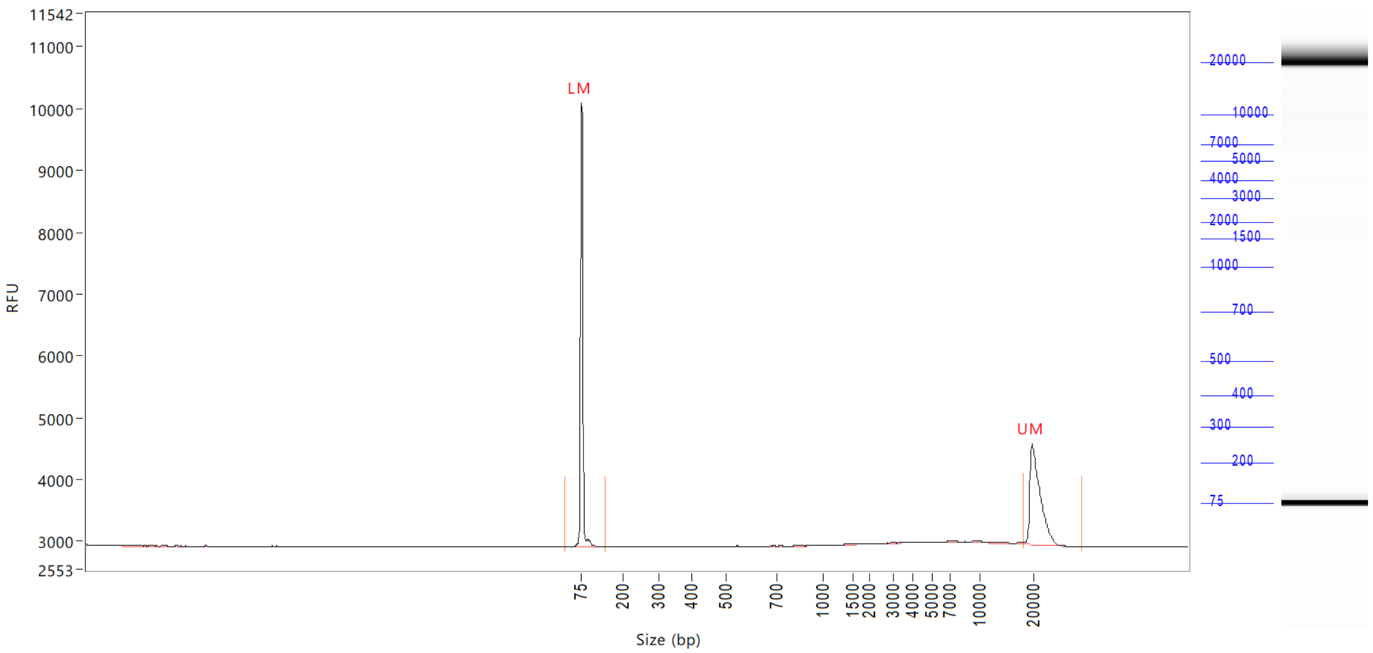

Peak	Size	Concentration	Relative concentration	Molarity	From	To	CV%	RFU
	(bp)	(ng/uL)	Percent	(nmole/L)	(bp)	(bp)		
1	75 (LM)	0.5000		11.0851	26	145	6.95	7187
2	20000 (UM)	0.2510		0.0199	18415	29430	5.54	1633

TIC:	0.0000	ng/uL
TIM:	0.0000	nmole/L
Total concentration:	0.5690	ng/uL

Sample peak width (sec): 5 Sample min peak height: 50 Sample baseline V to V?: Y Sample baseline V to V points: 3
Sample filter: Binomial Number of points for filter: 3 Sample start region (min): 0 Sample end region (min): 30
Marker peak width (sec): 5 Marker min peak height: 200 Marker baseline V to V?: Y Marker baseline V to V points: 3
Lower marker selection: First peak > 500 RFU Upper marker selection: Last peak > 200 RFU
Ladder size (bp)75, 200, 300, 400, 500, 700, 1000, 1500, 2000, 3000, 4000, 5000, 7000, 10000, 20000
Quantification using: Lower Marker Final concentration (ng/uL): 0.5000 Dilution factor: 12.0

Sample: SampH11
Well location: H11
Created: Thursday, October 21, 2021 9:39:00 PM

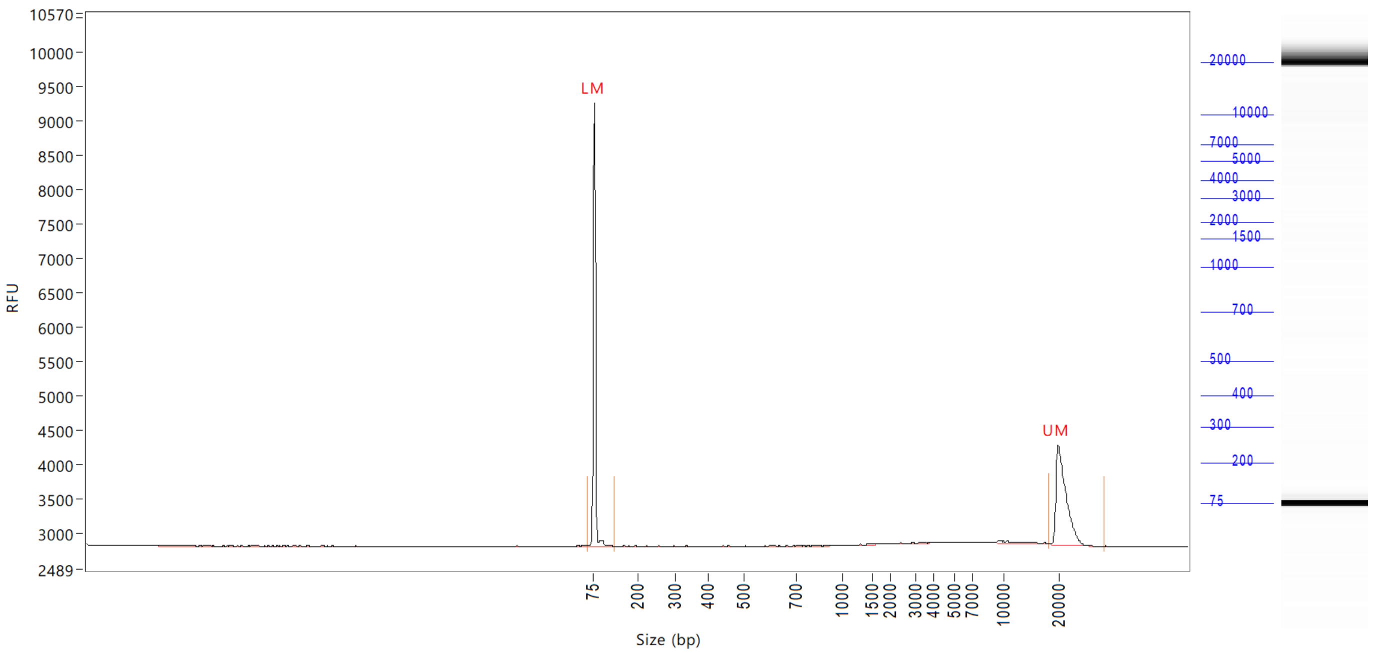

Peak	Size	Concentration	Relative concentration	Molarity	From	To	CV%	RFU
	(bp)	(ng/uL)	Percent	(nmole/L)	(bp)	(bp)		
1	75 (LM)	0.5000		11.0851	58	133	7.02	6458
2	20000 (UM)	0.2494		0.0198	18171	28342	5.31	1438

TIC:	0.0000	ng/uL
TIM:	0.0000	nmole/L
Total concentration:	0.6960	ng/uL

```
Sample peak width (sec): 5    Sample min peak height: 50    Sample baseline V to V?: Y    Sample baseline V to V points: 3
Sample filter: Binomial       Number of points for filter: 3  Sample start region (min): 0  Sample end region (min): 30
Marker peak width (sec): 5    Marker min peak height: 200   Marker baseline V to V?: Y    Marker baseline V to V points: 3
Lower marker selection: First peak > 500 RFU                Upper marker selection: Last peak > 200 RFU
Ladder size (bp)75, 200, 300, 400, 500, 700, 1000, 1500, 2000, 3000, 4000, 5000, 7000, 10000, 20000
Quantification using: Lower Marker          Final concentration (ng/uL): 0.5000          Dilution factor: 12.0
```

Sample: SampA12
Well location: A12
Created: Thursday, October 21, 2021 9:39:00 PM

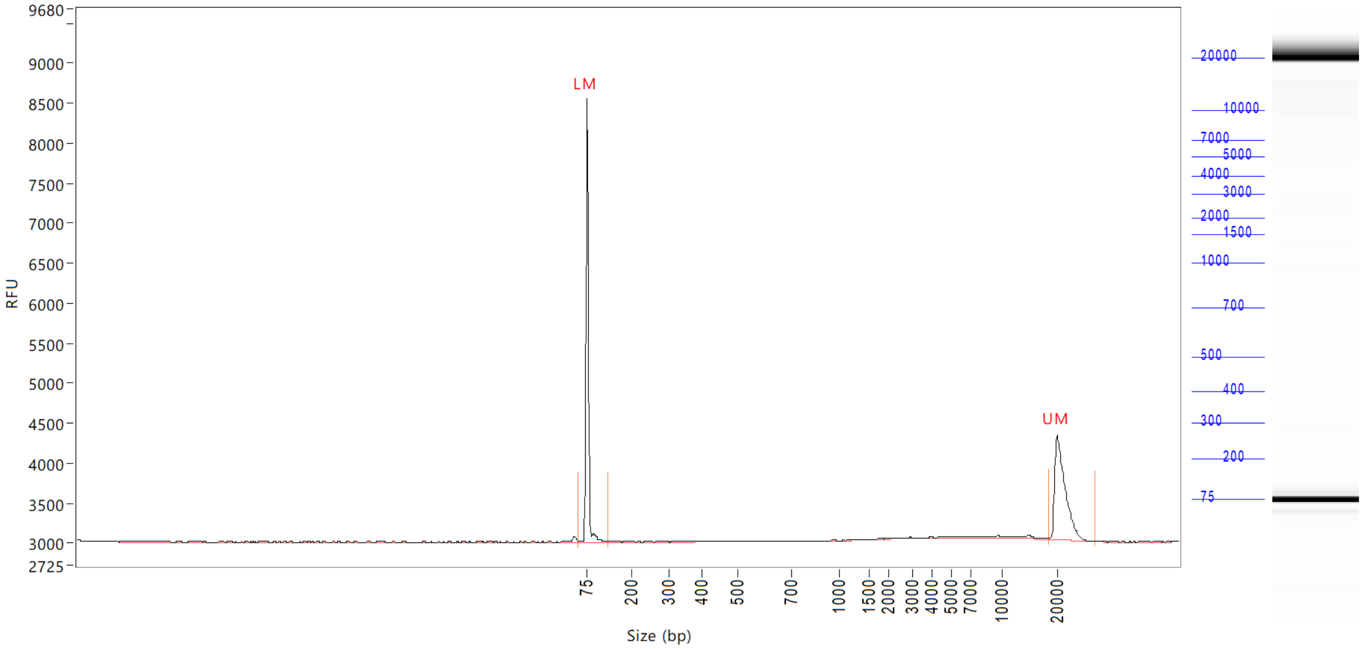

Peak	Size	Concentration	Relative concentration	Molarity	From	To	CV%	RFU
	(bp)	(ng/uL)	Percent	(nmole/L)	(bp)	(bp)		
1	75 (LM)	0.5000		11.2364	50	135	8.68	5556
2	20000 (UM)	0.2543		0.0201	18537	26891	5.37	1305

TIC:	0.0000	ng/uL
TIM:	0.0000	nmole/L
Total concentration:	0.8035	ng/uL

Sample peak width (sec): 5 Sample min peak height: 50 Sample baseline V to V?: Y Sample baseline V to V points: 3
Sample filter: Binomial Number of points for filter: 3 Sample start region (min): 0 Sample end region (min): 30
Marker peak width (sec): 5 Marker min peak height: 200 Marker baseline V to V?: Y Marker baseline V to V points: 3
Lower marker selection: First peak > 500 RFU Upper marker selection: Last peak > 200 RFU
Ladder size (bp)75, 200, 300, 400, 500, 700, 1000, 1500, 2000, 3000, 4000, 5000, 7000, 10000, 20000
Quantification using: Lower Marker Final concentration (ng/uL): 0.5000 Dilution factor: 12.0

Sample: SampB12
Well location: B12
Created: Thursday, October 21, 2021 9:39:00 PM

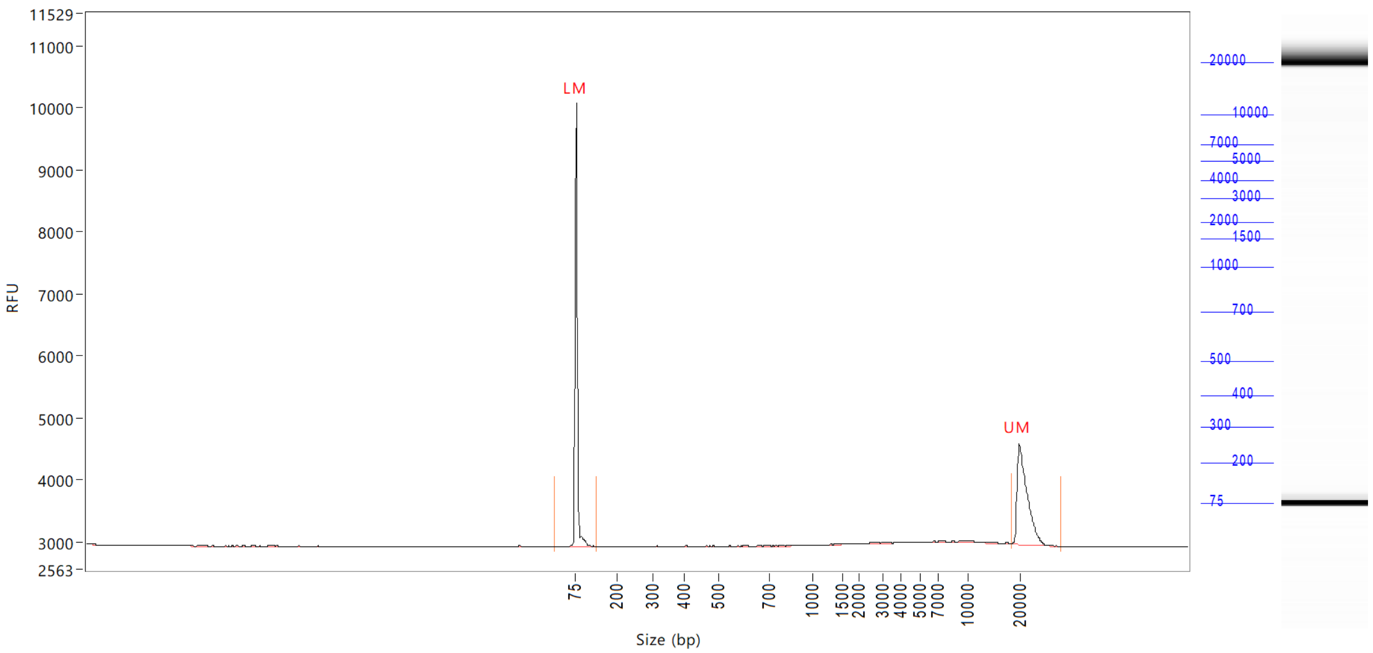

Peak	Size	Concentration	Relative concentration	Molarity	From	To	CV%	RFU
	(bp)	(ng/uL)	Percent	(nmole/L)	(bp)	(bp)		
1	75 (LM)	0.5000		11.0851	11	135	8.20	7168
2	20000 (UM)	0.2498		0.0198	18415	27859	5.49	1624

TIC:	0.0000	ng/uL
TIM:	0.0000	nmole/L
Total concentration:	0.5904	ng/uL

```
Sample peak width (sec): 5    Sample min peak height: 50    Sample baseline V to V?: Y    Sample baseline V to V points: 3
Sample filter: Binomial       Number of points for filter: 3  Sample start region (min): 0  Sample end region (min): 30
Marker peak width (sec): 5    Marker min peak height: 200   Marker baseline V to V?: Y    Marker baseline V to V points: 3
Lower marker selection: First peak > 500 RFU                Upper marker selection: Last peak > 200 RFU
Ladder size (bp)75, 200, 300, 400, 500, 700, 1000, 1500, 2000, 3000, 4000, 5000, 7000, 10000, 20000
Quantification using: Lower Marker          Final concentration (ng/uL): 0.5000          Dilution factor: 12.0
```

Sample: SampC12
Well location: C12
Created: Thursday, October 21, 2021 9:39:00 PM

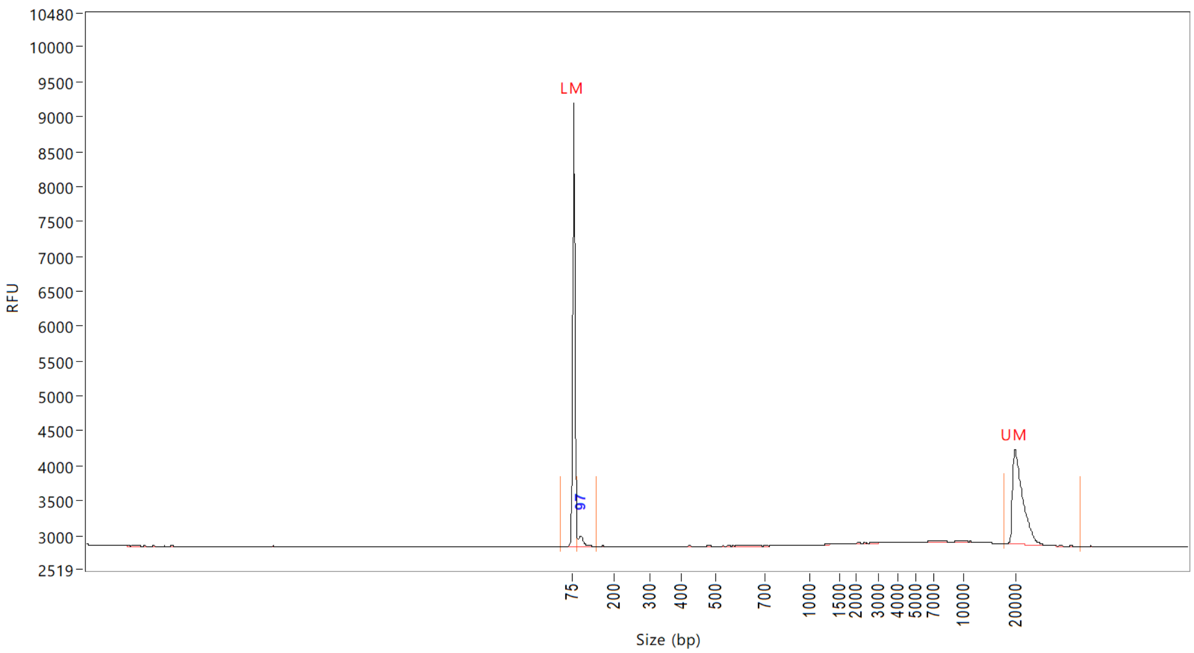

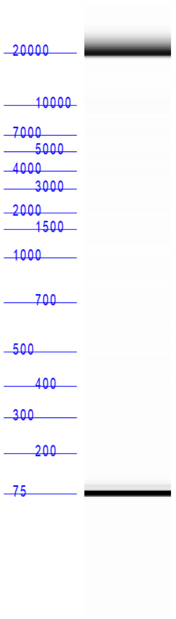

Peak	Size	Concentration	Relative concentration	Molarity	From	To	CV%	RFU
	(bp)	(ng/uL)	Percent	(nmole/L)	(bp)	(bp)		
1	75 (LM)	0.5000		11.2364	37	87	4.75	6365
2	97	0.3289	100.0	5.4556	87	143	10.92	148
3	20000 (UM)	0.2411		0.0191	17927	32574	6.39	1349

TIC:	0.3289	ng/uL
TIM:	5.4556	nmole/L
Total concentration:	0.7956	ng/uL

Sample peak width (sec): 5 Sample min peak height: 50 Sample baseline V to V?: Y Sample baseline V to V points: 3
Sample filter: Binomial Number of points for filter: 3 Sample start region (min): 0 Sample end region (min): 30
Marker peak width (sec): 5 Marker min peak height: 200 Marker baseline V to V?: Y Marker baseline V to V points: 3
Lower marker selection: First peak > 500 RFU Upper marker selection: Last peak > 200 RFU
Ladder size (bp)75, 200, 300, 400, 500, 700, 1000, 1500, 2000, 3000, 4000, 5000, 7000, 10000, 20000
Quantification using: Lower Marker Final concentration (ng/uL): 0.5000 Dilution factor: 12.0

Sample: SampD12
Well location: D12
Created: Thursday, October 21, 2021 9:39:00 PM

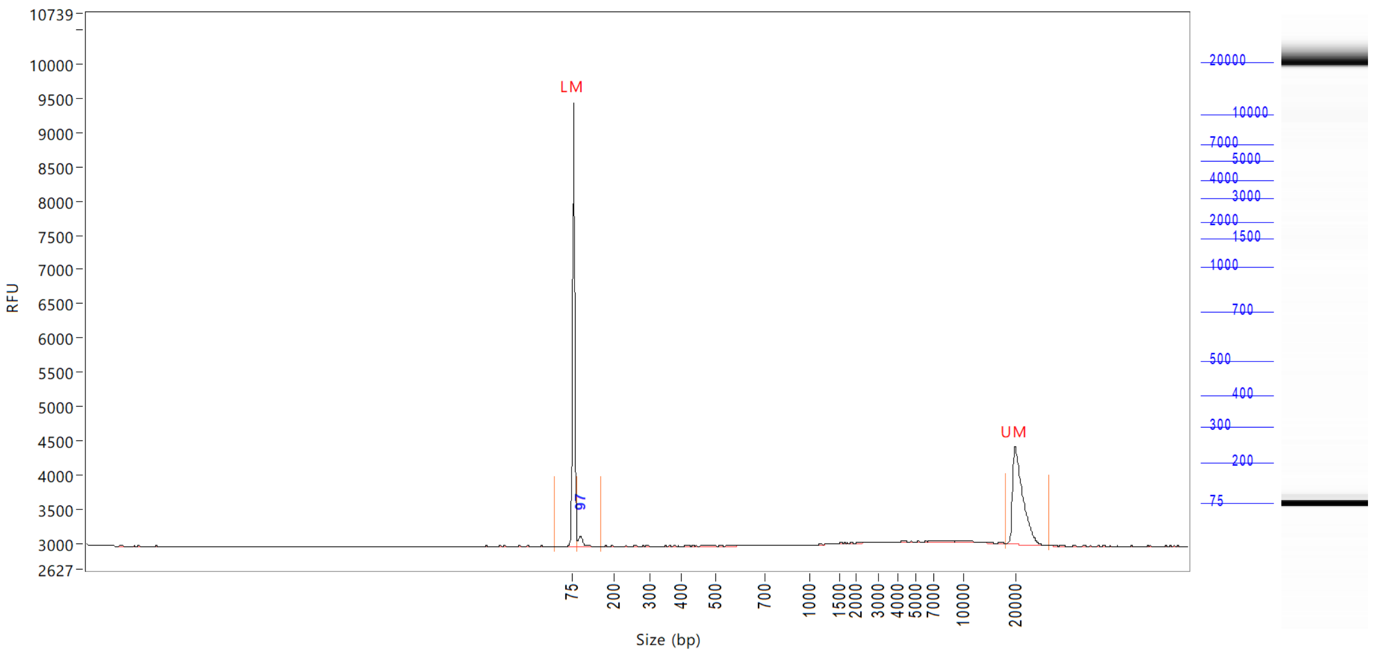

Peak	Size	Concentration	Relative concentration	Molarity	From	To	CV%	RFU
	(bp)	(ng/uL)	Percent	(nmole/L)	(bp)	(bp)		
1	75 (LM)	0.5000		11.2364	20	86	5.03	6484
2	97	0.3073	100.0	5.1485	86	158	12.97	142
3	20000 (UM)	0.2571		0.0204	18171	26529	5.55	1429

TIC:	0.3073	ng/uL
TIM:	5.1485	nmole/L
Total concentration:	0.9508	ng/uL

Sample peak width (sec): 5   Sample min peak height: 50   Sample baseline V to V?: Y   Sample baseline V to V points: 3
Sample filter: Binomial   Number of points for filter: 3 Sample start region (min): 0 Sample end region (min): 30
Marker peak width (sec): 5   Marker min peak height: 200   Marker baseline V to V?: Y   Marker baseline V to V points: 3
Lower marker selection: First peak > 500 RFU   Upper marker selection: Last peak > 200 RFU
Ladder size (bp)75, 200, 300, 400, 500, 700, 1000, 1500, 2000, 3000, 4000, 5000, 7000, 10000, 20000
Quantification using: Lower Marker   Final concentration (ng/uL): 0.5000   Dilution factor: 12.0

Sample: SampE12
Well location: E12
Created: Thursday, October 21, 2021 9:39:00 PM

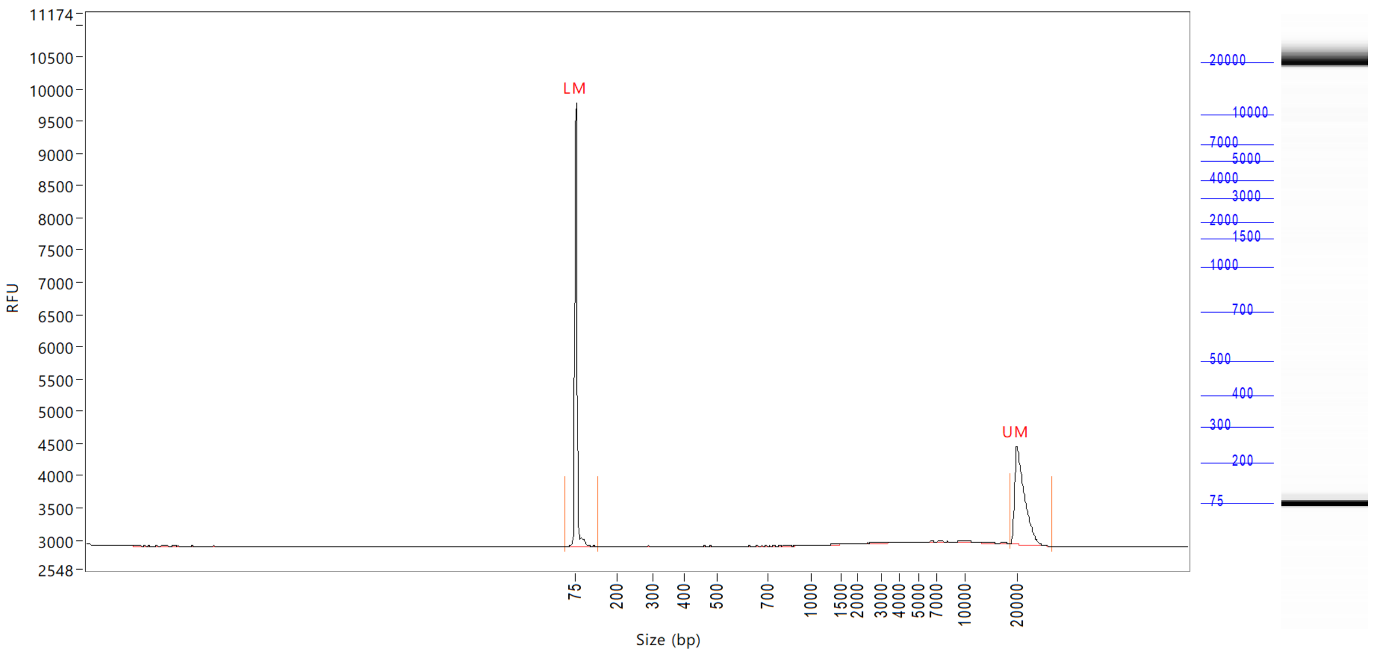

Peak	Size	Concentration	Relative concentration	Molarity	From	To	CV%	RFU
	(bp)	(ng/uL)	Percent	(nmole/L)	(bp)	(bp)		
1	75 (LM)	0.5000		11.2364	45	141	8.68	6899
2	20000 (UM)	0.2346		0.0186	18537	26770	5.24	1526

TIC:	0.0000	ng/uL
TIM:	0.0000	nmole/L
Total concentration:	0.5606	ng/uL

Sample peak width (sec): 5 Sample min peak height: 50 Sample baseline V to V?: Y Sample baseline V to V points: 3
Sample filter: Binomial Number of points for filter: 3 Sample start region (min): 0 Sample end region (min): 30
Marker peak width (sec): 5 Marker min peak height: 200 Marker baseline V to V?: Y Marker baseline V to V points: 3
Lower marker selection: First peak > 500 RFU Upper marker selection: Last peak > 200 RFU
Ladder size (bp)75, 200, 300, 400, 500, 700, 1000, 1500, 2000, 3000, 4000, 5000, 7000, 10000, 20000
Quantification using: Lower Marker Final concentration (ng/uL): 0.5000 Dilution factor: 12.0

Sample: SampF12
Well location: F12
Created: Thursday, October 21, 2021 9:39:00 PM

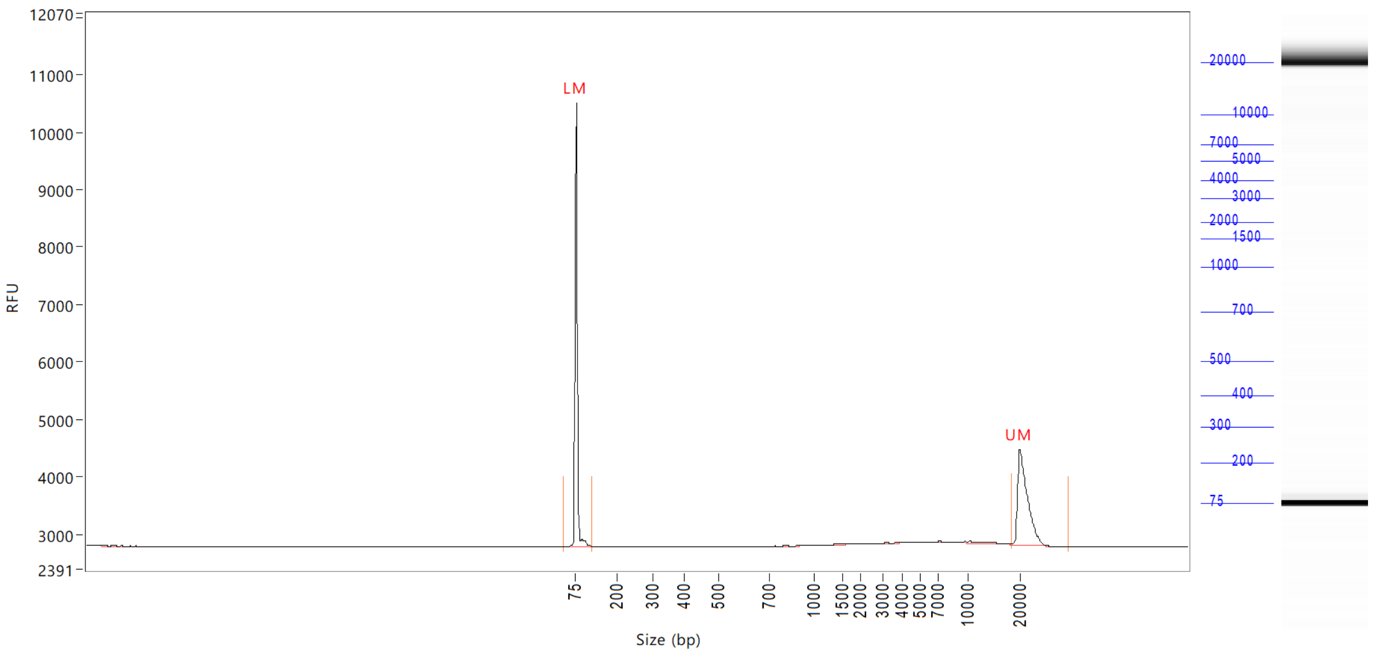

Peak	Size	Concentration	Relative concentration	Molarity	From	To	CV%	RFU
	(bp)	(ng/uL)	Percent	(nmole/L)	(bp)	(bp)		
1	75 (LM)	0.5000		11.0851	41	124	7.26	7740
2	20000 (UM)	0.2348		0.0187	18415	29068	5.59	1653

TIC:	0.0000	ng/uL
TIM:	0.0000	nmole/L
Total concentration:	0.5293	ng/uL

Sample peak width (sec): 5 Sample min peak height: 50 Sample baseline V to V?: Y Sample baseline V to V points: 3
Sample filter: Binomial Number of points for filter: 3 Sample start region (min): 0 Sample end region (min): 30
Marker peak width (sec): 5 Marker min peak height: 200 Marker baseline V to V?: Y Marker baseline V to V points: 3
Lower marker selection: First peak > 500 RFU Upper marker selection: Last peak > 200 RFU
Ladder size (bp)75, 200, 300, 400, 500, 700, 1000, 1500, 2000, 3000, 4000, 5000, 7000, 10000, 20000
Quantification using: Lower Marker Final concentration (ng/uL): 0.5000 Dilution factor: 12.0

Sample: SampG12
Well location: G12
Created: Thursday, October 21, 2021 9:39:00 PM

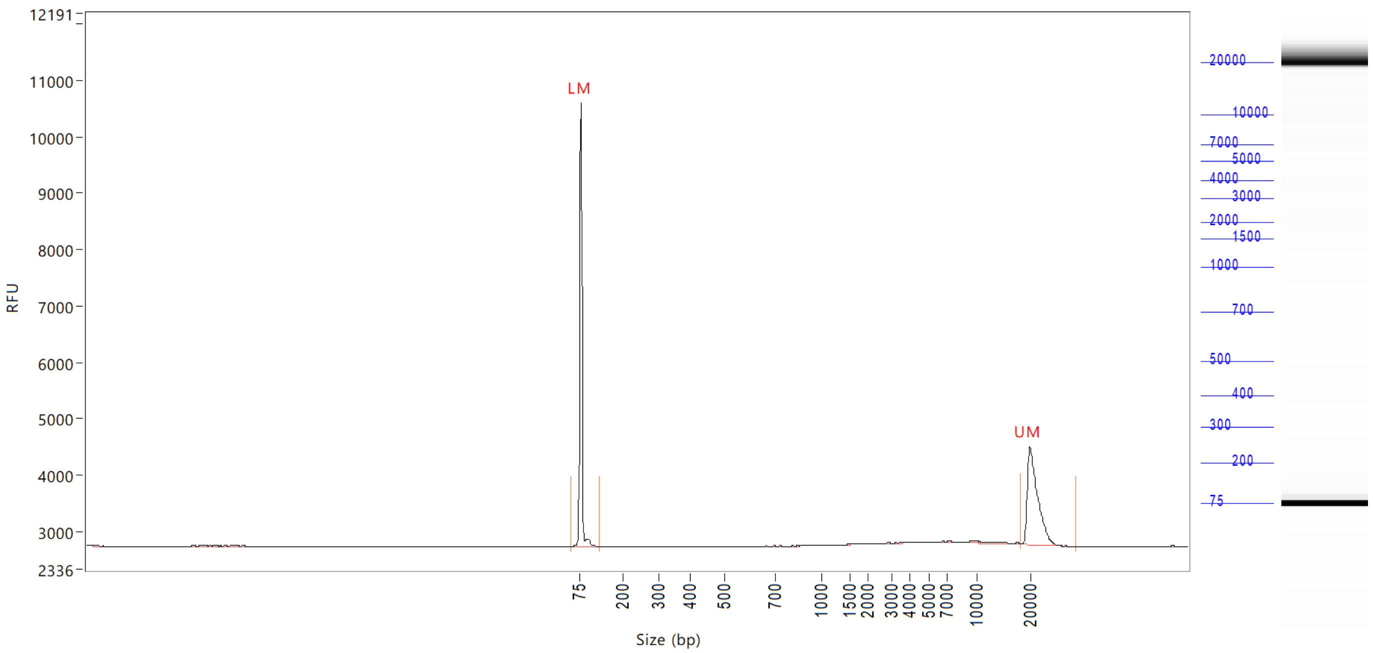

Peak	Size	Concentration	Relative concentration	Molarity	From	To	CV%	RFU
	(bp)	(ng/uL)	Percent	(nmole/L)	(bp)	(bp)		
1	75 (LM)	0.5000		11.0851	48	129	7.67	7881
2	20000 (UM)	0.2475		0.0196	18415	28584	5.56	1744

TIC:	0.0000	ng/uL
TIM:	0.0000	nmole/L
Total concentration:	0.5657	ng/uL

Sample peak width (sec): 5 Sample min peak height: 50 Sample baseline V to V?: Y Sample baseline V to V points: 3
Sample filter: Binomial Number of points for filter: 3 Sample start region (min): 0 Sample end region (min): 30
Marker peak width (sec): 5 Marker min peak height: 200 Marker baseline V to V?: Y Marker baseline V to V points: 3
Lower marker selection: First peak > 500 RFU Upper marker selection: Last peak > 200 RFU
Ladder size (bp)75, 200, 300, 400, 500, 700, 1000, 1500, 2000, 3000, 4000, 5000, 7000, 10000, 20000
Quantification using: Lower Marker Final concentration (ng/uL): 0.5000 Dilution factor: 12.0

Sample: SampH12
Well location: H12
Created: Thursday, October 21, 2021 9:39:00 PM

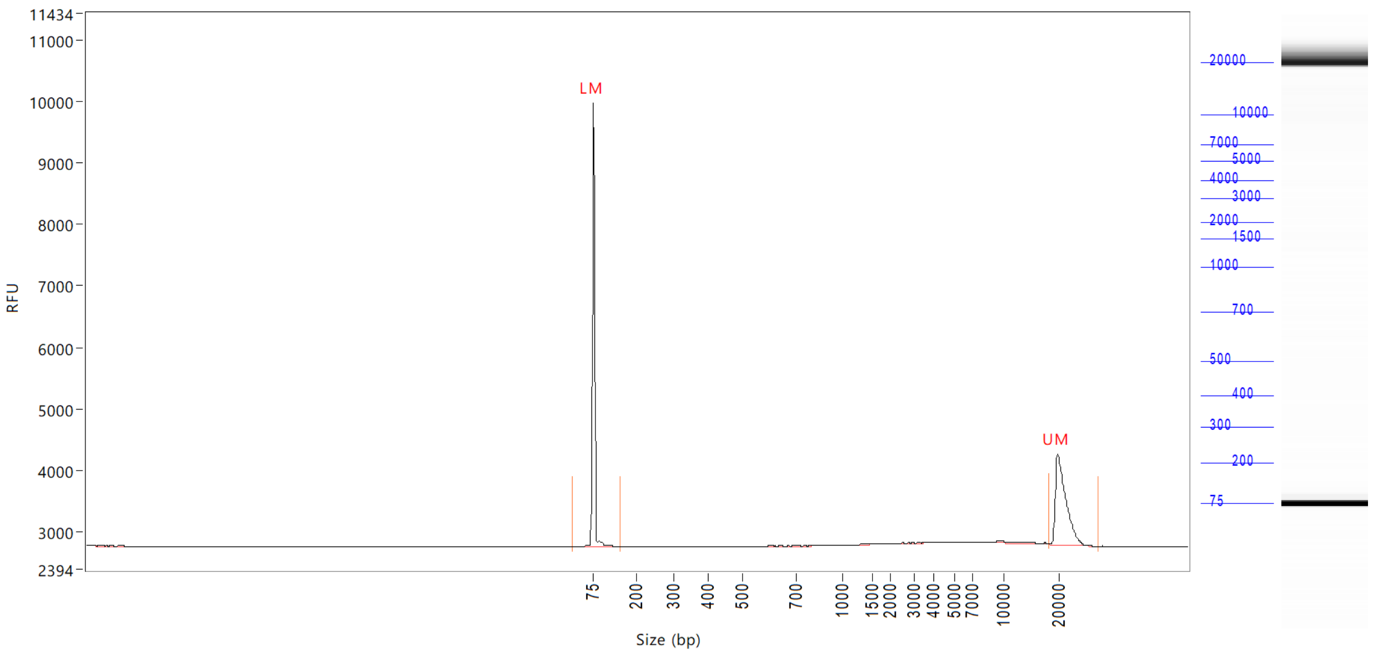

Peak	Size	Concentration	Relative concentration	Molarity	From	To	CV%	RFU
	(bp)	(ng/uL)	Percent	(nmole/L)	(bp)	(bp)		
1	75 (LM)	0.5000		11.2364	14	152	7.88	7225
2	20000 (UM)	0.2351		0.0187	18293	27375	5.41	1478

TIC:	0.0000	ng/uL
TIM:	0.0000	nmole/L
Total concentration:	0.5393	ng/uL

Sample peak width (sec): 5 Sample min peak height: 200 Sample baseline V to V?: Y Sample baseline V to V points: 3
Sample filter: Binomial Number of points for filter: 3 Sample start region (min): 0 Sample end region (min): 30
Marker peak width (sec): 5 Marker min peak height: 200 Marker baseline V to V?: Y Marker baseline V to V points: 3
Lower marker selection: First peak > 500 RFU Upper marker selection: Last peak > 200 RFU
Ladder size (bp)75, 200, 300, 400, 500, 700, 1000, 1500, 2000, 3000, 4000, 5000, 7000, 10000, 20000
Quantification using: Lower Marker Final concentration (ng/uL): 0.5000 Dilution factor: 12.0

Sample: DNA size ladder
Well location: A7
Created: Thursday, October 21, 2021 9:39:00 PM
Import from: C:\Users\admin\Desktop\210810_alberto_ladder.SCAL
Fit type: Point to point

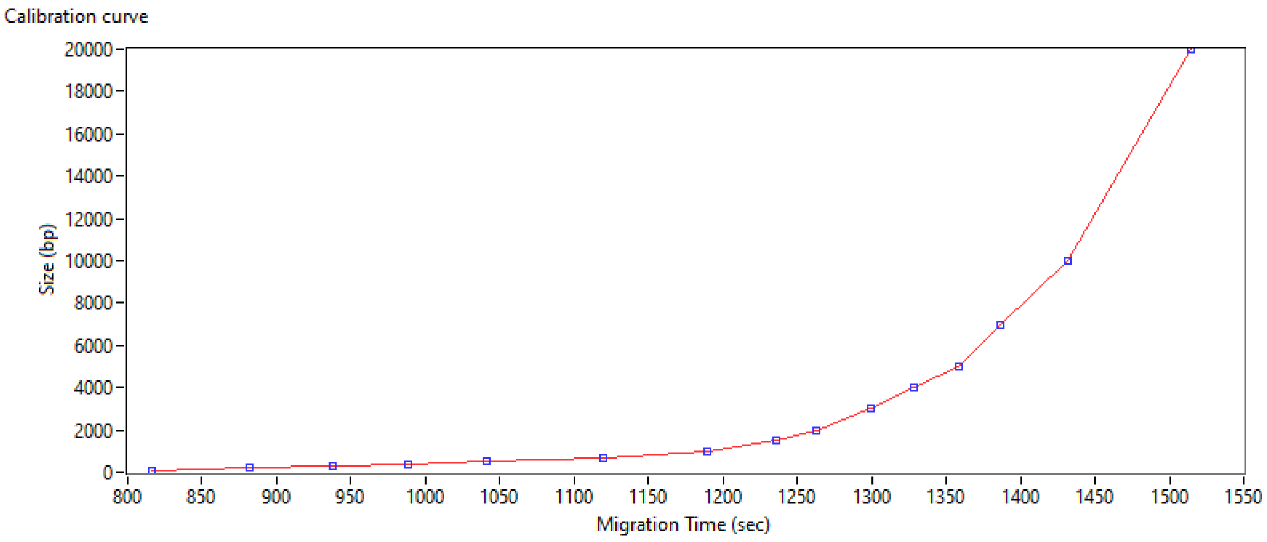

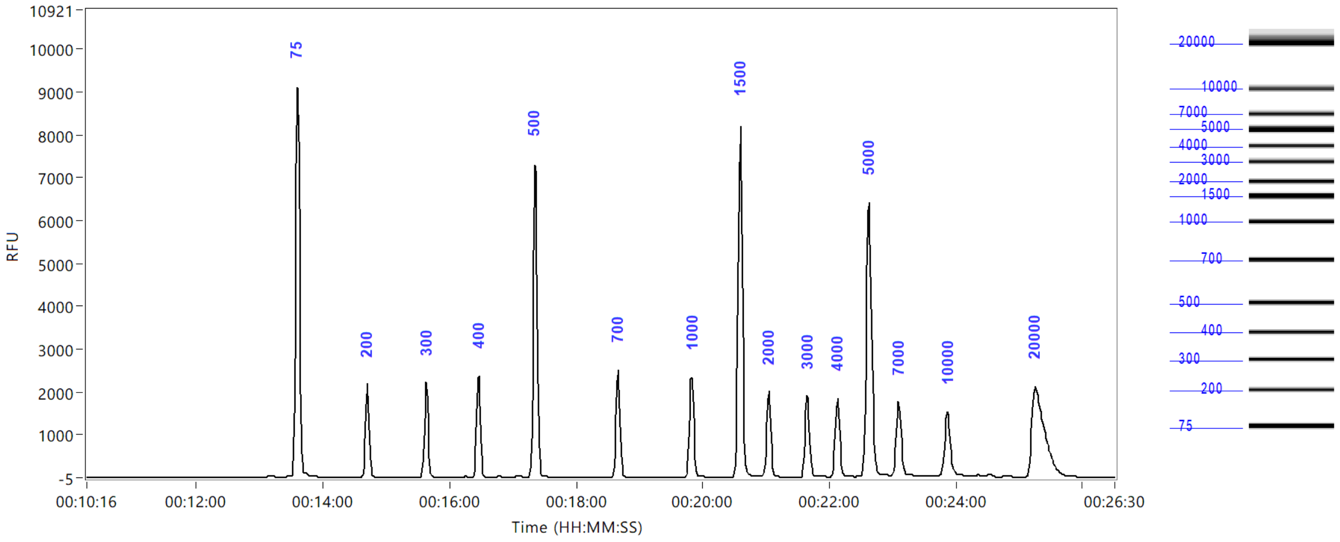

Supplement: Supplementary file 2 — sb3c00292_si_002.zip [file sb3c00292_si_002.zip › dnada_supplementary_material_pks_library_build/colony_pcr/ZAG/21-29-00_plate11/colonypcrplate_11.pdf]
